# New perspectives on the mechanisms and treatment of valvular heart disease: Mendelian randomization and systematic analysis based on plasma proteins

**DOI:** 10.1097/MD.0000000000048210

**Published:** 2026-04-03

**Authors:** Cheng Wang, Xiao Shao, Jia Wang, Rui Shi, Moxuan Han, Huiyan Feng, Hezeng Dong, Tengyue Zhang, Wenqian Yang, Yue Deng

**Affiliations:** a School of Traditional Chinese Medicine, Changchun University of Chinese Medicine, Changchun, Jilin, China; b Department of Cardiology, Affiliated Hospital of Changchun University of Chinese Medicine, Changchun, Jilin, China; c Department of General Internal Medicine, Affiliated Hospital of Changchun University of Chinese Medicine, Changchun, Jilin, China; d School of Integrated Traditional Chinese and Western Medicine, Changchun University of Chinese Medicine, Changchun, Jilin, China.

**Keywords:** Mendelian randomization, plasma proteins, valvular calcification, valvular fibrosis, valvular heart disease

## Abstract

Valvular heart disease (VHD) is a common cardiovascular disorder with insidious early symptoms and can progress to heart failure or sudden death. Pharmacological options remain limited, and severe disease often requires surgical intervention. This study aimed to identify key molecular targets and potential therapeutic candidates for VHD using Mendelian randomization (MR) and integrative analyses. A 2-sample MR design was used to evaluate the association between genetically predicted exposures and VHD using publicly available genome-wide association study summary statistics. Downstream analyses included functional enrichment, drug repurposing with molecular docking, protein–protein interaction network construction with hub-gene identification, and single-cell RNA sequencing-based analysis to examine the cell-type distribution of candidate gene expression. MR analysis identified 76 genes associated with VHD, including stathmin 1, ribosomal protein S5, and mitogen-activated protein kinase 8. Enrichment analysis suggested that these genes were involved in multiple signaling pathways potentially related to disease progression. Drug prediction and molecular docking prioritized razoxane, reserpine, and bisindolylmaleimide I as candidate compounds targeting key molecules. Protein–protein interaction network analysis further identified 10 hub genes, such as DEAD-box helicase 6 and apolipoprotein E. Single-cell sequencing showed high expression of these genes in cardiomyocytes, fibroblasts, and smooth muscle cells. This study identified candidate genes and potential drug leads for VHD through an MR-based integrative analysis, providing targets for subsequent mechanistic and experimental validation.

## 1. Introduction

Valvular heart disease (VHD) is a common cardiovascular condition characterized by injury or dysfunction of one or more heart valves, resulting in narrowing or incomplete closure of the valve openings.^[1]^ These valves regulate the flow of blood through the heart and the entire body, ensuring 1-way blood flow; their dysfunction can lead to impaired heart function.^[1,2]^ VHD affects tens of millions of people worldwide, and in the early stages of the disease, most patients have no symptoms or only mild symptoms, making it easy to be overlooked.^[3]^ Among the elderly population, especially those over 75 years old, the prevalence of moderate to severe VHD is as high as 22. 5%, and it is often accompanied by multiple valve lesions.^[4,5]^ VHD often leads to heart failure, atrial fibrillation, and other arrhythmias, seriously affecting patients’ quality of life.^[5,6]^ Due to high hospitalization rates and the need for repeated hospital treatments, this disease brings significant psychological stress and financial burden to patients.^[7]^ The pathogenesis of VHD is relatively complex and may involve the interaction of multiple factors. Degenerative diseases (most common in the elderly), infection, immune responses, oxidative stress, abnormal lipid metabolism, inborn errors, chronic kidney disease, and previous radiation exposure may all contribute to valvular fibrosis, calcification, endothelial injury, and dysfunction.^[8-10]^ In the treatment section, surgical options such as valve repair or replacement, along with transcatheter interventions, are the most common approaches. However, these methods have challenges and problems such as high cost, limited applicability, and lack of postoperative valve durability.^[11]^ In addition, there is currently no specific drug that can prevent valve calcification, fibrosis, or reverse valvular lesions. Pharmacologic therapy focuses more on the management of complications such as atrial fibrillation or heart failure.^[9]^ Therefore, it is of great interest to further explore the potential pathogenesis of VHD and find effective targets for drug therapy.

Proteins are important molecules in life activities and assume diverse and critical functions in organisms. They play a multifaceted role in the pathogenesis of VHD by regulating inflammatory responses, immune mechanisms, apoptosis, and lipid metabolism.^[12]^ Proteins form the fundamental structural components of heart valves, such as collagen, elastin, and laminin, which maintain the strength and flexibility of the valves.^[13]^ They also mediate key signaling pathways and participate in cellular and molecular processes that drive disease progression.^[14]^ Abnormalities or alterations in proteins can lead to valve damage. Plasma proteins play a particularly critical role in maintaining the health of the cardiovascular system. The structure and function of heart valves are directly dependent on specific plasma proteins. Existing studies indicate that plasma proteins are closely related to the pathogenesis of VHD and are important biomarkers of valve calcification.^[15-17]^ Although studies have suggested an association between some plasma proteins and VHD, the available evidence still lacks comprehensive systematic causal validation. For example, some proteins may only serve as concomitant markers of the disease process rather than direct drivers. Mendelian randomization (MR) can utilize genetic variations as instrumental variables to explore the causal relationship between plasma proteins and VHD, providing evidence-based support for disease mechanism exploration and drug target screening.

In recent years, there has been a rapid development of technologies related to plasma proteomics, and the available technologies are capable of quantifying thousands of plasma proteins simultaneously in large-scale cohorts, facilitating the exploration of correlations between plasma proteins and diseases.^[18,19]^ In addition, by combining plasma proteomics with genome-wide association studies (GWAS), researchers can identify protein quantitative trait loci, which can serve as robust instrumental variables for causal inference.^[19,20]^ MR uses these instrumental variables to minimize confounding and reverse causation bias by treating genetic variation as a natural experiment, thereby establishing a causal relationship between plasma proteins and VHD.^[20,21]^

This study systematically identified plasma proteins causally associated with VHD using MR. By combining drug prediction and molecular docking analysis of the screened proteins, it pinpointed target proteins and candidate drugs with clinical translational potential. Enrichment analysis combined with protein–protein interaction (PPI) networks was employed to elucidate the biological significance of the identified positive proteins and to uncover the pivotal role of core genes in the pathological progression of VHD. Through this multidimensional research approach, we have not only deepened our understanding of the pathogenesis of VHD, but also provided a reliable reference for VHD drug treatment and subsequent research.

## 2. Materials and methods

**Figure 1.**
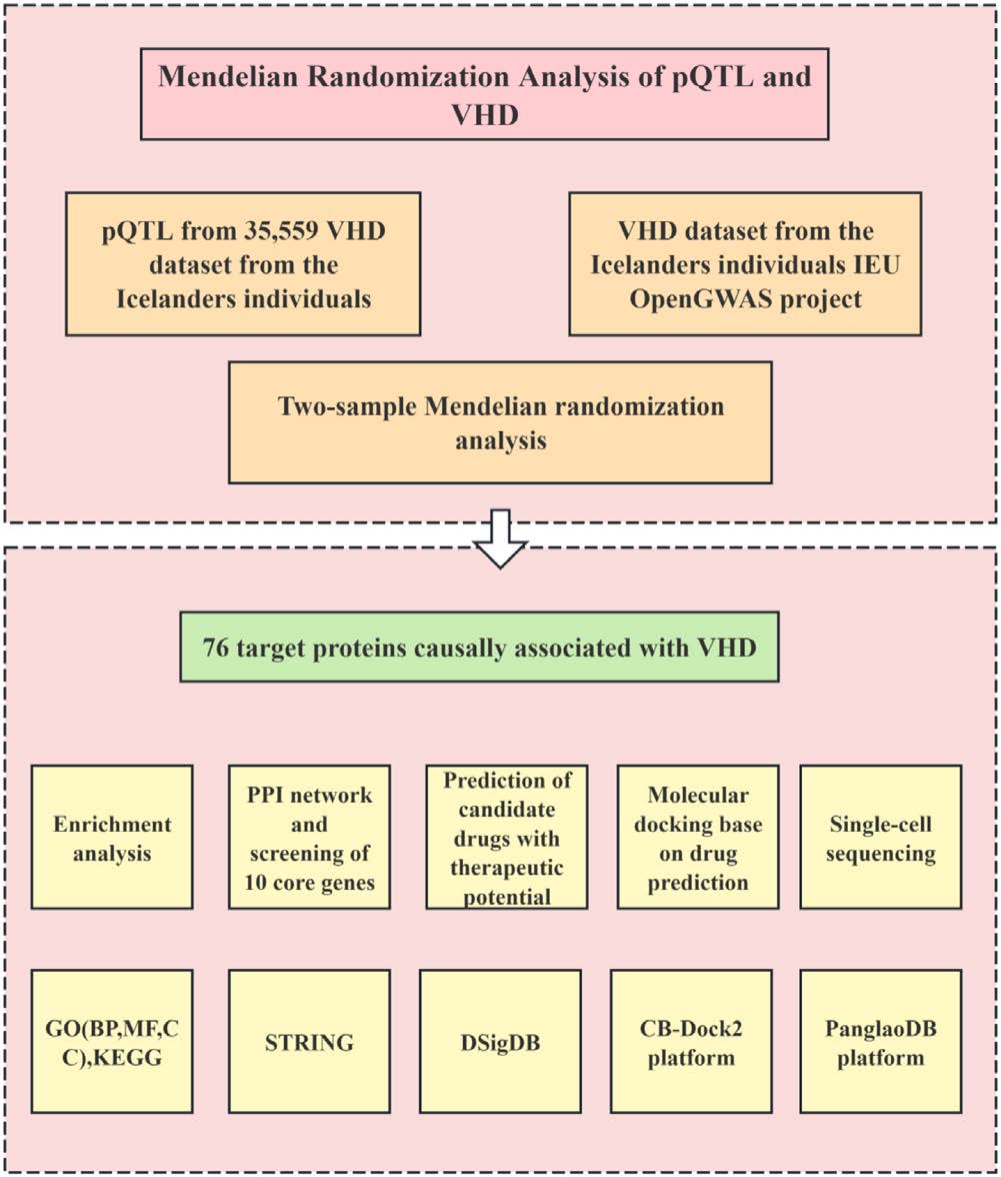
Overview of the study design. GWAS = genome-wide association study, MR = Mendelian randomization, PPI = protein–protein interaction, pQTL = protein quantitative trait locus, VHD = valvular heart disease.

This study employed an integrative strategy to evaluate the causal association between plasma proteins and VHD, while discussing the biological functions of these plasma proteins and their translational potential as therapeutic targets. The research framework is illustrated in Figure 1. This study is a secondary analysis based on publicly available database data and does not involve any direct intervention or contact with human subjects. Therefore, this study is exempt from requiring additional ethics committee review and patient informed consent.

### 2.1. Data sources

#### 2.1.1. Exposure data

Genetic instrumental variables for plasma protein levels were derived from the large-scale pQTL study conducted by Ferkingstad et al.^[19]^ This study employed aptamer-based assays to perform high-throughput profiling of the plasma proteome in a cohort of 35,559 individuals of Icelandic ancestry. Through genome-wide association analyses, the researchers identified 18,084 protein quantitative trait loci. The dataset, constructed by the deCODE Genetics team, provided reliable instrumental variables for the current study. The pre-processed exposure data are detailed in Table S1, Supplemental Digital Content, https://links.lww.com/MD/R610 in Supplementary Material.

#### 2.1.2. Outcome data

The genetic data for this investigation were obtained from the IEU OpenGWAS repository (GWAS ID: ebi-a-GCST90018811), a publicly available GWAS database containing VHD-related genetic variants.^[22]^ The dataset includes 25,070 VHD (referred to as “Cardiac valvular disease” in database terminology, it is conventionally termed VHD in academic literature) cases and 4,40,457 healthy controls, resulting in an aggregated sample size of 4,65,527 participants, with 2,41,76,672 single nucleotide polymorphisms (SNPs) analyzed. The populations in both the outcome and exposure datasets were of European descent.

### 2.2. Mendelian randomization analysis

This study used 2-sample MR to explore the causal association between plasma proteins and VHD. In the instrumental variable selection phase, we processed the exposure data (preprocessed pQTL data) as follows: to avoid statistical noise caused by low-frequency SNPs, only SNPs with an effect allele frequency >0. 01 were retained. In the association analysis, SNPs significantly associated with the exposure factor (*P* < 5 × 10^−8^) were selected, and the *F*-test statistic was calculated to exclude weak instruments with *F*-statistics < 10. In addition, to ensure data independence and reduce redundant signals, we used the clump_data function for clustering to eliminate linkage disequilibrium among SNPs, retaining only the SNPs most strongly associated with the exposure. The outcome data were integrated from the EBI database (ID: ebi-a-GCST90018811), with chromosomal position matching, allele direction correction, and palindromic SNP removal completed through a standardized harmonization process.^[23]^ To eliminate confounding bias, SNPs directly associated with VHD (outcome *P*-value > .05) were excluded. During the handling of missing data, we further processed the datasets using functions from the TwoSampleMR package (such as read_exposure_data and clump_data), which automatically exclude SNPs lacking essential information to ensure data completeness. For causal effect estimation, we employed the inverse-variance weighted method (primary approach), MR-Egger regression, weighted median method, simple mode method, and weighted mode method. All effect sizes were converted to odds ratios (OR) with 95% confidence intervals (95% CI). In the sensitivity analysis, the following components were included: Cochran’s *Q* test to assess heterogeneity among instrumental variables, MR-Egger intercept test to exclude horizontal pleiotropy interference (an intercept term *P* < .05 indicating systematic bias), and leave-one-out analysis to identify outlier SNPs with significant impact on causal estimates, thereby evaluating the robustness of the results. Based on the above content, we visualized the results of the MR analysis by generating 4 core diagnostic plots: scatter plot, funnel plot, forest plot, and leave-one-out plot (Figs. S1, S2, Supplemental Digital Content, https://links.lww.com/MD/R611, S3, and S4, Supplemental Digital Content, https://links.lww.com/MD/R612 Supplementary Material). Finally, we performed the final result screening and created a summarized forest plot, requiring that the inverse-variance weighted results show *P* < .05 (statistically significant) and that the OR values from all 5 methods maintain consistent directionality (all values >1 or all values <1). The results of pleiotropy analysis and heterogeneity analysis are provided in Tables S2 and S3, Supplemental Digital Content, https://links.lww.com/MD/R610 in Supplementary Material.

### 2.3. Enrichment analysis

To explore the biological functions of plasma proteins (proteins screened through MR analysis) as potential therapeutic targets, this study conducted gene ontology (GO) and kyoto encyclopedia of genes and genomes (KEGG) pathway enrichment analyses. The R package ClusterProfiler was used to perform GO enrichment analysis on the screened genes, covering 3 categories: biological process (BP), molecular function (MF), and cellular component (CC).^[24]^ KEGG pathway enrichment analysis was also performed on the same gene set. All the aforementioned enrichment analyses employed the Benjamini–Hochberg correction method, with the significance level set at *P*-value < .05, and the adjusted *q*-value also required to be <0.05. In the GO enrichment analysis, the results displayed significant biological functions under each GO category and their associated gene proportions. The KEGG pathway enrichment analysis primarily highlighted pathways related to key genes. The results of the 2 enrichment analyses were visualized using 2 methods (bubble plots and bar charts). Through these enrichment analyses, we revealed the biological functions of the potential targets of the positive proteins and their roles in different pathways. The visualization results are presented as bar charts in the Results section. Detailed statistical information (Table S4, Supplemental Digital Content, https://links.lww.com/MD/R613 and Table S5, Supplemental Digital Content, https://links.lww.com/MD/R614) and bubble plots (Figs. S5 and S6, Supplemental Digital Content, https://links.lww.com/MD/R612) are provided in Supplementary Material.

### 2.4. Drug prediction

In the early stages of new drug development, analyzing the connection between proteins and candidate drugs, and exploring whether their interaction has specificity and biological significance, is a crucial step in confirming their target potential. In this study, we used drug enrichment methods to analyze and investigate the interactions between positive proteins (statistically significant proteins) screened by MR and potential drugs. To explore whether positive proteins could serve as potential drug targets, we utilized the Drug Signature Database (DSigDB) for drug prediction.^[25]^ The DSigDB contains over 22,500 gene sets, more than 17,000 compounds, and nearly 20,000 genes, providing crucial informational support for research on gene-drug target correlations and new drug development.^[26]^ We downloaded drug and target information from the DSigDB database and performed drug enrichment analysis using the R package ClusterProfiler to identify candidate drugs significantly associated with positive genes. In the analysis phase, we set significance threshold parameters (both *P*-value and adjusted *P*-value at .05) and evaluated whether target genes were significantly enriched in specific drug target gene sets through hypergeometric testing. Based on the enrichment analysis results, a gene-drug interaction network diagram and a 3-line table were created, displaying the ranking of enriched drugs, corresponding gene names, and the interrelationships between core genes and drugs.

### 2.5. Molecular docking based on candidate drugs

To further investigate the effects of screened drugs on target proteins and their drug availability, we conducted molecular docking analysis to evaluate the binding affinity and interaction patterns between candidate drugs and their targets. Based on the results of drug enrichment analysis, we selected the top 5 enriched candidate drugs for analysis. The molecular docking was performed using the CB-Dock2 platform.^[27]^ The structural data of the drugs were obtained from the PubChem compound database, while the protein structural data were sourced from the Protein Data Bank.

### 2.6. Construction of protein–protein interaction network and screening of core genes

PPI are central to various BPs within the cell. In this study, a PPI network was constructed using the STRING database.^[28]^ During the analysis, a minimum interaction confidence score of 0.15 was set, and unconnected nodes were excluded. Other analysis parameters were kept at their default values. To further identify core genes with greater biological significance, the generated protein network data were uploaded to cytoscape v3. 10. 3.^[29]^ Within Cytoscape, the plugin named “cytoHubba” was selected, and the top 10 genes were screened as core genes based on the degree value of the nodes. During network visualization, the color of the nodes was proportional to their degree values, with nodes of higher degree displayed in a redder color.

### 2.7. Single-cell sequencing

Cardiac tissue exhibits high cellular heterogeneity, comprising diverse cell types. To further investigate the potential biological functions of the 10 identified core genes in the pathogenesis of VHD, we performed single-cell RNA sequencing to analyze their differential expression patterns across distinct cardiac cell populations at single-cell resolution. The single-cell sequencing component of this study utilized the PanglaoDB database.^[30]^ This database collects and integrates single-cell transcriptomic data from multiple studies, containing information from over 1054 single-cell experiments, 4 million cells from various tissues and organs, and more than 6000 gene-cell type associations.^[31]^ Furthermore, this platform can assist users in automated cell type annotation. We obtained a single-cell RNA sequencing dataset of mouse heart tissue (SRA762414: SRS3703557) from the PanglaoDB platform, which contains gene expression profiles at the single-cell level. Using the t-SNE (interactive) dimensionality reduction method, we analyzed and visually demonstrated the expression differences and distribution patterns of core genes across different cardiac cell populations.^[32]^

## 3. Results

### 3.1. Results of MR analysis

**Figure 2.**
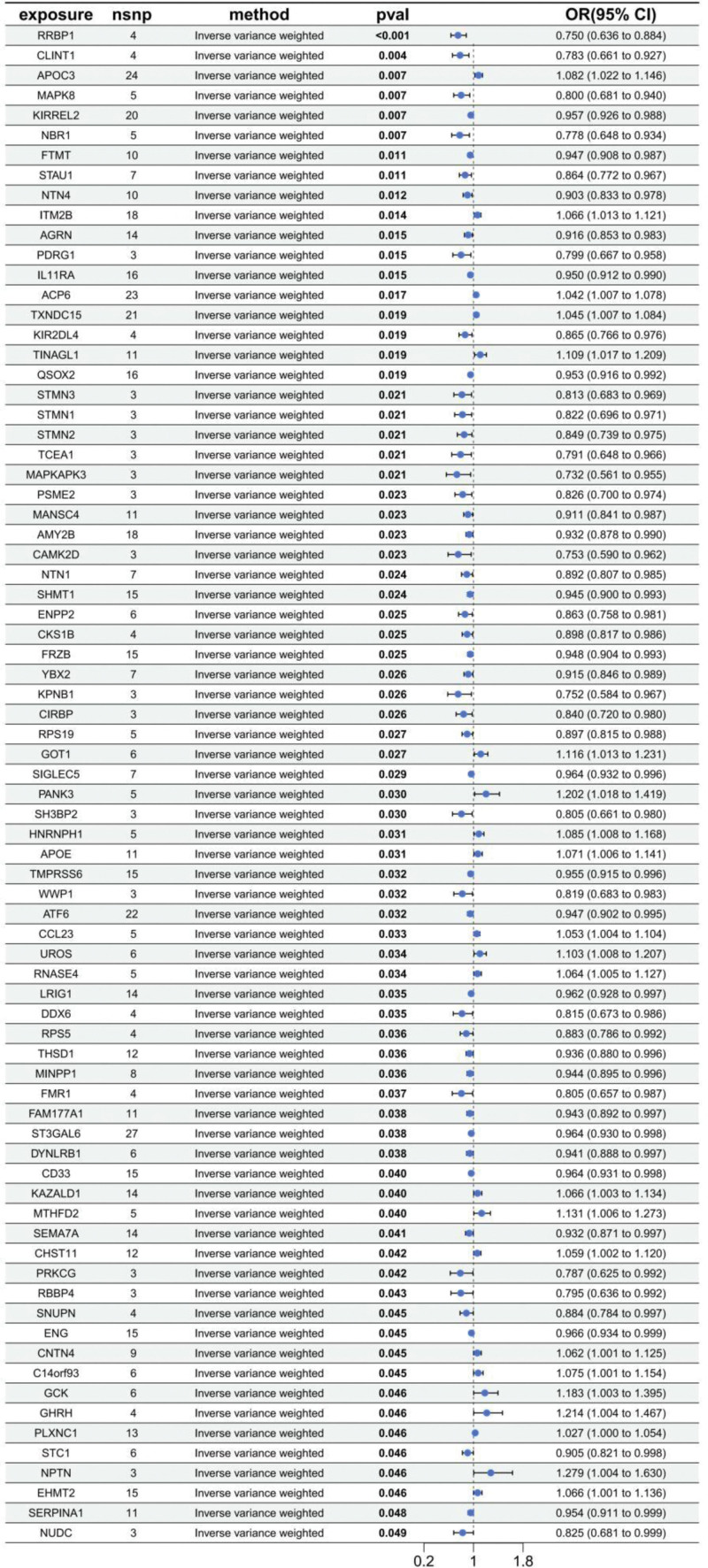
MR analysis results of pQTL on VHD. Points denote SNP-specific causal estimates and lines show 95% CIs. CI = confidence interval, IVW = inverse-variance weighted, MR = Mendelian randomization, OR = odds ra, pQTL = protein quantitative trait locus, SNP = single-nucleotide polymorphismtio, VHD = valvular heart disease.

The MR analysis results presented in the forest plot (Fig. 2) demonstrate that a total of 76 genetic loci showed significant associations with VHD. For instance, loci APOC3 (*P* = 0. 007, OR = 1.082, CI [1.022–1.146]) and GOT1 (*P* = .027, OR = 1.116, CI [1.013–1.231]) exhibited positive correlations with VHD. Conversely, RRBP1 (*P* < .001, OR = 0.750, 95% CI [0.636–0.884]) and MAPKAPK3 (*P* = .021, OR = 0.732, CI [0.561–0.955]) showed significant negative associations with VHD. The analysis results indicate that these genetic loci include both protective sites and risk-associated sites. The aforementioned proteins with significant statistical significance may serve as potential therapeutic targets and important biomarkers in future VHD research.

### 3.2. Enrichment analysis results

**Figure 3.**
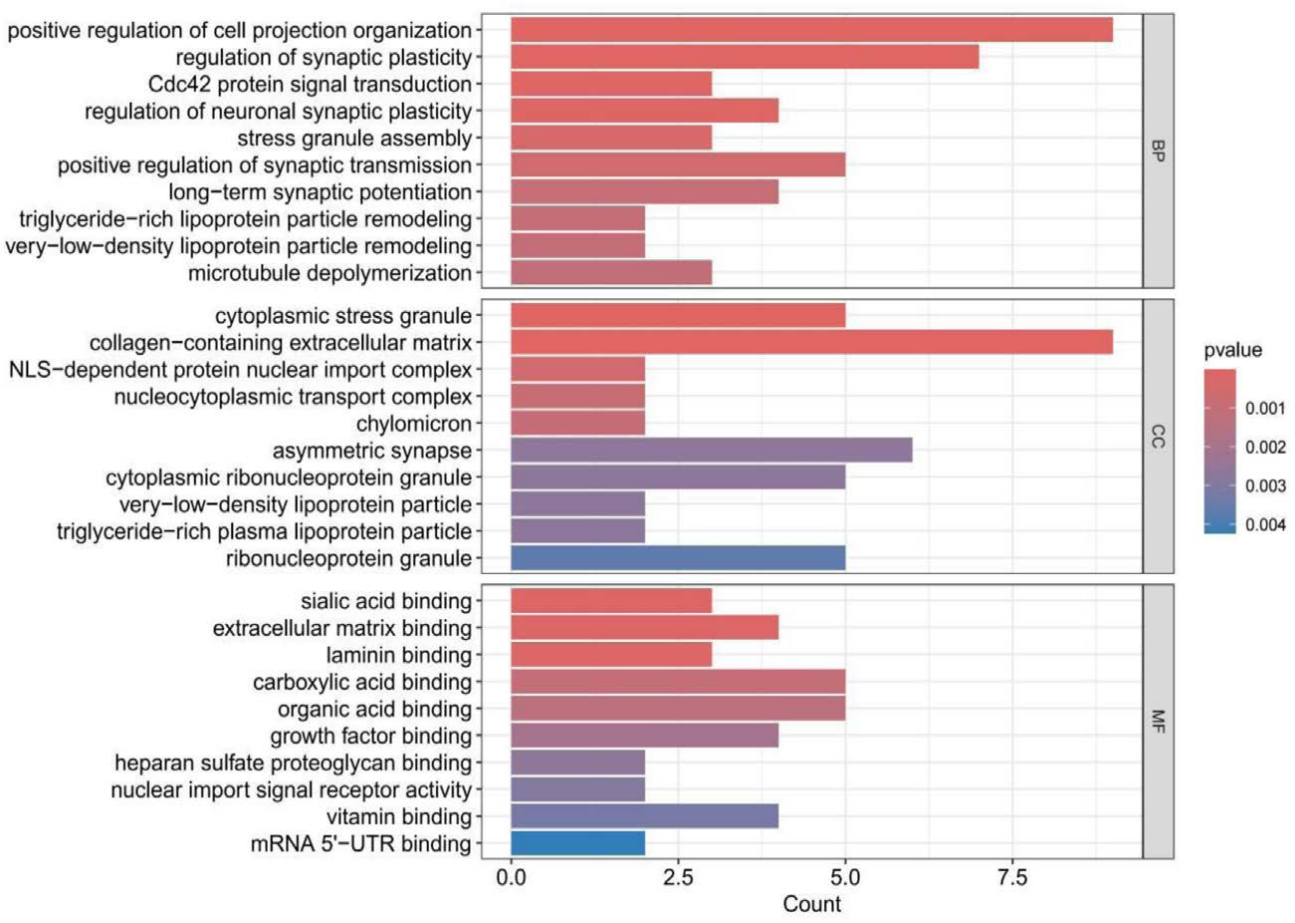
GO enrichment analysis results. BP = biological process, CC = cellular component, GO = gene ontology, MF = molecular function, Padj = Benjamini–Hochberg adjusted *P*-value.

**Figure 4.**
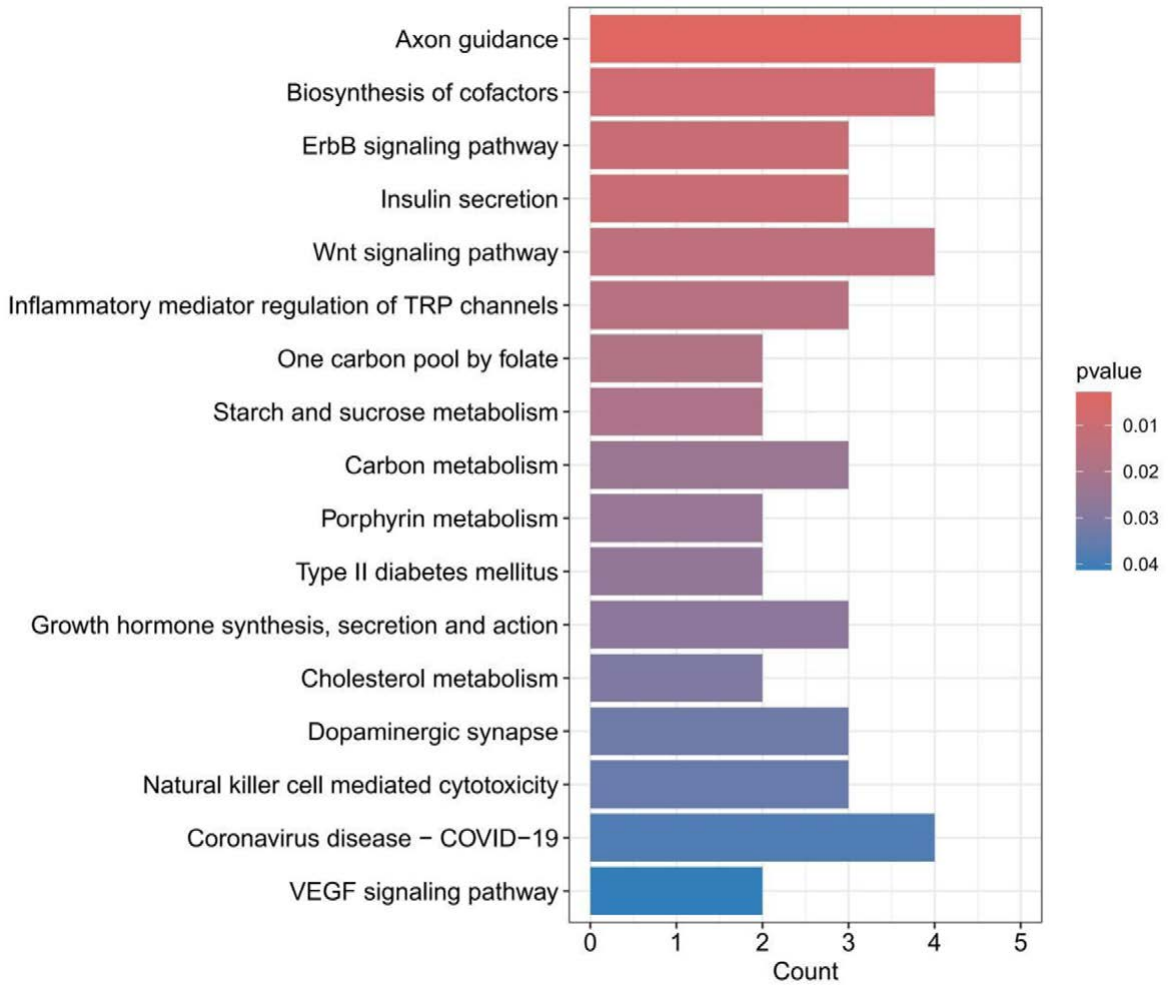
KEGG enrichment analysis results. KEGG = kyoto encyclopedia of genes and genomes, Padj = Benjamini–Hochberg adjusted *P*-value.

GO enrichment analysis systematically annotates the functional attributes of positive genes through 3 hierarchical categories: BP, CC, and MF (Fig. 3). For instance, the BP category showed significant enrichment in regulation of synaptic plasticity, long-term synaptic potentiation, and very-low-density lipoprotein particle remodeling, involving neural signal transmission and metabolic regulation. The CC category included cytoplasmic stress granule, collagen-containing extracellular matrix, and nucleocytoplasmic transport complex, reflecting stress response and transmembrane transport. The MF category featured Growth factor binding, mRNA 5’-UTR binding, and nuclear import signal receptor activity, indicating key functions in molecular interactions and gene expression regulation. KEGG enrichment analysis focuses on elucidating the synergistic mechanisms of genes within complex biological networks such as signal transduction and metabolic pathways (Fig. 4). We identified the top 5 significant biological pathways as axon guidance, biosynthesis of cofactors, ErbB signaling pathway, insulin secretion, and Wnt signaling pathway.

### 3.3. Drug prediction

**Figure 5.**
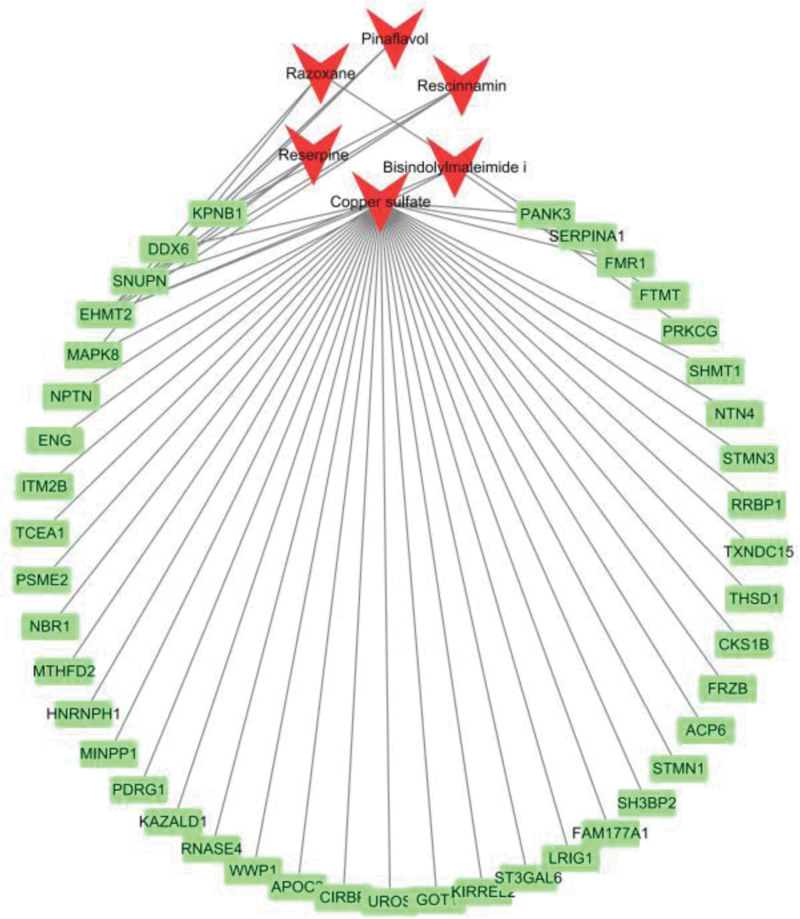
Gene-drug interaction network diagram.

Based on the information from the DSigDB database and using the R software package ClusterProfiler for drug enrichment analysis, we predicted significantly enriched candidate drugs. The results showed that razoxane, rescinnamin, pinaflavol, reserpine, bisindolylmaleimide I, and other key drugs are associated with positive genes (Fig. 5, Table 1). The drug prediction summary table is shown in Table S6, Supplemental Digital Content, https://links.lww.com/MD/R610 in Supplementary Material.

**Table 1:** DSigDB term enrichment analysis.

	Term	Count	Padj	Odds ratio	Combined score	Genes
1	Razoxane	3	0.009	102.308	1194.353	MAPK8; FTMT; EHMT2
2	Rescinnamin	3	0.009	74.395	810.238	EHMT2; SNUPN; KPNB1
3	Pinaflavol	3	0.018	51.134	507.533	EHMT2; SNUPN; KPNB1
4	Reserpine	3	0.025	40.899	381.728	EHMT2; SNUPN; KPNB1
5	Bisindolylmaleimide i	3	0.030	34.075	301.318	PRKCG; MAPK8; EHMT2
6	Copper sulfate	38	0.038	2.333	19.757	DDX6; PANK3; SERPINA1; FMR1; EHMT2; SHMT1; NTN4; STMN3; RRBP1; TXNDC15; THSD1; CKS1B; FRZB; ACP6; STMN1; SH3BP2; FAM177A1; NPTN; LRIG1; ST3GAL6; KIRREL2; GOT1; UROS; CIRBP; APOC3; WWP1; RNASE4; KAZALD1; SNUPN; PDRG1; MINPP1; HNRNPH1; MTHFD2; NBR1; PSME2; TCEA1; ITM2B; ENG

CI = confidence interval, DSigDB = Drug Signature Database, OR = odds ratio, Padj = Benjamini–Hochberg adjusted *P*-value.

### 3.4. Molecular docking

**Figure 6.**
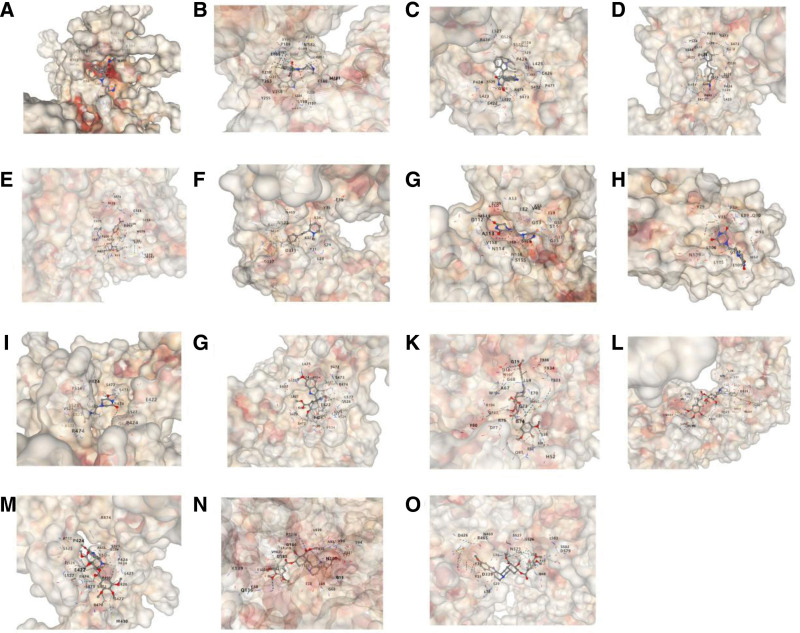
Docking results of available proteins with small molecules. (A) PRKCG docking bisindolylmaleimide I. (B) MAPK8 docking bisindolylmaleimide I. (C) EHMT2 docking bisindolylmaleimide I. (D) EHMT2 docking pinaflavol. (E) SNUPN docking pinaflavol. (F) KPNB1 docking pinaflavol. (G) MAPK8 docking razoxane. (H) FTMT docking razoxane. (I) EHMT2 docking razoxane. (J) EHMT2 docking rescinnamin. (K) SNUPN docking rescinnamin. (L) KPNB1 docking rescinnamin. (M) EHMT2 docking reserpine. (N) SNUPN docking reserpine. (O) KPNB1 docking reserpine. Gene symbols: PRKCG, protein kinase C gamma; MAPK8, mitogen‑activated protein kinase 8; EHMT2, euchromatic histone-lysine *N*-methyltransferase 2; FTMT, mitochondrial ferritin; KPNB1, karyopherin subunit beta 1; SNUPN, snurportin 1.

To explore the prediction of interactions between drugs and target proteins, their binding affinity, and the potential druggability between them, we conducted molecular docking studies. Based on drug enrichment results, we selected the top 5 most significantly enriched candidate drugs for systematic analysis on the CB-Dock2 platform (results shown in Fig. 6). Research data indicate that all 5 drugs exhibit favorable and stable binding affinity with their corresponding target proteins. Overall, the binding energy ranges from −5.9 kcal/mol to −10. 2 kcal/mol. The binding interaction between bisindolylmaleimide I and mitogen-activated protein kinase 8 (MAPK8) is particularly stable, demonstrating the lowest binding energy (results shown in Table 2).

**Table 2:** Docking results of available proteins with small molecules.

Drug	PubChem ID	Target	UniProt ID	Binding energy (kcal/mol)
Bisindolylmaleimide I	2396	PRKCG	P05129	−8.7
Bisindolylmaleimide I	2396	MAPK8	P45983	−10.2
Bisindolylmaleimide I	2396	EHMT2	Q96KQ7	−9.0
Pinaflavol	53,53,565	EHMT2	Q96KQ7	−6.3
Pinaflavol	53,53,565	SNUPN	O95149	−7.7
Pinaflavol	53,53,565	KPNB1	Q14974	−6.1
Razoxane	30,623	MAPK8	P45983	−7.3
Razoxane	30,623	FTMT	Q8N4E7	−5.9
Razoxane	30,623	EHMT2	Q96KQ7	−6.8
Rescinnamin	52,80,954	EHMT2	Q96KQ7	−8.7
Rescinnamin	52,80,954	SNUPN	O95149	−10.0
Rescinnamin	52,80,954	KPNB1	Q14974	−8.5
Reserpine	5770	EHMT2	Q96KQ7	−8.3
Reserpine	5770	SNUPN	O95149	−10.0
Reserpine	5770	KPNB1	Q14974	−7.9

EHMT2 = euchromatic histone-lysine *N*-methyltransferase 2, FTMT = mitochondrial ferritin, KPNB1 = karyopherin subunit beta 1, MAPK8 = mitogen‑activated protein kinase 8, PRKCG = protein kinase C gamma, SNUPN = snurportin 1, UniProt ID = Universal Protein Resource identifier.

### 3.5. PPI network and core genes

**Figure 7.**
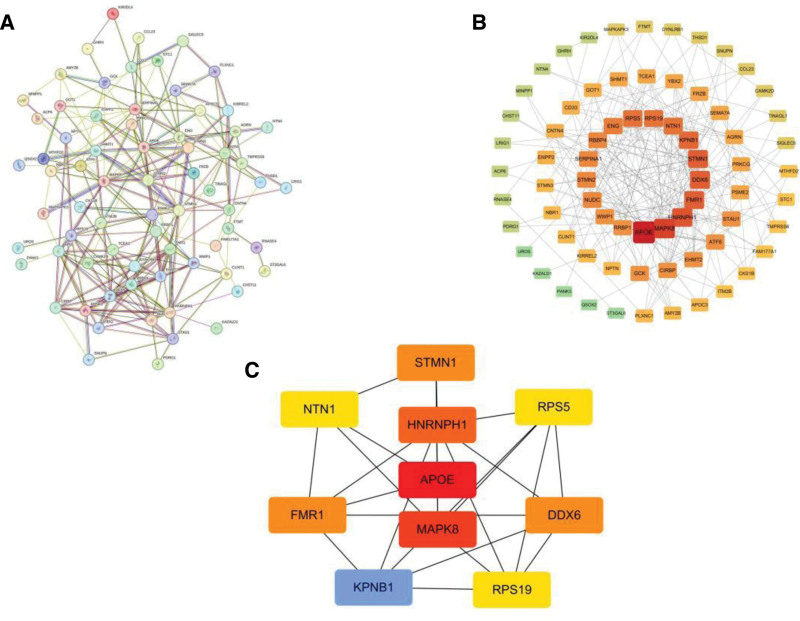
PPI network construction diagram. (A) PPI network built with STRING. (B) Full PPI network of selected genes. Key clusters with hub genes highlighted in red. (C) The subnetwork demonstrates the interaction relationships among 10 core genes.

We uploaded 76 positive gene proteins to the STRING database for network construction. The generated data were imported into Cytoscape, and a complete PPI network diagram was plotted. The screening of 10 hub genes was completed using the cytoHubba plugin. As shown in Figure 7, the PPI network contained 71 nodes and 226 edges. The identified hub genes were DEAD-box helicase 6 (DDX6), heterogeneous nuclear ribonucleoprotein H1 (HNRNPH1), Netrin 1 (NTN1), fragile X mental retardation 1 (FMR1), ribosomal protein S19 (RPS19), stathmin 1 (STMN1), ribosomal protein S5 (RPS5), MAPK8, apolipoprotein E (APOE), and karyopherin subunit beta 1.

### 3.6. Single-cell sequencing

**Figure 8.**
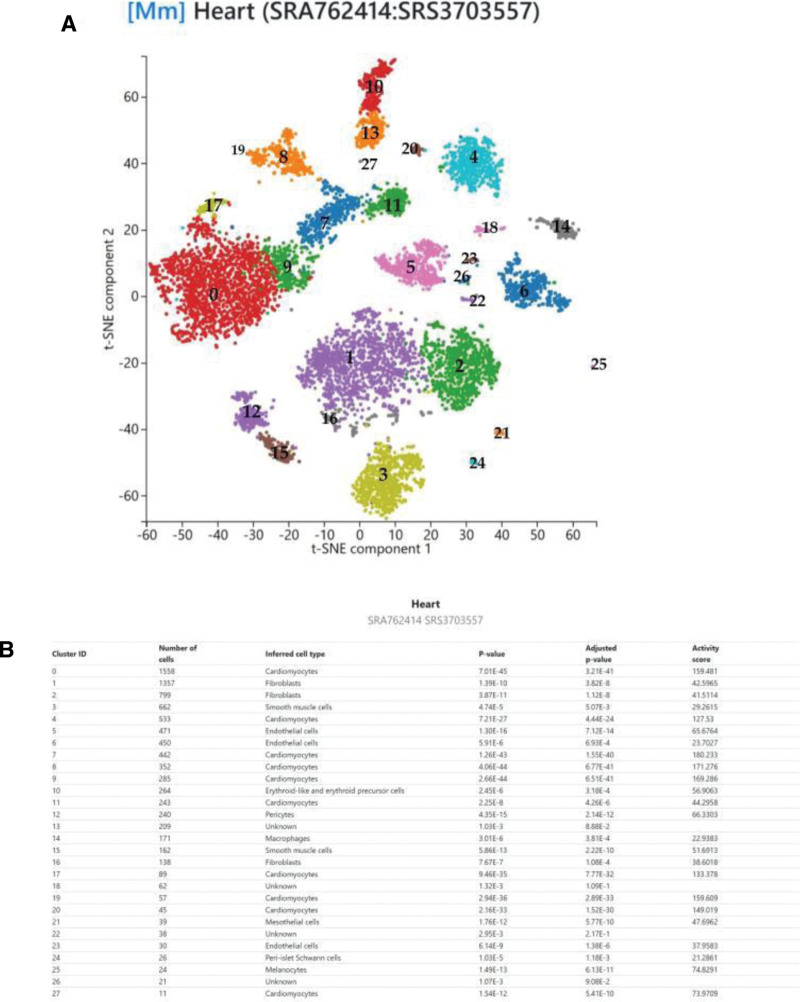
(A) t-SNE cell distribution map. Based on single-cell RNA sequencing data, the t-SNE dimensionality reduction plot illustrates the heterogeneity of different cell types in heart tissue. Different colors represent distinct cell sub-populations. (B) Main information of 27 cell clusters. RNA-seq = RNA sequencing, t-SNE = t-distributed stochastic neighbor embedding.

**Figure 9.**
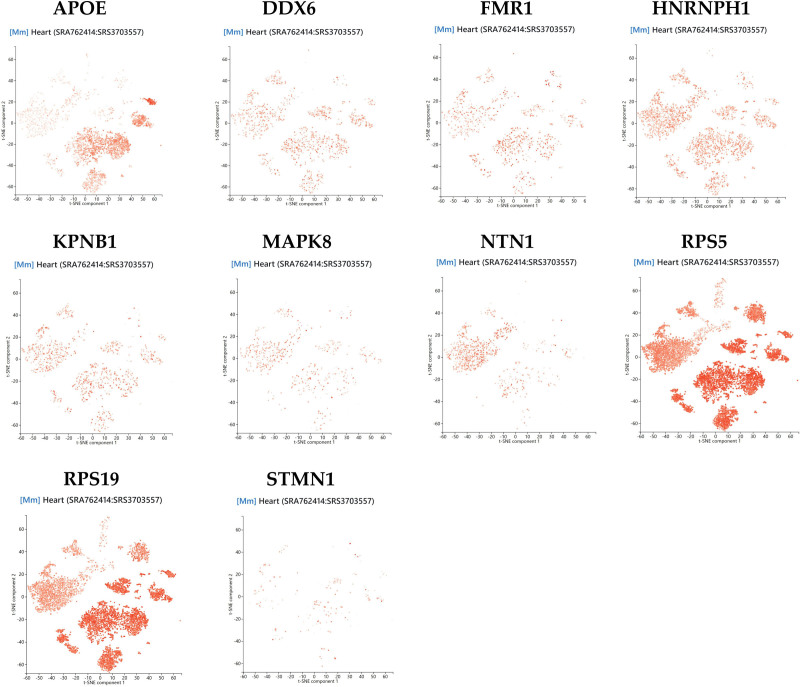
The scatter plot shows the expression of core genes in heart tissue cells.

In the single-cell sequencing session, we used the dataset of mouse heart tissues in the PanglaoDB platform for single-cell RNA sequencing analysis. Based on the analysis results, we can see the expression of core genes in different cell types (Figs. 8 and 9). For example, the APOE gene exhibits high expression in macrophages, endothelial cells, fibroblasts, and smooth muscle cells. The NTN1 gene shows high expression in cardiomyocytes. The RPS5 gene is expressed across all cell types but demonstrates significant overexpression in fibroblasts, smooth muscle cells, endothelial cells, pericytes, macrophages, and cardiomyocytes. These single-cell sequencing results further indicate that these core genes may play important roles in the pathogenesis and treatment of VHD.

## 4. Discussion

The plasma proteome contains various types of proteins that perform a wide range of biological functions, such as cell signal transduction, substance transport, and hormone regulation.^[33]^ In addition, the plasma proteome is characterized by a certain degree of stability and specificity and predictability.^[34]^ Therefore, this omics approach holds significant value for conducting research related to VHD. In this study, we screened 76 positive proteins related to VHD in the plasma proteome through MR analysis methods. In VHD, regardless of the subtype, valvular fibrosis and calcification are the primary factors contributing to disease progression and valvular dysfunction. The results indicate that most predicted drugs and identified core genes function through mechanisms such as inflammation, oxidative stress, endothelial injury, and cellular proliferation, exerting either positive or negative effects on fibrosis and calcification. These findings hold significant implications for future pharmacological treatments and the exploration of pathogenic mechanisms in VHD.

In the KEGG enrichment results, the significantly enriched axon guidance signaling pathway has traditionally been considered to primarily regulate nervous system development. However, recent studies have revealed its potential connection with cardiac valve development and pathological remodeling. During valve development, axon guidance molecules regulate the migration of cardiac neural crest cells and the differentiation of valve interstitial cells (VICs), thereby influencing the formation and functional maintenance of valve structures.^[35]^ In calcific aortic valve disease, the downregulation of netrin-1 (a key molecule in the axon guidance pathway) may reduce M2 macrophage polarization, exacerbate local inflammation, and promote the osteogenic phenotypic transformation of valvular interstitial cells.^[36]^ In adult cardiac tissue, the ErbB signaling pathway regulates the survival, anti-fibrotic, and anti-apoptotic processes of cardiac fibroblasts, and inhibition of this pathway exacerbates cardiac fibrosis.^[37]^ Animal experiments have shown that the absence of ErbB family members leads to abnormal cardiac valve development and cardiac defects.^[38]^ The VEGF signaling pathway is not only crucial in angiogenesis but also directly involved in cardiac valve repair.^[39]^ VEGF helps maintain the stability of the vascular network in valves and myocardium while participating in antioxidant and antifibrotic regulation. Dysregulation of this pathway may lead to localized ischemia in valve tissues or accelerate the progression of valvular degeneration.^[40]^

Razoxane, as an antineoplastic agent, is primarily used to prevent chemotherapy-induced cardiotoxicity, and its potential antifibrotic effects may confer protective benefits in VHD.^[41]^ Excessive fibrosis of valvular tissue can lead to loss of valve function and calcification, subsequently affecting cardiac hemodynamics. Transforming growth factor beta (TGF-β) is a key regulatory factor in cardiac valve fibrosis, capable of inducing fibroblast proliferation and excessive deposition of extracellular matrix (ECM) components such as collagen, leading to valve sclerosis and dysfunction.^[42]^ Razoxane can reduce collagen synthesis by interfering with the TGF-β/Smad pathway, particularly by inhibiting the activation of Smad2 and Smad3, thereby slowing the fibrotic process.^[42,43]^ Furthermore, the metabolites of this drug possess iron-chelating properties, which can reduce iron-mediated free radical generation and mitigate oxidative stress-induced damage to cardiac tissue. Although this mechanism primarily serves to prevent anthracycline-induced cardiotoxicity, its antioxidant effects may also indirectly protect valvular tissue from oxidative damage.^[41,44]^ Reserpine is an alkaloid extracted from *Rauwolfia serpentina* that functions as an adrenergic blocking agent. The drug’s potential effects on VHD may operate through multiple pathways including antifibrotic activity, calcium metabolism modulation, anti-inflammatory effects, and antioxidant properties. From an anti-fibrotic perspective, reserpine activates nuclear factor erythroid 2-related factor 2 by inhibiting Kelch-like ECH-associated protein 1, thereby reducing oxidative stress-mediated fibrosis and significantly decreasing collagen deposition and fibrosis-related markers in liver fibrosis models.^[45]^ Furthermore, 5-hydroxytryptamine (5-HT, also known as serotonin) is closely related to valvular fibrosis, and this drug can deplete serotonin, thereby reducing its mediation of valvular fibrosis.^[46,47]^ Recent studies have also shown that serotonin is one of the important factors driving valvular disease.^[48]^ Bisindolylmaleimide I is a protein kinase C (PKC) inhibitor known to possess potential therapeutic value in inflammatory-related diseases.^[49]^ The potential association of this drug with VHD may be related to PKC. PKC is implicated in various cardiovascular diseases, including hypertension, coronary artery disease, and cardiac ischemia.^[50]^ PKC may intervene in vascular remodeling by affecting vascular fibroblasts and ECM generation, ultimately regulating valve fibrosis. Evidence suggests that inhibiting PKC can alleviate cardiac fibrosis induced by hypertension in heart failure.^[51]^ Since PKC is also involved in inflammatory responses, we hypothesize that bisindolylmaleimide I may ultimately affect valvular endothelial function by suppressing inflammatory reactions. In the molecular docking results, we can observe that the drug exhibits highly stable binding with PRKCG (PRKCG is the gene encoding protein kinase C gamma (PKCγ), a neuron-specific subtype of the PKC family). This further suggests that the drug may exert potential effects on VHD by regulating PRKCG expression.

Among the screened core genes, DDX6 suppresses negative regulators of the BMP signaling pathway (bone morphogenetic protein signaling pathway) through miRNA-mediated gene silencing, ensuring proper BMP signal activation.^[52]^ The BMP signaling pathway plays a crucial role in cardiac valve development and can coordinately regulate valve endothelial-mesenchymal transition.^[53]^ Deficiency of DDX6 leads to abnormalBMP signaling, subsequently affecting mesoderm differentiation and cardiac tissue development.^[54]^ In addition, as an RNA helicase, DDX6 affects the mRNA stability of fibrosis-related genes such as collagen and metalloproteinases.^[55]^ HNRNPH1 is a crucial RNA-binding protein involved in multiple aspects of RNA metabolism. It can bind to PTPN6 mRNA, activate the PI3K/AKT signaling pathway, promote valve interstitial cell proliferation, and inhibit apoptosis, which may exacerbate valvular fibrosis.^[56]^ The PI3K/AKT pathway participates in cell proliferation, apoptosis, and fibrosis, serving as a key driver in the progression of VHD. Another study suggests that HNRNPH1 also regulates the TGF-β signaling pathway, ultimately affecting valvular fibrosis and calcification.^[57]^ NTN1 is a laminin-associated secreted protein that may serve as a protective factor for cardiac valves. It is well established that the progression of valvular diseases typically involves ventricular remodeling secondary to pressure overload. In a mouse model of transverse aortic constriction, netrin-1 treatment alleviated ventricular remodeling and DNA damage while improving cardiac function through inhibition of MAPK-related signaling pathways.^[58]^ Chronic inflammation and endothelial injury are key factors leading to valvular dysfunction. NTN1 also reduces the expression of endothelial adhesion molecules induced by pro-inflammatory cytokines and blocks the NF-κB signaling pathway, thereby decreasing leukocyte and T-cell recruitment.^[59]^ Individuals with a full mutation of the FMR1 gene are characterized by the absence of FMRP protein expression. This protein is closely associated with connective tissue, and its deficiency leads to structural abnormalities in connective tissue (such as shortened and fragmented elastic fibers), resulting in mitral valve prolapse, aortic root dilation, and other valvular abnormalities.^[60,61]^ There is clinical evidence showing that FMR1 premutation carriers may experience mitral valve prolapse or regurgitation, tricuspid valve dysfunction, and accompanying cardiovascular manifestations such as blood pressure instability.^[61]^ RPS19 has been employed as a reference gene (to standardize gene expression levels) in studies of mitral VICs. Therefore, it can be inferred that the RPS19 gene is stably expressed in valve tissue; nevertheless, its association with VHD remains unclear in current research. STMN1 belongs to the Stathmin gene family and is widely present in the cytoplasm, with its core function being the regulation of microtubule dynamics within the cytoskeletal system.^[62]^ Single-cell sequencing studies have found that in the population of fibrosis-activated VICs, STMN1 is highly expressed, and other microtubule-related genes also show increased expression.^[63]^ Valvular endothelial cells perceive hemodynamic shear stress through the microtubule network. Microtubule depolymerization may disrupt force signal transduction (mechanical stress sensing dysfunction), leading to abnormal ECM deposition.^[64,65]^ The integrity of the microtubule network is closely associated with matrix vesicle release, and aberrant STMN1 expression may accelerate the extracellular deposition of calcification precursors.^[66,67]^ The RPS5 gene, as a crucial component of ribosome biogenesis, exhibits haploinsufficiency that leads to structural abnormalities in the heart.^[68]^ In mouse models of cardiac fibrosis, this gene promotes myocardial fibroblast proliferation and collagen deposition through activation of the p38 MAPK signaling pathway.^[69]^ MAPK8 belongs to the mitogen-activated protein kinase (MAPK) family and is involved in various BPs such as cell proliferation, differentiation, and transcriptional regulation.^[70]^ This gene co-expresses with multiple valvular-affectinggenes and participates in inflammatory and fibrotic signaling pathways including TGFβand p38 MAPK.^[71]^ Research indicates that the MAPK8/JNK1 signaling pathway is activated in pressure overload-induced cardiac fibrosis, regulating cardiomyocyte apoptosis, inflammatory responses, and the expression of intercellular junction proteins, thereby indirectly affecting cardiac diastolic function and electrophysiological stability.^[70,72]^ The APOE gene, as an important gene influencing lipid metabolism and inflammatory regulation, has garnered widespread attention in recent years for its association with VHD. In VICs, APOE promotes lipid core formation by regulating lipoprotein receptor (such as LRP1)-mediated cholesterol uptake.^[73]^ A study of 802 patients undergoing echocardiography found that individuals carrying the APOE4 allele had a significantly increased risk of calcific aortic valve stenosis.^[74]^ In addition, another study demonstrated that APOE is highly expressed in elastin-producing cells of human fetal heart valves, and its reduced expression is directly associated with elastin fragmentation.^[75]^ The protein encoded by the Karyopherin subunit beta 1 gene is a crucial component of the nucleocytoplasmic transport system, responsible for mediating the nuclear import of multiple important signaling pathways (such as NF-κB, Notch, and Wnt signaling). This regulates cell proliferation, differentiation, and chronic inflammatory responses, processes that may significantly influence cardiac valve fibrosis and calcification.^[76,77]^ From the above, it can be seen that the protective or risk effects exhibited by the selected core genes in existing evidence are generally consistent with the results of MR in this study. However, some genes have limited research evidence or show certain contradictions or controversies in different studies, which still require further in-depth exploration in subsequent research.

In summary, the positive proteins identified and the predicted drug targets in this study are all closely related to VHD, providing a reliable theoretical reference for subsequent mechanism research, drug development, and optimization of clinical treatment protocols.

However, while this study has made certain contributions to the further in-depth exploration of VHD, it must be acknowledged that there are still some shortcomings and limitations. Firstly, this study focuses on VHD as a whole, placing greater emphasis on the impact of plasma proteins on pathological changes and functional abnormalities of cardiac valves. Although this represents one of the innovative aspects of this paper, it also constitutes a limitation of the study. Due to the failure to incorporate different subtypes of VHD into a stratified analysis framework, the study results may be subject to confounding bias risks caused by subtype heterogeneity. Secondly, although this study employed a multidimensional sensitivity analysis framework (including MR-Egger regression, weighted median/mode methods, Cochran’s *Q* test, and Mendelian Randomization Pleiotropy RESidual Sum and Outlier pleiotropy tests) to systematically evaluate the assumed validity of instrumental variables, it is still necessary to acknowledge the inherent limitations of MR – including potential residual pleiotropic effects and unmeasured confounding factors not explained by the instrumental variables. The current conclusions have not yet undergone external validation in independent cohort studies such as the UK Biobank, which to some extent limits the generalizability of the findings. Future research could establish a validation framework by integrating multi-center pQTL data resources, which would be of significant importance for enhancing the reproducibility of causal inference. Furthermore, the exposure and outcome data in this study were collected from a sample of people of European descent, which may also affect the generalizability of the findings. Therefore, similar analyses need to be expanded to more diverse populations in future studies. Another limitation of this study is that the predictive efficacy of molecular docking is constrained by the accuracy of receptor structure modeling and the reliability of ligand conformational space. Insufficient precision and reliability may affect the biological relevance of target screening. In the single-cell sequencing component, variations in dataset selection and analytical platforms may lead to incomplete representation of phenotypic and functional characteristics in specific cell types or subpopulations. Due to data limitations, this study utilized murine cardiac tissue datasets, which may exhibit certain discrepancies compared to human tissues. Consequently, although candidate drugs and their targets were preliminarily identified through bioinformatics approaches, their therapeutic translation potential requires confirmation through a multidimensional experimental validation system, including organoid models and preclinical pharmacodynamic evaluations.

Based on the findings and limitations of this study, we propose several recommendations for future research and outline potential directions. First, subsequent studies should include populations from diverse regions (such as Asia and Africa) and ethnic backgrounds to identify more protein variants and enhance the generalizability of results. Second, different subtypes of VHD (such as aortic stenosis and mitral regurgitation) should be incorporated into stratified analyses to reduce confounding bias caused by subtype heterogeneity and to further investigate whether subtype-specific findings could provide novel therapeutic targets for different types of VHD. Additionally, with future technological advancements, new platforms or algorithms should be introduced, leveraging AI technology to optimize molecular docking and minimize computational bias. Furthermore, researchers should integrate large-scale, multicenter independent cohort data for external validation to strengthen the credibility of the conclusions. Finally, future studies need to account for the impact of comorbidities such as chronic kidney disease on circulating protein metabolism by employing multivariate adjustments or stratified analyses to reduce the influence of confounding factors on the final results. In conclusion, the treatment and pathogenesis of VHD require further in-depth understanding in future research, along with the exploration of more optimized therapeutic strategies to improve efficacy and alleviate the burden on patients and society.

## 5. Conclusions

In summary, this study, based on a 2-sample MR approach, identified 76 therapeutic targets potentially associated with VHD and conducted GO and KEGG enrichment analyses to explore their biological functions and underlying pathway mechanisms (such as axon guidance, biosynthesis of cofactors). Through drug prediction and docking analysis validation, potential therapeutic candidates such as Razoxane, Reserpine, and Bisindolylmaleimide I were identified. Additionally, the construction of the PPI network, screening of 10 key genes, and single-cell sequencing results have provided new insights for future research on VHD. However, these findings still require further validation through subsequent basic research and clinical studies to explore the therapeutic potential of these drugs and targets, as well as their efficacy and safety in clinical applications.

## Acknowledgments

All research data in this study were obtained from publicly available open databases, for which we express our gratitude. We also extend our sincere thanks to various analysis platforms, such as molecular docking. Reference styles.

## Author contributions

**Conceptualization:** Cheng Wang, Yue Deng.

**Data curation:** Cheng Wang, Xiao Shao, Jia Wang, Moxuan Han, Huiyan Feng.

**Formal analysis:** Xiao Shao, Jia Wang, Huiyan Feng, Hezeng Dong.

**Funding acquisition:** Rui Shi.

**Investigation:** Moxuan Han, Wenqian Yang.

**Methodology:** Cheng Wang, Rui Shi, Tengyue Zhang.

**Project administration:** Yue Deng.

**Software:** Cheng Wang, Xiao Shao, Jia Wang.

**Supervision:** Rui Shi, Yue Deng.

**Validation:** Moxuan Han, Hezeng Dong.

**Visualization:** Cheng Wang, Xiao Shao, Jia Wang, Huiyan Feng, Tengyue Zhang.

**Writing – original draft:** Cheng Wang, Xiao Shao, Moxuan Han.

**Writing – review & editing:** Yue Deng.

## Supplementary Material
