# Supplementary material for: New perspectives on the mechanisms and treatment of valvular heart disease: Mendelian randomization and systematic analysis based on plasma proteins
Source: Medicine (Baltimore). 2026 Apr 3;105(14):e48210. doi: 10.1097/MD.0000000000048210 (PMC13052936; doi:10.1097/MD.0000000000048210)
Supplement: Supplementary file 2 [file medi-105-e48210-s002.pdf]

Figure **S1**: MR analysis funnel plot;

Figure **S2**: MR analysis forest plot;

Figure **S3**: MR analysis scatter plot;

Figure **S4**: MR analysis leave-one-out plot;

Figure **S5**: Bubble plot of GO enrichment analysis results;

Figure **S6**: Bubble plot of KEGG enrichment analysis results;

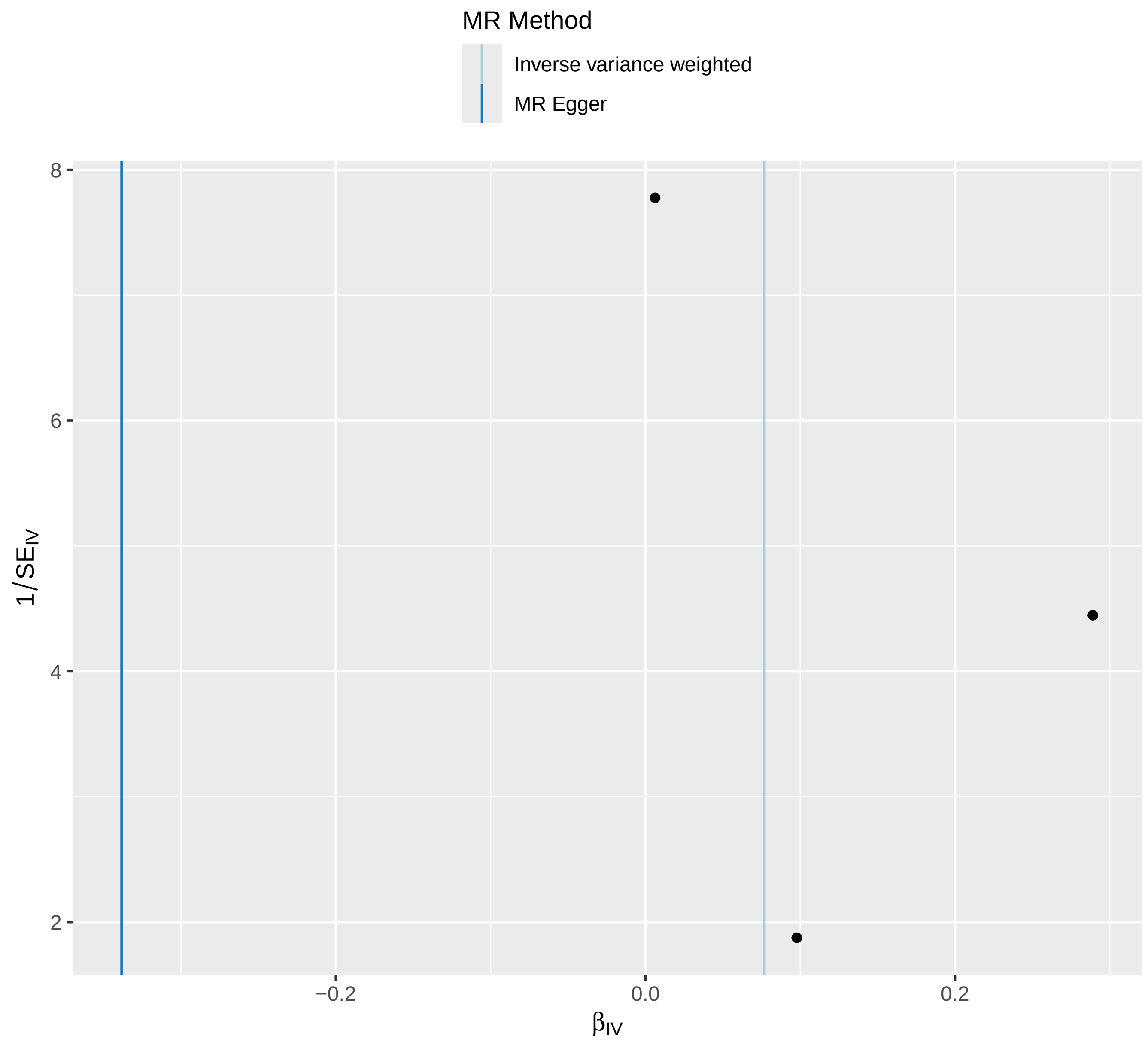

MR Method
Inverse variance weighted
MR Egger
8
6
4
2
1/SE_IV
−0.2
0.0
0.2
β_IV

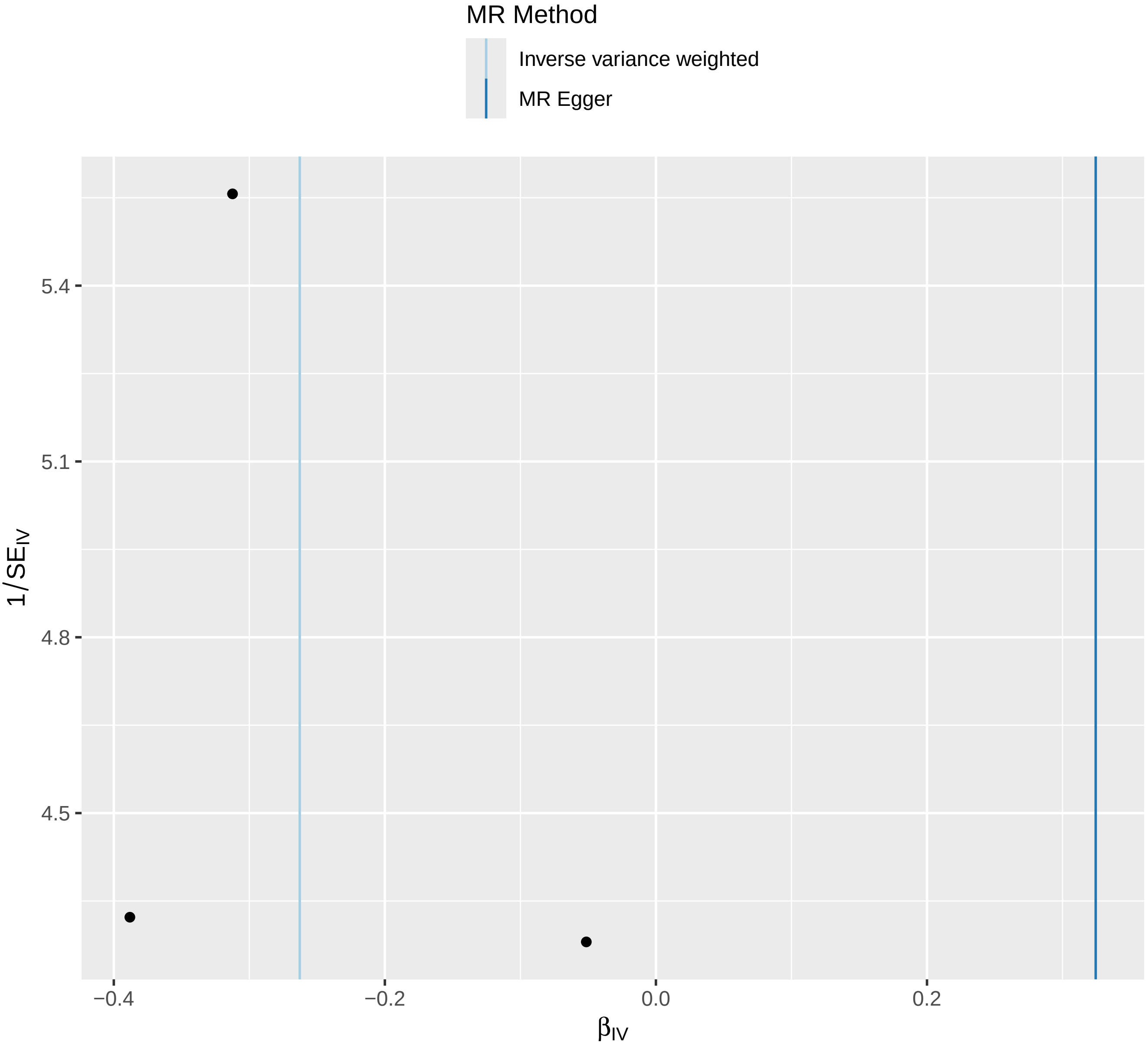
MR Method
Inverse variance weighted
MR Egger
5.4
5.1
4.8
4.5
1/SE$_{IV}$
−0.4
−0.2
0.0
0.2
β$_{IV}$

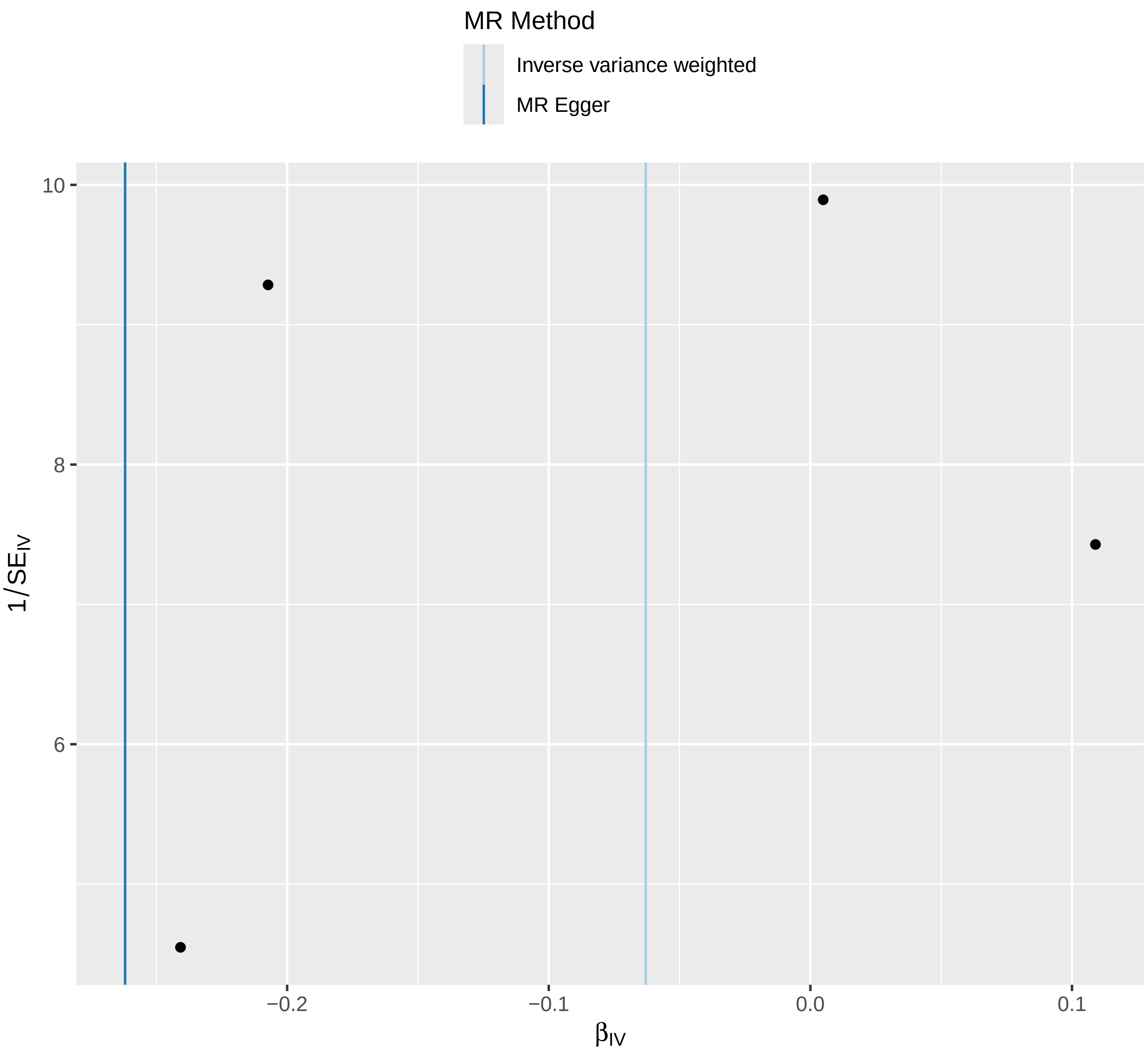

MR Method
Inverse variance weighted
MR Egger
10
8
6
1/SE$_{IV}$
−0.2
−0.1
0.0
0.1
β$_{IV}$

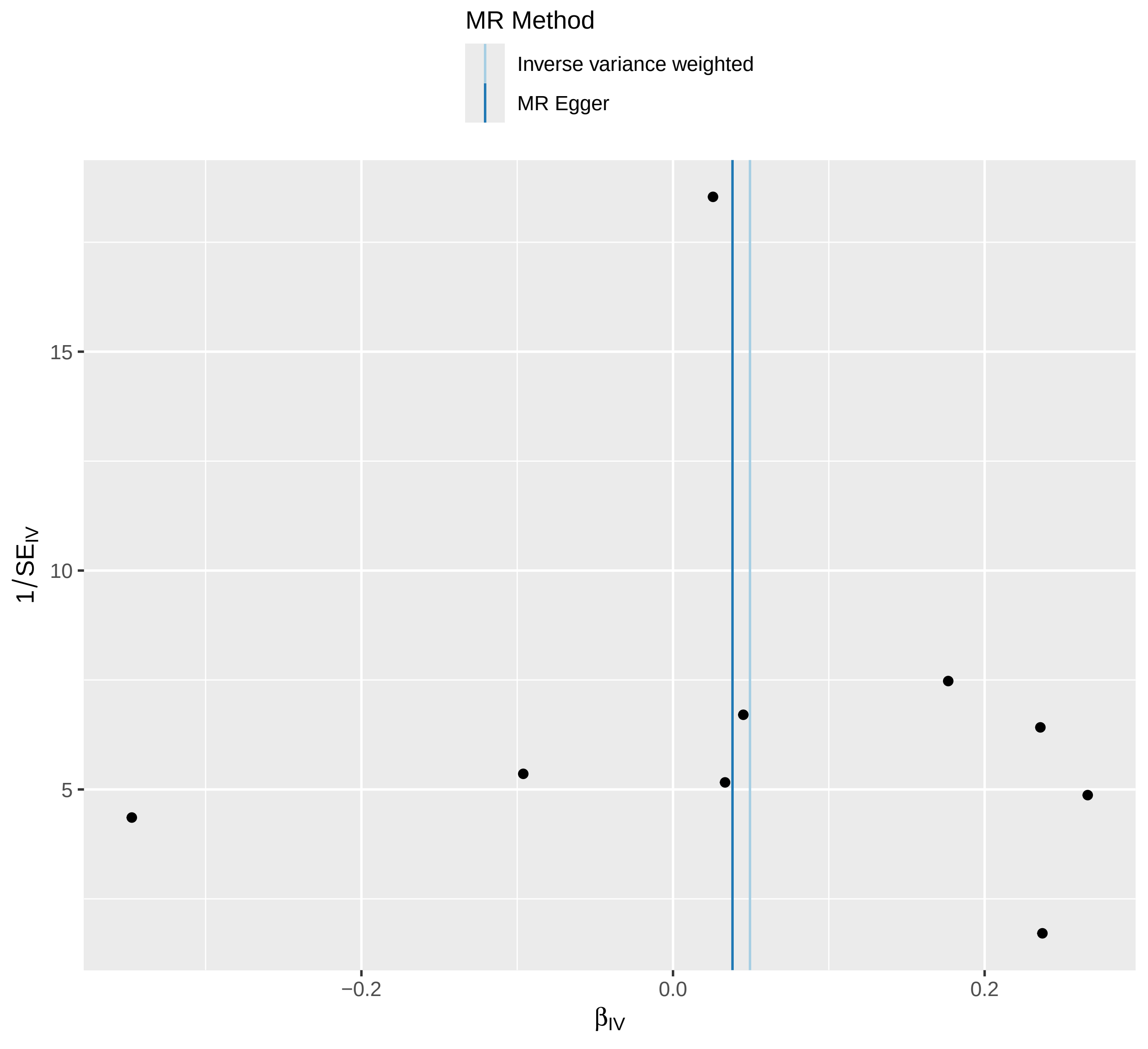
MR Method
Inverse variance weighted
MR Egger
15
10
5
1/SE_IV
−0.2
0.0
0.2
β_IV

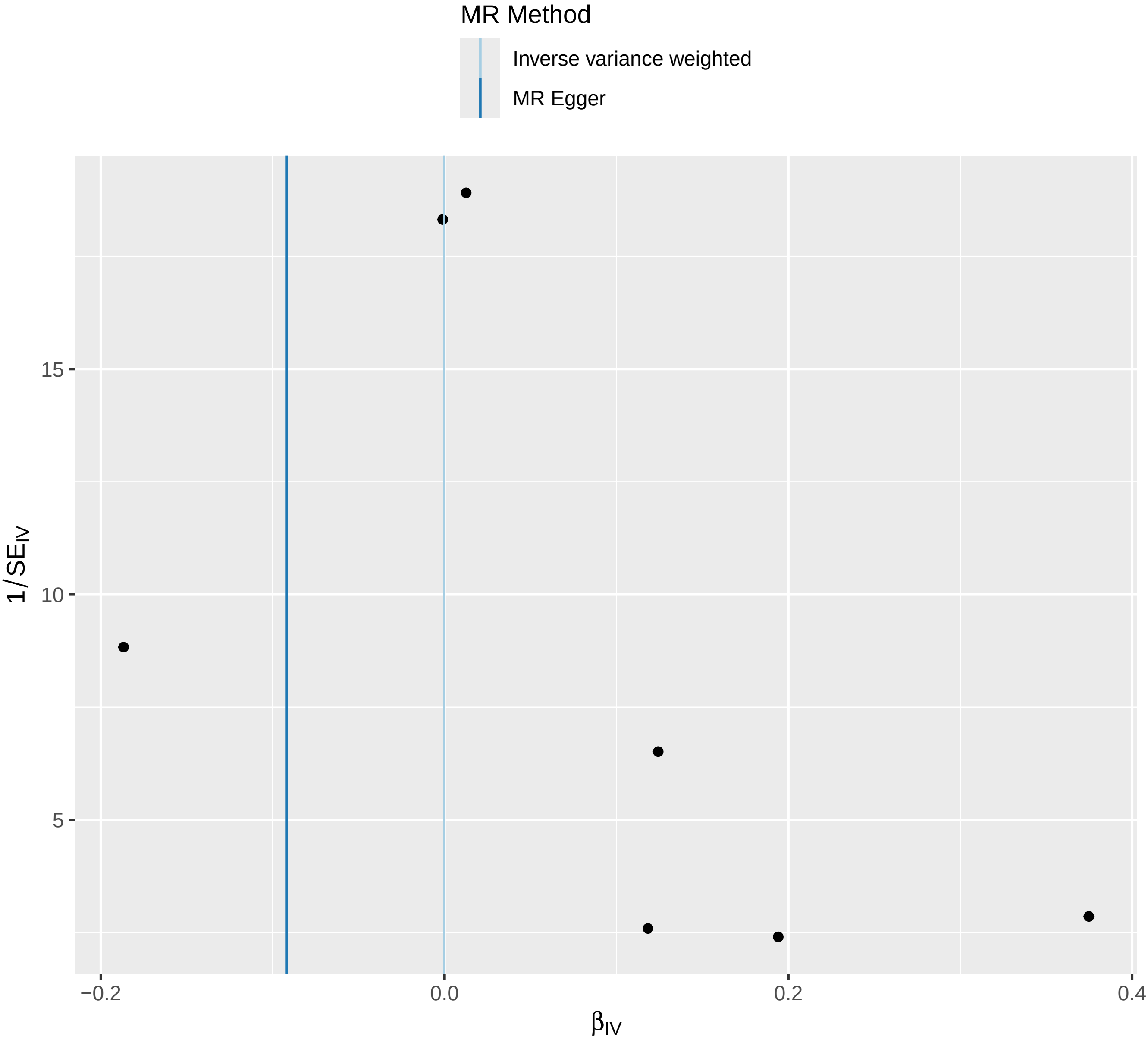
MR Method
Inverse variance weighted
MR Egger
15
10
5
1/SE$_{IV}$
−0.2
0.0
0.2
0.4
β$_{IV}$

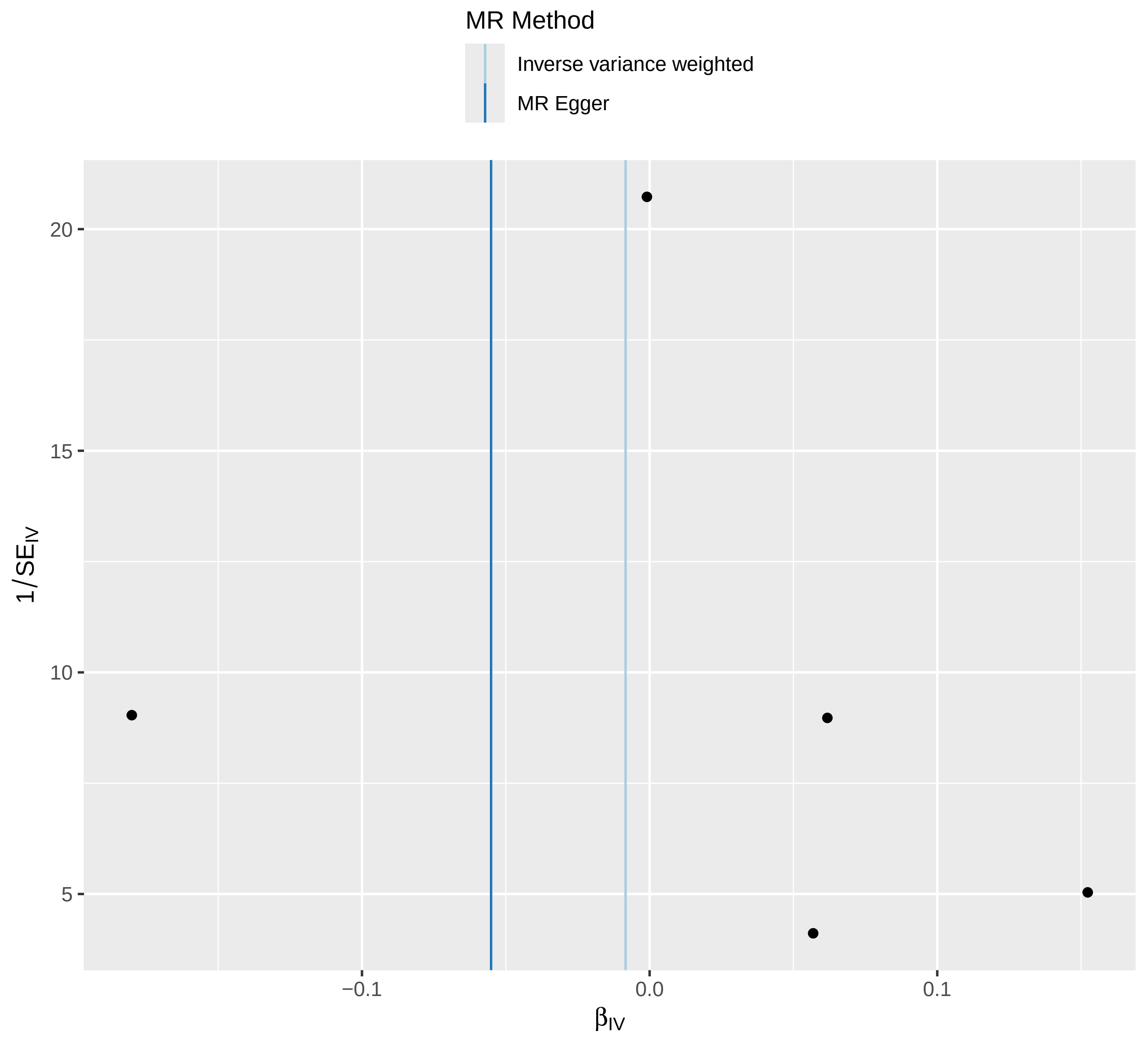

MR Method
Inverse variance weighted
MR Egger
20
15
10
5
1/SE_IV
−0.1
0.0
0.1
β_IV

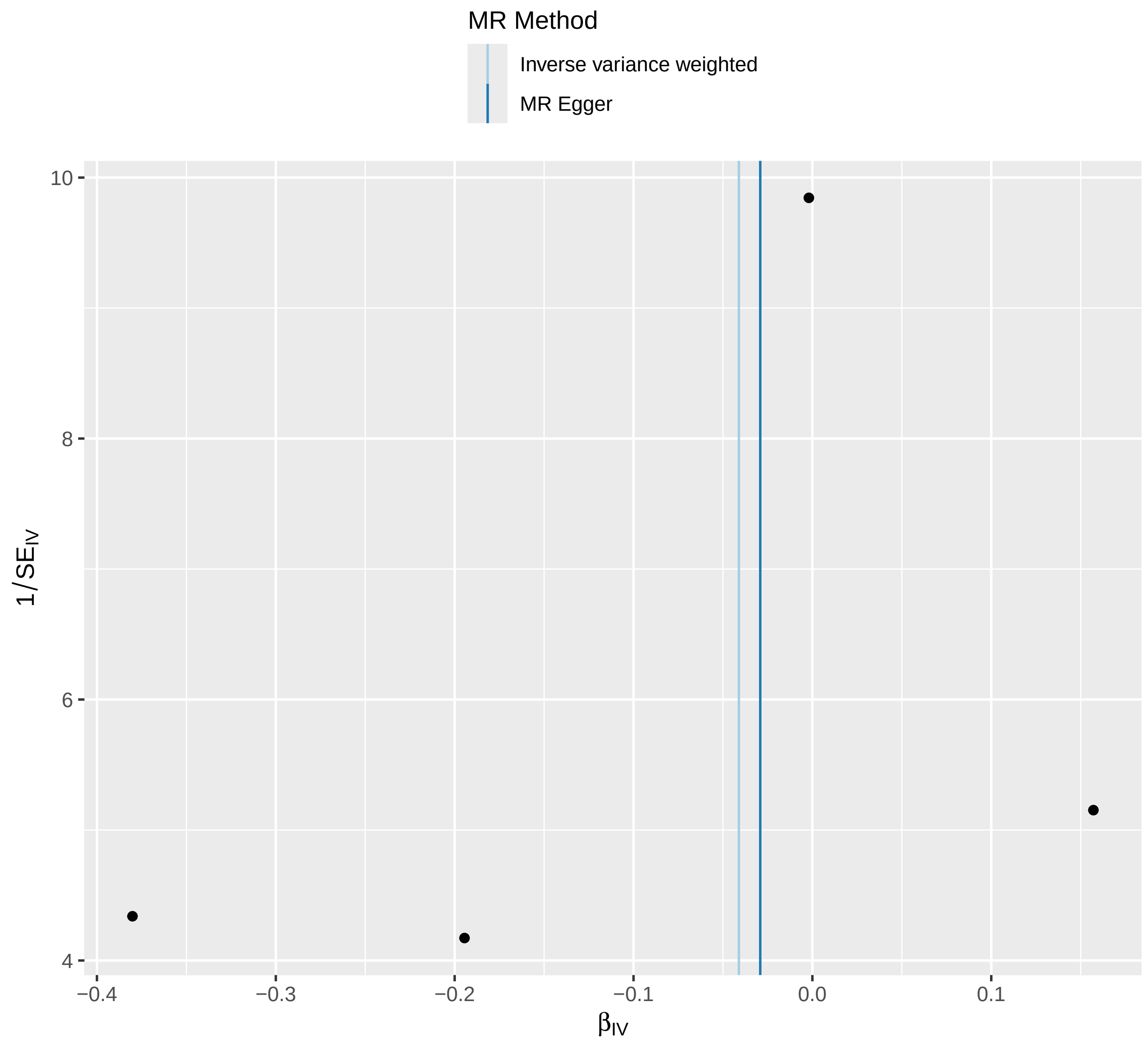

MR Method
Inverse variance weighted
MR Egger
10
8
6
4
1/SE_IV
−0.4
−0.3
−0.2
−0.1
0.0
0.1
β_IV

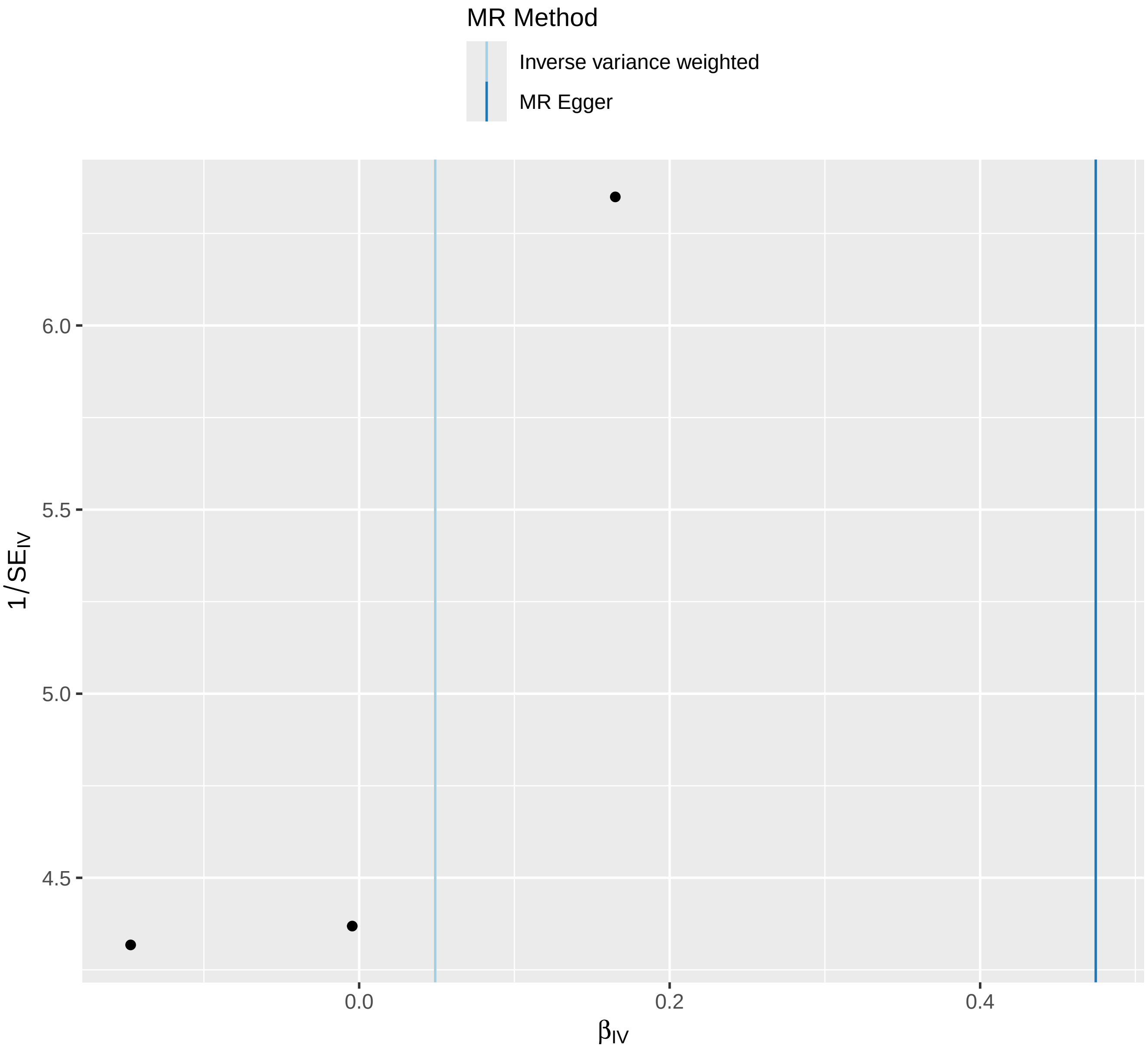
MR Method
Inverse variance weighted
MR Egger
6.0
5.5
5.0
4.5
$1/SE_{IV}$
0.0
0.2
0.4
$\beta_{IV}$

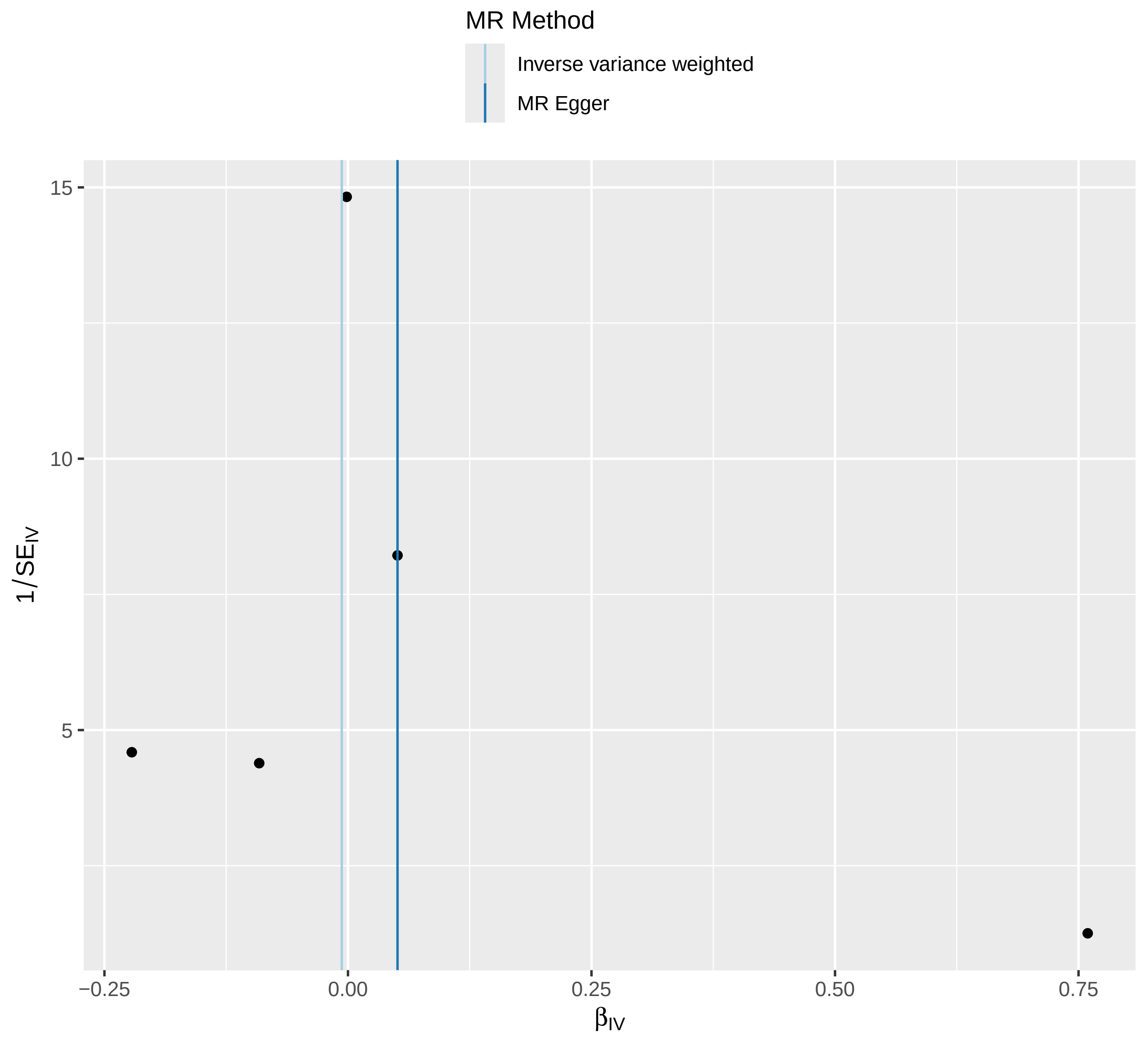
MR Method
Inverse variance weighted
MR Egger
15
10
5
1/SE_IV
−0.25
0.00
0.25
0.50
0.75
β_IV

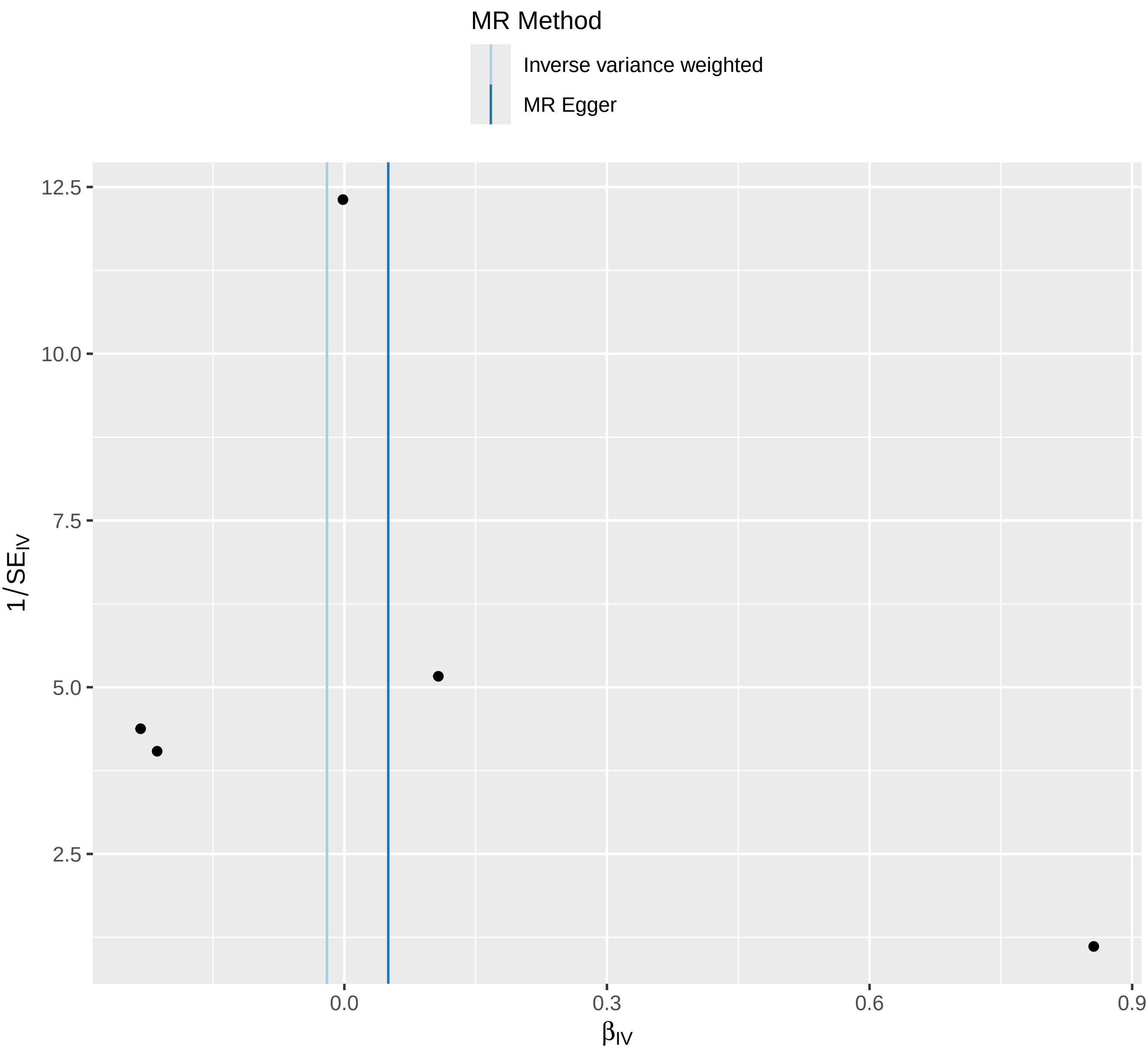

MR Method
Inverse variance weighted
MR Egger
12.5
10.0
7.5
5.0
2.5
1/SE_IV
0.0
0.3
0.6
0.9
β_IV

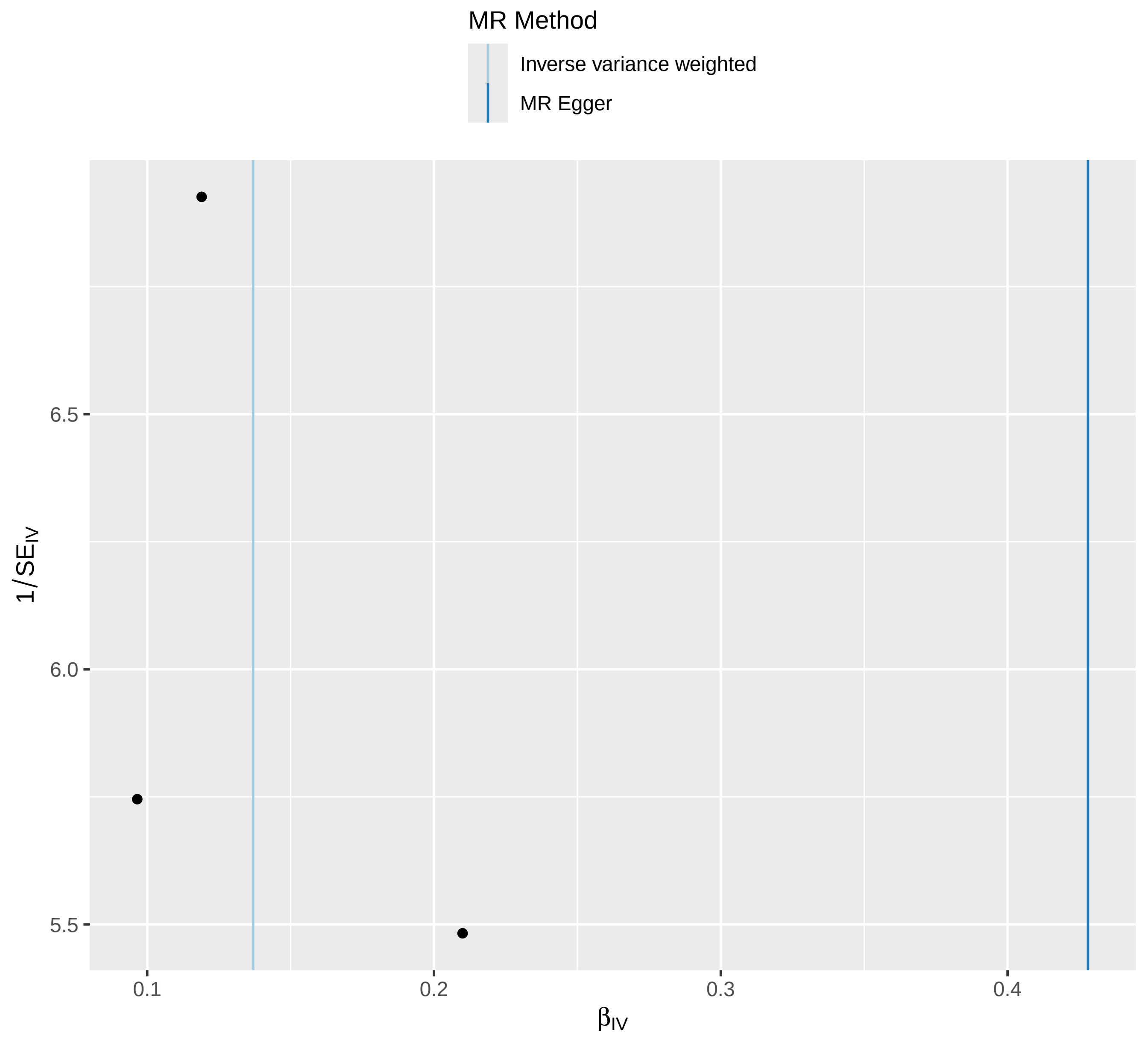
MR Method
Inverse variance weighted
MR Egger
6.5
6.0
5.5
0.1
0.2
0.3
0.4
1/SE$_{IV}$
β$_{IV}$

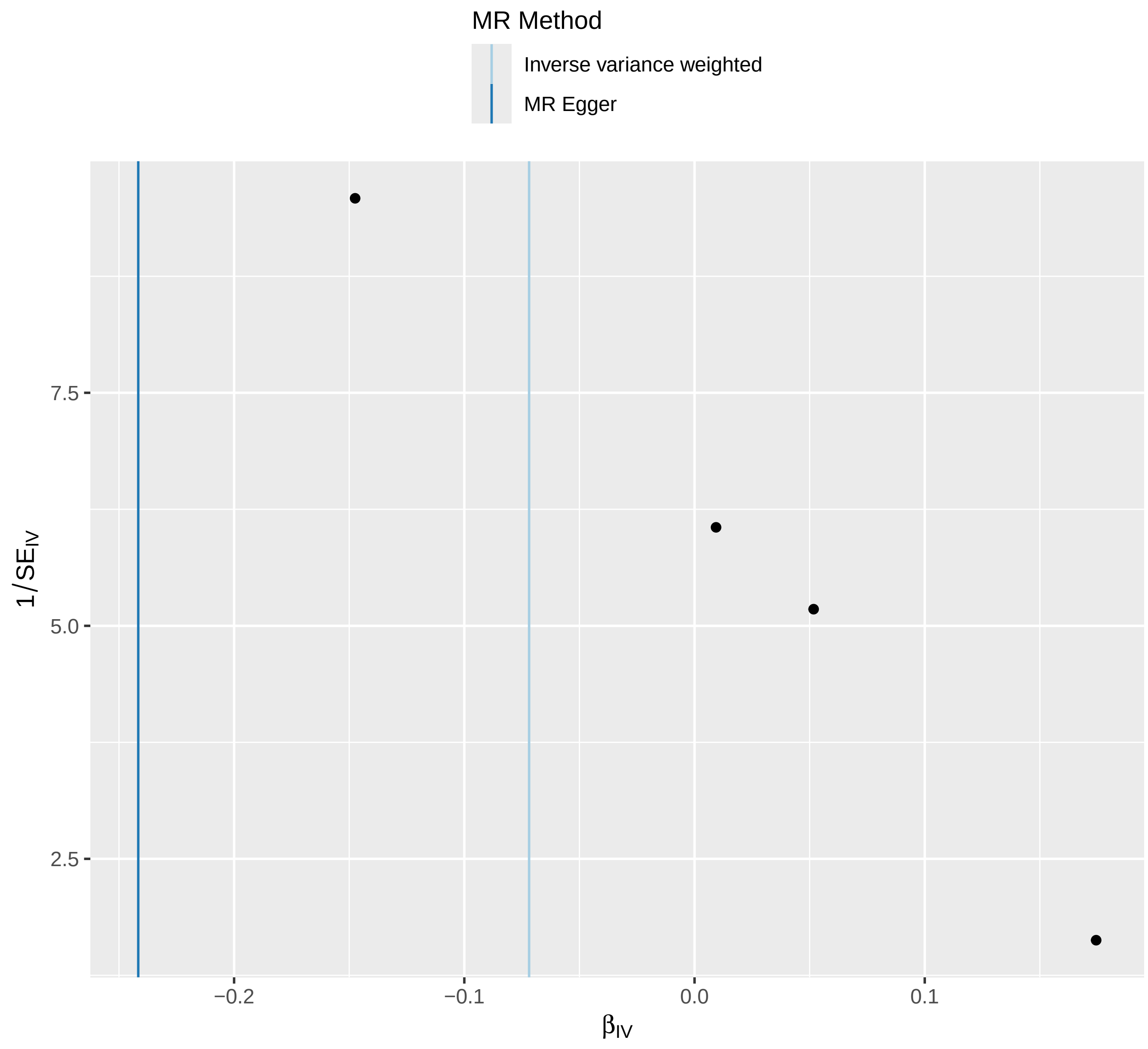
MR Method
Inverse variance weighted
MR Egger
7.5
5.0
2.5
1/SE$_{IV}$
−0.2
−0.1
0.0
0.1
β$_{IV}$

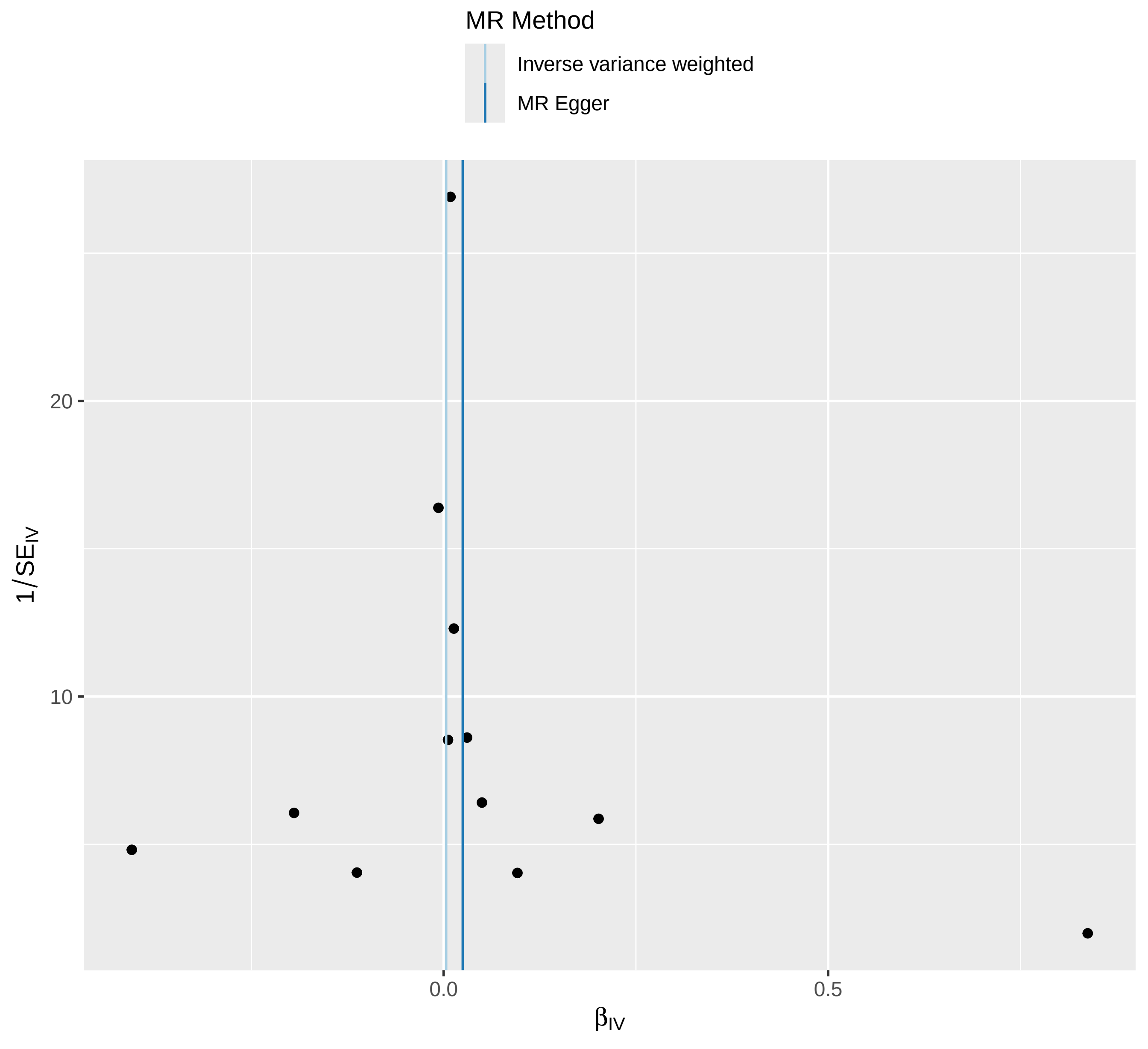
MR Method
Inverse variance weighted
MR Egger
20
10
1/SE_IV
0.0
0.5
β_IV

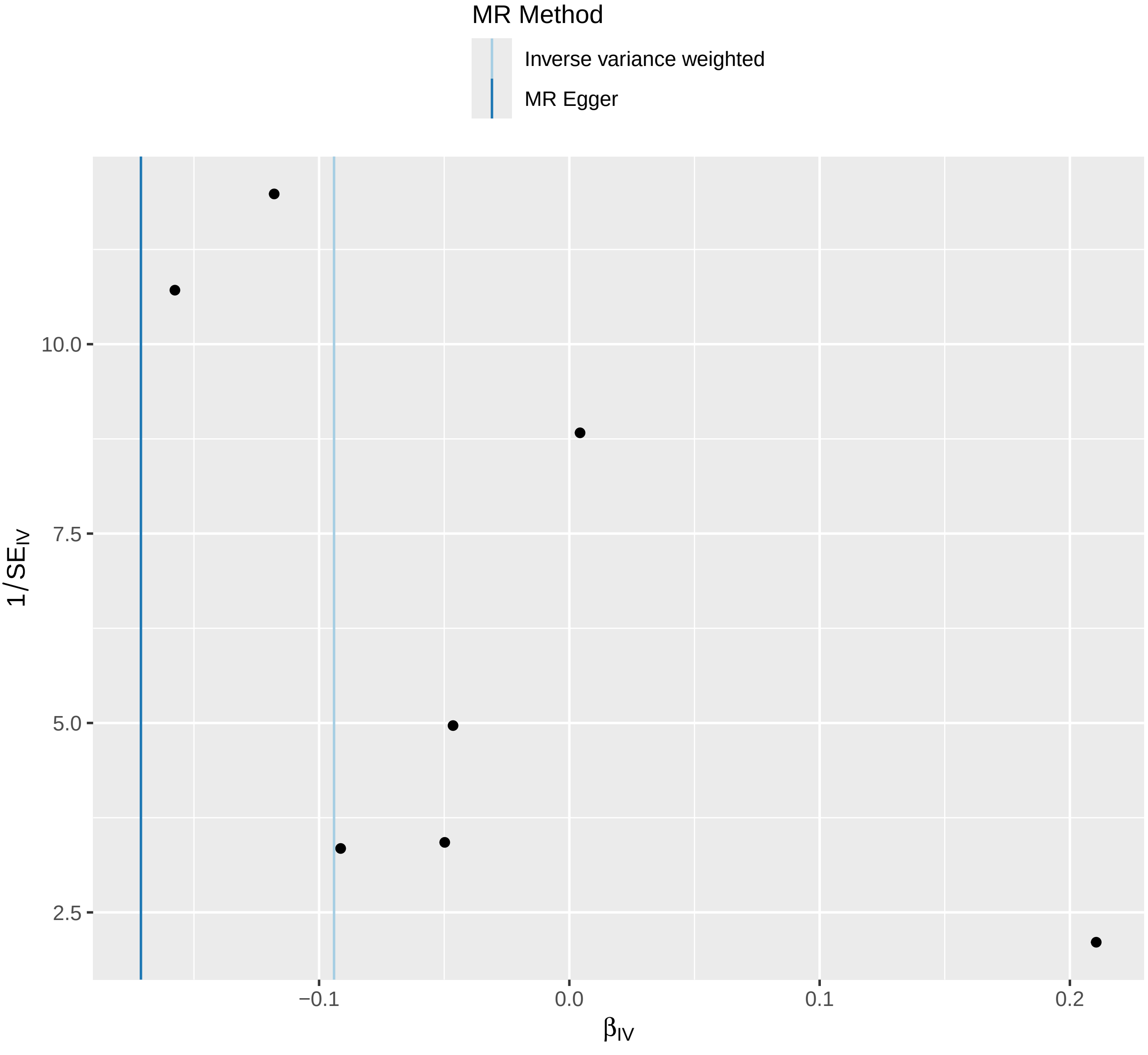
MR Method
Inverse variance weighted
MR Egger
10.0
7.5
5.0
2.5
1/SE_IV
−0.1
0.0
0.1
0.2
β_IV

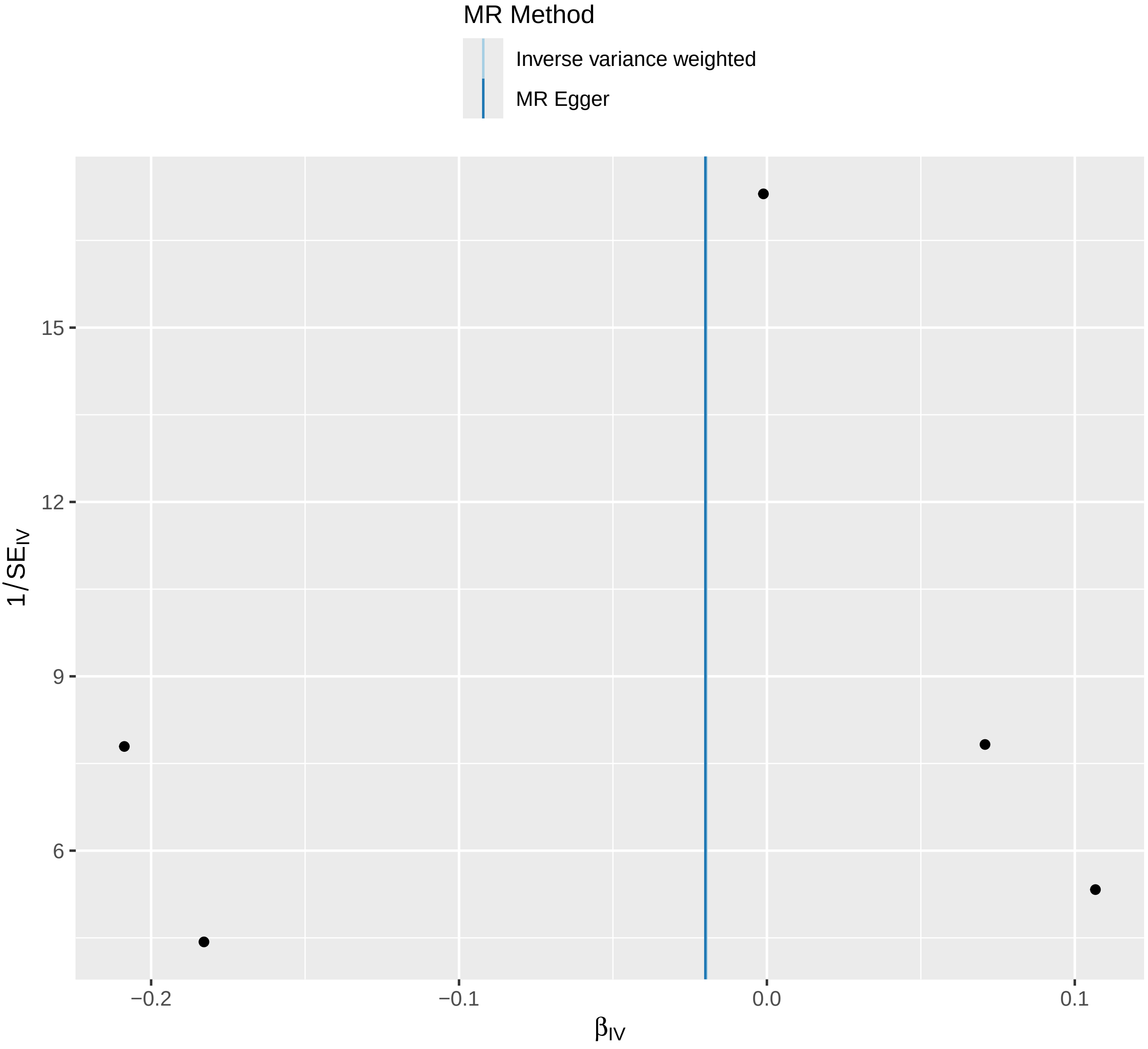
MR Method
Inverse variance weighted
MR Egger
15
12
9
6
−0.2
−0.1
0.0
0.1
$1/SE_{IV}$
$\beta_{IV}$

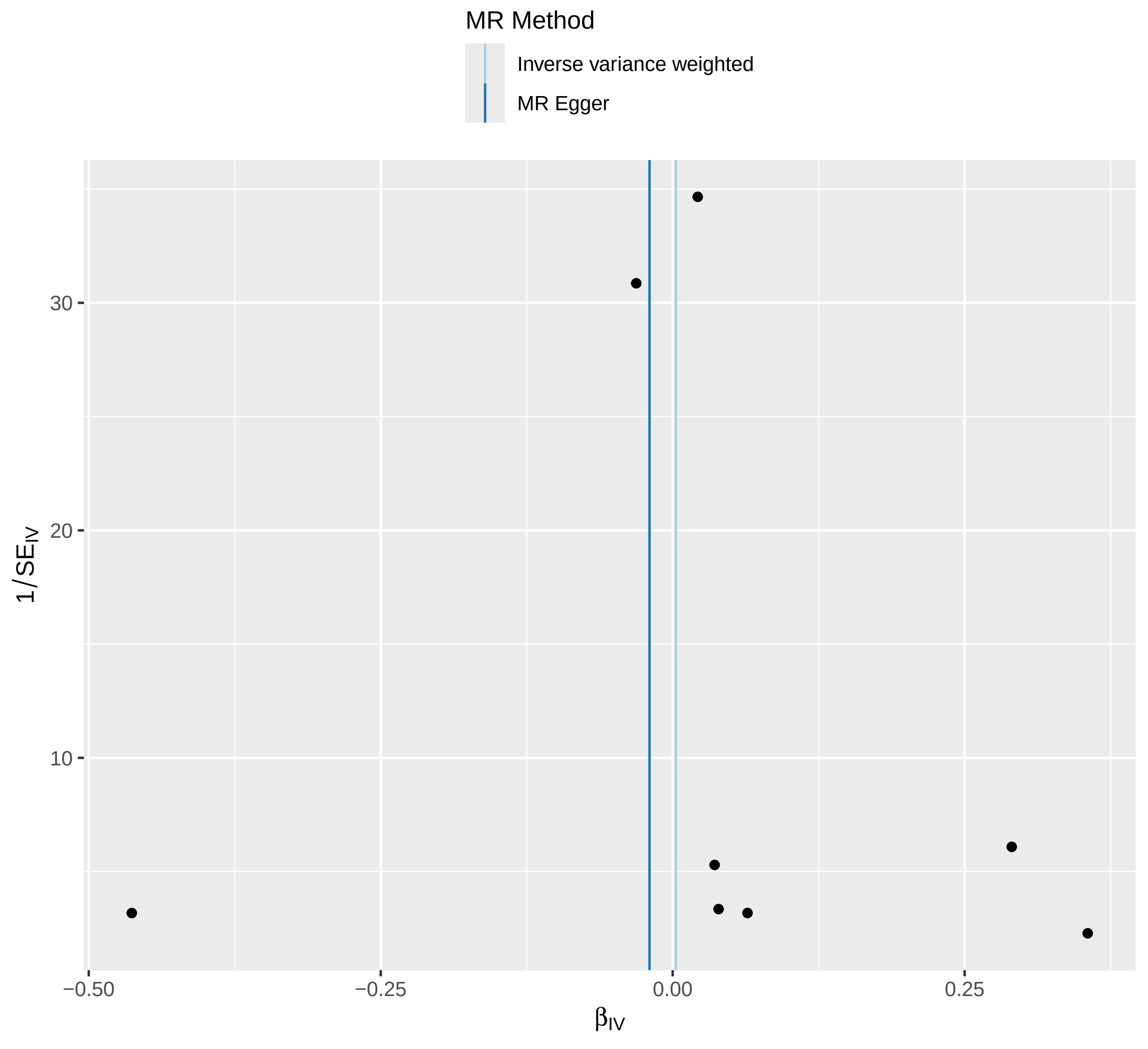

MR Method
Inverse variance weighted
MR Egger
1/SE_IV
30
20
10
−0.50
−0.25
0.00
0.25
β_IV

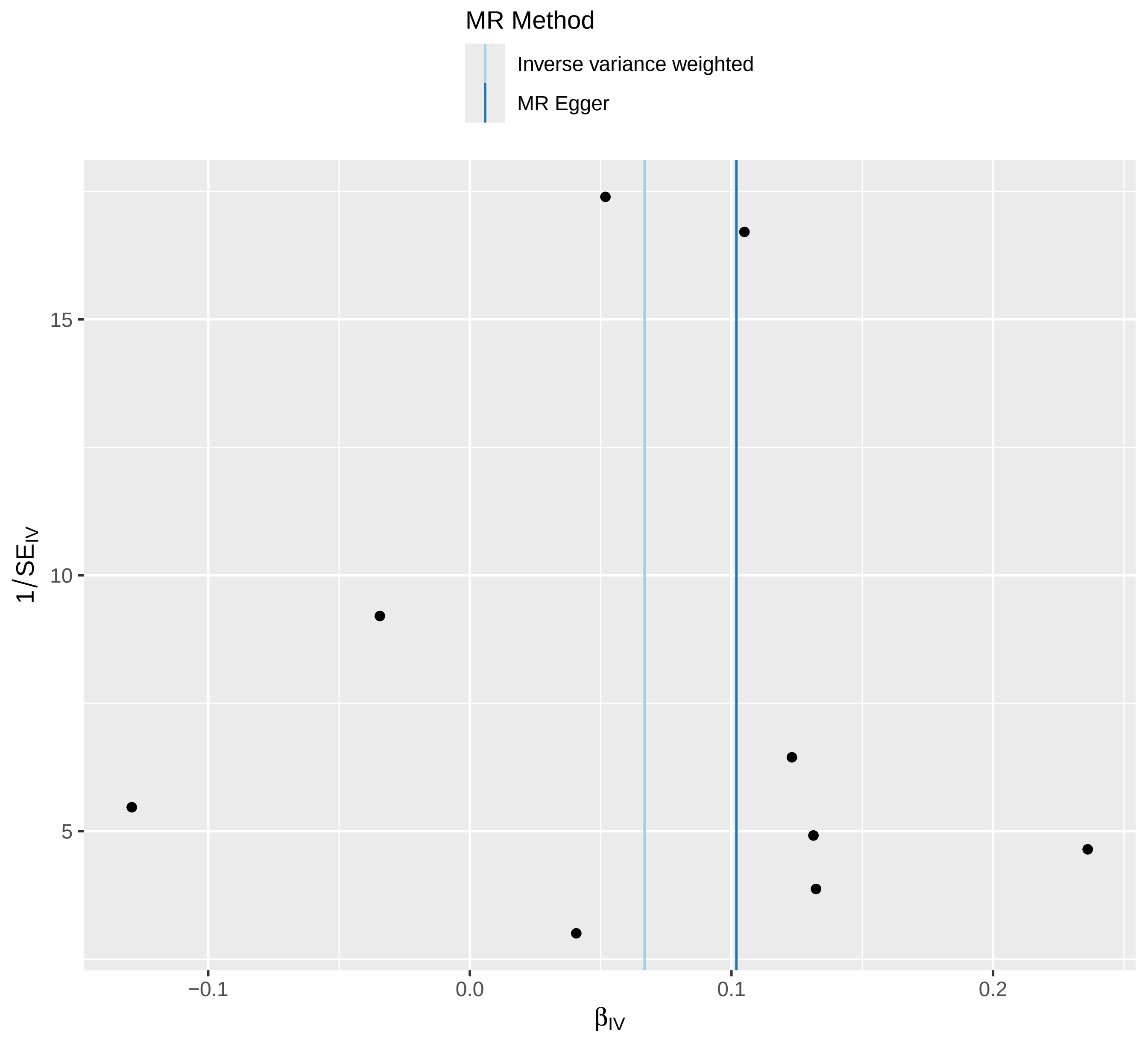

MR Method
Inverse variance weighted
MR Egger
15
10
5
1/SE$_{IV}$
−0.1
0.0
0.1
0.2
β$_{IV}$

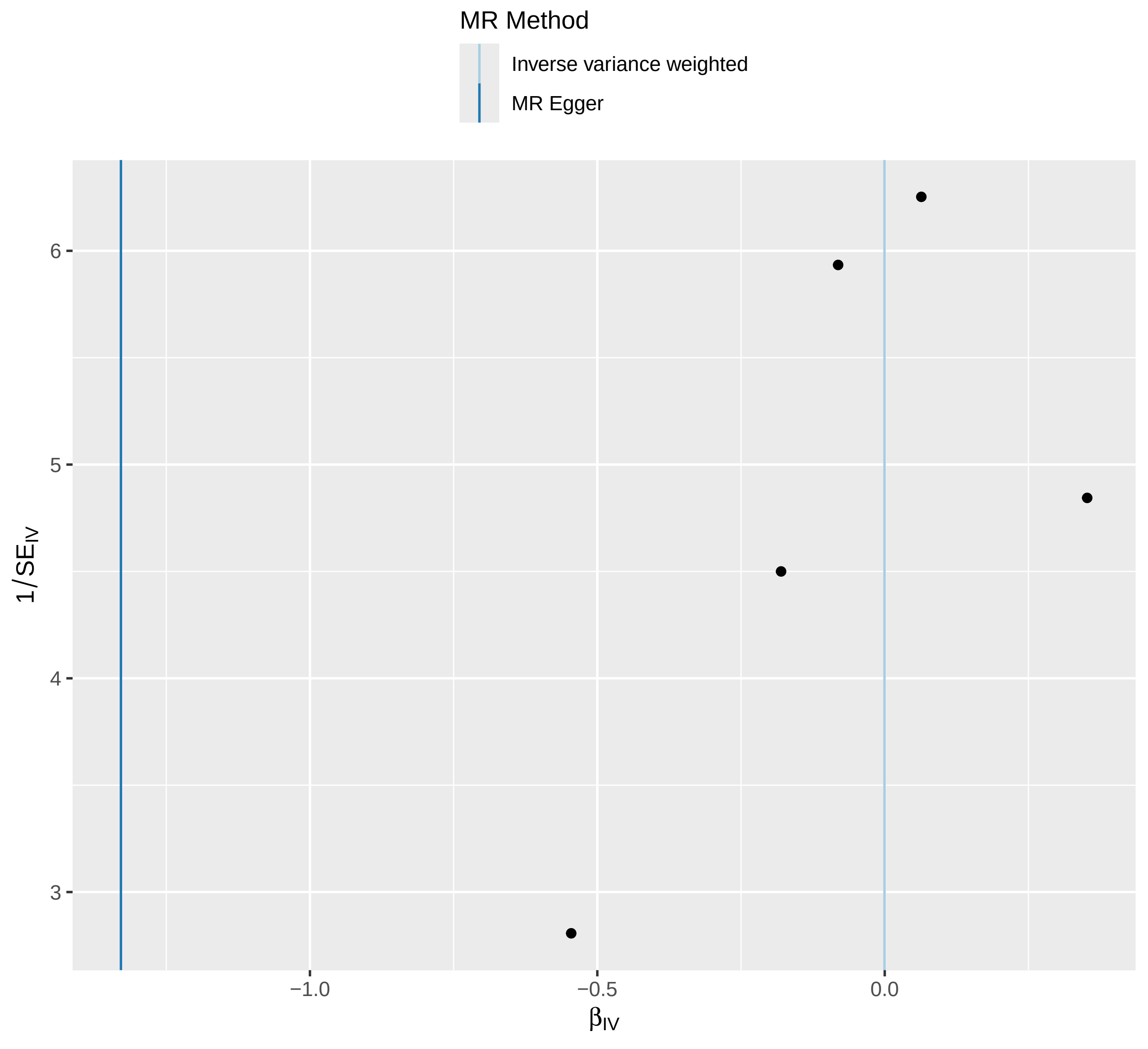
MR Method
Inverse variance weighted
MR Egger
6
5
4
3
1/SE_IV
−1.0
−0.5
0.0
β_IV

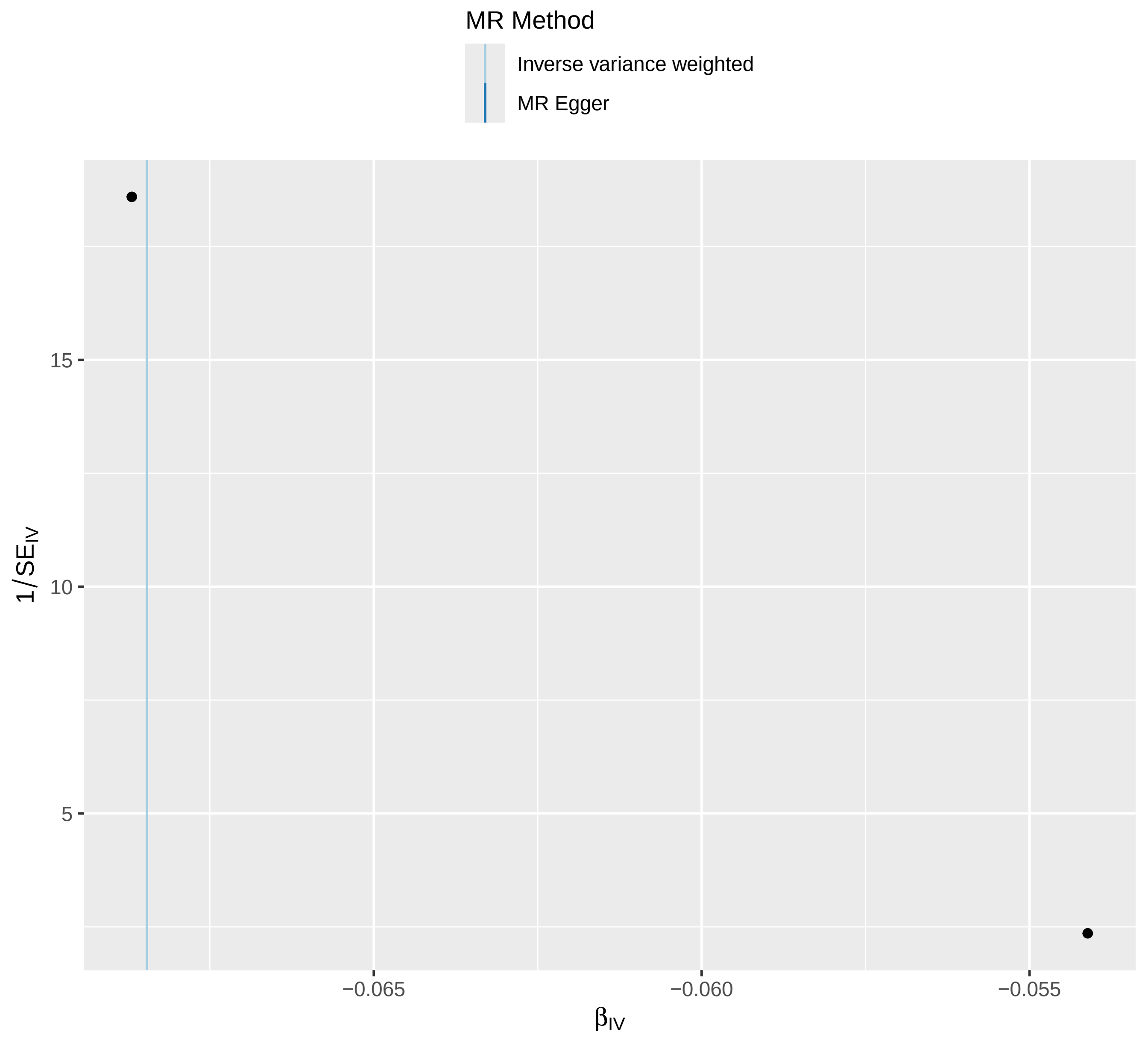
MR Method
Inverse variance weighted
MR Egger
15
10
5
1/SE$_{IV}$
−0.065
−0.060
−0.055
β$_{IV}$

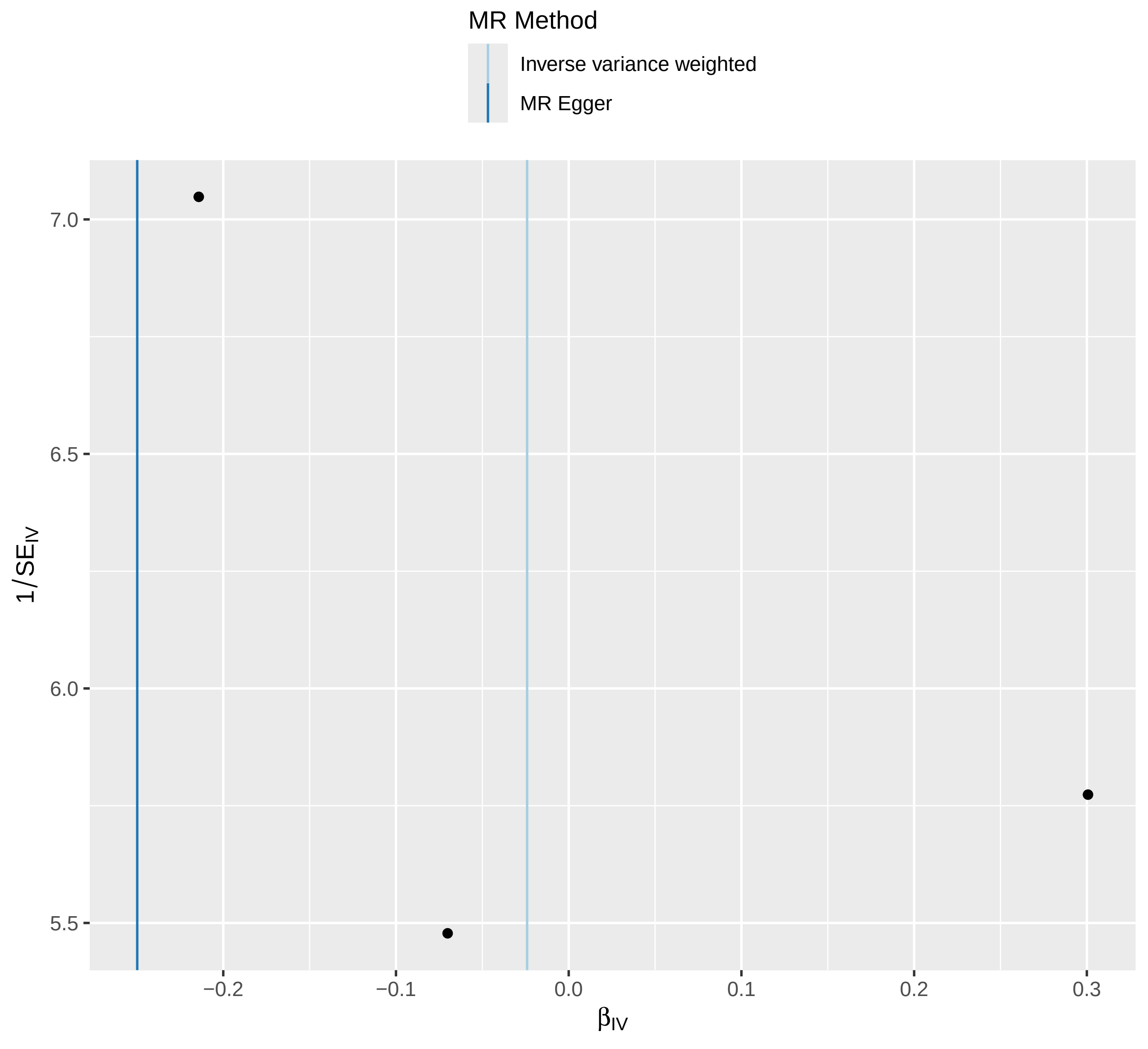

MR Method
Inverse variance weighted
MR Egger
7.0
6.5
6.0
5.5
$1/SE_{IV}$
−0.2
−0.1
0.0
0.1
0.2
0.3
$\beta_{IV}$

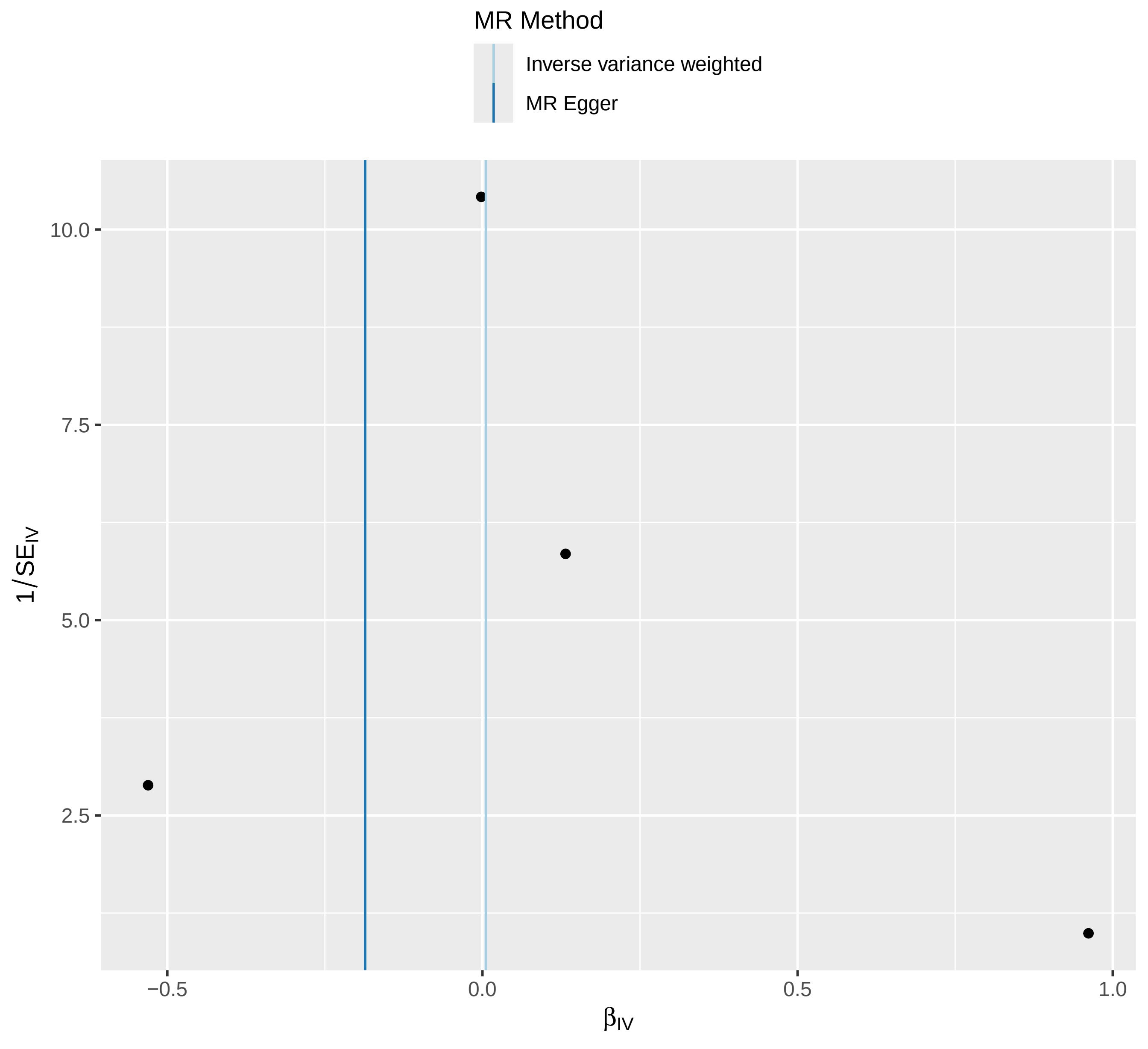

MR Method
Inverse variance weighted
MR Egger
10.0
7.5
5.0
2.5
1/SE_IV
−0.5
0.0
0.5
1.0
β_IV

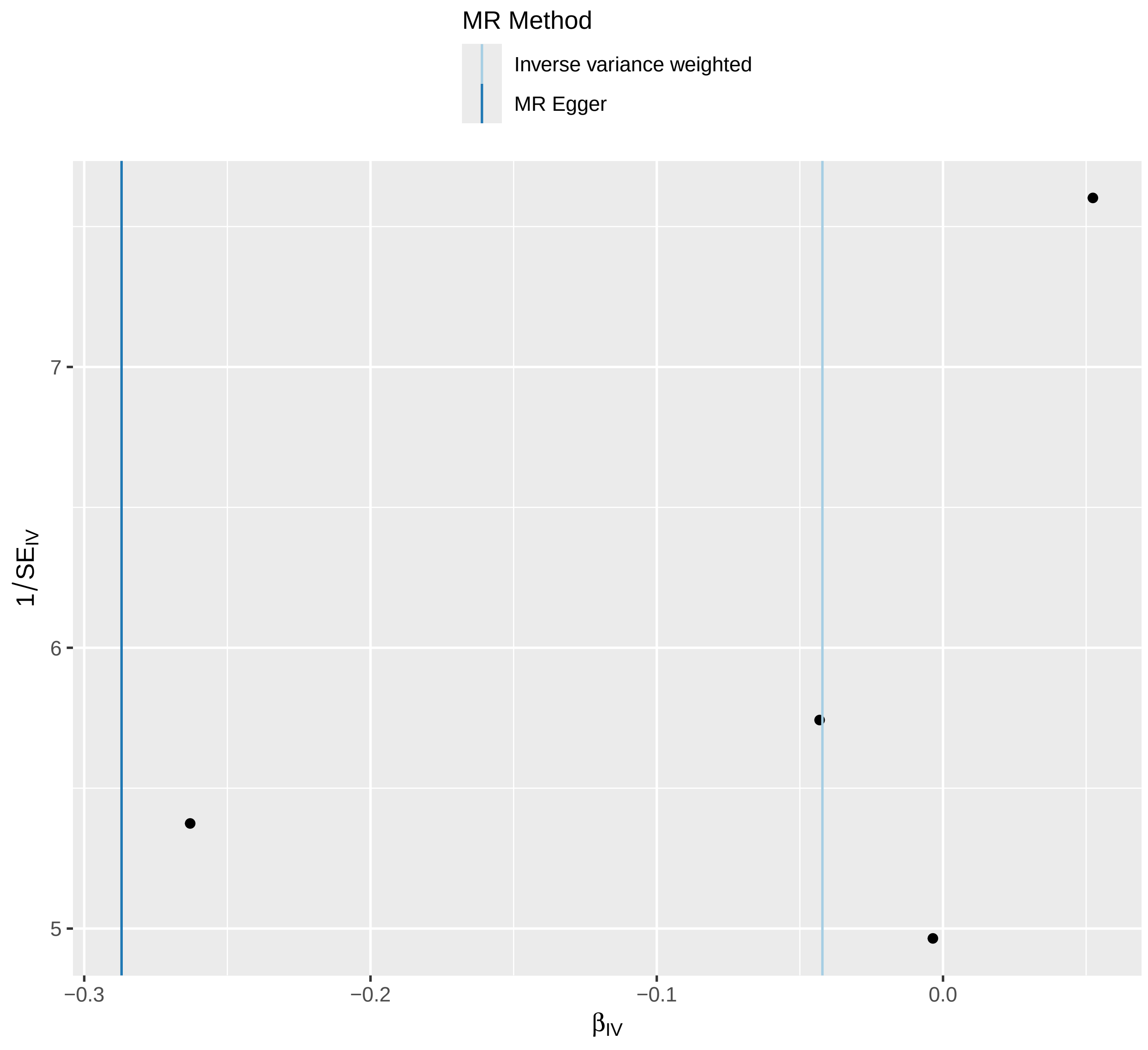

MR Method
Inverse variance weighted
MR Egger
1/SE_IV
β_IV
7
6
5
−0.3
−0.2
−0.1
0.0

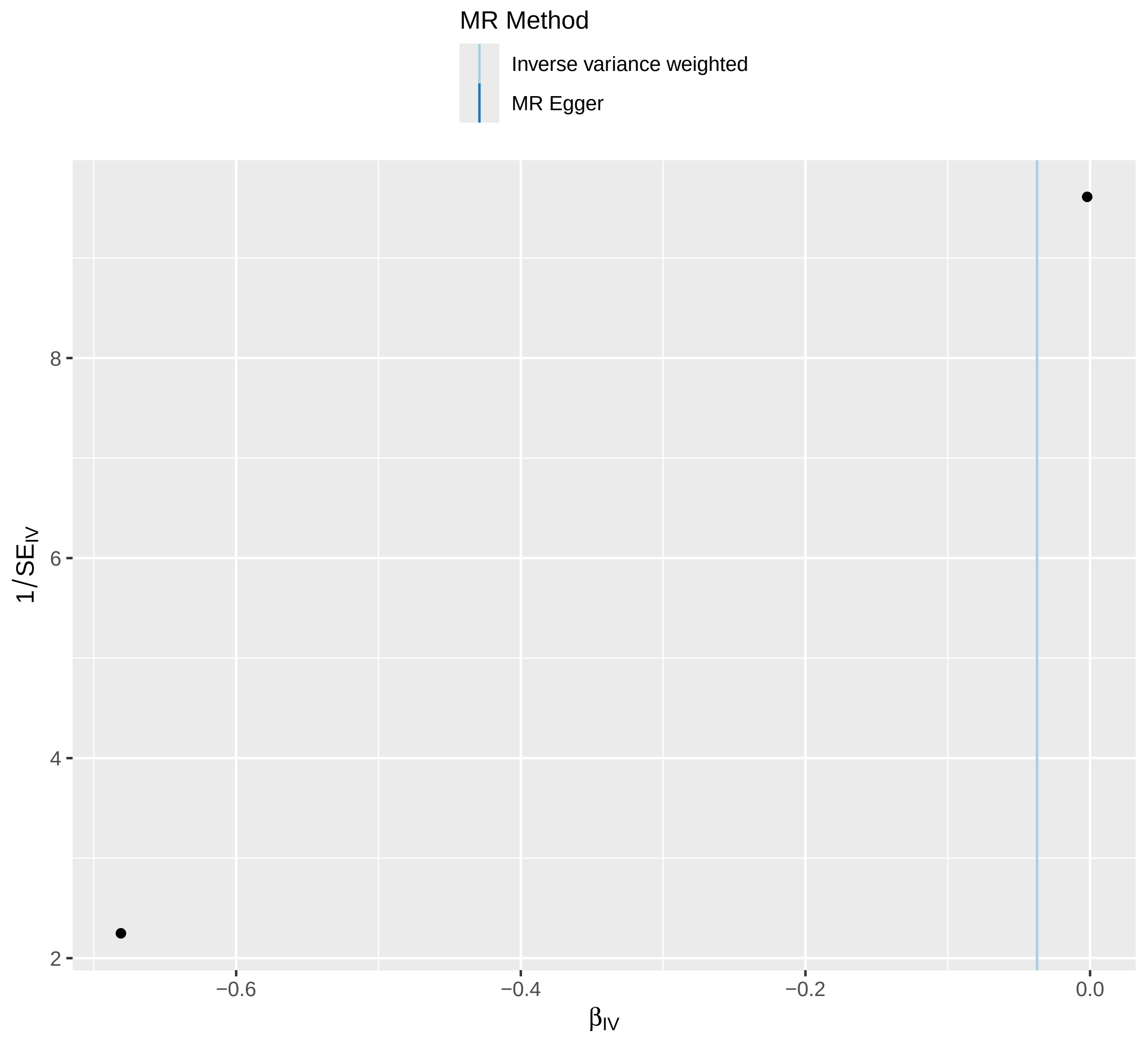

MR Method
Inverse variance weighted
MR Egger
8
6
4
2
1/SE_IV
−0.6
−0.4
−0.2
0.0
β_IV

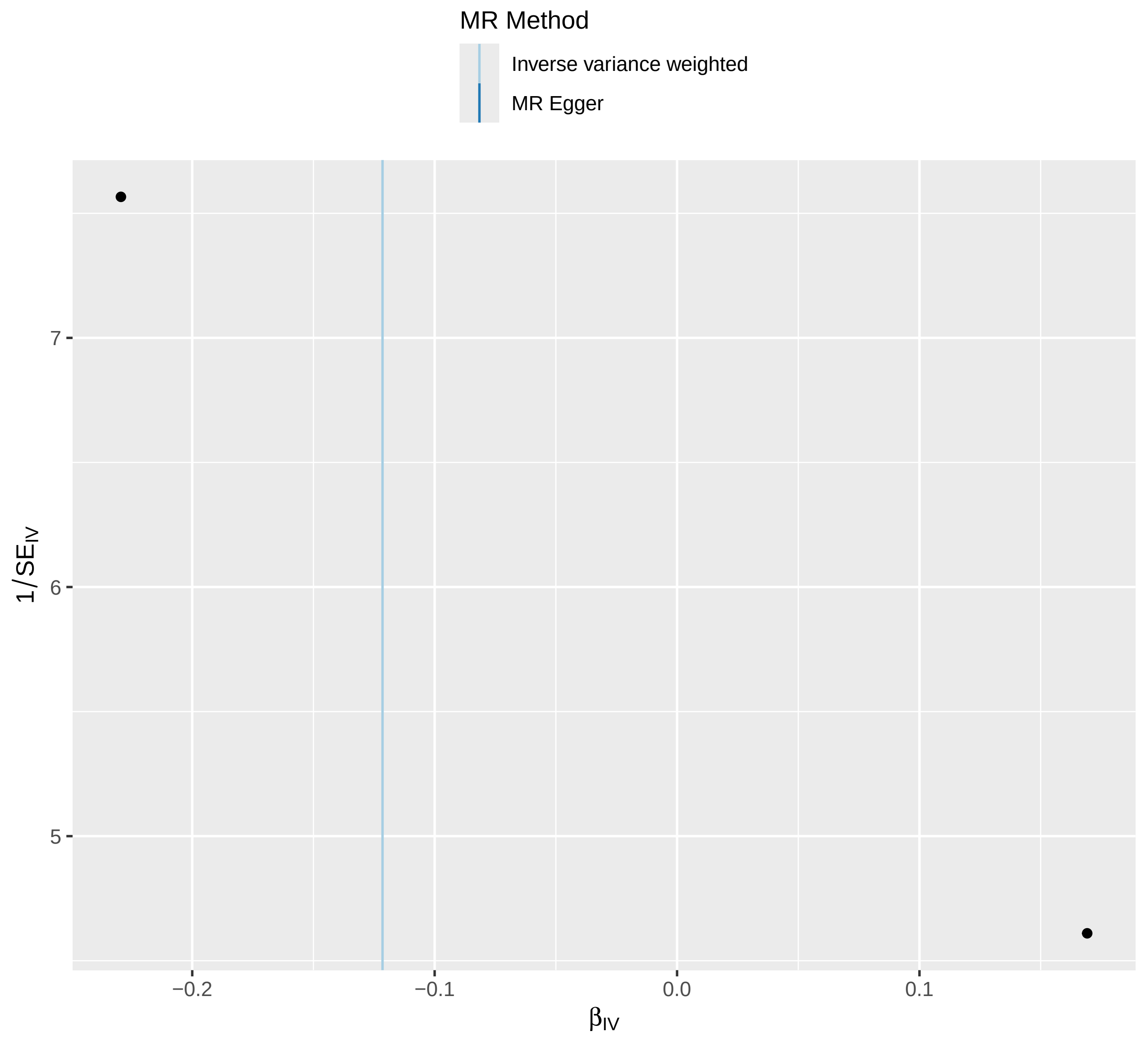
MR Method
Inverse variance weighted
MR Egger
7
6
5
1/SE$_{IV}$
−0.2
−0.1
0.0
0.1
β$_{IV}$

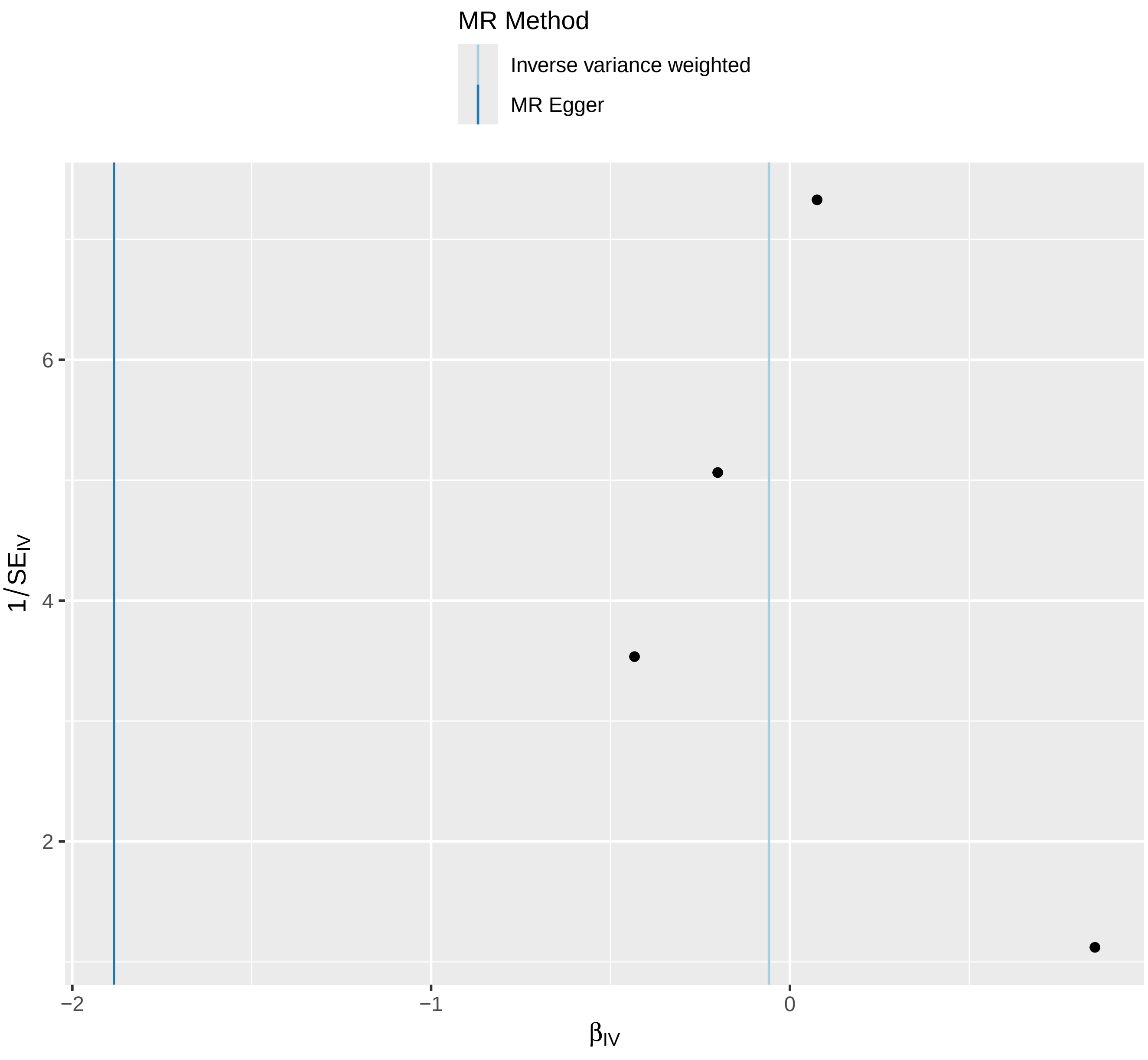

MR Method
Inverse variance weighted
MR Egger
6
4
2
1/$SE_{IV}$
−2
−1
0
$\beta_{IV}$

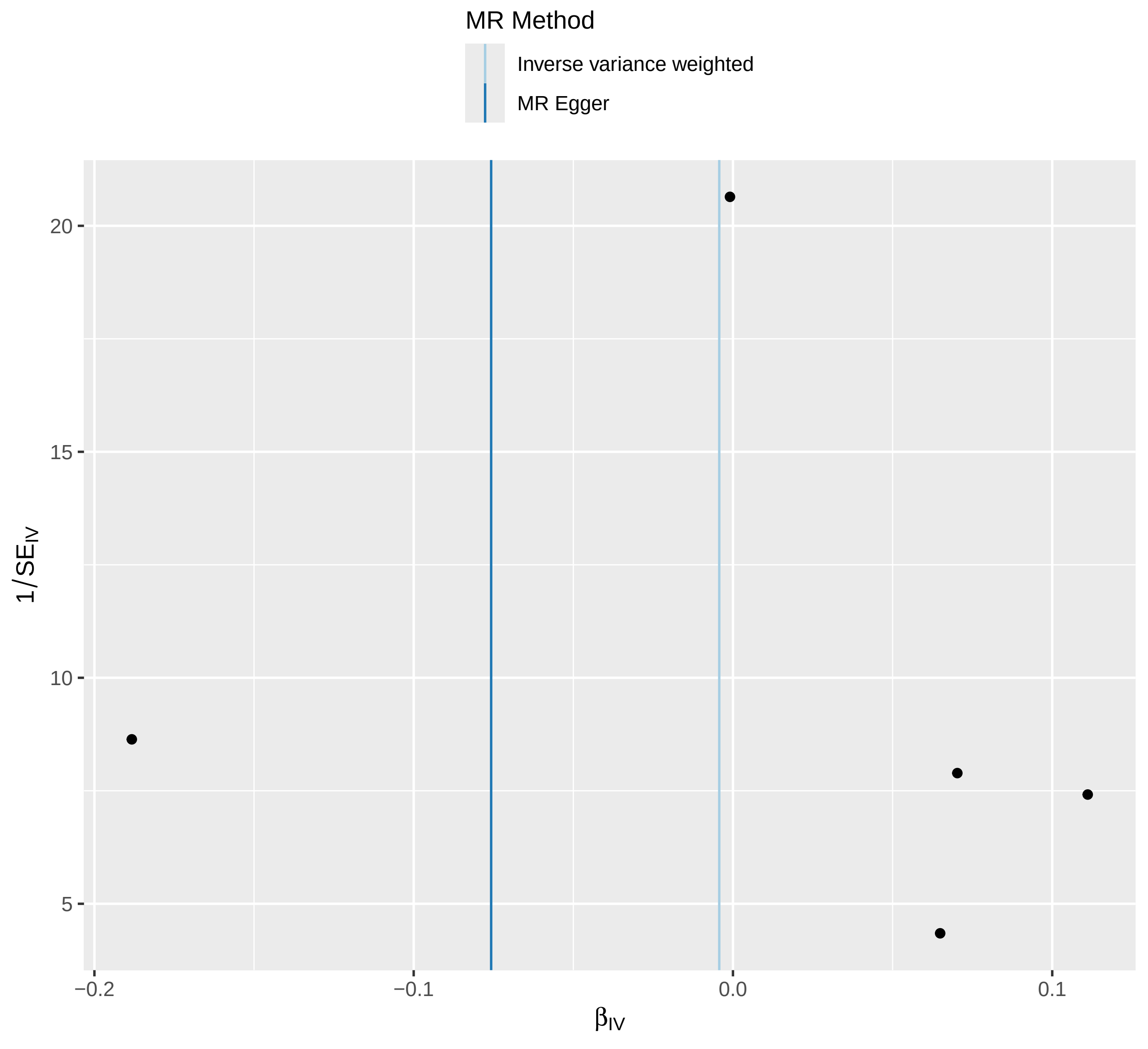

MR Method
Inverse variance weighted
MR Egger
20
15
10
5
1/SE_IV
−0.2
−0.1
0.0
0.1
β_IV

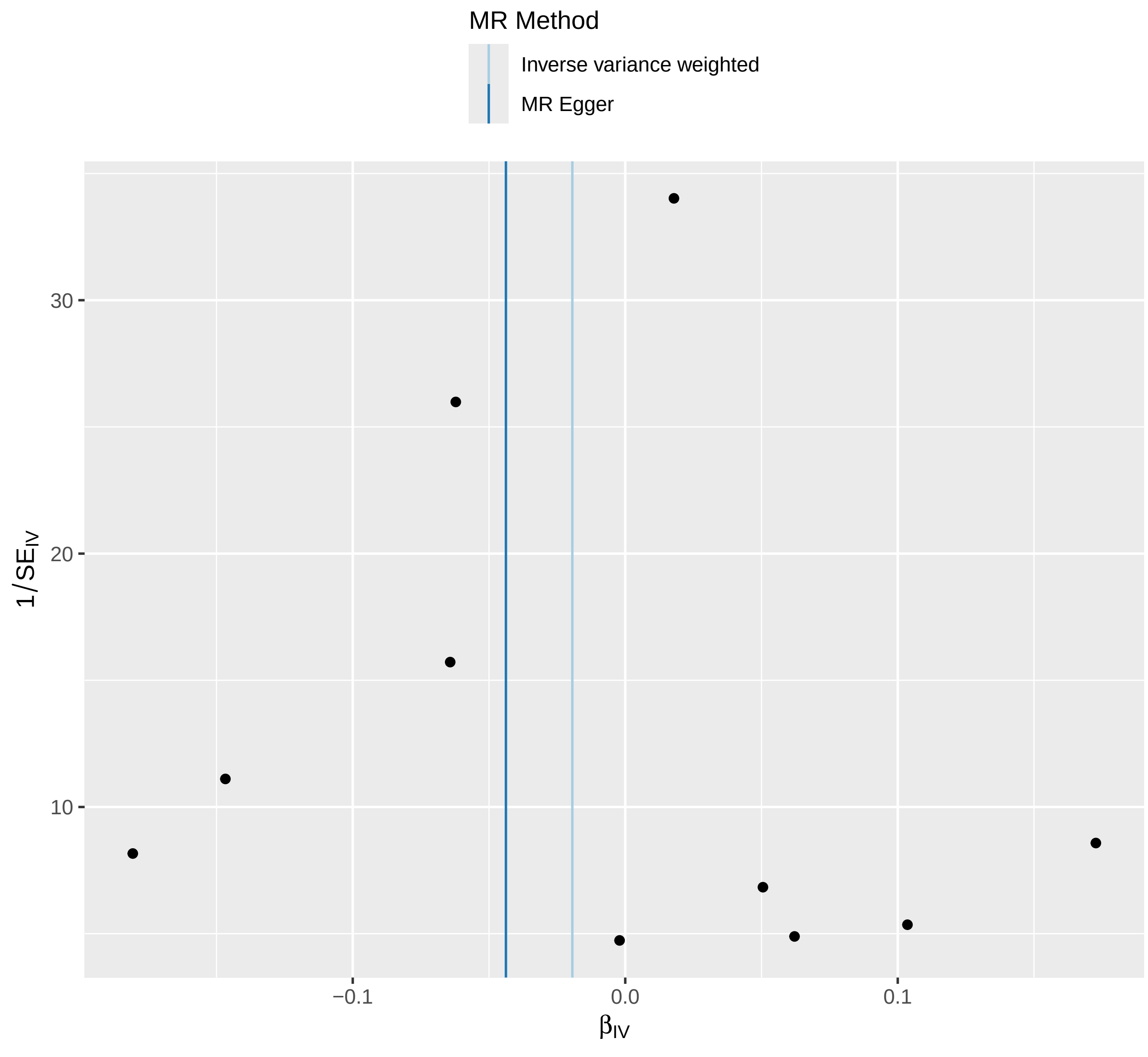

MR Method
Inverse variance weighted
MR Egger
30
20
10
1/SE_IV
−0.1
0.0
0.1
β_IV

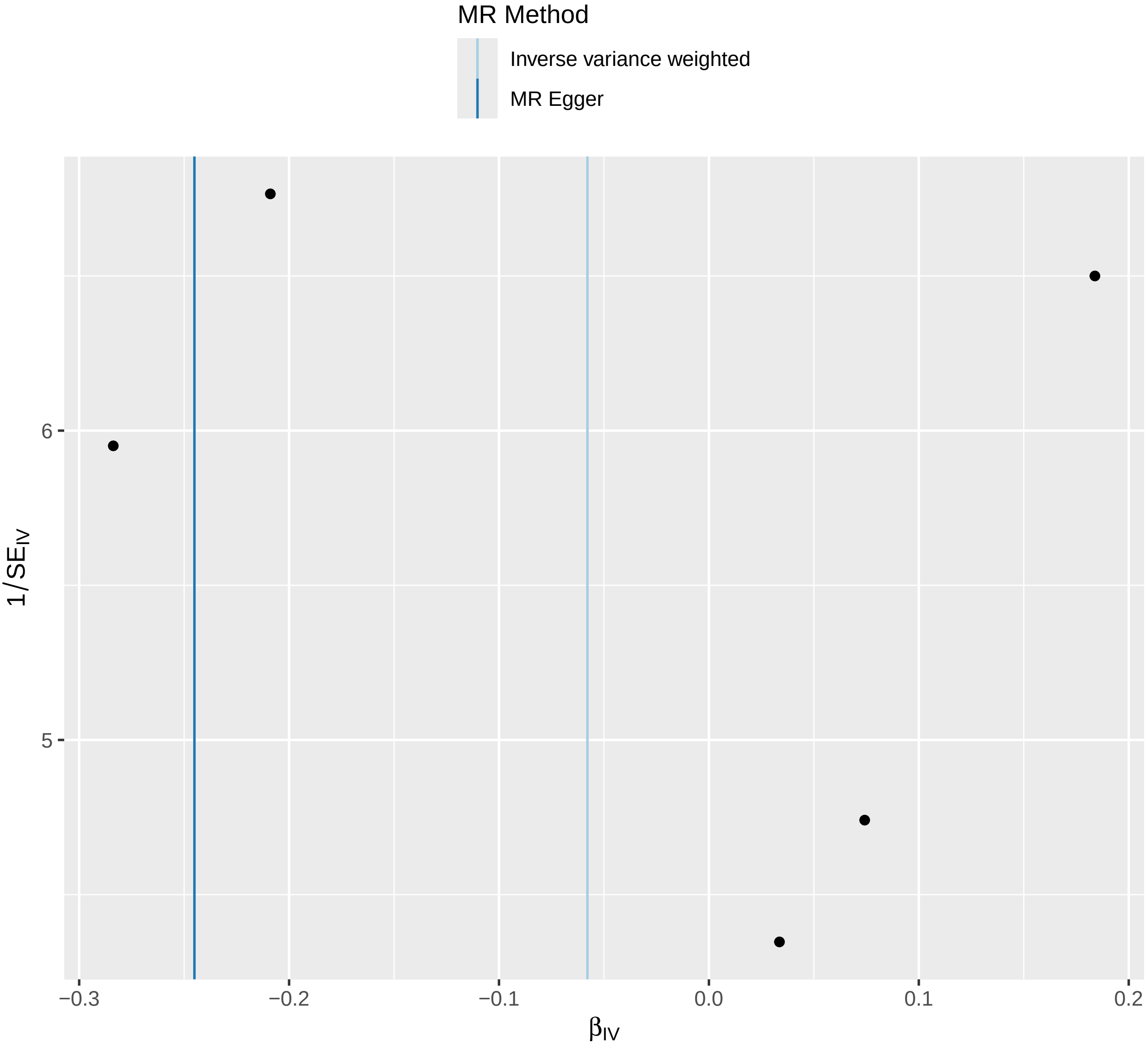
MR Method
Inverse variance weighted
MR Egger
6
5
1/SE$_{IV}$
−0.3
−0.2
−0.1
0.0
0.1
0.2
β$_{IV}$

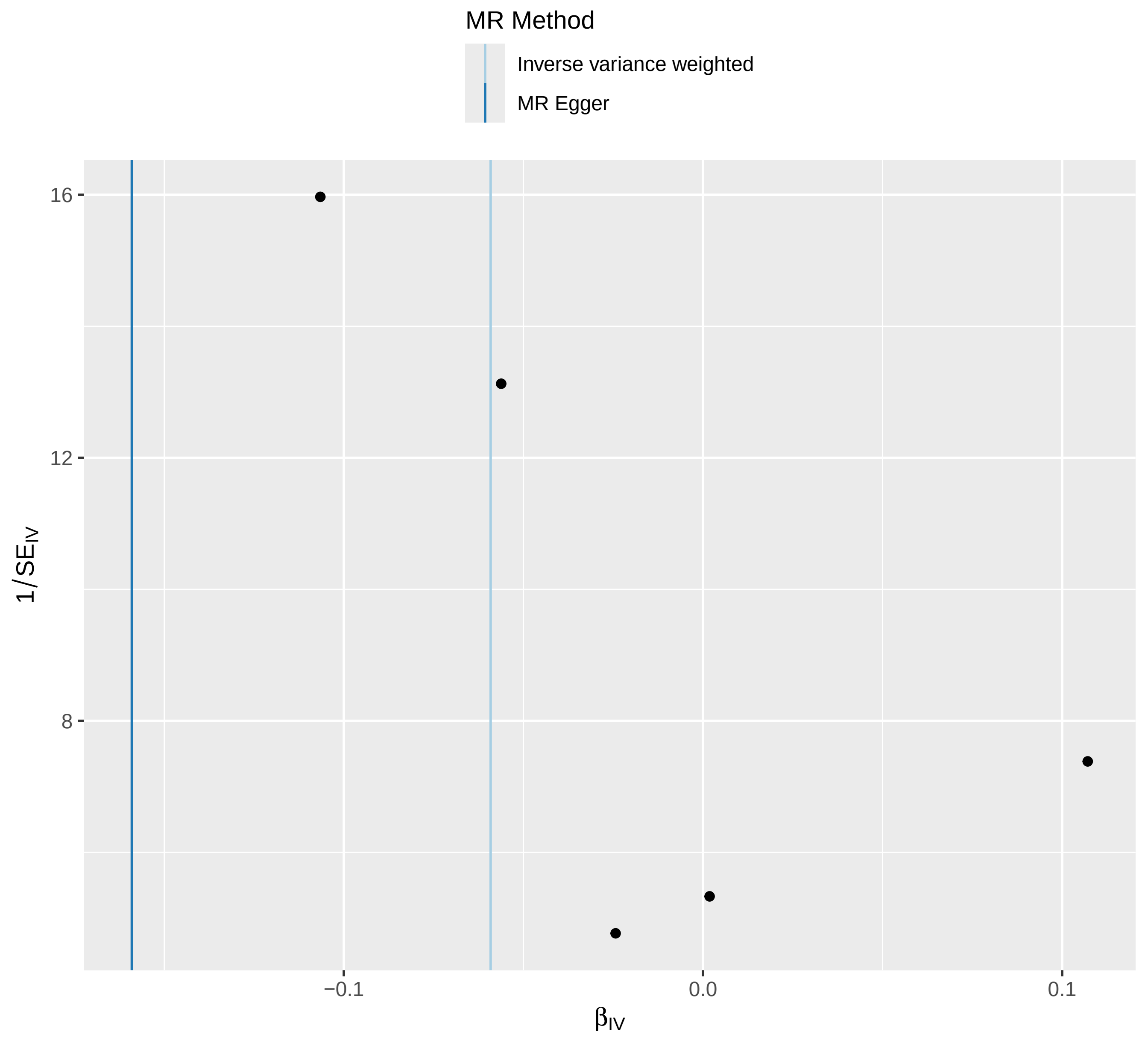

MR Method
Inverse variance weighted
MR Egger
16
12
8
1/SE_IV
−0.1
0.0
0.1
β_IV

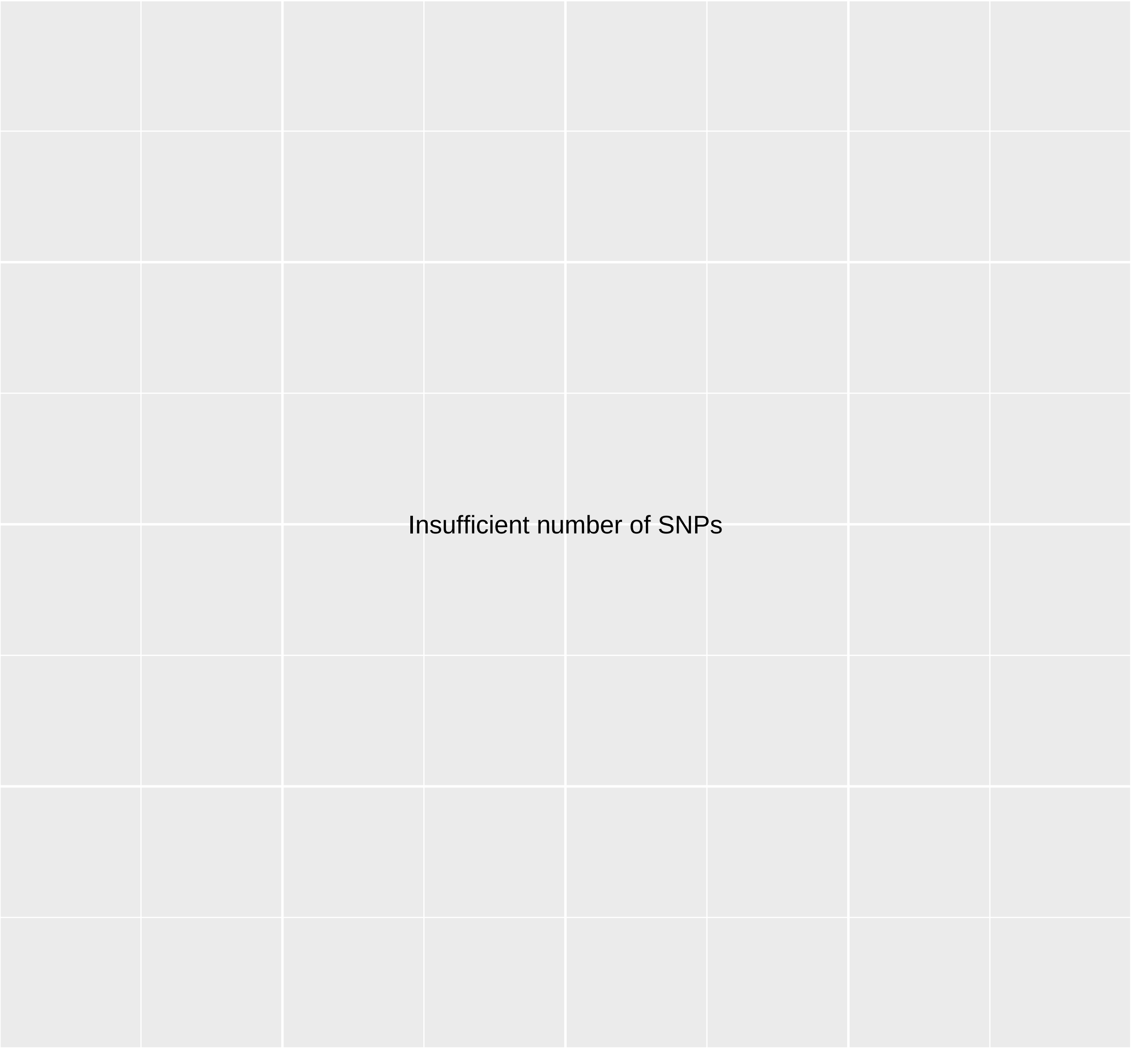
Insufficient number of SNPs

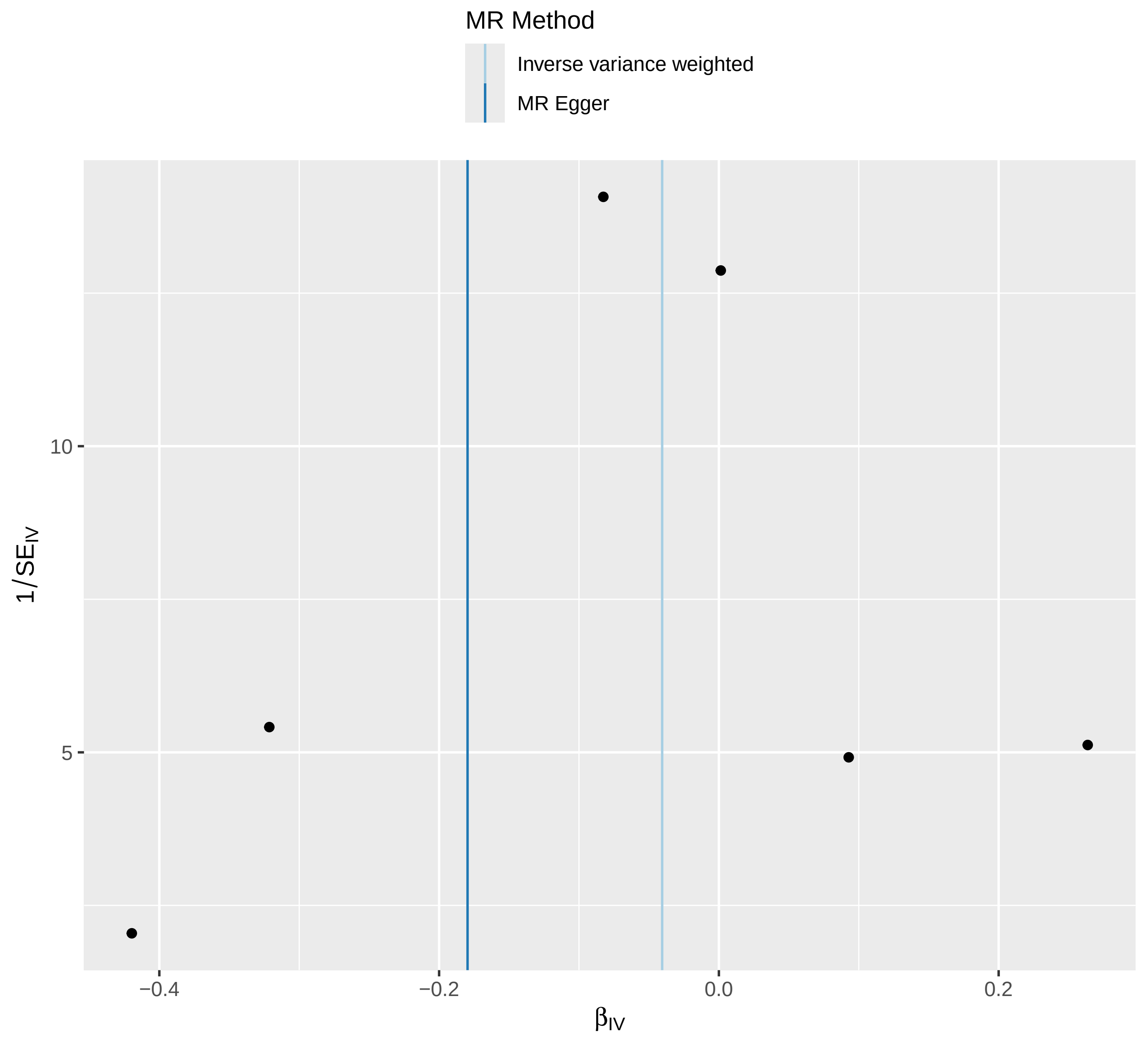
MR Method
Inverse variance weighted
MR Egger
10
5
1/SE$_{IV}$
−0.4
−0.2
0.0
0.2
β$_{IV}$

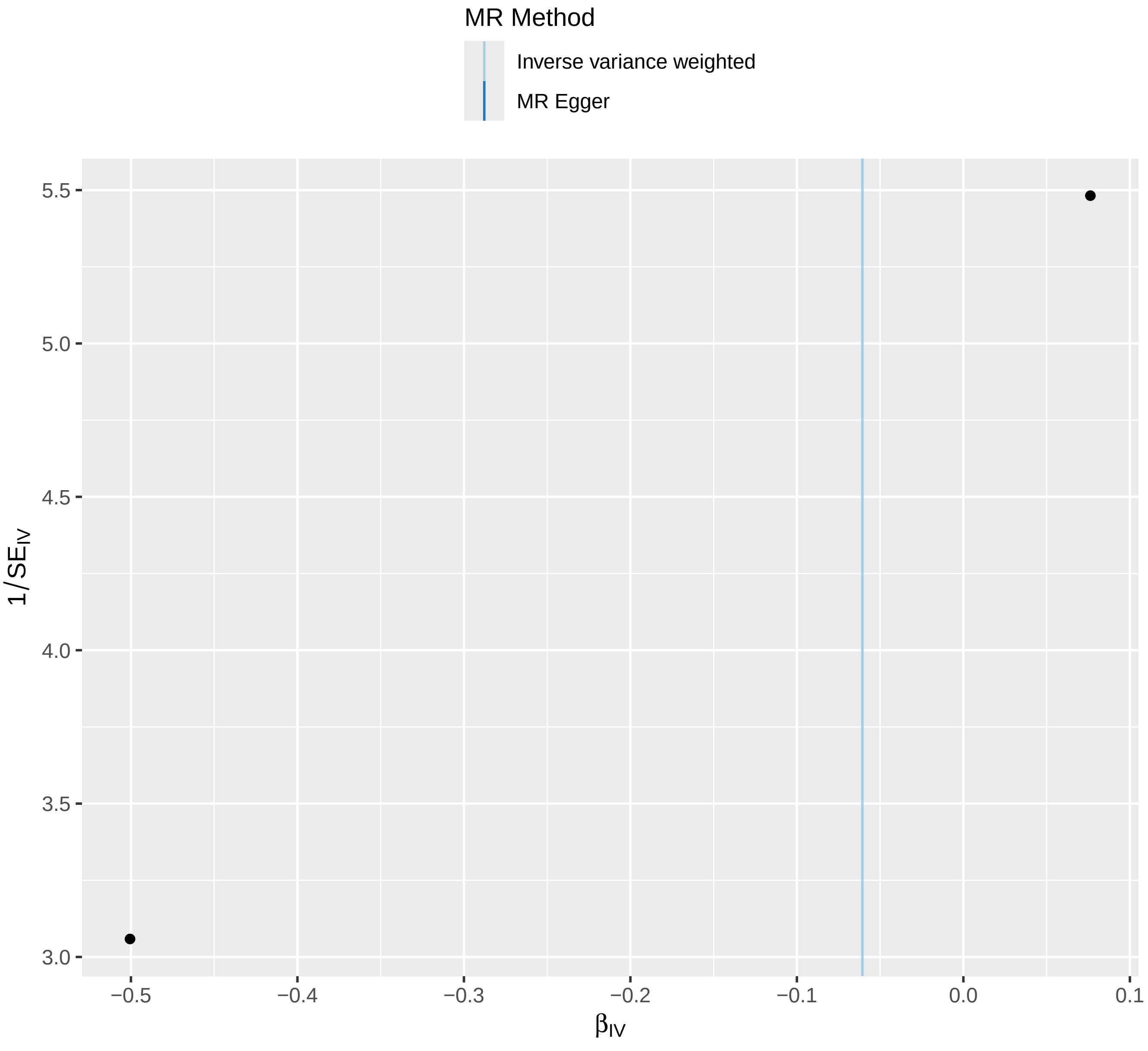
MR Method
Inverse variance weighted
MR Egger
5.5
5.0
4.5
4.0
3.5
3.0
1/SE_IV
−0.5
−0.4
−0.3
−0.2
−0.1
0.0
0.1
β_IV

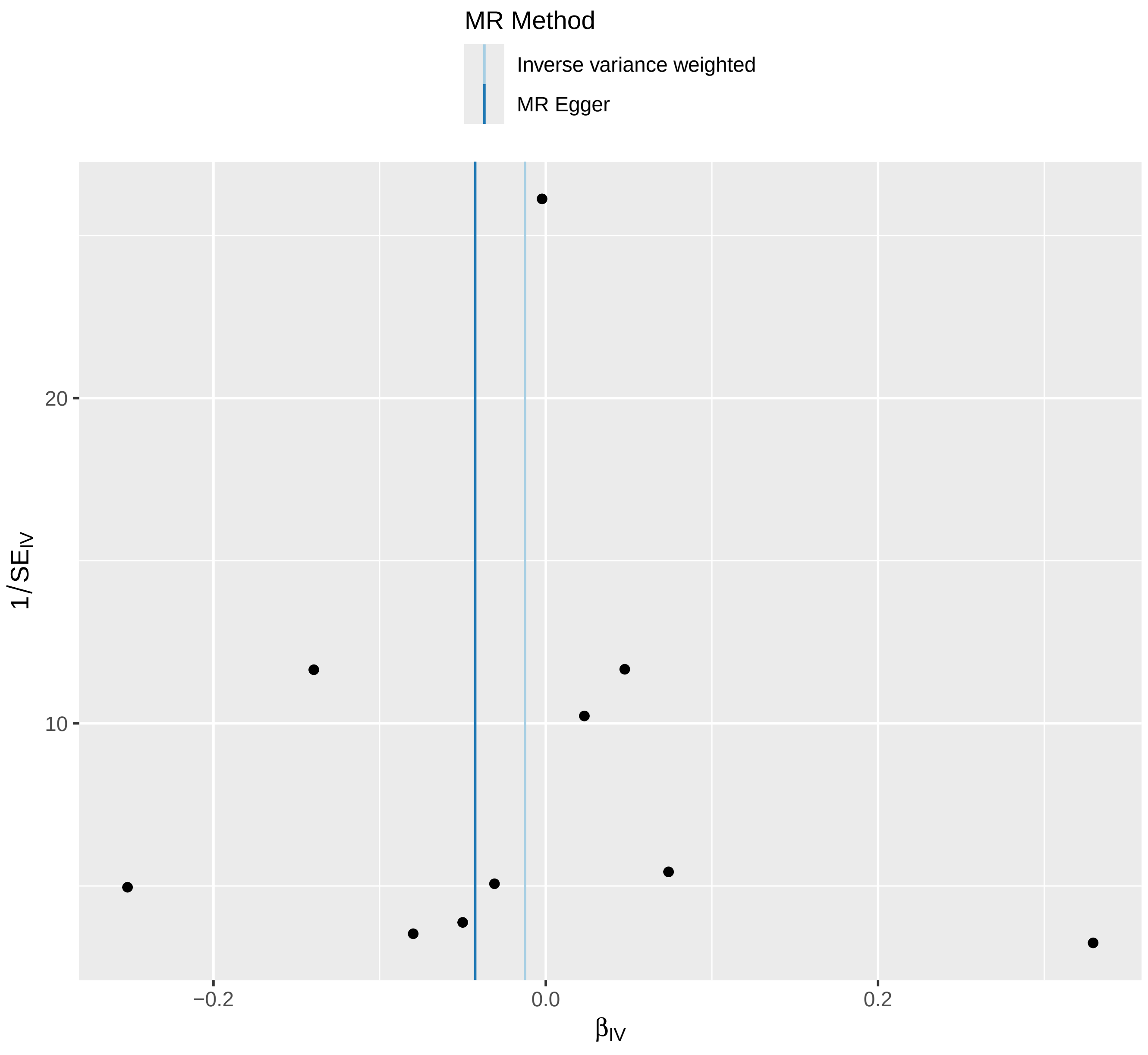

MR Method
Inverse variance weighted
MR Egger
20
10
1/SE_IV
−0.2
0.0
0.2
β_IV

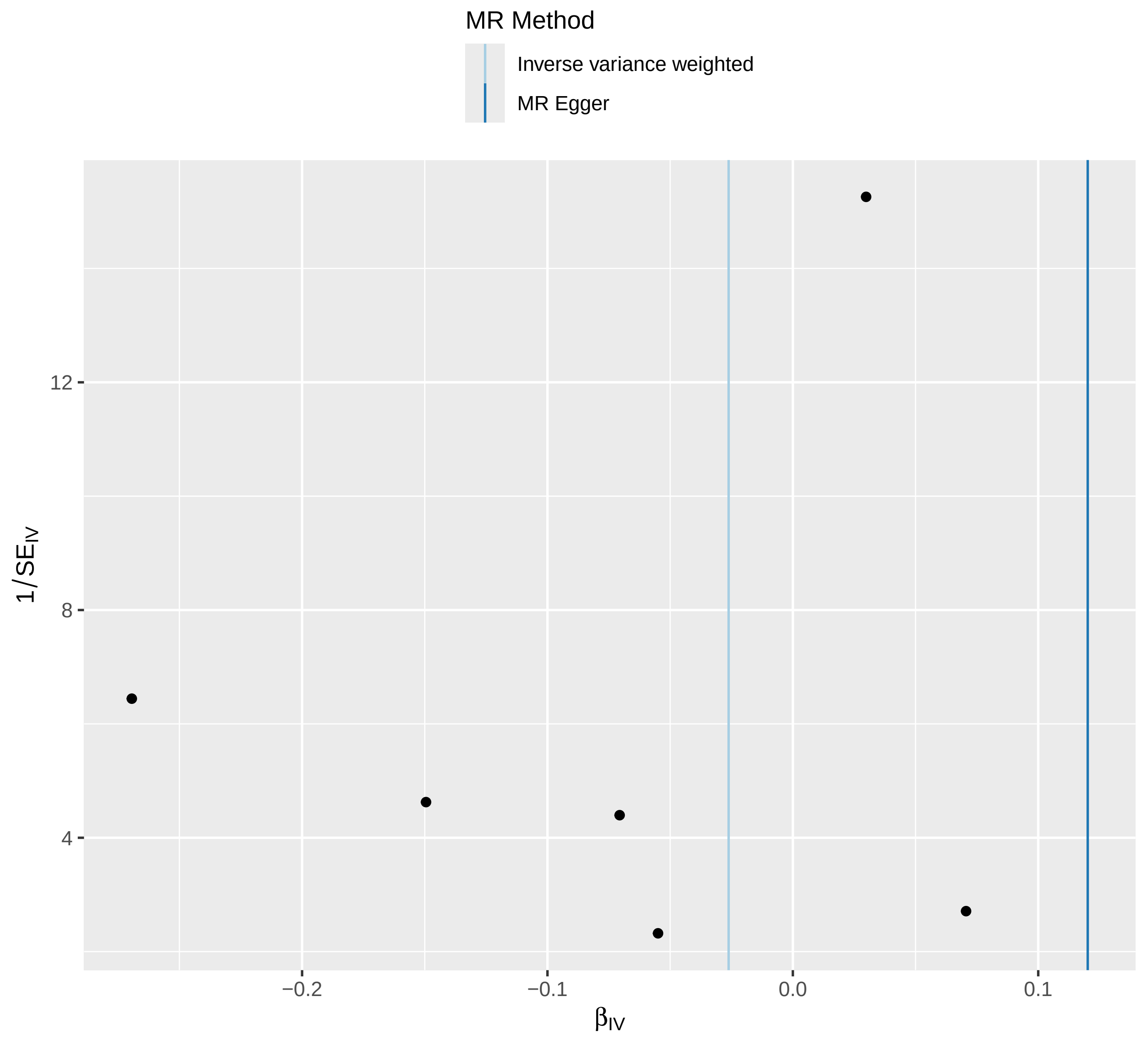
MR Method
Inverse variance weighted
MR Egger
12
8
4
$1/SE_{IV}$
−0.2
−0.1
0.0
0.1
$\beta_{IV}$

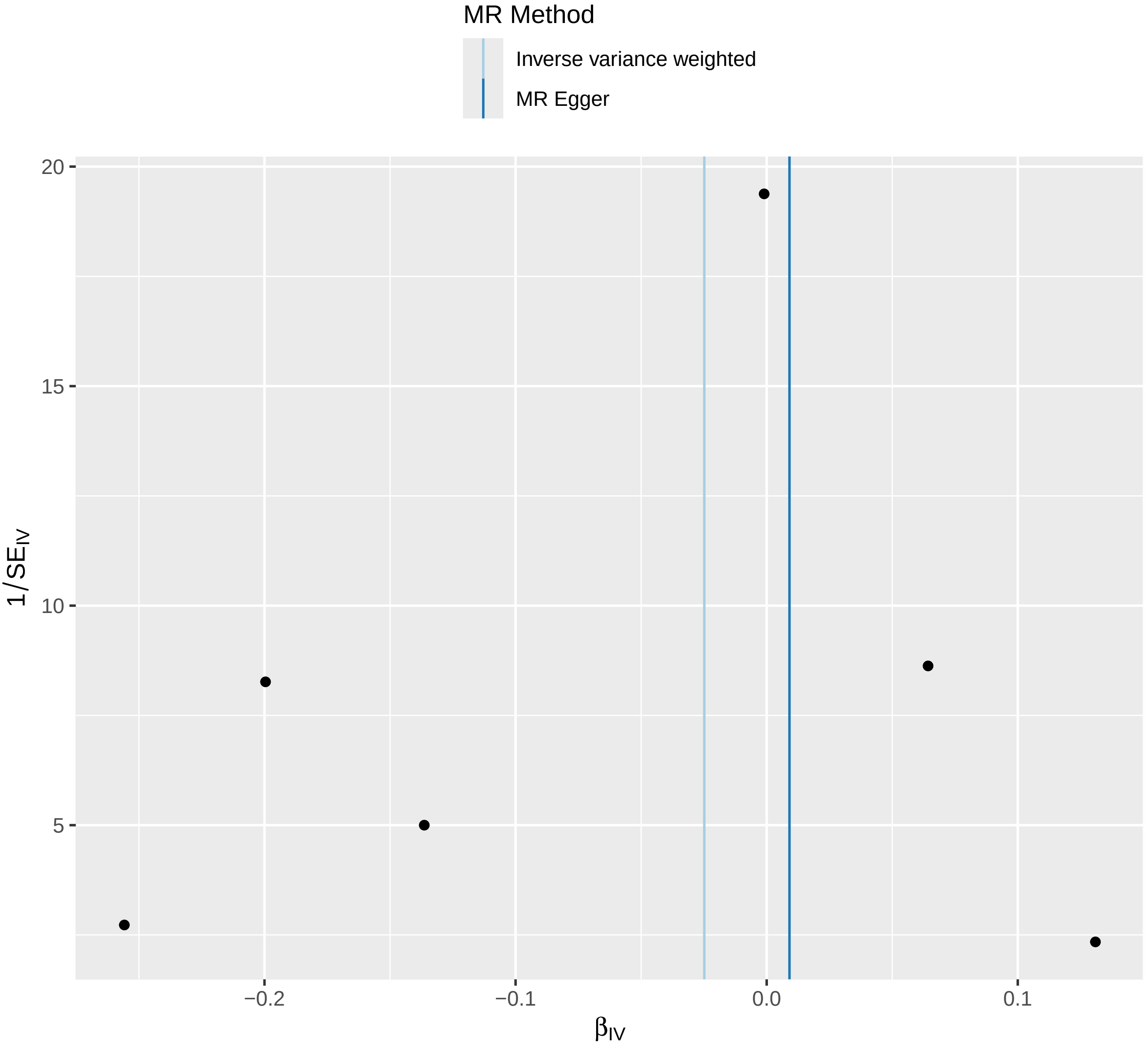
MR Method
Inverse variance weighted
MR Egger
20
15
10
5
1/SE_IV
−0.2
−0.1
0.0
0.1
β_IV

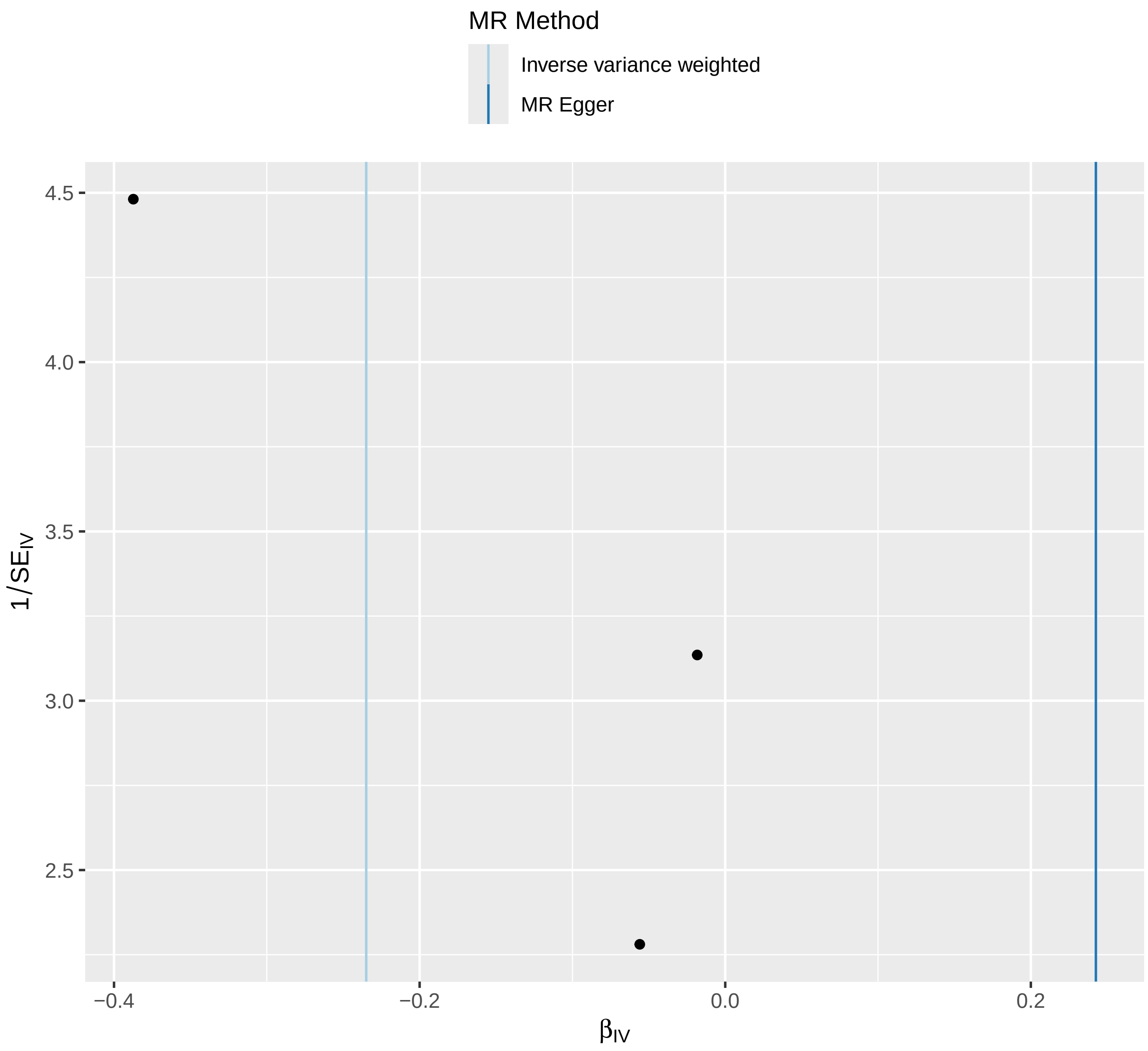
MR Method
Inverse variance weighted
MR Egger
4.5
4.0
3.5
3.0
2.5
1/SE_IV
−0.4
−0.2
0.0
0.2
β_IV

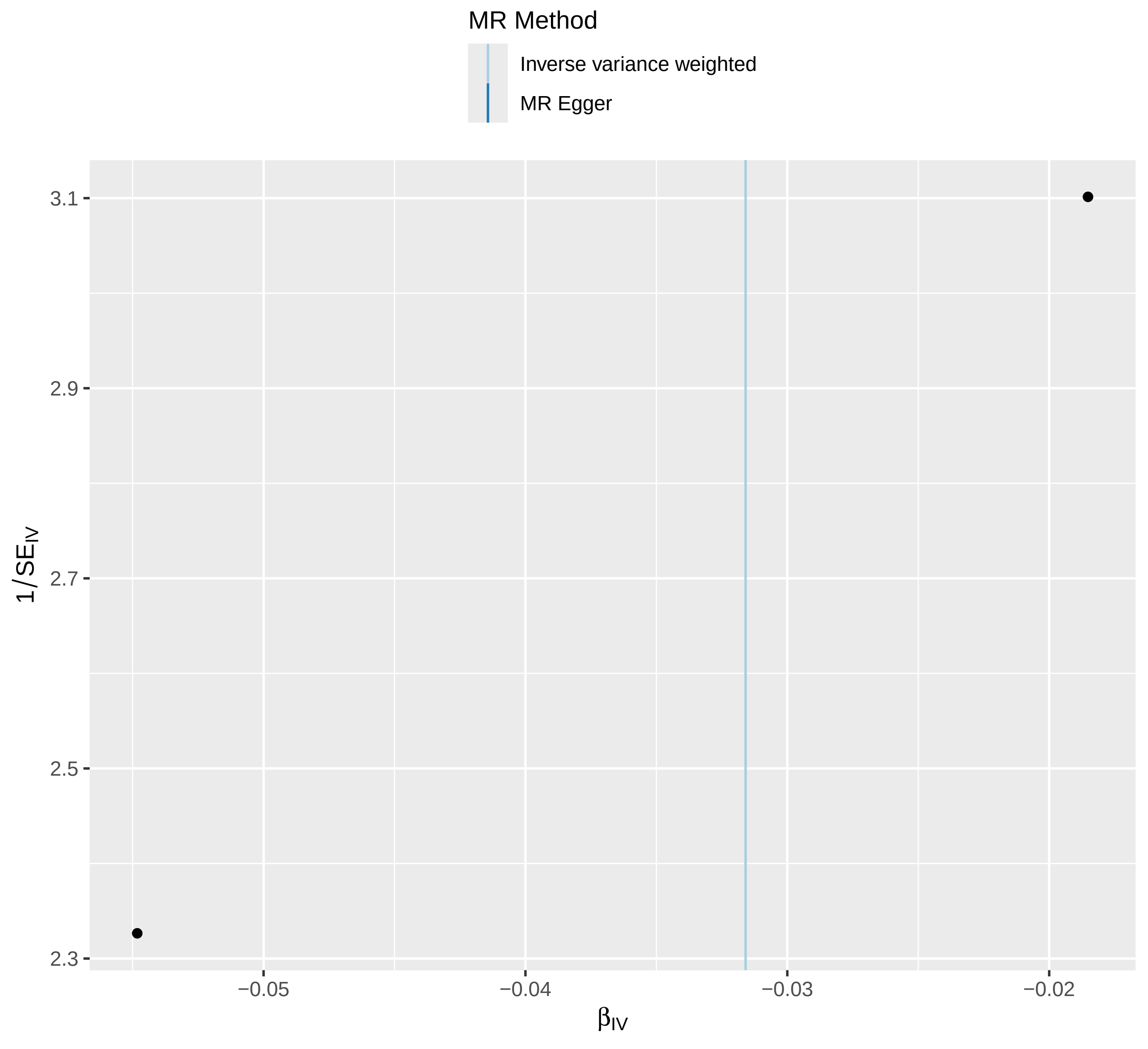
MR Method
Inverse variance weighted
MR Egger
3.1
2.9
2.7
2.5
2.3
1/SE_IV
−0.05
−0.04
−0.03
−0.02
β_IV

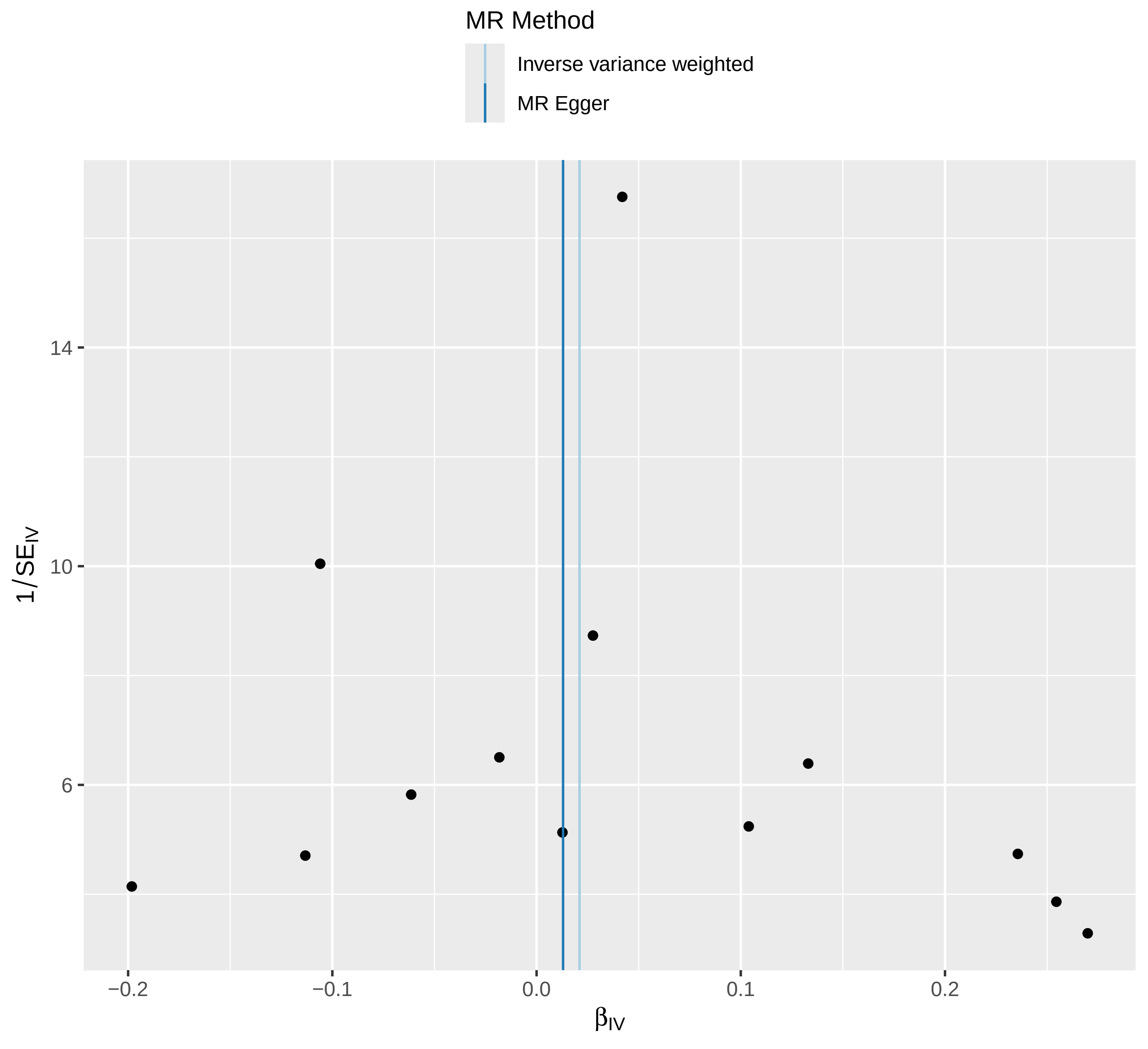

MR Method
Inverse variance weighted
MR Egger
14
10
6
1/SE_IV
−0.2
−0.1
0.0
0.1
0.2
β_IV

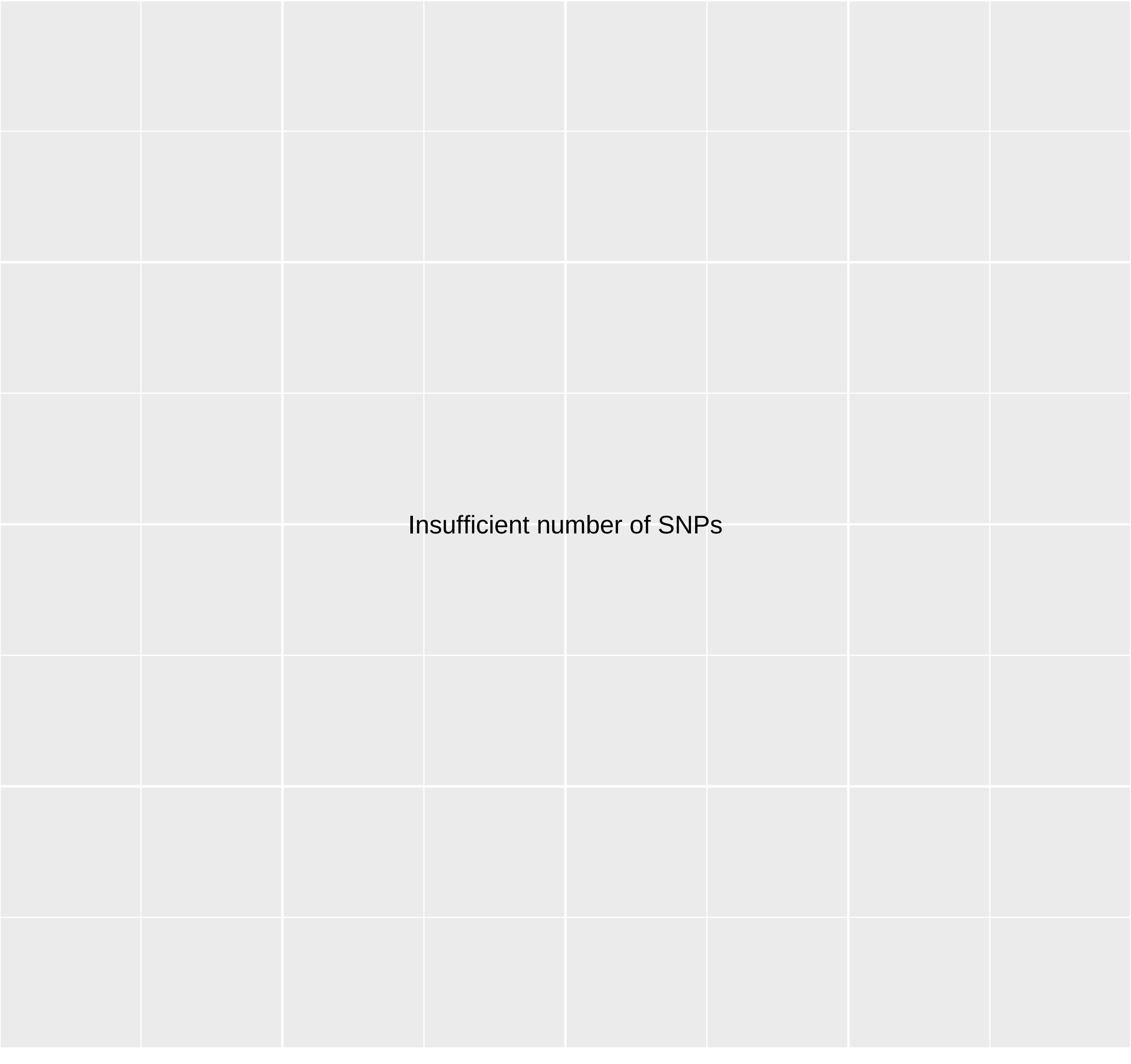
Insufficient number of SNPs

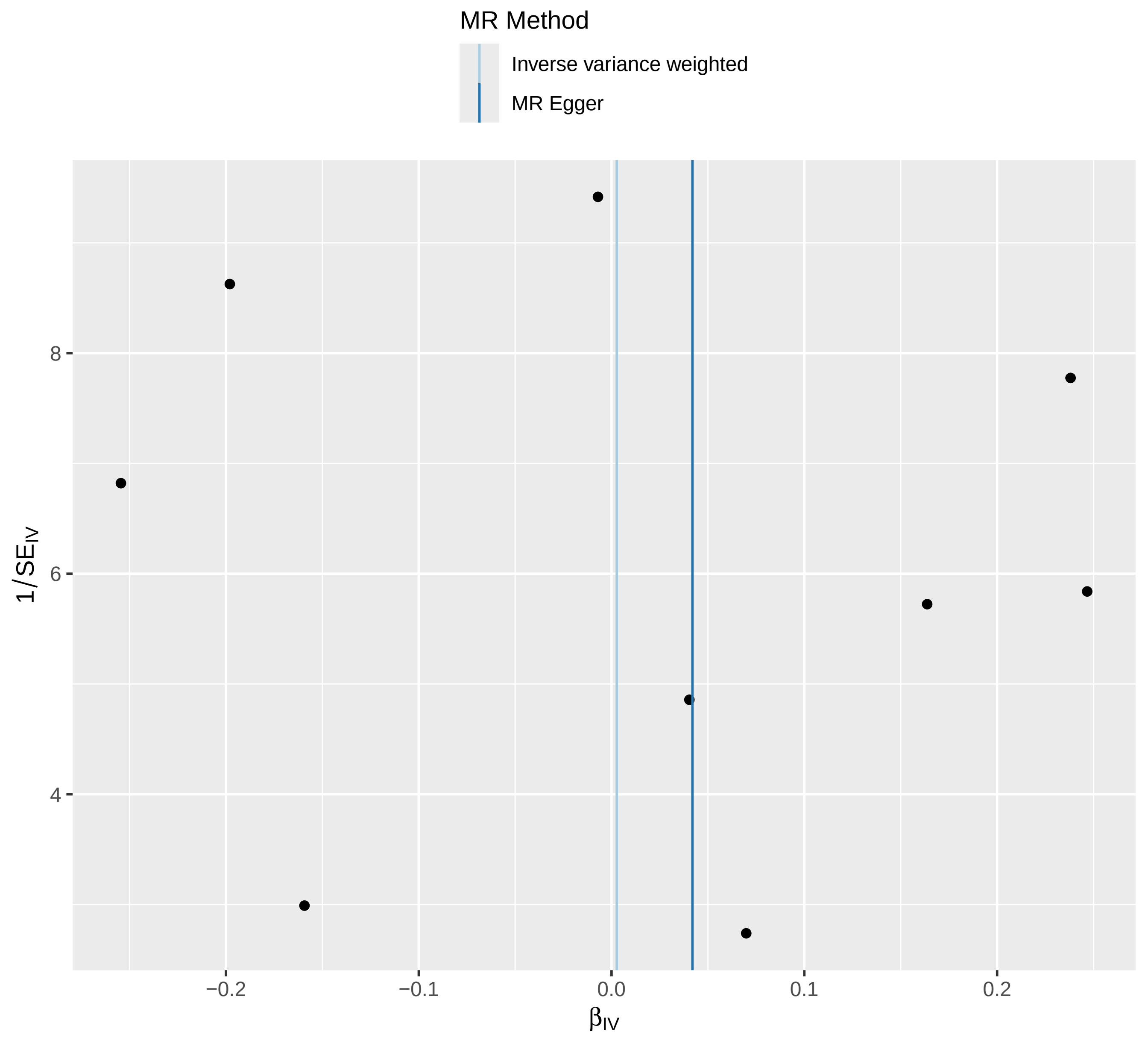

MR Method
Inverse variance weighted
MR Egger
8
6
4
1/SE$_{IV}$
−0.2
−0.1
0.0
0.1
0.2
β$_{IV}$

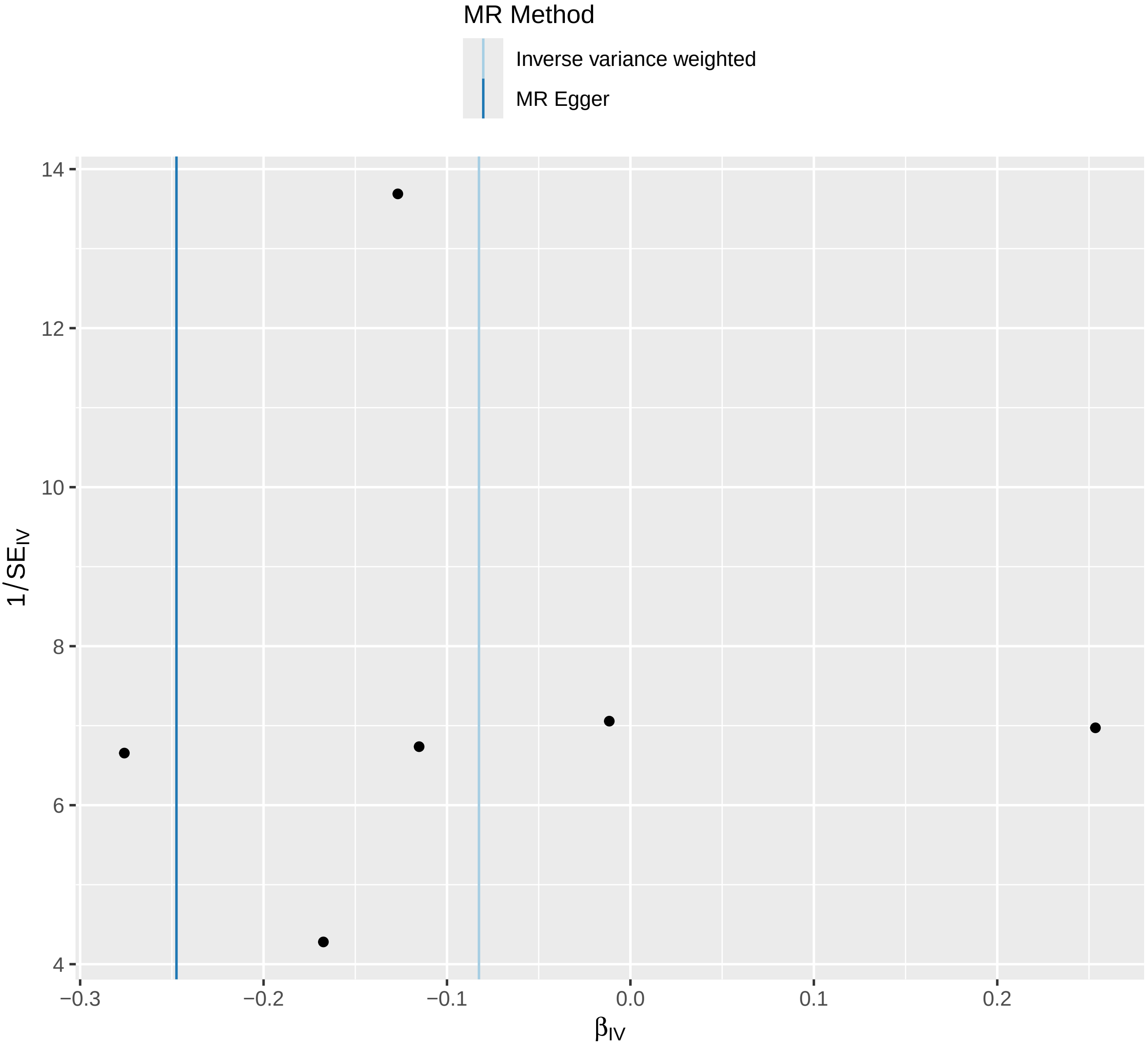

MR Method
Inverse variance weighted
MR Egger
14
12
10
8
6
4
1/SE$_{IV}$
−0.3
−0.2
−0.1
0.0
0.1
0.2
β$_{IV}$

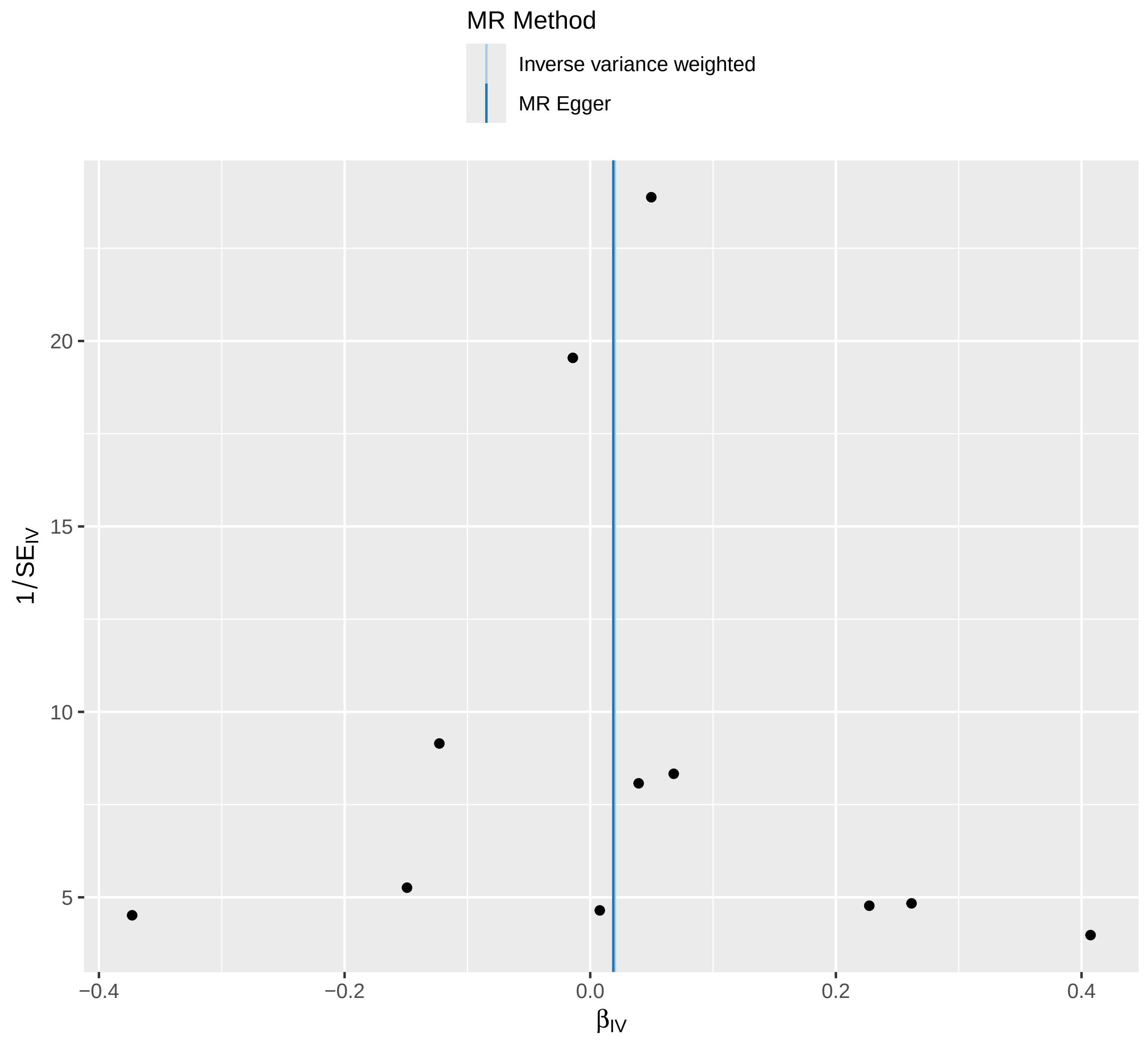
MR Method
Inverse variance weighted
MR Egger
20
15
10
5
1/SE$_{IV}$
−0.4
−0.2
0.0
0.2
0.4
β$_{IV}$

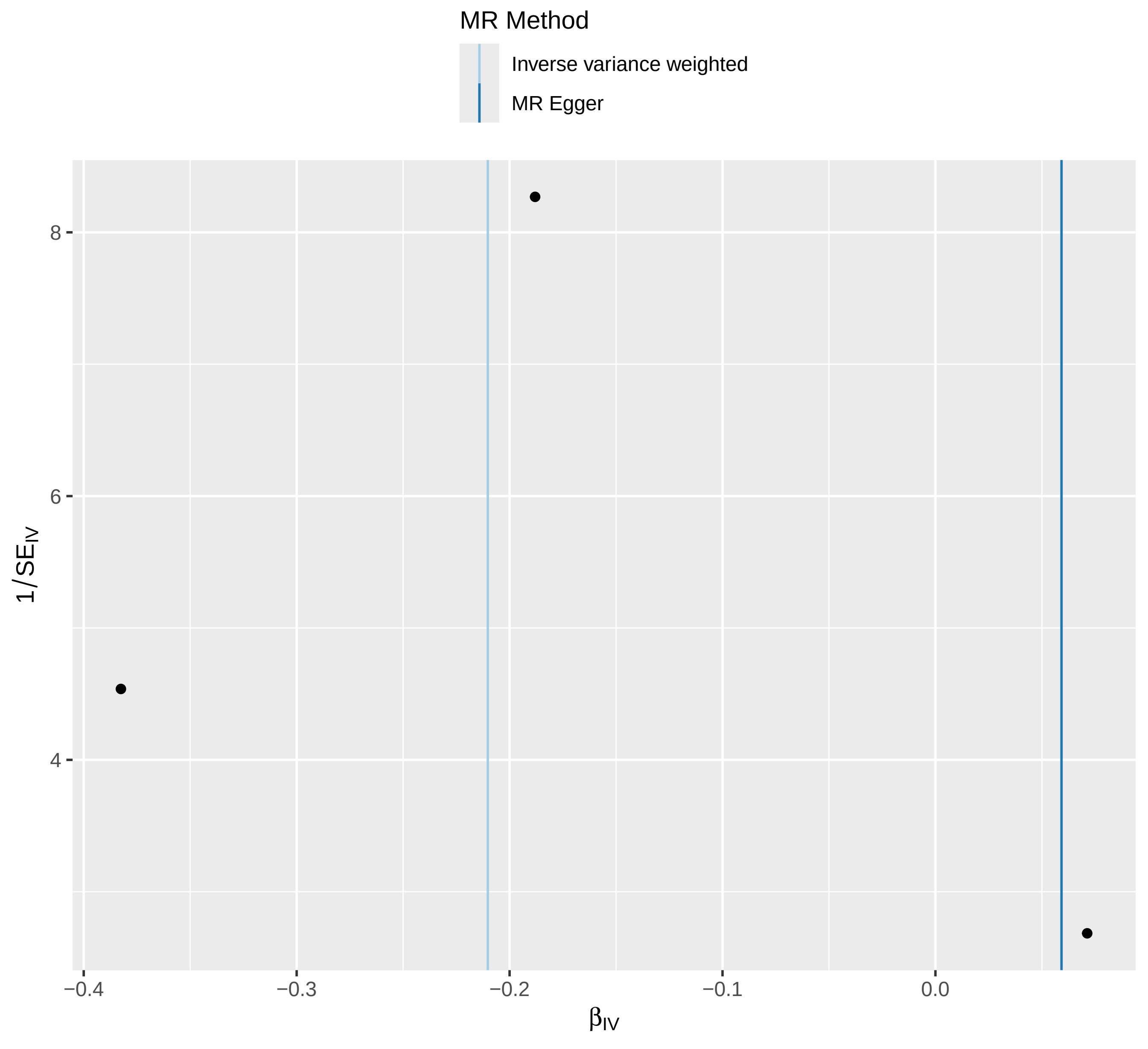

MR Method
Inverse variance weighted
MR Egger
8
6
4
1/SE$_{IV}$
−0.4
−0.3
−0.2
−0.1
0.0
β$_{IV}$

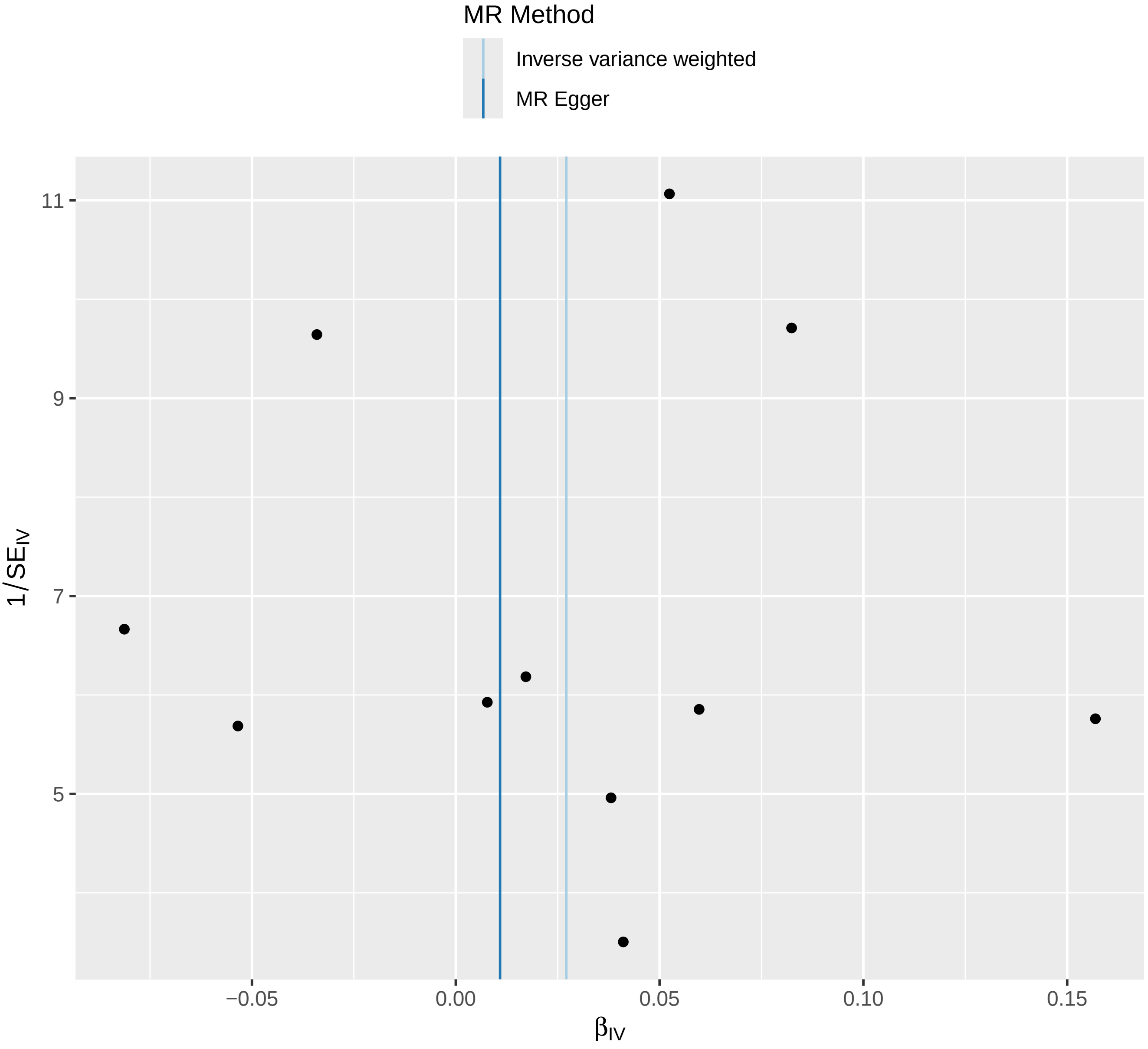

MR Method
Inverse variance weighted
MR Egger
11
9
7
5
1/SE$_{IV}$
−0.05
0.00
0.05
0.10
0.15
β$_{IV}$

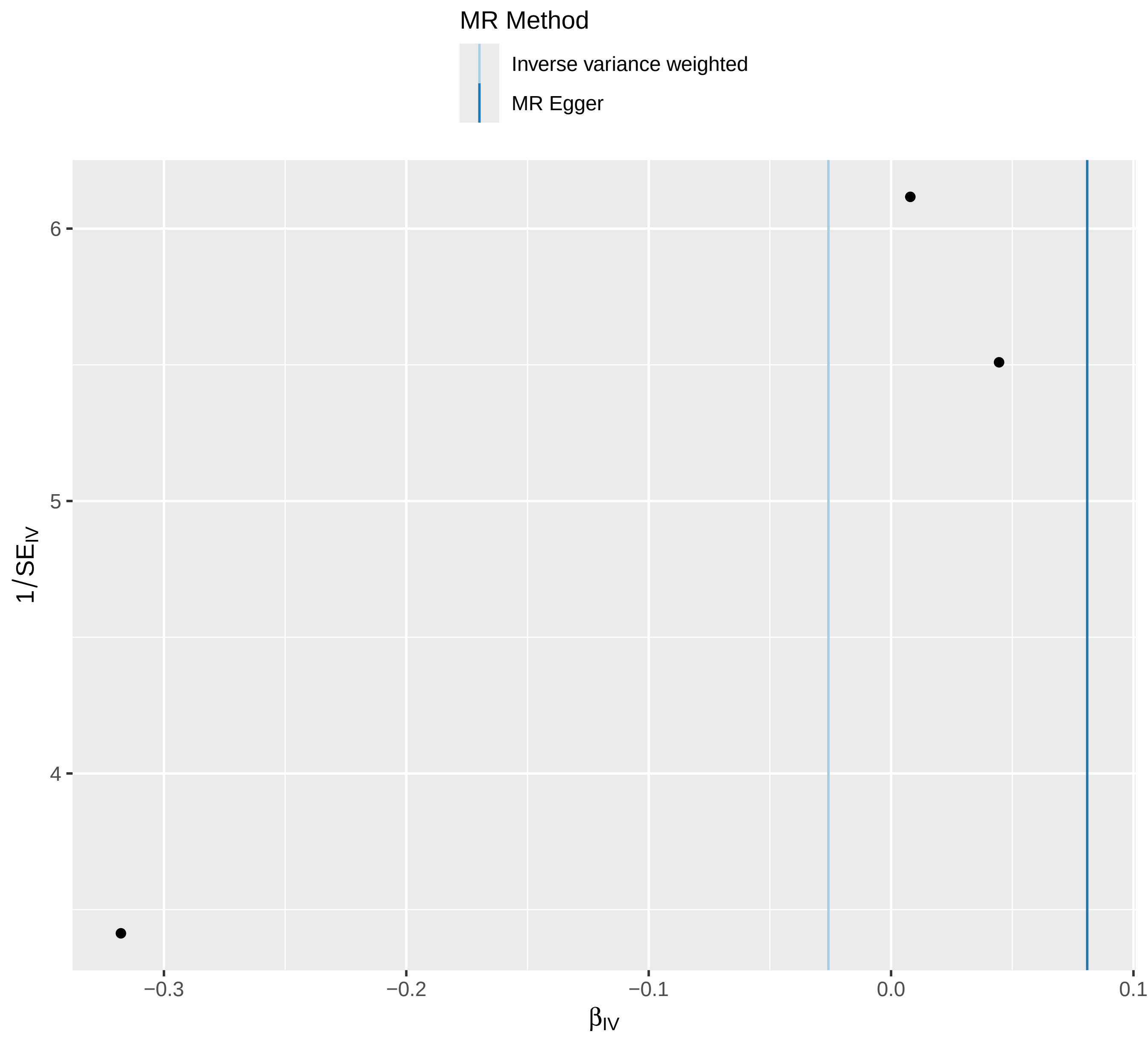
MR Method
Inverse variance weighted
MR Egger
6
5
4
−0.3
−0.2
−0.1
0.0
0.1
$1/SE_{IV}$
$\beta_{IV}$

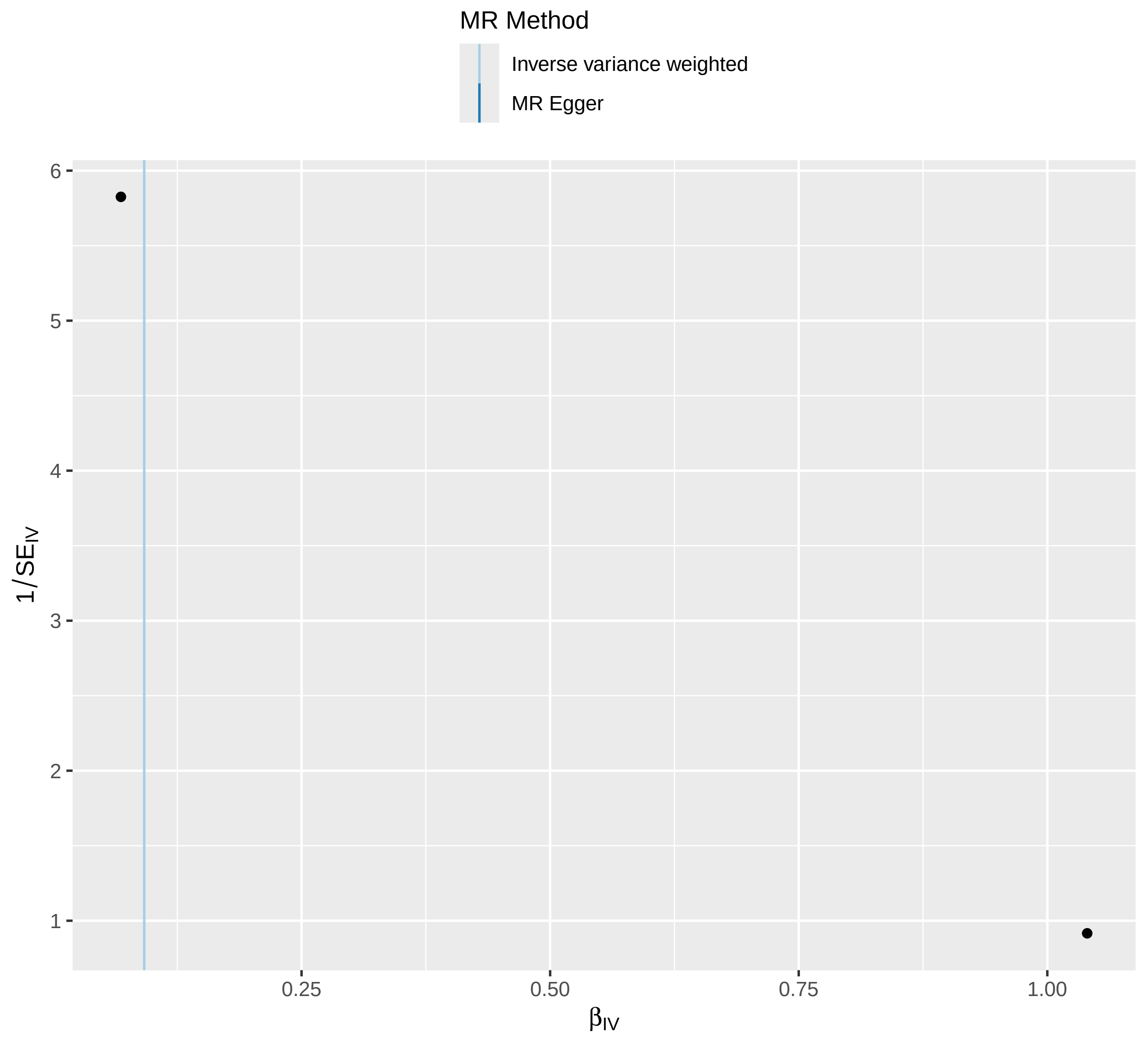

MR Method
Inverse variance weighted
MR Egger
6
5
4
3
2
1
0.25
0.50
0.75
1.00
1/SE_IV
β_IV

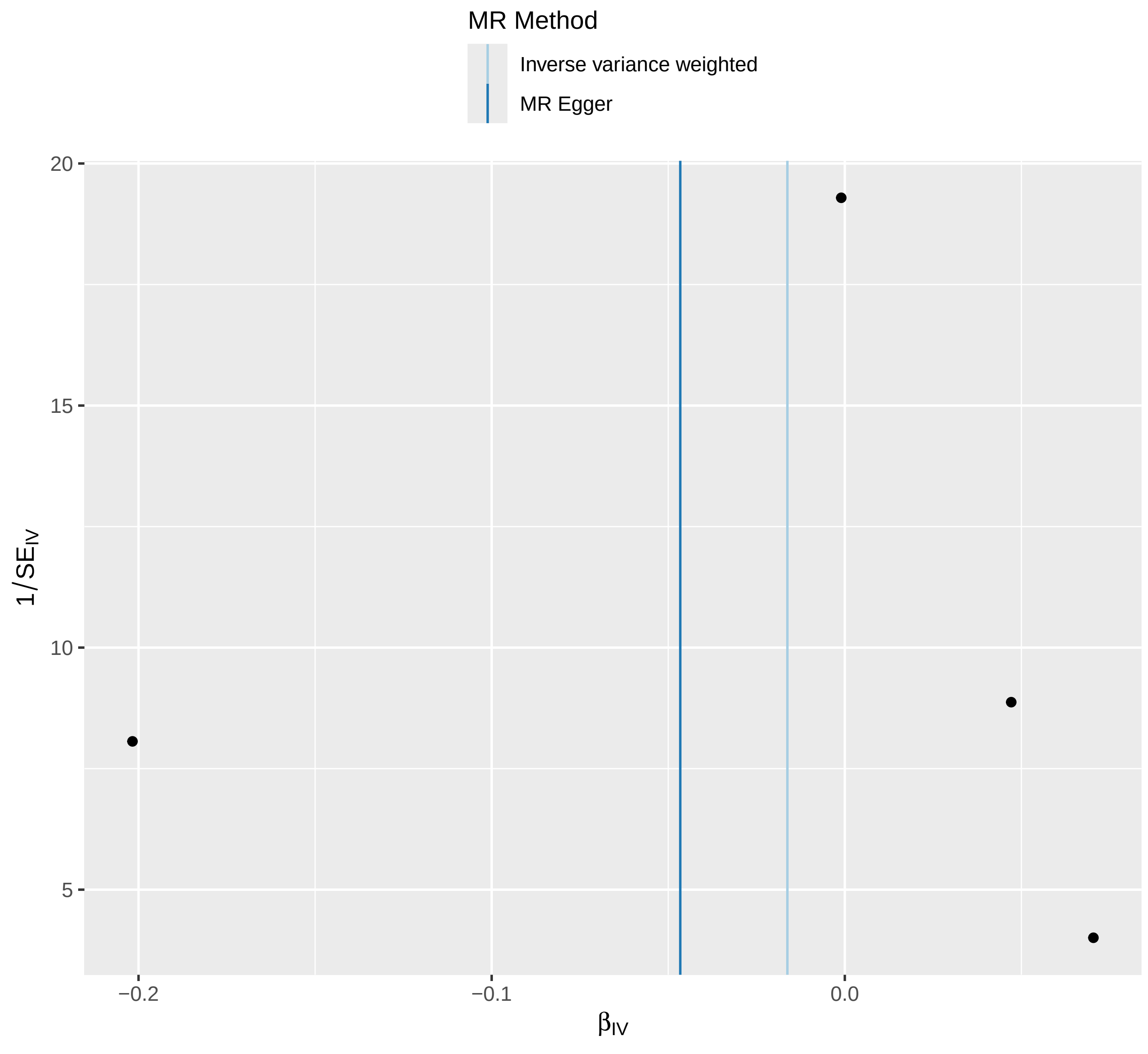
MR Method
Inverse variance weighted
MR Egger
20
15
10
5
1/SE$_{IV}$
−0.2
−0.1
0.0
β$_{IV}$

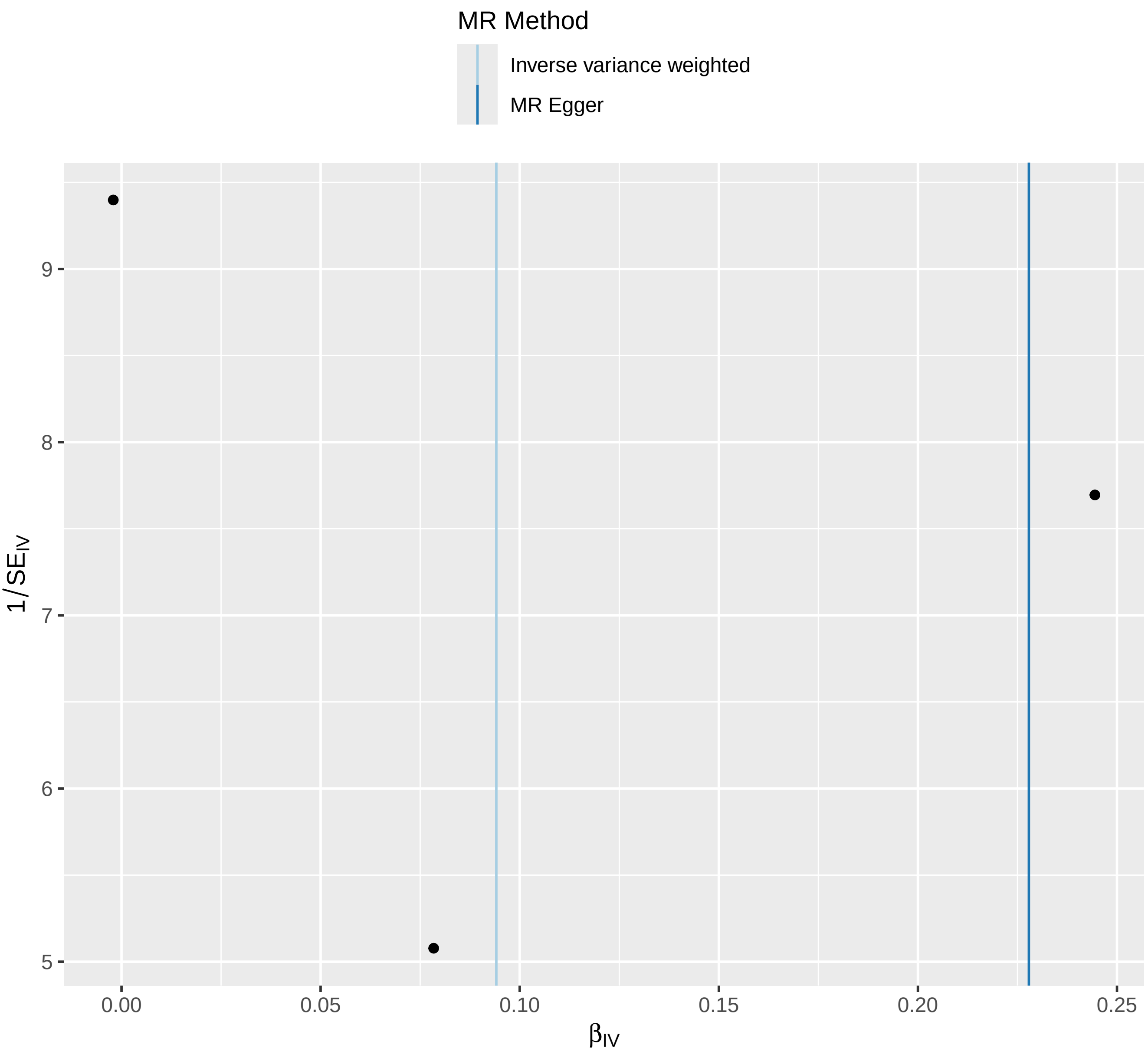
MR Method
Inverse variance weighted
MR Egger
1/SE_IV
9
8
7
6
5
0.00
0.05
0.10
0.15
0.20
0.25
β_IV

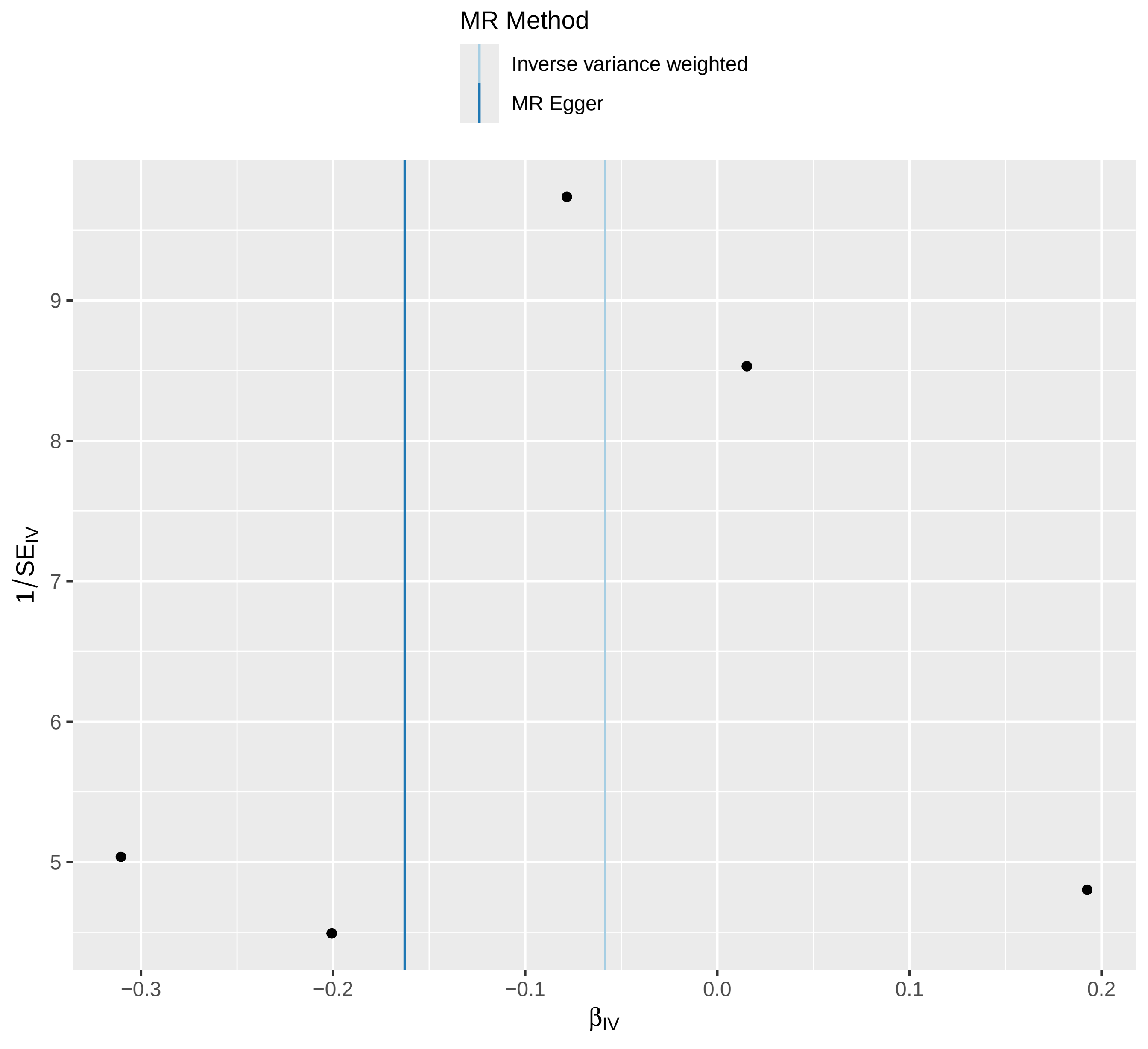

MR Method
Inverse variance weighted
MR Egger
9
8
7
6
5
1/SE_IV
−0.3
−0.2
−0.1
0.0
0.1
0.2
β_IV

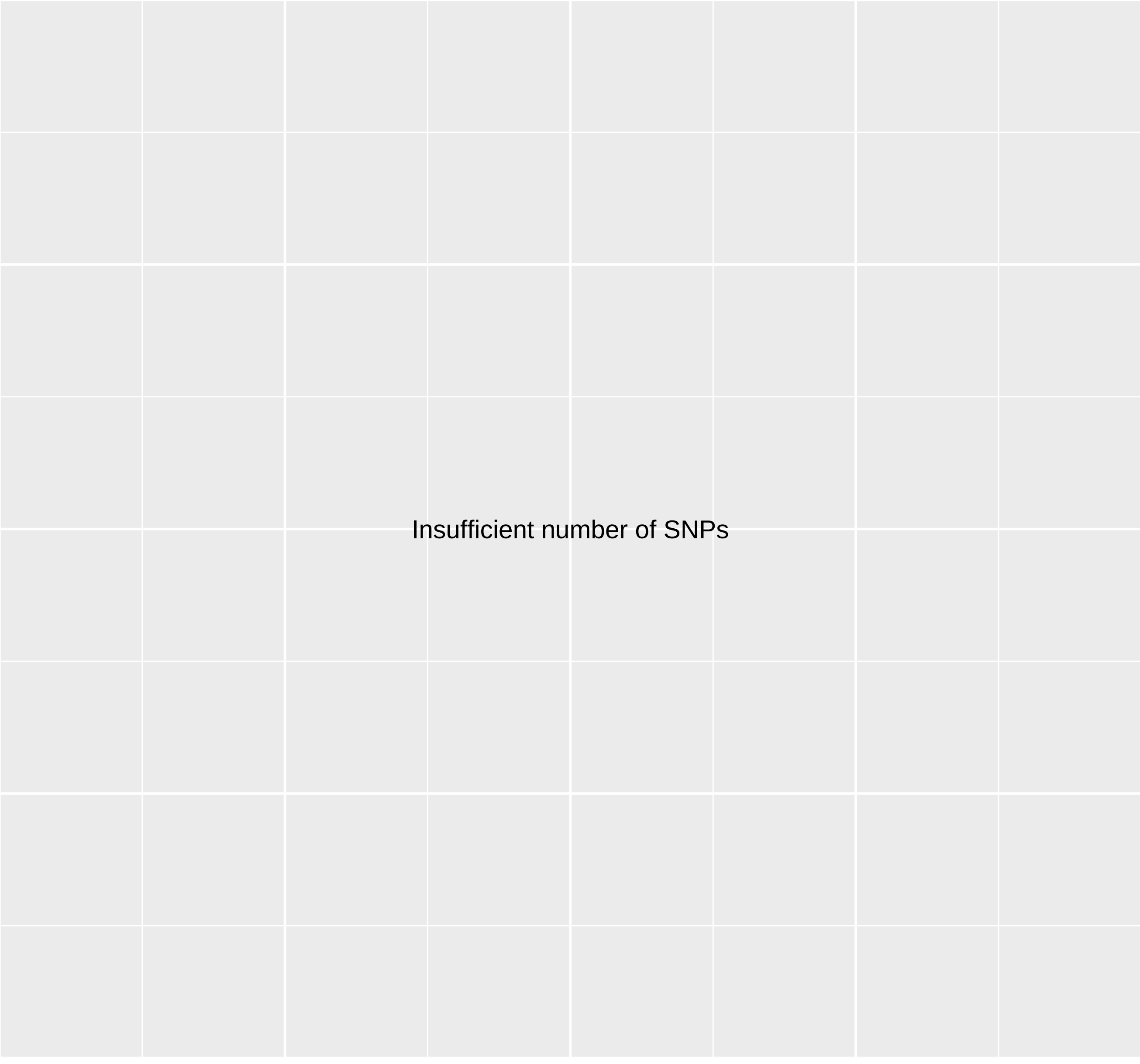
Insufficient number of SNPs

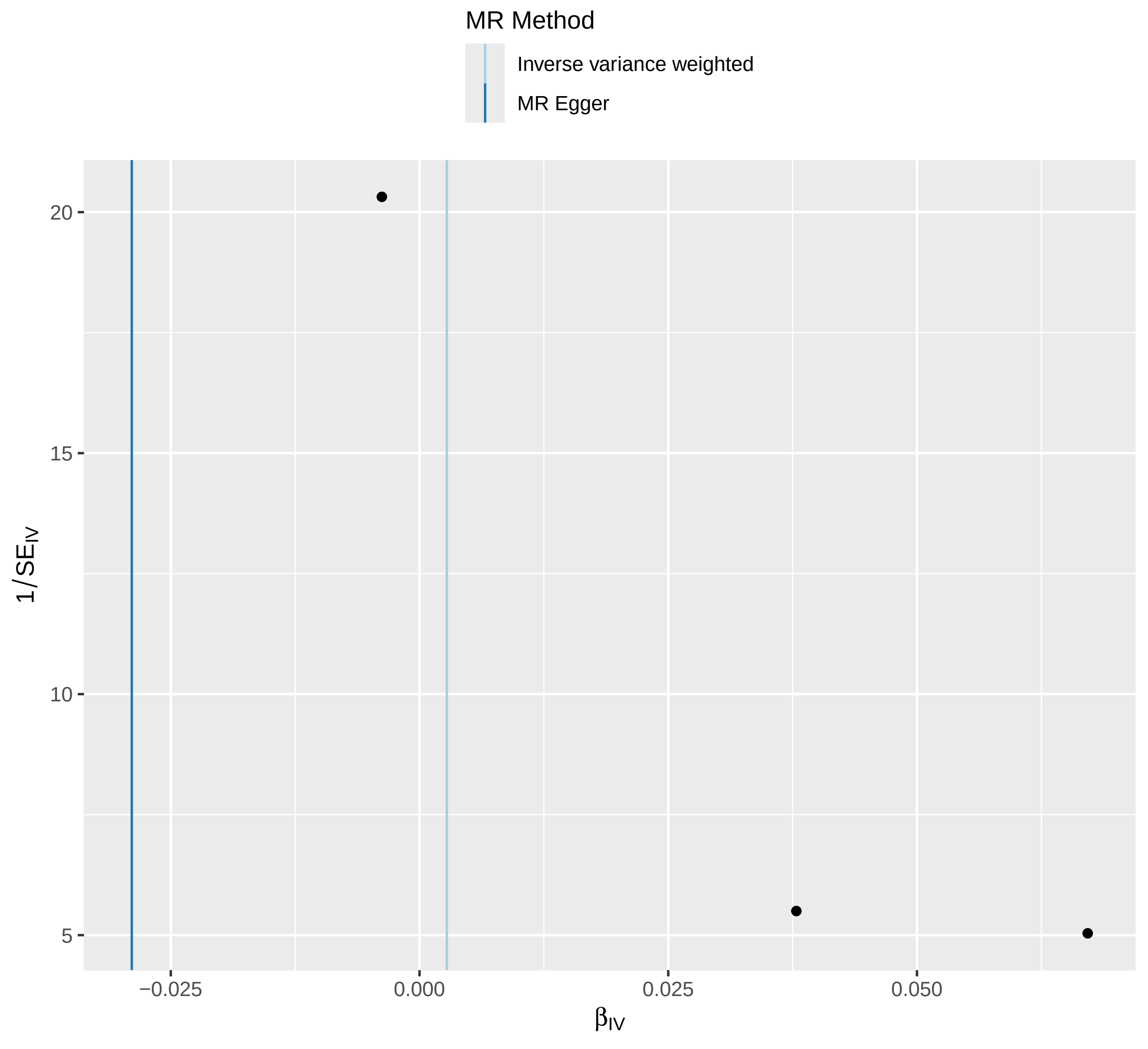

MR Method
Inverse variance weighted
MR Egger
20
15
10
5
1/SE$_{IV}$
−0.025
0.000
0.025
0.050
β$_{IV}$

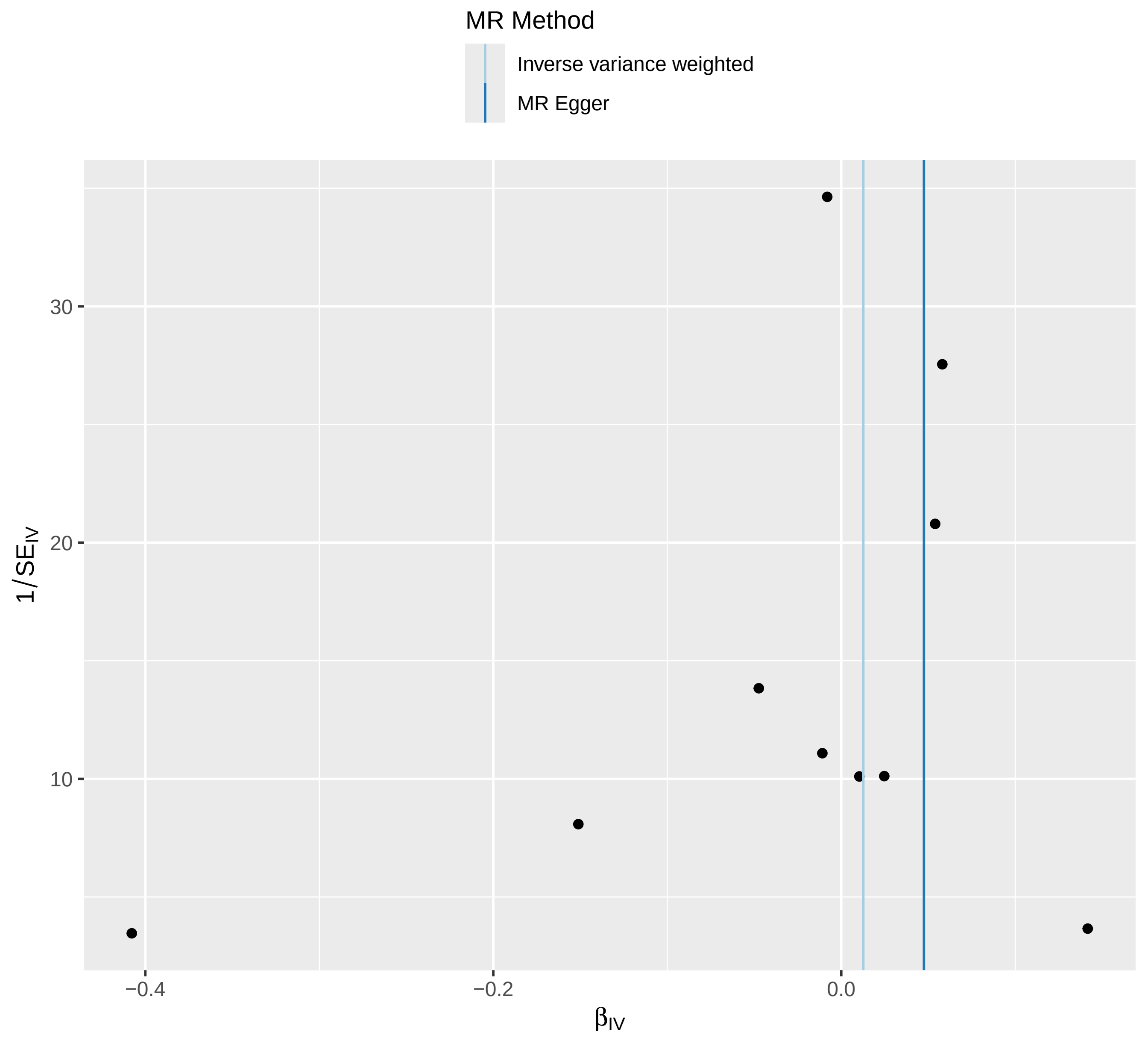
MR Method
Inverse variance weighted
MR Egger
30
20
10
1/SE$_{IV}$
−0.4
−0.2
0.0
β$_{IV}$

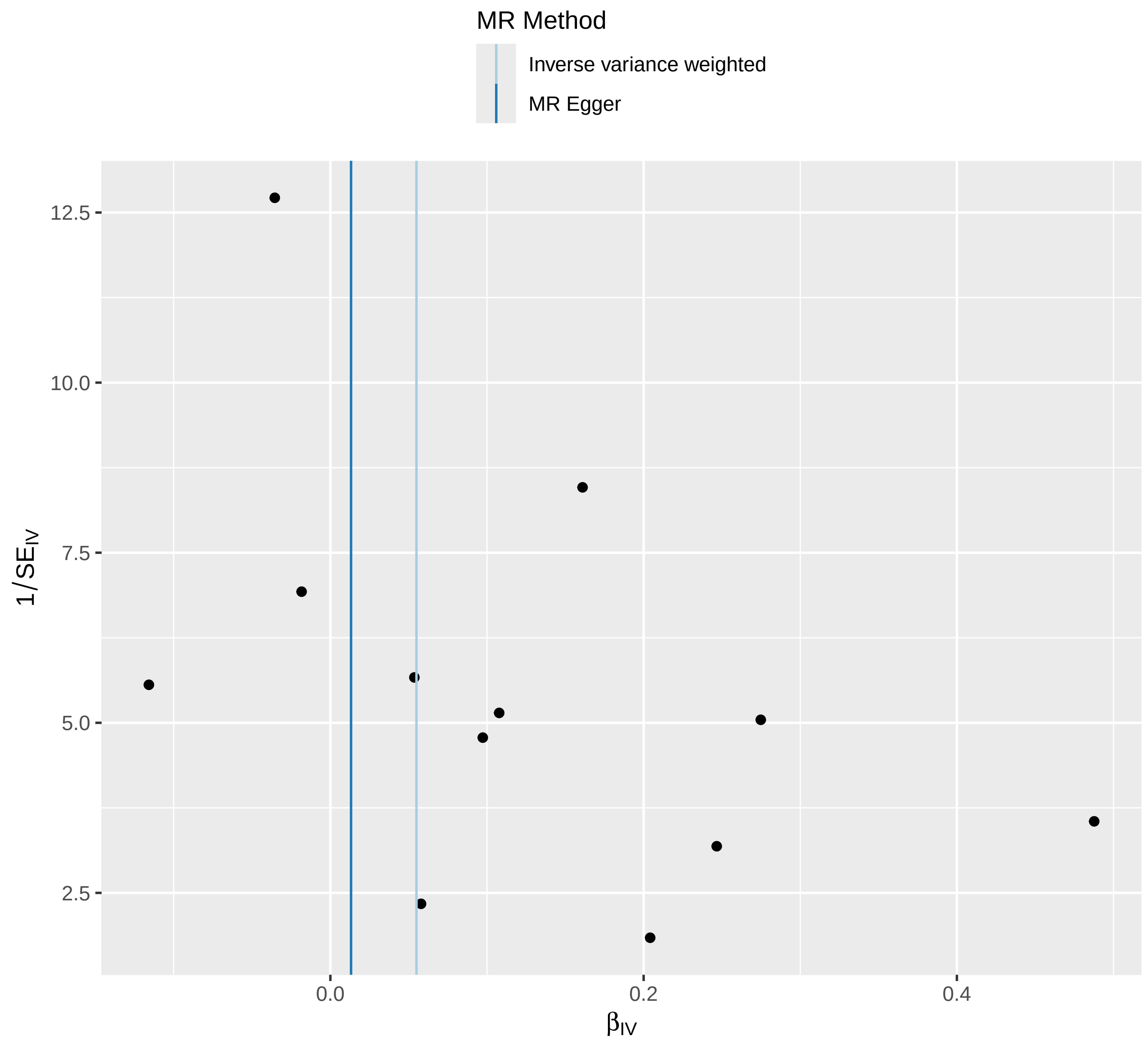
MR Method
Inverse variance weighted
MR Egger
12.5
10.0
7.5
5.0
2.5
$1/SE_{IV}$
0.0
0.2
0.4
$\beta_{IV}$

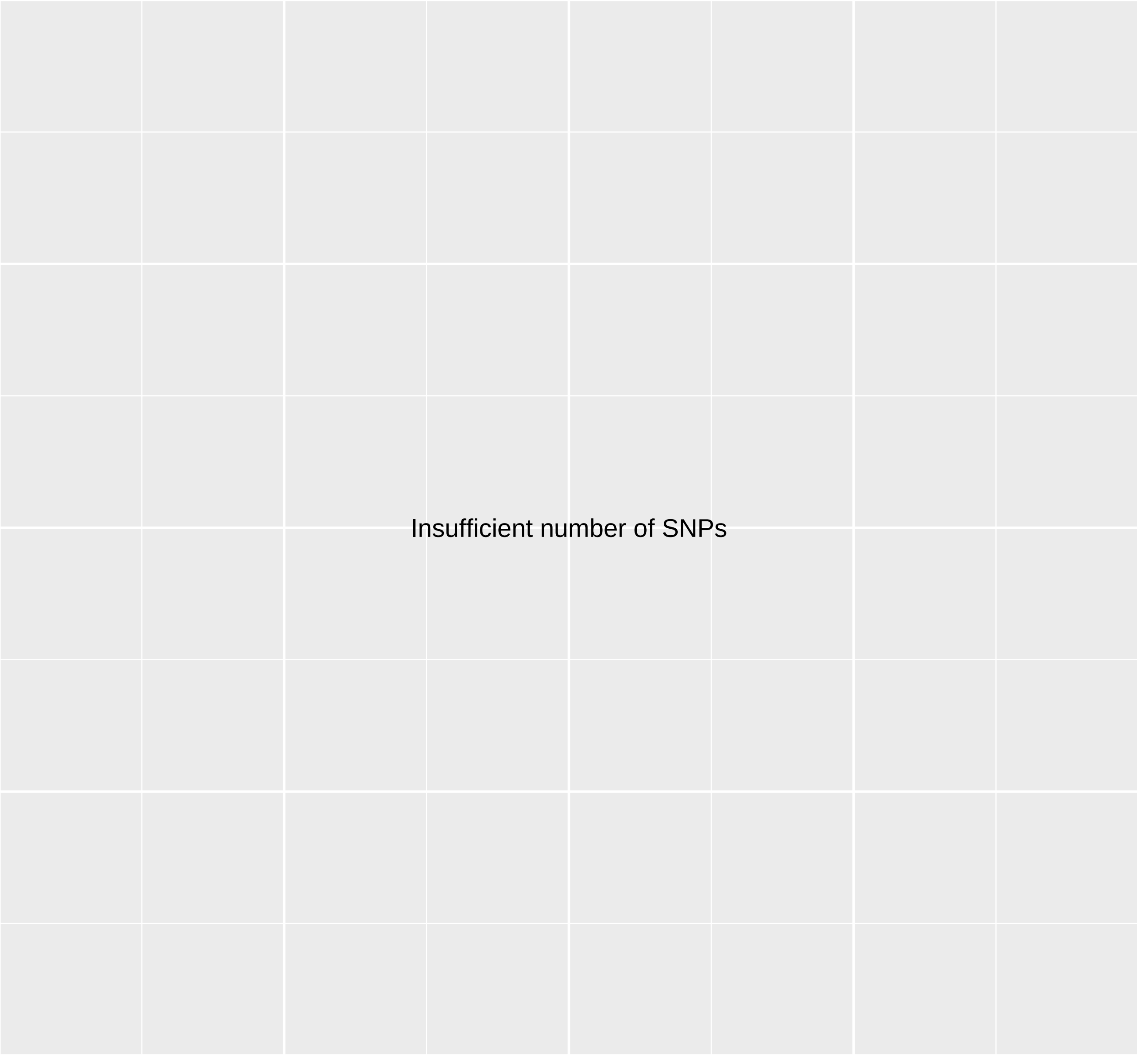
Insufficient number of SNPs

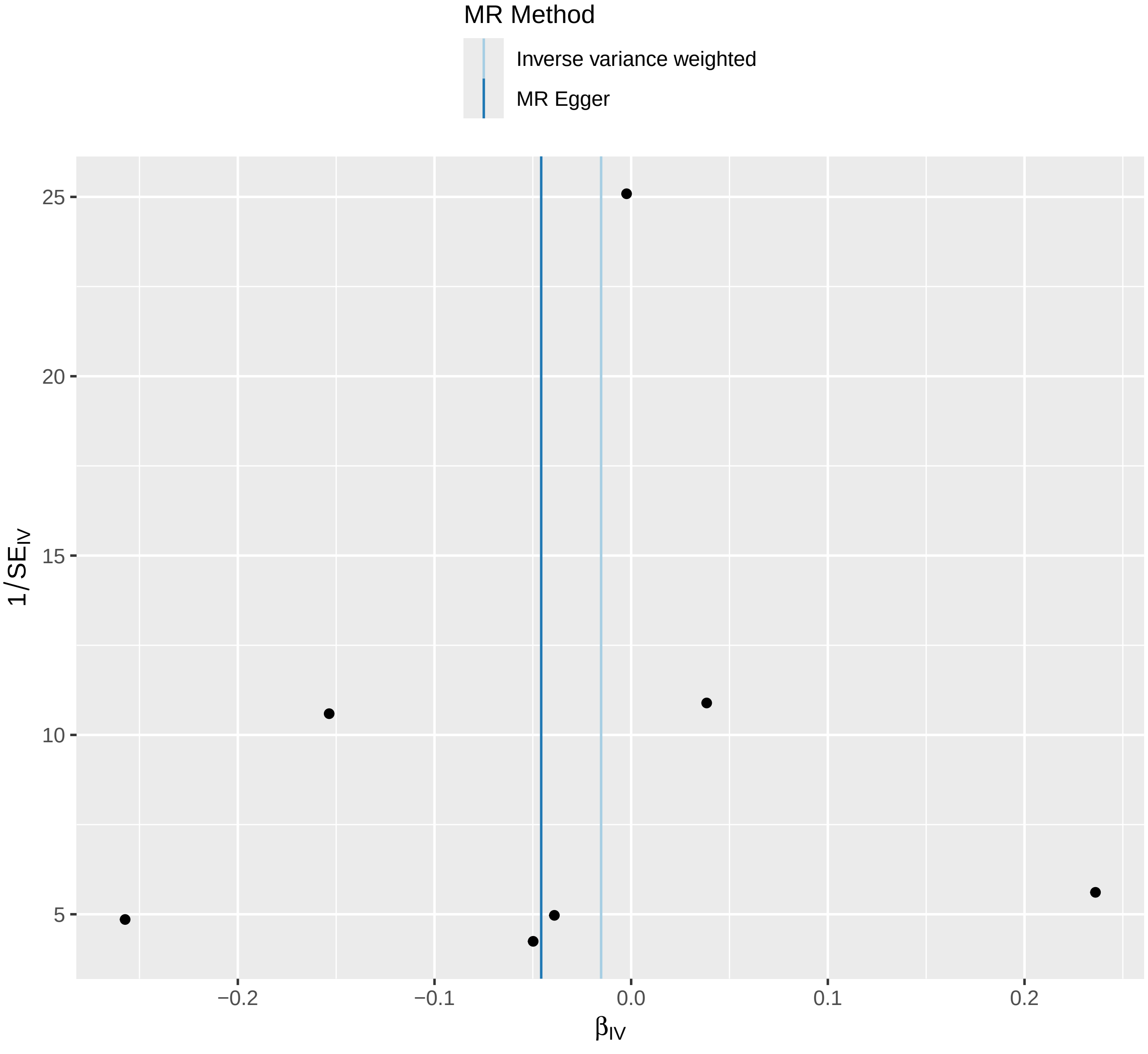
MR Method
Inverse variance weighted
MR Egger
25
20
15
10
5
1/SE_IV
−0.2
−0.1
0.0
0.1
0.2
β_IV

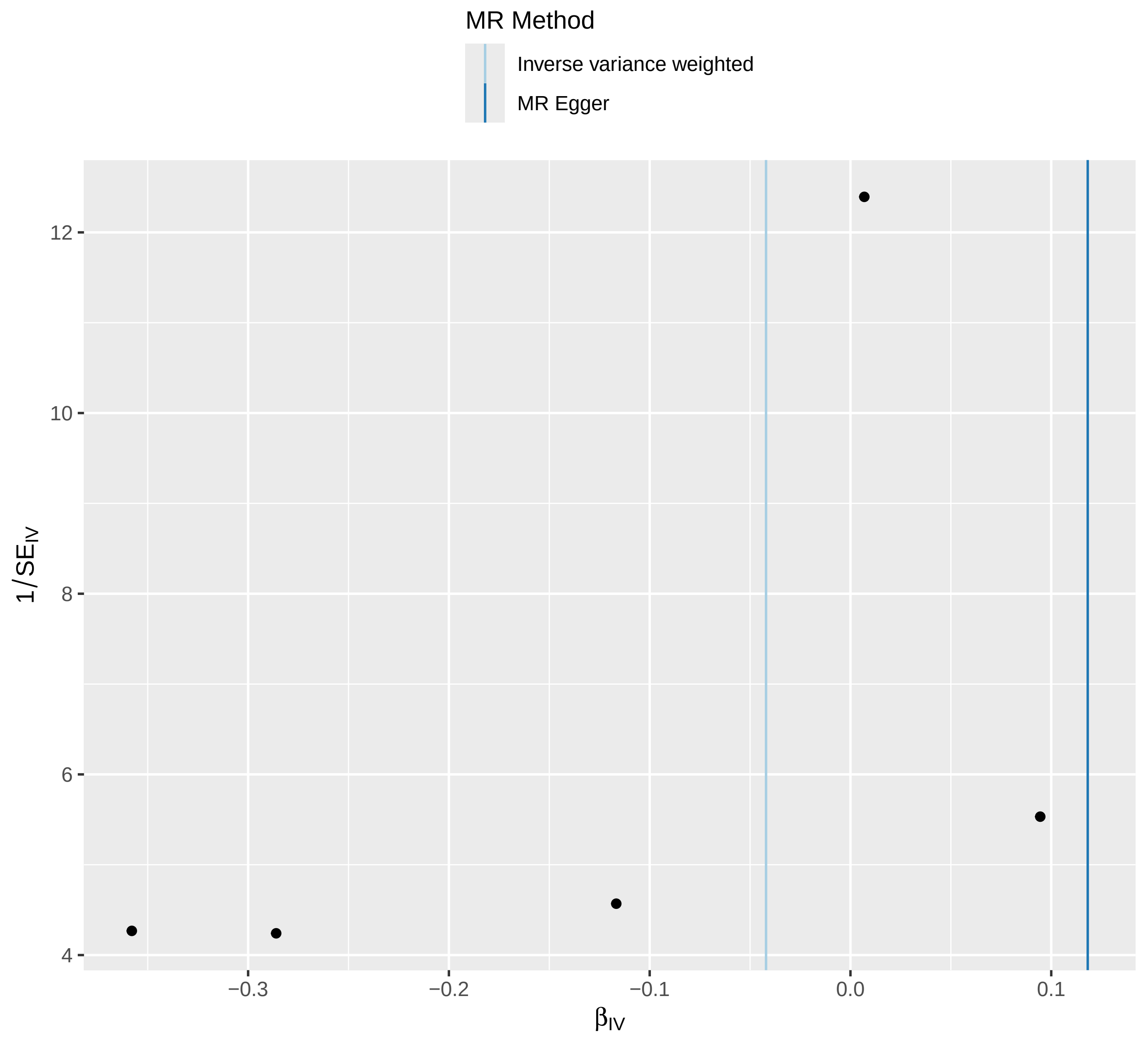

MR Method
Inverse variance weighted
MR Egger
12
10
8
6
4
1/SE$_{IV}$
−0.3
−0.2
−0.1
0.0
0.1
β$_{IV}$

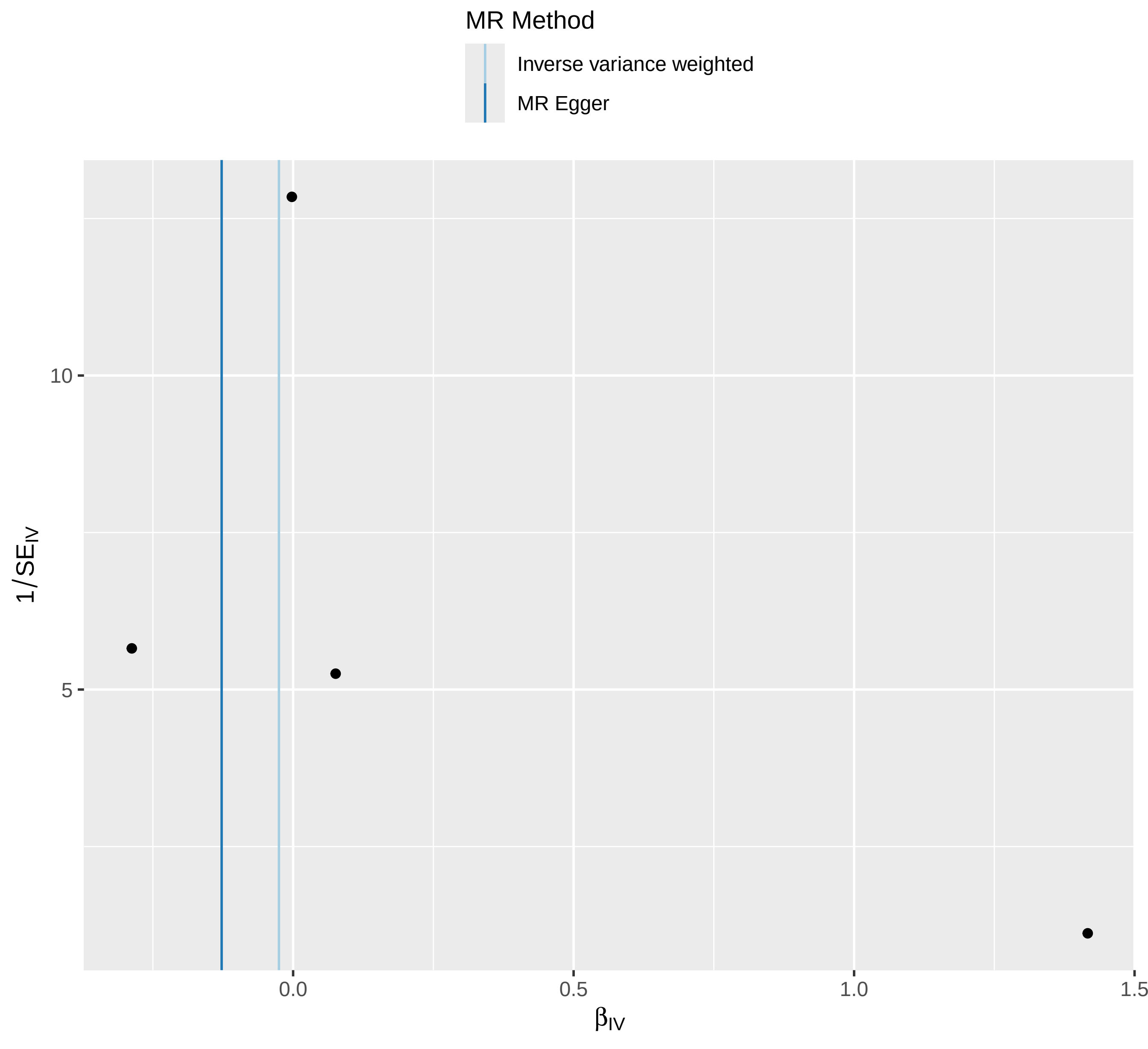

MR Method
Inverse variance weighted
MR Egger
1/SE_IV
β_IV
10
5
0.0
0.5
1.0
1.5

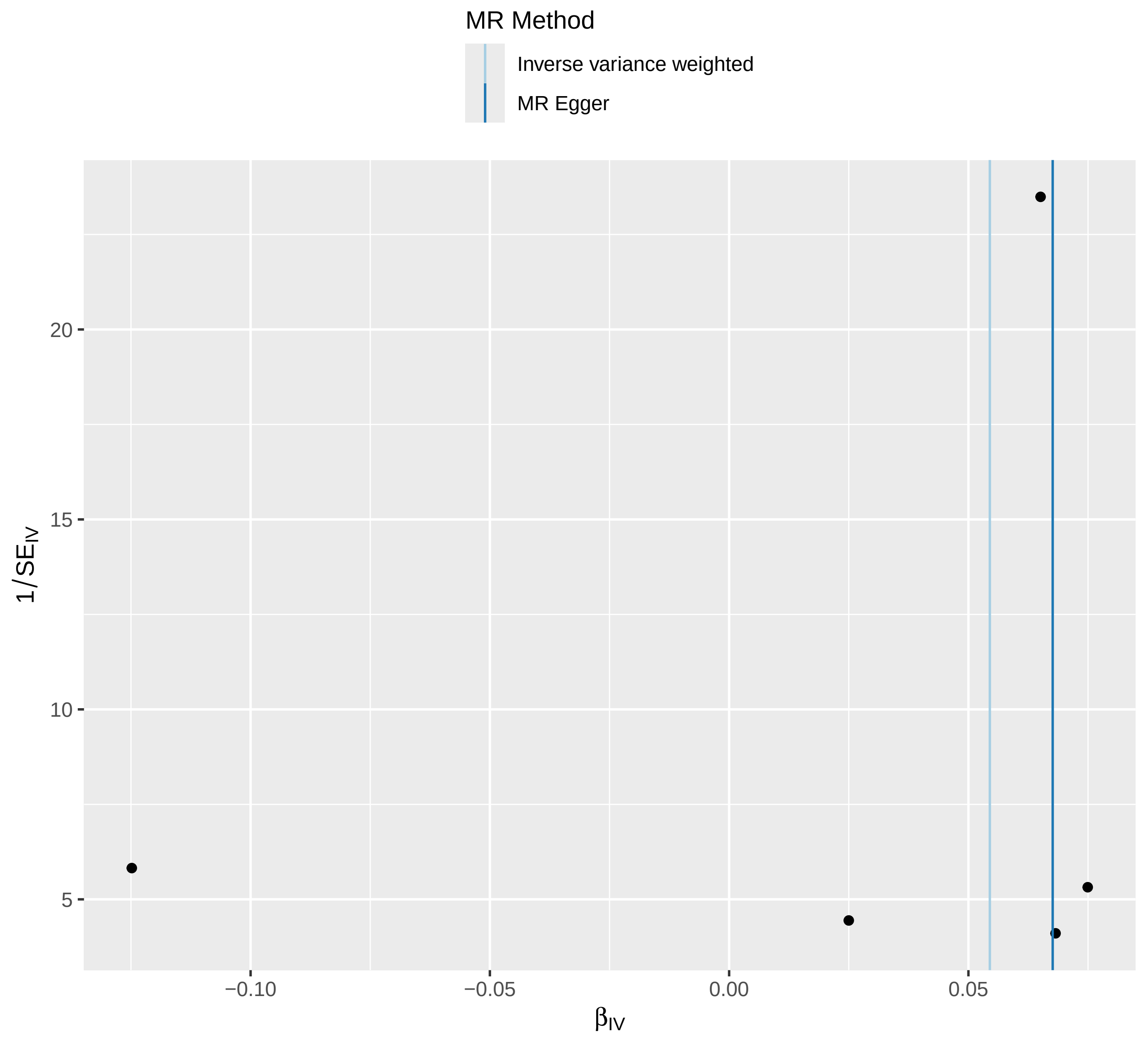

MR Method
Inverse variance weighted
MR Egger
20
15
10
5
1/SE$_{IV}$
−0.10
−0.05
0.00
0.05
β$_{IV}$

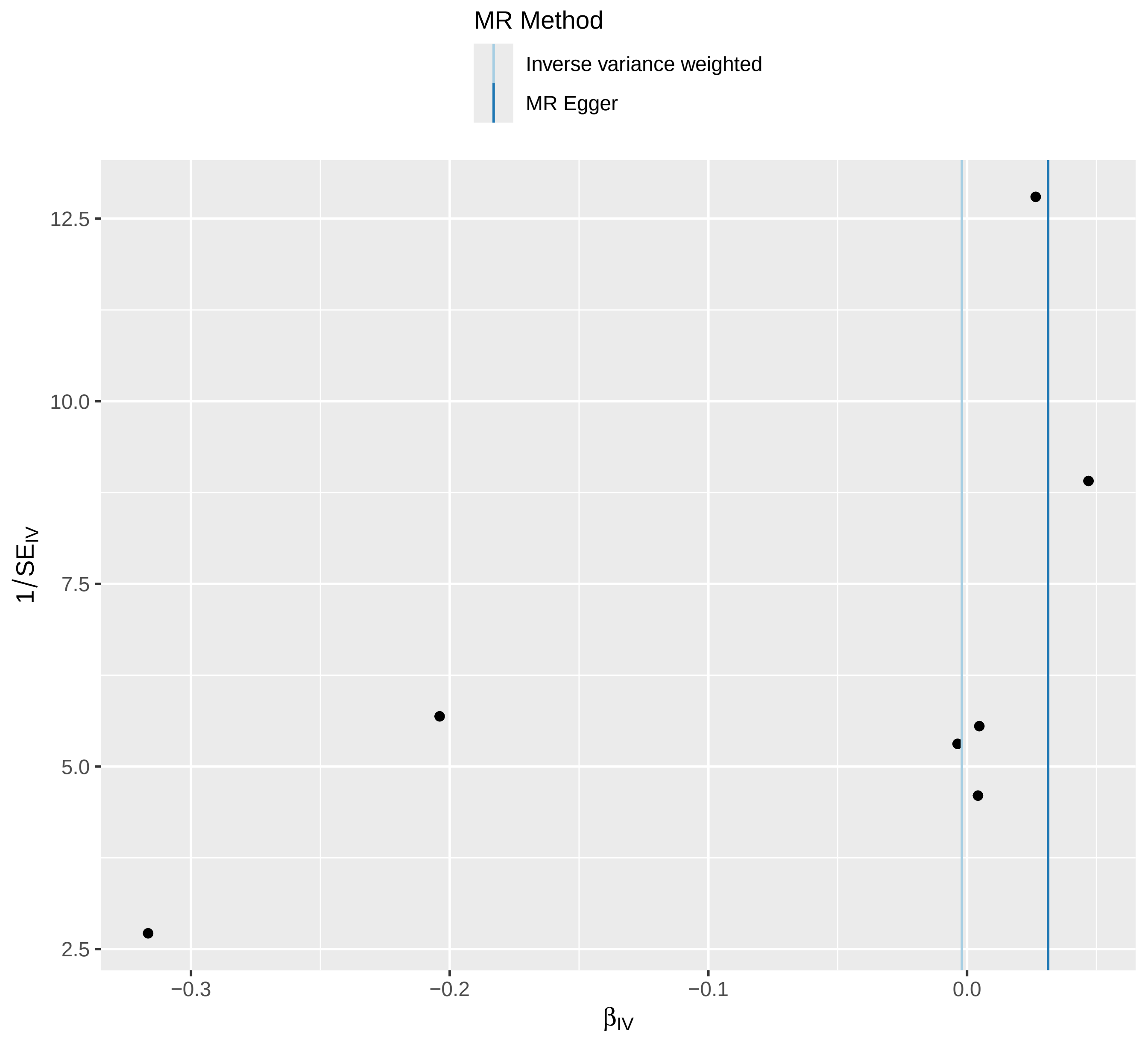

MR Method
Inverse variance weighted
MR Egger
12.5
10.0
7.5
5.0
2.5
1/SE_IV
−0.3
−0.2
−0.1
0.0
β_IV

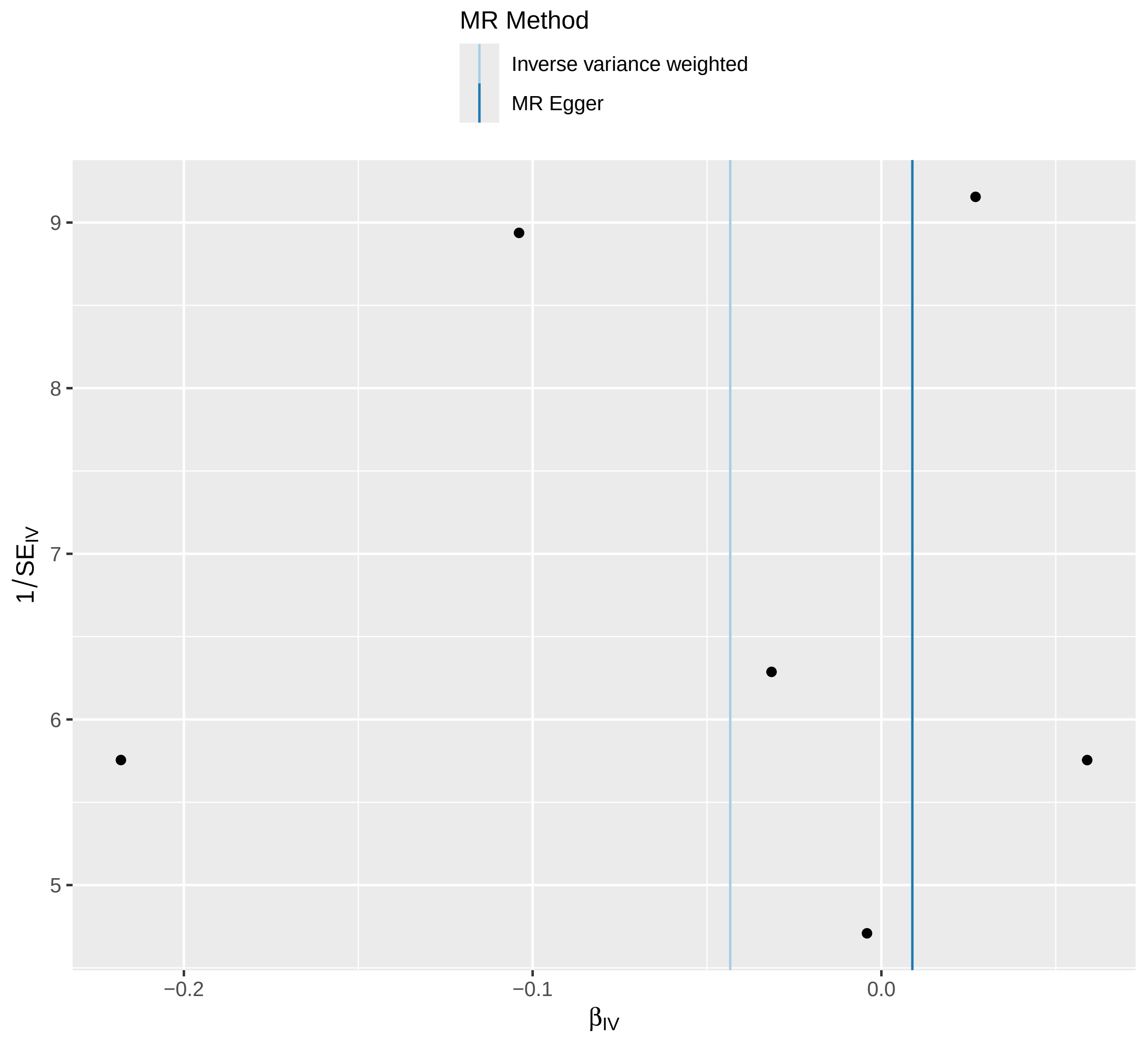
MR Method
Inverse variance weighted
MR Egger
1/SE$_{IV}$
9
8
7
6
5
−0.2
−0.1
0.0
β$_{IV}$

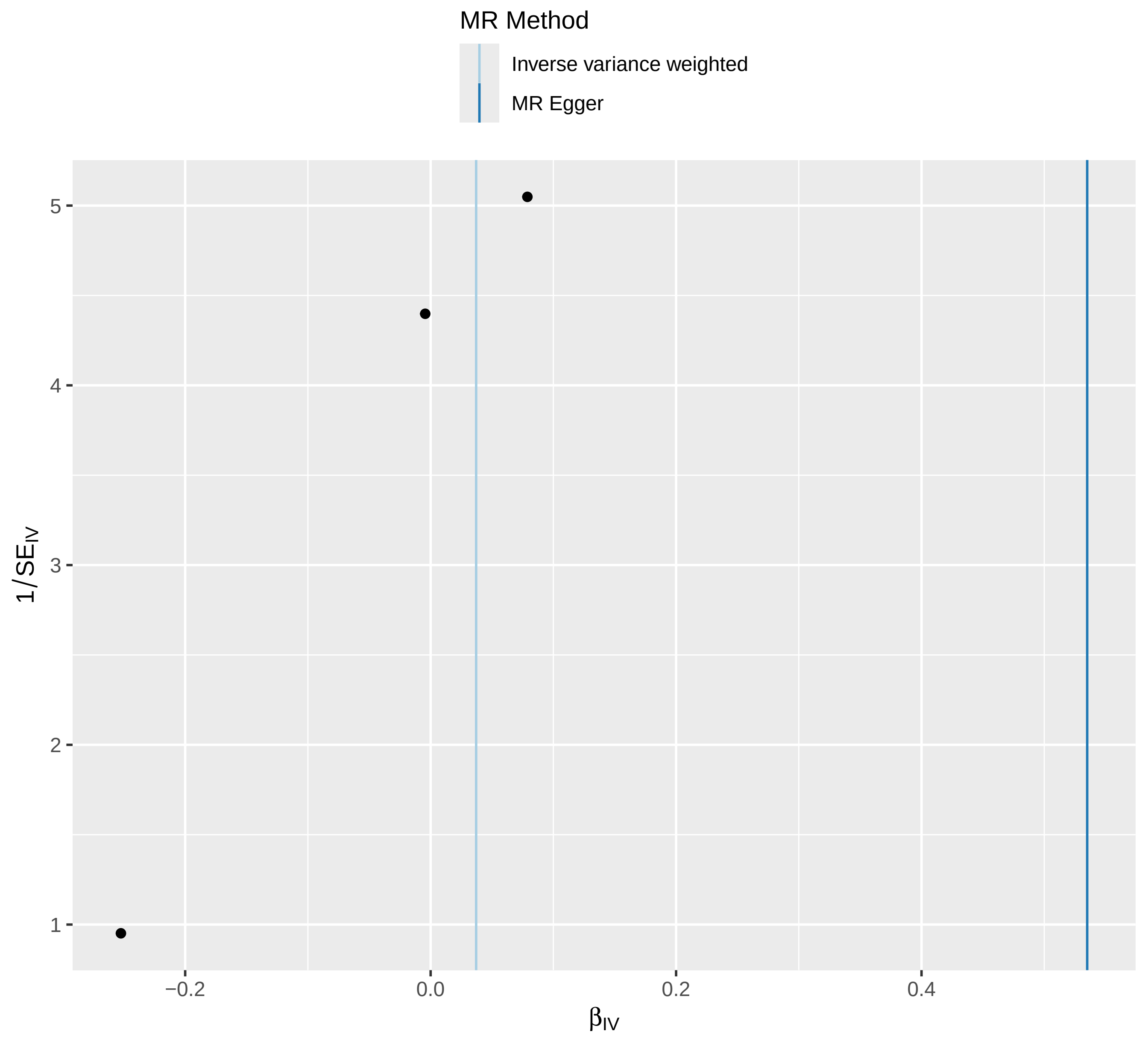
MR Method
Inverse variance weighted
MR Egger
5
4
3
2
1
$1/SE_{IV}$
−0.2
0.0
0.2
0.4
$\beta_{IV}$

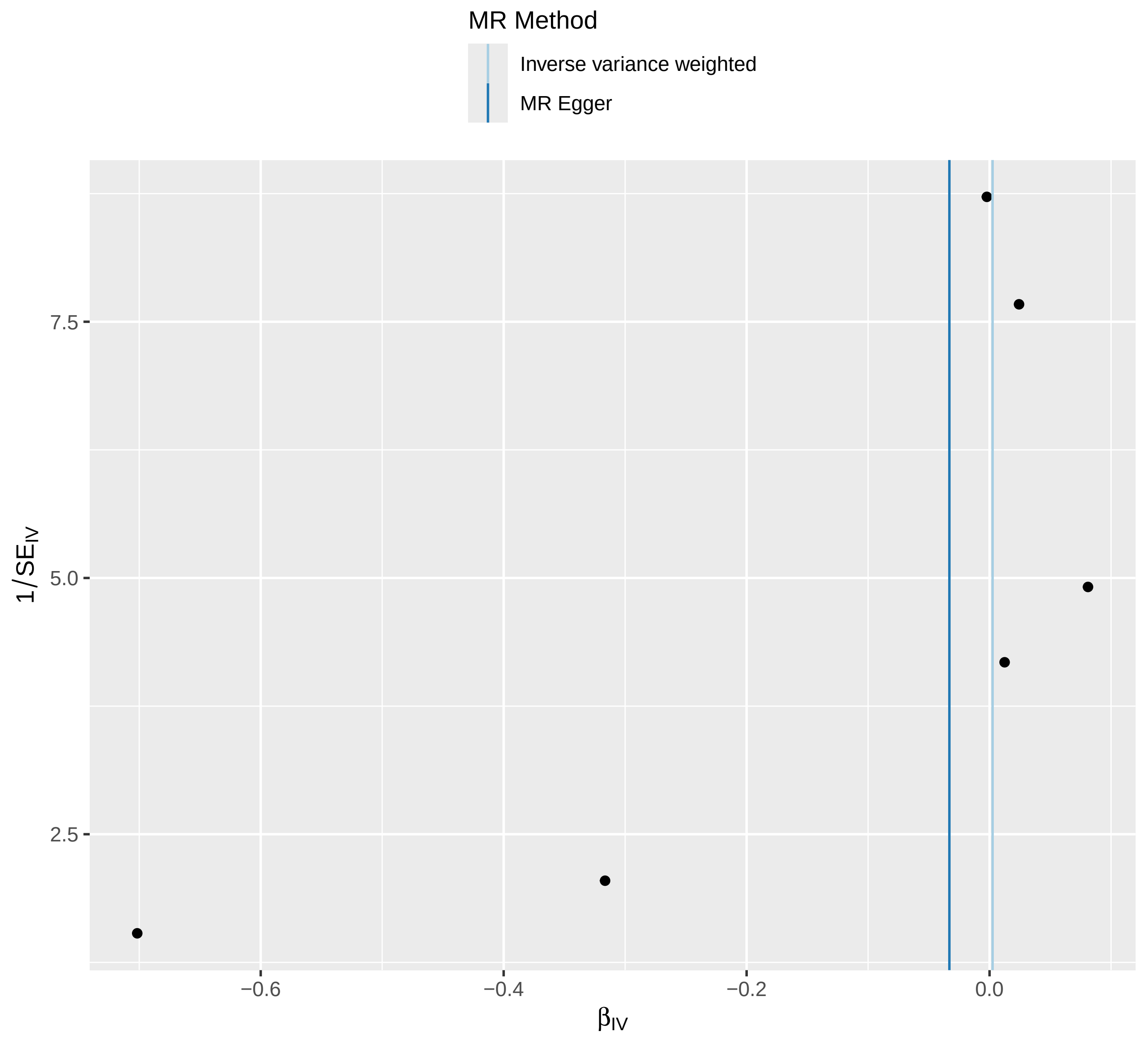
MR Method
Inverse variance weighted
MR Egger
1/$SE_{IV}$
7.5
5.0
2.5
−0.6
−0.4
−0.2
0.0
$\beta_{IV}$

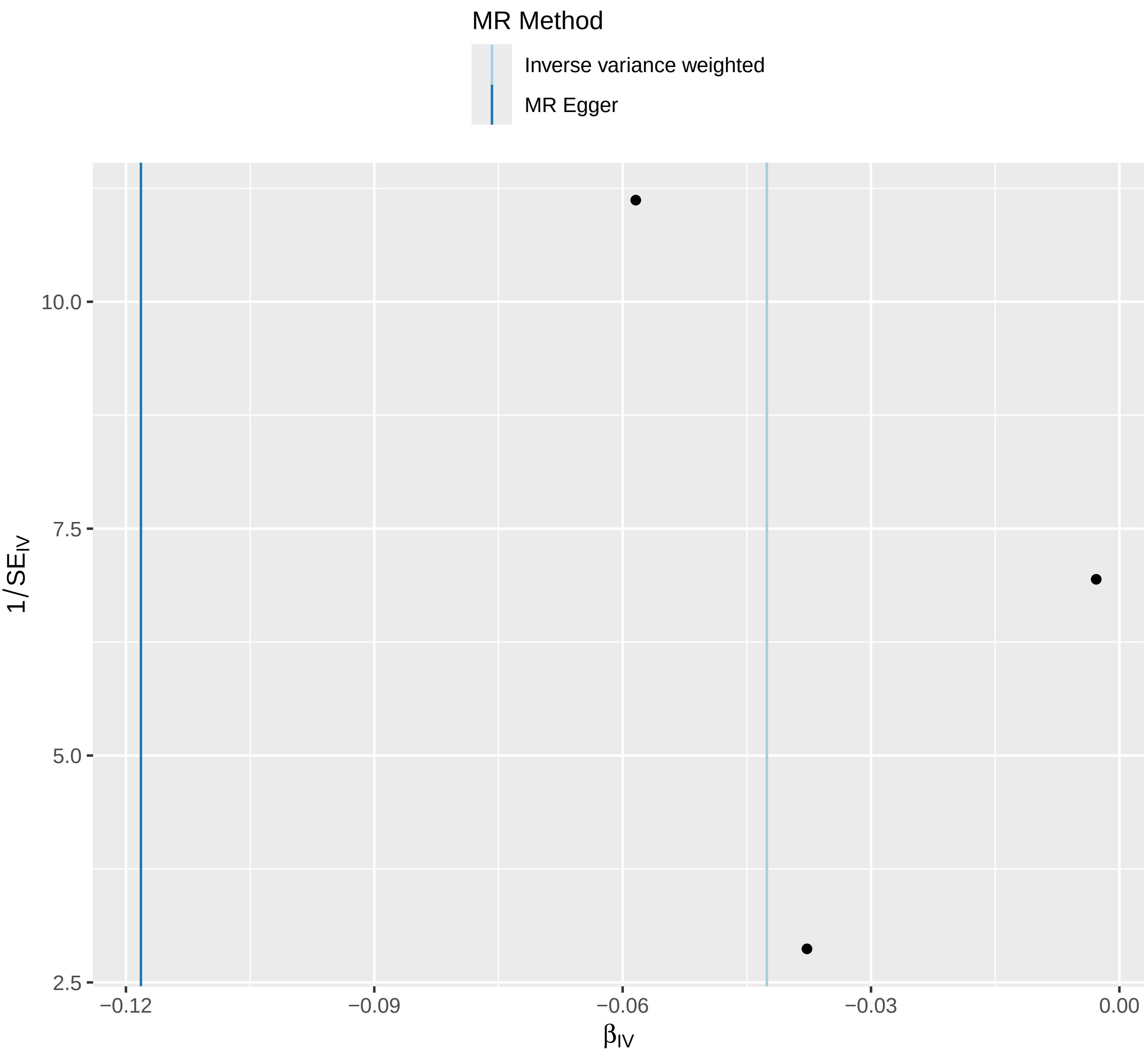
MR Method
Inverse variance weighted
MR Egger
10.0
7.5
5.0
2.5
1/SE_IV
−0.12
−0.09
−0.06
−0.03
0.00
β_IV

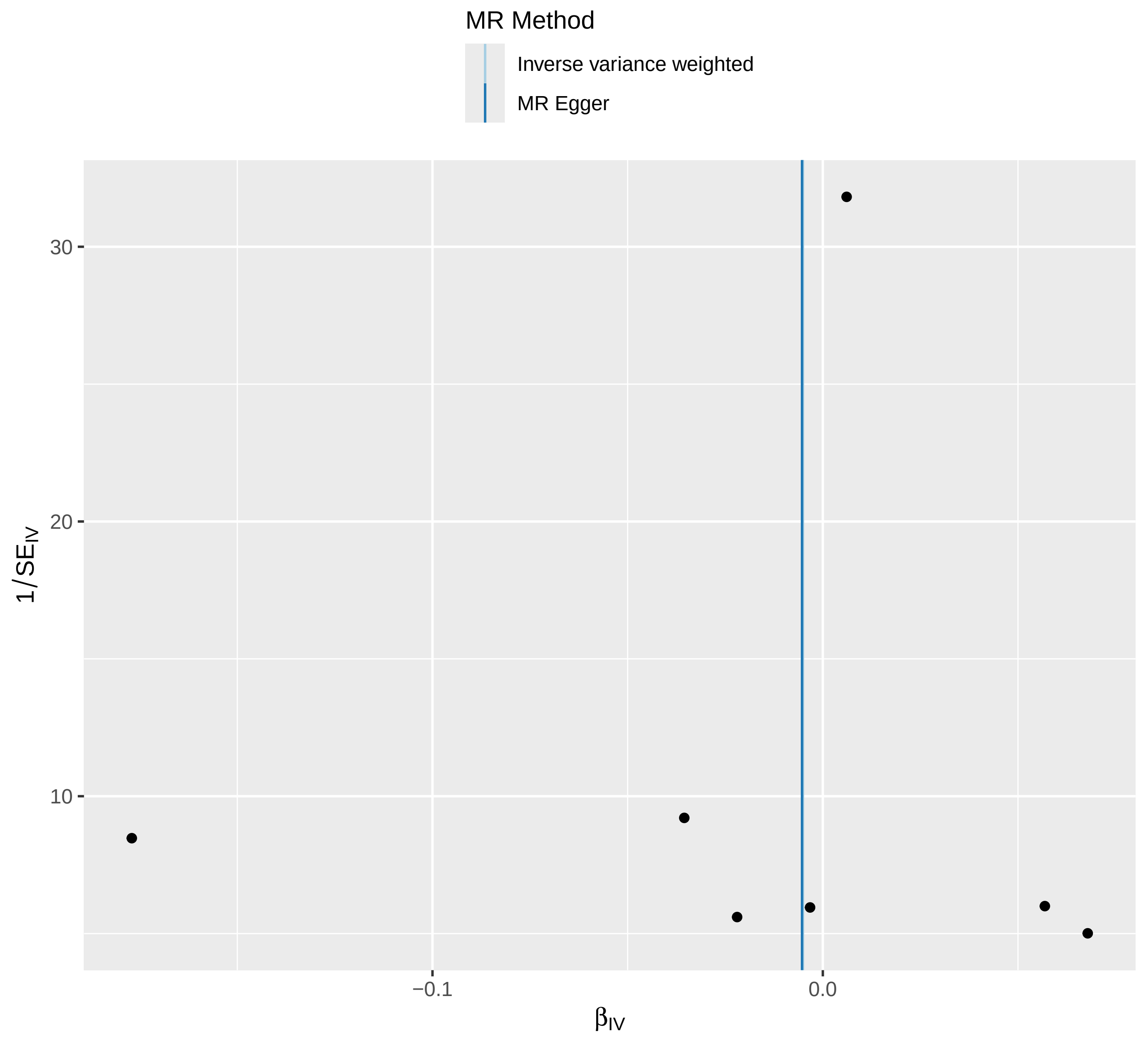

MR Method
Inverse variance weighted
MR Egger
30
20
10
1/SE_IV
−0.1
0.0
β_IV

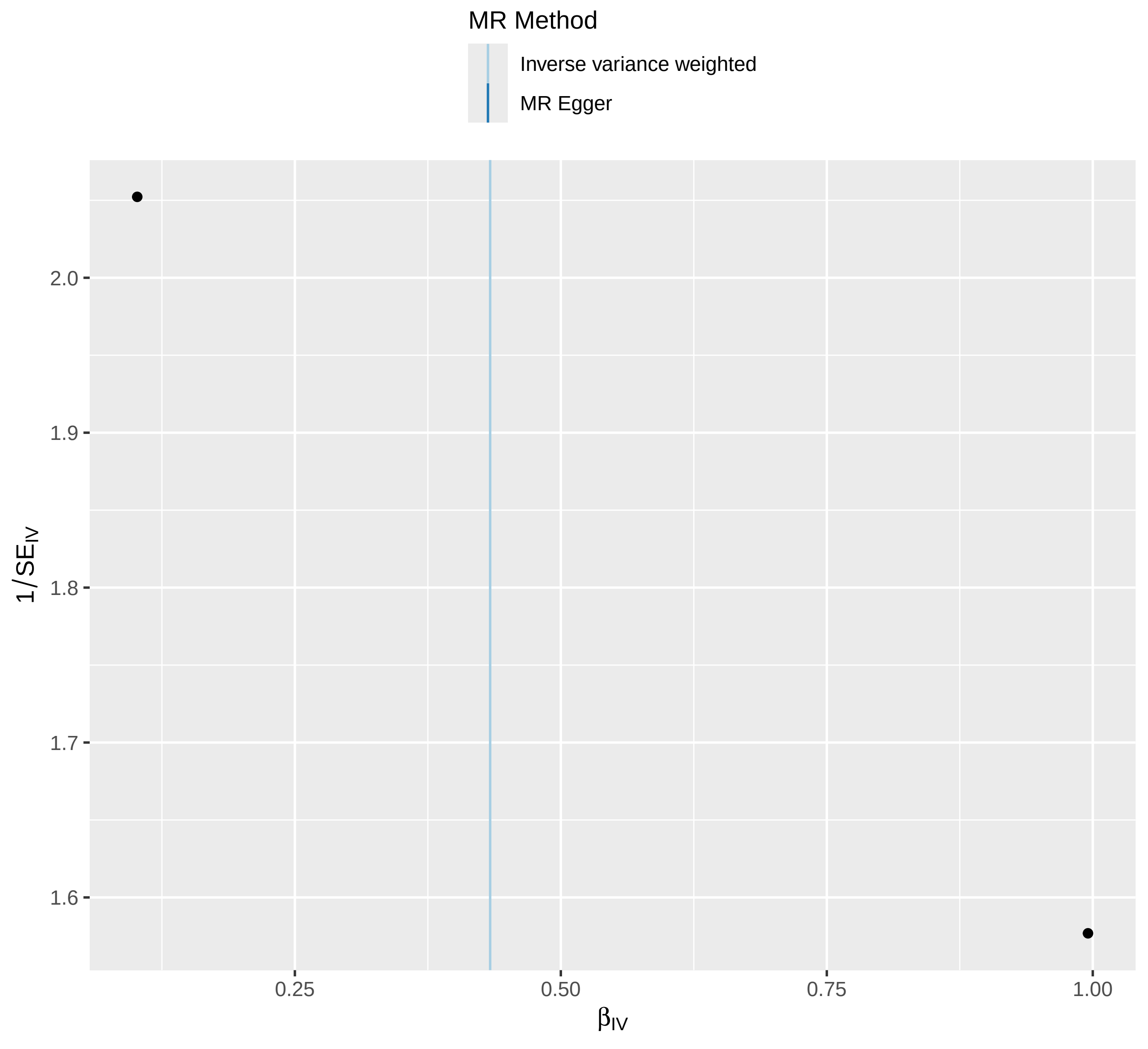
MR Method
Inverse variance weighted
MR Egger
2.0
1.9
1.8
1.7
1.6
$1/SE_{IV}$
0.25
0.50
0.75
1.00
$\beta_{IV}$

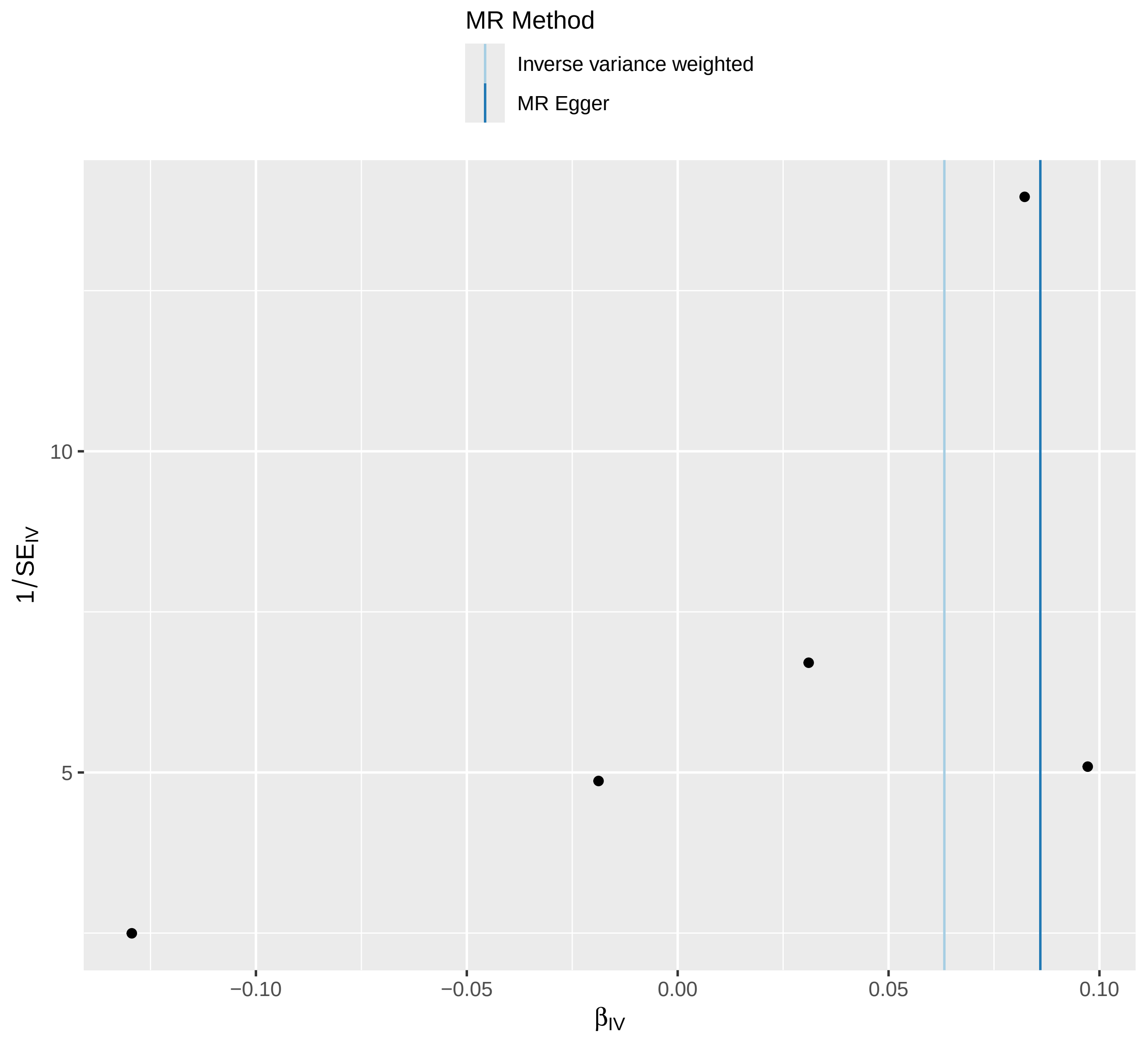

MR Method
Inverse variance weighted
MR Egger
10
5
$1/SE_{IV}$
−0.10
−0.05
0.00
0.05
0.10
$\beta_{IV}$

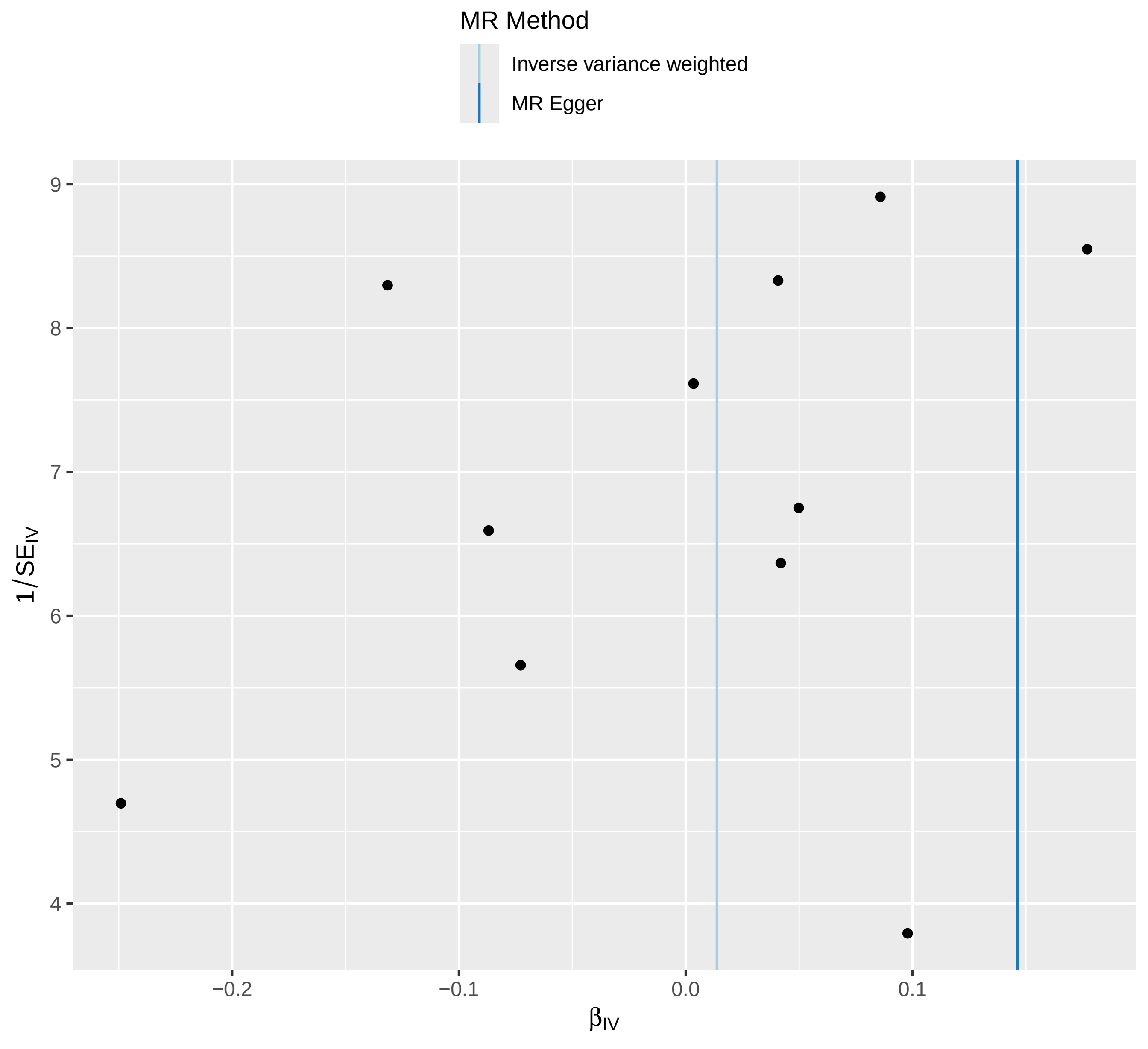

MR Method
Inverse variance weighted
MR Egger
9
8
7
6
5
4
1/SE_IV
−0.2
−0.1
0.0
0.1
β_IV

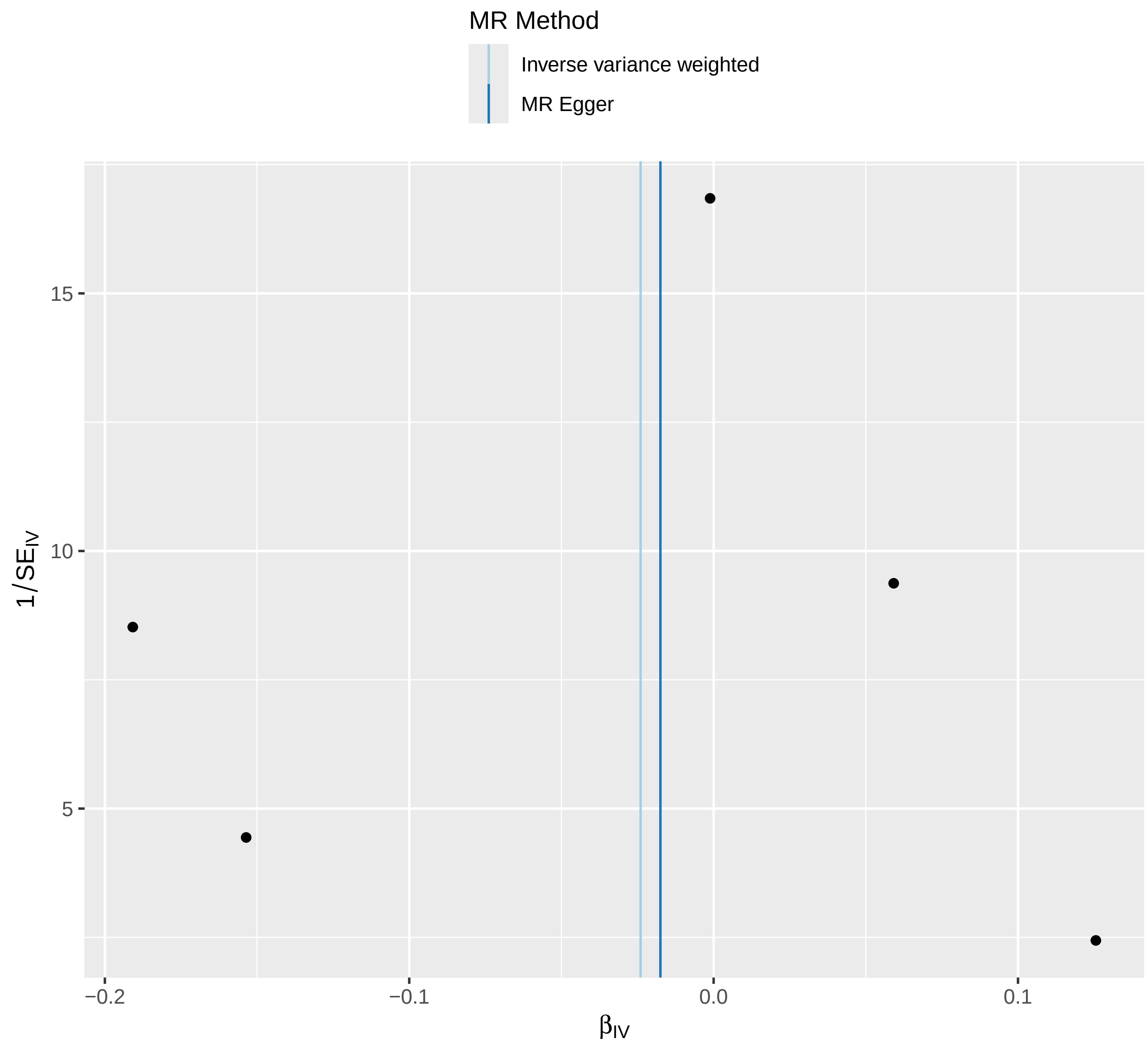
MR Method
Inverse variance weighted
MR Egger
15
10
5
$1/SE_{IV}$
−0.2
−0.1
0.0
0.1
$\beta_{IV}$

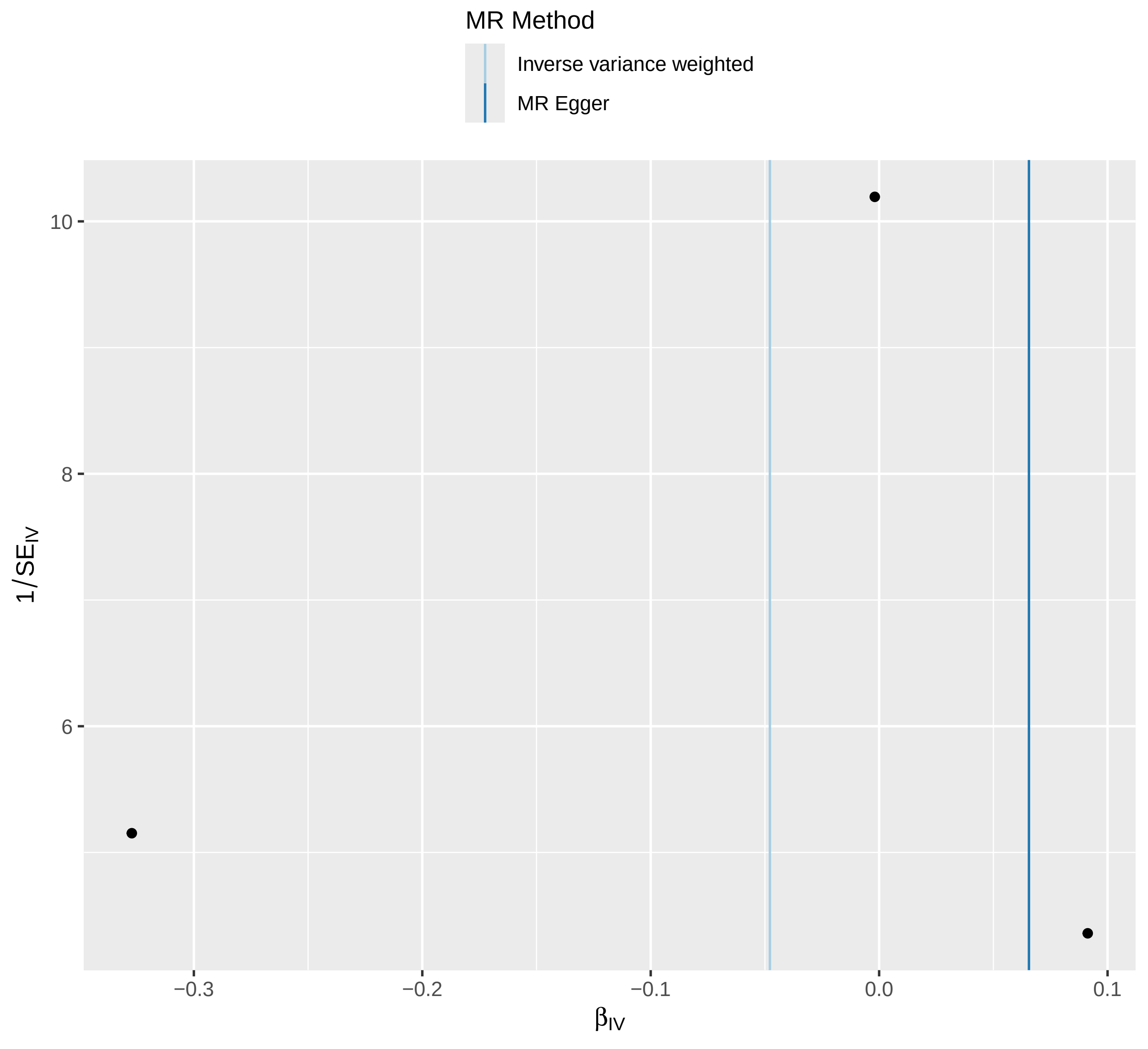

MR Method
Inverse variance weighted
MR Egger
10
8
6
$1/SE_{IV}$
−0.3
−0.2
−0.1
0.0
0.1
$\beta_{IV}$

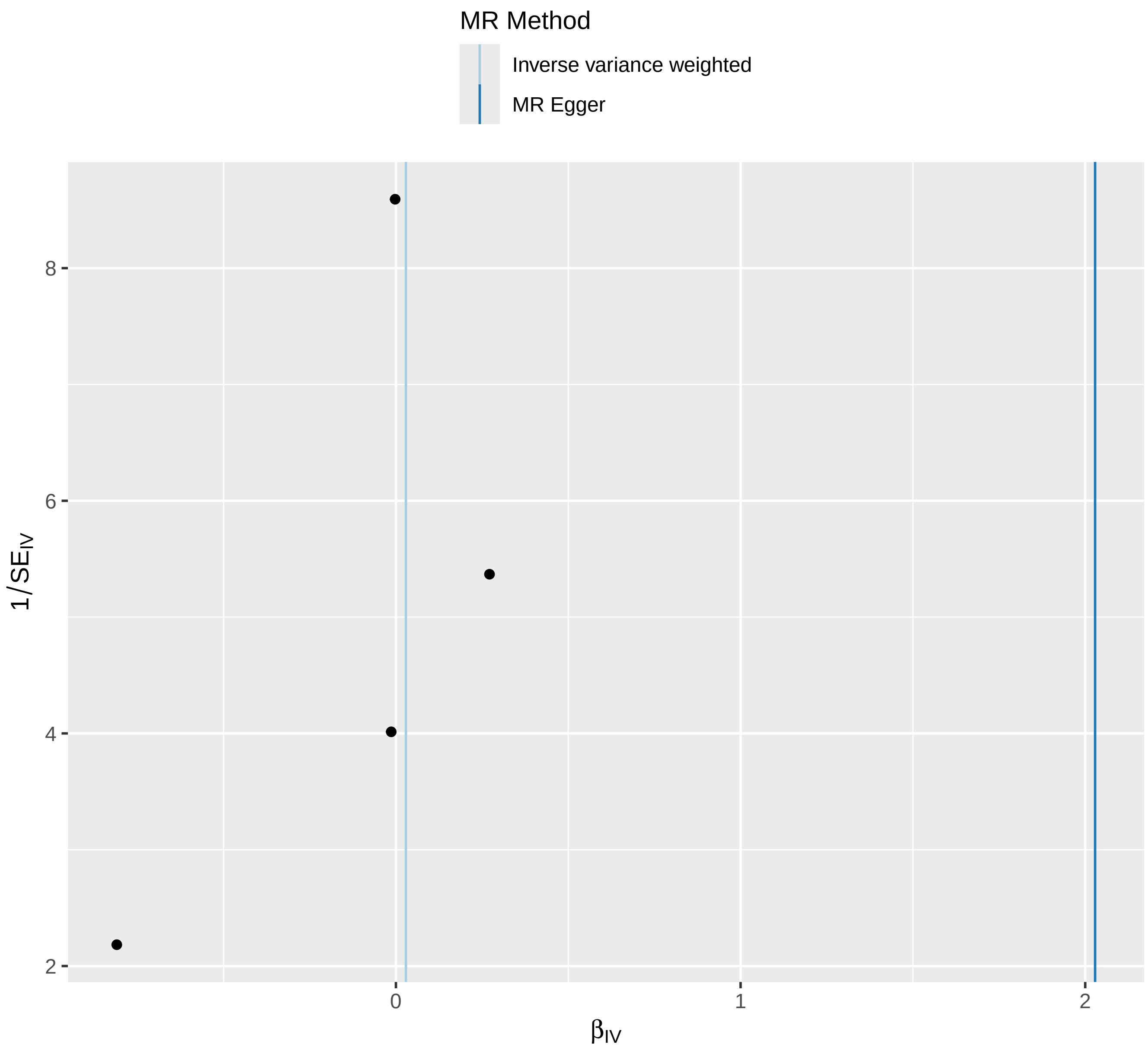

MR Method
Inverse variance weighted
MR Egger
8
6
4
2
0
1
2
$1/SE_{IV}$
$\beta_{IV}$

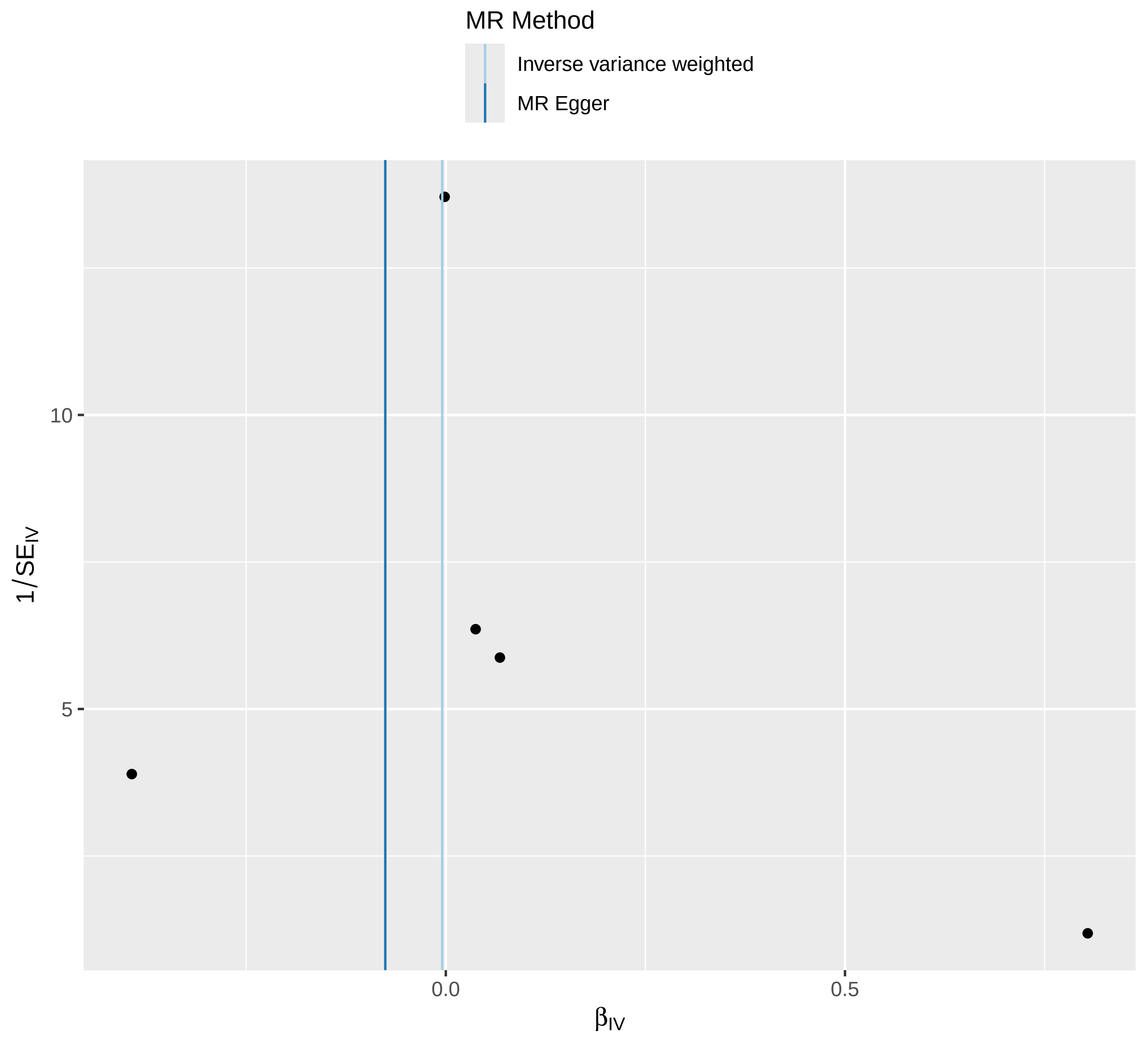

MR Method
Inverse variance weighted
MR Egger
10
5
1/SE_IV
0.0
0.5
β_IV

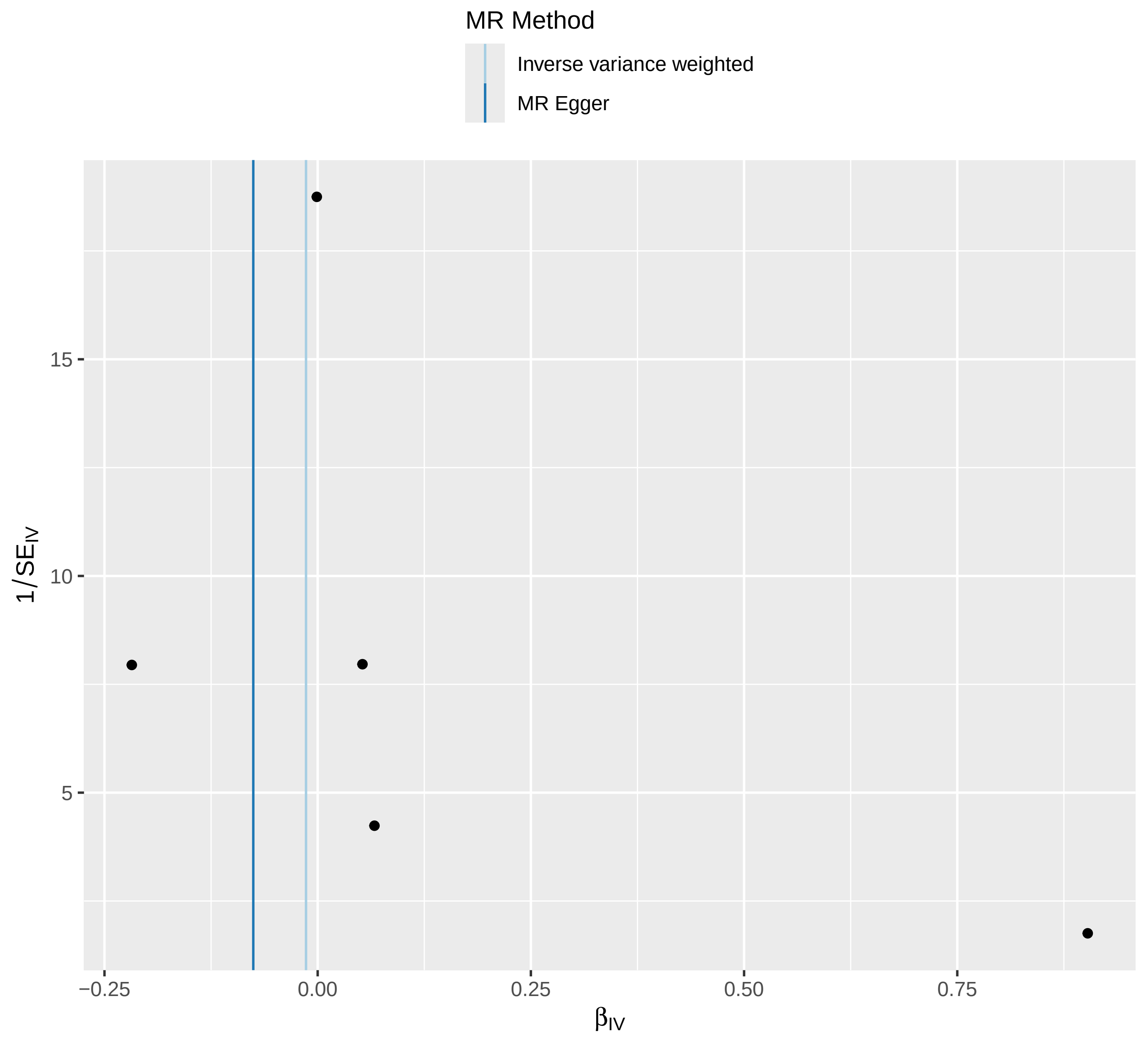
MR Method
Inverse variance weighted
MR Egger
15
10
5
$1/SE_{IV}$
−0.25
0.00
0.25
0.50
0.75
$\beta_{IV}$

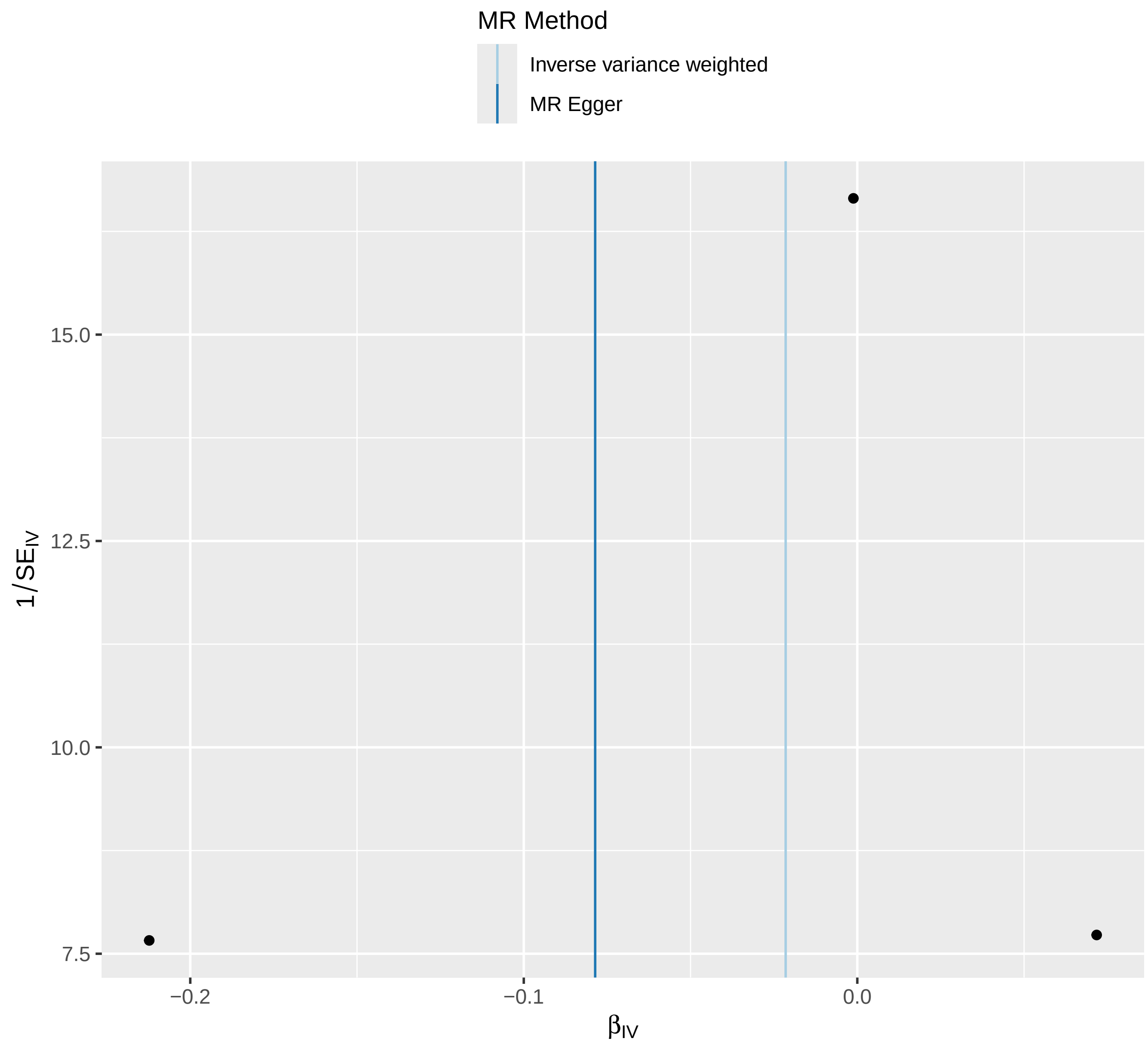
MR Method
Inverse variance weighted
MR Egger
15.0
12.5
10.0
7.5
1/SE$_{IV}$
−0.2
−0.1
0.0
β$_{IV}$

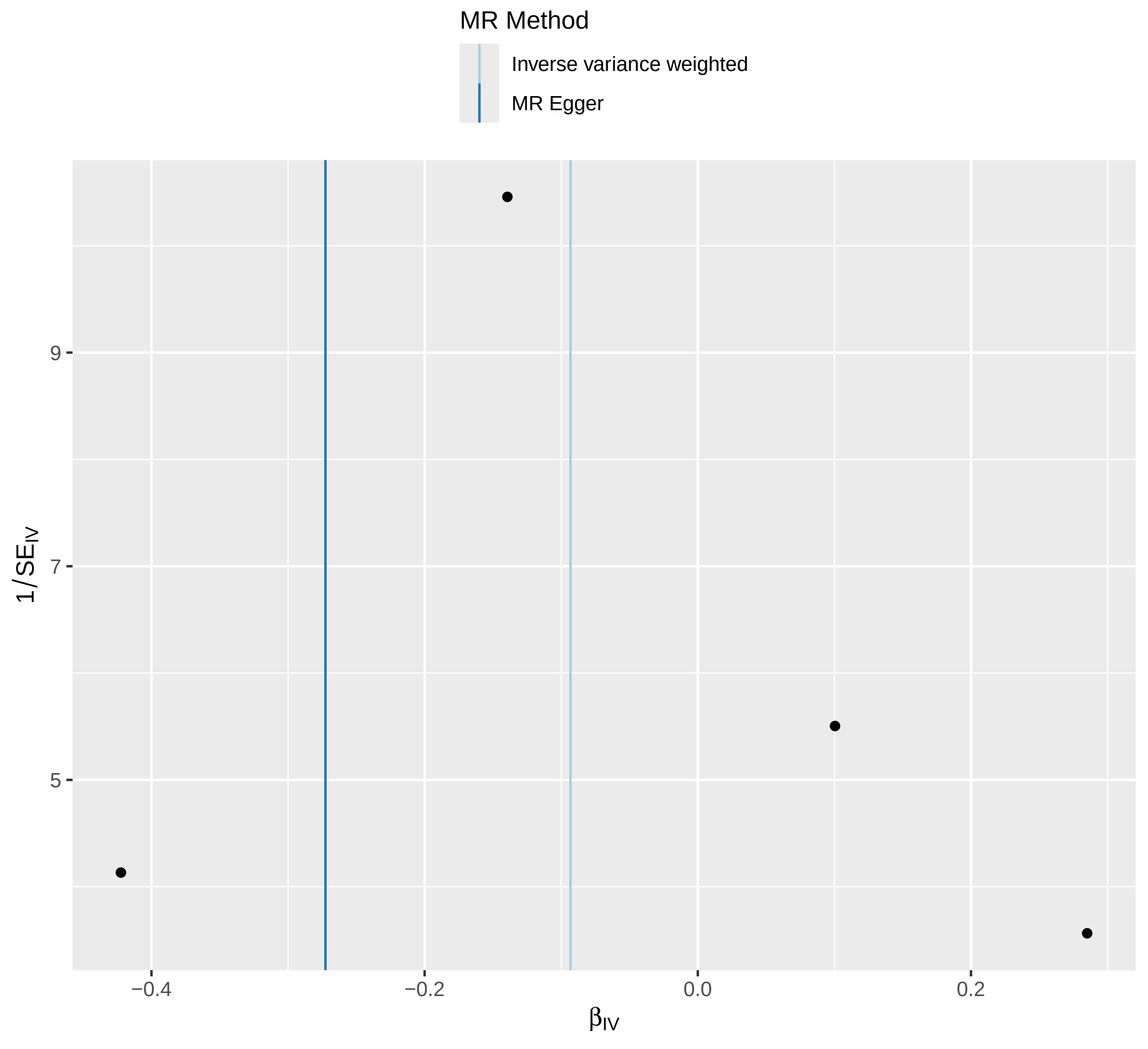
MR Method
Inverse variance weighted
MR Egger
9
7
5
1/SE$_{IV}$
−0.4
−0.2
0.0
0.2
β$_{IV}$

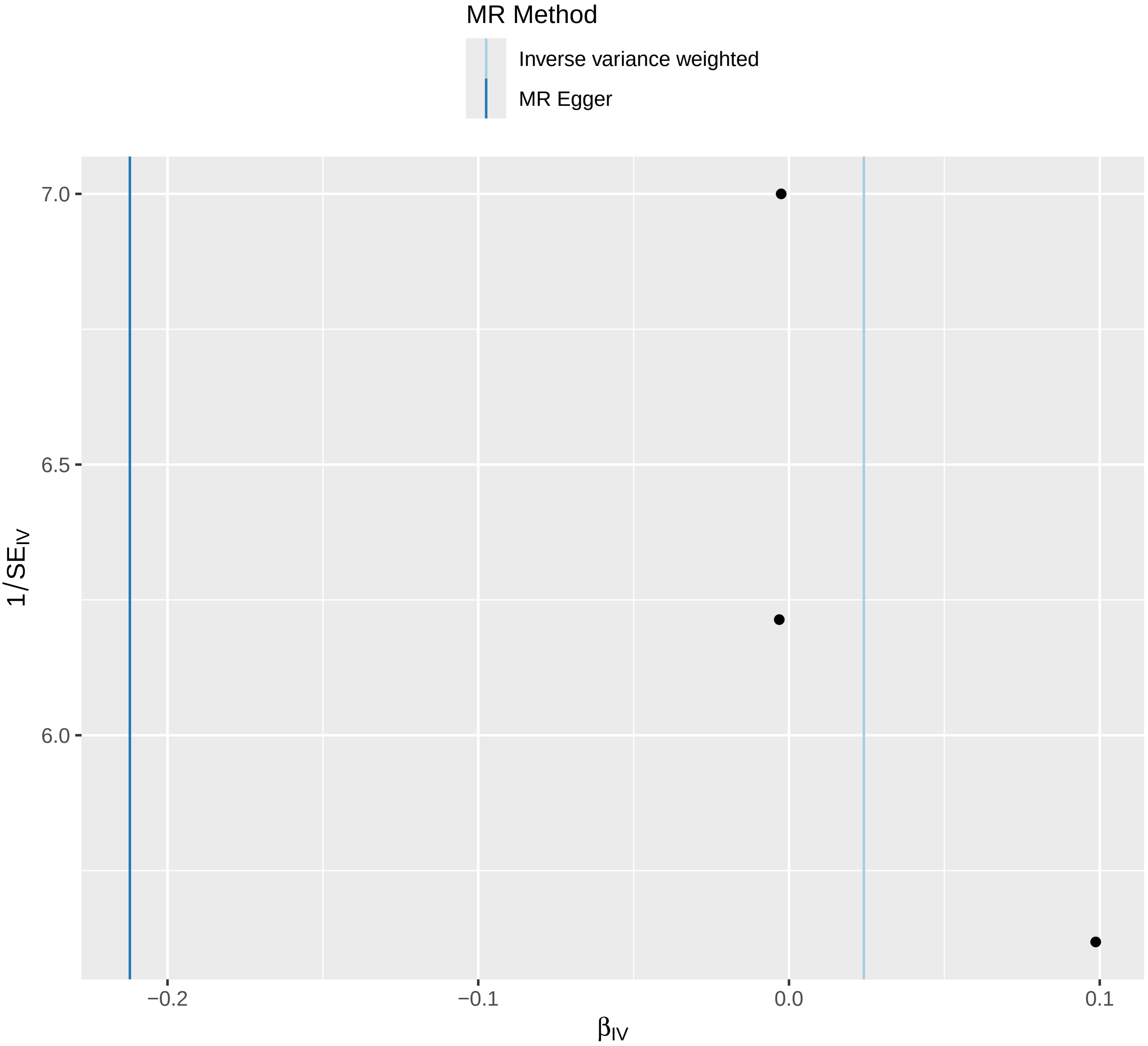
MR Method
Inverse variance weighted
MR Egger
7.0
6.5
6.0
1/$SE_{IV}$
−0.2
−0.1
0.0
0.1
$\beta_{IV}$

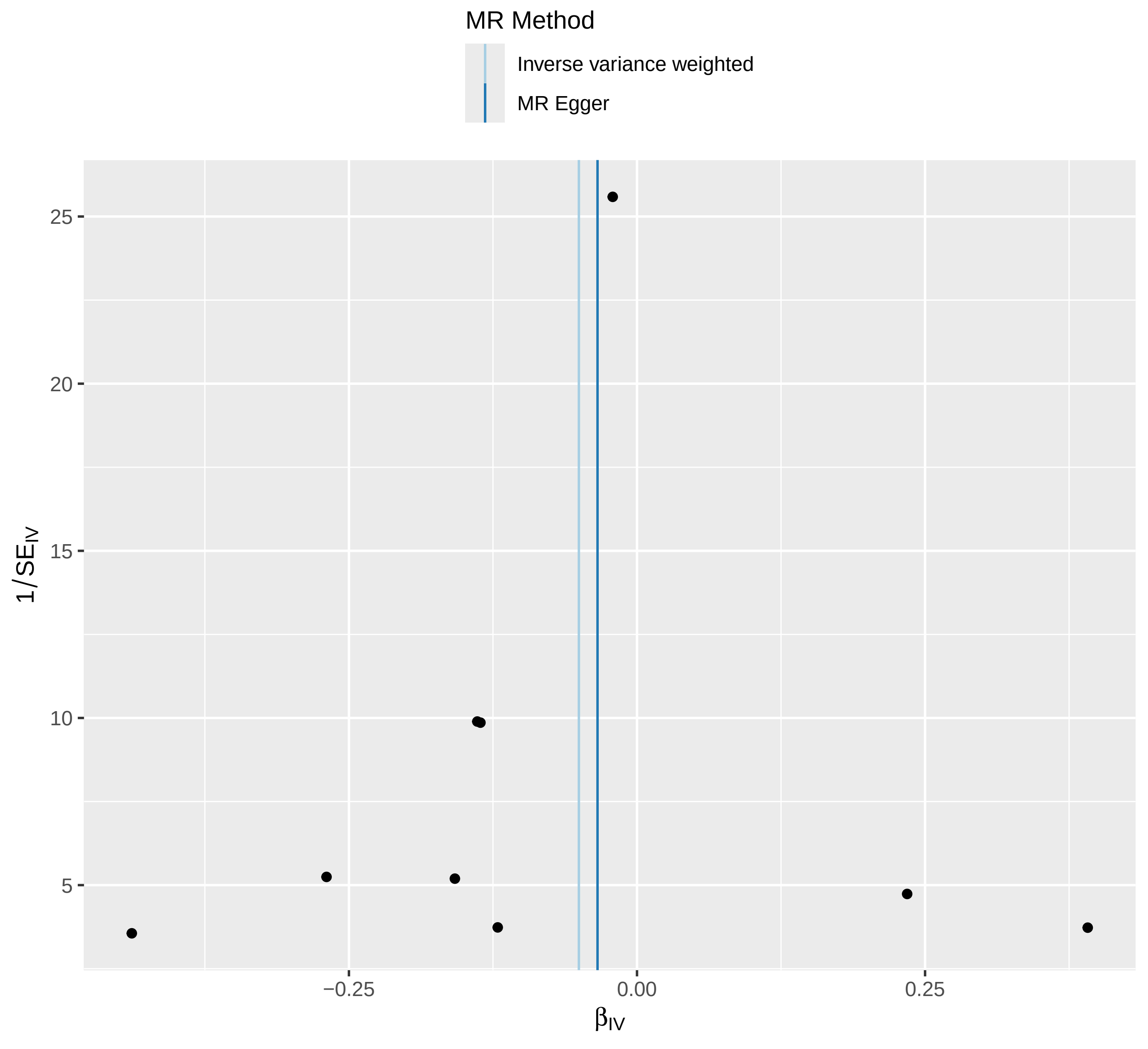
MR Method
Inverse variance weighted
MR Egger
25
20
15
10
5
−0.25
0.00
0.25
$1/SE_{IV}$
$\beta_{IV}$

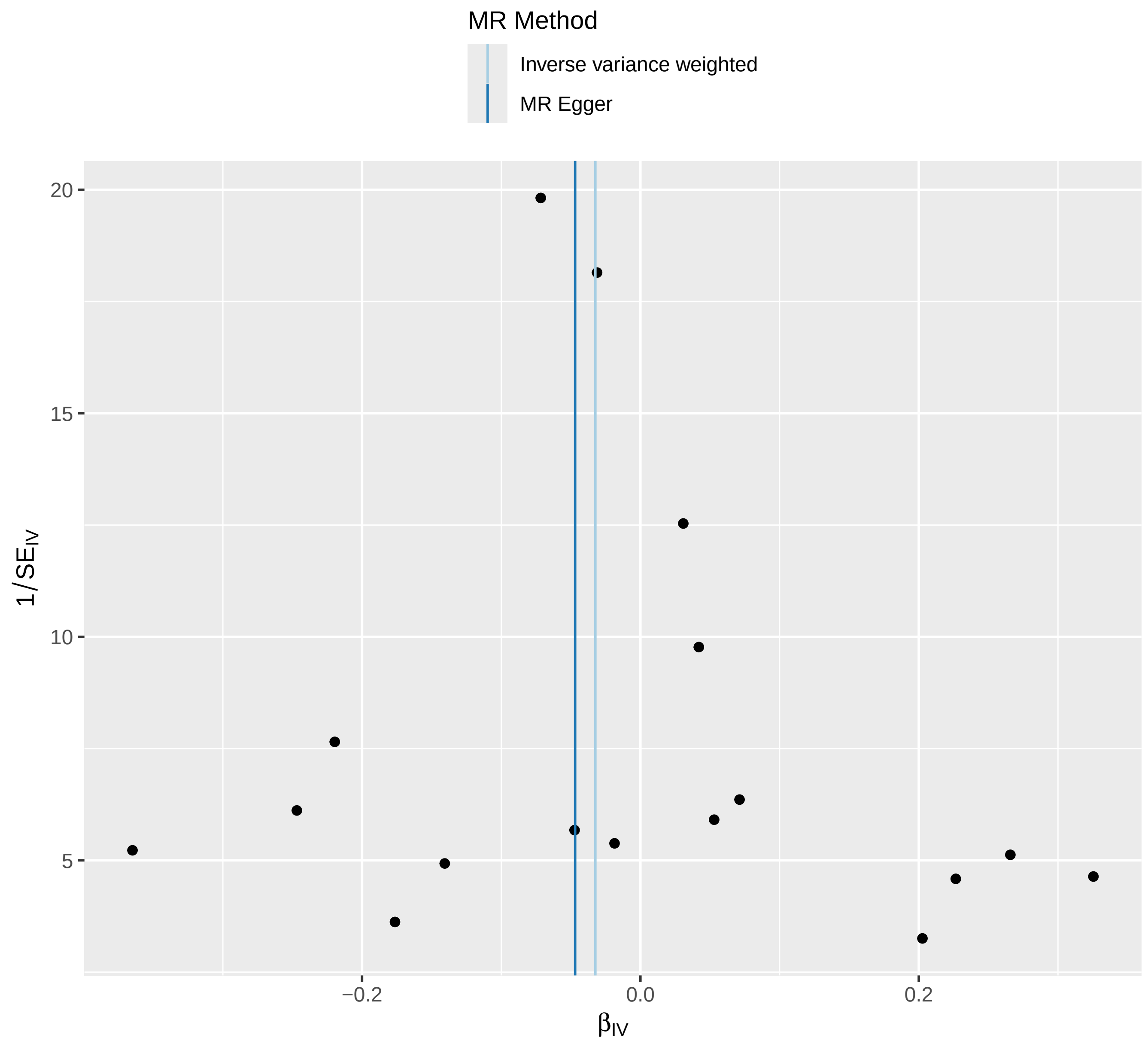
MR Method
Inverse variance weighted
MR Egger
20
15
10
5
1/SE$_{IV}$
−0.2
0.0
0.2
β$_{IV}$

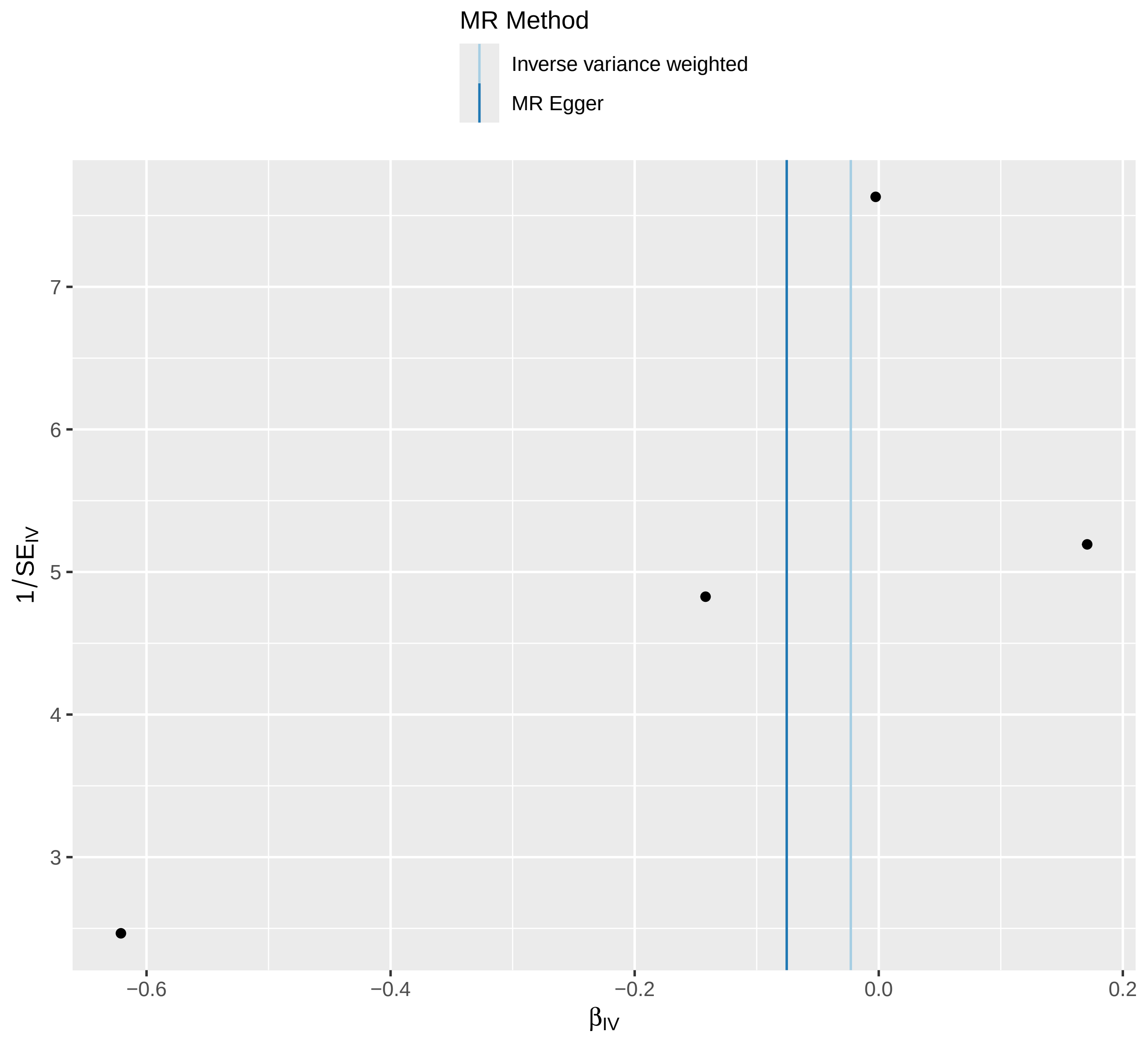
MR Method
Inverse variance weighted
MR Egger
7
6
5
4
3
1/$SE_{IV}$
−0.6
−0.4
−0.2
0.0
0.2
$\beta_{IV}$

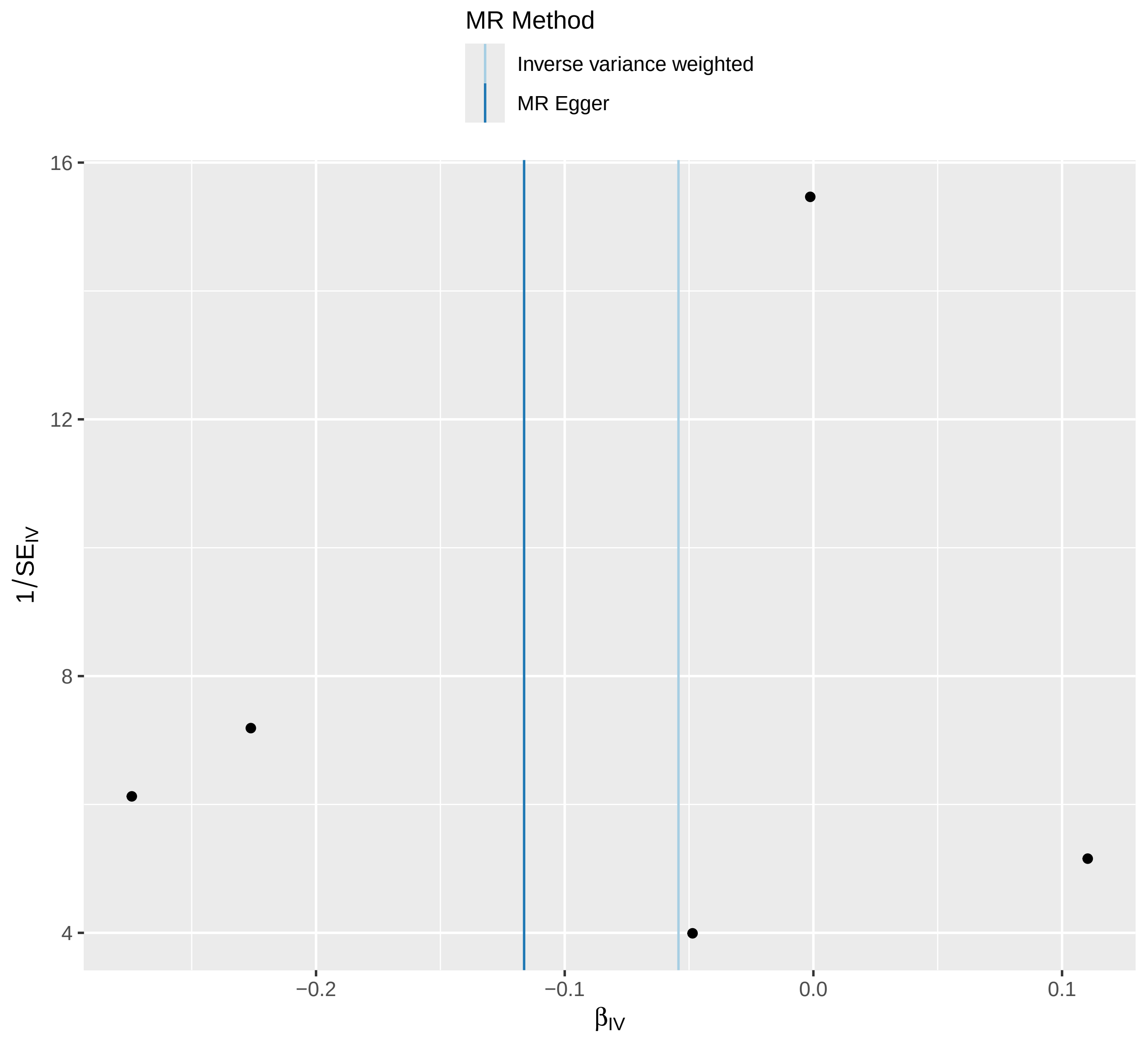

MR Method
Inverse variance weighted
MR Egger
16
12
8
4
1/SE$_{IV}$
−0.2
−0.1
0.0
0.1
β$_{IV}$

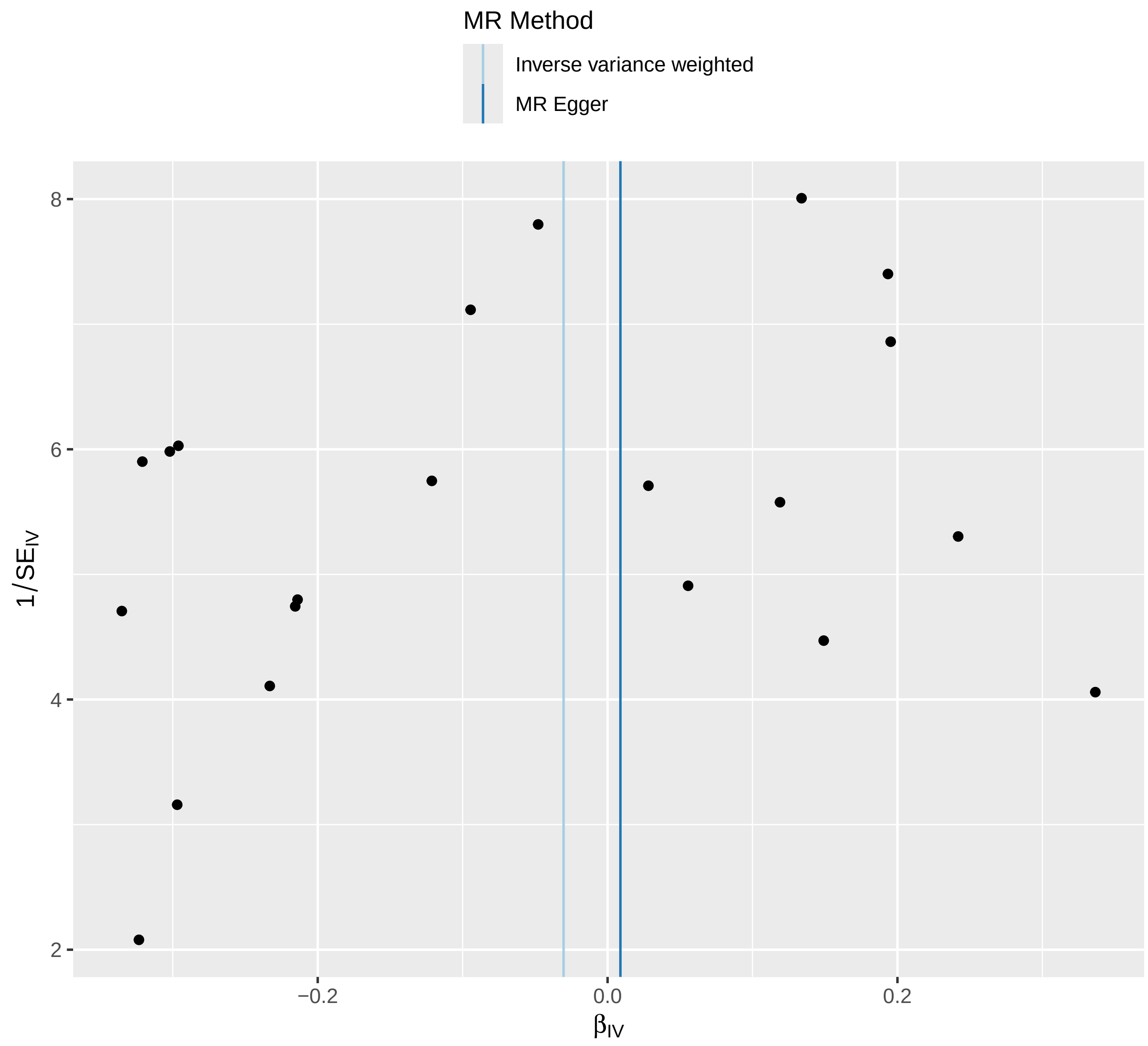

MR Method
Inverse variance weighted
MR Egger
8
6
4
2
1/SE_IV
−0.2
0.0
0.2
β_IV

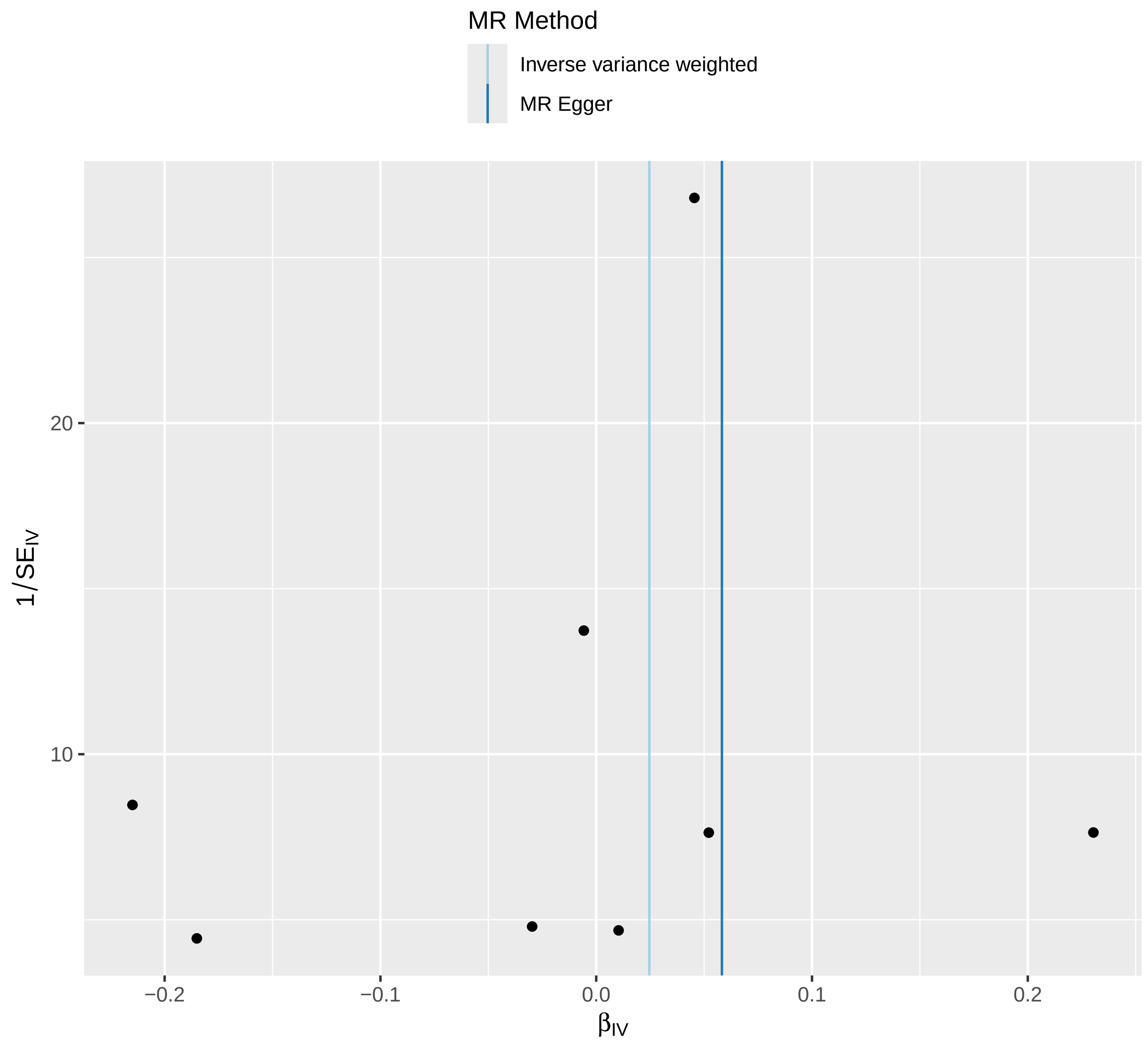

MR Method
Inverse variance weighted
MR Egger
20
10
1/SE_IV
−0.2
−0.1
0.0
0.1
0.2
β_IV

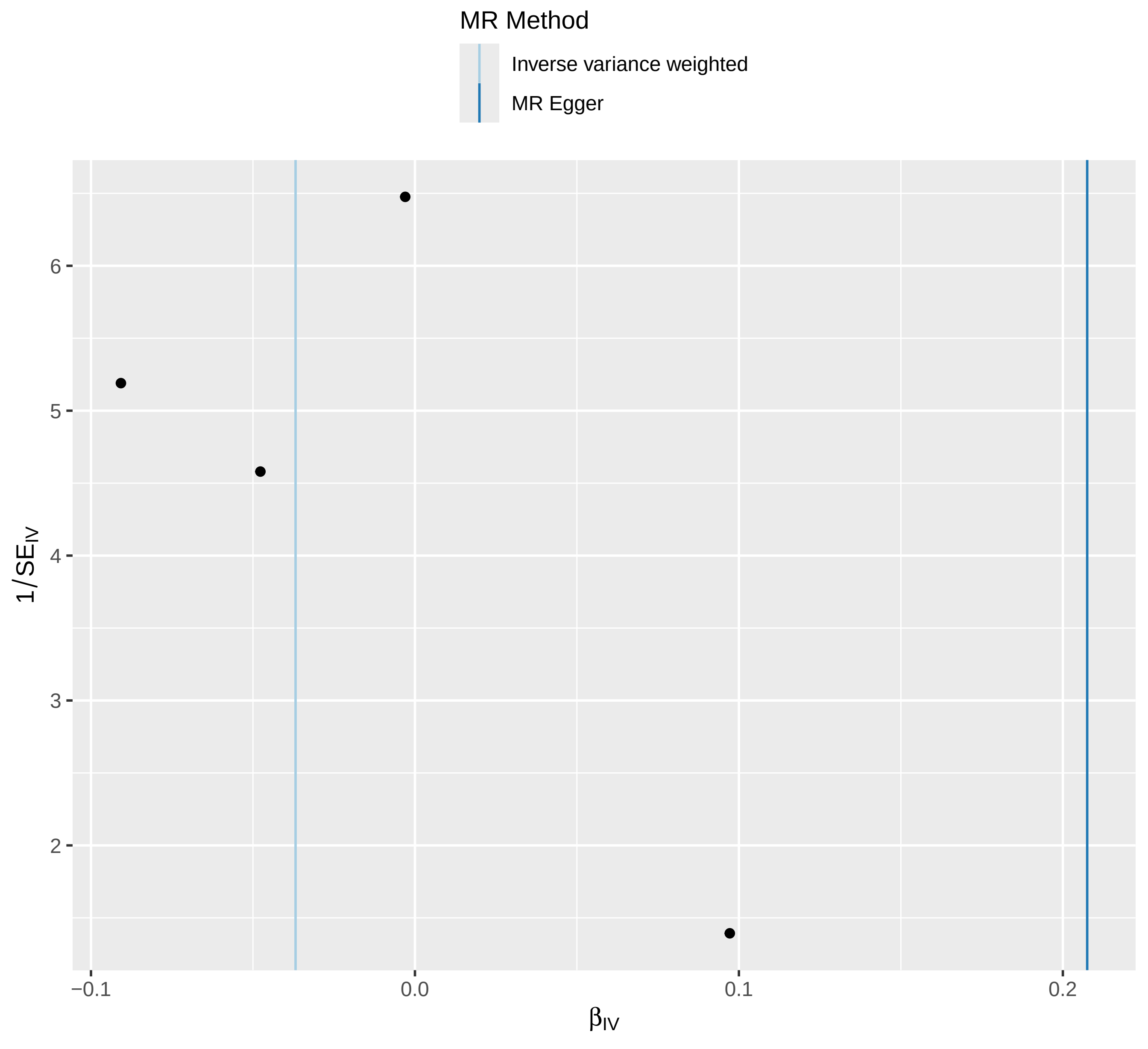

MR Method
Inverse variance weighted
MR Egger
6
5
4
3
2
1/SE_IV
−0.1
0.0
0.1
0.2
β_IV

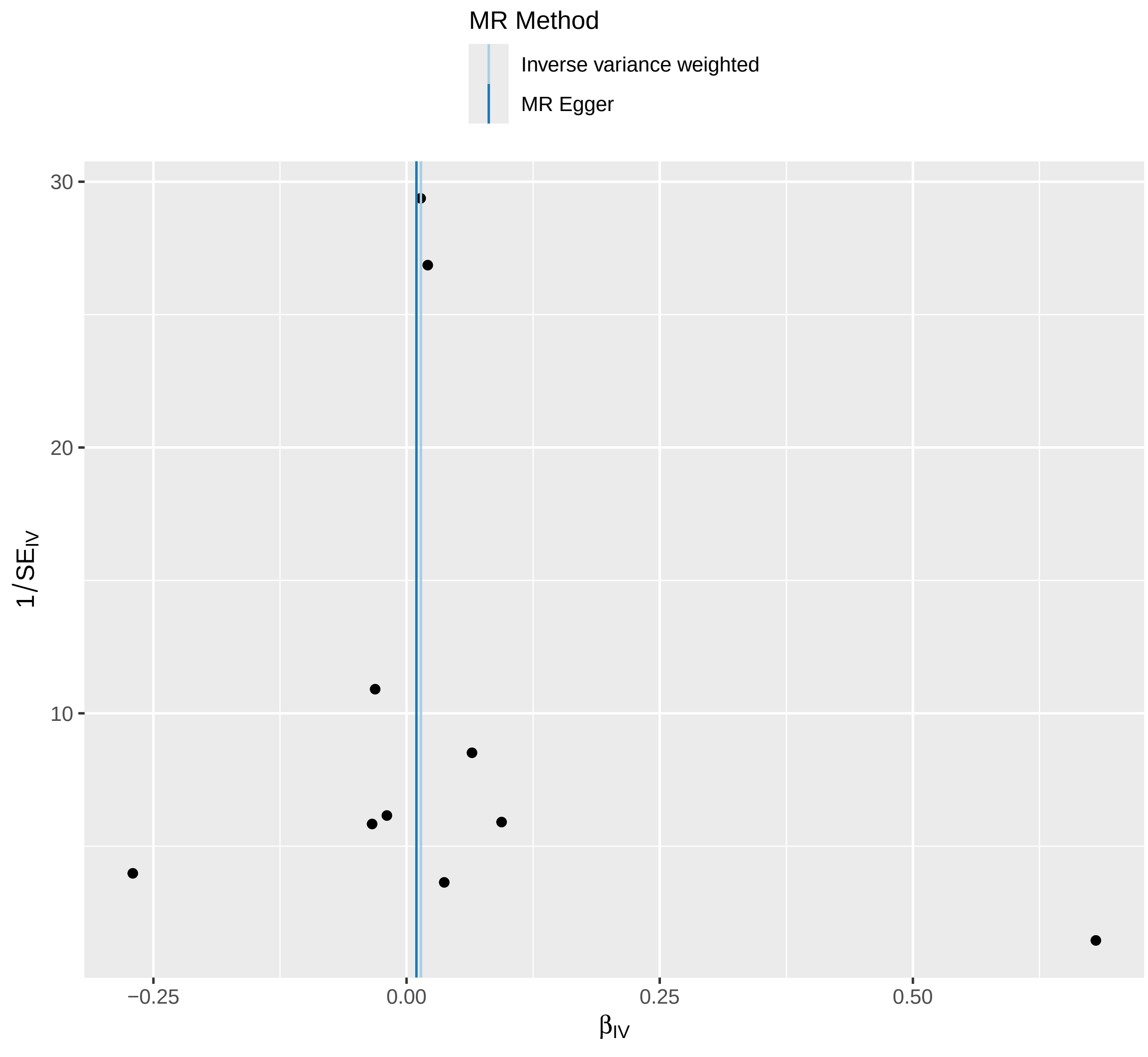
MR Method
Inverse variance weighted
MR Egger
30
20
10
1/$SE_{IV}$
−0.25
0.00
0.25
0.50
$\beta_{IV}$

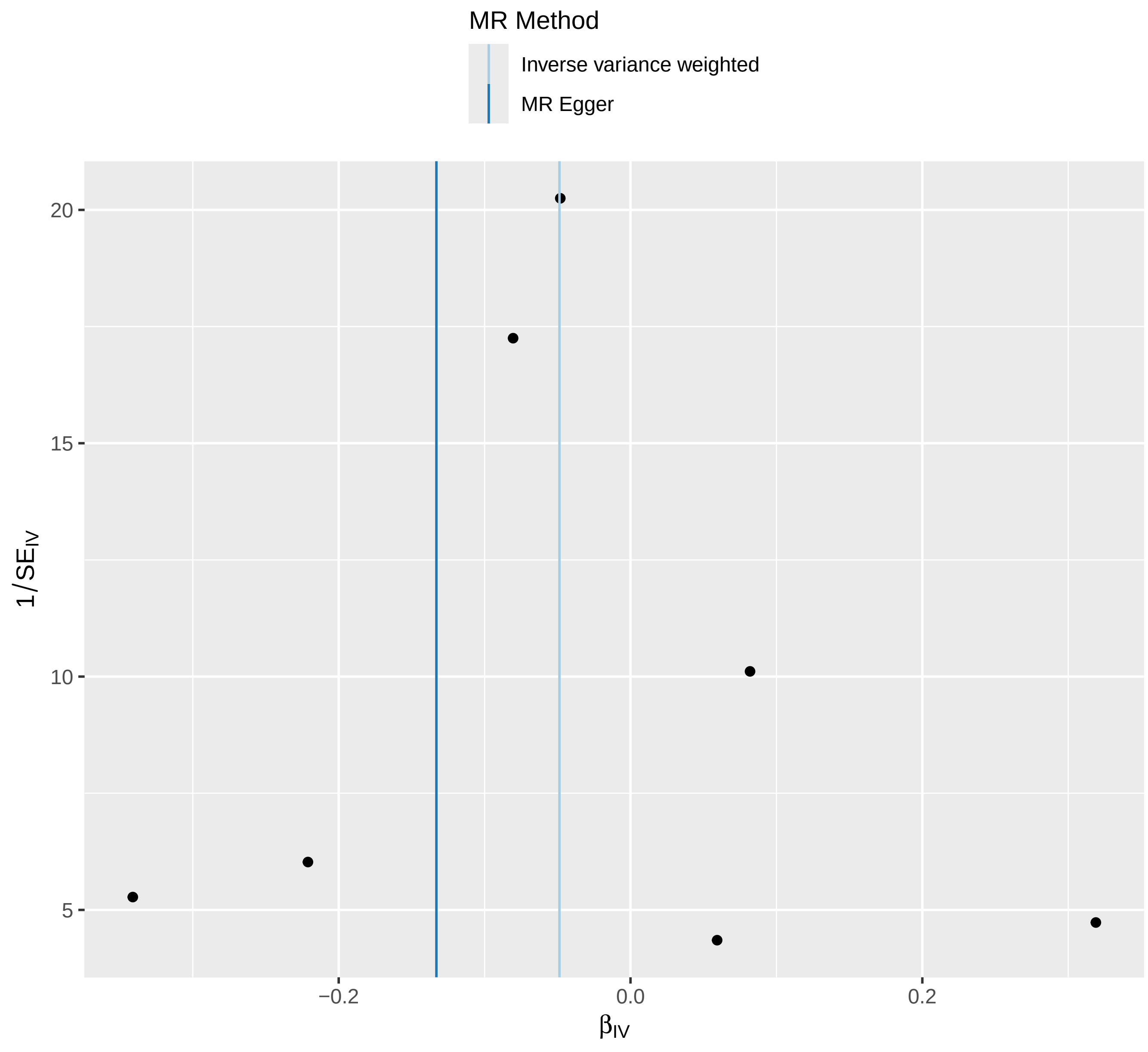

MR Method
Inverse variance weighted
MR Egger
20
15
10
5
1/SE$_{IV}$
−0.2
0.0
0.2
β$_{IV}$

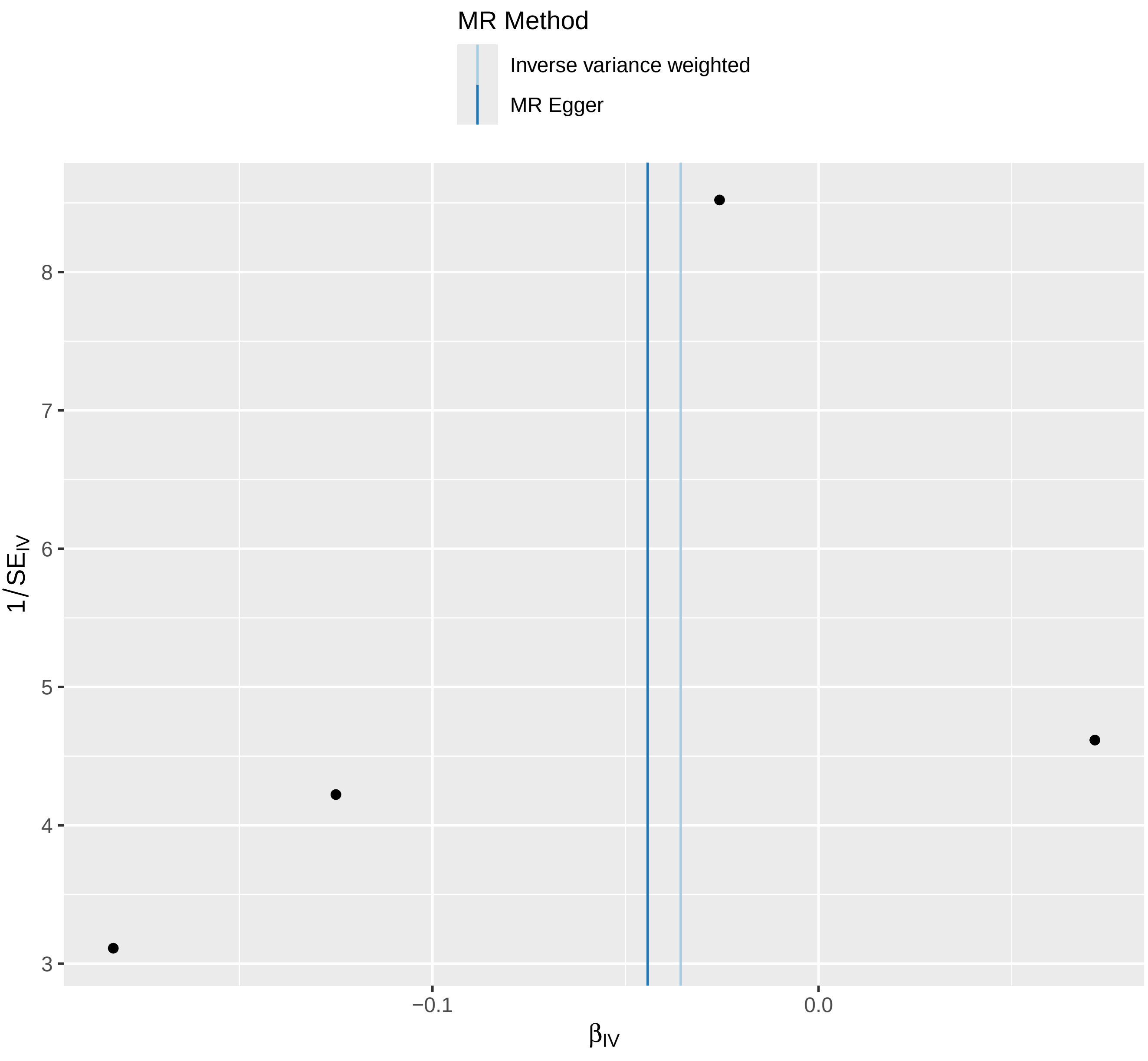
MR Method
Inverse variance weighted
MR Egger
1/SE_IV
8
7
6
5
4
3
−0.1
0.0
β_IV

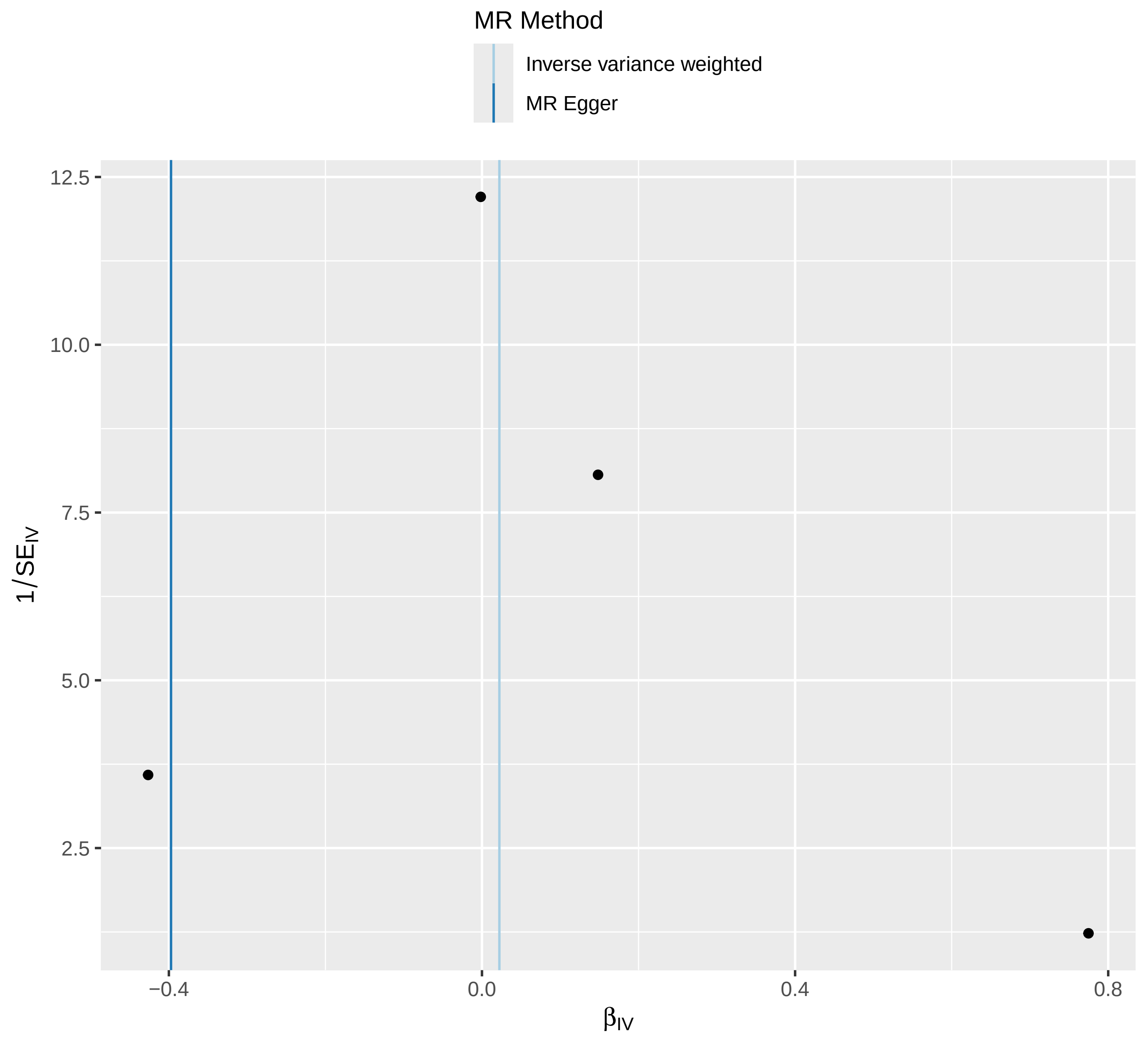

MR Method
Inverse variance weighted
MR Egger
12.5
10.0
7.5
5.0
2.5
1/SE$_{IV}$
−0.4
0.0
0.4
0.8
β$_{IV}$

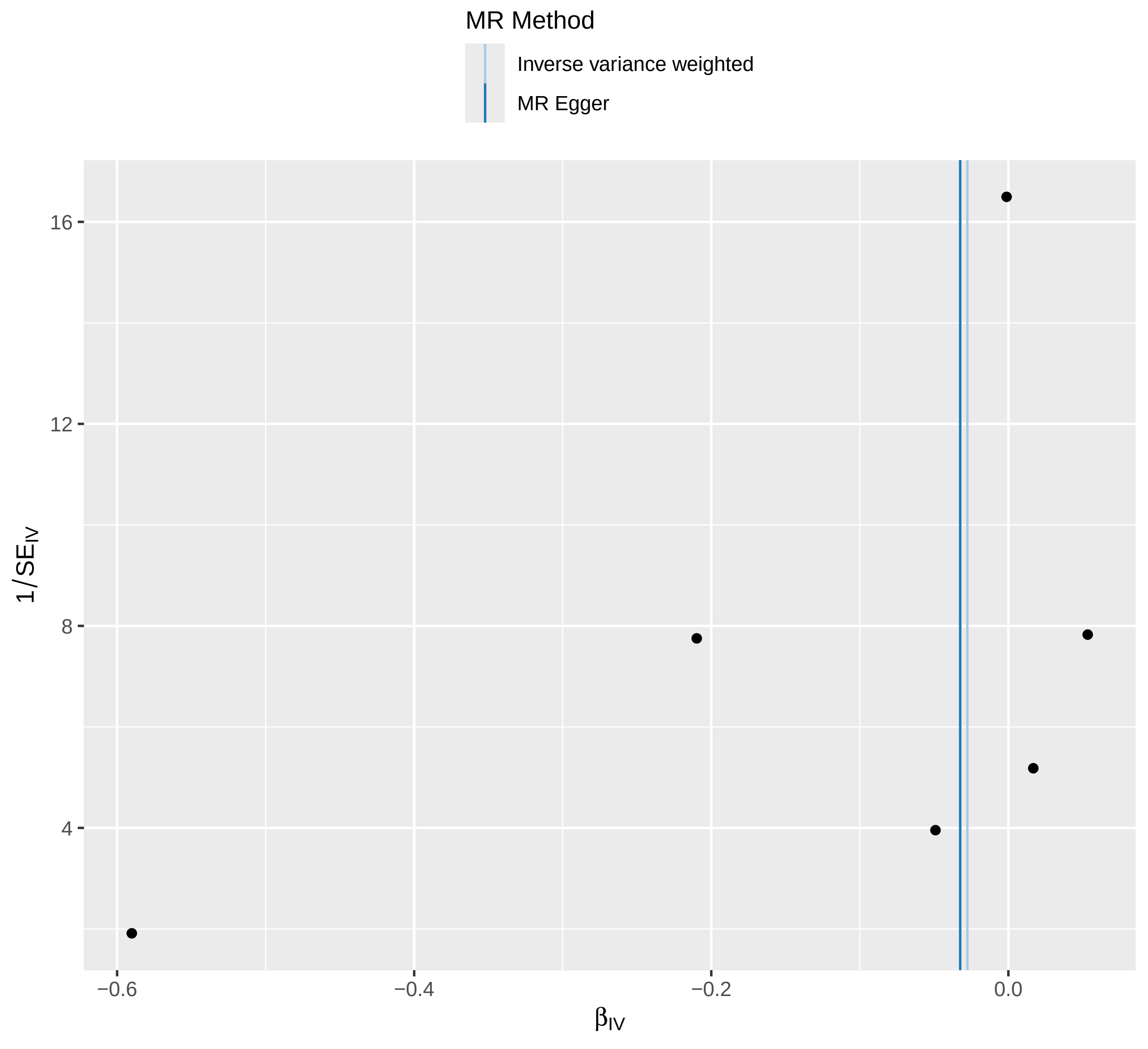

MR Method
Inverse variance weighted
MR Egger
16
12
8
4
1/SE$_{IV}$
−0.6
−0.4
−0.2
0.0
β$_{IV}$

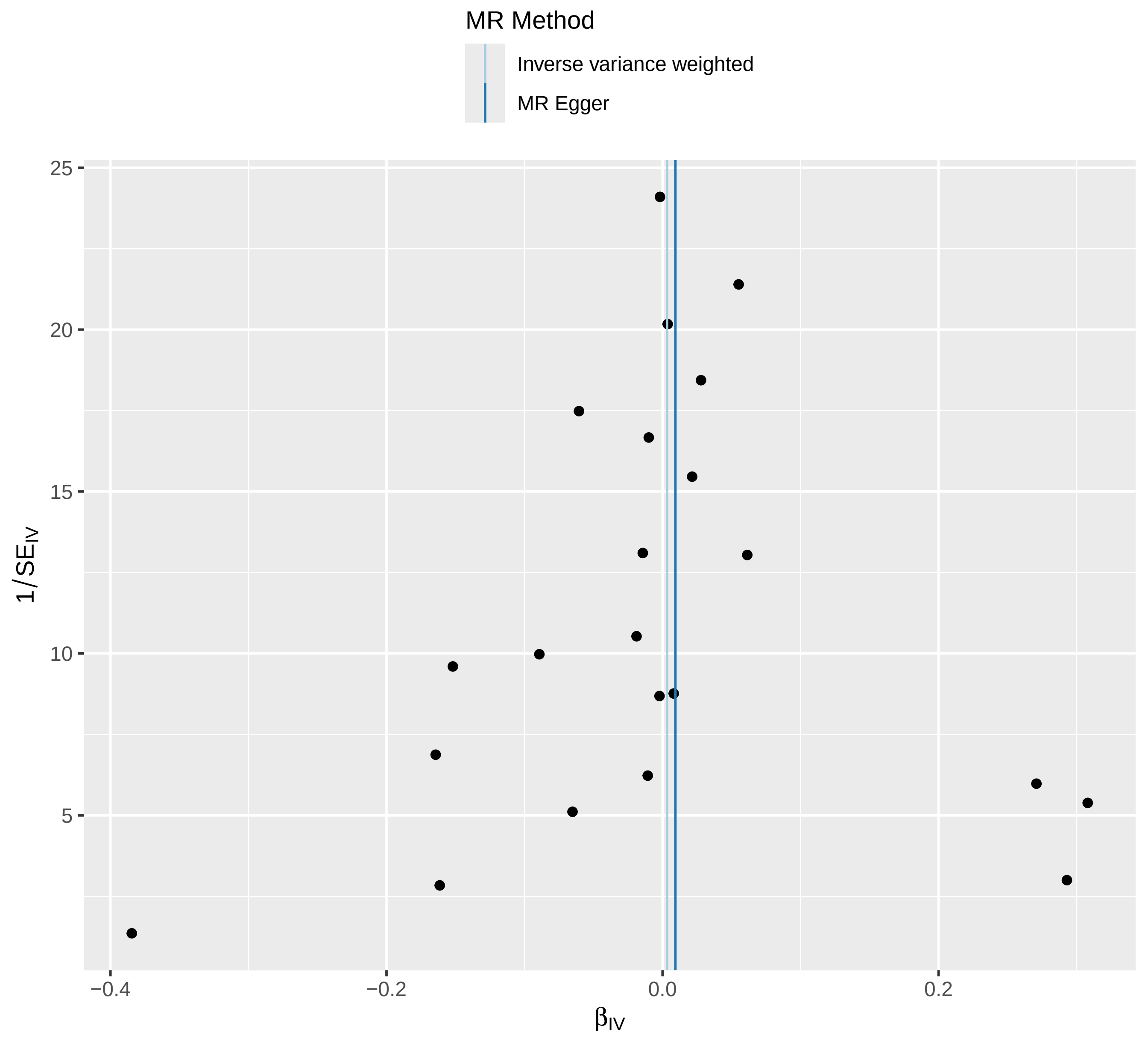

MR Method
Inverse variance weighted
MR Egger
25
20
15
10
5
1/SE_IV
−0.4
−0.2
0.0
0.2
β_IV

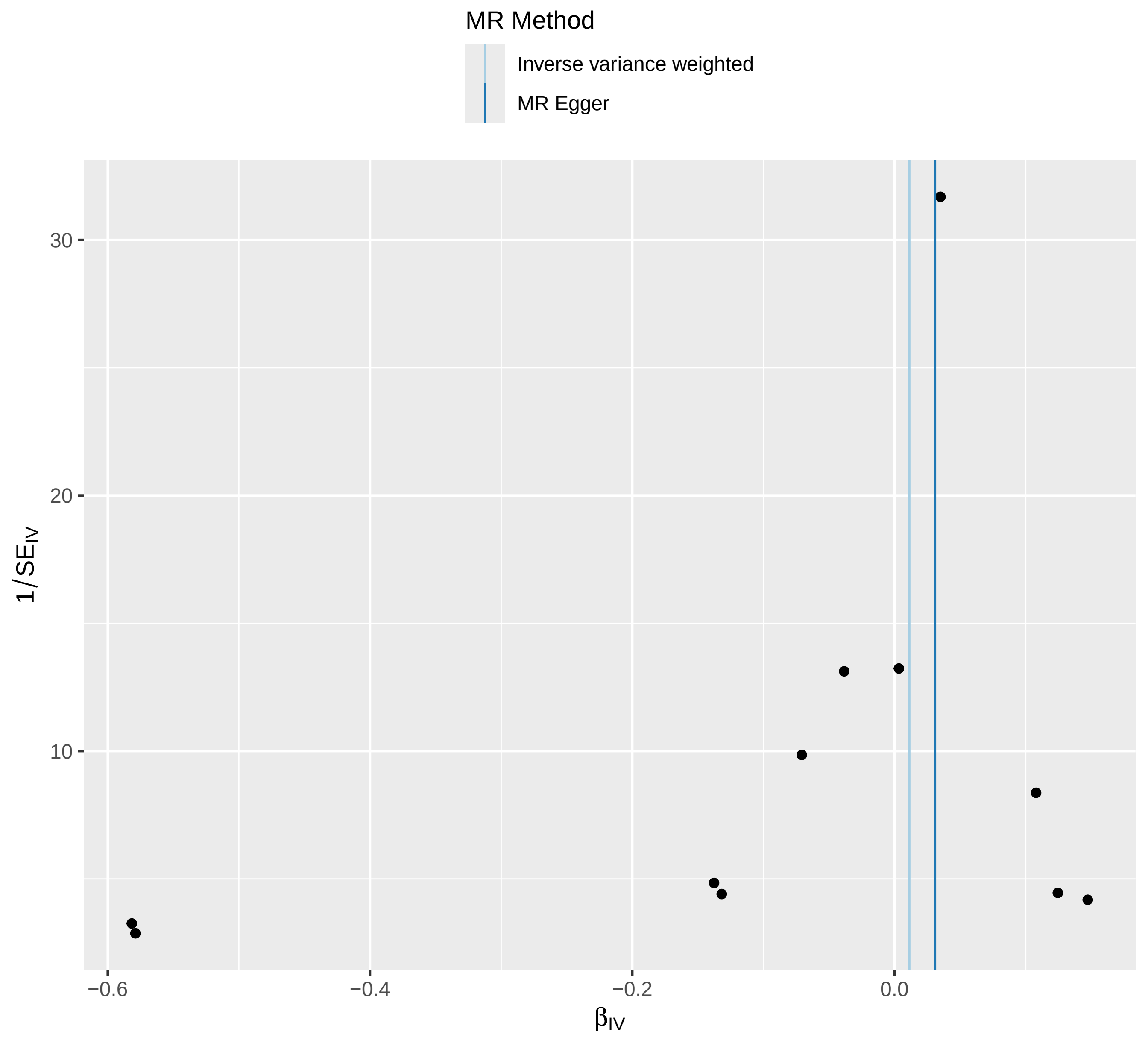

MR Method
Inverse variance weighted
MR Egger
30
20
10
1/SE$_{IV}$
−0.6
−0.4
−0.2
0.0
β$_{IV}$

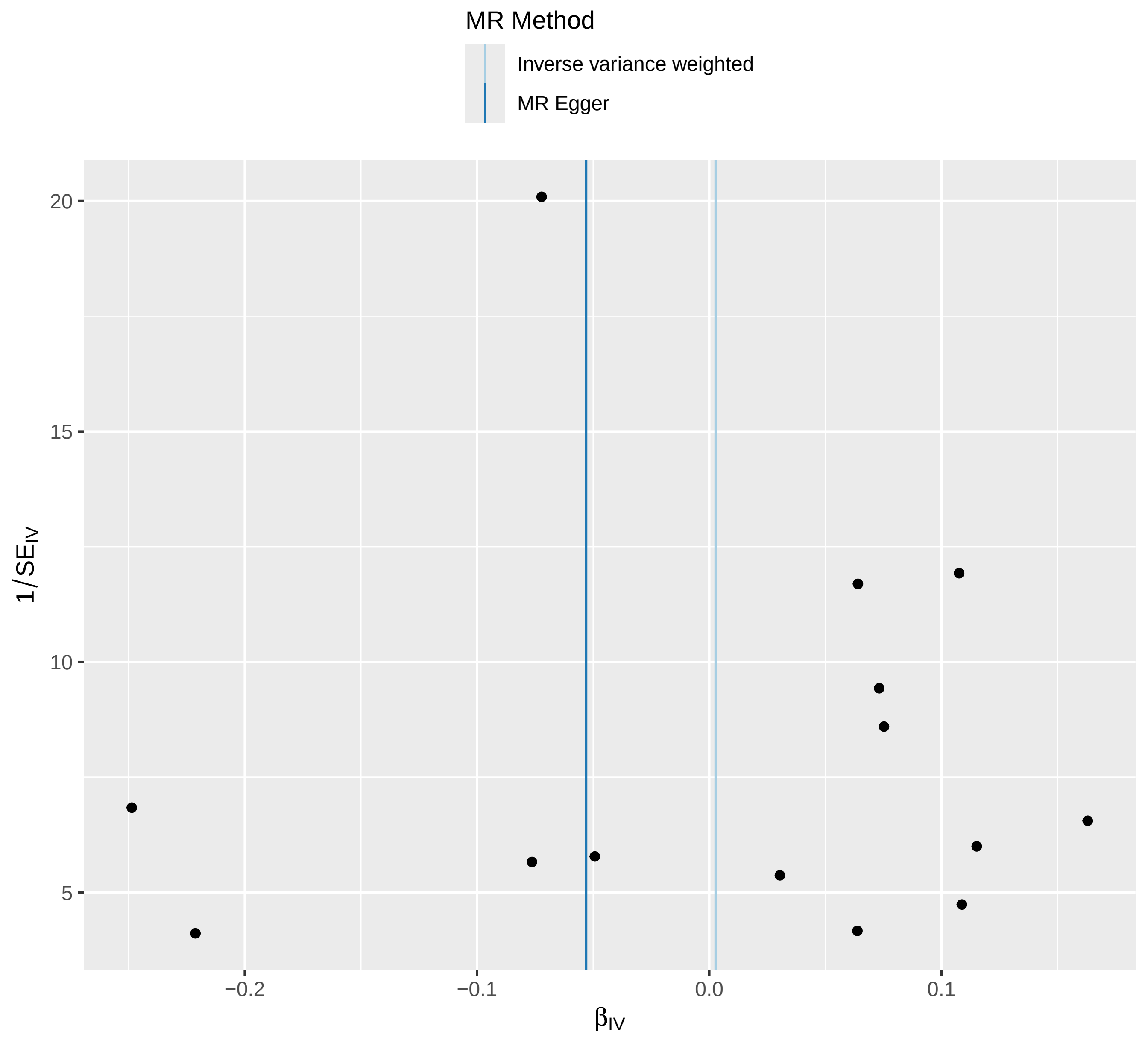

MR Method
Inverse variance weighted
MR Egger
20
15
10
5
1/SE$_{IV}$
−0.2
−0.1
0.0
0.1
β$_{IV}$

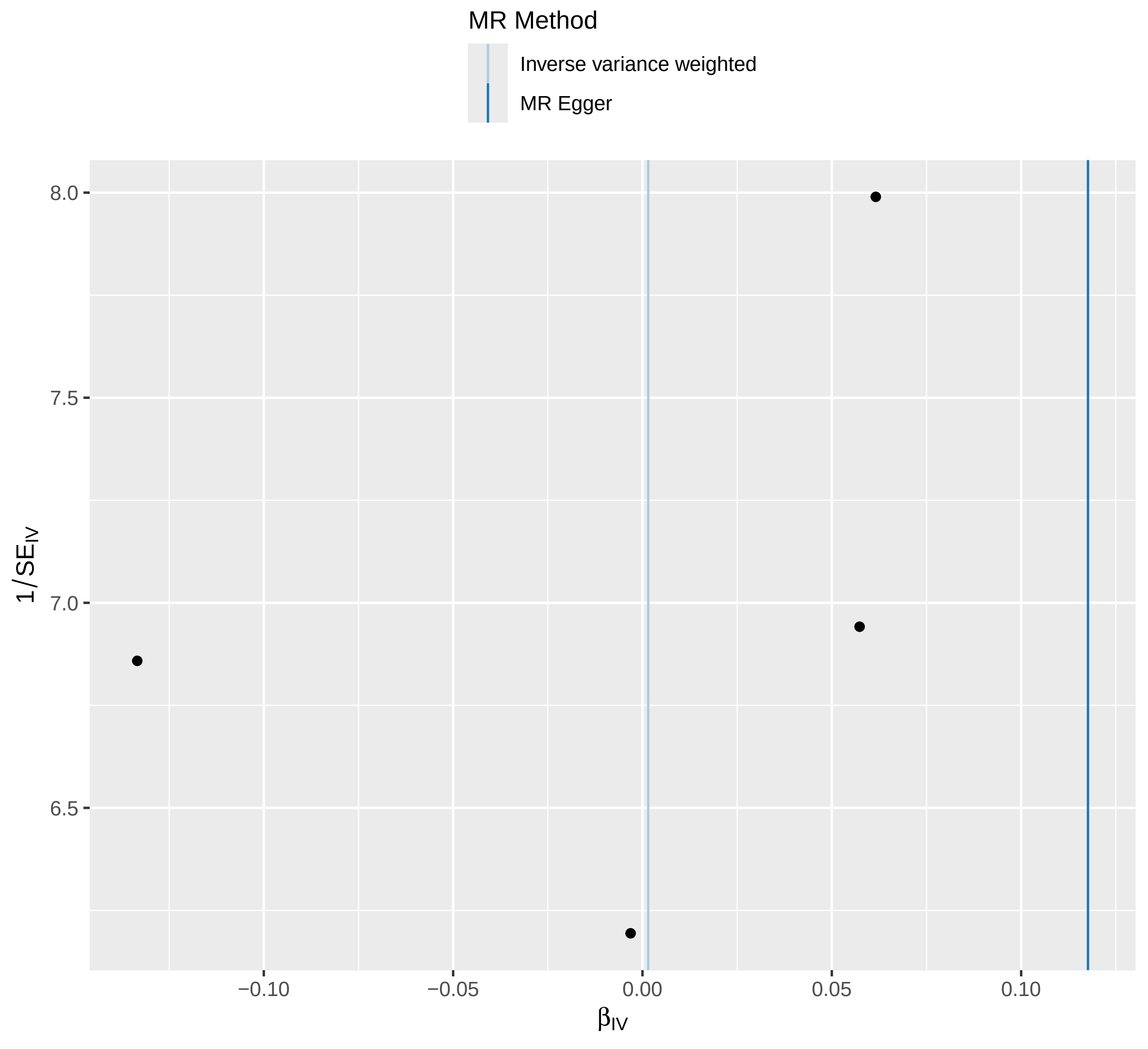
MR Method
Inverse variance weighted
MR Egger
8.0
7.5
7.0
6.5
1/SE_IV
−0.10
−0.05
0.00
0.05
0.10
β_IV

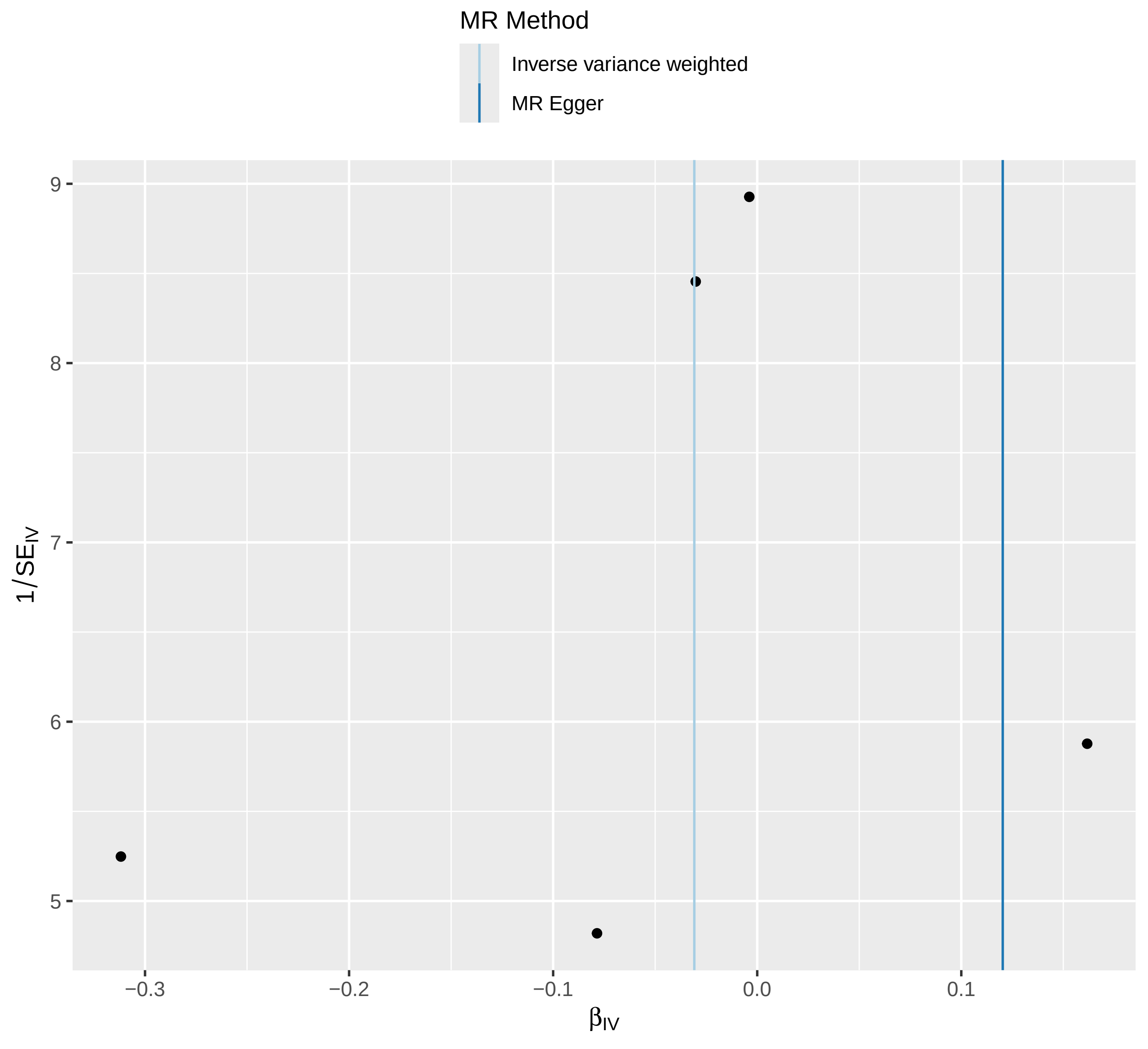

MR Method
Inverse variance weighted
MR Egger
9
8
7
6
5
1/SE_IV
−0.3
−0.2
−0.1
0.0
0.1
β_IV

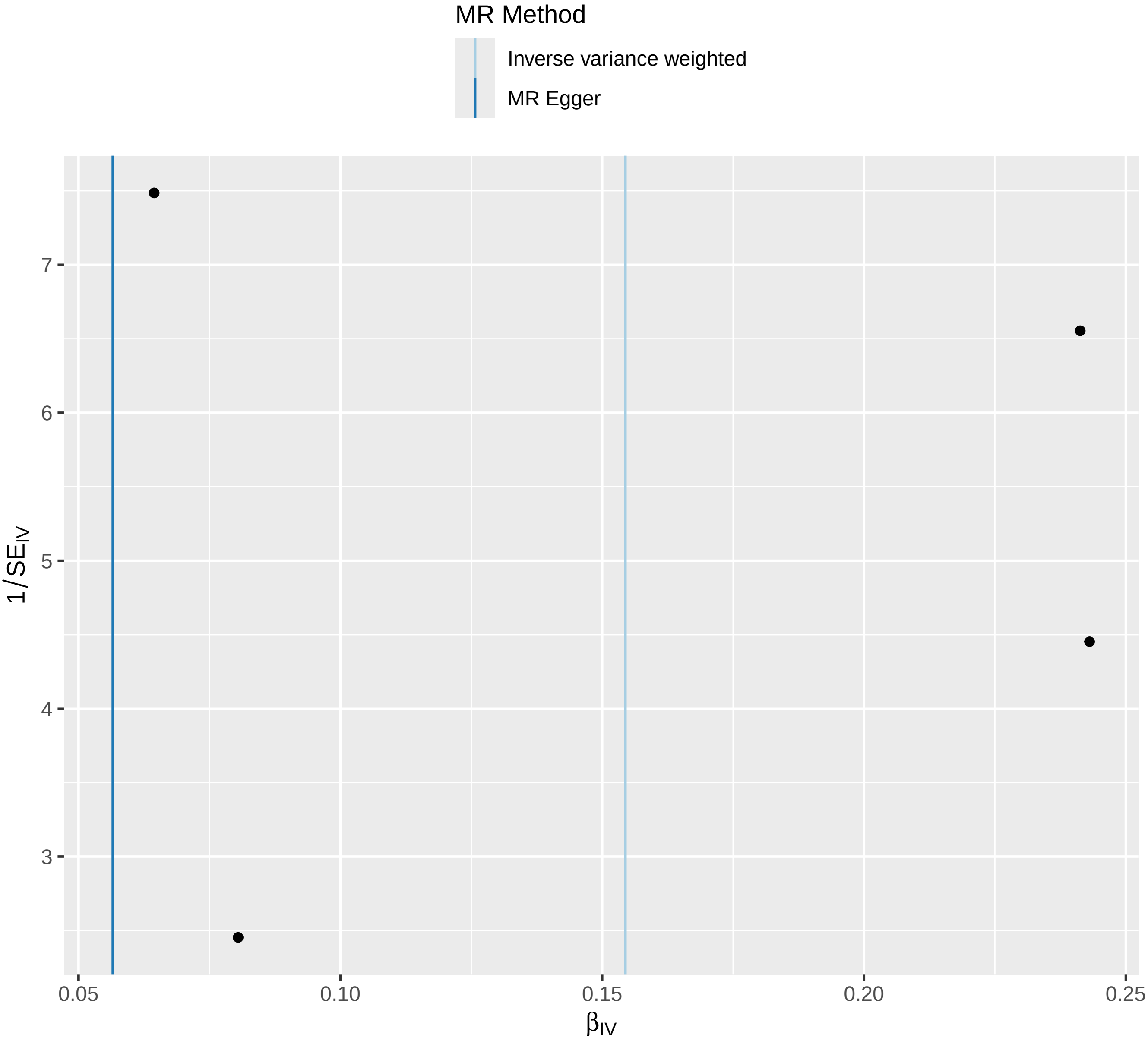

MR Method
Inverse variance weighted
MR Egger
7
6
5
4
3
$1/SE_{IV}$
0.05
0.10
0.15
0.20
0.25
$\beta_{IV}$

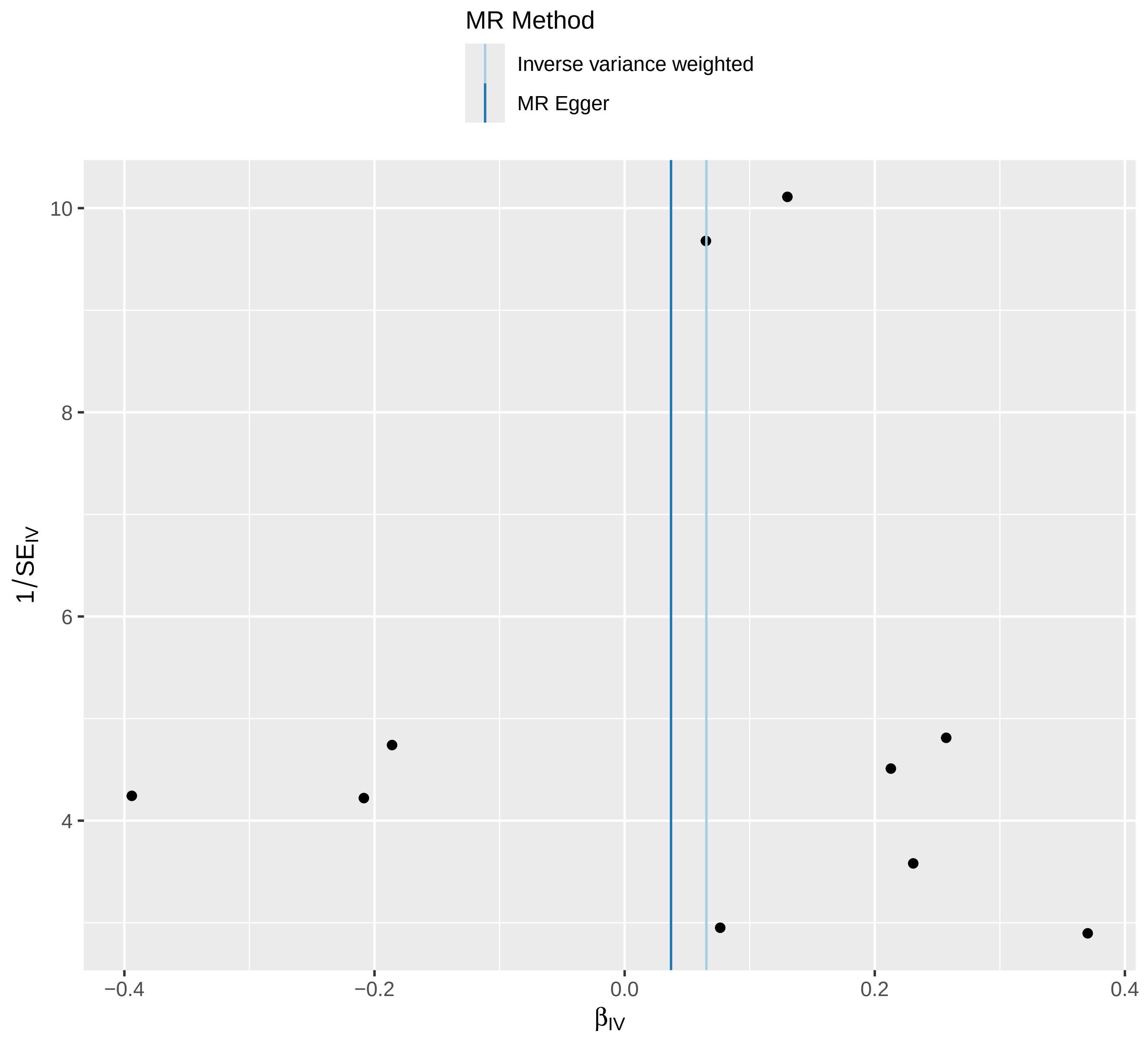

MR Method
Inverse variance weighted
MR Egger
10
8
6
4
1/SE_IV
−0.4
−0.2
0.0
0.2
0.4
β_IV

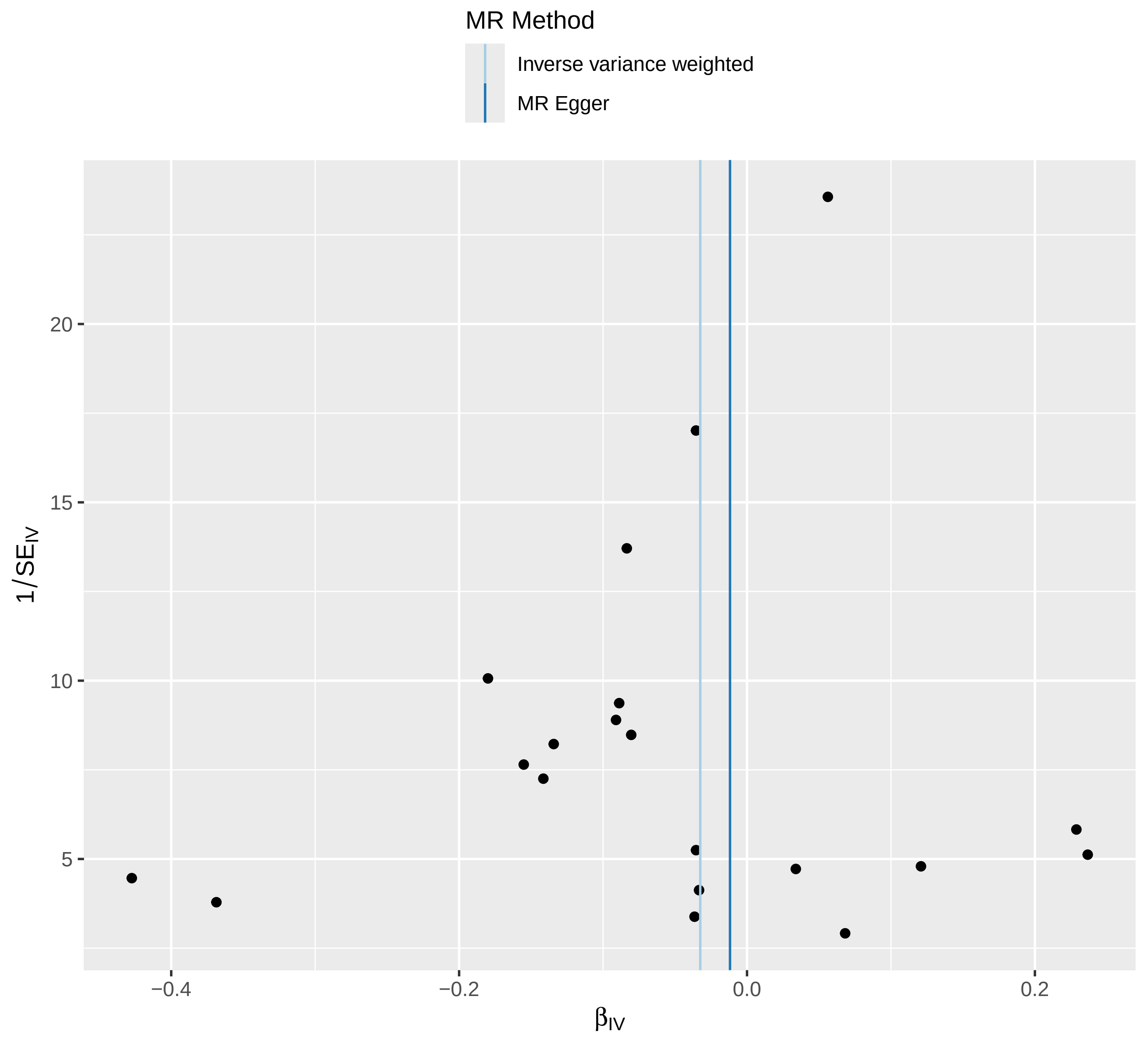
MR Method
Inverse variance weighted
MR Egger
20
15
10
5
1/SE$_{IV}$
−0.4
−0.2
0.0
0.2
β$_{IV}$

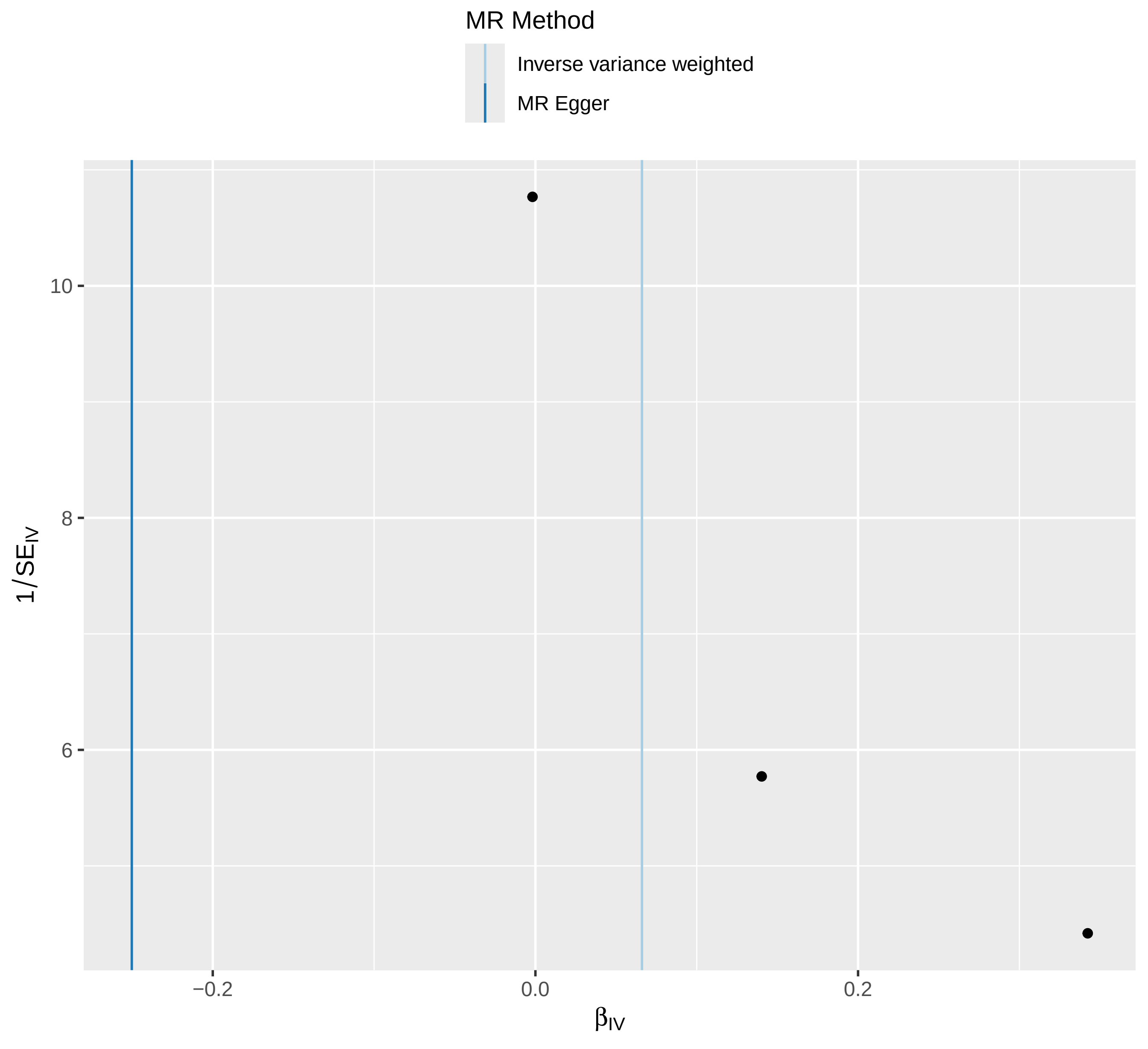
MR Method
Inverse variance weighted
MR Egger
10
8
6
1/SE_IV
−0.2
0.0
0.2
β_IV

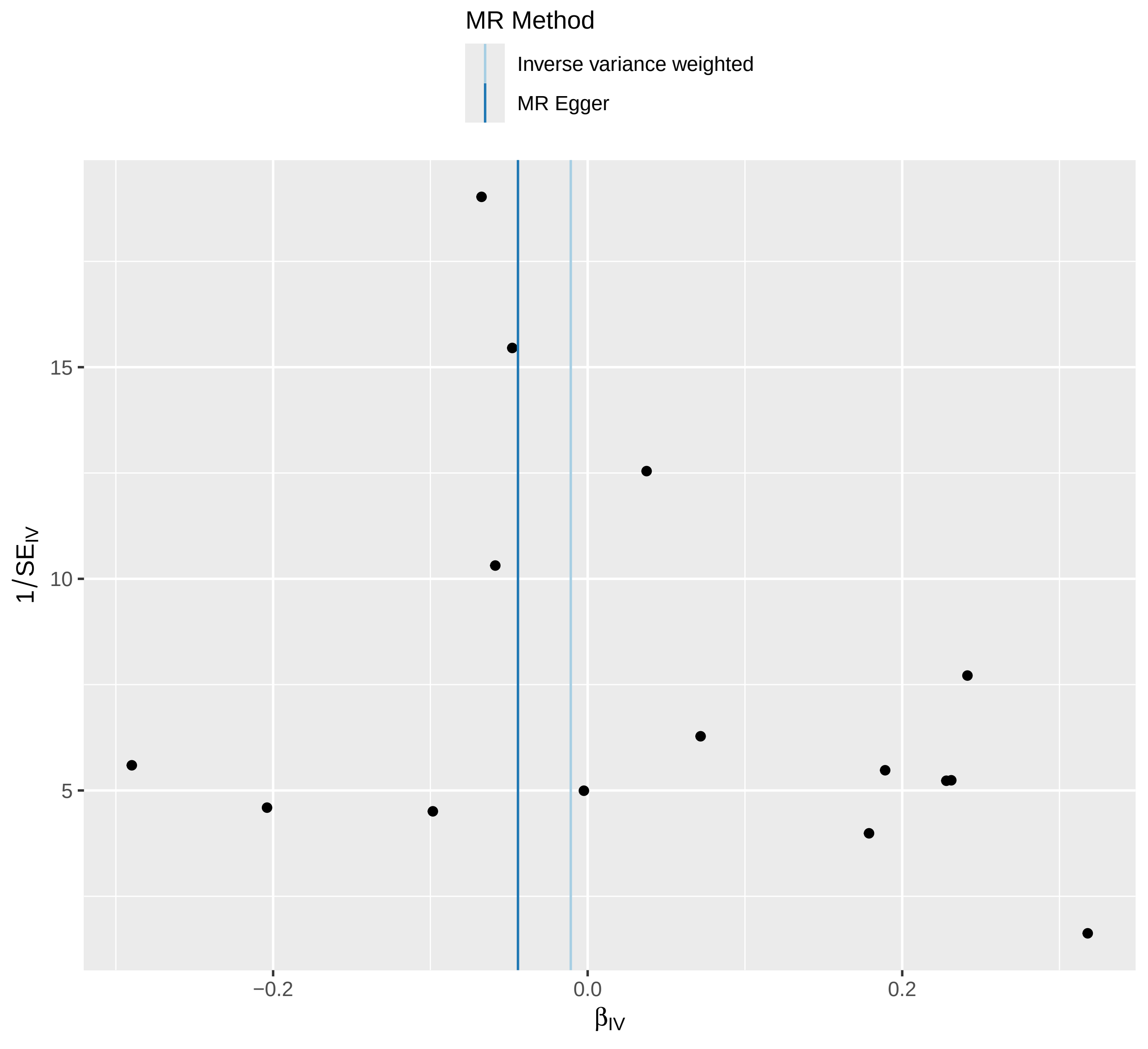
MR Method
Inverse variance weighted
MR Egger
15
10
5
1/SE$_{IV}$
−0.2
0.0
0.2
β$_{IV}$

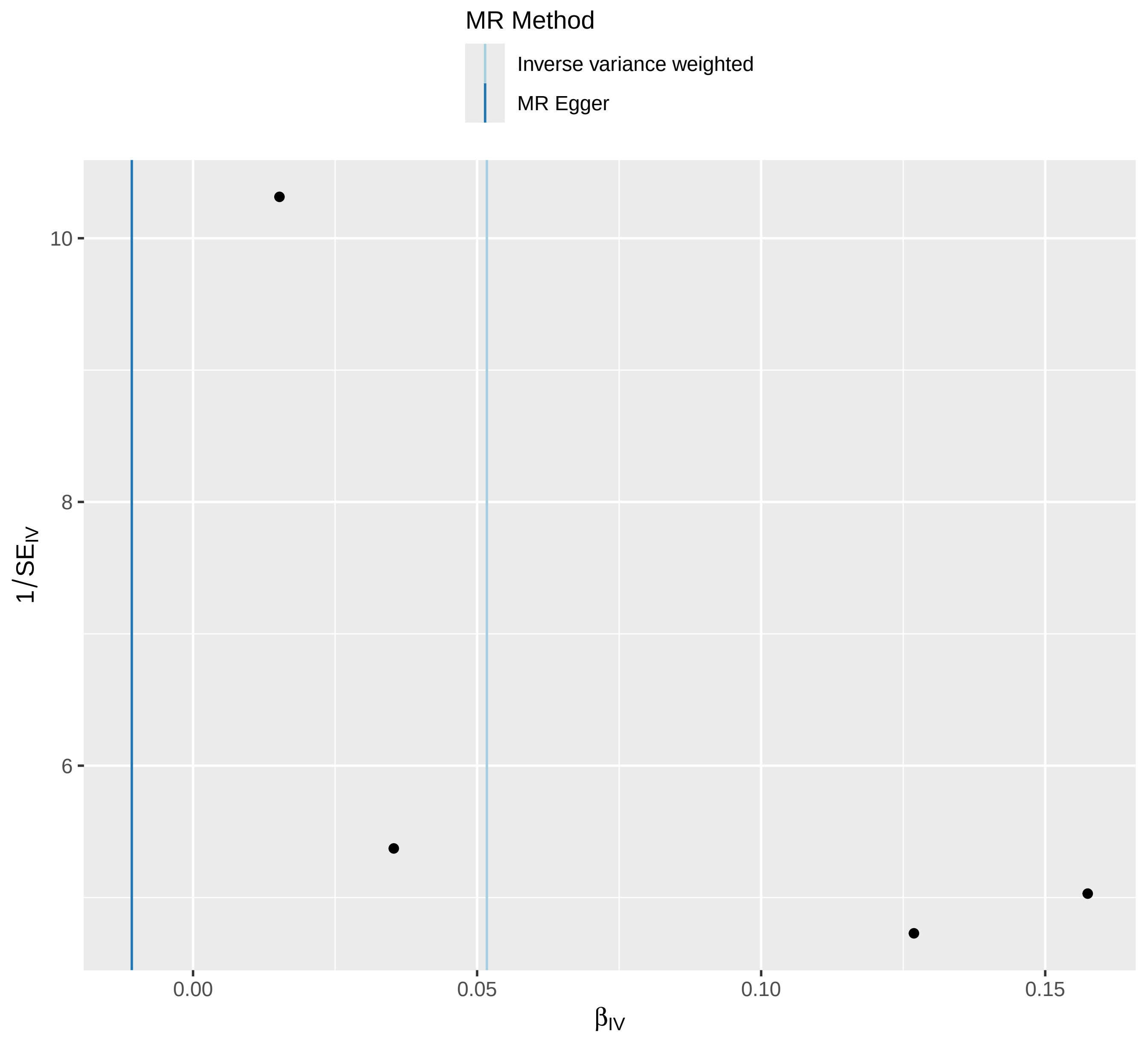
MR Method
Inverse variance weighted
MR Egger
10
8
6
1/SE_IV
0.00
0.05
0.10
0.15
β_IV

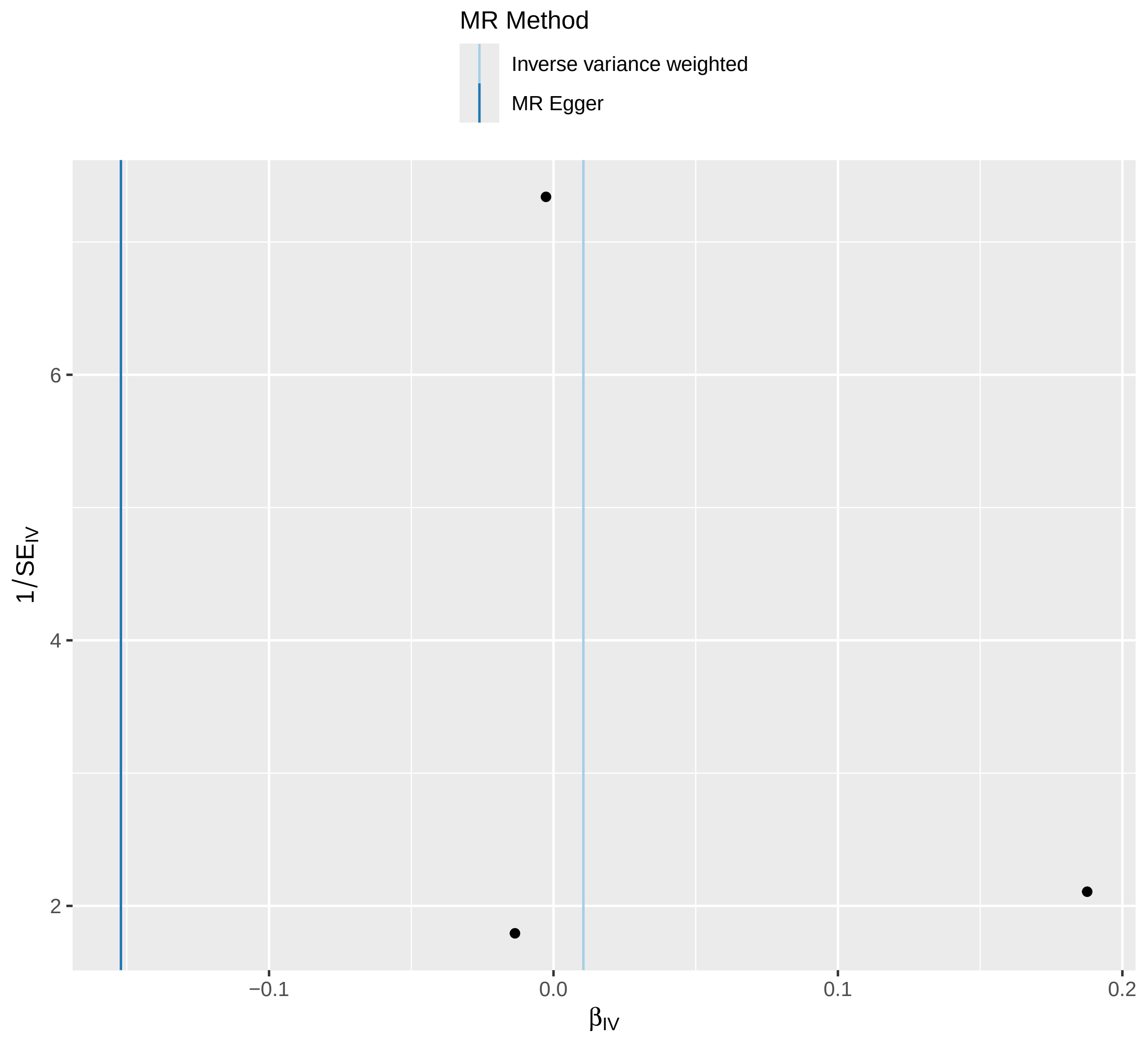
MR Method
Inverse variance weighted
MR Egger
6
4
2
1/SE_IV
−0.1
0.0
0.1
0.2
β_IV

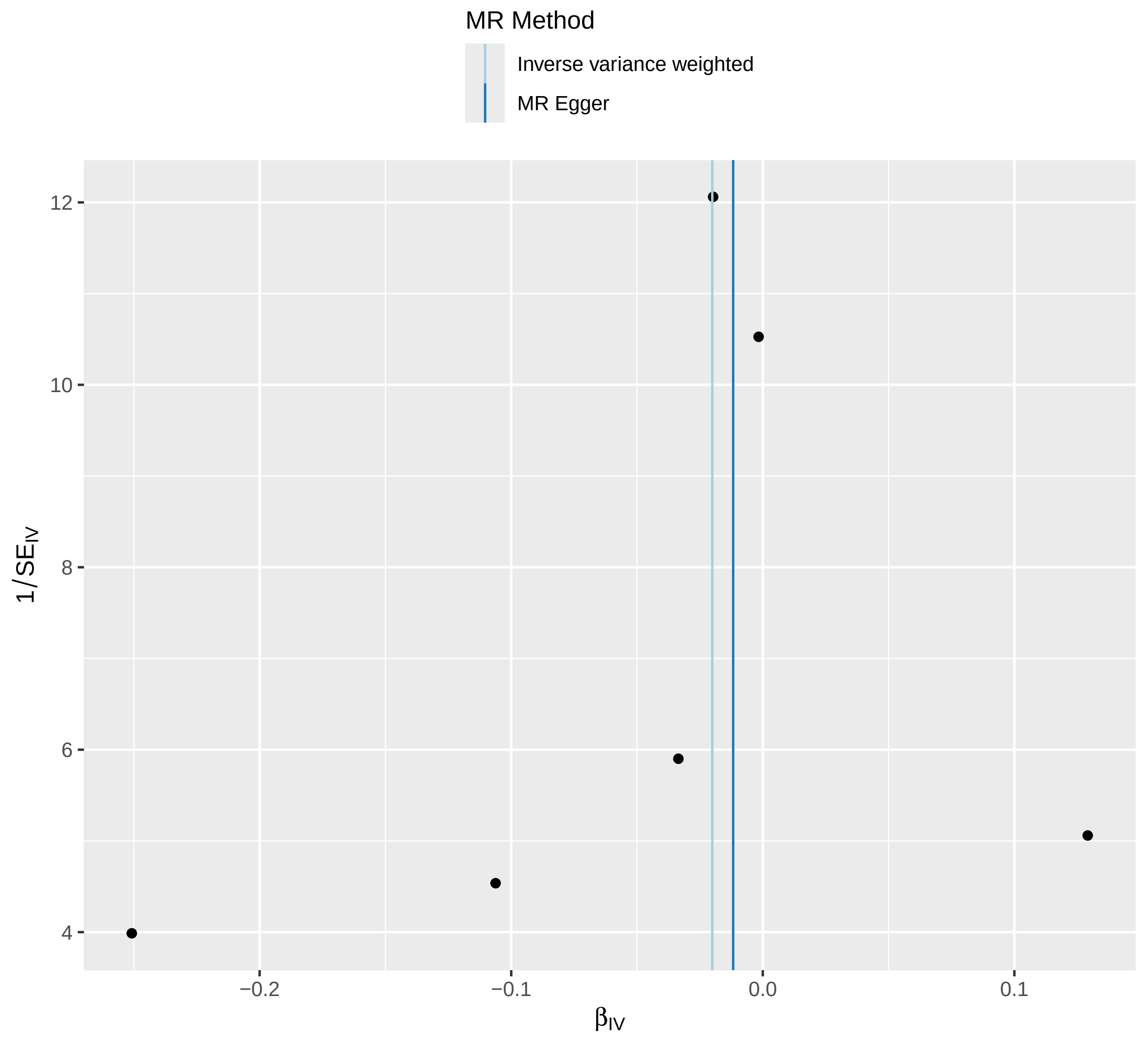
MR Method
Inverse variance weighted
MR Egger
12
10
8
6
4
1/SE$_{IV}$
−0.2
−0.1
0.0
0.1
β$_{IV}$

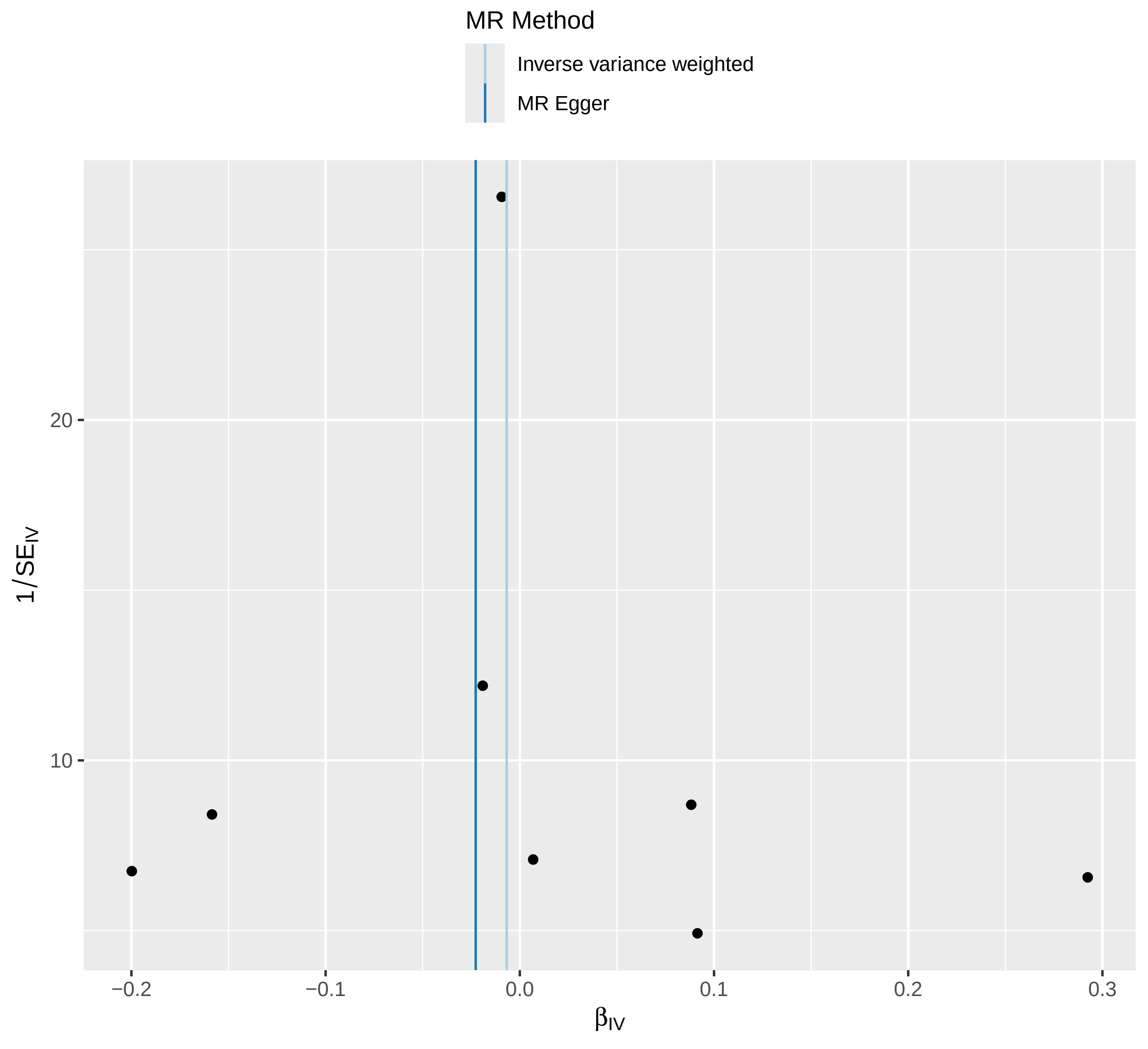
MR Method
Inverse variance weighted
MR Egger
20
10
1/SE_IV
−0.2
−0.1
0.0
0.1
0.2
0.3
β_IV

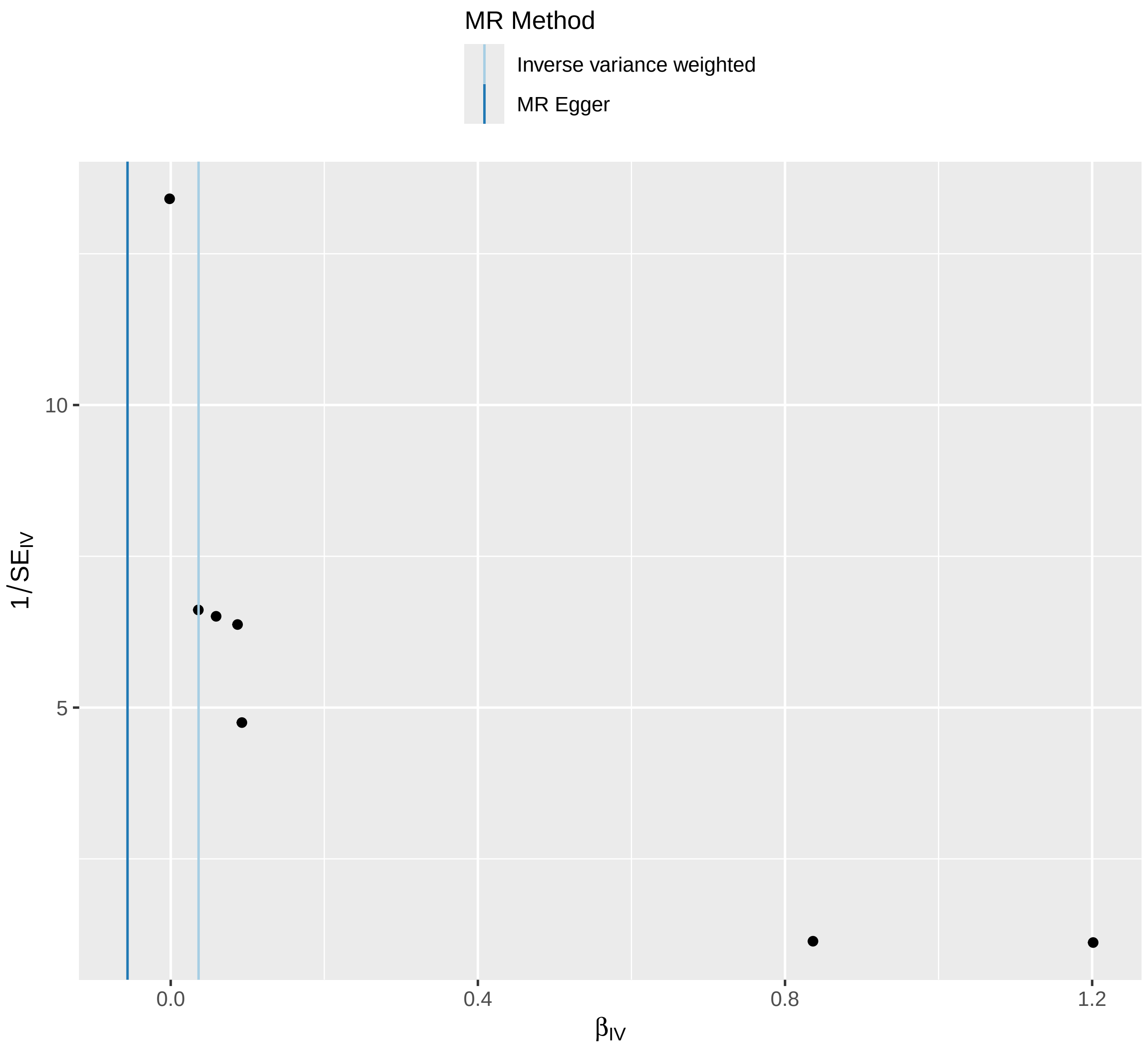
MR Method
Inverse variance weighted
MR Egger
10
5
0.0
0.4
0.8
1.2
$1/SE_{IV}$
$\beta_{IV}$

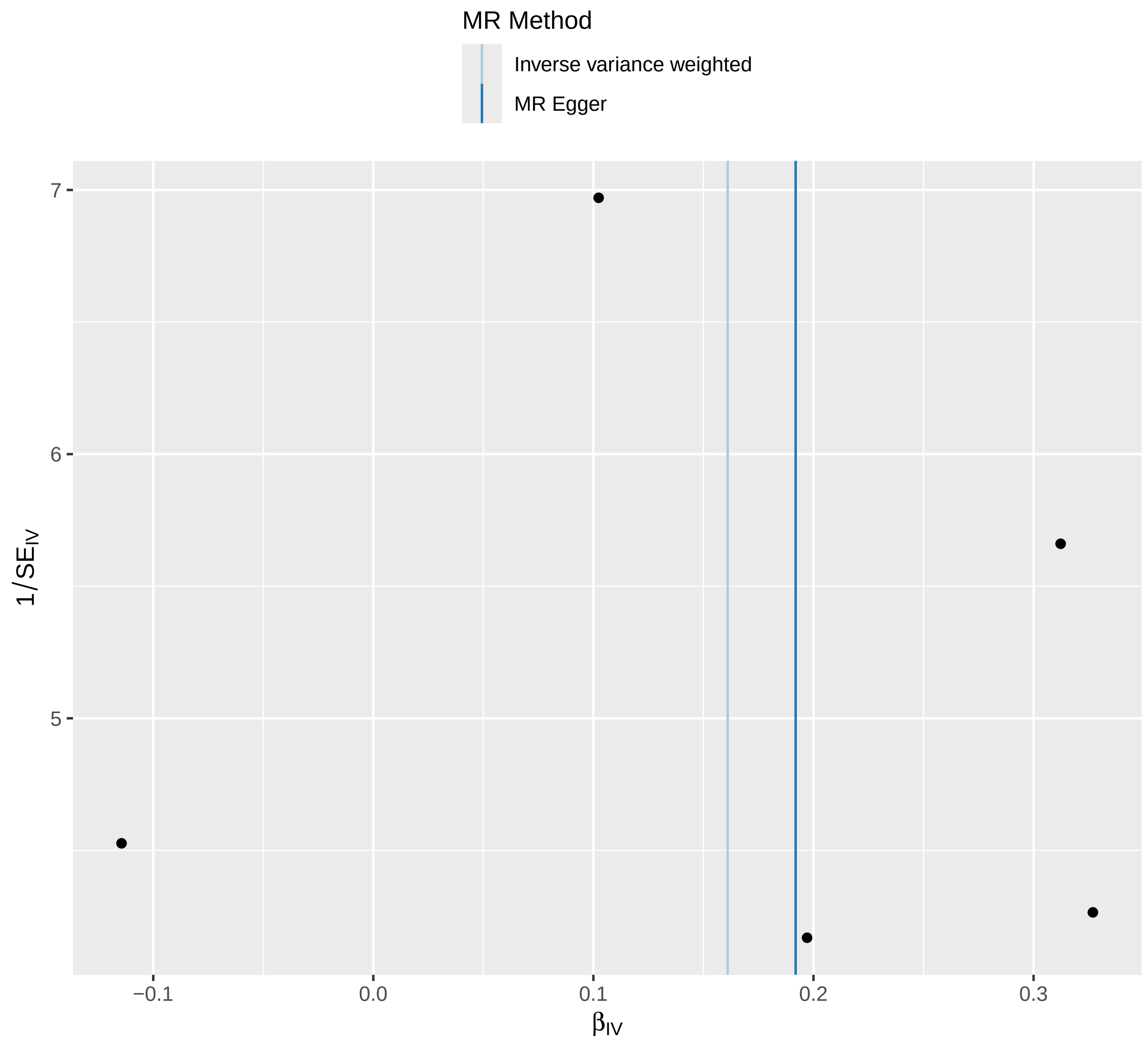
MR Method
Inverse variance weighted
MR Egger
7
6
5
1/SE_IV
−0.1
0.0
0.1
0.2
0.3
β_IV

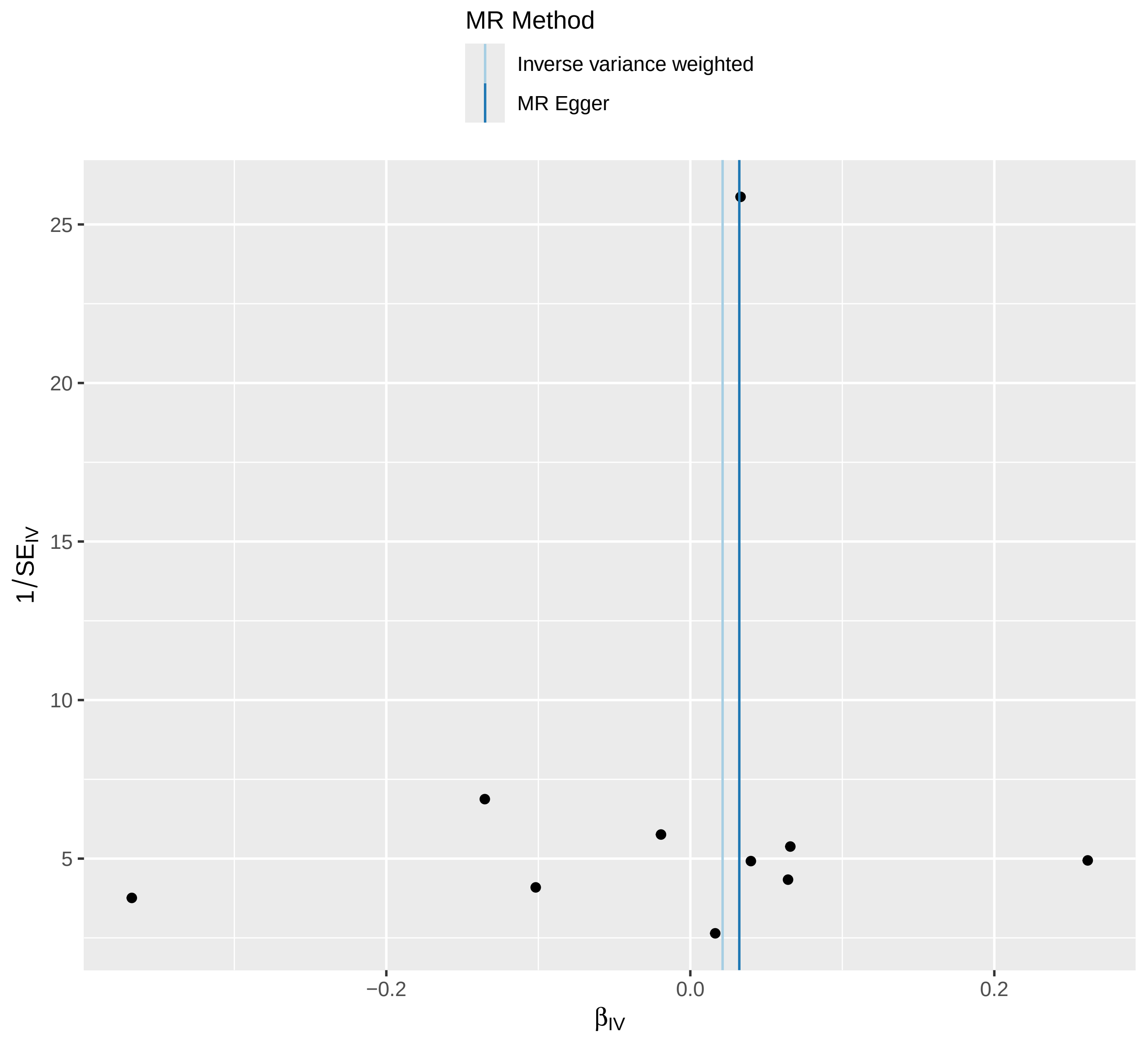

MR Method
Inverse variance weighted
MR Egger
25
20
15
10
5
1/SE_IV
−0.2
0.0
0.2
β_IV

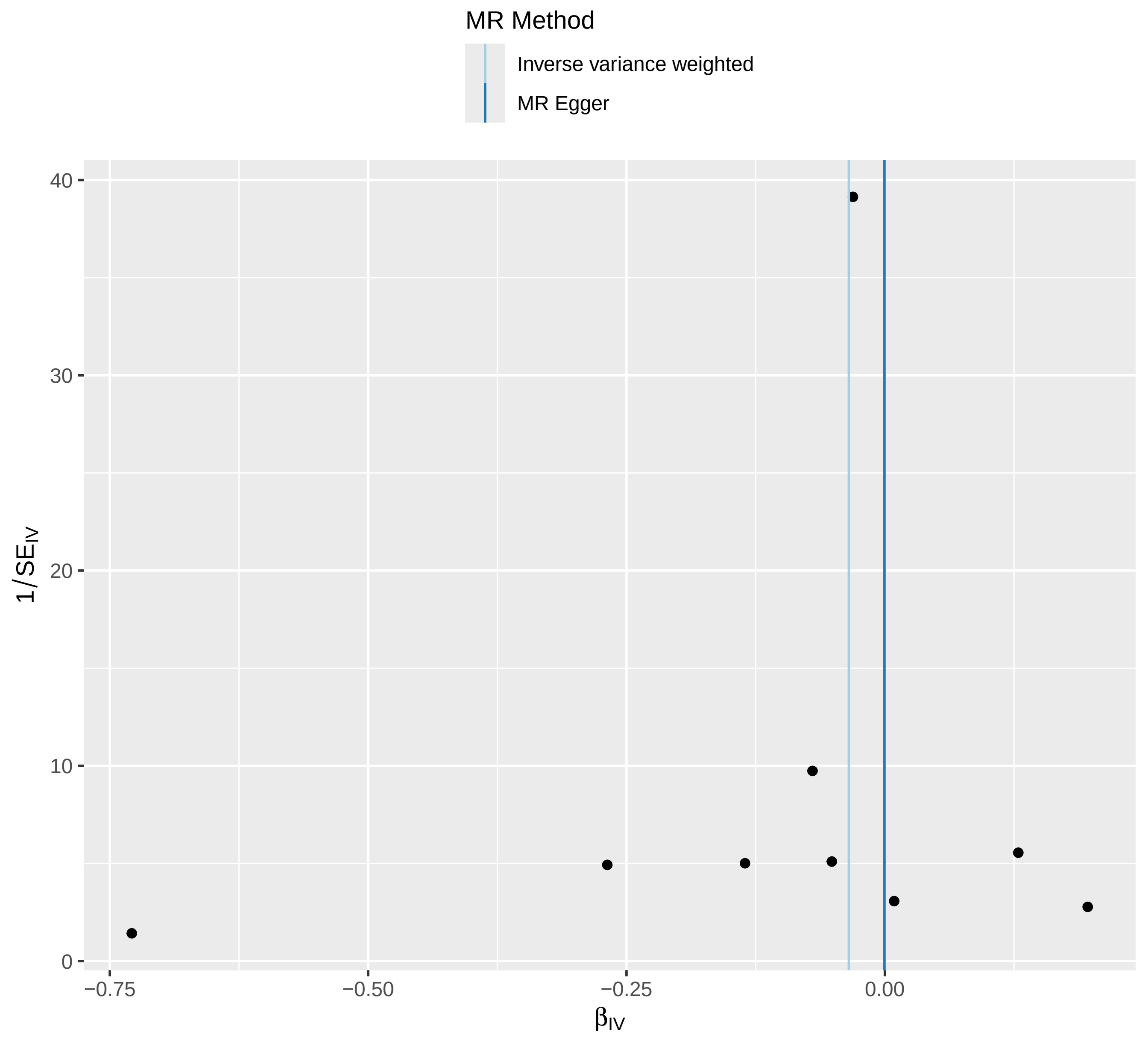

MR Method
Inverse variance weighted
MR Egger
40
30
20
10
0
$1/SE_{IV}$
−0.75
−0.50
−0.25
0.00
$\beta_{IV}$

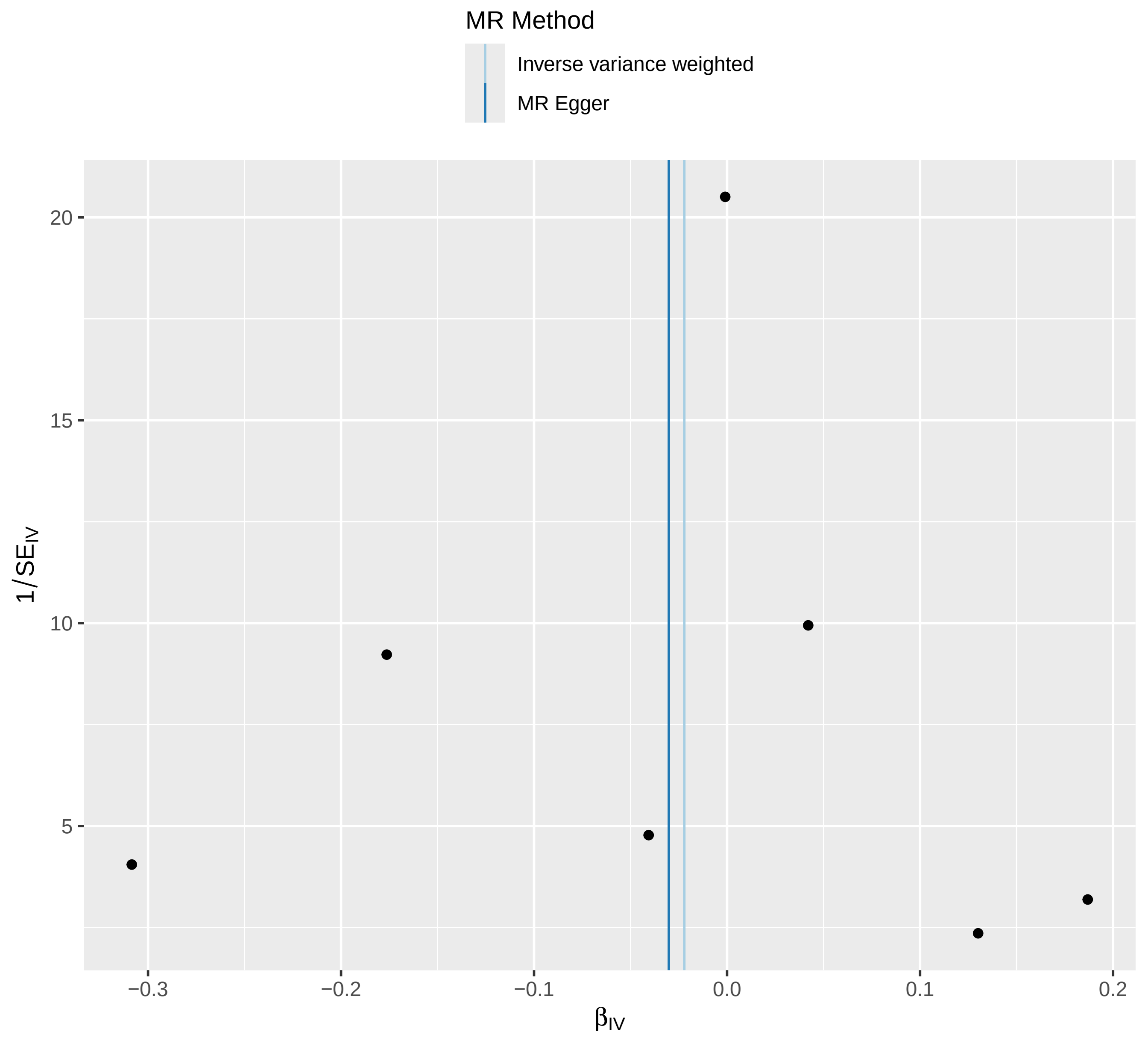
MR Method
Inverse variance weighted
MR Egger
20
15
10
5
$1/SE_{IV}$
−0.3
−0.2
−0.1
0.0
0.1
0.2
$\beta_{IV}$

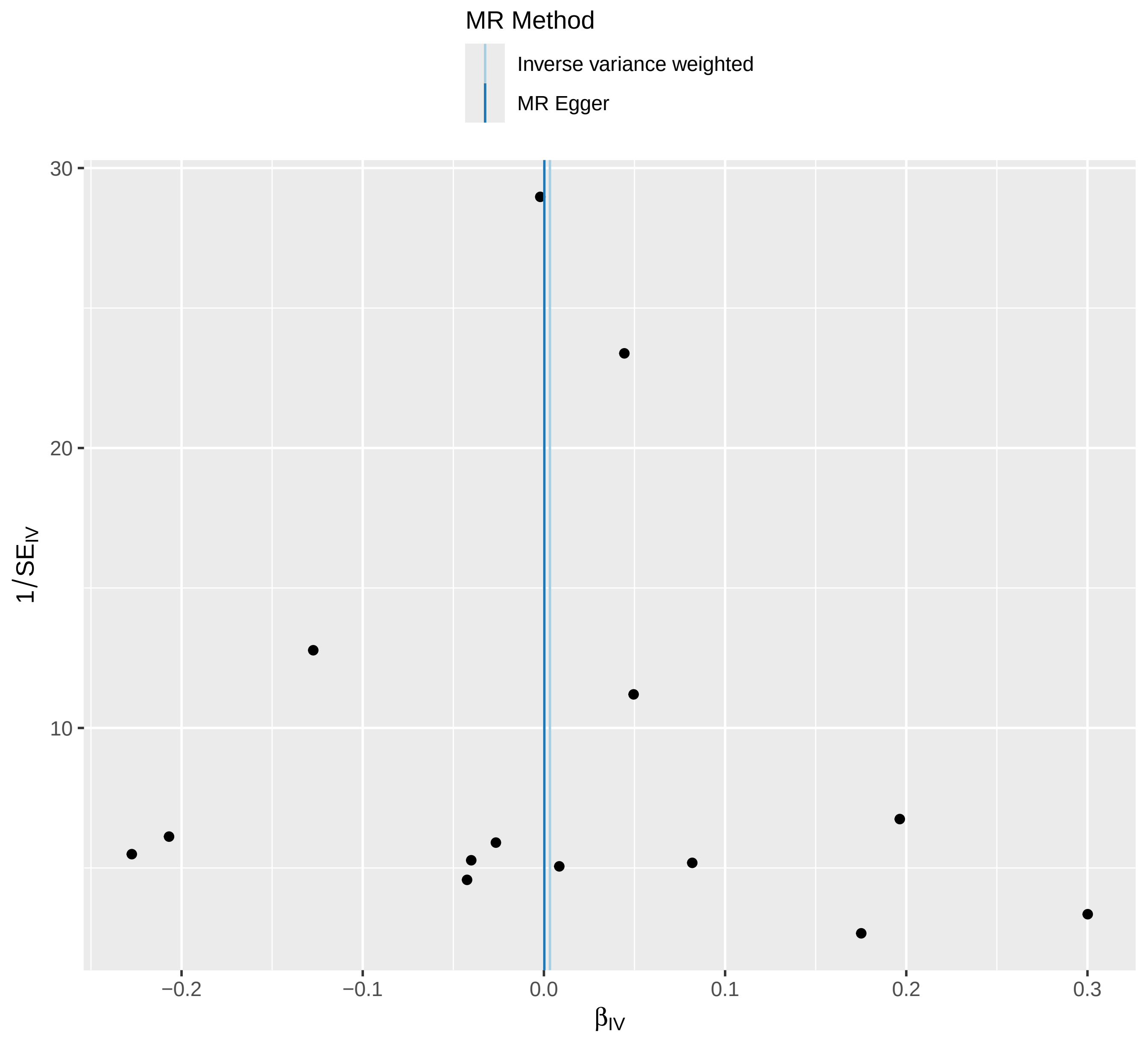

MR Method
Inverse variance weighted
MR Egger
30
20
10
1/SE_IV
−0.2
−0.1
0.0
0.1
0.2
0.3
β_IV

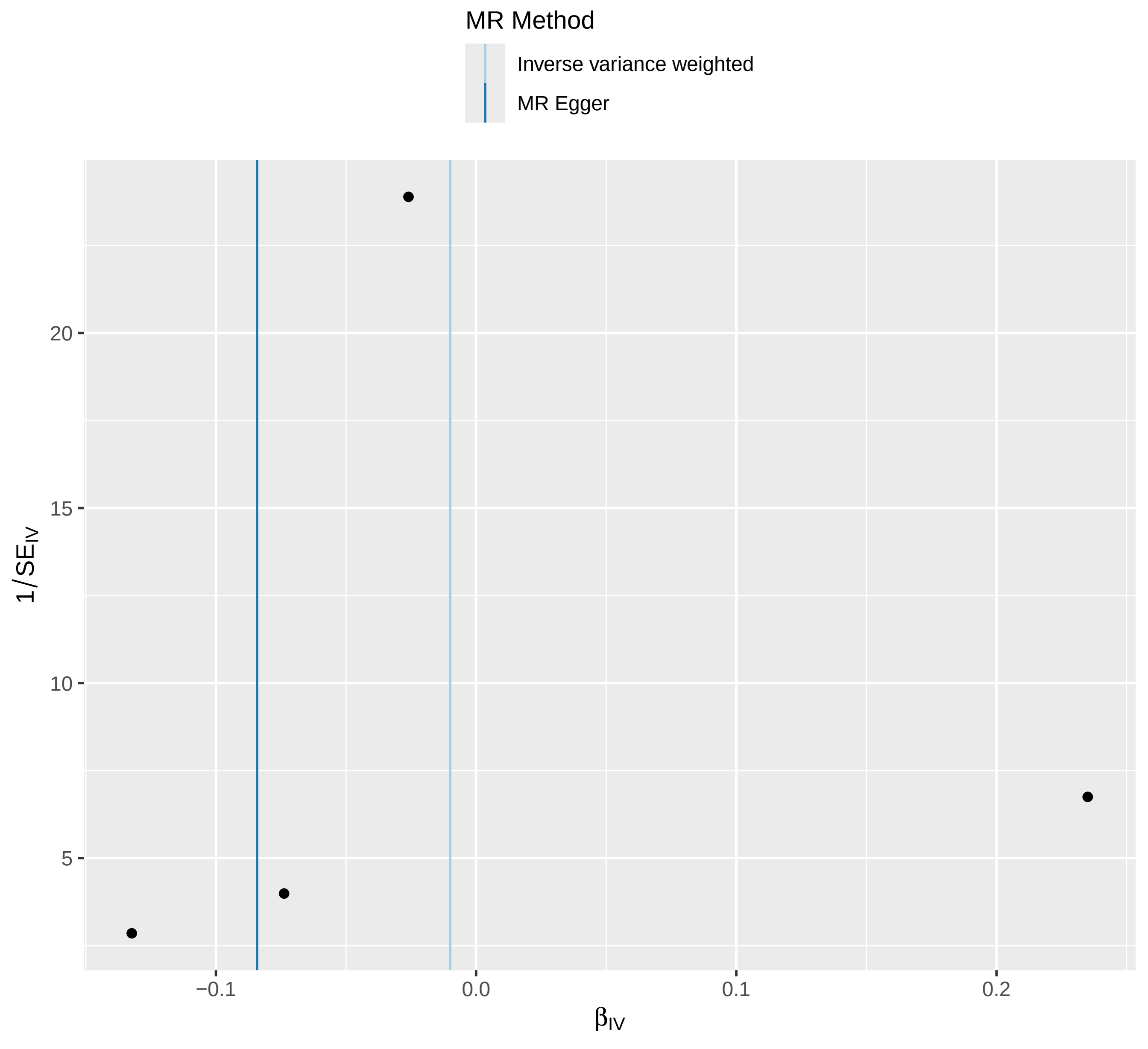

MR Method
Inverse variance weighted
MR Egger
20
15
10
5
1/SE$_{IV}$
−0.1
0.0
0.1
0.2
β$_{IV}$

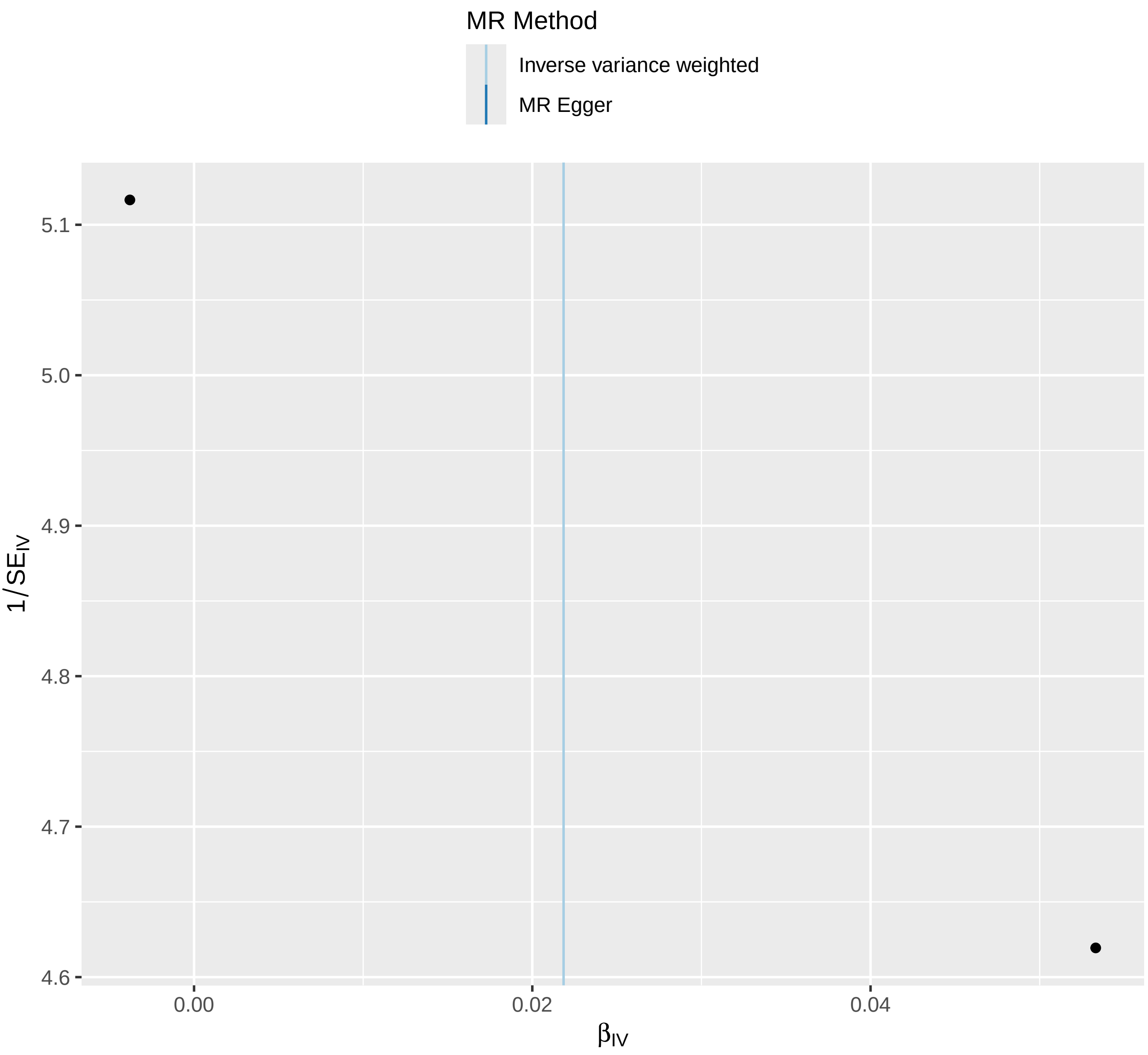

MR Method
Inverse variance weighted
MR Egger
5.1
5.0
4.9
4.8
4.7
4.6
$1/SE_{IV}$
0.00
0.02
0.04
$\beta_{IV}$

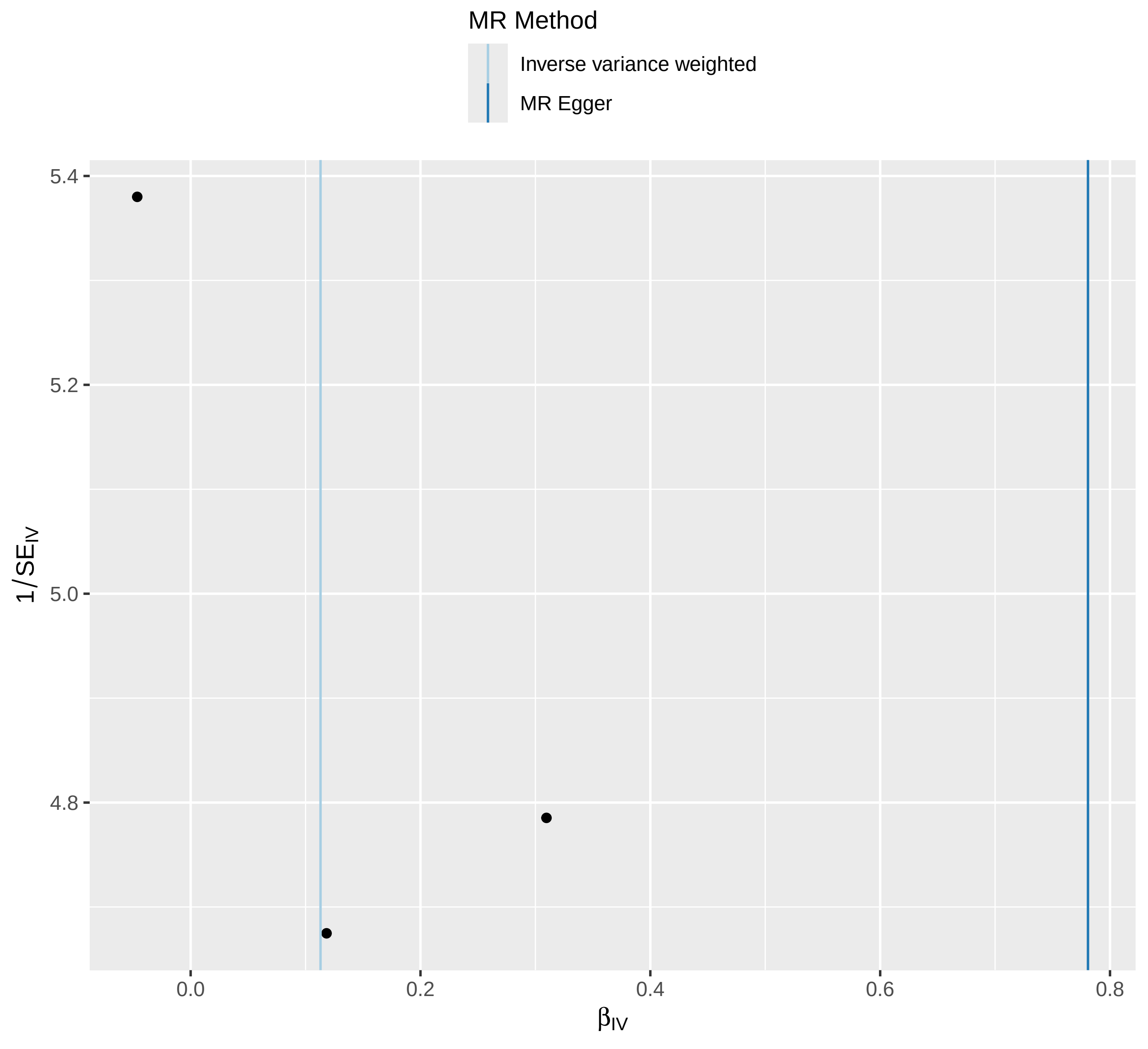

MR Method
Inverse variance weighted
MR Egger
5.4
5.2
5.0
4.8
1/SE$_{IV}$
0.0
0.2
0.4
0.6
0.8
$\beta_{IV}$

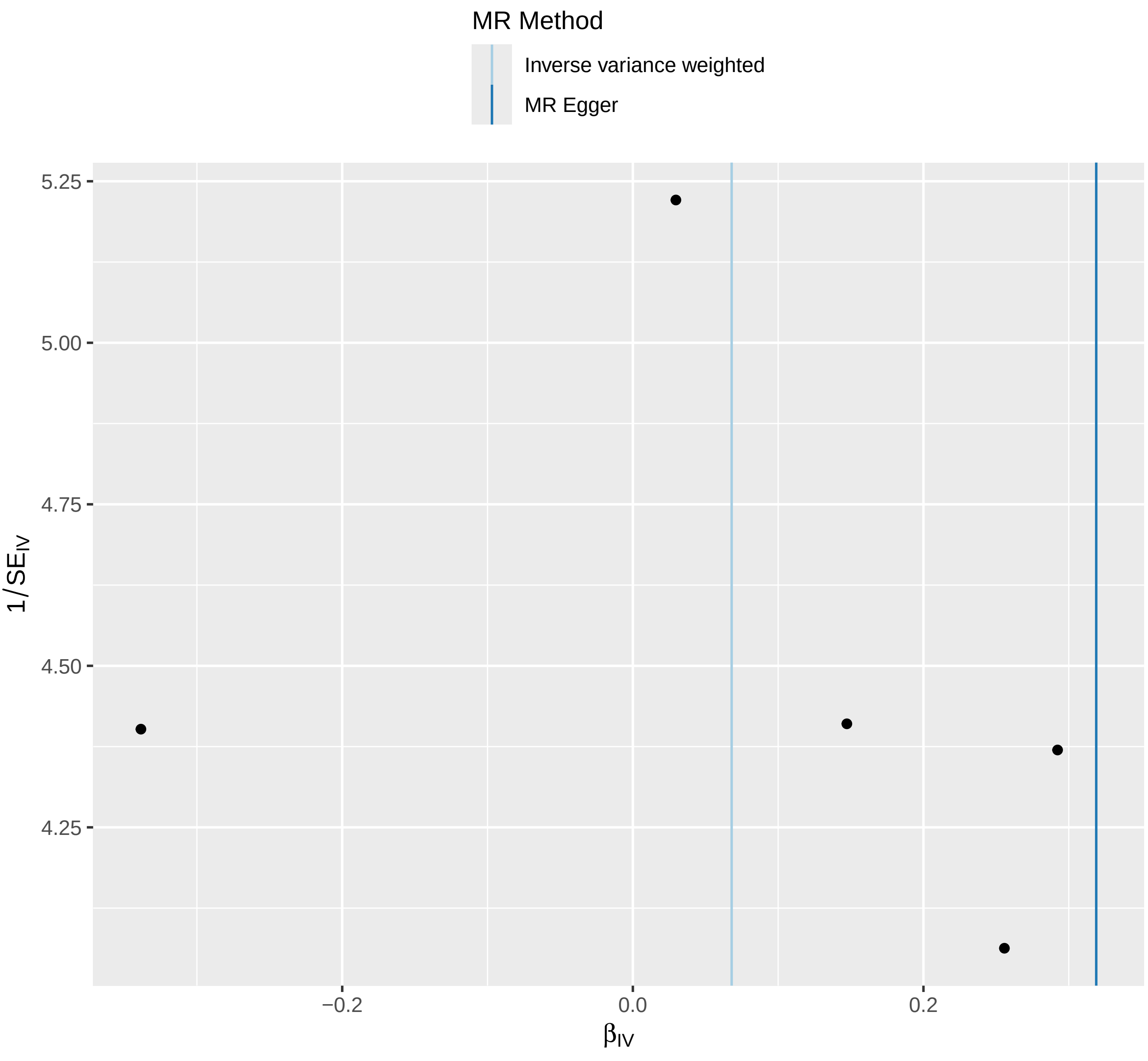
MR Method
Inverse variance weighted
MR Egger
5.25
5.00
4.75
4.50
4.25
$1/SE_{IV}$
−0.2
0.0
0.2
$\beta_{IV}$

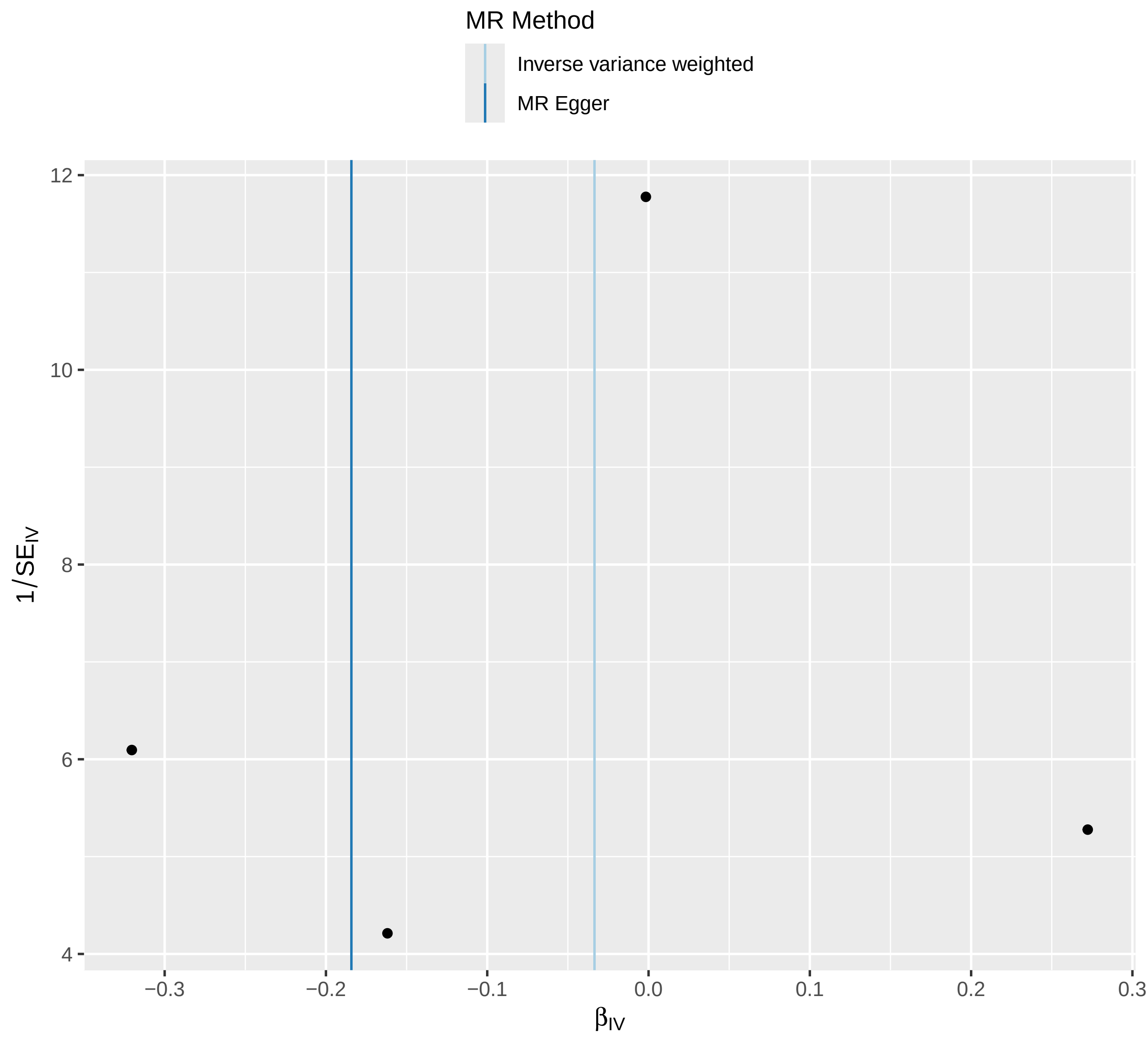

MR Method
Inverse variance weighted
MR Egger
12
10
8
6
4
1/SE$_{IV}$
−0.3
−0.2
−0.1
0.0
0.1
0.2
0.3
β$_{IV}$

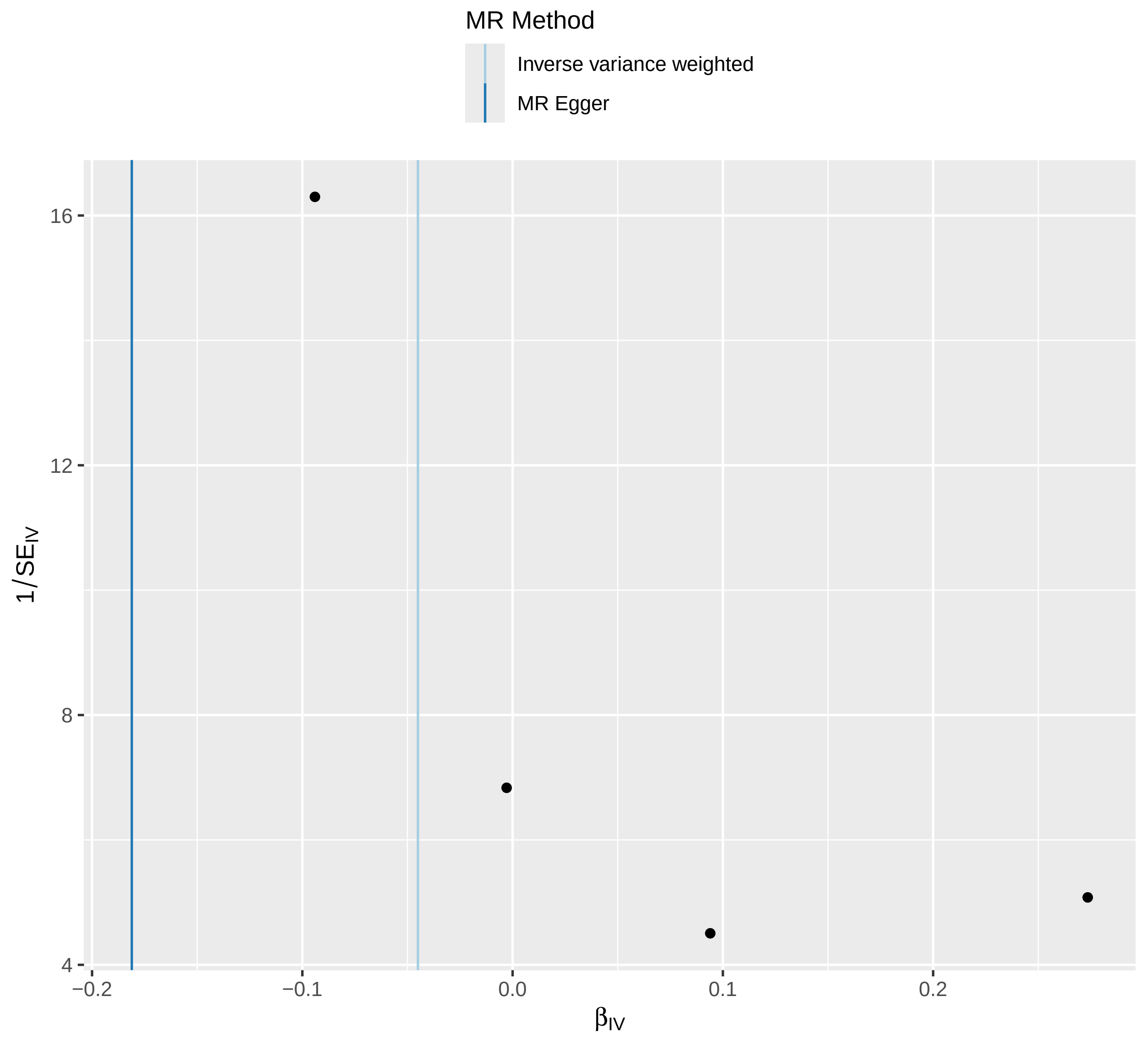
MR Method
Inverse variance weighted
MR Egger
16
12
8
4
−0.2
−0.1
0.0
0.1
0.2
$1/SE_{IV}$
$\beta_{IV}$

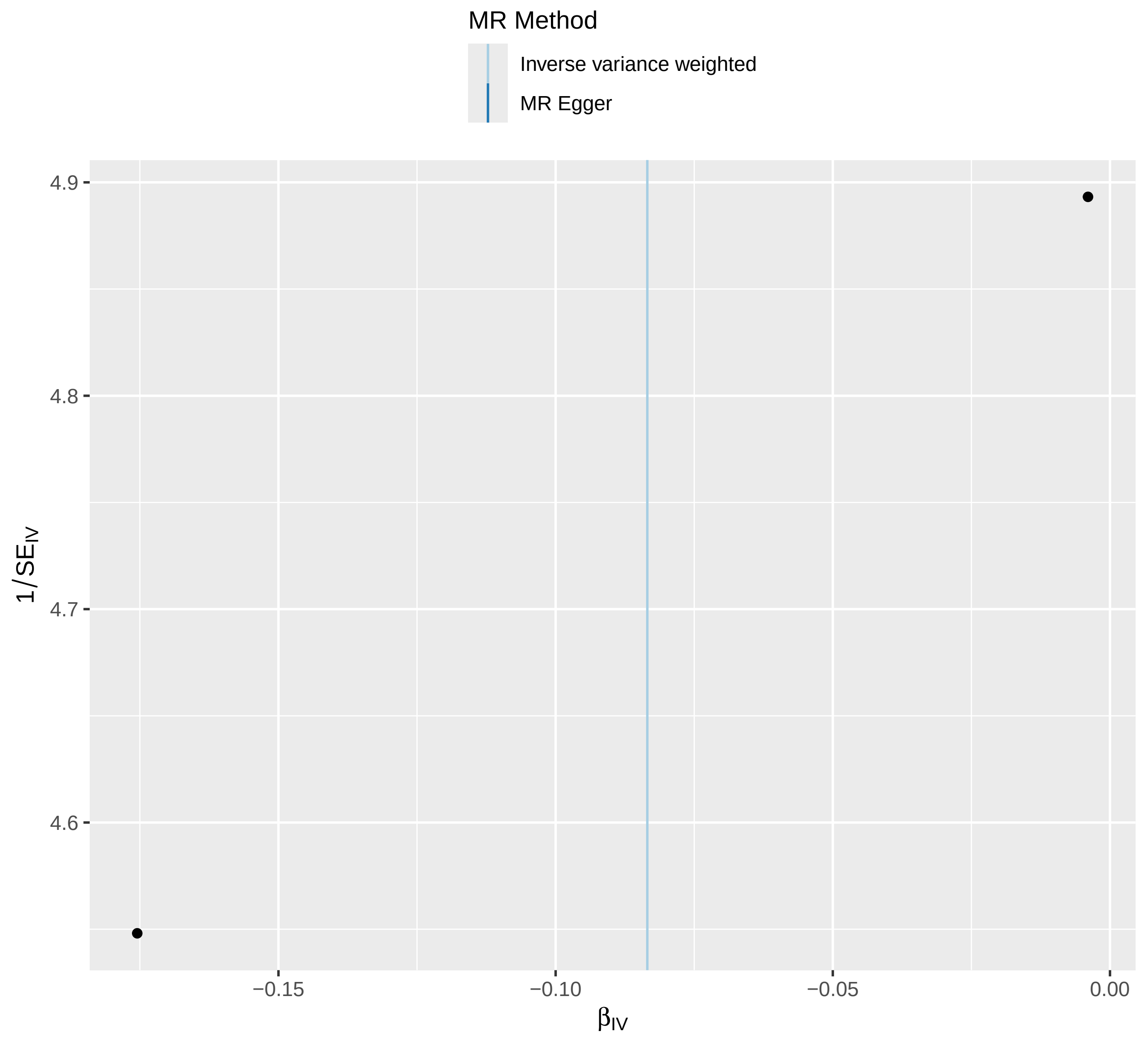
MR Method
Inverse variance weighted
MR Egger
4.9
4.8
4.7
4.6
1/SE_IV
−0.15
−0.10
−0.05
0.00
β_IV

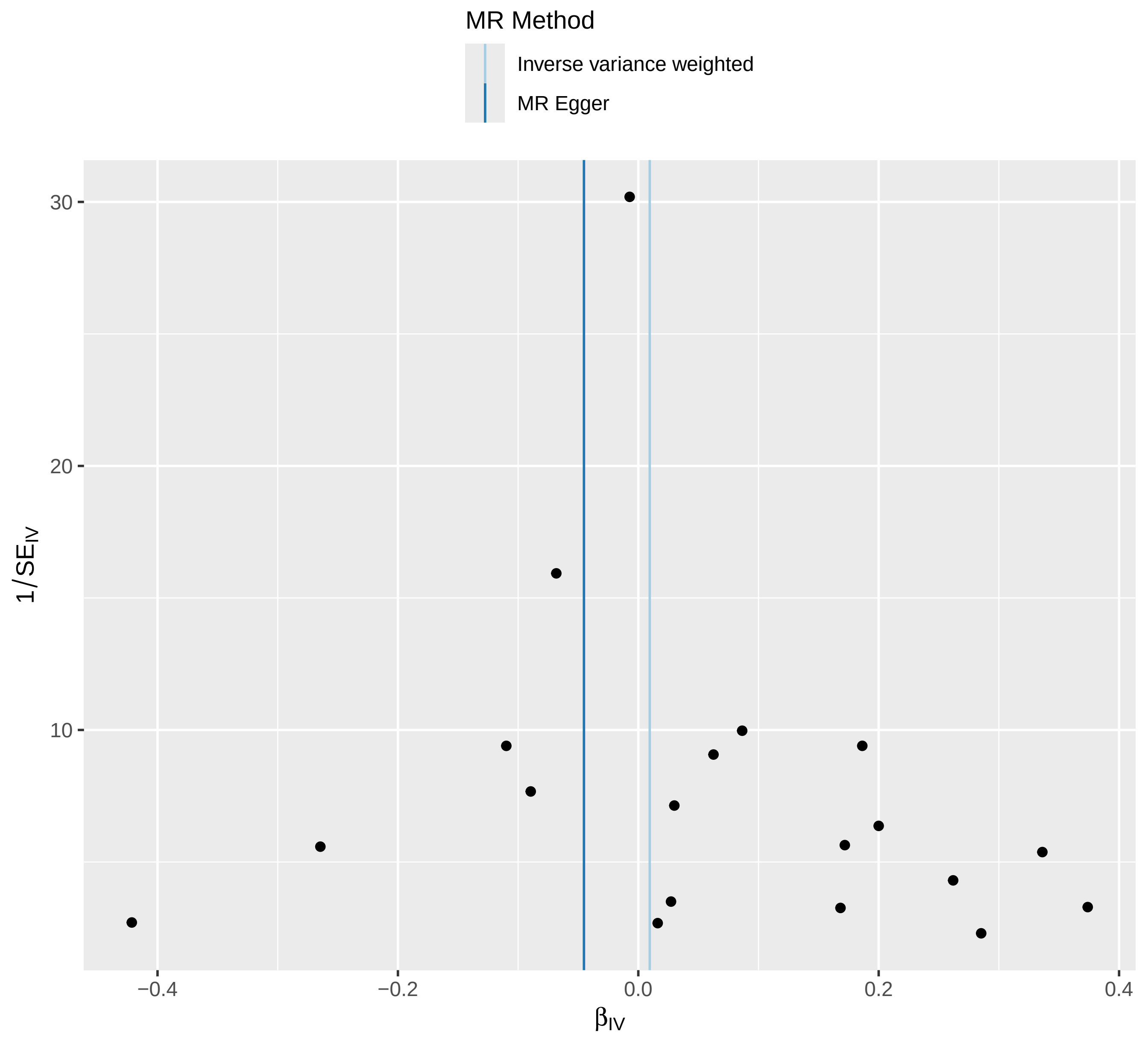
MR Method
Inverse variance weighted
MR Egger
30
20
10
1/SE_IV
−0.4
−0.2
0.0
0.2
0.4
β_IV

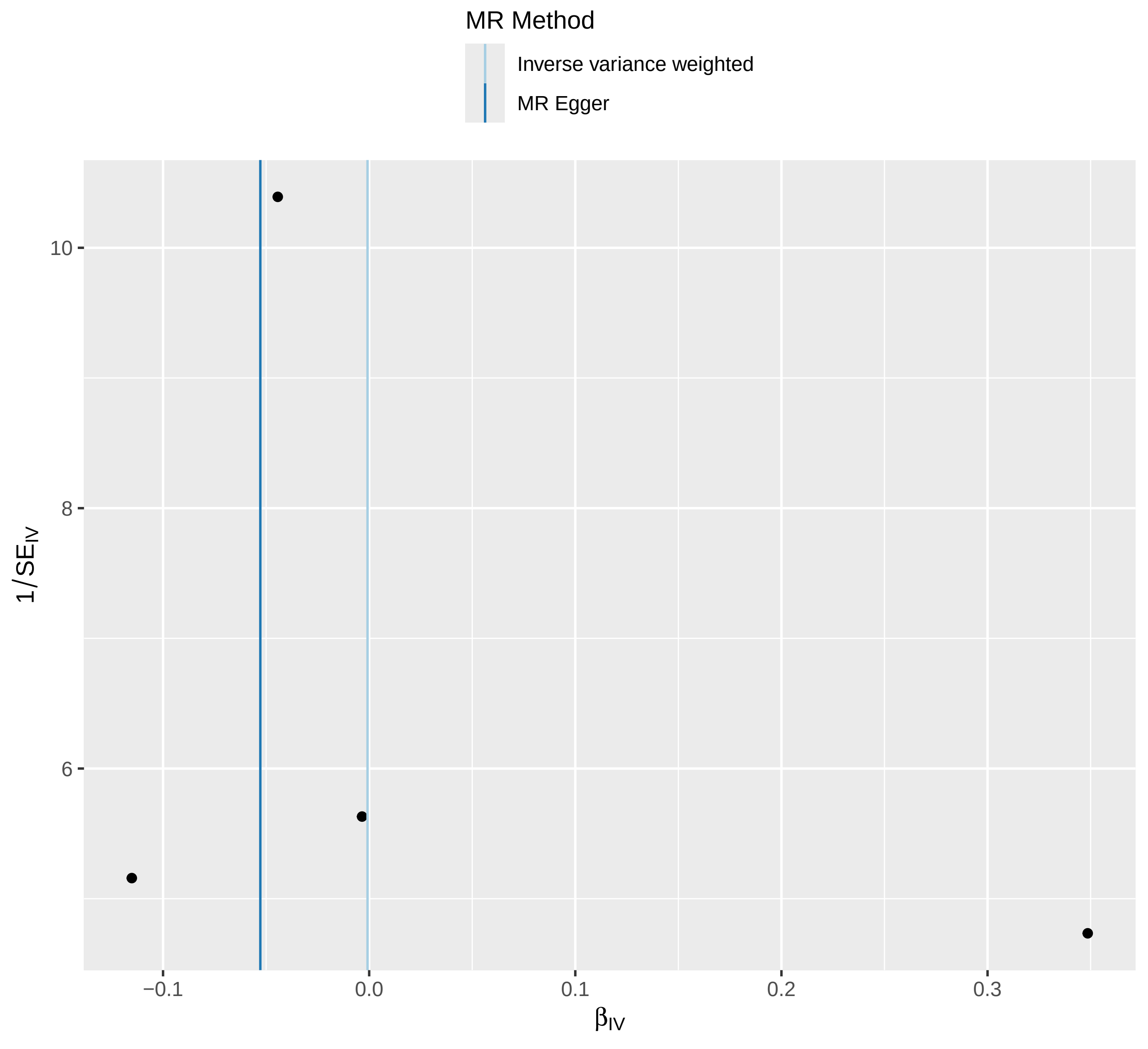
MR Method
Inverse variance weighted
MR Egger
10
8
6
1/SE$_{IV}$
−0.1
0.0
0.1
0.2
0.3
β$_{IV}$

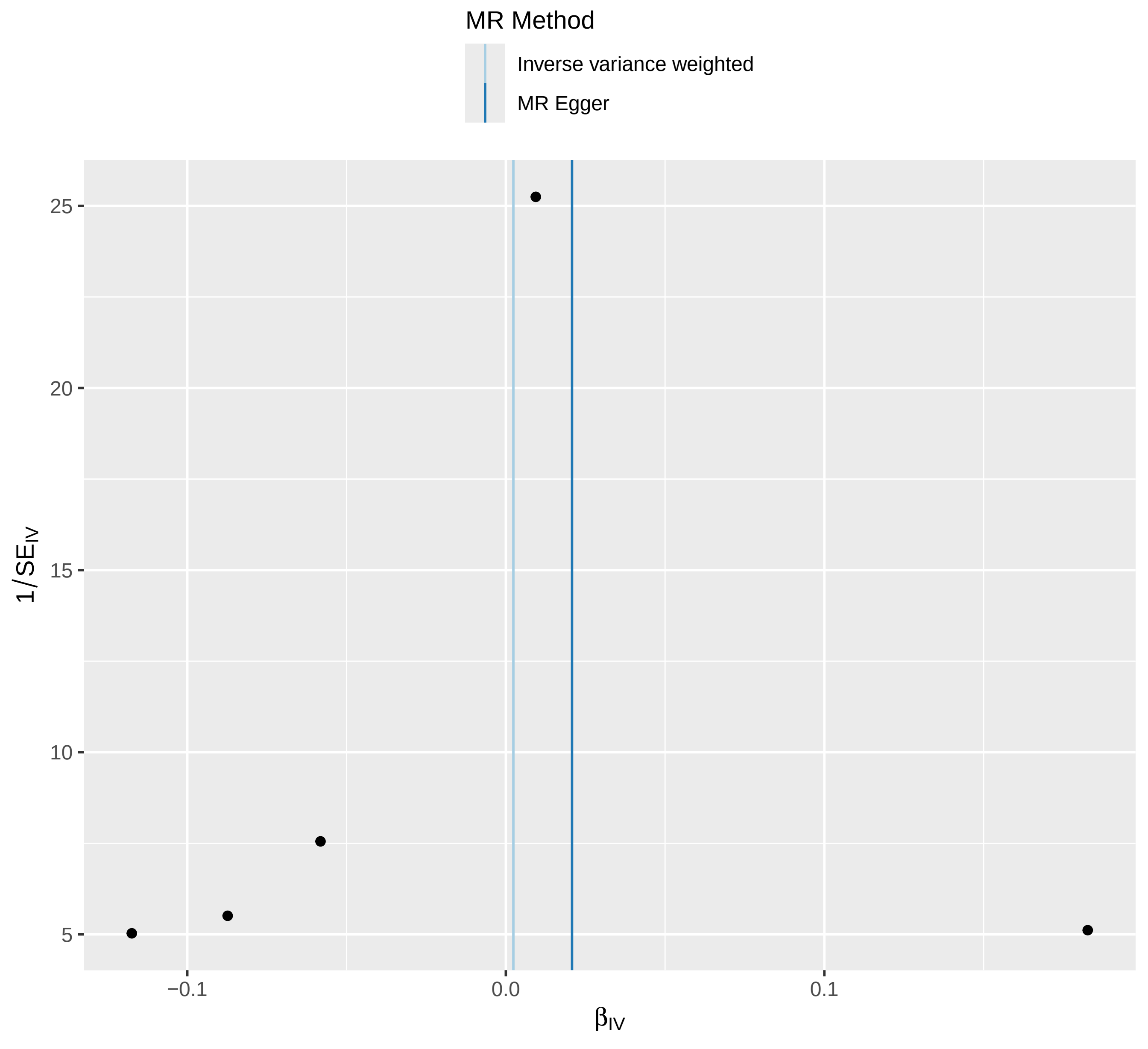

MR Method
Inverse variance weighted
MR Egger
25
20
15
10
5
1/SE_IV
−0.1
0.0
0.1
β_IV

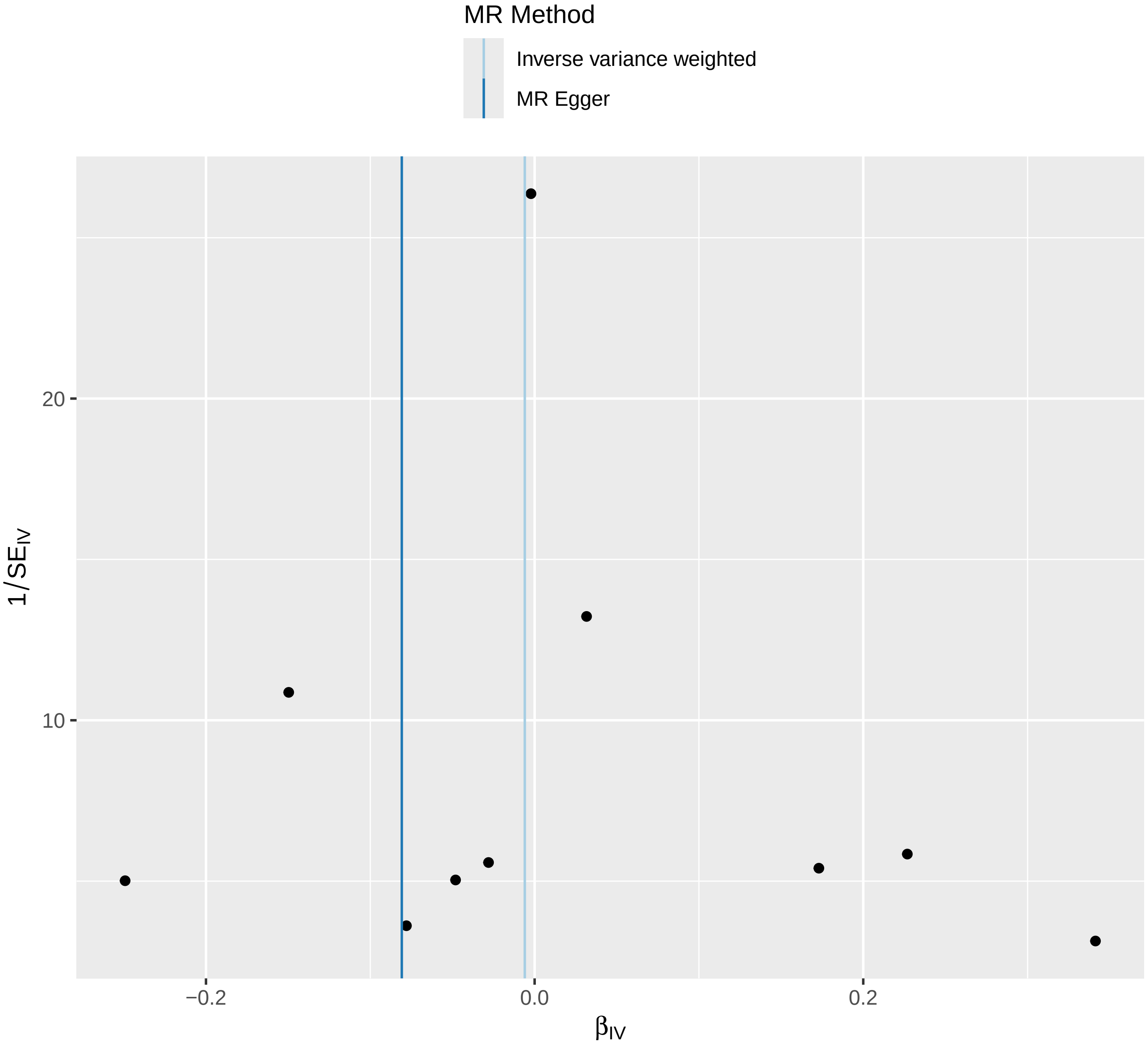
MR Method
Inverse variance weighted
MR Egger
20
10
1/$SE_{IV}$
−0.2
0.0
0.2
$\beta_{IV}$

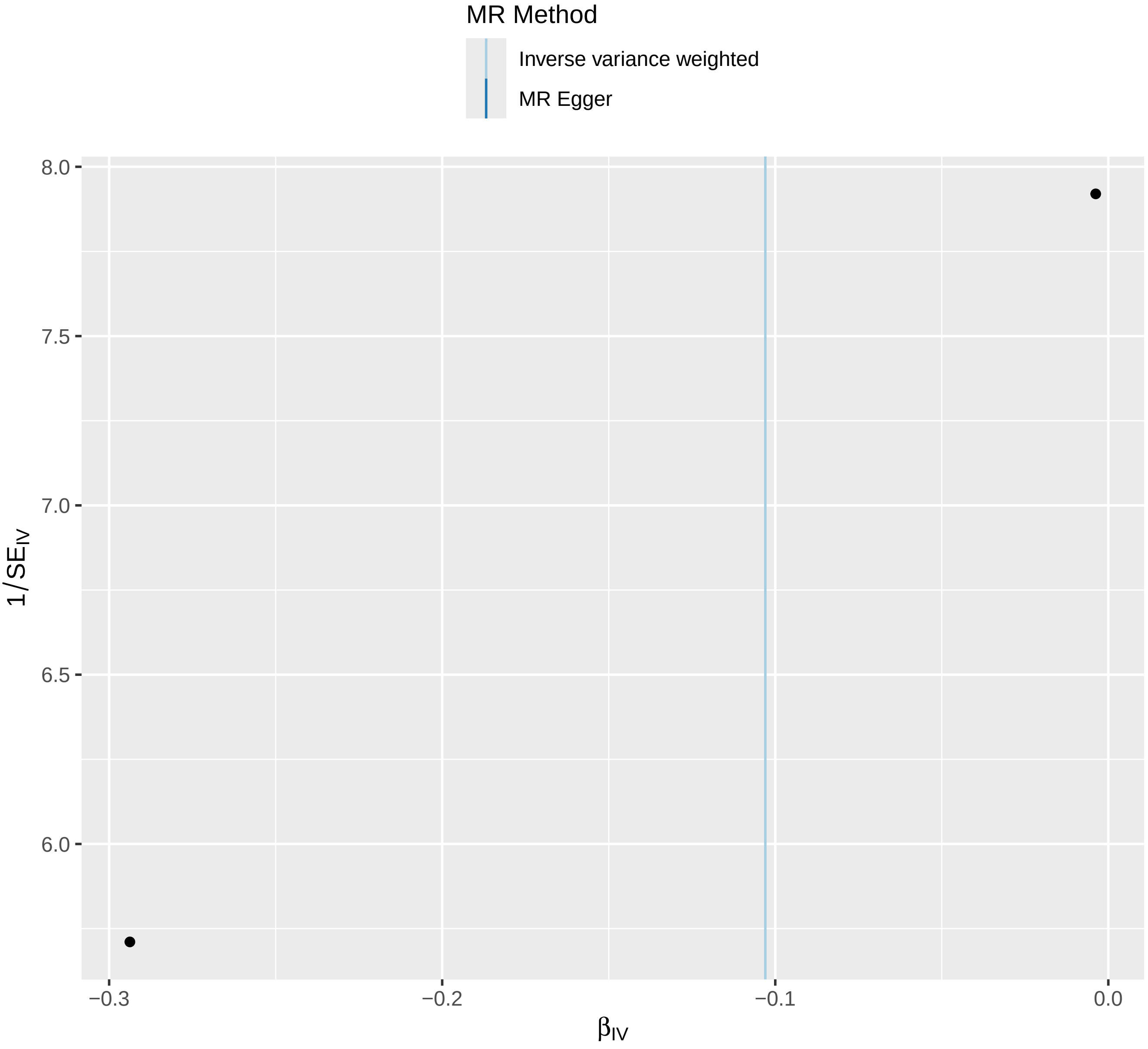
MR Method
Inverse variance weighted
MR Egger
8.0
7.5
7.0
6.5
6.0
1/SE_IV
−0.3
−0.2
−0.1
0.0
β_IV

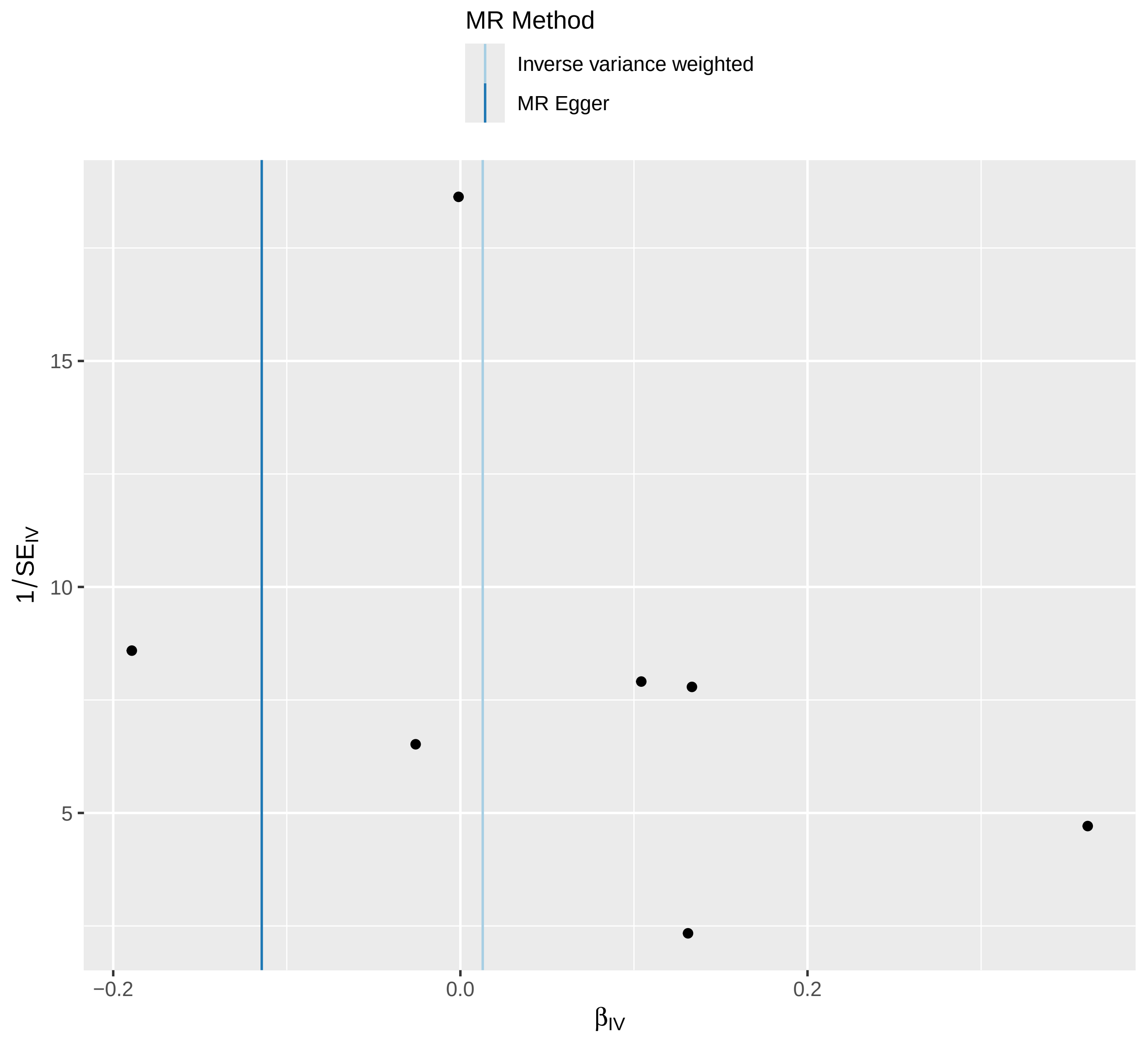

MR Method
Inverse variance weighted
MR Egger
15
10
5
1/SE_IV
−0.2
0.0
0.2
β_IV

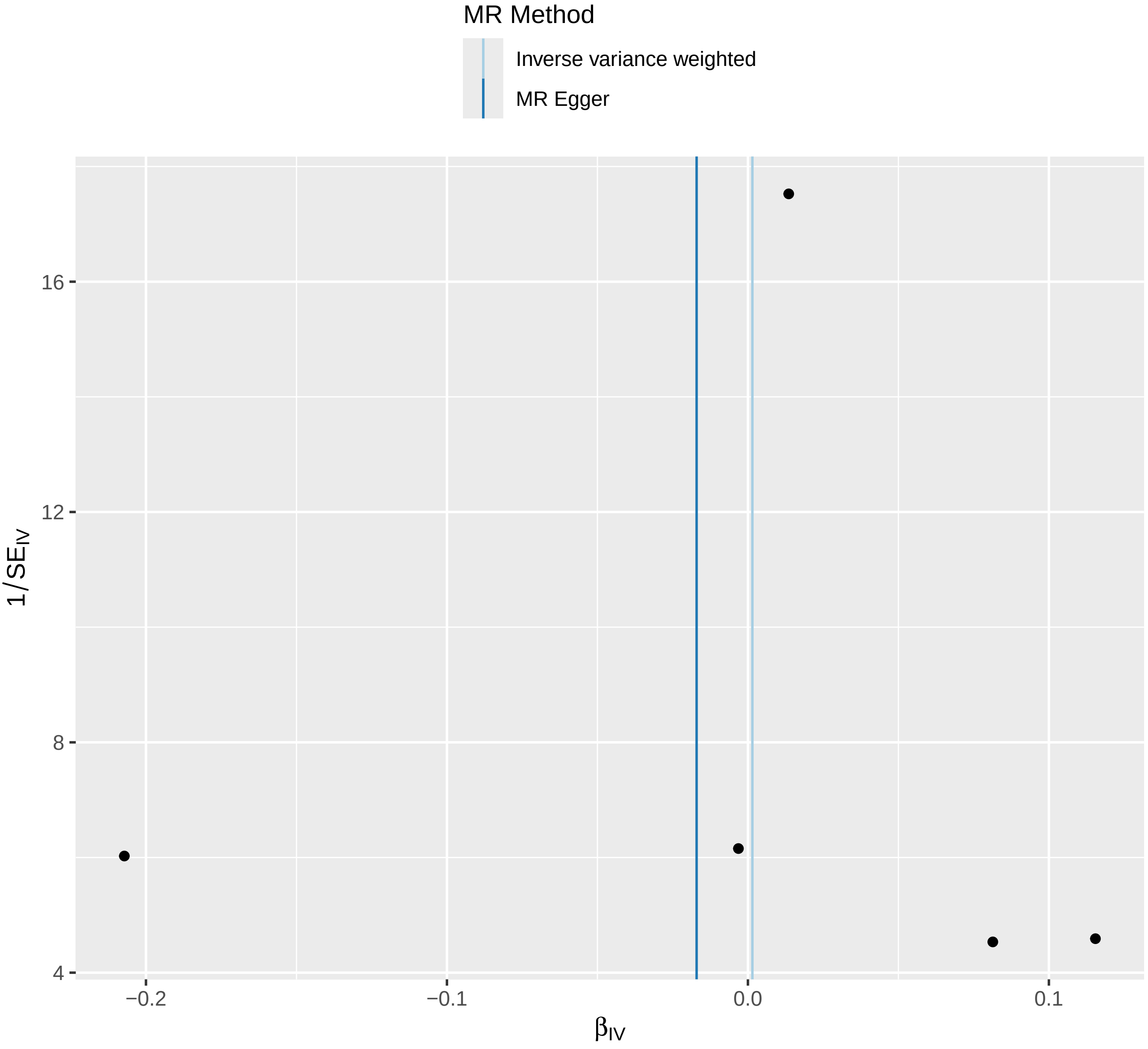

MR Method
Inverse variance weighted
MR Egger
16
12
8
4
1/SE$_{IV}$
−0.2
−0.1
0.0
0.1
β$_{IV}$

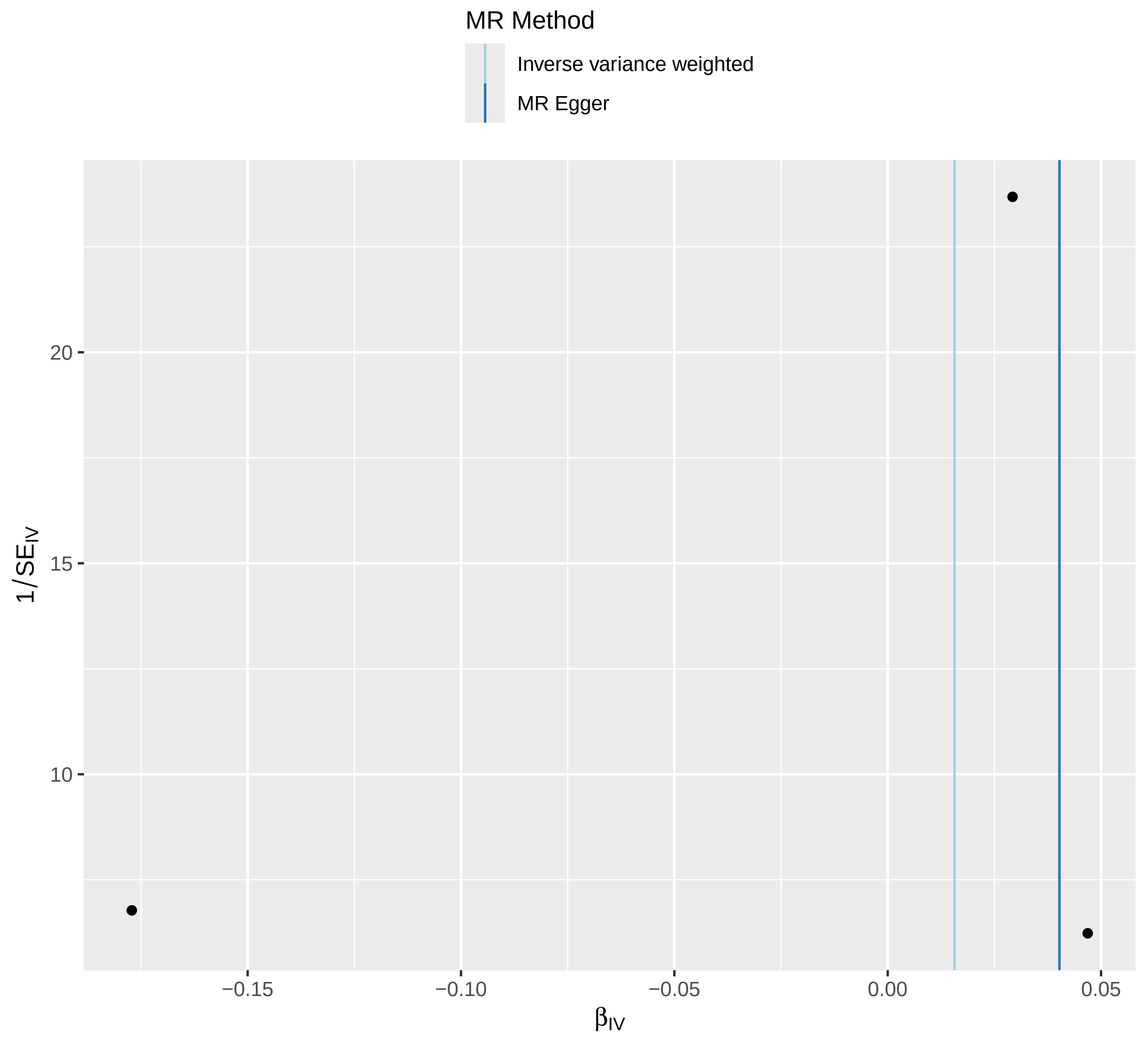

MR Method
Inverse variance weighted
MR Egger
20
15
10
1/SE_IV
−0.15
−0.10
−0.05
0.00
0.05
β_IV

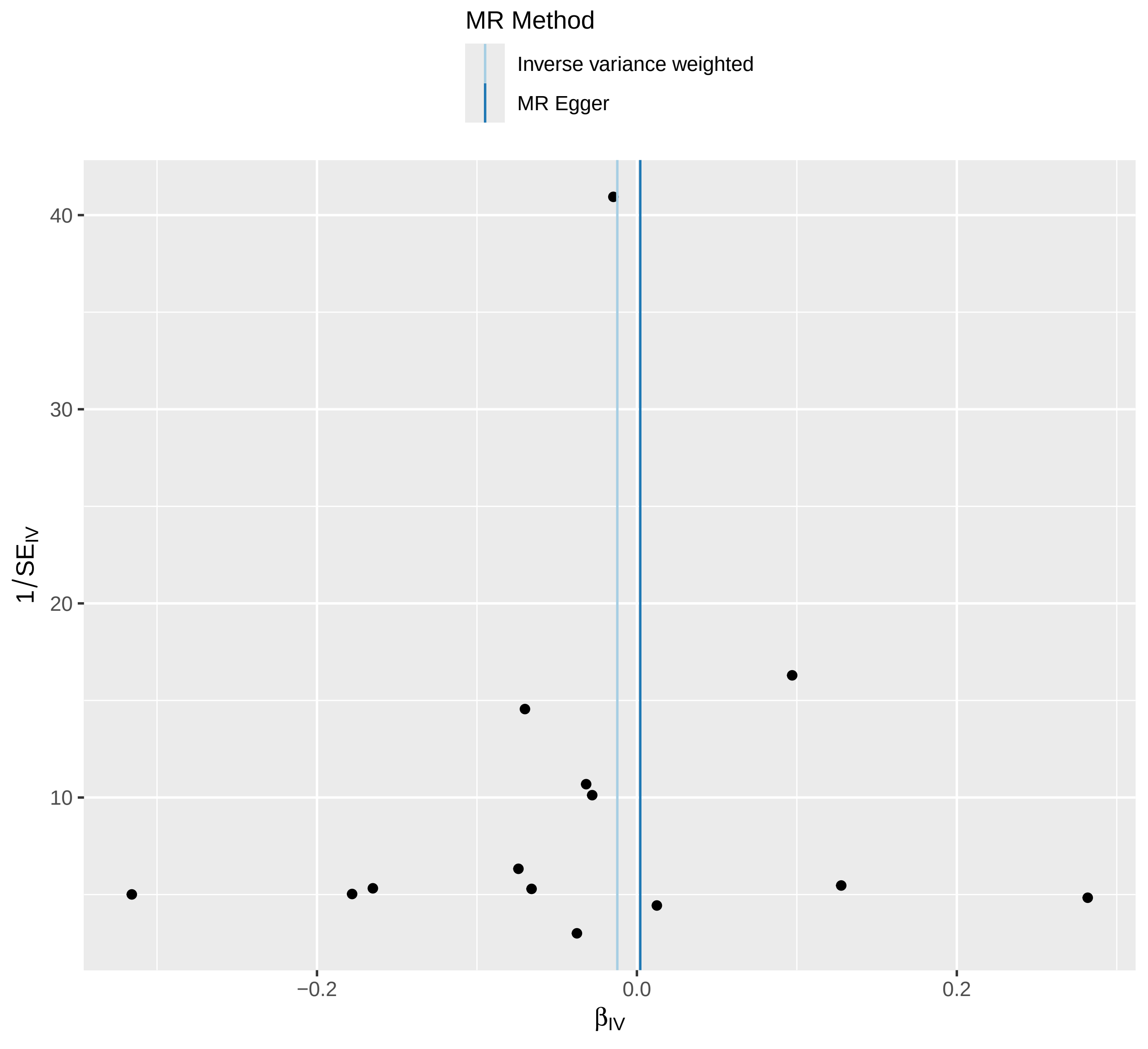

MR Method
Inverse variance weighted
MR Egger
40
30
20
10
1/SE$_{IV}$
−0.2
0.0
0.2
β$_{IV}$

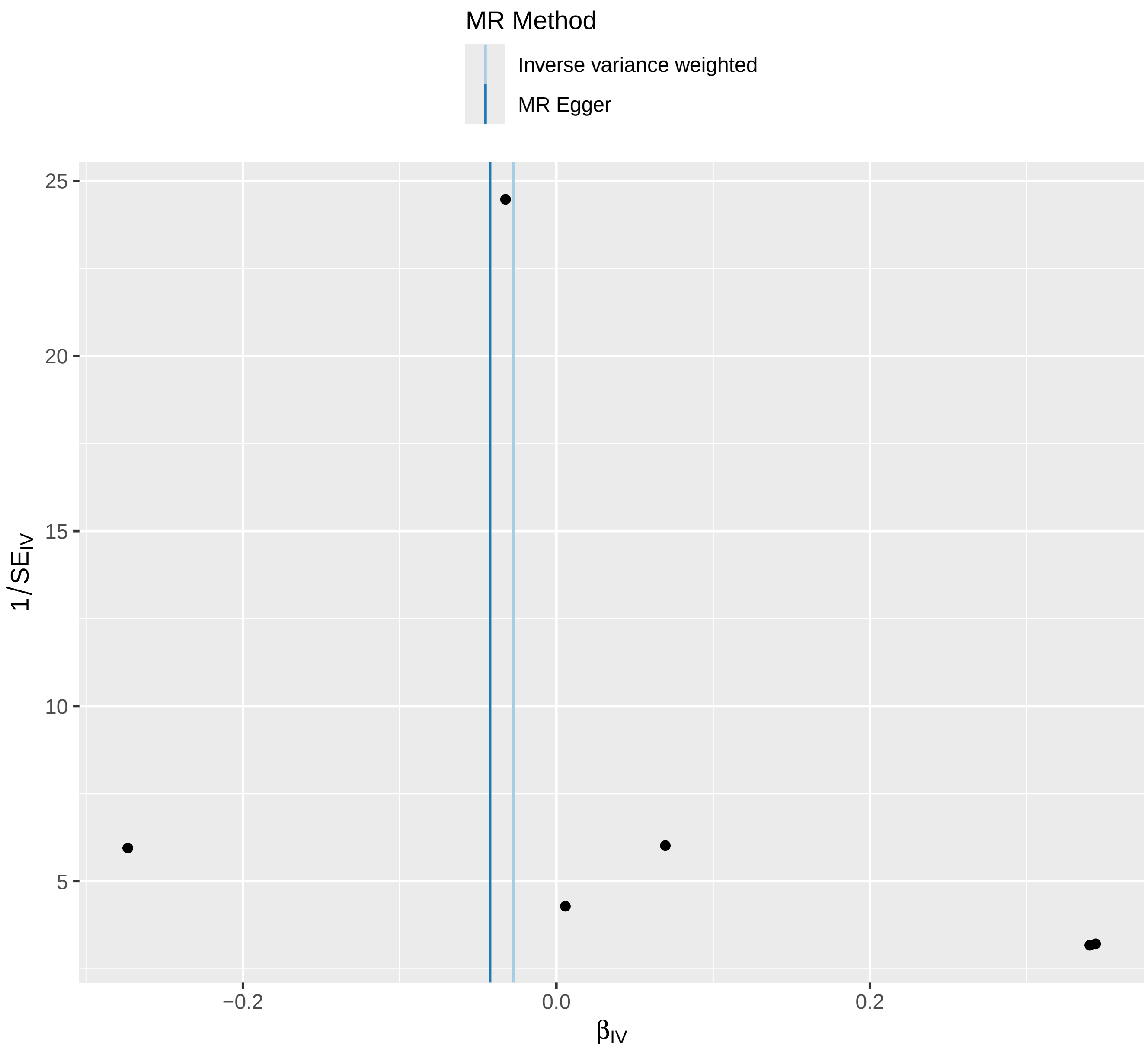
MR Method
Inverse variance weighted
MR Egger
25
20
15
10
5
$1/SE_{IV}$
−0.2
0.0
0.2
$\beta_{IV}$

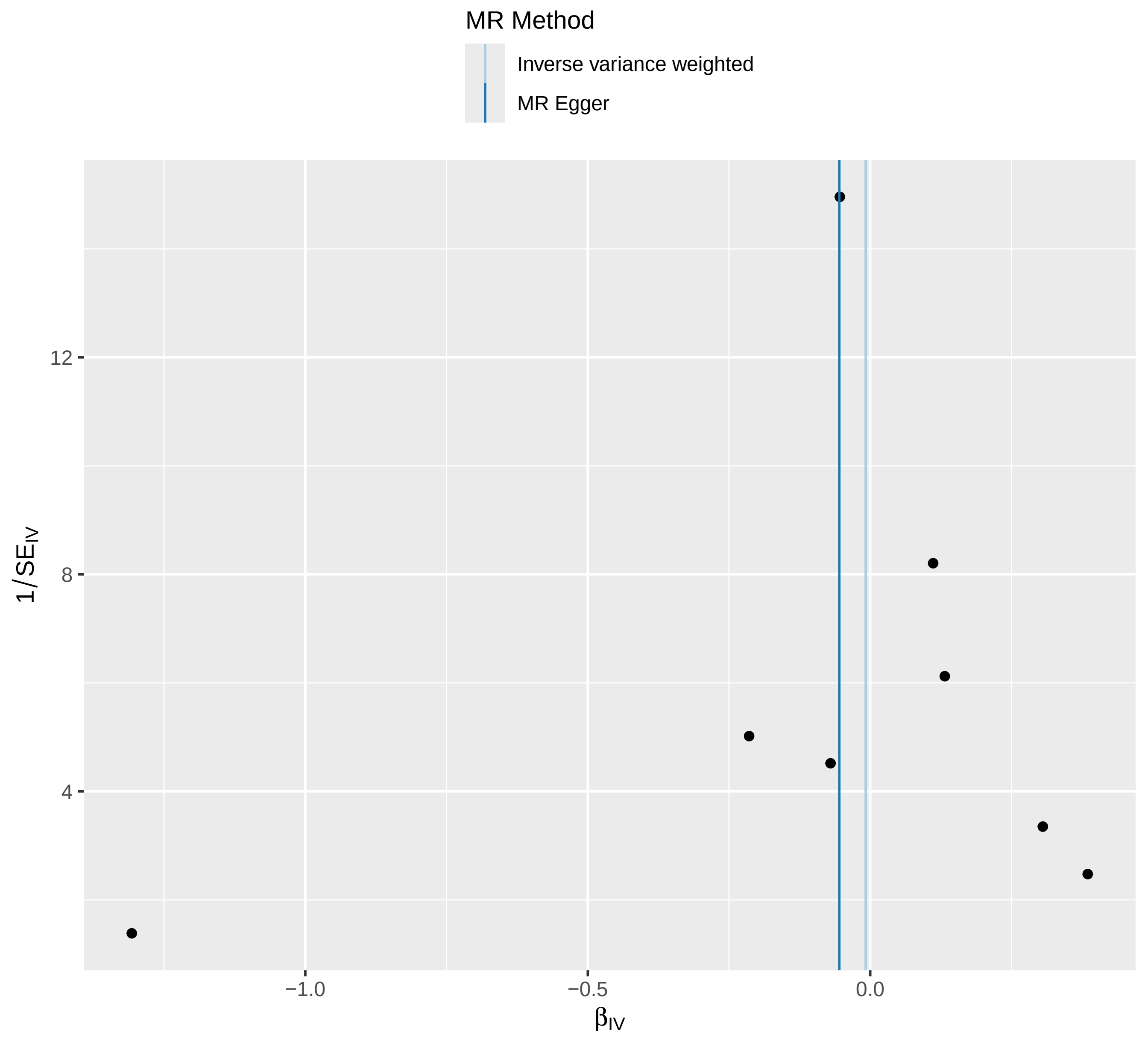
MR Method
Inverse variance weighted
MR Egger
12
8
4
1/SE_IV
−1.0
−0.5
0.0
β_IV

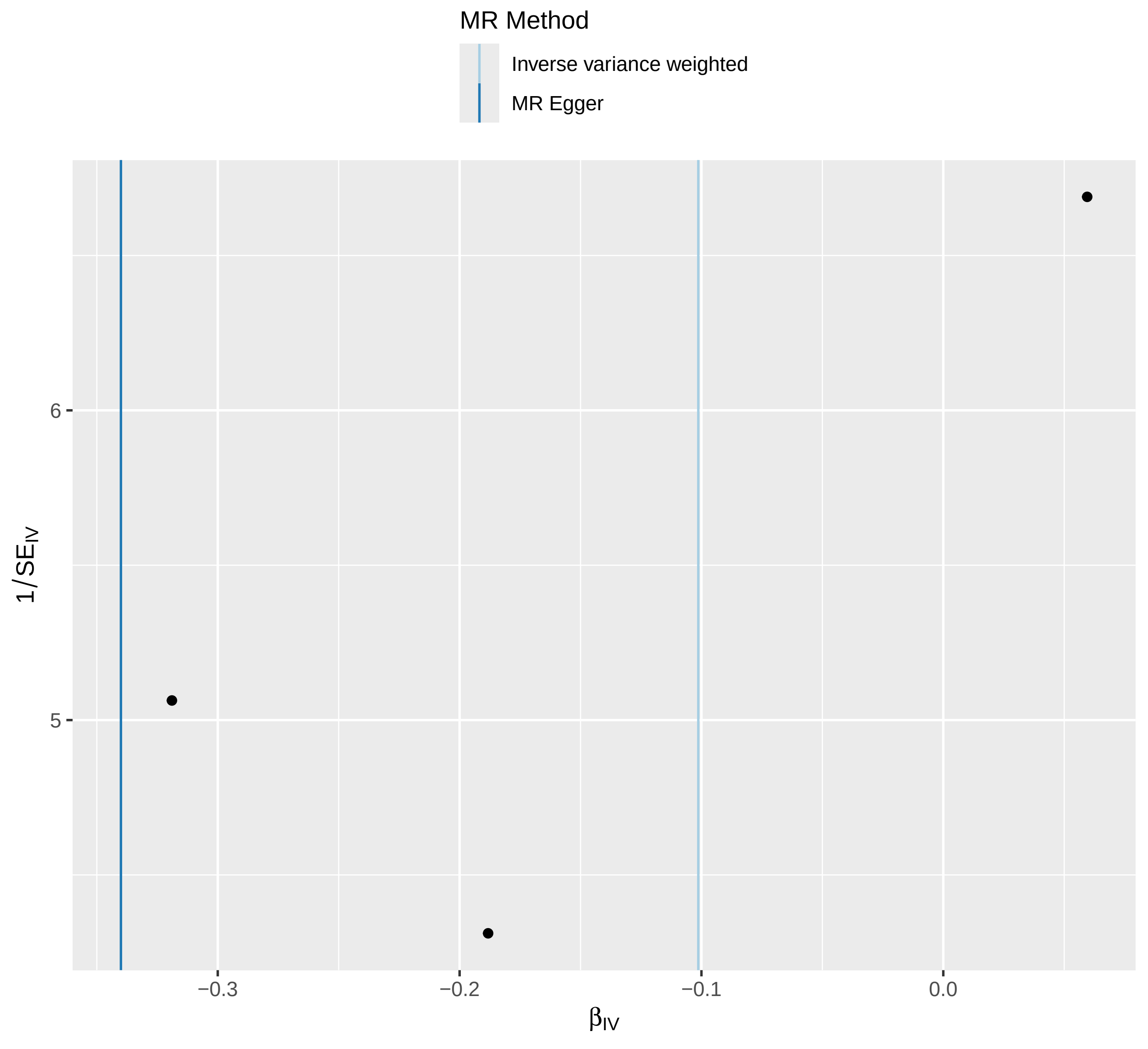
MR Method
Inverse variance weighted
MR Egger
1/SE_IV
6
5
−0.3
−0.2
−0.1
0.0
β_IV

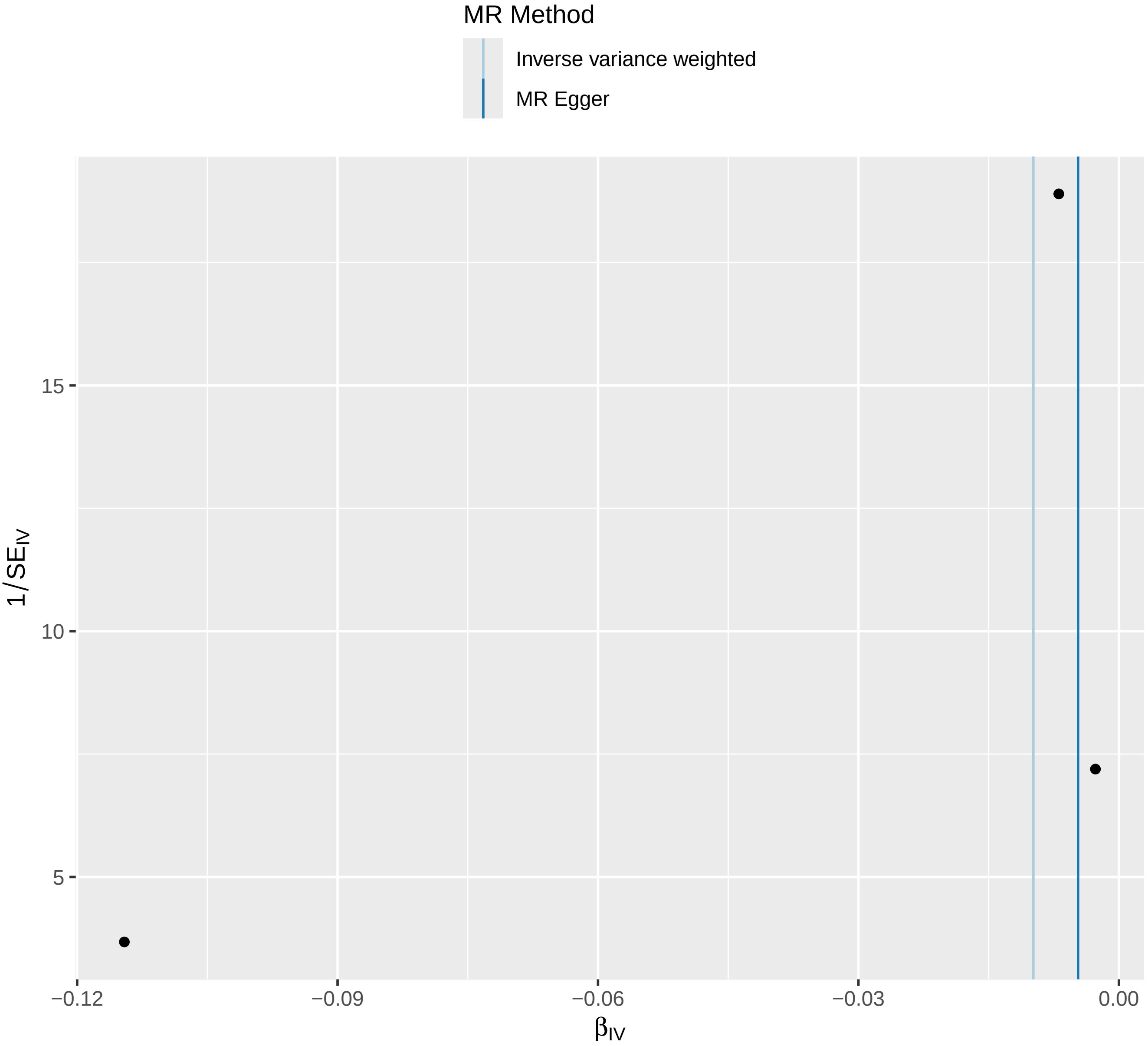

MR Method
Inverse variance weighted
MR Egger
15
10
5
1/SE$_{IV}$
−0.12
−0.09
−0.06
−0.03
0.00
β$_{IV}$

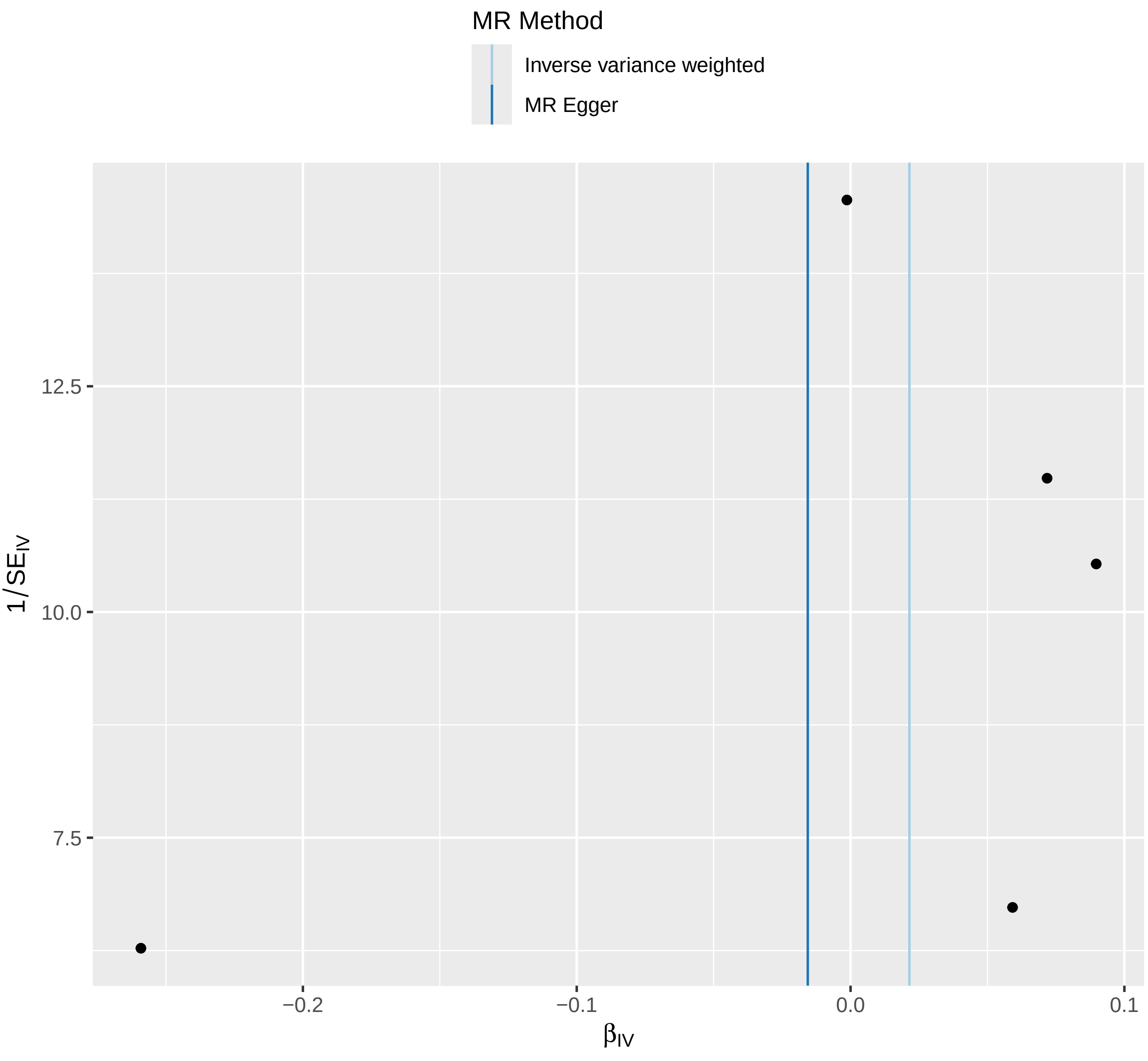
MR Method
Inverse variance weighted
MR Egger
12.5
10.0
7.5
1/SE_IV
−0.2
−0.1
0.0
0.1
β_IV

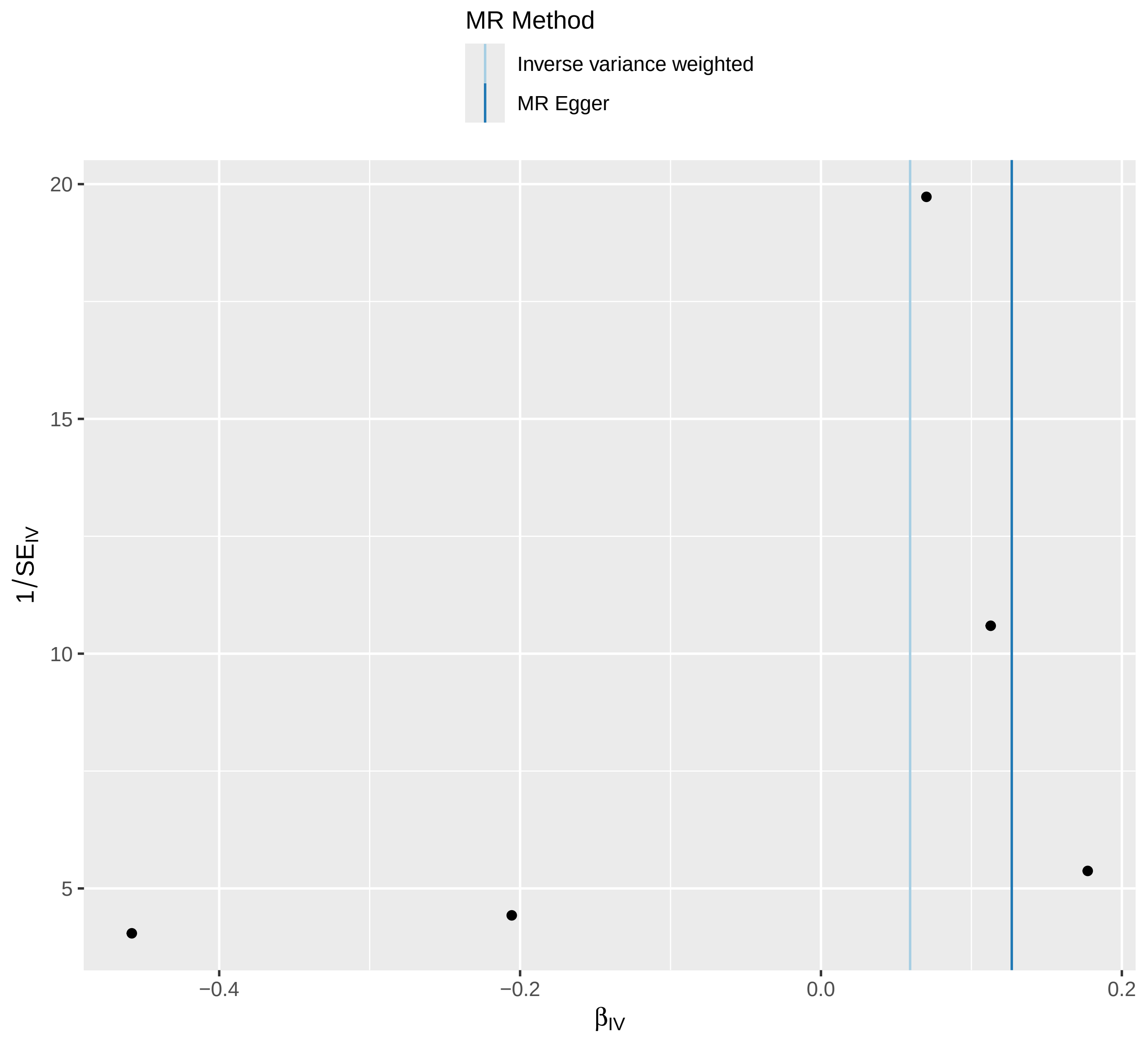

MR Method
Inverse variance weighted
MR Egger
20
15
10
5
$1/SE_{IV}$
−0.4
−0.2
0.0
0.2
$\beta_{IV}$

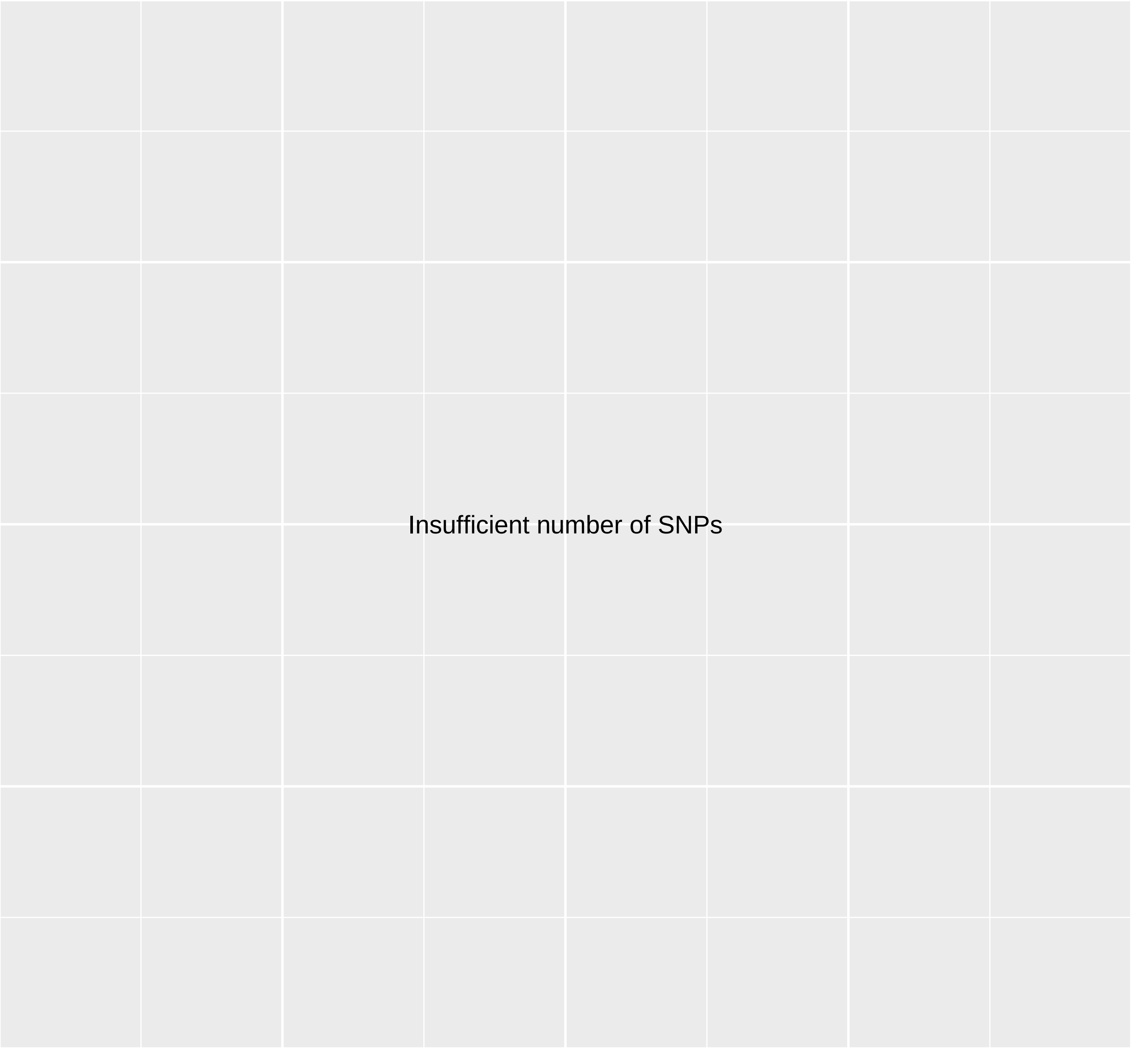
Insufficient number of SNPs

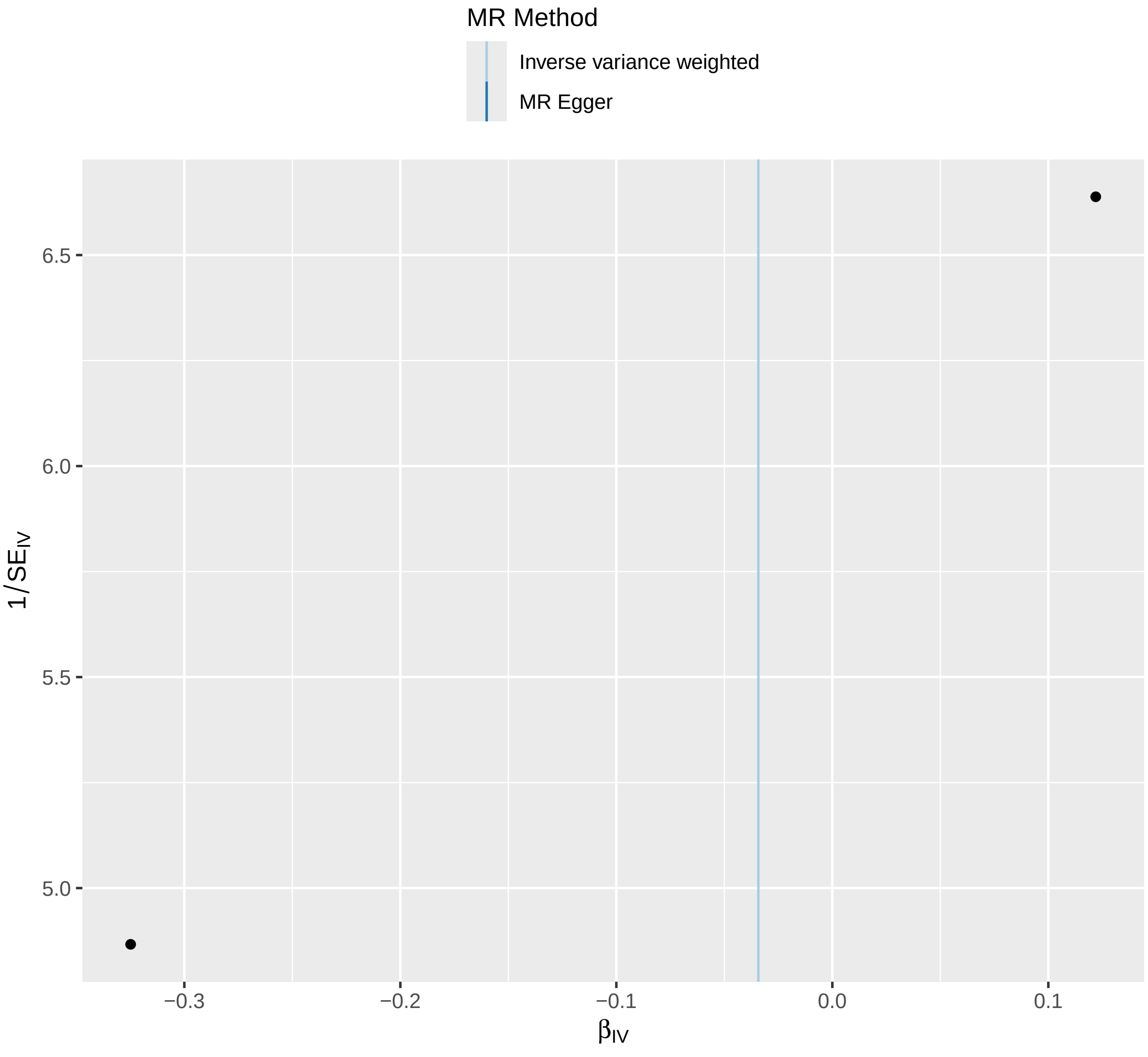

MR Method
Inverse variance weighted
MR Egger
6.5
6.0
5.5
5.0
1/SE$_{IV}$
−0.3
−0.2
−0.1
0.0
0.1
β$_{IV}$

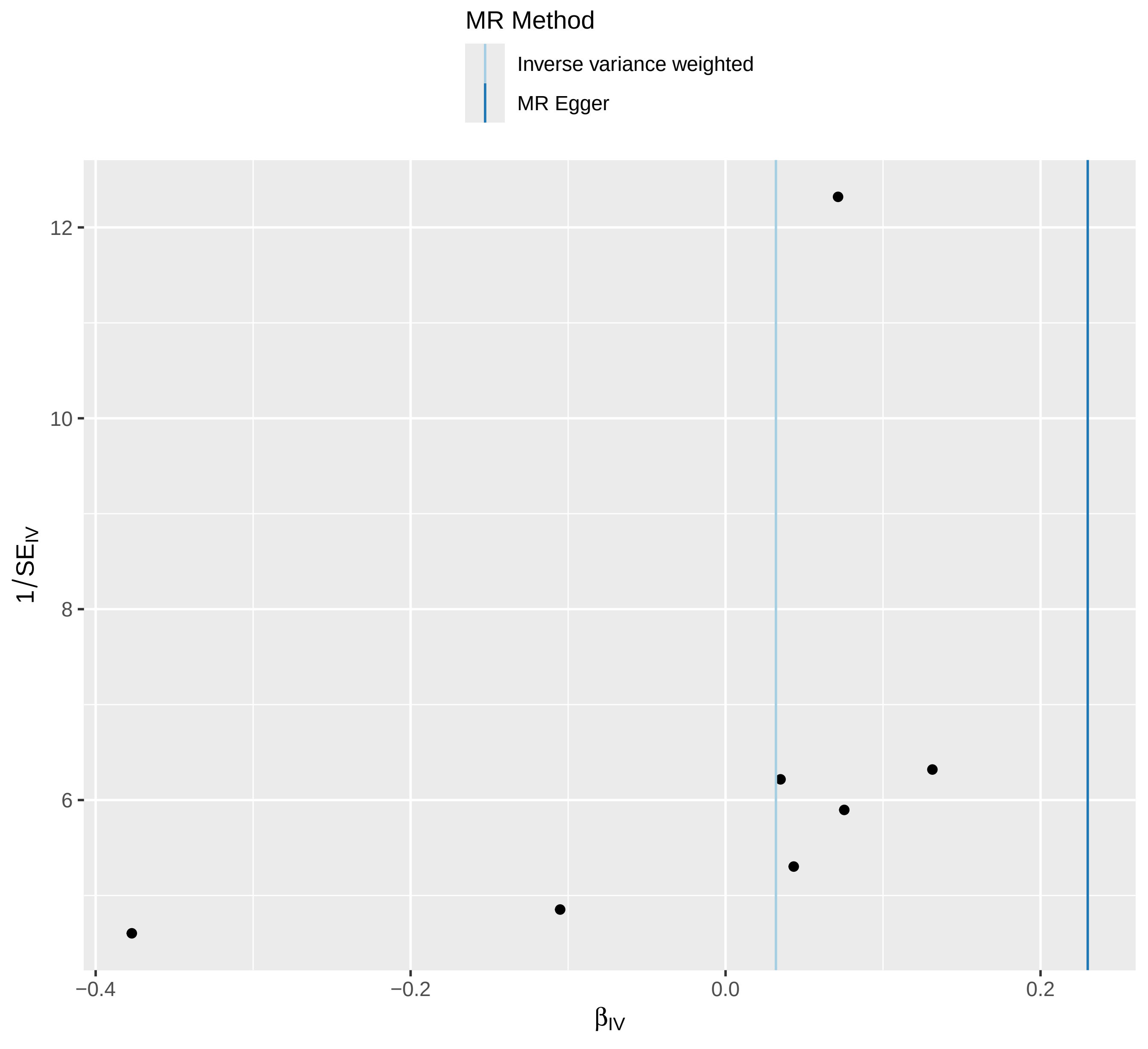
MR Method
Inverse variance weighted
MR Egger
12
10
8
6
−0.4
−0.2
0.0
0.2
1/SE_IV
β_IV

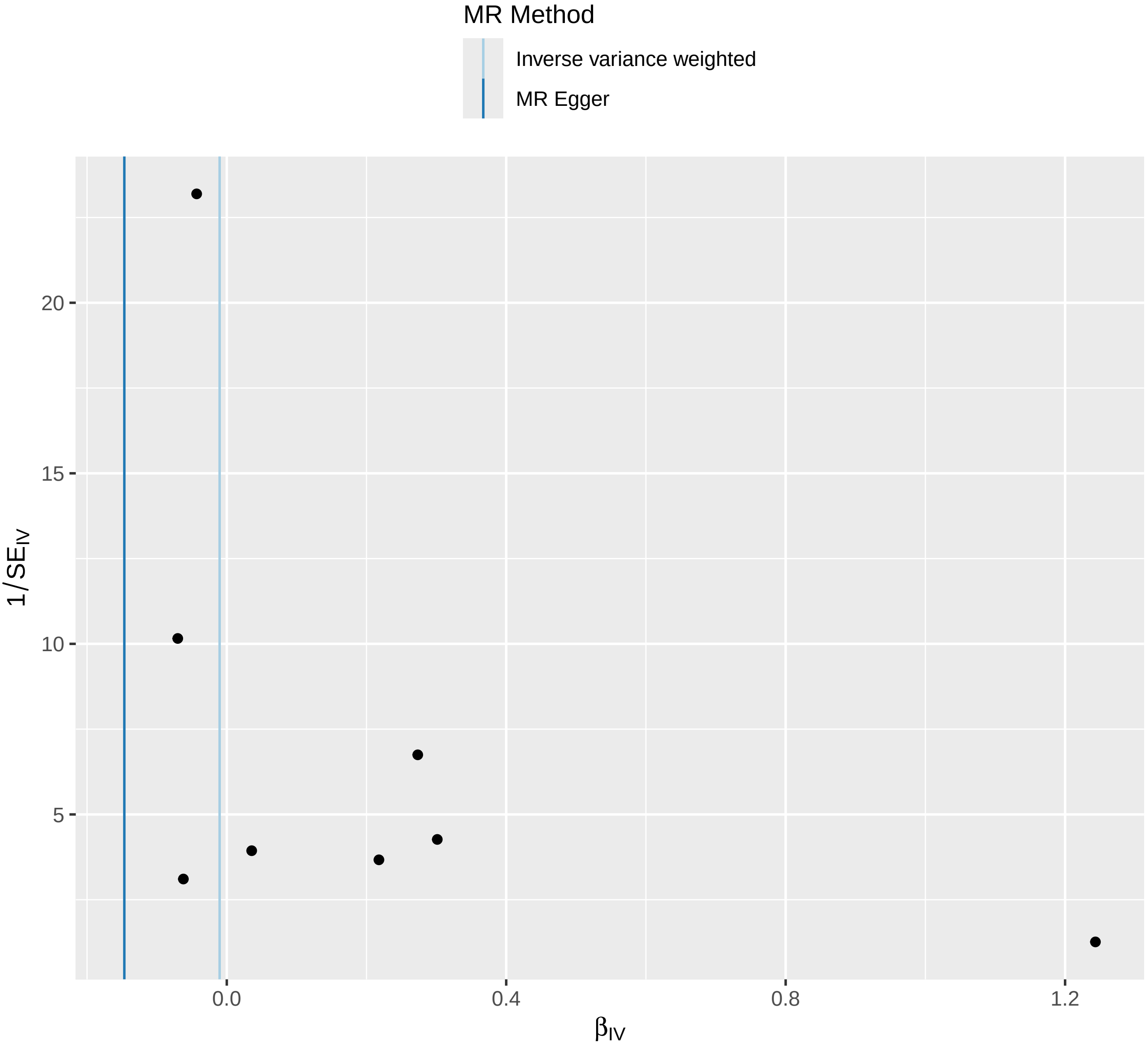
MR Method
Inverse variance weighted
MR Egger
20
15
10
5
$1/SE_{IV}$
0.0
0.4
0.8
1.2
$\beta_{IV}$

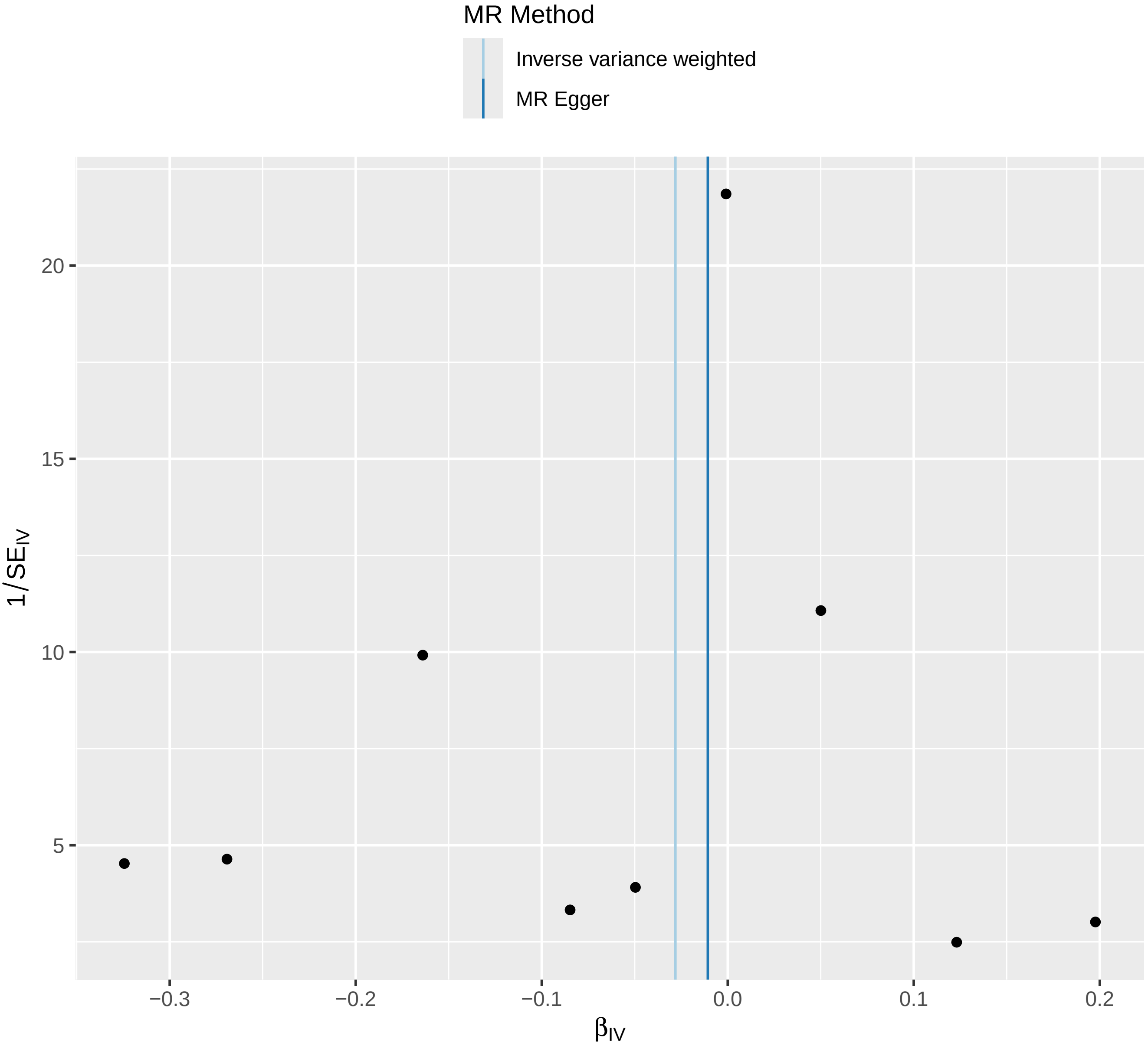
MR Method
Inverse variance weighted
MR Egger
20
15
10
5
1/SE$_{IV}$
−0.3
−0.2
−0.1
0.0
0.1
0.2
β$_{IV}$

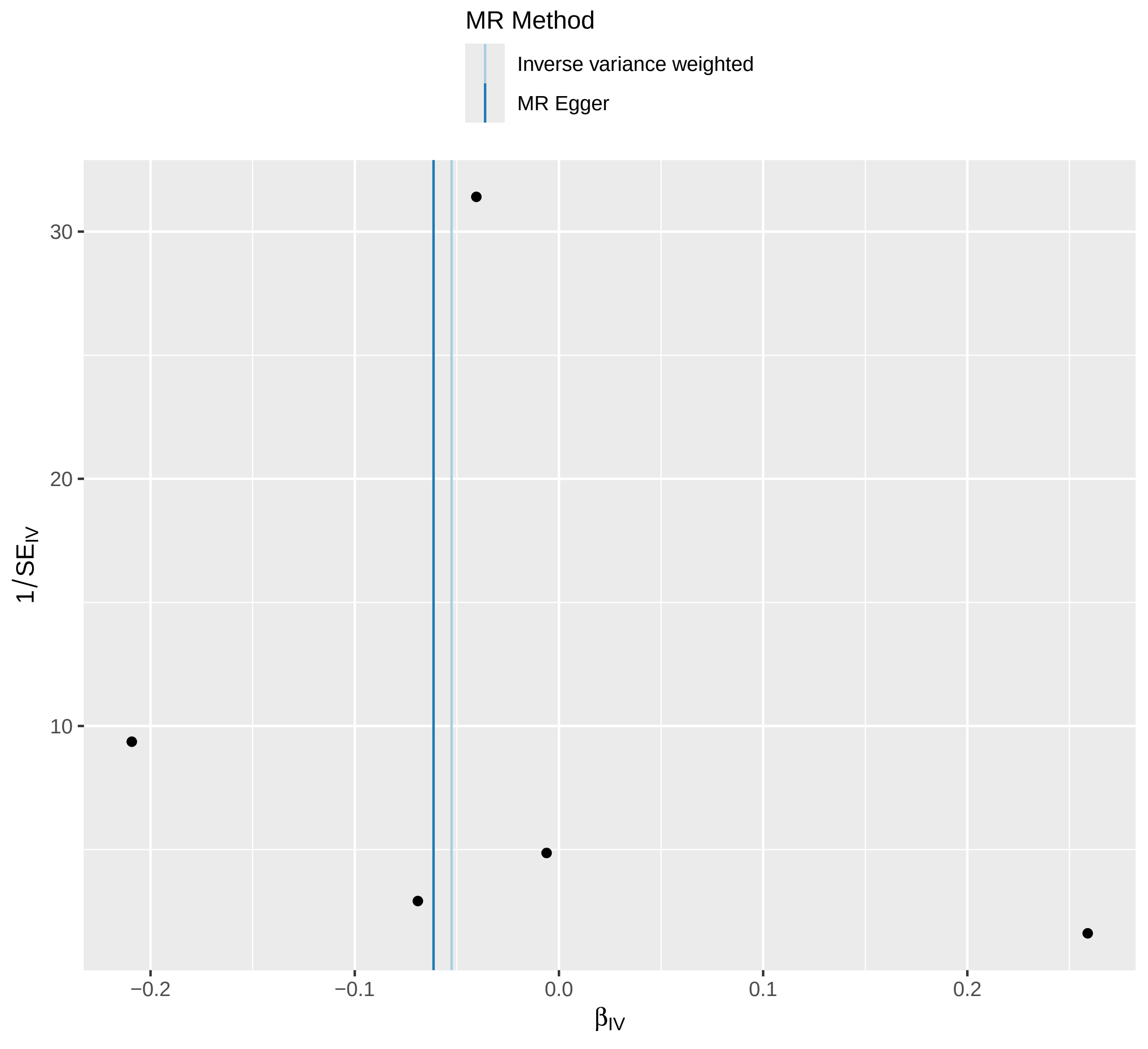

MR Method
Inverse variance weighted
MR Egger
30
20
10
1/SE_IV
−0.2
−0.1
0.0
0.1
0.2
β_IV

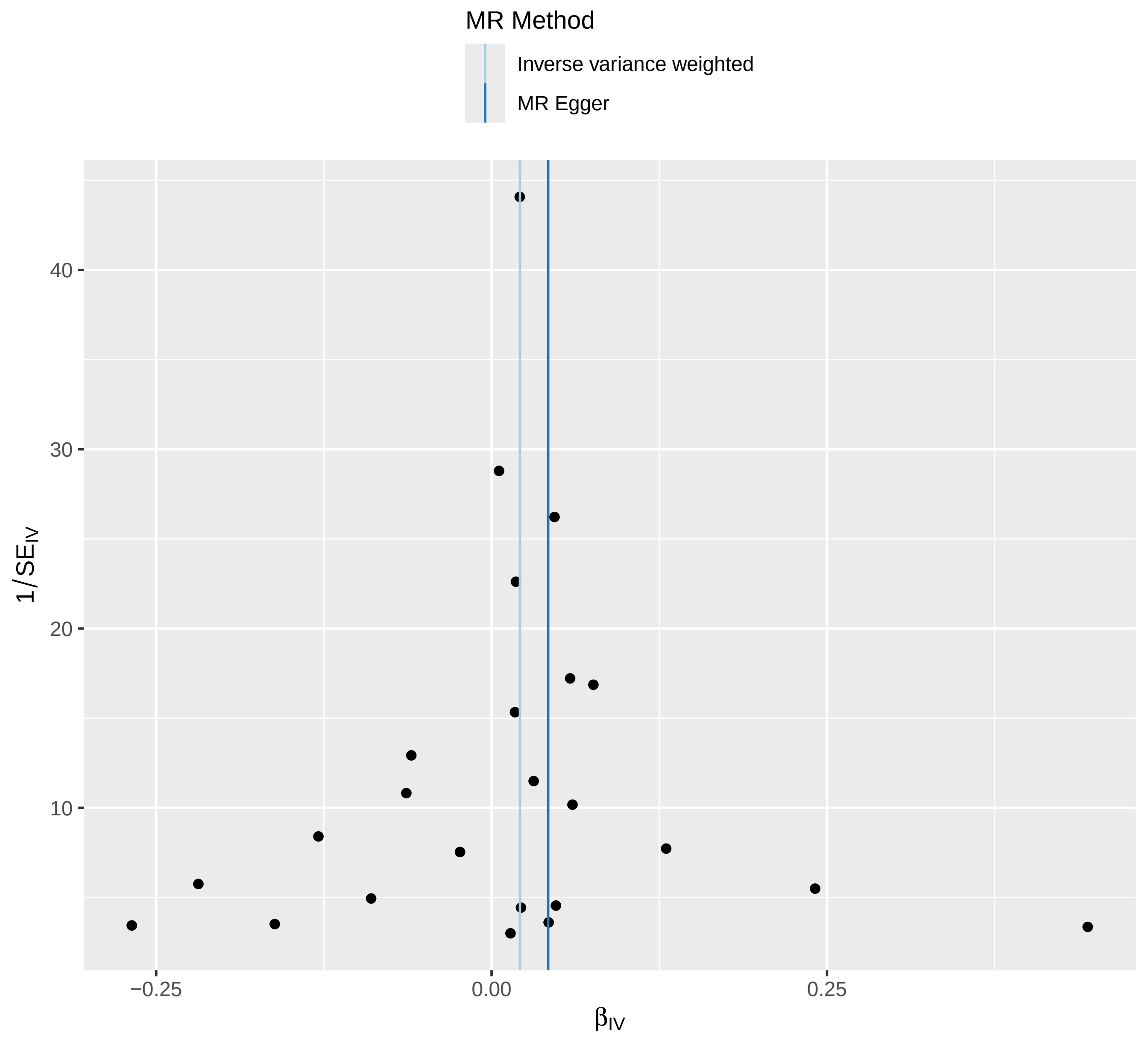

MR Method
Inverse variance weighted
MR Egger
40
30
20
10
1/SE_IV
−0.25
0.00
0.25
β_IV

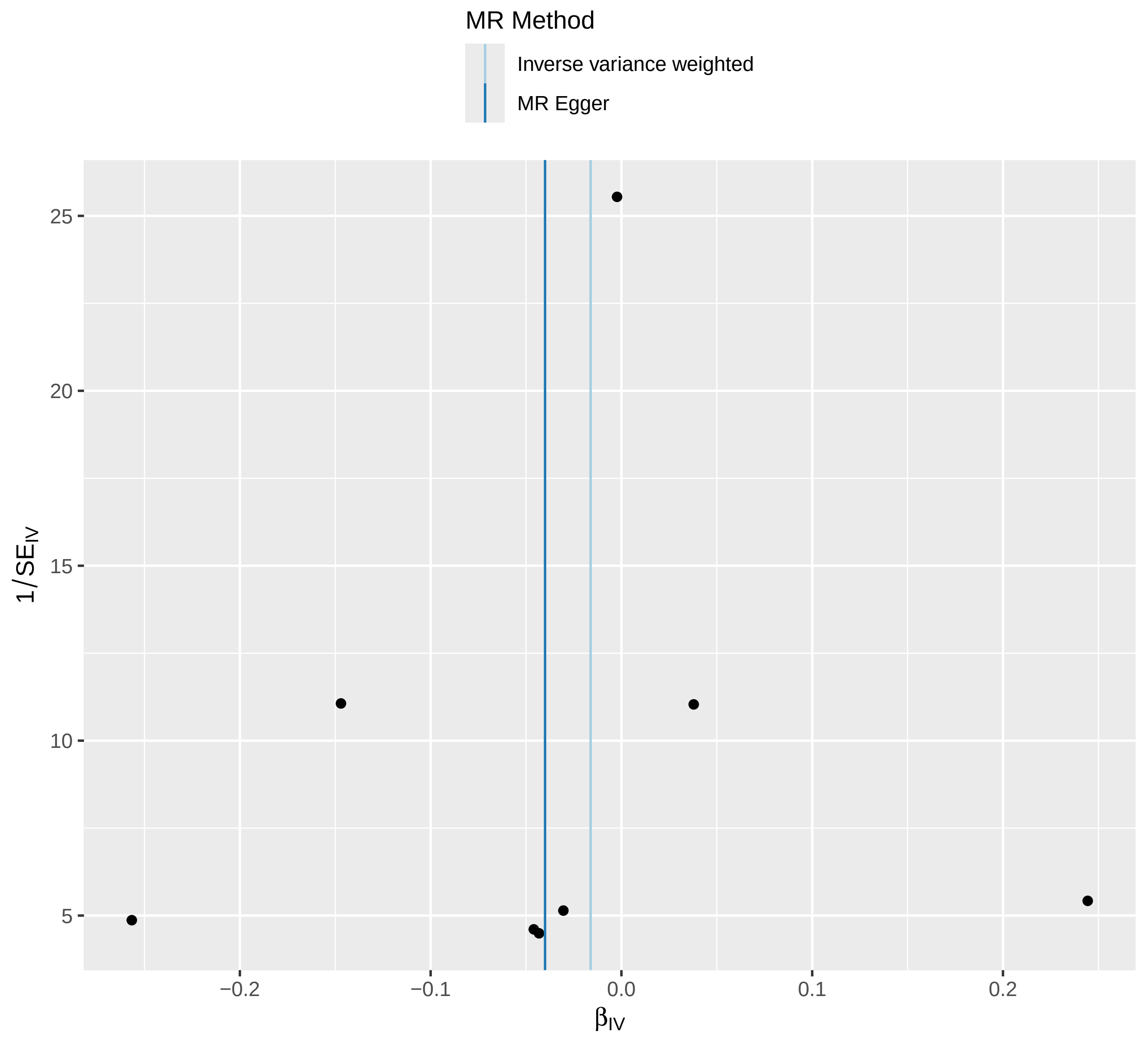

MR Method
Inverse variance weighted
MR Egger
25
20
15
10
5
$1/SE_{IV}$
−0.2
−0.1
0.0
0.1
0.2
$\beta_{IV}$

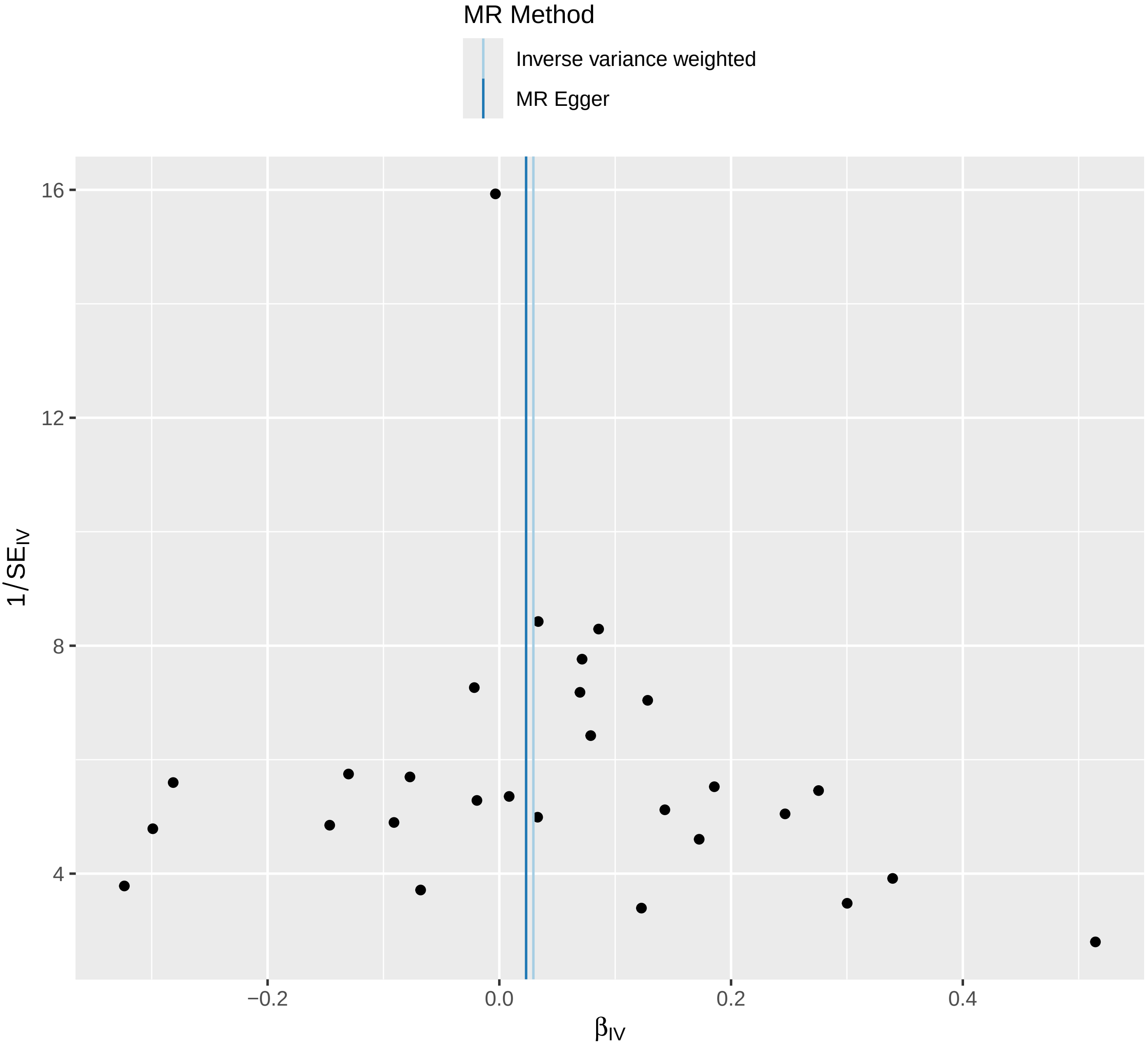

MR Method
Inverse variance weighted
MR Egger
16
12
8
4
1/SE_IV
−0.2
0.0
0.2
0.4
β_IV

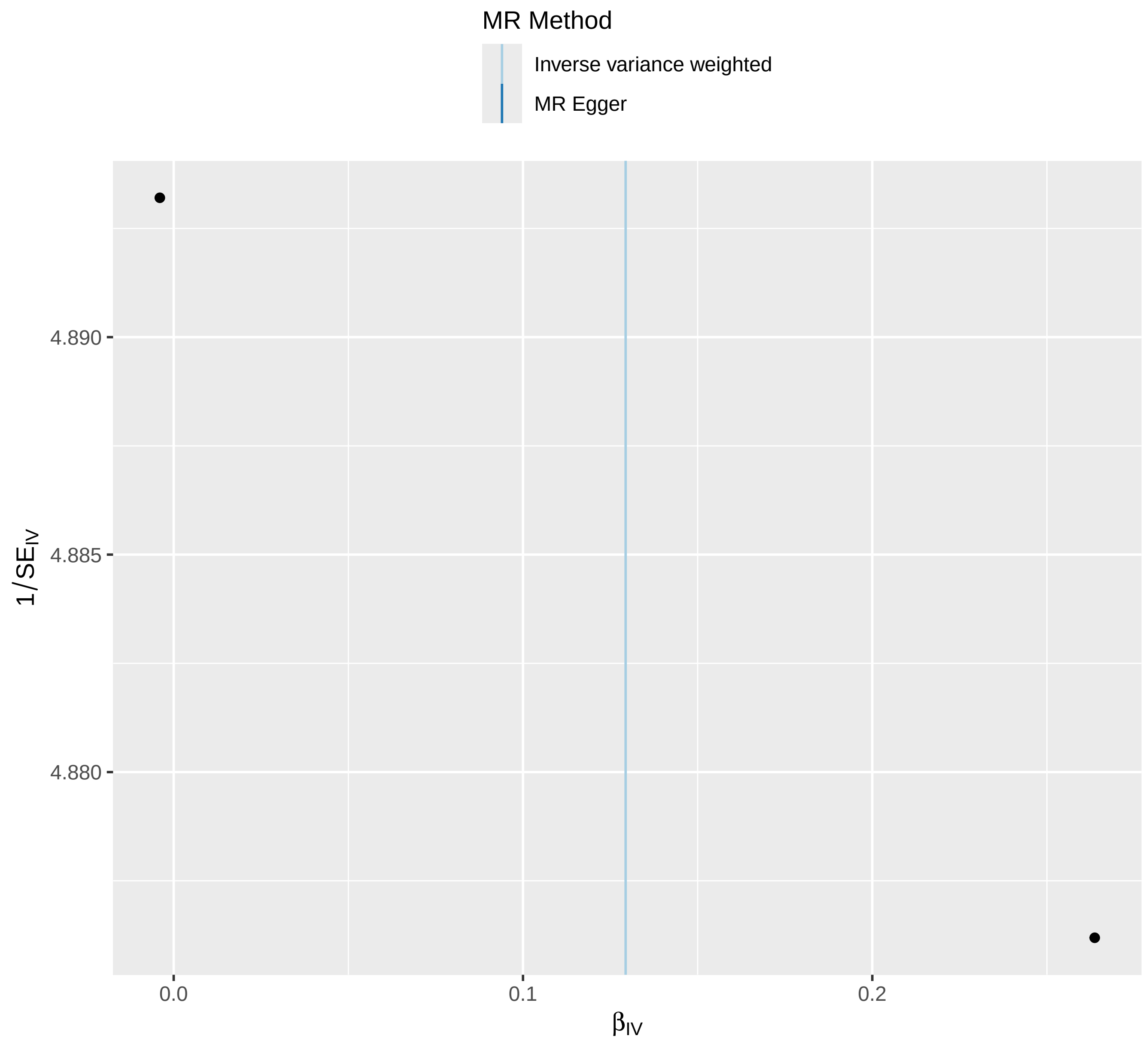

MR Method
Inverse variance weighted
MR Egger
4.890
4.885
4.880
1/SE$_{IV}$
0.0
0.1
0.2
β$_{IV}$

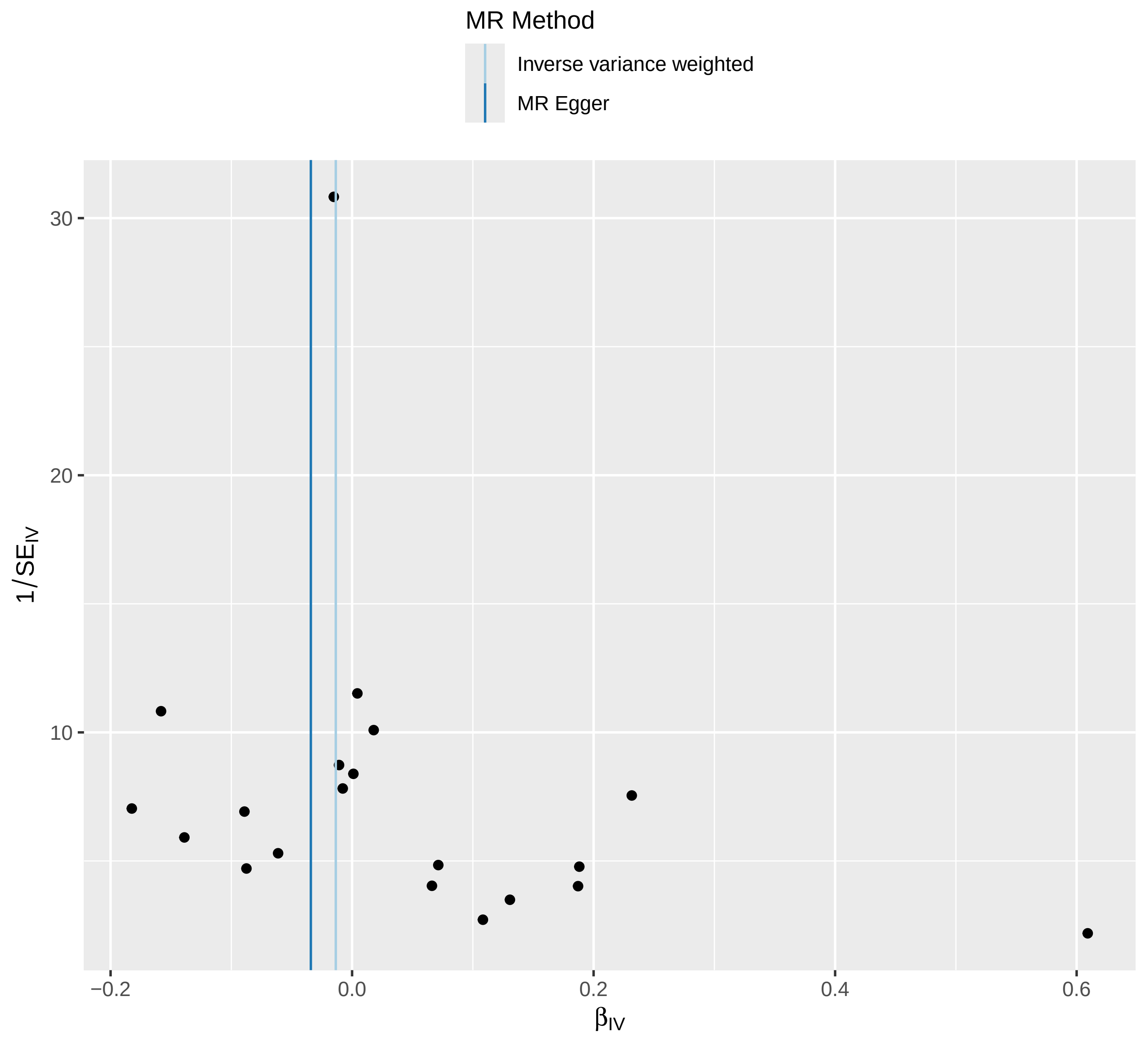

MR Method
Inverse variance weighted
MR Egger
30
20
10
1/SE_IV
−0.2
0.0
0.2
0.4
0.6
β_IV

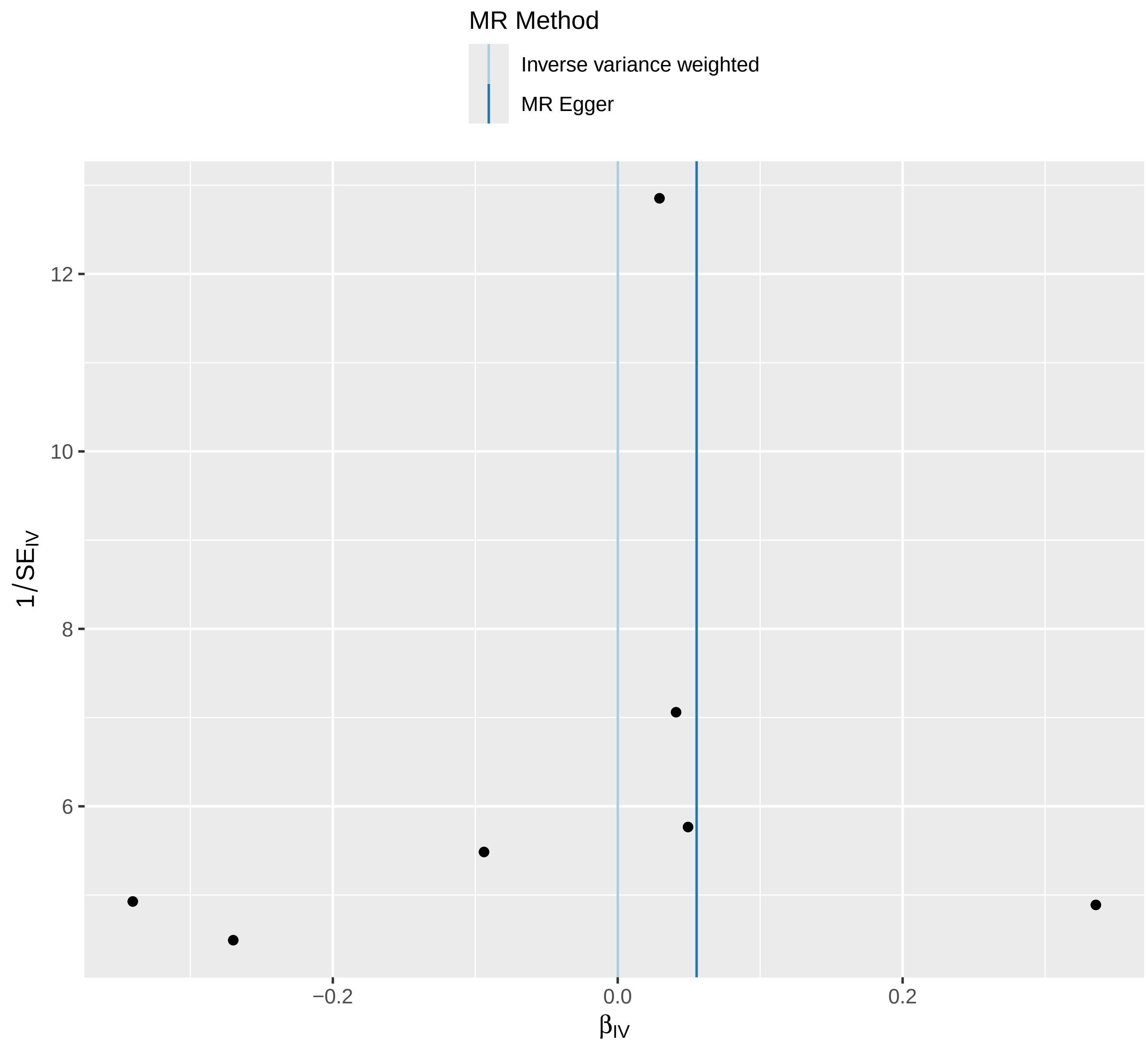

MR Method
Inverse variance weighted
MR Egger
12
10
8
6
1/SE$_{IV}$
−0.2
0.0
0.2
β$_{IV}$

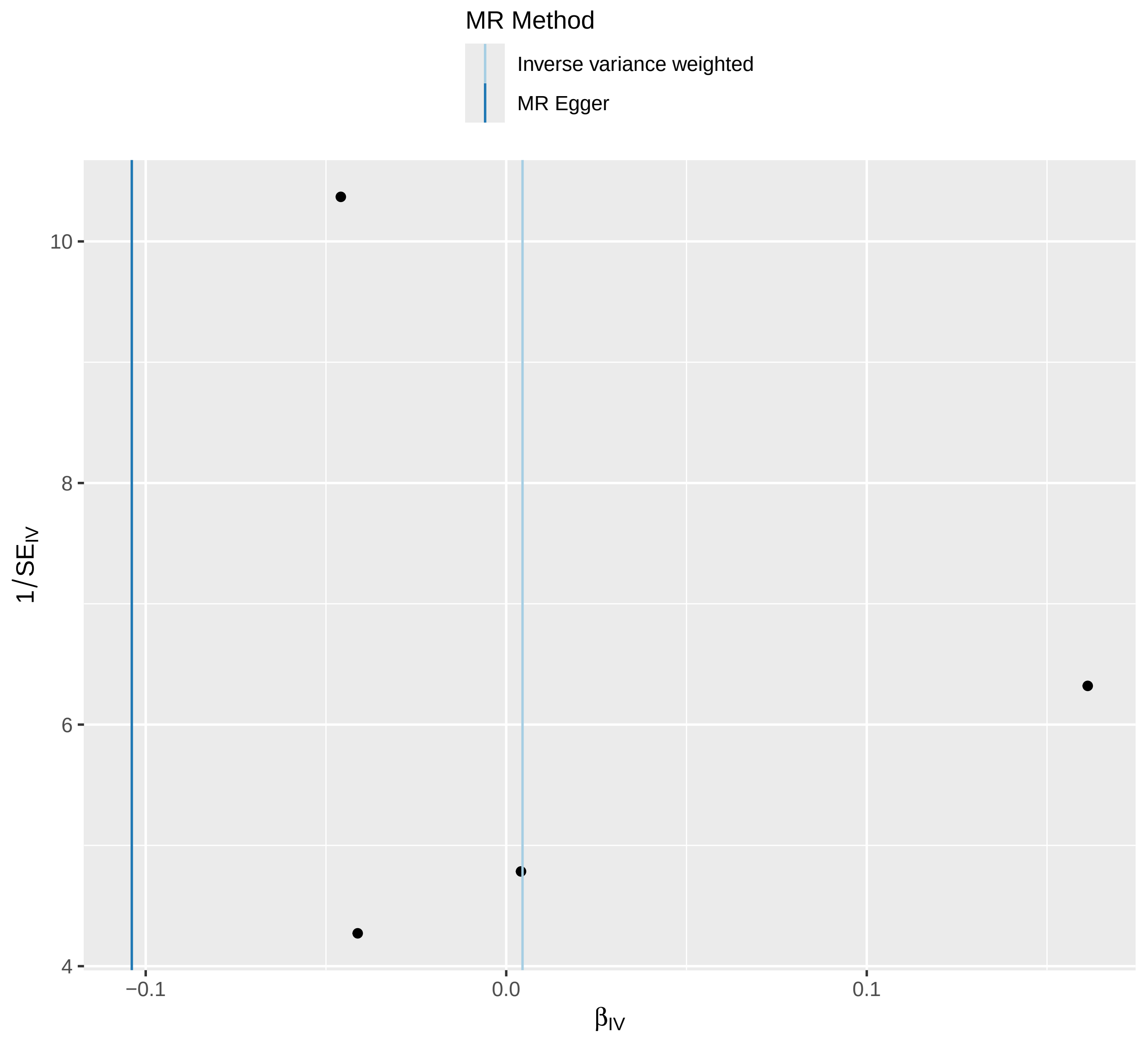
MR Method
Inverse variance weighted
MR Egger
10
8
6
4
1/SE$_{IV}$
−0.1
0.0
0.1
$\beta_{IV}$

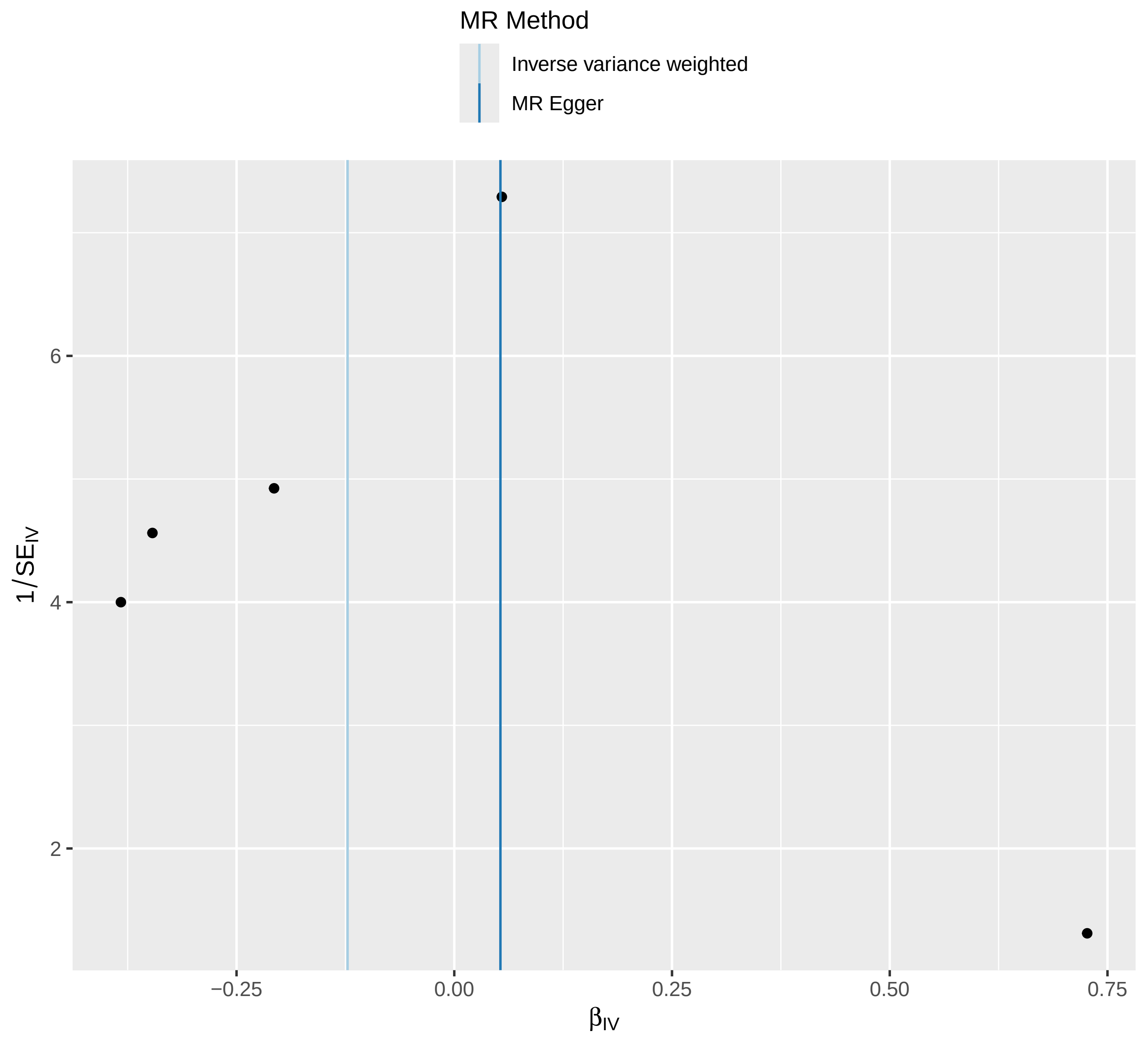

MR Method
Inverse variance weighted
MR Egger
6
4
2
−0.25
0.00
0.25
0.50
0.75
$\beta_{IV}$
$1/SE_{IV}$

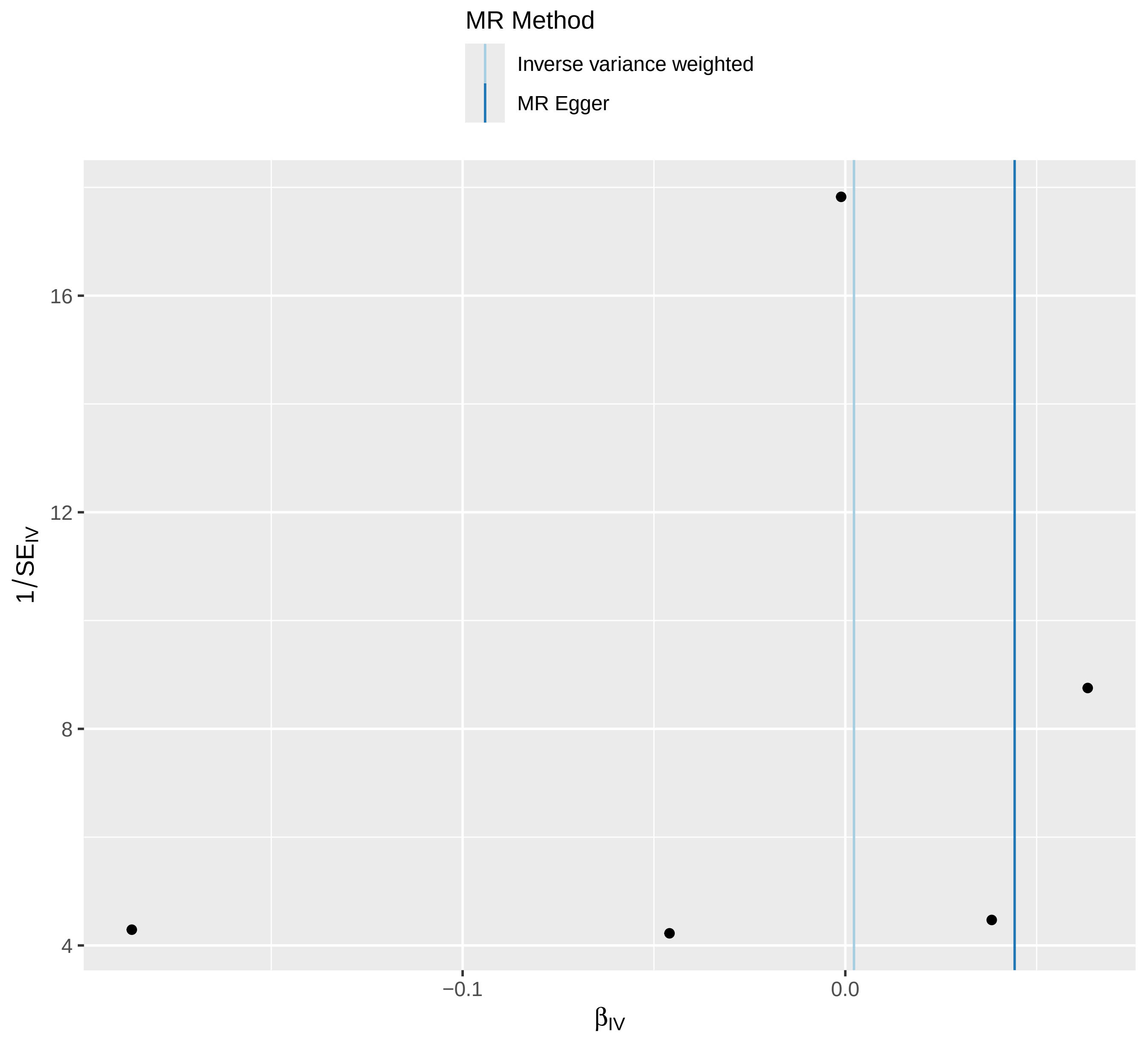

MR Method
Inverse variance weighted
MR Egger
16
12
8
4
1/SE$_{IV}$
−0.1
0.0
β$_{IV}$

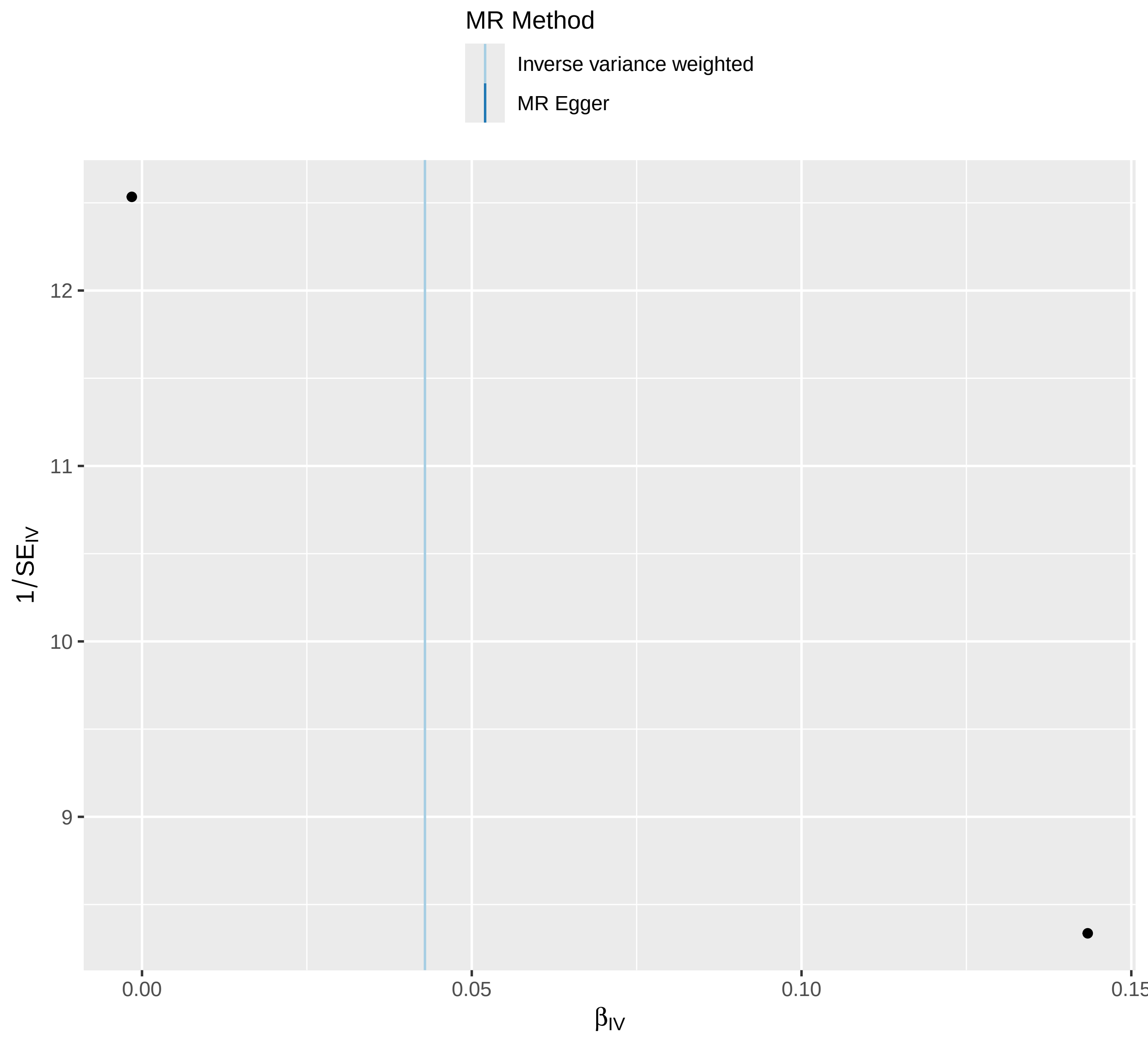
MR Method
Inverse variance weighted
MR Egger
12
11
10
9
1/SE_IV
0.00
0.05
0.10
0.15
β_IV

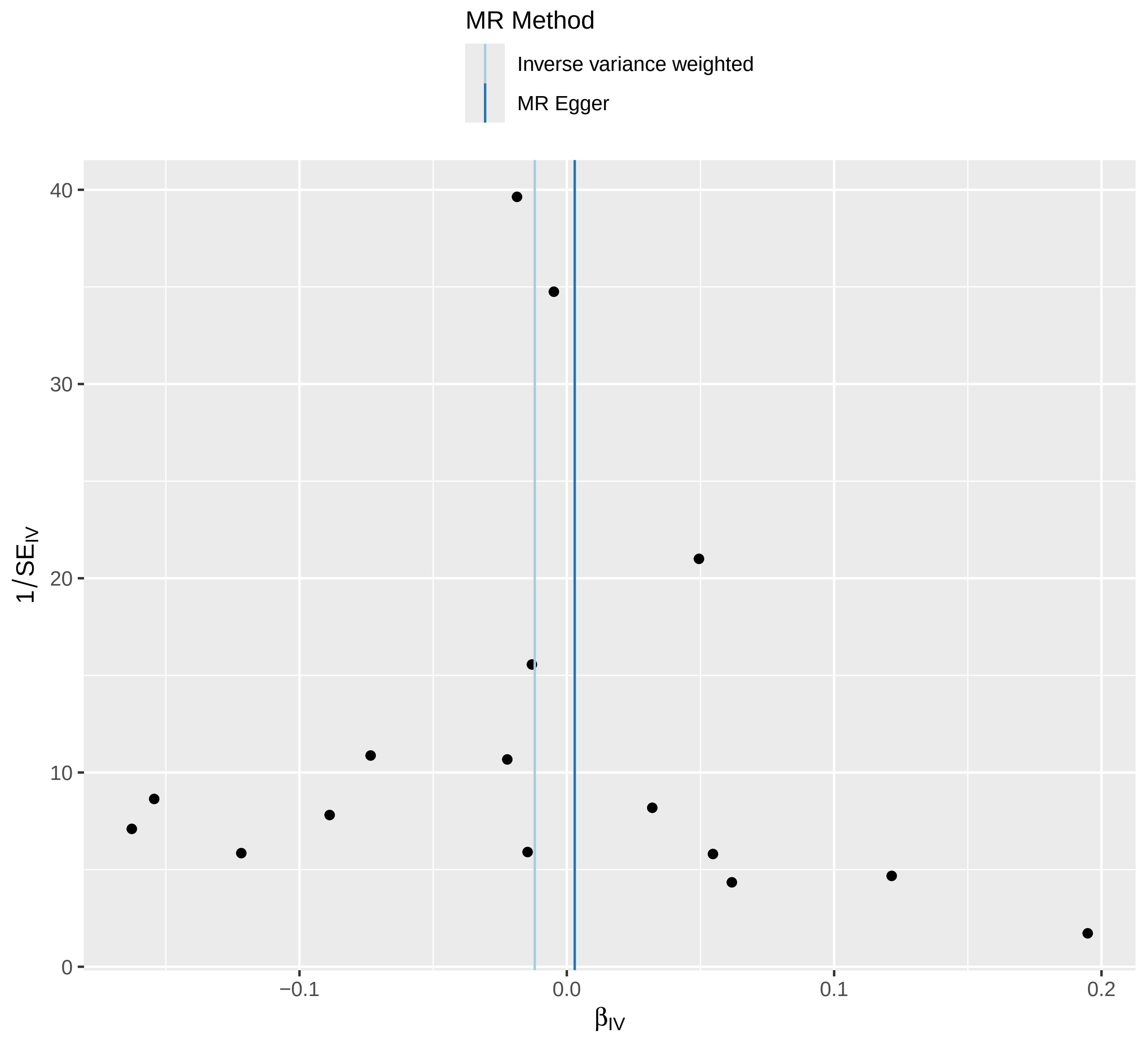

MR Method
Inverse variance weighted
MR Egger
40
30
20
10
0
$1/SE_{IV}$
−0.1
0.0
0.1
0.2
$\beta_{IV}$

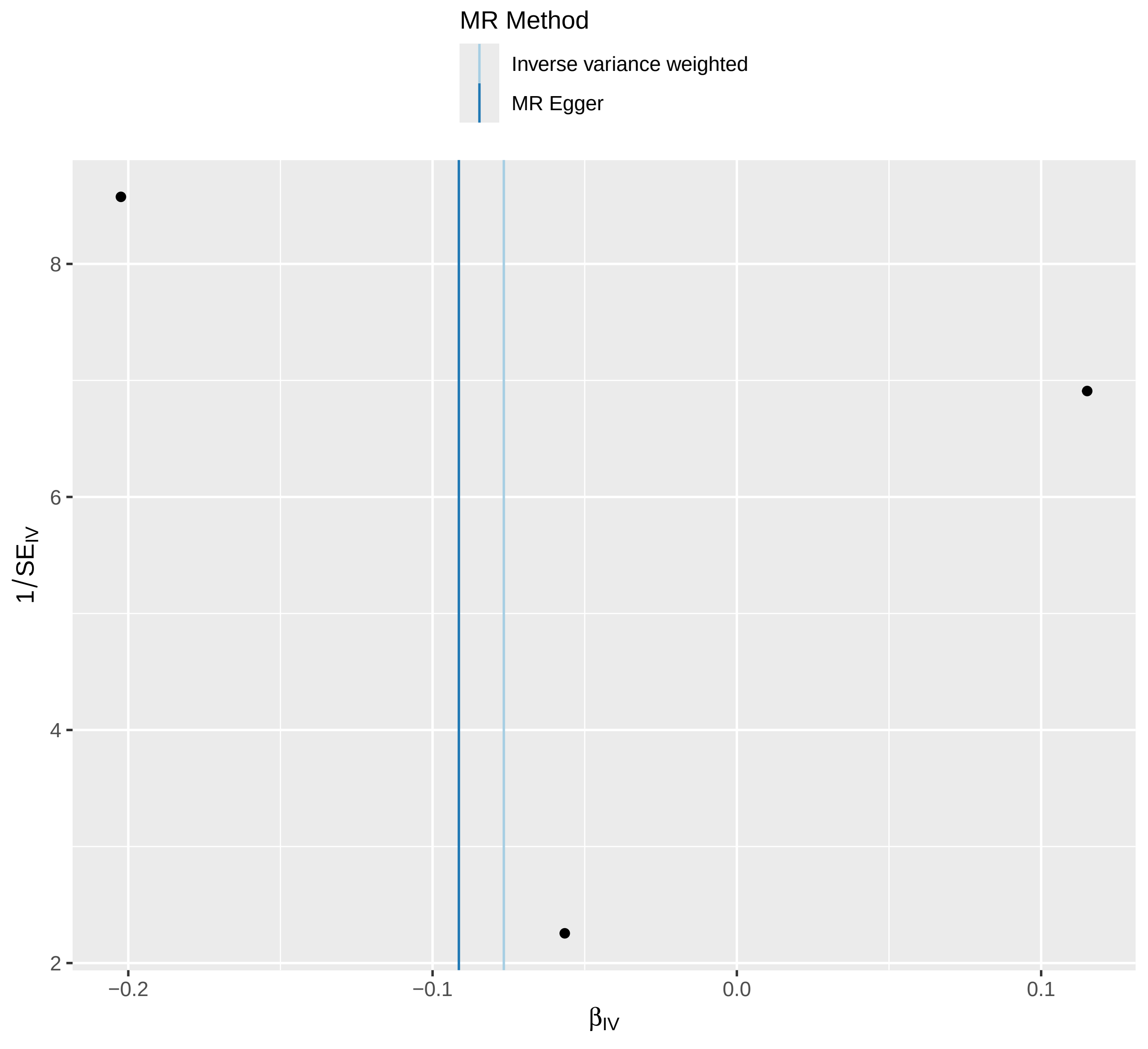

MR Method
Inverse variance weighted
MR Egger
8
6
4
2
1/SE_IV
−0.2
−0.1
0.0
0.1
β_IV

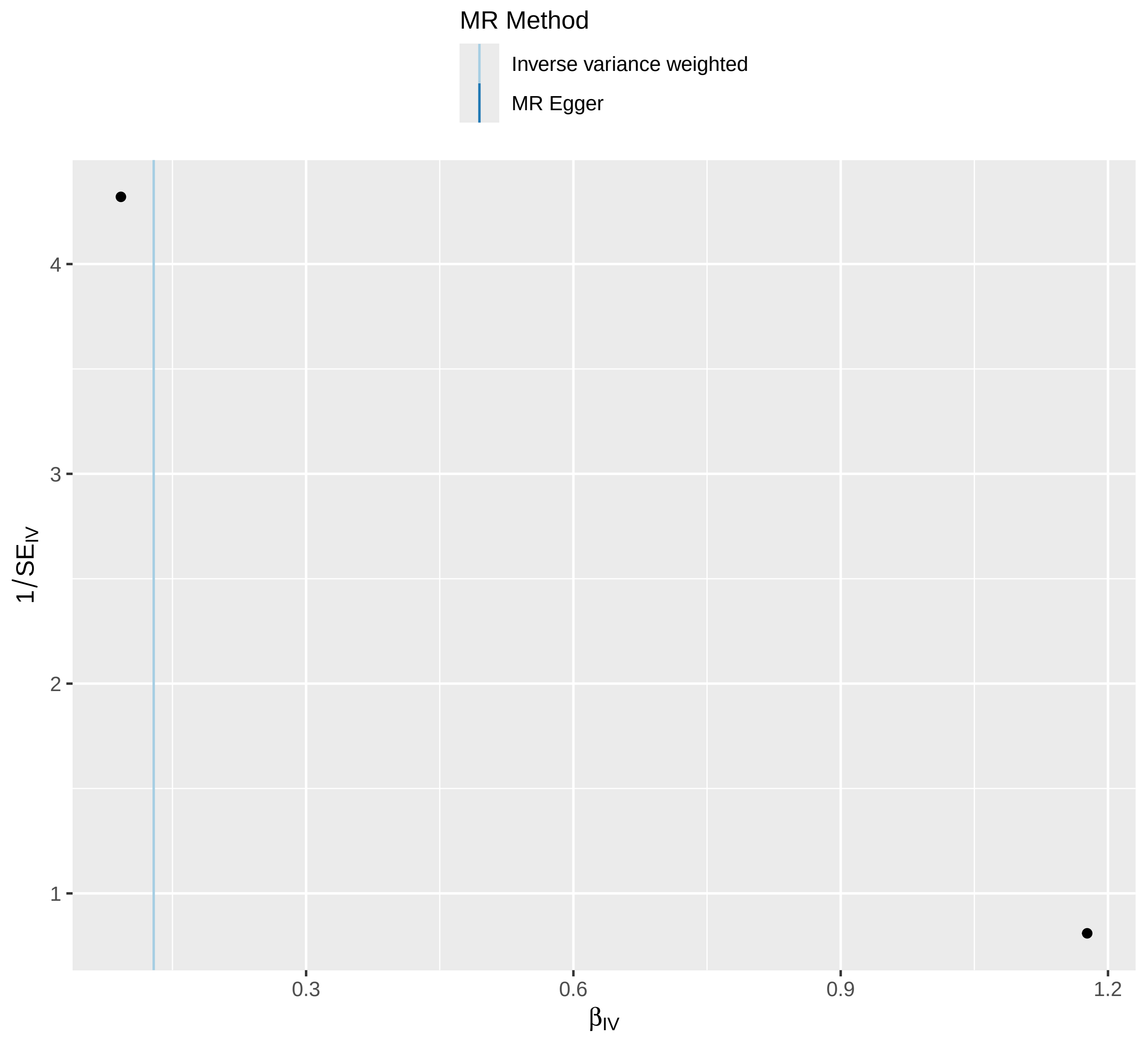
MR Method
Inverse variance weighted
MR Egger
4
3
2
1
$1/SE_{IV}$
0.3
0.6
0.9
1.2
$\beta_{IV}$

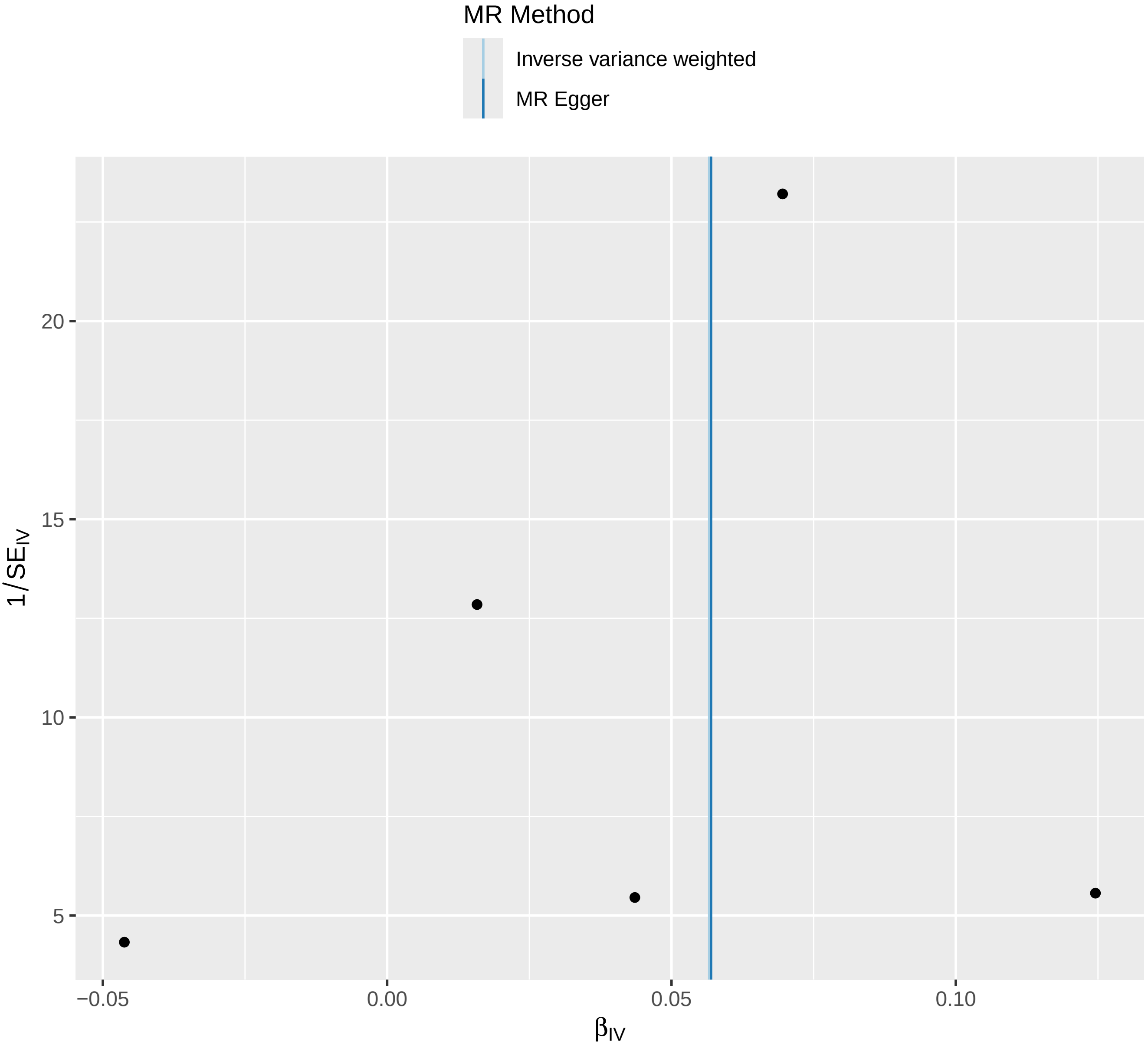
MR Method
Inverse variance weighted
MR Egger
20
15
10
5
1/SE_IV
−0.05
0.00
0.05
0.10
β_IV

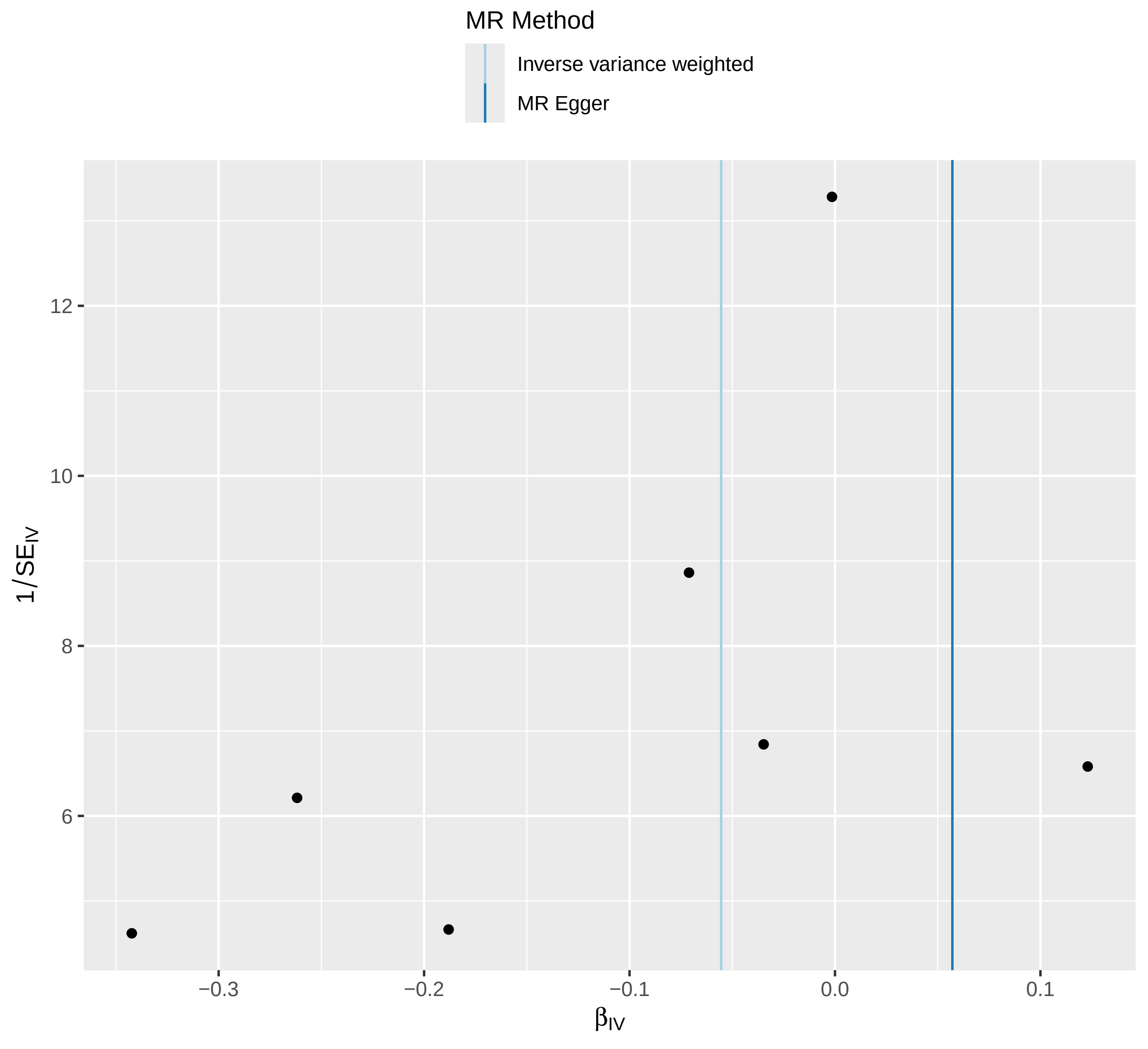

MR Method
Inverse variance weighted
MR Egger
12
10
8
6
1/SE$_{IV}$
−0.3
−0.2
−0.1
0.0
0.1
β$_{IV}$

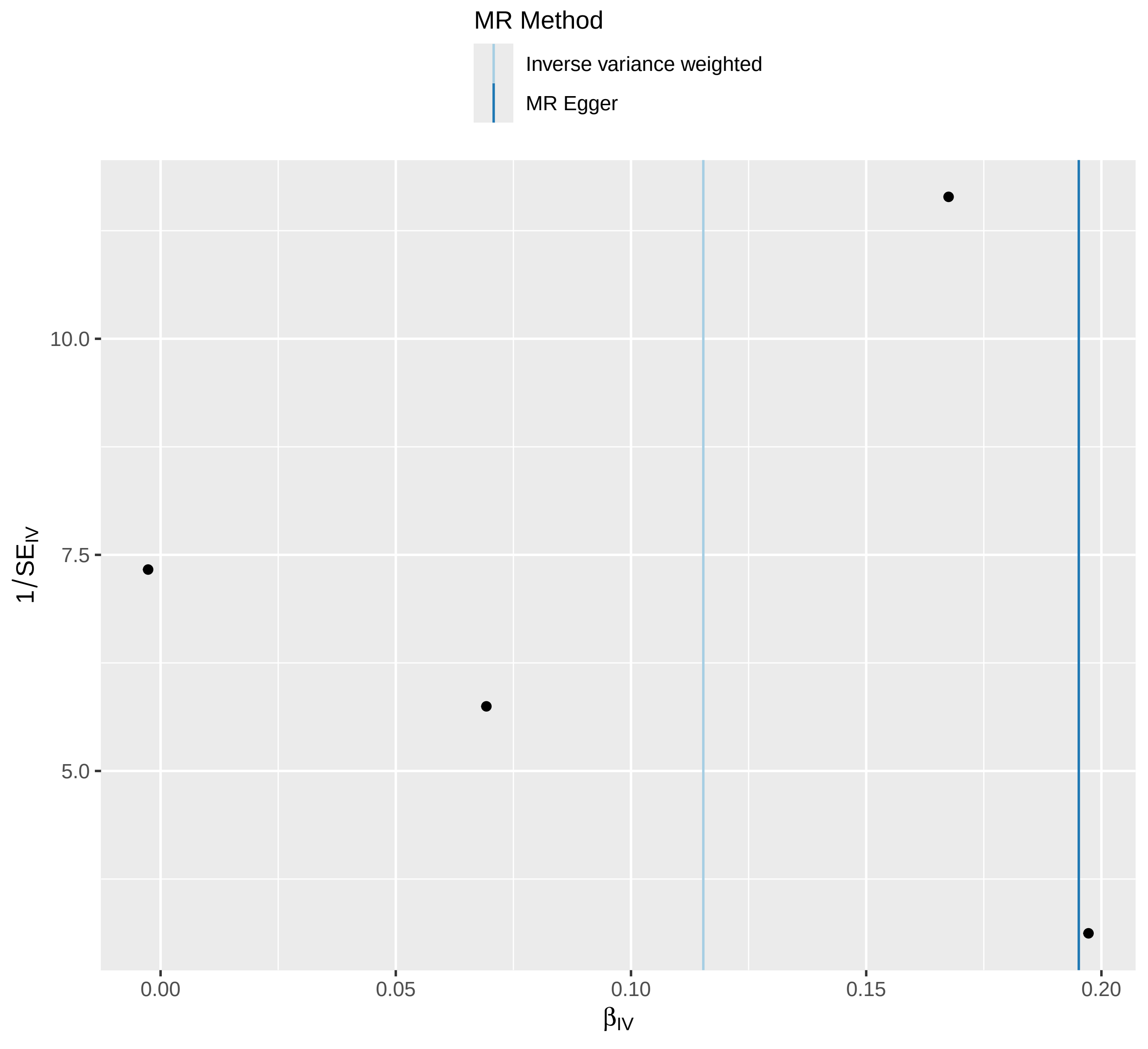
MR Method
Inverse variance weighted
MR Egger
10.0
7.5
5.0
0.00
0.05
0.10
0.15
0.20
$1/SE_{IV}$
$\beta_{IV}$

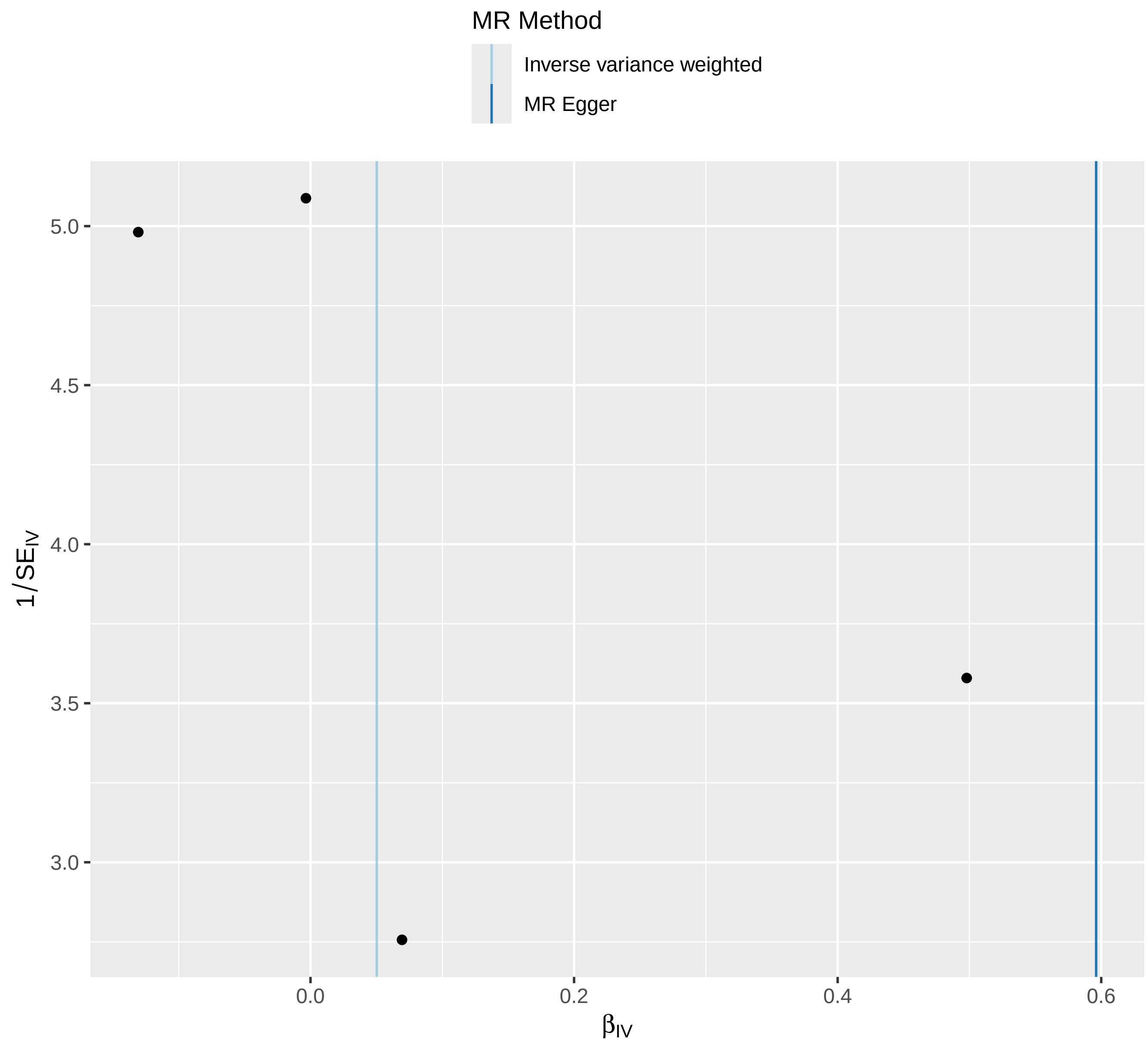
MR Method
Inverse variance weighted
MR Egger
5.0
4.5
4.0
3.5
3.0
$1/SE_{IV}$
0.0
0.2
0.4
0.6
$\beta_{IV}$

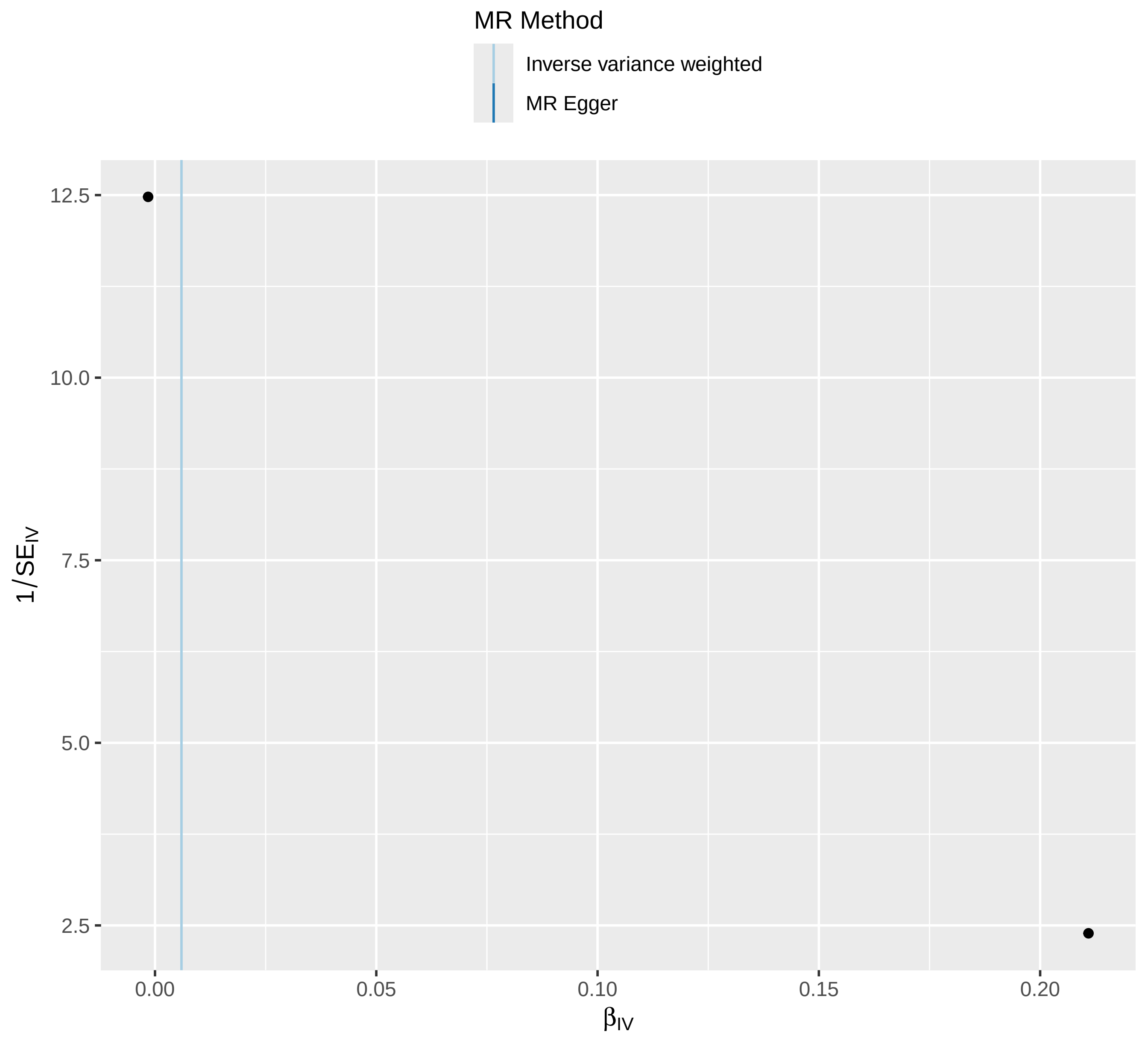

MR Method
Inverse variance weighted
MR Egger
12.5
10.0
7.5
5.0
2.5
$1/SE_{IV}$
0.00
0.05
0.10
0.15
0.20
$\beta_{IV}$

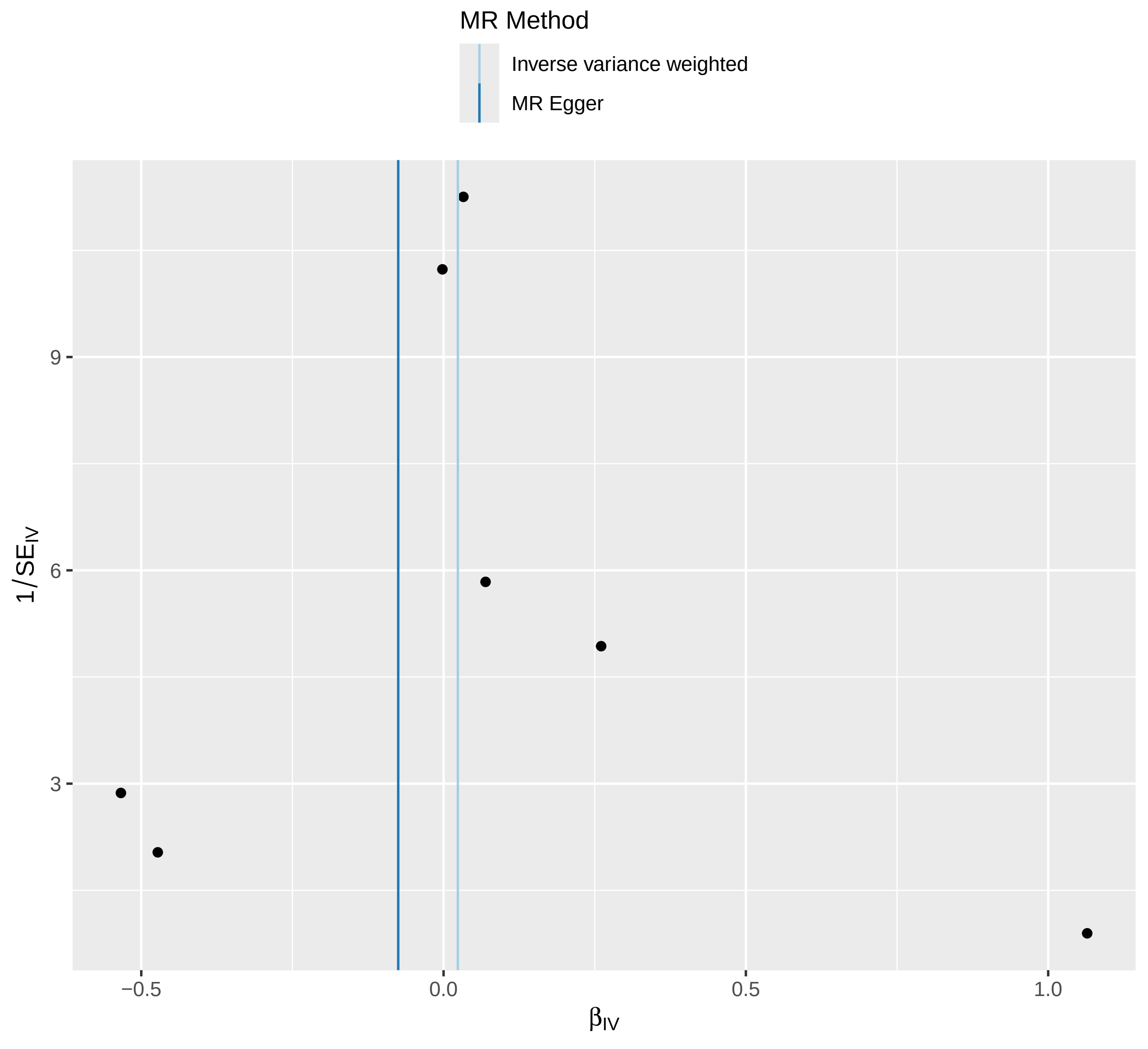
MR Method
Inverse variance weighted
MR Egger
9
6
3
1/SE_IV
−0.5
0.0
0.5
1.0
β_IV

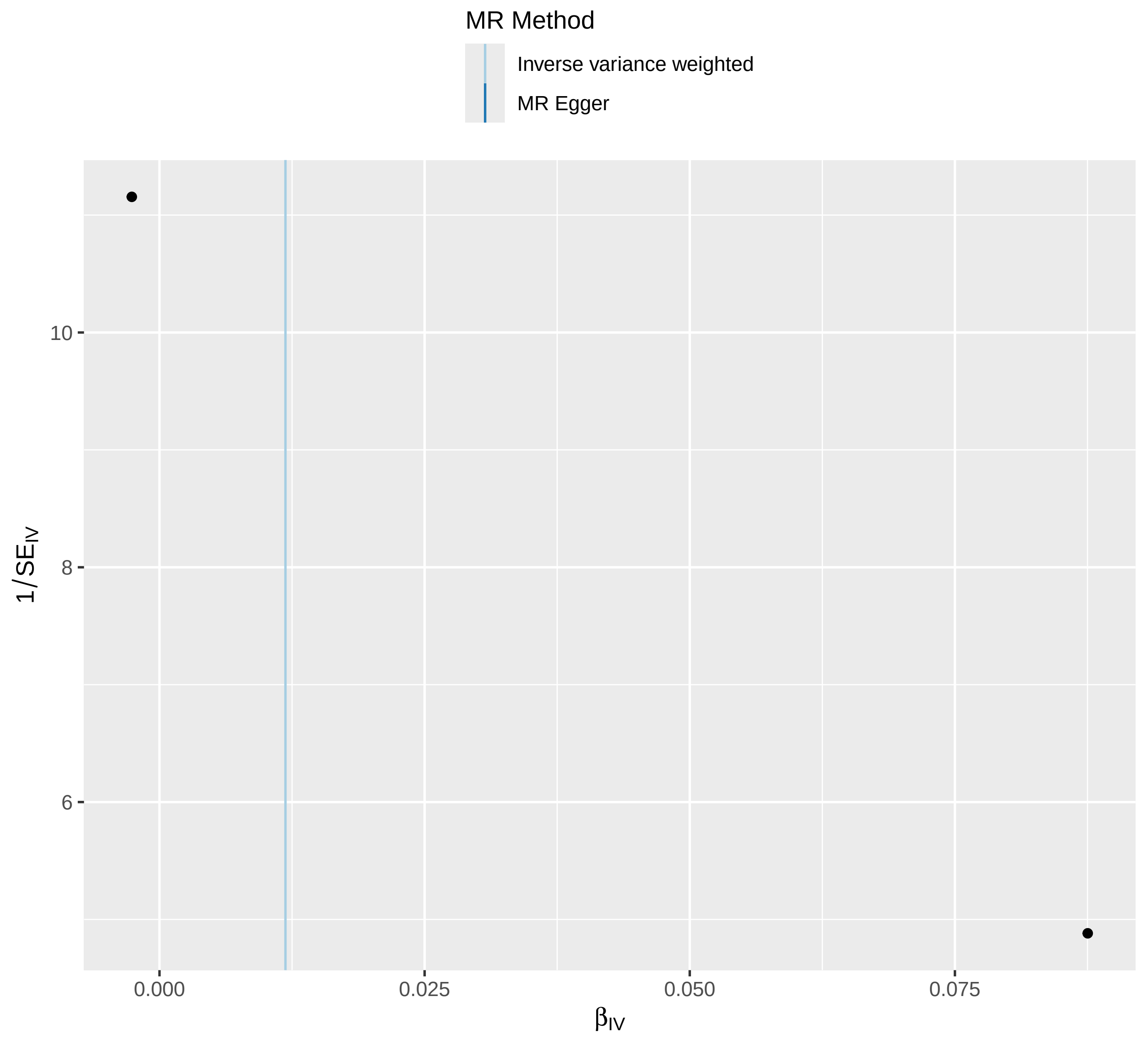

MR Method
Inverse variance weighted
MR Egger
10
8
6
1/SE_IV
0.000
0.025
0.050
0.075
β_IV

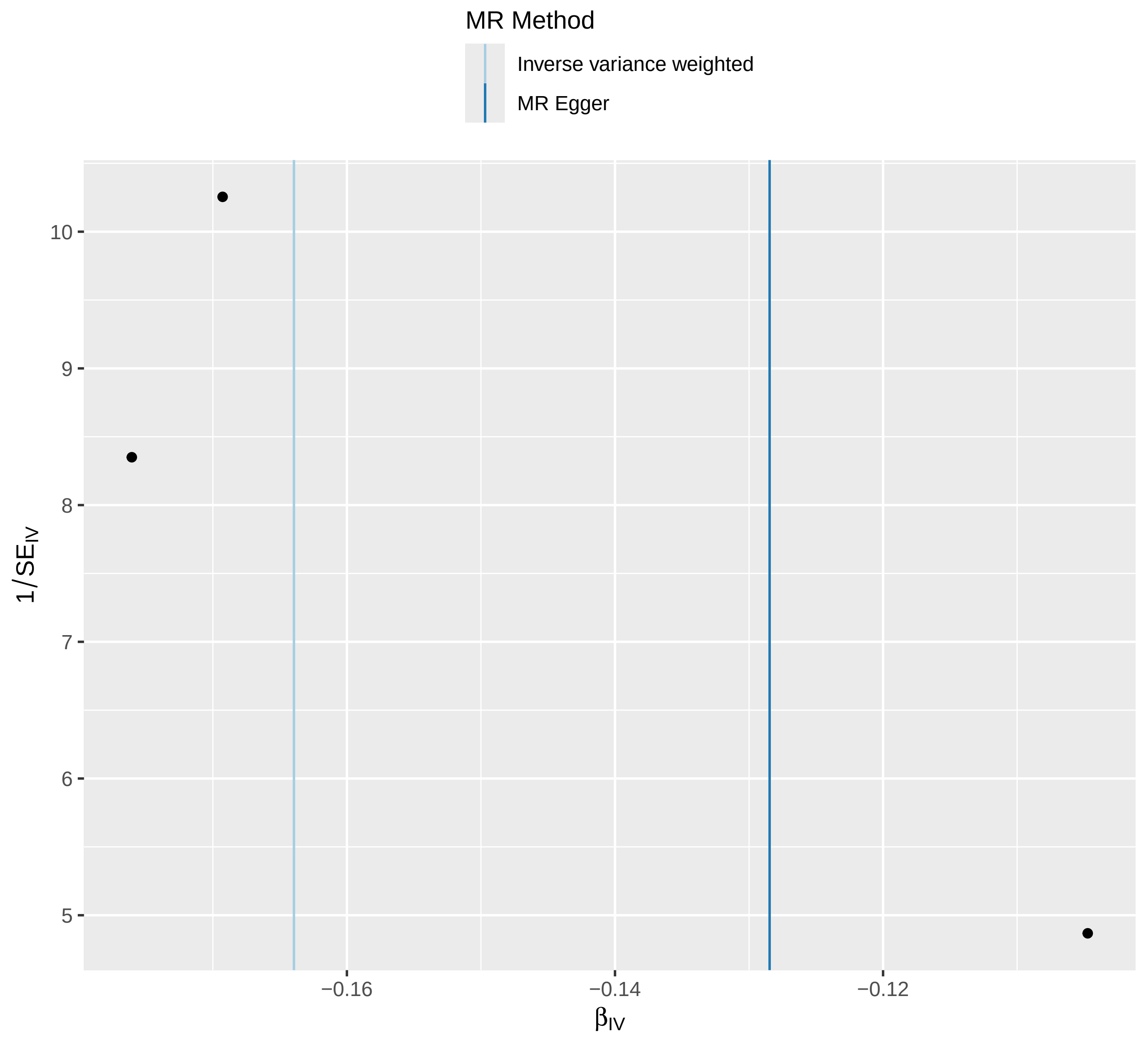

MR Method
Inverse variance weighted
MR Egger
10
9
8
7
6
5
1/SE$_{IV}$
−0.16
−0.14
−0.12
β$_{IV}$

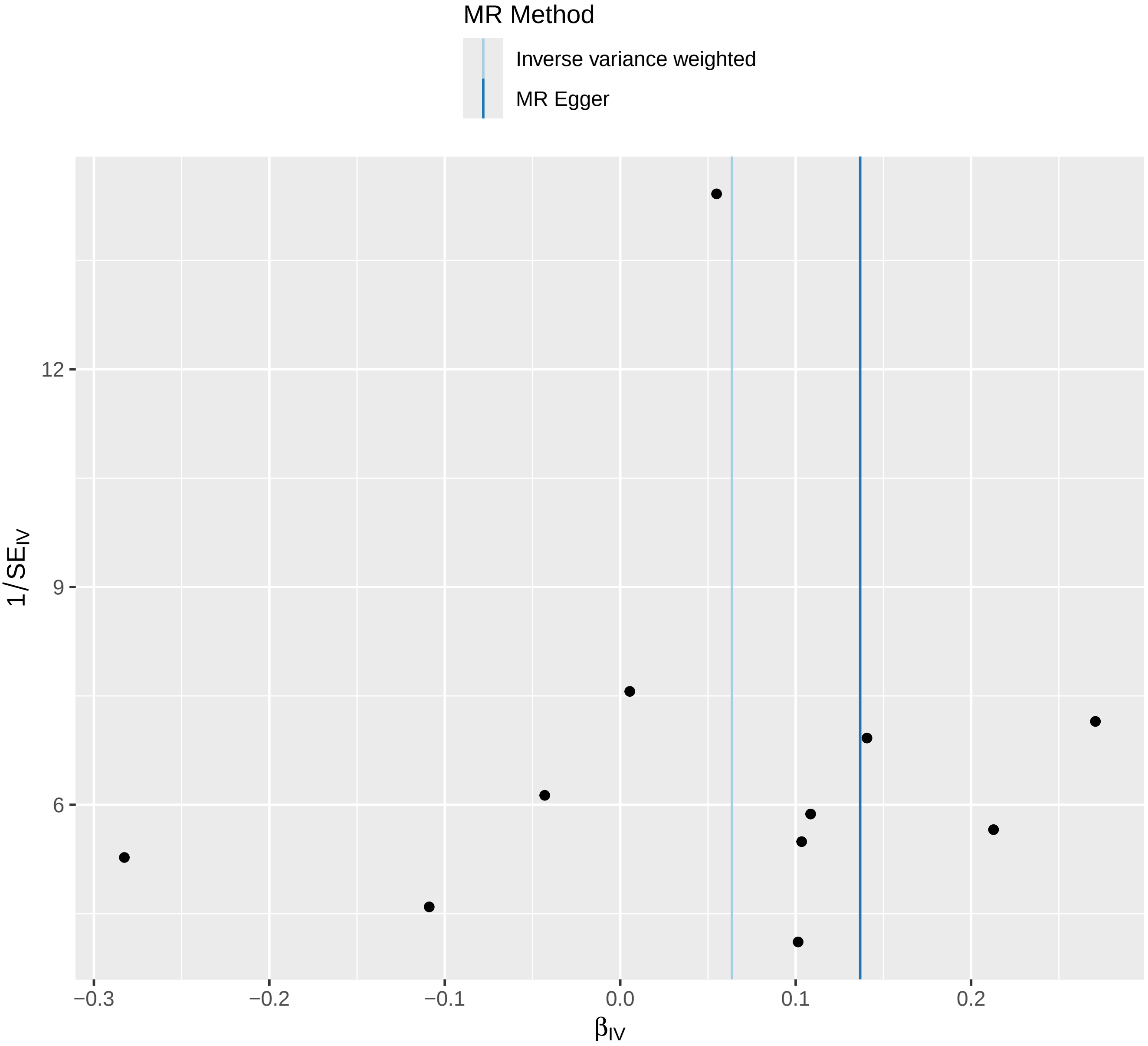

MR Method
Inverse variance weighted
MR Egger
1/SE$_{IV}$
12
9
6
−0.3
−0.2
−0.1
0.0
0.1
0.2
β$_{IV}$

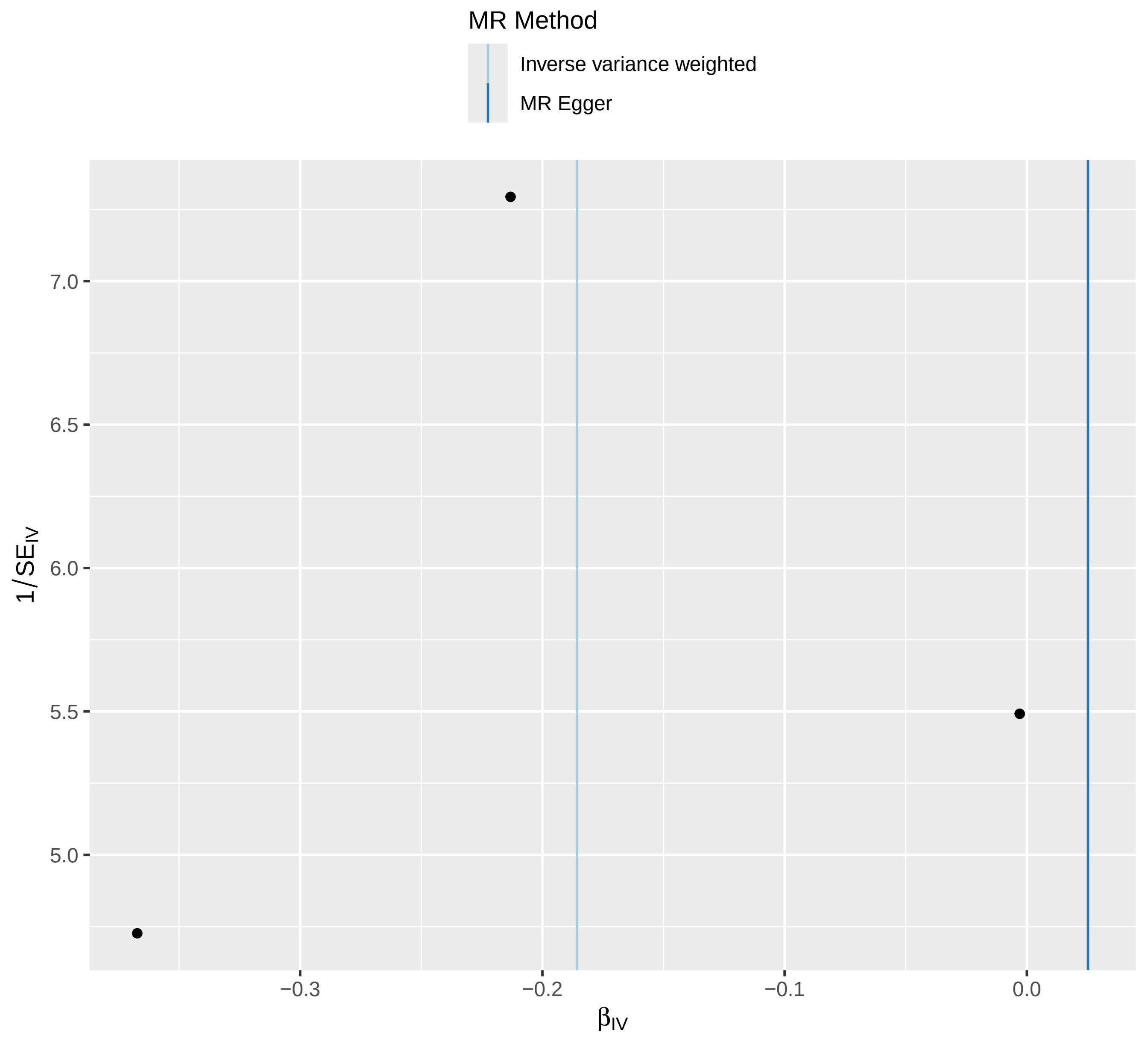
MR Method
Inverse variance weighted
MR Egger
7.0
6.5
6.0
5.5
5.0
$1/SE_{IV}$
−0.3
−0.2
−0.1
0.0
$\beta_{IV}$

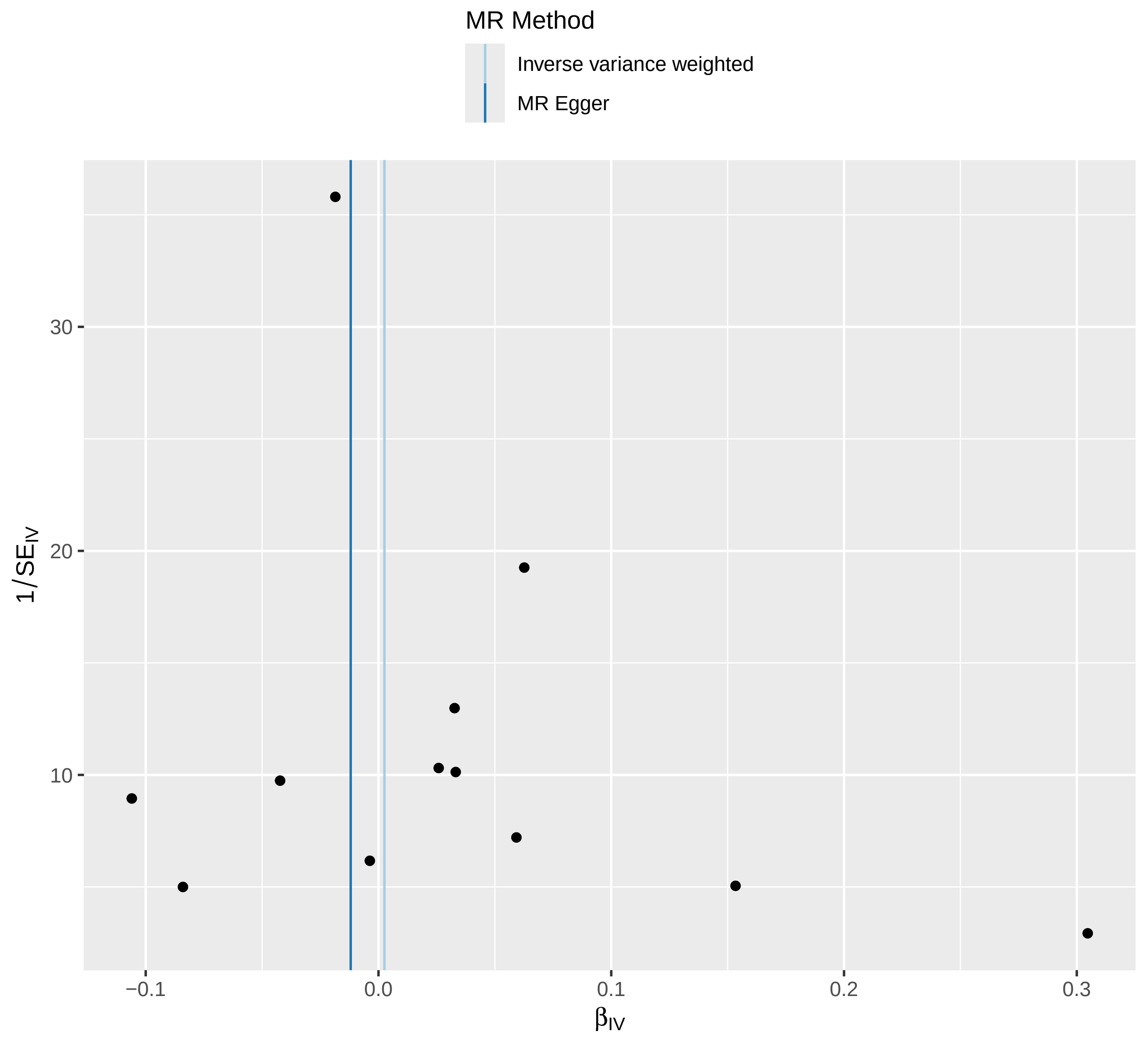

MR Method
Inverse variance weighted
MR Egger
30
20
10
1/SE$_{IV}$
−0.1
0.0
0.1
0.2
0.3
β$_{IV}$

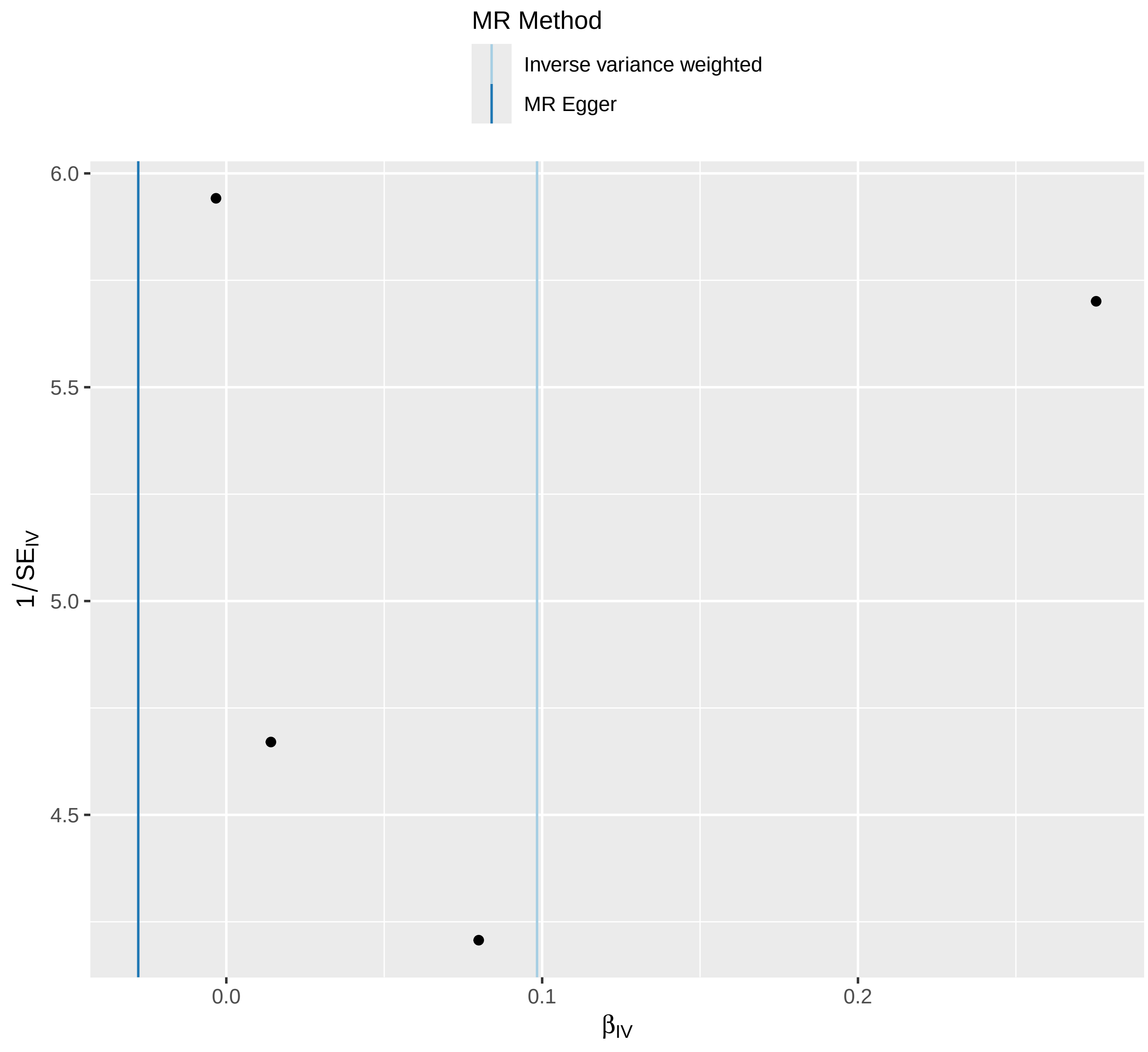

MR Method
Inverse variance weighted
MR Egger
6.0
5.5
5.0
4.5
1/SE$_{IV}$
0.0
0.1
0.2
β$_{IV}$

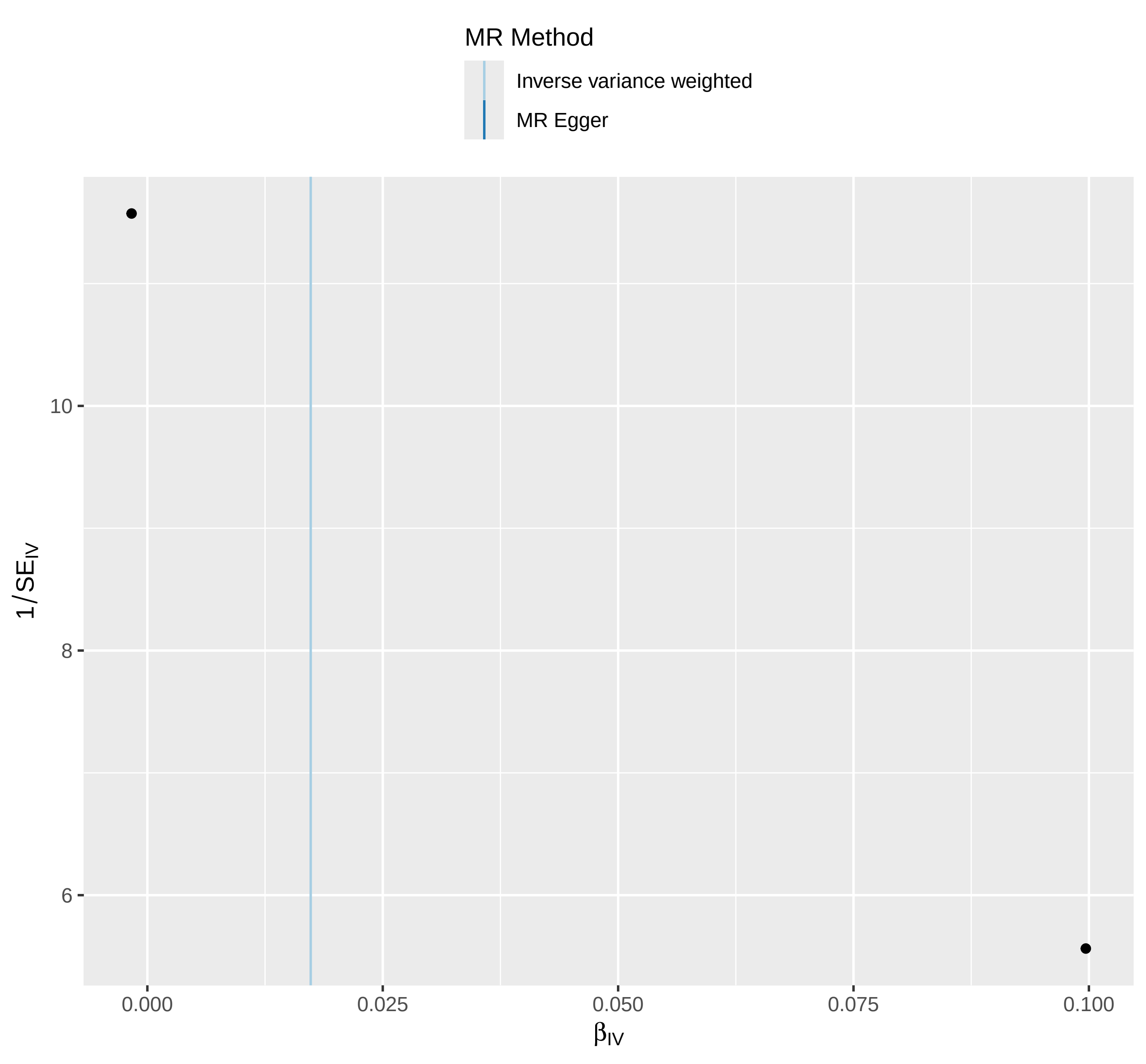
MR Method
Inverse variance weighted
MR Egger
$1/SE_{IV}$
10
8
6
0.000
0.025
0.050
0.075
0.100
$\beta_{IV}$

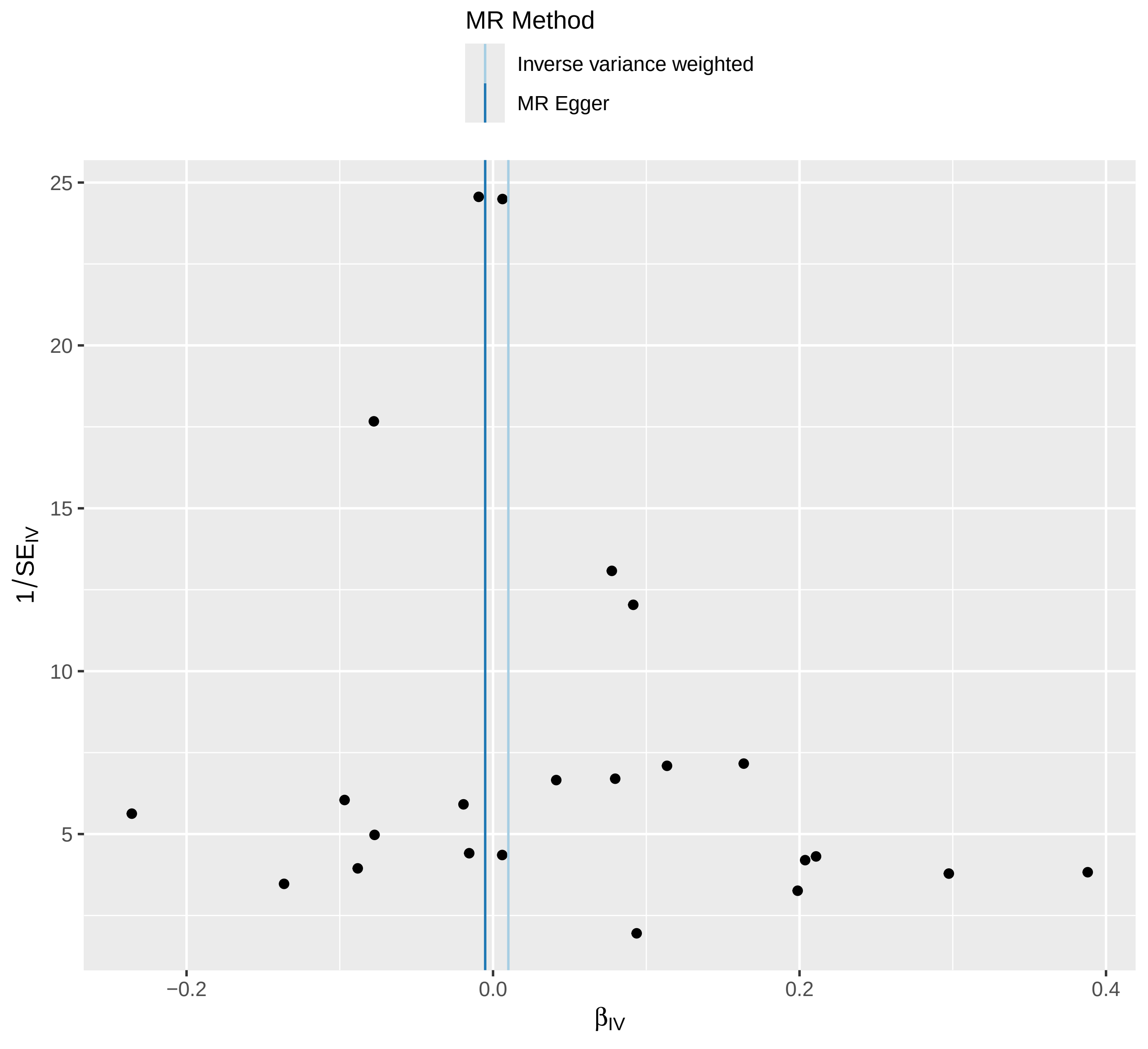

MR Method
Inverse variance weighted
MR Egger
25
20
15
10
5
1/SE_IV
−0.2
0.0
0.2
0.4
β_IV

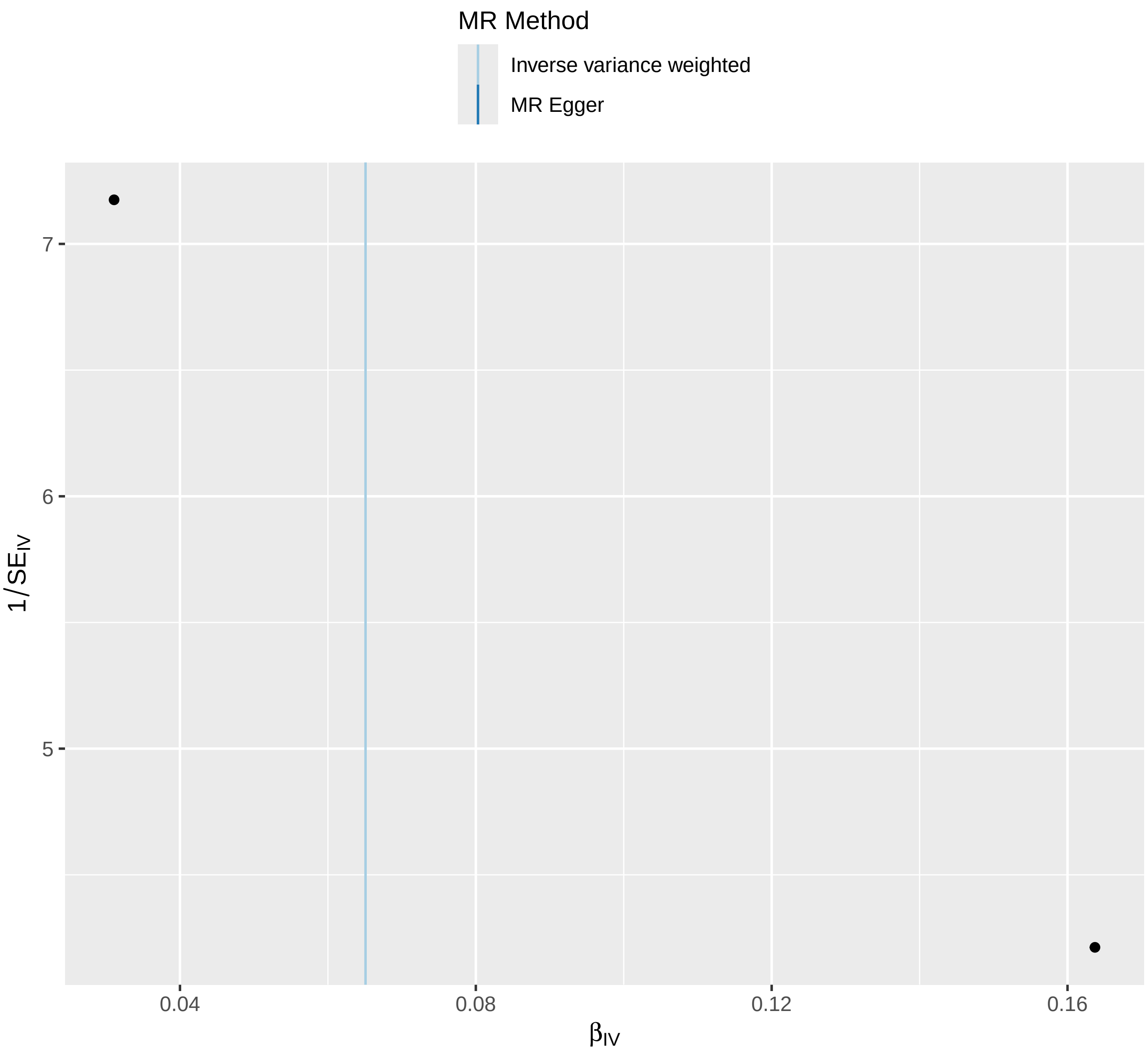
MR Method
Inverse variance weighted
MR Egger
7
6
5
$1/SE_{IV}$
0.04
0.08
0.12
0.16
$\beta_{IV}$

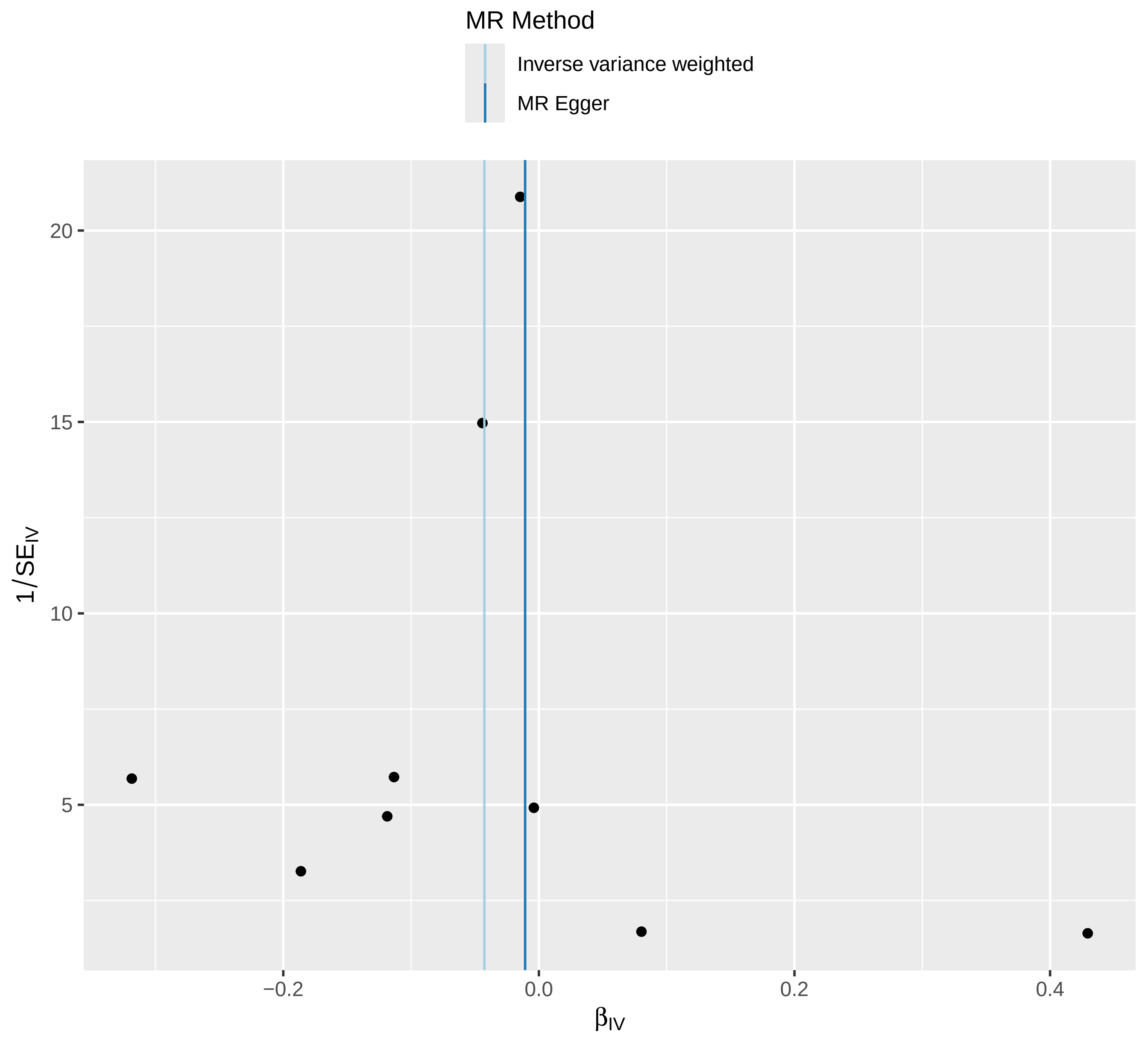
MR Method
Inverse variance weighted
MR Egger
1/SE_IV
20
15
10
5
−0.2
0.0
0.2
0.4
β_IV

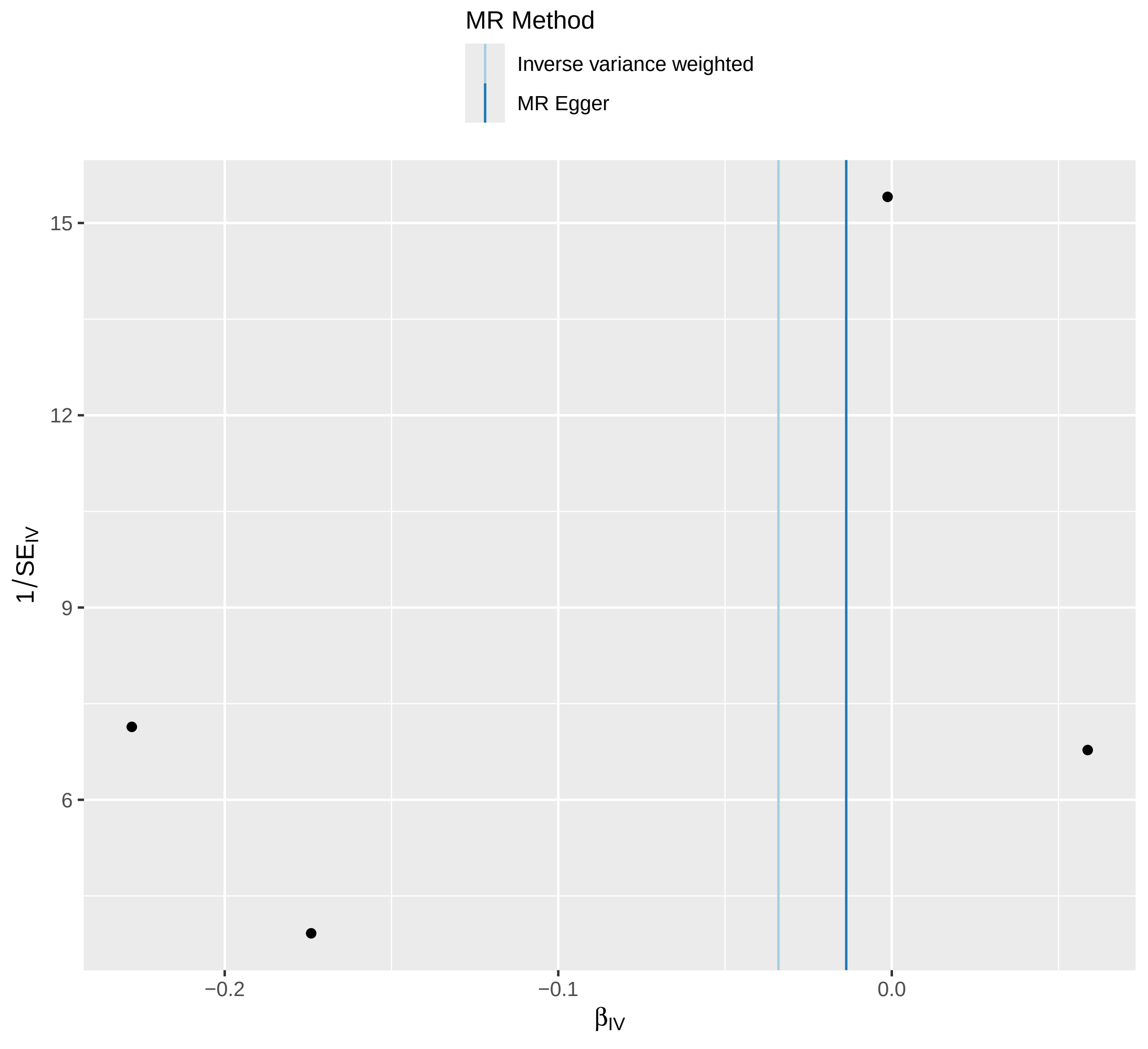

MR Method
Inverse variance weighted
MR Egger
15
12
9
6
1/SE$_{IV}$
−0.2
−0.1
0.0
$\beta_{IV}$

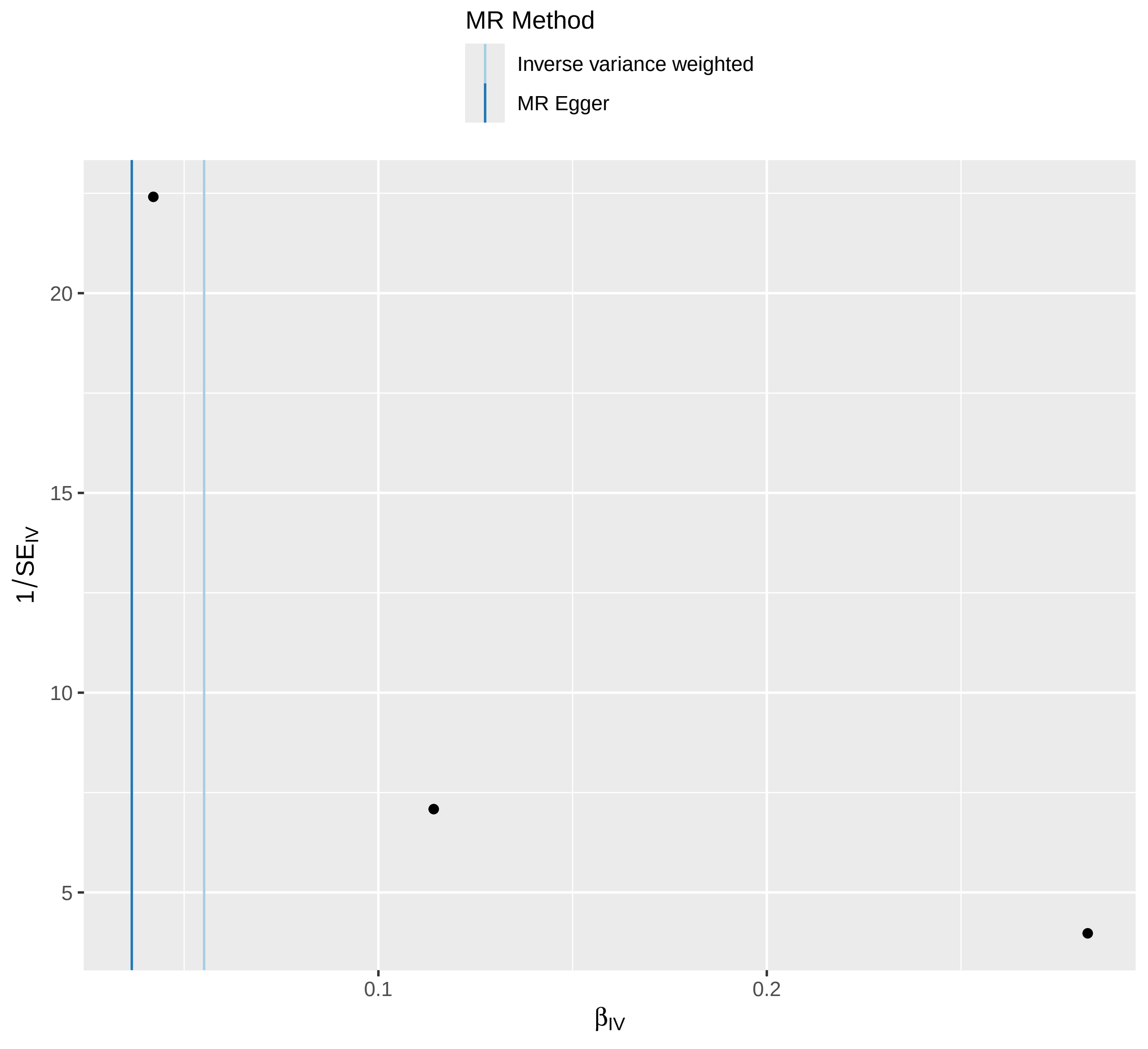

MR Method
Inverse variance weighted
MR Egger
20
15
10
5
0.1
0.2
1/SE_IV
β_IV

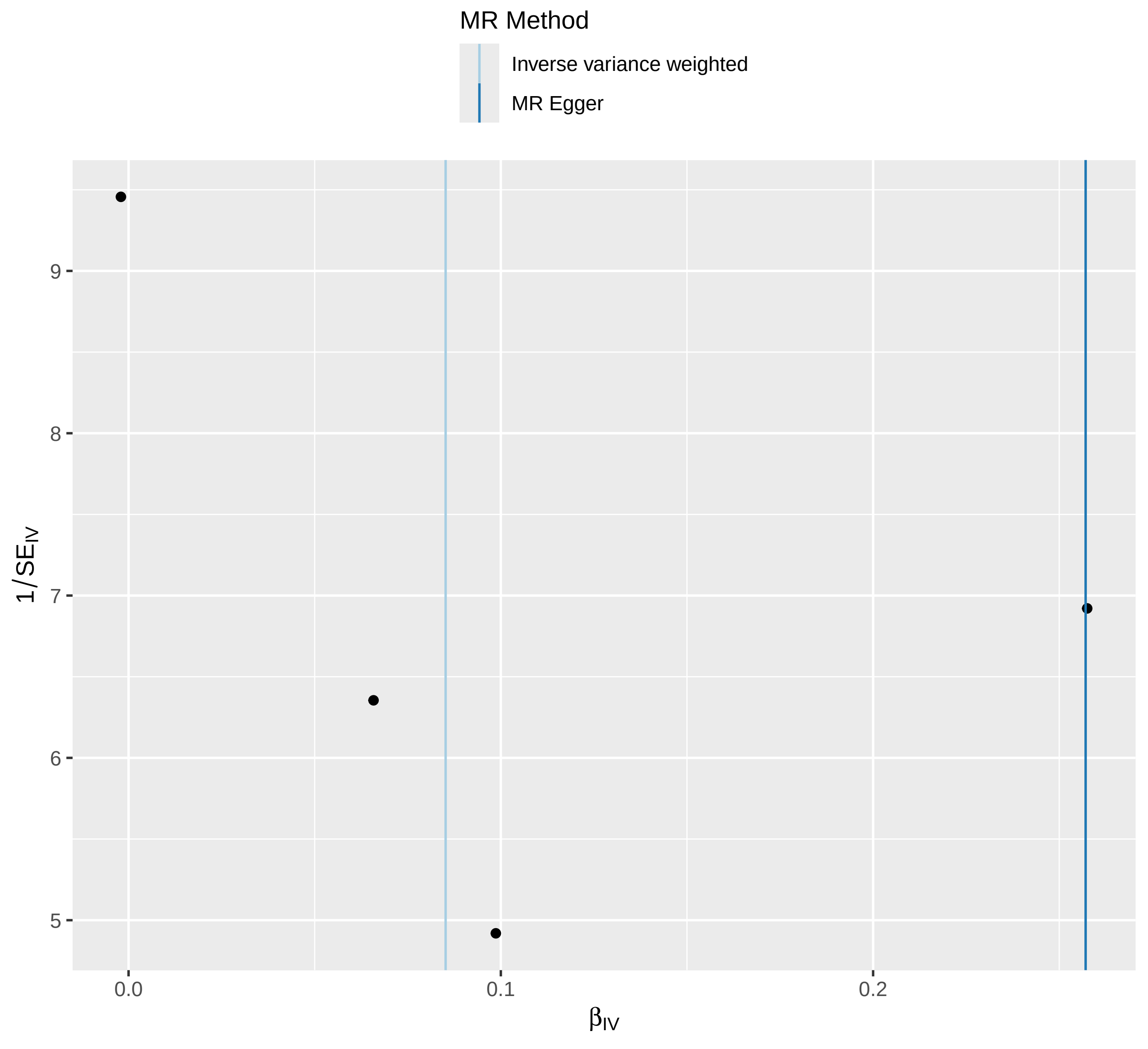
MR Method
Inverse variance weighted
MR Egger
9
8
7
6
5
0.0
0.1
0.2
1/SE_IV
β_IV

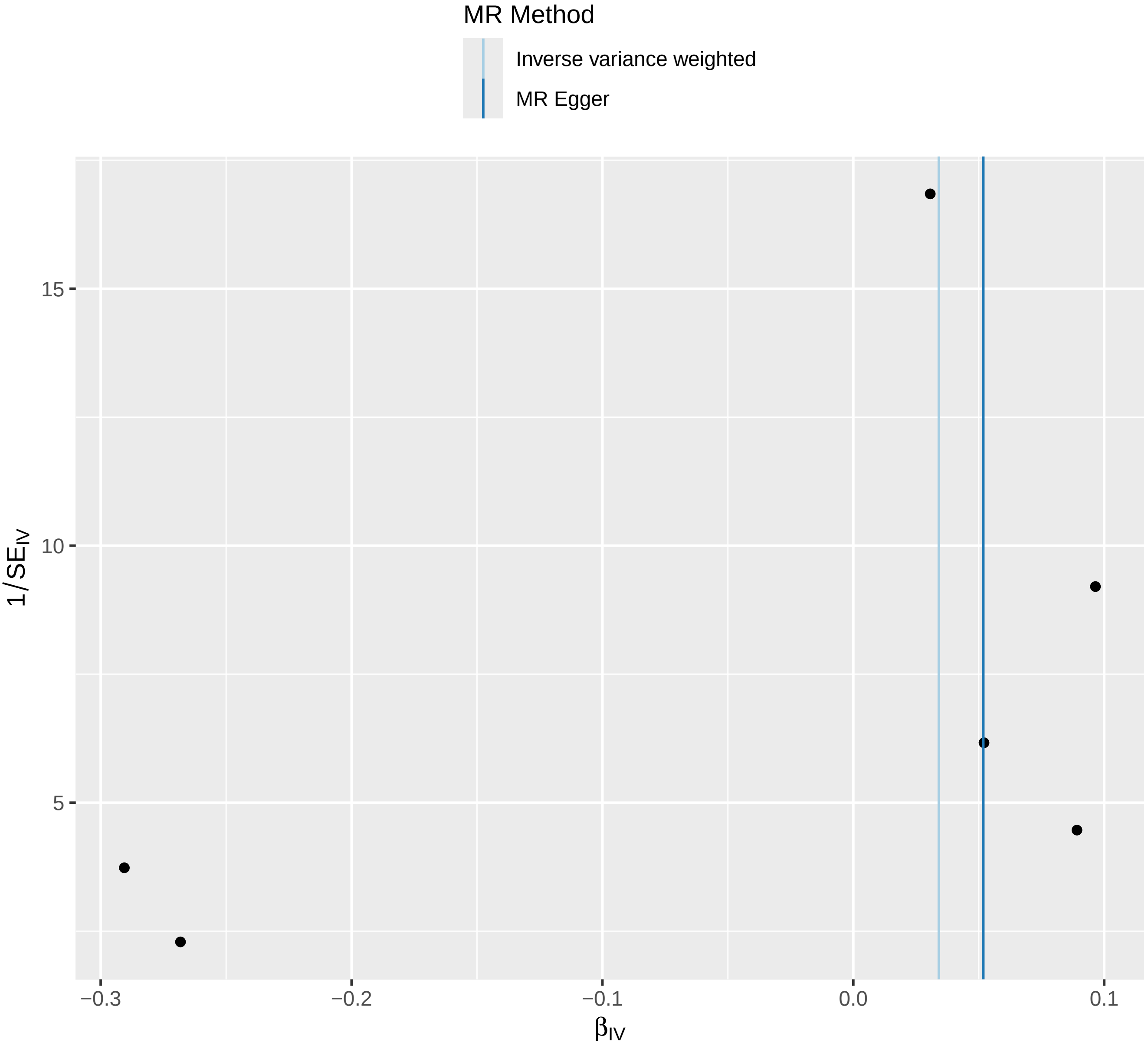
MR Method
Inverse variance weighted
MR Egger
15
10
5
1/SE_IV
−0.3
−0.2
−0.1
0.0
0.1
β_IV

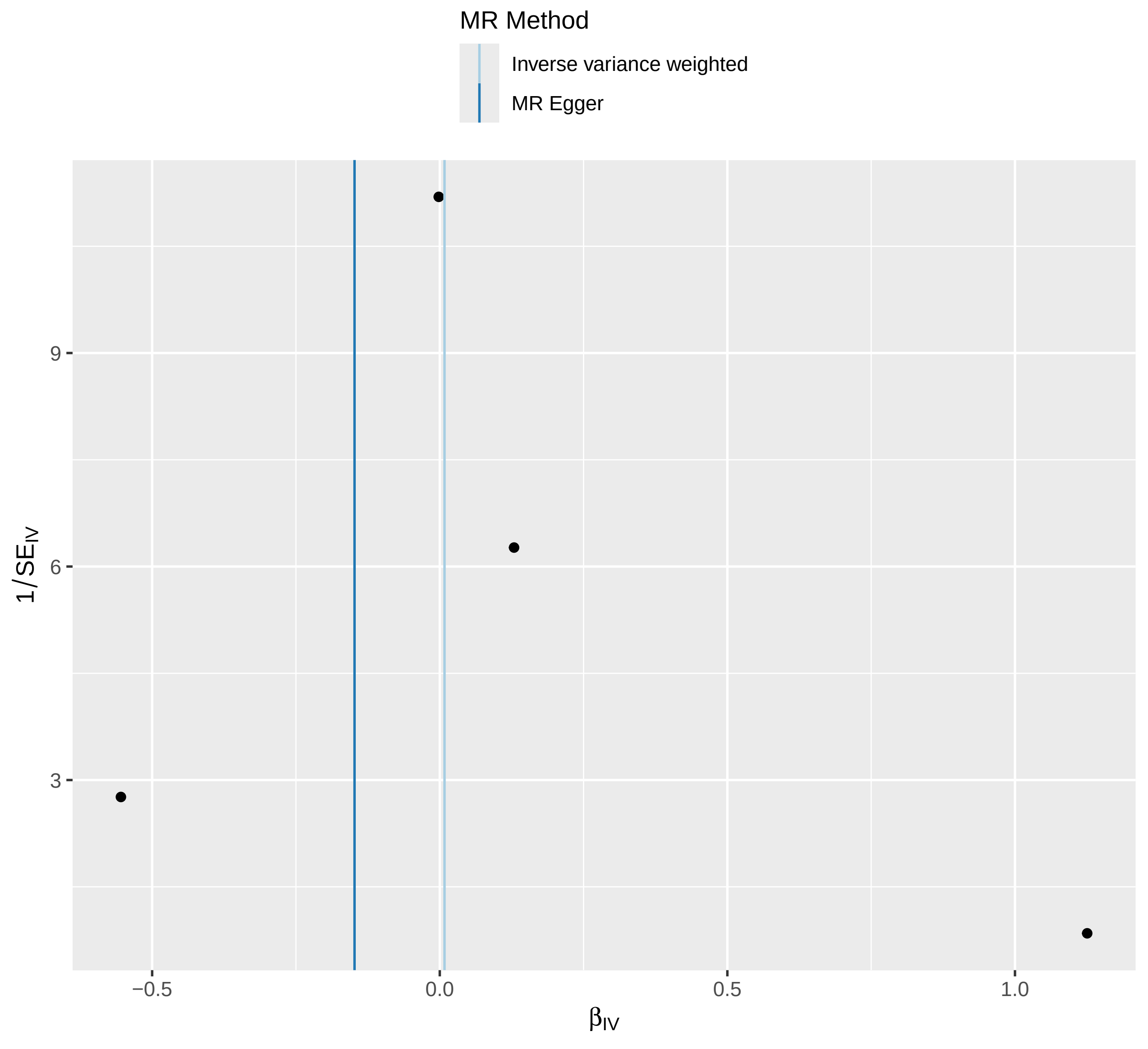
MR Method
Inverse variance weighted
MR Egger
1/$SE_{IV}$
9
6
3
−0.5
0.0
0.5
1.0
$\beta_{IV}$

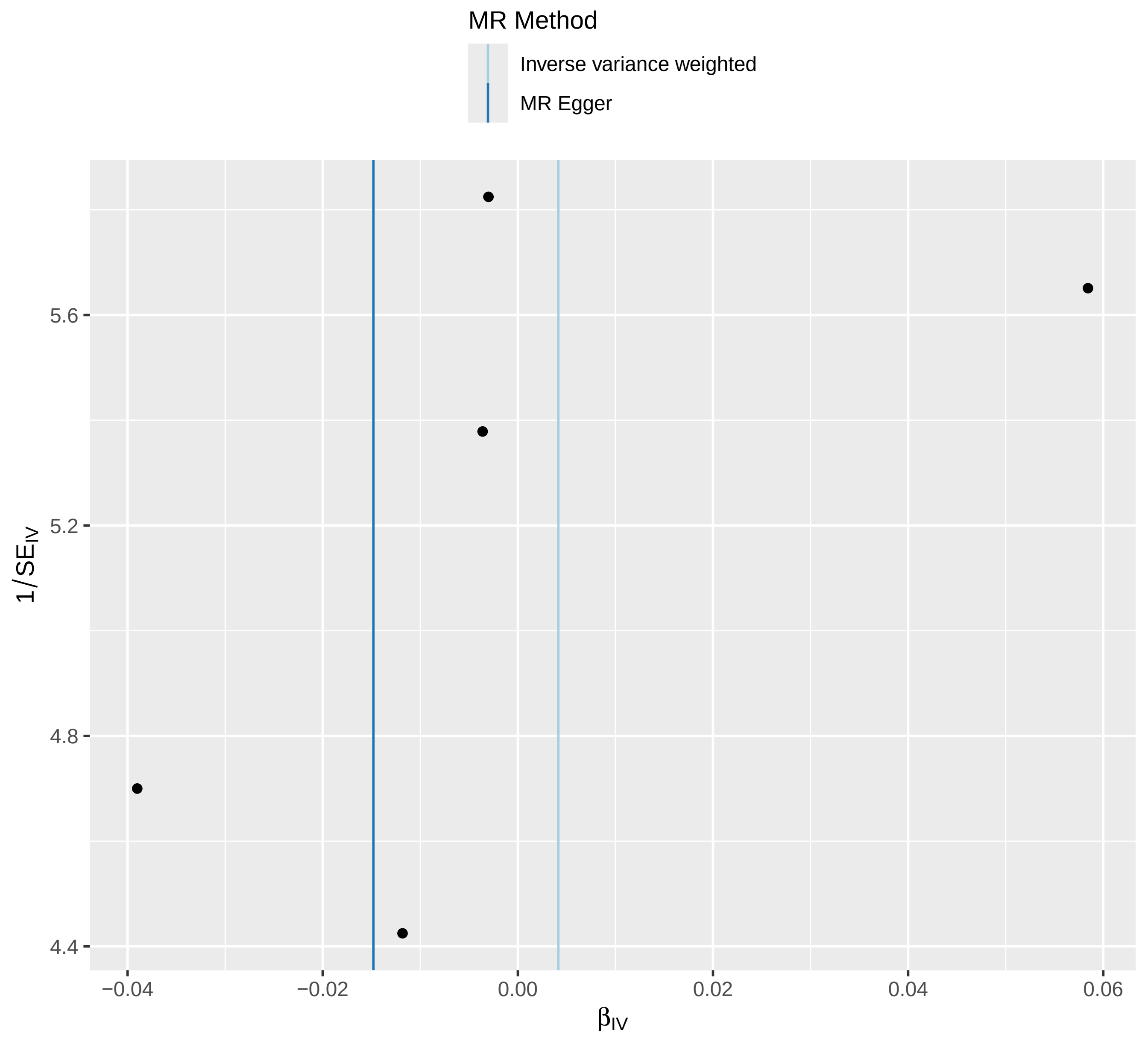

MR Method
Inverse variance weighted
MR Egger
5.6
5.2
4.8
4.4
1/SE_IV
−0.04
−0.02
0.00
0.02
0.04
0.06
β_IV

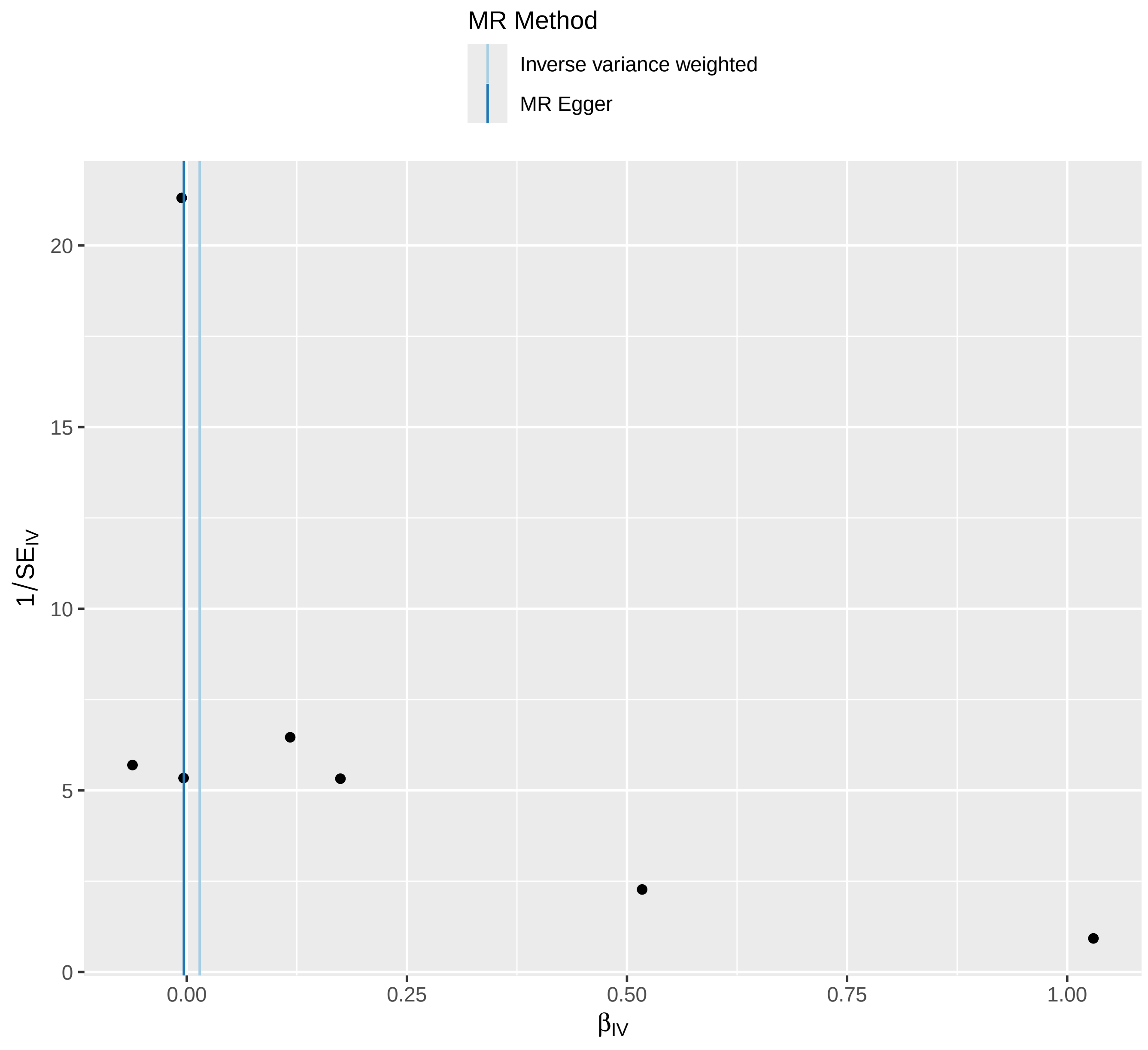

MR Method
Inverse variance weighted
MR Egger
20
15
10
5
0
1/SE$_{IV}$
0.00
0.25
0.50
0.75
1.00
β$_{IV}$

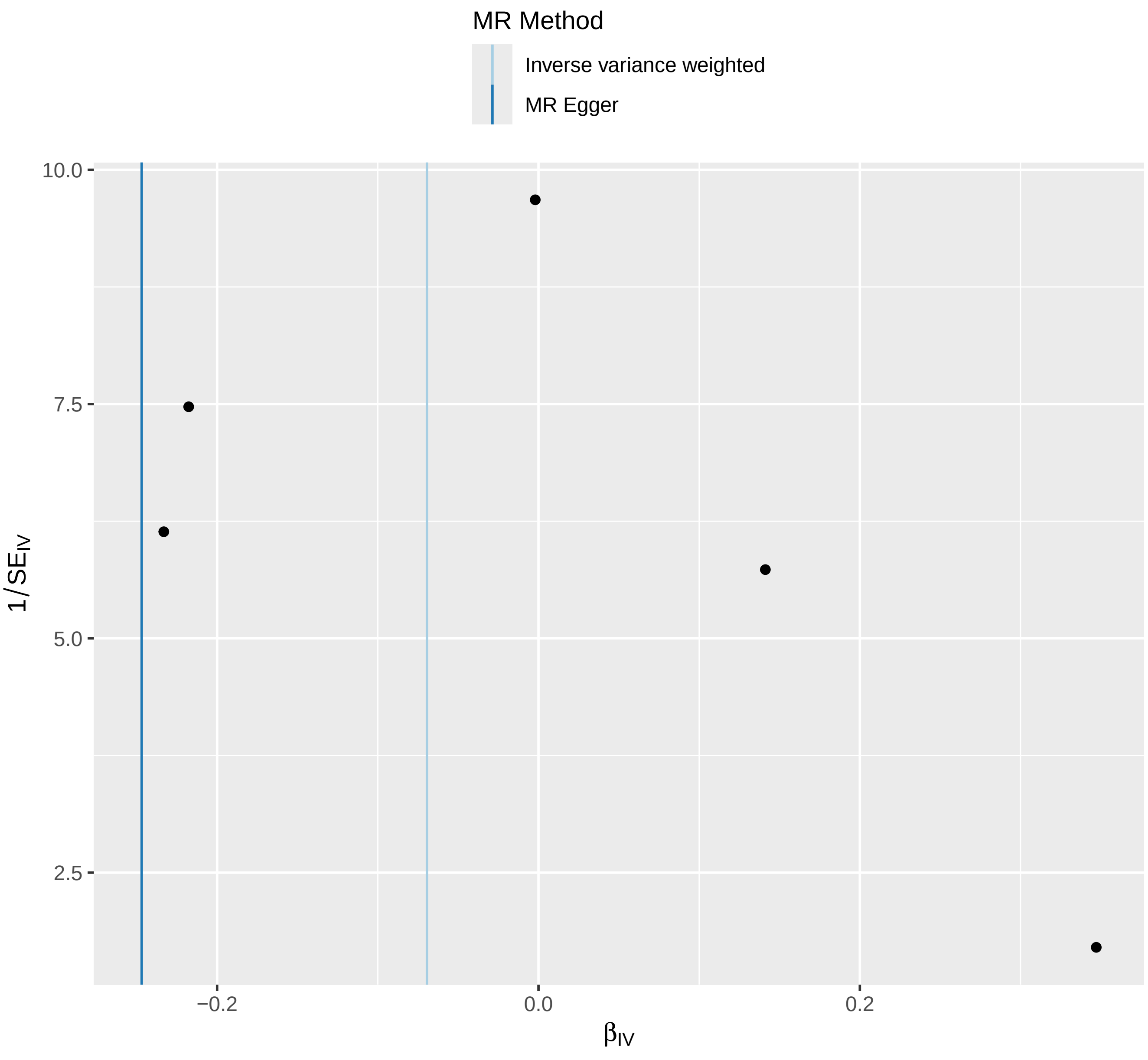
MR Method
Inverse variance weighted
MR Egger
10.0
7.5
5.0
2.5
$1/SE_{IV}$
−0.2
0.0
0.2
$\beta_{IV}$

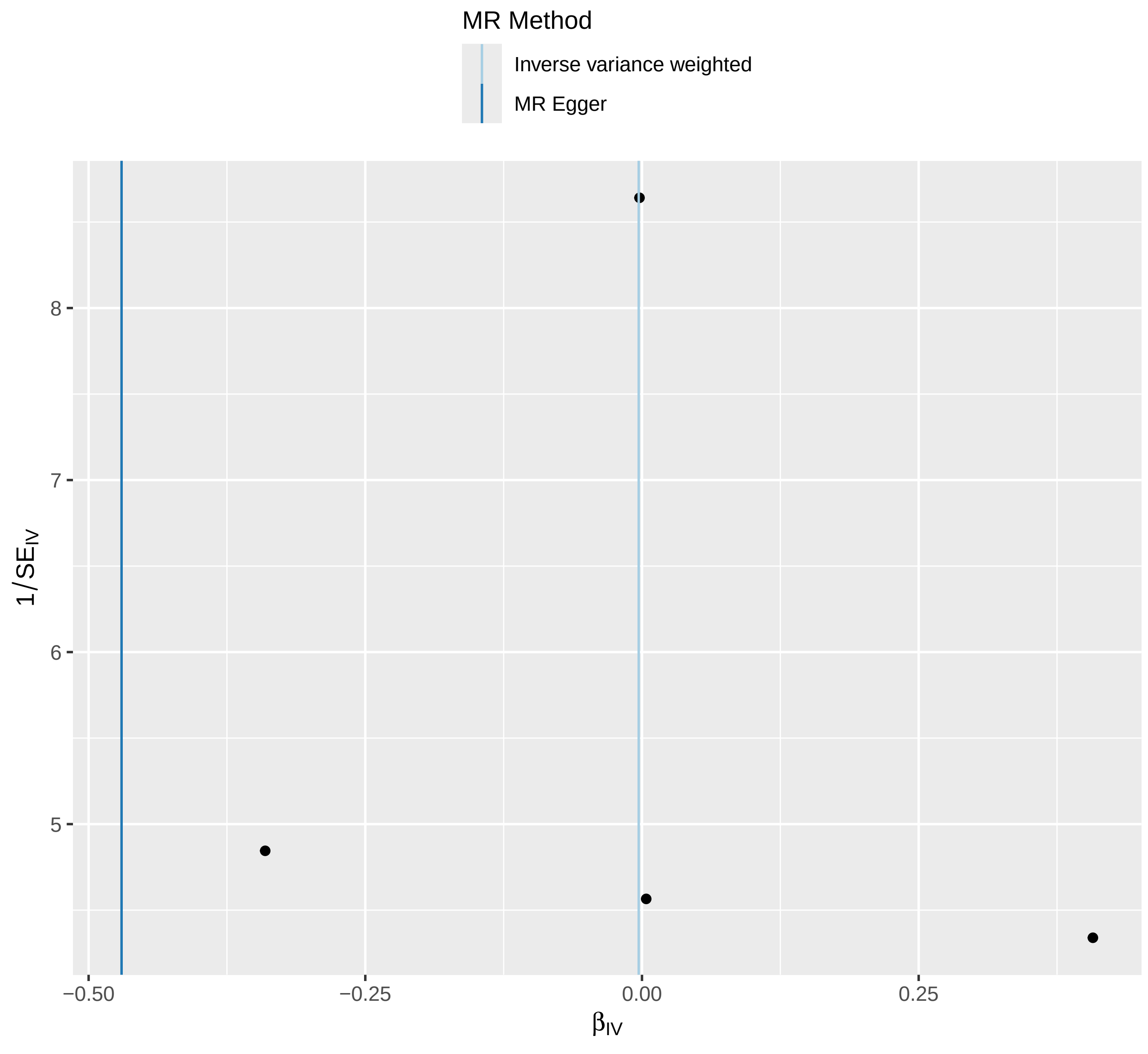
MR Method
Inverse variance weighted
MR Egger
8
7
6
5
1/SE$_{IV}$
−0.50
−0.25
0.00
0.25
β$_{IV}$

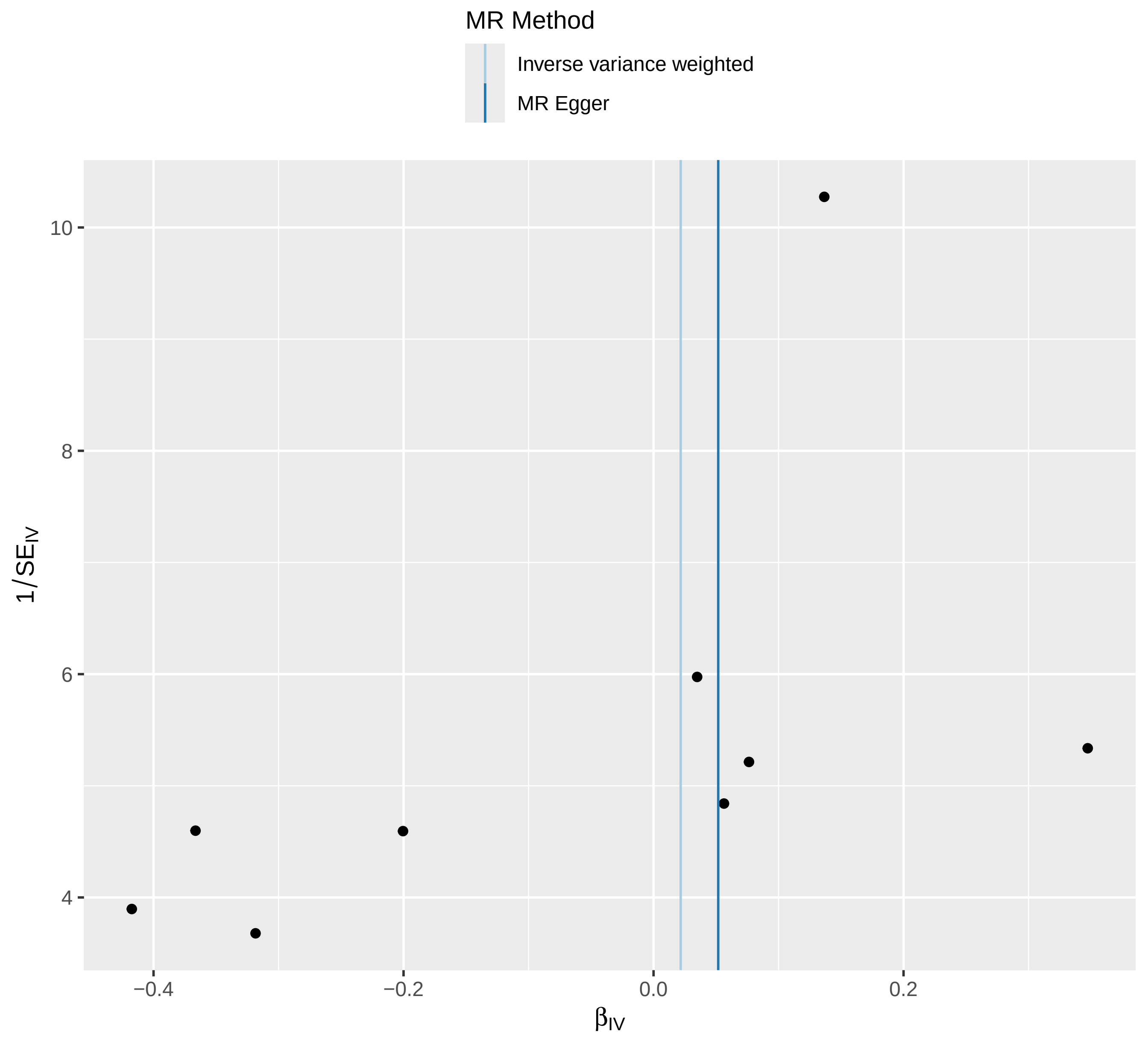

MR Method
Inverse variance weighted
MR Egger
1/SE$_{IV}$
β$_{IV}$
10
8
6
4
−0.4
−0.2
0.0
0.2

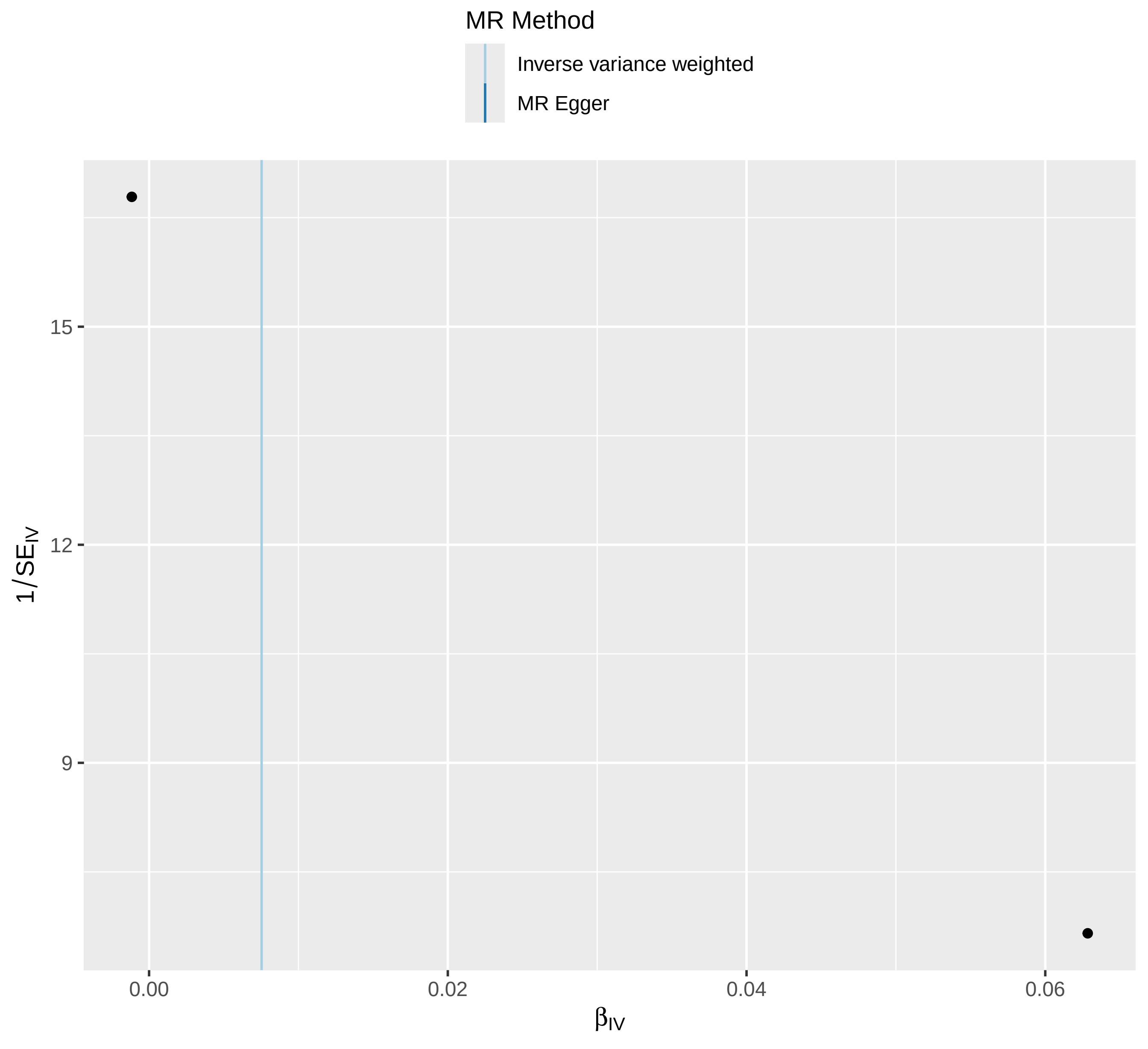
MR Method
Inverse variance weighted
MR Egger
15
12
9
1/SE_IV
0.00
0.02
0.04
0.06
β_IV

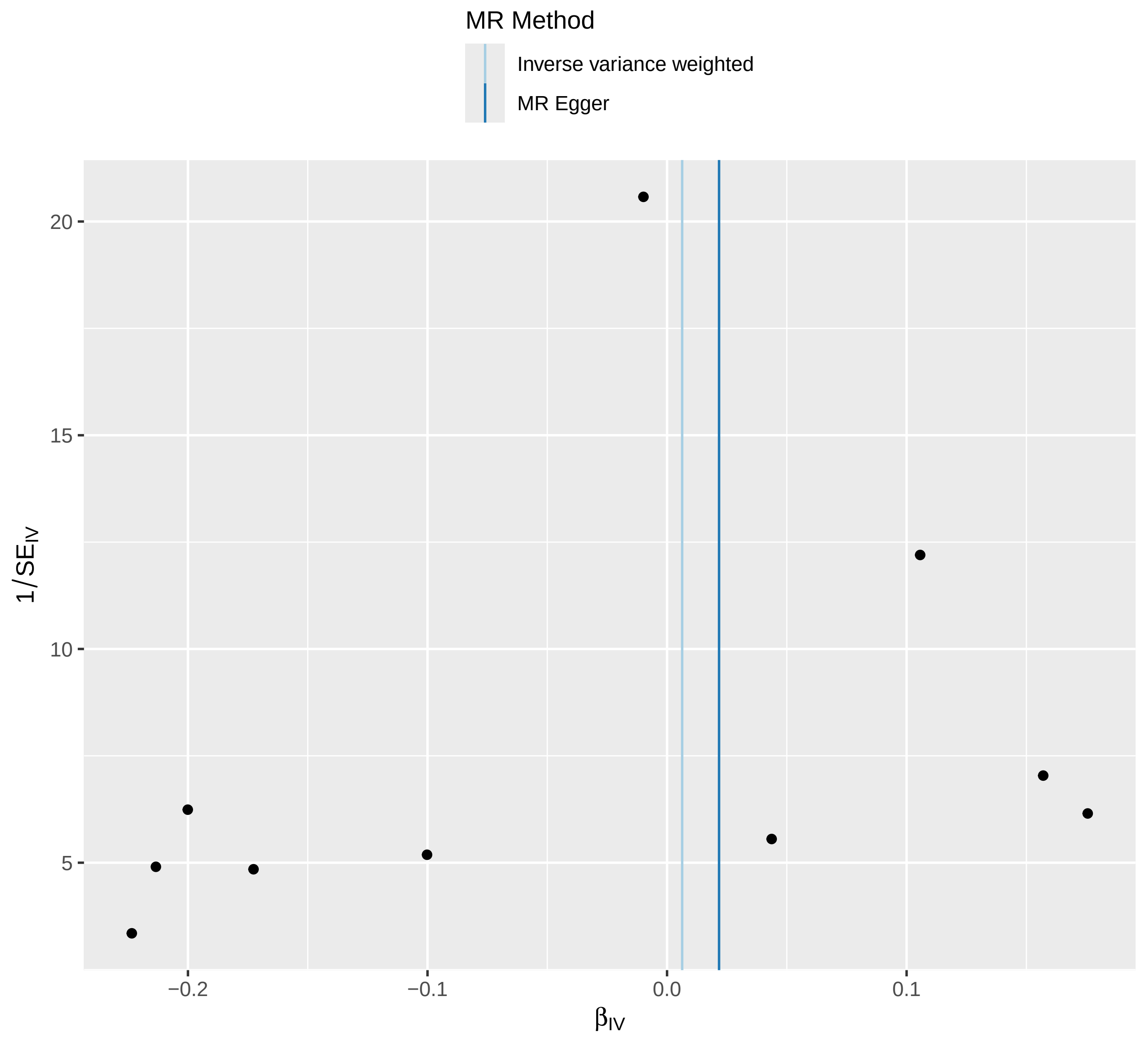
MR Method
Inverse variance weighted
MR Egger
1/SE_IV
20
15
10
5
−0.2
−0.1
0.0
0.1
β_IV

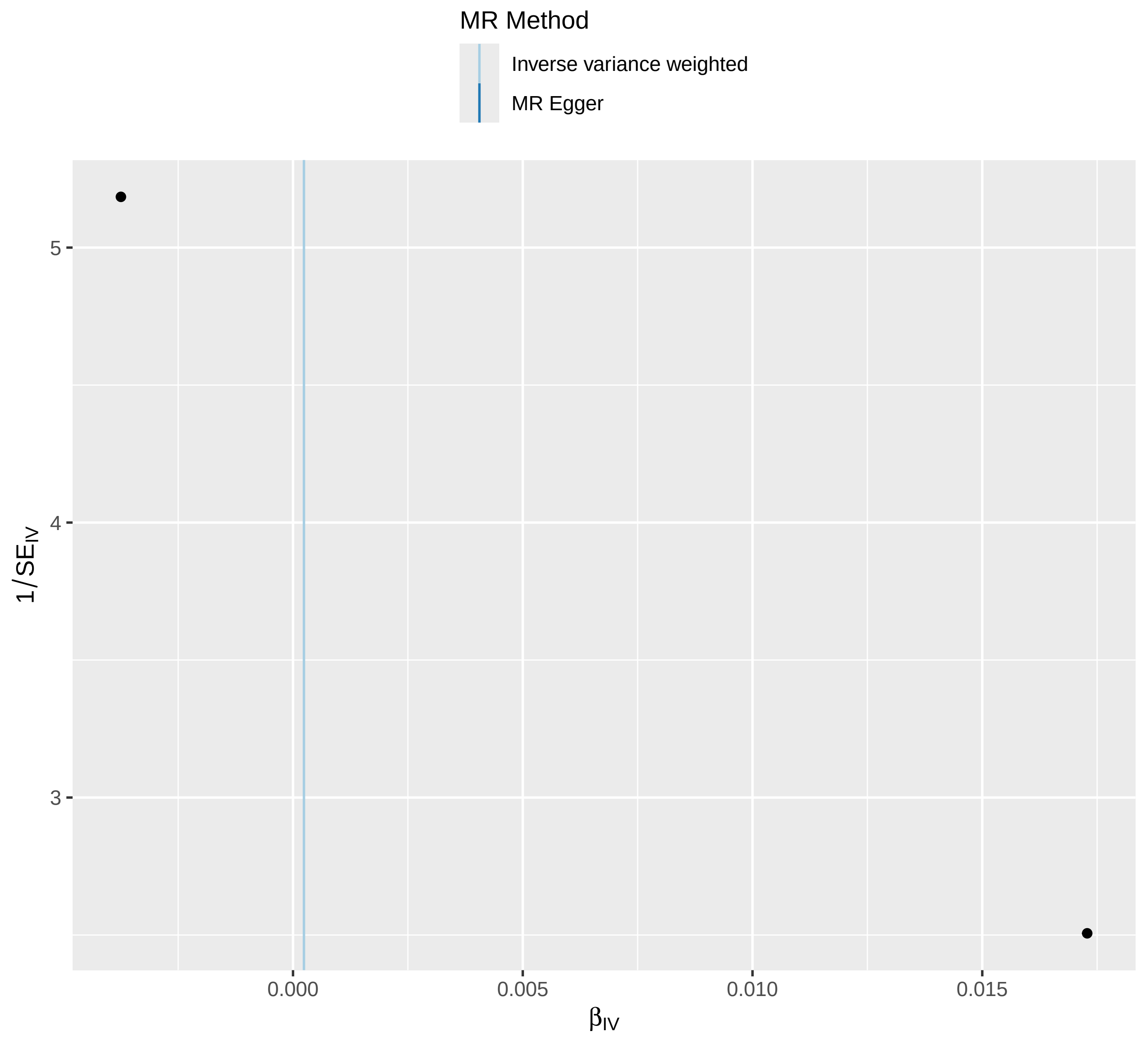

MR Method
Inverse variance weighted
MR Egger
5
4
3
$1/SE_{IV}$
0.000
0.005
0.010
0.015
$\beta_{IV}$

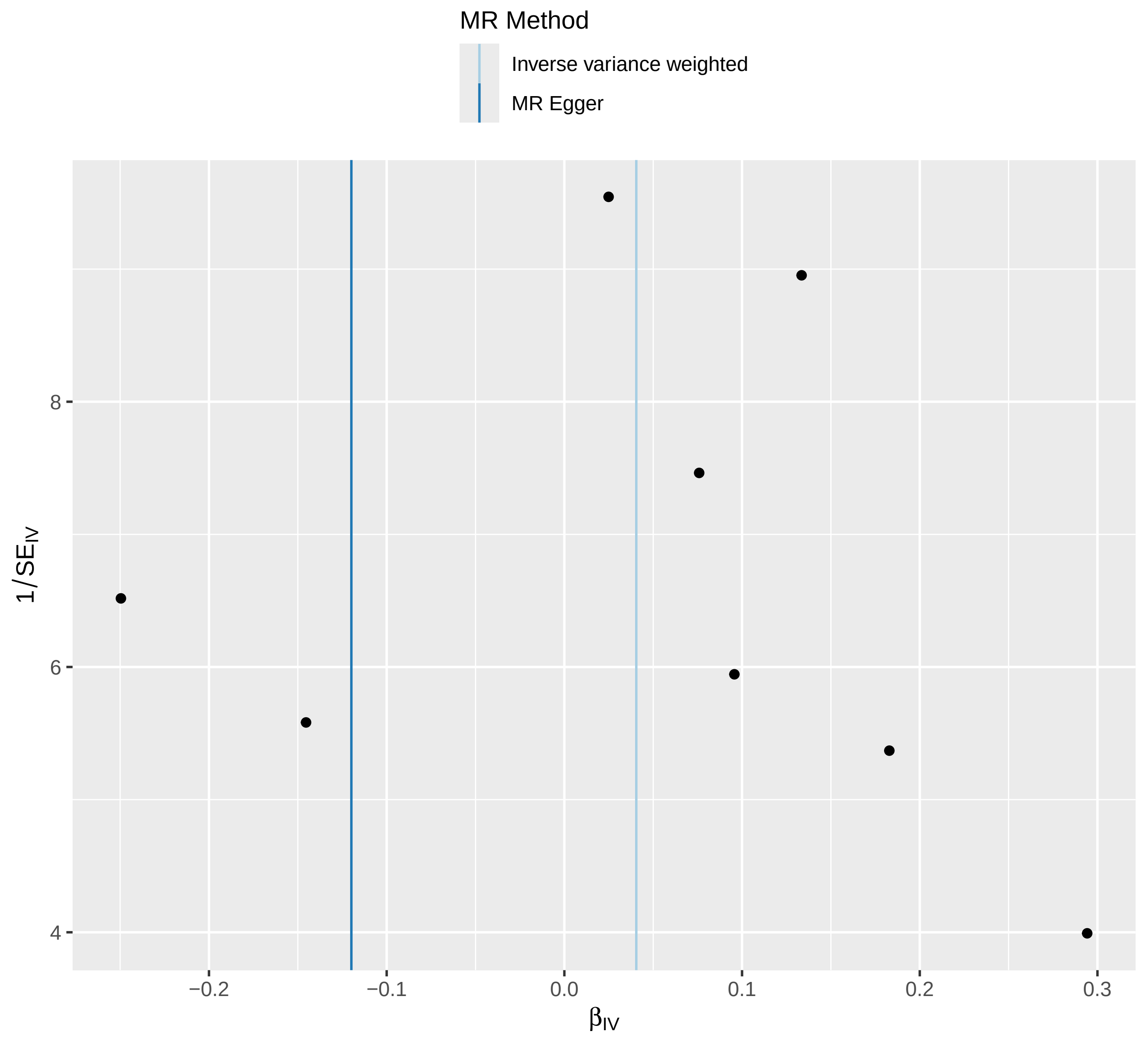
MR Method
Inverse variance weighted
MR Egger
1/SE_IV
8
6
4
−0.2
−0.1
0.0
0.1
0.2
0.3
β_IV

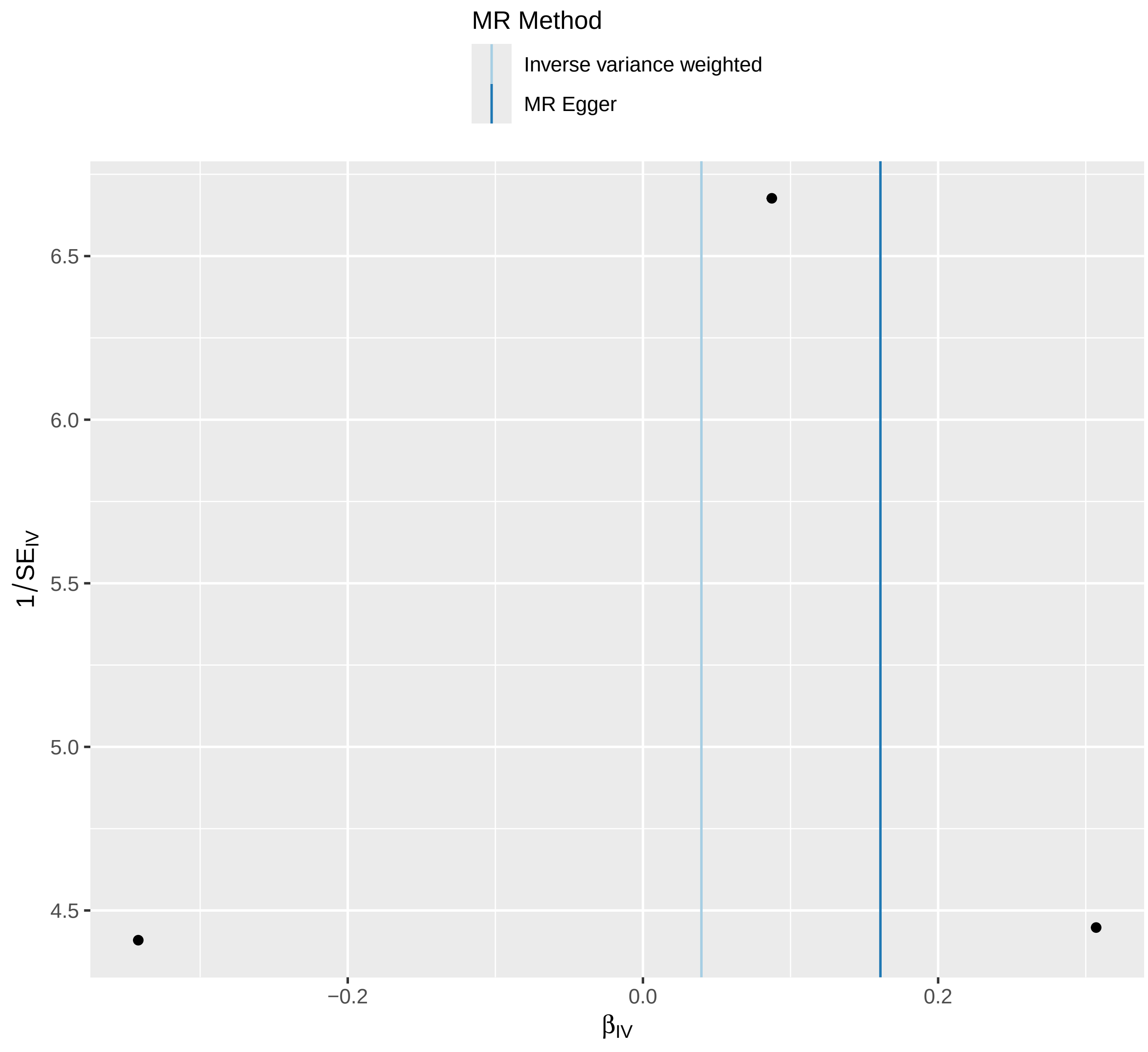

MR Method
Inverse variance weighted
MR Egger
6.5
6.0
5.5
5.0
4.5
1/SE$_{IV}$
−0.2
0.0
0.2
β$_{IV}$

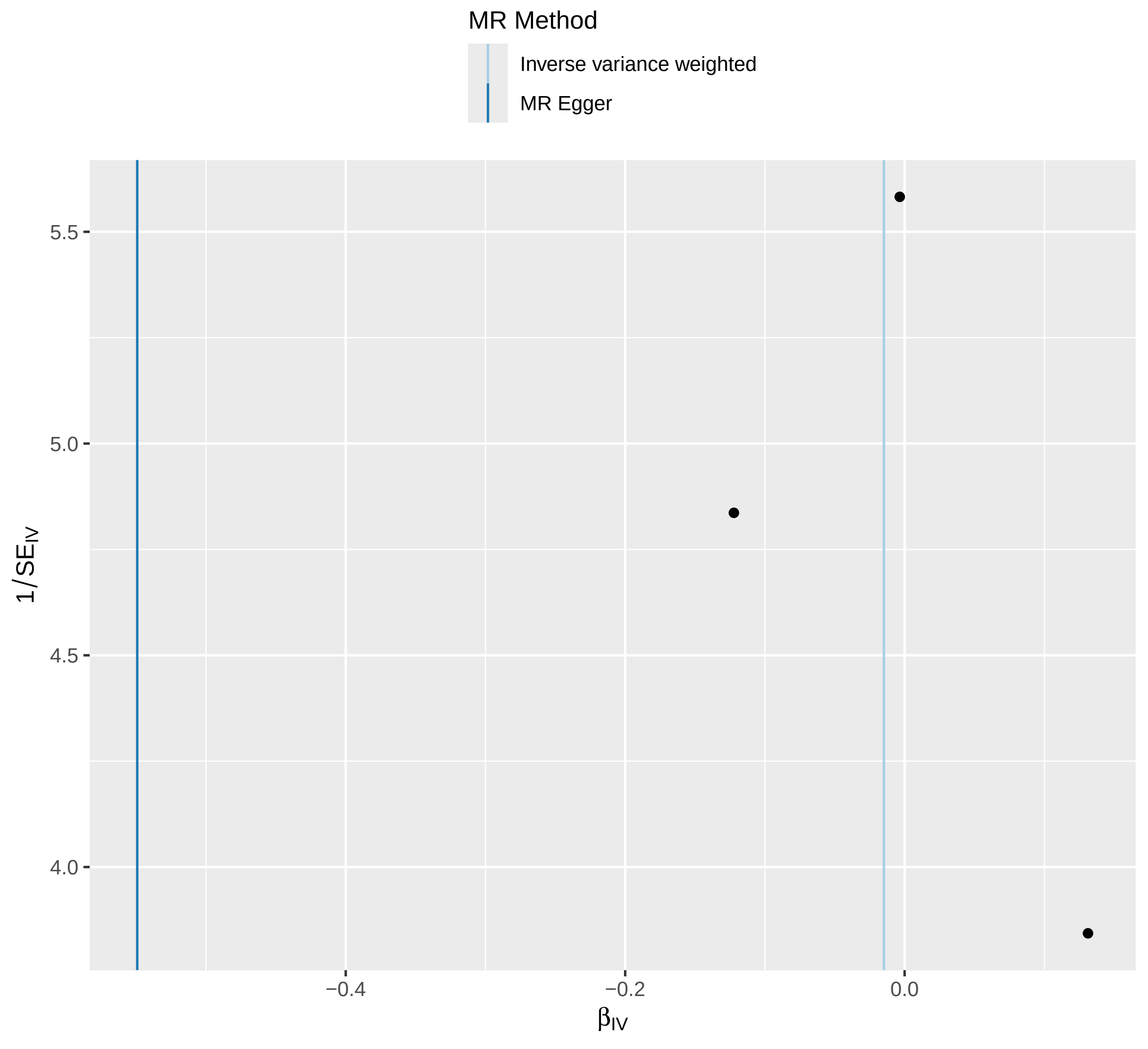

MR Method
Inverse variance weighted
MR Egger
5.5
5.0
4.5
4.0
1/SE$_{IV}$
−0.4
−0.2
0.0
β$_{IV}$

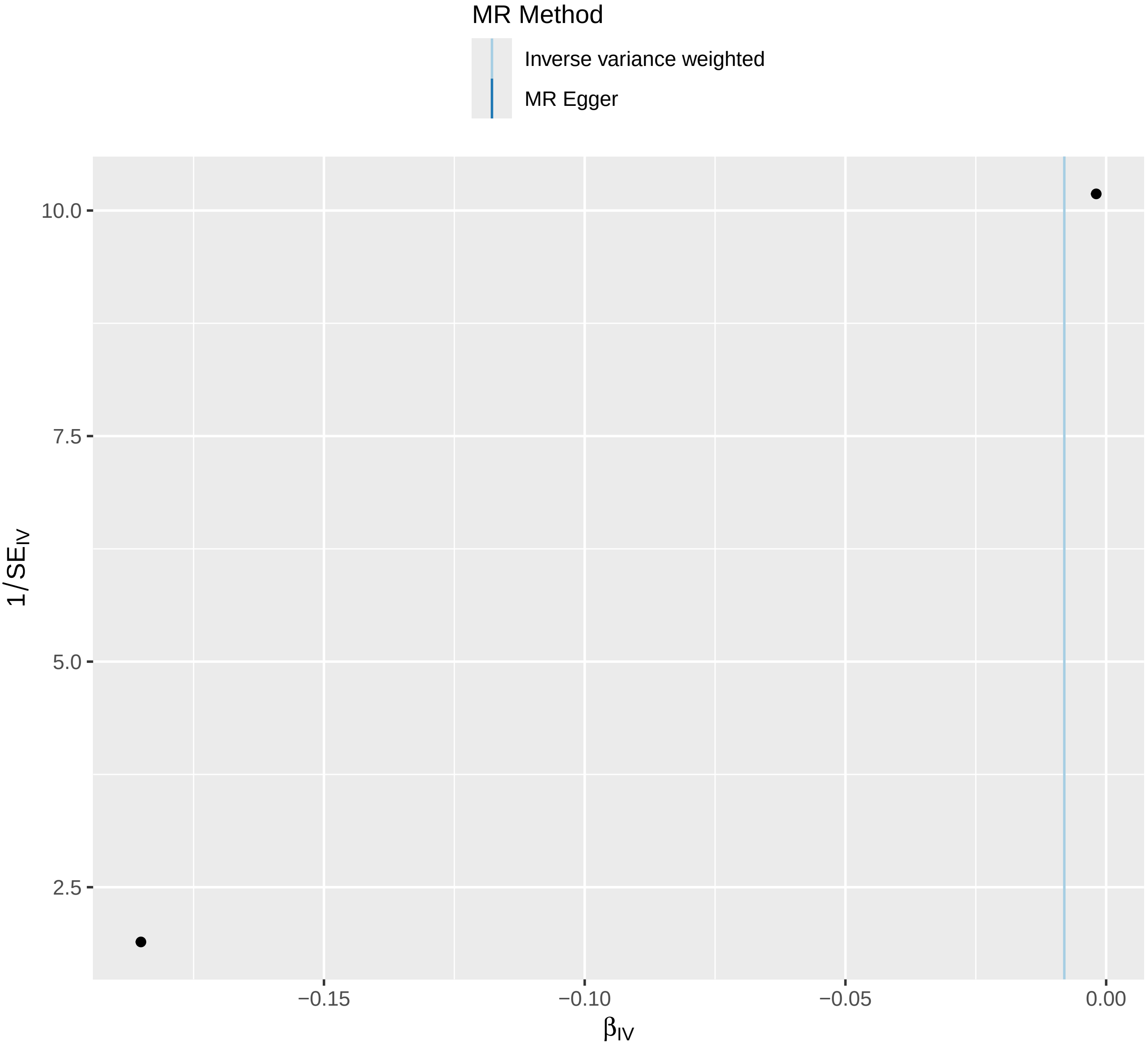
MR Method
Inverse variance weighted
MR Egger
10.0
7.5
5.0
2.5
$1/SE_{IV}$
−0.15
−0.10
−0.05
0.00
$\beta_{IV}$

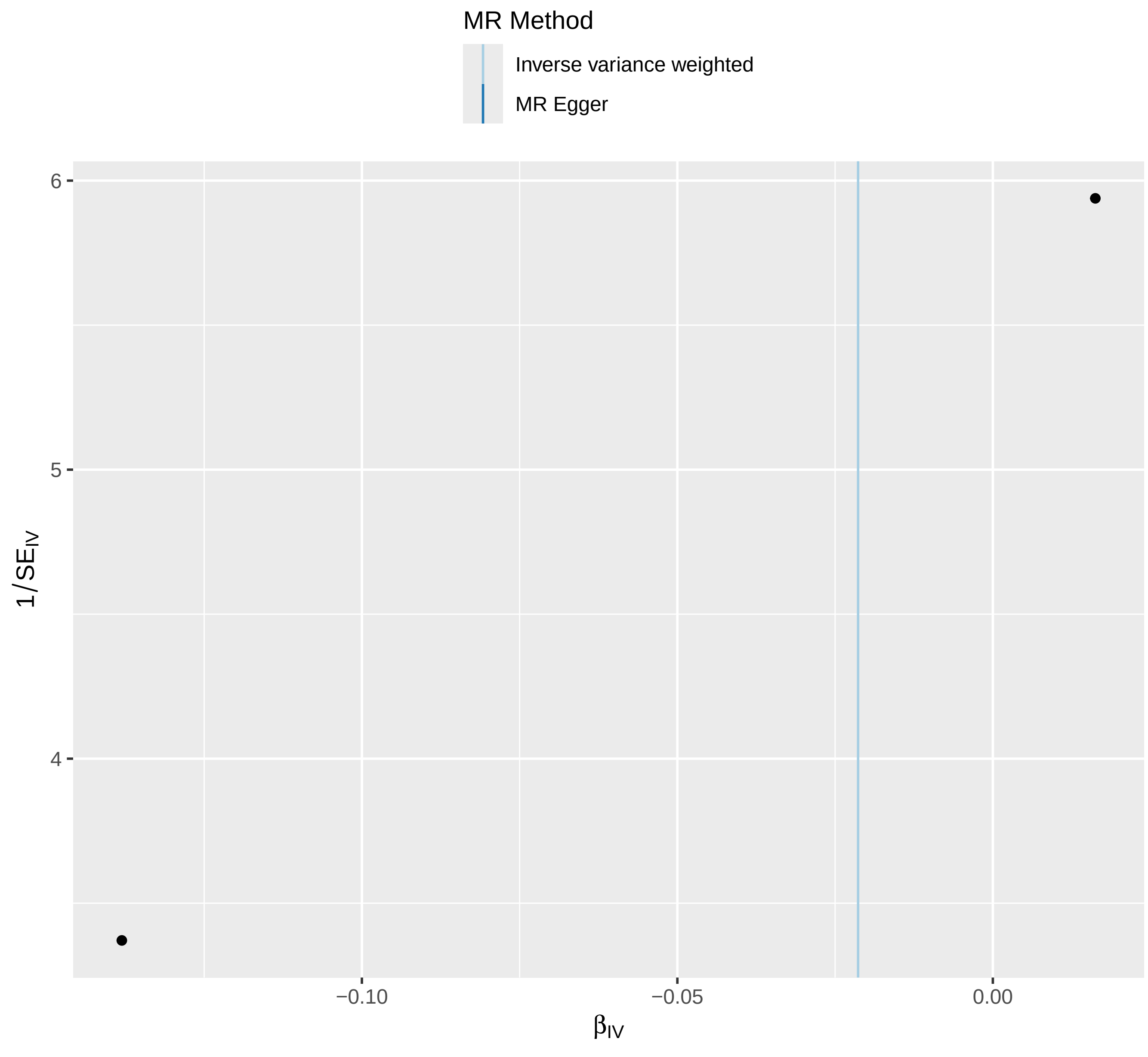

MR Method
Inverse variance weighted
MR Egger
6
5
4
1/SE_IV
−0.10
−0.05
0.00
β_IV

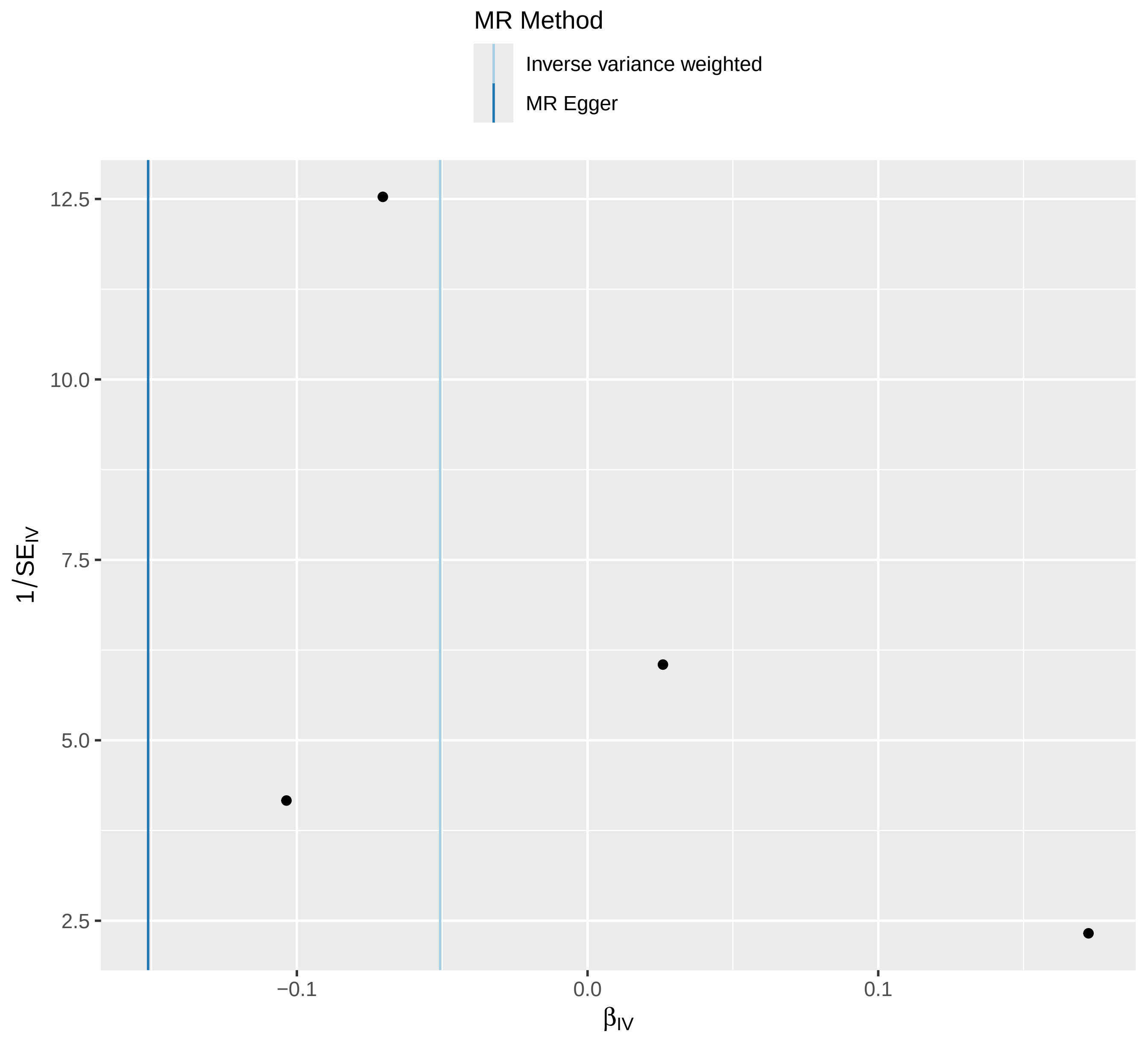

MR Method
Inverse variance weighted
MR Egger
12.5
10.0
7.5
5.0
2.5
1/SE_IV
−0.1
0.0
0.1
β_IV

Insufficient number of SNPs

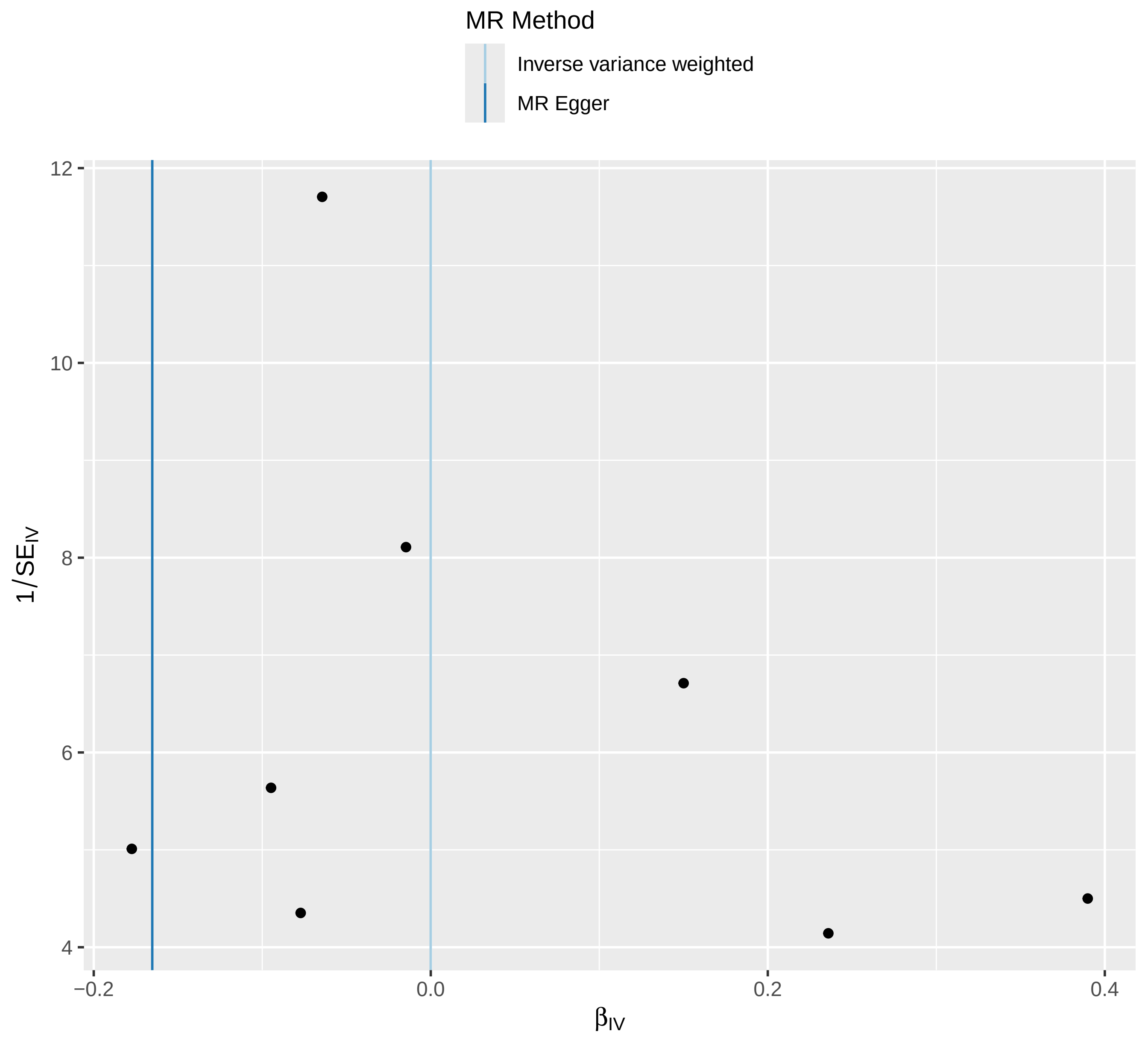

MR Method
Inverse variance weighted
MR Egger
12
10
8
6
4
1/SE$_{IV}$
−0.2
0.0
0.2
0.4
β$_{IV}$

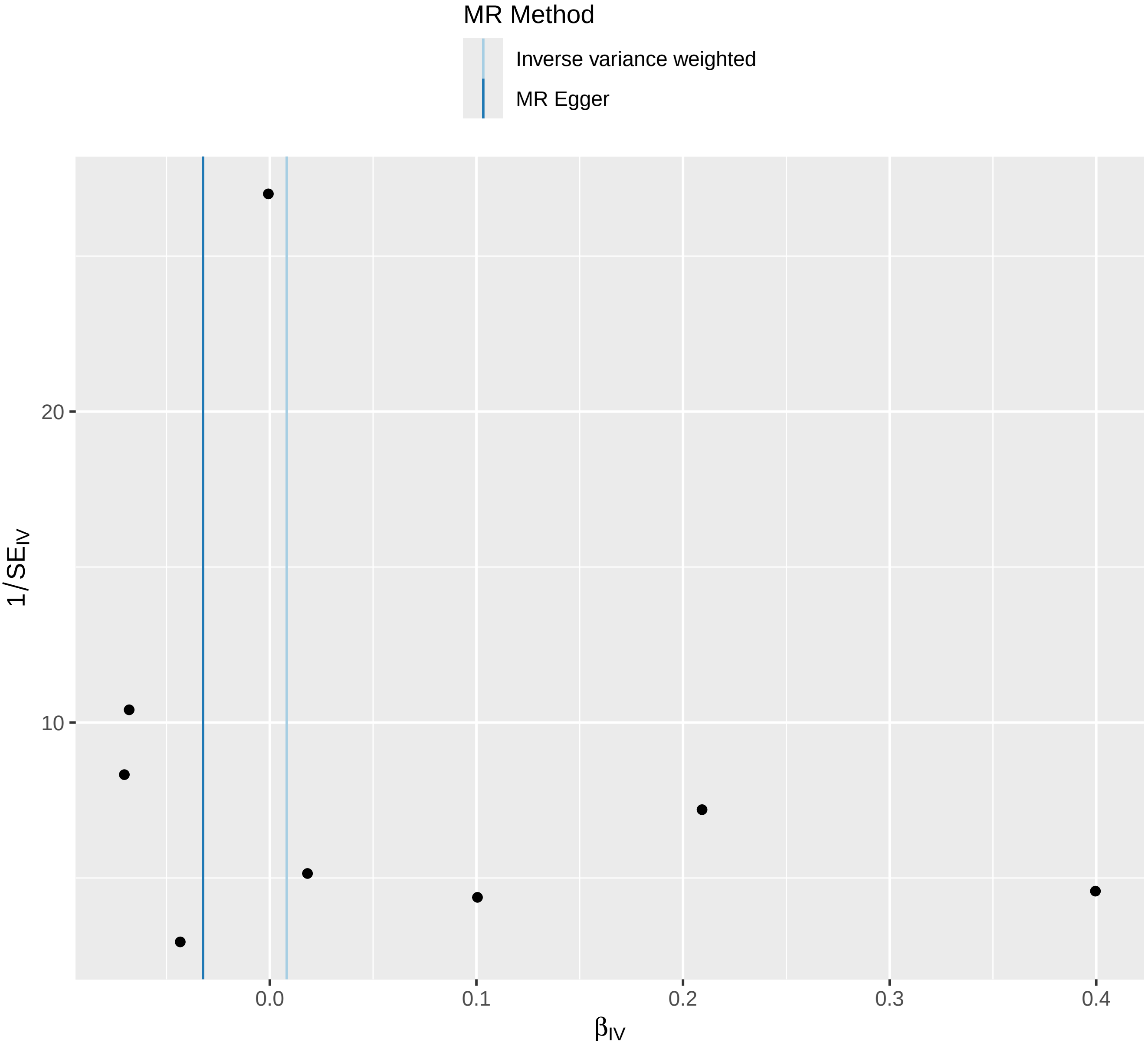
MR Method
Inverse variance weighted
MR Egger
20
10
0.0
0.1
0.2
0.3
0.4
$1/SE_{IV}$
$\beta_{IV}$

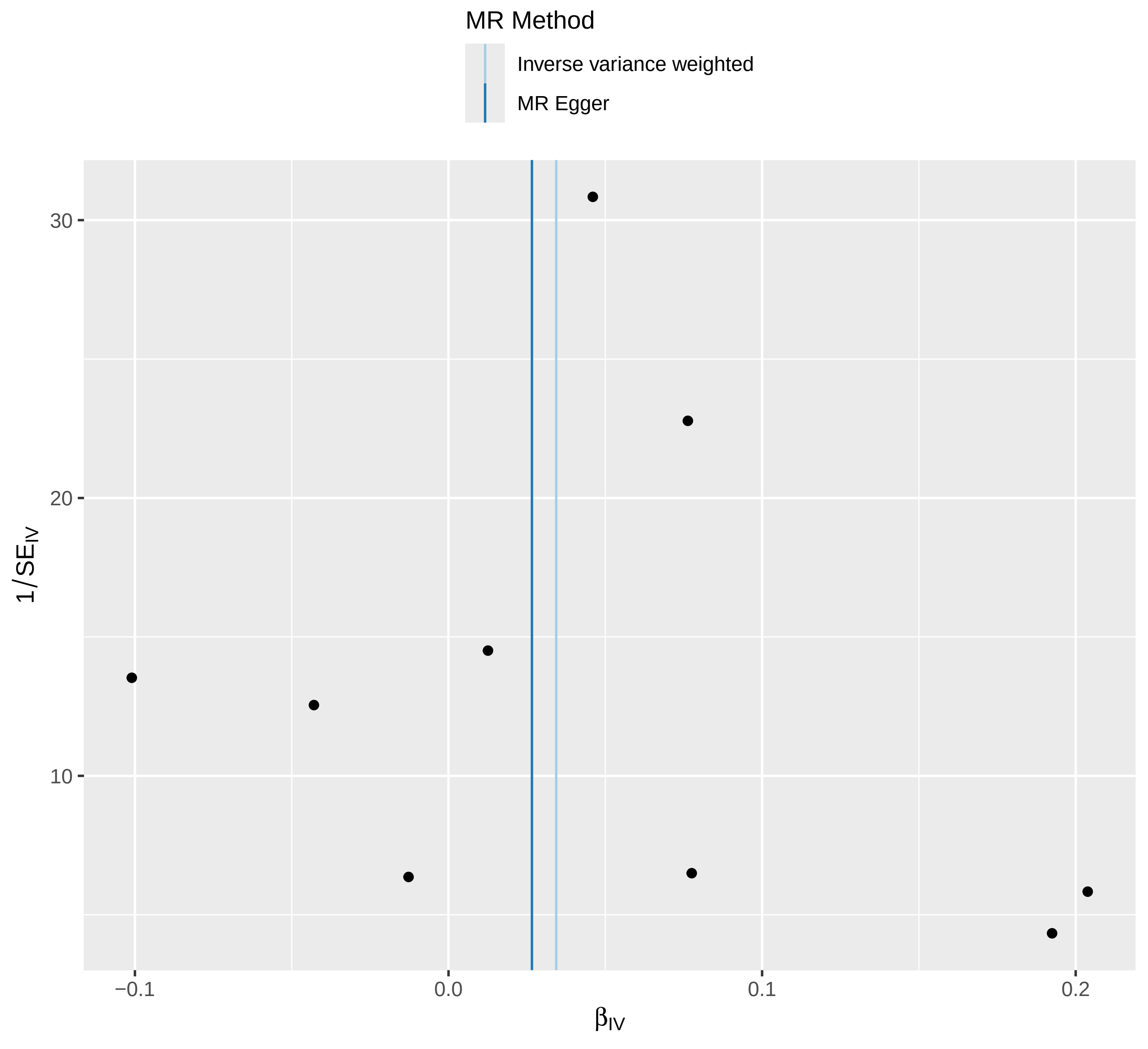
MR Method
Inverse variance weighted
MR Egger
30
20
10
1/SE_IV
−0.1
0.0
0.1
0.2
β_IV

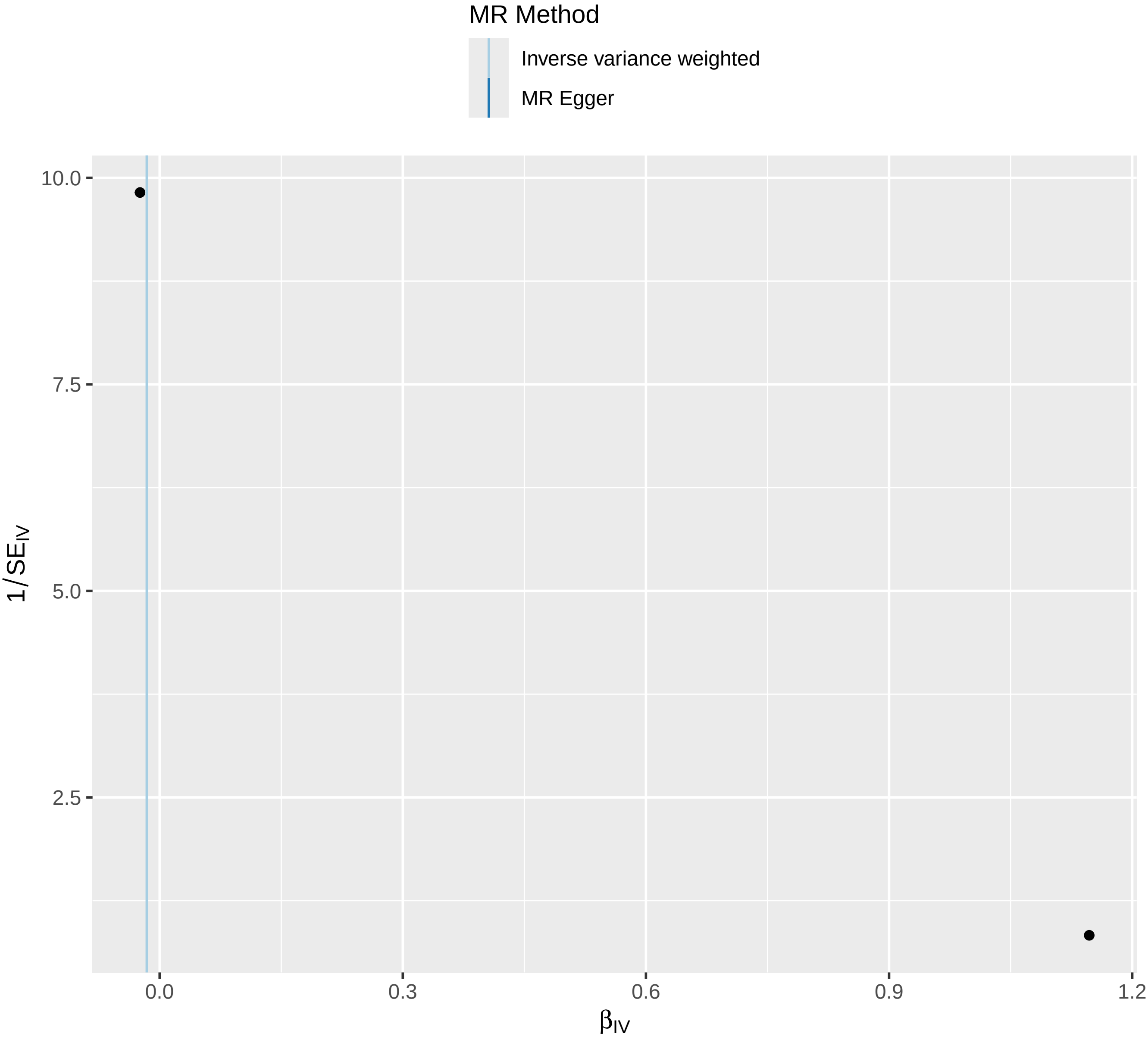

MR Method
Inverse variance weighted
MR Egger
10.0
7.5
5.0
2.5
1/SE$_{IV}$
0.0
0.3
0.6
0.9
1.2
$\beta_{IV}$

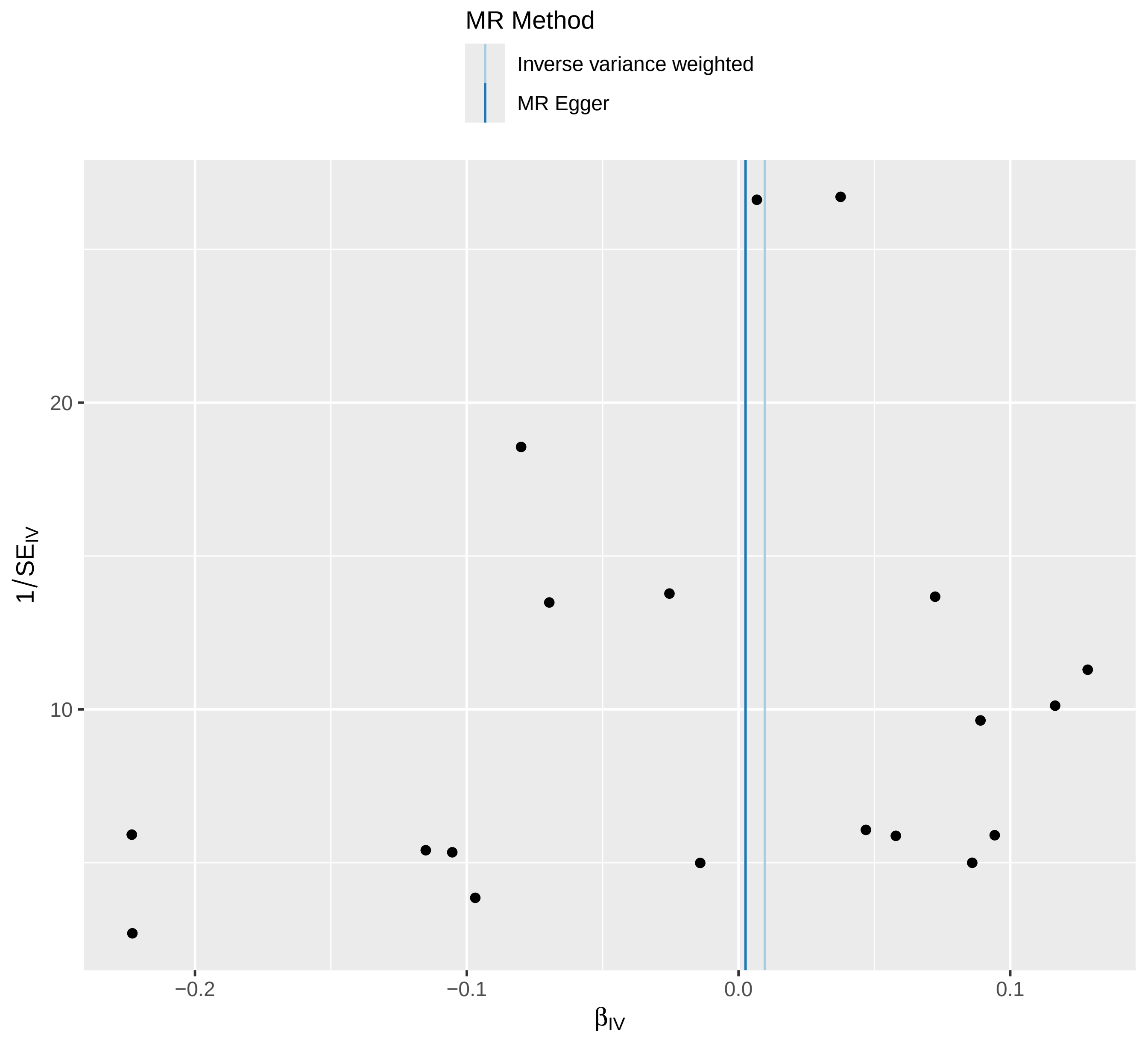
MR Method
Inverse variance weighted
MR Egger
20
10
1/SE$_{IV}$
−0.2
−0.1
0.0
0.1
β$_{IV}$

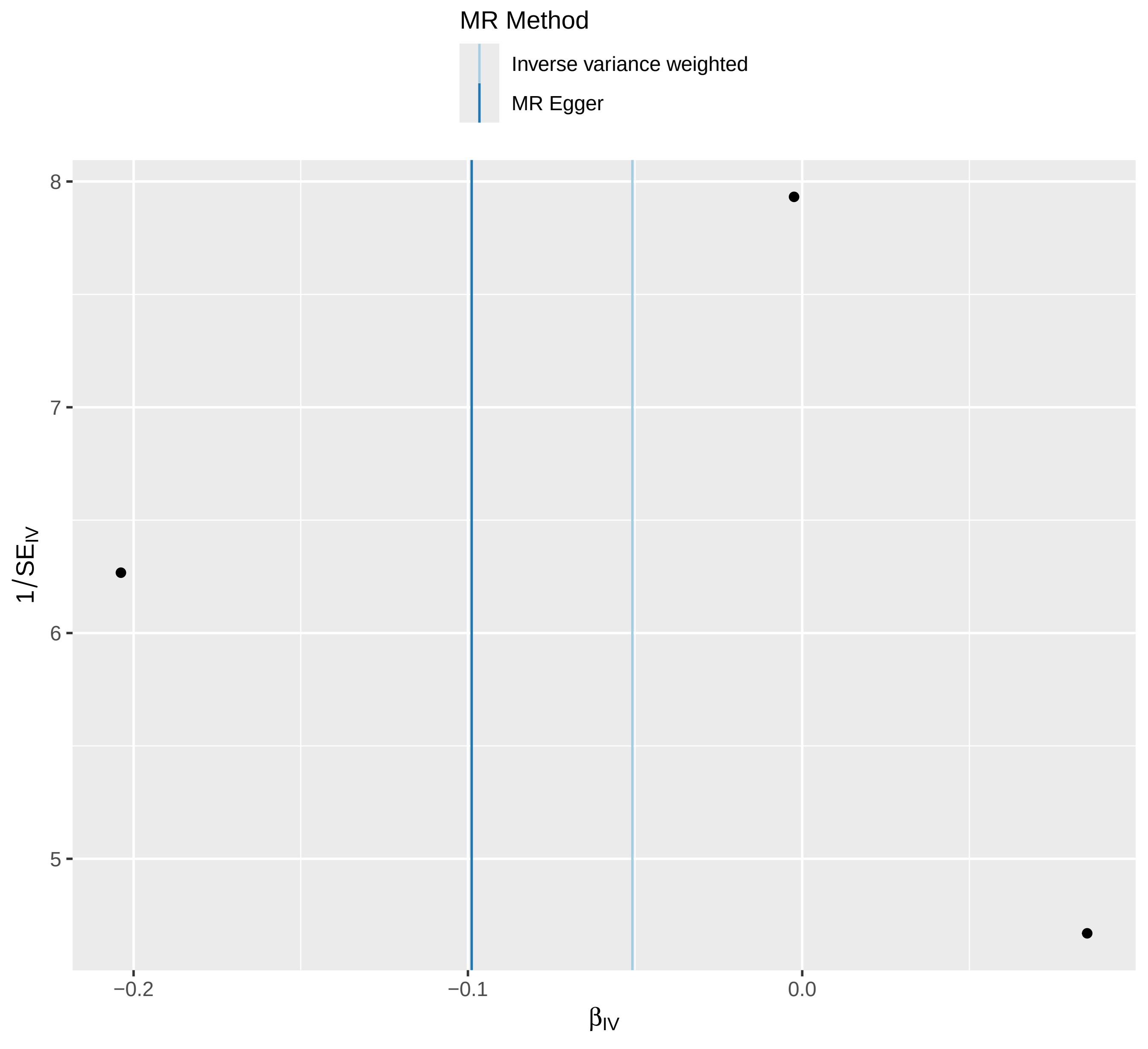

MR Method
Inverse variance weighted
MR Egger
8
7
6
5
1/SE_IV
−0.2
−0.1
0.0
β_IV

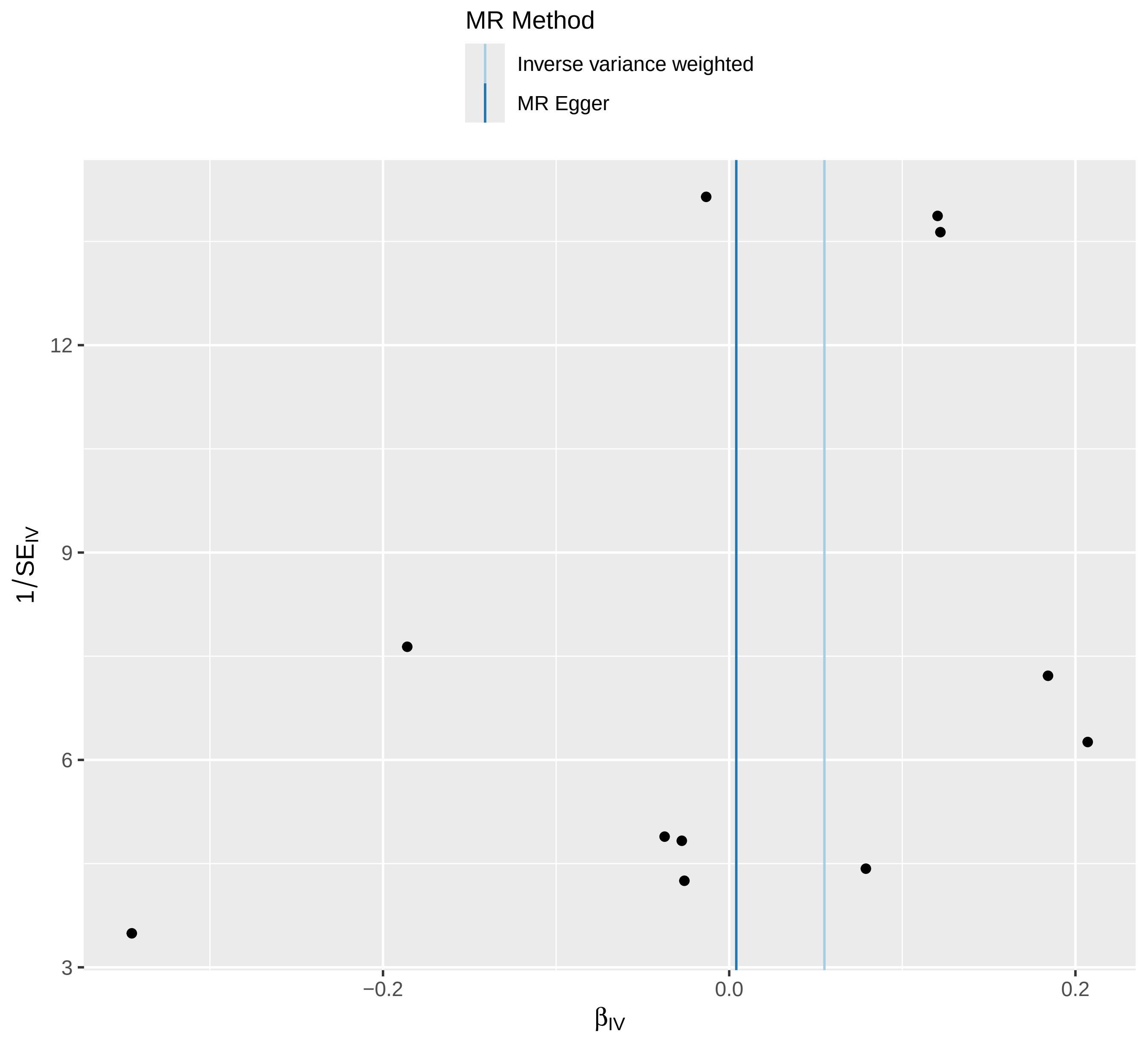
MR Method
Inverse variance weighted
MR Egger
12
9
6
3
−0.2
0.0
0.2
$1/SE_{IV}$
$\beta_{IV}$

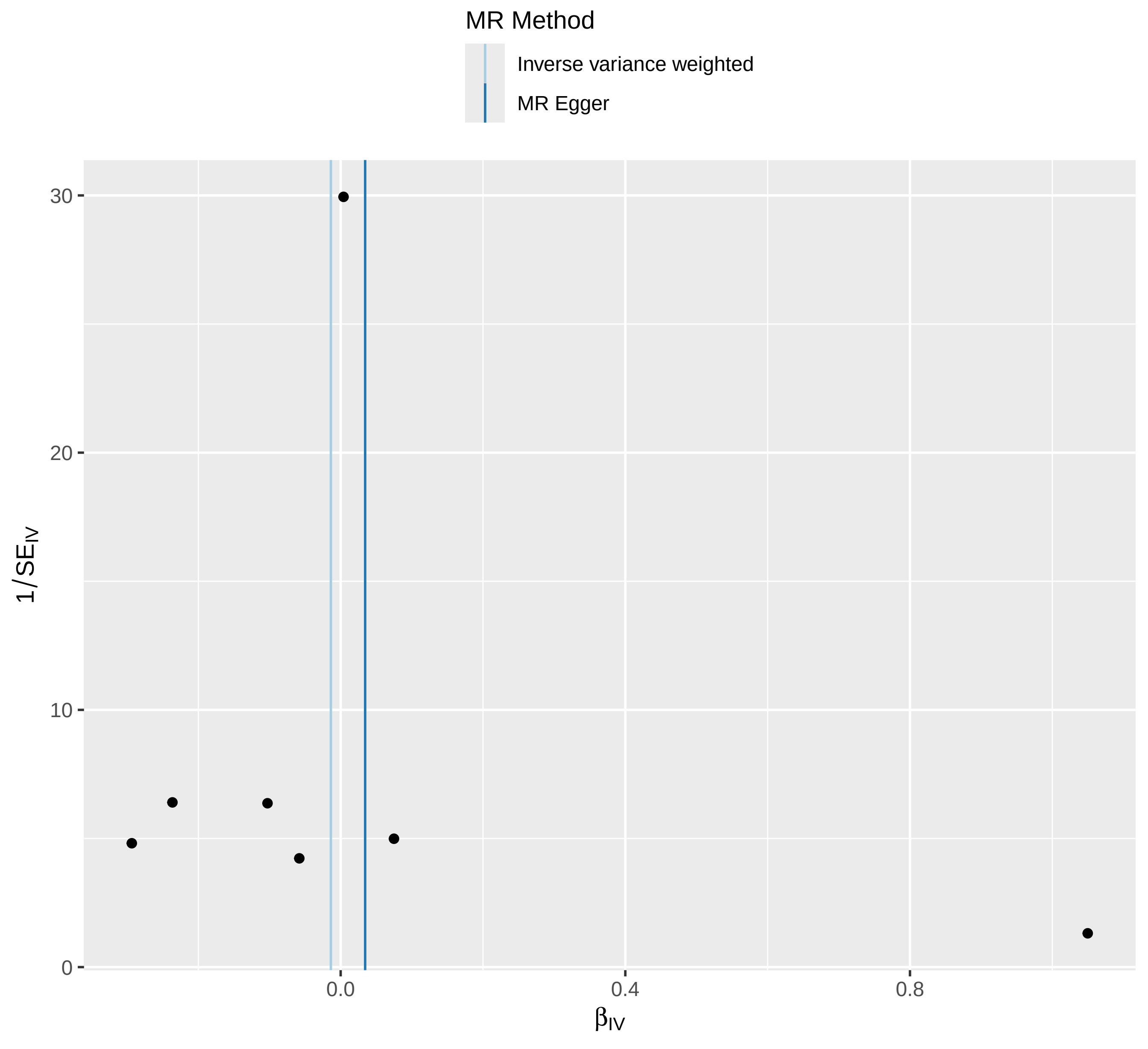
MR Method
Inverse variance weighted
MR Egger
30
20
10
0
0.0
0.4
0.8
$1/SE_{IV}$
$\beta_{IV}$

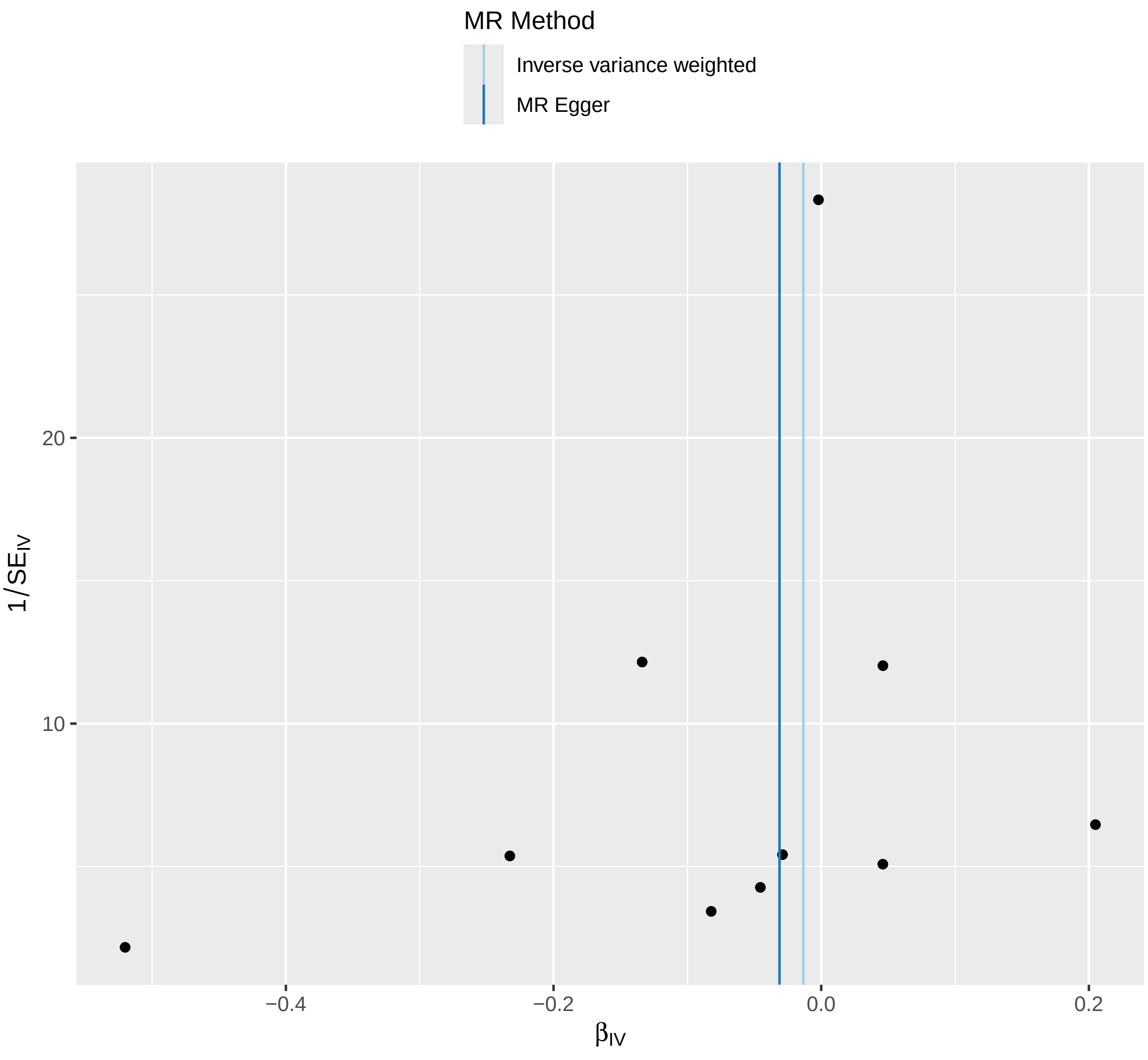
MR Method
Inverse variance weighted
MR Egger
20
10
1/SE$_{IV}$
−0.4
−0.2
0.0
0.2
β$_{IV}$

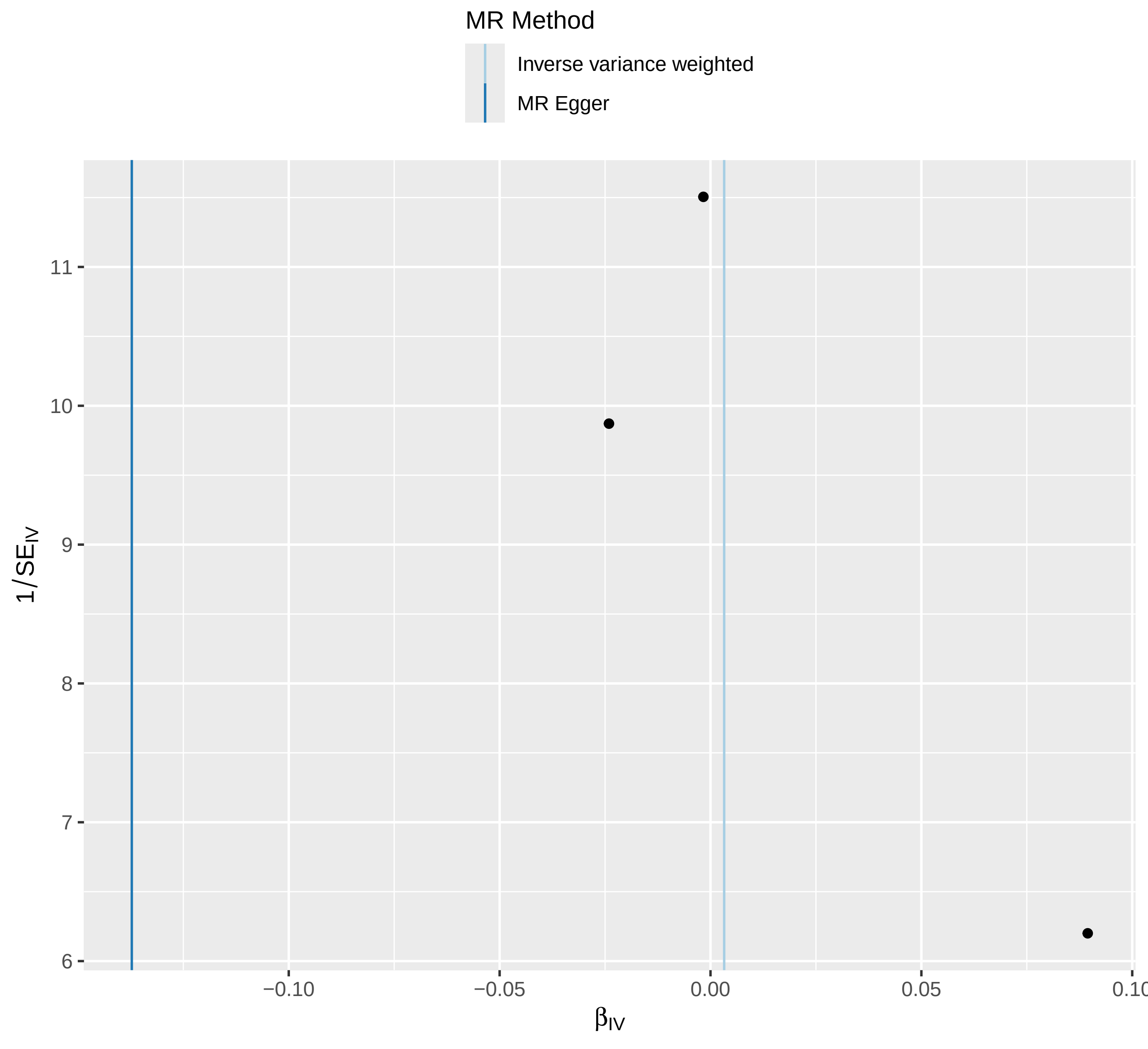

MR Method
Inverse variance weighted
MR Egger
11
10
9
8
7
6
1/SE$_{IV}$
−0.10
−0.05
0.00
0.05
0.10
β$_{IV}$

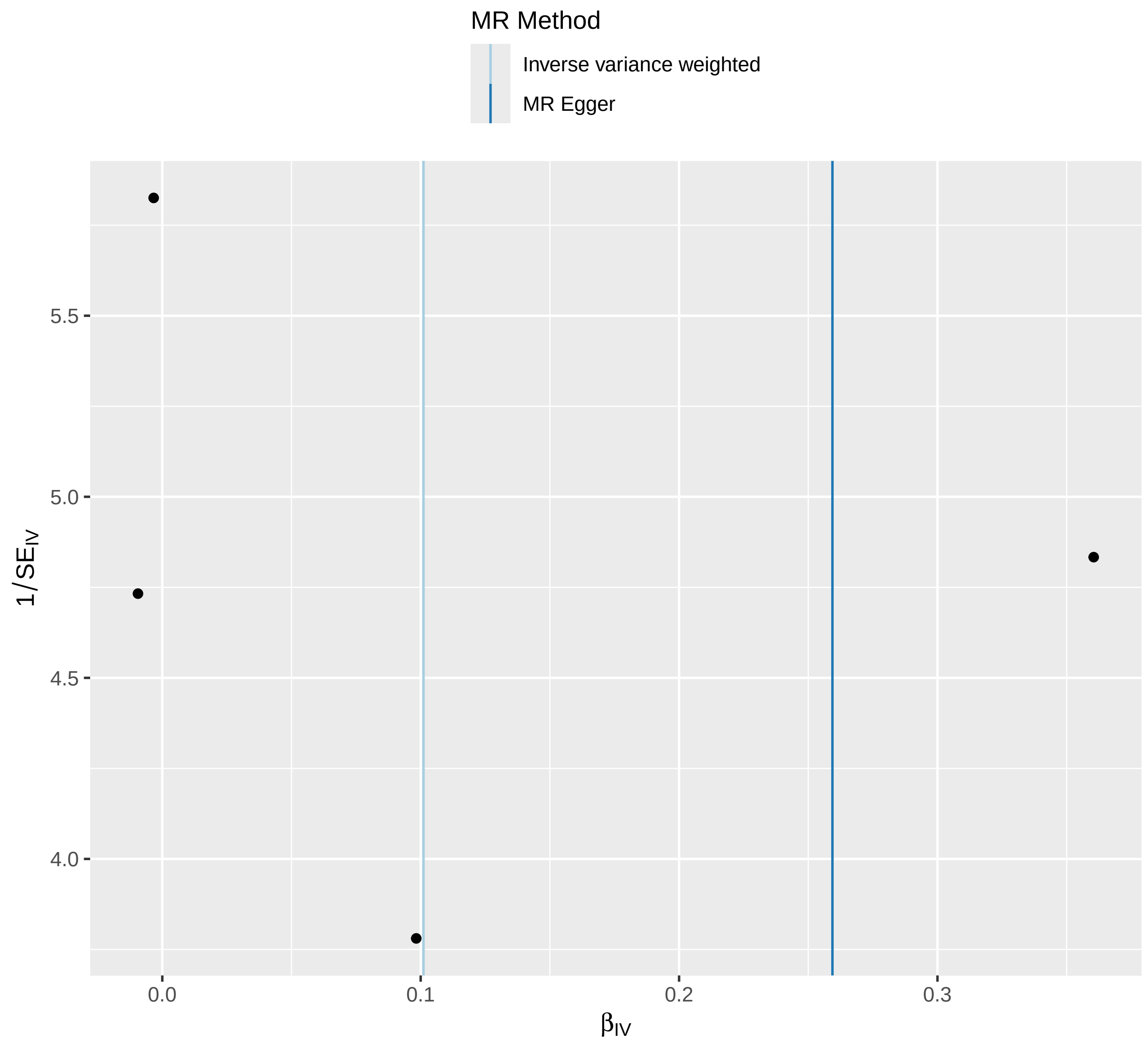

MR Method
Inverse variance weighted
MR Egger
5.5
5.0
4.5
4.0
1/SE$_{IV}$
0.0
0.1
0.2
0.3
β$_{IV}$

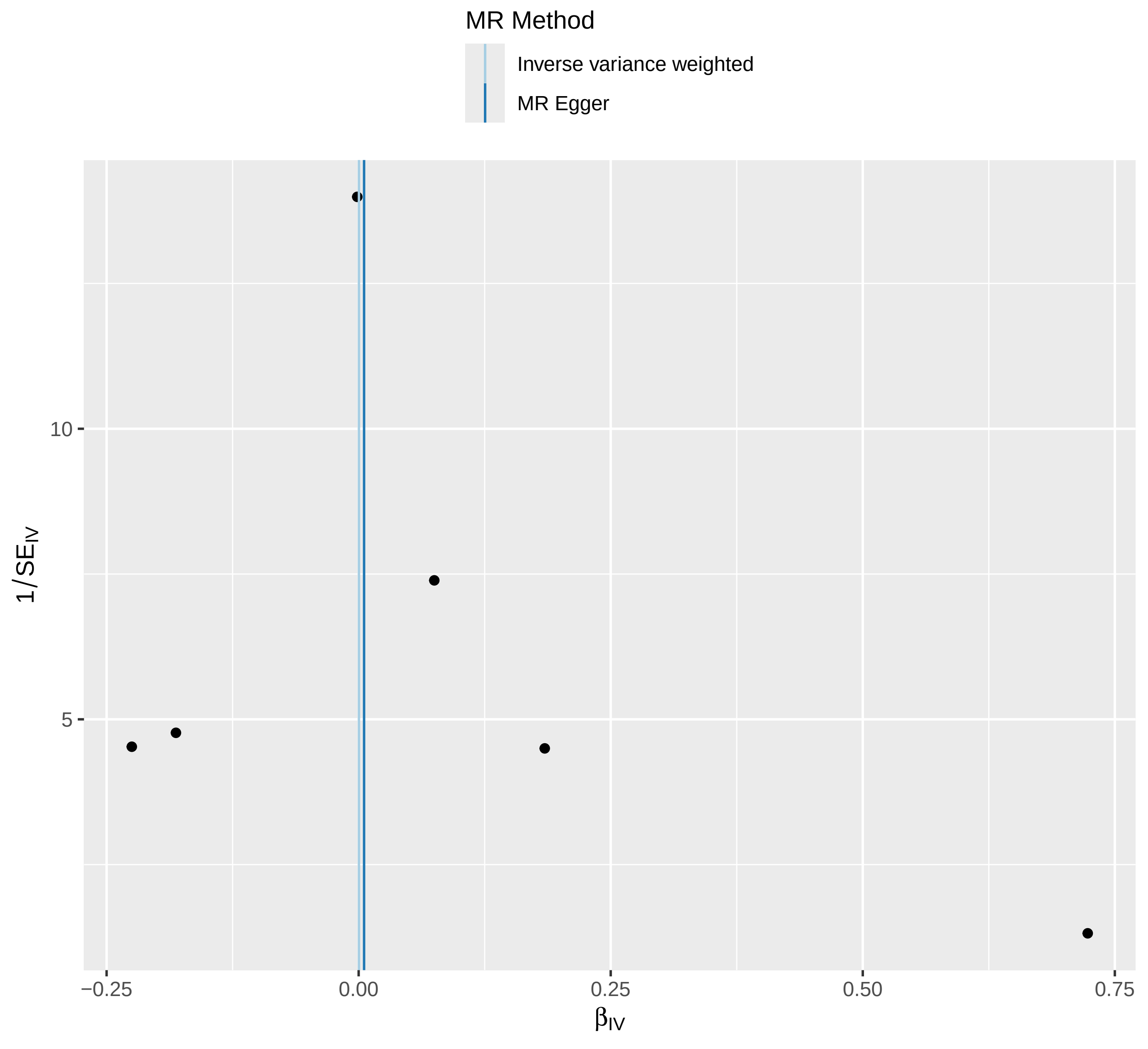
MR Method
Inverse variance weighted
MR Egger
10
5
1/SE$_{IV}$
−0.25
0.00
0.25
0.50
0.75
β$_{IV}$

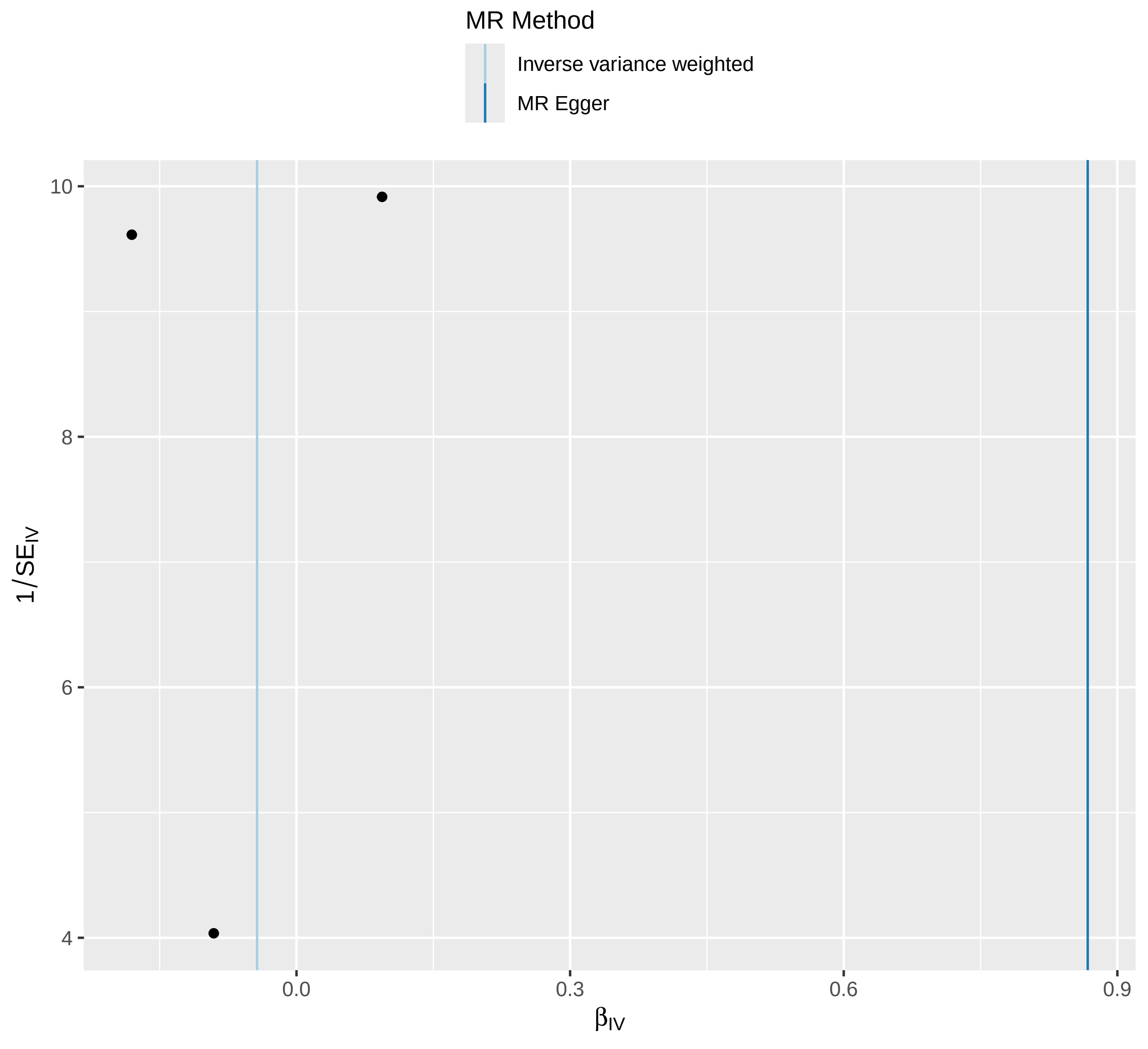

MR Method
Inverse variance weighted
MR Egger
10
8
6
4
1/SE_IV
0.0
0.3
0.6
0.9
β_IV

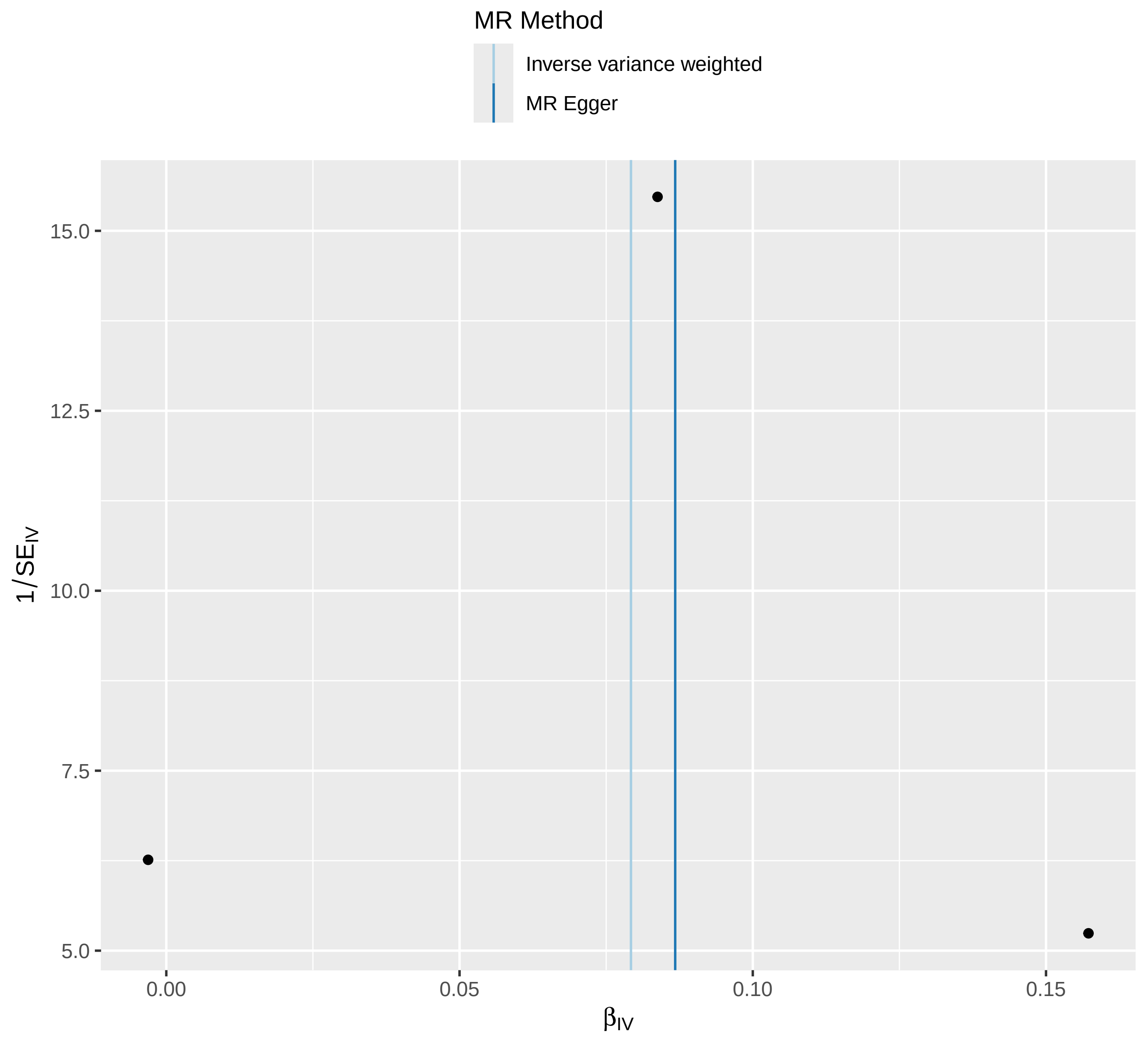

MR Method
Inverse variance weighted
MR Egger
15.0
12.5
10.0
7.5
5.0
$1/SE_{IV}$
0.00
0.05
0.10
0.15
$\beta_{IV}$

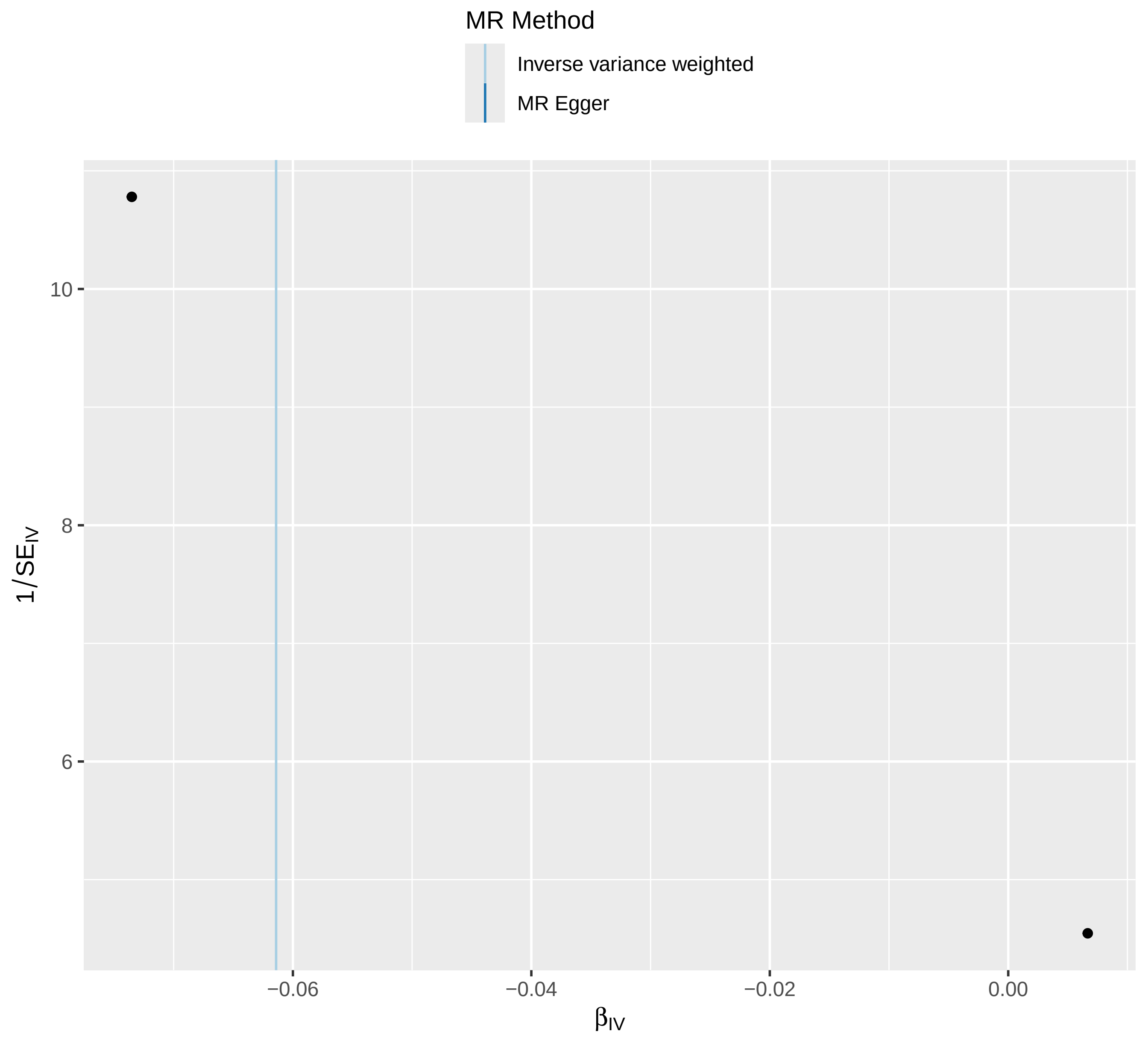

MR Method
Inverse variance weighted
MR Egger
10
8
6
1/SE_IV
−0.06
−0.04
−0.02
0.00
β_IV

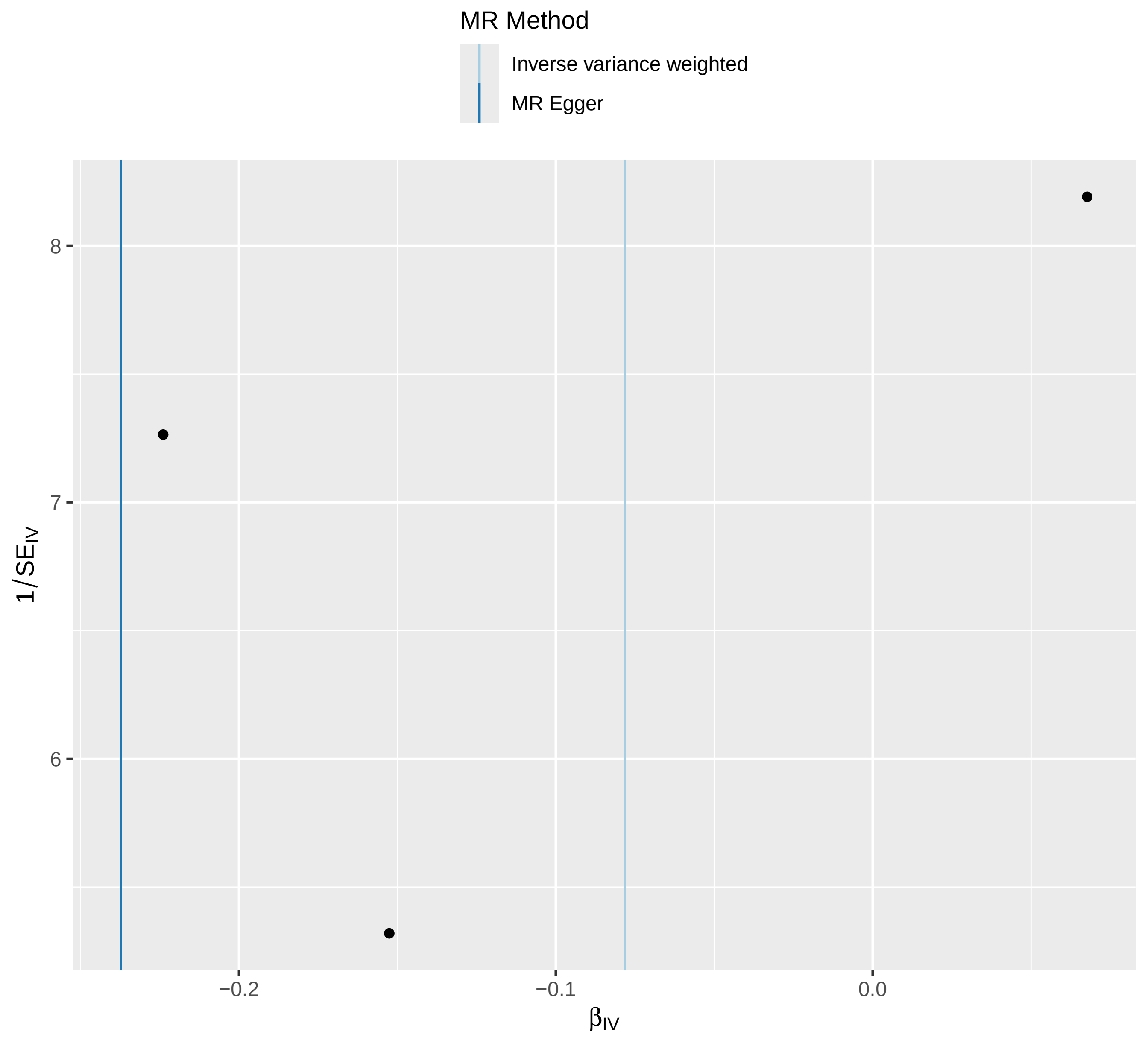
MR Method
Inverse variance weighted
MR Egger
8
7
6
1/SE$_{IV}$
−0.2
−0.1
0.0
$\beta_{IV}$

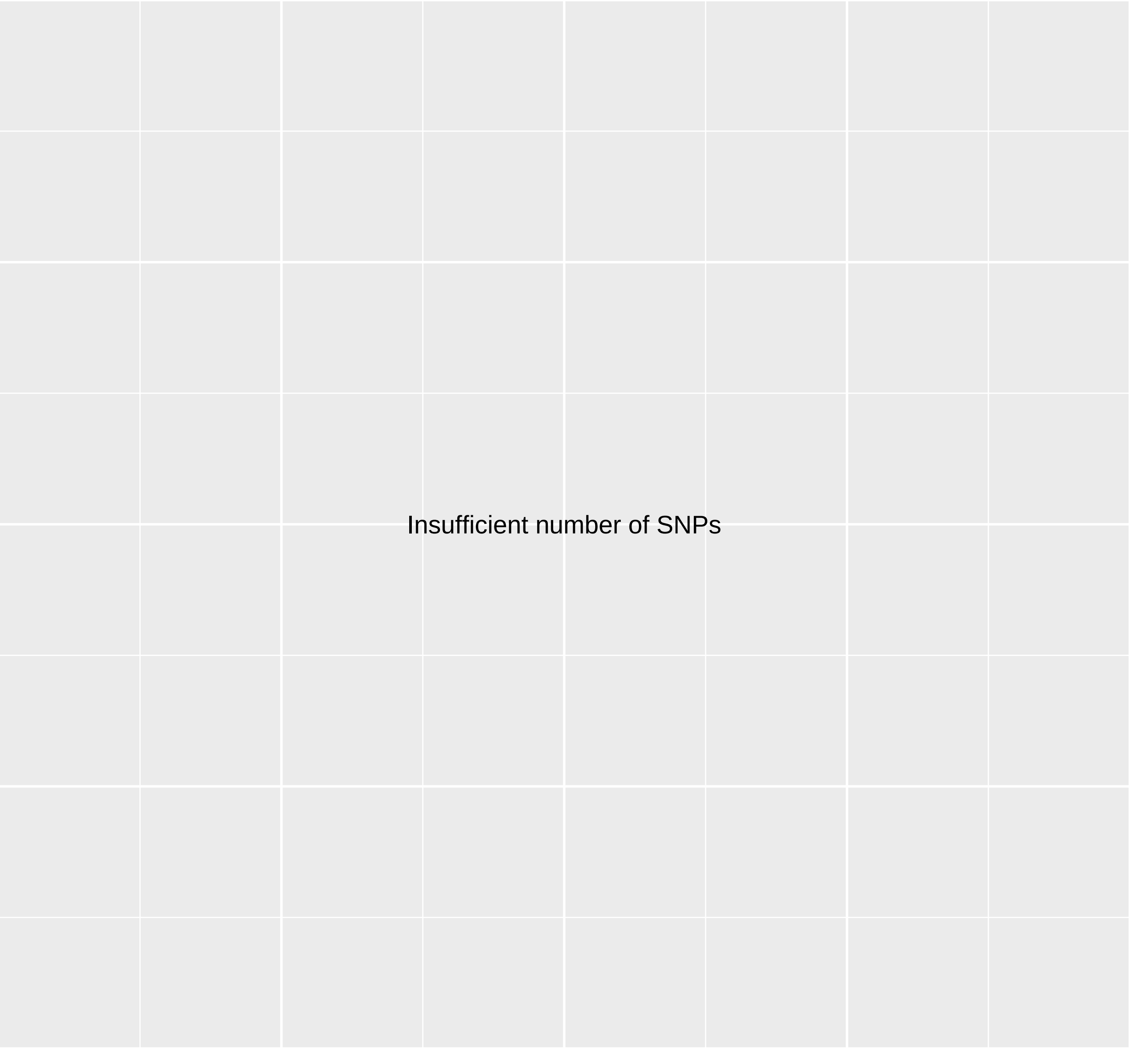
Insufficient number of SNPs

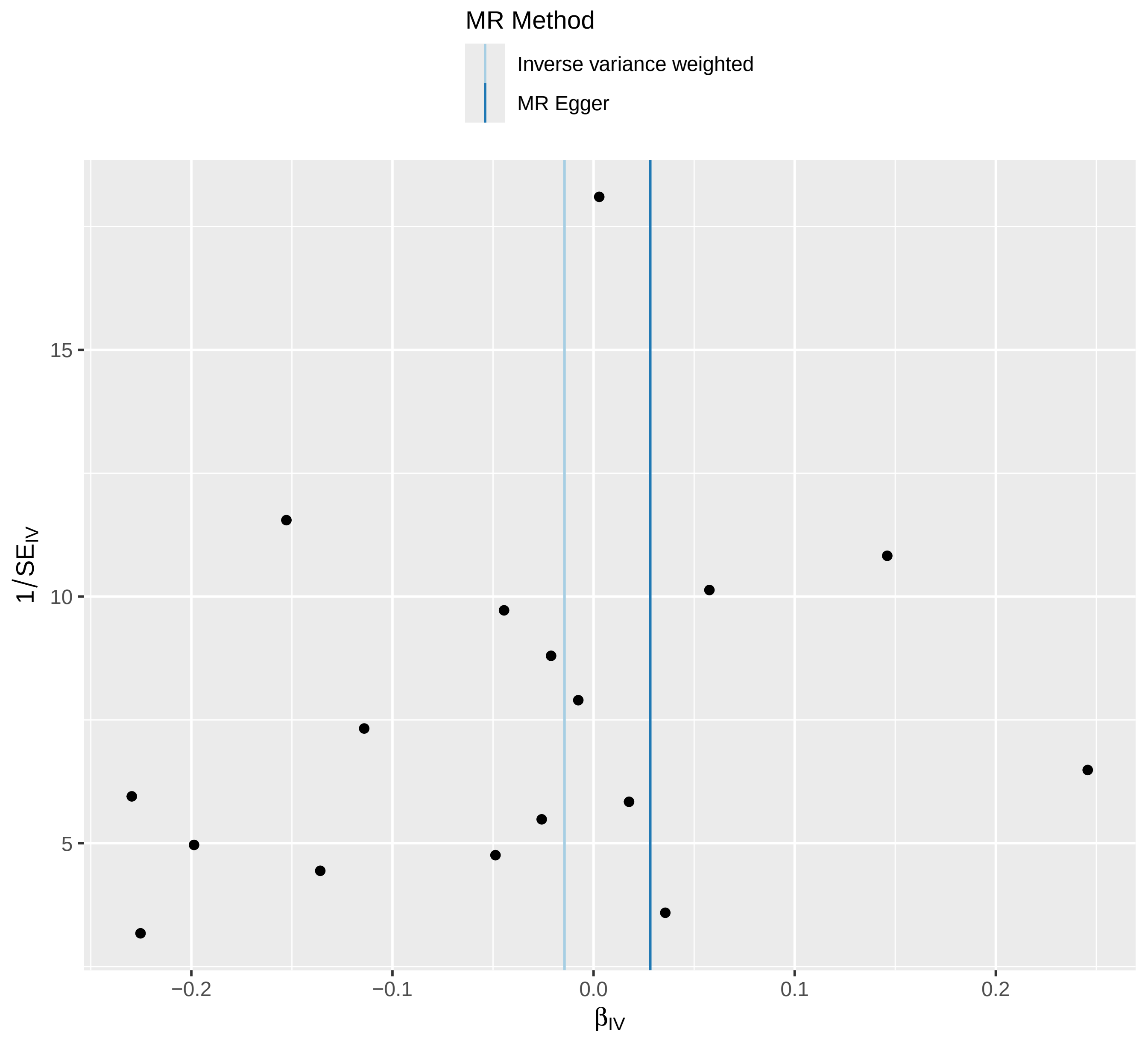
MR Method
Inverse variance weighted
MR Egger
15
10
5
$1/SE_{IV}$
−0.2
−0.1
0.0
0.1
0.2
$\beta_{IV}$

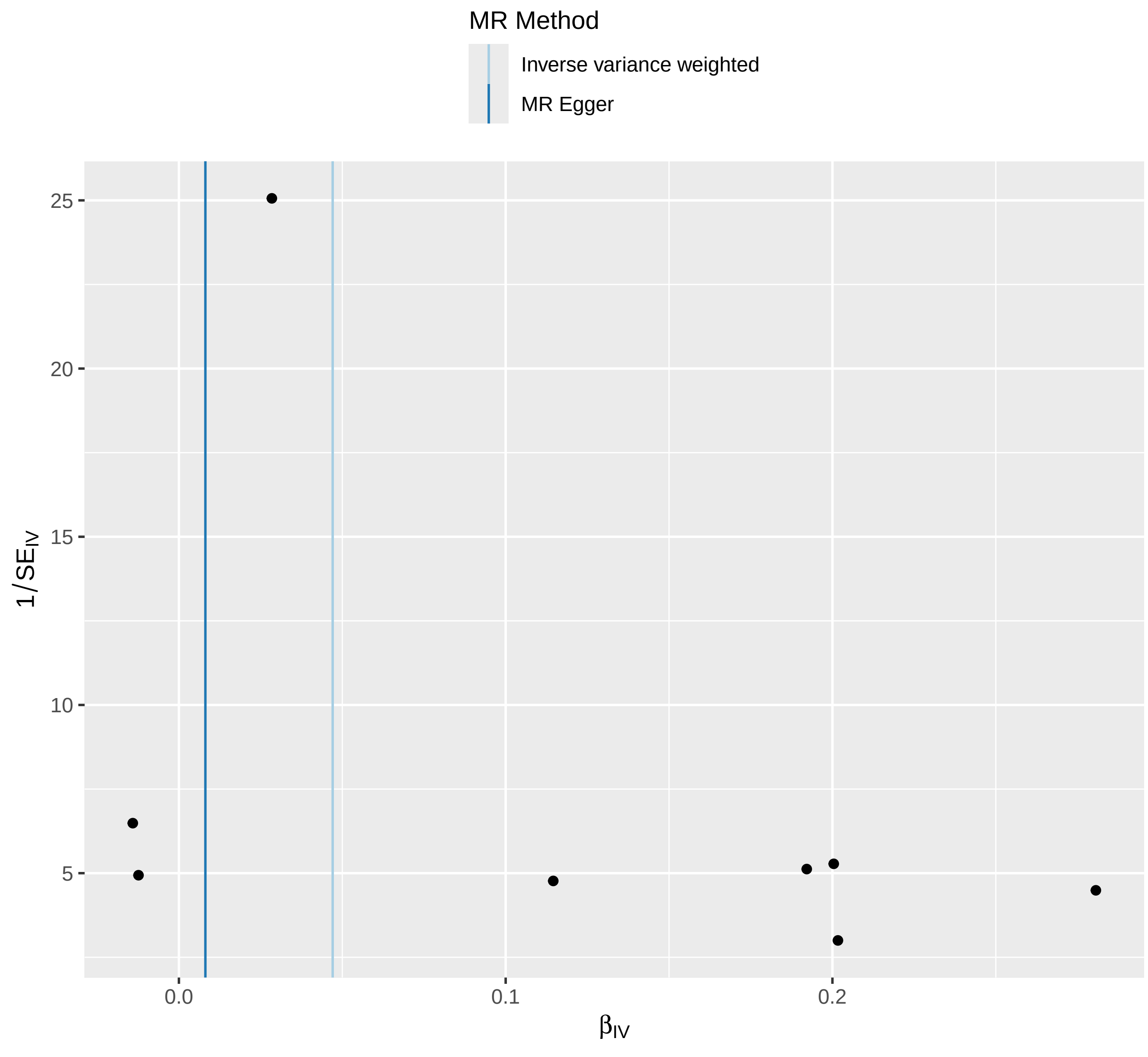

MR Method
Inverse variance weighted
MR Egger
25
20
15
10
5
0.0
0.1
0.2
$1/SE_{IV}$
$\beta_{IV}$

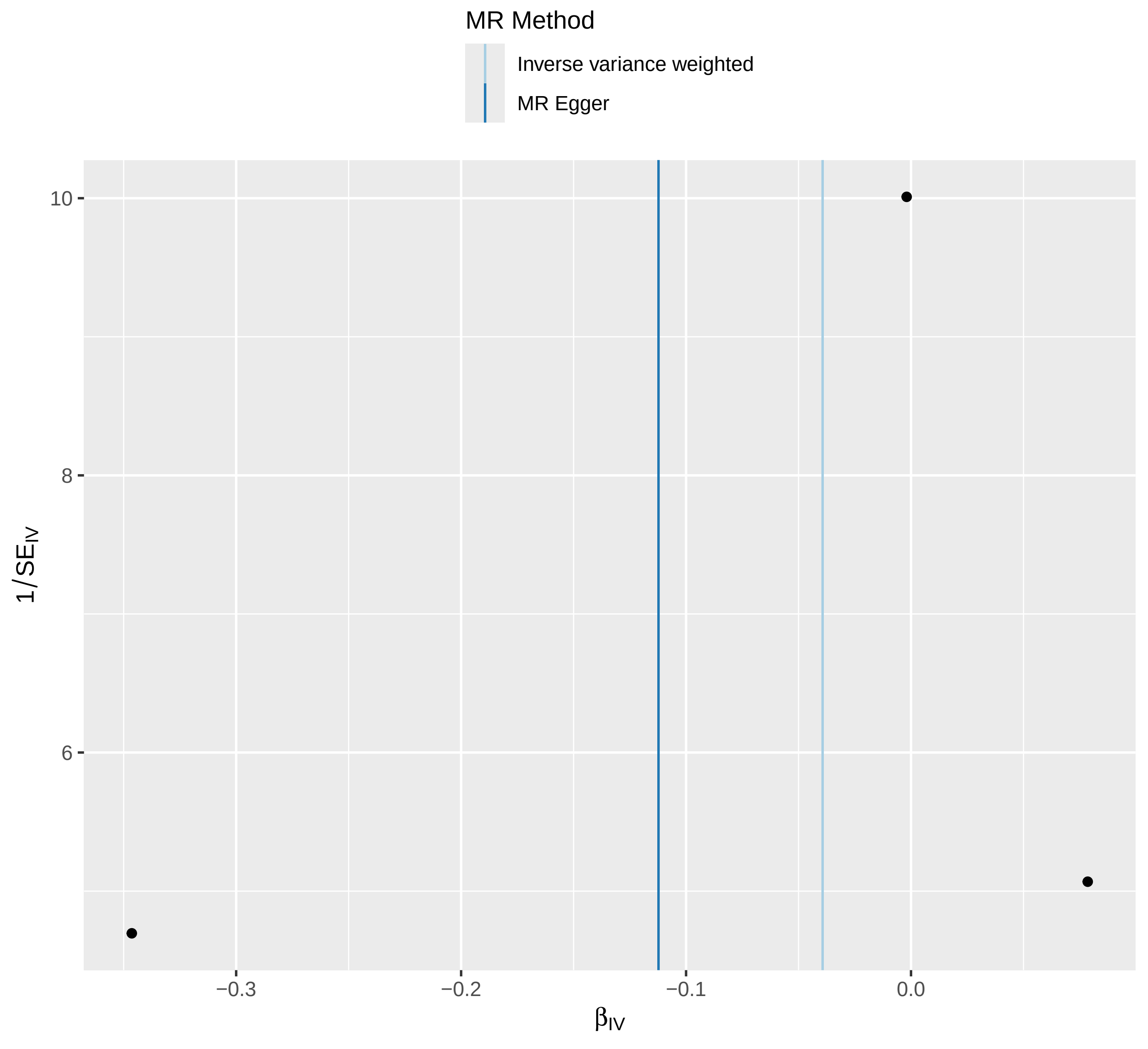
MR Method
Inverse variance weighted
MR Egger
10
8
6
$1/SE_{IV}$
−0.3
−0.2
−0.1
0.0
$\beta_{IV}$

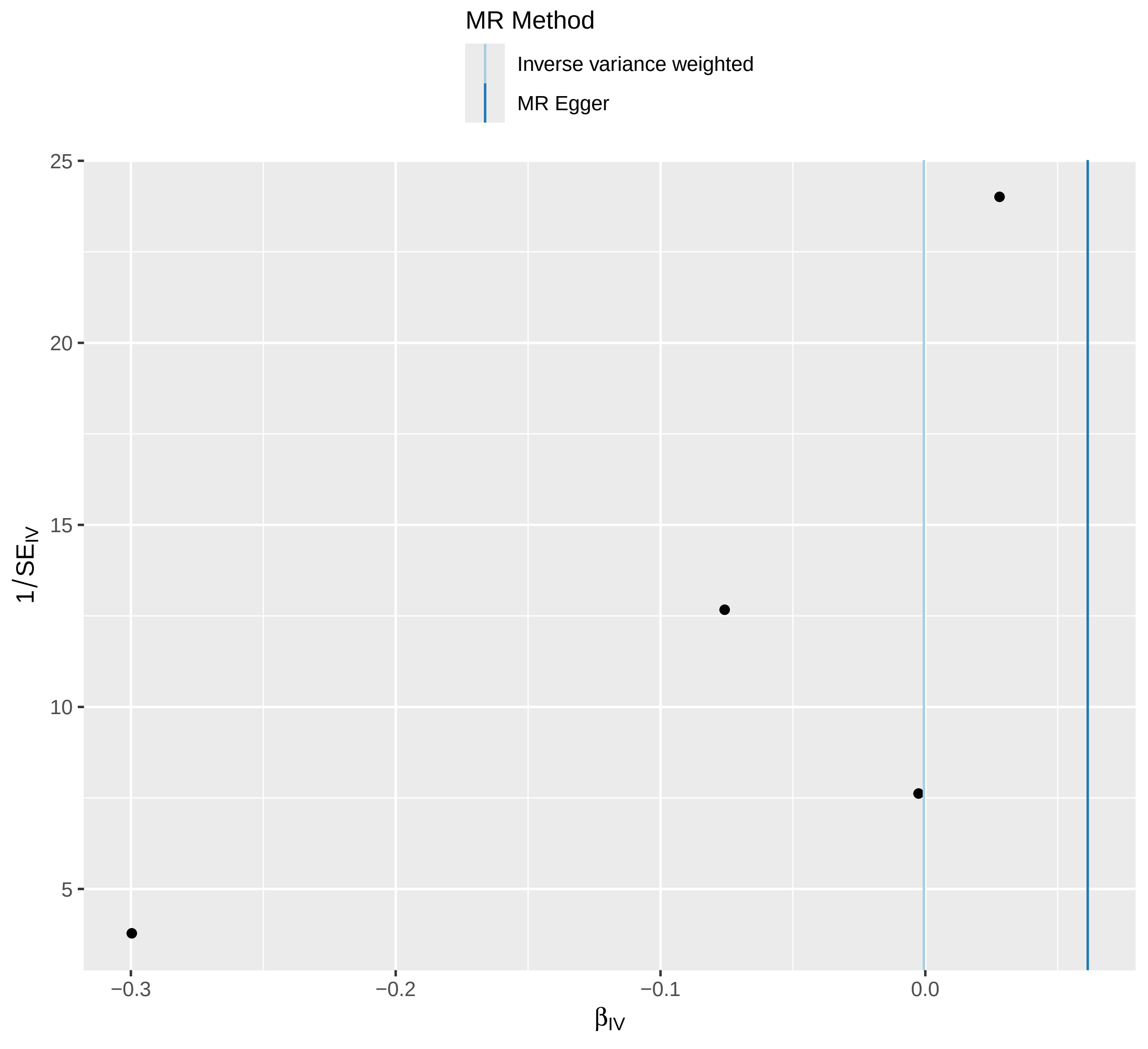

MR Method
Inverse variance weighted
MR Egger
25
20
15
10
5
1/SE$_{IV}$
−0.3
−0.2
−0.1
0.0
β$_{IV}$

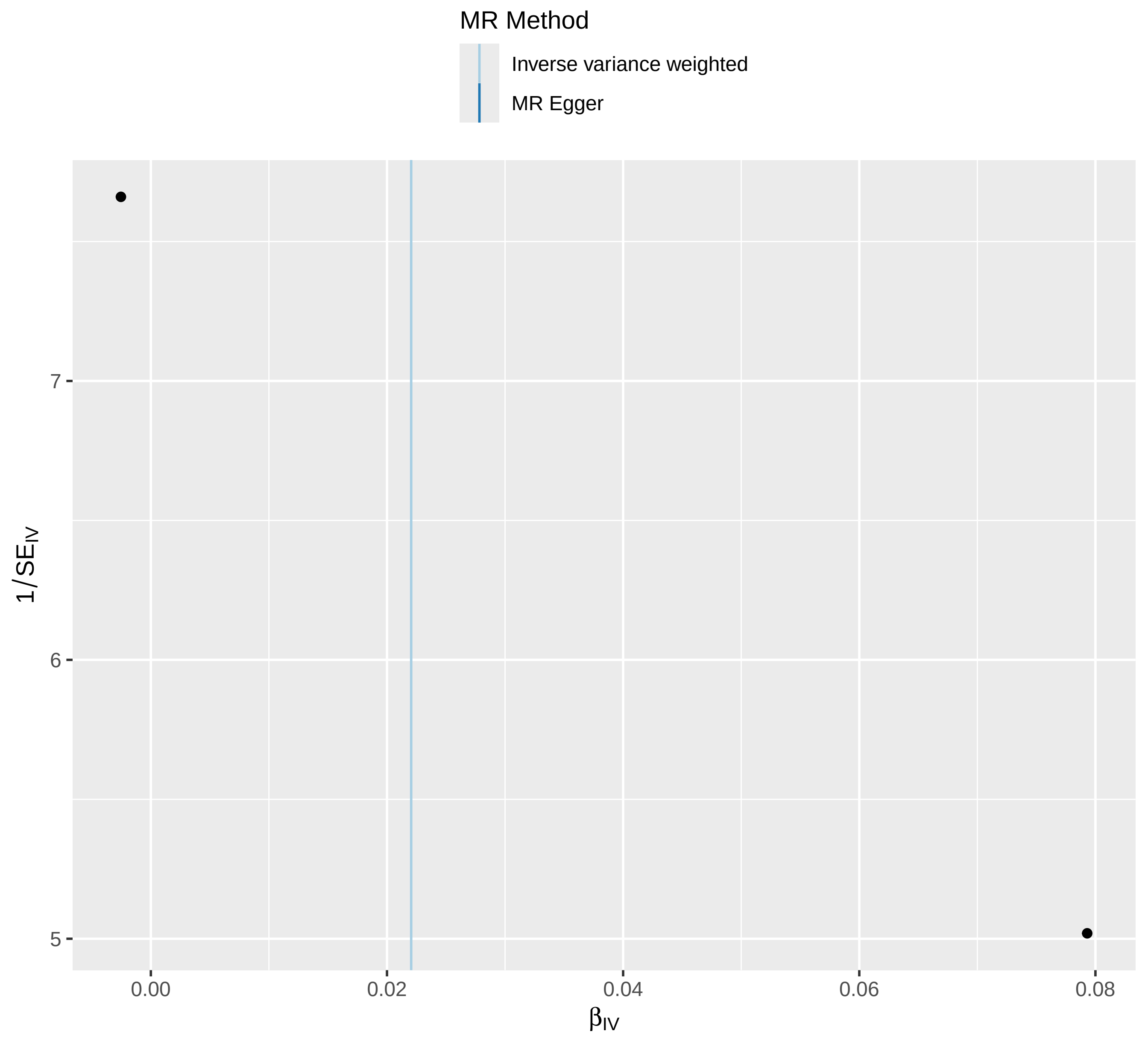
MR Method
Inverse variance weighted
MR Egger
1/SE_IV
7
6
5
0.00
0.02
0.04
0.06
0.08
β_IV

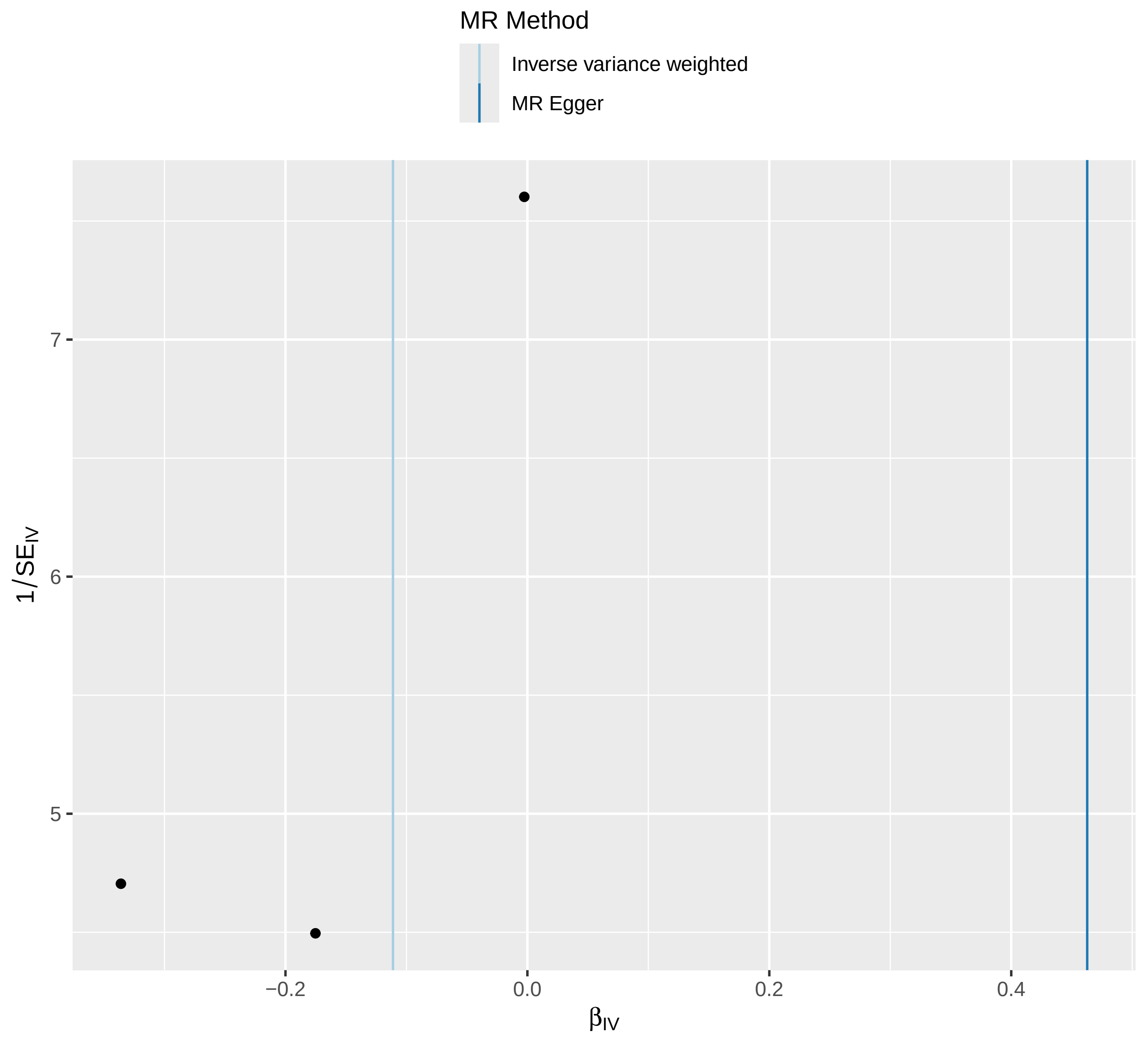

MR Method
Inverse variance weighted
MR Egger
7
6
5
1/SE$_{IV}$
−0.2
0.0
0.2
0.4
$\beta_{IV}$

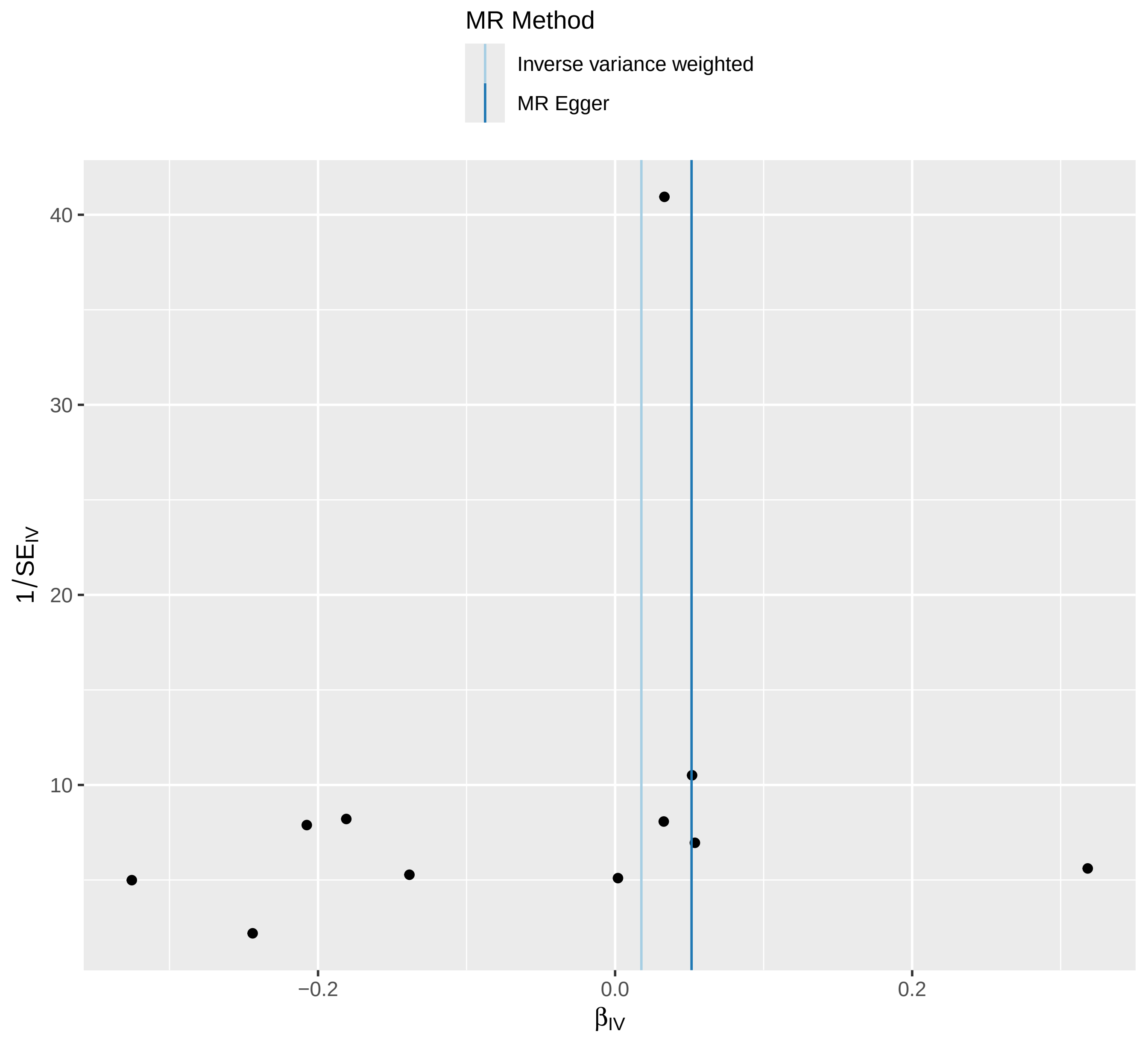
MR Method
Inverse variance weighted
MR Egger
1/SE_IV
40
30
20
10
−0.2
0.0
0.2
β_IV

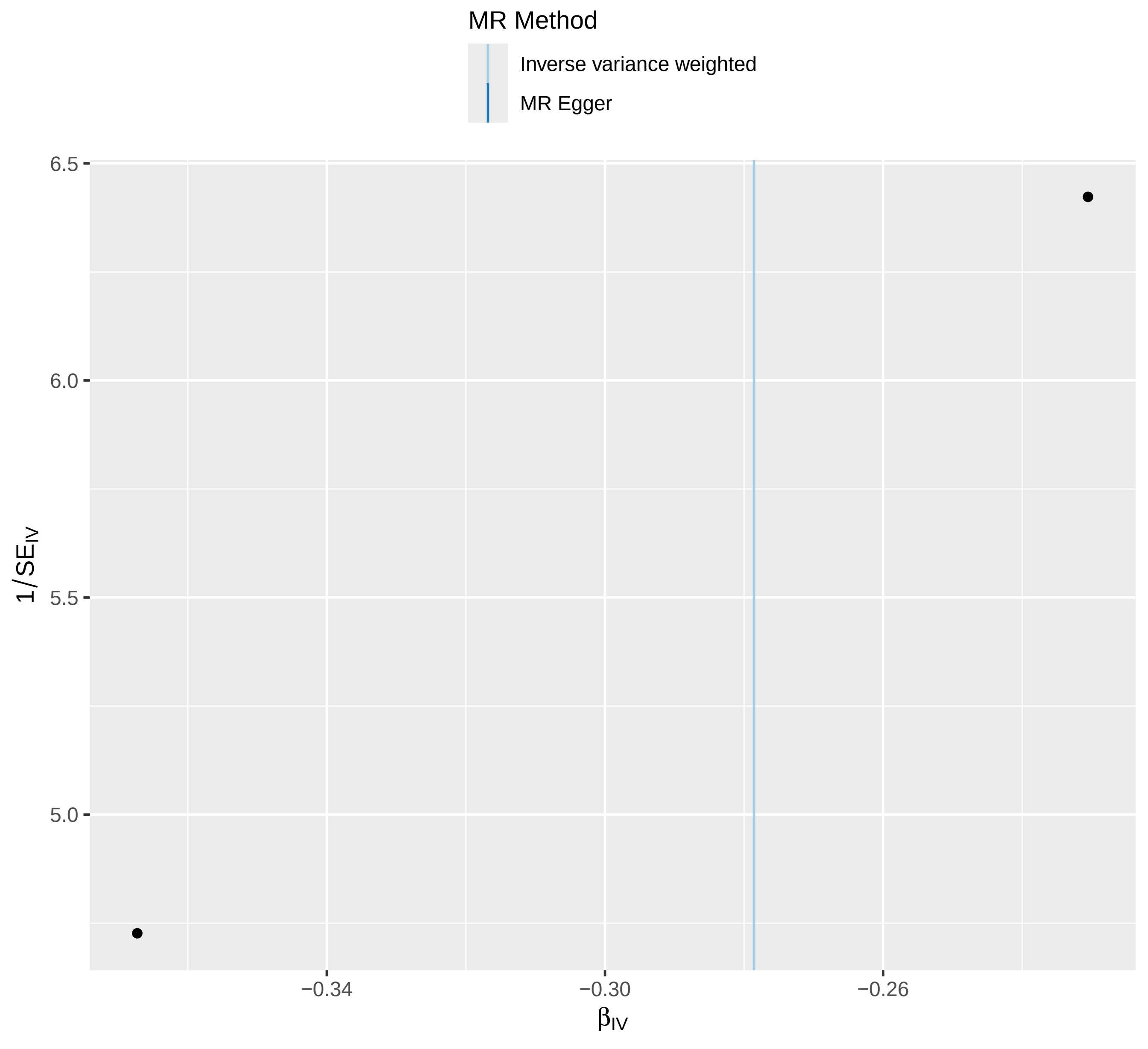

MR Method
Inverse variance weighted
MR Egger
6.5
6.0
5.5
5.0
1/SE$_{IV}$
−0.34
−0.30
−0.26
β$_{IV}$

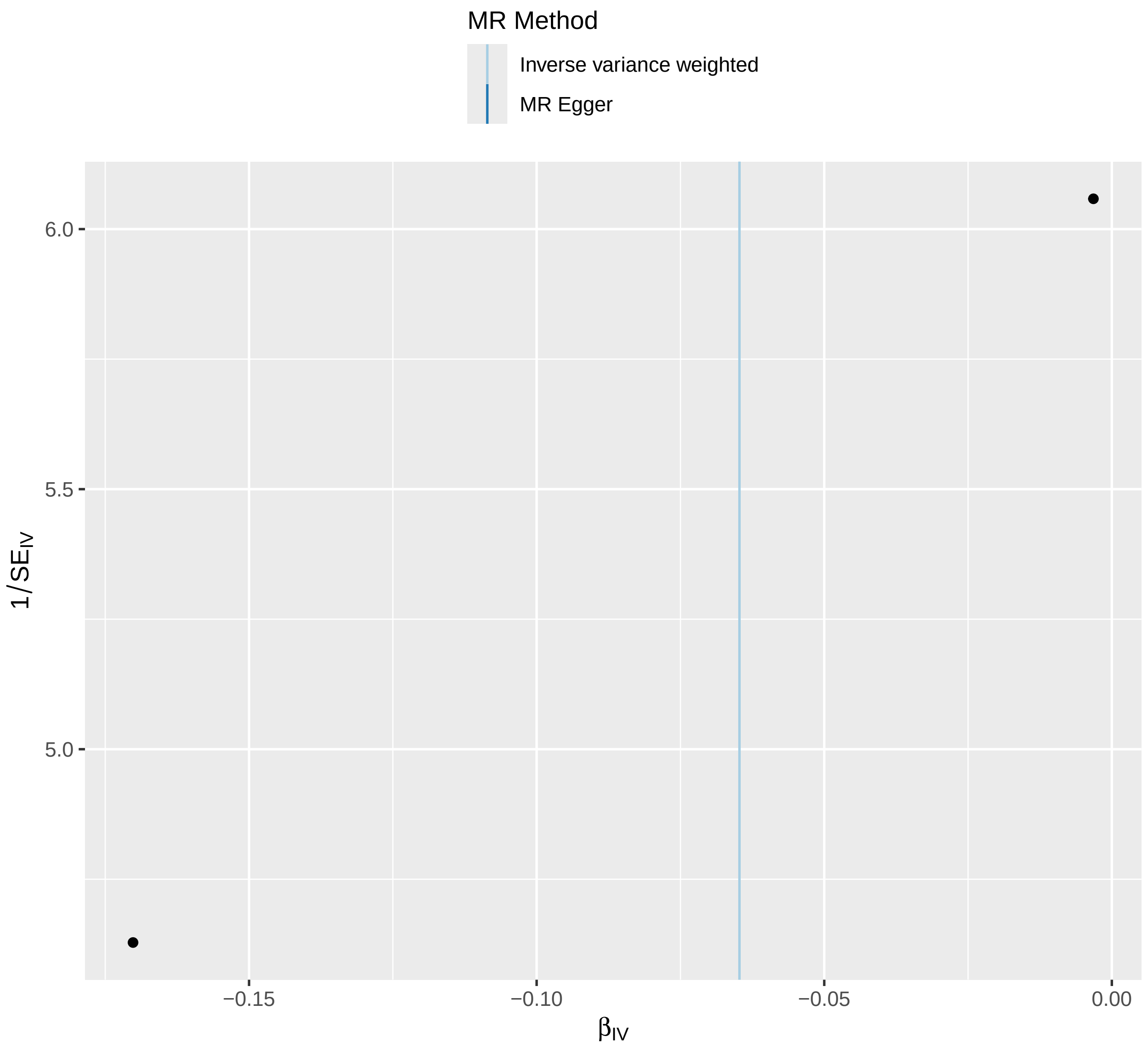
MR Method
Inverse variance weighted
MR Egger
6.0
5.5
5.0
$1/SE_{IV}$
−0.15
−0.10
−0.05
0.00
$\beta_{IV}$

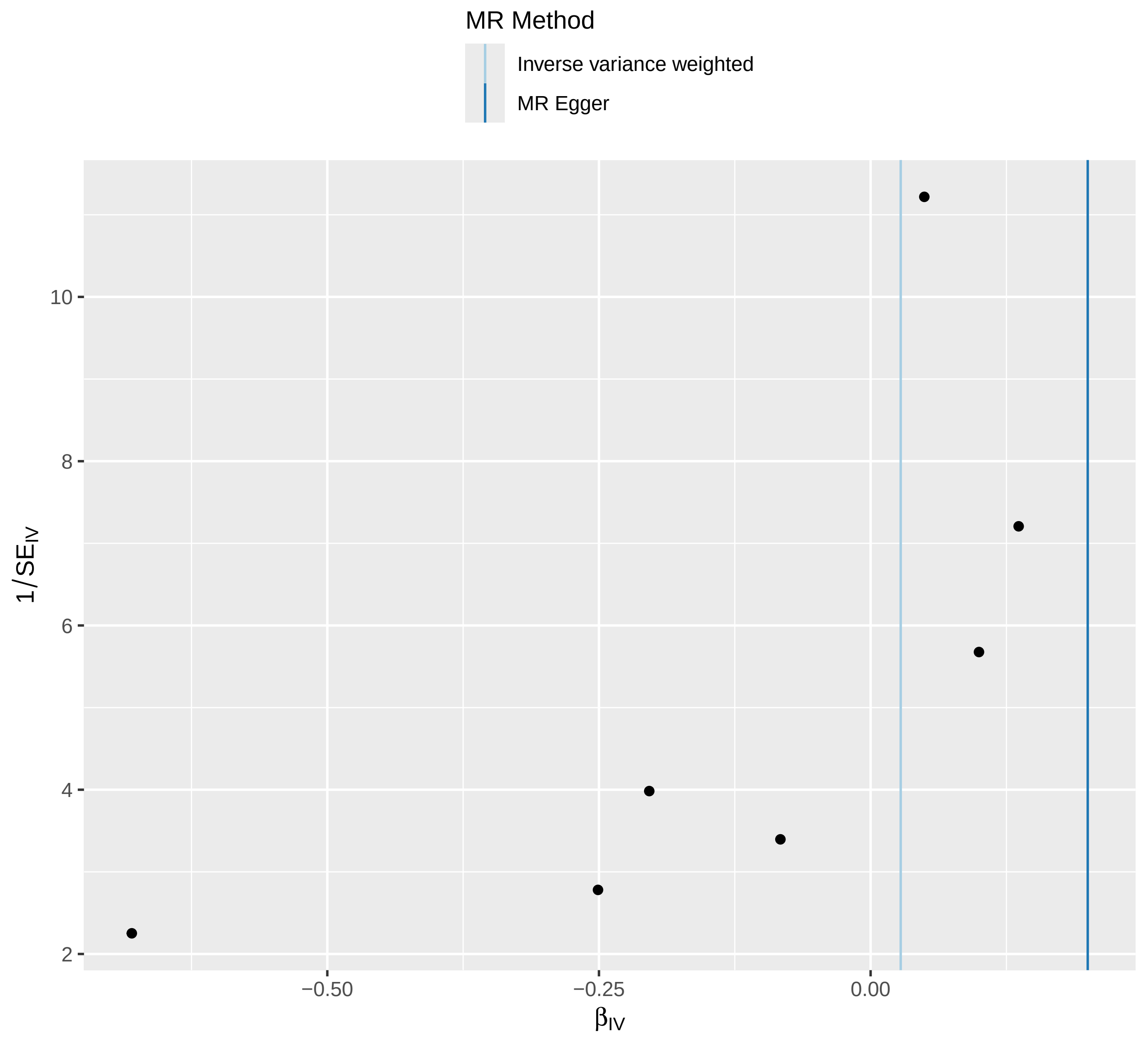
MR Method
Inverse variance weighted
MR Egger
1/SE_IV
10
8
6
4
2
−0.50
−0.25
0.00
β_IV

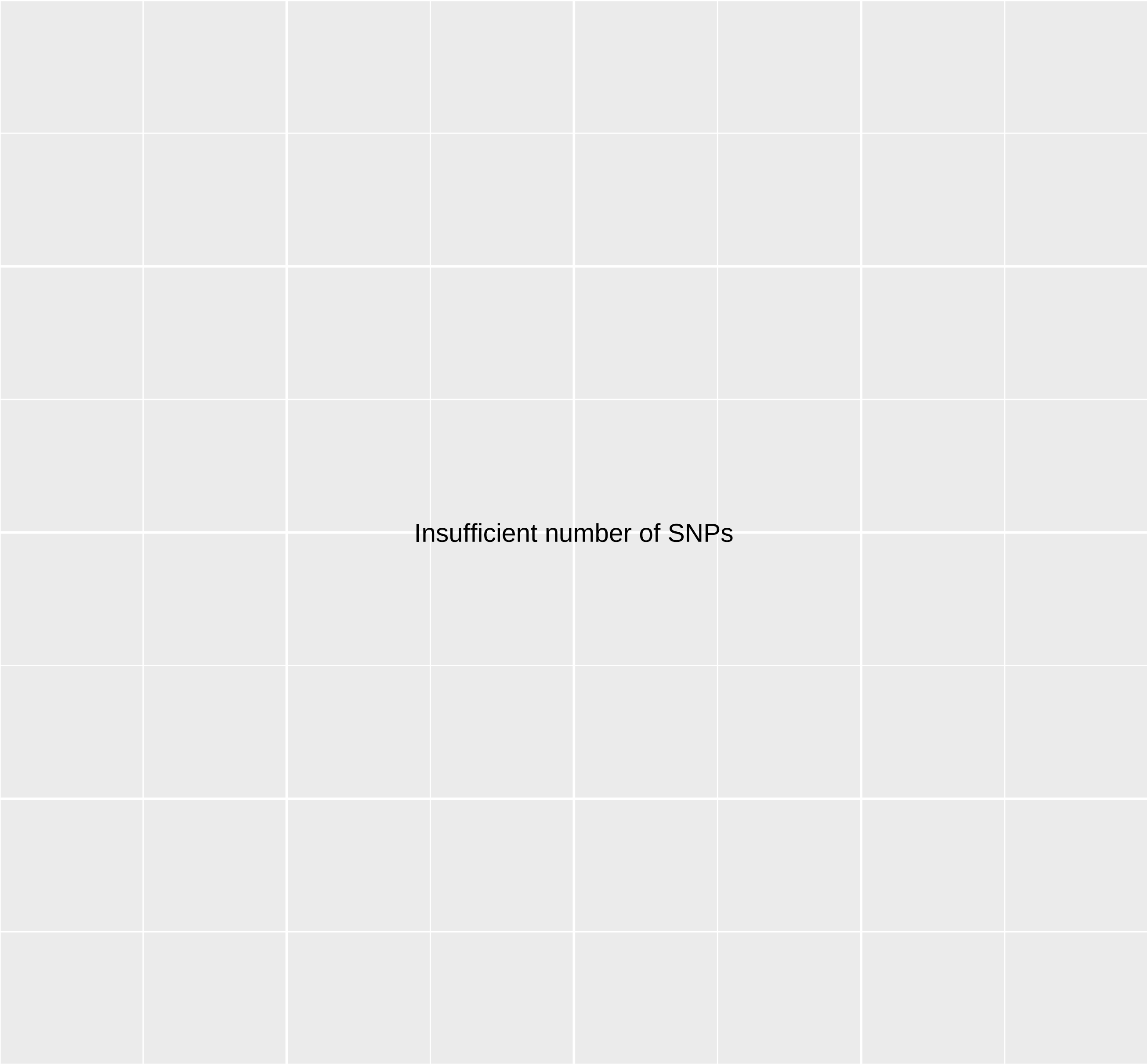
Insufficient number of SNPs

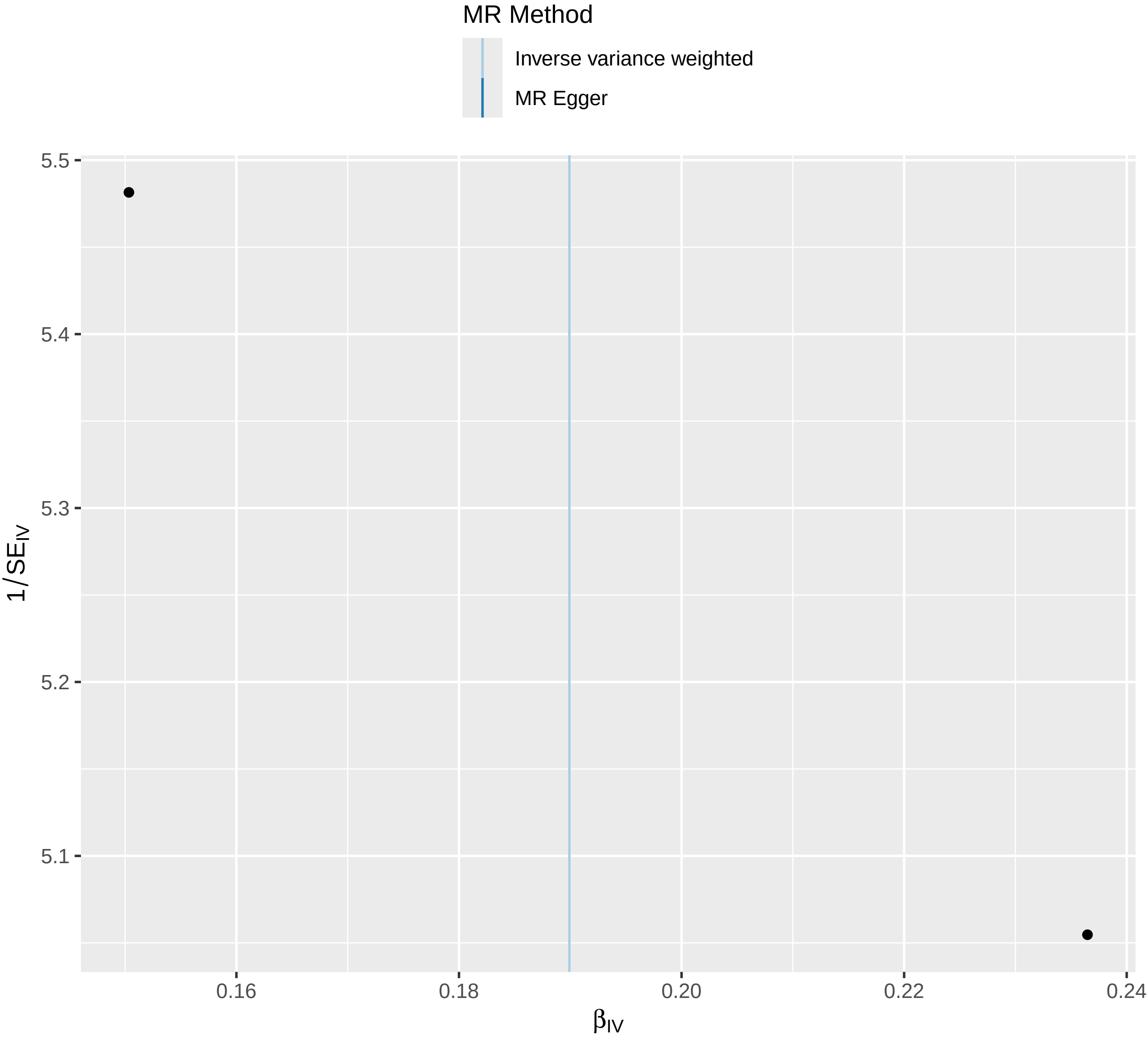

MR Method
Inverse variance weighted
MR Egger
5.5
5.4
5.3
5.2
5.1
1/SE$_{IV}$
0.16
0.18
0.20
0.22
0.24
$\beta_{IV}$

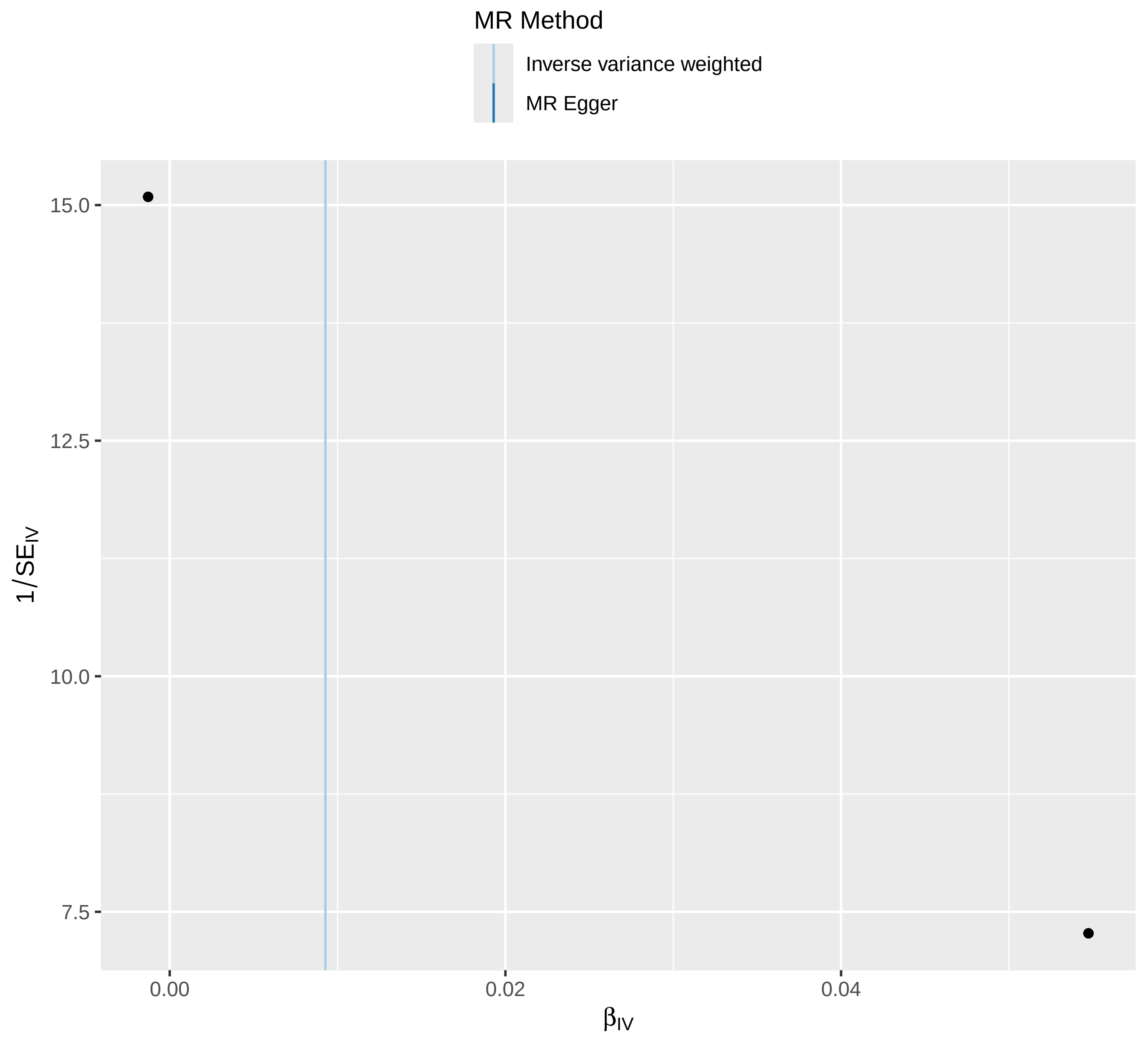

MR Method
Inverse variance weighted
MR Egger
15.0
12.5
10.0
7.5
1/SE_IV
0.00
0.02
0.04
β_IV

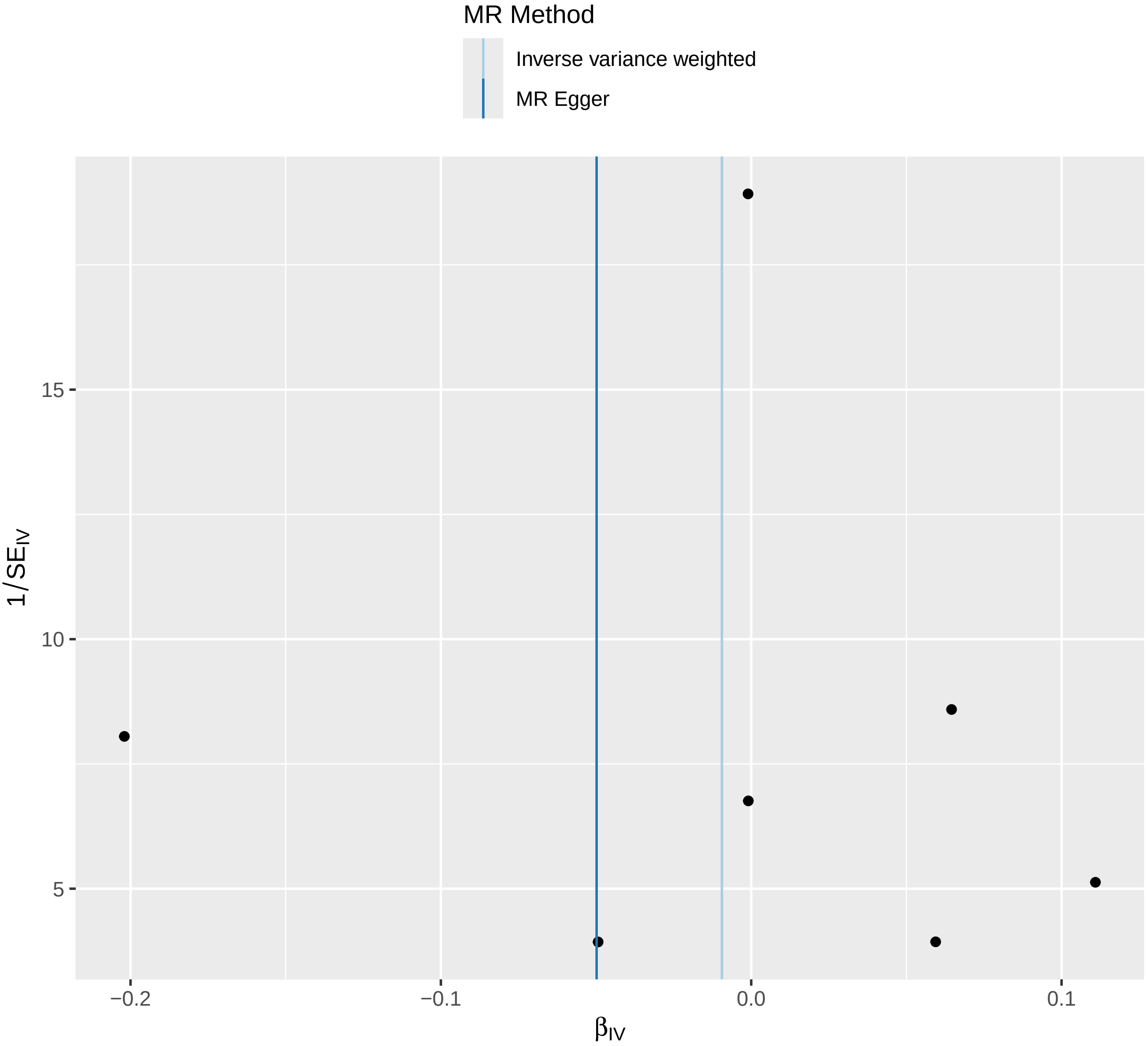

MR Method
Inverse variance weighted
MR Egger
15
10
5
1/SE$_{IV}$
−0.2
−0.1
0.0
0.1
β$_{IV}$

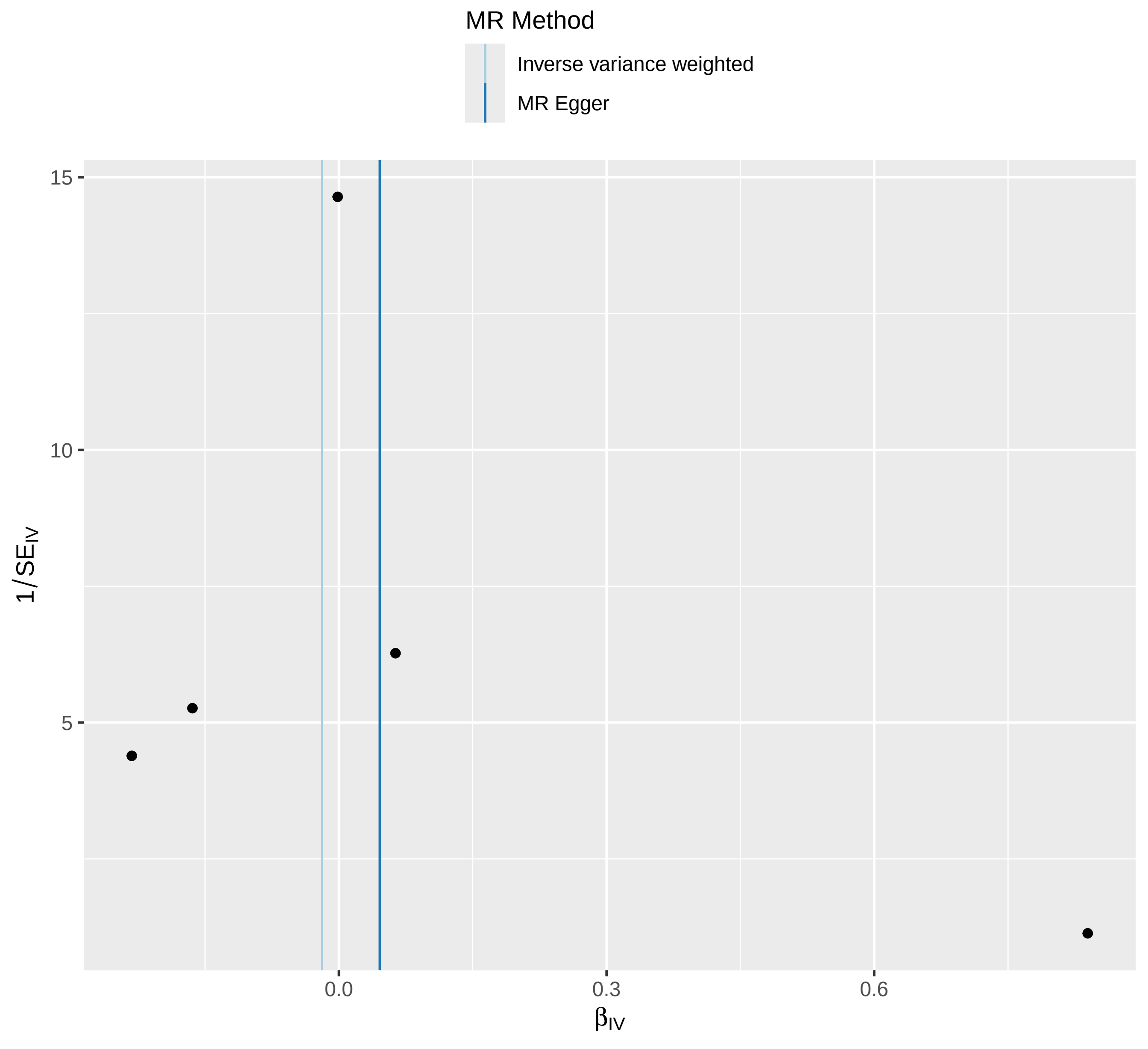
MR Method
Inverse variance weighted
MR Egger
15
10
5
1/SE_IV
0.0
0.3
0.6
β_IV

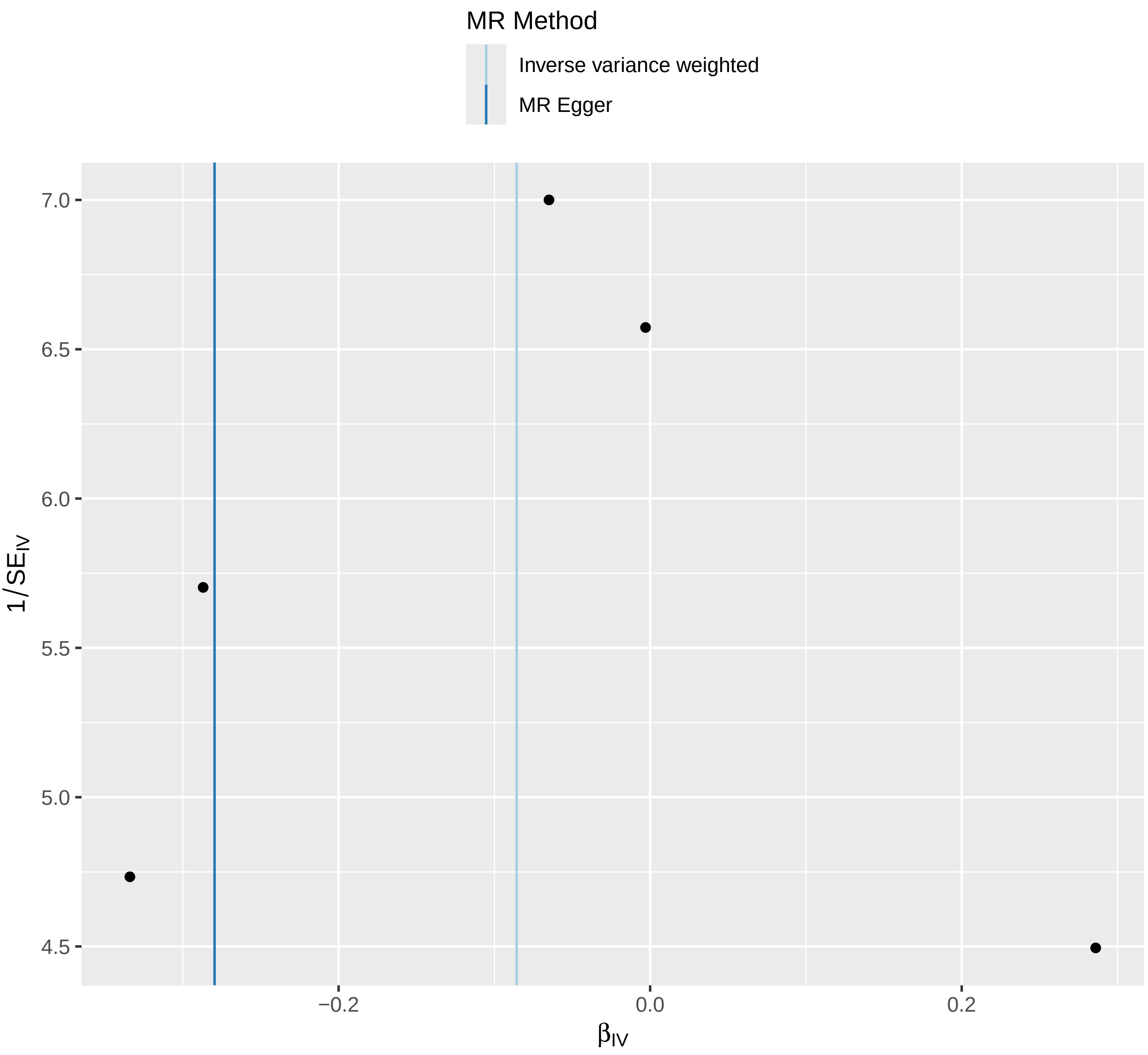
MR Method
Inverse variance weighted
MR Egger
7.0
6.5
6.0
5.5
5.0
4.5
1/SE$_{IV}$
−0.2
0.0
0.2
β$_{IV}$

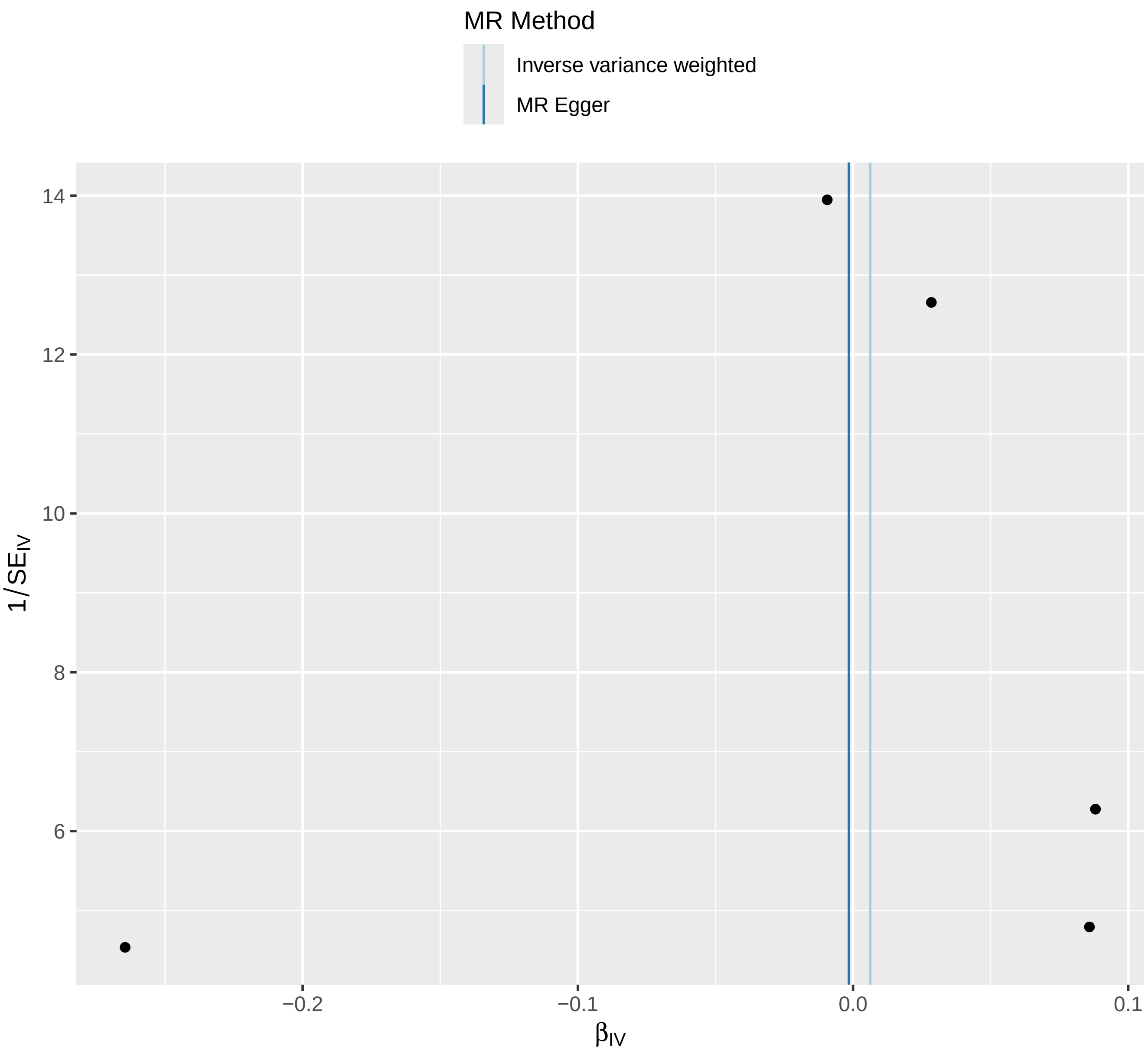
MR Method
Inverse variance weighted
MR Egger
14
12
10
8
6
1/SE$_{IV}$
−0.2
−0.1
0.0
0.1
β$_{IV}$

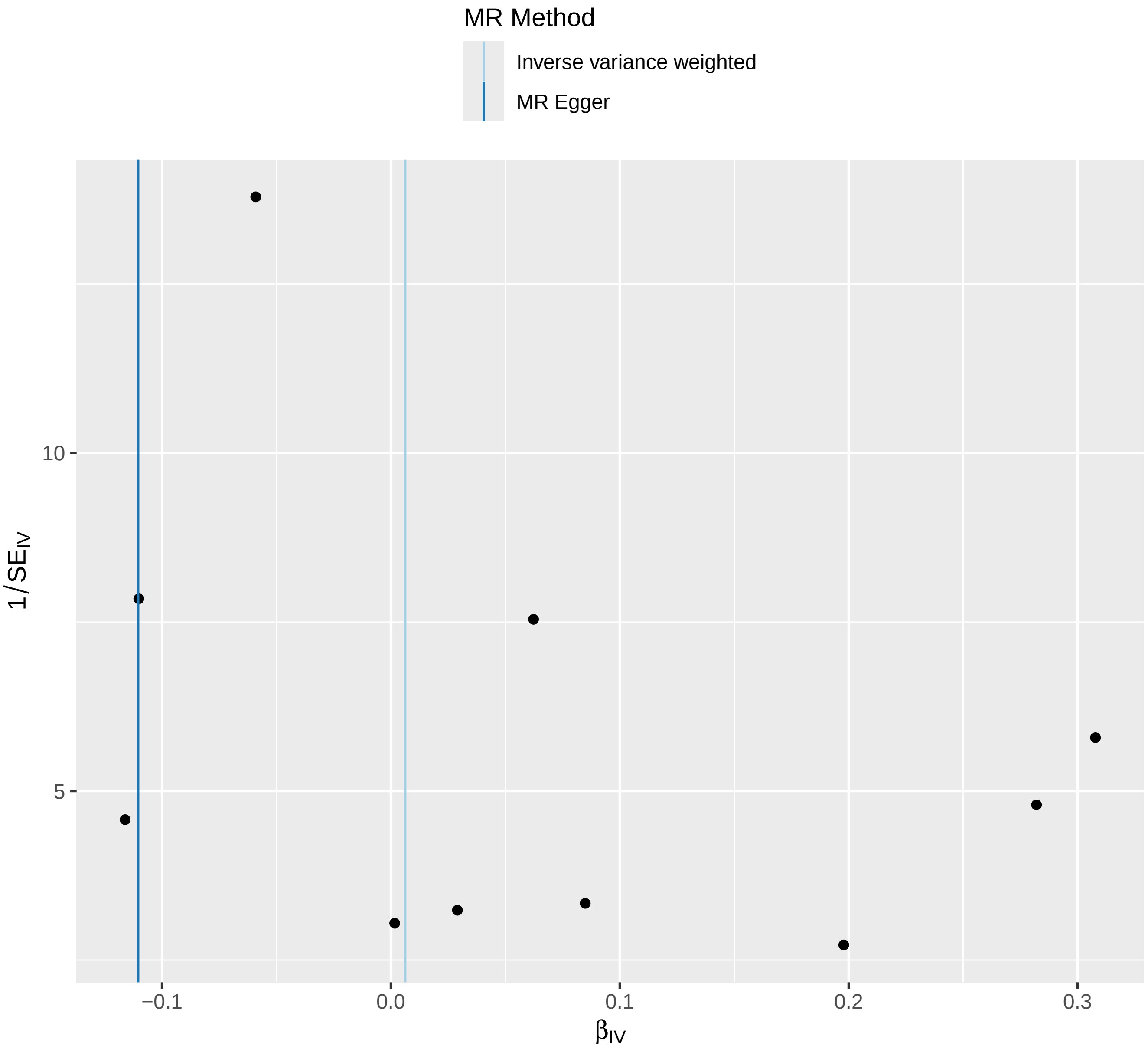

MR Method
Inverse variance weighted
MR Egger
10
5
$1/SE_{IV}$
−0.1
0.0
0.1
0.2
0.3
$\beta_{IV}$

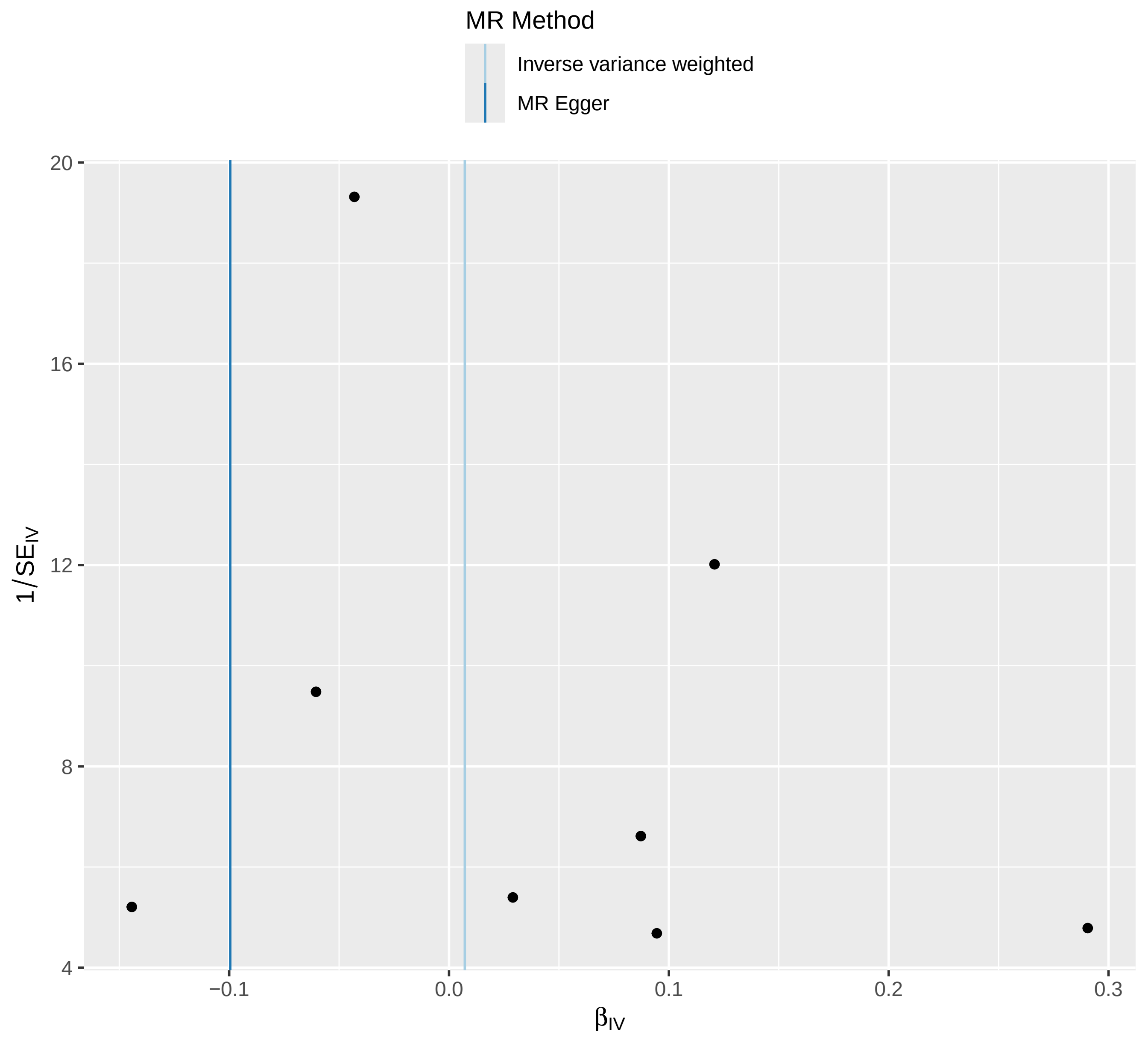
MR Method
Inverse variance weighted
MR Egger
20
16
12
8
4
1/SE_IV
−0.1
0.0
0.1
0.2
0.3
β_IV

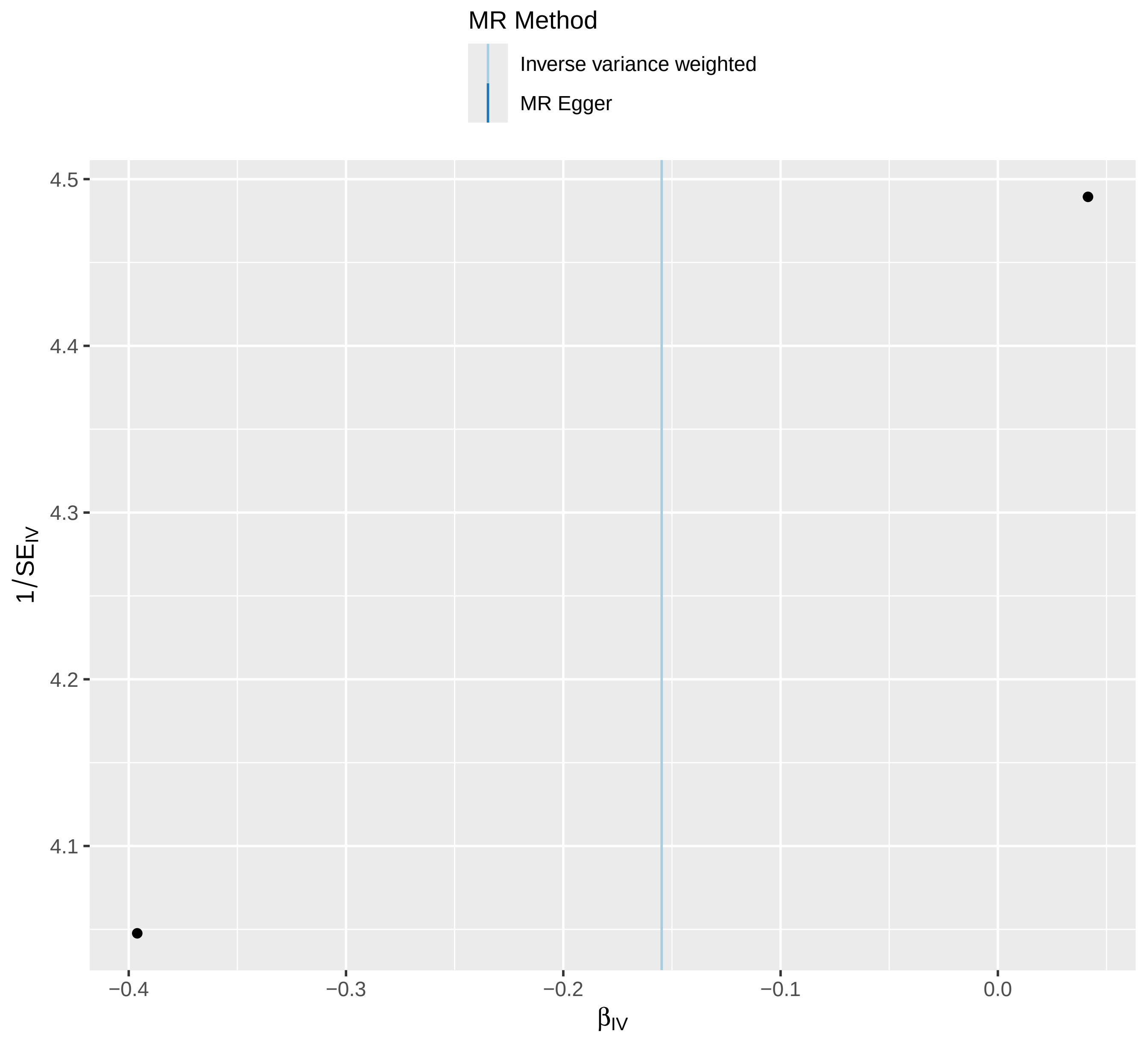

MR Method
Inverse variance weighted
MR Egger
4.5
4.4
4.3
4.2
4.1
1/SE$_{IV}$
−0.4
−0.3
−0.2
−0.1
0.0
β$_{IV}$

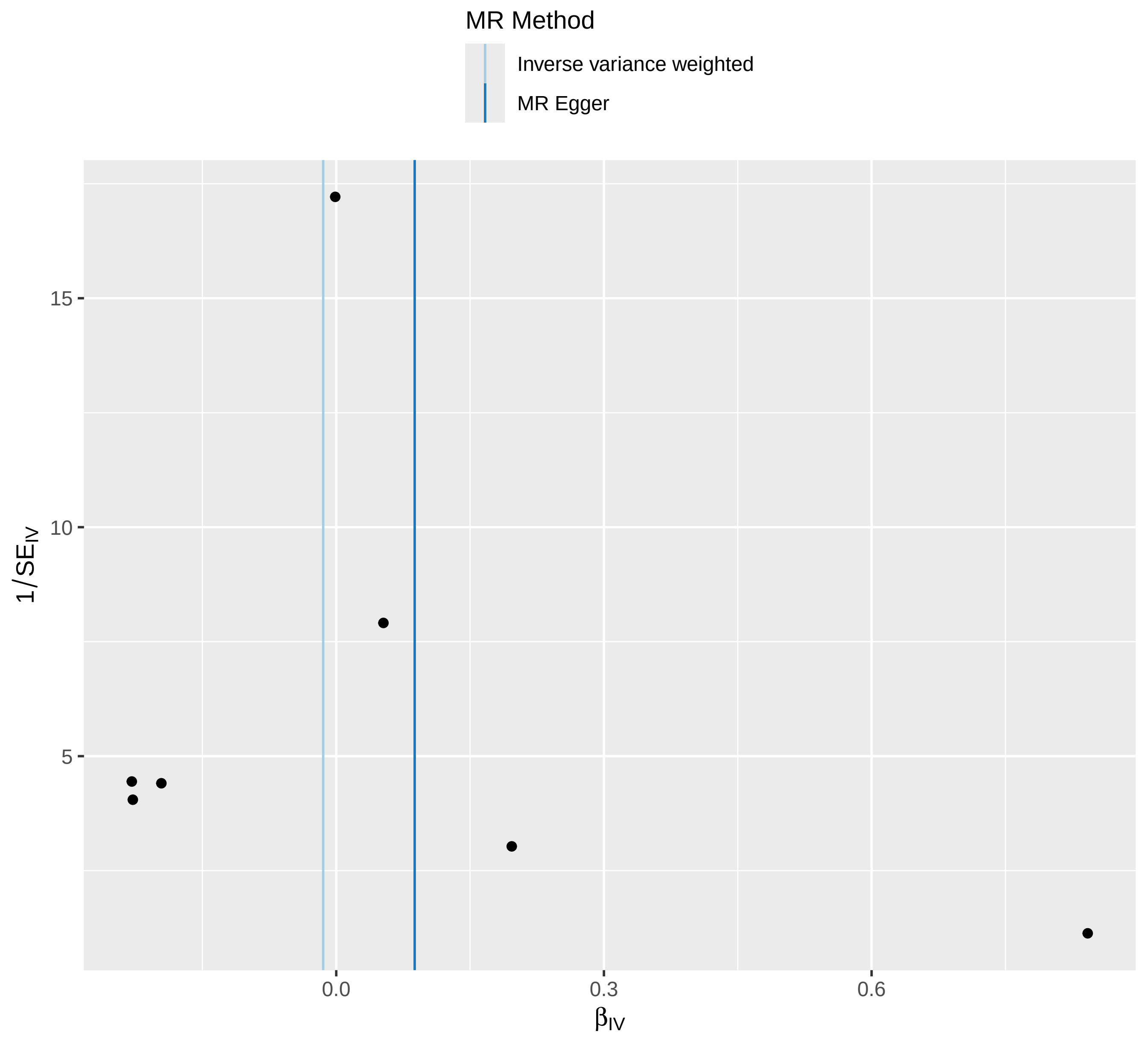

MR Method
Inverse variance weighted
MR Egger
15
10
5
1/SE_IV
0.0
0.3
0.6
β_IV

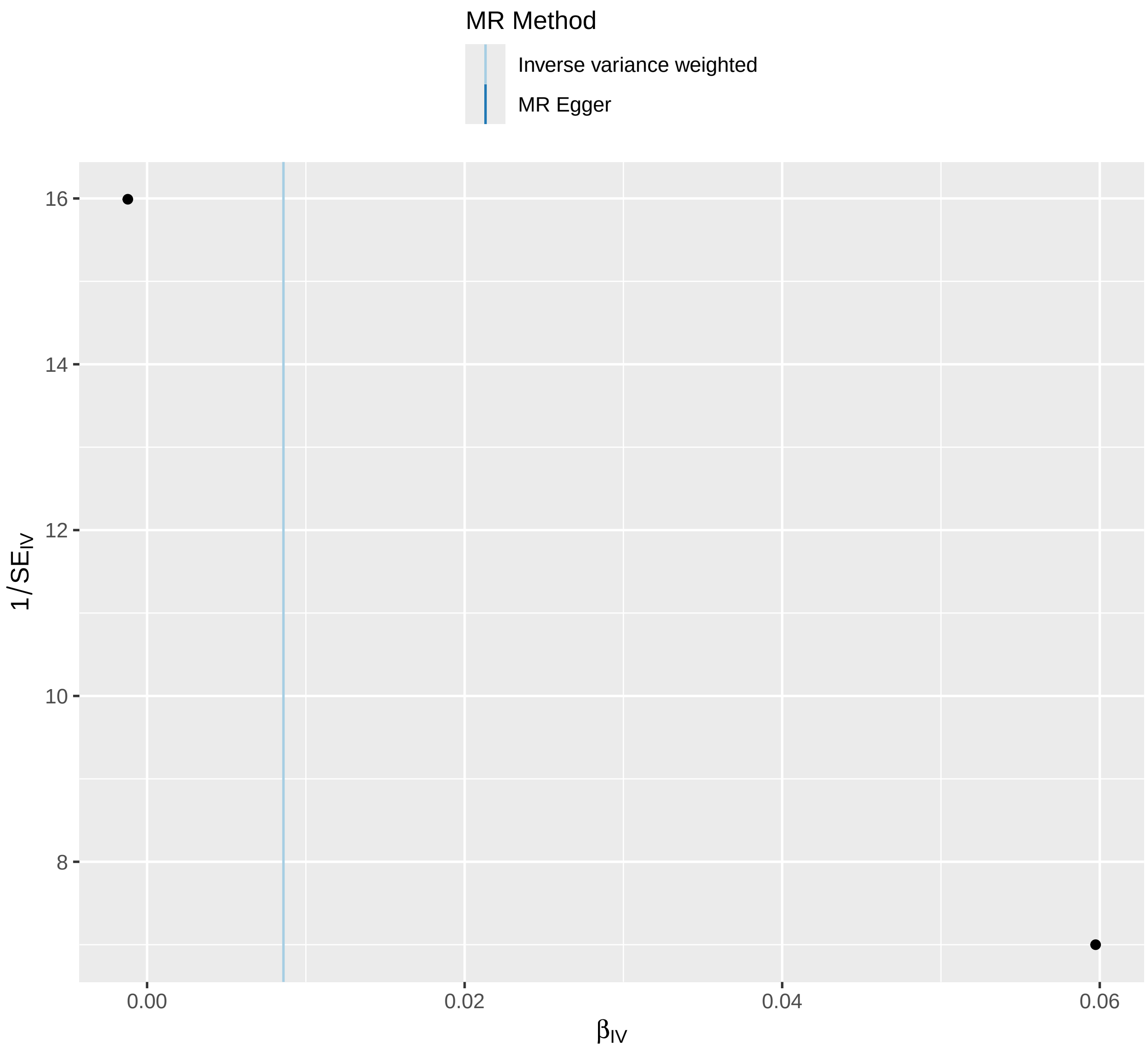

MR Method
Inverse variance weighted
MR Egger
16
14
12
10
8
1/SE$_{IV}$
0.00
0.02
0.04
0.06
β$_{IV}$

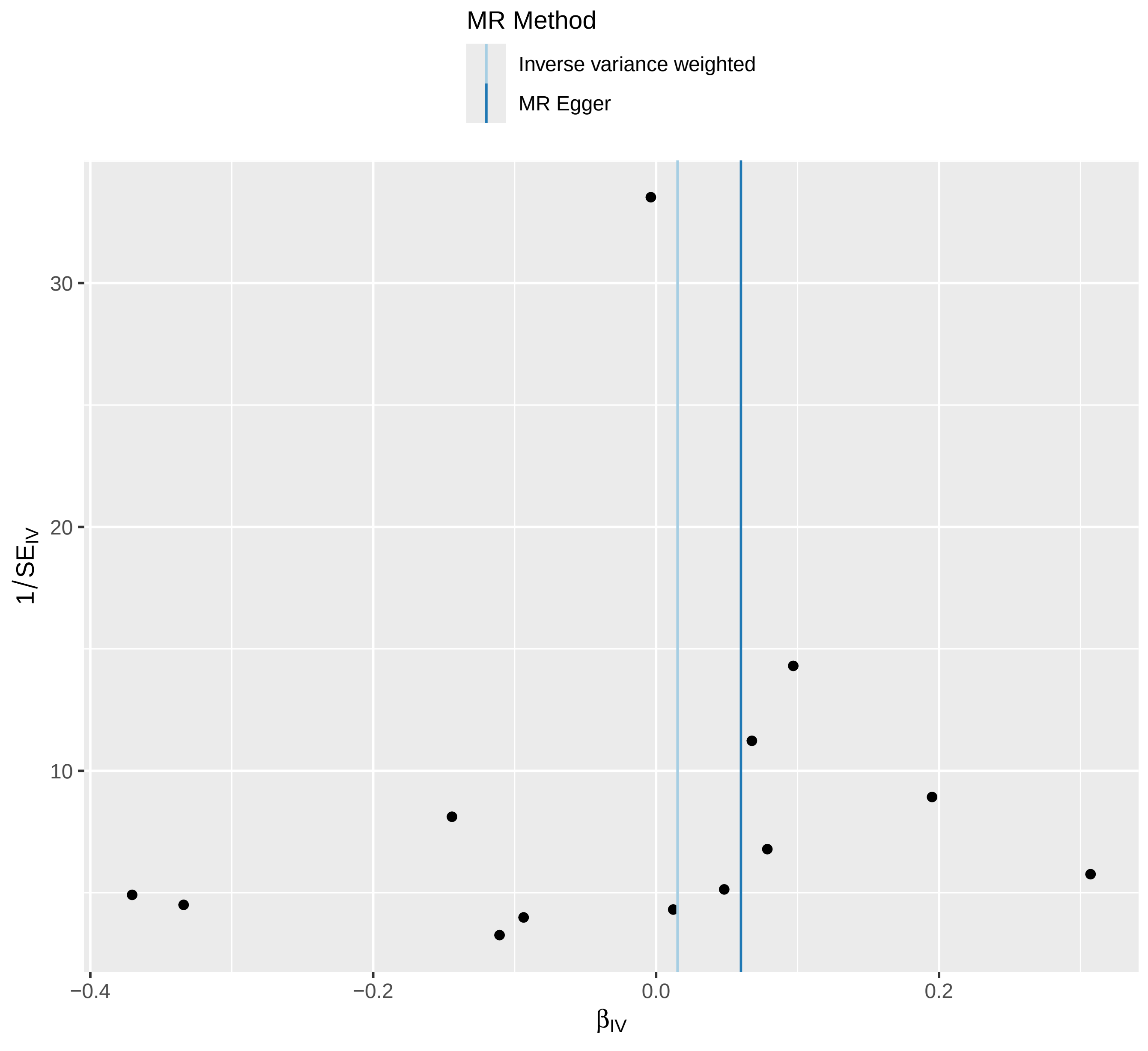

MR Method
Inverse variance weighted
MR Egger
30
20
10
$1/SE_{IV}$
−0.4
−0.2
0.0
0.2
$\beta_{IV}$

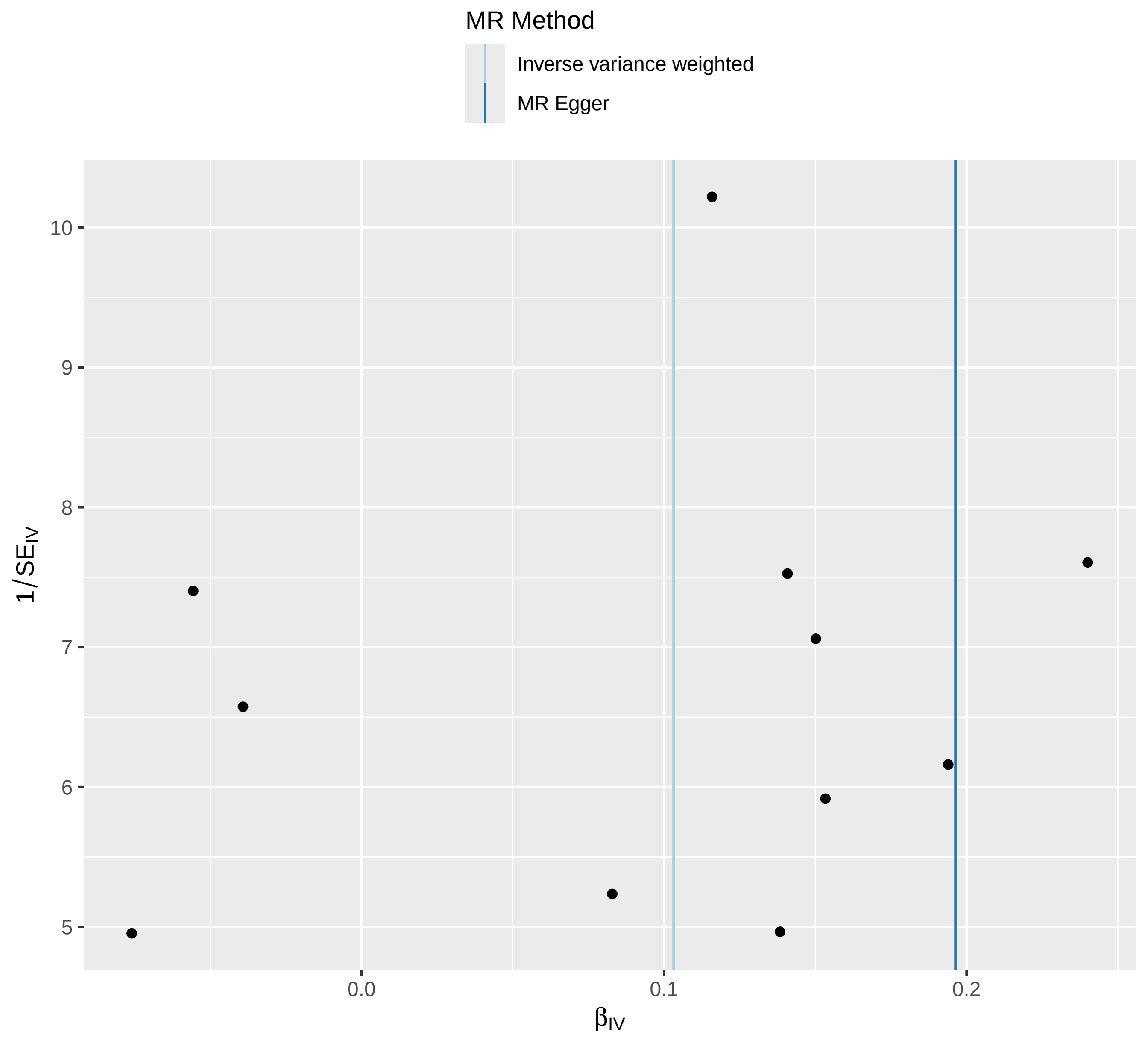
MR Method
Inverse variance weighted
MR Egger
10
9
8
7
6
5
1/SE_IV
0.0
0.1
0.2
β_IV

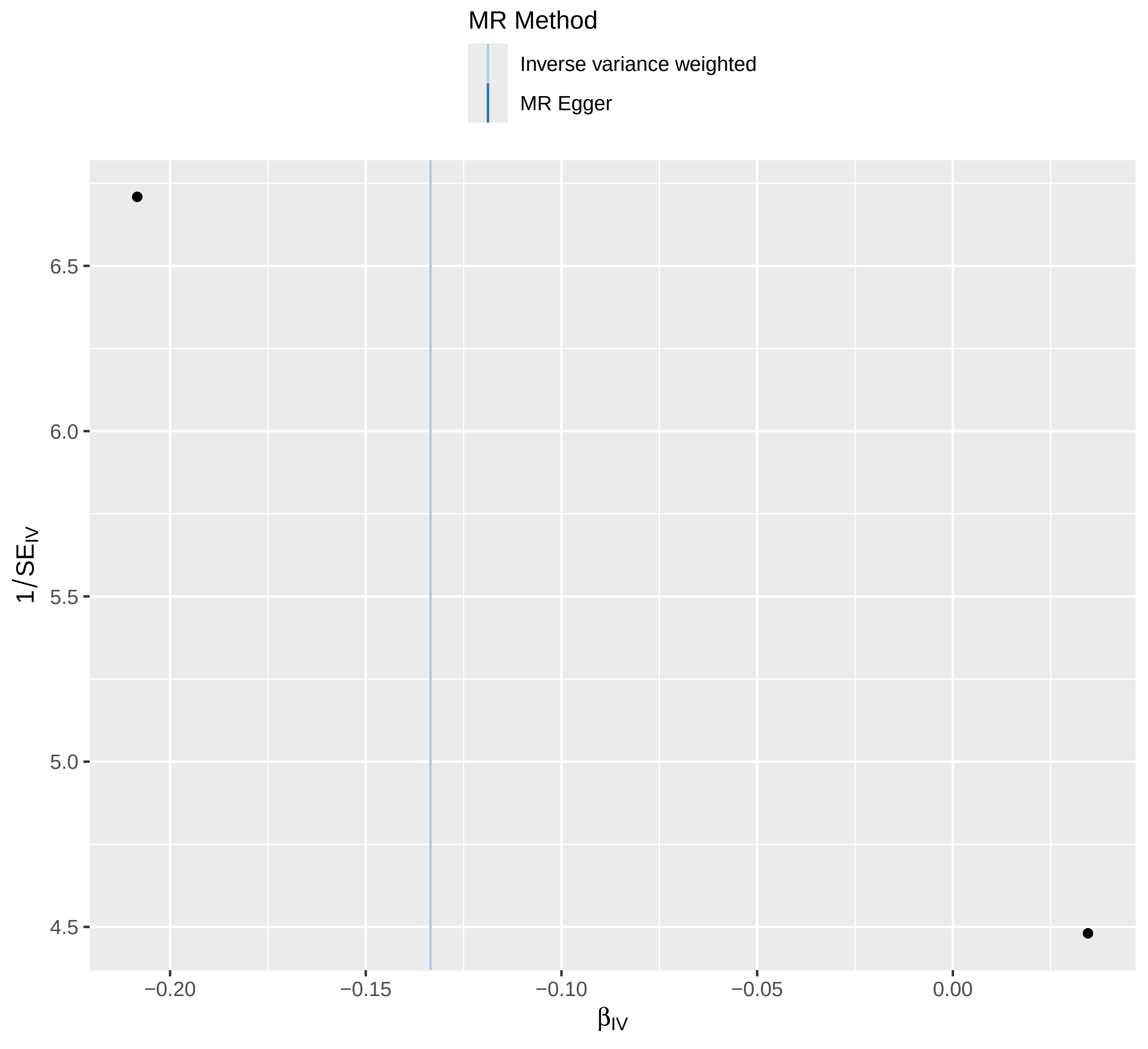
MR Method
Inverse variance weighted
MR Egger
6.5
6.0
5.5
5.0
4.5
1/SE$_{IV}$
−0.20
−0.15
−0.10
−0.05
0.00
β$_{IV}$

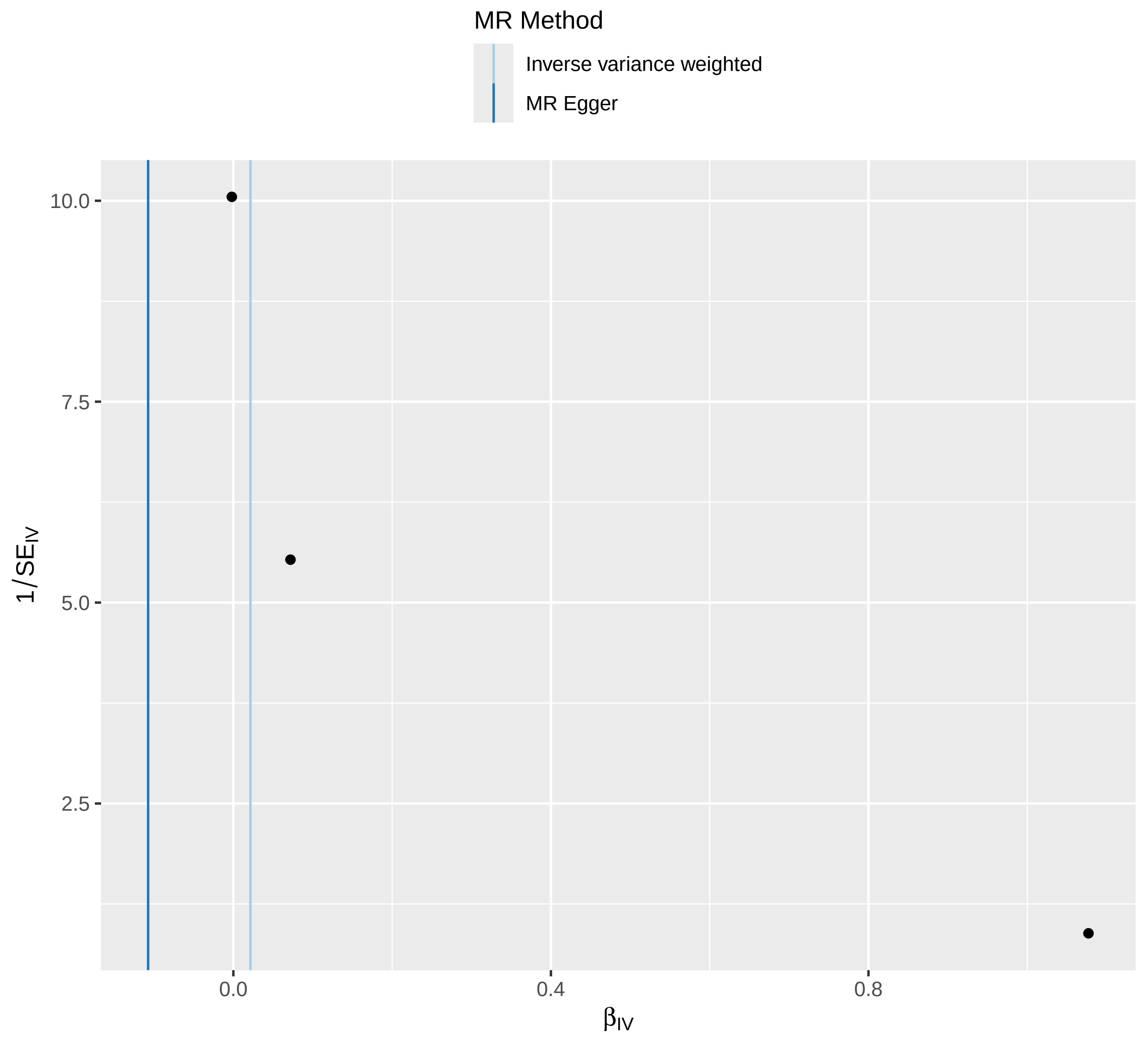

MR Method
Inverse variance weighted
MR Egger
10.0
7.5
5.0
2.5
1/SE_IV
0.0
0.4
0.8
β_IV

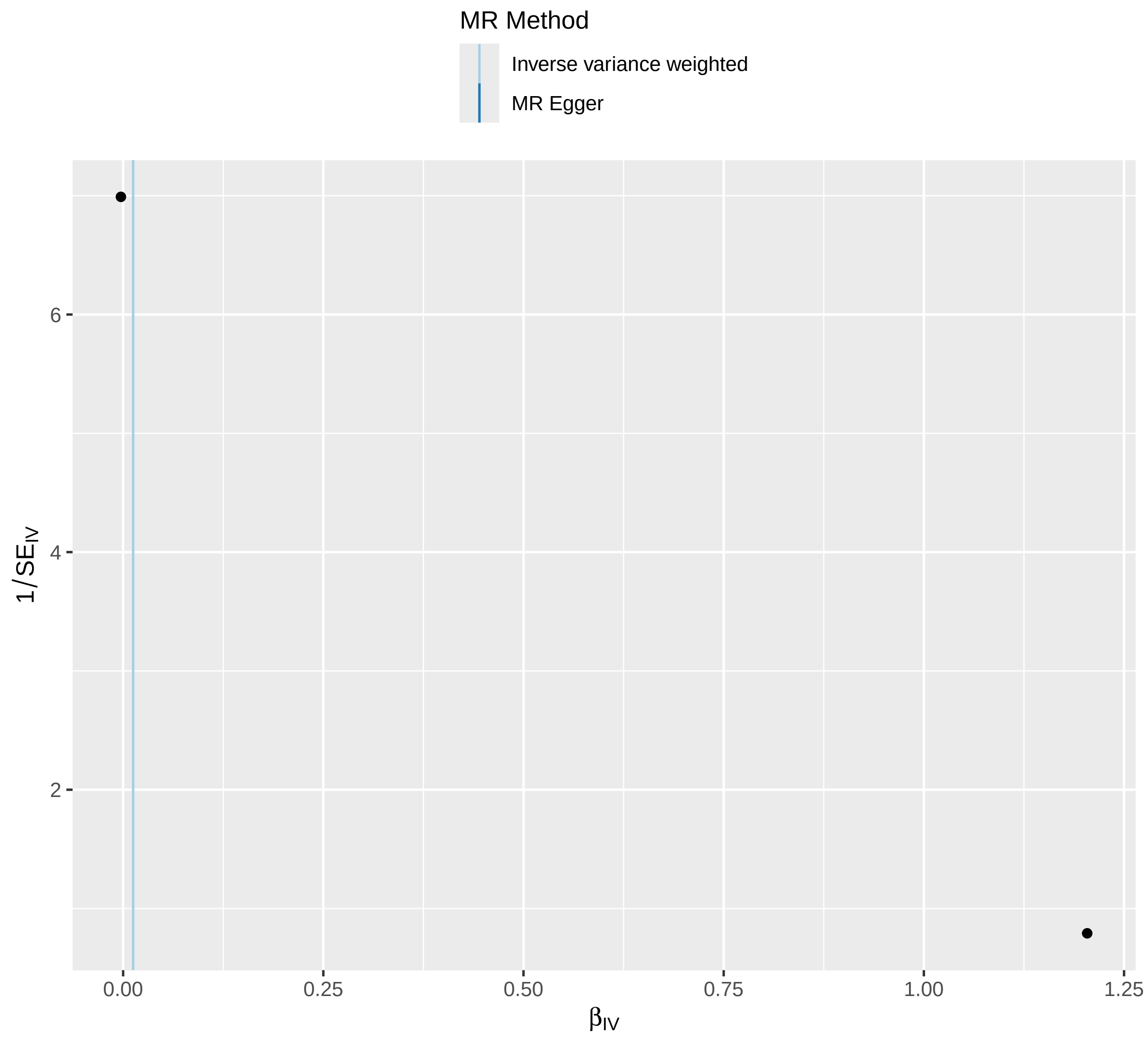
MR Method
Inverse variance weighted
MR Egger
6
4
2
1/SE_IV
0.00
0.25
0.50
0.75
1.00
1.25
β_IV

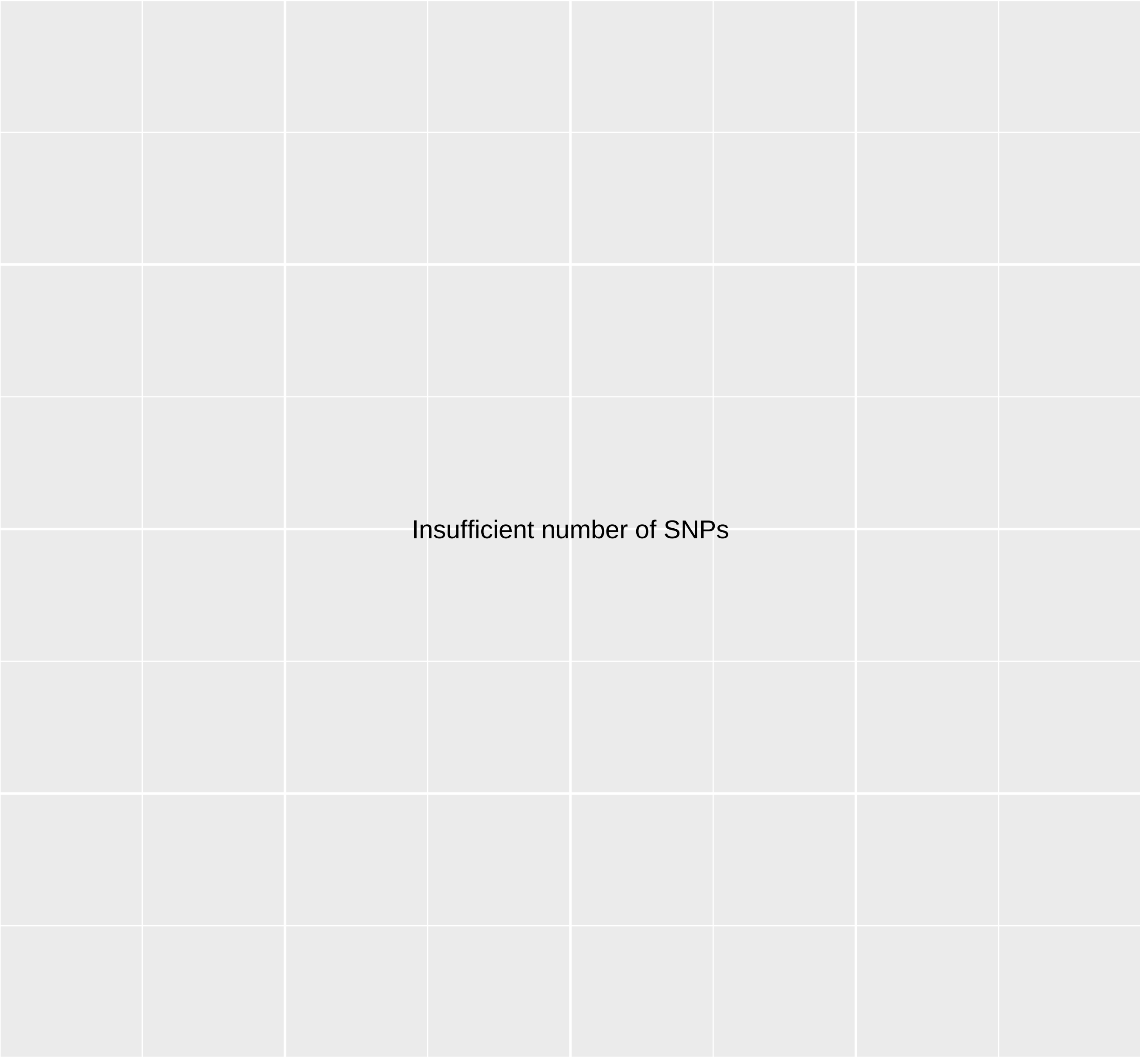
Insufficient number of SNPs

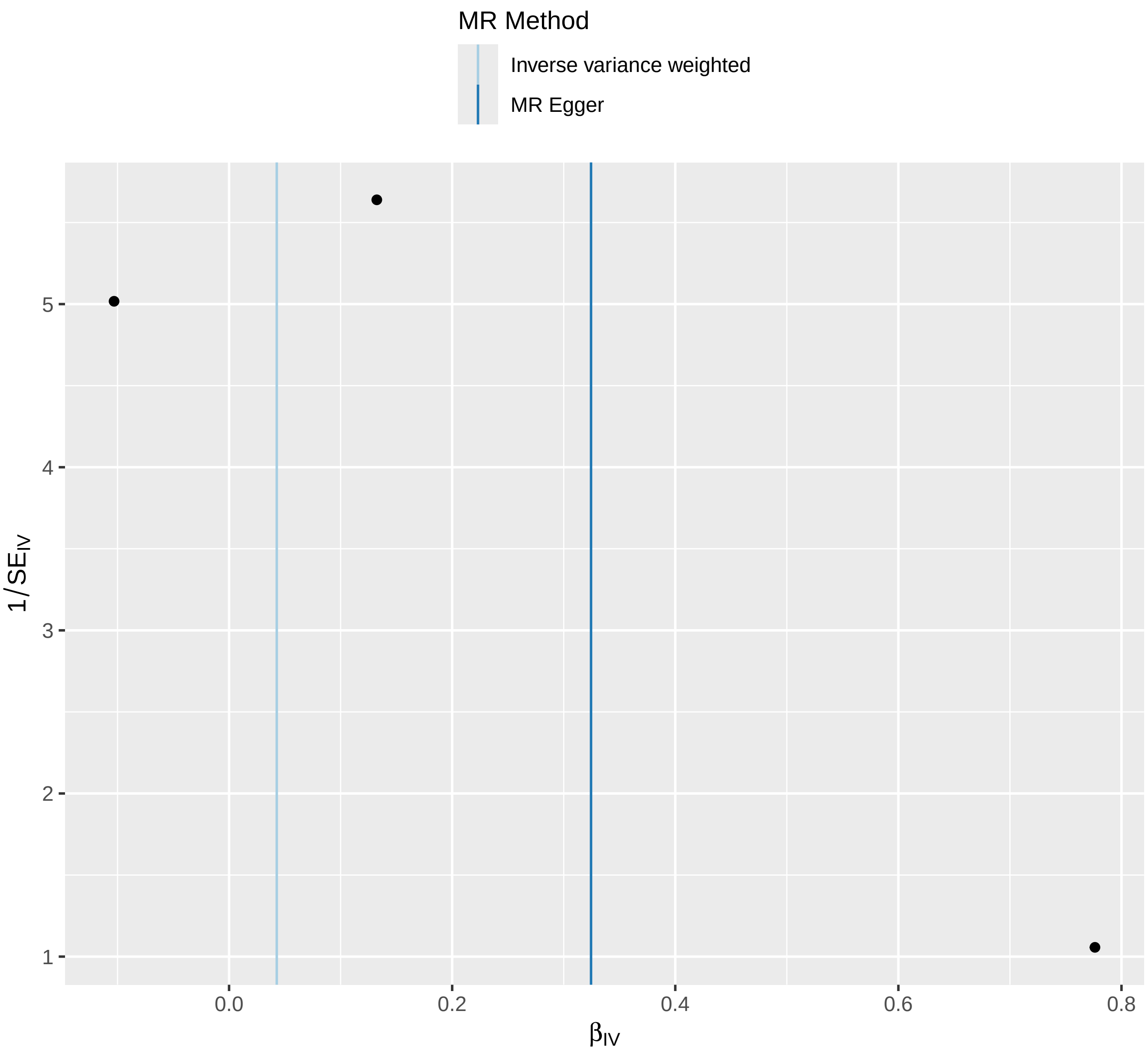

MR Method
Inverse variance weighted
MR Egger
1/SE_IV
5
4
3
2
1
0.0
0.2
0.4
0.6
0.8
β_IV

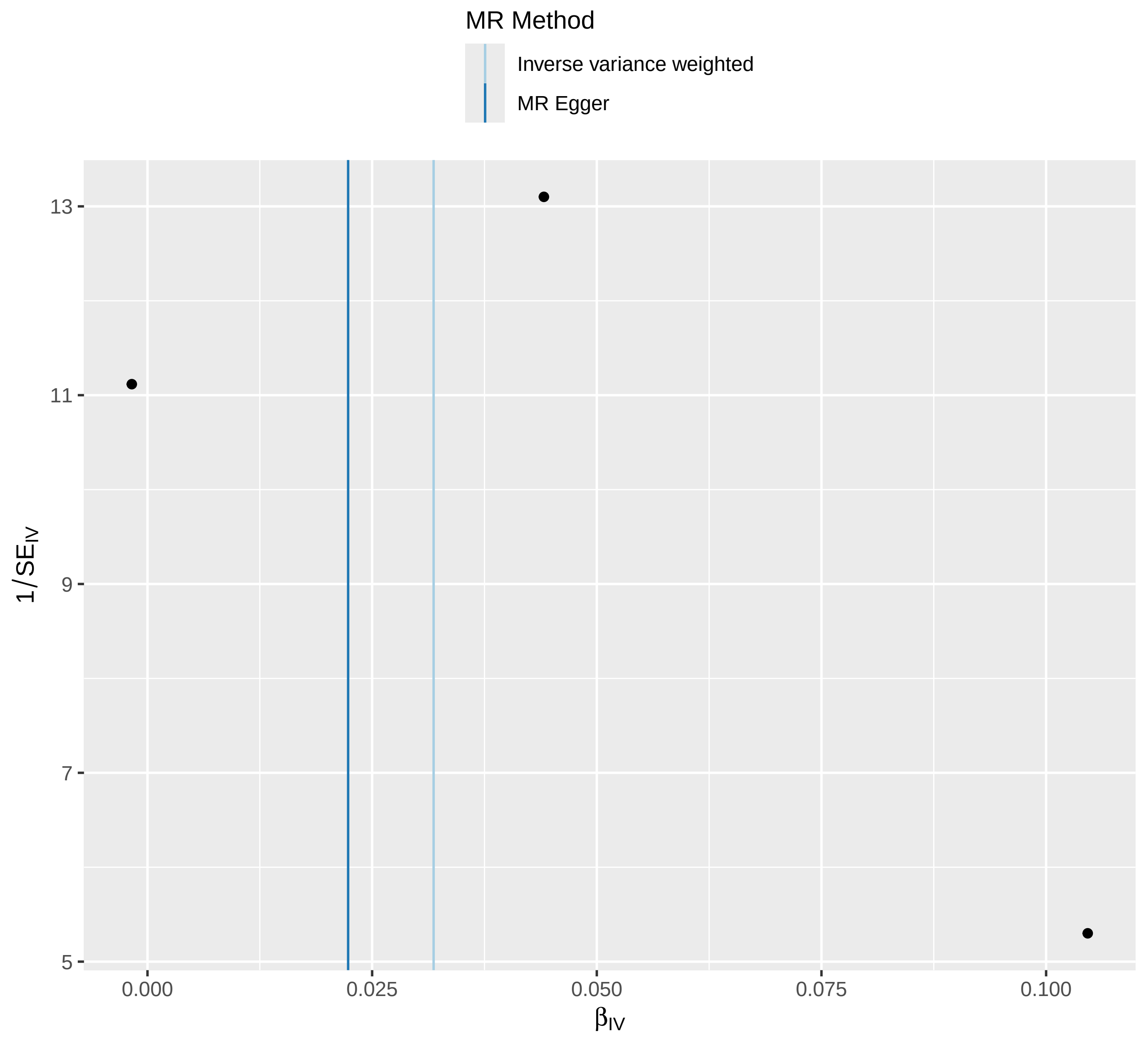

MR Method
Inverse variance weighted
MR Egger
13
11
9
7
5
1/SE_IV
0.000
0.025
0.050
0.075
0.100
β_IV

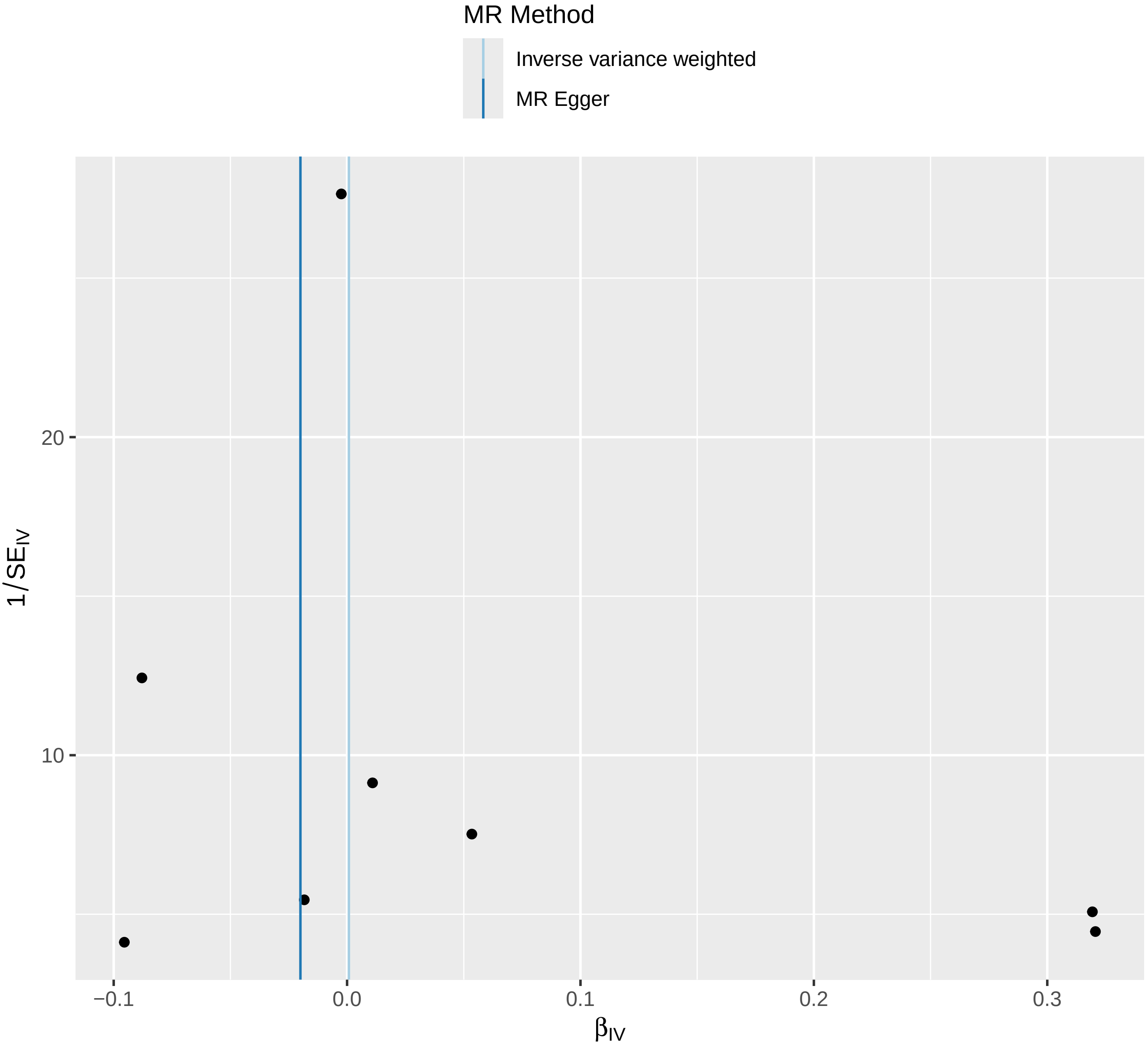

MR Method
Inverse variance weighted
MR Egger
20
10
$1/SE_{IV}$
−0.1
0.0
0.1
0.2
0.3
$\beta_{IV}$

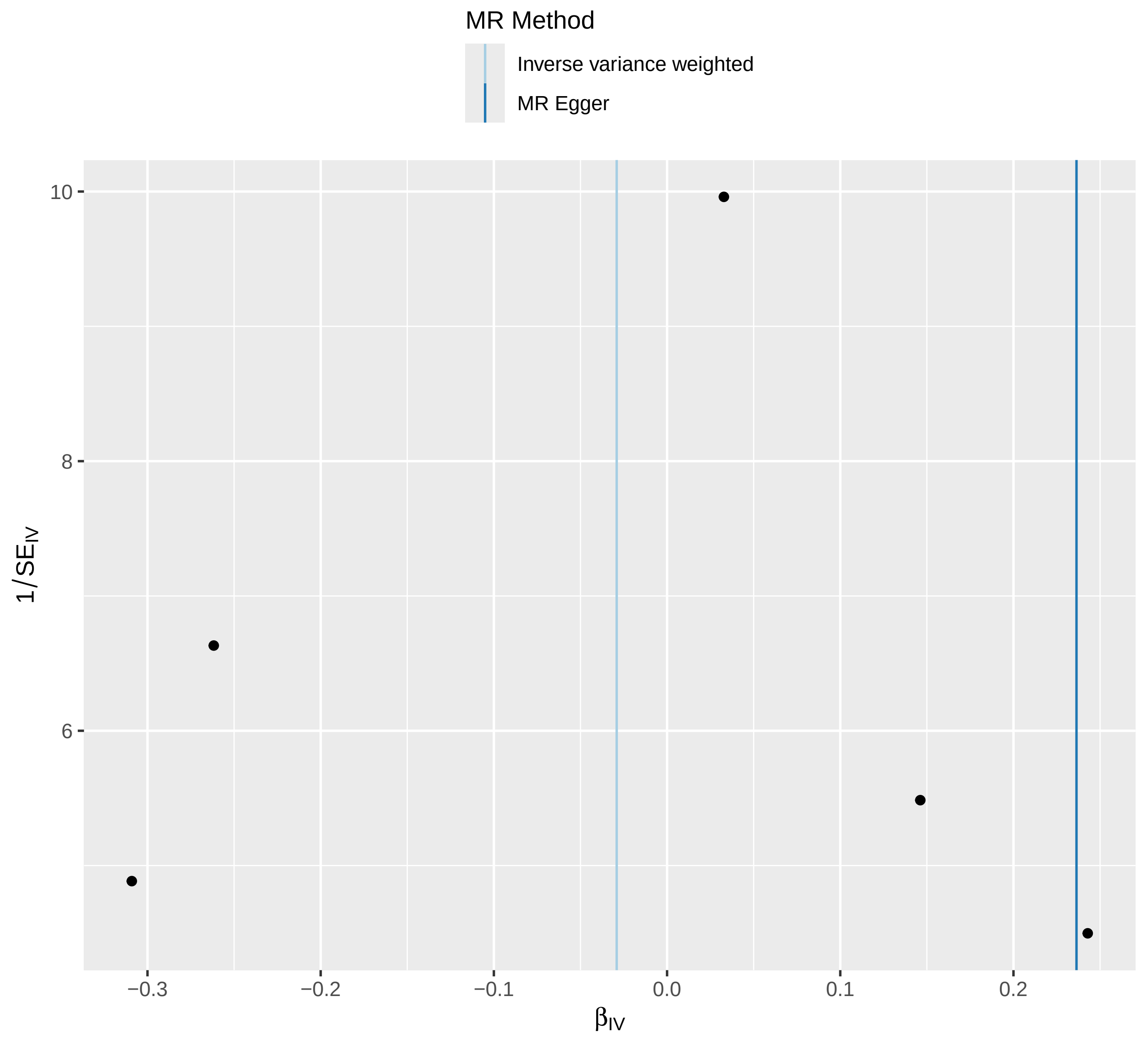
MR Method
Inverse variance weighted
MR Egger
10
8
6
1/SE$_{IV}$
−0.3
−0.2
−0.1
0.0
0.1
0.2
β$_{IV}$

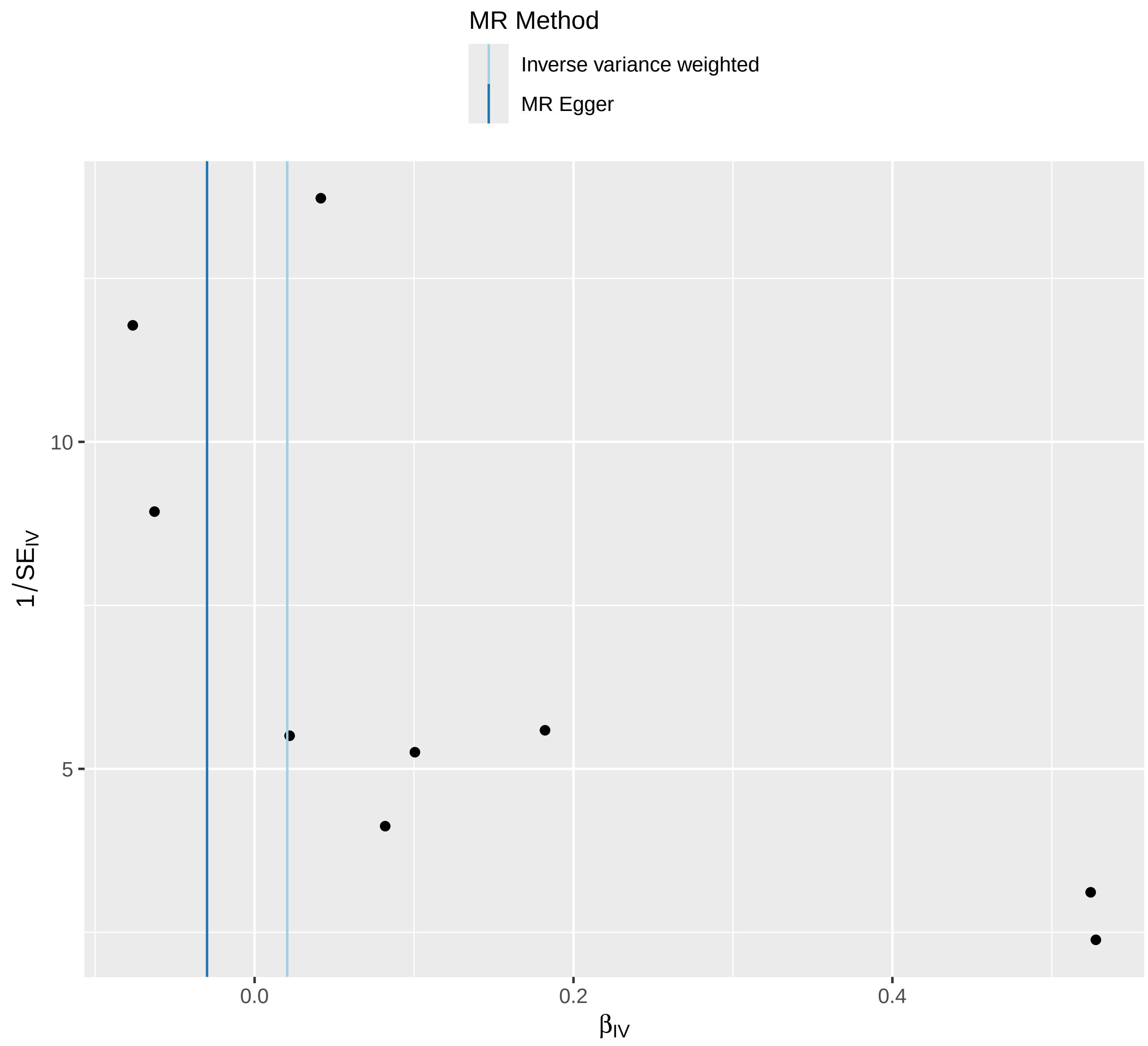

MR Method
Inverse variance weighted
MR Egger
10
5
1/SE$_{IV}$
0.0
0.2
0.4
β$_{IV}$

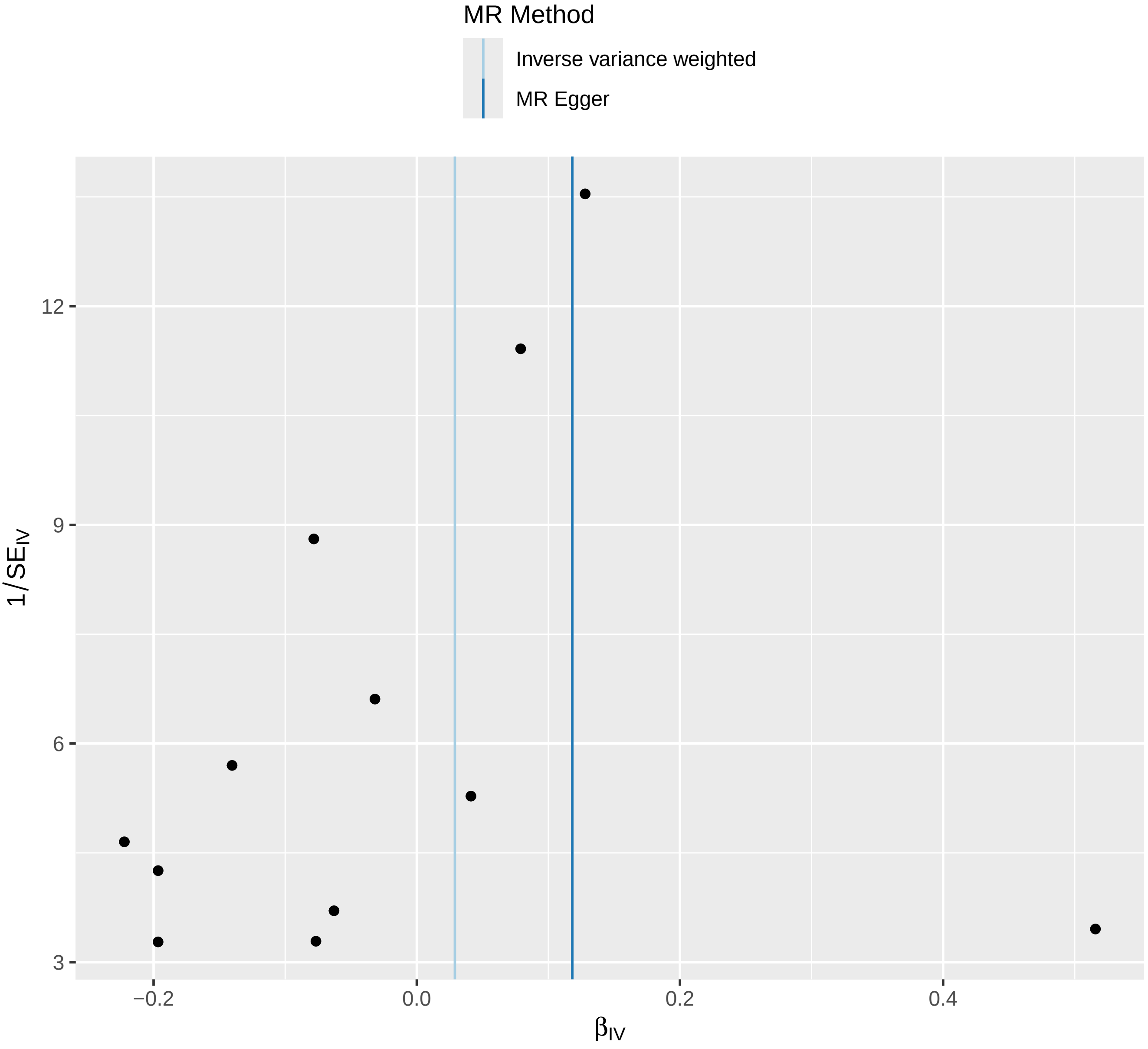
MR Method
Inverse variance weighted
MR Egger
12
9
6
3
1/SE$_{IV}$
−0.2
0.0
0.2
0.4
β$_{IV}$

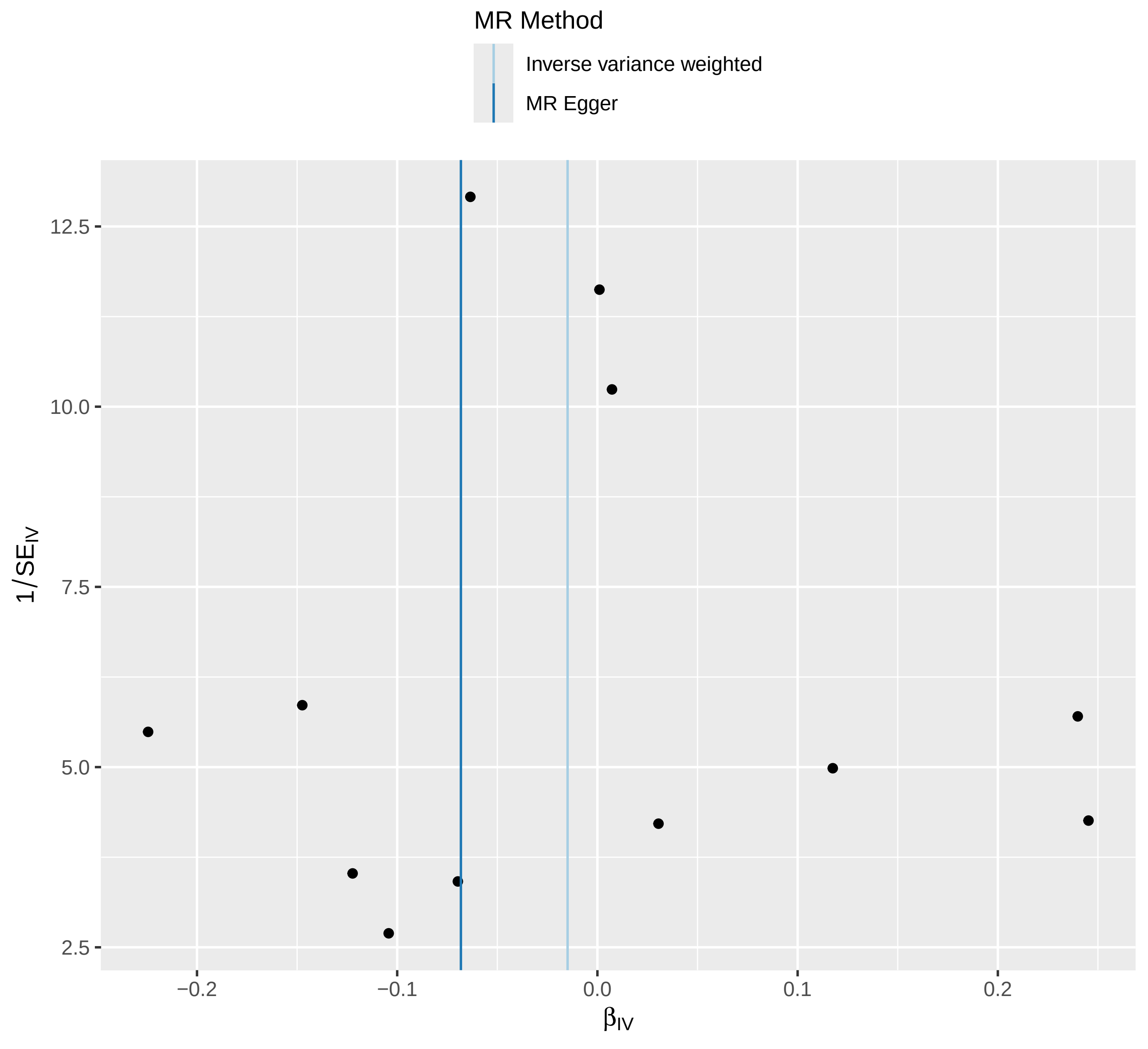
MR Method
Inverse variance weighted
MR Egger
1/SE$_{IV}$
12.5
10.0
7.5
5.0
2.5
−0.2
−0.1
0.0
0.1
0.2
$\beta_{IV}$

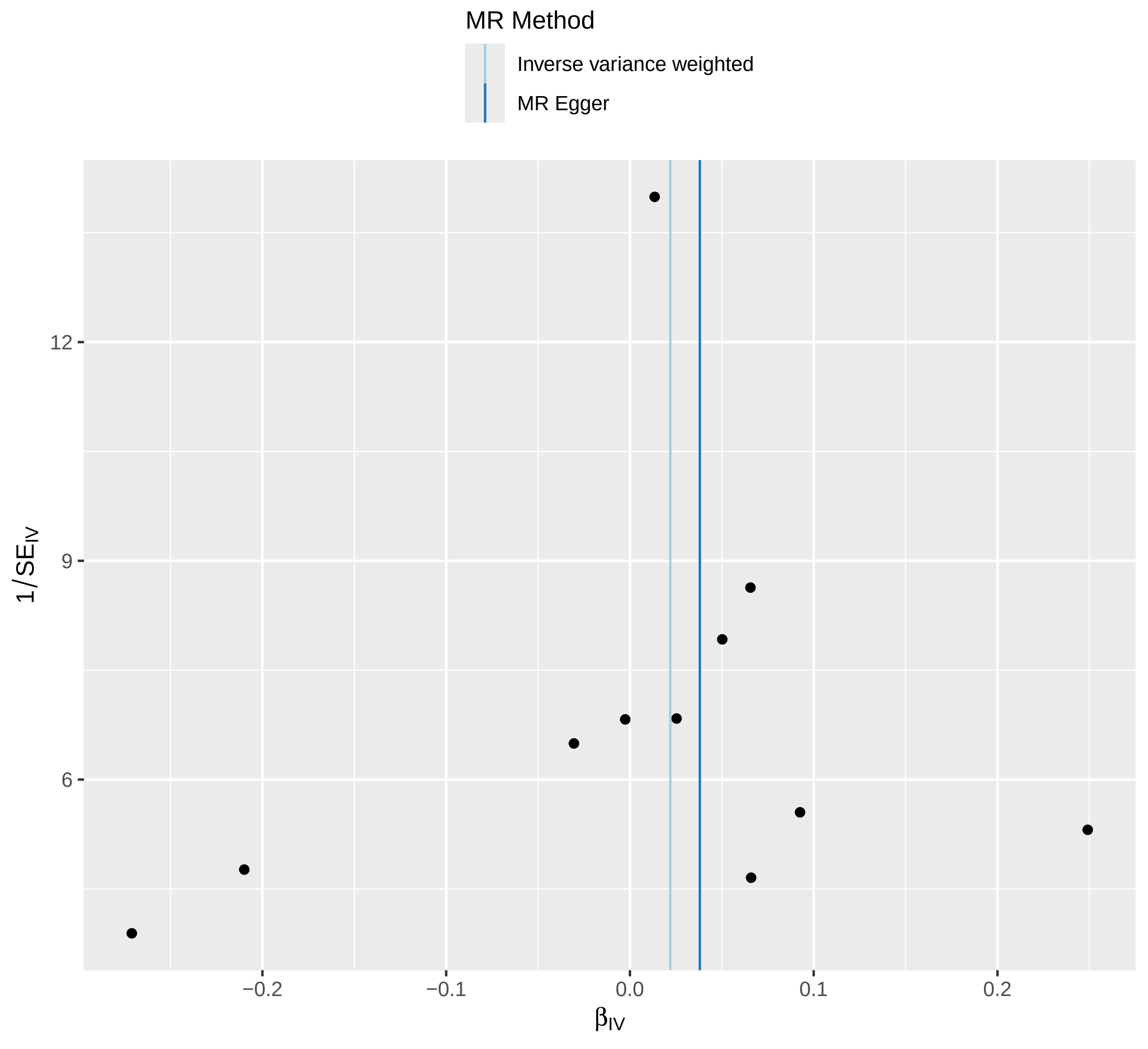
MR Method
Inverse variance weighted
MR Egger
12
9
6
−0.2
−0.1
0.0
0.1
0.2
$1/SE_{IV}$
$\beta_{IV}$

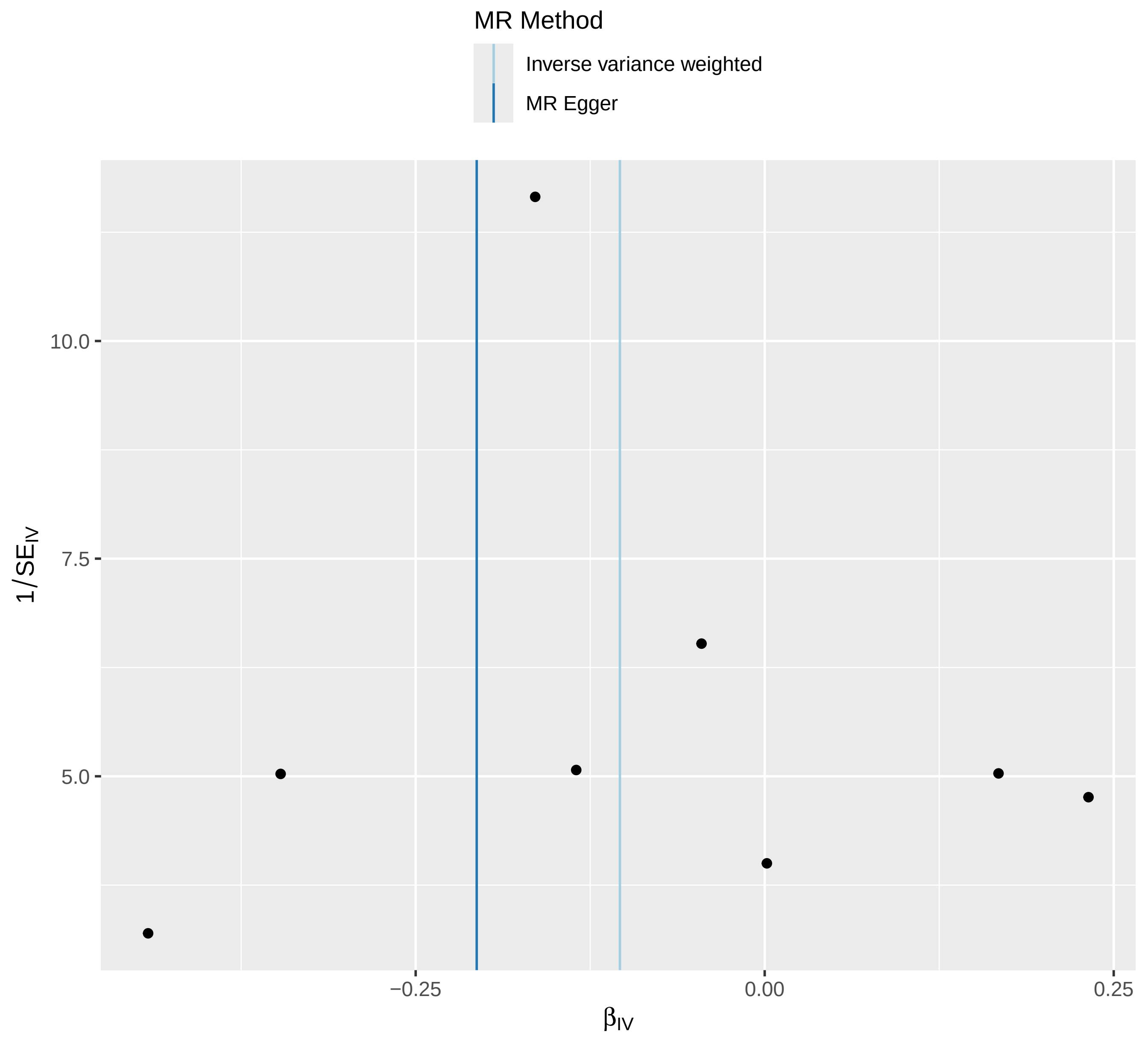

MR Method
Inverse variance weighted
MR Egger
10.0
7.5
5.0
1/SE_IV
−0.25
0.00
0.25
β_IV

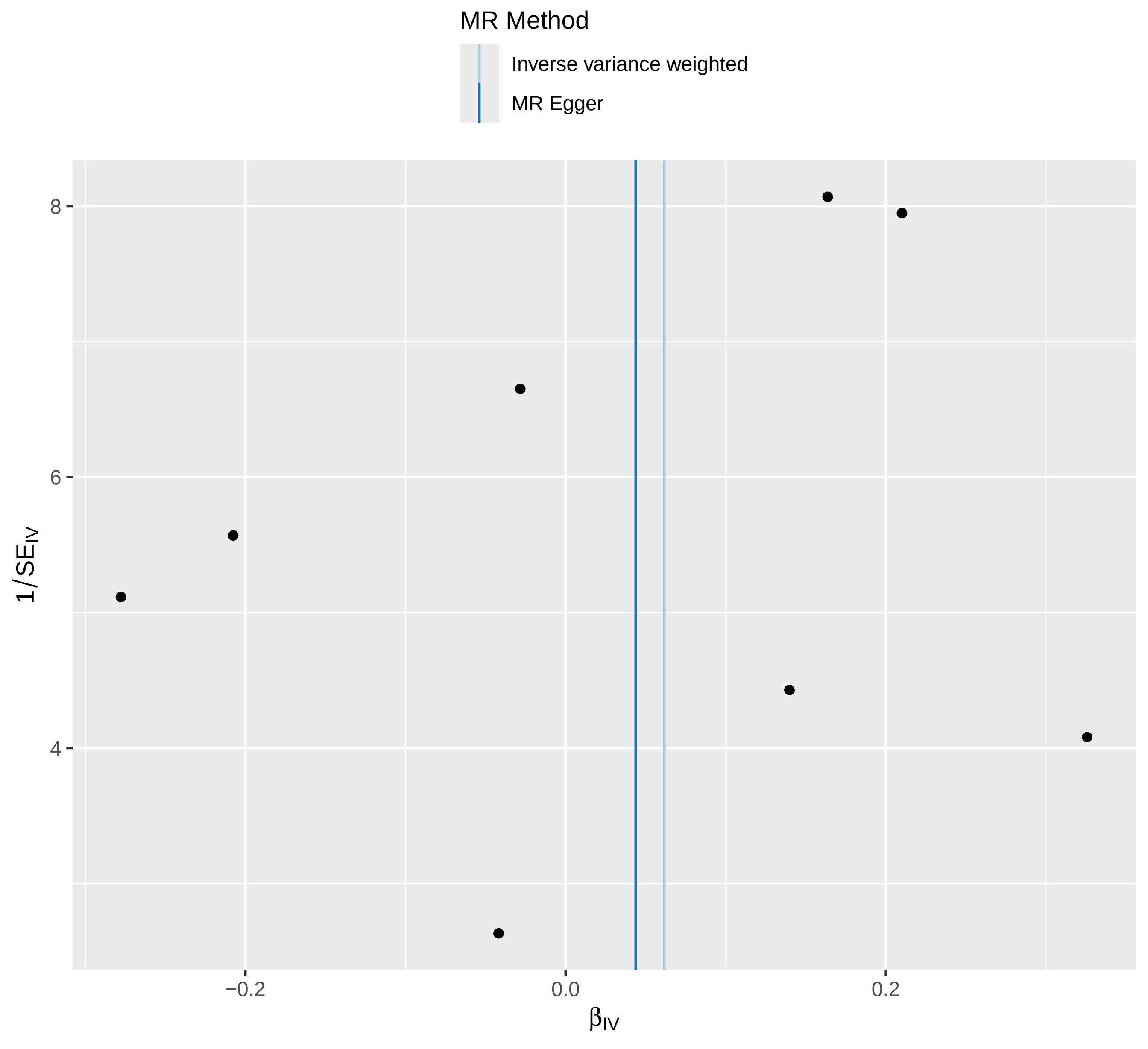
MR Method
Inverse variance weighted
MR Egger
8
6
4
−0.2
0.0
0.2
1/SE$_{IV}$
β$_{IV}$

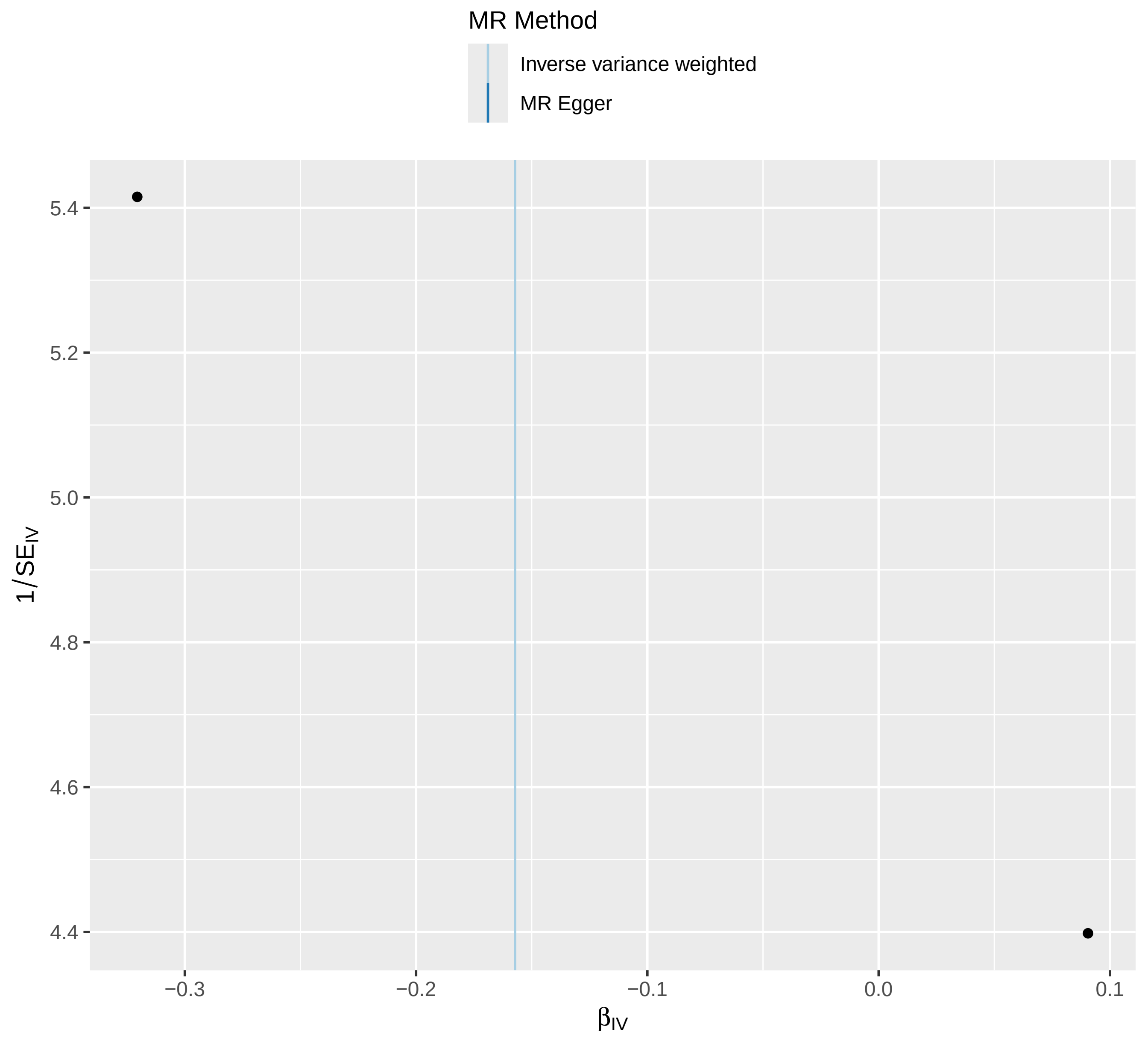

MR Method
Inverse variance weighted
MR Egger
5.4
5.2
5.0
4.8
4.6
4.4
1/SE$_{IV}$
−0.3
−0.2
−0.1
0.0
0.1
β$_{IV}$

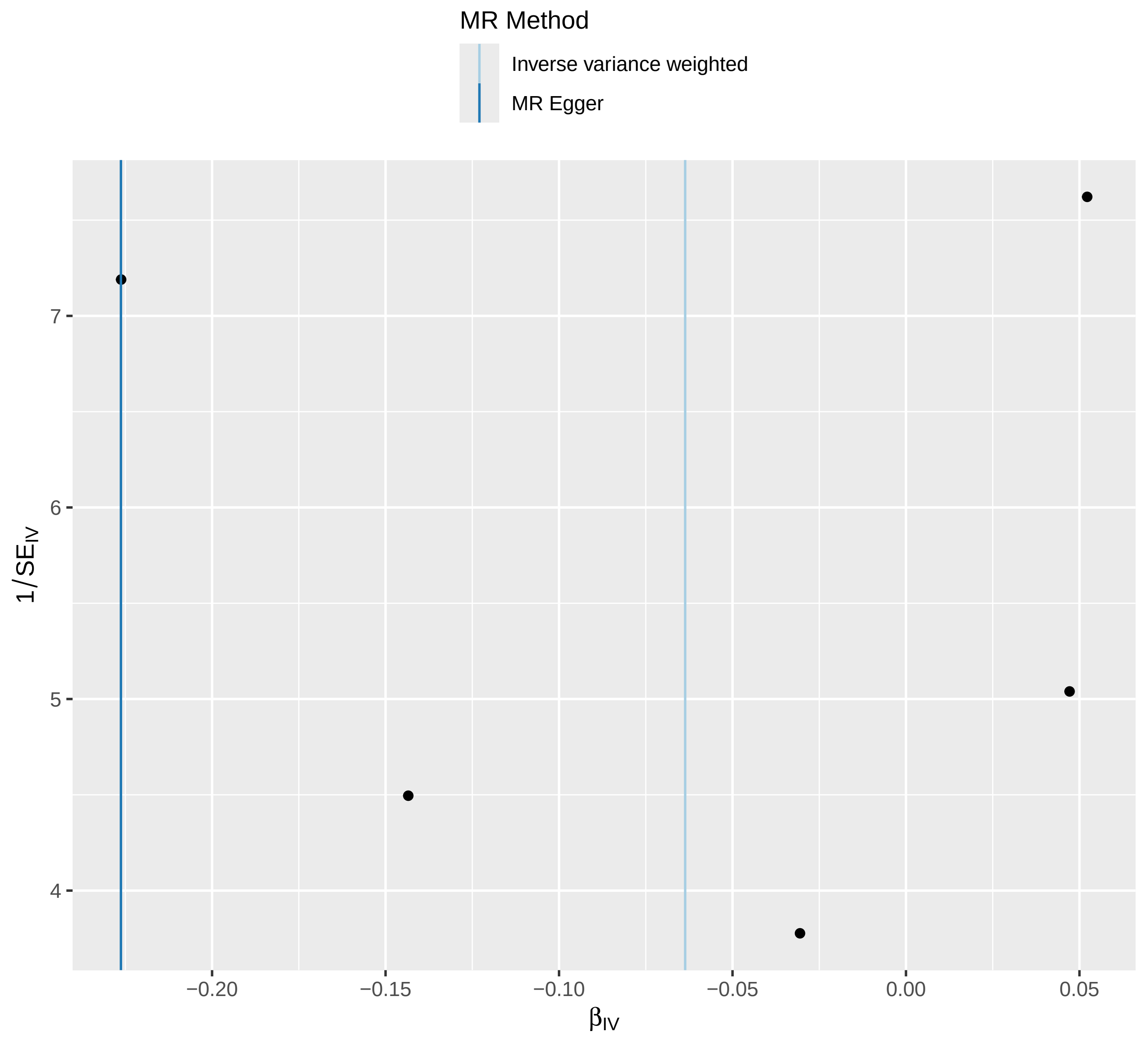
MR Method
Inverse variance weighted
MR Egger
7
6
5
4
1/SE$_{IV}$
−0.20
−0.15
−0.10
−0.05
0.00
0.05
β$_{IV}$

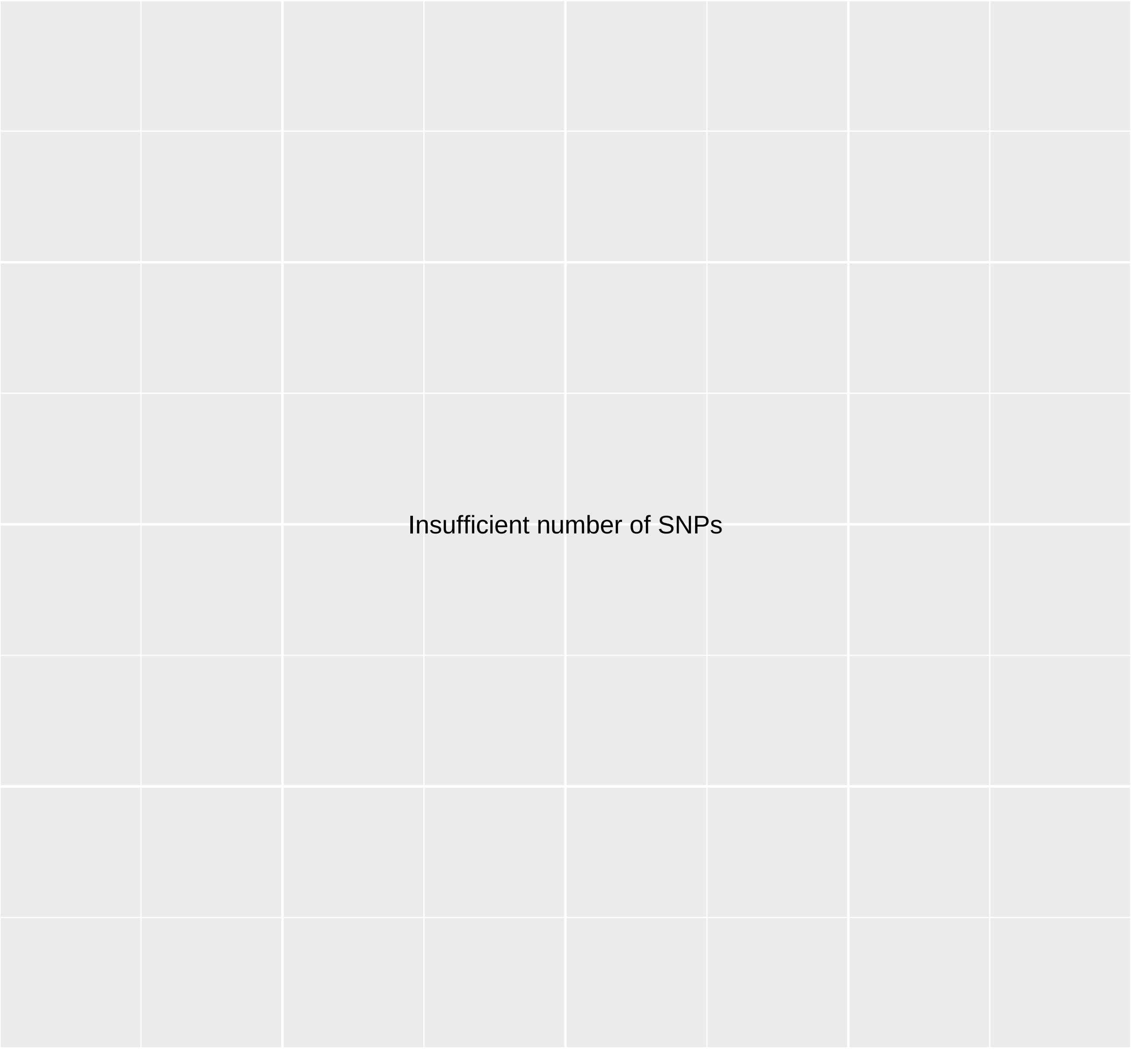
Insufficient number of SNPs

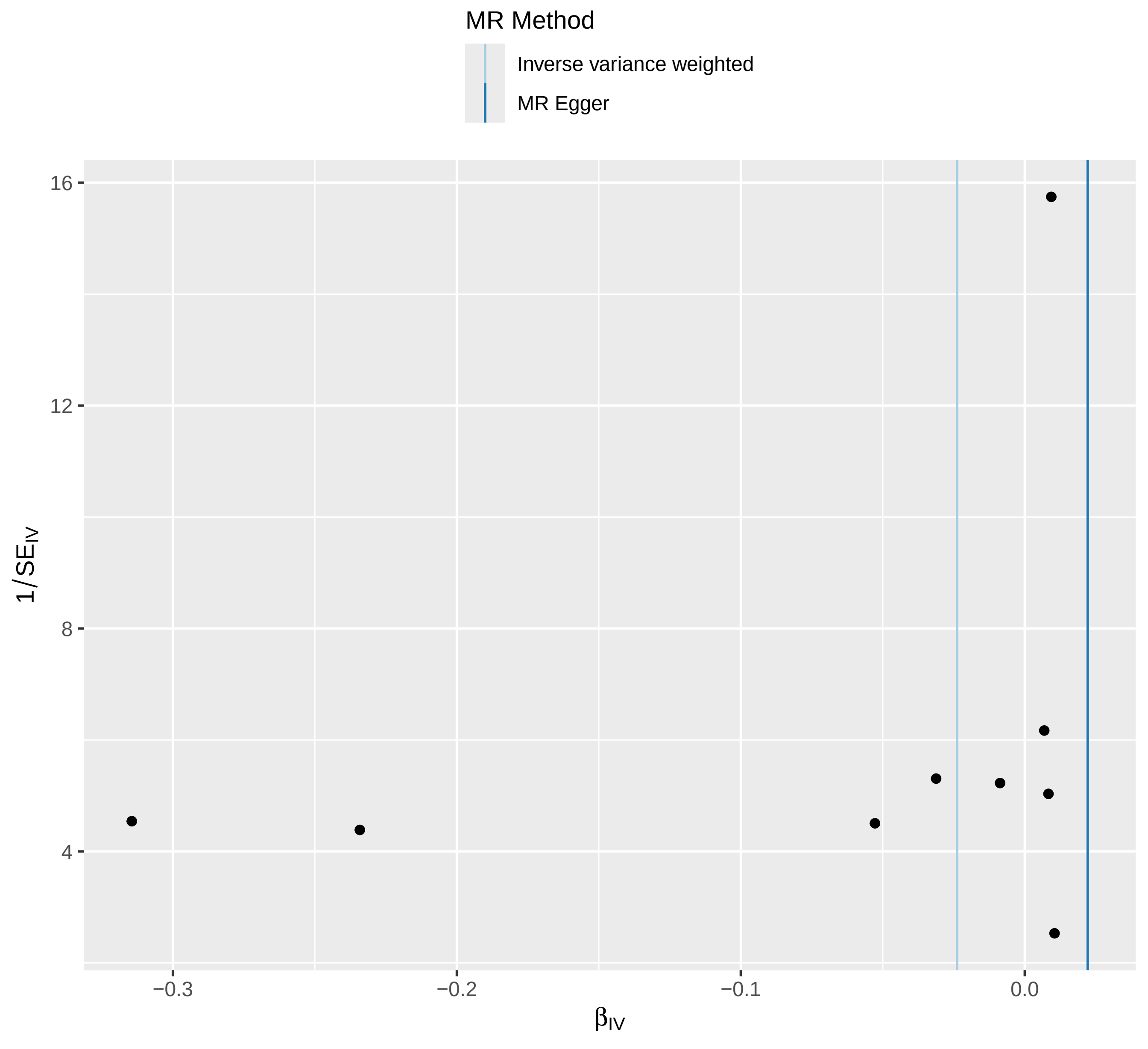
MR Method
Inverse variance weighted
MR Egger
16
12
8
4
$1/SE_{IV}$
−0.3
−0.2
−0.1
0.0
$\beta_{IV}$

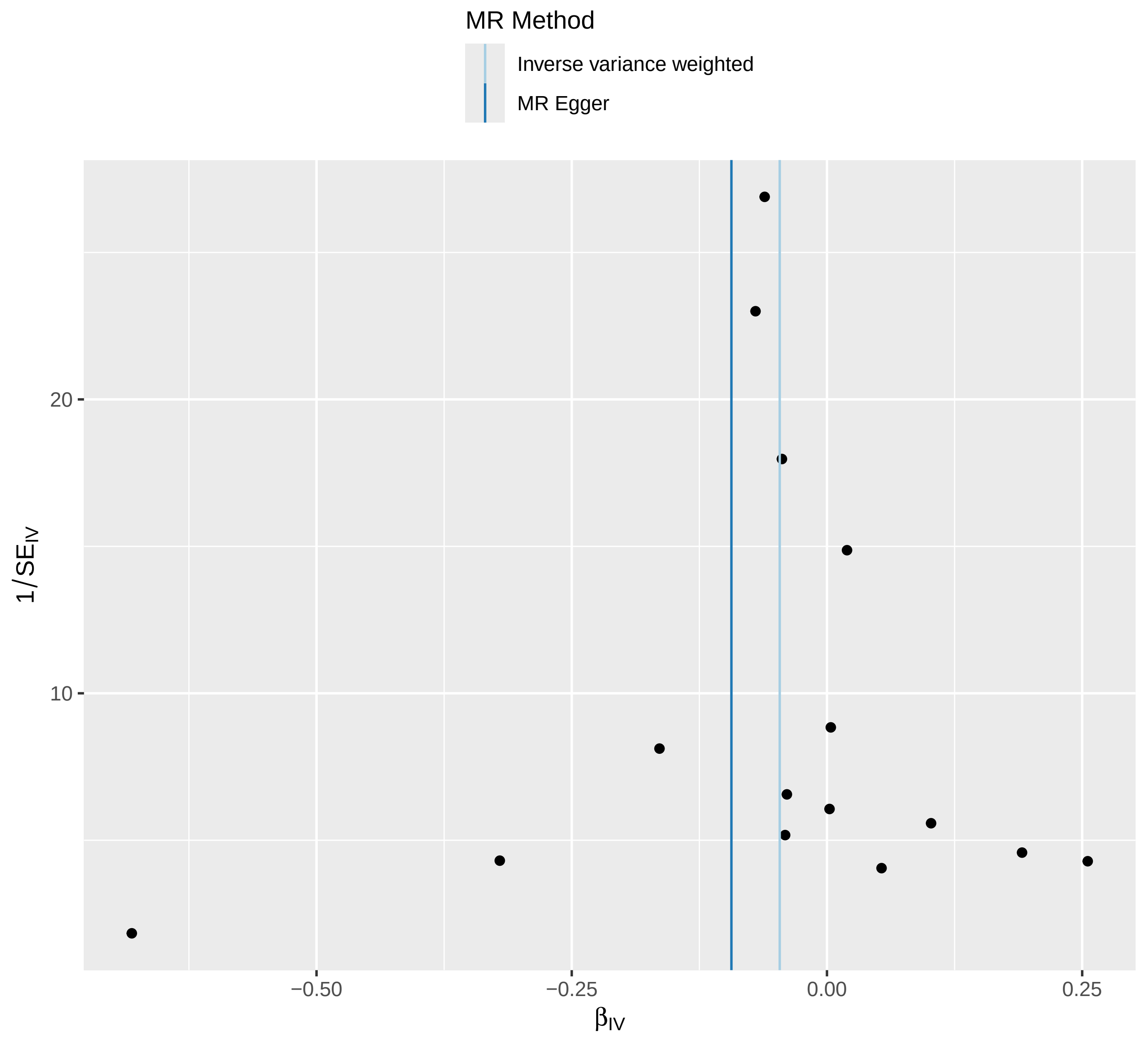

MR Method
Inverse variance weighted
MR Egger
20
10
$1/SE_{IV}$
−0.50
−0.25
0.00
0.25
$\beta_{IV}$

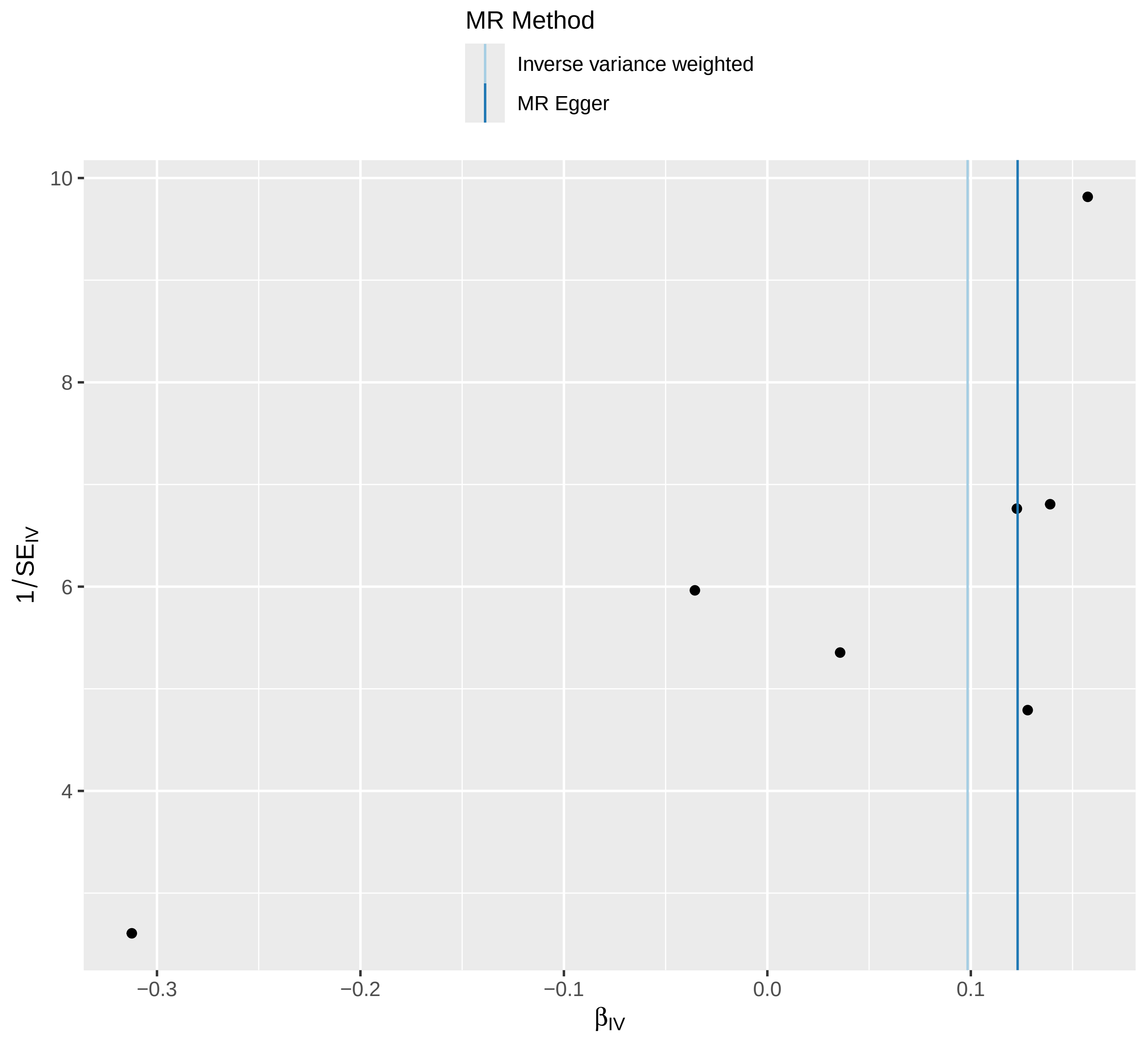
MR Method
Inverse variance weighted
MR Egger
10
8
6
4
1/SE_IV
−0.3
−0.2
−0.1
0.0
0.1
β_IV

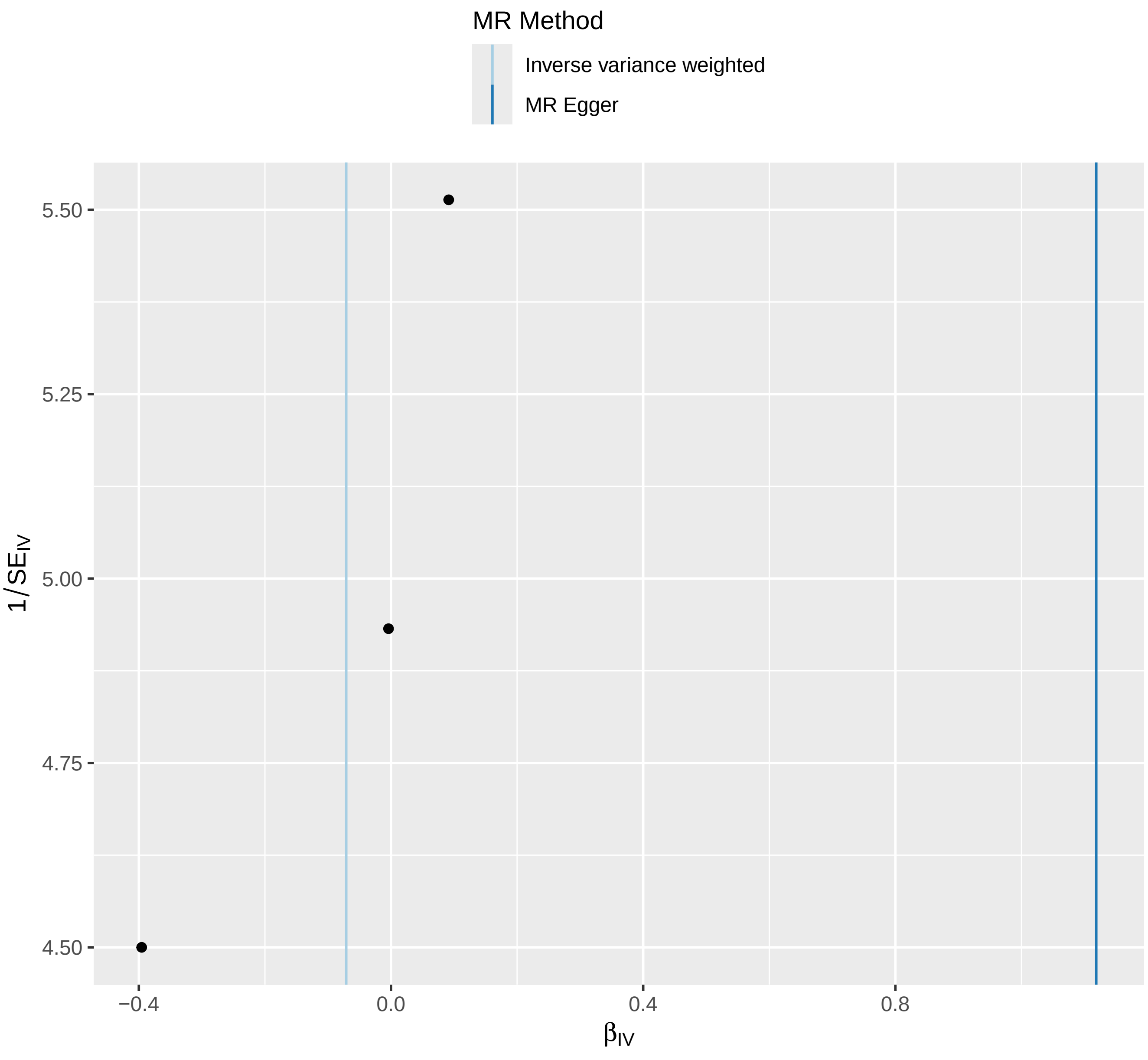
MR Method
Inverse variance weighted
MR Egger
5.50
5.25
5.00
4.75
4.50
1/$SE_{IV}$
−0.4
0.0
0.4
0.8
$\beta_{IV}$

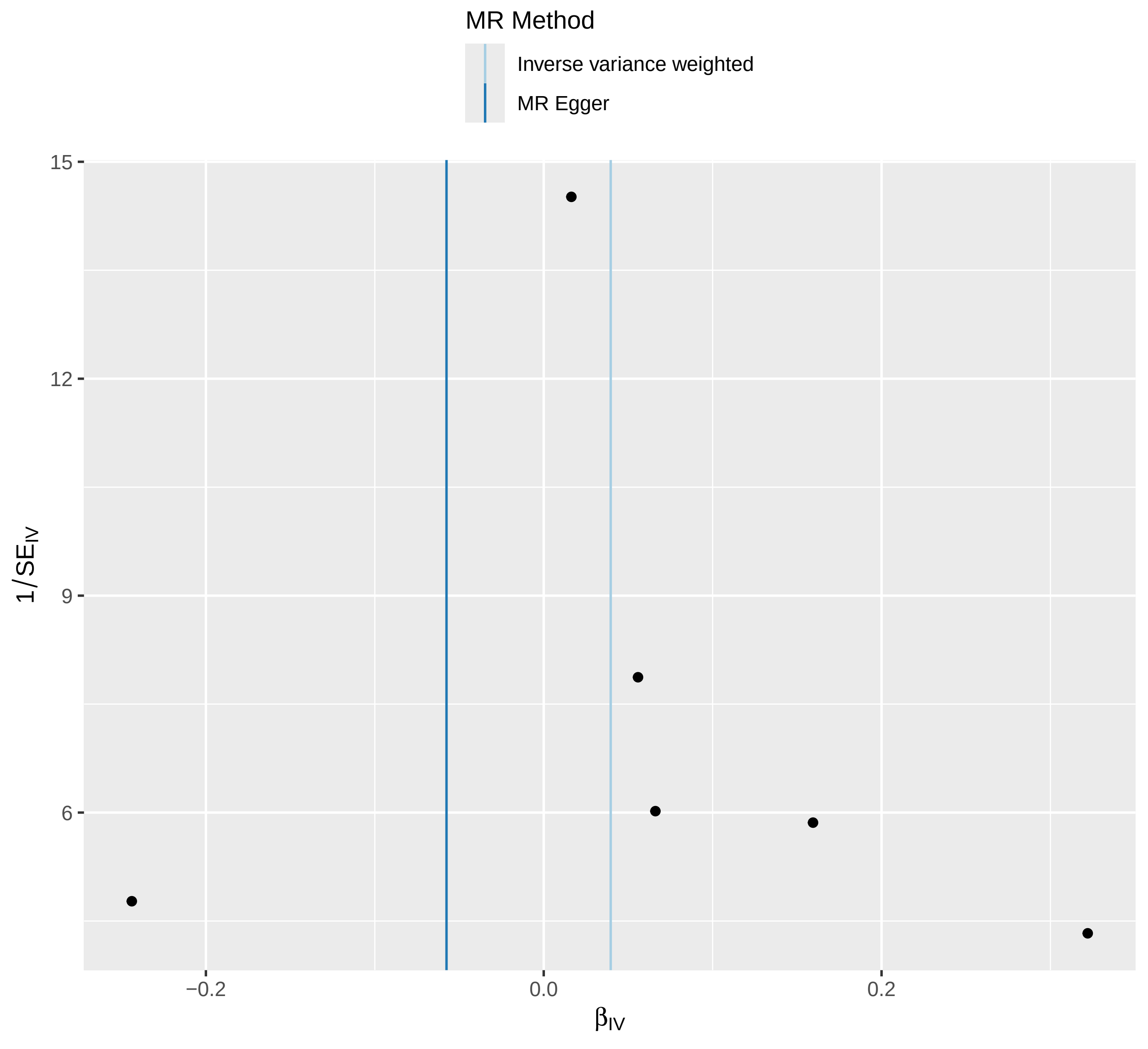
MR Method
Inverse variance weighted
MR Egger
15
12
9
6
1/SE_IV
−0.2
0.0
0.2
β_IV

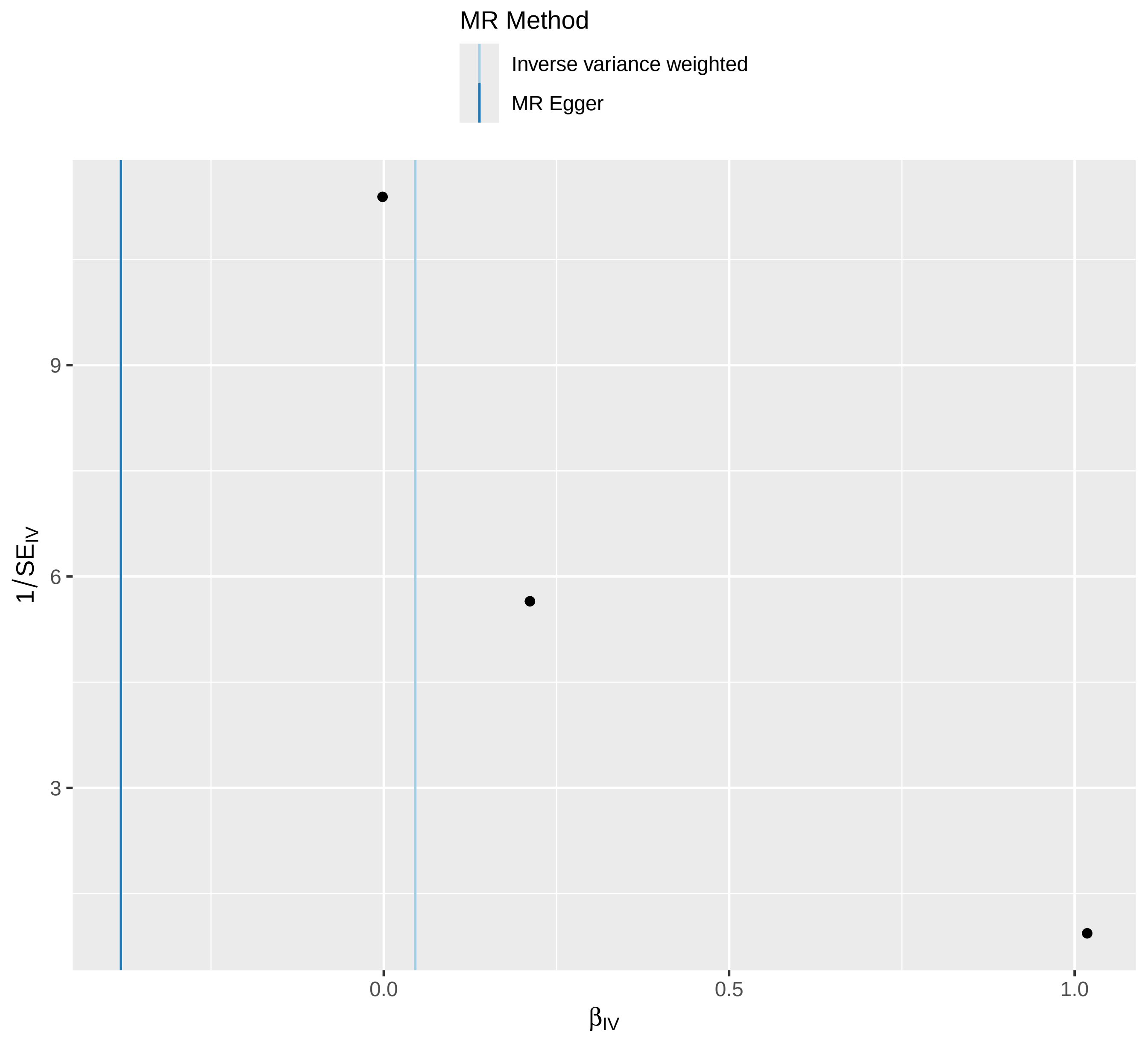
MR Method
Inverse variance weighted
MR Egger
9
6
3
0.0
0.5
1.0
$1/SE_{IV}$
$\beta_{IV}$

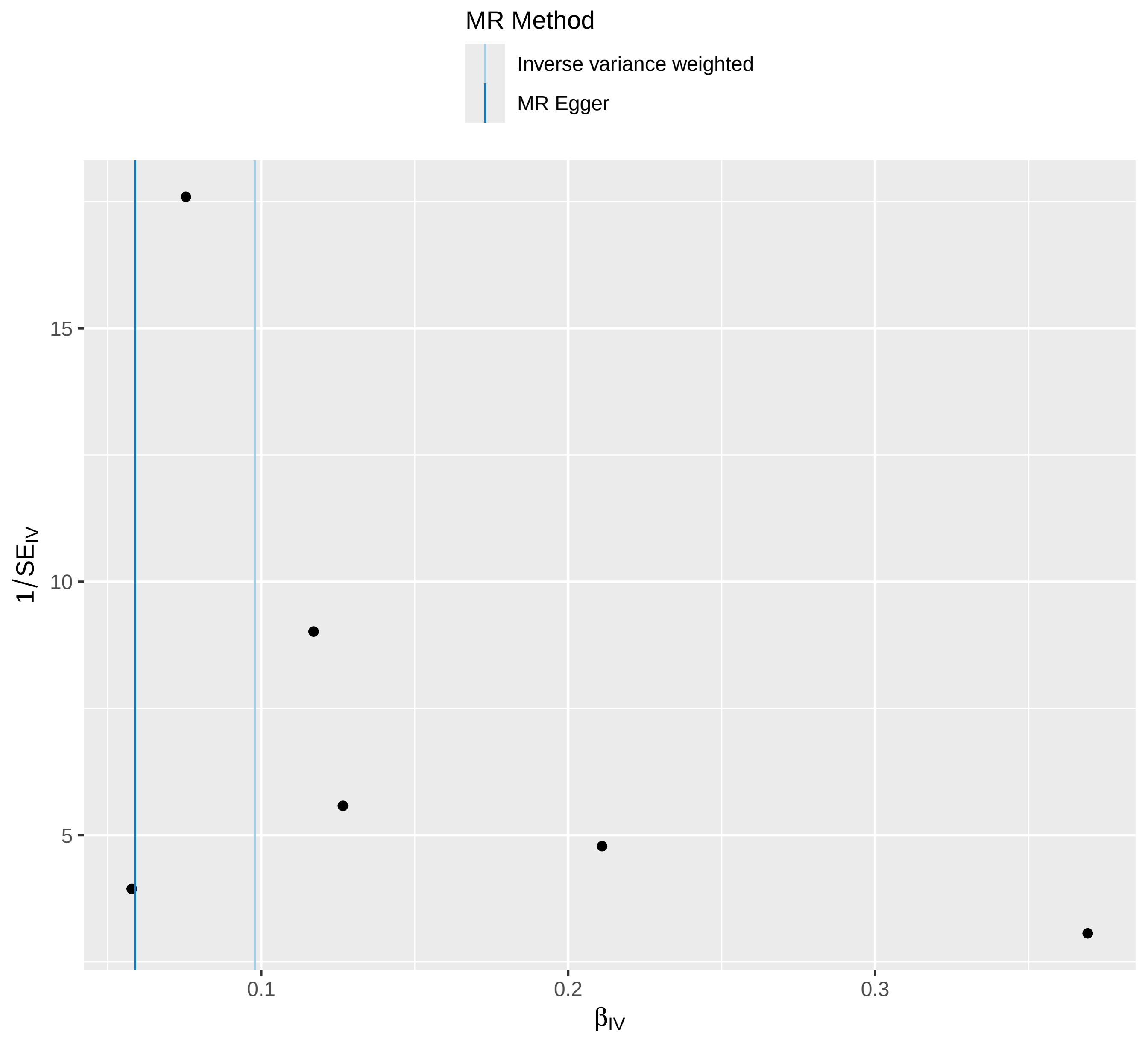

MR Method
Inverse variance weighted
MR Egger
15
10
5
1/SE$_{IV}$
0.1
0.2
0.3
β$_{IV}$

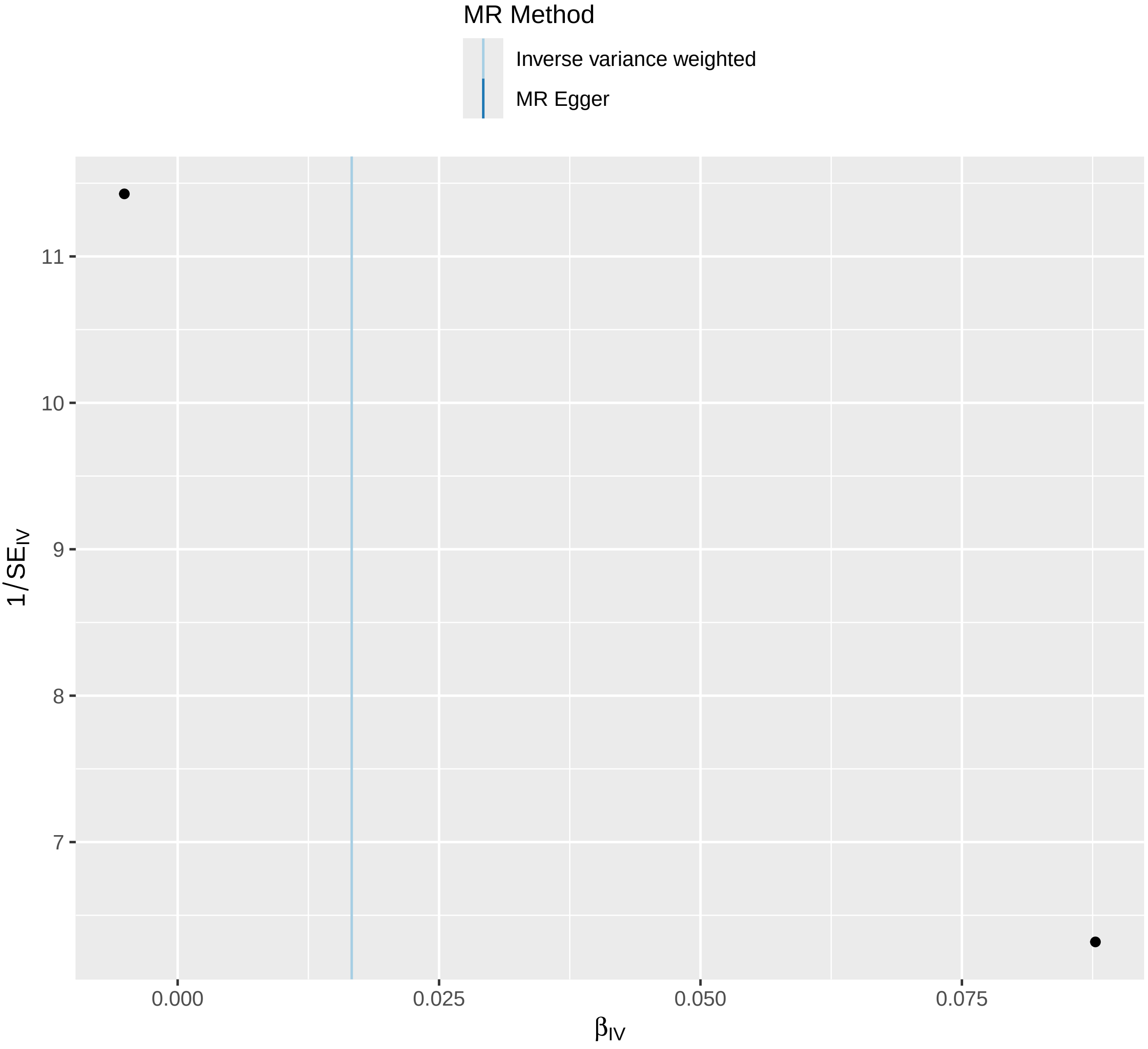

MR Method
Inverse variance weighted
MR Egger
11
10
9
8
7
1/SE_IV
0.000
0.025
0.050
0.075
β_IV

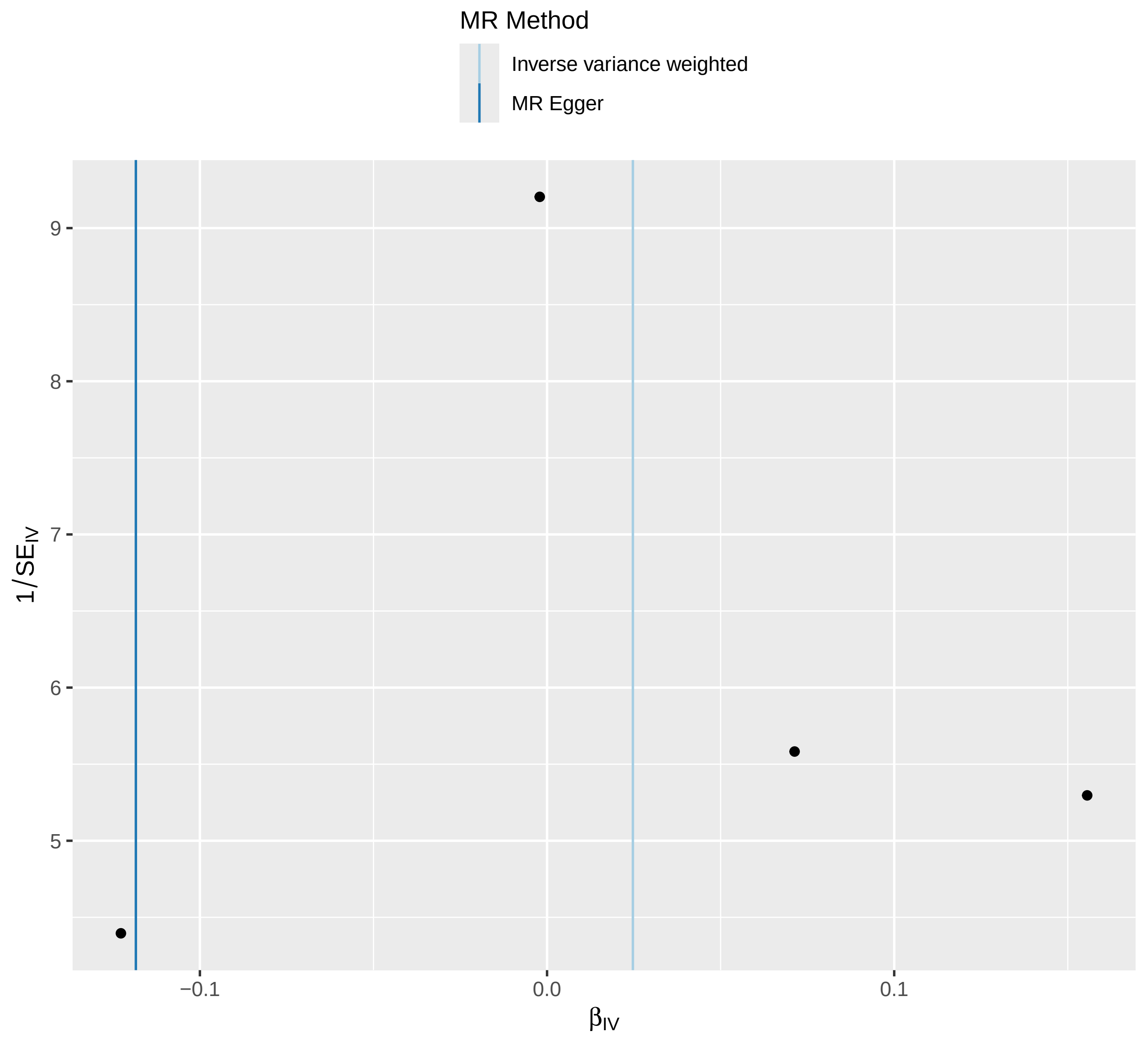
MR Method
Inverse variance weighted
MR Egger
9
8
7
6
5
1/SE_IV
−0.1
0.0
0.1
β_IV

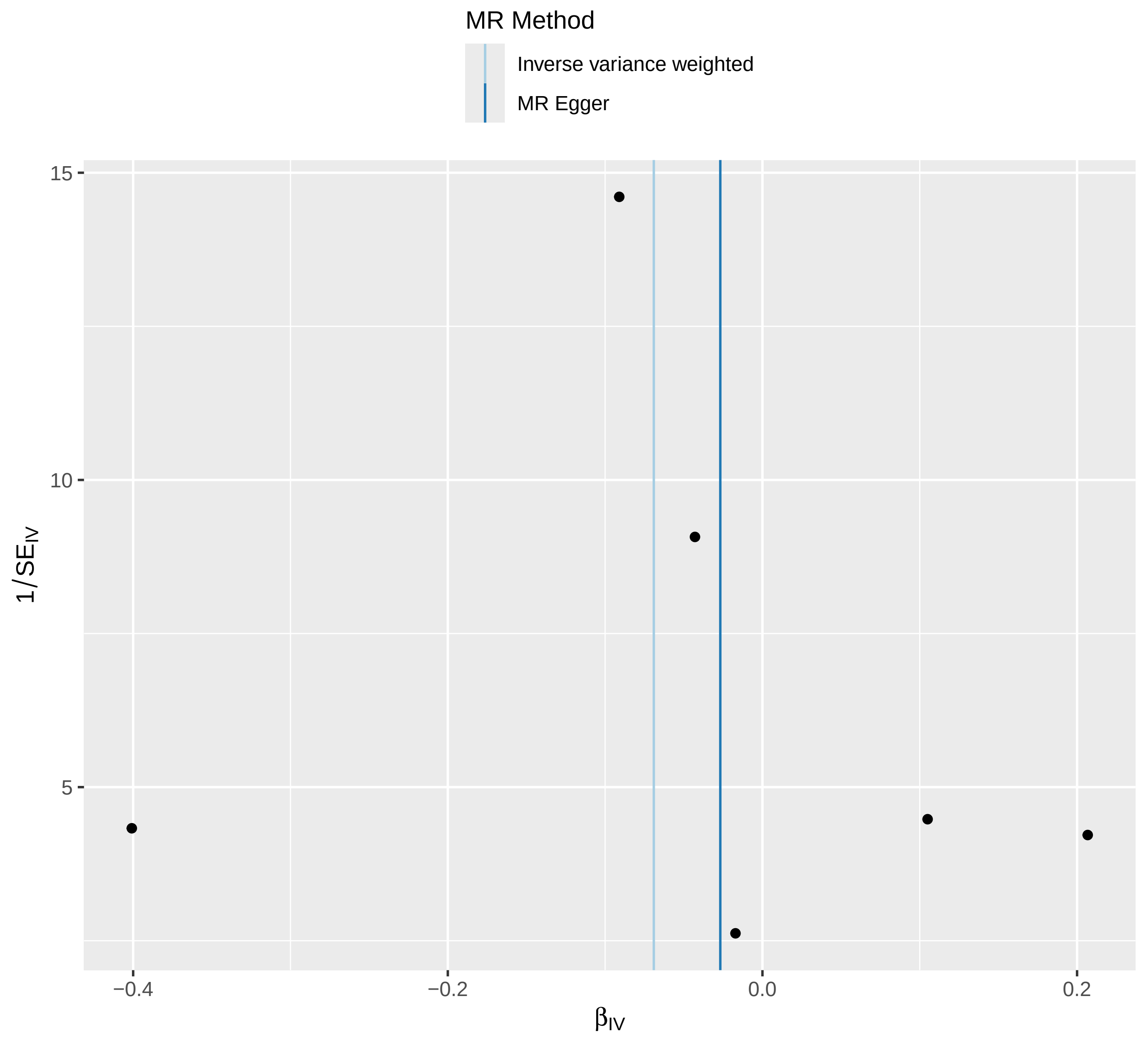
MR Method
Inverse variance weighted
MR Egger
15
10
5
$1/SE_{IV}$
−0.4
−0.2
0.0
0.2
$\beta_{IV}$

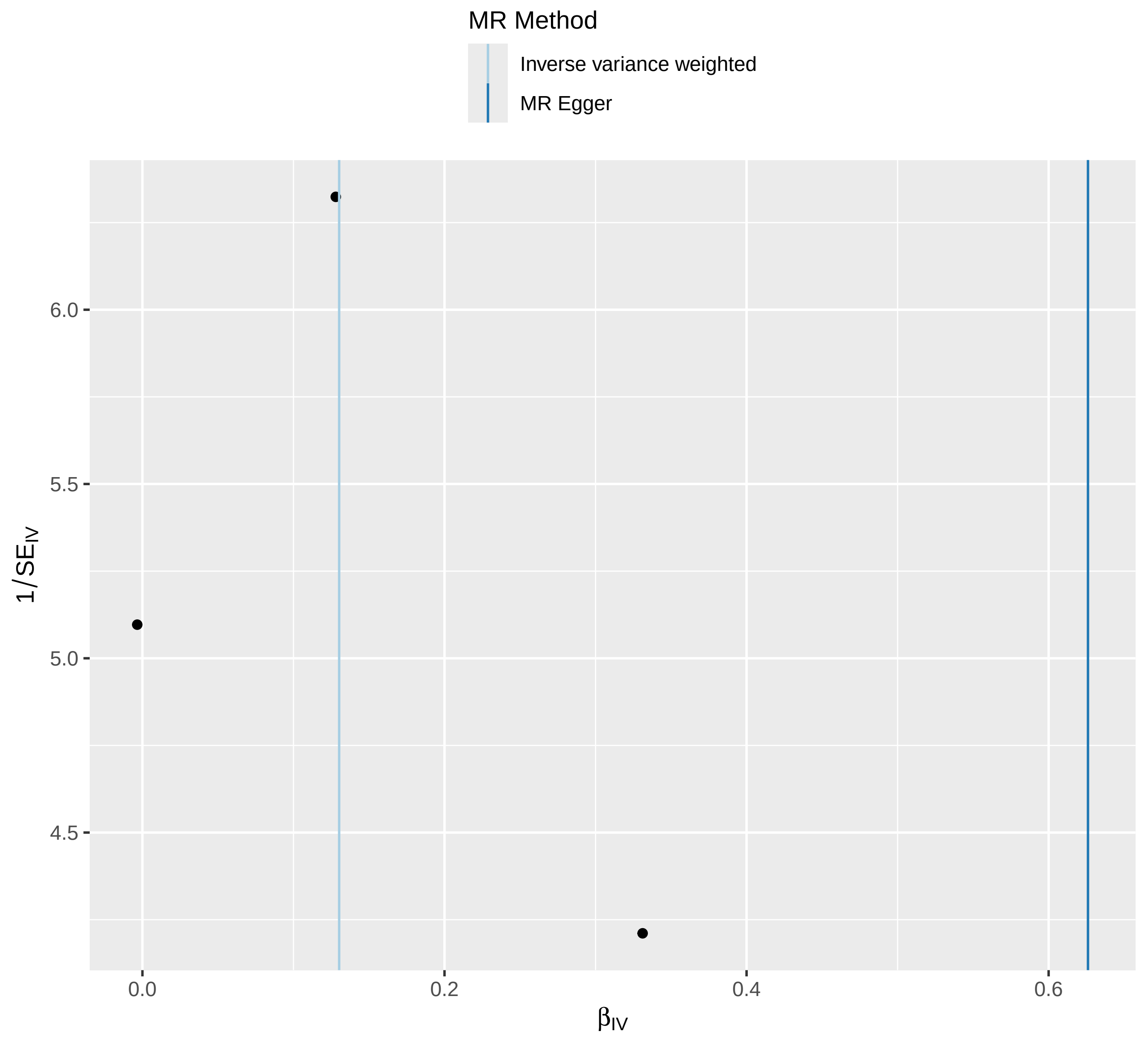
MR Method
Inverse variance weighted
MR Egger
6.0
5.5
5.0
4.5
1/SE$_{IV}$
0.0
0.2
0.4
0.6
β$_{IV}$

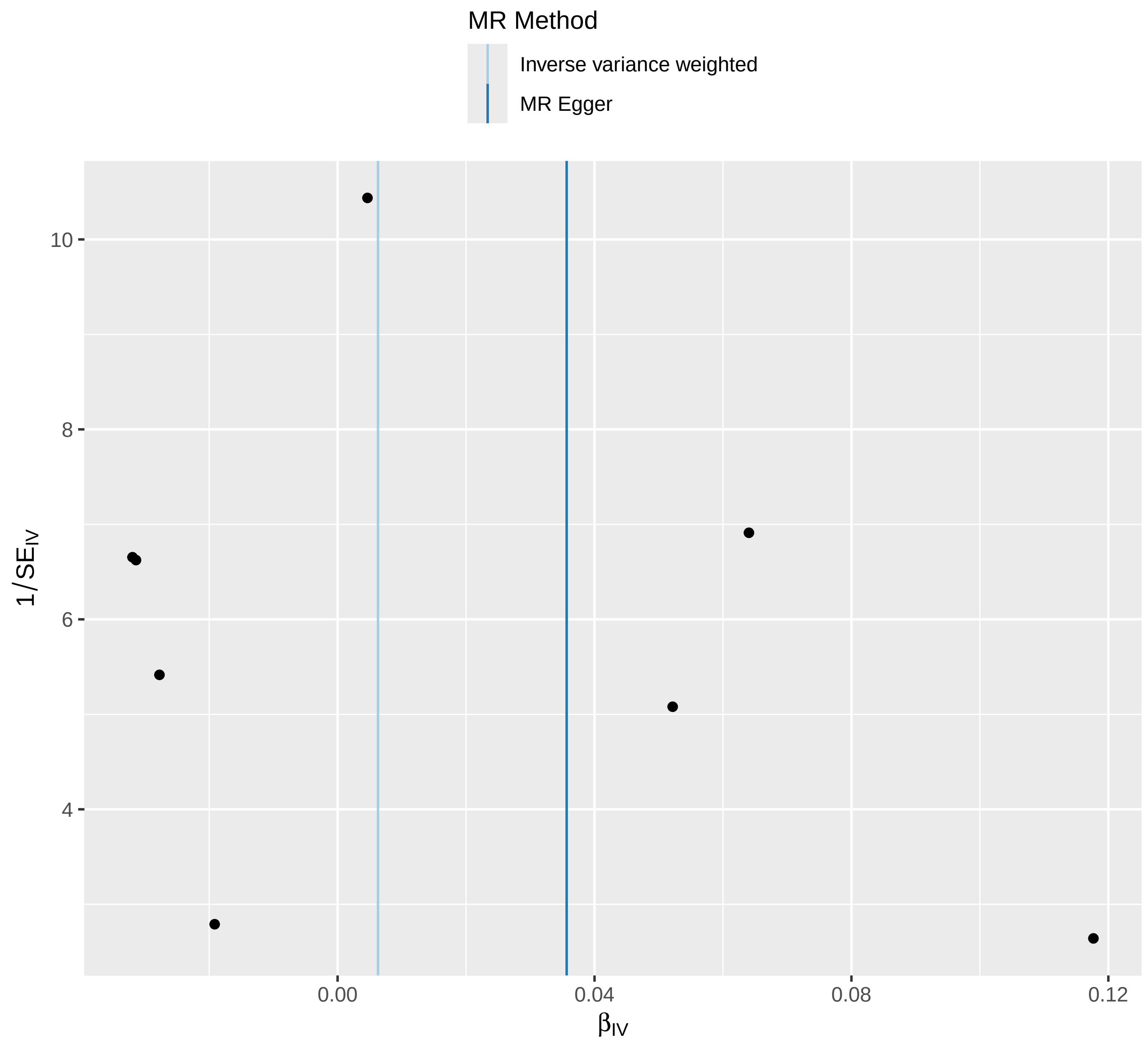
MR Method
Inverse variance weighted
MR Egger
10
8
6
4
1/SE_IV
0.00
0.04
0.08
0.12
β_IV

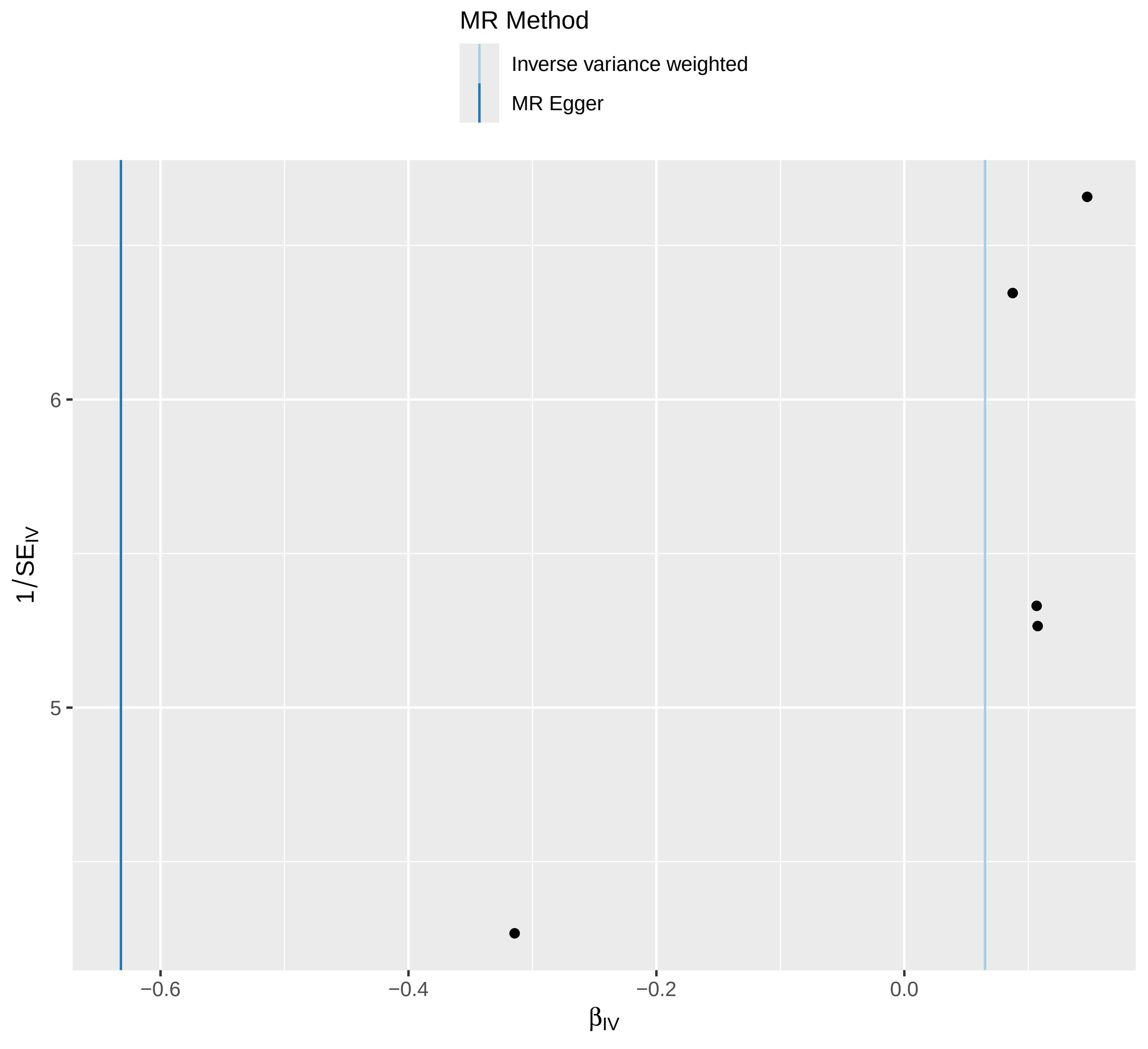
MR Method
Inverse variance weighted
MR Egger
6
5
1/SE_IV
−0.6
−0.4
−0.2
0.0
β_IV

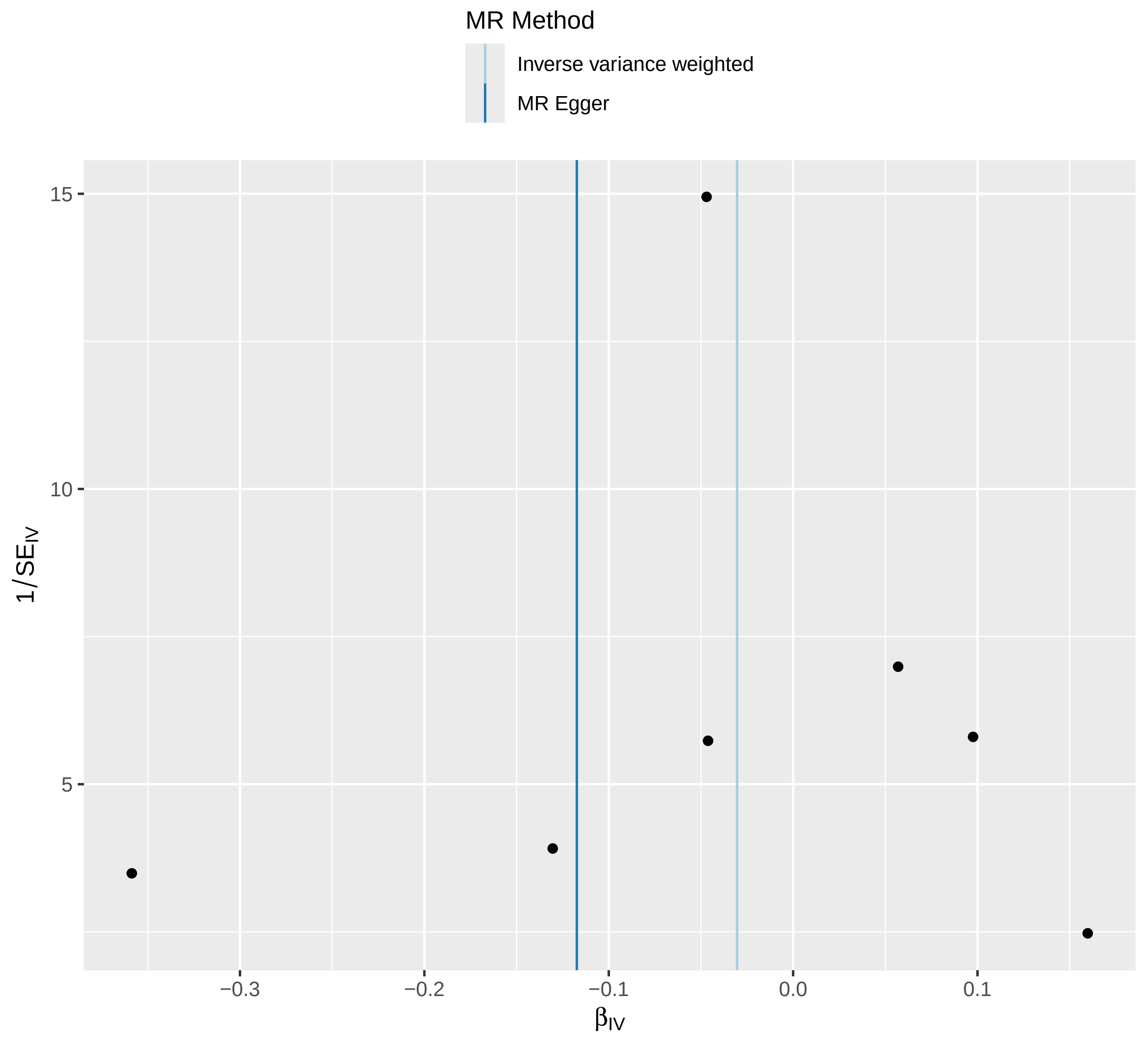

MR Method
Inverse variance weighted
MR Egger
15
10
5
1/SE_IV
−0.3
−0.2
−0.1
0.0
0.1
β_IV

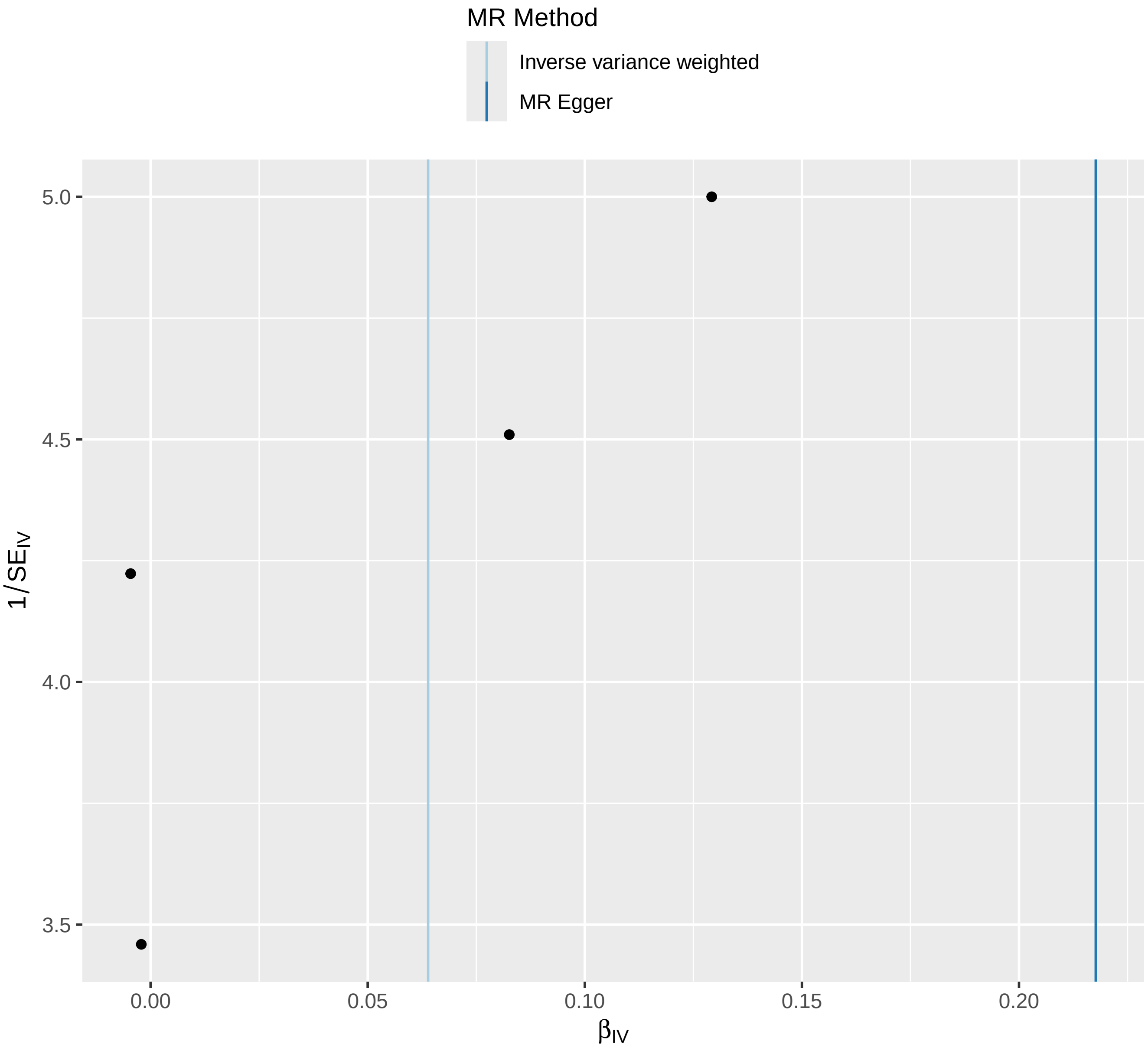
MR Method
Inverse variance weighted
MR Egger
5.0
4.5
4.0
3.5
1/SE_IV
0.00
0.05
0.10
0.15
0.20
β_IV

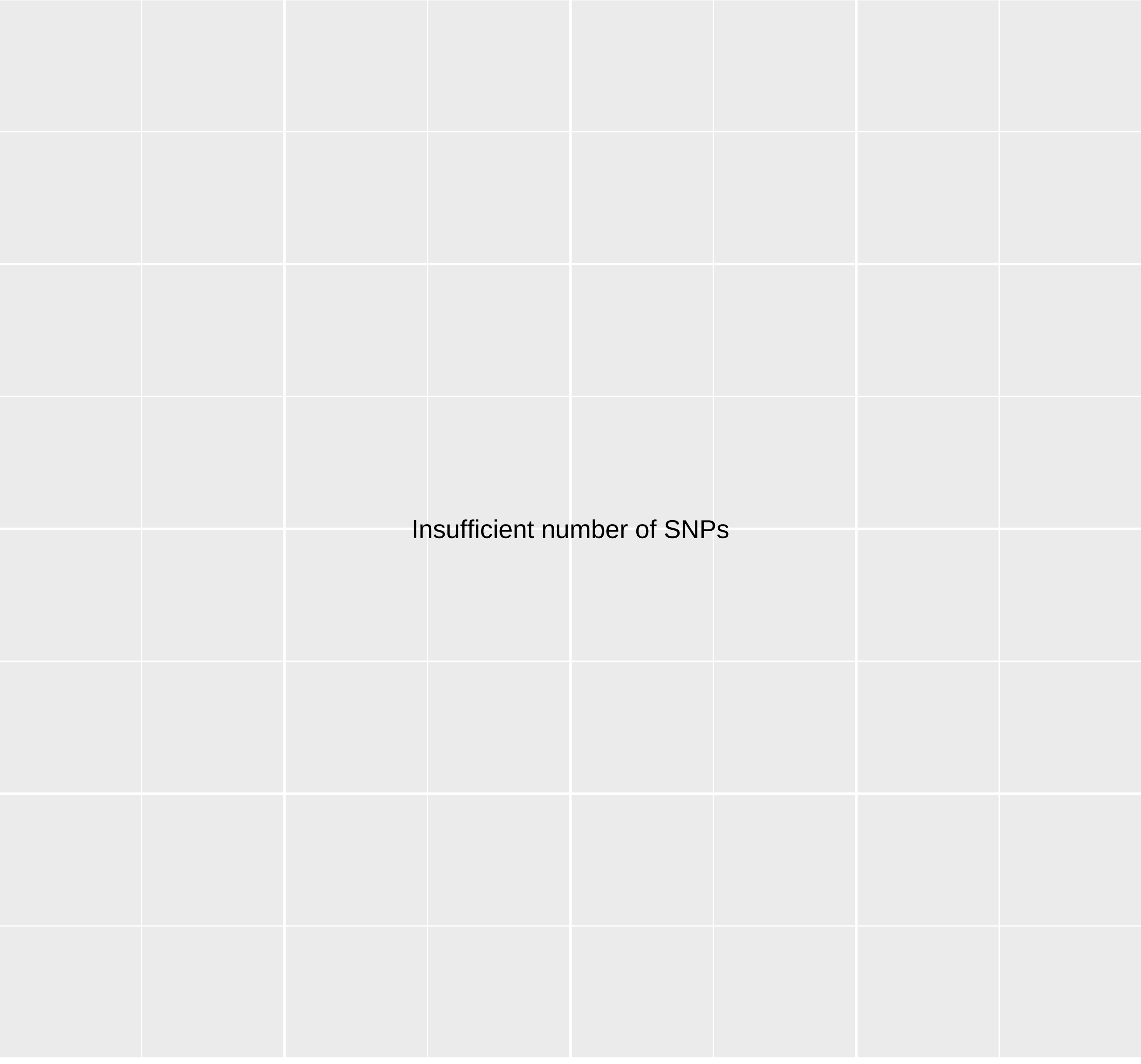
Insufficient number of SNPs

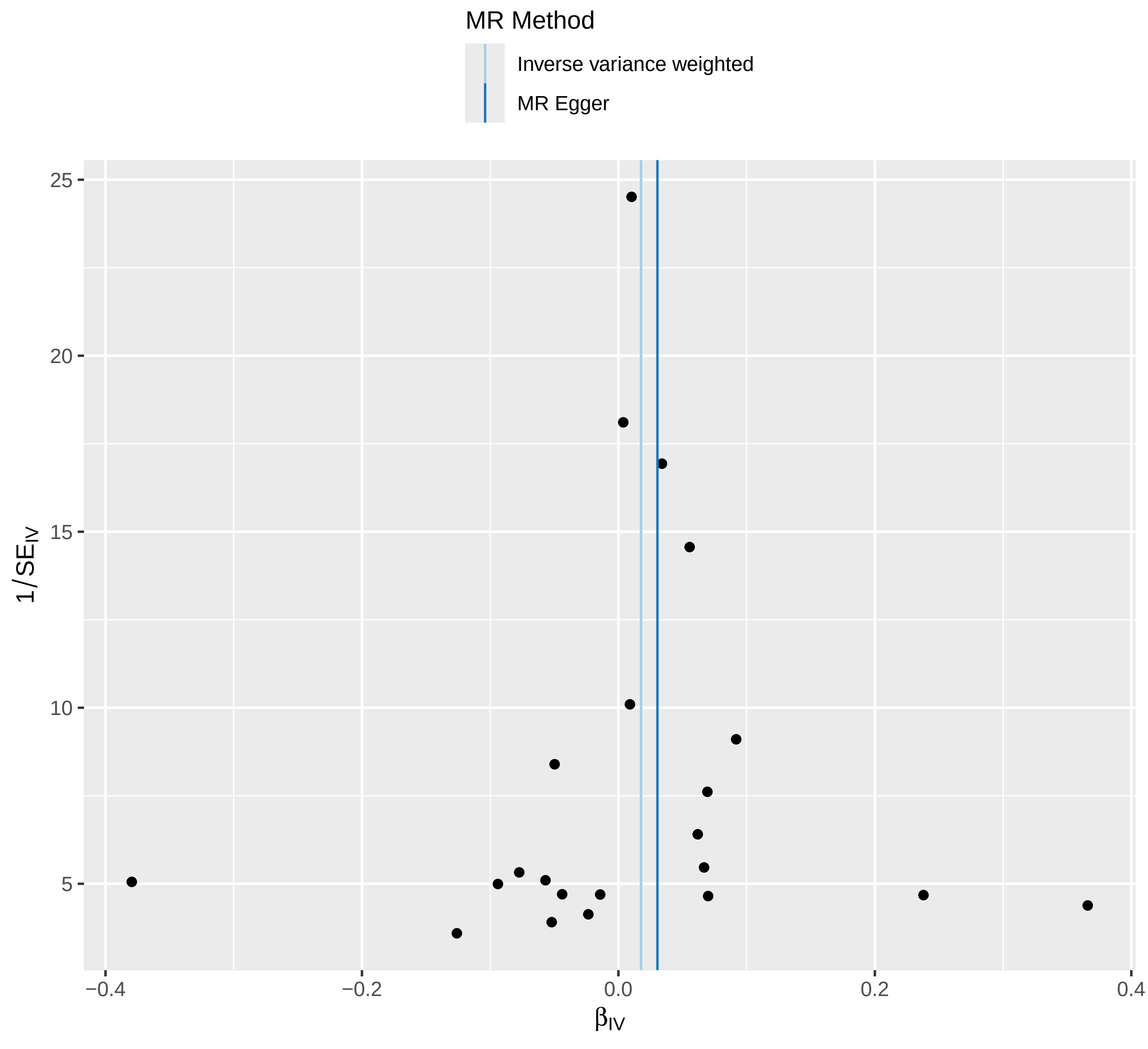

MR Method
Inverse variance weighted
MR Egger
25
20
15
10
5
1/SE$_{IV}$
−0.4
−0.2
0.0
0.2
0.4
β$_{IV}$

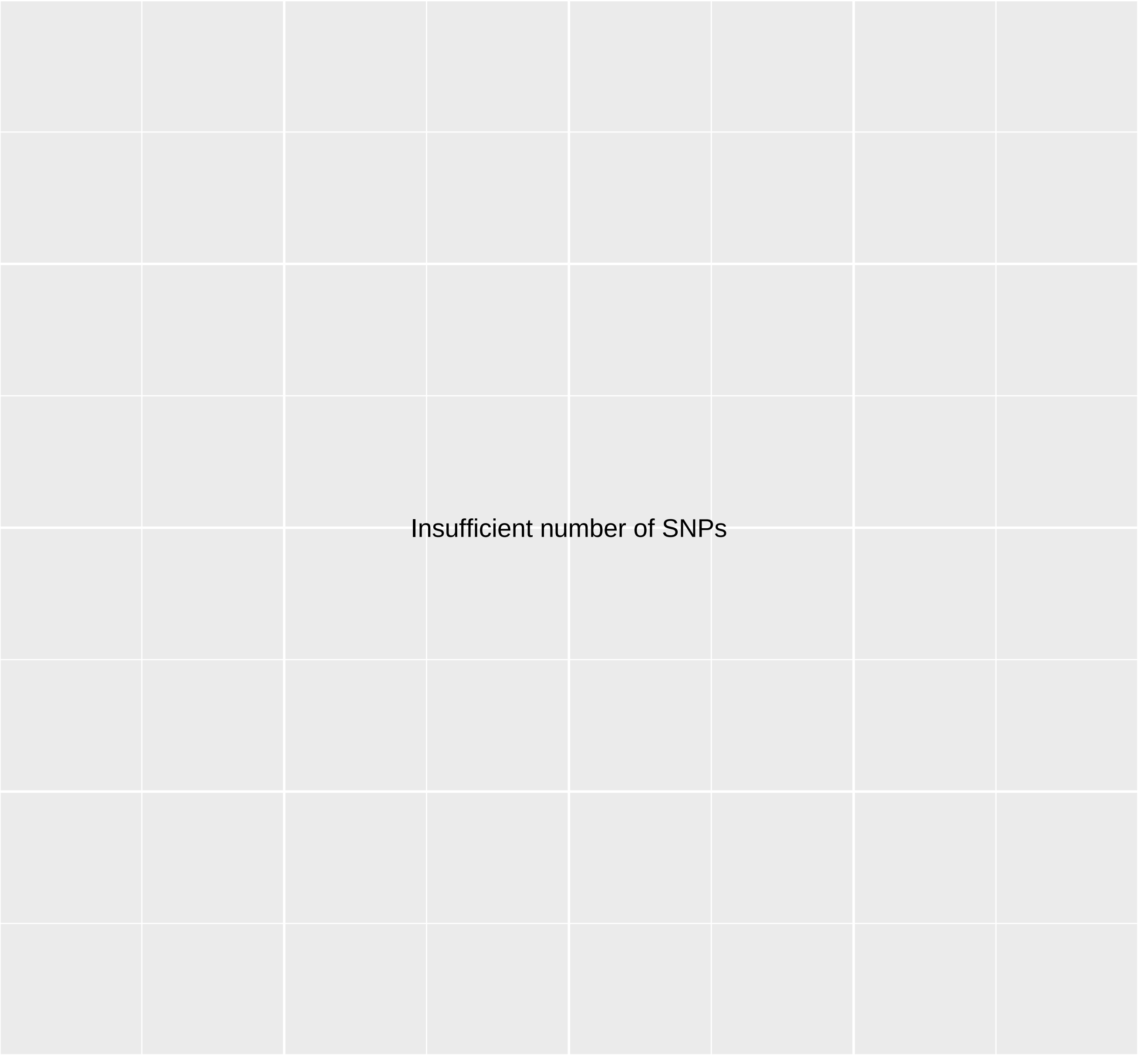
Insufficient number of SNPs

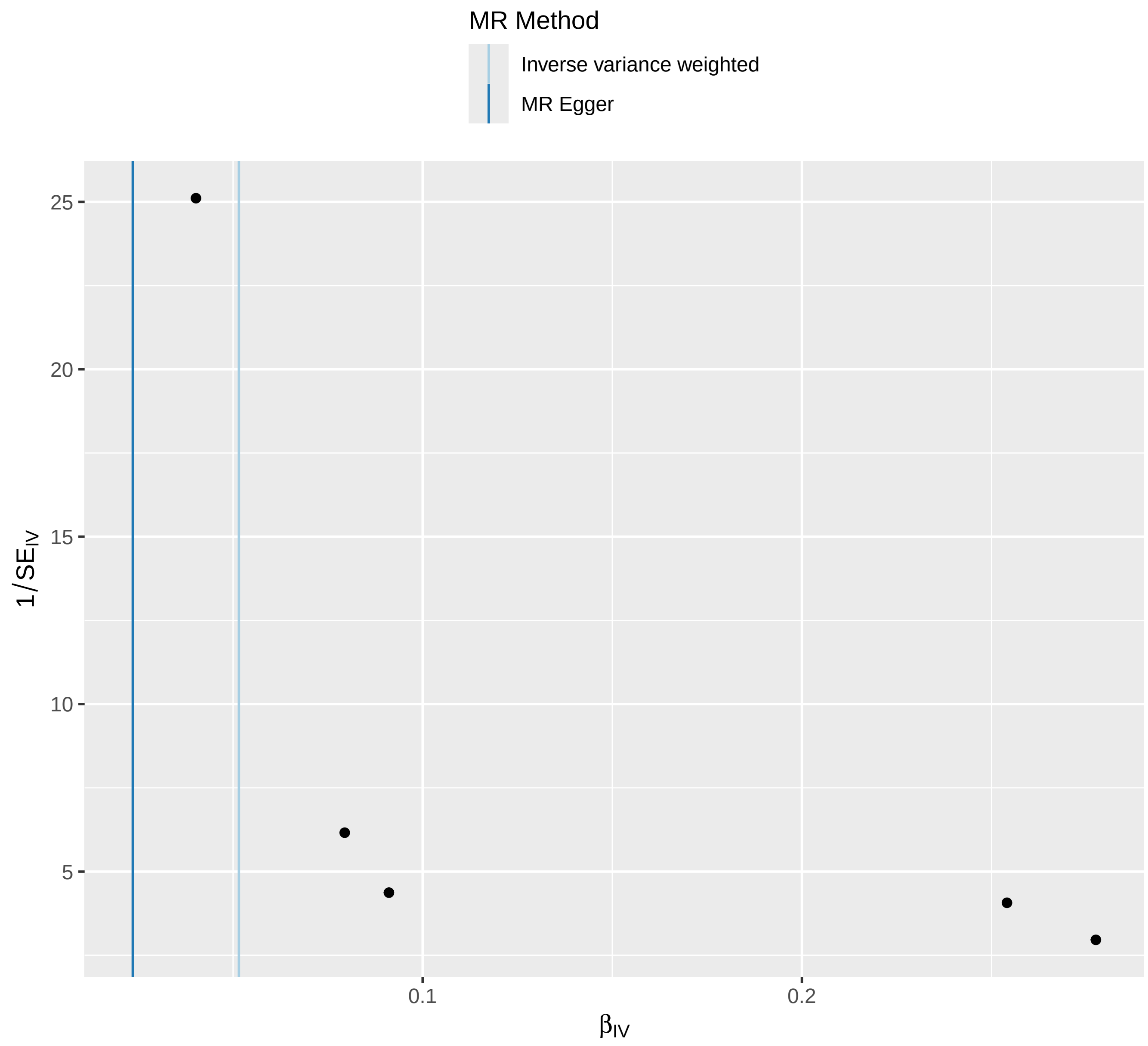

MR Method
Inverse variance weighted
MR Egger
25
20
15
10
5
1/SE_IV
0.1
0.2
β_IV

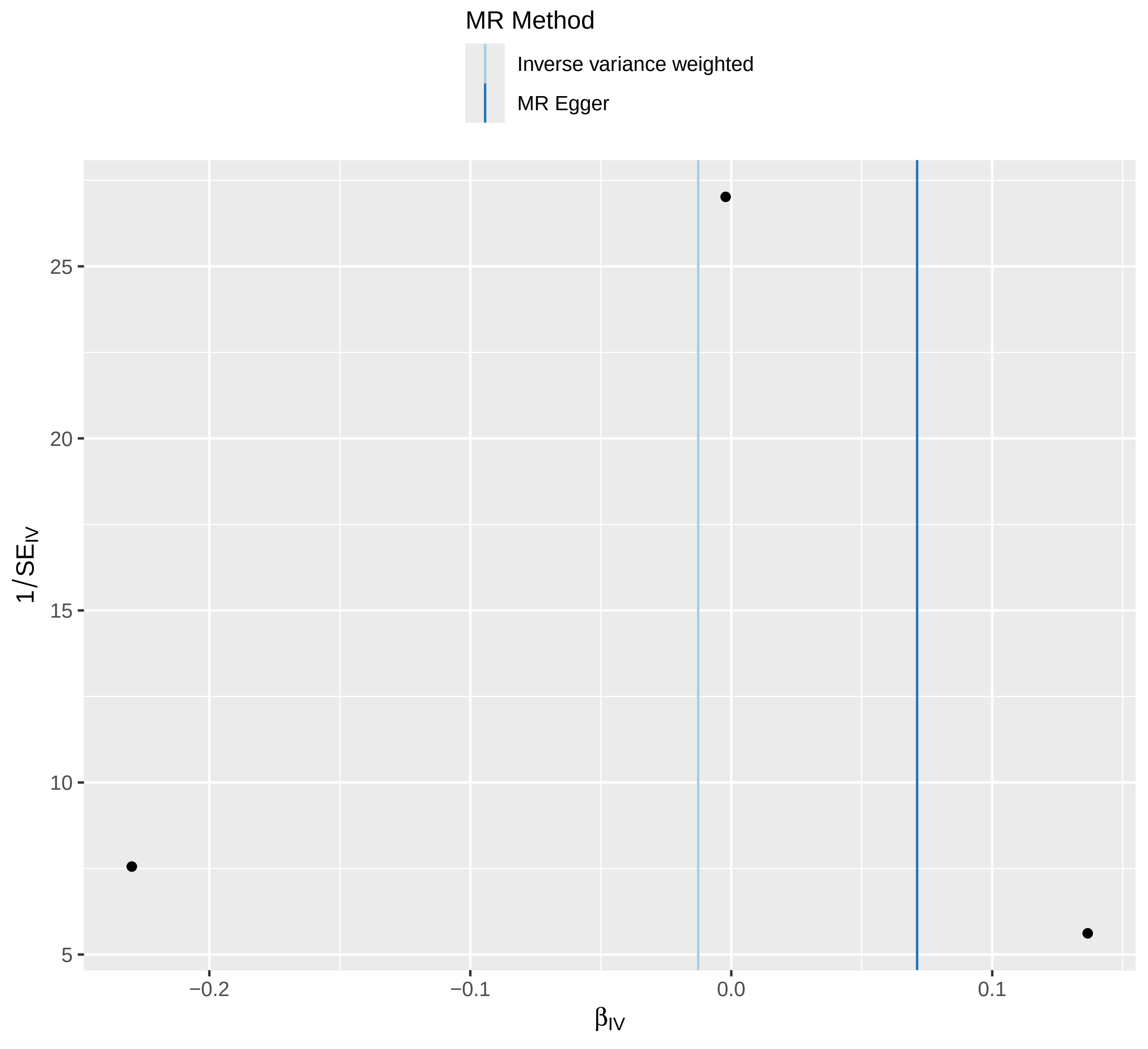
MR Method
Inverse variance weighted
MR Egger
25
20
15
10
5
1/SE_IV
−0.2
−0.1
0.0
0.1
β_IV

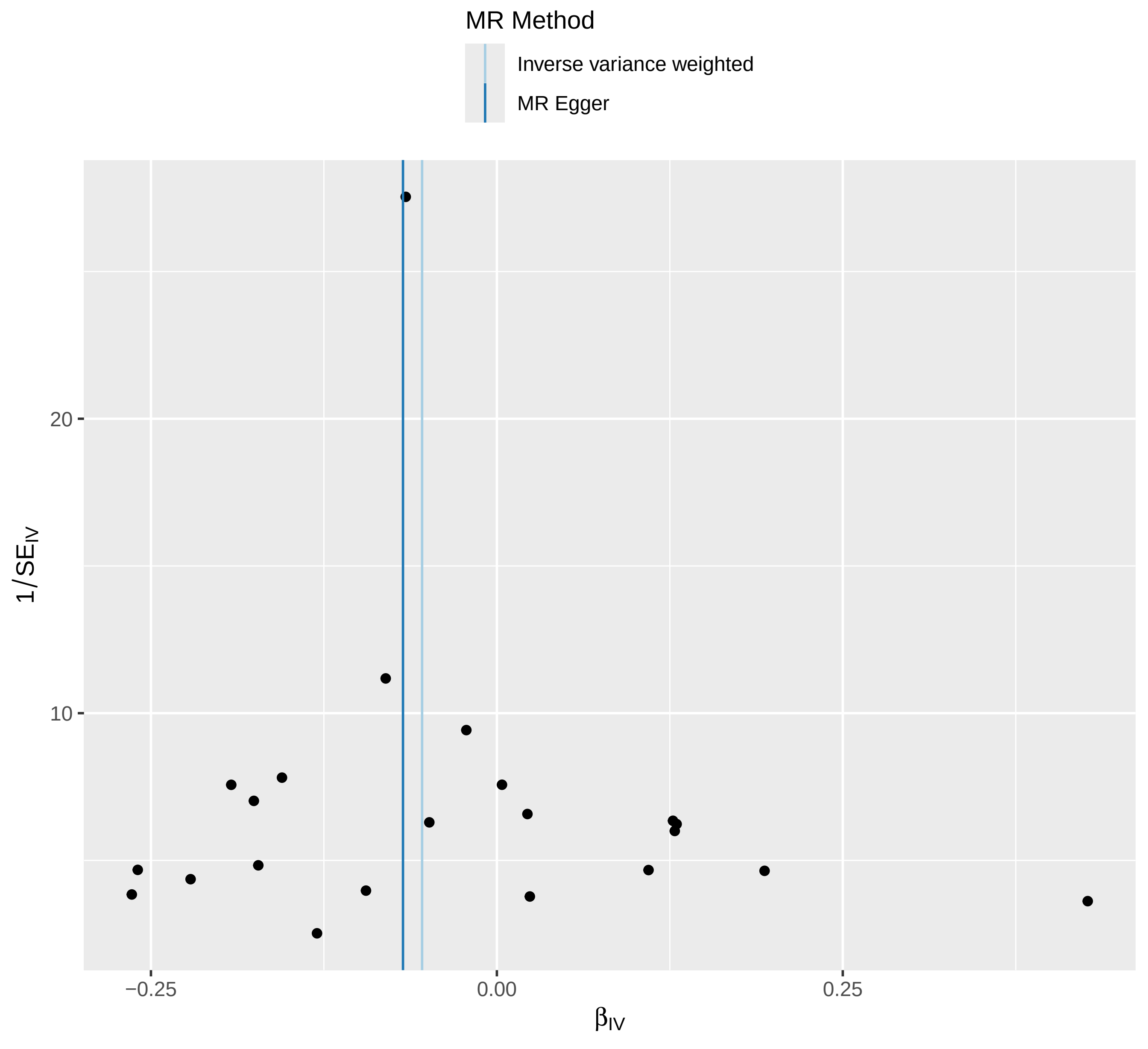
MR Method
Inverse variance weighted
MR Egger
20
10
1/SE_IV
−0.25
0.00
0.25
β_IV

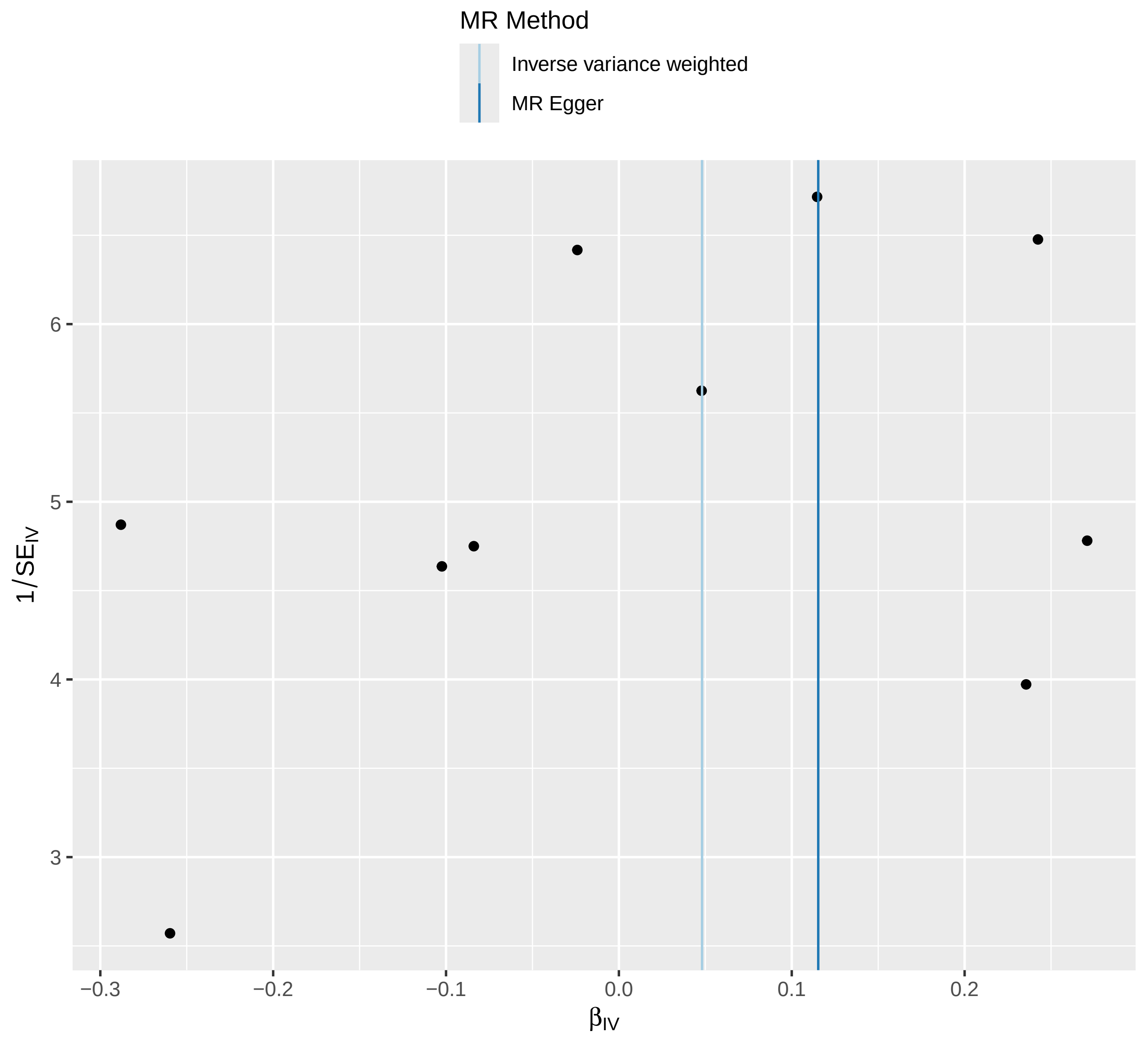

MR Method
Inverse variance weighted
MR Egger
1/SE$_{IV}$
β$_{IV}$
6
5
4
3
−0.3
−0.2
−0.1
0.0
0.1
0.2

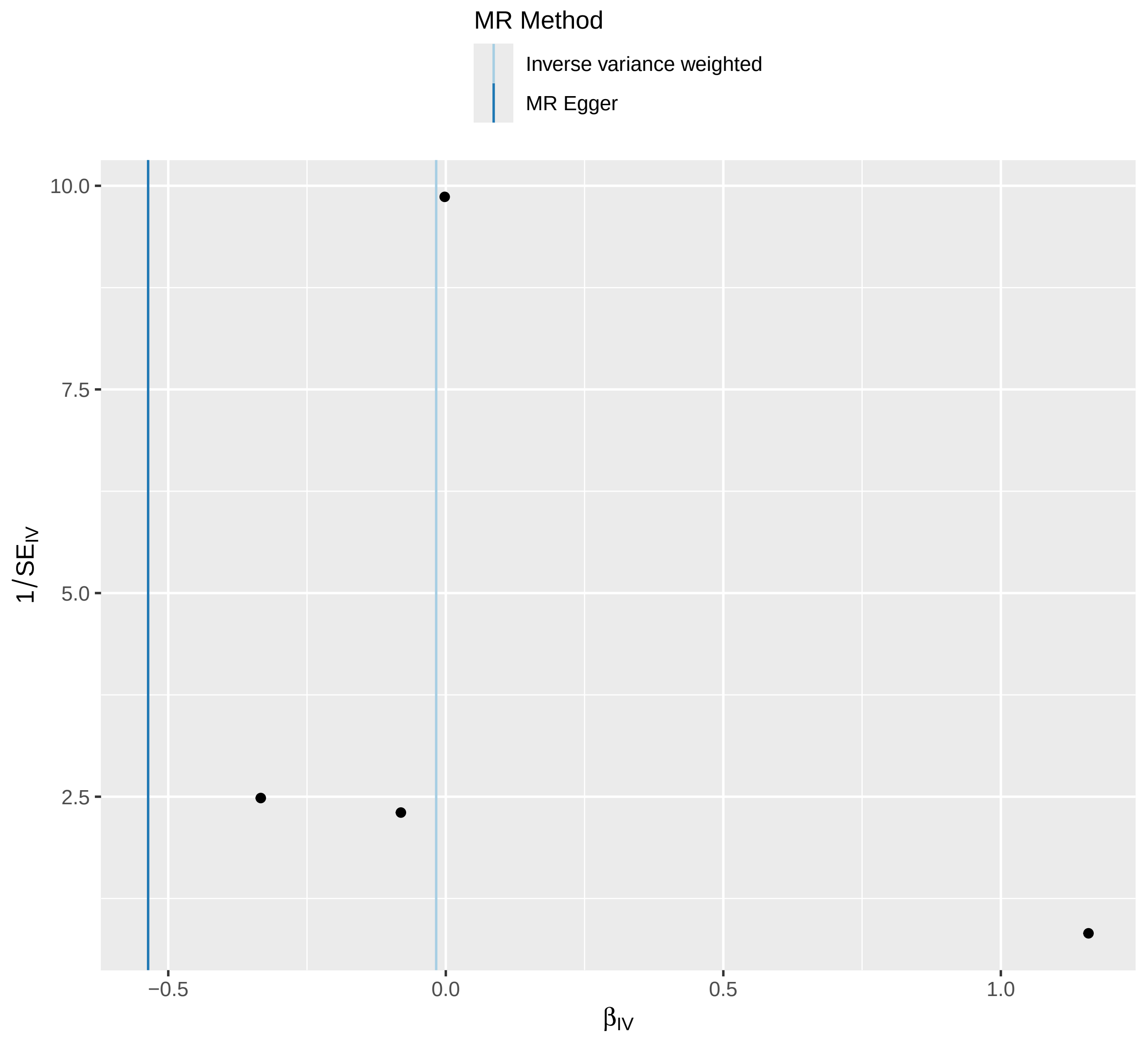
MR Method
Inverse variance weighted
MR Egger
10.0
7.5
5.0
2.5
1/SE$_{IV}$
−0.5
0.0
0.5
1.0
β$_{IV}$

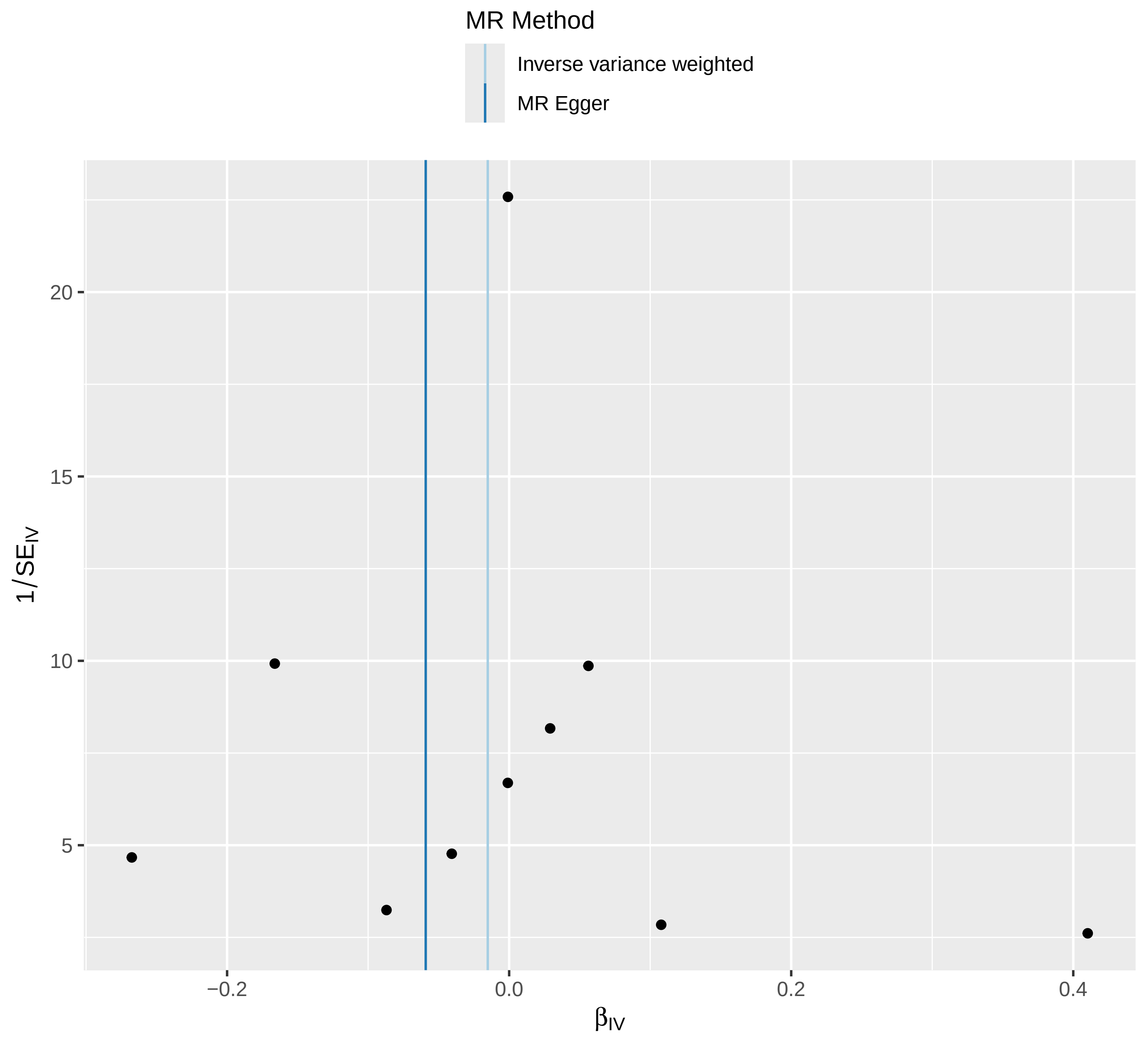

MR Method
Inverse variance weighted
MR Egger
20
15
10
5
1/SE$_{IV}$
−0.2
0.0
0.2
0.4
β$_{IV}$

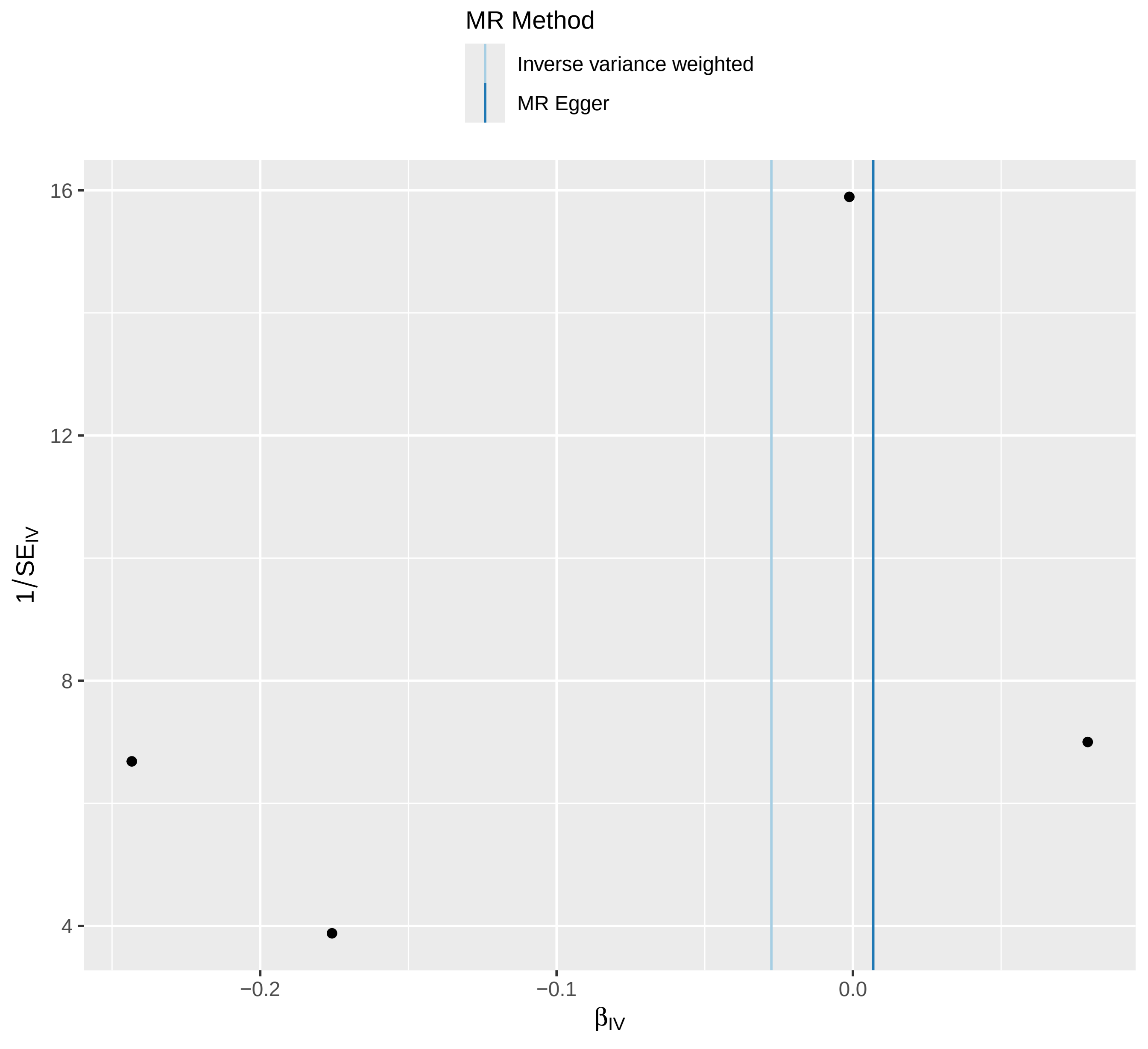
MR Method
Inverse variance weighted
MR Egger
16
12
8
4
1/SE$_{IV}$
−0.2
−0.1
0.0
β$_{IV}$

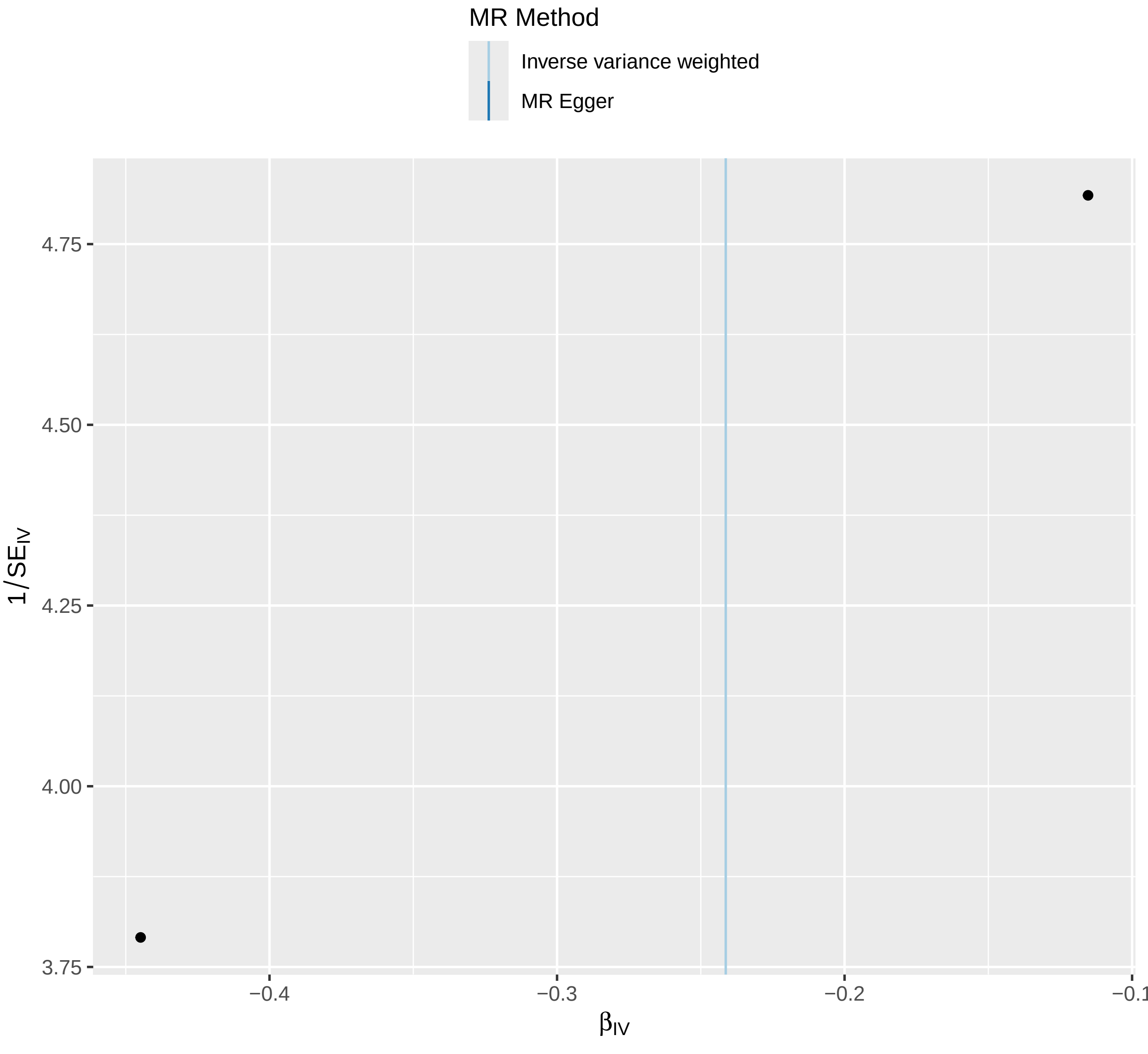
MR Method
Inverse variance weighted
MR Egger
1/SE$_{IV}$
4.75
4.50
4.25
4.00
3.75
−0.4
−0.3
−0.2
−0.1
β$_{IV}$

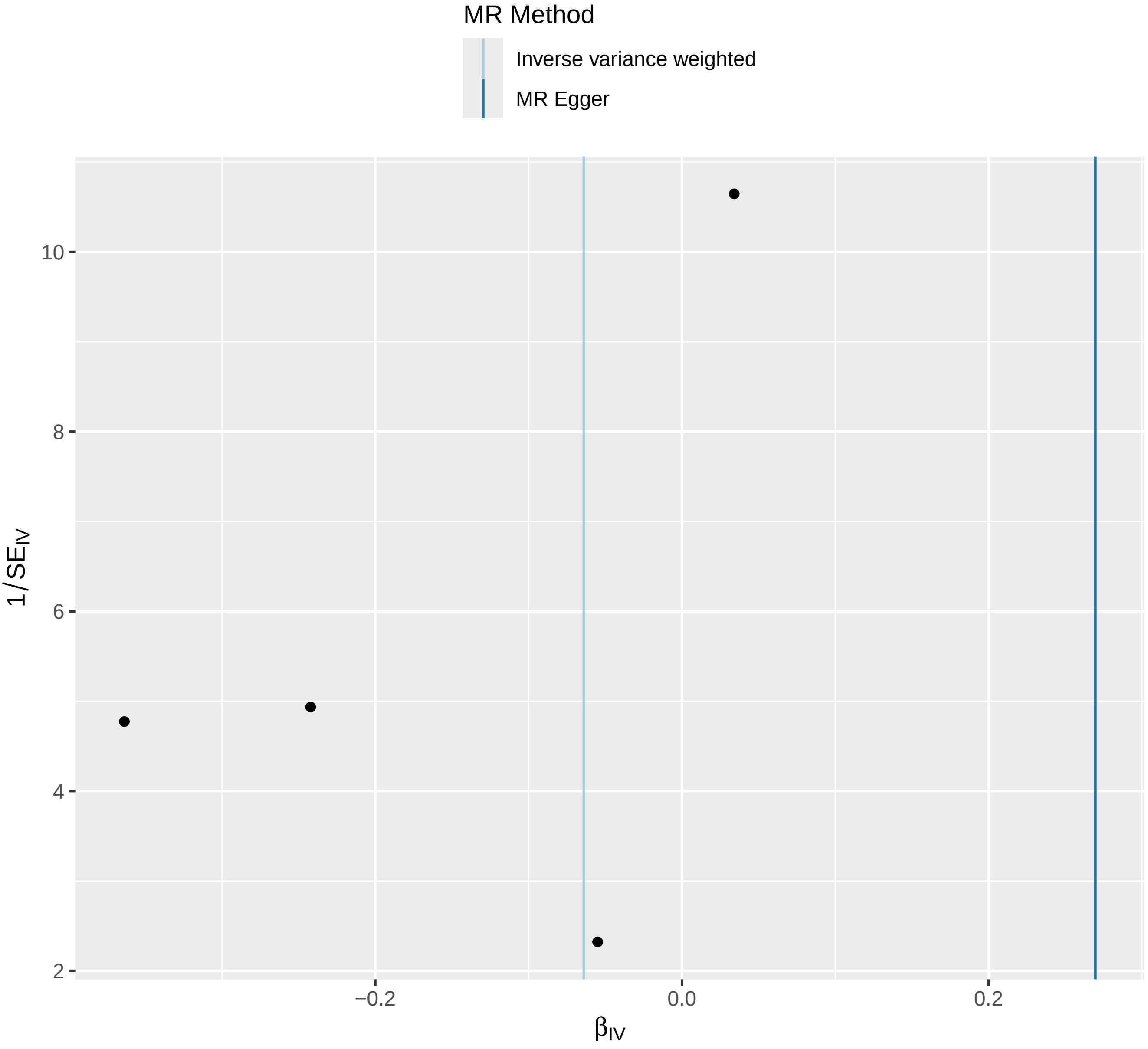
MR Method
Inverse variance weighted
MR Egger
10
8
6
4
2
−0.2
0.0
0.2
$1/SE_{IV}$
$\beta_{IV}$

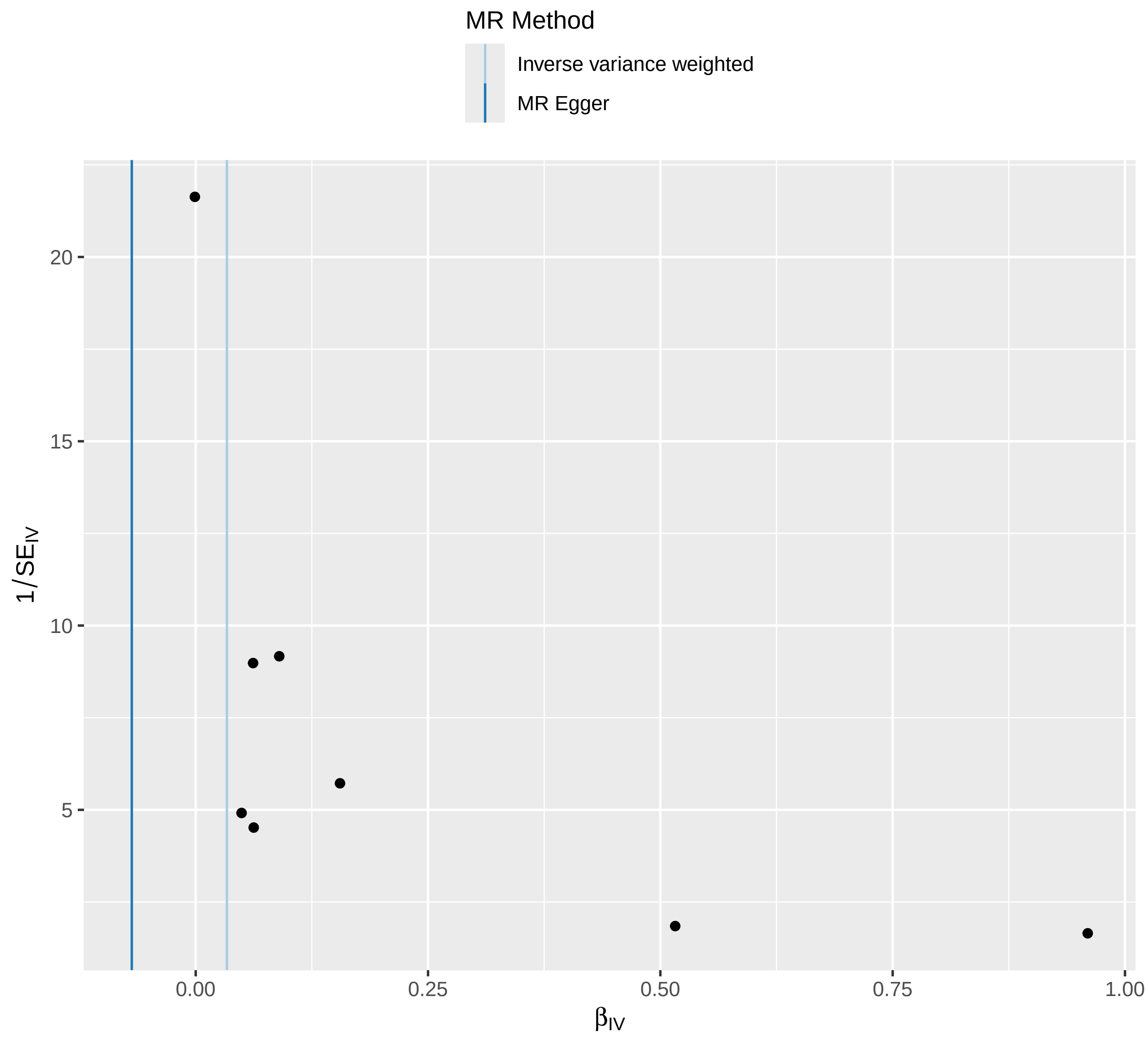
MR Method
Inverse variance weighted
MR Egger
20
15
10
5
1/SE_IV
0.00
0.25
0.50
0.75
1.00
β_IV

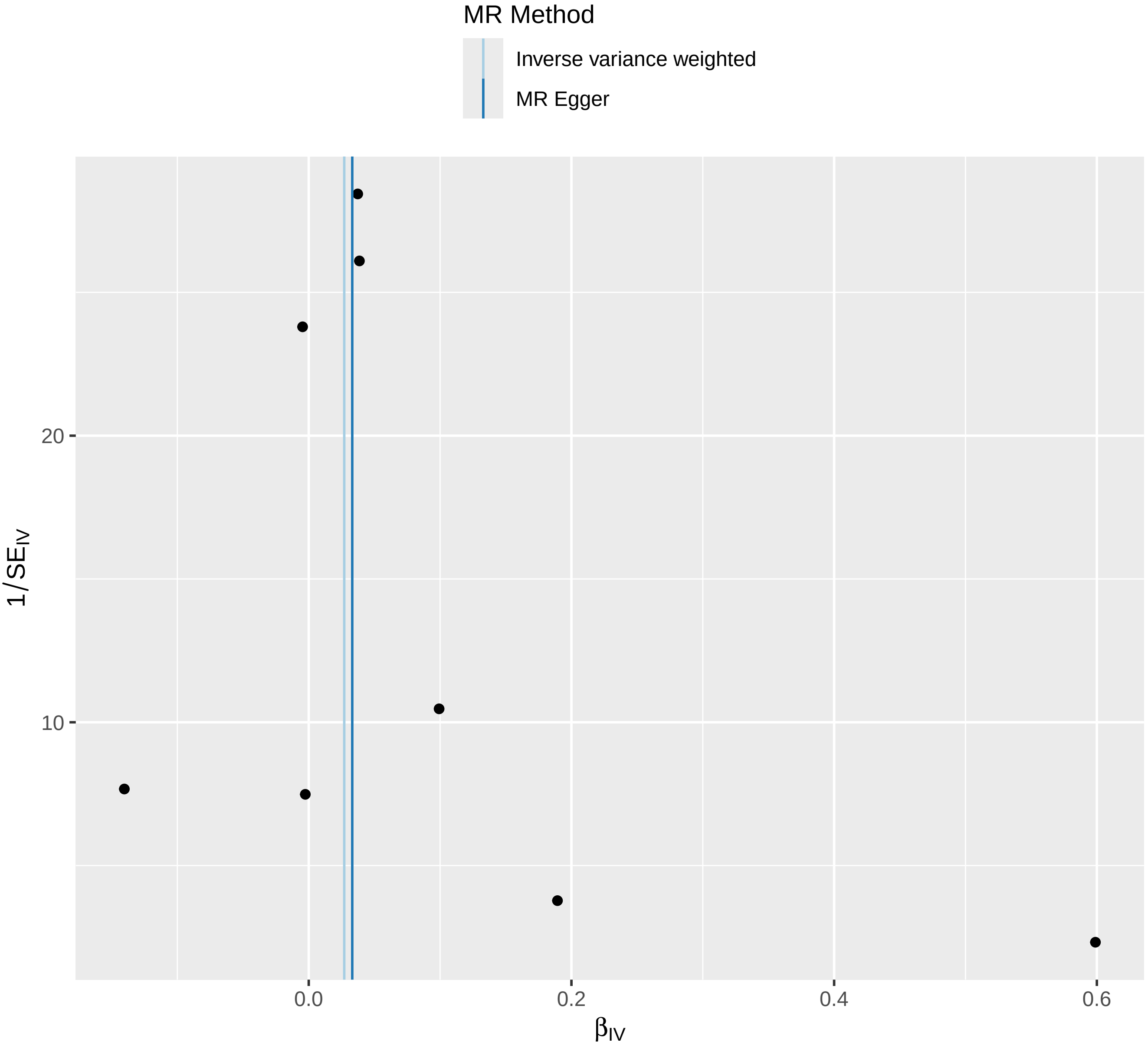
MR Method
Inverse variance weighted
MR Egger
20
10
$1/SE_{IV}$
0.0
0.2
0.4
0.6
$\beta_{IV}$

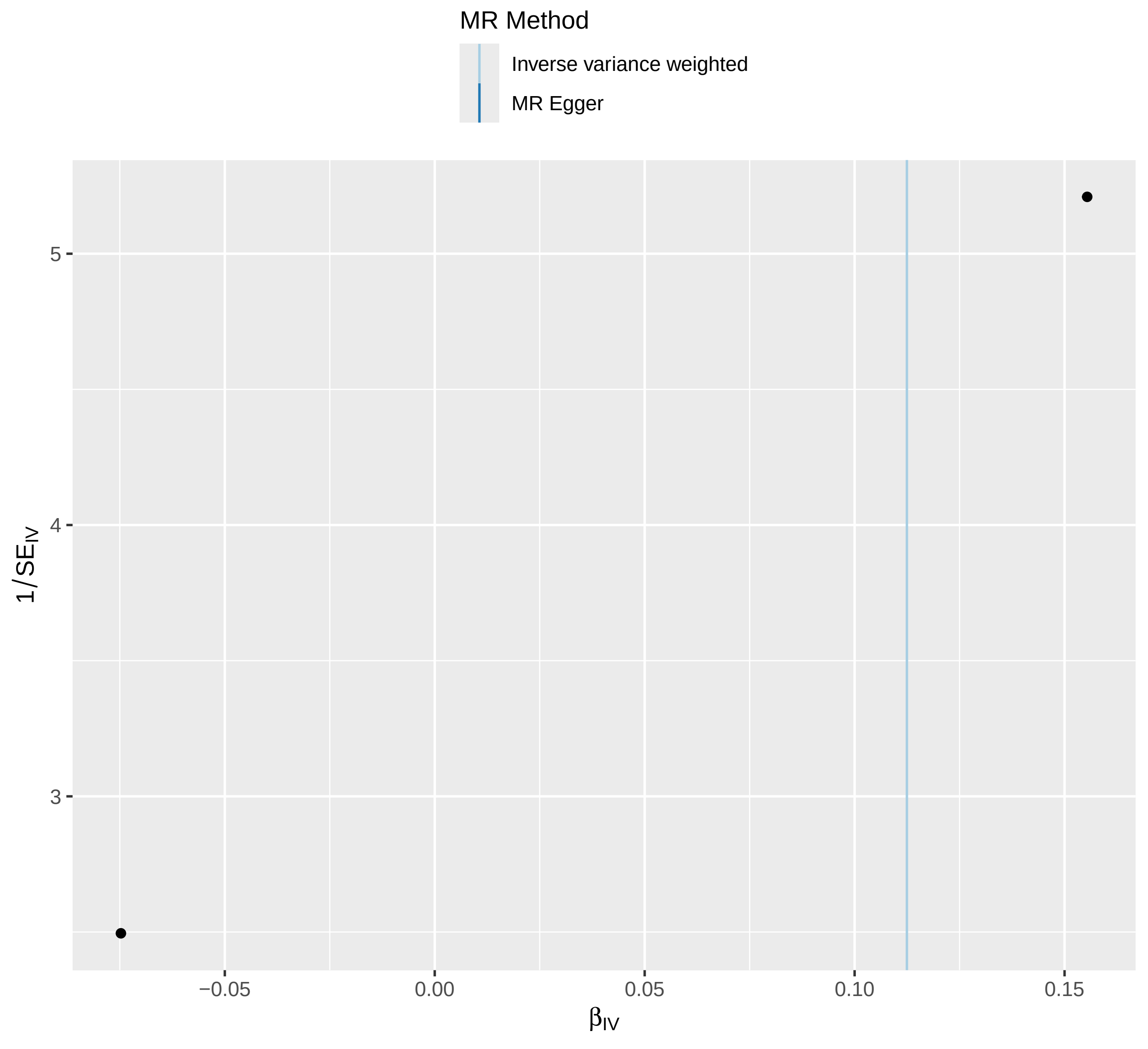

MR Method
Inverse variance weighted
MR Egger
5
4
3
$1/SE_{IV}$
−0.05
0.00
0.05
0.10
0.15
$\beta_{IV}$

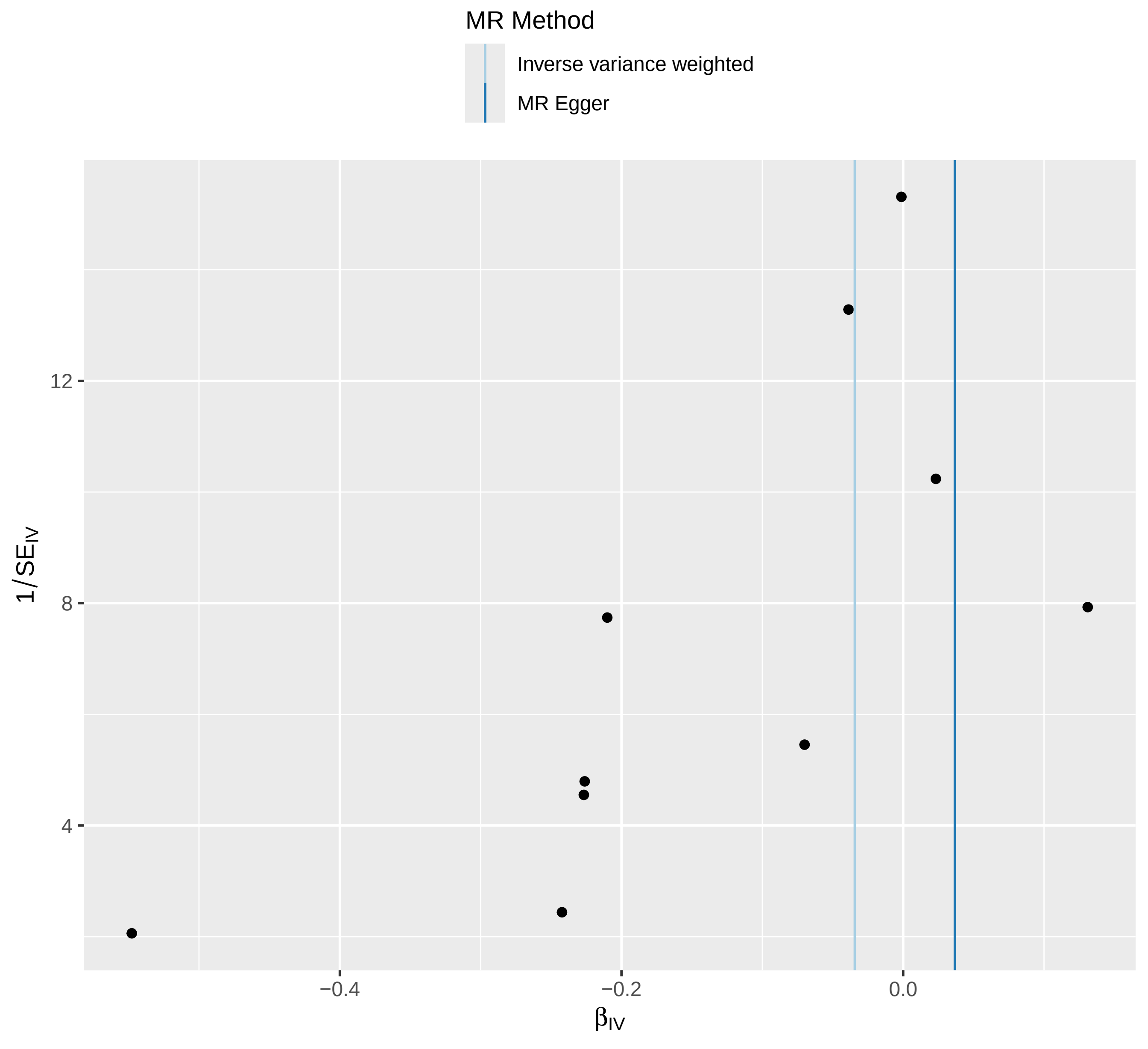
MR Method
Inverse variance weighted
MR Egger
12
8
4
1/$SE_{IV}$
−0.4
−0.2
0.0
$\beta_{IV}$

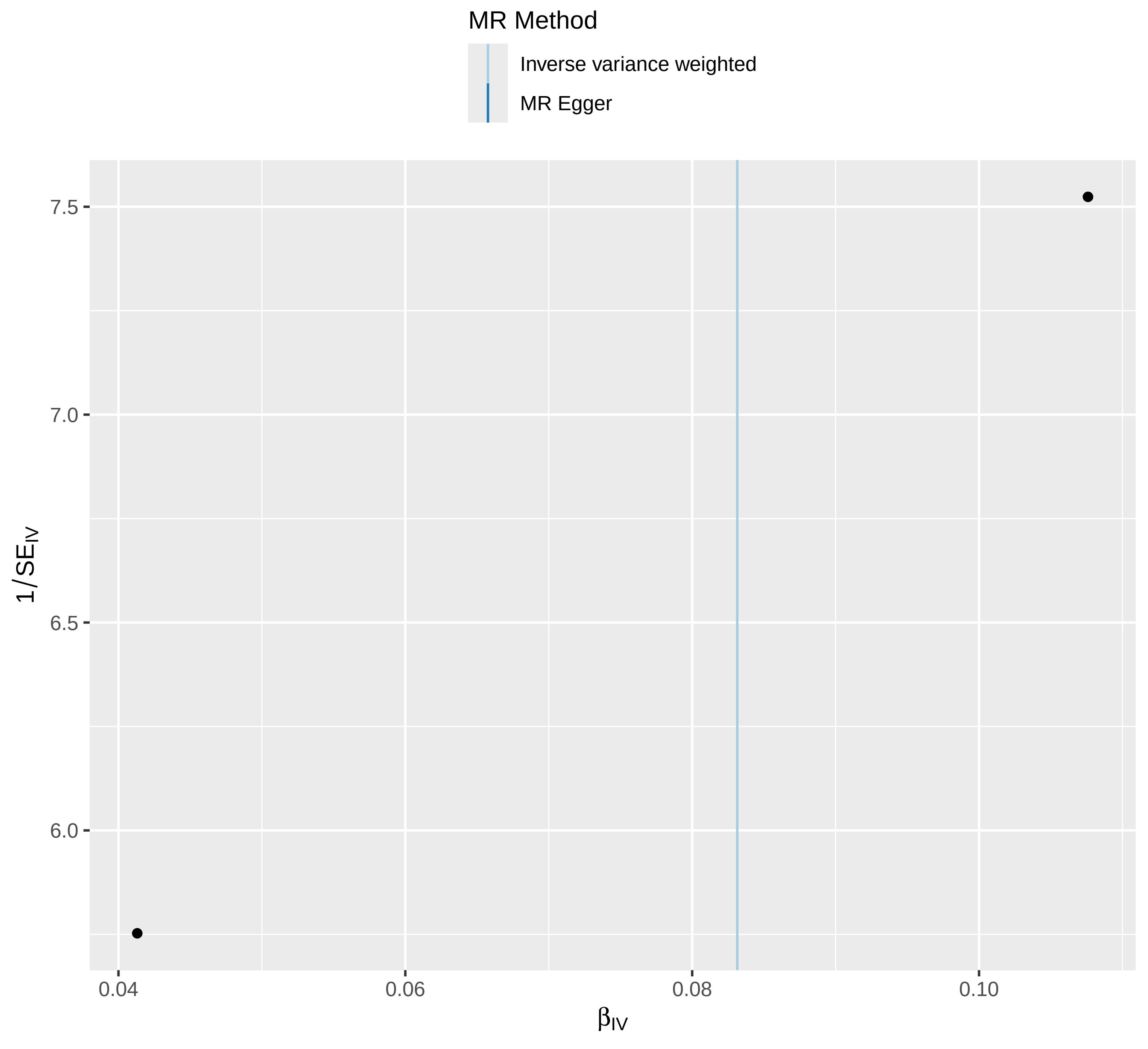
MR Method
Inverse variance weighted
MR Egger
7.5
7.0
6.5
6.0
1/SE_IV
0.04
0.06
0.08
0.10
β_IV

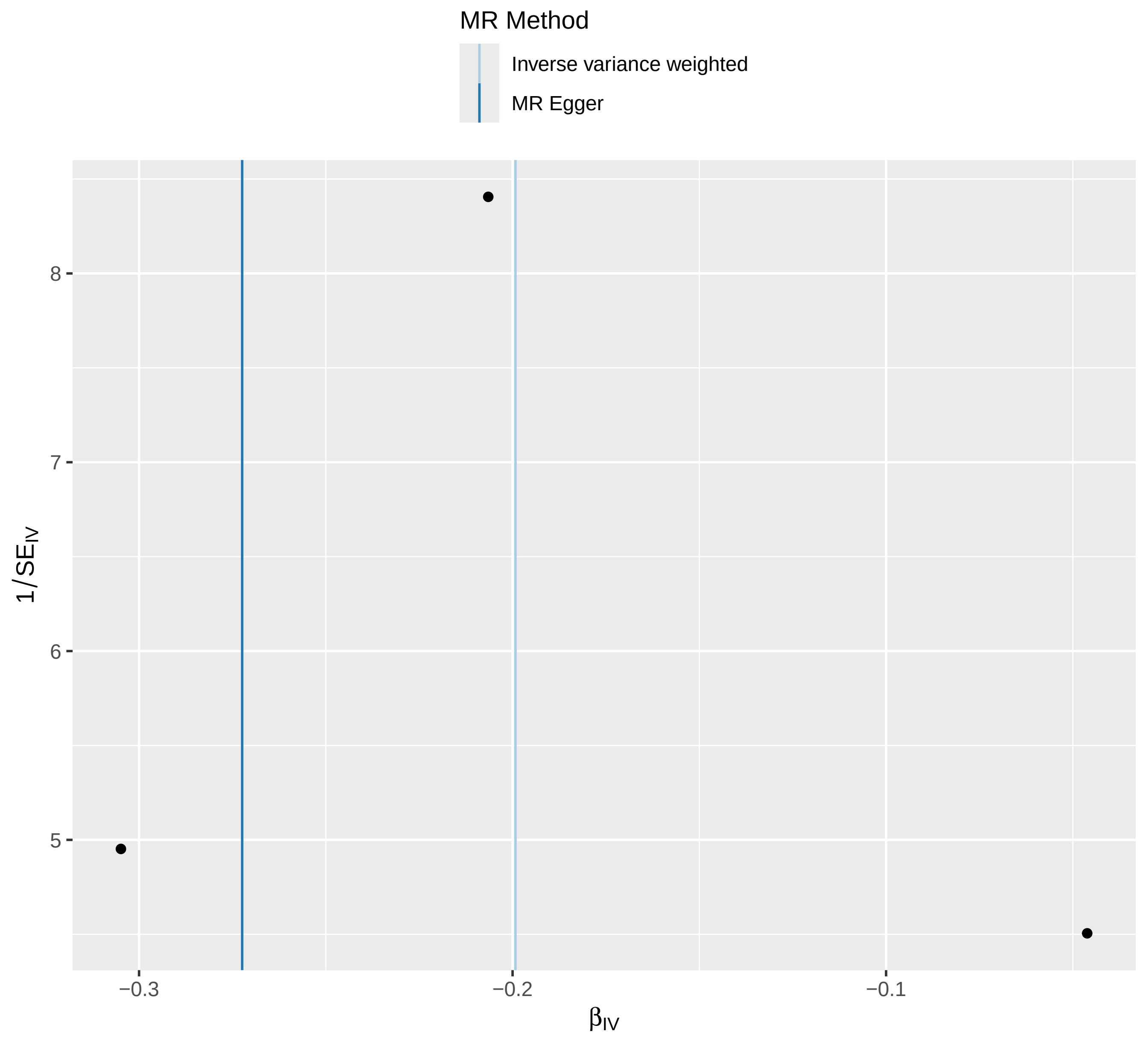
MR Method
Inverse variance weighted
MR Egger
8
7
6
5
1/SE_IV
−0.3
−0.2
−0.1
β_IV

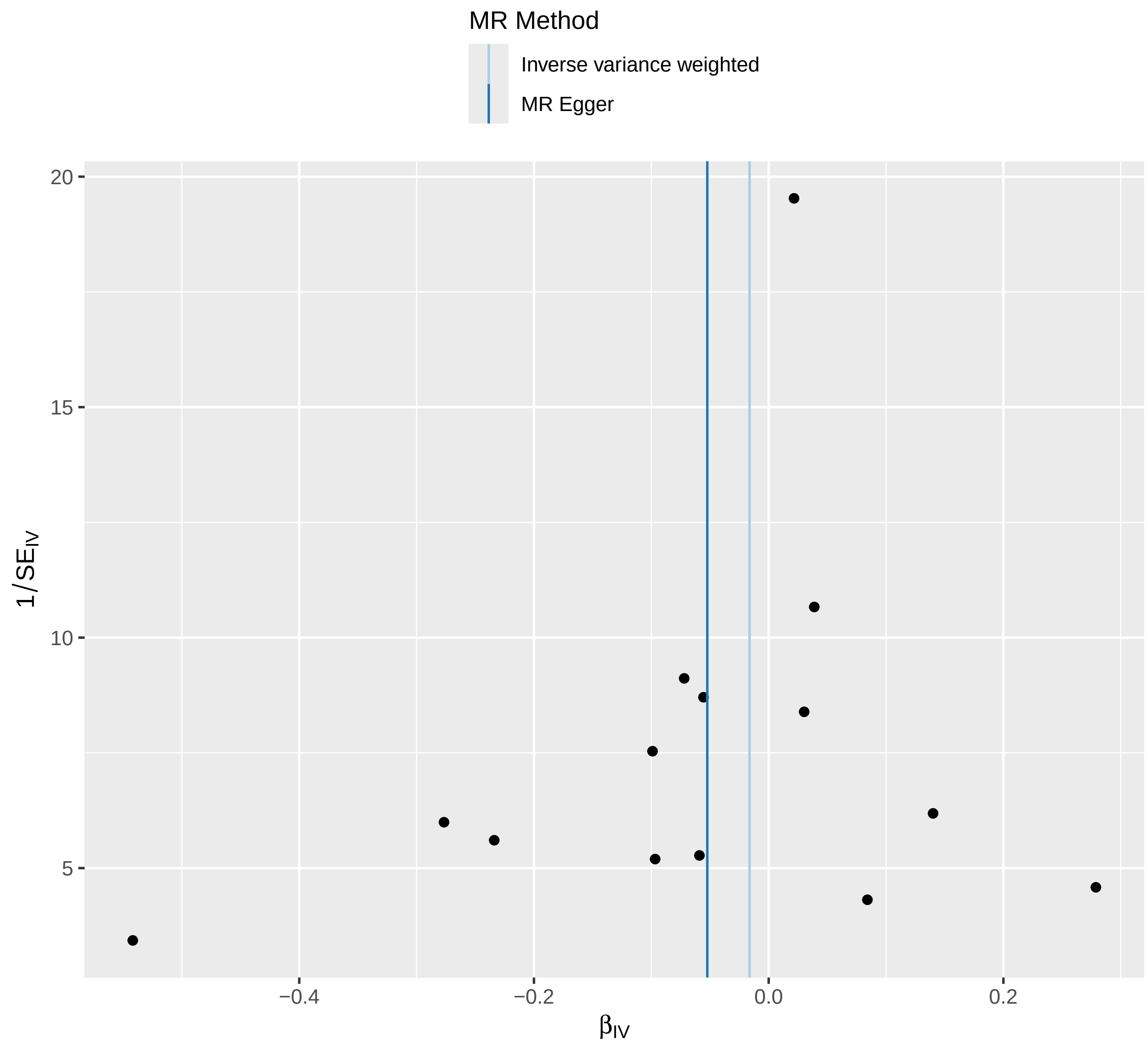

MR Method
Inverse variance weighted
MR Egger
20
15
10
5
$1/SE_{IV}$
−0.4
−0.2
0.0
0.2
$\beta_{IV}$

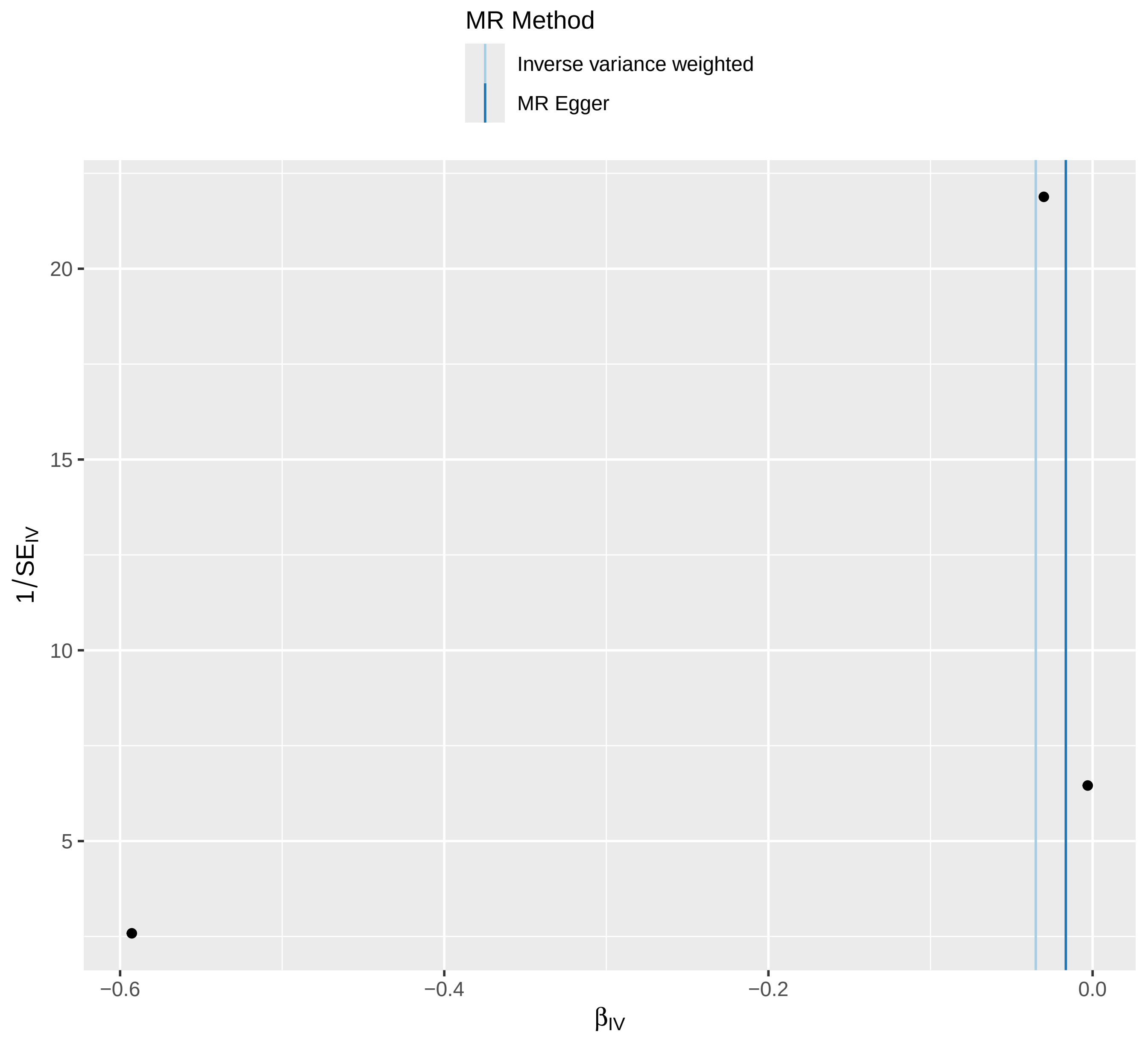

MR Method
Inverse variance weighted
MR Egger
20
15
10
5
1/$SE_{IV}$
−0.6
−0.4
−0.2
0.0
$\beta_{IV}$

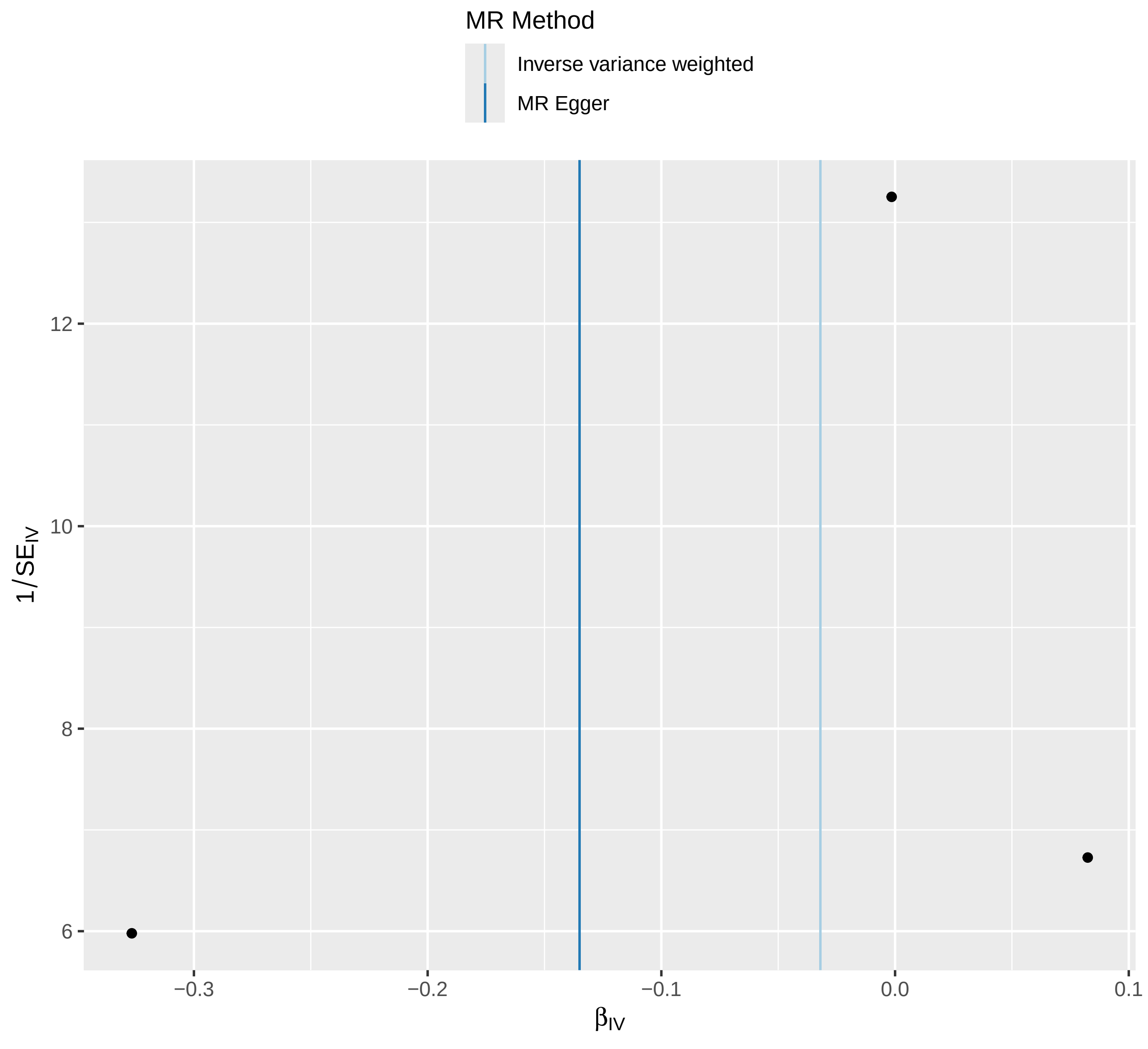

MR Method
Inverse variance weighted
MR Egger
12
10
8
6
$1/SE_{IV}$
−0.3
−0.2
−0.1
0.0
0.1
$\beta_{IV}$

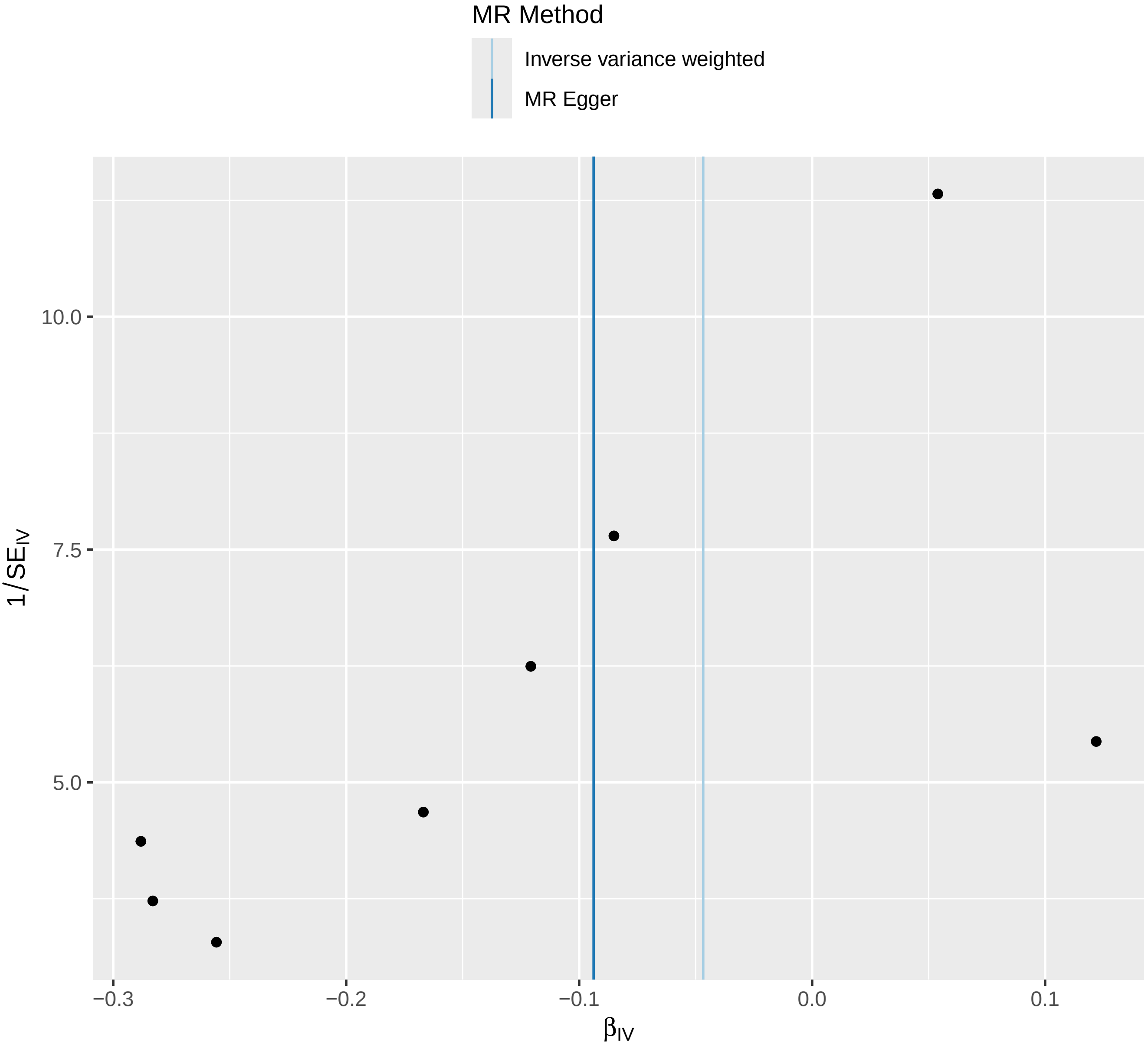

MR Method
Inverse variance weighted
MR Egger
10.0
7.5
5.0
1/SE$_{IV}$
−0.3
−0.2
−0.1
0.0
0.1
β$_{IV}$

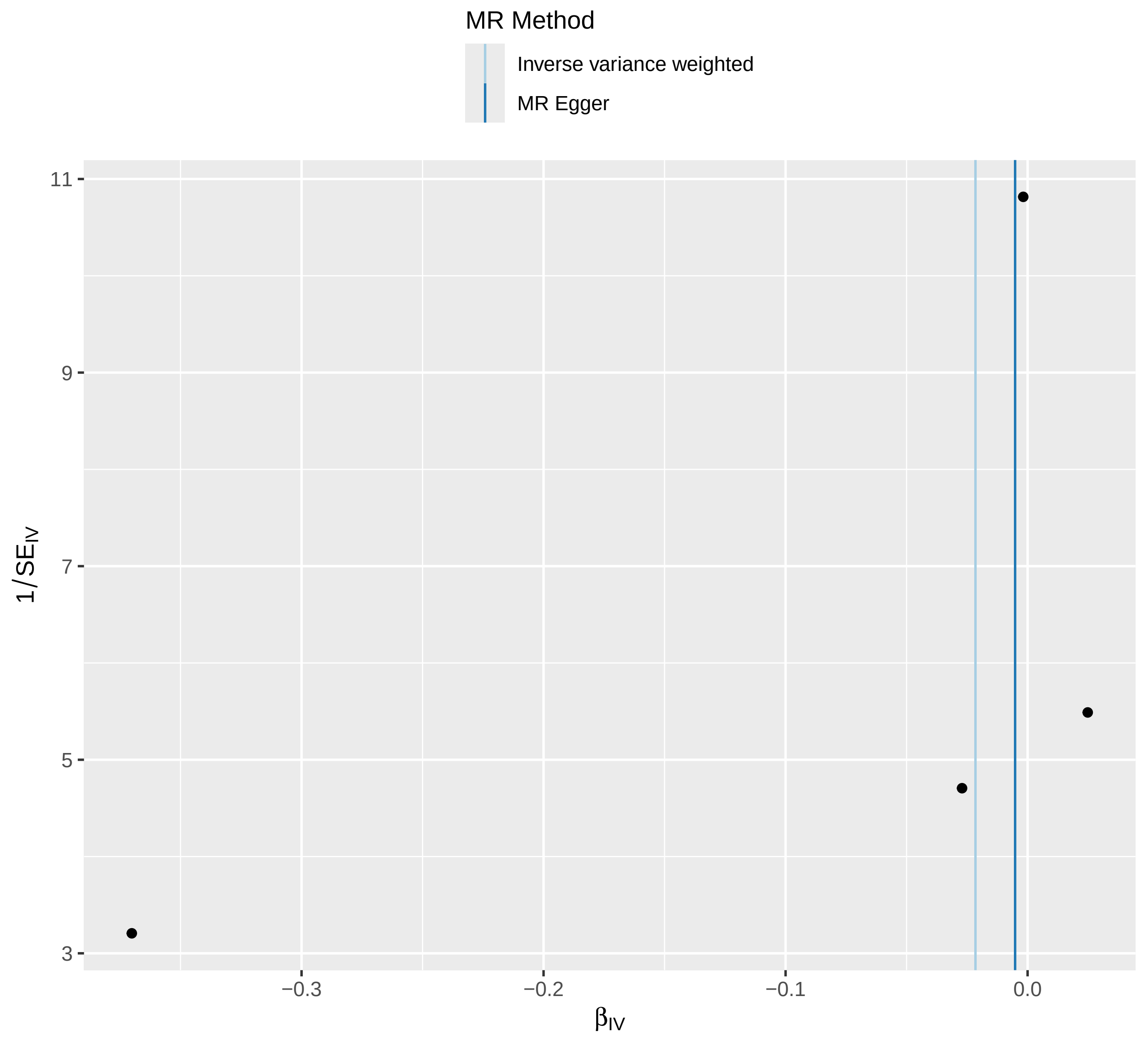
MR Method
Inverse variance weighted
MR Egger
11
9
7
5
3
1/SE_IV
−0.3
−0.2
−0.1
0.0
β_IV

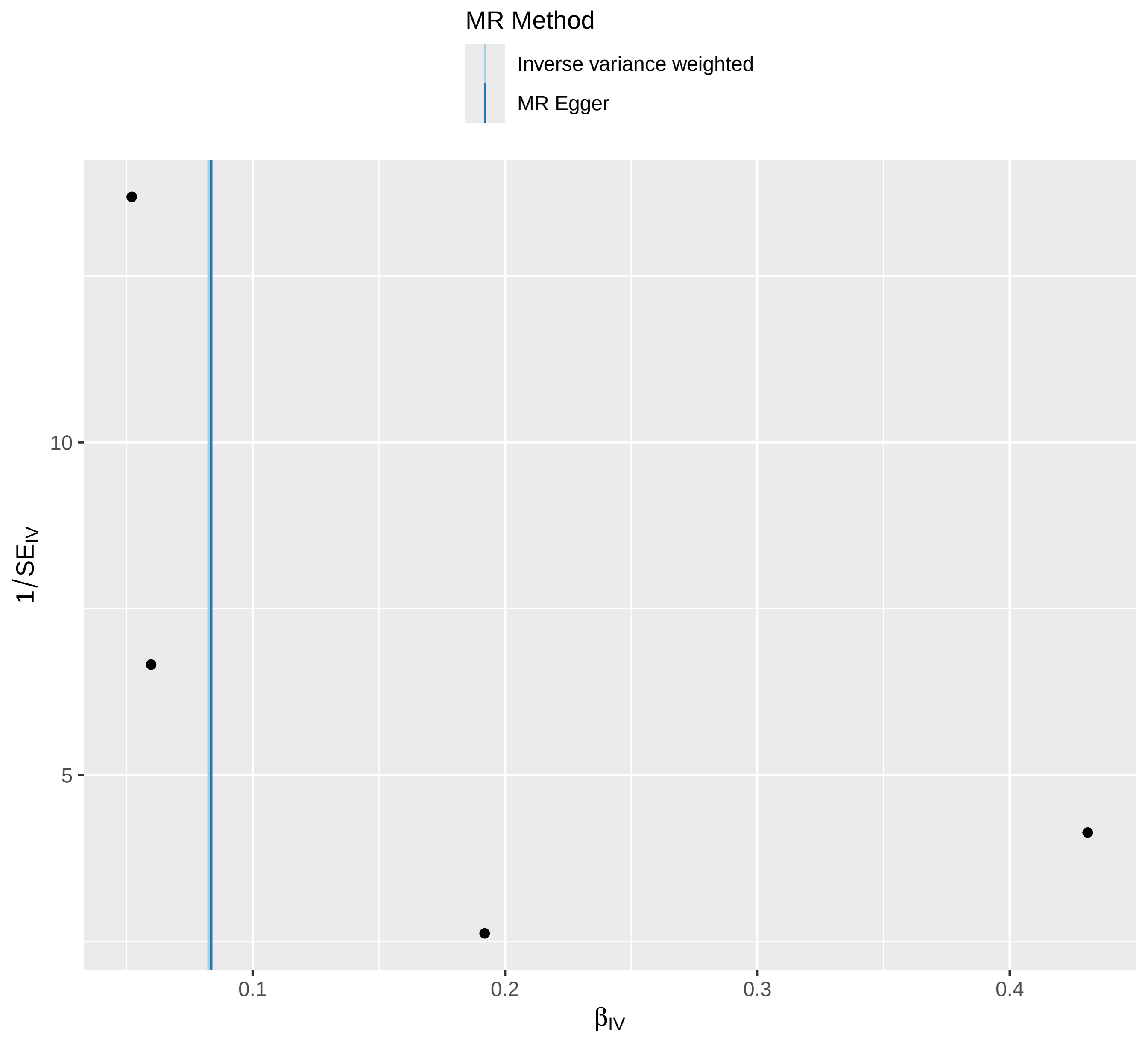

MR Method
Inverse variance weighted
MR Egger
10
5
$1/SE_{IV}$
0.1
0.2
0.3
0.4
$\beta_{IV}$

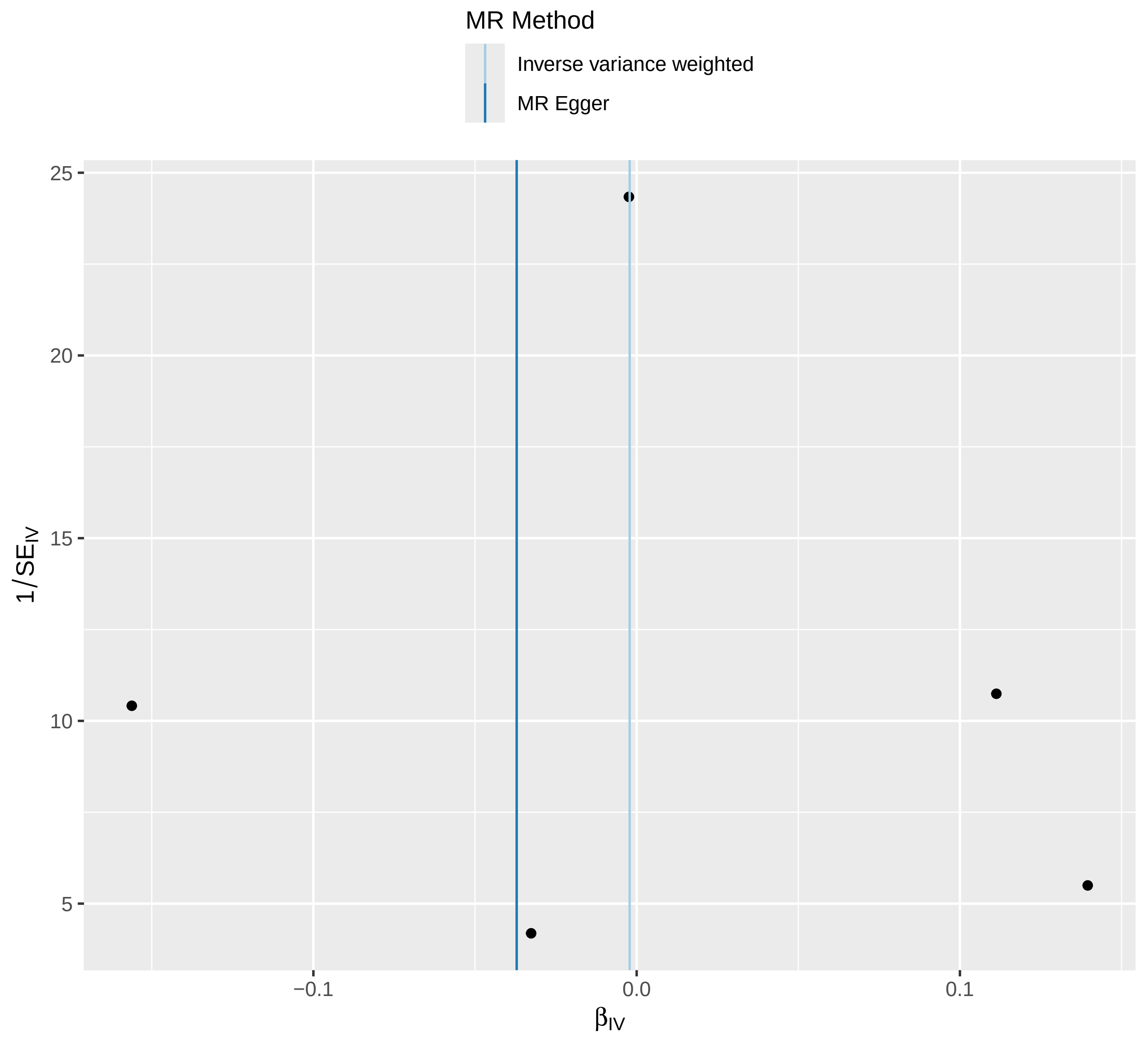

MR Method
Inverse variance weighted
MR Egger
25
20
15
10
5
1/SE_IV
−0.1
0.0
0.1
β_IV

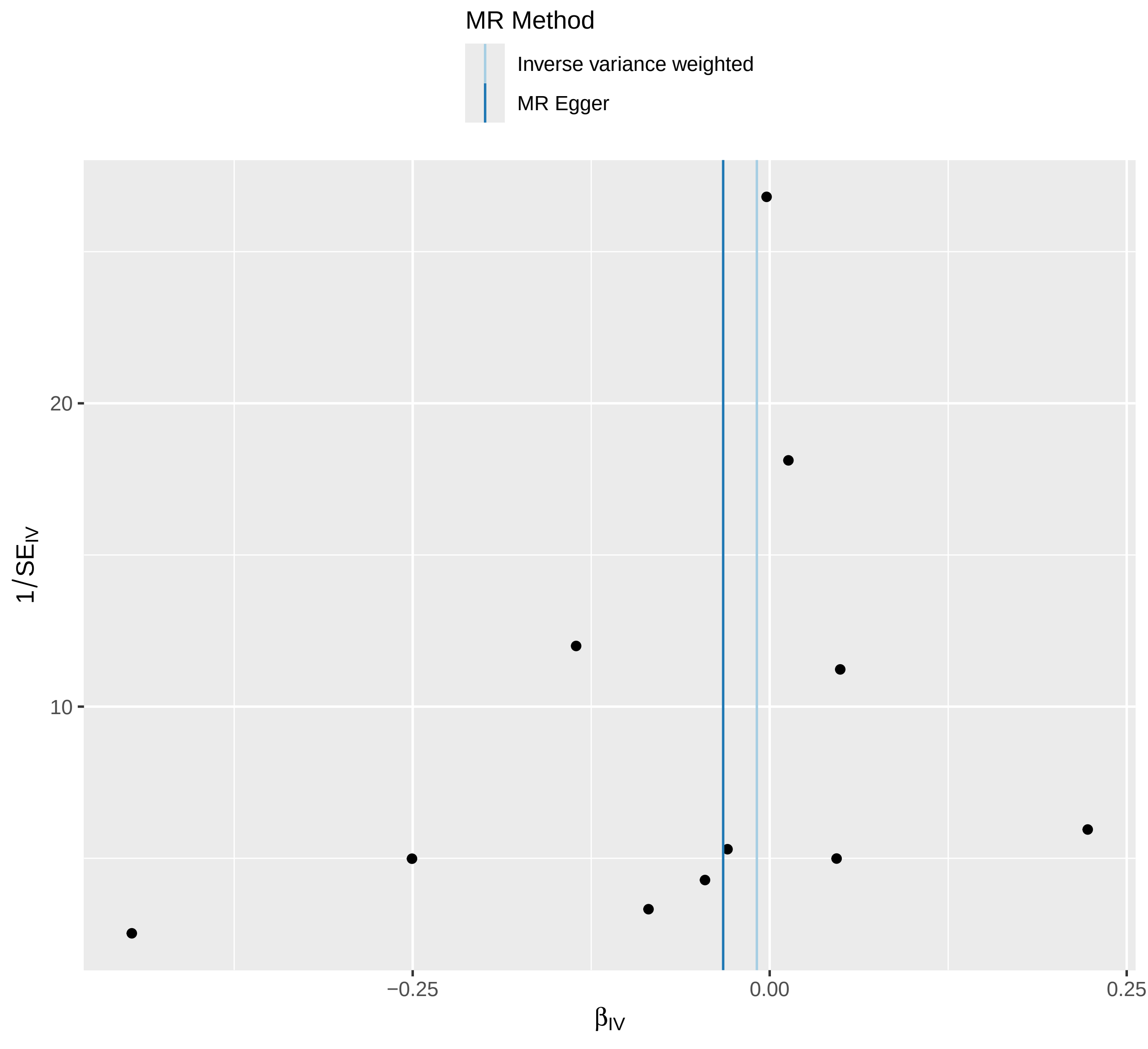

MR Method
Inverse variance weighted
MR Egger
20
10
1/SE$_{IV}$
−0.25
0.00
0.25
β$_{IV}$

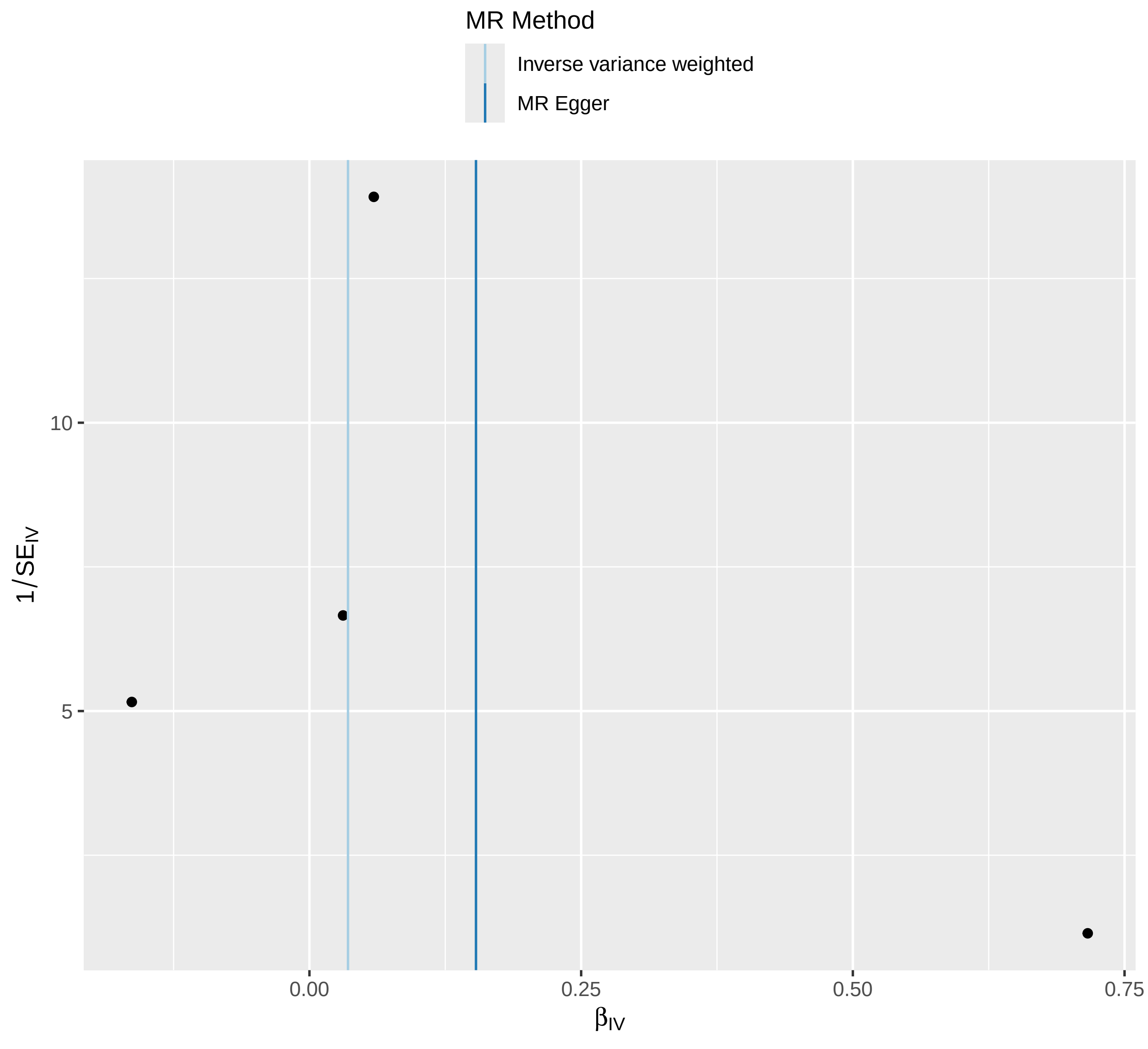

MR Method
Inverse variance weighted
MR Egger
10
5
0.00
0.25
0.50
0.75
1/SE_IV
β_IV

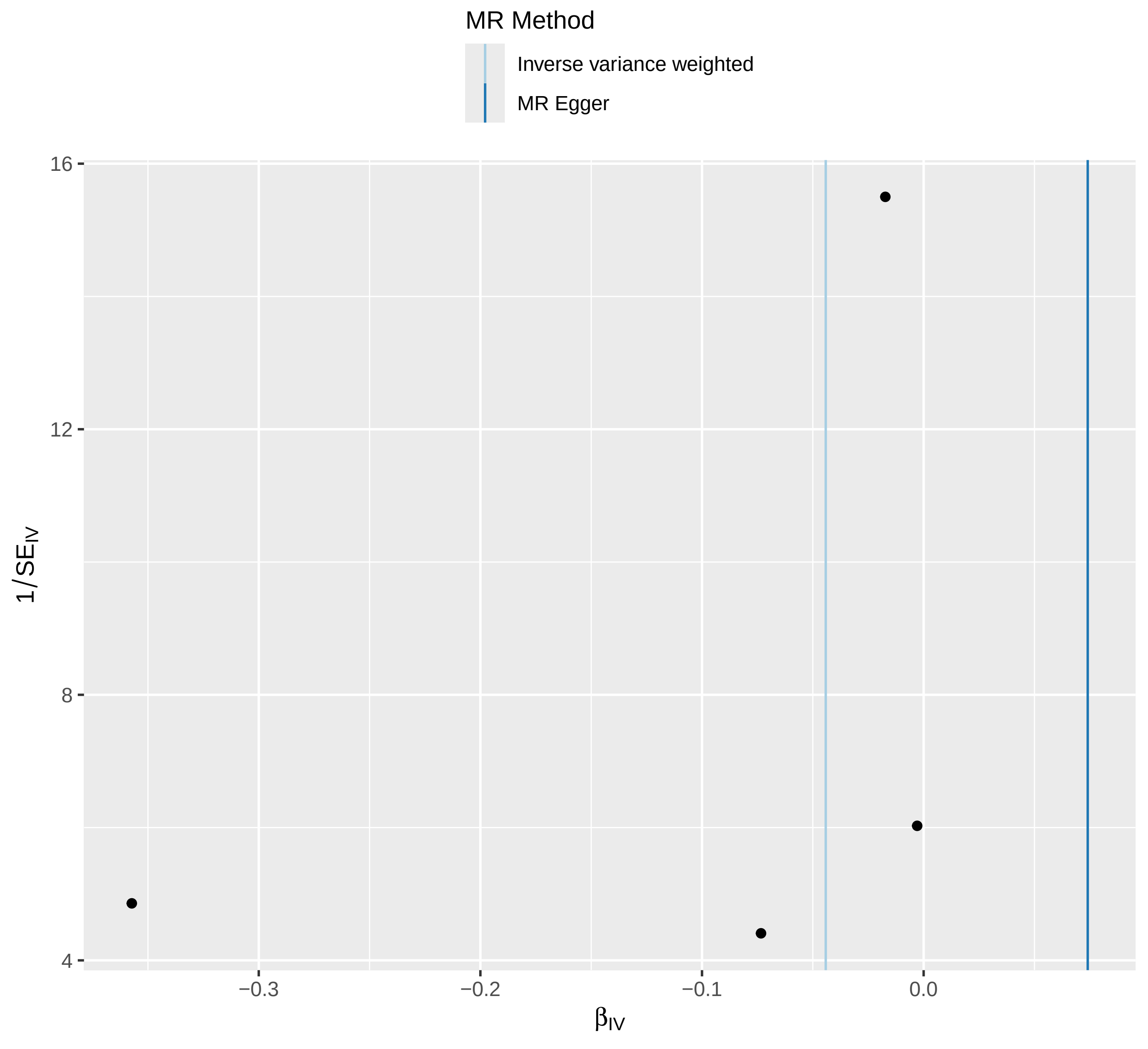

MR Method
Inverse variance weighted
MR Egger
16
12
8
4
1/SE_IV
−0.3
−0.2
−0.1
0.0
β_IV

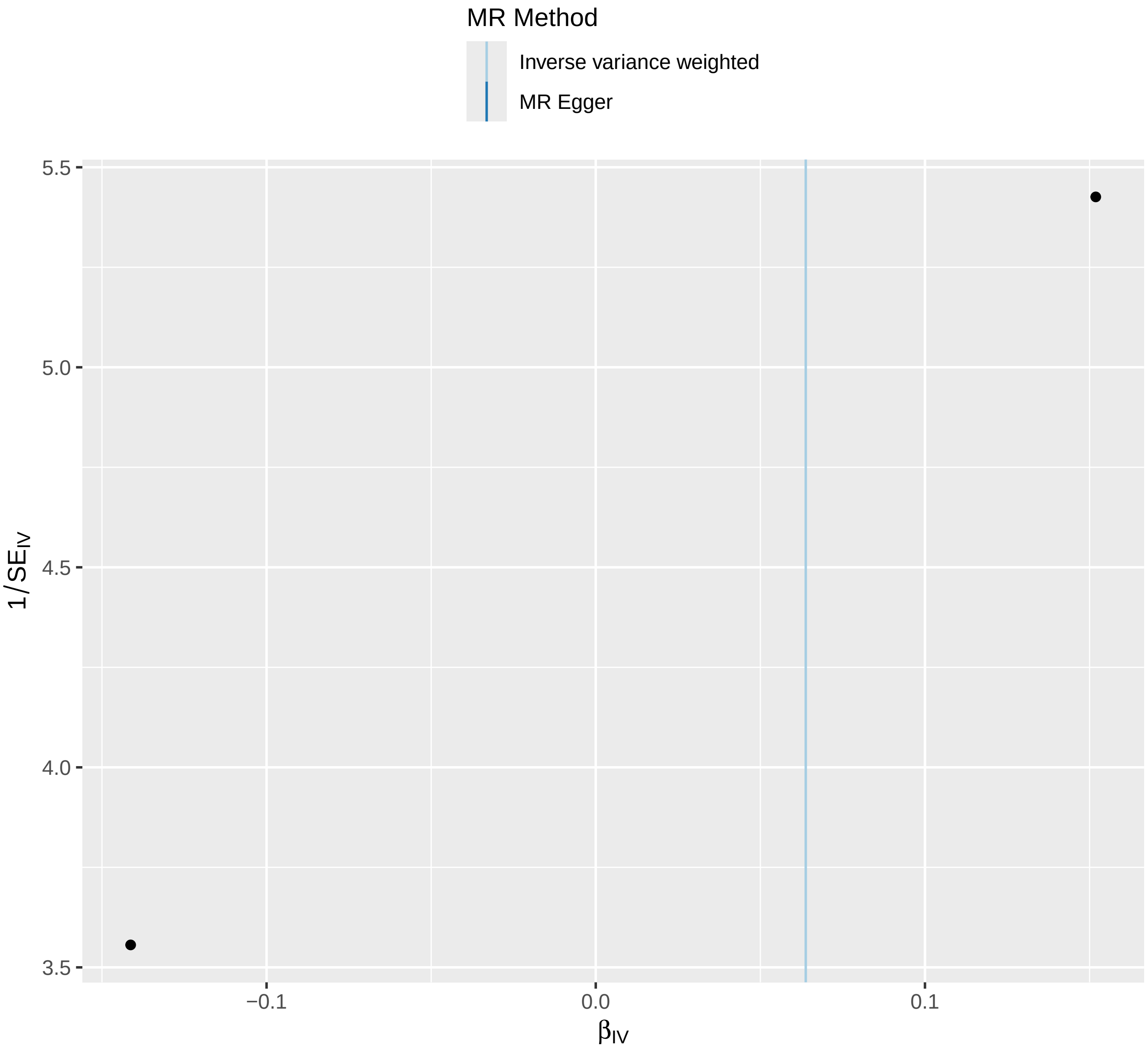

MR Method
Inverse variance weighted
MR Egger
5.5
5.0
4.5
4.0
3.5
1/SE$_{IV}$
−0.1
0.0
0.1
β$_{IV}$

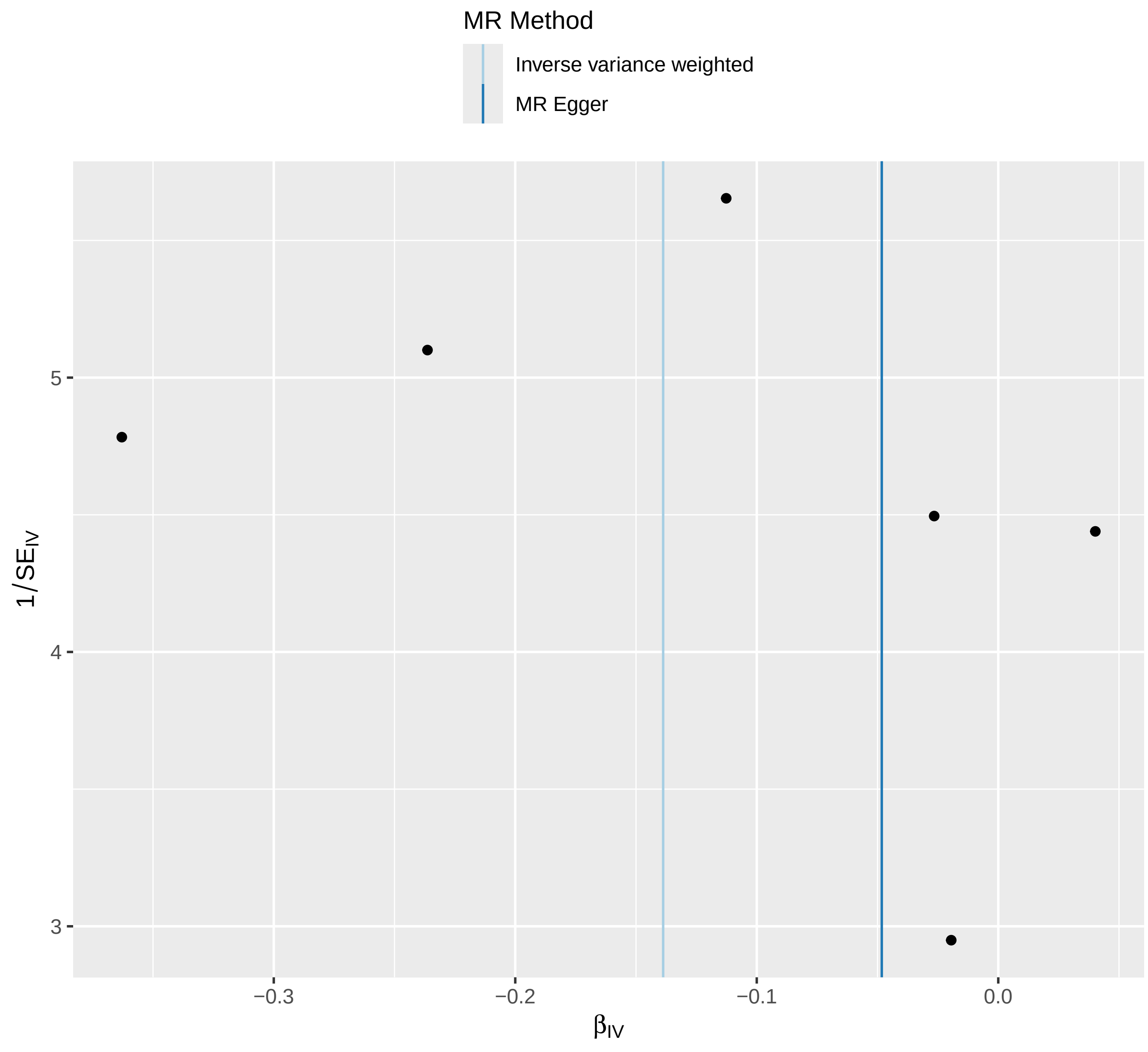

MR Method
Inverse variance weighted
MR Egger
5
4
3
1/SE$_{IV}$
−0.3
−0.2
−0.1
0.0
β$_{IV}$

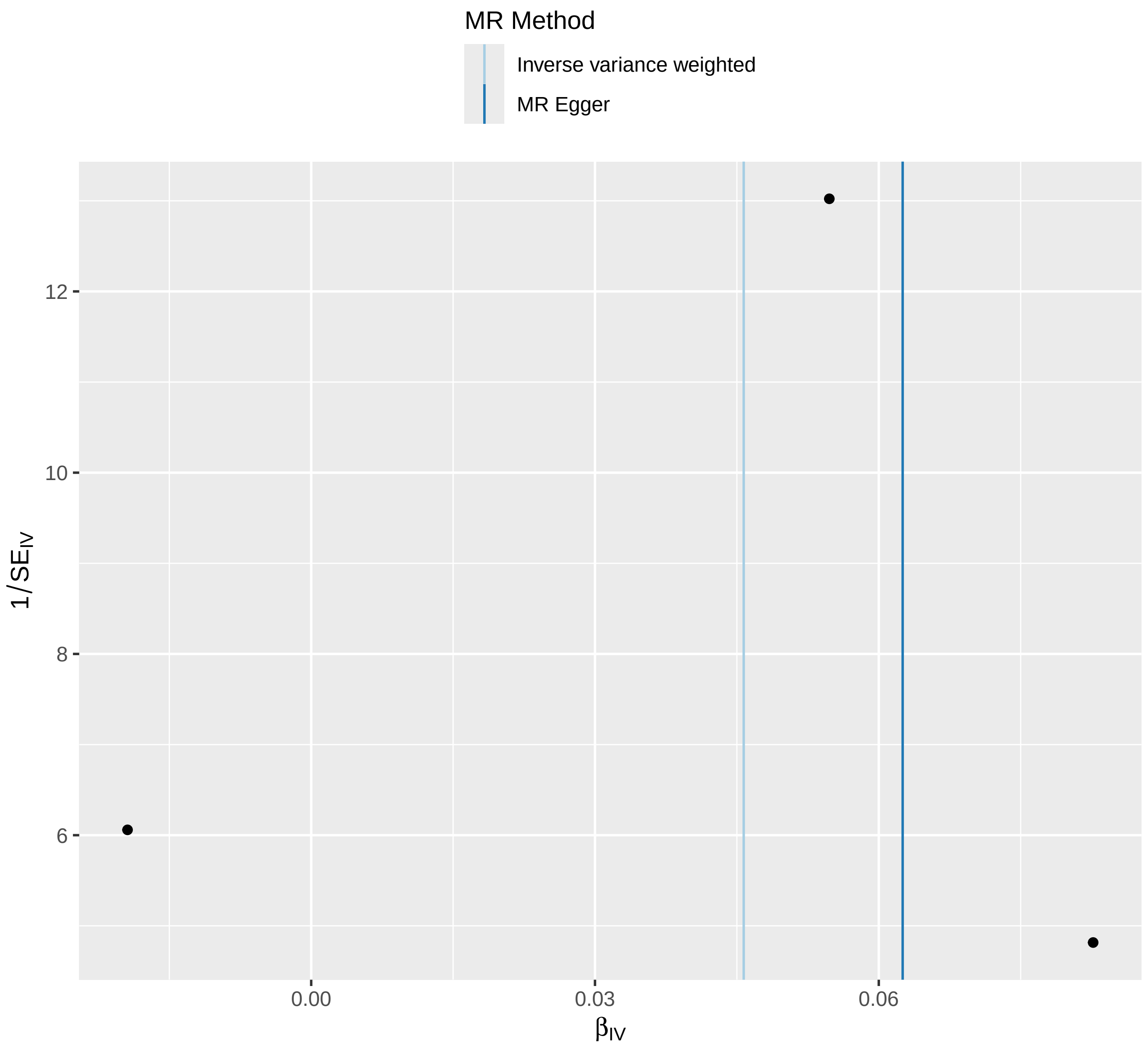

MR Method
Inverse variance weighted
MR Egger
12
10
8
6
0.00
0.03
0.06
$1/SE_{IV}$
$\beta_{IV}$

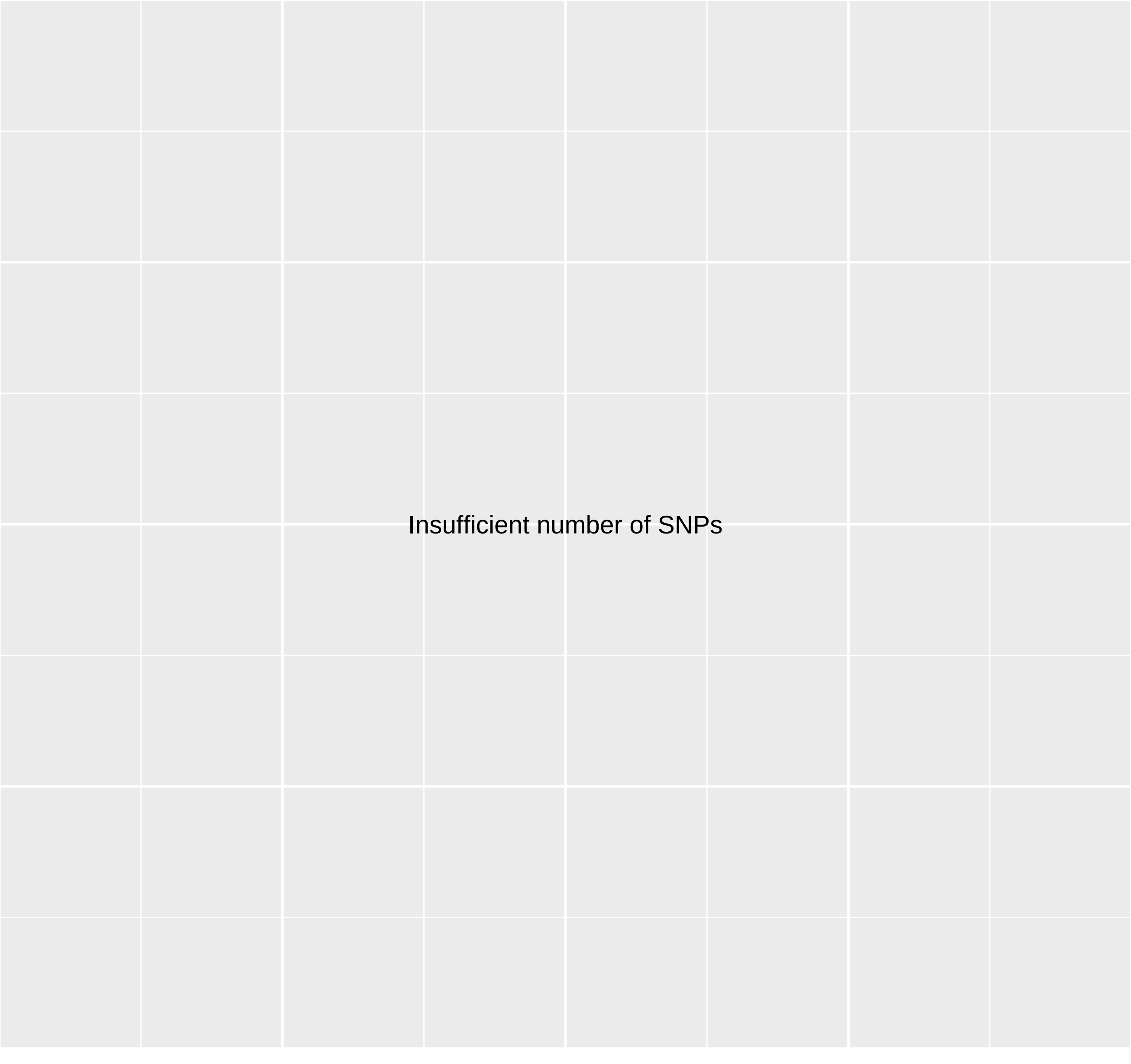
Insufficient number of SNPs

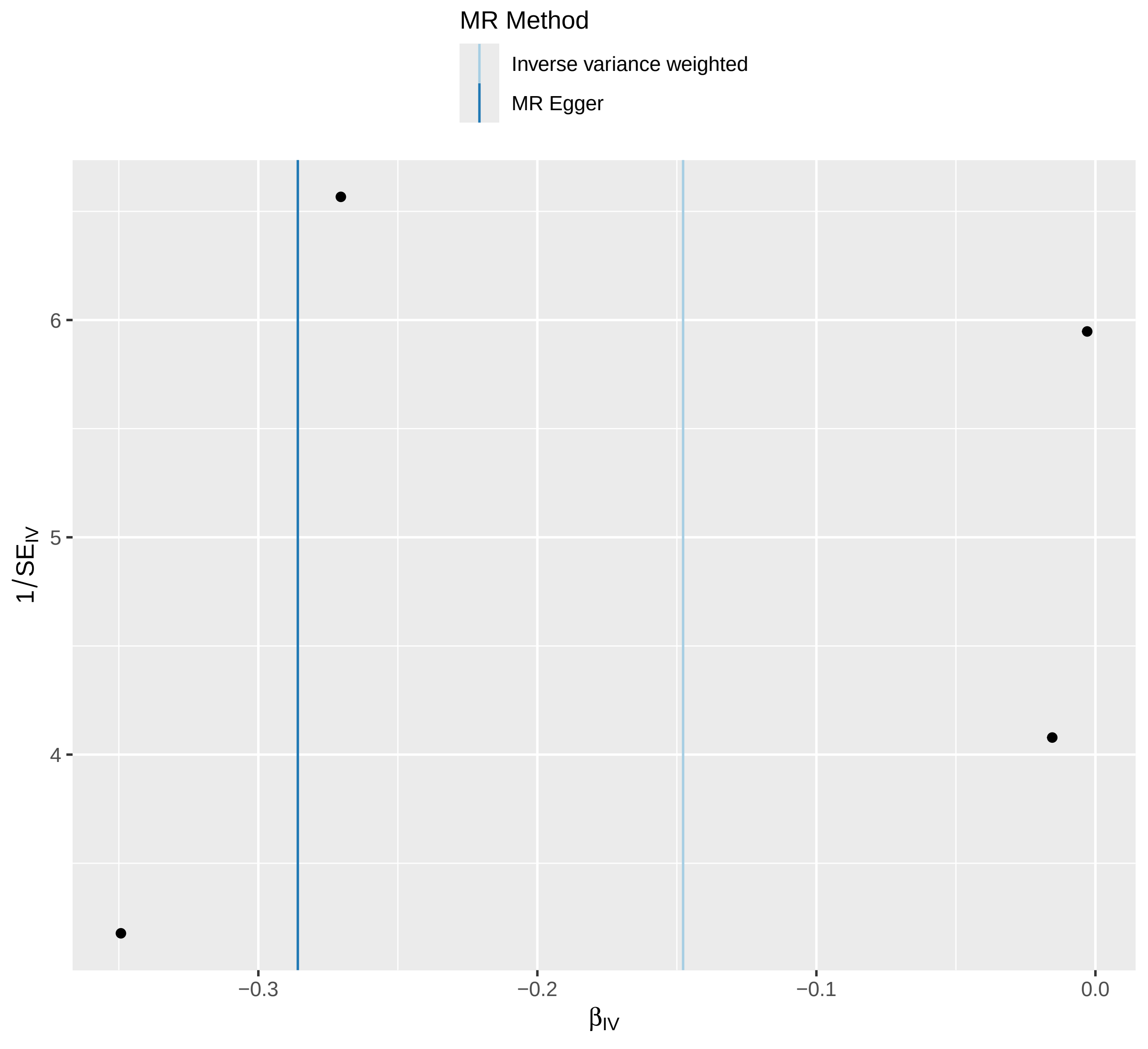
MR Method
Inverse variance weighted
MR Egger
6
5
4
1/SE_IV
−0.3
−0.2
−0.1
0.0
β_IV

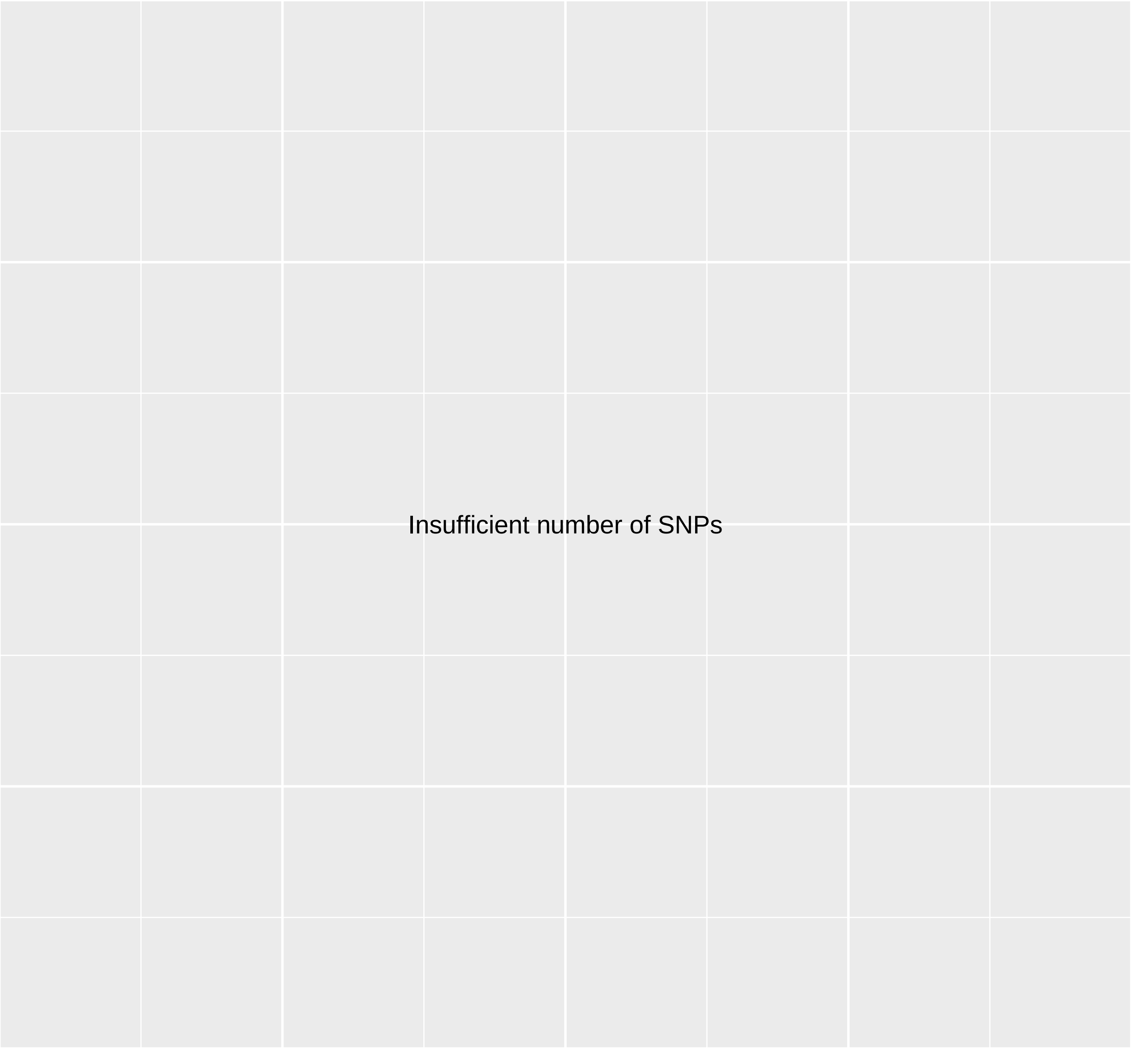
Insufficient number of SNPs

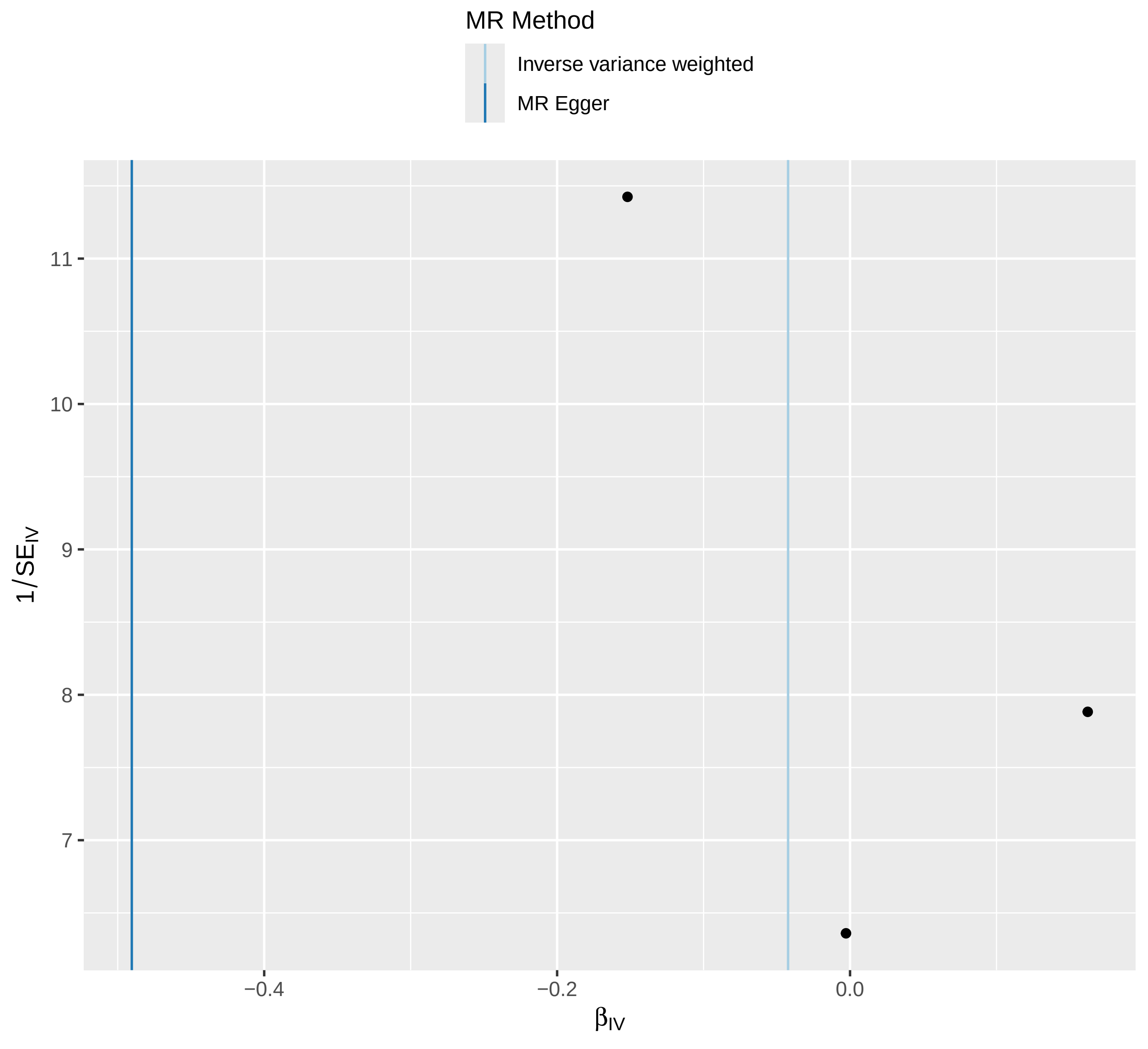
MR Method
Inverse variance weighted
MR Egger
11
10
9
8
7
$1/SE_{IV}$
−0.4
−0.2
0.0
$\beta_{IV}$

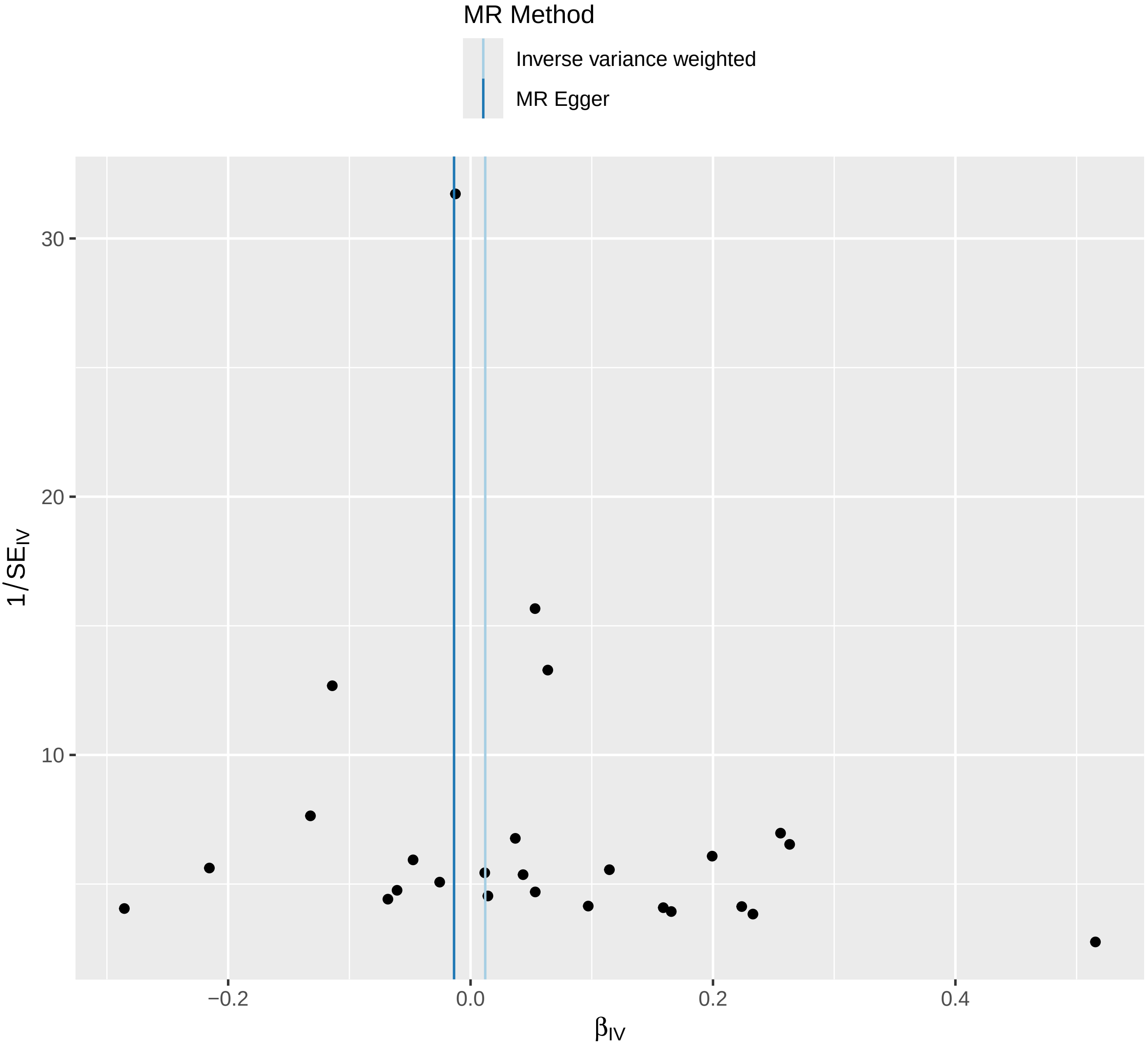
MR Method
Inverse variance weighted
MR Egger
30
20
10
1/SE$_{IV}$
−0.2
0.0
0.2
0.4
β$_{IV}$

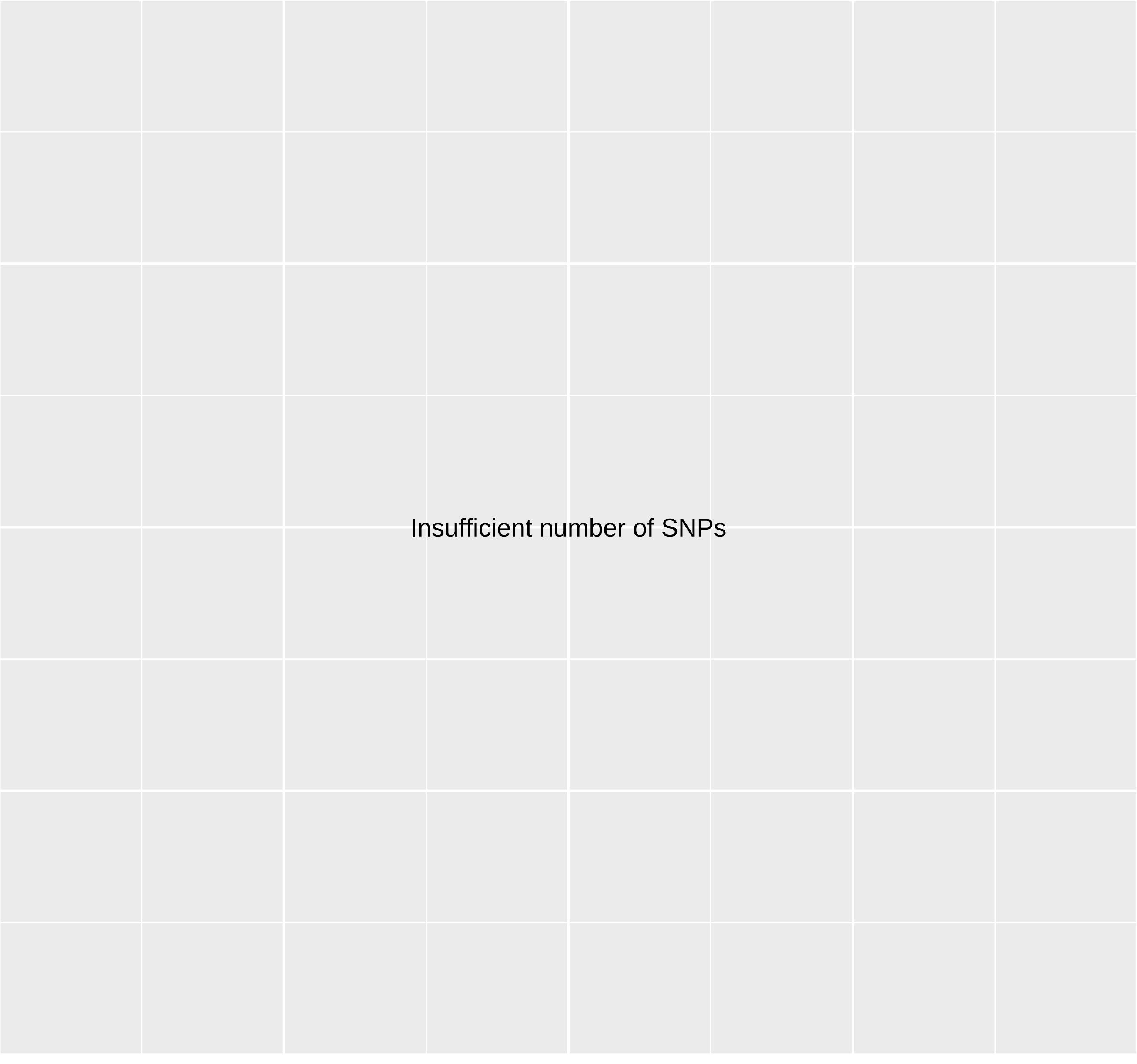
Insufficient number of SNPs

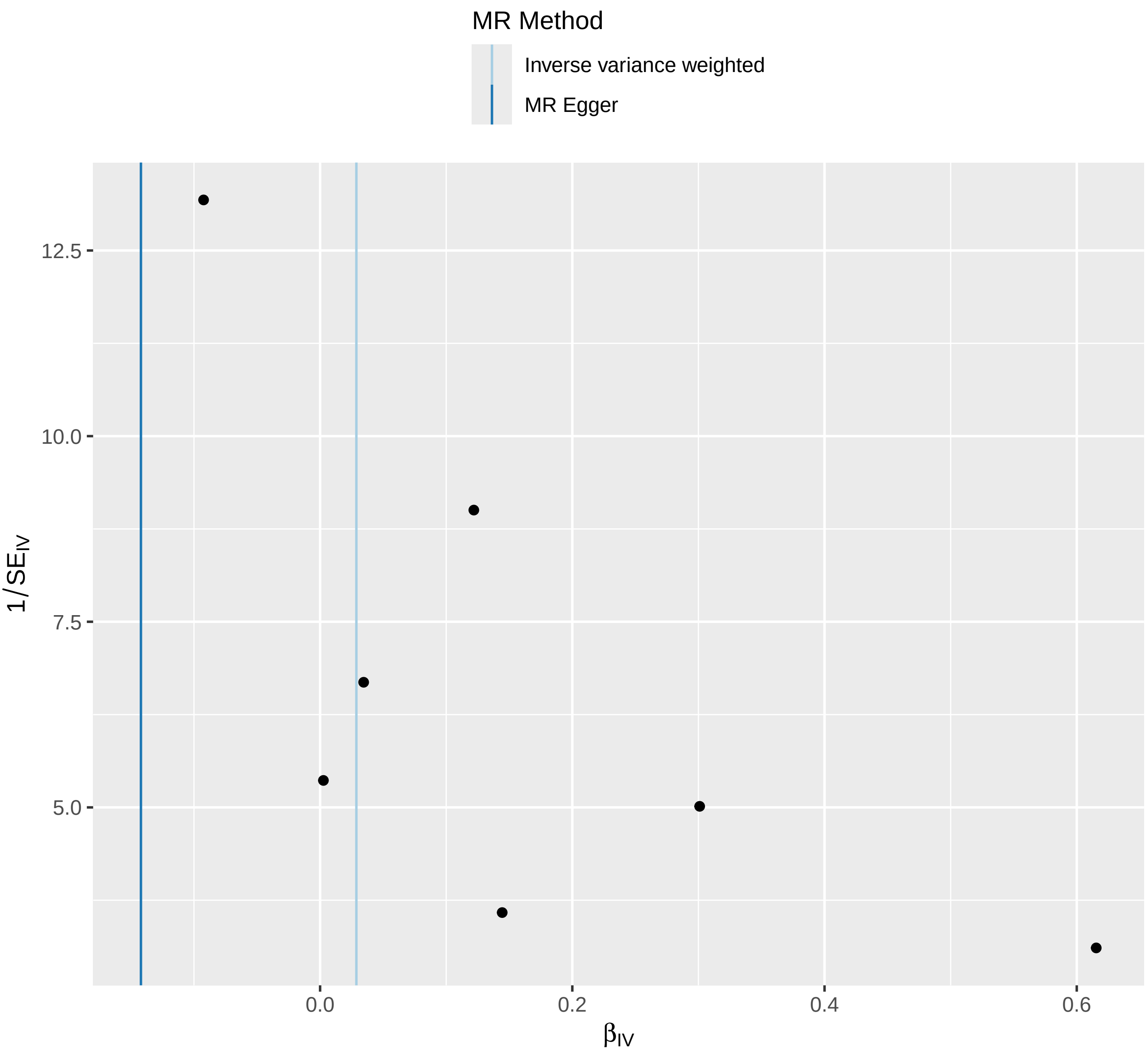

MR Method
Inverse variance weighted
MR Egger
12.5
10.0
7.5
5.0
0.0
0.2
0.4
0.6
1/SE_IV
β_IV

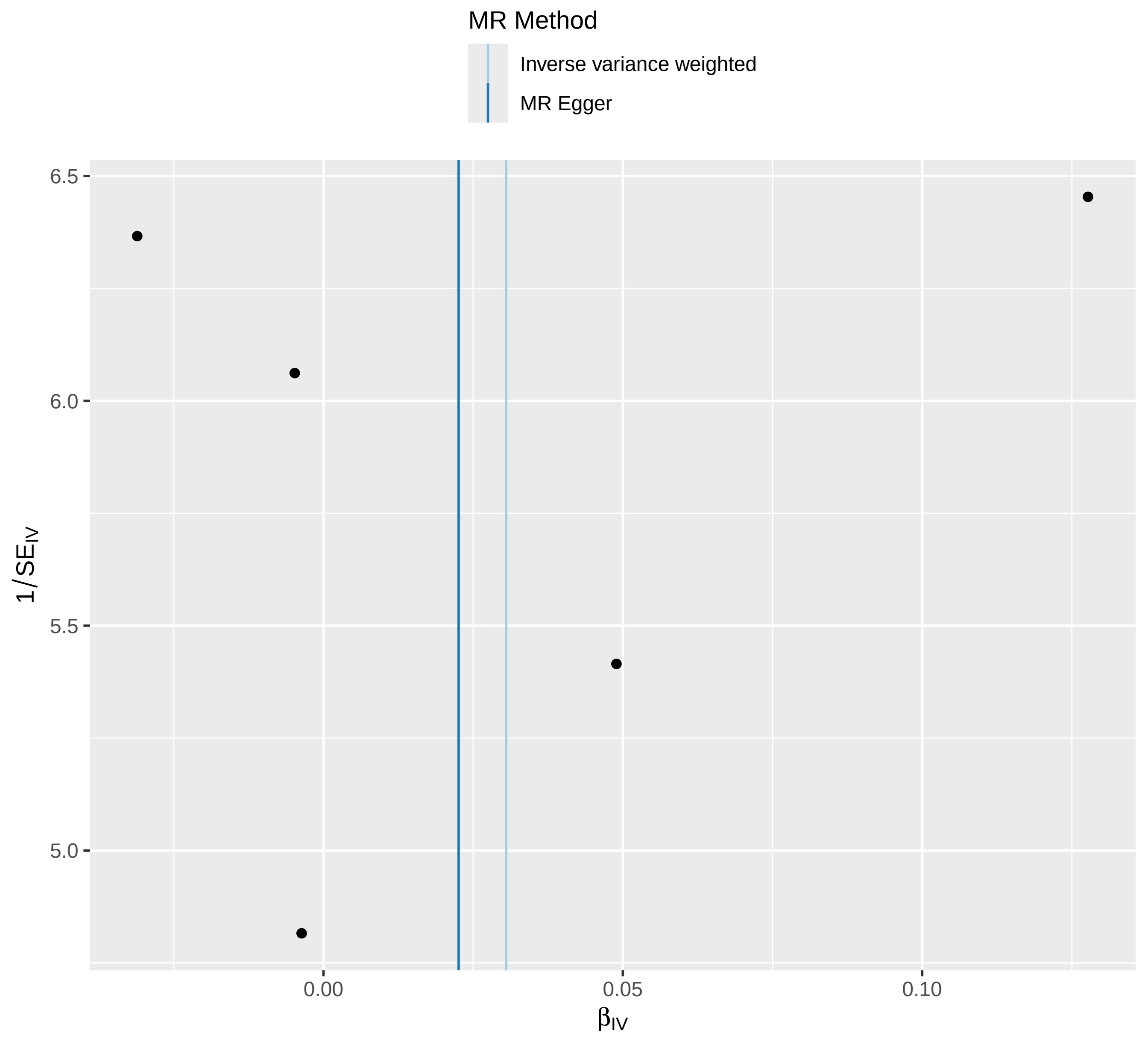

MR Method
Inverse variance weighted
MR Egger
6.5
6.0
5.5
5.0
1/SE_IV
0.00
0.05
0.10
β_IV

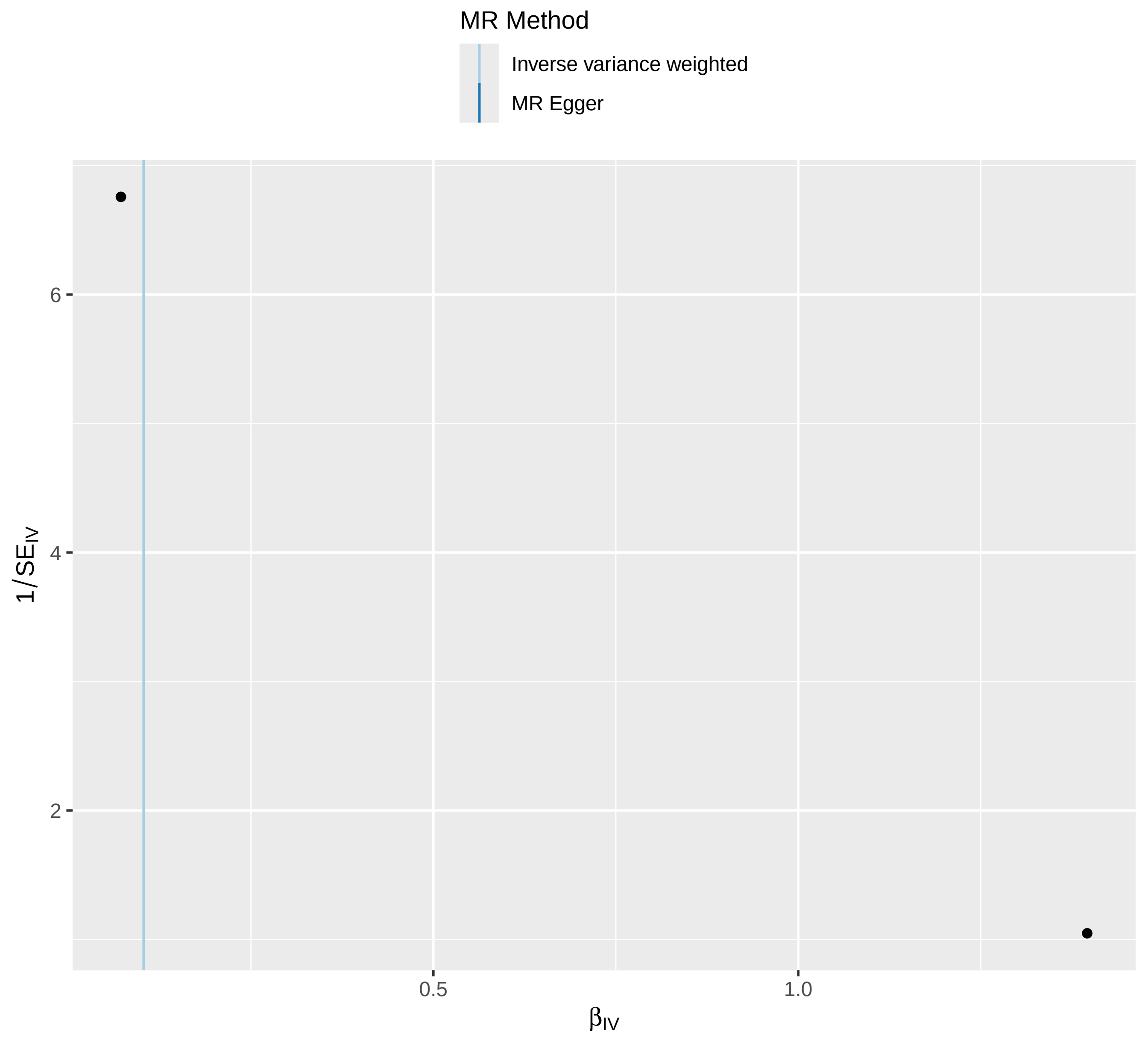

MR Method
Inverse variance weighted
MR Egger
6
4
2
0.5
1.0
$\beta_{IV}$
$1/SE_{IV}$

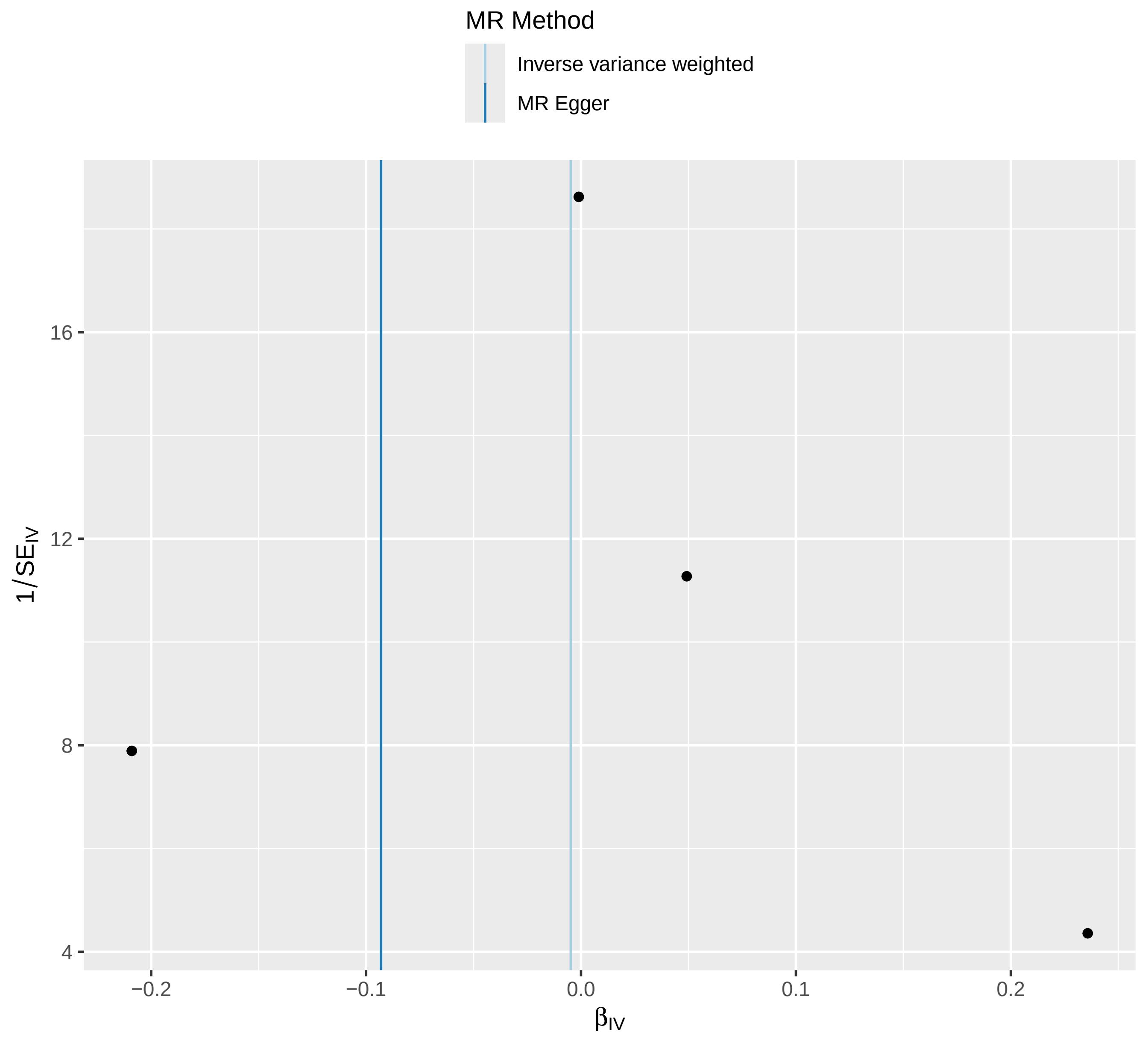
MR Method
Inverse variance weighted
MR Egger
16
12
8
4
1/SE_IV
−0.2
−0.1
0.0
0.1
0.2
β_IV

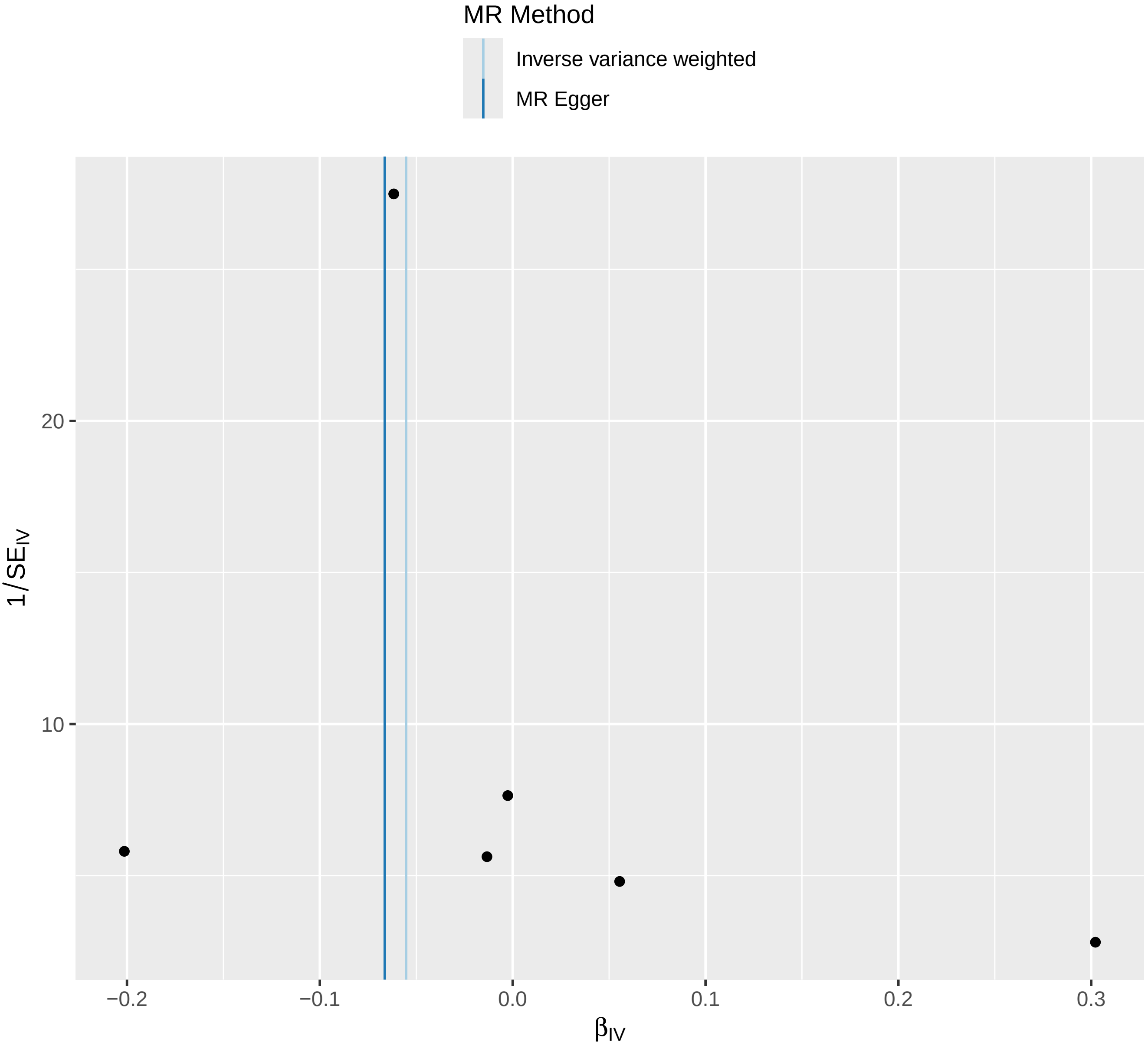
MR Method
Inverse variance weighted
MR Egger
20
10
−0.2
−0.1
0.0
0.1
0.2
0.3
$1/SE_{IV}$
$\beta_{IV}$

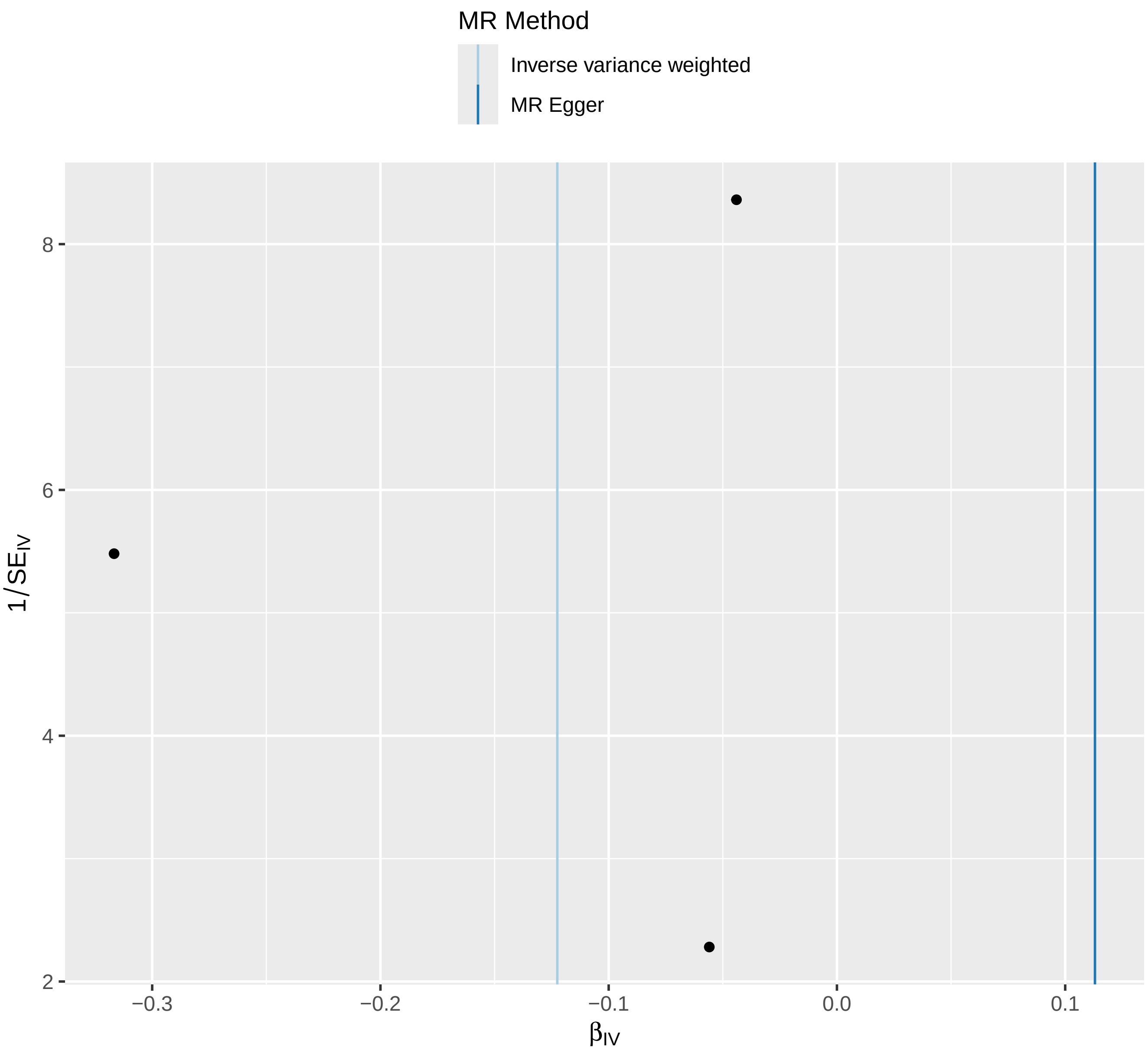
MR Method
Inverse variance weighted
MR Egger
8
6
4
2
−0.3
−0.2
−0.1
0.0
0.1
$1/SE_{IV}$
$\beta_{IV}$

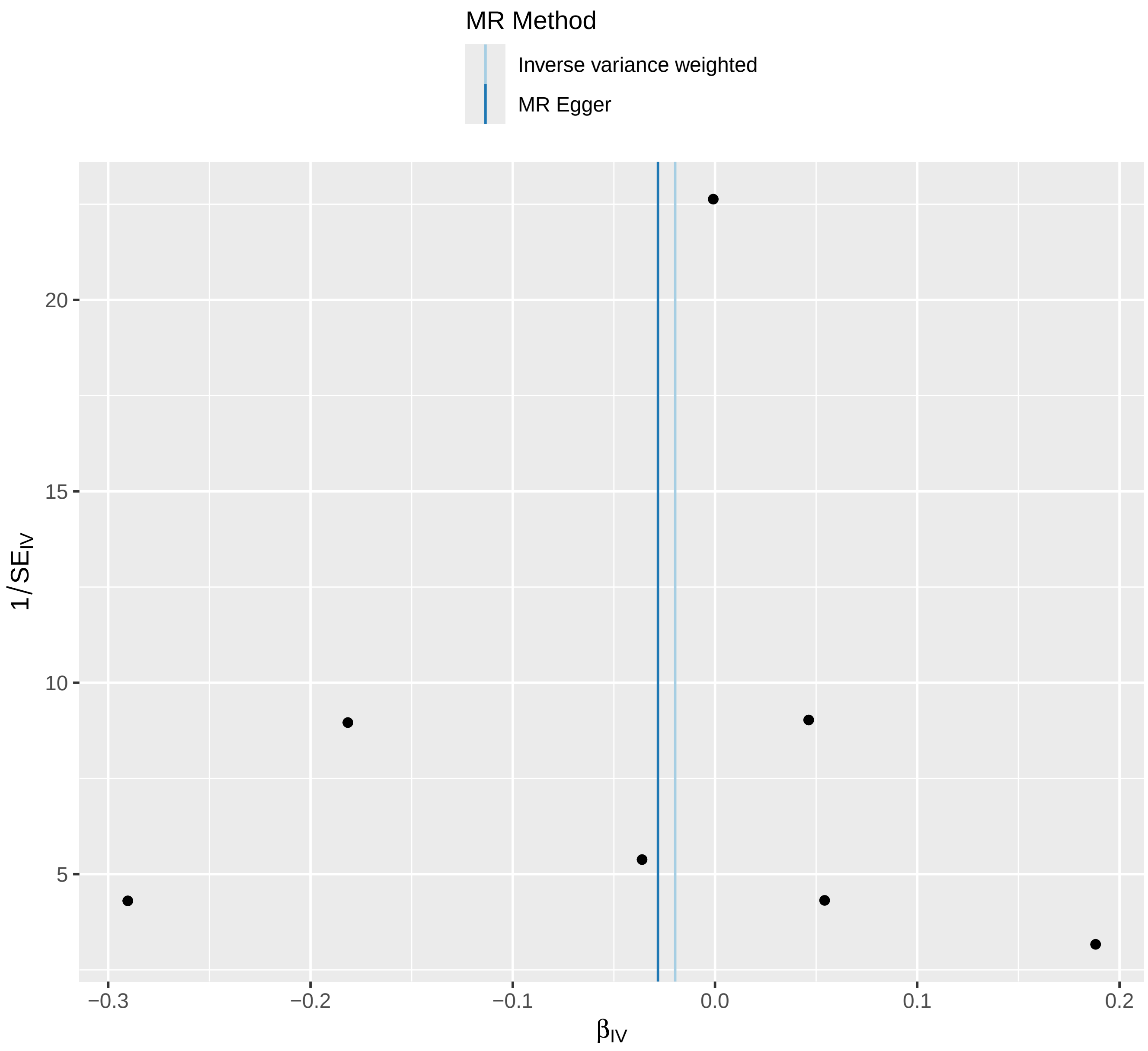
MR Method
Inverse variance weighted
MR Egger
20
15
10
5
1/SE_IV
−0.3
−0.2
−0.1
0.0
0.1
0.2
β_IV

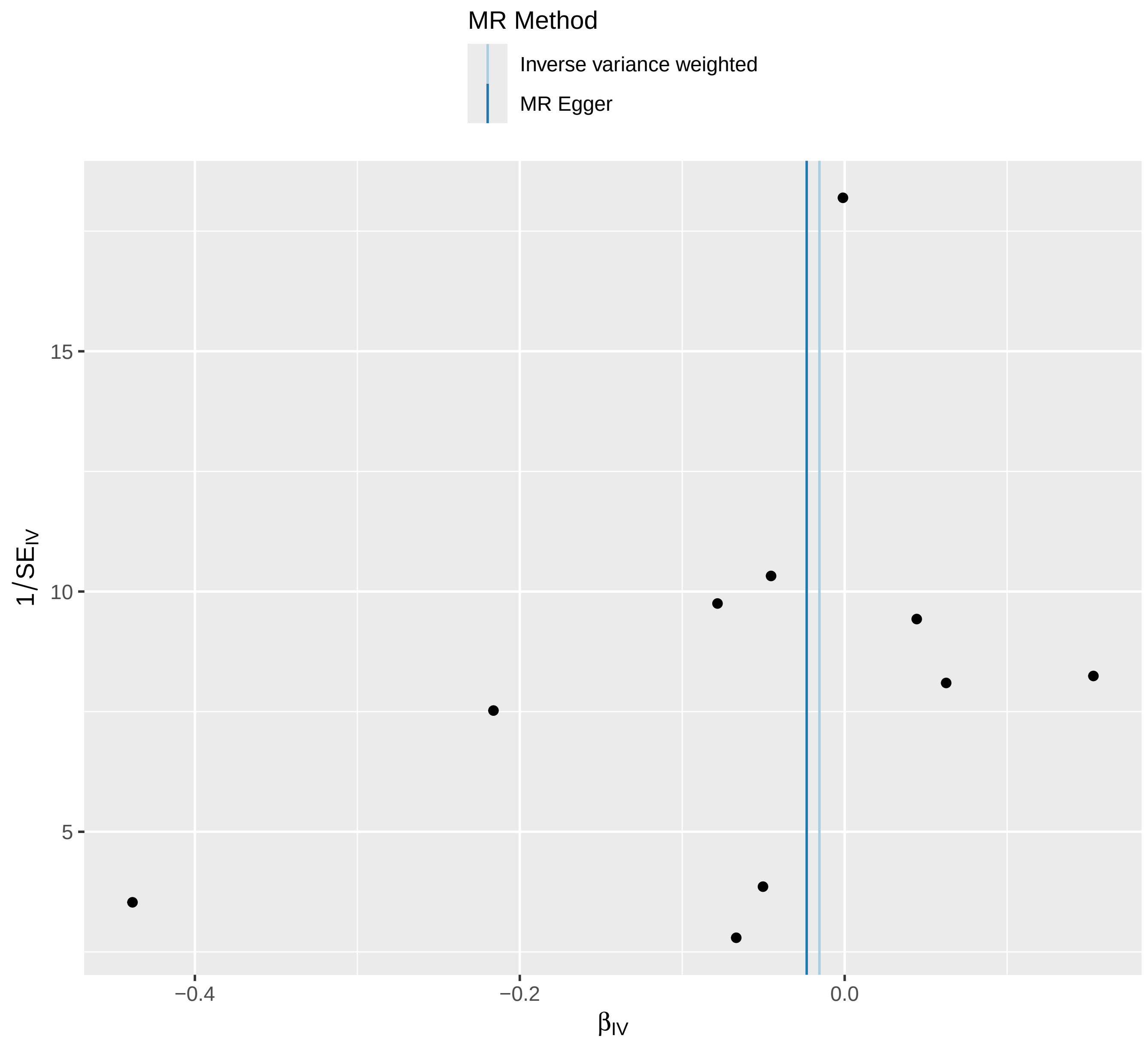
MR Method
Inverse variance weighted
MR Egger
15
10
5
1/SE$_{IV}$
−0.4
−0.2
0.0
β$_{IV}$

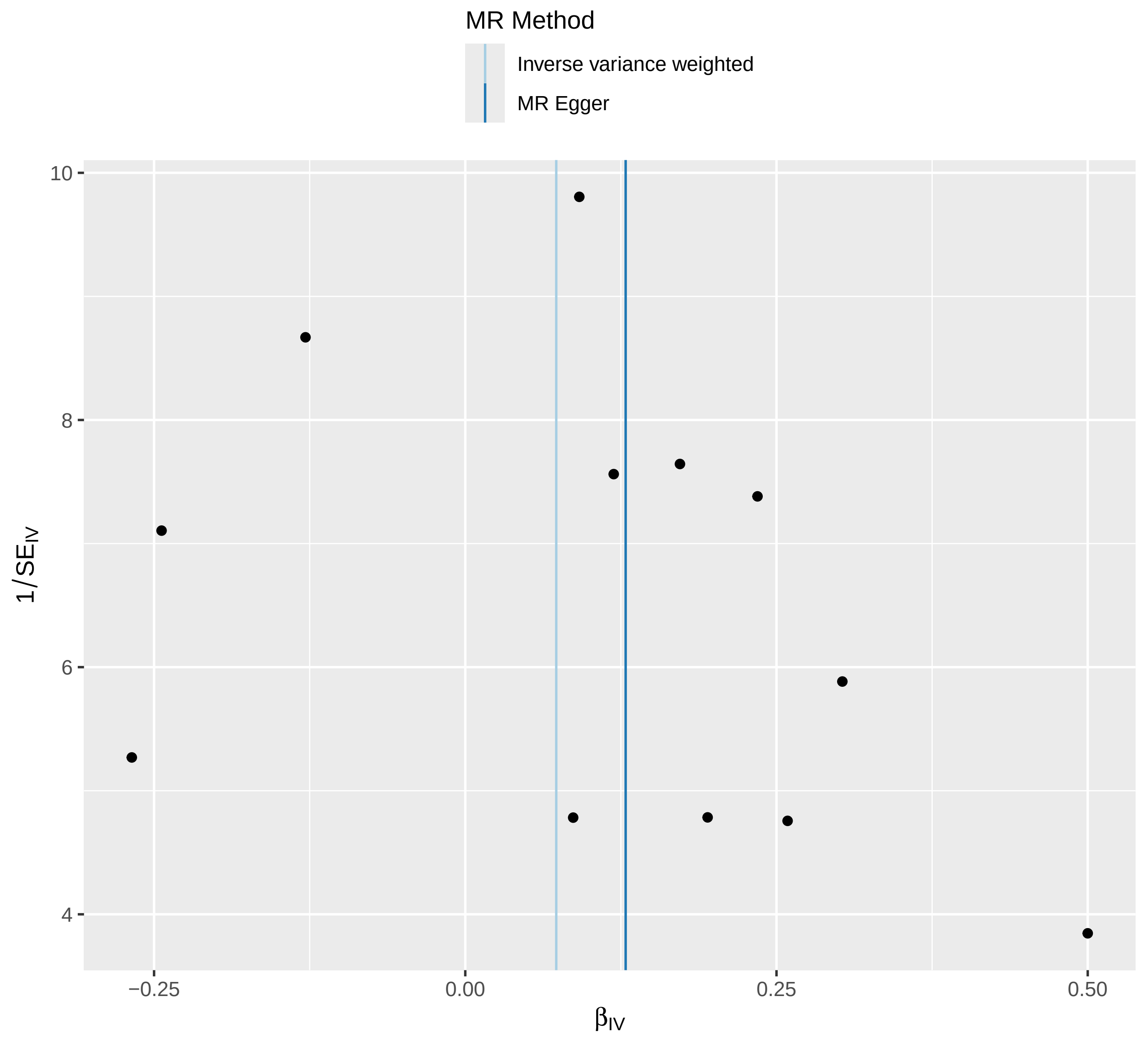
MR Method
Inverse variance weighted
MR Egger
10
8
6
4
1/SE$_{IV}$
−0.25
0.00
0.25
0.50
β$_{IV}$

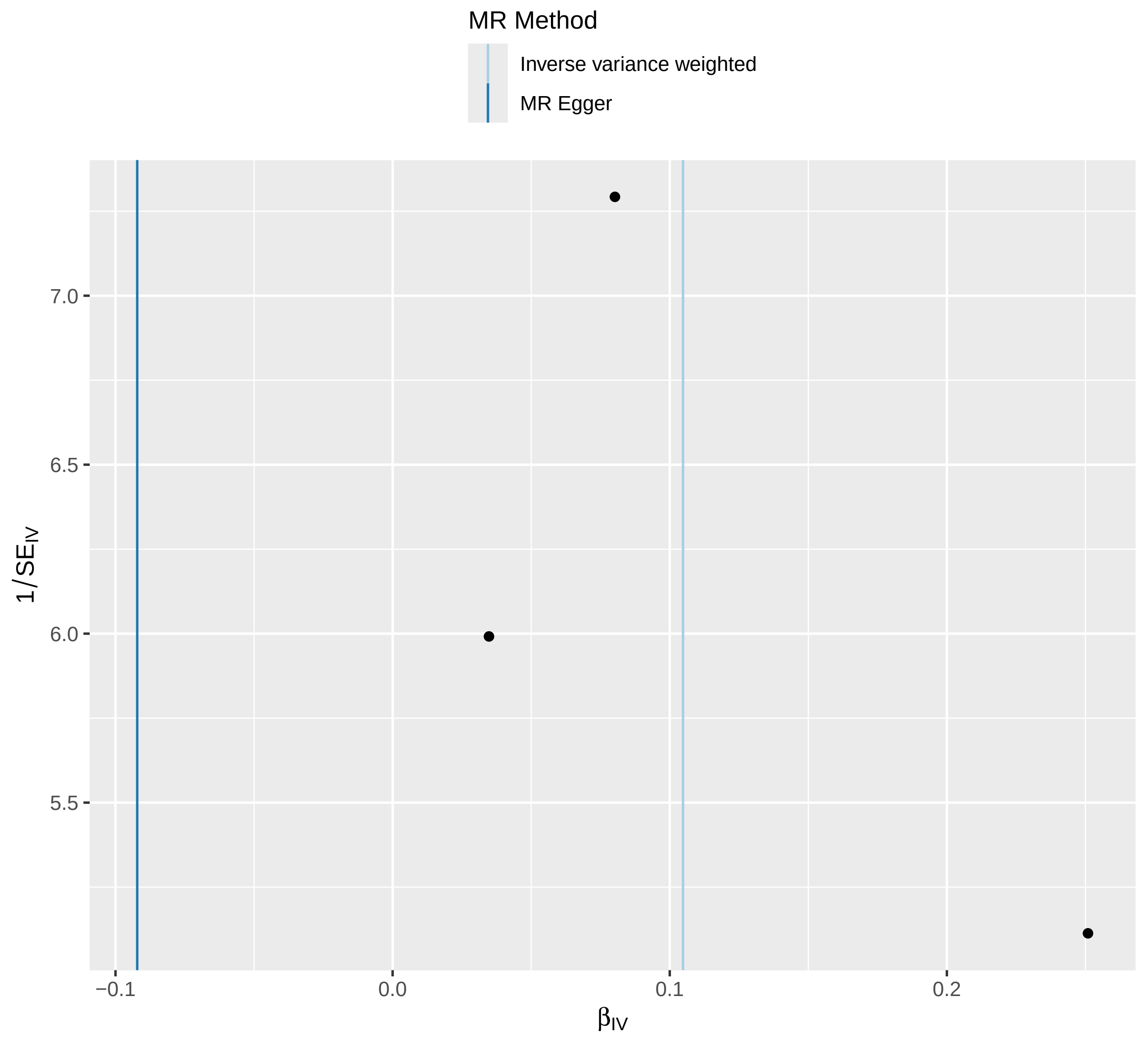
MR Method
Inverse variance weighted
MR Egger
7.0
6.5
6.0
5.5
1/SE_IV
−0.1
0.0
0.1
0.2
β_IV

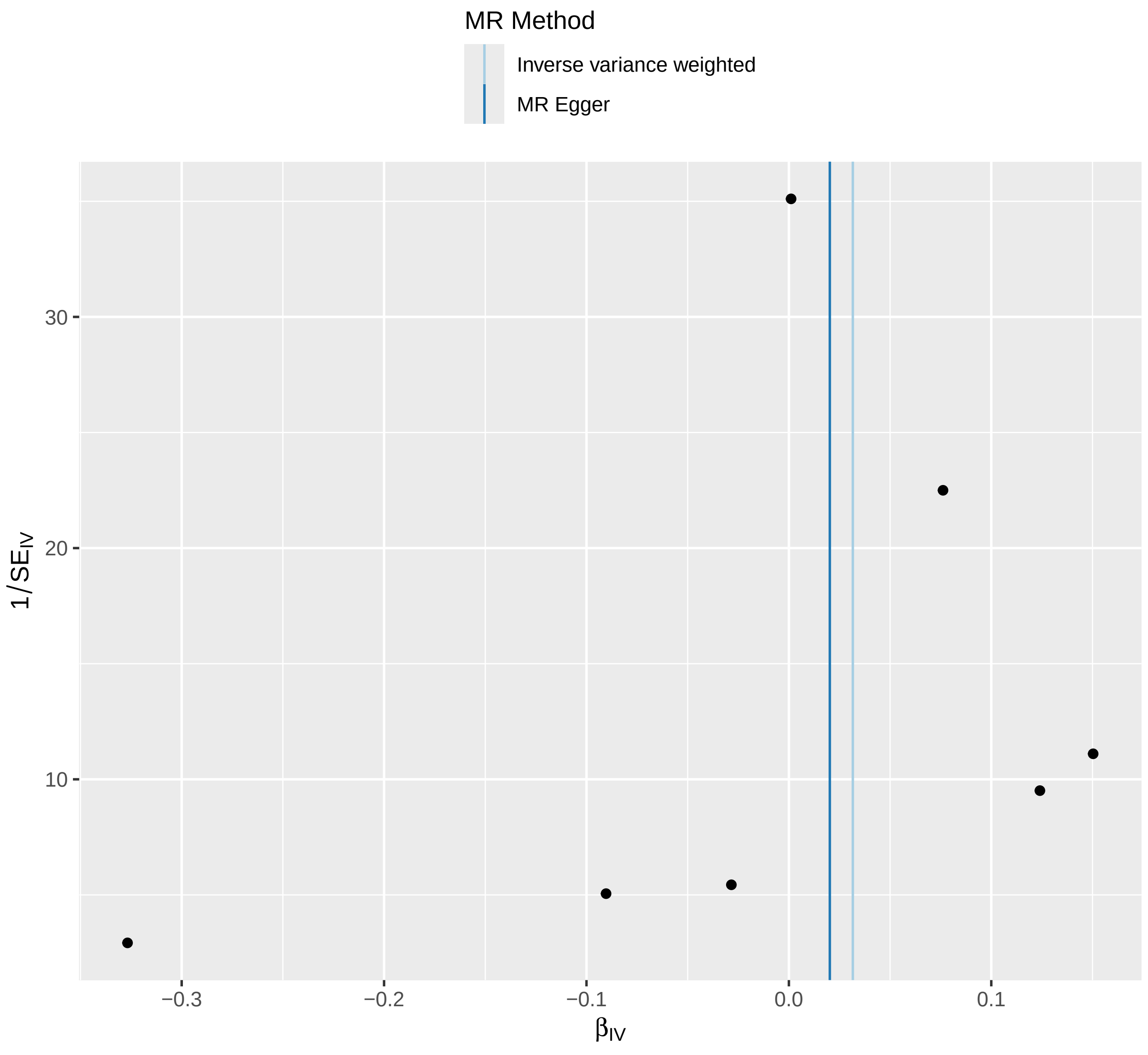

MR Method
Inverse variance weighted
MR Egger
30
20
10
1/$SE_{IV}$
−0.3
−0.2
−0.1
0.0
0.1
$β_{IV}$

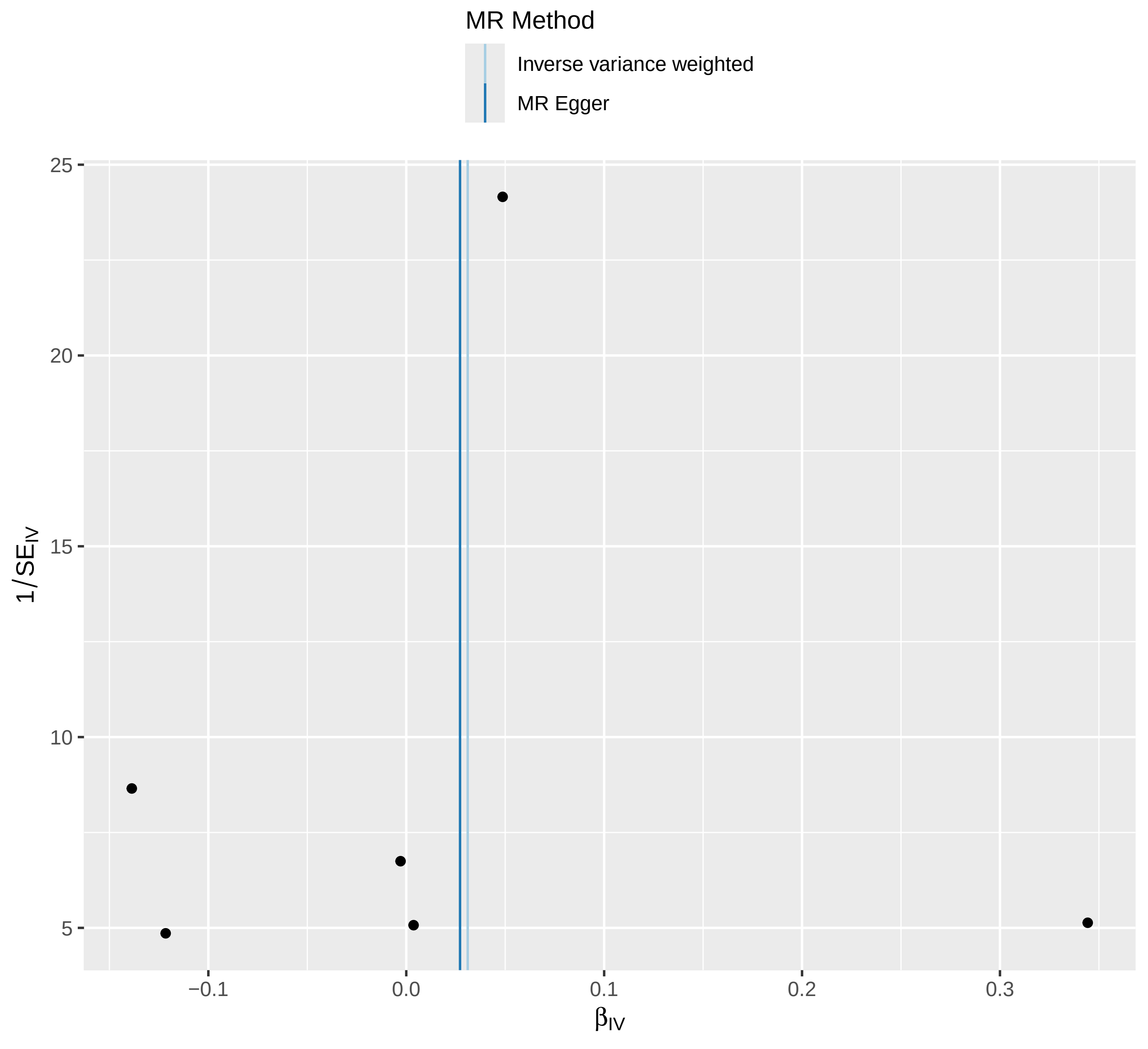
MR Method
Inverse variance weighted
MR Egger
25
20
15
10
5
1/SE_IV
−0.1
0.0
0.1
0.2
0.3
β_IV

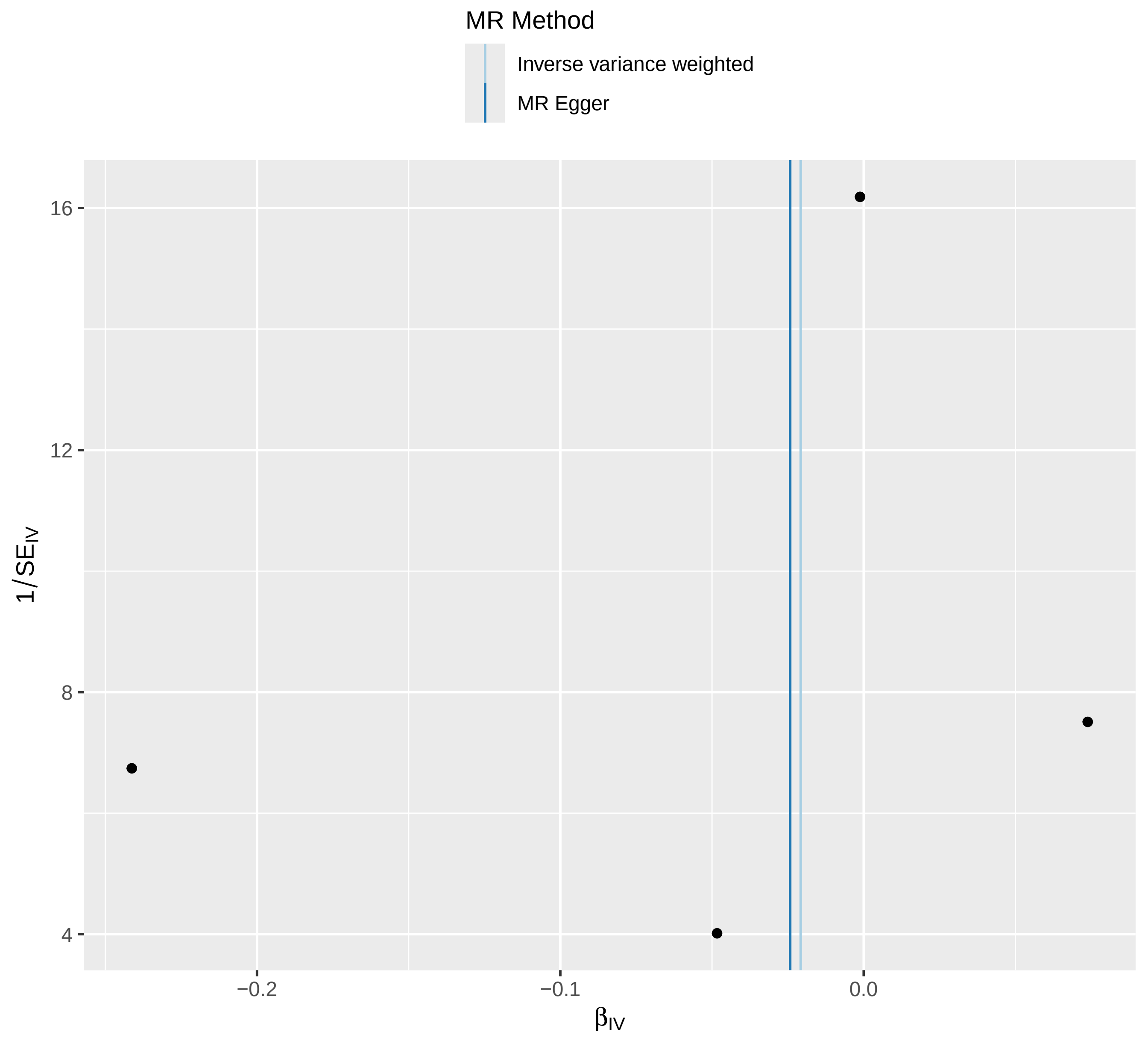
MR Method
Inverse variance weighted
MR Egger
16
12
8
4
−0.2
−0.1
0.0
$\beta_{IV}$
$1/SE_{IV}$

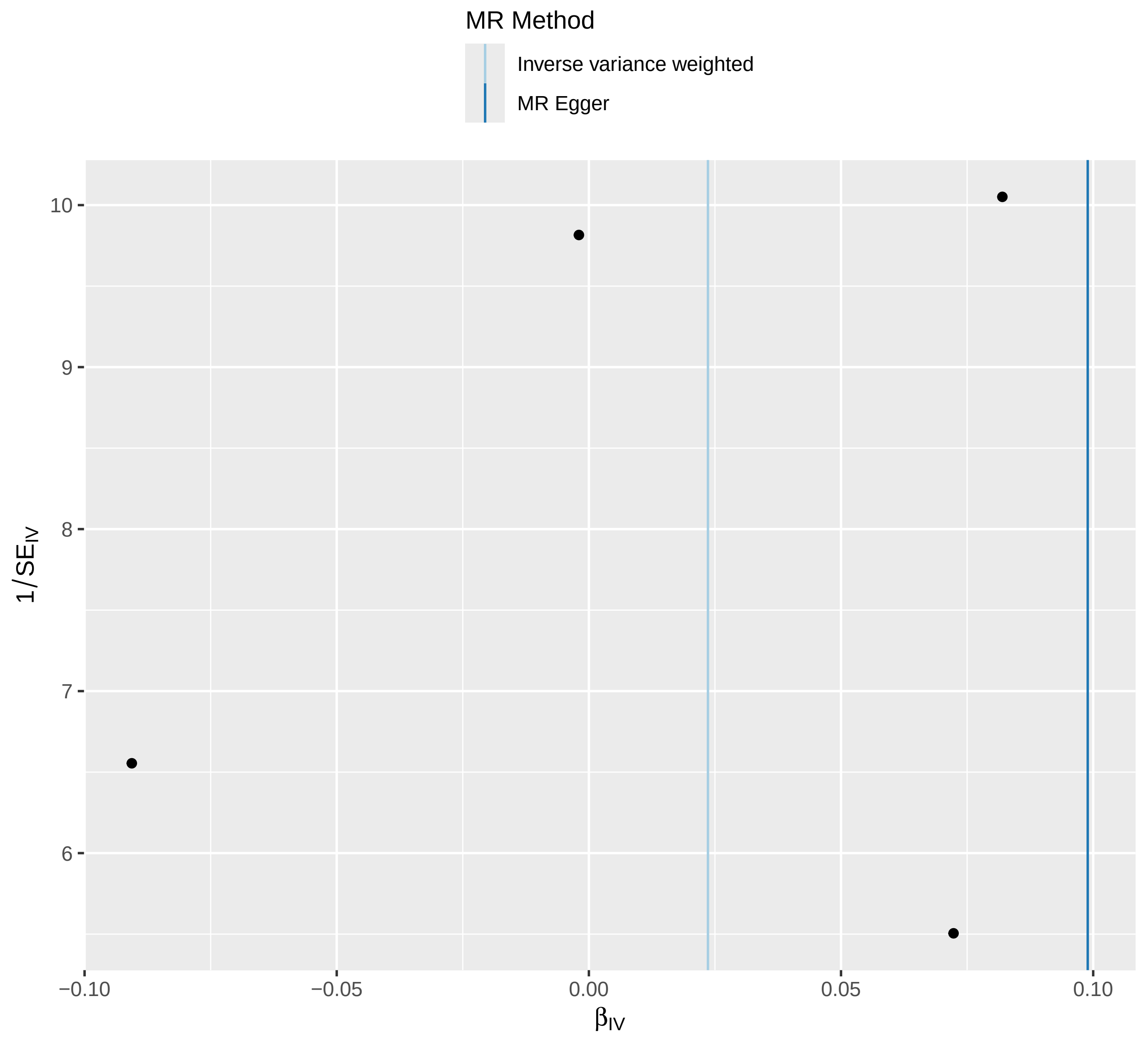
MR Method
Inverse variance weighted
MR Egger
10
9
8
7
6
$1/SE_{IV}$
−0.10
−0.05
0.00
0.05
0.10
$\beta_{IV}$

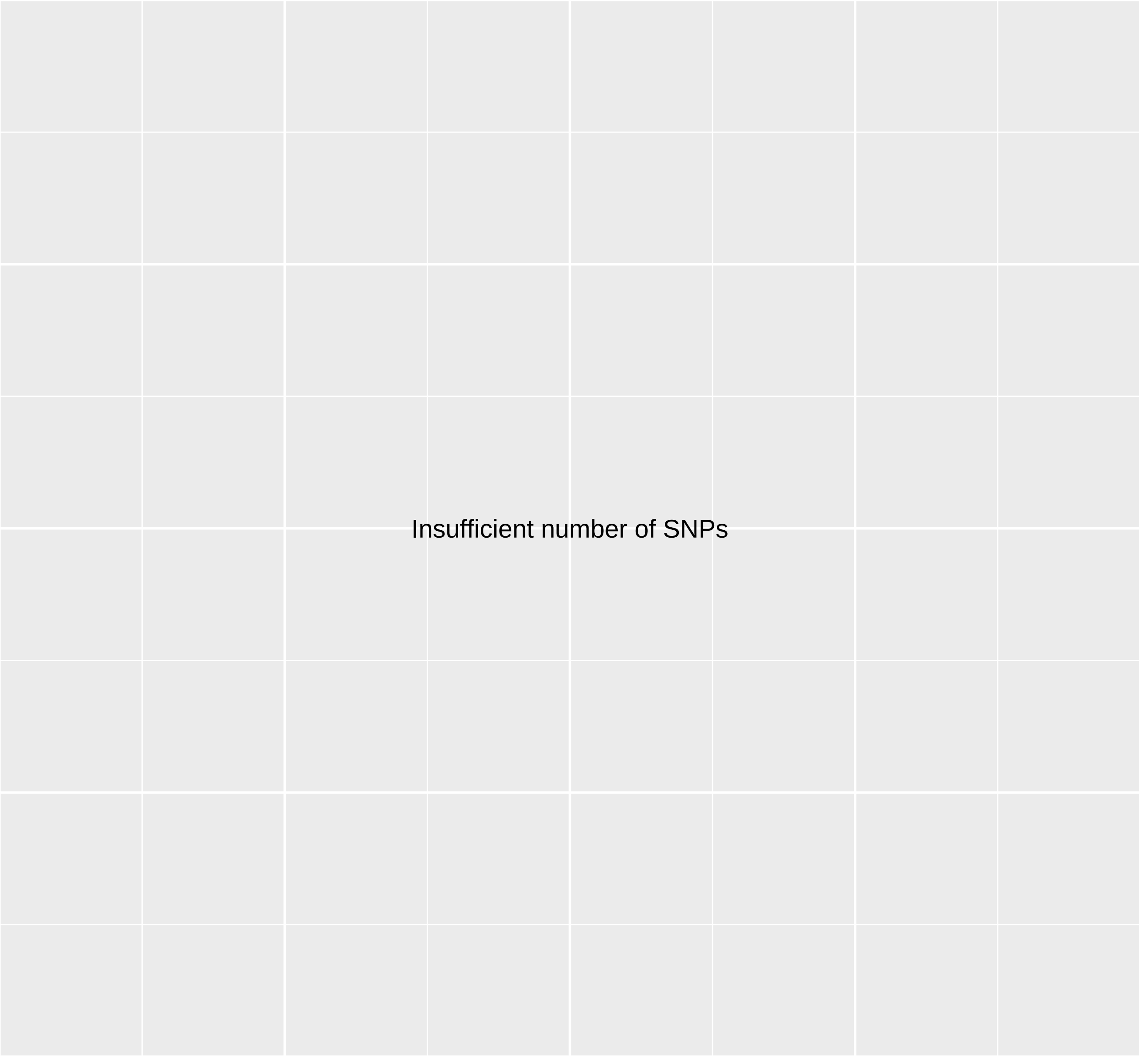
Insufficient number of SNPs

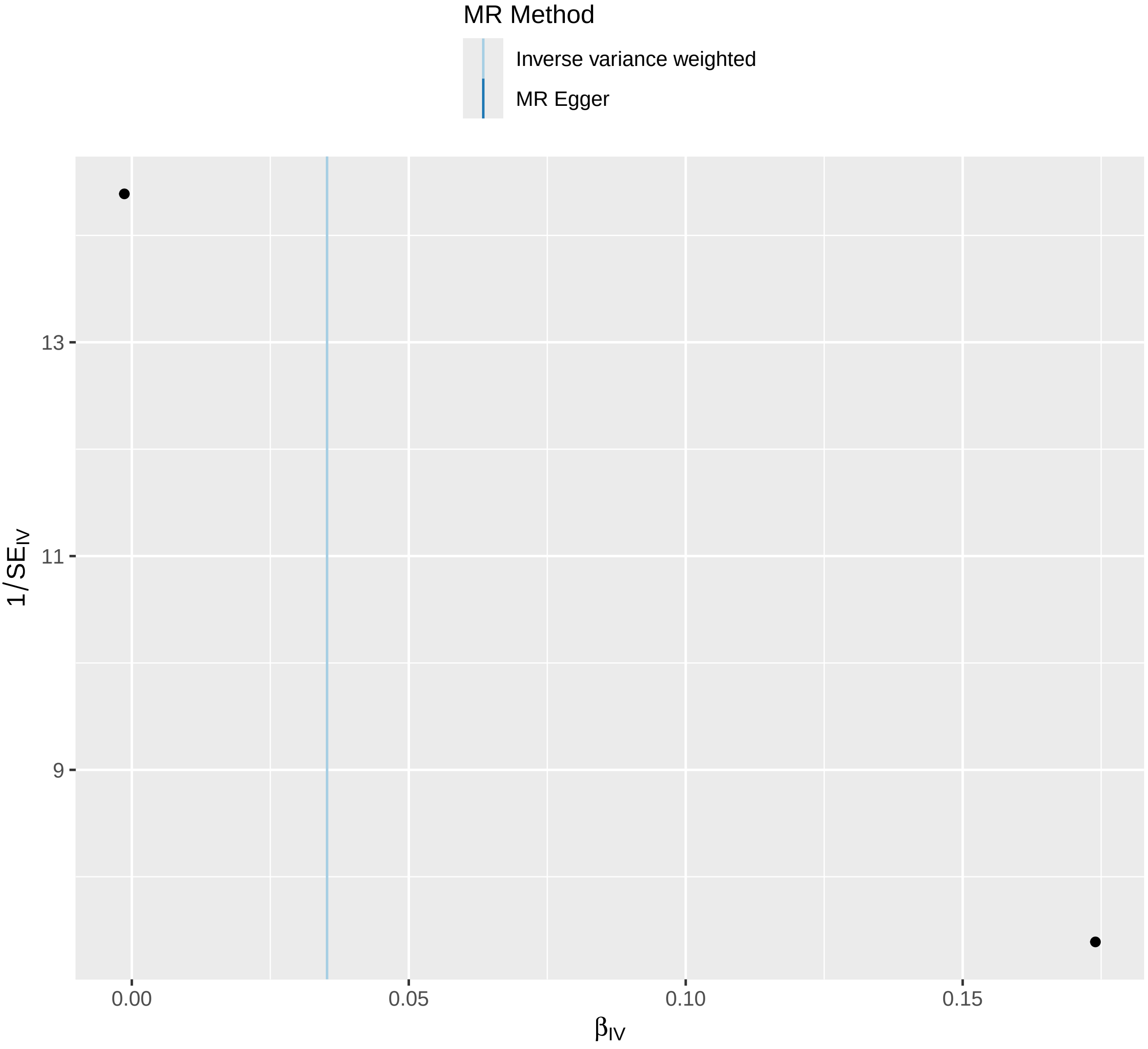
MR Method
Inverse variance weighted
MR Egger
13
11
9
1/SE$_{IV}$
0.00
0.05
0.10
0.15
β$_{IV}$

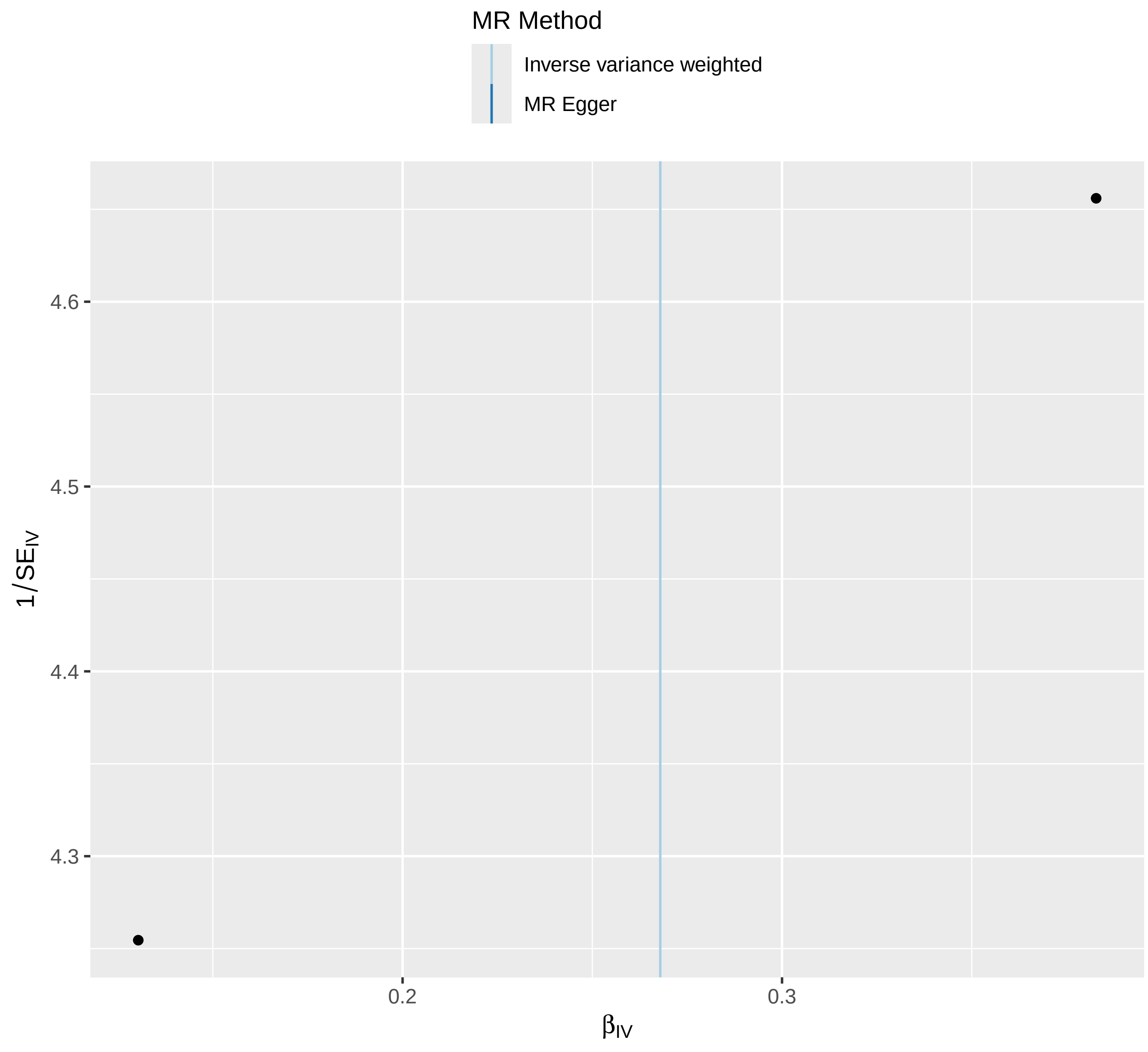

MR Method
Inverse variance weighted
MR Egger
4.6
4.5
4.4
4.3
1/SE_IV
0.2
0.3
β_IV

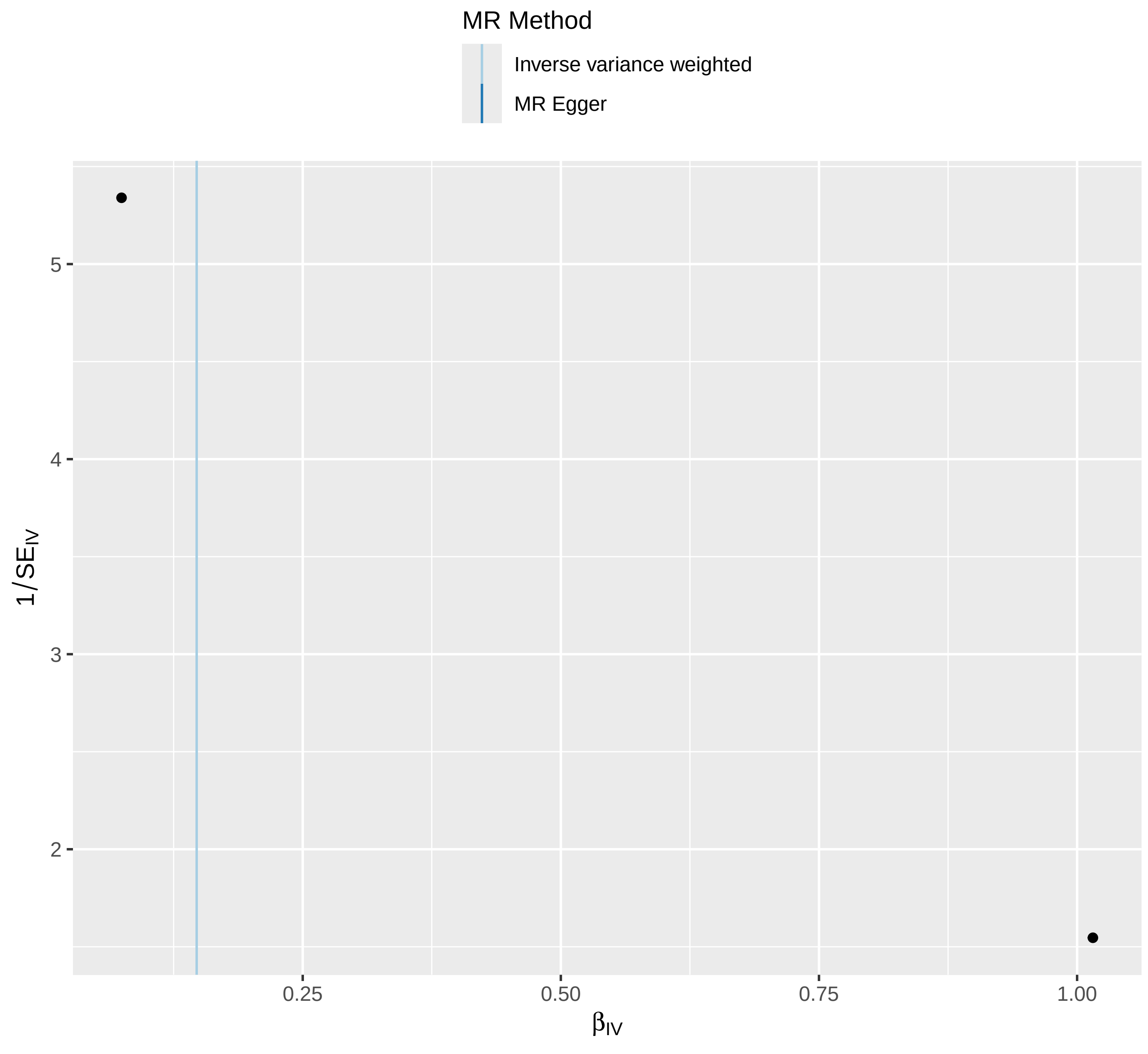
MR Method
Inverse variance weighted
MR Egger
5
4
3
2
0.25
0.50
0.75
1.00
$\beta_{IV}$
$1/SE_{IV}$

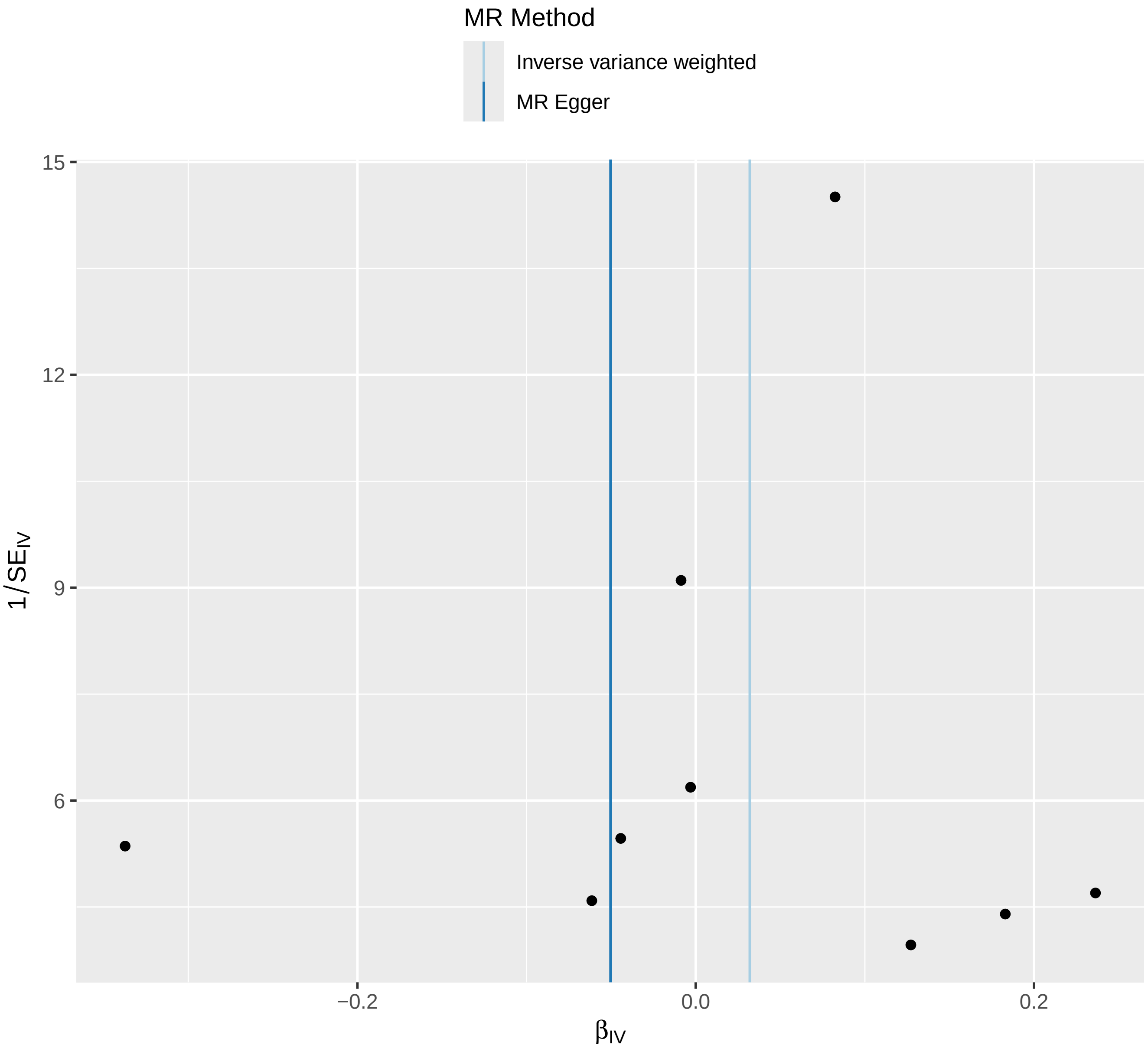
MR Method
Inverse variance weighted
MR Egger
15
12
9
6
1/SE$_{IV}$
−0.2
0.0
0.2
β$_{IV}$

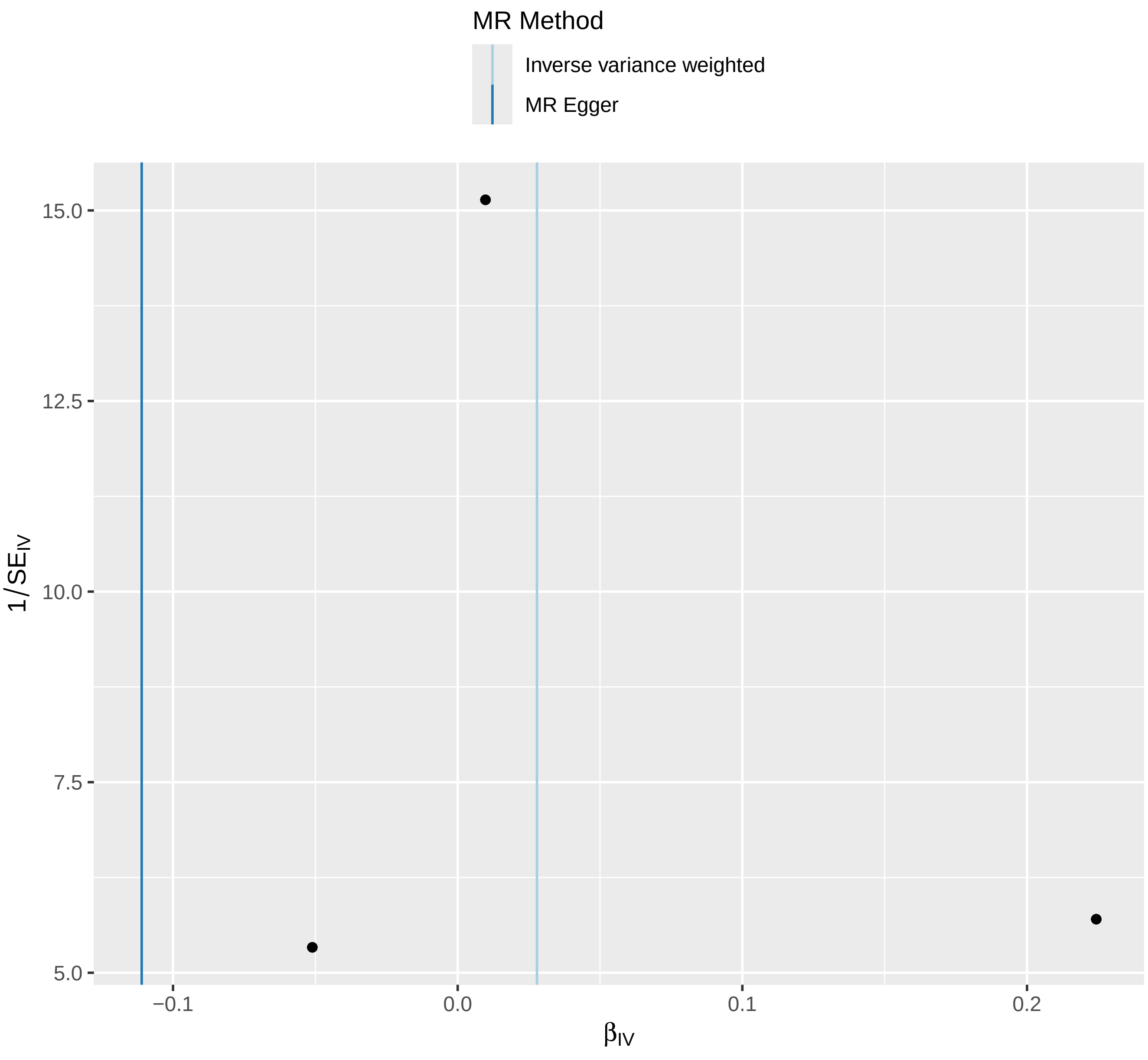
MR Method
Inverse variance weighted
MR Egger
15.0
12.5
10.0
7.5
5.0
$1/SE_{IV}$
−0.1
0.0
0.1
0.2
$\beta_{IV}$

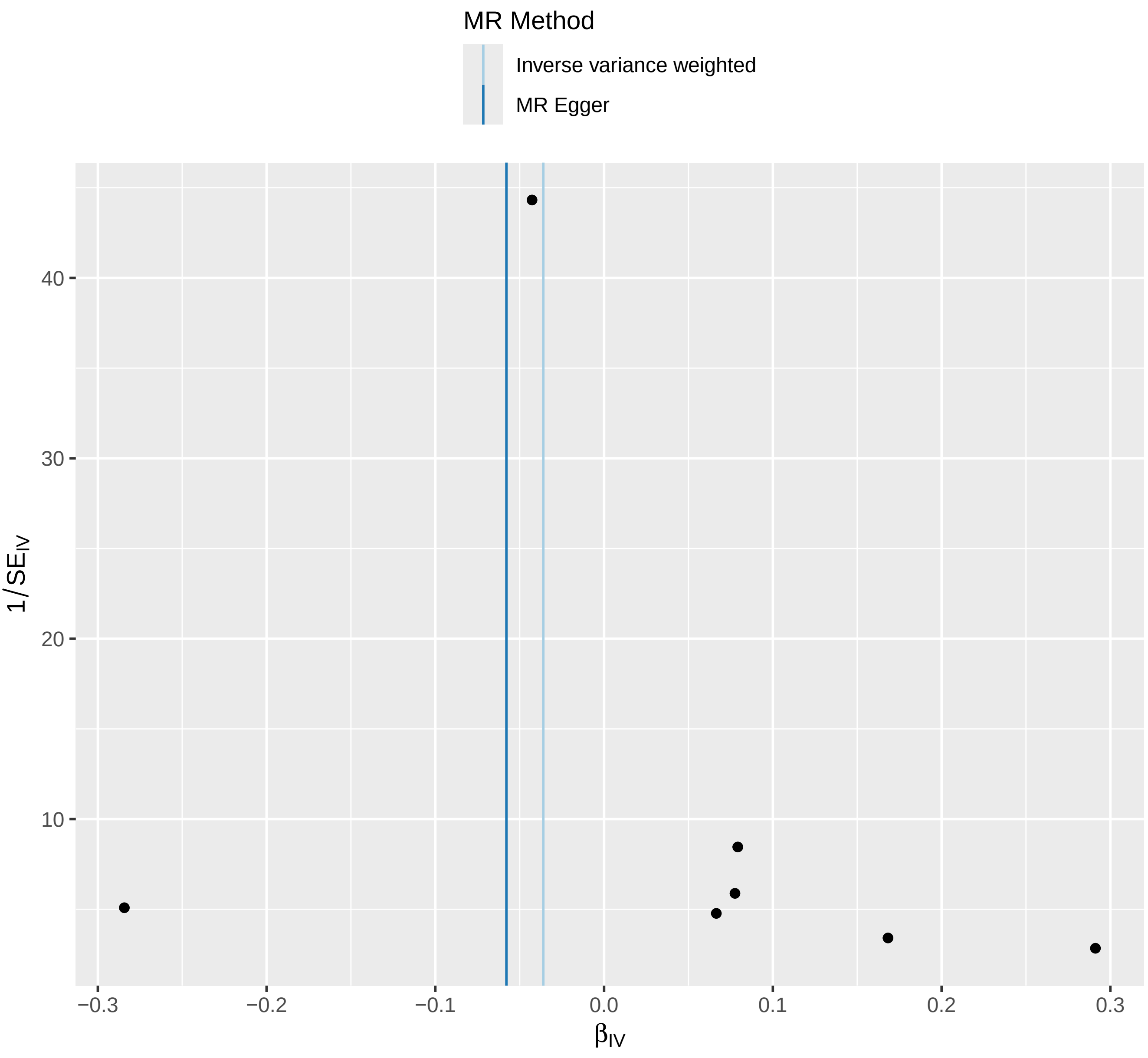
MR Method
Inverse variance weighted
MR Egger
40
30
20
10
1/SE$_{IV}$
−0.3
−0.2
−0.1
0.0
0.1
0.2
0.3
$\beta_{IV}$

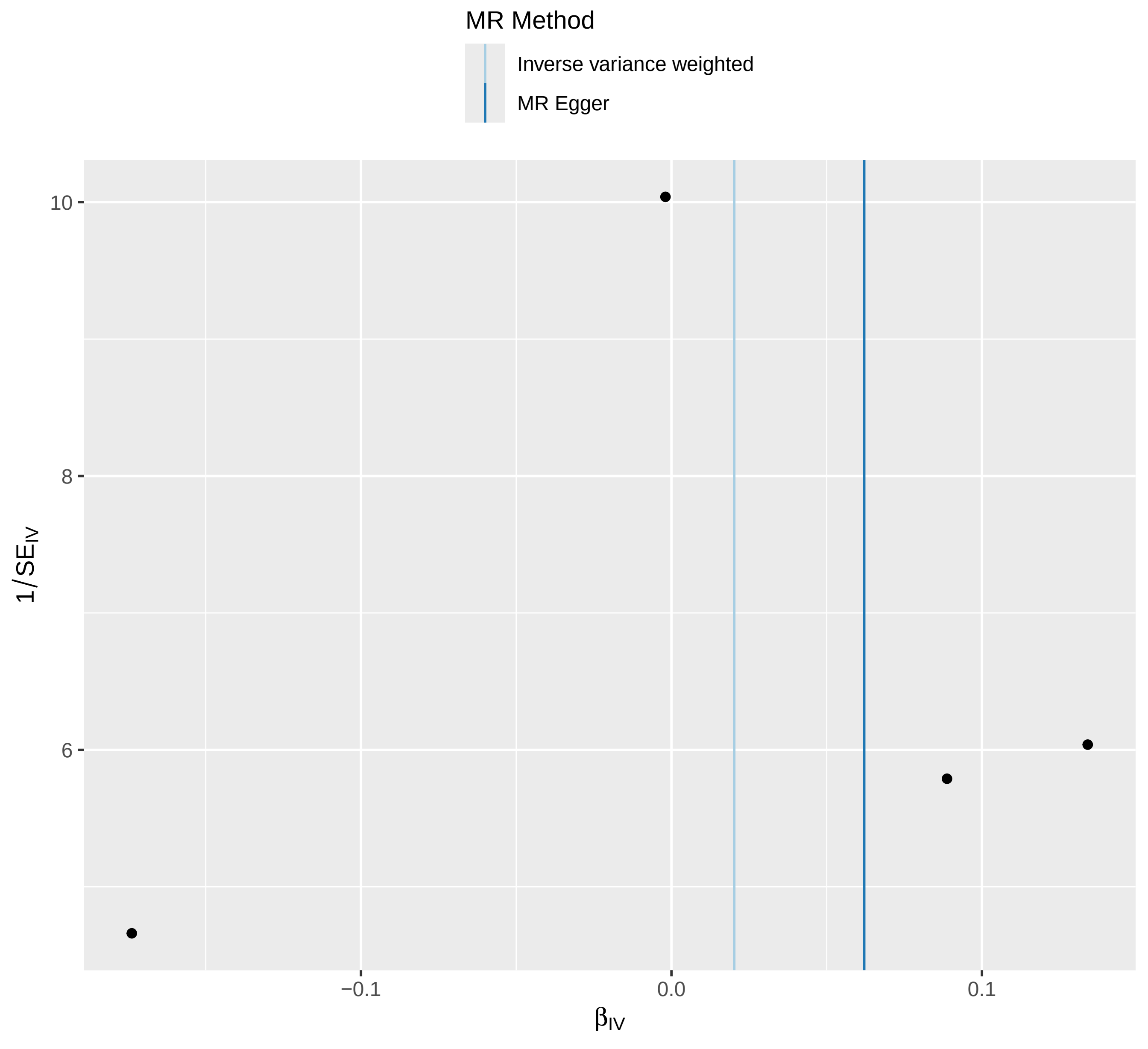
MR Method
Inverse variance weighted
MR Egger
10
8
6
1/SE_IV
−0.1
0.0
0.1
β_IV

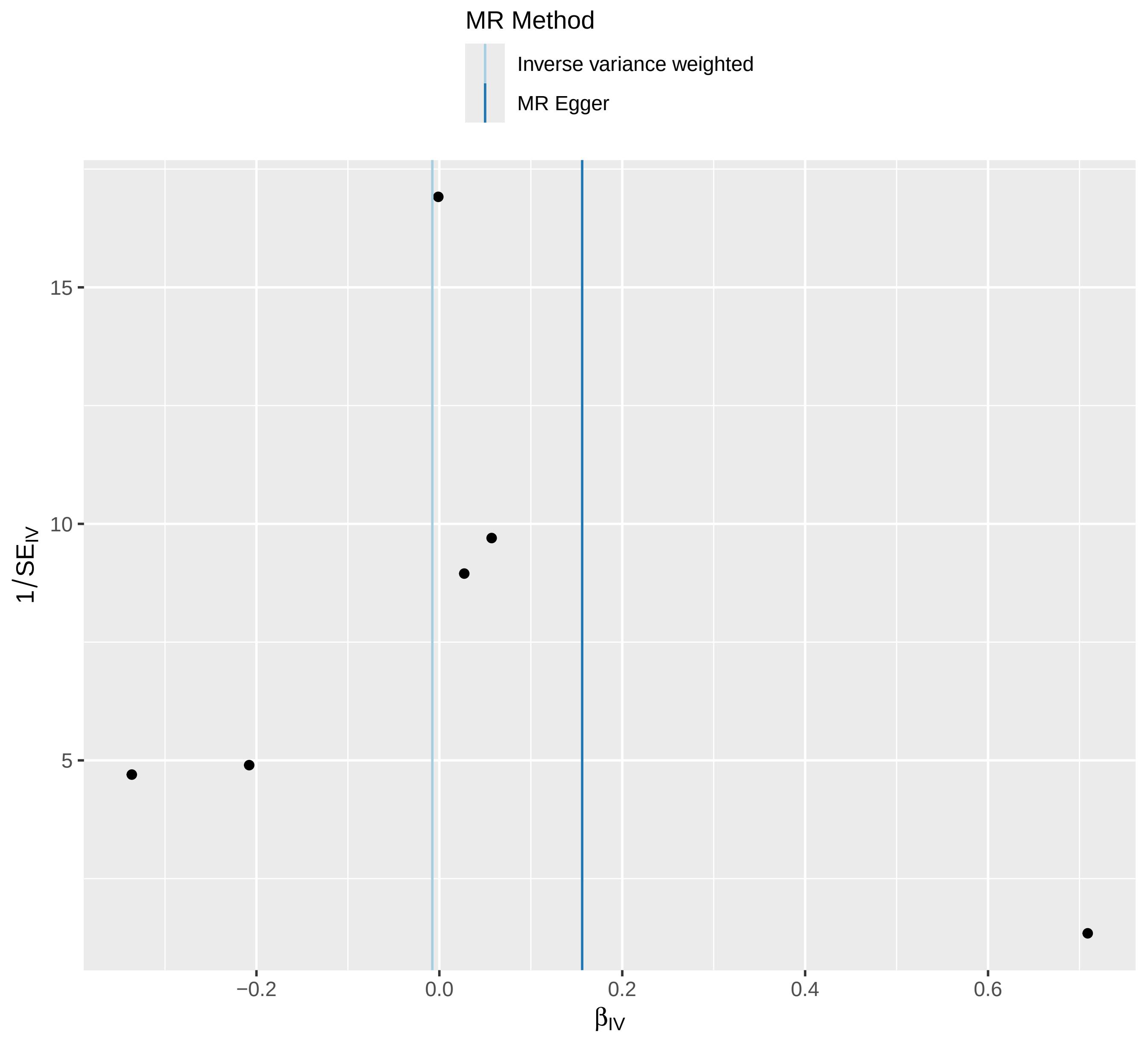

MR Method
Inverse variance weighted
MR Egger
15
10
5
$1/SE_{IV}$
−0.2
0.0
0.2
0.4
0.6
$\beta_{IV}$

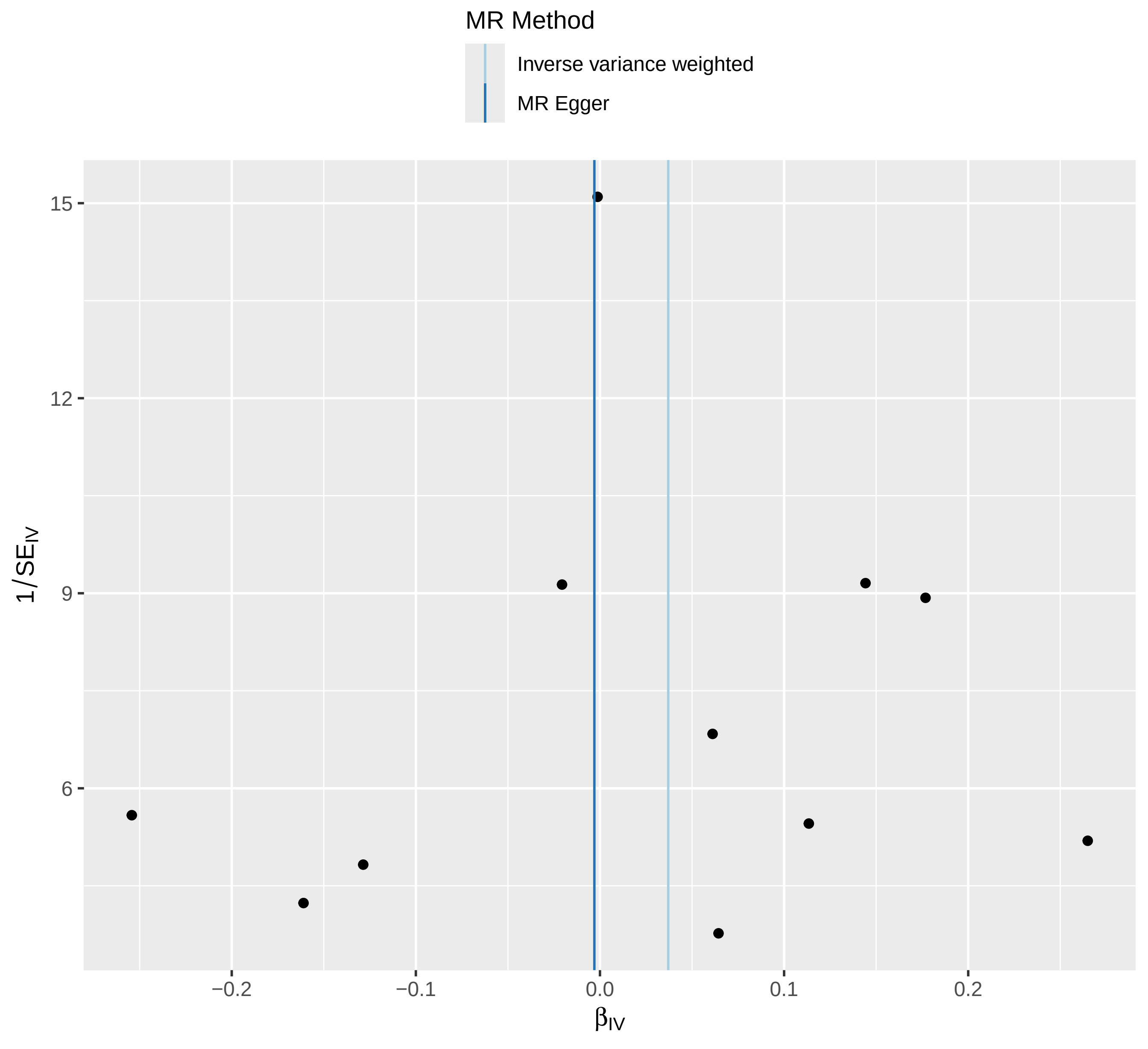

MR Method
Inverse variance weighted
MR Egger
15
12
9
6
1/SE_IV
−0.2
−0.1
0.0
0.1
0.2
β_IV

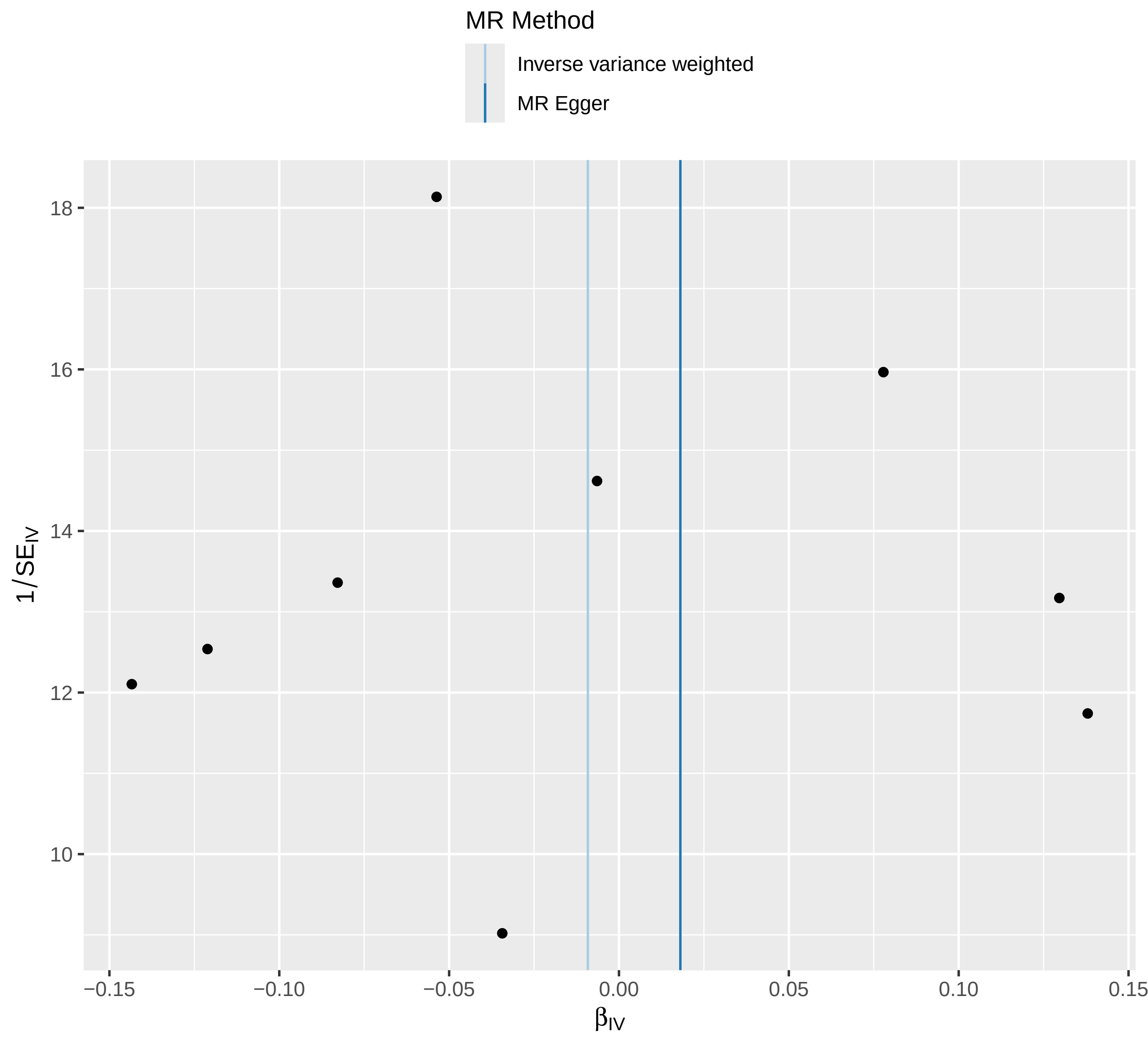

MR Method
Inverse variance weighted
MR Egger
18
16
14
12
10
1/SE_IV
−0.15
−0.10
−0.05
0.00
0.05
0.10
0.15
β_IV

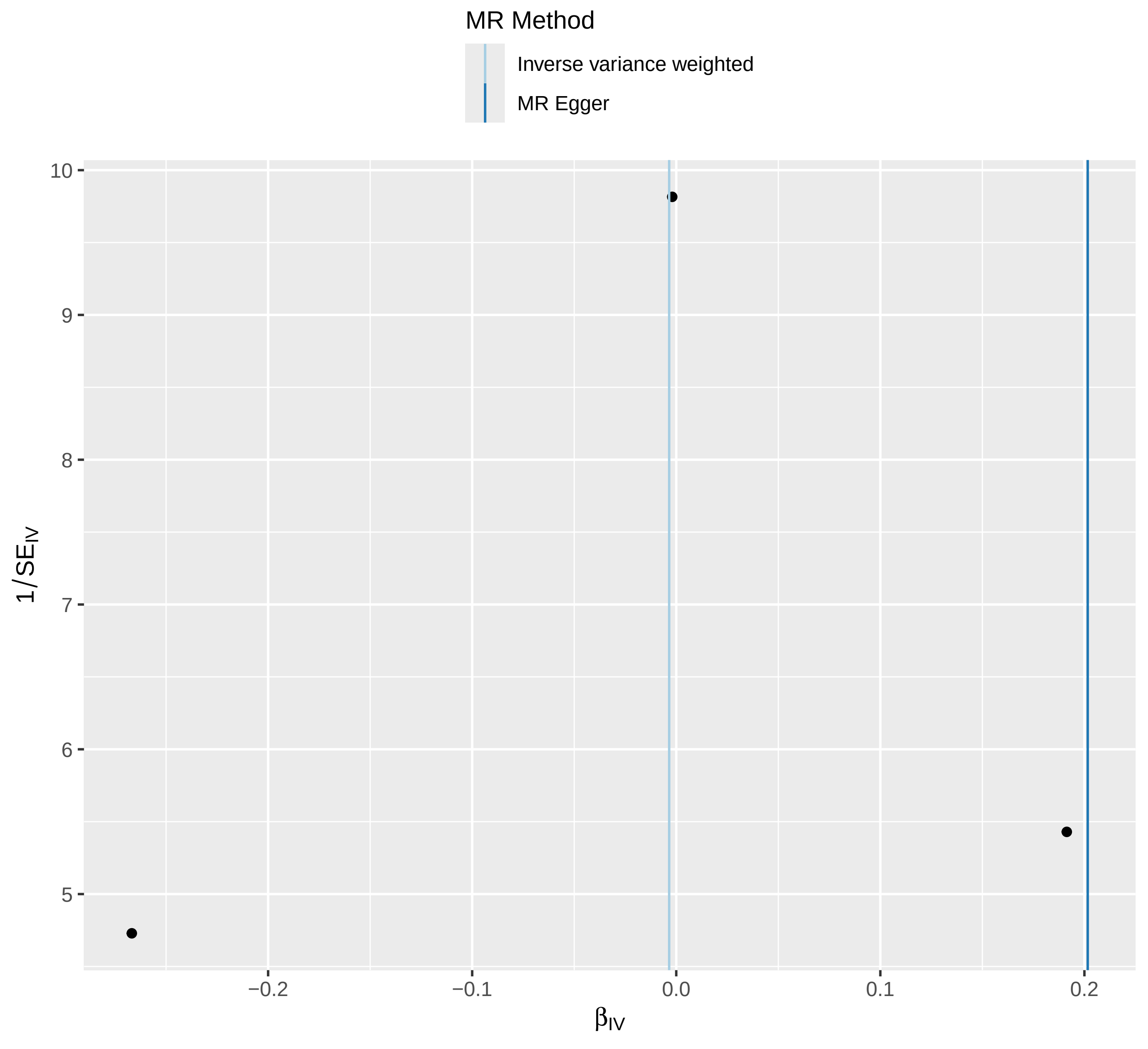
MR Method
Inverse variance weighted
MR Egger
1/SE_IV
10
9
8
7
6
5
−0.2
−0.1
0.0
0.1
0.2
β_IV

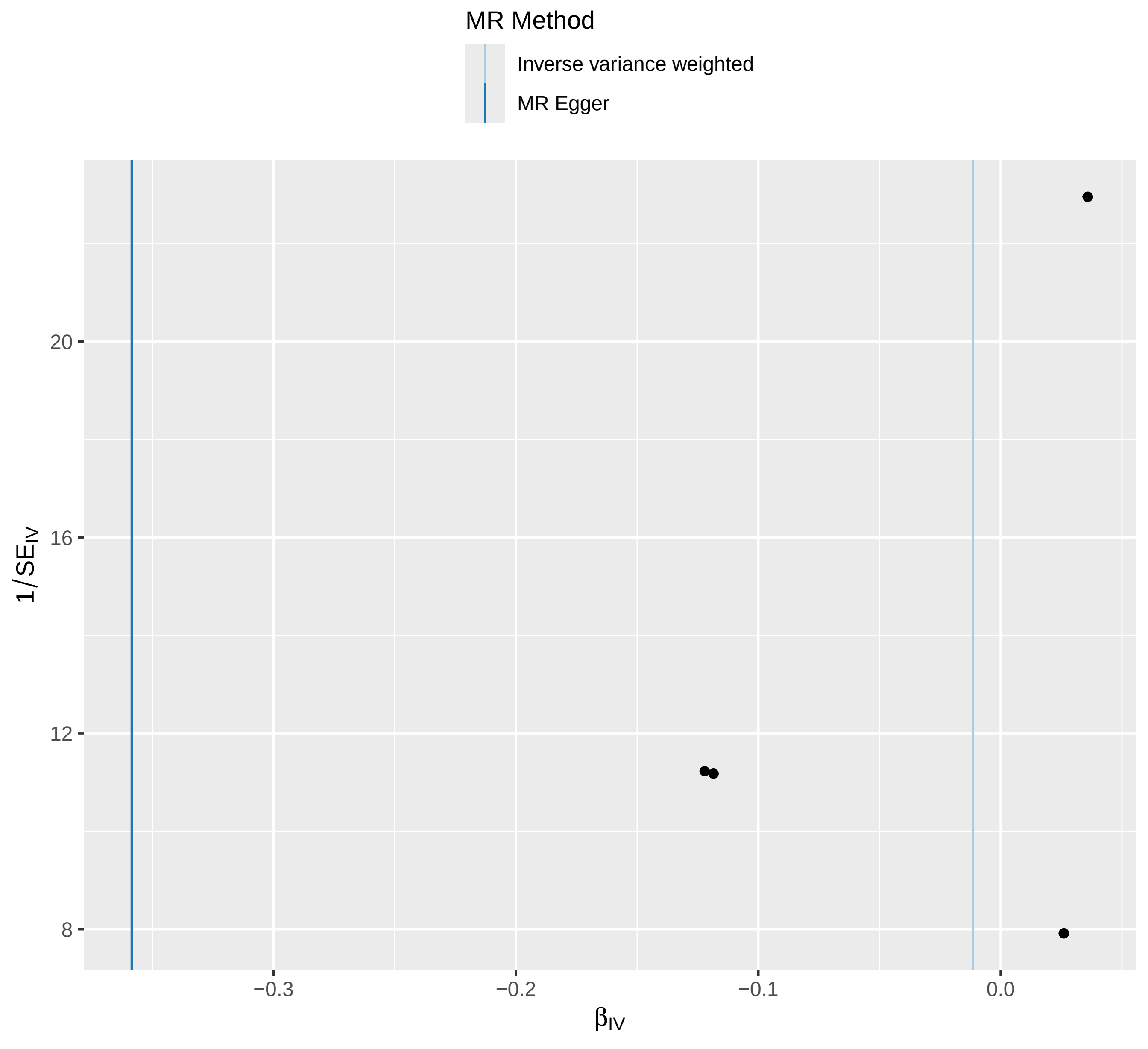

MR Method
Inverse variance weighted
MR Egger
20
16
12
8
1/$SE_{IV}$
−0.3
−0.2
−0.1
0.0
$\beta_{IV}$

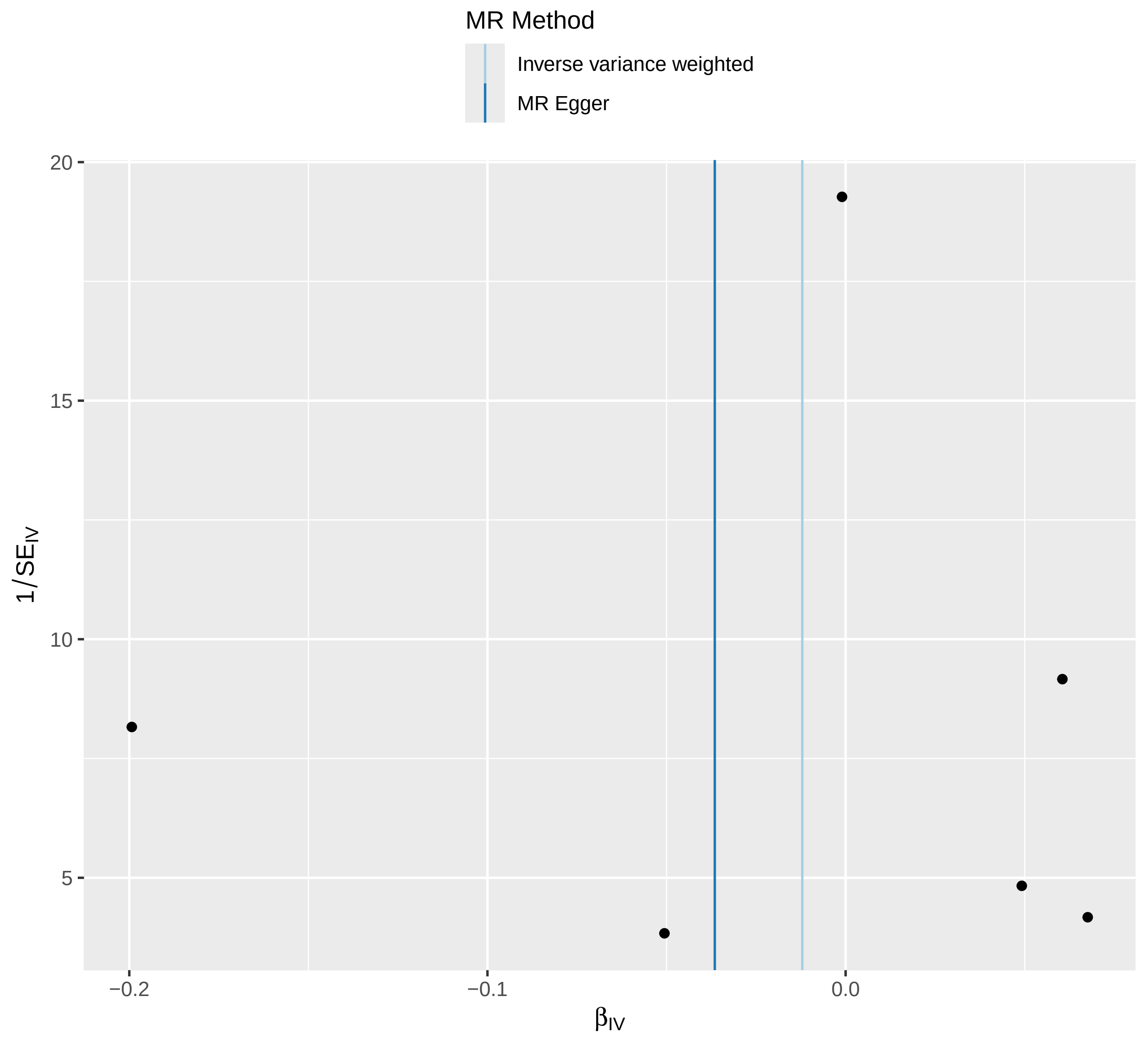

MR Method
Inverse variance weighted
MR Egger
20
15
10
5
1/$SE_{IV}$
−0.2
−0.1
0.0
$\beta_{IV}$

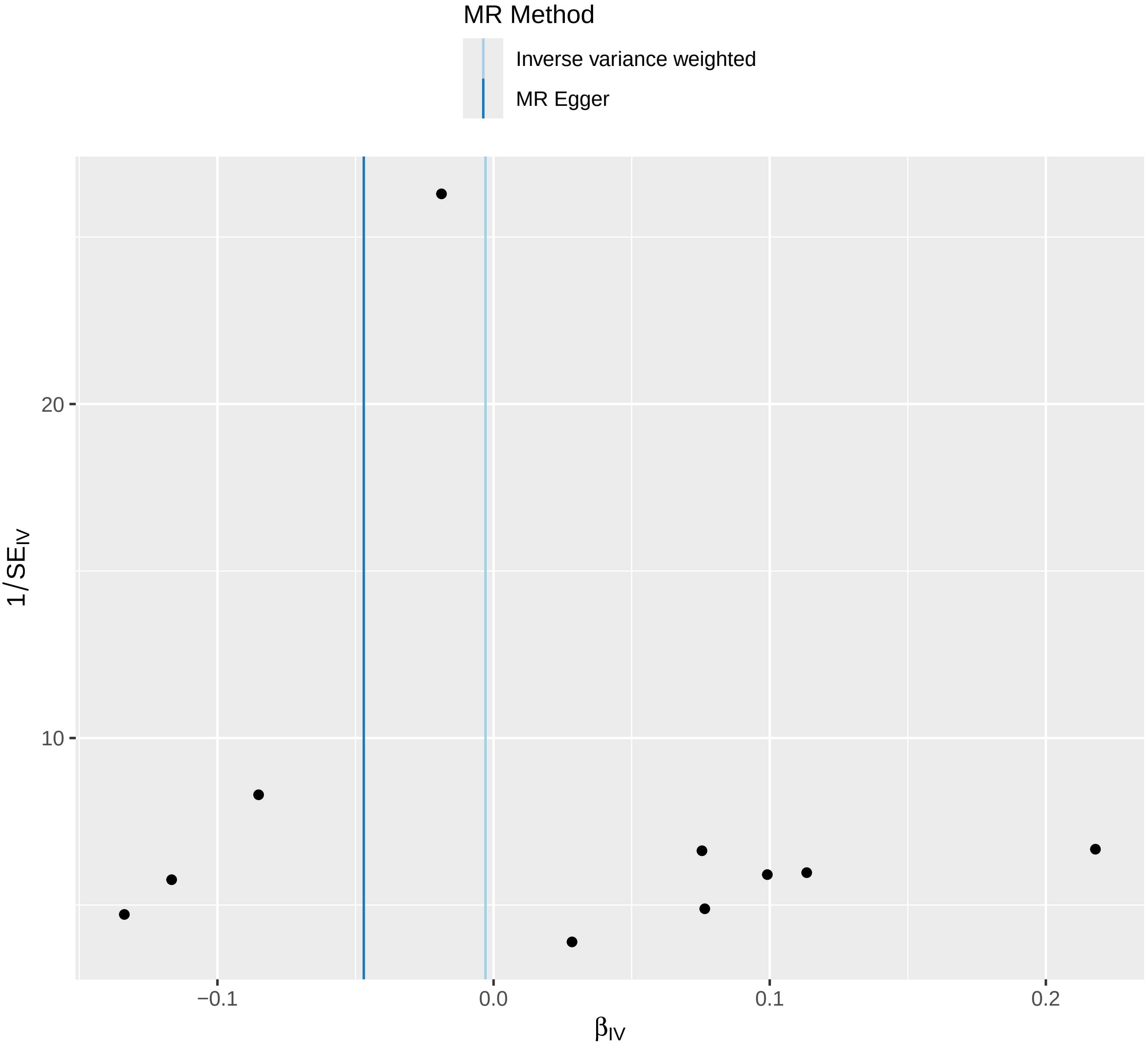

MR Method
Inverse variance weighted
MR Egger
20
10
1/SE$_{IV}$
−0.1
0.0
0.1
0.2
β$_{IV}$

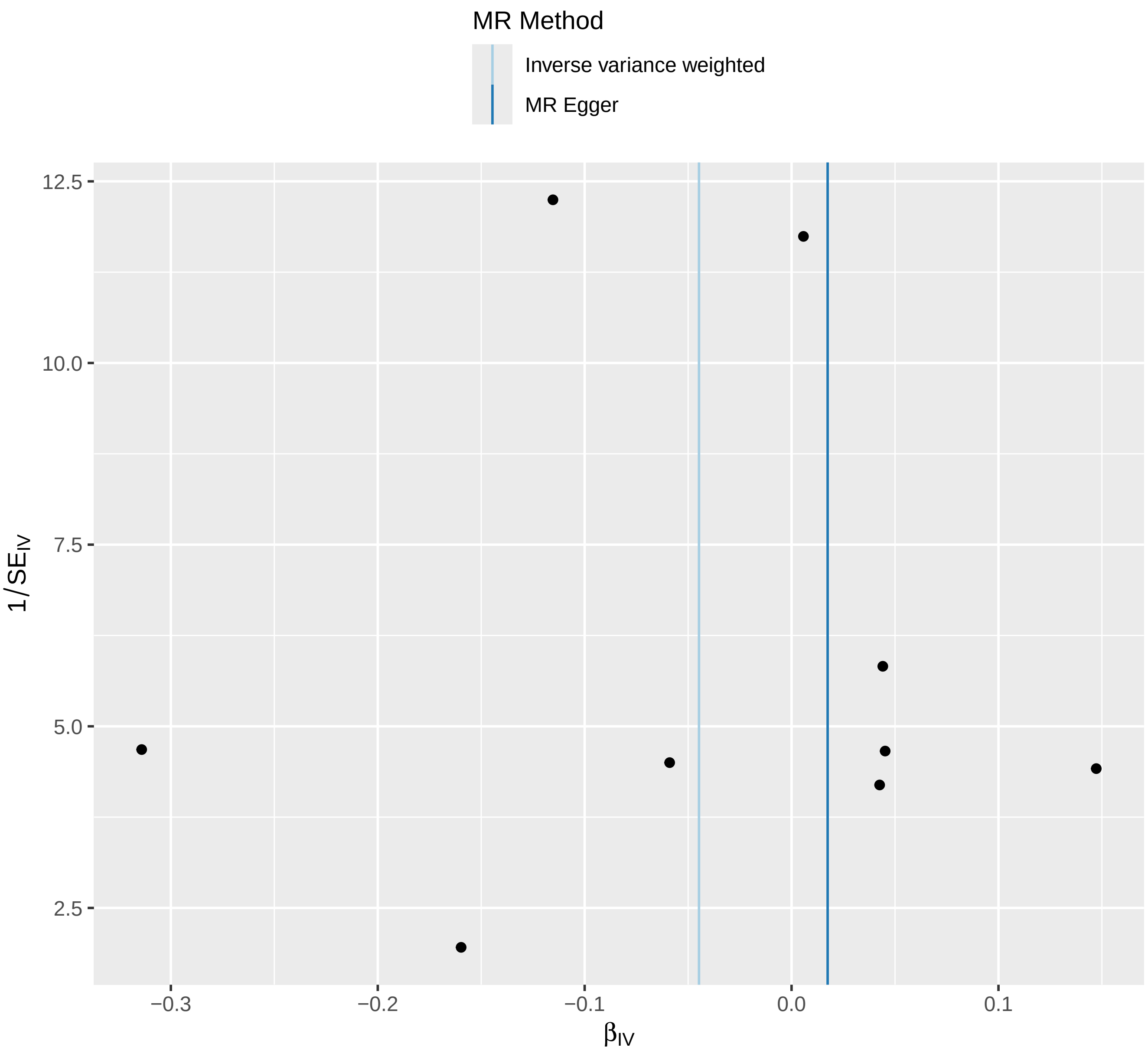

MR Method
Inverse variance weighted
MR Egger
12.5
10.0
7.5
5.0
2.5
1/SE_IV
−0.3
−0.2
−0.1
0.0
0.1
β_IV

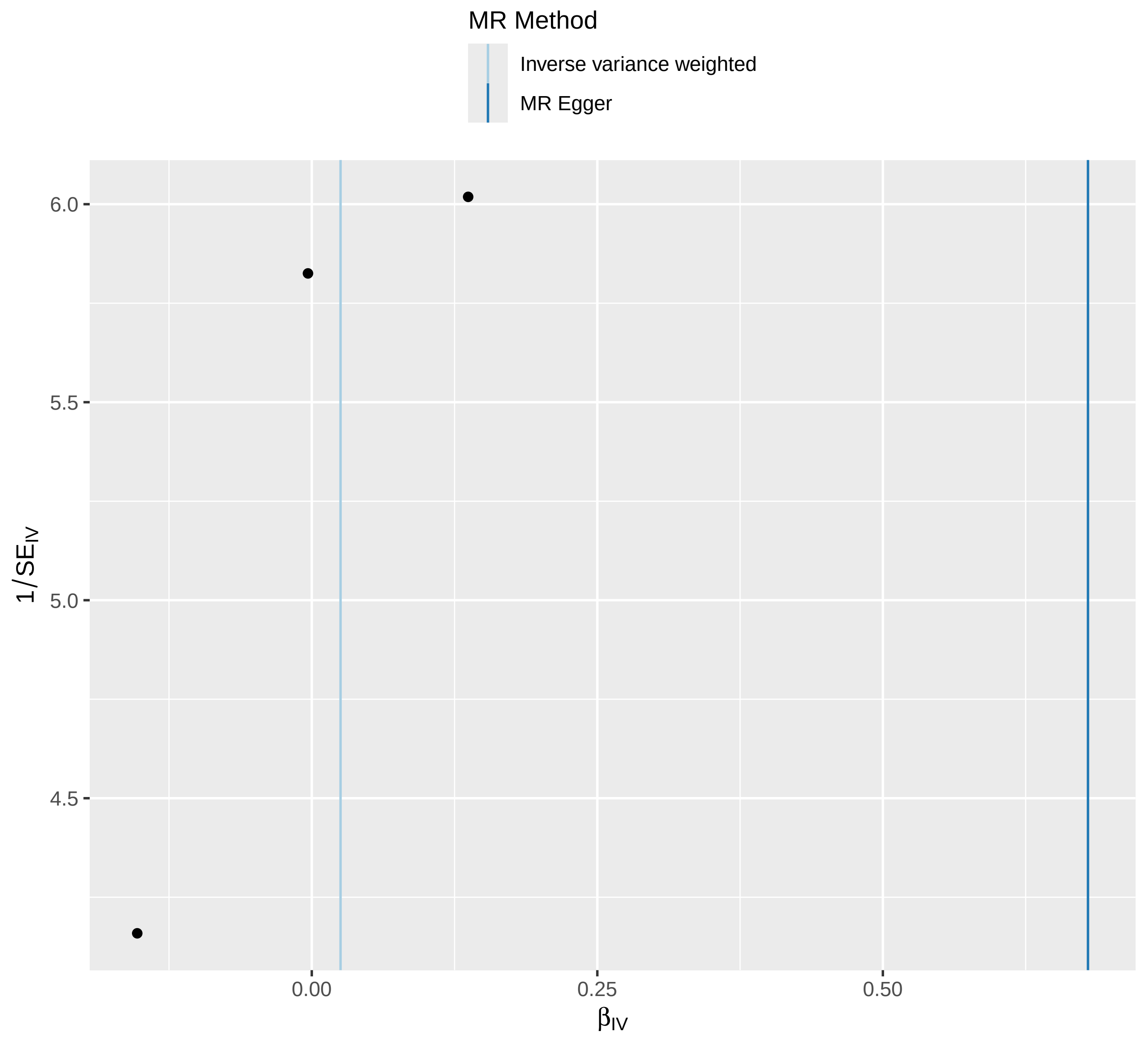

MR Method
Inverse variance weighted
MR Egger
6.0
5.5
5.0
4.5
1/SE_IV
0.00
0.25
0.50
β_IV

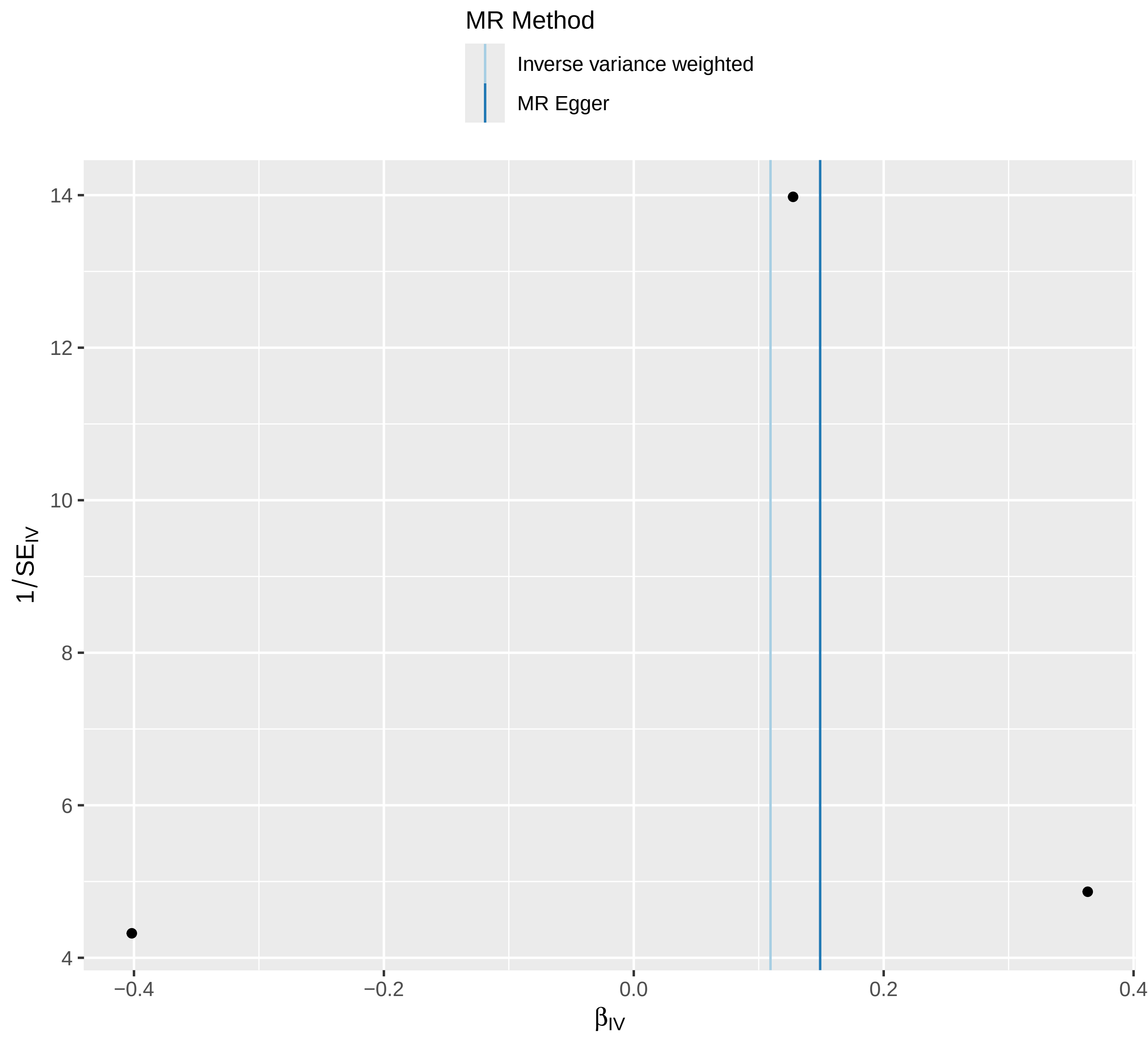
MR Method
Inverse variance weighted
MR Egger
1/SE_IV
14
12
10
8
6
4
−0.4
−0.2
0.0
0.2
0.4
β_IV

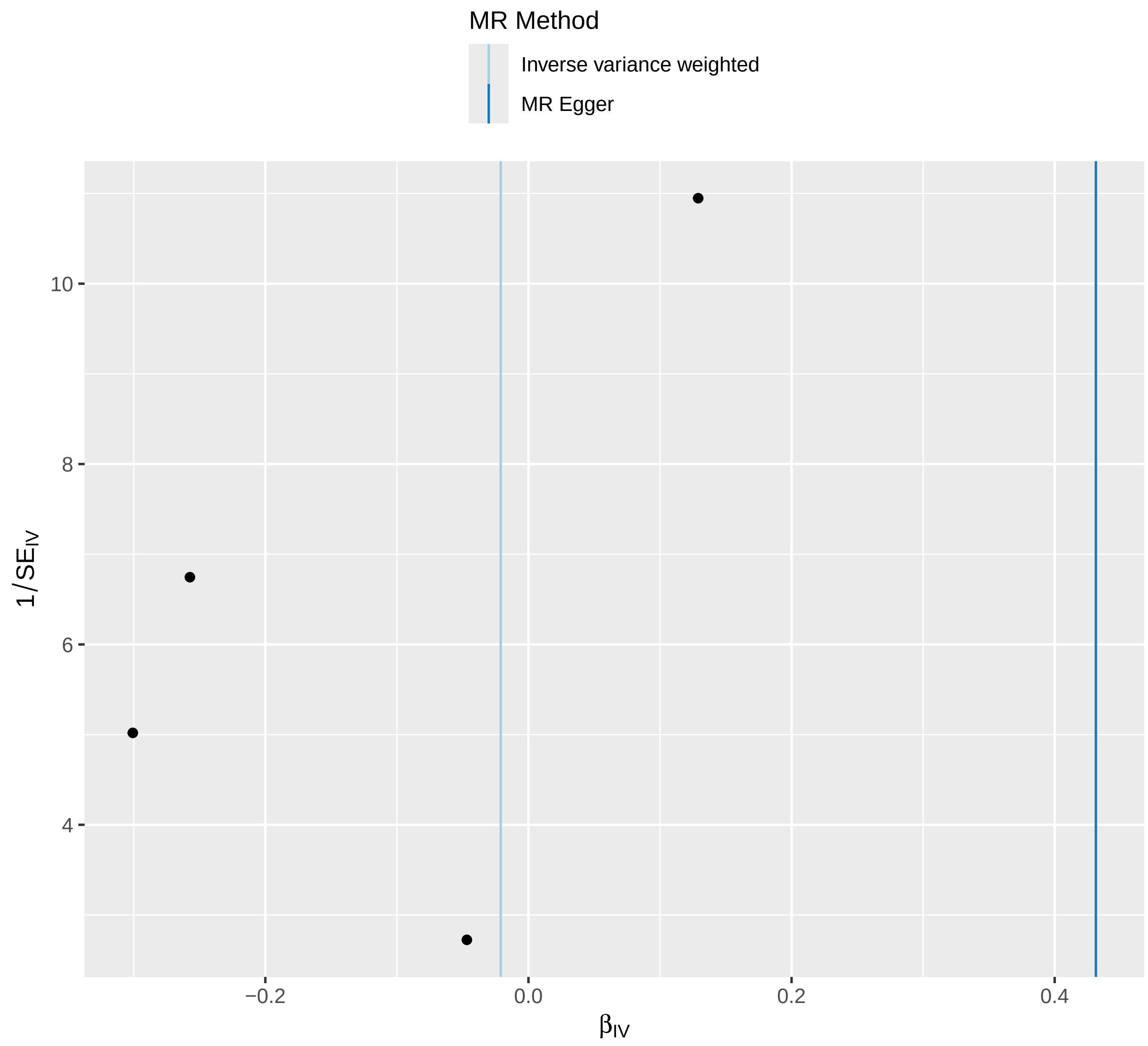

MR Method
Inverse variance weighted
MR Egger
10
8
6
4
−0.2
0.0
0.2
0.4
$1/SE_{IV}$
$\beta_{IV}$

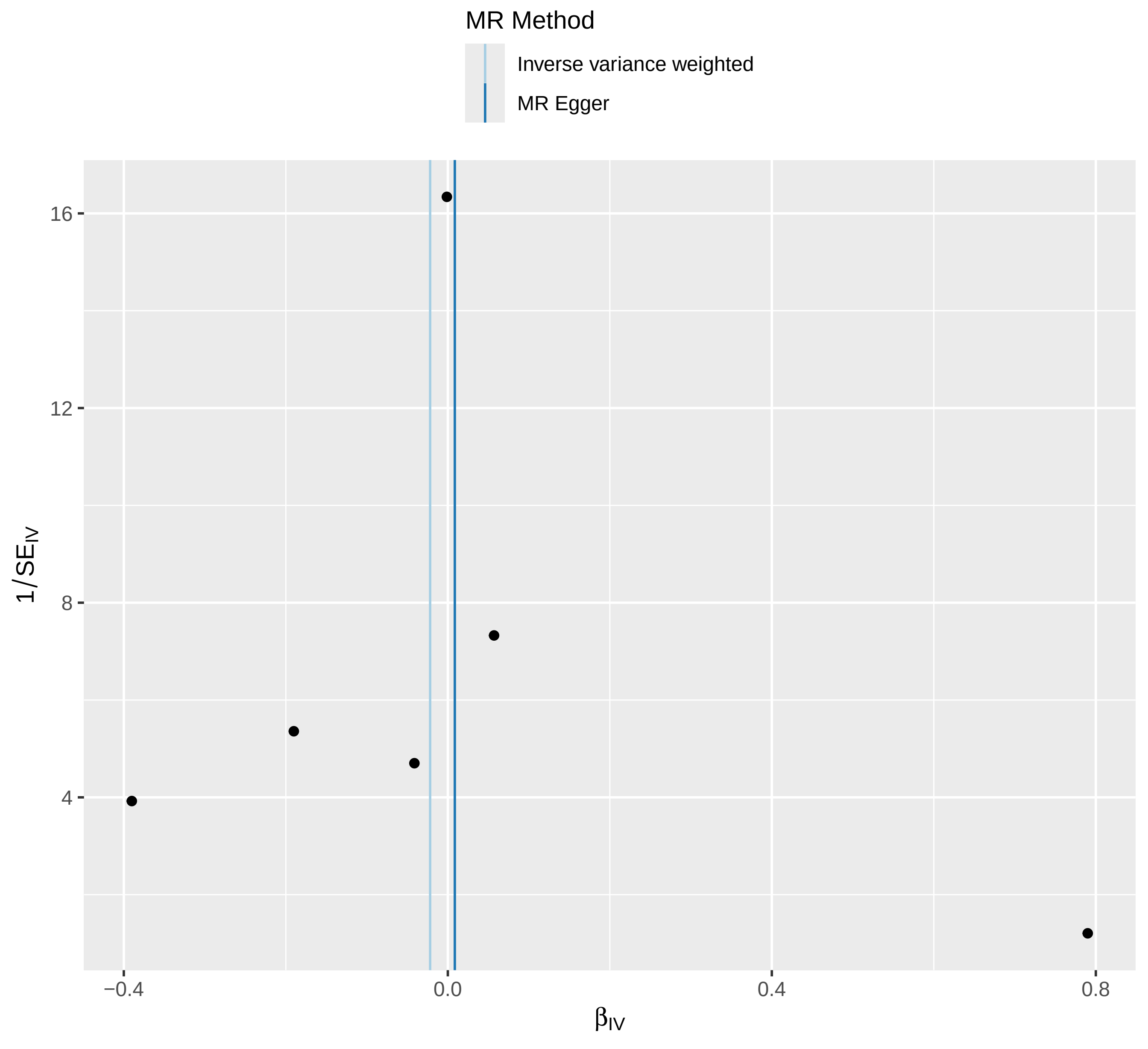
MR Method
Inverse variance weighted
MR Egger
16
12
8
4
1/SE_IV
−0.4
0.0
0.4
0.8
β_IV

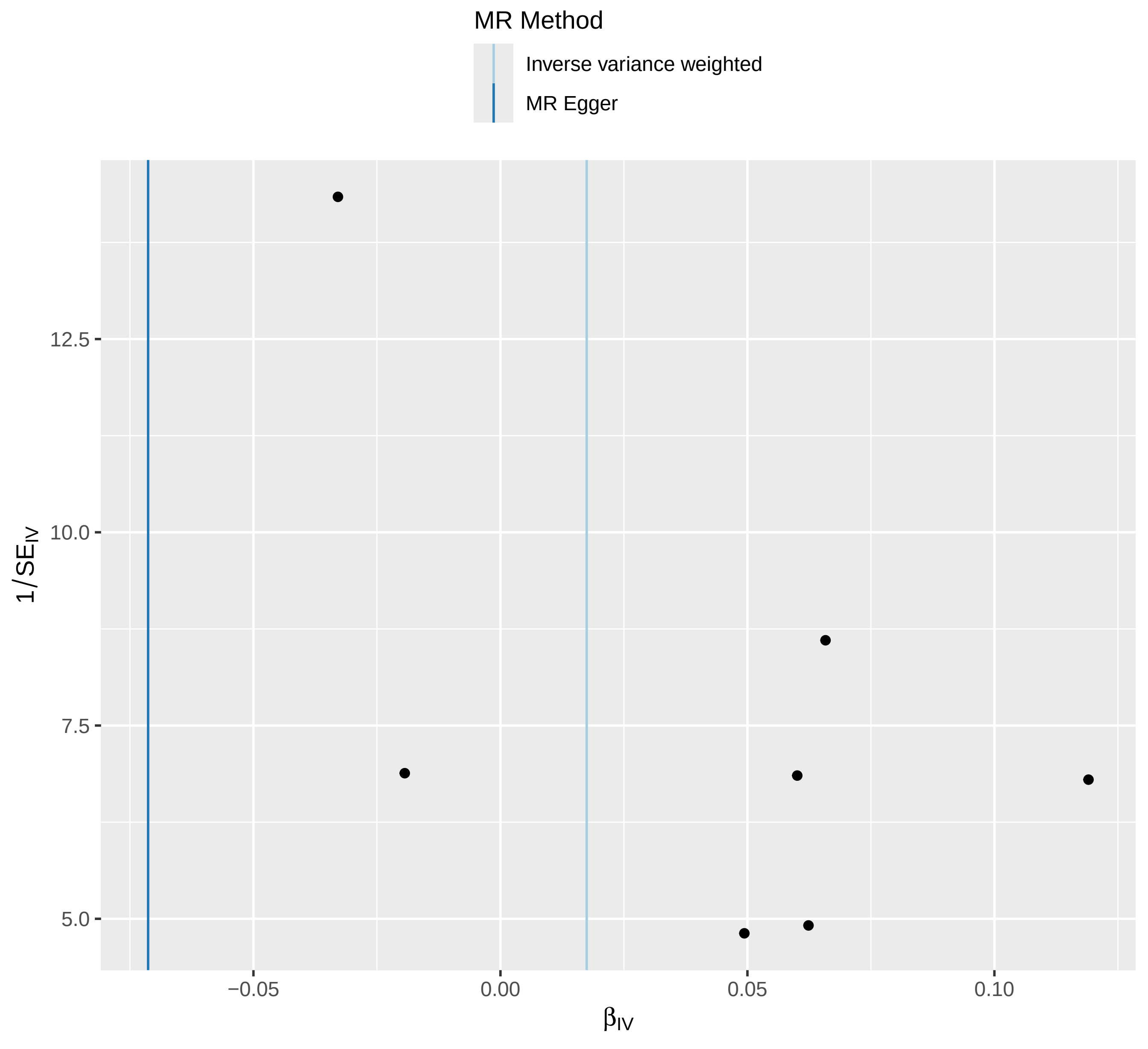
MR Method
Inverse variance weighted
MR Egger
12.5
10.0
7.5
5.0
1/SE_IV
−0.05
0.00
0.05
0.10
β_IV

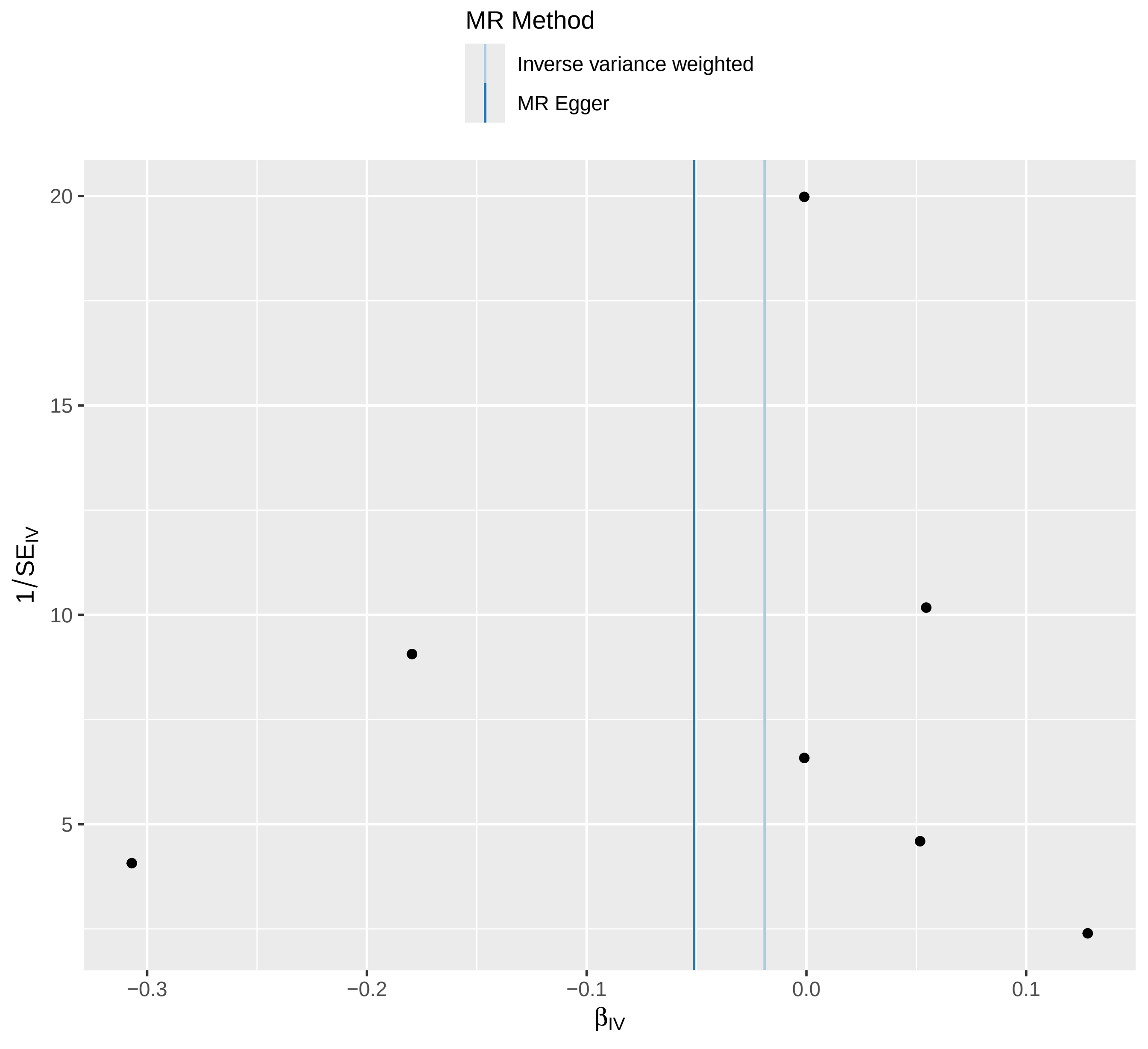
MR Method
Inverse variance weighted
MR Egger
20
15
10
5
1/SE_IV
−0.3
−0.2
−0.1
0.0
0.1
β_IV

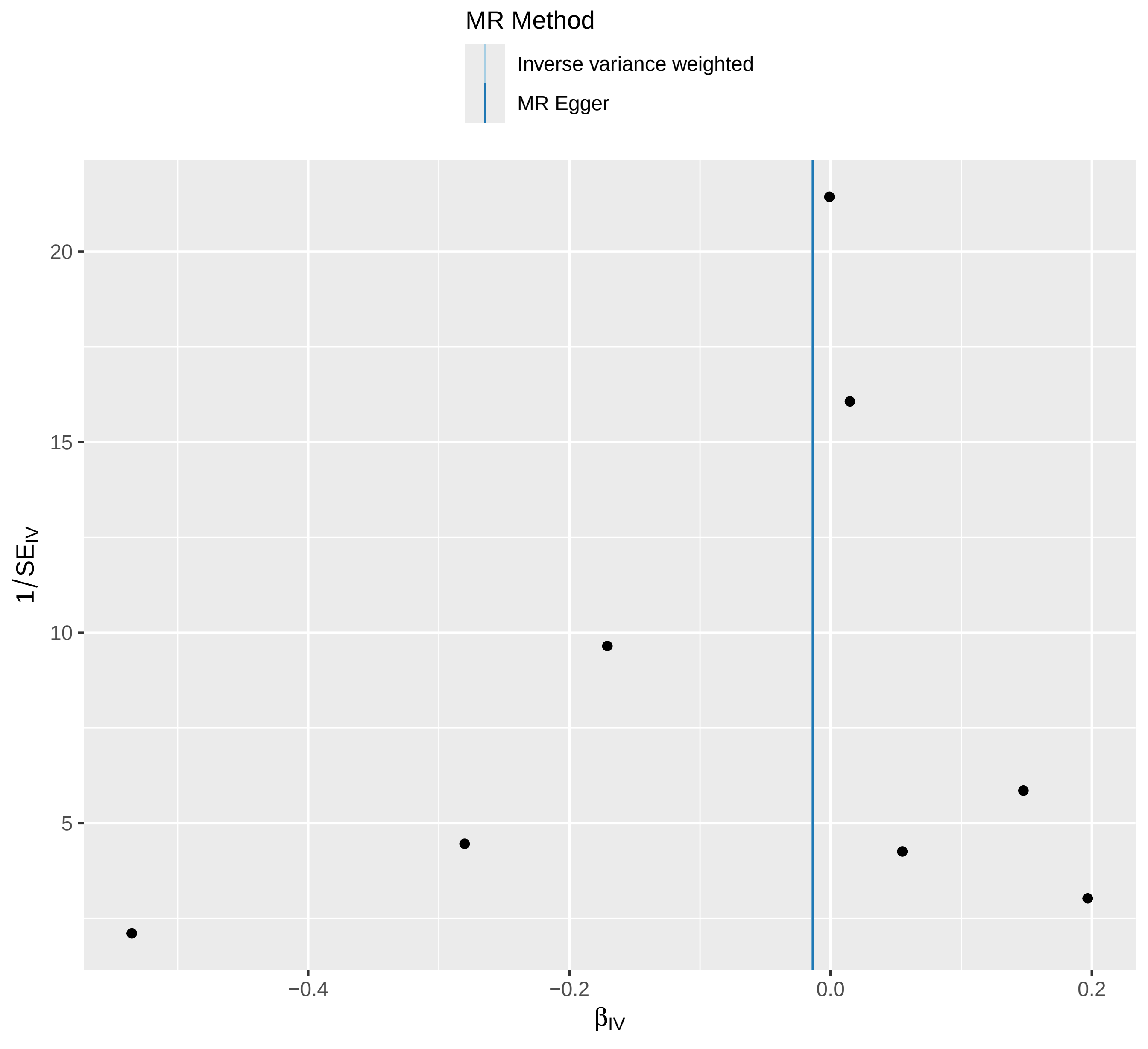

MR Method
Inverse variance weighted
MR Egger
20
15
10
5
1/SE$_{IV}$
−0.4
−0.2
0.0
0.2
β$_{IV}$

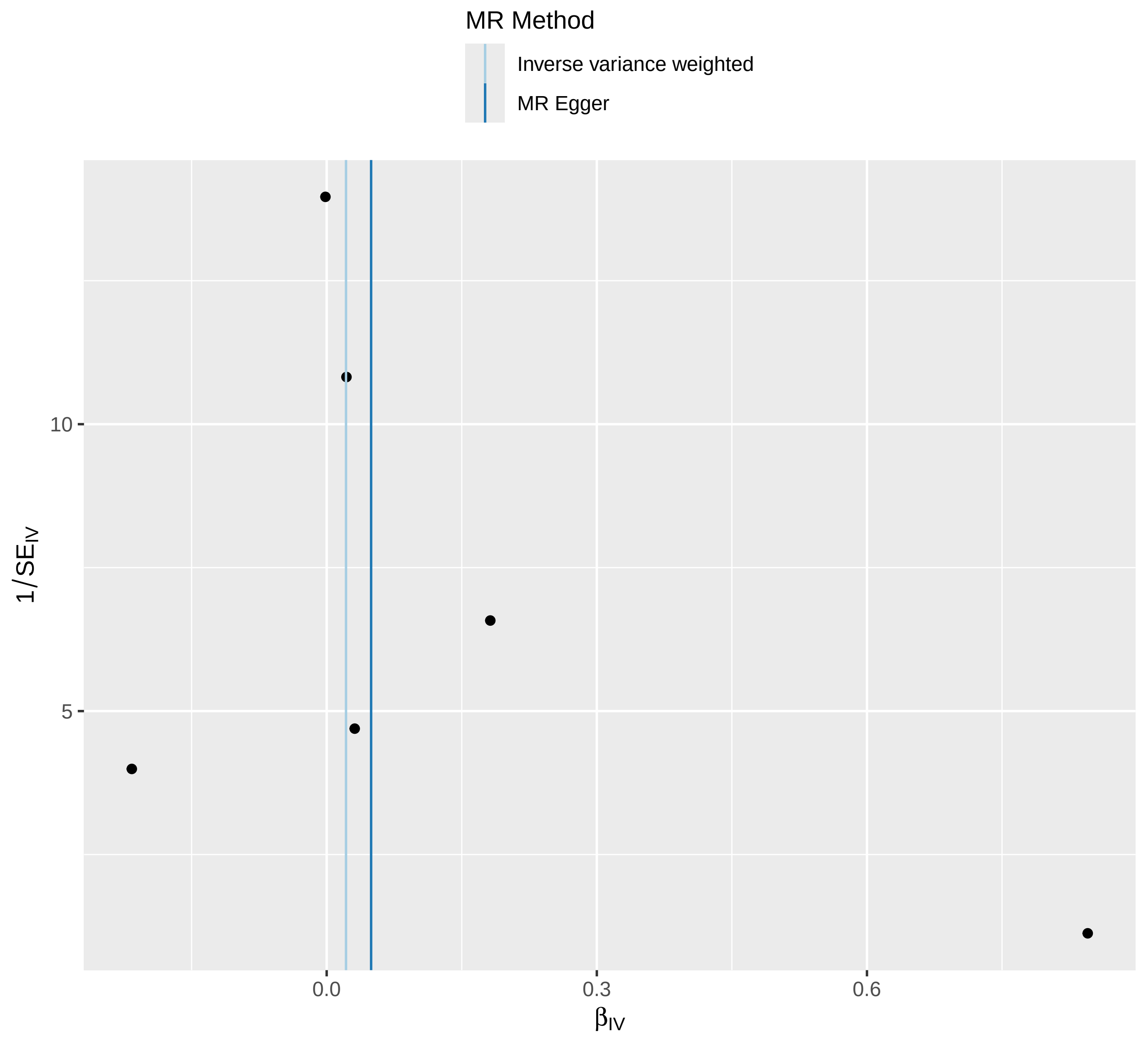

MR Method
Inverse variance weighted
MR Egger
10
5
1/SE_IV
0.0
0.3
0.6
β_IV

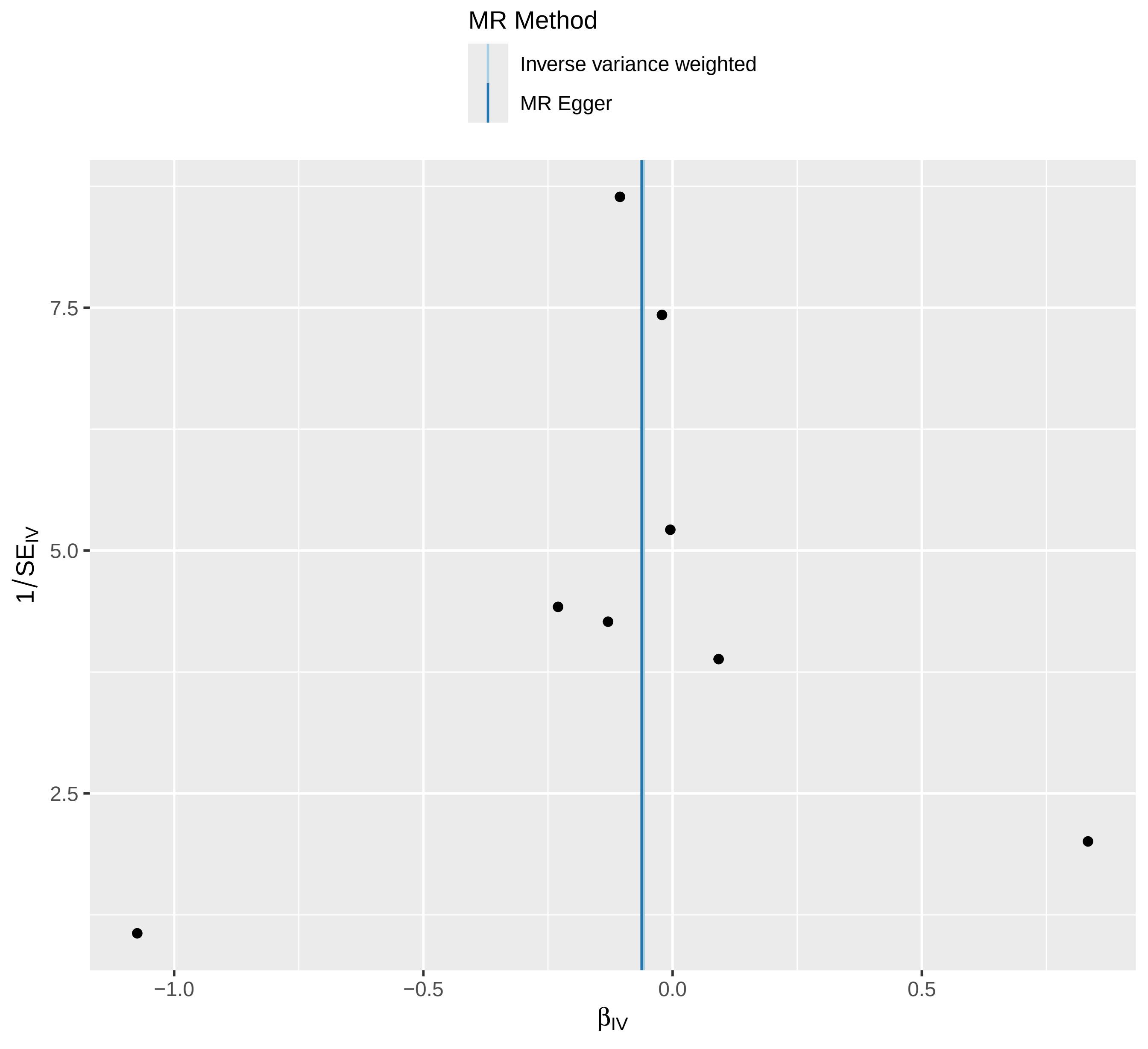

MR Method
Inverse variance weighted
MR Egger
7.5
5.0
2.5
1/SE_IV
−1.0
−0.5
0.0
0.5
β_IV

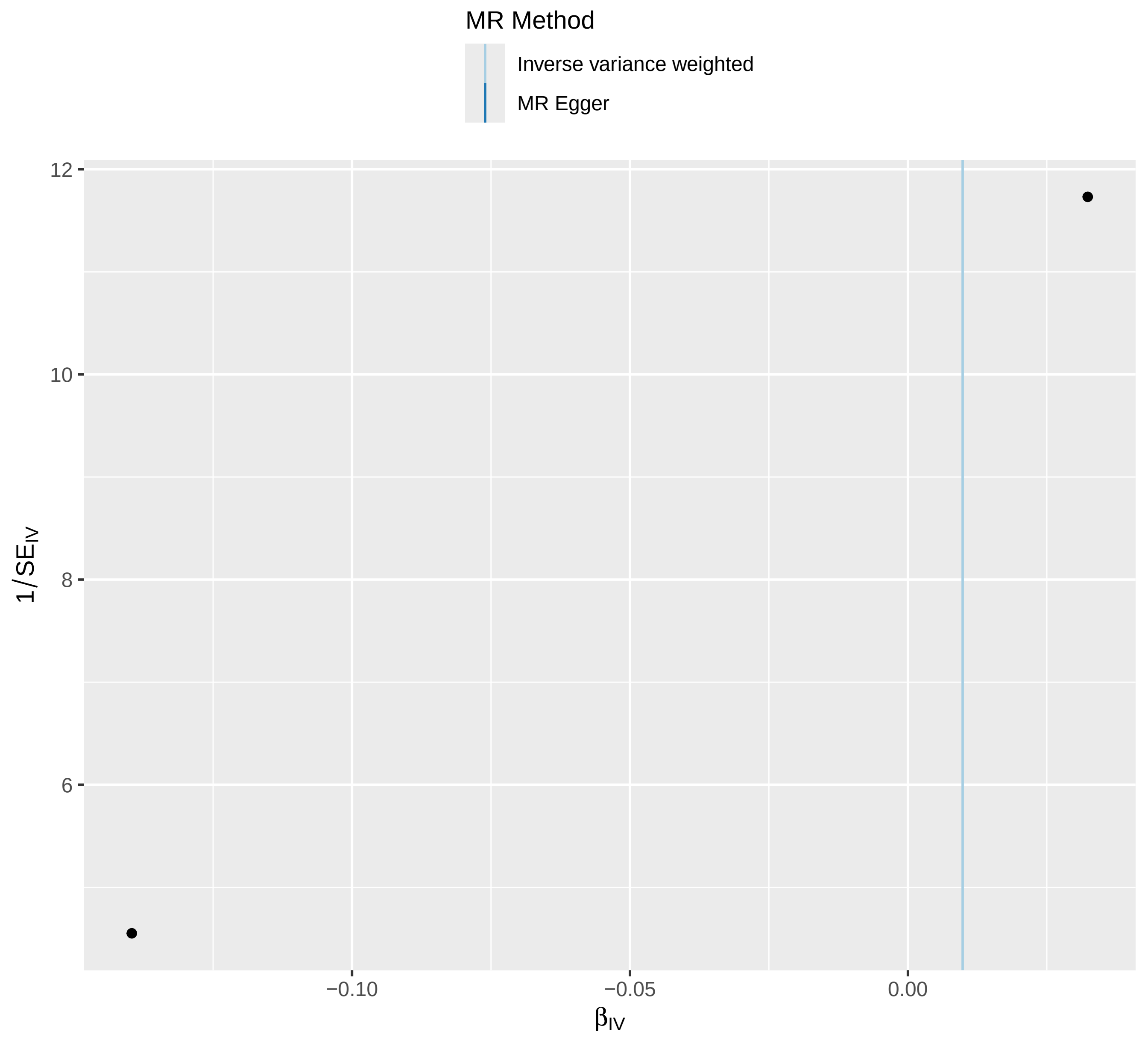

MR Method
Inverse variance weighted
MR Egger
12
10
8
6
1/$SE_{IV}$
−0.10
−0.05
0.00
$\beta_{IV}$

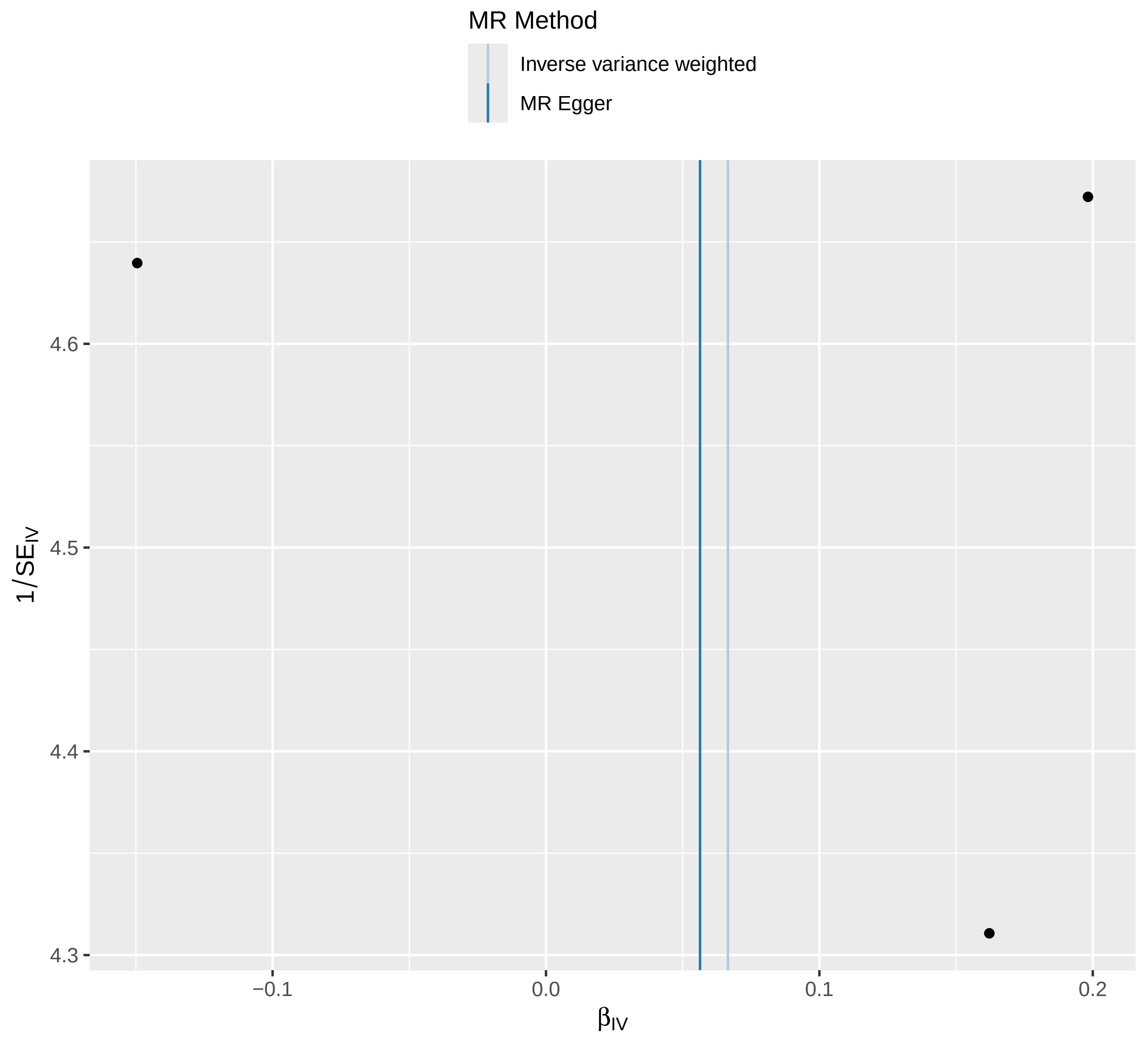
MR Method
Inverse variance weighted
MR Egger
1/SE$_{IV}$
4.6
4.5
4.4
4.3
−0.1
0.0
0.1
0.2
β$_{IV}$

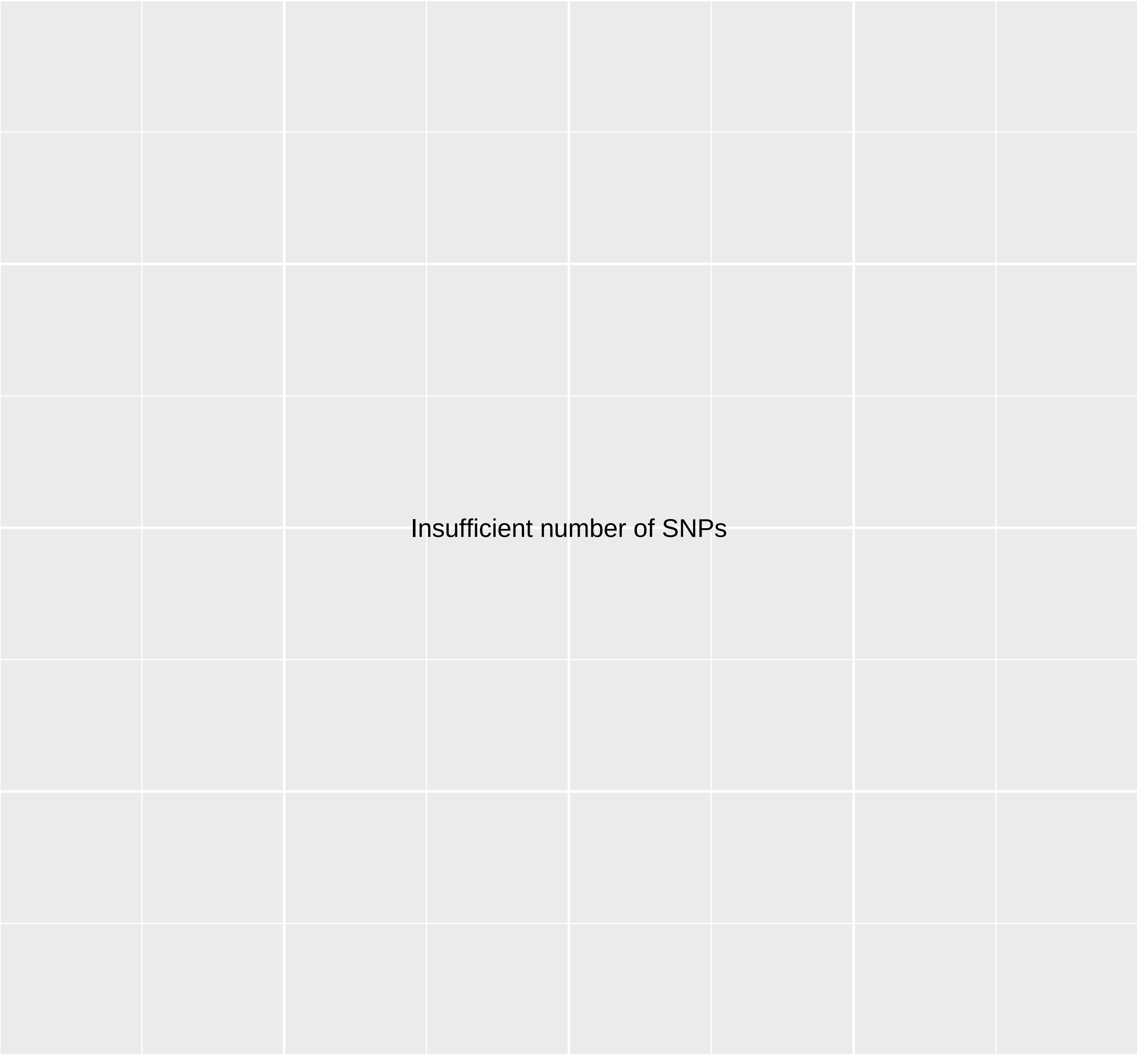
Insufficient number of SNPs

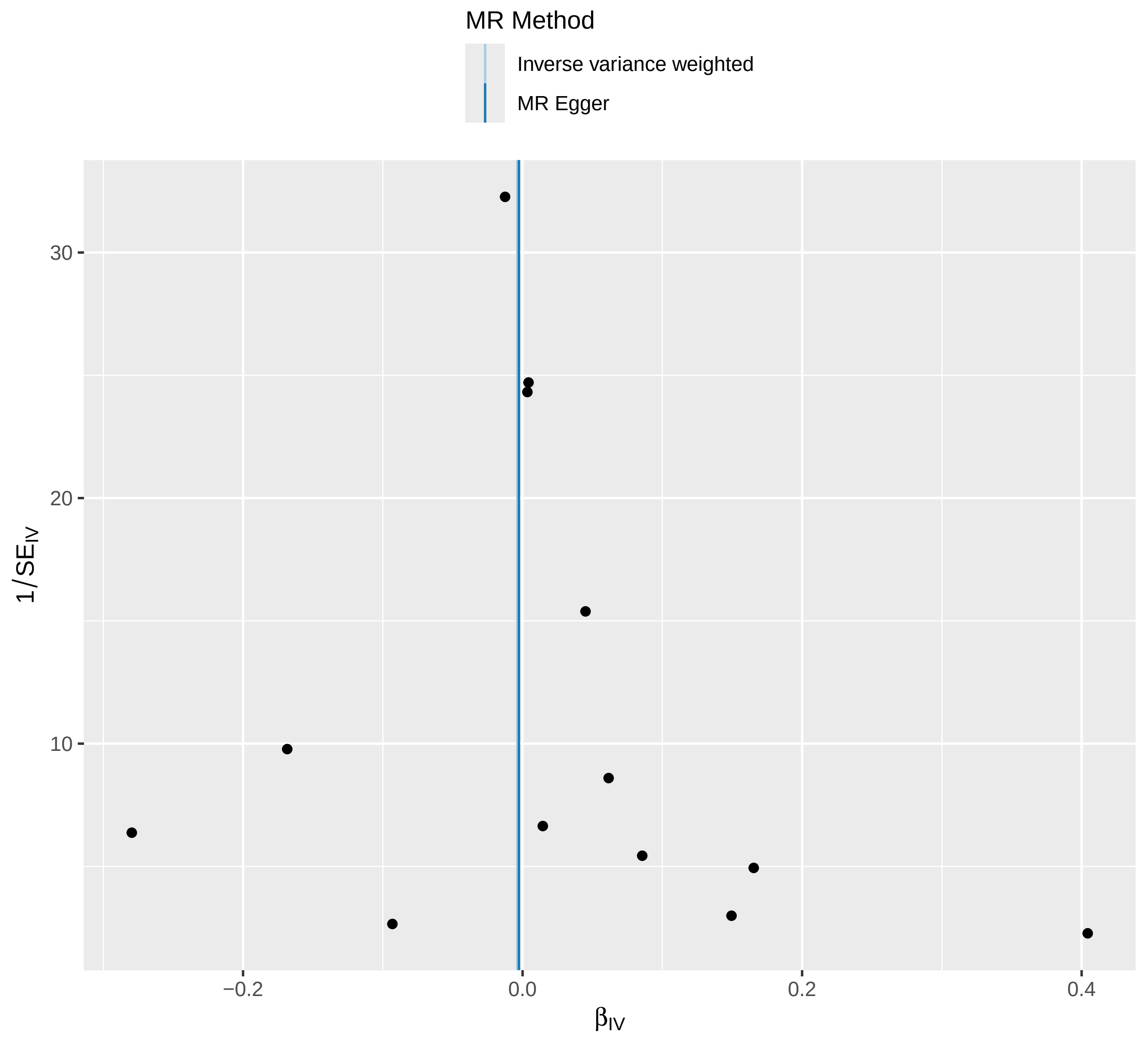

MR Method
Inverse variance weighted
MR Egger
30
20
10
1/SE_IV
−0.2
0.0
0.2
0.4
β_IV

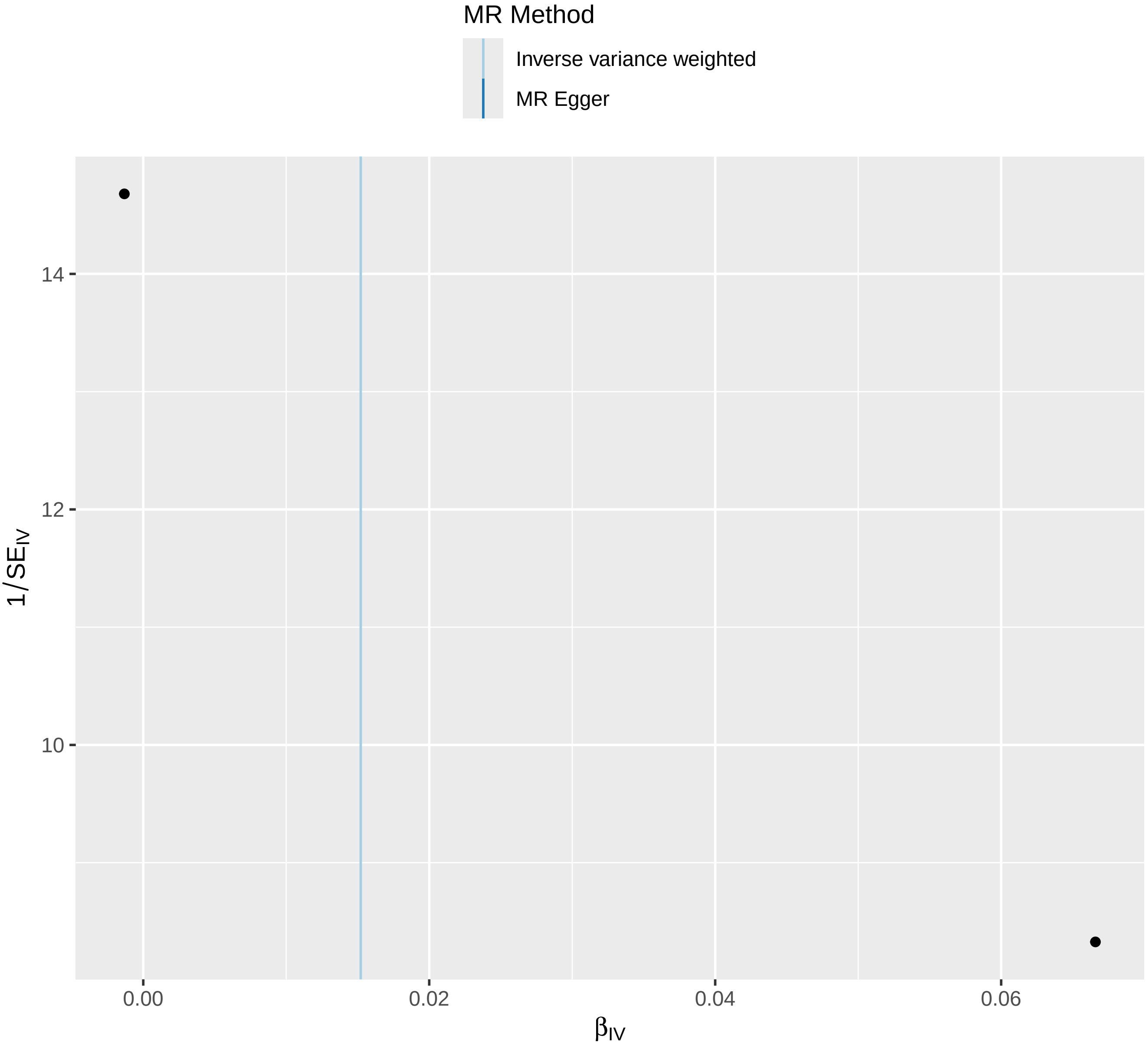
MR Method
Inverse variance weighted
MR Egger
14
12
10
1/SE$_{IV}$
0.00
0.02
0.04
0.06
β$_{IV}$

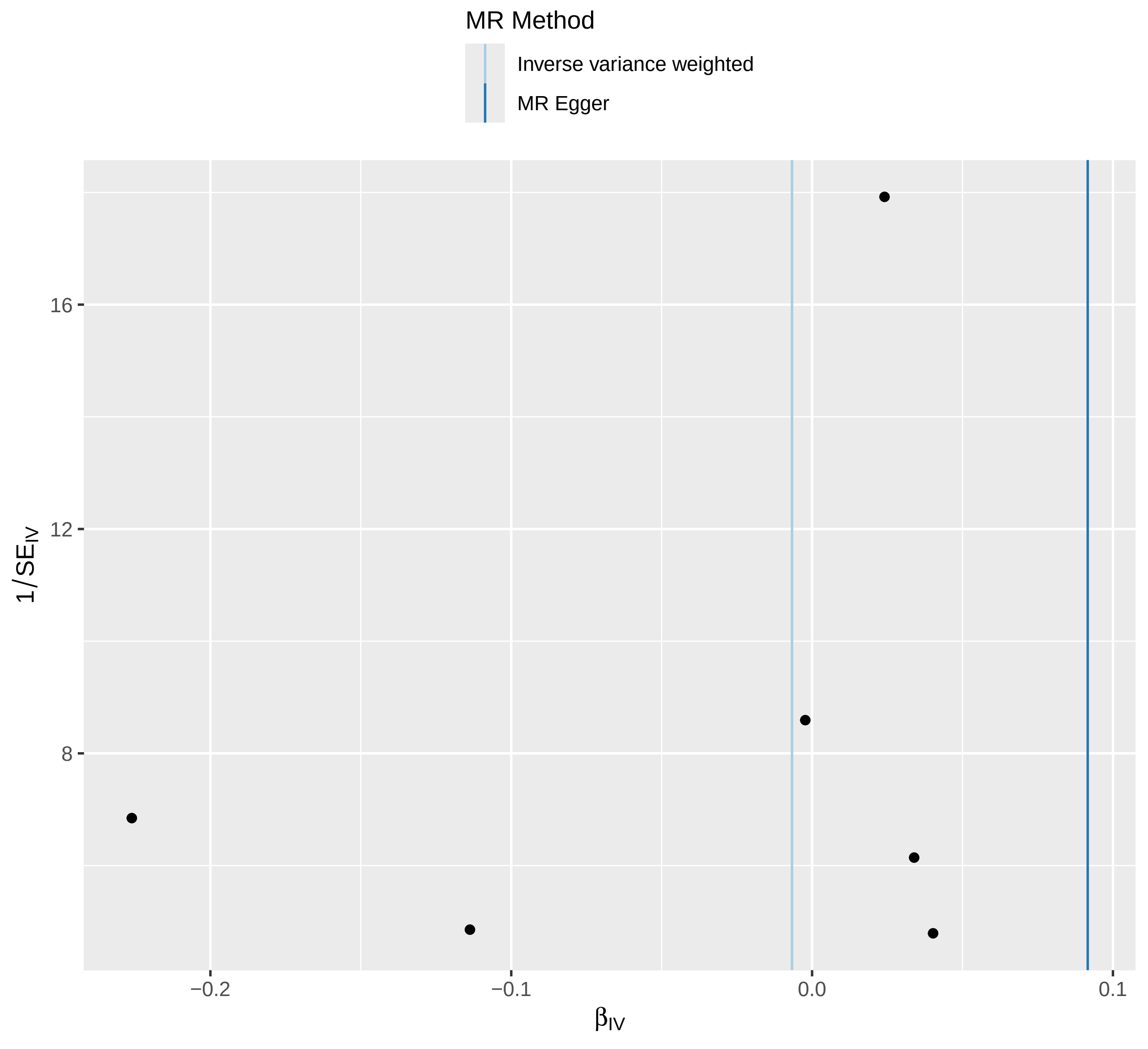
MR Method
Inverse variance weighted
MR Egger
16
12
8
1/SE_IV
−0.2
−0.1
0.0
0.1
β_IV

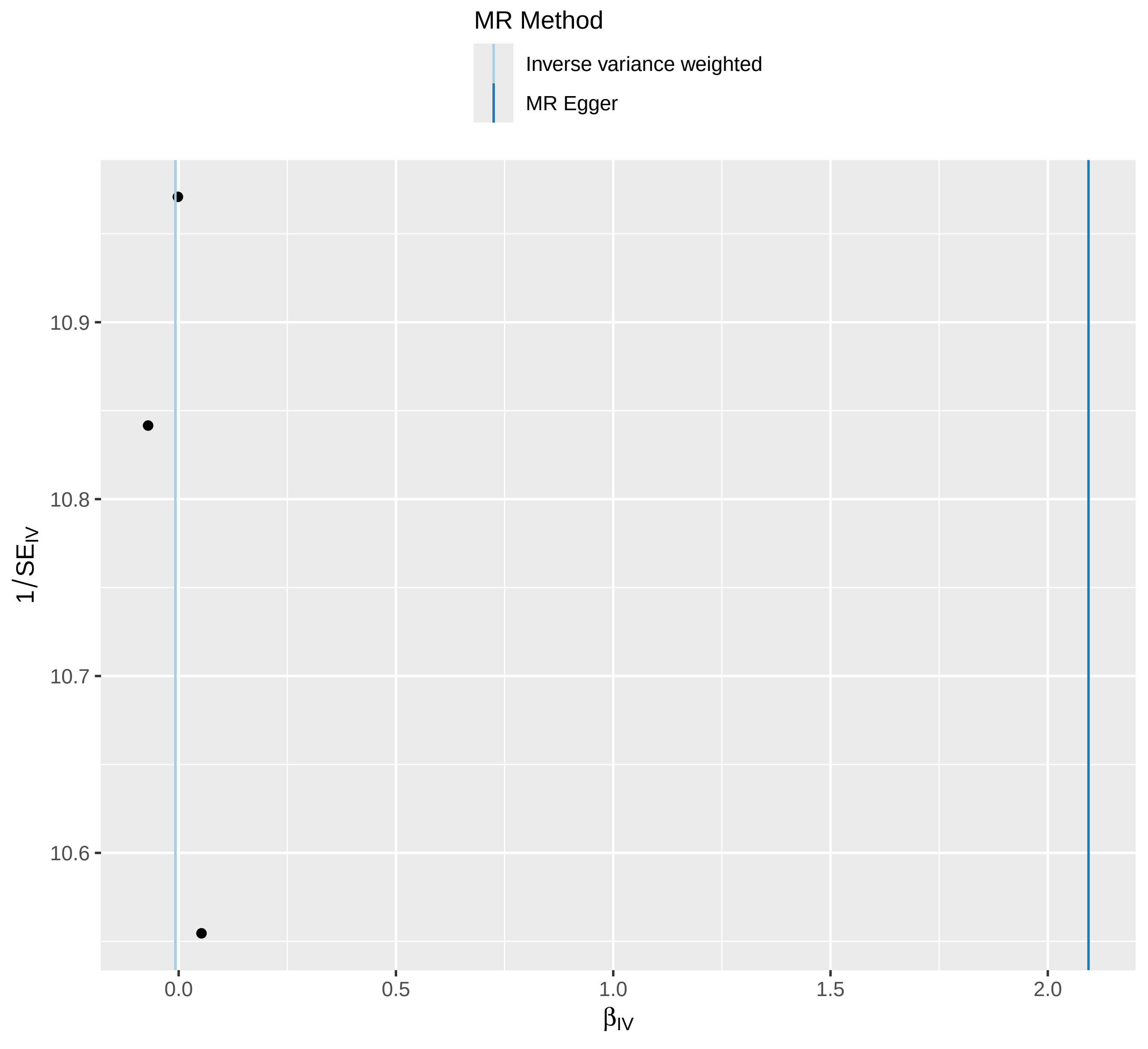

MR Method
Inverse variance weighted
MR Egger
10.9
10.8
10.7
10.6
$1/SE_{IV}$
0.0
0.5
1.0
1.5
2.0
$\beta_{IV}$

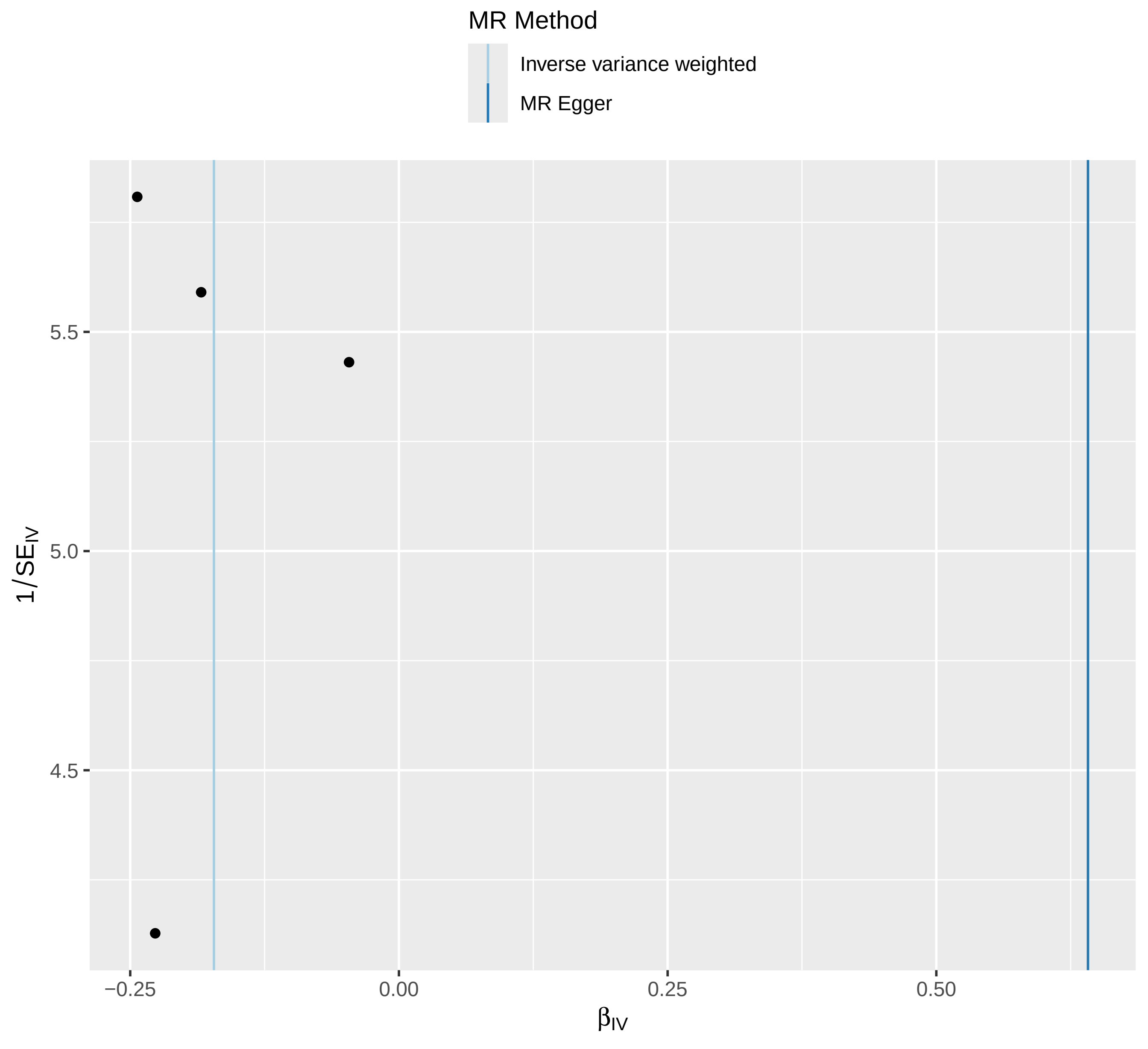
MR Method
Inverse variance weighted
MR Egger
5.5
5.0
4.5
1/SE_IV
−0.25
0.00
0.25
0.50
β_IV

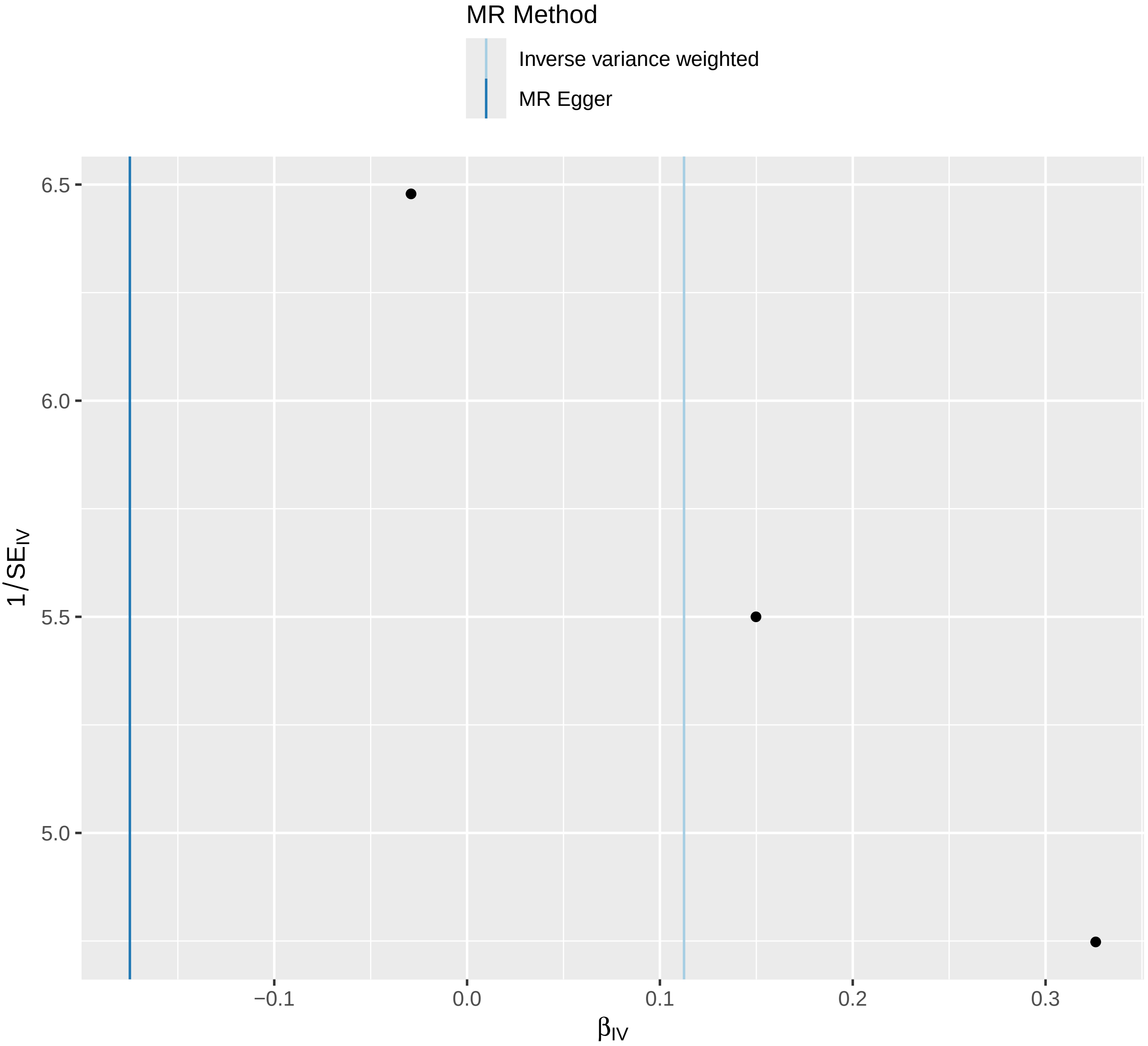
MR Method
Inverse variance weighted
MR Egger
6.5
6.0
5.5
5.0
1/SE_IV
−0.1
0.0
0.1
0.2
0.3
β_IV

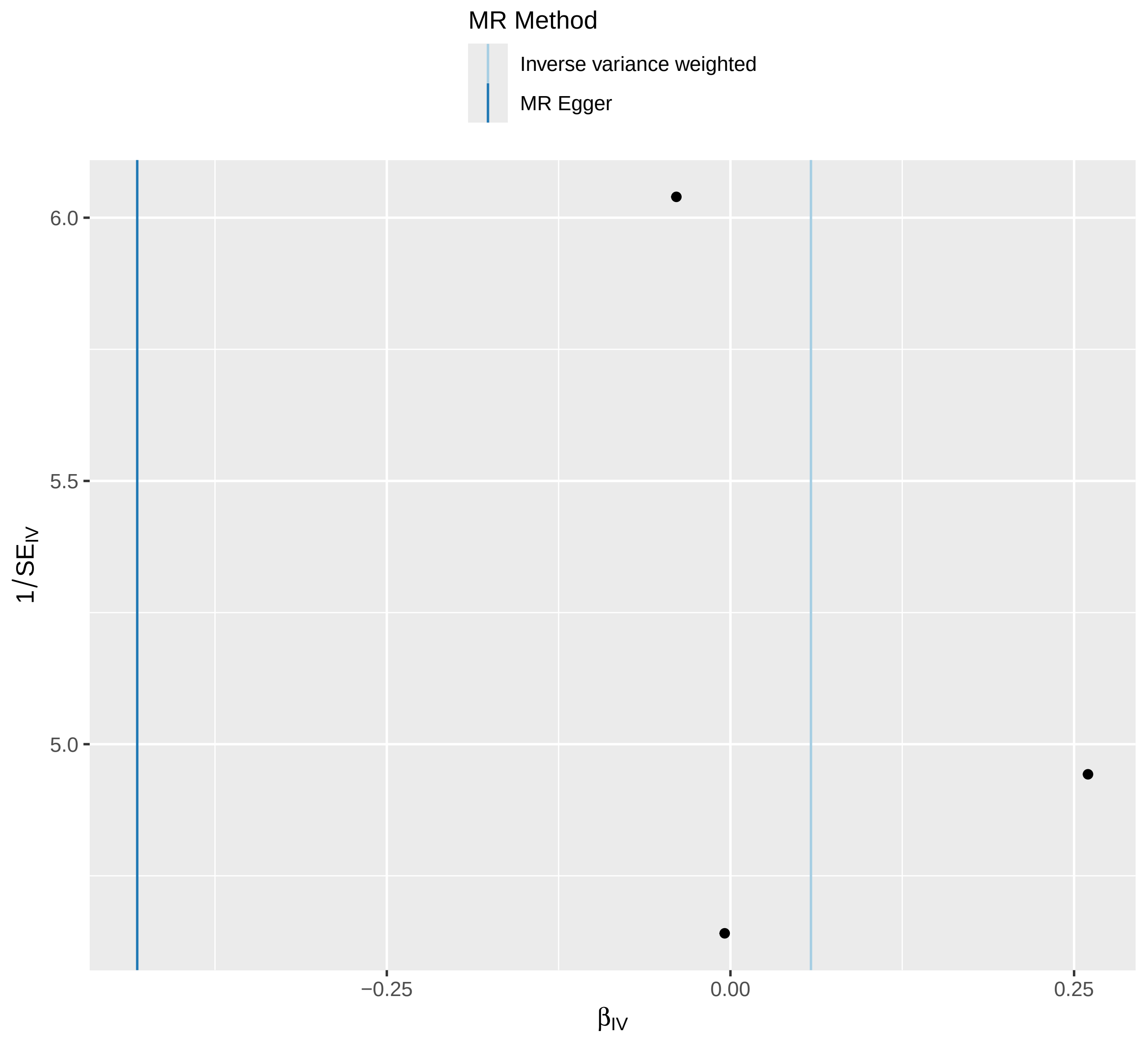
MR Method
Inverse variance weighted
MR Egger
6.0
5.5
5.0
1/SE$_{IV}$
−0.25
0.00
0.25
β$_{IV}$

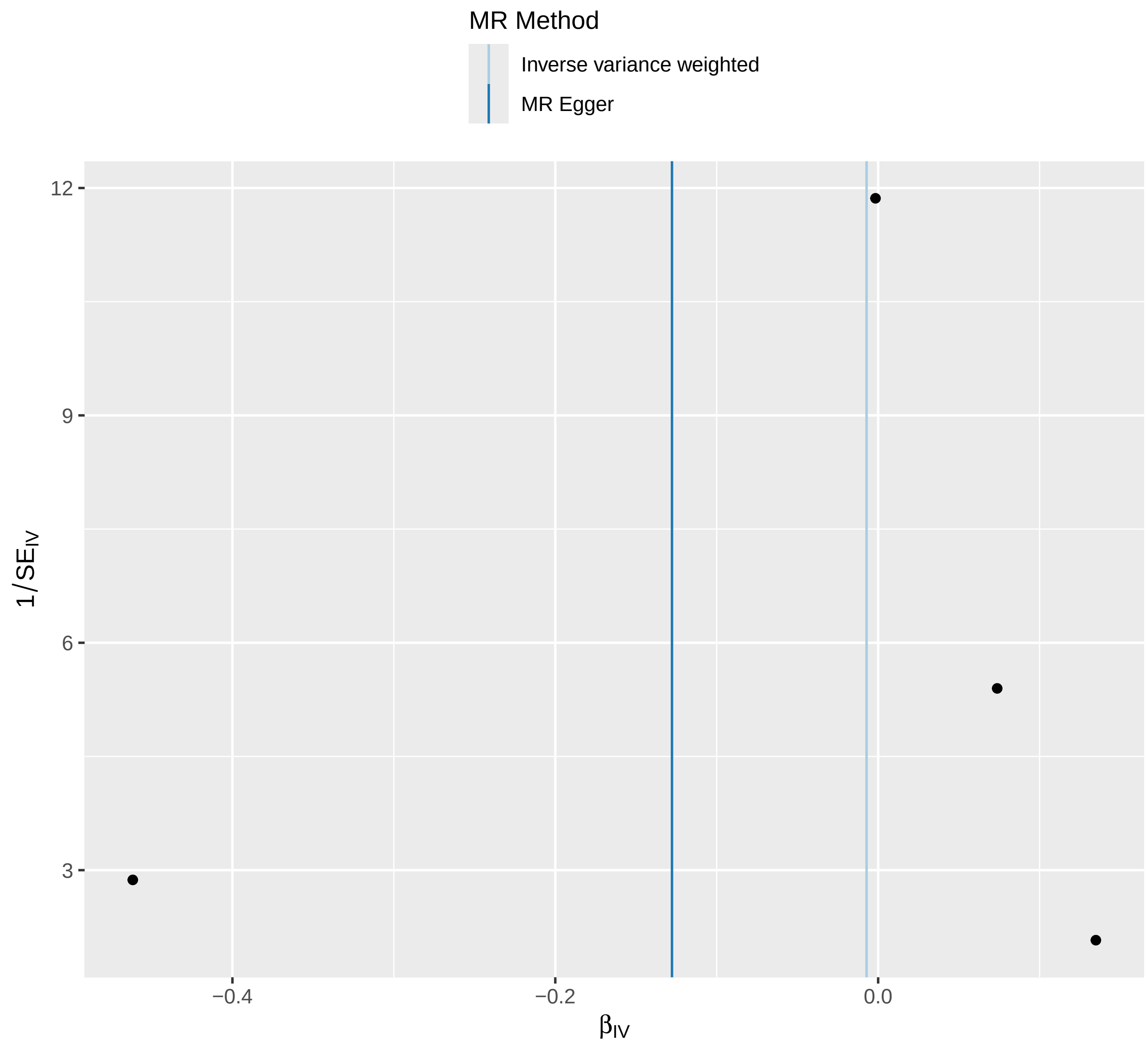
MR Method
Inverse variance weighted
MR Egger
12
9
6
3
1/SE_IV
−0.4
−0.2
0.0
β_IV

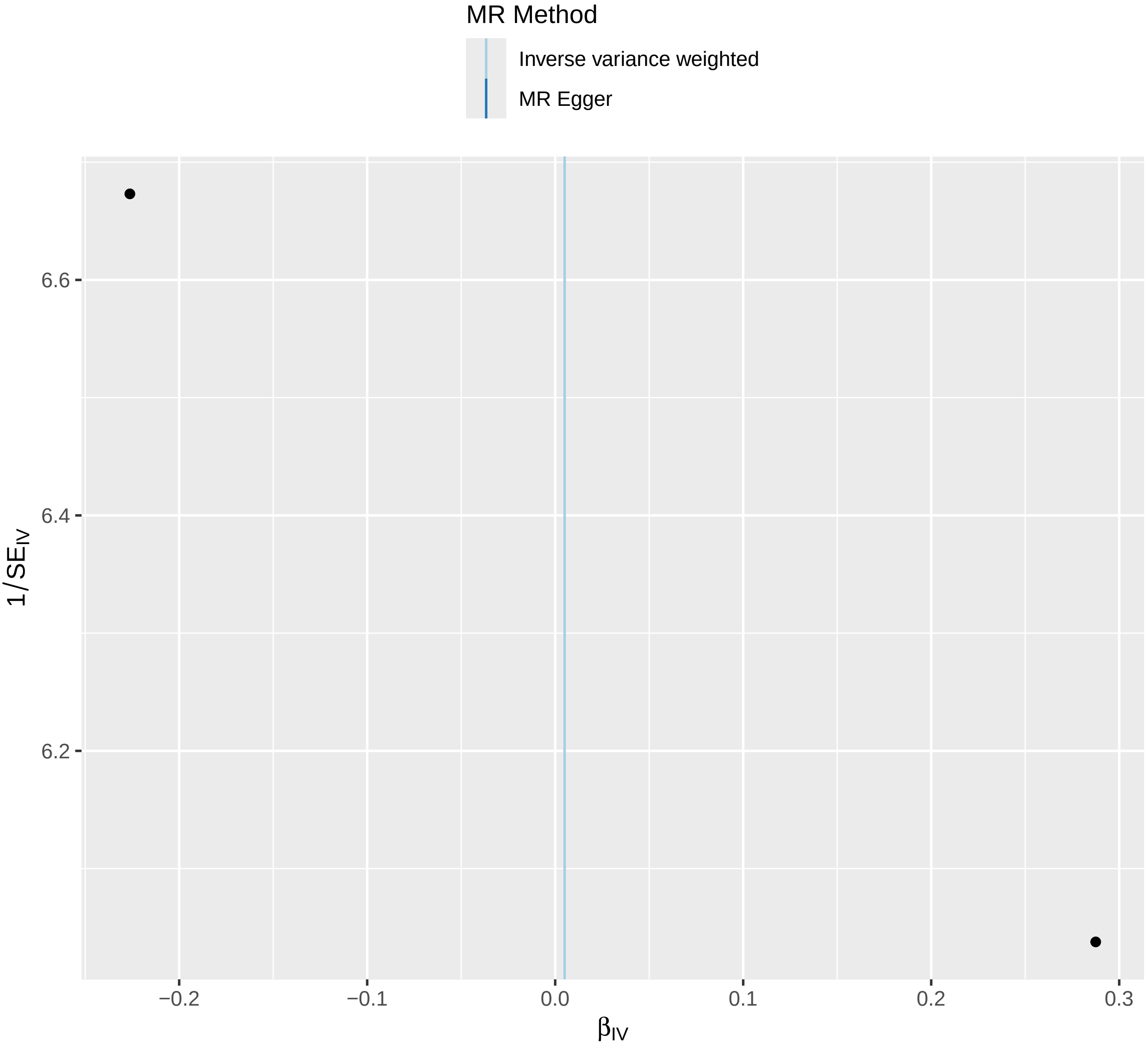

MR Method
Inverse variance weighted
MR Egger
6.6
6.4
6.2
1/SE$_{IV}$
−0.2
−0.1
0.0
0.1
0.2
0.3
β$_{IV}$

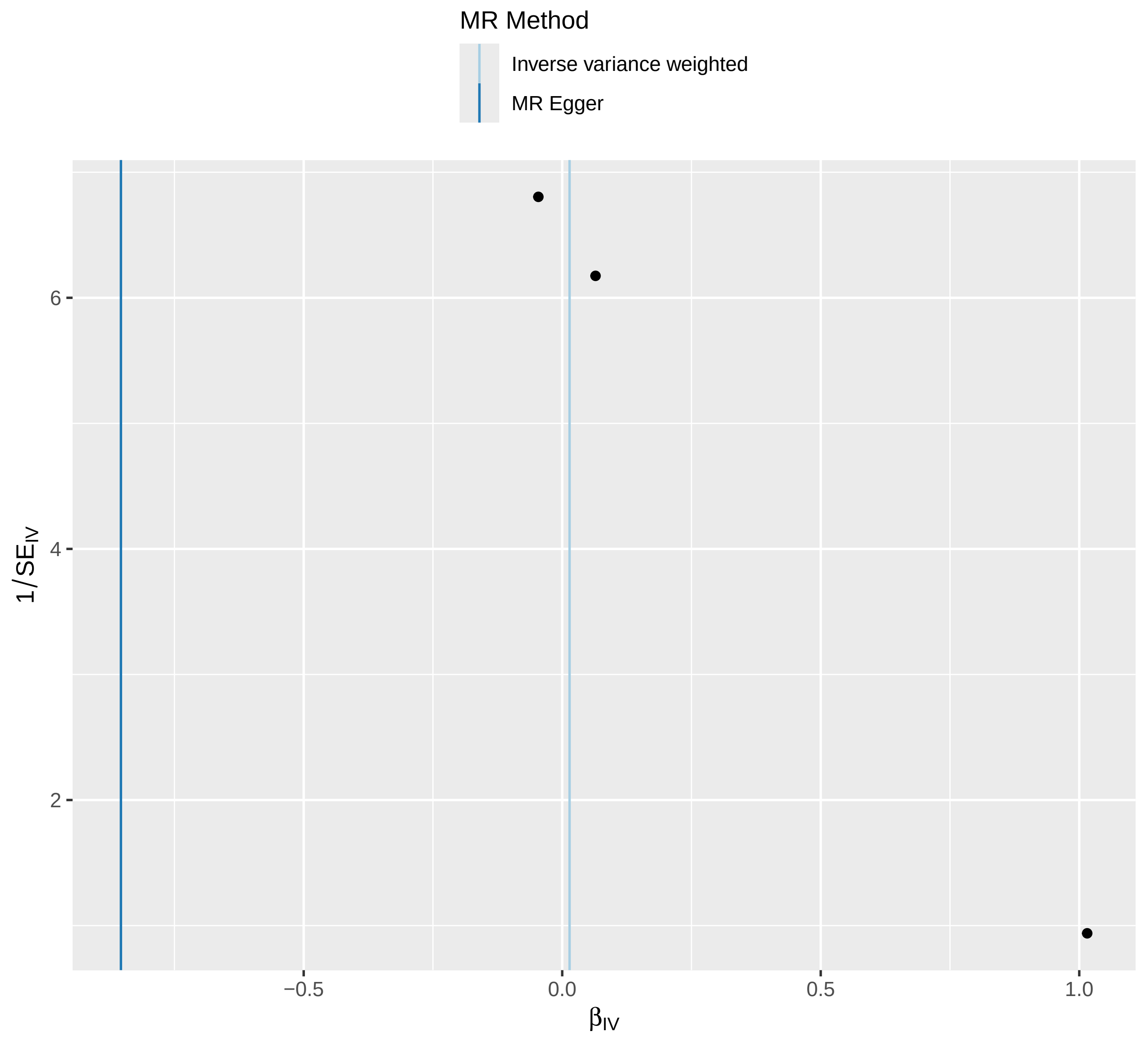

MR Method
Inverse variance weighted
MR Egger
6
4
2
1/SE$_{IV}$
−0.5
0.0
0.5
1.0
β$_{IV}$

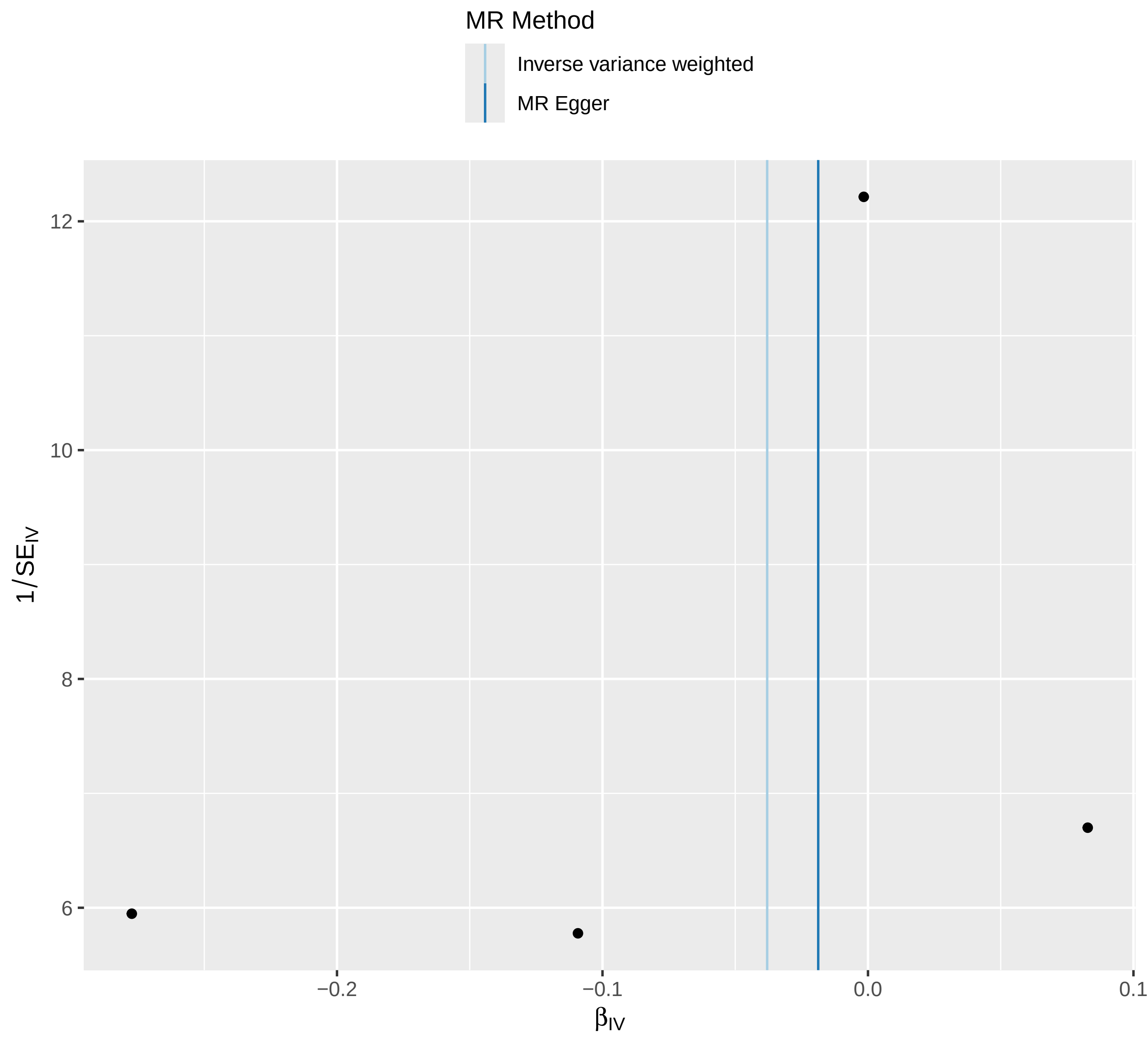

MR Method
Inverse variance weighted
MR Egger
12
10
8
6
1/SE_IV
−0.2
−0.1
0.0
0.1
β_IV

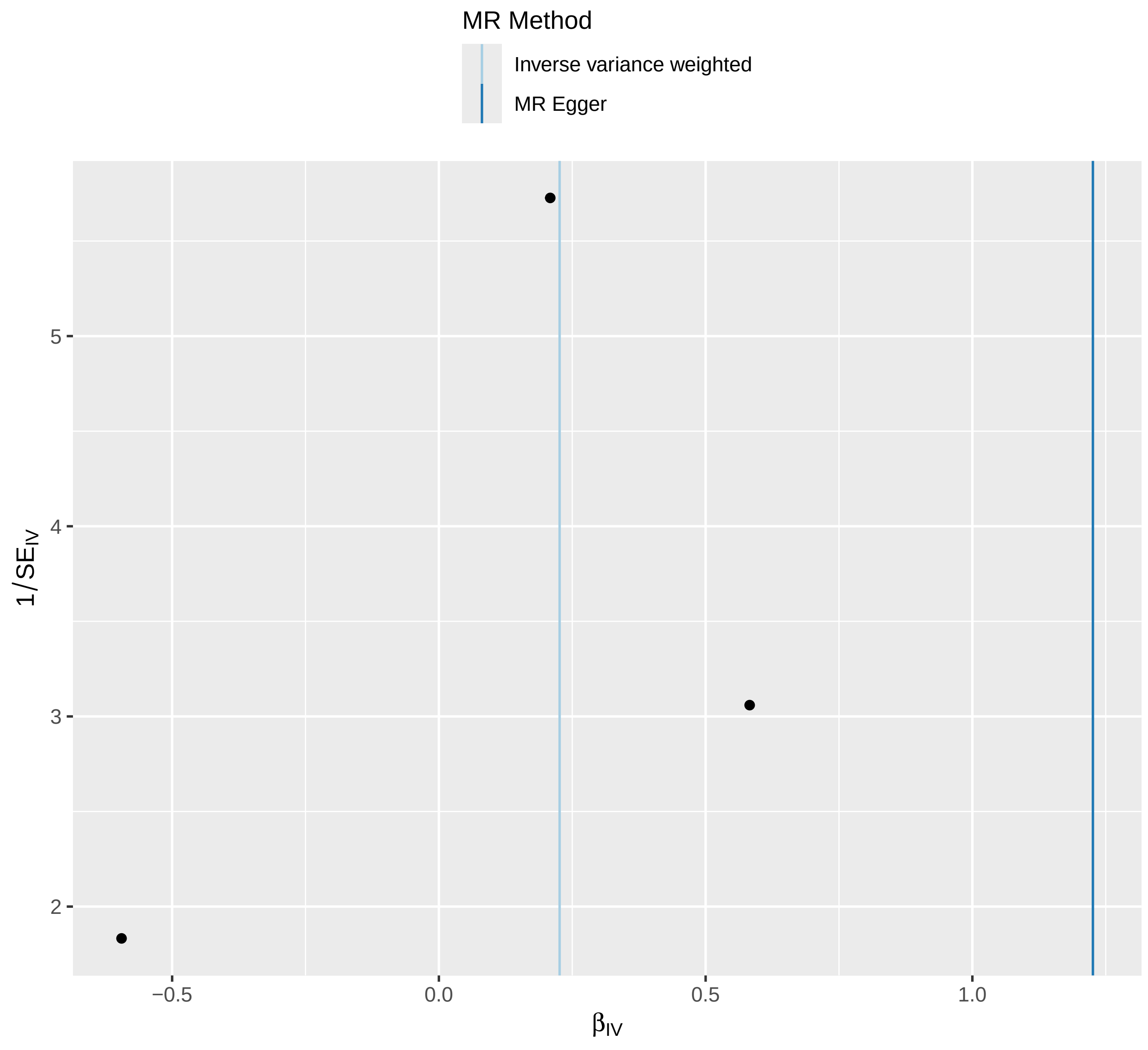
MR Method
Inverse variance weighted
MR Egger
5
4
3
2
1/SE_IV
−0.5
0.0
0.5
1.0
β_IV

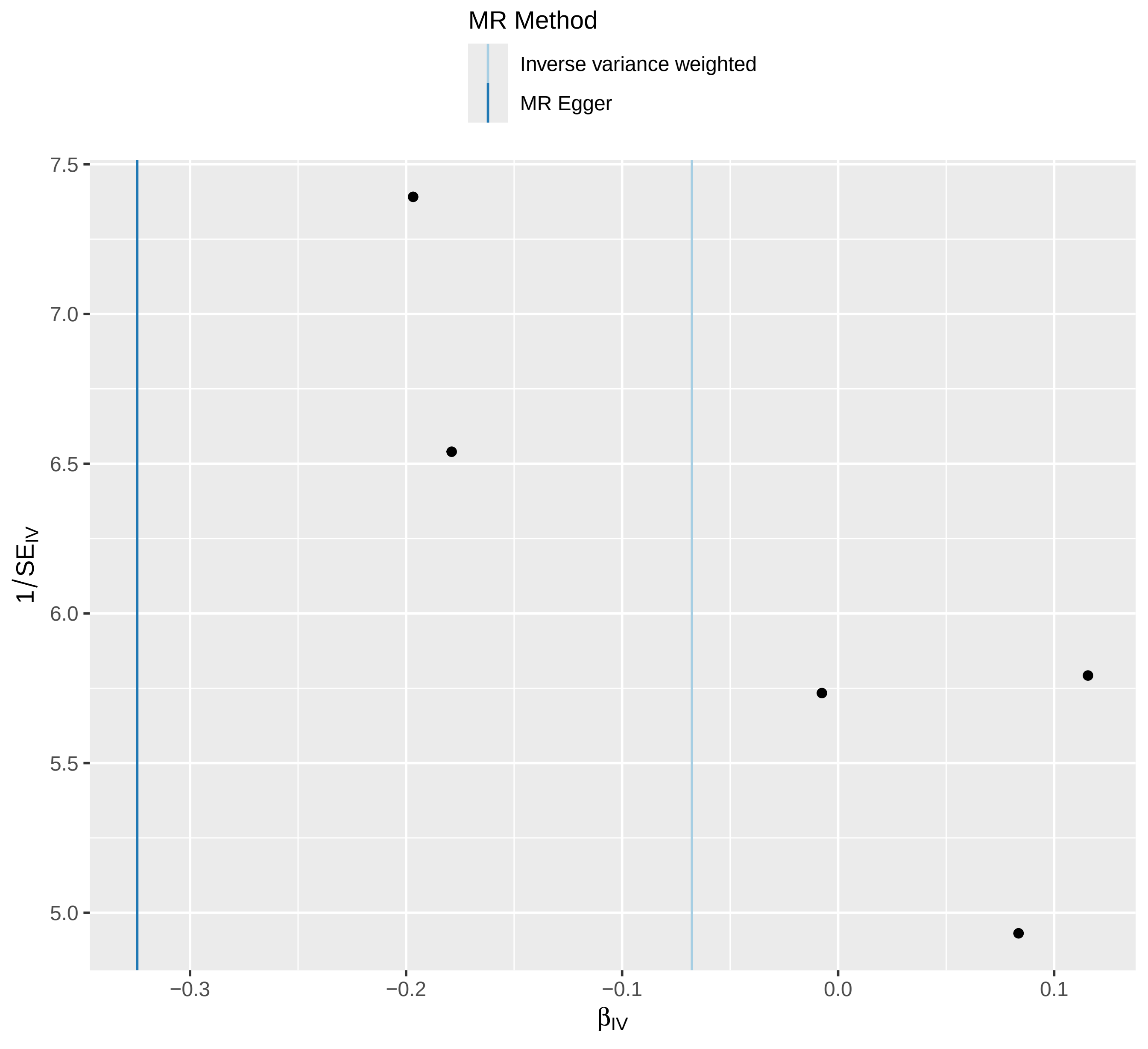
MR Method
Inverse variance weighted
MR Egger
7.5
7.0
6.5
6.0
5.5
5.0
1/SE_IV
−0.3
−0.2
−0.1
0.0
0.1
β_IV

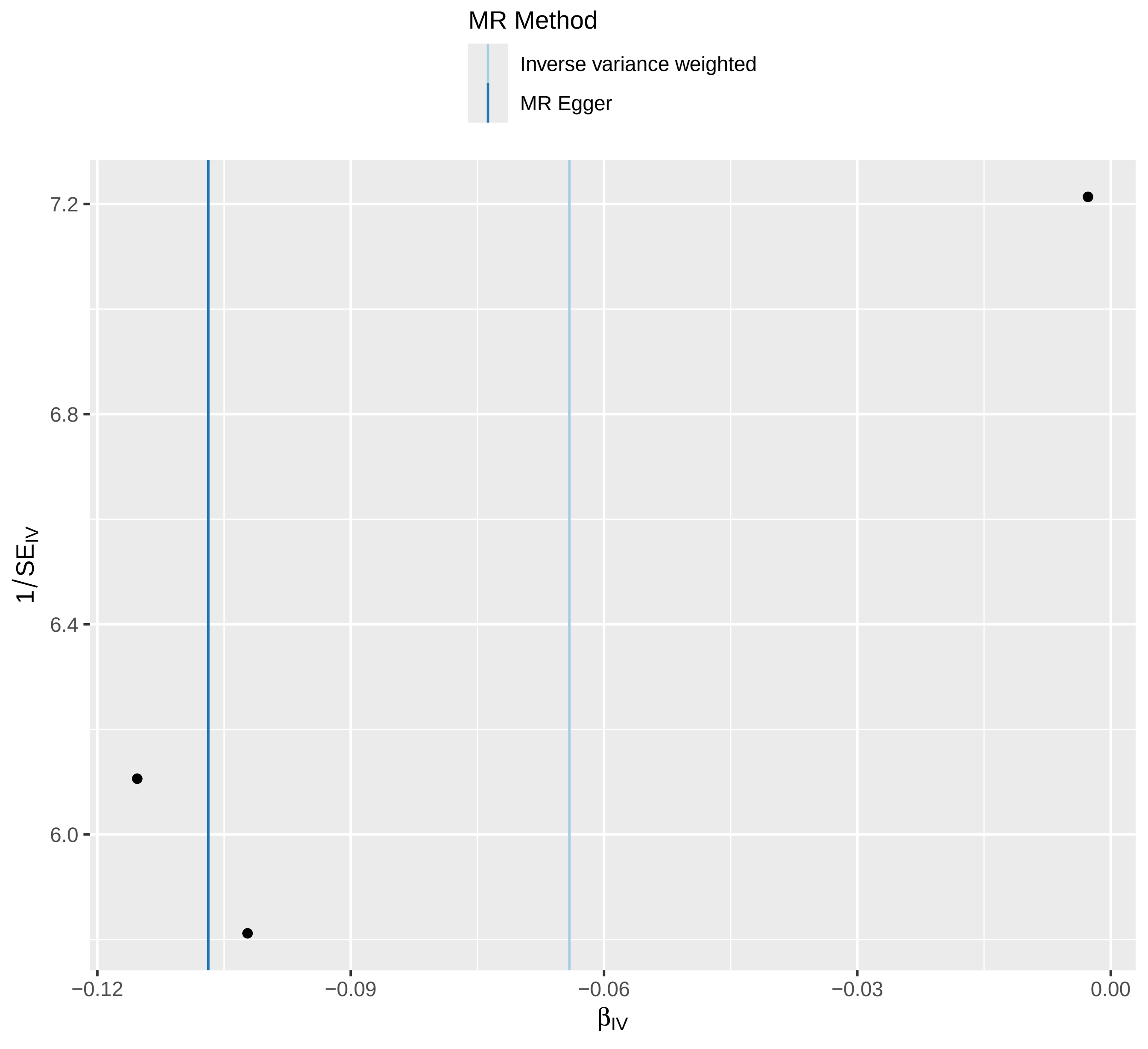
MR Method
Inverse variance weighted
MR Egger
7.2
6.8
6.4
6.0
1/SE$_{IV}$
−0.12
−0.09
−0.06
−0.03
0.00
β$_{IV}$

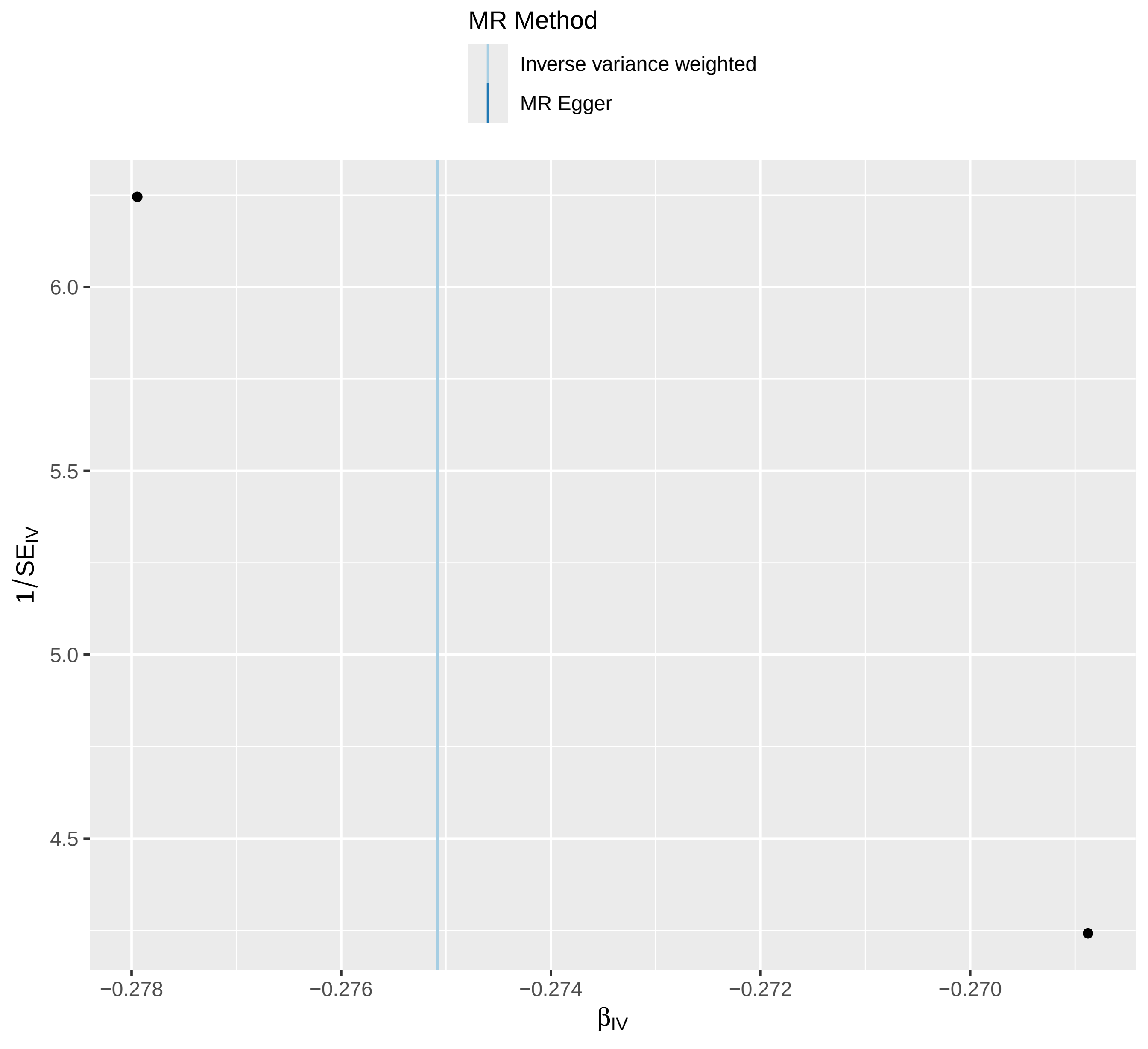

MR Method
Inverse variance weighted
MR Egger
6.0
5.5
5.0
4.5
1/SE_IV
−0.278
−0.276
−0.274
−0.272
−0.270
β_IV

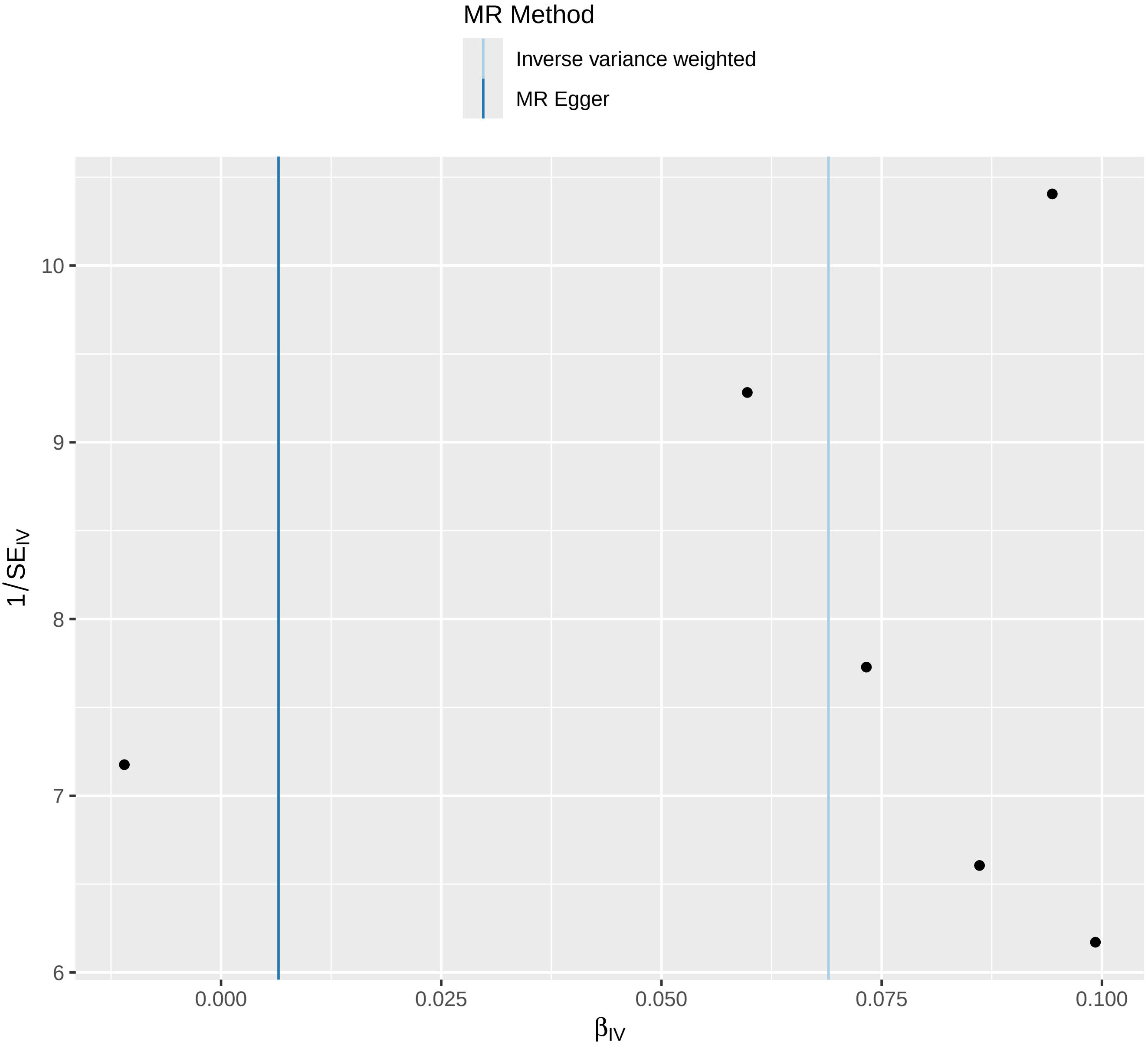

MR Method
Inverse variance weighted
MR Egger
10
9
8
7
6
1/SE_IV
0.000
0.025
0.050
0.075
0.100
β_IV

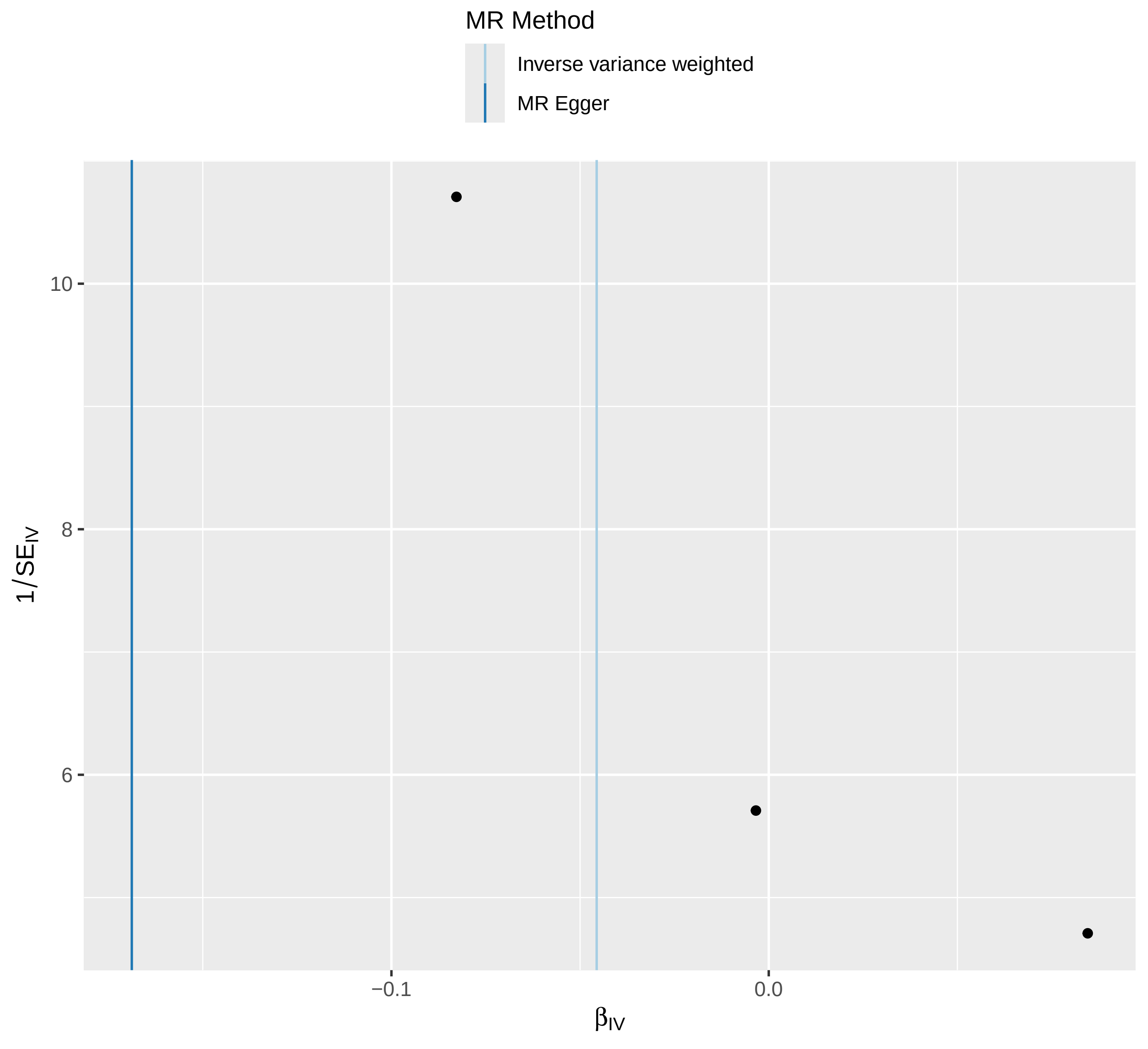

MR Method
Inverse variance weighted
MR Egger
10
8
6
1/SE_IV
−0.1
0.0
β_IV

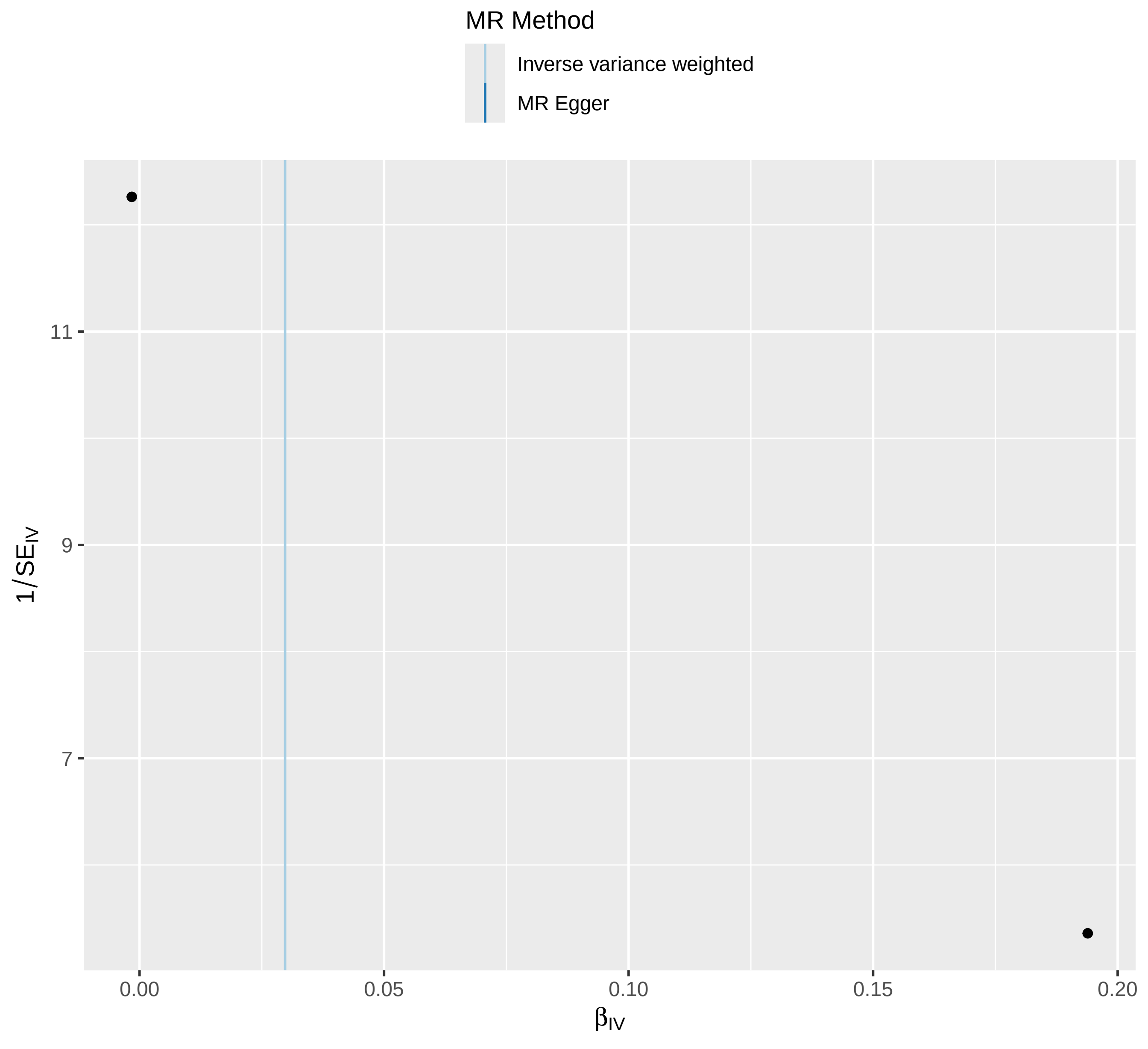
MR Method
Inverse variance weighted
MR Egger
11
9
7
0.00
0.05
0.10
0.15
0.20
$1/SE_{IV}$
$\beta_{IV}$

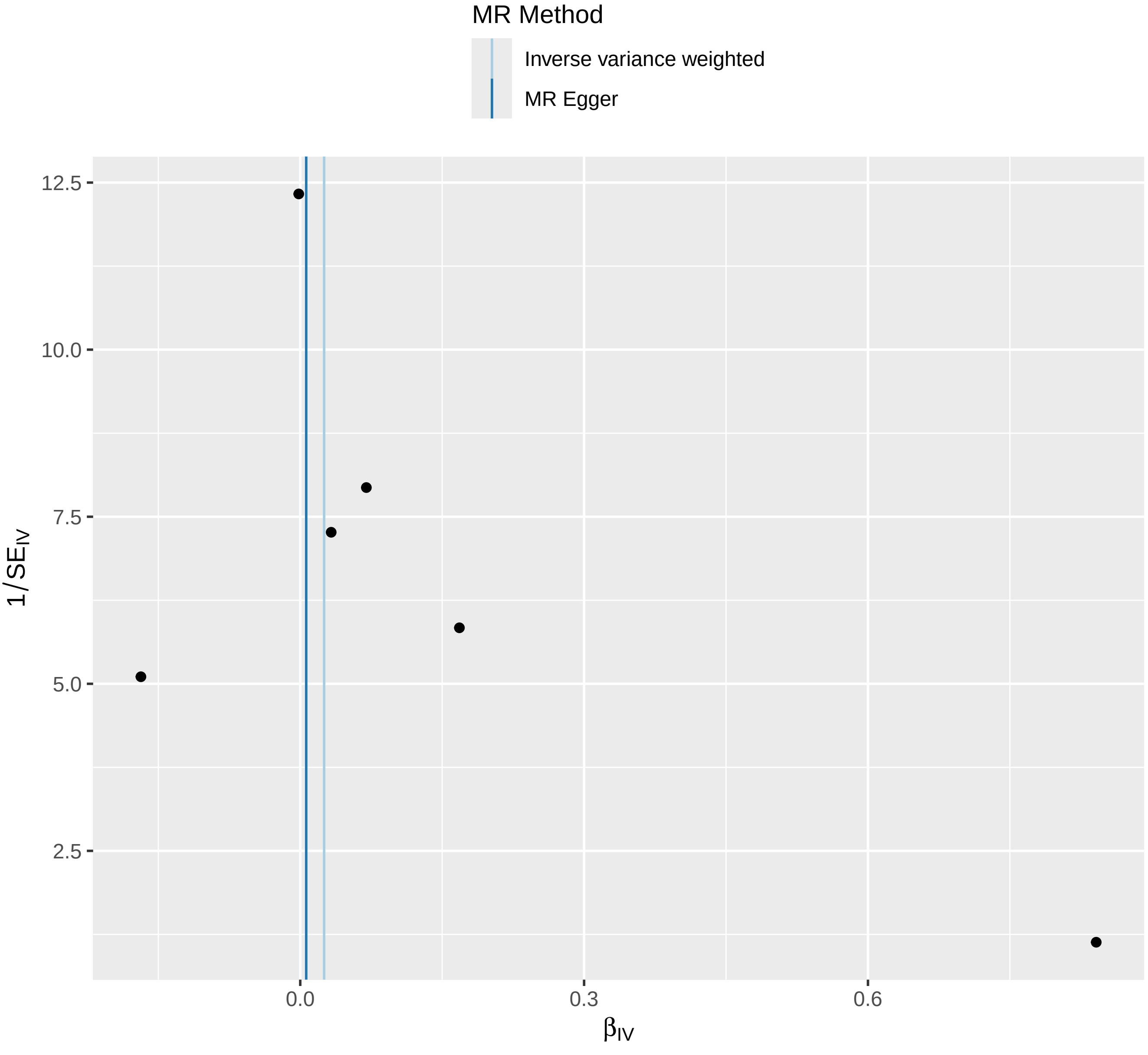
MR Method
Inverse variance weighted
MR Egger
12.5
10.0
7.5
5.0
2.5
1/SE_IV
0.0
0.3
0.6
β_IV

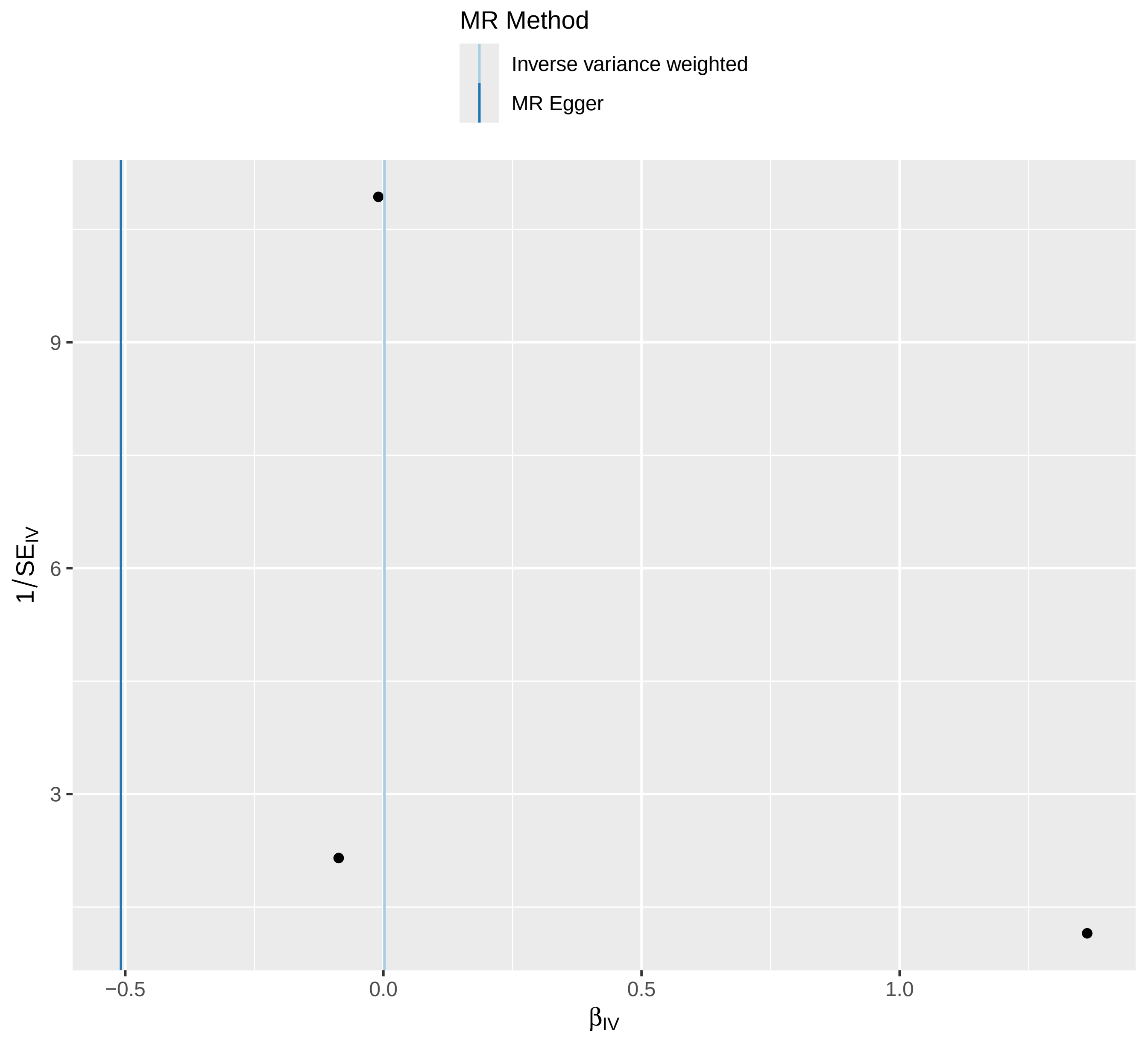
MR Method
Inverse variance weighted
MR Egger
9
6
3
1/SE_IV
−0.5
0.0
0.5
1.0
β_IV

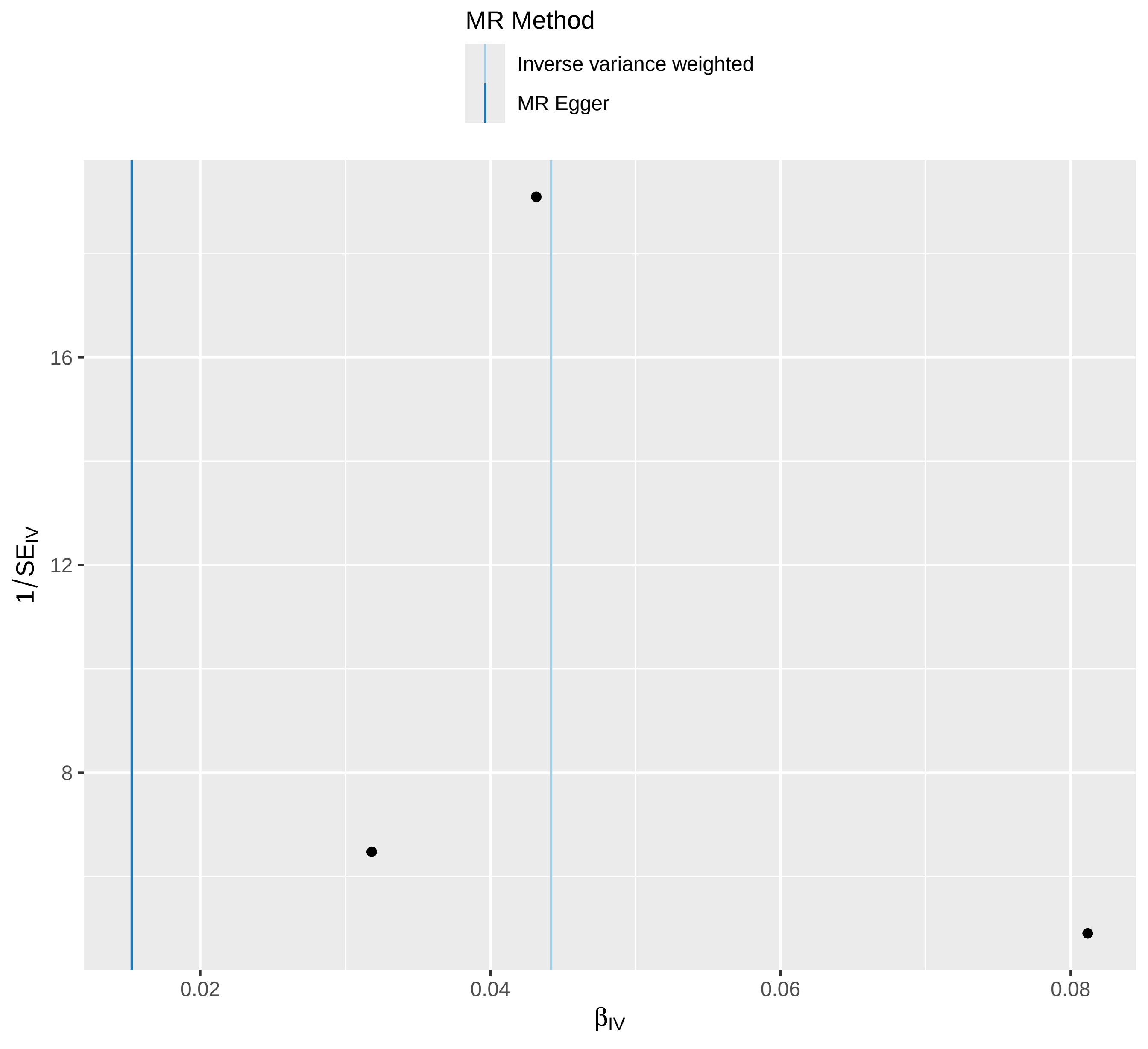
MR Method
Inverse variance weighted
MR Egger
16
12
8
0.02
0.04
0.06
0.08
1/SE_IV
β_IV

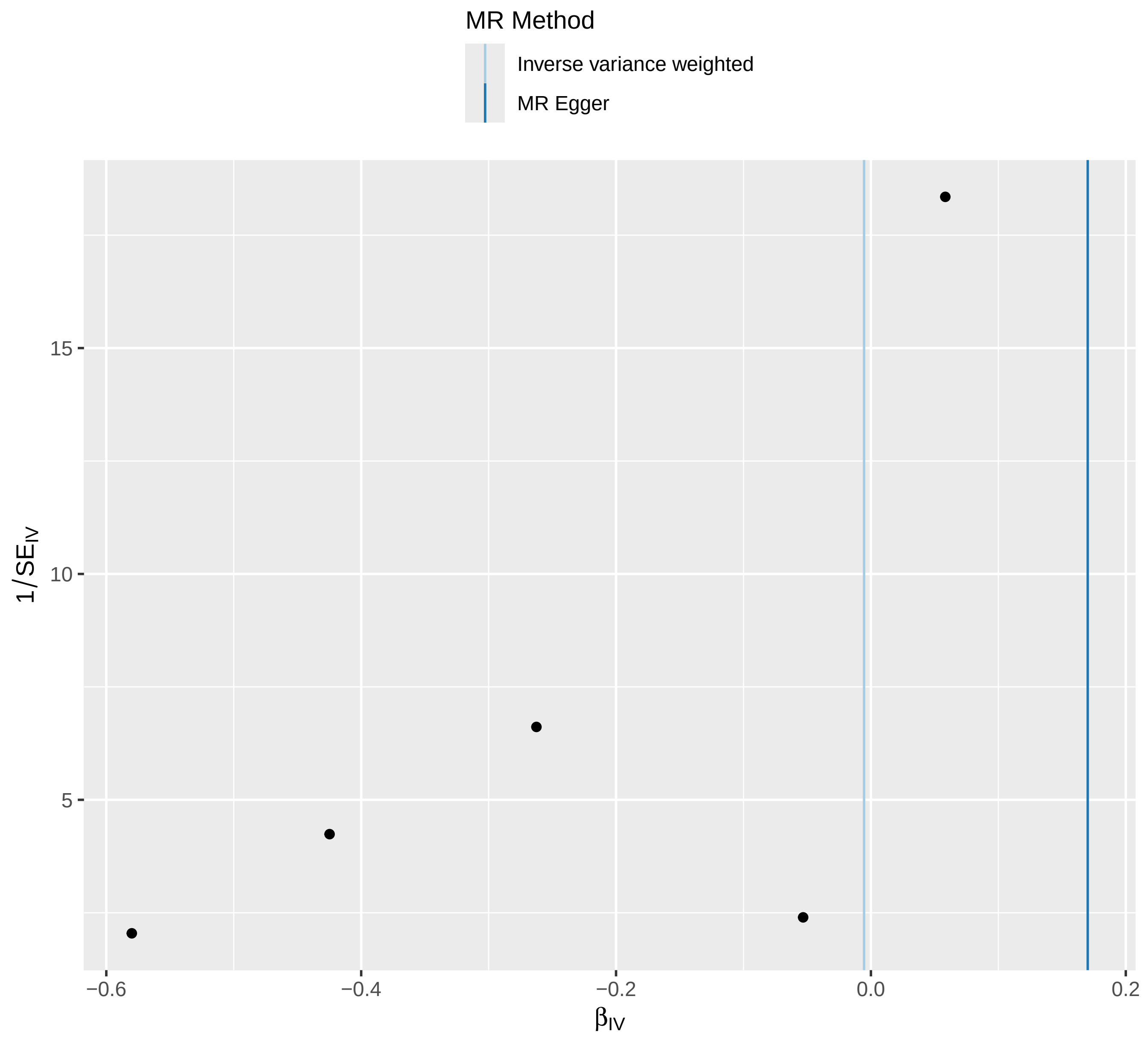
MR Method
Inverse variance weighted
MR Egger
1/SE$_{IV}$
15
10
5
−0.6
−0.4
−0.2
0.0
0.2
β$_{IV}$

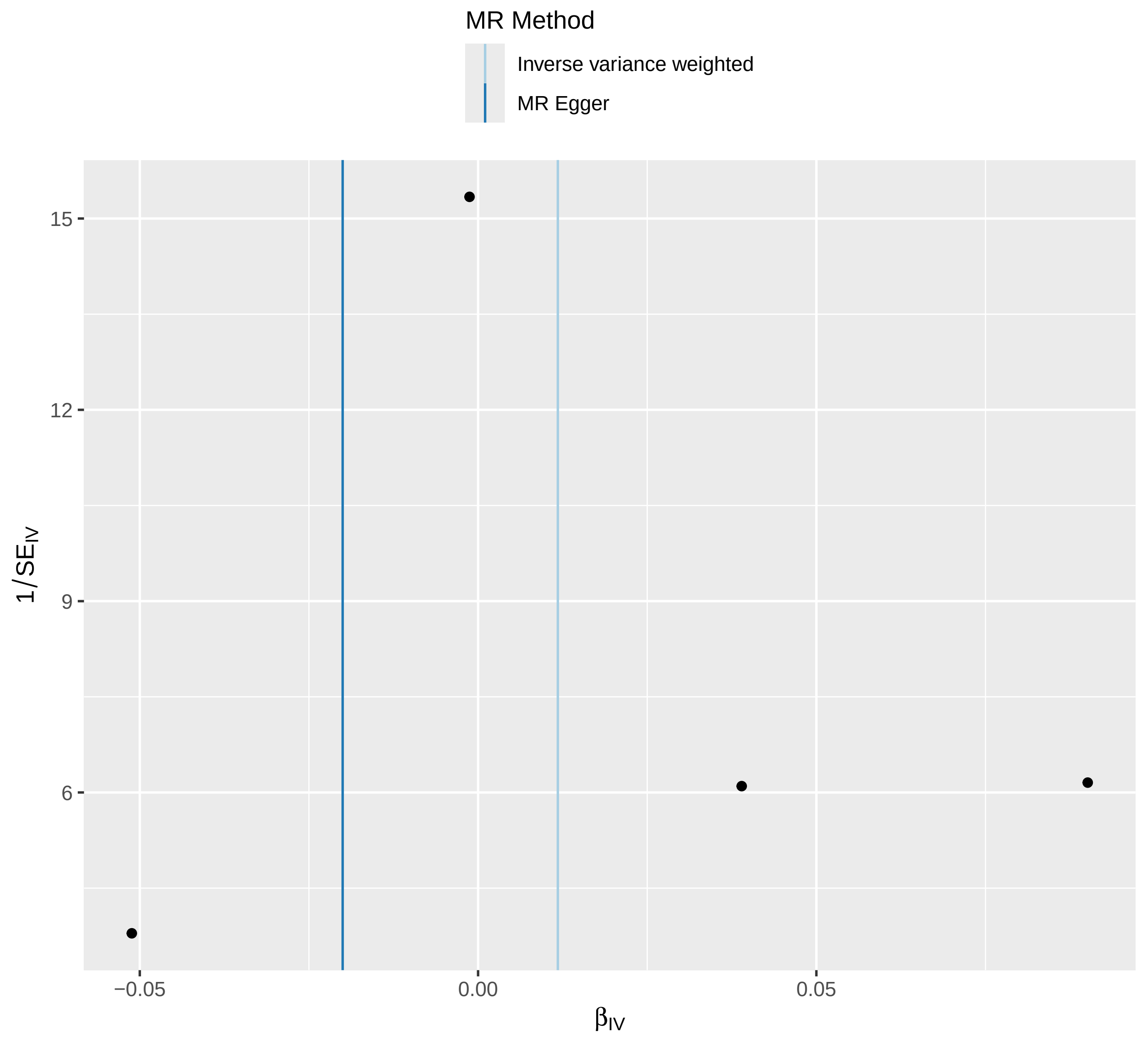
MR Method
Inverse variance weighted
MR Egger
15
12
9
6
−0.05
0.00
0.05
1/SE_IV
β_IV

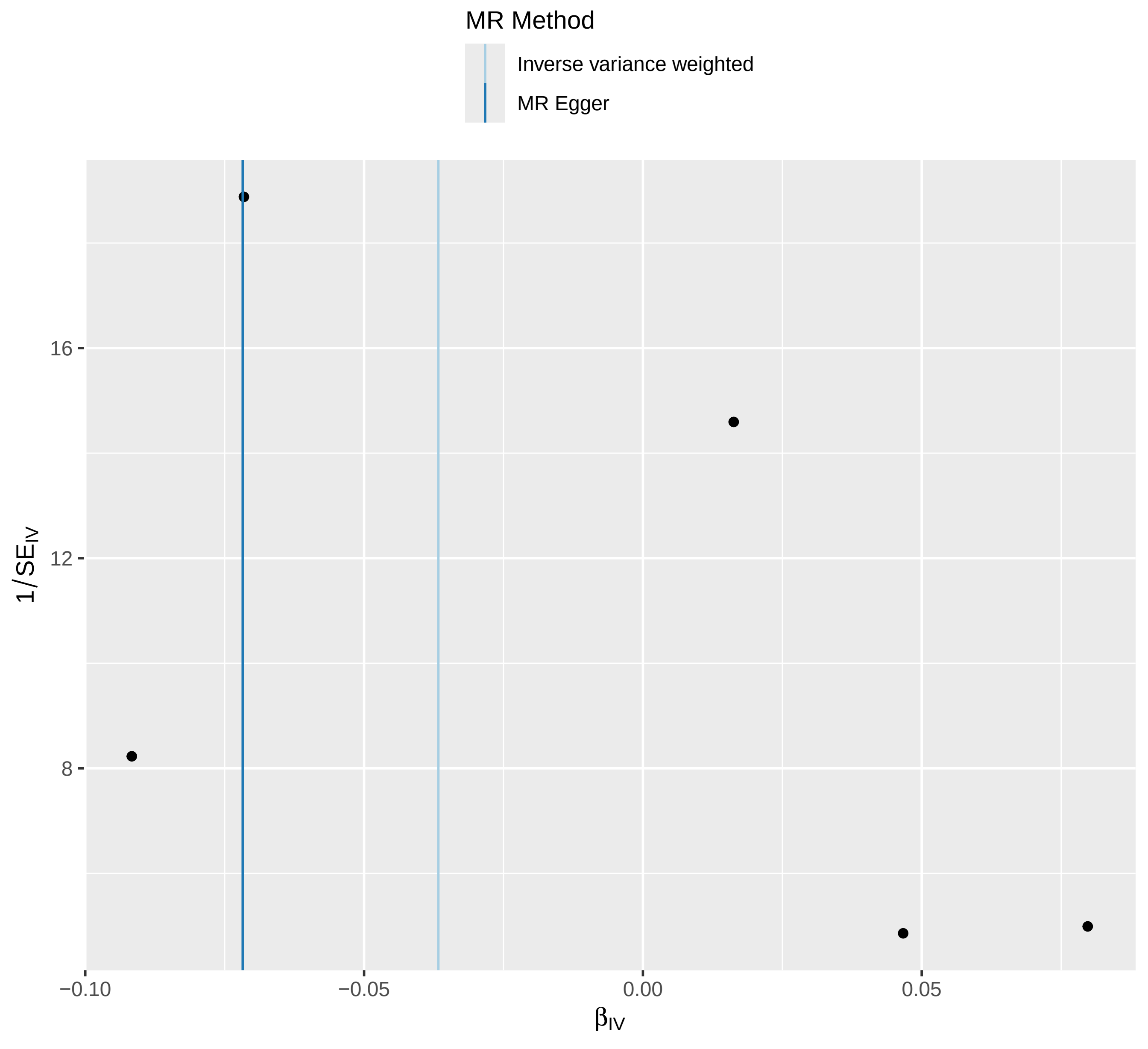
MR Method
Inverse variance weighted
MR Egger
16
12
8
1/SE_IV
−0.10
−0.05
0.00
0.05
β_IV

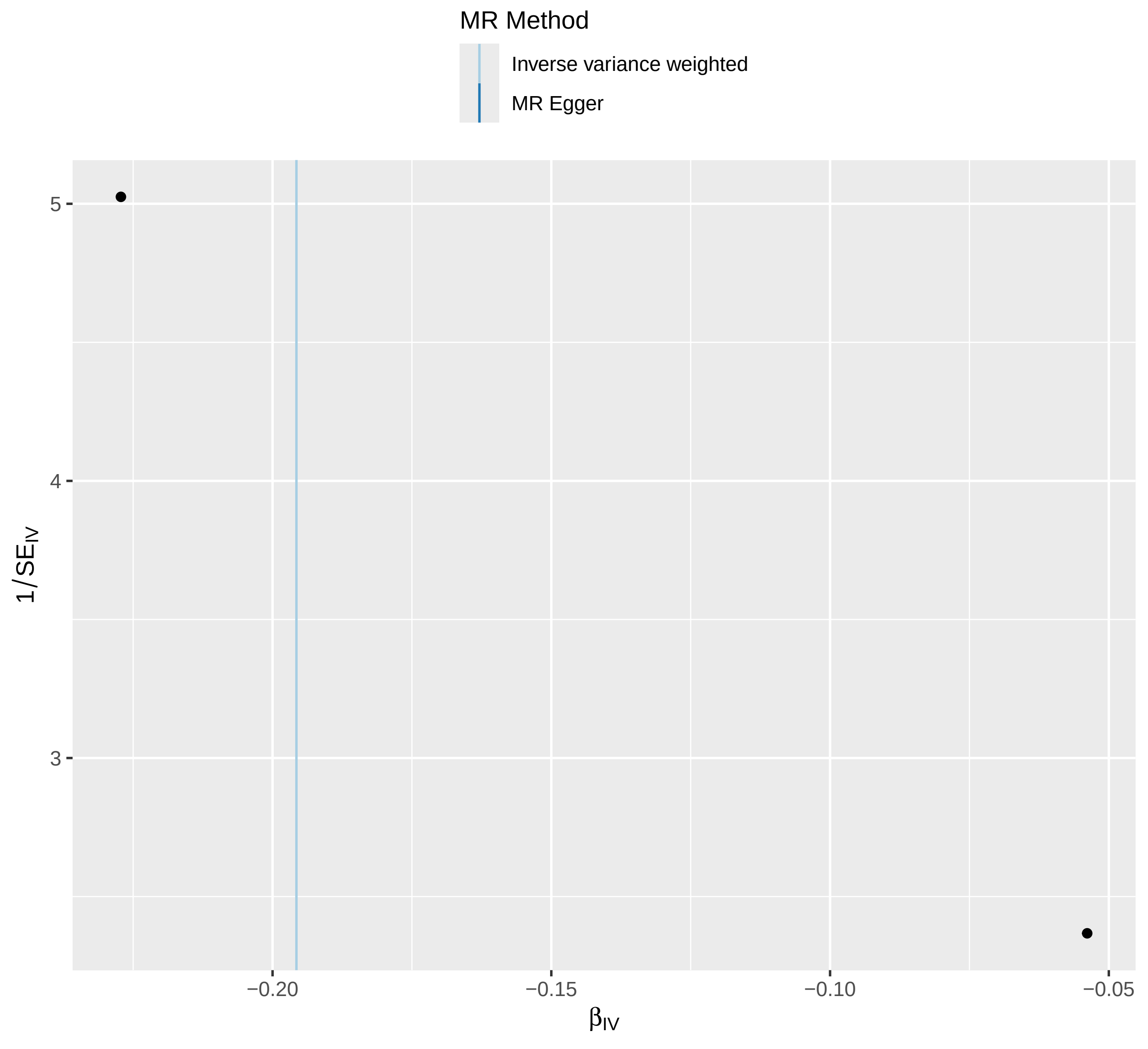

MR Method
Inverse variance weighted
MR Egger
5
4
3
1/$SE_{IV}$
−0.20
−0.15
−0.10
−0.05
$\beta_{IV}$

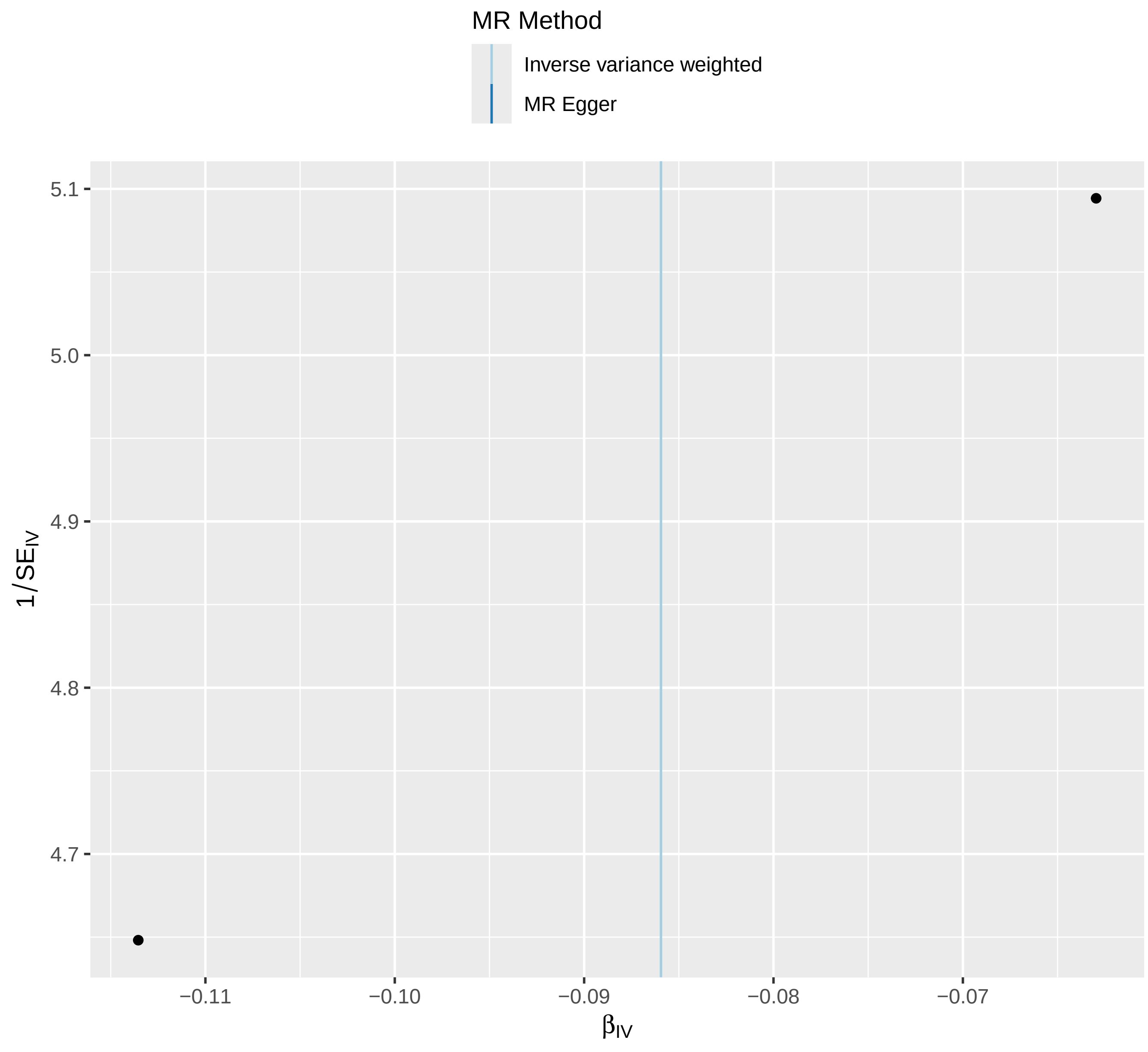

MR Method
Inverse variance weighted
MR Egger
5.1
5.0
4.9
4.8
4.7
1/SE_IV
−0.11
−0.10
−0.09
−0.08
−0.07
β_IV

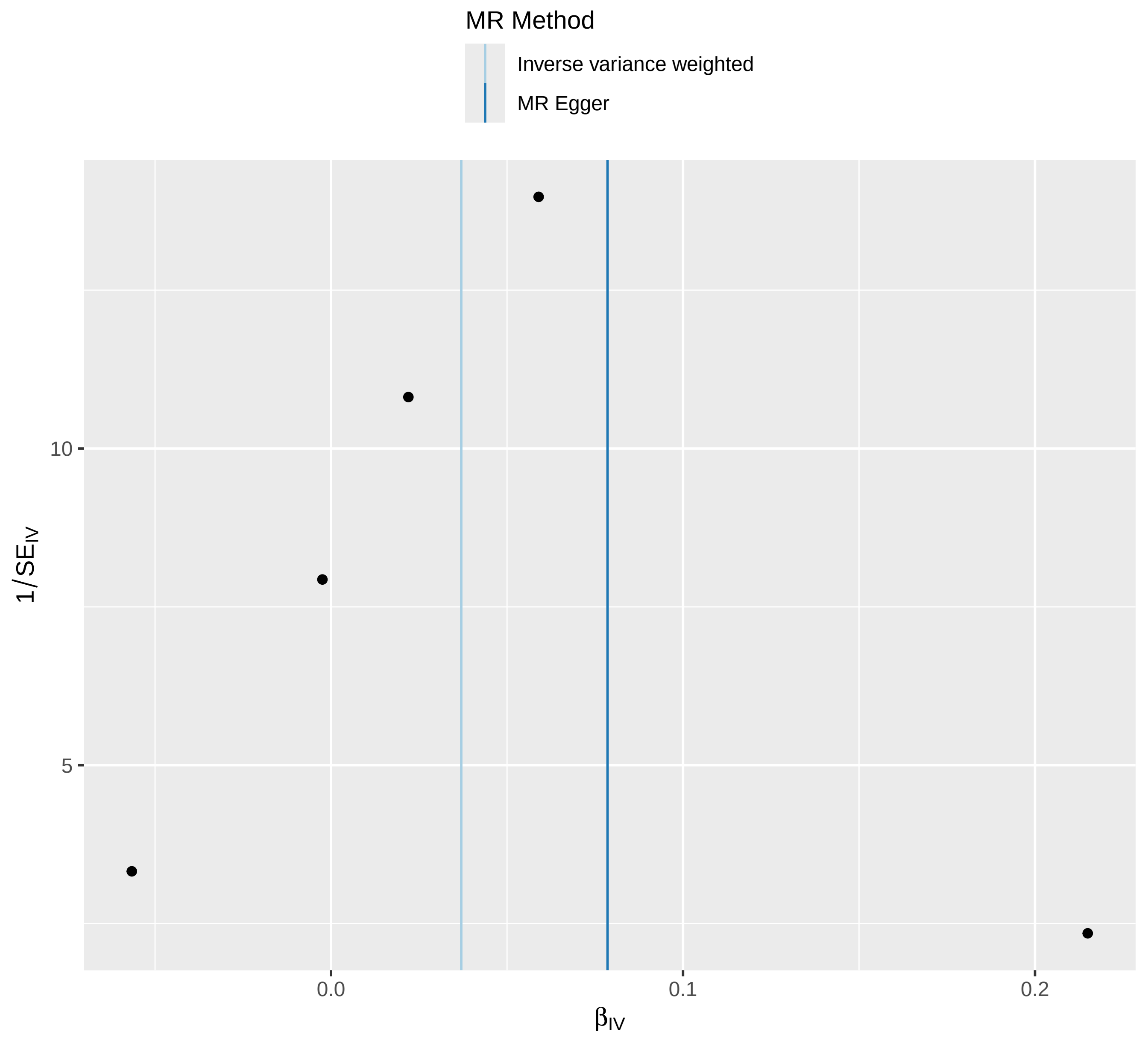
MR Method
Inverse variance weighted
MR Egger
10
5
$1/SE_{IV}$
0.0
0.1
0.2
$\beta_{IV}$

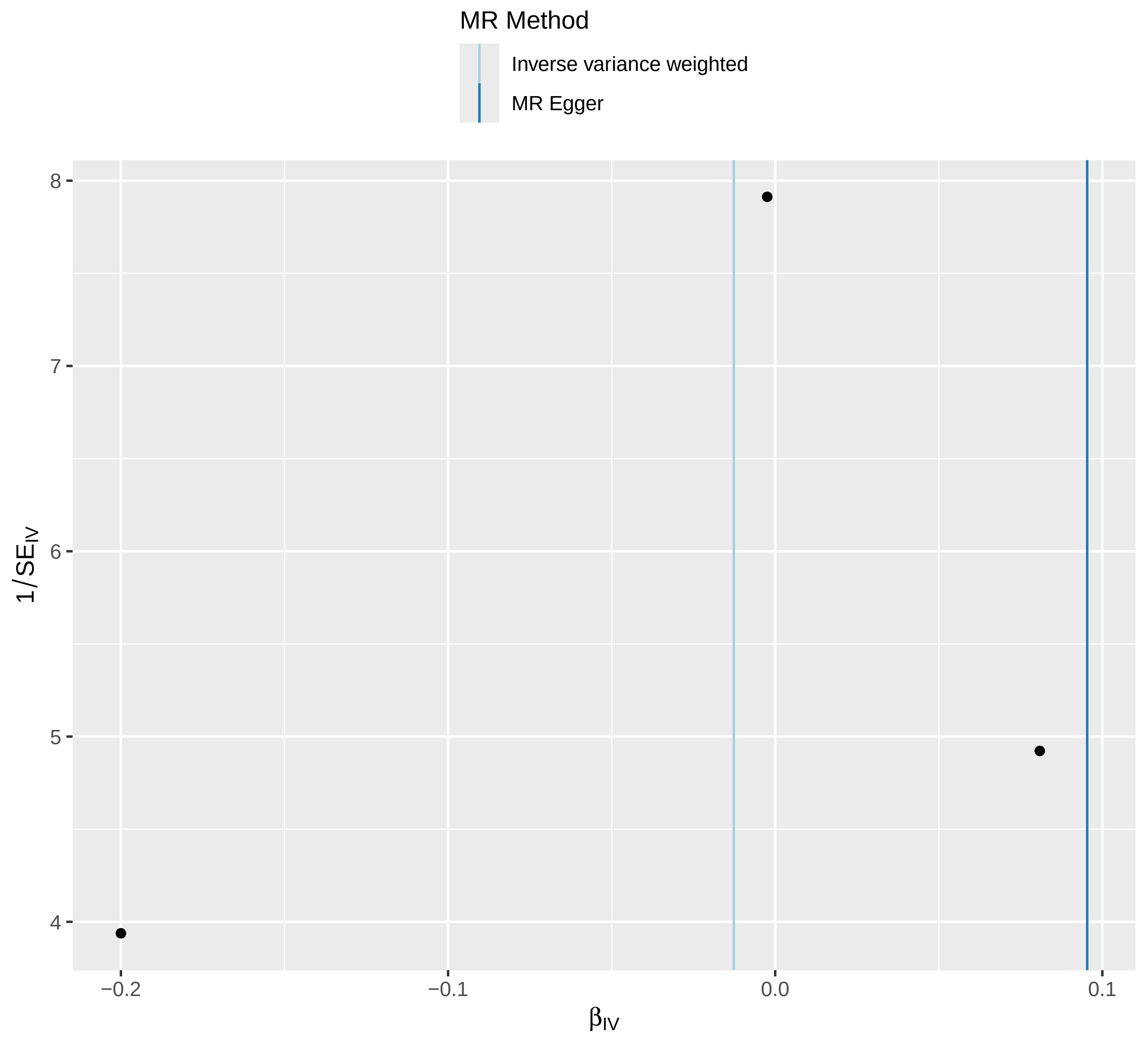
MR Method
Inverse variance weighted
MR Egger
8
7
6
5
4
1/SE_IV
−0.2
−0.1
0.0
0.1
β_IV

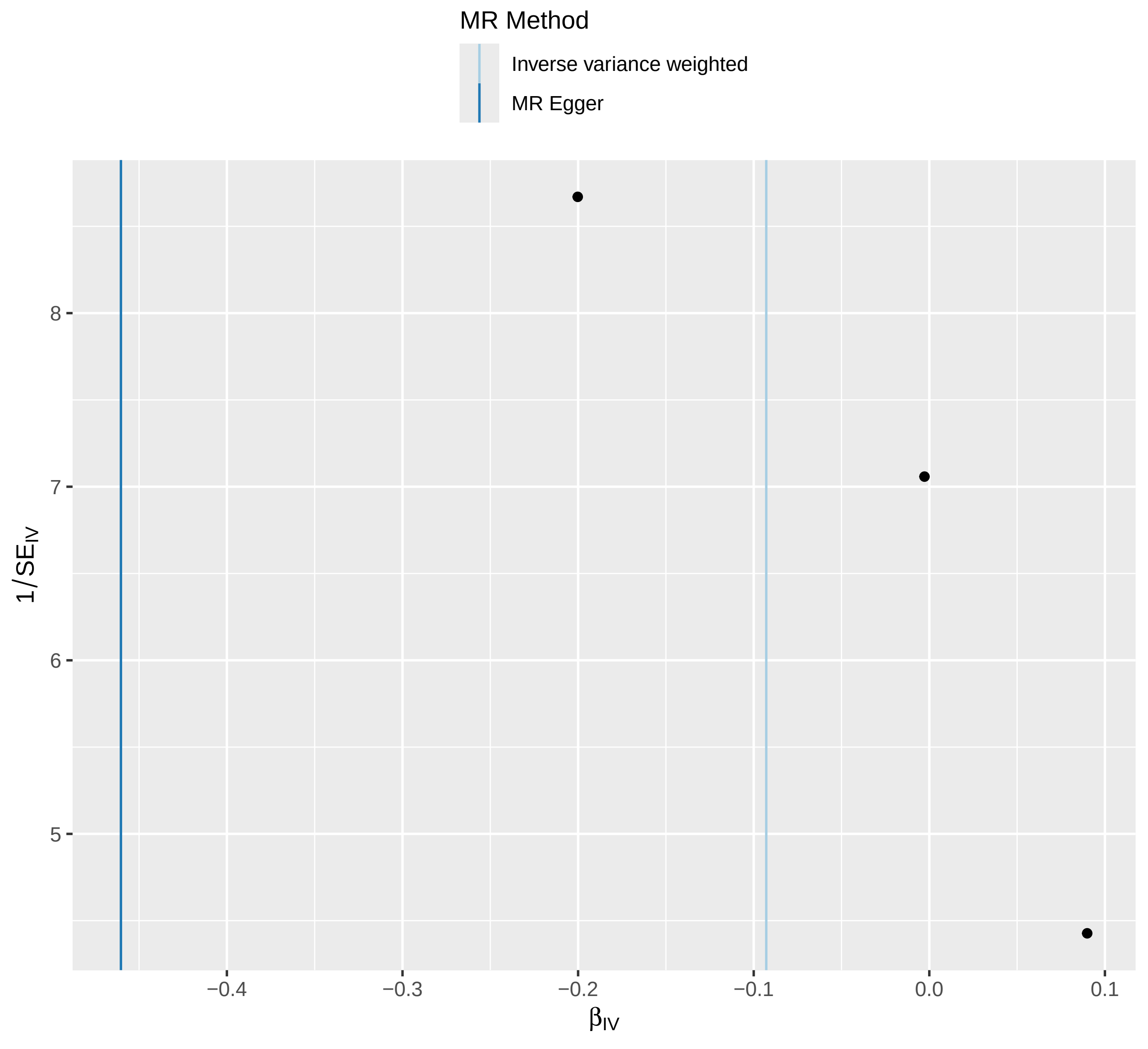

MR Method
Inverse variance weighted
MR Egger
8
7
6
5
1/SE_IV
−0.4
−0.3
−0.2
−0.1
0.0
0.1
β_IV

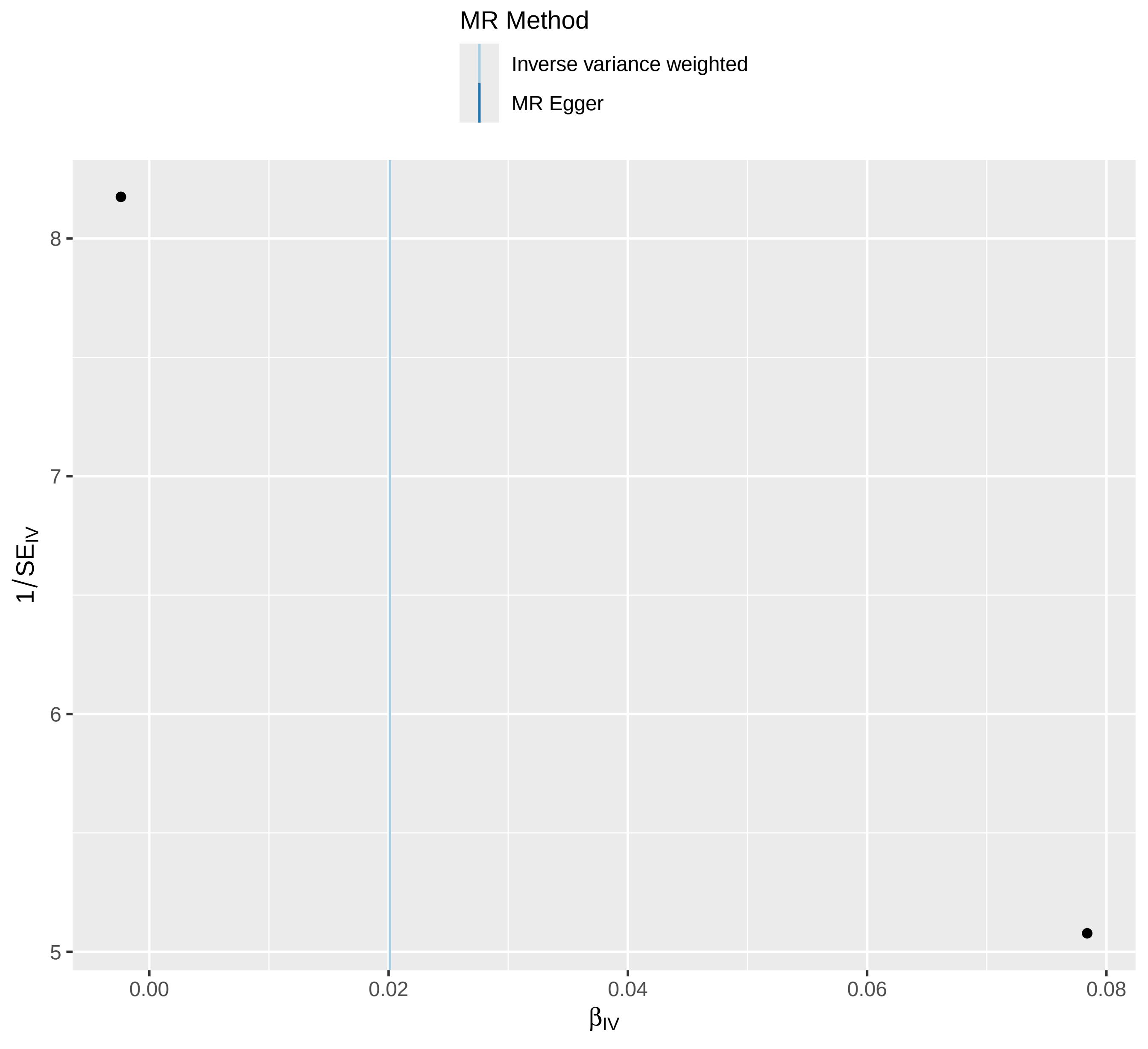

MR Method
Inverse variance weighted
MR Egger
1/SE$_{IV}$
8
7
6
5
0.00
0.02
0.04
0.06
0.08
β$_{IV}$

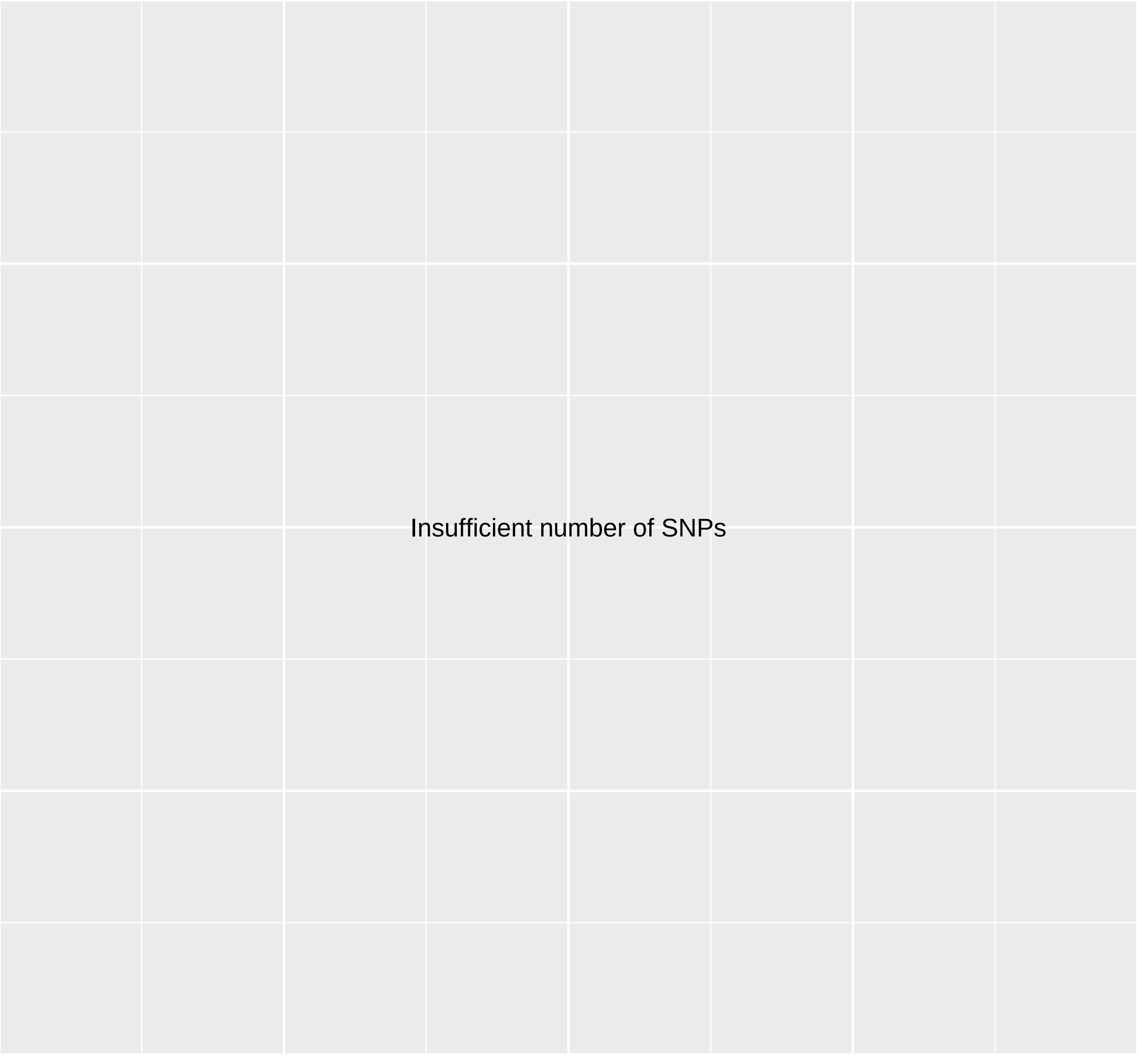
Insufficient number of SNPs

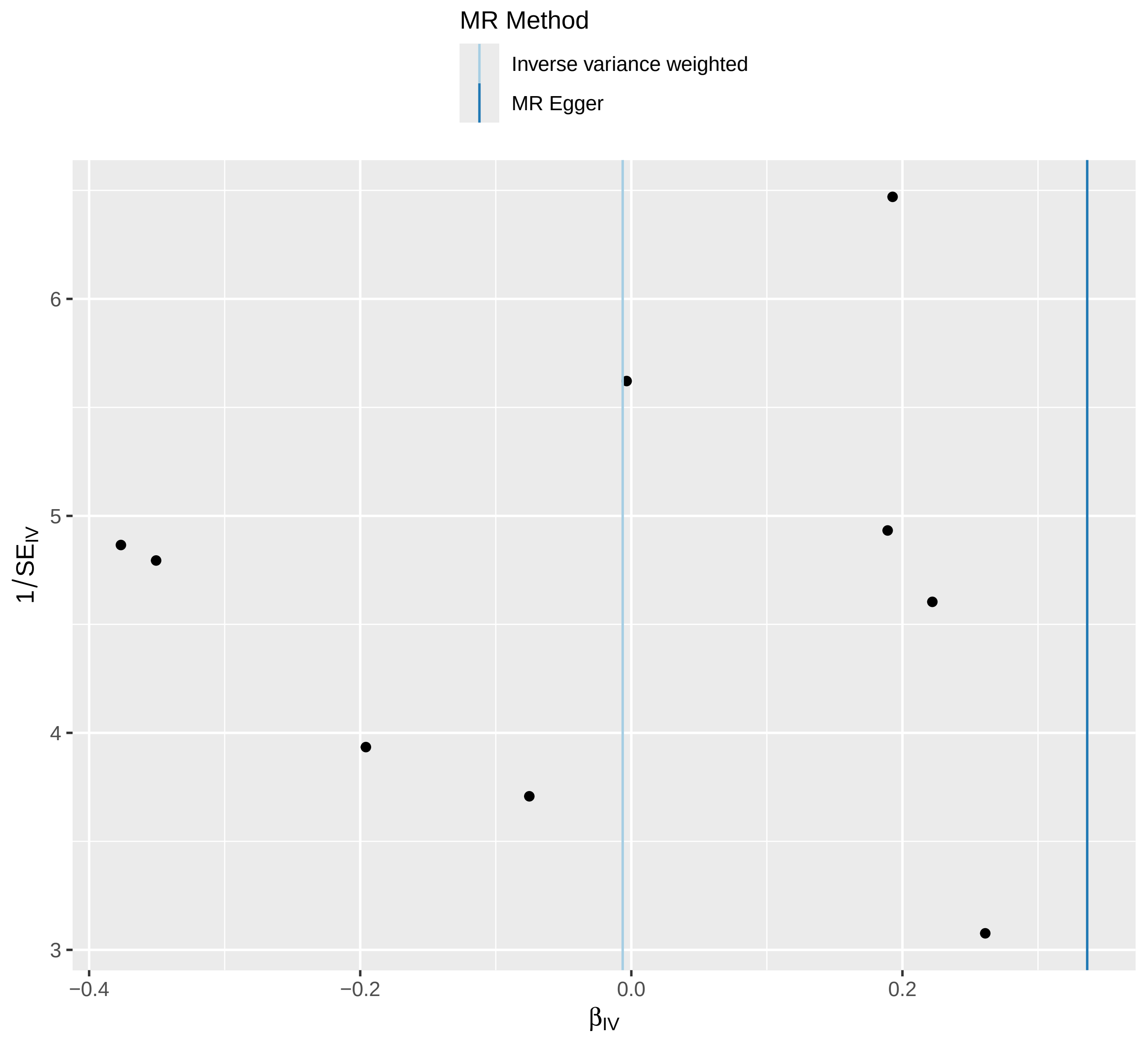
MR Method
Inverse variance weighted
MR Egger
6
5
4
3
1/SE$_{IV}$
−0.4
−0.2
0.0
0.2
β$_{IV}$

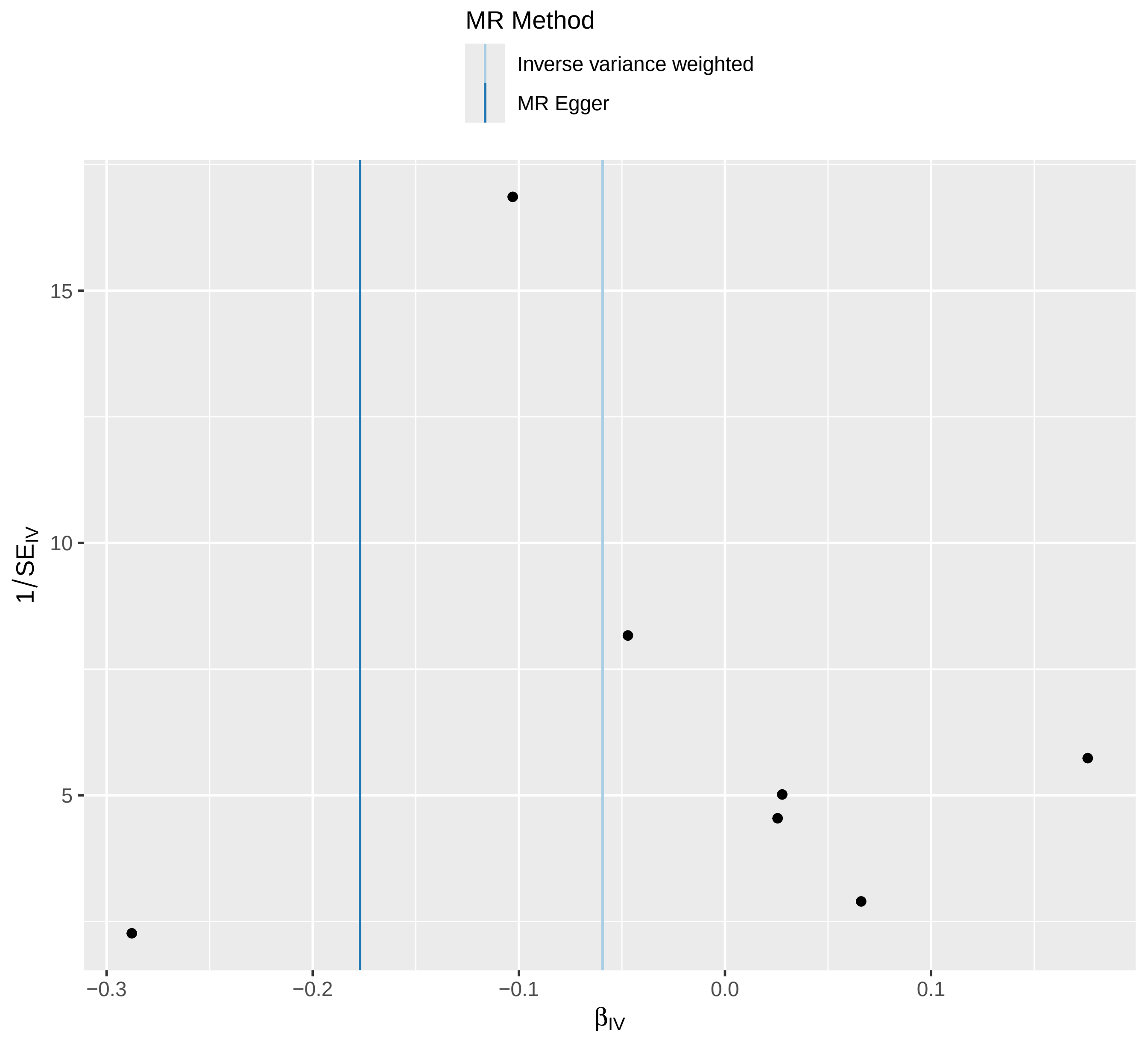
MR Method
Inverse variance weighted
MR Egger
15
10
5
1/SE_IV
−0.3
−0.2
−0.1
0.0
0.1
β_IV

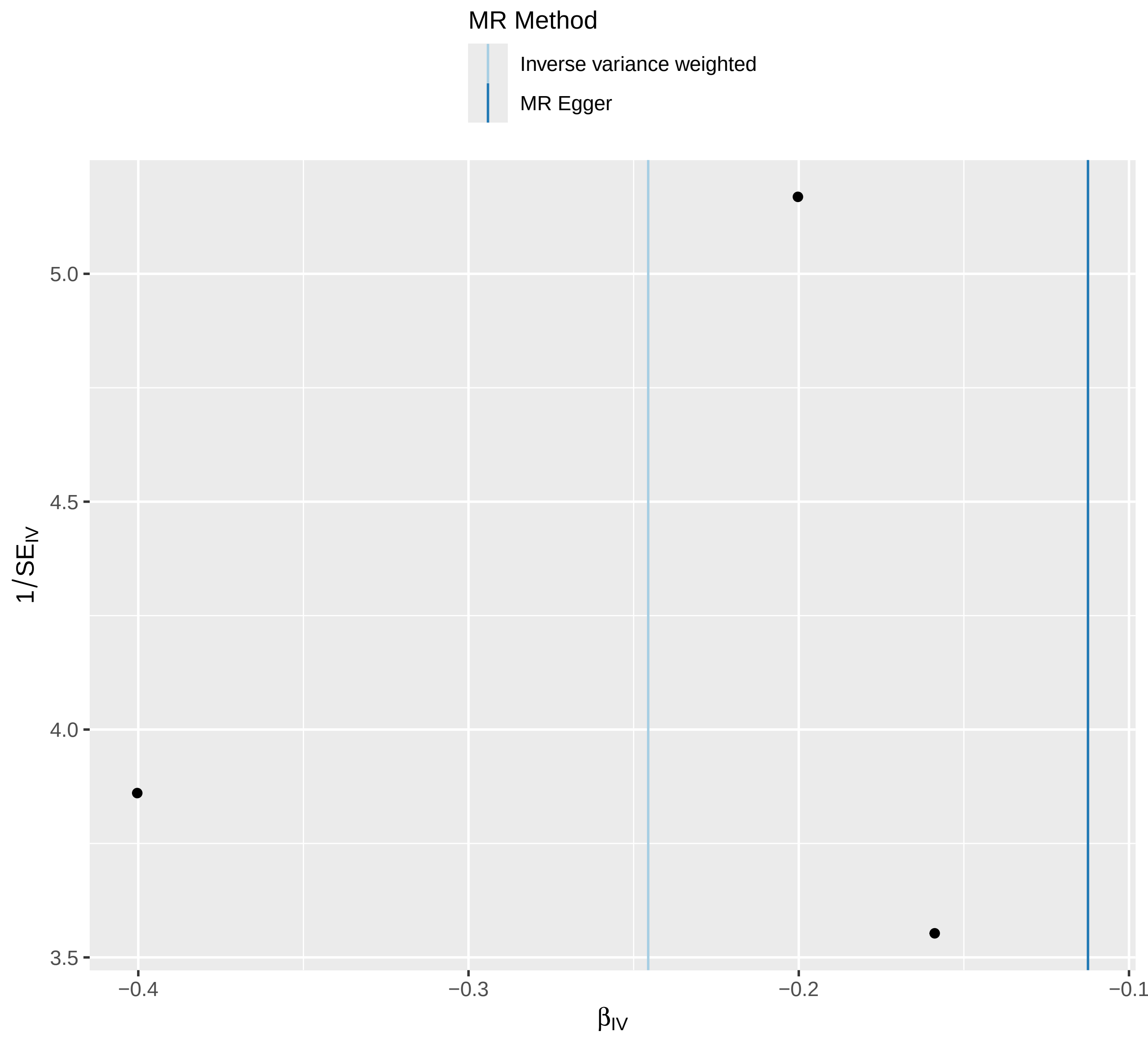

MR Method
Inverse variance weighted
MR Egger
5.0
4.5
4.0
3.5
1/SE$_{IV}$
−0.4
−0.3
−0.2
−0.1
β$_{IV}$

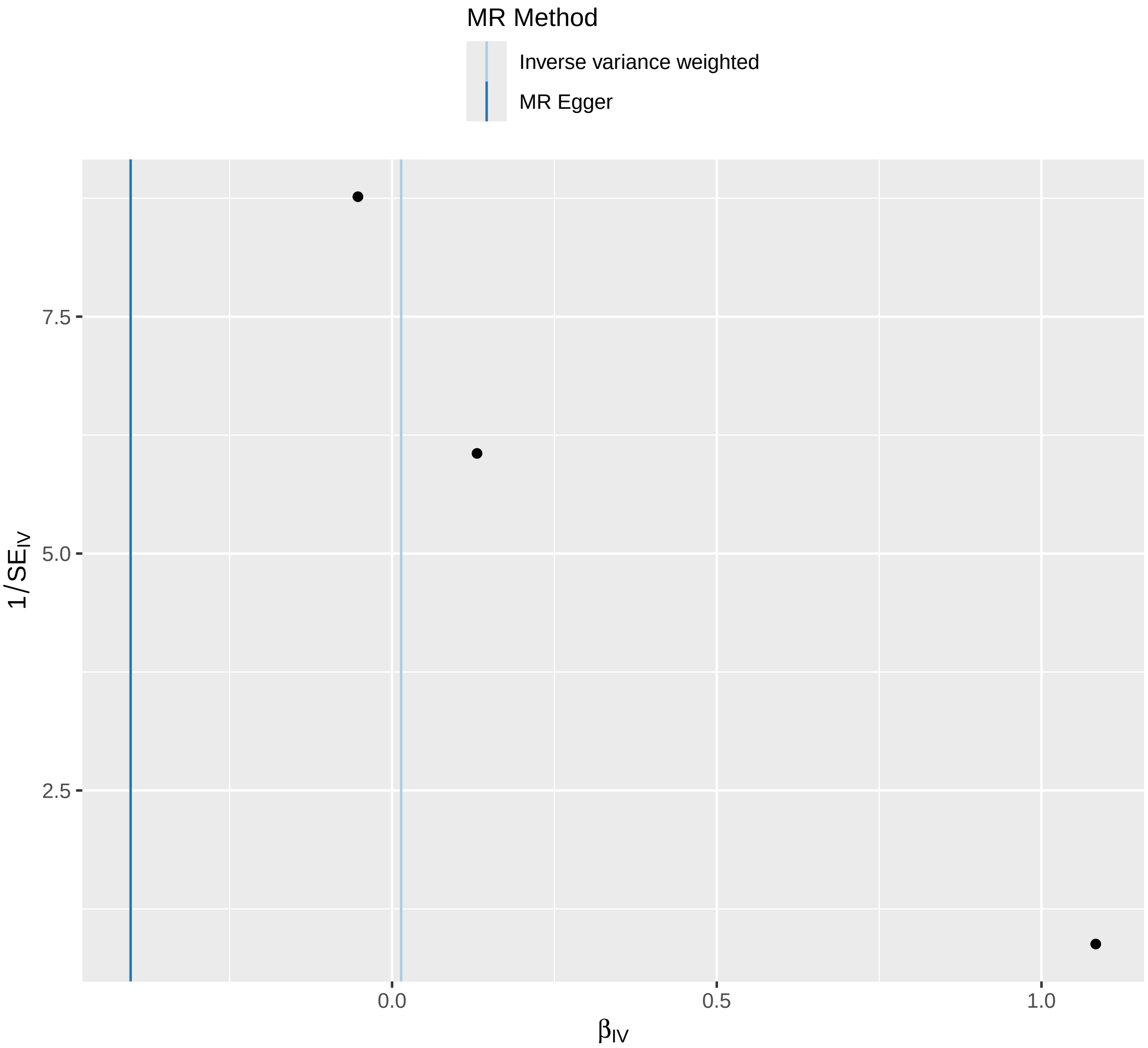
MR Method
Inverse variance weighted
MR Egger
1/SE_IV
7.5
5.0
2.5
0.0
0.5
1.0
β_IV

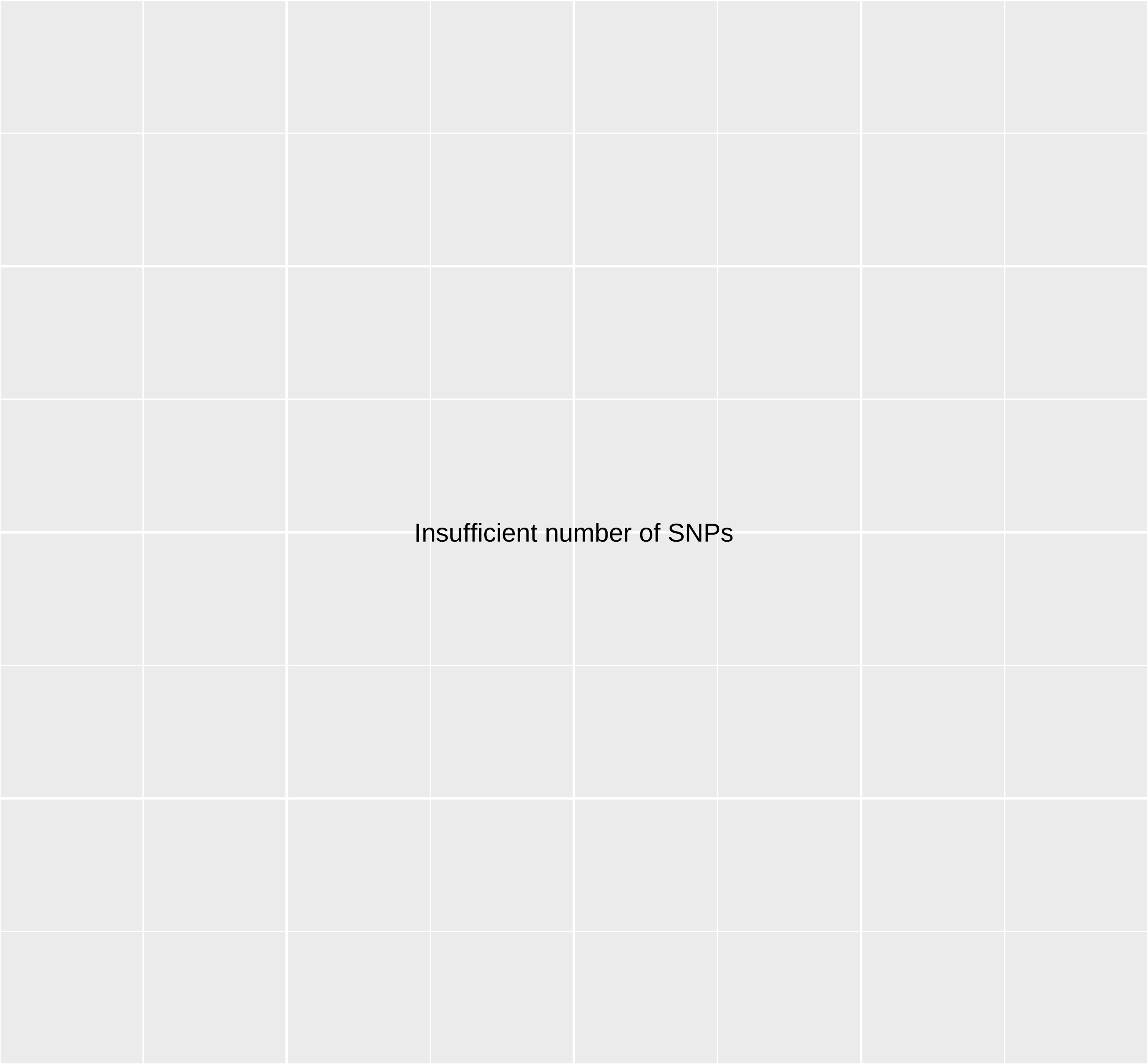
Insufficient number of SNPs

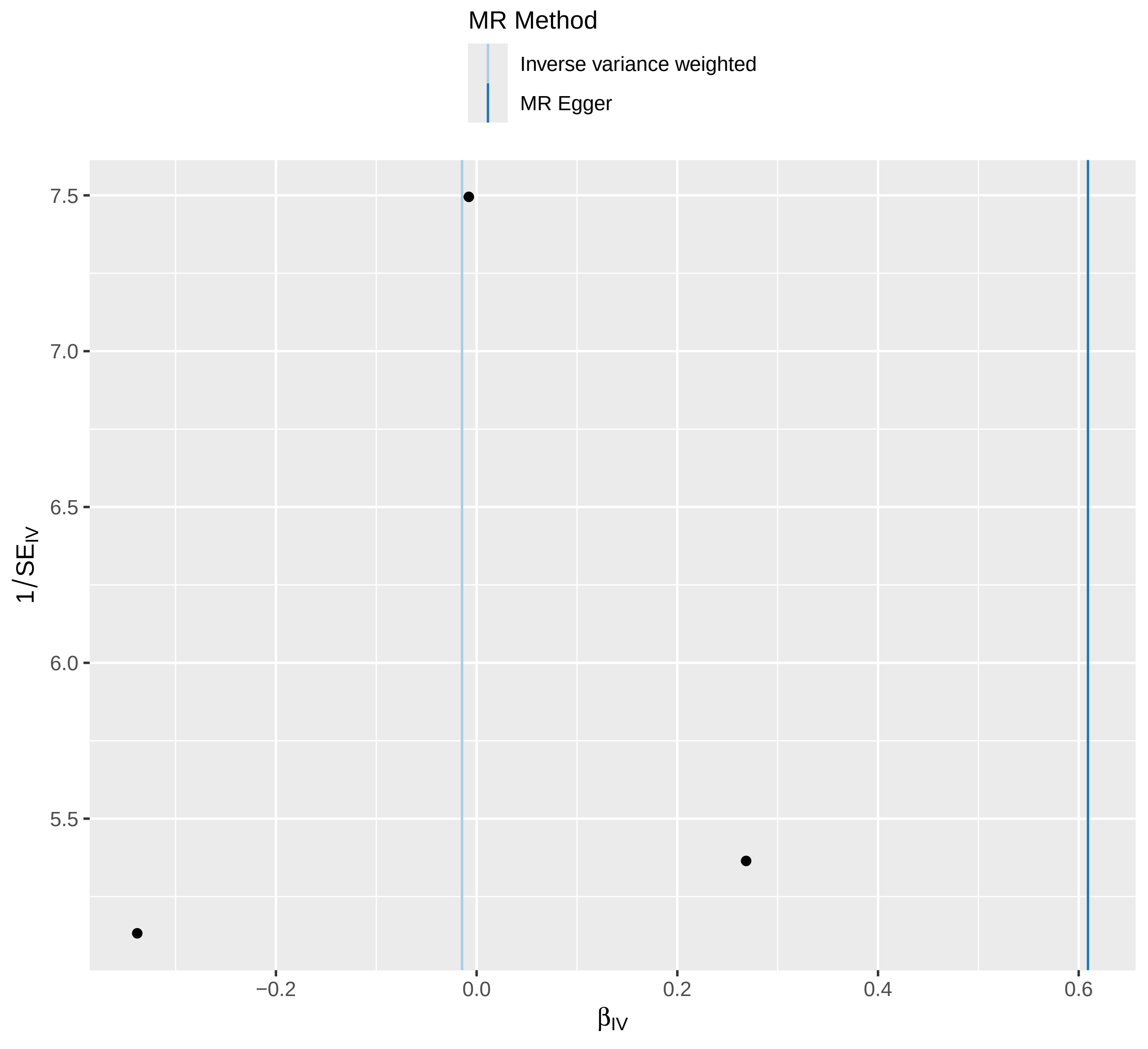

MR Method
Inverse variance weighted
MR Egger
7.5
7.0
6.5
6.0
5.5
1/SE_IV
−0.2
0.0
0.2
0.4
0.6
β_IV

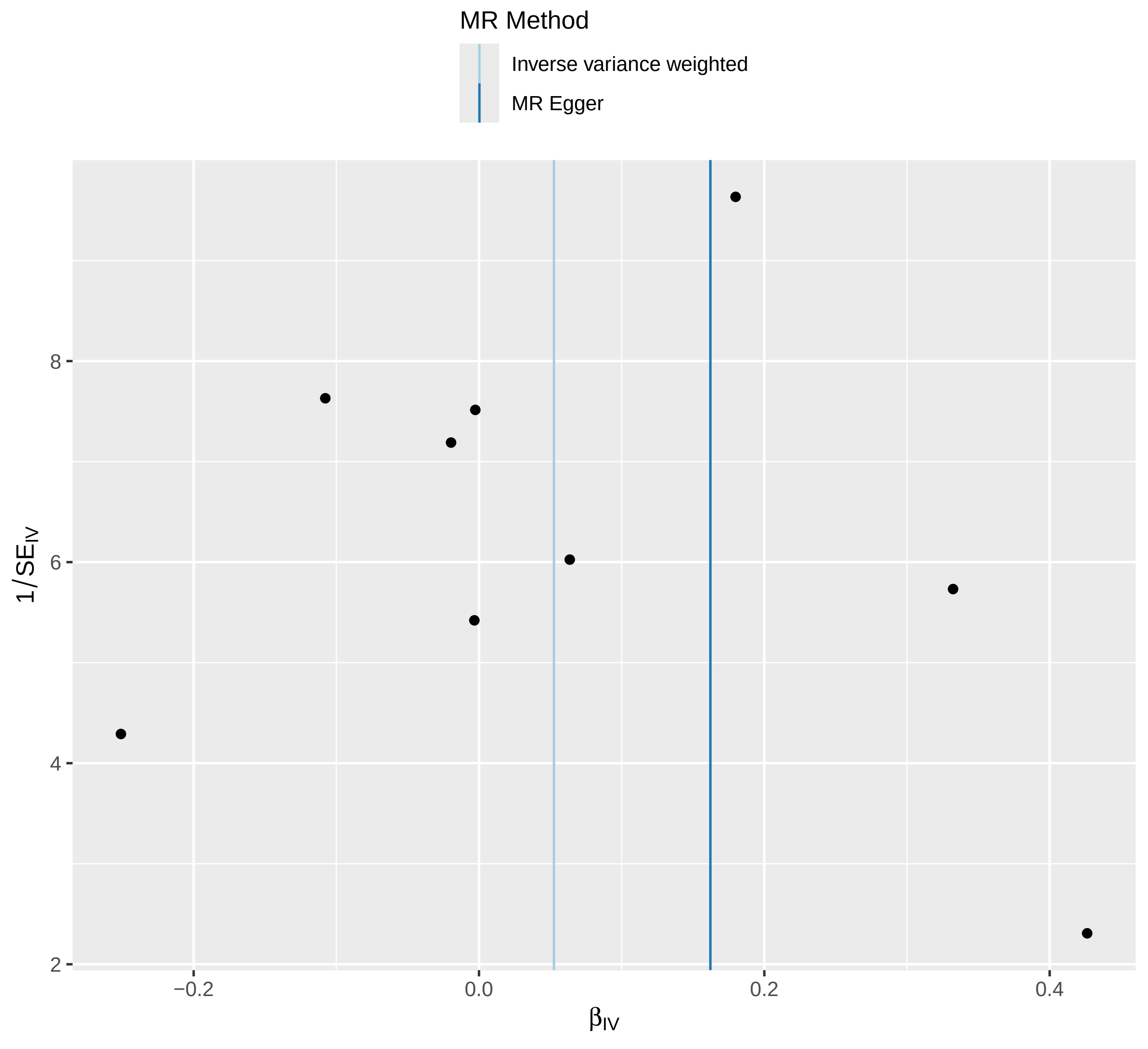

MR Method
Inverse variance weighted
MR Egger
1/SE$_{IV}$
8
6
4
2
−0.2
0.0
0.2
0.4
β$_{IV}$

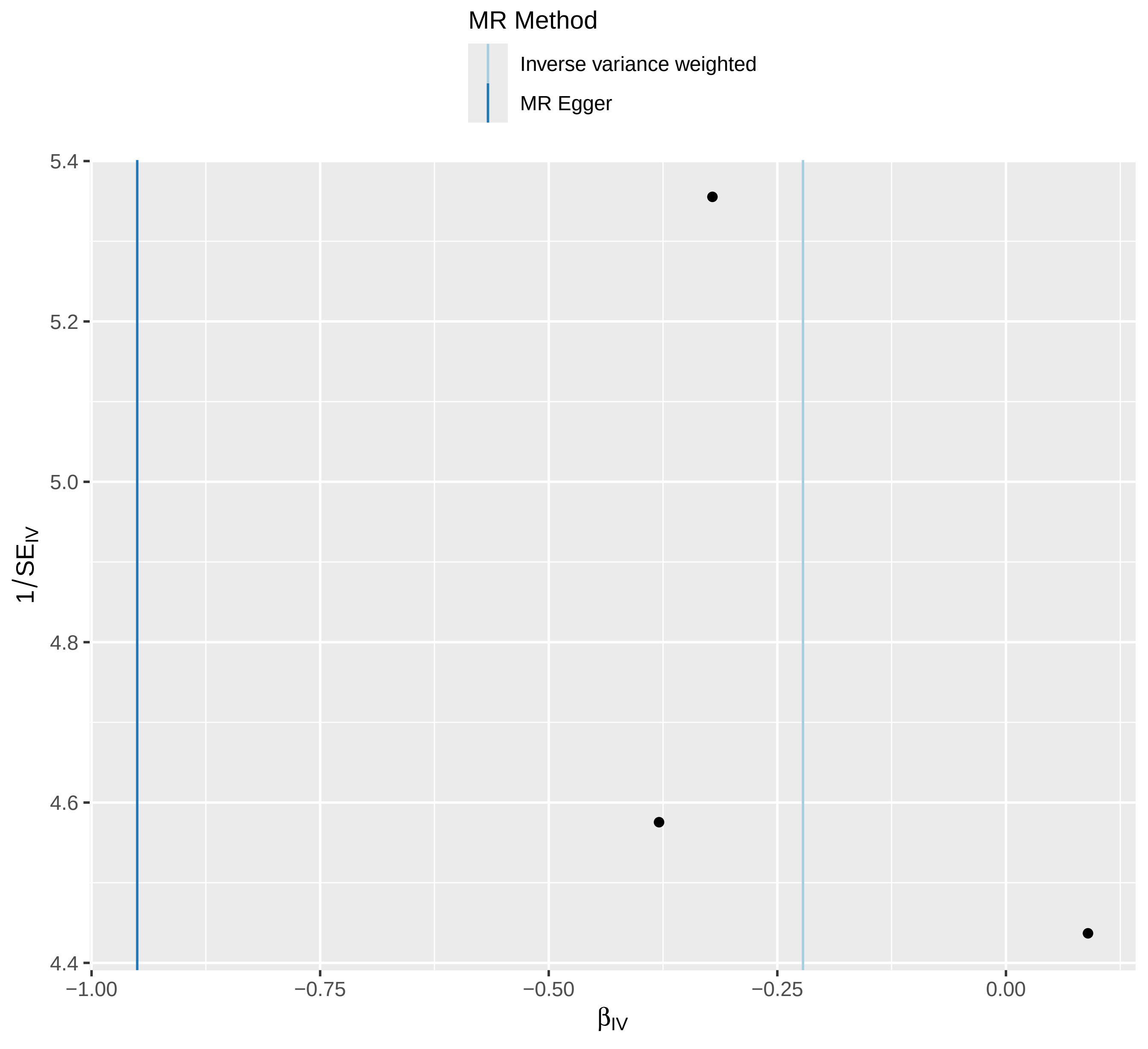
MR Method
Inverse variance weighted
MR Egger
5.4
5.2
5.0
4.8
4.6
4.4
1/SE$_{IV}$
−1.00
−0.75
−0.50
−0.25
0.00
β$_{IV}$

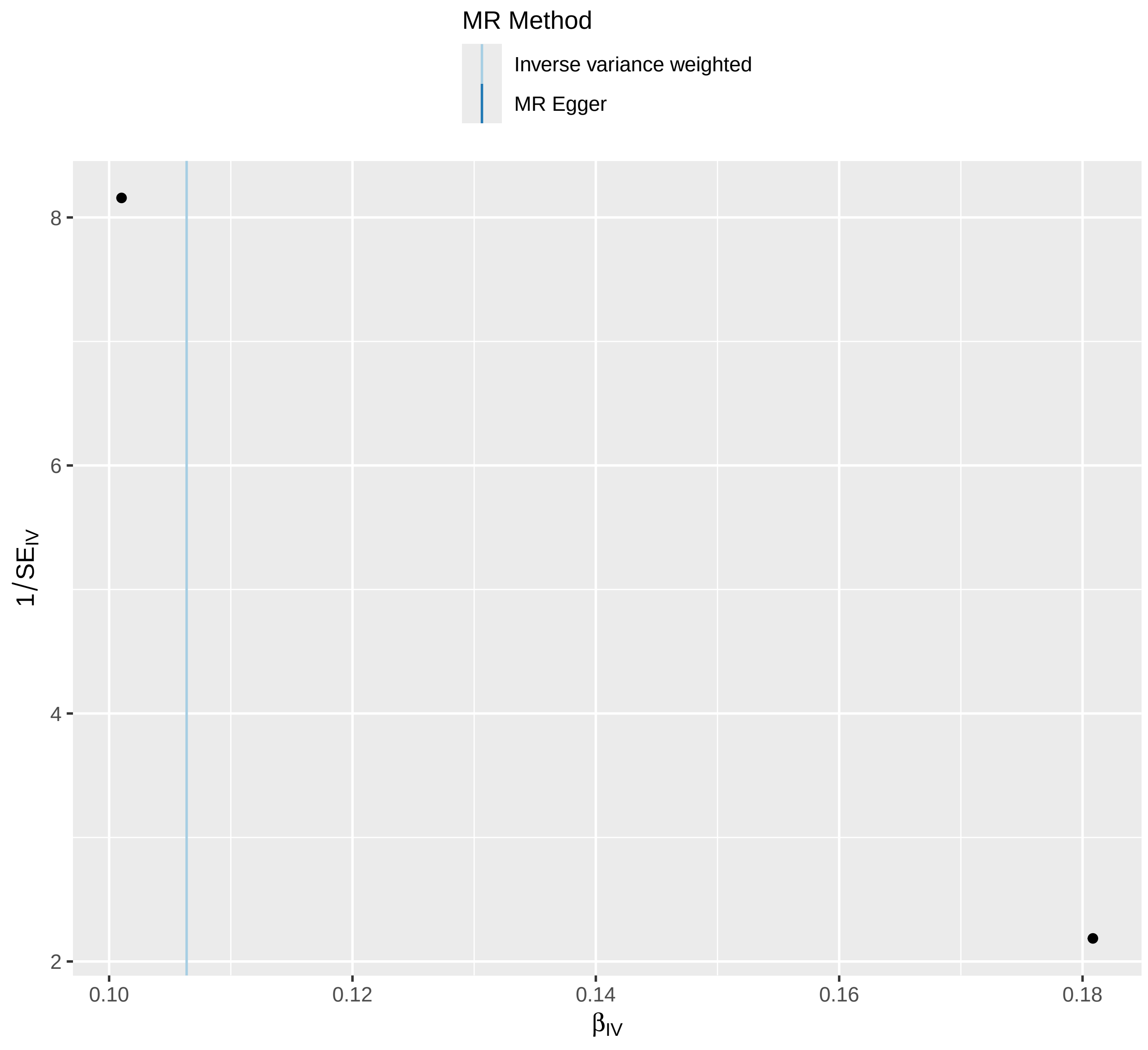

MR Method
Inverse variance weighted
MR Egger
8
6
4
2
$1/SE_{IV}$
0.10
0.12
0.14
0.16
0.18
$\beta_{IV}$

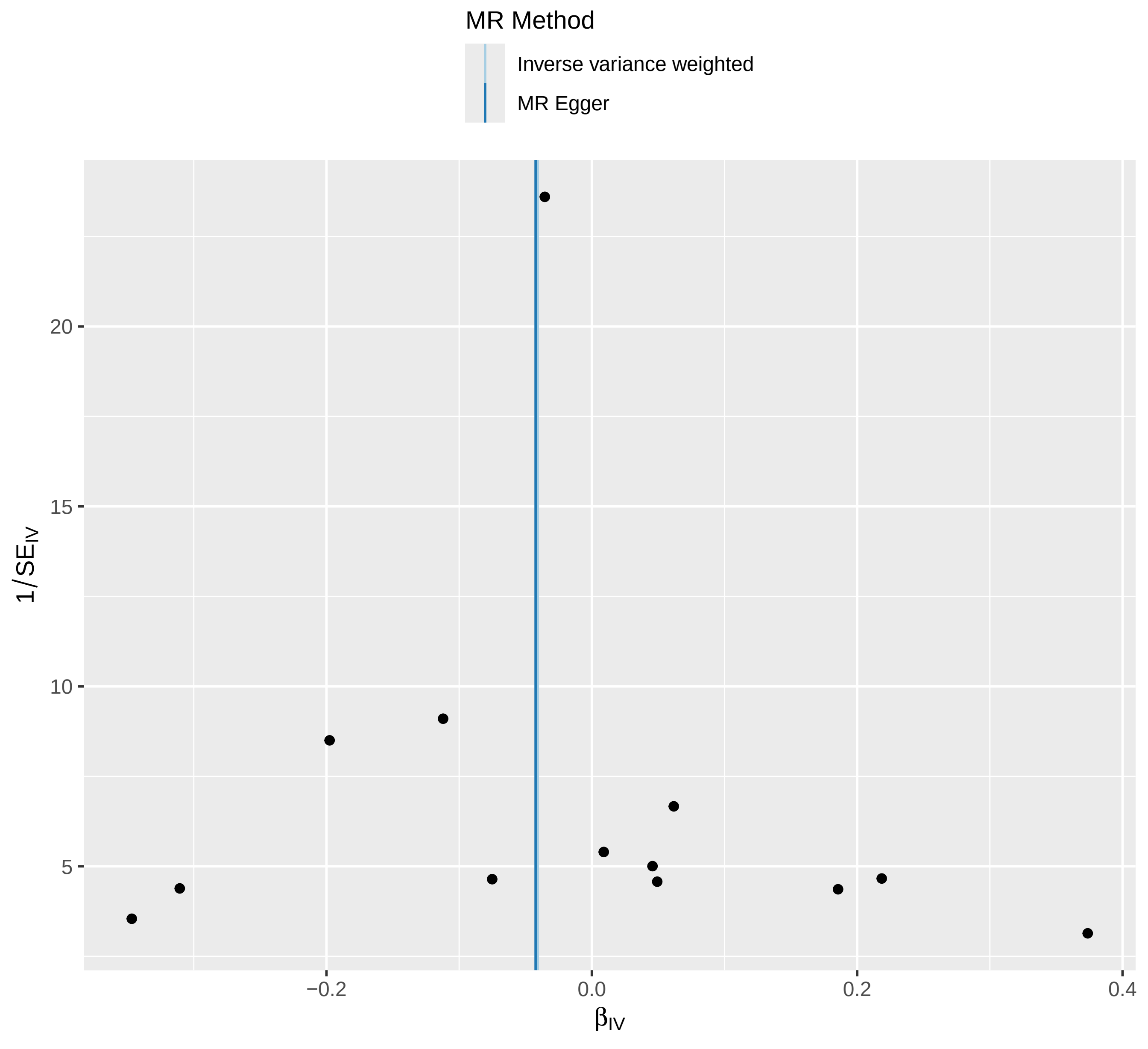
MR Method
Inverse variance weighted
MR Egger
20
15
10
5
1/SE_IV
−0.2
0.0
0.2
0.4
β_IV

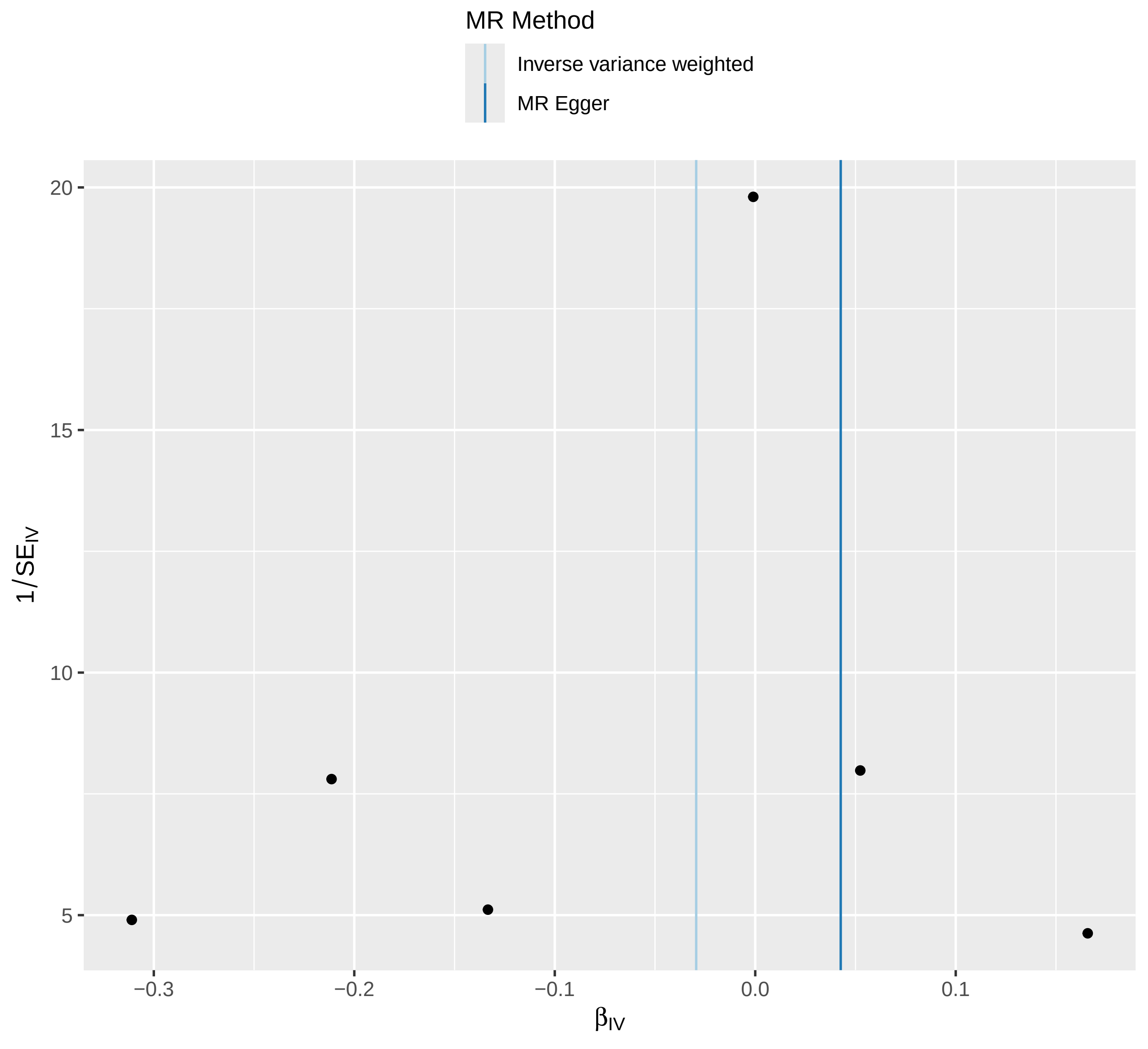

MR Method
Inverse variance weighted
MR Egger
20
15
10
5
1/SE$_{IV}$
−0.3
−0.2
−0.1
0.0
0.1
β$_{IV}$

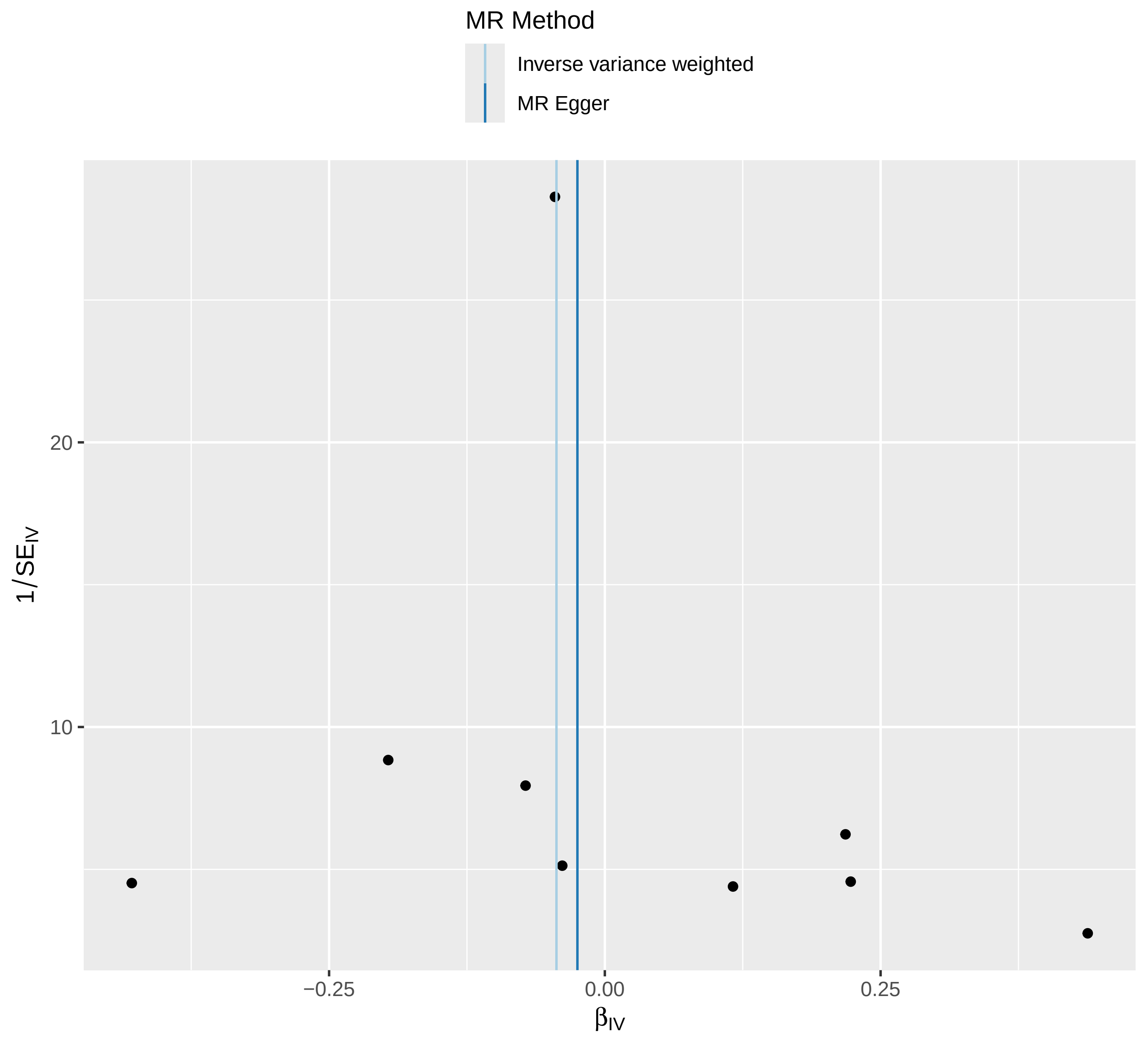
MR Method
Inverse variance weighted
MR Egger
20
10
1/SE_IV
−0.25
0.00
0.25
β_IV

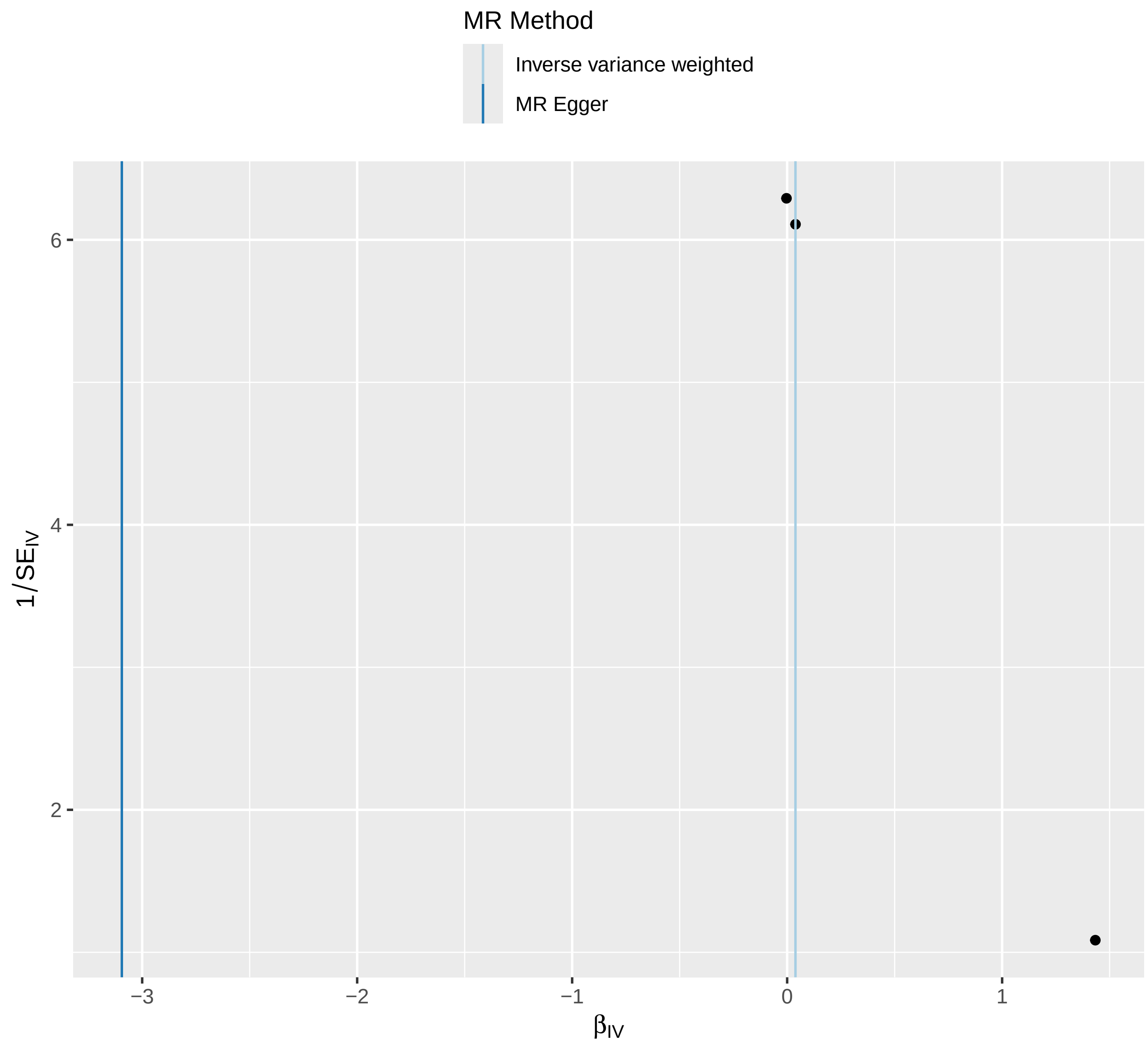

MR Method
Inverse variance weighted
MR Egger
6
4
2
1/SE_IV
−3
−2
−1
0
1
β_IV

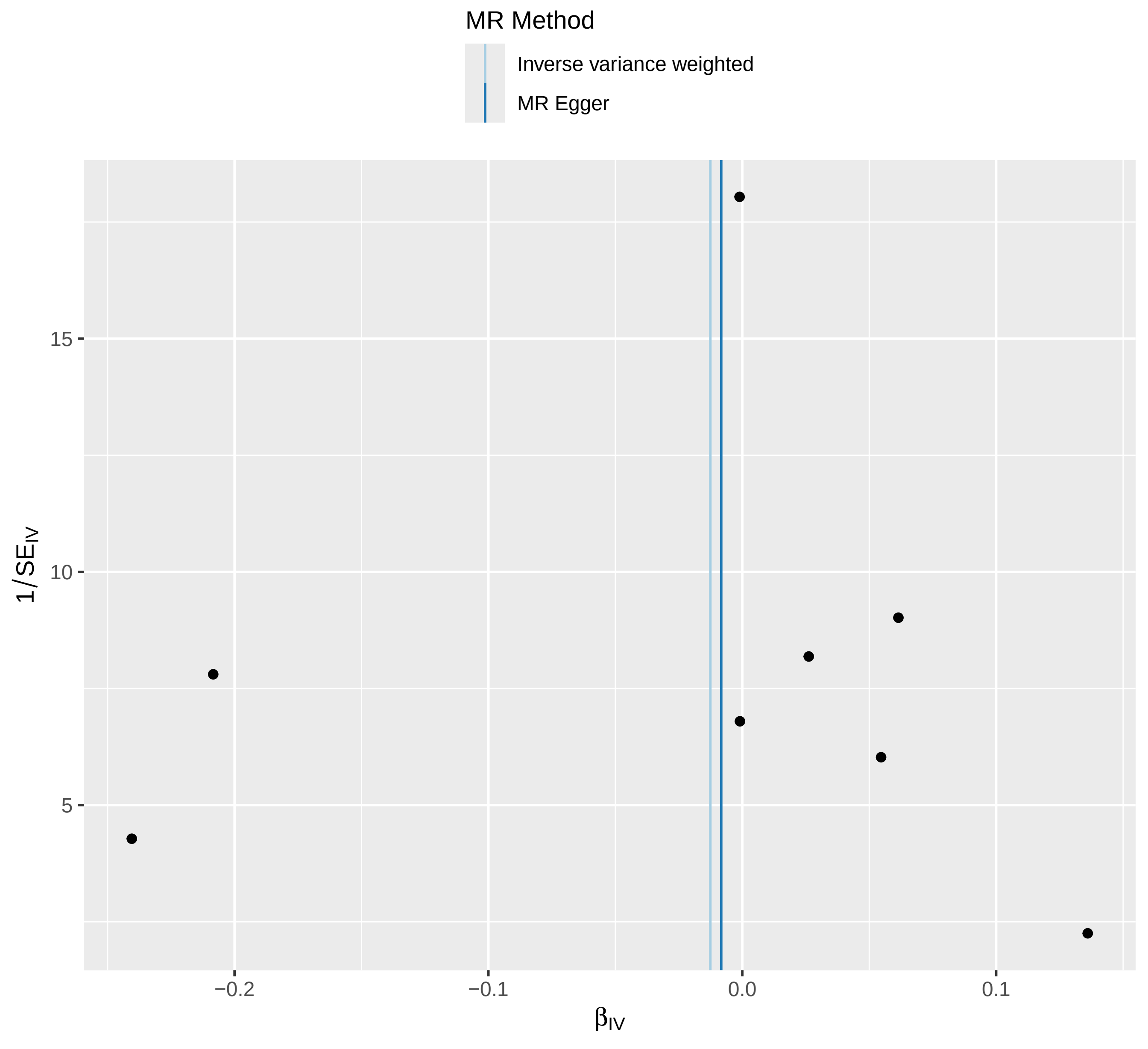

MR Method
Inverse variance weighted
MR Egger
15
10
5
1/SE$_{IV}$
−0.2
−0.1
0.0
0.1
β$_{IV}$

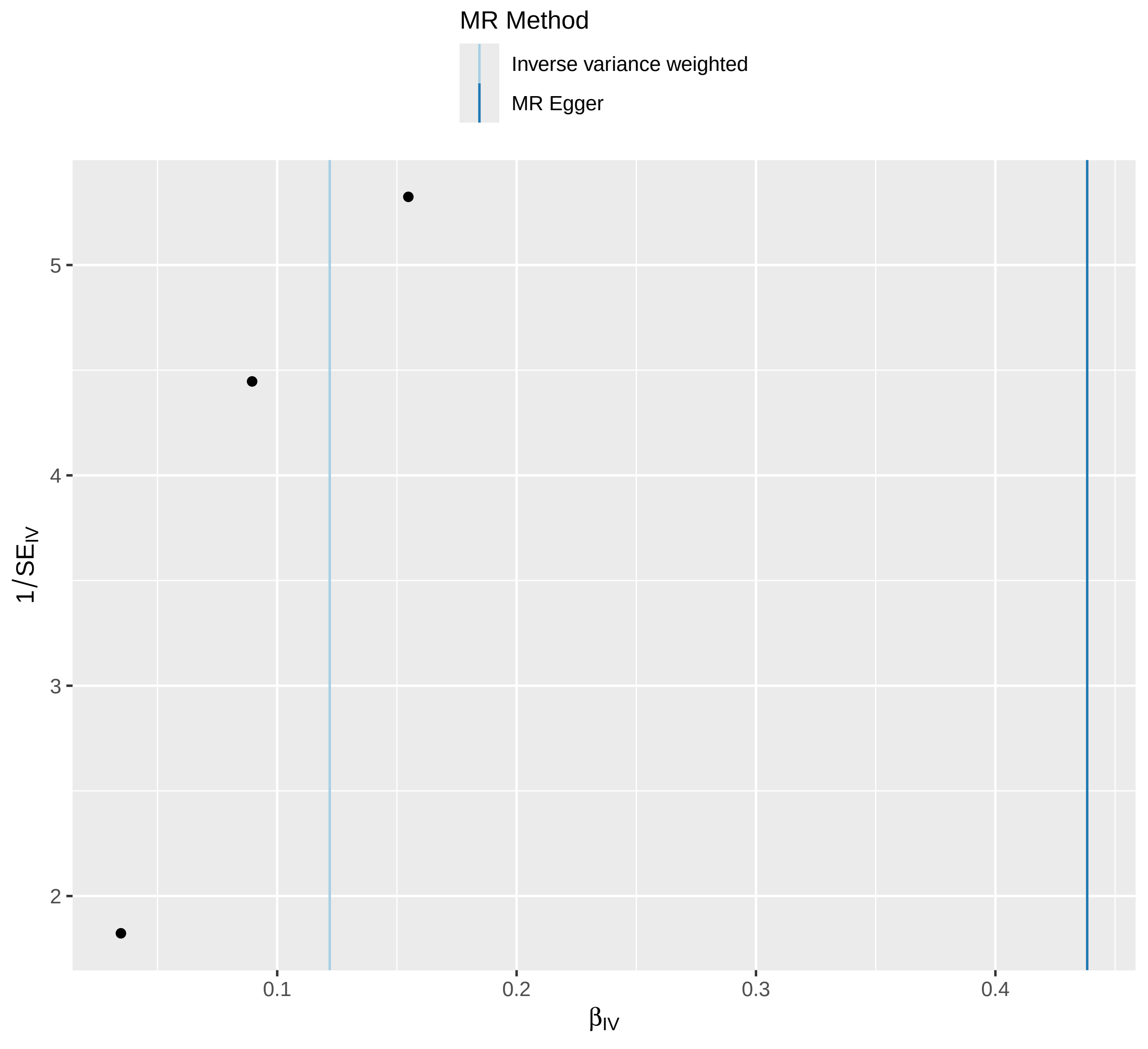
MR Method
Inverse variance weighted
MR Egger
5
4
3
2
0.1
0.2
0.3
0.4
$\beta_{IV}$
$1/SE_{IV}$

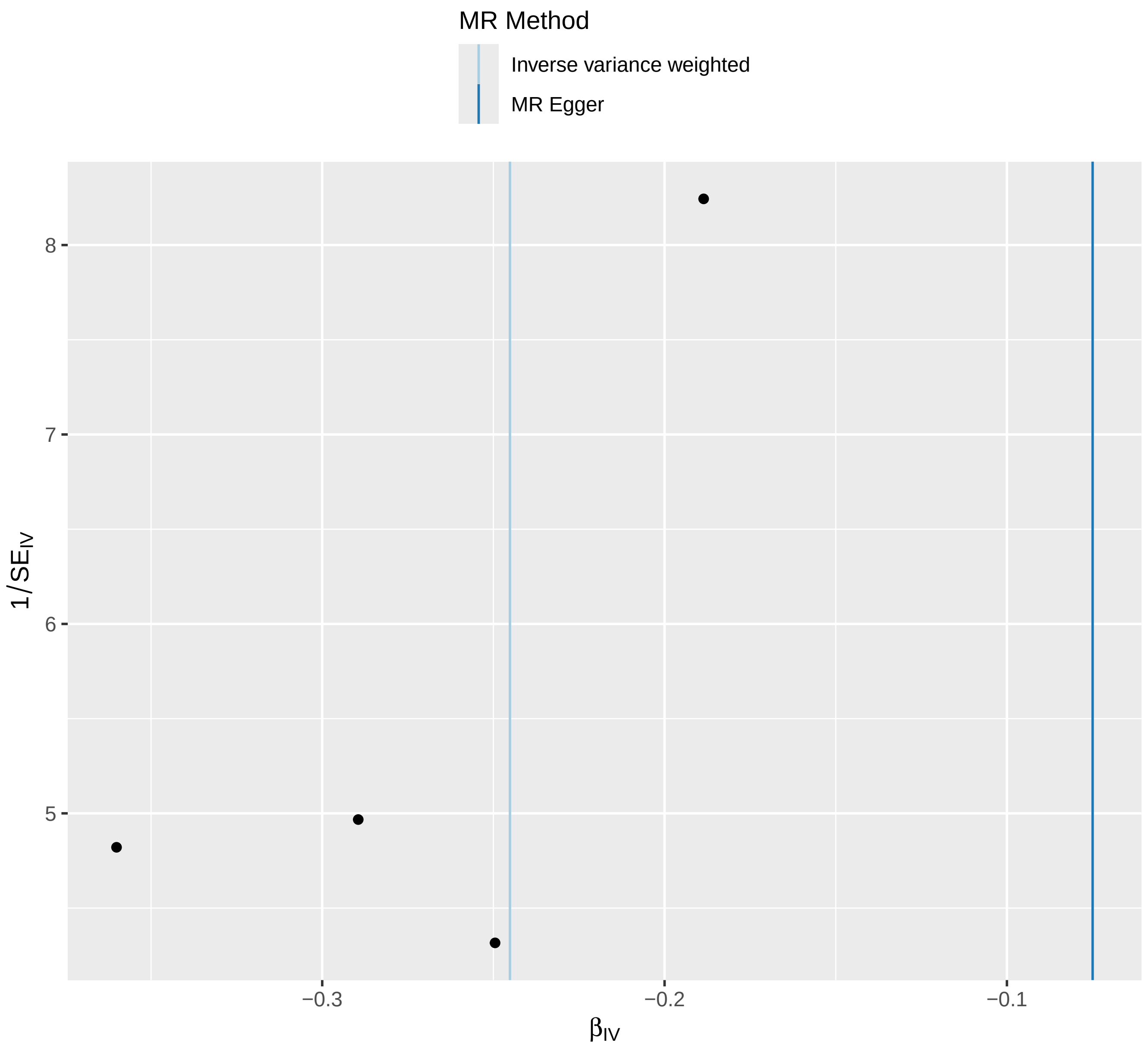

MR Method
Inverse variance weighted
MR Egger
8
7
6
5
1/SE$_{IV}$
−0.3
−0.2
−0.1
β$_{IV}$

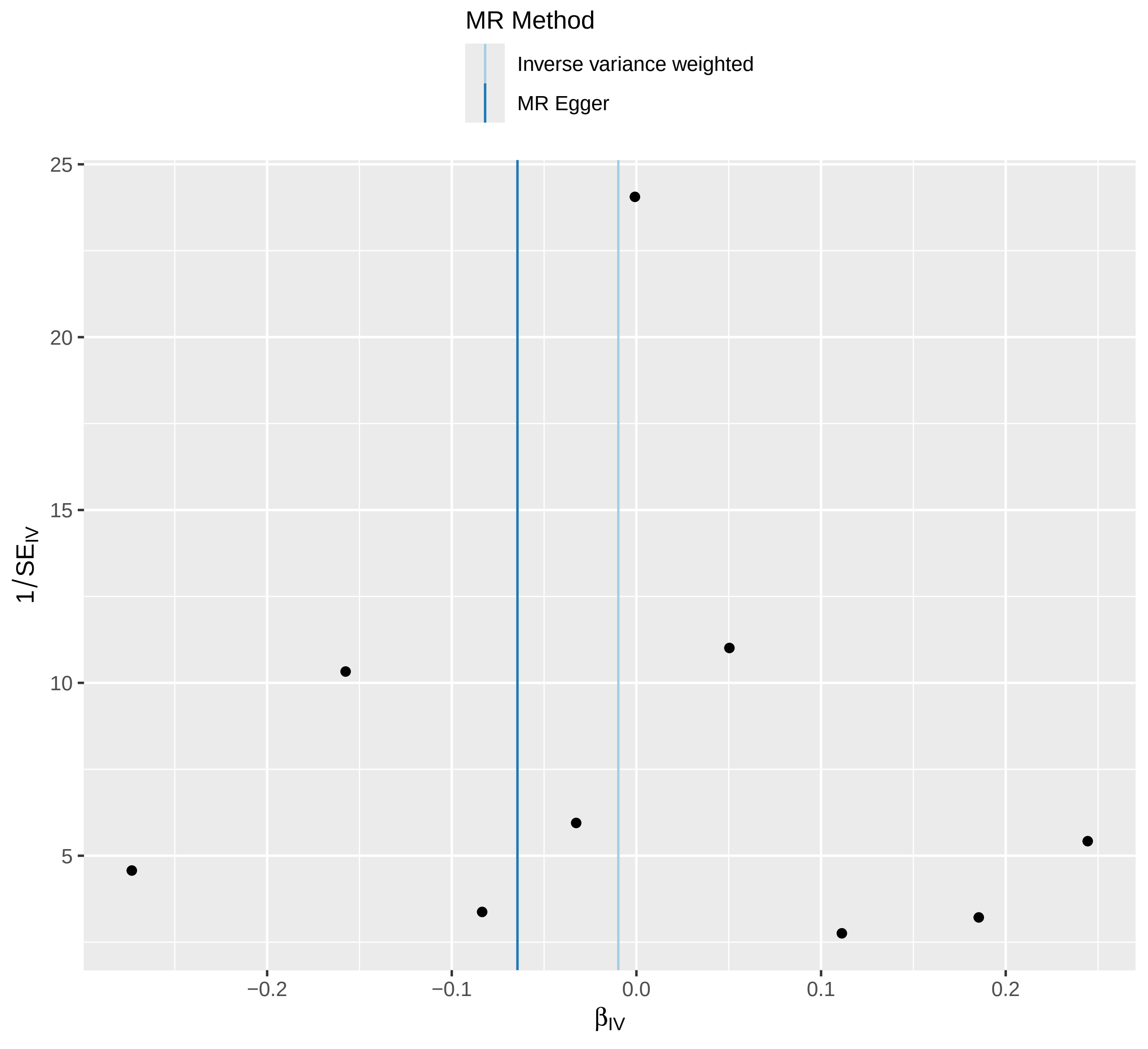
MR Method
Inverse variance weighted
MR Egger
25
20
15
10
5
1/SE_IV
−0.2
−0.1
0.0
0.1
0.2
β_IV

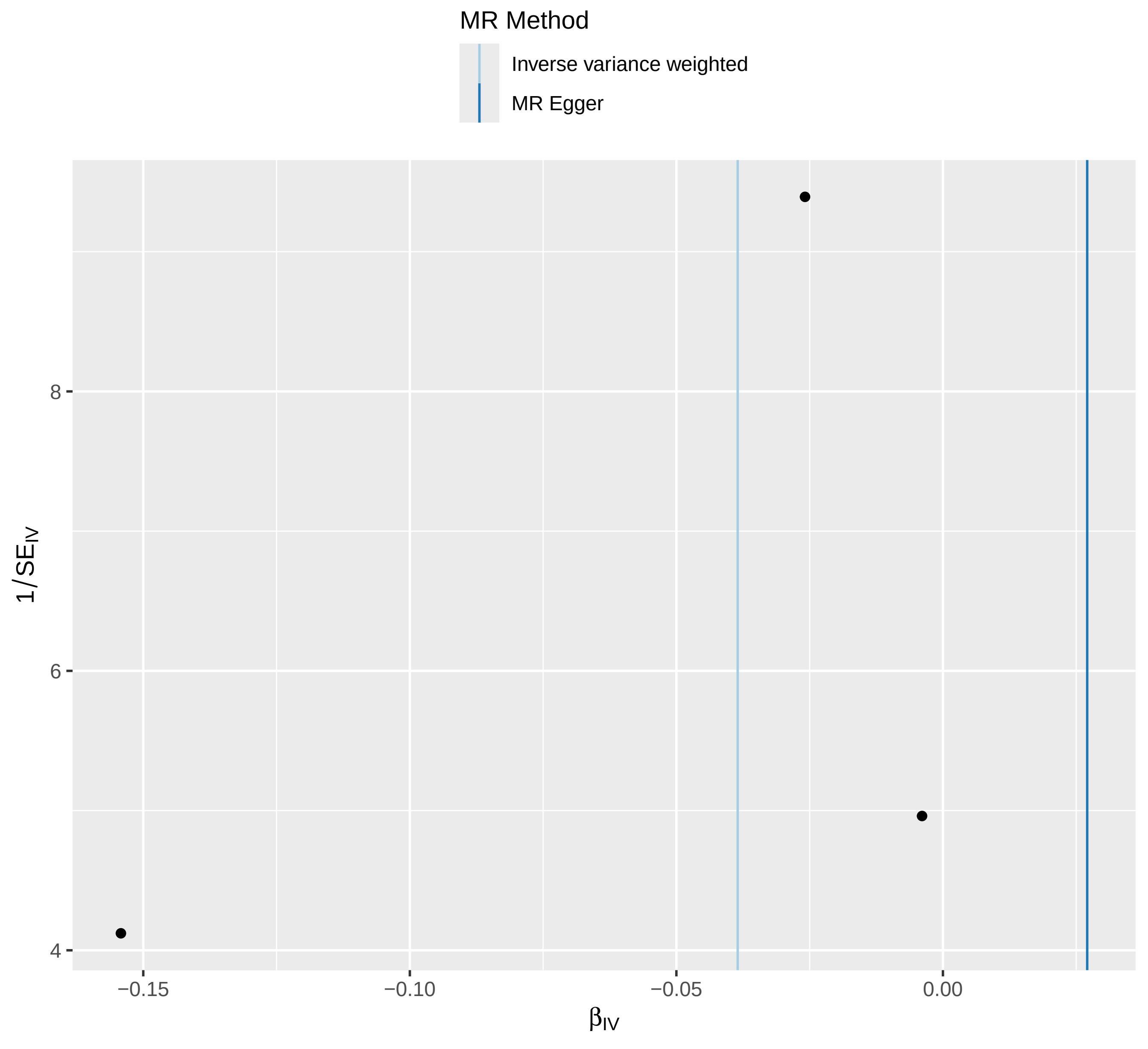
MR Method
Inverse variance weighted
MR Egger
1/SE_IV
8
6
4
−0.15
−0.10
−0.05
0.00
β_IV

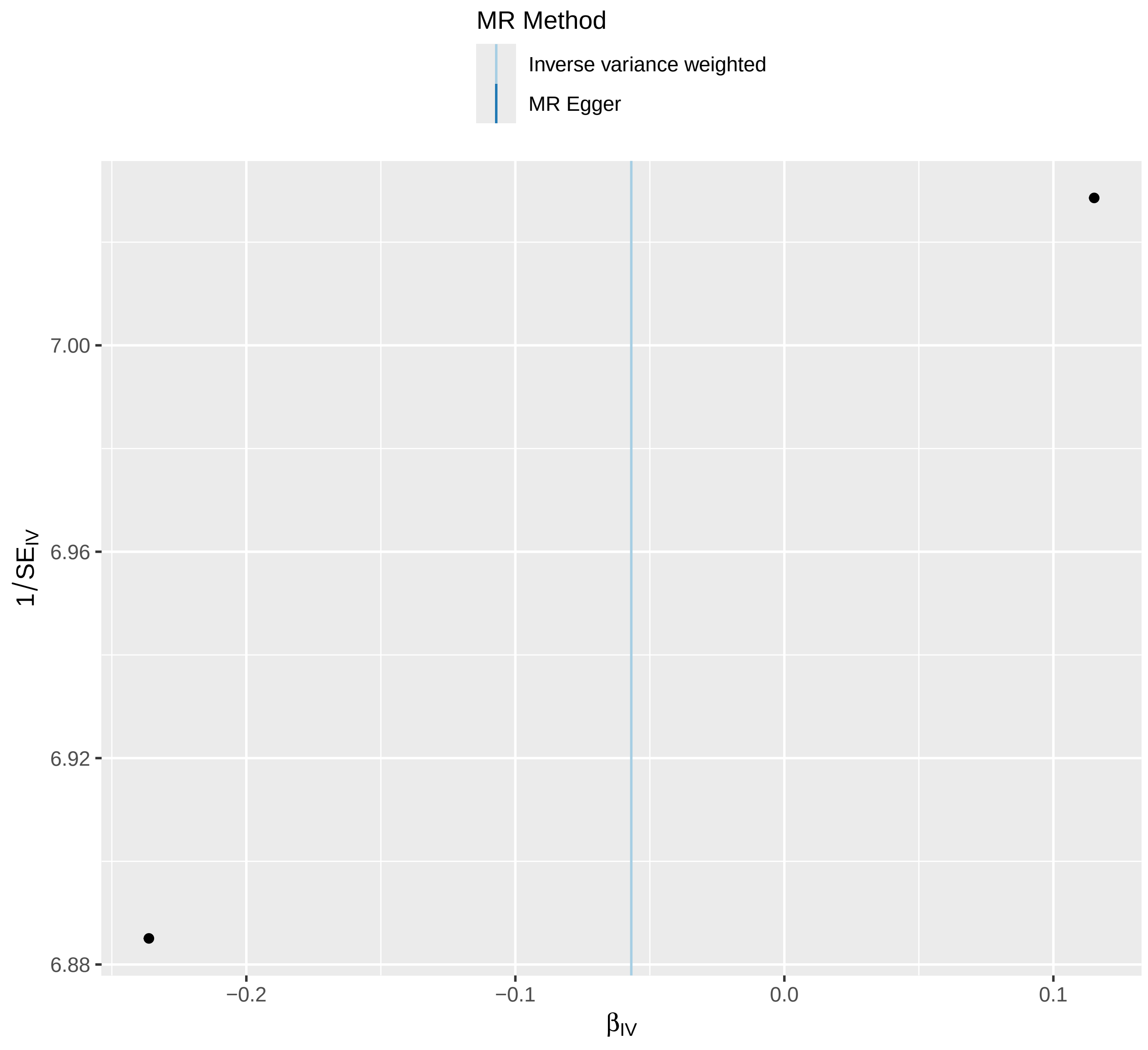
MR Method
Inverse variance weighted
MR Egger
7.00
6.96
6.92
6.88
1/SE$_{IV}$
−0.2
−0.1
0.0
0.1
β$_{IV}$

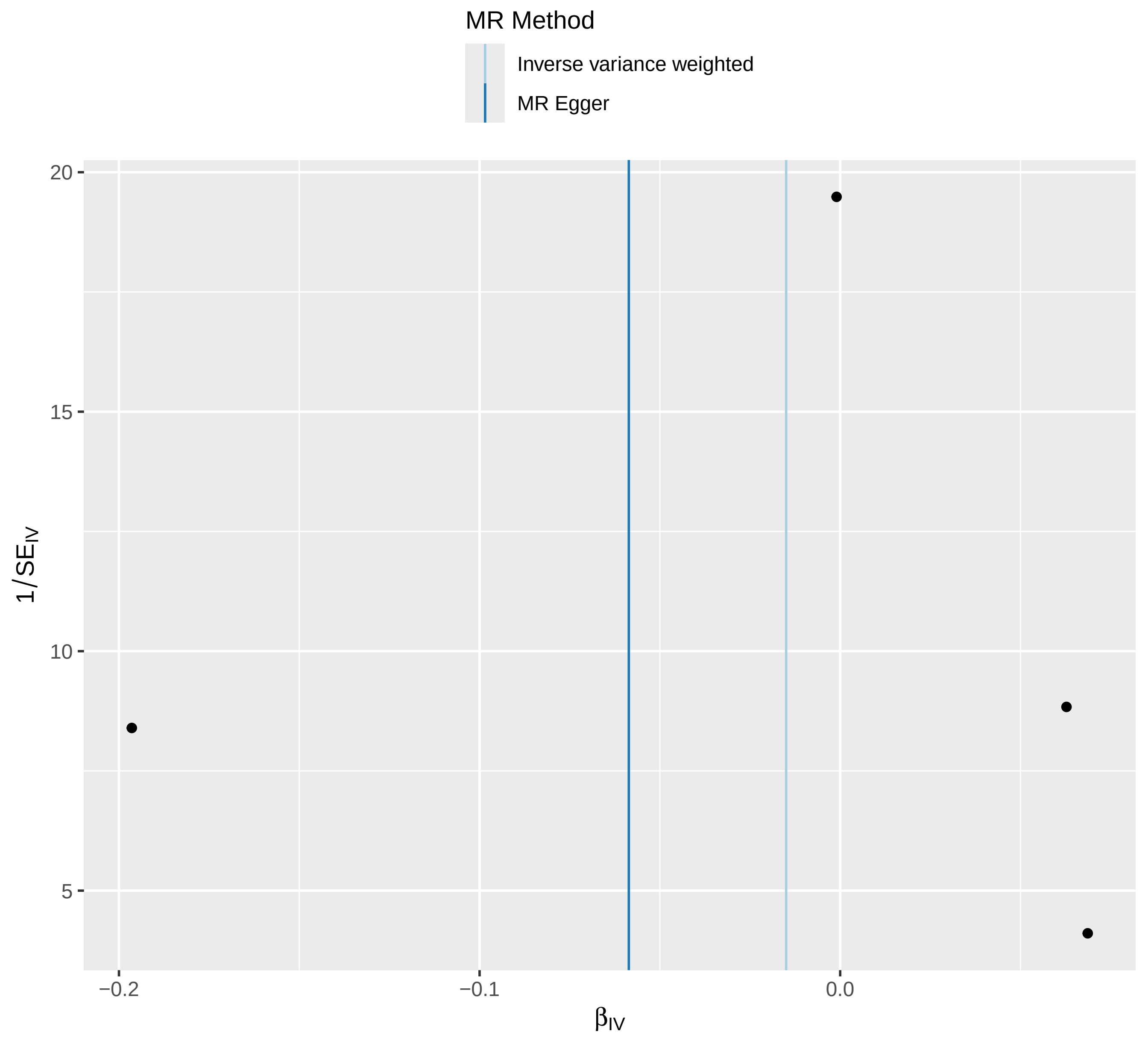
MR Method
Inverse variance weighted
MR Egger
20
15
10
5
1/SE_IV
−0.2
−0.1
0.0
β_IV

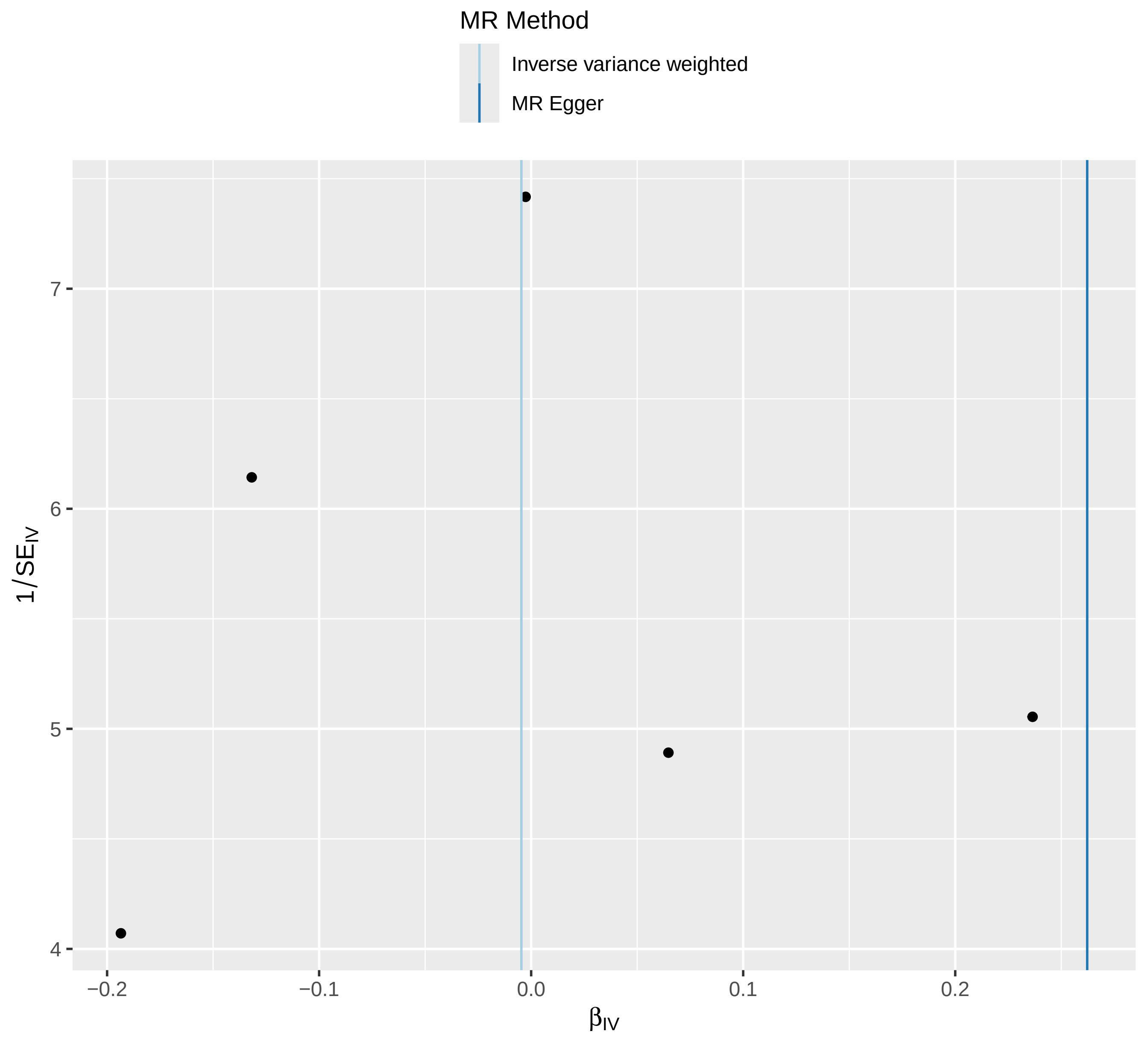
MR Method
Inverse variance weighted
MR Egger
1/SE_IV
β_IV
7
6
5
4
−0.2
−0.1
0.0
0.1
0.2

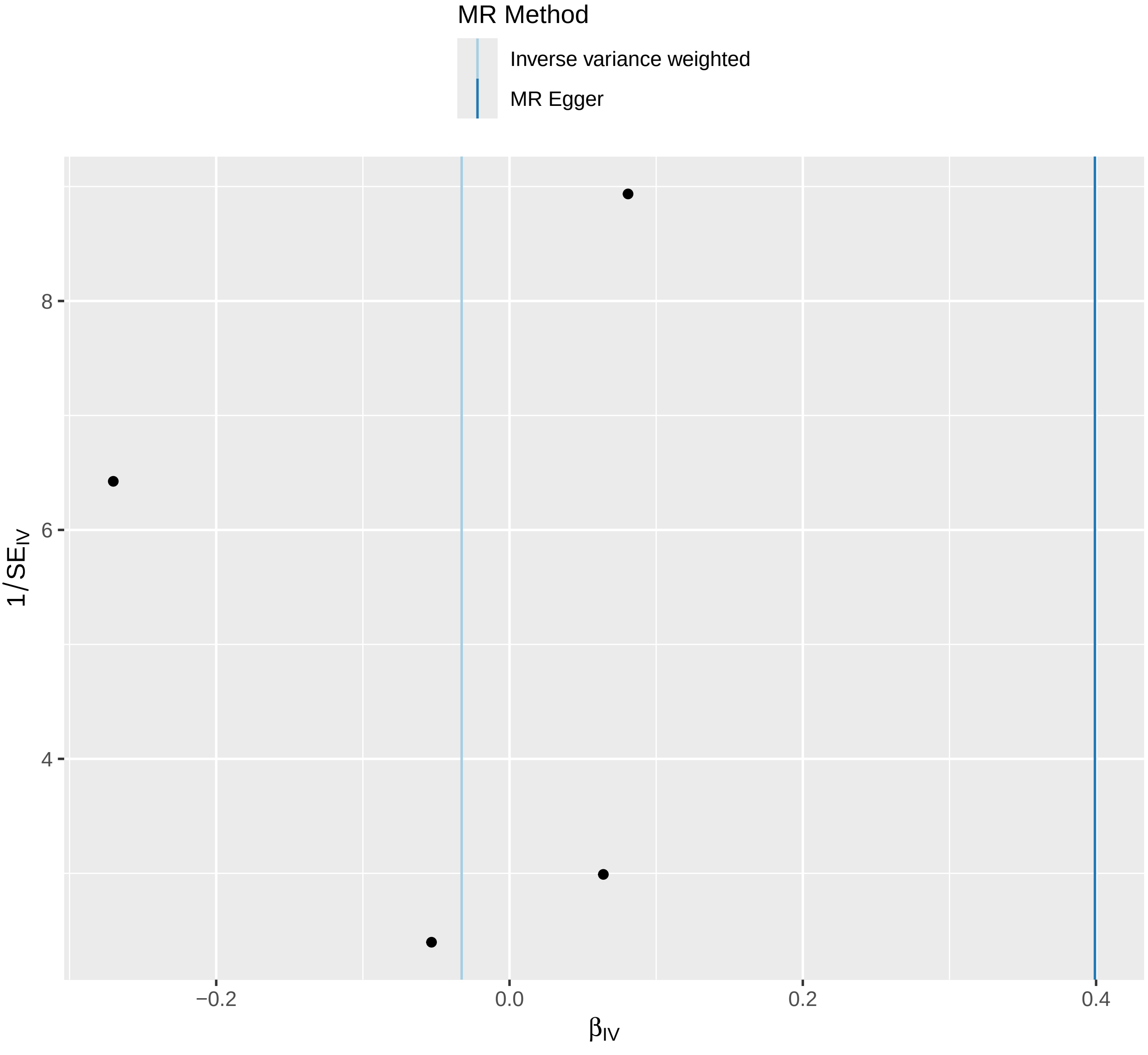
MR Method
Inverse variance weighted
MR Egger
8
6
4
1/SE_IV
−0.2
0.0
0.2
0.4
β_IV

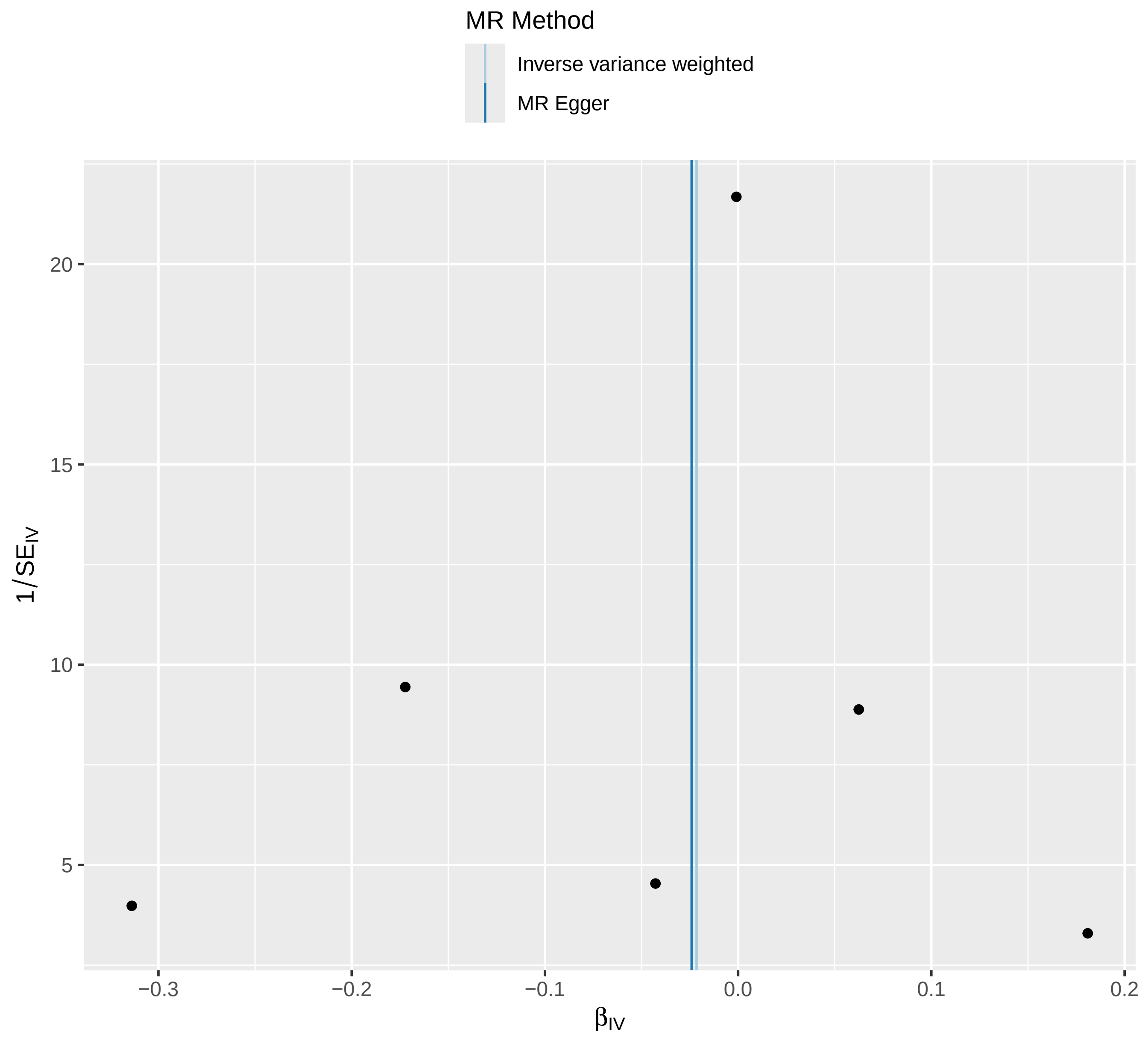

MR Method
Inverse variance weighted
MR Egger
20
15
10
5
$1/SE_{IV}$
−0.3
−0.2
−0.1
0.0
0.1
0.2
$\beta_{IV}$

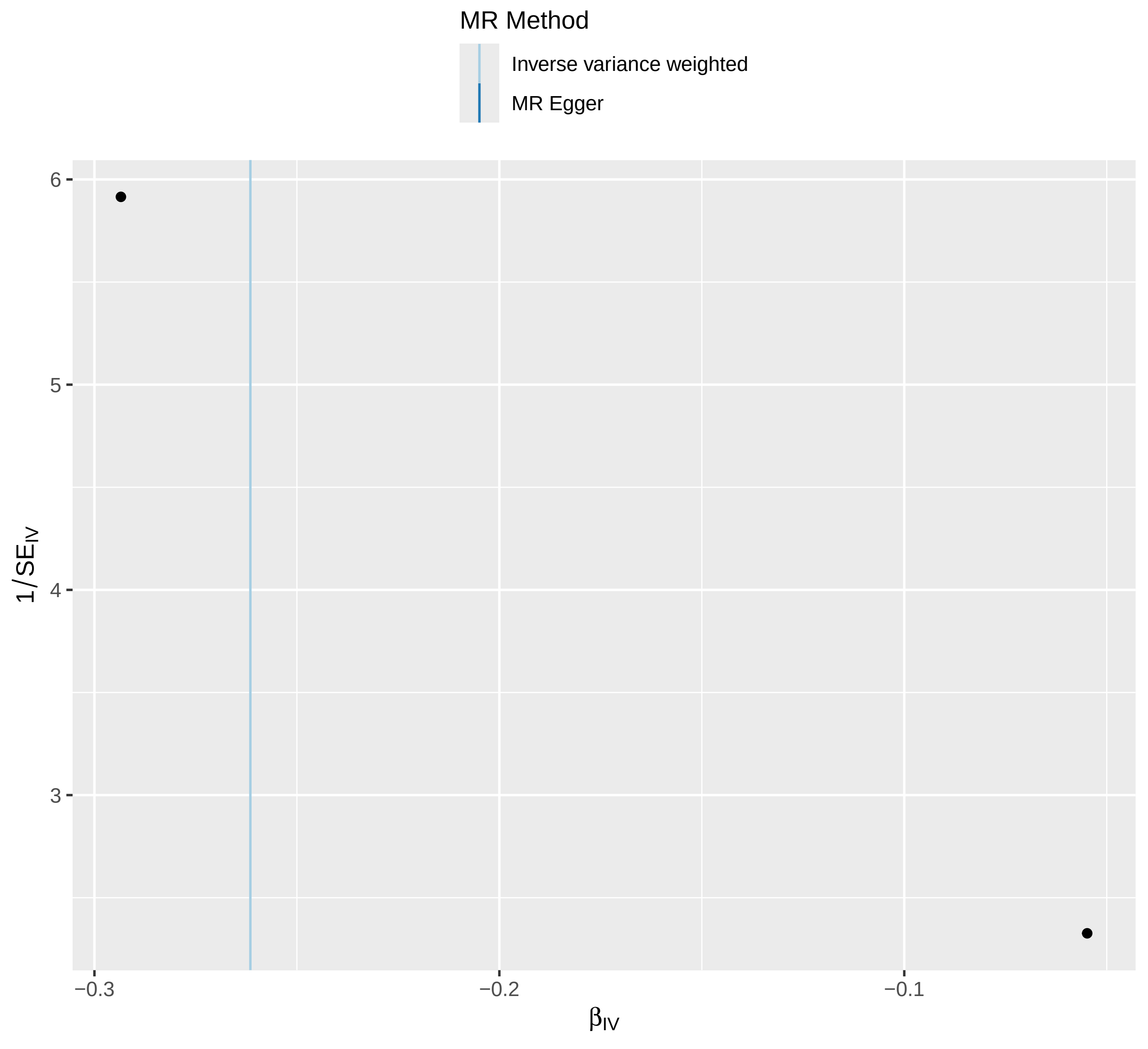

MR Method
Inverse variance weighted
MR Egger
6
5
4
3
1/SE_IV
−0.3
−0.2
−0.1
β_IV

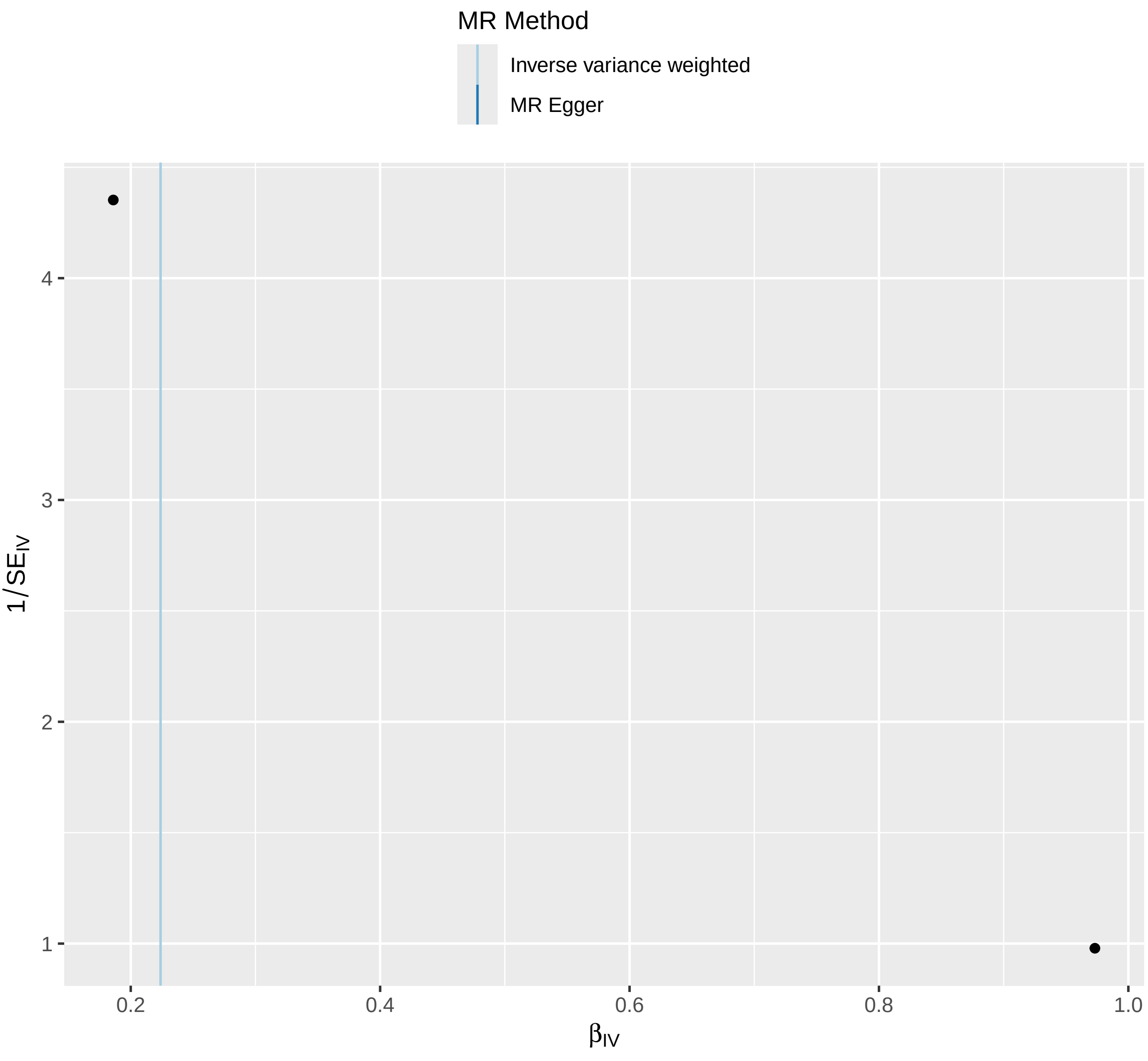

MR Method
Inverse variance weighted
MR Egger
4
3
2
1
1/SE$_{IV}$
0.2
0.4
0.6
0.8
1.0
β$_{IV}$

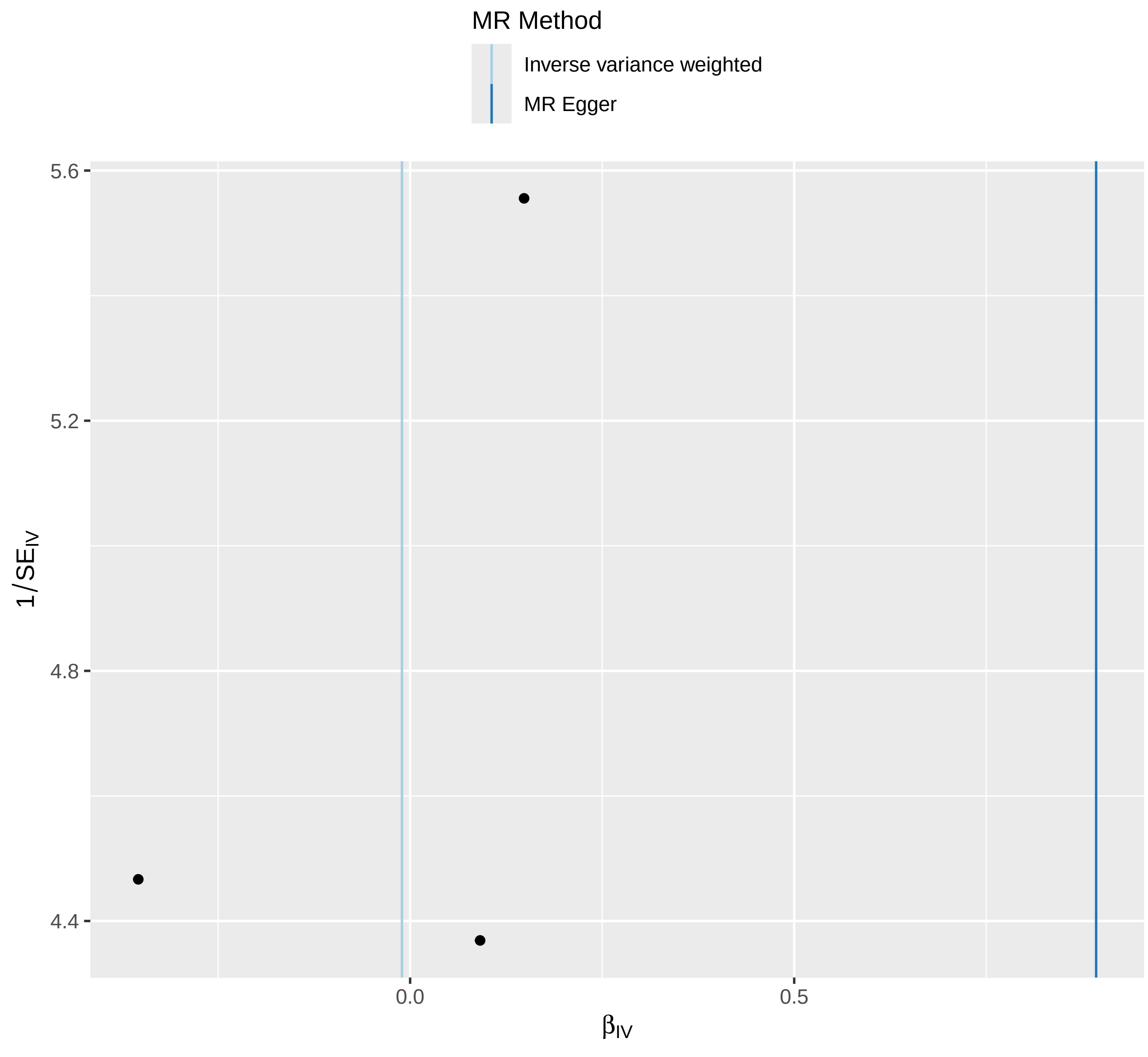

MR Method
Inverse variance weighted
MR Egger
5.6
5.2
4.8
4.4
1/SE_IV
0.0
0.5
β_IV

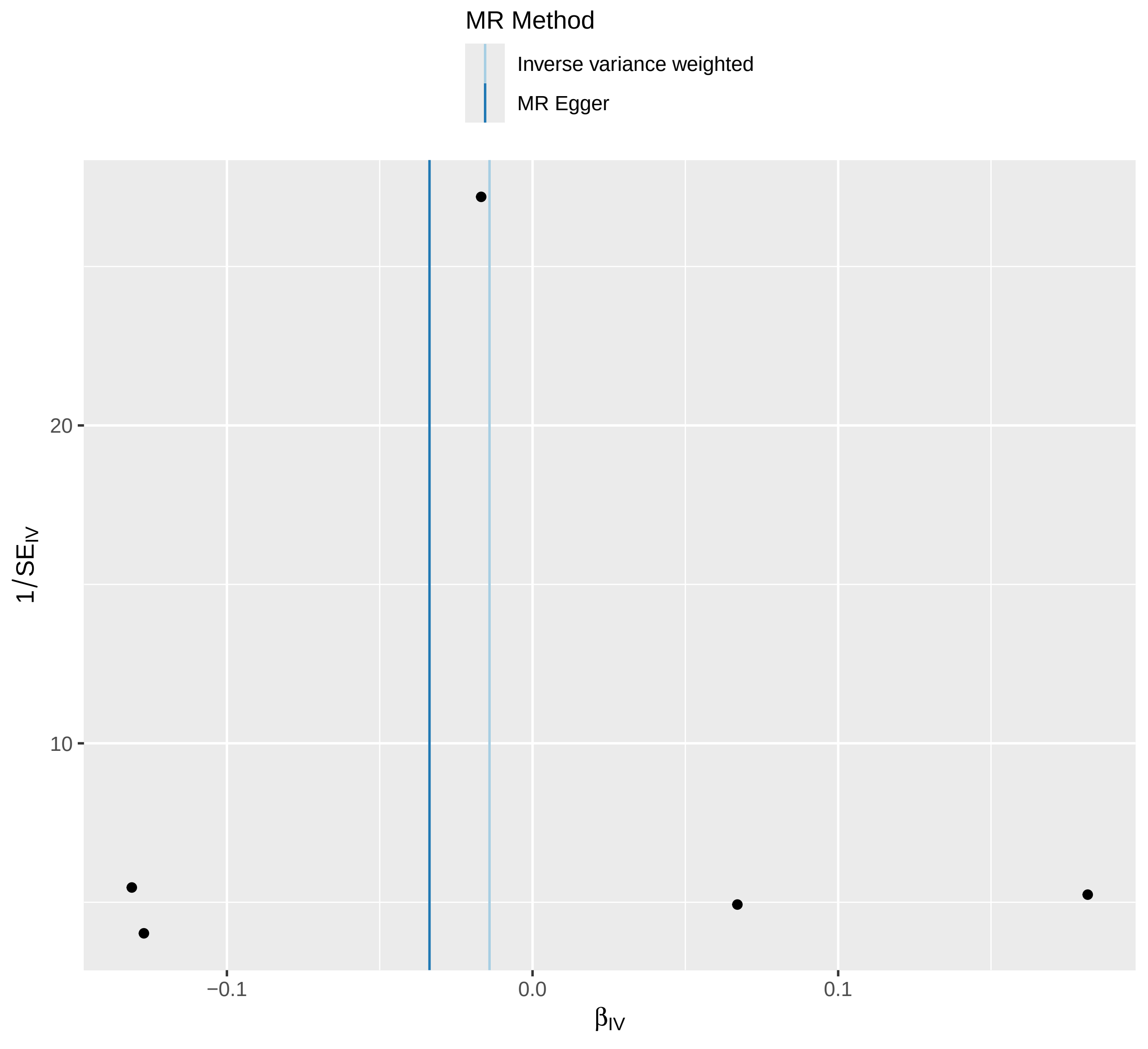

MR Method
Inverse variance weighted
MR Egger
20
10
1/SE_IV
−0.1
0.0
0.1
β_IV

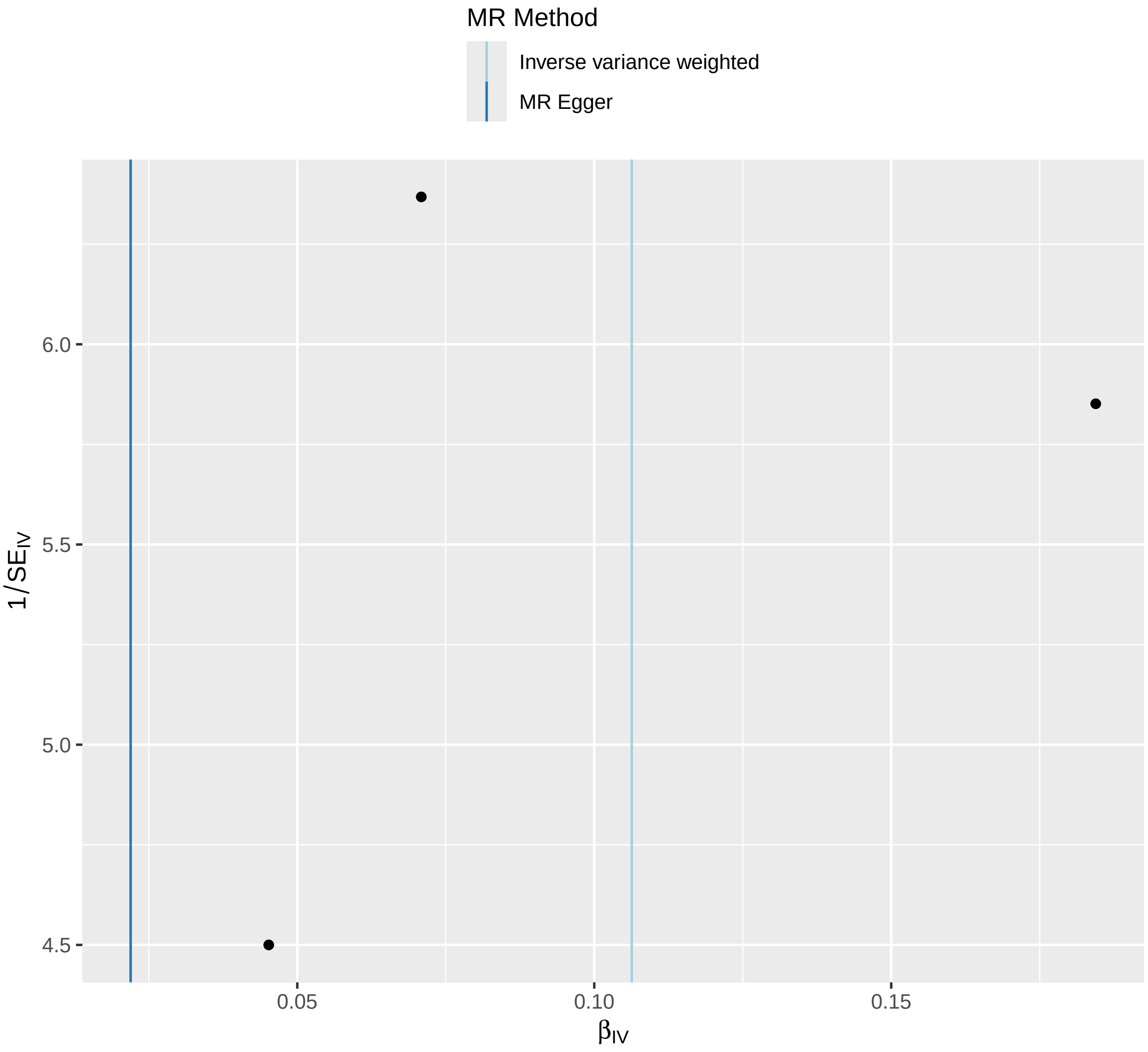
MR Method
Inverse variance weighted
MR Egger
6.0
5.5
5.0
4.5
$1/SE_{IV}$
0.05
0.10
0.15
$\beta_{IV}$

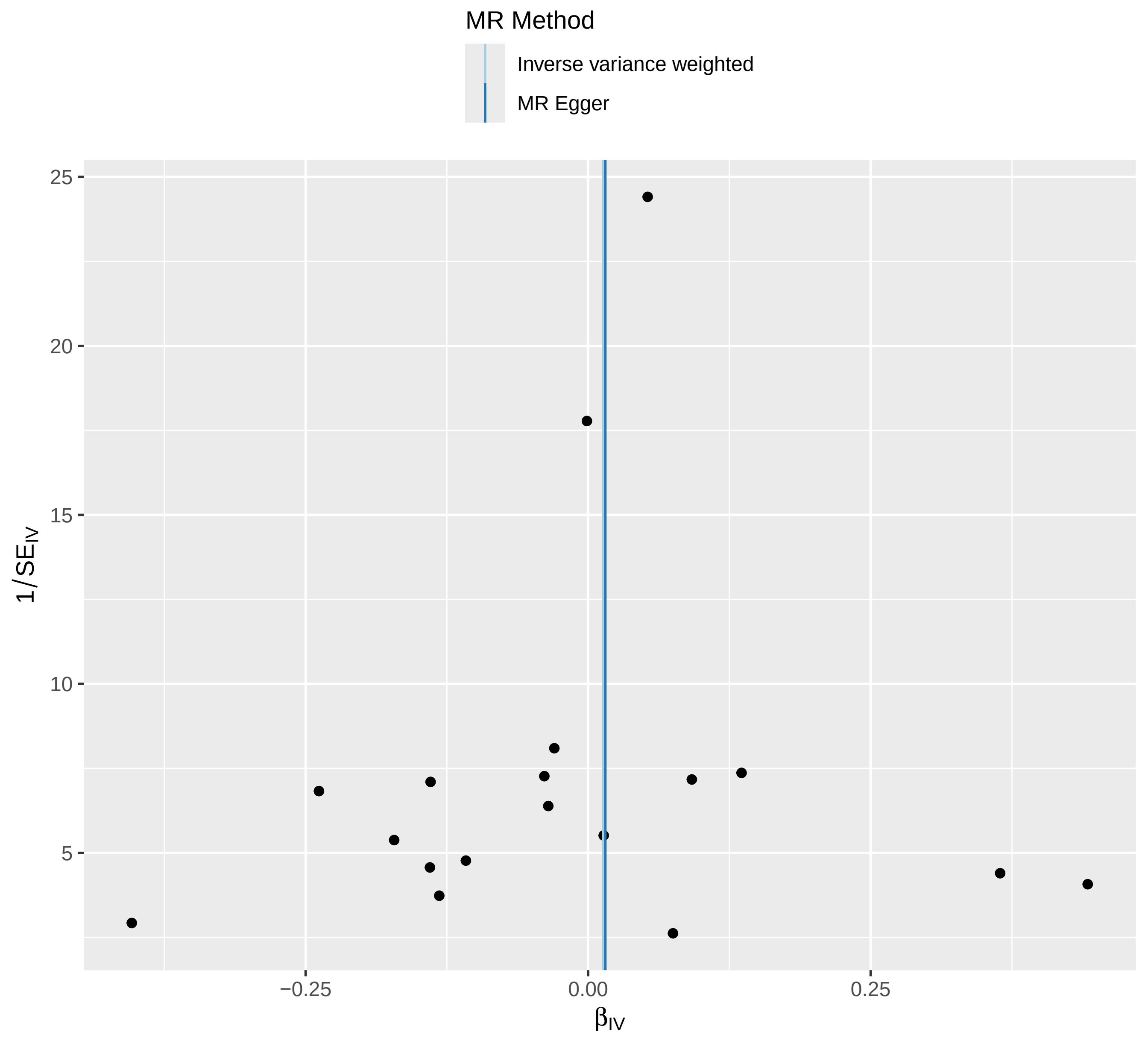
MR Method
Inverse variance weighted
MR Egger
25
20
15
10
5
1/SE_IV
−0.25
0.00
0.25
β_IV

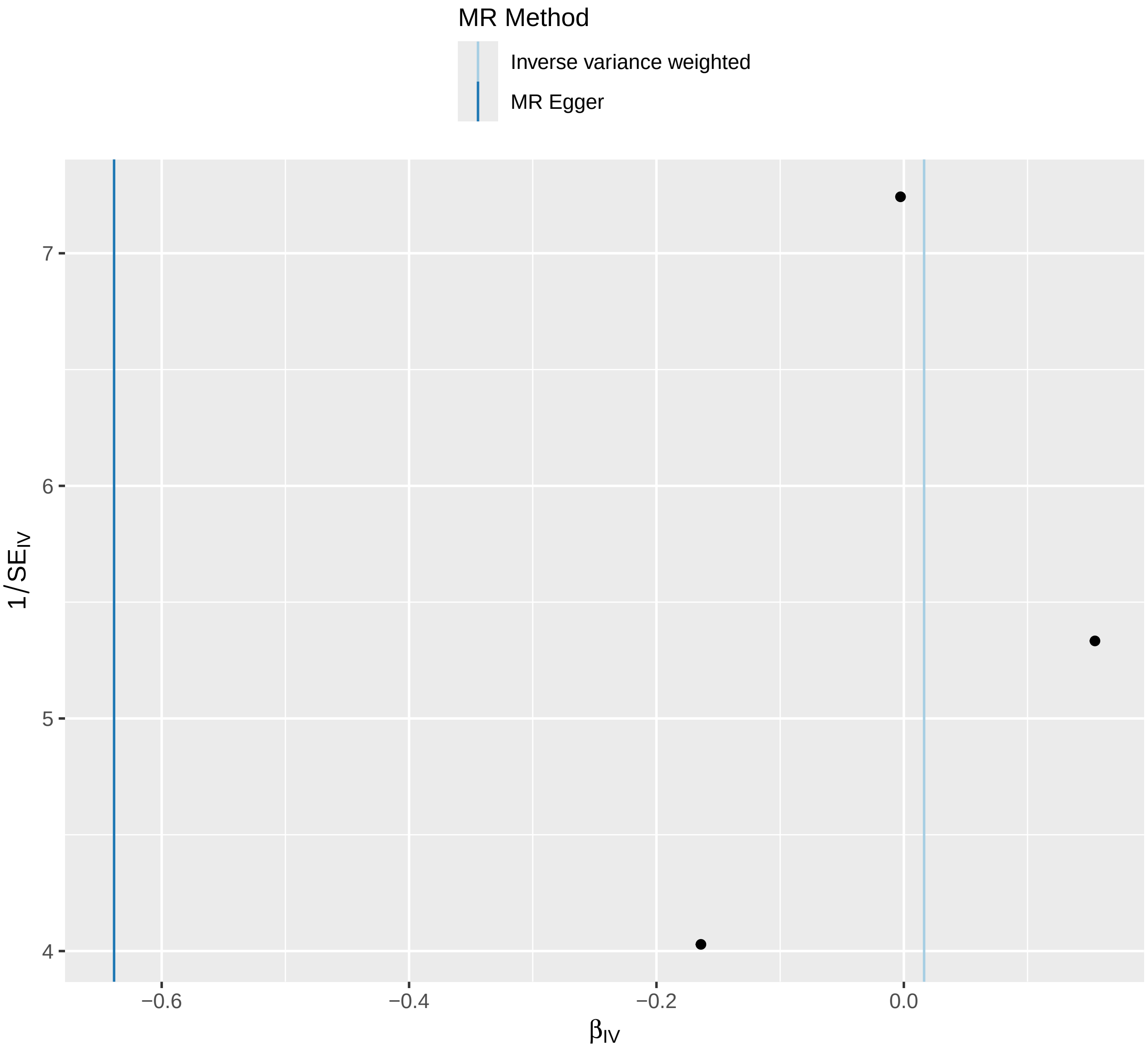
MR Method
Inverse variance weighted
MR Egger
7
6
5
4
1/SE_IV
−0.6
−0.4
−0.2
0.0
β_IV

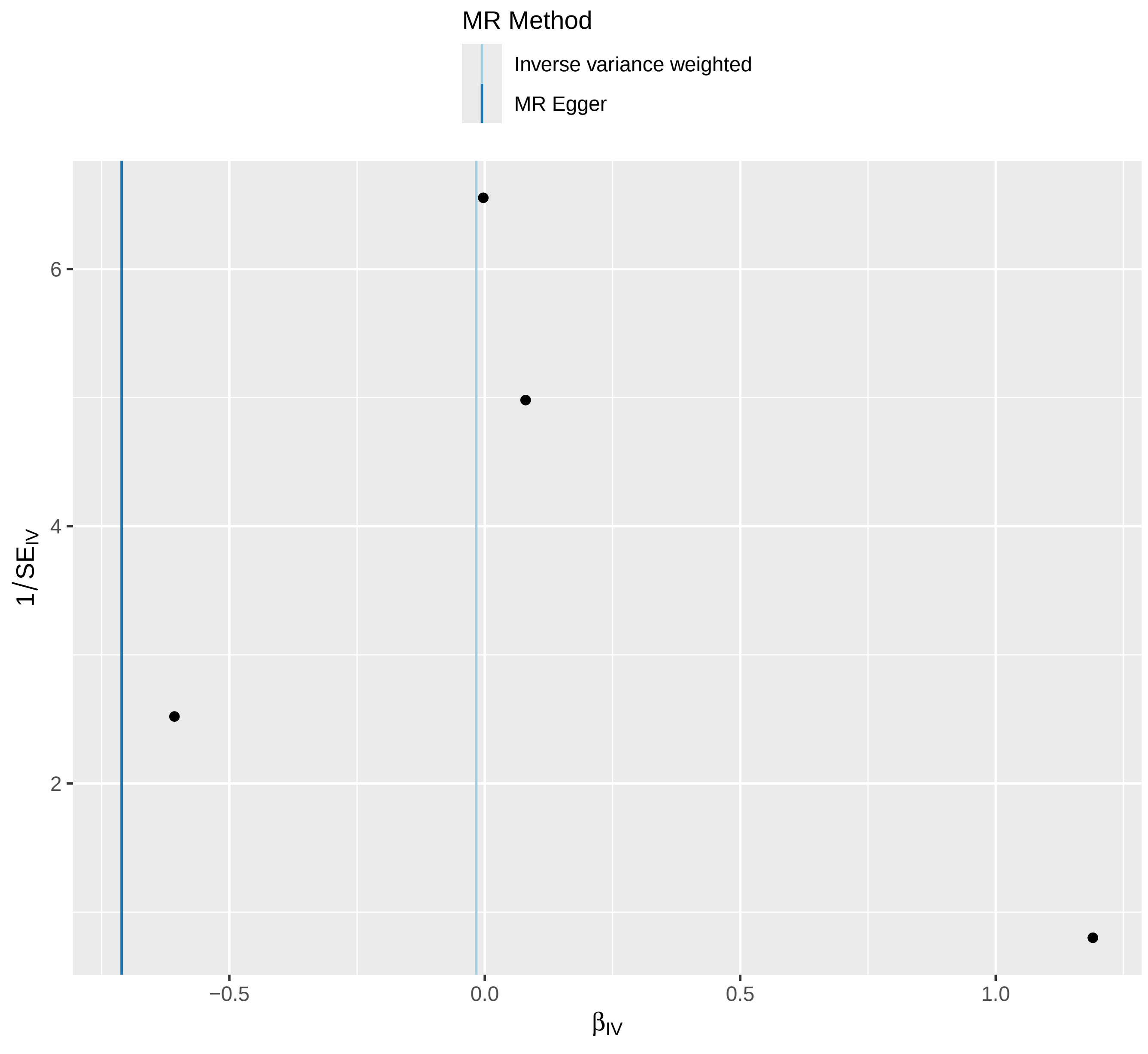

MR Method
Inverse variance weighted
MR Egger
6
4
2
1/SE$_{IV}$
−0.5
0.0
0.5
1.0
β$_{IV}$

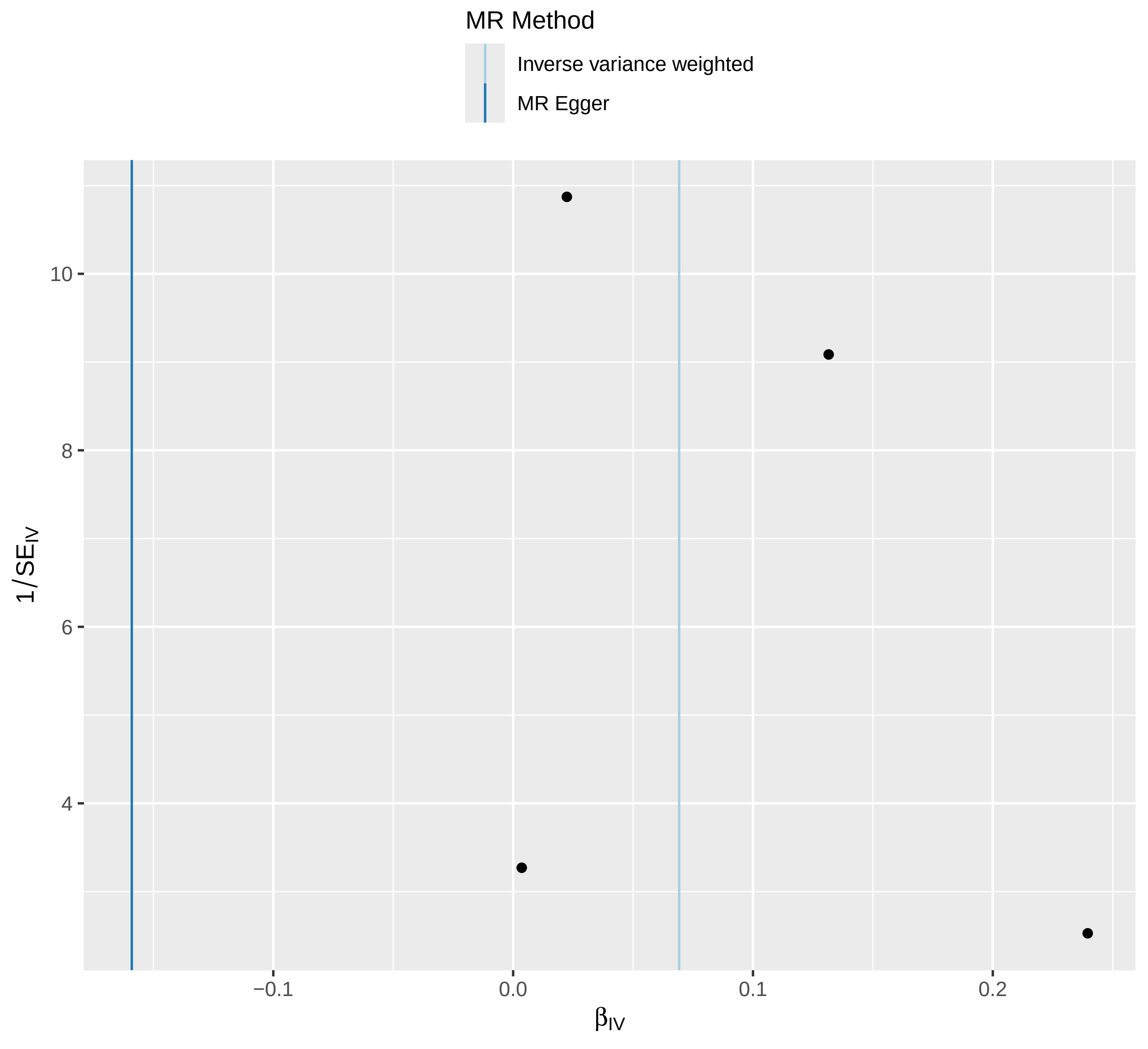
MR Method
Inverse variance weighted
MR Egger
10
8
6
4
1/SE_IV
−0.1
0.0
0.1
0.2
β_IV

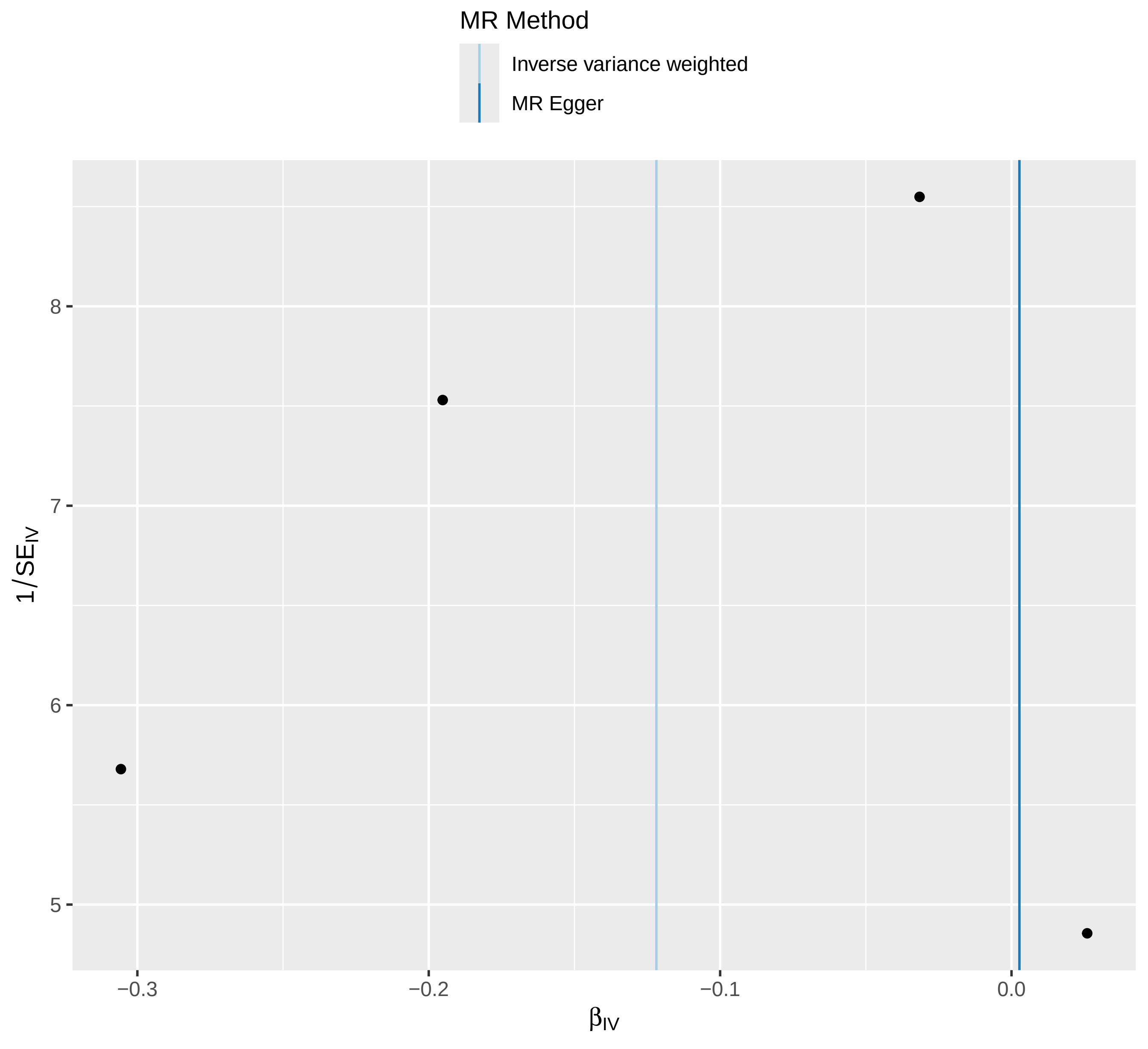

MR Method
Inverse variance weighted
MR Egger
8
7
6
5
1/SE_IV
−0.3
−0.2
−0.1
0.0
β_IV

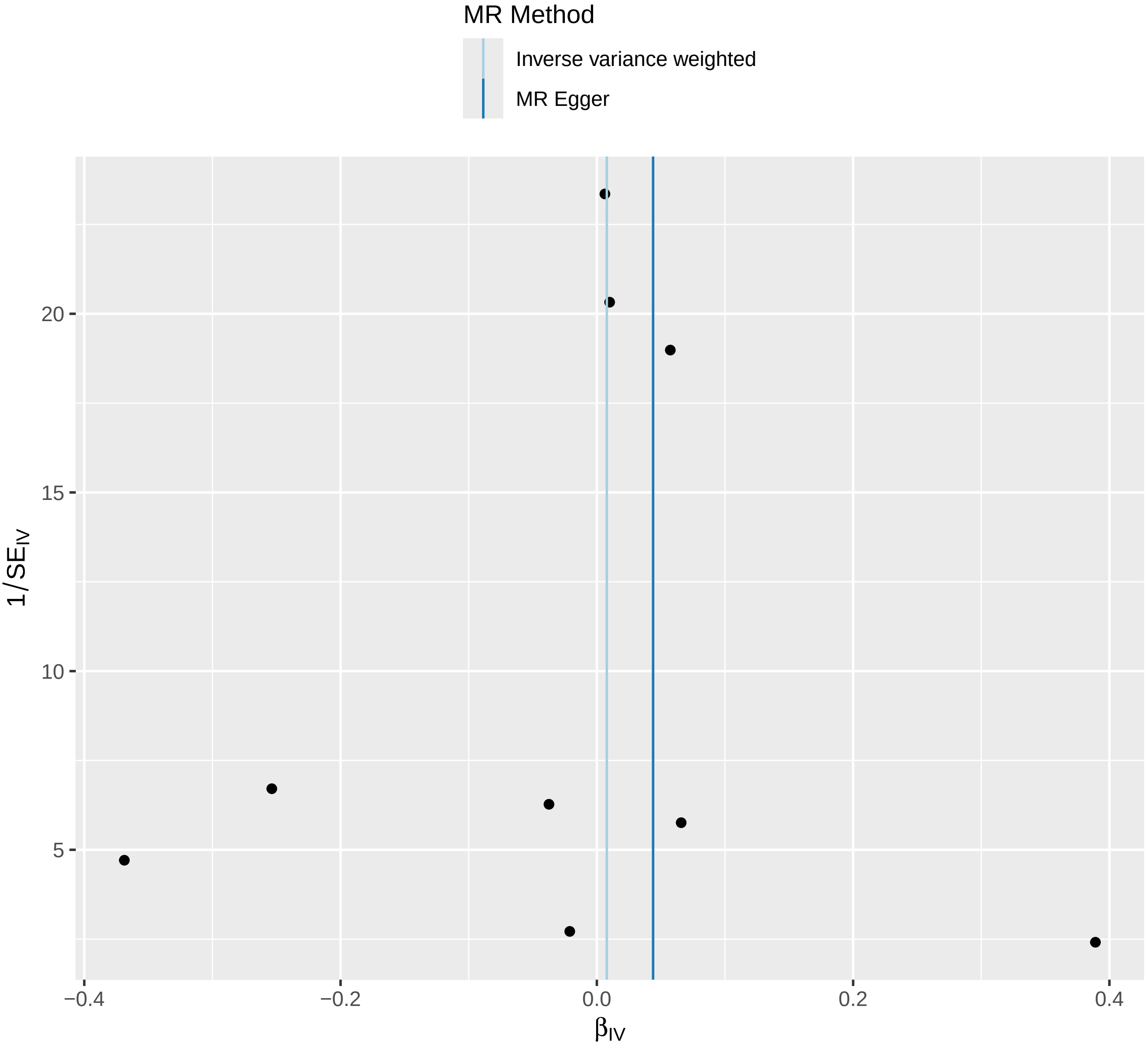
MR Method
Inverse variance weighted
MR Egger
20
15
10
5
−0.4
−0.2
0.0
0.2
0.4
$1/SE_{IV}$
$\beta_{IV}$

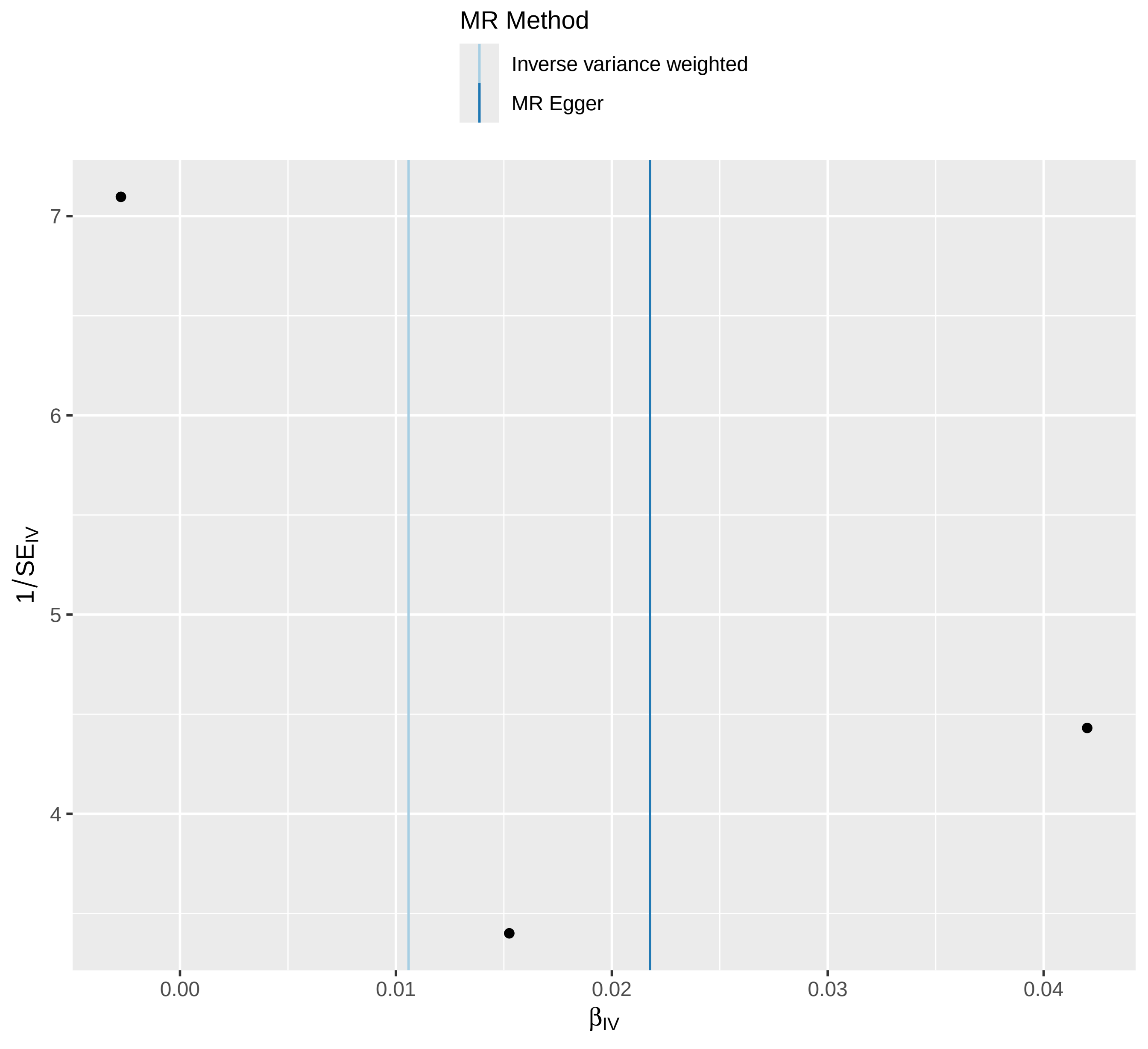

MR Method
Inverse variance weighted
MR Egger
7
6
5
4
1/SE_IV
0.00
0.01
0.02
0.03
0.04
β_IV

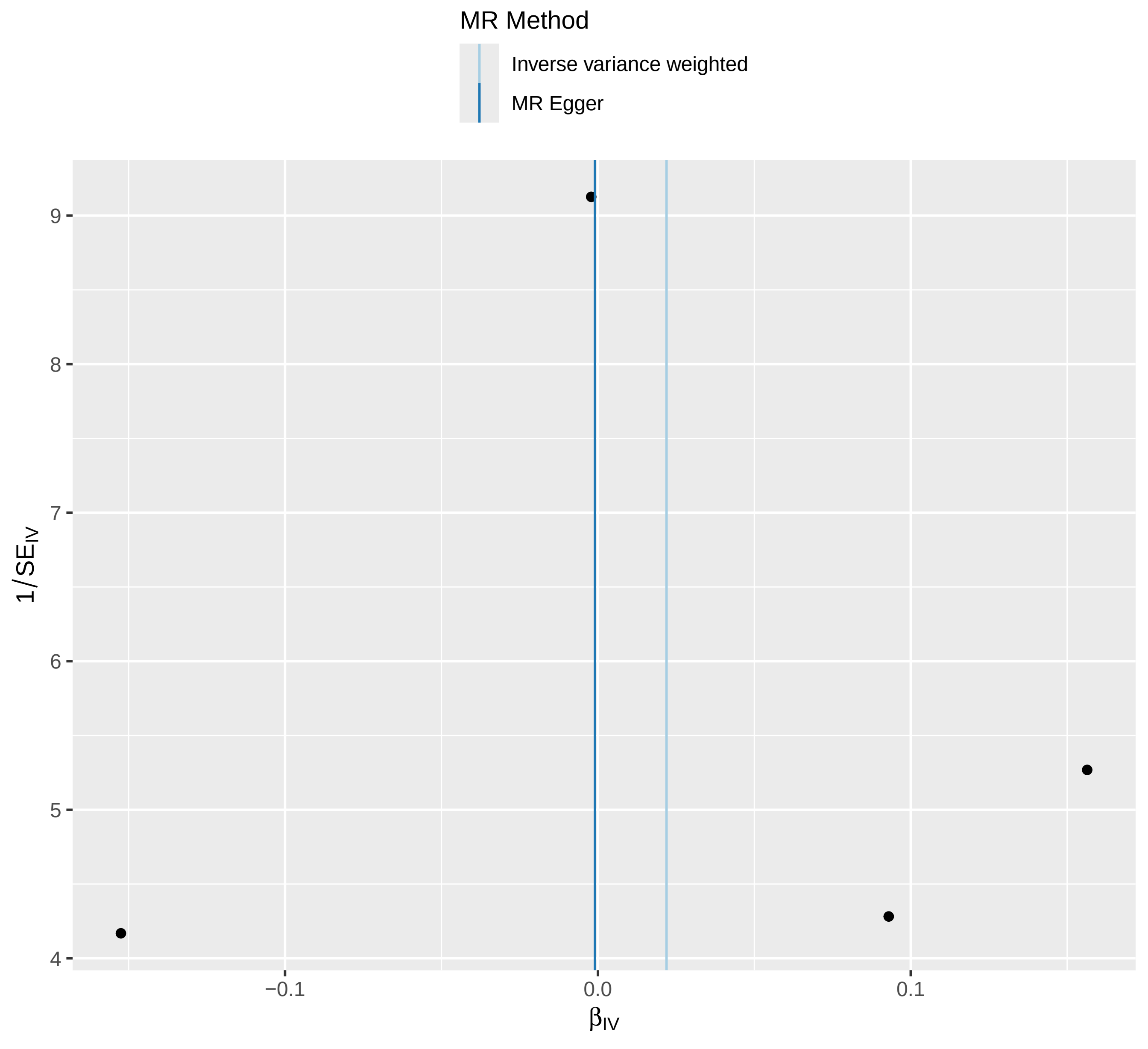

MR Method
Inverse variance weighted
MR Egger
1/SE_IV
9
8
7
6
5
4
−0.1
0.0
0.1
β_IV

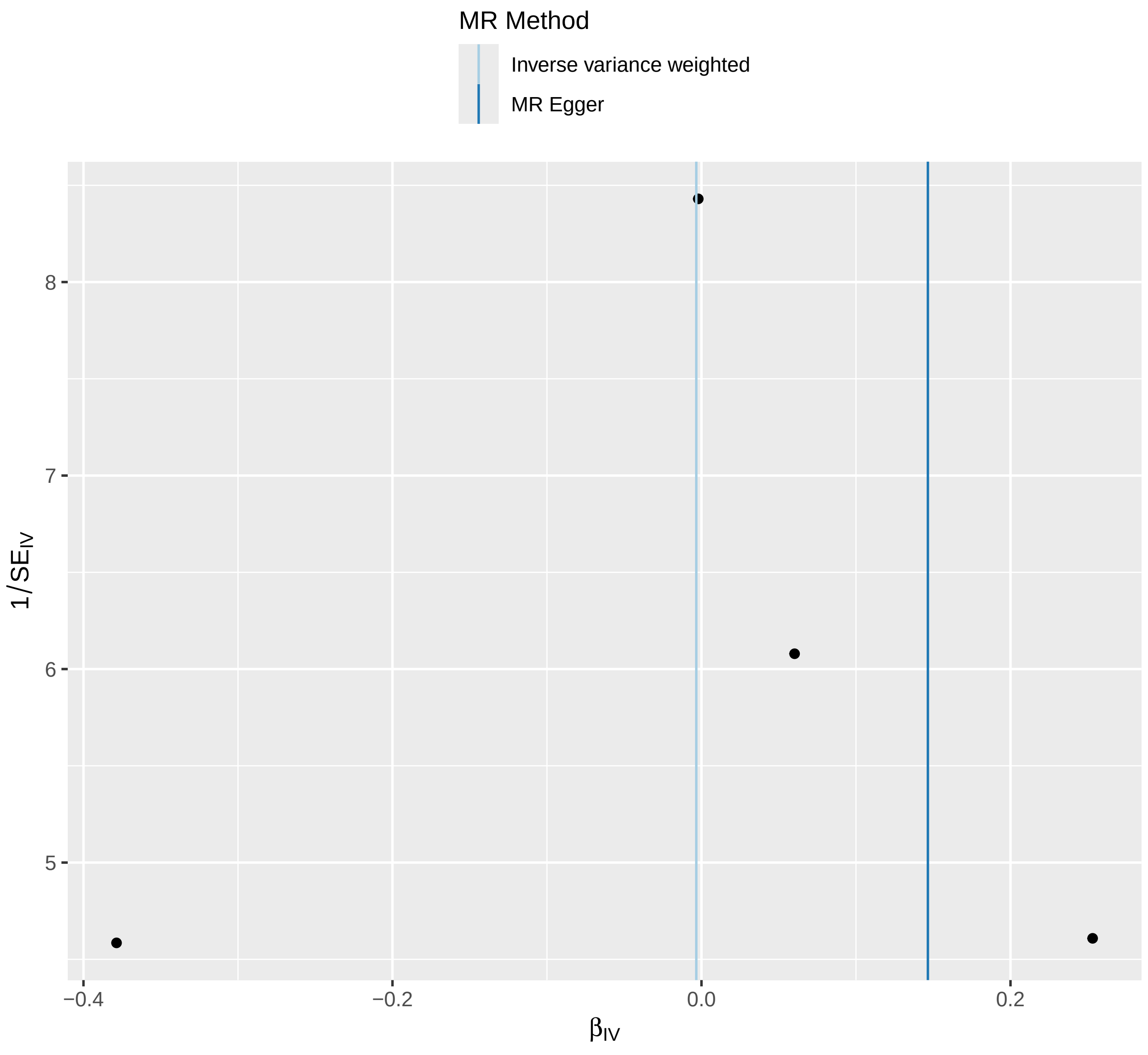
MR Method
Inverse variance weighted
MR Egger
8
7
6
5
1/SE_IV
−0.4
−0.2
0.0
0.2
β_IV

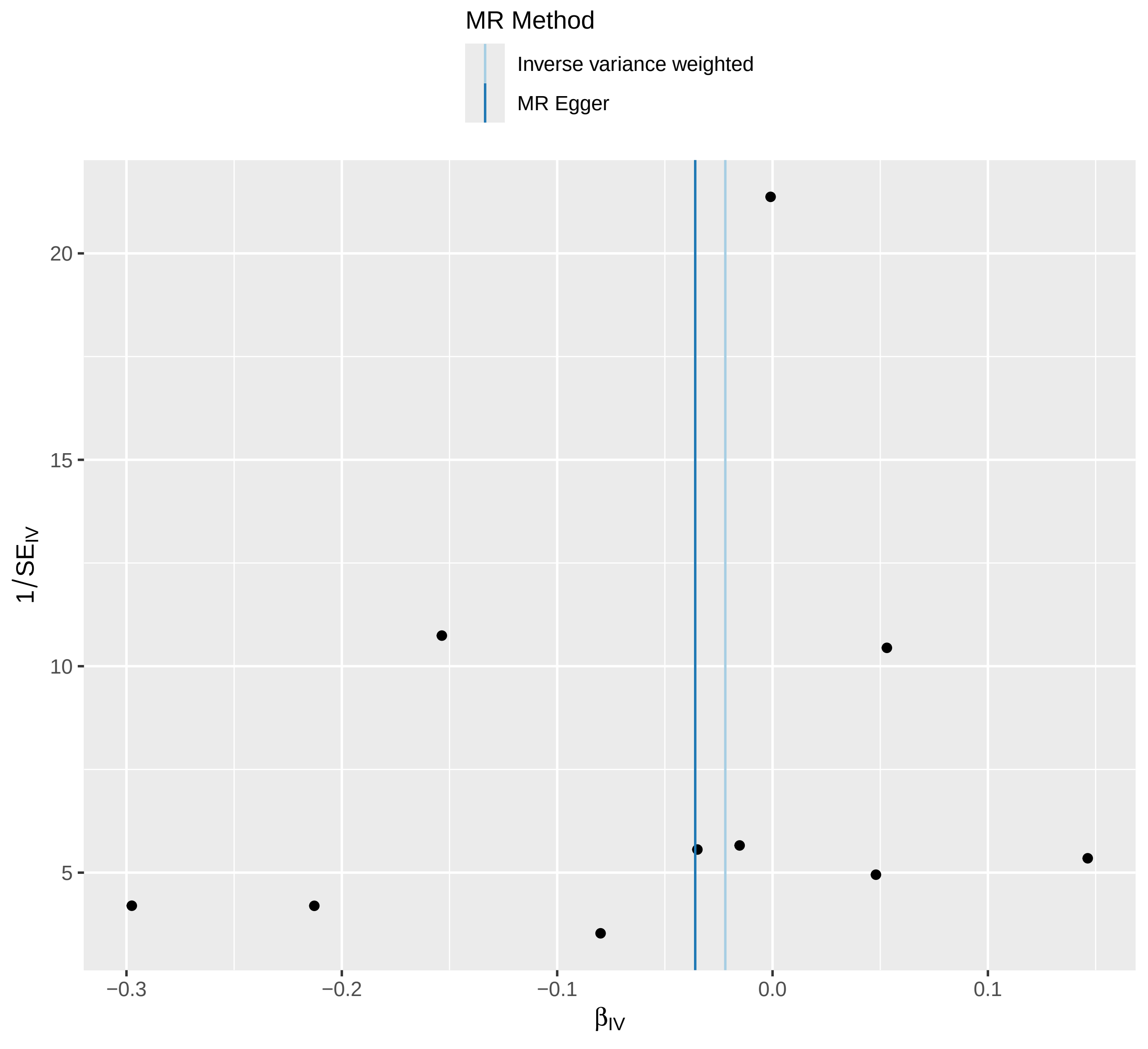

MR Method
Inverse variance weighted
MR Egger
20
15
10
5
1/SE_IV
−0.3
−0.2
−0.1
0.0
0.1
β_IV

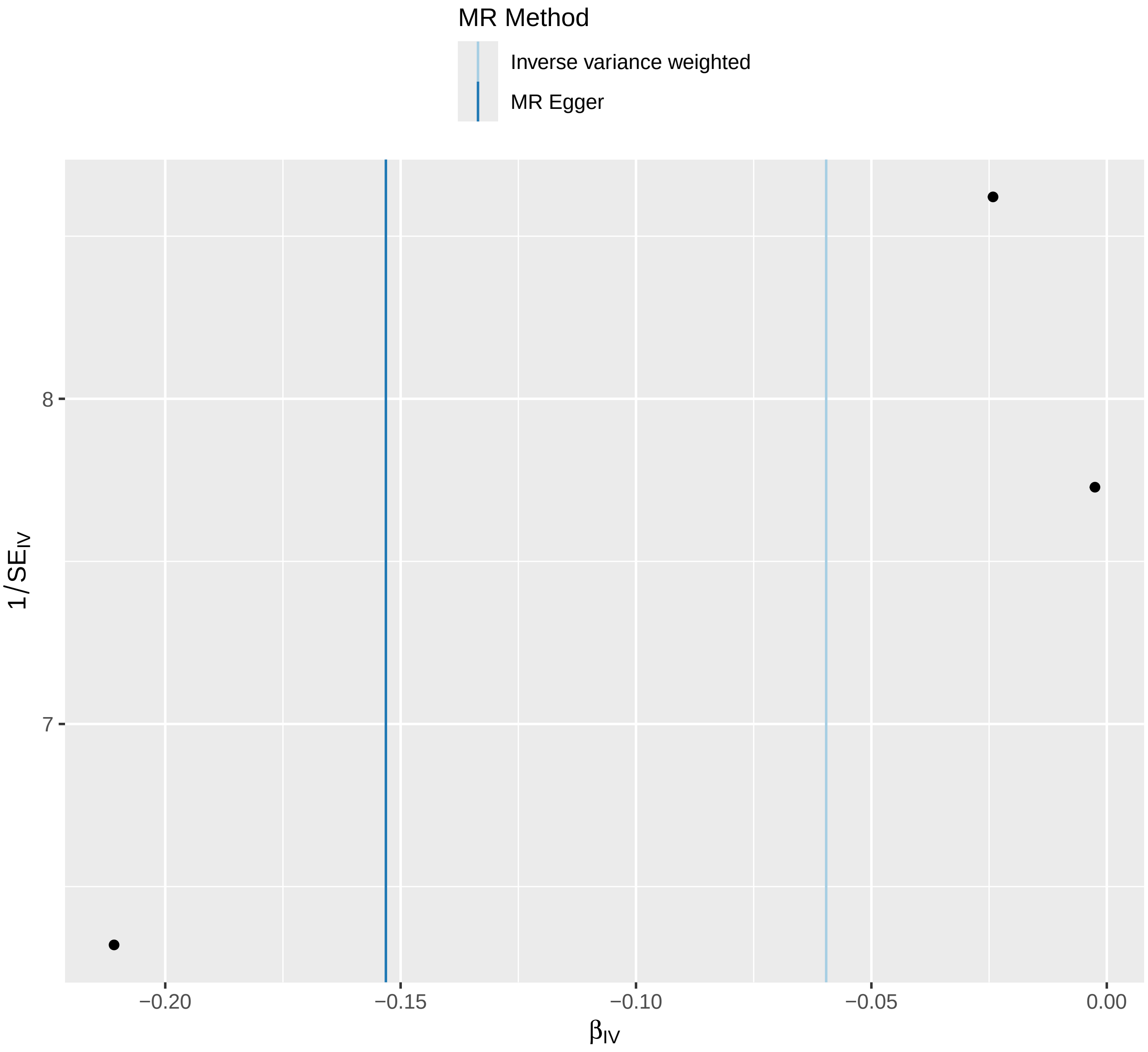
MR Method
Inverse variance weighted
MR Egger
8
7
1/SE$_{IV}$
−0.20
−0.15
−0.10
−0.05
0.00
β$_{IV}$

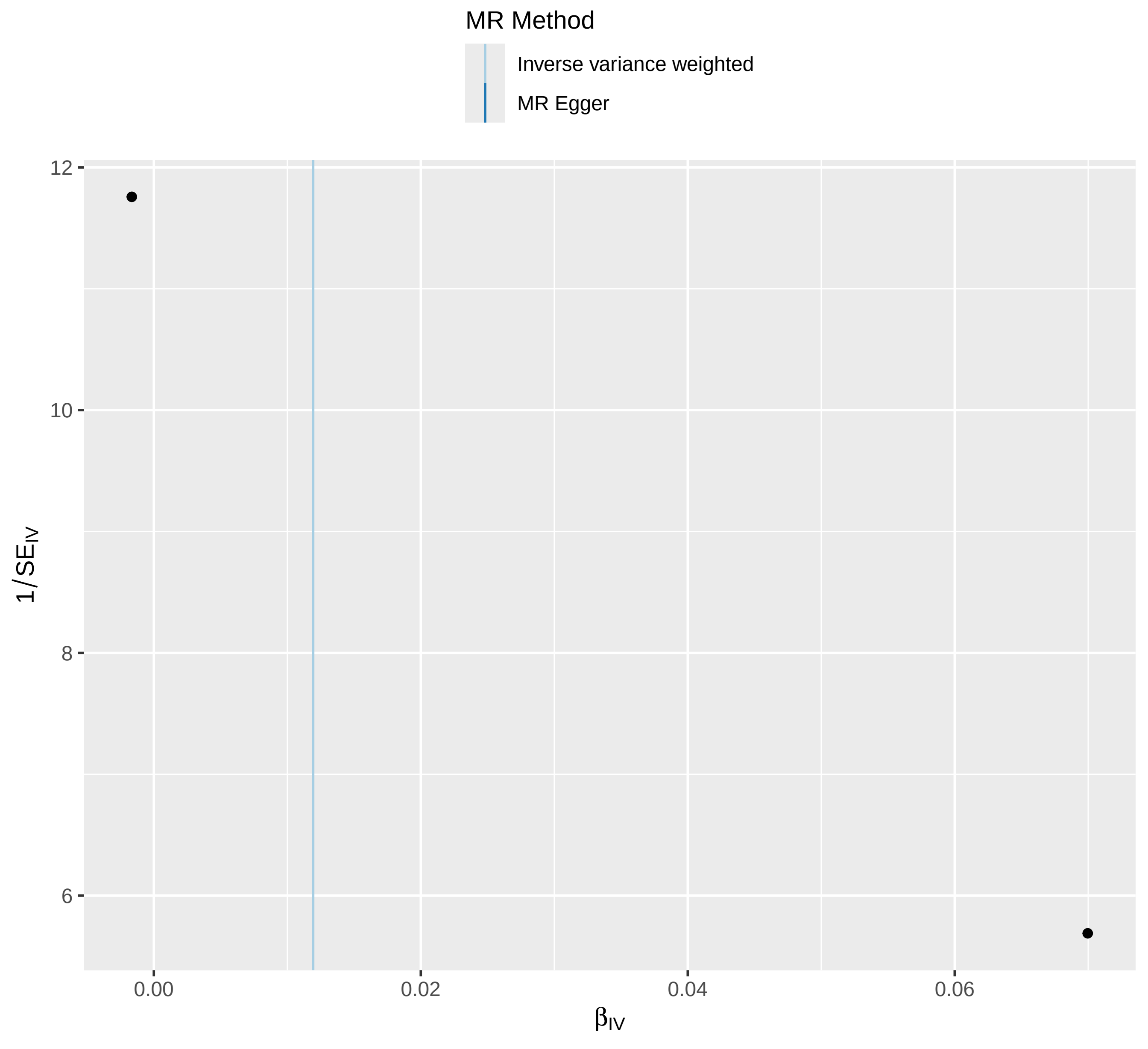

MR Method
Inverse variance weighted
MR Egger
12
10
8
6
$1/SE_{IV}$
0.00
0.02
0.04
0.06
$\beta_{IV}$

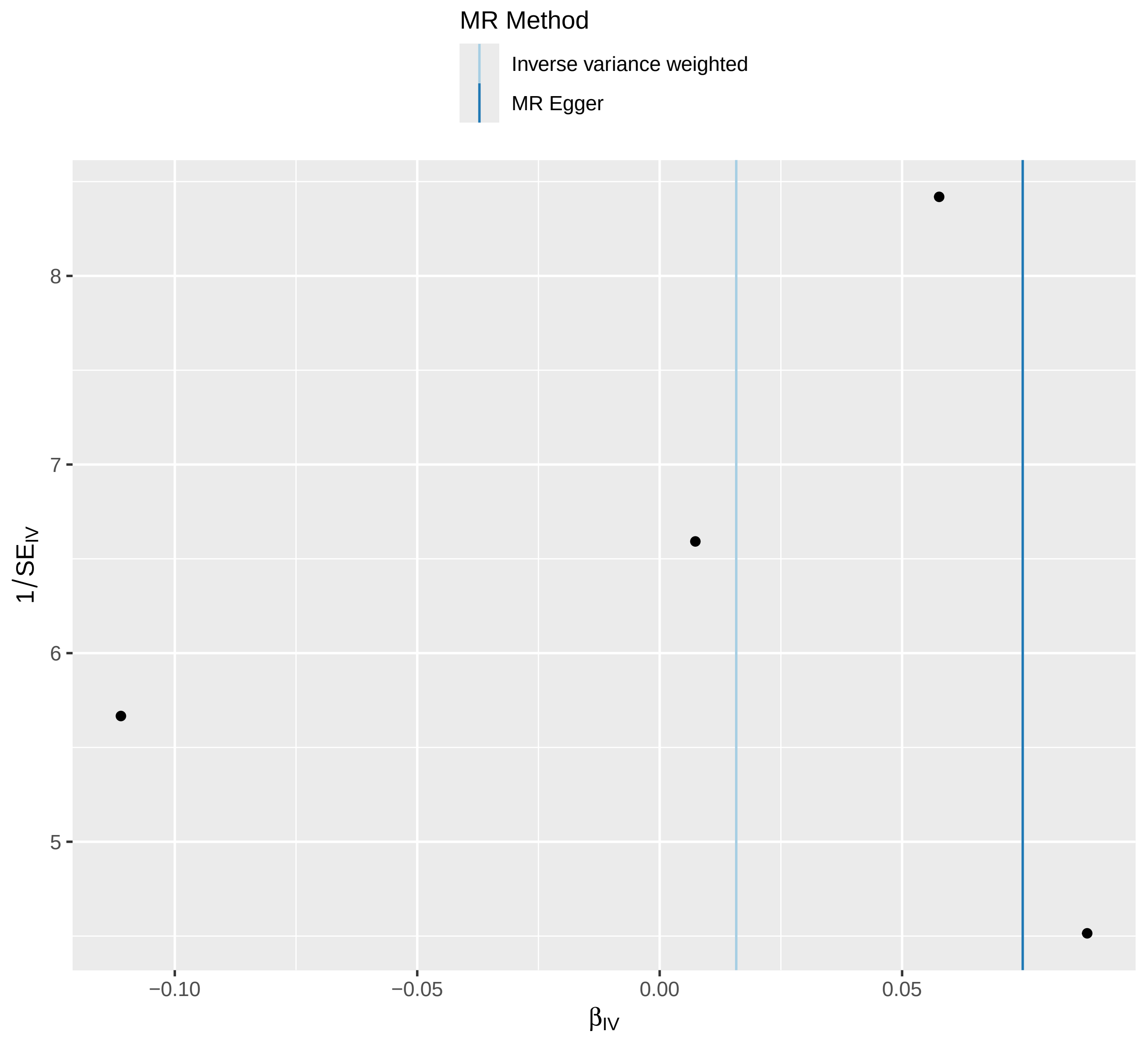
MR Method
Inverse variance weighted
MR Egger
8
7
6
5
1/SE_IV
−0.10
−0.05
0.00
0.05
β_IV

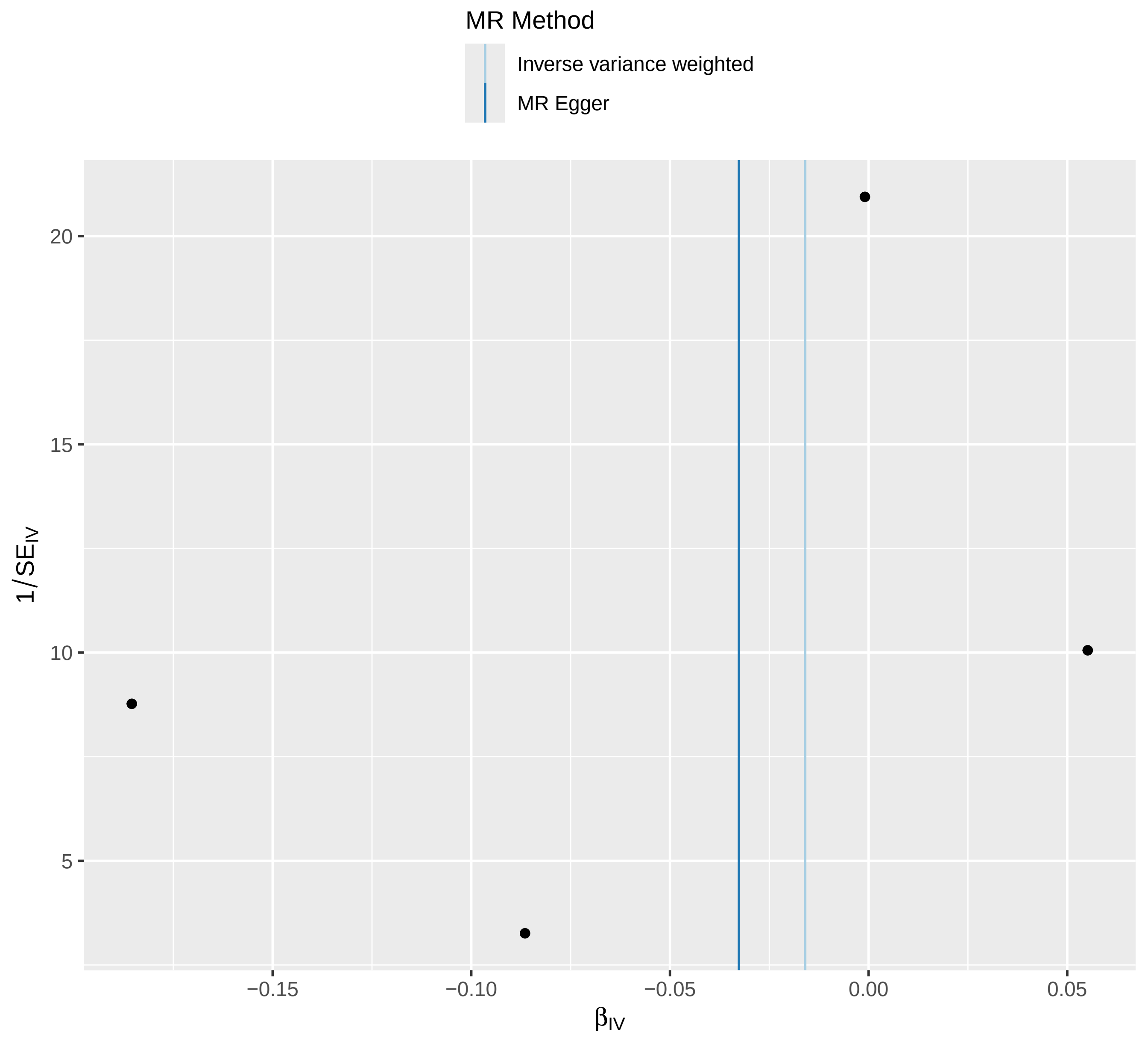

MR Method
Inverse variance weighted
MR Egger
1/SE$_{IV}$
20
15
10
5
−0.15
−0.10
−0.05
0.00
0.05
β$_{IV}$

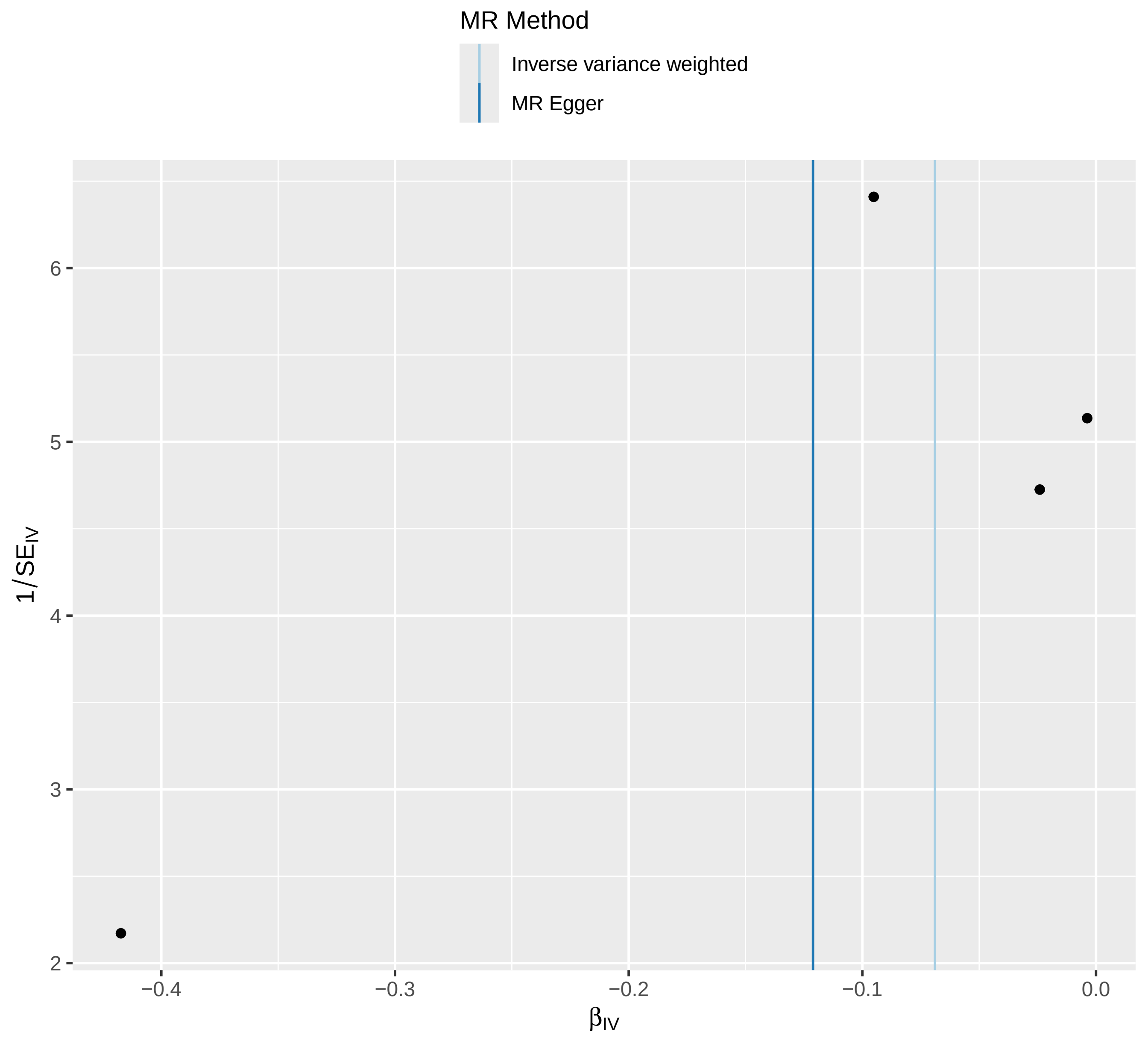
MR Method
Inverse variance weighted
MR Egger
6
5
4
3
2
1/SE_IV
−0.4
−0.3
−0.2
−0.1
0.0
β_IV

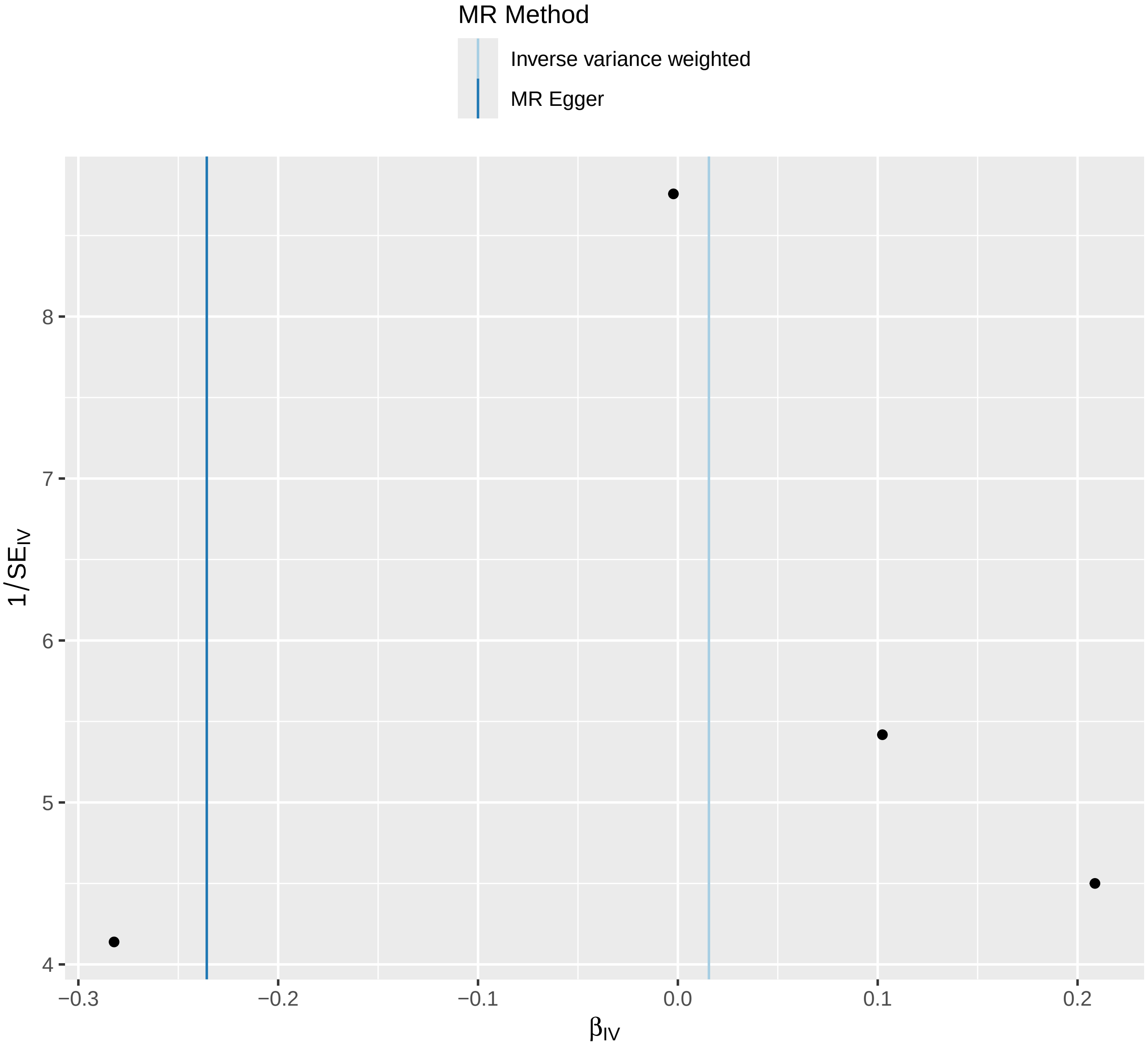

MR Method
Inverse variance weighted
MR Egger
1/SE_IV
8
7
6
5
4
−0.3
−0.2
−0.1
0.0
0.1
0.2
β_IV

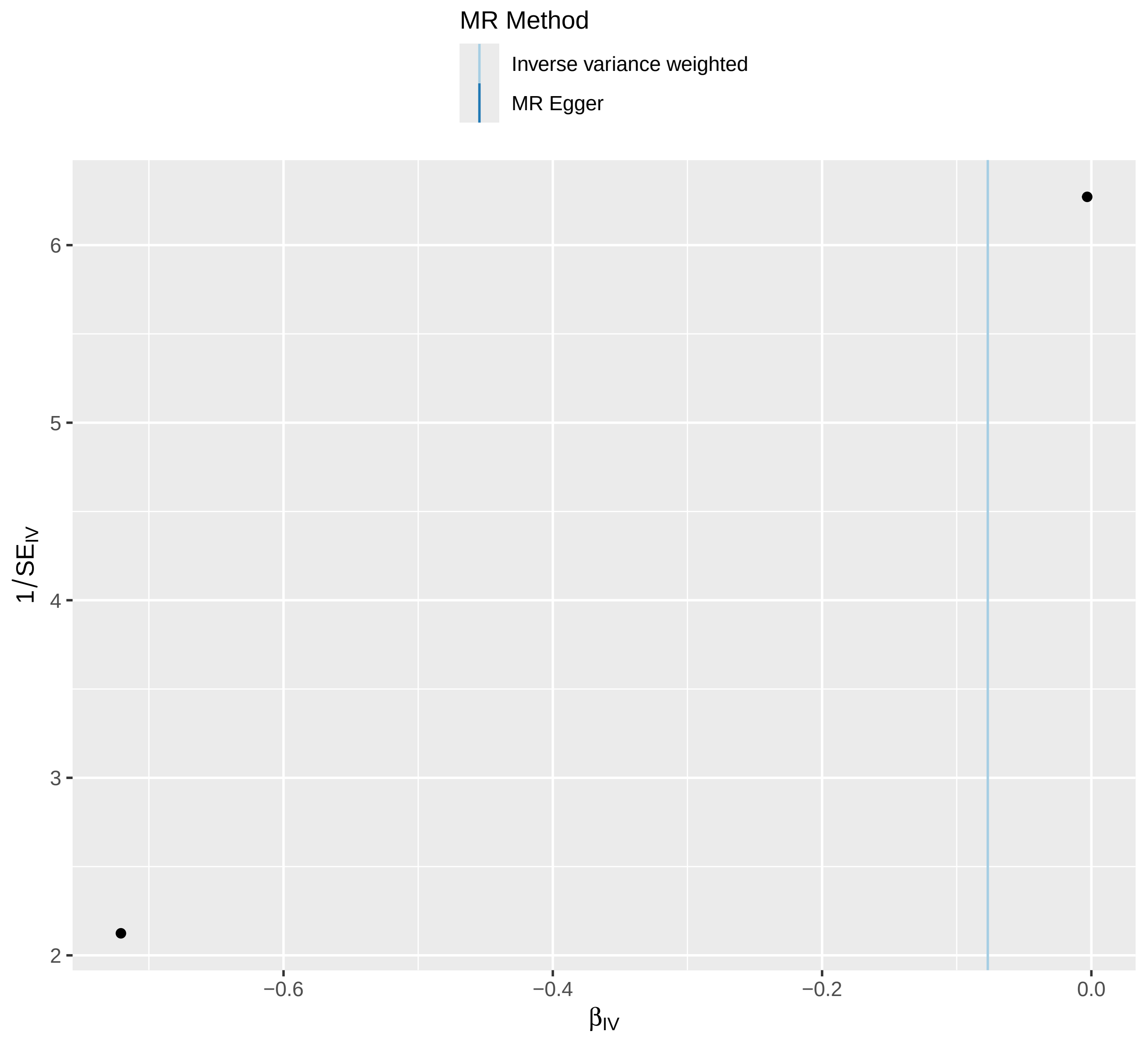

MR Method
Inverse variance weighted
MR Egger
6
5
4
3
2
1/SE_IV
−0.6
−0.4
−0.2
0.0
β_IV

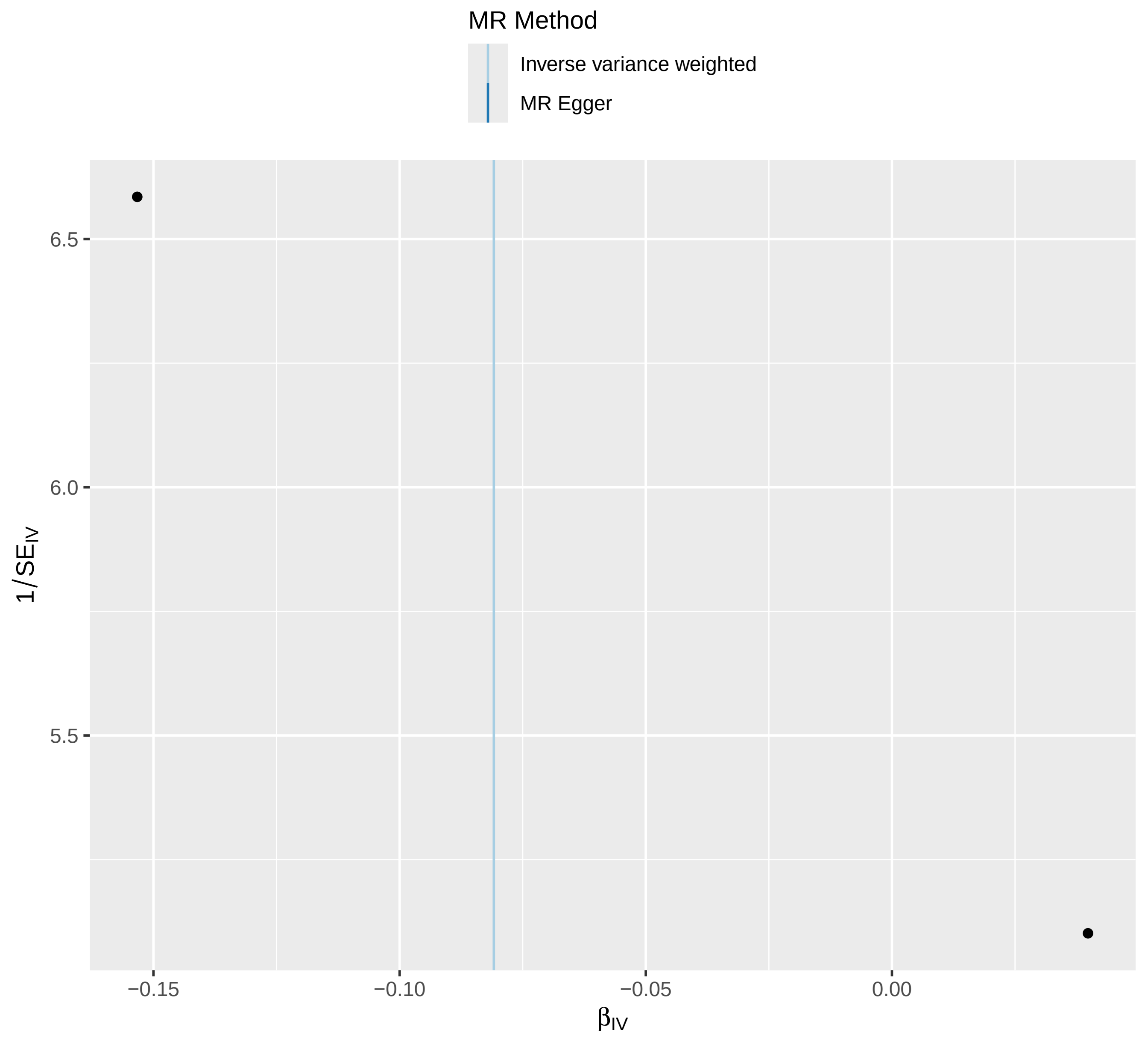
MR Method
Inverse variance weighted
MR Egger
6.5
6.0
5.5
1/SE_IV
−0.15
−0.10
−0.05
0.00
β_IV

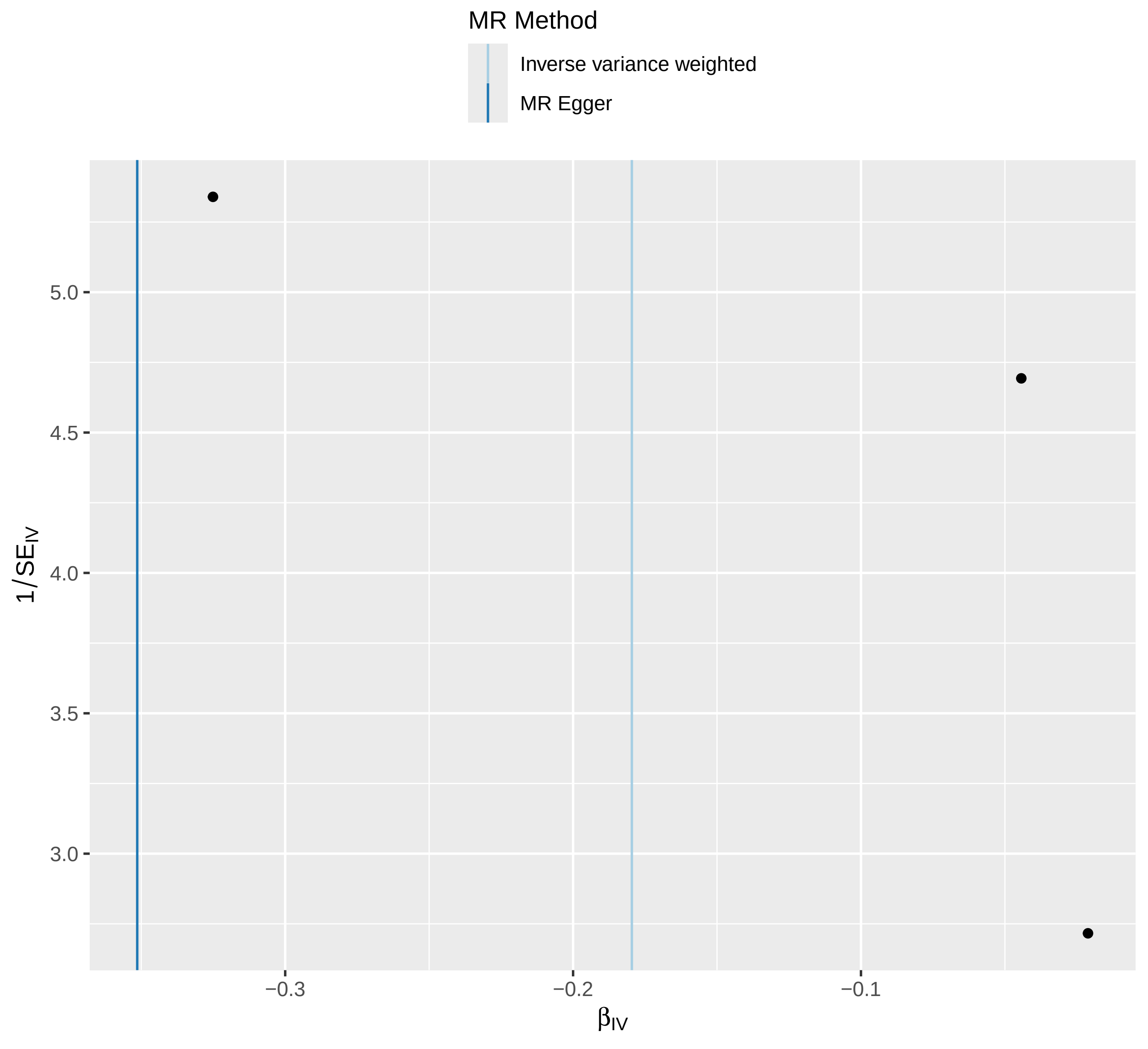

MR Method
Inverse variance weighted
MR Egger
5.0
4.5
4.0
3.5
3.0
1/SE_IV
−0.3
−0.2
−0.1
β_IV

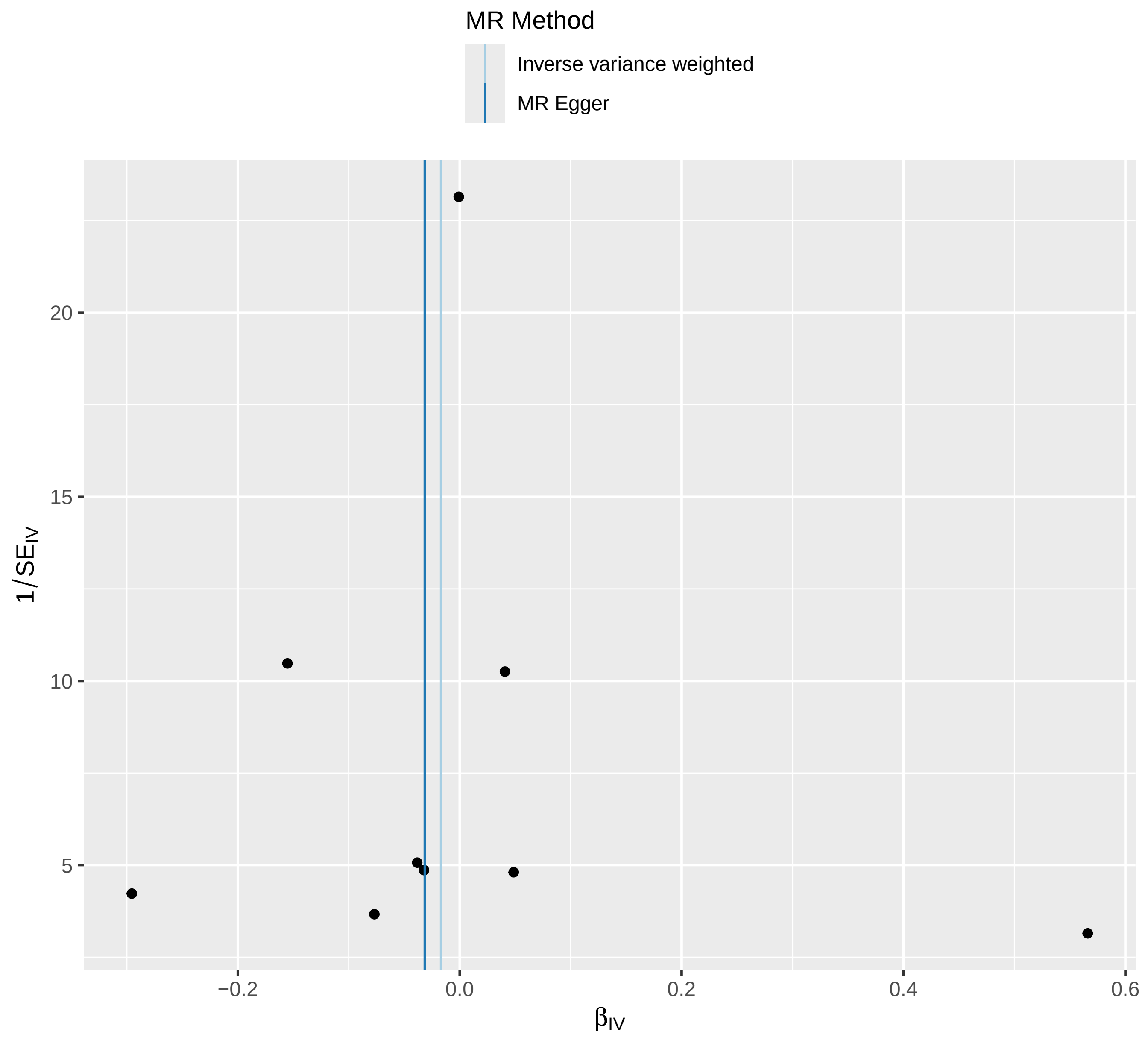

MR Method
Inverse variance weighted
MR Egger
1/SE_IV
β_IV
20
15
10
5
−0.2
0.0
0.2
0.4
0.6

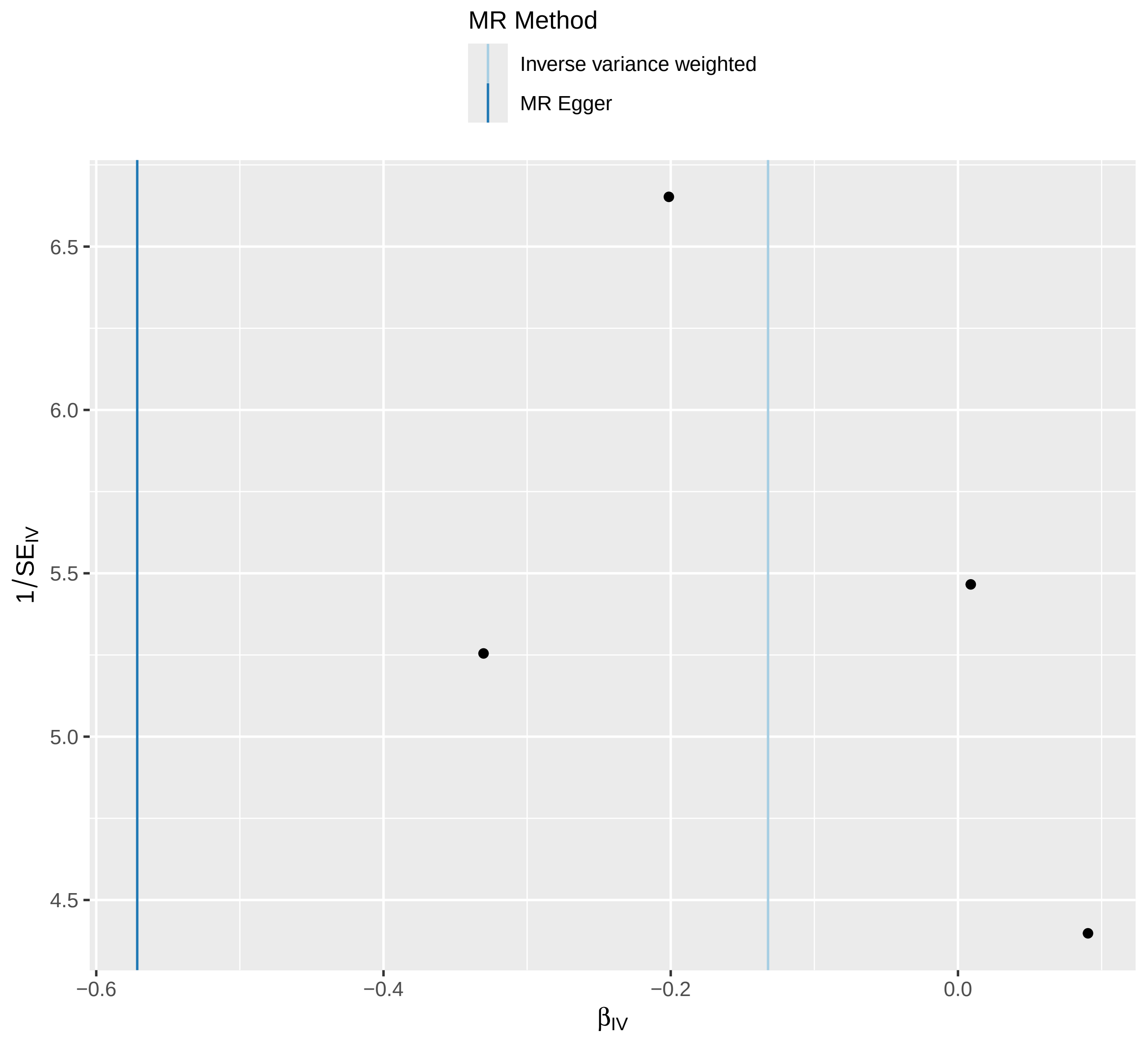
MR Method
Inverse variance weighted
MR Egger
6.5
6.0
5.5
5.0
4.5
1/SE_IV
−0.6
−0.4
−0.2
0.0
β_IV

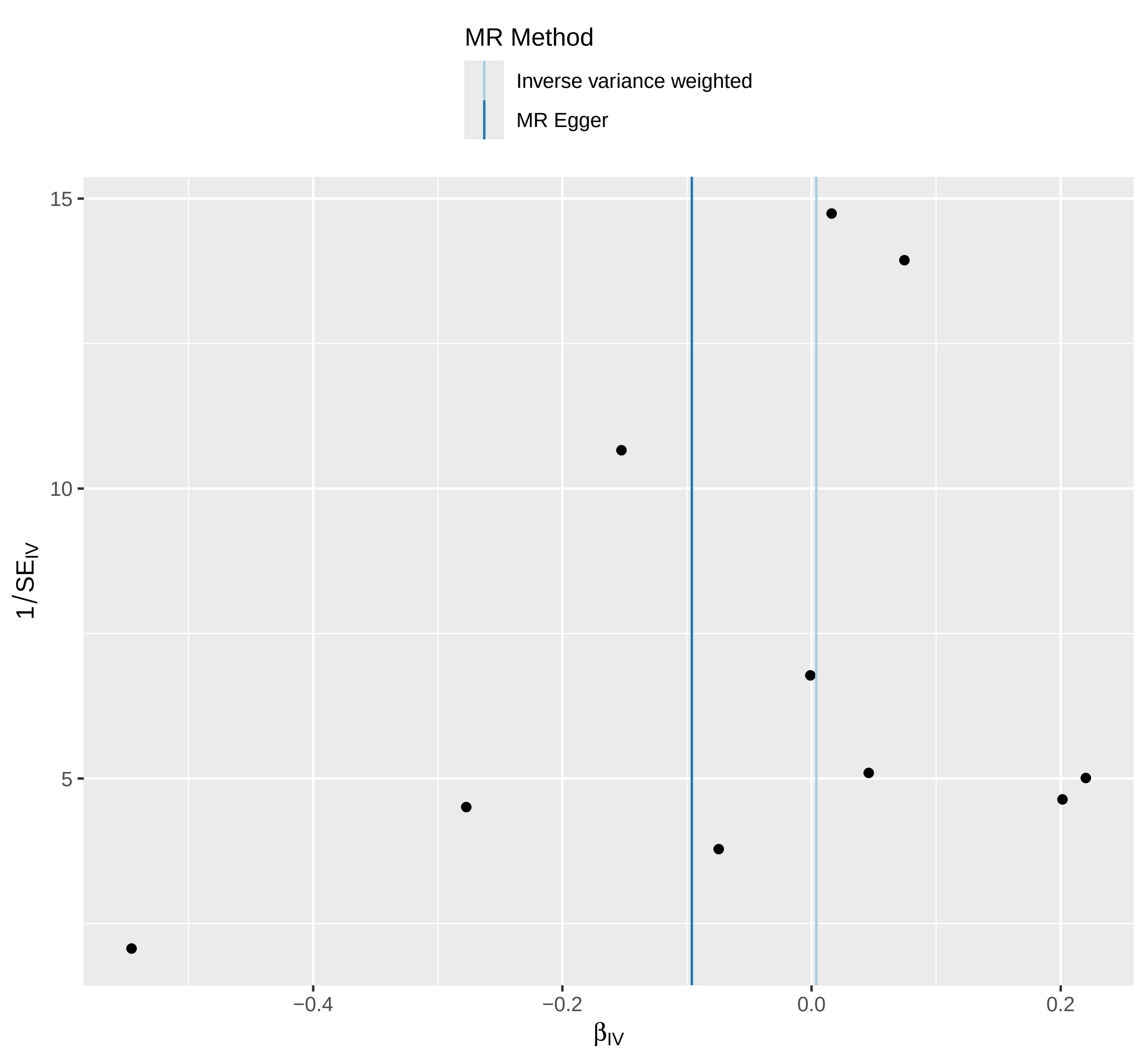

MR Method
Inverse variance weighted
MR Egger
15
10
5
1/SE$_{IV}$
−0.4
−0.2
0.0
0.2
β$_{IV}$

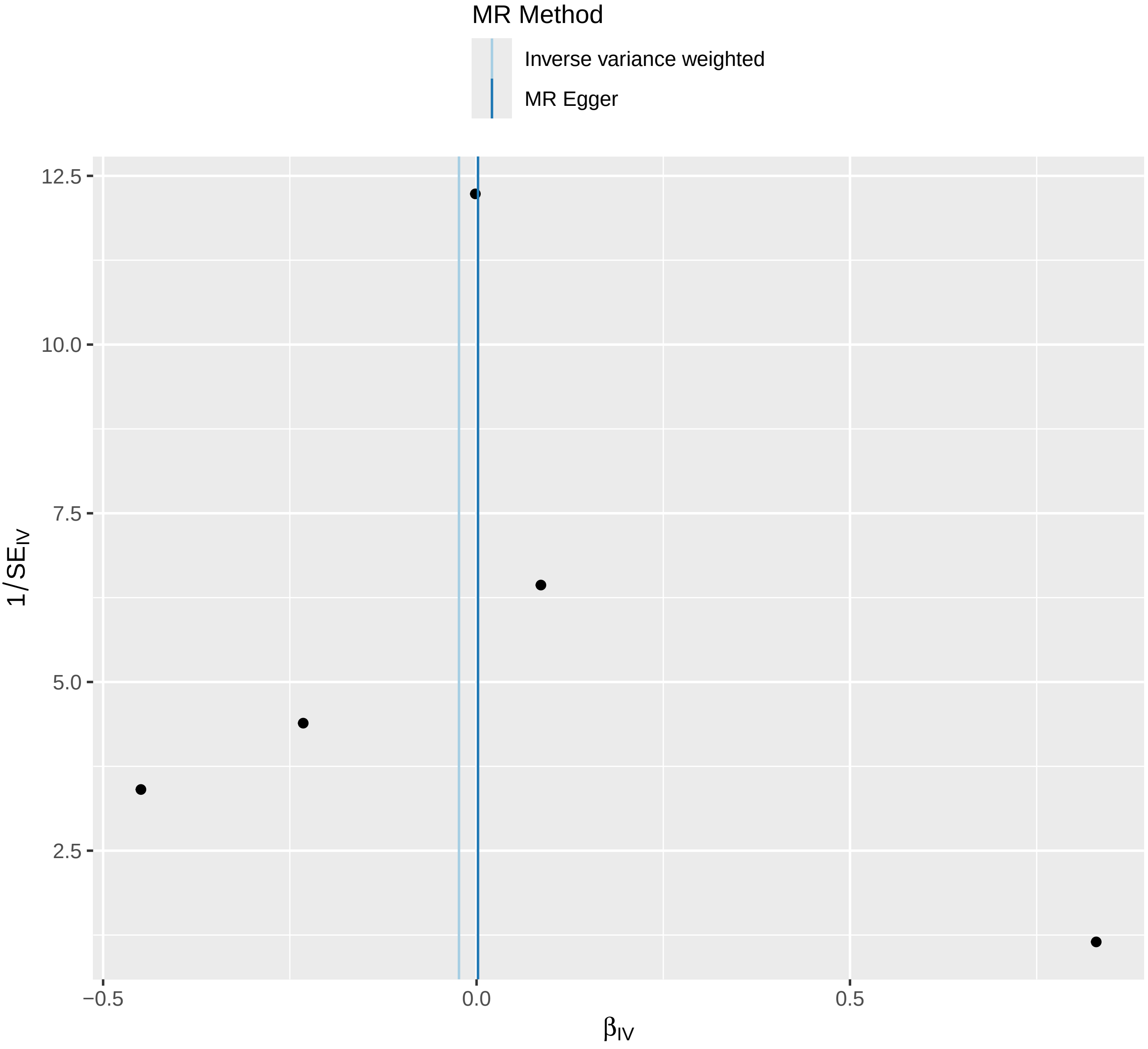

MR Method
Inverse variance weighted
MR Egger
12.5
10.0
7.5
5.0
2.5
$1/SE_{IV}$
−0.5
0.0
0.5
$\beta_{IV}$

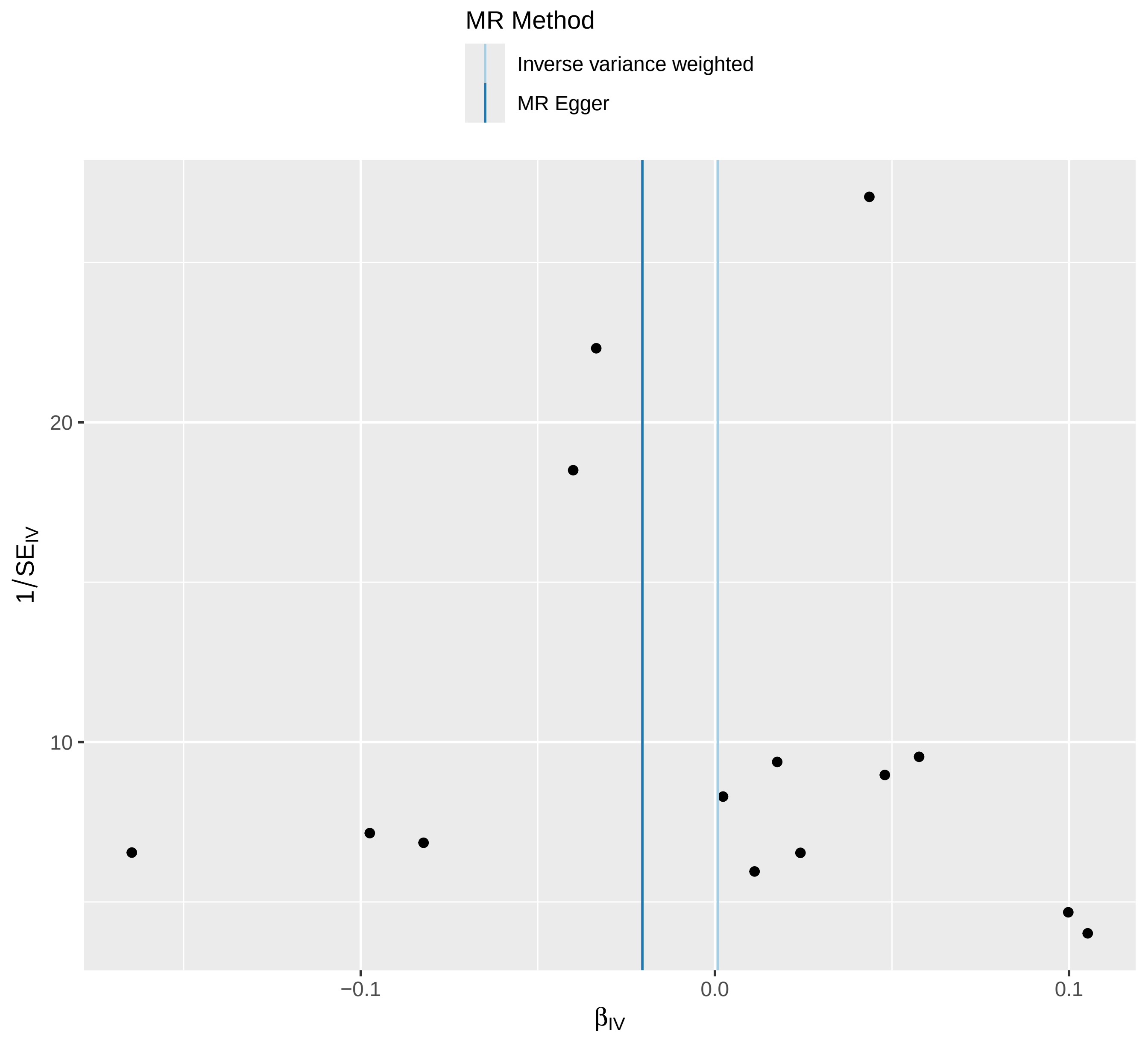

MR Method
Inverse variance weighted
MR Egger
20
10
1/SE_IV
−0.1
0.0
0.1
β_IV

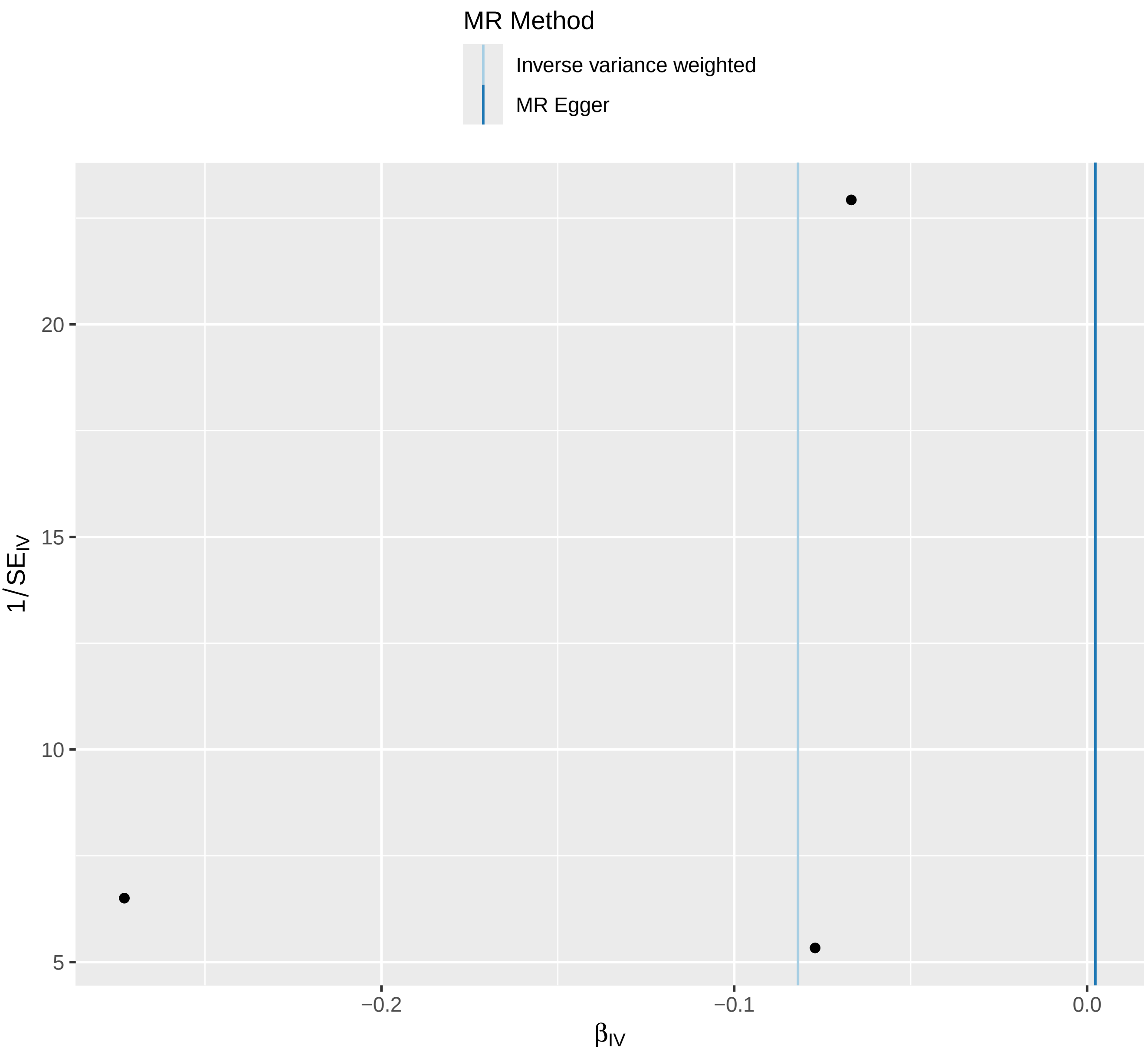

MR Method
Inverse variance weighted
MR Egger
20
15
10
5
1/SE_IV
−0.2
−0.1
0.0
β_IV

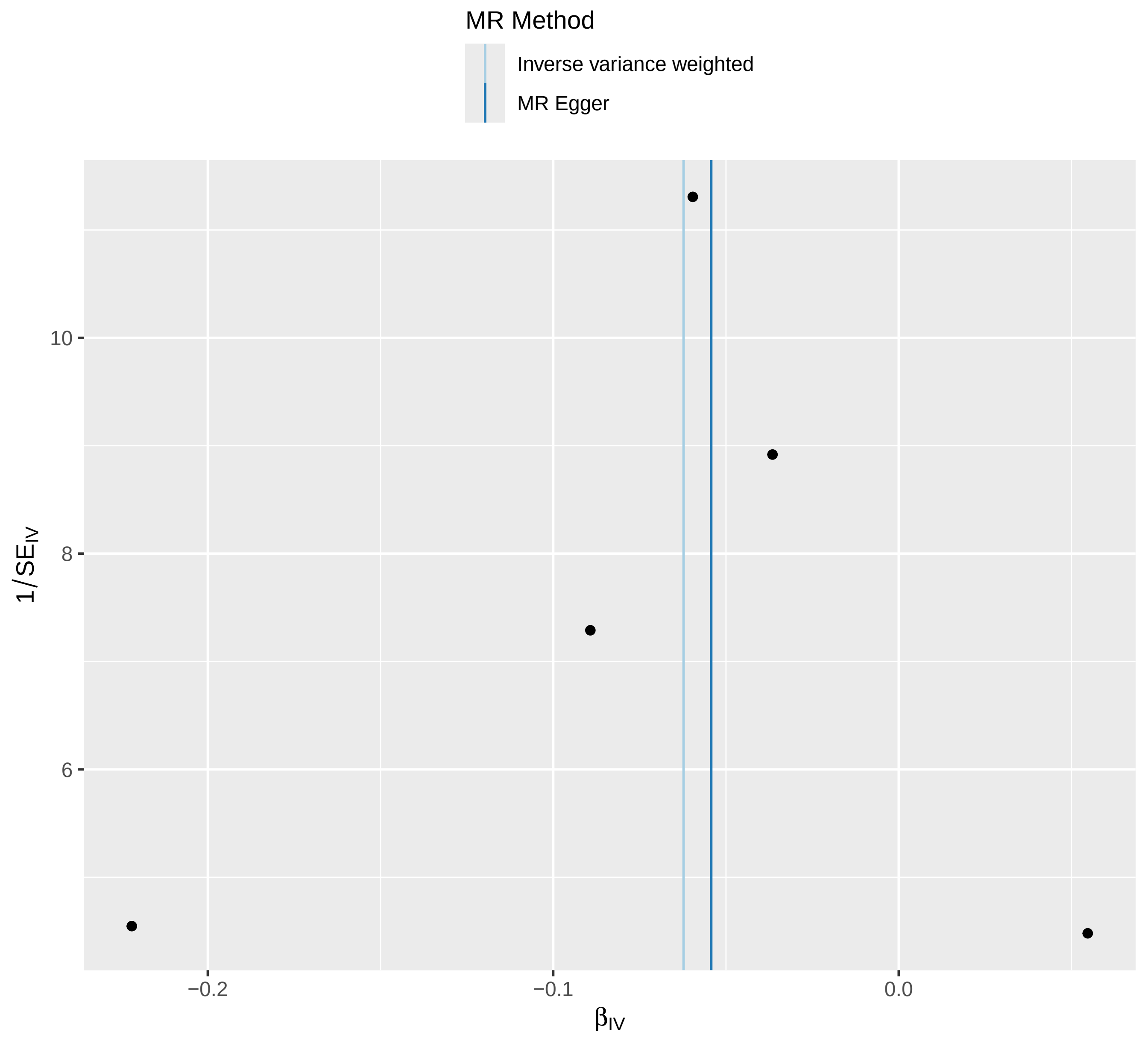
MR Method
Inverse variance weighted
MR Egger
10
8
6
1/SE_IV
−0.2
−0.1
0.0
β_IV

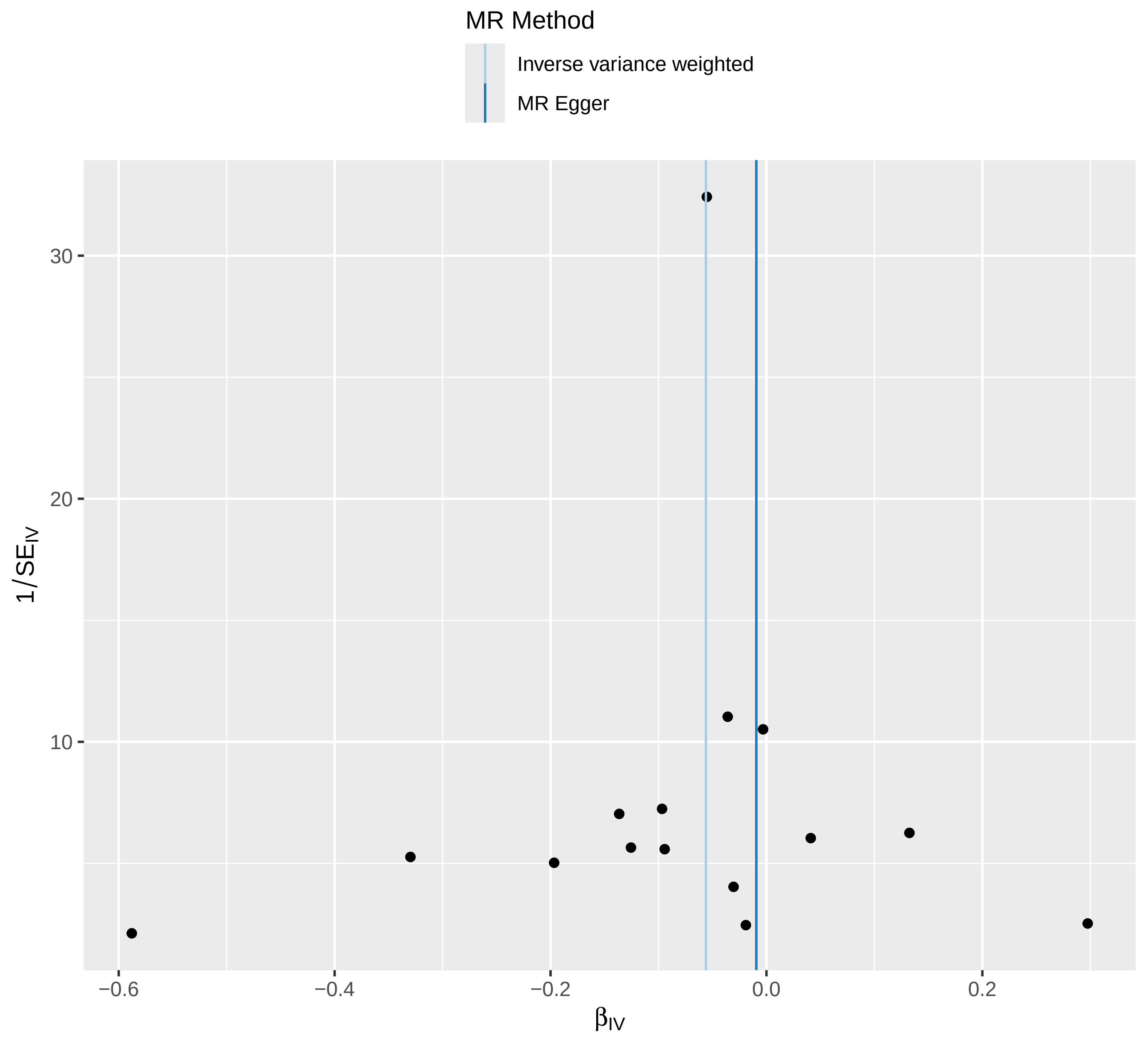
MR Method
Inverse variance weighted
MR Egger
30
20
10
1/SE$_{IV}$
−0.6
−0.4
−0.2
0.0
0.2
β$_{IV}$

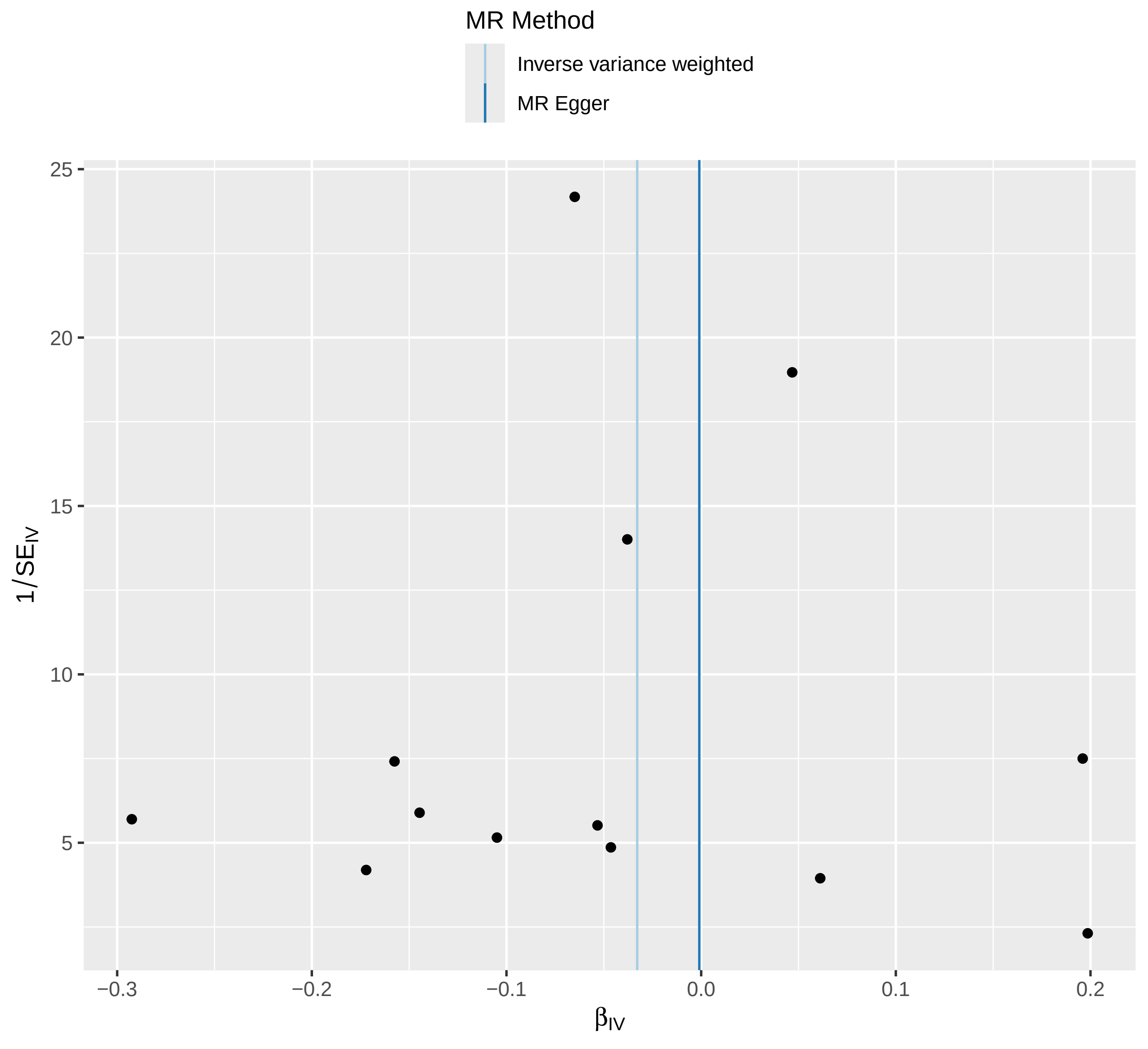
MR Method
Inverse variance weighted
MR Egger
25
20
15
10
5
1/SE_IV
−0.3
−0.2
−0.1
0.0
0.1
0.2
β_IV

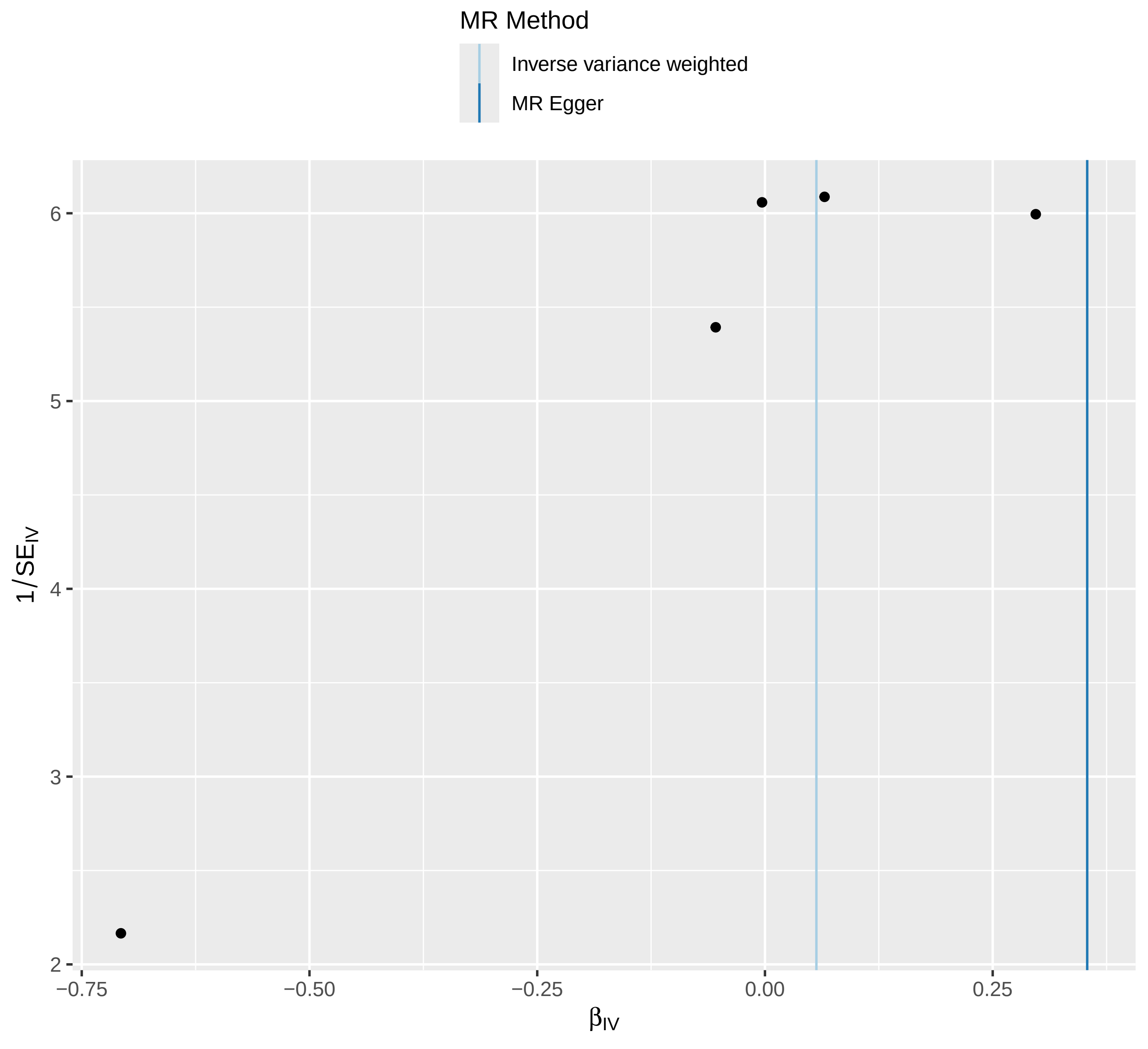
MR Method
Inverse variance weighted
MR Egger
6
5
4
3
2
$1/SE_{IV}$
−0.75
−0.50
−0.25
0.00
0.25
$\beta_{IV}$

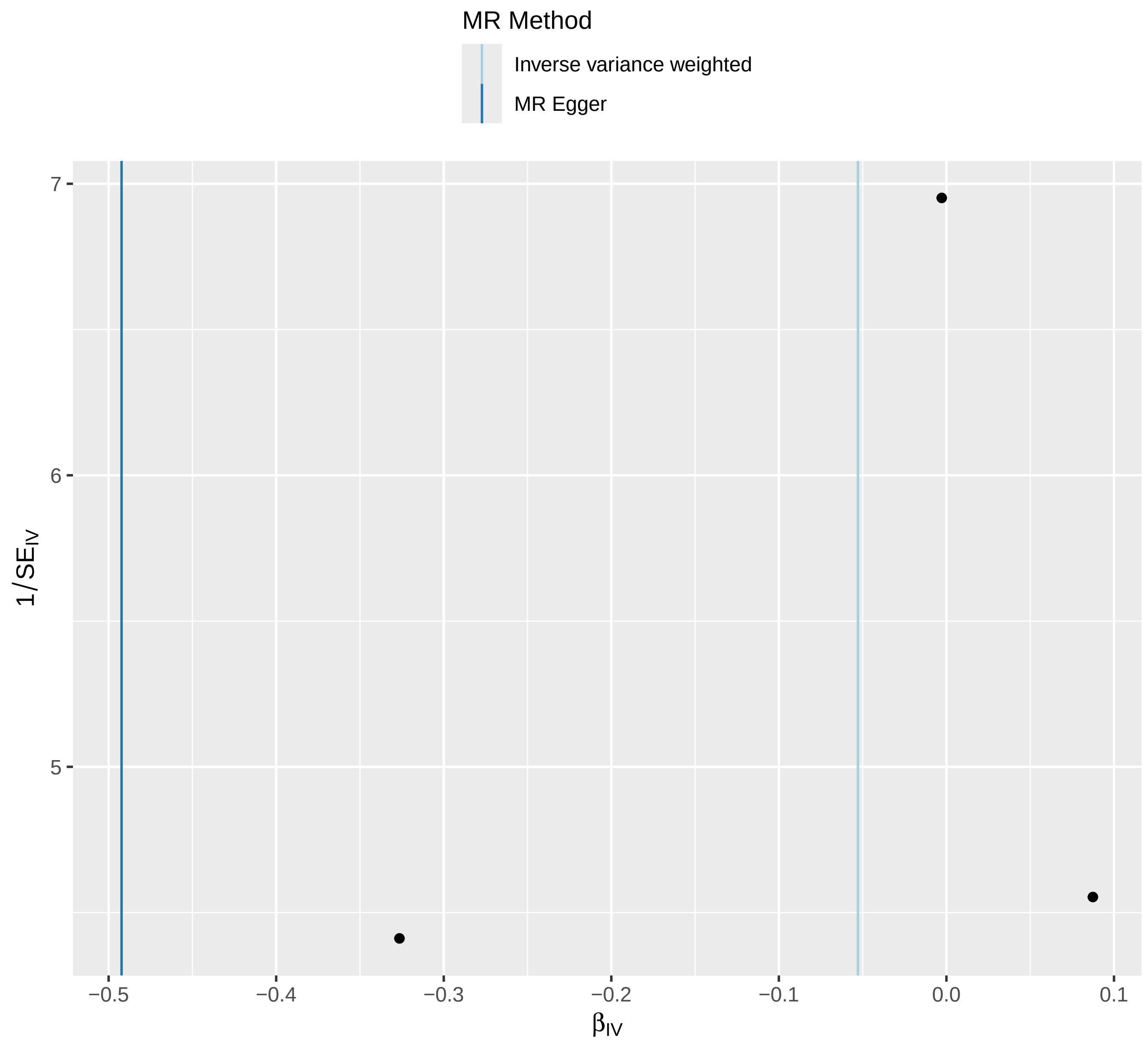
MR Method
Inverse variance weighted
MR Egger
7
6
5
1/SE_IV
−0.5
−0.4
−0.3
−0.2
−0.1
0.0
0.1
β_IV

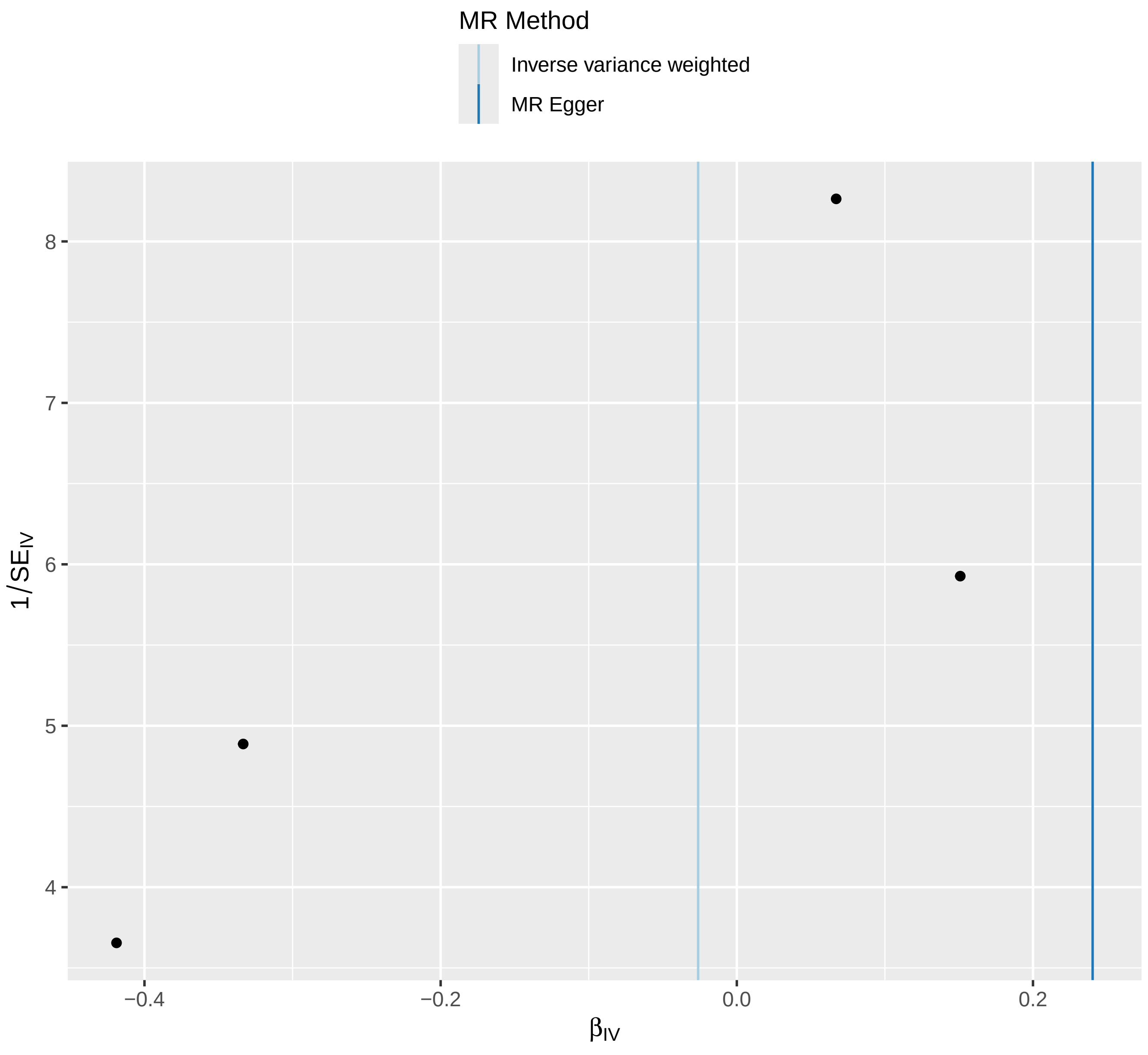
MR Method
Inverse variance weighted
MR Egger
8
7
6
5
4
1/SE_IV
−0.4
−0.2
0.0
0.2
β_IV

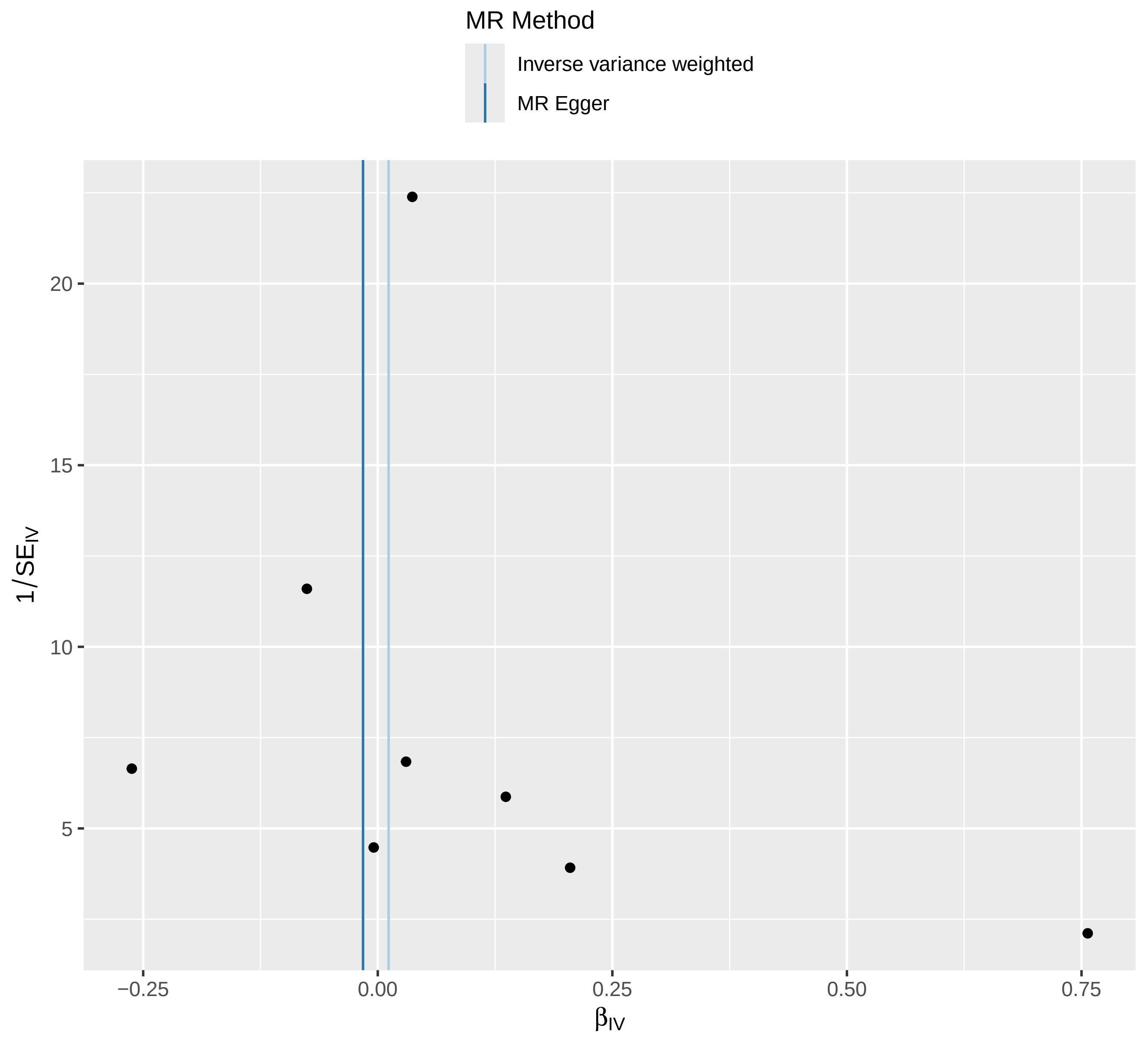

MR Method
Inverse variance weighted
MR Egger
20
15
10
5
−0.25
0.00
0.25
0.50
0.75
$1/SE_{IV}$
$\beta_{IV}$

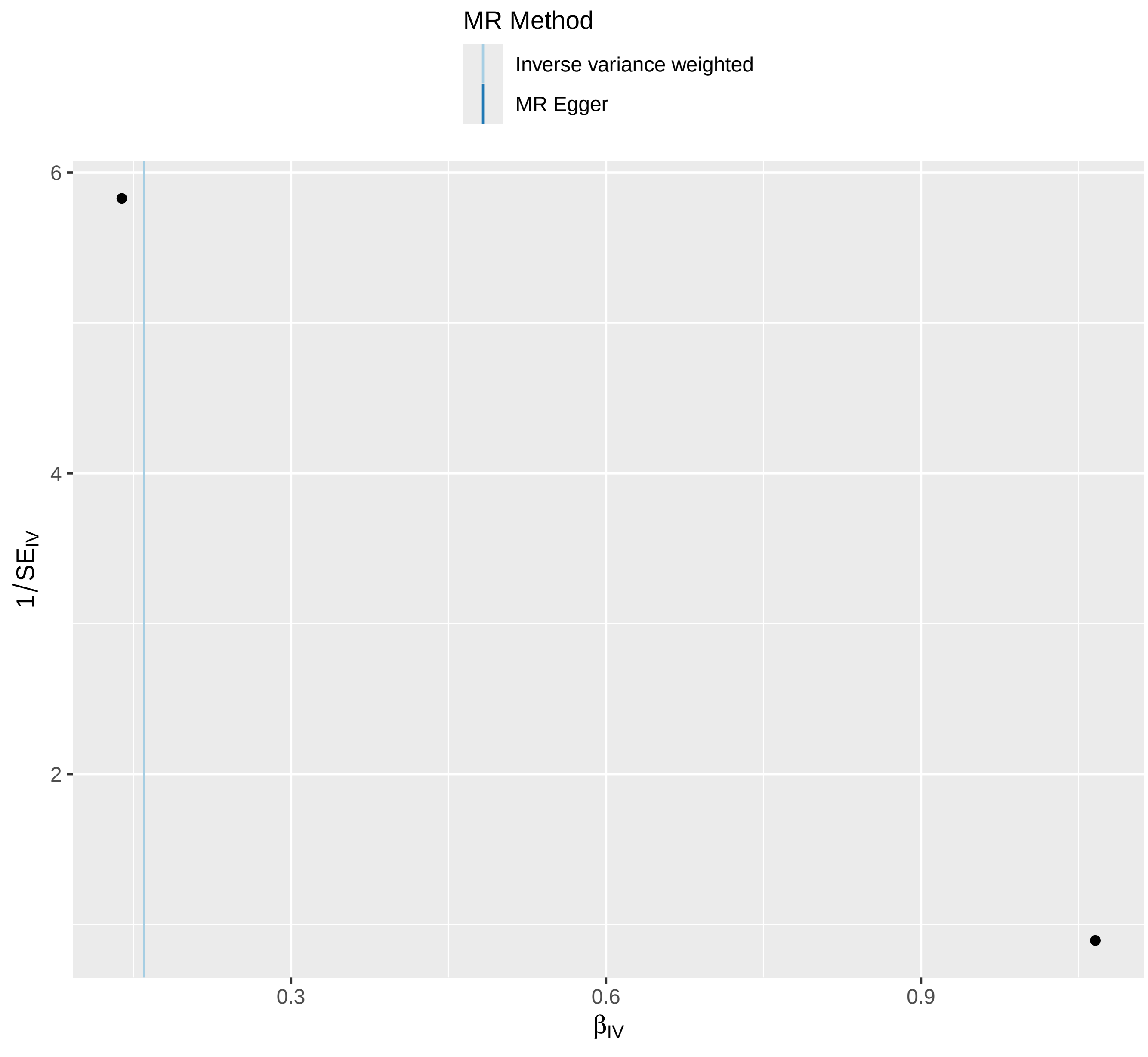
MR Method
Inverse variance weighted
MR Egger
6
4
2
0.3
0.6
0.9
$1/SE_{IV}$
$\beta_{IV}$

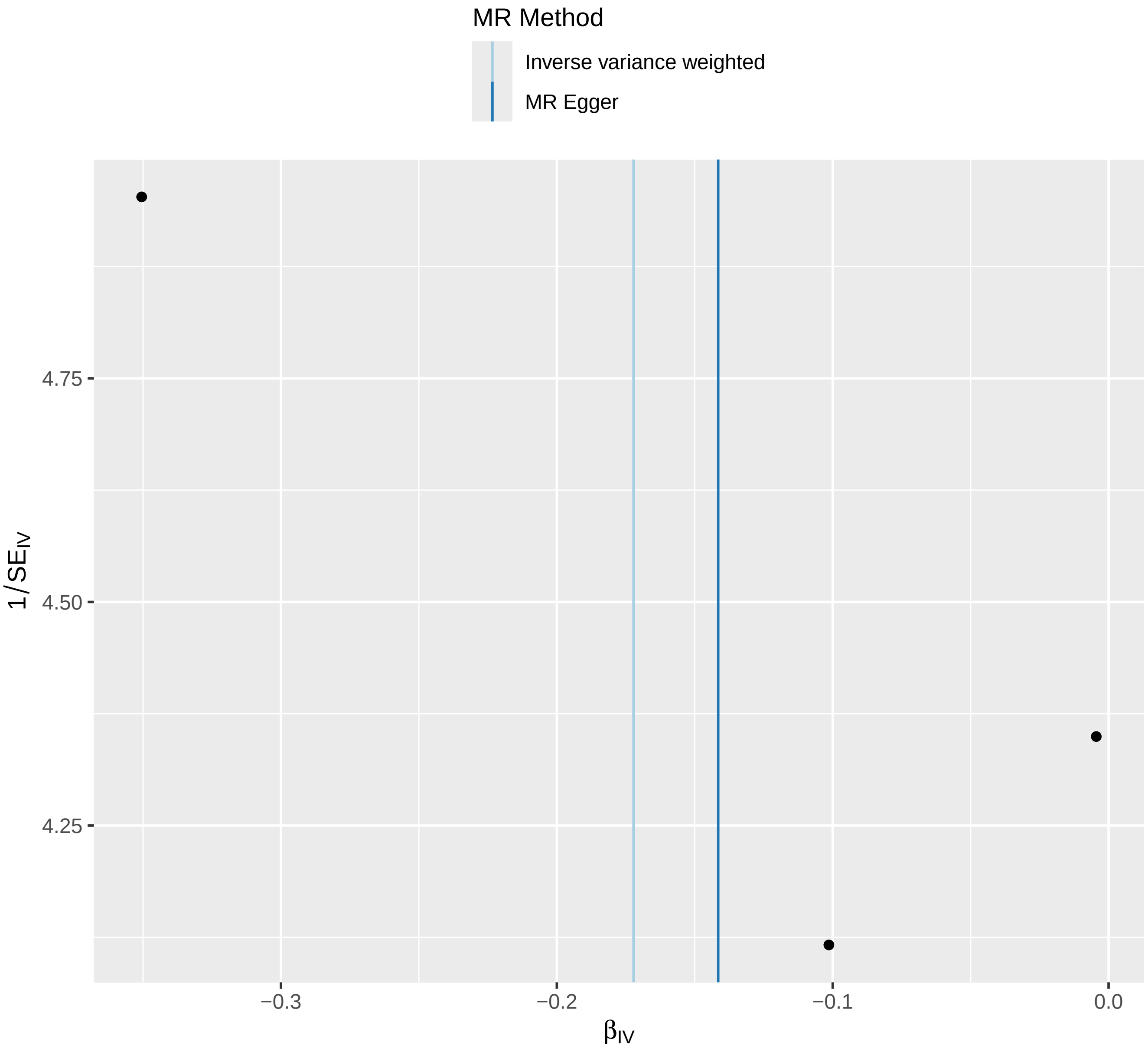

MR Method
Inverse variance weighted
MR Egger
4.75
4.50
4.25
1/SE_IV
−0.3
−0.2
−0.1
0.0
β_IV

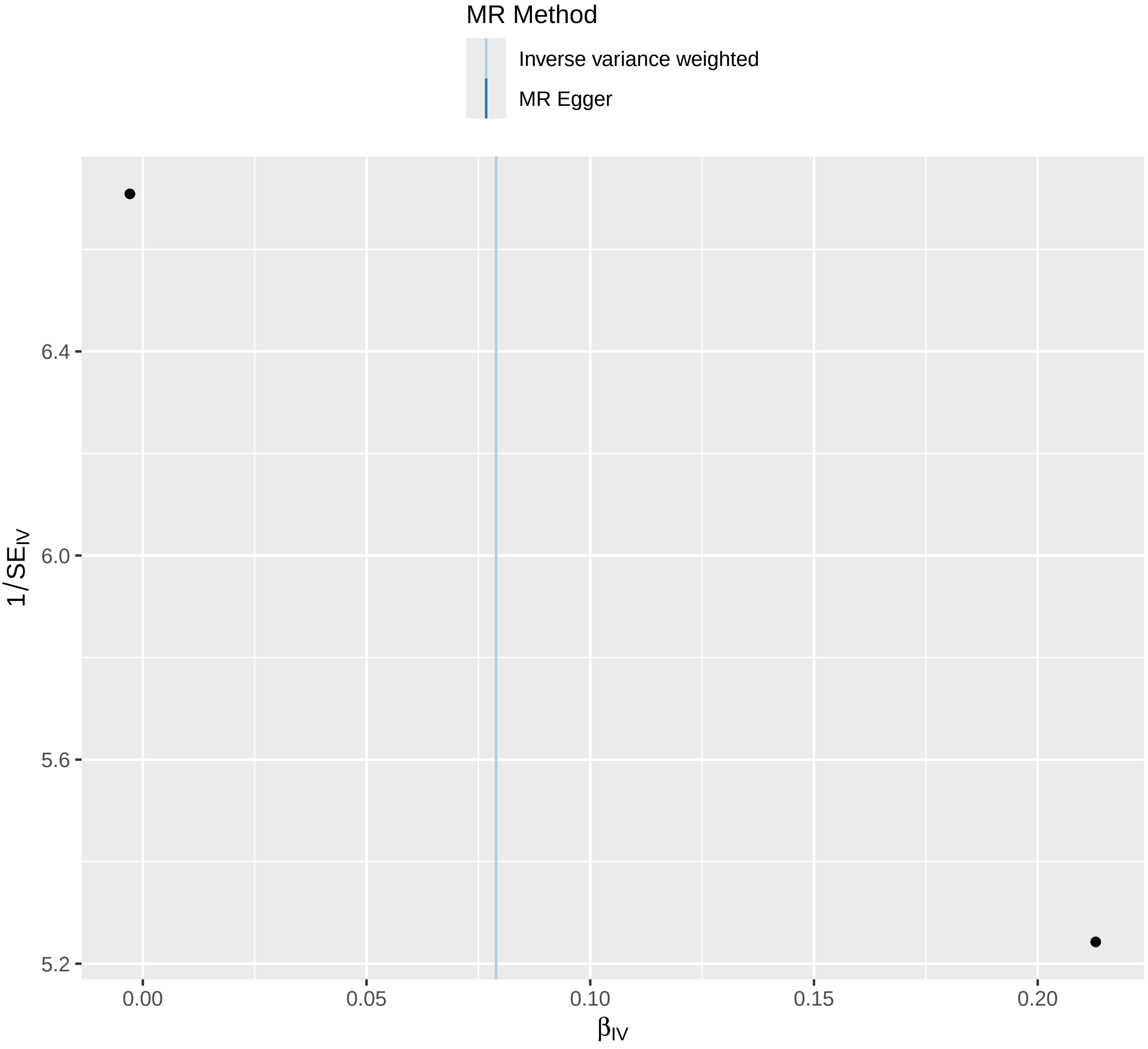
MR Method
Inverse variance weighted
MR Egger
6.4
6.0
5.6
5.2
$1/SE_{IV}$
0.00
0.05
0.10
0.15
0.20
$\beta_{IV}$

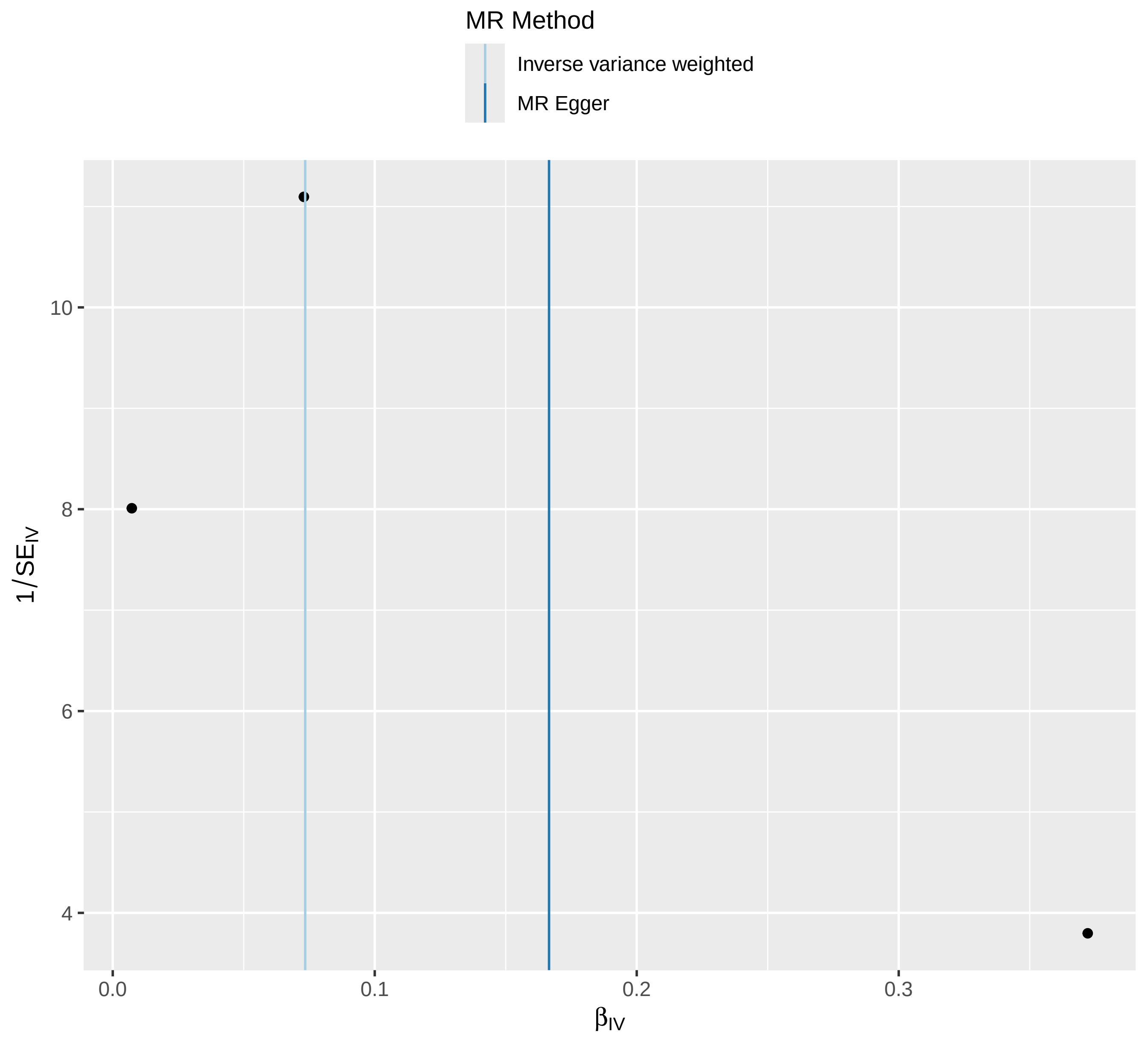
MR Method
Inverse variance weighted
MR Egger
10
8
6
4
$1/SE_{IV}$
0.0
0.1
0.2
0.3
$\beta_{IV}$

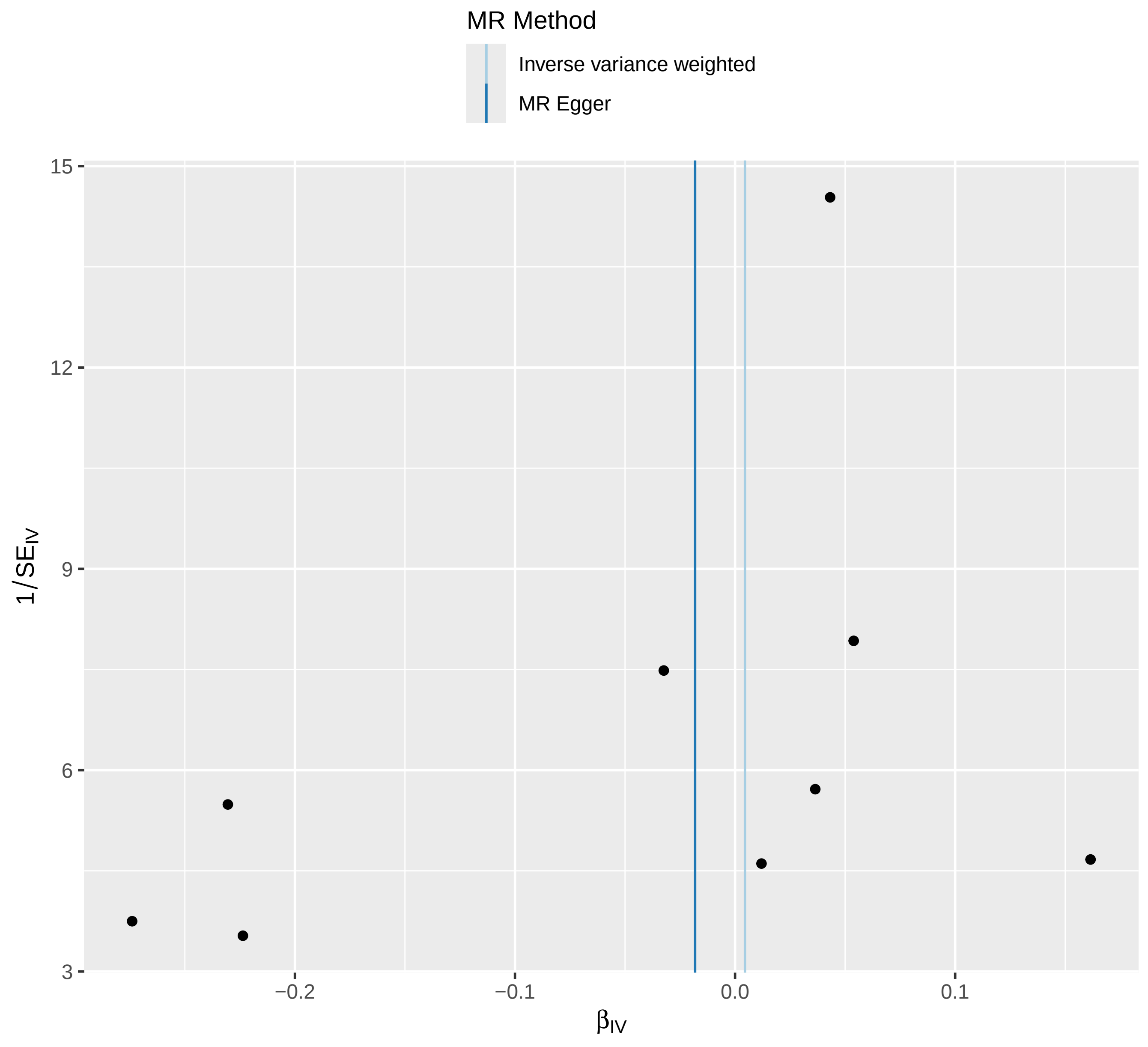

MR Method
Inverse variance weighted
MR Egger
15
12
9
6
3
1/SE_IV
−0.2
−0.1
0.0
0.1
β_IV

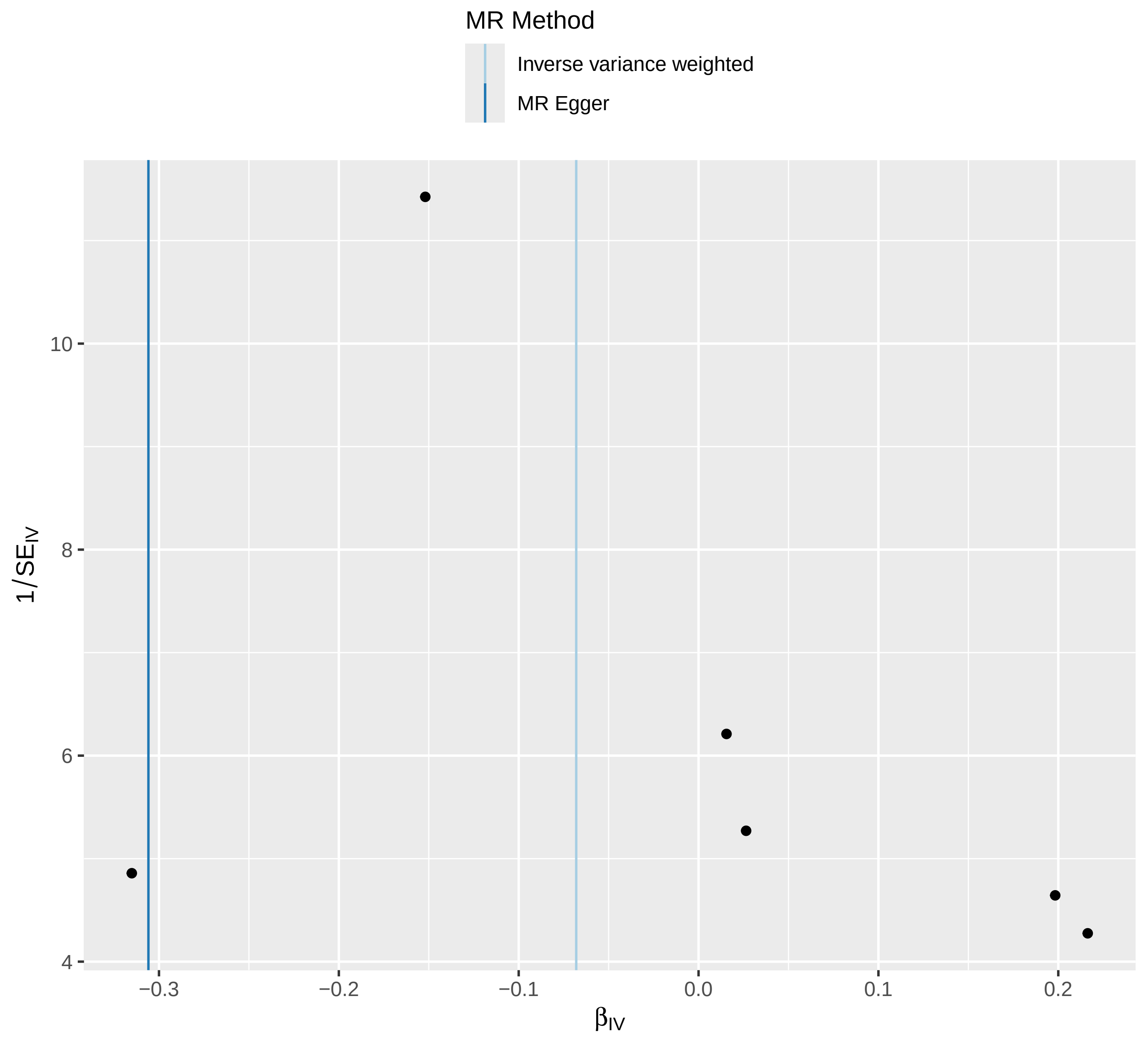
MR Method
Inverse variance weighted
MR Egger
10
8
6
4
1/SE_IV
−0.3
−0.2
−0.1
0.0
0.1
0.2
β_IV

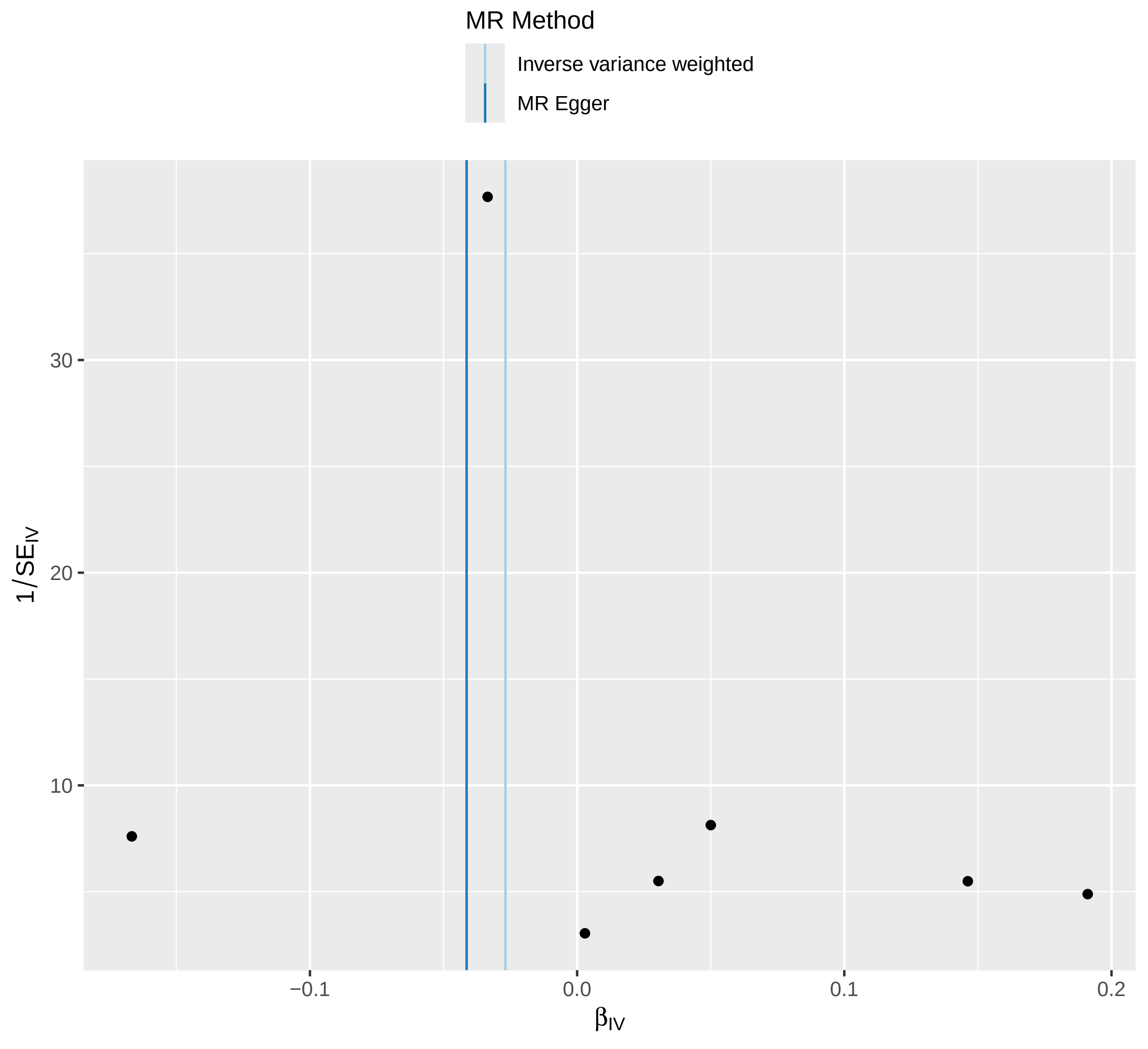

MR Method
Inverse variance weighted
MR Egger
30
20
10
1/SE$_{IV}$
−0.1
0.0
0.1
0.2
β$_{IV}$

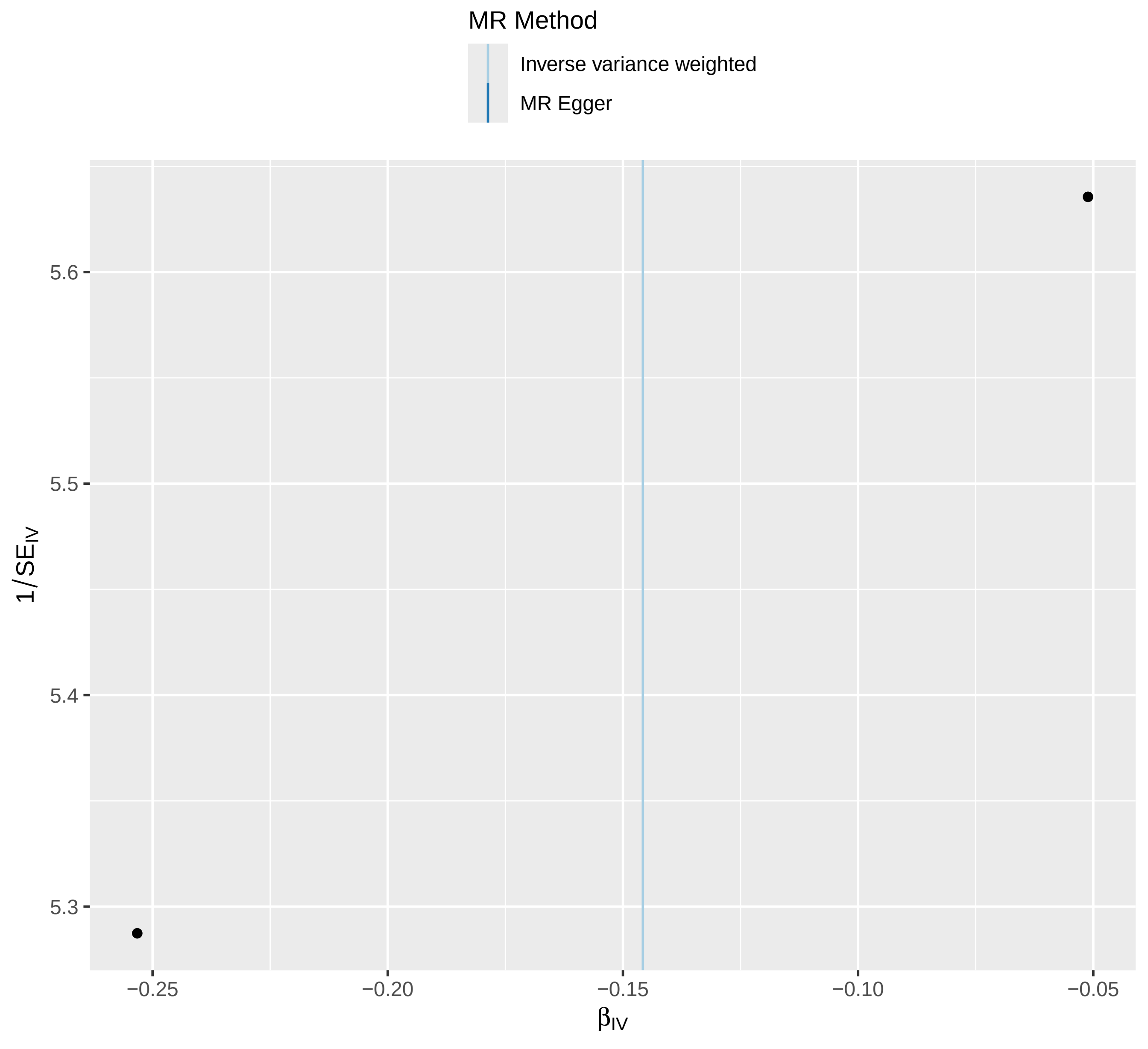
MR Method
Inverse variance weighted
MR Egger
5.6
5.5
5.4
5.3
1/SE_IV
−0.25
−0.20
−0.15
−0.10
−0.05
β_IV

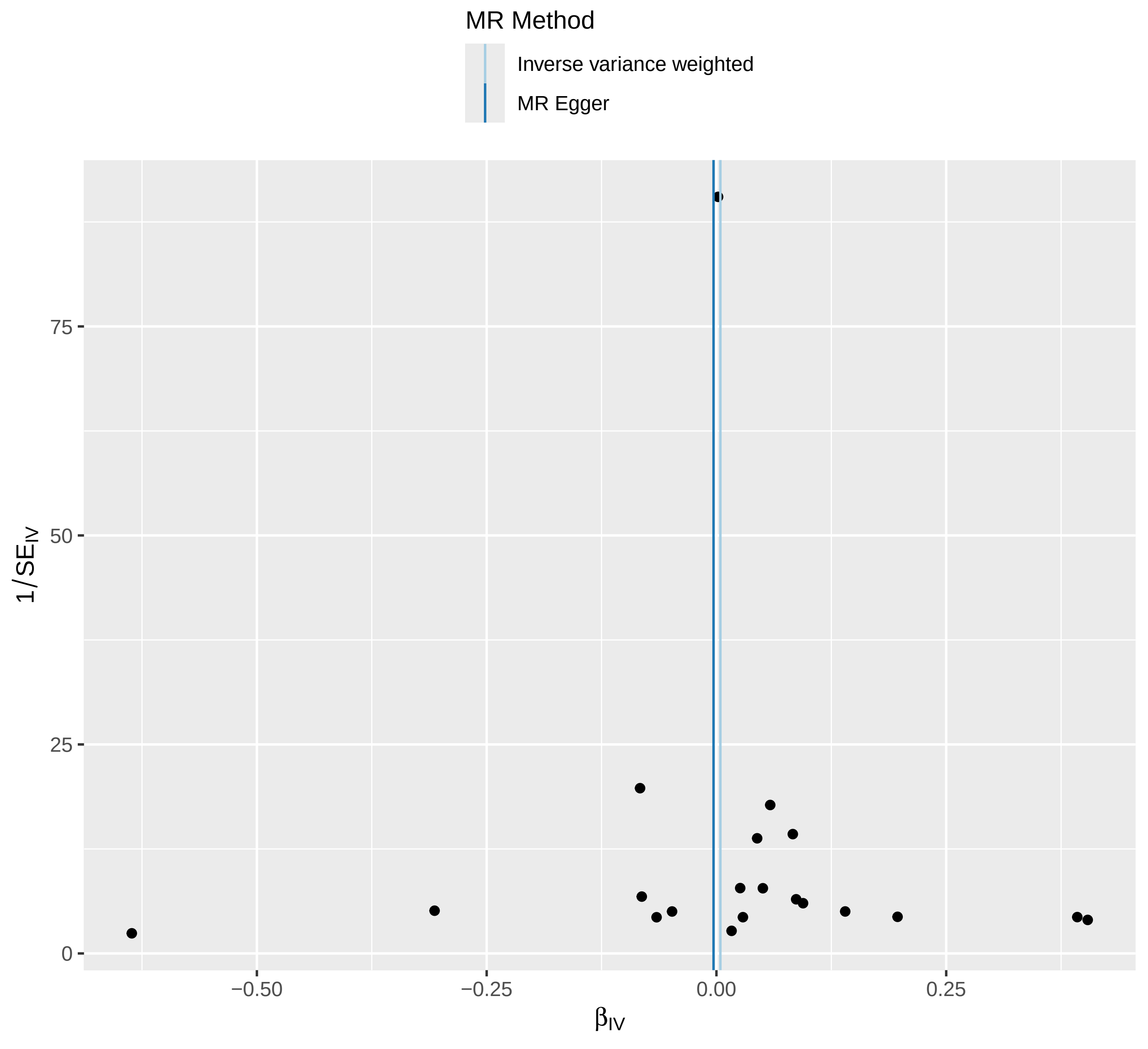

MR Method
Inverse variance weighted
MR Egger
75
50
25
0
1/SE_IV
−0.50
−0.25
0.00
0.25
β_IV

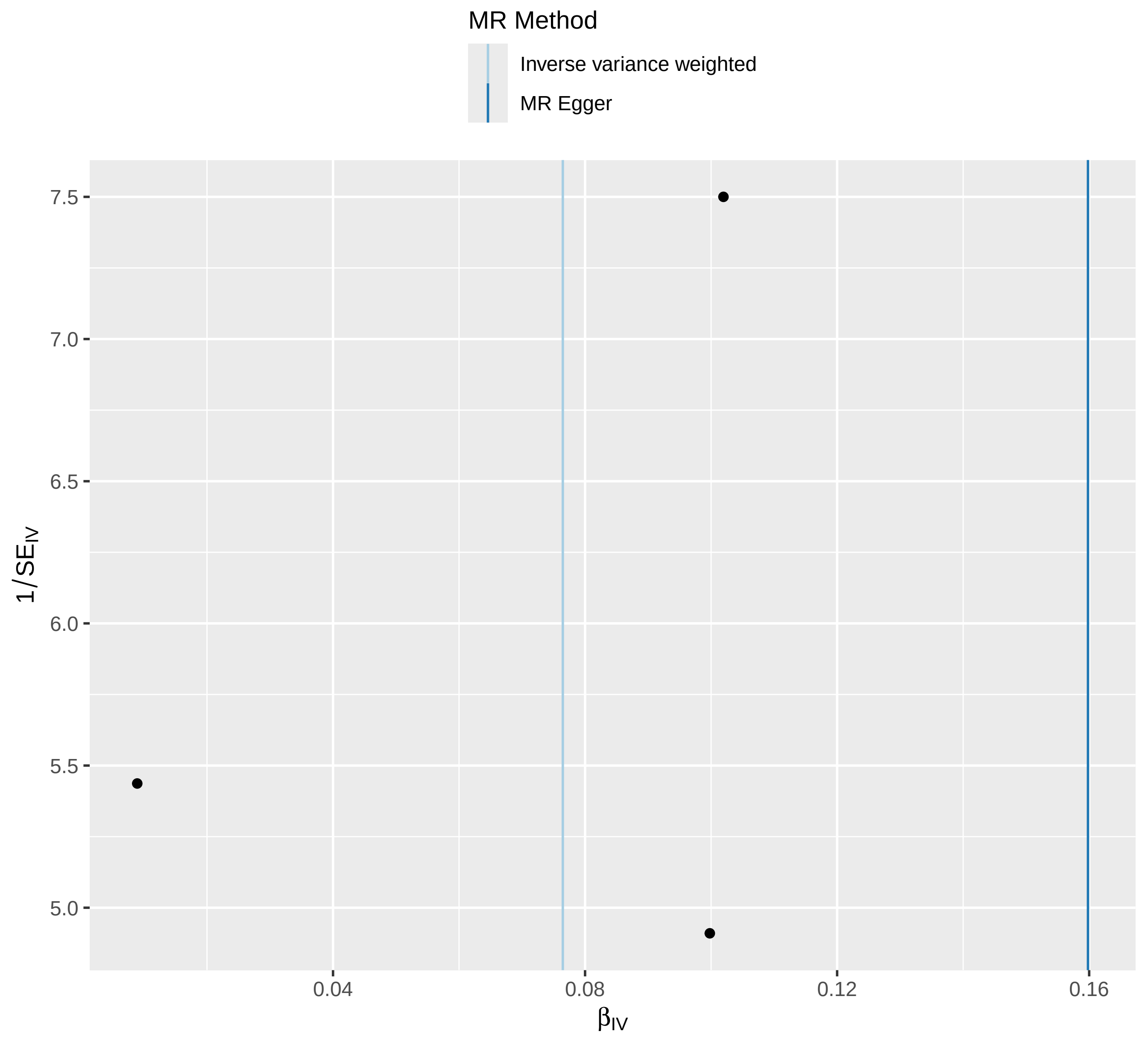
MR Method
Inverse variance weighted
MR Egger
7.5
7.0
6.5
6.0
5.5
5.0
$1/SE_{IV}$
0.04
0.08
0.12
0.16
$\beta_{IV}$

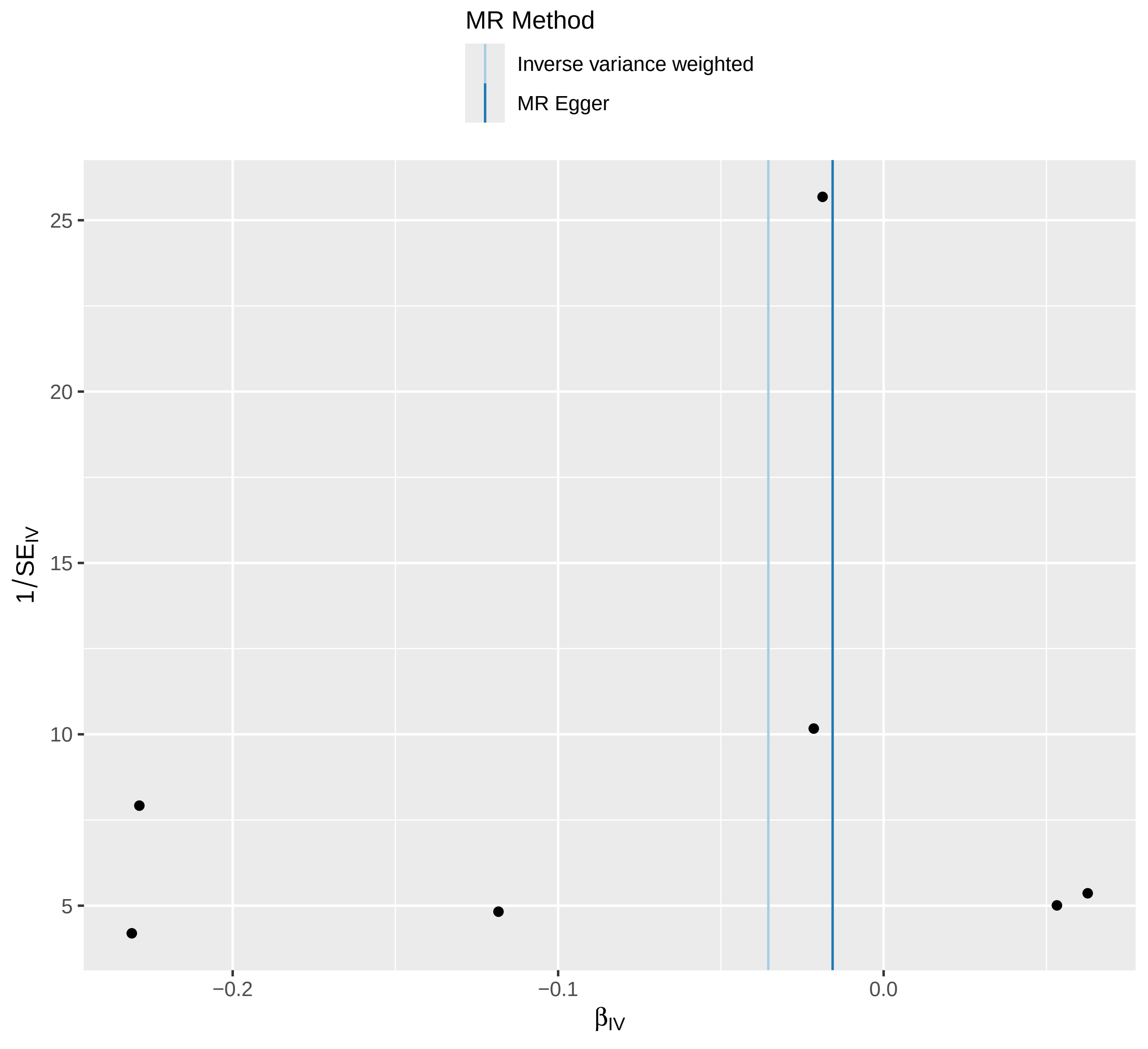

MR Method
Inverse variance weighted
MR Egger
25
20
15
10
5
1/SE$_{IV}$
−0.2
−0.1
0.0
β$_{IV}$

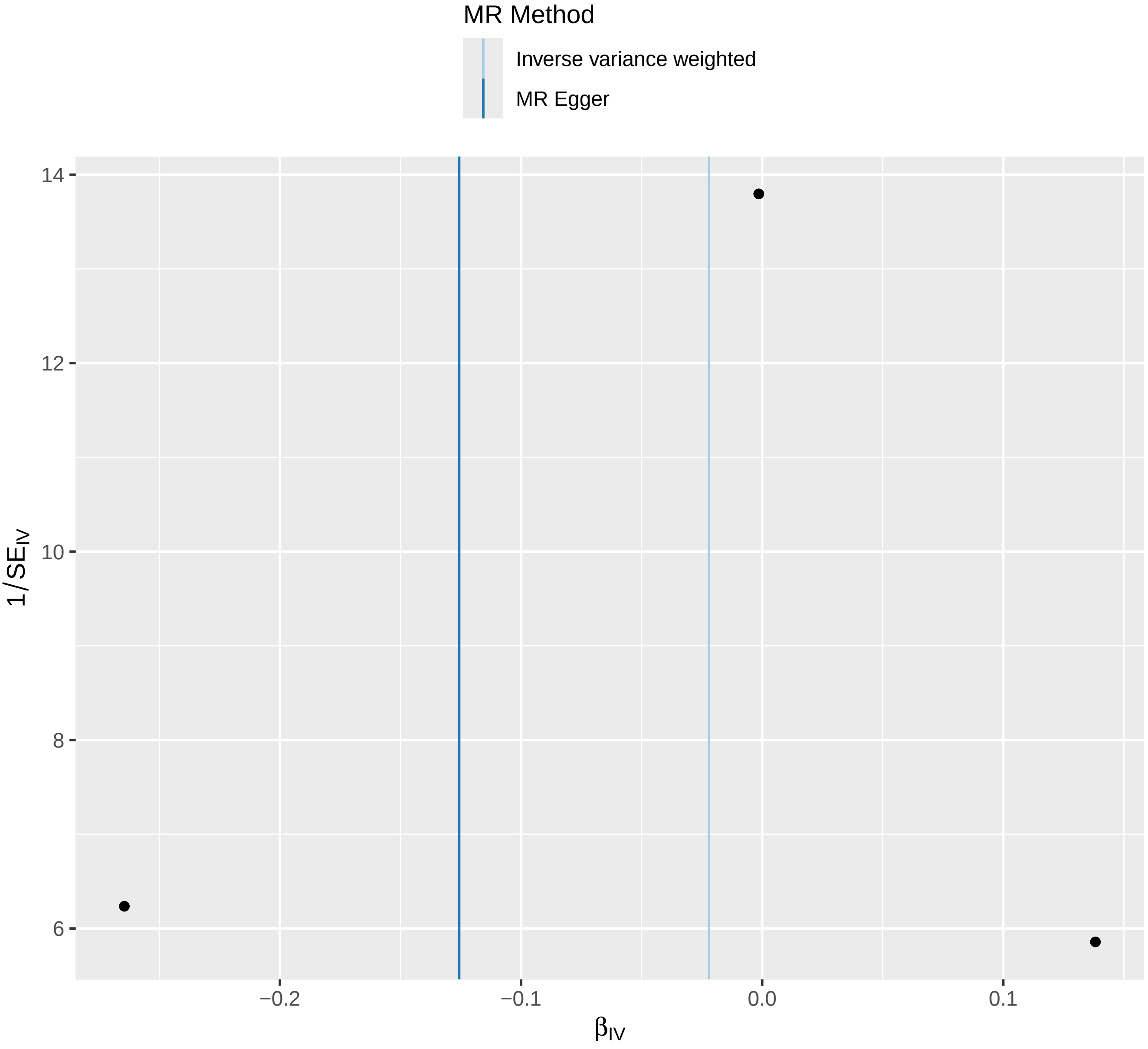
MR Method
Inverse variance weighted
MR Egger
1/$SE_{IV}$
$\beta_{IV}$
14
12
10
8
6
−0.2
−0.1
0.0
0.1

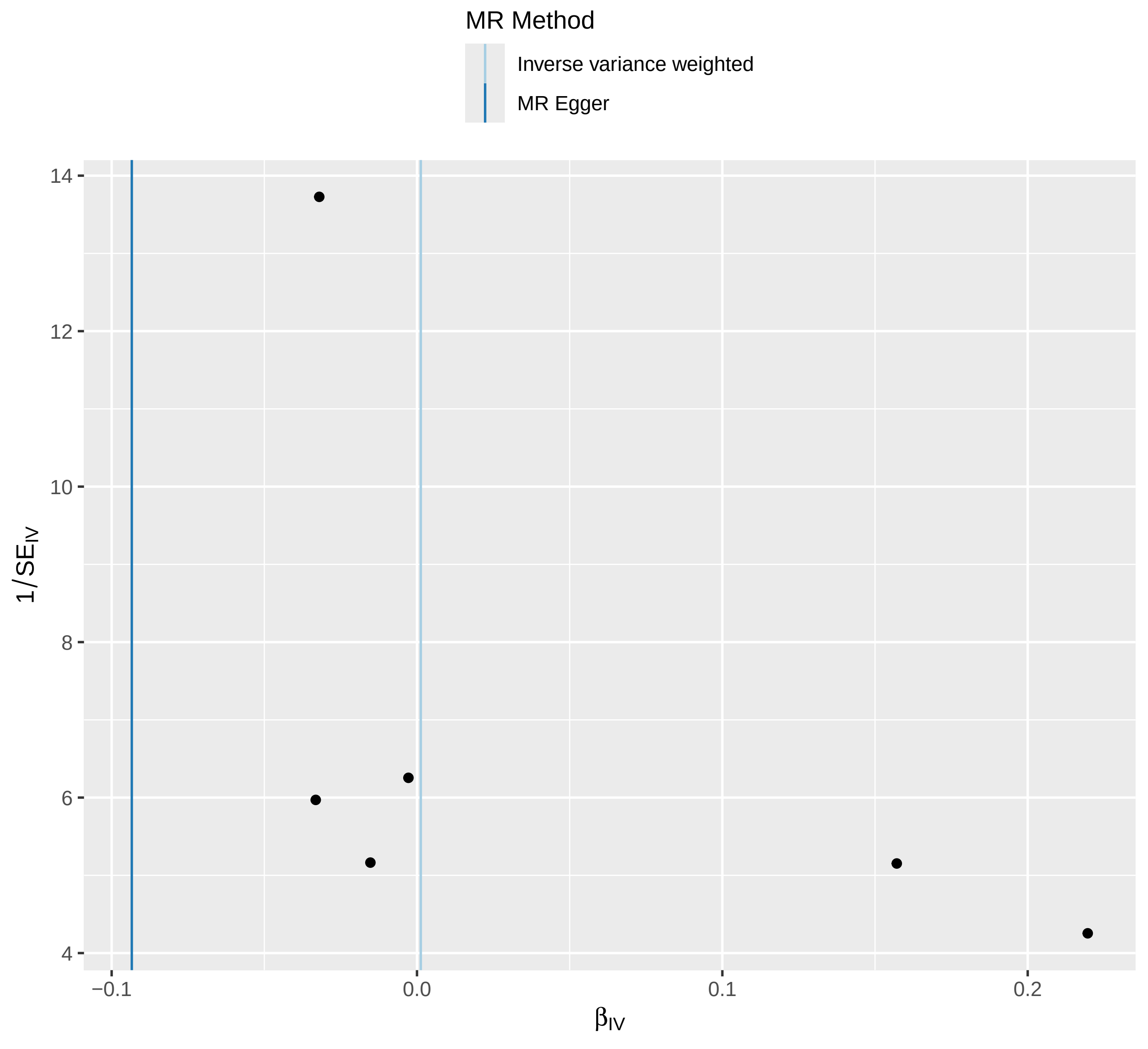

MR Method
Inverse variance weighted
MR Egger
14
12
10
8
6
4
1/SE$_{IV}$
−0.1
0.0
0.1
0.2
β$_{IV}$

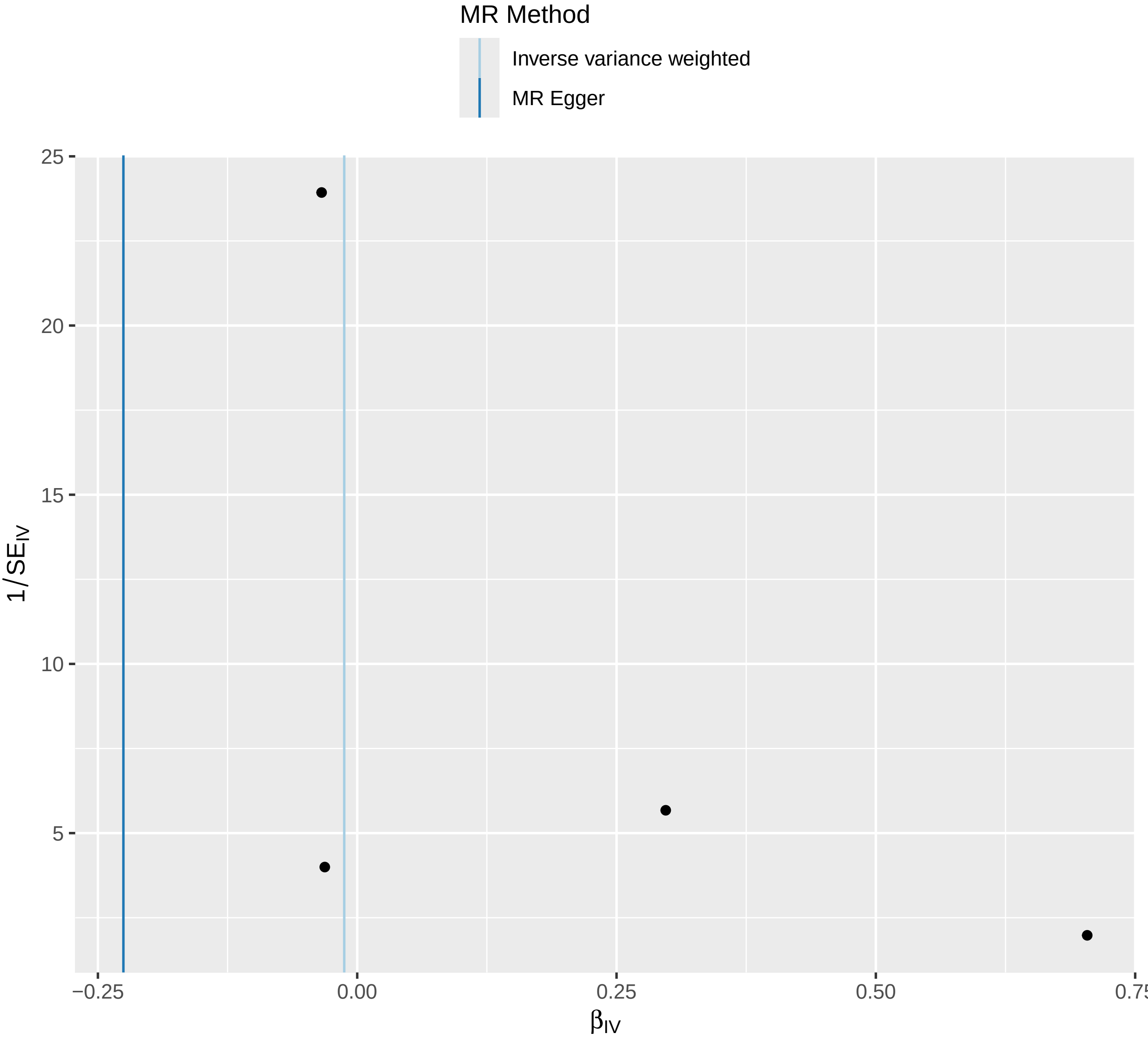

MR Method
Inverse variance weighted
MR Egger
25
20
15
10
5
1/SE$_{IV}$
−0.25
0.00
0.25
0.50
0.75
β$_{IV}$

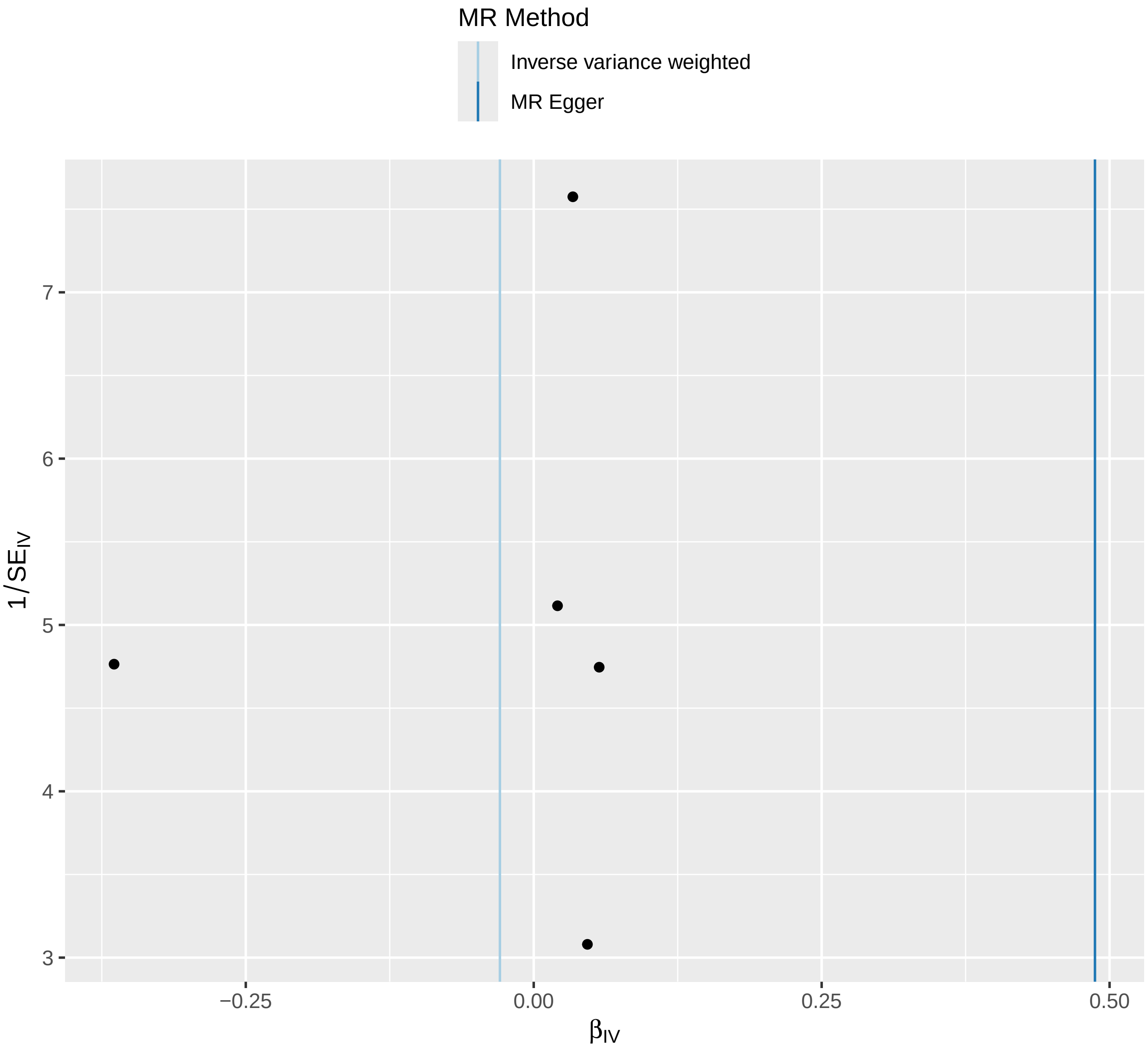
MR Method
Inverse variance weighted
MR Egger
7
6
5
4
3
1/SE$_{IV}$
−0.25
0.00
0.25
0.50
β$_{IV}$

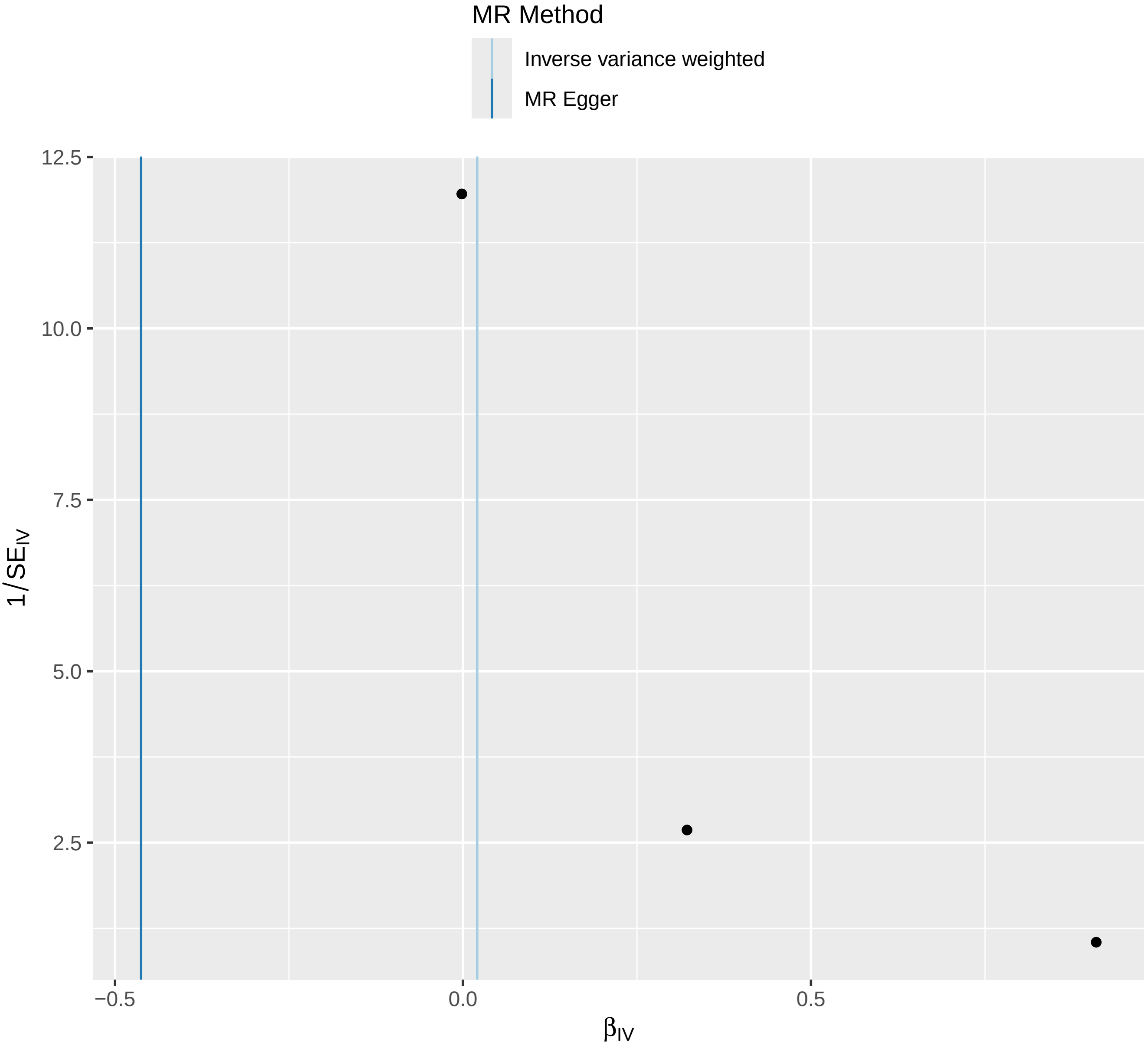

MR Method
Inverse variance weighted
MR Egger
12.5
10.0
7.5
5.0
2.5
1/SE$_{IV}$
−0.5
0.0
0.5
β$_{IV}$

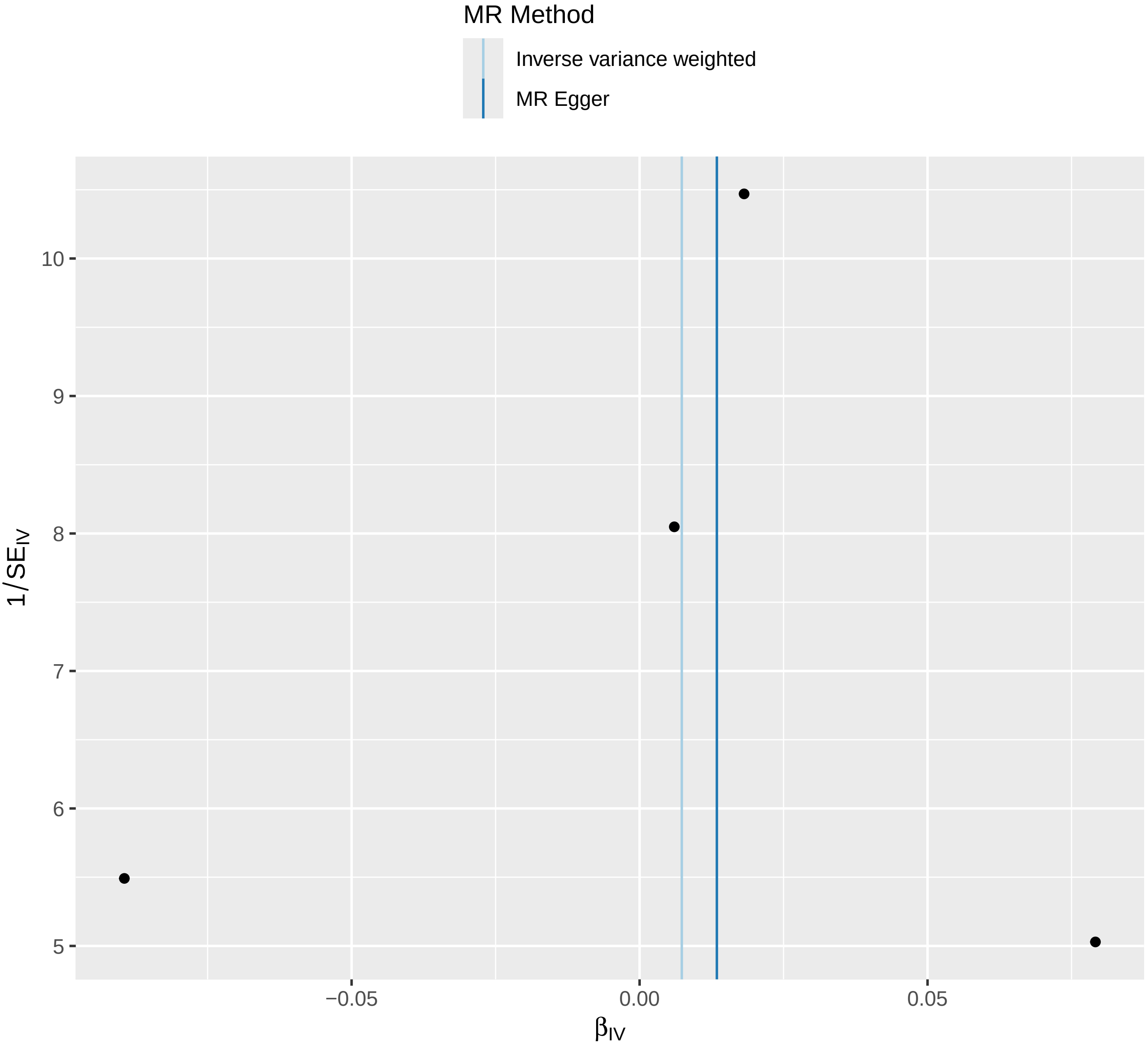

MR Method
Inverse variance weighted
MR Egger
10
9
8
7
6
5
1/SE_IV
−0.05
0.00
0.05
β_IV

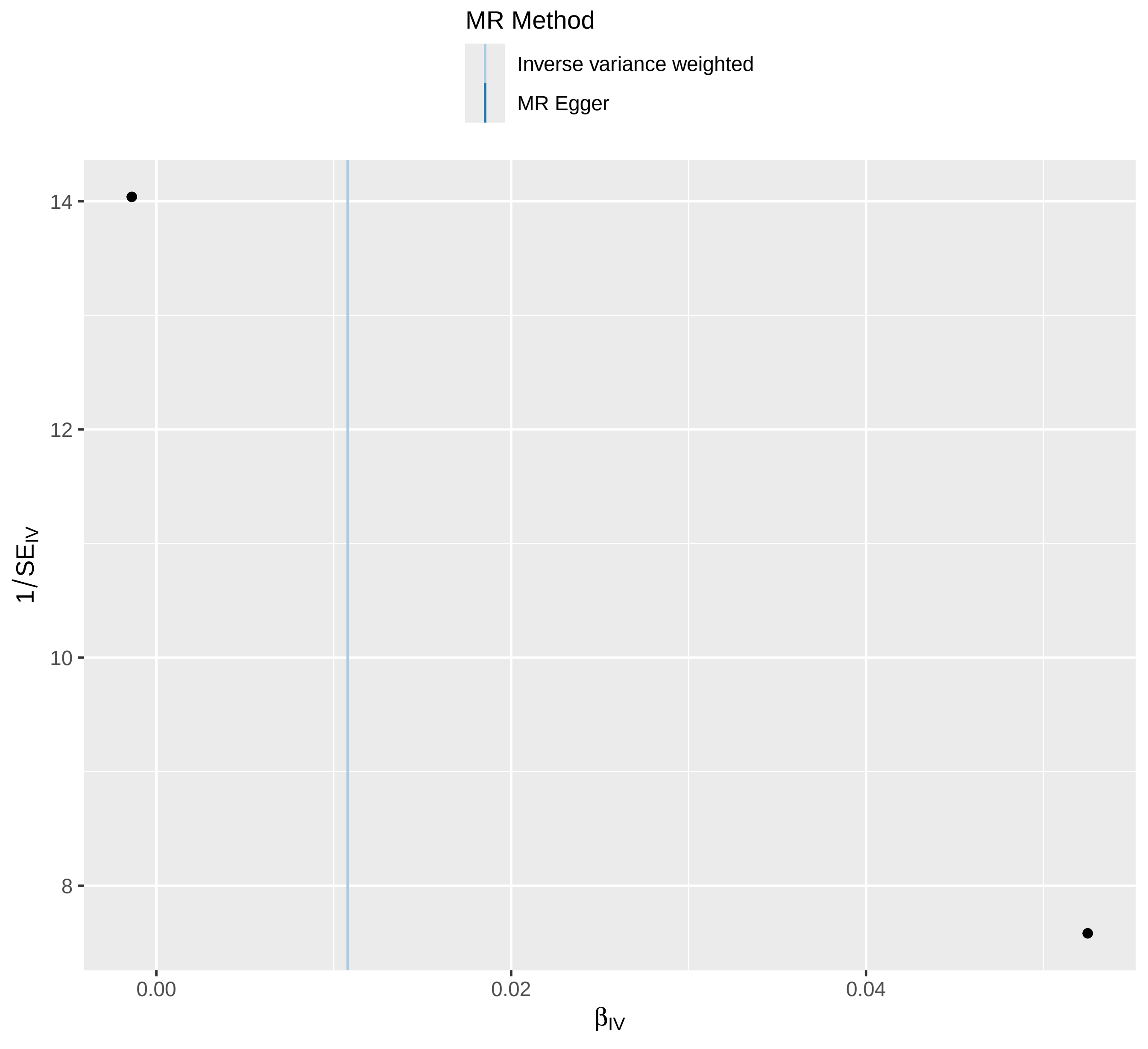

MR Method
Inverse variance weighted
MR Egger
14
12
10
8
1/SE_IV
0.00
0.02
0.04
β_IV

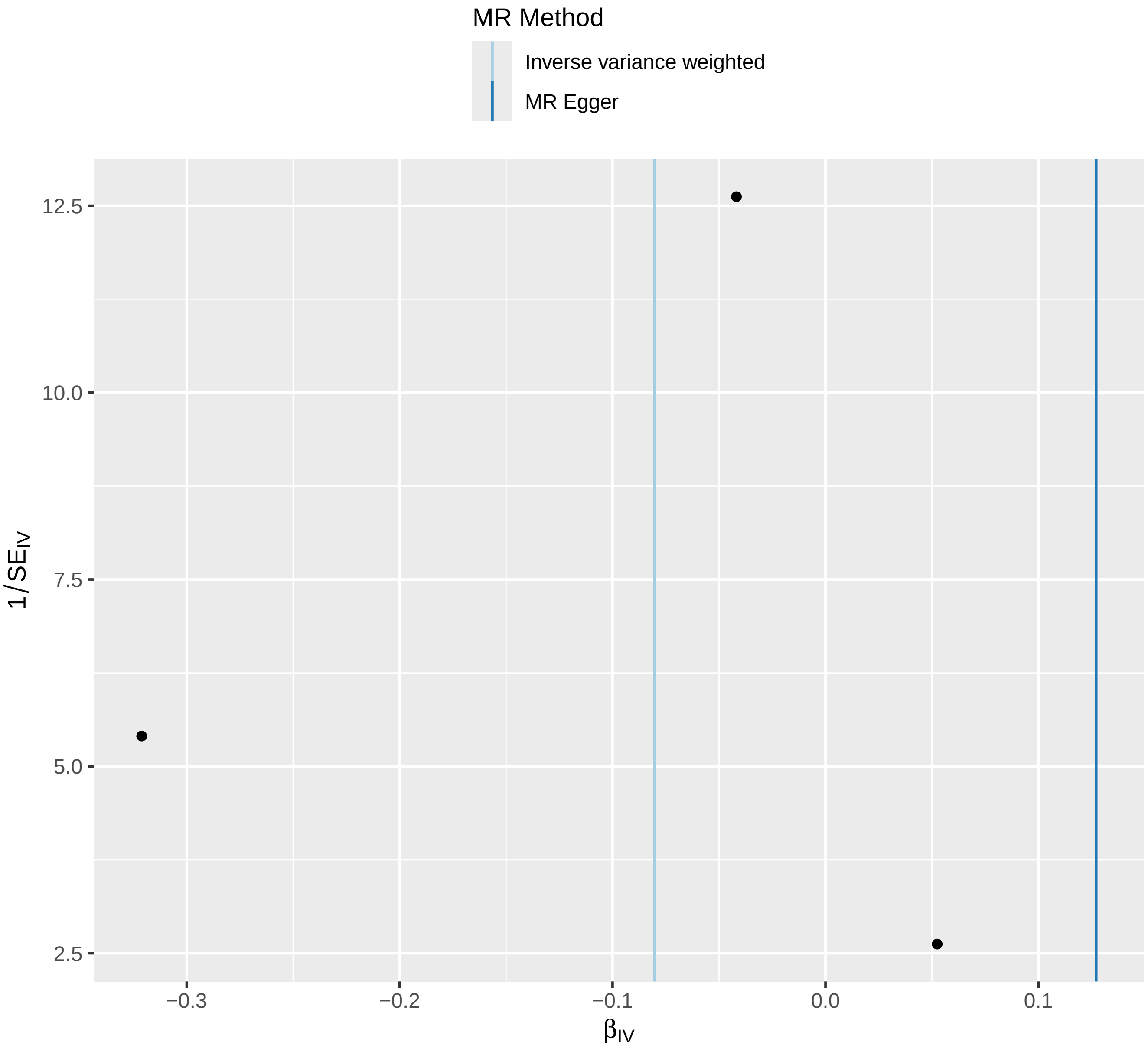

MR Method
Inverse variance weighted
MR Egger
12.5
10.0
7.5
5.0
2.5
1/SE_IV
−0.3
−0.2
−0.1
0.0
0.1
β_IV

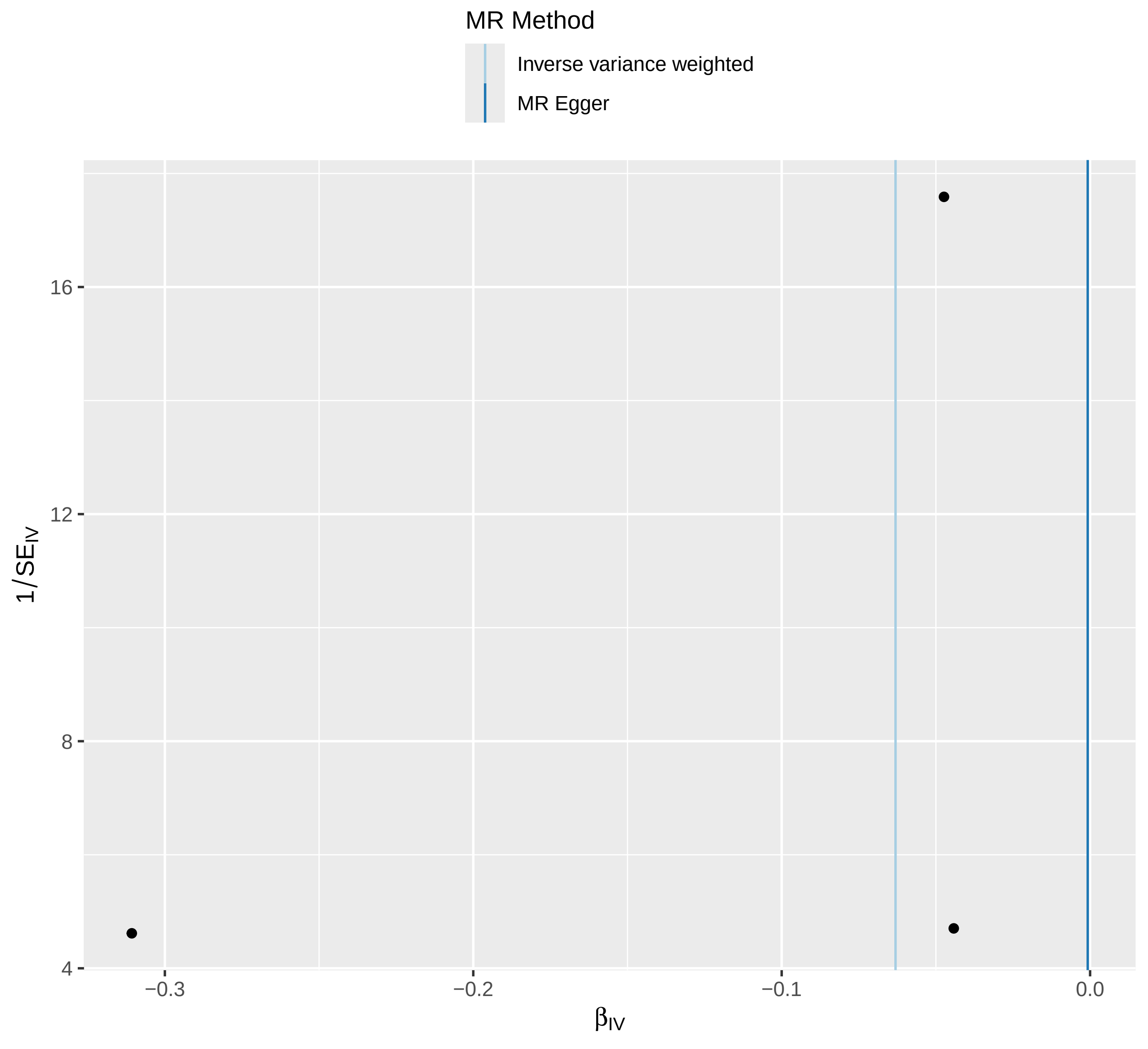

MR Method
Inverse variance weighted
MR Egger
16
12
8
4
1/SE_IV
−0.3
−0.2
−0.1
0.0
β_IV

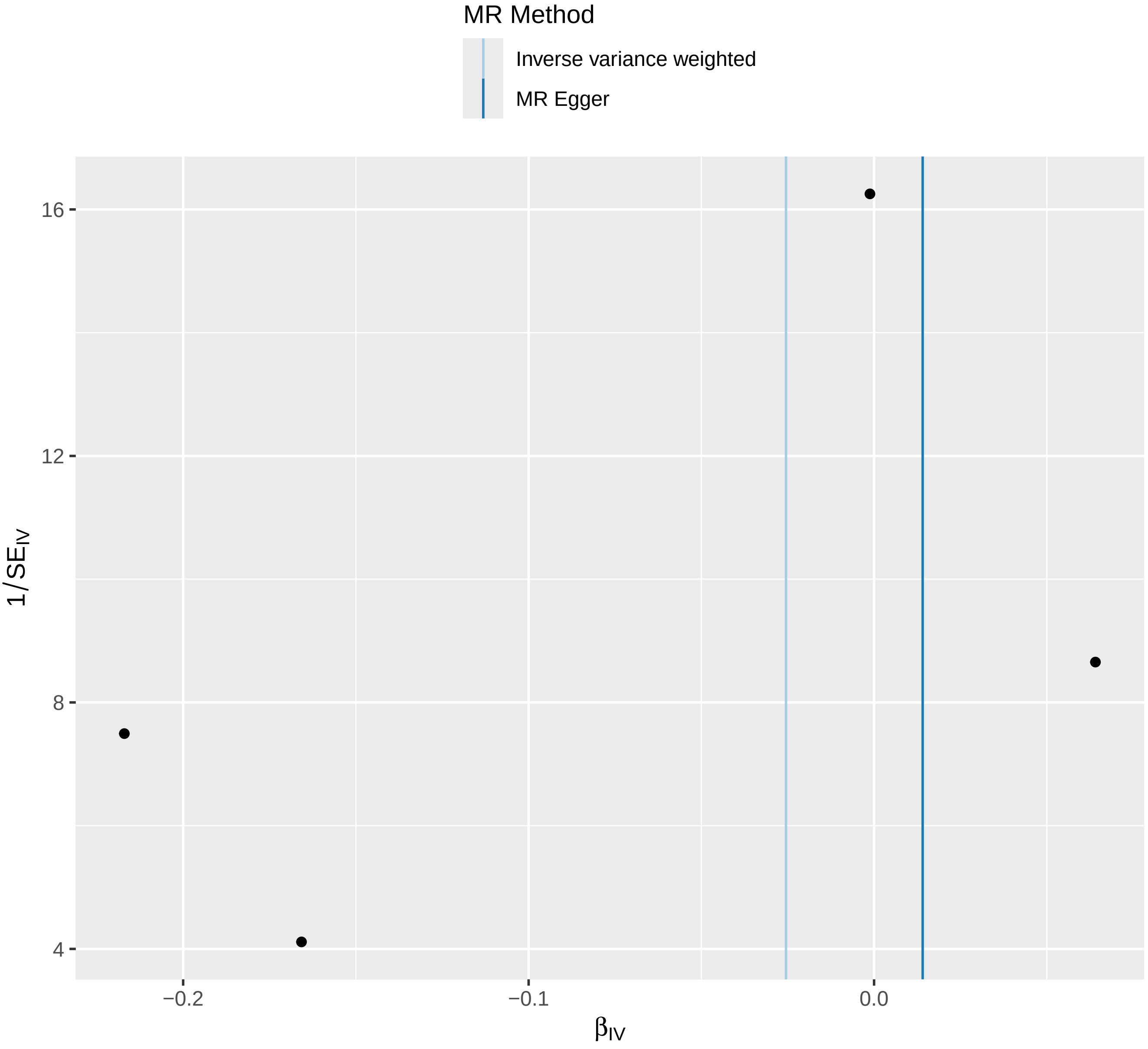

MR Method
Inverse variance weighted
MR Egger
16
12
8
4
−0.2
−0.1
0.0
$\beta_{IV}$
$1/SE_{IV}$

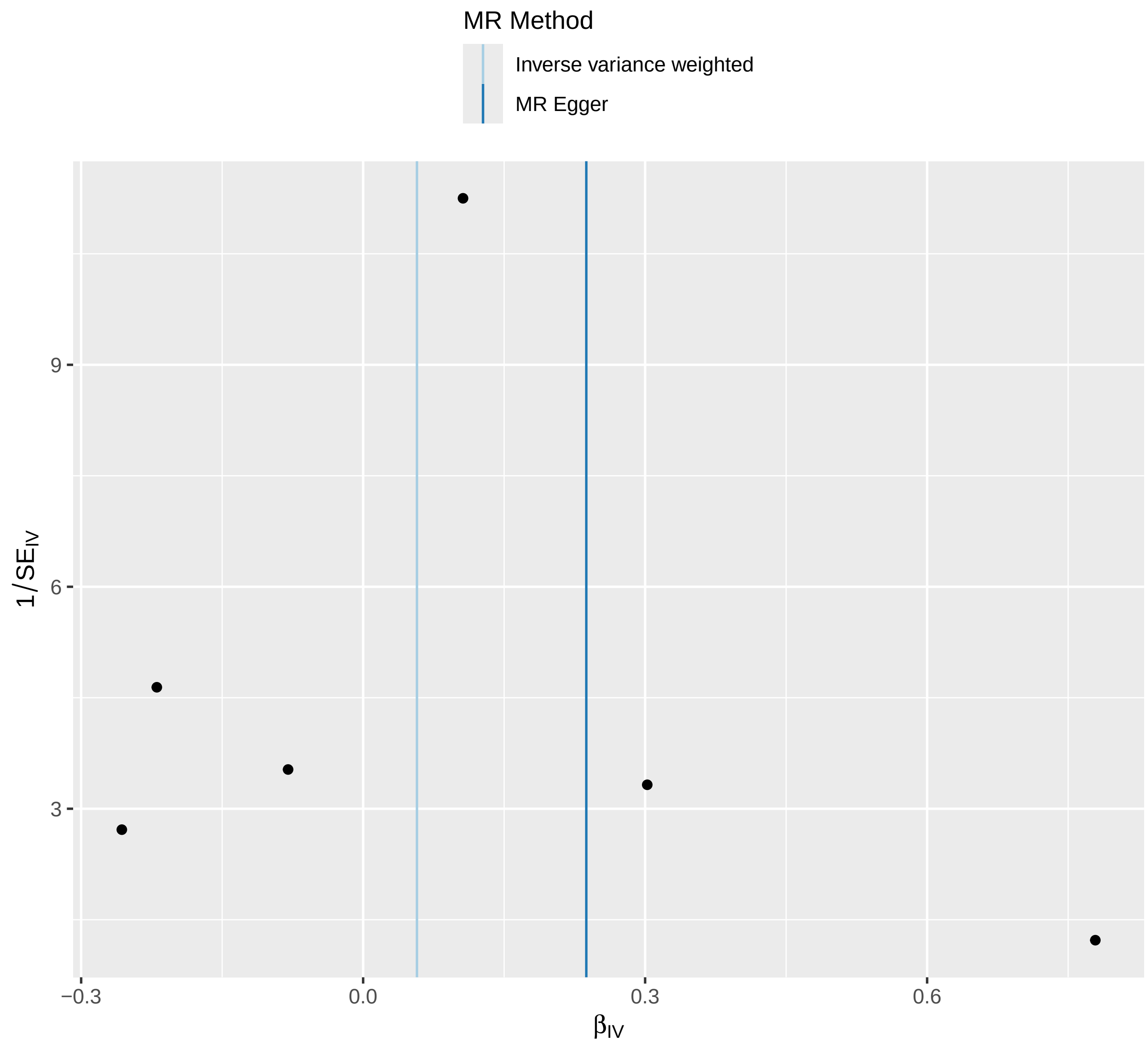
MR Method
Inverse variance weighted
MR Egger
1/SE_IV
9
6
3
−0.3
0.0
0.3
0.6
β_IV

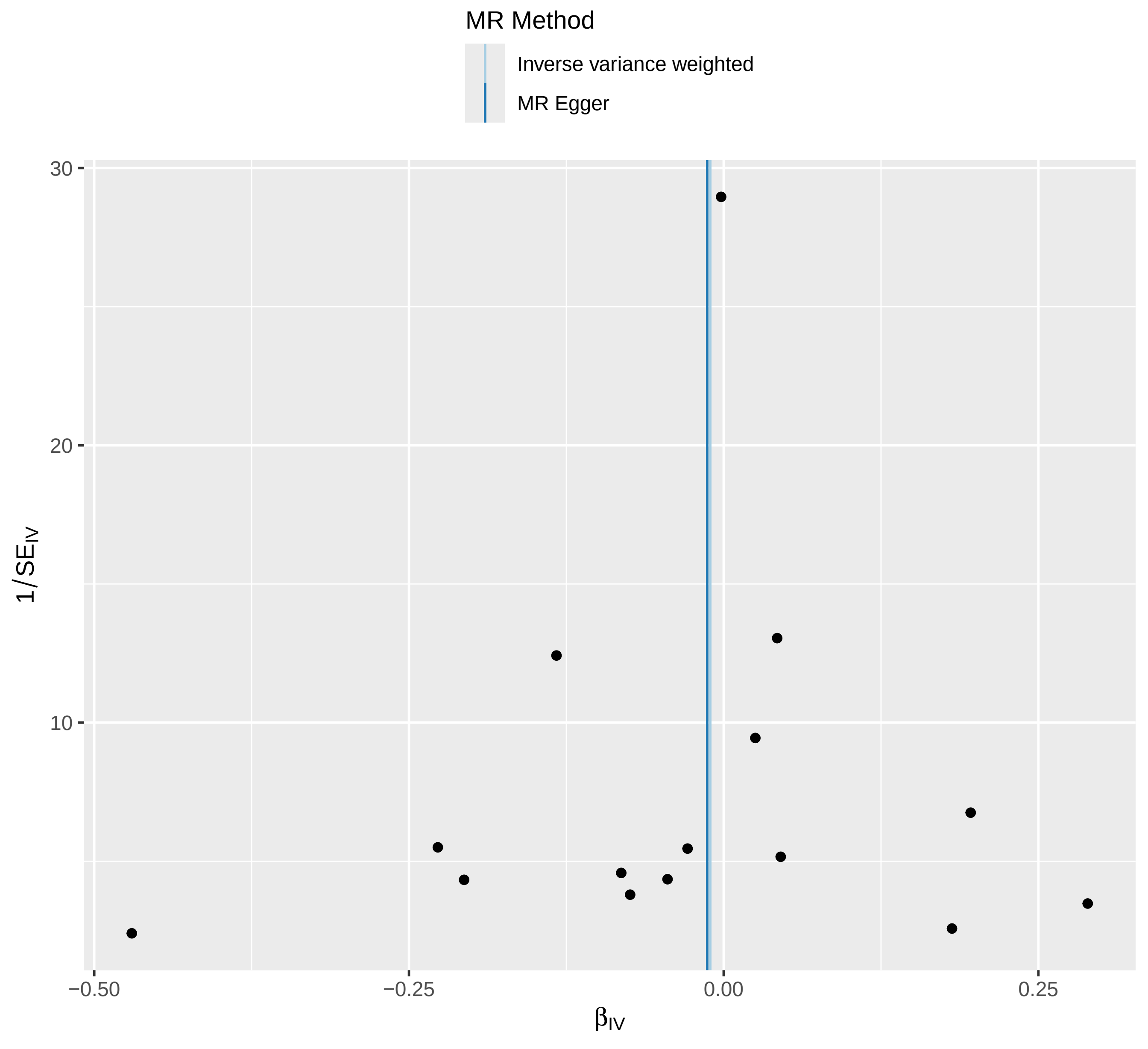

MR Method
Inverse variance weighted
MR Egger
30
20
10
1/SE$_{IV}$
−0.50
−0.25
0.00
0.25
β$_{IV}$

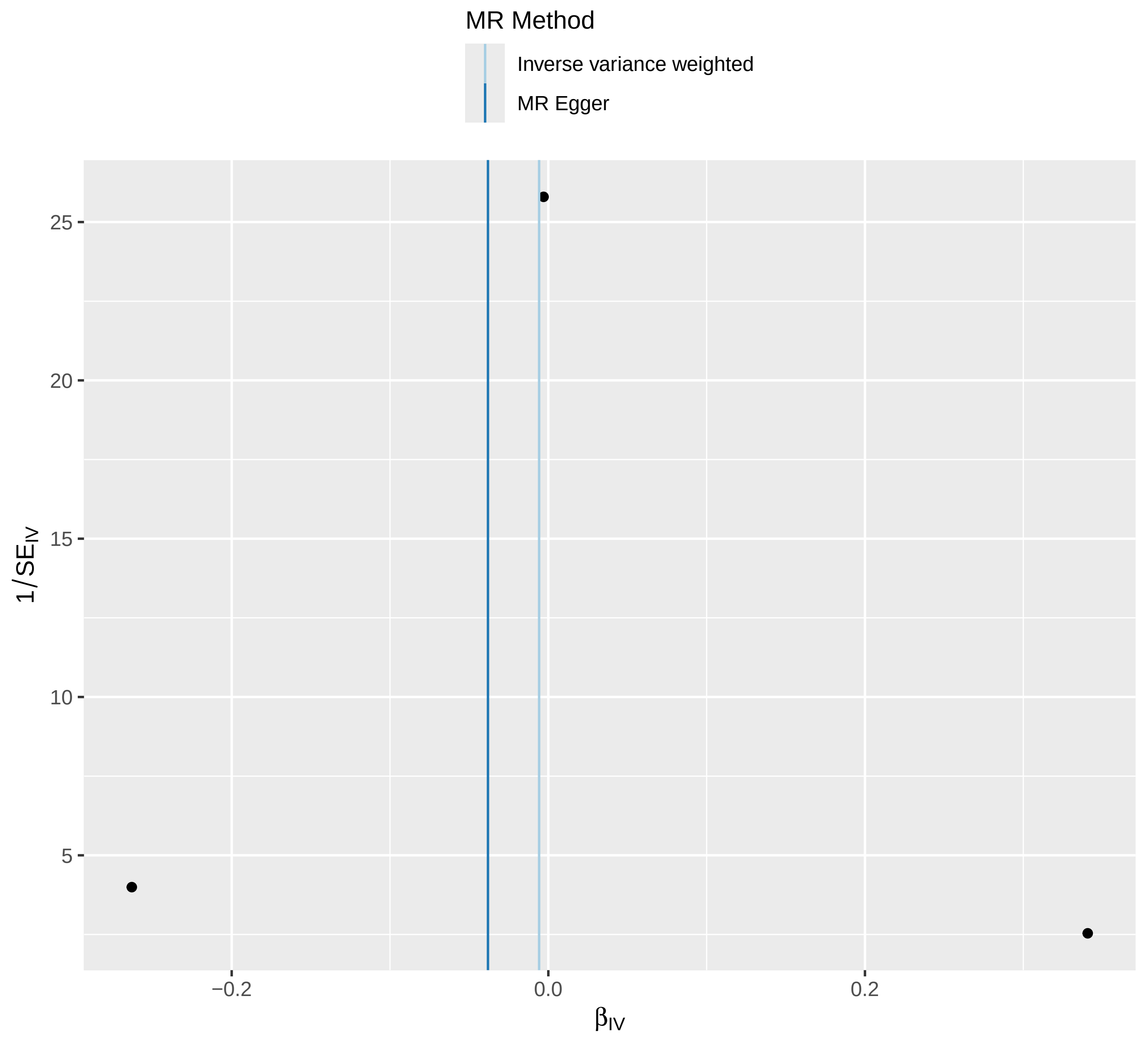

MR Method
Inverse variance weighted
MR Egger
25
20
15
10
5
1/SE_IV
−0.2
0.0
0.2
β_IV

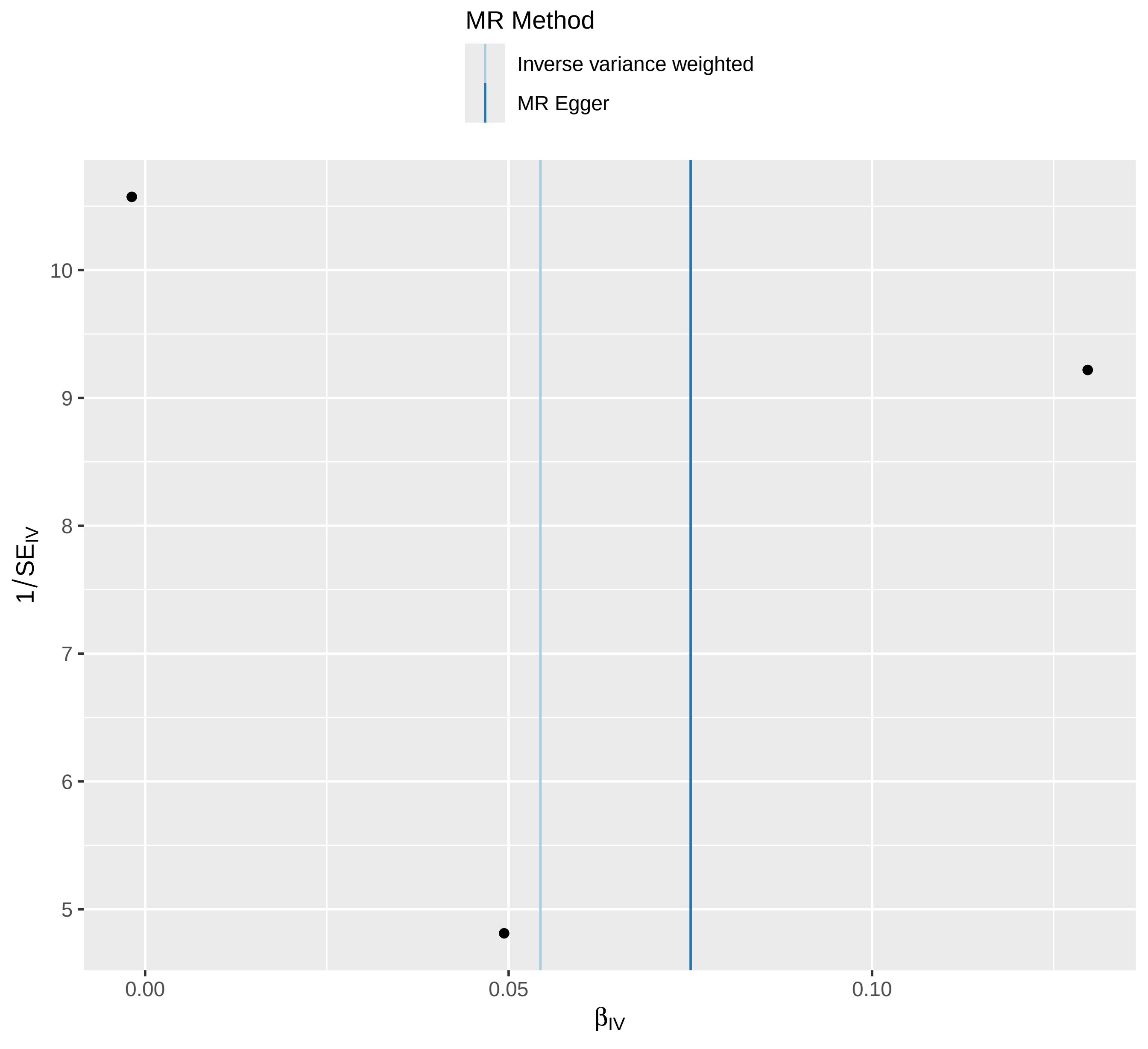

MR Method
Inverse variance weighted
MR Egger
10
9
8
7
6
5
1/SE_IV
0.00
0.05
0.10
β_IV

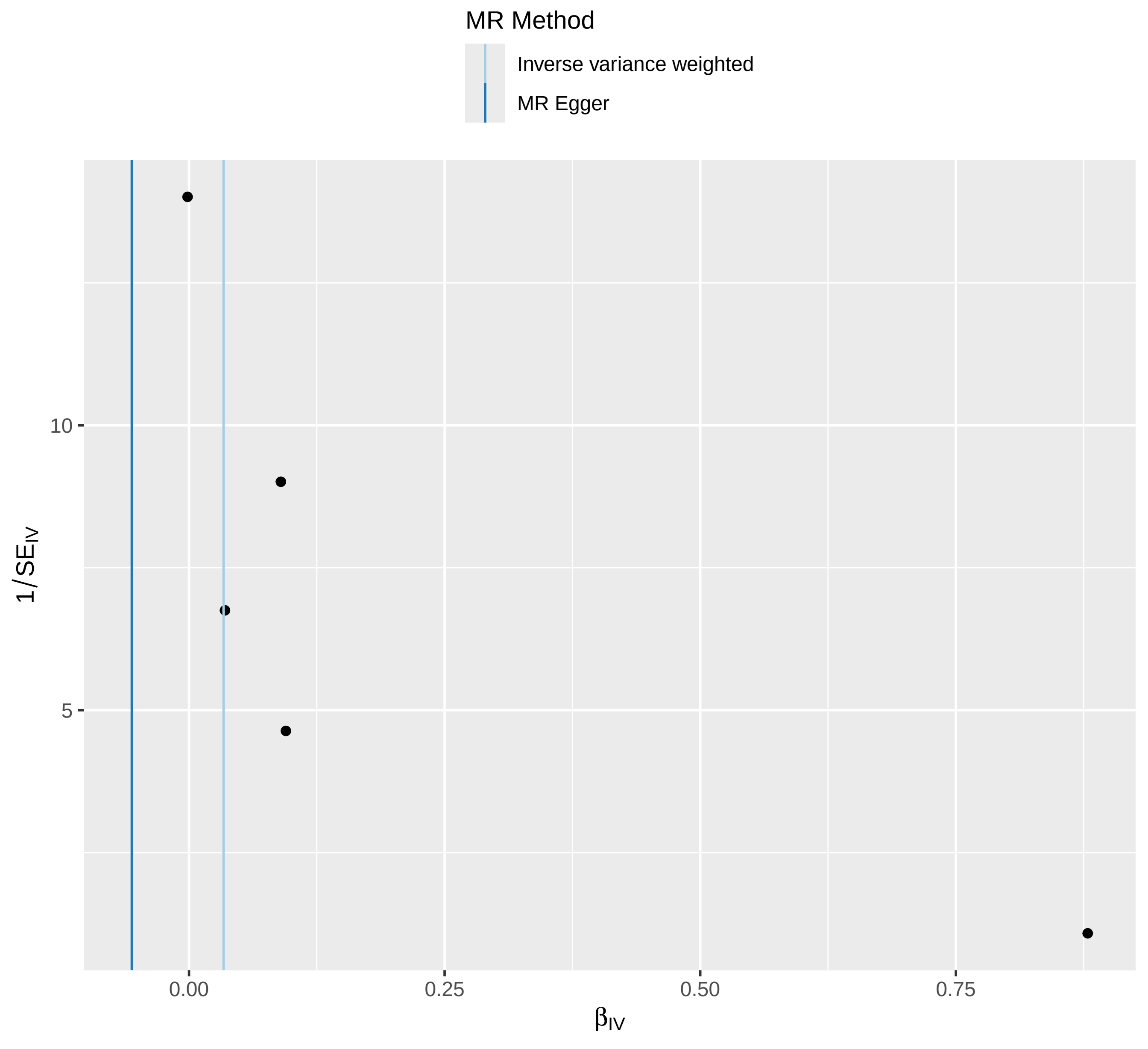

MR Method
Inverse variance weighted
MR Egger
10
5
1/SE_IV
0.00
0.25
0.50
0.75
β_IV

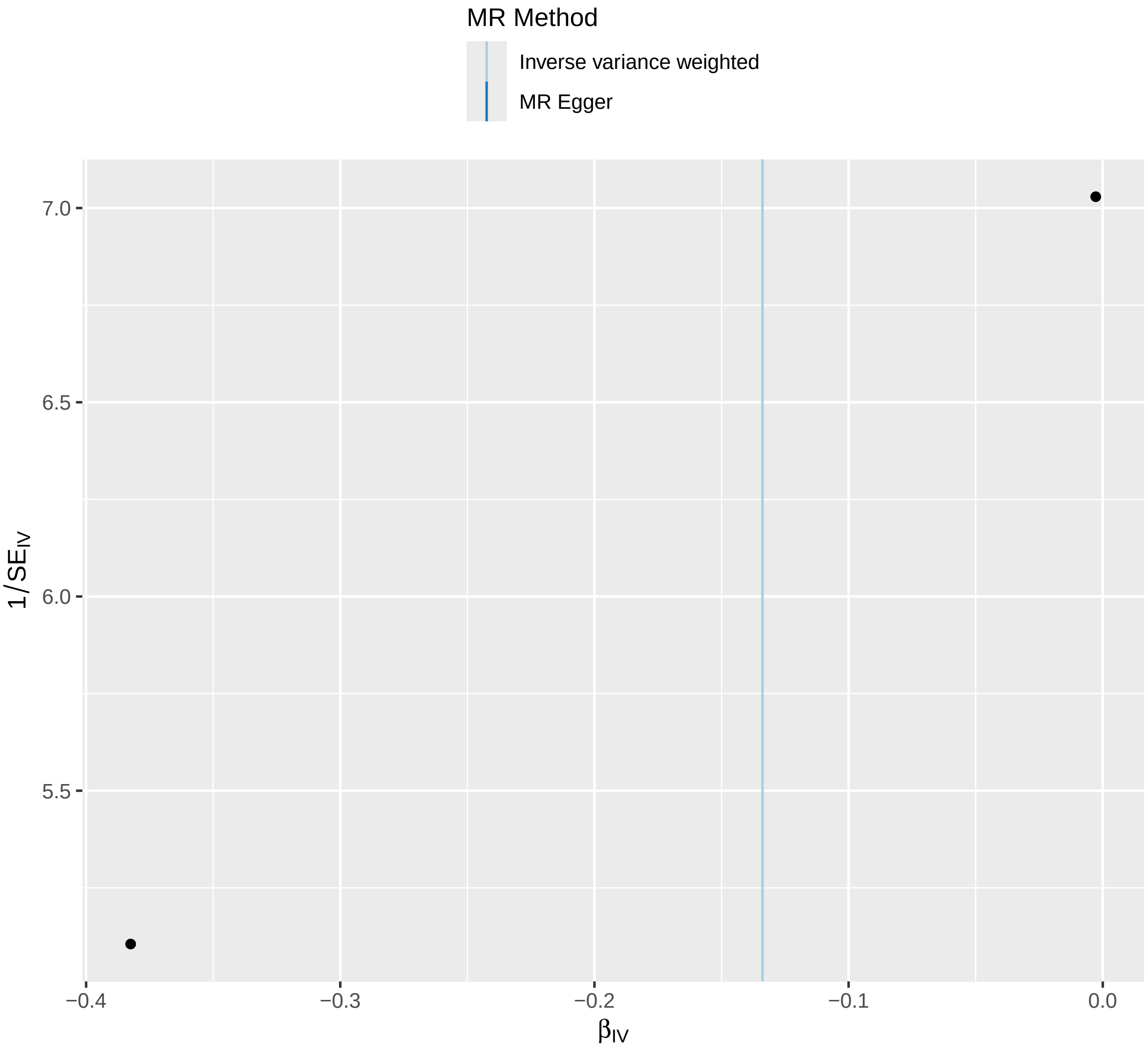
MR Method
Inverse variance weighted
MR Egger
7.0
6.5
6.0
5.5
1/SE_IV
−0.4
−0.3
−0.2
−0.1
0.0
β_IV

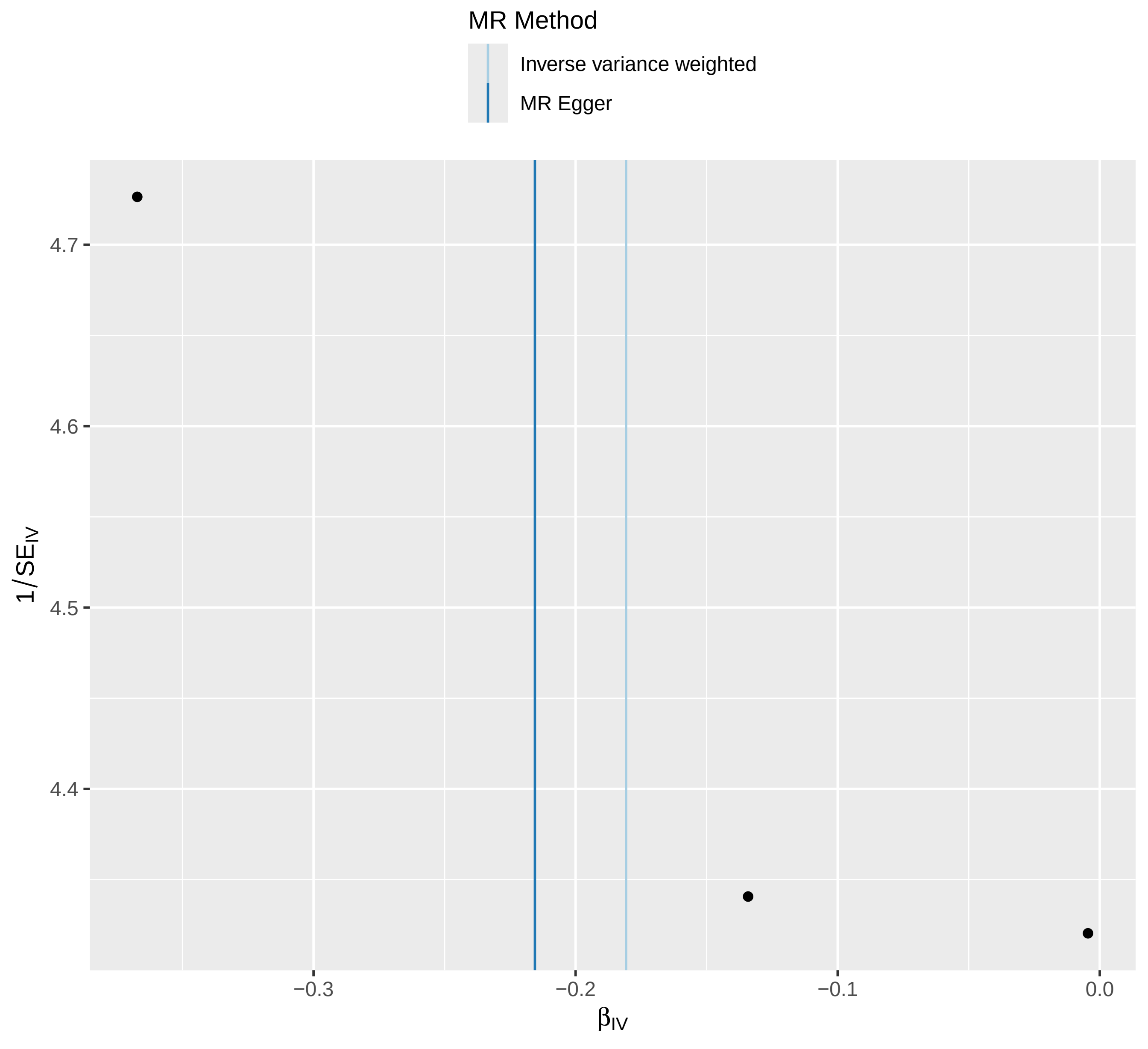
MR Method
Inverse variance weighted
MR Egger
4.7
4.6
4.5
4.4
1/SE_IV
−0.3
−0.2
−0.1
0.0
β_IV

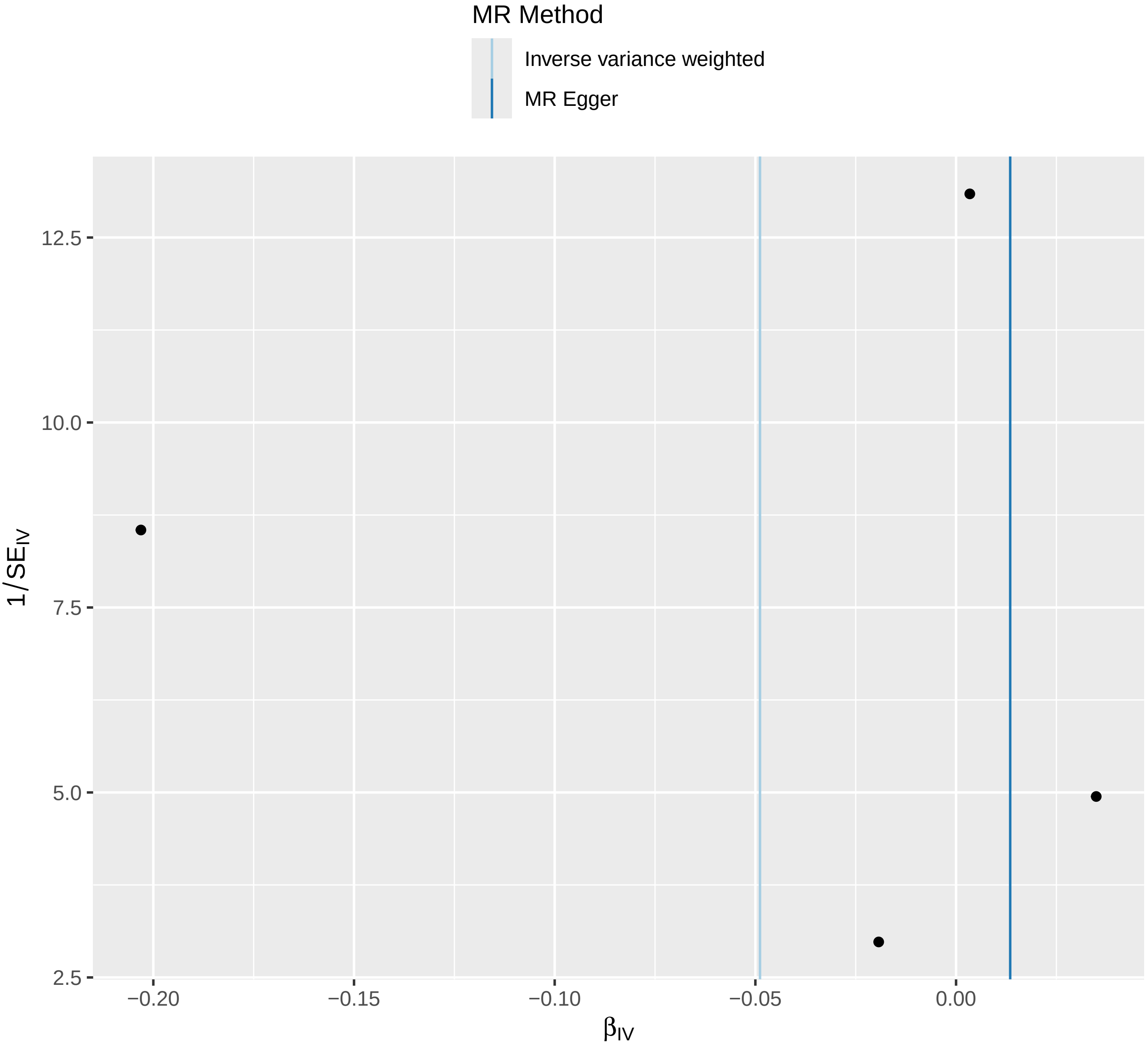

MR Method
Inverse variance weighted
MR Egger
12.5
10.0
7.5
5.0
2.5
1/SE_IV
−0.20
−0.15
−0.10
−0.05
0.00
β_IV

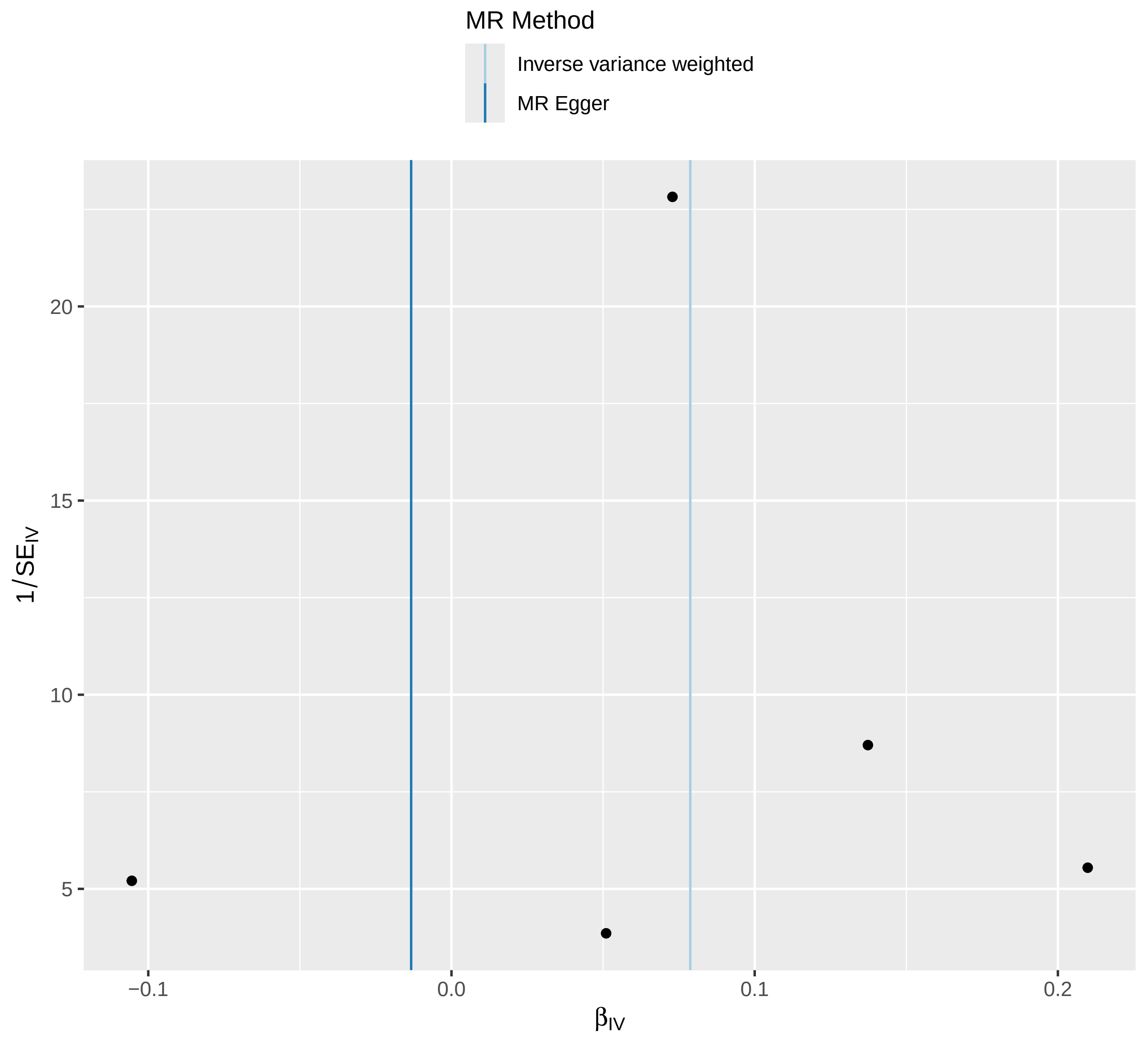
MR Method
Inverse variance weighted
MR Egger
20
15
10
5
1/SE$_{IV}$
−0.1
0.0
0.1
0.2
β$_{IV}$

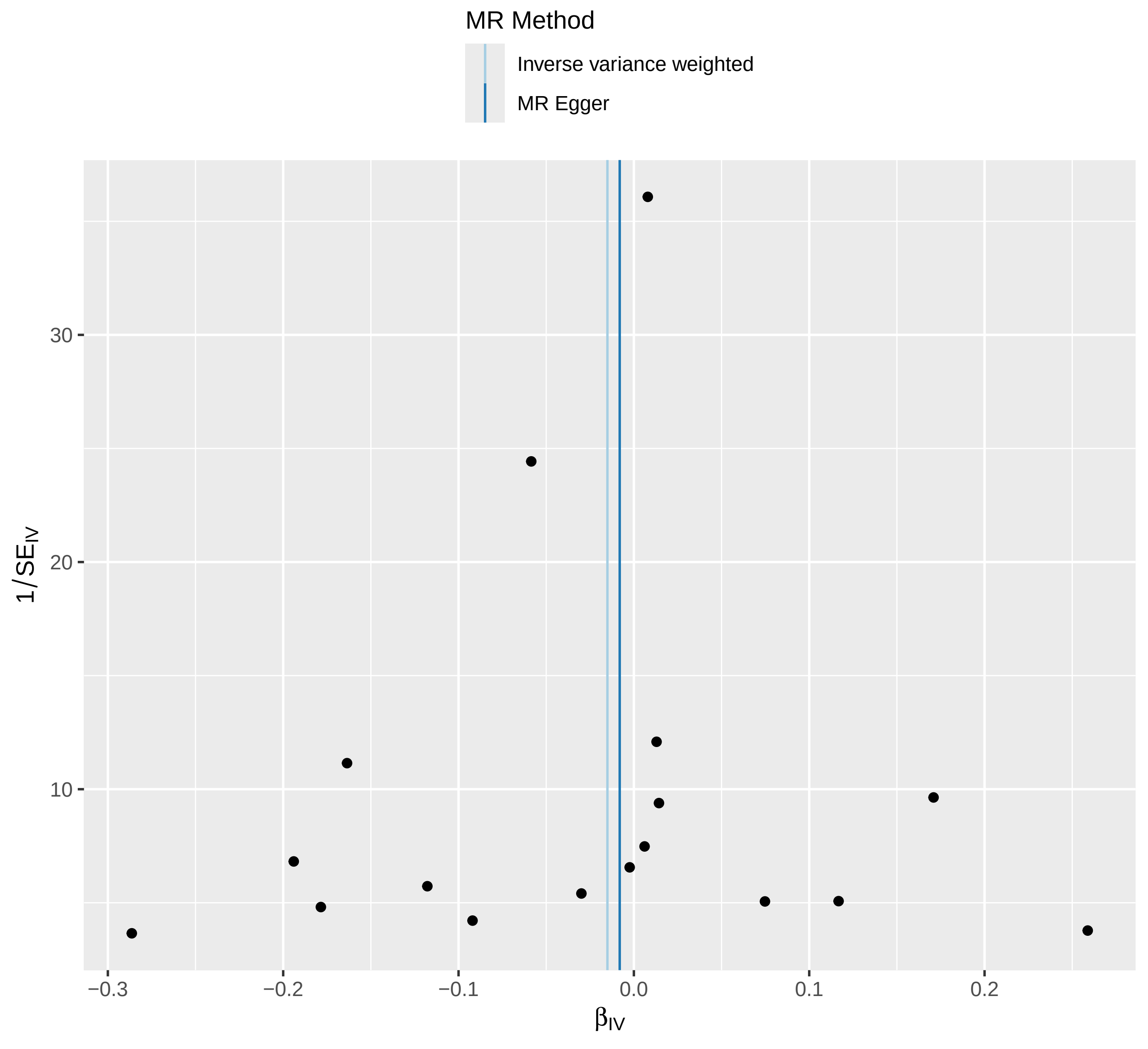

MR Method
Inverse variance weighted
MR Egger
30
20
10
1/SE$_{IV}$
−0.3
−0.2
−0.1
0.0
0.1
0.2
β$_{IV}$

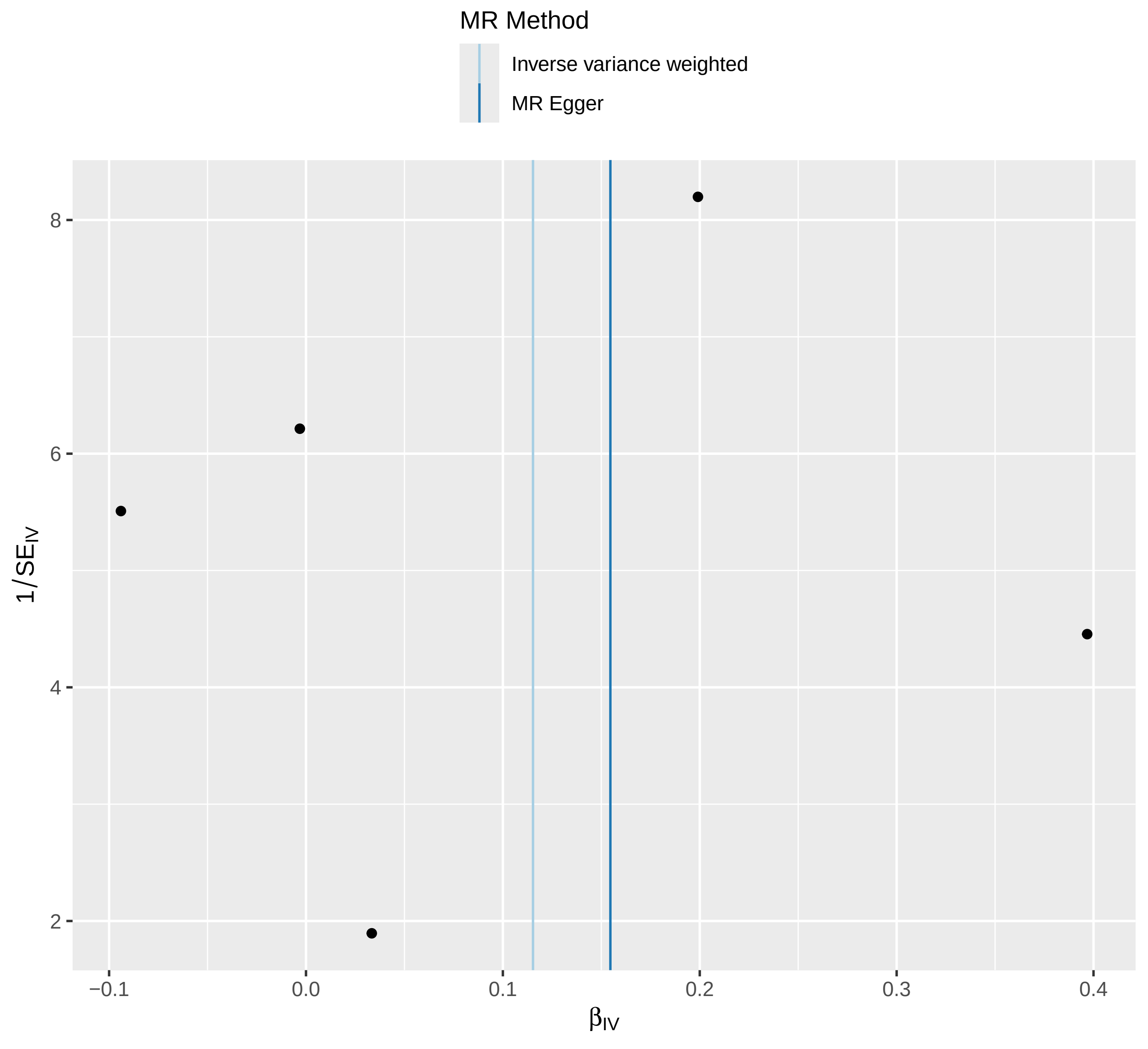

MR Method
Inverse variance weighted
MR Egger
8
6
4
2
1/SE_IV
−0.1
0.0
0.1
0.2
0.3
0.4
β_IV

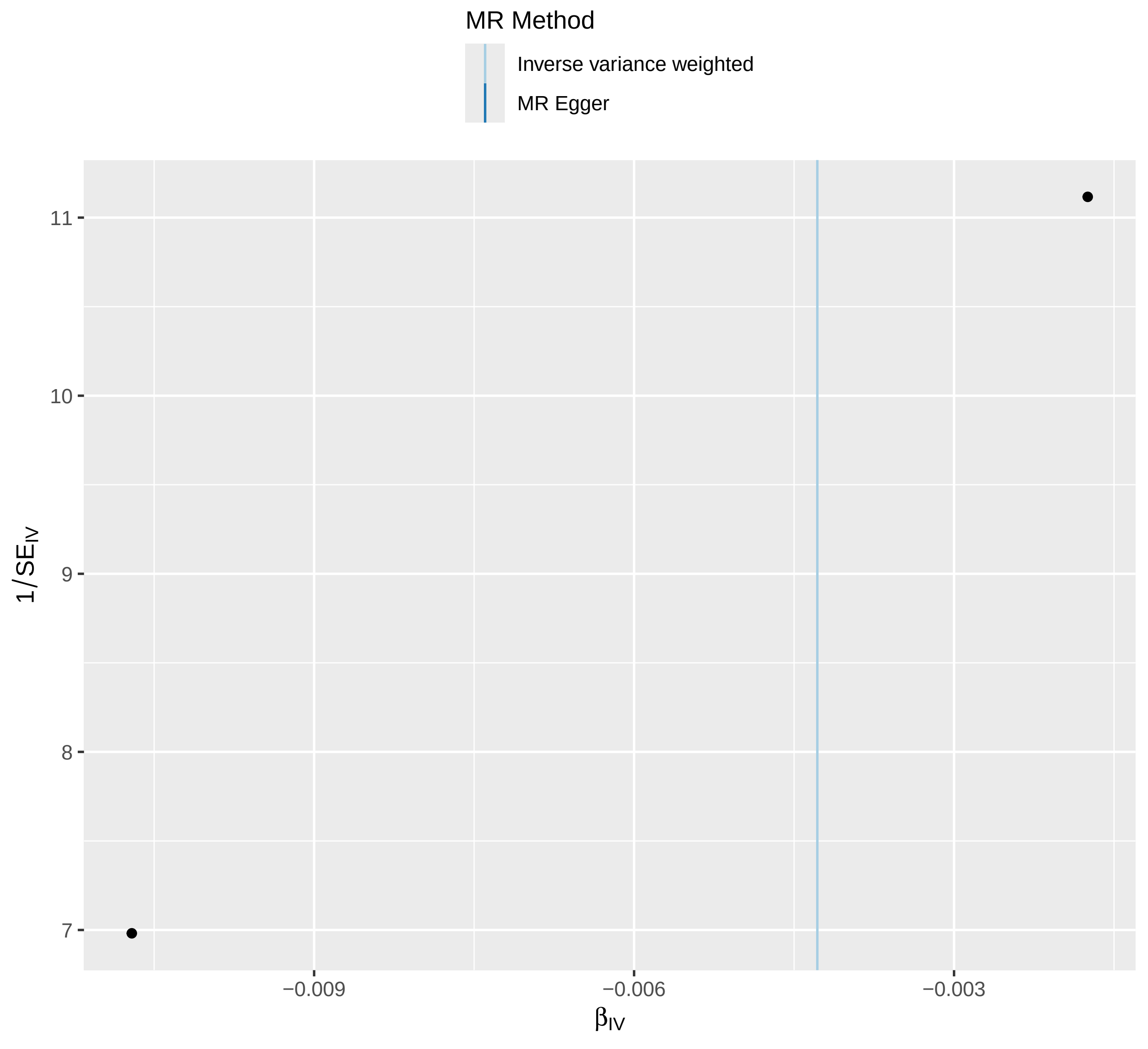
MR Method
Inverse variance weighted
MR Egger
11
10
9
8
7
1/SE_IV
−0.009
−0.006
−0.003
β_IV

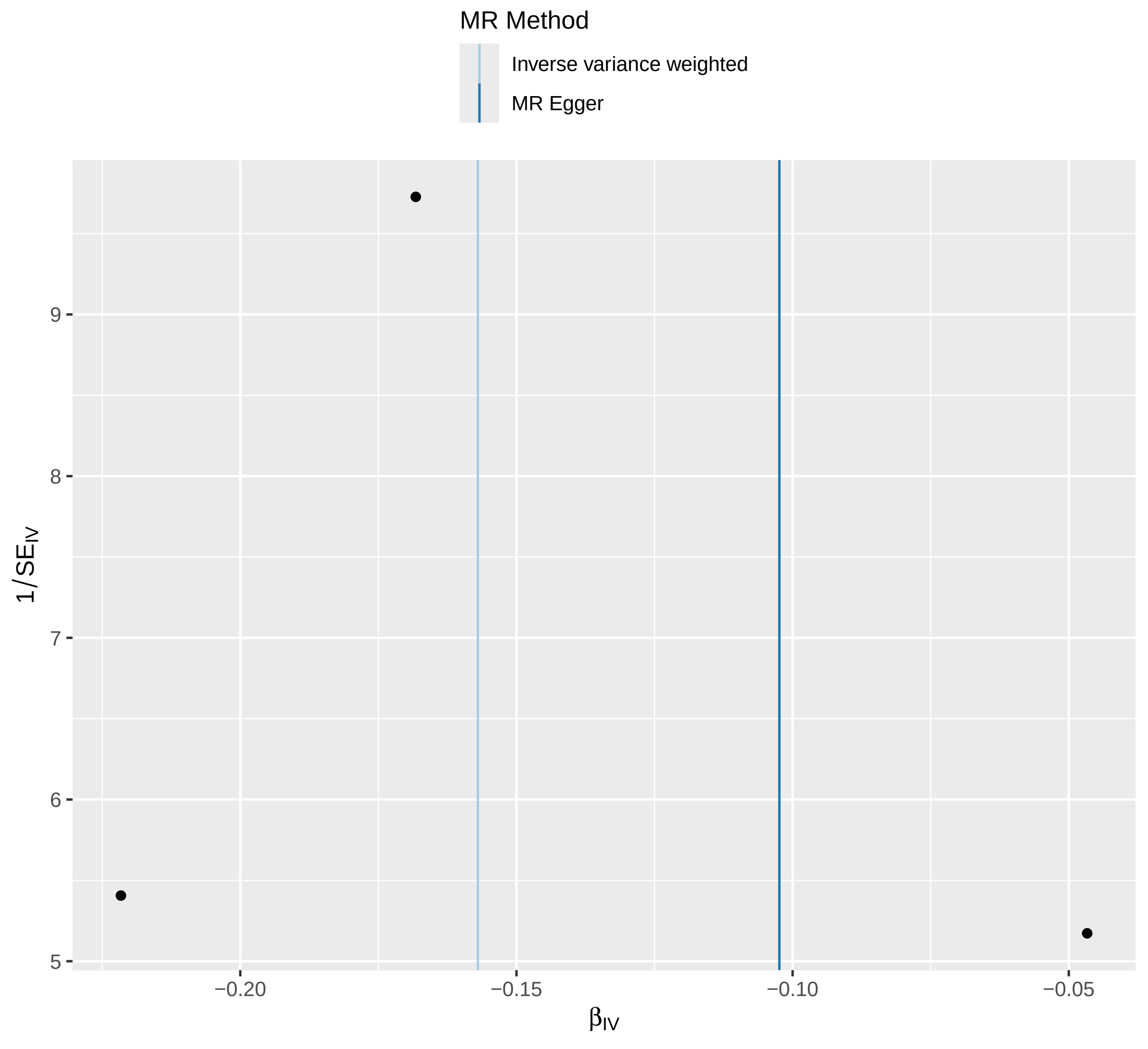
MR Method
Inverse variance weighted
MR Egger
9
8
7
6
5
1/SE$_{IV}$
−0.20
−0.15
−0.10
−0.05
β$_{IV}$

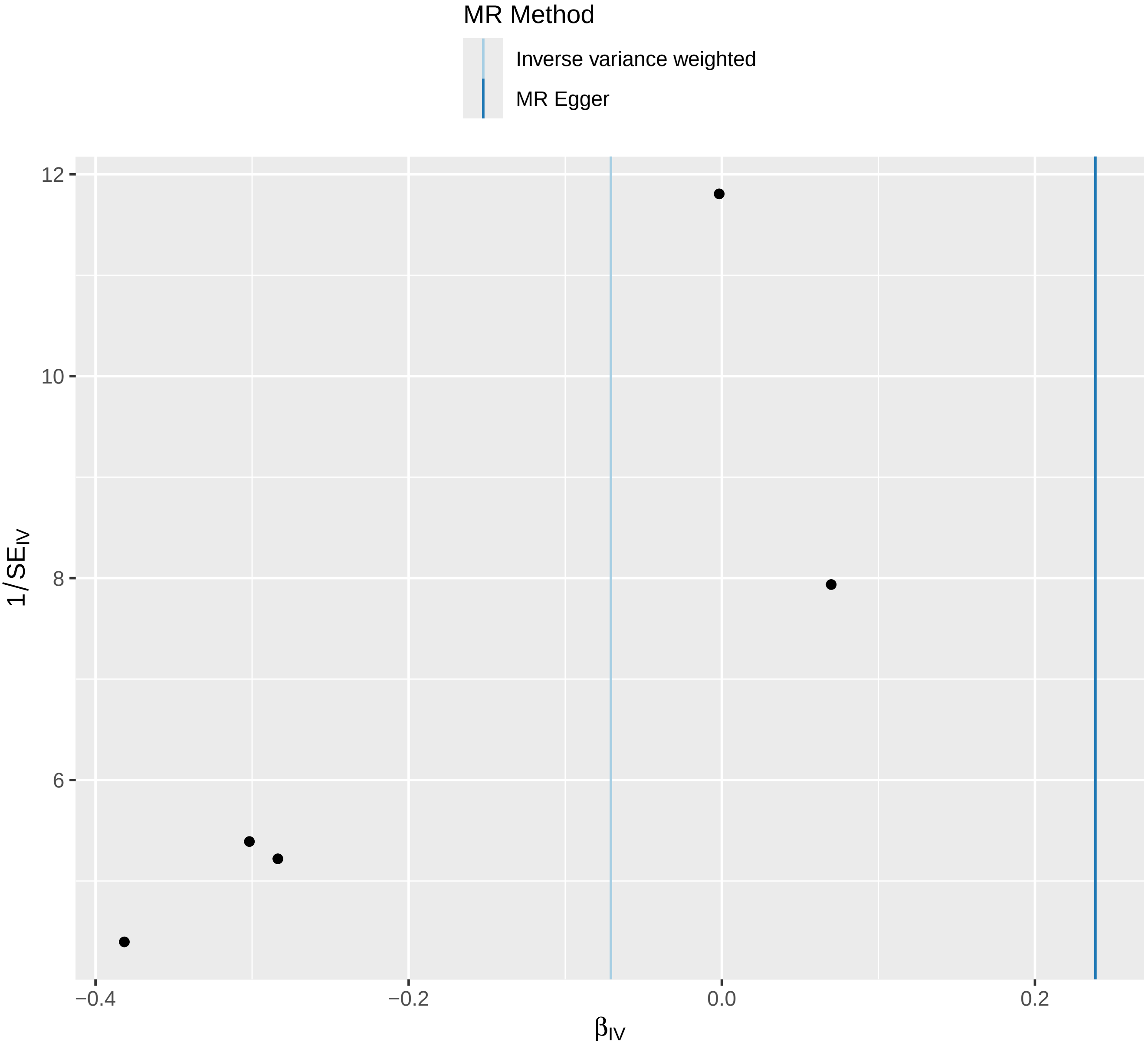
MR Method
Inverse variance weighted
MR Egger
12
10
8
6
1/SE_IV
−0.4
−0.2
0.0
0.2
β_IV

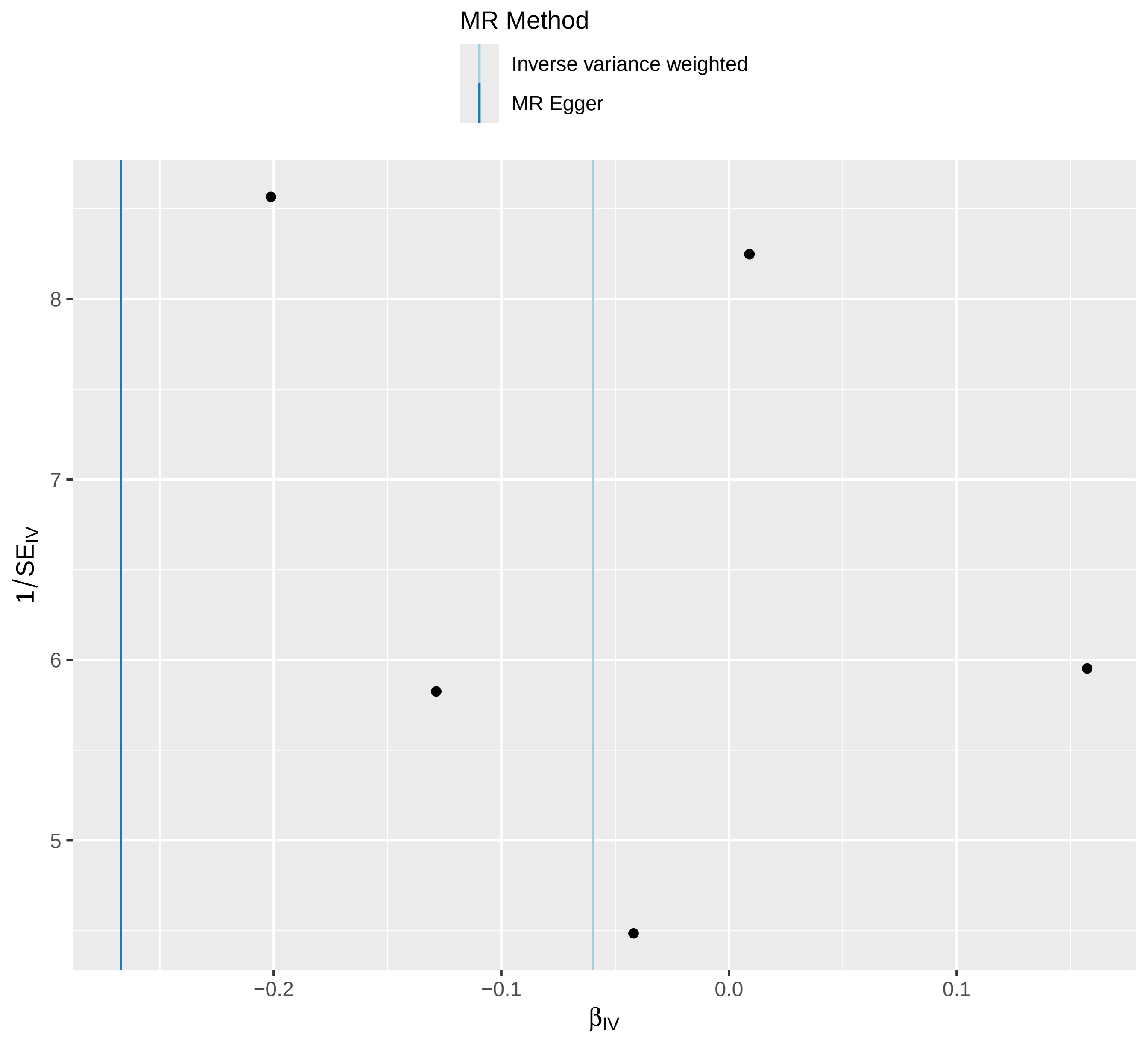
MR Method
Inverse variance weighted
MR Egger
8
7
6
5
−0.2
−0.1
0.0
0.1
$\beta_{IV}$
$1/SE_{IV}$

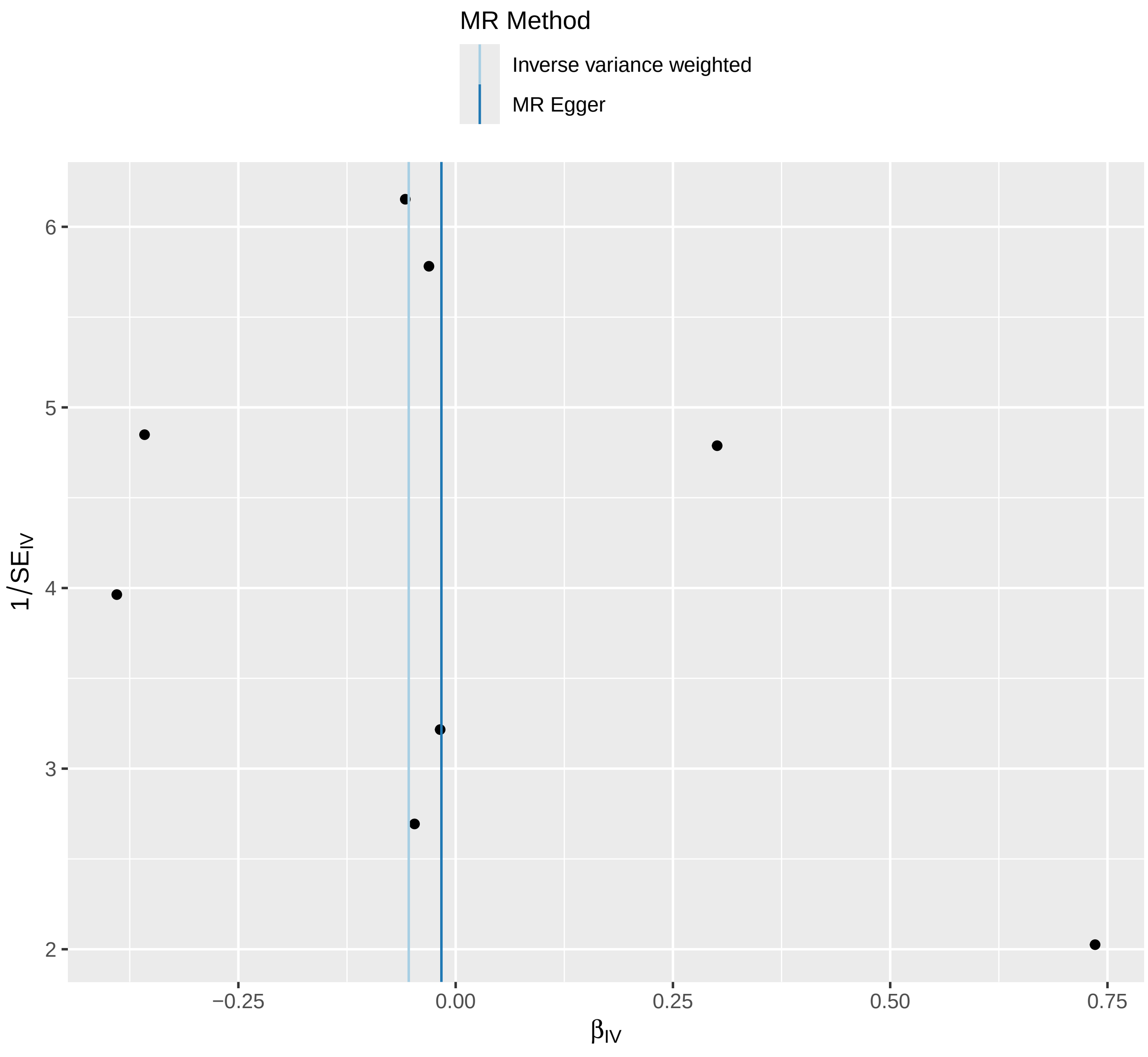

MR Method
Inverse variance weighted
MR Egger
1/SE$_{IV}$
6
5
4
3
2
−0.25
0.00
0.25
0.50
0.75
β$_{IV}$

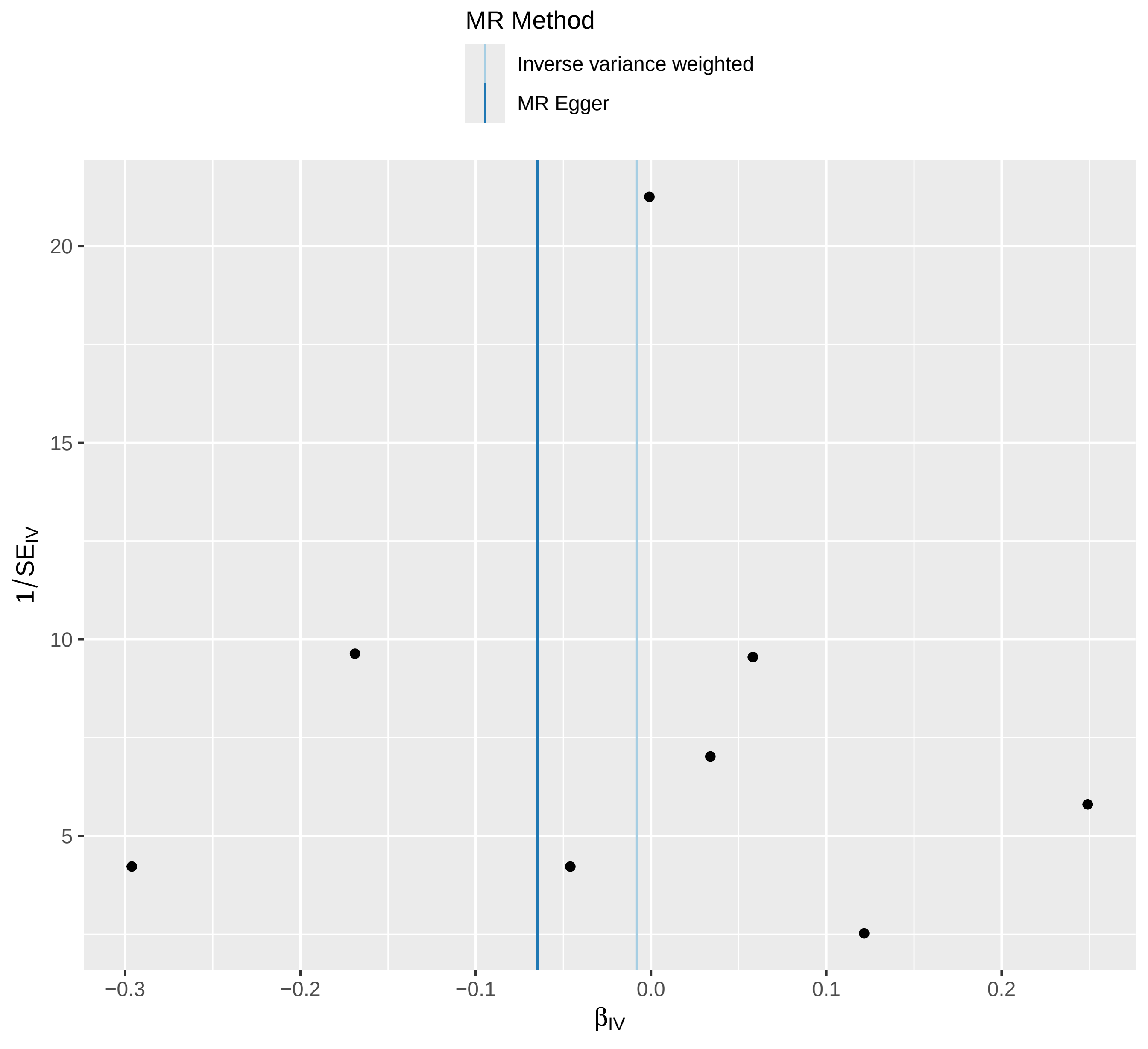

MR Method
Inverse variance weighted
MR Egger
1/SE_IV
20
15
10
5
−0.3
−0.2
−0.1
0.0
0.1
0.2
β_IV

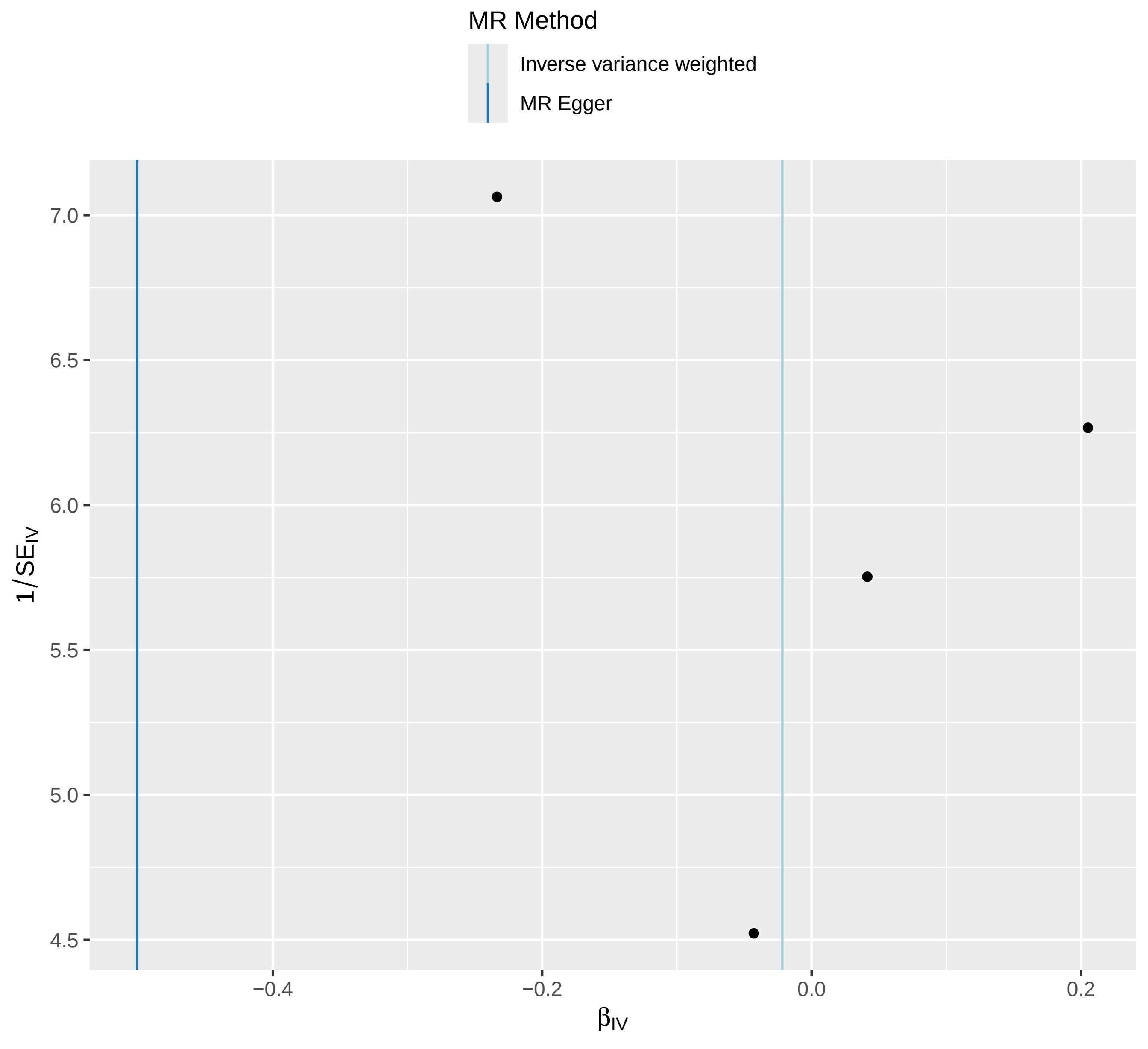
MR Method
Inverse variance weighted
MR Egger
7.0
6.5
6.0
5.5
5.0
4.5
1/SE_IV
−0.4
−0.2
0.0
0.2
β_IV

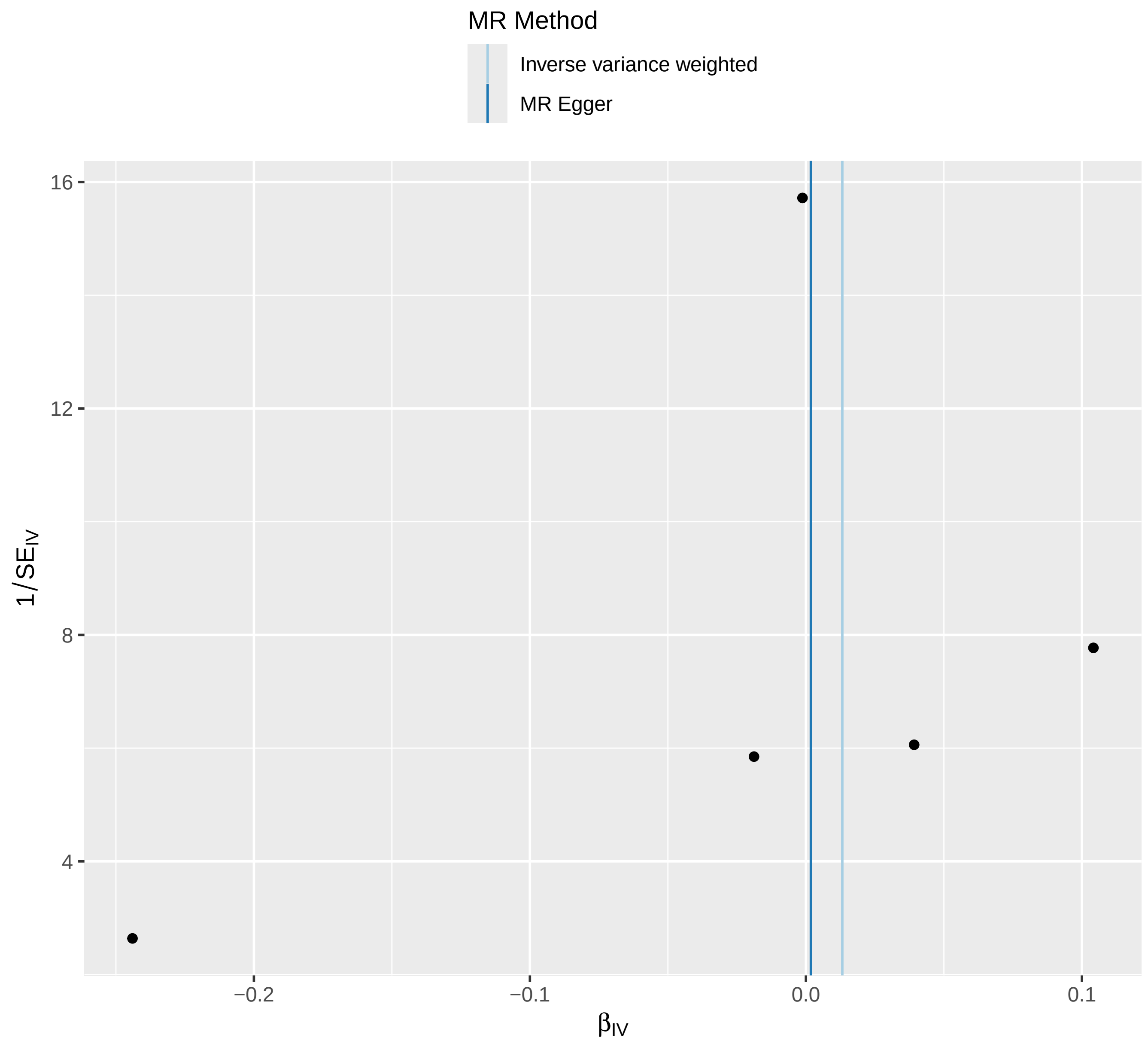

MR Method
Inverse variance weighted
MR Egger
16
12
8
4
$1/SE_{IV}$
−0.2
−0.1
0.0
0.1
$\beta_{IV}$

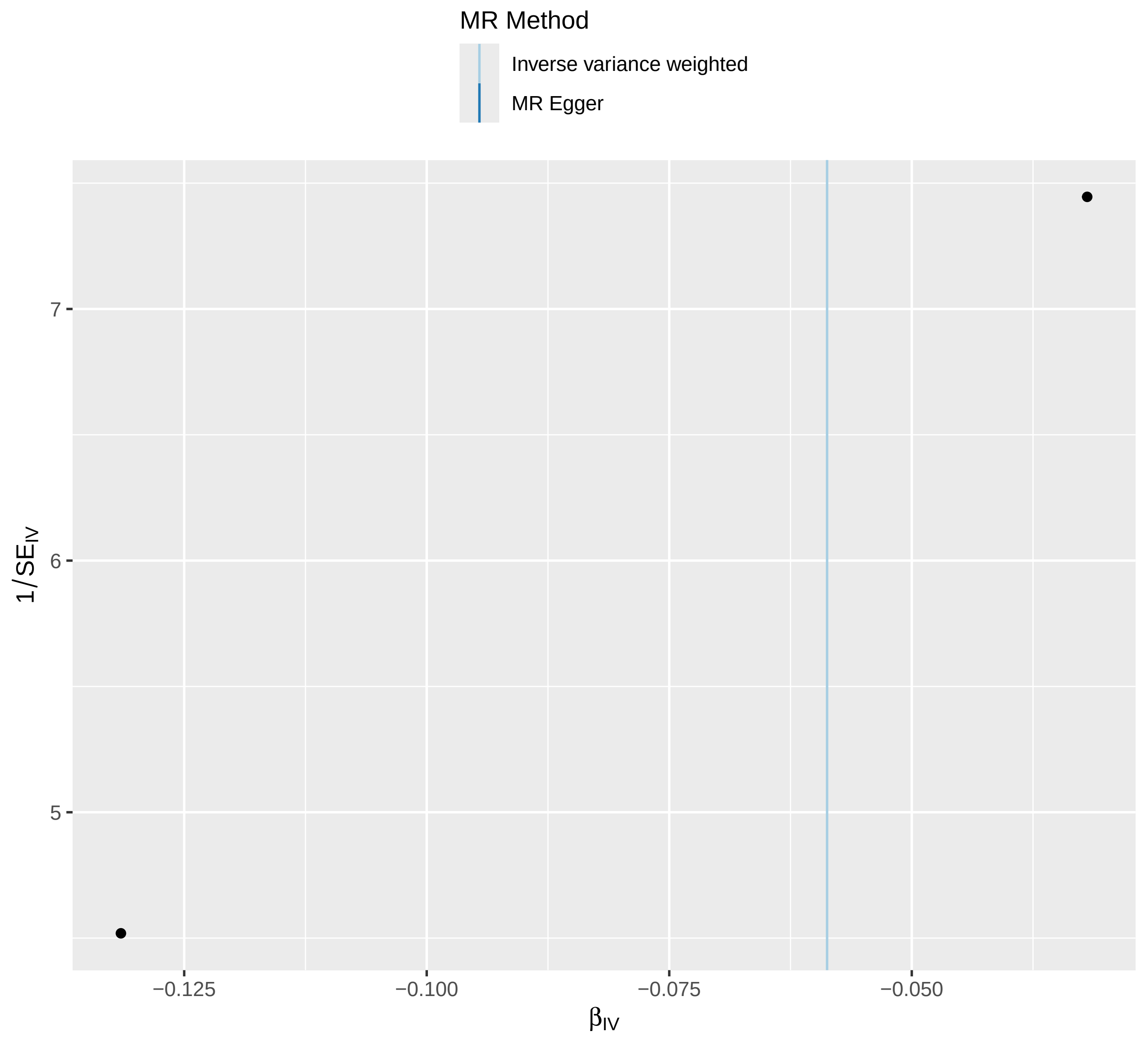
MR Method
Inverse variance weighted
MR Egger
7
6
5
1/SE_IV
−0.125
−0.100
−0.075
−0.050
β_IV

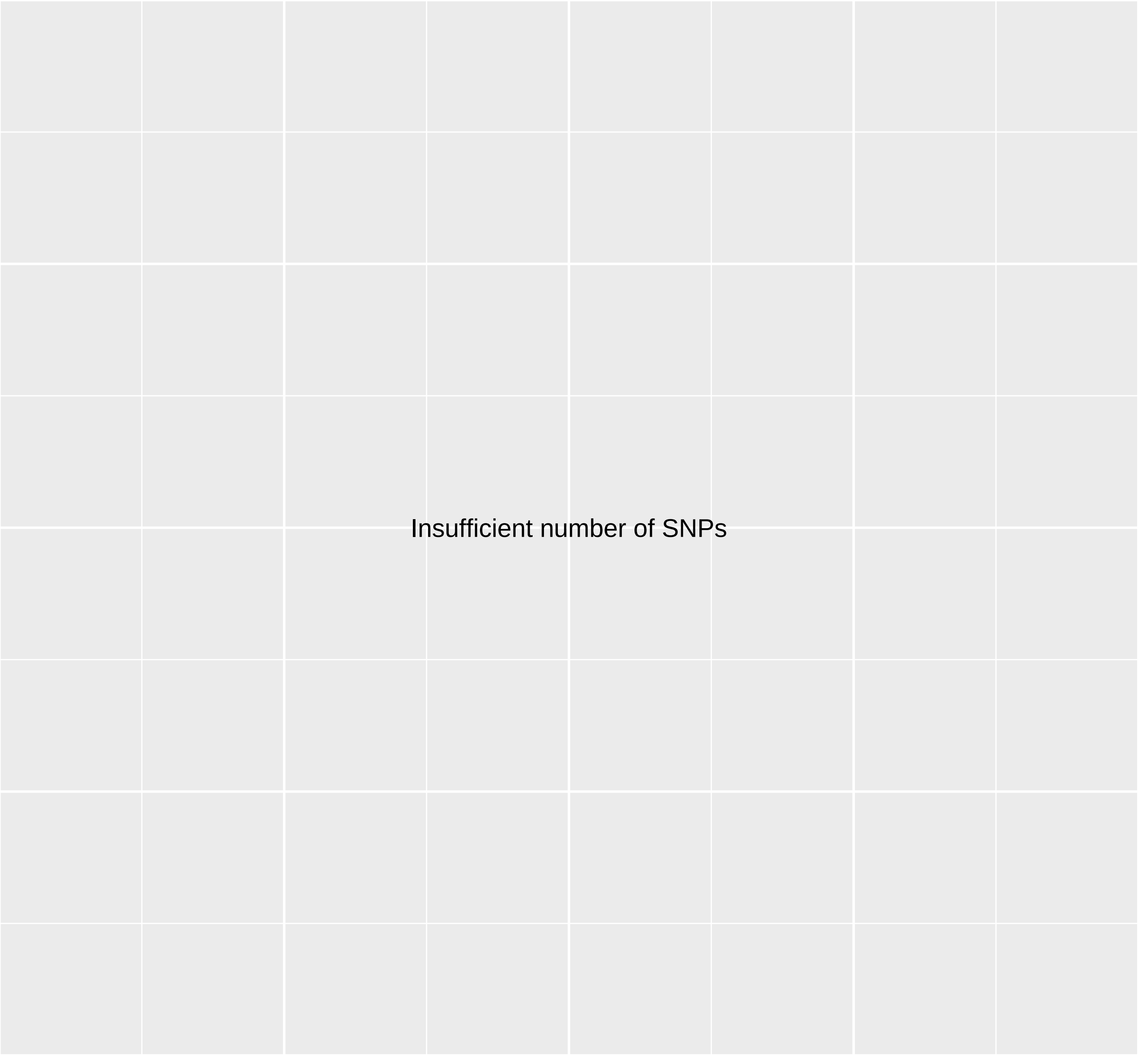
Insufficient number of SNPs

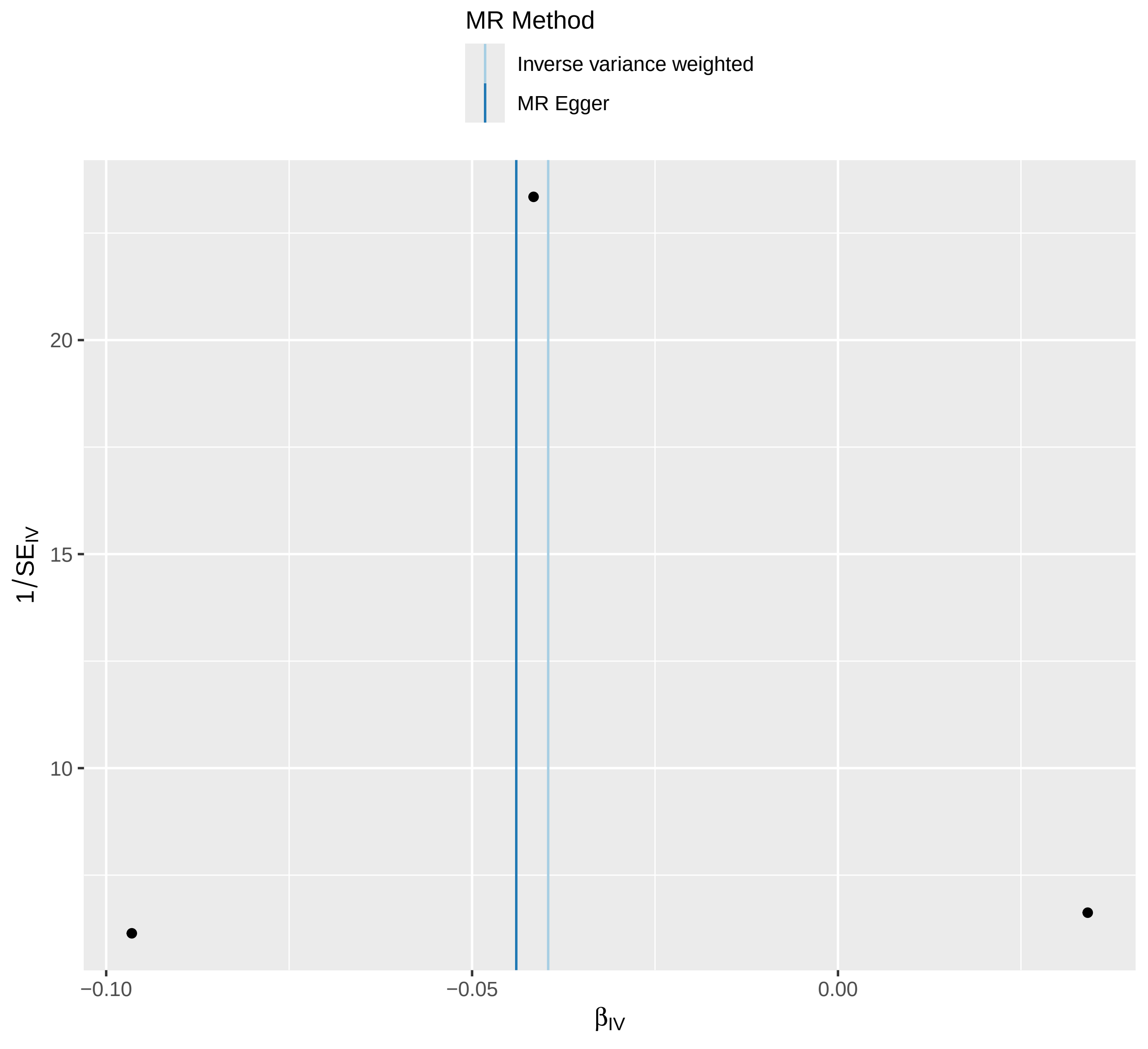
MR Method
Inverse variance weighted
MR Egger
20
15
10
1/SE$_{IV}$
−0.10
−0.05
0.00
β$_{IV}$

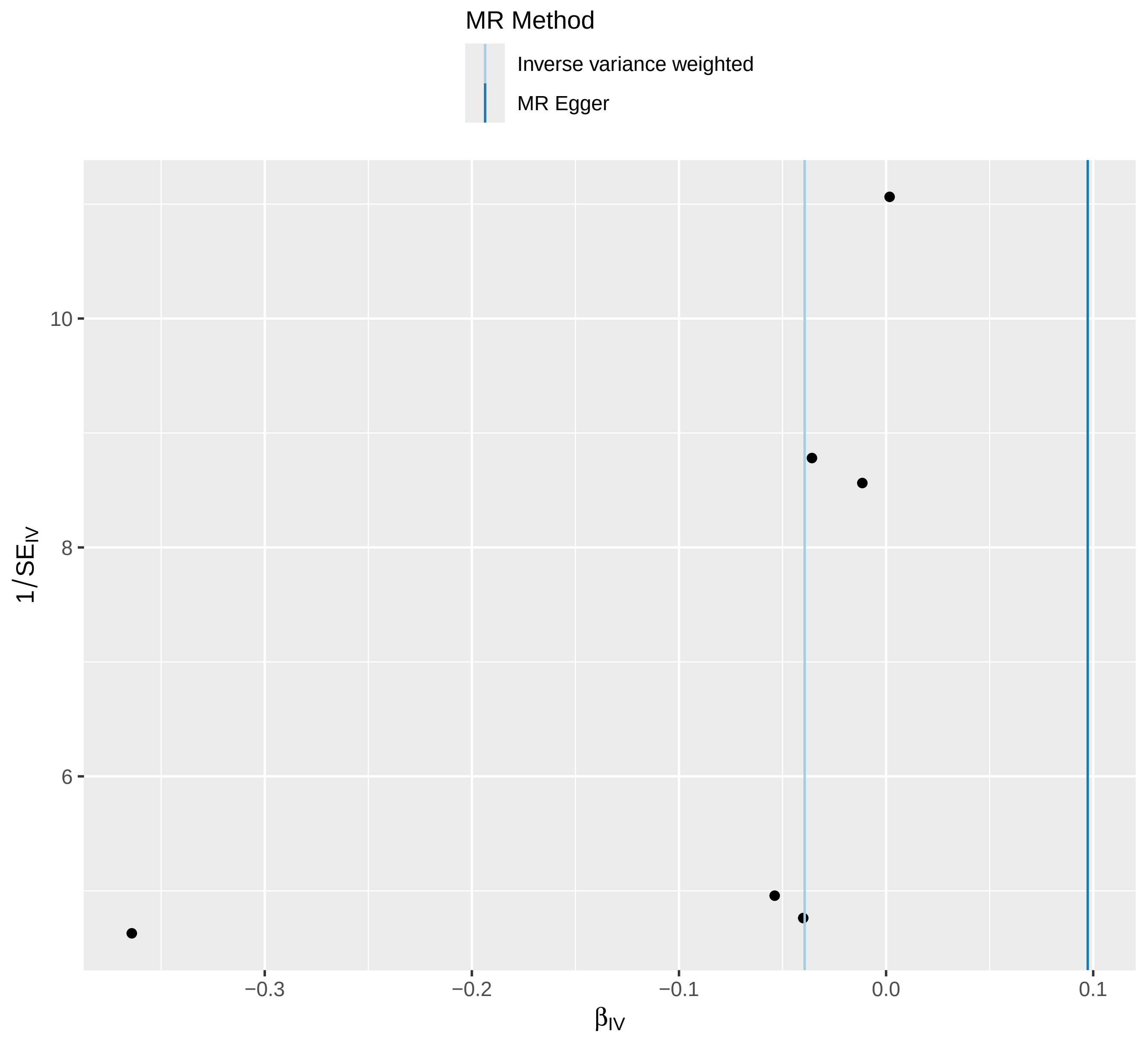
MR Method
Inverse variance weighted
MR Egger
10
8
6
1/SE$_{IV}$
−0.3
−0.2
−0.1
0.0
0.1
β$_{IV}$

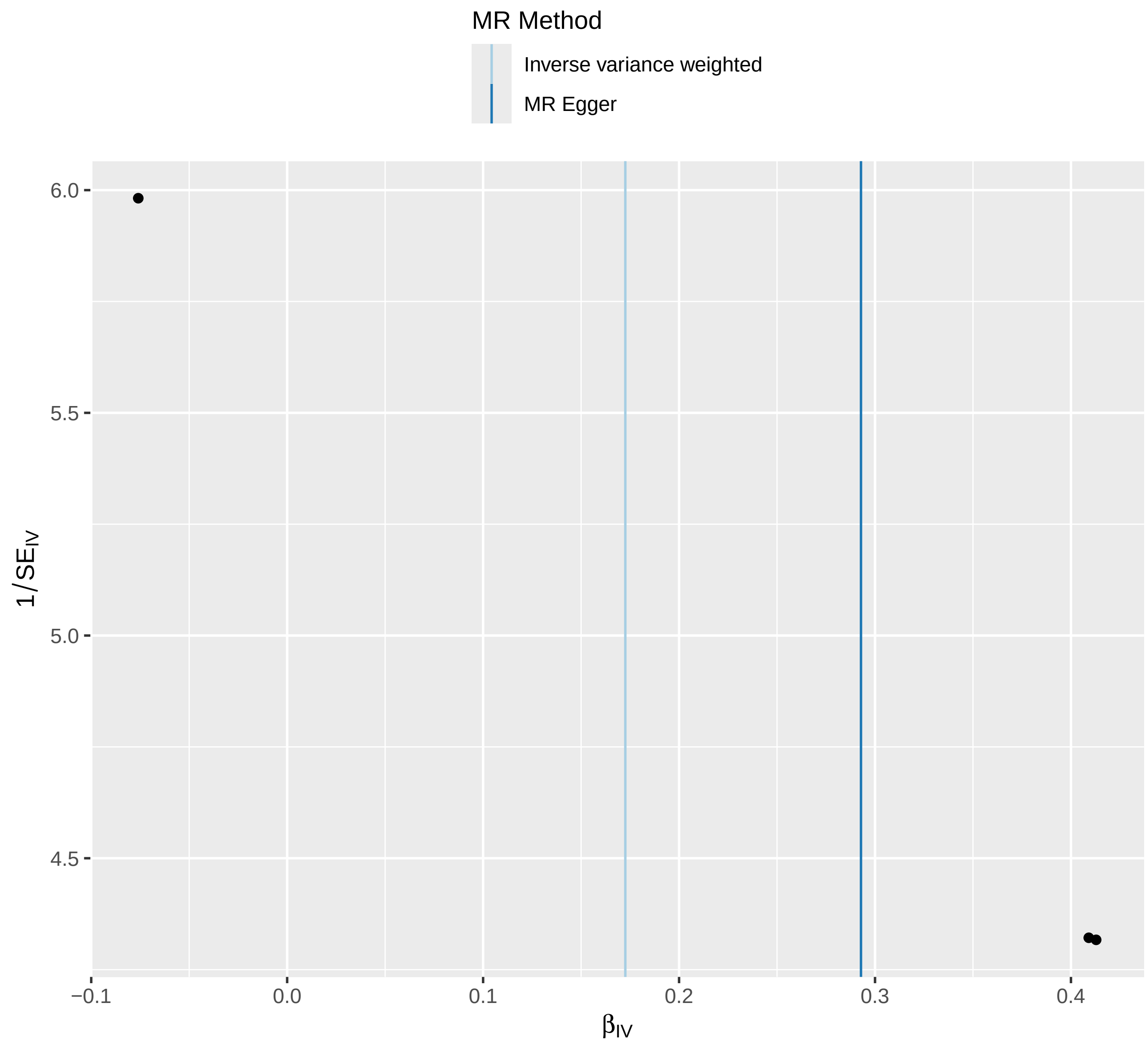

MR Method
Inverse variance weighted
MR Egger
6.0
5.5
5.0
4.5
$1/SE_{IV}$
−0.1
0.0
0.1
0.2
0.3
0.4
$\beta_{IV}$

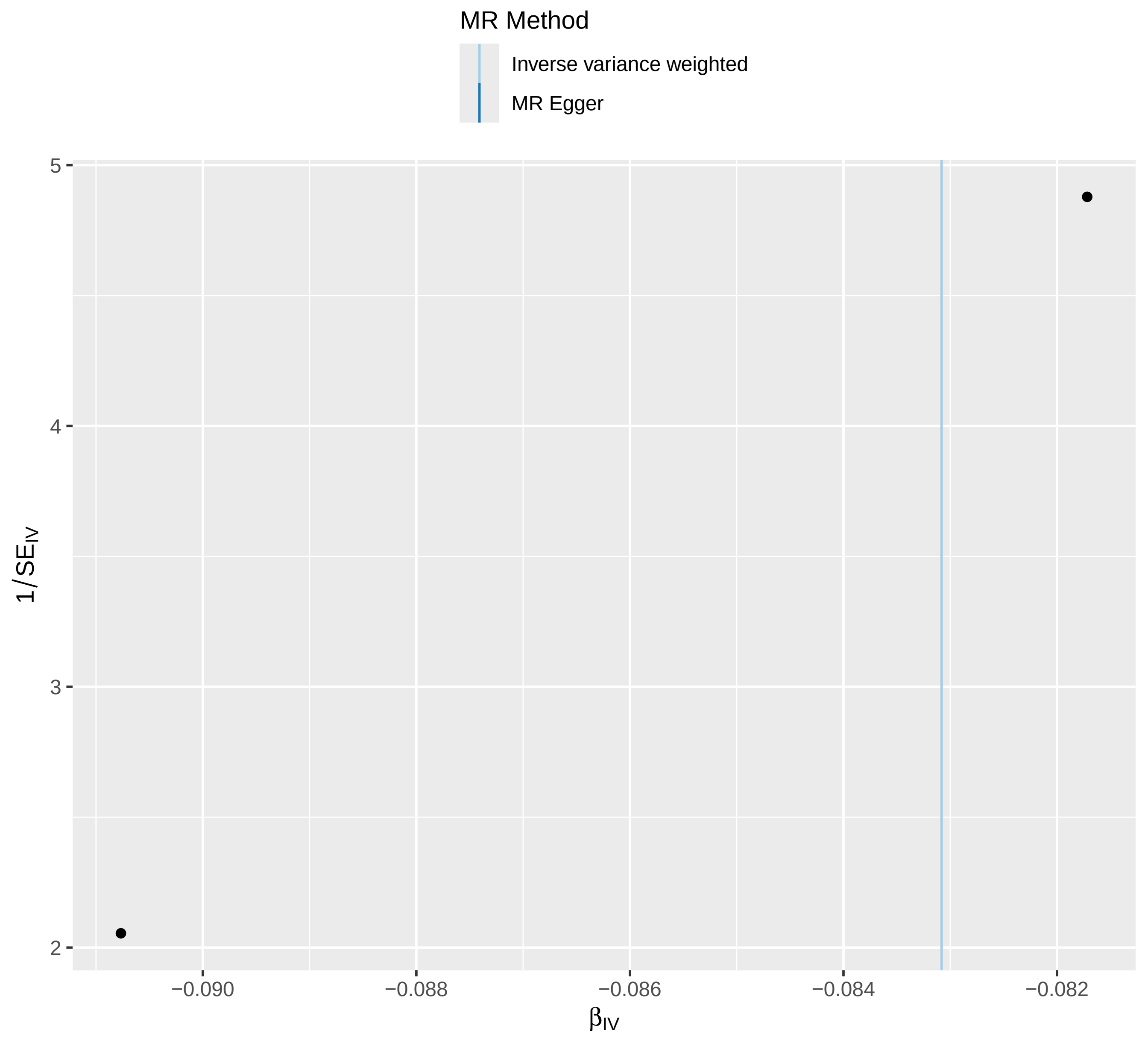

MR Method
Inverse variance weighted
MR Egger
5
4
3
2
1/SE$_{IV}$
−0.090
−0.088
−0.086
−0.084
−0.082
β$_{IV}$

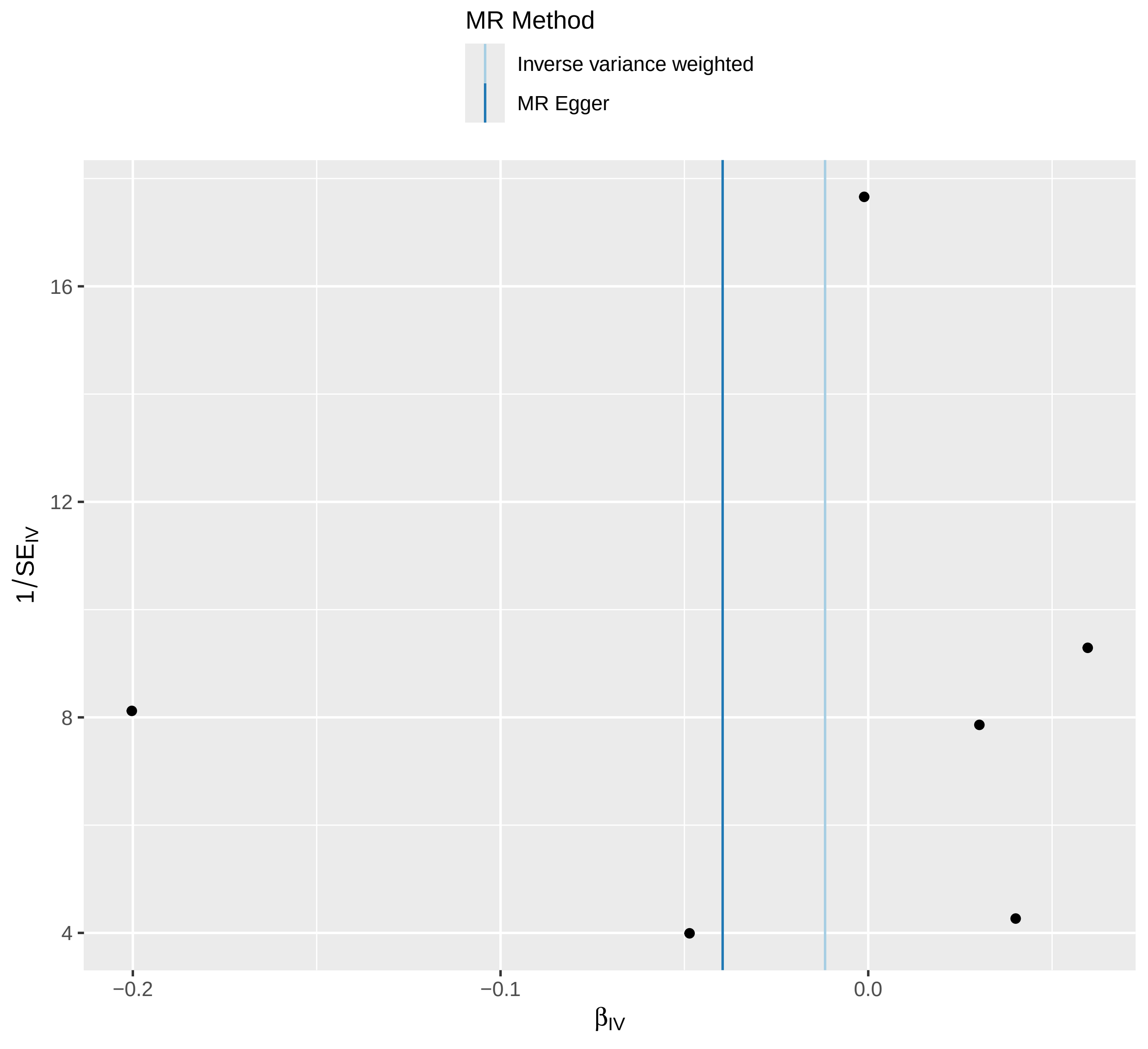

MR Method
Inverse variance weighted
MR Egger
16
12
8
4
1/SE$_{IV}$
−0.2
−0.1
0.0
β$_{IV}$

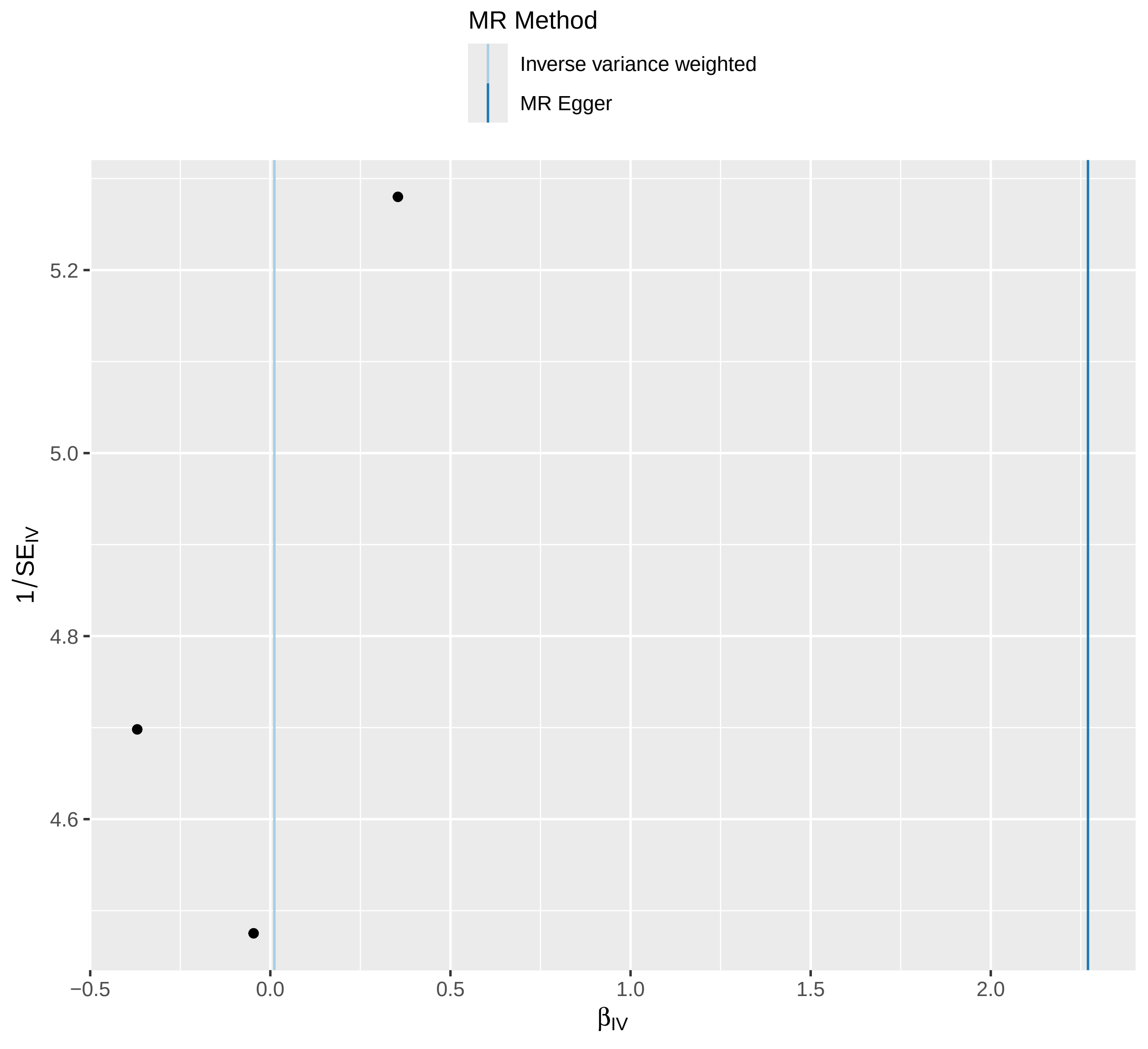
MR Method
Inverse variance weighted
MR Egger
5.2
5.0
4.8
4.6
$1/SE_{IV}$
−0.5
0.0
0.5
1.0
1.5
2.0
$\beta_{IV}$

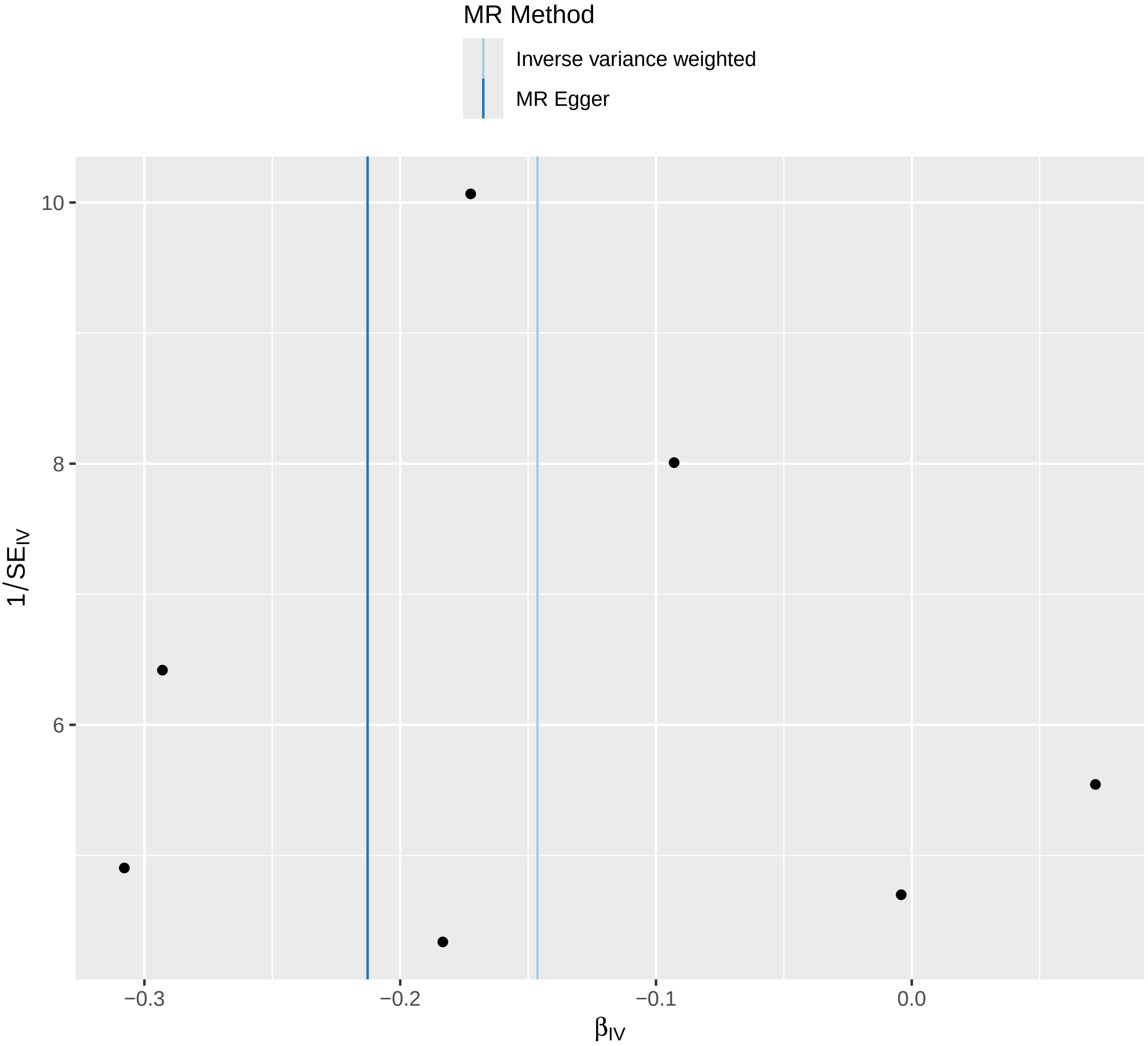
MR Method
Inverse variance weighted
MR Egger
10
8
6
$1/SE_{IV}$
−0.3
−0.2
−0.1
0.0
$\beta_{IV}$

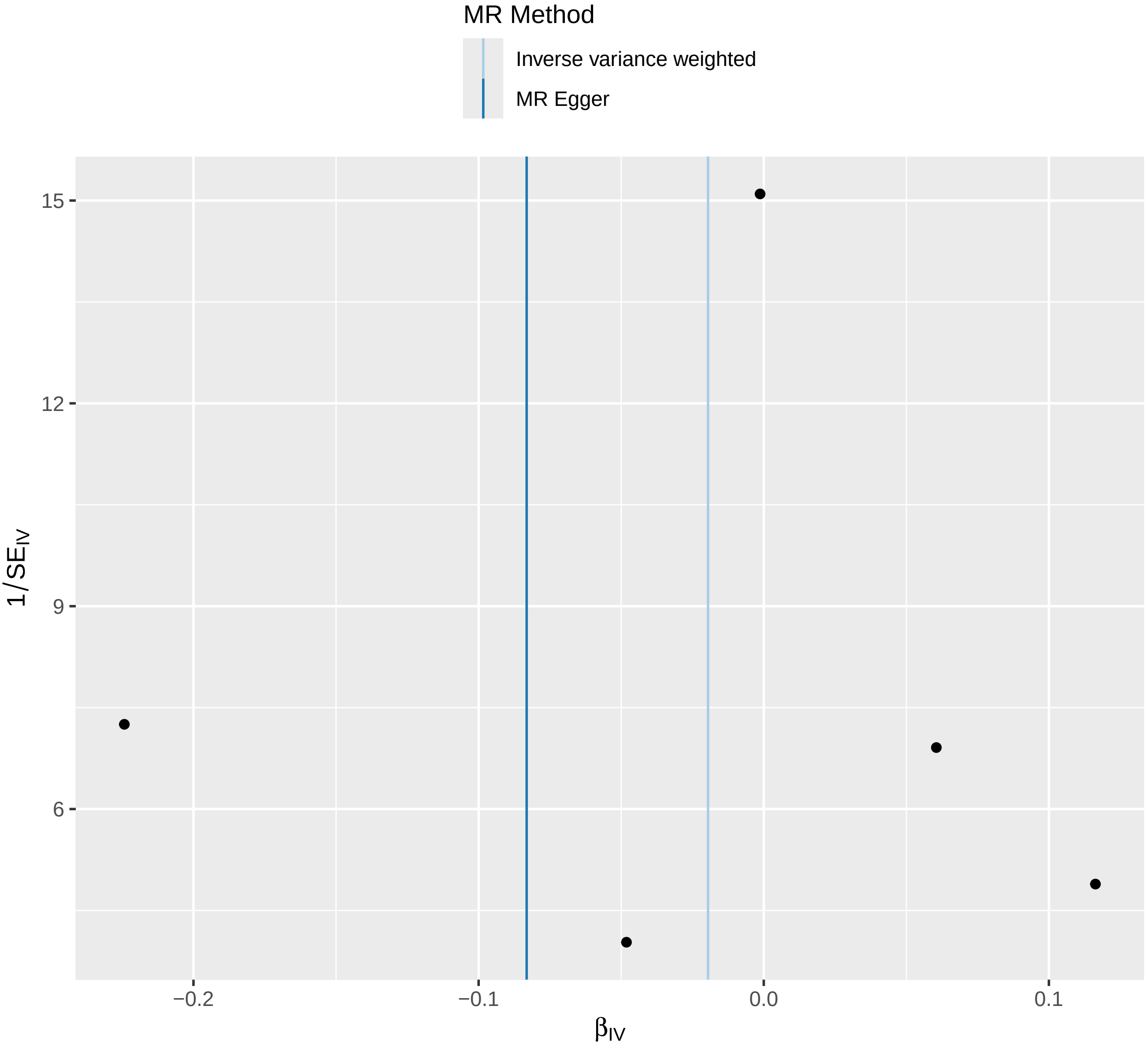

MR Method
Inverse variance weighted
MR Egger
15
12
9
6
1/SE_IV
−0.2
−0.1
0.0
0.1
β_IV

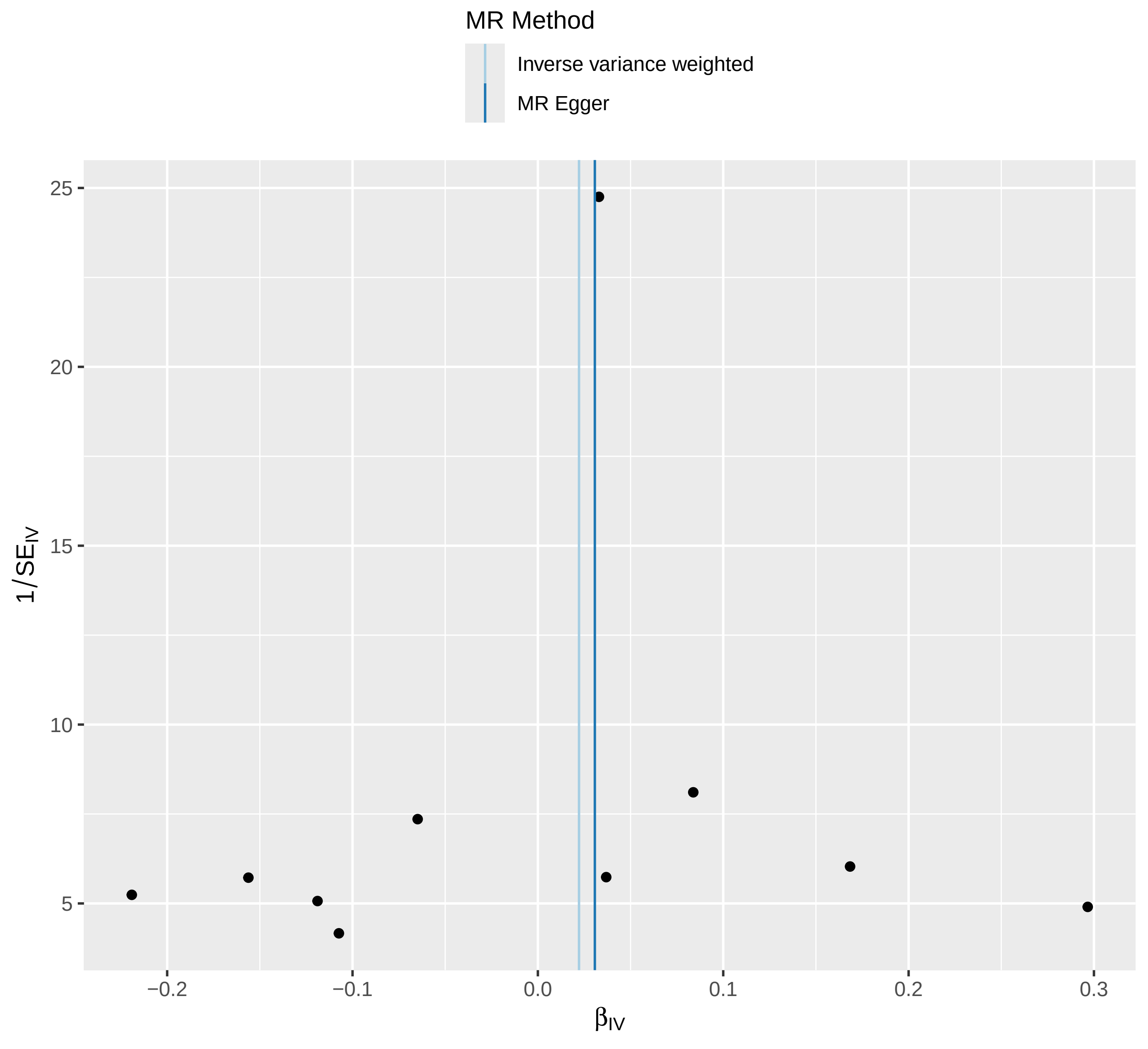

MR Method
Inverse variance weighted
MR Egger
25
20
15
10
5
$1/SE_{IV}$
−0.2
−0.1
0.0
0.1
0.2
0.3
$\beta_{IV}$

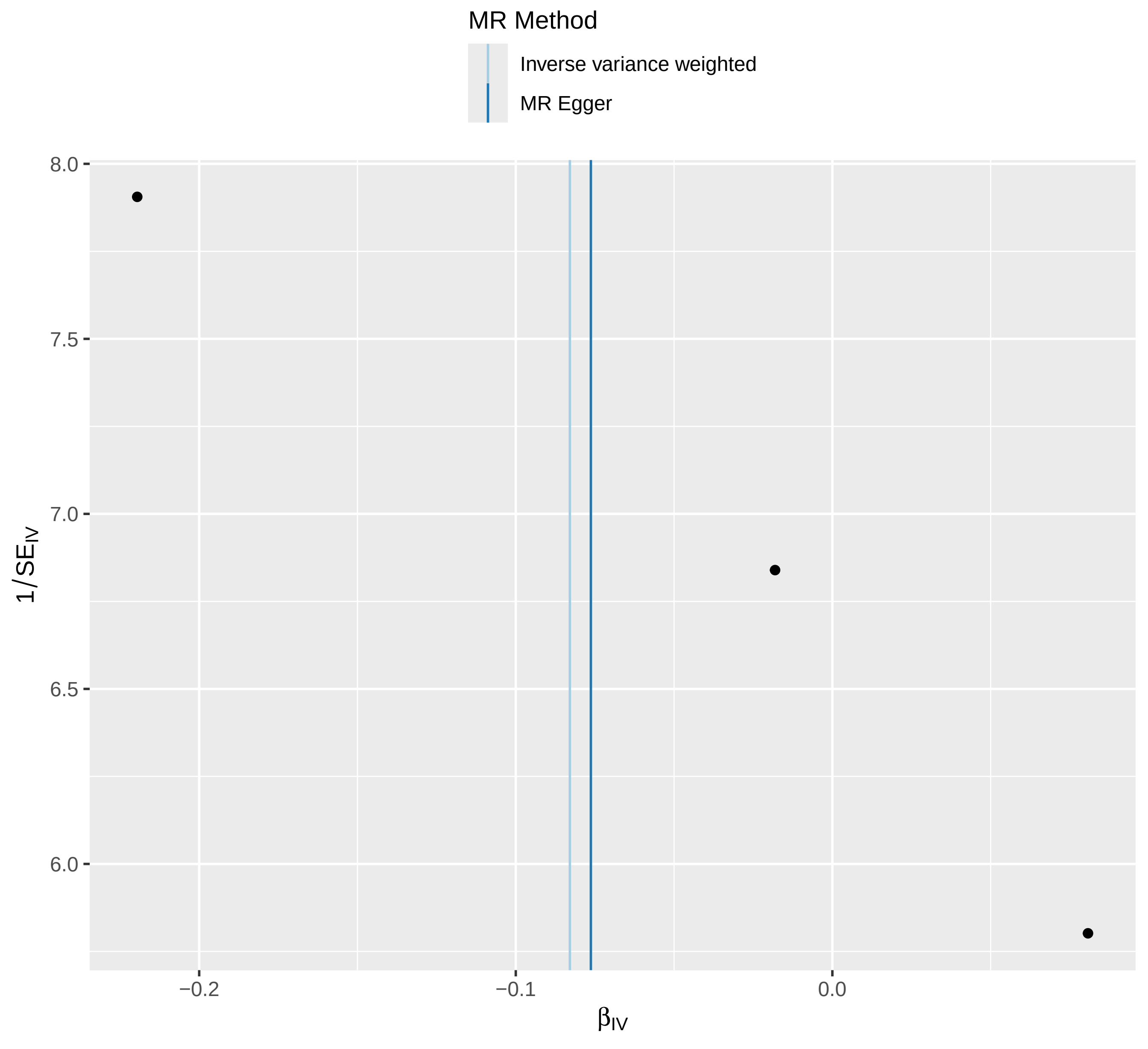
MR Method
Inverse variance weighted
MR Egger
8.0
7.5
7.0
6.5
6.0
1/SE_IV
−0.2
−0.1
0.0
β_IV

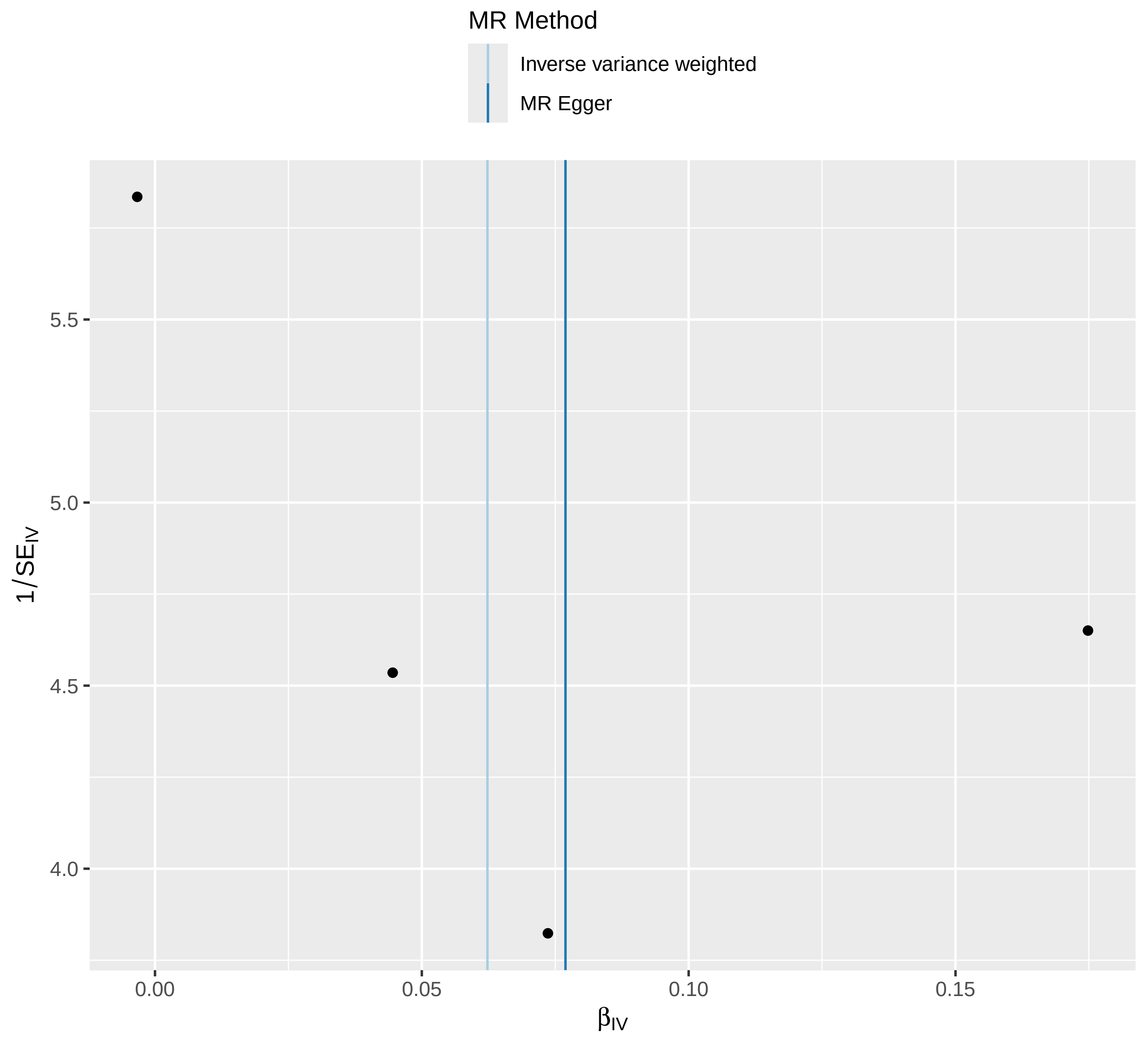

MR Method
Inverse variance weighted
MR Egger
5.5
5.0
4.5
4.0
1/SE_IV
0.00
0.05
0.10
0.15
β_IV

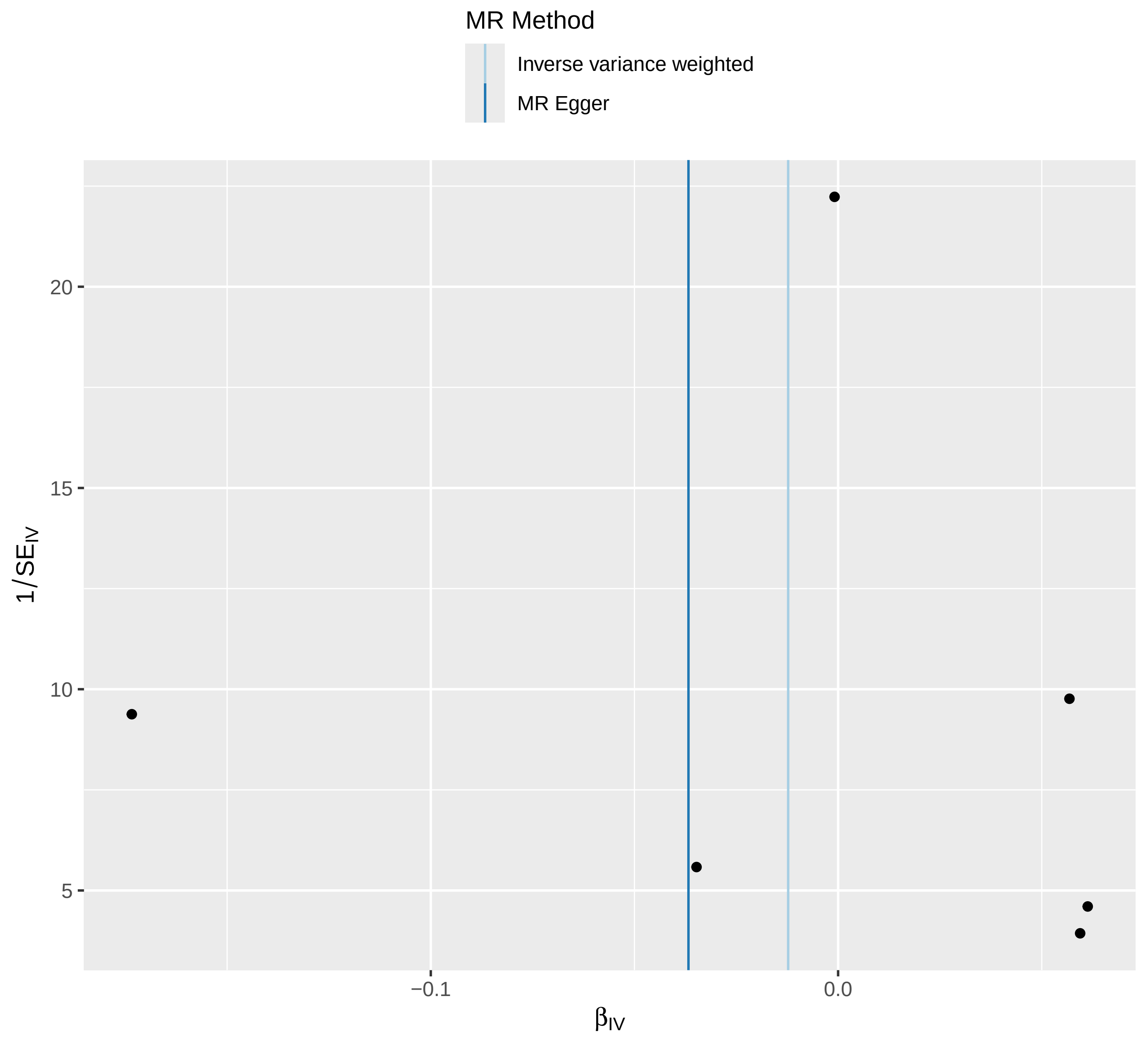
MR Method
Inverse variance weighted
MR Egger
20
15
10
5
1/SE_IV
−0.1
0.0
β_IV

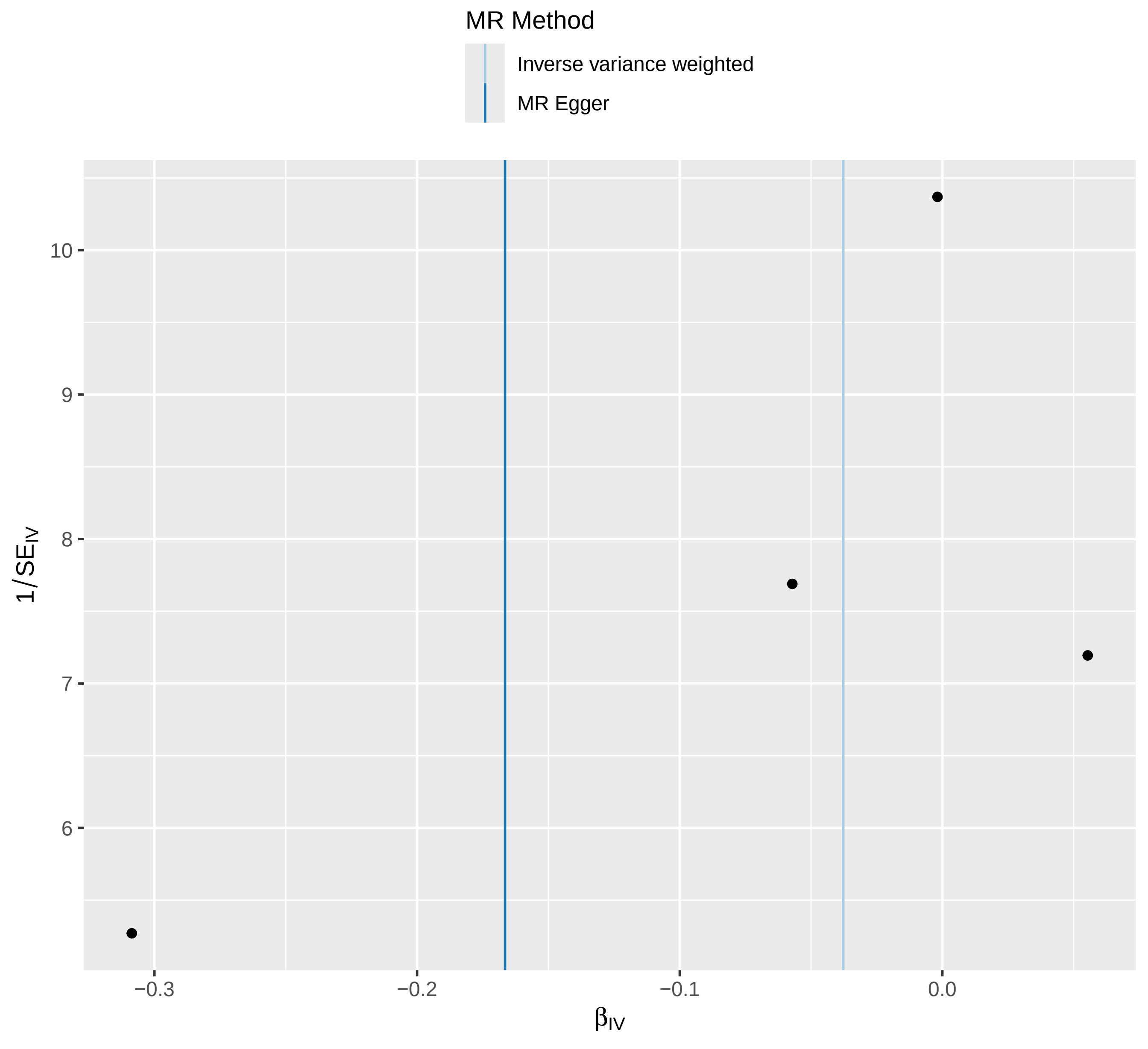
MR Method
Inverse variance weighted
MR Egger
10
9
8
7
6
1/SE_IV
−0.3
−0.2
−0.1
0.0
β_IV

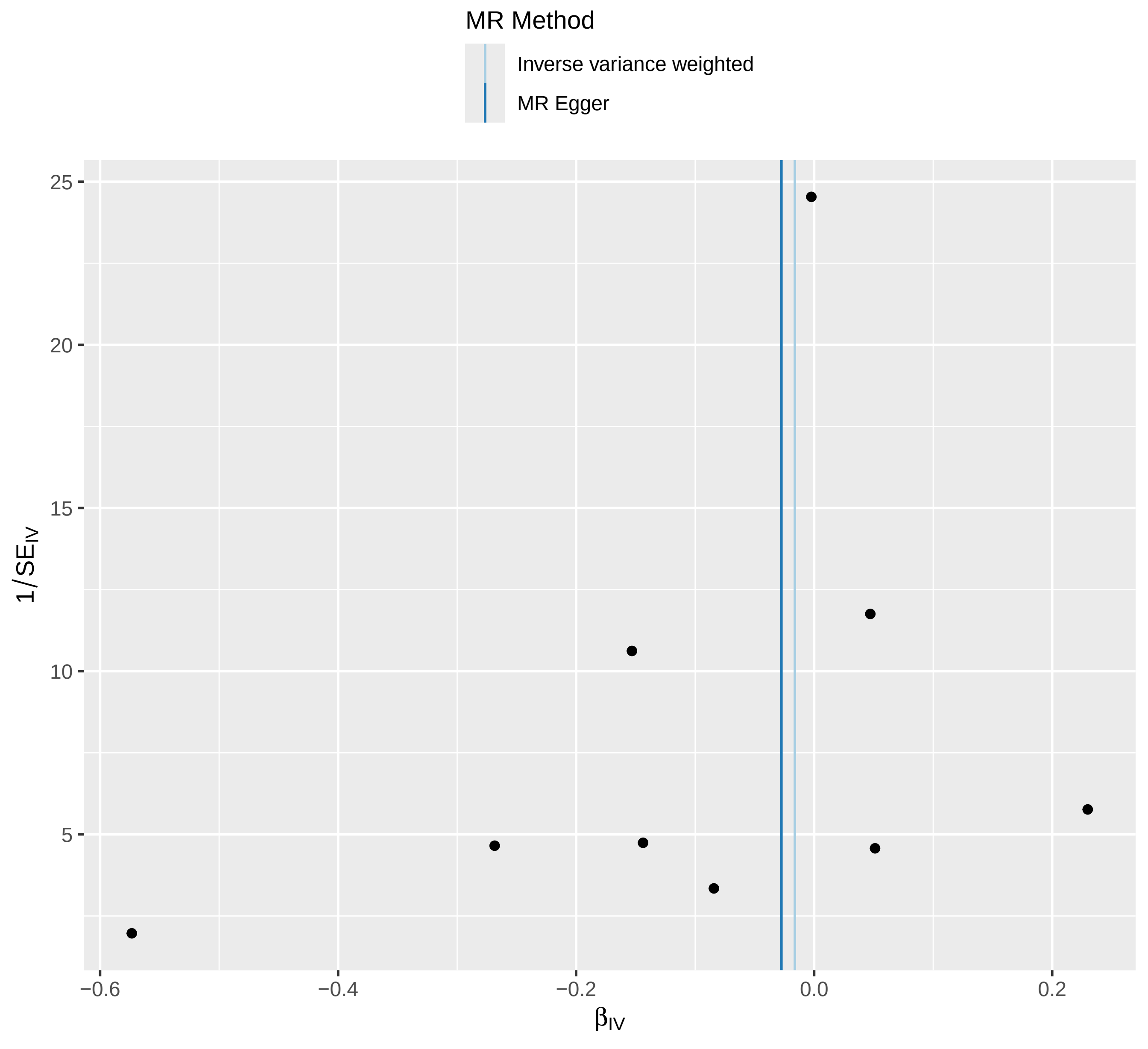
MR Method
Inverse variance weighted
MR Egger
25
20
15
10
5
$1/SE_{IV}$
−0.6
−0.4
−0.2
0.0
0.2
$\beta_{IV}$

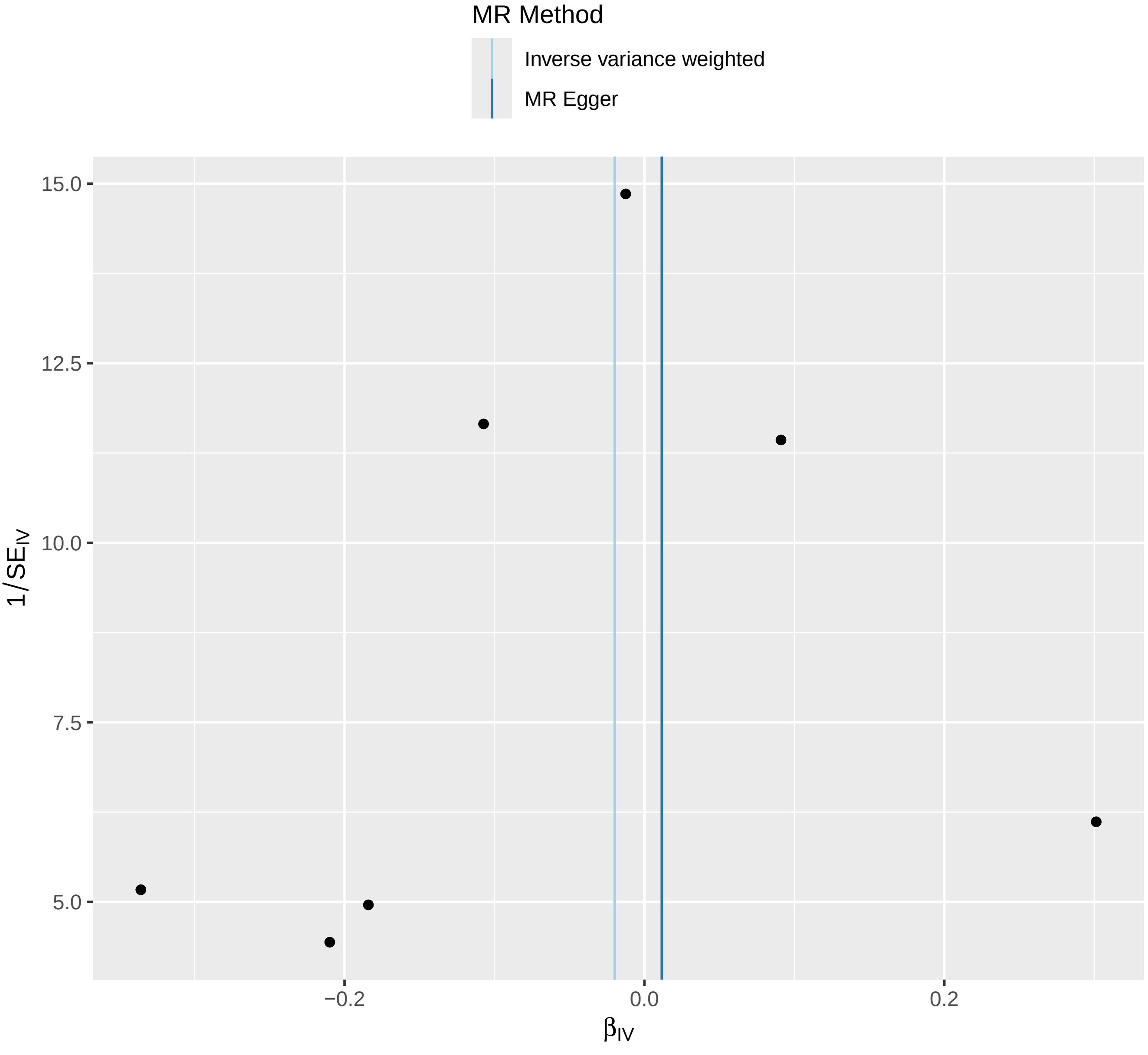
MR Method
Inverse variance weighted
MR Egger
15.0
12.5
10.0
7.5
5.0
$1/SE_{IV}$
−0.2
0.0
0.2
$\beta_{IV}$

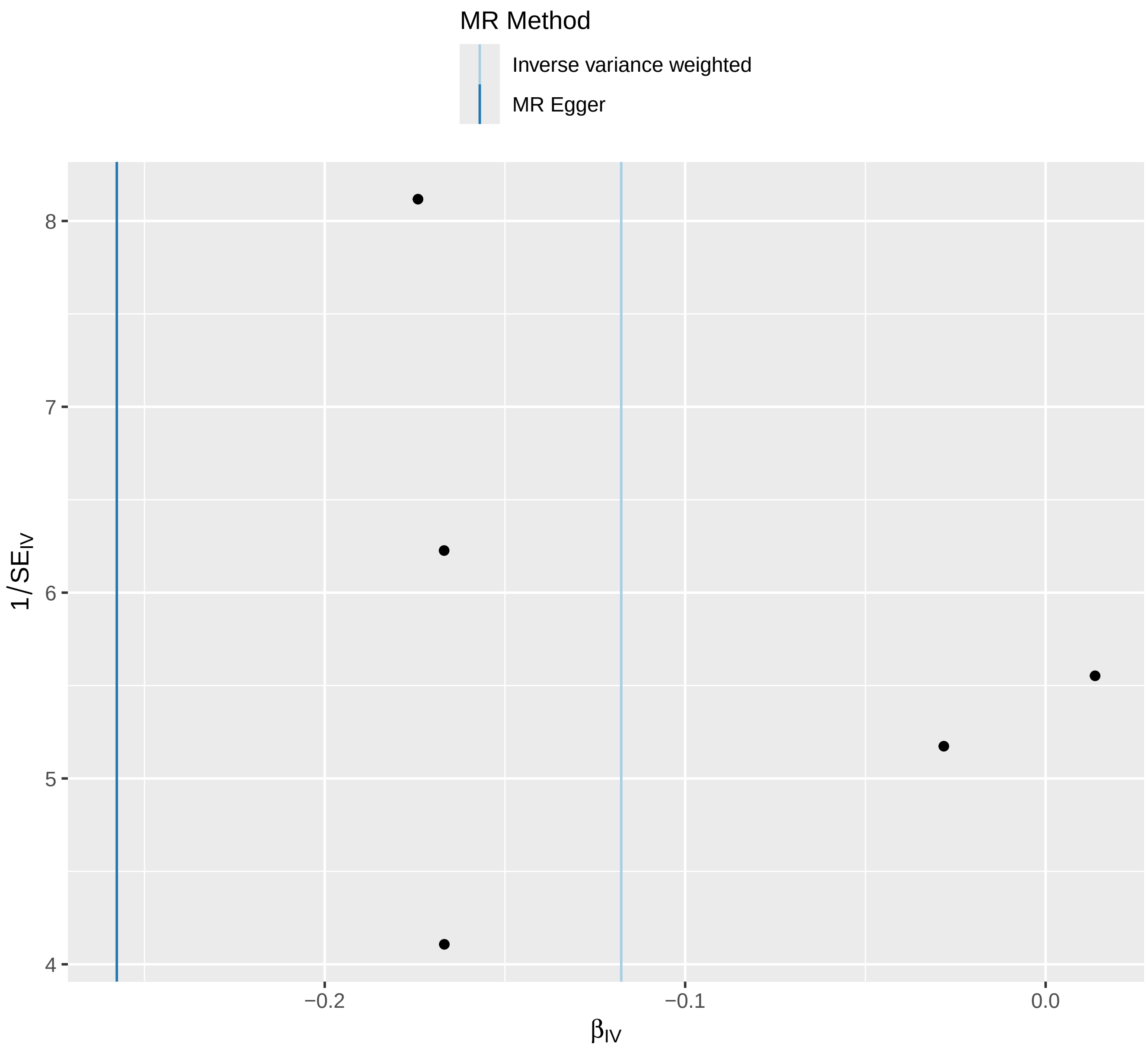

MR Method
Inverse variance weighted
MR Egger
8
7
6
5
4
$1/SE_{IV}$
−0.2
−0.1
0.0
$\beta_{IV}$

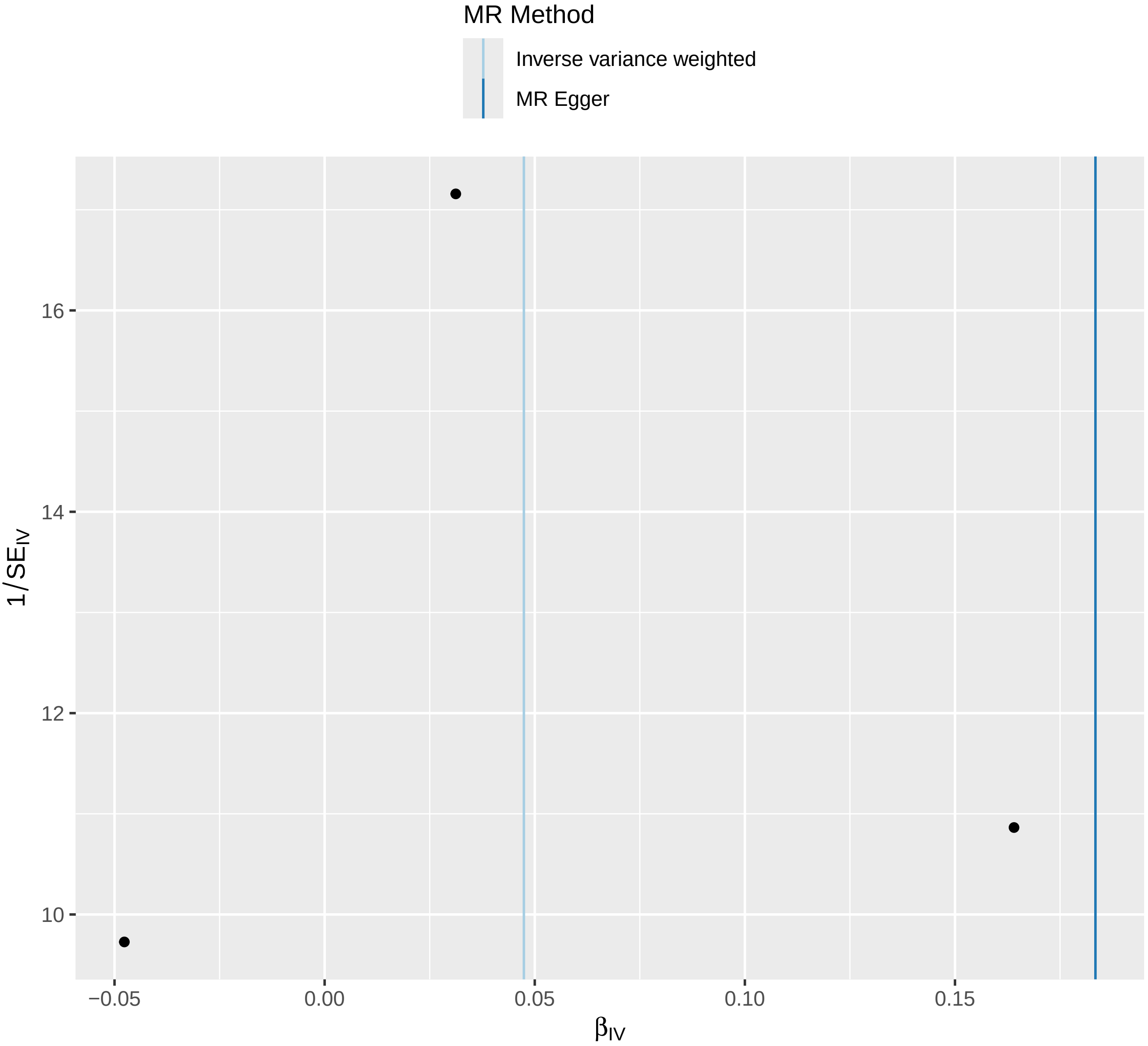

MR Method
Inverse variance weighted
MR Egger
16
14
12
10
1/SE_IV
−0.05
0.00
0.05
0.10
0.15
β_IV

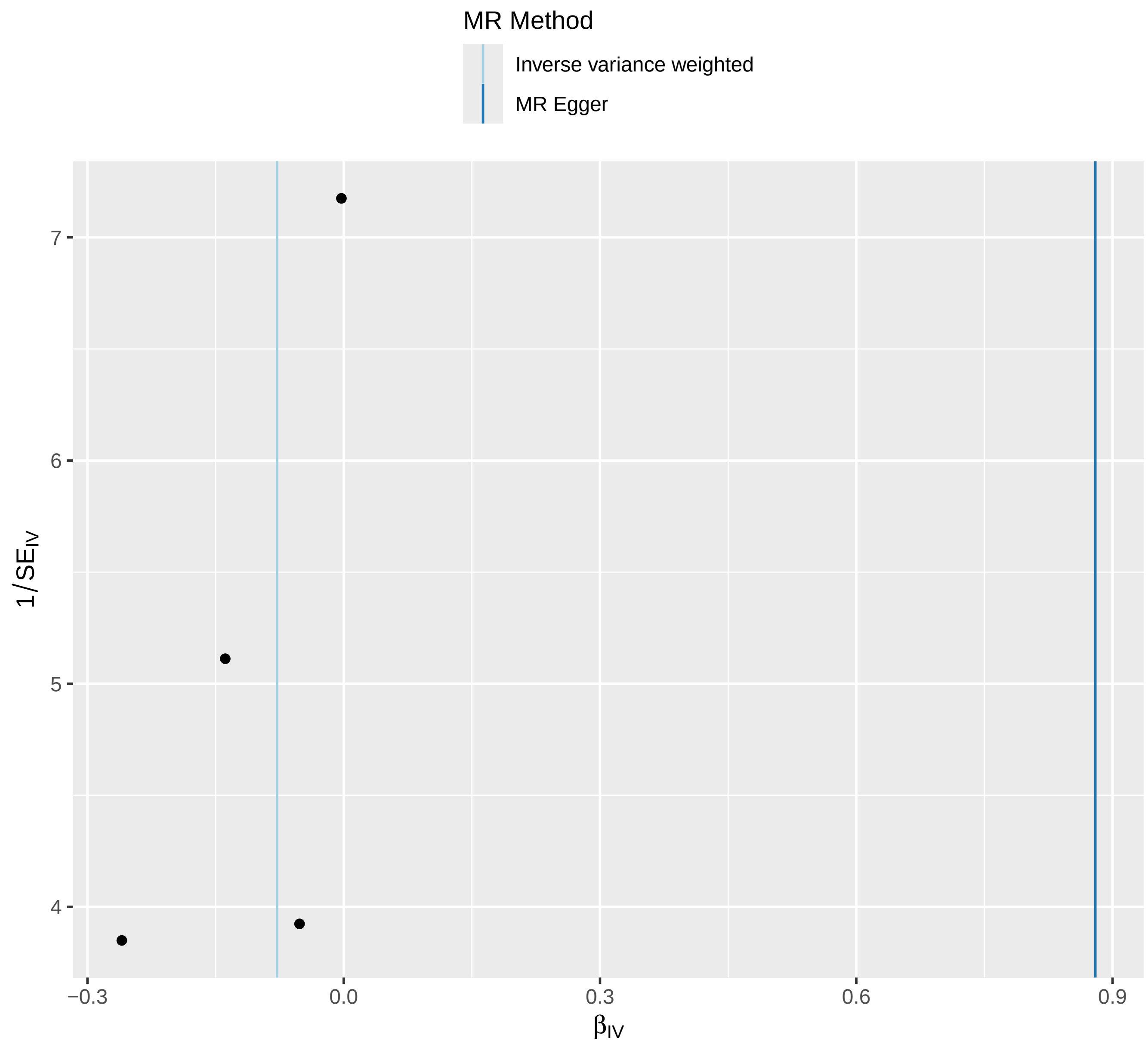

MR Method
Inverse variance weighted
MR Egger
7
6
5
4
$1/SE_{IV}$
−0.3
0.0
0.3
0.6
0.9
$\beta_{IV}$

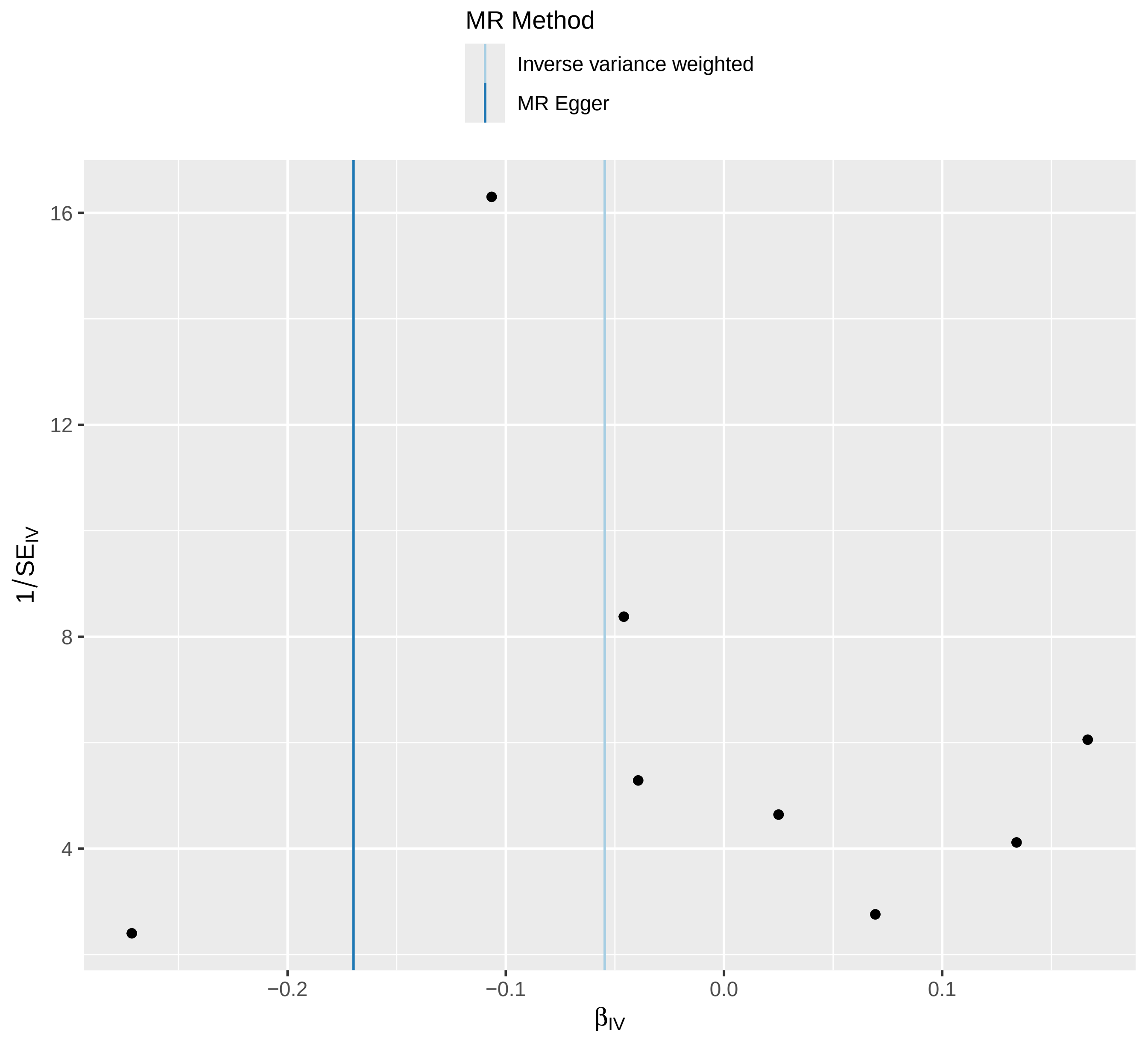
MR Method
Inverse variance weighted
MR Egger
16
12
8
4
1/SE_IV
−0.2
−0.1
0.0
0.1
β_IV

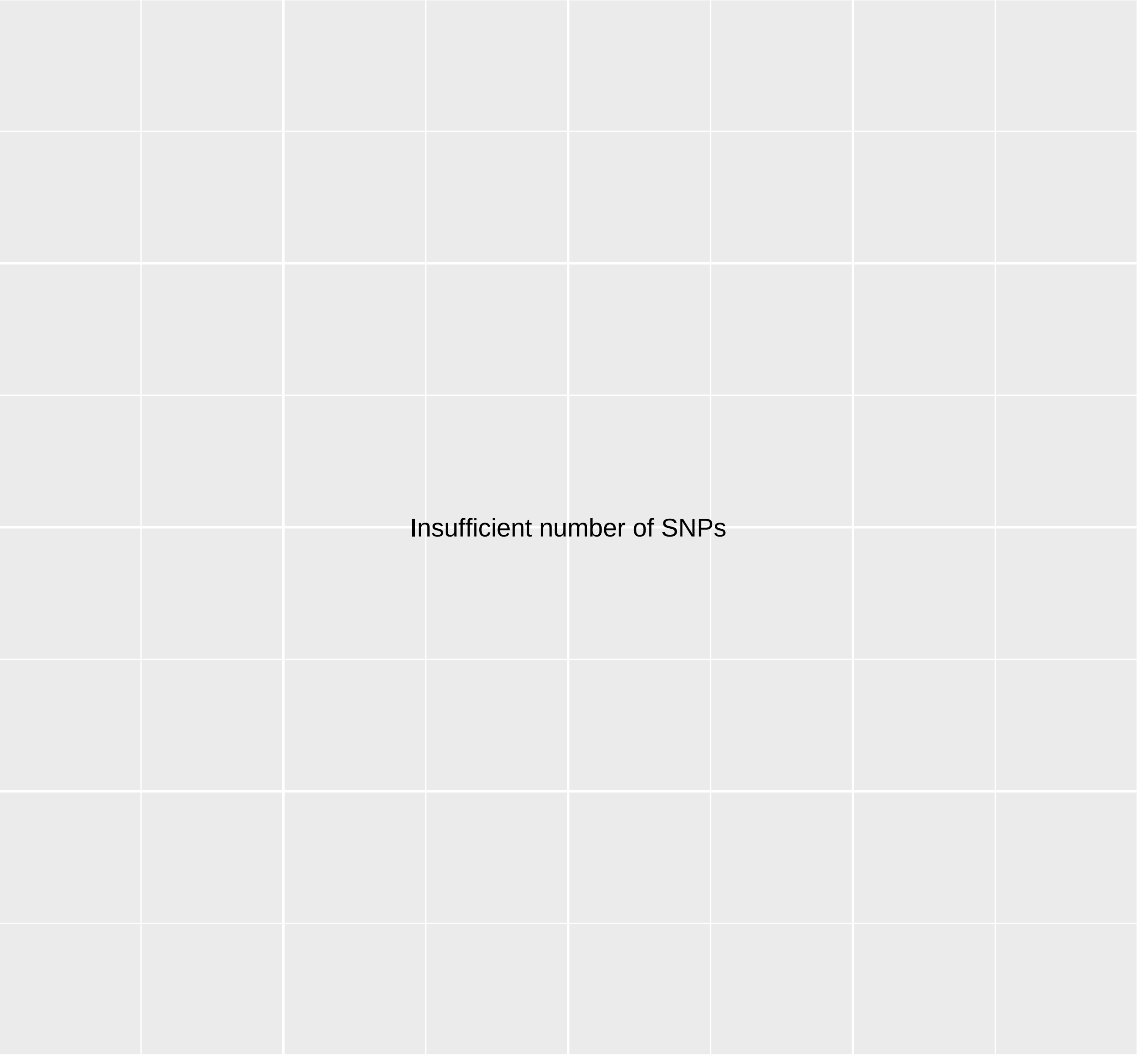
Insufficient number of SNPs

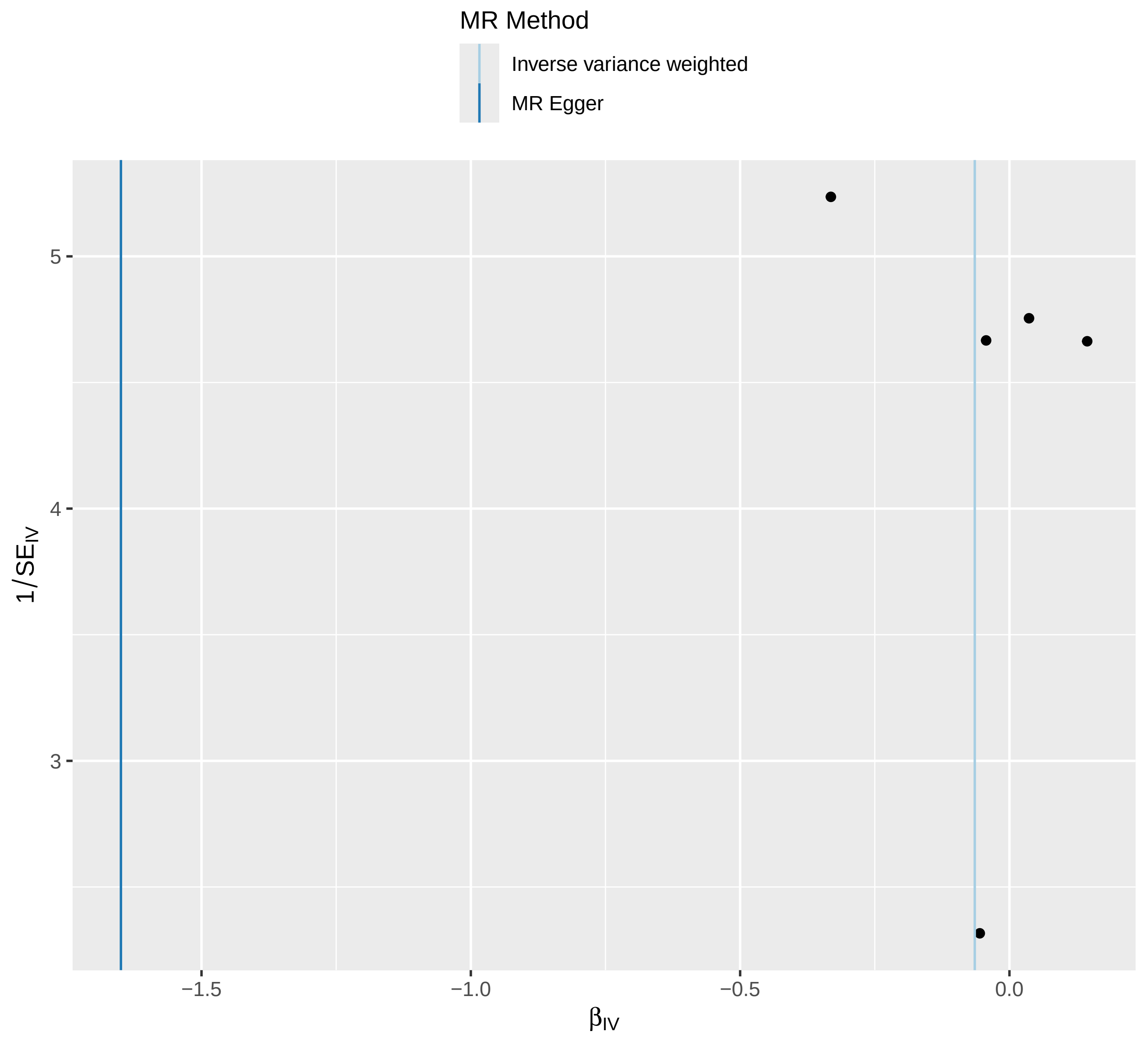
MR Method
Inverse variance weighted
MR Egger
5
4
3
1/SE_IV
−1.5
−1.0
−0.5
0.0
β_IV

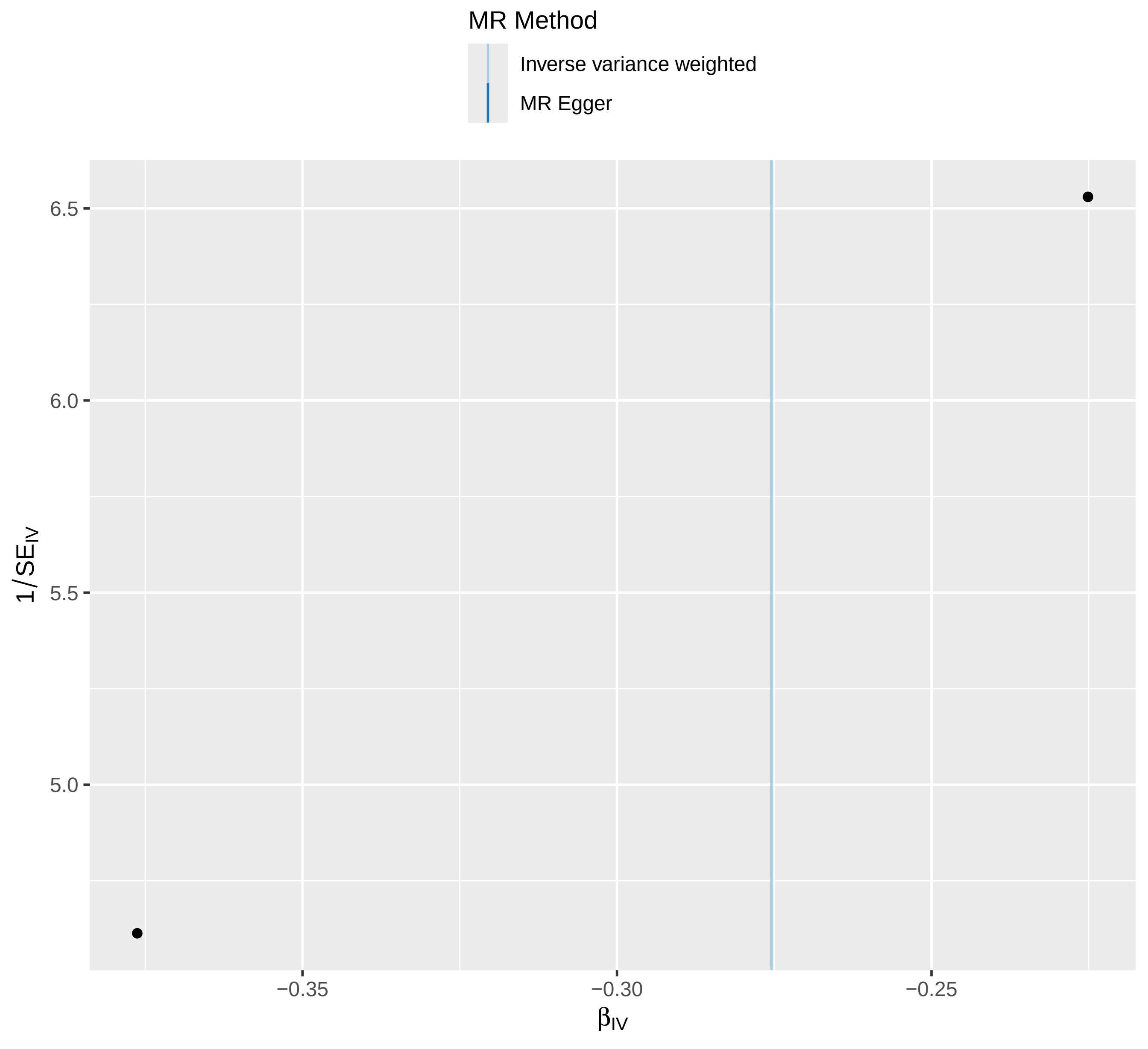

MR Method
Inverse variance weighted
MR Egger
6.5
6.0
5.5
5.0
1/SE_IV
−0.35
−0.30
−0.25
β_IV

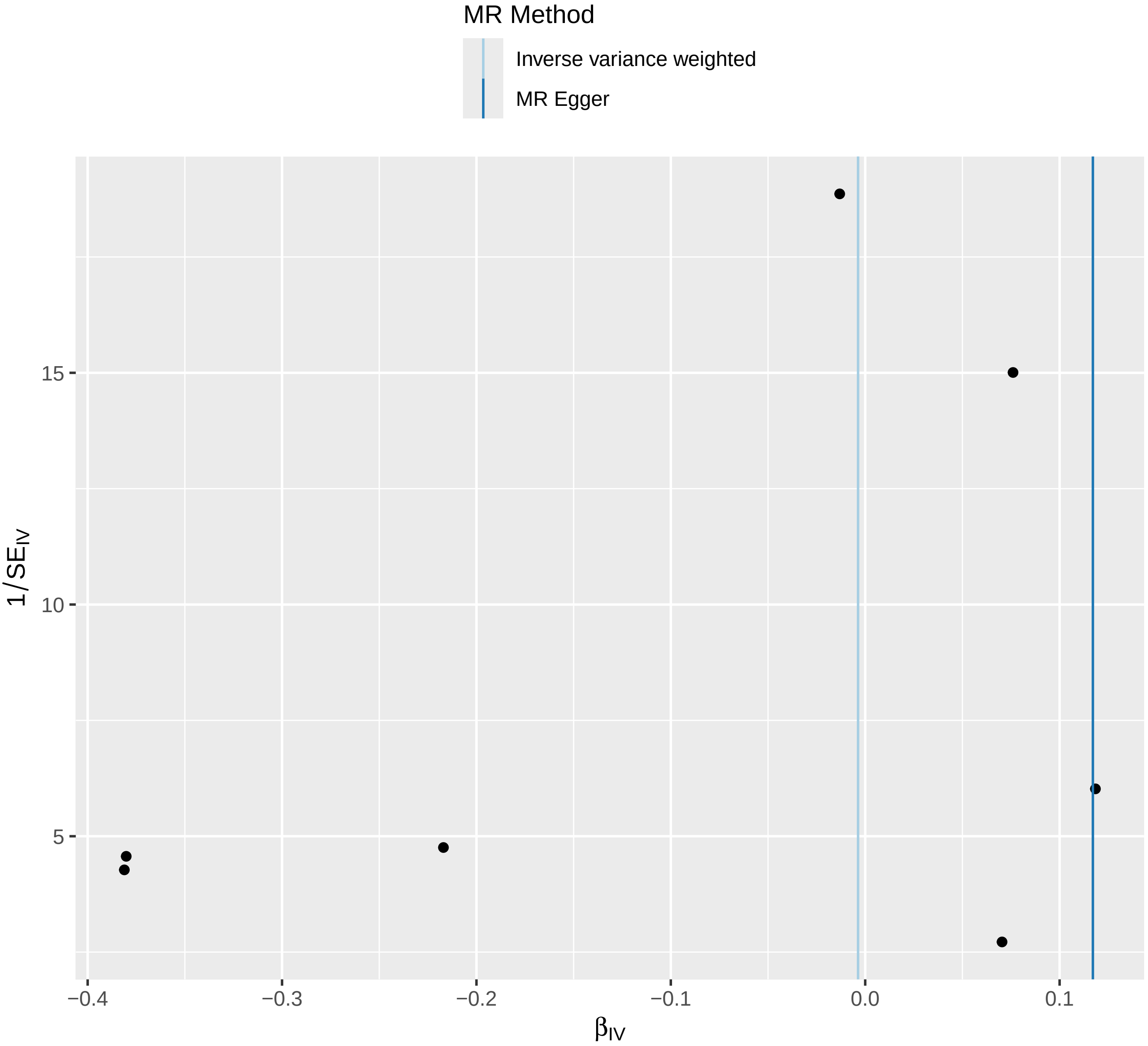

MR Method
Inverse variance weighted
MR Egger
15
10
5
1/SE_IV
−0.4
−0.3
−0.2
−0.1
0.0
0.1
β_IV

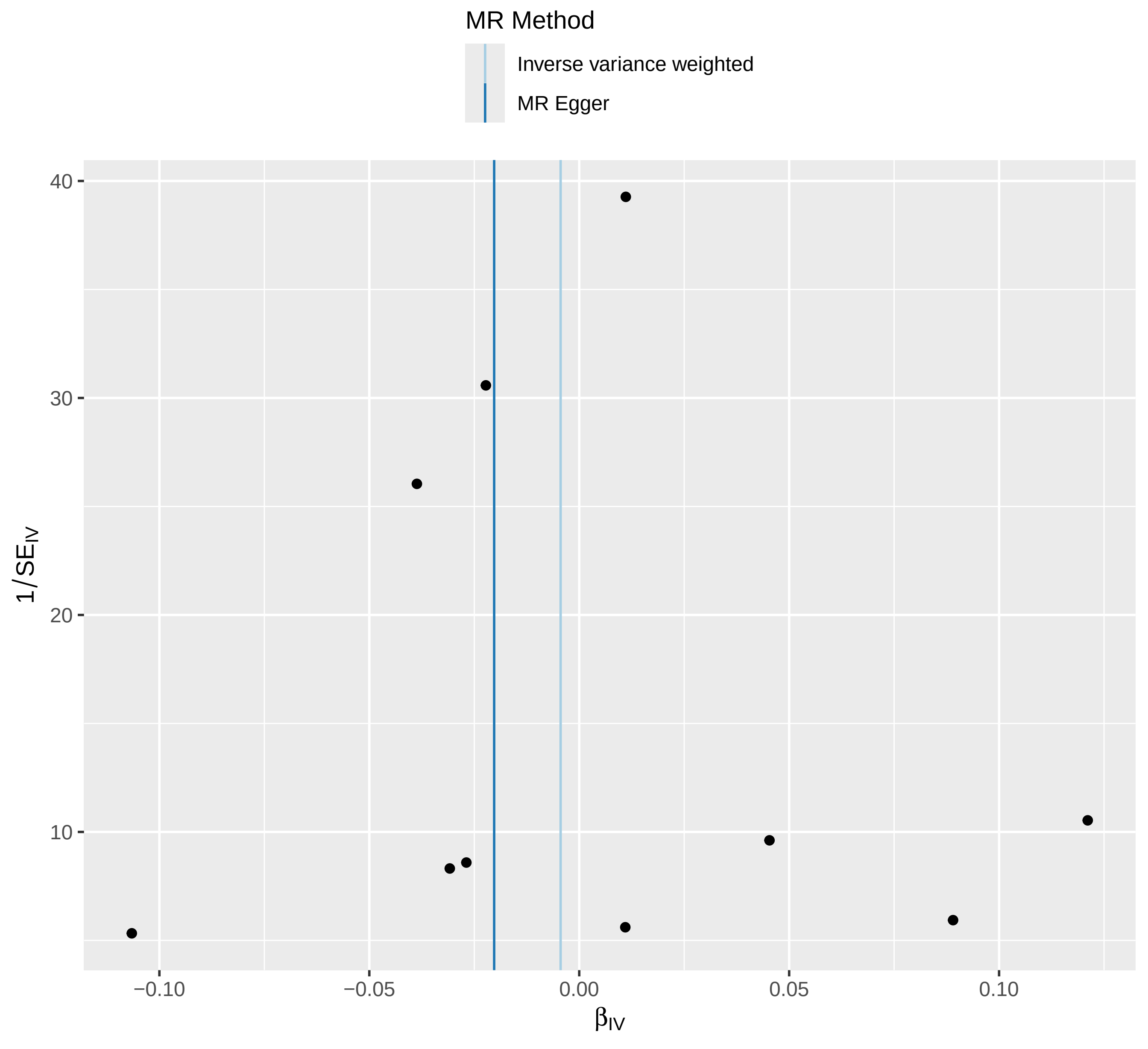
MR Method
Inverse variance weighted
MR Egger
40
30
20
10
1/SE_IV
−0.10
−0.05
0.00
0.05
0.10
β_IV

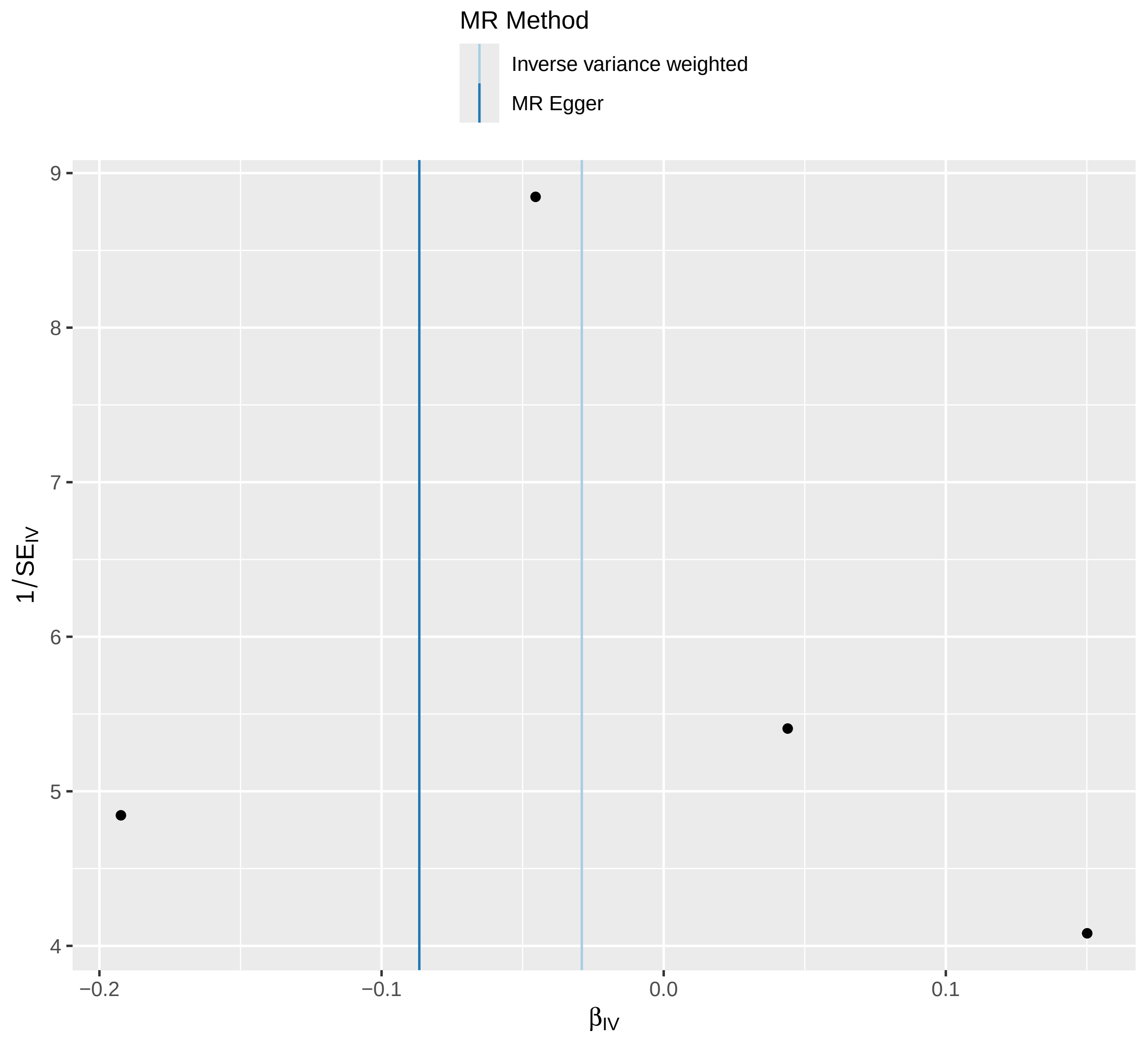
MR Method
Inverse variance weighted
MR Egger
9
8
7
6
5
4
1/SE_IV
−0.2
−0.1
0.0
0.1
β_IV

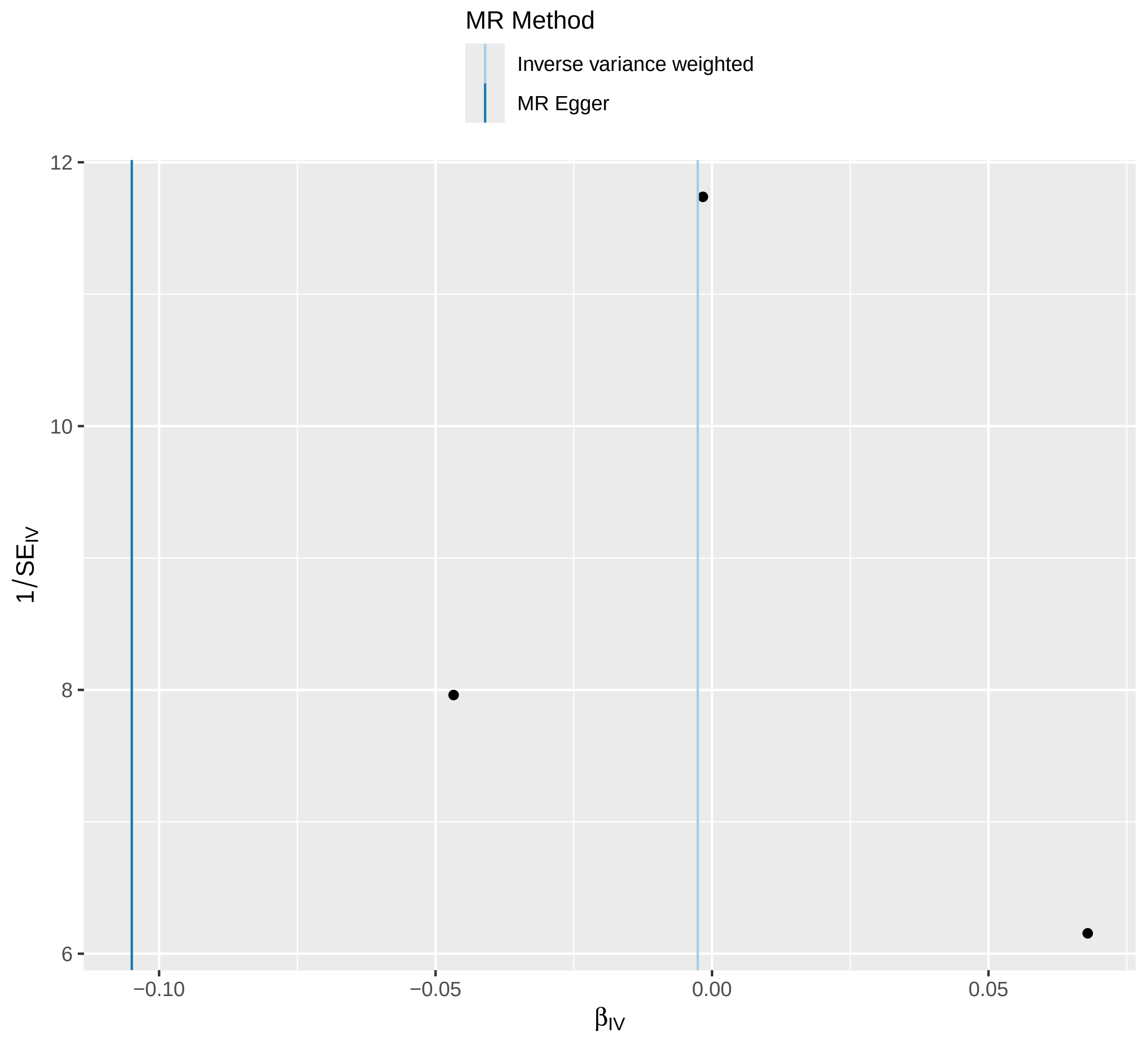
MR Method
Inverse variance weighted
MR Egger
12
10
8
6
1/SE_IV
−0.10
−0.05
0.00
0.05
β_IV

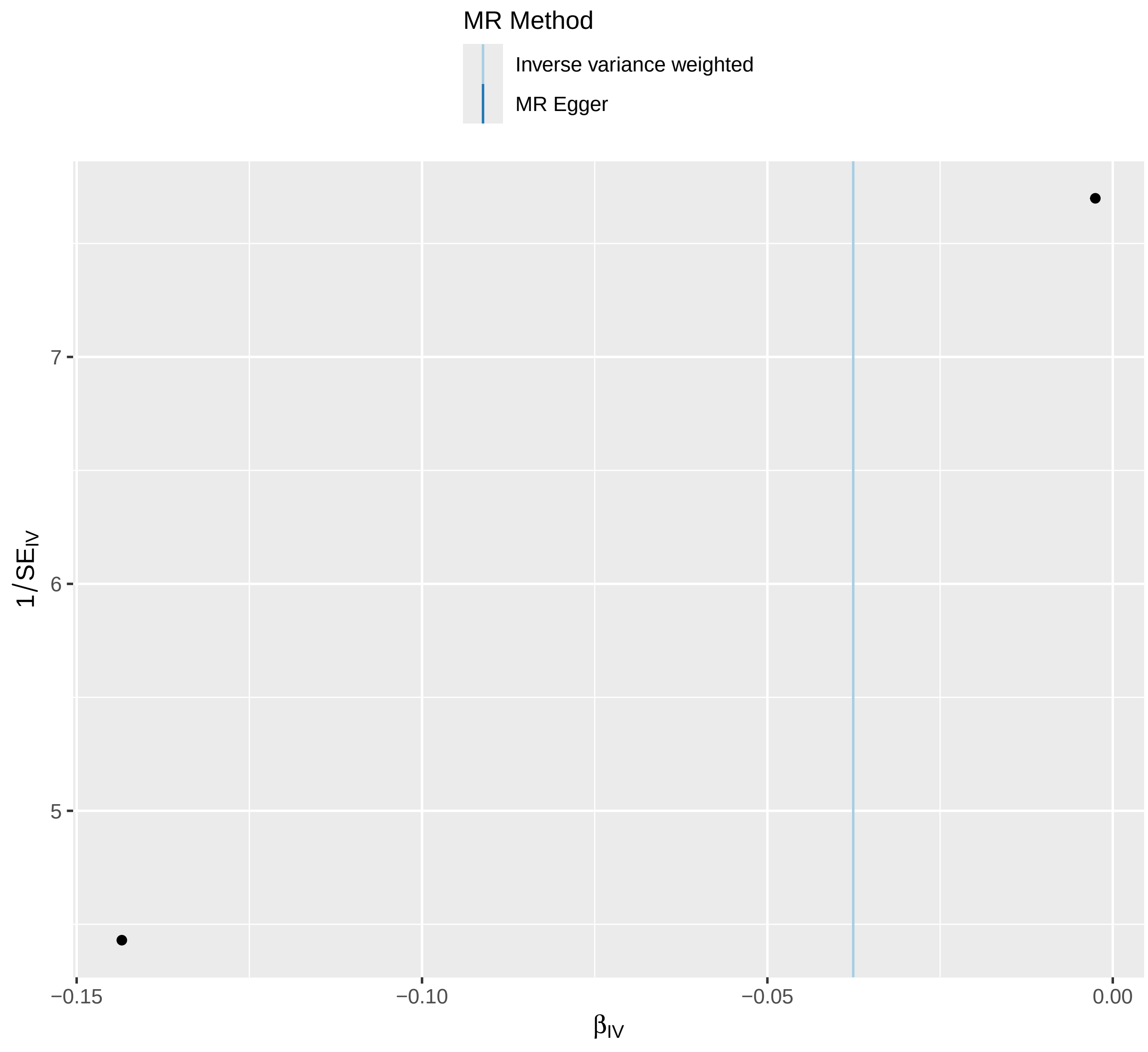

MR Method
Inverse variance weighted
MR Egger
7
6
5
1/SE$_{IV}$
−0.15
−0.10
−0.05
0.00
$\beta_{IV}$

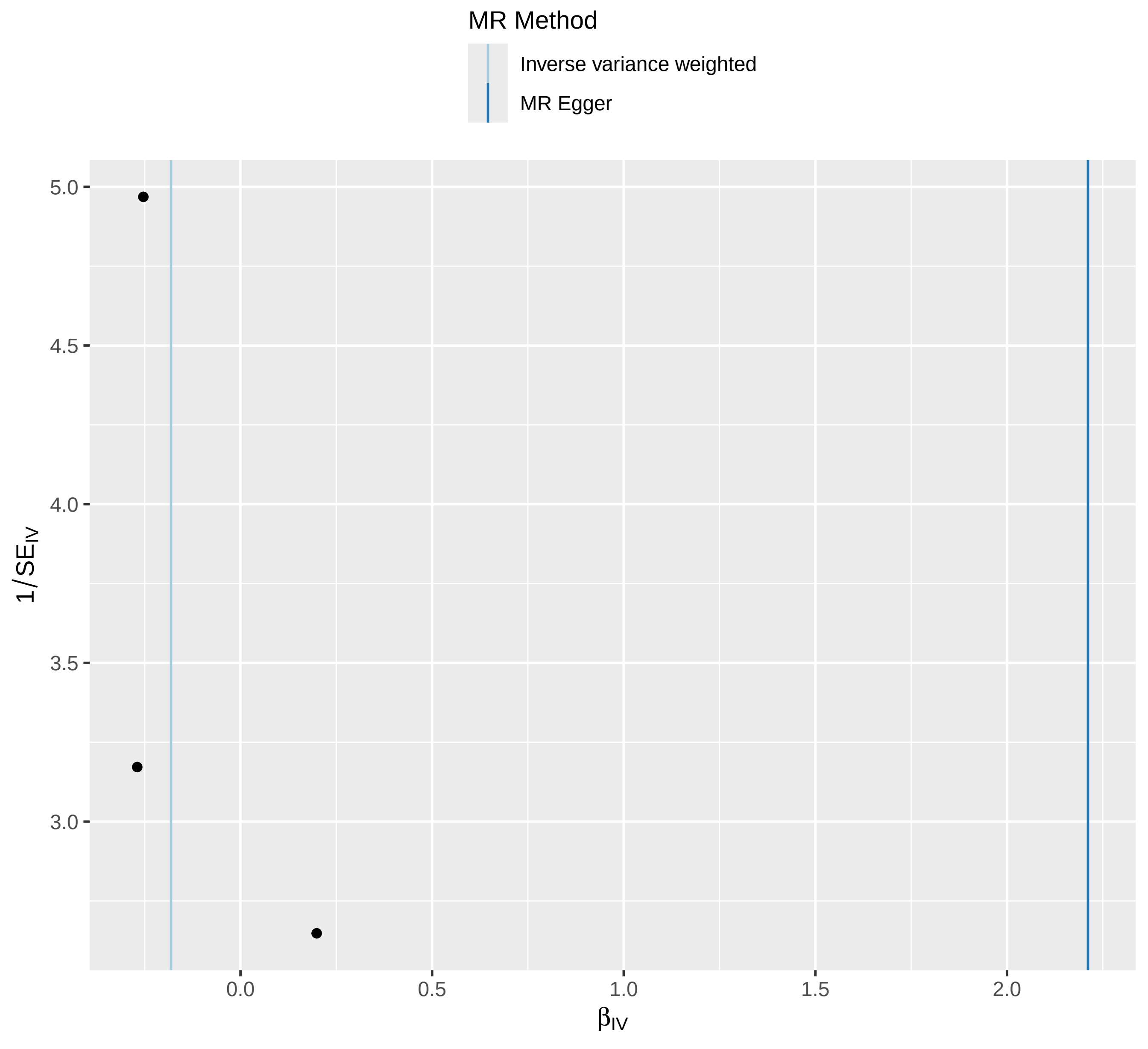
MR Method
Inverse variance weighted
MR Egger
5.0
4.5
4.0
3.5
3.0
1/SE_IV
0.0
0.5
1.0
1.5
2.0
β_IV

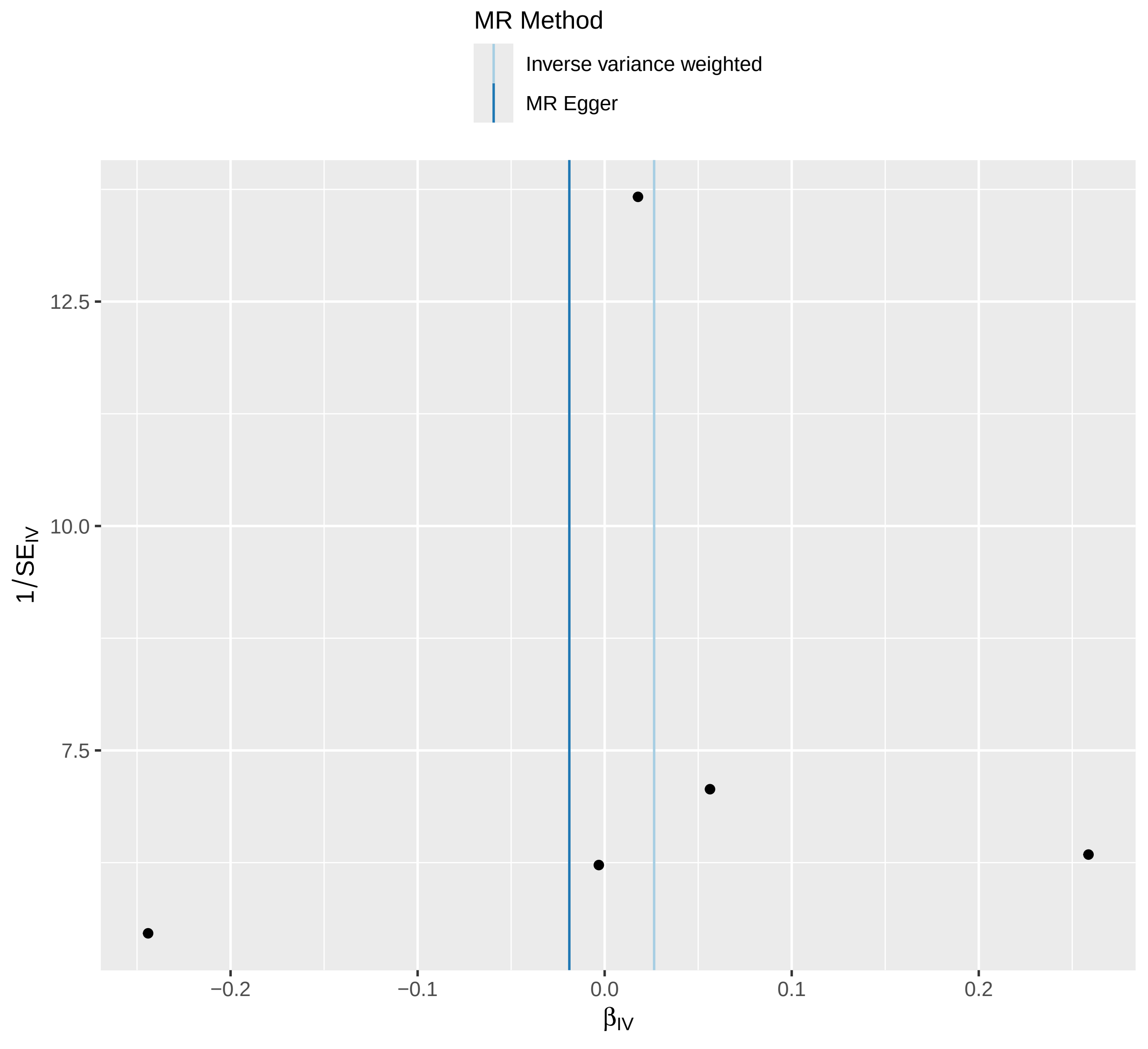
MR Method
Inverse variance weighted
MR Egger
12.5
10.0
7.5
1/SE$_{IV}$
−0.2
−0.1
0.0
0.1
0.2
β$_{IV}$

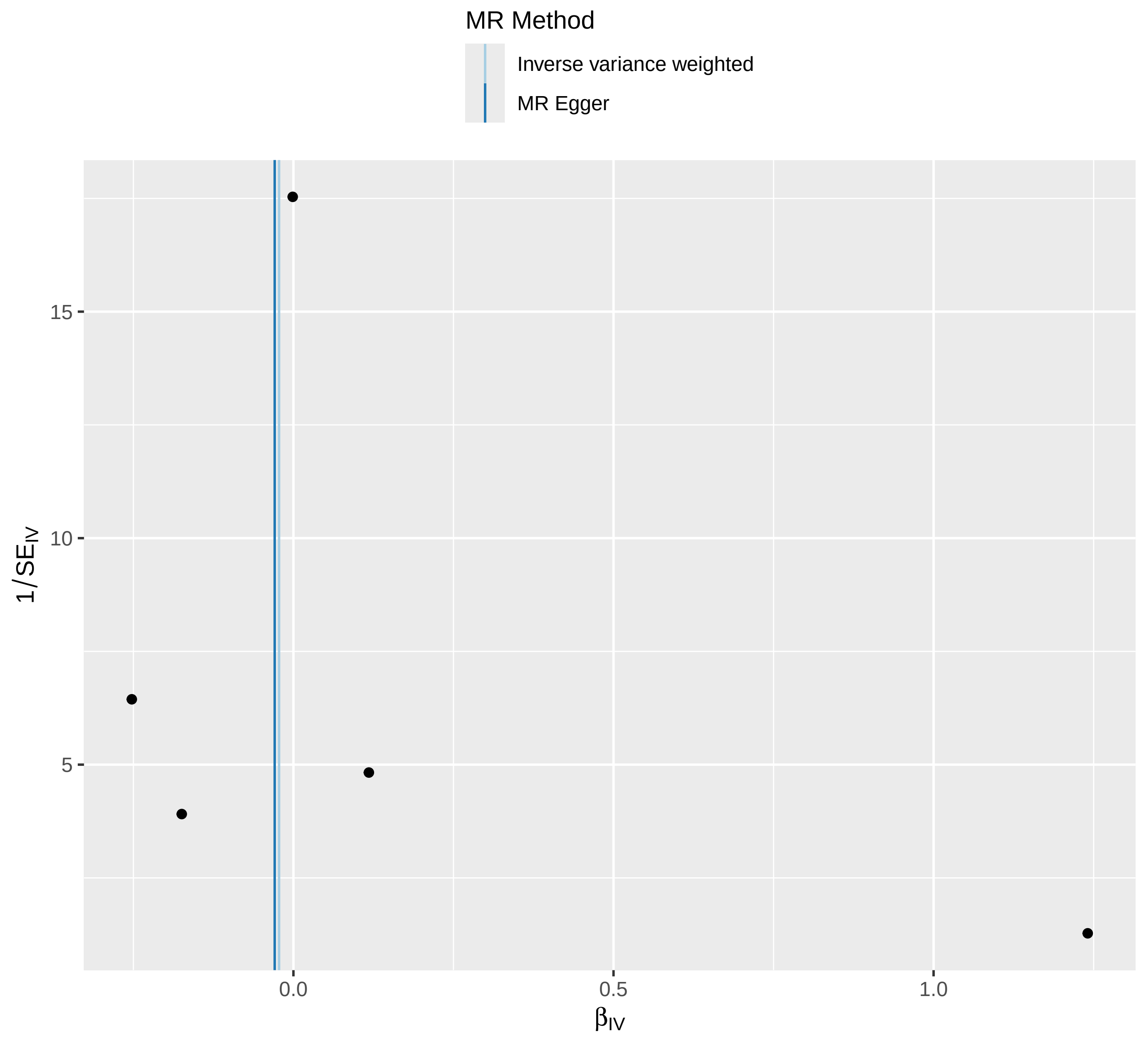

MR Method
Inverse variance weighted
MR Egger
15
10
5
1/SE_IV
0.0
0.5
1.0
β_IV

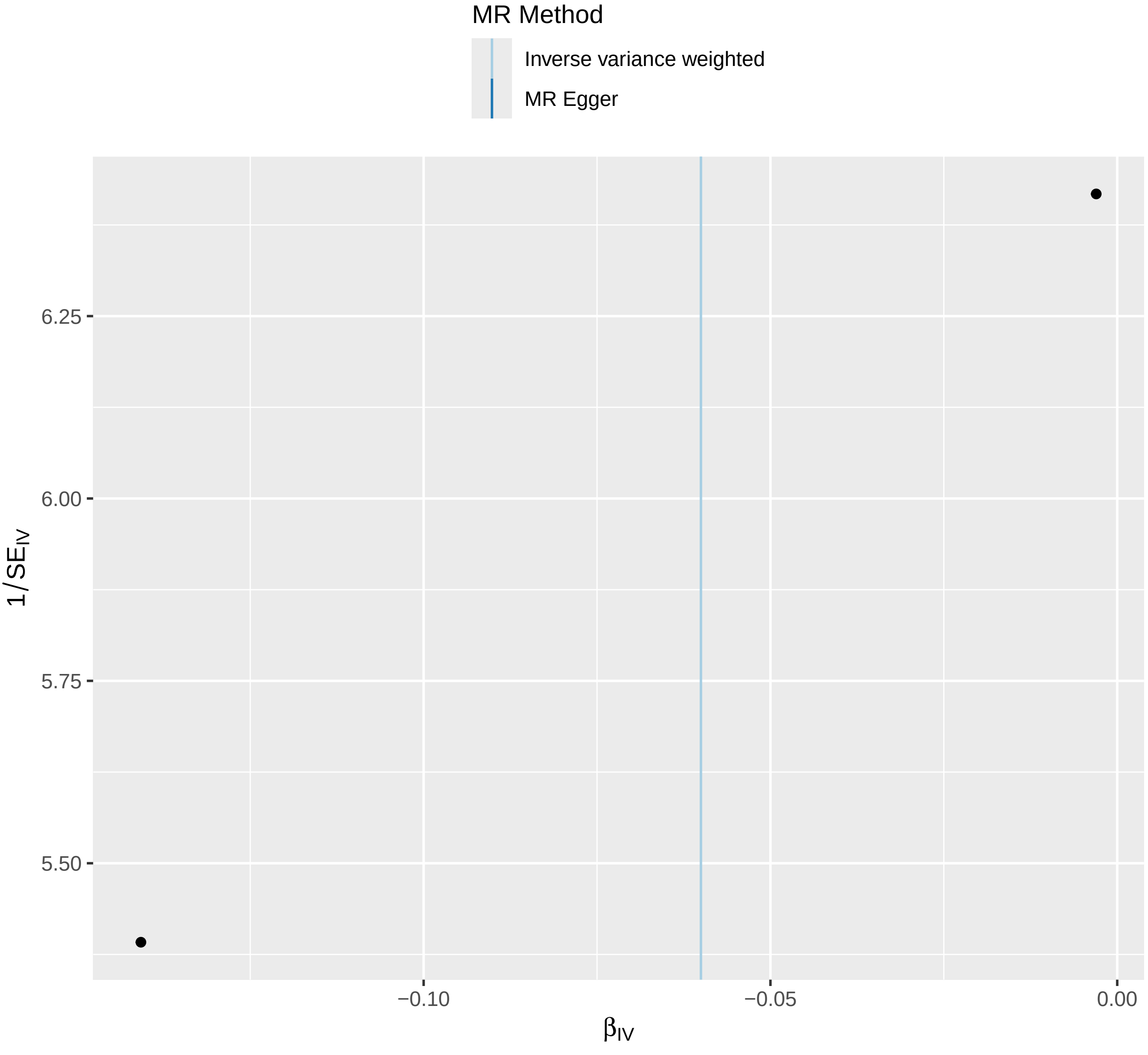

MR Method
Inverse variance weighted
MR Egger
6.25
6.00
5.75
5.50
1/SE$_{IV}$
−0.10
−0.05
0.00
$\beta_{IV}$

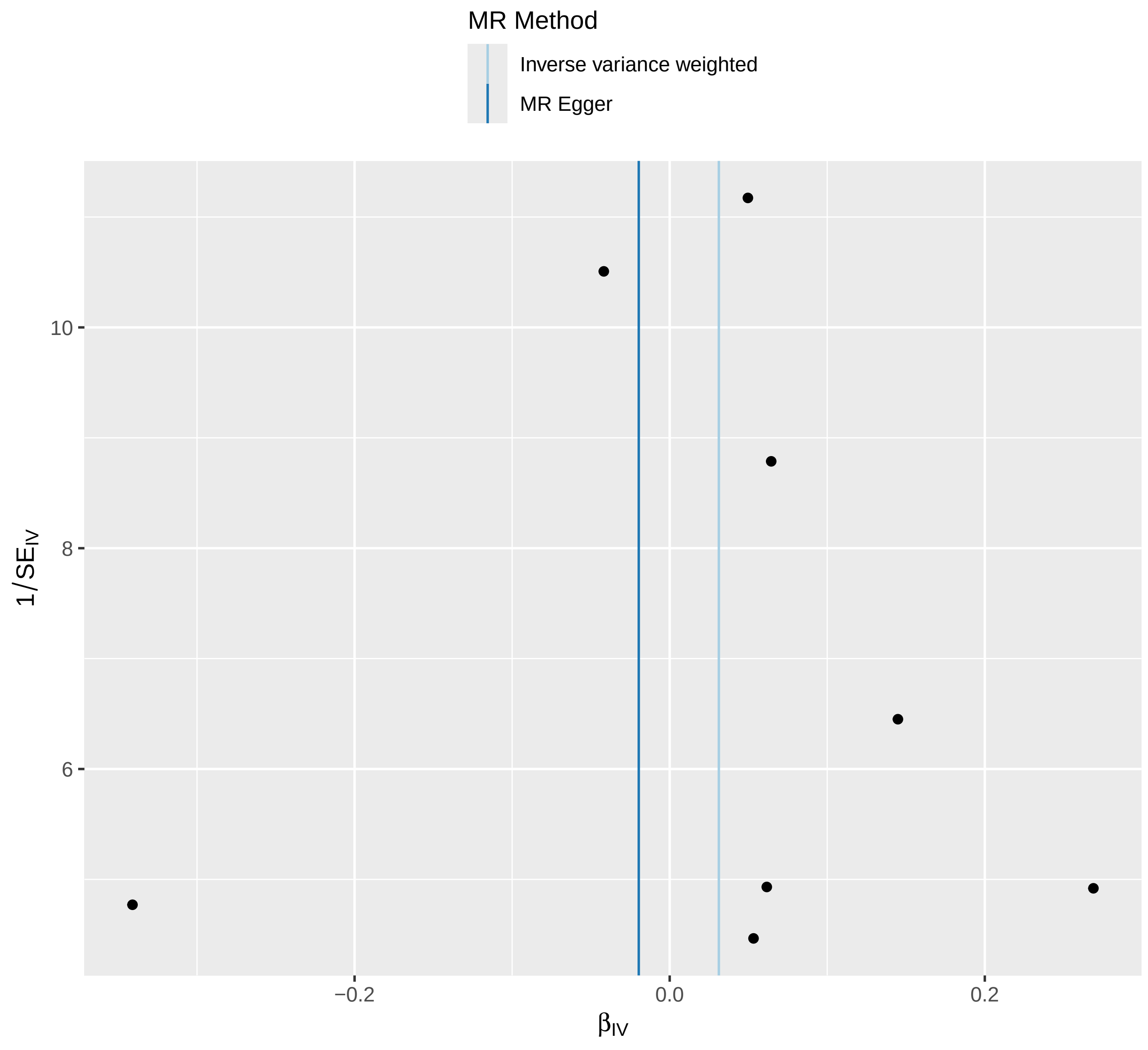
MR Method
Inverse variance weighted
MR Egger
10
8
6
−0.2
0.0
0.2
$\beta_{IV}$
$1/SE_{IV}$

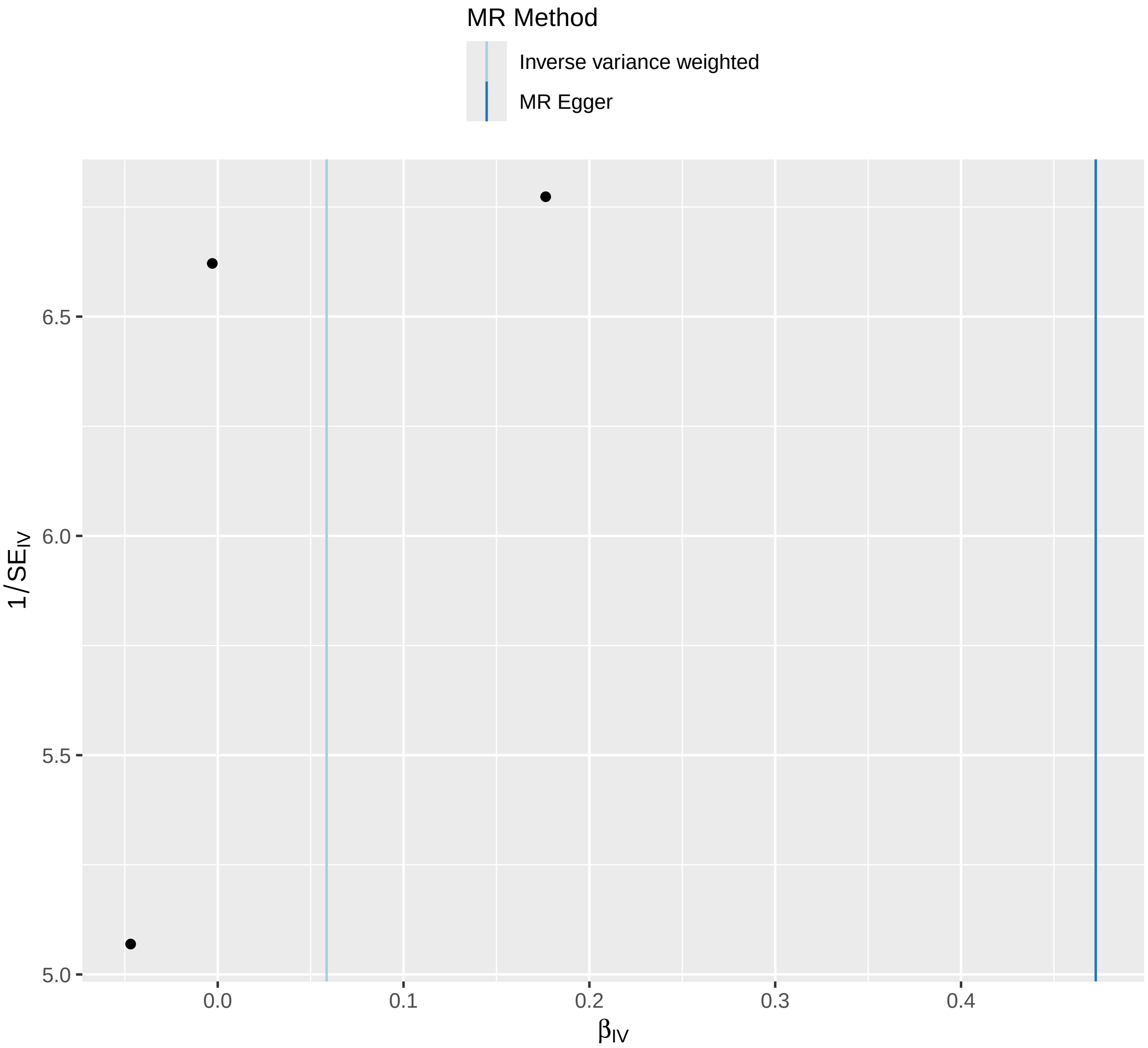
MR Method
Inverse variance weighted
MR Egger
1/SE_IV
6.5
6.0
5.5
5.0
0.0
0.1
0.2
0.3
0.4
β_IV

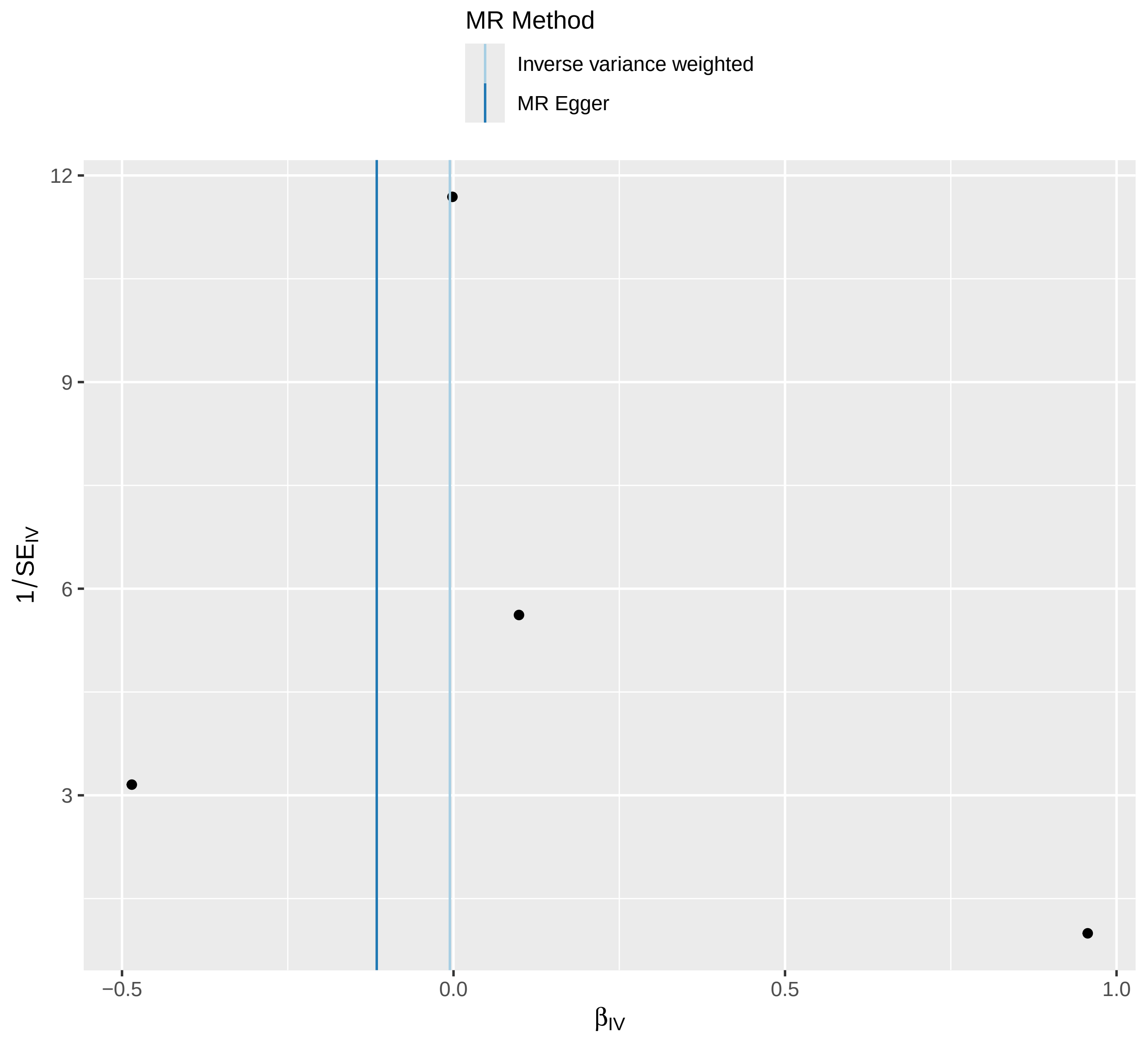
MR Method
Inverse variance weighted
MR Egger
12
9
6
3
1/SE$_{IV}$
−0.5
0.0
0.5
1.0
β$_{IV}$

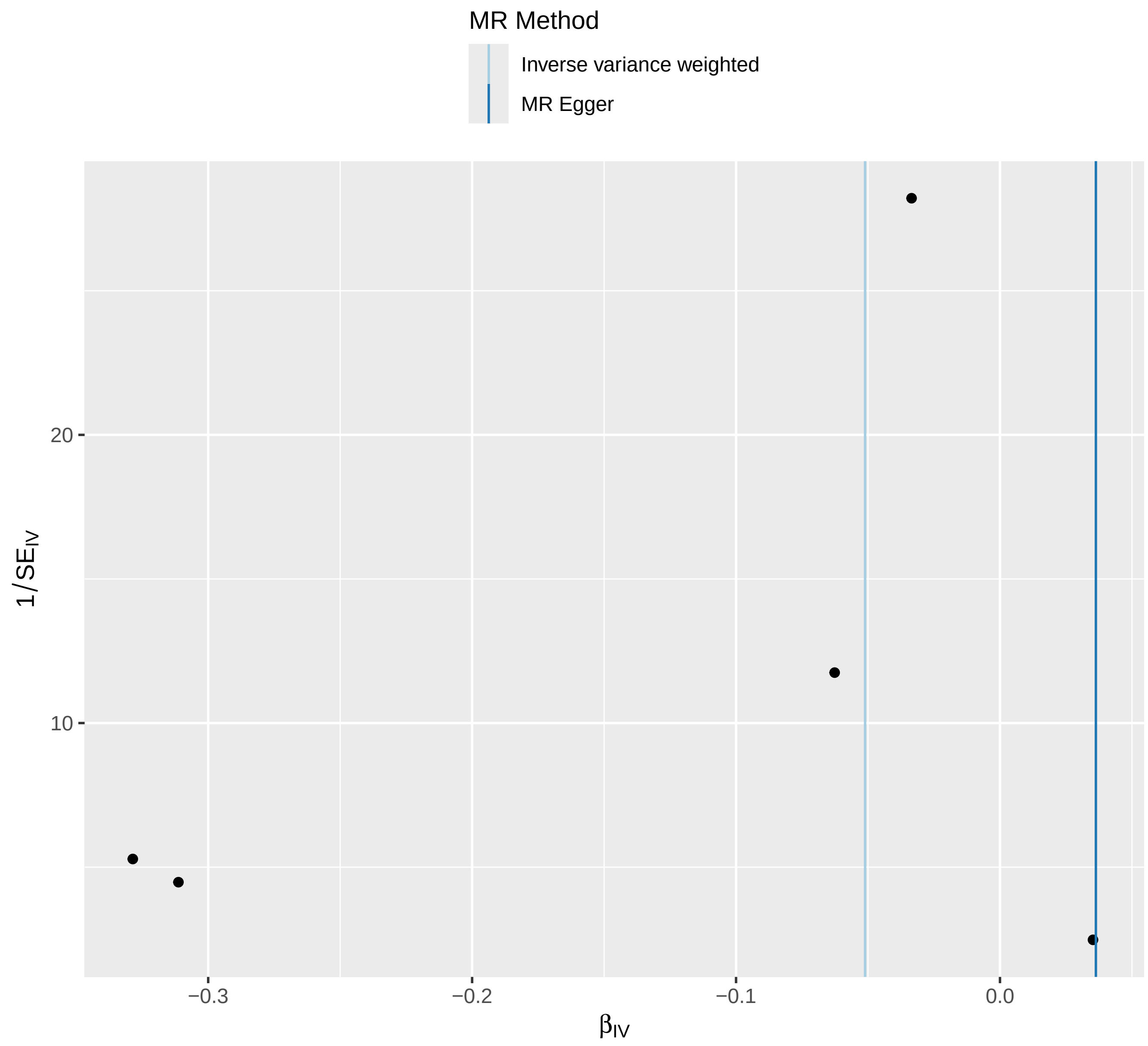
MR Method
Inverse variance weighted
MR Egger
20
10
1/SE$_{IV}$
−0.3
−0.2
−0.1
0.0
β$_{IV}$

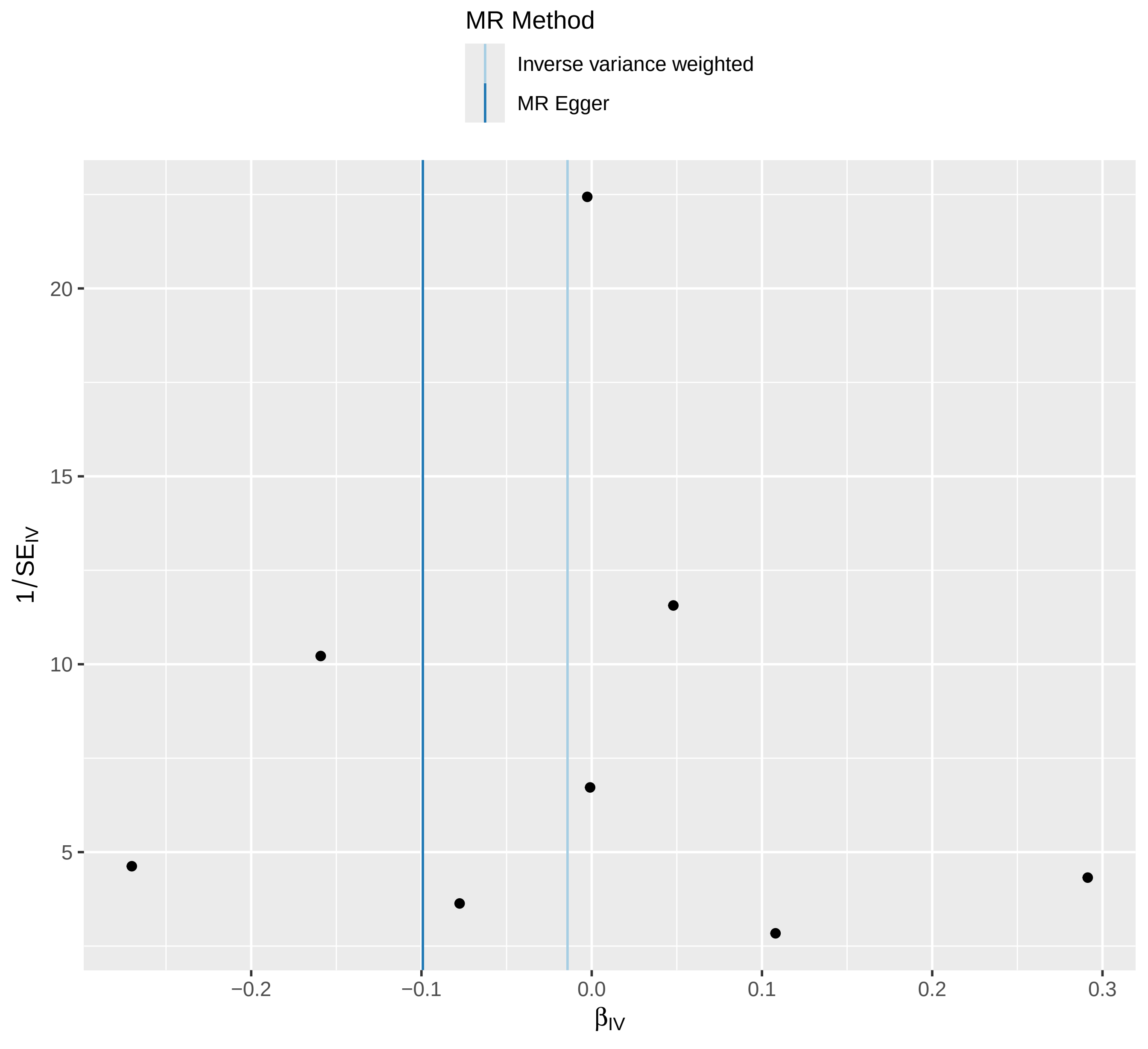
MR Method
Inverse variance weighted
MR Egger
20
15
10
5
1/$SE_{IV}$
−0.2
−0.1
0.0
0.1
0.2
0.3
$\beta_{IV}$

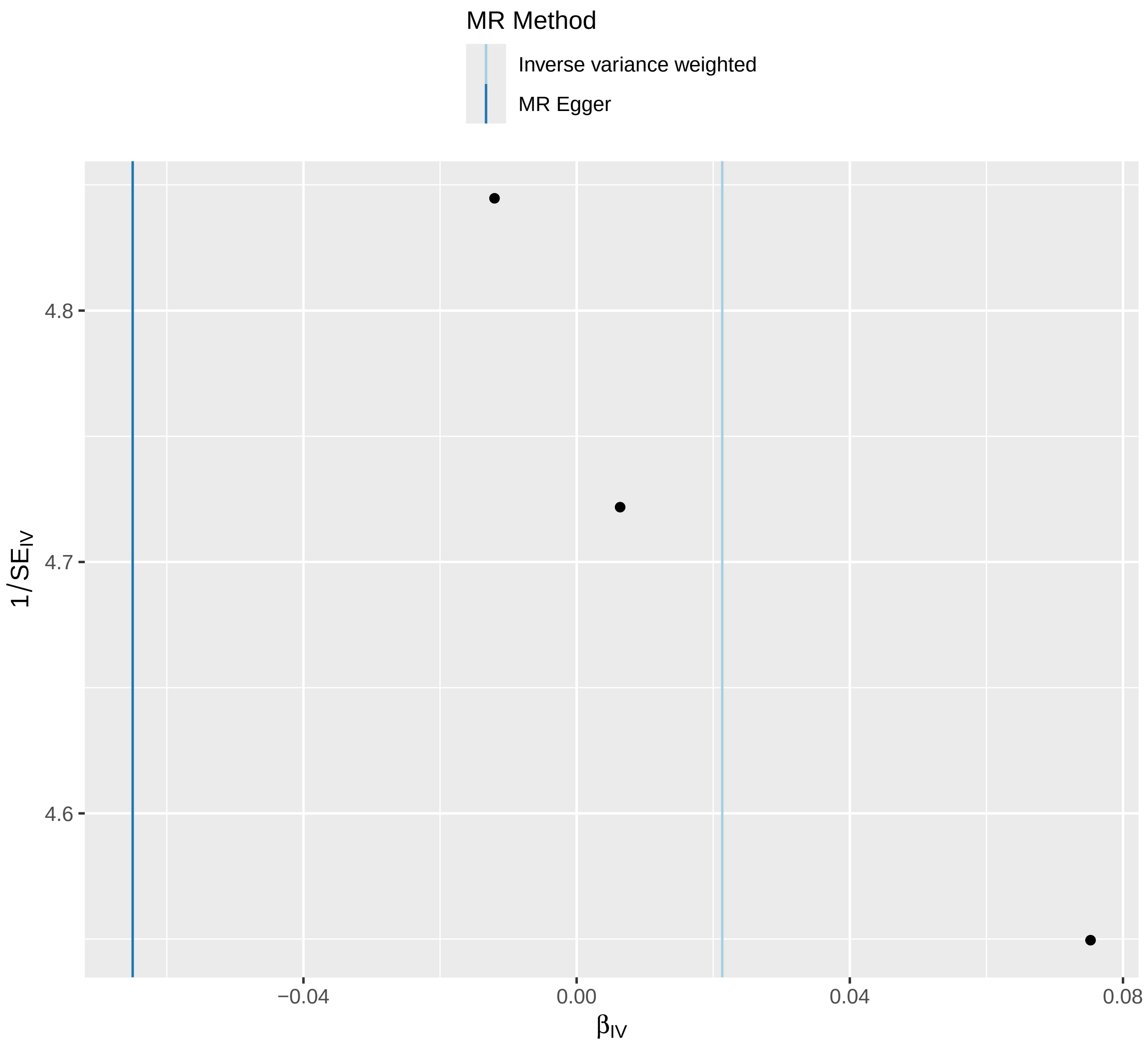

MR Method
Inverse variance weighted
MR Egger
4.8
4.7
4.6
1/SE$_{IV}$
−0.04
0.00
0.04
0.08
β$_{IV}$

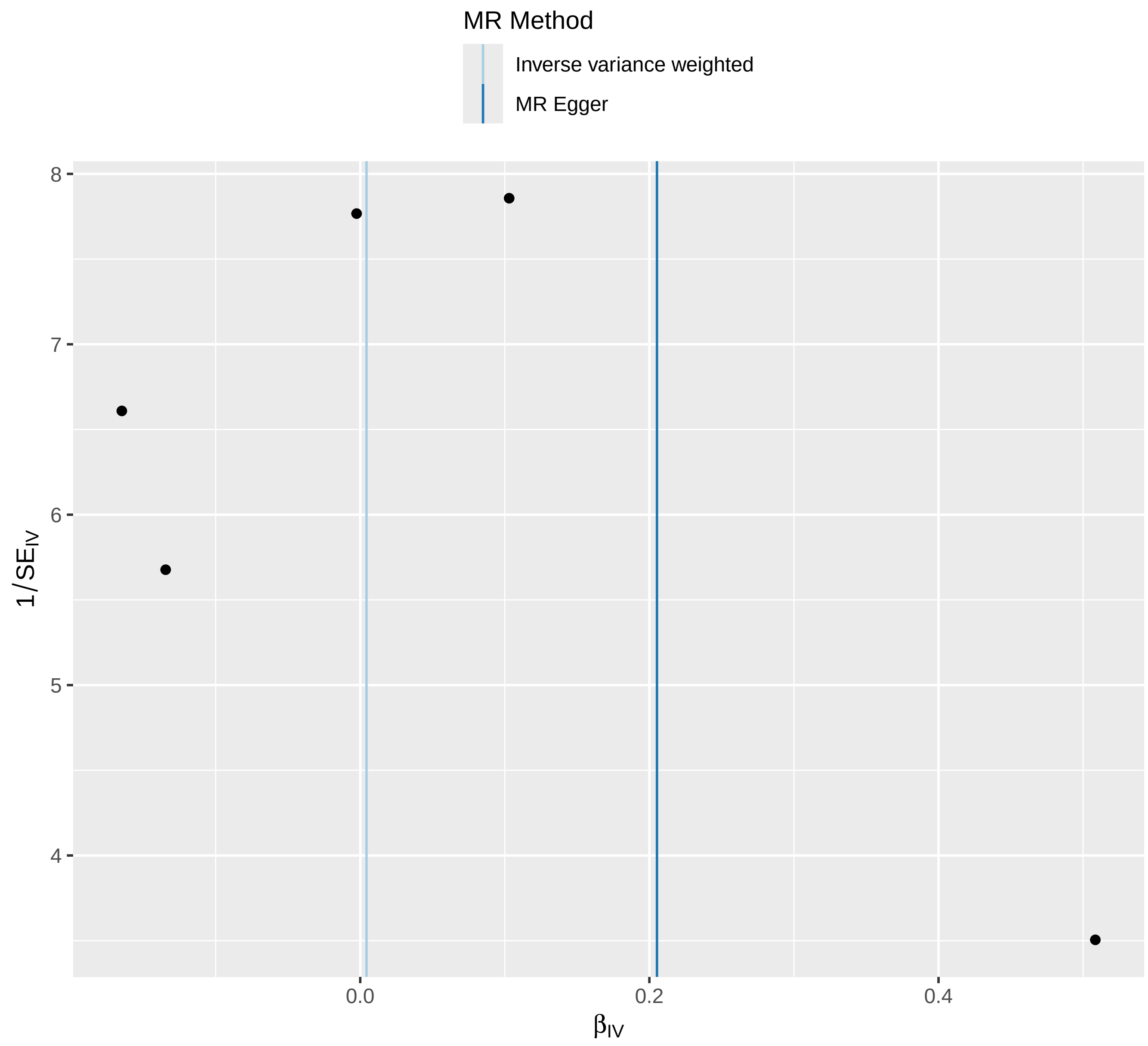
MR Method
Inverse variance weighted
MR Egger
8
7
6
5
4
1/SE_IV
0.0
0.2
0.4
β_IV

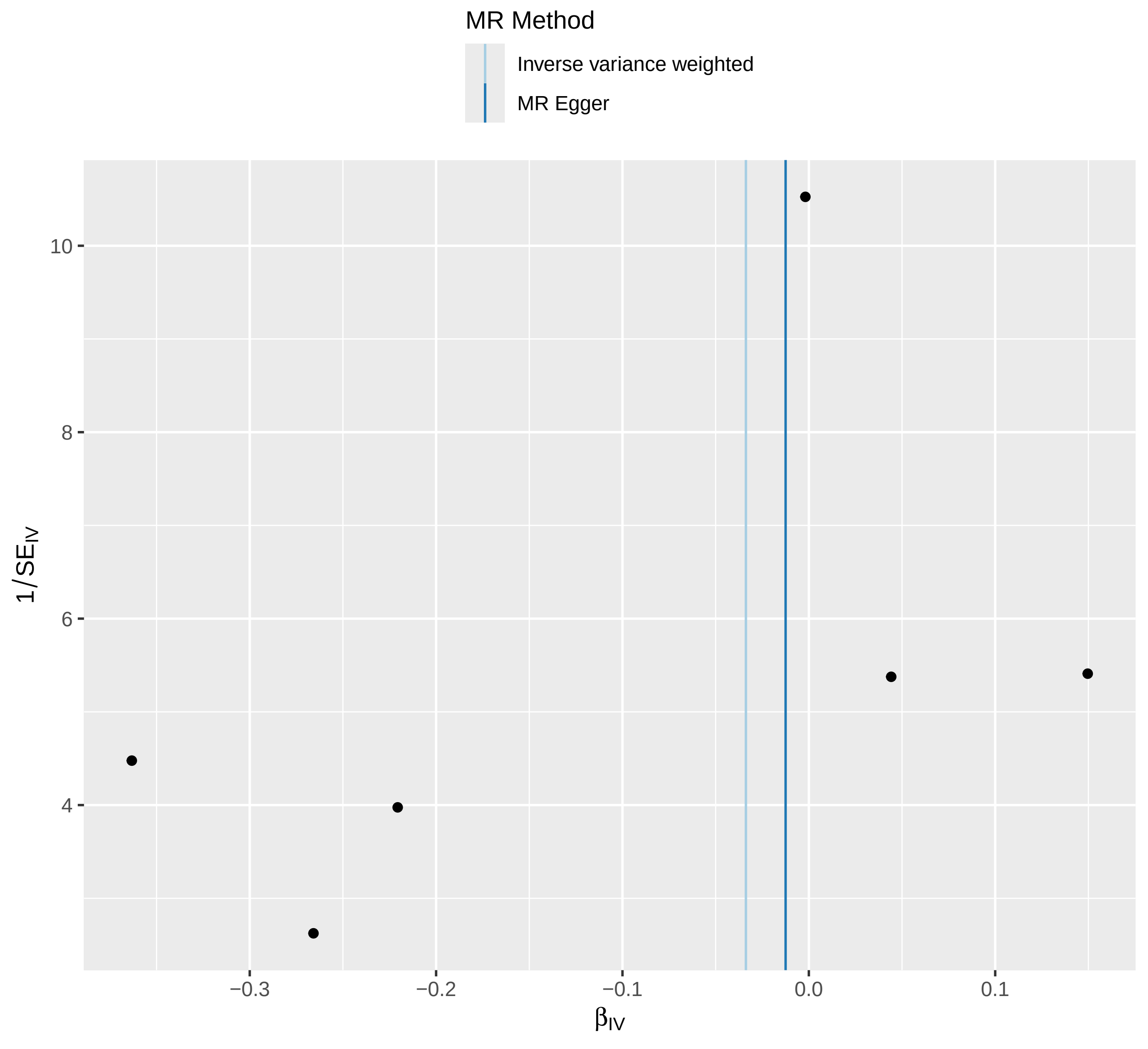

MR Method
Inverse variance weighted
MR Egger
10
8
6
4
1/SE_IV
−0.3
−0.2
−0.1
0.0
0.1
β_IV

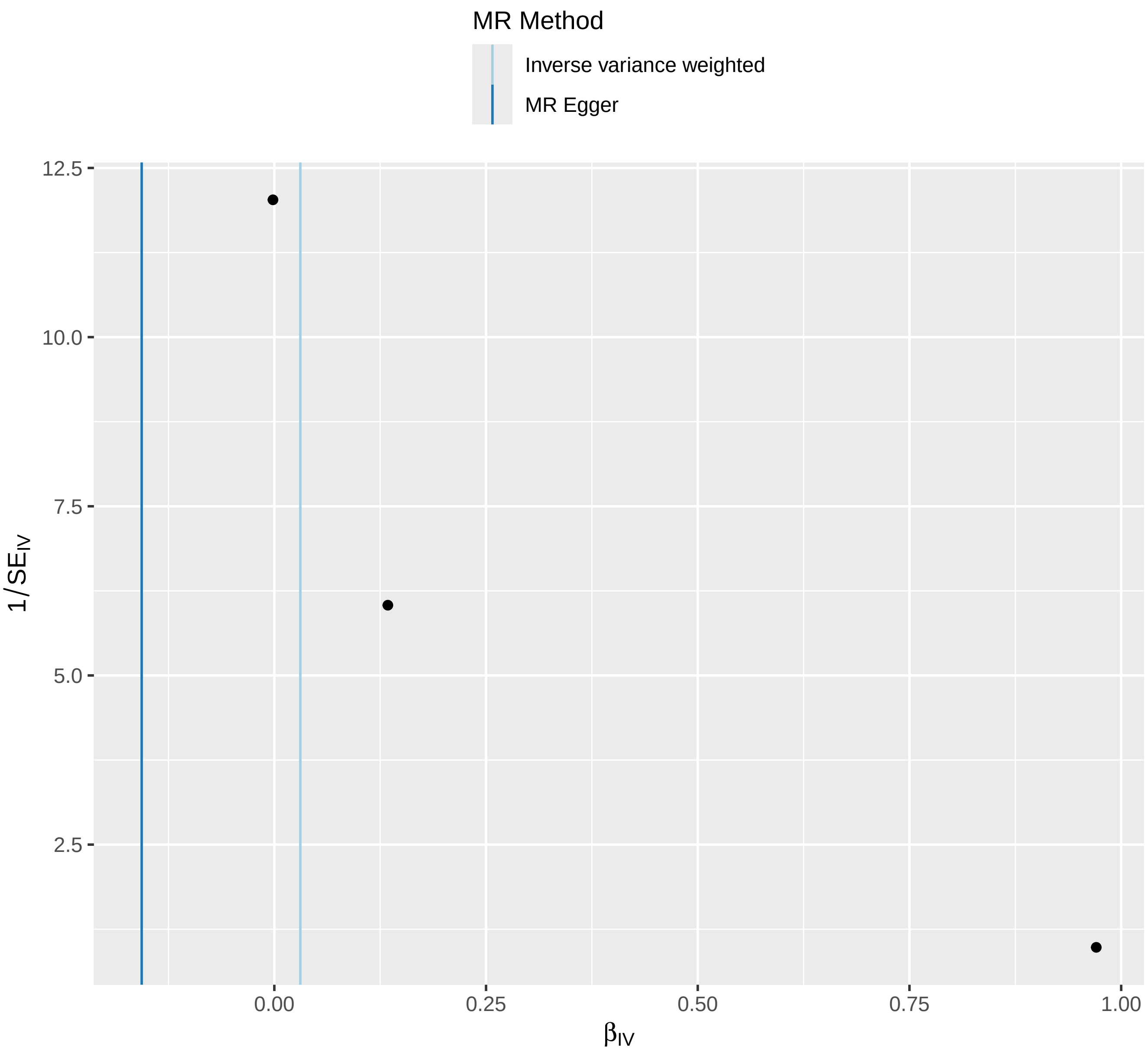
MR Method
Inverse variance weighted
MR Egger
12.5
10.0
7.5
5.0
2.5
1/SE_IV
0.00
0.25
0.50
0.75
1.00
β_IV

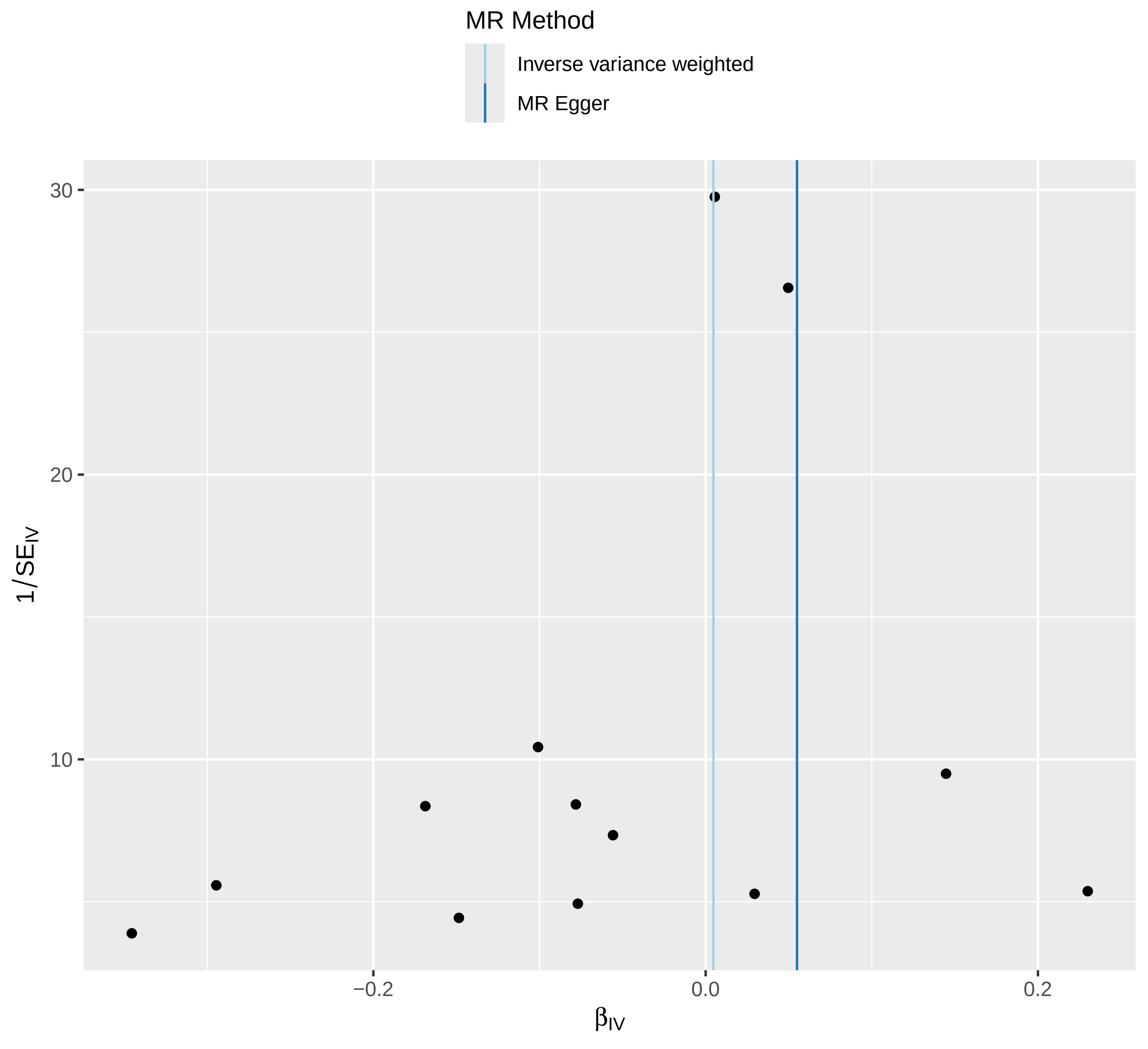
MR Method
Inverse variance weighted
MR Egger
30
20
10
−0.2
0.0
0.2
$1/SE_{IV}$
$\beta_{IV}$

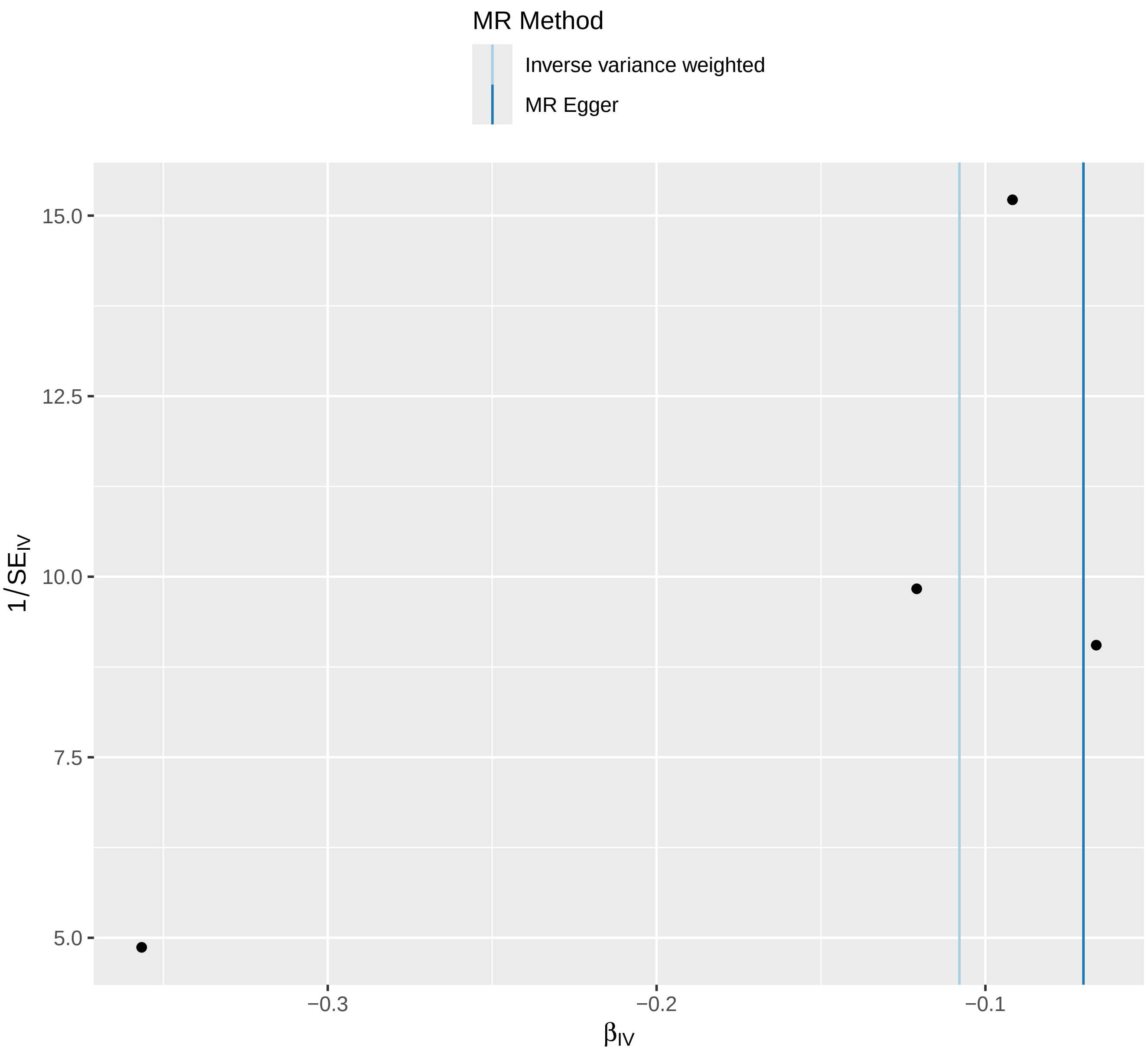
MR Method
Inverse variance weighted
MR Egger
15.0
12.5
10.0
7.5
5.0
$1/SE_{IV}$
−0.3
−0.2
−0.1
$\beta_{IV}$

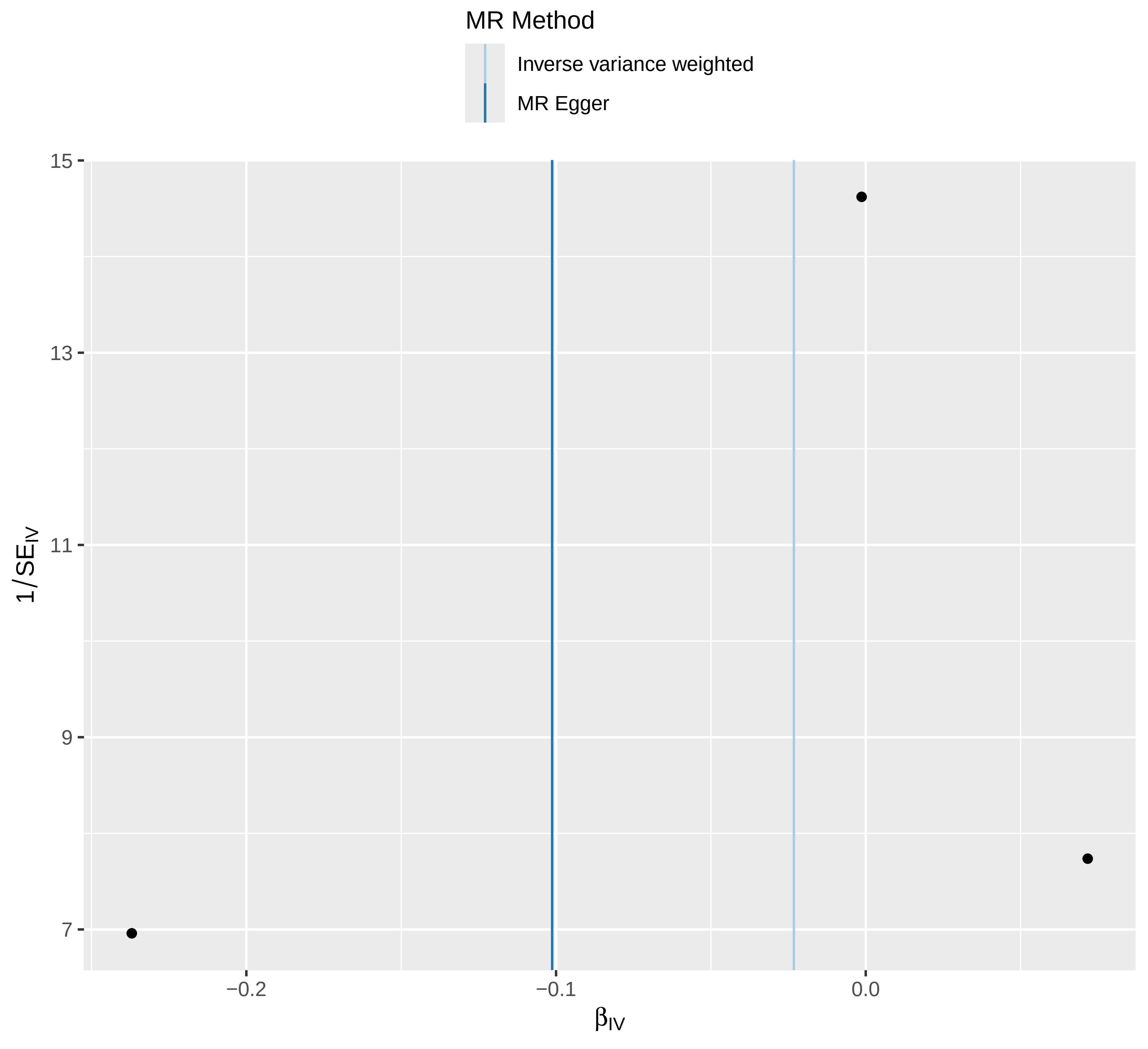
MR Method
Inverse variance weighted
MR Egger
15
13
11
9
7
1/SE_IV
−0.2
−0.1
0.0
β_IV

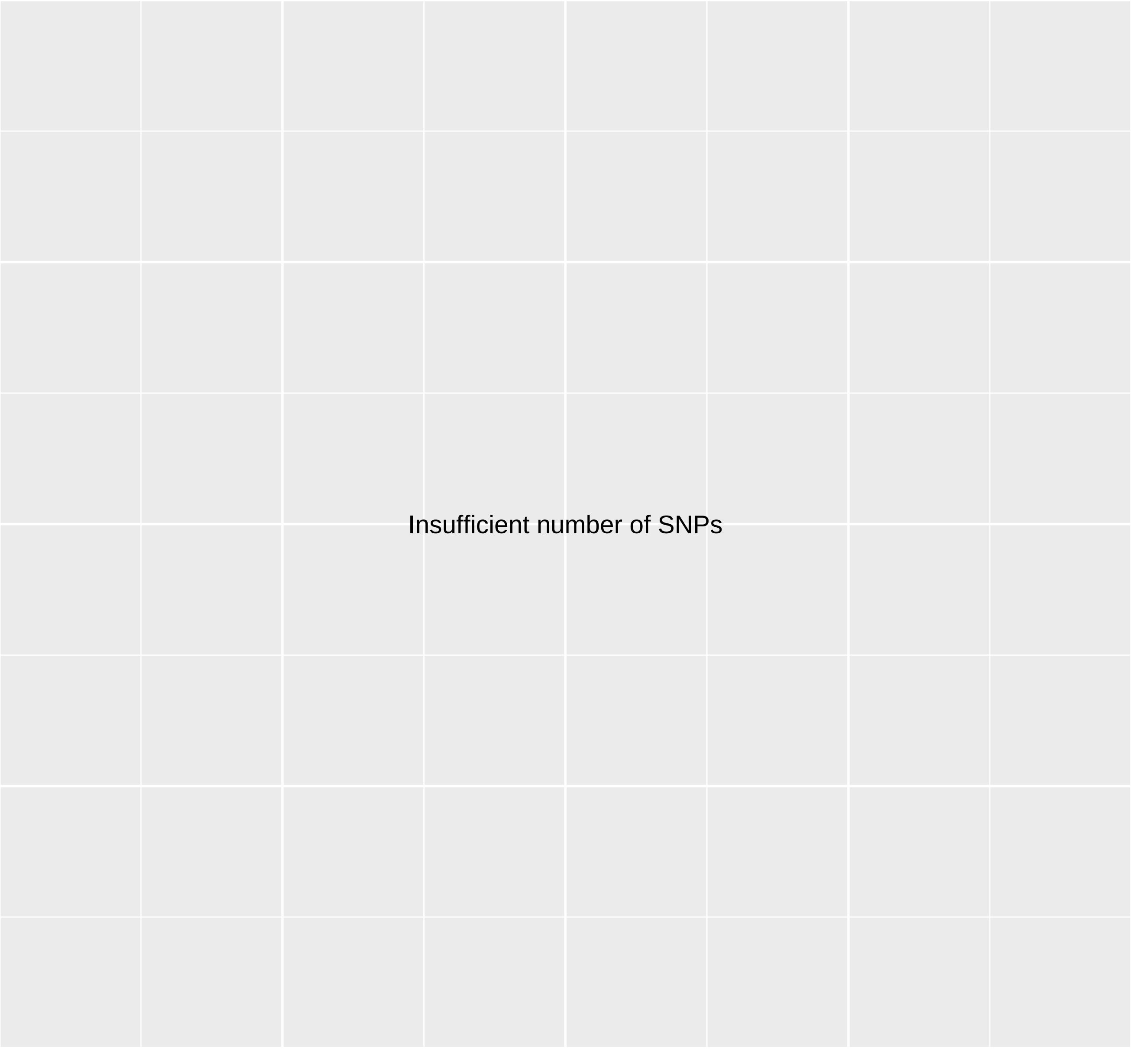
Insufficient number of SNPs

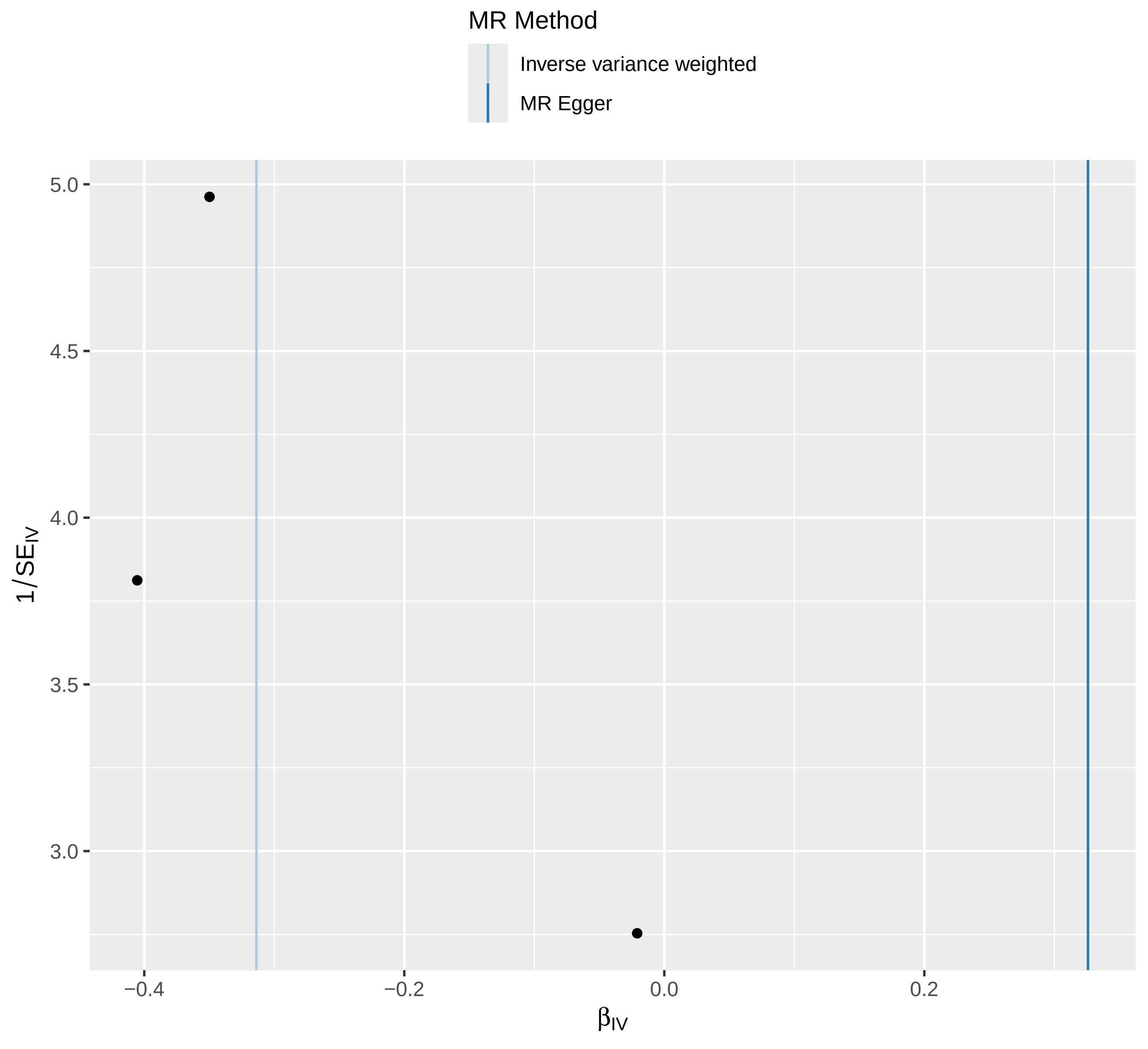
MR Method
Inverse variance weighted
MR Egger
5.0
4.5
4.0
3.5
3.0
1/SE_IV
−0.4
−0.2
0.0
0.2
β_IV

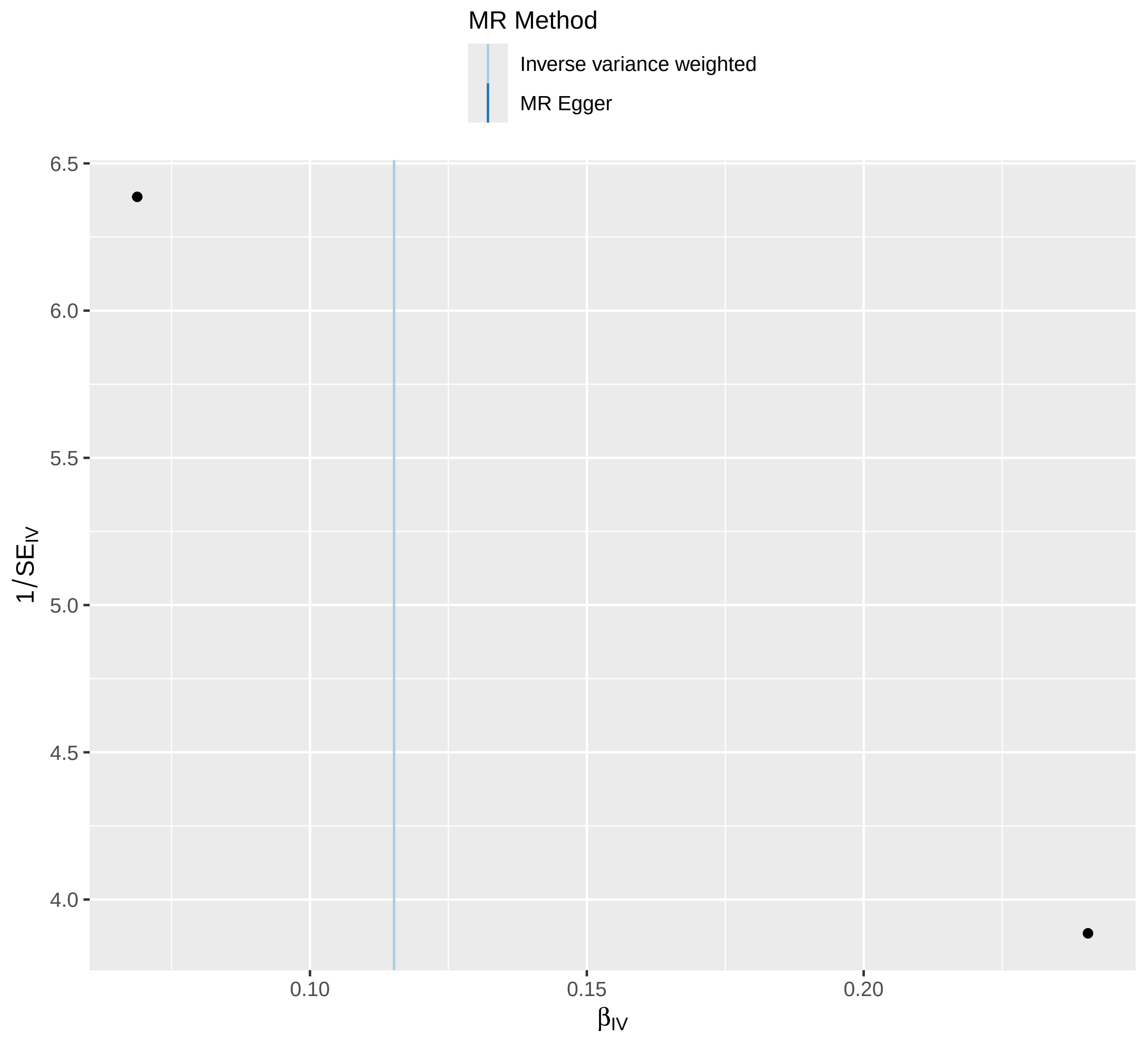
MR Method
Inverse variance weighted
MR Egger
6.5
6.0
5.5
5.0
4.5
4.0
$1/SE_{IV}$
0.10
0.15
0.20
$\beta_{IV}$

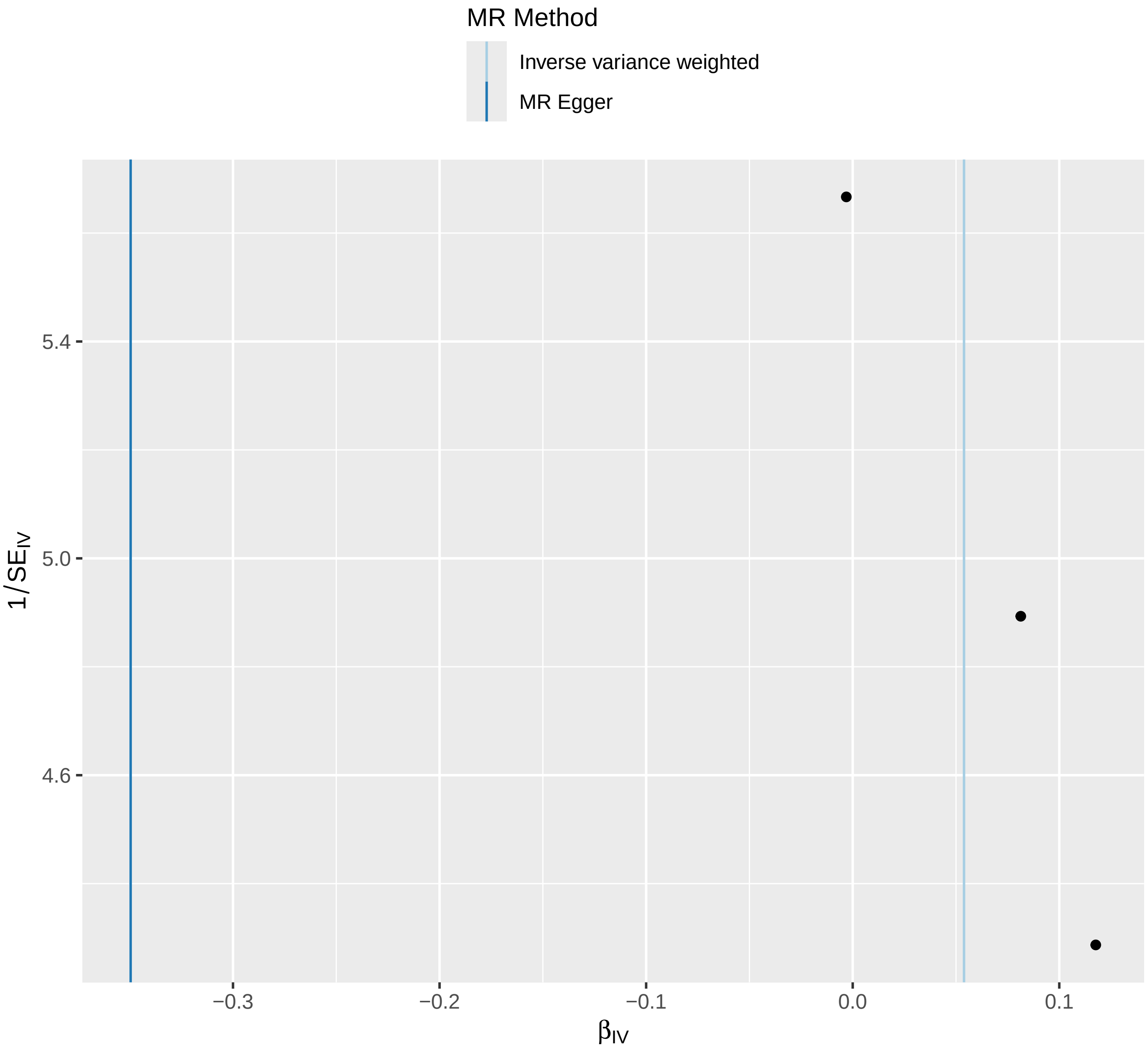

MR Method
Inverse variance weighted
MR Egger
5.4
5.0
4.6
1/SE$_{IV}$
−0.3
−0.2
−0.1
0.0
0.1
$\beta_{IV}$

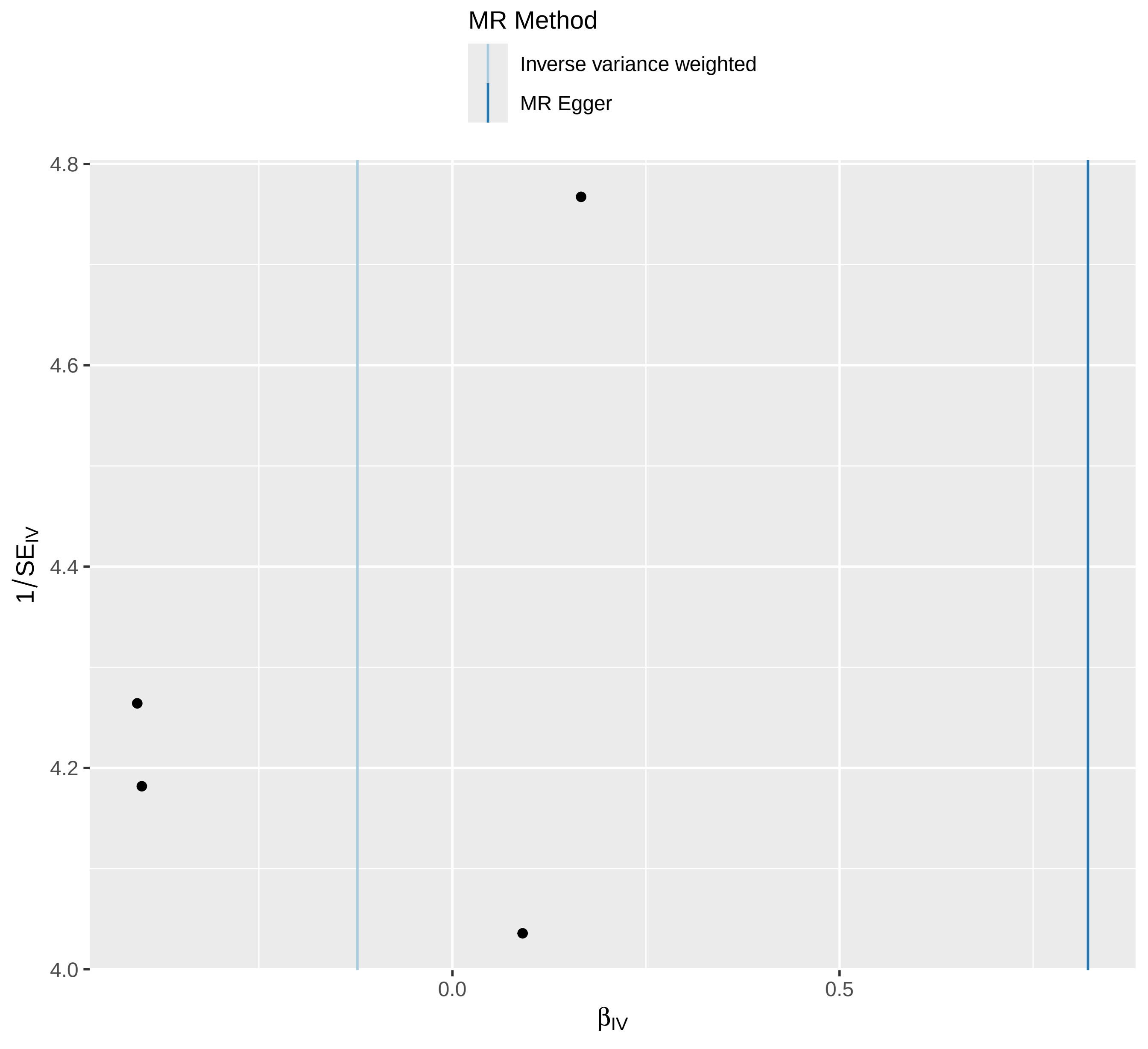

MR Method
Inverse variance weighted
MR Egger
4.8
4.6
4.4
4.2
4.0
1/SE$_{IV}$
0.0
0.5
$\beta_{IV}$

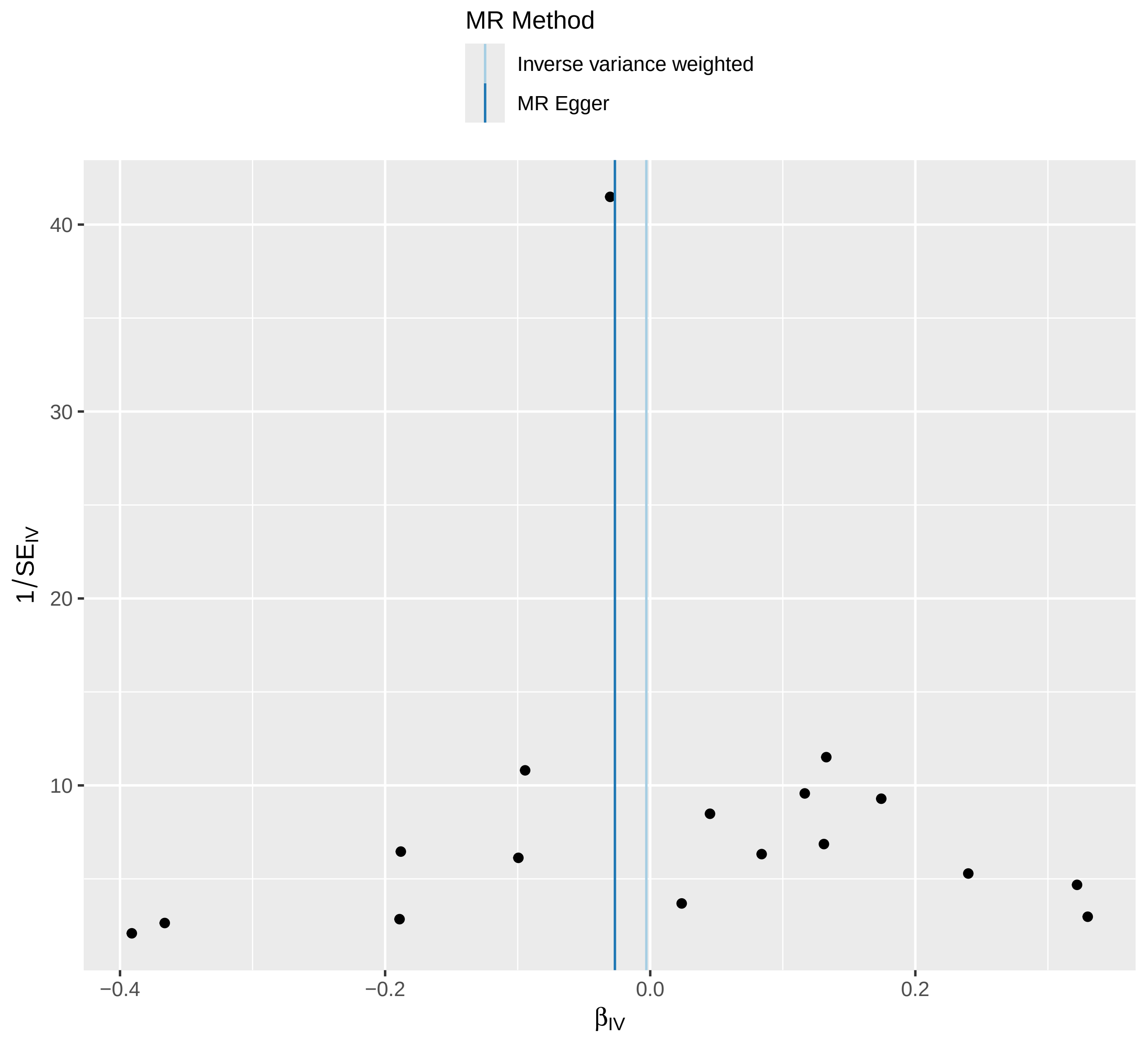

MR Method
Inverse variance weighted
MR Egger
40
30
20
10
1/SE_IV
−0.4
−0.2
0.0
0.2
β_IV

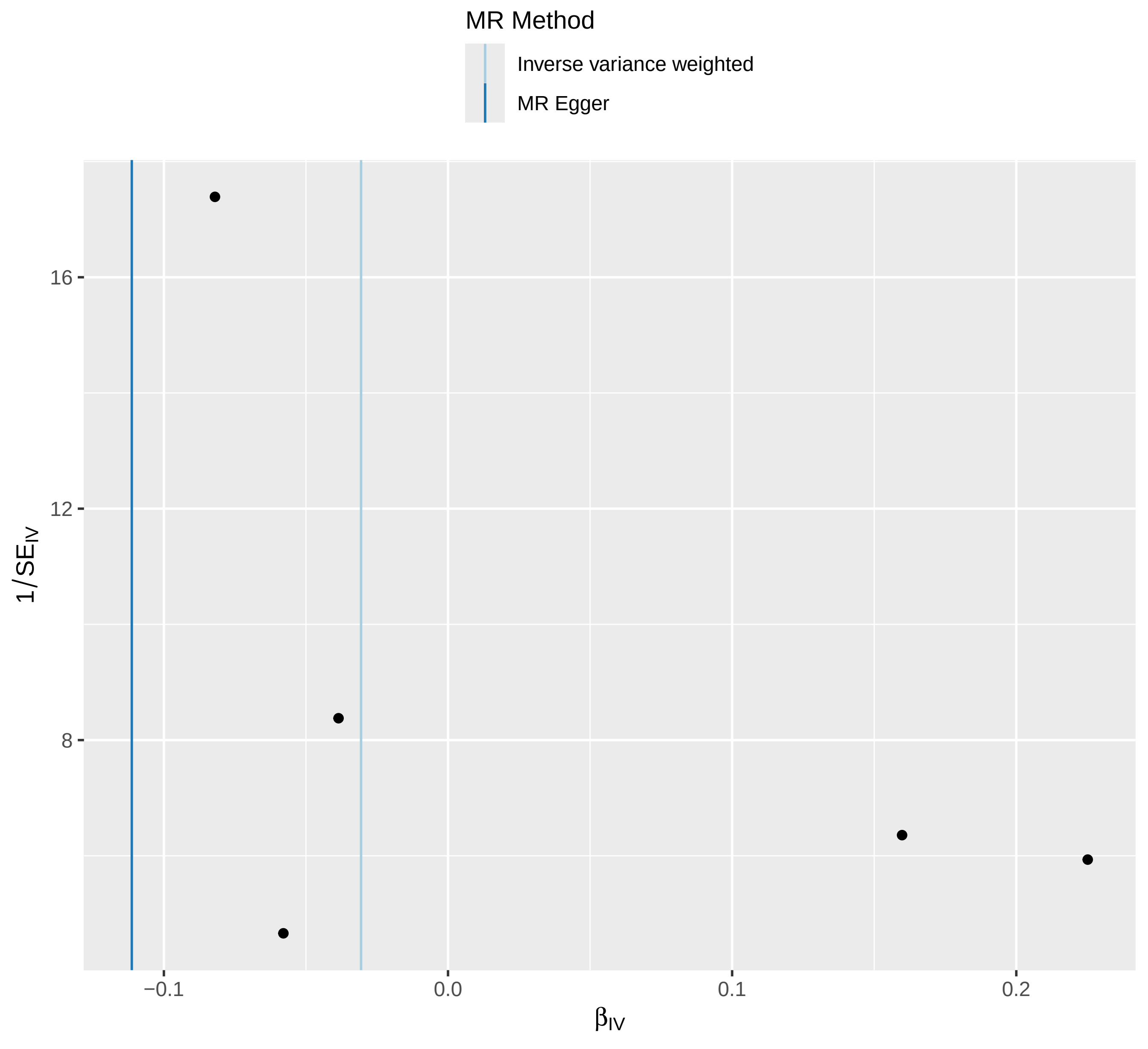

MR Method
Inverse variance weighted
MR Egger
16
12
8
1/SE$_{IV}$
−0.1
0.0
0.1
0.2
β$_{IV}$

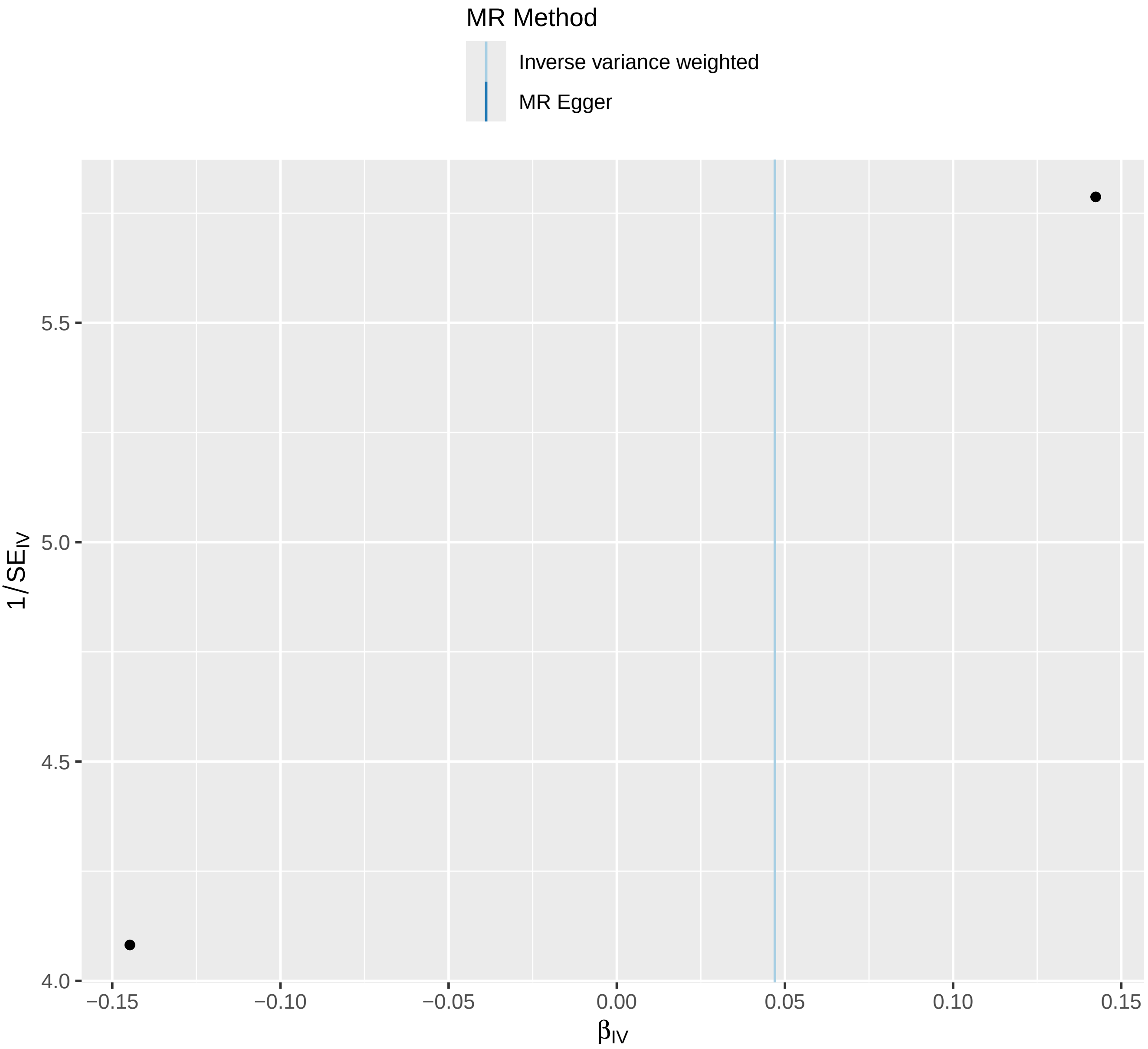
MR Method
Inverse variance weighted
MR Egger
5.5
5.0
4.5
4.0
1/SE_IV
−0.15
−0.10
−0.05
0.00
0.05
0.10
0.15
β_IV

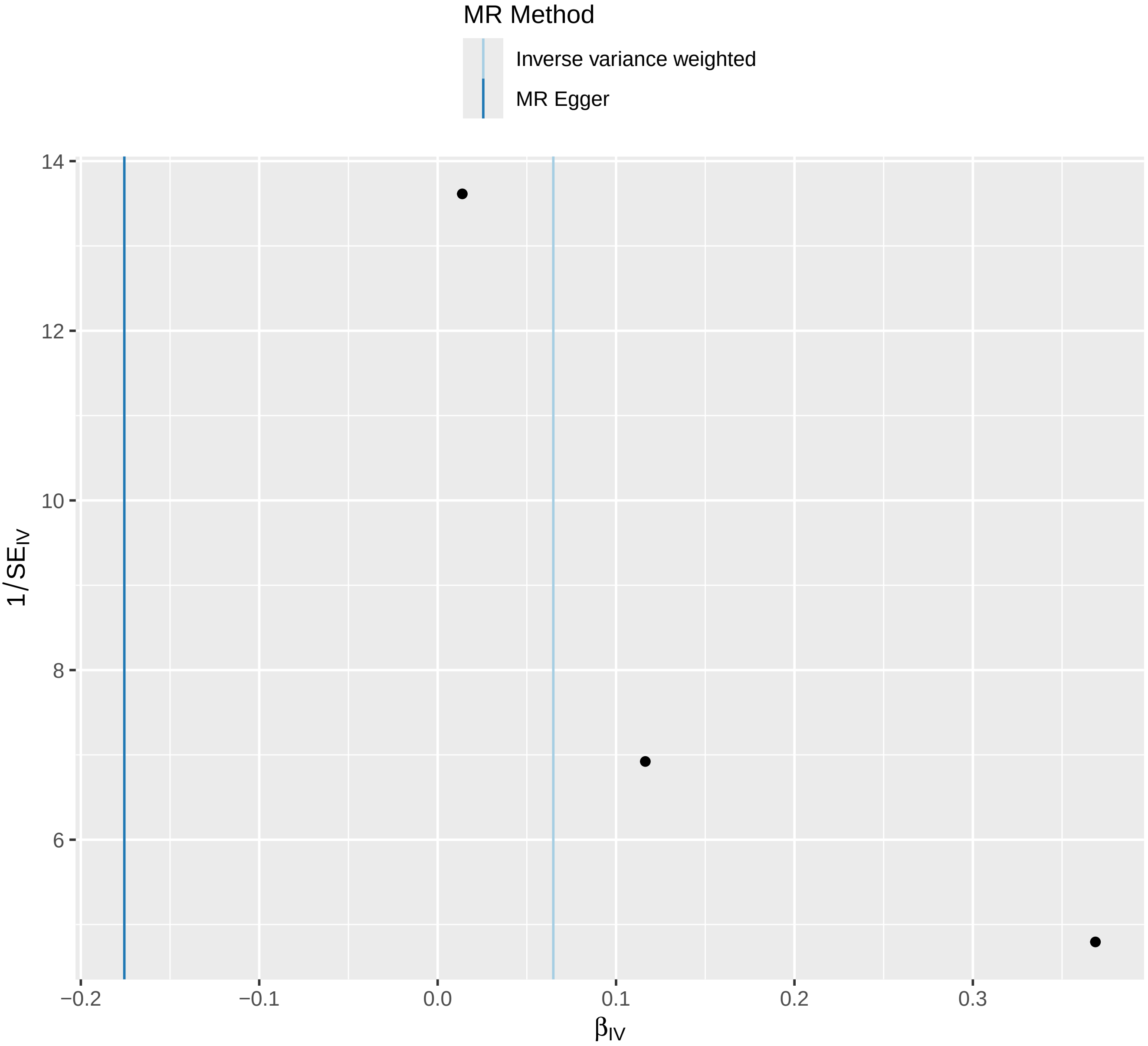

MR Method
Inverse variance weighted
MR Egger
14
12
10
8
6
1/SE$_{IV}$
−0.2
−0.1
0.0
0.1
0.2
0.3
β$_{IV}$

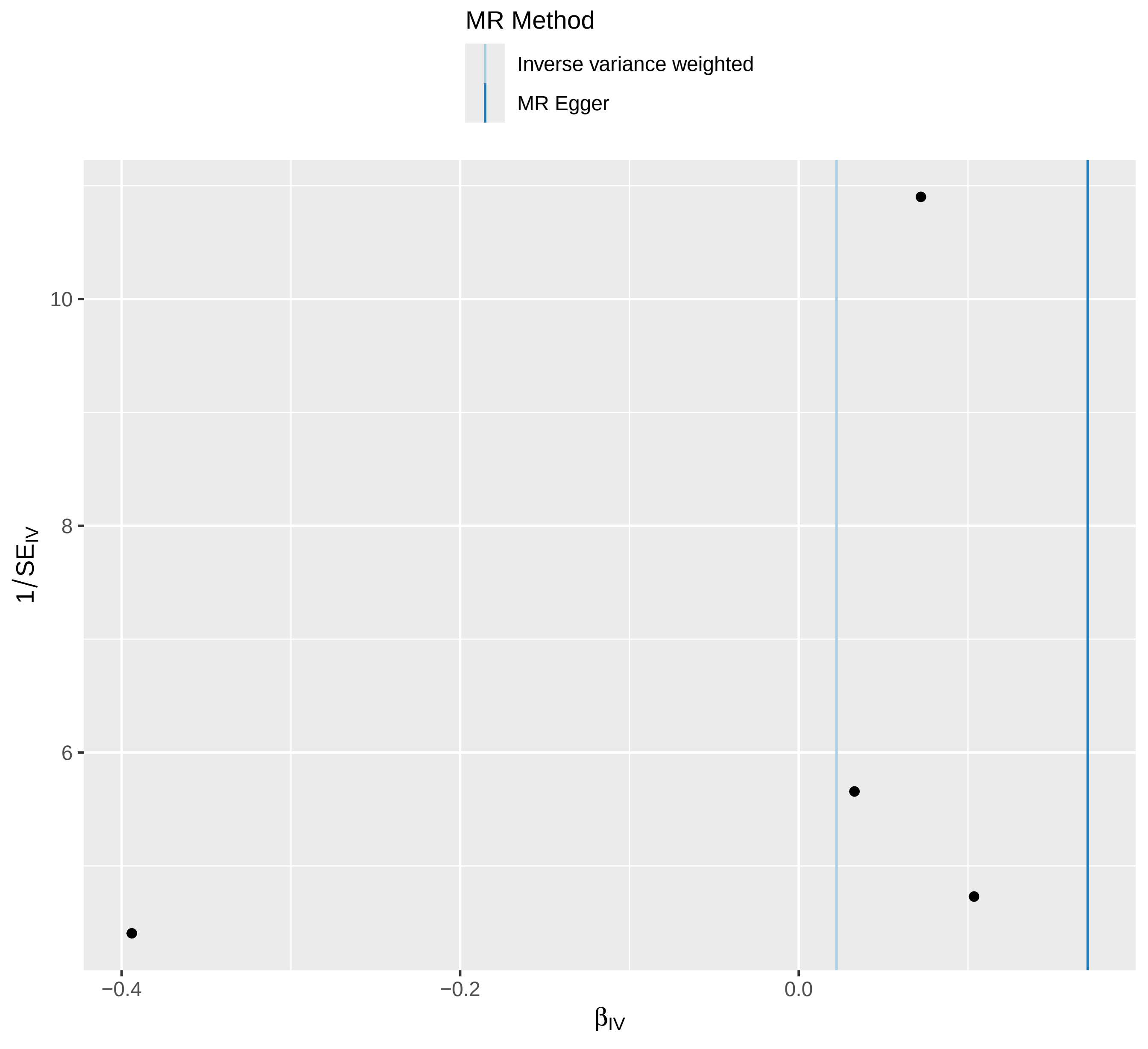

MR Method
Inverse variance weighted
MR Egger
10
8
6
1/SE$_{IV}$
−0.4
−0.2
0.0
β$_{IV}$

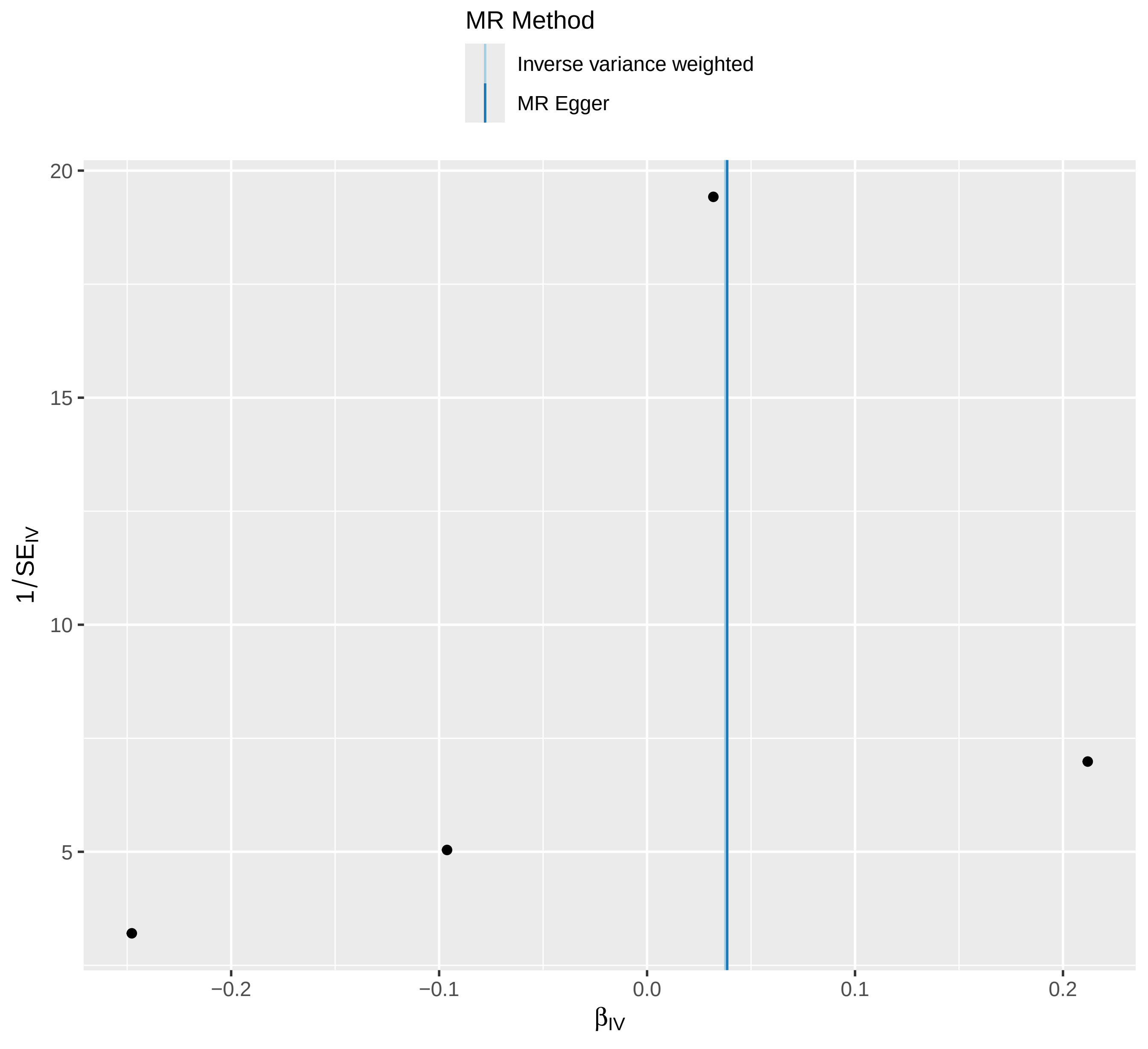
MR Method
Inverse variance weighted
MR Egger
20
15
10
5
$1/SE_{IV}$
−0.2
−0.1
0.0
0.1
0.2
$\beta_{IV}$

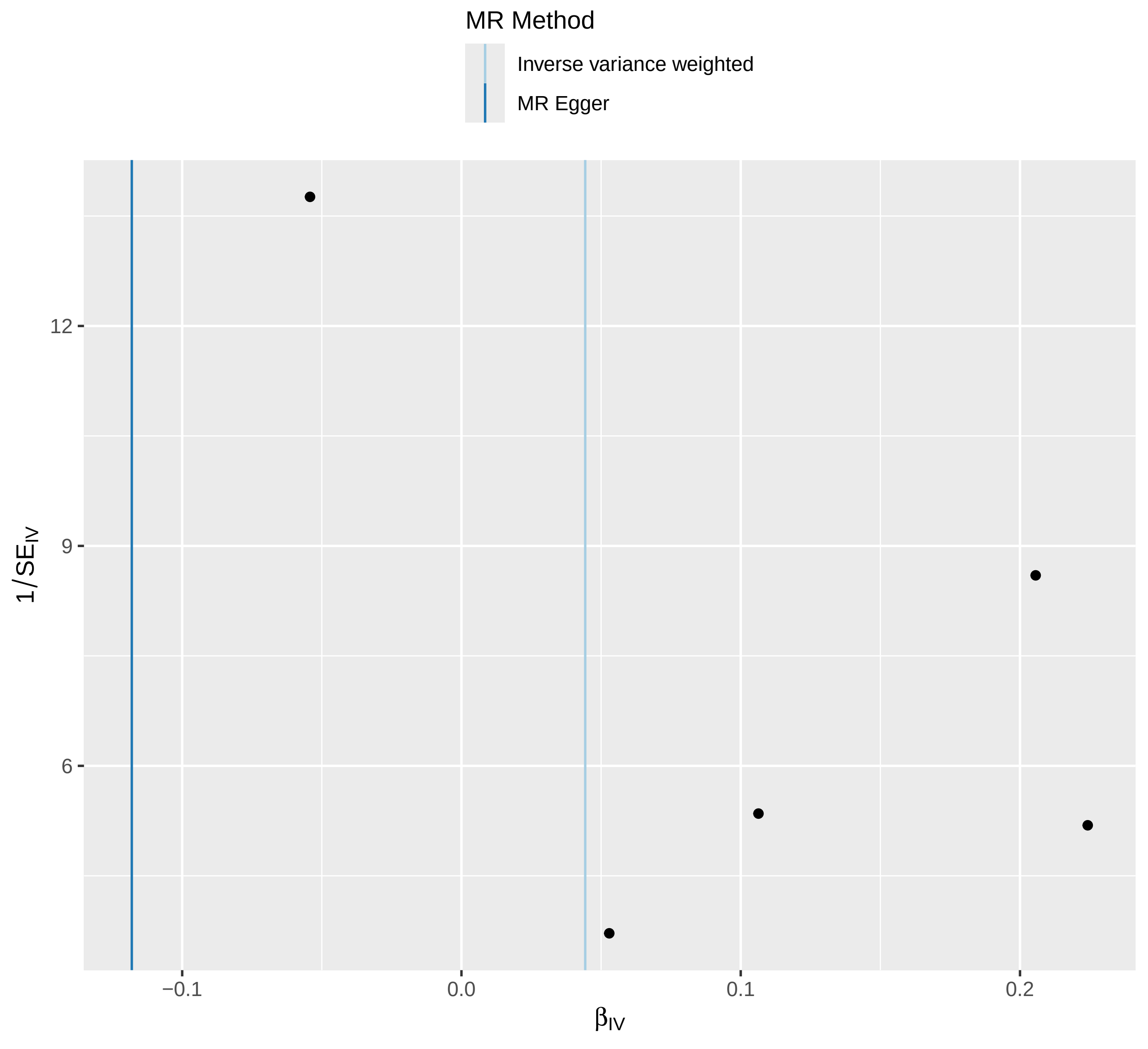

MR Method
Inverse variance weighted
MR Egger
12
9
6
$1/SE_{IV}$
−0.1
0.0
0.1
0.2
$\beta_{IV}$

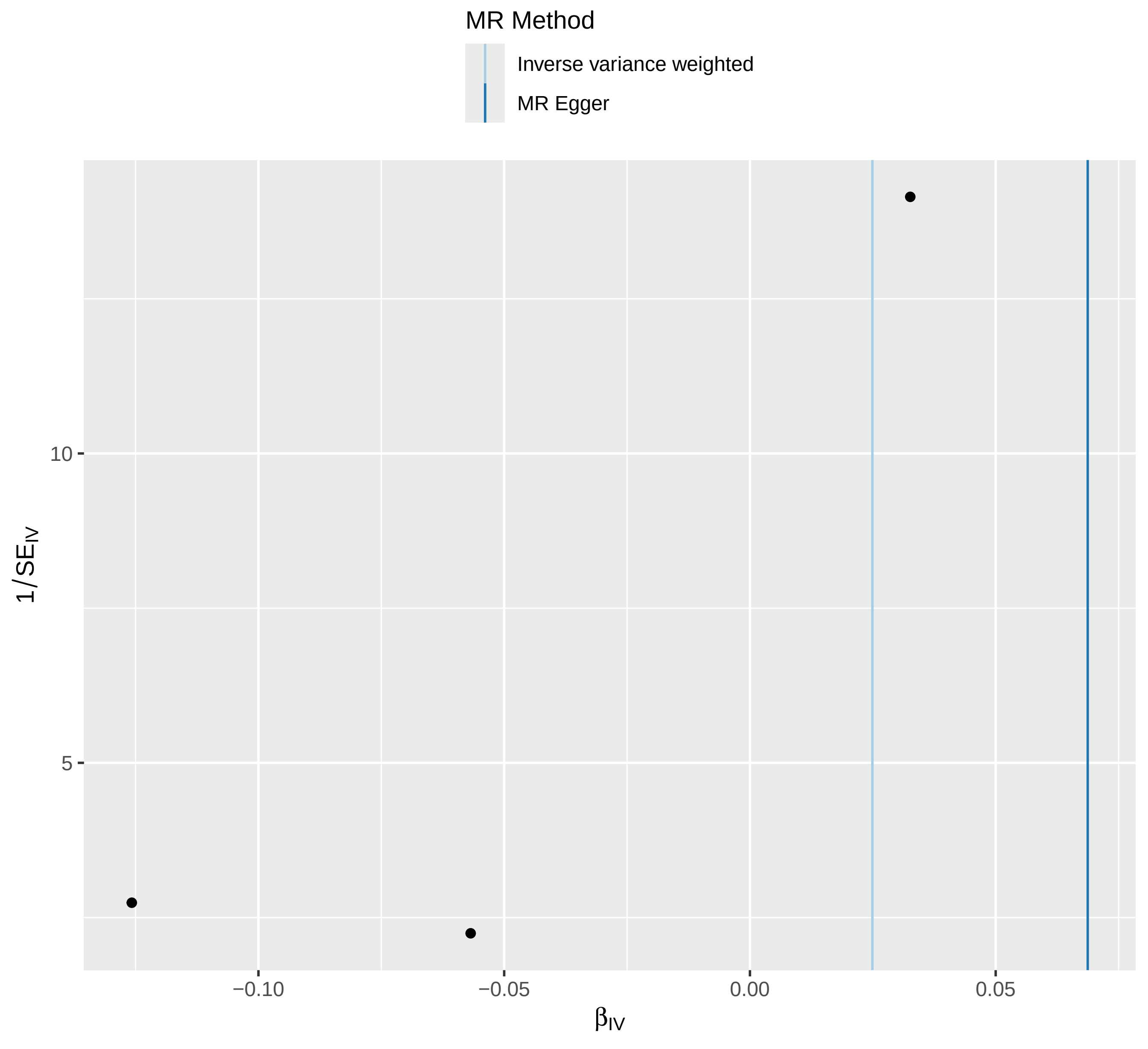
MR Method
Inverse variance weighted
MR Egger
10
5
1/SE$_{IV}$
−0.10
−0.05
0.00
0.05
β$_{IV}$

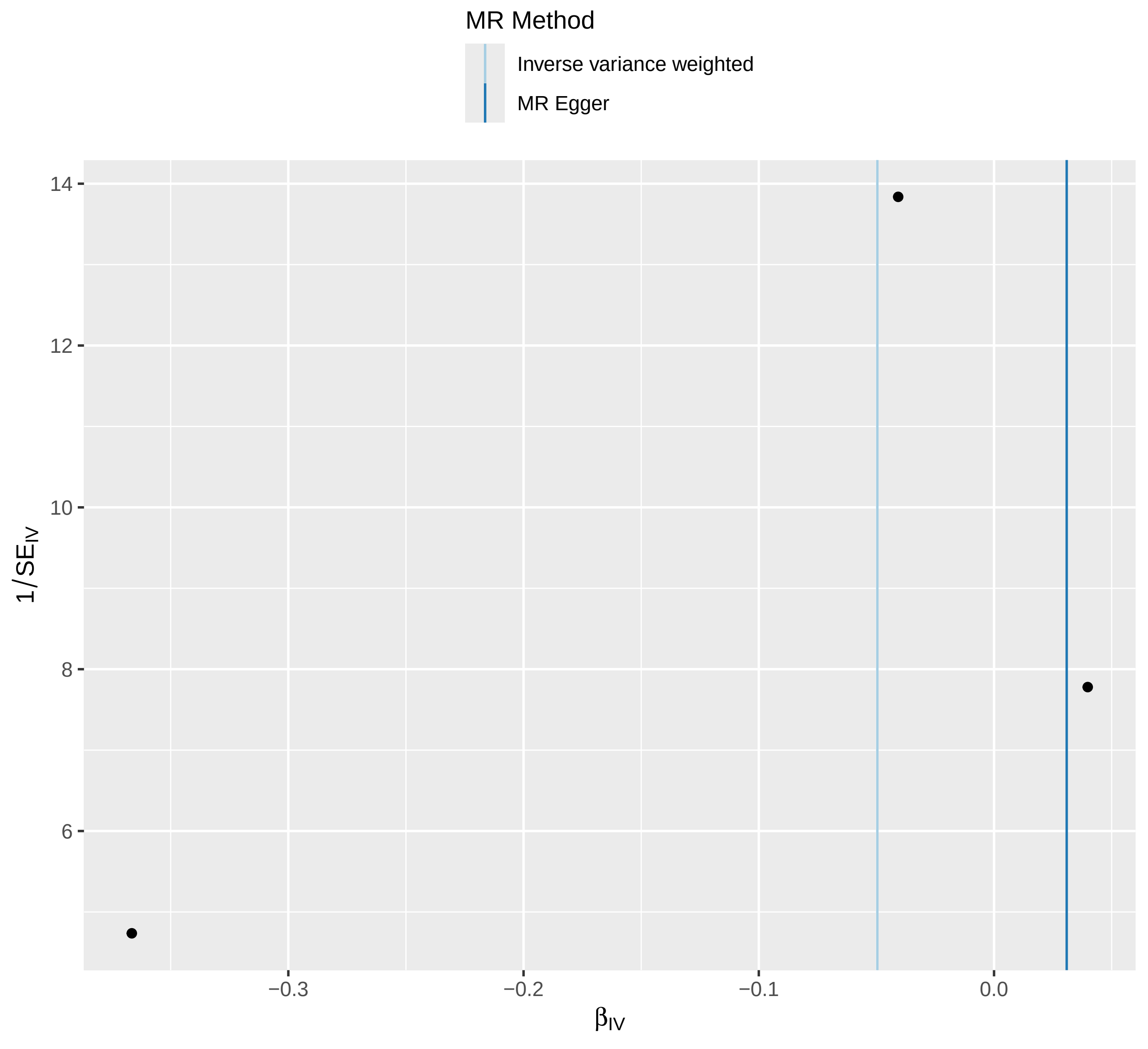
MR Method
Inverse variance weighted
MR Egger
14
12
10
8
6
1/SE_IV
−0.3
−0.2
−0.1
0.0
β_IV

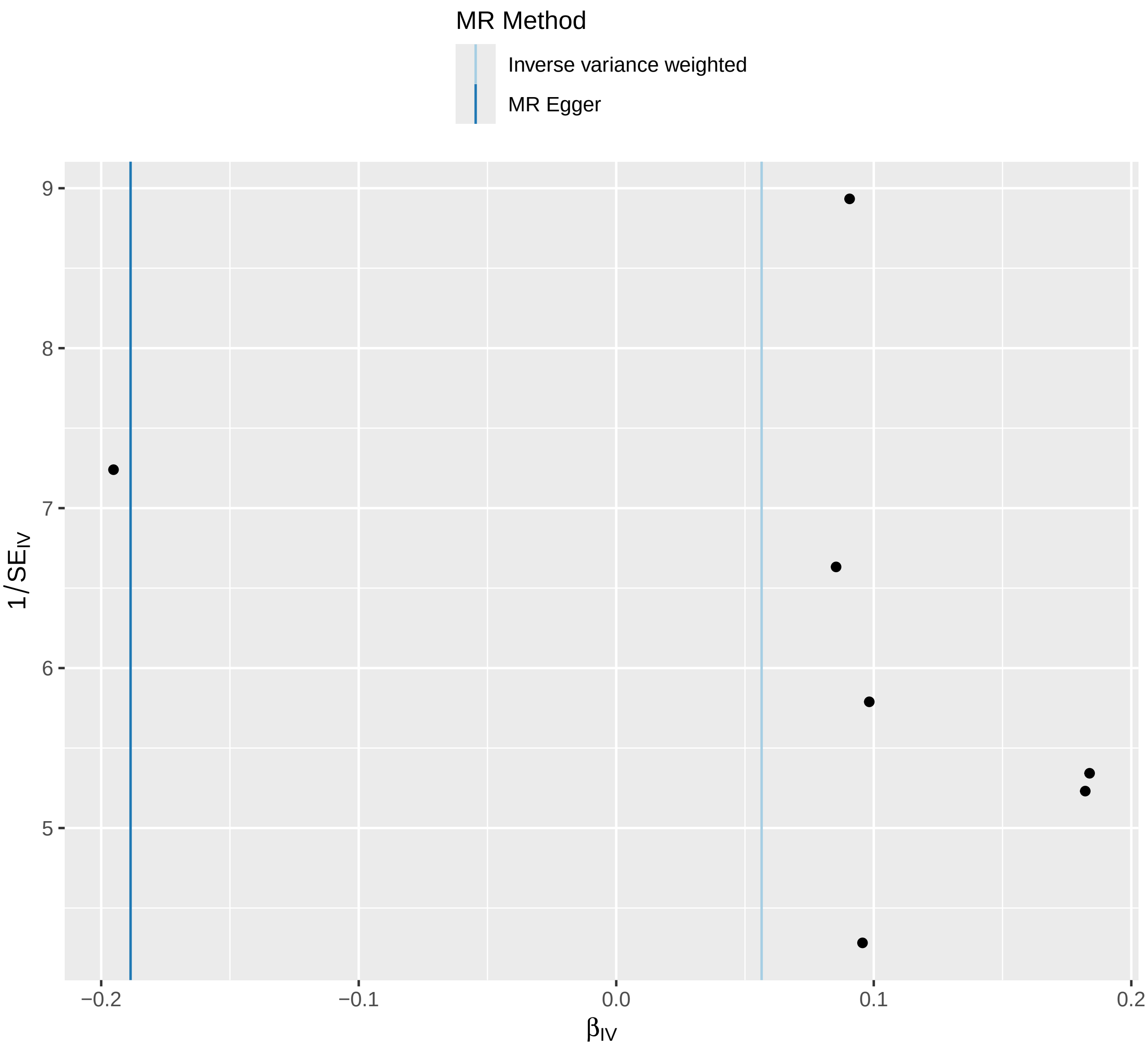
MR Method
Inverse variance weighted
MR Egger
9
8
7
6
5
1/SE_IV
−0.2
−0.1
0.0
0.1
0.2
β_IV

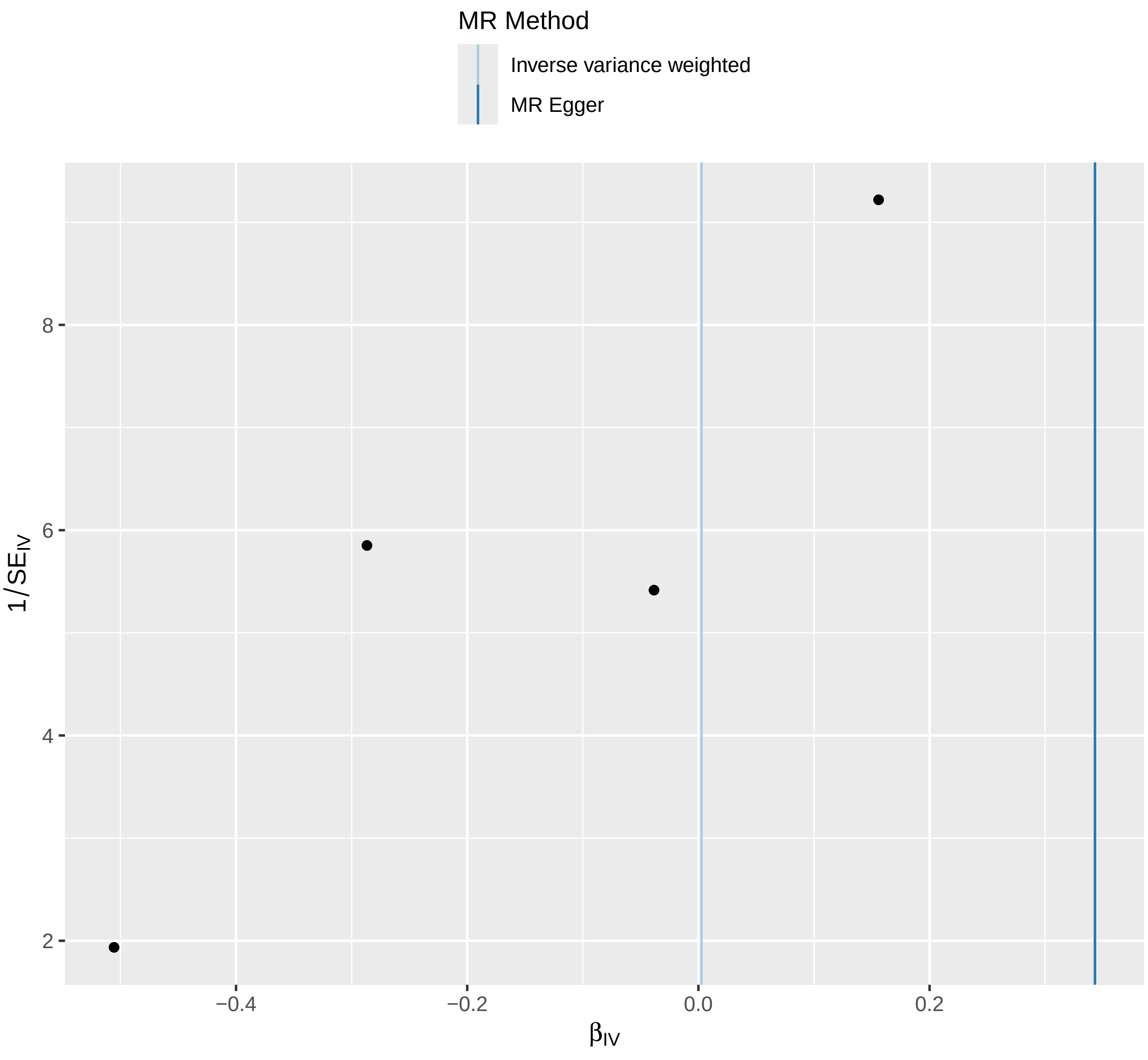
MR Method
Inverse variance weighted
MR Egger
8
6
4
2
1/SE_IV
−0.4
−0.2
0.0
0.2
β_IV

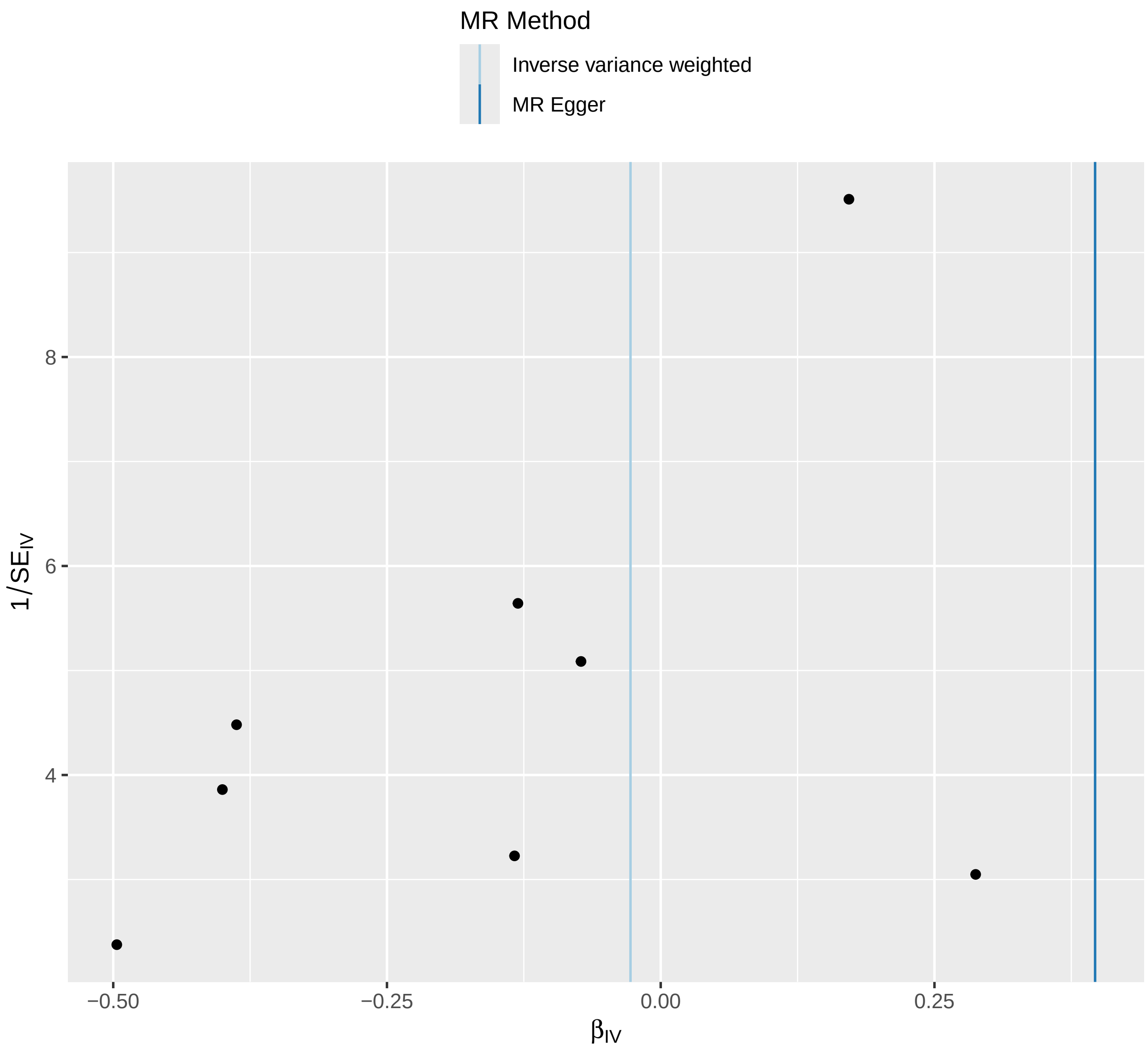

MR Method
Inverse variance weighted
MR Egger
1/SE_IV
8
6
4
−0.50
−0.25
0.00
0.25
β_IV

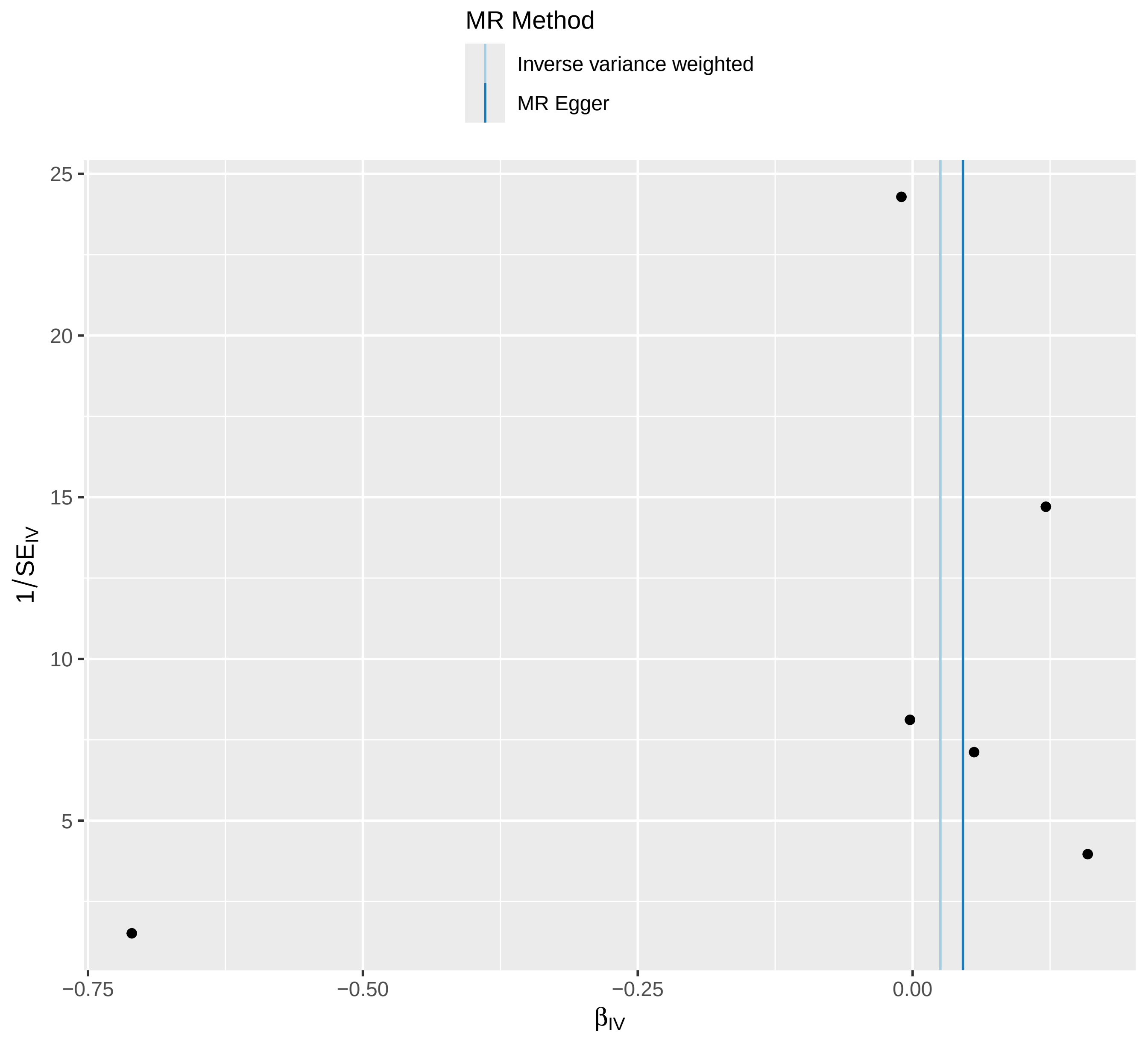
MR Method
Inverse variance weighted
MR Egger
1/SE_IV
25
20
15
10
5
−0.75
−0.50
−0.25
0.00
β_IV

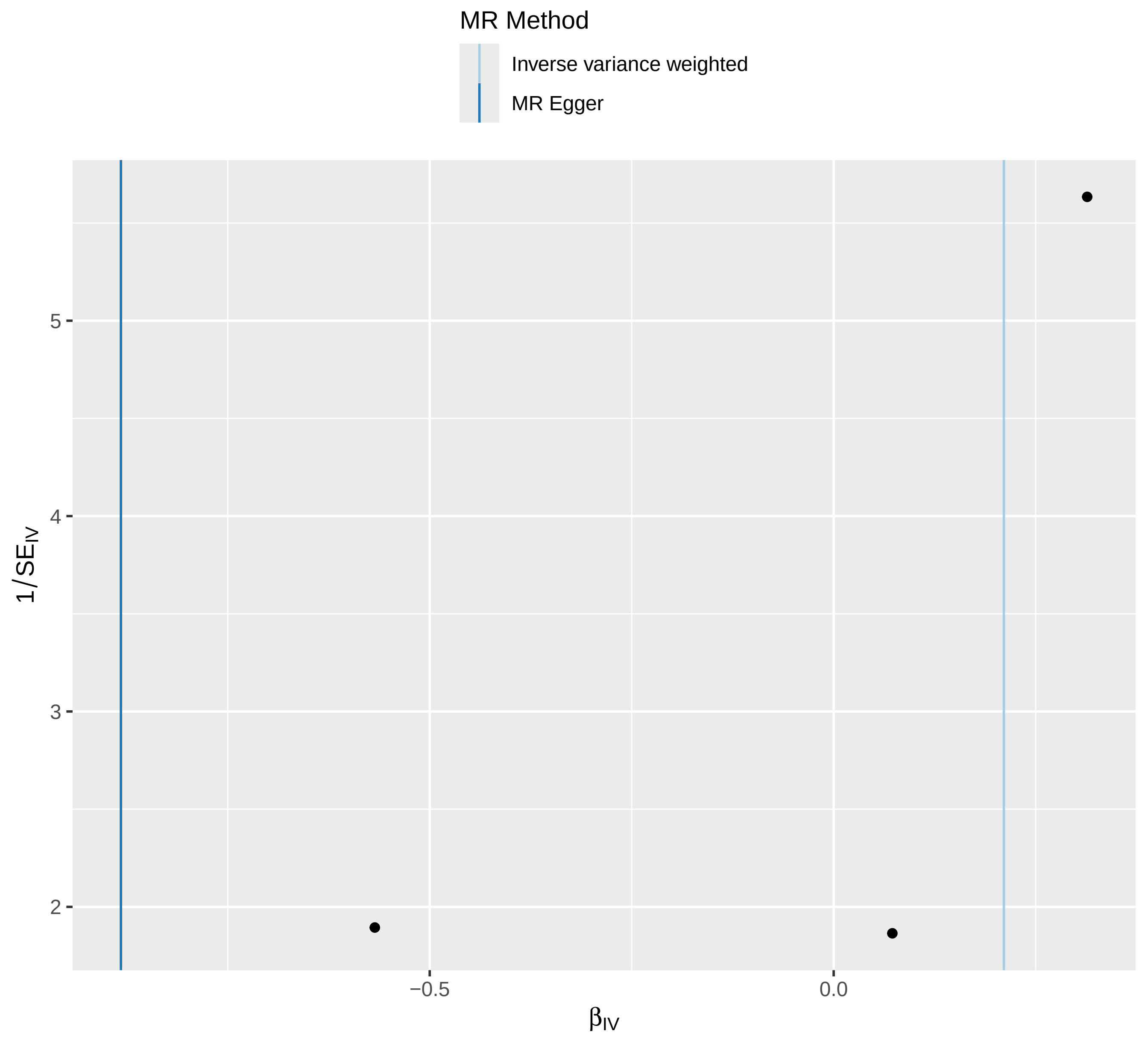
MR Method
Inverse variance weighted
MR Egger
5
4
3
2
1/SE_IV
−0.5
0.0
β_IV

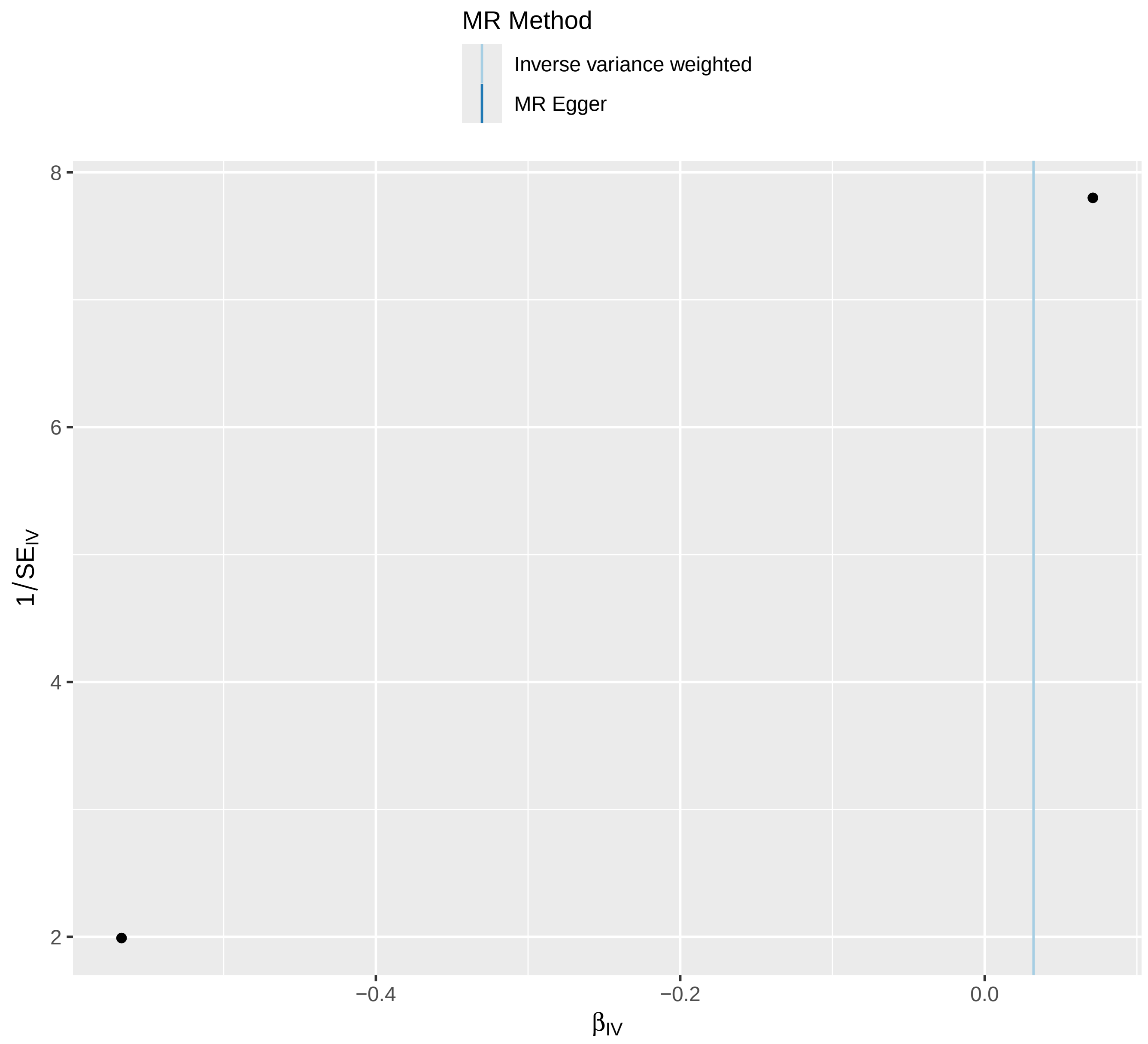
MR Method
Inverse variance weighted
MR Egger
8
6
4
2
1/SE$_{IV}$
−0.4
−0.2
0.0
β$_{IV}$

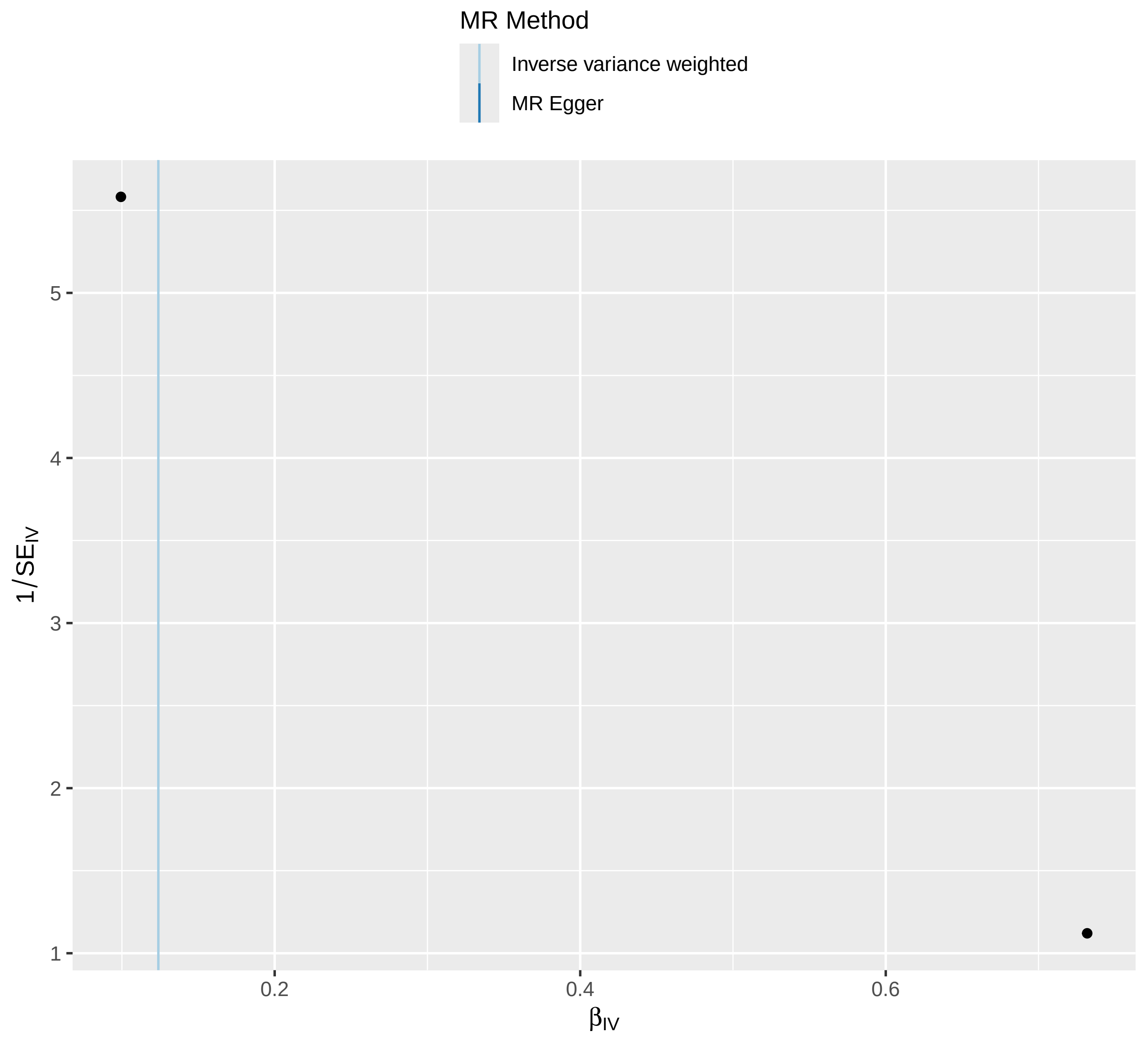

MR Method
Inverse variance weighted
MR Egger
1/SE$_{IV}$
5
4
3
2
1
0.2
0.4
0.6
β$_{IV}$

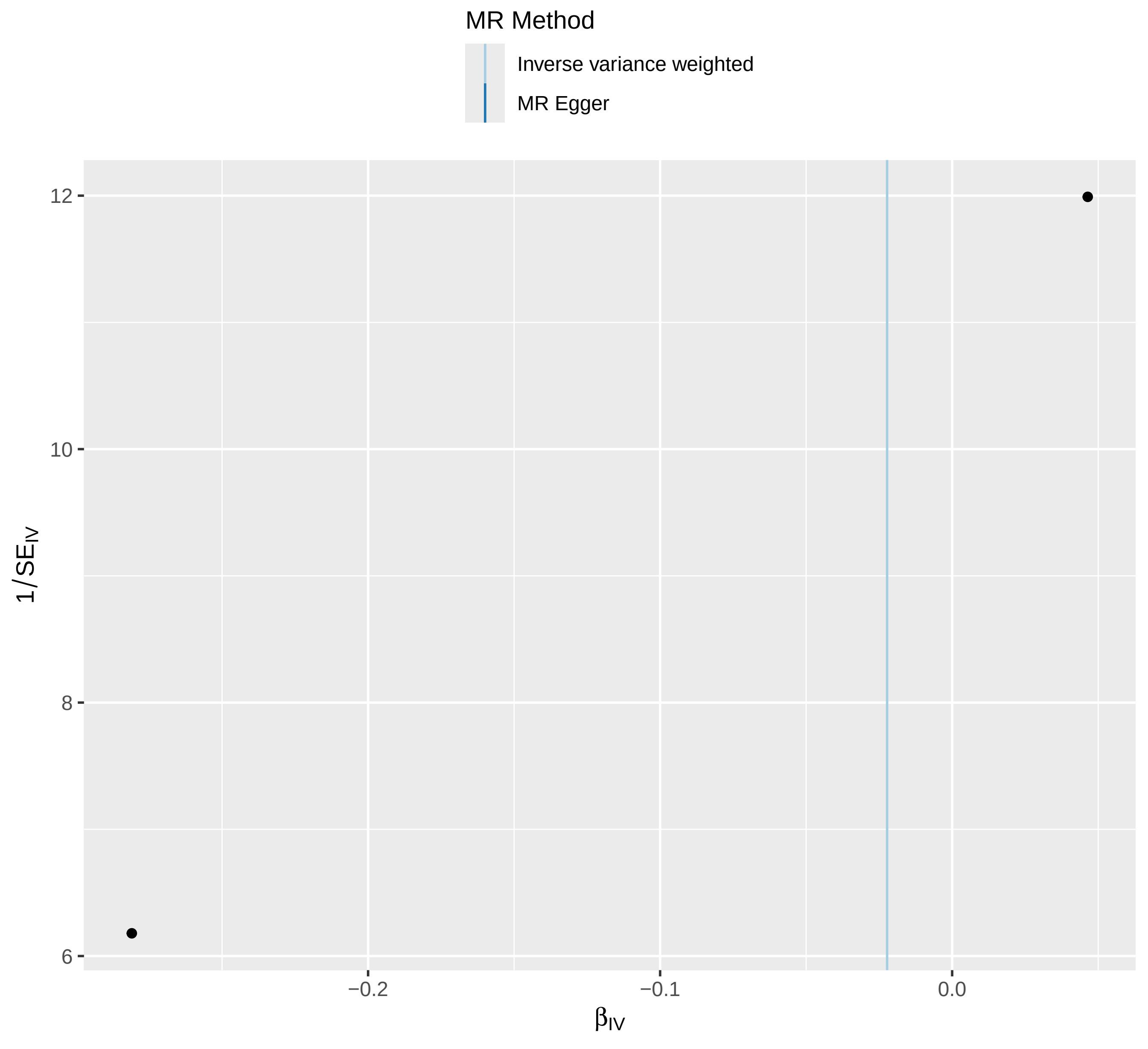
MR Method
Inverse variance weighted
MR Egger
12
10
8
6
1/SE_IV
−0.2
−0.1
0.0
β_IV

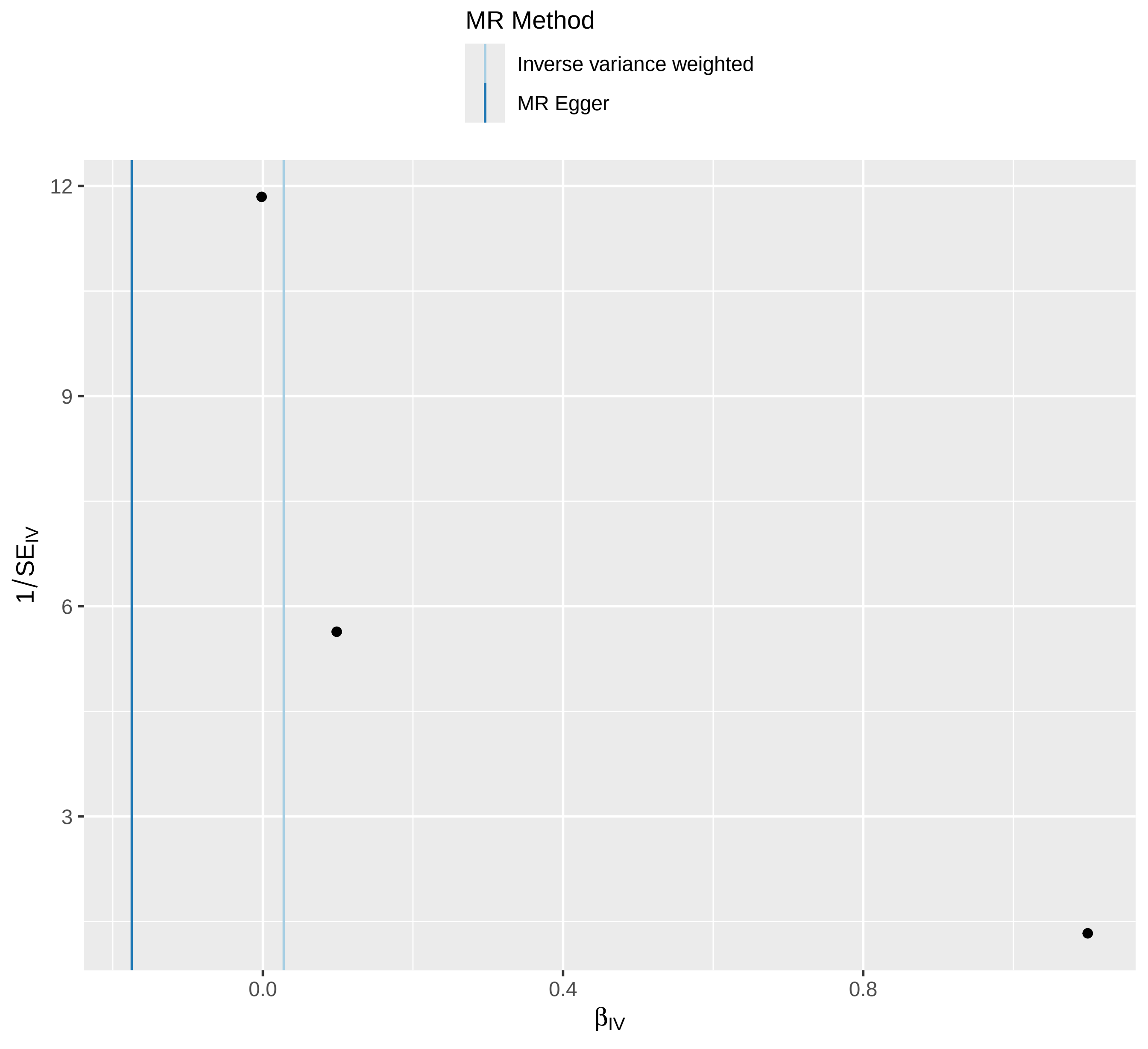
MR Method
Inverse variance weighted
MR Egger
12
9
6
3
1/SE_IV
0.0
0.4
0.8
β_IV

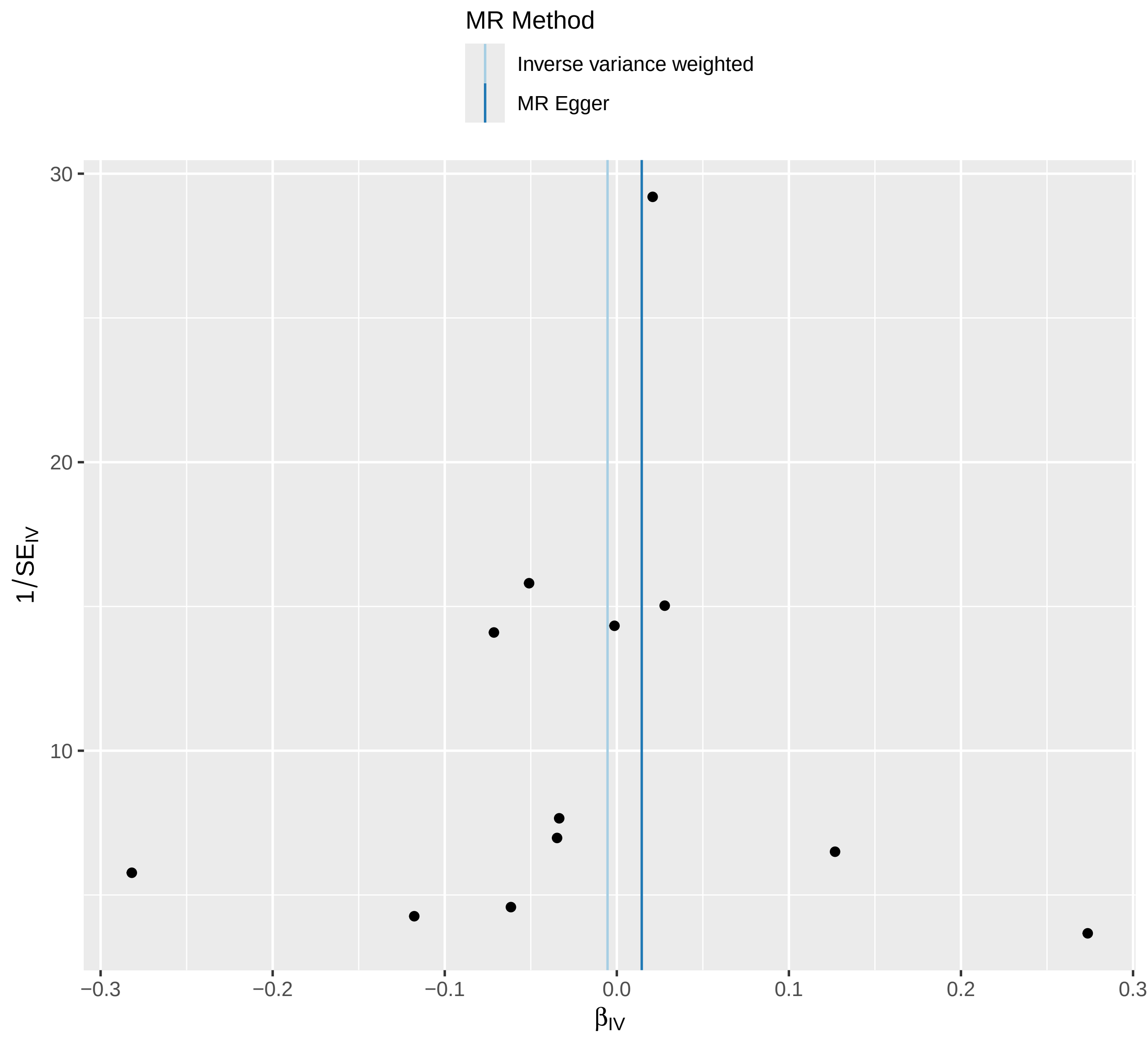

MR Method
Inverse variance weighted
MR Egger
30
20
10
1/SE_IV
−0.3
−0.2
−0.1
0.0
0.1
0.2
0.3
β_IV

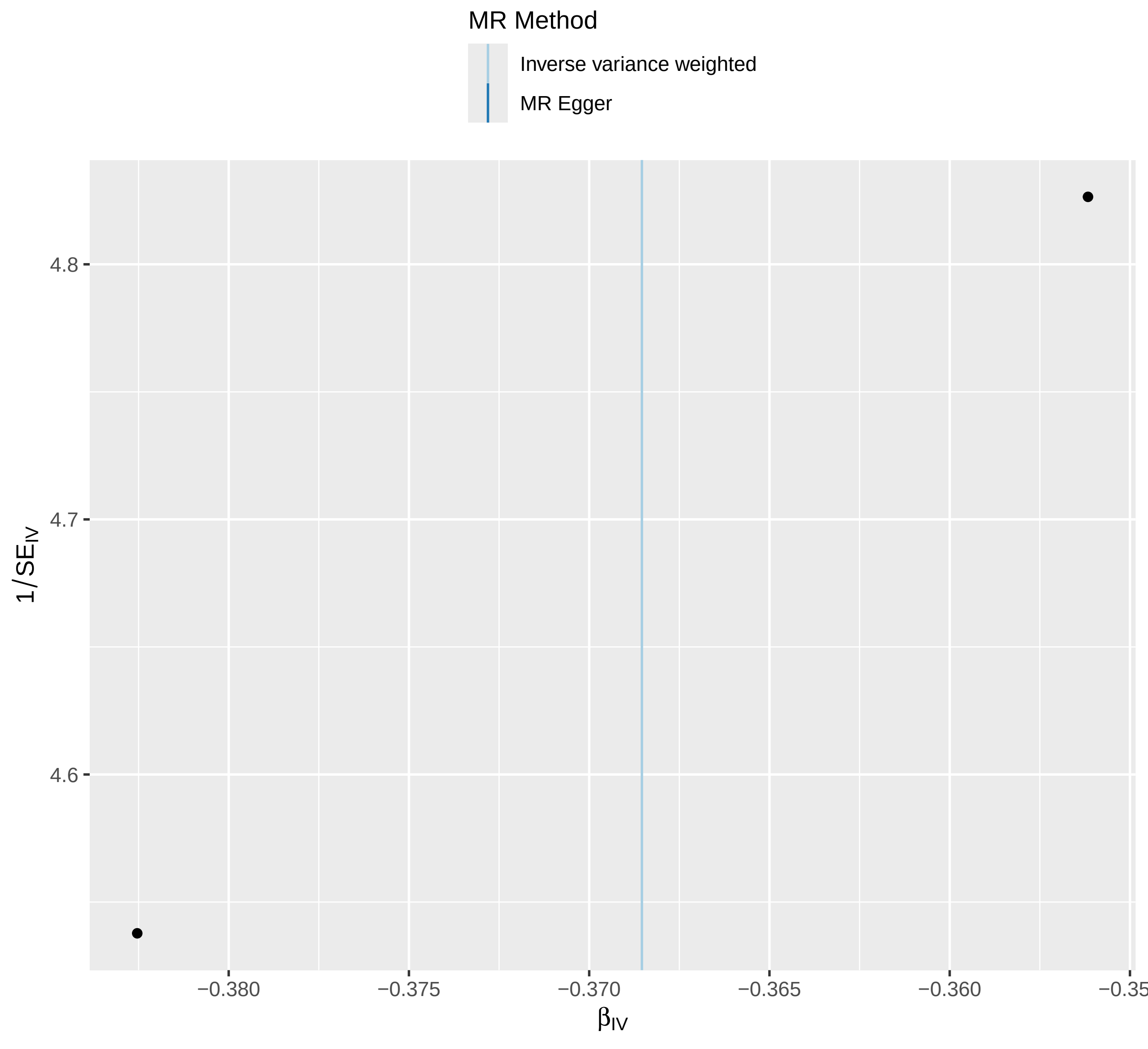

MR Method
Inverse variance weighted
MR Egger
4.8
4.7
4.6
1/SE_IV
−0.380
−0.375
−0.370
−0.365
−0.360
−0.35
β_IV

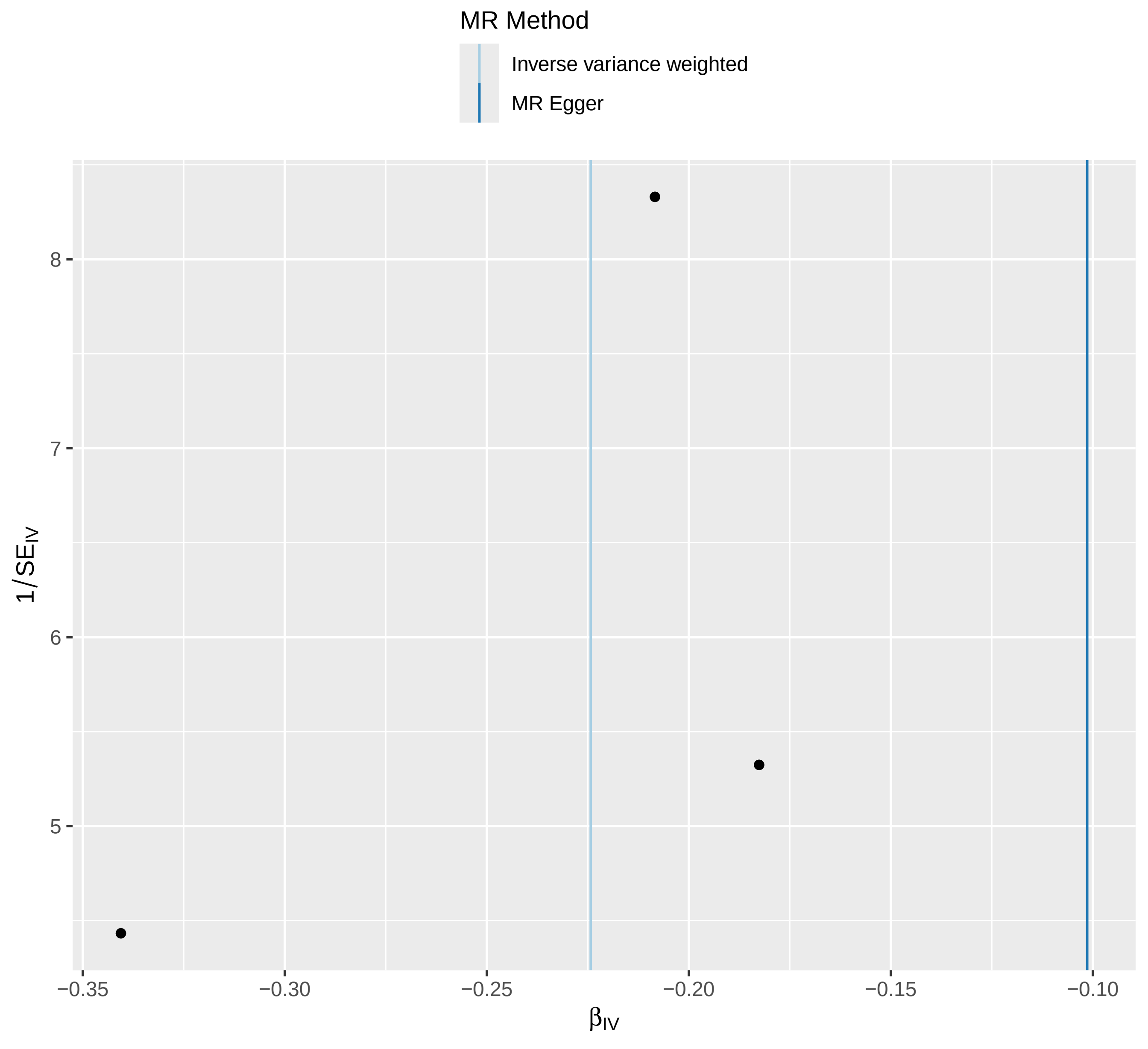

MR Method
Inverse variance weighted
MR Egger
8
7
6
5
1/SE_IV
−0.35
−0.30
−0.25
−0.20
−0.15
−0.10
β_IV

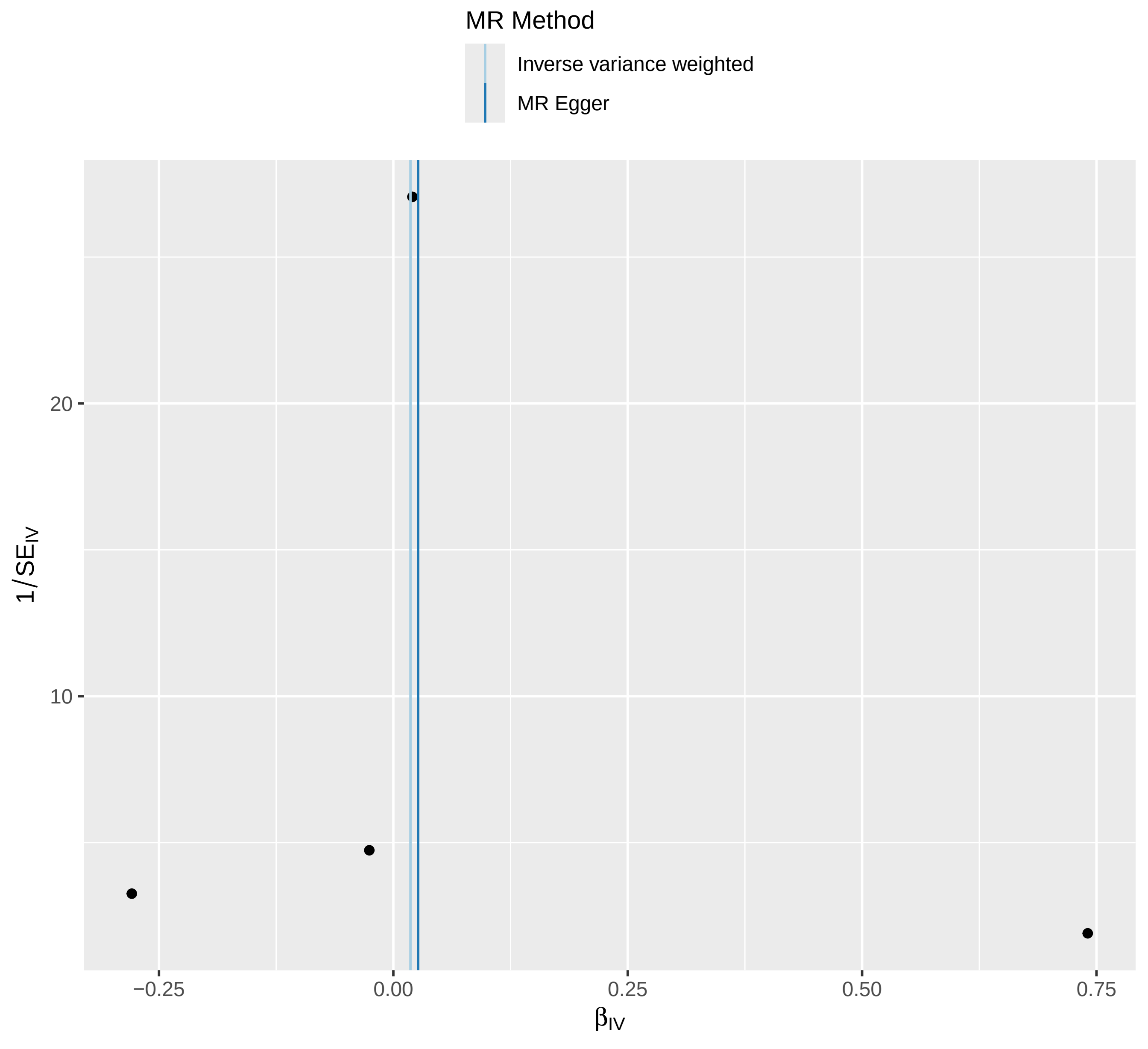

MR Method
Inverse variance weighted
MR Egger
20
10
1/SE$_{IV}$
−0.25
0.00
0.25
0.50
0.75
β$_{IV}$

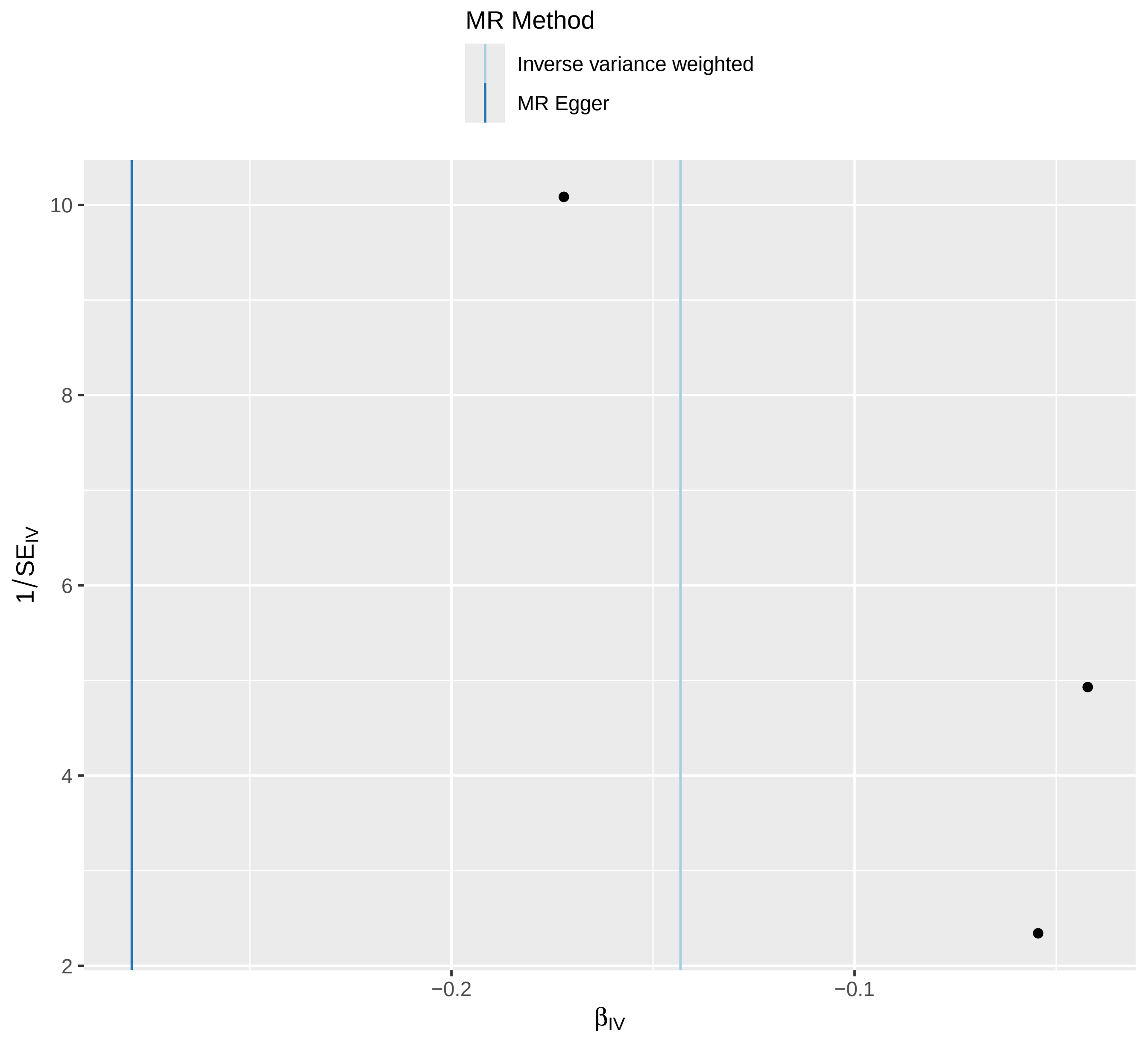
MR Method
Inverse variance weighted
MR Egger
10
8
6
4
2
−0.2
−0.1
$1/SE_{IV}$
$\beta_{IV}$

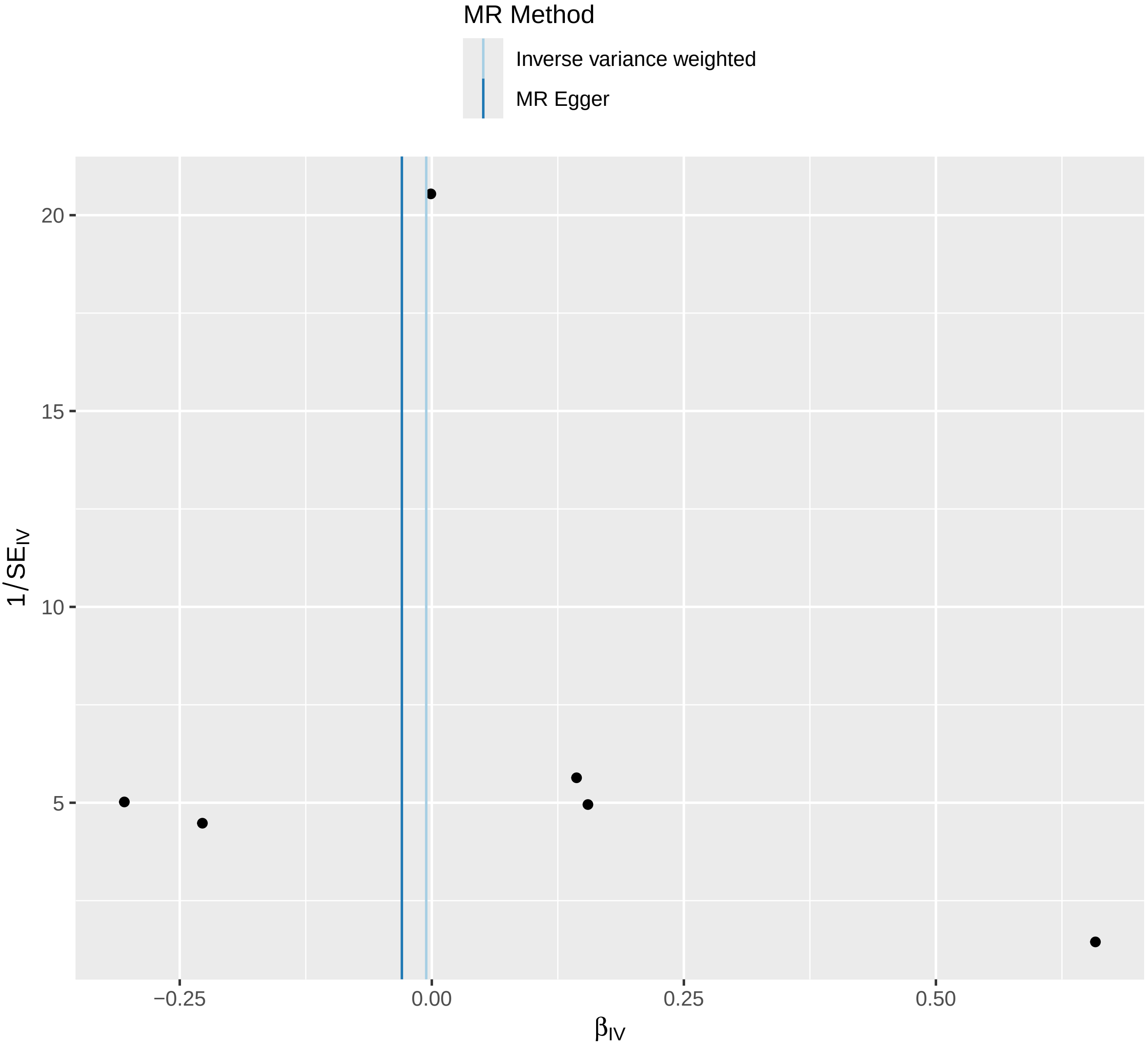
MR Method
Inverse variance weighted
MR Egger
20
15
10
5
1/SE_IV
−0.25
0.00
0.25
0.50
β_IV

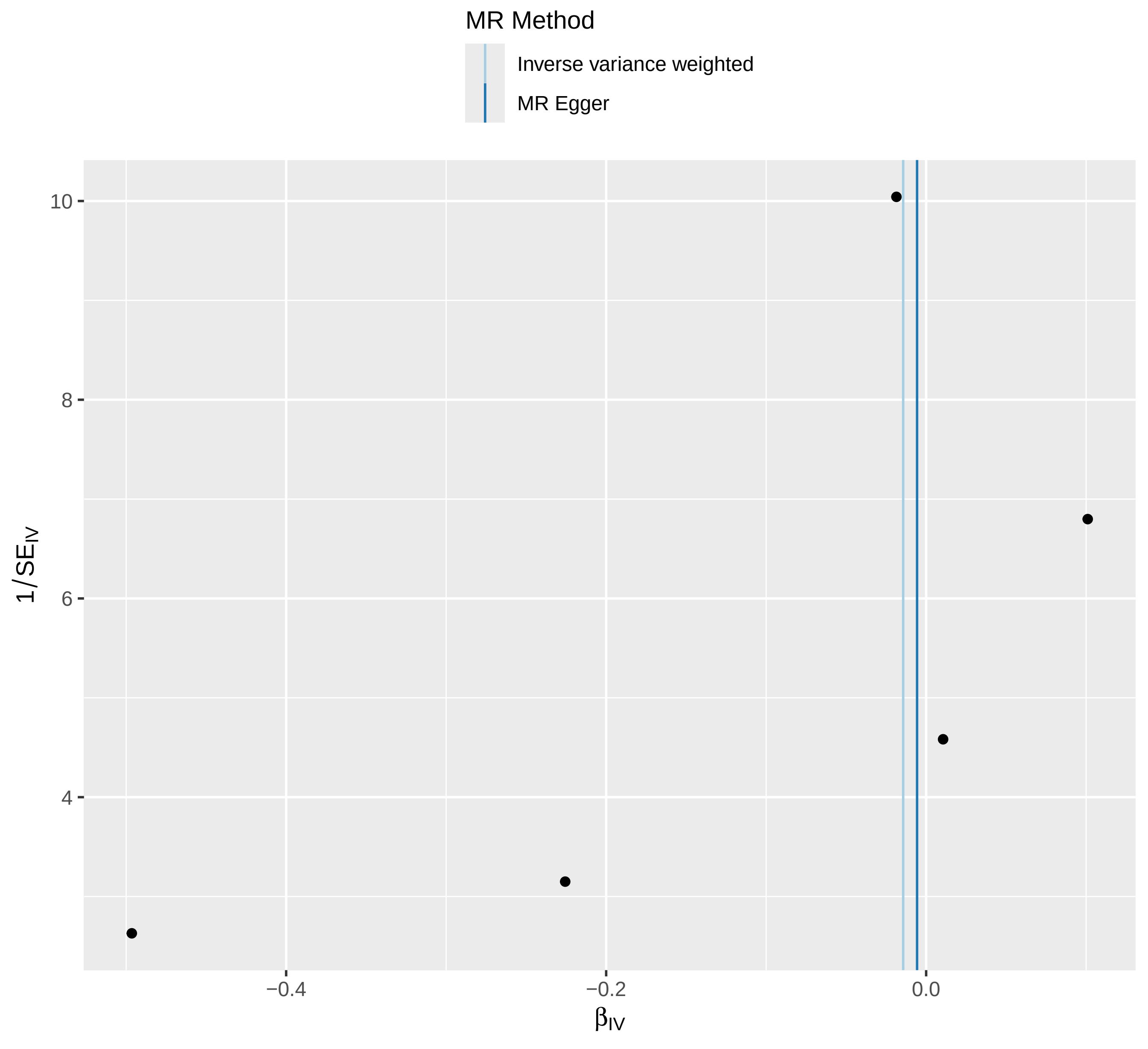
MR Method
Inverse variance weighted
MR Egger
10
8
6
4
1/SE$_{IV}$
−0.4
−0.2
0.0
β$_{IV}$

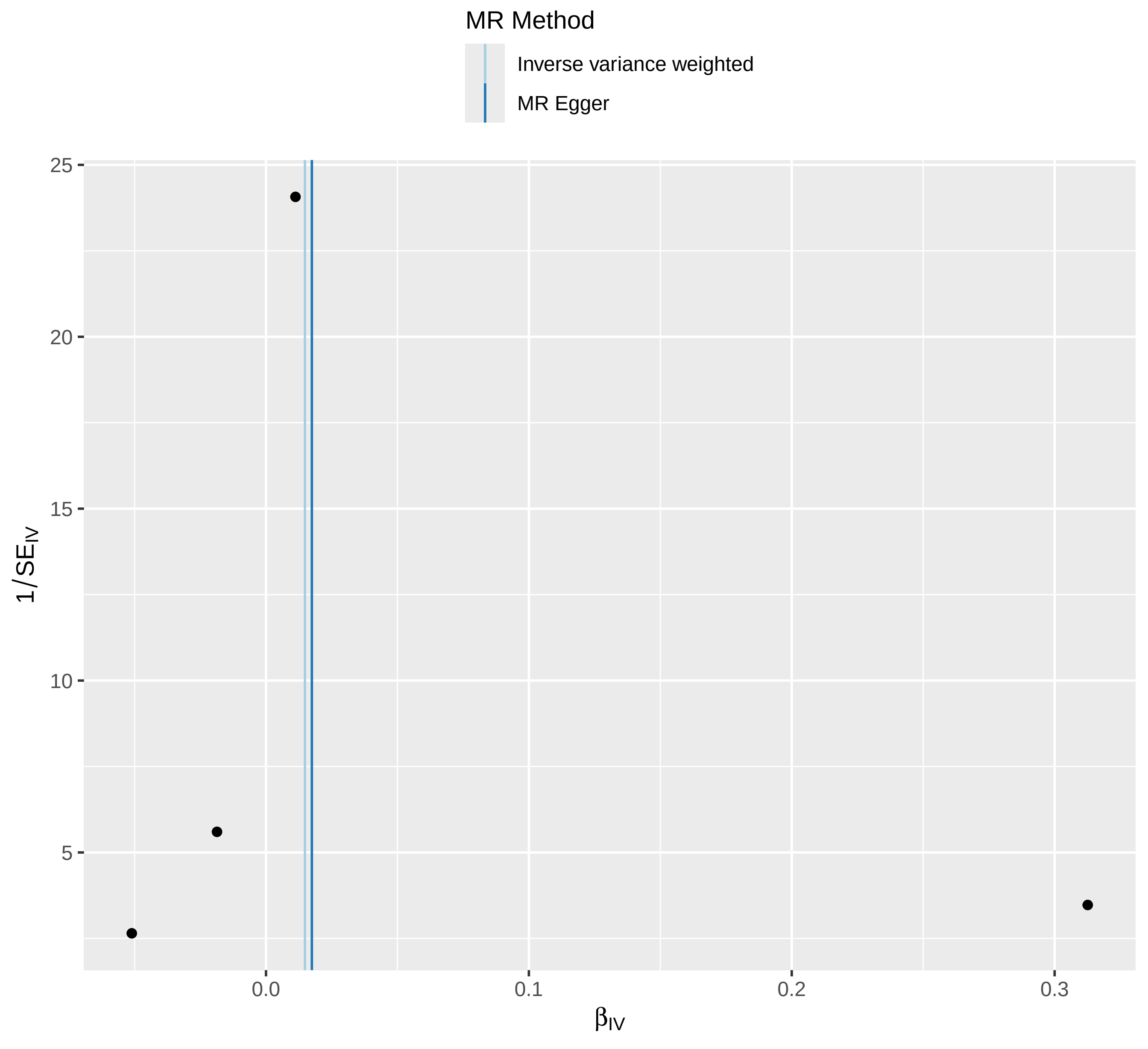

MR Method
Inverse variance weighted
MR Egger
25
20
15
10
5
1/SE$_{IV}$
0.0
0.1
0.2
0.3
β$_{IV}$

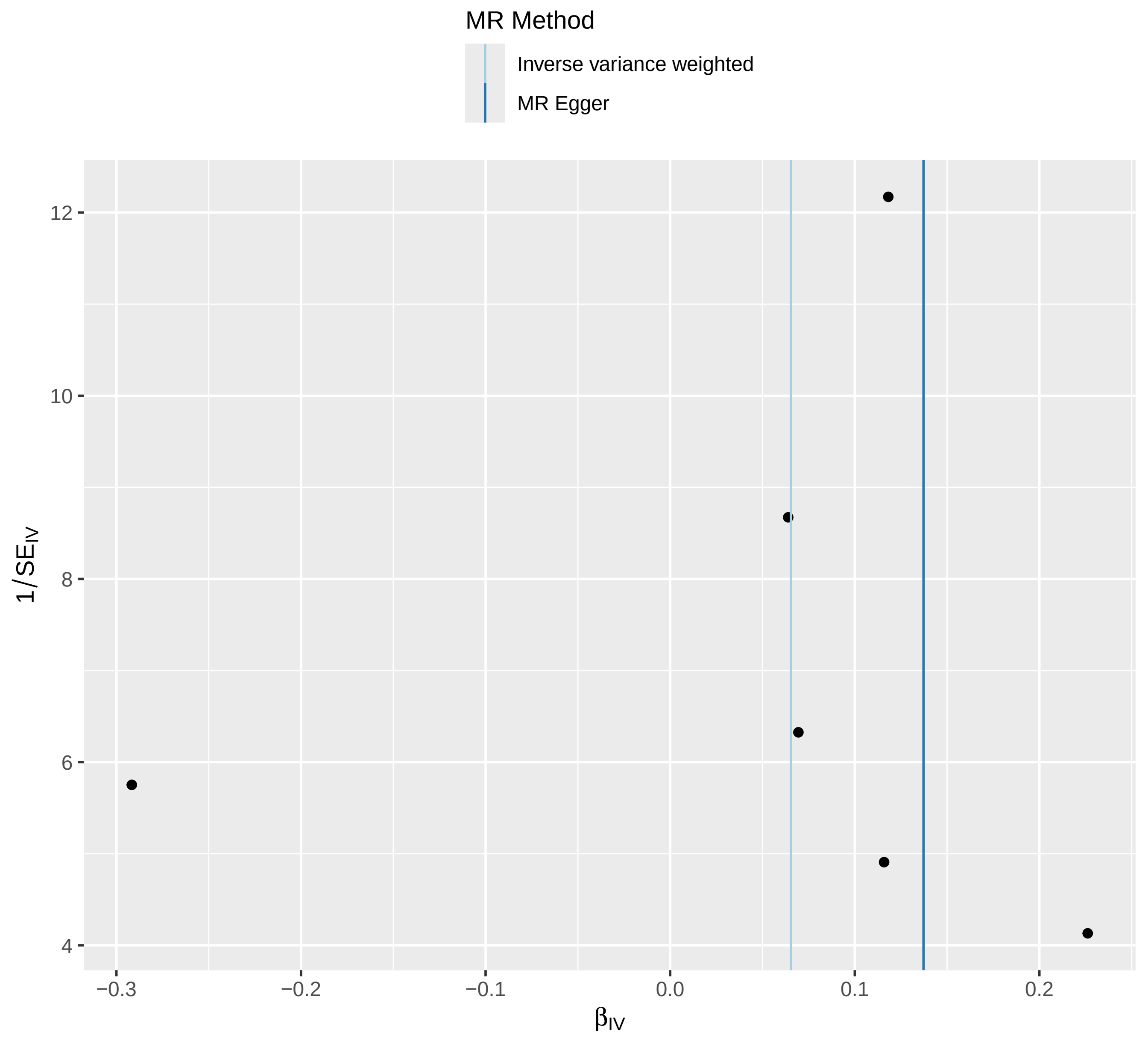
MR Method
Inverse variance weighted
MR Egger
12
10
8
6
4
1/SE_IV
−0.3
−0.2
−0.1
0.0
0.1
0.2
β_IV

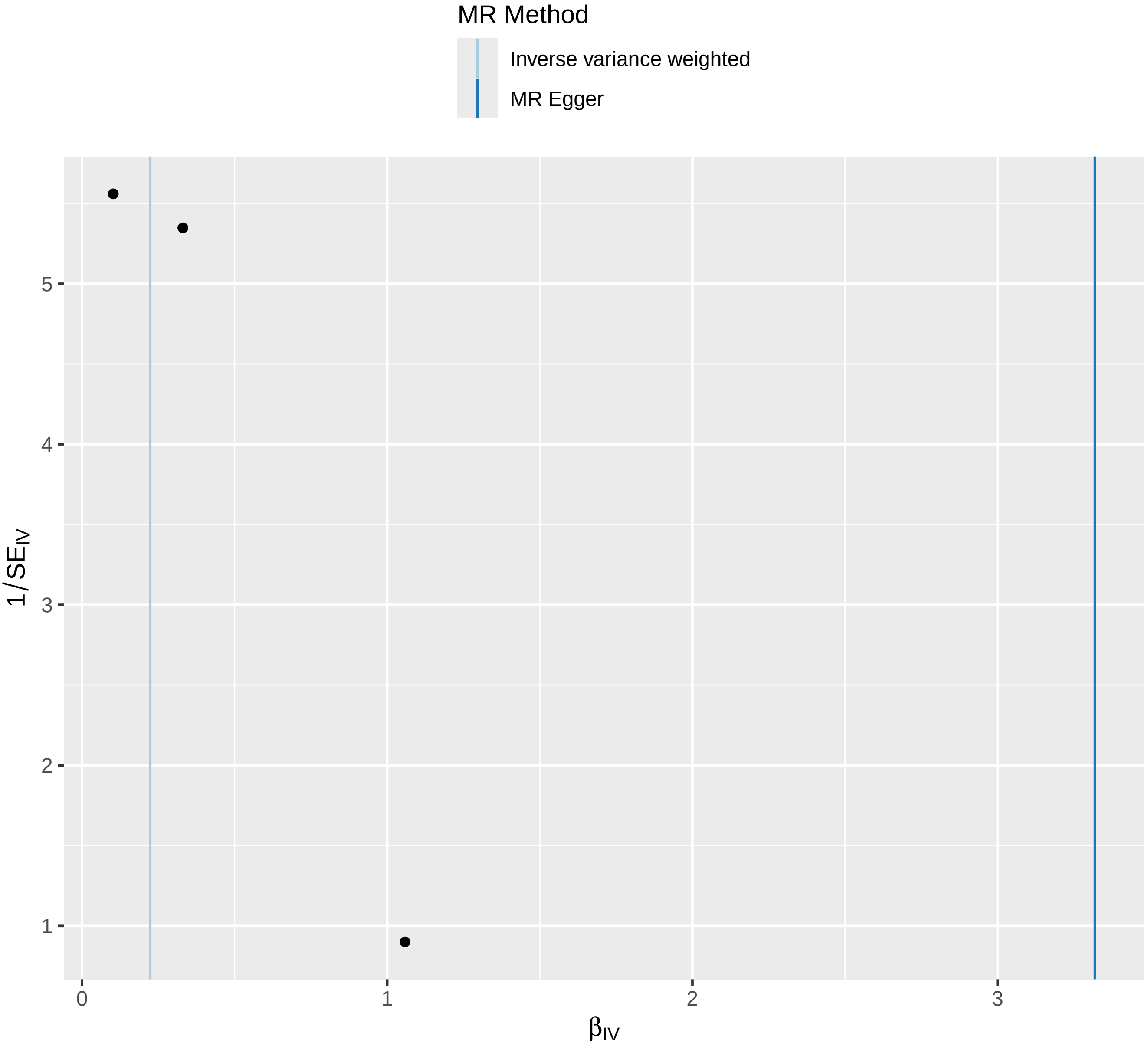
MR Method
Inverse variance weighted
MR Egger
1/SE$_{IV}$
5
4
3
2
1
0
1
2
3
β$_{IV}$

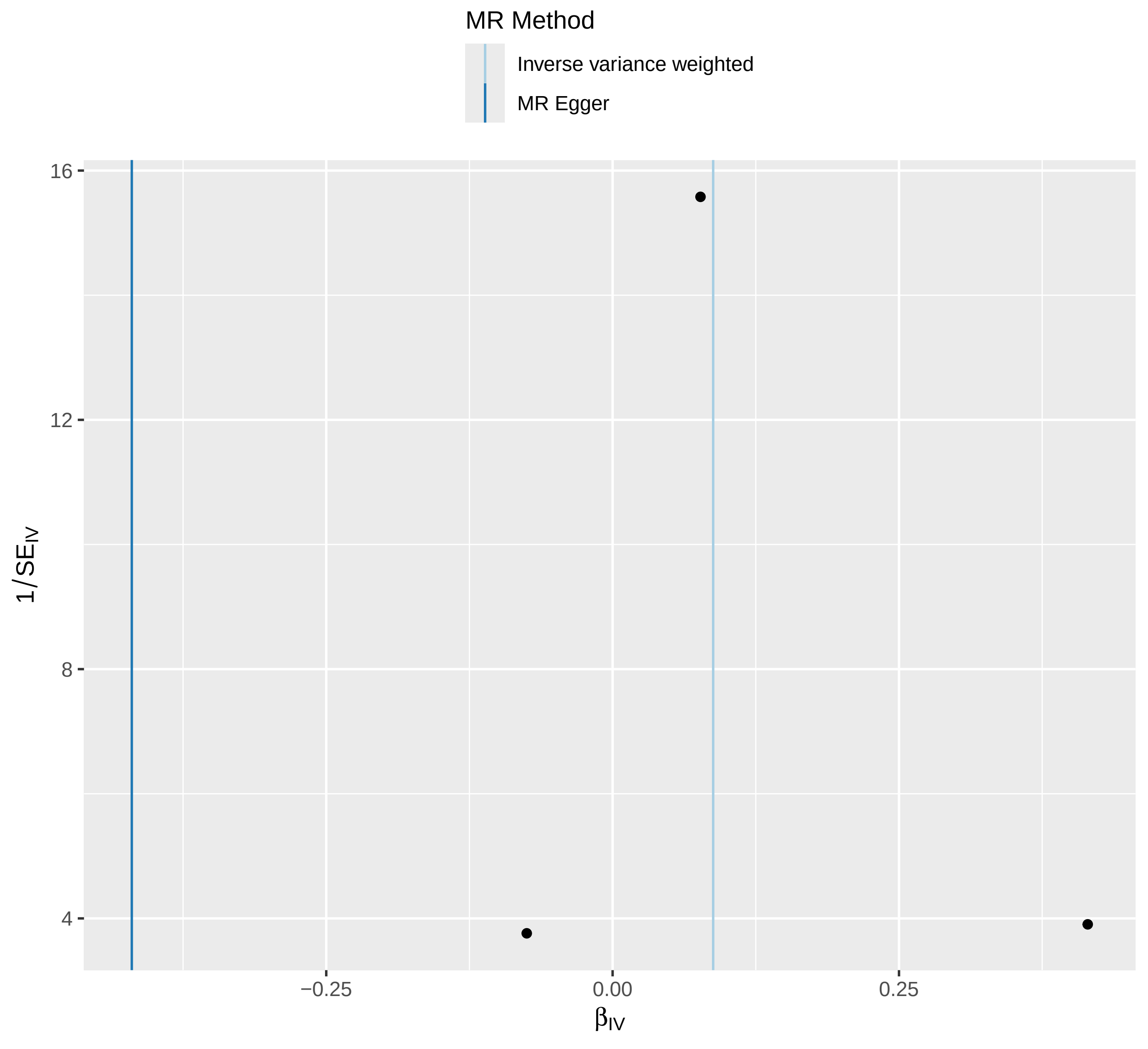
MR Method
Inverse variance weighted
MR Egger
16
12
8
4
1/SE_IV
−0.25
0.00
0.25
β_IV

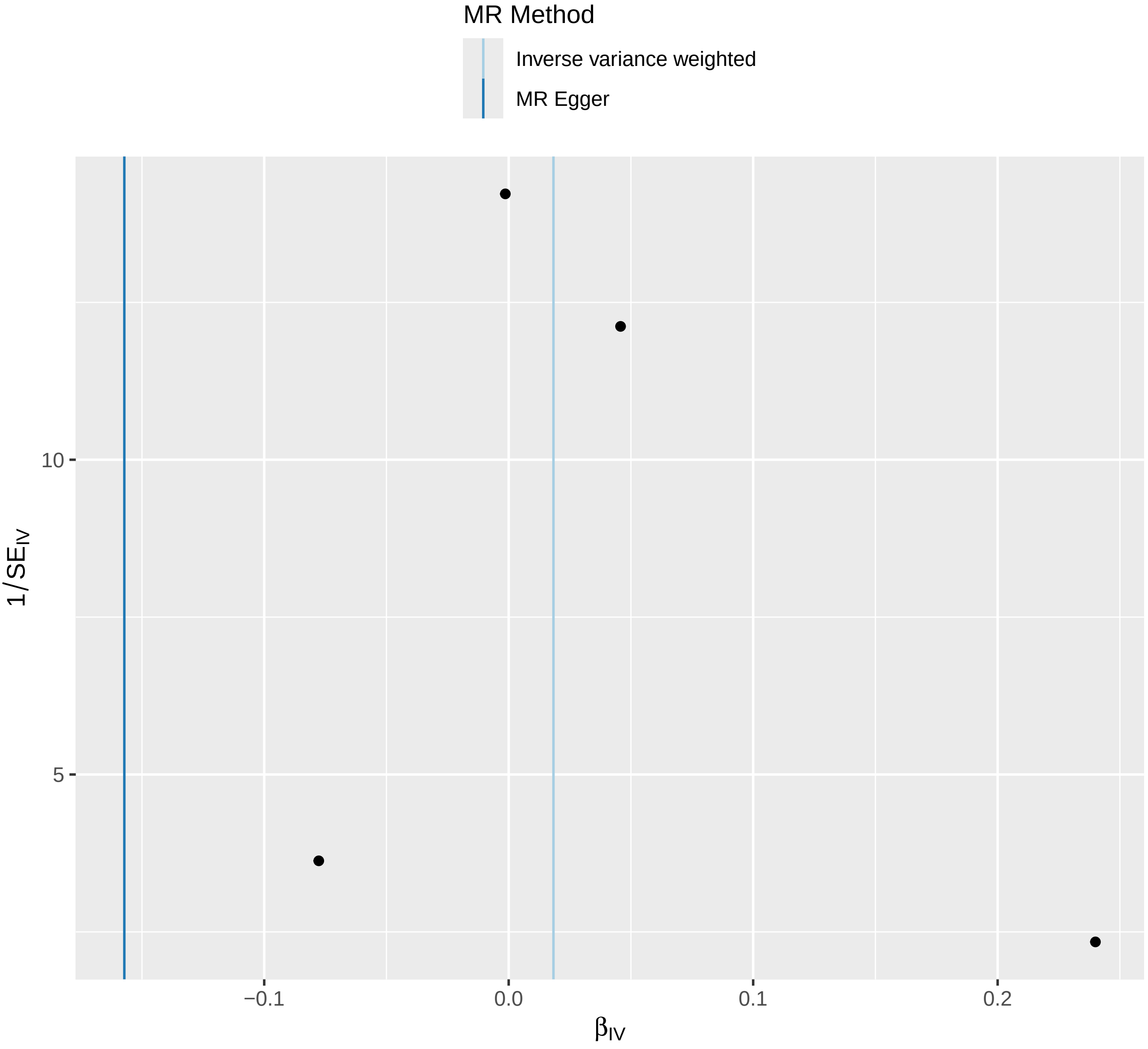
MR Method
Inverse variance weighted
MR Egger
10
5
1/SE$_{IV}$
−0.1
0.0
0.1
0.2
β$_{IV}$

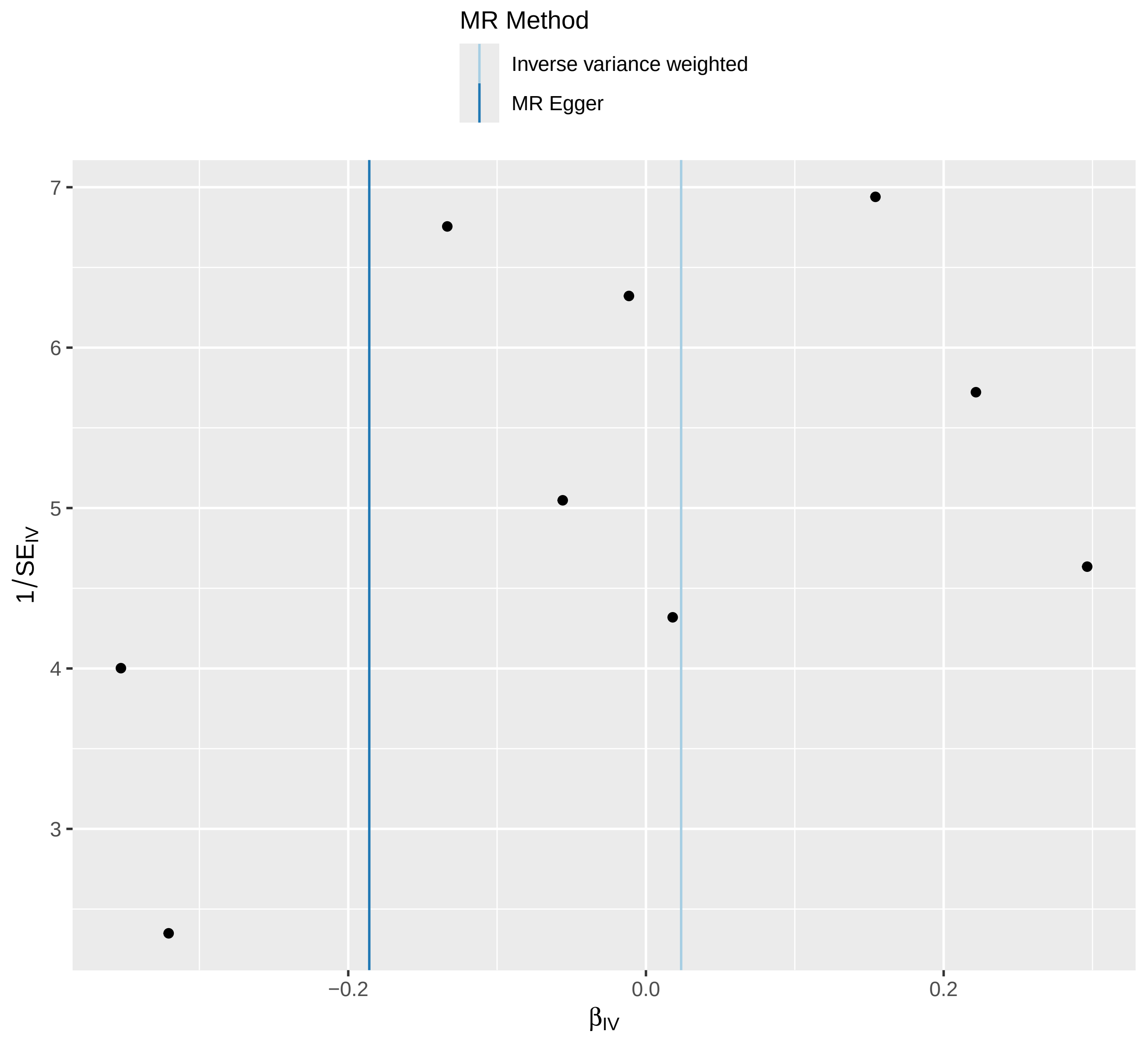

MR Method
Inverse variance weighted
MR Egger
7
6
5
4
3
1/SE_IV
−0.2
0.0
0.2
β_IV

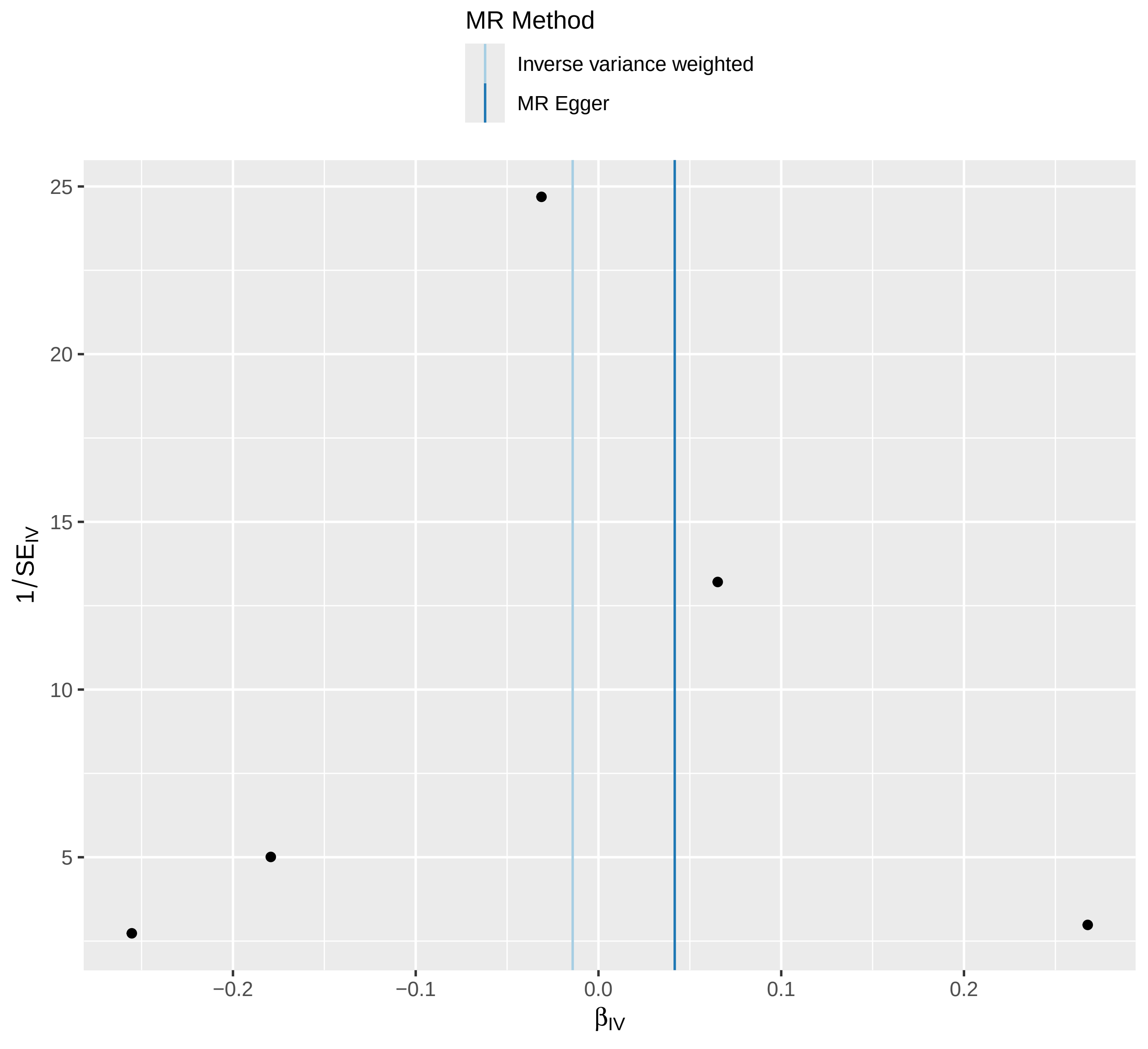

MR Method
Inverse variance weighted
MR Egger
25
20
15
10
5
1/SE_IV
−0.2
−0.1
0.0
0.1
0.2
β_IV

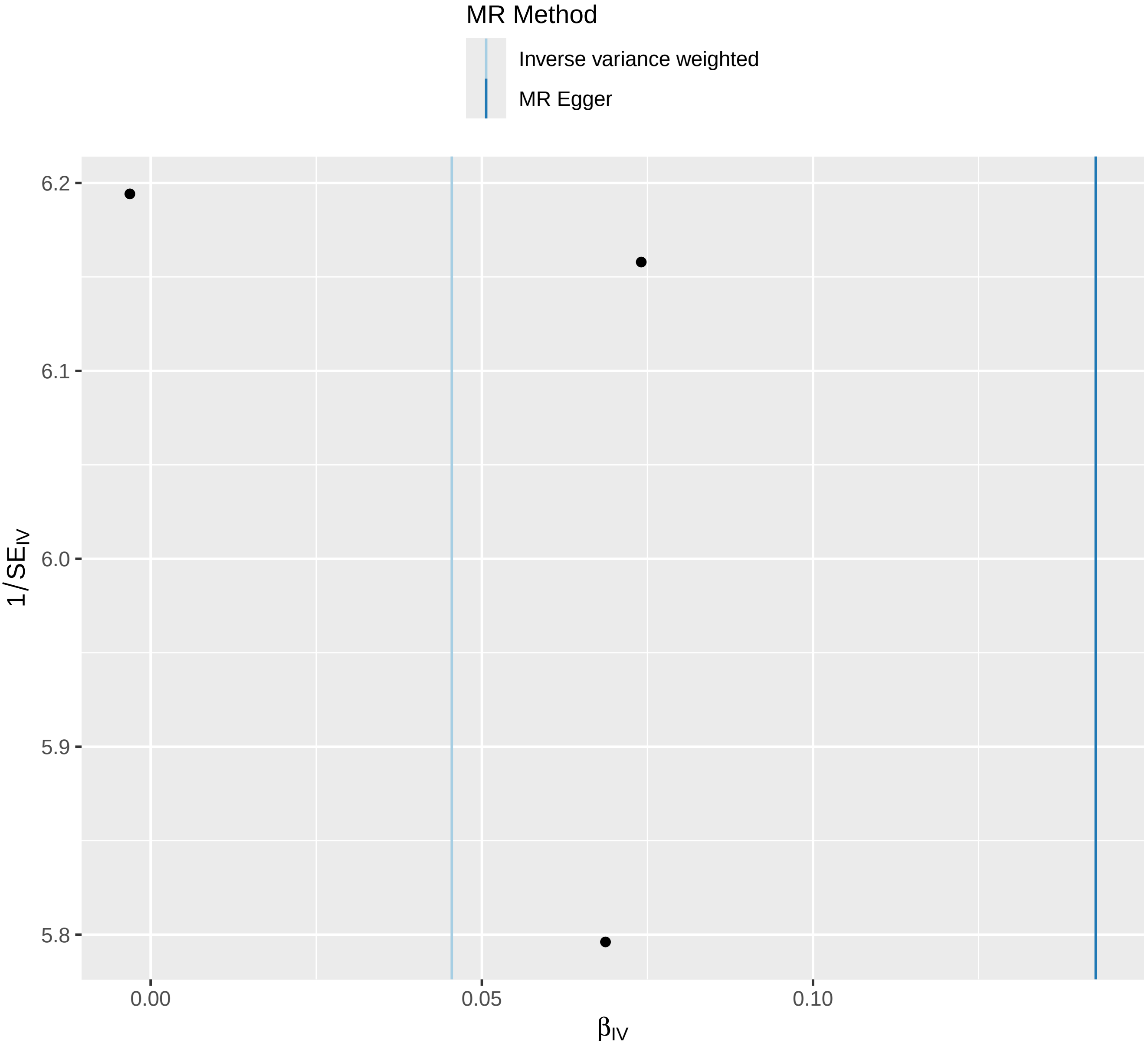

MR Method
Inverse variance weighted
MR Egger
6.2
6.1
6.0
5.9
5.8
1/SE_IV
0.00
0.05
0.10
β_IV

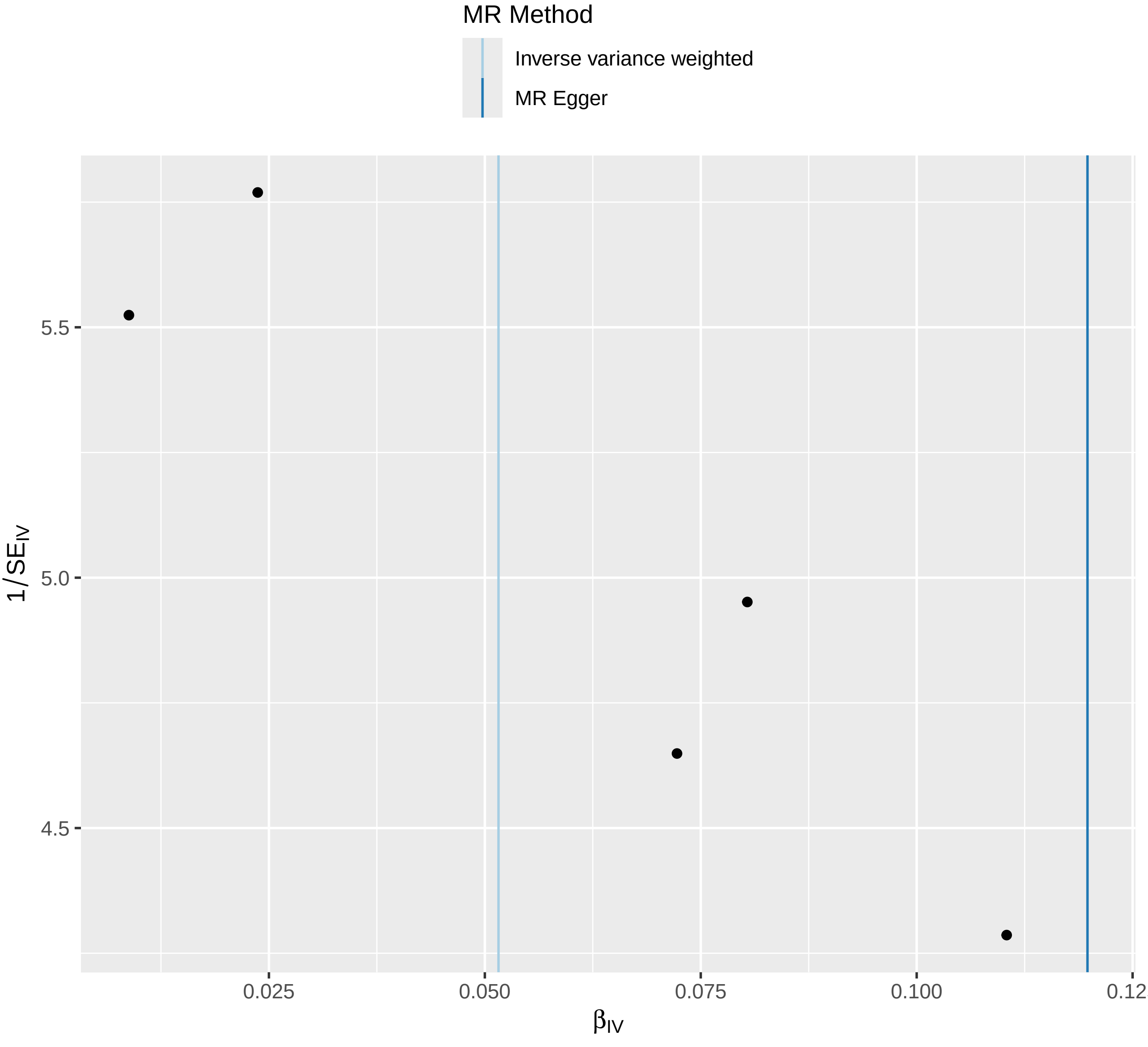

MR Method
Inverse variance weighted
MR Egger
5.5
5.0
4.5
1/SE$_{IV}$
0.025
0.050
0.075
0.100
0.12
β$_{IV}$

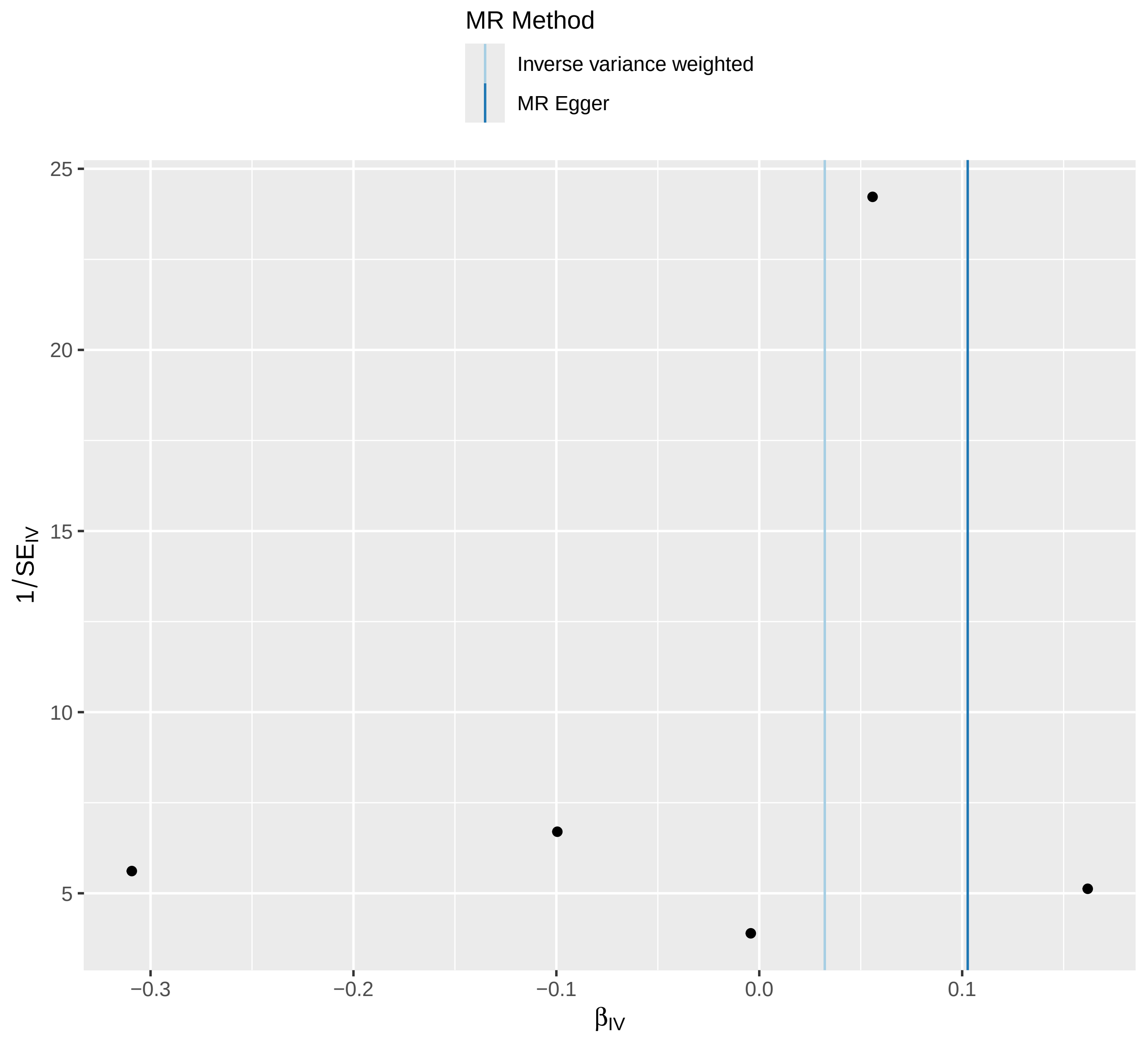
MR Method
Inverse variance weighted
MR Egger
25
20
15
10
5
$1/SE_{IV}$
−0.3
−0.2
−0.1
0.0
0.1
$\beta_{IV}$

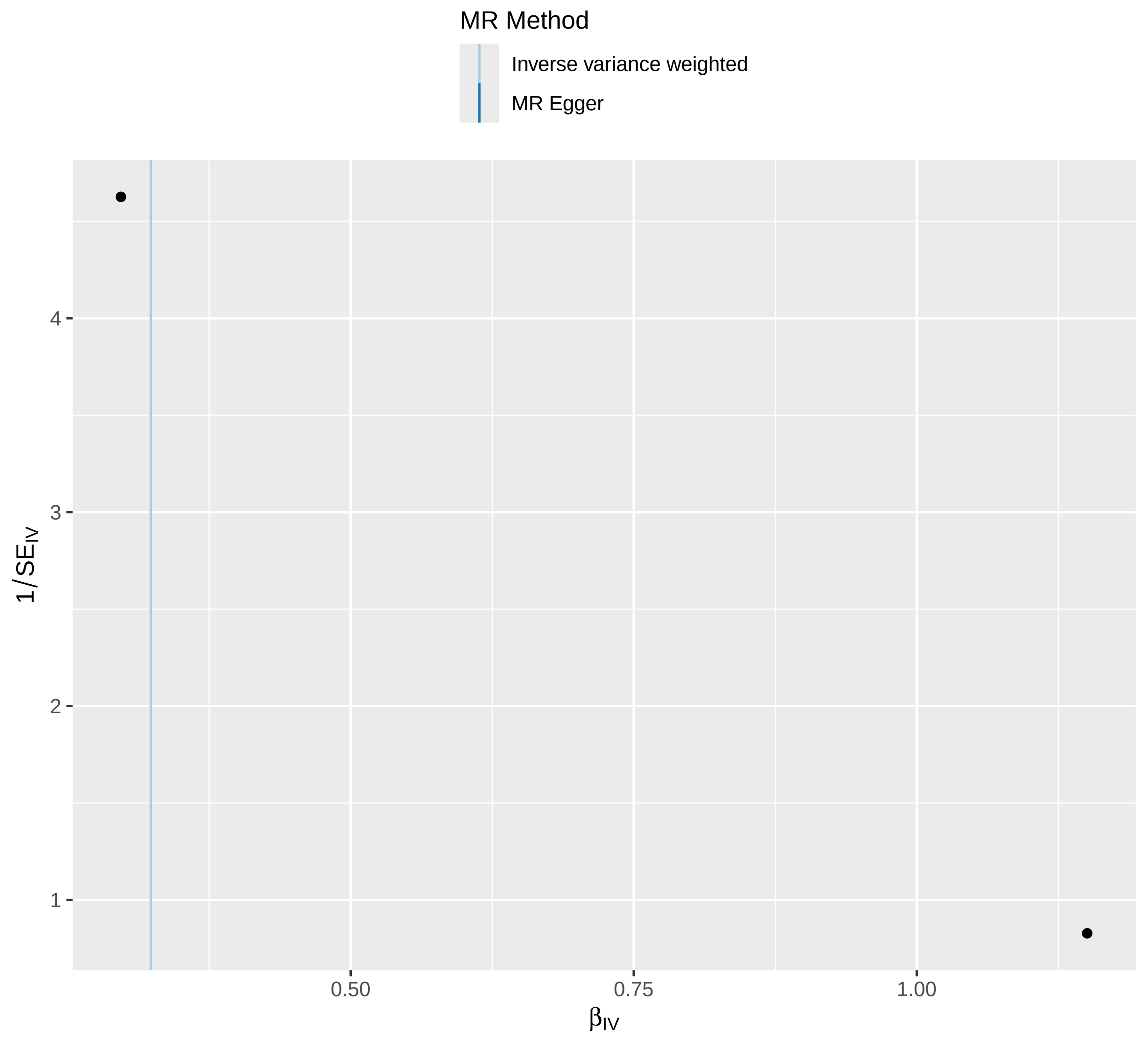

MR Method
Inverse variance weighted
MR Egger
1/SE$_{IV}$
4
3
2
1
0.50
0.75
1.00
β$_{IV}$

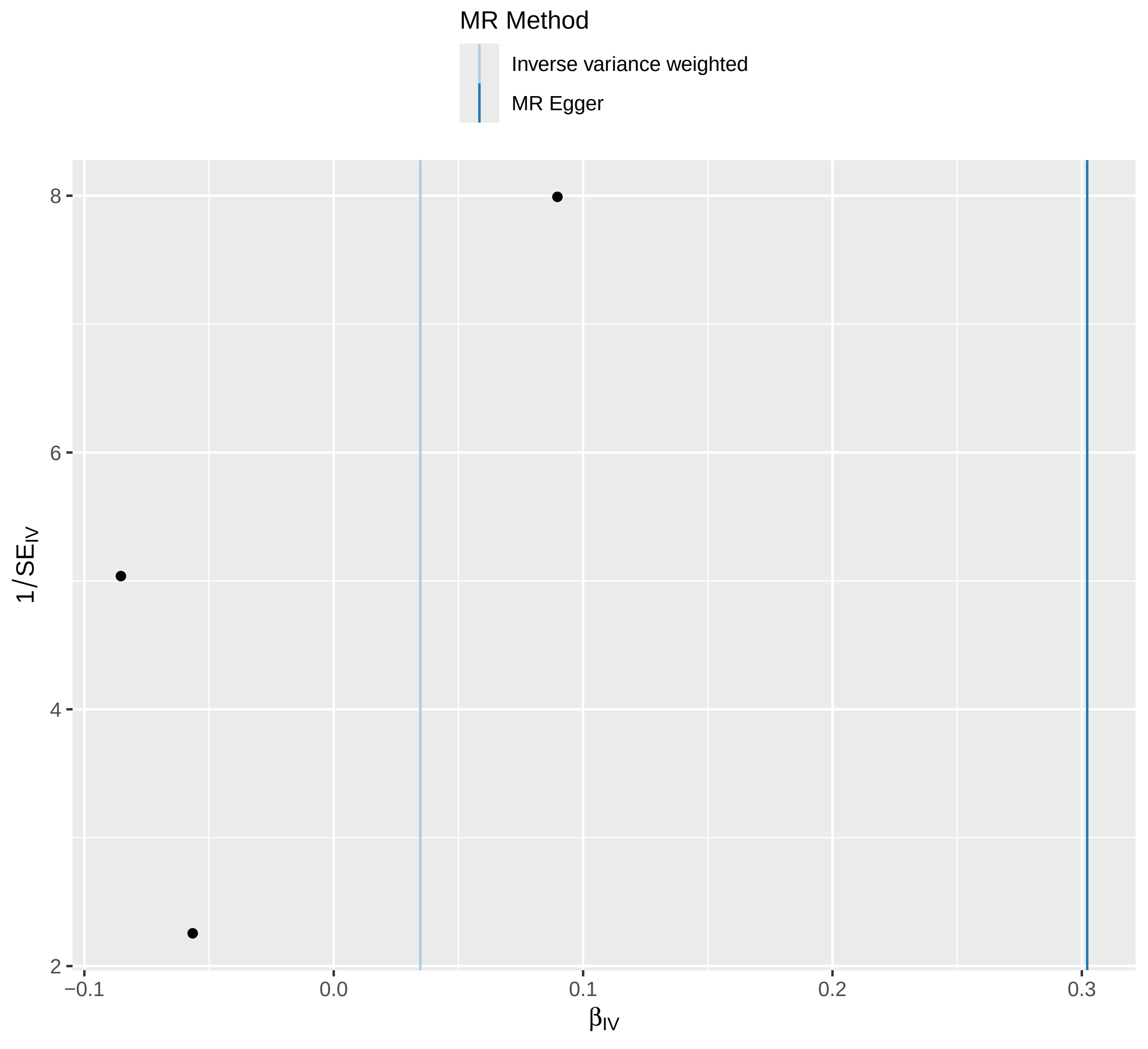
MR Method
Inverse variance weighted
MR Egger
8
6
4
2
$1/SE_{IV}$
−0.1
0.0
0.1
0.2
0.3
$\beta_{IV}$

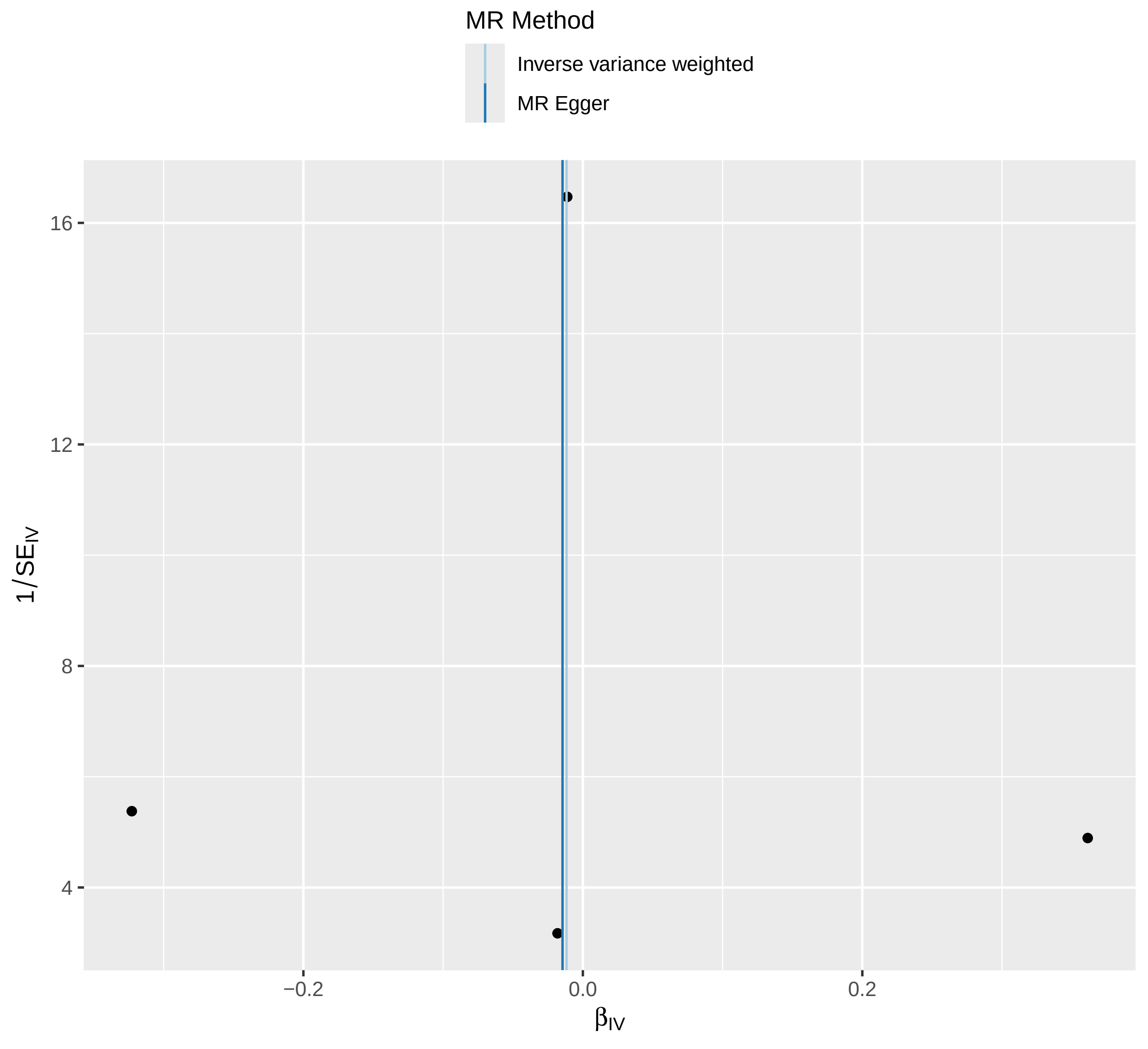

MR Method
Inverse variance weighted
MR Egger
16
12
8
4
1/SE_IV
−0.2
0.0
0.2
β_IV

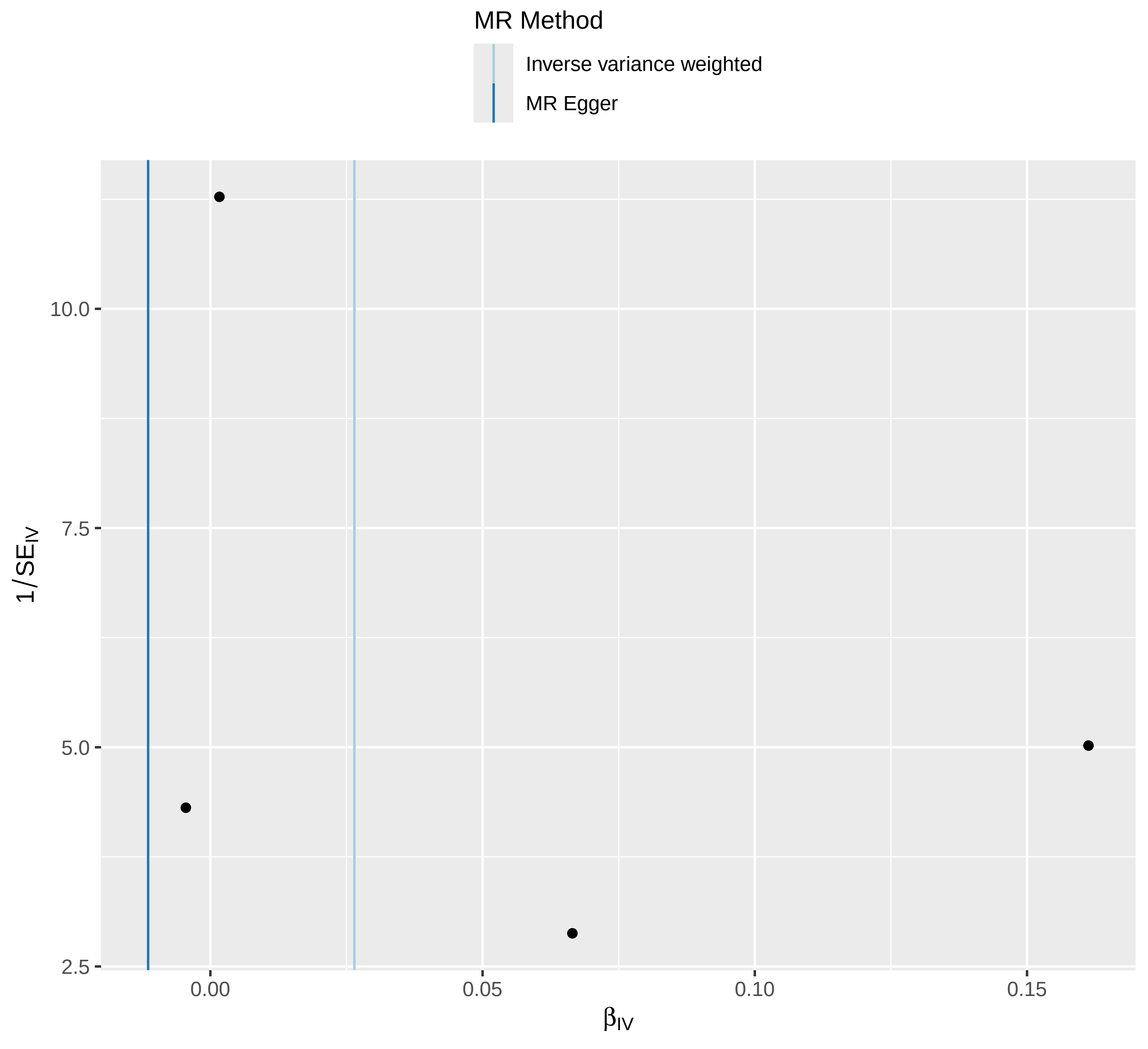
MR Method
Inverse variance weighted
MR Egger
10.0
7.5
5.0
2.5
1/SE$_{IV}$
0.00
0.05
0.10
0.15
β$_{IV}$

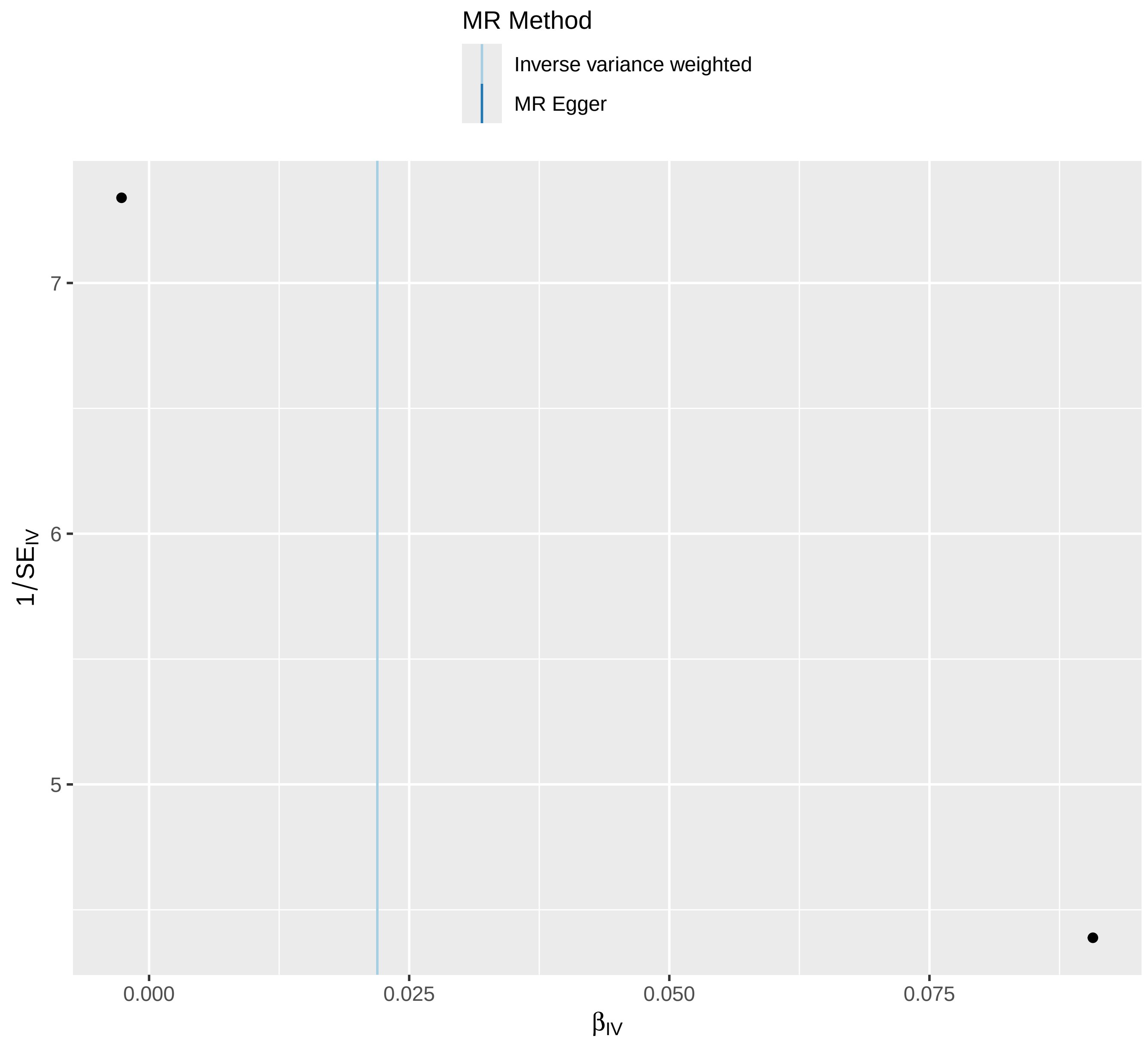
MR Method
Inverse variance weighted
MR Egger
7
6
5
0.000
0.025
0.050
0.075
1/SE_IV
β_IV

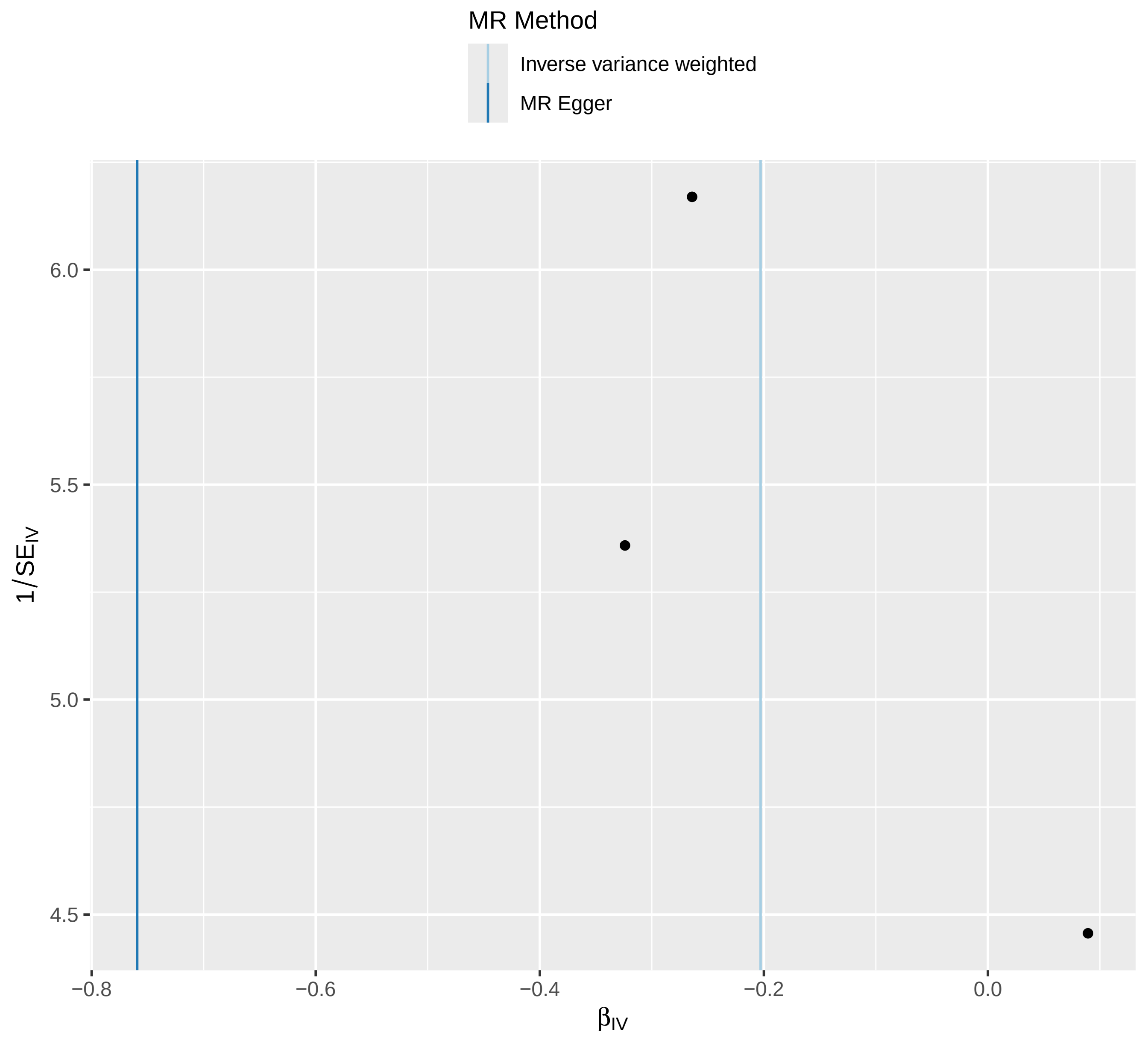
MR Method
Inverse variance weighted
MR Egger
6.0
5.5
5.0
4.5
1/SE_IV
−0.8
−0.6
−0.4
−0.2
0.0
β_IV

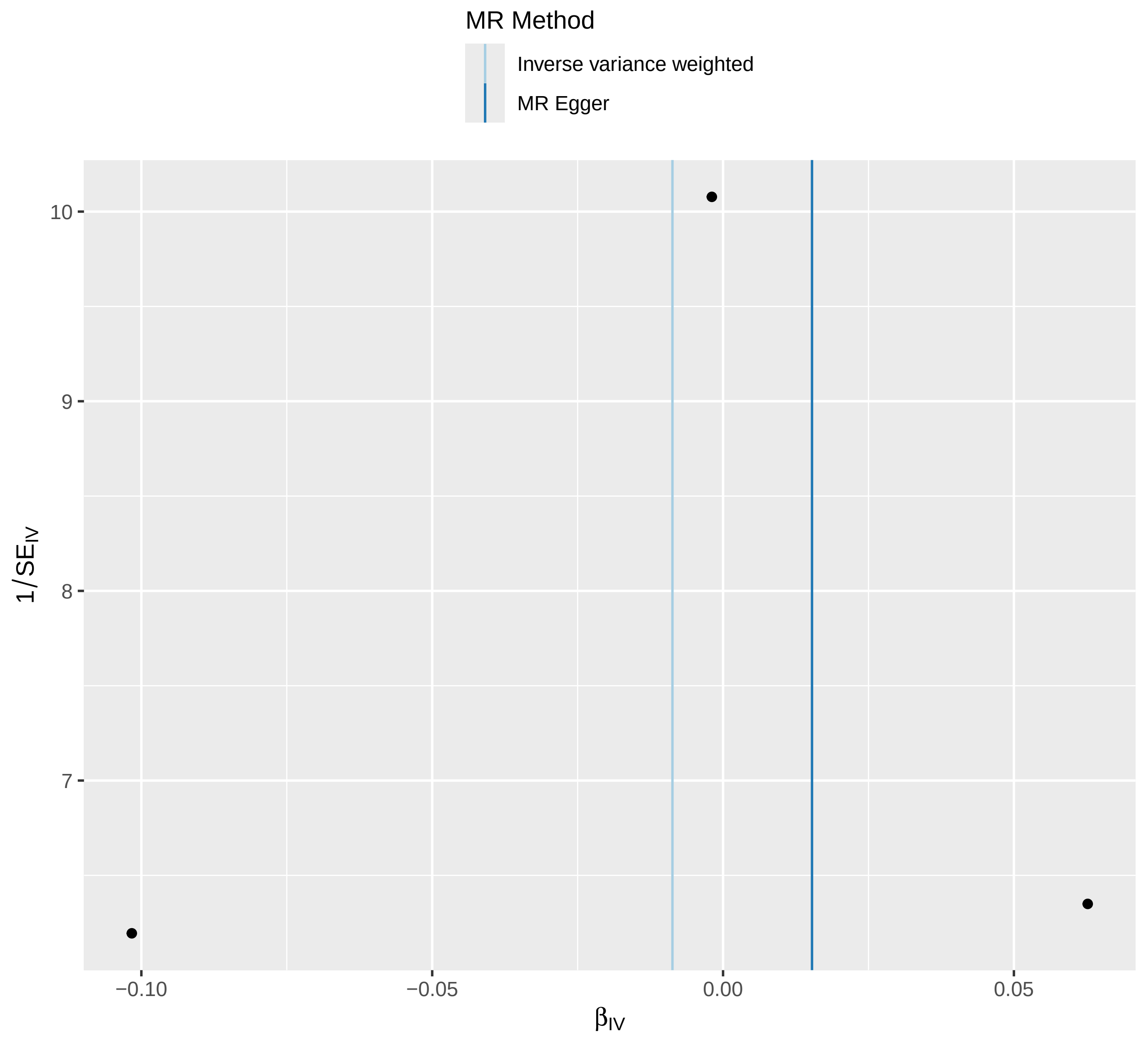
MR Method
Inverse variance weighted
MR Egger
10
9
8
7
$1/SE_{IV}$
−0.10
−0.05
0.00
0.05
$\beta_{IV}$

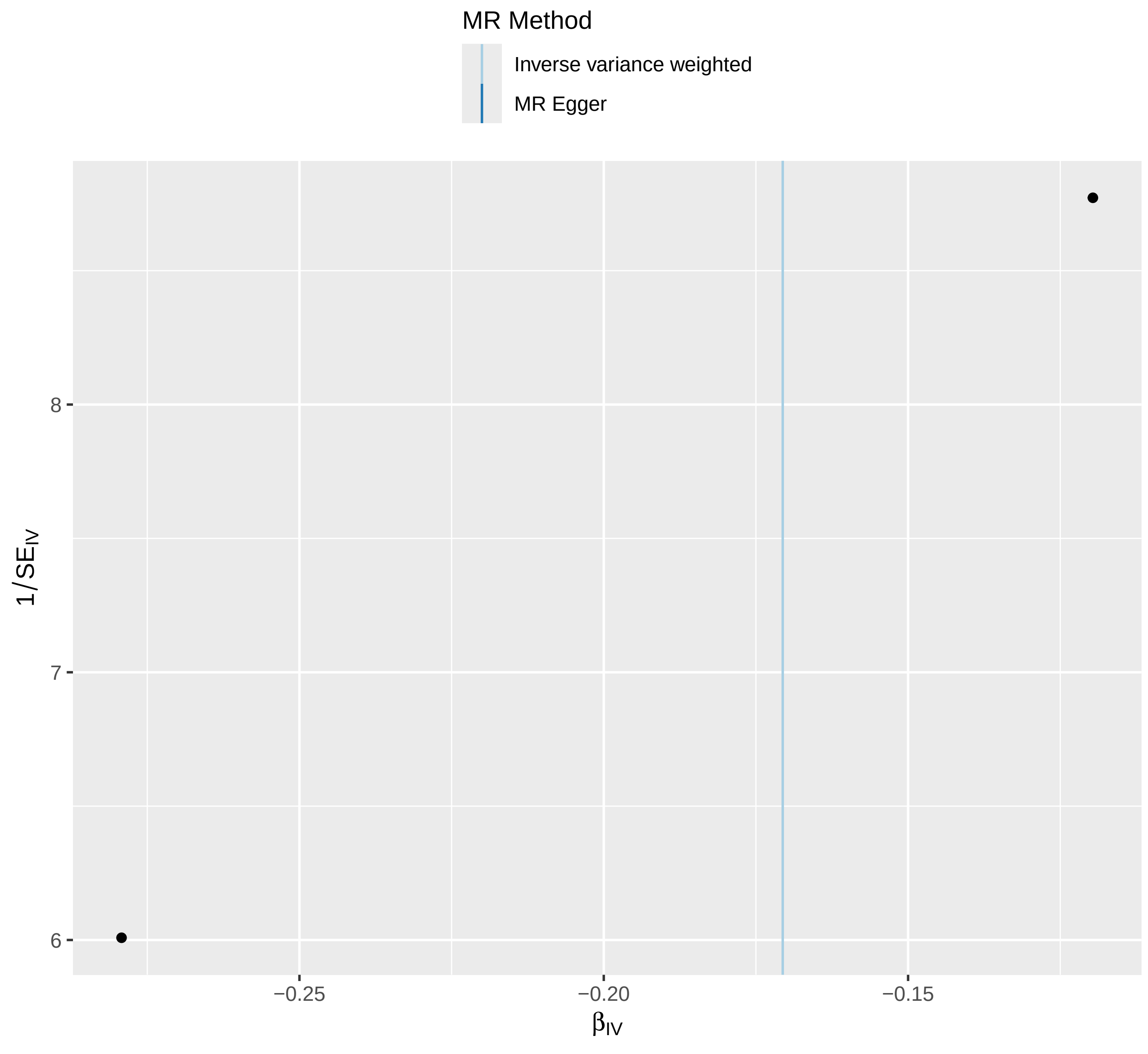

MR Method
Inverse variance weighted
MR Egger
8
7
6
1/SE_IV
−0.25
−0.20
−0.15
β_IV

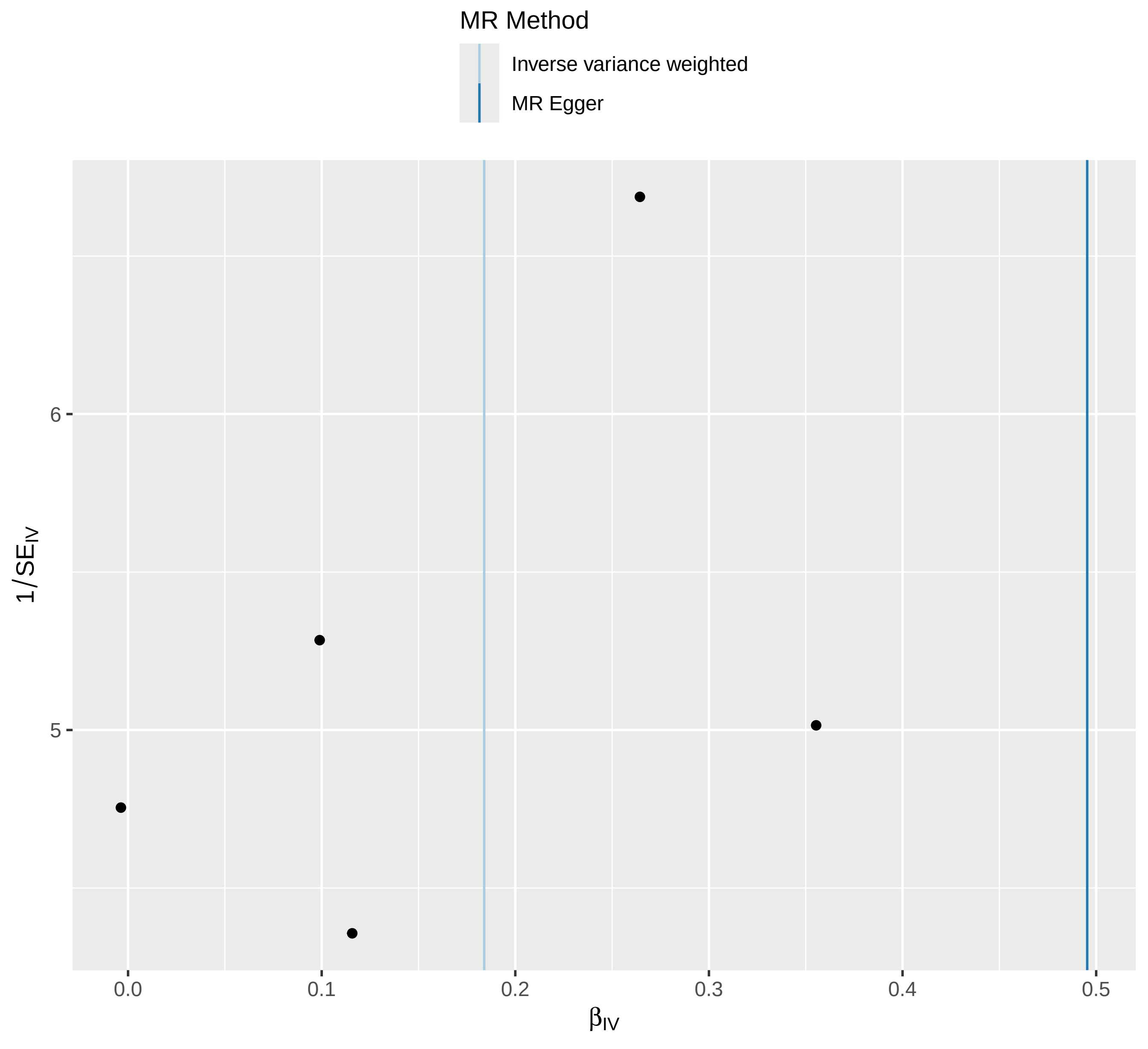

MR Method
Inverse variance weighted
MR Egger
6
5
1/SE_IV
0.0
0.1
0.2
0.3
0.4
0.5
β_IV

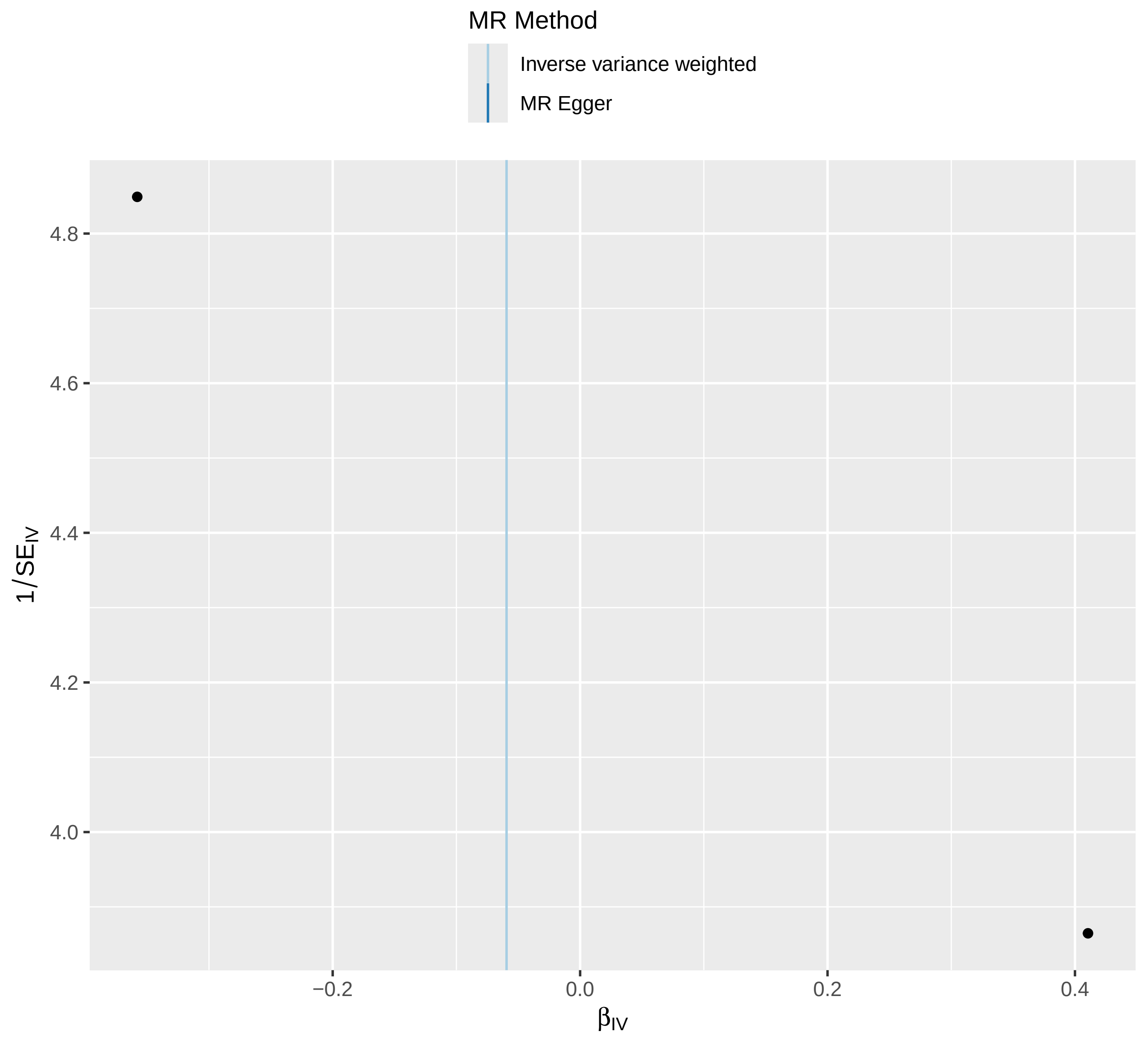

MR Method
Inverse variance weighted
MR Egger
4.8
4.6
4.4
4.2
4.0
1/SE_IV
−0.2
0.0
0.2
0.4
β_IV

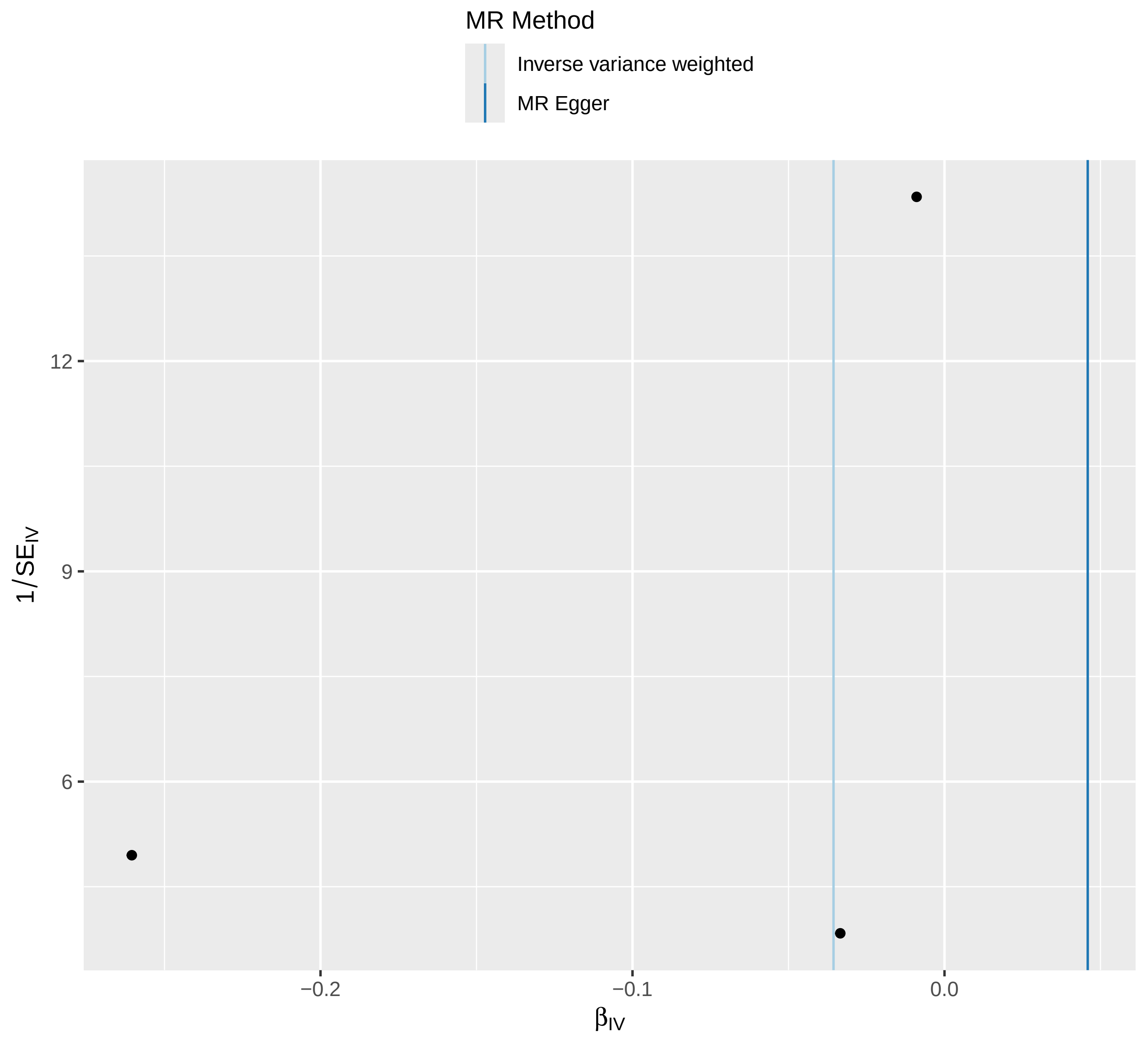
MR Method
Inverse variance weighted
MR Egger
12
9
6
−0.2
−0.1
0.0
1/SE$_{IV}$
β$_{IV}$

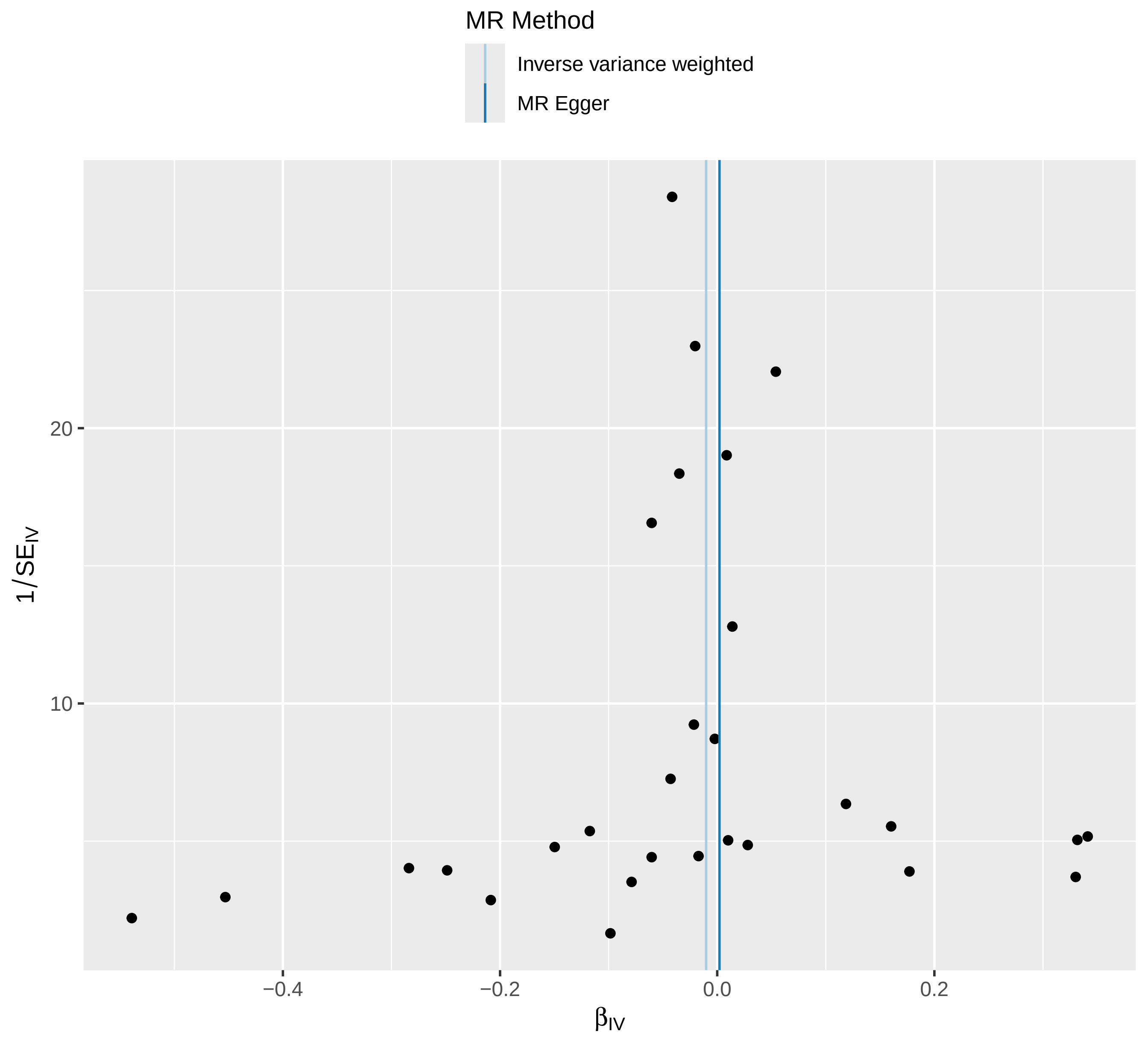
MR Method
Inverse variance weighted
MR Egger
20
10
1/SE_IV
−0.4
−0.2
0.0
0.2
β_IV

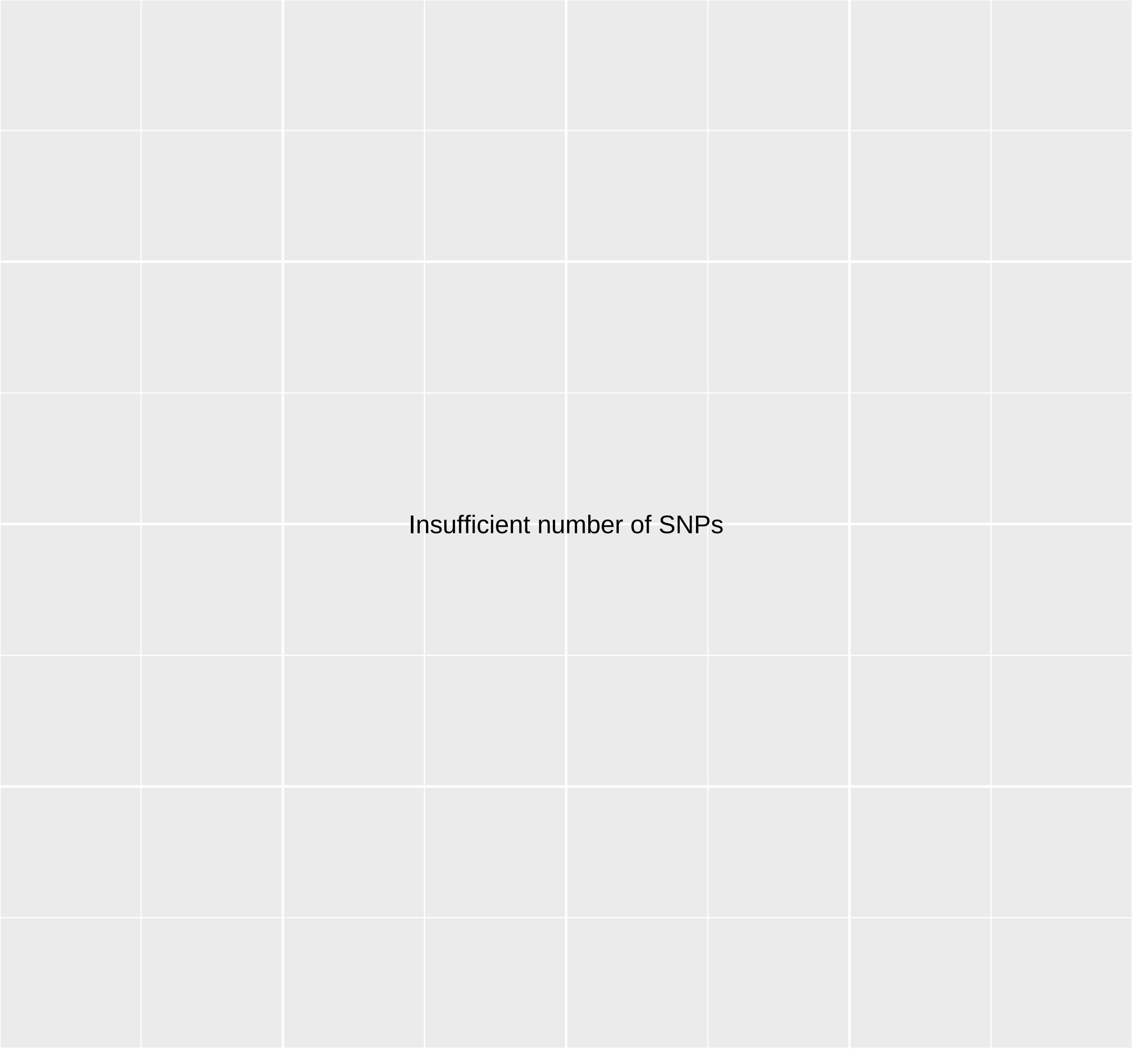
Insufficient number of SNPs

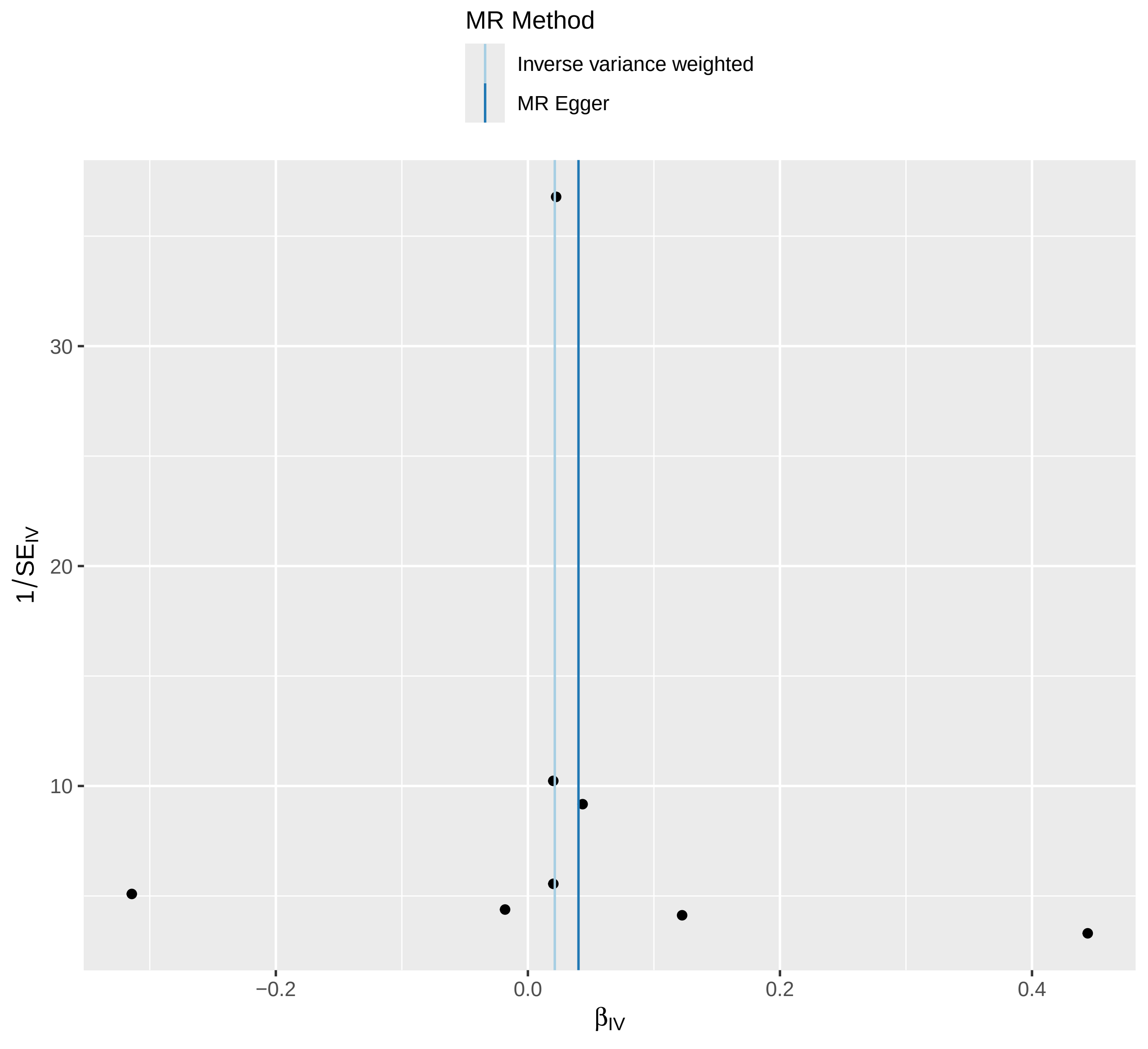

MR Method
Inverse variance weighted
MR Egger
1/SE_IV
30
20
10
−0.2
0.0
0.2
0.4
β_IV

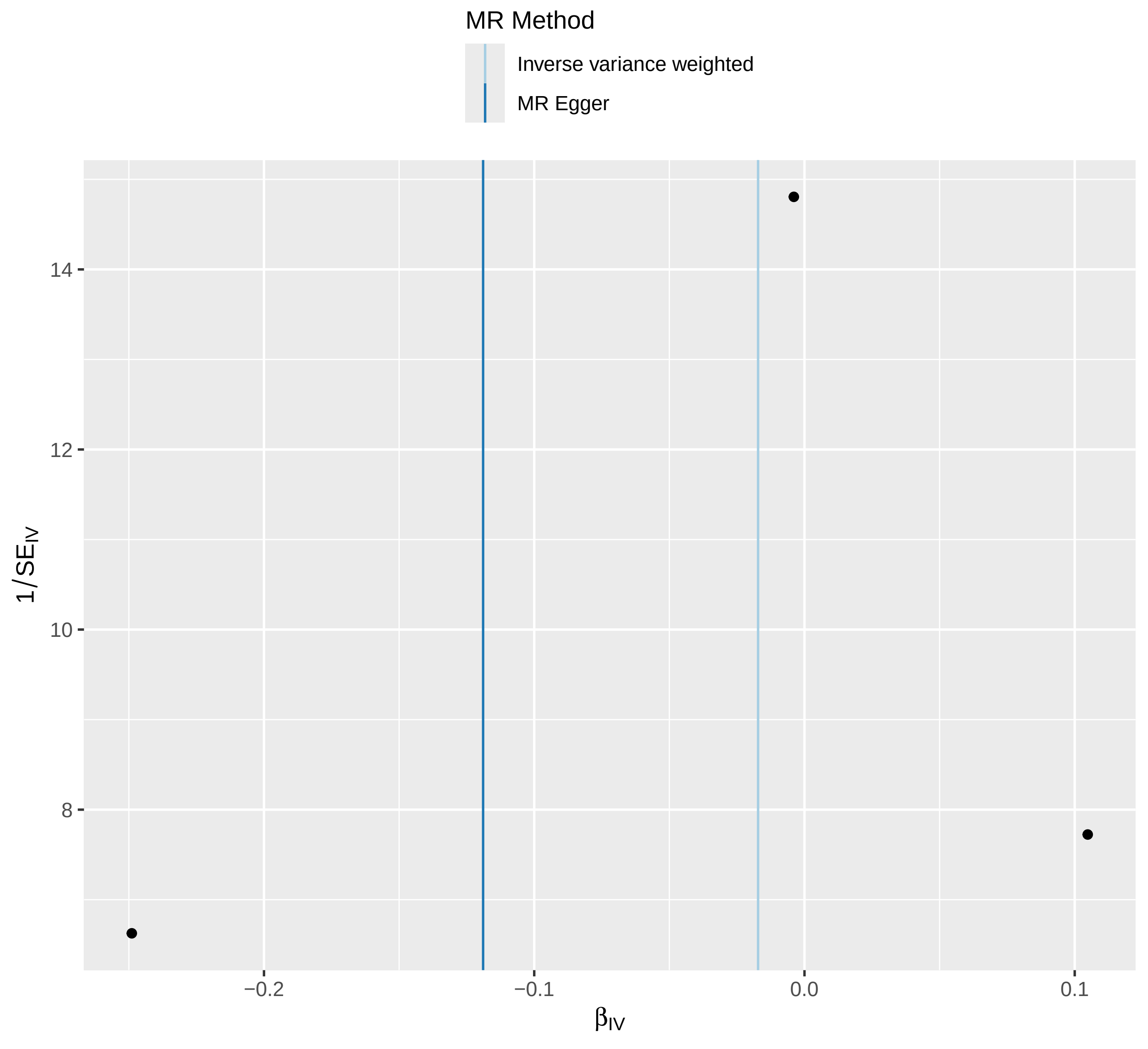
MR Method
Inverse variance weighted
MR Egger
14
12
10
8
1/SE$_{IV}$
−0.2
−0.1
0.0
0.1
β$_{IV}$

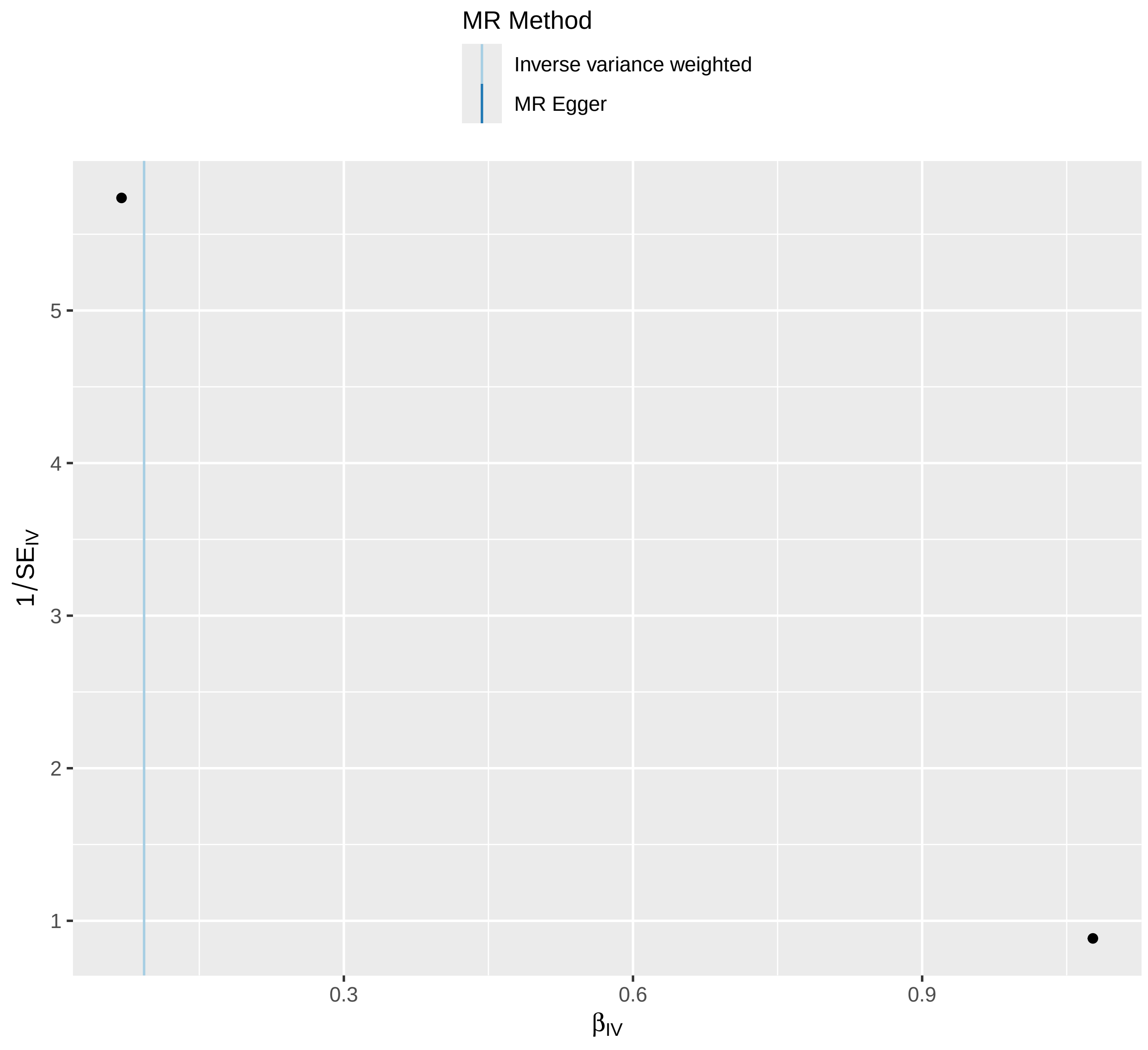
MR Method
Inverse variance weighted
MR Egger
5
4
3
2
1
1/SE_IV
0.3
0.6
0.9
β_IV

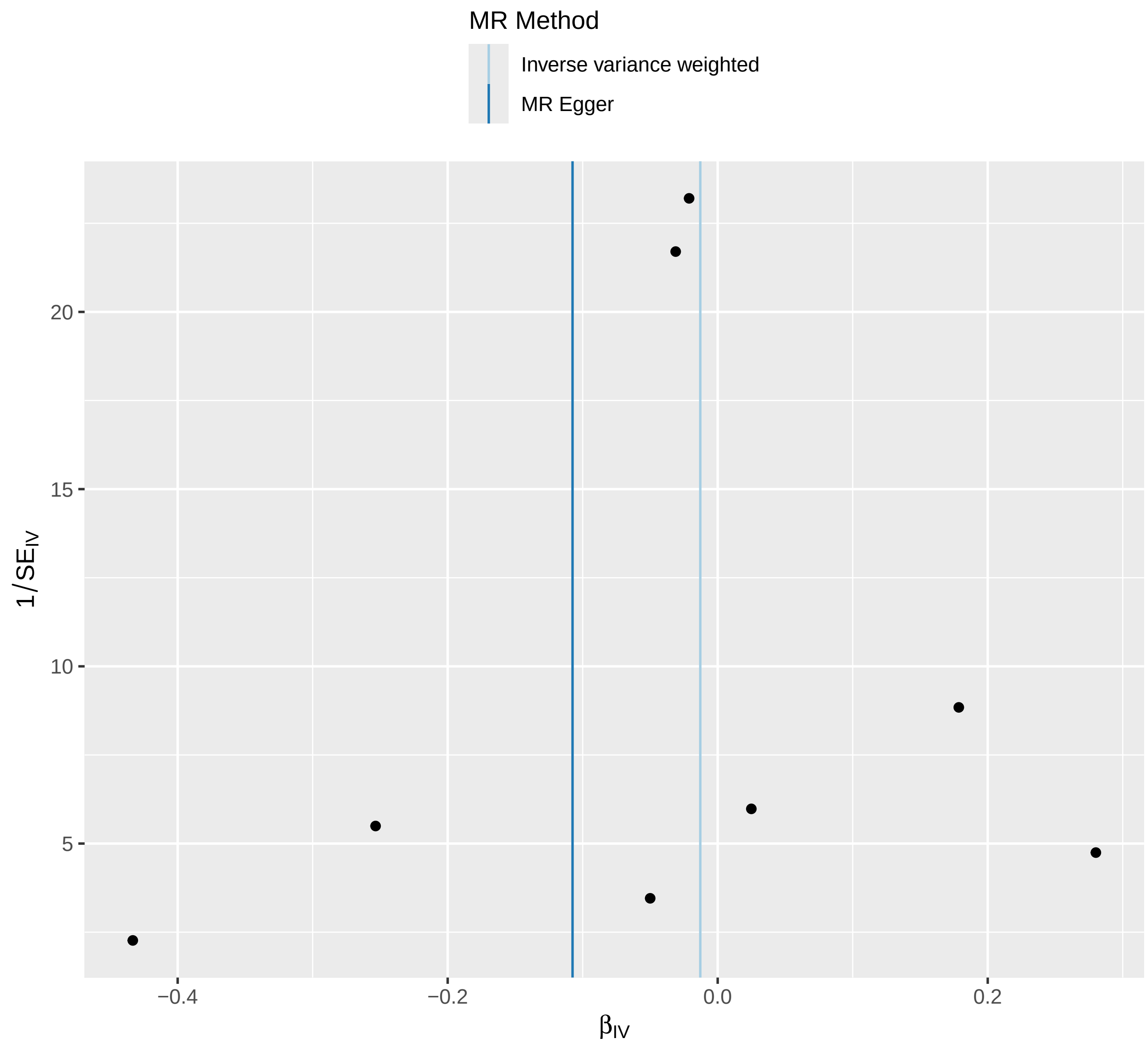

MR Method
Inverse variance weighted
MR Egger
20
15
10
5
1/SE$_{IV}$
−0.4
−0.2
0.0
0.2
β$_{IV}$

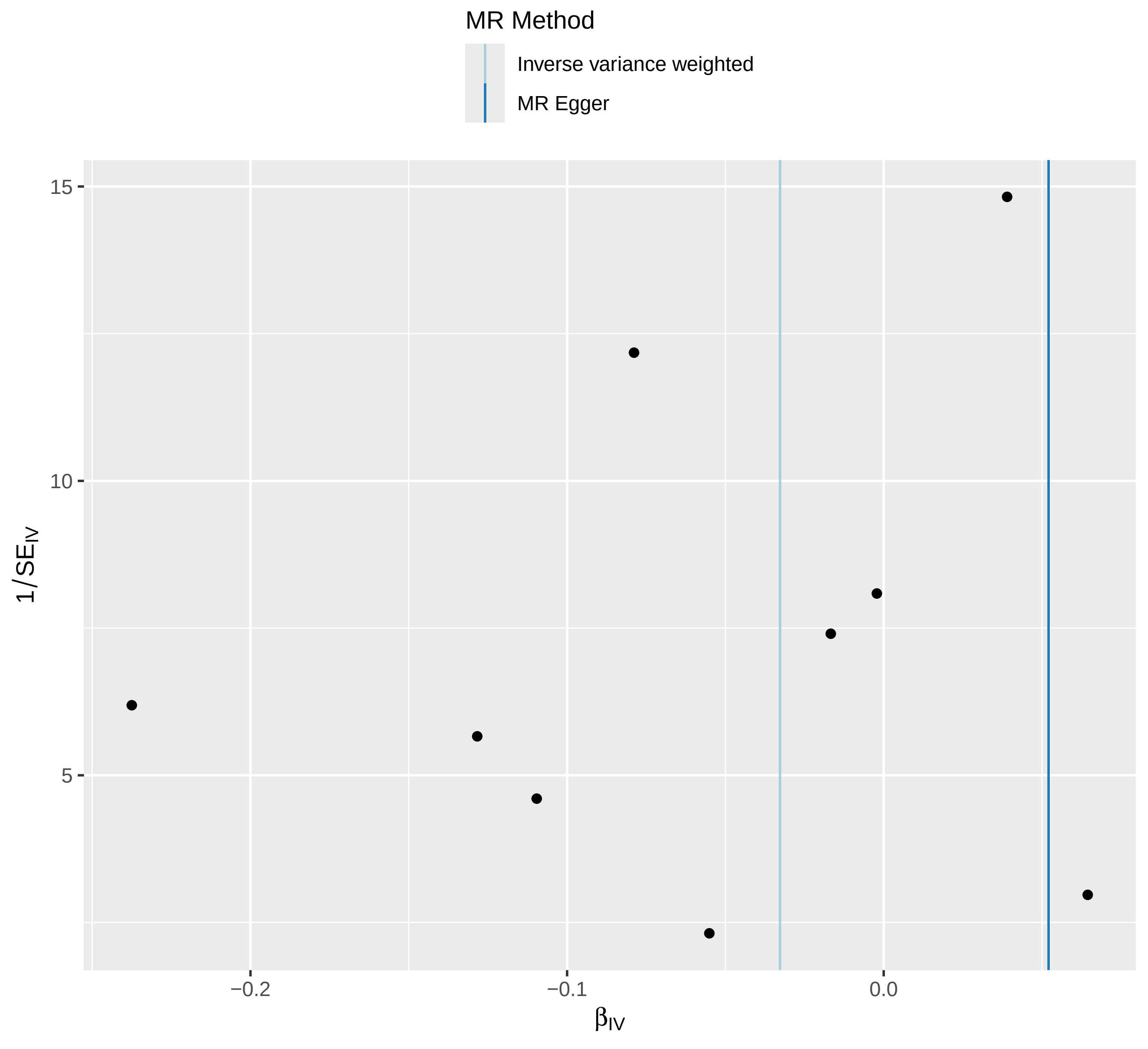

MR Method
Inverse variance weighted
MR Egger
15
10
5
1/SE_IV
−0.2
−0.1
0.0
β_IV

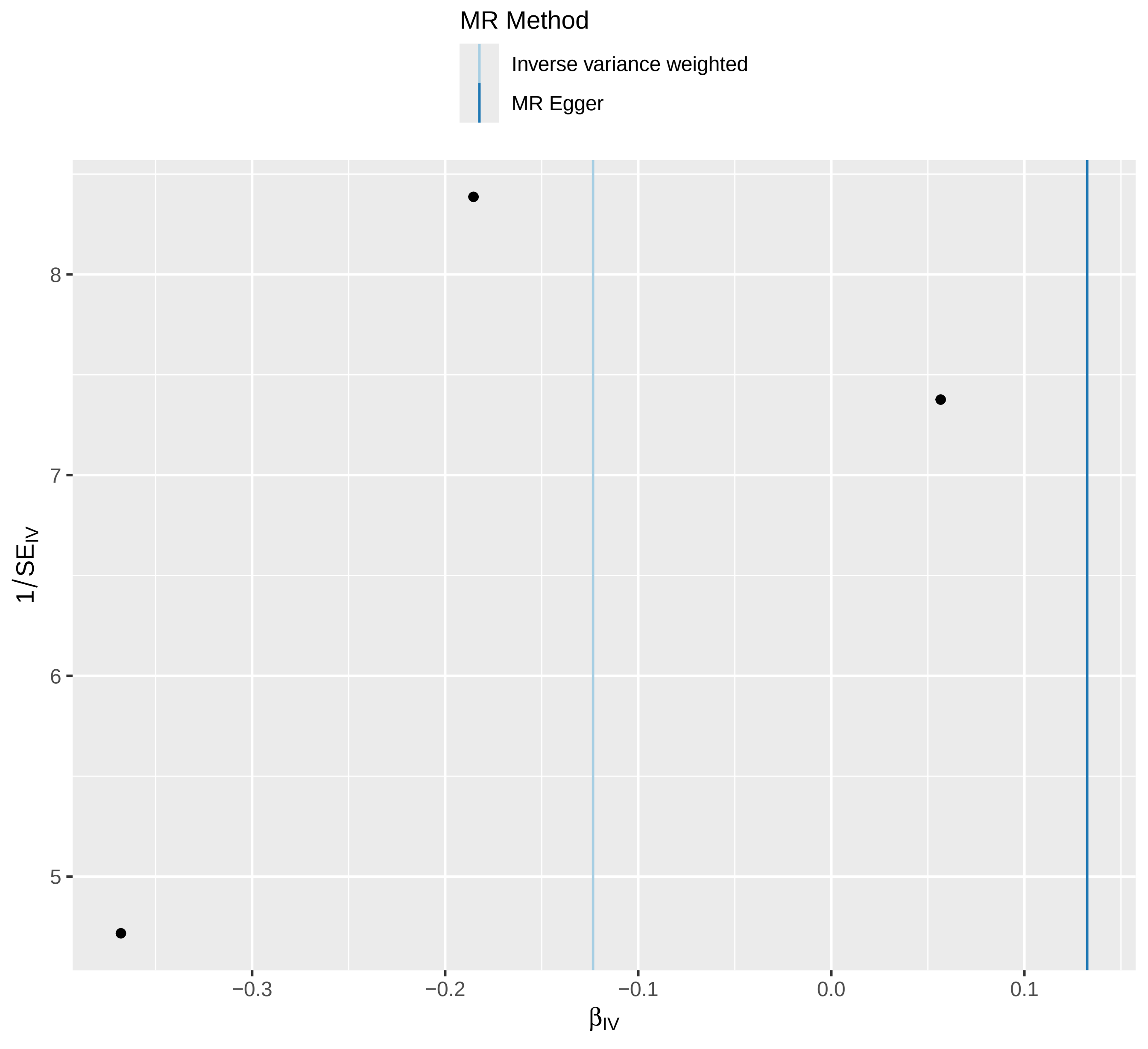

MR Method
Inverse variance weighted
MR Egger
8
7
6
5
1/SE_IV
−0.3
−0.2
−0.1
0.0
0.1
β_IV

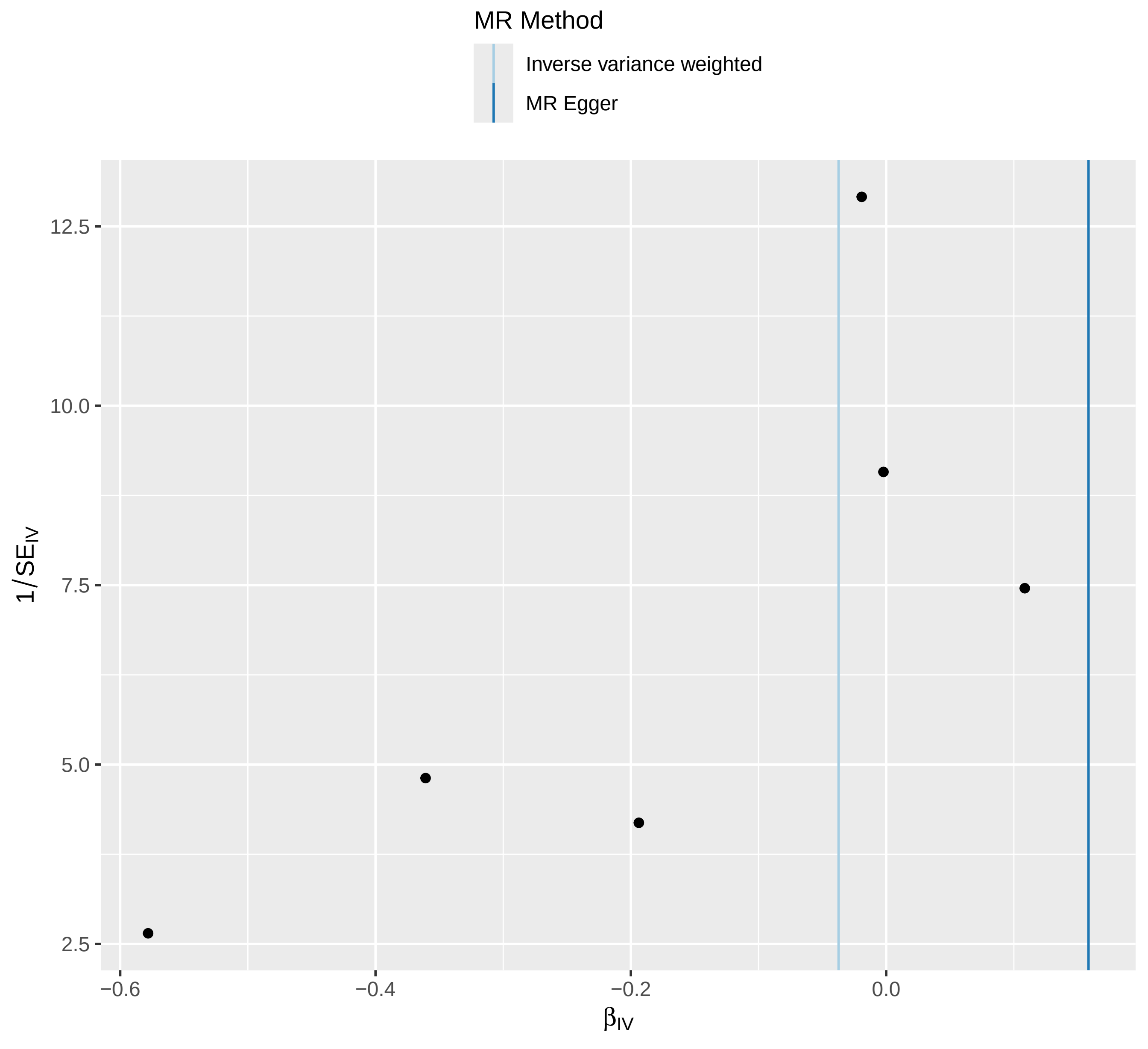

MR Method
Inverse variance weighted
MR Egger
12.5
10.0
7.5
5.0
2.5
1/SE_IV
−0.6
−0.4
−0.2
0.0
β_IV

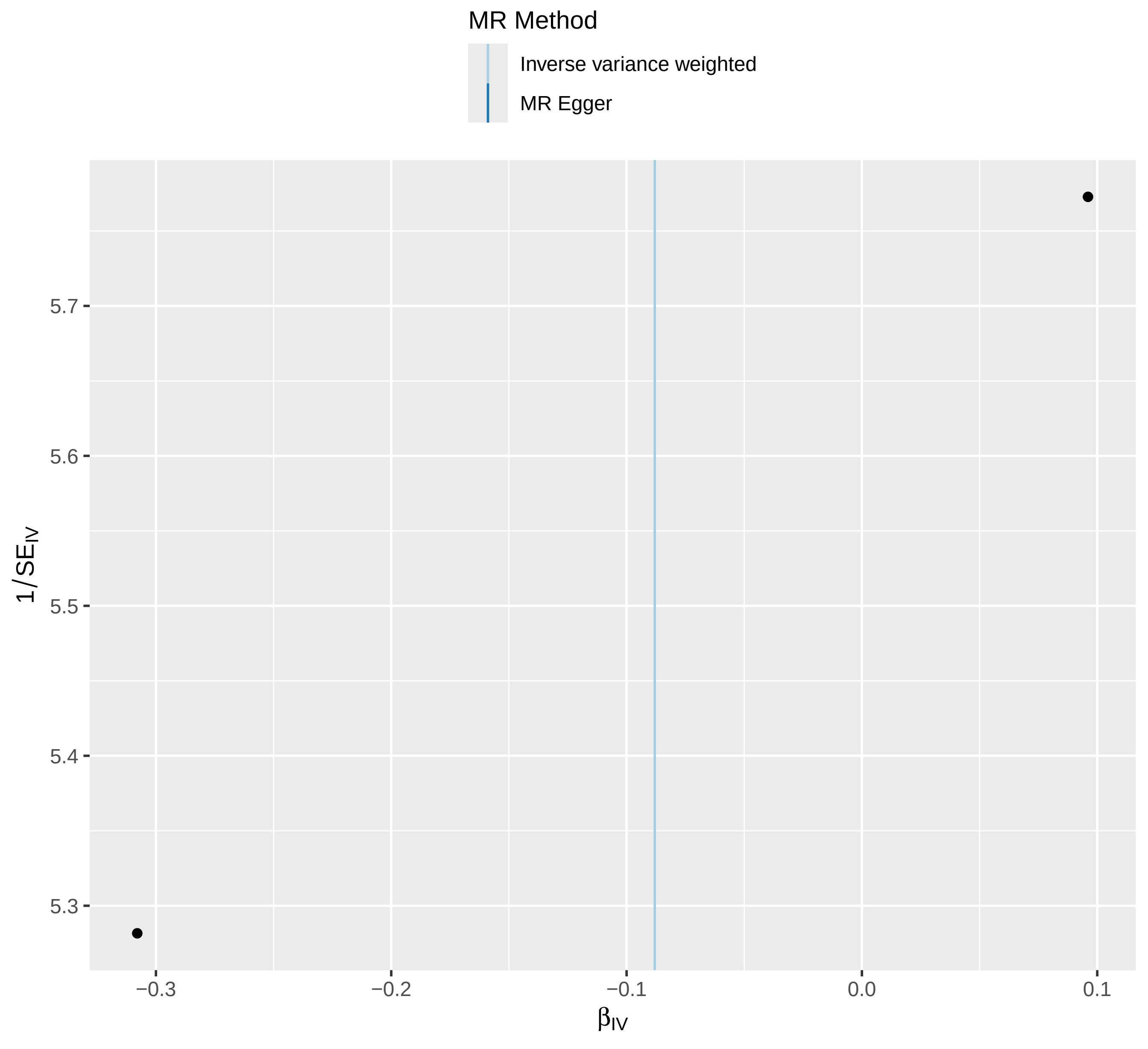

MR Method
Inverse variance weighted
MR Egger
1/SE$_{IV}$
5.7
5.6
5.5
5.4
5.3
−0.3
−0.2
−0.1
0.0
0.1
β$_{IV}$

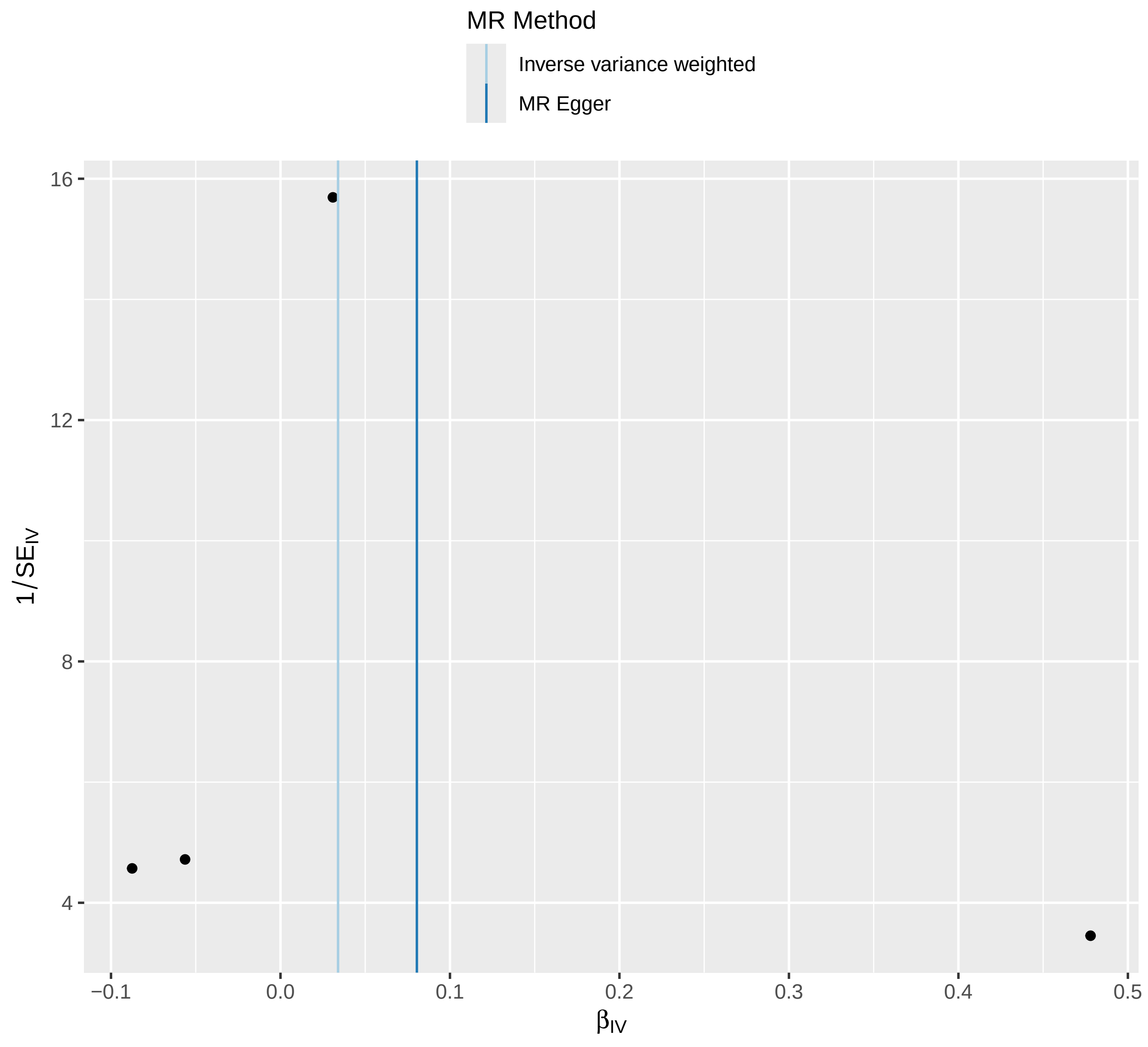
MR Method
Inverse variance weighted
MR Egger
16
12
8
4
1/SE_IV
−0.1
0.0
0.1
0.2
0.3
0.4
0.5
β_IV

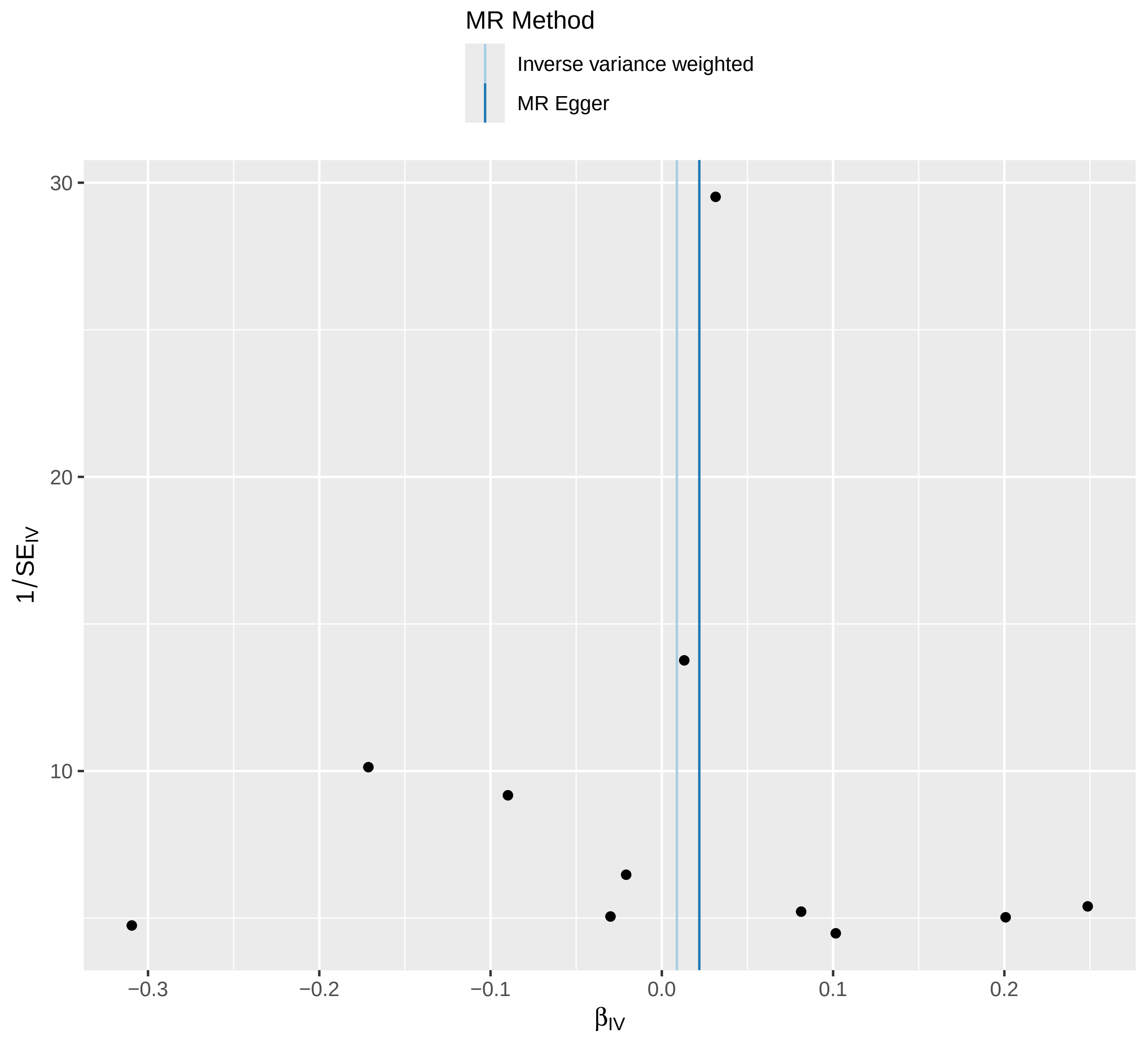
MR Method
Inverse variance weighted
MR Egger
1/SE_IV
β_IV
30
20
10
−0.3
−0.2
−0.1
0.0
0.1
0.2

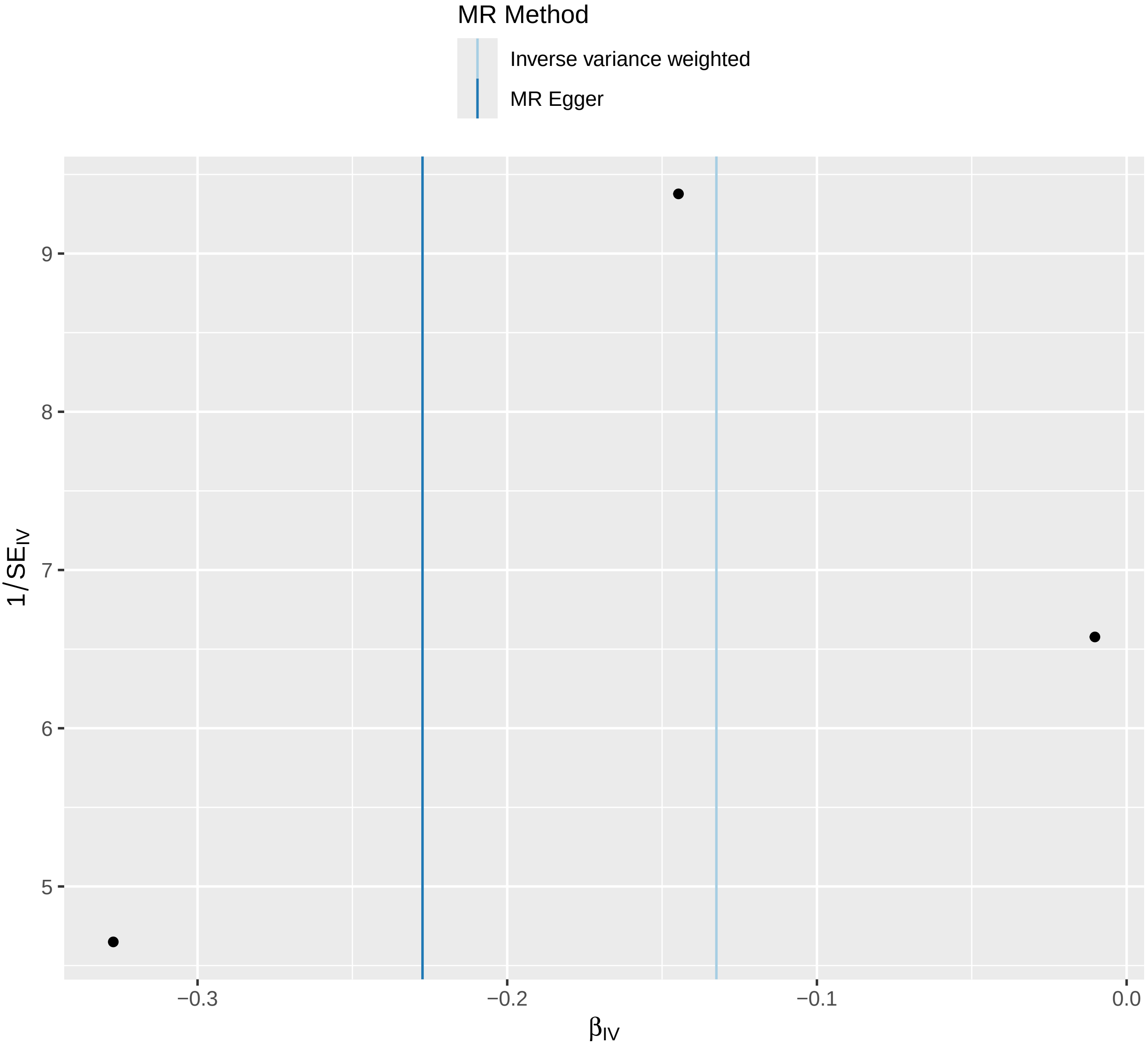
MR Method
Inverse variance weighted
MR Egger
9
8
7
6
5
1/SE_IV
−0.3
−0.2
−0.1
0.0
β_IV

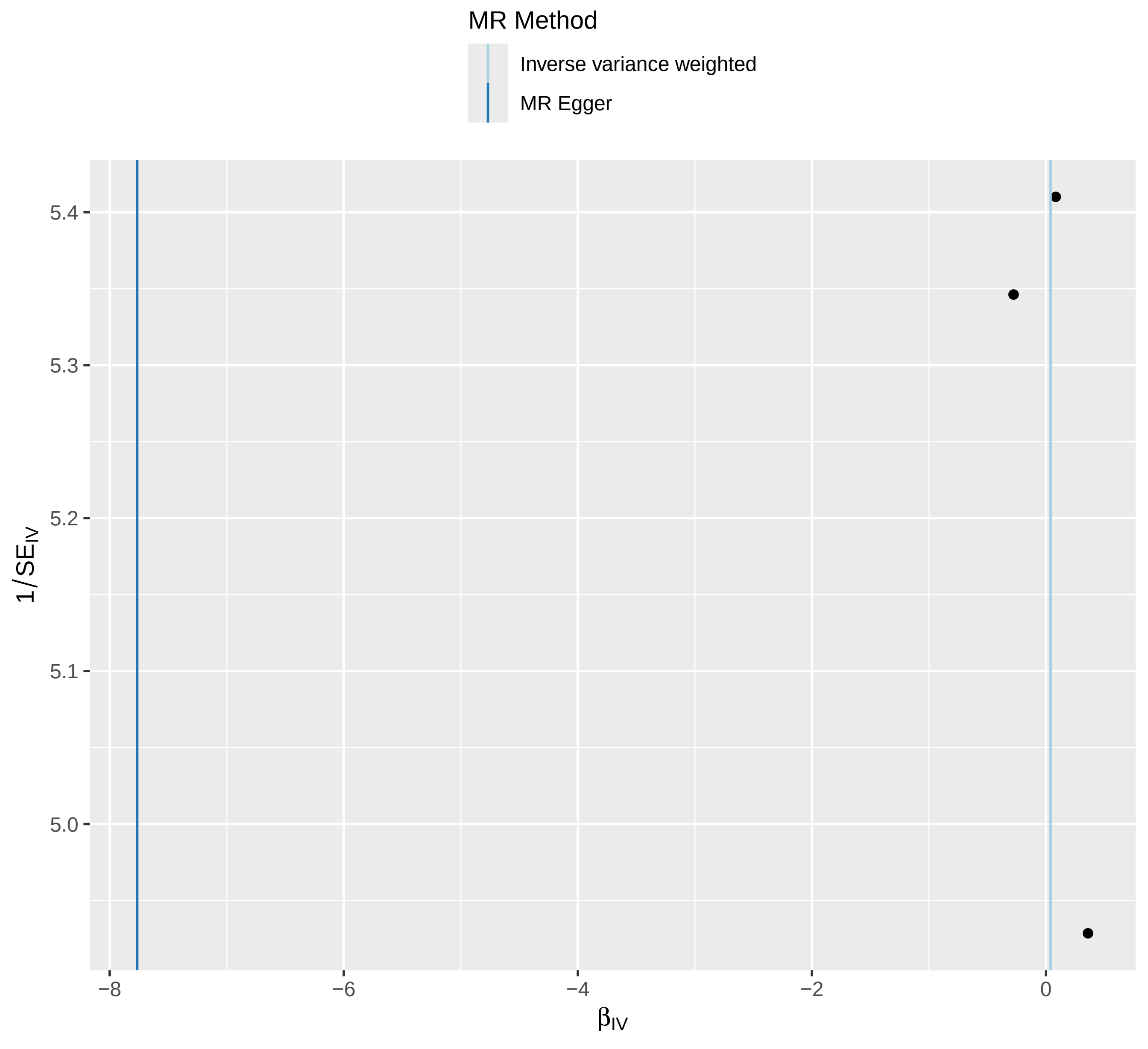

MR Method
Inverse variance weighted
MR Egger
5.4
5.3
5.2
5.1
5.0
$1/SE_{IV}$
−8
−6
−4
−2
0
$\beta_{IV}$

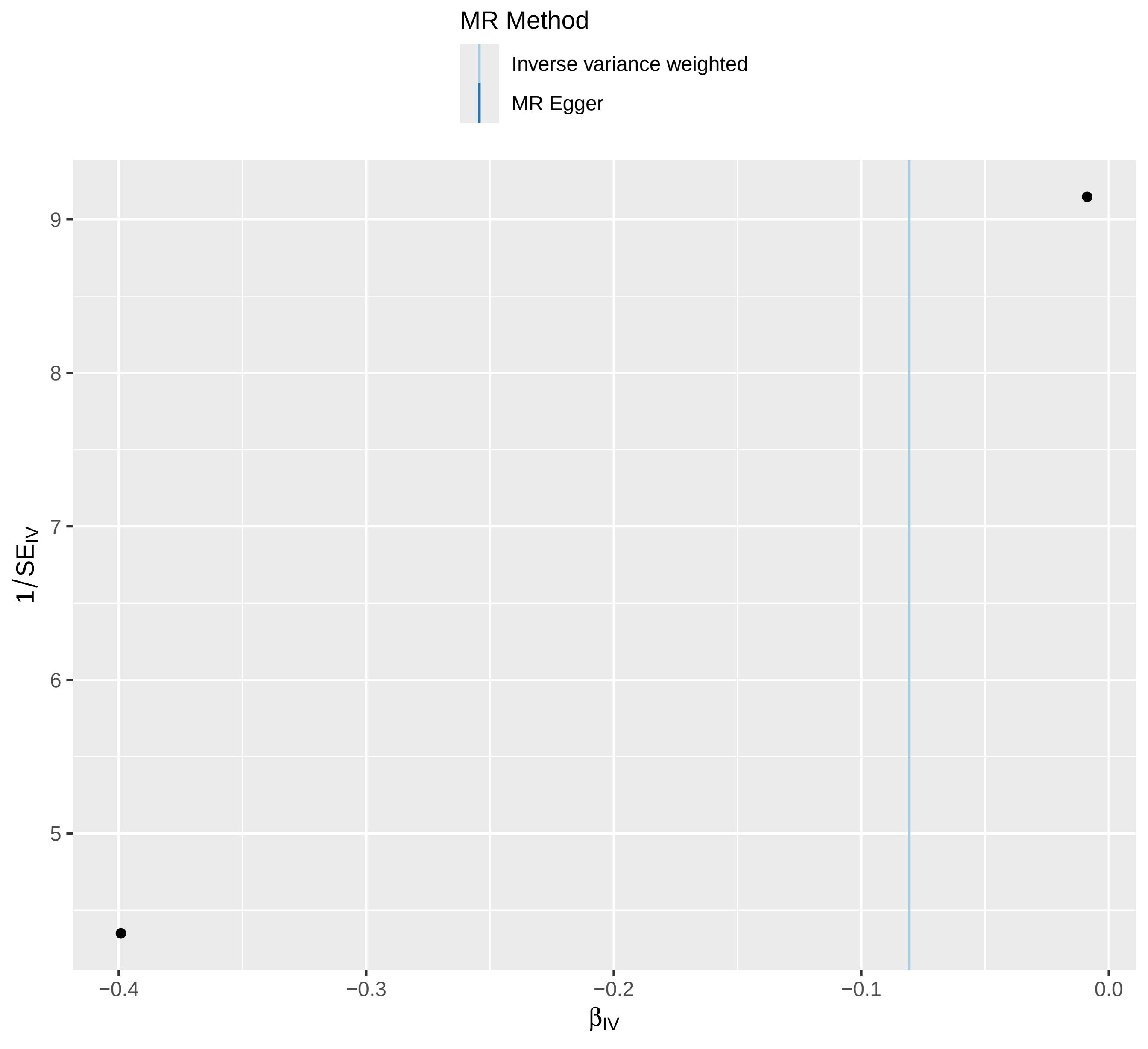

MR Method
Inverse variance weighted
MR Egger
1/SE_IV
β_IV
9
8
7
6
5
−0.4
−0.3
−0.2
−0.1
0.0

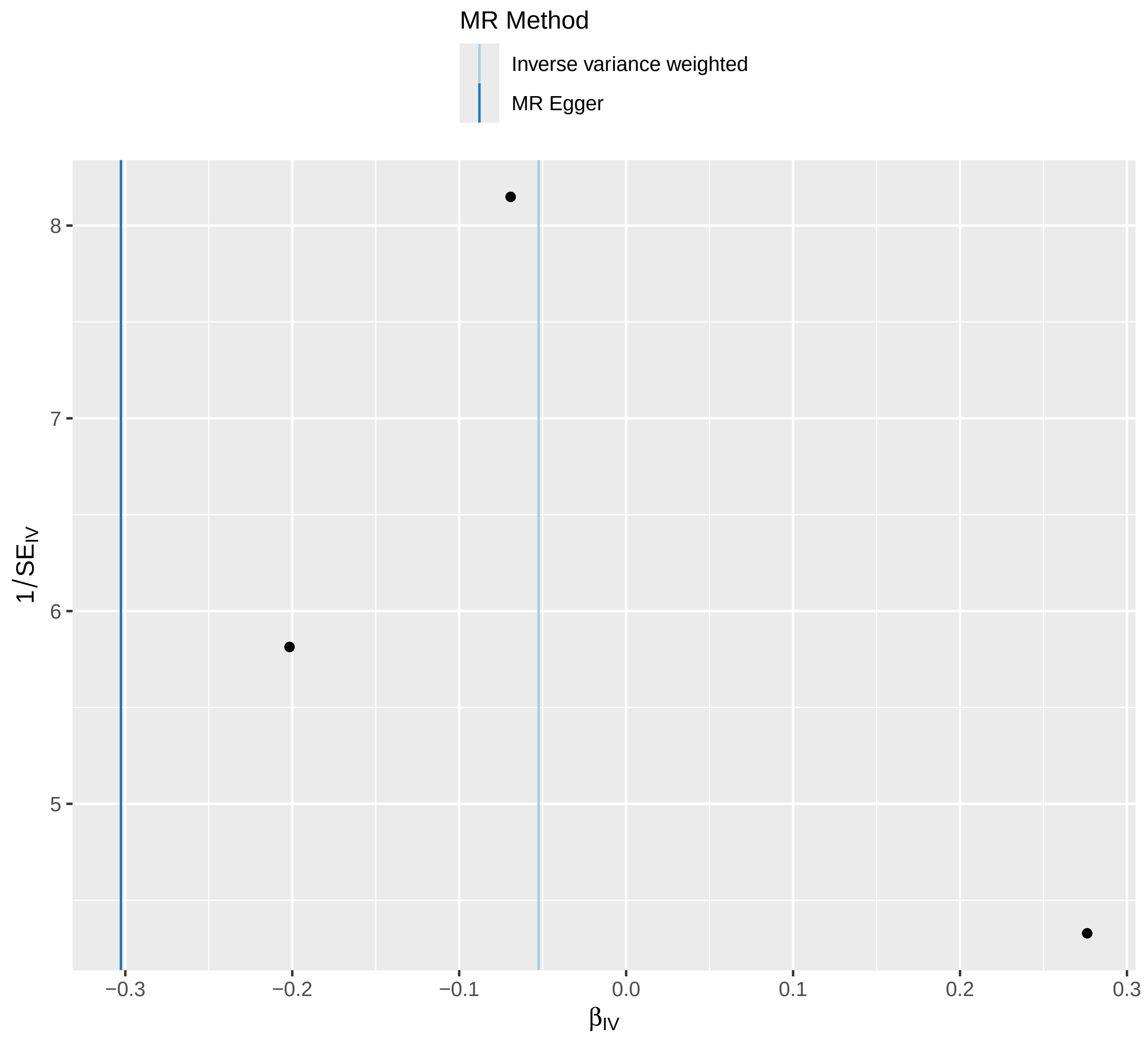

MR Method
Inverse variance weighted
MR Egger
8
7
6
5
1/SE$_{IV}$
−0.3
−0.2
−0.1
0.0
0.1
0.2
0.3
$\beta_{IV}$

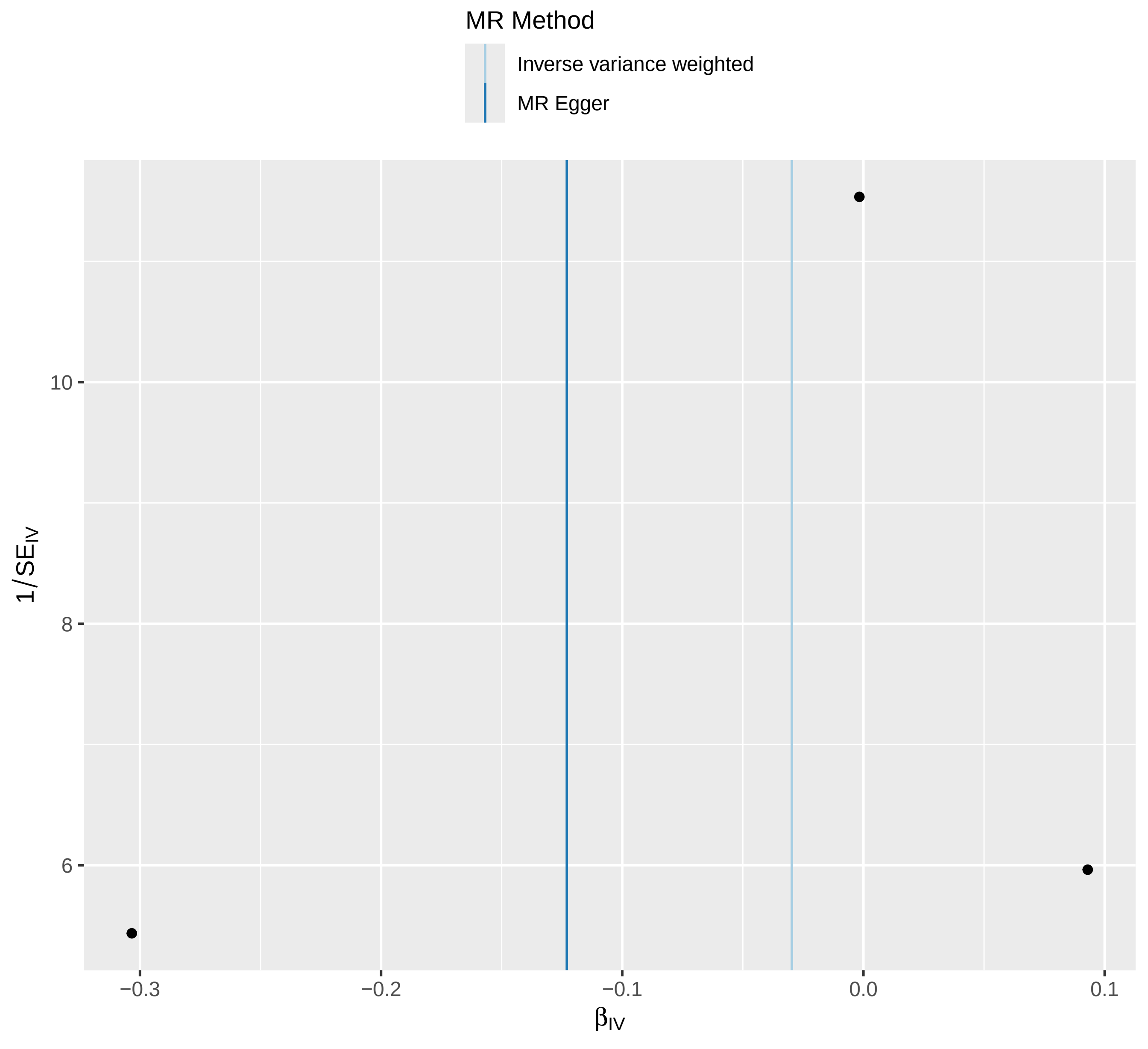
MR Method
Inverse variance weighted
MR Egger
1/SE_IV
10
8
6
−0.3
−0.2
−0.1
0.0
0.1
β_IV

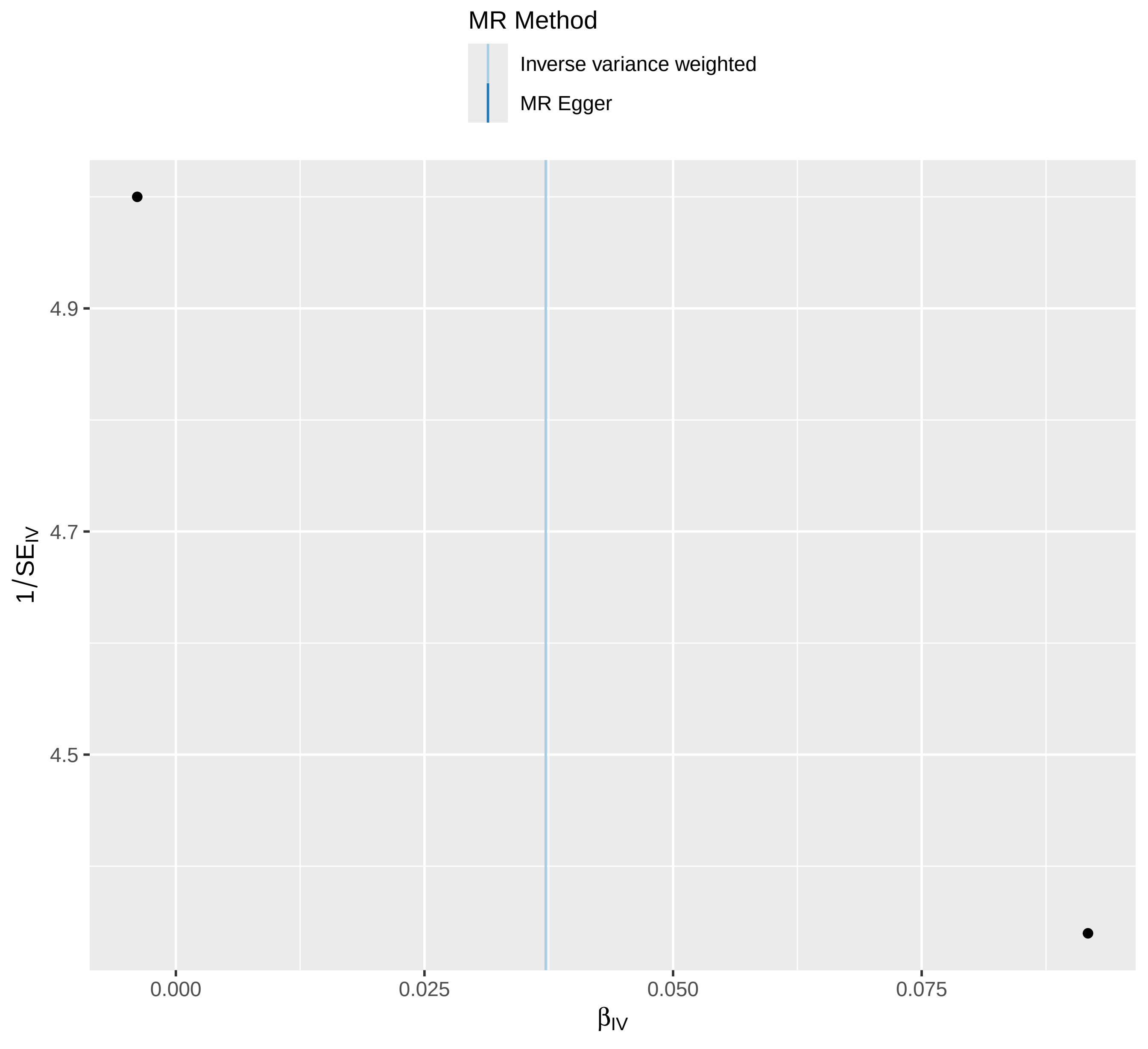

MR Method
Inverse variance weighted
MR Egger
4.9
4.7
4.5
1/SE_IV
0.000
0.025
0.050
0.075
β_IV

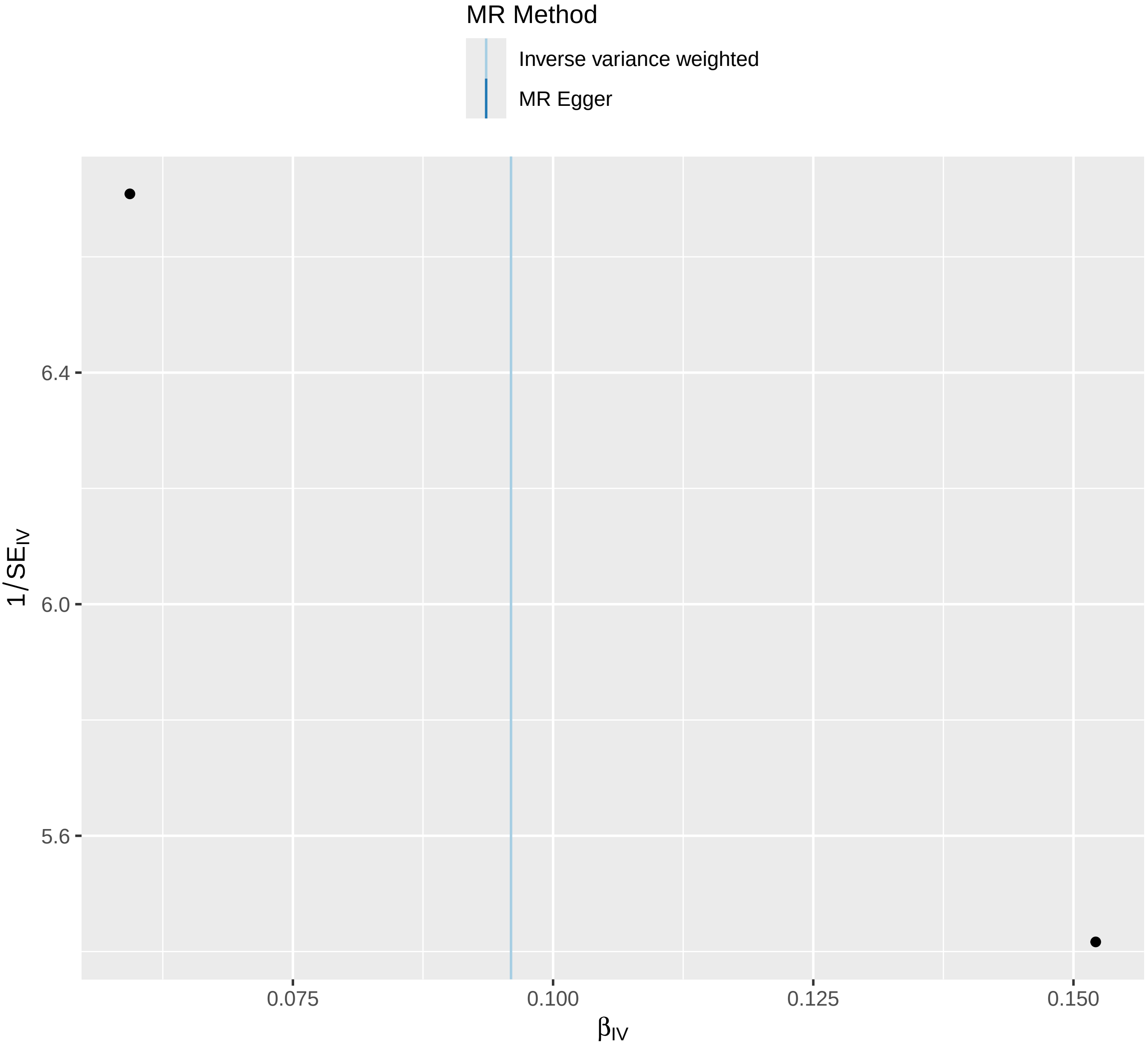
MR Method
Inverse variance weighted
MR Egger
6.4
6.0
5.6
$1/SE_{IV}$
0.075
0.100
0.125
0.150
$\beta_{IV}$

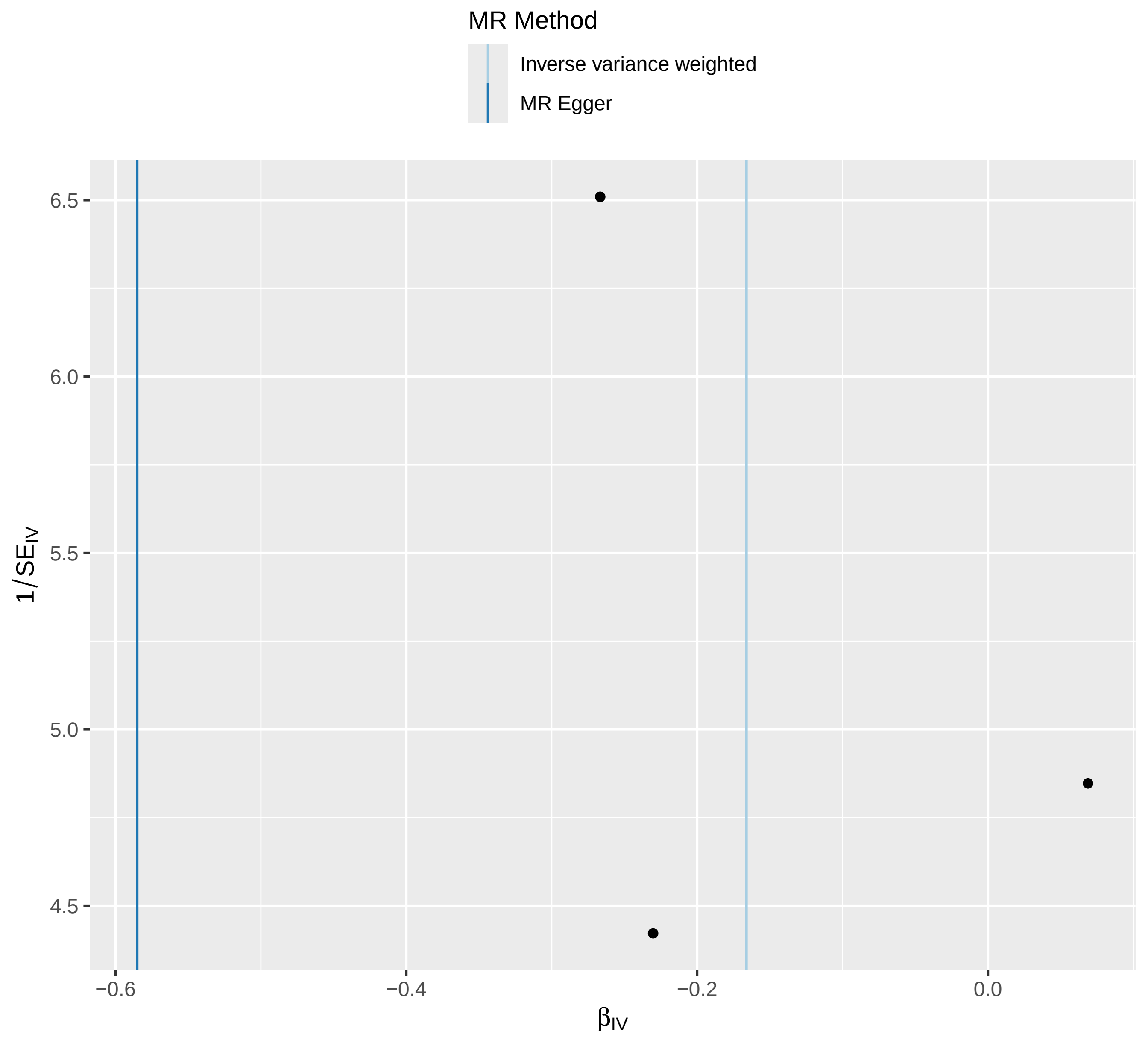

MR Method
Inverse variance weighted
MR Egger
6.5
6.0
5.5
5.0
4.5
1/SE$_{IV}$
−0.6
−0.4
−0.2
0.0
β$_{IV}$

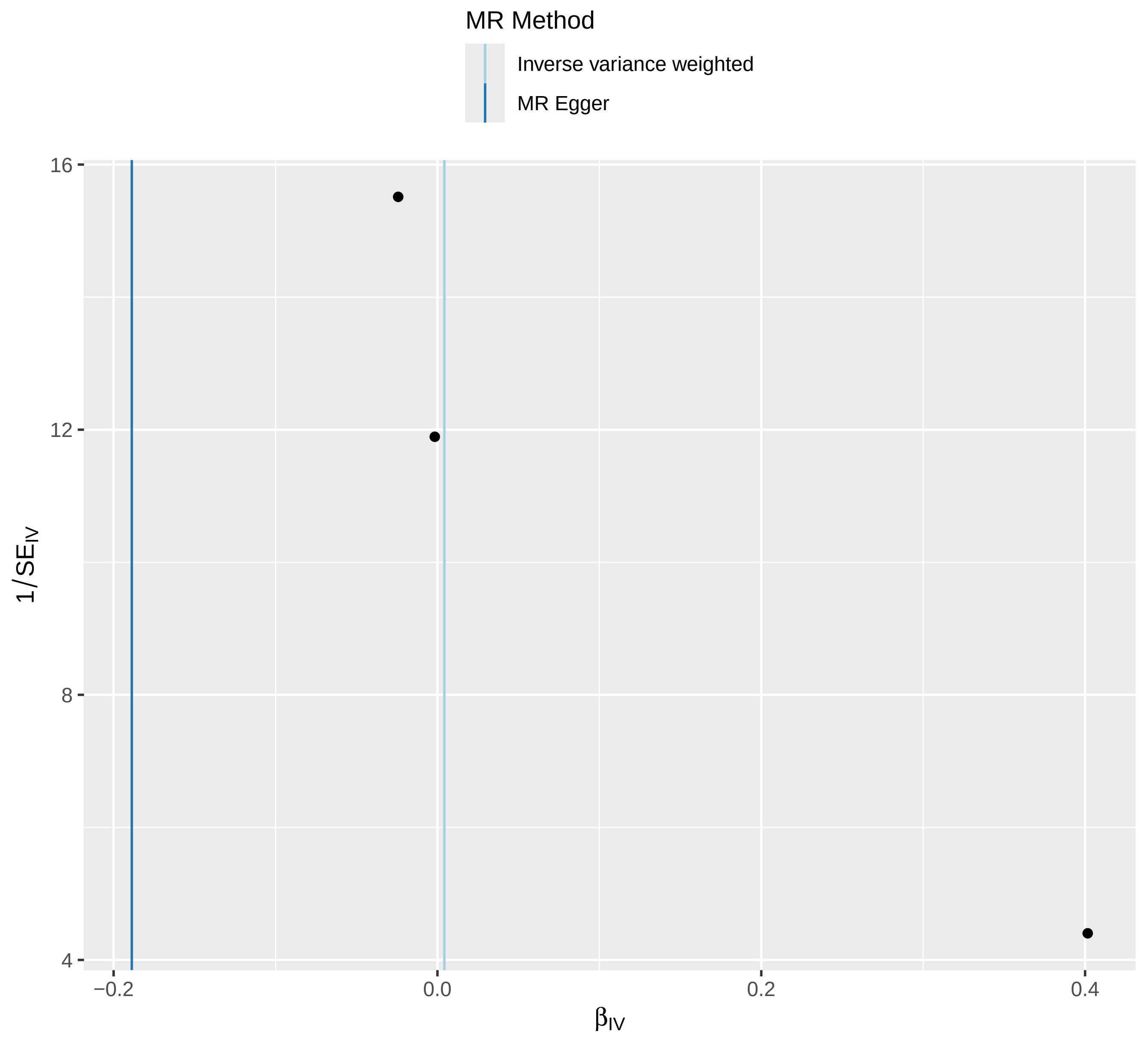
MR Method
Inverse variance weighted
MR Egger
16
12
8
4
−0.2
0.0
0.2
0.4
$1/SE_{IV}$
$\beta_{IV}$

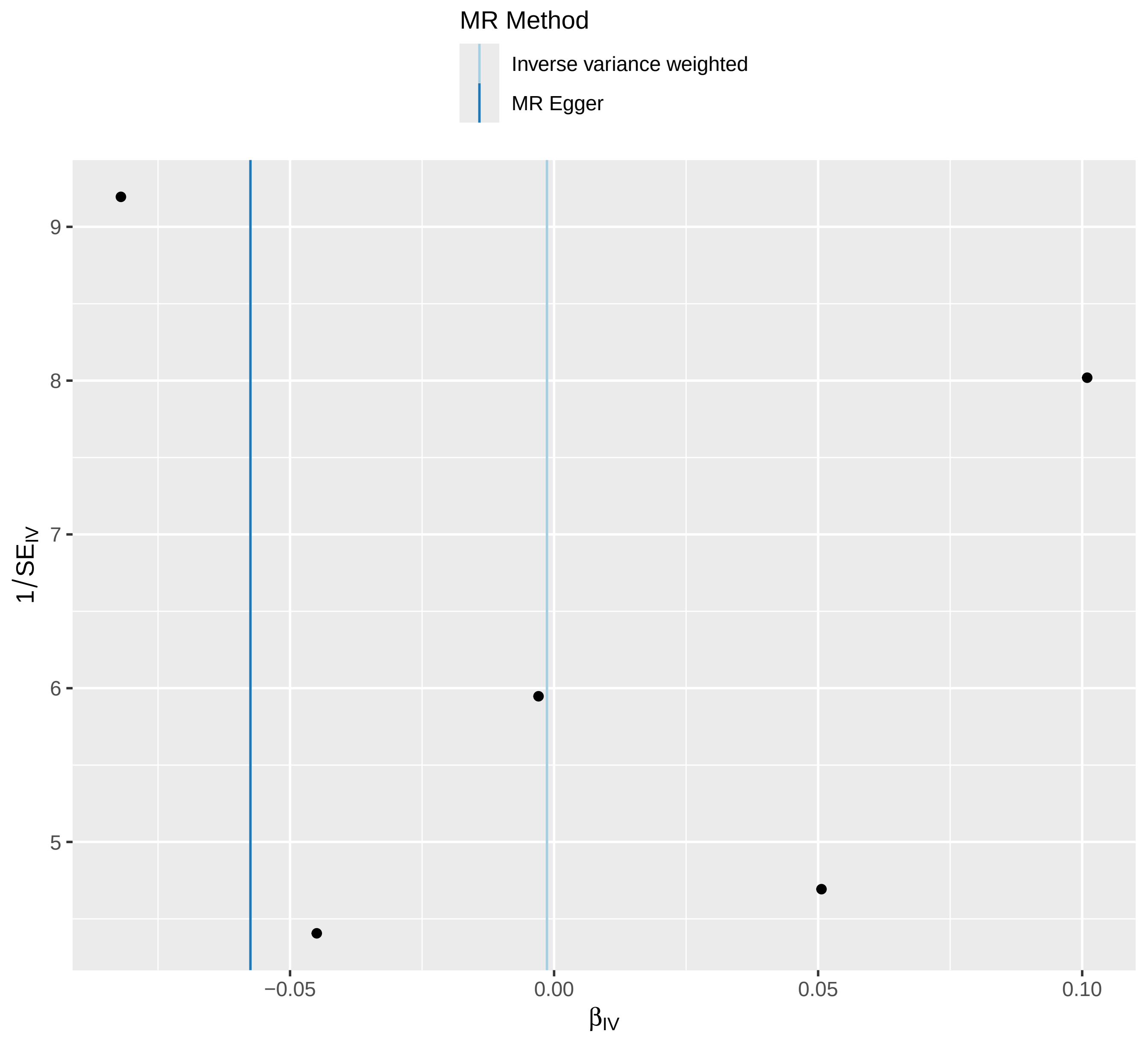
MR Method
Inverse variance weighted
MR Egger
9
8
7
6
5
1/SE_IV
−0.05
0.00
0.05
0.10
β_IV

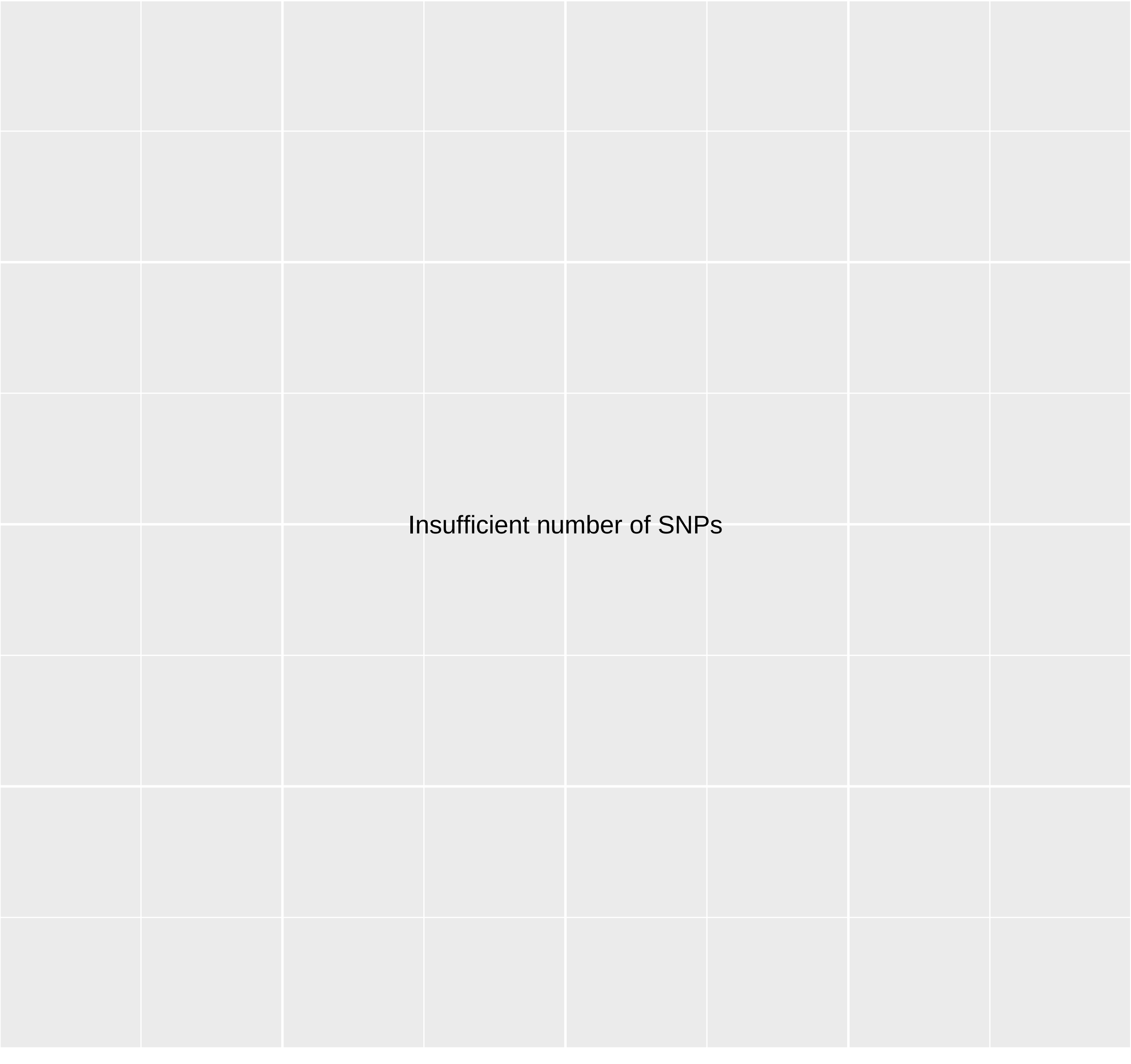
Insufficient number of SNPs

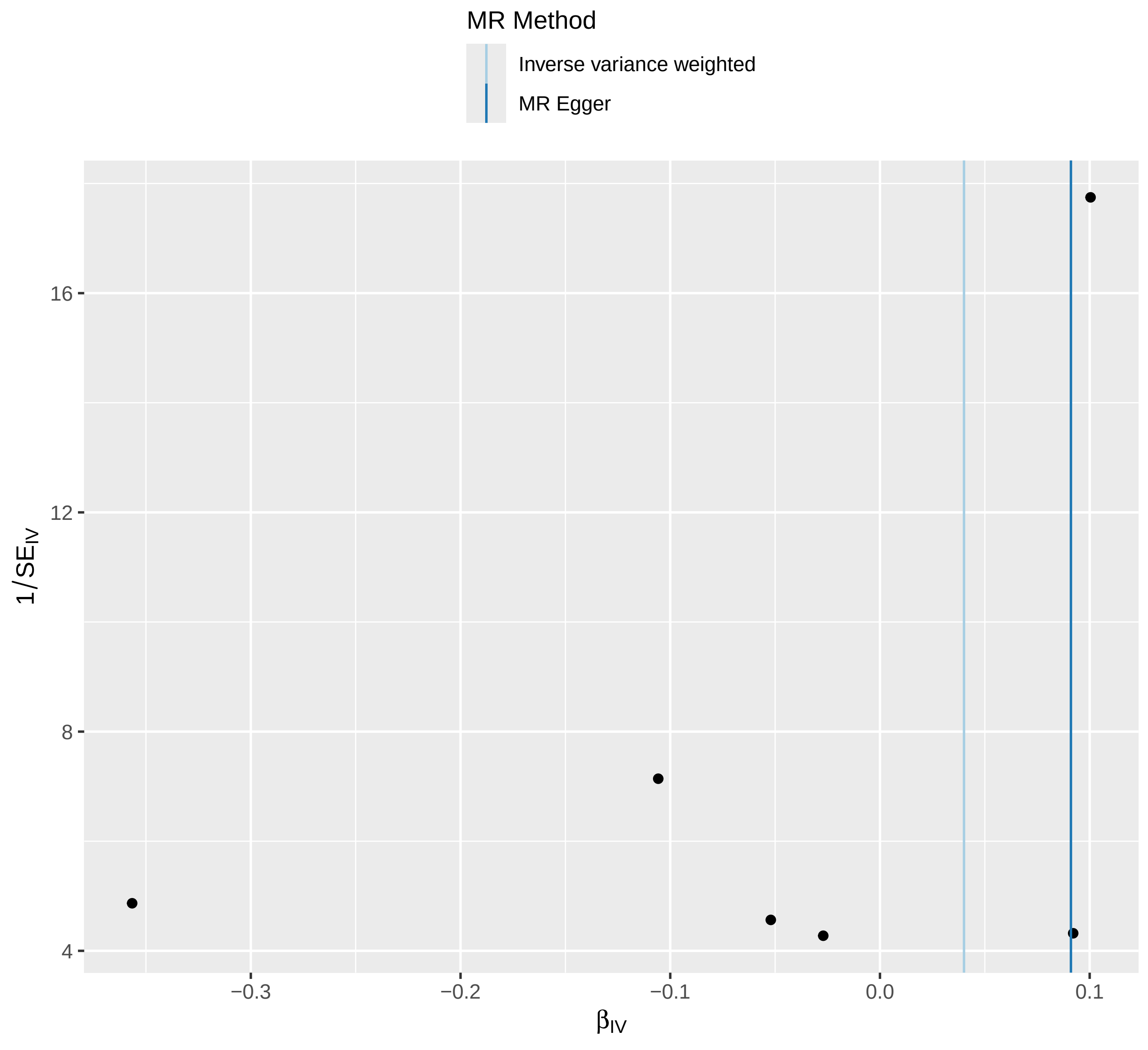

MR Method
Inverse variance weighted
MR Egger
16
12
8
4
1/SE_IV
−0.3
−0.2
−0.1
0.0
0.1
β_IV

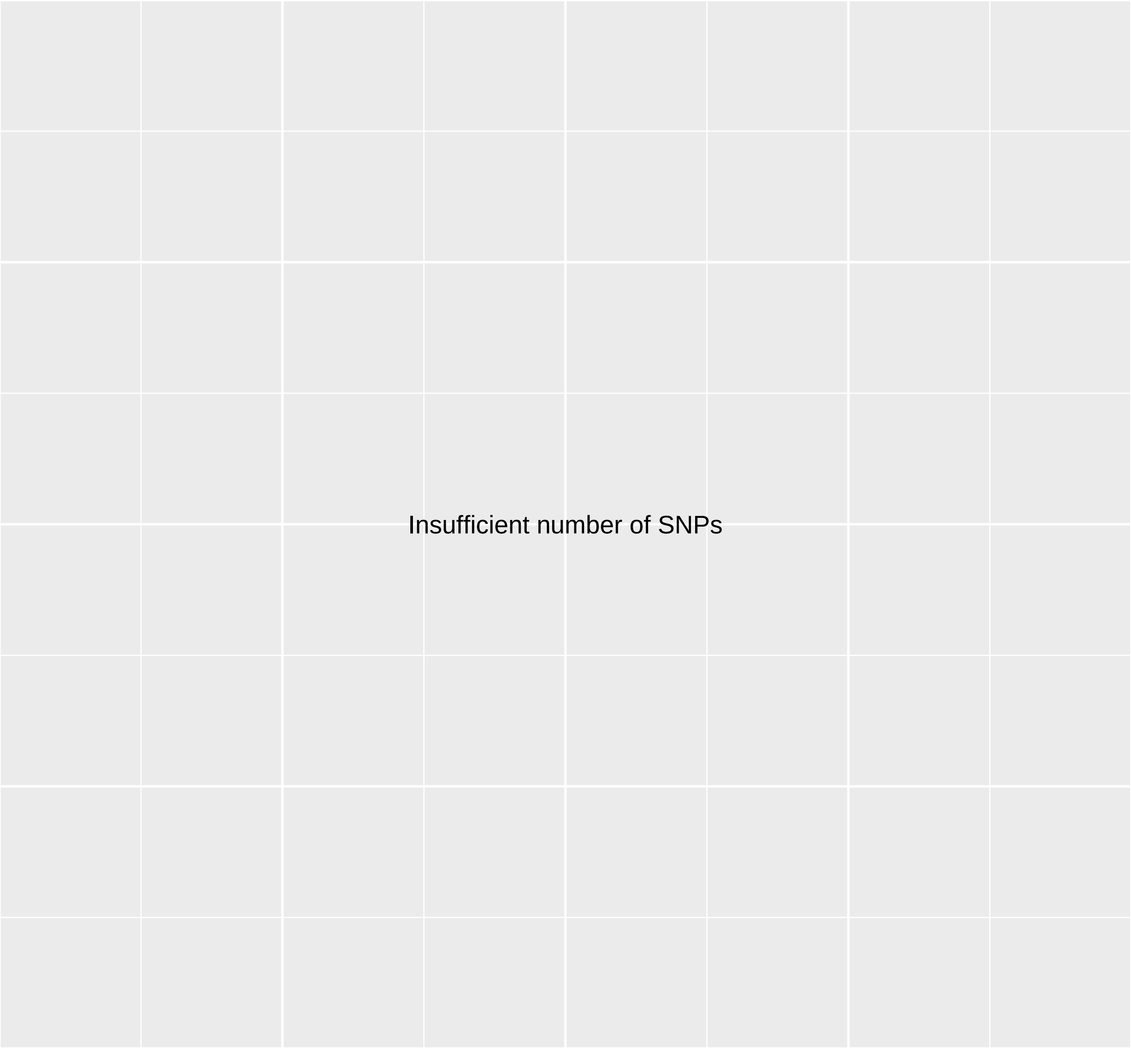
Insufficient number of SNPs

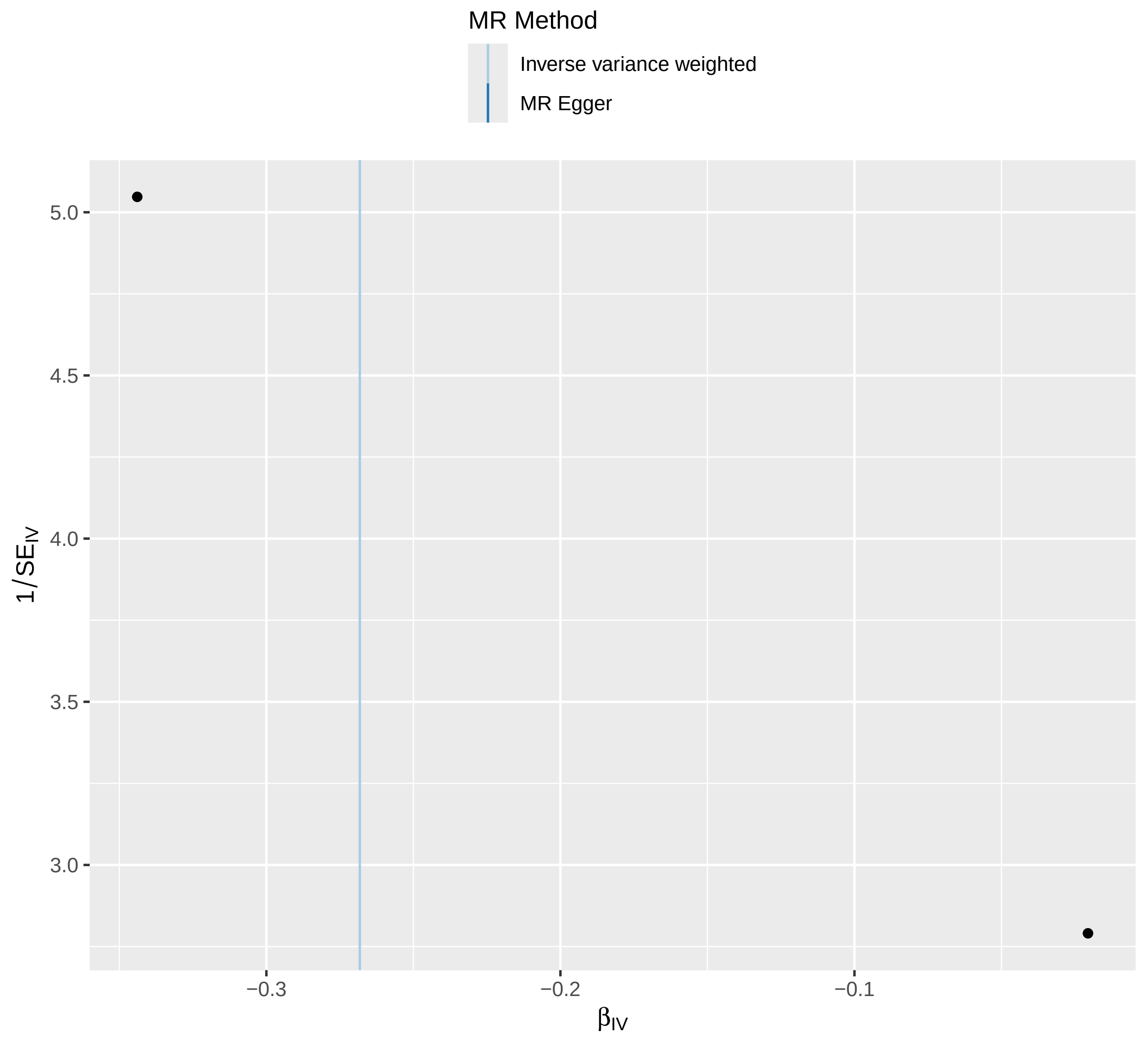

MR Method
Inverse variance weighted
MR Egger
5.0
4.5
4.0
3.5
3.0
1/SE_IV
−0.3
−0.2
−0.1
β_IV

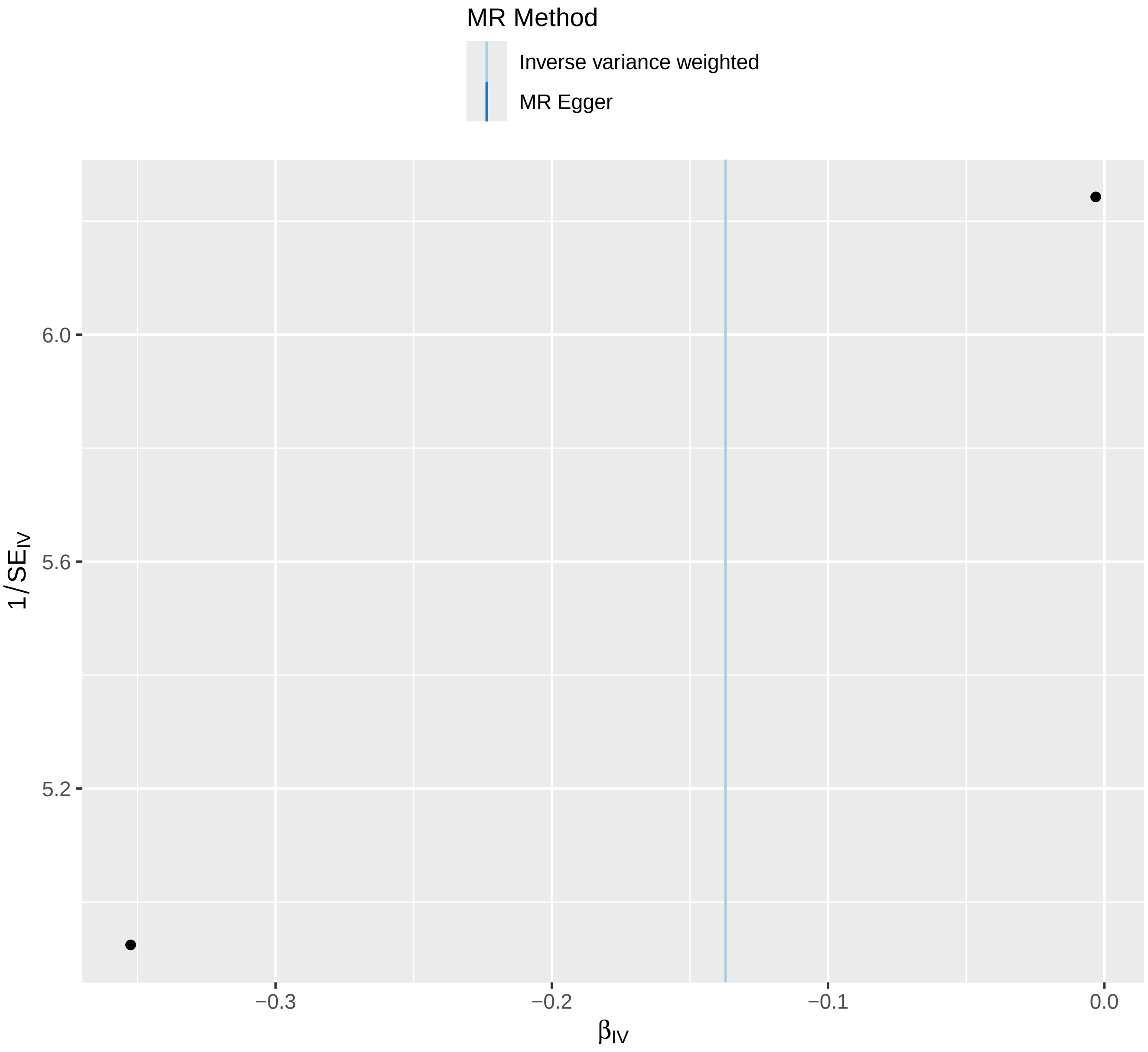
MR Method
Inverse variance weighted
MR Egger
6.0
5.6
5.2
1/SE_IV
−0.3
−0.2
−0.1
0.0
β_IV

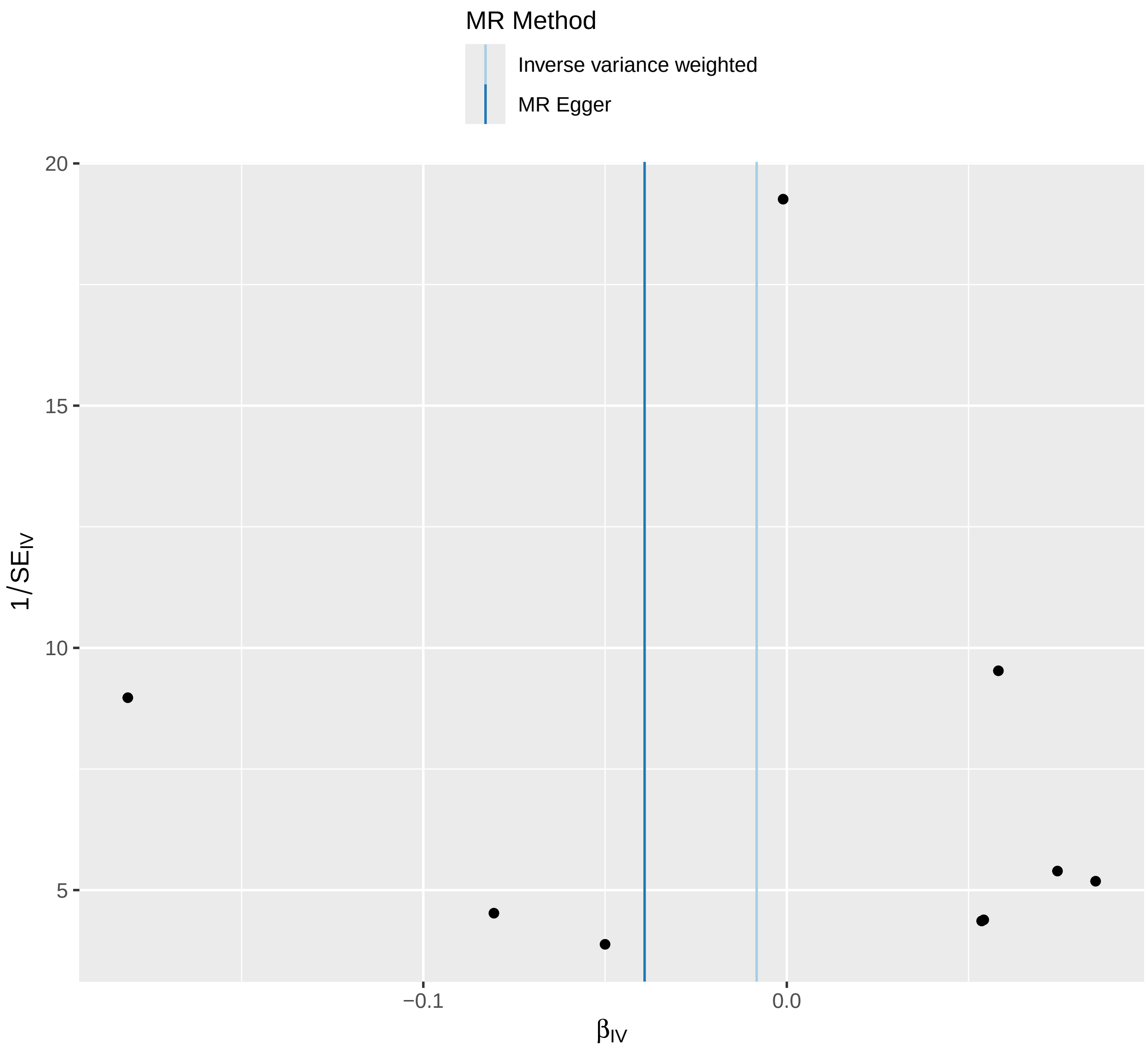
MR Method
Inverse variance weighted
MR Egger
20
15
10
5
1/$SE_{IV}$
−0.1
0.0
$\beta_{IV}$

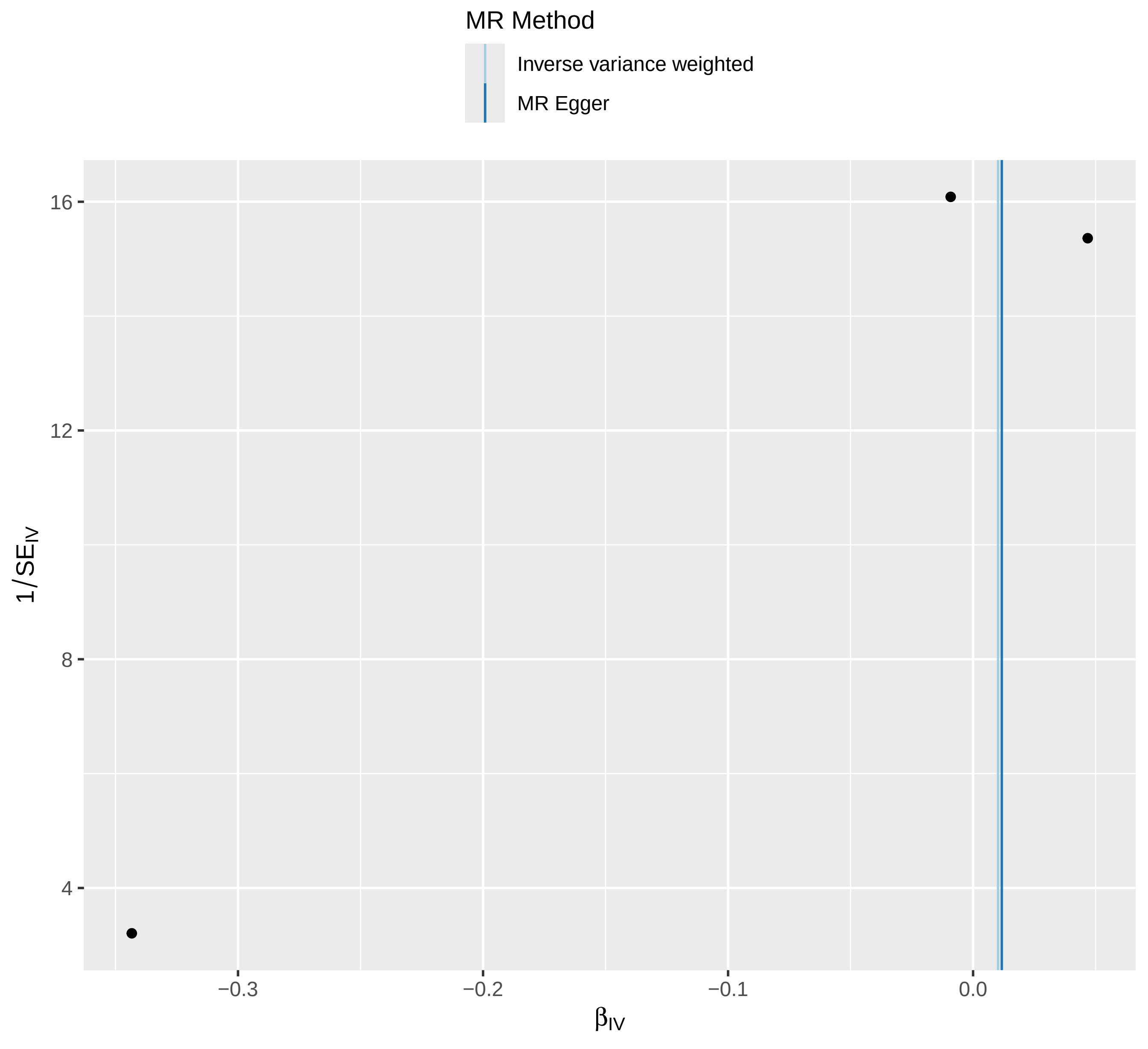
MR Method
Inverse variance weighted
MR Egger
16
12
8
4
1/SE$_{IV}$
−0.3
−0.2
−0.1
0.0
β$_{IV}$

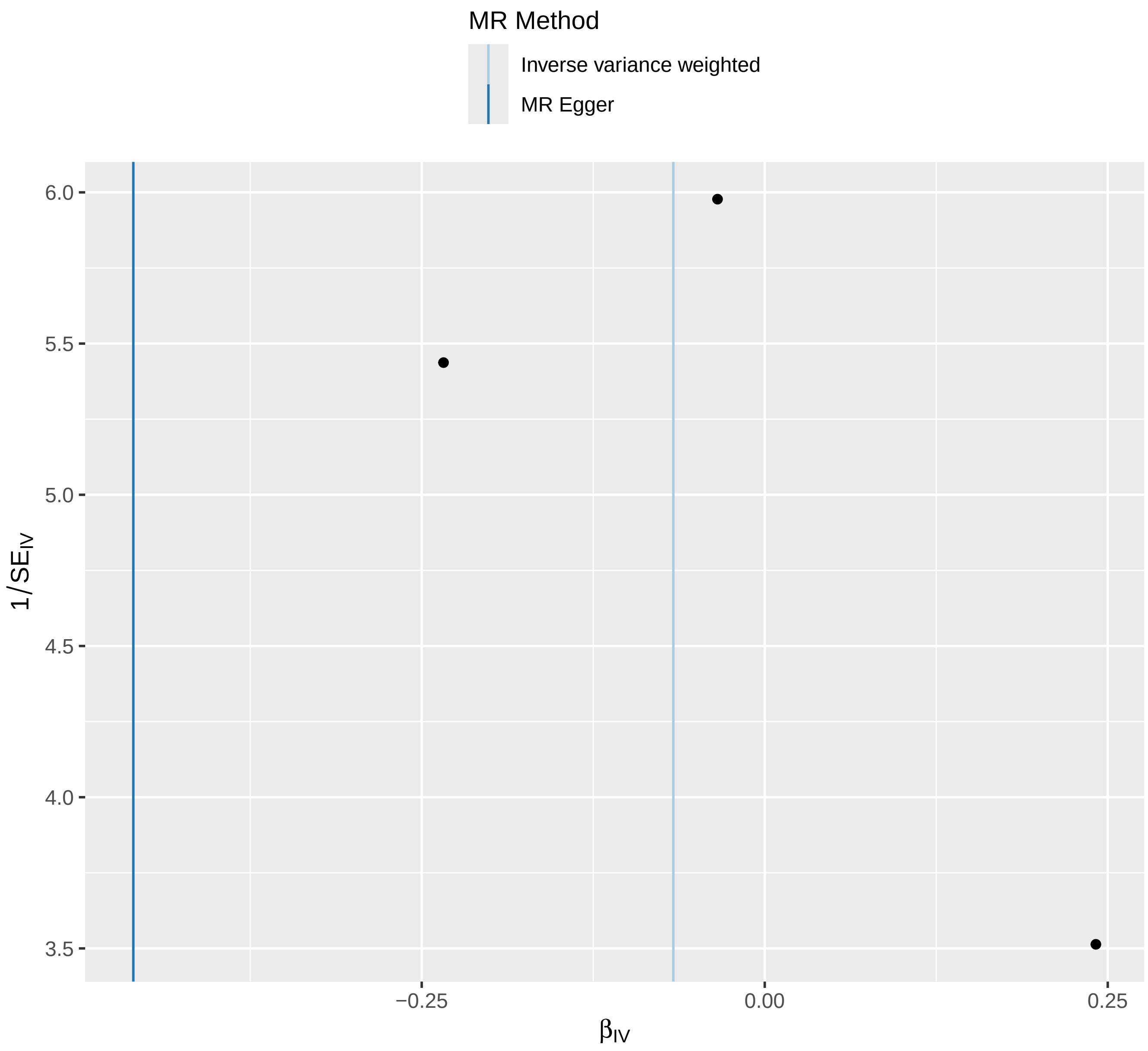
MR Method
Inverse variance weighted
MR Egger
6.0
5.5
5.0
4.5
4.0
3.5
$1/SE_{IV}$
−0.25
0.00
0.25
$\beta_{IV}$

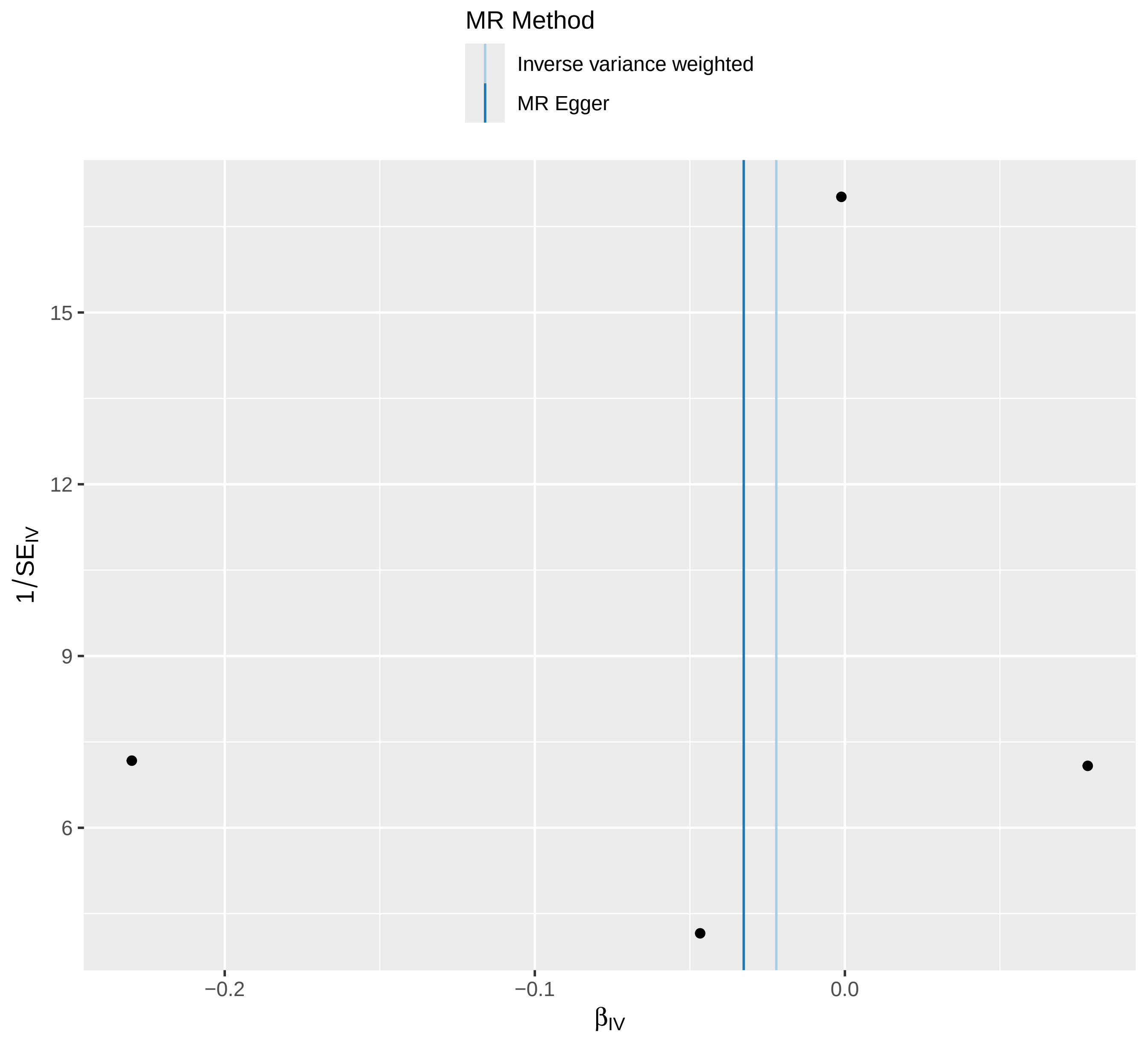
MR Method
Inverse variance weighted
MR Egger
15
12
9
6
1/SE_IV
−0.2
−0.1
0.0
β_IV

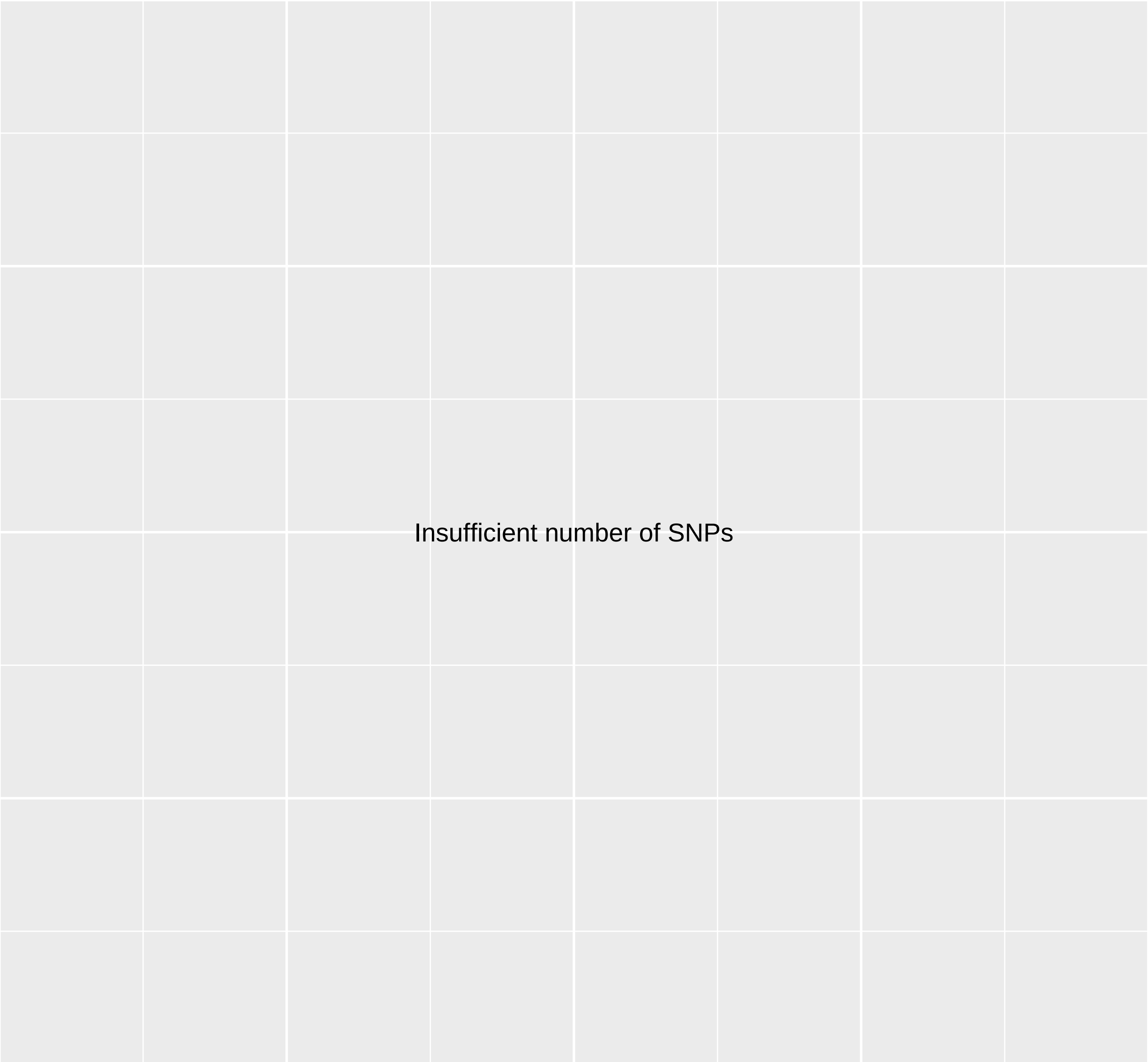
Insufficient number of SNPs

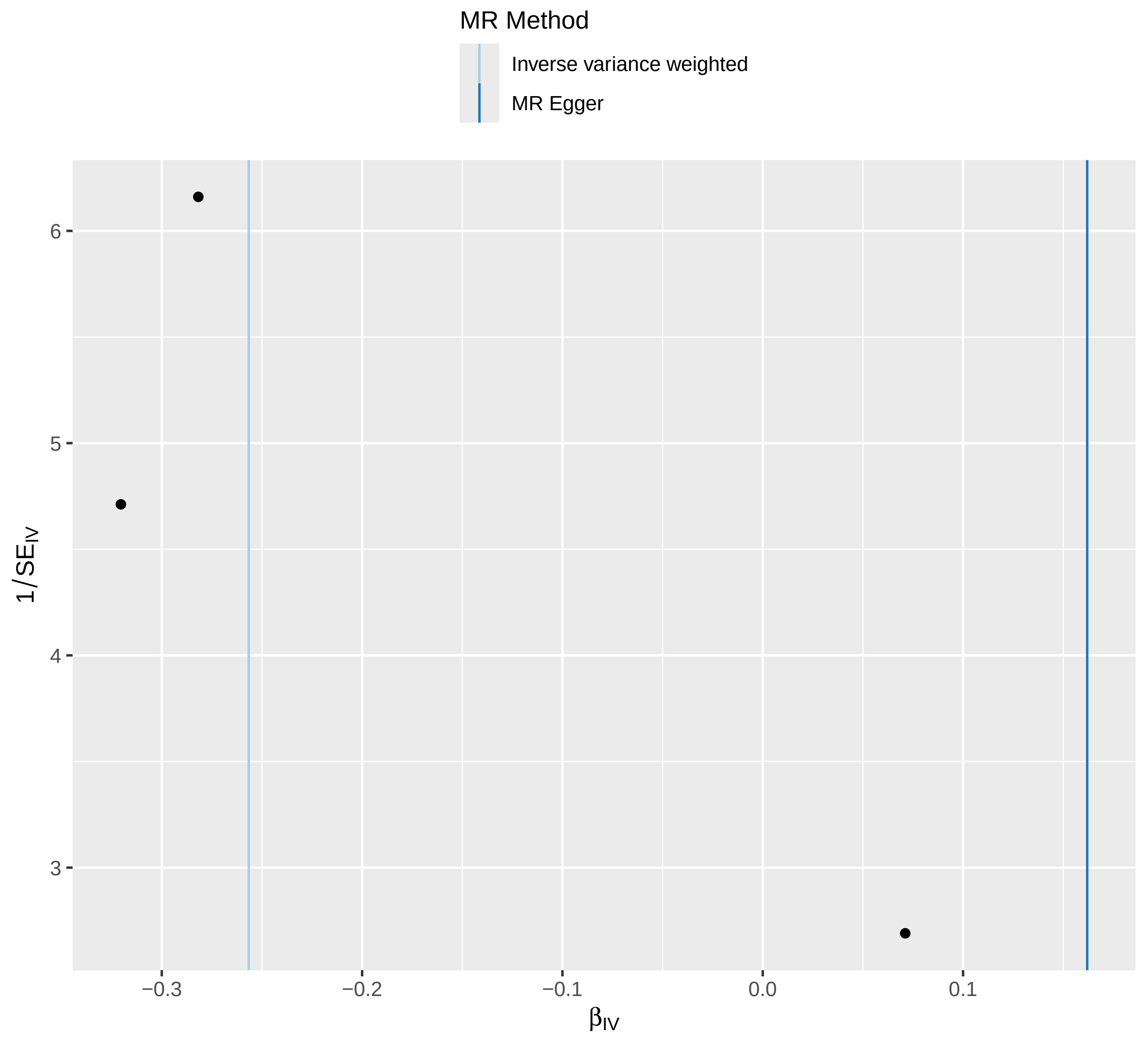
MR Method
Inverse variance weighted
MR Egger
6
5
4
3
1/SE_IV
−0.3
−0.2
−0.1
0.0
0.1
β_IV

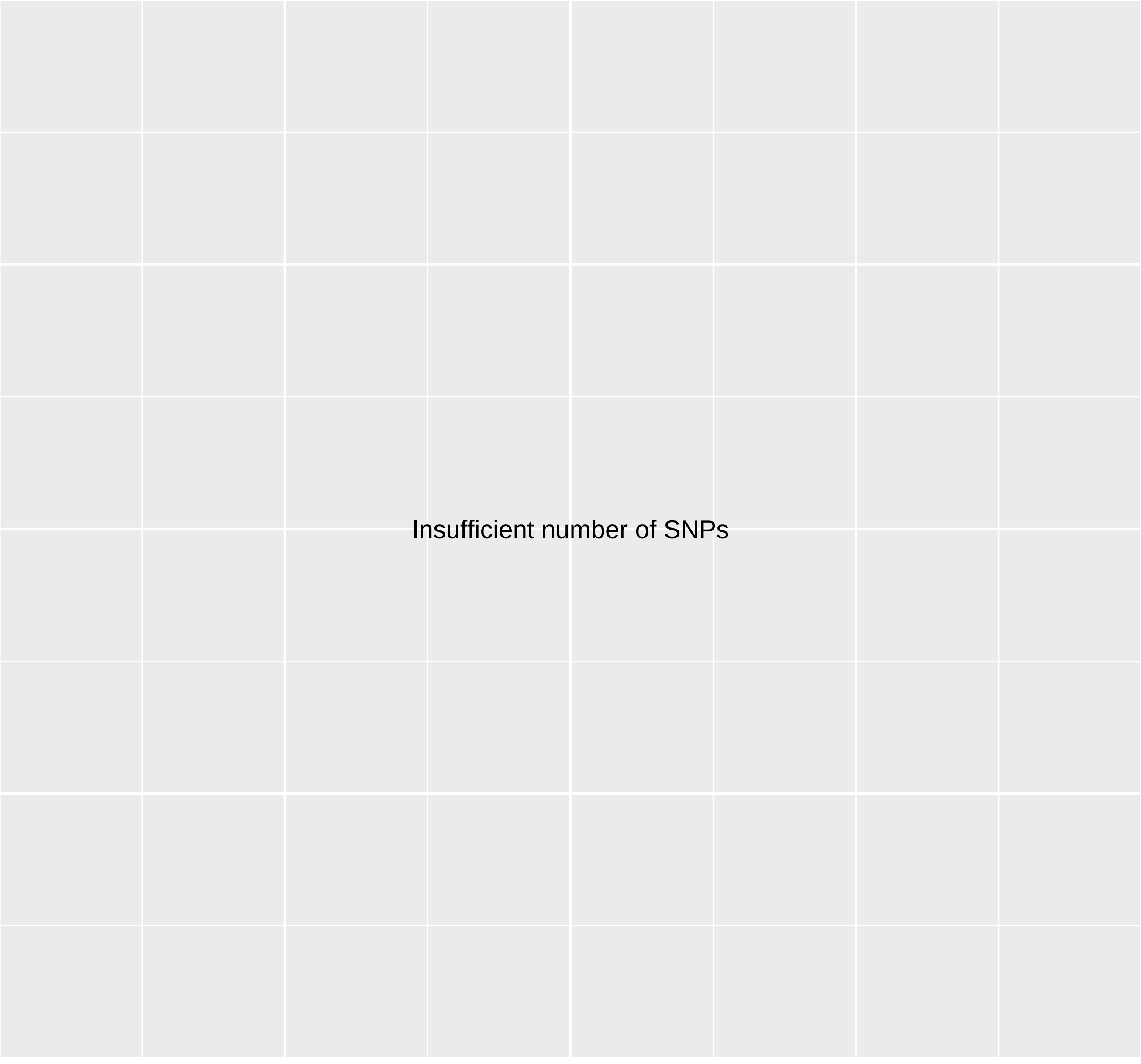
Insufficient number of SNPs

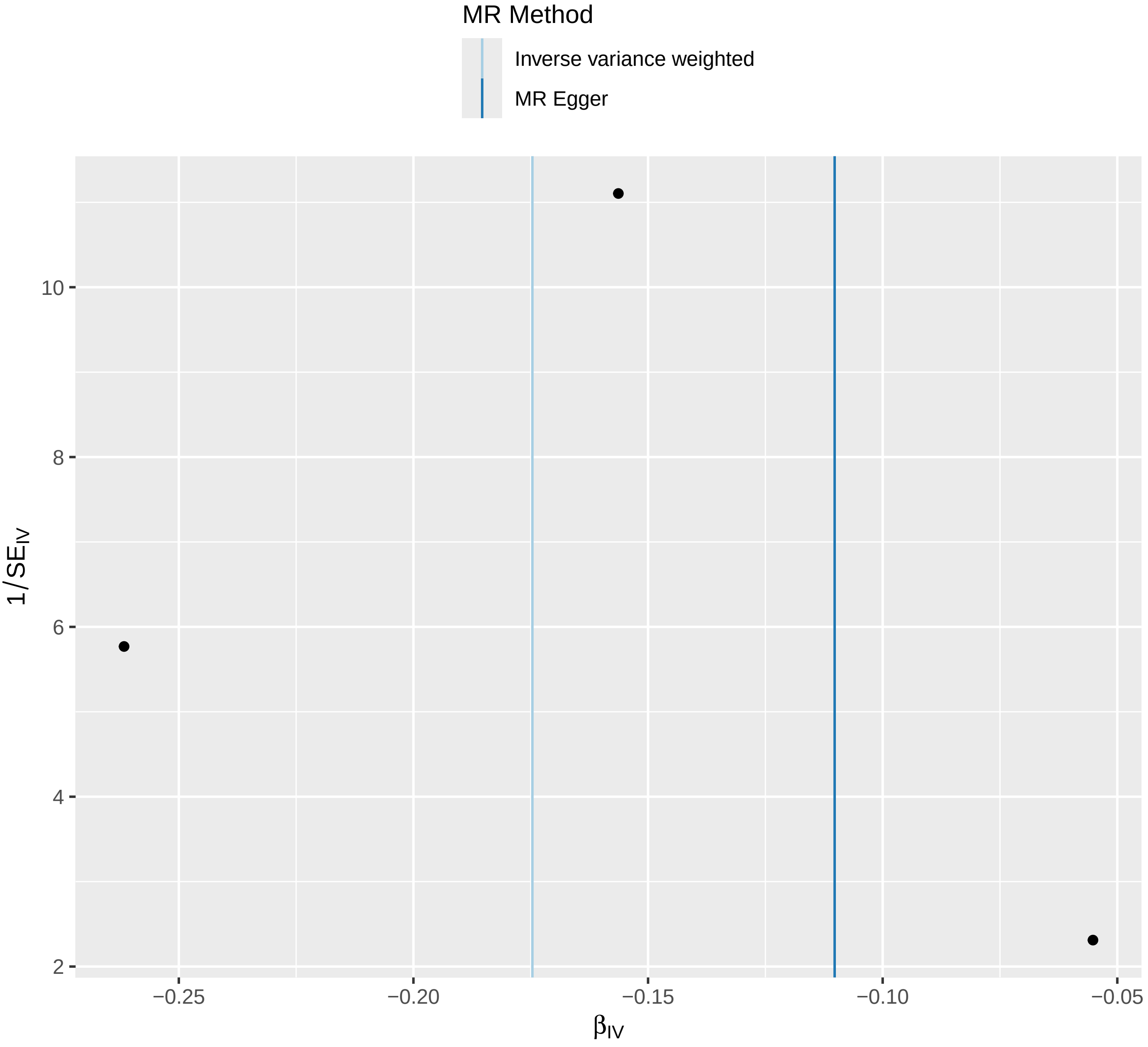

MR Method
Inverse variance weighted
MR Egger
10
8
6
4
2
1/SE_IV
−0.25
−0.20
−0.15
−0.10
−0.05
β_IV

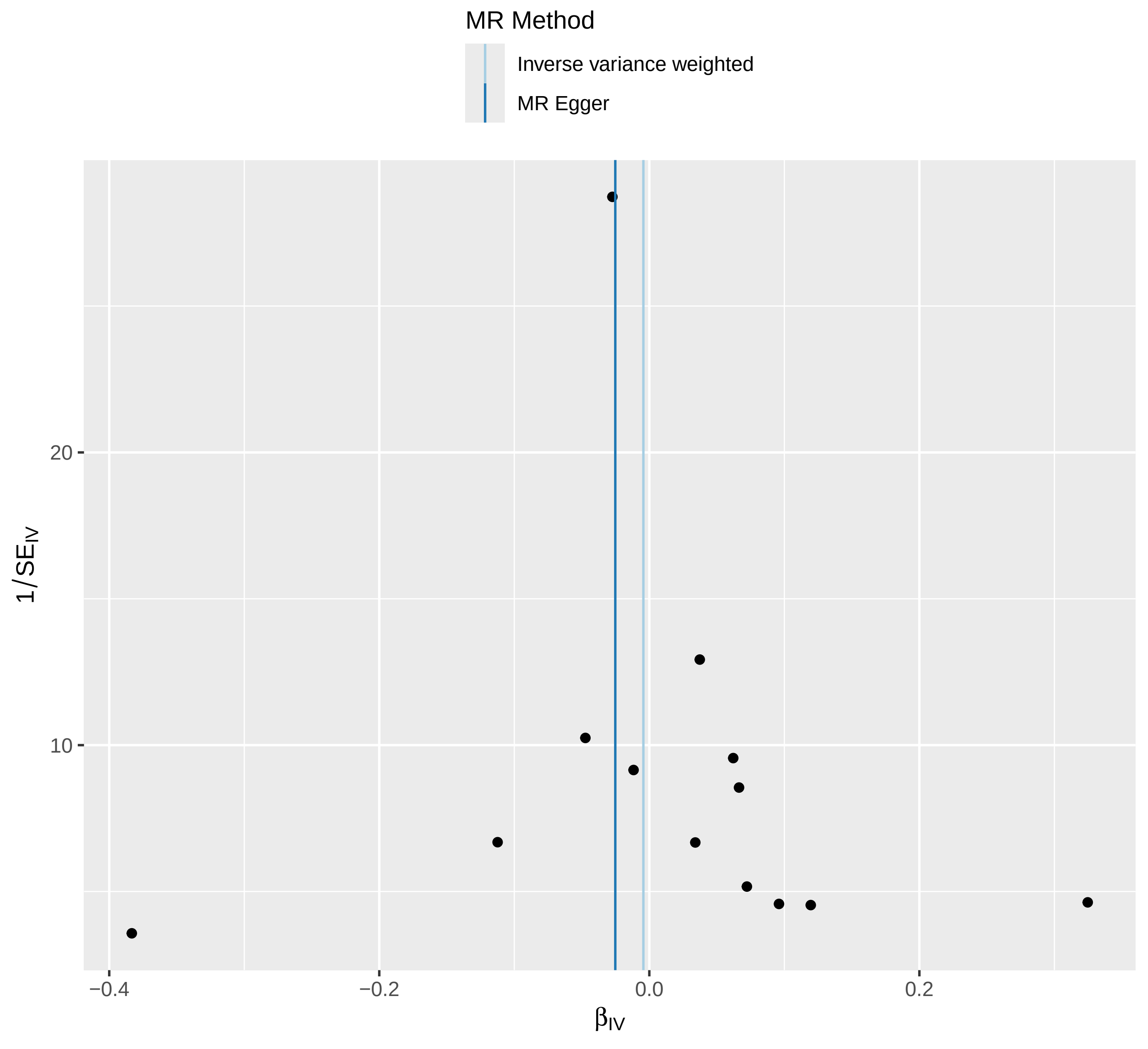
MR Method
Inverse variance weighted
MR Egger
20
10
1/SE_IV
−0.4
−0.2
0.0
0.2
β_IV

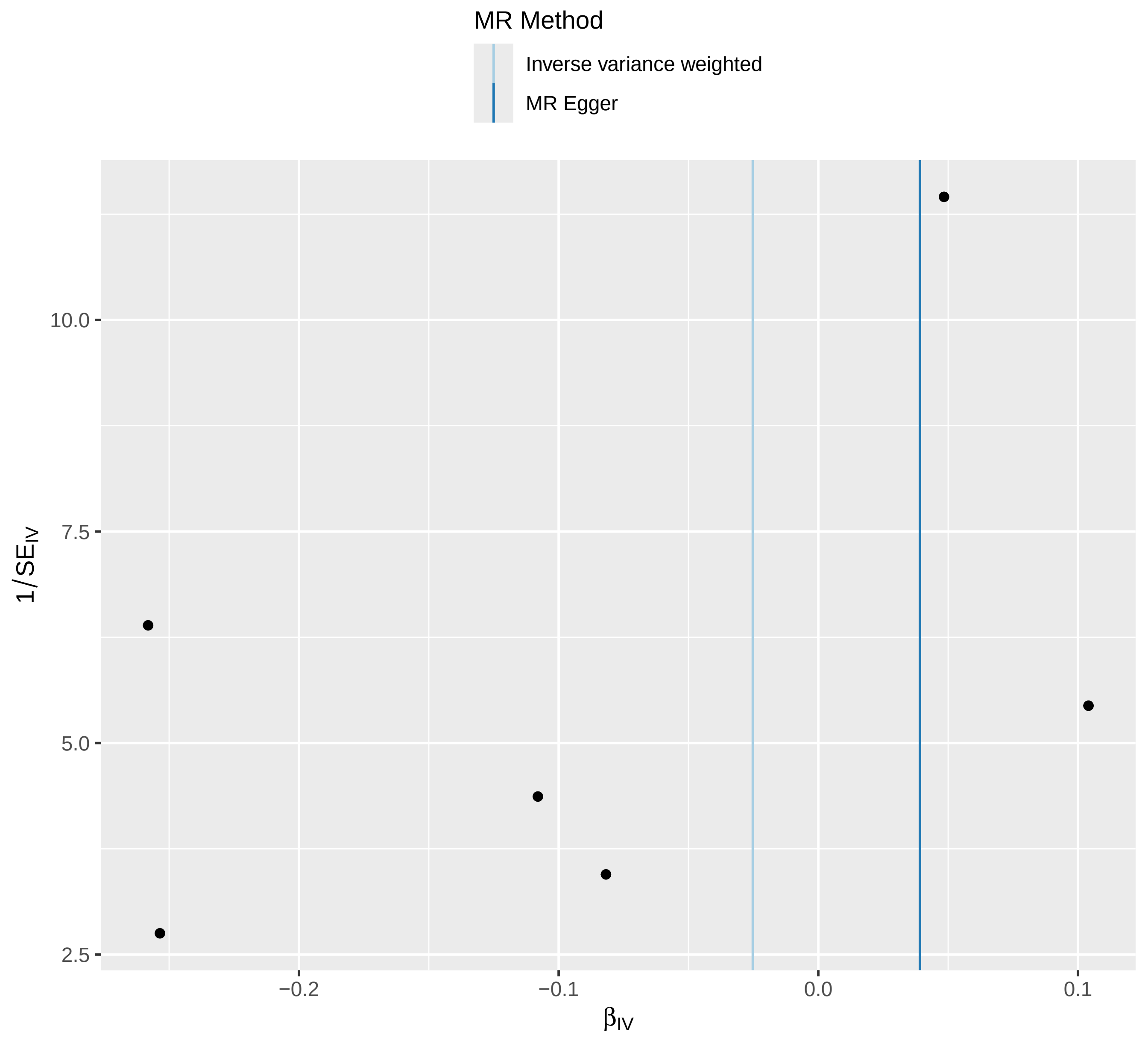

MR Method
Inverse variance weighted
MR Egger
10.0
7.5
5.0
2.5
1/SE$_{IV}$
−0.2
−0.1
0.0
0.1
β$_{IV}$

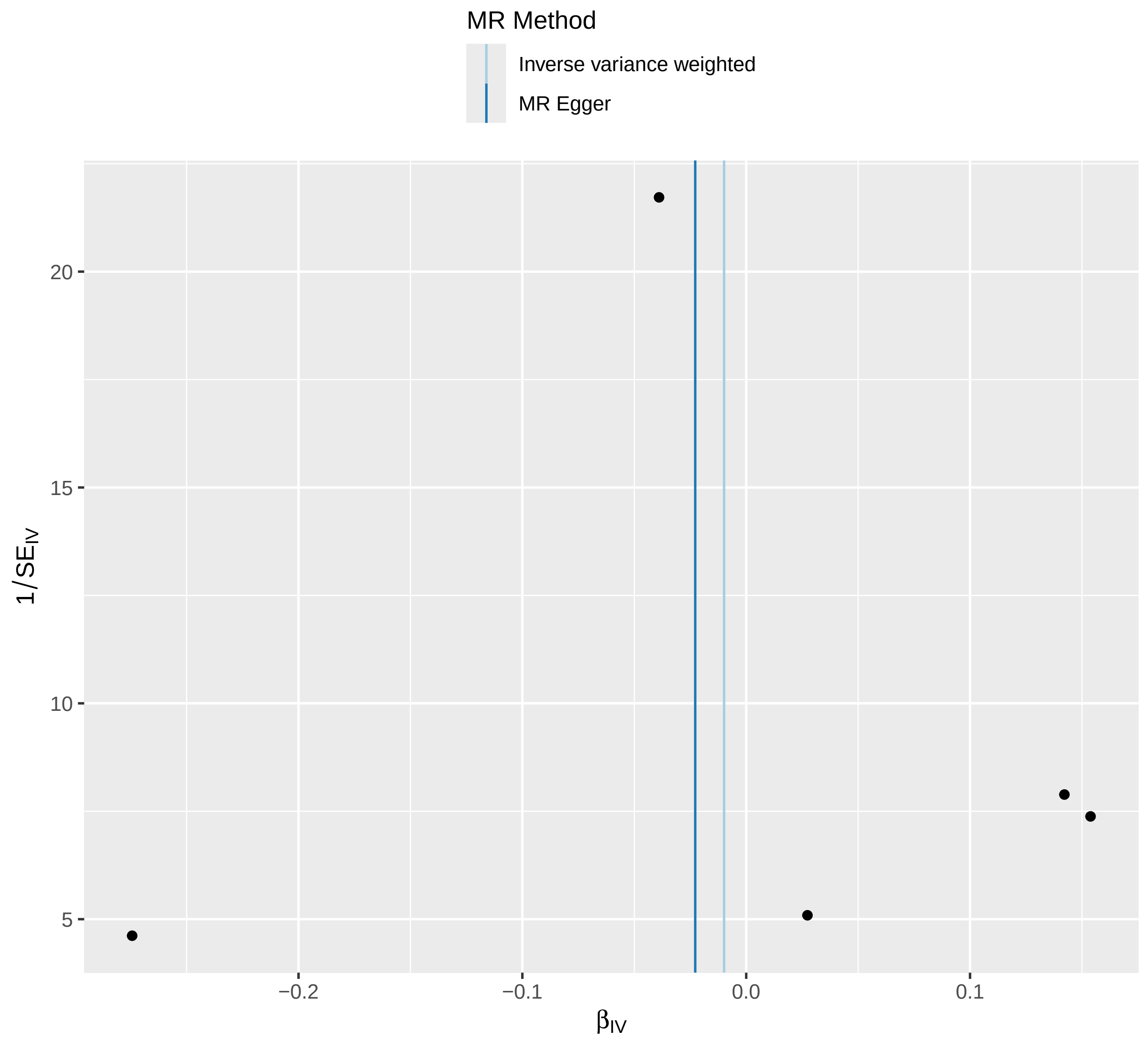

MR Method
Inverse variance weighted
MR Egger
20
15
10
5
1/SE$_{IV}$
−0.2
−0.1
0.0
0.1
β$_{IV}$

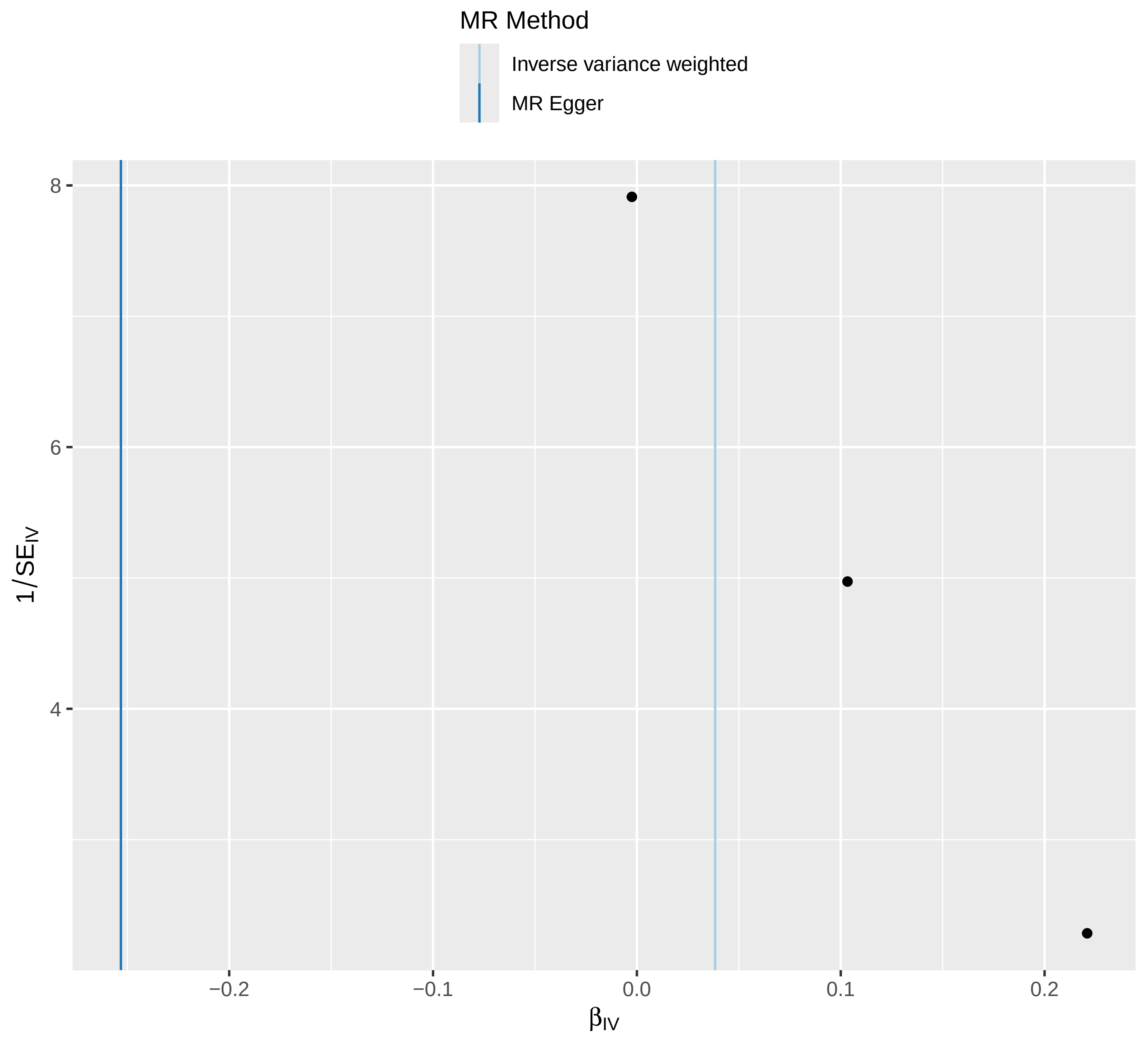
MR Method
Inverse variance weighted
MR Egger
8
6
4
1/SE$_{IV}$
−0.2
−0.1
0.0
0.1
0.2
β$_{IV}$

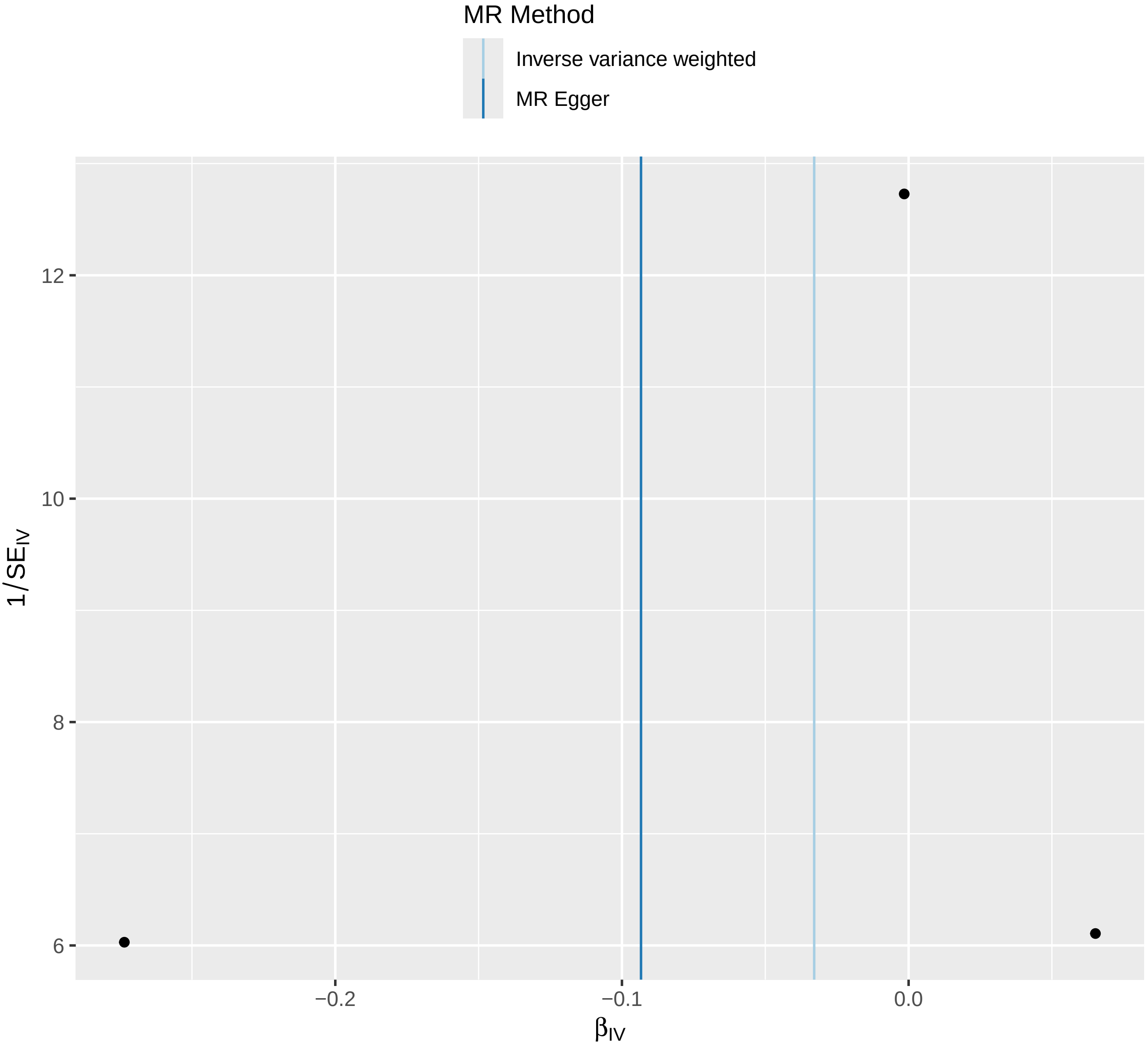
MR Method
Inverse variance weighted
MR Egger
12
10
8
6
1/SE$_{IV}$
−0.2
−0.1
0.0
β$_{IV}$

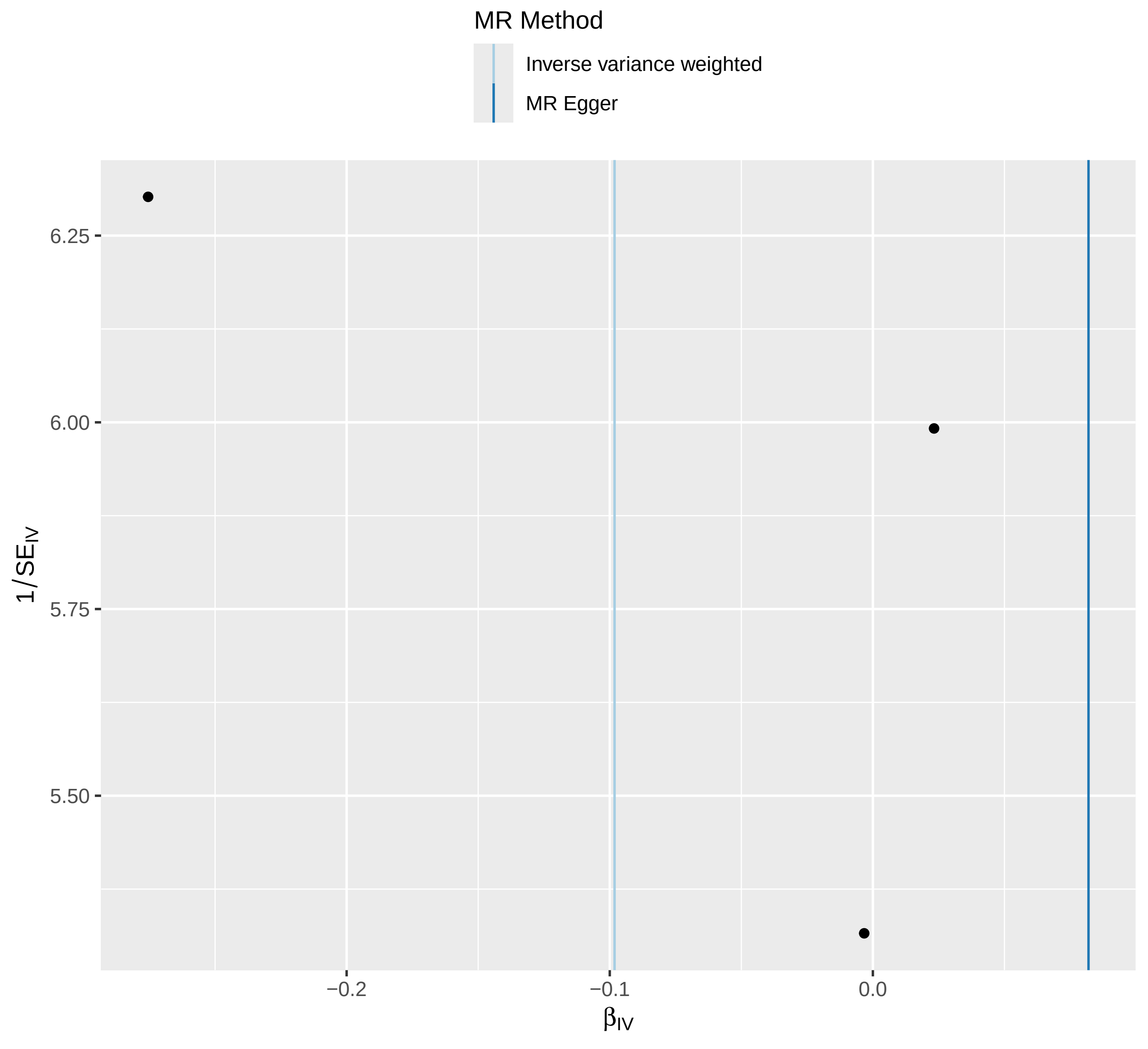

MR Method
Inverse variance weighted
MR Egger
6.25
6.00
5.75
5.50
1/SE$_{IV}$
−0.2
−0.1
0.0
β$_{IV}$

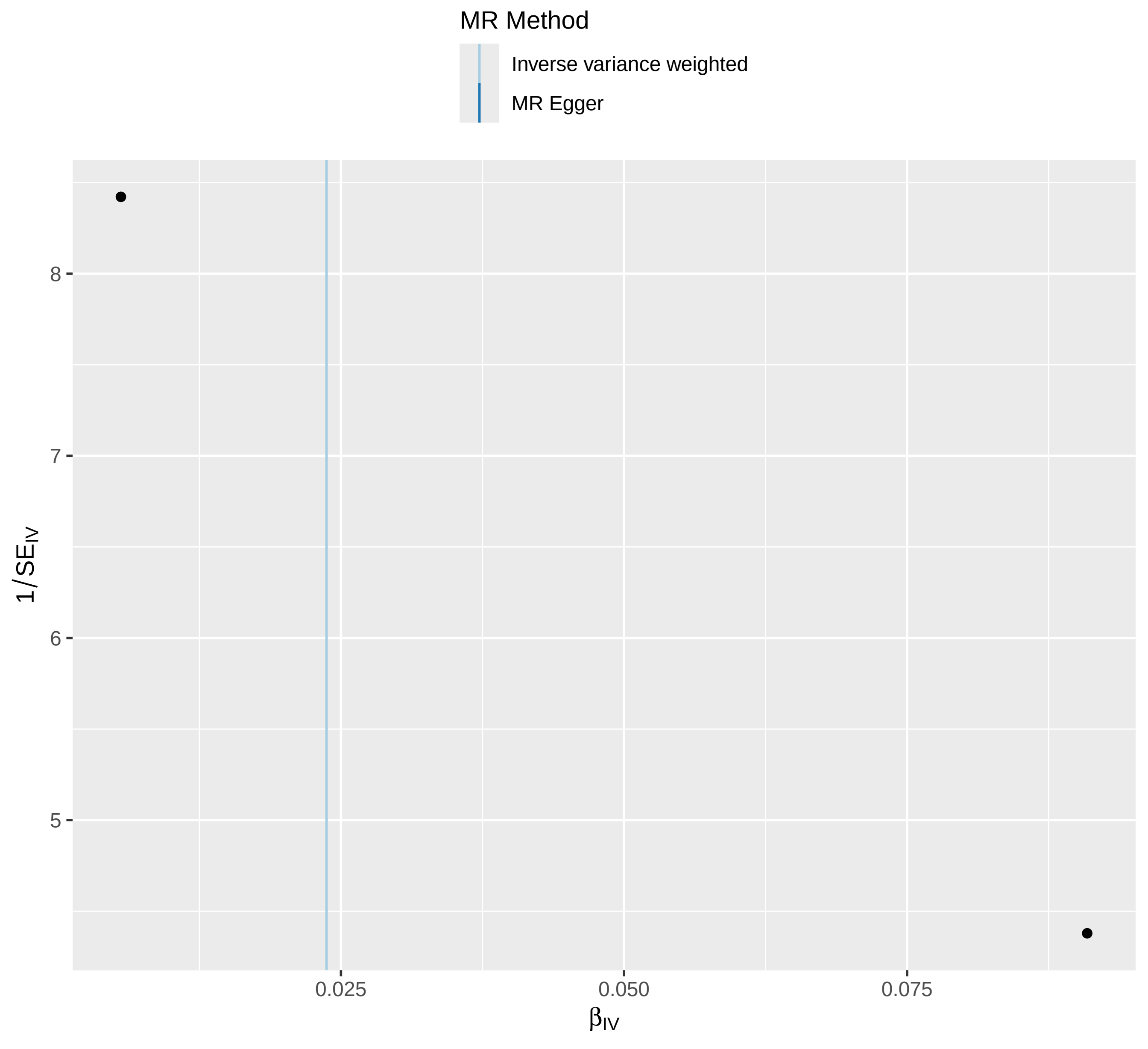
MR Method
Inverse variance weighted
MR Egger
8
7
6
5
$1/SE_{IV}$
0.025
0.050
0.075
$\beta_{IV}$

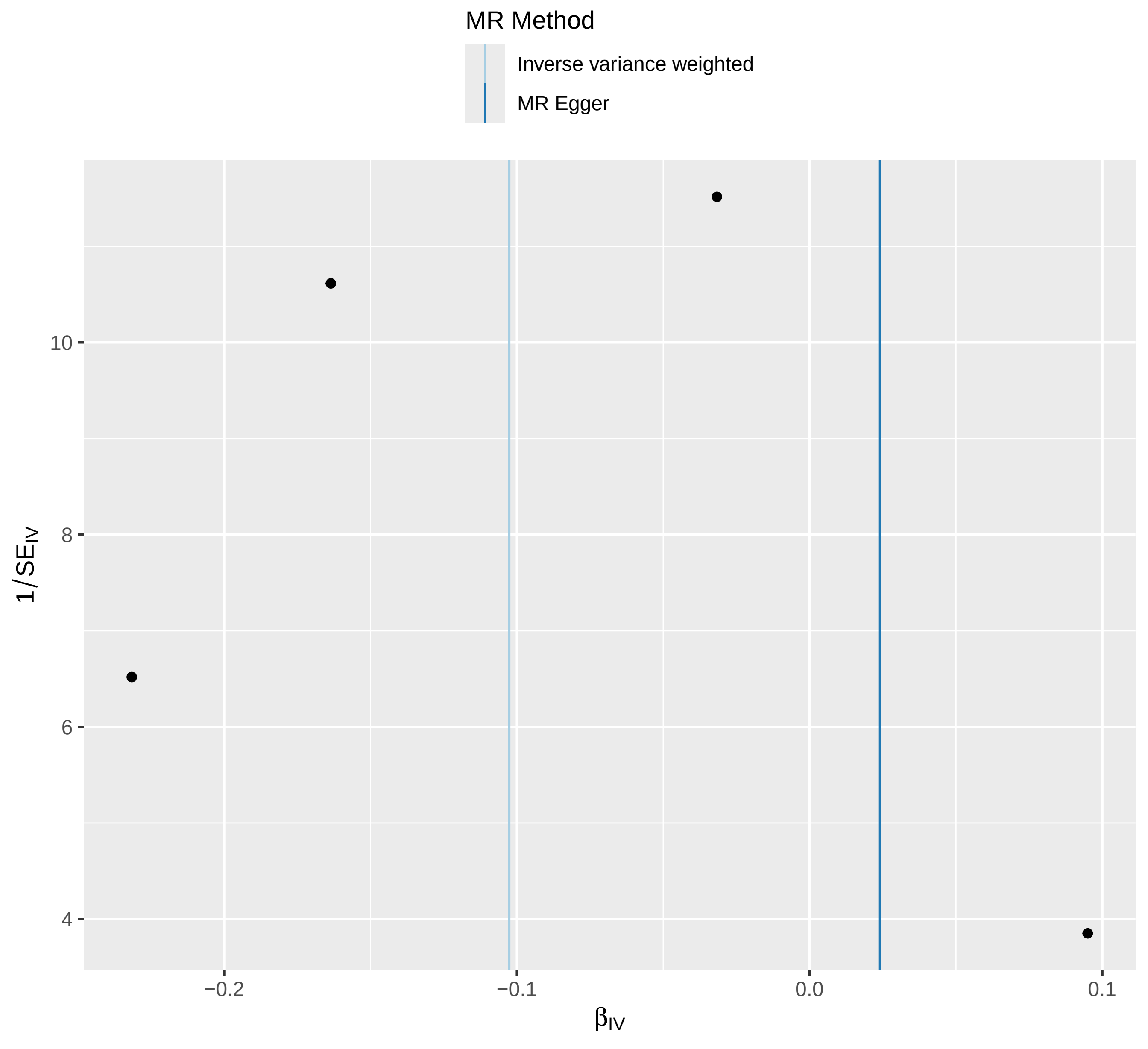
MR Method
Inverse variance weighted
MR Egger
10
8
6
4
1/SE_IV
−0.2
−0.1
0.0
0.1
β_IV

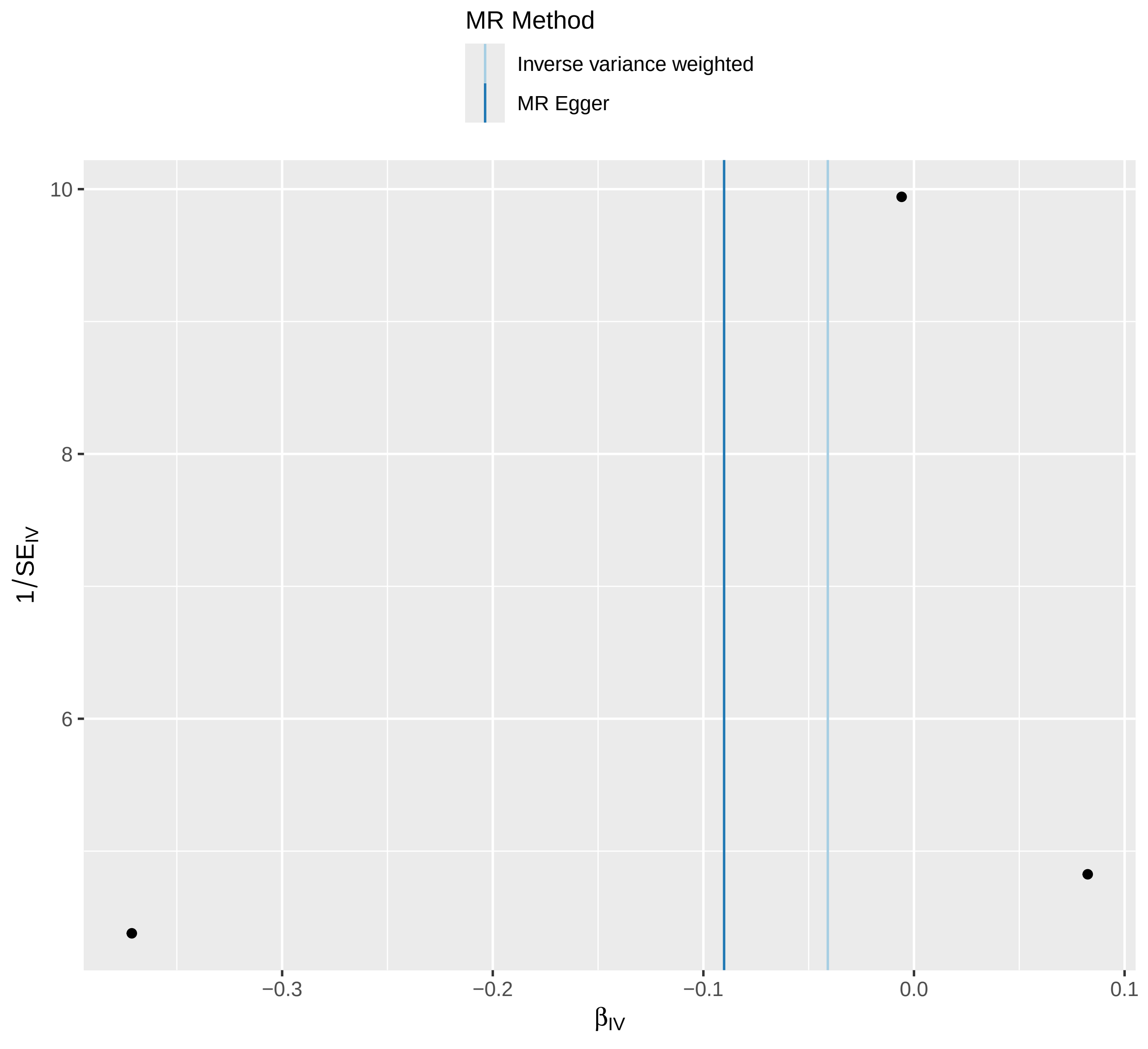

MR Method
Inverse variance weighted
MR Egger
10
8
6
$1/SE_{IV}$
−0.3
−0.2
−0.1
0.0
0.1
$\beta_{IV}$

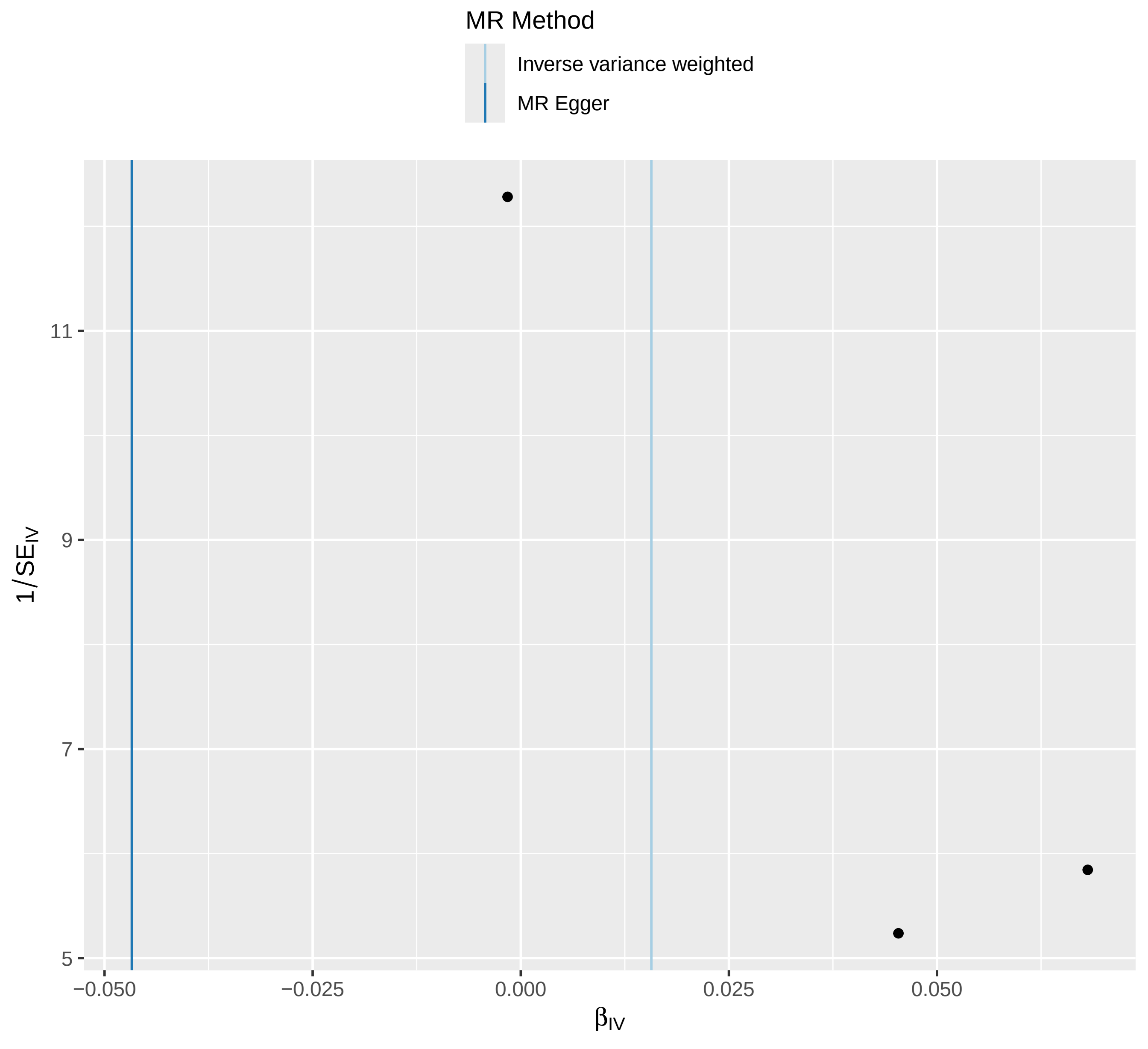

MR Method
Inverse variance weighted
MR Egger
1/SE_IV
11
9
7
5
−0.050
−0.025
0.000
0.025
0.050
β_IV

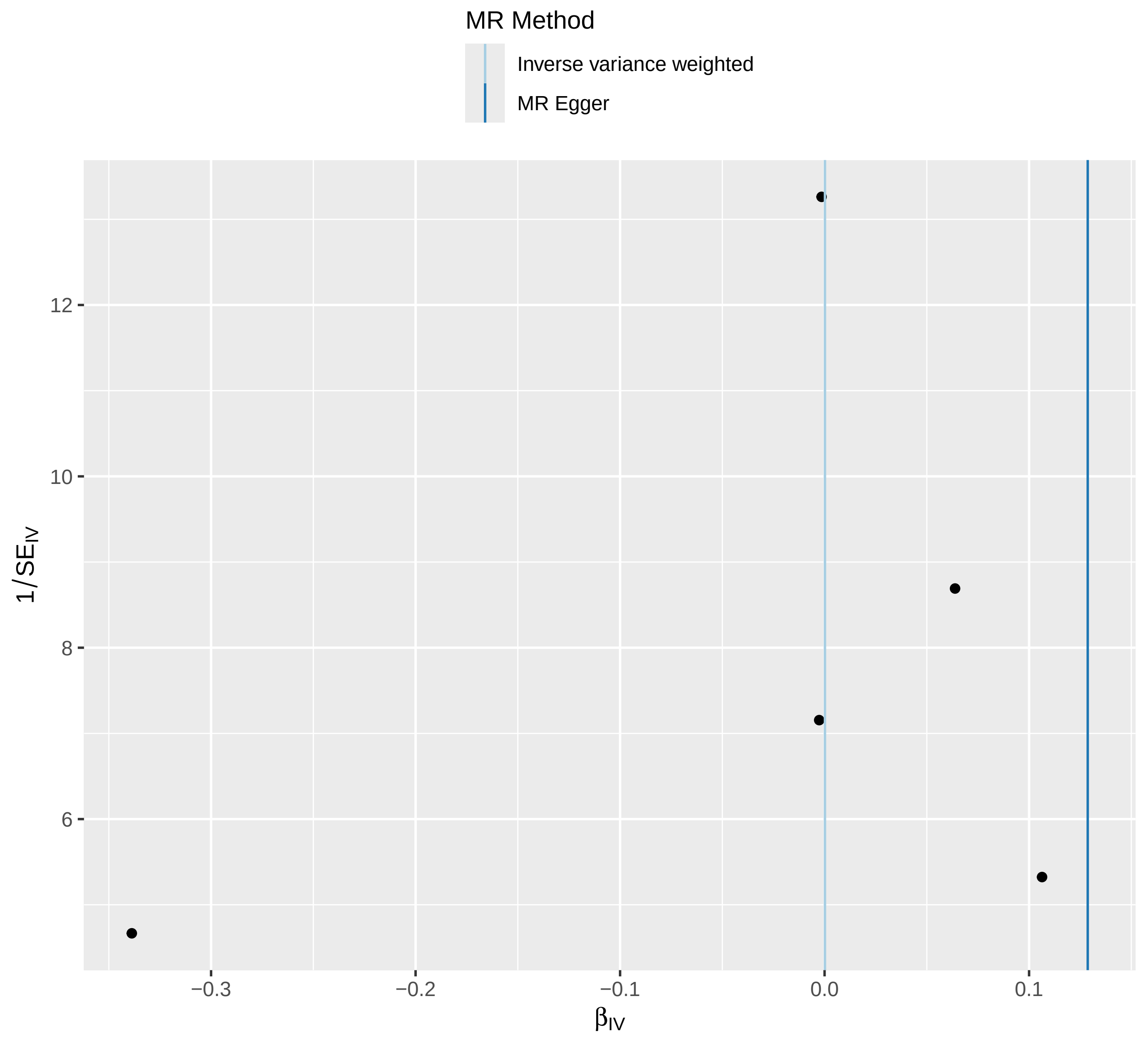

MR Method
Inverse variance weighted
MR Egger
12
10
8
6
1/SE$_{IV}$
−0.3
−0.2
−0.1
0.0
0.1
β$_{IV}$

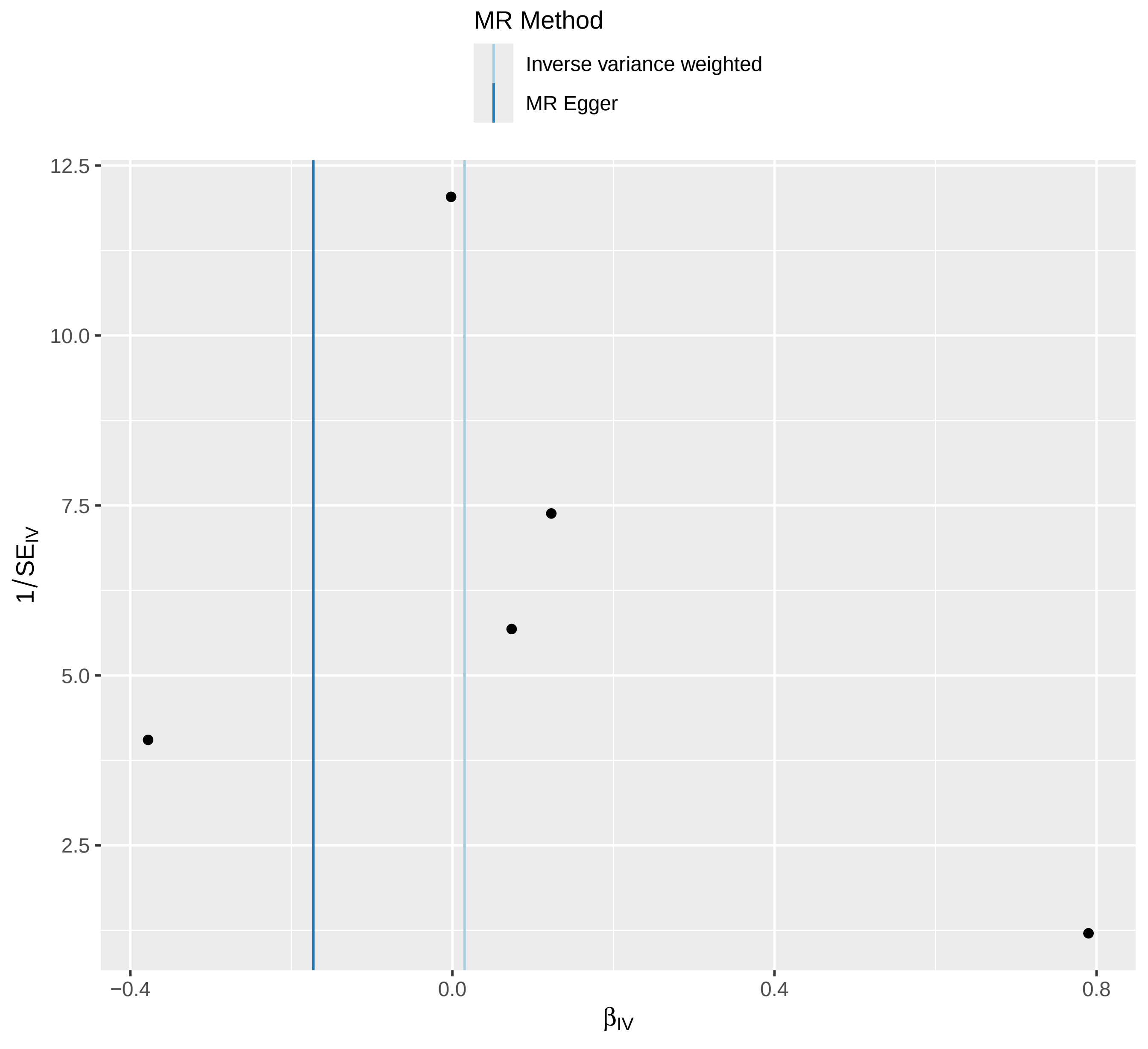
MR Method
Inverse variance weighted
MR Egger
12.5
10.0
7.5
5.0
2.5
1/SE_IV
−0.4
0.0
0.4
0.8
β_IV

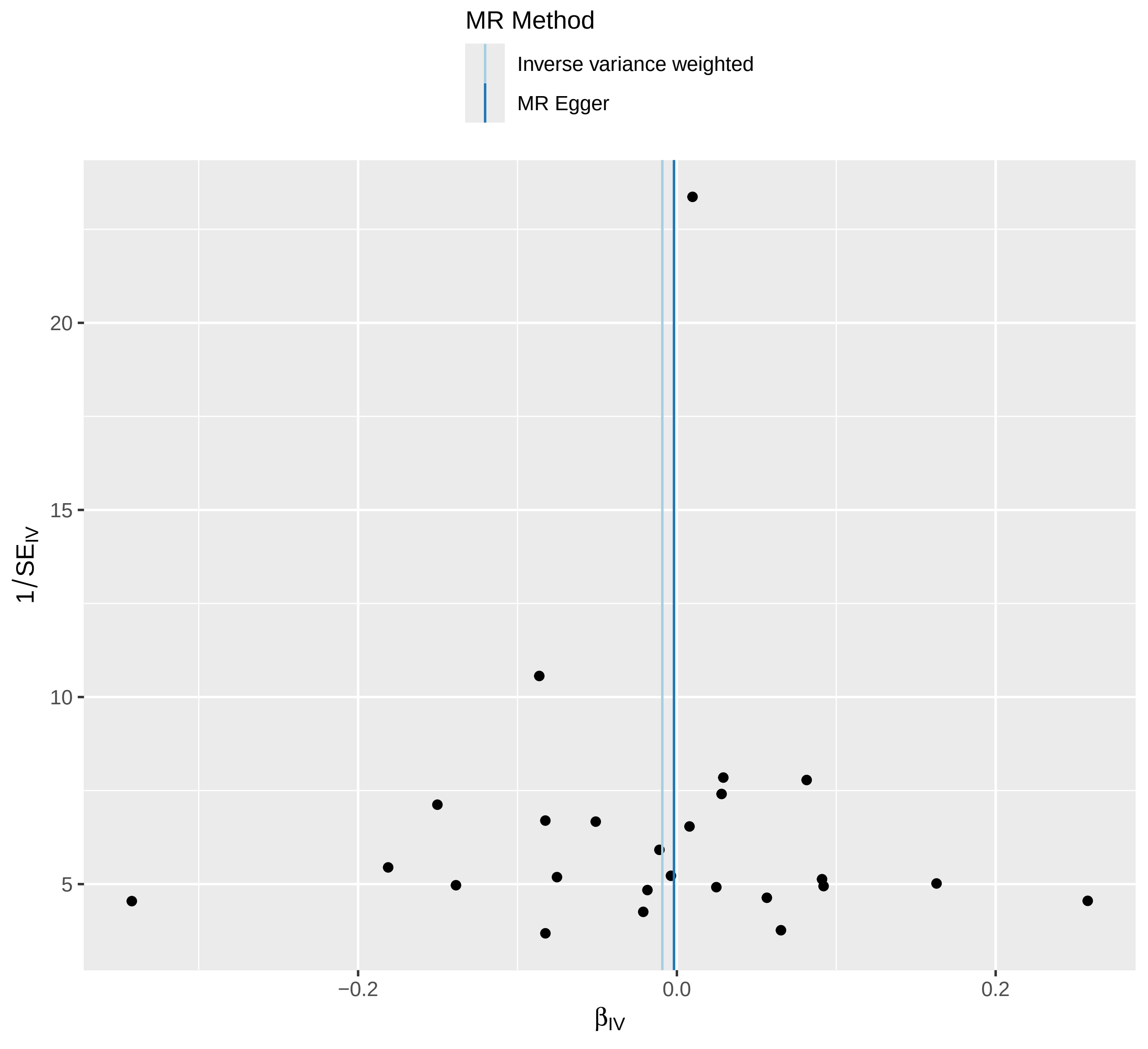
MR Method
Inverse variance weighted
MR Egger
20
15
10
5
1/SE_IV
−0.2
0.0
0.2
β_IV

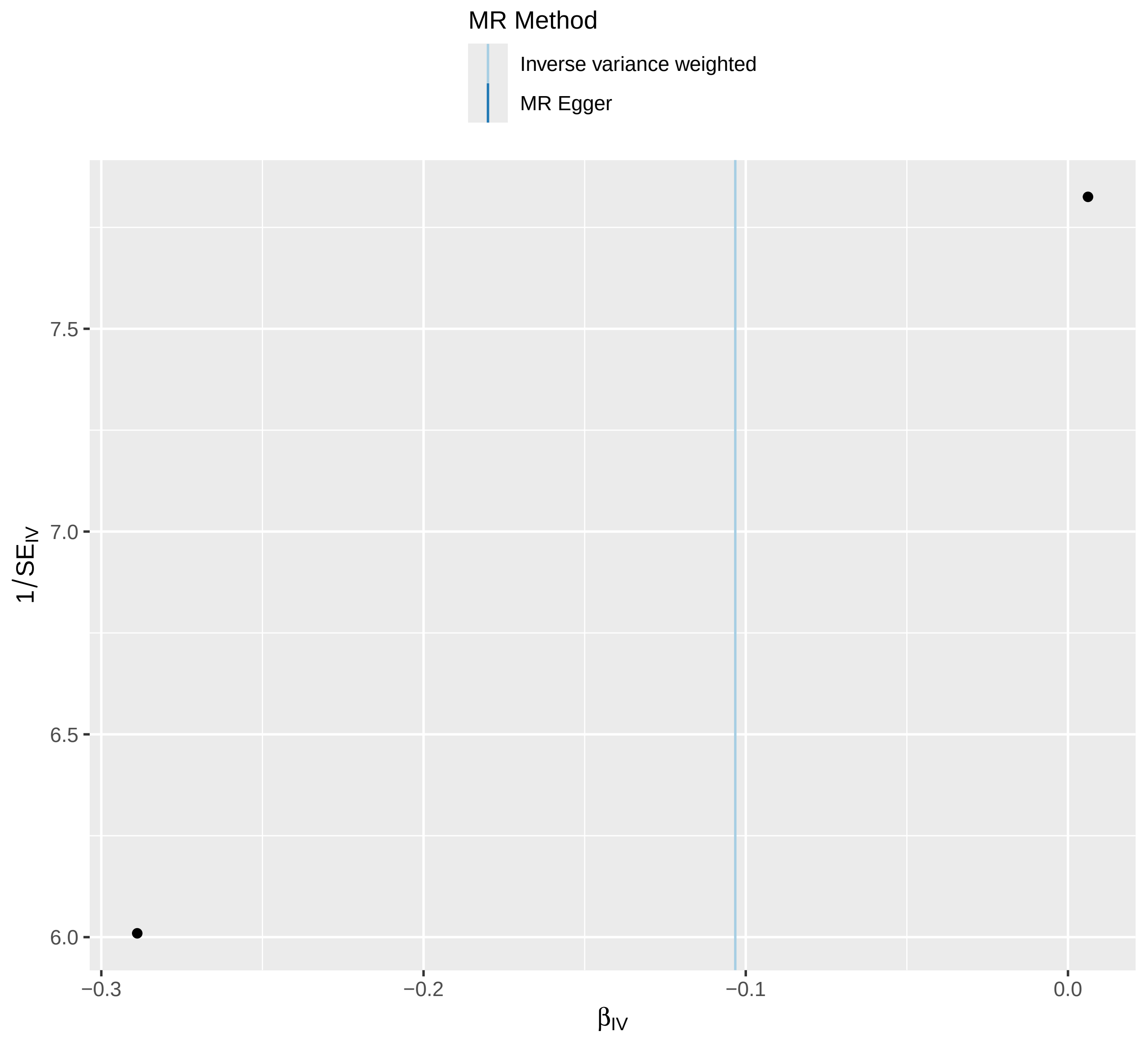

MR Method
Inverse variance weighted
MR Egger
7.5
7.0
6.5
6.0
1/SE$_{IV}$
−0.3
−0.2
−0.1
0.0
β$_{IV}$

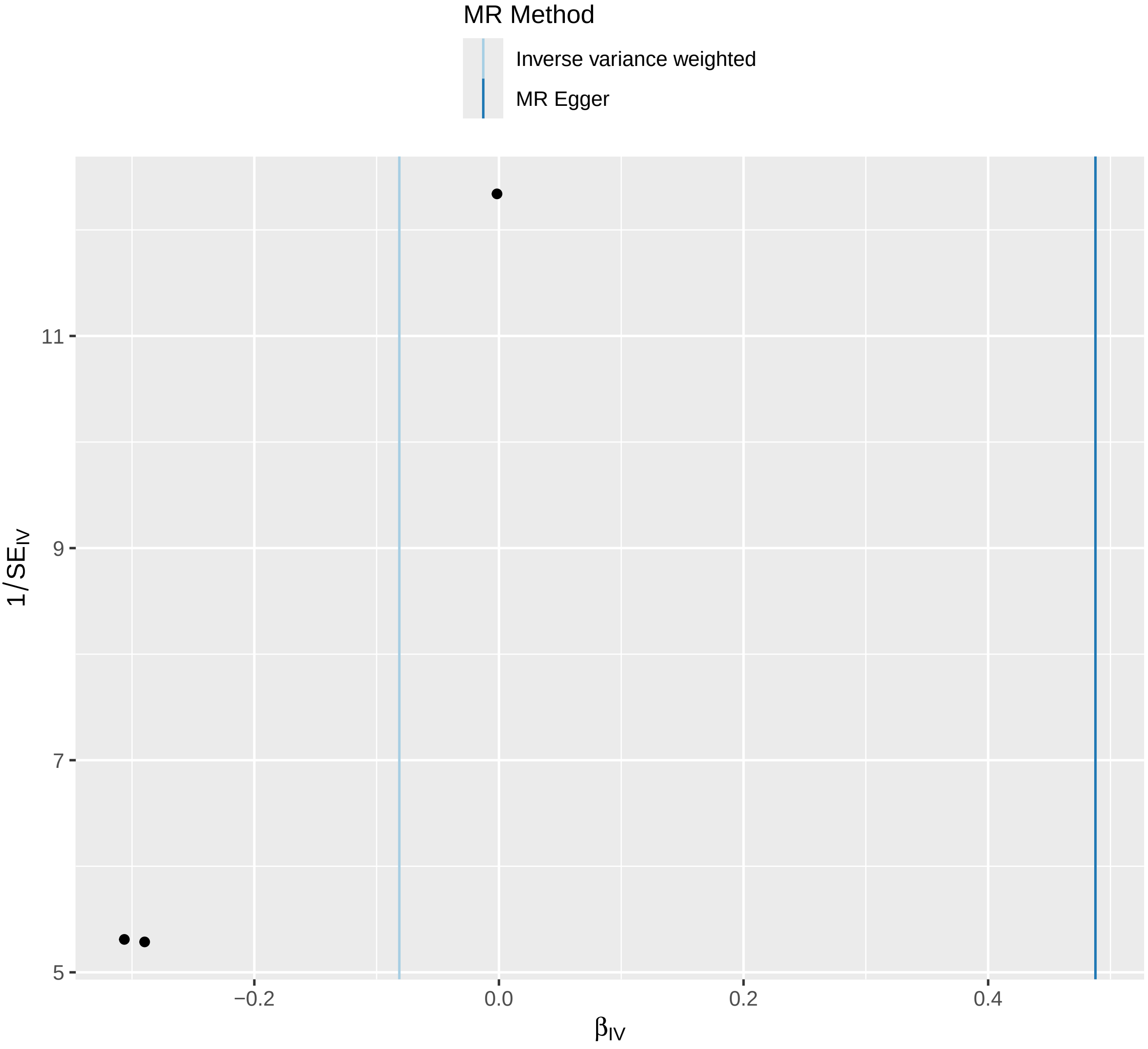

MR Method
Inverse variance weighted
MR Egger
11
9
7
5
−0.2
0.0
0.2
0.4
1/SE_IV
β_IV

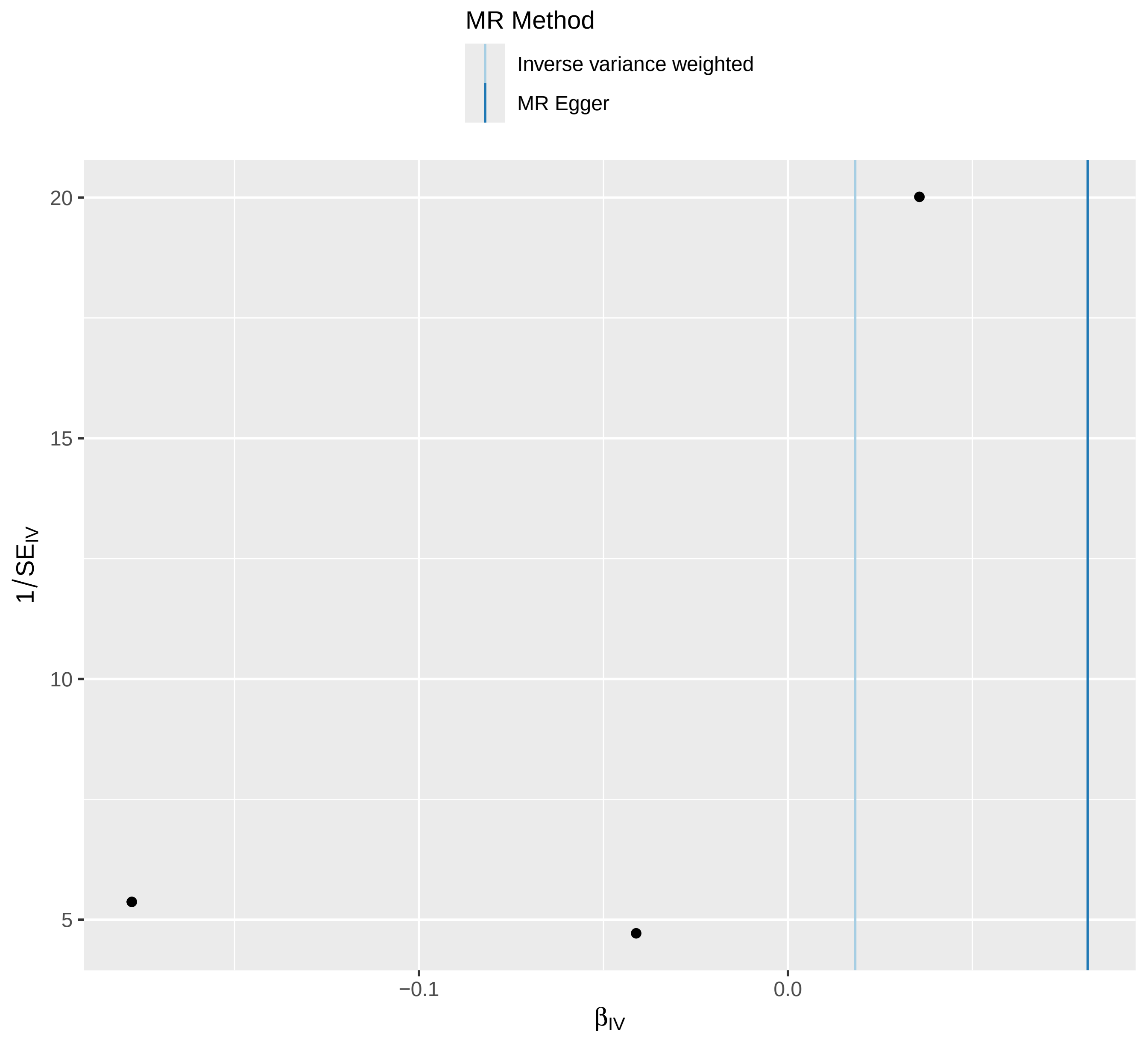
MR Method
Inverse variance weighted
MR Egger
20
15
10
5
1/SE$_{IV}$
−0.1
0.0
β$_{IV}$

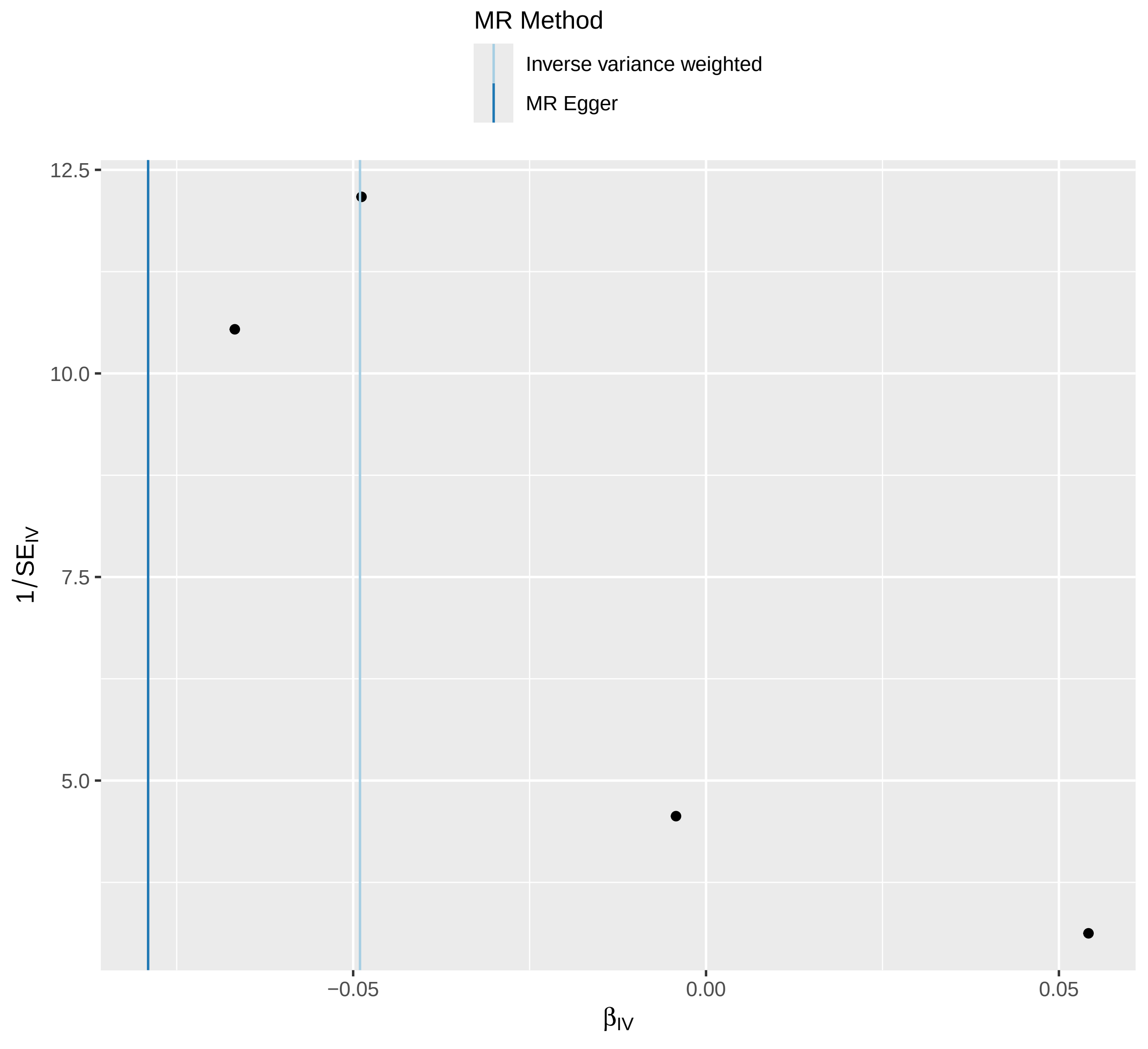
MR Method
Inverse variance weighted
MR Egger
12.5
10.0
7.5
5.0
$1/SE_{IV}$
−0.05
0.00
0.05
$\beta_{IV}$

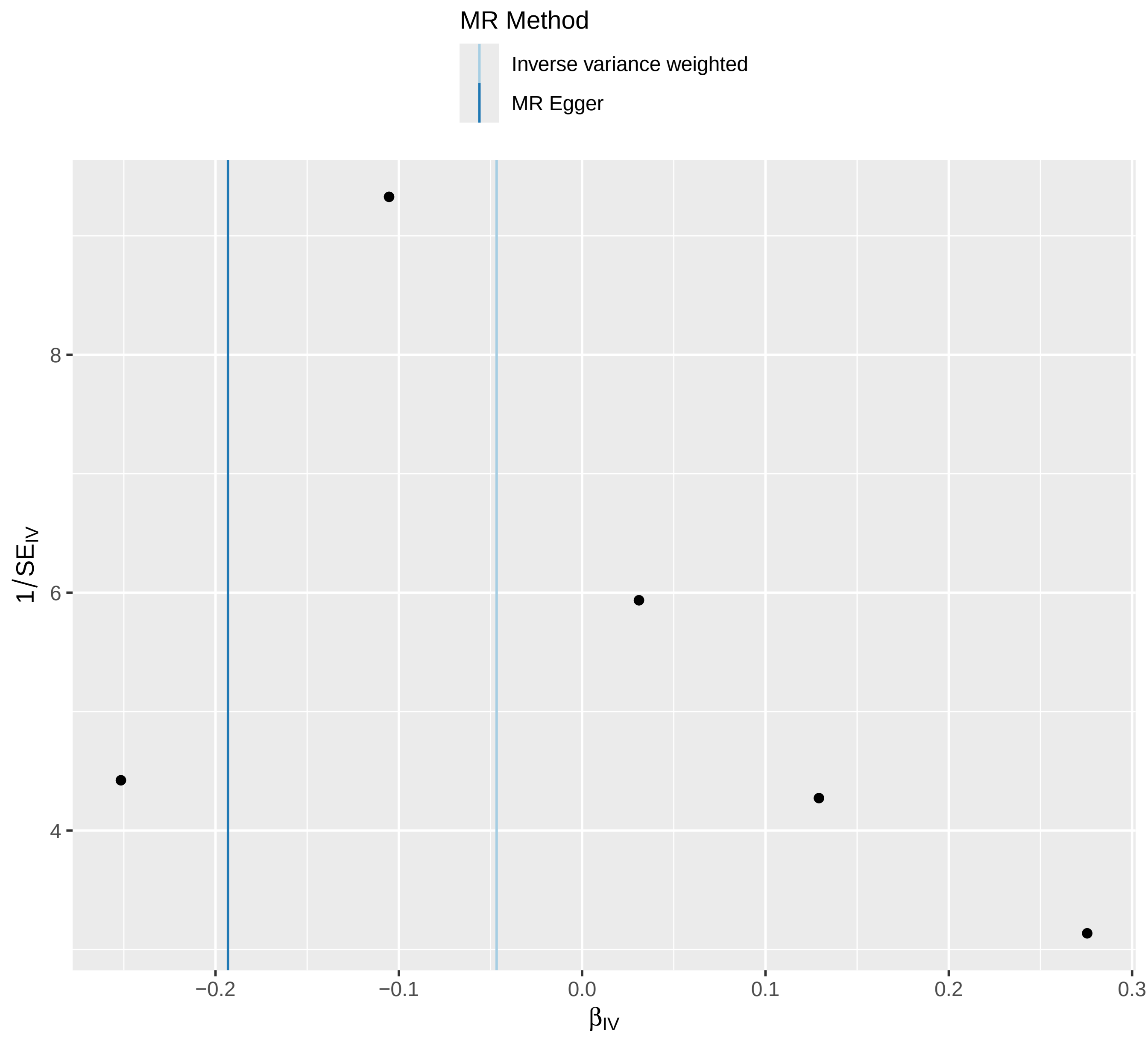
MR Method
Inverse variance weighted
MR Egger
8
6
4
1/SE$_{IV}$
−0.2
−0.1
0.0
0.1
0.2
0.3
β$_{IV}$

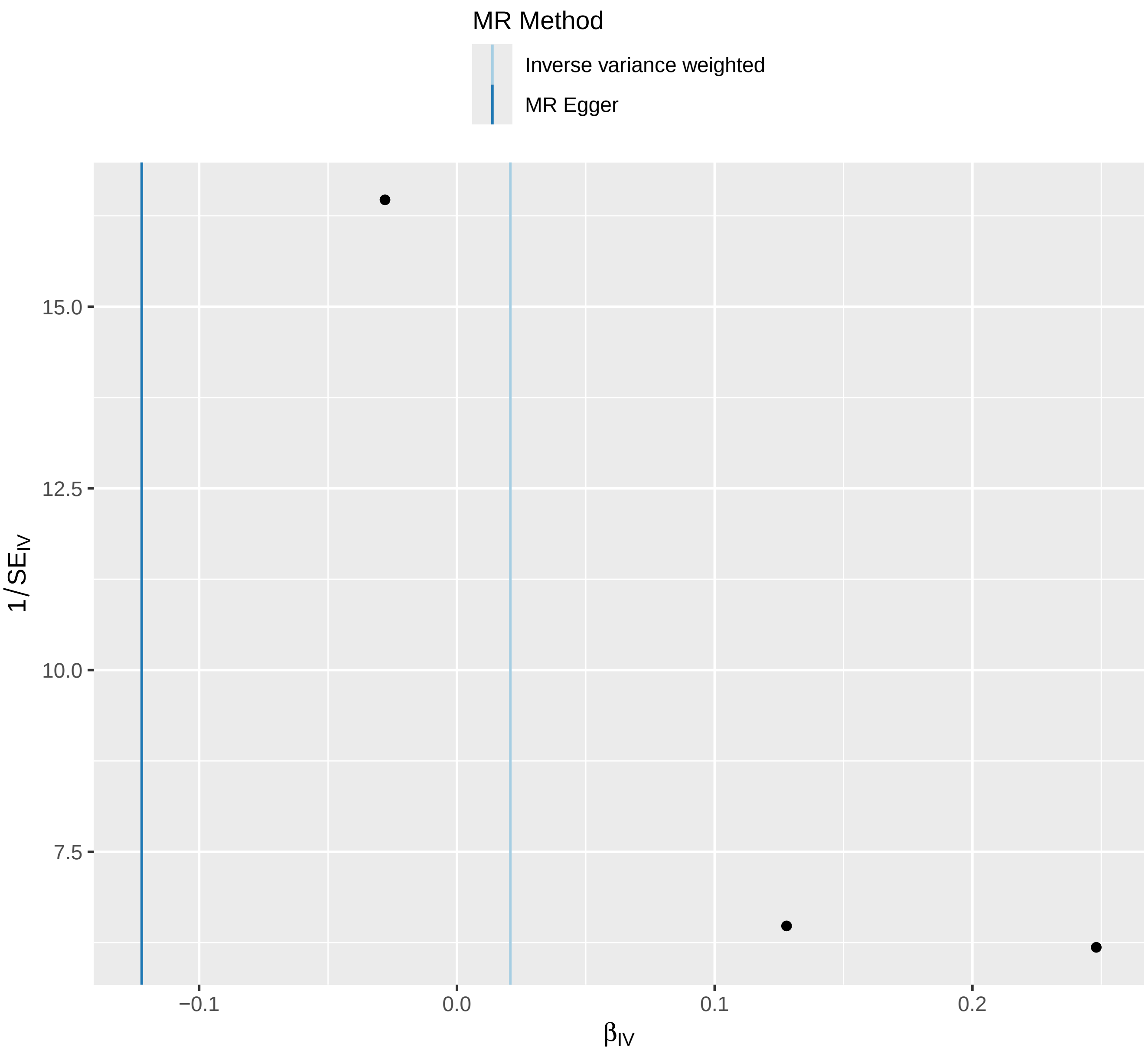
MR Method
Inverse variance weighted
MR Egger
15.0
12.5
10.0
7.5
−0.1
0.0
0.1
0.2
$1/SE_{IV}$
$\beta_{IV}$

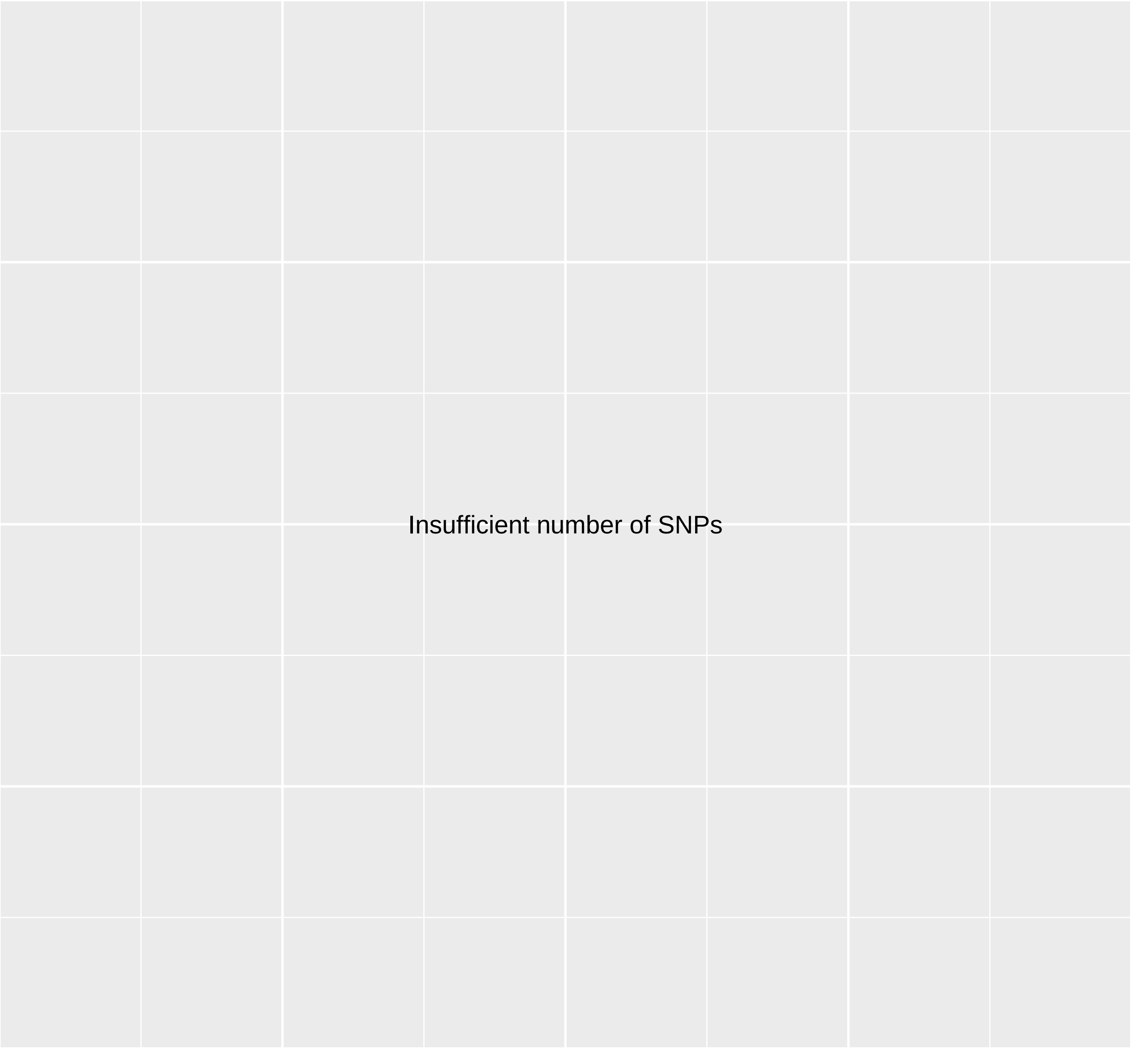
Insufficient number of SNPs

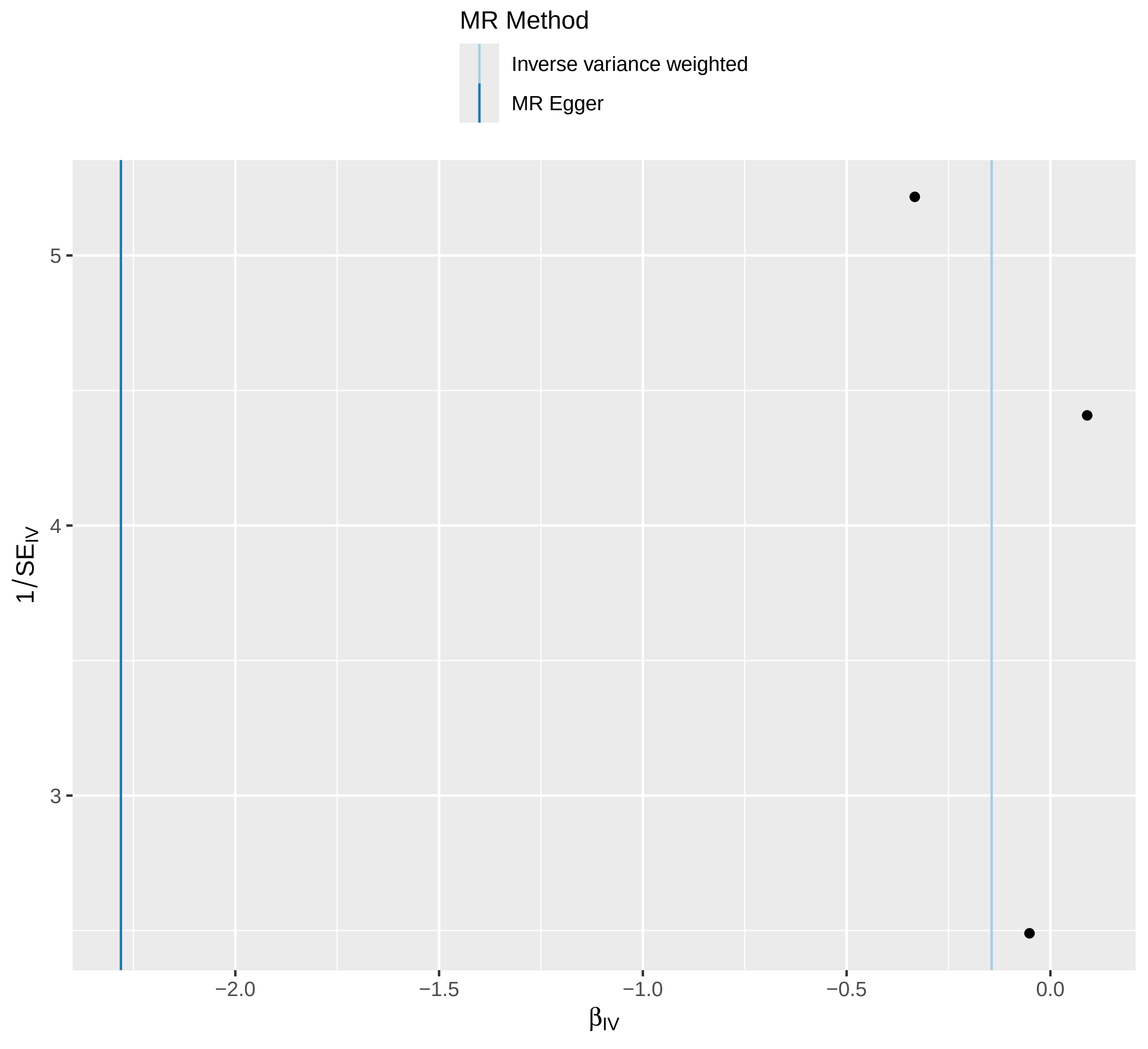
MR Method
Inverse variance weighted
MR Egger
1/SE$_{IV}$
5
4
3
−2.0
−1.5
−1.0
−0.5
0.0
β$_{IV}$

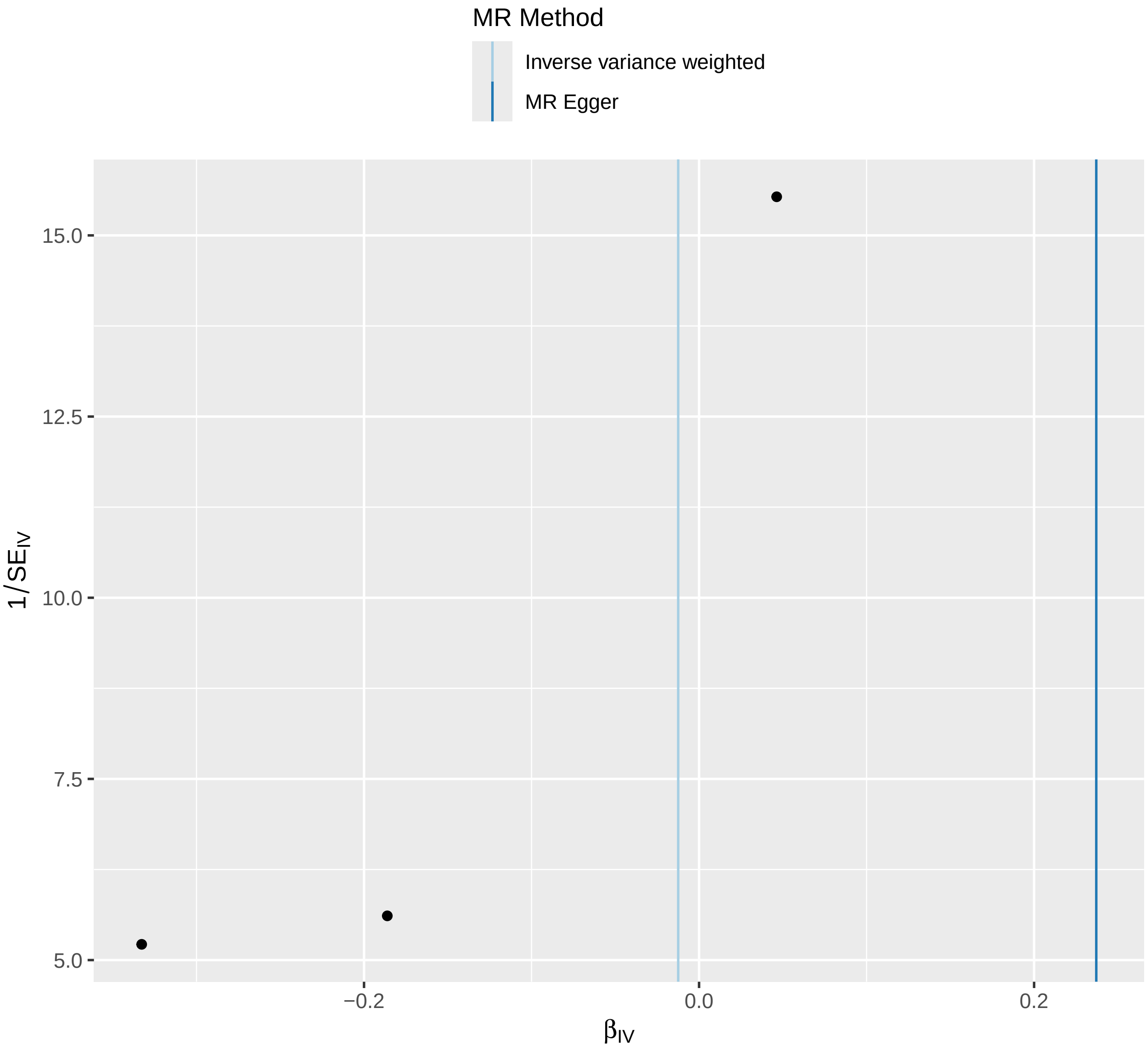

MR Method
Inverse variance weighted
MR Egger
15.0
12.5
10.0
7.5
5.0
1/SE_IV
−0.2
0.0
0.2
β_IV

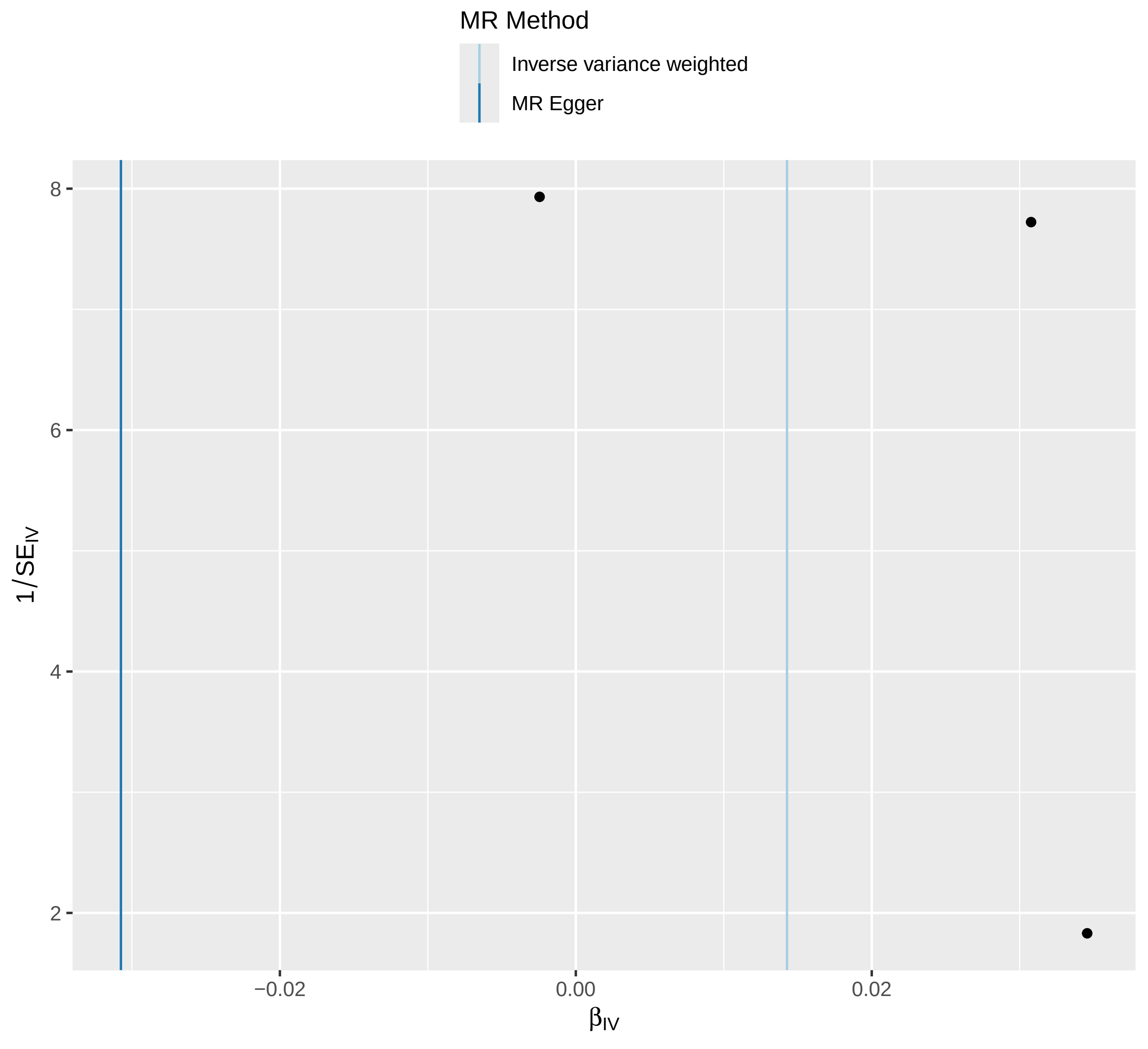
MR Method
Inverse variance weighted
MR Egger
8
6
4
2
1/SE$_{IV}$
−0.02
0.00
0.02
β$_{IV}$

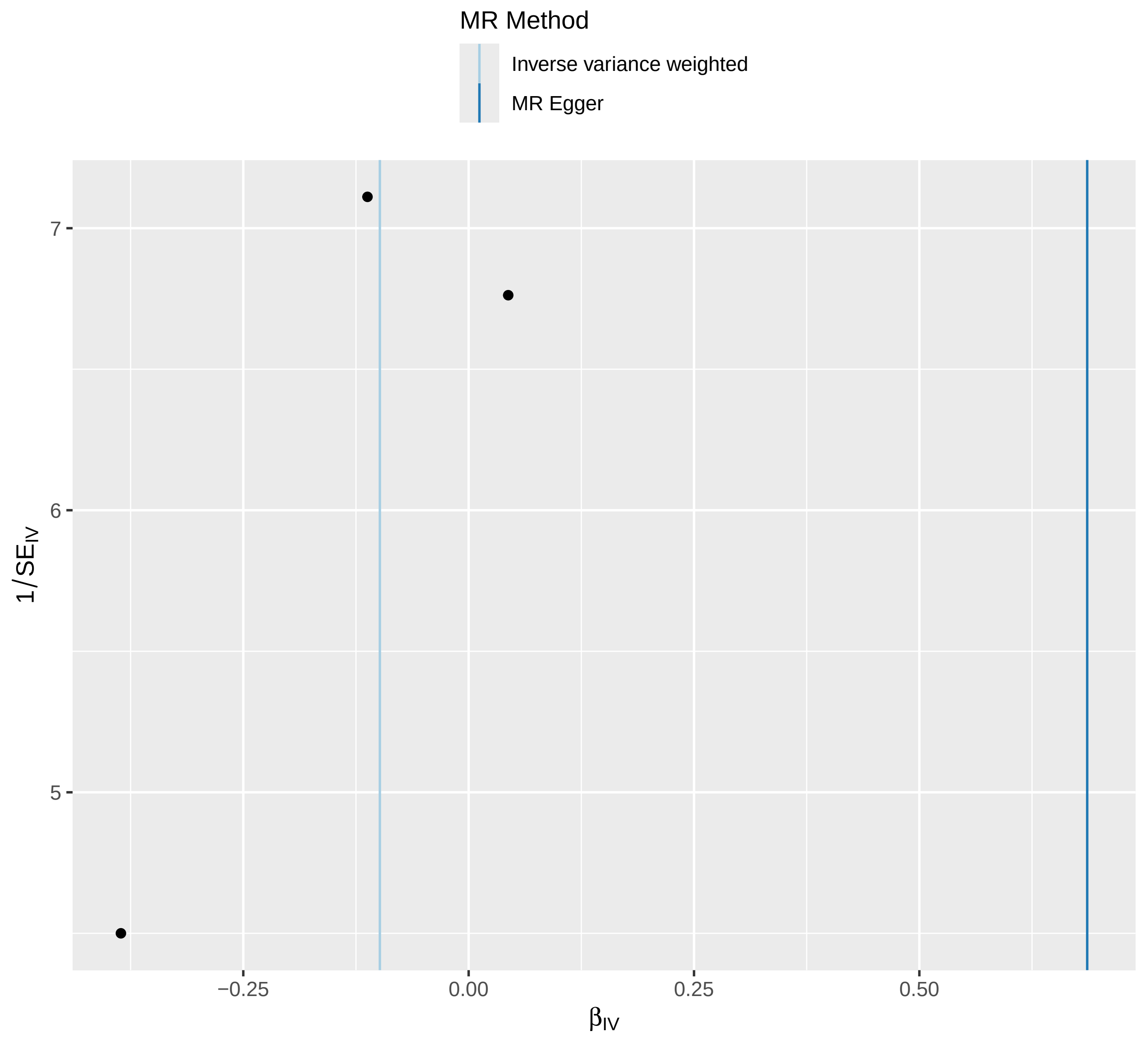

MR Method
Inverse variance weighted
MR Egger
7
6
5
1/SE_IV
−0.25
0.00
0.25
0.50
β_IV

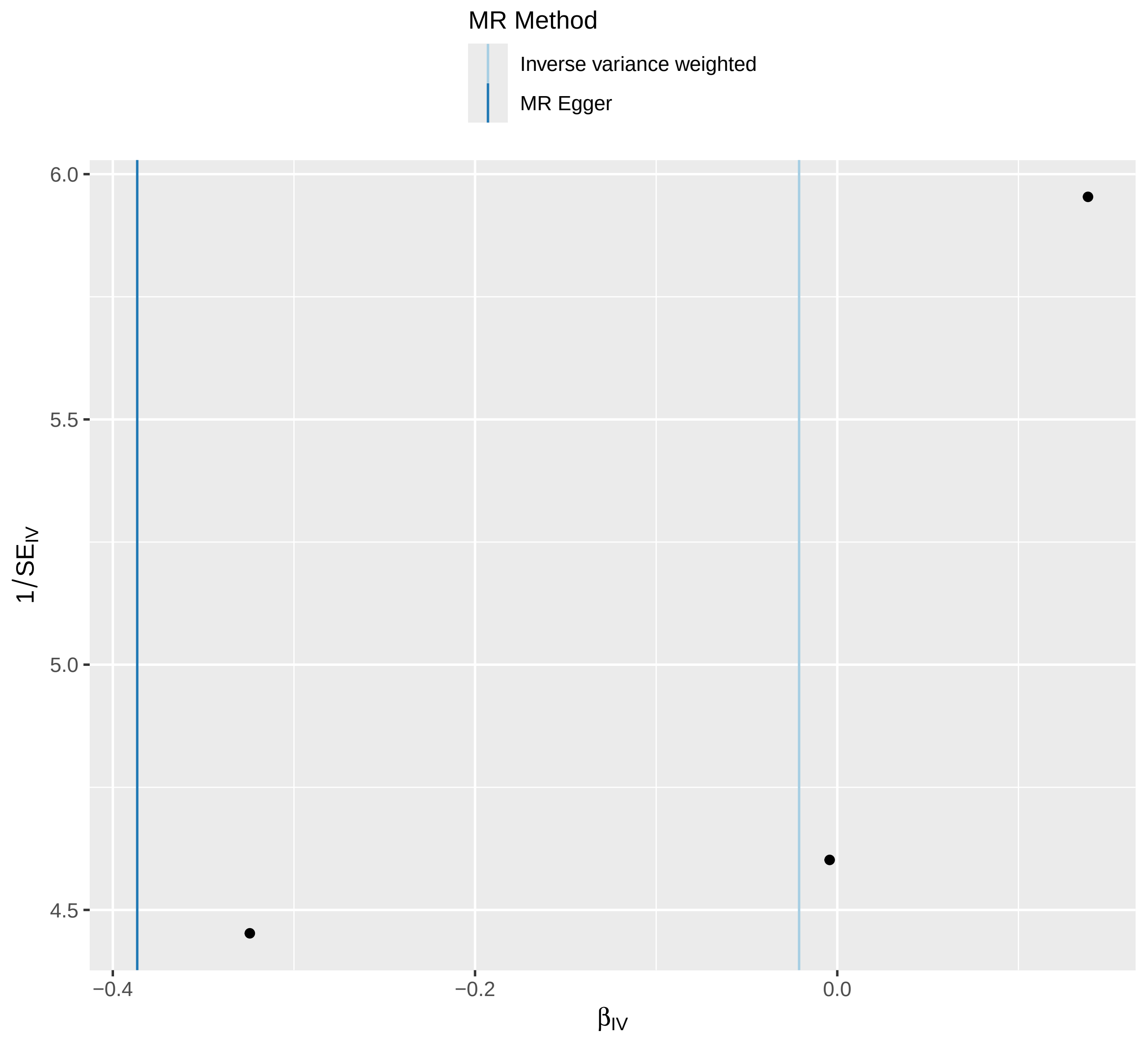
MR Method
Inverse variance weighted
MR Egger
6.0
5.5
5.0
4.5
1/SE_IV
−0.4
−0.2
0.0
β_IV

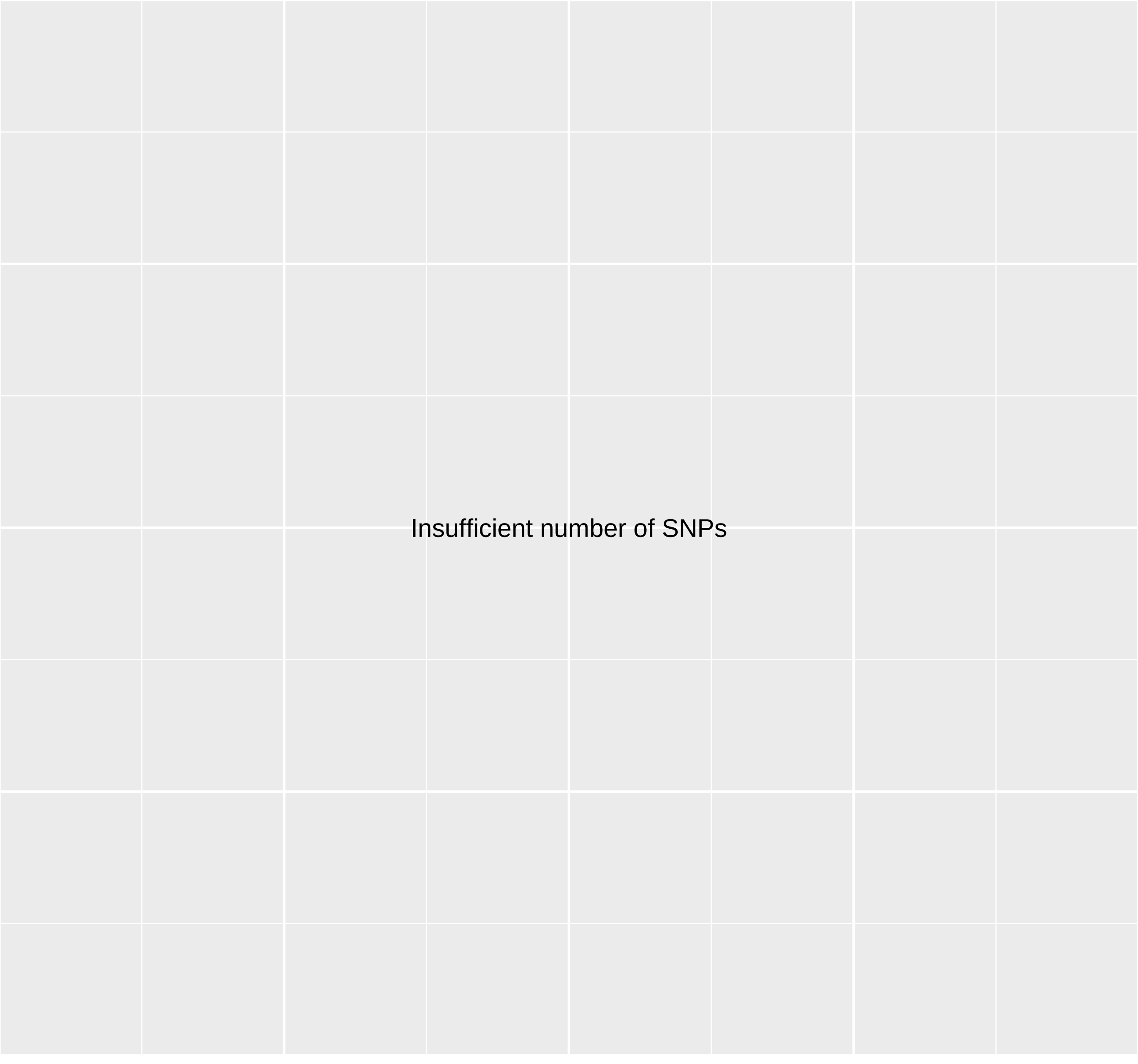
Insufficient number of SNPs

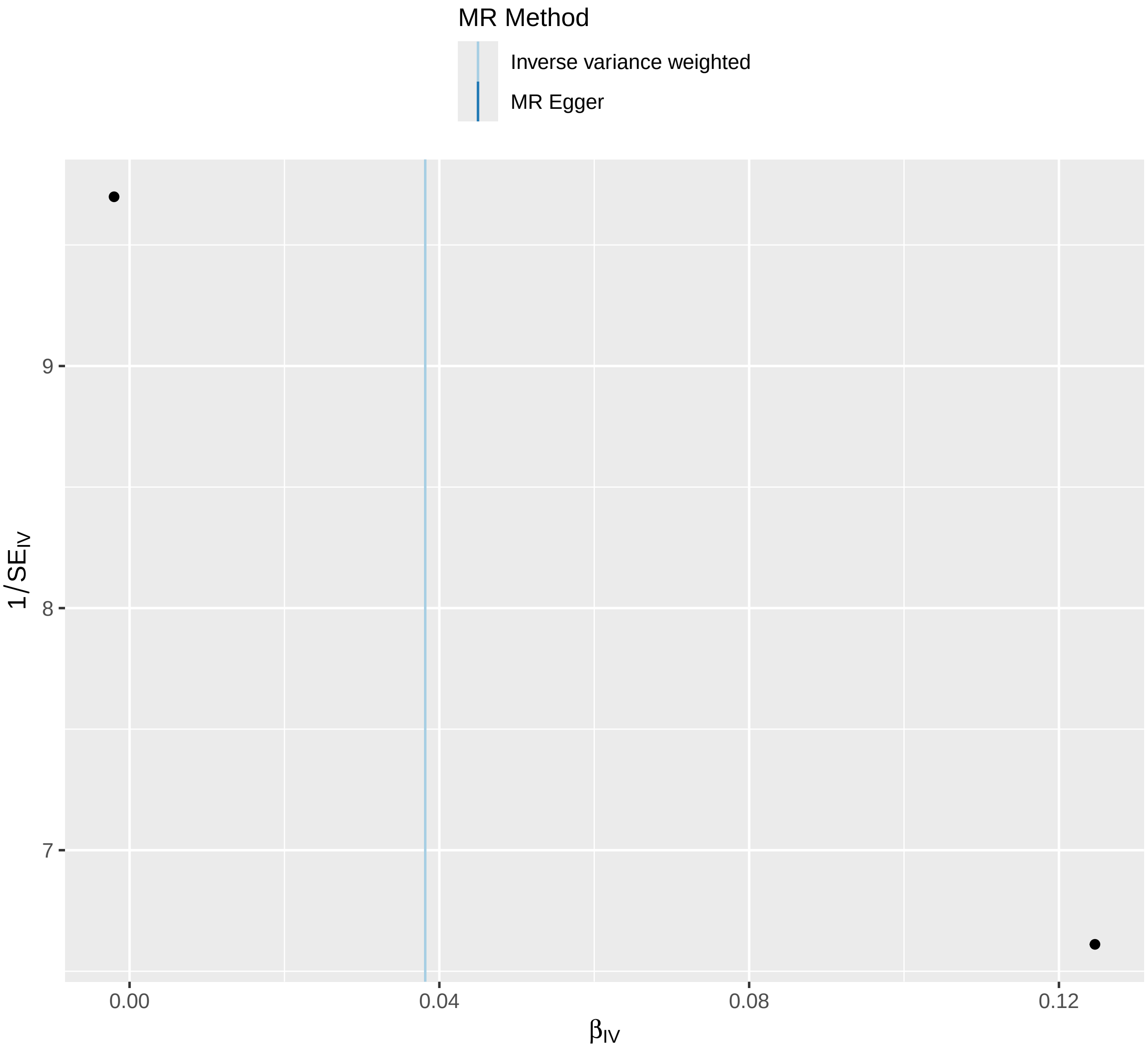
MR Method
Inverse variance weighted
MR Egger
9
8
7
1/SE_IV
0.00
0.04
0.08
0.12
β_IV

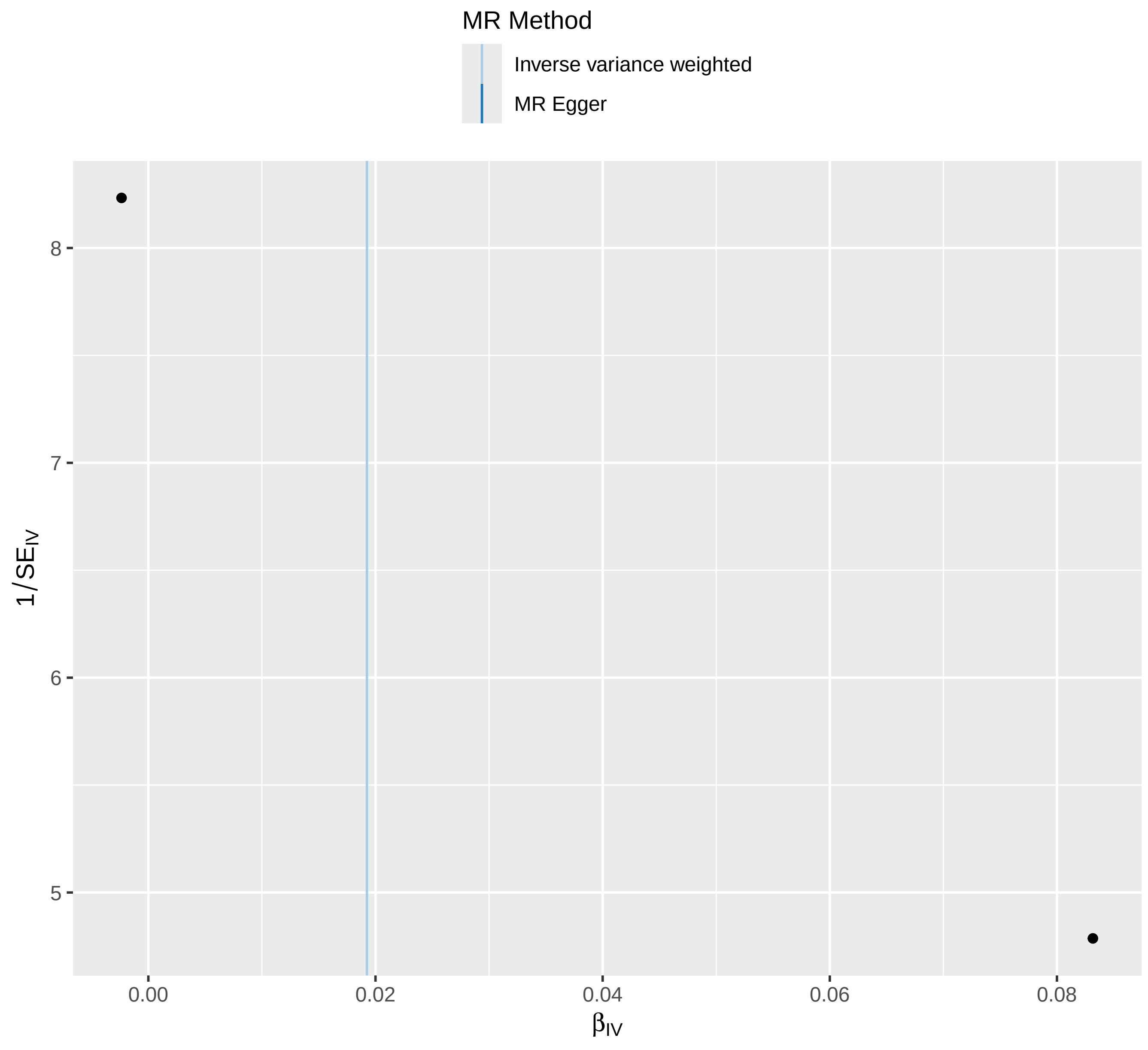
MR Method
Inverse variance weighted
MR Egger
8
7
6
5
1/SE_IV
0.00
0.02
0.04
0.06
0.08
β_IV

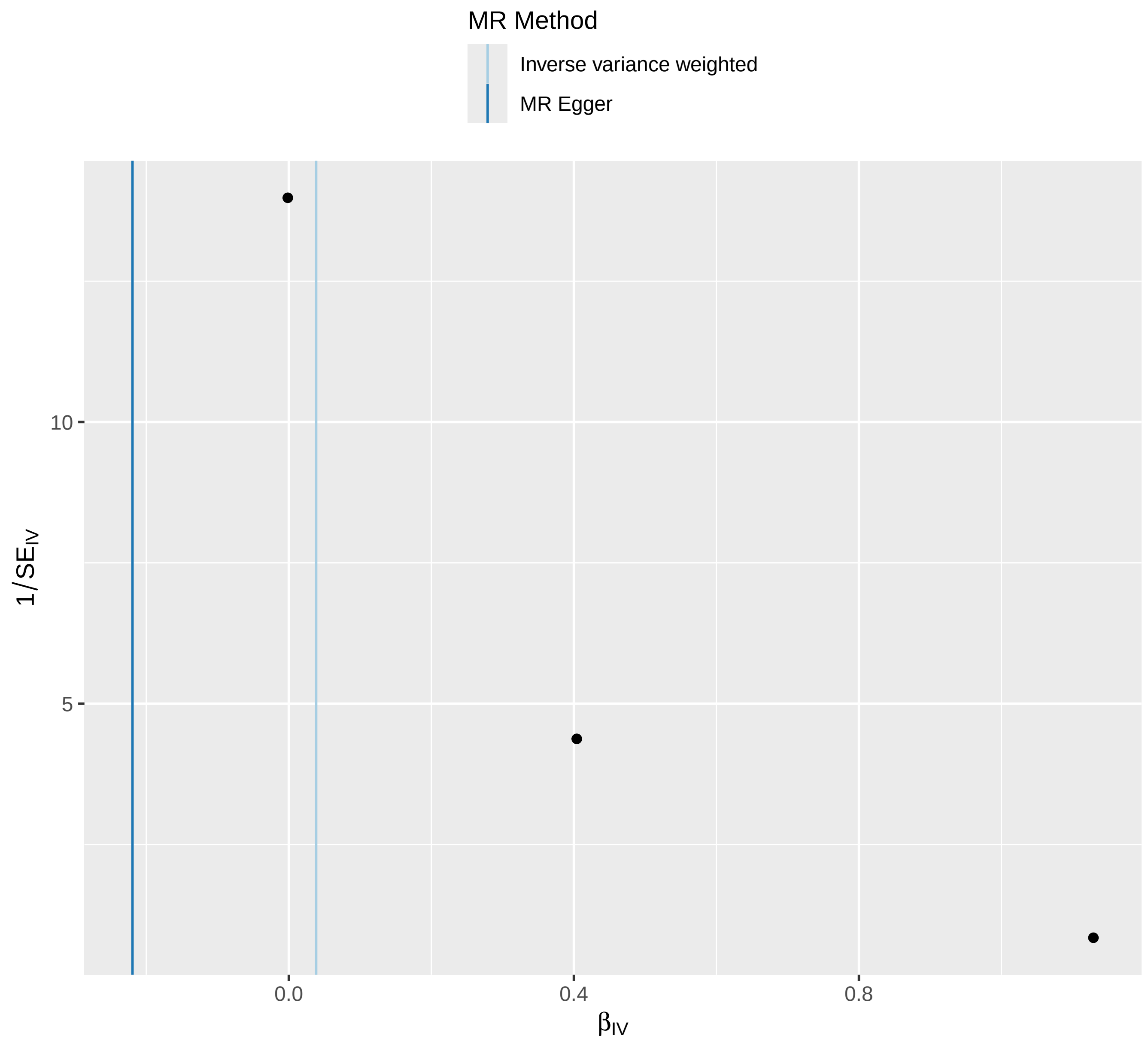
MR Method
Inverse variance weighted
MR Egger
10
5
1/SE_IV
0.0
0.4
0.8
β_IV

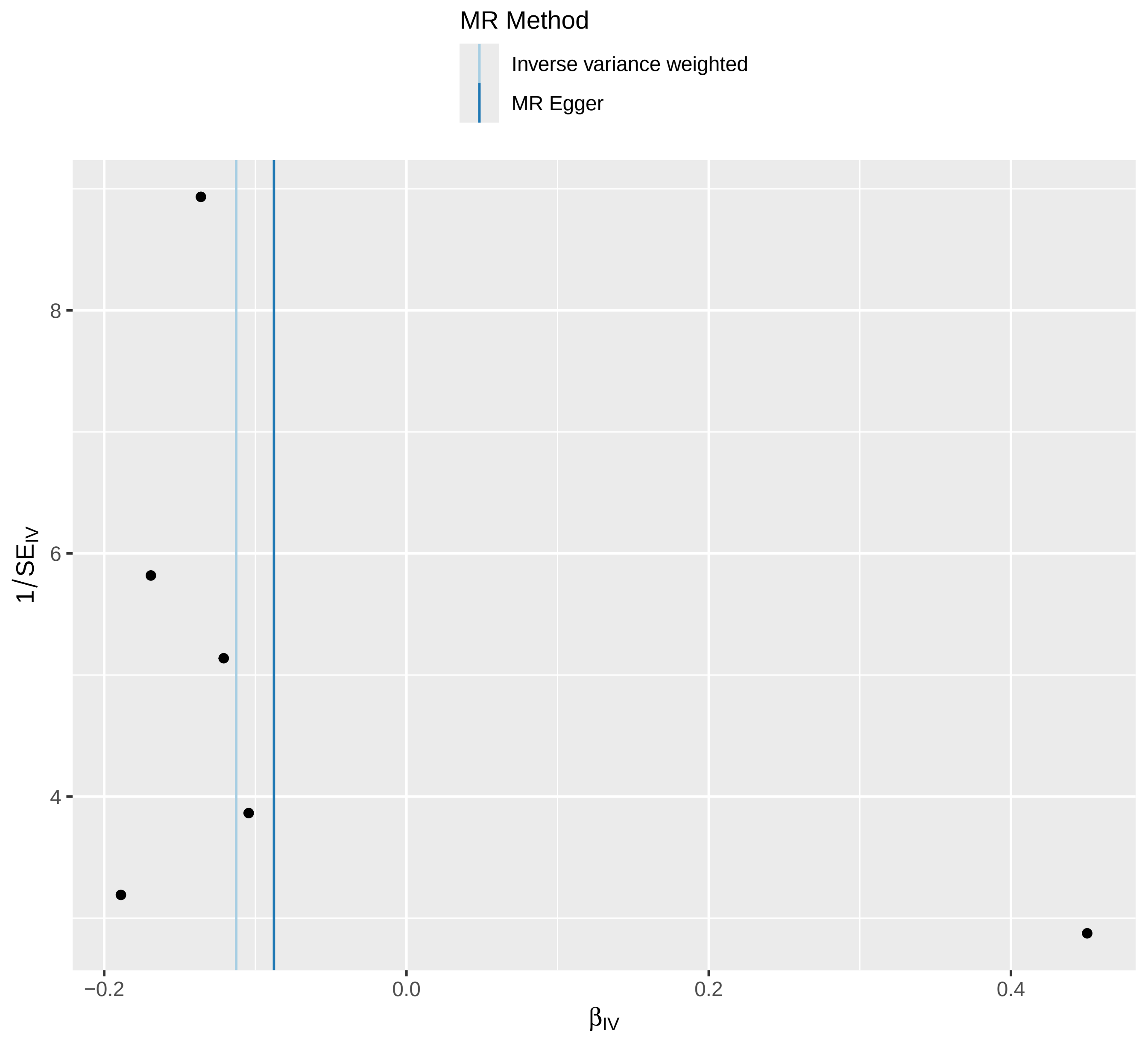

MR Method
Inverse variance weighted
MR Egger
8
6
4
1/SE$_{IV}$
−0.2
0.0
0.2
0.4
β$_{IV}$

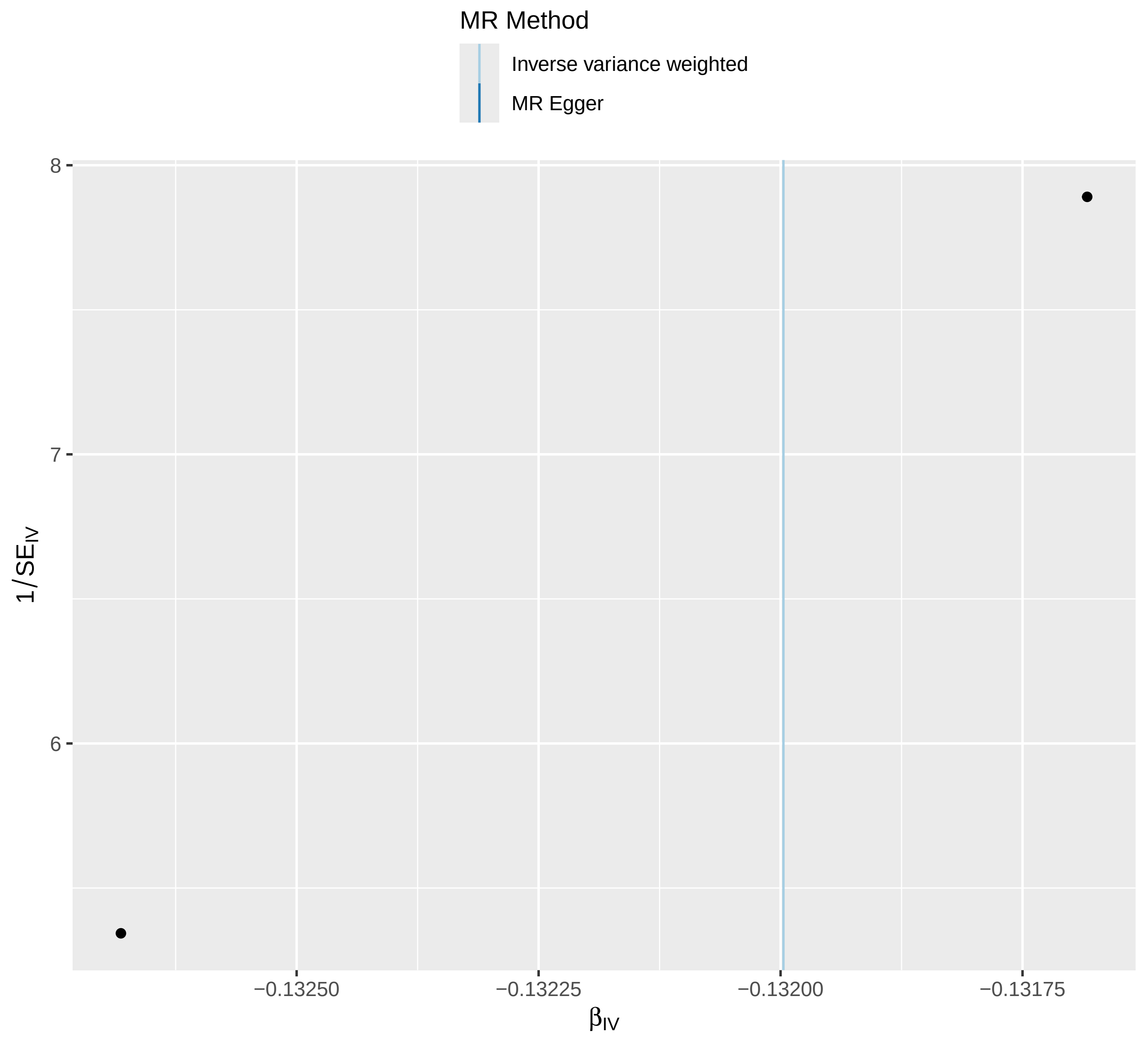
MR Method
Inverse variance weighted
MR Egger
8
7
6
1/SE$_{IV}$
−0.13250
−0.13225
−0.13200
−0.13175
β$_{IV}$

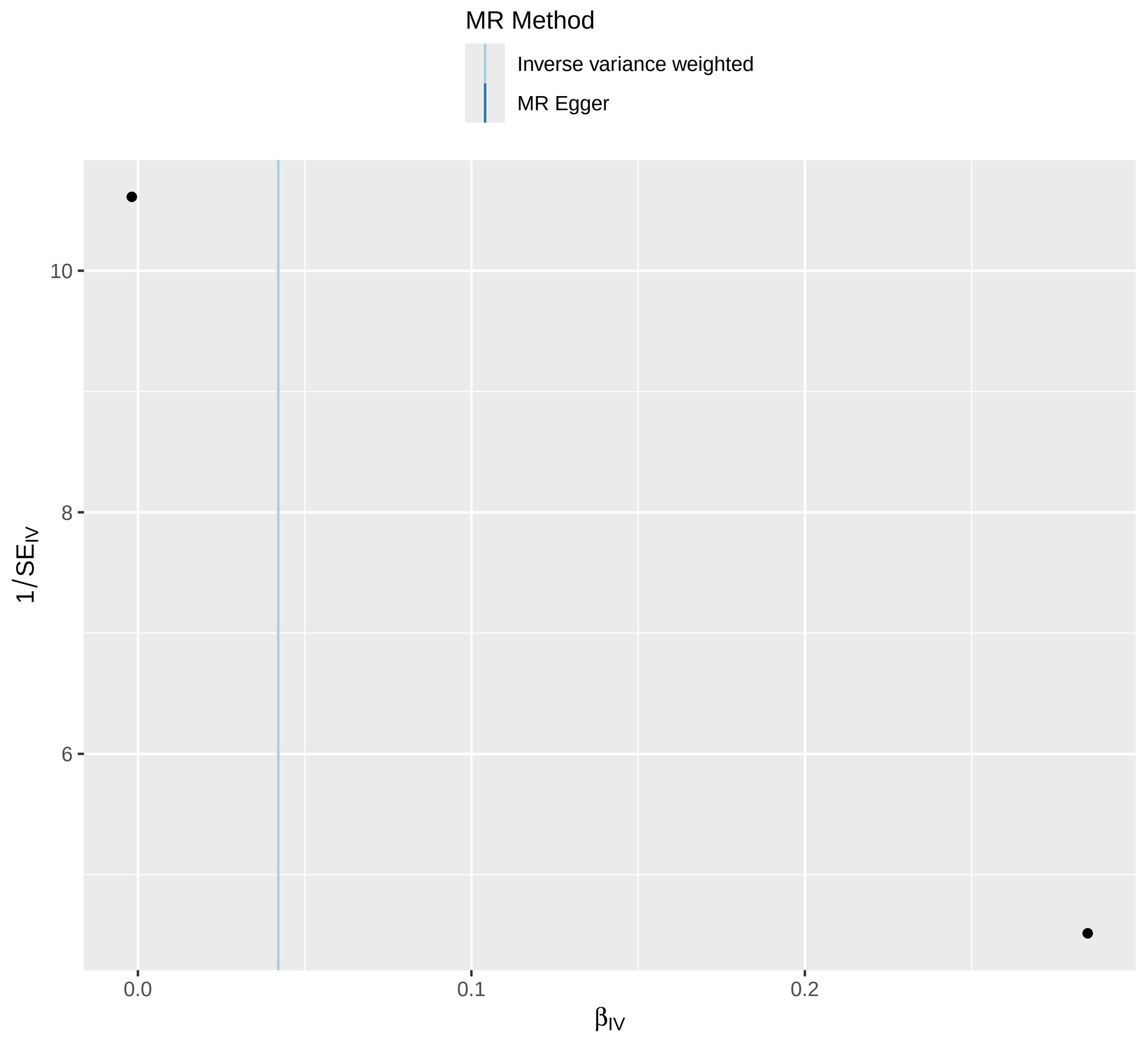

MR Method
Inverse variance weighted
MR Egger
10
8
6
1/SE_IV
0.0
0.1
0.2
β_IV

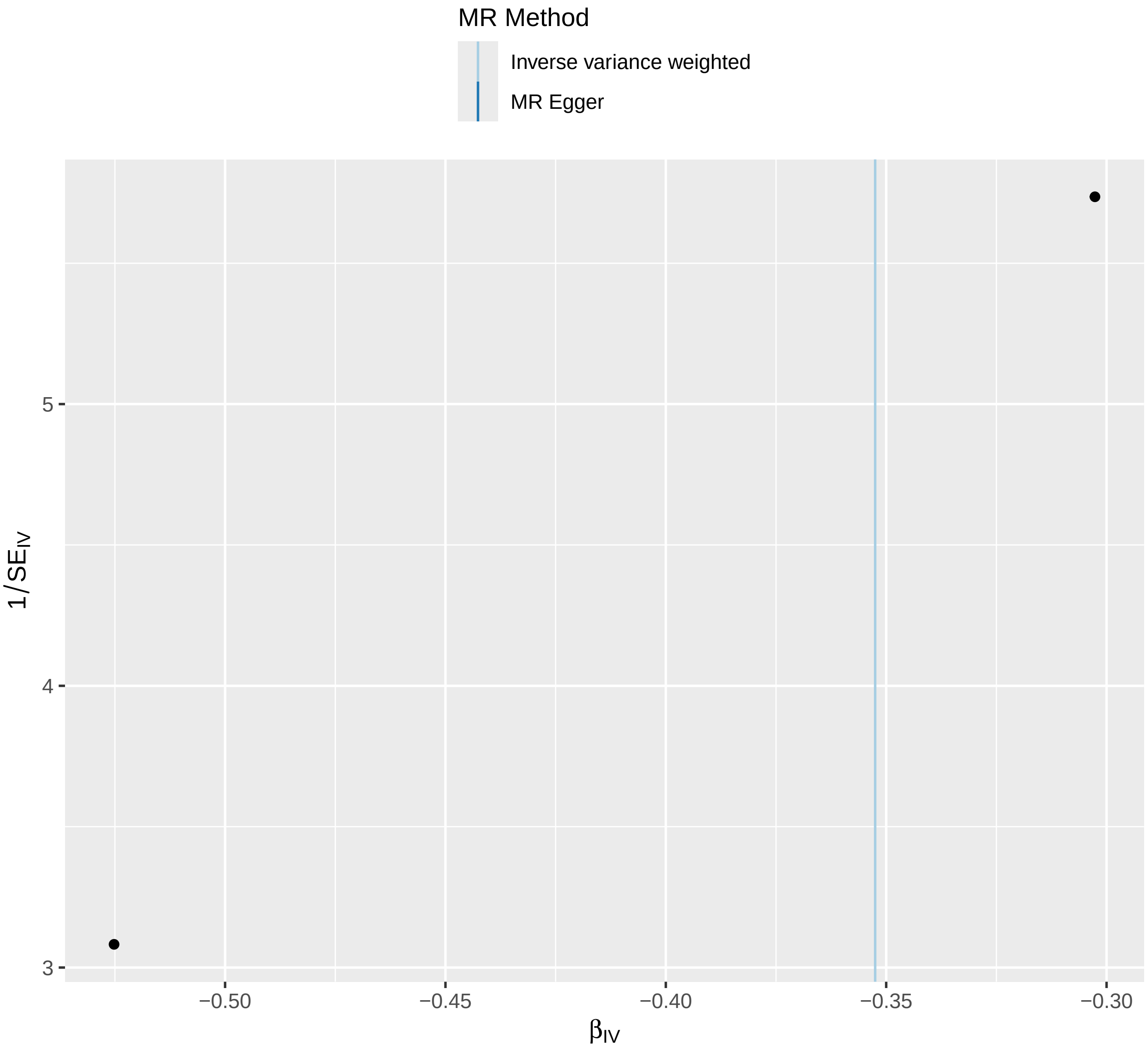
MR Method
Inverse variance weighted
MR Egger
5
4
3
1/SE_IV
−0.50
−0.45
−0.40
−0.35
−0.30
β_IV

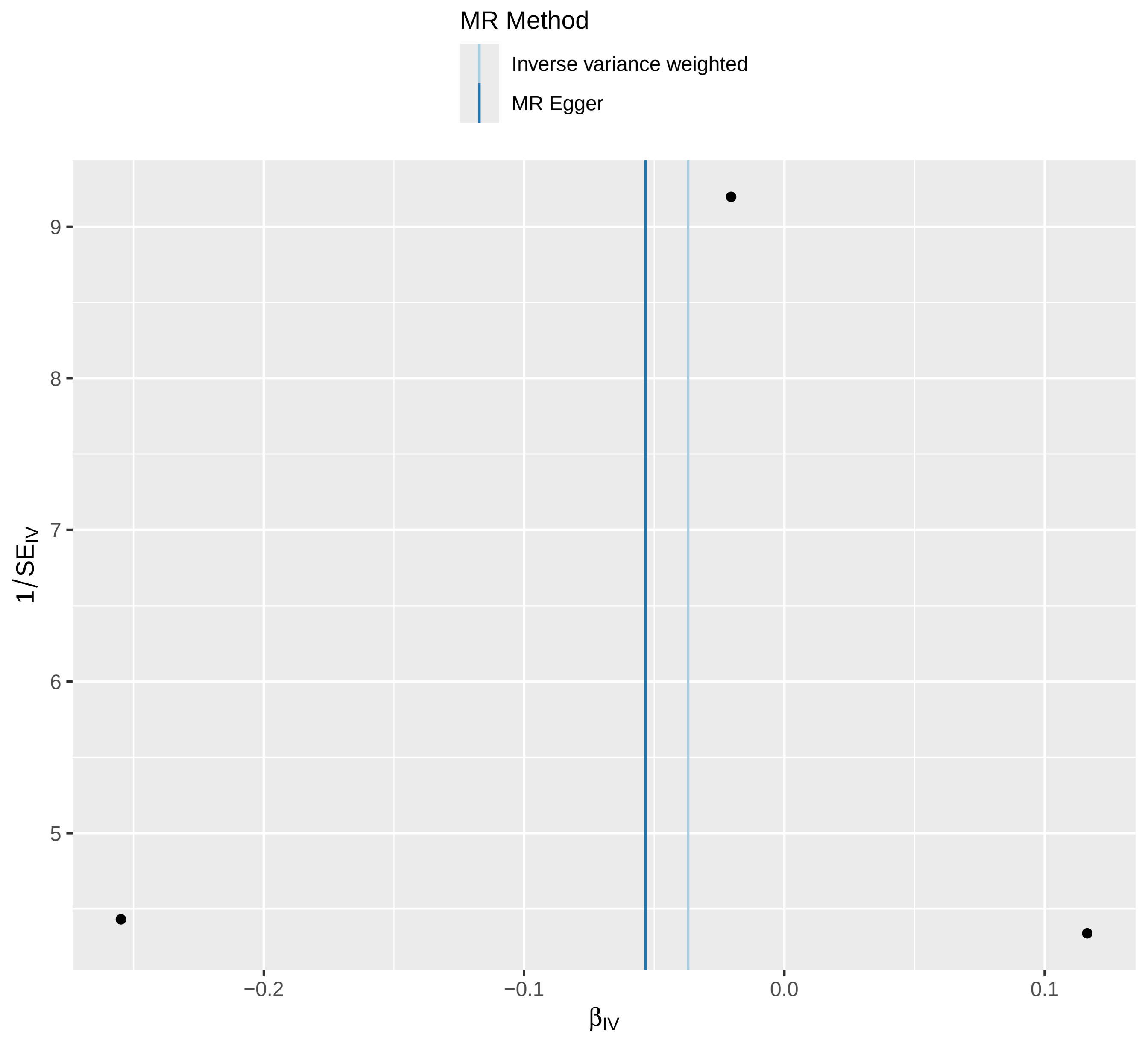
MR Method
Inverse variance weighted
MR Egger
9
8
7
6
5
1/SE_IV
−0.2
−0.1
0.0
0.1
β_IV

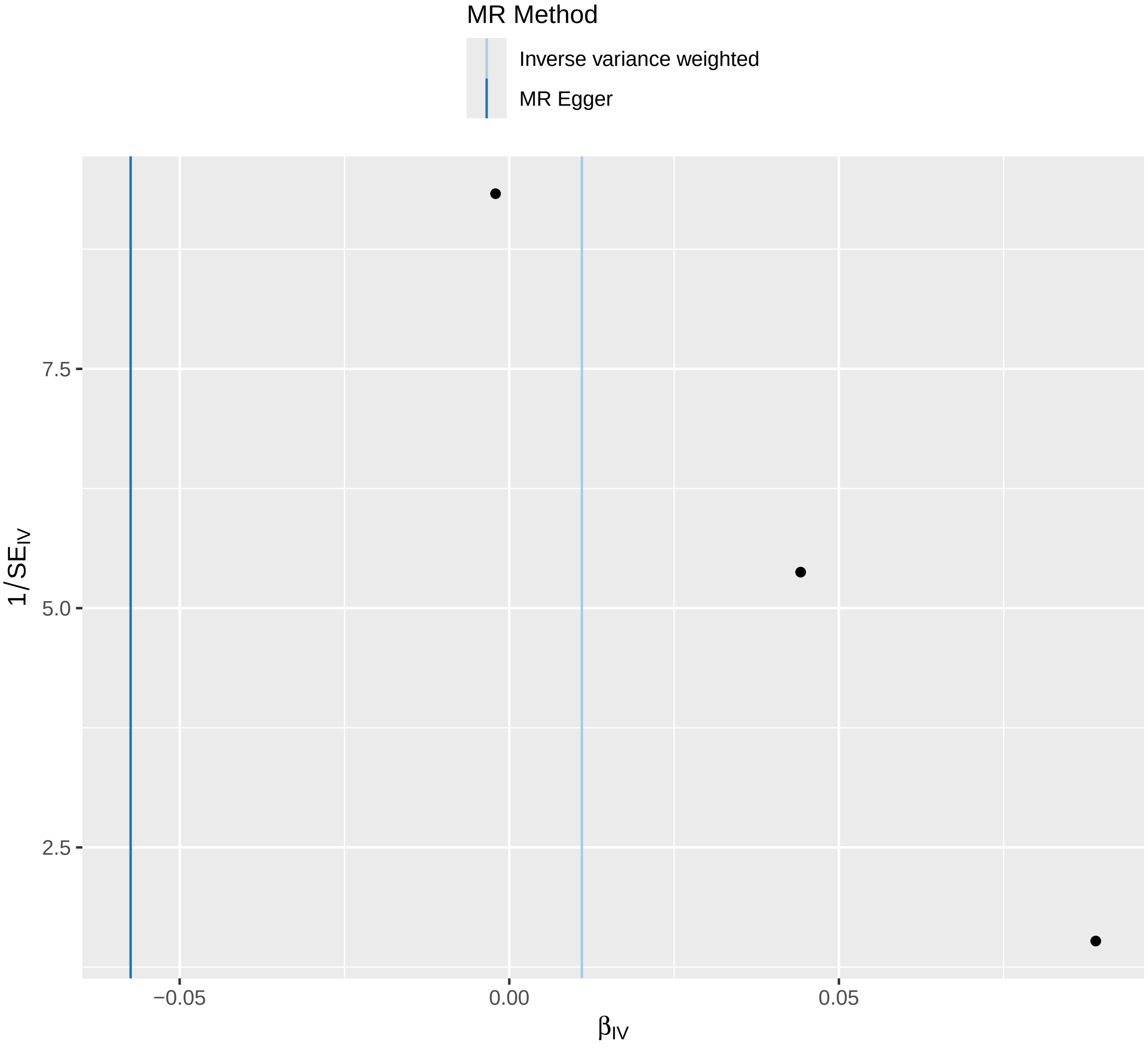
MR Method
Inverse variance weighted
MR Egger
7.5
5.0
2.5
1/SE_IV
−0.05
0.00
0.05
β_IV

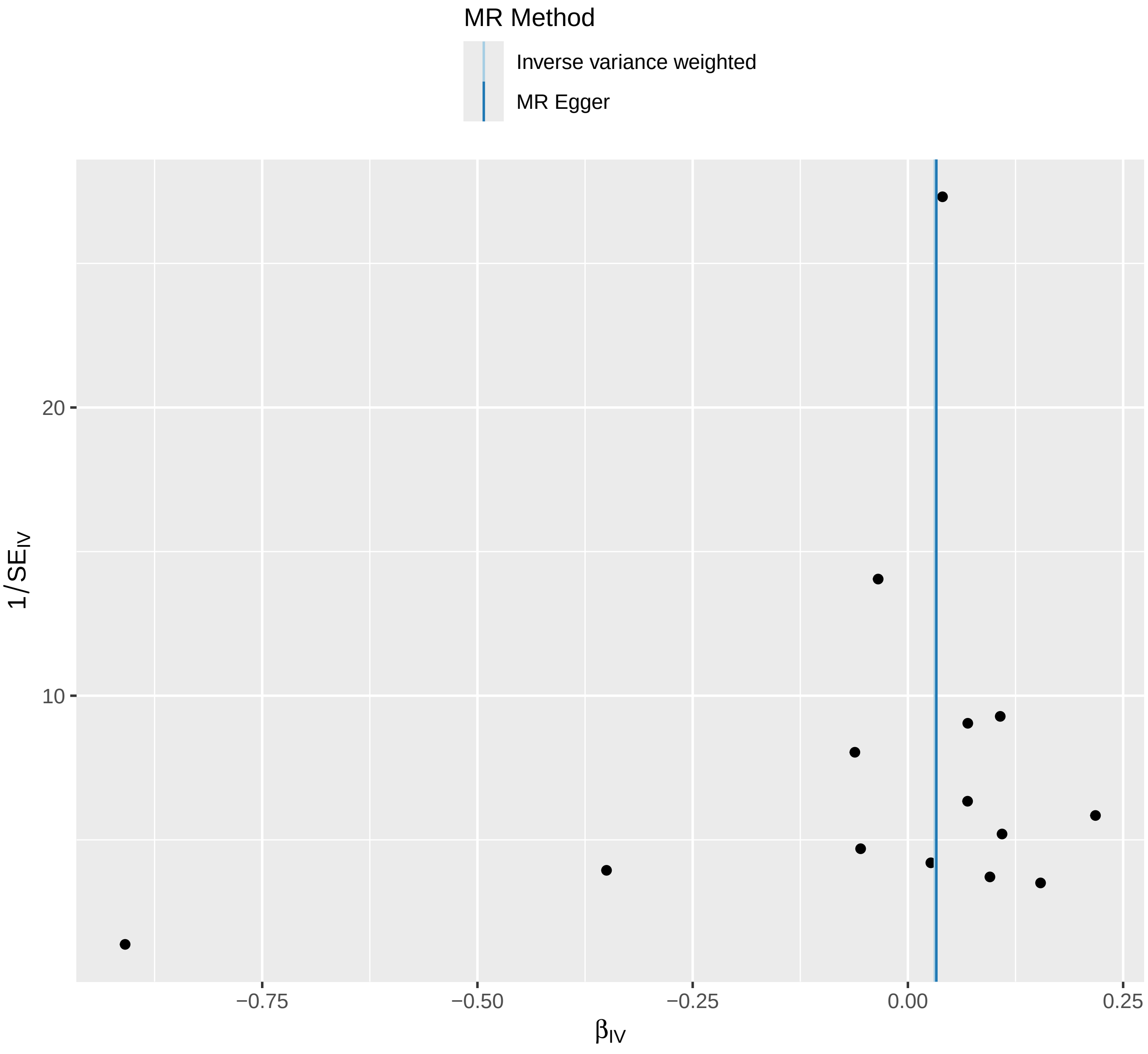

MR Method
Inverse variance weighted
MR Egger
20
10
1/SE_IV
−0.75
−0.50
−0.25
0.00
0.25
β_IV

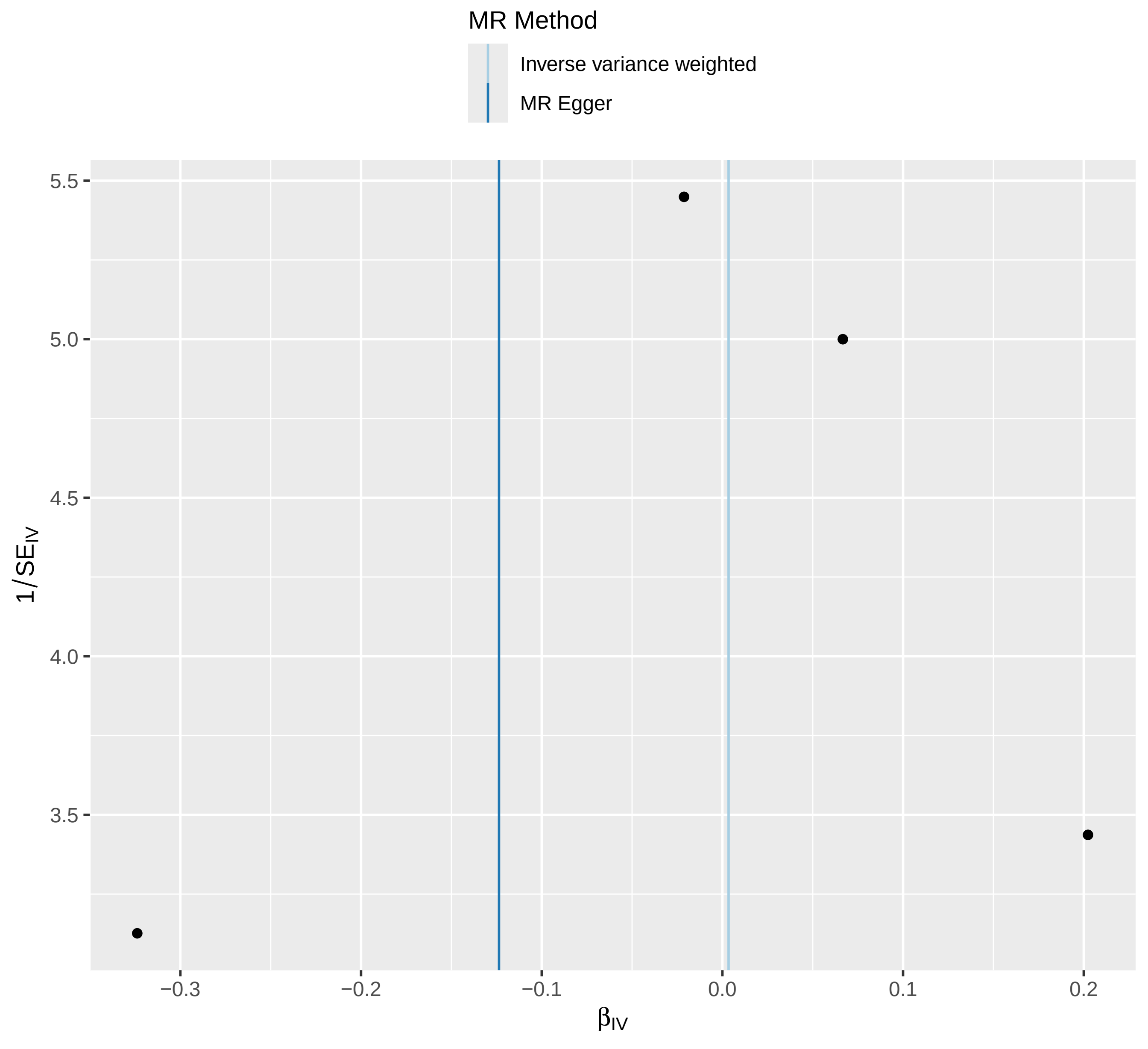

MR Method
Inverse variance weighted
MR Egger
5.5
5.0
4.5
4.0
3.5
1/SE_IV
−0.3
−0.2
−0.1
0.0
0.1
0.2
β_IV

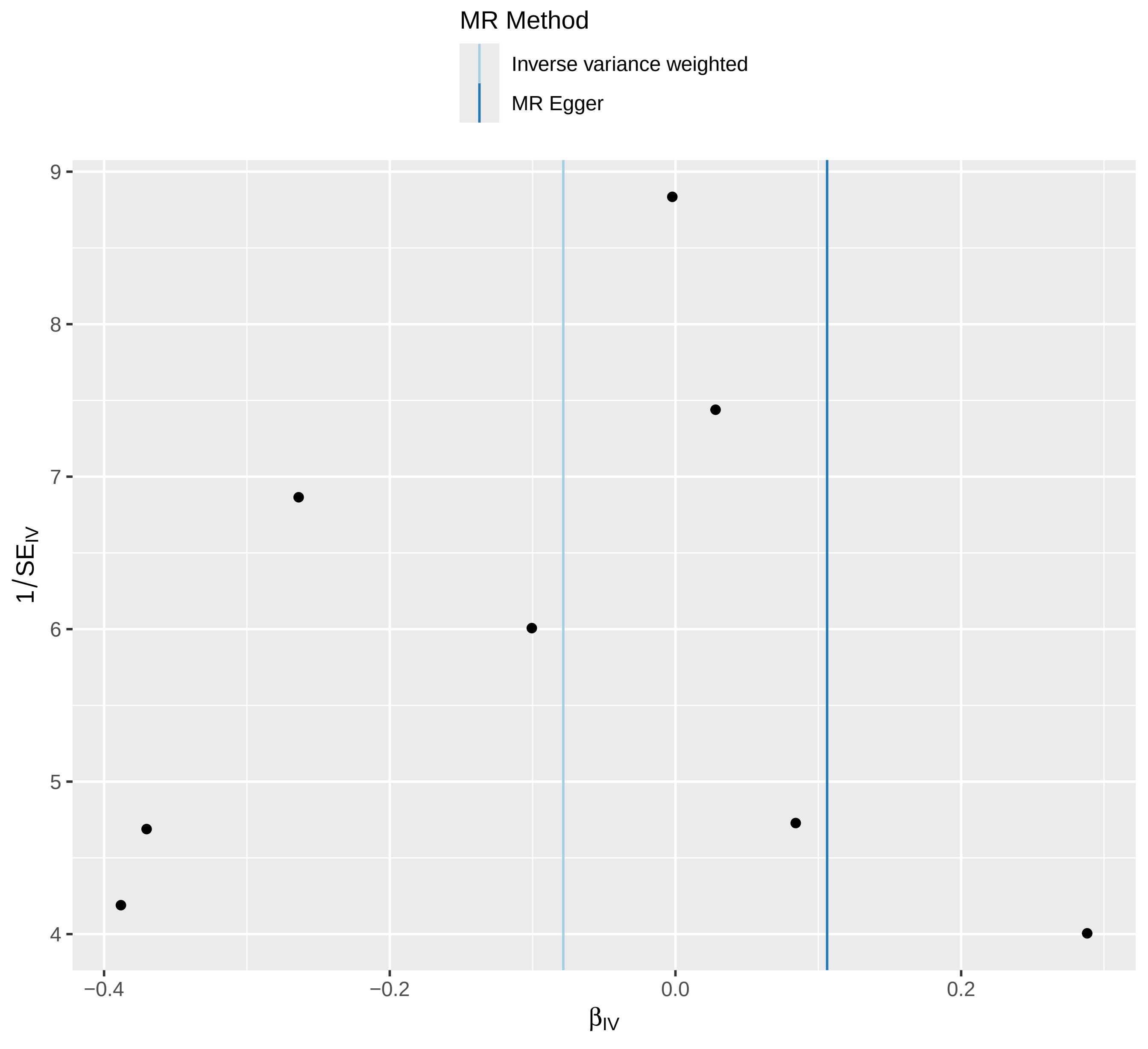

MR Method
Inverse variance weighted
MR Egger
9
8
7
6
5
4
1/SE$_{IV}$
−0.4
−0.2
0.0
0.2
β$_{IV}$

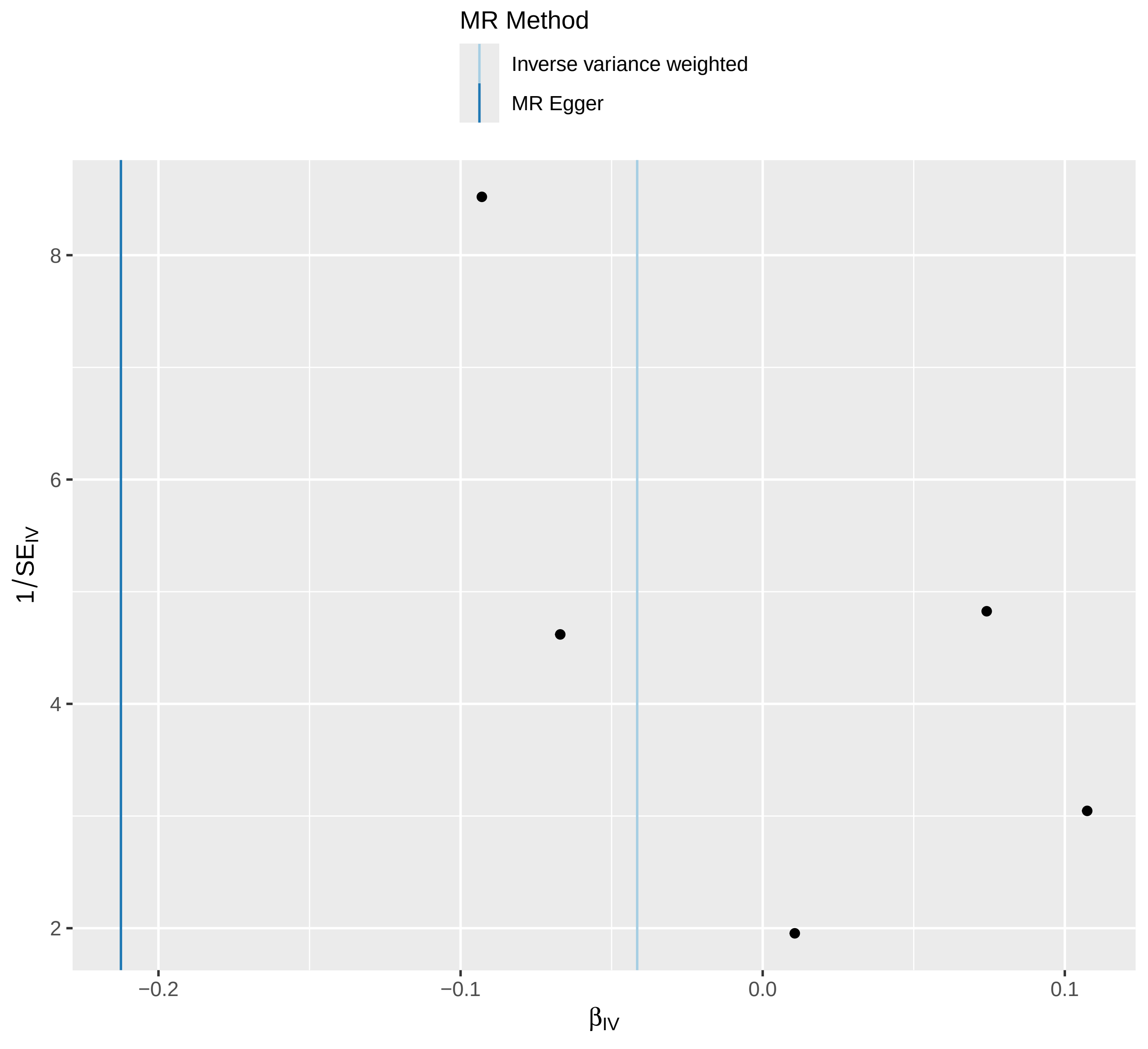
MR Method
Inverse variance weighted
MR Egger
8
6
4
2
1/SE_IV
−0.2
−0.1
0.0
0.1
β_IV

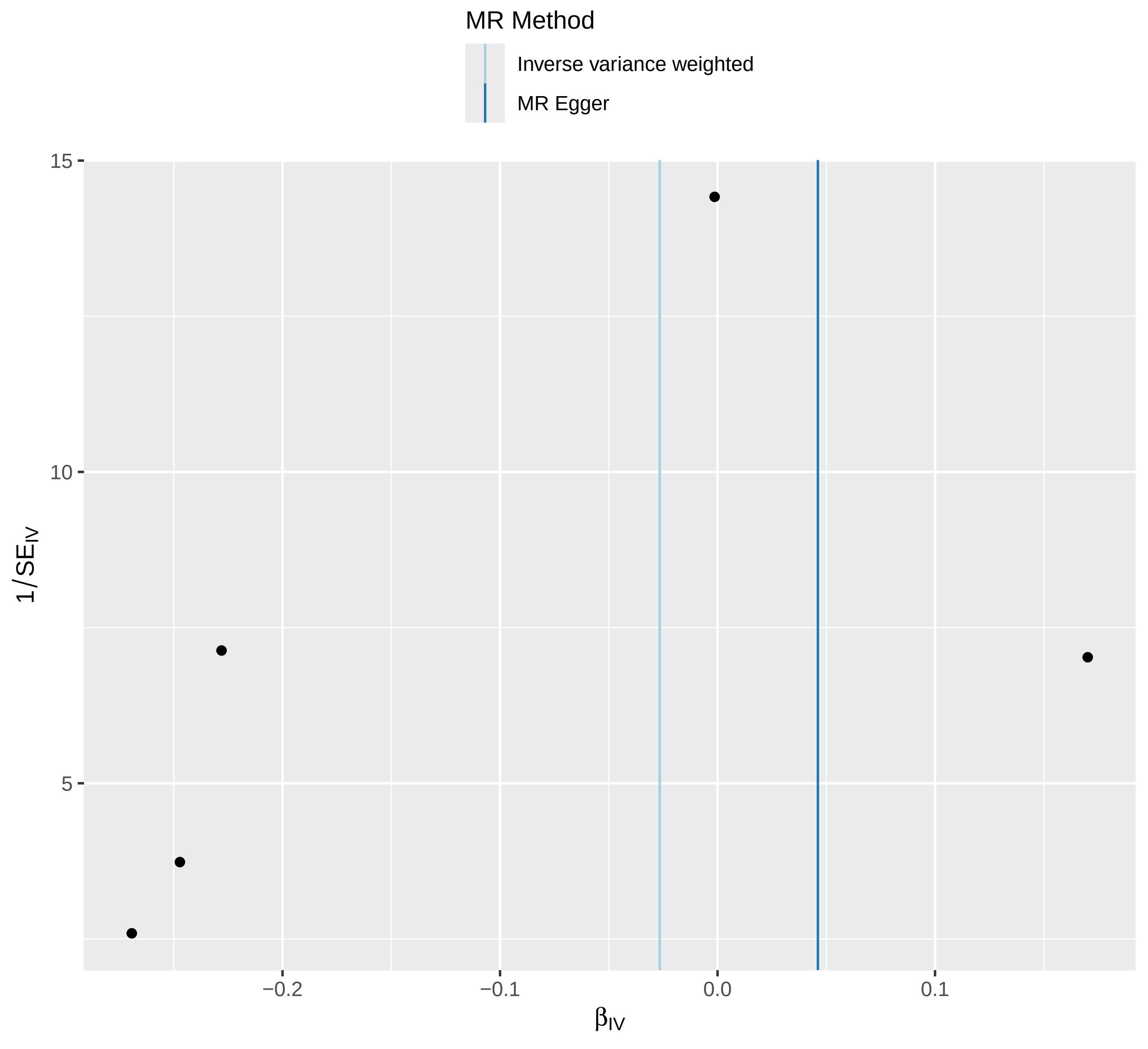
MR Method
Inverse variance weighted
MR Egger
15
10
5
$1/SE_{IV}$
−0.2
−0.1
0.0
0.1
$\beta_{IV}$

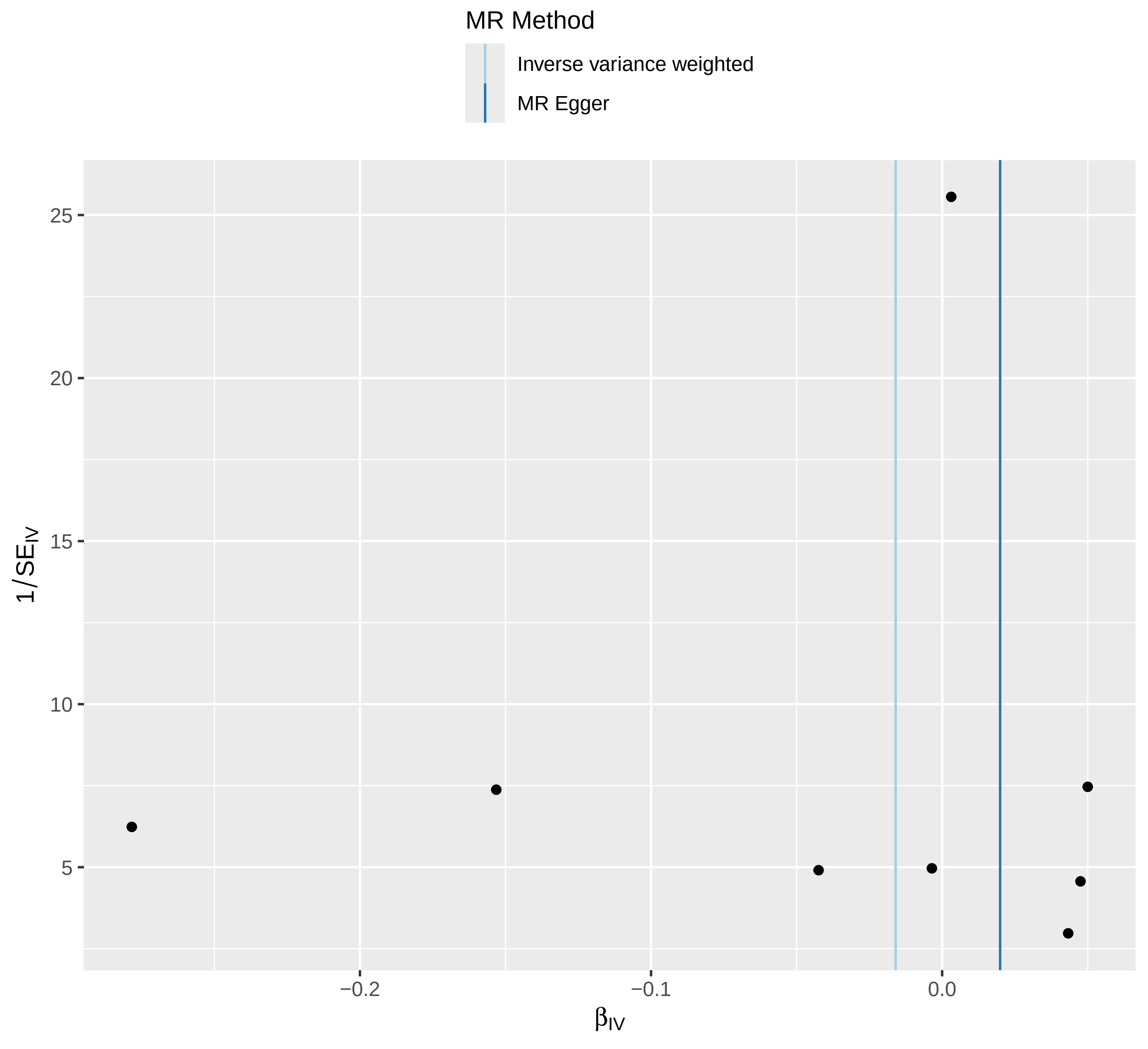
MR Method
Inverse variance weighted
MR Egger
1/SE_IV
β_IV
25
20
15
10
5
−0.2
−0.1
0.0

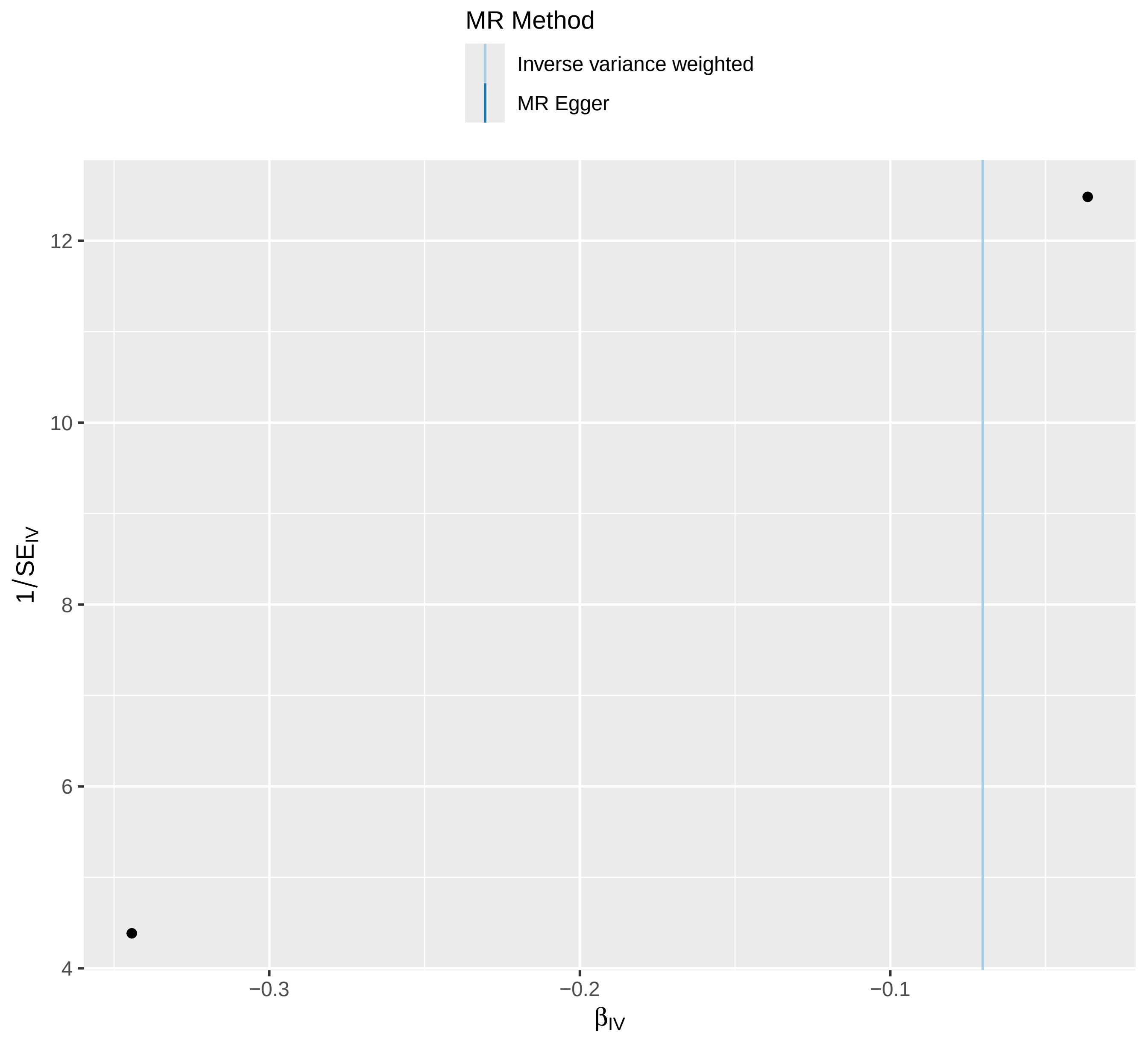

MR Method
Inverse variance weighted
MR Egger
12
10
8
6
4
−0.3
−0.2
−0.1
1/SE_IV
β_IV

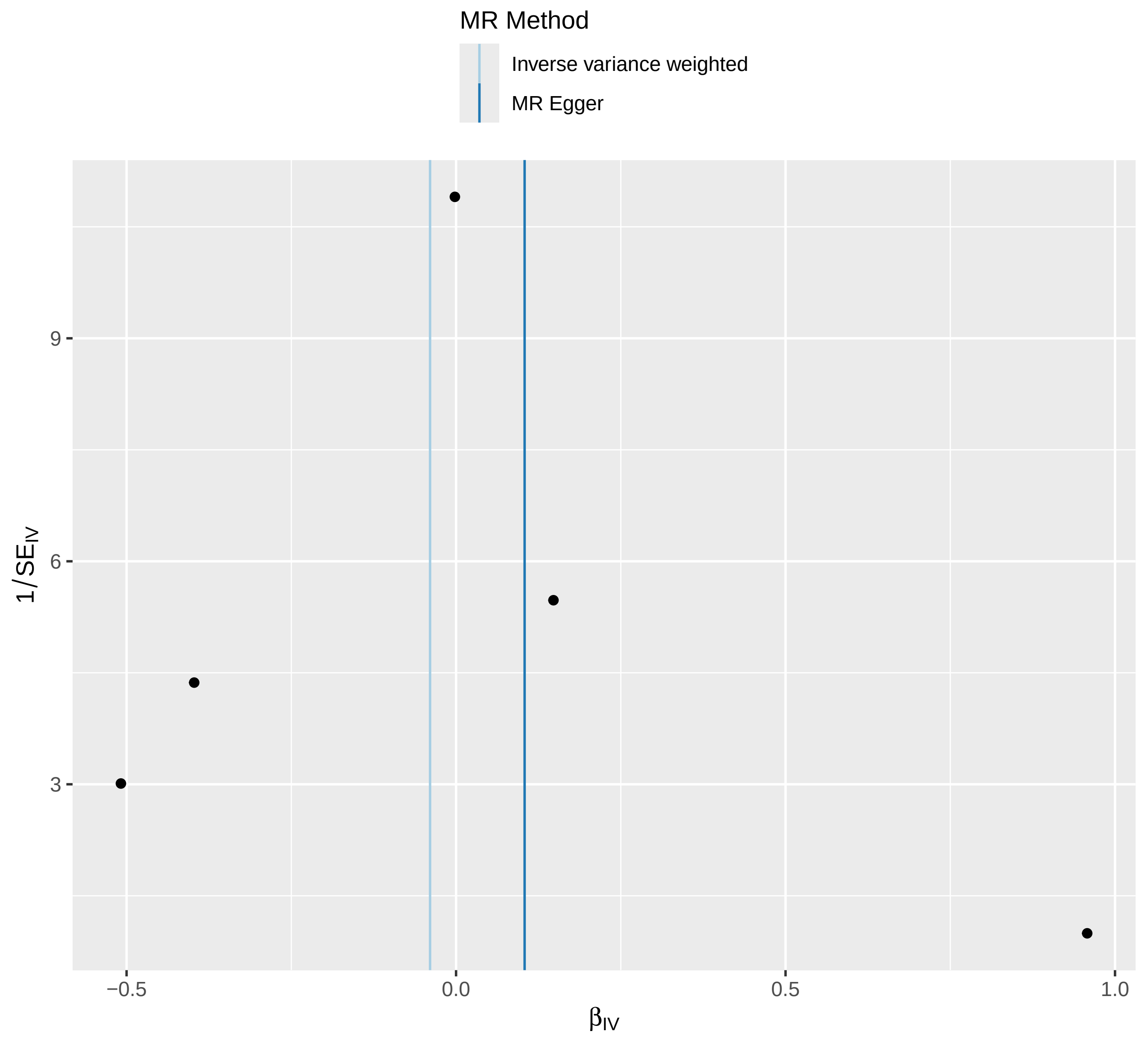

MR Method
Inverse variance weighted
MR Egger
1/SE$_{IV}$
9
6
3
−0.5
0.0
0.5
1.0
β$_{IV}$

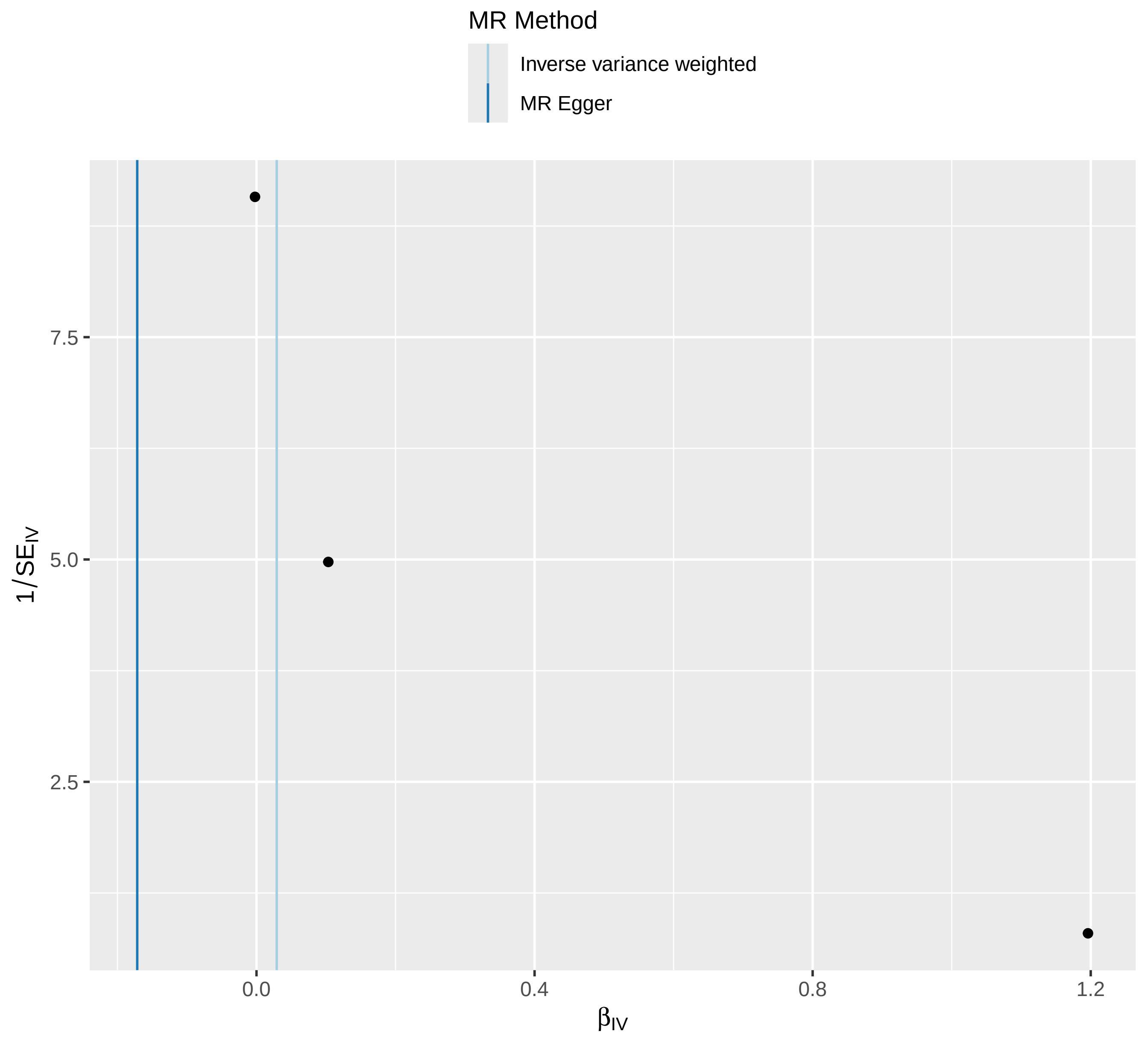

MR Method
Inverse variance weighted
MR Egger
7.5
5.0
2.5
$1/SE_{IV}$
0.0
0.4
0.8
1.2
$\beta_{IV}$

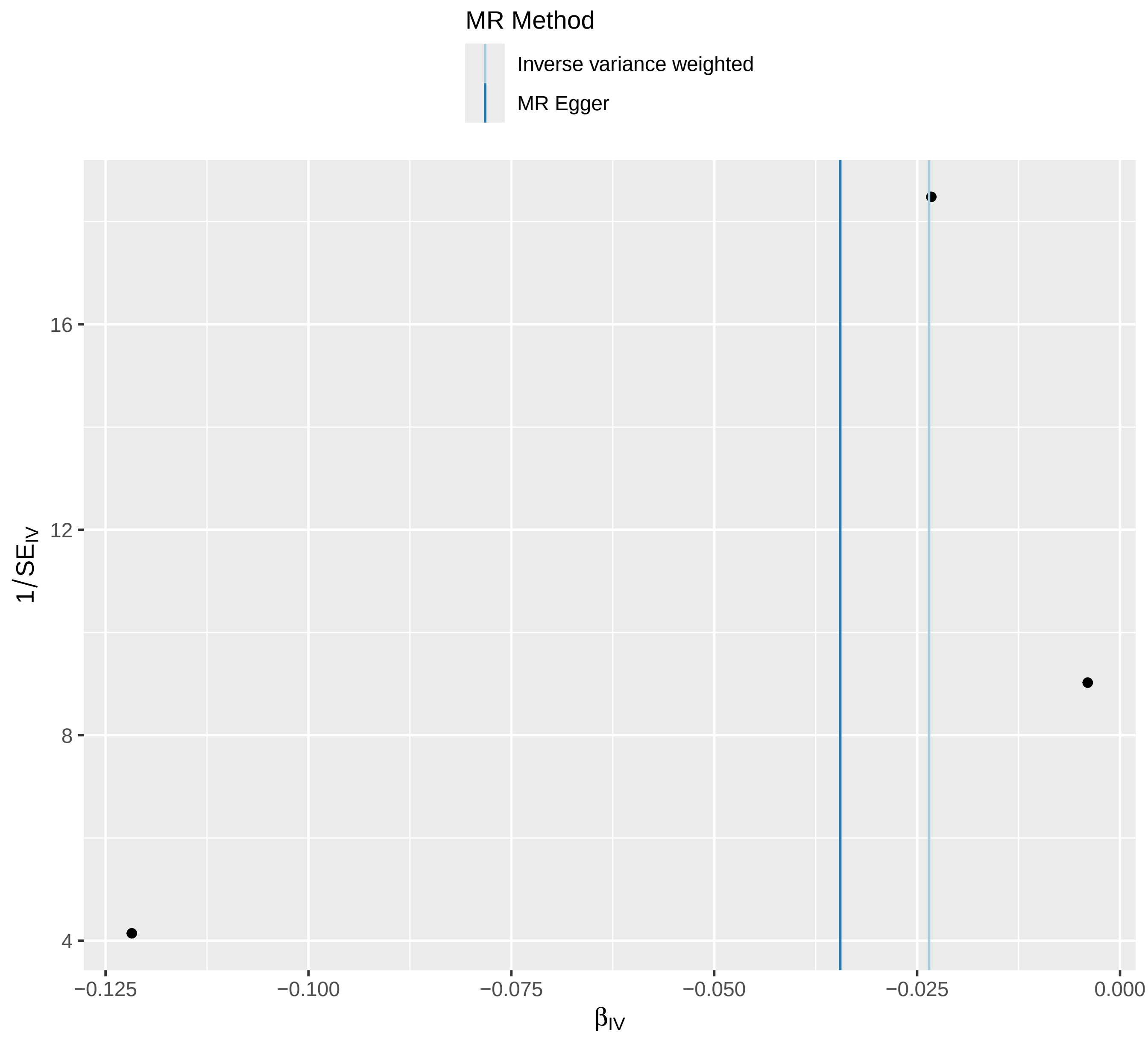
MR Method
Inverse variance weighted
MR Egger
16
12
8
4
1/SE$_{IV}$
−0.125
−0.100
−0.075
−0.050
−0.025
0.000
β$_{IV}$

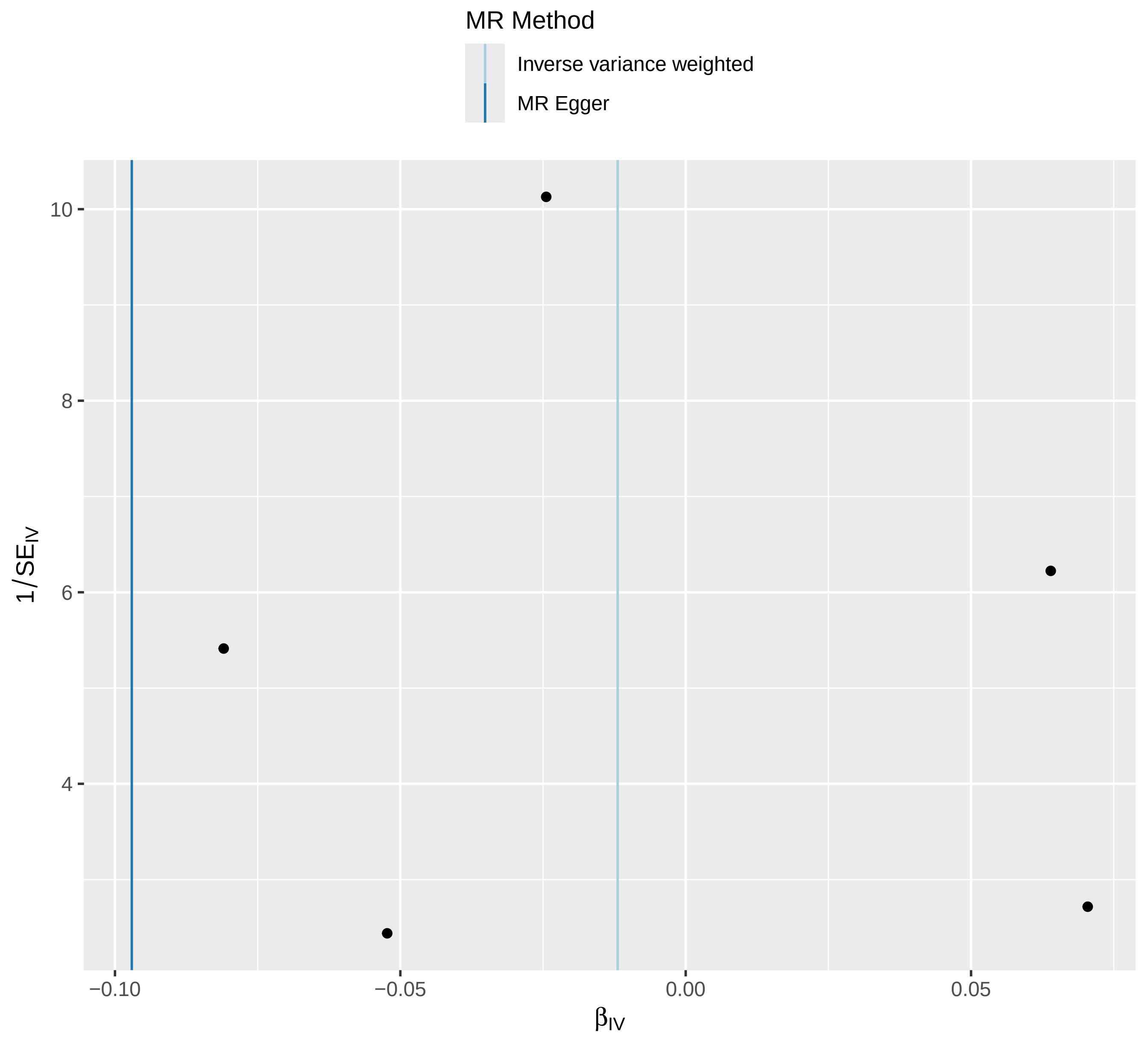

MR Method
Inverse variance weighted
MR Egger
10
8
6
4
1/SE_IV
−0.10
−0.05
0.00
0.05
β_IV

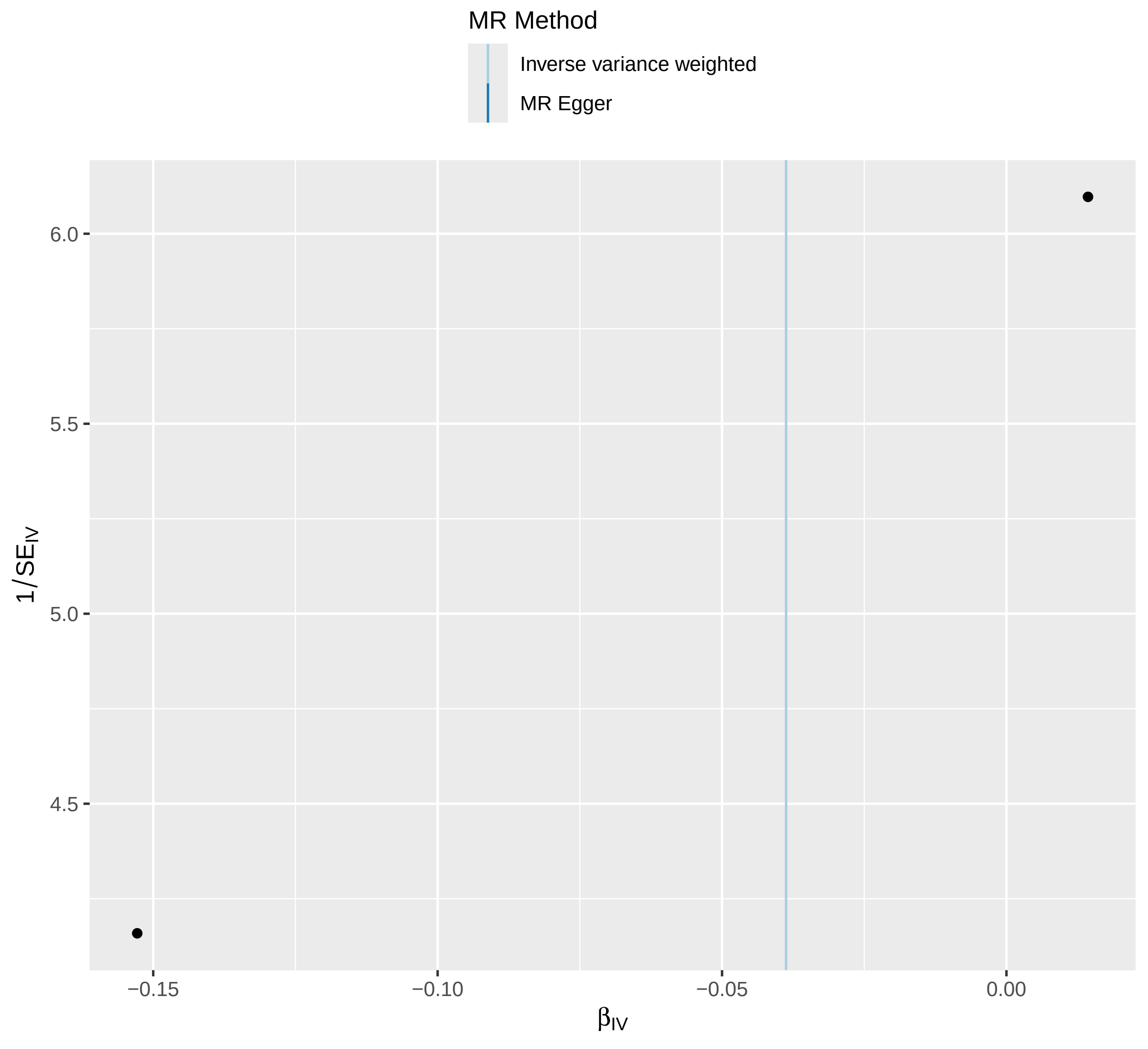

MR Method
Inverse variance weighted
MR Egger
6.0
5.5
5.0
4.5
1/SE$_{IV}$
−0.15
−0.10
−0.05
0.00
β$_{IV}$

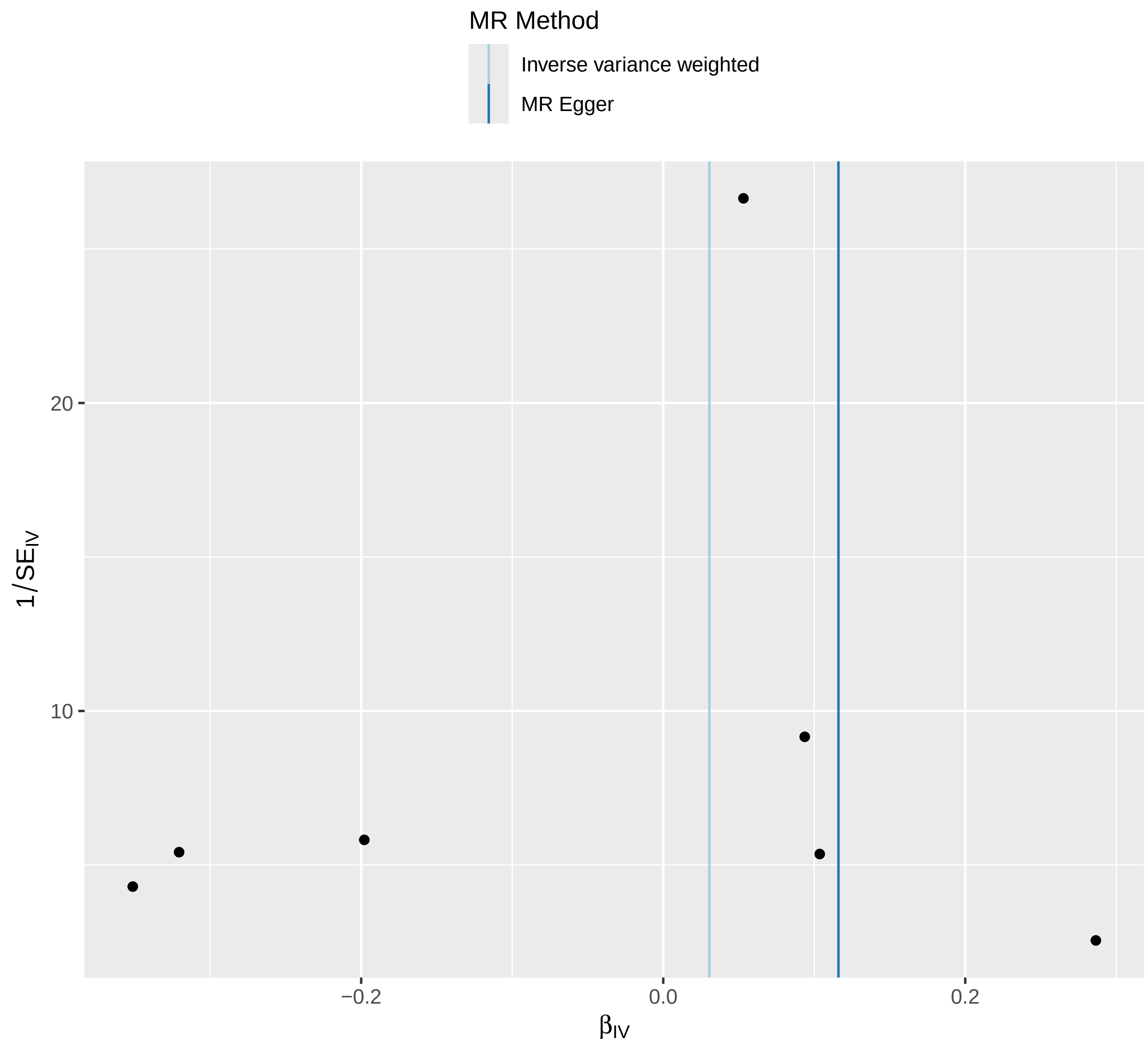
MR Method
Inverse variance weighted
MR Egger
20
10
1/SE_IV
−0.2
0.0
0.2
β_IV

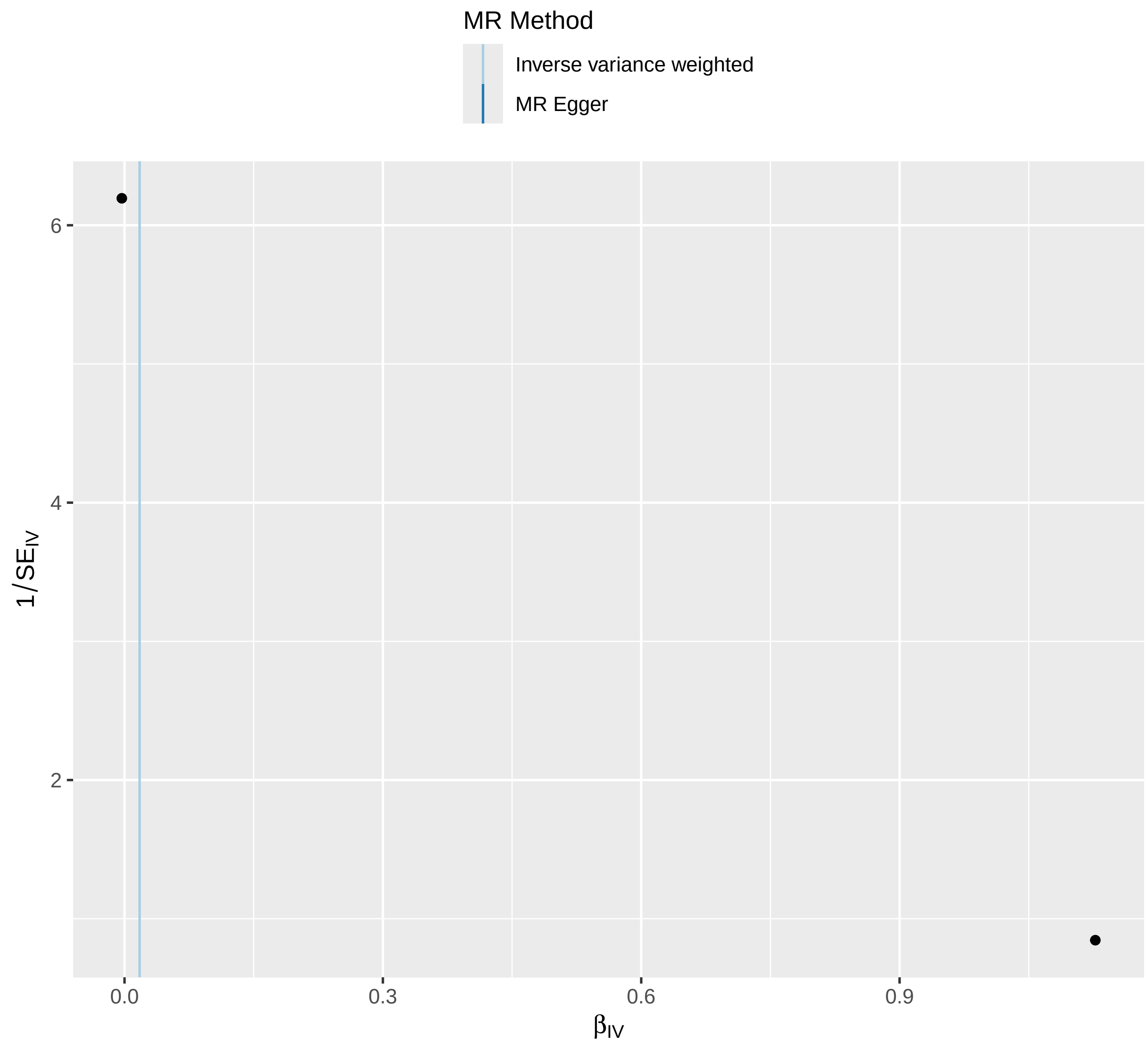
MR Method
Inverse variance weighted
MR Egger
6
4
2
$1/SE_{IV}$
0.0
0.3
0.6
0.9
$\beta_{IV}$

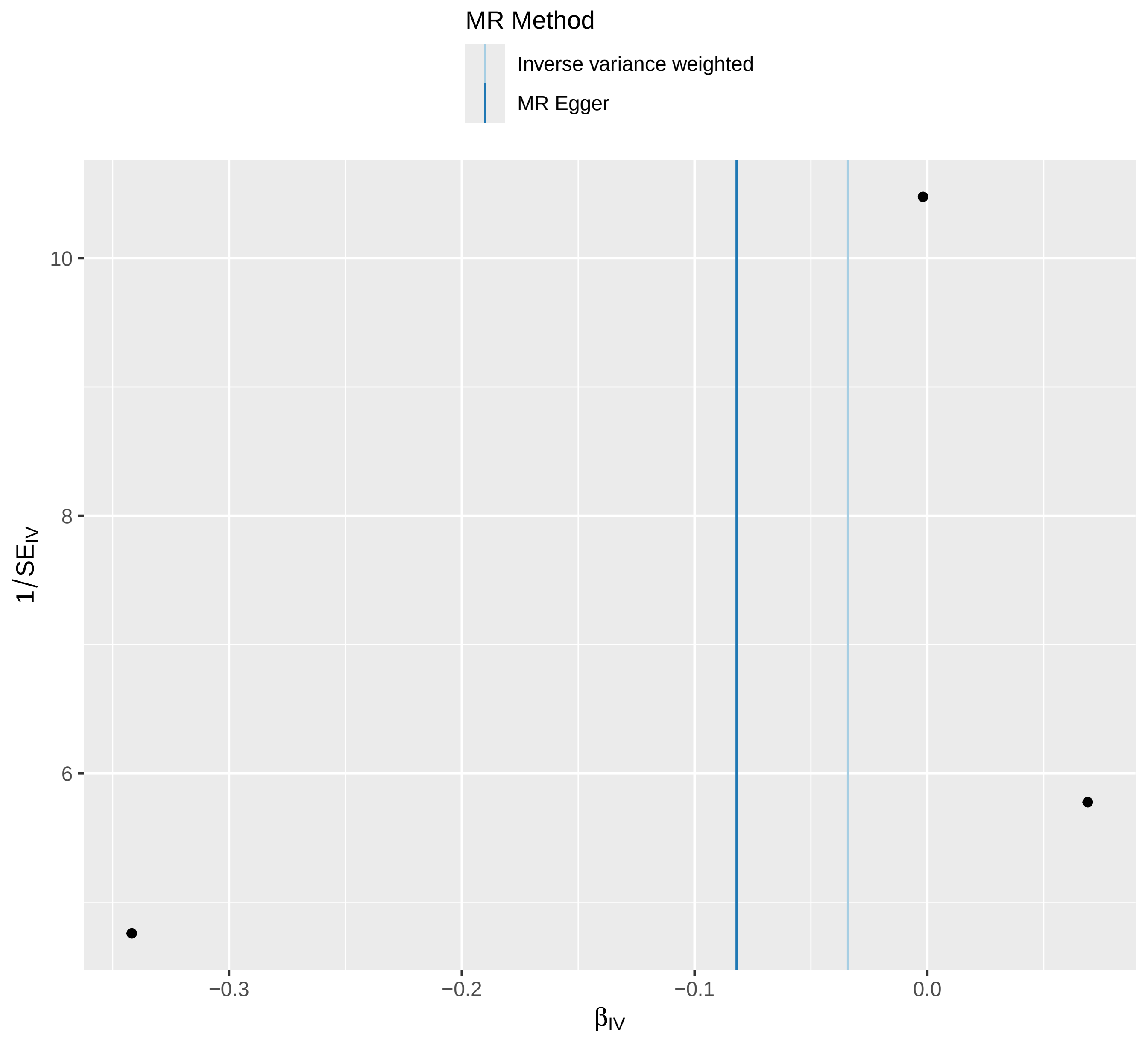
MR Method
Inverse variance weighted
MR Egger
10
8
6
−0.3
−0.2
−0.1
0.0
$1/SE_{IV}$
$\beta_{IV}$

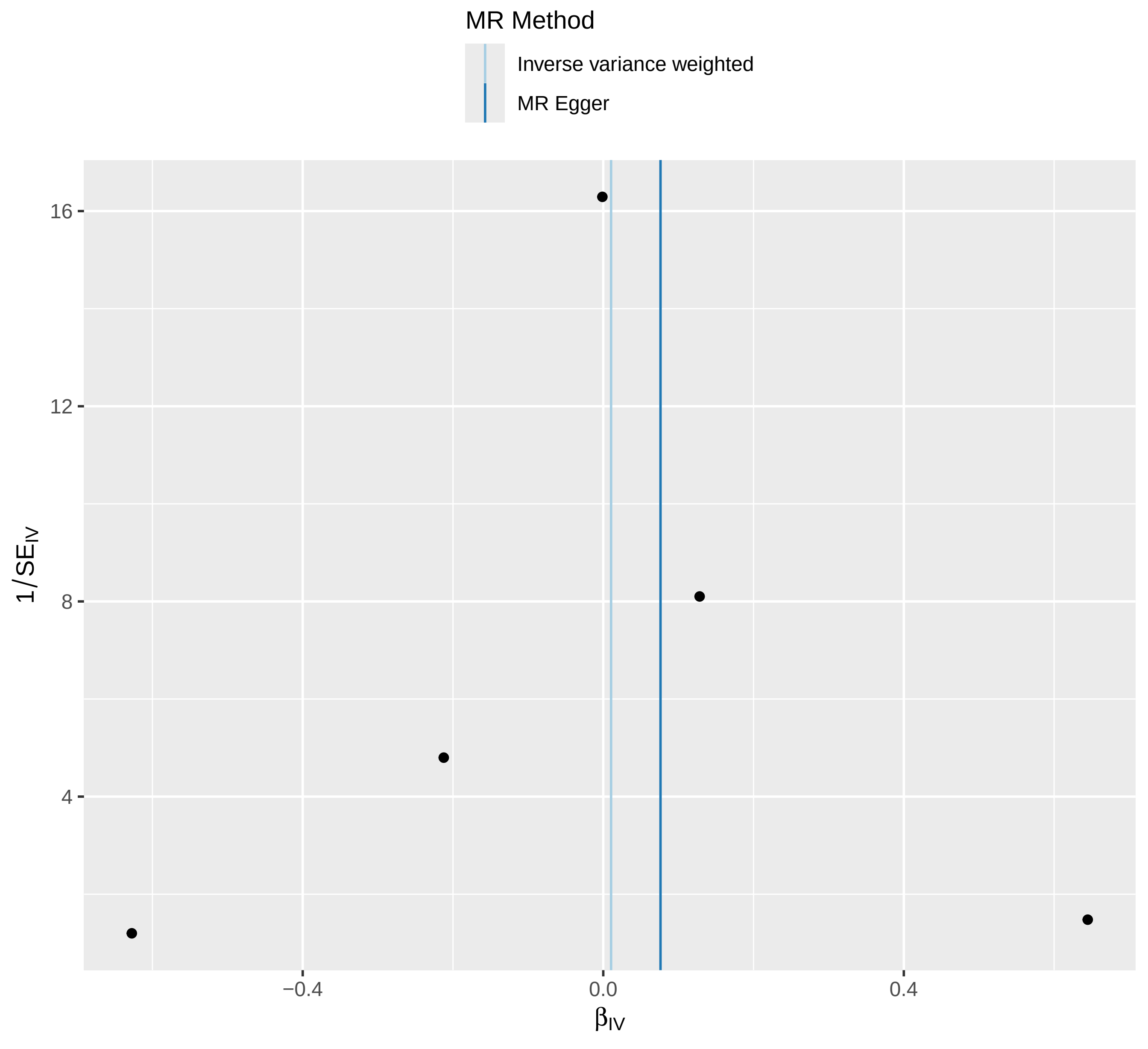
MR Method
Inverse variance weighted
MR Egger
16
12
8
4
1/SE_IV
−0.4
0.0
0.4
β_IV

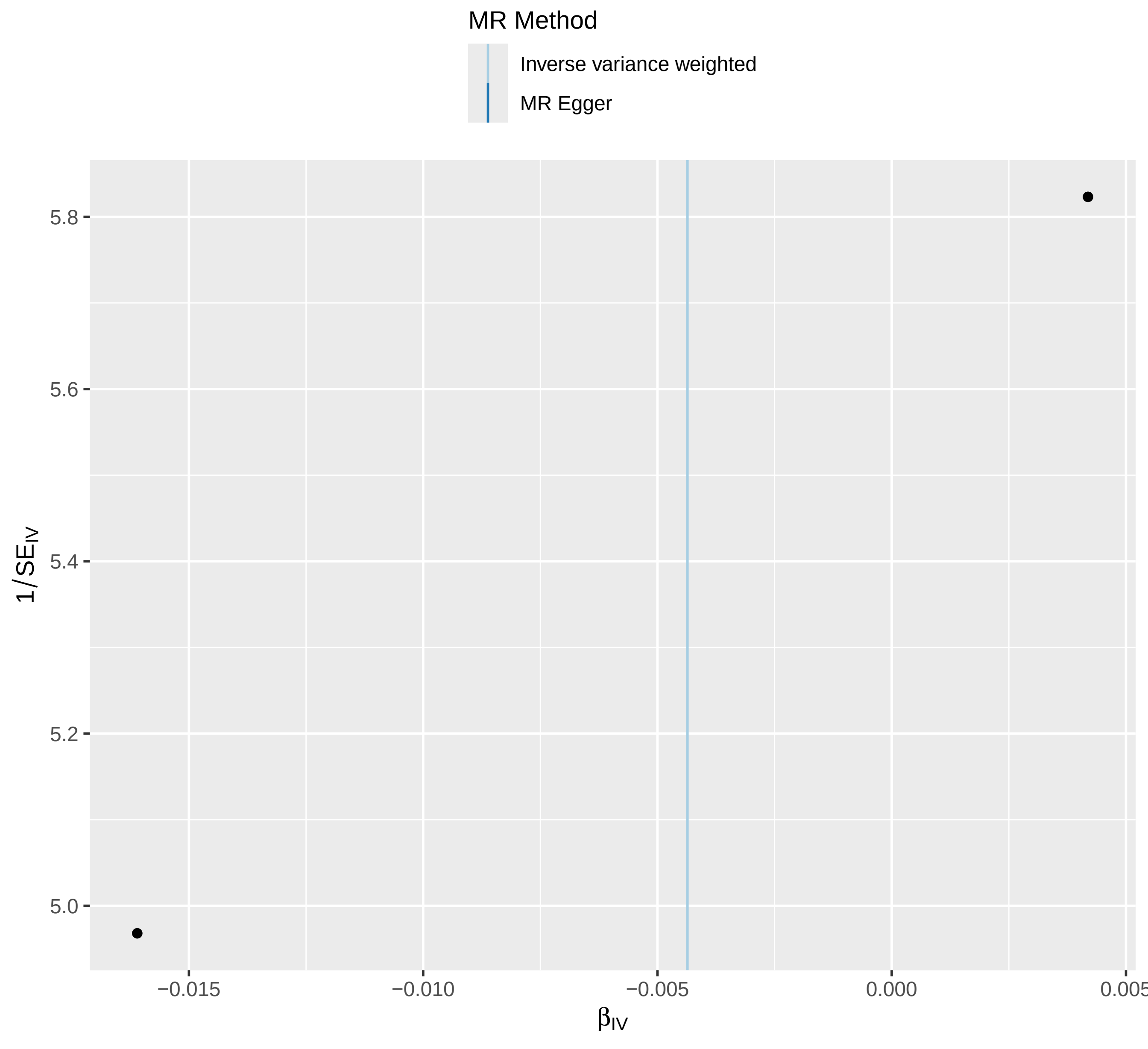
MR Method
Inverse variance weighted
MR Egger
5.8
5.6
5.4
5.2
5.0
$1/SE_{IV}$
−0.015
−0.010
−0.005
0.000
0.005
$\beta_{IV}$

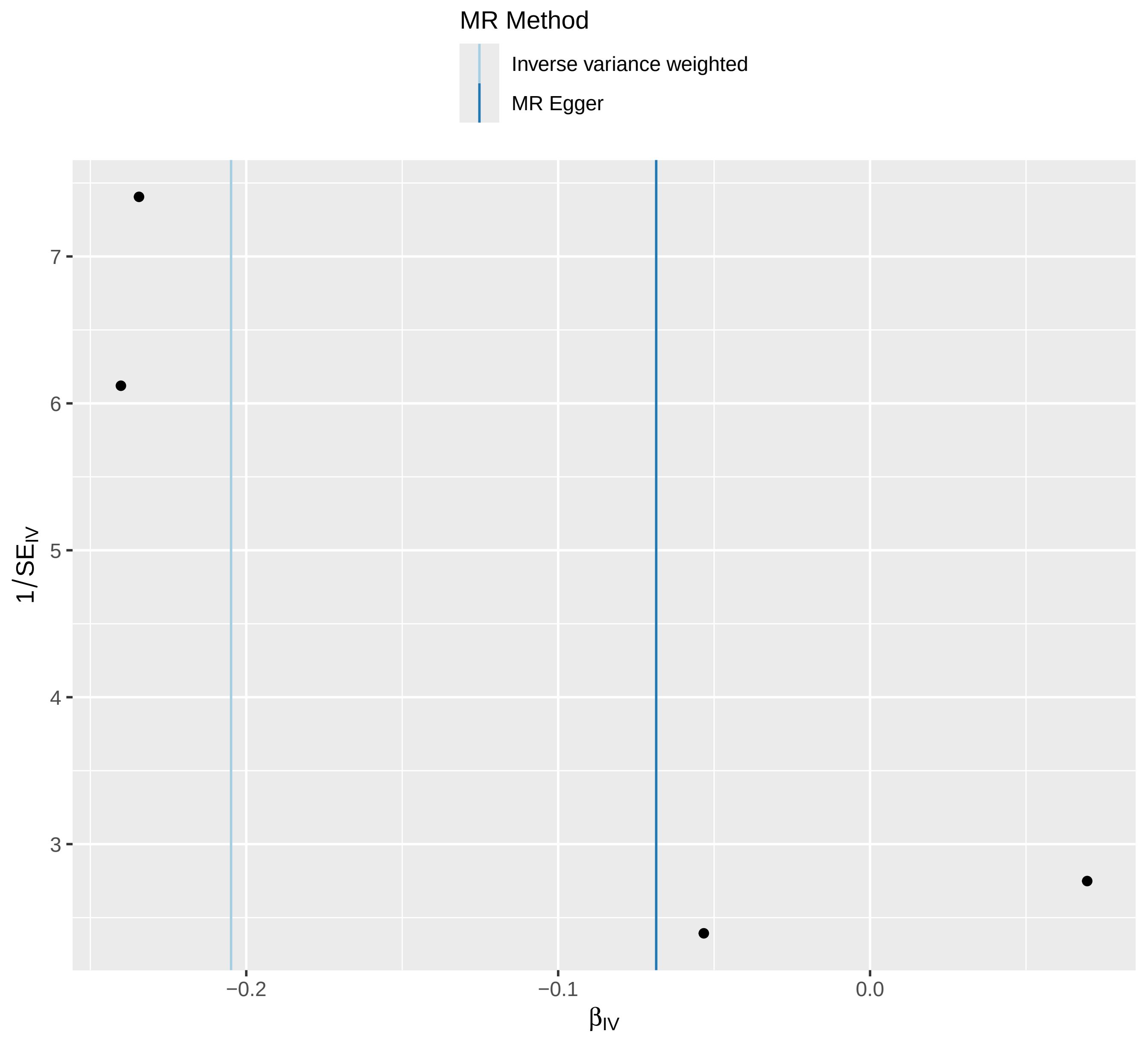

MR Method
Inverse variance weighted
MR Egger
7
6
5
4
3
1/SE$_{IV}$
−0.2
−0.1
0.0
β$_{IV}$

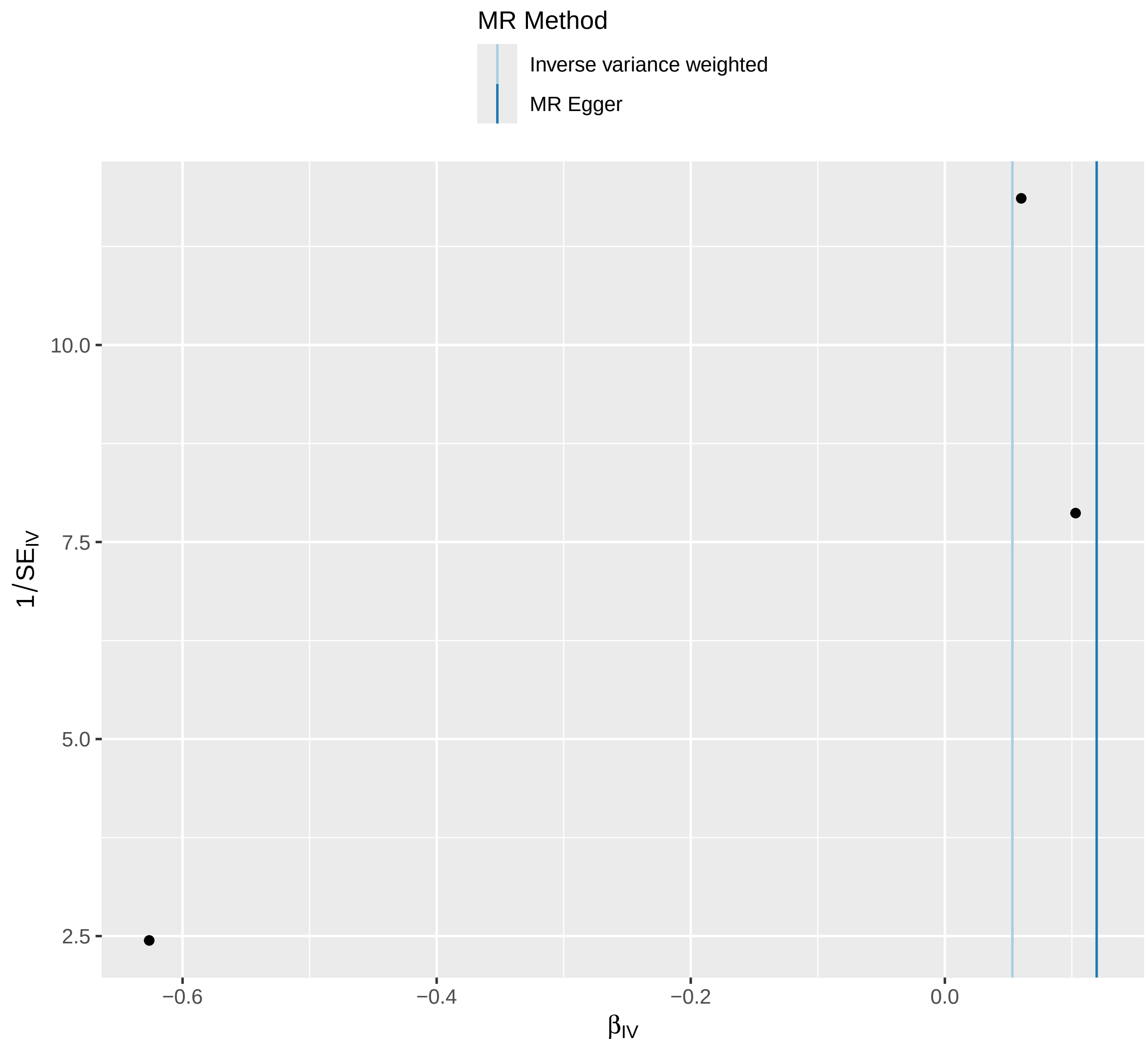

MR Method
Inverse variance weighted
MR Egger
10.0
7.5
5.0
2.5
1/SE_IV
−0.6
−0.4
−0.2
0.0
β_IV

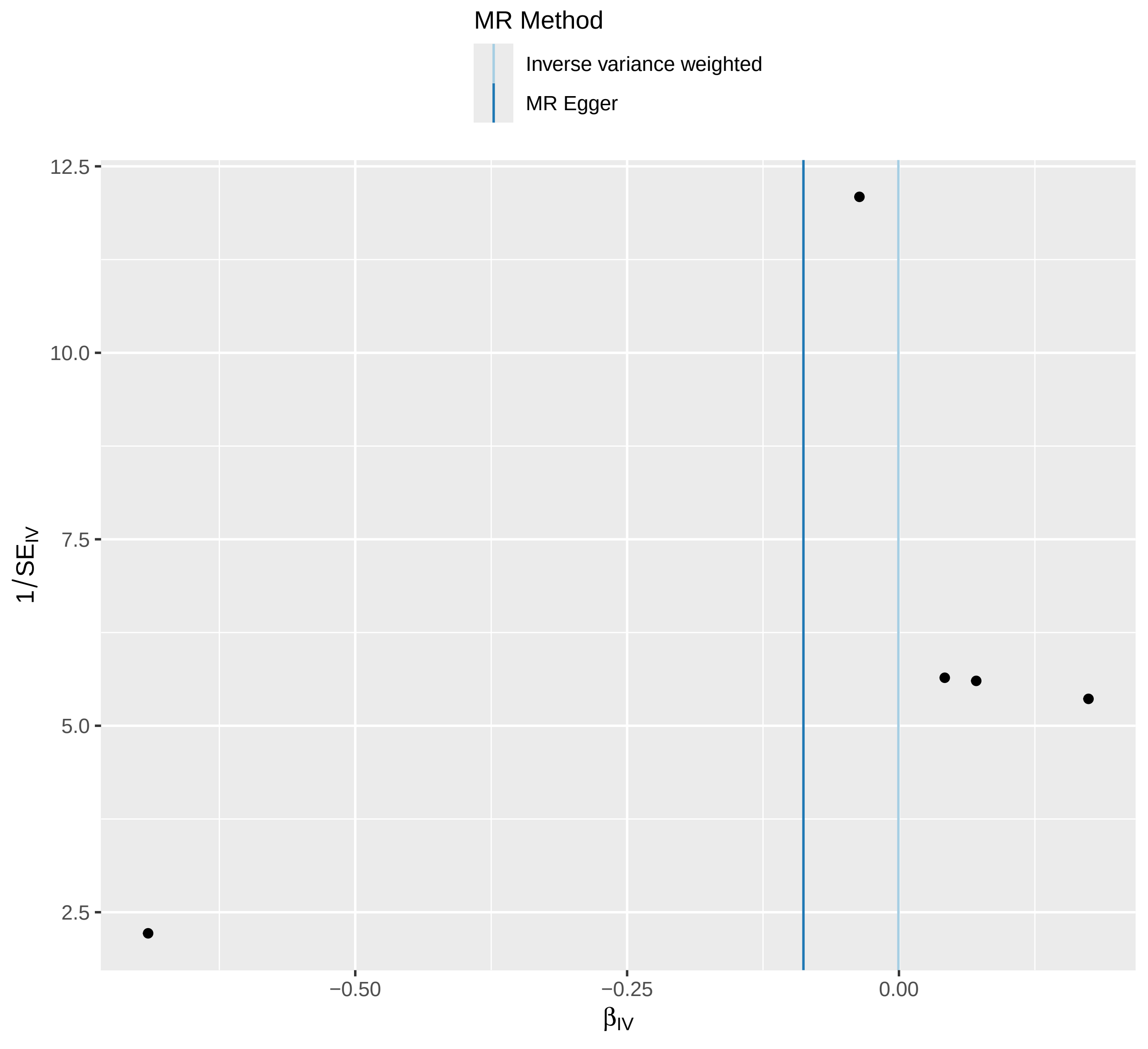
MR Method
Inverse variance weighted
MR Egger
12.5
10.0
7.5
5.0
2.5
1/SE_IV
−0.50
−0.25
0.00
β_IV

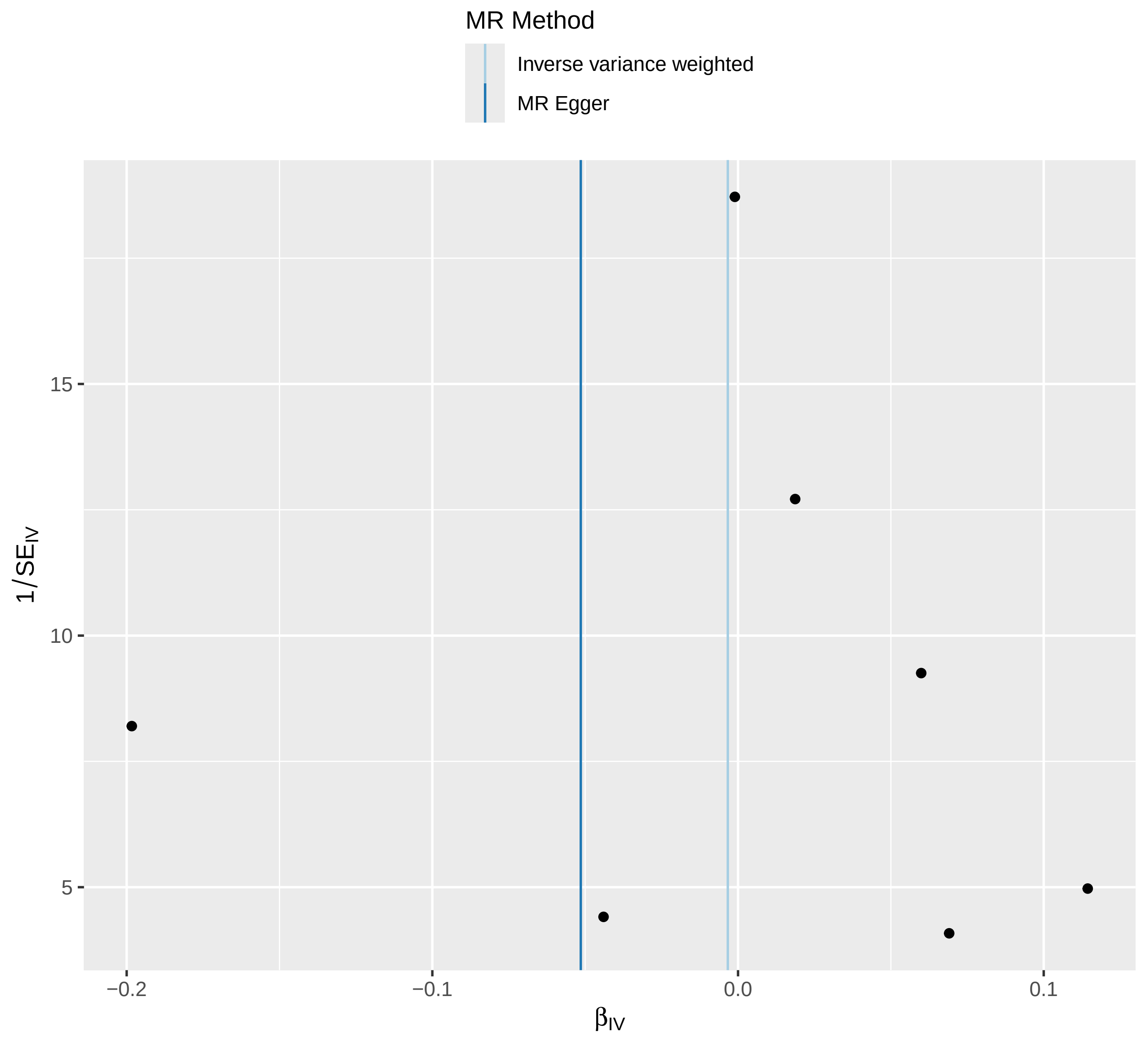
MR Method
Inverse variance weighted
MR Egger
15
10
5
1/SE_IV
−0.2
−0.1
0.0
0.1
β_IV

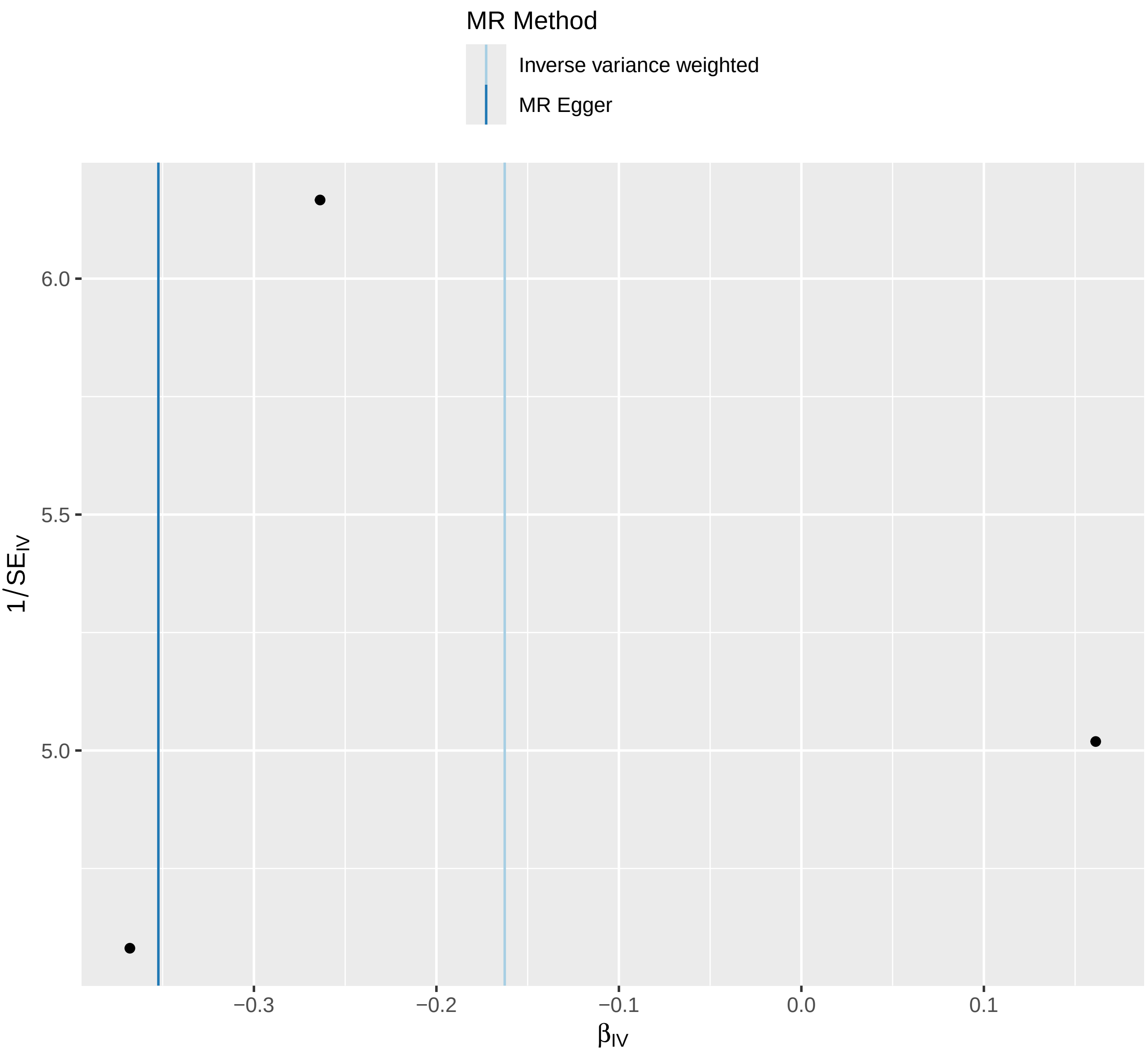

MR Method
Inverse variance weighted
MR Egger
6.0
5.5
5.0
1/SE$_{IV}$
−0.3
−0.2
−0.1
0.0
0.1
β$_{IV}$

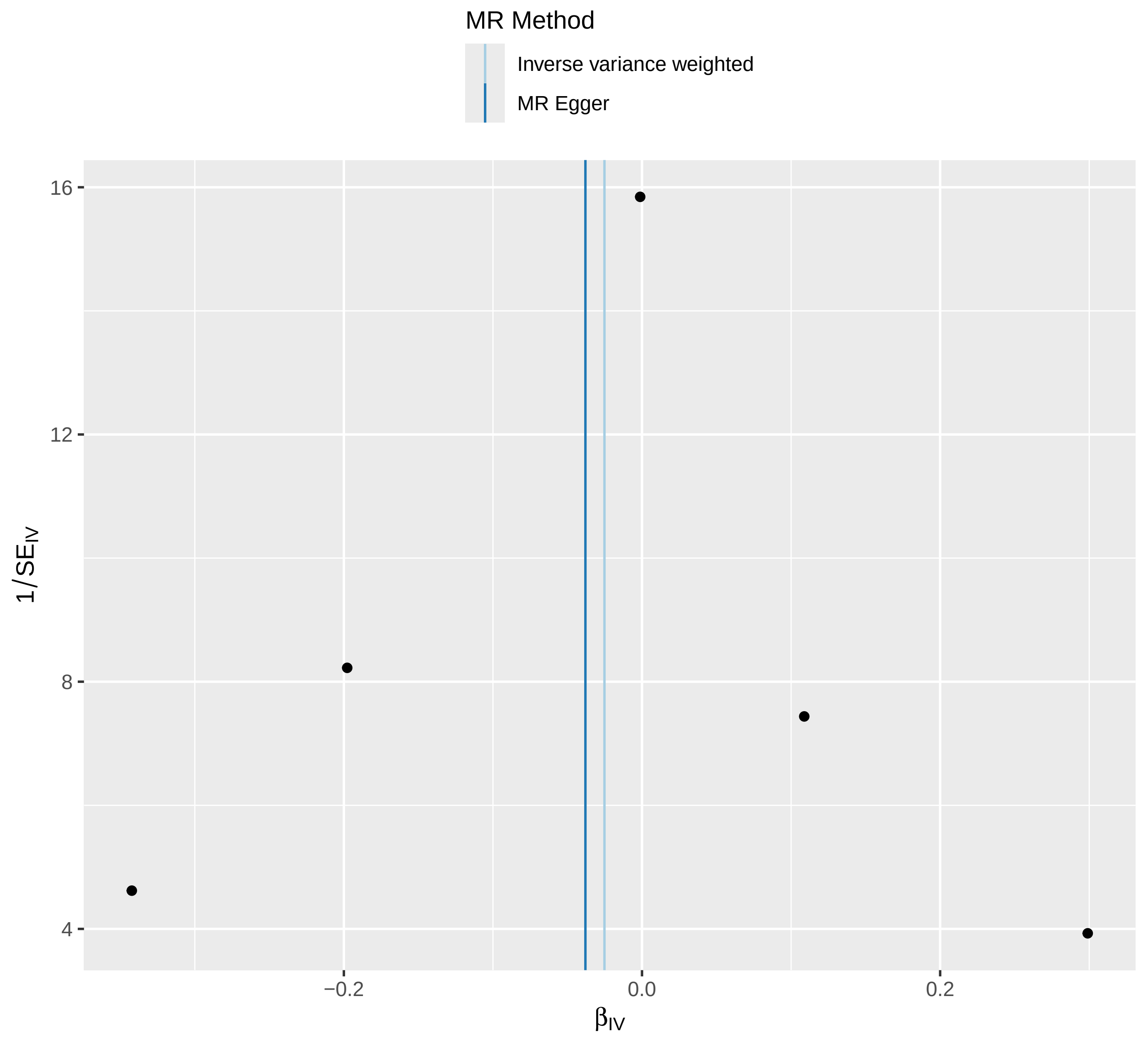
MR Method
Inverse variance weighted
MR Egger
16
12
8
4
1/SE$_{IV}$
−0.2
0.0
0.2
β$_{IV}$

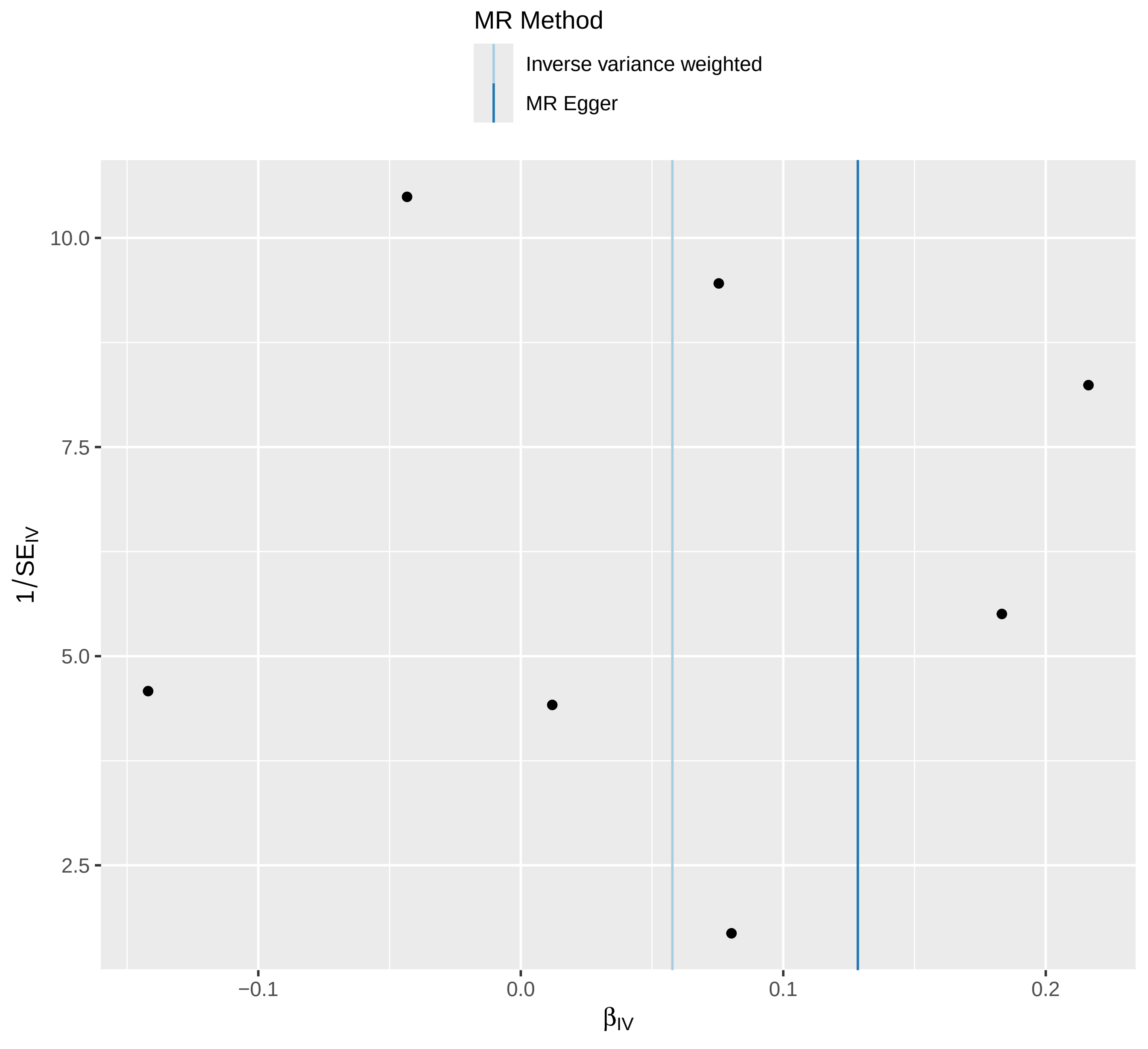
MR Method
Inverse variance weighted
MR Egger
10.0
7.5
5.0
2.5
$1/SE_{IV}$
−0.1
0.0
0.1
0.2
$\beta_{IV}$

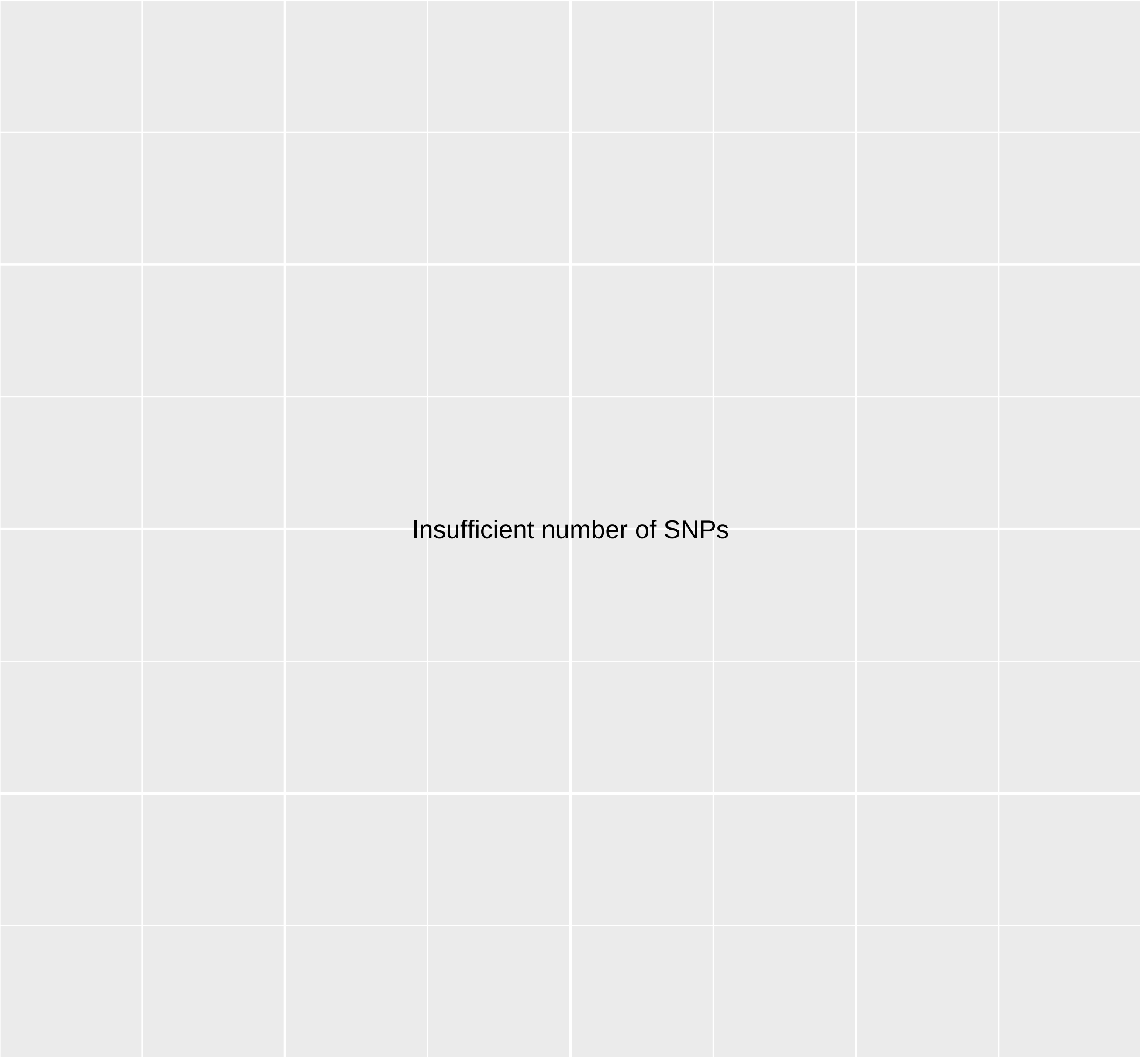
Insufficient number of SNPs

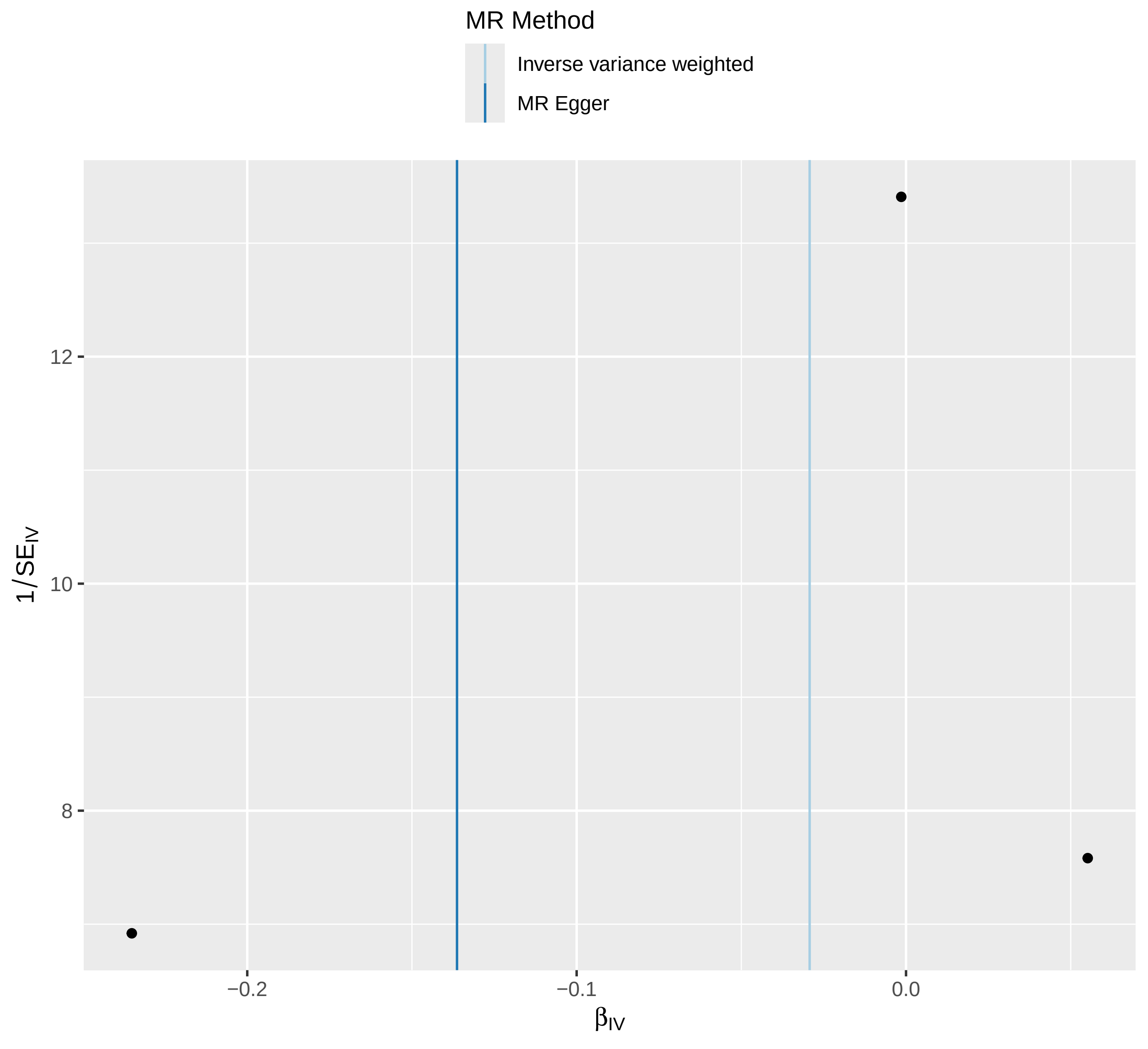
MR Method
Inverse variance weighted
MR Egger
12
10
8
1/SE$_{IV}$
−0.2
−0.1
0.0
β$_{IV}$

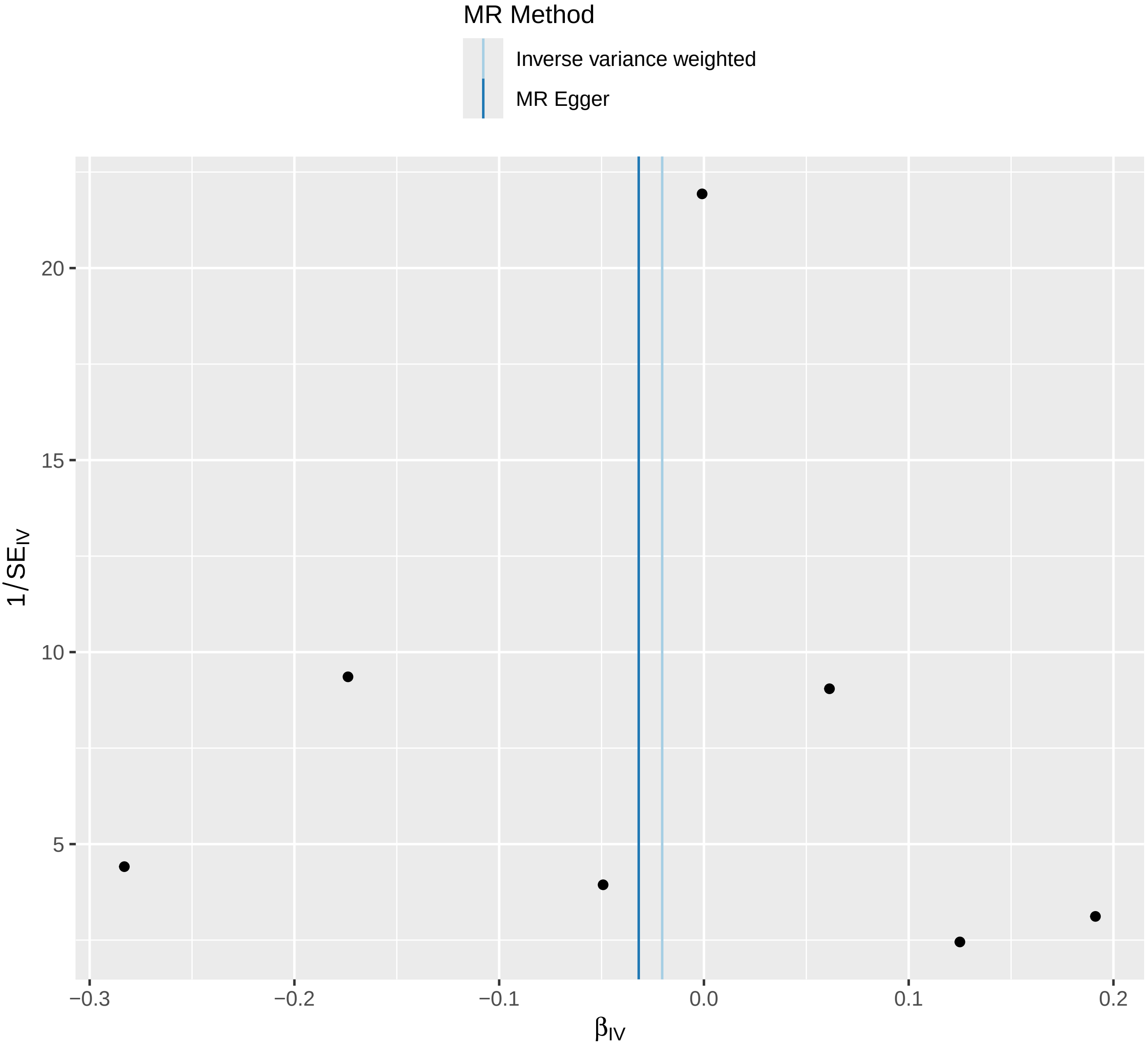
MR Method
Inverse variance weighted
MR Egger
1/SE$_{IV}$
β$_{IV}$
20
15
10
5
−0.3
−0.2
−0.1
0.0
0.1
0.2

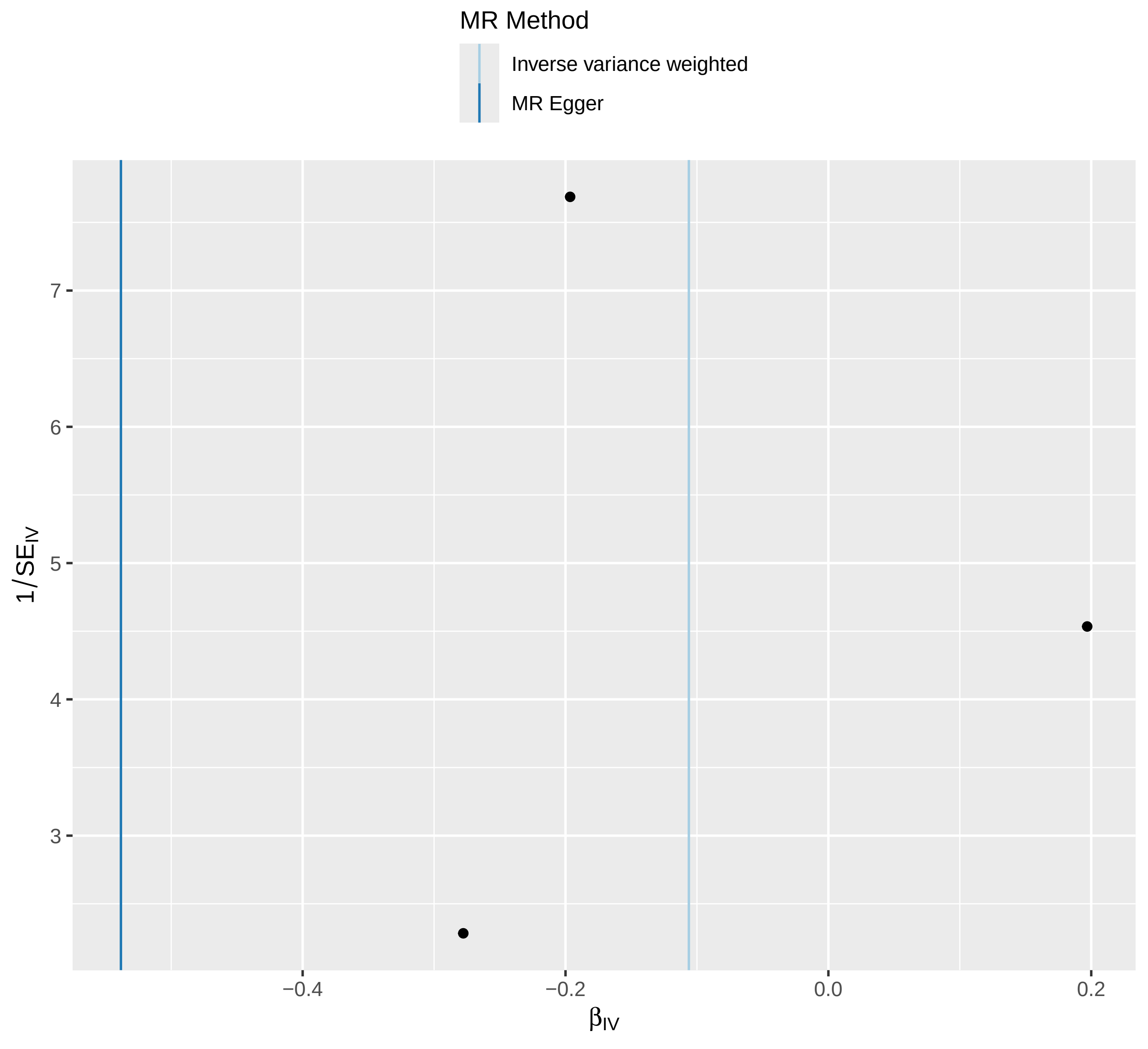
MR Method
Inverse variance weighted
MR Egger
7
6
5
4
3
1/SE$_{IV}$
−0.4
−0.2
0.0
0.2
β$_{IV}$

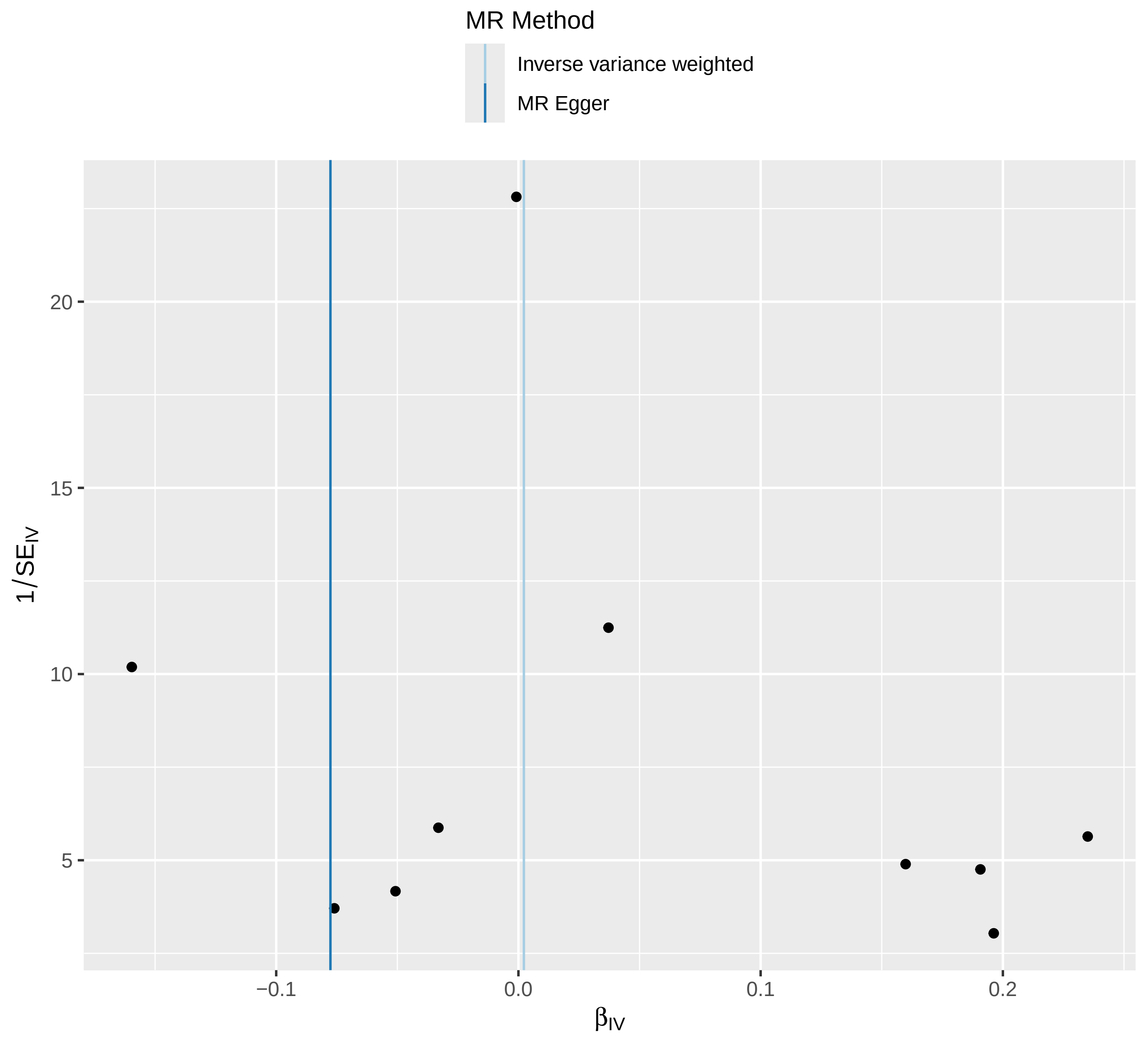

MR Method
Inverse variance weighted
MR Egger
20
15
10
5
1/SE$_{IV}$
−0.1
0.0
0.1
0.2
β$_{IV}$

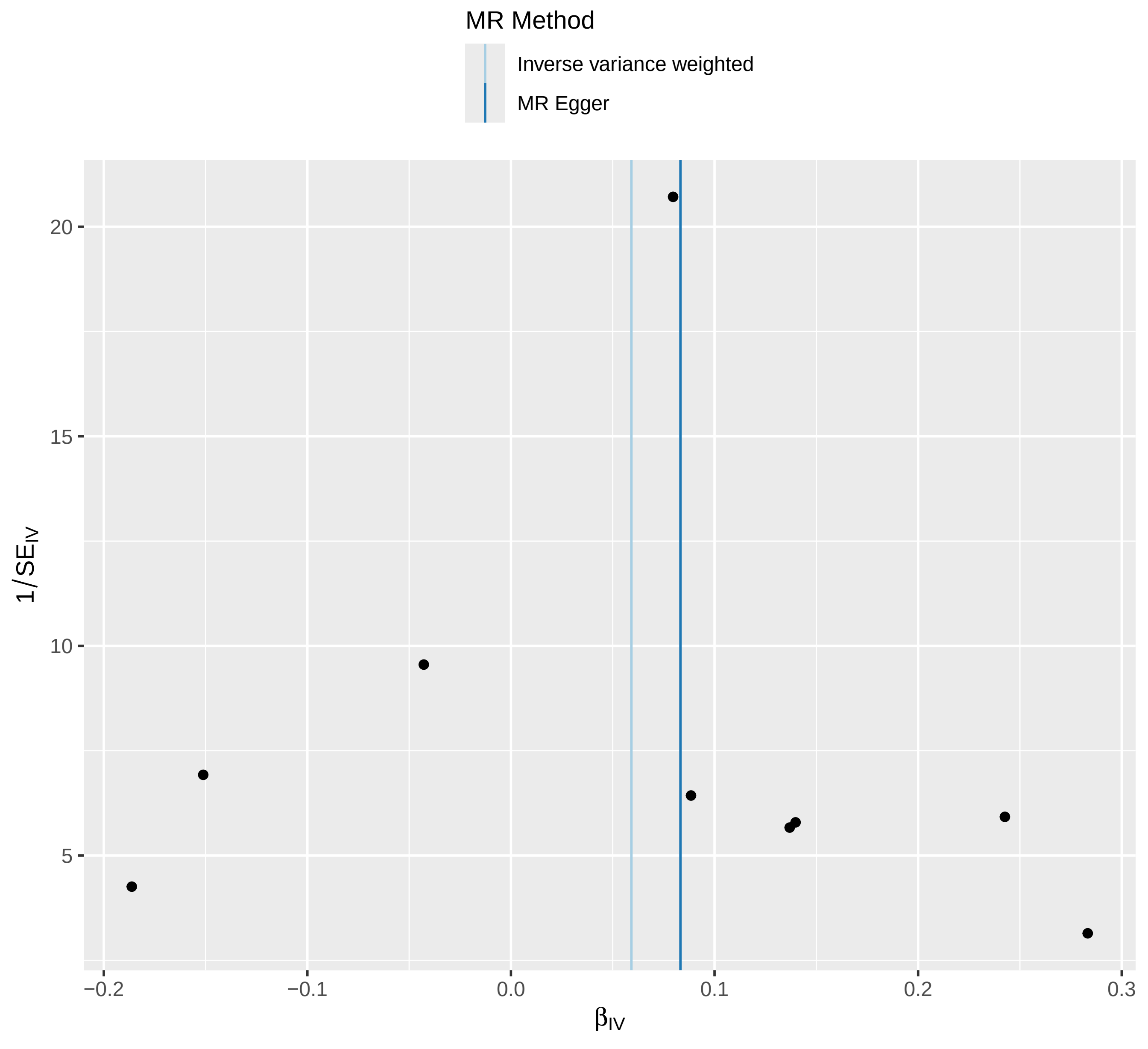
MR Method
Inverse variance weighted
MR Egger
$1/SE_{IV}$
20
15
10
5
−0.2
−0.1
0.0
0.1
0.2
0.3
$\beta_{IV}$

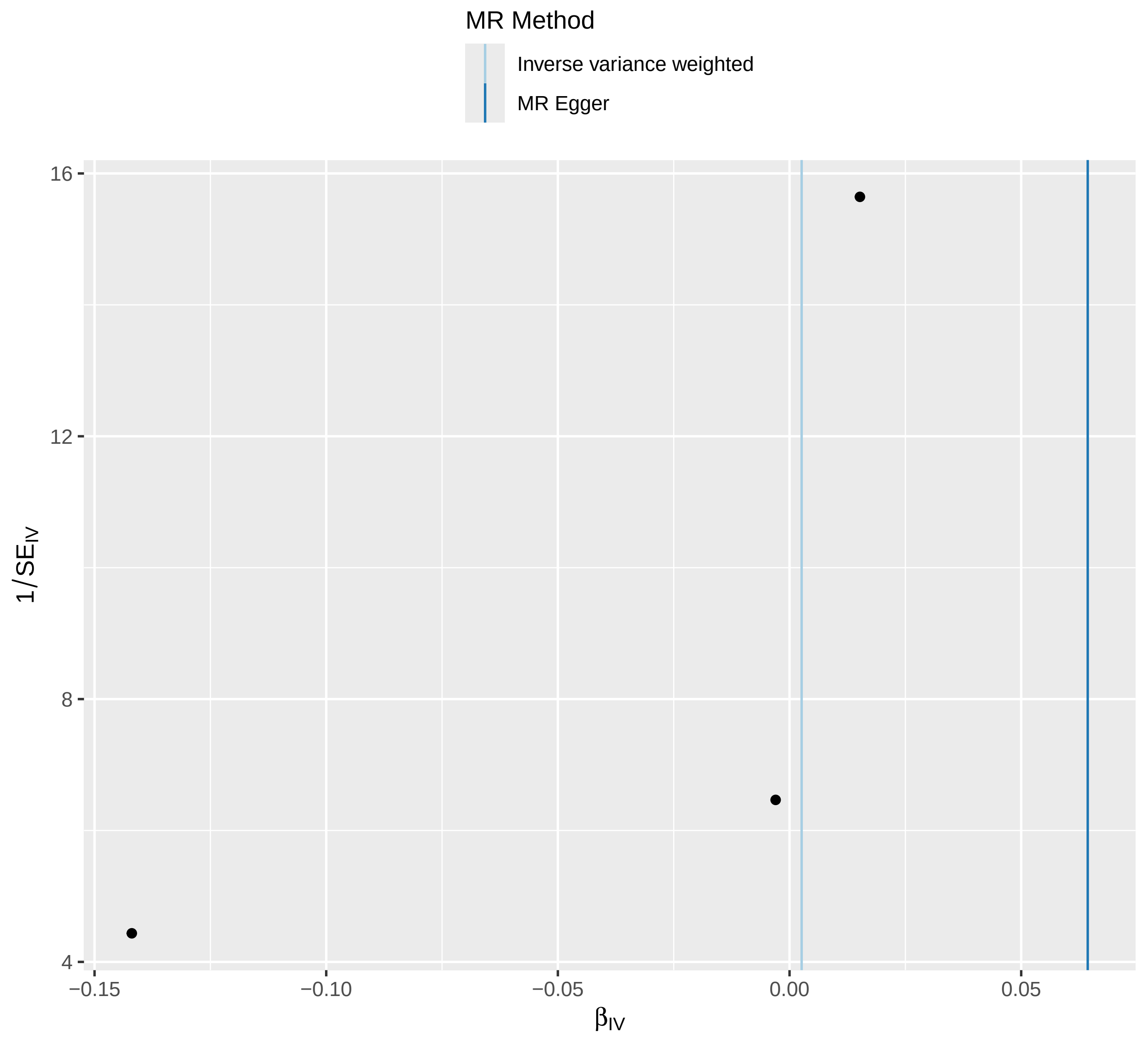

MR Method
Inverse variance weighted
MR Egger
16
12
8
4
$1/SE_{IV}$
−0.15
−0.10
−0.05
0.00
0.05
$\beta_{IV}$

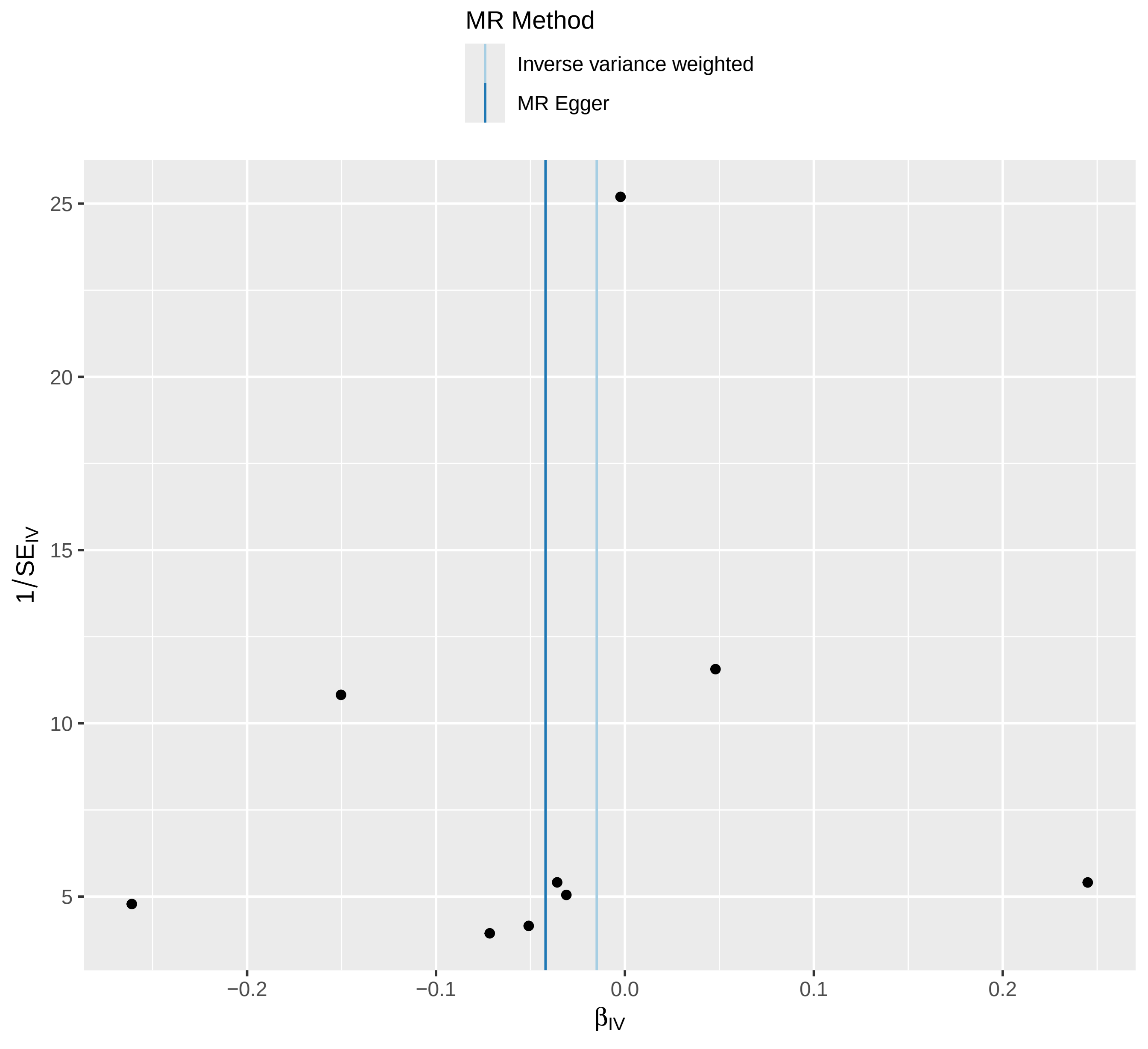

MR Method
Inverse variance weighted
MR Egger
25
20
15
10
5
$1/SE_{IV}$
−0.2
−0.1
0.0
0.1
0.2
$\beta_{IV}$

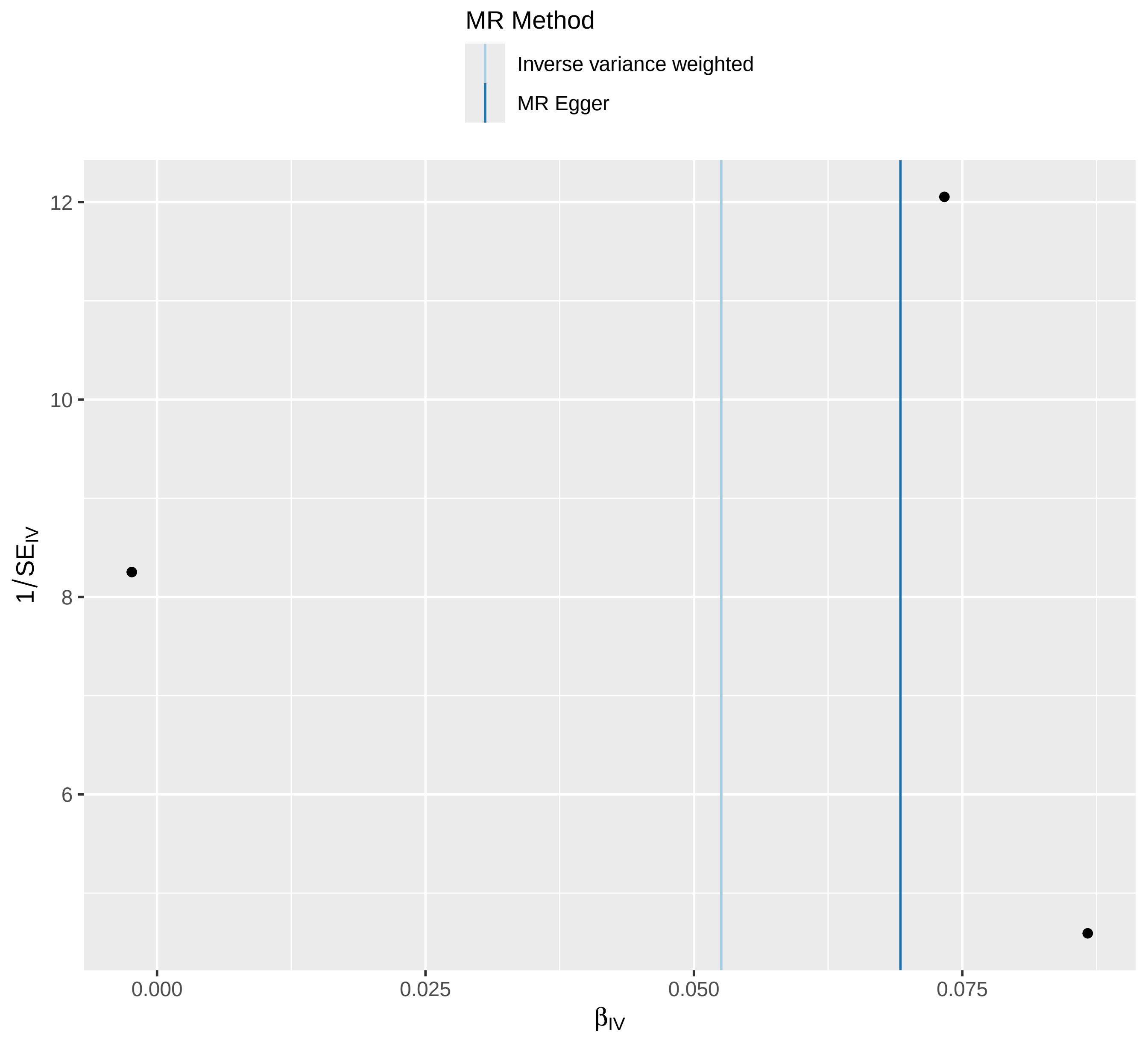
MR Method
Inverse variance weighted
MR Egger
12
10
8
6
0.000
0.025
0.050
0.075
$1/SE_{IV}$
$\beta_{IV}$

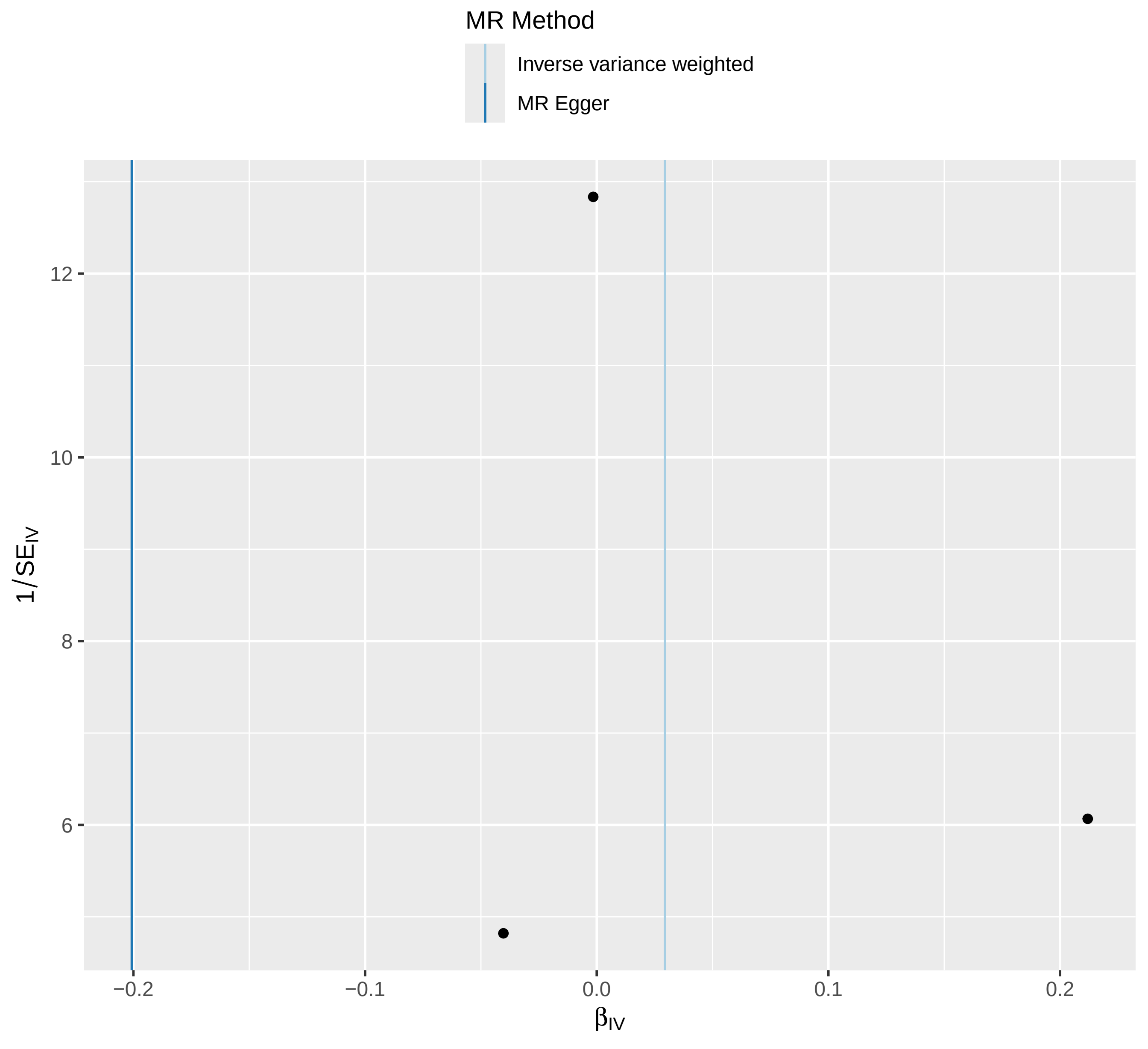
MR Method
Inverse variance weighted
MR Egger
1/SE$_{IV}$
12
10
8
6
−0.2
−0.1
0.0
0.1
0.2
β$_{IV}$

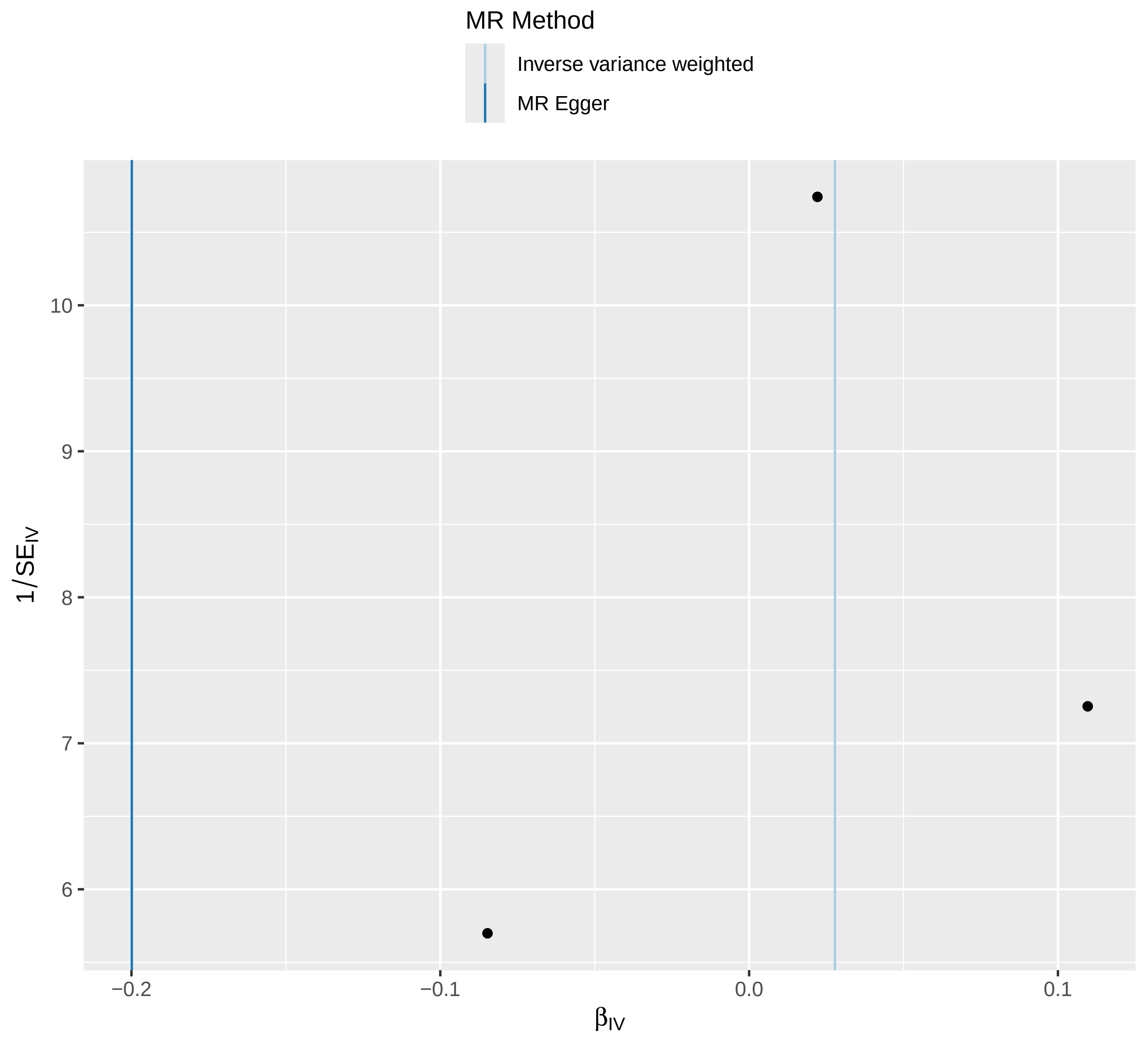
MR Method
Inverse variance weighted
MR Egger
10
9
8
7
6
−0.2
−0.1
0.0
0.1
$1/SE_{IV}$
$\beta_{IV}$

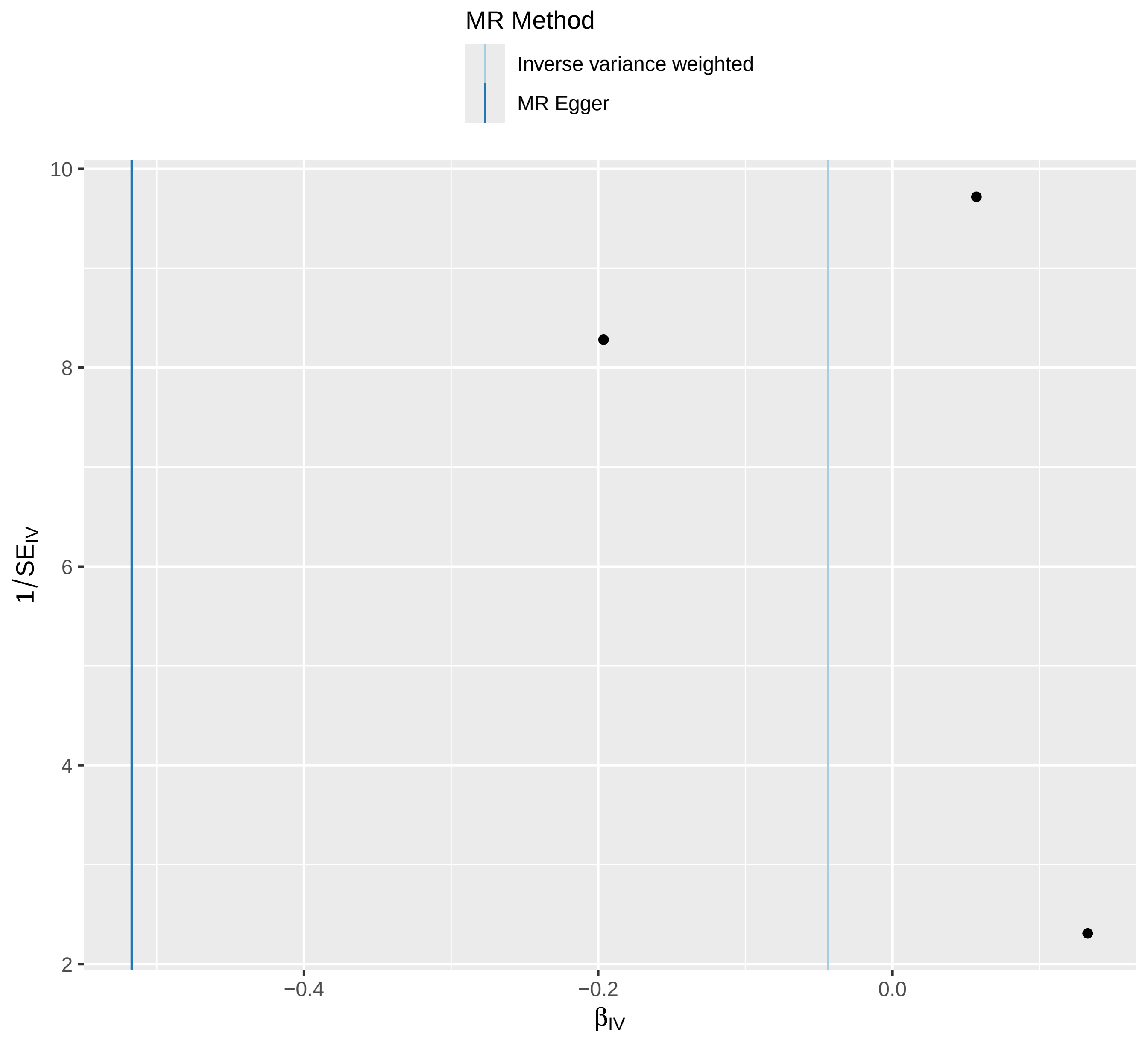
MR Method
Inverse variance weighted
MR Egger
10
8
6
4
2
$1/SE_{IV}$
−0.4
−0.2
0.0
$\beta_{IV}$

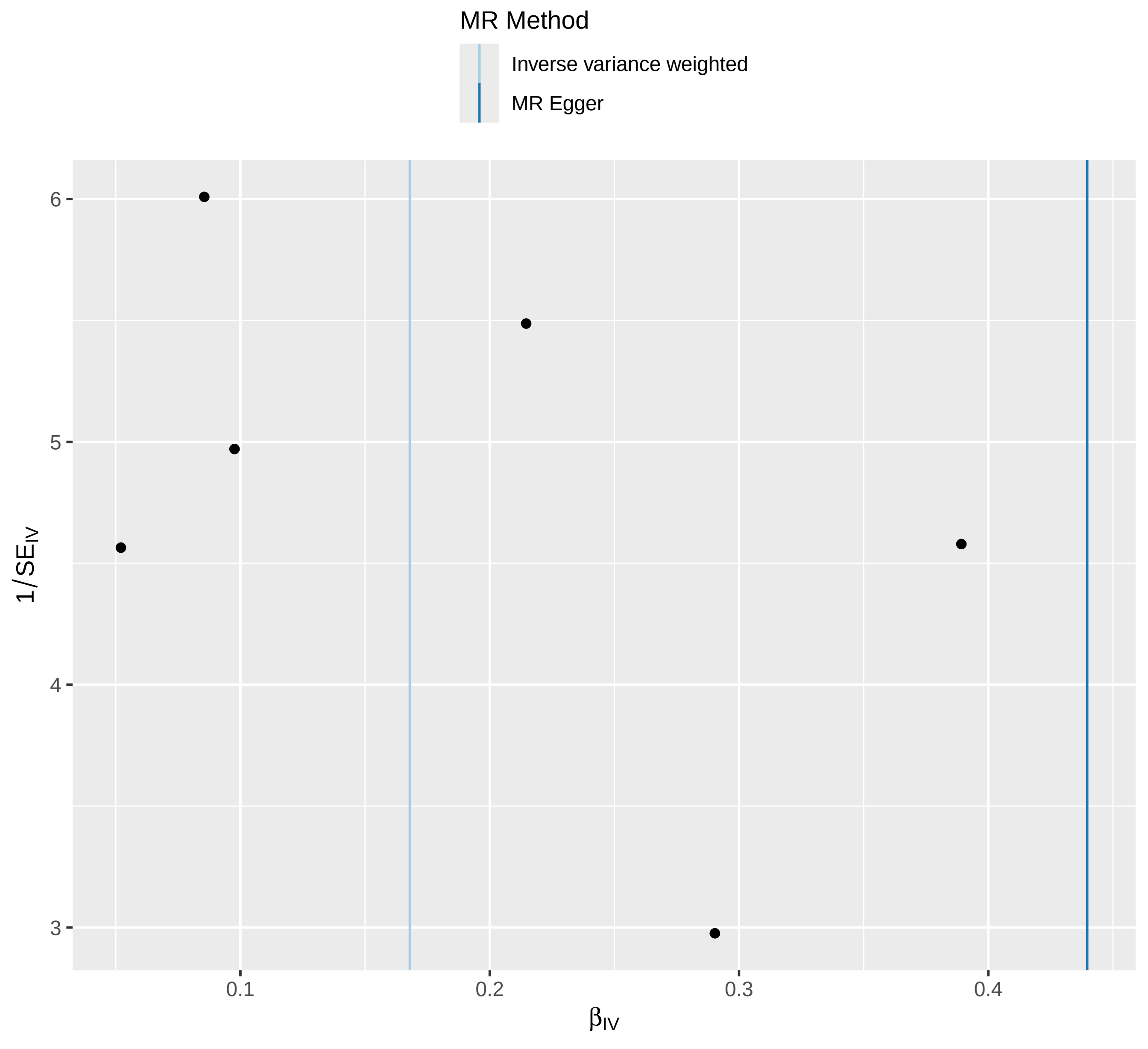
MR Method
Inverse variance weighted
MR Egger
6
5
4
3
0.1
0.2
0.3
0.4
$1/SE_{IV}$
$\beta_{IV}$

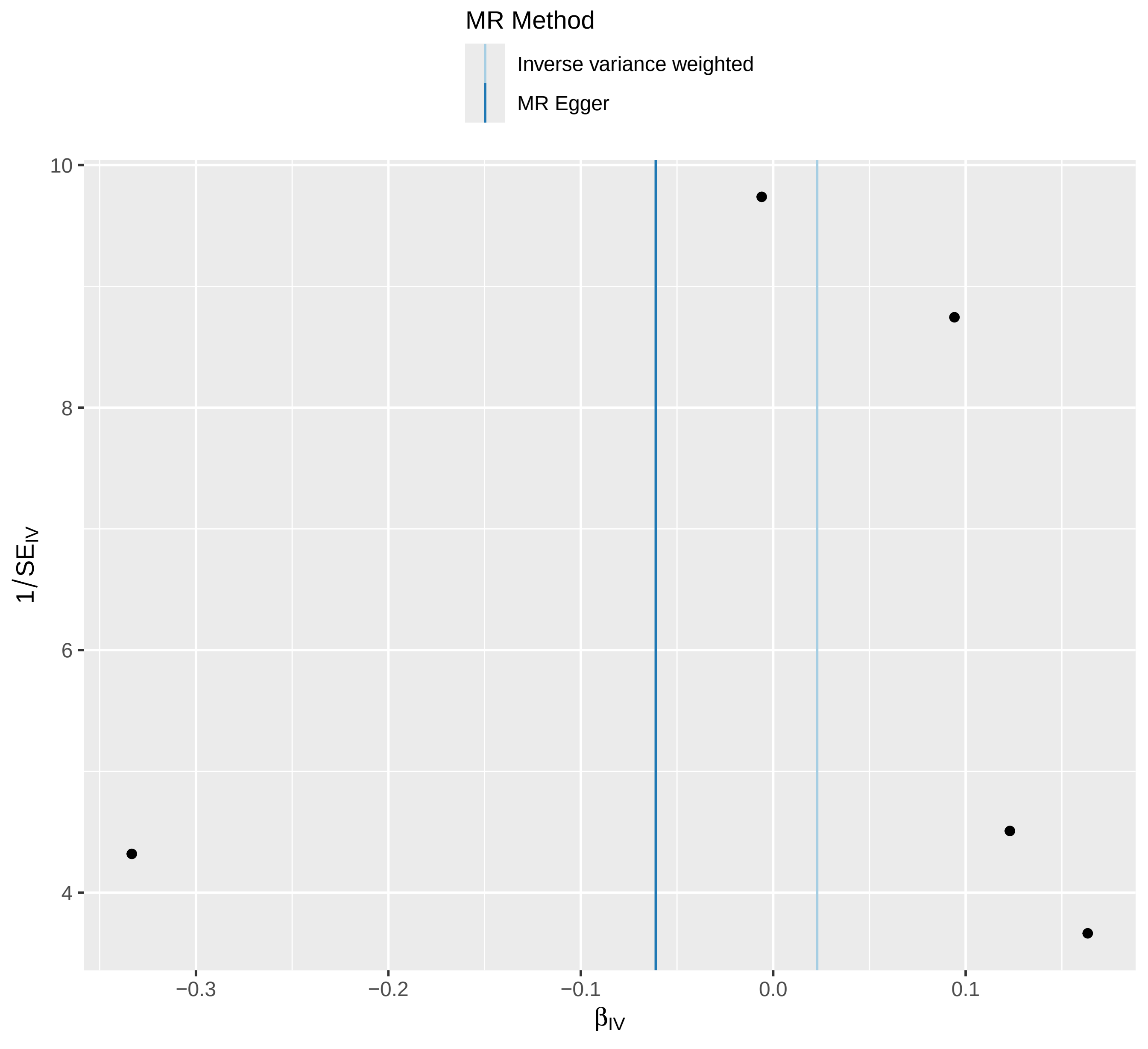

MR Method
Inverse variance weighted
MR Egger
10
8
6
4
1/SE_IV
−0.3
−0.2
−0.1
0.0
0.1
β_IV

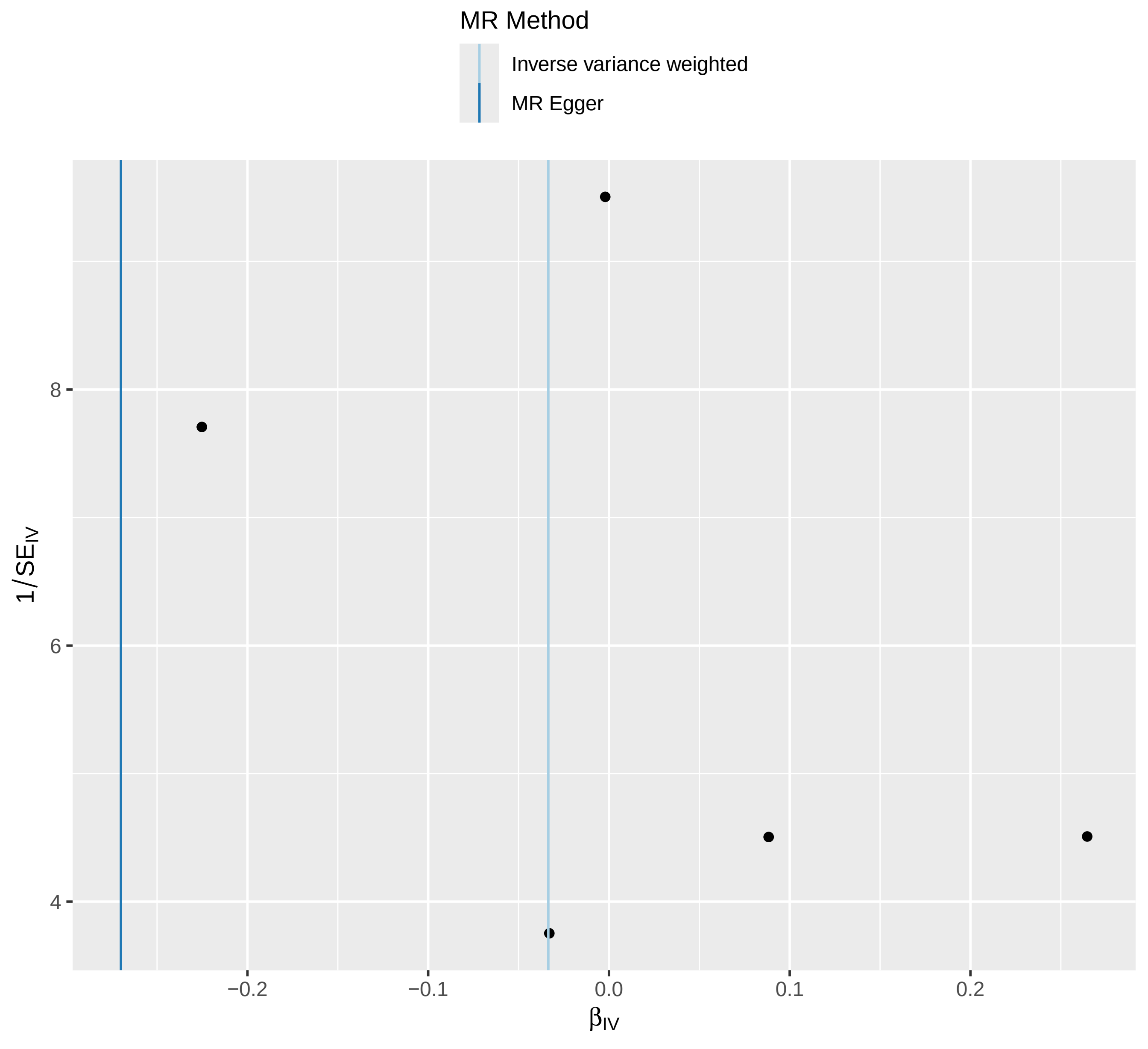

MR Method
Inverse variance weighted
MR Egger
8
6
4
1/SE$_{IV}$
−0.2
−0.1
0.0
0.1
0.2
β$_{IV}$

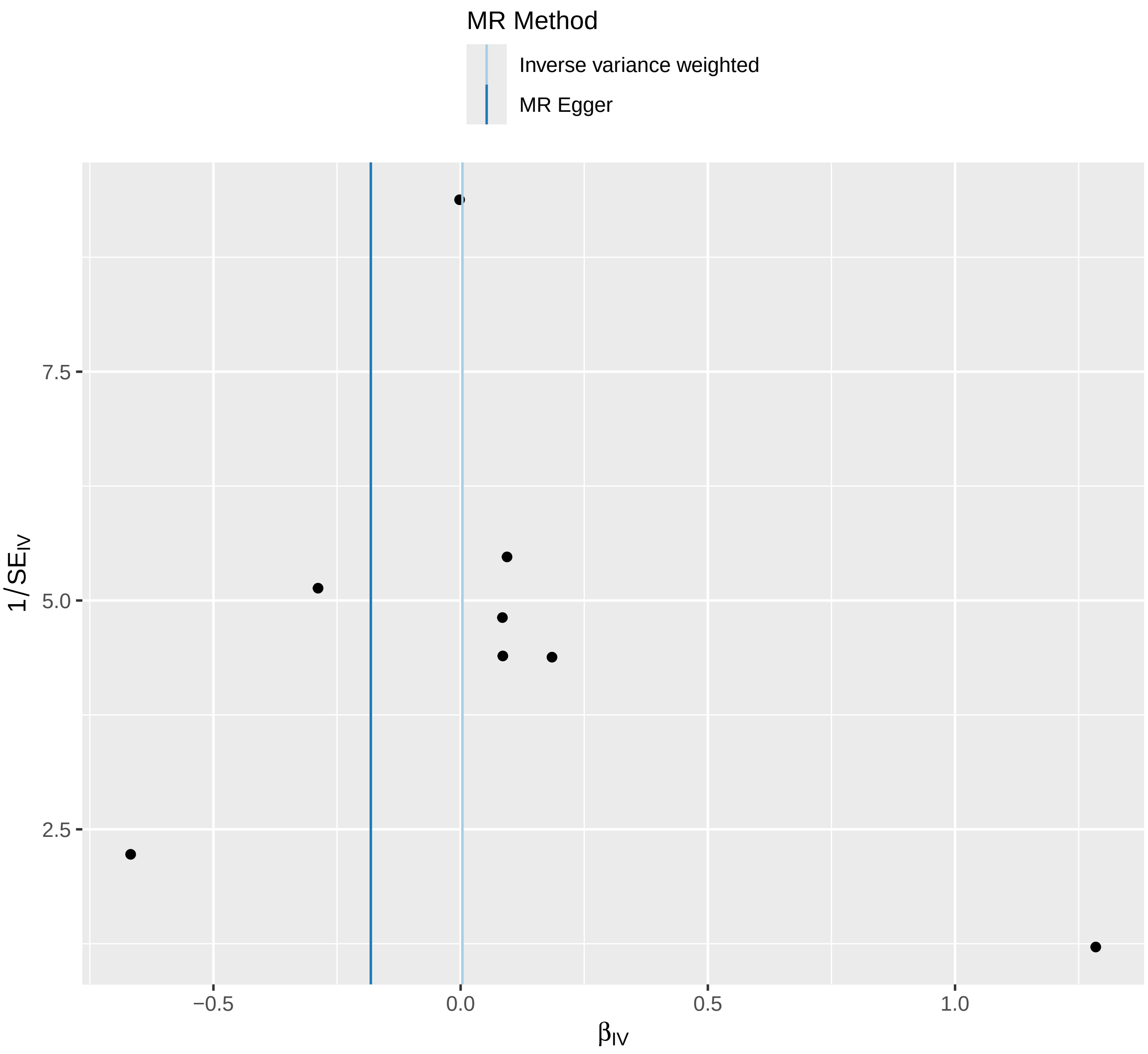
MR Method
Inverse variance weighted
MR Egger
7.5
5.0
2.5
1/SE$_{IV}$
−0.5
0.0
0.5
1.0
β$_{IV}$

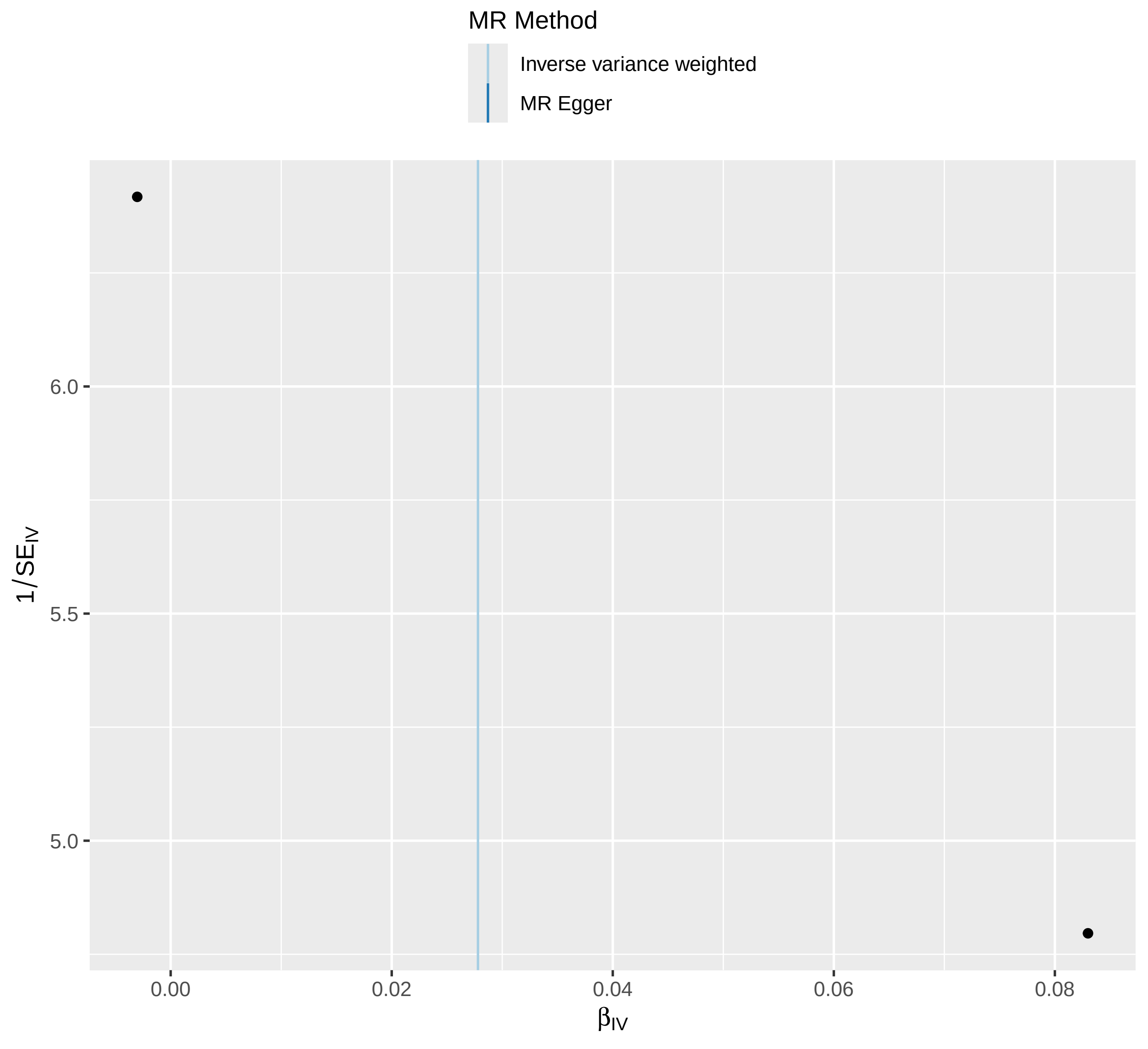
MR Method
Inverse variance weighted
MR Egger
6.0
5.5
5.0
0.00
0.02
0.04
0.06
0.08
$1/SE_{IV}$
$\beta_{IV}$

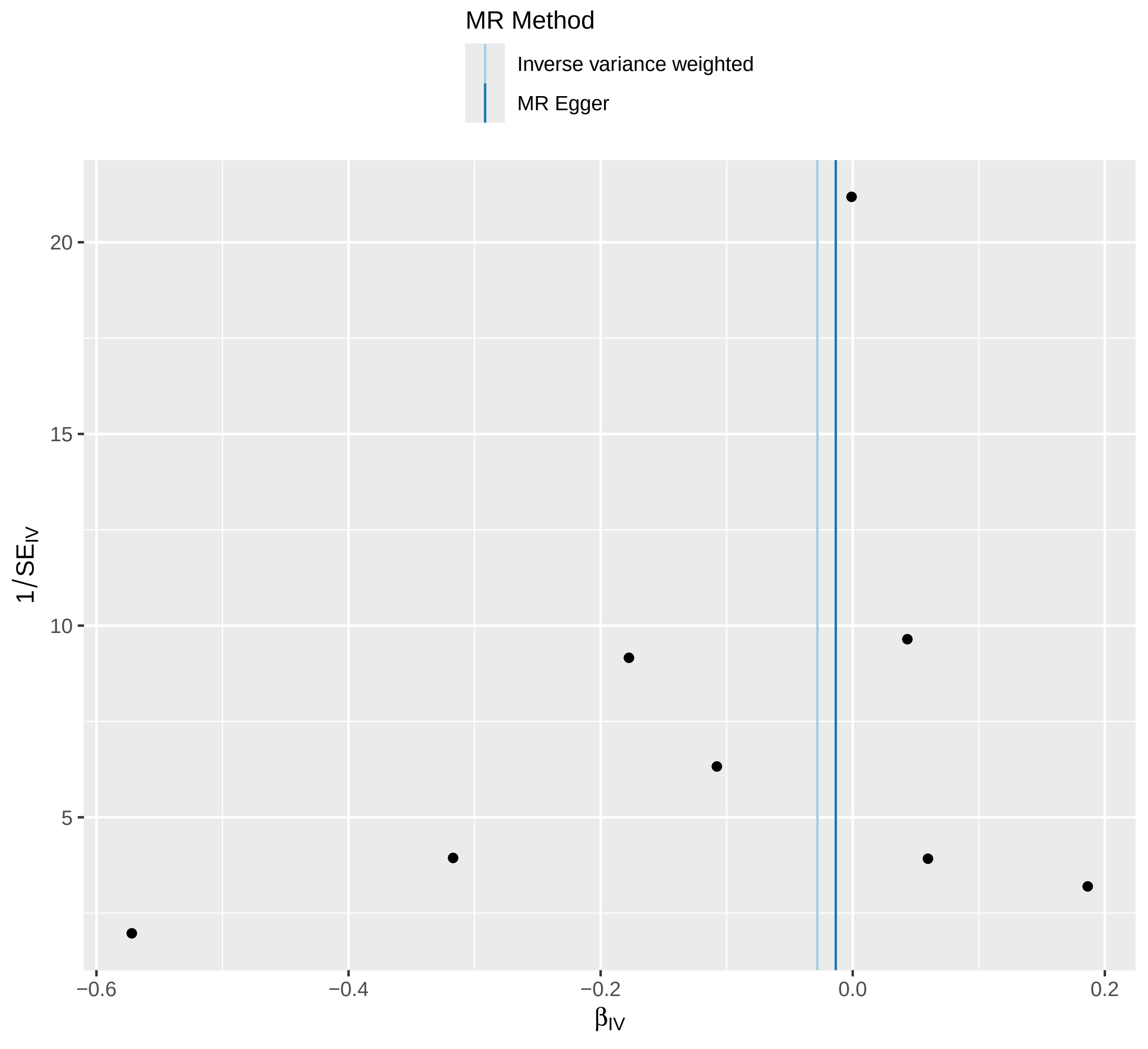

MR Method
Inverse variance weighted
MR Egger
20
15
10
5
1/SE_IV
−0.6
−0.4
−0.2
0.0
0.2
β_IV

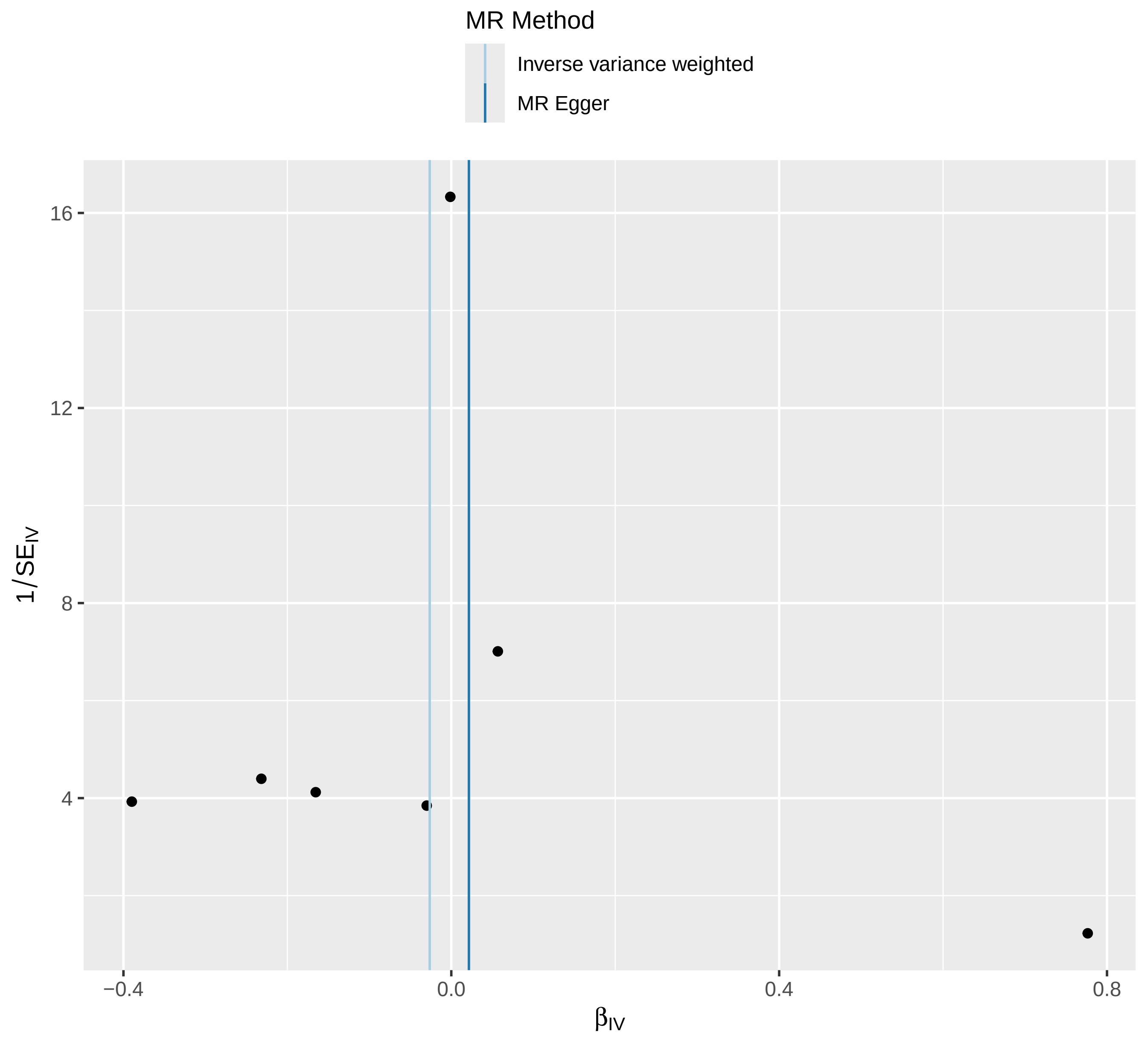
MR Method
Inverse variance weighted
MR Egger
16
12
8
4
1/SE_IV
−0.4
0.0
0.4
0.8
β_IV

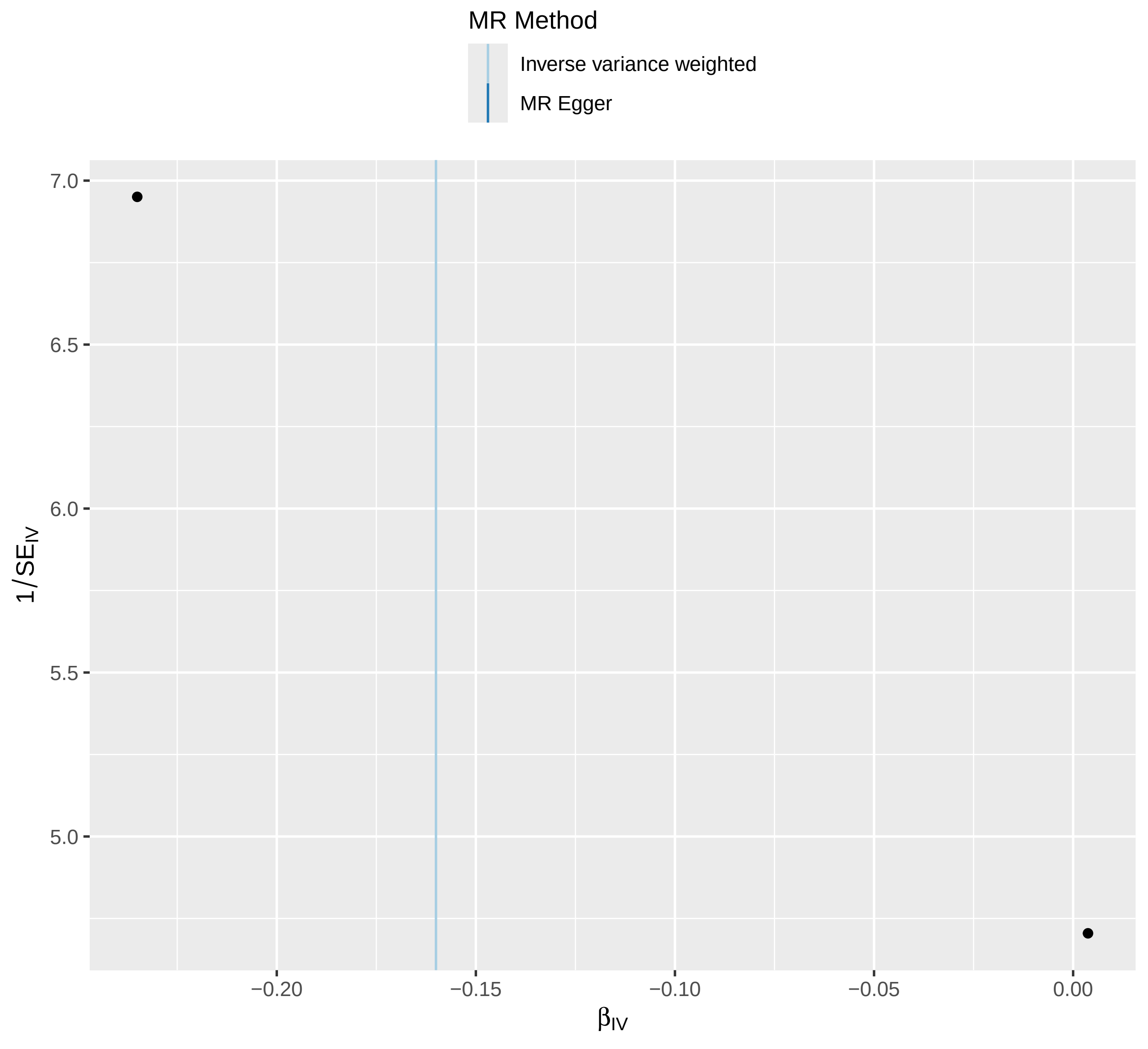
MR Method
Inverse variance weighted
MR Egger
7.0
6.5
6.0
5.5
5.0
1/SE$_{IV}$
−0.20
−0.15
−0.10
−0.05
0.00
β$_{IV}$

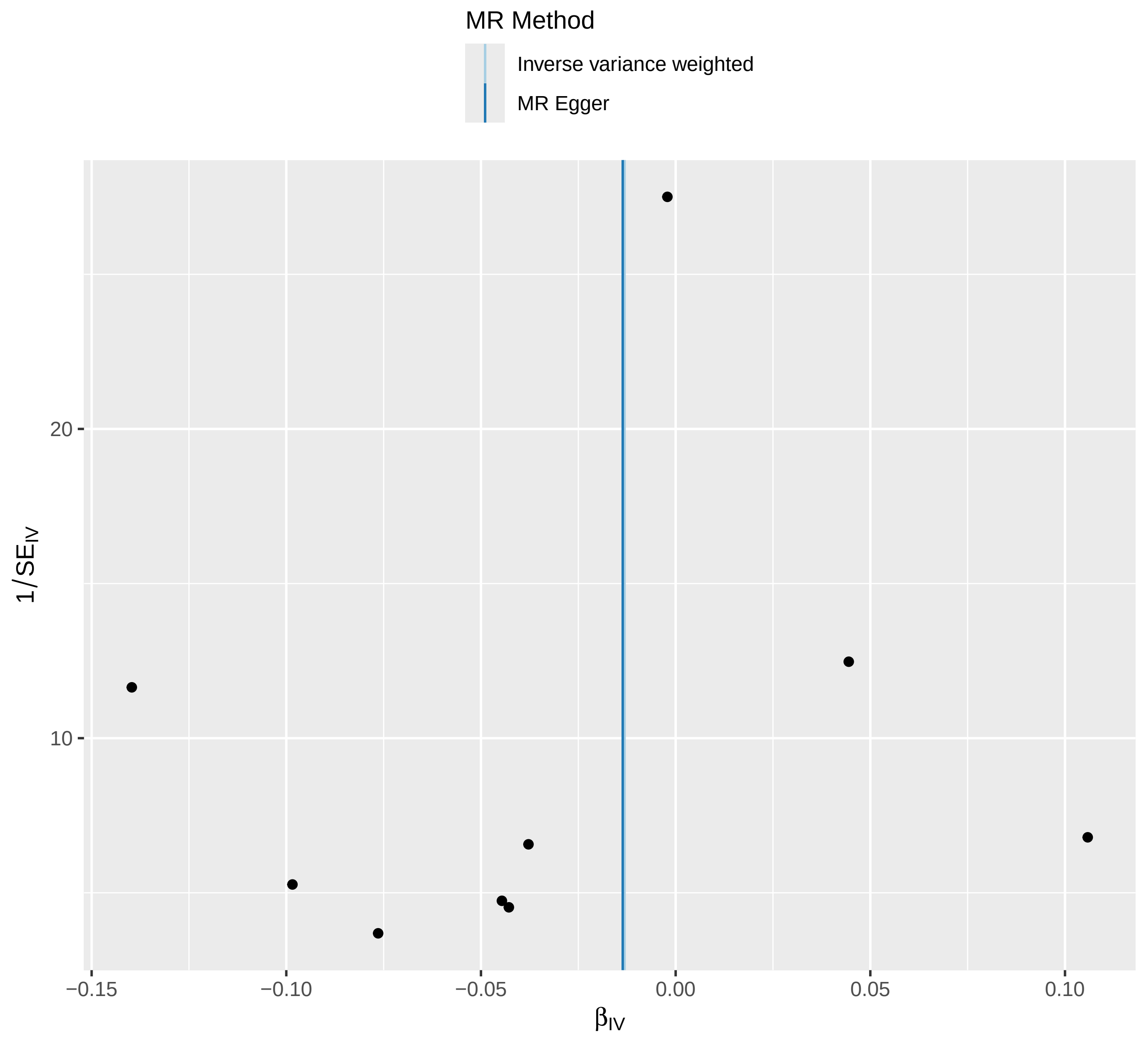

MR Method
Inverse variance weighted
MR Egger
20
10
$1/SE_{IV}$
−0.15
−0.10
−0.05
0.00
0.05
0.10
$\beta_{IV}$

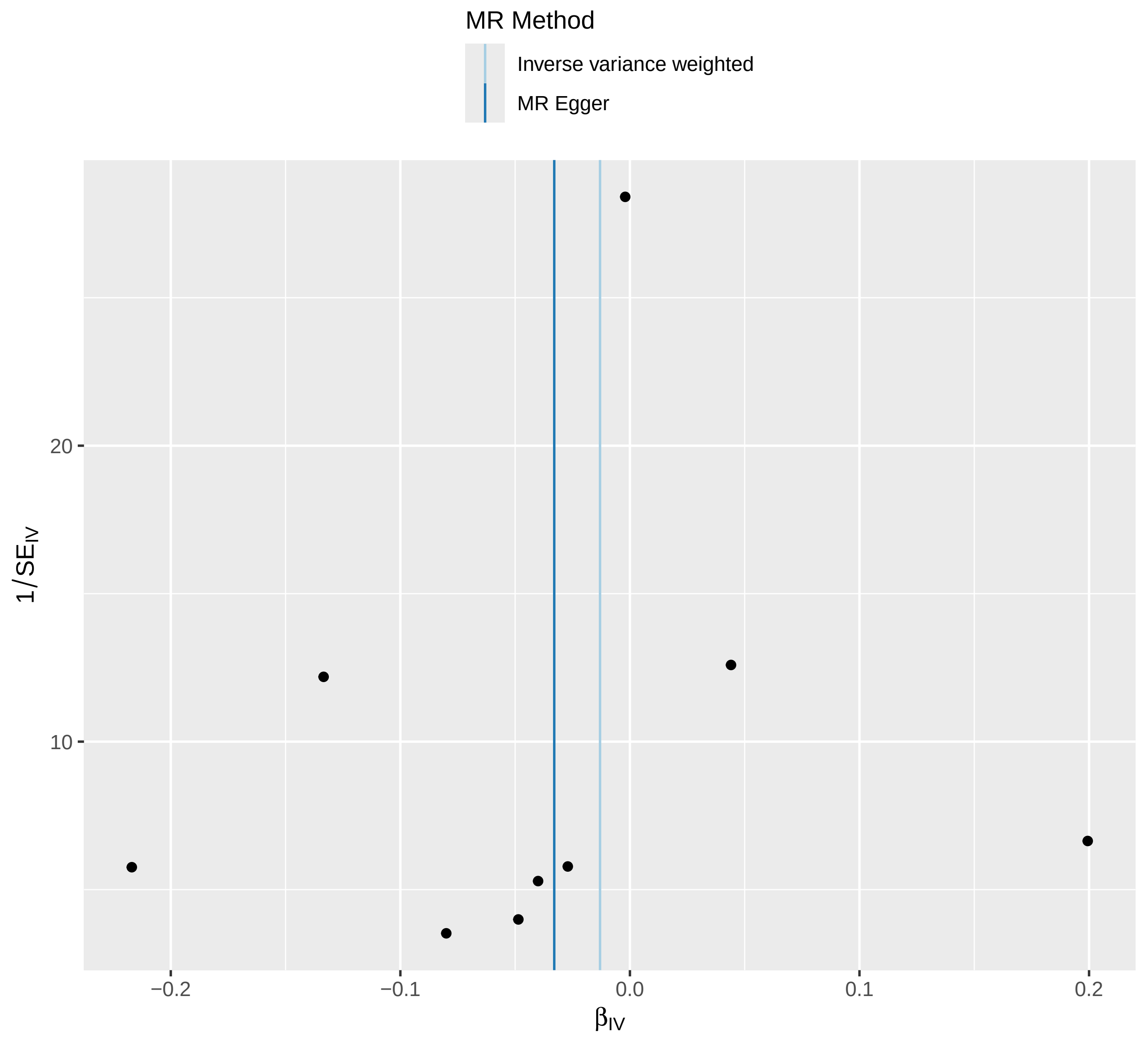

MR Method
Inverse variance weighted
MR Egger
20
10
1/SE$_{IV}$
−0.2
−0.1
0.0
0.1
0.2
β$_{IV}$

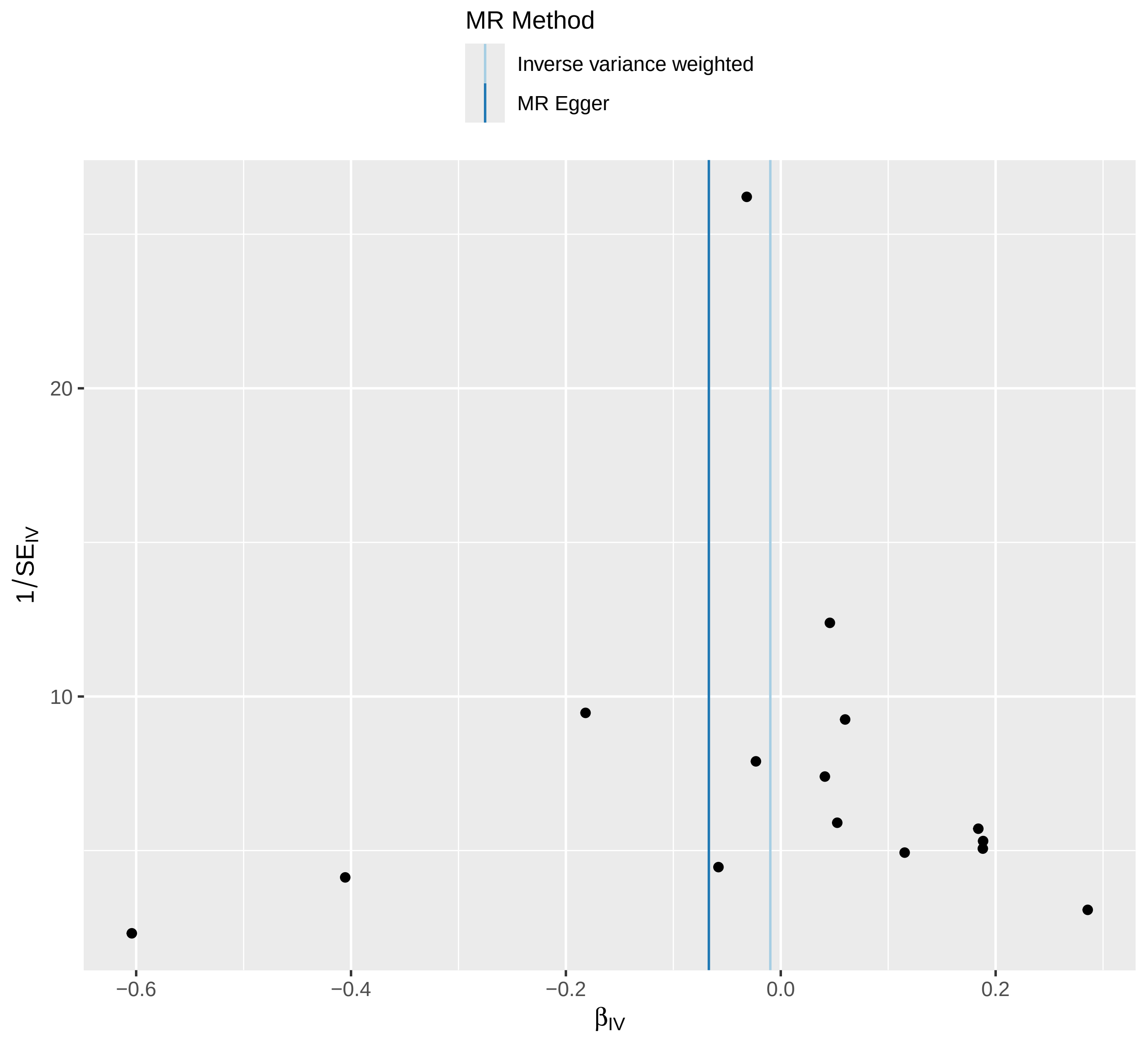
MR Method
Inverse variance weighted
MR Egger
20
10
1/SE$_{IV}$
−0.6
−0.4
−0.2
0.0
0.2
β$_{IV}$

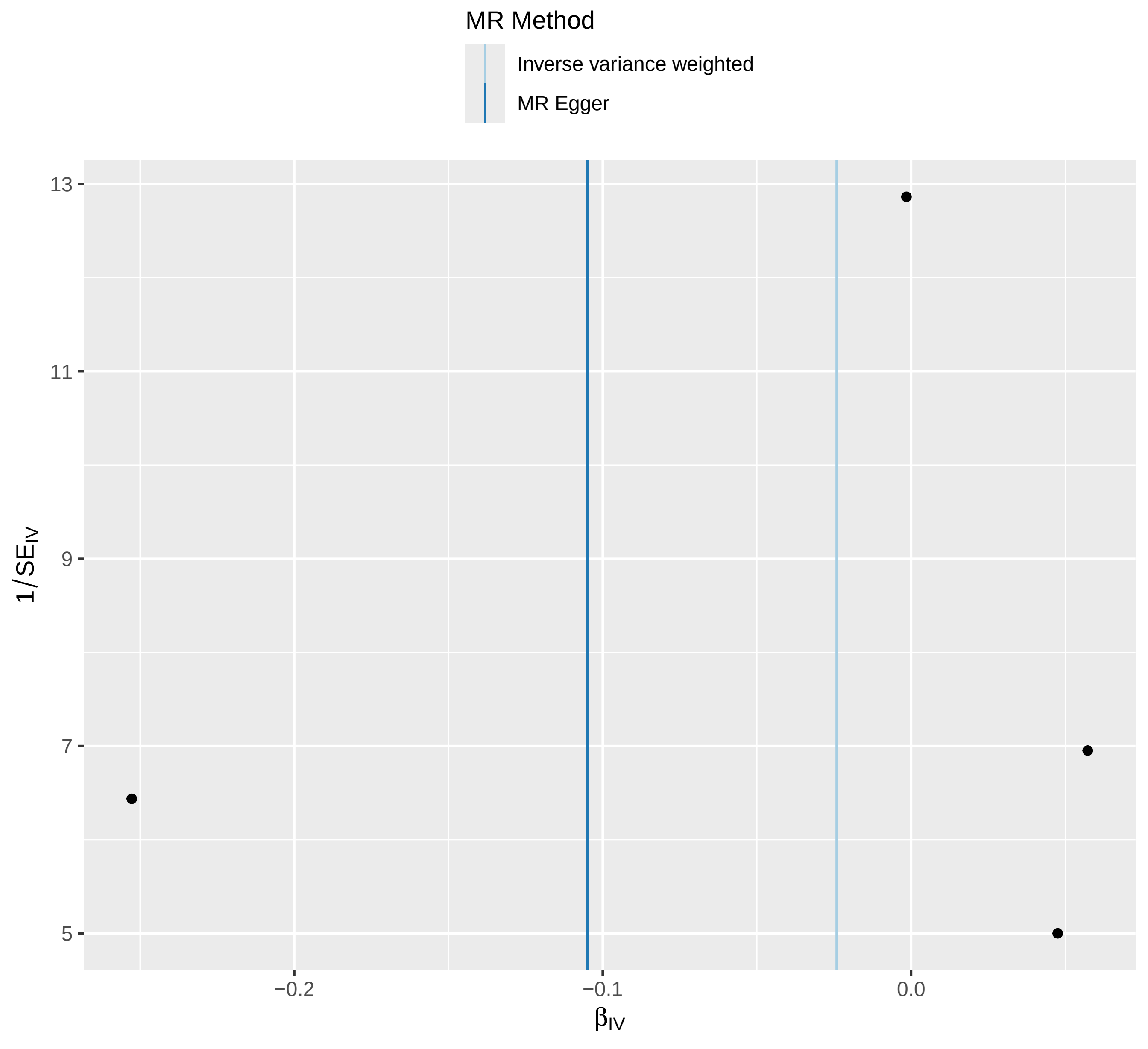

MR Method
Inverse variance weighted
MR Egger
13
11
9
7
5
1/SE_IV
−0.2
−0.1
0.0
β_IV

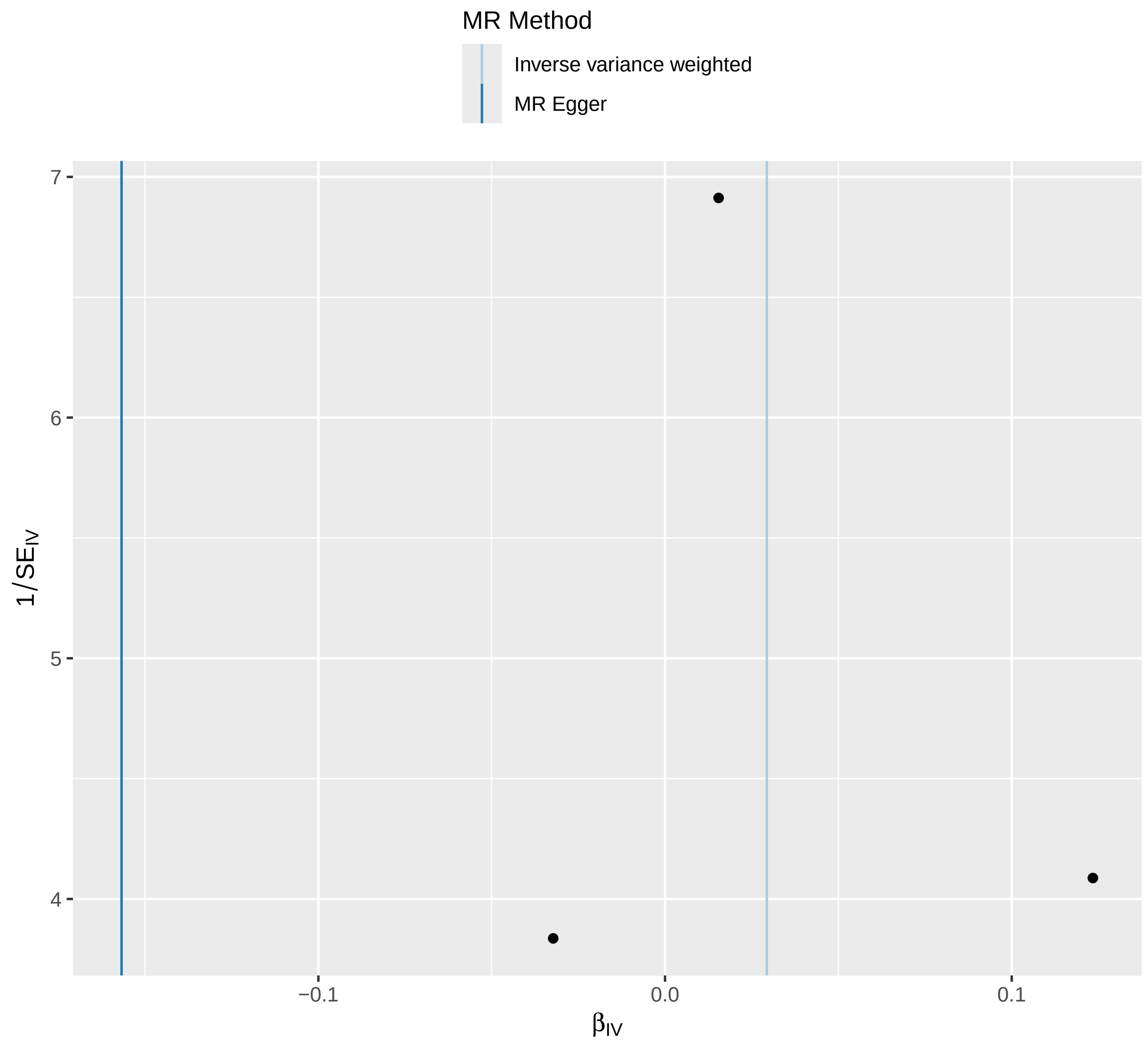

MR Method
Inverse variance weighted
MR Egger
7
6
5
4
1/SE_IV
−0.1
0.0
0.1
β_IV

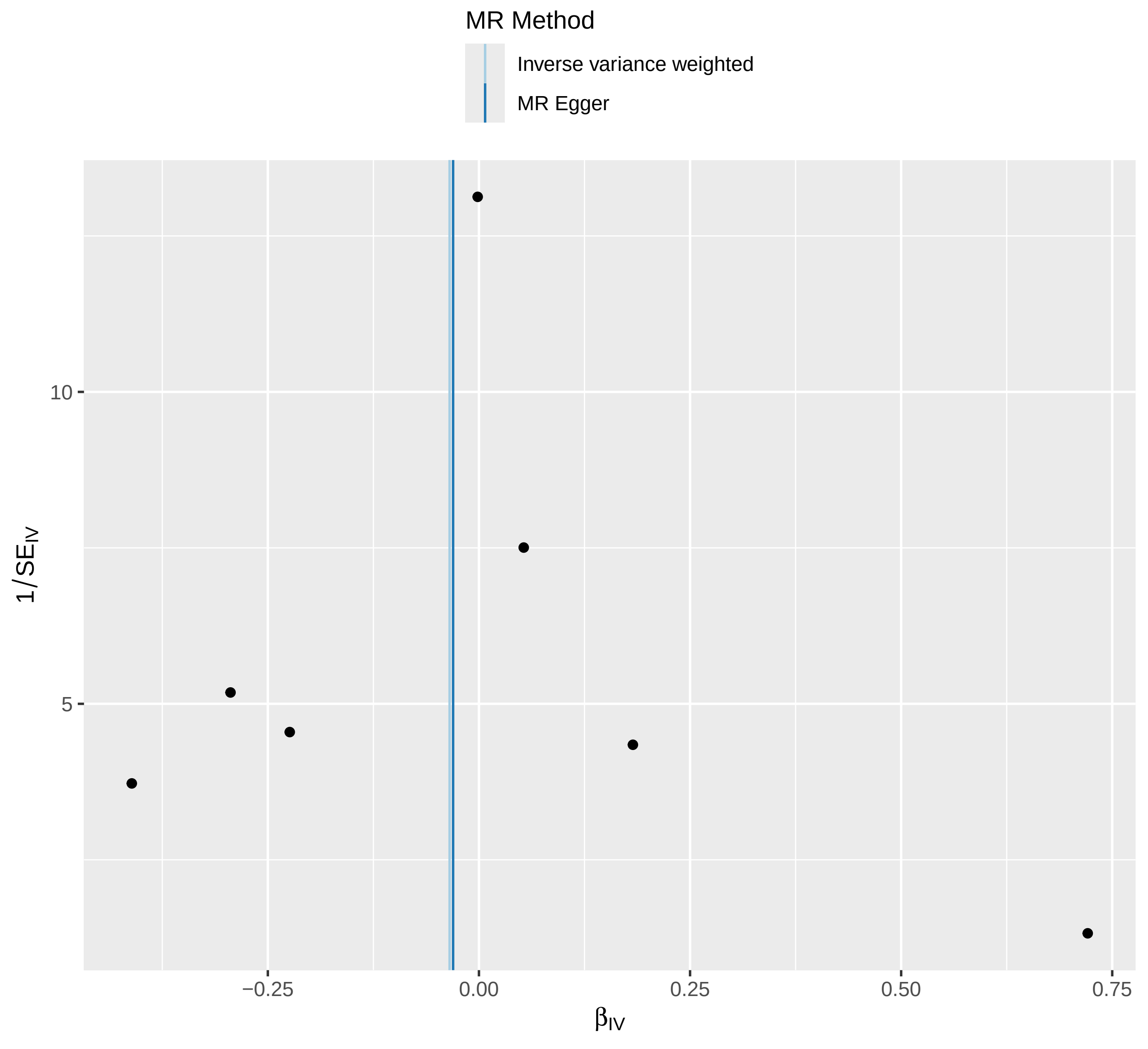
MR Method
Inverse variance weighted
MR Egger
10
5
1/SE_IV
−0.25
0.00
0.25
0.50
0.75
β_IV

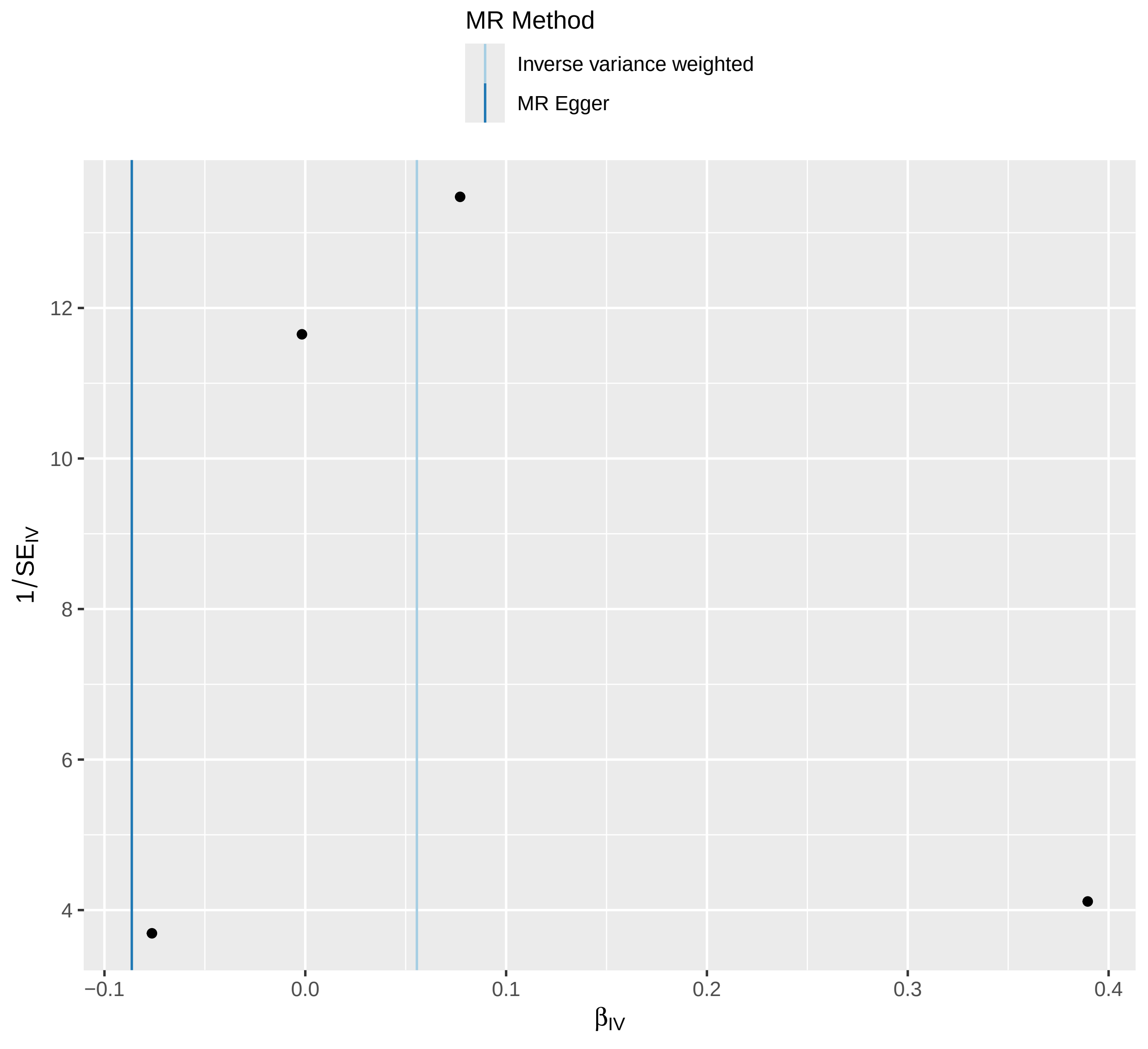

MR Method
Inverse variance weighted
MR Egger
1/$SE_{IV}$
12
10
8
6
4
−0.1
0.0
0.1
0.2
0.3
0.4
$\beta_{IV}$

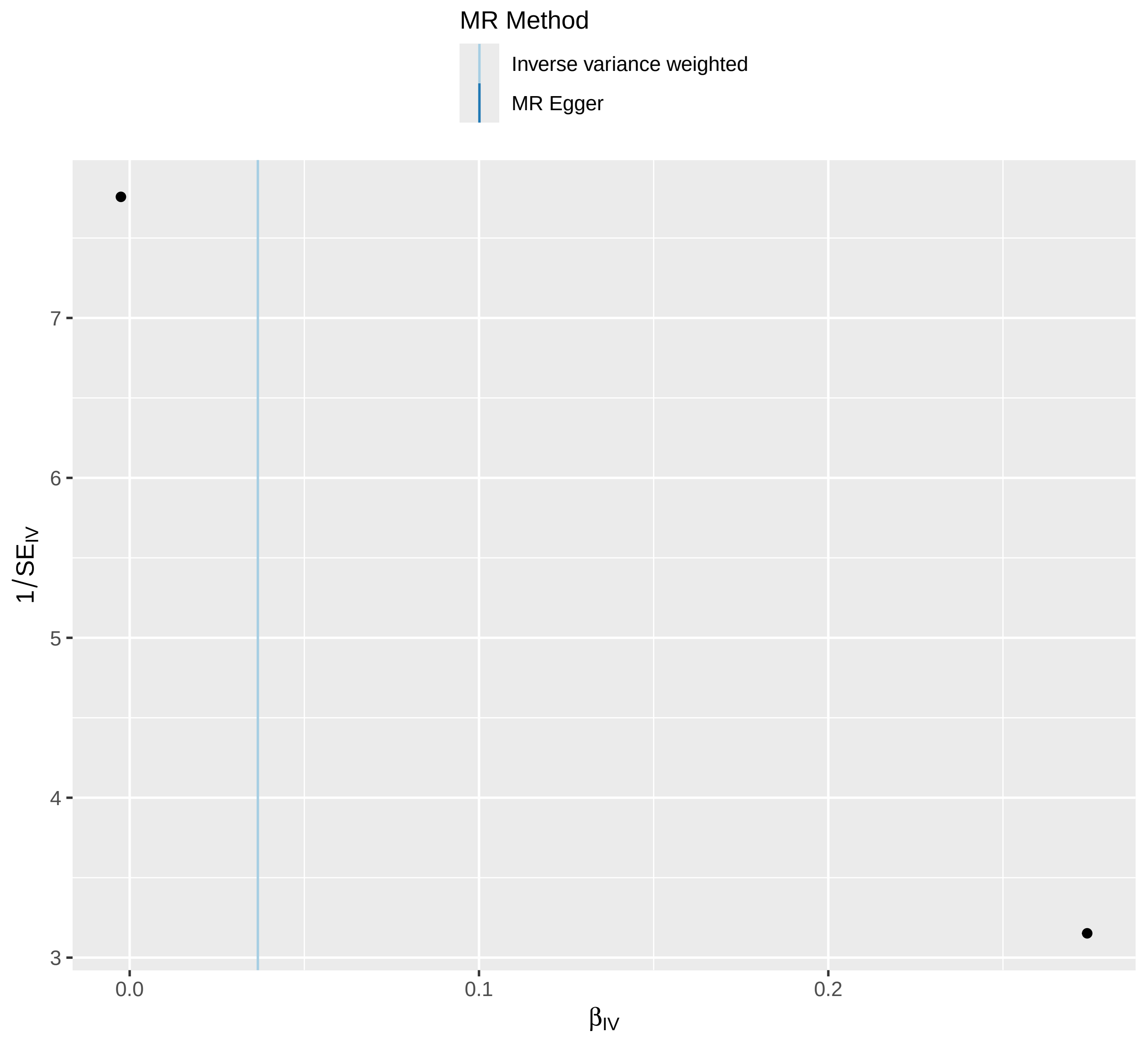
MR Method
Inverse variance weighted
MR Egger
1/SE_IV
7
6
5
4
3
0.0
0.1
0.2
β_IV

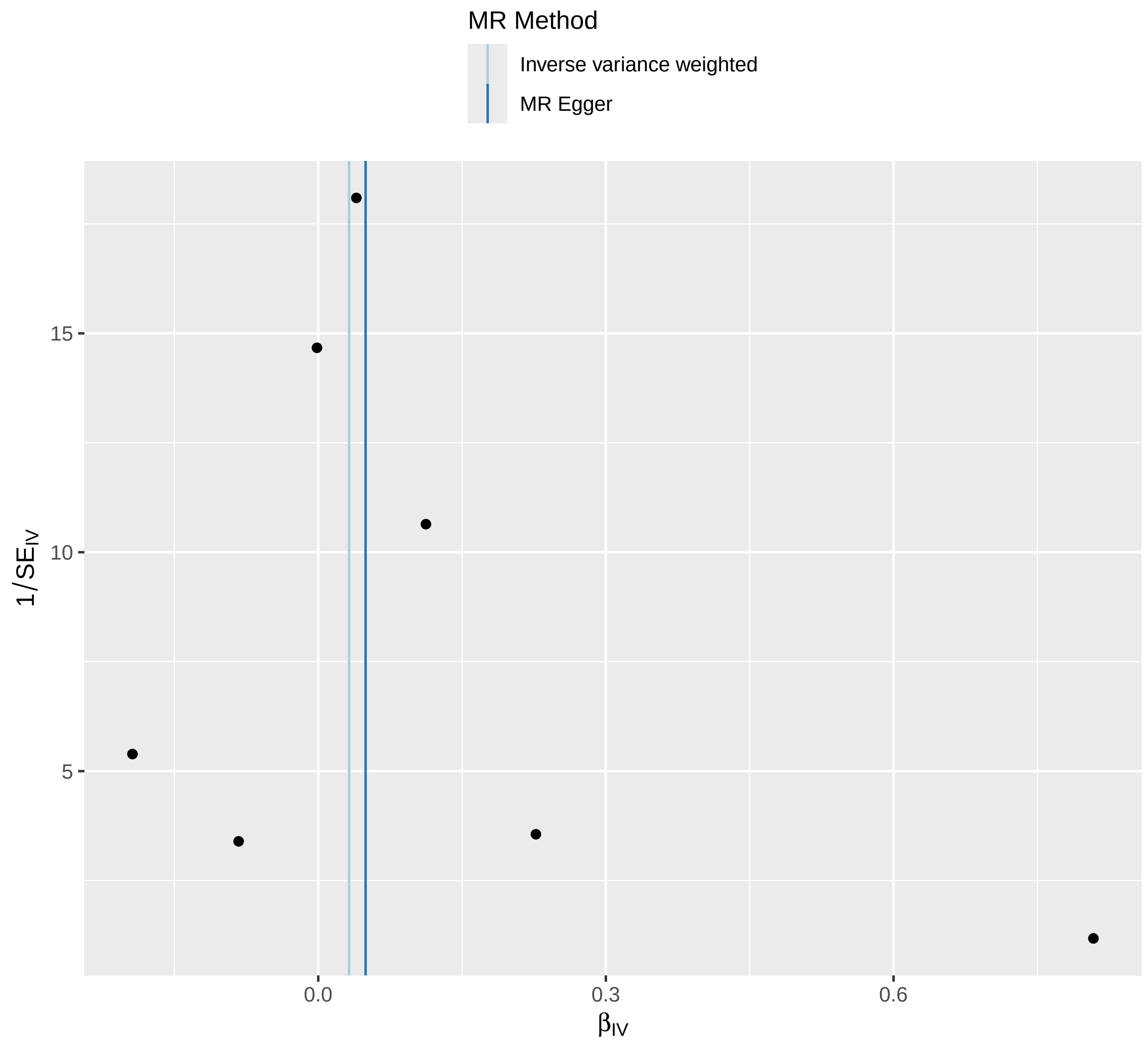

MR Method
Inverse variance weighted
MR Egger
15
10
5
0.0
0.3
0.6
$1/SE_{IV}$
$\beta_{IV}$

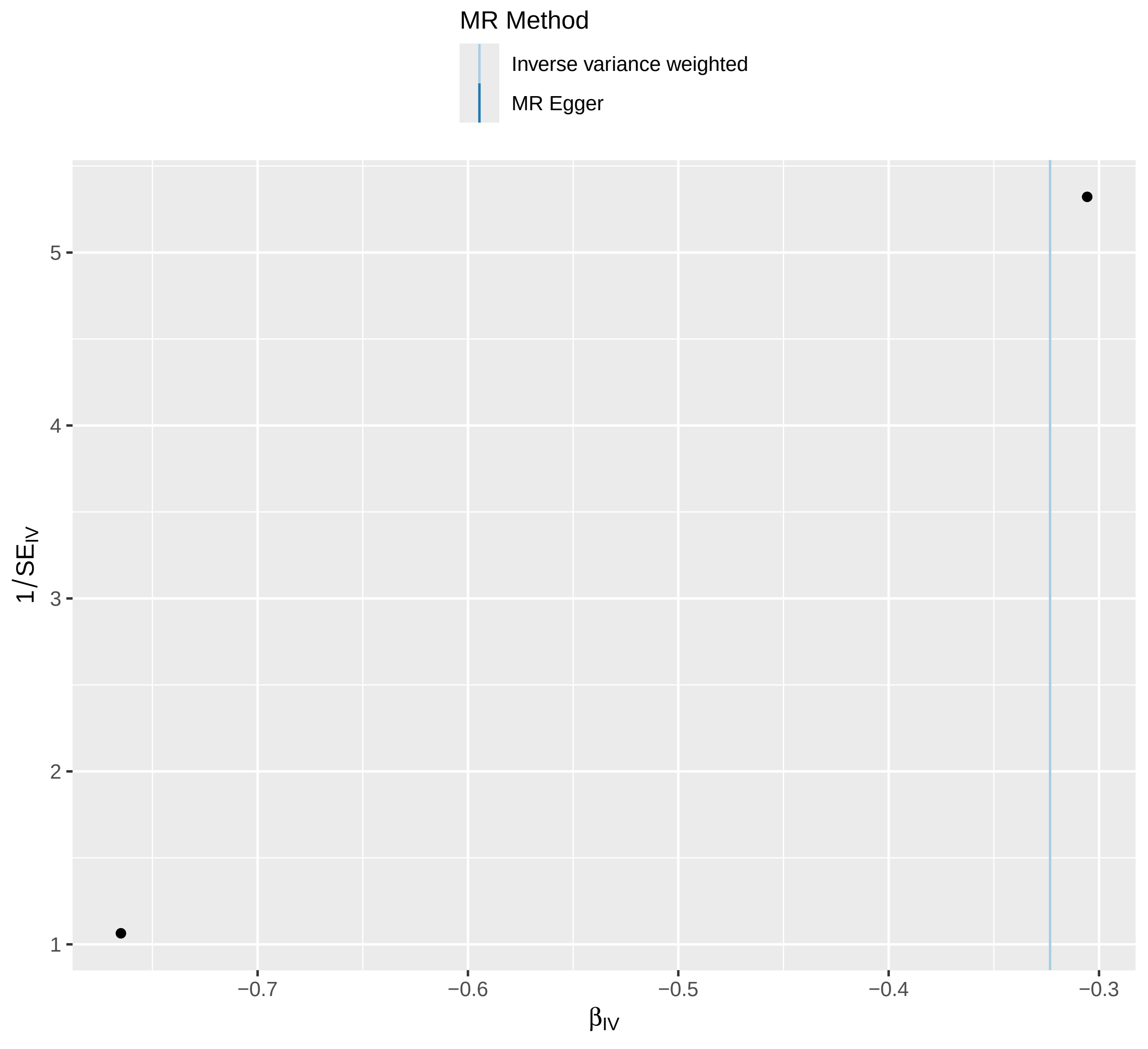
MR Method
Inverse variance weighted
MR Egger
1/SE_IV
5
4
3
2
1
−0.7
−0.6
−0.5
−0.4
−0.3
β_IV

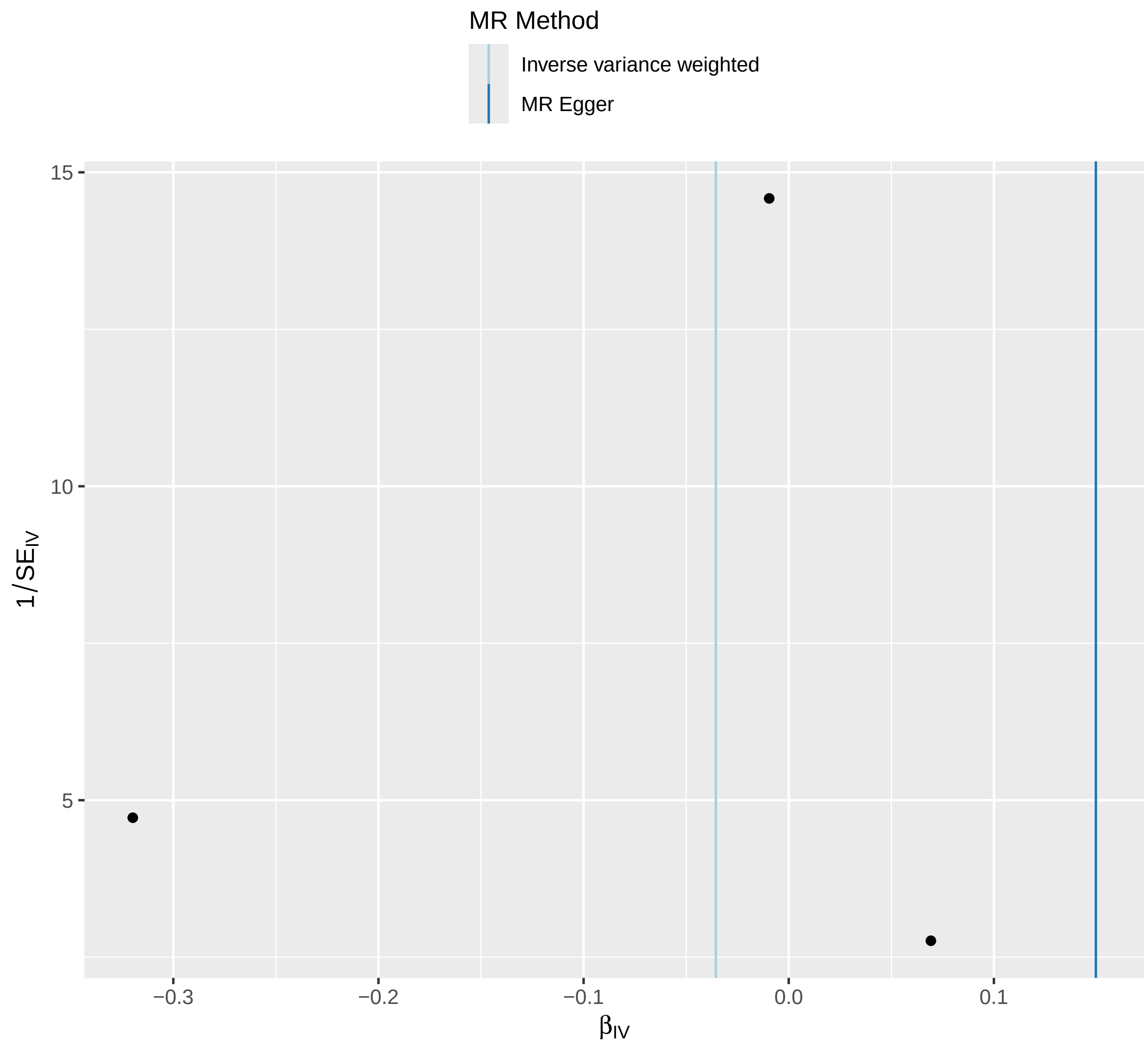
MR Method
Inverse variance weighted
MR Egger
15
10
5
−0.3
−0.2
−0.1
0.0
0.1
$1/SE_{IV}$
$\beta_{IV}$

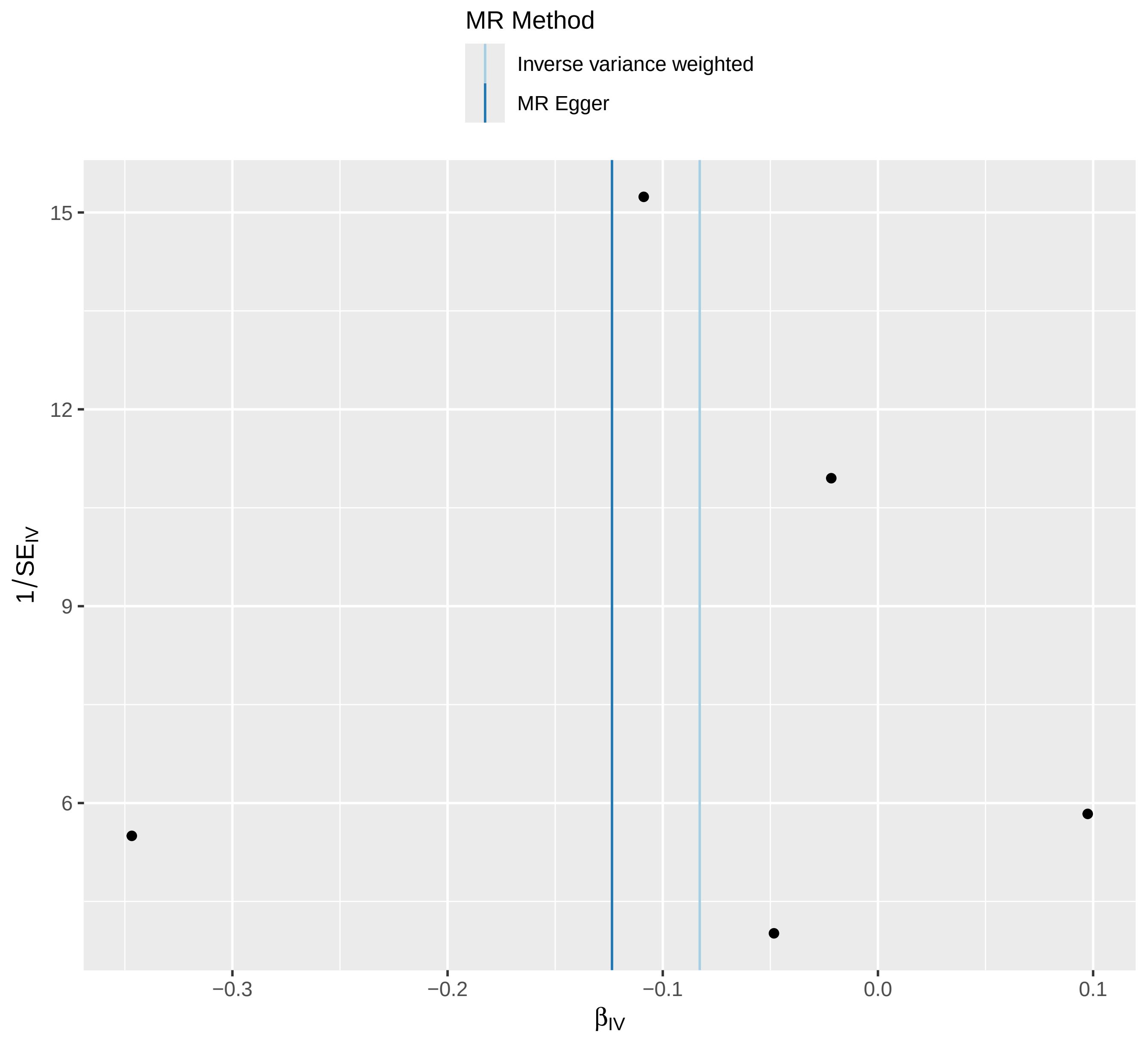
MR Method
Inverse variance weighted
MR Egger
15
12
9
6
$1/SE_{IV}$
−0.3
−0.2
−0.1
0.0
0.1
$\beta_{IV}$

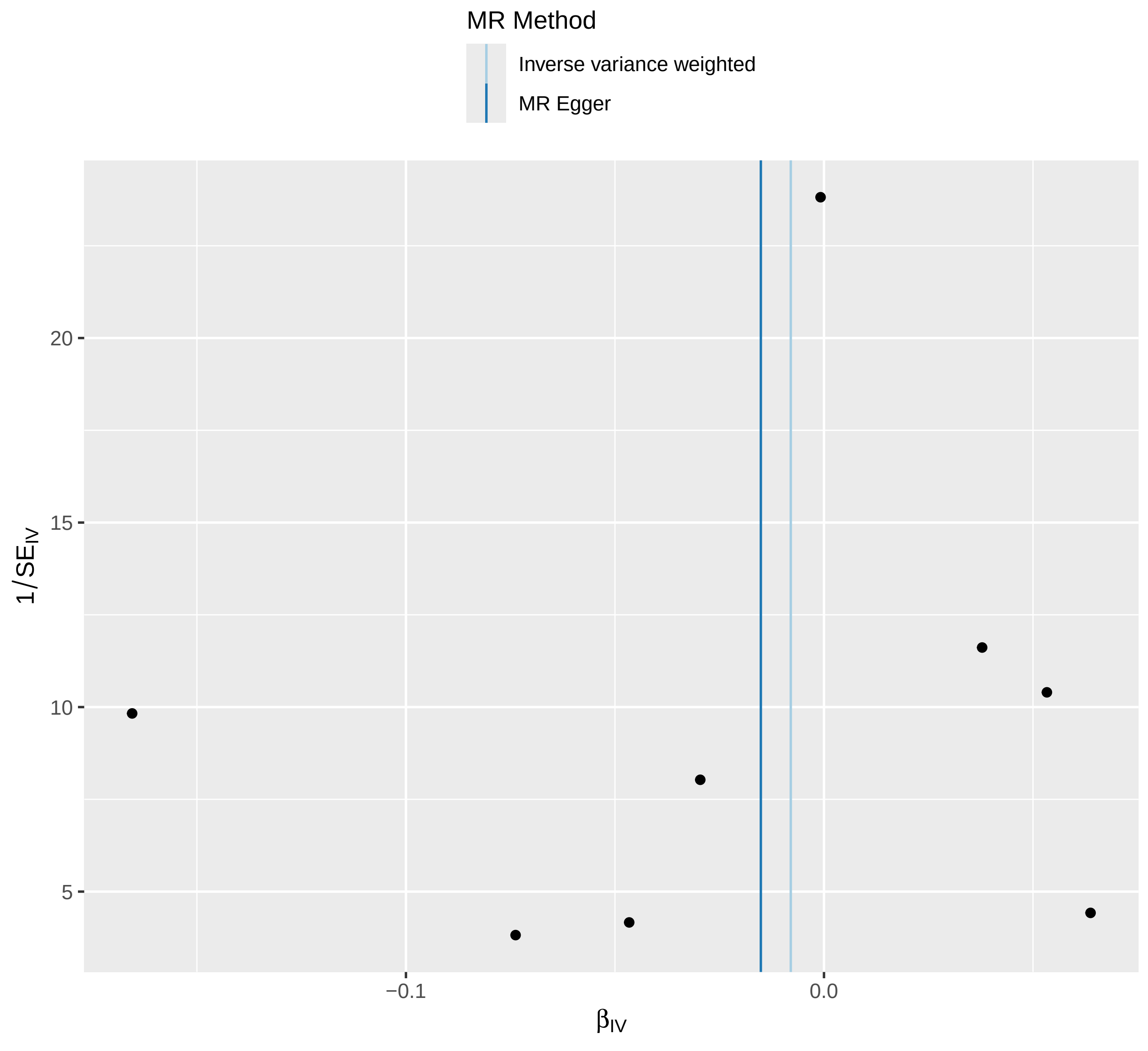
MR Method
Inverse variance weighted
MR Egger
20
15
10
5
1/SE_IV
−0.1
0.0
β_IV

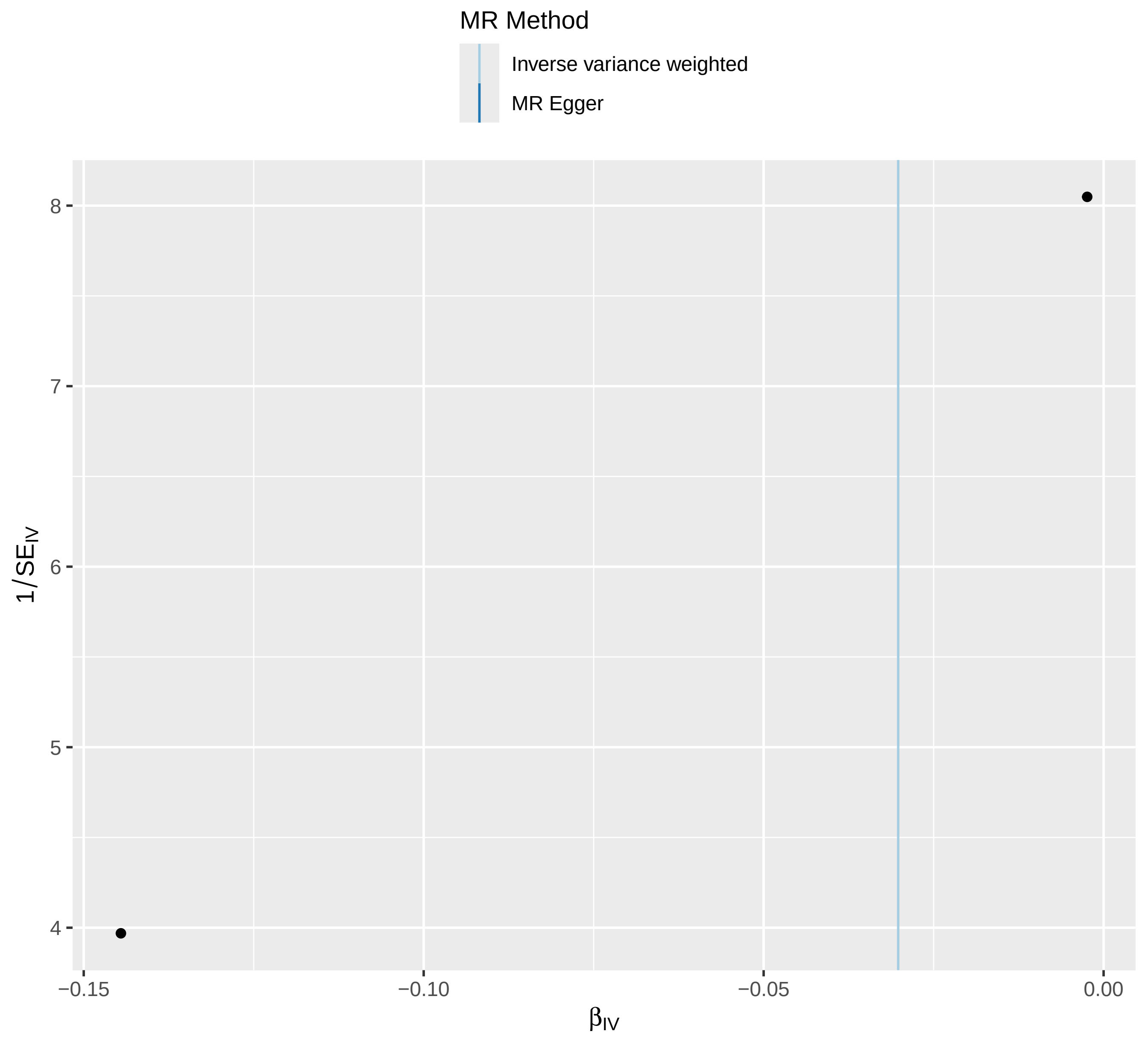
MR Method
Inverse variance weighted
MR Egger
8
7
6
5
4
1/SE_IV
−0.15
−0.10
−0.05
0.00
β_IV

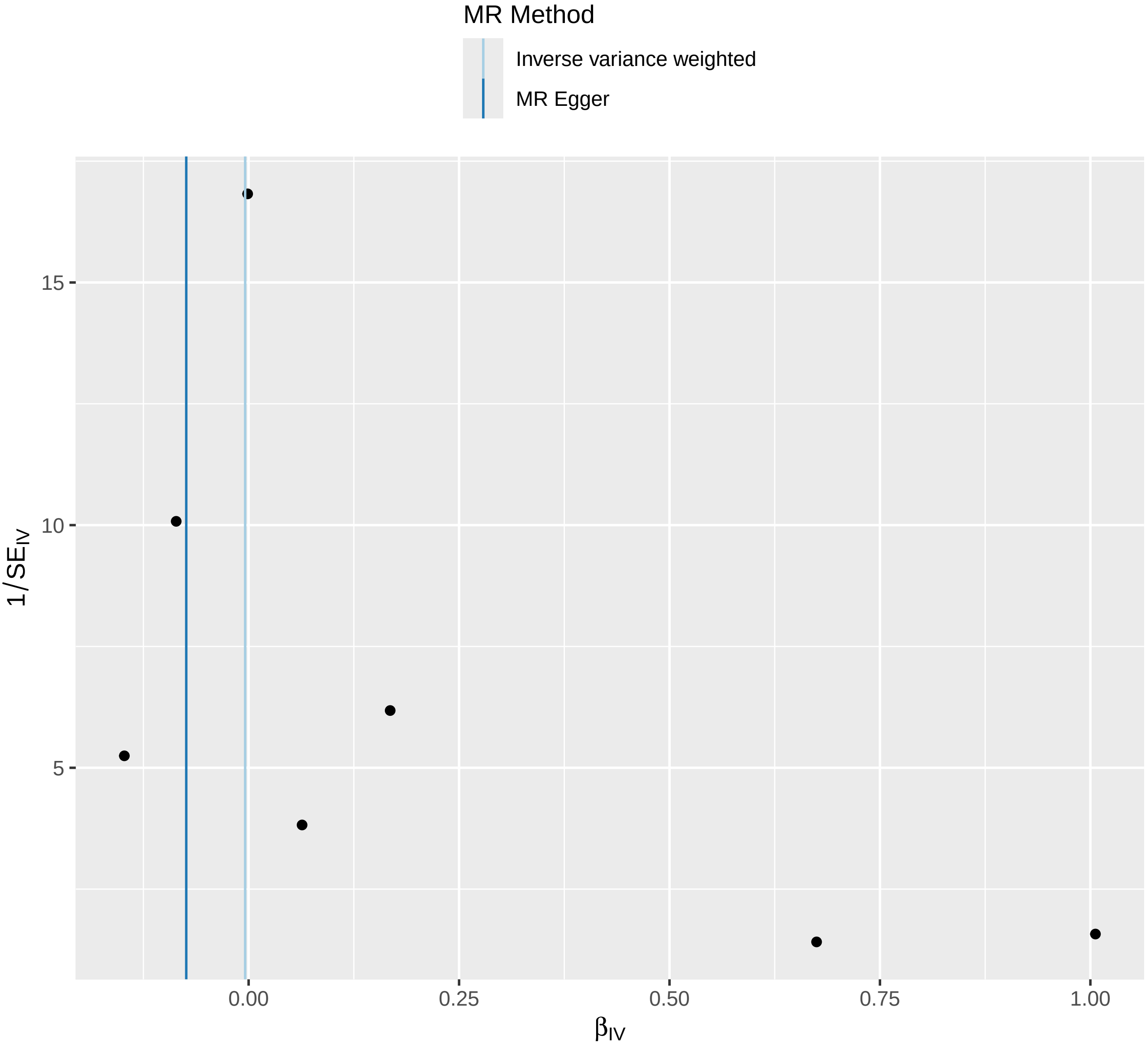

MR Method
Inverse variance weighted
MR Egger
15
10
5
1/SE_IV
0.00
0.25
0.50
0.75
1.00
β_IV

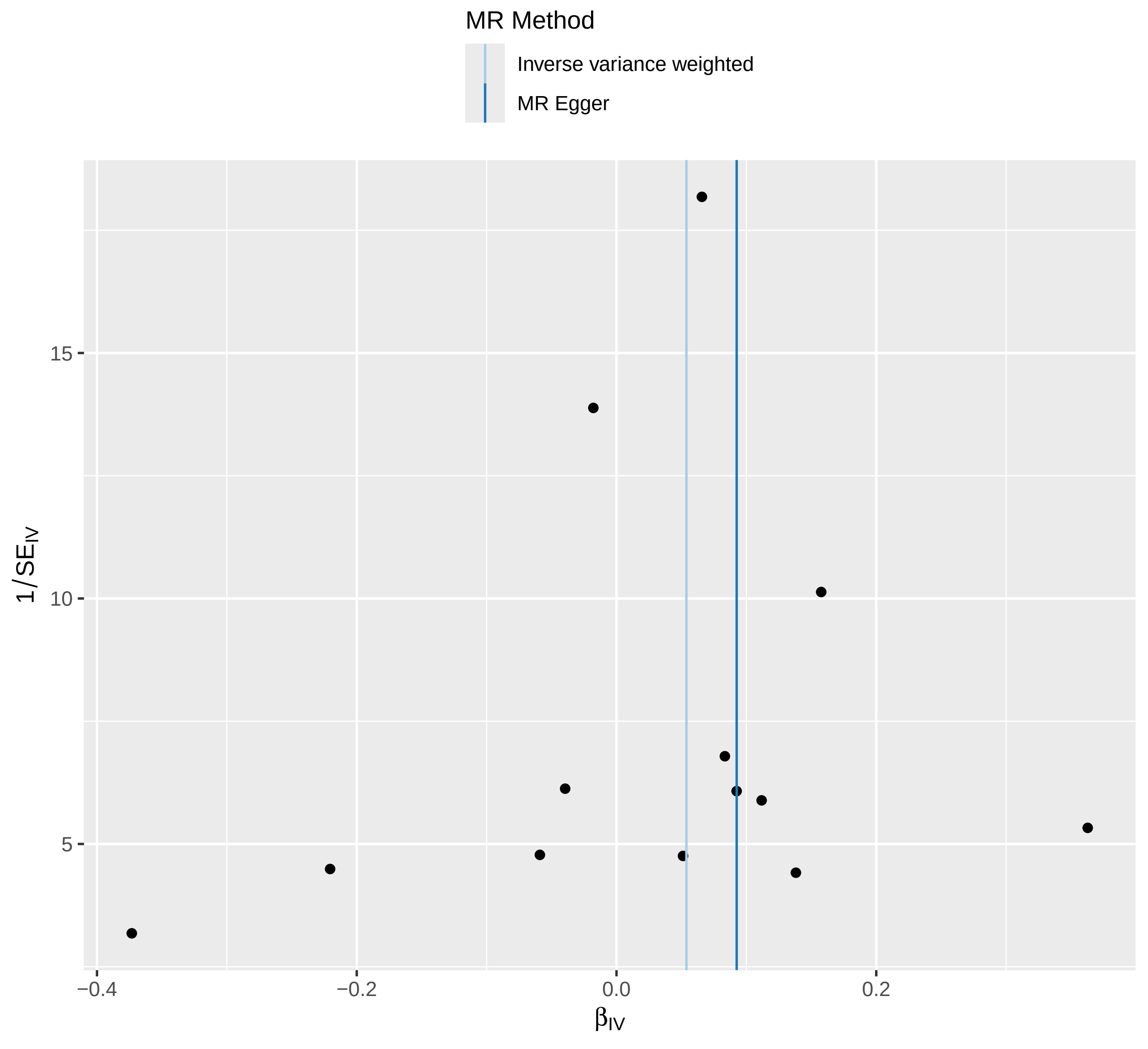
MR Method
Inverse variance weighted
MR Egger
1/SE_IV
15
10
5
−0.4
−0.2
0.0
0.2
β_IV

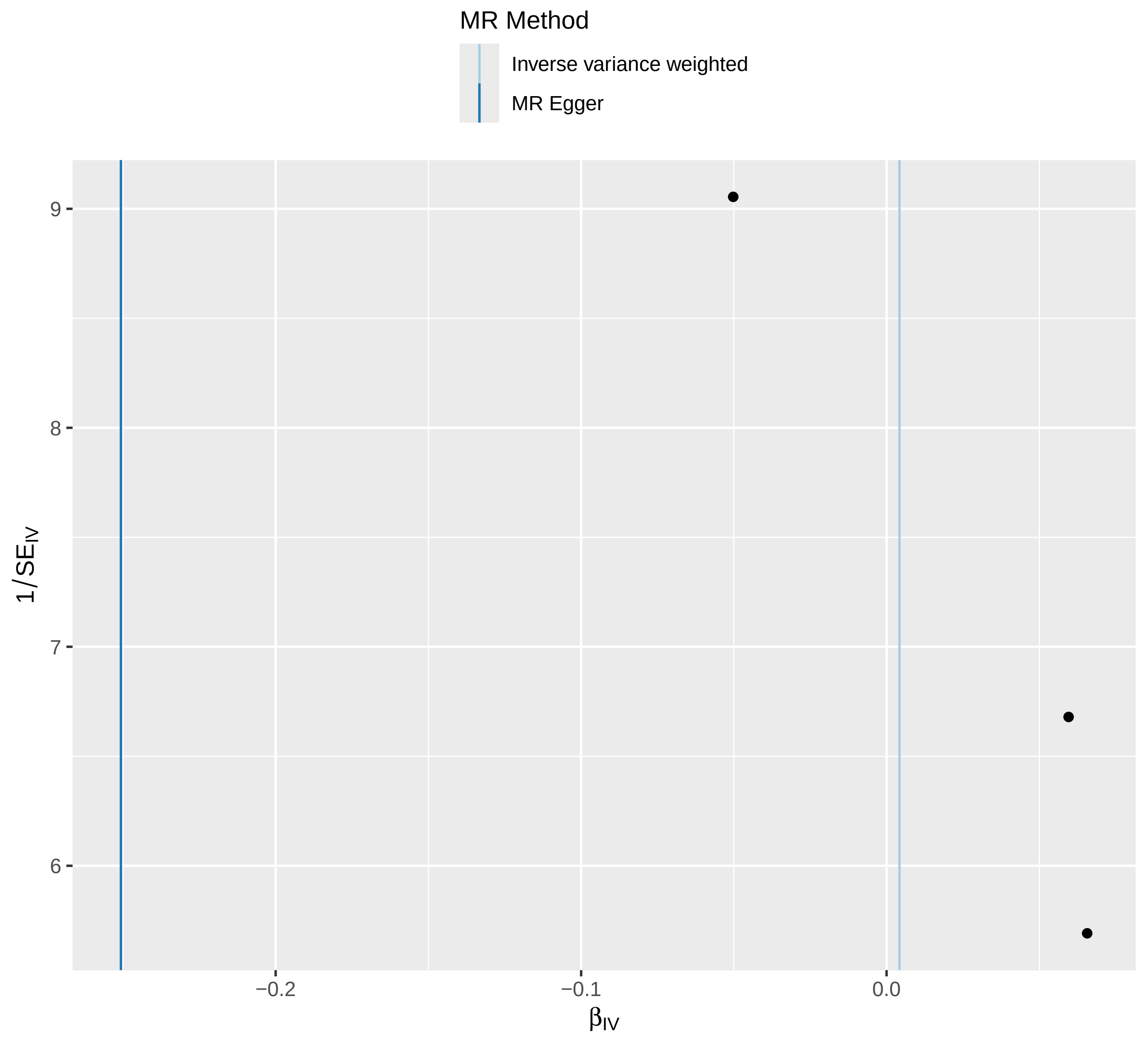
MR Method
Inverse variance weighted
MR Egger
9
8
7
6
−0.2
−0.1
0.0
$1/SE_{IV}$
$\beta_{IV}$

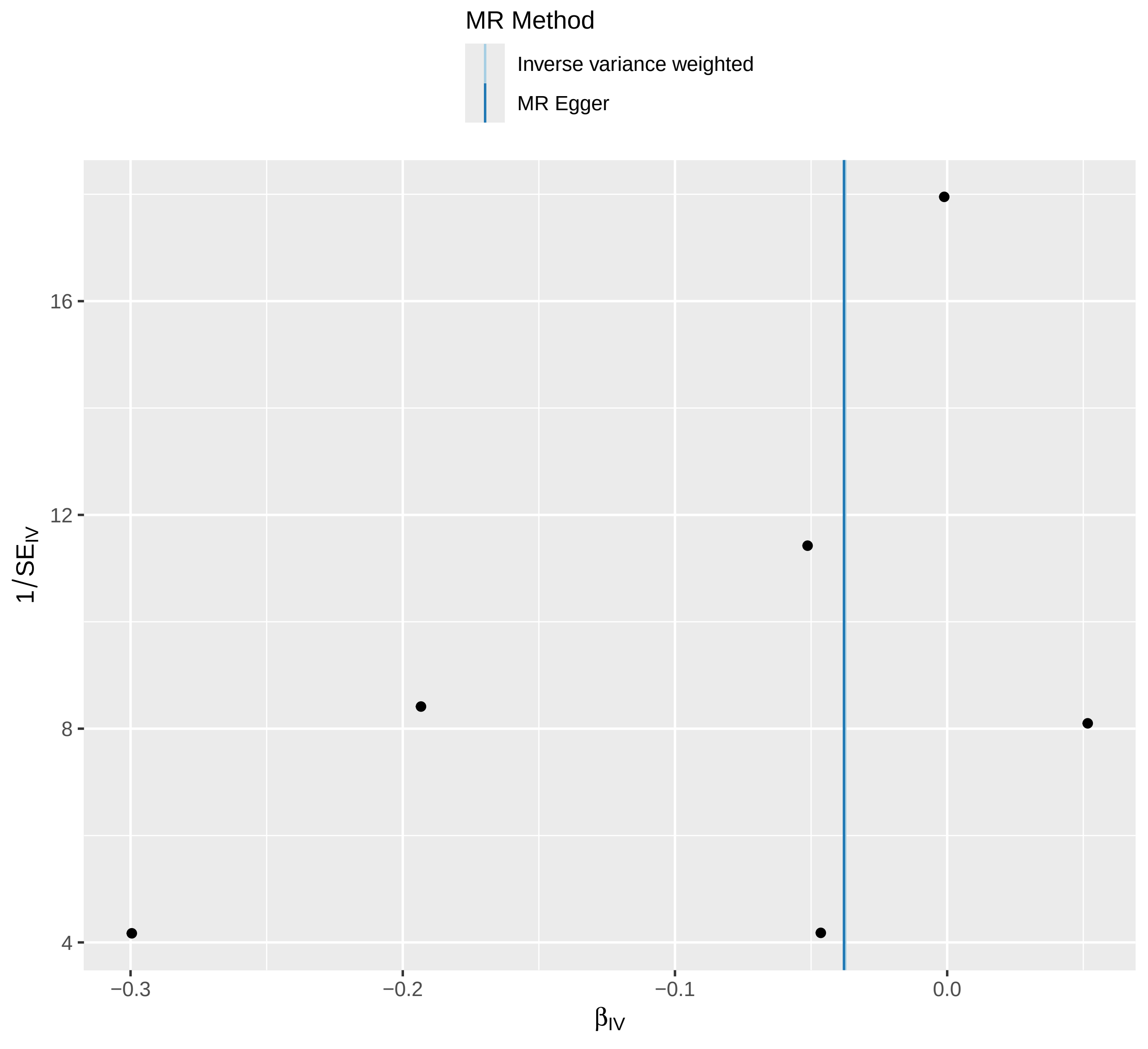

MR Method
Inverse variance weighted
MR Egger
16
12
8
4
1/SE_IV
−0.3
−0.2
−0.1
0.0
β_IV

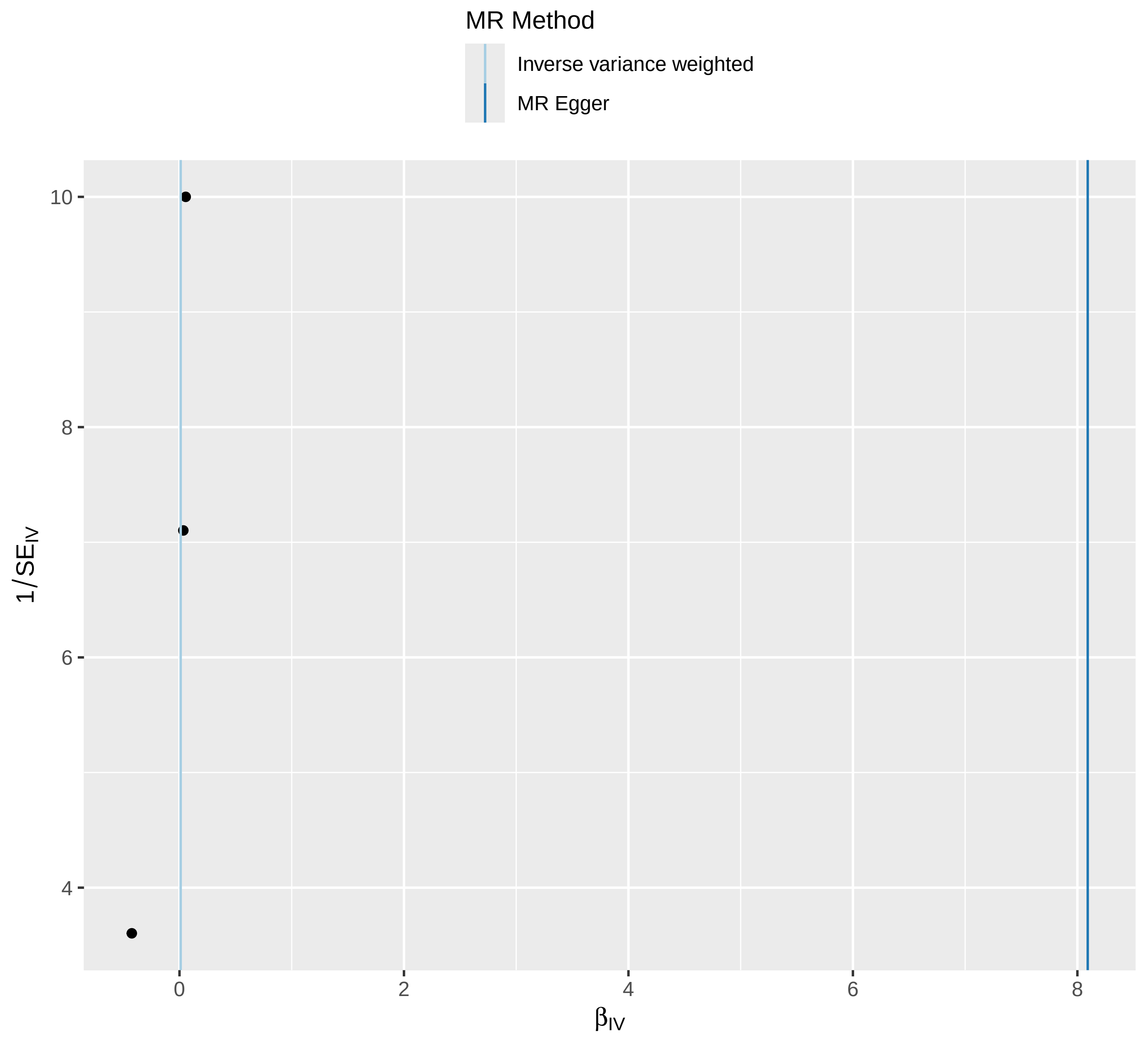

MR Method
Inverse variance weighted
MR Egger
10
8
6
4
0
2
4
6
8
1/SE_IV
β_IV

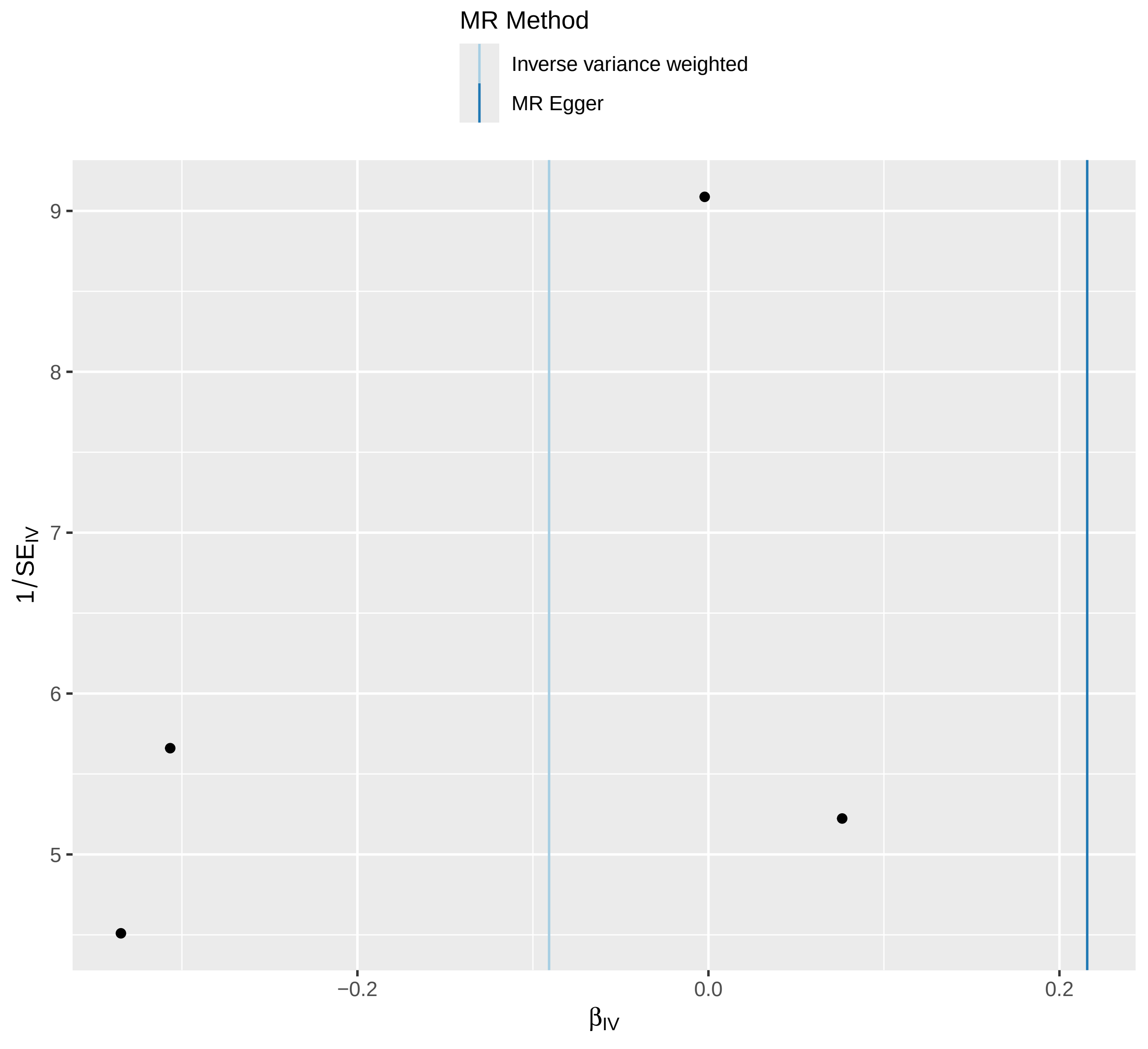

MR Method
Inverse variance weighted
MR Egger
9
8
7
6
5
1/SE_IV
−0.2
0.0
0.2
β_IV

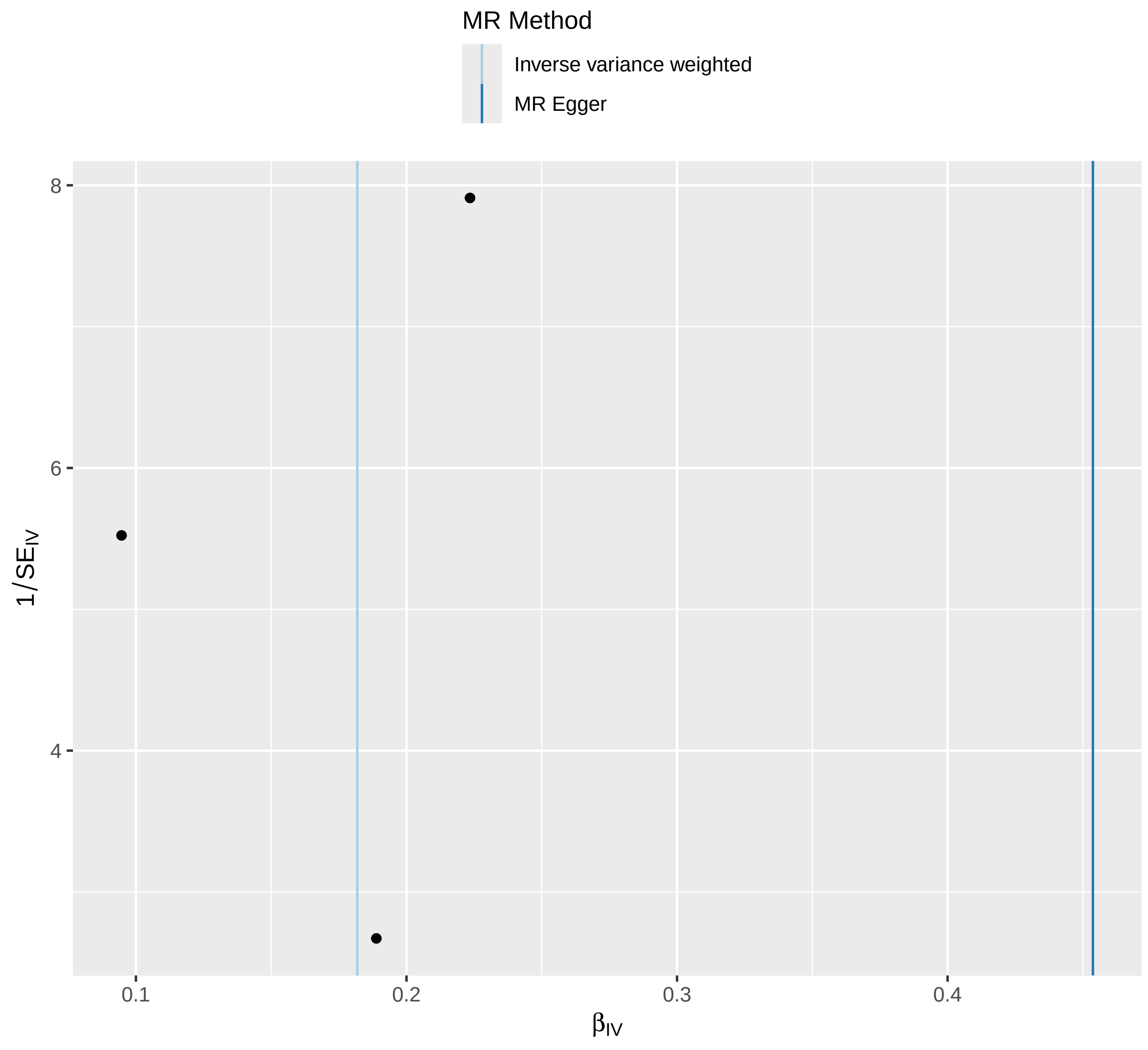

MR Method
Inverse variance weighted
MR Egger
8
6
4
1/SE_IV
0.1
0.2
0.3
0.4
β_IV

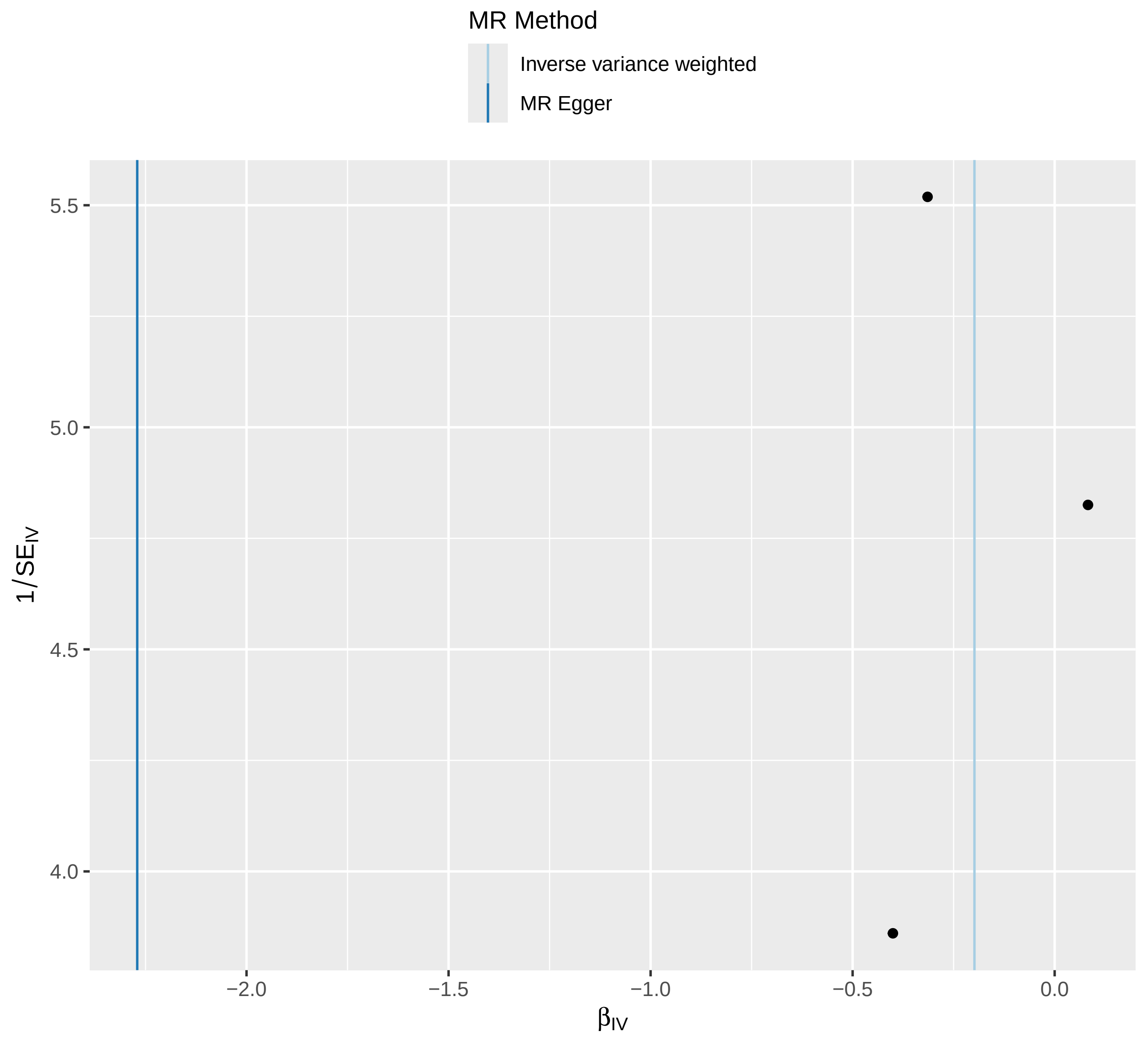
MR Method
Inverse variance weighted
MR Egger
5.5
5.0
4.5
4.0
1/SE_IV
−2.0
−1.5
−1.0
−0.5
0.0
β_IV

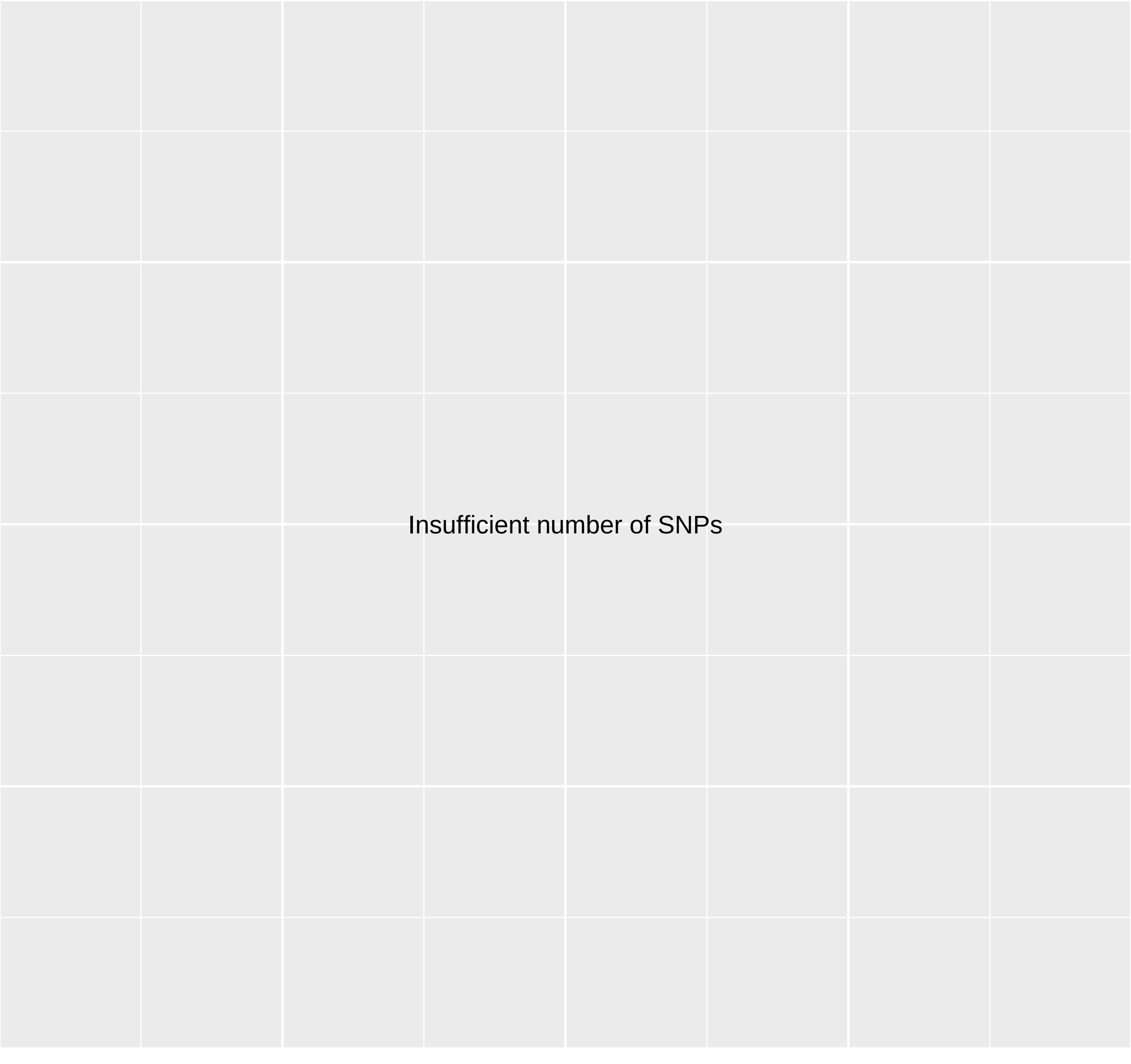
Insufficient number of SNPs

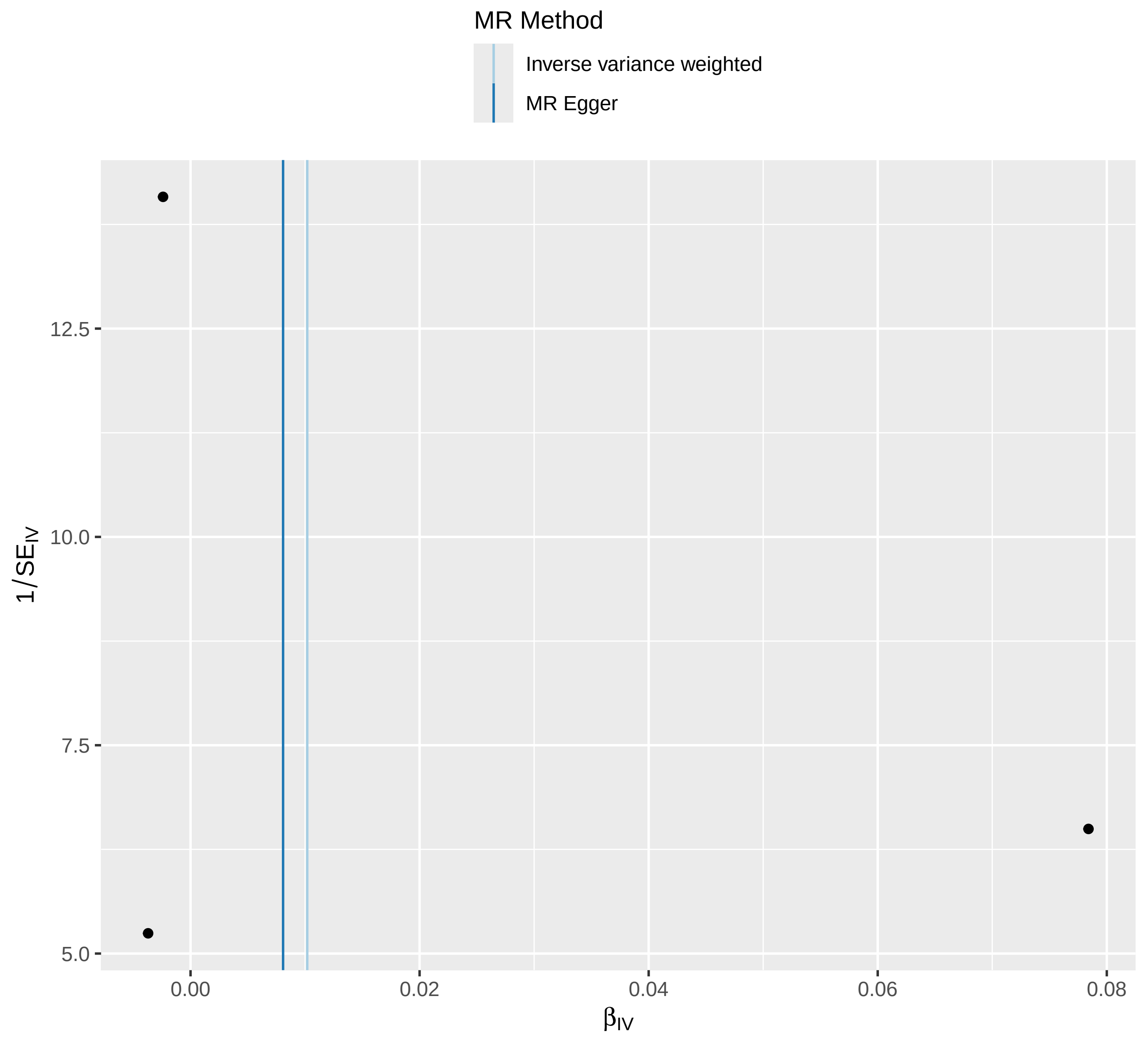
MR Method
Inverse variance weighted
MR Egger
12.5
10.0
7.5
5.0
1/SE$_{IV}$
0.00
0.02
0.04
0.06
0.08
β$_{IV}$

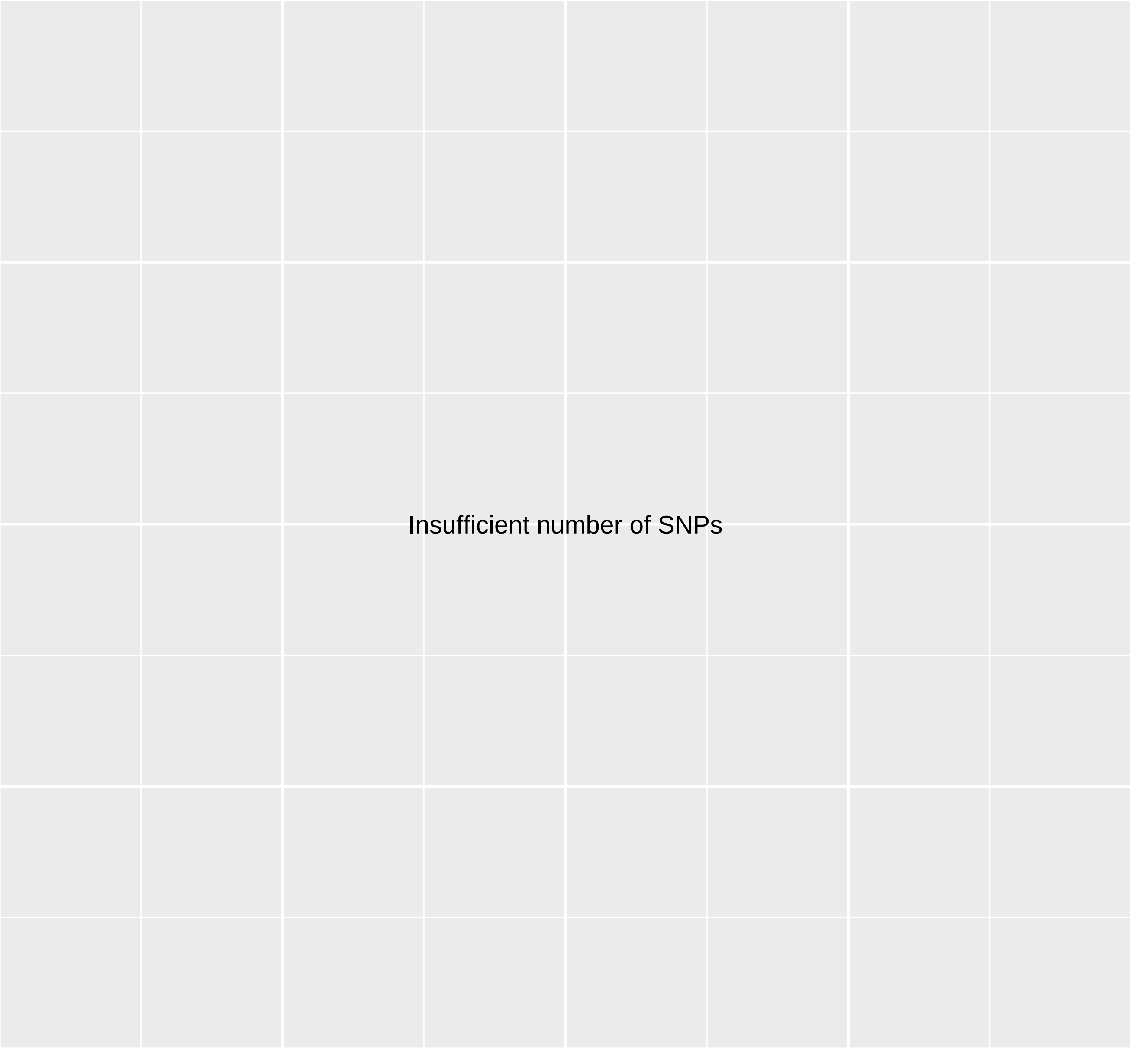
Insufficient number of SNPs

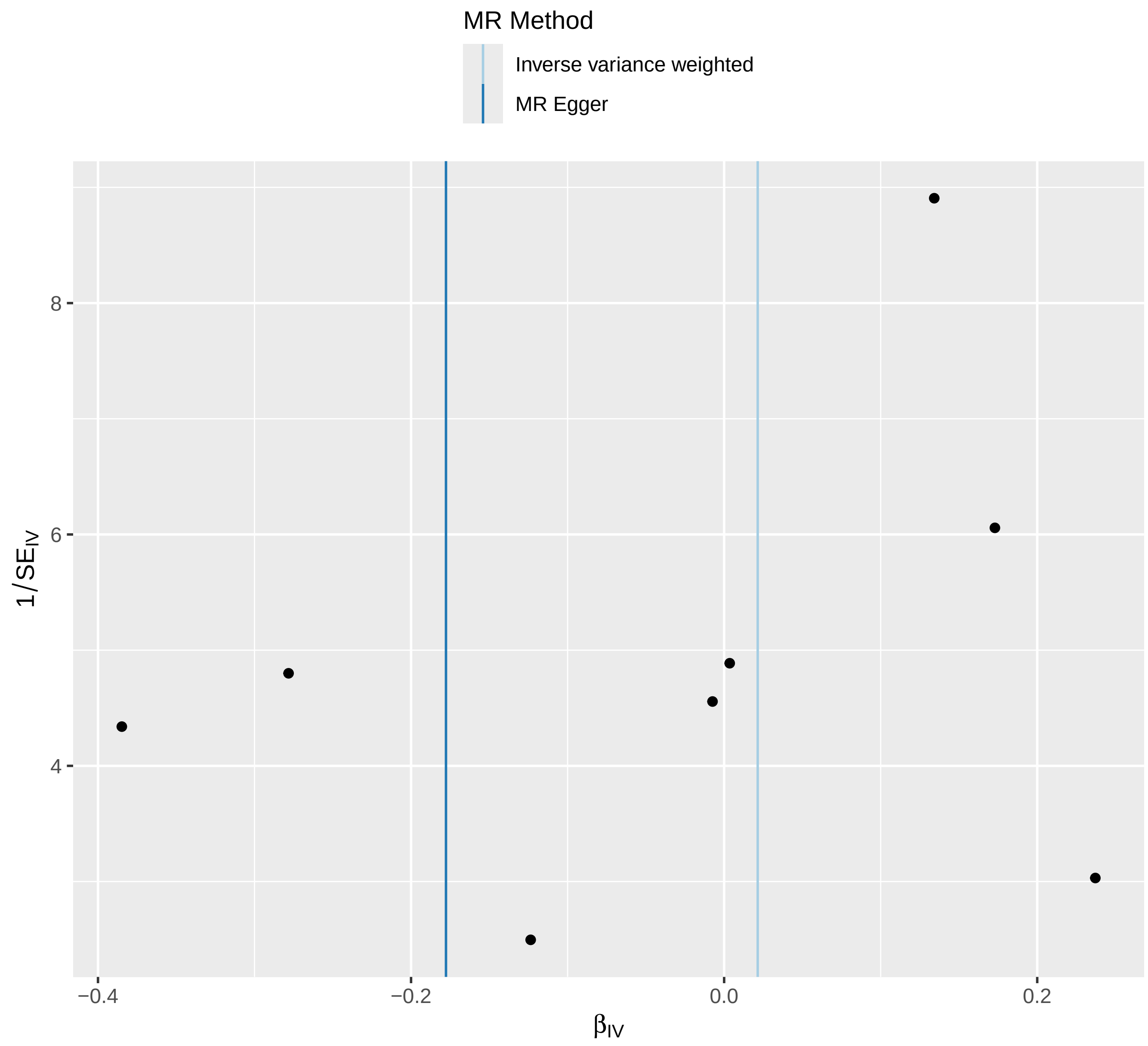
MR Method
Inverse variance weighted
MR Egger
8
6
4
1/SE_IV
−0.4
−0.2
0.0
0.2
β_IV

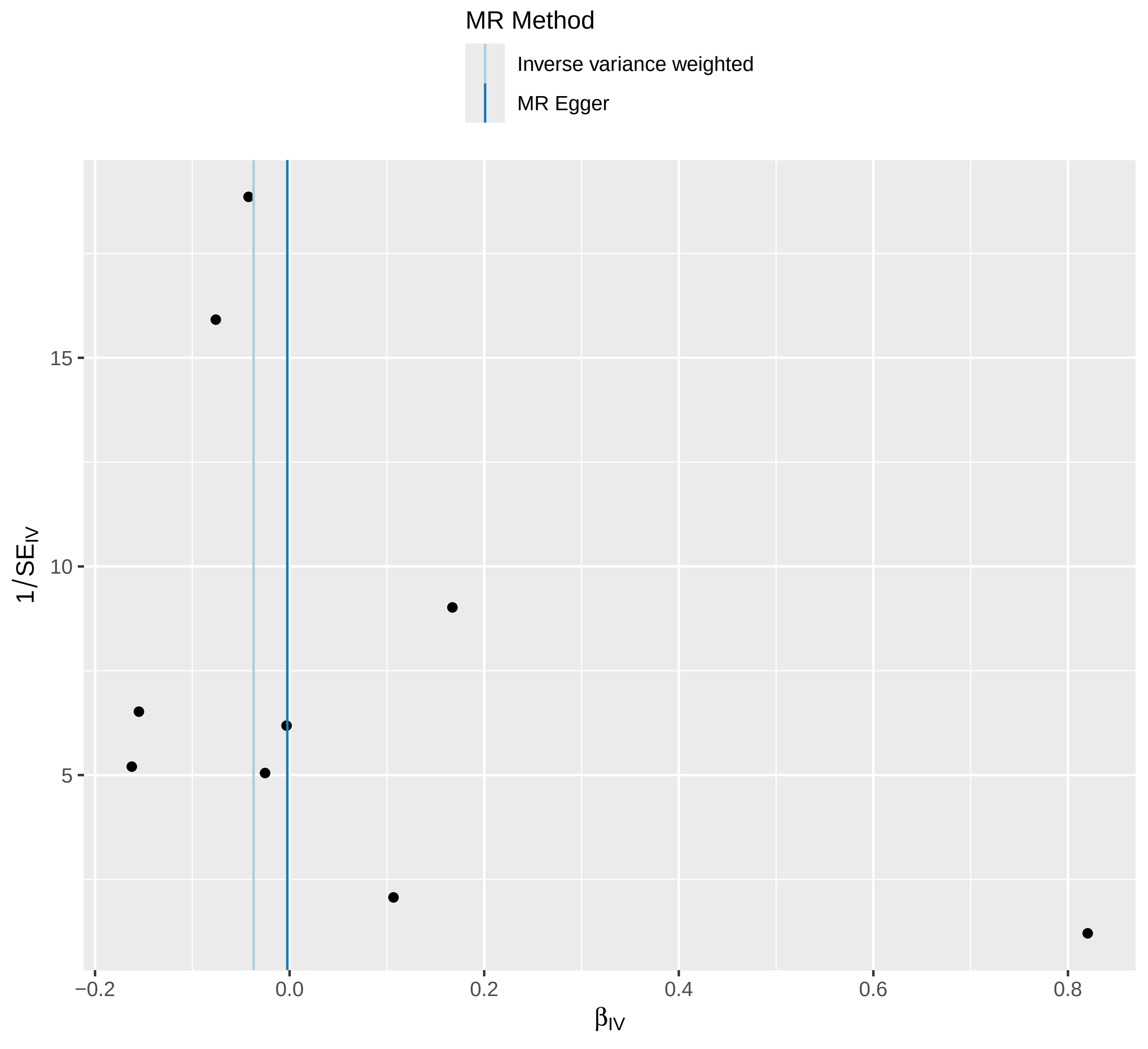

MR Method
Inverse variance weighted
MR Egger
15
10
5
1/SE_IV
−0.2
0.0
0.2
0.4
0.6
0.8
β_IV

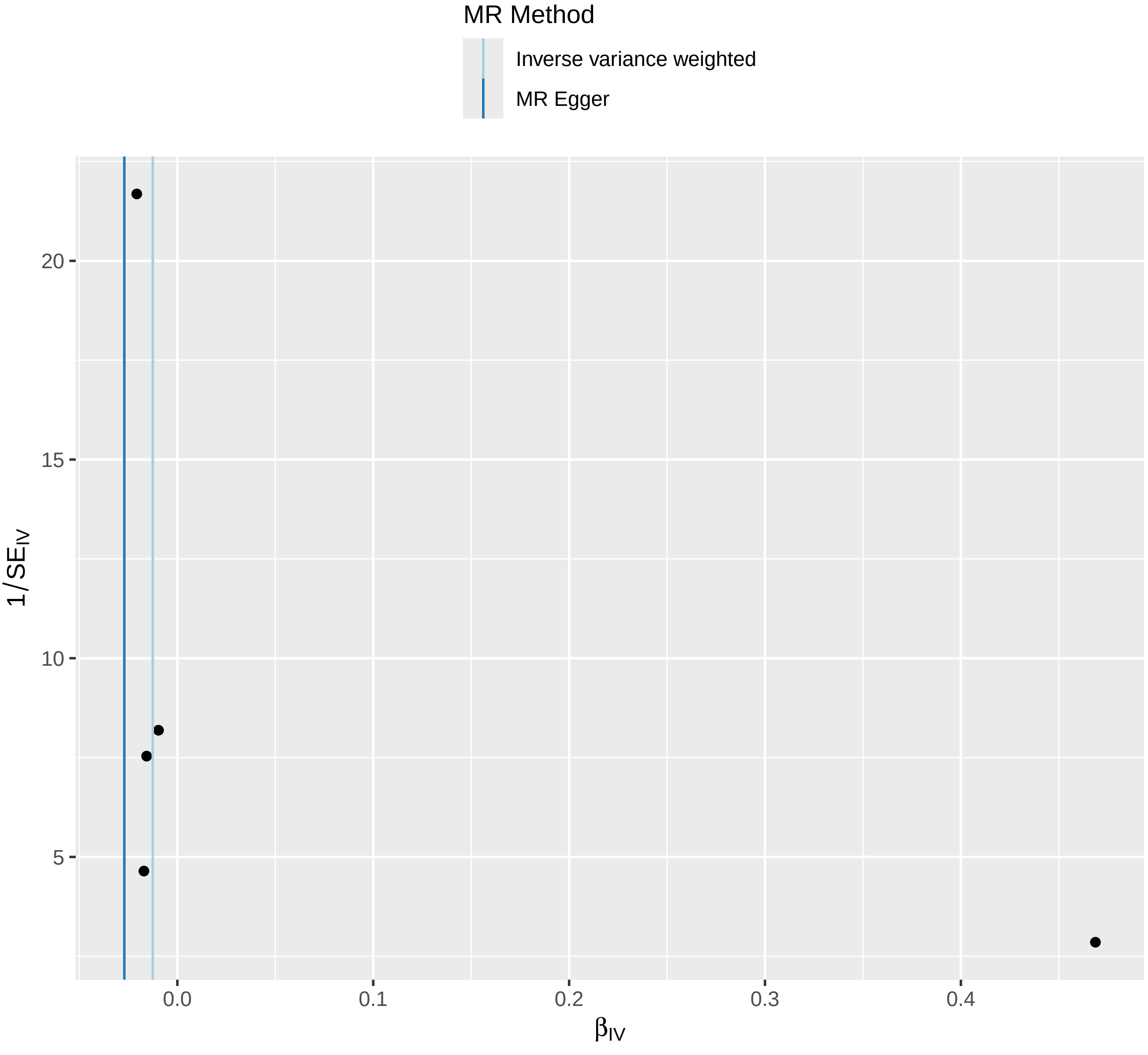

MR Method
Inverse variance weighted
MR Egger
20
15
10
5
0.0
0.1
0.2
0.3
0.4
$1/SE_{IV}$
$\beta_{IV}$

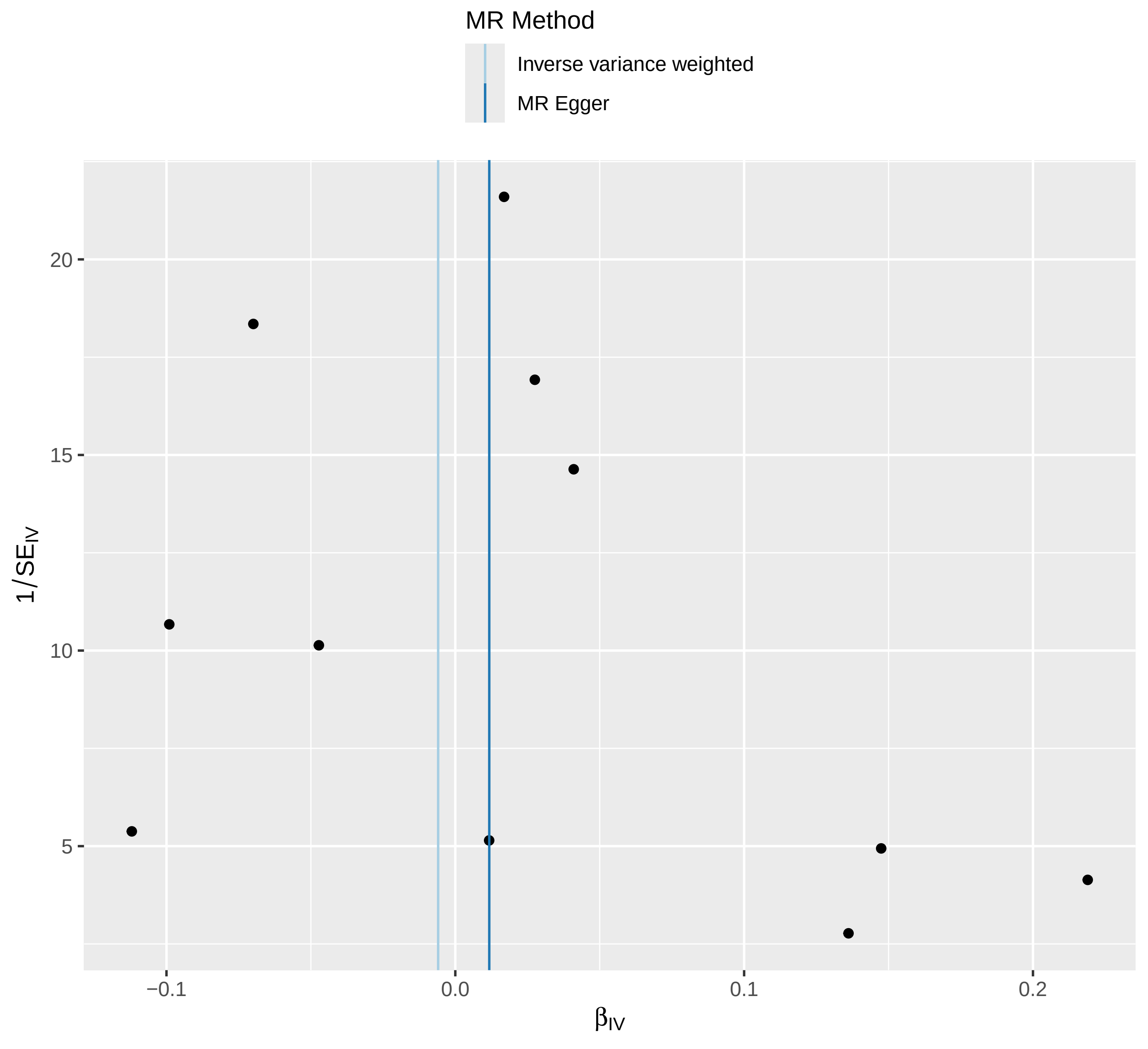

MR Method
Inverse variance weighted
MR Egger
1/SE_IV
20
15
10
5
−0.1
0.0
0.1
0.2
β_IV

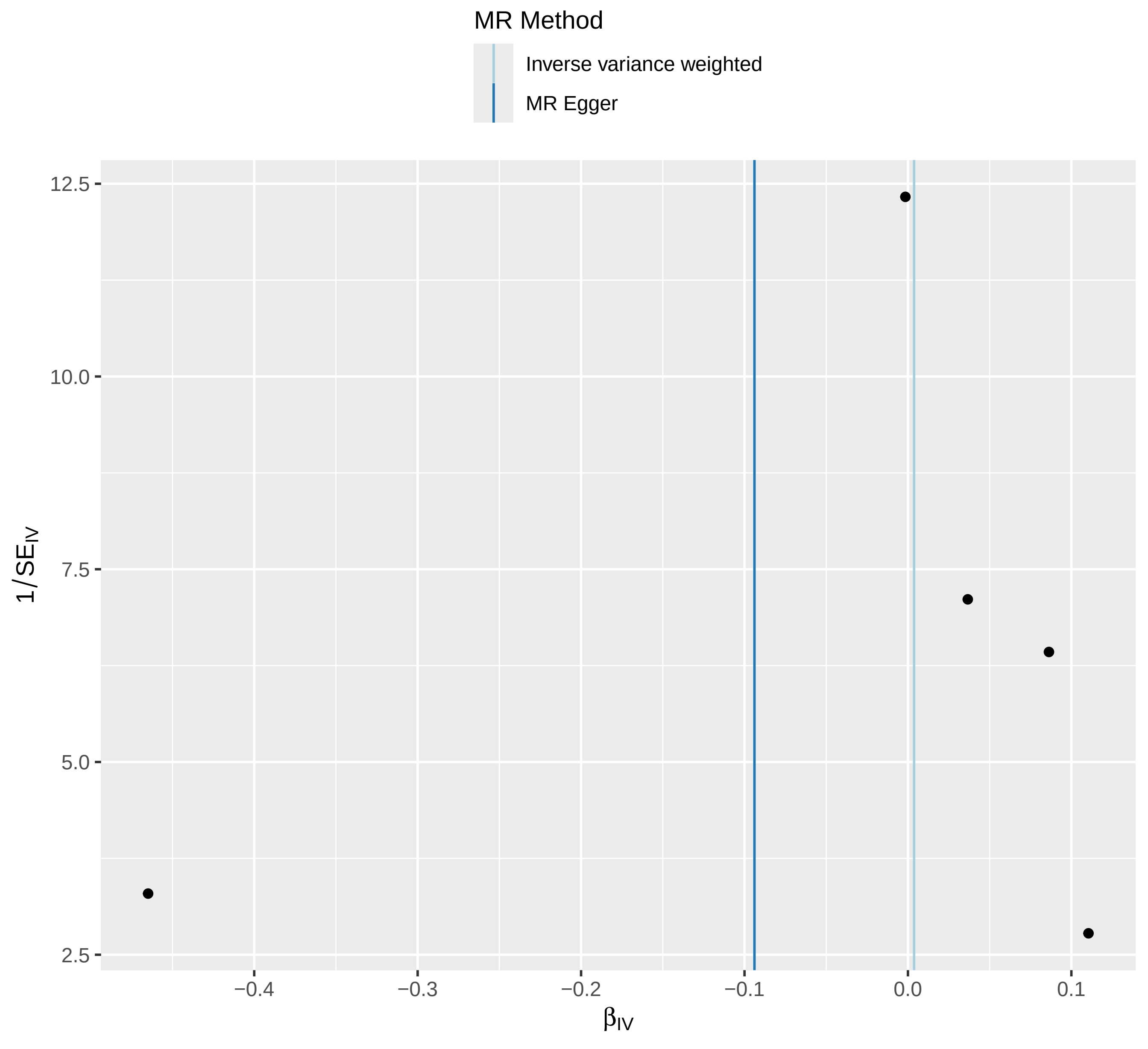
MR Method
Inverse variance weighted
MR Egger
12.5
10.0
7.5
5.0
2.5
$1/SE_{IV}$
−0.4
−0.3
−0.2
−0.1
0.0
0.1
$\beta_{IV}$

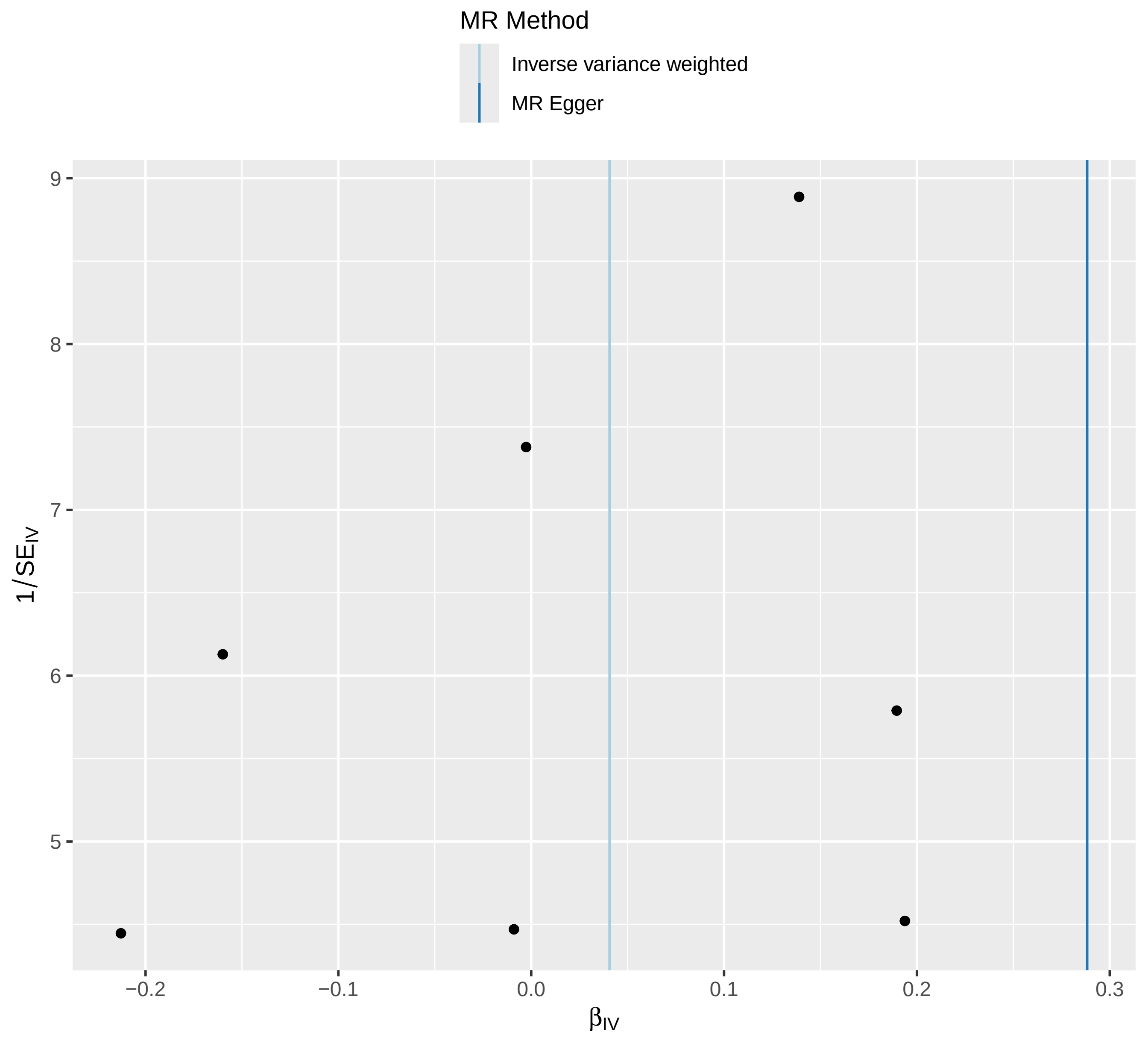

MR Method
Inverse variance weighted
MR Egger
9
8
7
6
5
1/SE_IV
−0.2
−0.1
0.0
0.1
0.2
0.3
β_IV

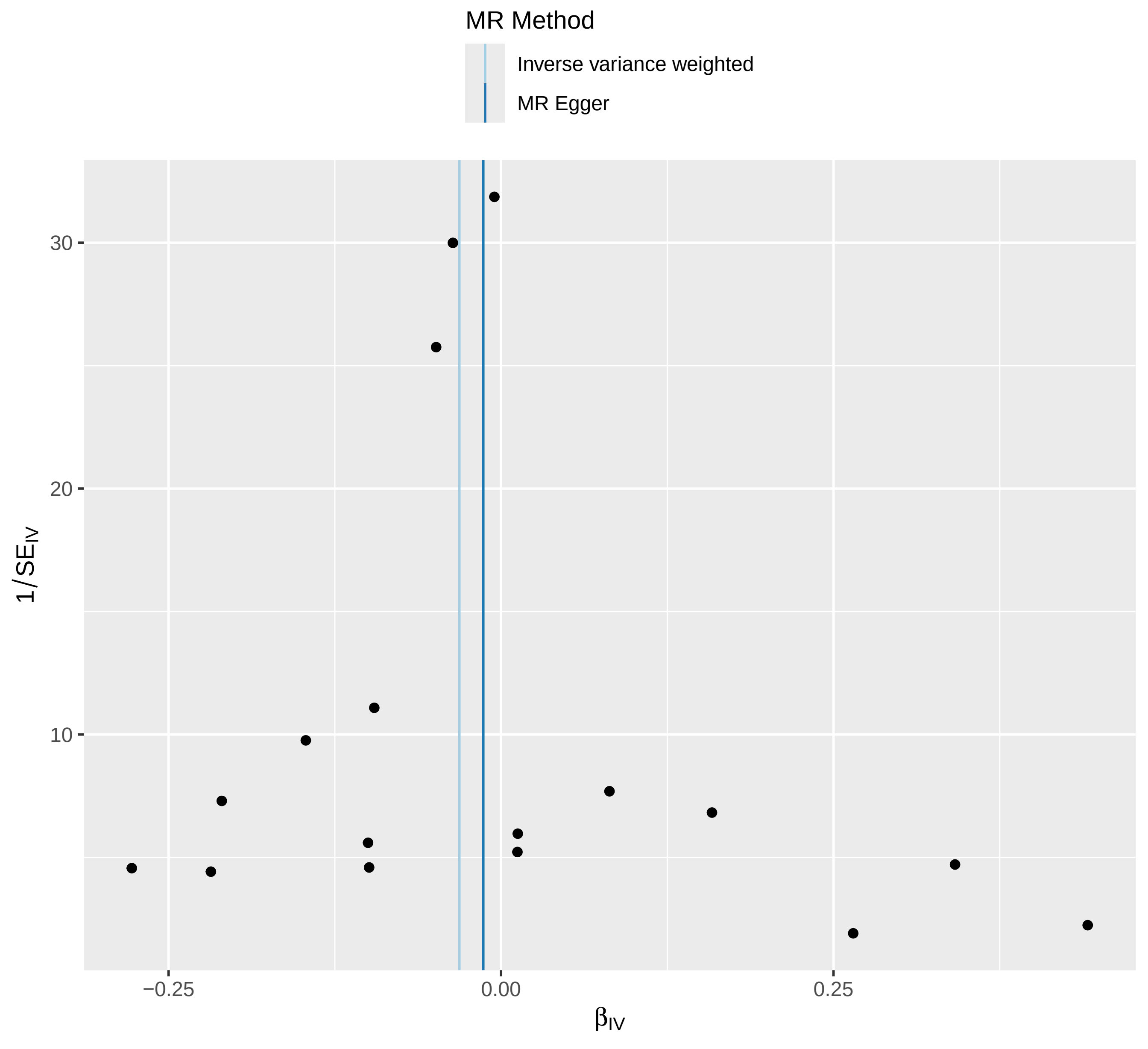

MR Method
Inverse variance weighted
MR Egger
30
20
10
−0.25
0.00
0.25
1/SE_IV
β_IV

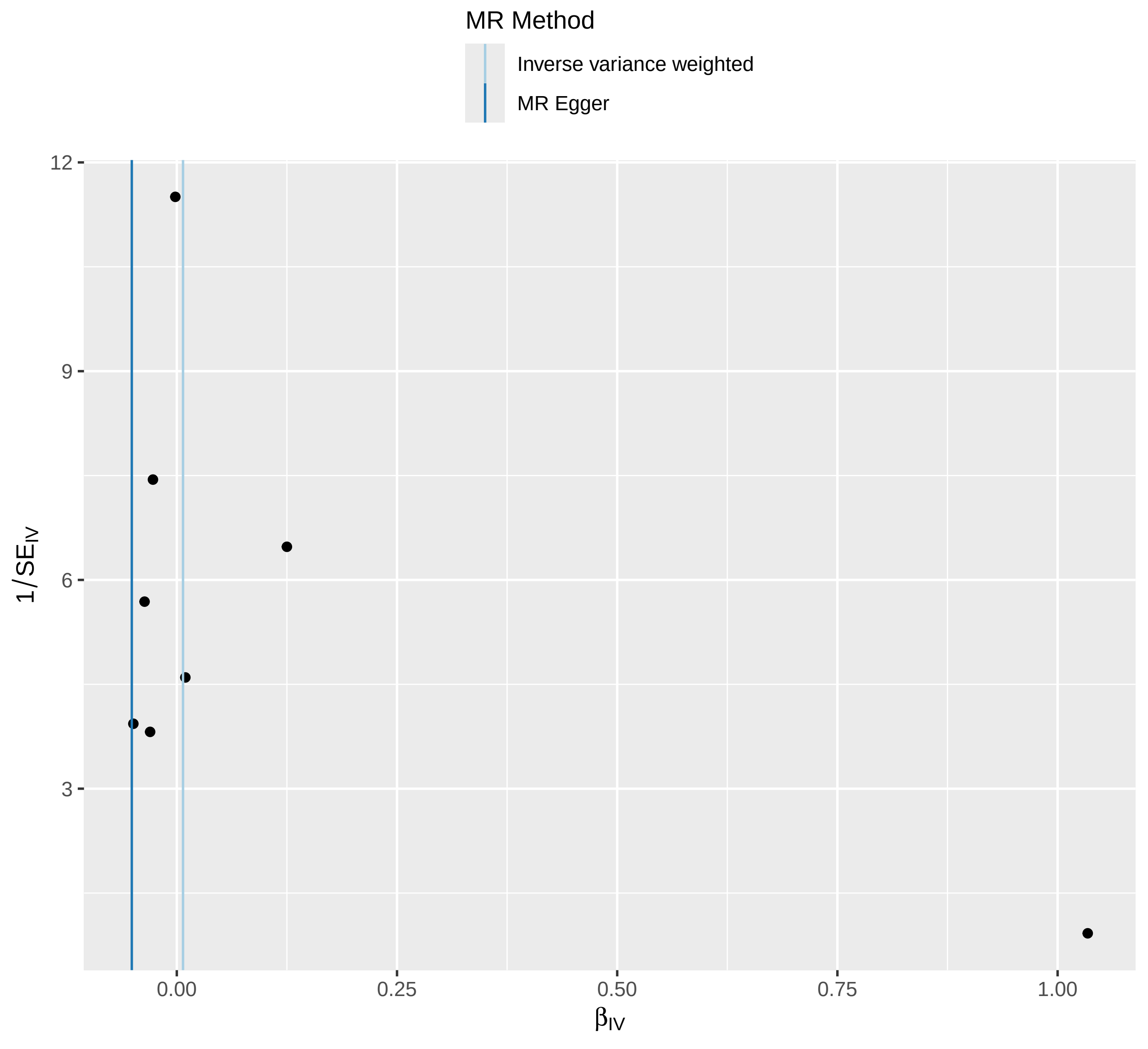

MR Method
Inverse variance weighted
MR Egger
12
9
6
3
1/SE_IV
0.00
0.25
0.50
0.75
1.00
β_IV

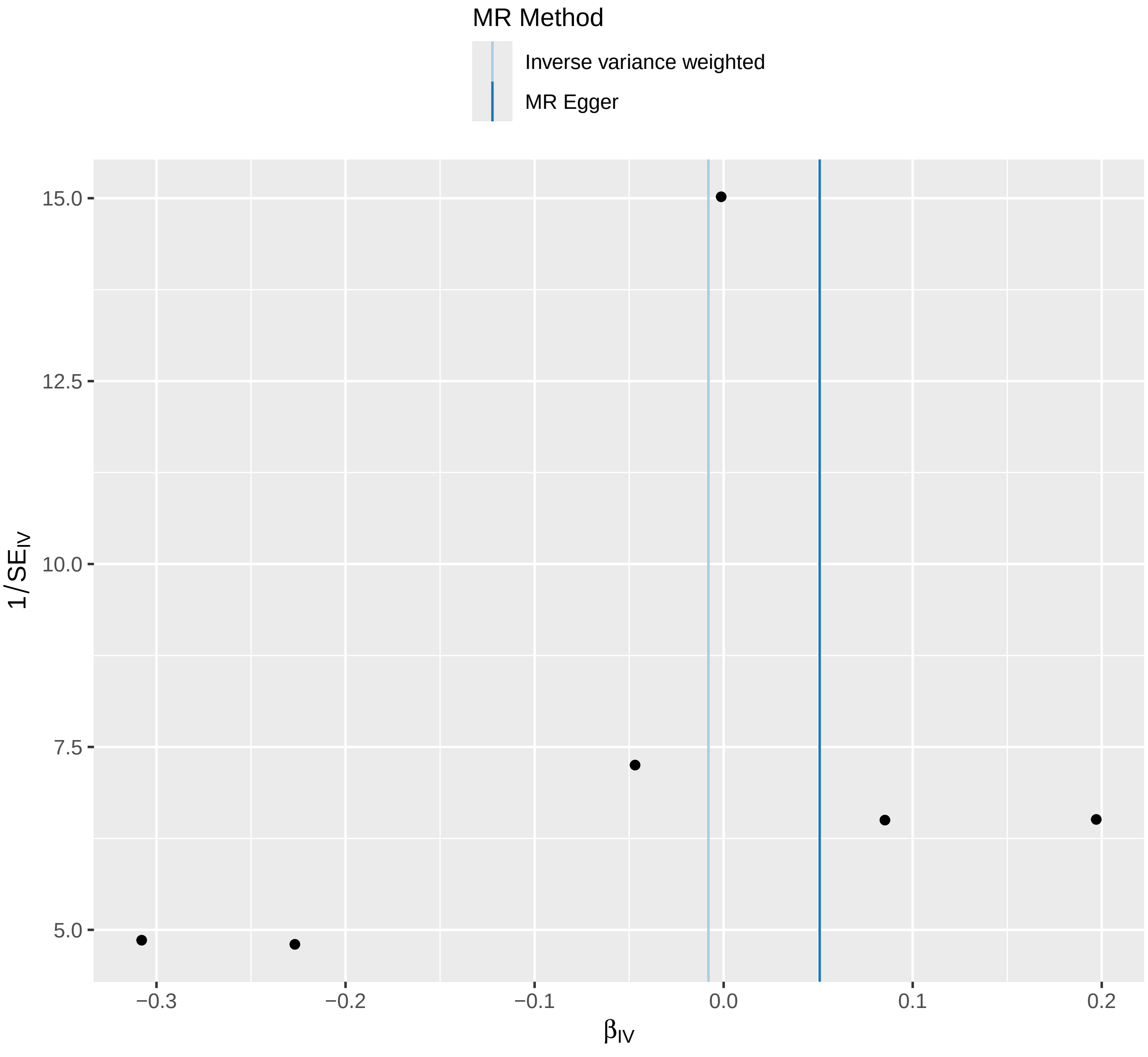
MR Method
Inverse variance weighted
MR Egger
15.0
12.5
10.0
7.5
5.0
1/SE_IV
−0.3
−0.2
−0.1
0.0
0.1
0.2
β_IV

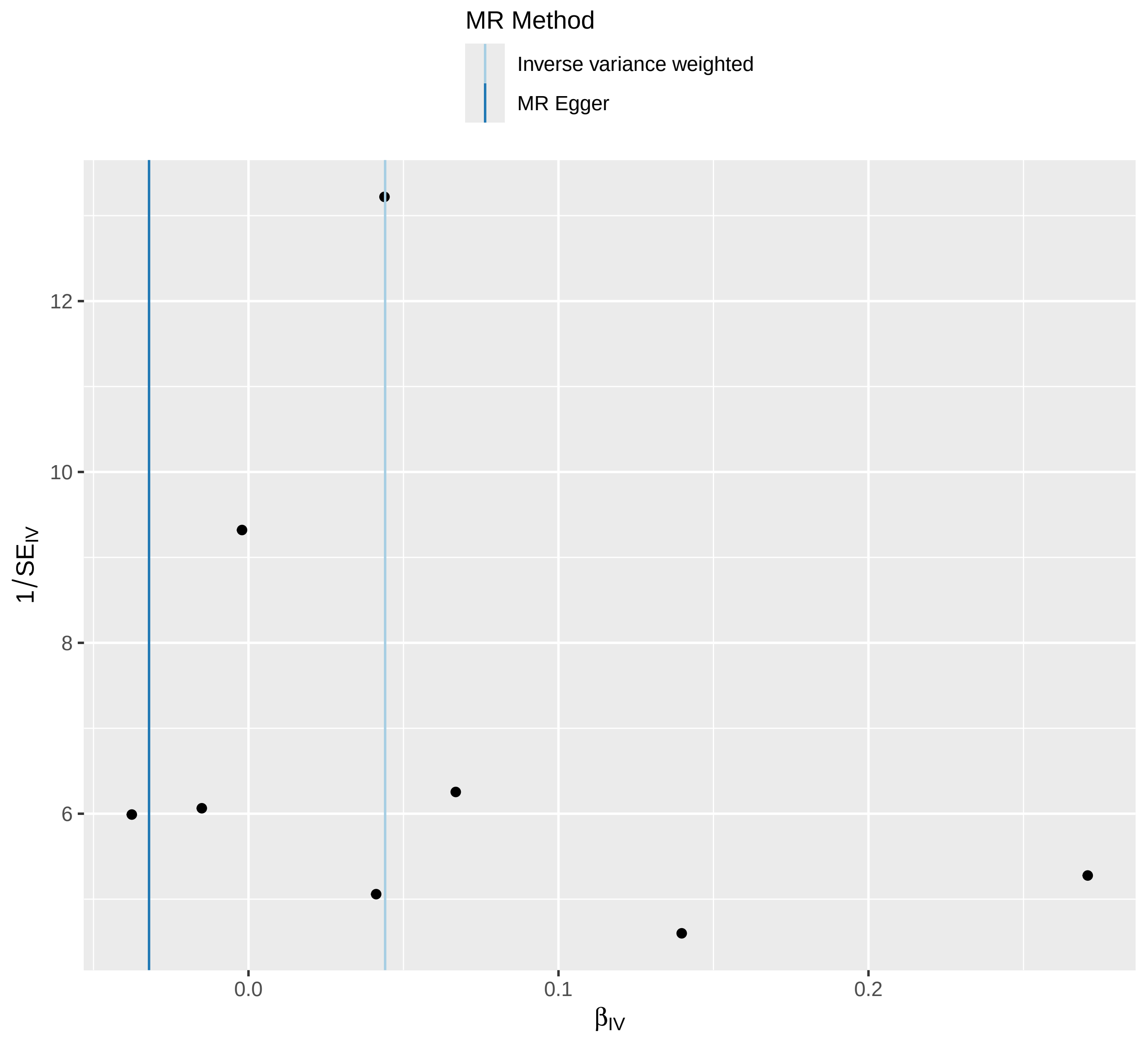

MR Method
Inverse variance weighted
MR Egger
12
10
8
6
1/SE_IV
0.0
0.1
0.2
β_IV

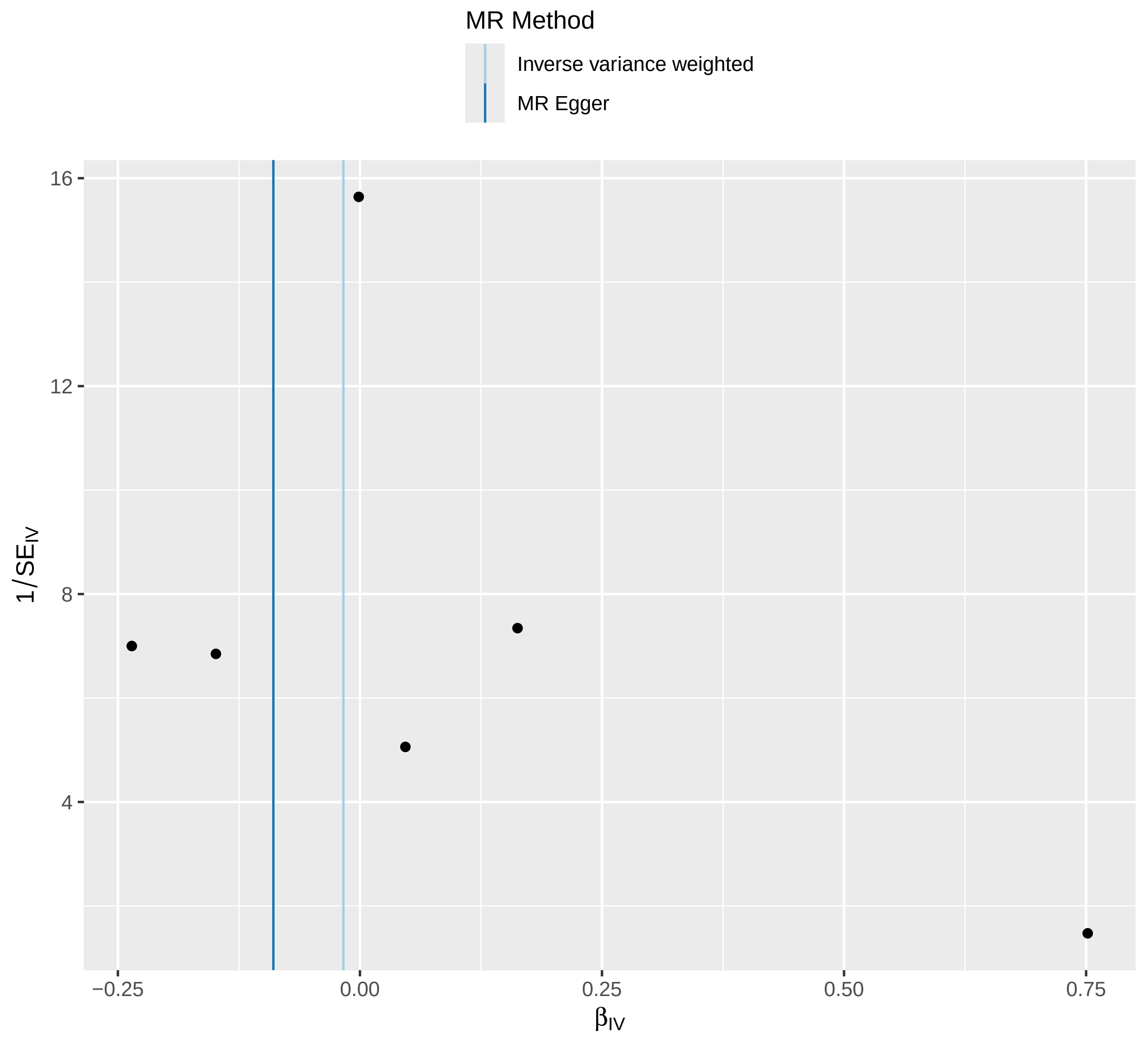
MR Method
Inverse variance weighted
MR Egger
16
12
8
4
1/SE_IV
−0.25
0.00
0.25
0.50
0.75
β_IV

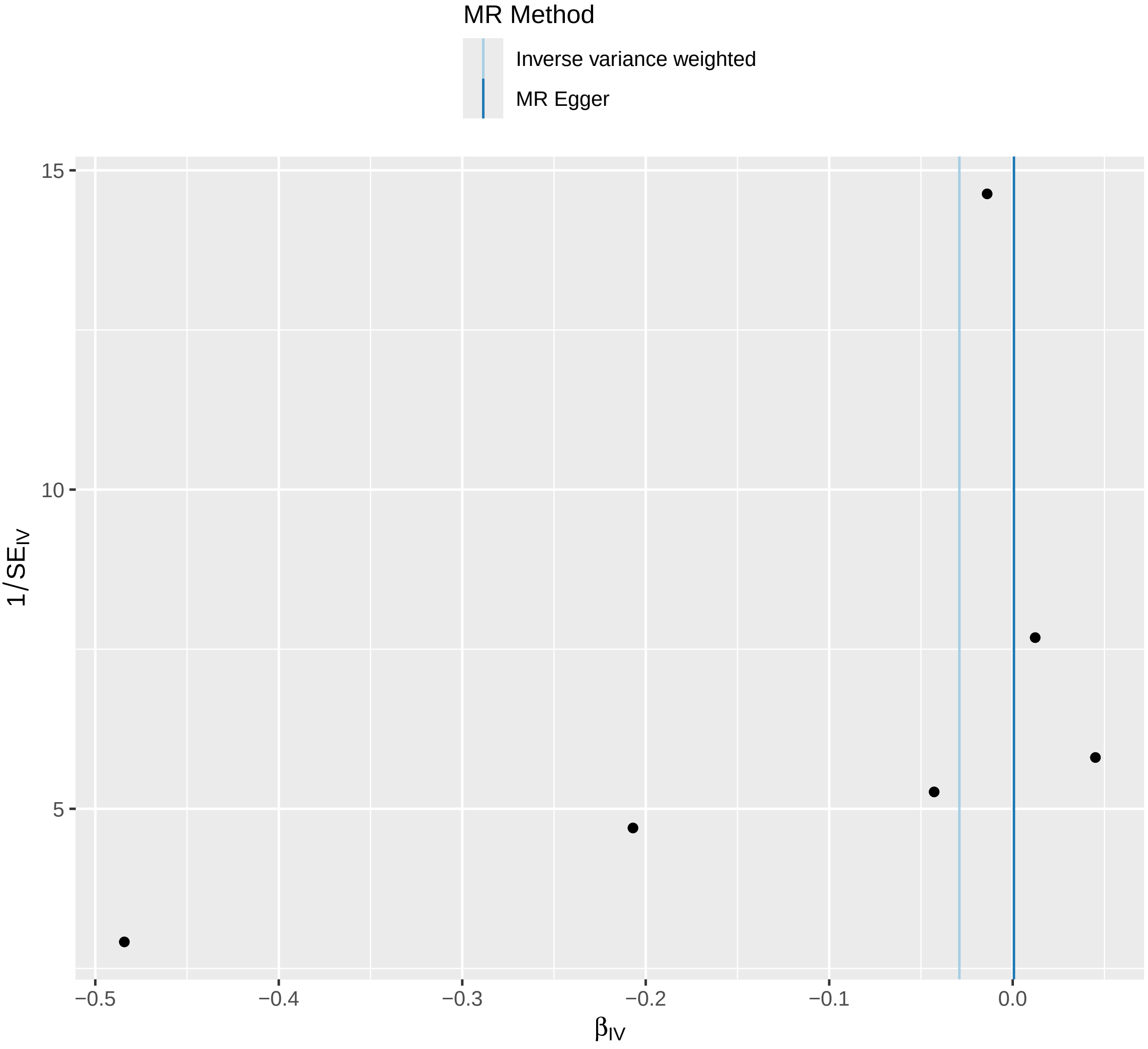

MR Method
Inverse variance weighted
MR Egger
15
10
5
1/SE$_{IV}$
−0.5
−0.4
−0.3
−0.2
−0.1
0.0
β$_{IV}$

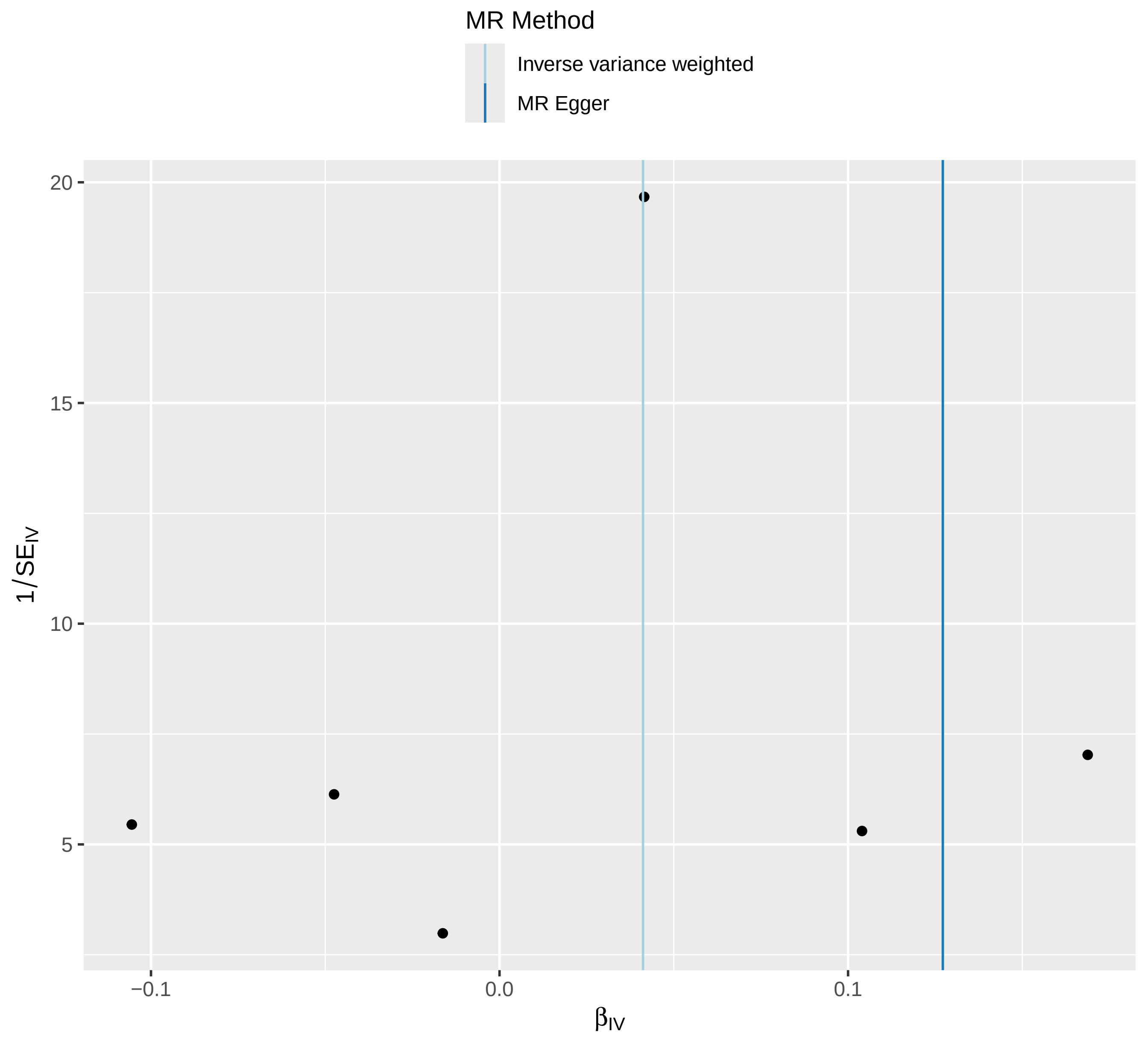
MR Method
Inverse variance weighted
MR Egger
20
15
10
5
1/SE$_{IV}$
−0.1
0.0
0.1
β$_{IV}$

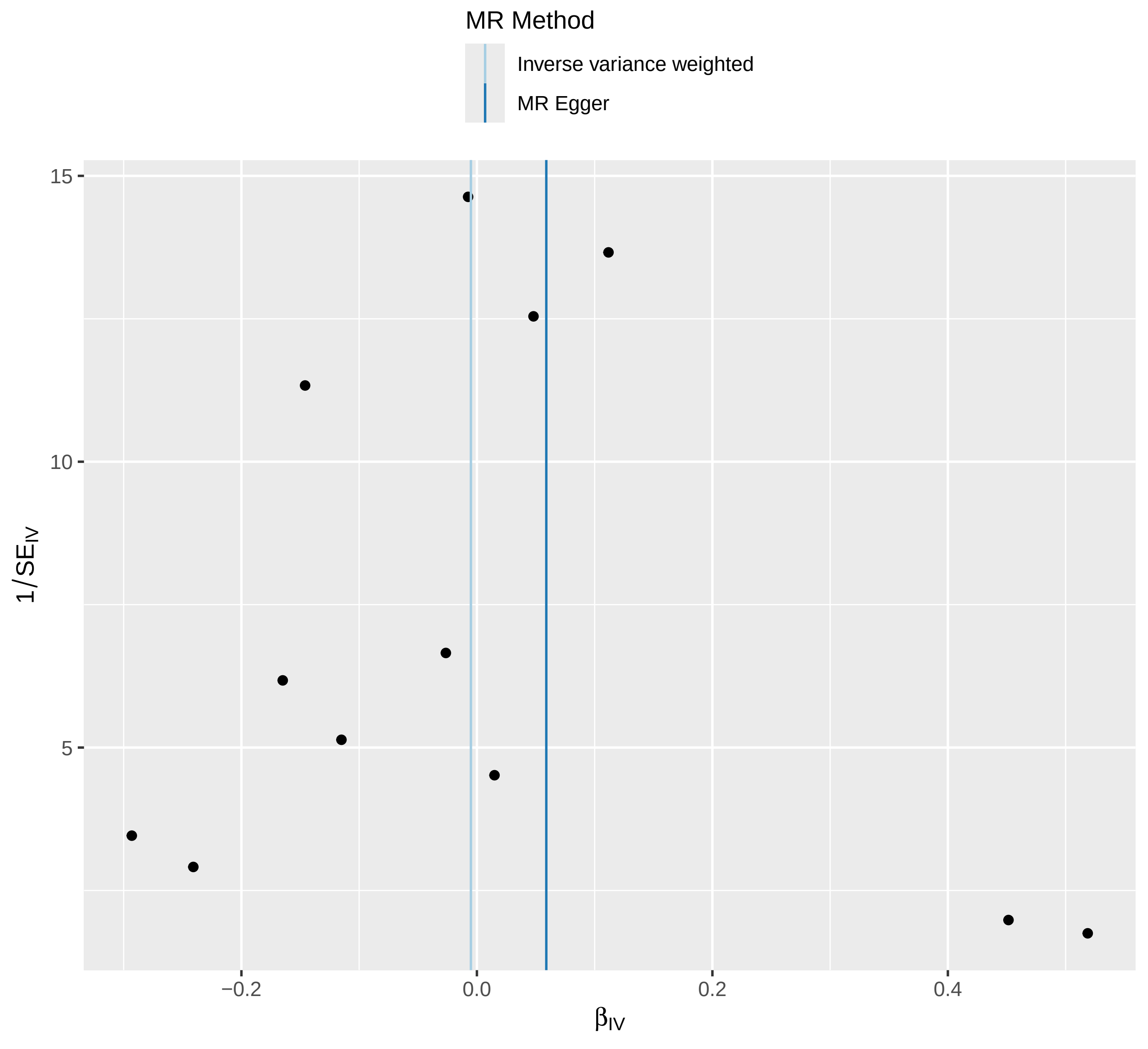

MR Method
Inverse variance weighted
MR Egger
15
10
5
1/SE$_{IV}$
−0.2
0.0
0.2
0.4
$\beta_{IV}$

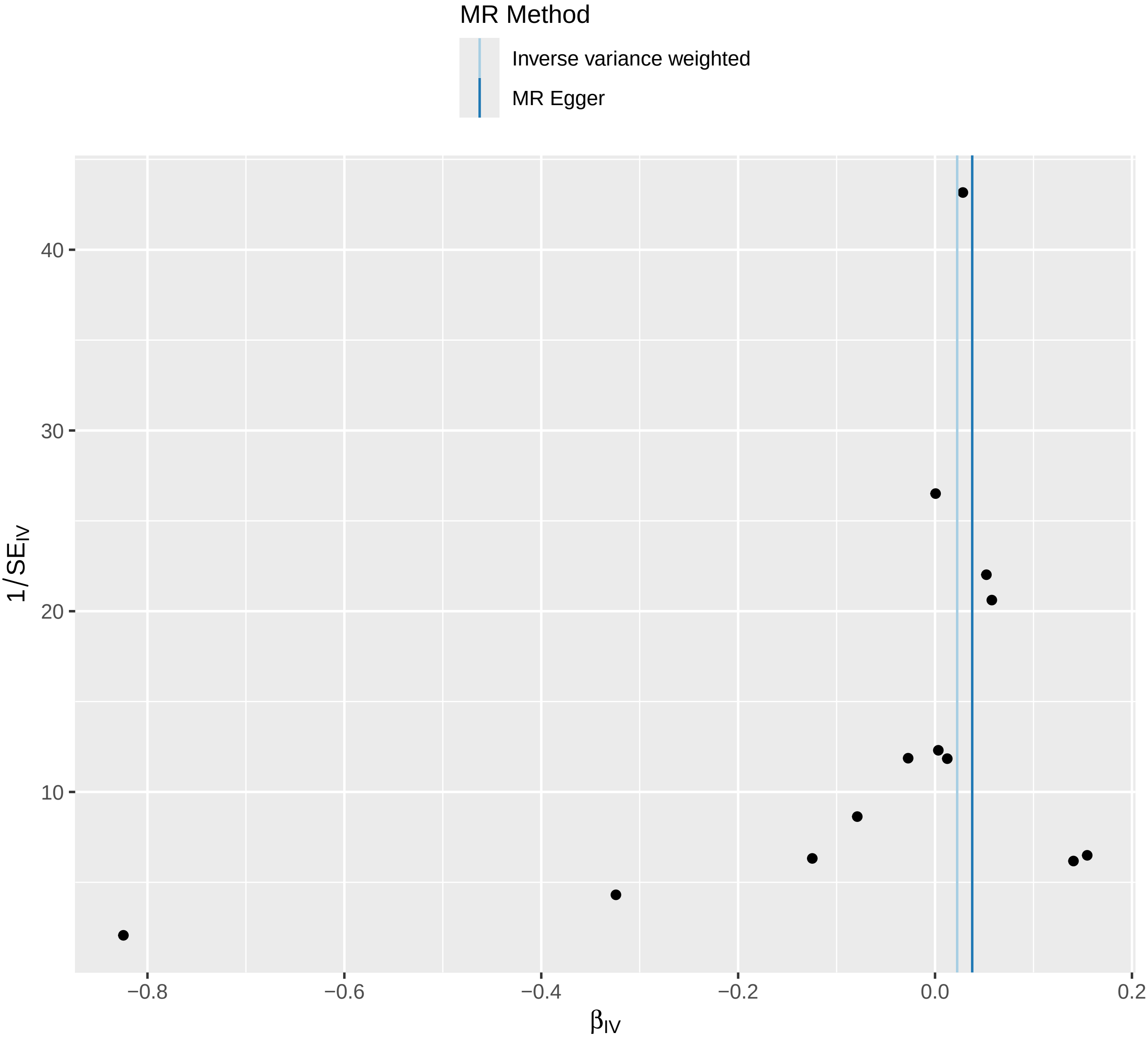
MR Method
Inverse variance weighted
MR Egger
40
30
20
10
1/SE$_{IV}$
−0.8
−0.6
−0.4
−0.2
0.0
0.2
β$_{IV}$

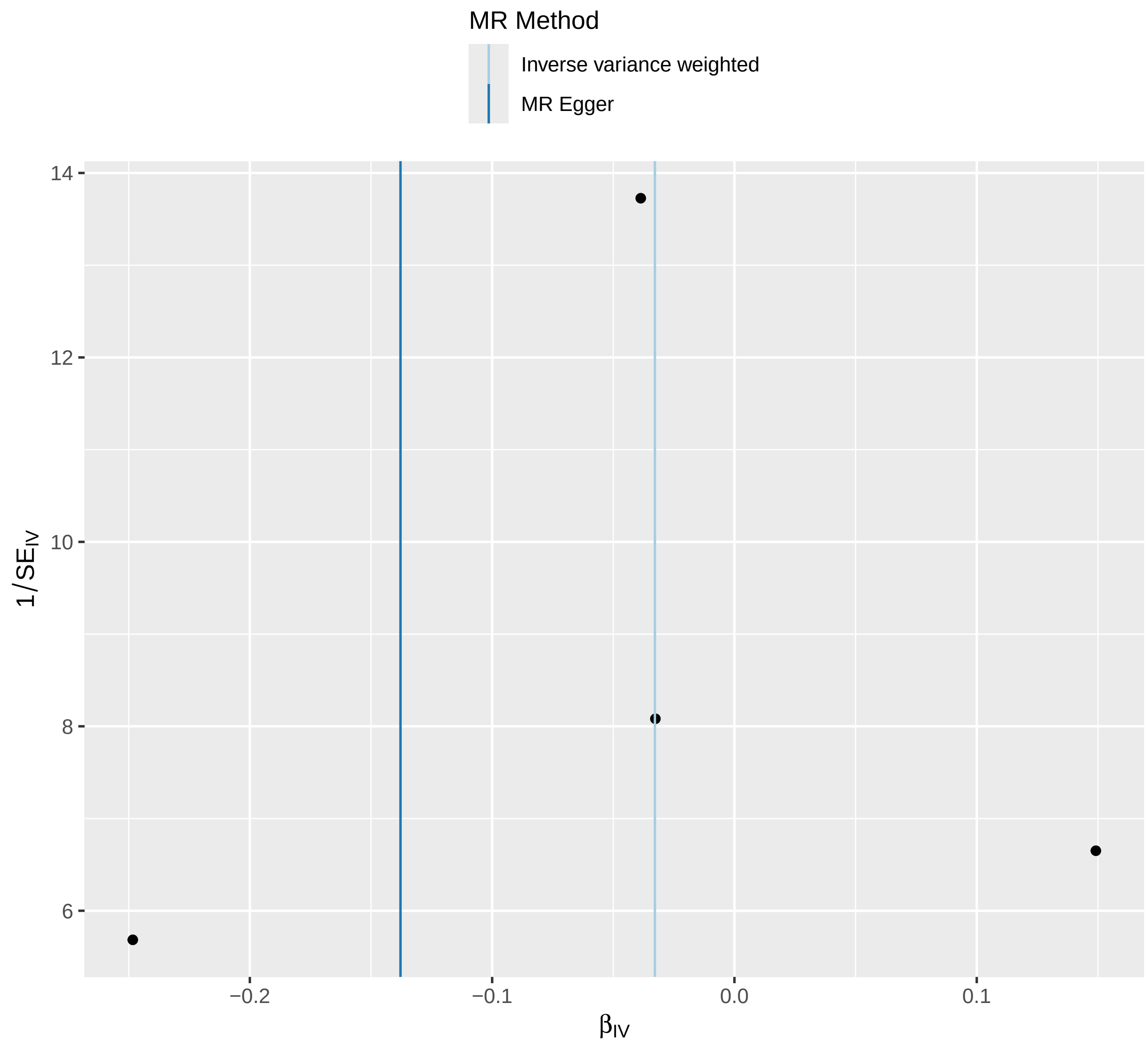

MR Method
Inverse variance weighted
MR Egger
14
12
10
8
6
1/SE$_{IV}$
−0.2
−0.1
0.0
0.1
β$_{IV}$

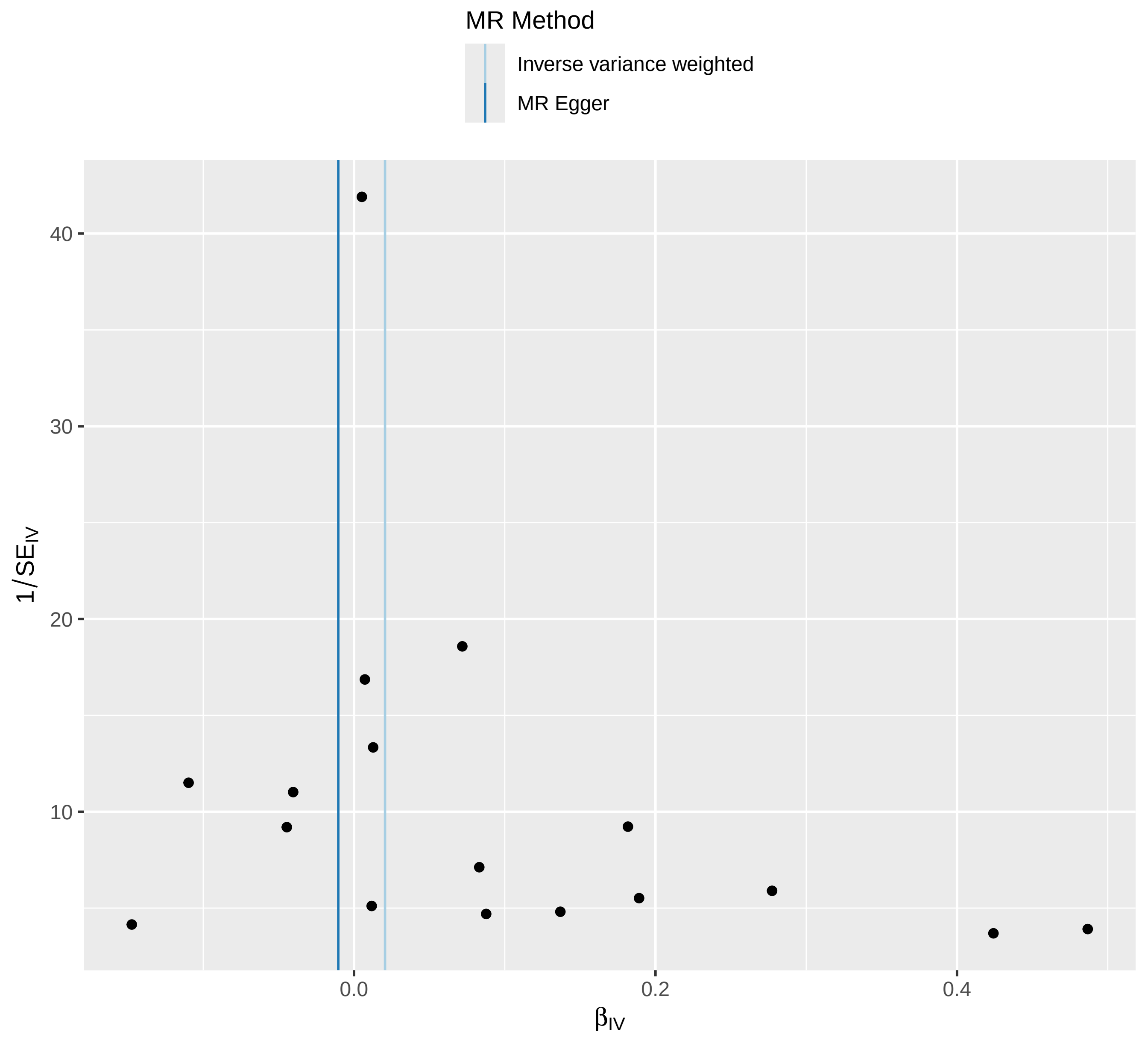

MR Method
Inverse variance weighted
MR Egger
40
30
20
10
1/SE_IV
0.0
0.2
0.4
β_IV

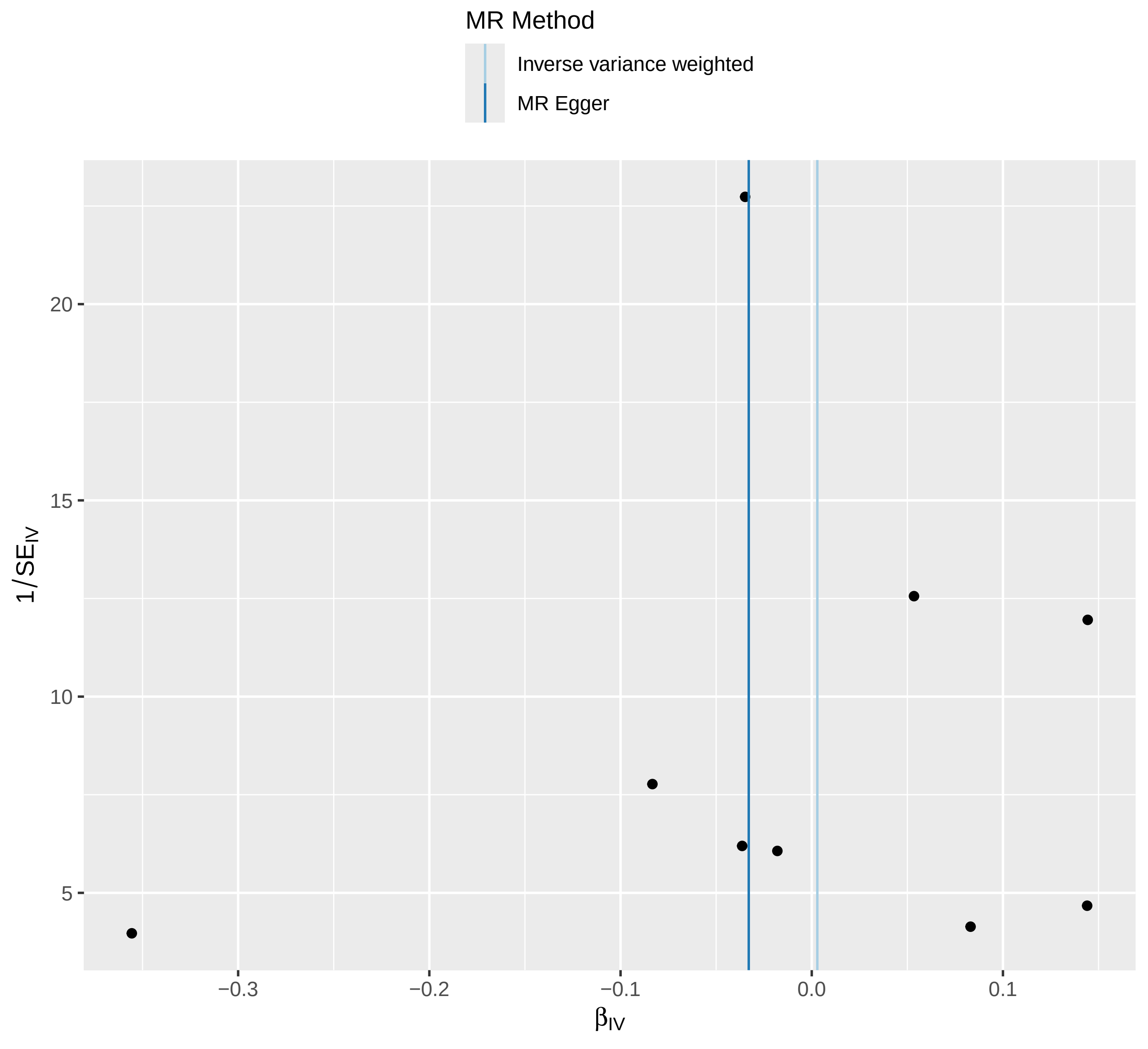

MR Method
Inverse variance weighted
MR Egger
20
15
10
5
1/SE$_{IV}$
−0.3
−0.2
−0.1
0.0
0.1
β$_{IV}$

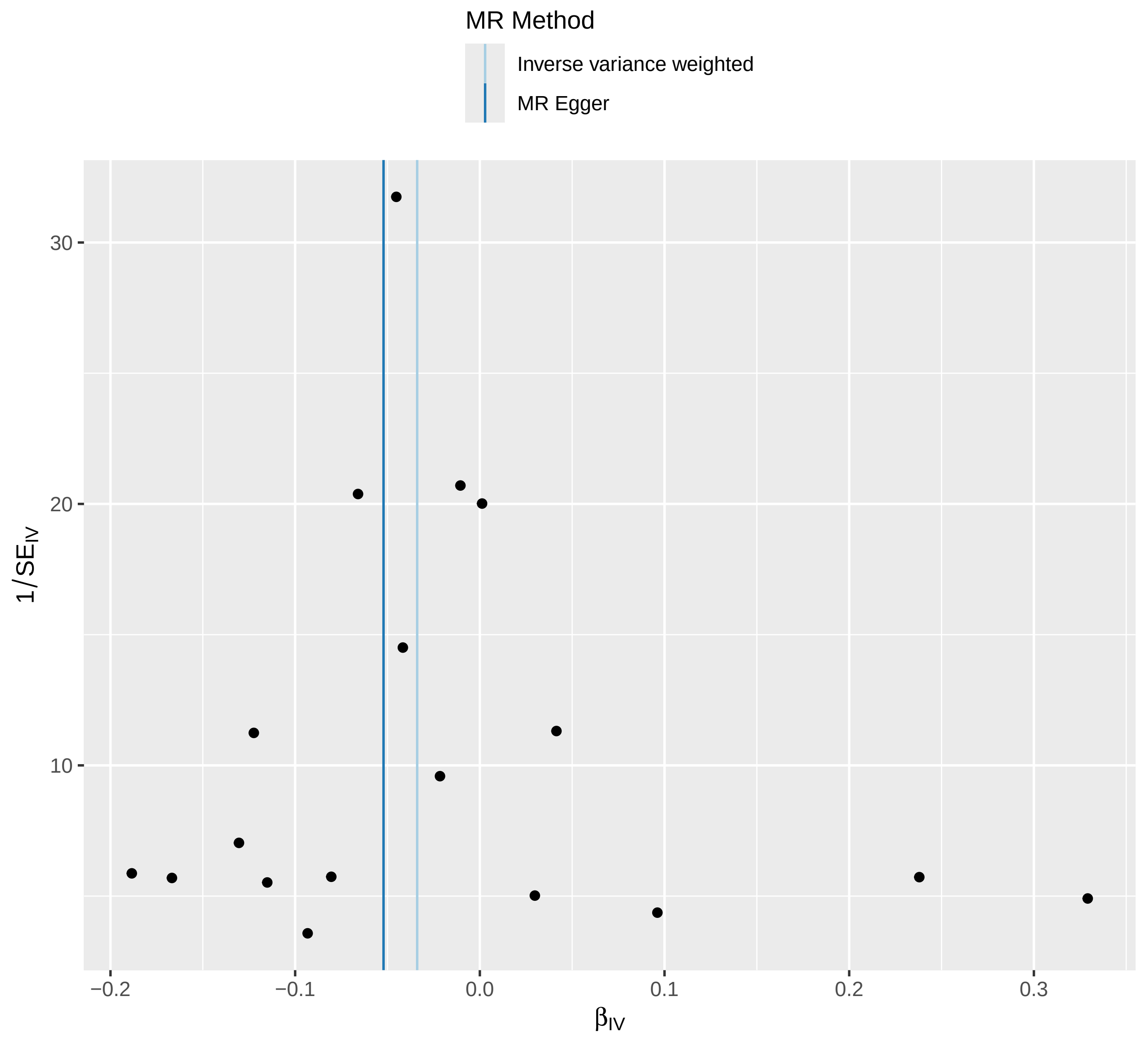
MR Method
Inverse variance weighted
MR Egger
30
20
10
1/SE_IV
−0.2
−0.1
0.0
0.1
0.2
0.3
β_IV

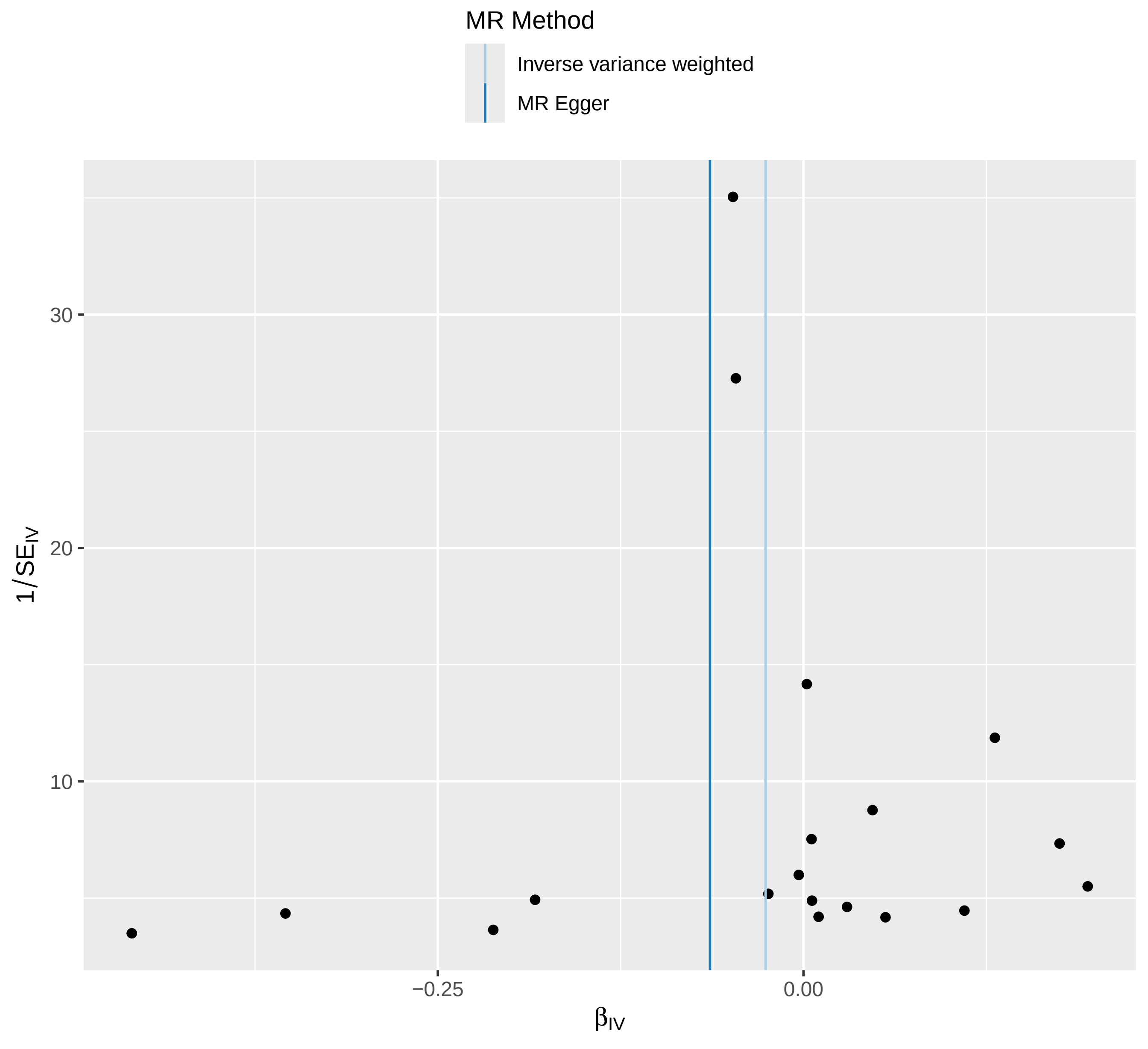
MR Method
Inverse variance weighted
MR Egger
30
20
10
1/SE_IV
−0.25
0.00
β_IV

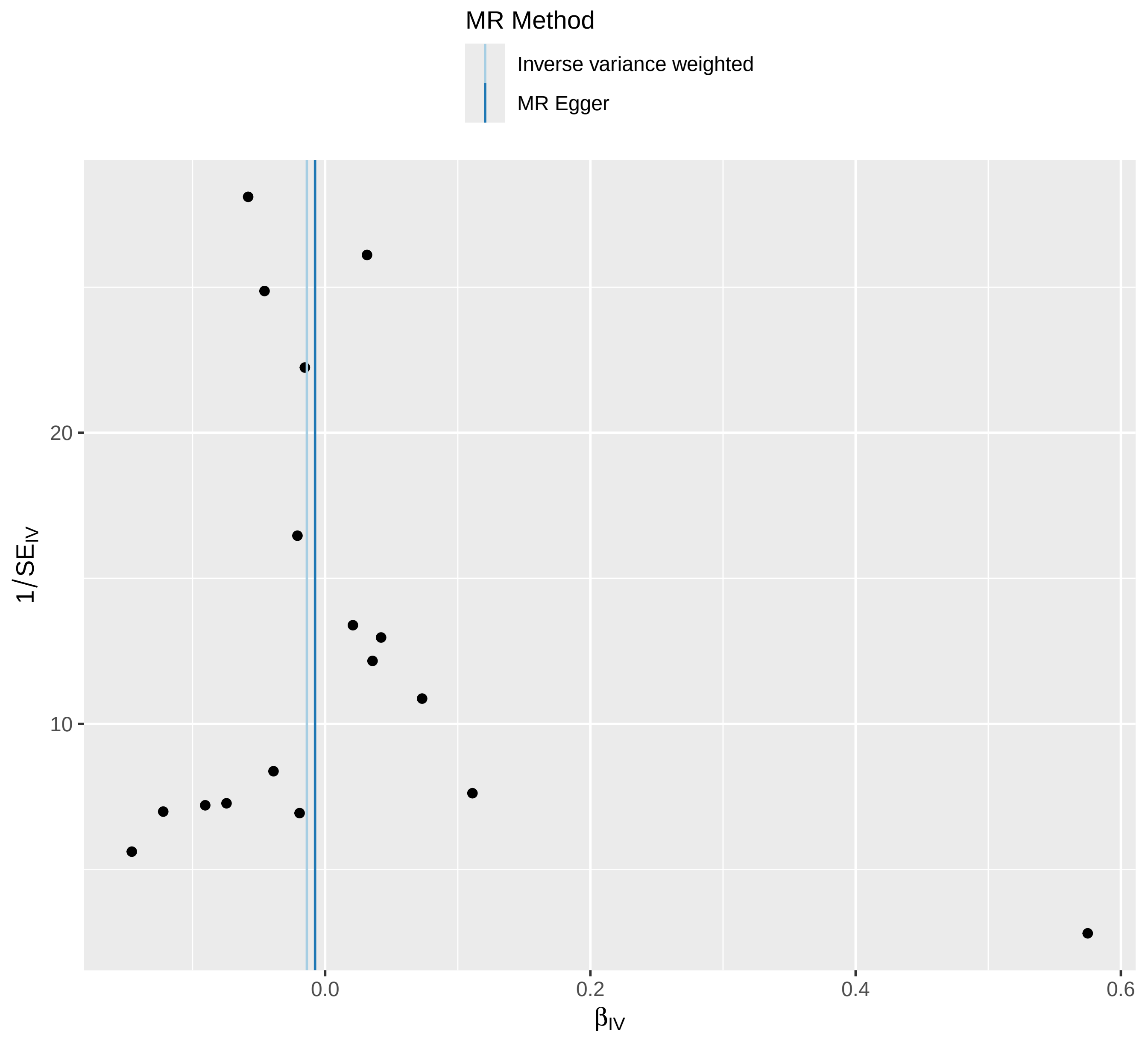

MR Method
Inverse variance weighted
MR Egger
20
10
1/SE_IV
0.0
0.2
0.4
0.6
β_IV

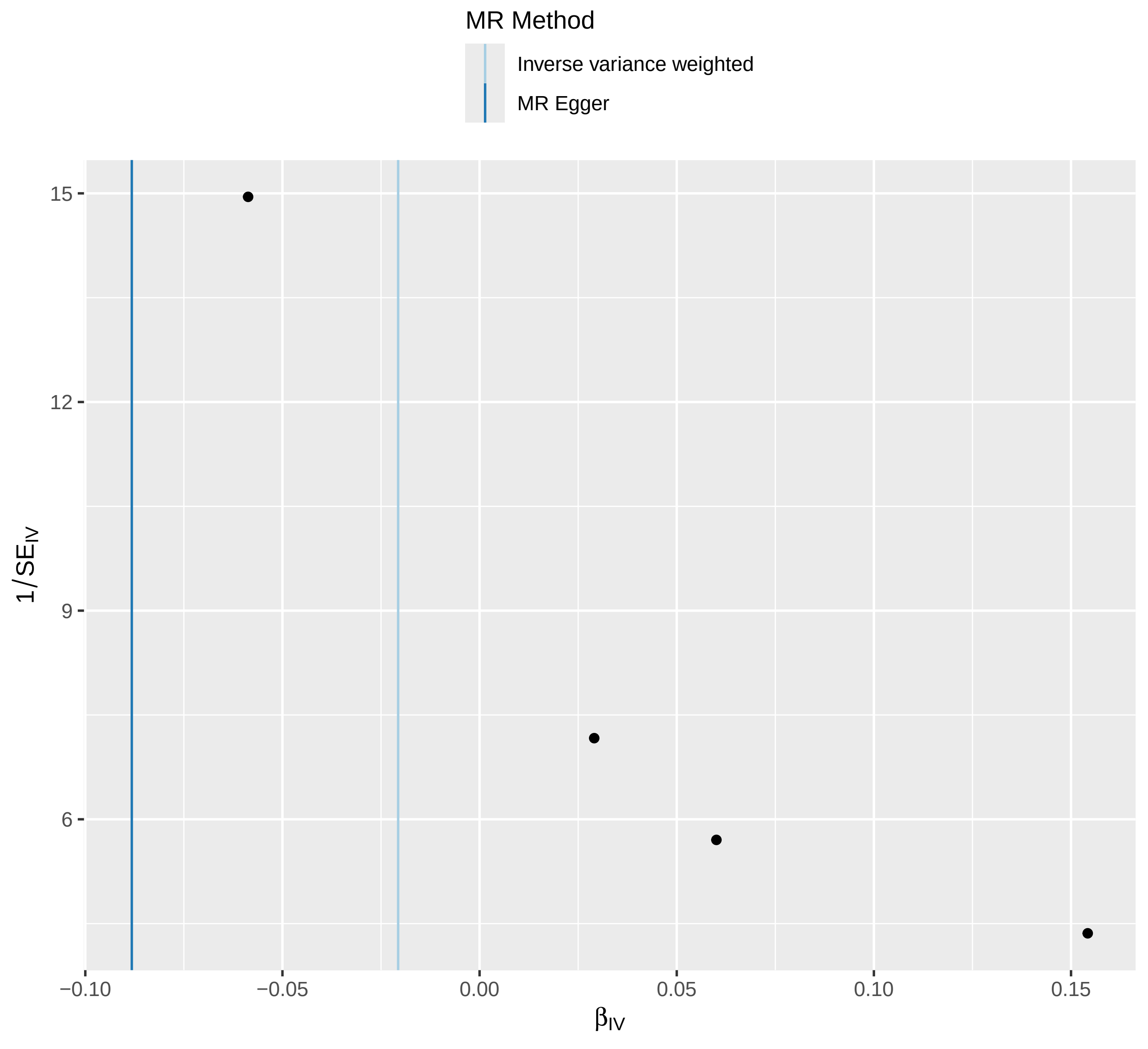

MR Method
Inverse variance weighted
MR Egger
15
12
9
6
1/SE$_{IV}$
−0.10
−0.05
0.00
0.05
0.10
0.15
β$_{IV}$

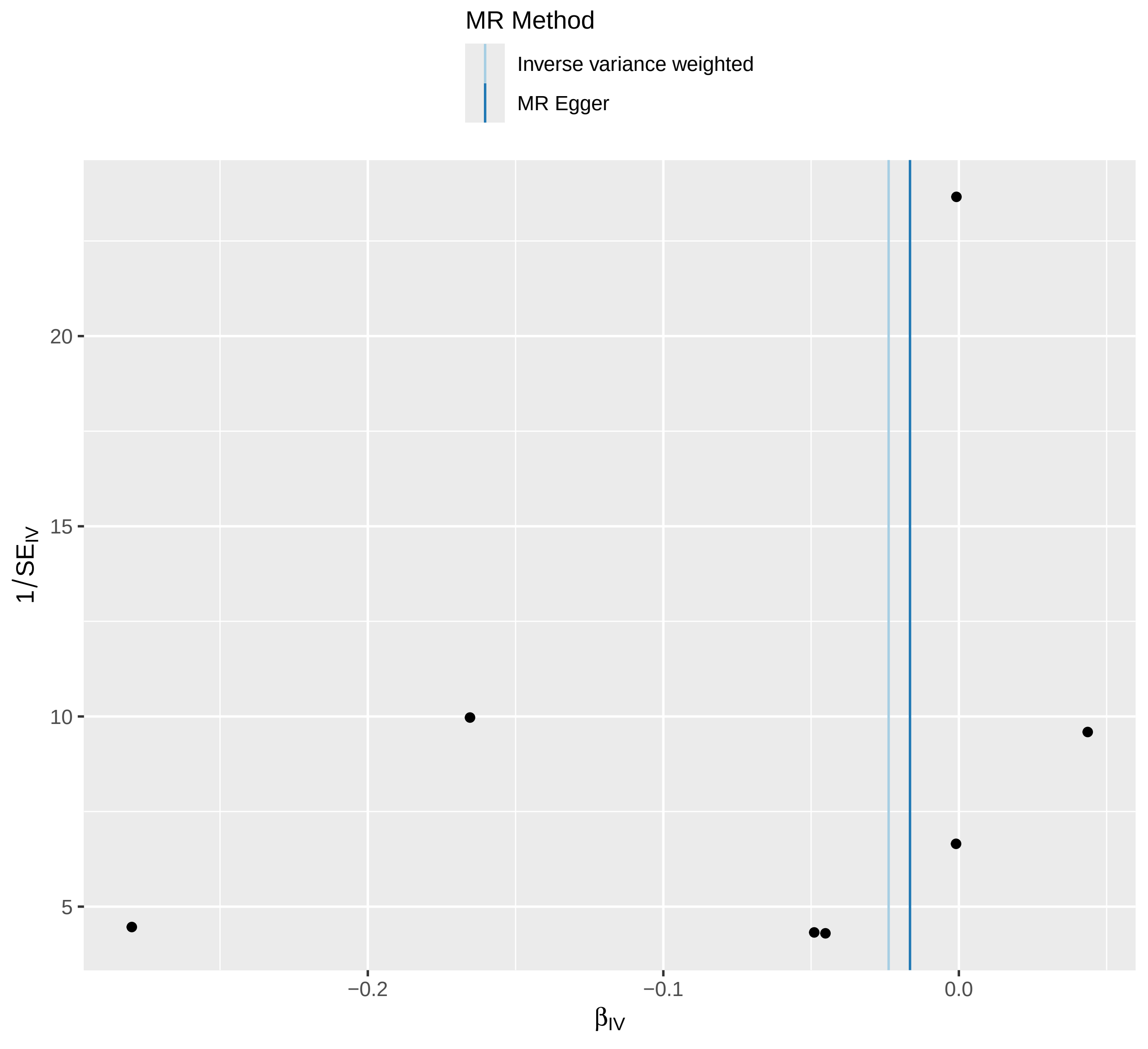
MR Method
Inverse variance weighted
MR Egger
20
15
10
5
1/SE_IV
−0.2
−0.1
0.0
β_IV

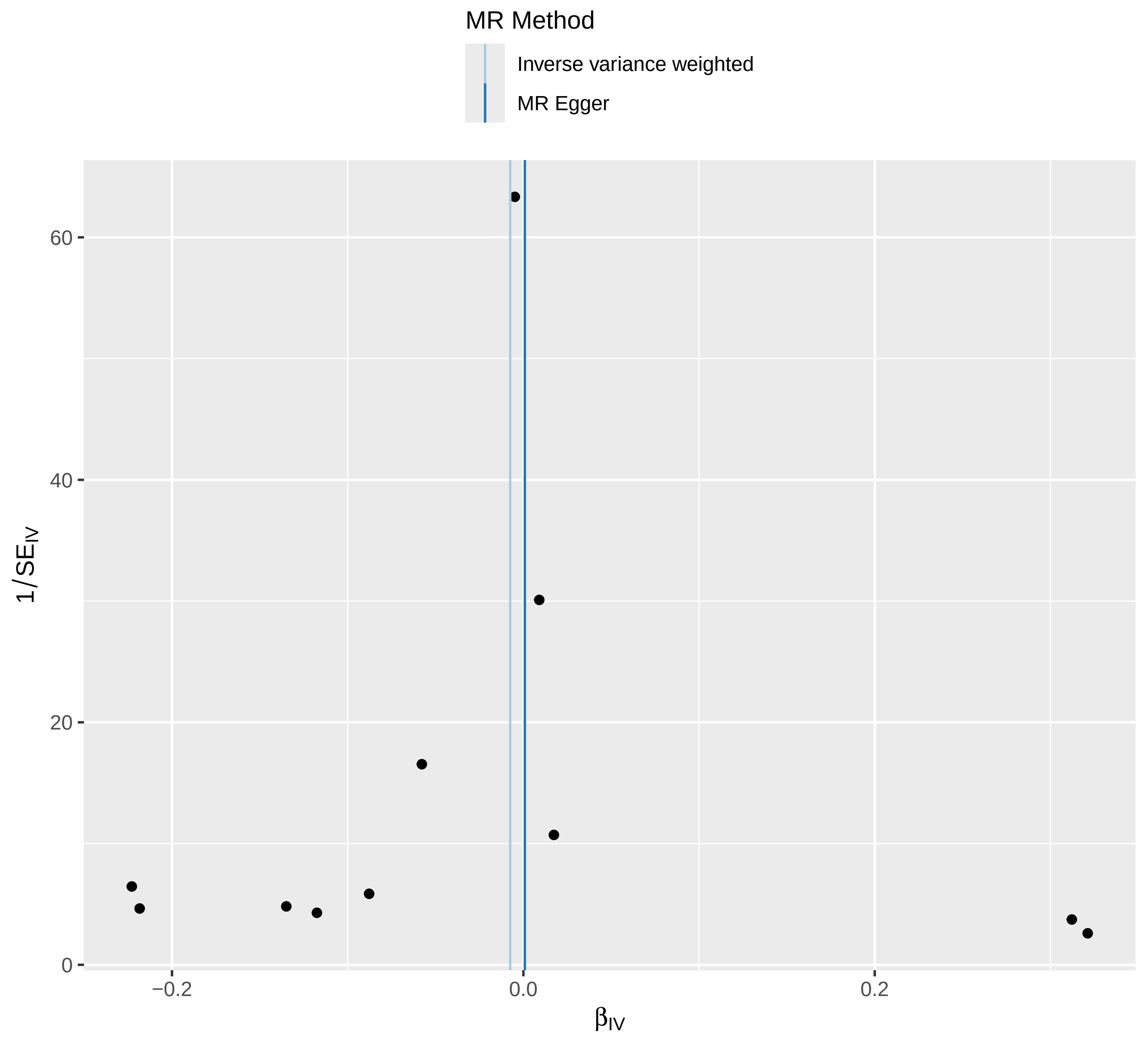
MR Method
Inverse variance weighted
MR Egger
60
40
20
0
1/SE$_{IV}$
−0.2
0.0
0.2
β$_{IV}$

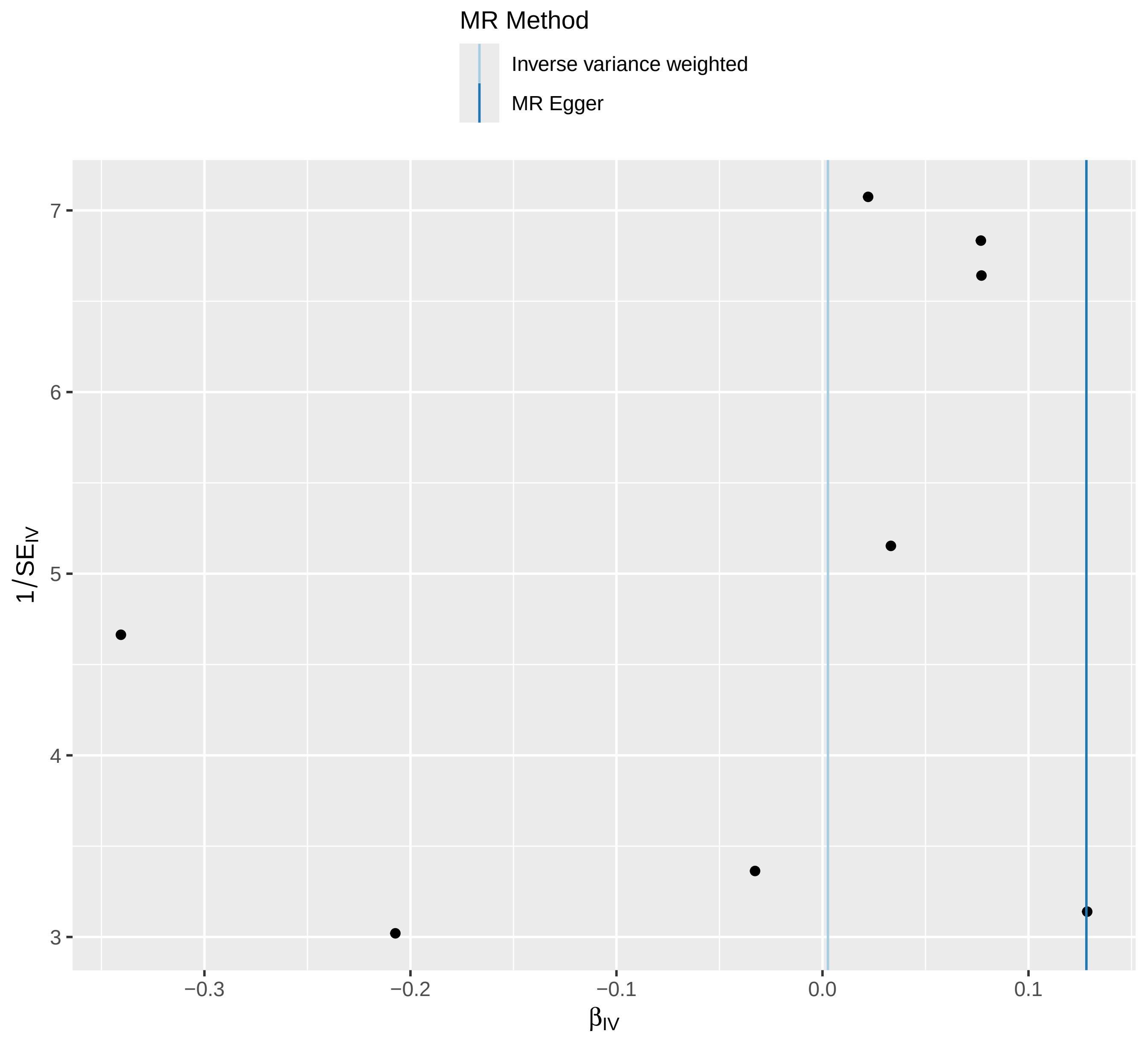

MR Method
Inverse variance weighted
MR Egger
7
6
5
4
3
1/SE_IV
−0.3
−0.2
−0.1
0.0
0.1
β_IV

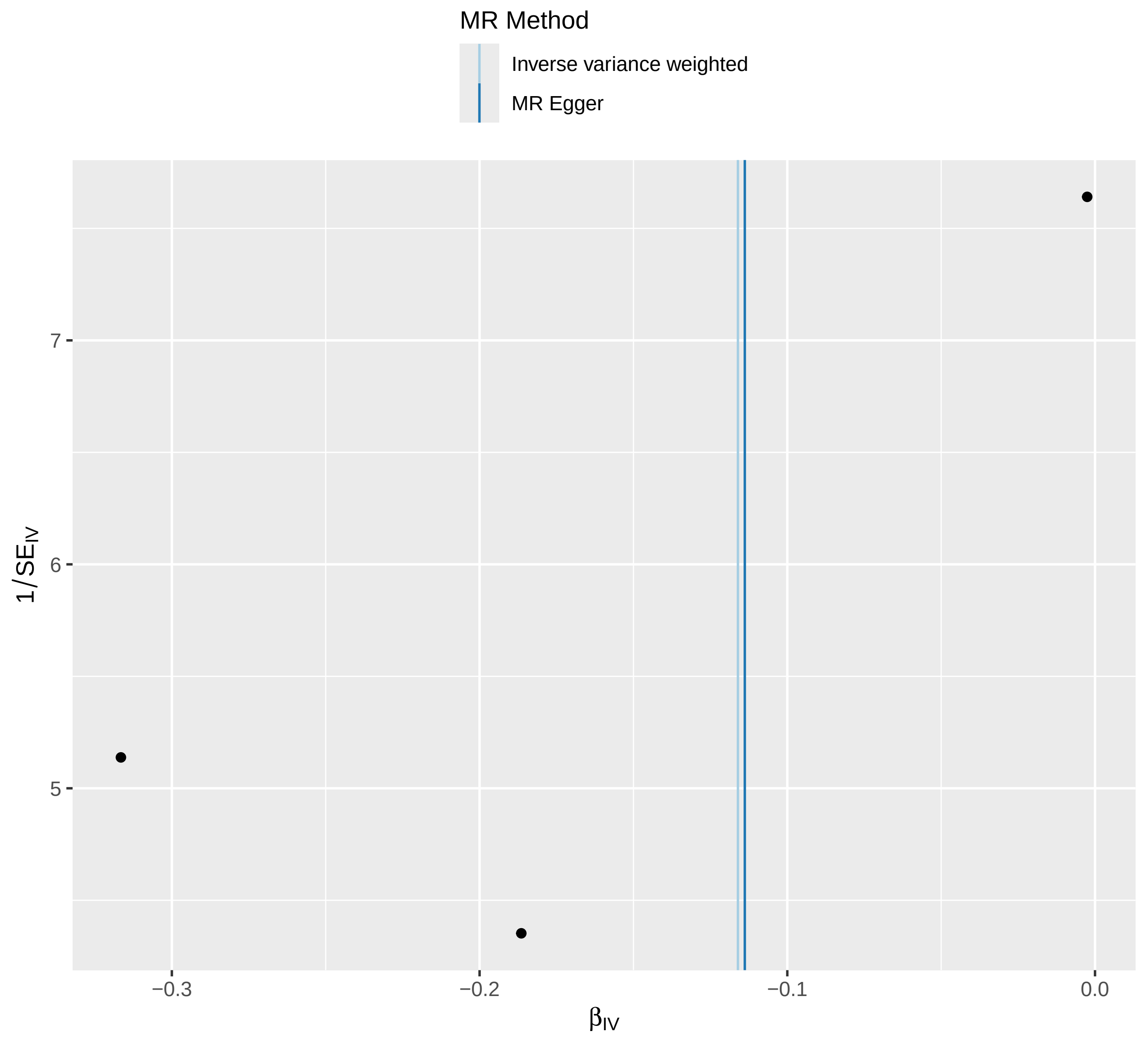
MR Method
Inverse variance weighted
MR Egger
7
6
5
$1/SE_{IV}$
−0.3
−0.2
−0.1
0.0
$\beta_{IV}$

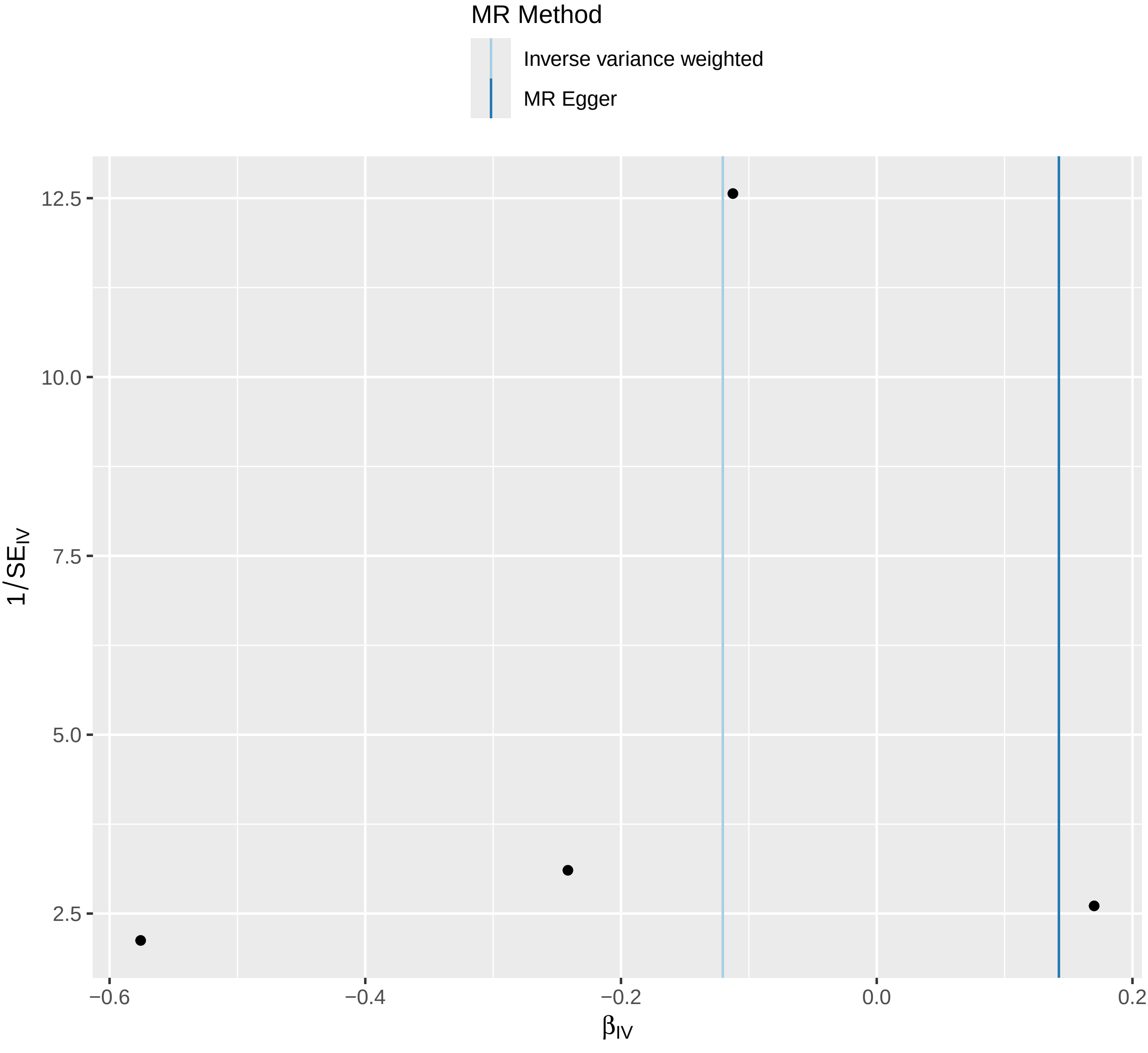

MR Method
Inverse variance weighted
MR Egger
12.5
10.0
7.5
5.0
2.5
1/SE$_{IV}$
−0.6
−0.4
−0.2
0.0
0.2
β$_{IV}$

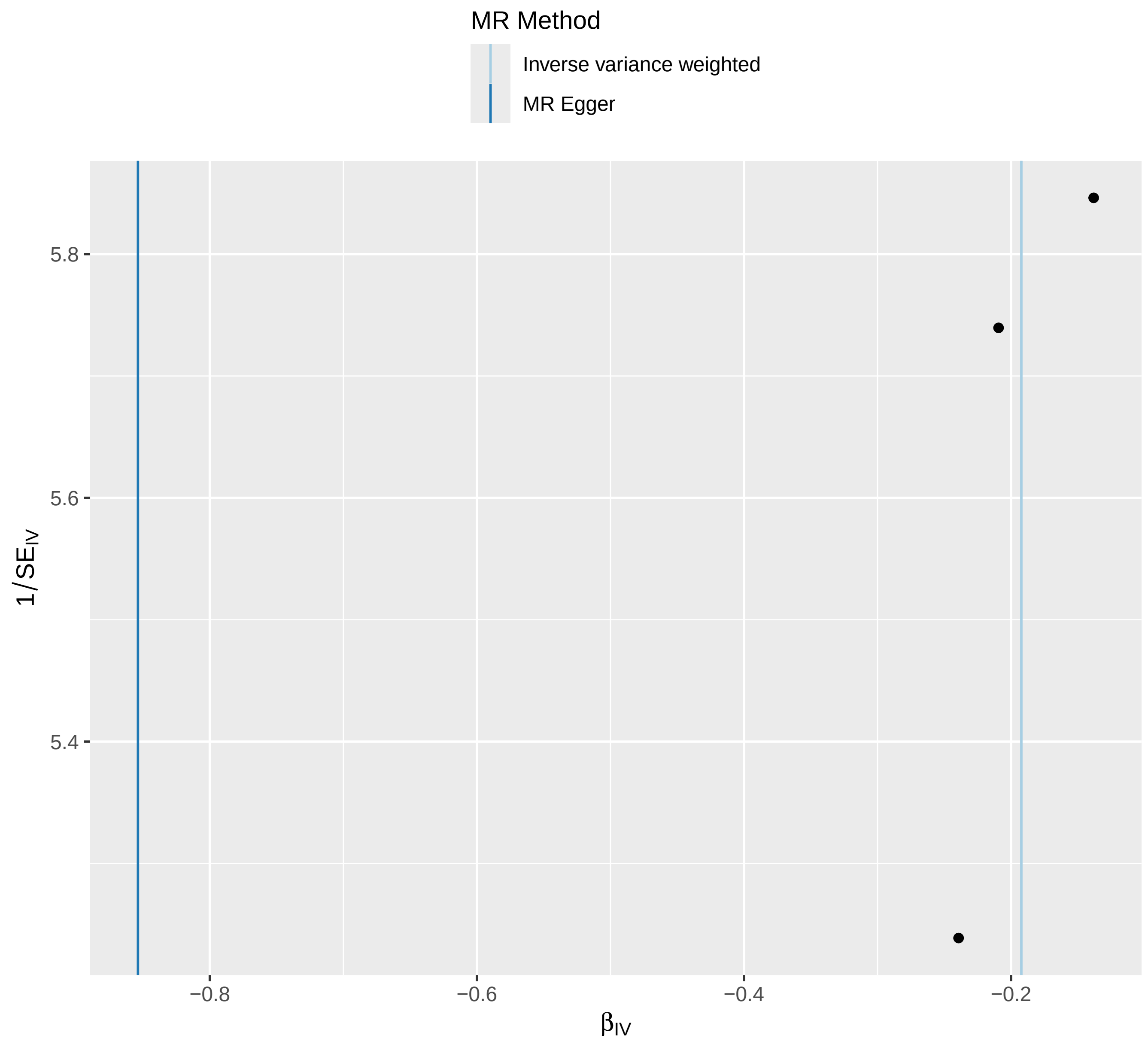
MR Method
Inverse variance weighted
MR Egger
5.8
5.6
5.4
$1/SE_{IV}$
−0.8
−0.6
−0.4
−0.2
$\beta_{IV}$

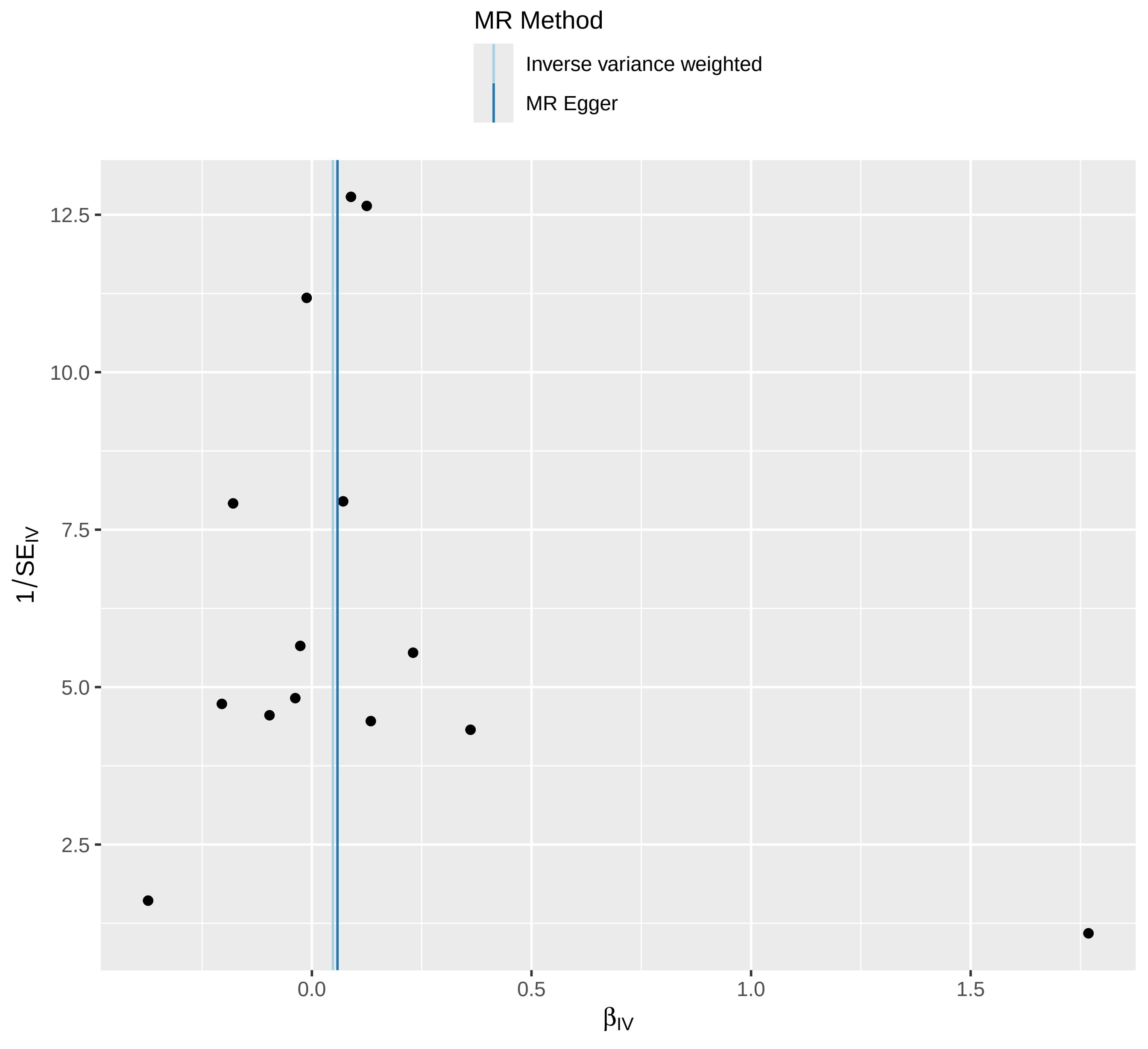
MR Method
Inverse variance weighted
MR Egger
12.5
10.0
7.5
5.0
2.5
1/SE_IV
0.0
0.5
1.0
1.5
β_IV

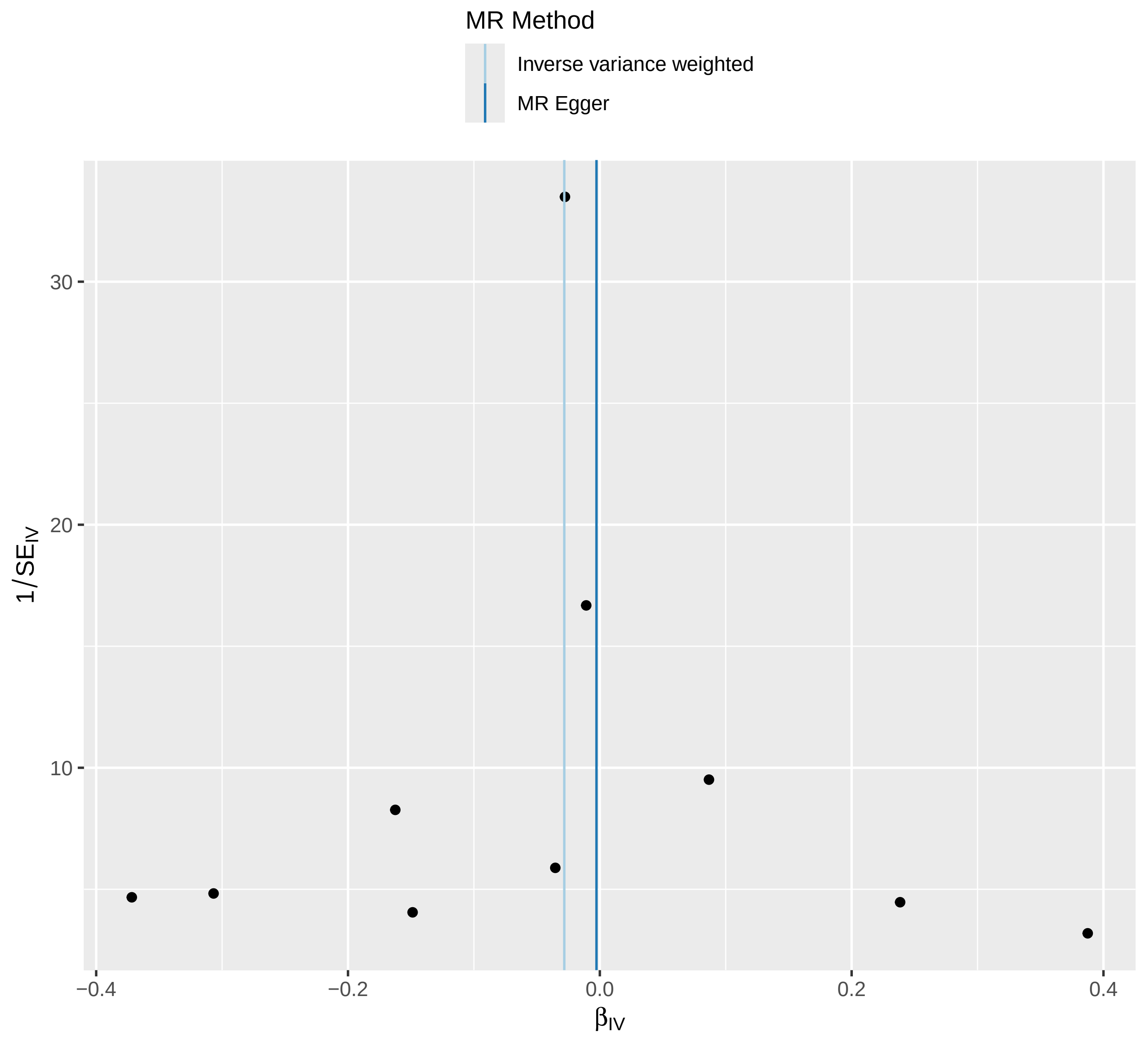

MR Method
Inverse variance weighted
MR Egger
30
20
10
1/SE_IV
−0.4
−0.2
0.0
0.2
0.4
β_IV

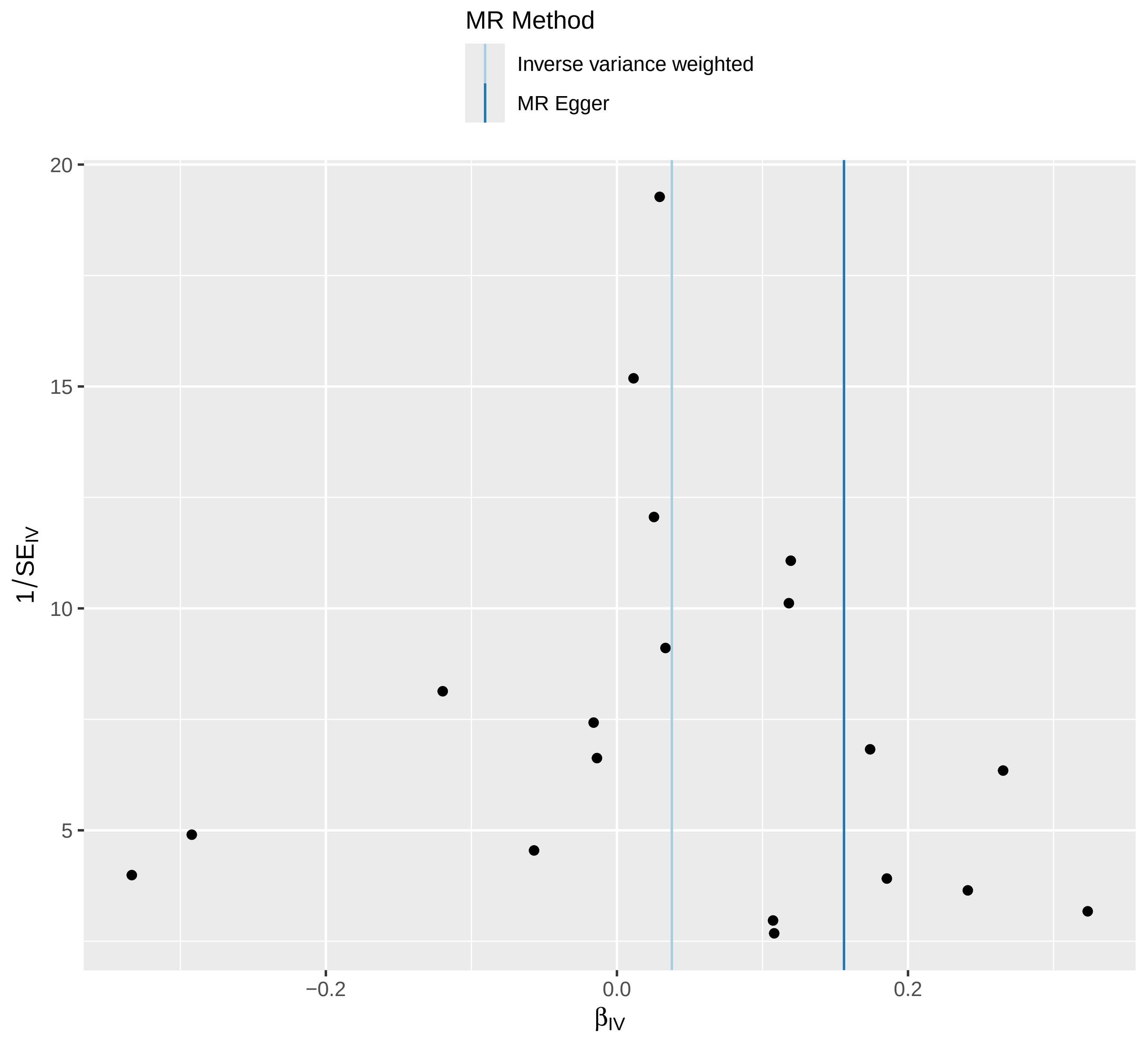
MR Method
Inverse variance weighted
MR Egger
20
15
10
5
1/SE_IV
−0.2
0.0
0.2
β_IV

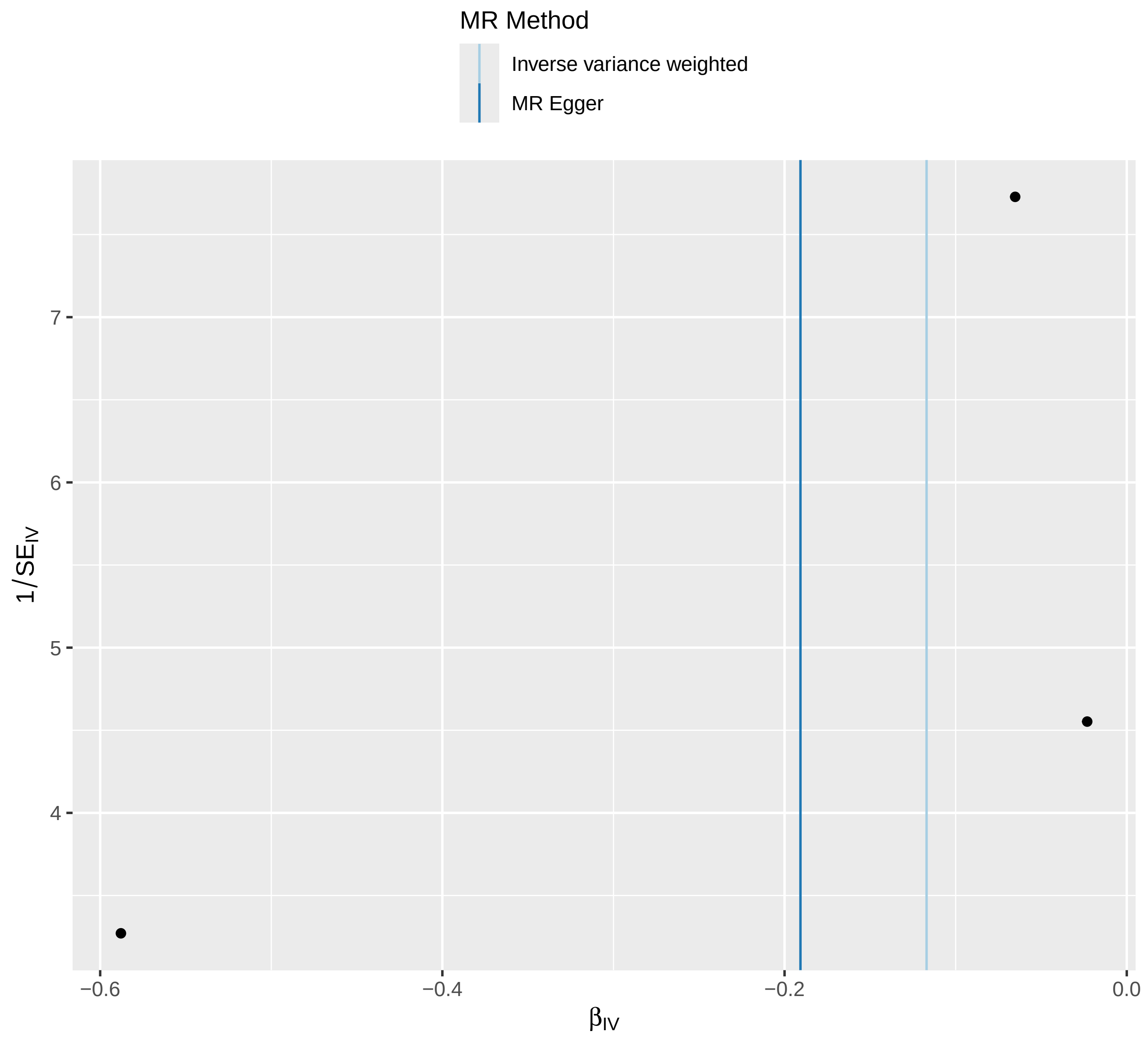
MR Method
Inverse variance weighted
MR Egger
7
6
5
4
1/SE_IV
−0.6
−0.4
−0.2
0.0
β_IV

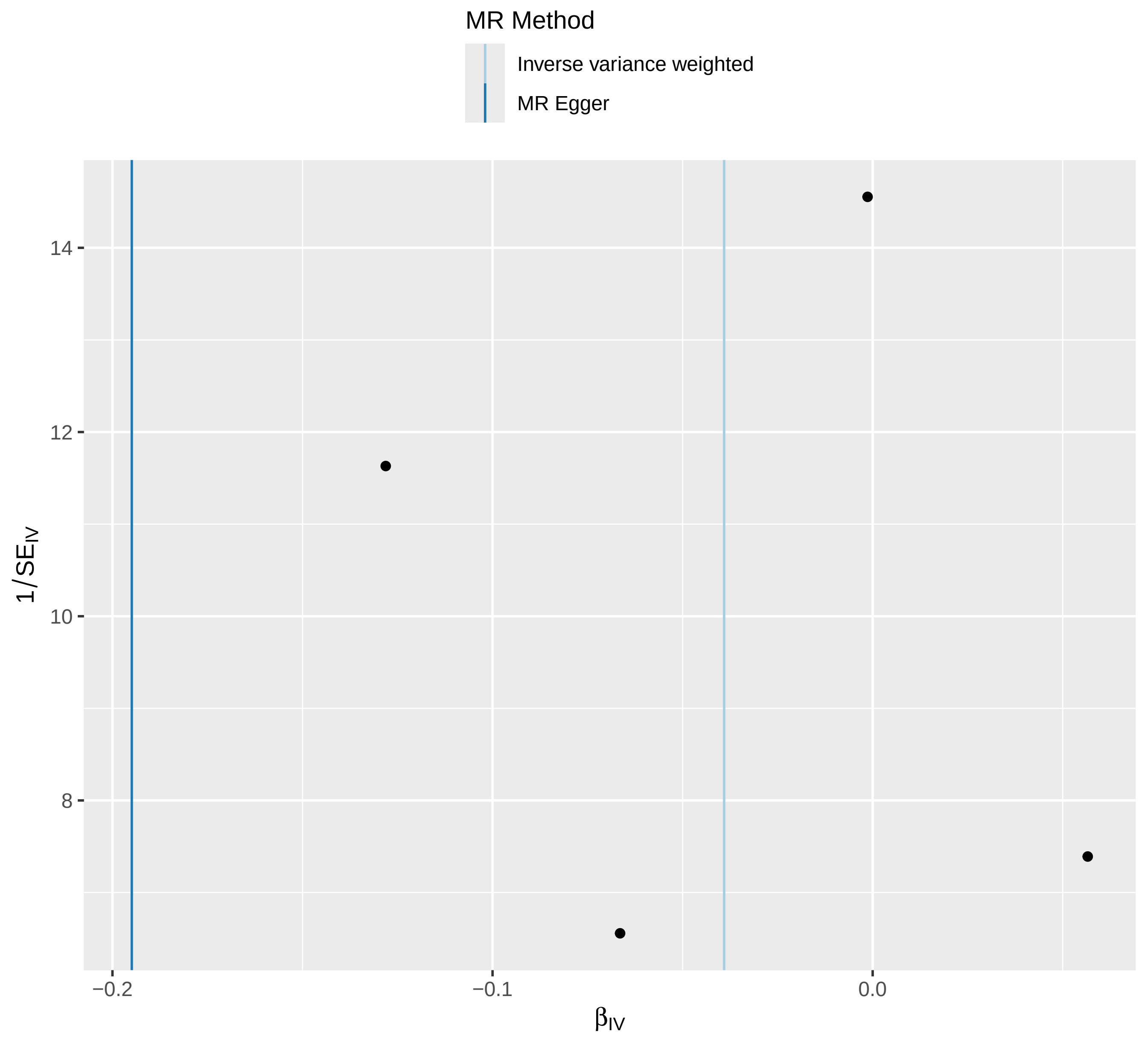
MR Method
Inverse variance weighted
MR Egger
1/SE$_{IV}$
14
12
10
8
−0.2
−0.1
0.0
β$_{IV}$

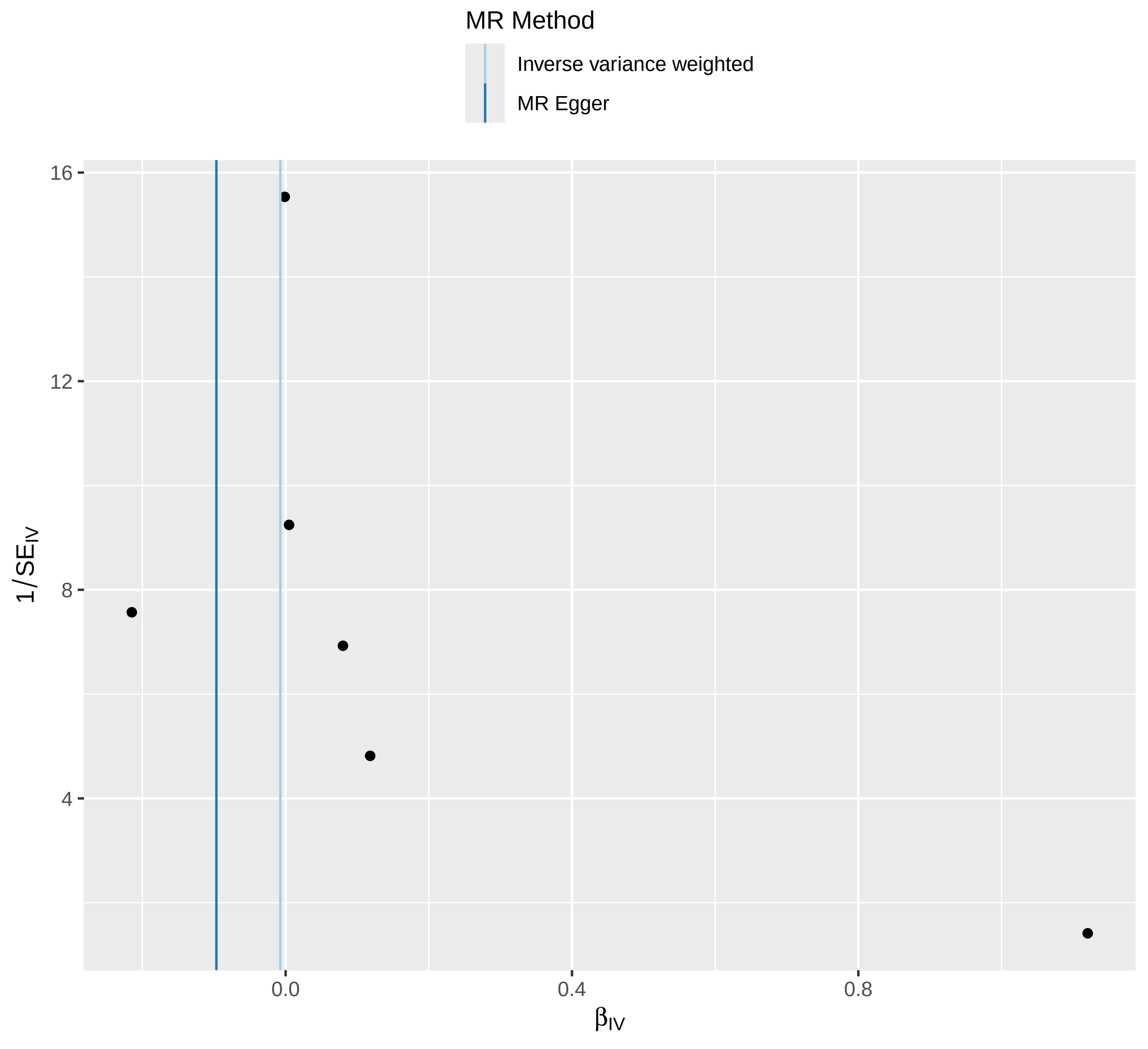

MR Method
Inverse variance weighted
MR Egger
16
12
8
4
1/SE_IV
0.0
0.4
0.8
β_IV

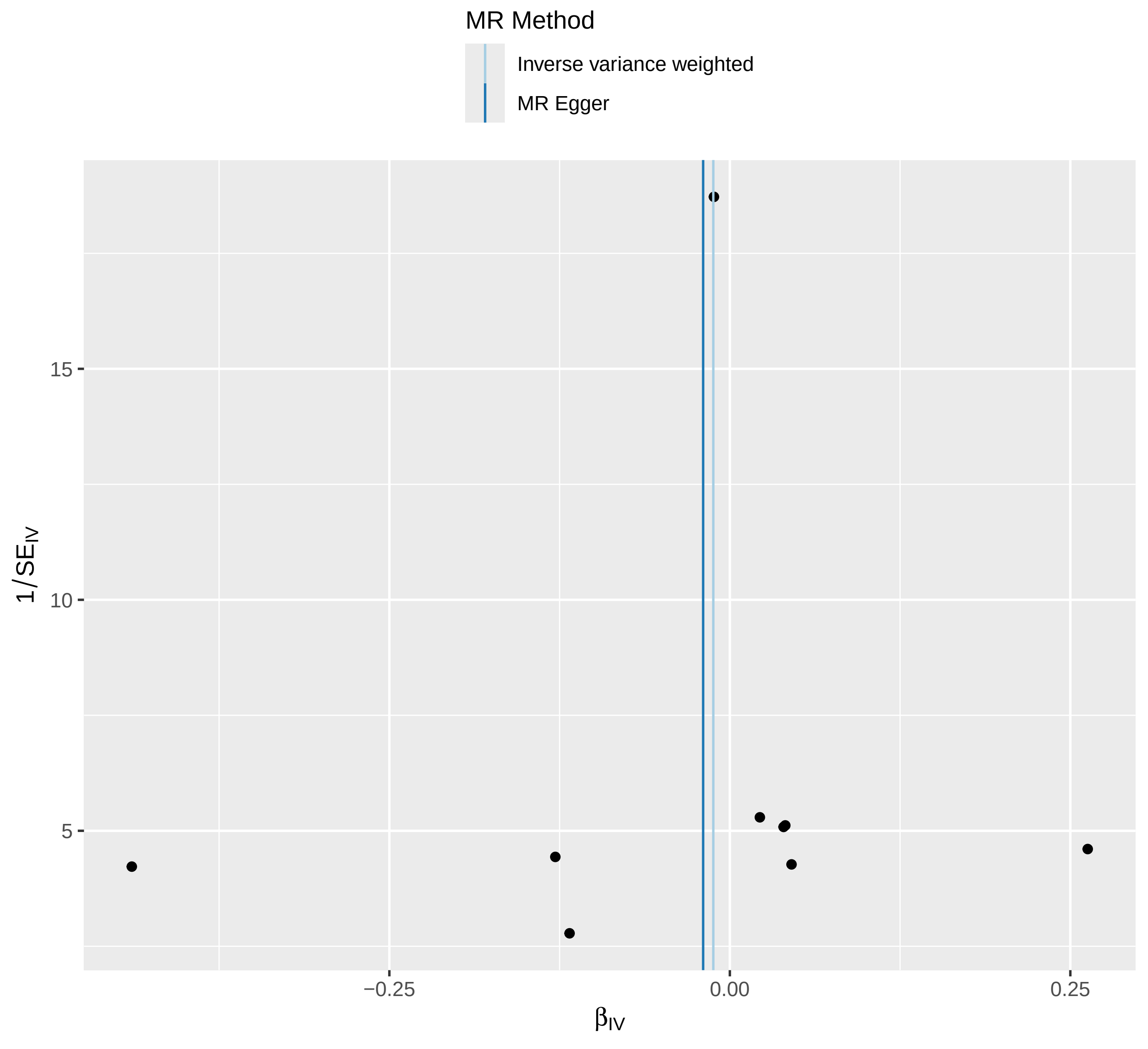

MR Method
Inverse variance weighted
MR Egger
15
10
5
1/SE_IV
−0.25
0.00
0.25
β_IV

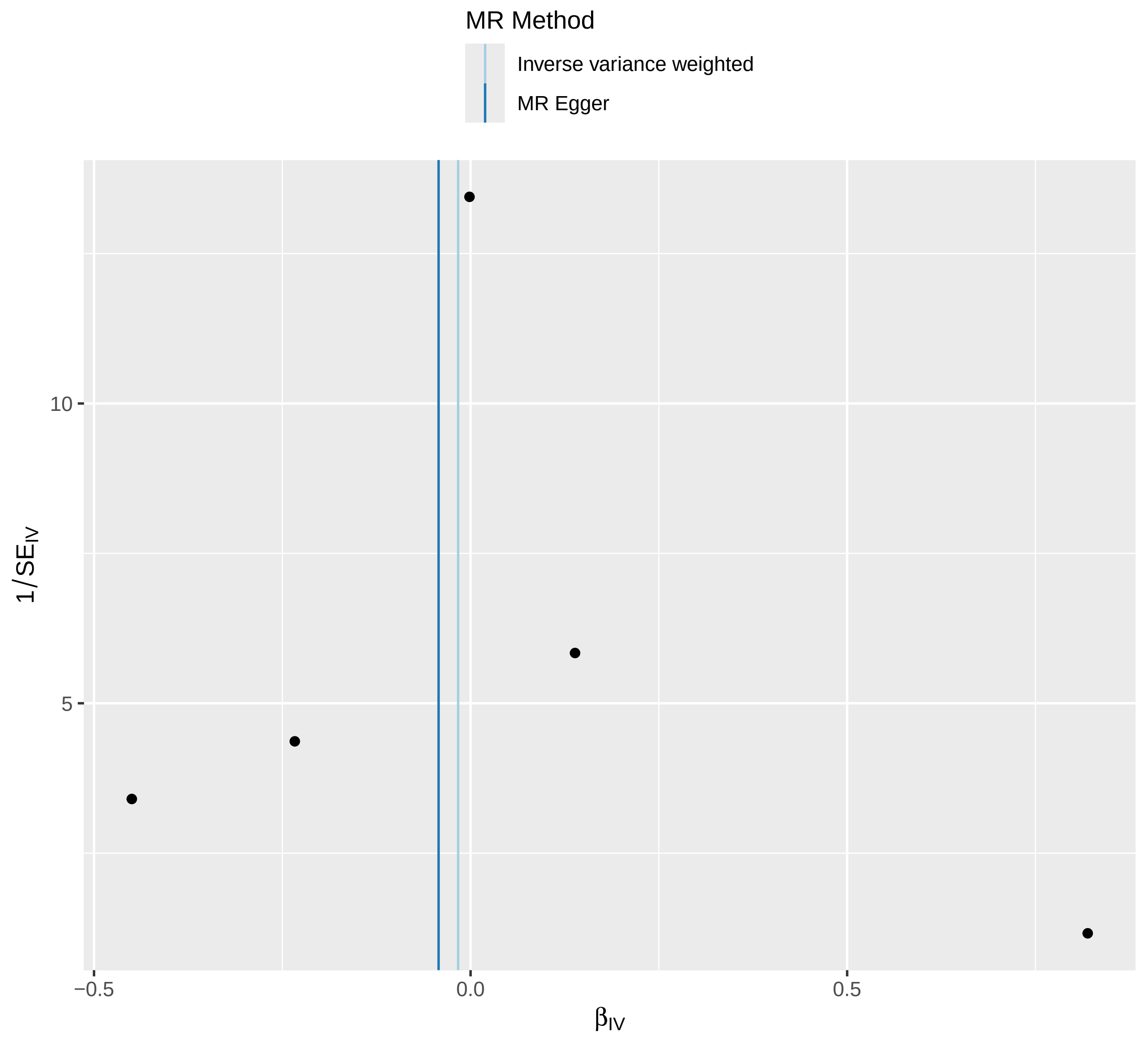

MR Method
Inverse variance weighted
MR Egger
10
5
1/SE$_{IV}$
−0.5
0.0
0.5
β$_{IV}$

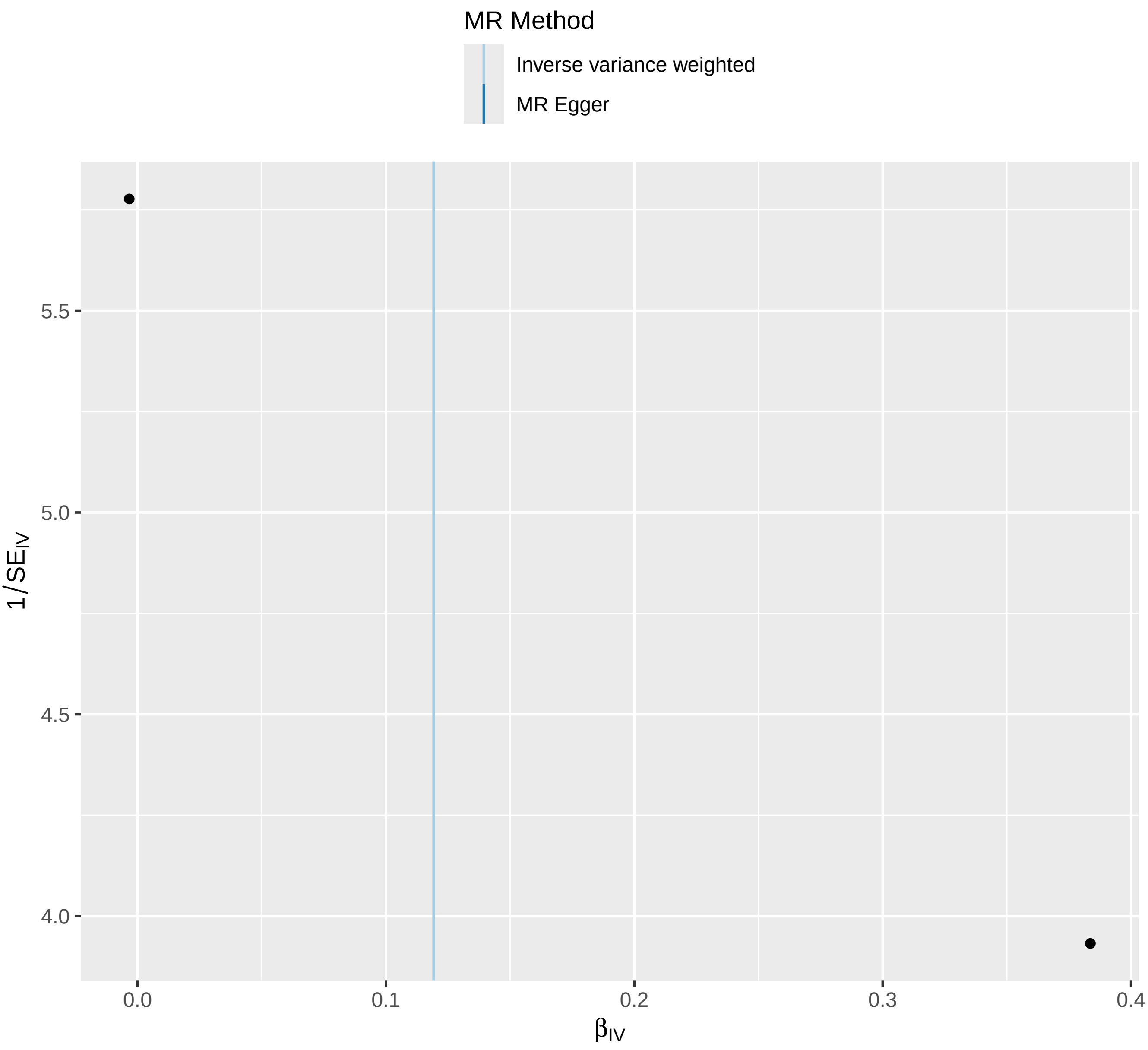
MR Method
Inverse variance weighted
MR Egger
5.5
5.0
4.5
4.0
1/SE_IV
0.0
0.1
0.2
0.3
0.4
β_IV

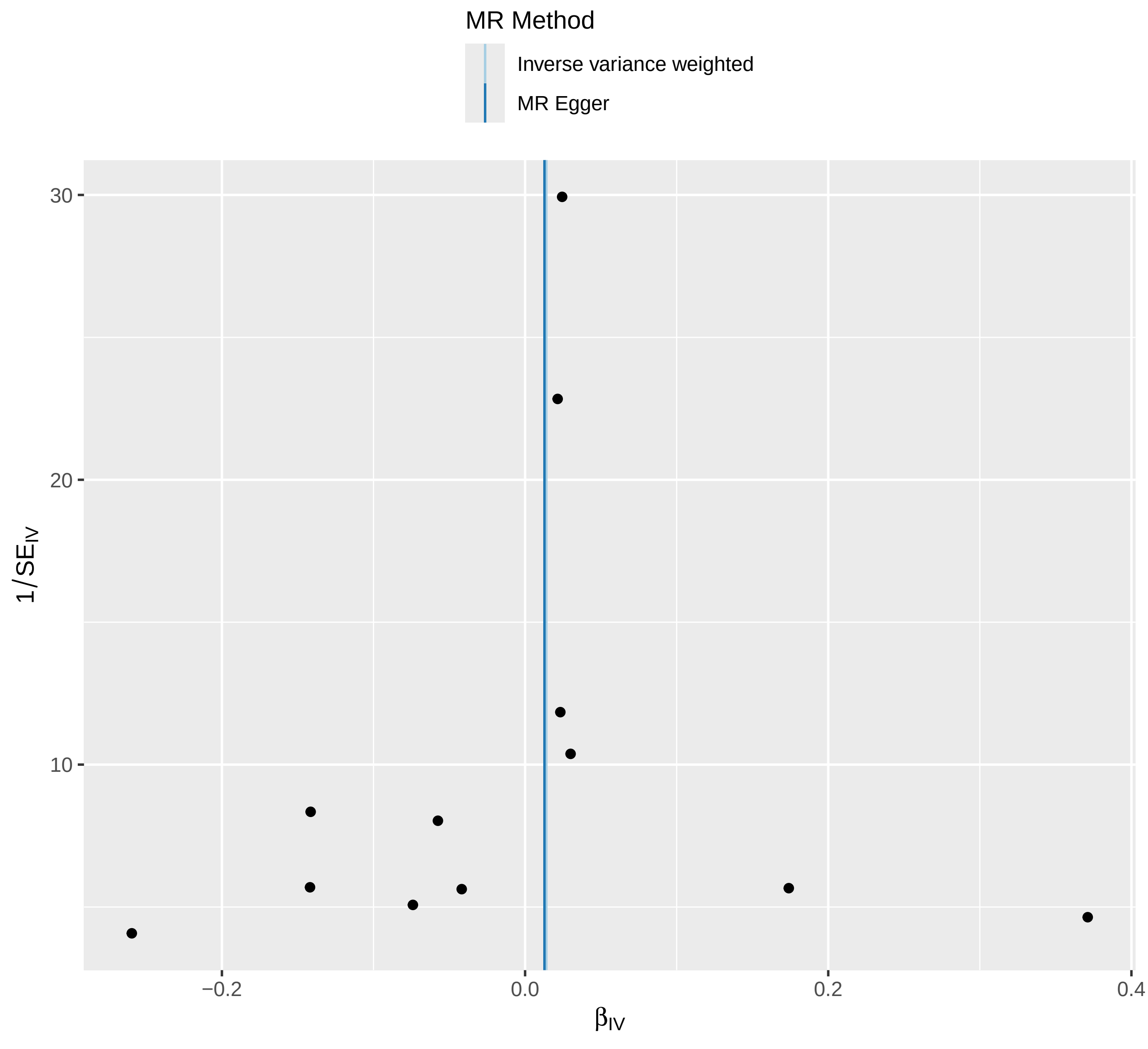

MR Method
Inverse variance weighted
MR Egger
30
20
10
1/SE$_{IV}$
−0.2
0.0
0.2
0.4
β$_{IV}$

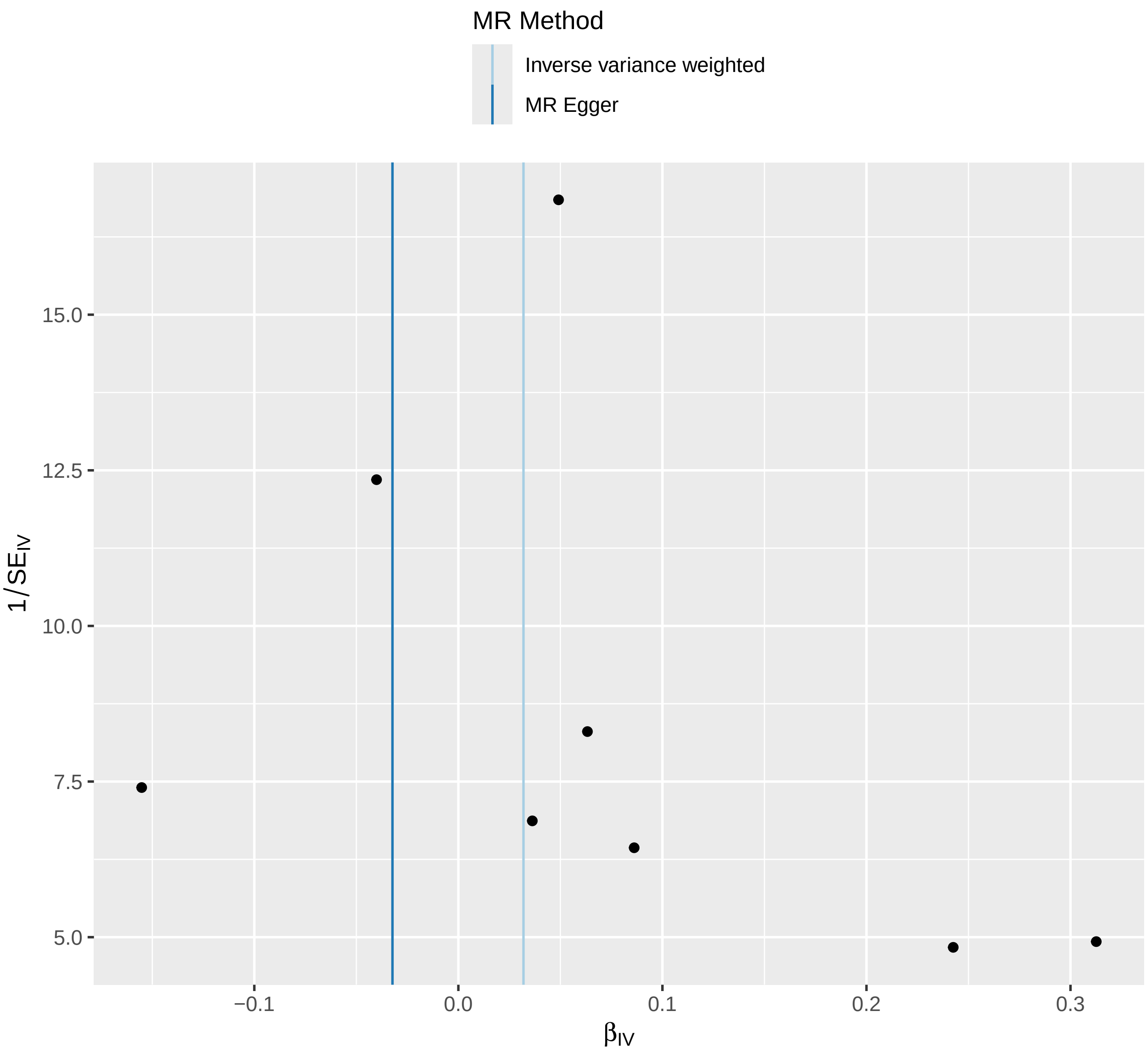

MR Method
Inverse variance weighted
MR Egger
15.0
12.5
10.0
7.5
5.0
1/SE$_{IV}$
−0.1
0.0
0.1
0.2
0.3
β$_{IV}$

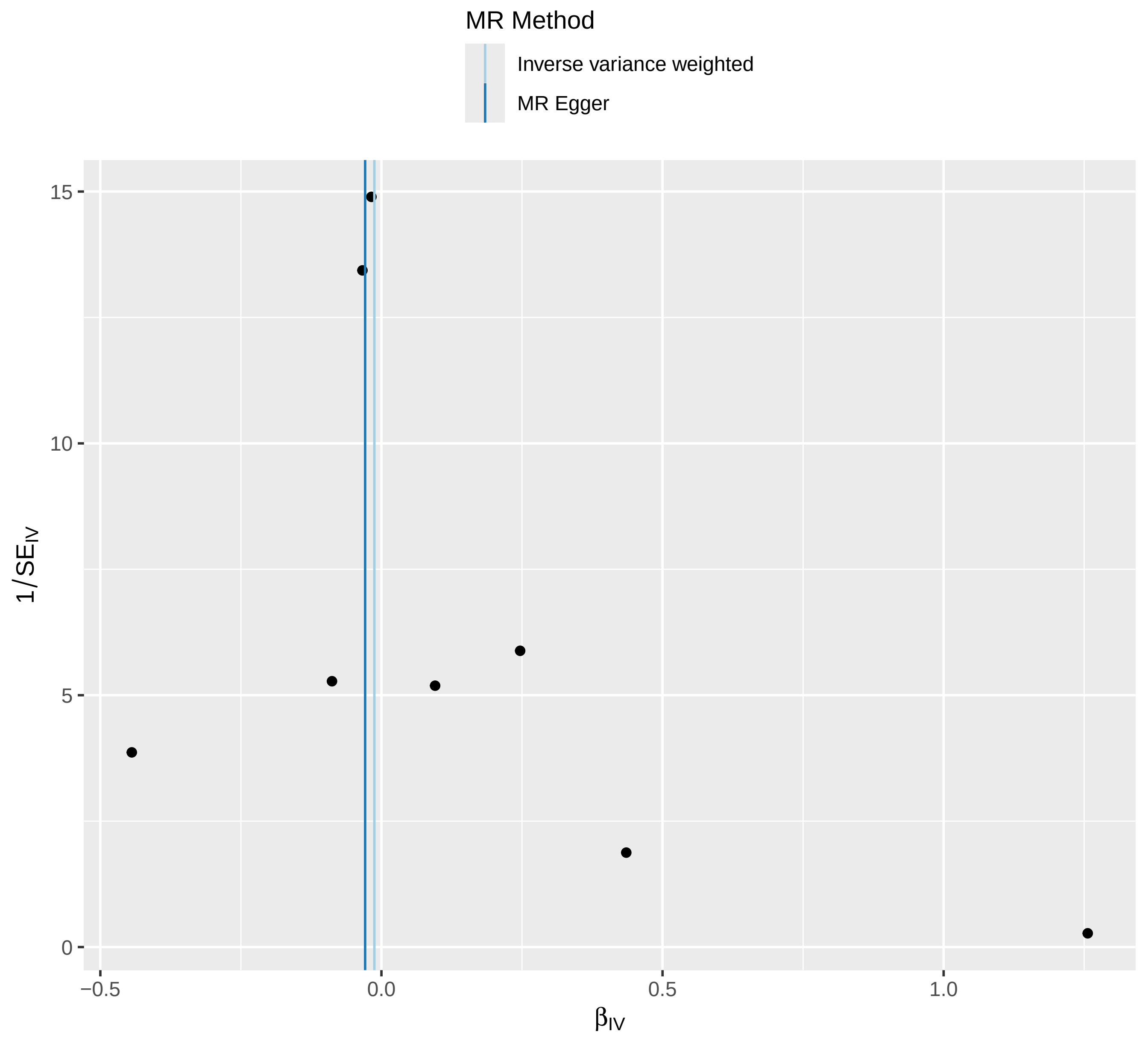
MR Method
Inverse variance weighted
MR Egger
15
10
5
0
1/SE$_{IV}$
−0.5
0.0
0.5
1.0
β$_{IV}$

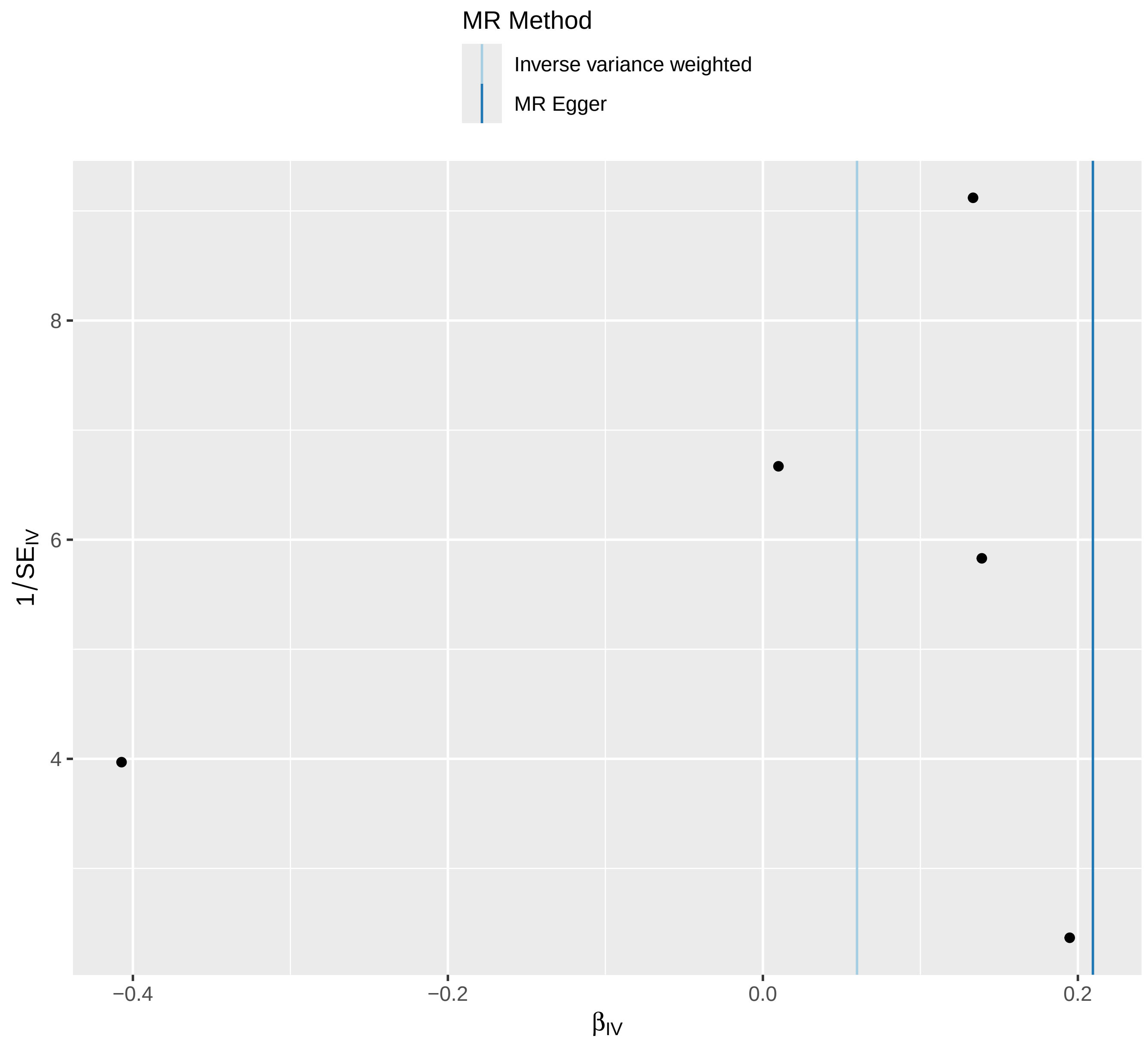

MR Method
Inverse variance weighted
MR Egger
8
6
4
1/SE$_{IV}$
−0.4
−0.2
0.0
0.2
β$_{IV}$

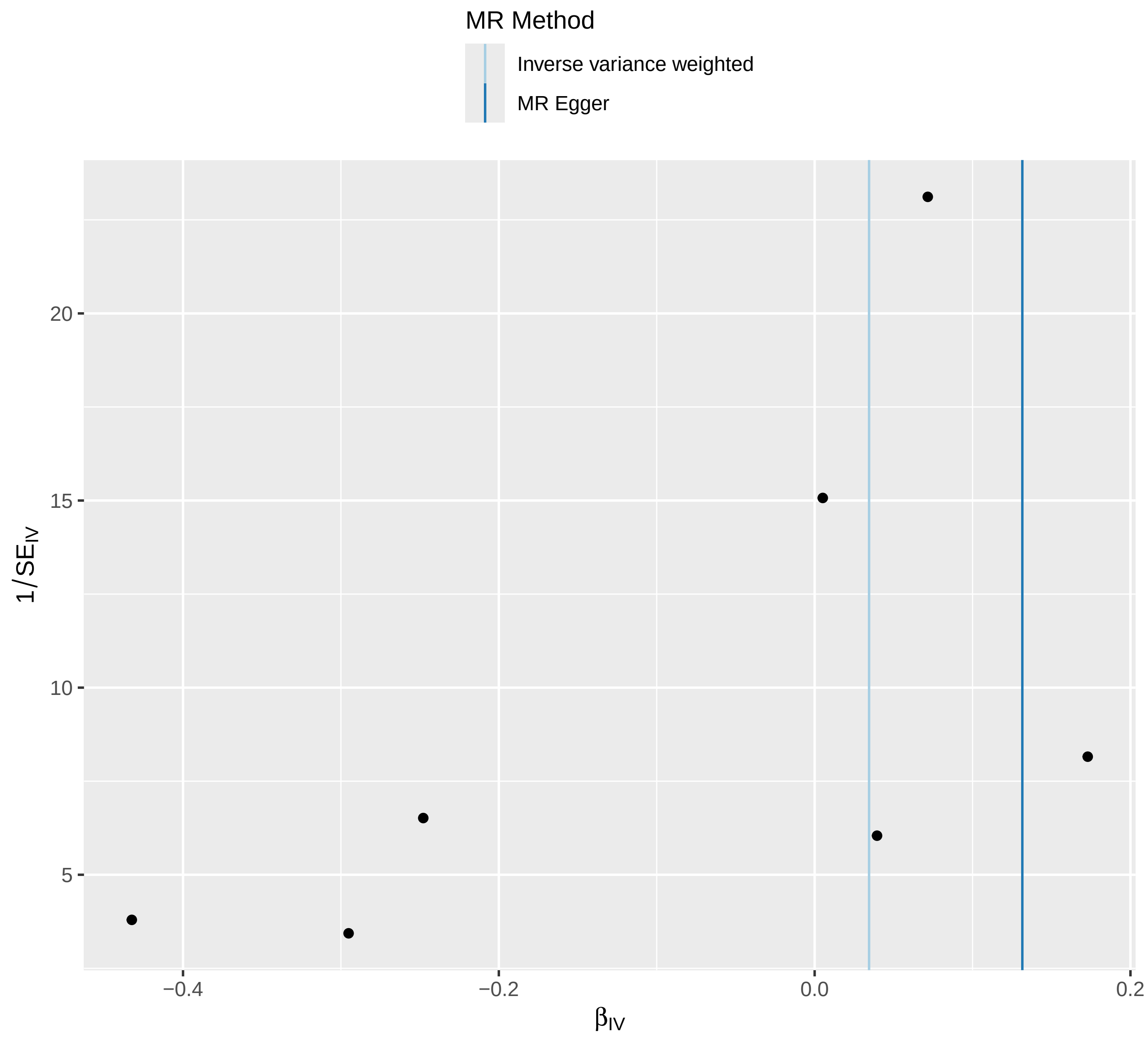

MR Method
Inverse variance weighted
MR Egger
20
15
10
5
1/SE$_{IV}$
−0.4
−0.2
0.0
0.2
β$_{IV}$

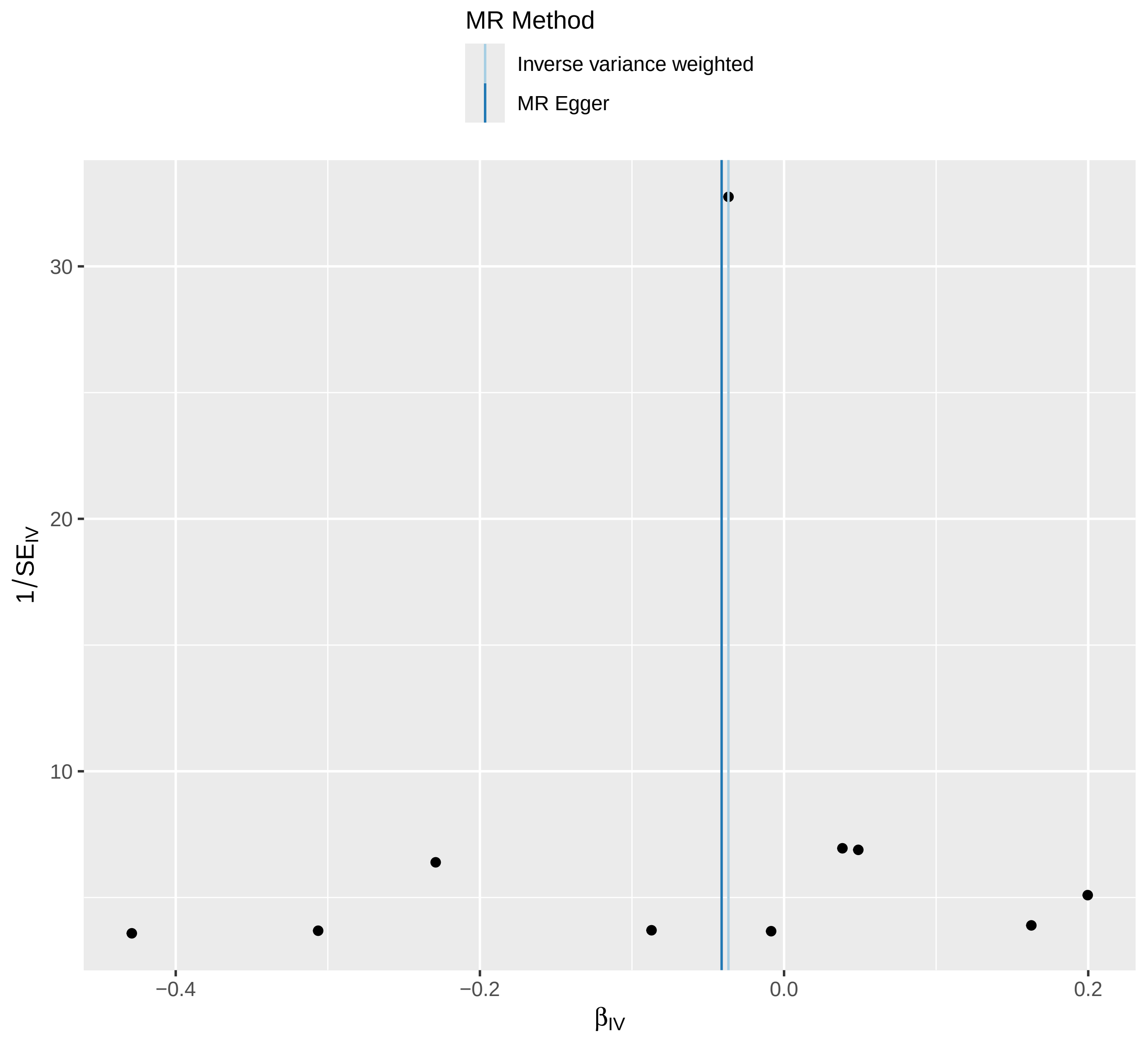

MR Method
Inverse variance weighted
MR Egger
30
20
10
$1/SE_{IV}$
−0.4
−0.2
0.0
0.2
$\beta_{IV}$

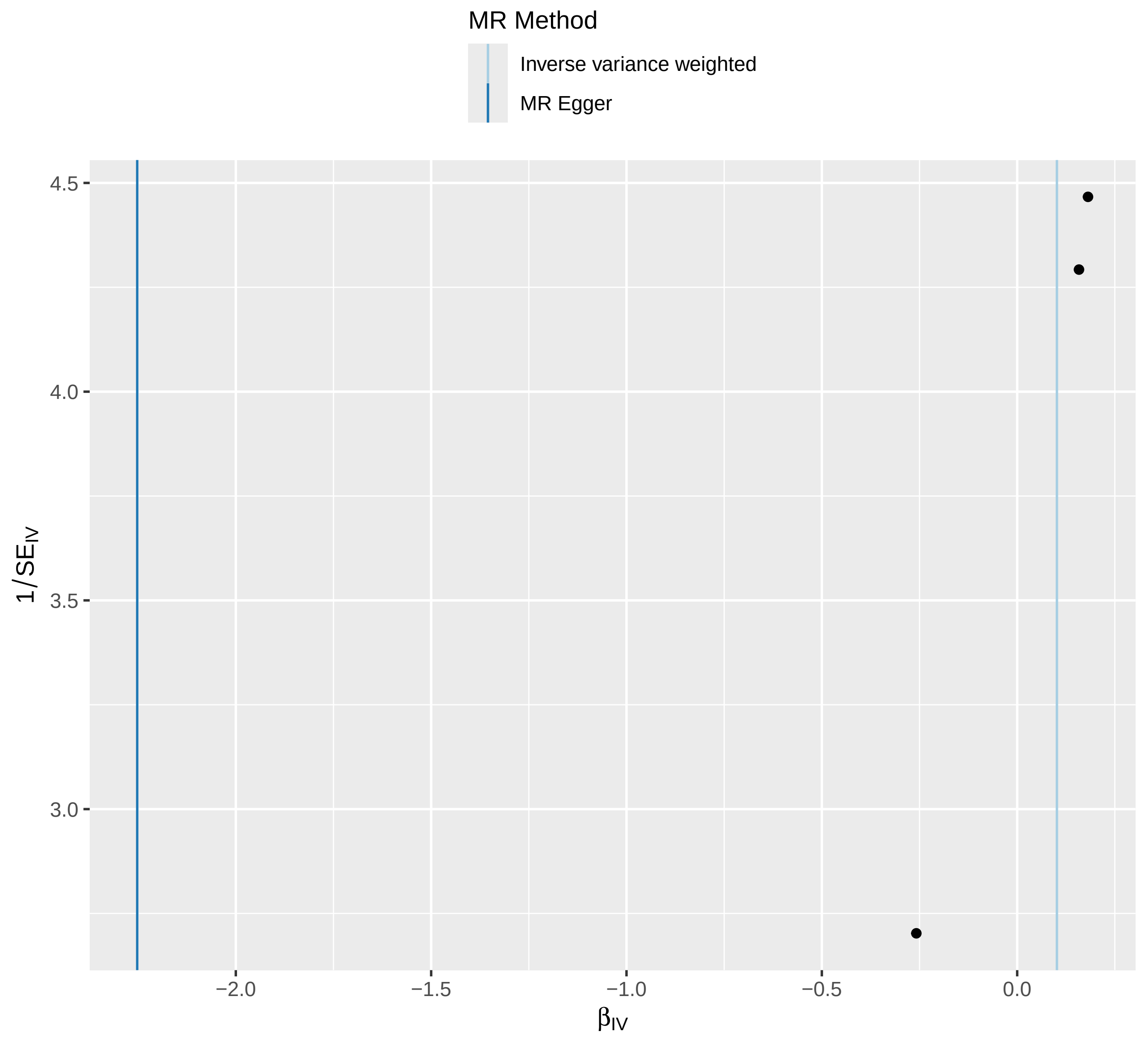

MR Method
Inverse variance weighted
MR Egger
4.5
4.0
3.5
3.0
$1/SE_{IV}$
−2.0
−1.5
−1.0
−0.5
0.0
$\beta_{IV}$

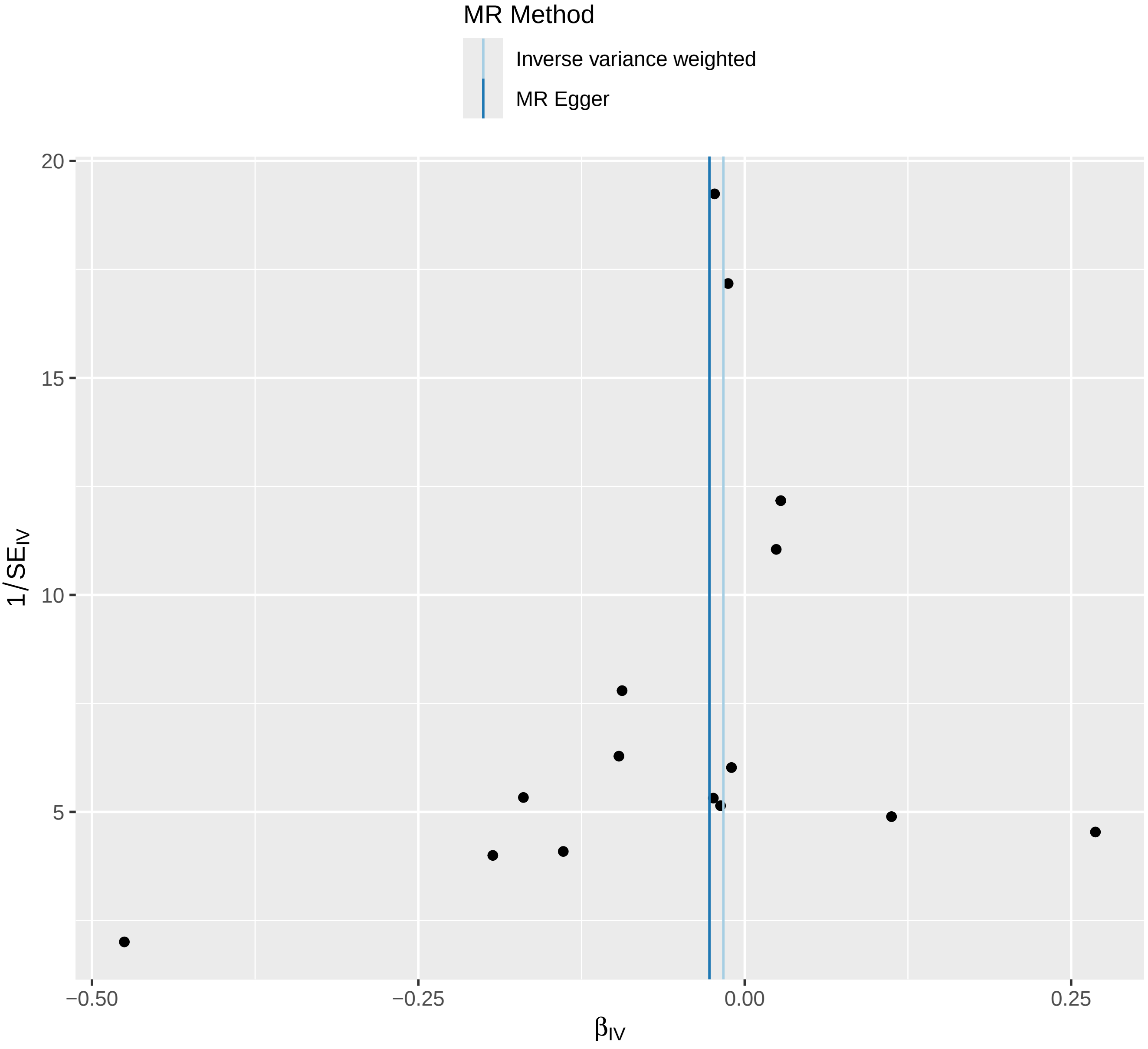
MR Method
Inverse variance weighted
MR Egger
20
15
10
5
1/SE_IV
−0.50
−0.25
0.00
0.25
β_IV

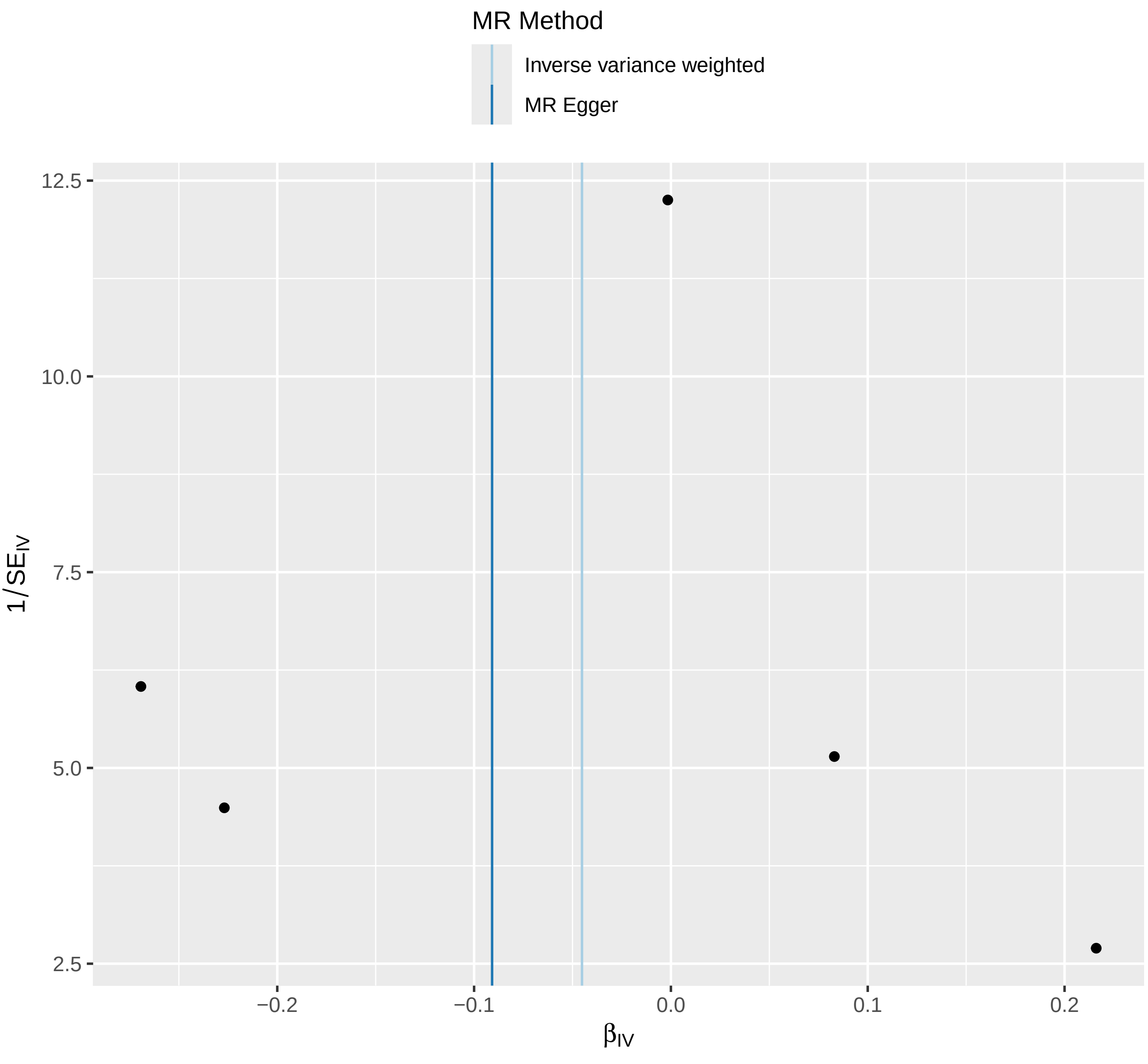
MR Method
Inverse variance weighted
MR Egger
12.5
10.0
7.5
5.0
2.5
1/SE_IV
−0.2
−0.1
0.0
0.1
0.2
β_IV

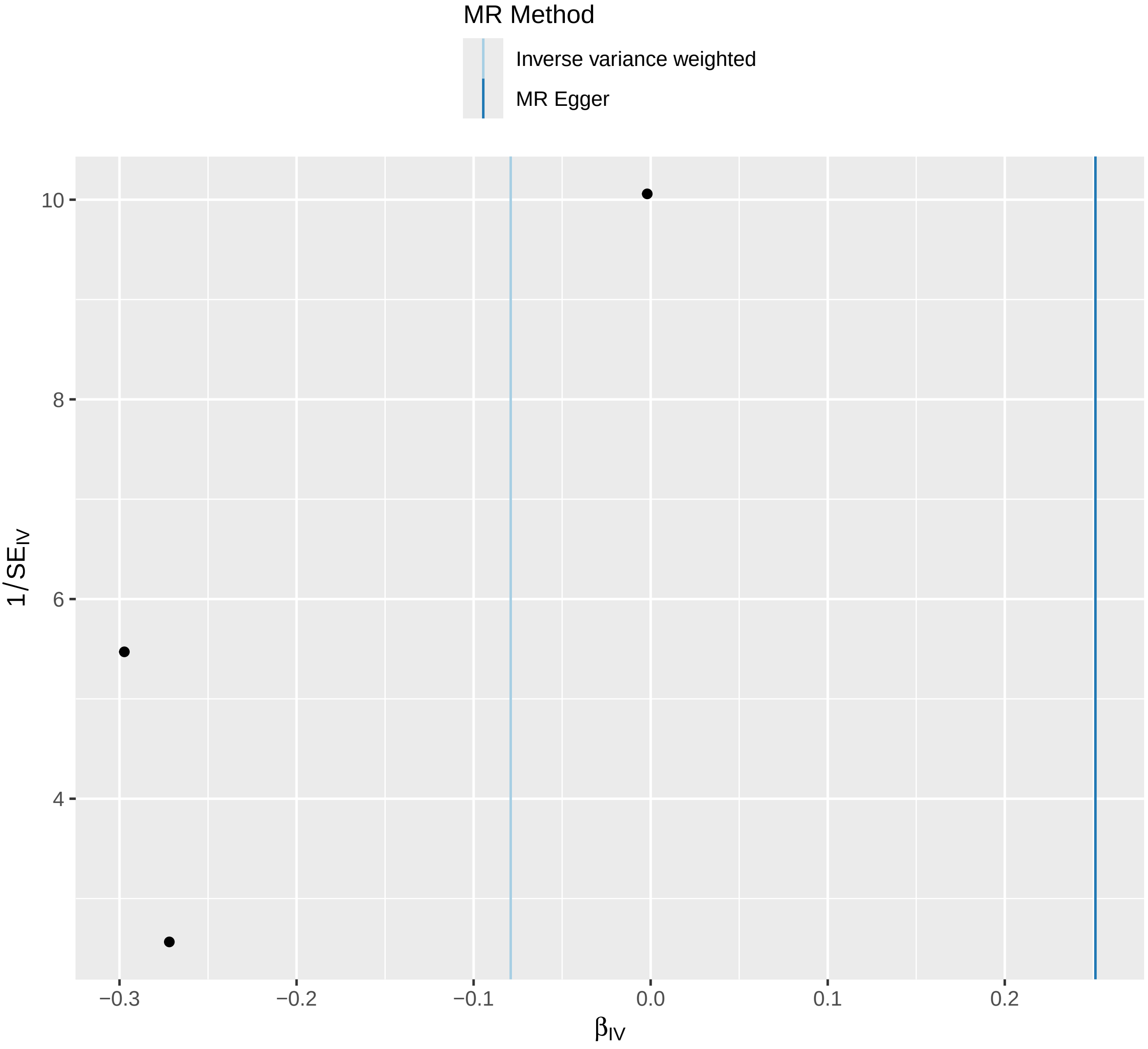
MR Method
Inverse variance weighted
MR Egger
10
8
6
4
1/SE_IV
−0.3
−0.2
−0.1
0.0
0.1
0.2
β_IV

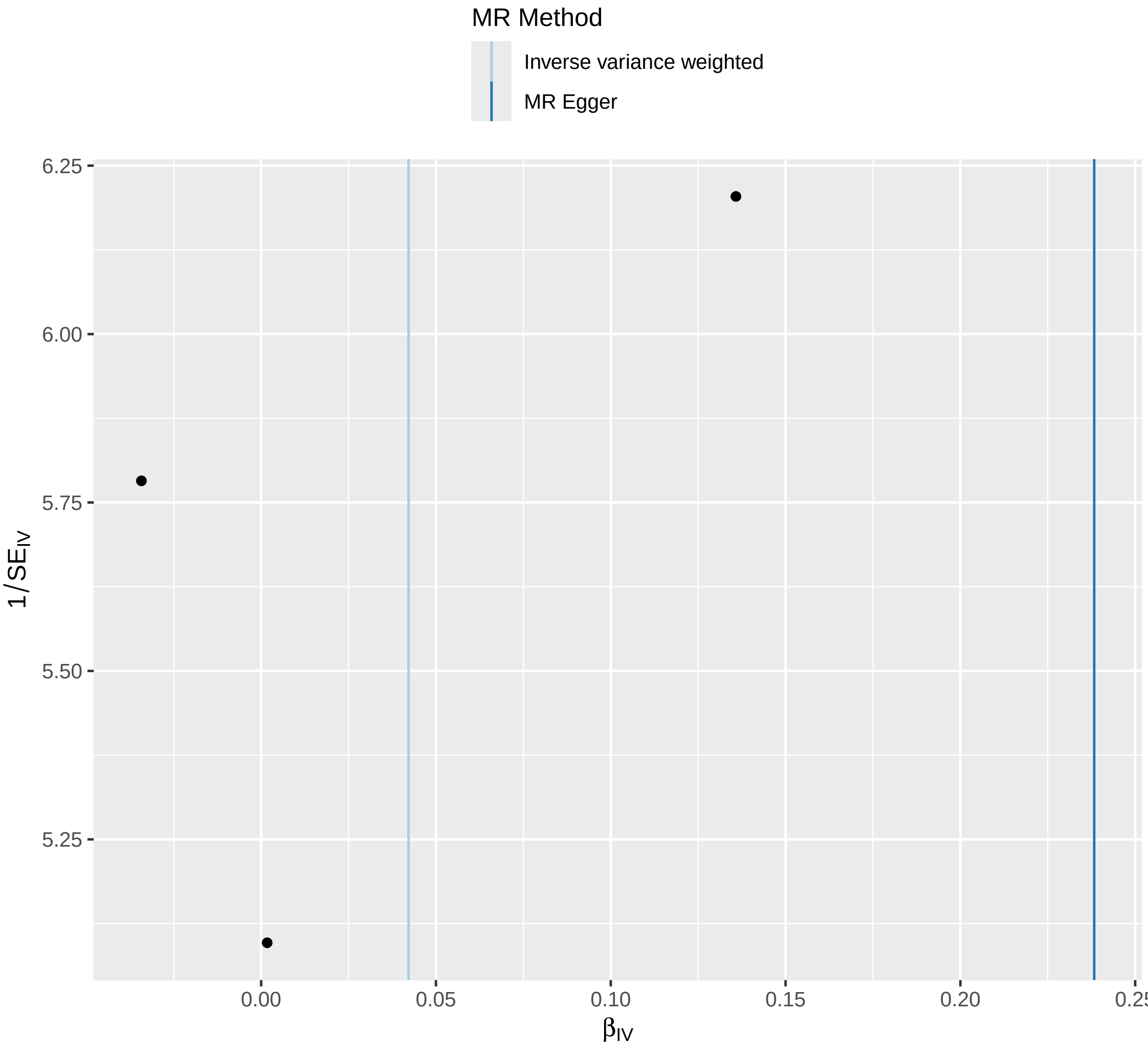
MR Method
Inverse variance weighted
MR Egger
6.25
6.00
5.75
5.50
5.25
0.00
0.05
0.10
0.15
0.20
0.25
1/SE_IV
β_IV

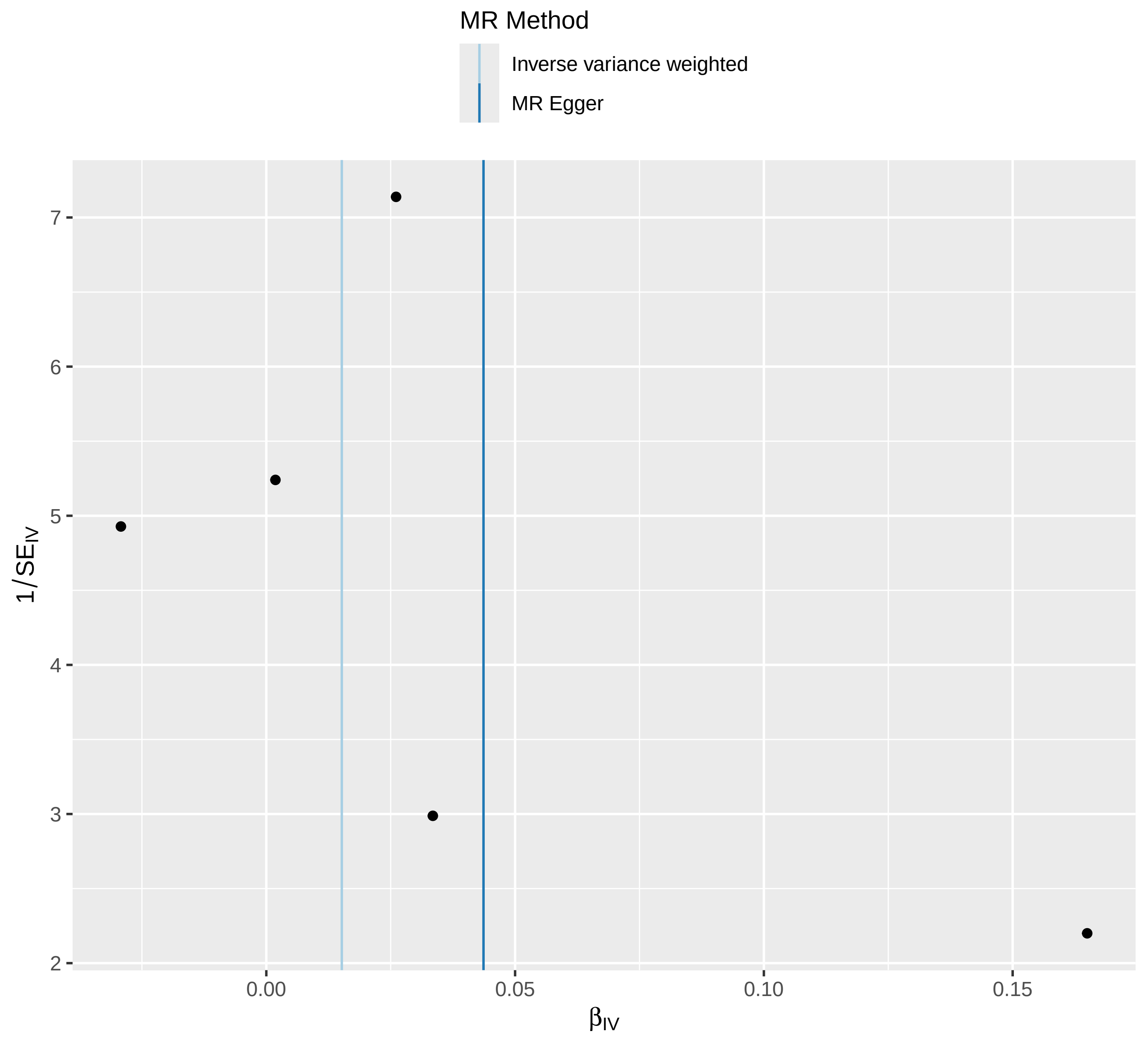

MR Method
Inverse variance weighted
MR Egger
1/SE_IV
β_IV
7
6
5
4
3
2
0.00
0.05
0.10
0.15

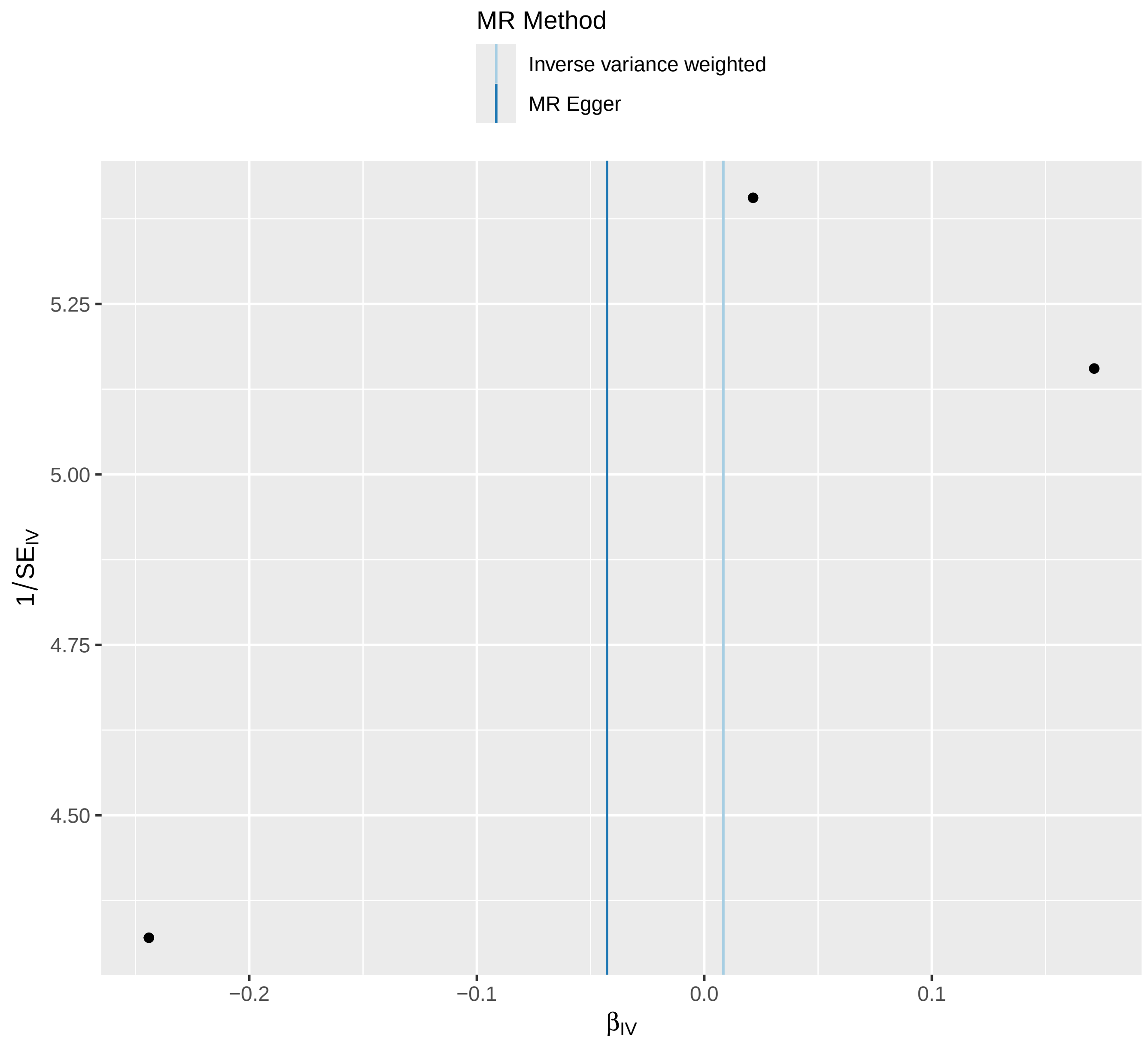
MR Method
Inverse variance weighted
MR Egger
5.25
5.00
4.75
4.50
1/SE_IV
−0.2
−0.1
0.0
0.1
β_IV

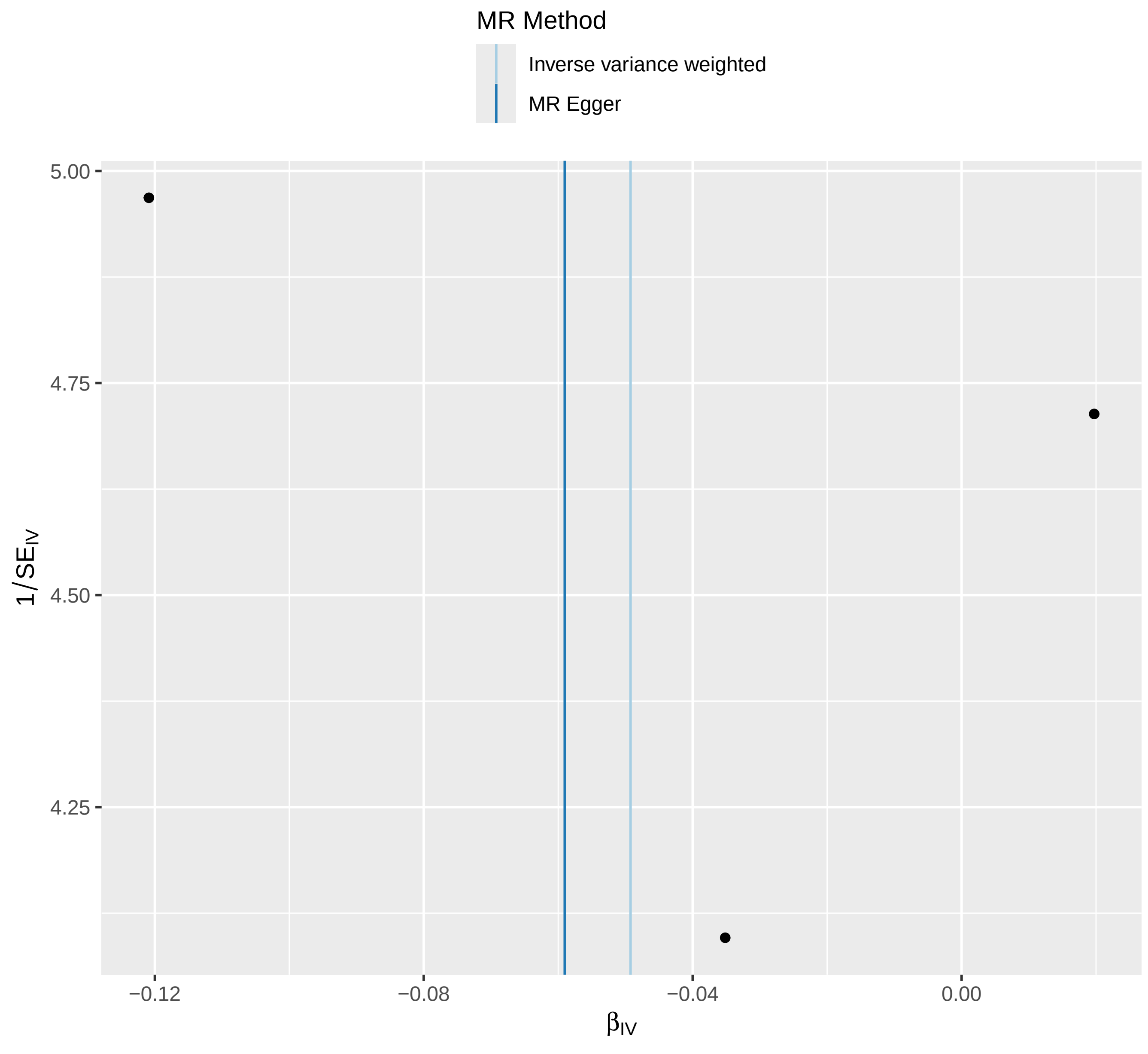

MR Method
Inverse variance weighted
MR Egger
5.00
4.75
4.50
4.25
1/SE_IV
−0.12
−0.08
−0.04
0.00
β_IV

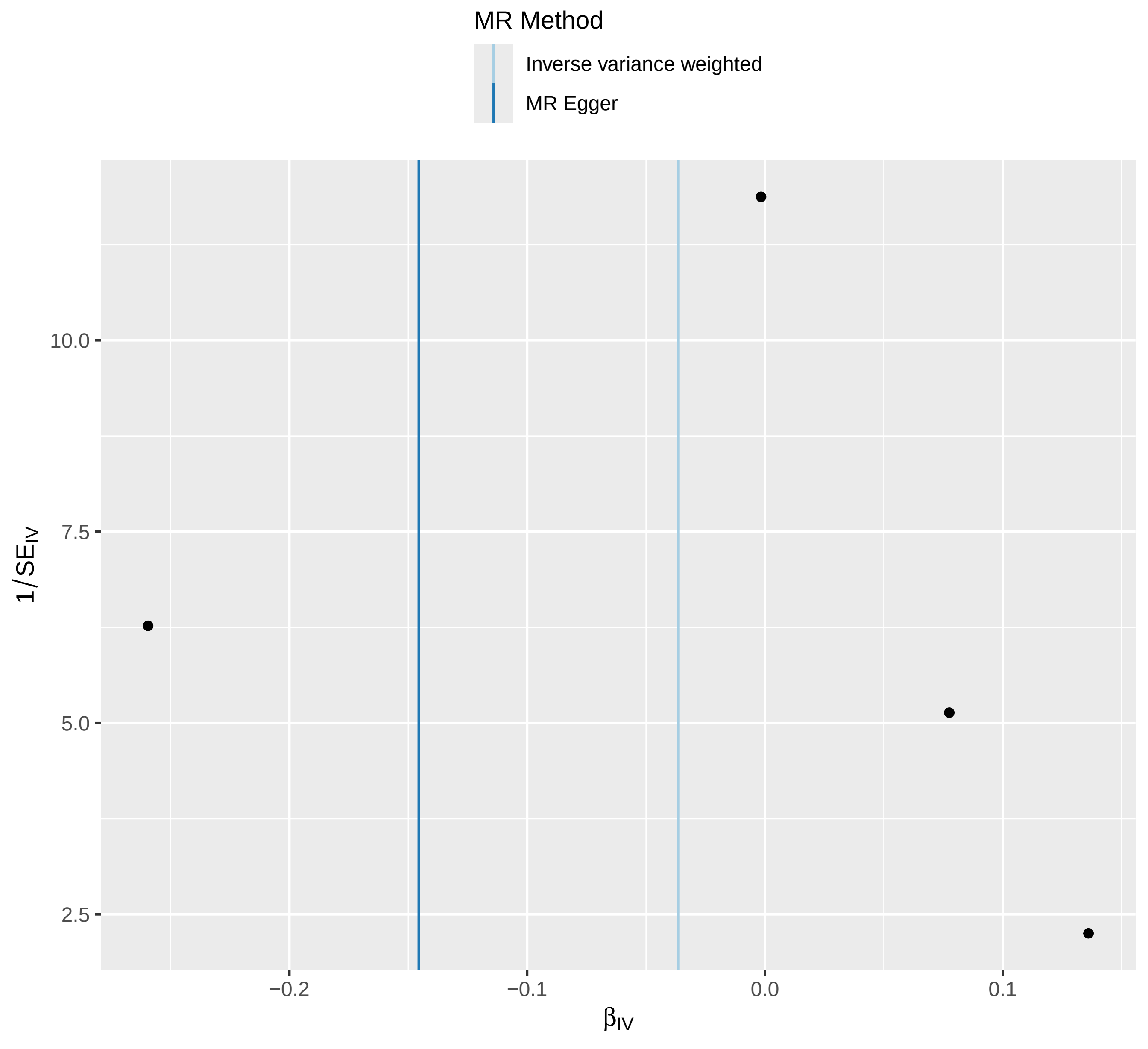

MR Method
Inverse variance weighted
MR Egger
10.0
7.5
5.0
2.5
1/SE$_{IV}$
−0.2
−0.1
0.0
0.1
β$_{IV}$

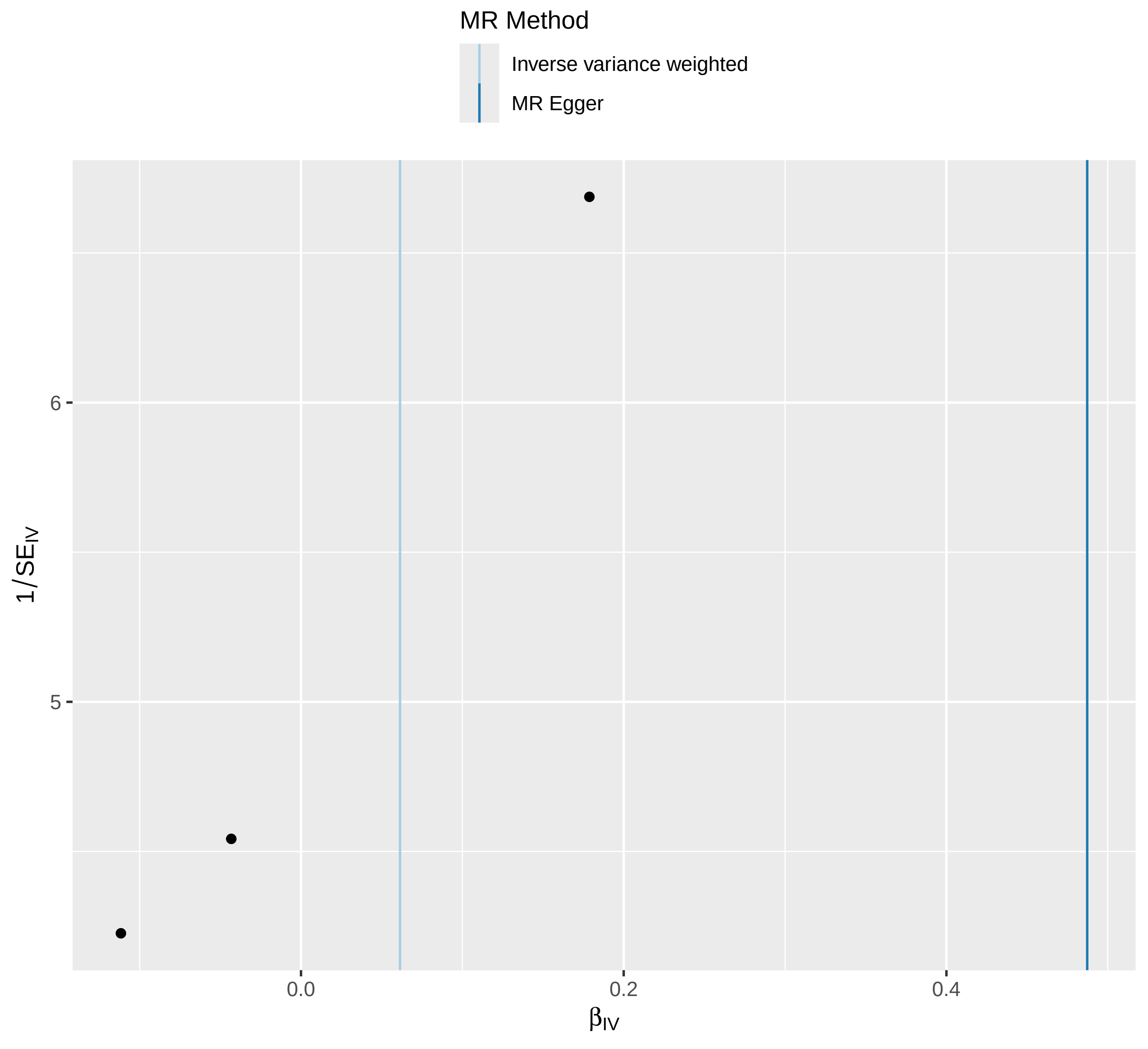

MR Method
Inverse variance weighted
MR Egger
6
5
1/SE_IV
0.0
0.2
0.4
β_IV

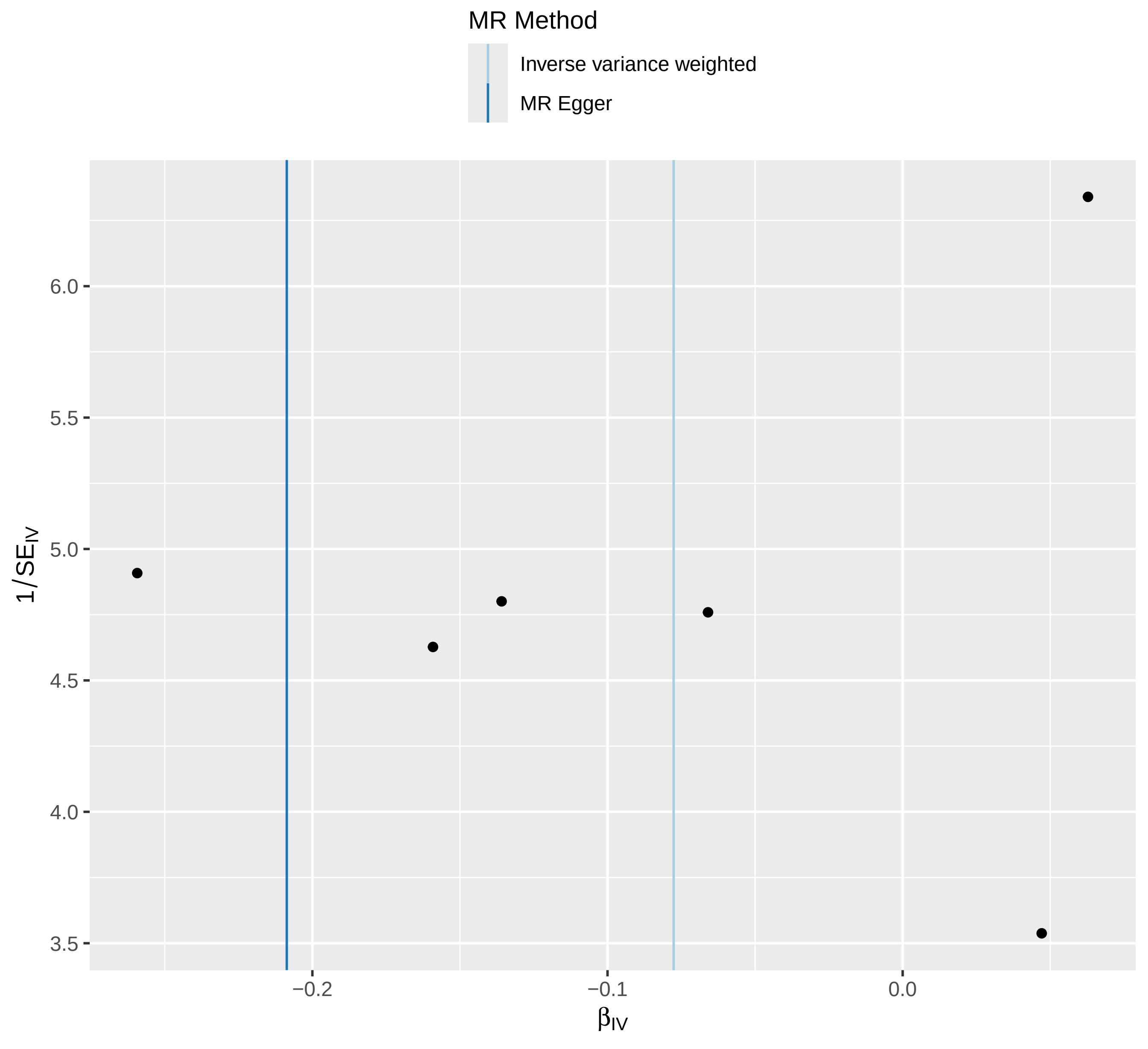

MR Method
Inverse variance weighted
MR Egger
6.0
5.5
5.0
4.5
4.0
3.5
$1/SE_{IV}$
−0.2
−0.1
0.0
$\beta_{IV}$

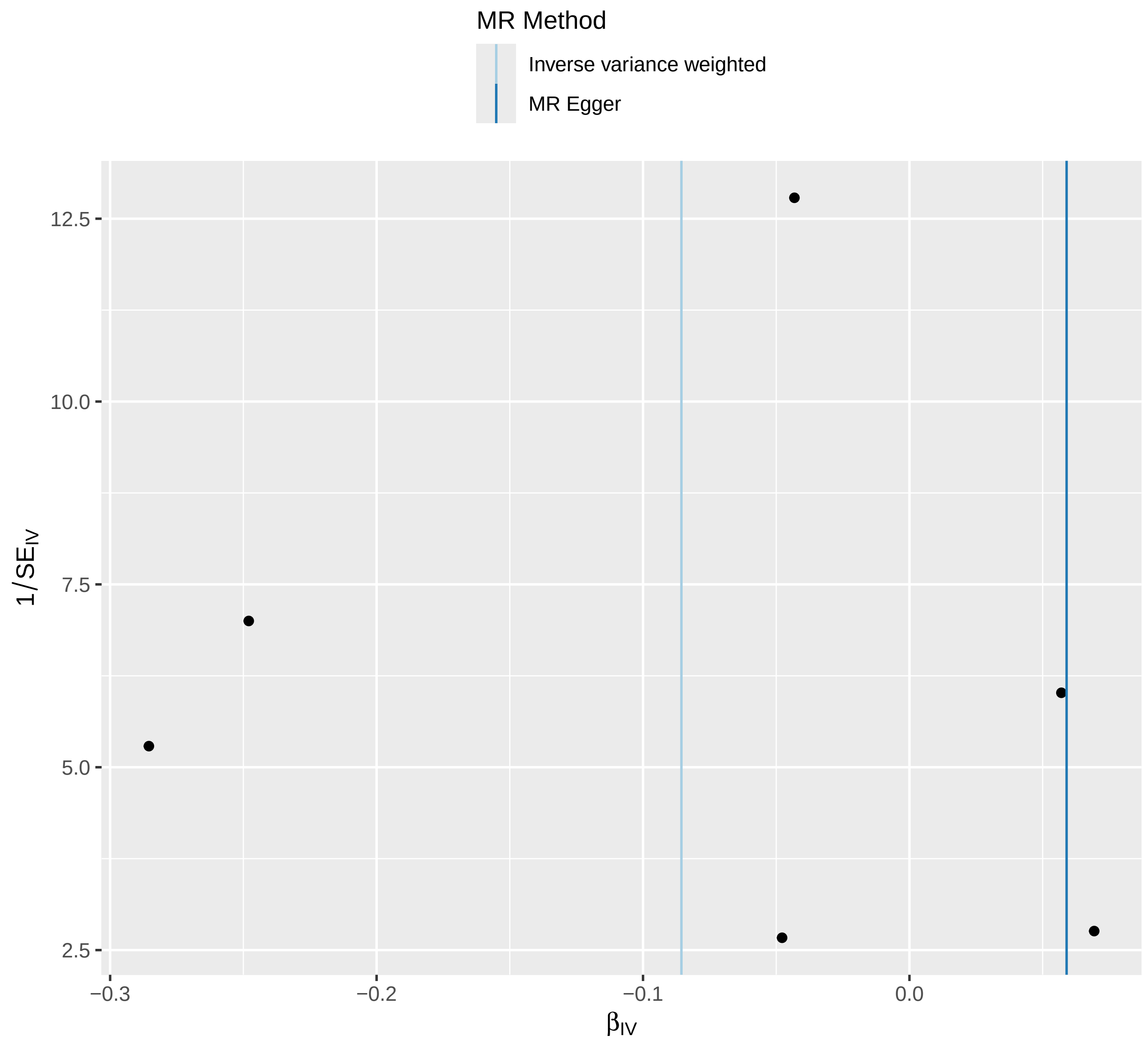

MR Method
Inverse variance weighted
MR Egger
12.5
10.0
7.5
5.0
2.5
1/SE_IV
−0.3
−0.2
−0.1
0.0
β_IV

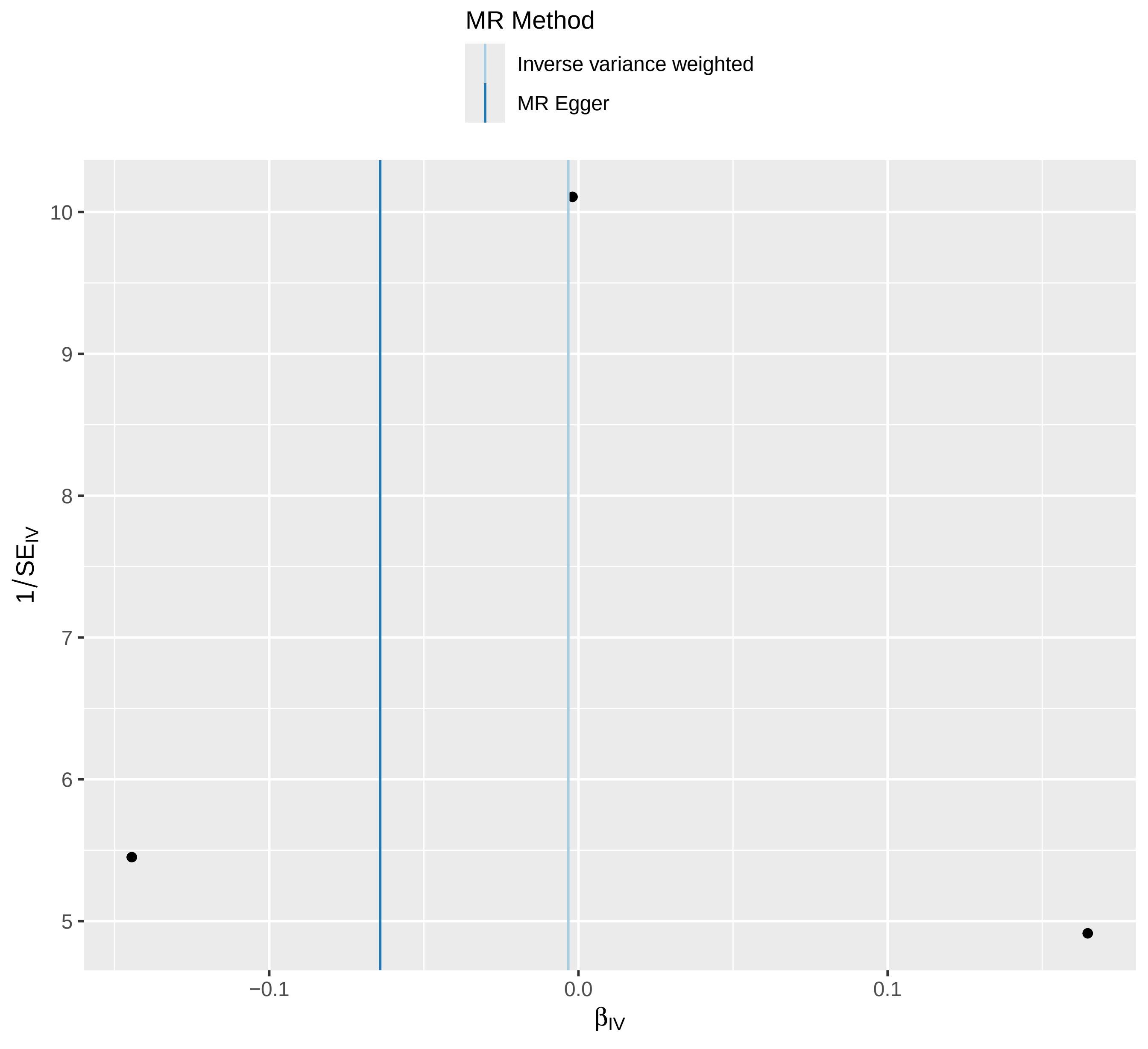
MR Method
Inverse variance weighted
MR Egger
10
9
8
7
6
5
1/SE$_{IV}$
−0.1
0.0
0.1
β$_{IV}$

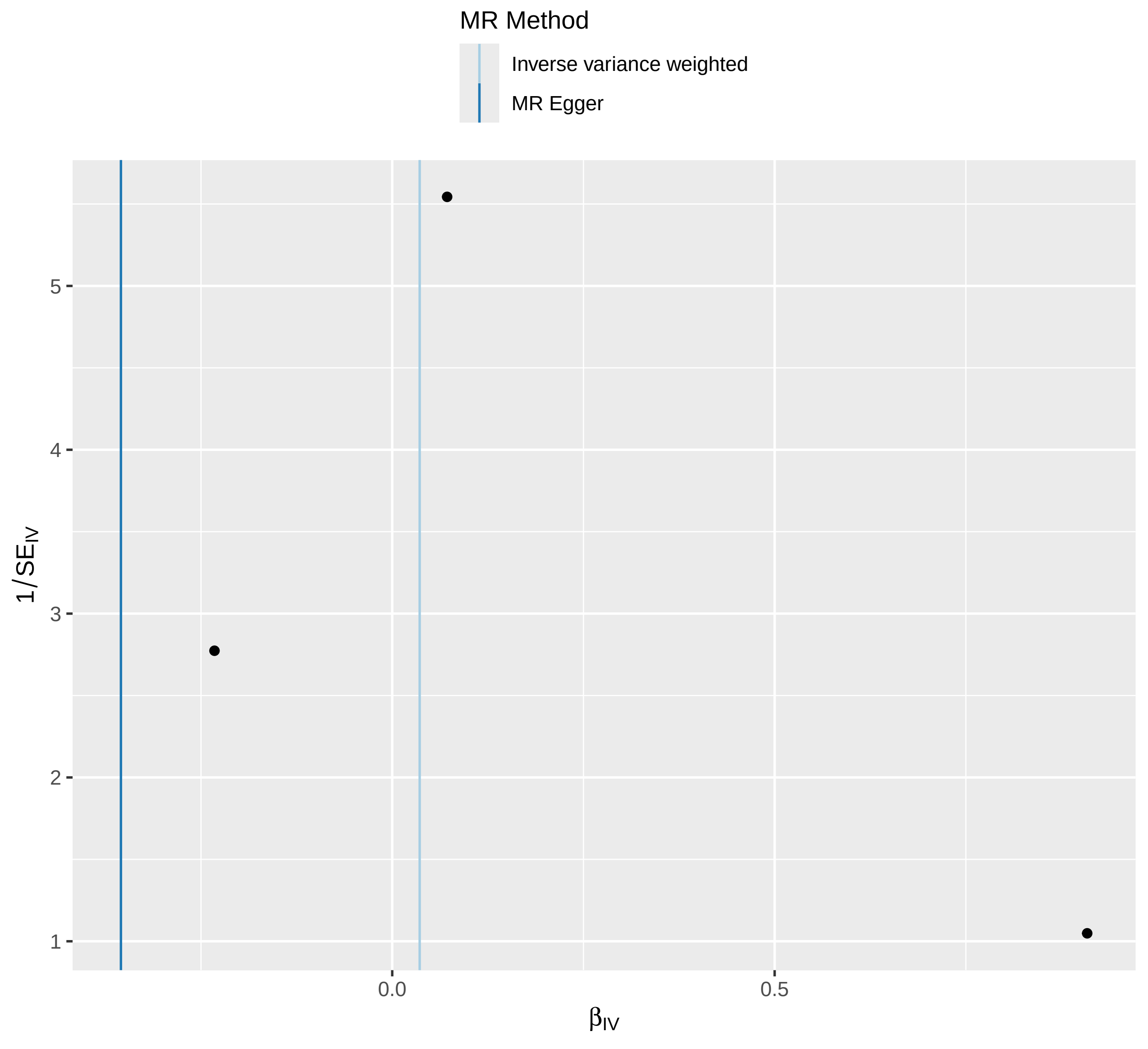

MR Method
Inverse variance weighted
MR Egger
5
4
3
2
1
0.0
0.5
1/SE_IV
β_IV

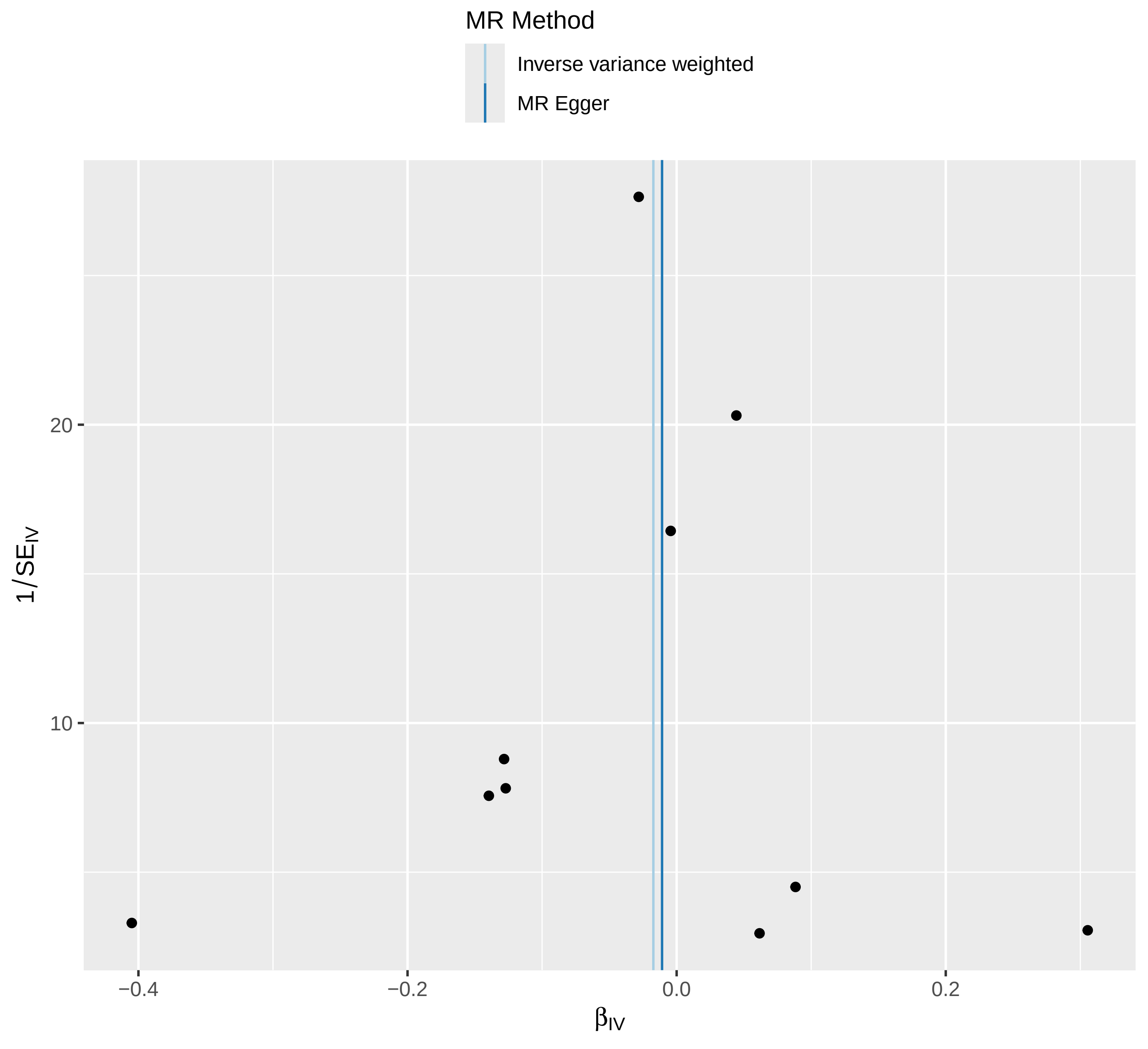

MR Method
Inverse variance weighted
MR Egger
20
10
1/SE$_{IV}$
−0.4
−0.2
0.0
0.2
β$_{IV}$

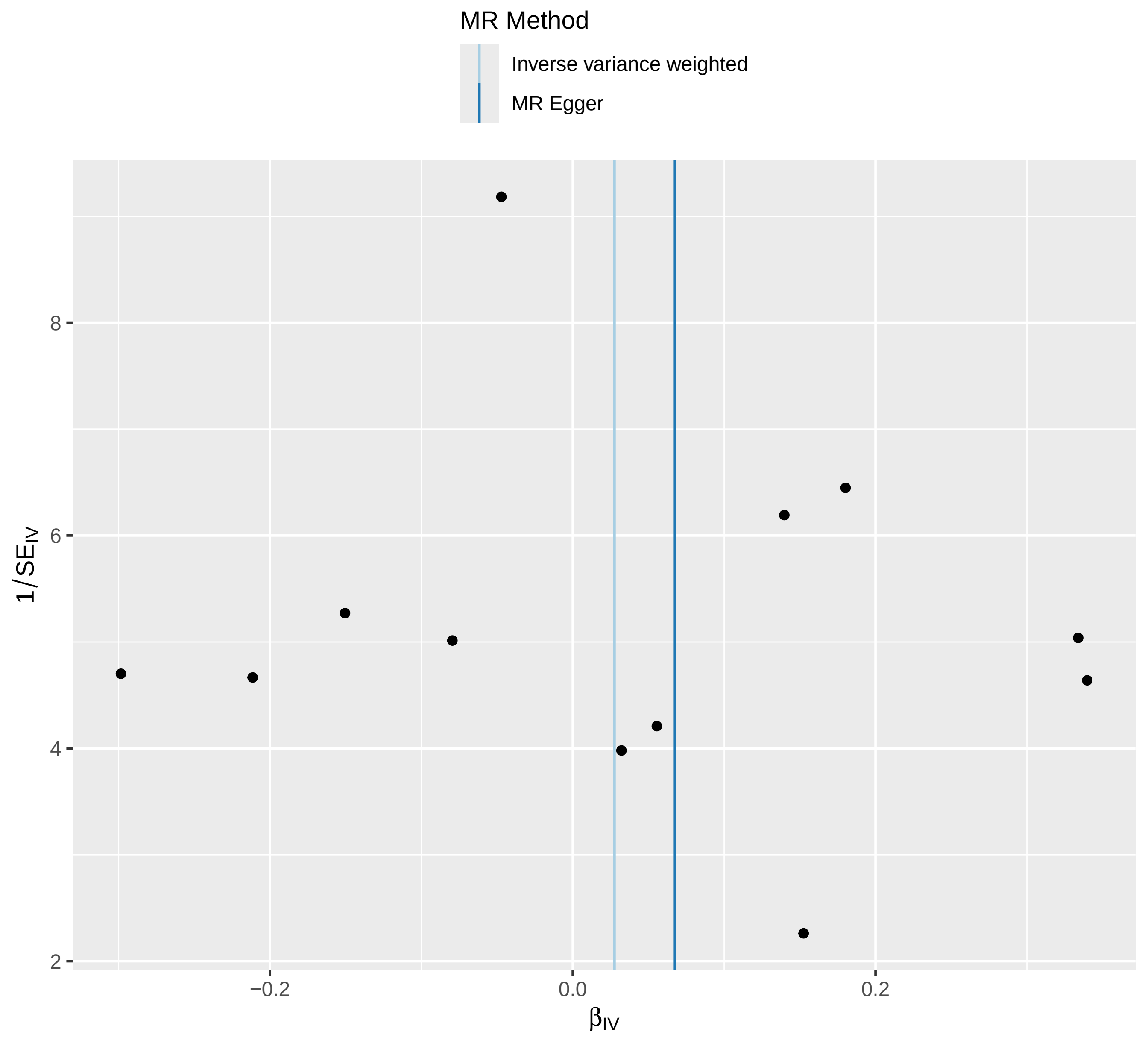
MR Method
Inverse variance weighted
MR Egger
1/SE$_{IV}$
8
6
4
2
−0.2
0.0
0.2
β$_{IV}$

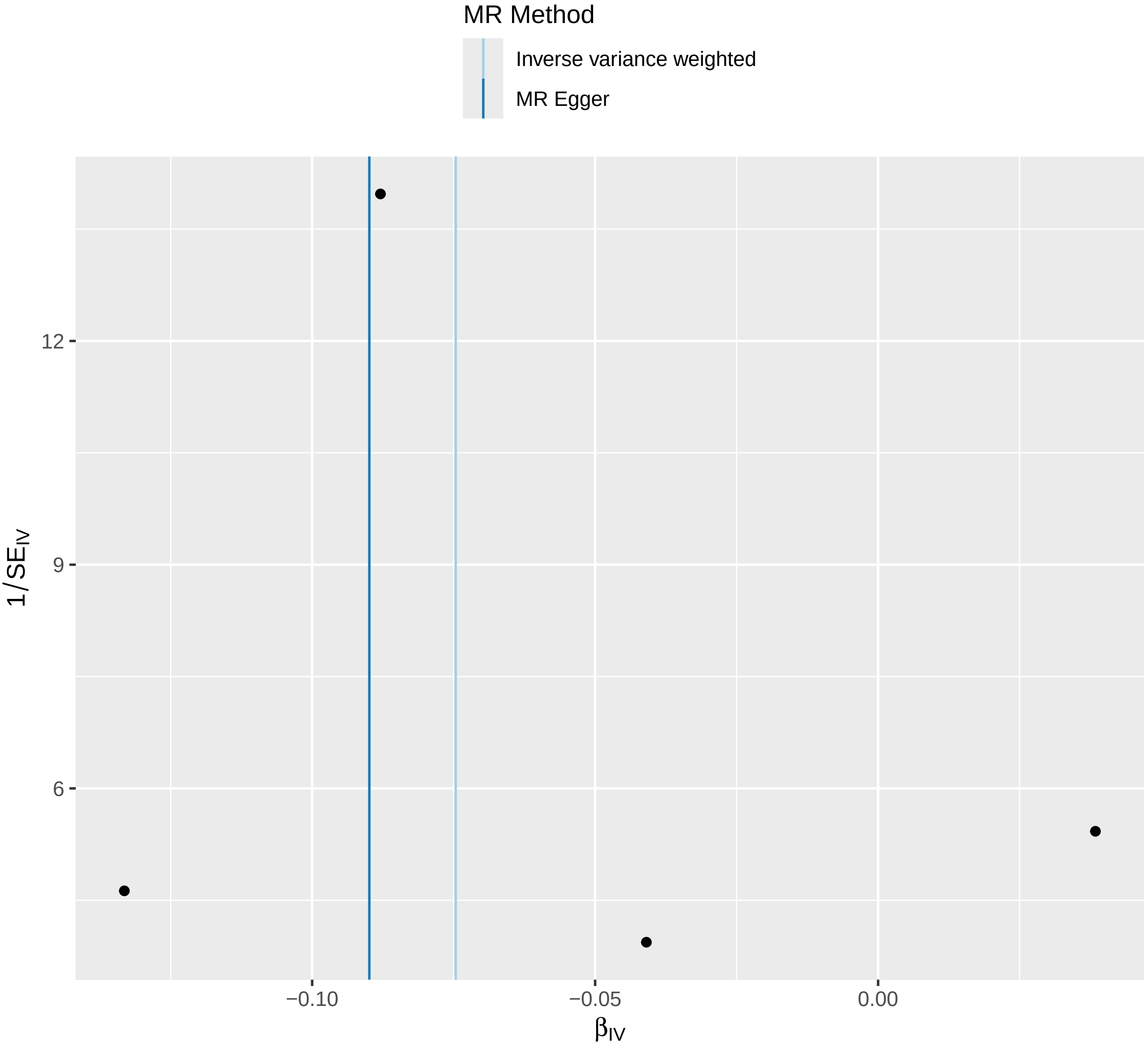
MR Method
Inverse variance weighted
MR Egger
12
9
6
$1/SE_{IV}$
−0.10
−0.05
0.00
$\beta_{IV}$

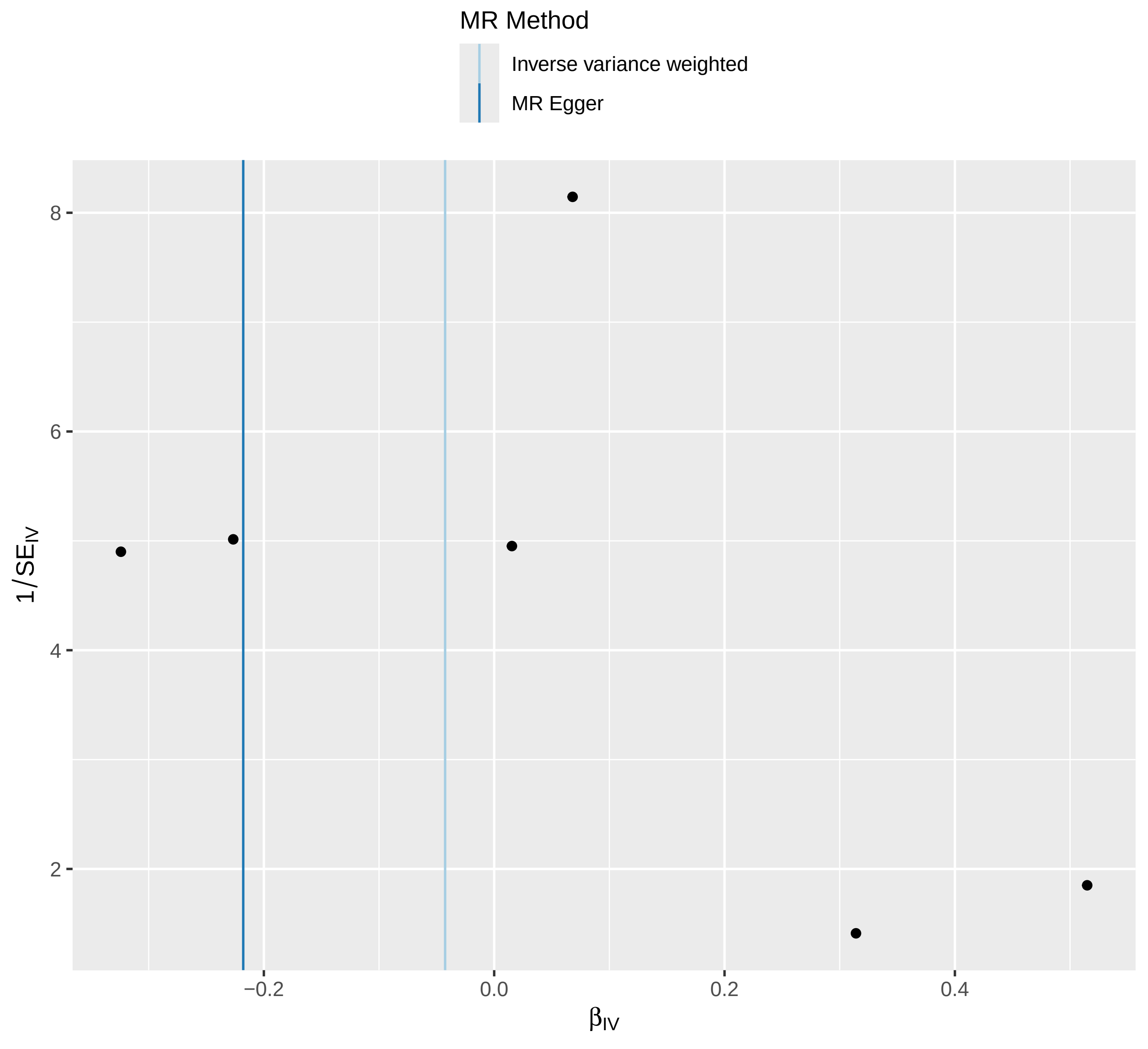

MR Method
Inverse variance weighted
MR Egger
8
6
4
2
1/SE$_{IV}$
−0.2
0.0
0.2
0.4
β$_{IV}$

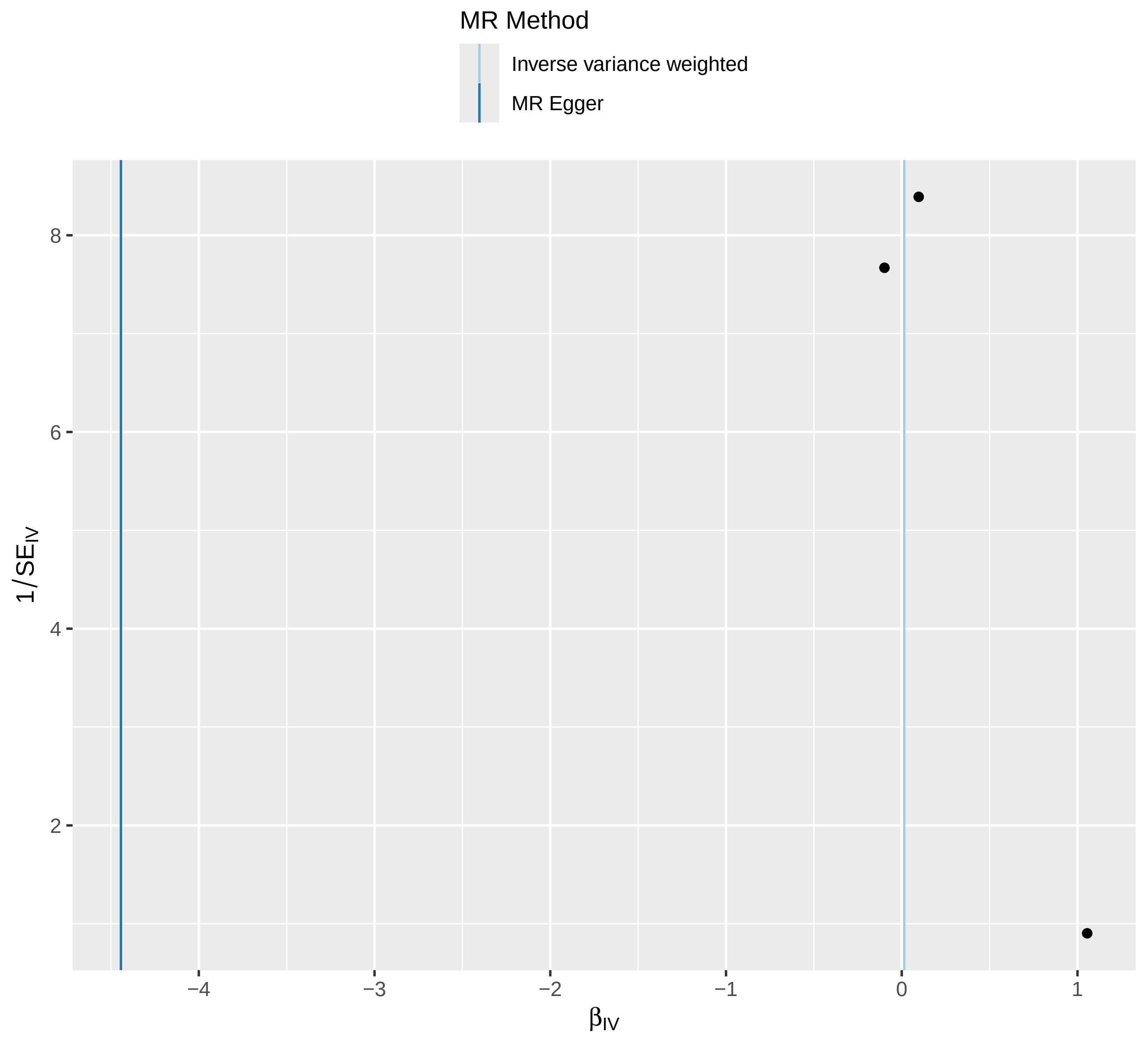
MR Method
Inverse variance weighted
MR Egger
8
6
4
2
1/SE$_{IV}$
−4
−3
−2
−1
0
1
β$_{IV}$

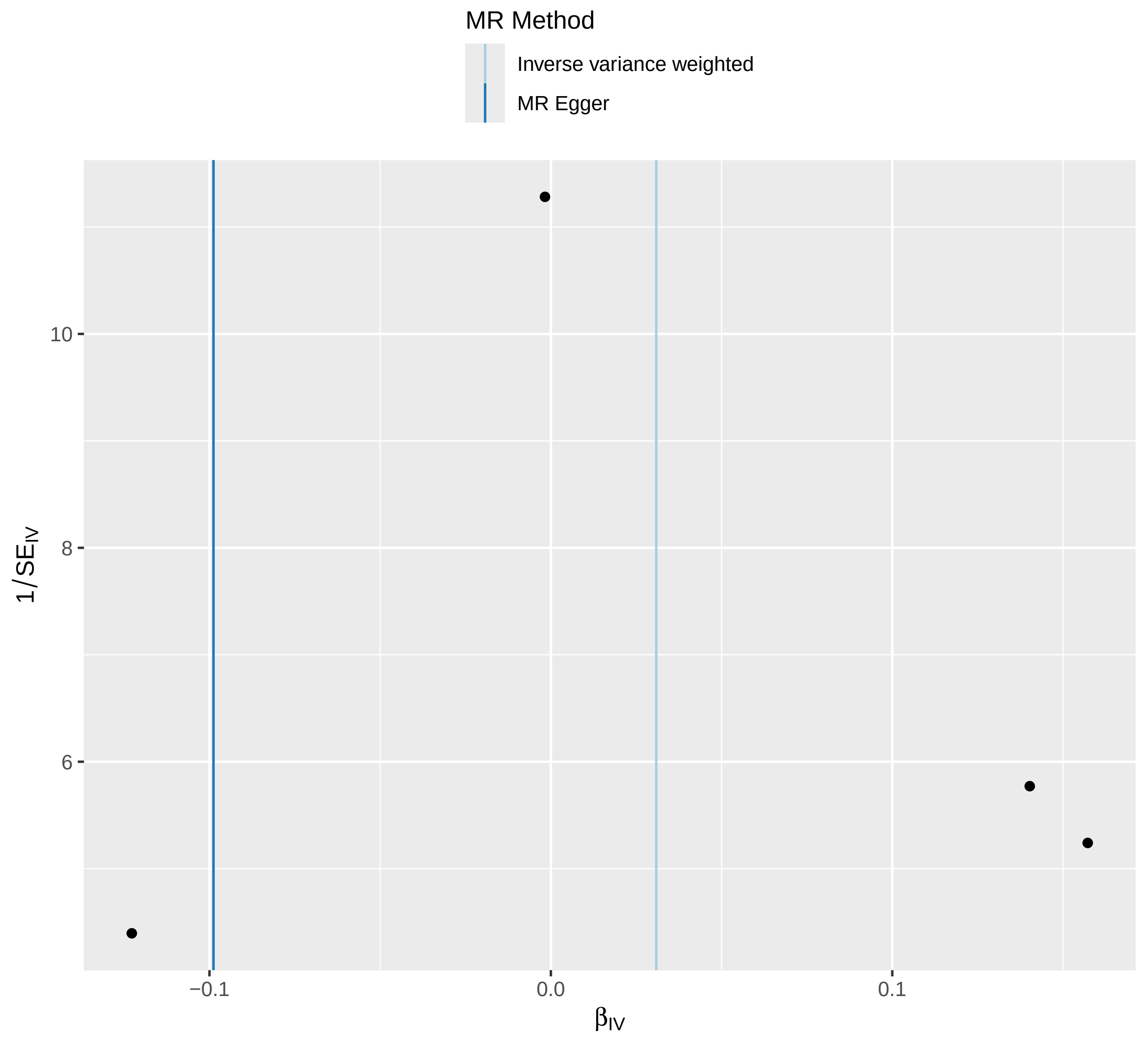
MR Method
Inverse variance weighted
MR Egger
10
8
6
1/SE$_{IV}$
−0.1
0.0
0.1
β$_{IV}$

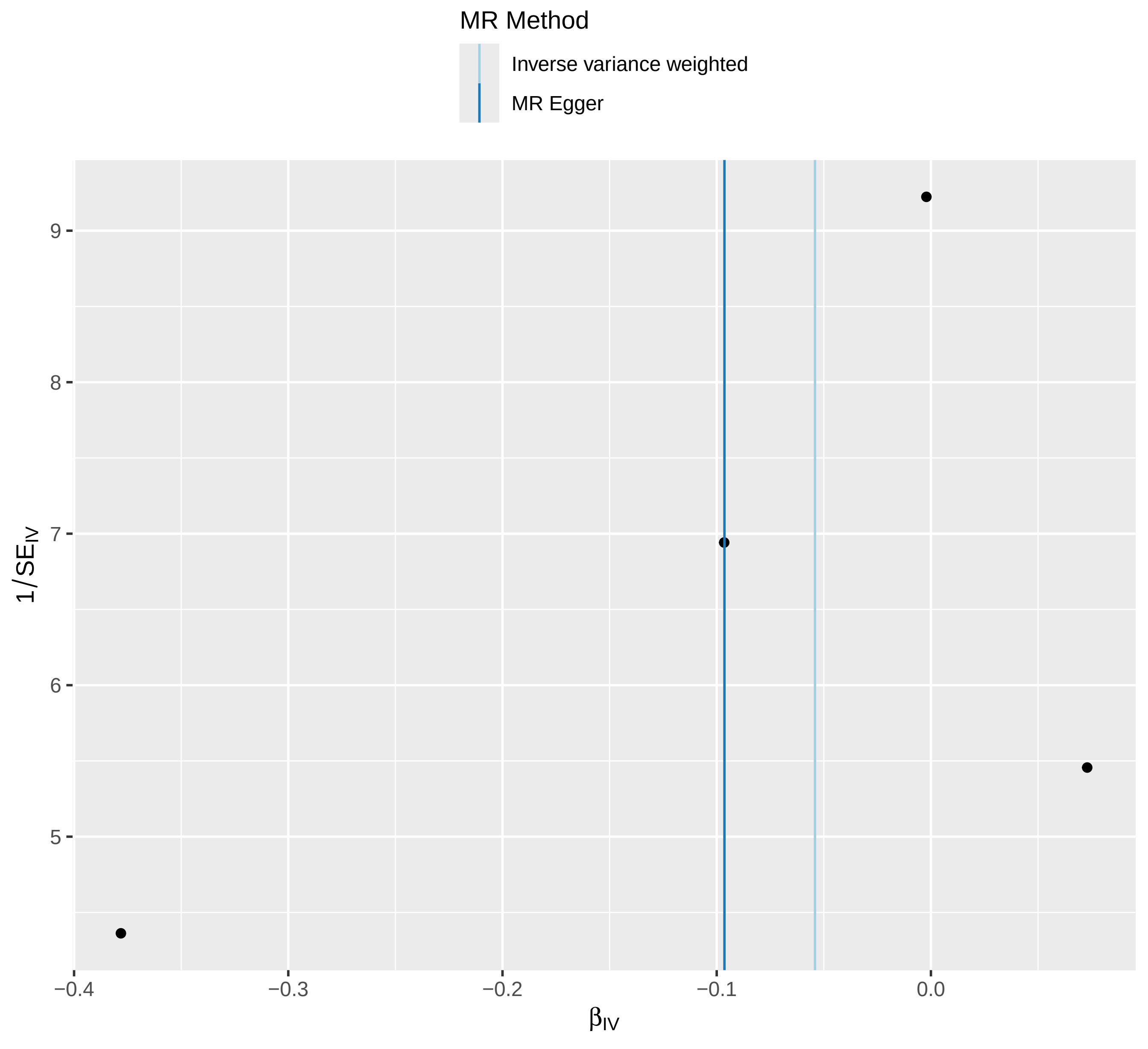
MR Method
Inverse variance weighted
MR Egger
9
8
7
6
5
$1/SE_{IV}$
−0.4
−0.3
−0.2
−0.1
0.0
$\beta_{IV}$

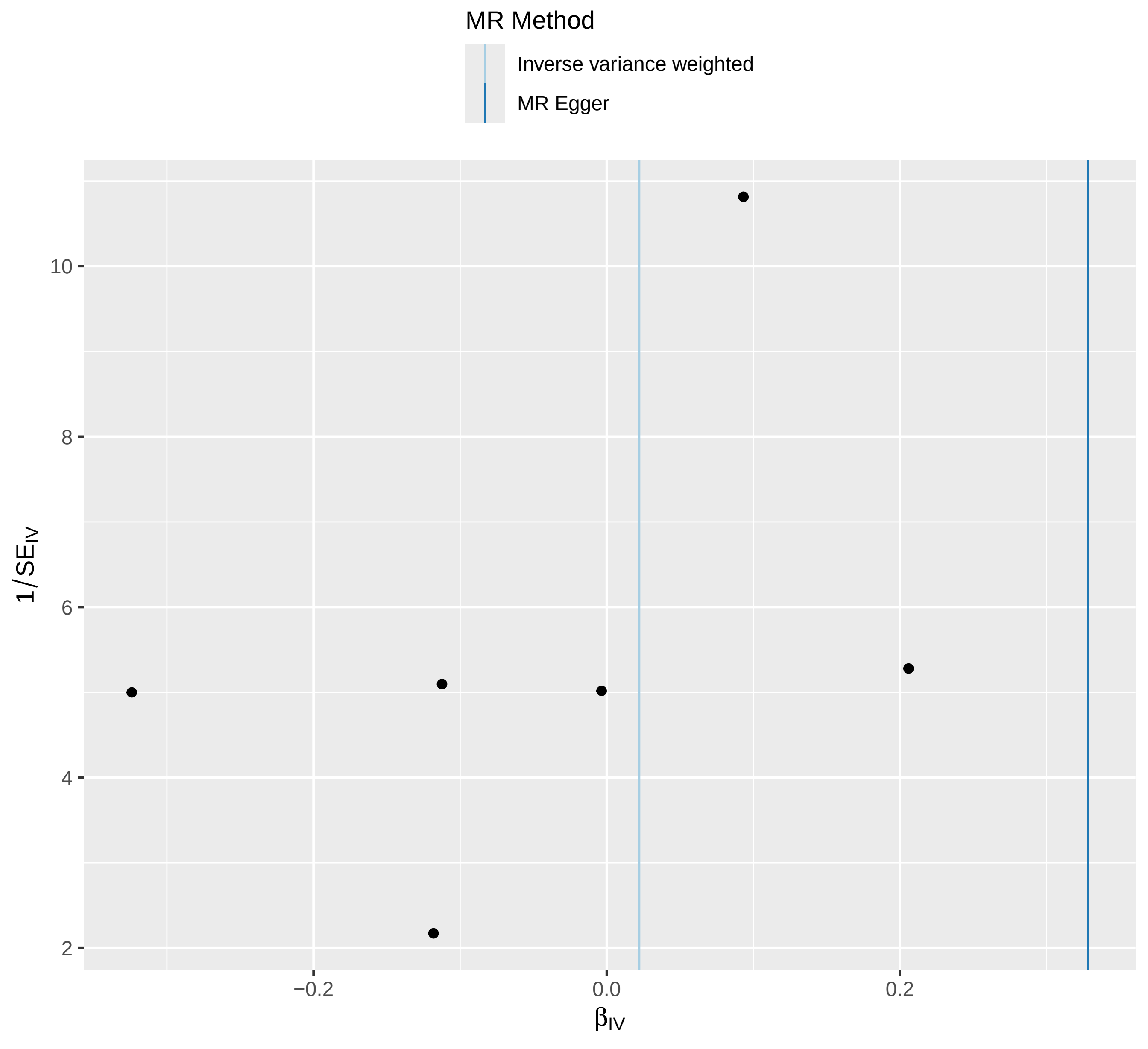
MR Method
Inverse variance weighted
MR Egger
$1/SE_{IV}$
10
8
6
4
2
−0.2
0.0
0.2
$\beta_{IV}$

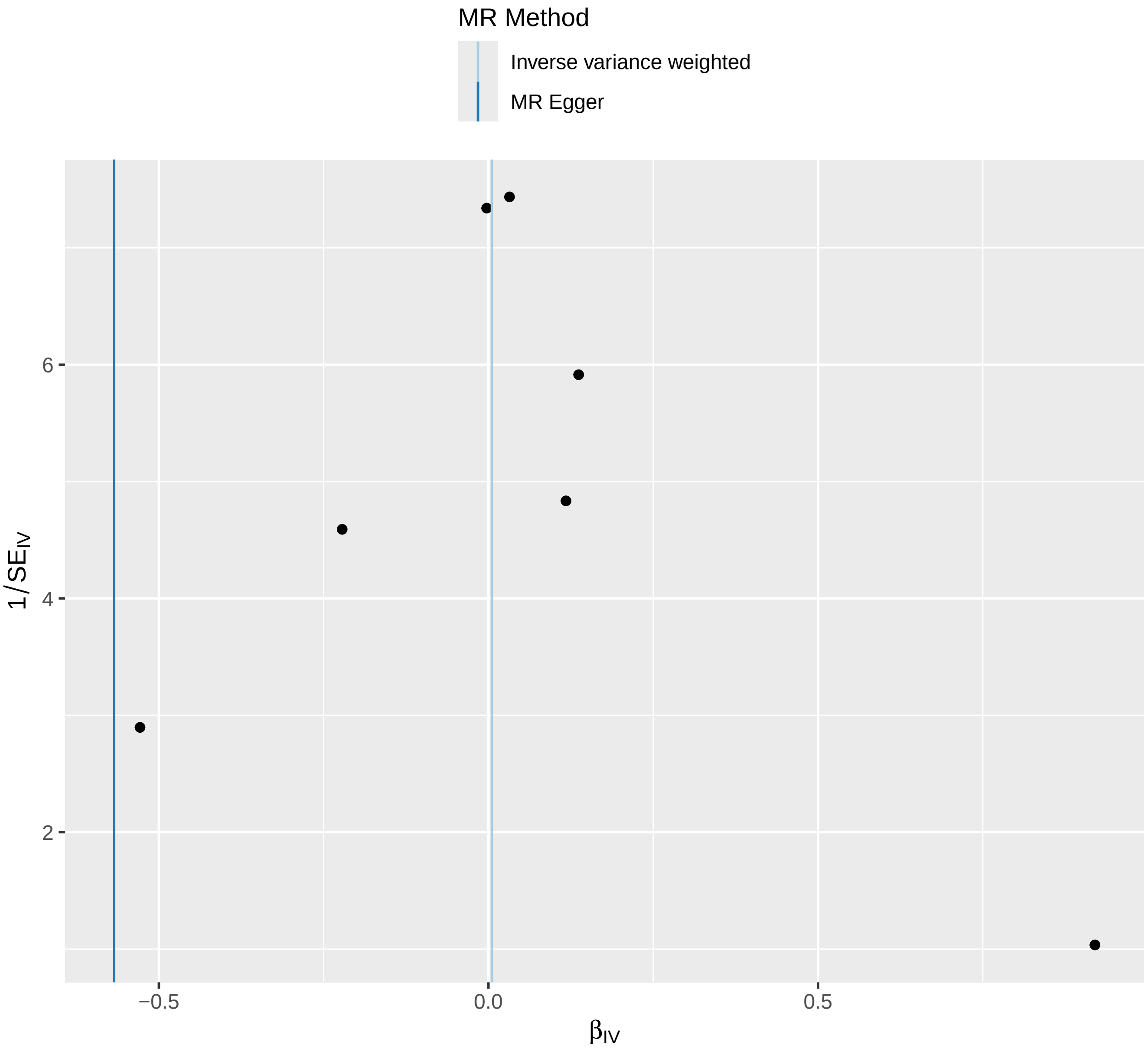
MR Method
Inverse variance weighted
MR Egger
1/SE$_{IV}$
2
4
6
−0.5
0.0
0.5
β$_{IV}$

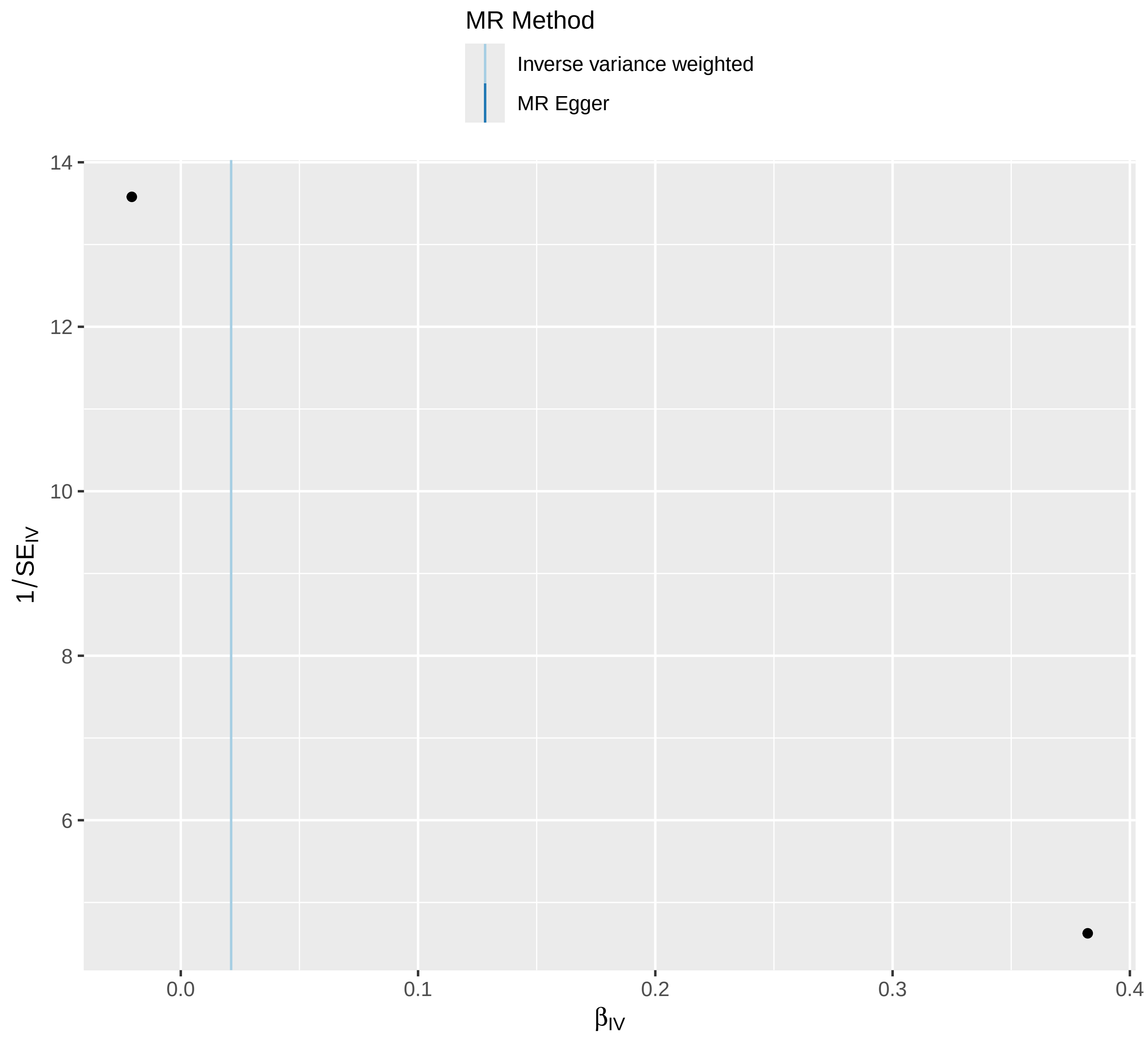
MR Method
Inverse variance weighted
MR Egger
14
12
10
8
6
0.0
0.1
0.2
0.3
0.4
$1/SE_{IV}$
$\beta_{IV}$

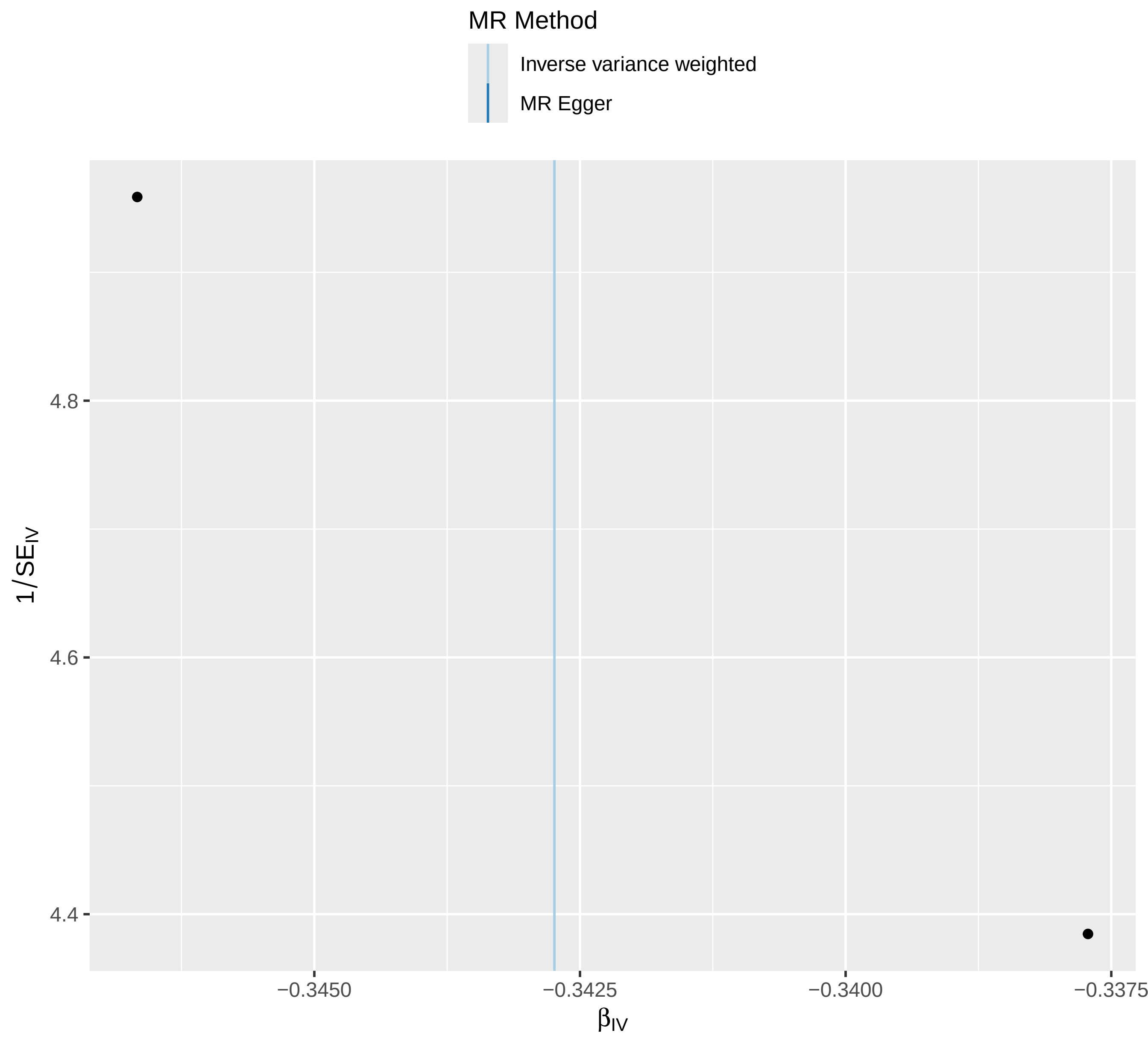

MR Method
Inverse variance weighted
MR Egger
4.8
4.6
4.4
1/SE$_{IV}$
−0.3450
−0.3425
−0.3400
−0.3375
β$_{IV}$

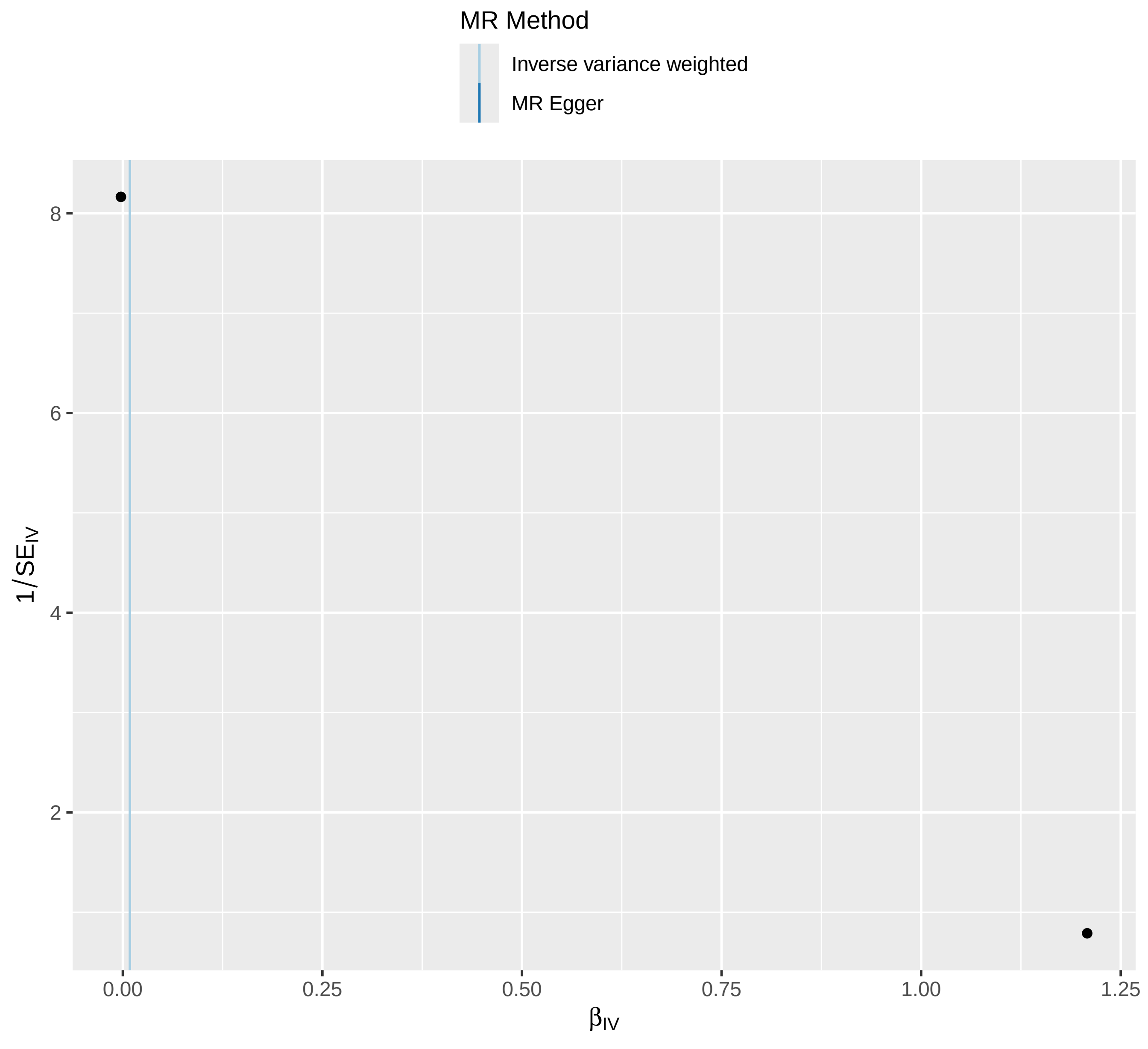
MR Method
Inverse variance weighted
MR Egger
$1/SE_{IV}$
8
6
4
2
0.00
0.25
0.50
0.75
1.00
1.25
$\beta_{IV}$

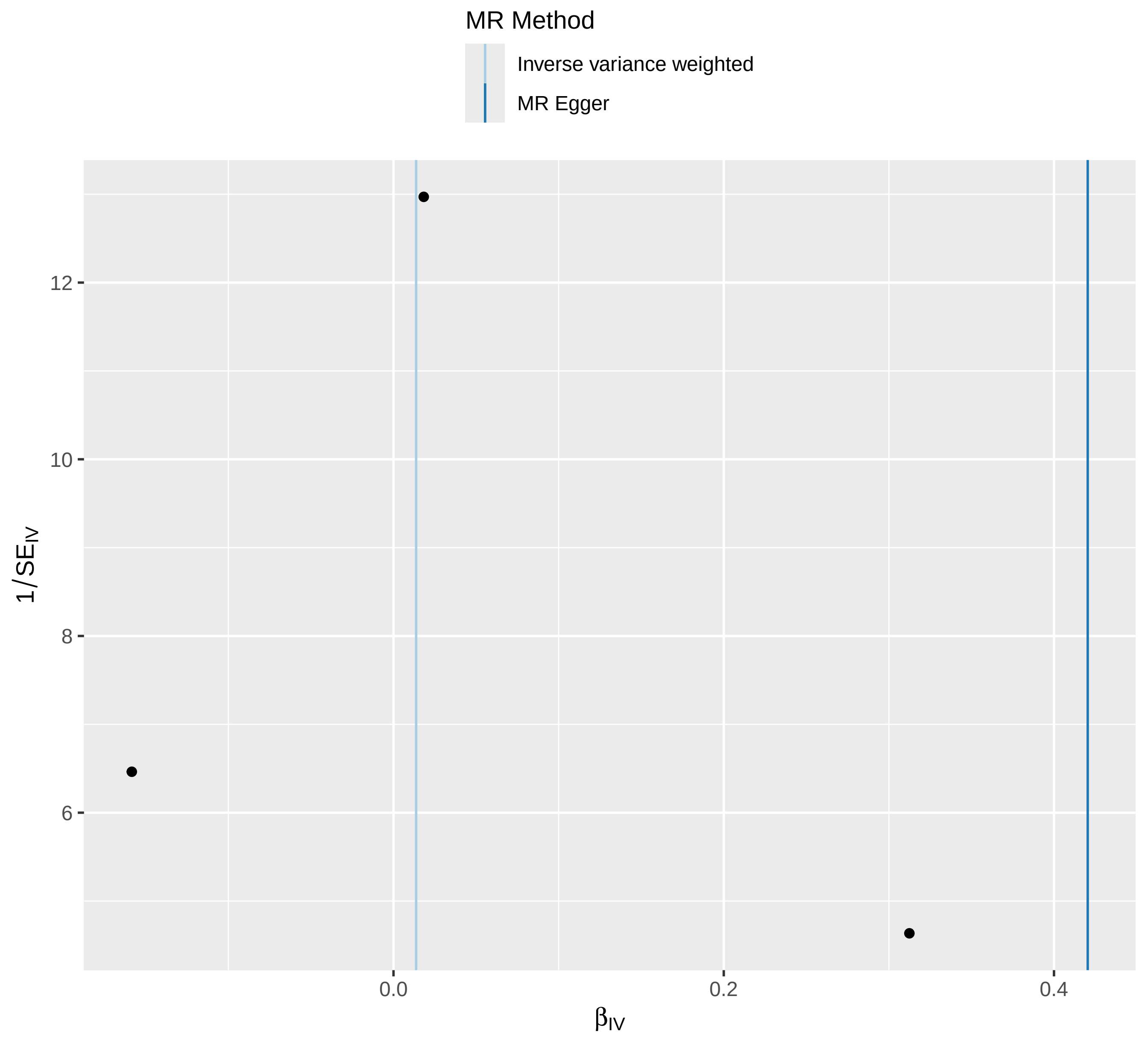

MR Method
Inverse variance weighted
MR Egger
12
10
8
6
1/SE_IV
0.0
0.2
0.4
β_IV

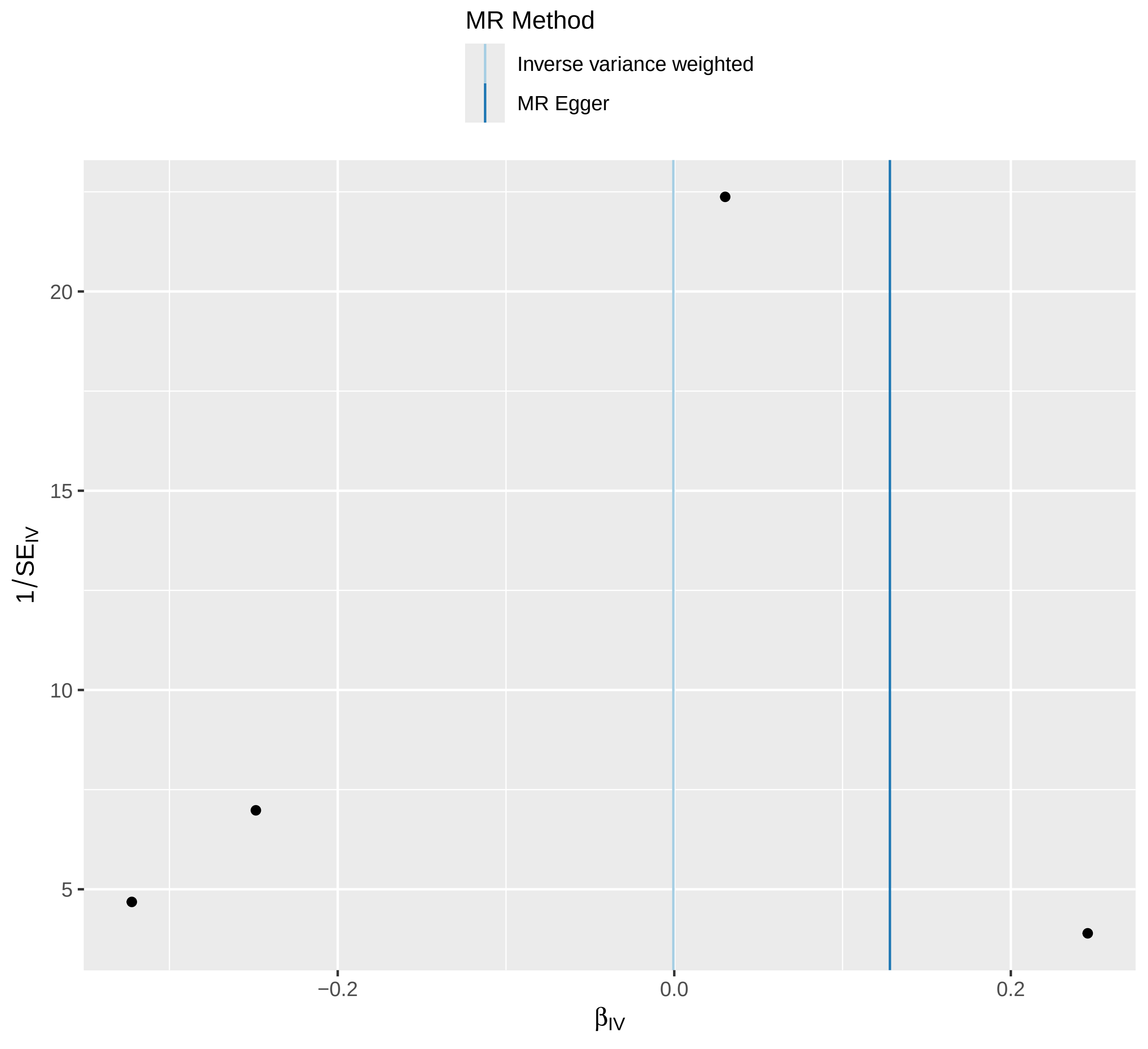
MR Method
Inverse variance weighted
MR Egger
20
15
10
5
1/SE_IV
−0.2
0.0
0.2
β_IV

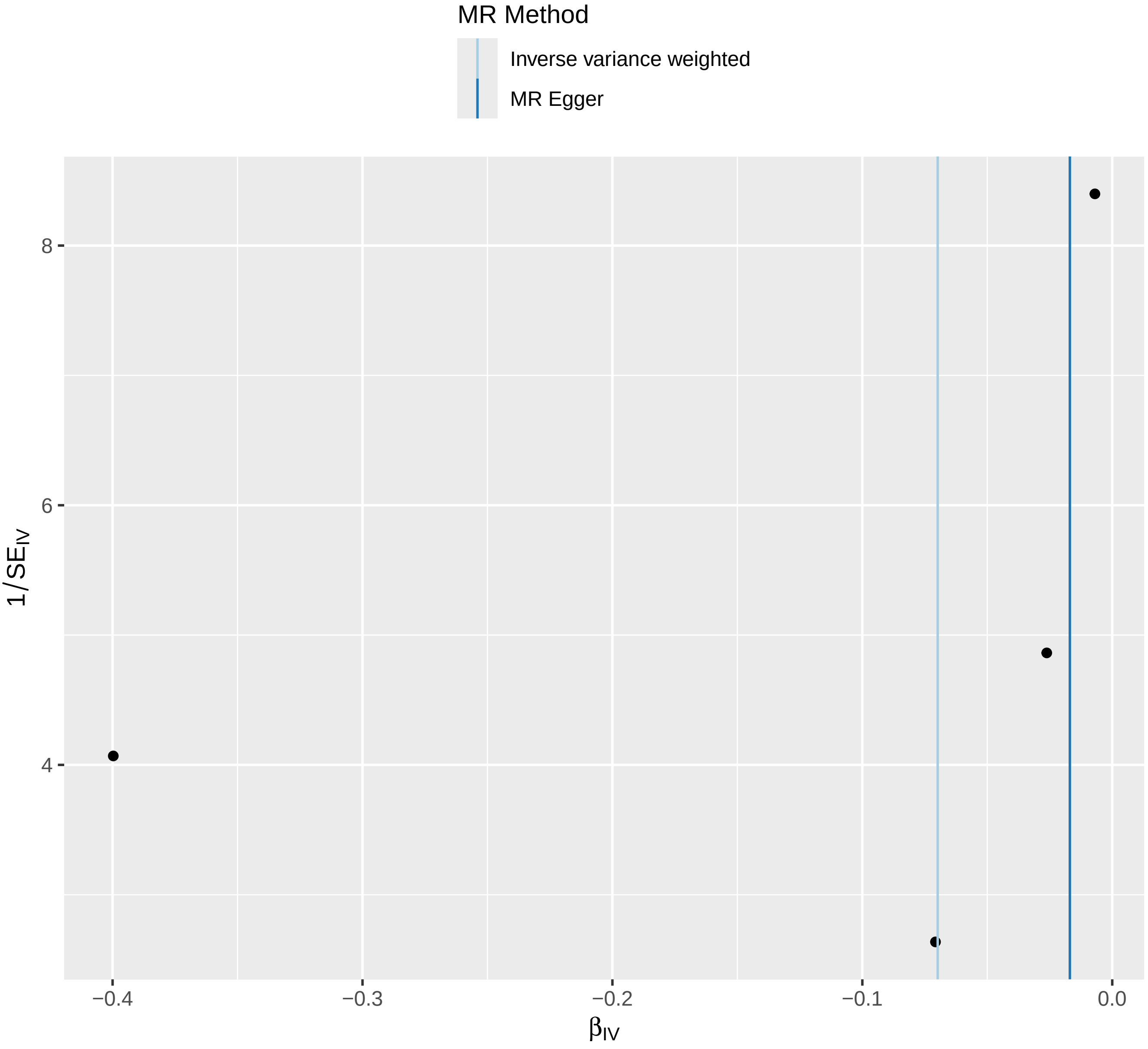
MR Method
Inverse variance weighted
MR Egger
8
6
4
1/SE_IV
−0.4
−0.3
−0.2
−0.1
0.0
β_IV

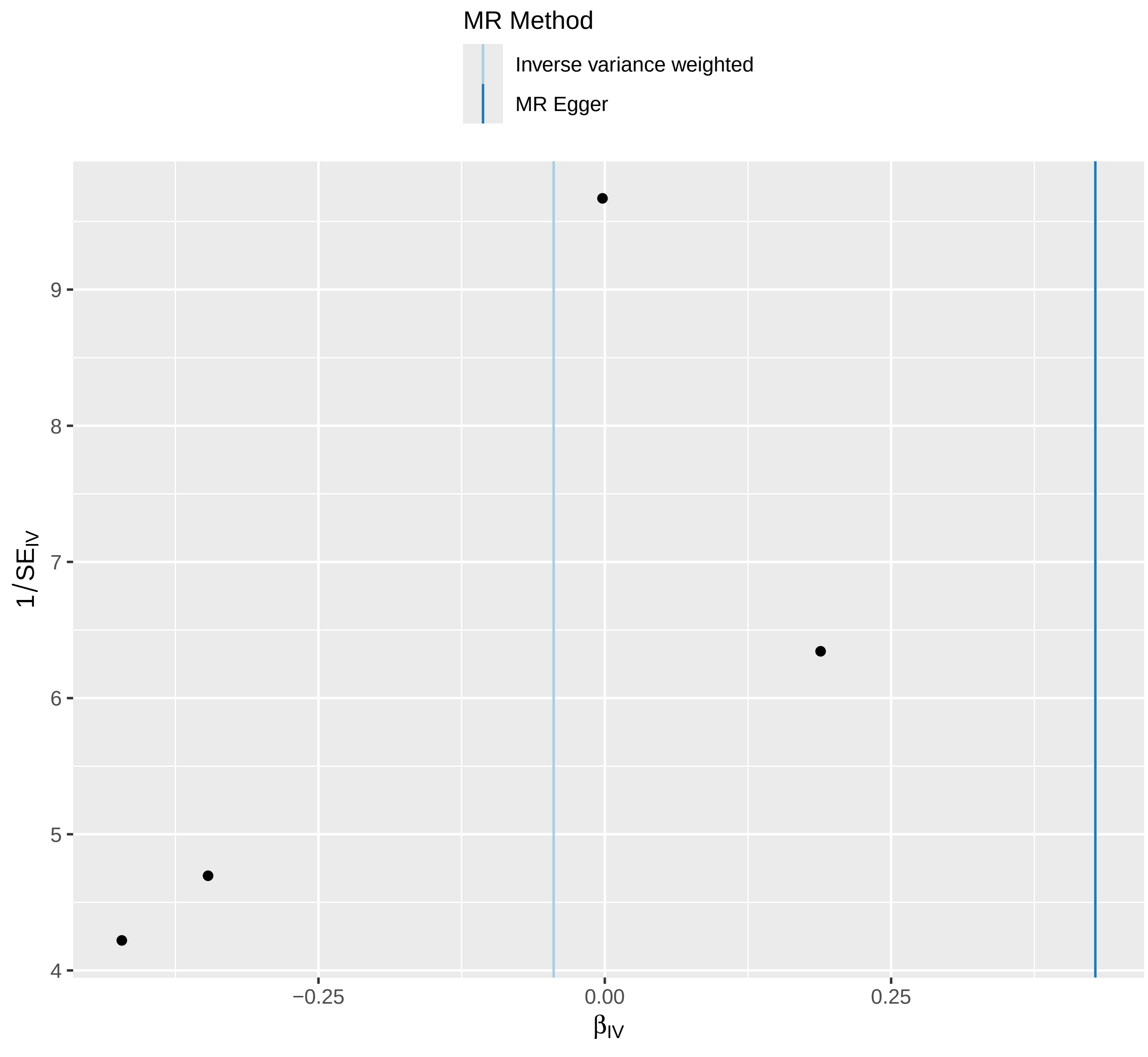
MR Method
Inverse variance weighted
MR Egger
9
8
7
6
5
4
$1/SE_{IV}$
−0.25
0.00
0.25
$\beta_{IV}$

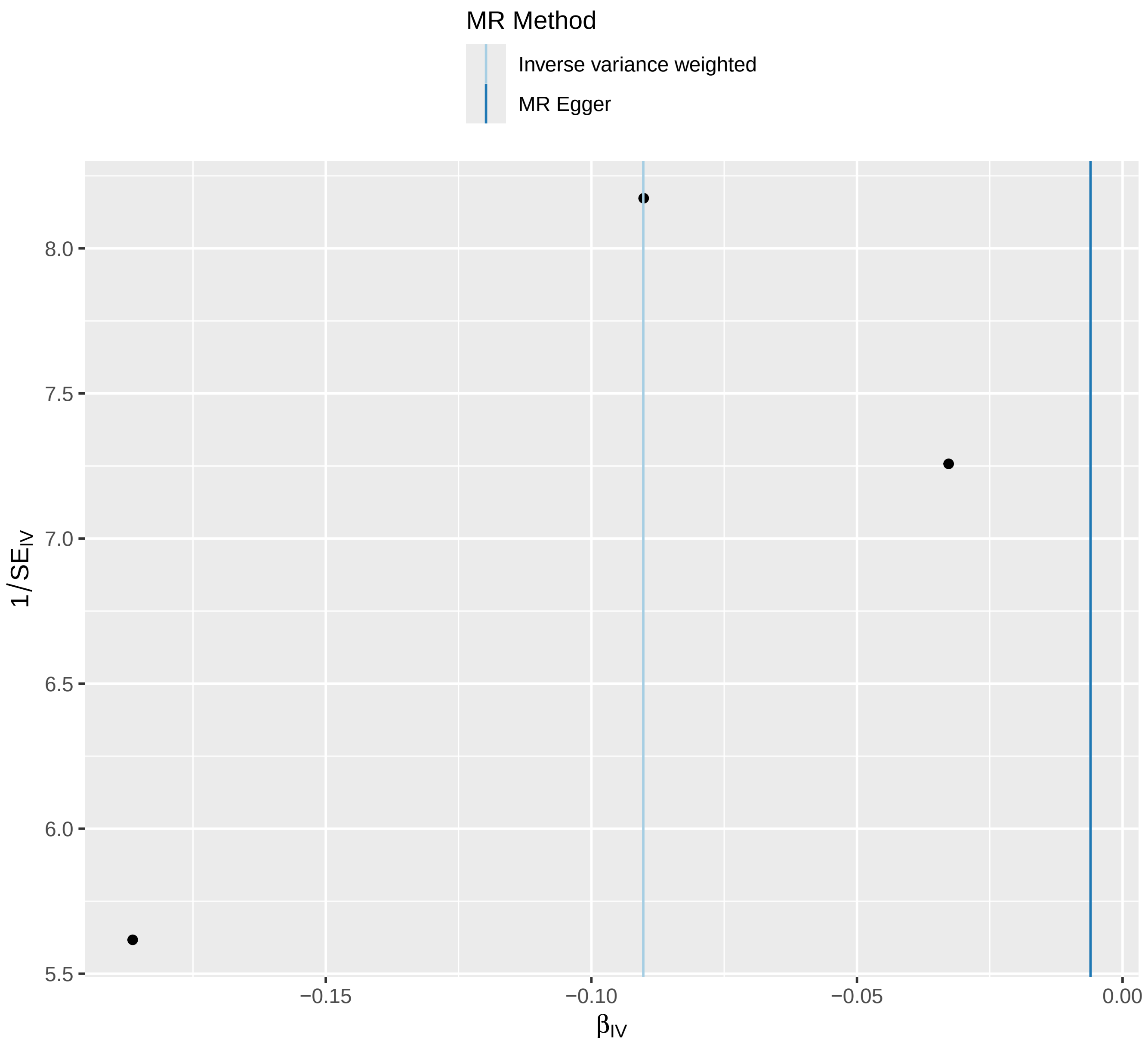

MR Method
Inverse variance weighted
MR Egger
8.0
7.5
7.0
6.5
6.0
5.5
1/SE$_{IV}$
−0.15
−0.10
−0.05
0.00
β$_{IV}$

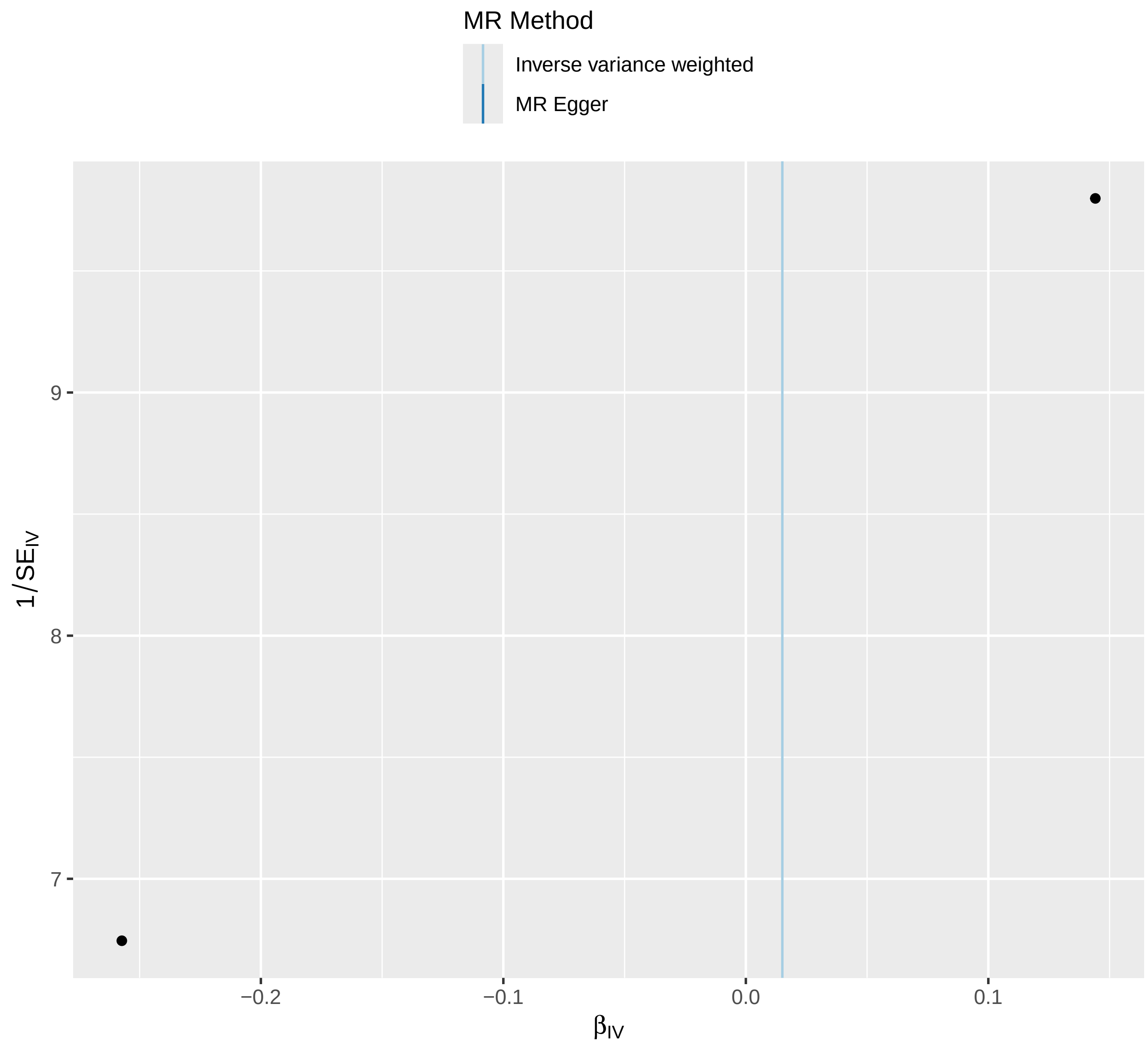
MR Method
Inverse variance weighted
MR Egger
9
8
7
−0.2
−0.1
0.0
0.1
$\beta_{IV}$
$1/SE_{IV}$

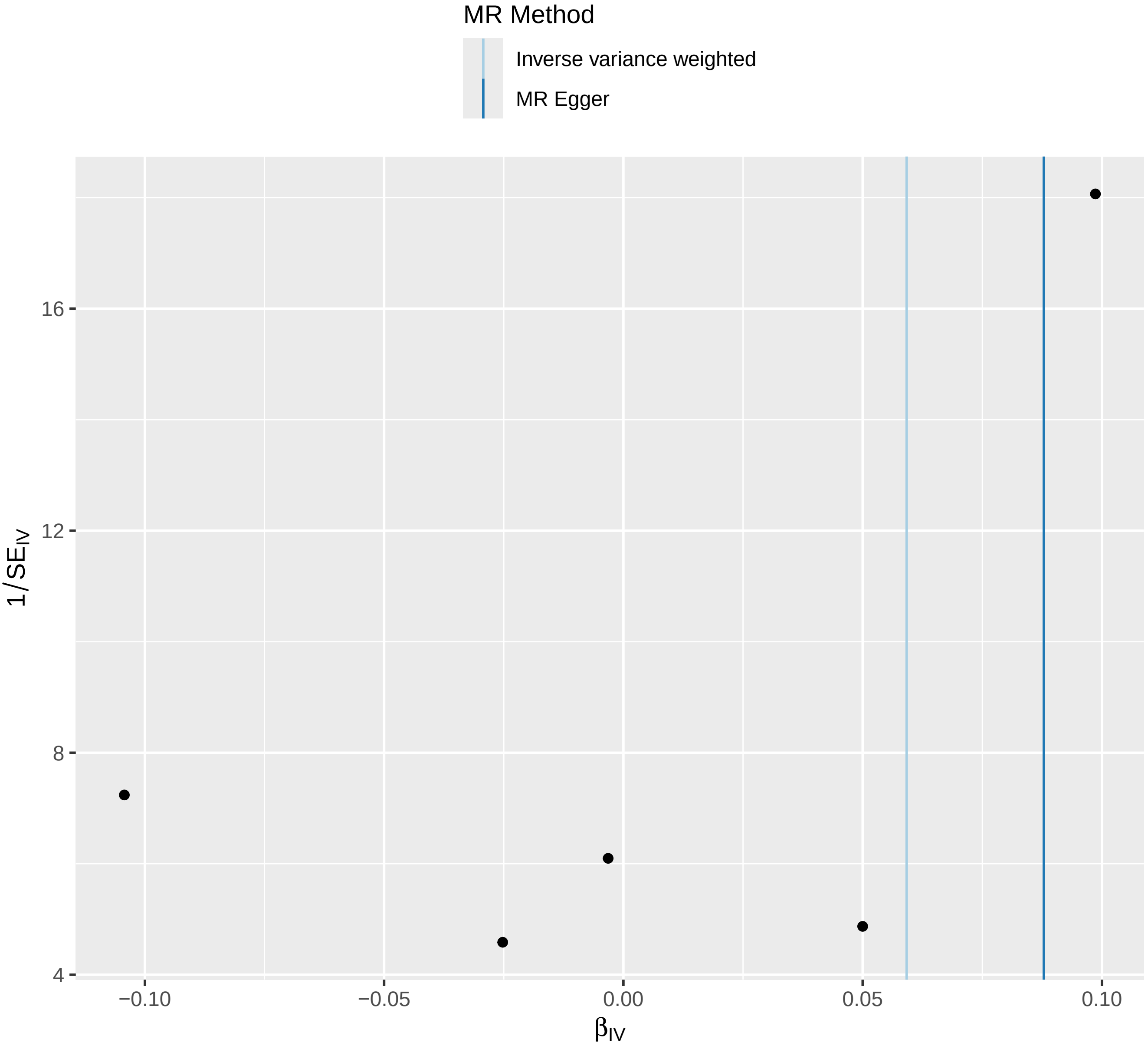
MR Method
Inverse variance weighted
MR Egger
16
12
8
4
1/SE$_{IV}$
−0.10
−0.05
0.00
0.05
0.10
β$_{IV}$

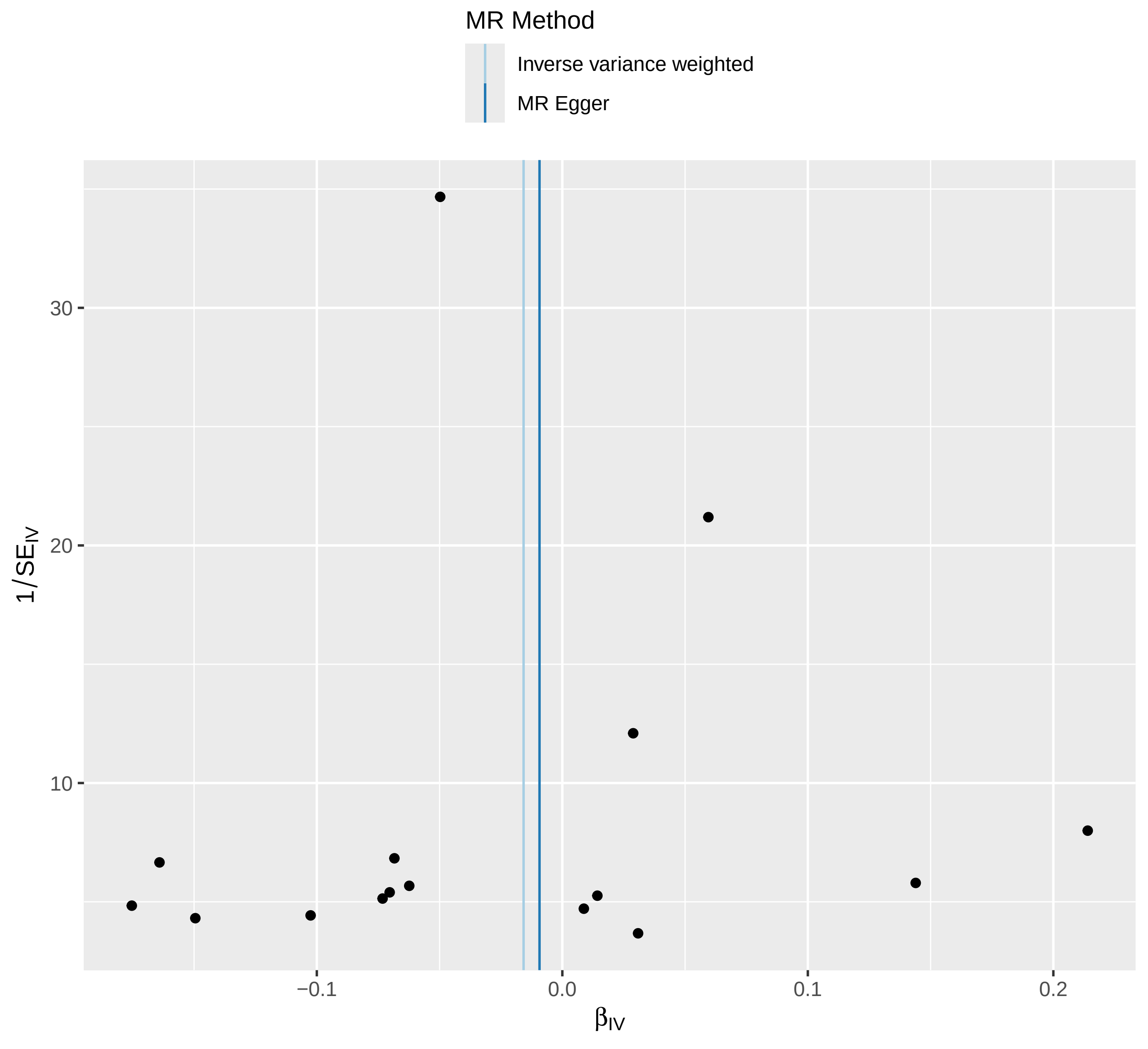
MR Method
Inverse variance weighted
MR Egger
30
20
10
$1/SE_{IV}$
−0.1
0.0
0.1
0.2
$\beta_{IV}$

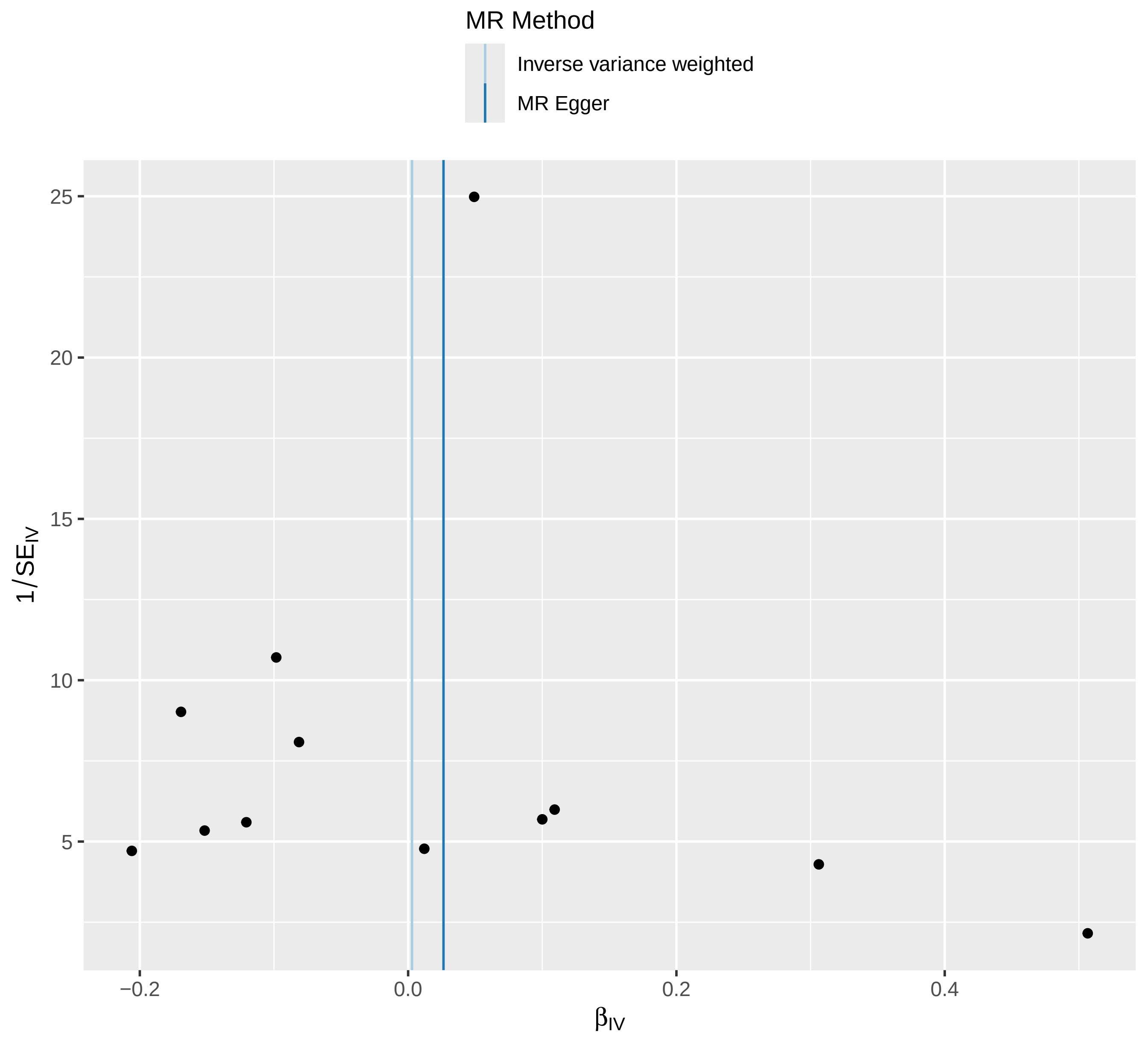
MR Method
Inverse variance weighted
MR Egger
25
20
15
10
5
1/SE$_{IV}$
−0.2
0.0
0.2
0.4
β$_{IV}$

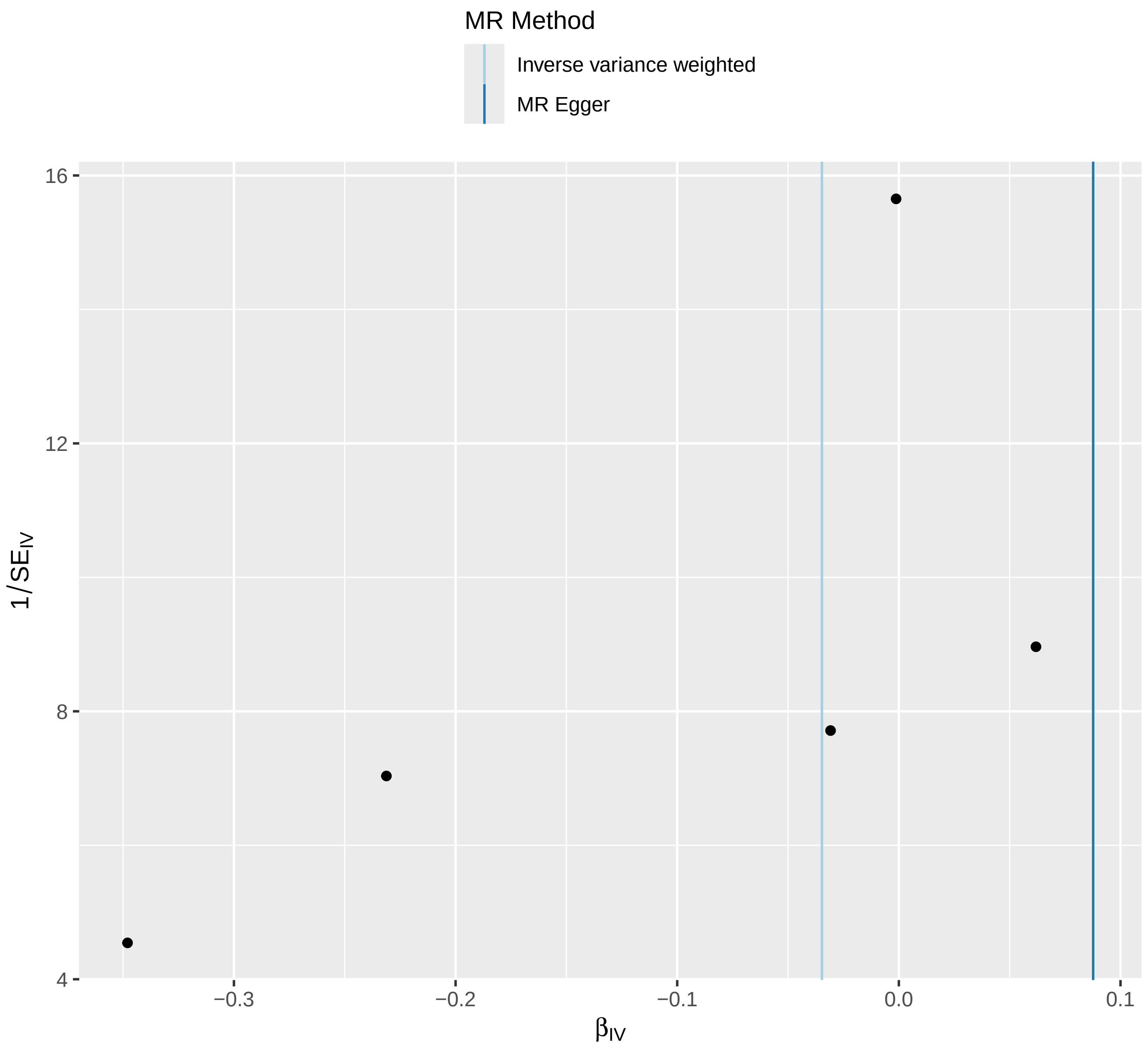
MR Method
Inverse variance weighted
MR Egger
16
12
8
4
1/SE$_{IV}$
−0.3
−0.2
−0.1
0.0
0.1
β$_{IV}$

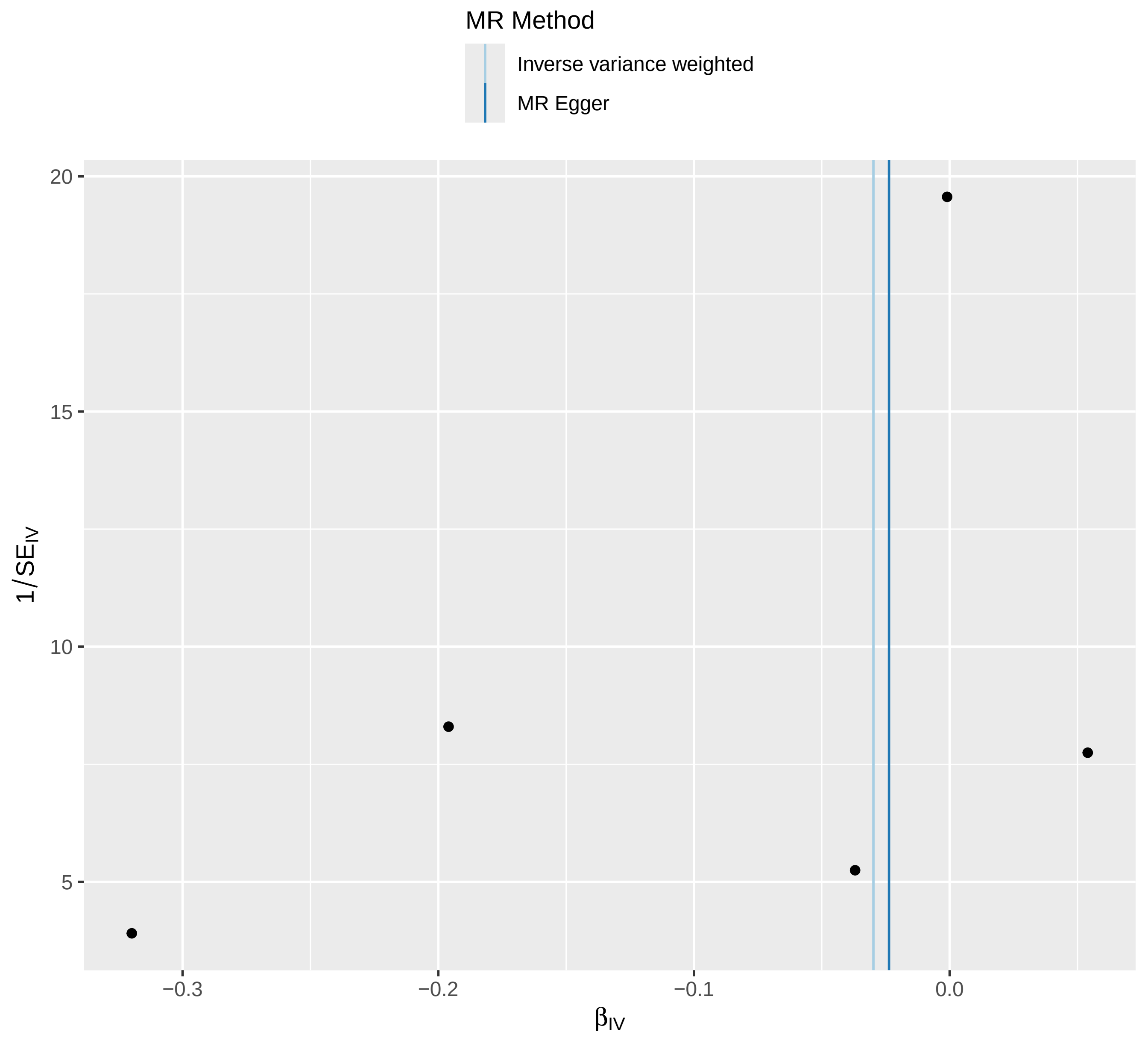
MR Method
Inverse variance weighted
MR Egger
20
15
10
5
−0.3
−0.2
−0.1
0.0
$1/SE_{IV}$
$\beta_{IV}$

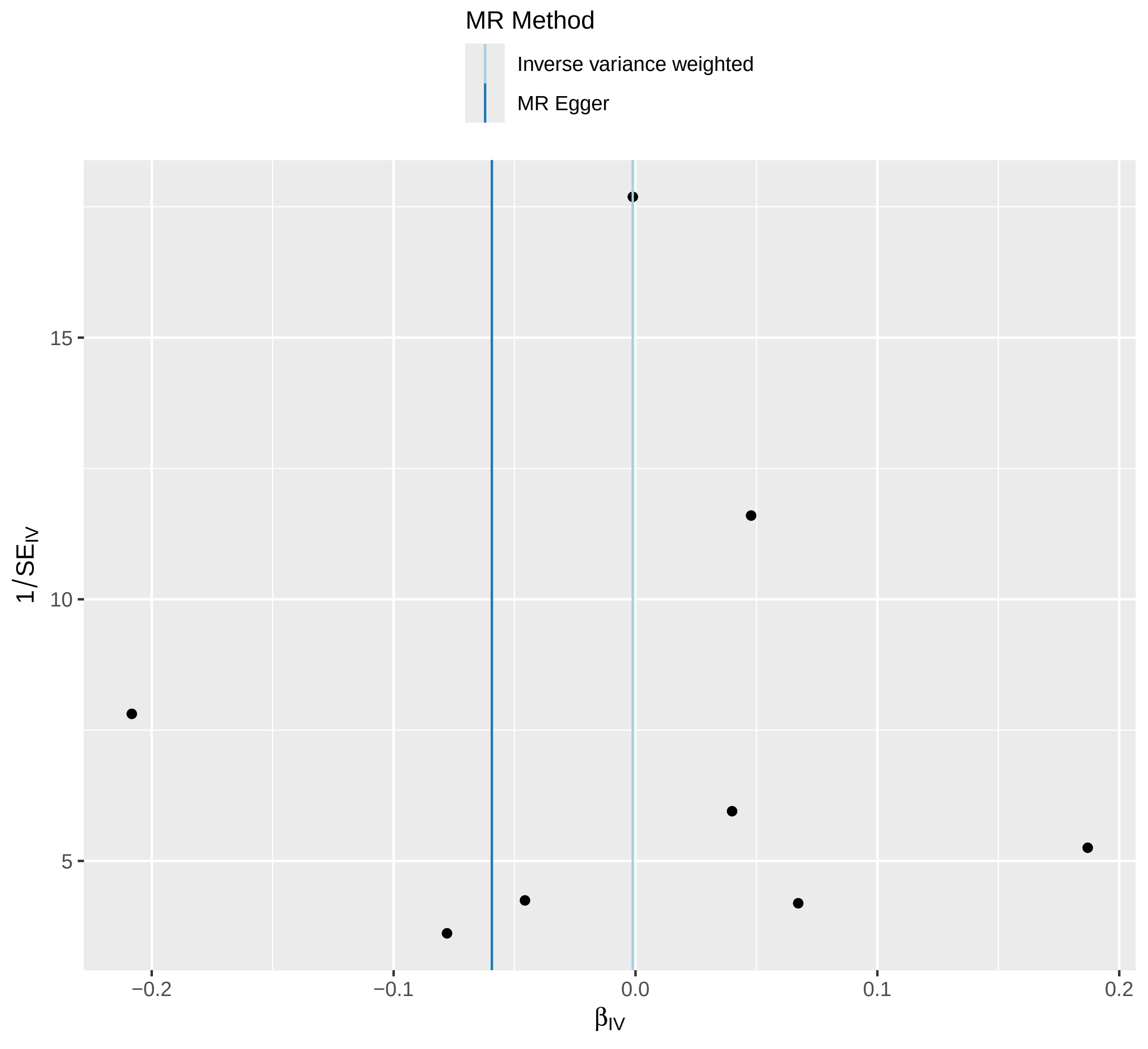
MR Method
Inverse variance weighted
MR Egger
15
10
5
1/SE_IV
−0.2
−0.1
0.0
0.1
0.2
β_IV

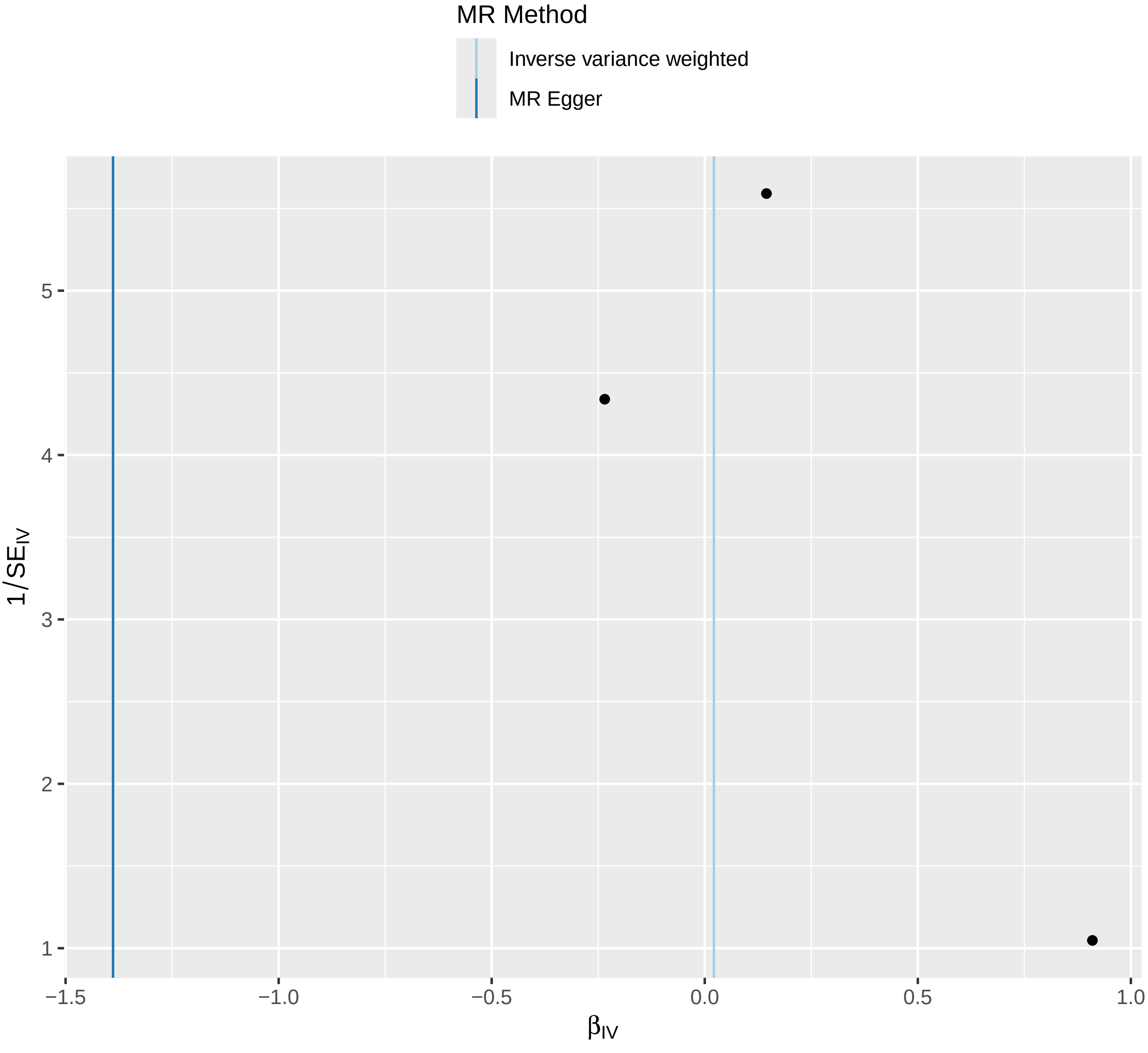
MR Method
Inverse variance weighted
MR Egger
1/$SE_{IV}$
5
4
3
2
1
−1.5
−1.0
−0.5
0.0
0.5
1.0
$\beta_{IV}$

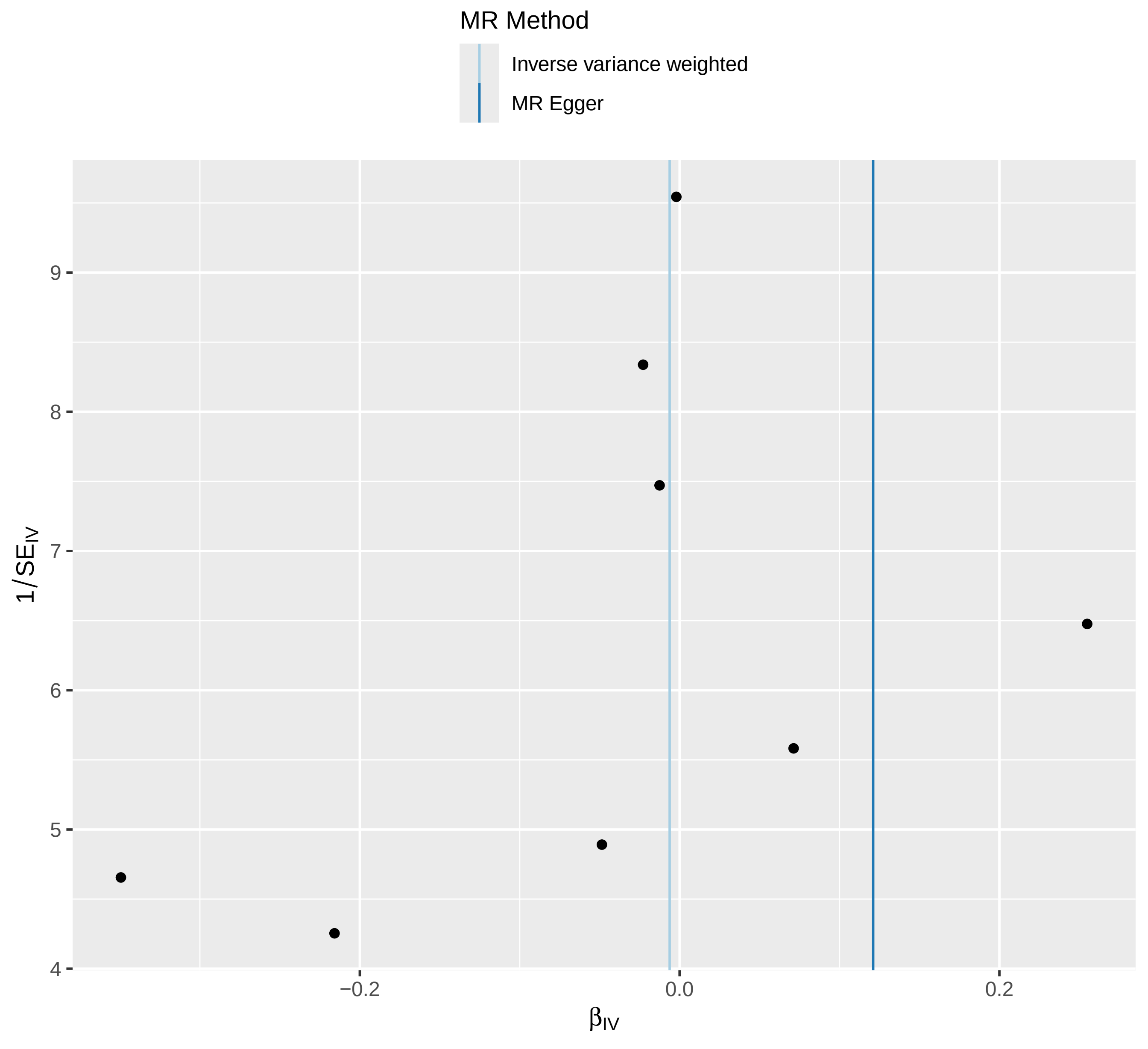

MR Method
Inverse variance weighted
MR Egger
9
8
7
6
5
4
1/SE$_{IV}$
−0.2
0.0
0.2
β$_{IV}$

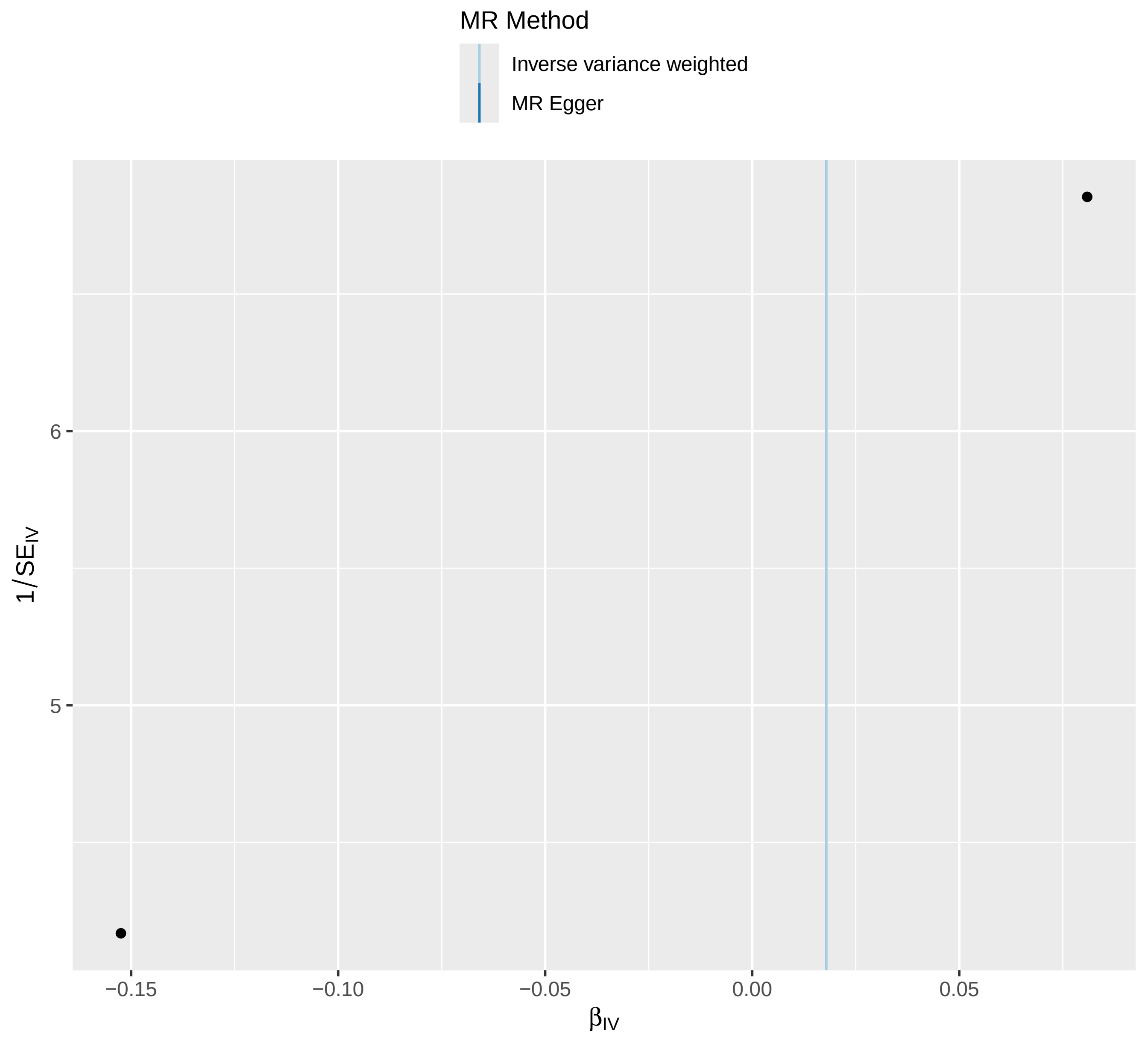

MR Method
Inverse variance weighted
MR Egger
6
5
1/SE$_{IV}$
−0.15
−0.10
−0.05
0.00
0.05
$\beta_{IV}$

Insufficient number of SNPs

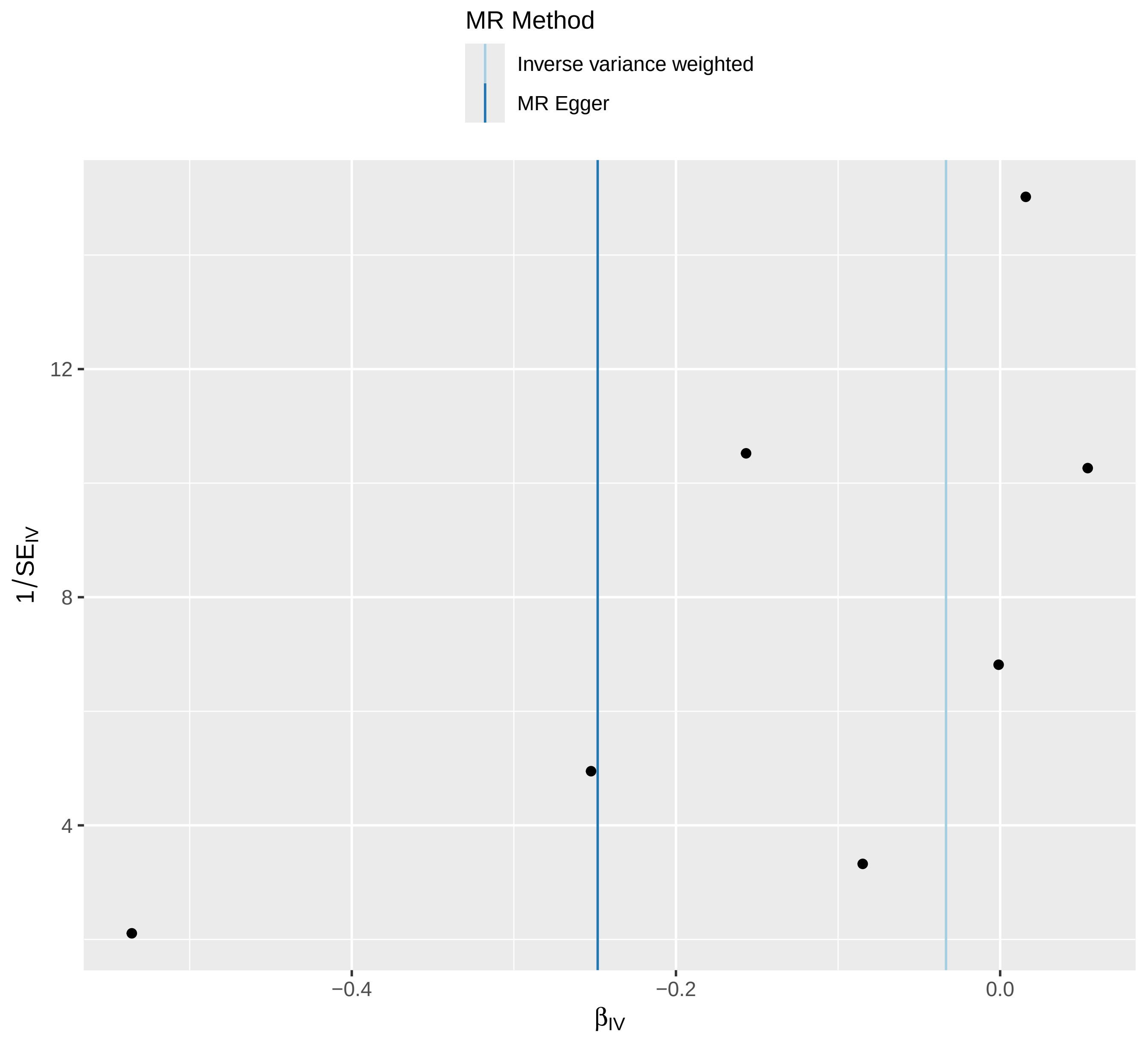

MR Method
Inverse variance weighted
MR Egger
1/SE$_{IV}$
12
8
4
−0.4
−0.2
0.0
β$_{IV}$

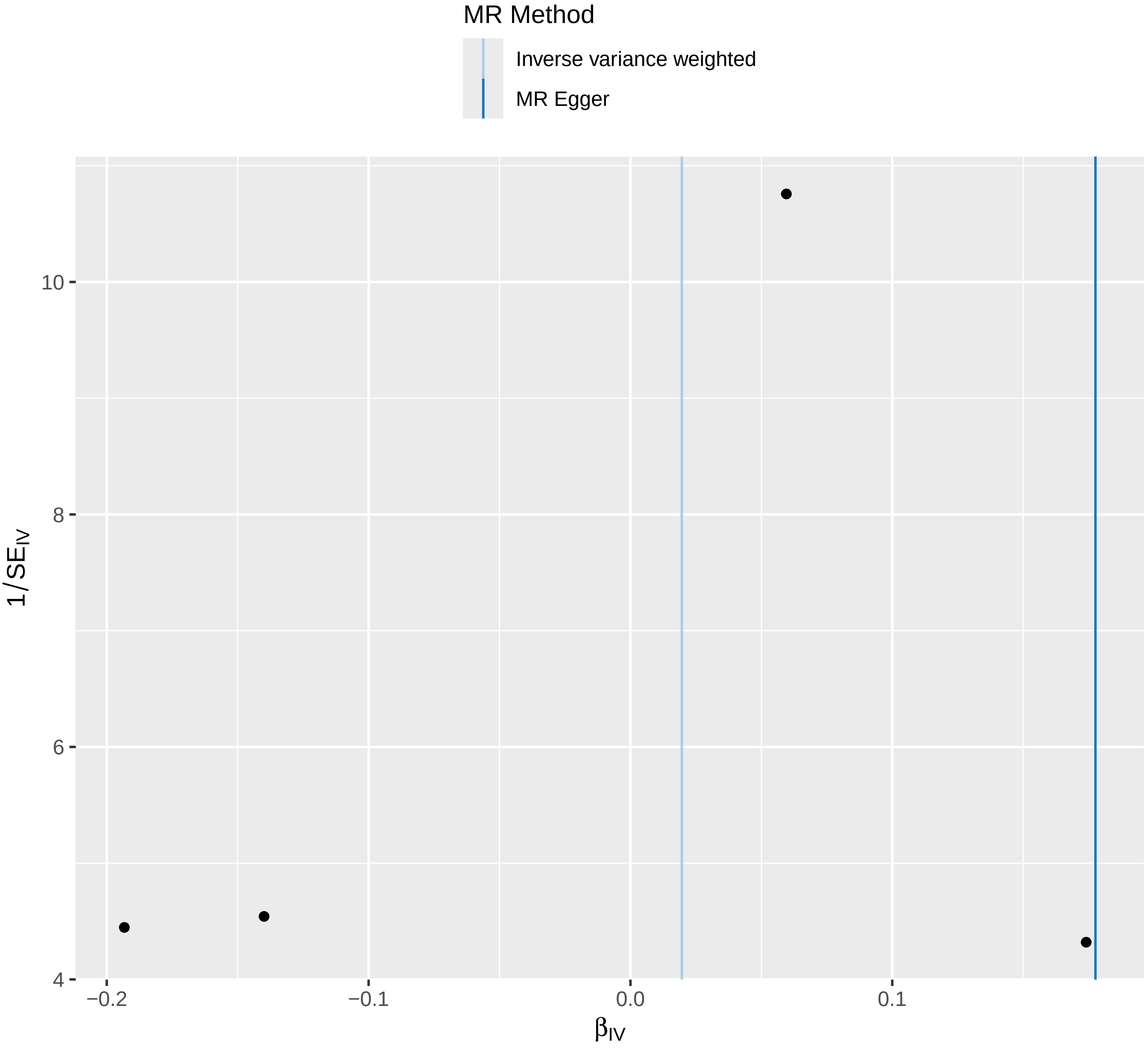
MR Method
Inverse variance weighted
MR Egger
10
8
6
4
1/SE$_{IV}$
−0.2
−0.1
0.0
0.1
β$_{IV}$

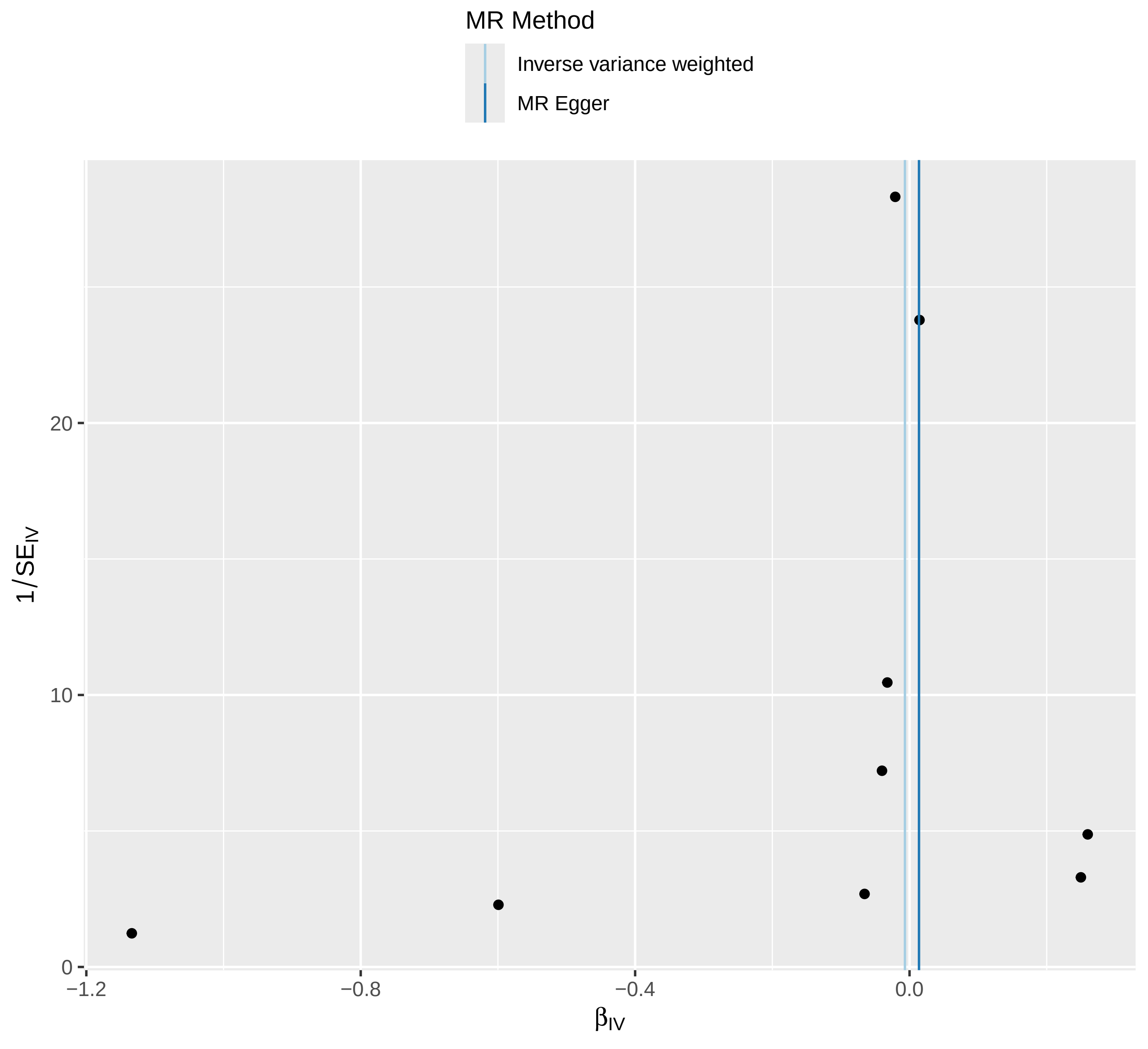
MR Method
Inverse variance weighted
MR Egger
1/SE$_{IV}$
20
10
0
−1.2
−0.8
−0.4
0.0
β$_{IV}$

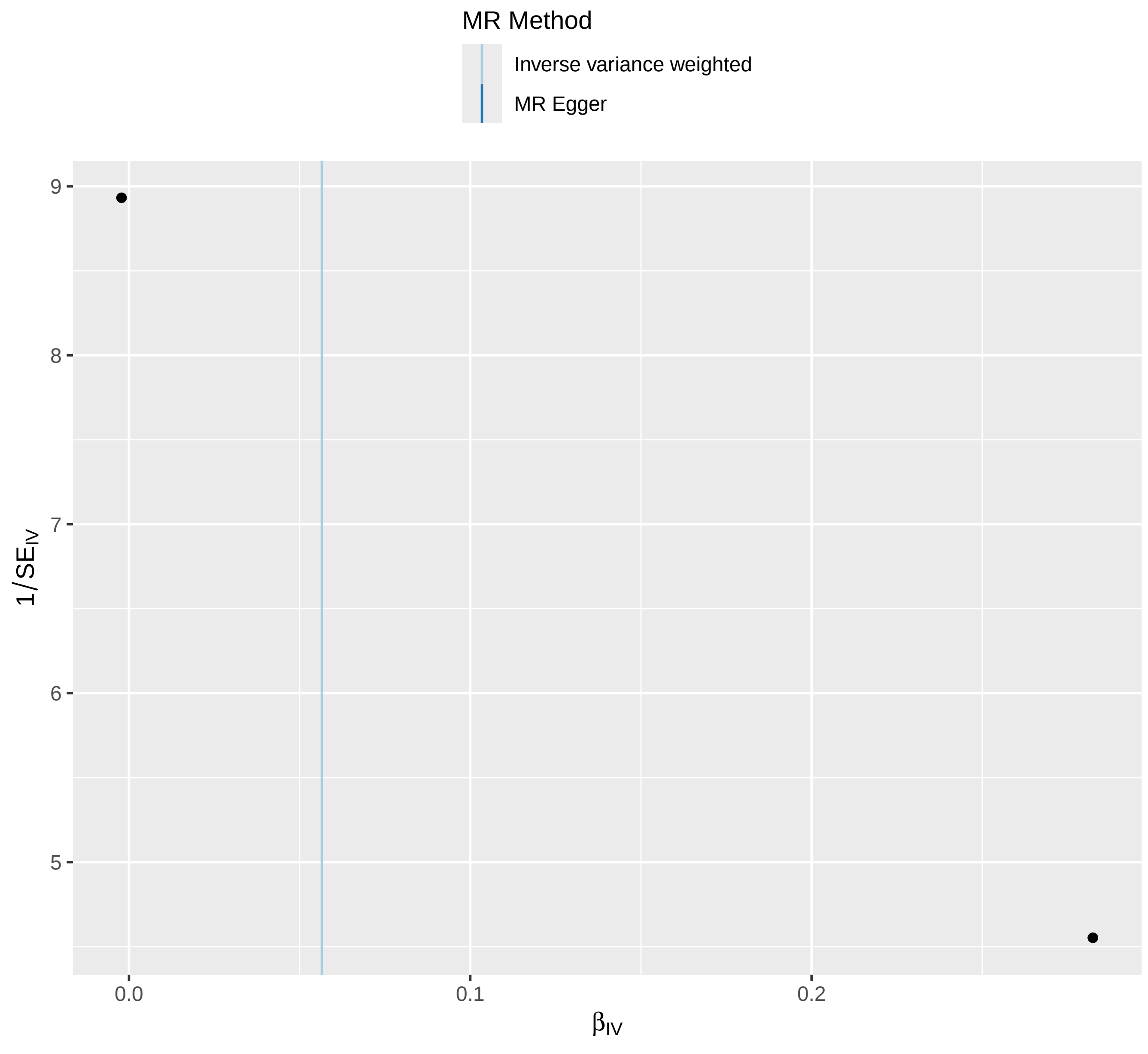
MR Method
Inverse variance weighted
MR Egger
9
8
7
6
5
$1/SE_{IV}$
0.0
0.1
0.2
$\beta_{IV}$

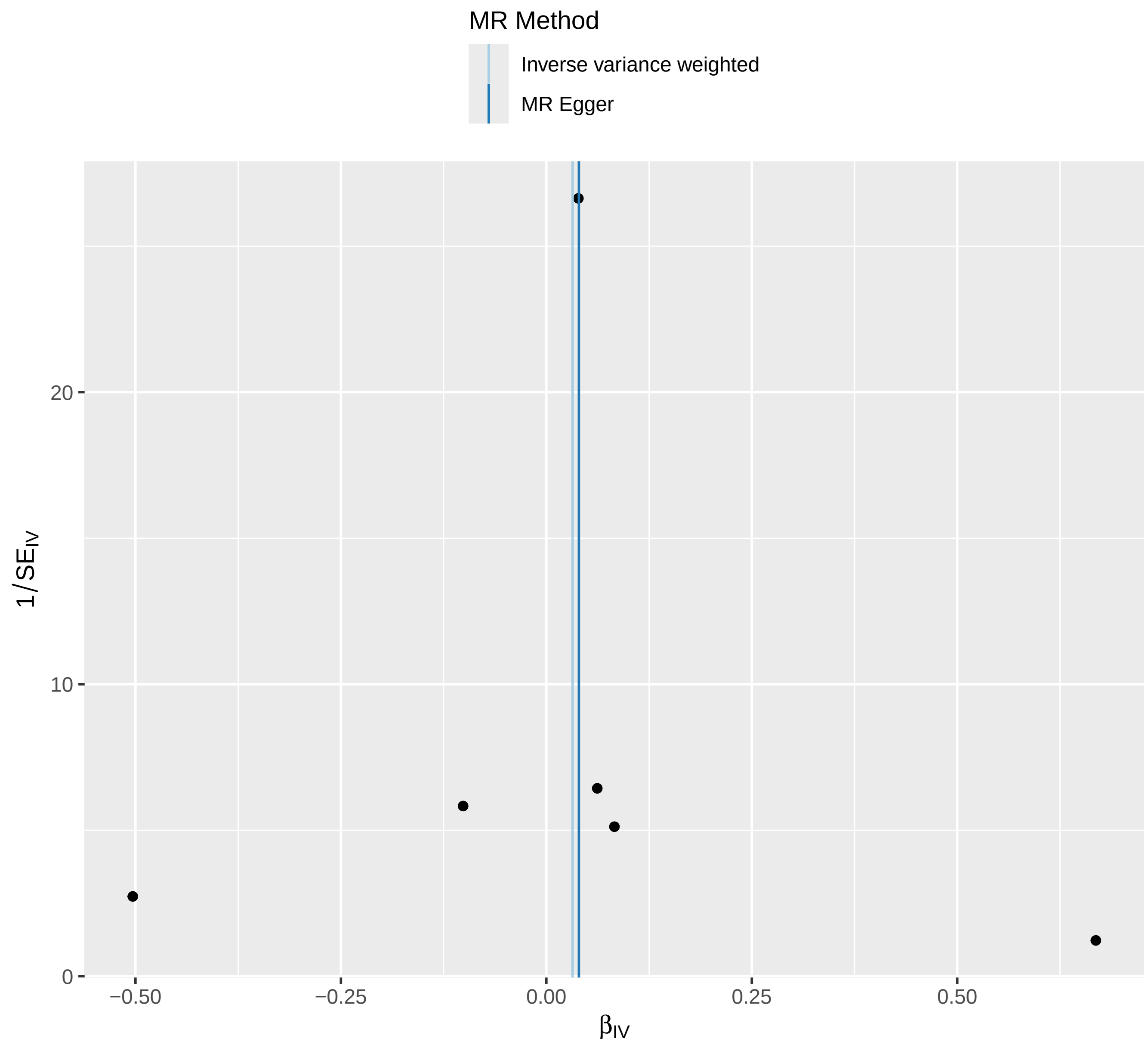
MR Method
Inverse variance weighted
MR Egger
1/SE_IV
20
10
0
−0.50
−0.25
0.00
0.25
0.50
β_IV

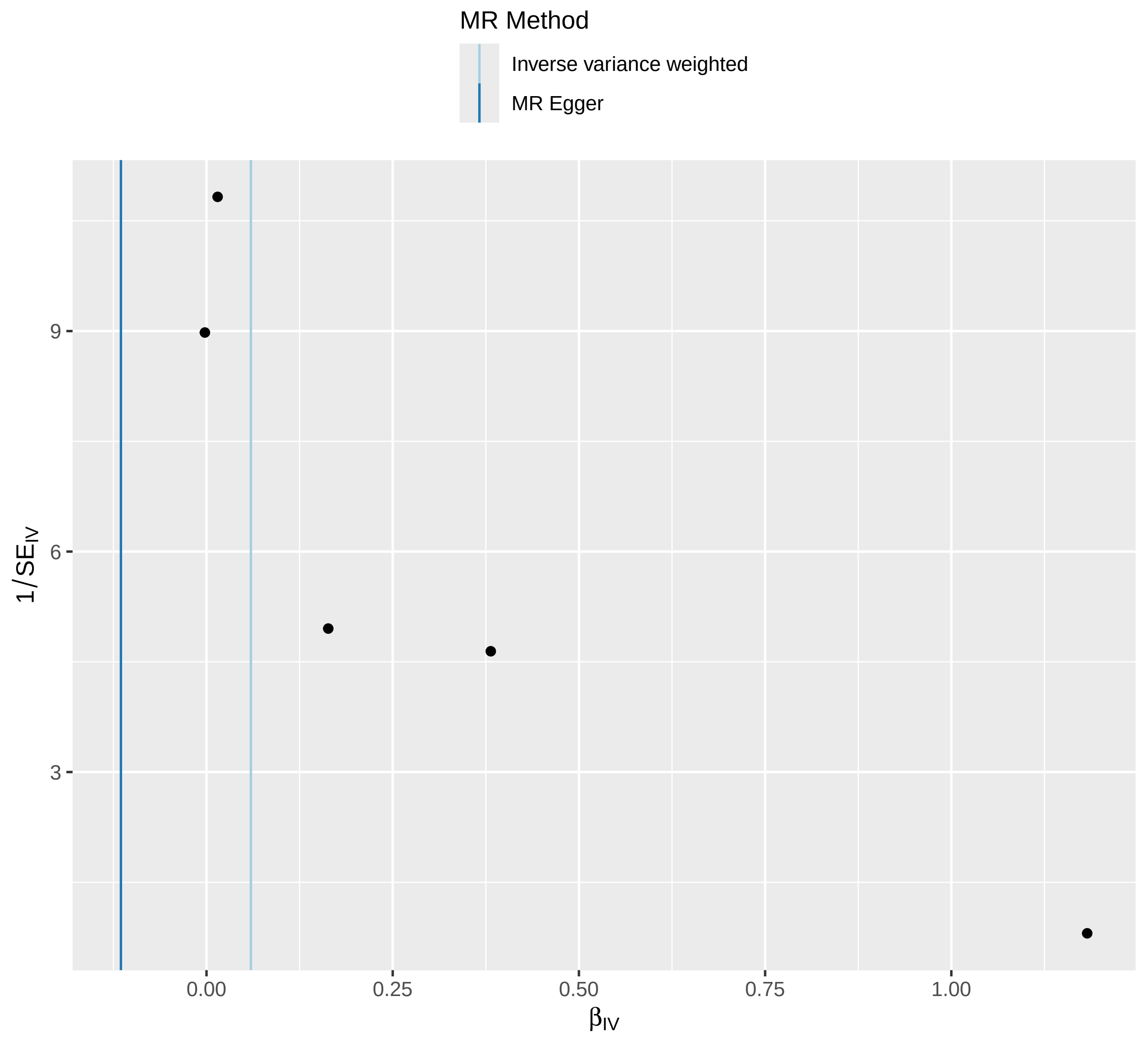
MR Method
Inverse variance weighted
MR Egger
9
6
3
1/$SE_{IV}$
0.00
0.25
0.50
0.75
1.00
$\beta_{IV}$

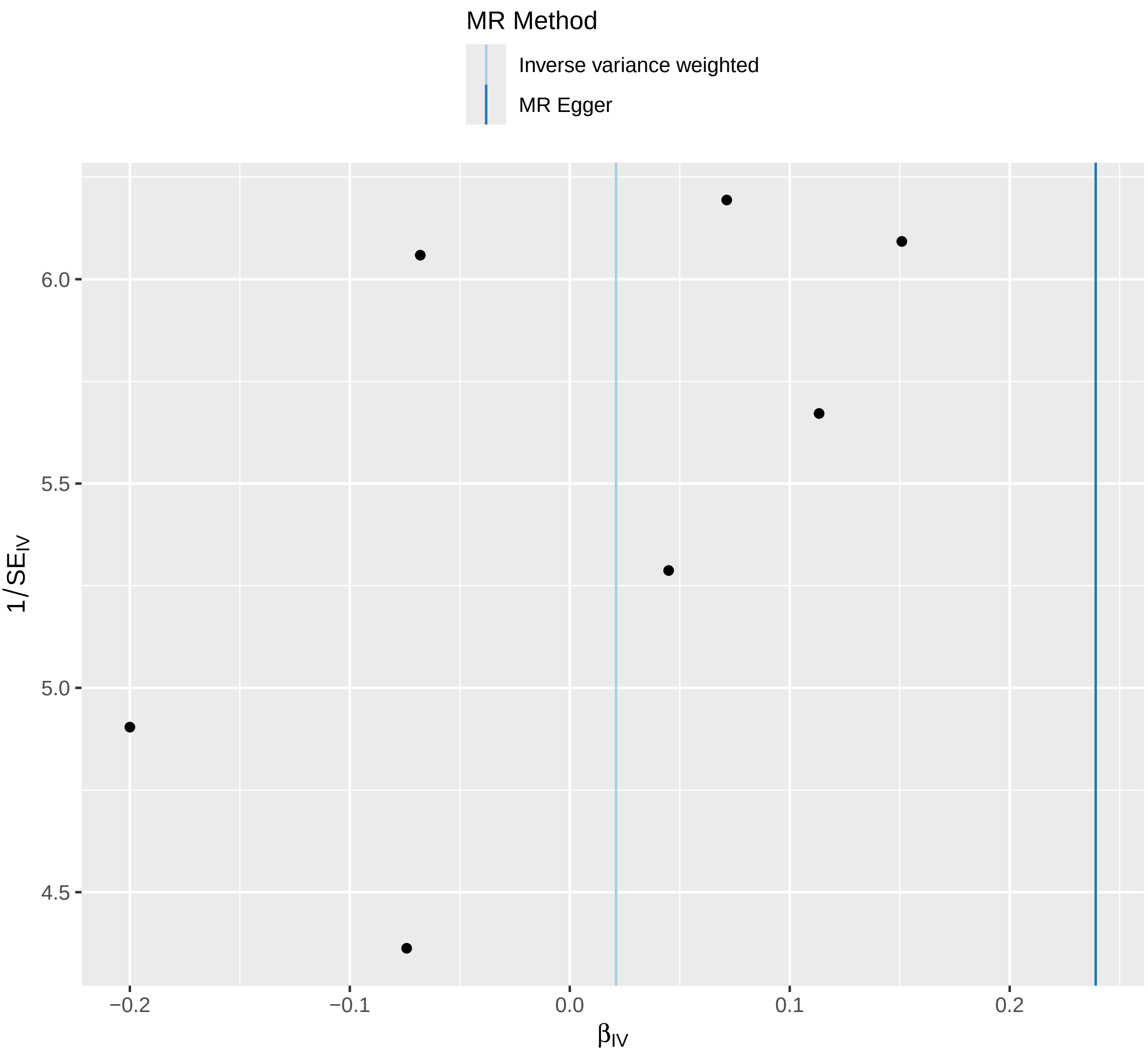
MR Method
Inverse variance weighted
MR Egger
6.0
5.5
5.0
4.5
$1/SE_{IV}$
−0.2
−0.1
0.0
0.1
0.2
$\beta_{IV}$

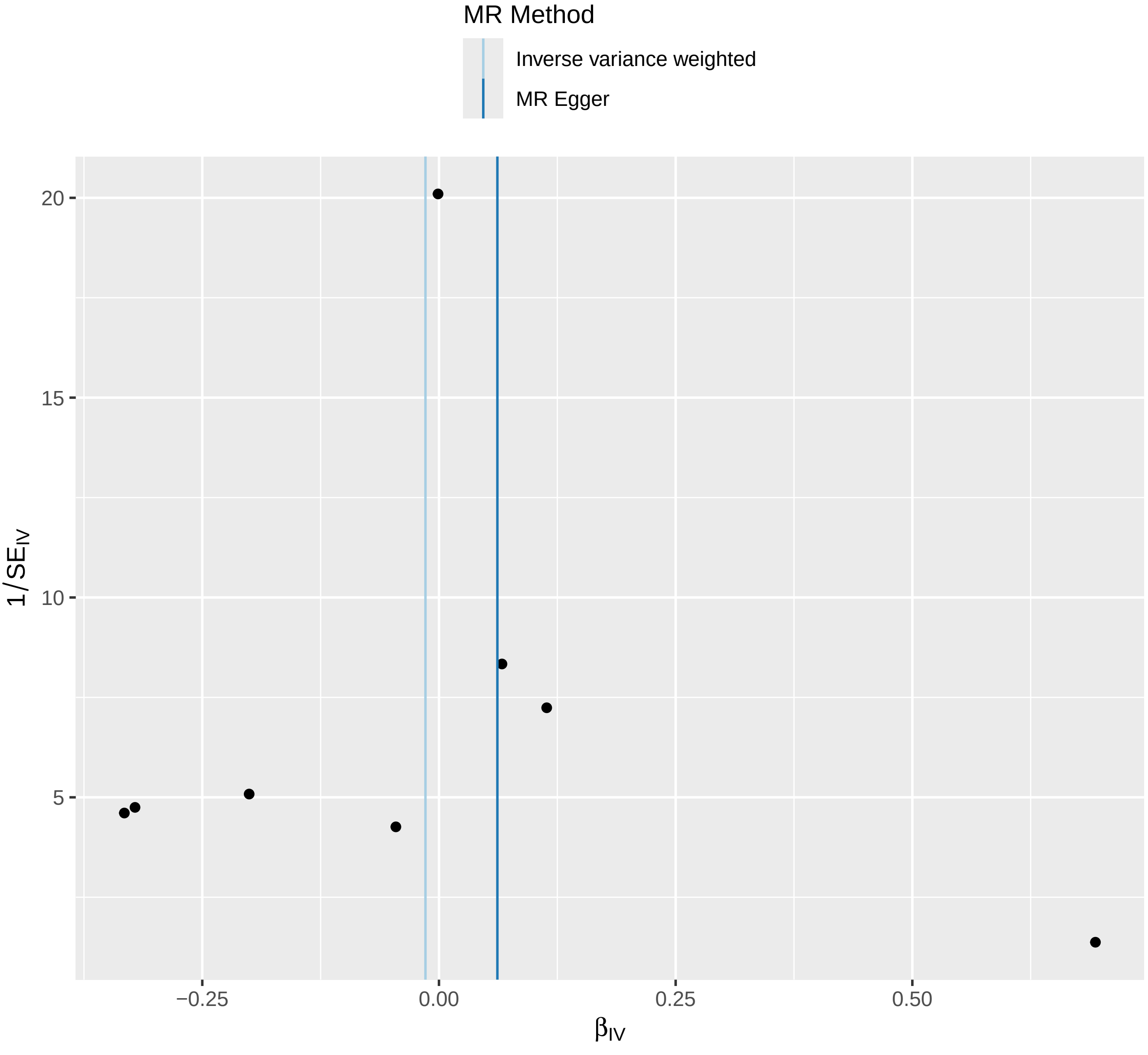
MR Method
Inverse variance weighted
MR Egger
20
15
10
5
1/SE_IV
−0.25
0.00
0.25
0.50
β_IV

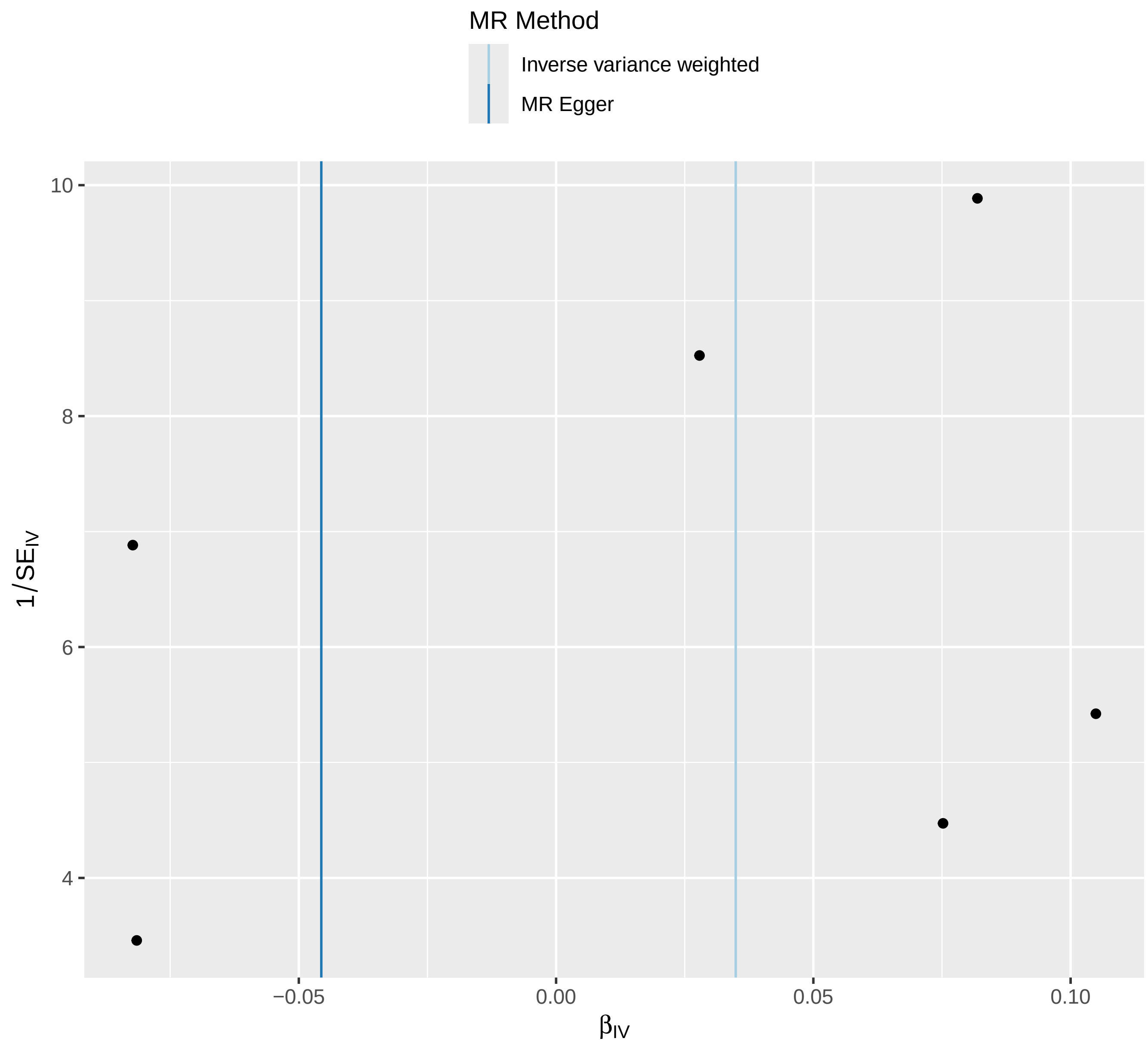
MR Method
Inverse variance weighted
MR Egger
10
8
6
4
1/SE_IV
−0.05
0.00
0.05
0.10
β_IV

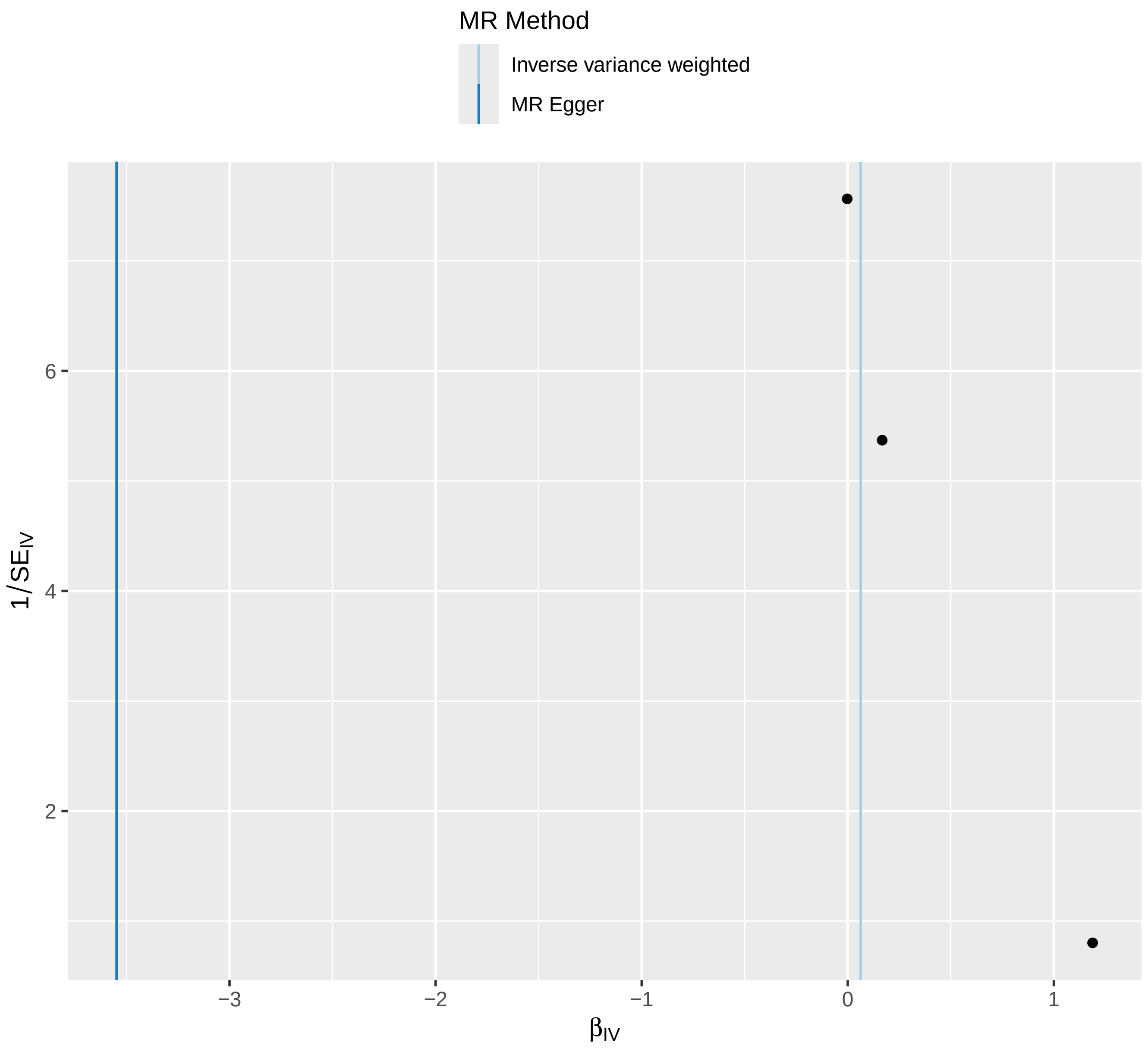
MR Method
Inverse variance weighted
MR Egger
6
4
2
1/SE_IV
−3
−2
−1
0
1
β_IV

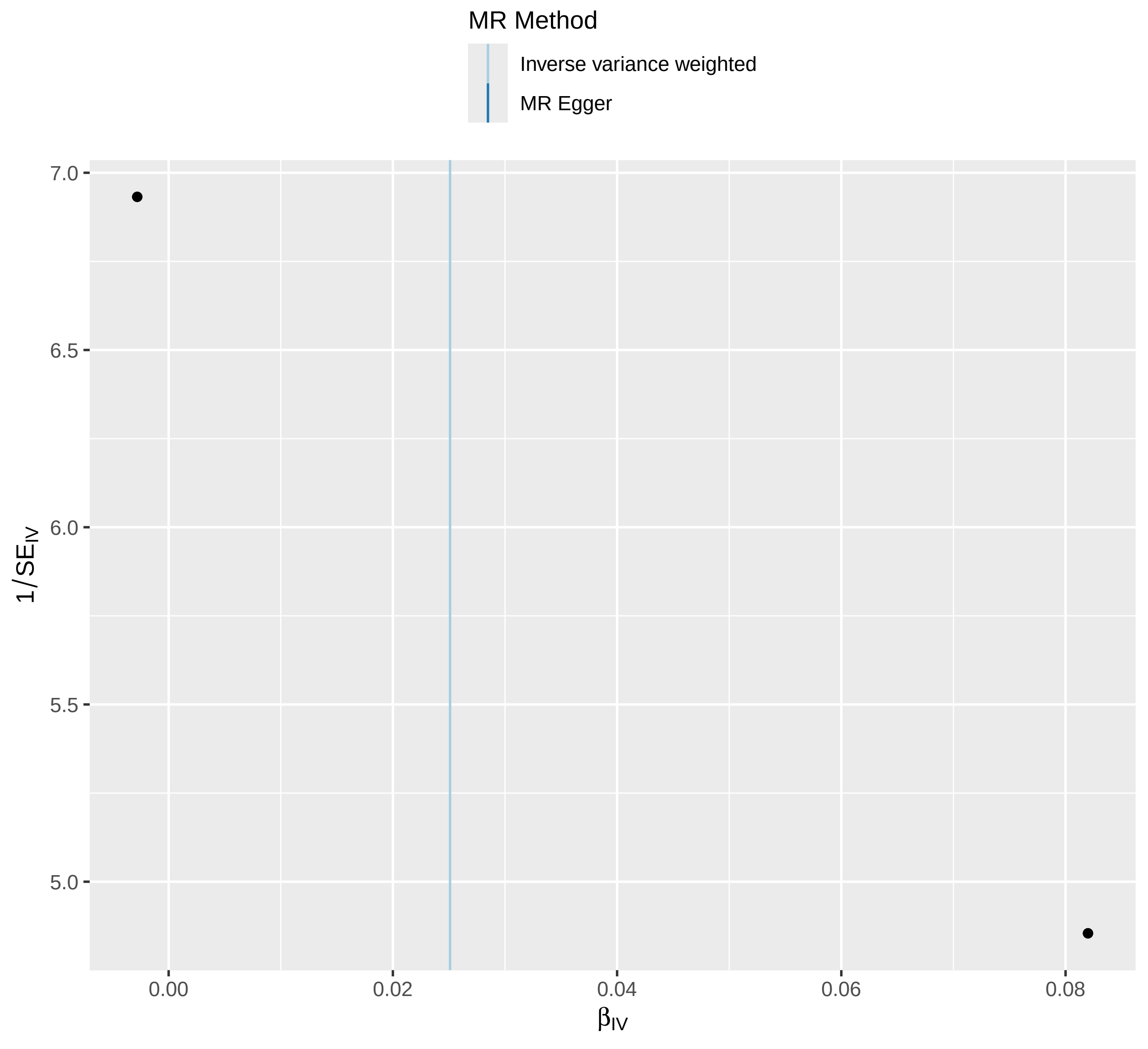
MR Method
Inverse variance weighted
MR Egger
7.0
6.5
6.0
5.5
5.0
1/SE_IV
0.00
0.02
0.04
0.06
0.08
β_IV

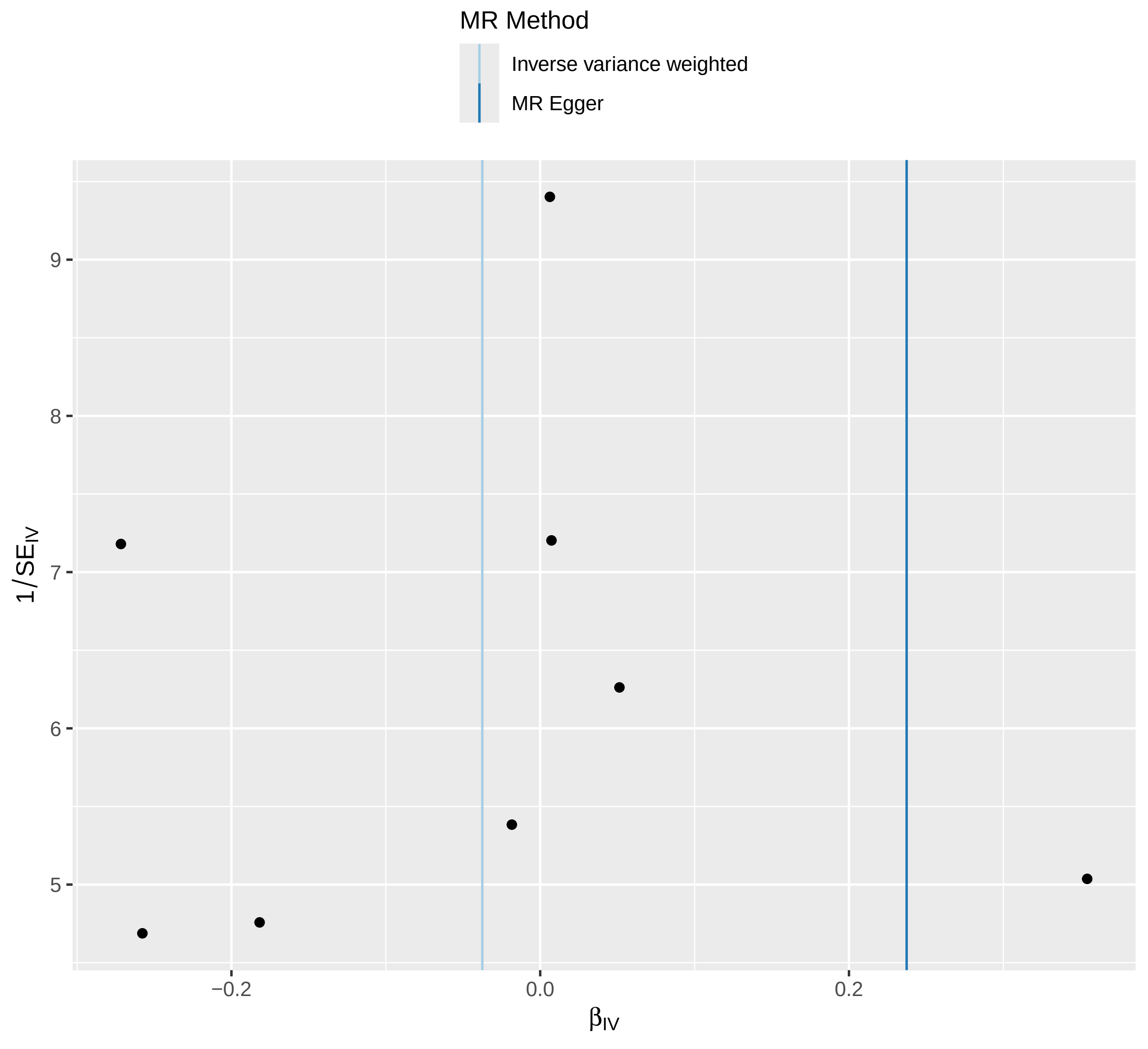
MR Method
Inverse variance weighted
MR Egger
1/SE_IV
9
8
7
6
5
−0.2
0.0
0.2
β_IV

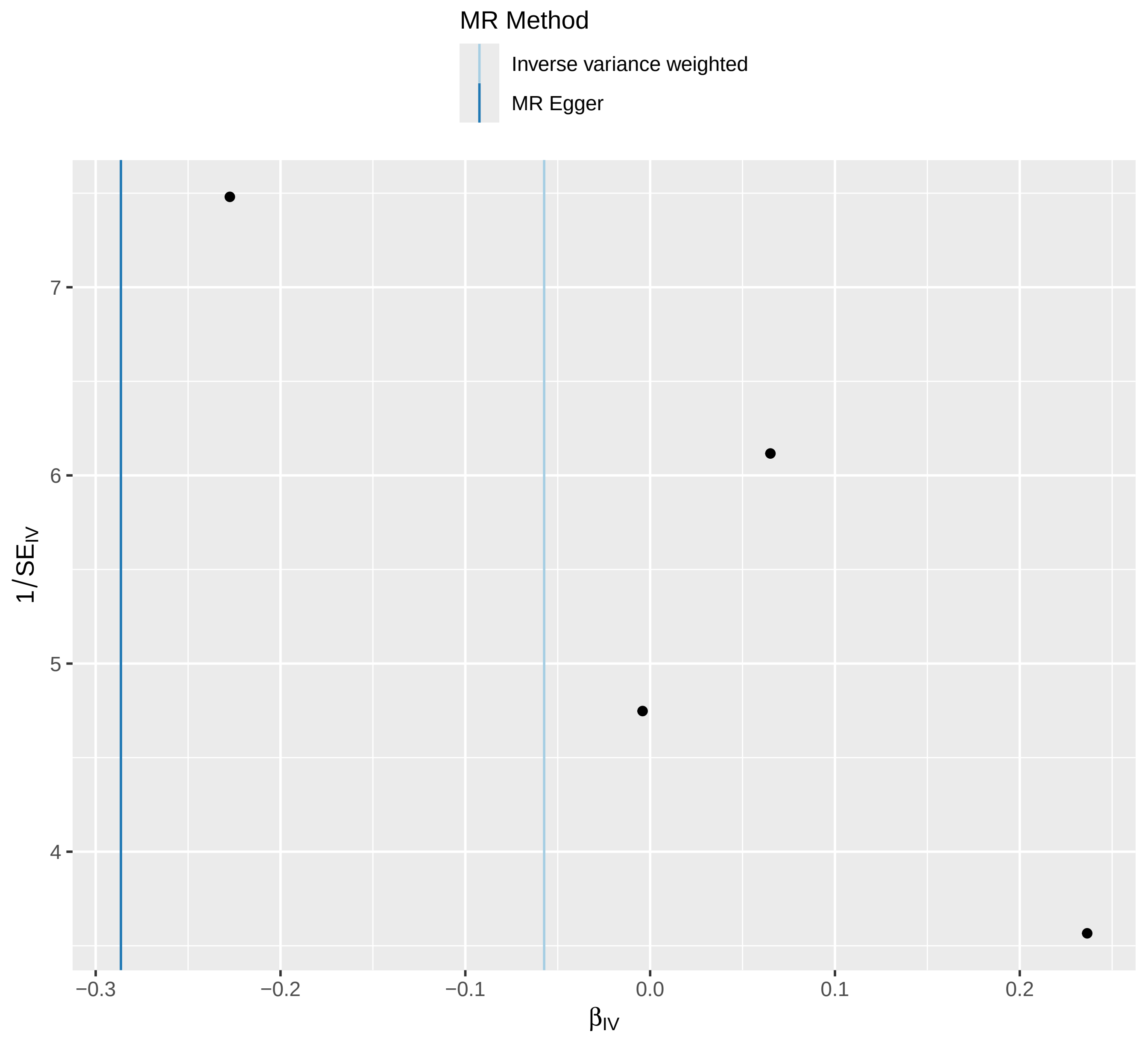

MR Method
Inverse variance weighted
MR Egger
7
6
5
4
$1/SE_{IV}$
−0.3
−0.2
−0.1
0.0
0.1
0.2
$\beta_{IV}$

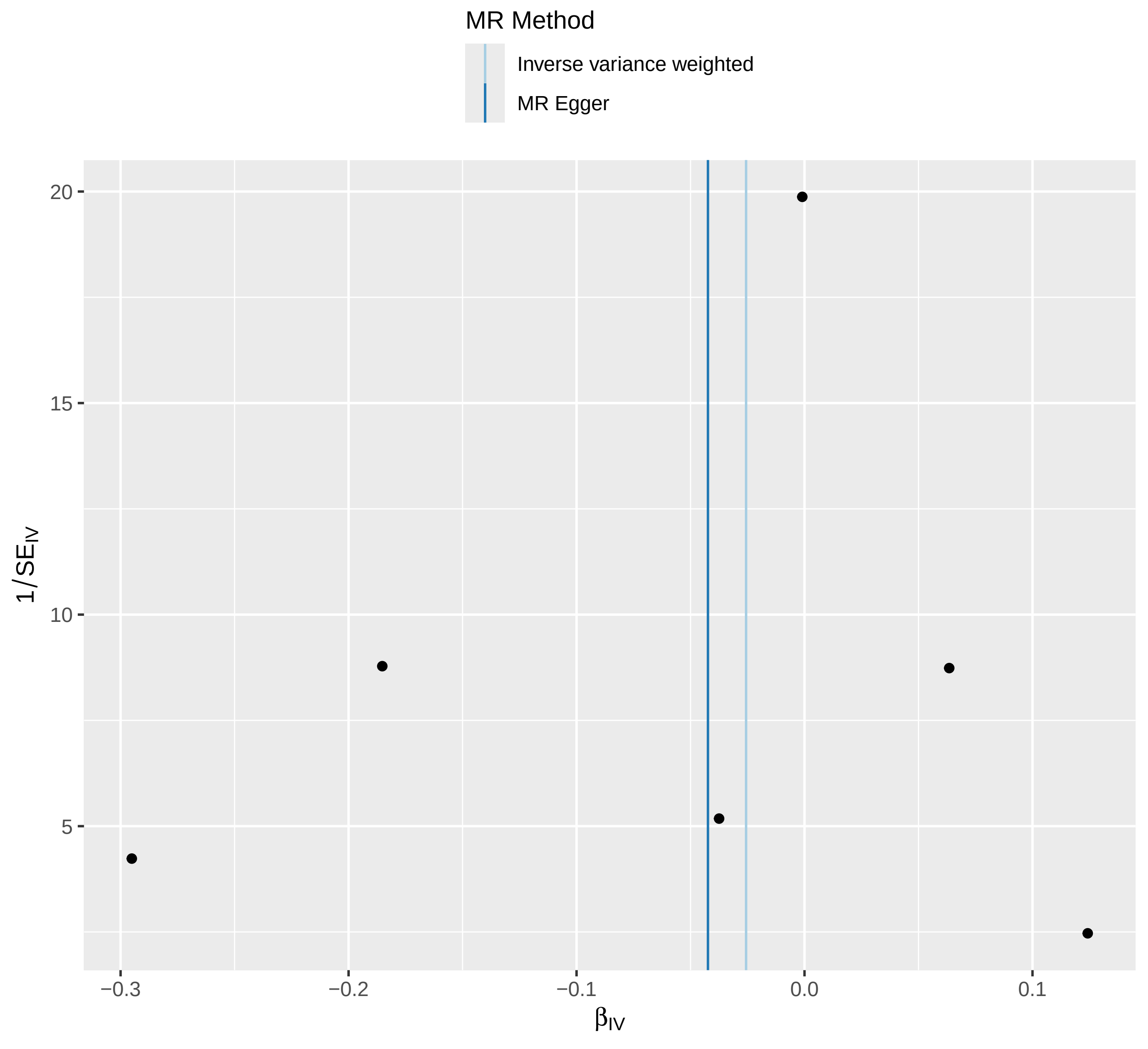

MR Method
Inverse variance weighted
MR Egger
20
15
10
5
1/SE_IV
−0.3
−0.2
−0.1
0.0
0.1
β_IV

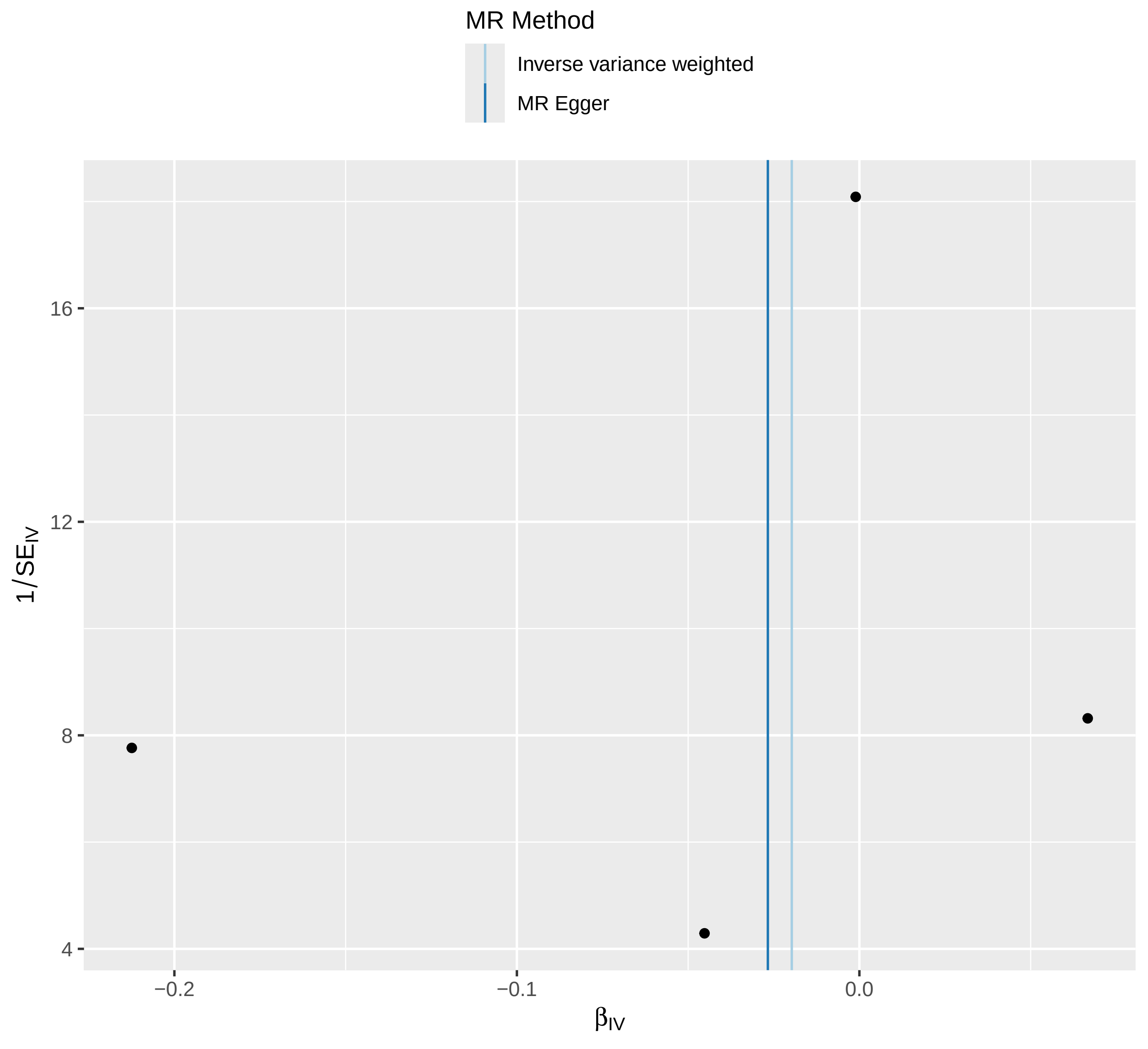

MR Method
Inverse variance weighted
MR Egger
16
12
8
4
1/SE_IV
−0.2
−0.1
0.0
β_IV

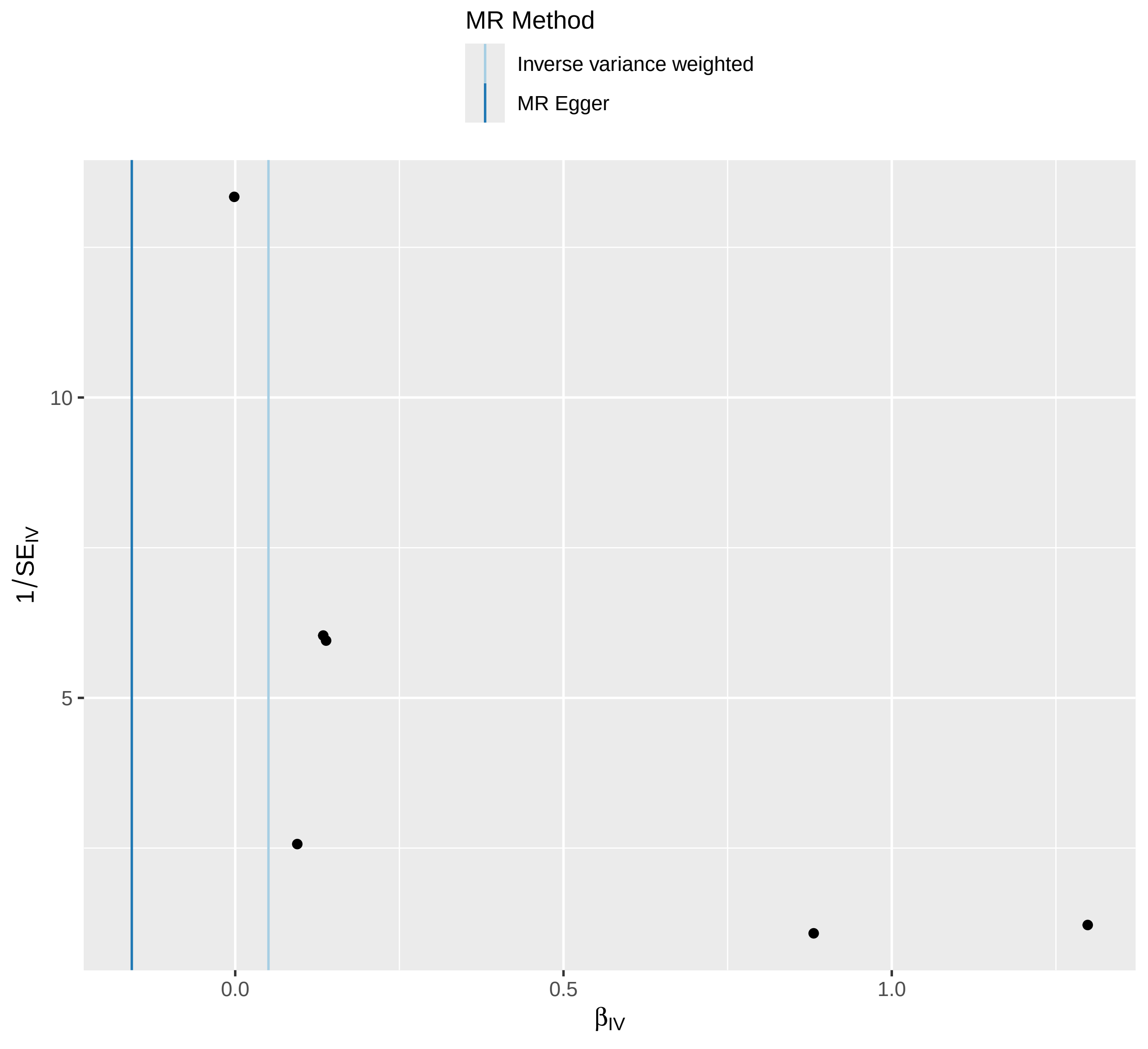
MR Method
Inverse variance weighted
MR Egger
10
5
1/SE_IV
0.0
0.5
1.0
β_IV

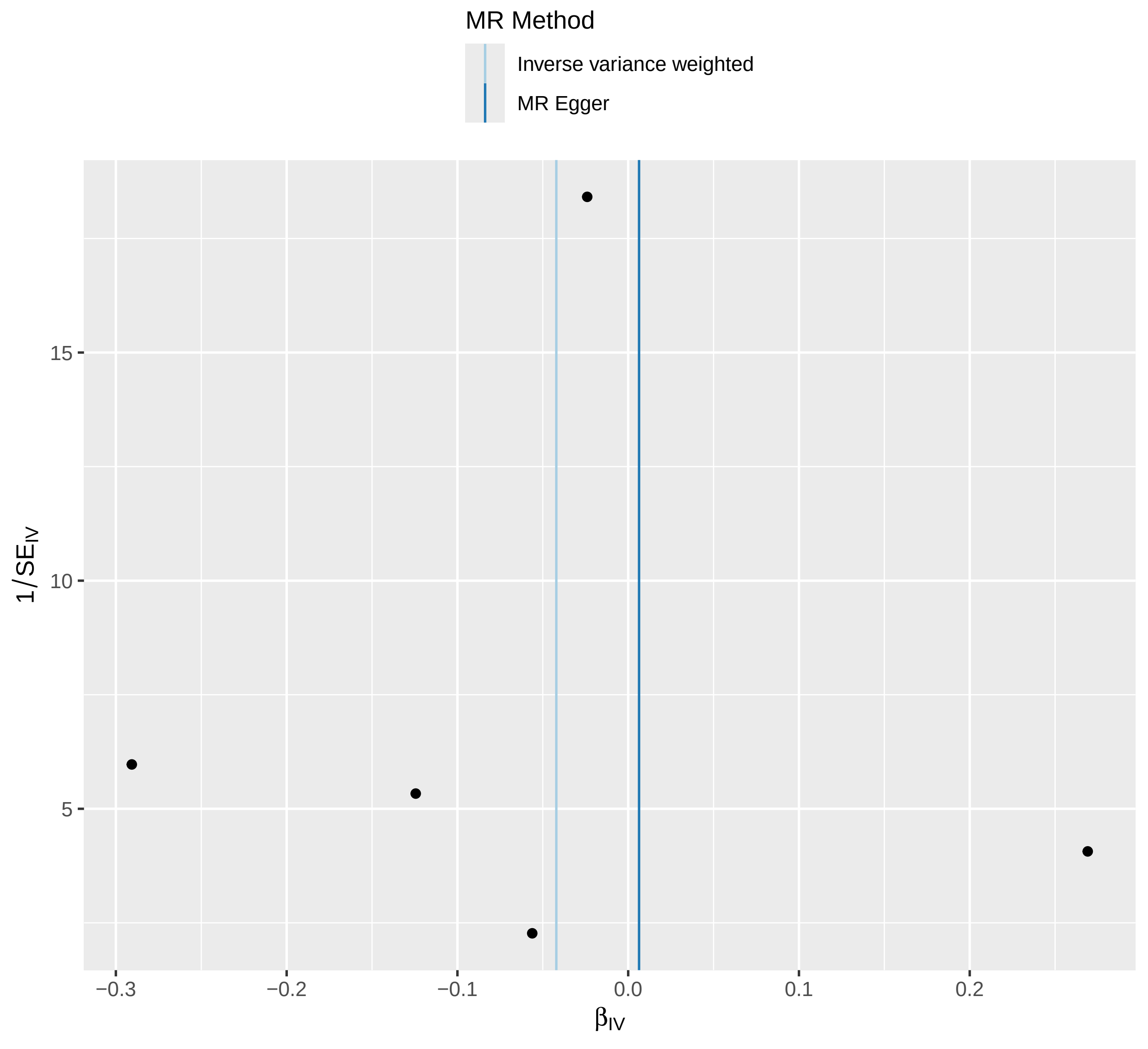

MR Method
Inverse variance weighted
MR Egger
15
10
5
$1/SE_{IV}$
−0.3
−0.2
−0.1
0.0
0.1
0.2
$\beta_{IV}$

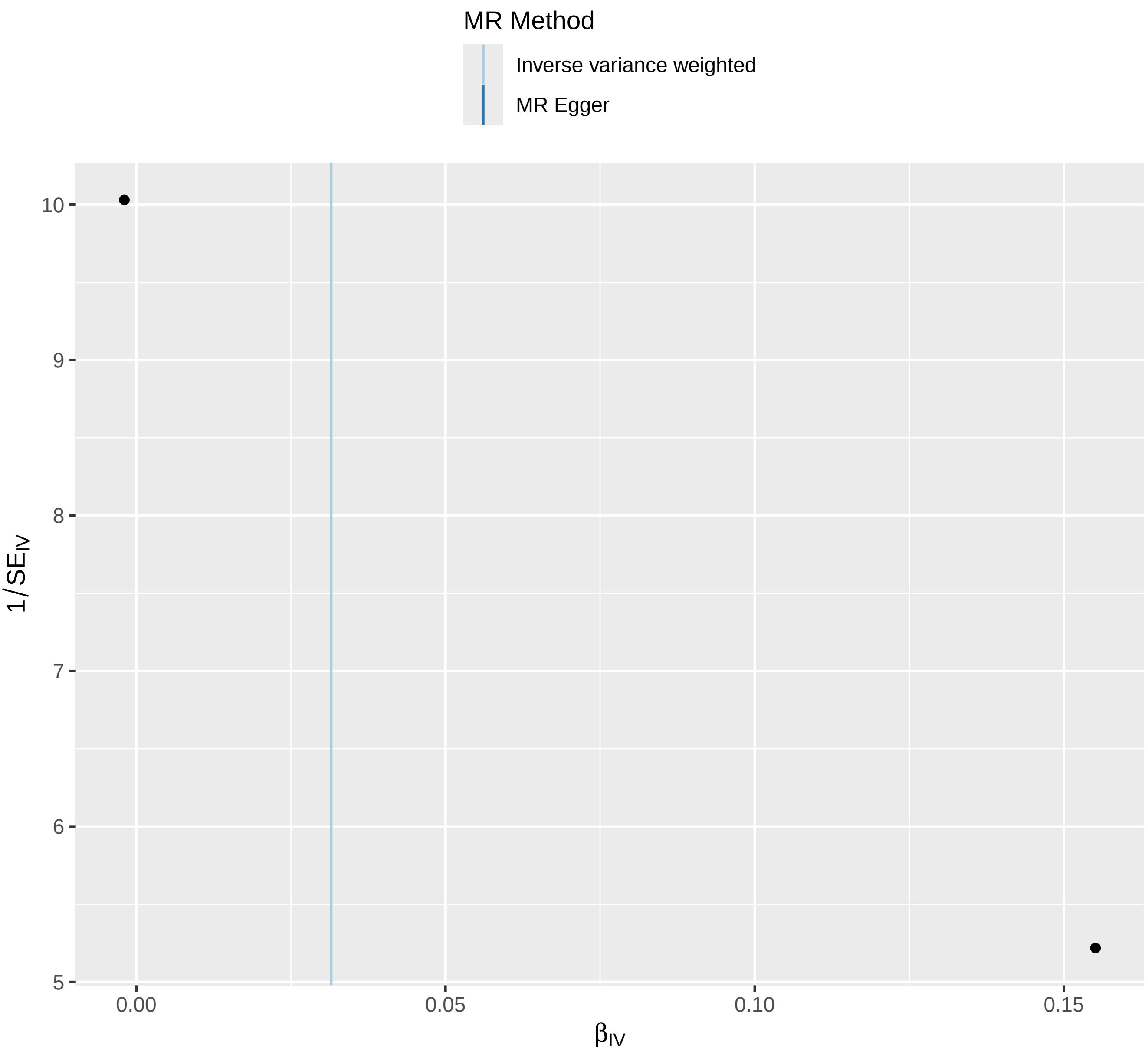

MR Method
Inverse variance weighted
MR Egger
10
9
8
7
6
5
1/SE$_{IV}$
0.00
0.05
0.10
0.15
$\beta_{IV}$

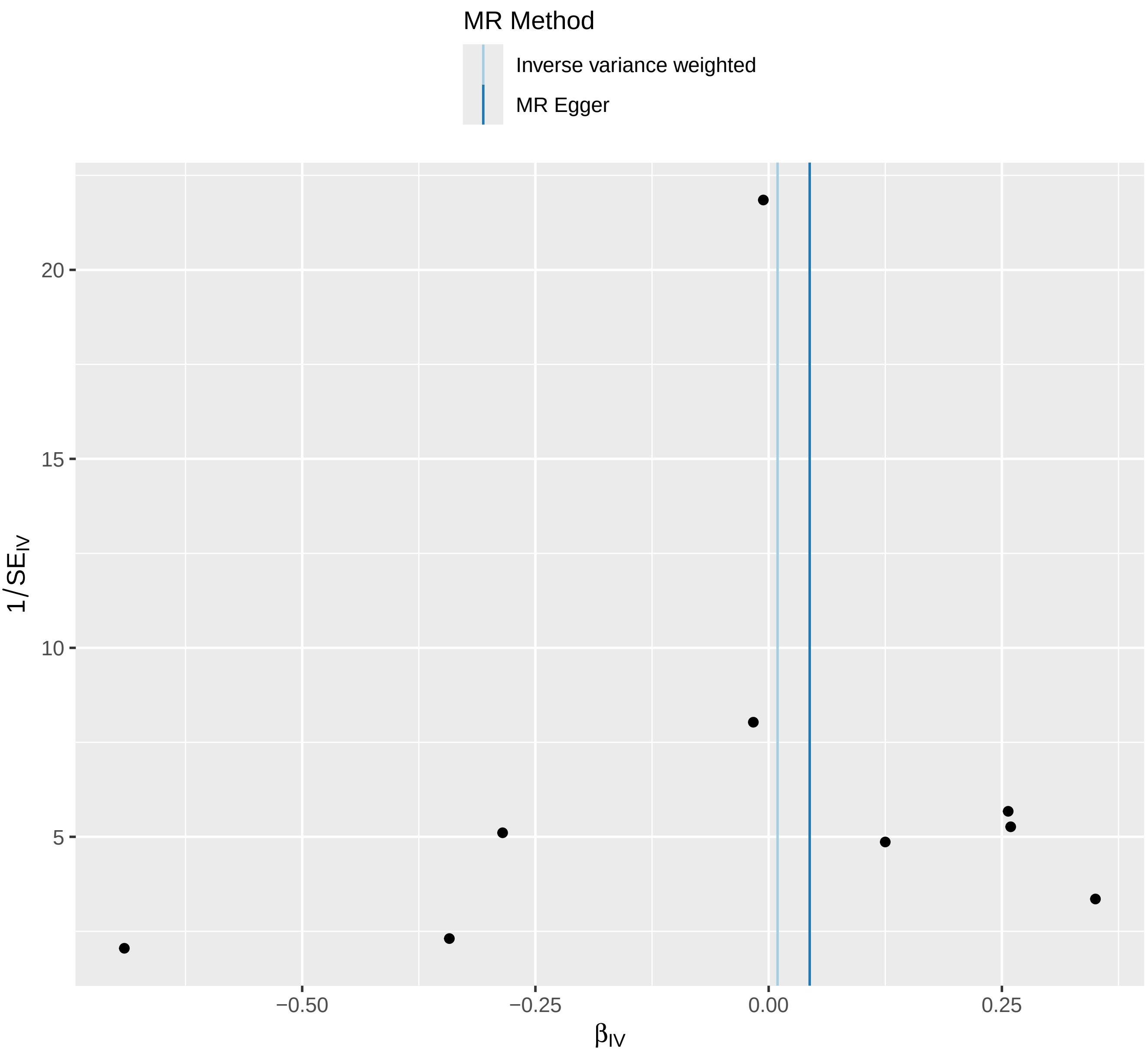
MR Method
Inverse variance weighted
MR Egger
20
15
10
5
$1/SE_{IV}$
−0.50
−0.25
0.00
0.25
$\beta_{IV}$

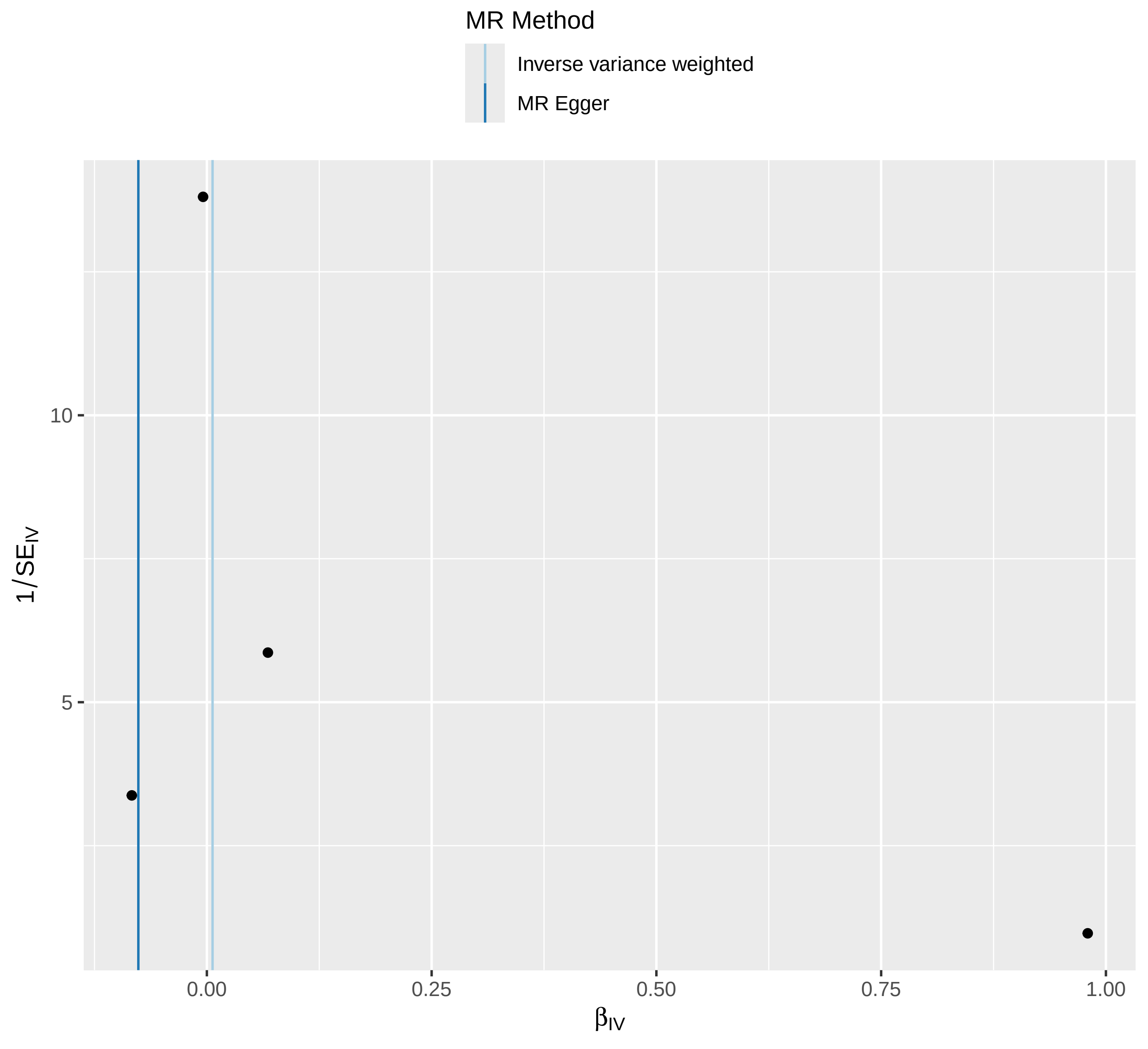
MR Method
Inverse variance weighted
MR Egger
10
5
1/SE$_{IV}$
0.00
0.25
0.50
0.75
1.00
$\beta_{IV}$

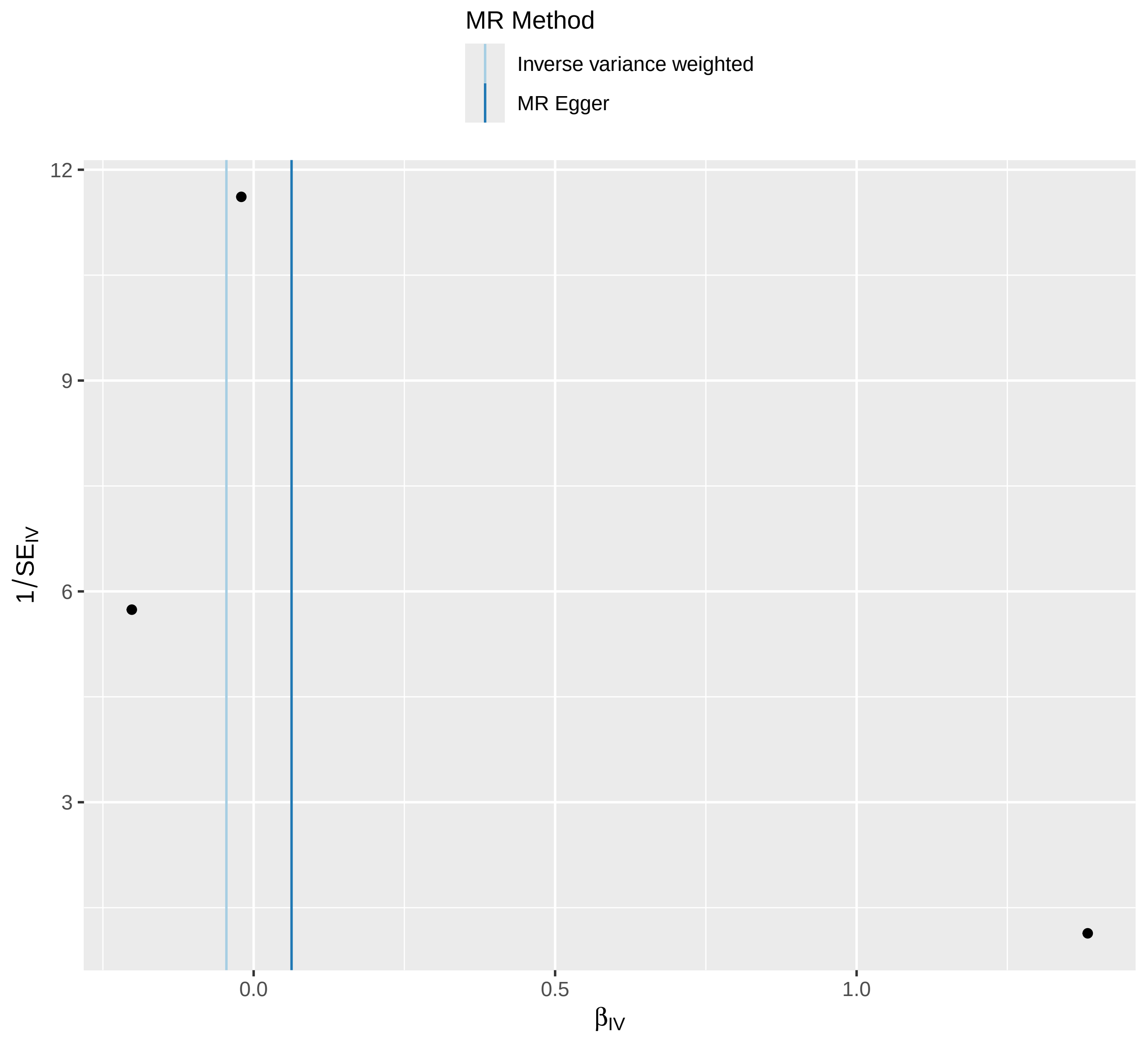

MR Method
Inverse variance weighted
MR Egger
12
9
6
3
1/SE_IV
0.0
0.5
1.0
β_IV

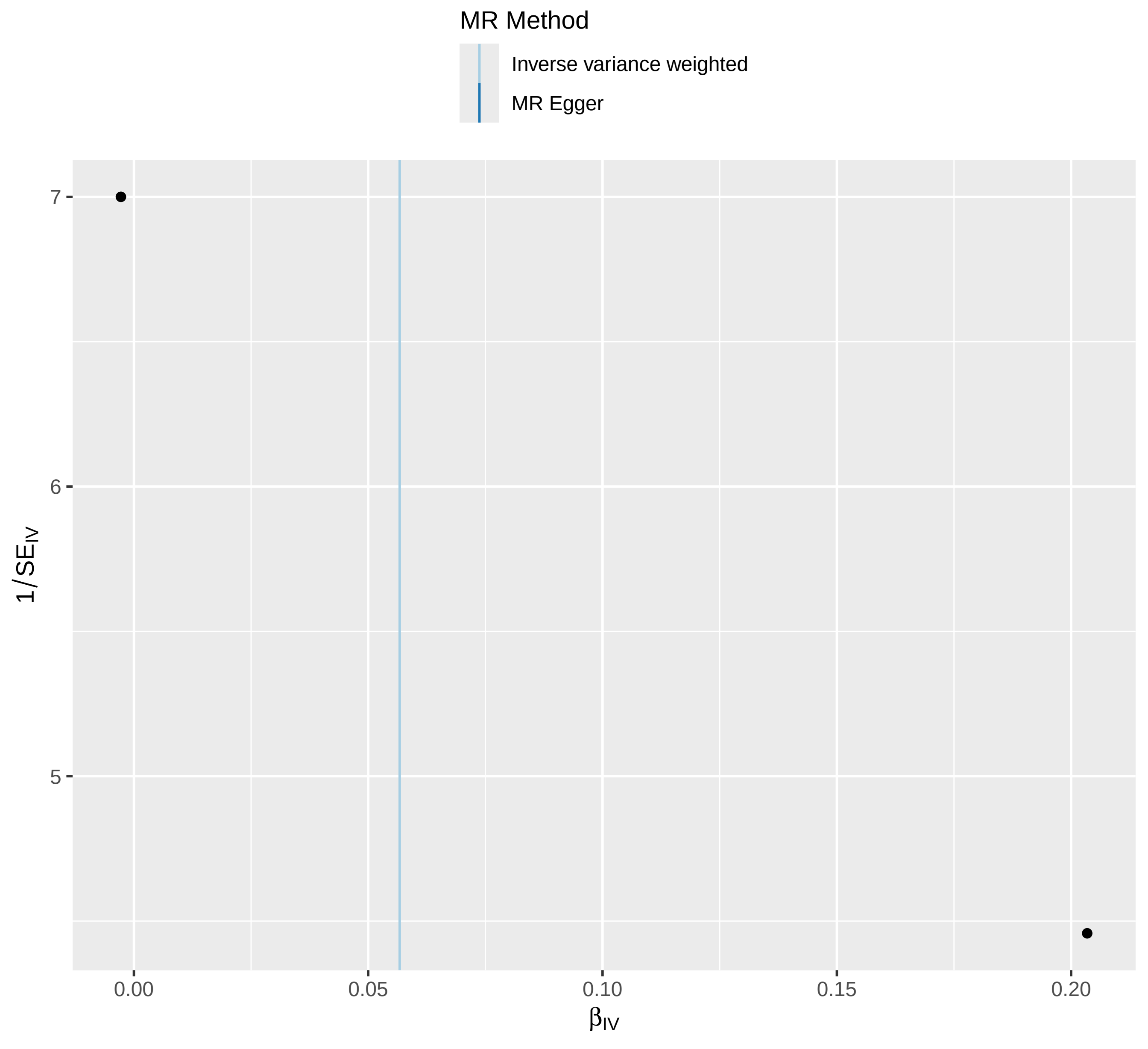
MR Method
Inverse variance weighted
MR Egger
7
6
5
$1/SE_{IV}$
0.00
0.05
0.10
0.15
0.20
$\beta_{IV}$

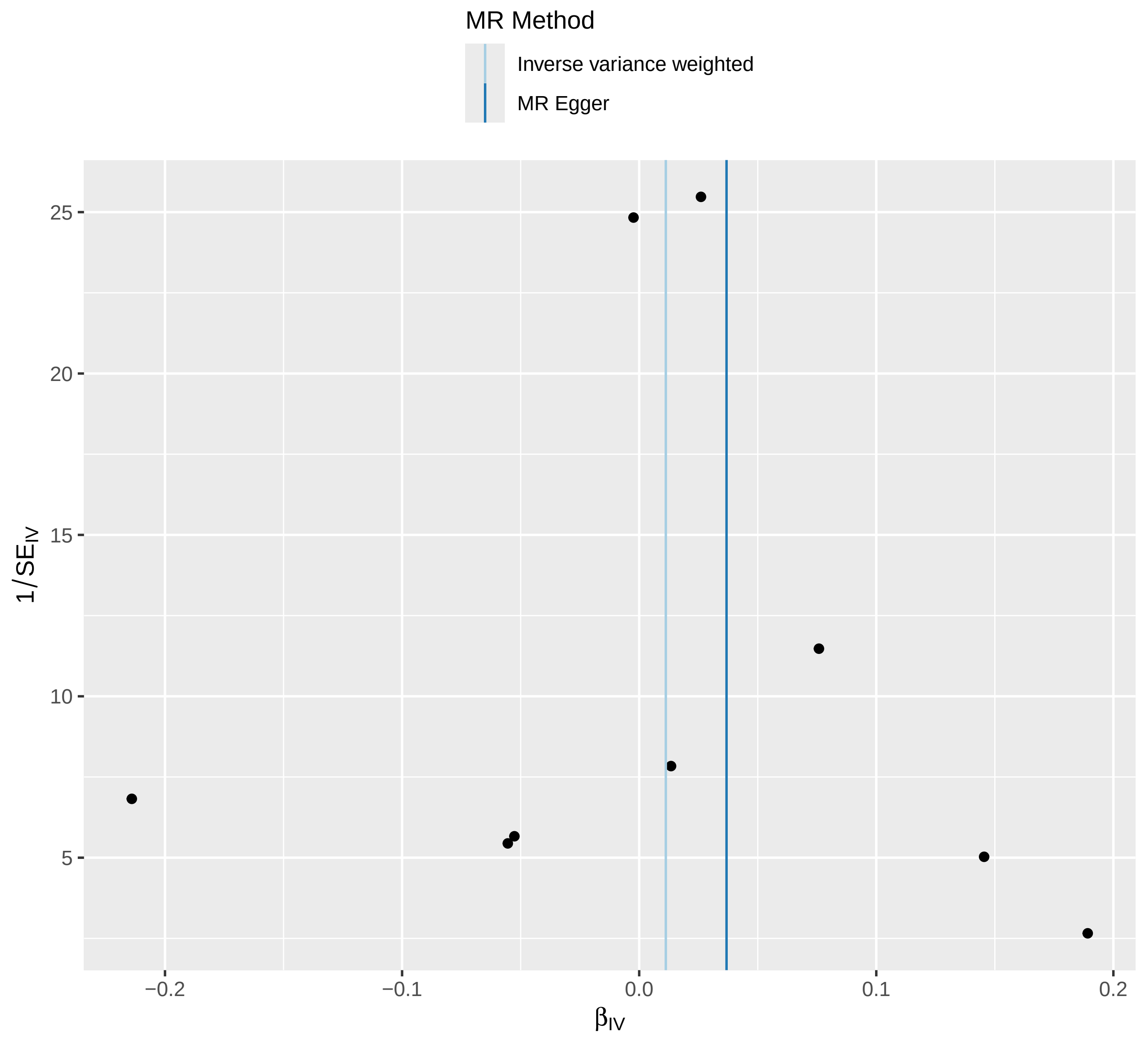

MR Method
Inverse variance weighted
MR Egger
25
20
15
10
5
1/SE_IV
−0.2
−0.1
0.0
0.1
0.2
β_IV

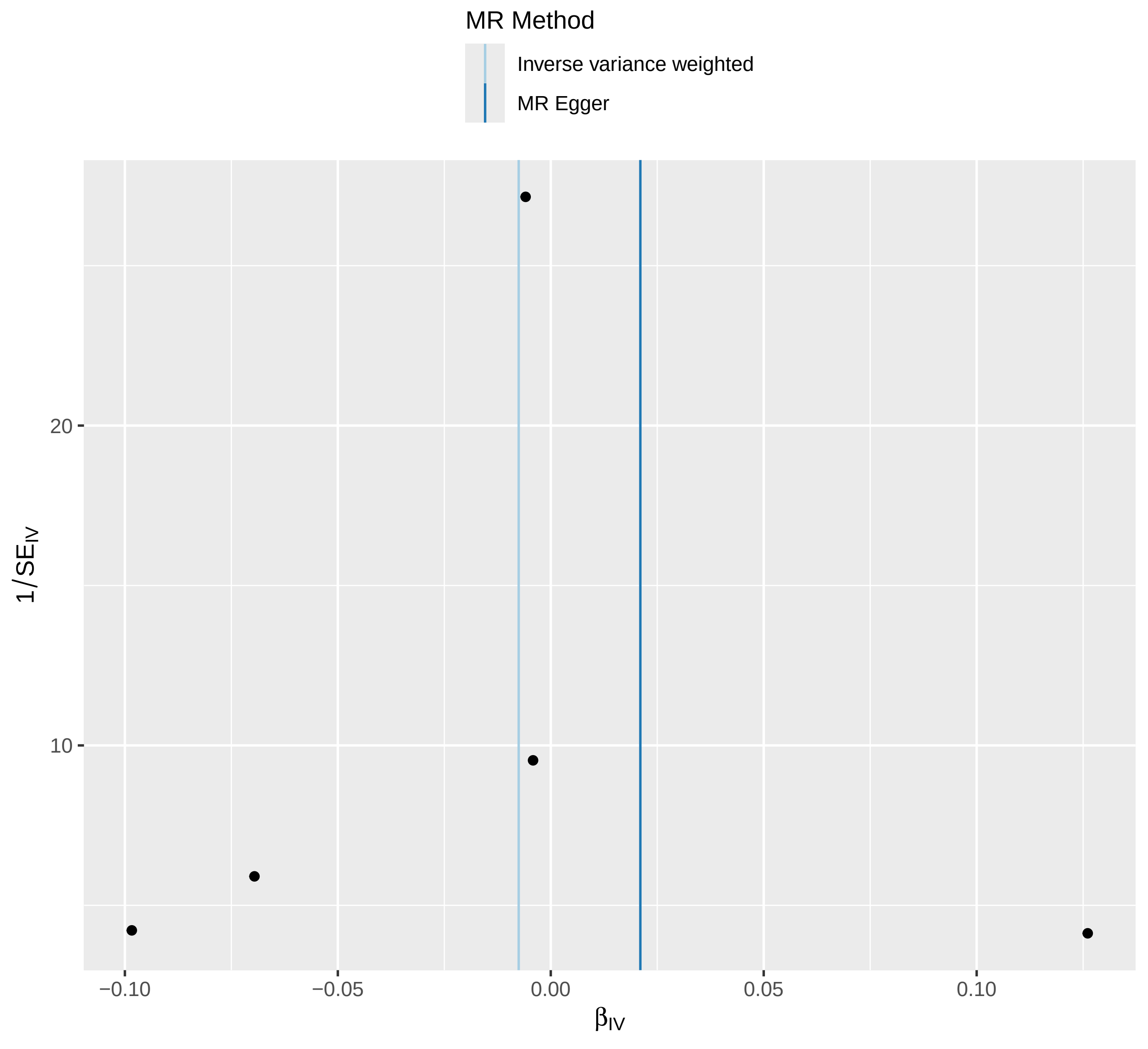
MR Method
Inverse variance weighted
MR Egger
20
10
1/$SE_{IV}$
−0.10
−0.05
0.00
0.05
0.10
$\beta_{IV}$

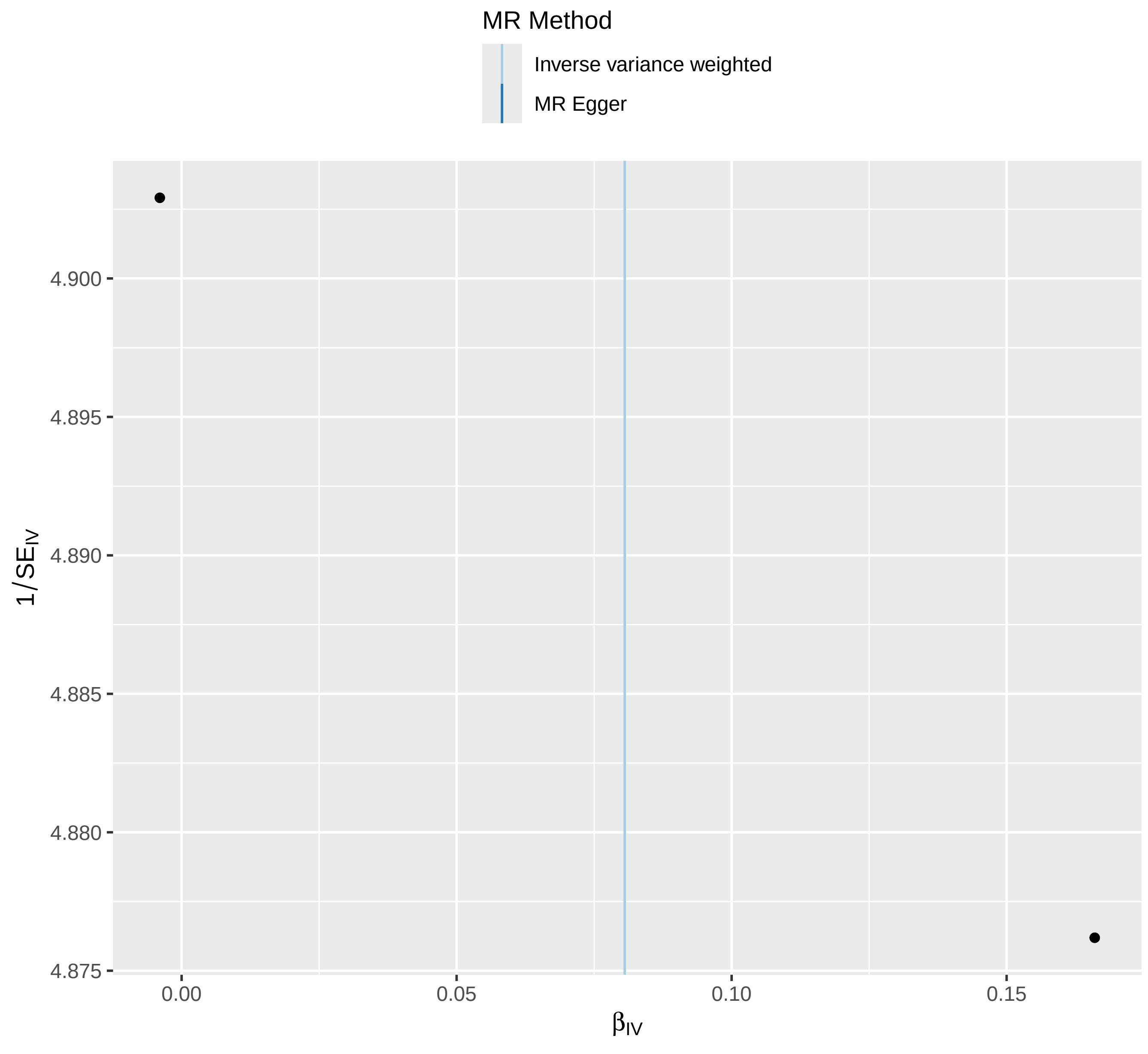

MR Method
Inverse variance weighted
MR Egger
4.900
4.895
4.890
4.885
4.880
4.875
1/SE$_{IV}$
0.00
0.05
0.10
0.15
β$_{IV}$

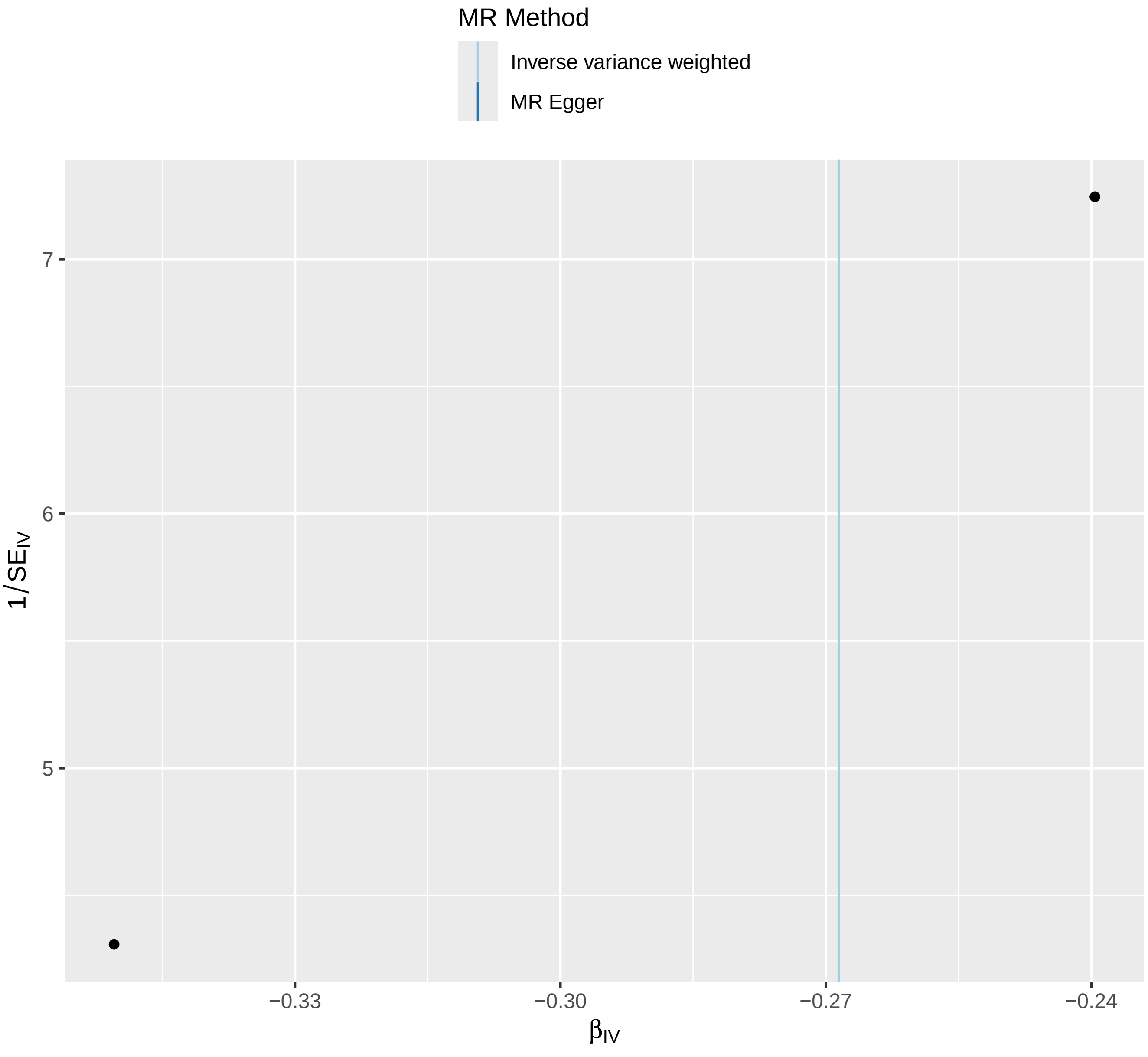

MR Method
Inverse variance weighted
MR Egger
7
6
5
1/SE$_{IV}$
−0.33
−0.30
−0.27
−0.24
β$_{IV}$

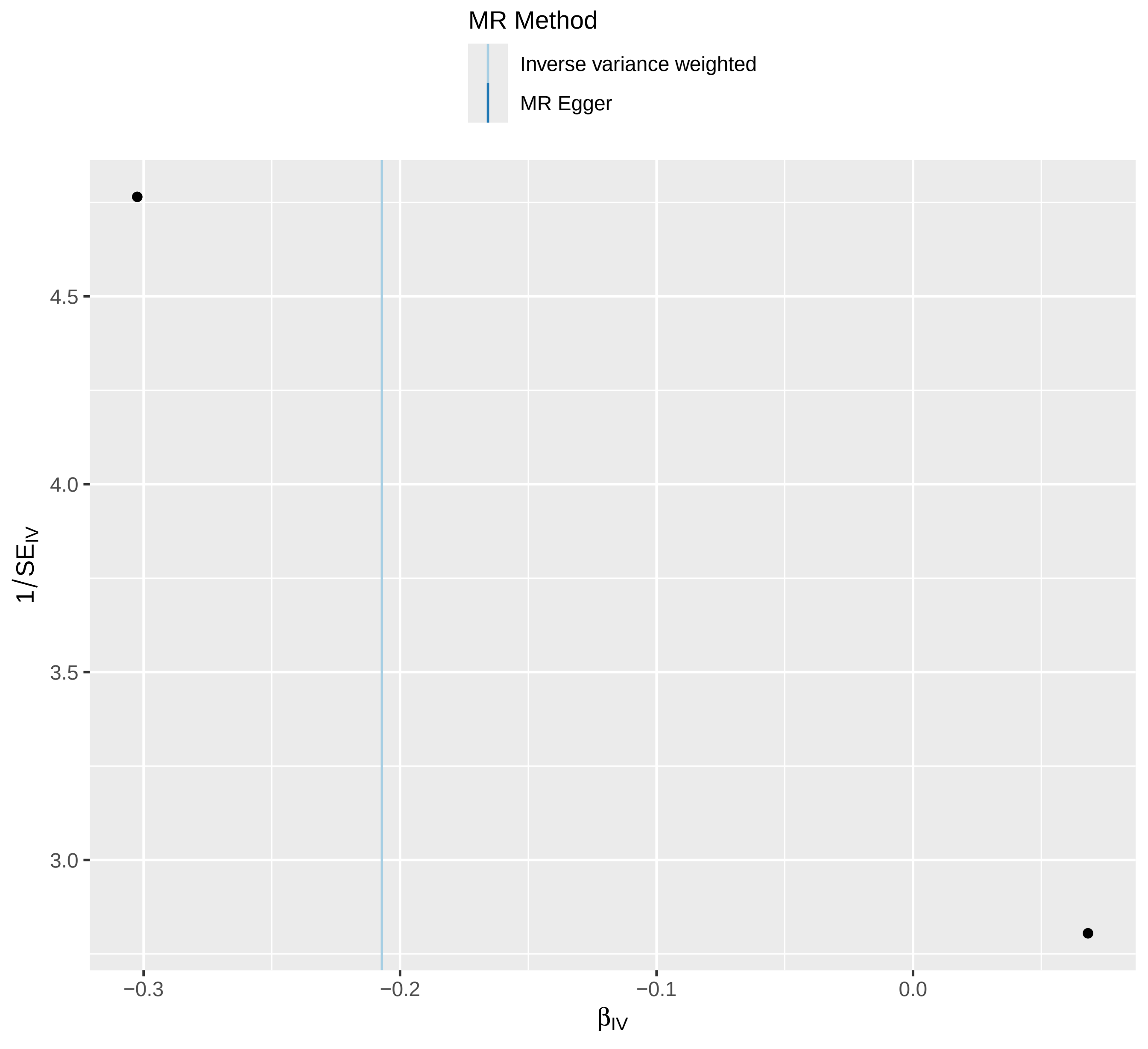

MR Method
Inverse variance weighted
MR Egger
4.5
4.0
3.5
3.0
$1/SE_{IV}$
−0.3
−0.2
−0.1
0.0
$\beta_{IV}$

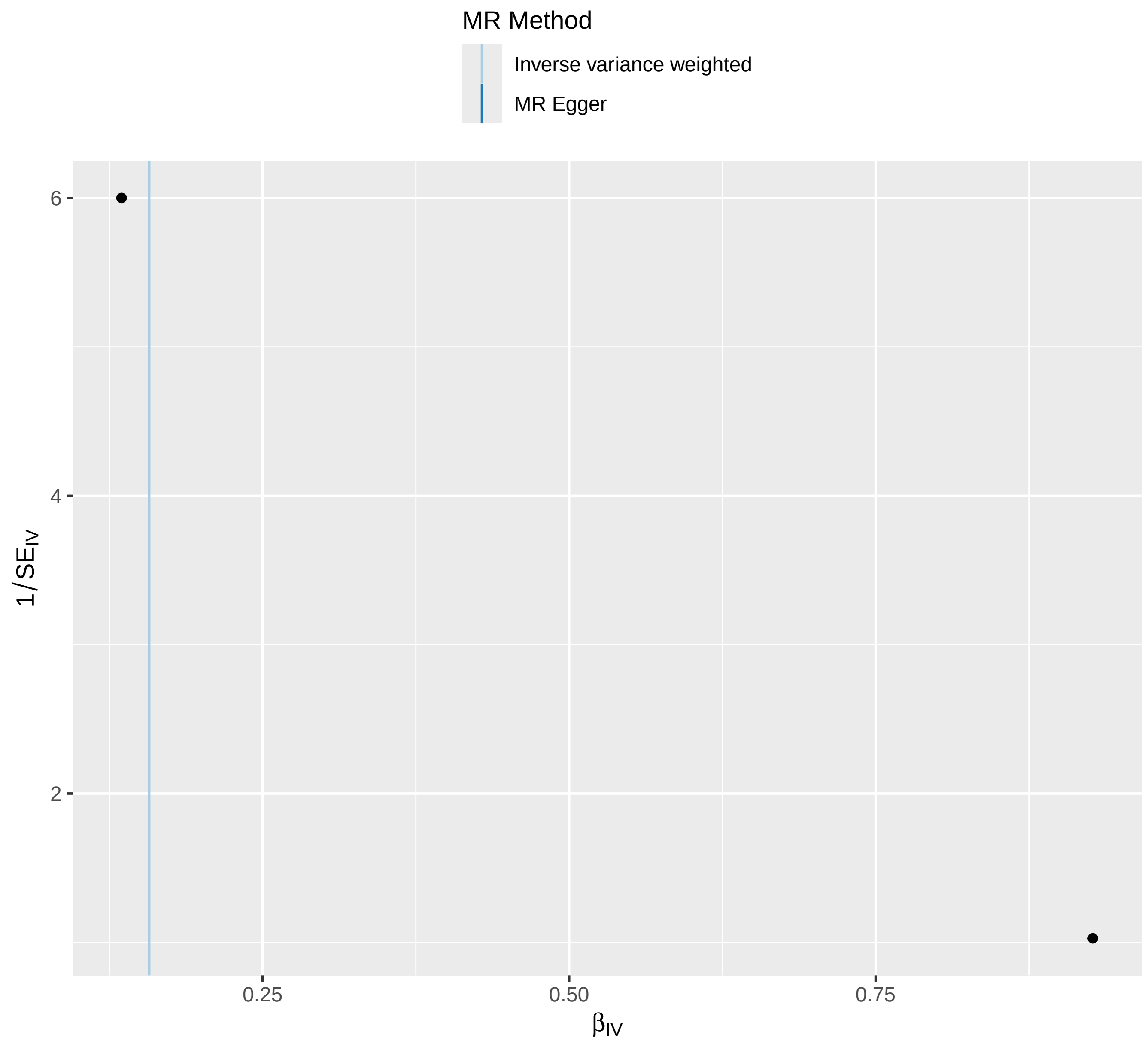

MR Method
Inverse variance weighted
MR Egger
6
4
2
$1/SE_{IV}$
0.25
0.50
0.75
$\beta_{IV}$

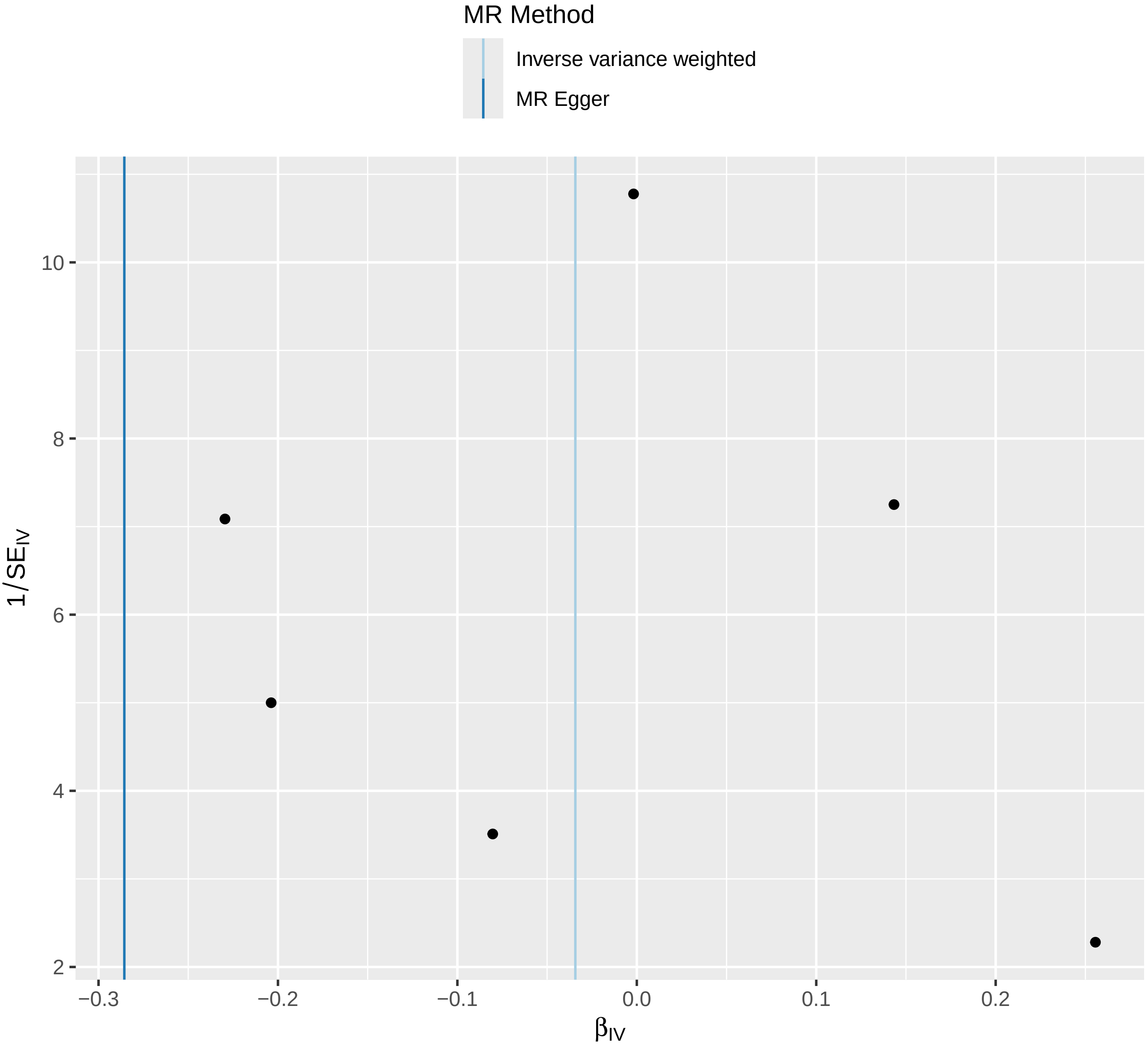
MR Method
Inverse variance weighted
MR Egger
1/SE$_{IV}$
10
8
6
4
2
−0.3
−0.2
−0.1
0.0
0.1
0.2
β$_{IV}$

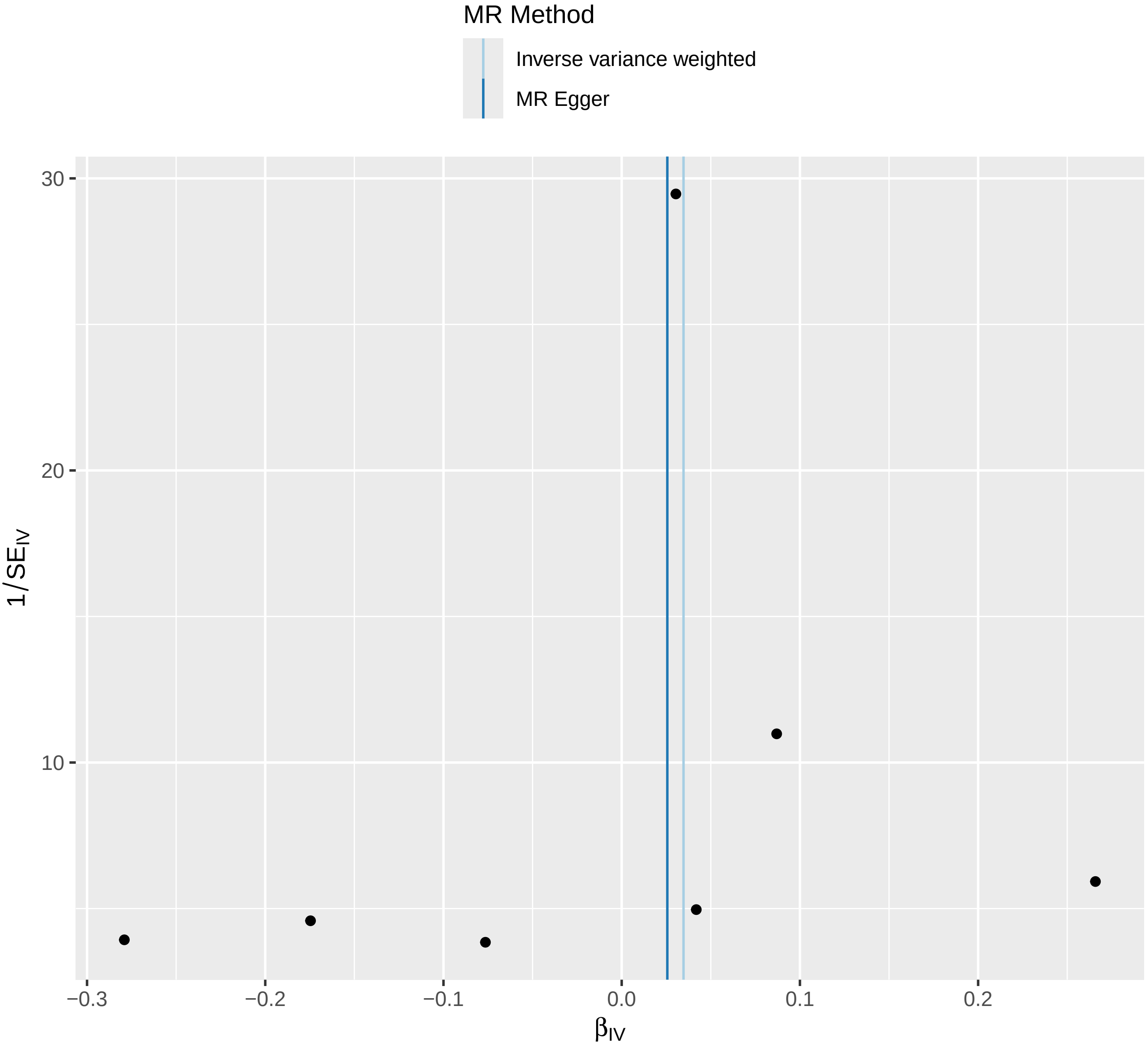

MR Method
Inverse variance weighted
MR Egger
30
20
10
1/SE$_{IV}$
−0.3
−0.2
−0.1
0.0
0.1
0.2
β$_{IV}$

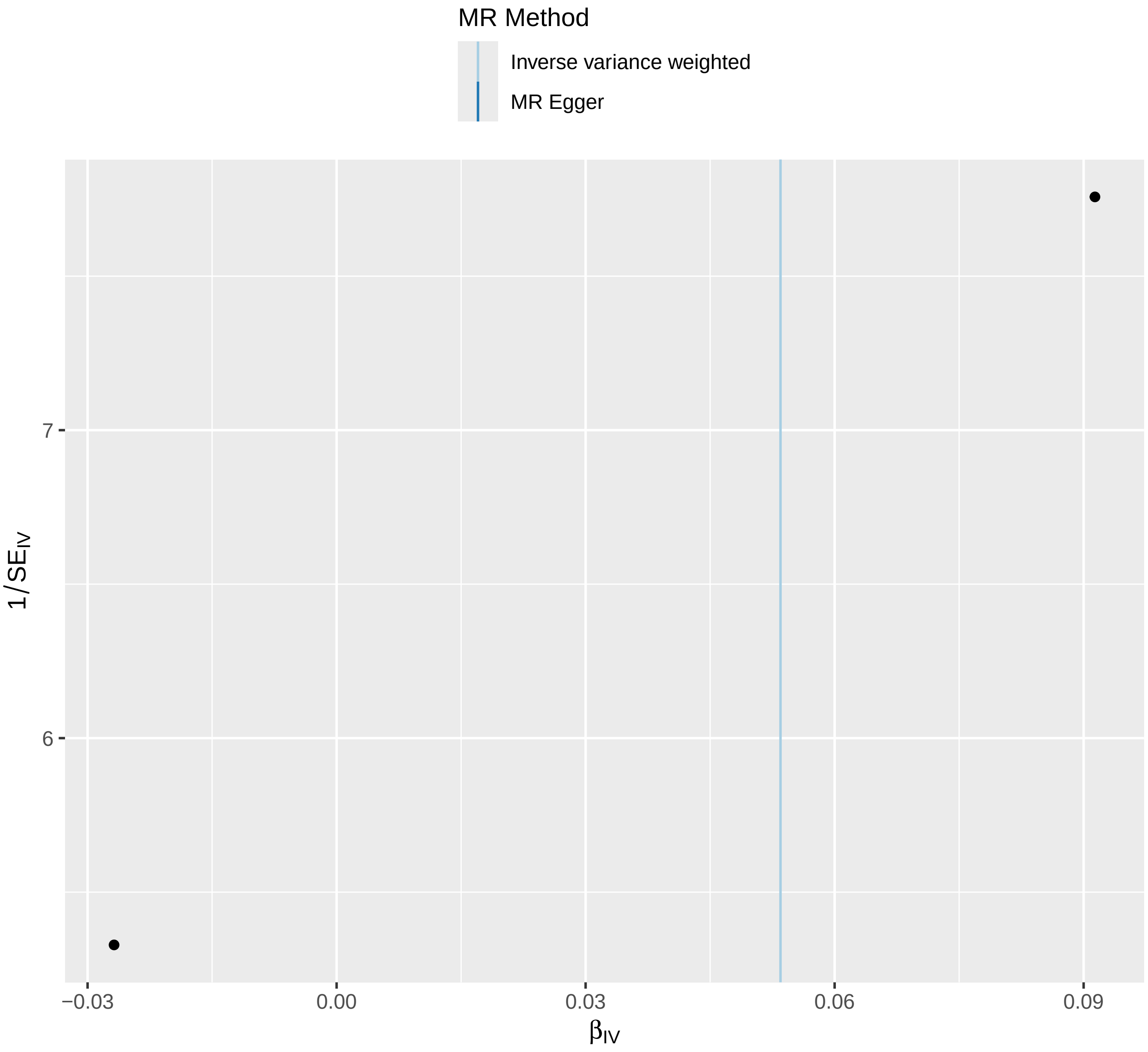
MR Method
Inverse variance weighted
MR Egger
1/SE_IV
7
6
−0.03
0.00
0.03
0.06
0.09
β_IV

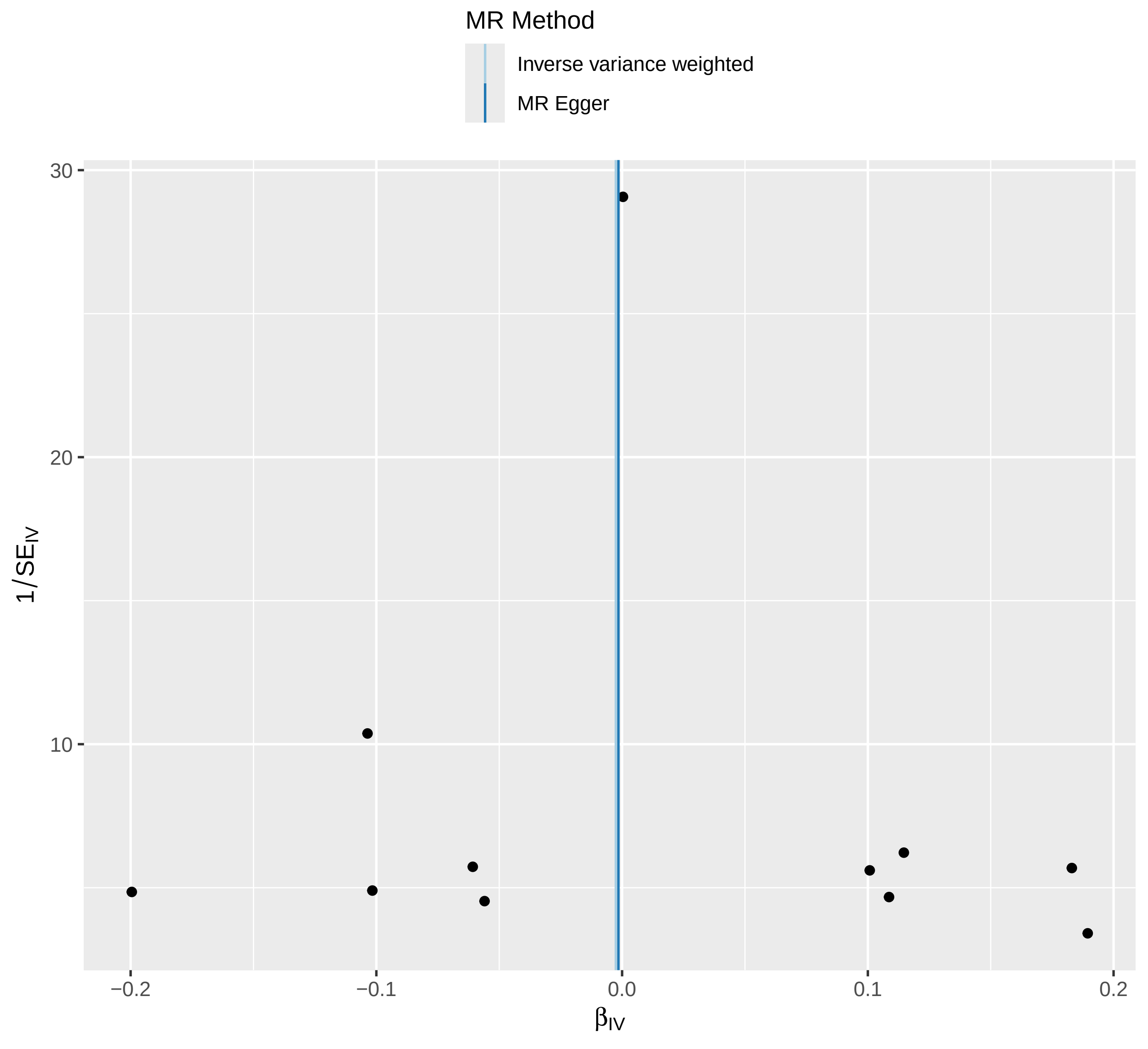

MR Method
Inverse variance weighted
MR Egger
30
20
10
1/SE$_{IV}$
−0.2
−0.1
0.0
0.1
0.2
β$_{IV}$

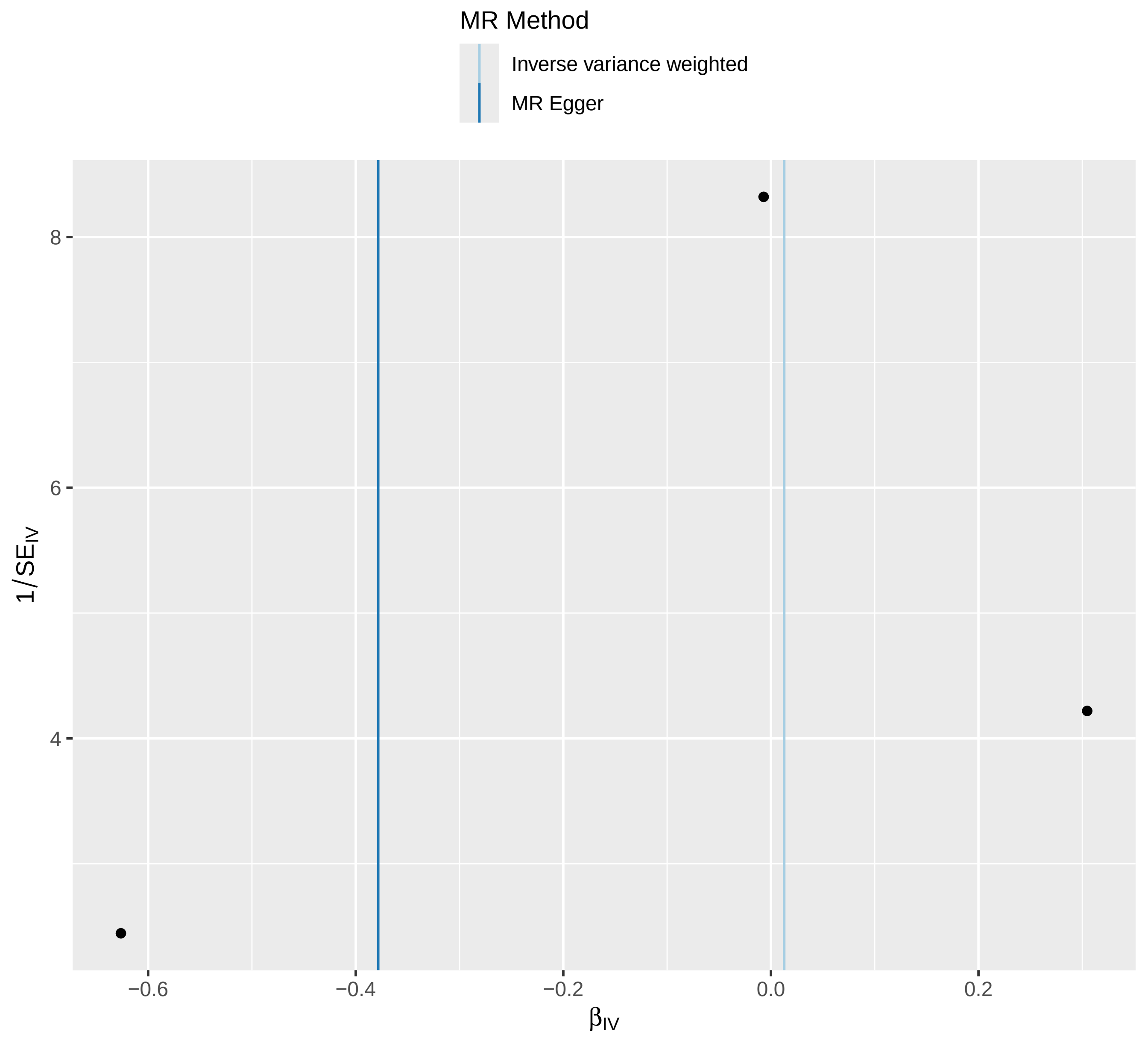
MR Method
Inverse variance weighted
MR Egger
8
6
4
1/SE$_{IV}$
−0.6
−0.4
−0.2
0.0
0.2
β$_{IV}$

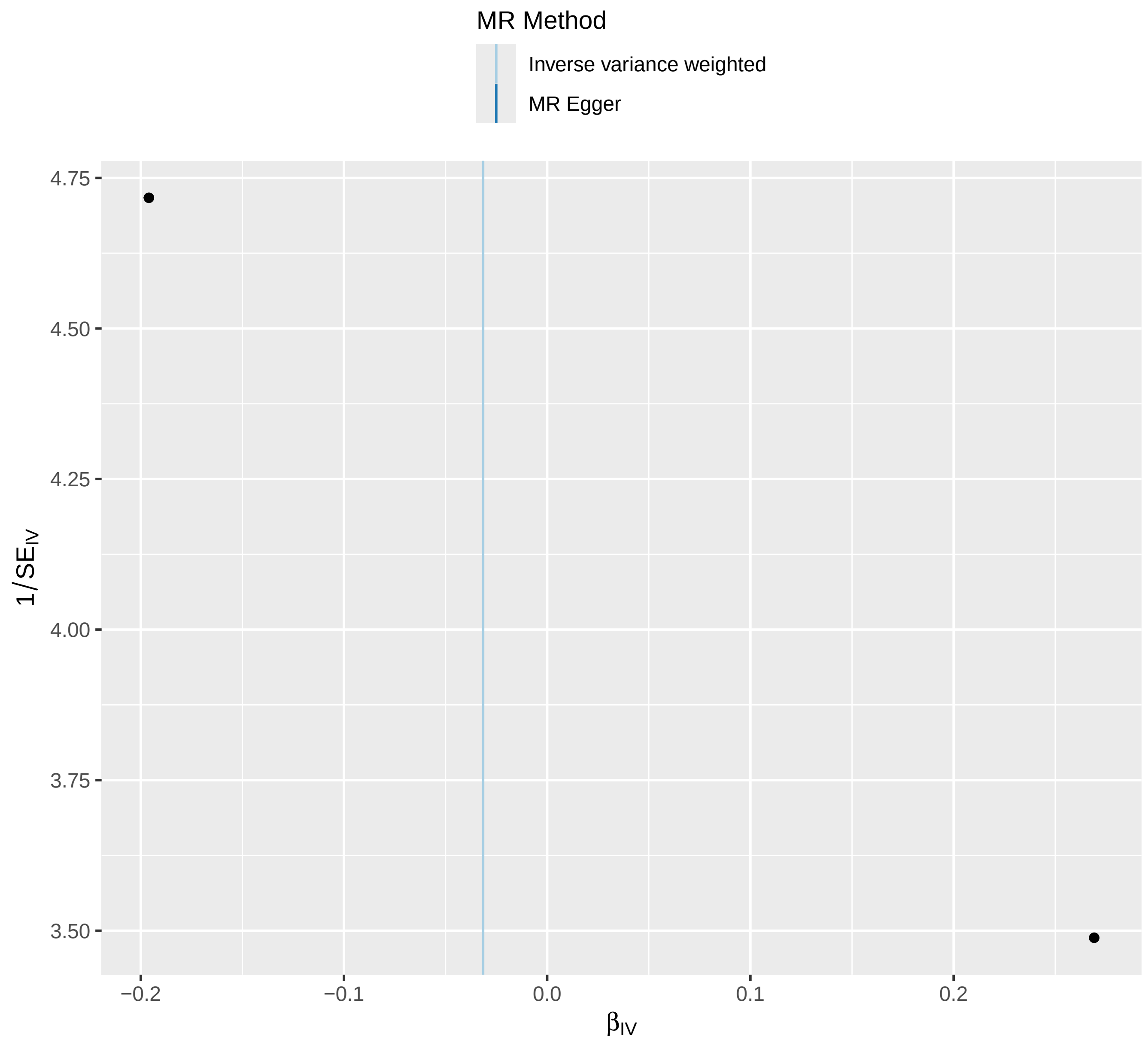
MR Method
Inverse variance weighted
MR Egger
4.75
4.50
4.25
4.00
3.75
3.50
1/SE$_{IV}$
−0.2
−0.1
0.0
0.1
0.2
β$_{IV}$

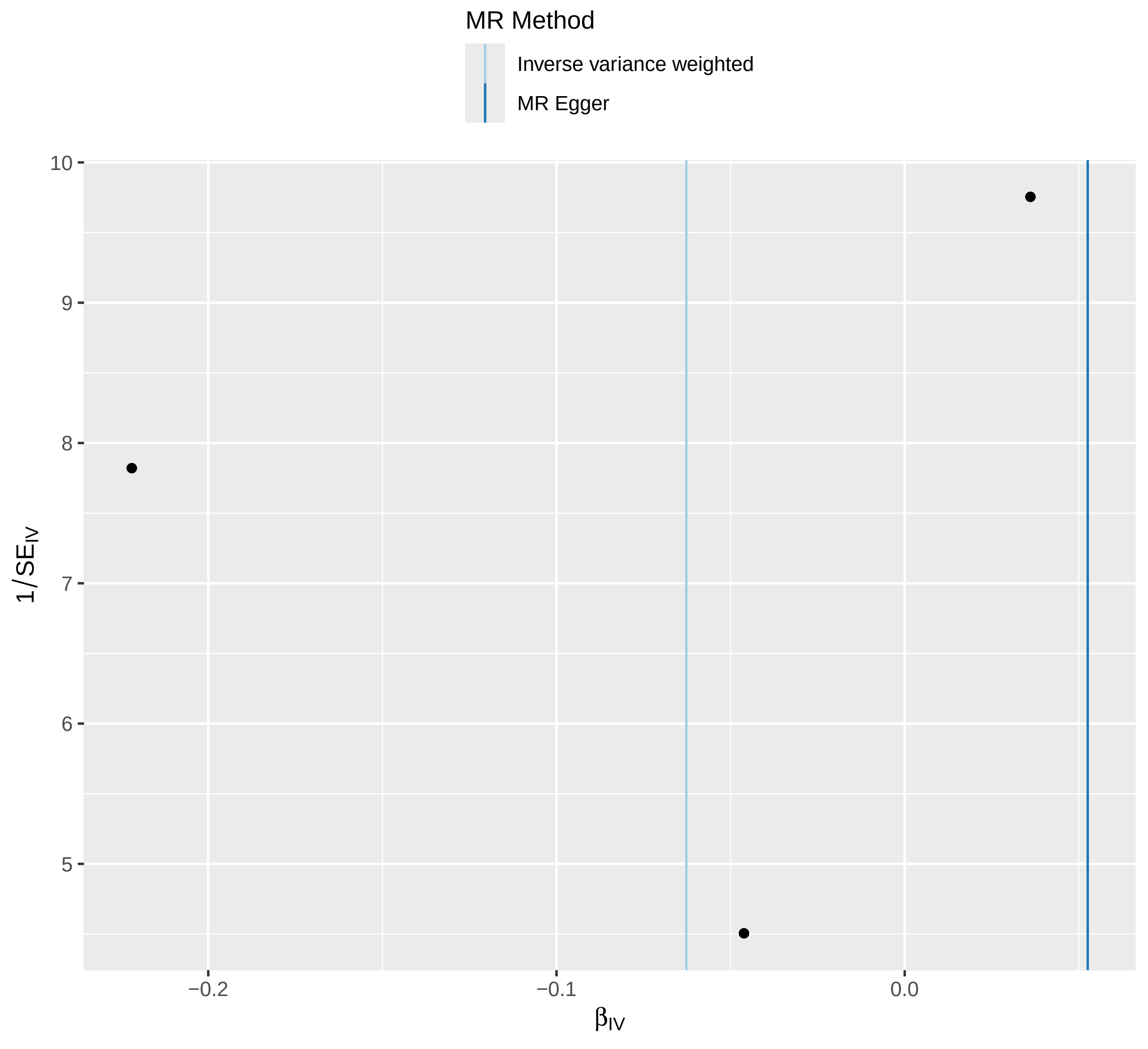

MR Method
Inverse variance weighted
MR Egger
10
9
8
7
6
5
1/SE$_{IV}$
−0.2
−0.1
0.0
β$_{IV}$

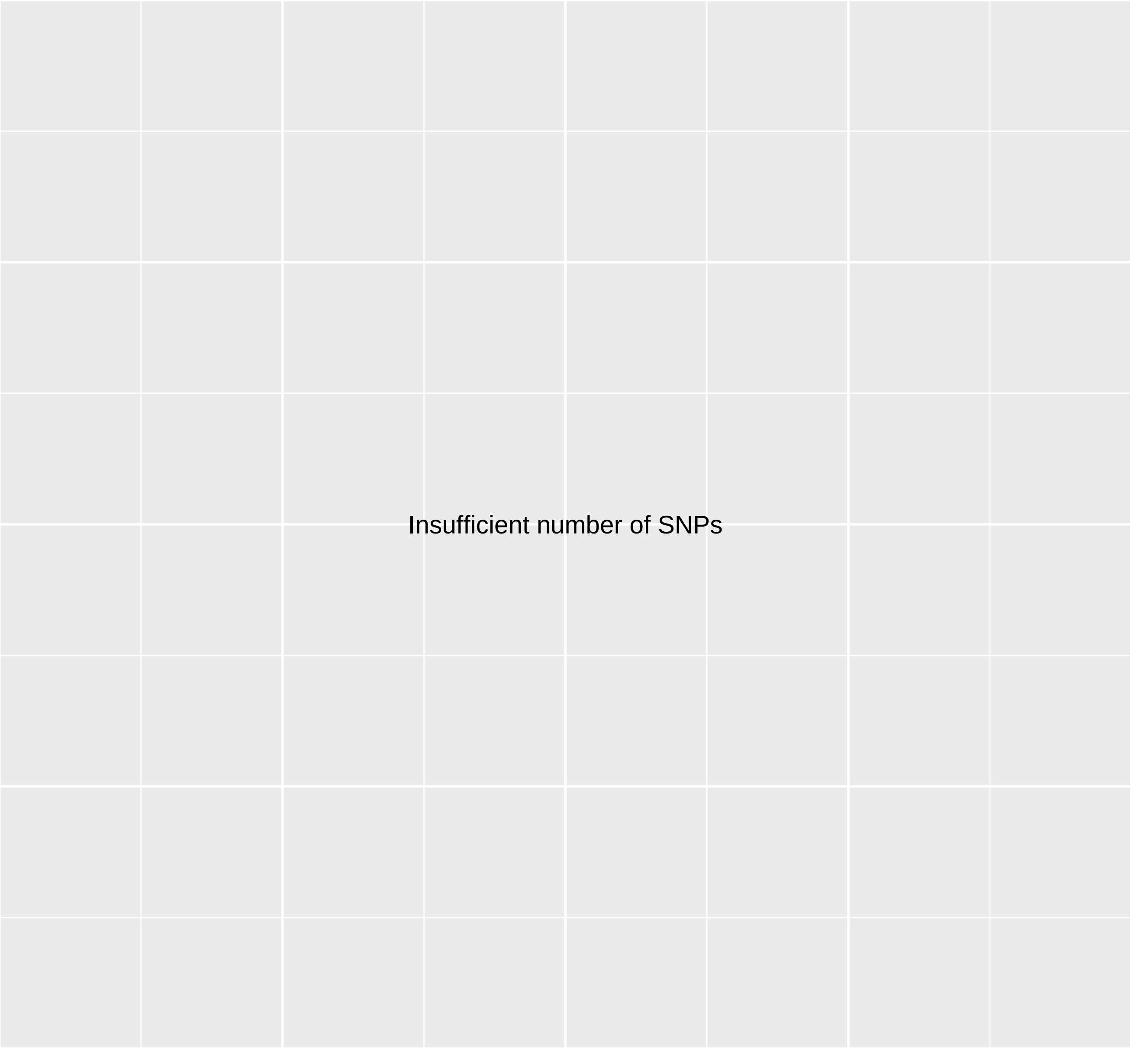
Insufficient number of SNPs

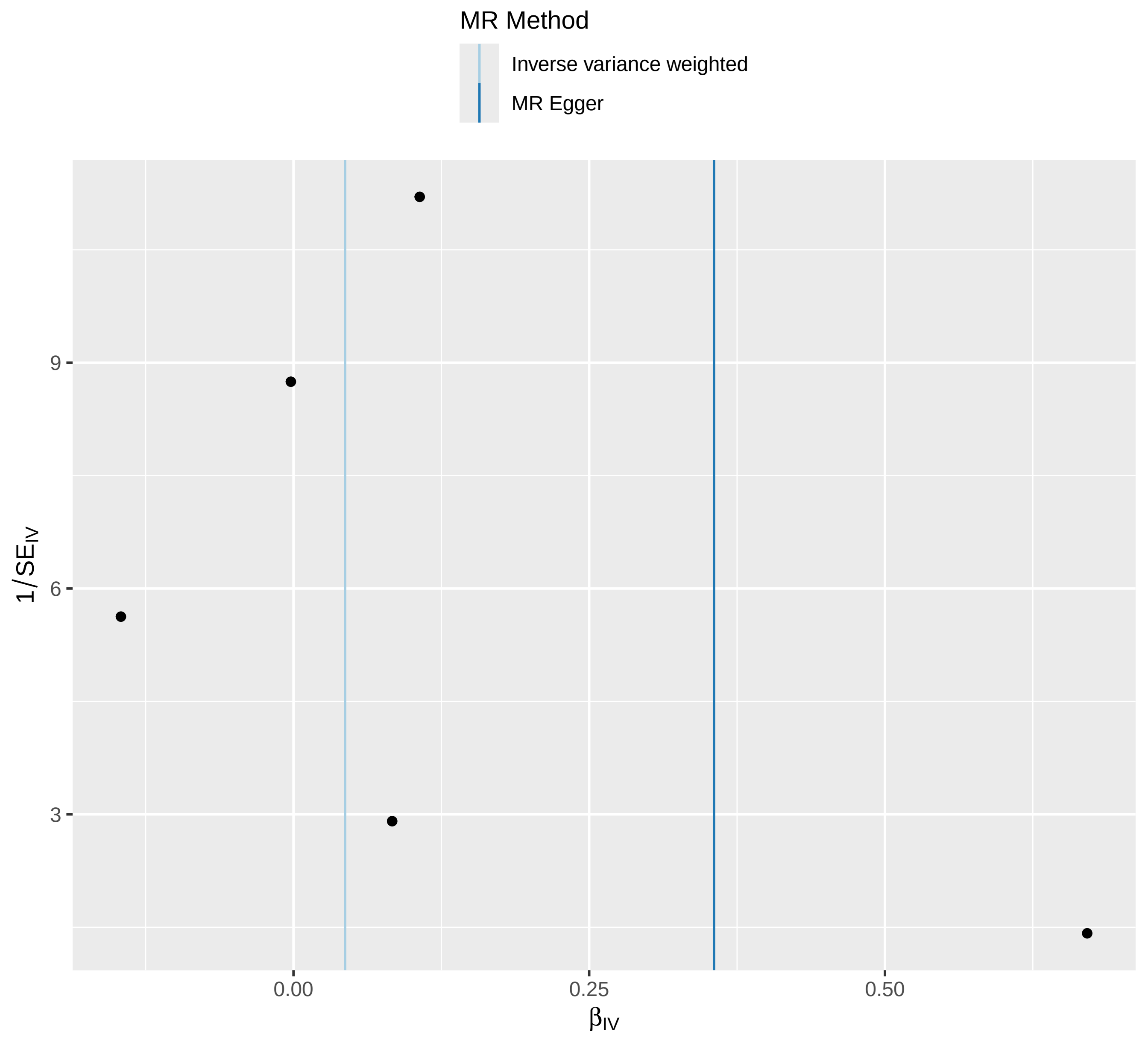

MR Method
Inverse variance weighted
MR Egger
9
6
3
$1/SE_{IV}$
0.00
0.25
0.50
$\beta_{IV}$

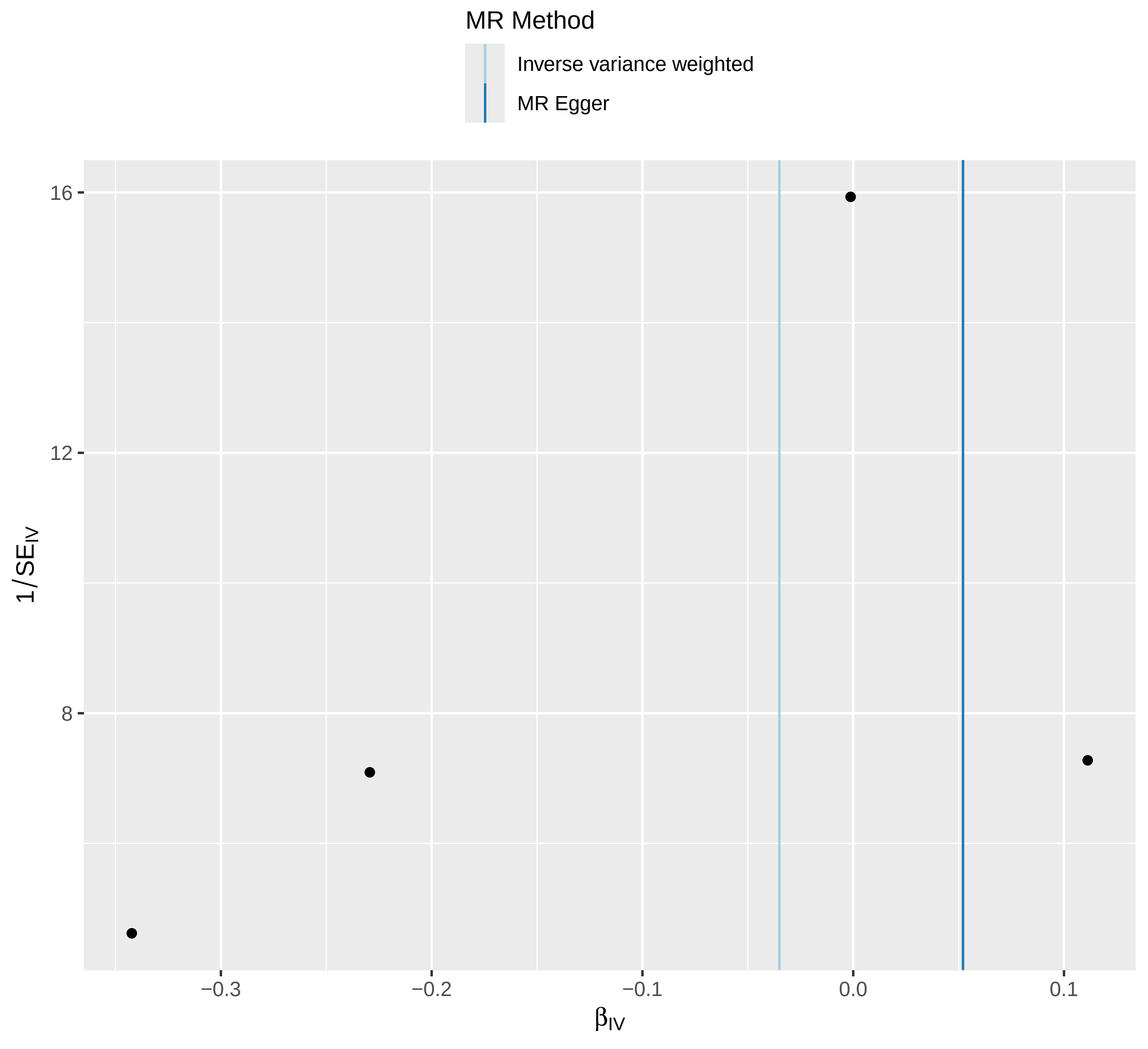

MR Method
Inverse variance weighted
MR Egger
16
12
8
1/SE_IV
−0.3
−0.2
−0.1
0.0
0.1
β_IV

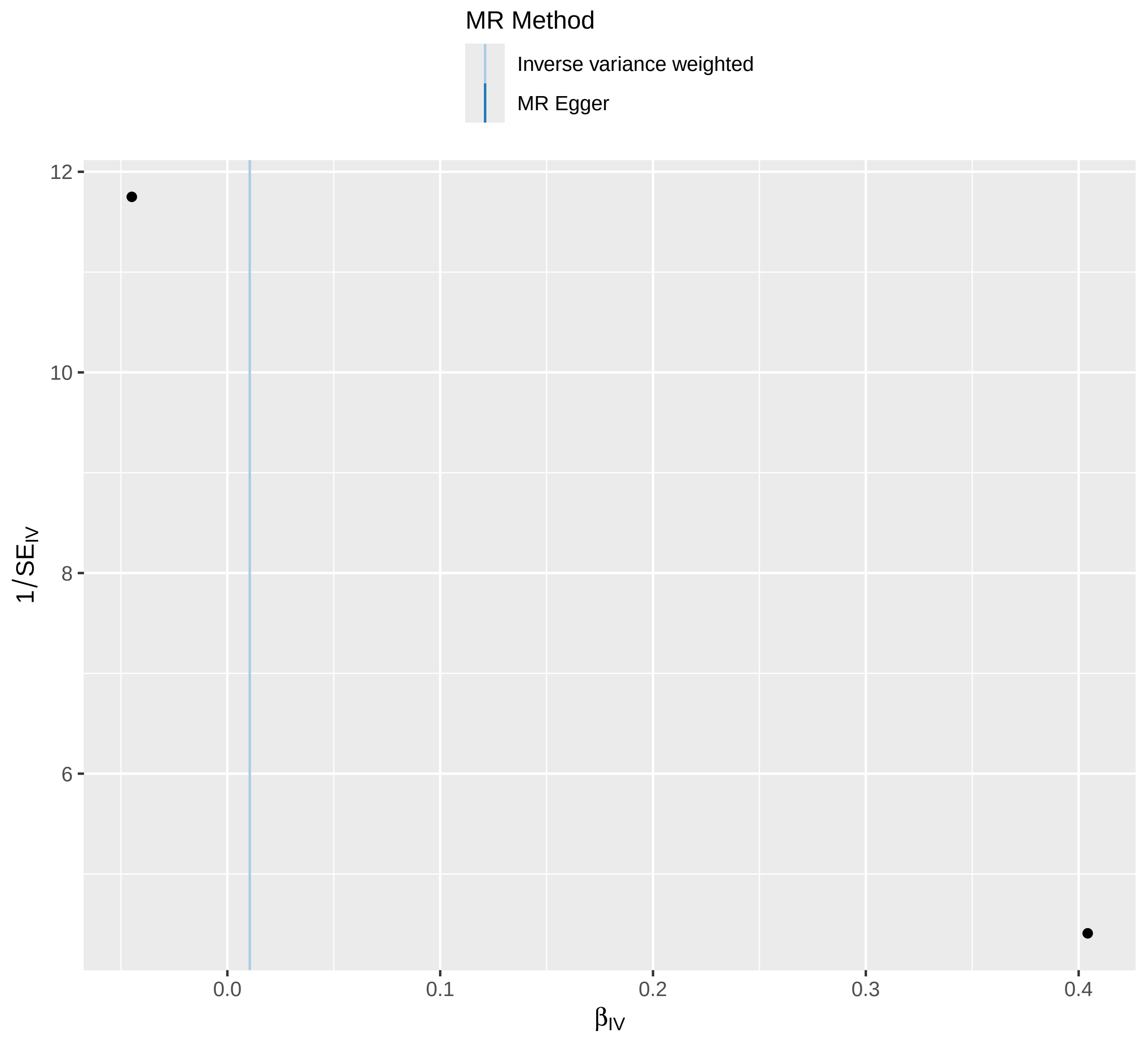
MR Method
Inverse variance weighted
MR Egger
12
10
8
6
1/SE_IV
0.0
0.1
0.2
0.3
0.4
β_IV

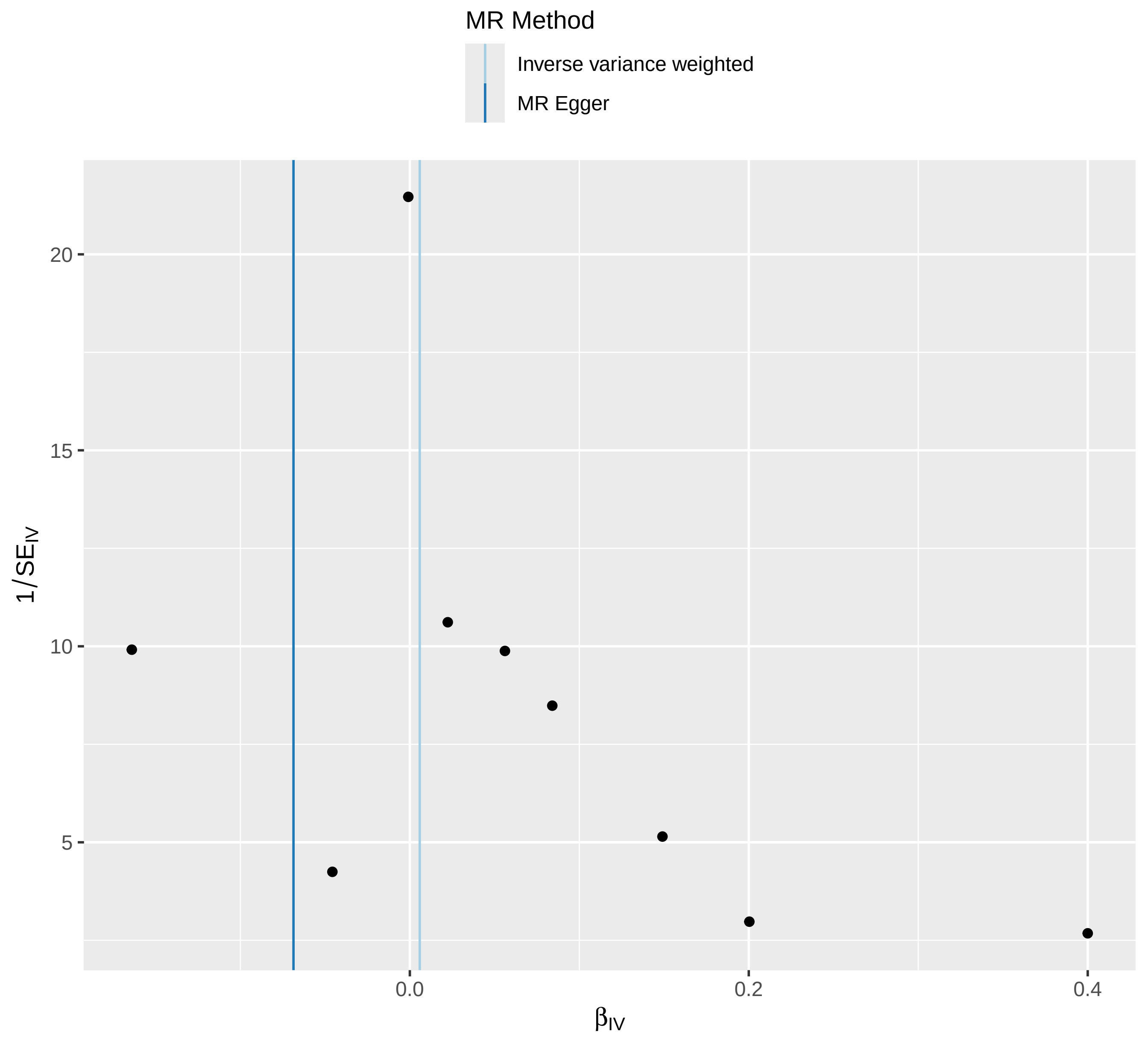
MR Method
Inverse variance weighted
MR Egger
20
15
10
5
0.0
0.2
0.4
1/SE$_{IV}$
β$_{IV}$

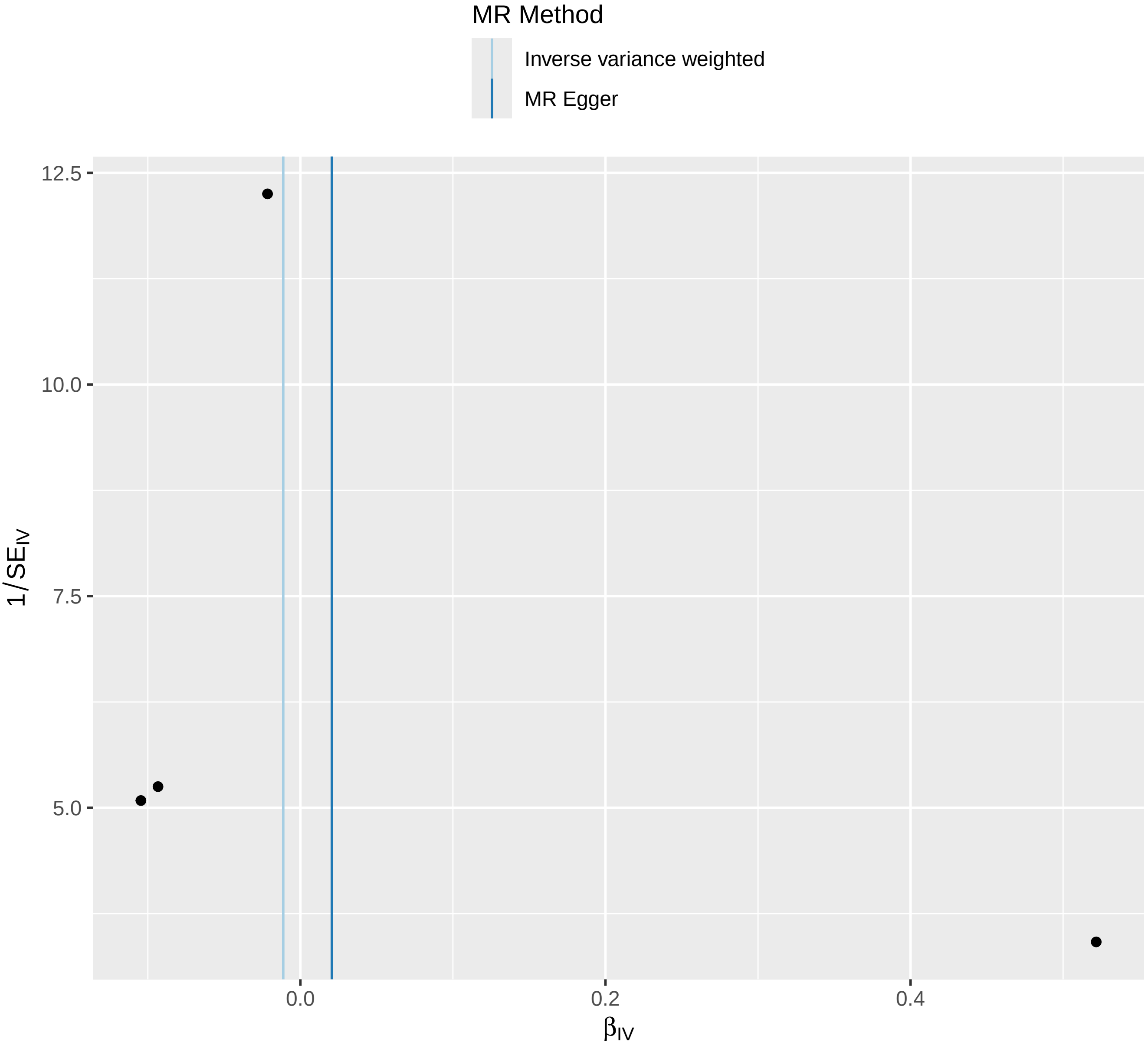
MR Method
Inverse variance weighted
MR Egger
12.5
10.0
7.5
5.0
$1/SE_{IV}$
0.0
0.2
0.4
$\beta_{IV}$

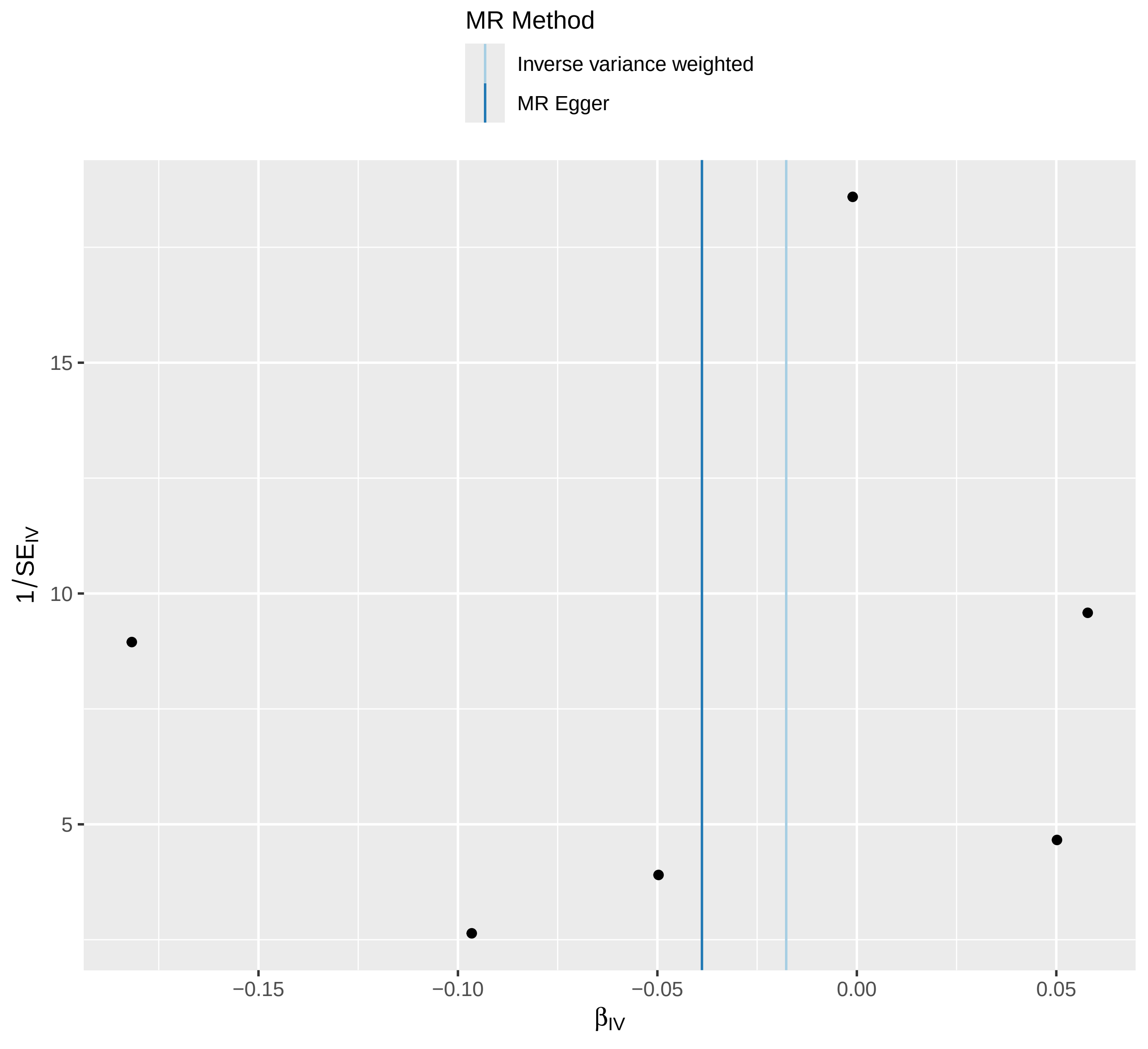

MR Method
Inverse variance weighted
MR Egger
15
10
5
1/SE_IV
−0.15
−0.10
−0.05
0.00
0.05
β_IV

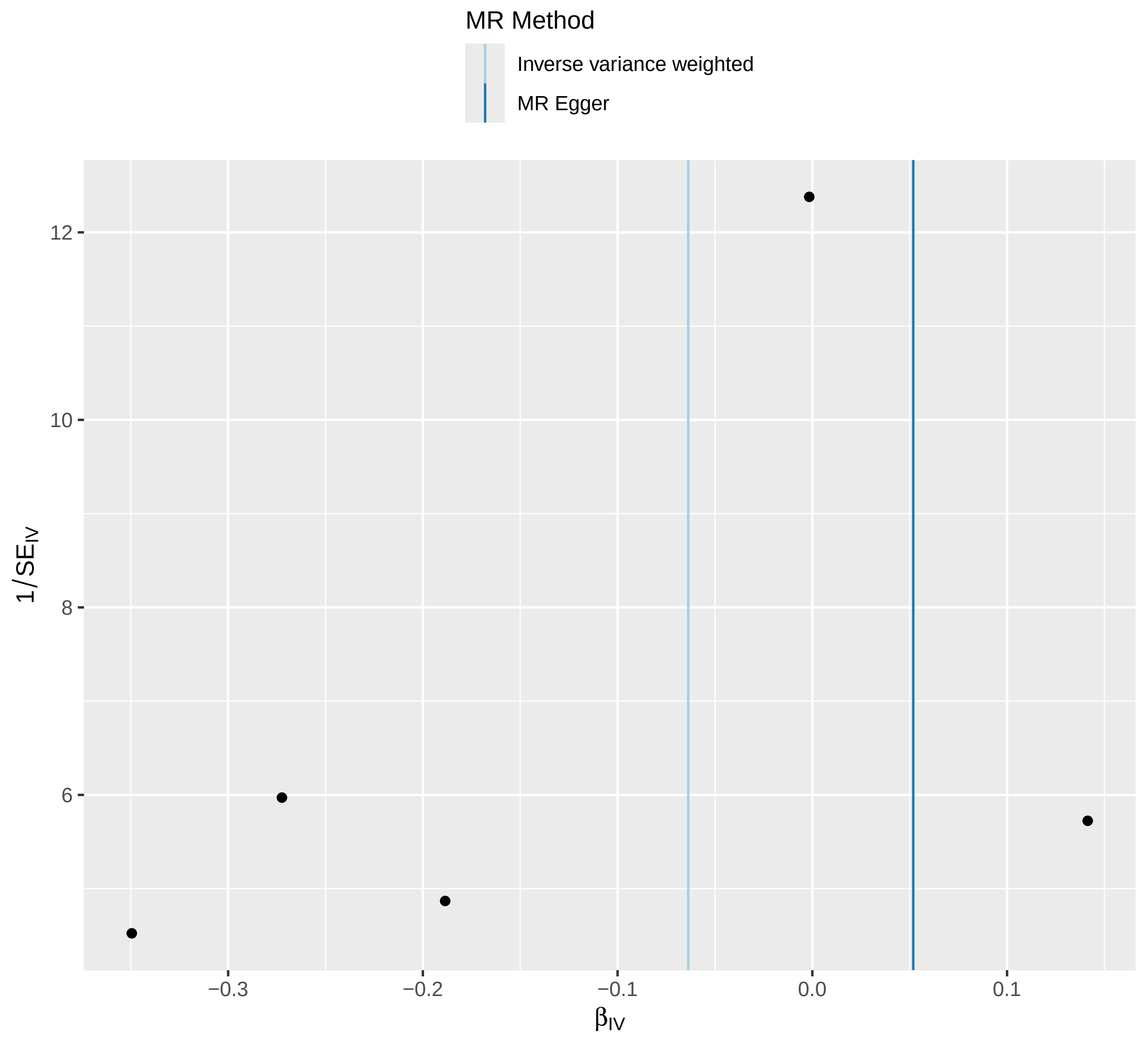
MR Method
Inverse variance weighted
MR Egger
12
10
8
6
$1/SE_{IV}$
−0.3
−0.2
−0.1
0.0
0.1
$\beta_{IV}$

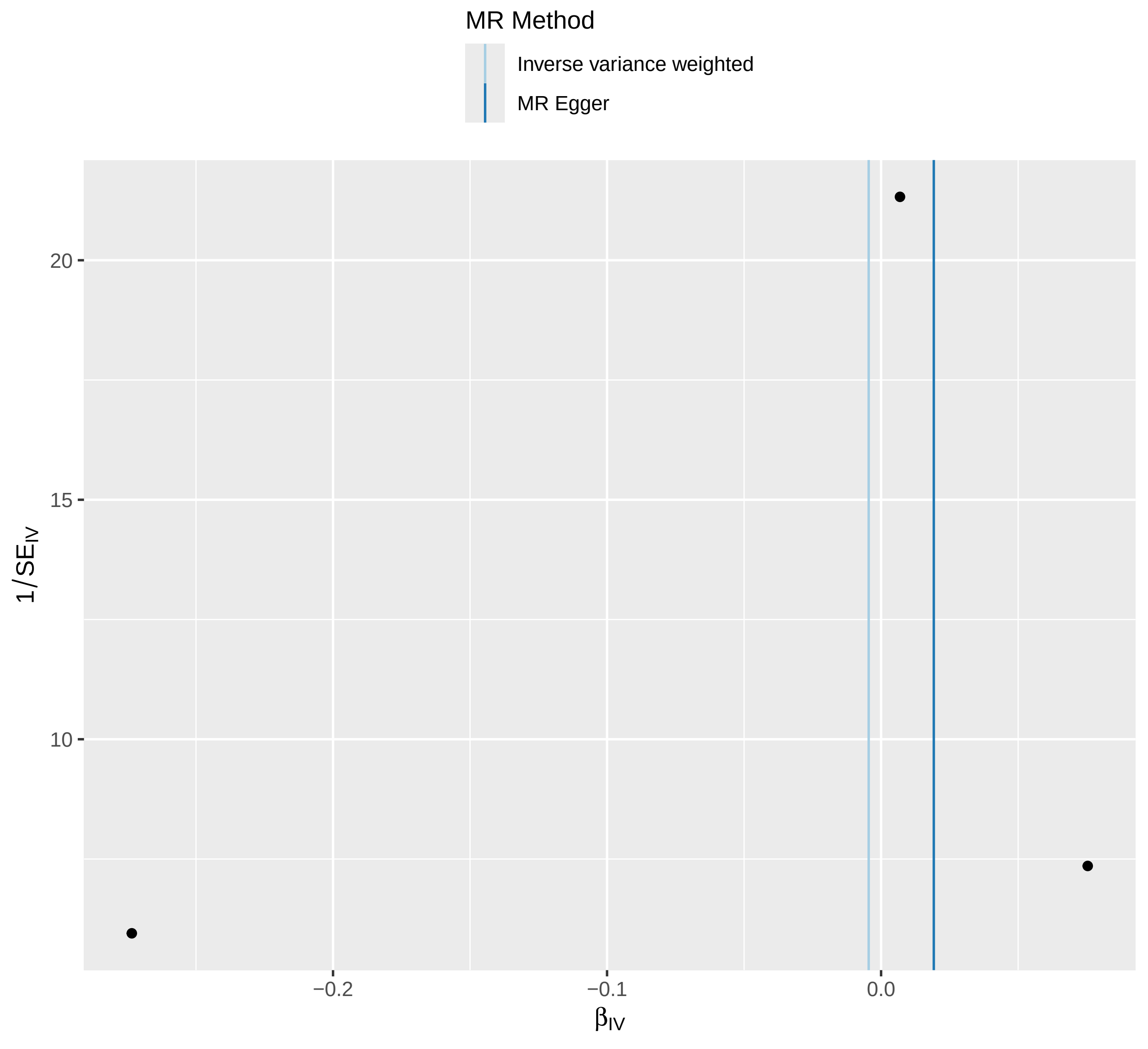
MR Method
Inverse variance weighted
MR Egger
20
15
10
1/SE_IV
−0.2
−0.1
0.0
β_IV

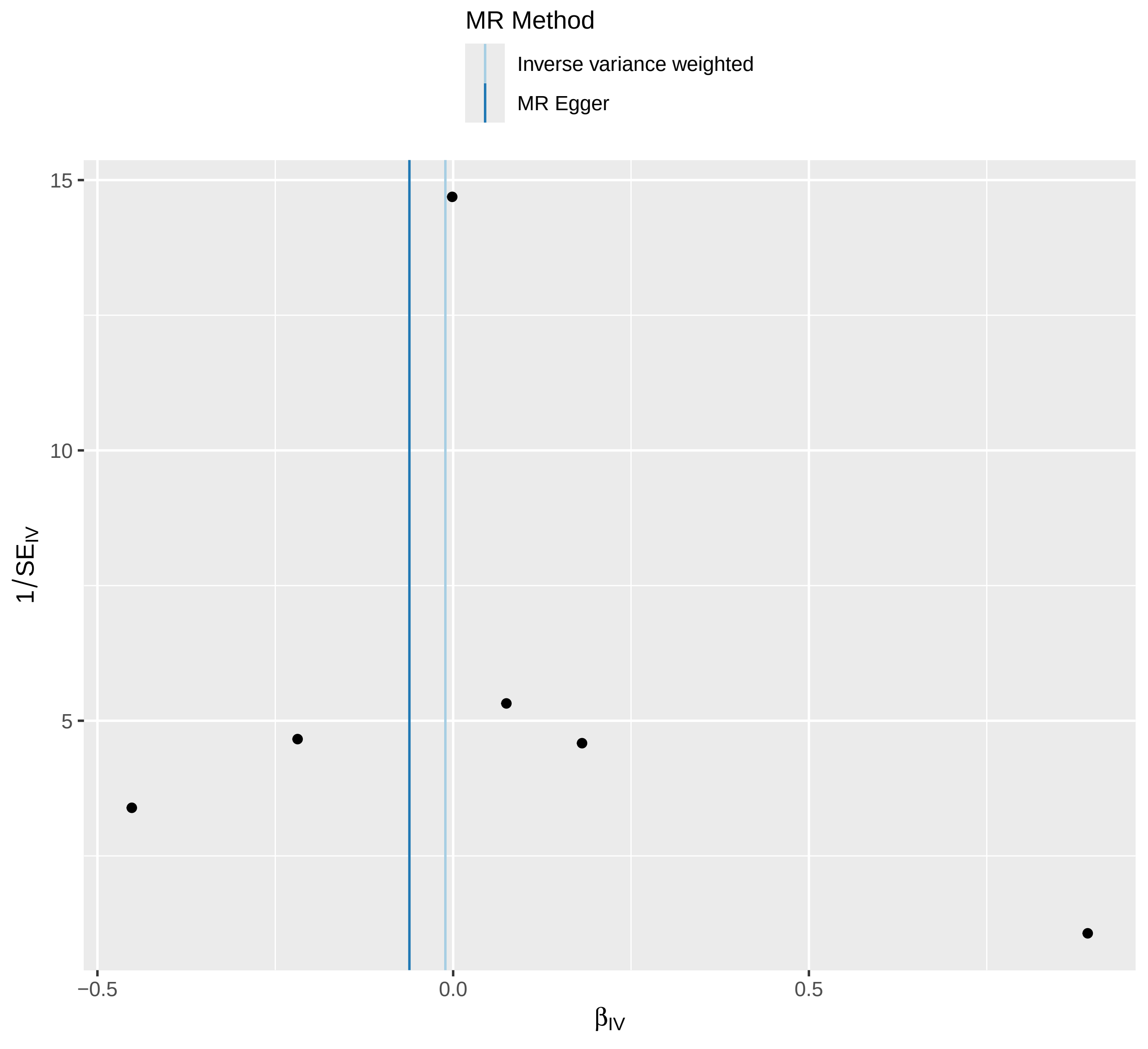

MR Method
Inverse variance weighted
MR Egger
15
10
5
1/SE_IV
−0.5
0.0
0.5
β_IV

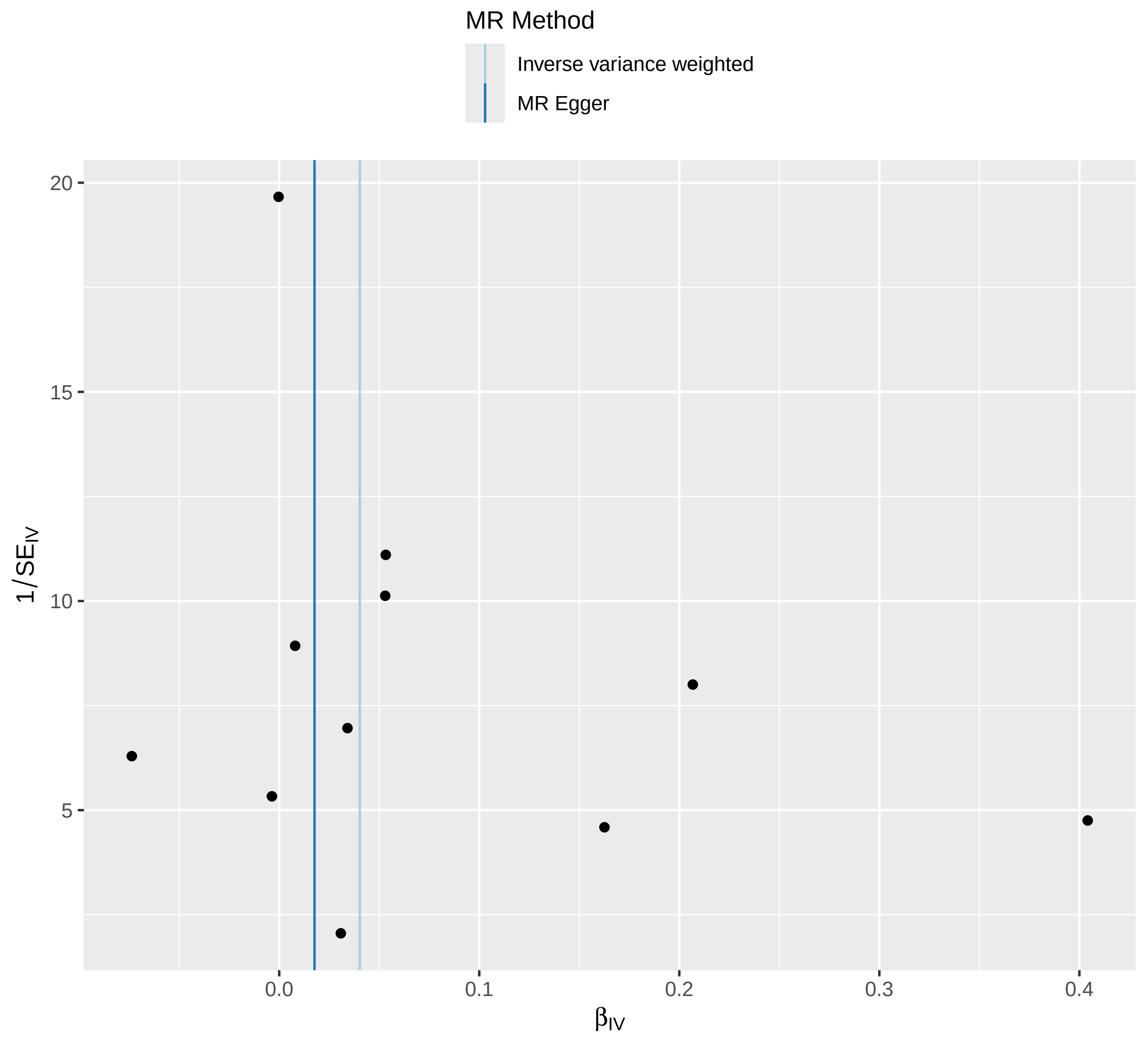

MR Method
Inverse variance weighted
MR Egger
20
15
10
5
1/SE_IV
0.0
0.1
0.2
0.3
0.4
β_IV

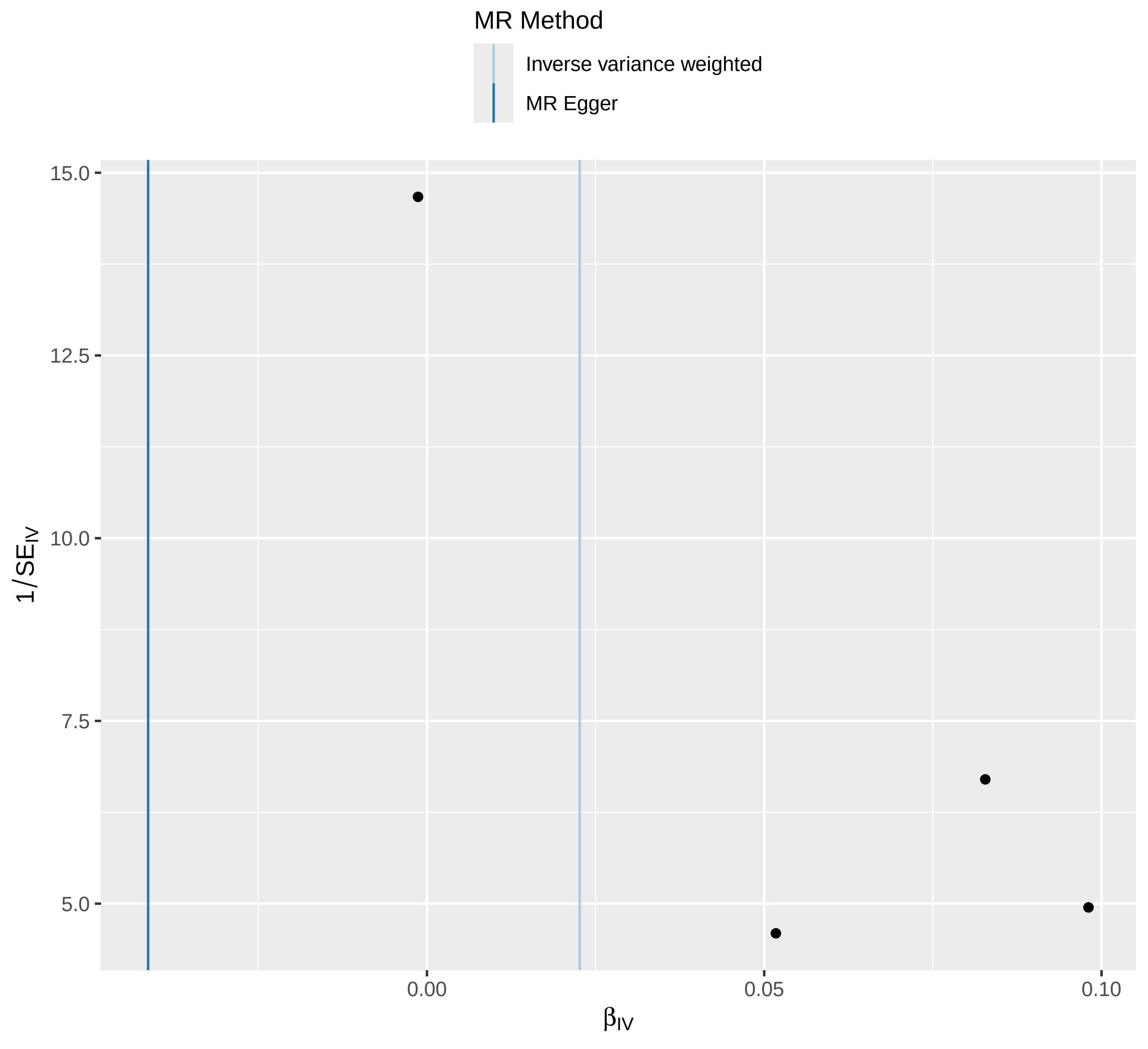
MR Method
Inverse variance weighted
MR Egger
15.0
12.5
10.0
7.5
5.0
1/SE_IV
0.00
0.05
0.10
β_IV

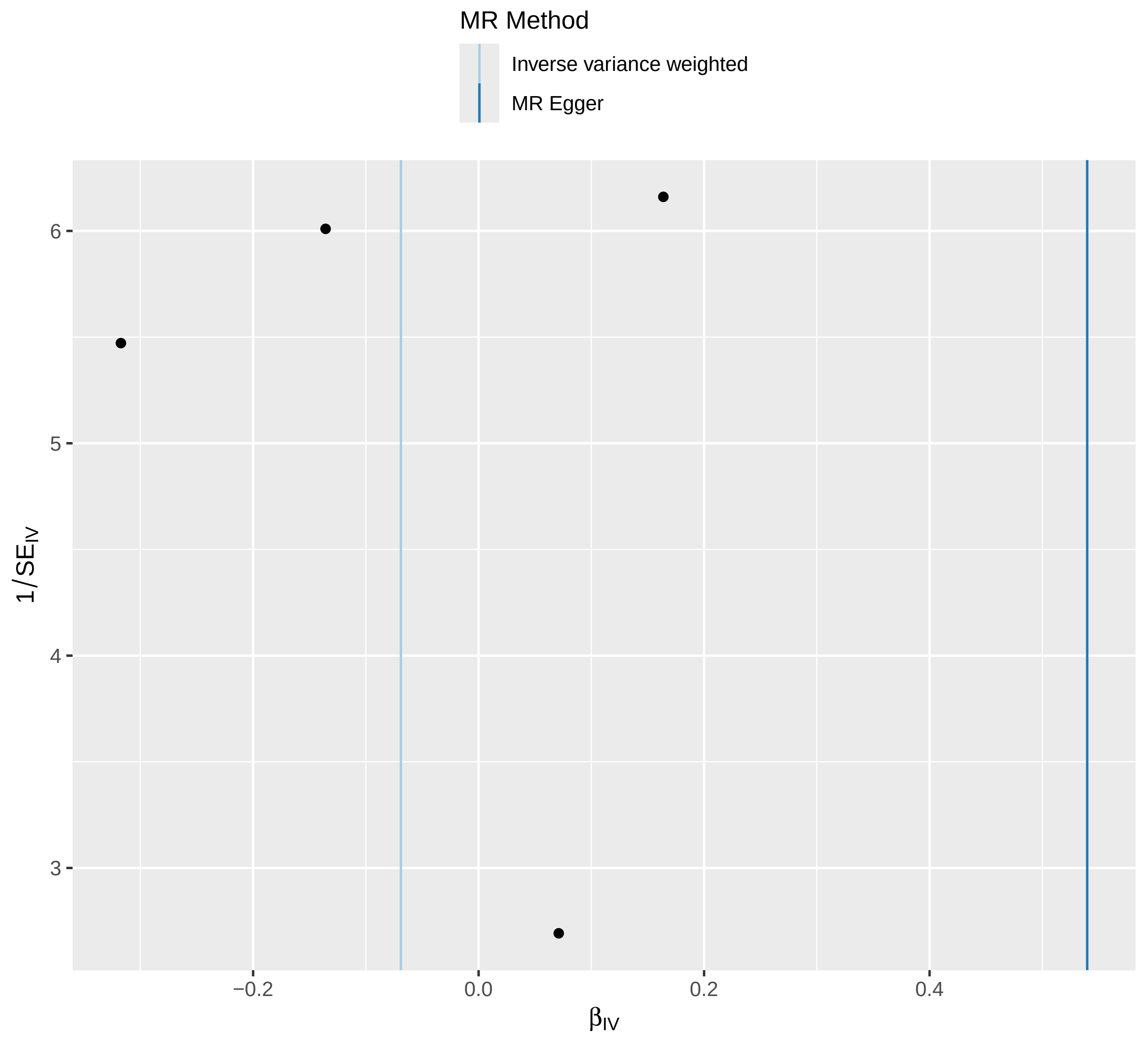
MR Method
Inverse variance weighted
MR Egger
6
5
4
3
1/SE$_{IV}$
−0.2
0.0
0.2
0.4
β$_{IV}$

Insufficient number of SNPs

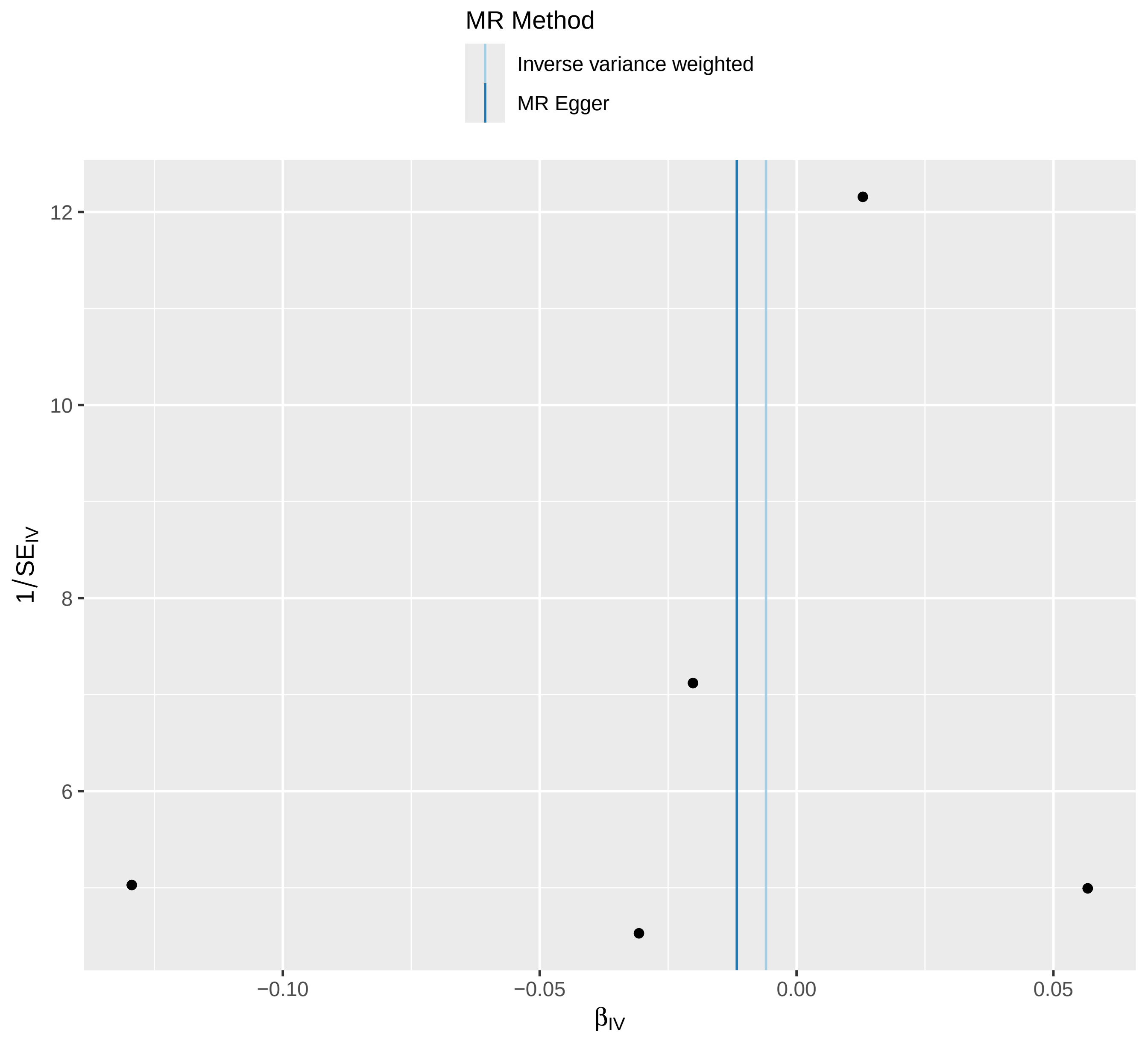
MR Method
Inverse variance weighted
MR Egger
1/$SE_{IV}$
12
10
8
6
−0.10
−0.05
0.00
0.05
$\beta_{IV}$

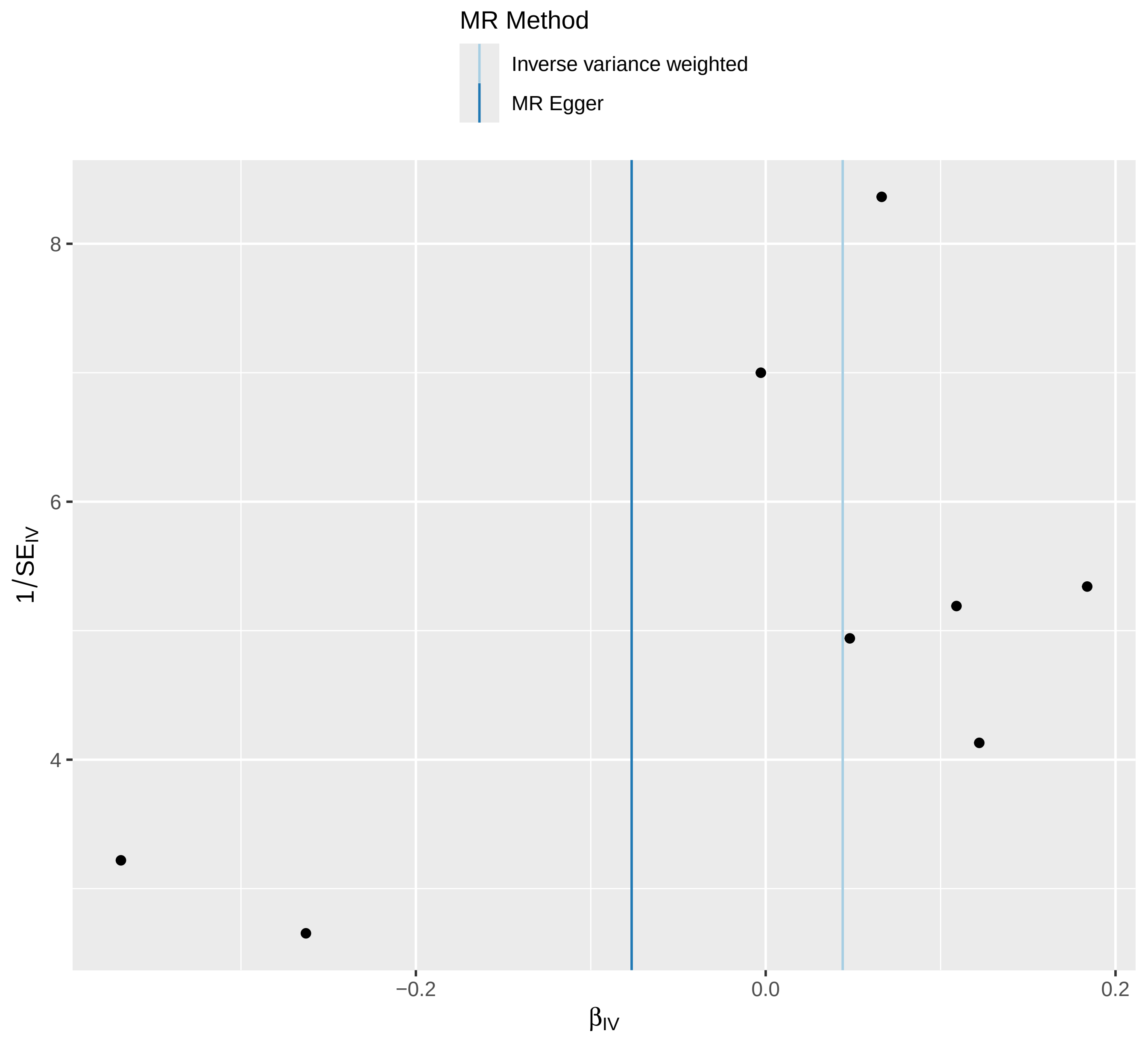

MR Method
Inverse variance weighted
MR Egger
8
6
4
1/SE$_{IV}$
−0.2
0.0
0.2
β$_{IV}$

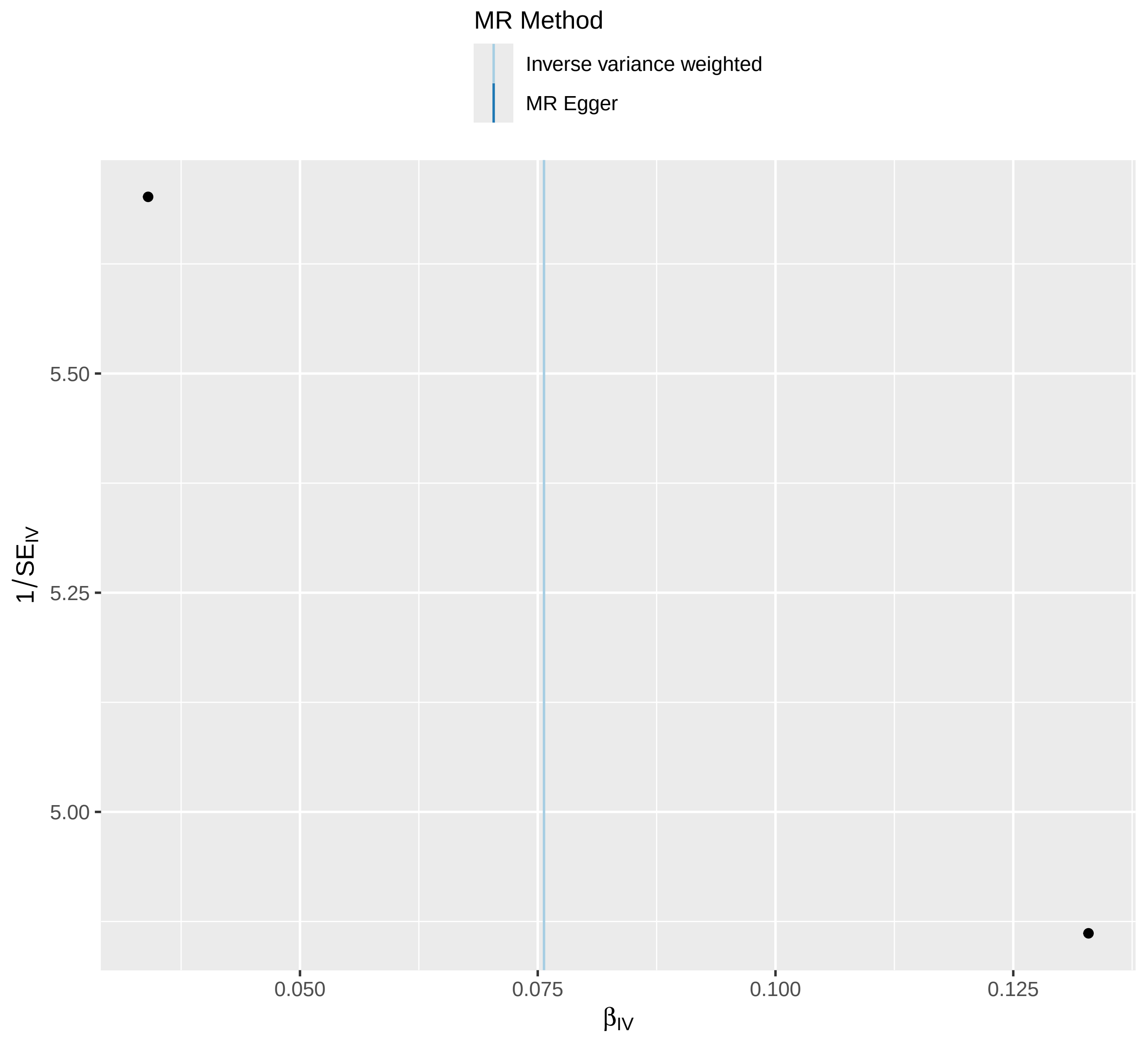
MR Method
Inverse variance weighted
MR Egger
5.50
5.25
5.00
1/SE$_{IV}$
0.050
0.075
0.100
0.125
β$_{IV}$

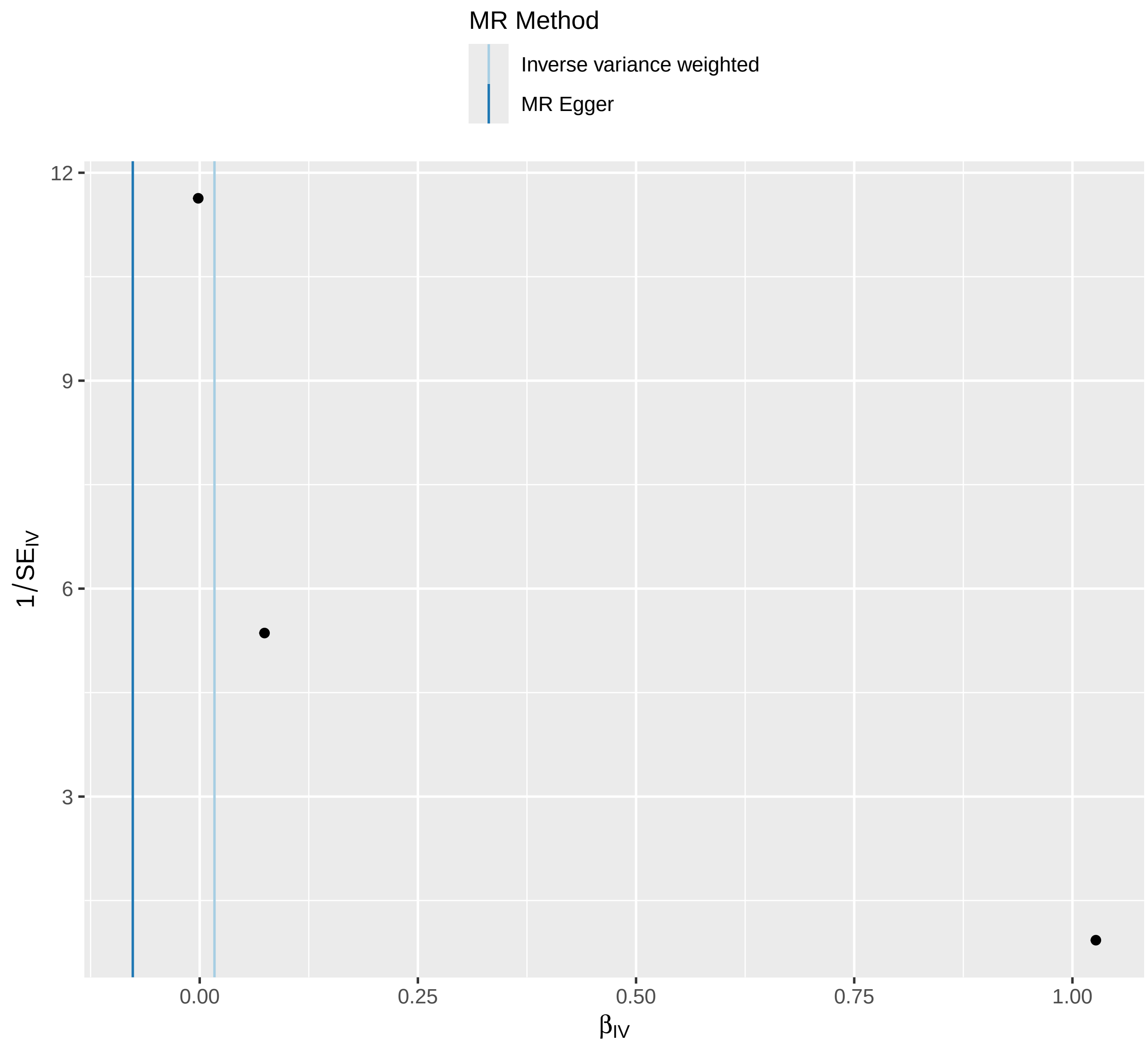
MR Method
Inverse variance weighted
MR Egger
12
9
6
3
1/SE_IV
0.00
0.25
0.50
0.75
1.00
β_IV

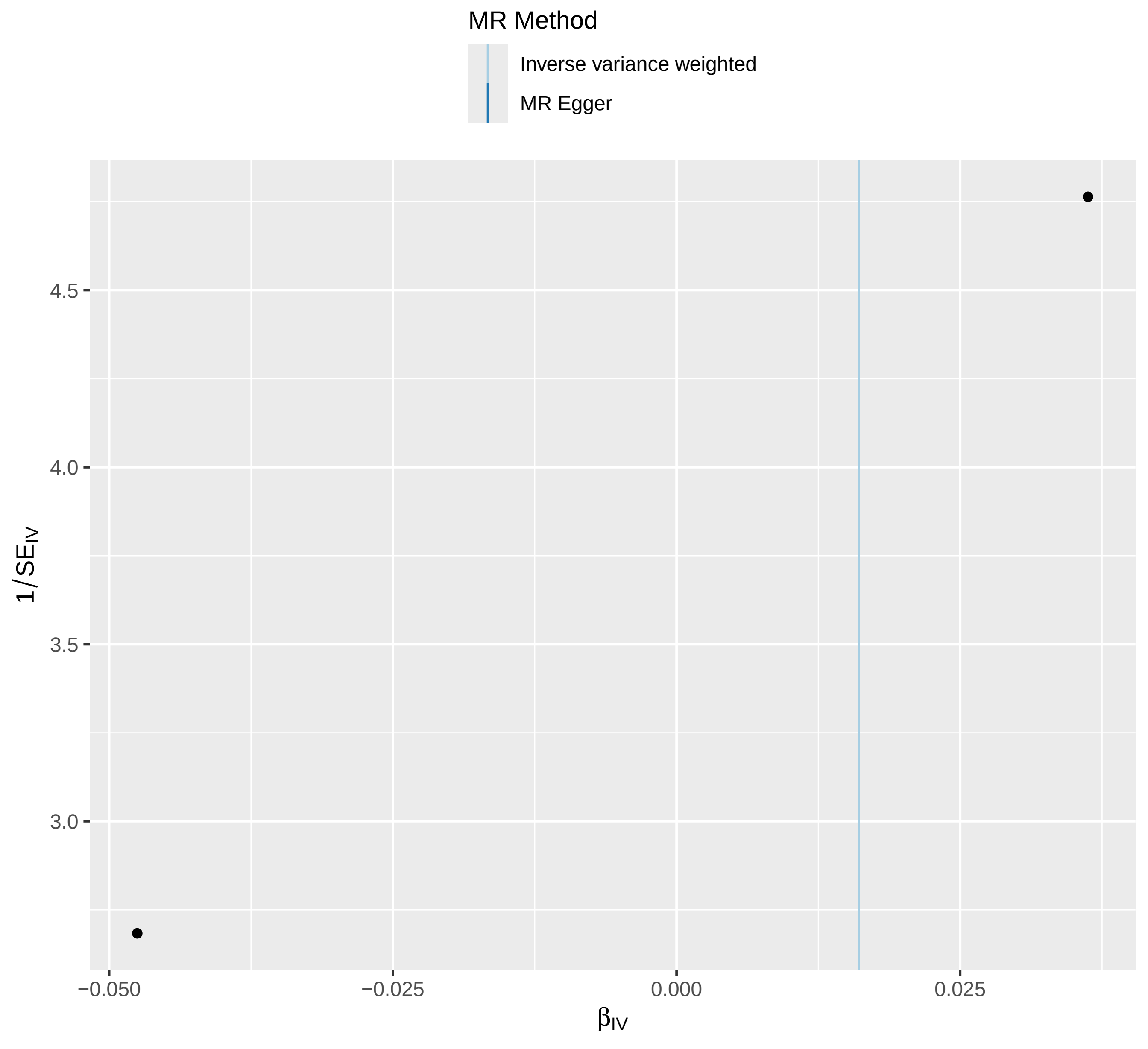

MR Method
Inverse variance weighted
MR Egger
4.5
4.0
3.5
3.0
1/SE$_{IV}$
−0.050
−0.025
0.000
0.025
β$_{IV}$

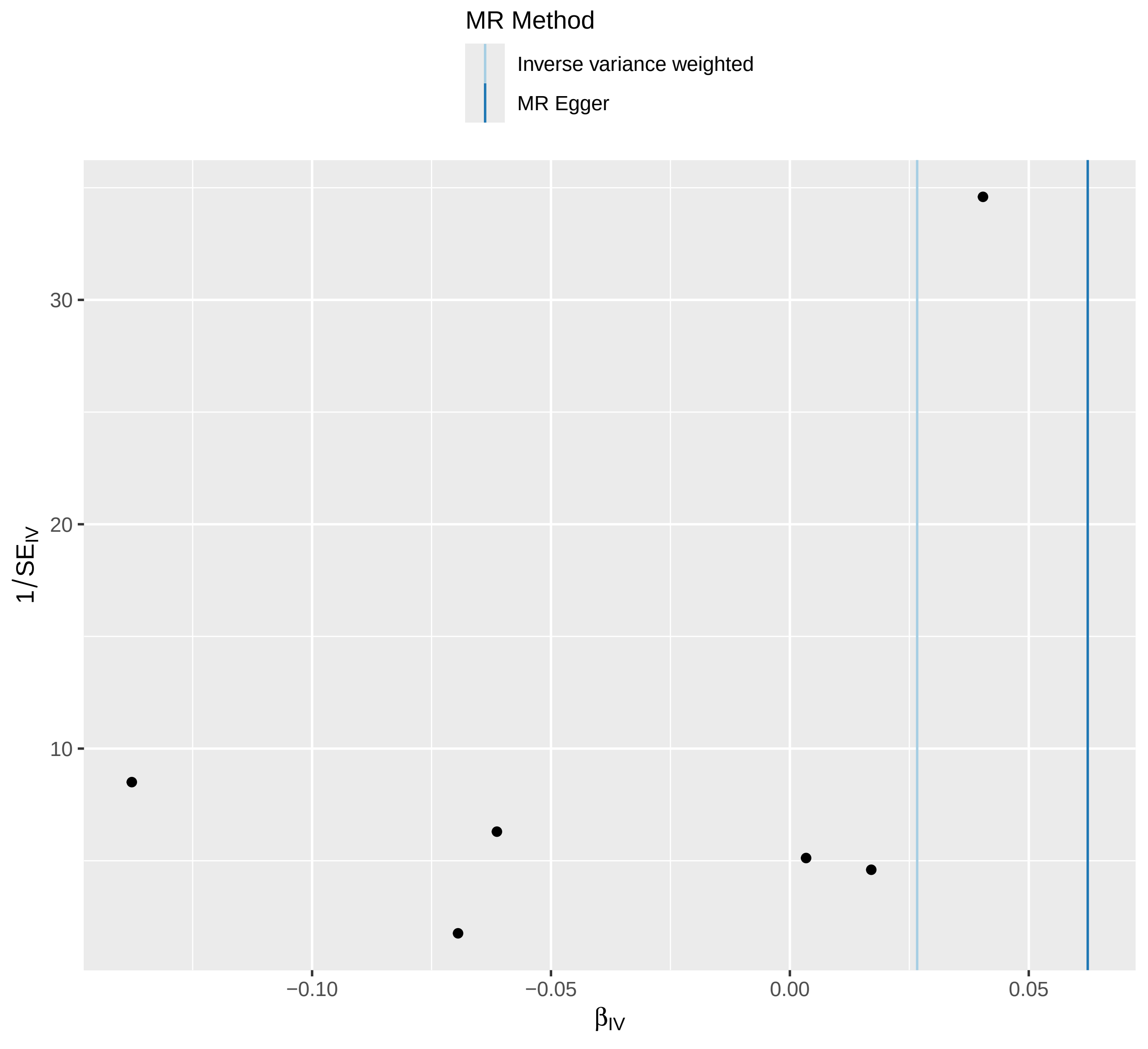

MR Method
Inverse variance weighted
MR Egger
30
20
10
1/SE$_{IV}$
−0.10
−0.05
0.00
0.05
β$_{IV}$

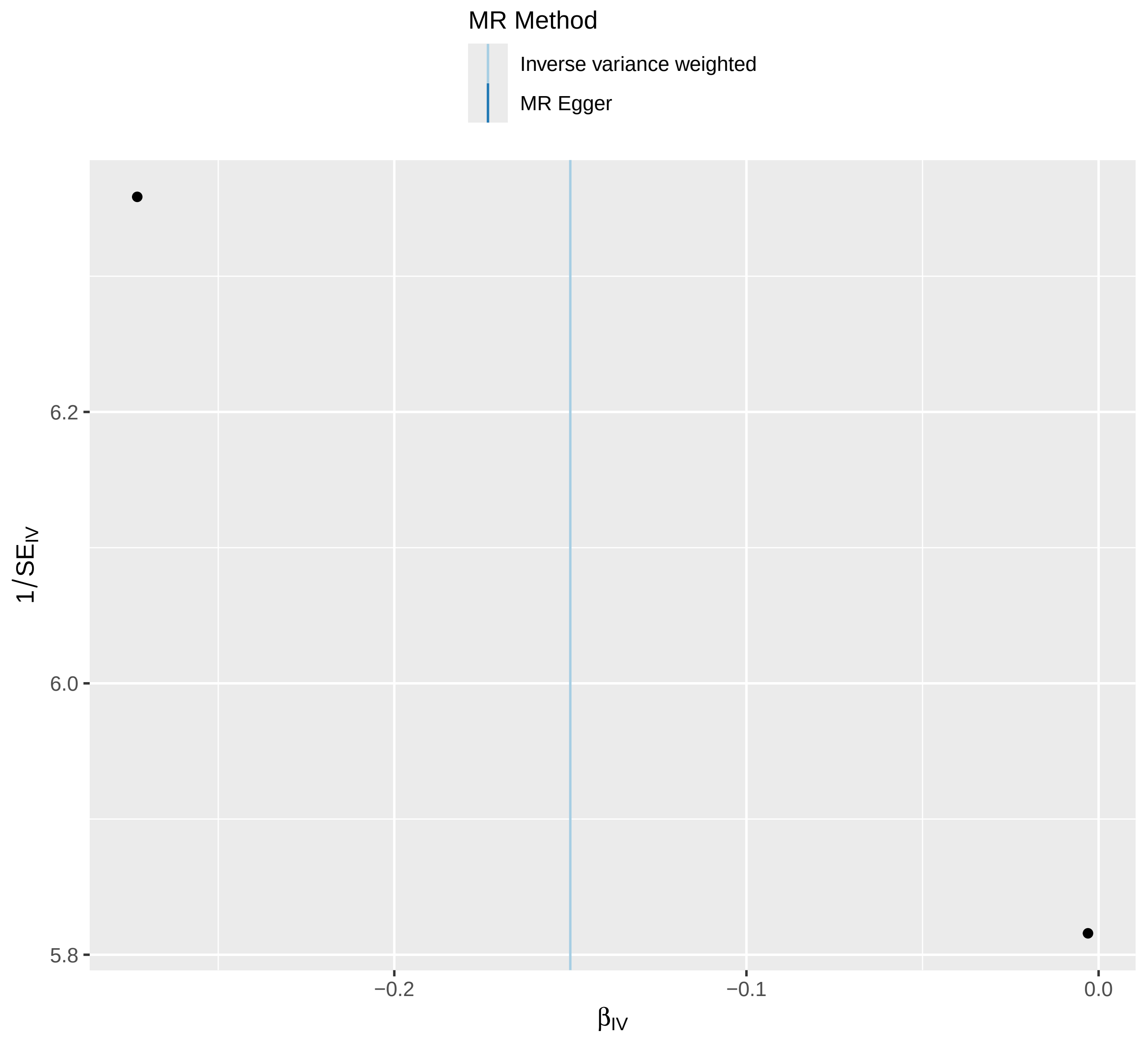

MR Method
Inverse variance weighted
MR Egger
6.2
6.0
5.8
1/SE$_{IV}$
−0.2
−0.1
0.0
β$_{IV}$

Insufficient number of SNPs

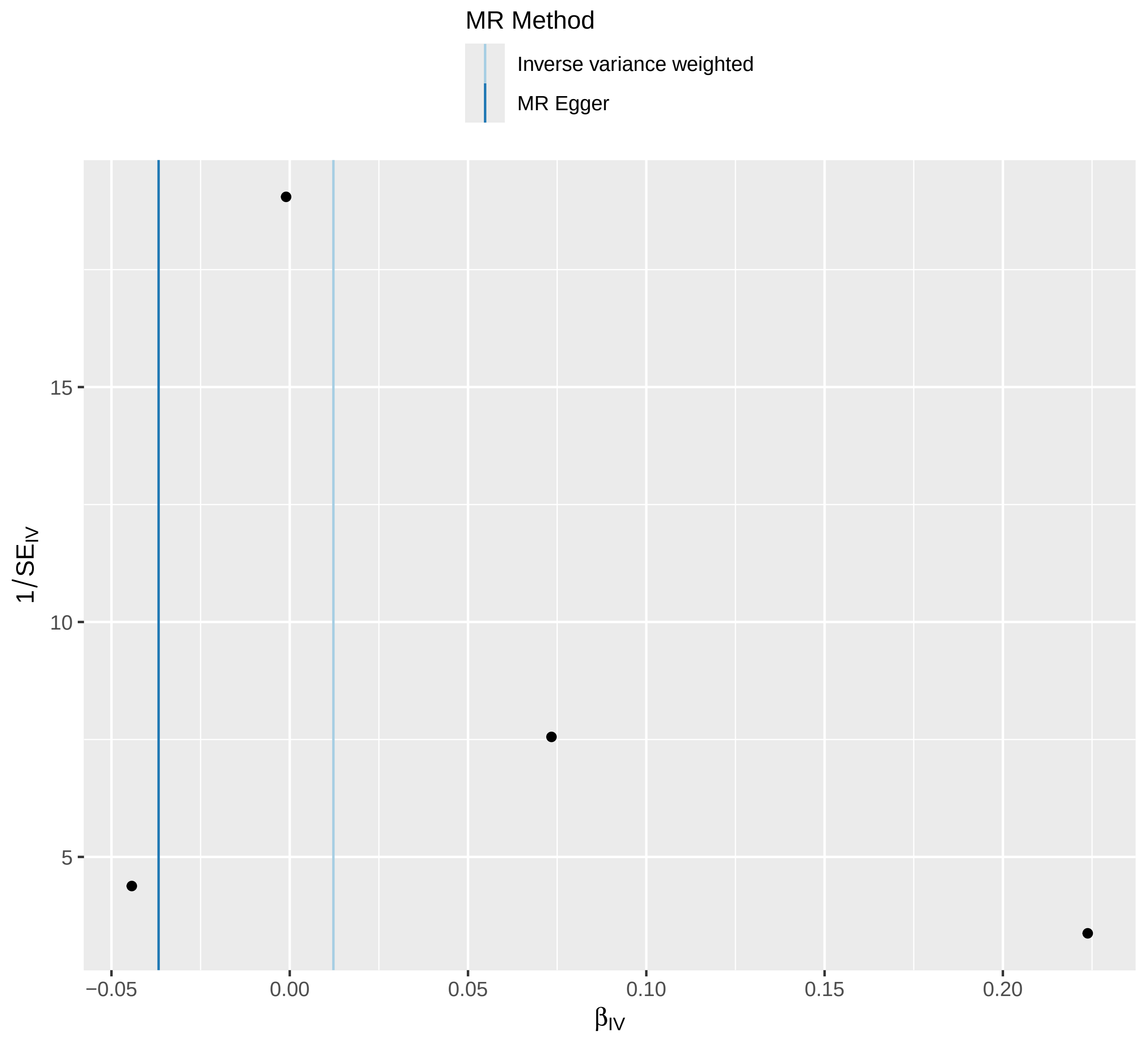
MR Method
Inverse variance weighted
MR Egger
1/SE_IV
15
10
5
−0.05
0.00
0.05
0.10
0.15
0.20
β_IV

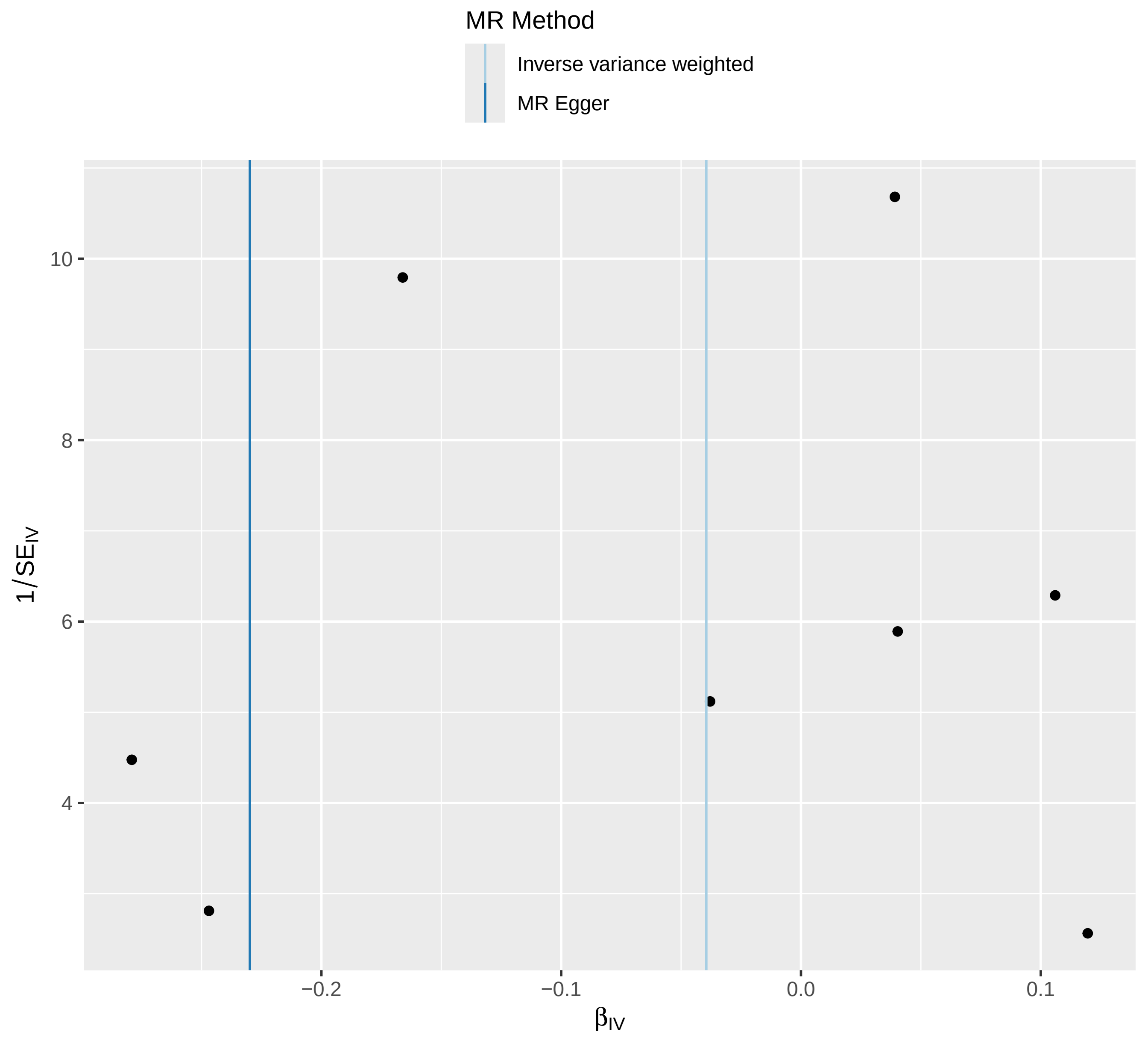

MR Method
Inverse variance weighted
MR Egger
10
8
6
4
1/SE_IV
−0.2
−0.1
0.0
0.1
β_IV

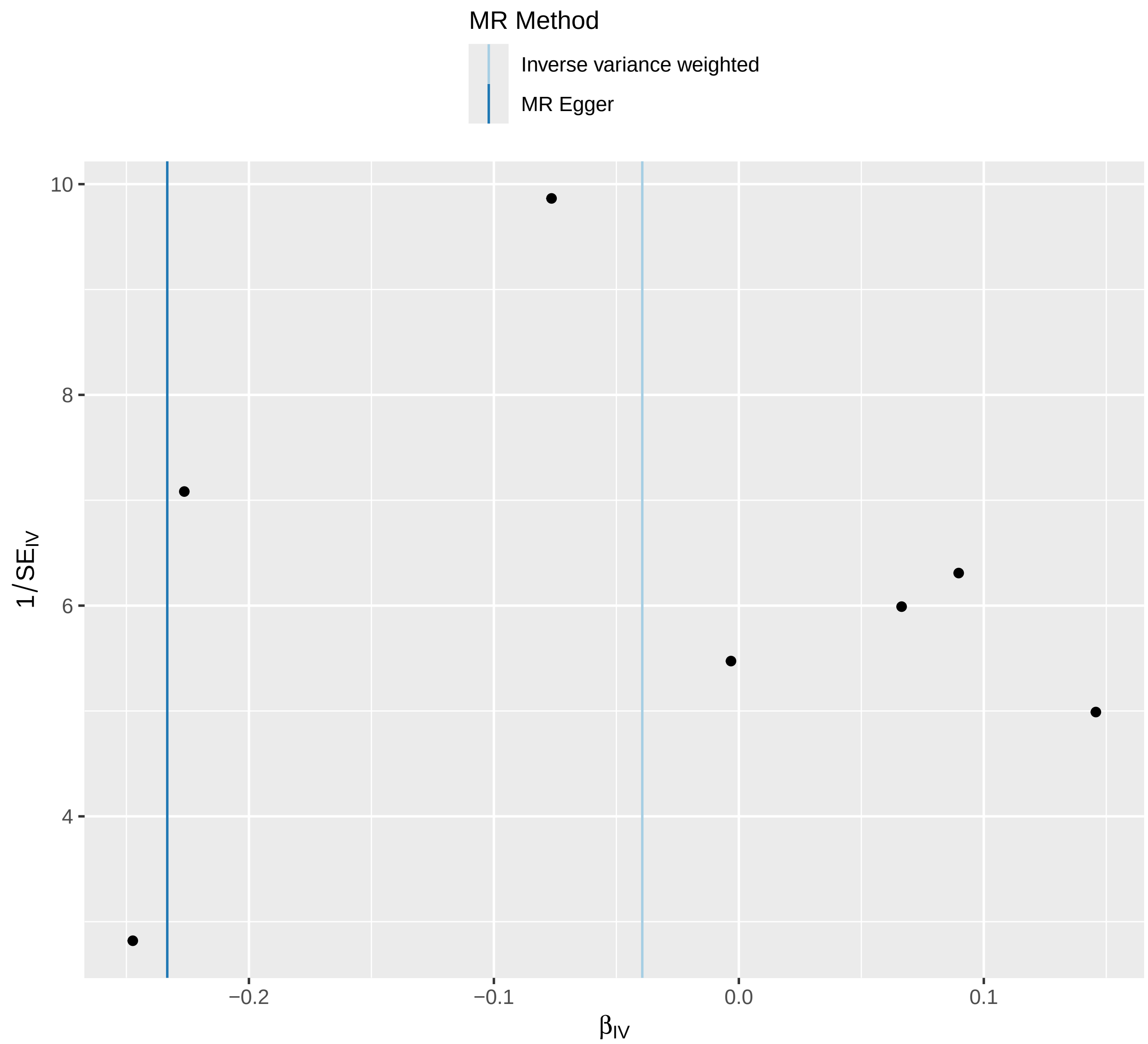

MR Method
Inverse variance weighted
MR Egger
10
8
6
4
1/SE$_{IV}$
−0.2
−0.1
0.0
0.1
β$_{IV}$

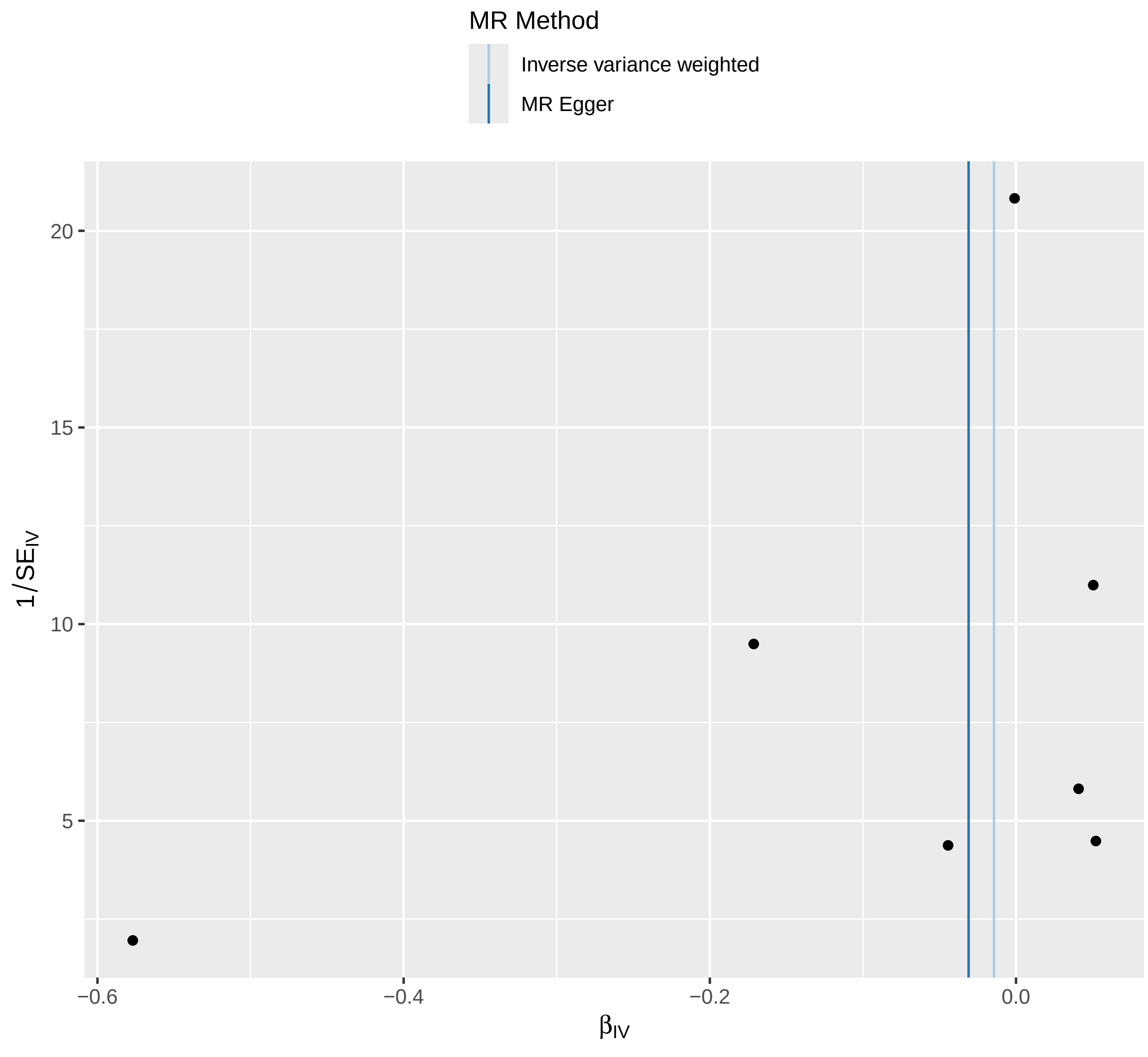
MR Method
Inverse variance weighted
MR Egger
20
15
10
5
1/SE_IV
−0.6
−0.4
−0.2
0.0
β_IV

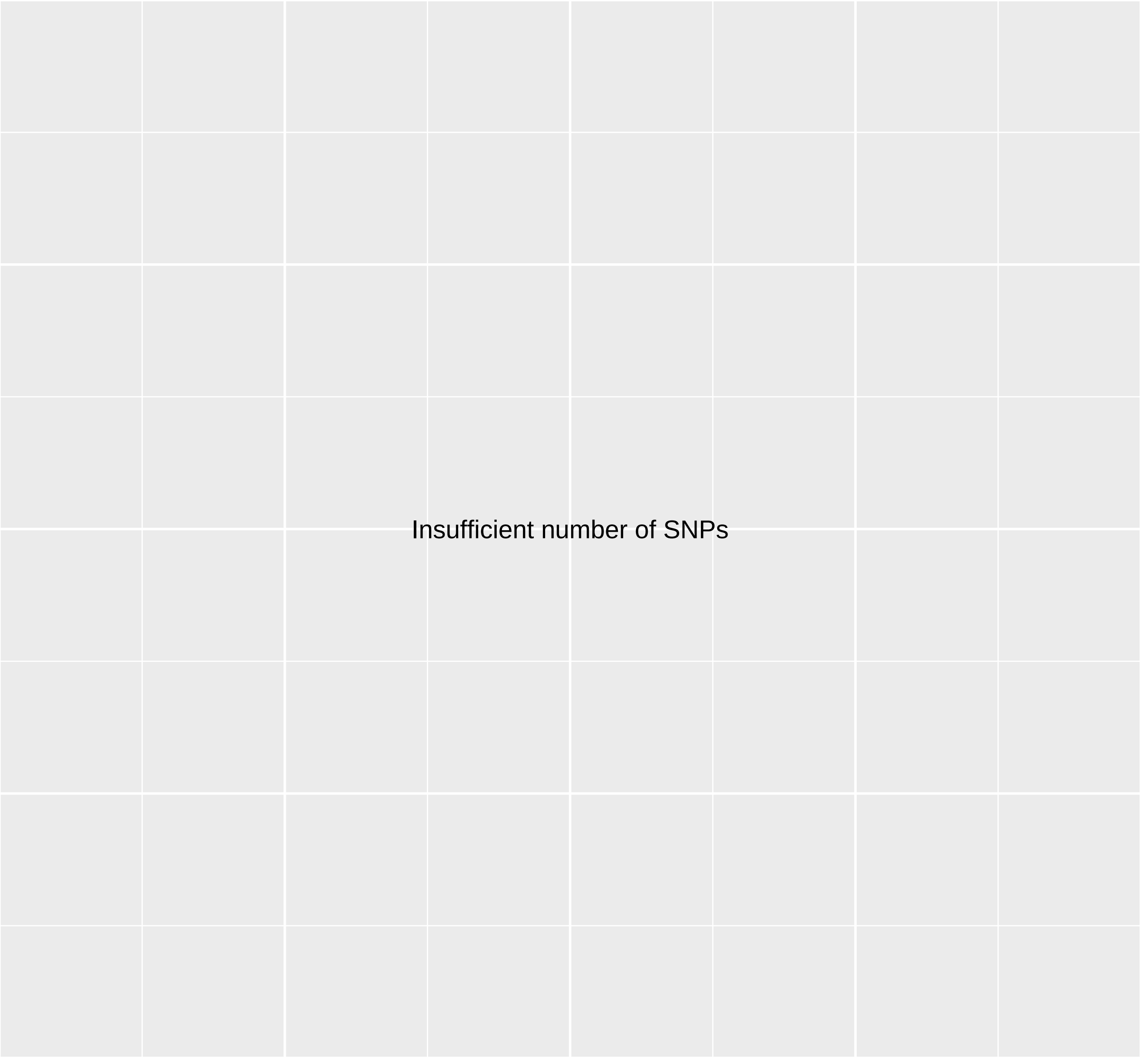
Insufficient number of SNPs

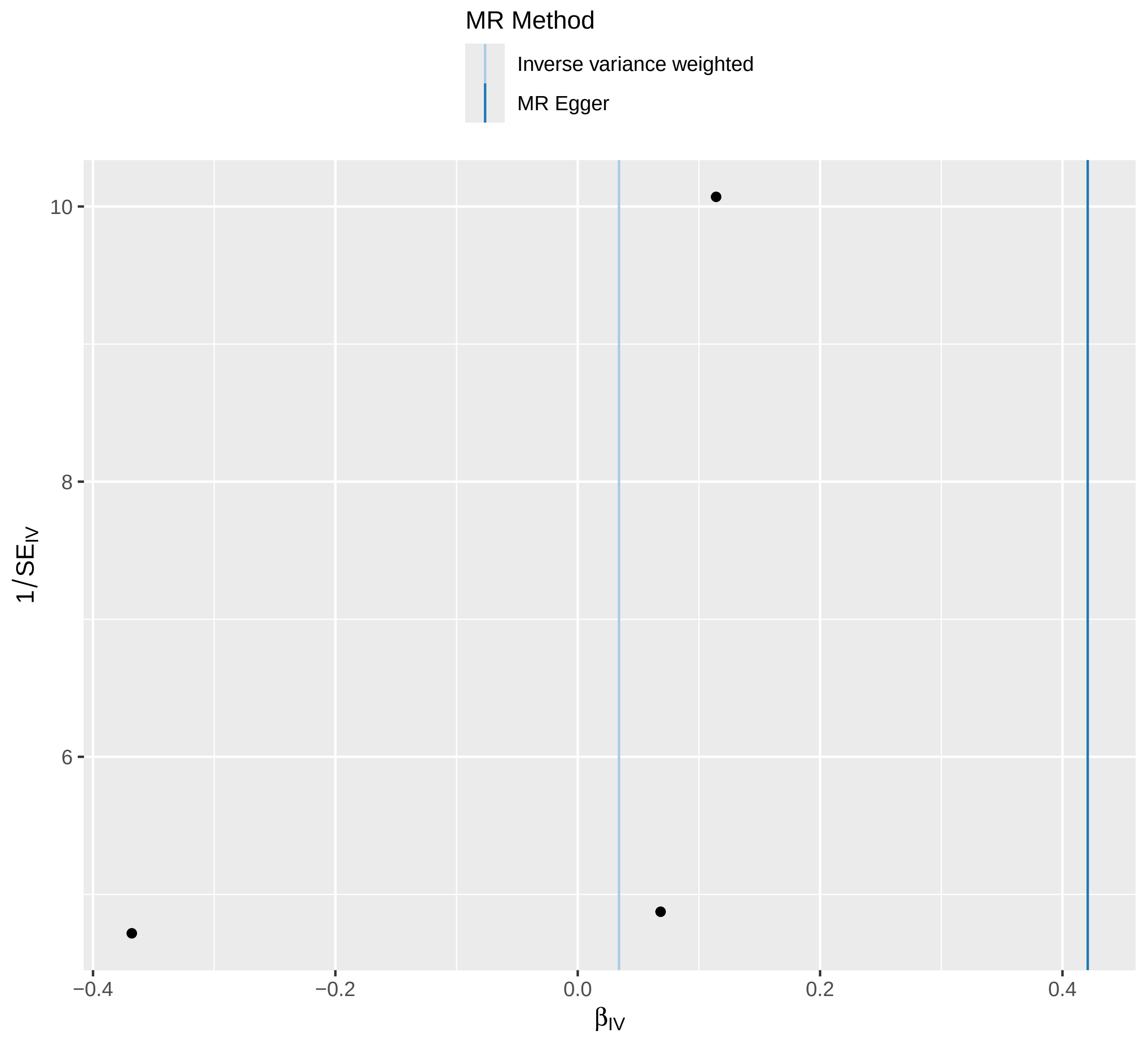
MR Method
Inverse variance weighted
MR Egger
10
8
6
$1/SE_{IV}$
−0.4
−0.2
0.0
0.2
0.4
$\beta_{IV}$

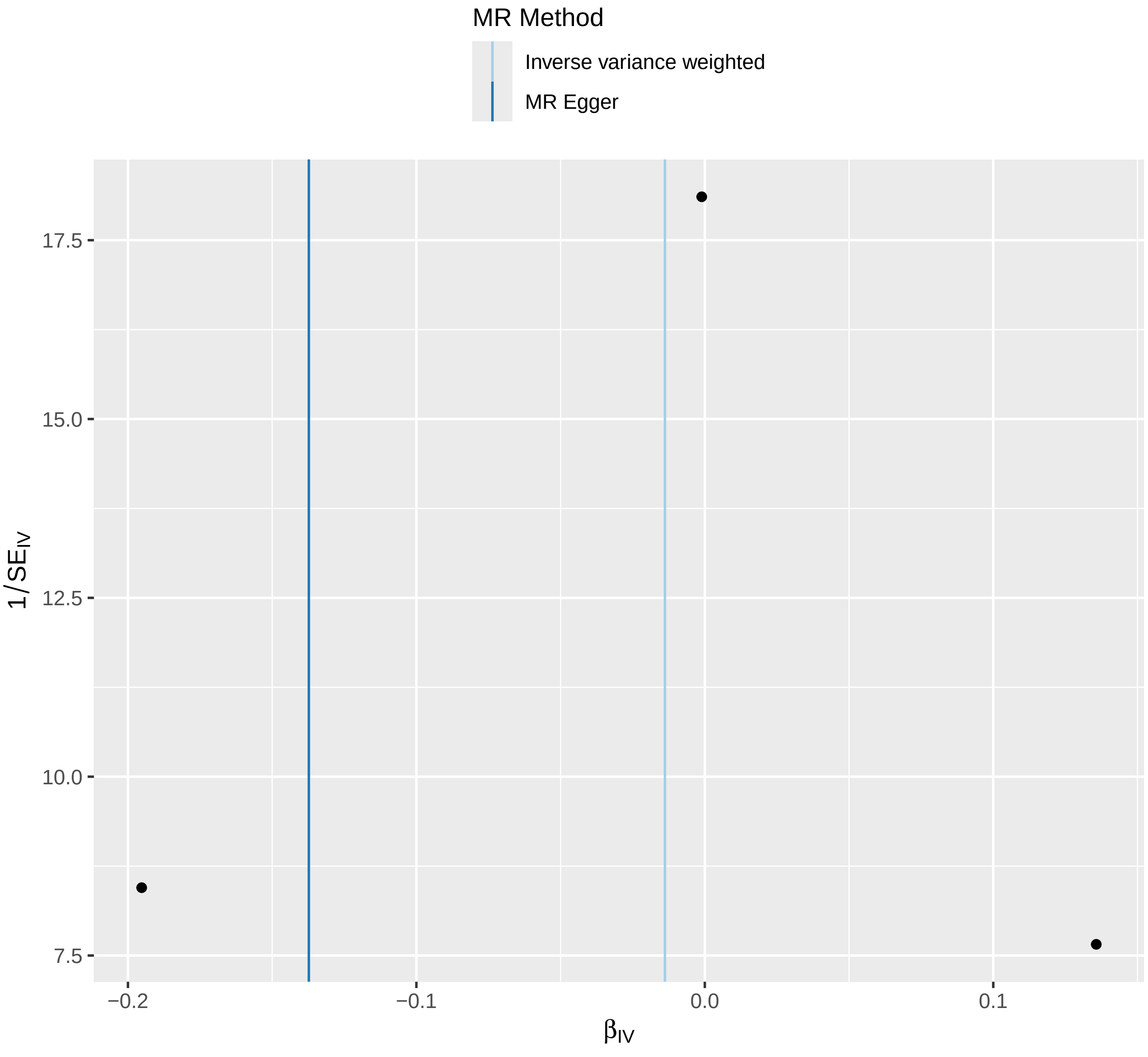
MR Method
Inverse variance weighted
MR Egger
17.5
15.0
12.5
10.0
7.5
$1/SE_{IV}$
−0.2
−0.1
0.0
0.1
$\beta_{IV}$

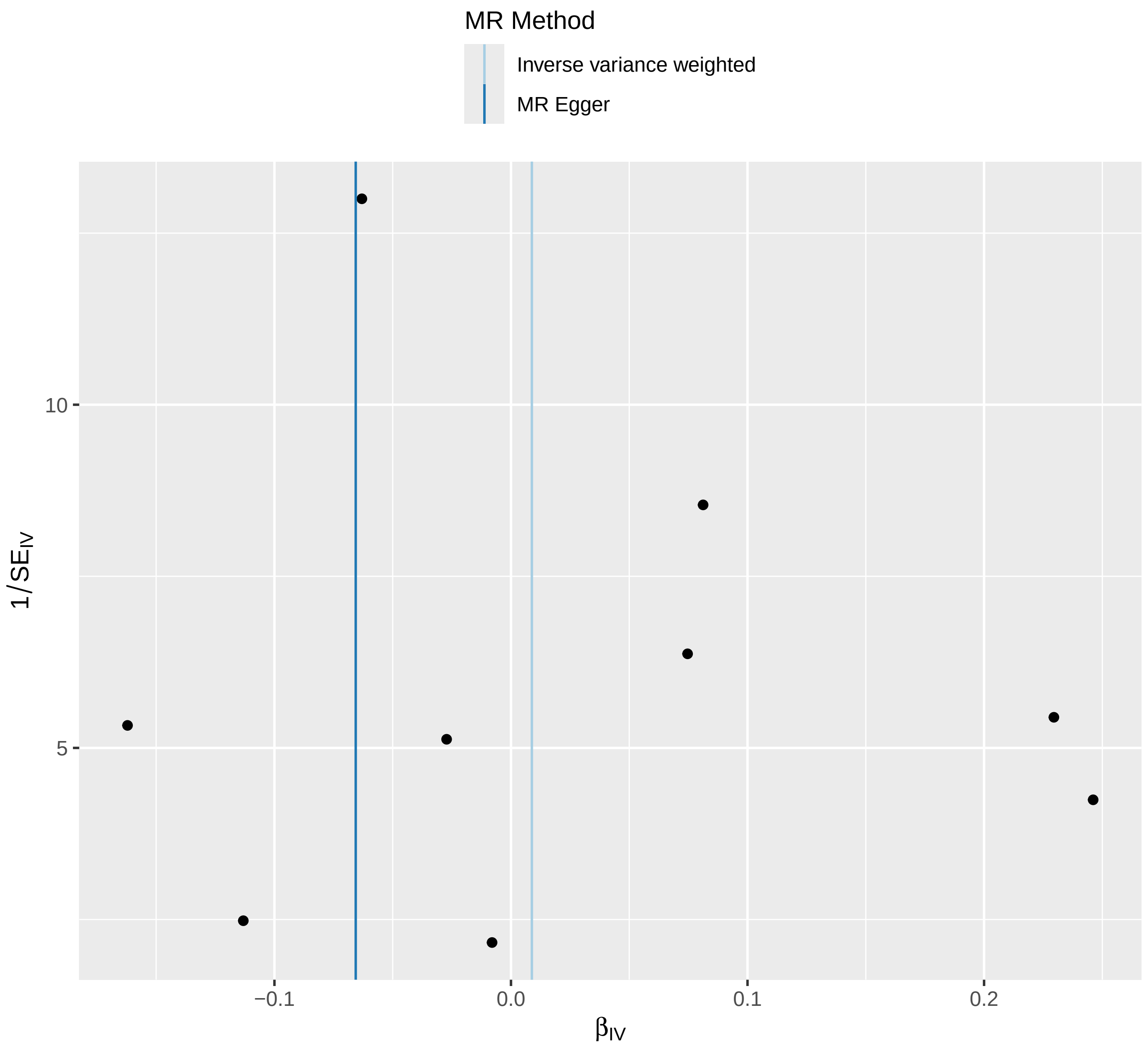

MR Method
Inverse variance weighted
MR Egger
10
5
−0.1
0.0
0.1
0.2
$\beta_{IV}$
$1/SE_{IV}$

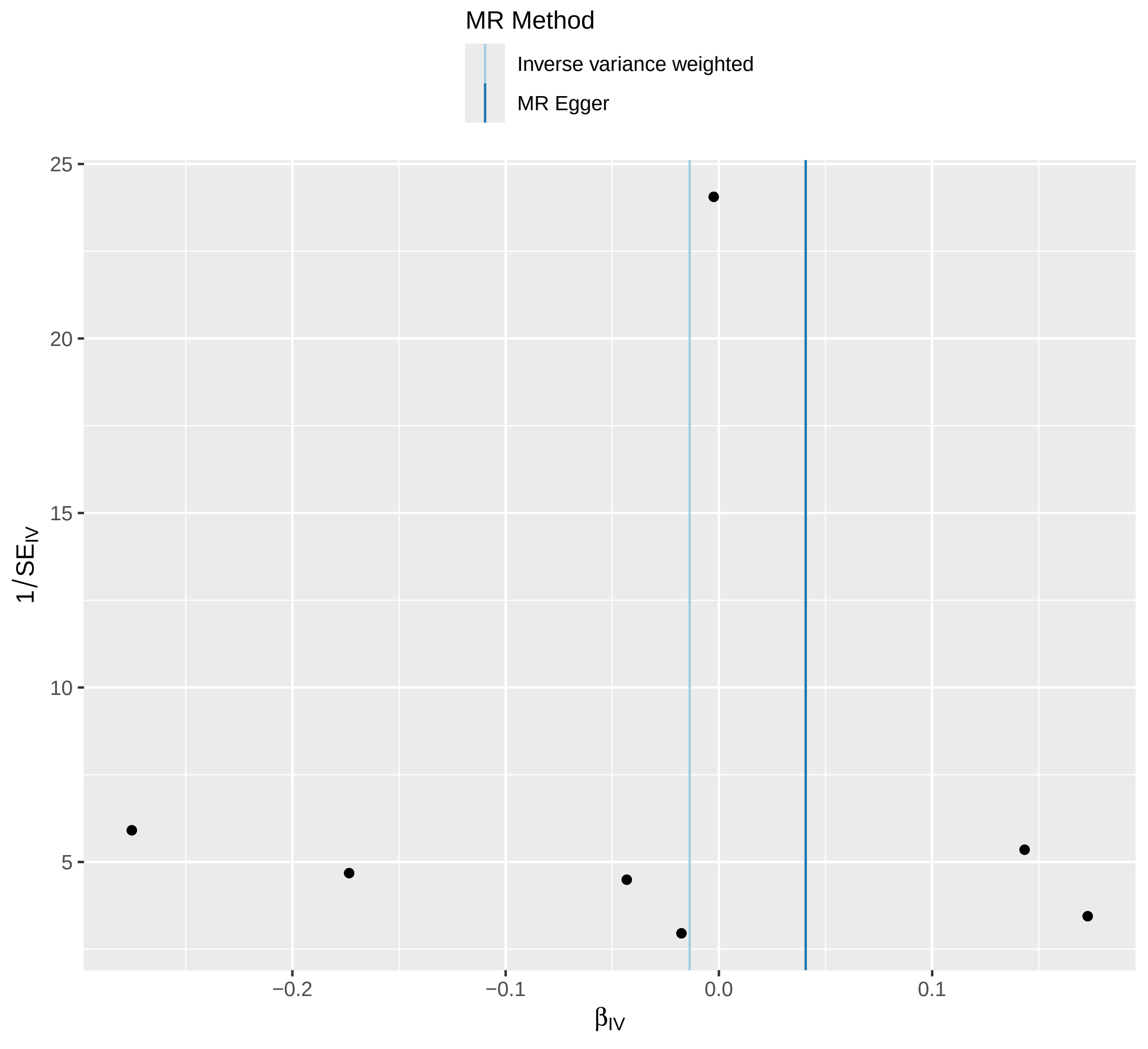
MR Method
Inverse variance weighted
MR Egger
25
20
15
10
5
1/SE$_{IV}$
−0.2
−0.1
0.0
0.1
β$_{IV}$

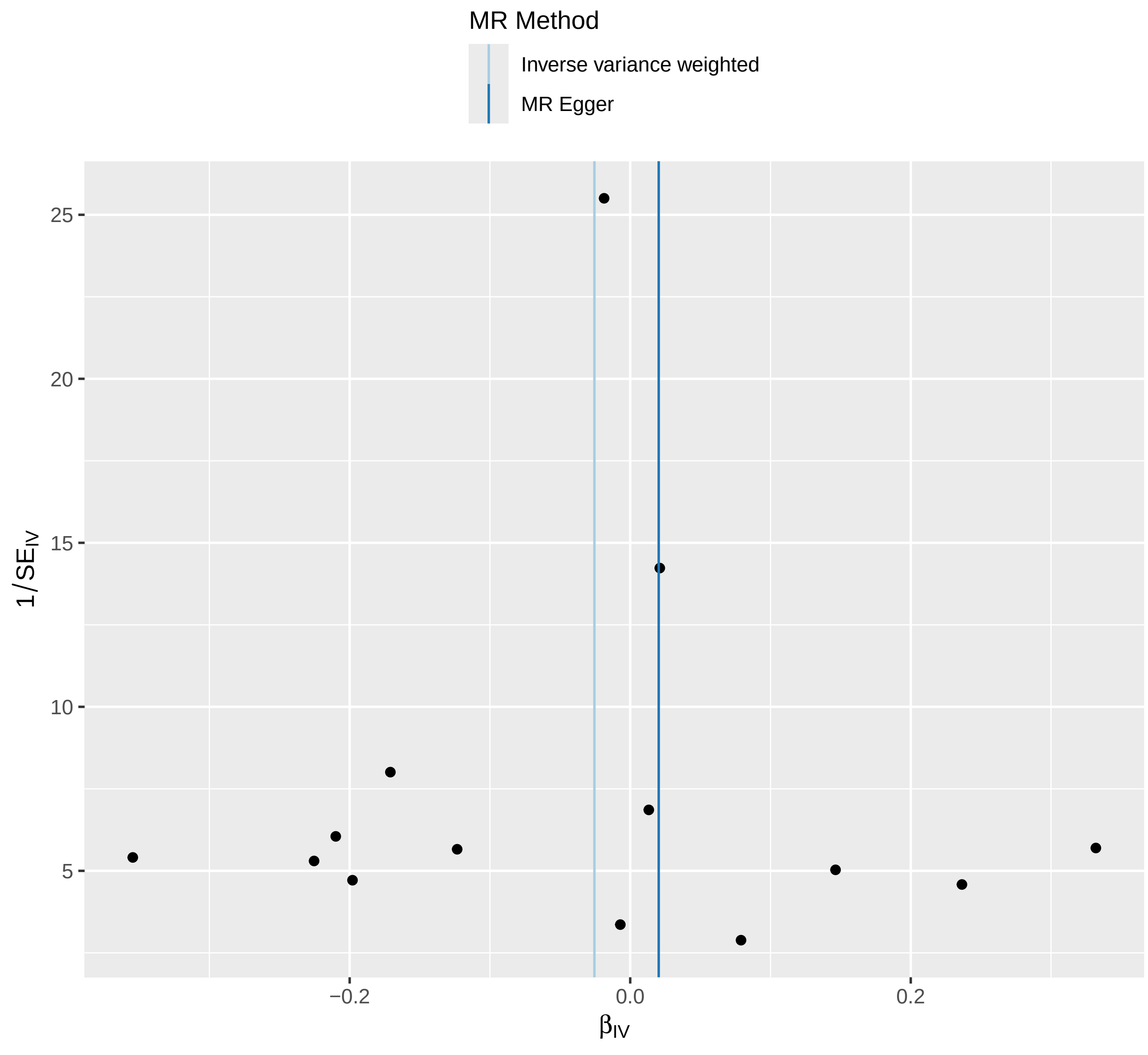
MR Method
Inverse variance weighted
MR Egger
25
20
15
10
5
1/SE$_{IV}$
−0.2
0.0
0.2
β$_{IV}$

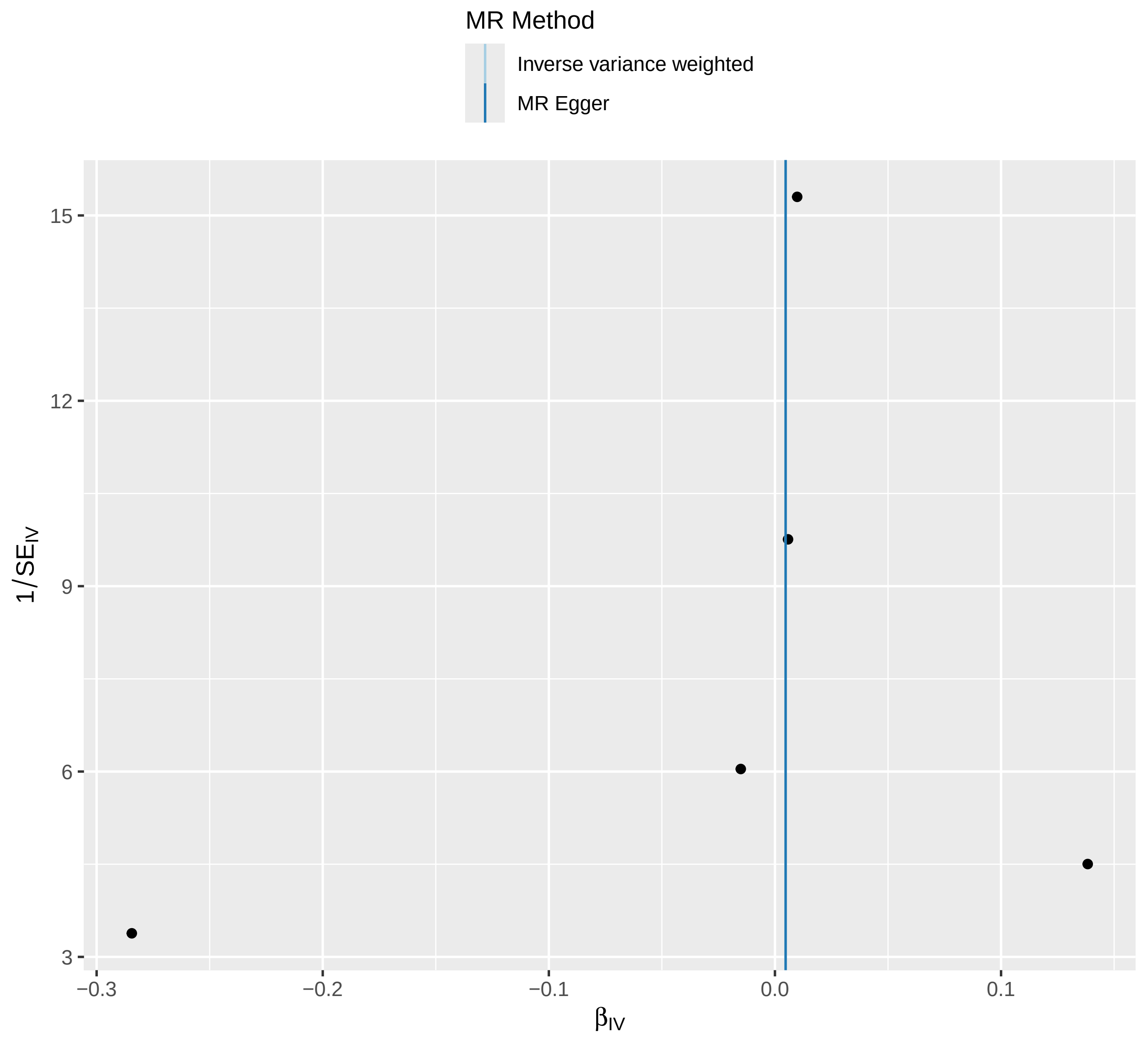
MR Method
Inverse variance weighted
MR Egger
1/SE$_{IV}$
15
12
9
6
3
−0.3
−0.2
−0.1
0.0
0.1
β$_{IV}$

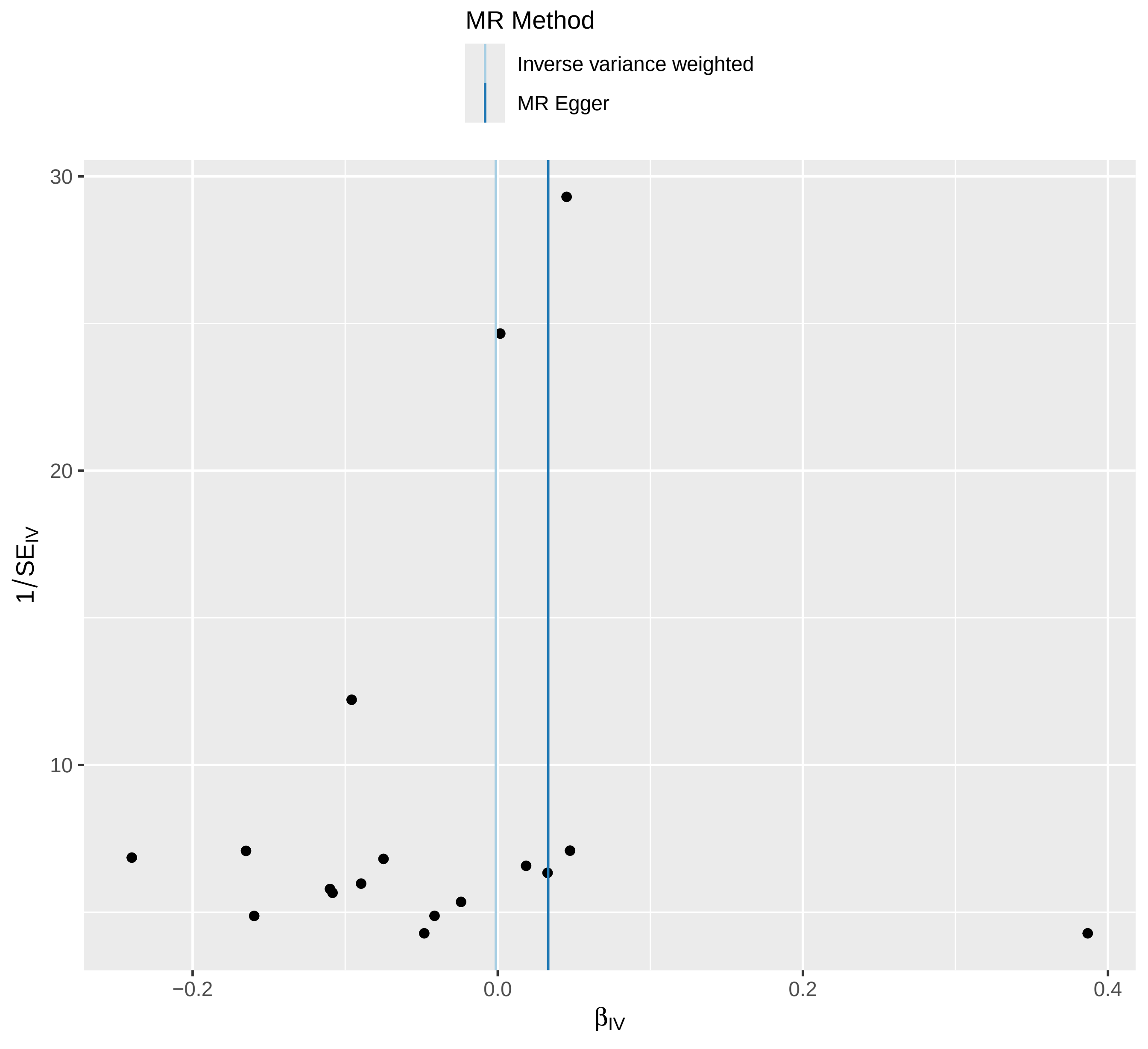
MR Method
Inverse variance weighted
MR Egger
30
20
10
1/SE_IV
−0.2
0.0
0.2
0.4
β_IV

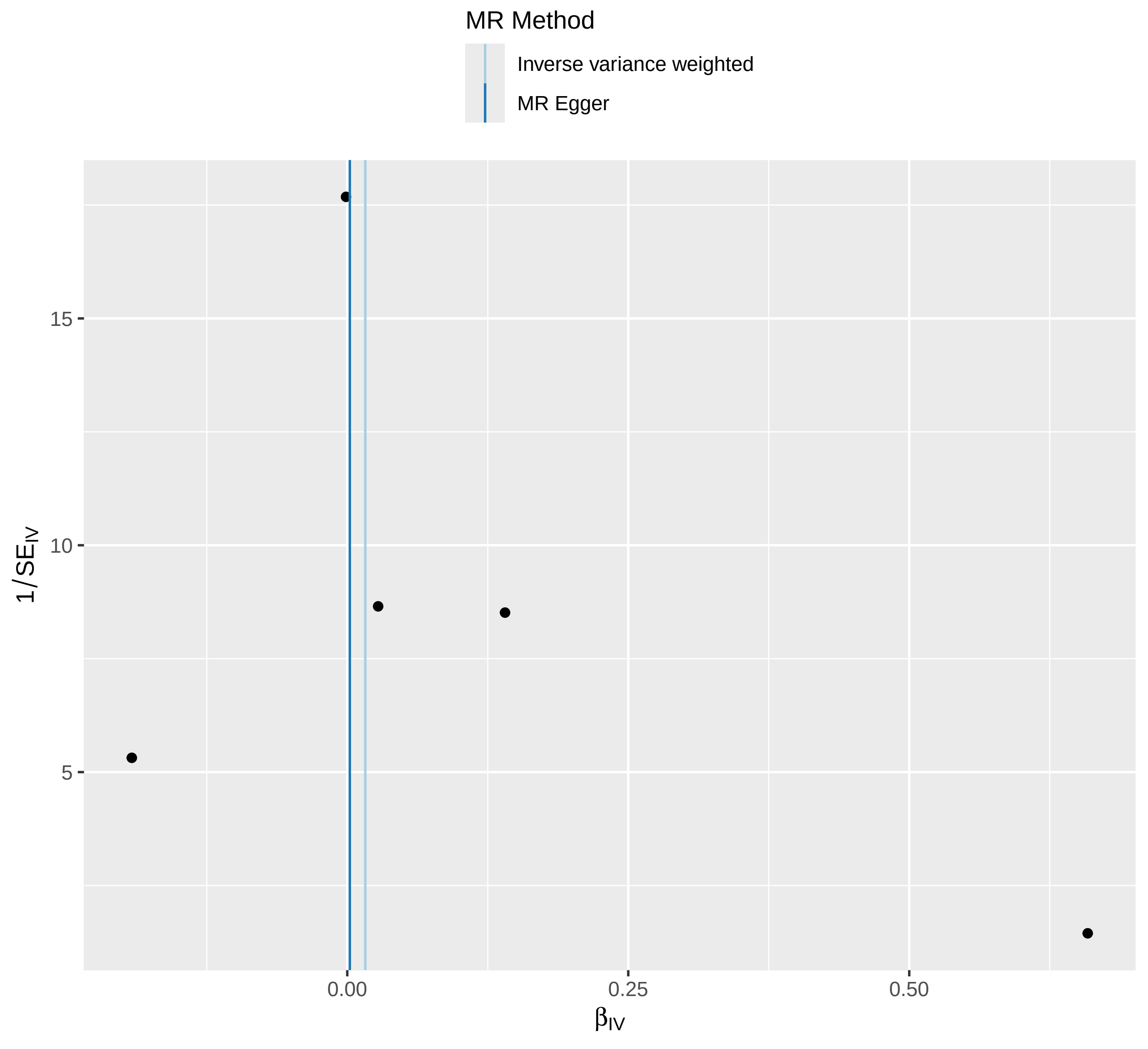
MR Method
Inverse variance weighted
MR Egger
15
10
5
1/SE$_{IV}$
0.00
0.25
0.50
β$_{IV}$

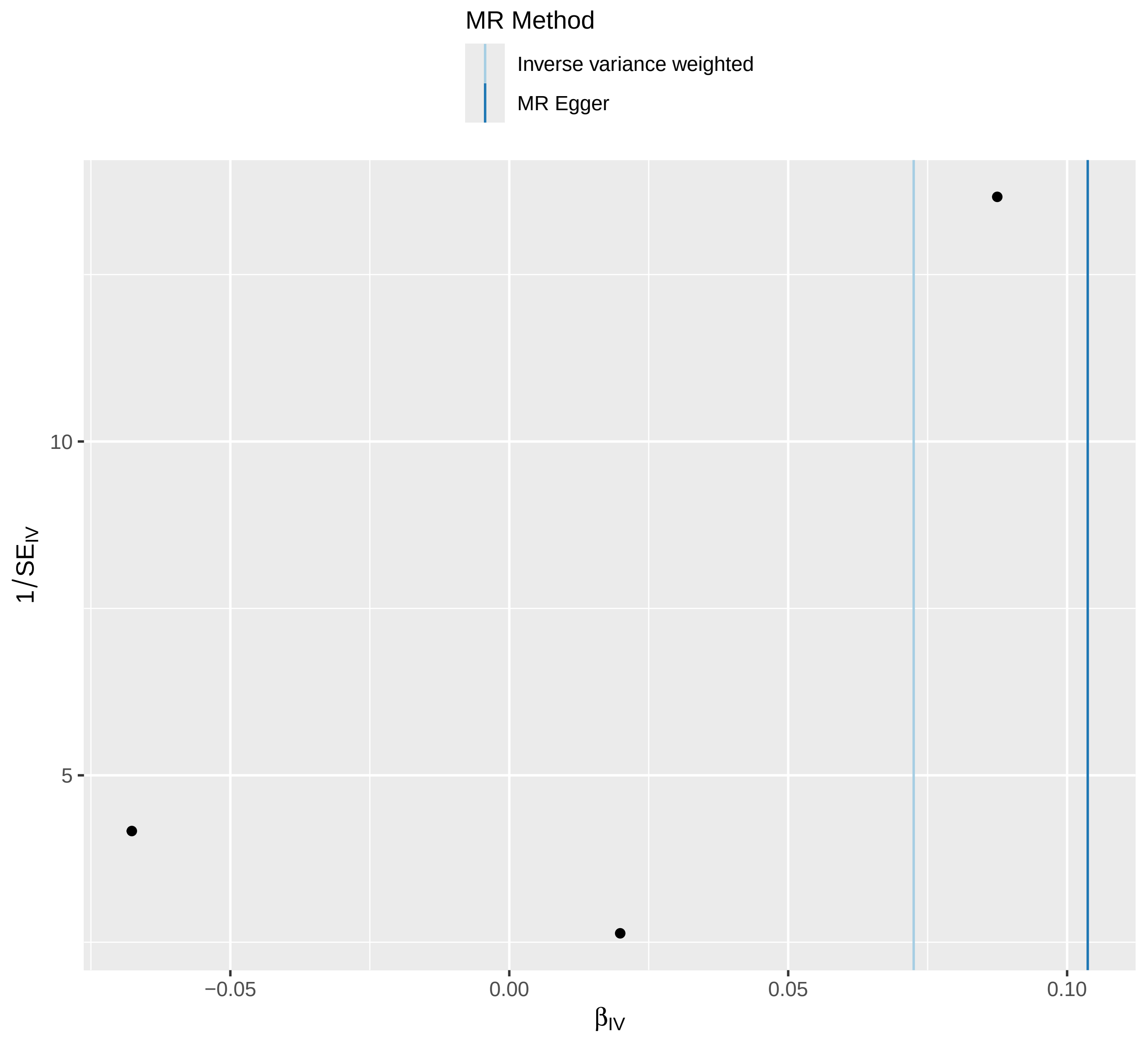
MR Method
Inverse variance weighted
MR Egger
10
5
$1/SE_{IV}$
−0.05
0.00
0.05
0.10
$\beta_{IV}$

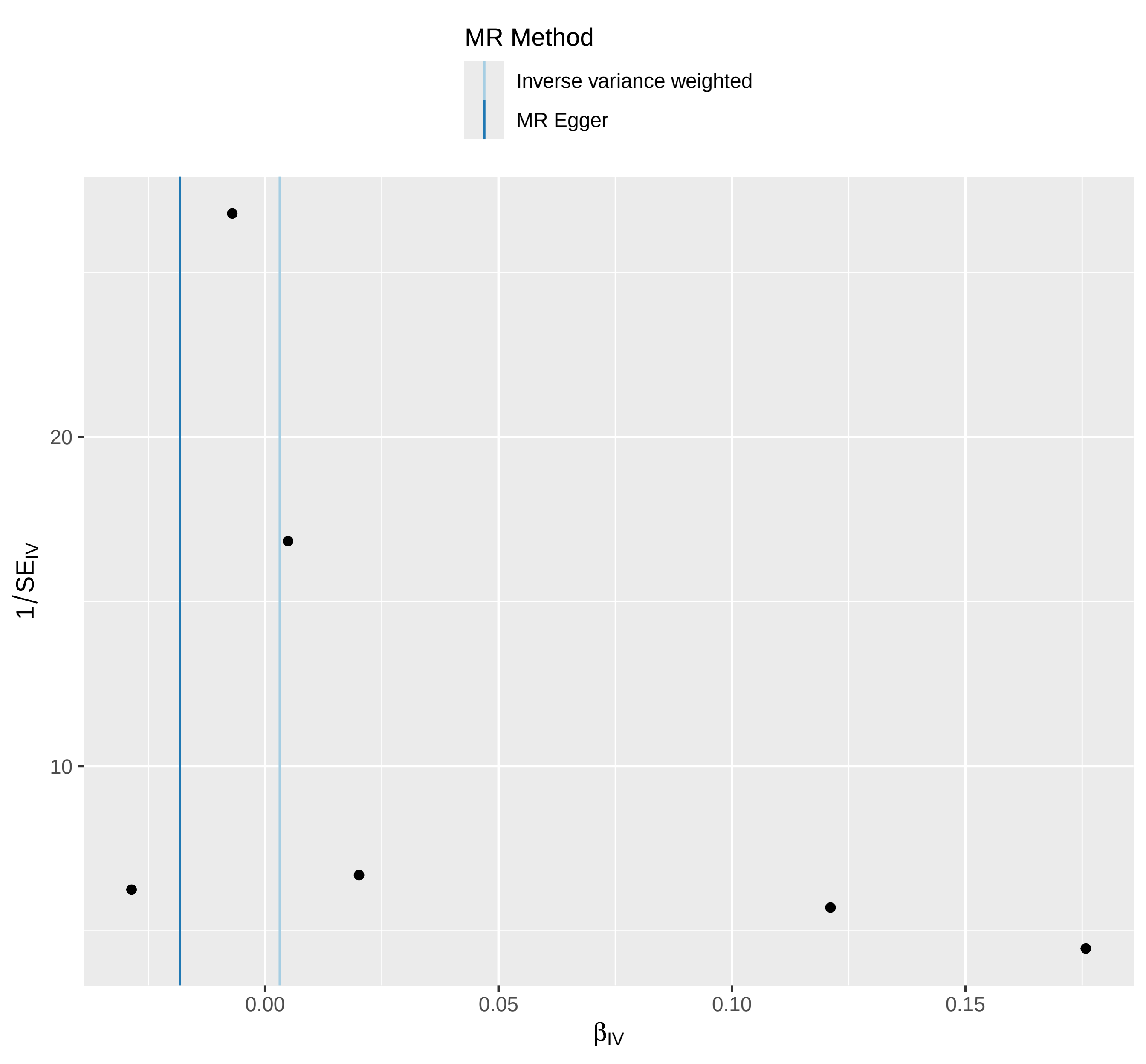
MR Method
Inverse variance weighted
MR Egger
20
10
$1/SE_{IV}$
0.00
0.05
0.10
0.15
$\beta_{IV}$

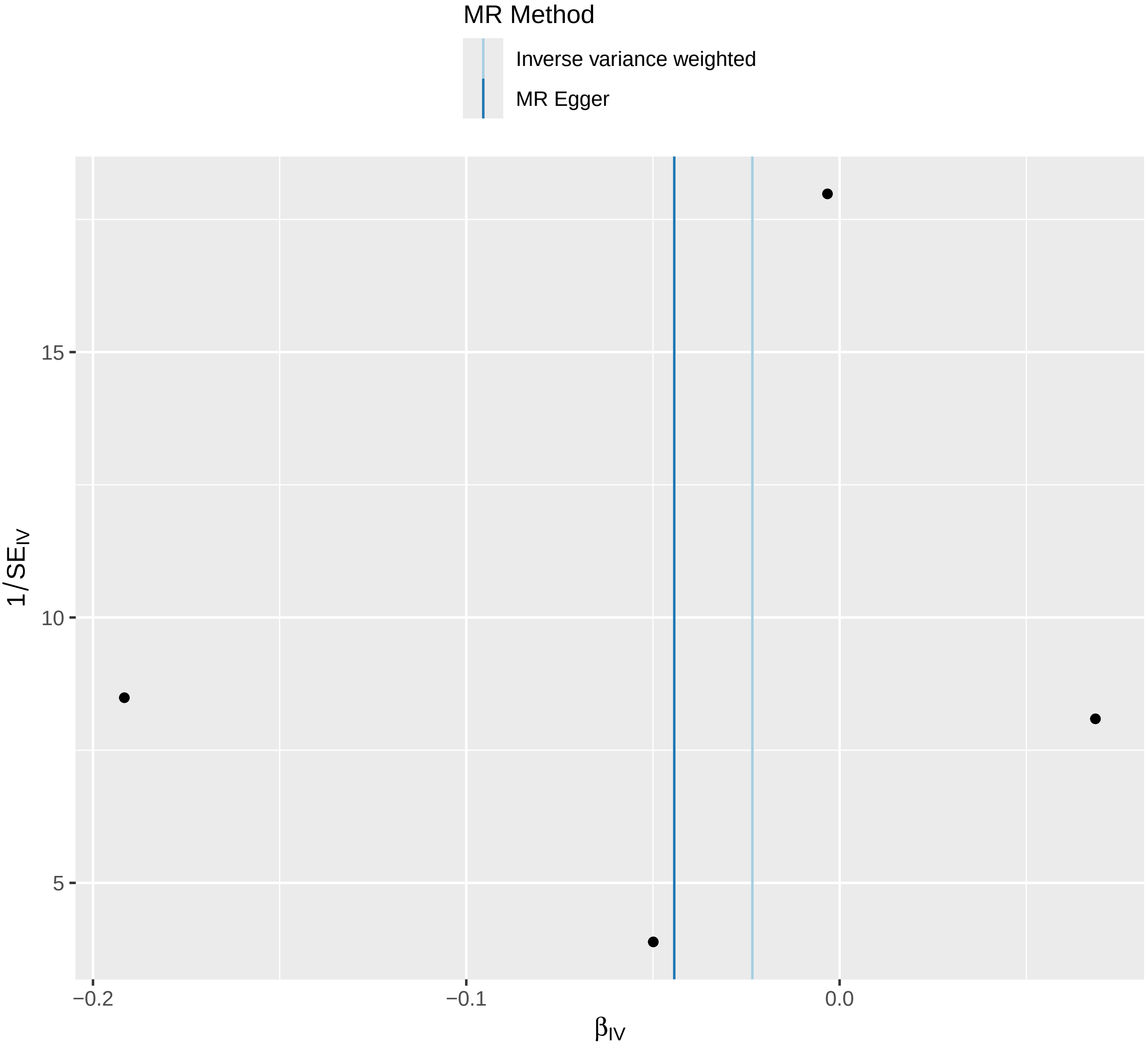

MR Method
Inverse variance weighted
MR Egger
15
10
5
1/SE$_{IV}$
−0.2
−0.1
0.0
β$_{IV}$

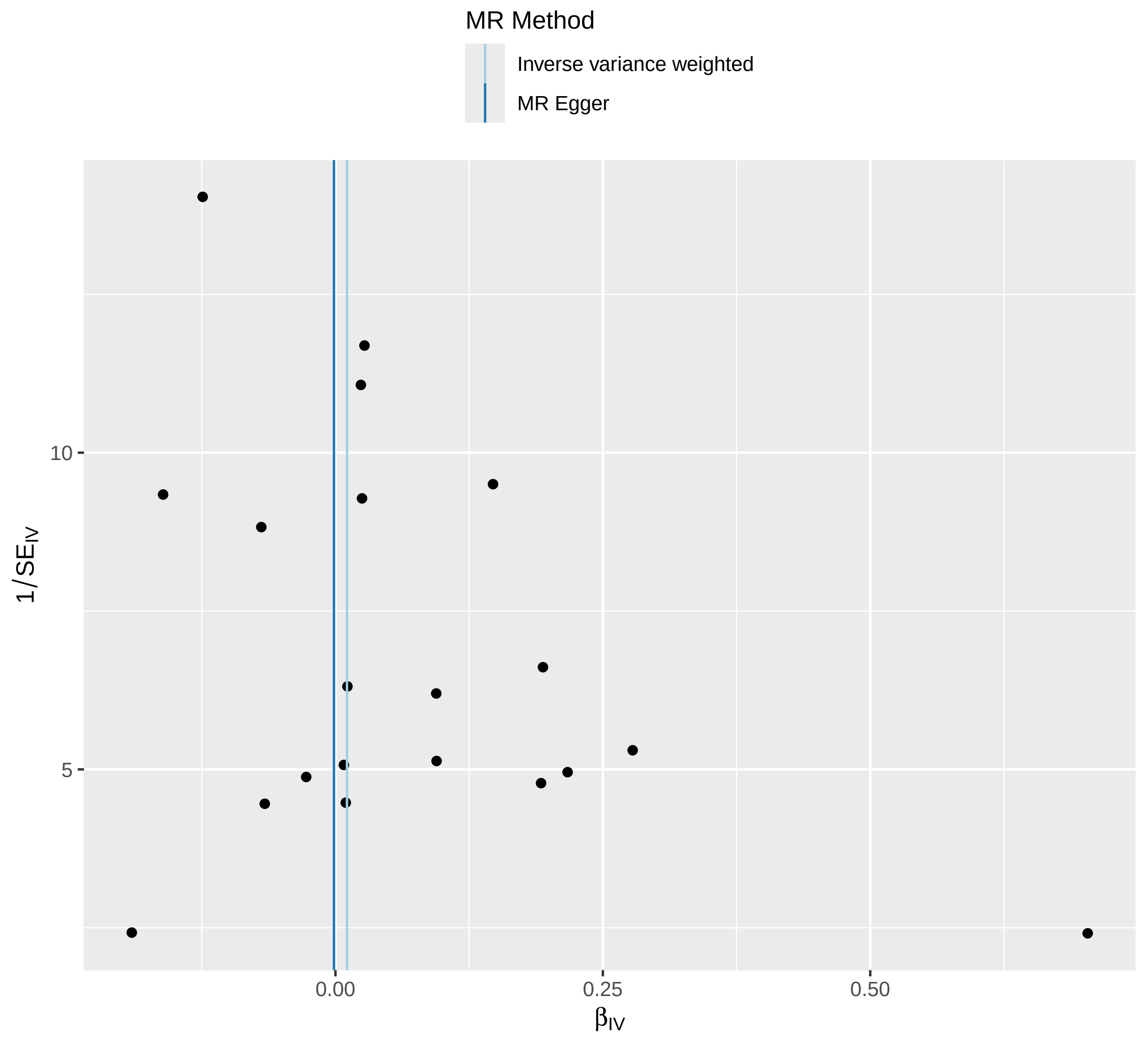
MR Method
Inverse variance weighted
MR Egger
10
5
1/SE_IV
0.00
0.25
0.50
β_IV

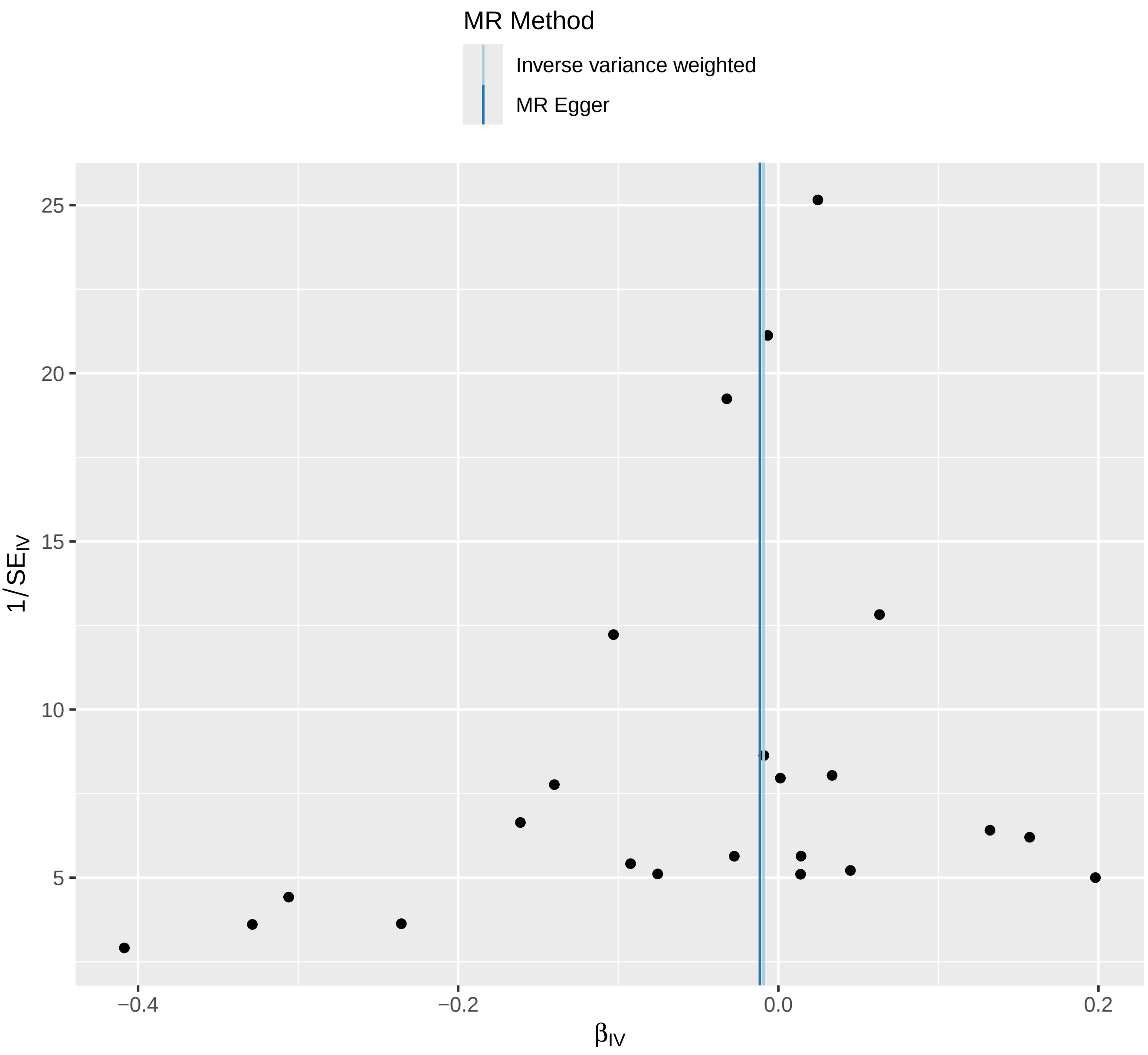
MR Method
Inverse variance weighted
MR Egger
25
20
15
10
5
1/SE_IV
−0.4
−0.2
0.0
0.2
β_IV

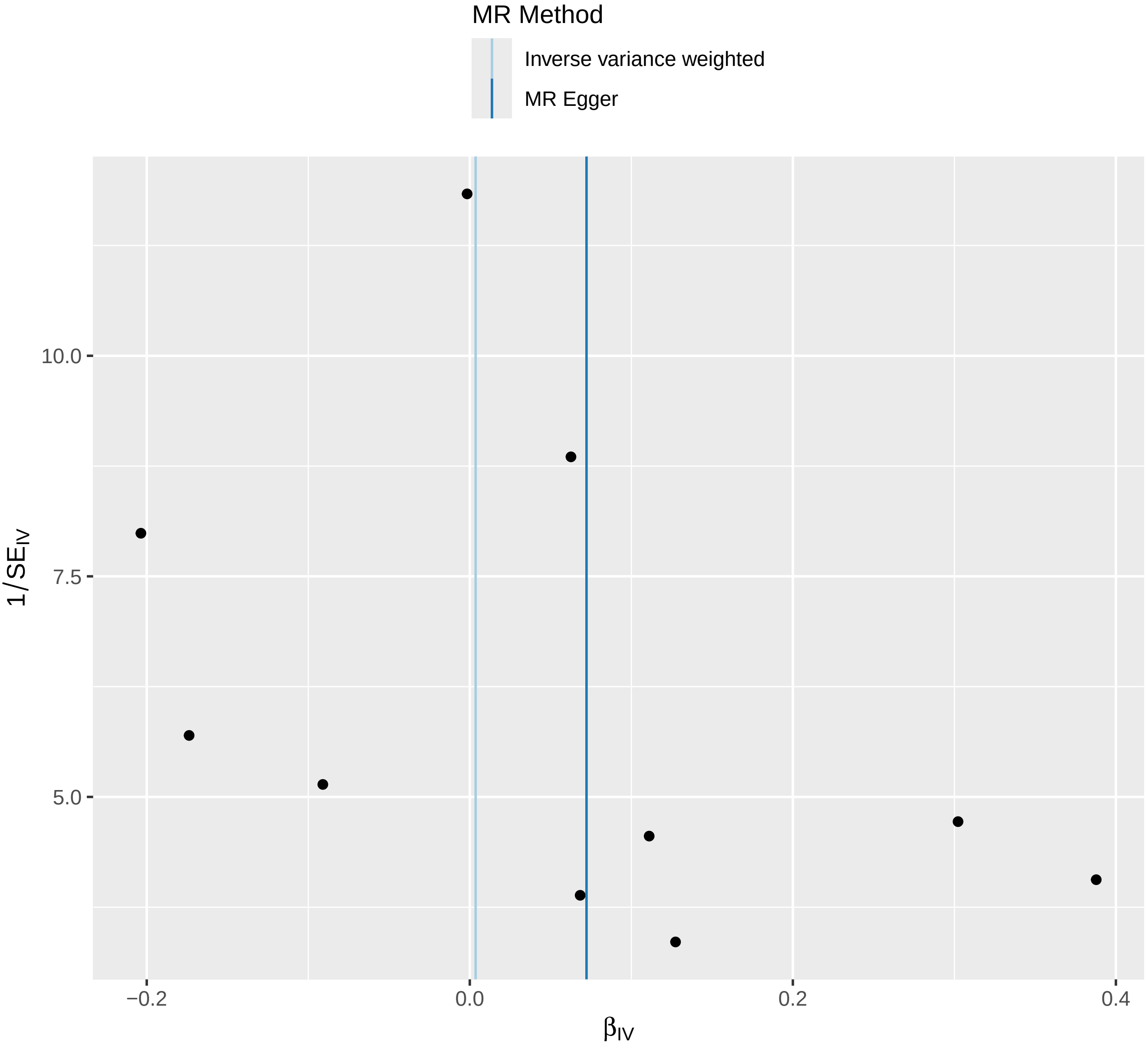

MR Method
Inverse variance weighted
MR Egger
10.0
7.5
5.0
1/SE_IV
−0.2
0.0
0.2
0.4
β_IV

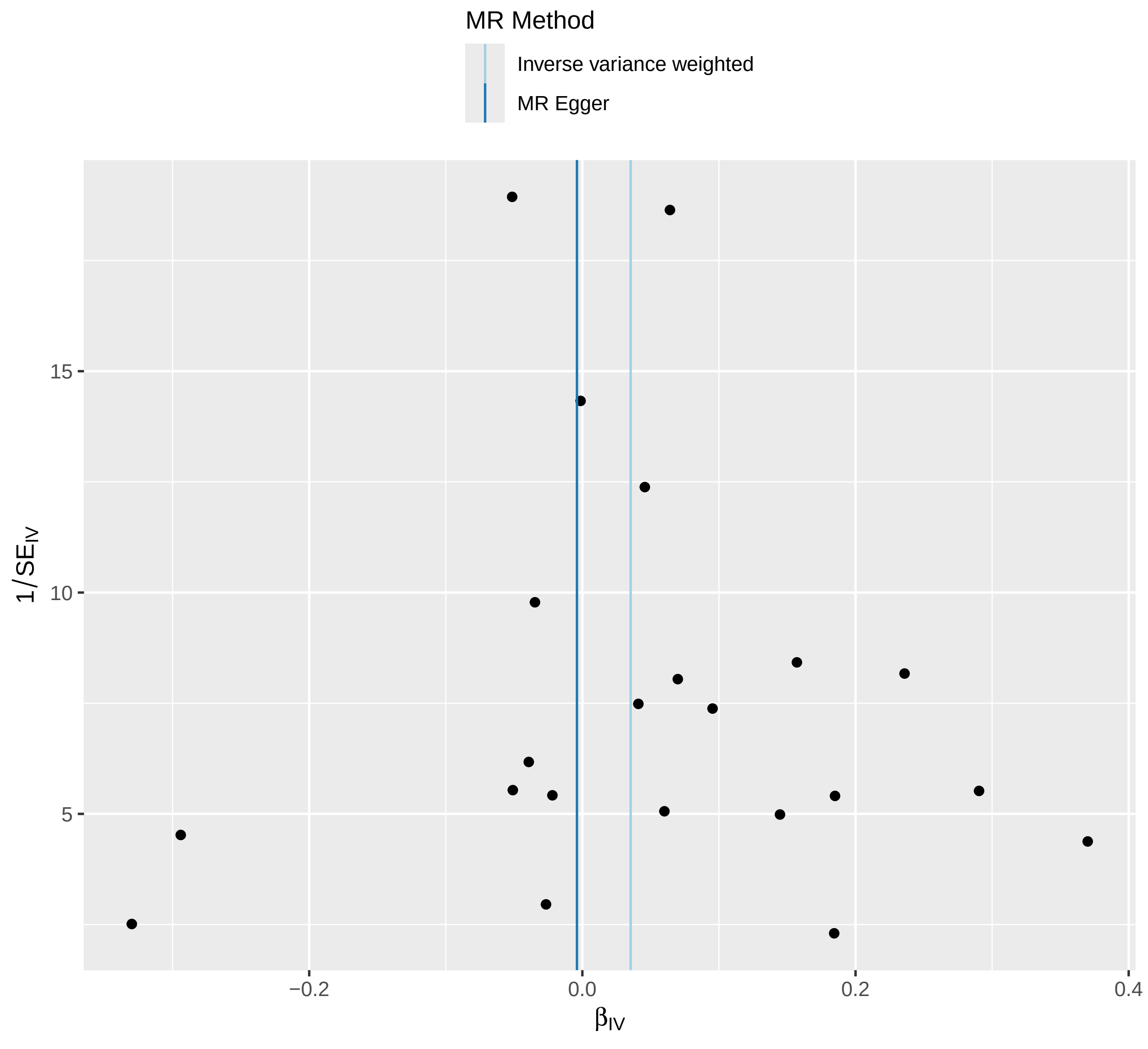

MR Method
Inverse variance weighted
MR Egger
15
10
5
1/SE$_{IV}$
−0.2
0.0
0.2
0.4
β$_{IV}$

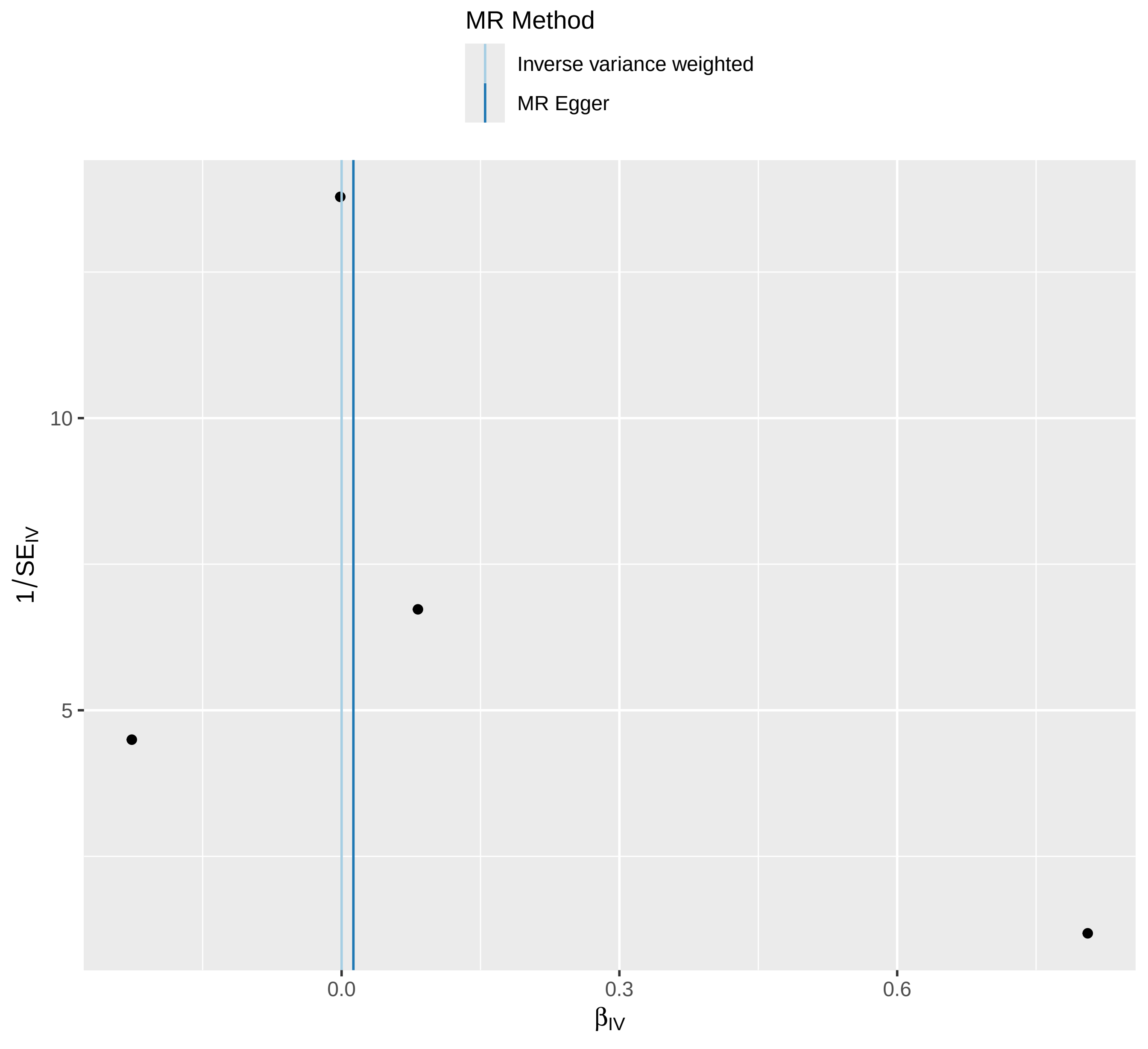

MR Method
Inverse variance weighted
MR Egger
10
5
$1/SE_{IV}$
0.0
0.3
0.6
$\beta_{IV}$

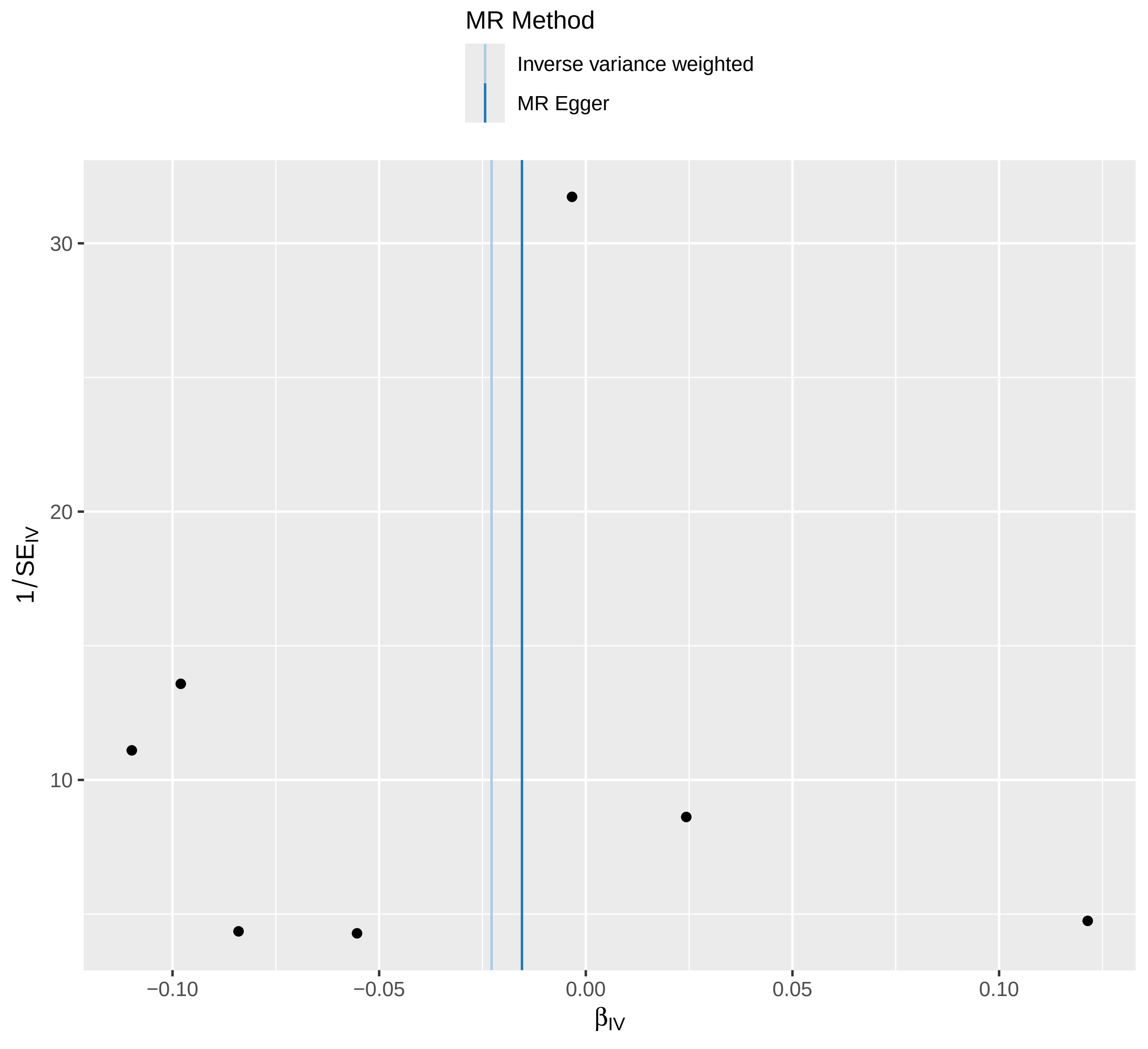

MR Method
Inverse variance weighted
MR Egger
30
20
10
$1/SE_{IV}$
−0.10
−0.05
0.00
0.05
0.10
$\beta_{IV}$

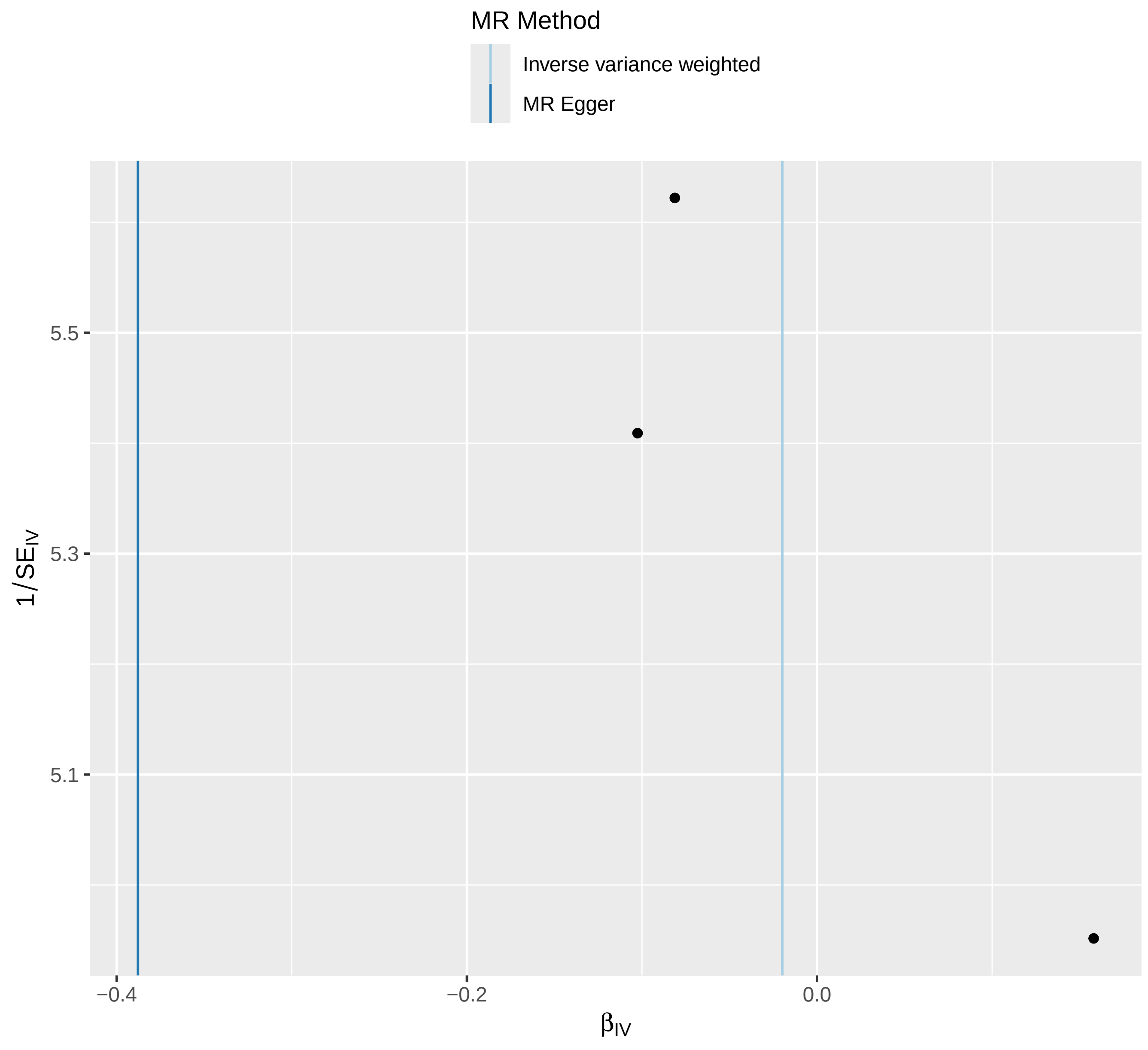
MR Method
Inverse variance weighted
MR Egger
5.5
5.3
5.1
1/SE_IV
−0.4
−0.2
0.0
β_IV

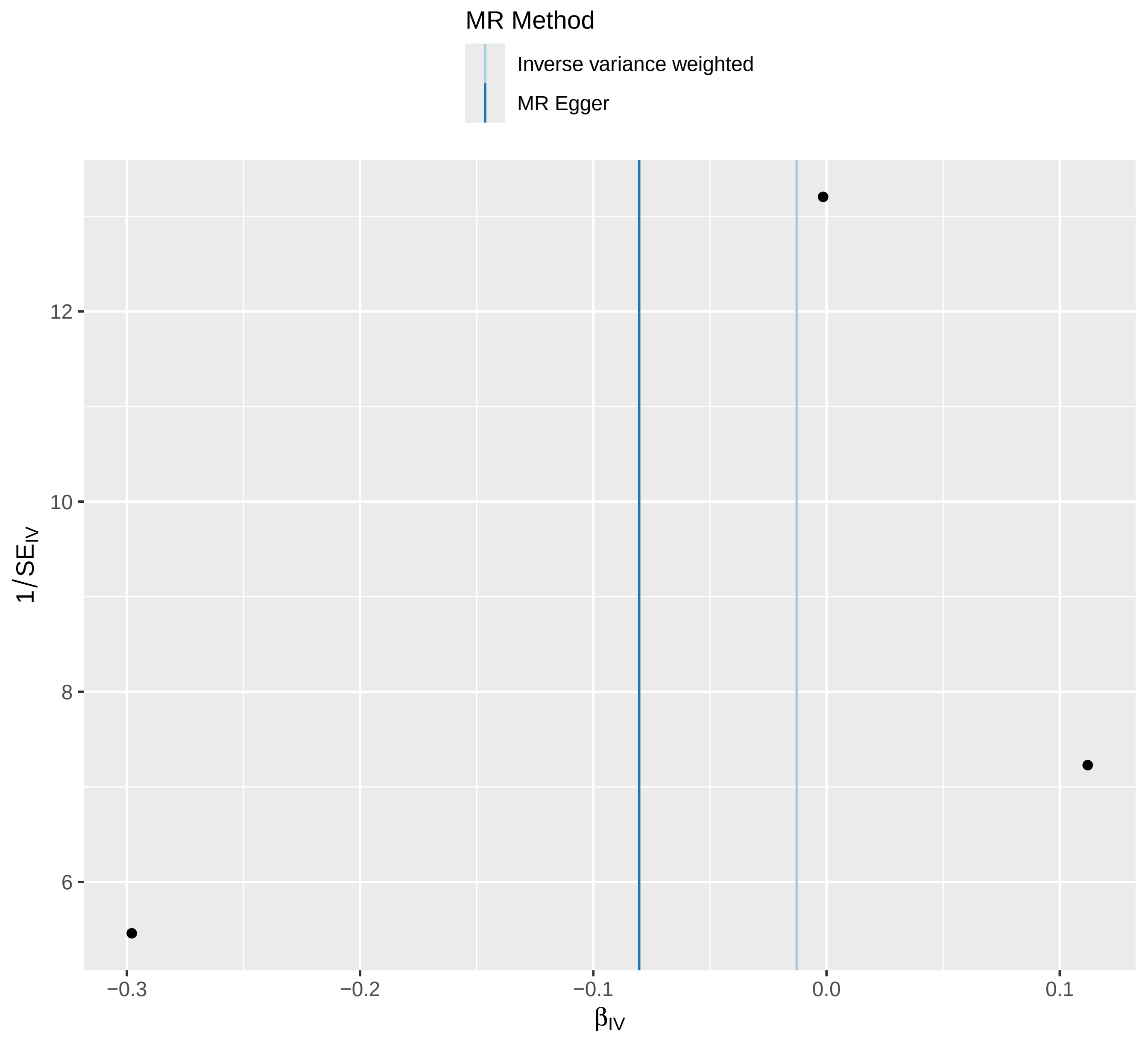
MR Method
Inverse variance weighted
MR Egger
12
10
8
6
−0.3
−0.2
−0.1
0.0
0.1
$1/SE_{IV}$
$\beta_{IV}$

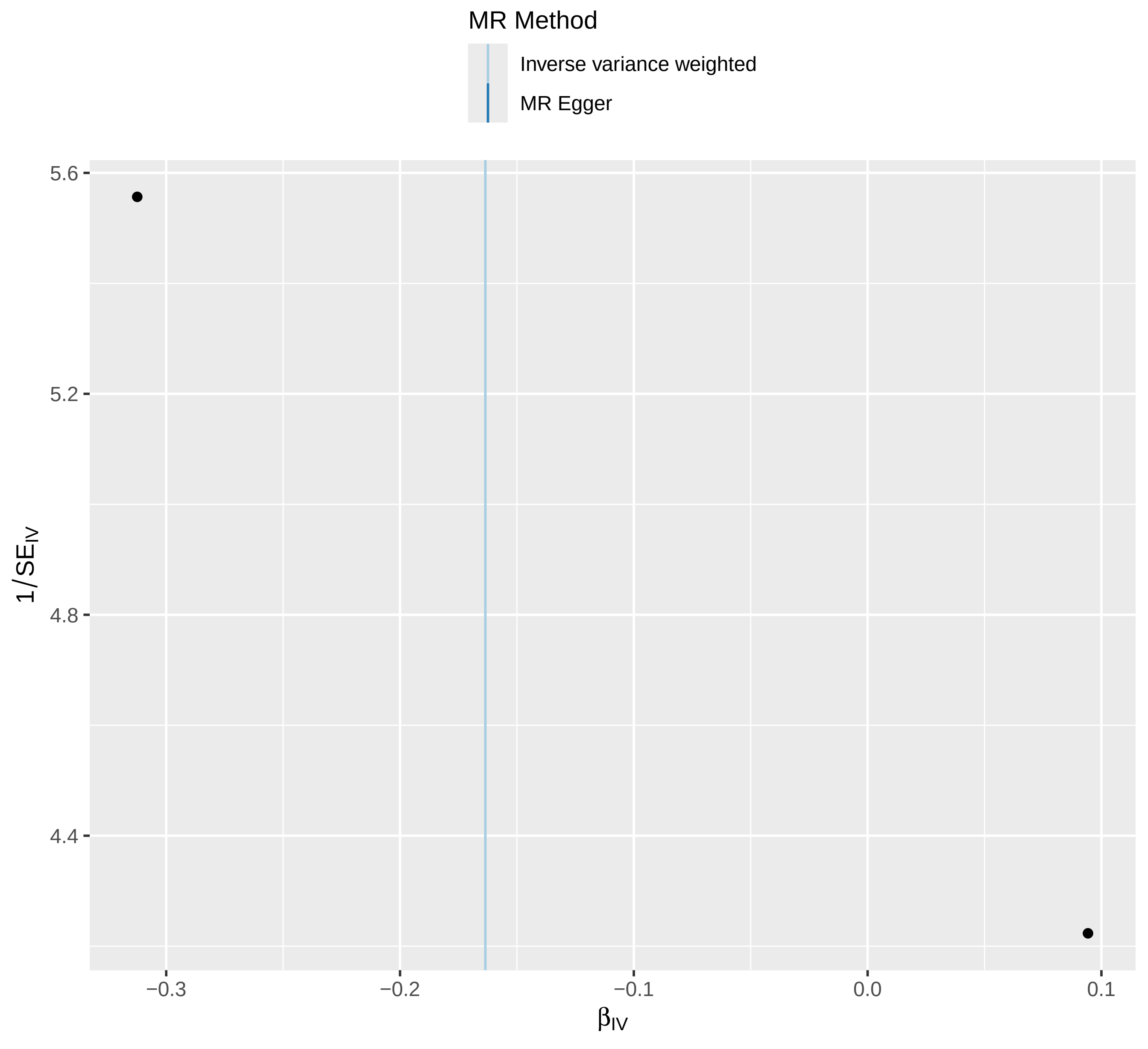

MR Method
Inverse variance weighted
MR Egger
5.6
5.2
4.8
4.4
1/SE_IV
−0.3
−0.2
−0.1
0.0
0.1
β_IV

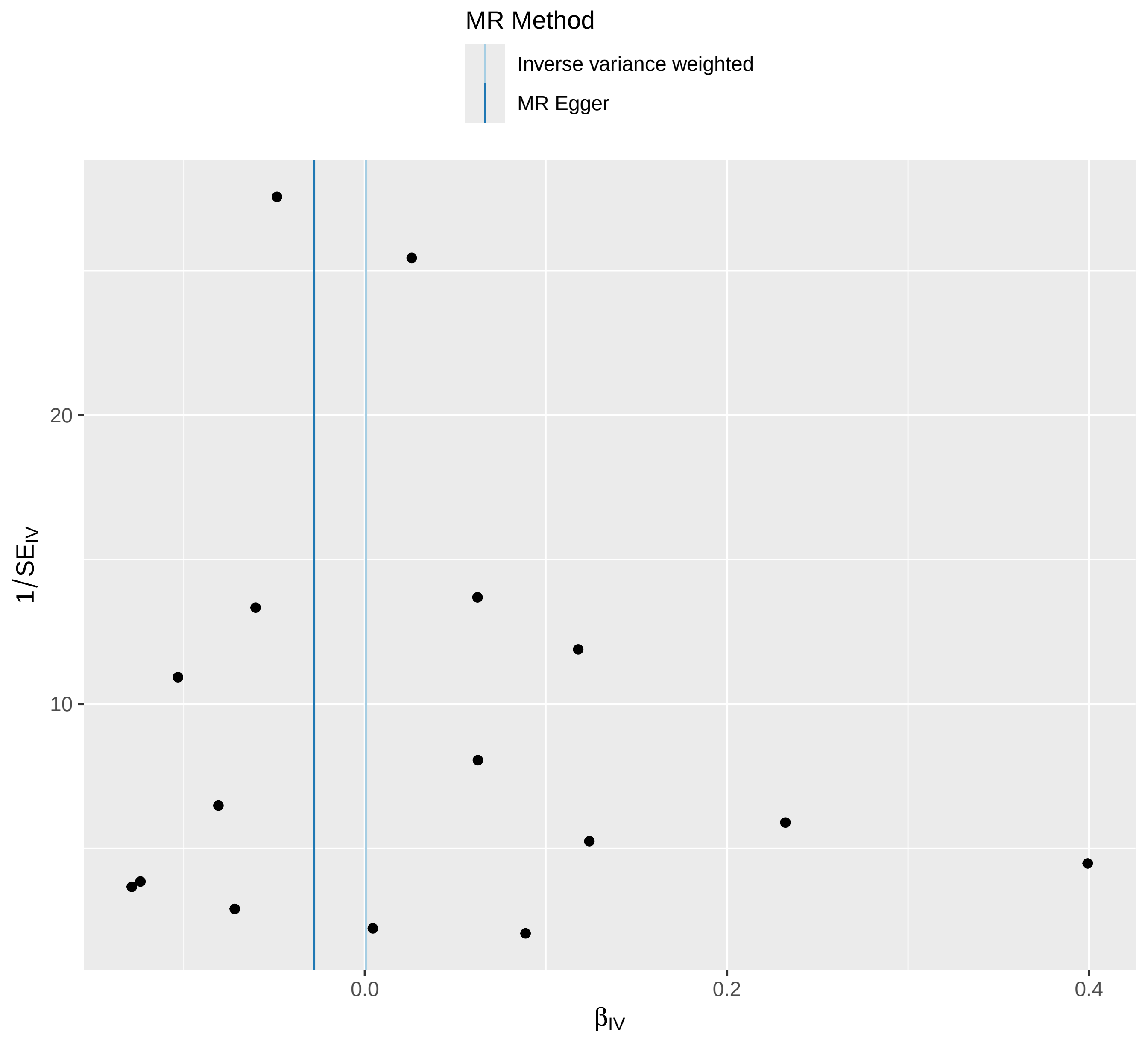
MR Method
Inverse variance weighted
MR Egger
20
10
1/SE_IV
0.0
0.2
0.4
β_IV

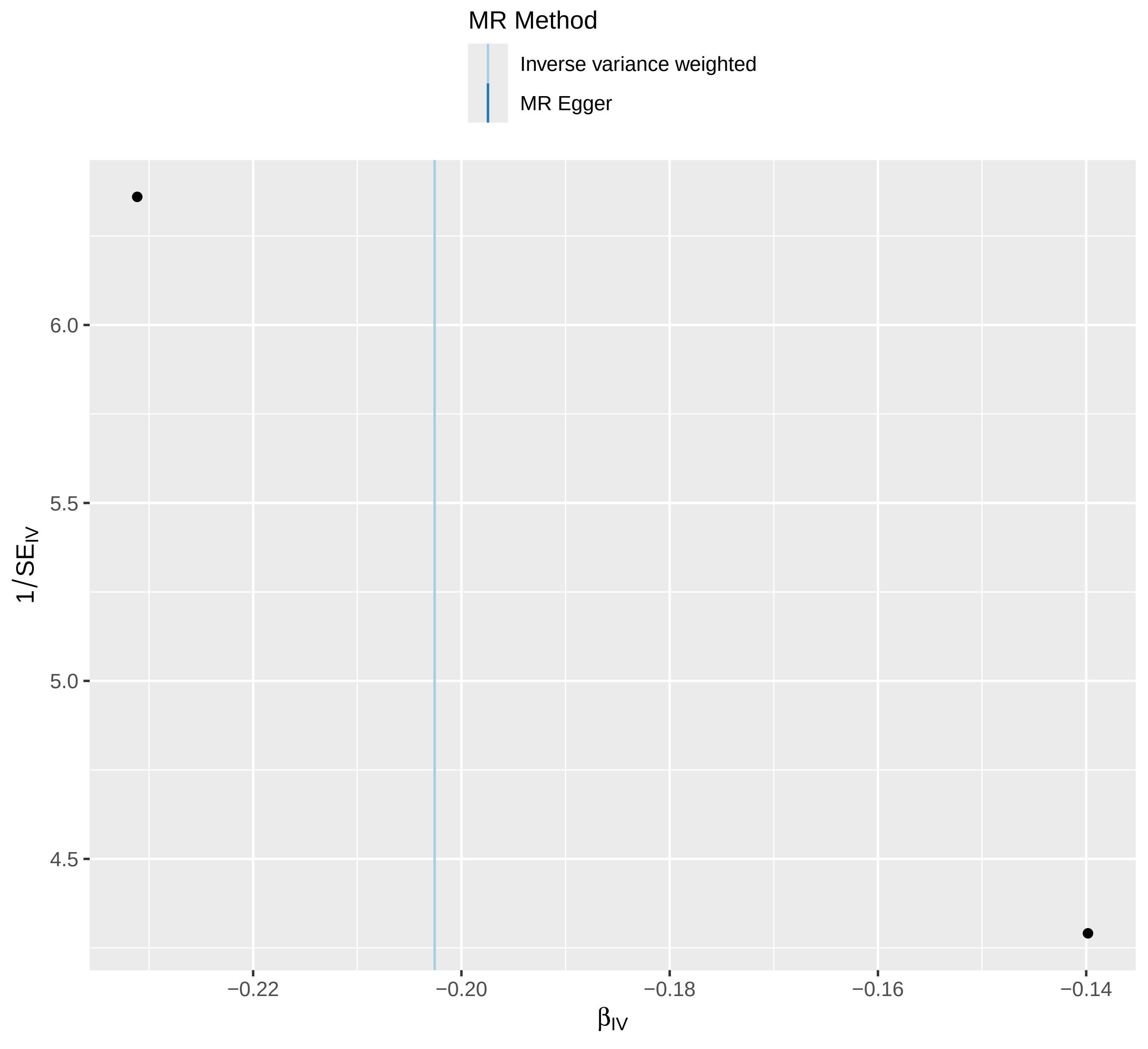

MR Method
Inverse variance weighted
MR Egger
6.0
5.5
5.0
4.5
1/SE_IV
−0.22
−0.20
−0.18
−0.16
−0.14
β_IV

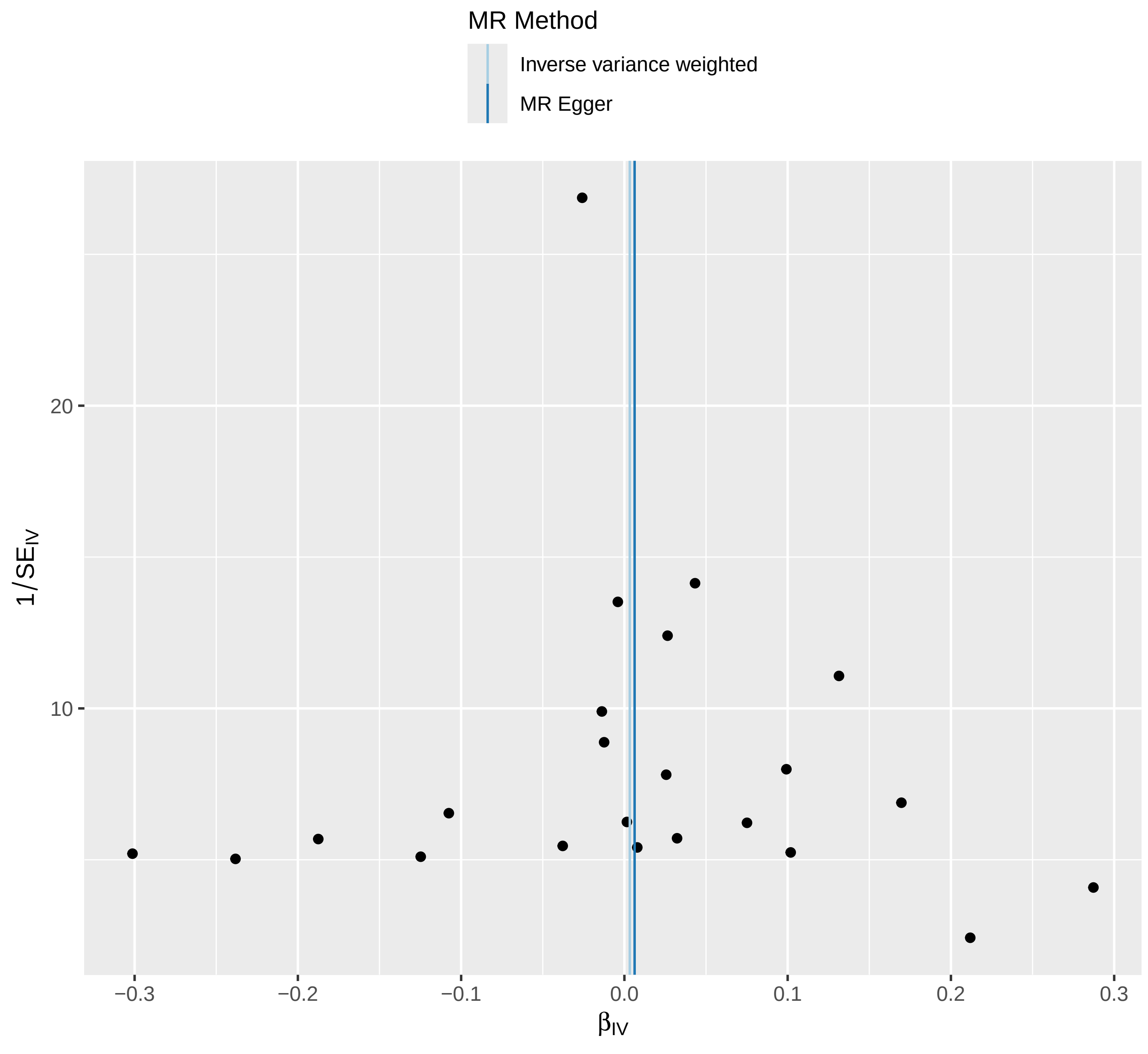
MR Method
Inverse variance weighted
MR Egger
20
10
1/SE$_{IV}$
−0.3
−0.2
−0.1
0.0
0.1
0.2
0.3
β$_{IV}$

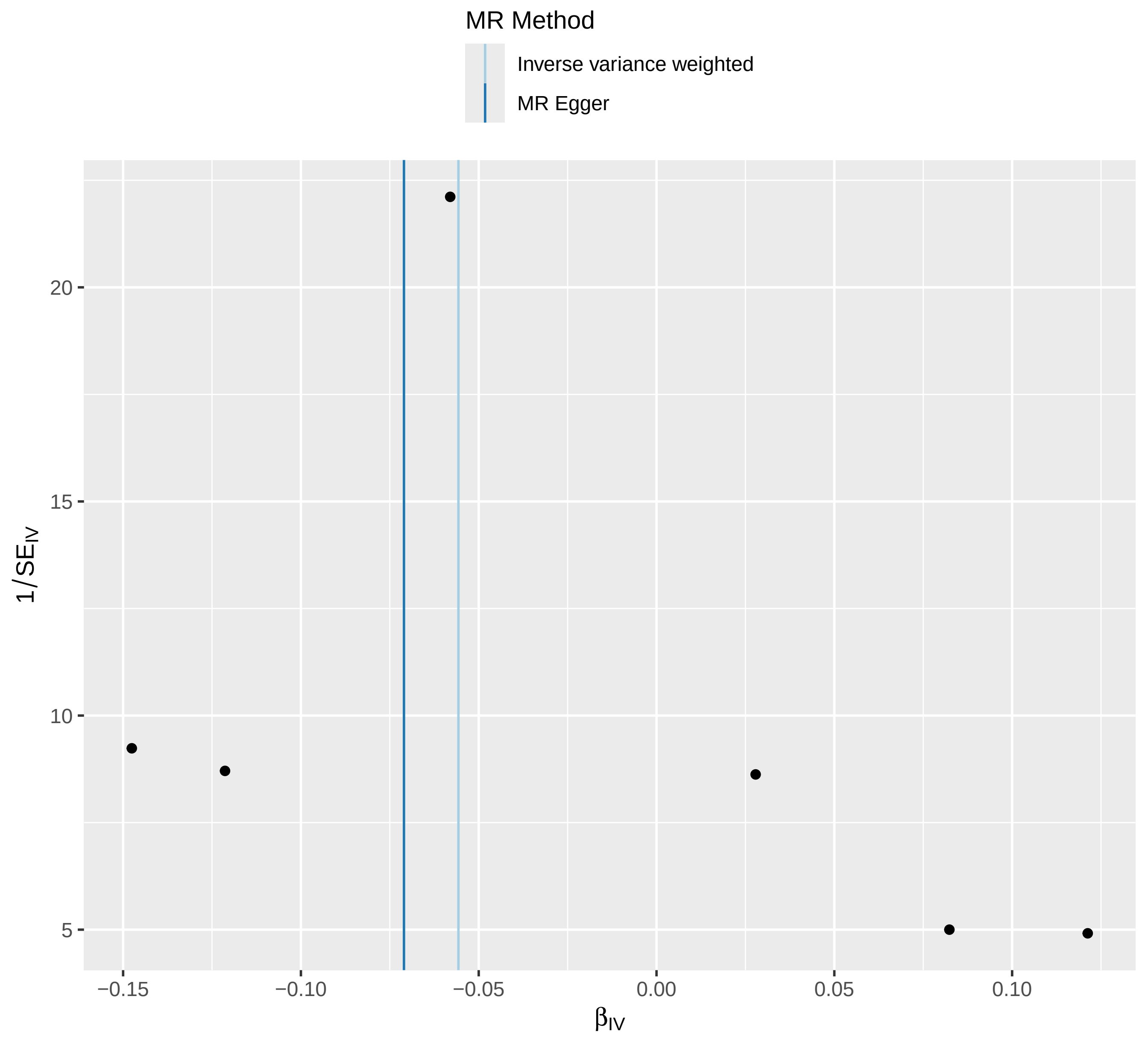
MR Method
Inverse variance weighted
MR Egger
20
15
10
5
1/SE_IV
−0.15
−0.10
−0.05
0.00
0.05
0.10
β_IV

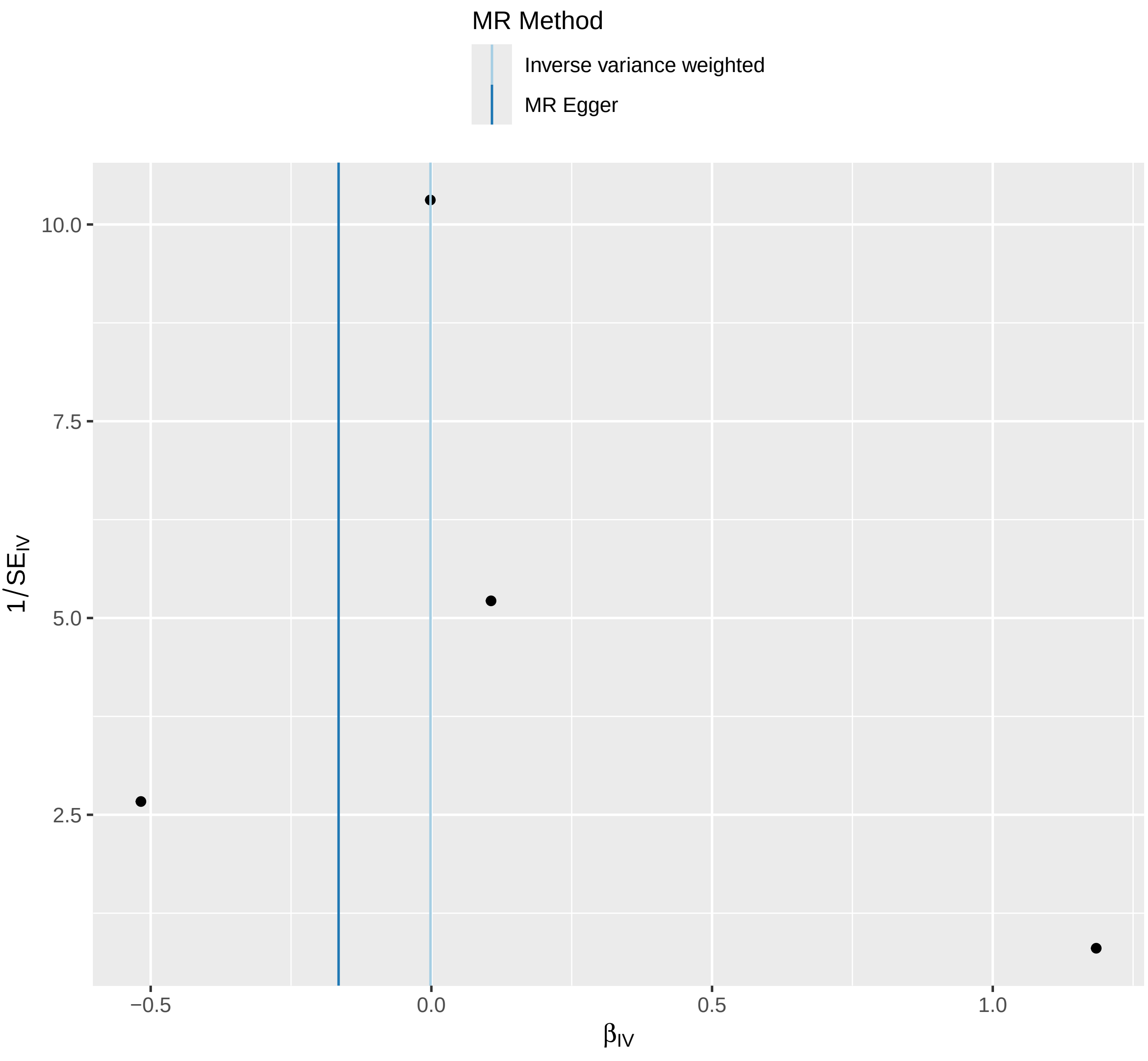
MR Method
Inverse variance weighted
MR Egger
10.0
7.5
5.0
2.5
1/SE_IV
−0.5
0.0
0.5
1.0
β_IV

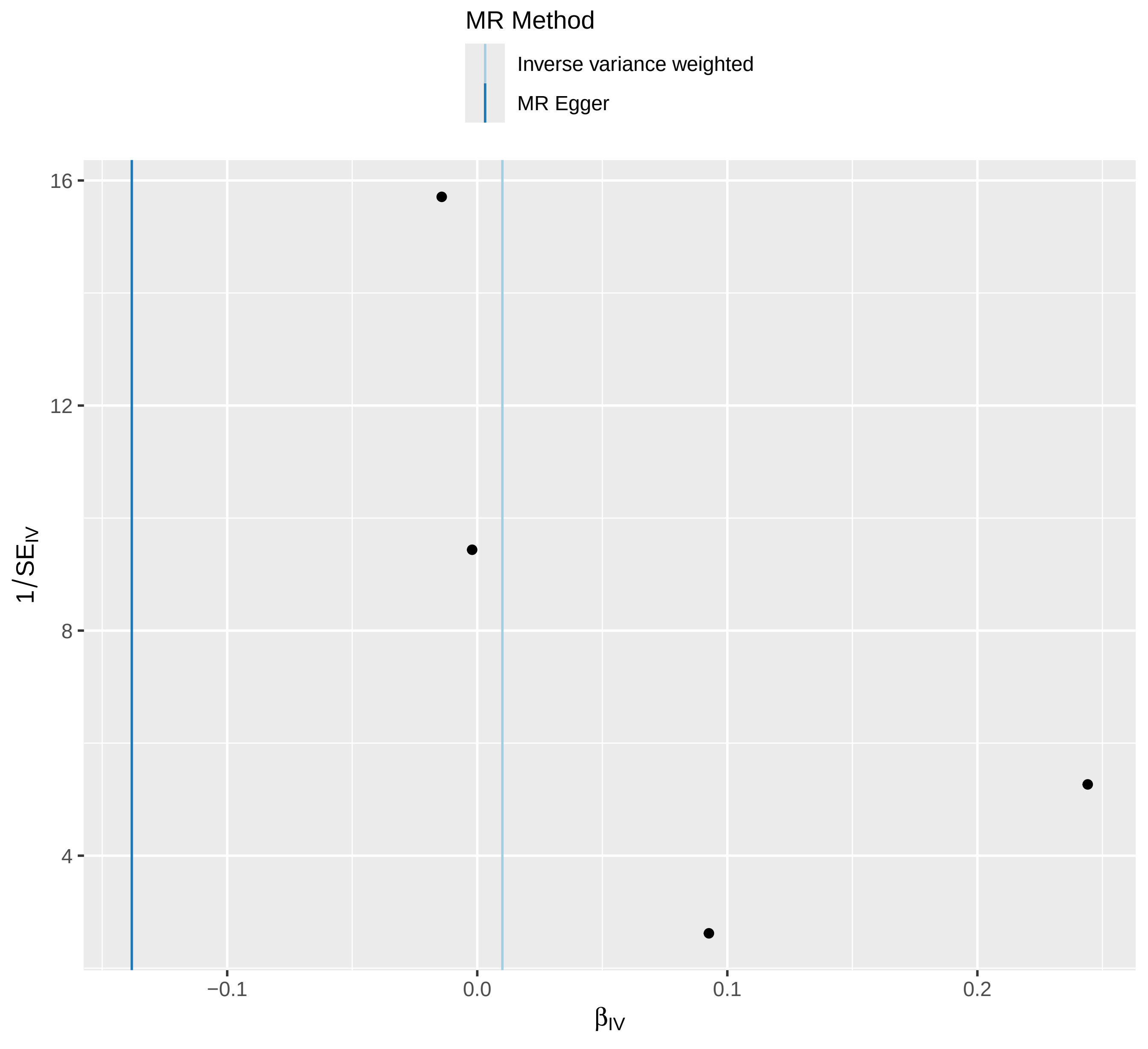
MR Method
Inverse variance weighted
MR Egger
16
12
8
4
1/SE_IV
−0.1
0.0
0.1
0.2
β_IV

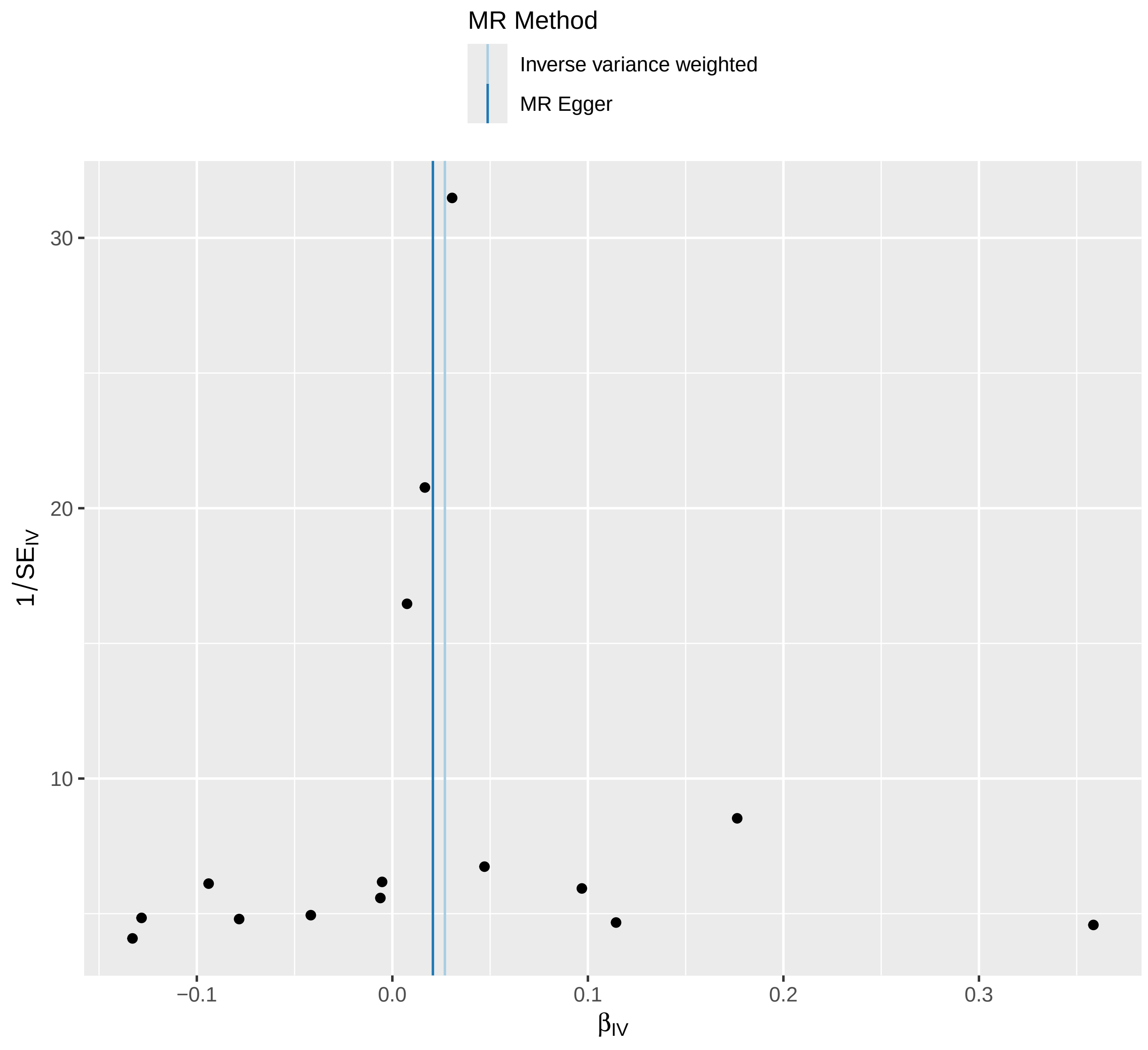
MR Method
Inverse variance weighted
MR Egger
30
20
10
1/SE$_{IV}$
−0.1
0.0
0.1
0.2
0.3
β$_{IV}$

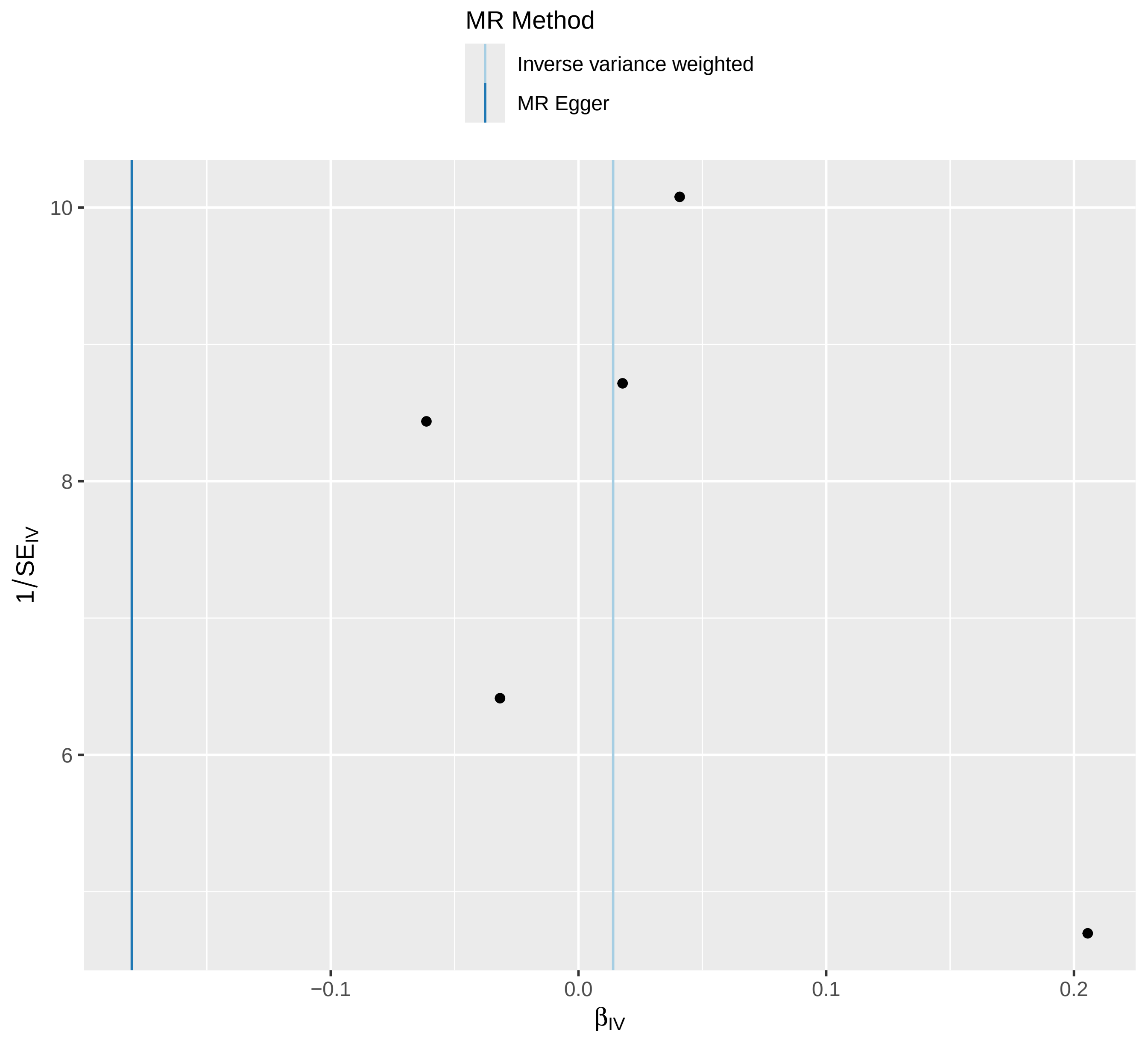

MR Method
Inverse variance weighted
MR Egger
10
8
6
1/SE_IV
−0.1
0.0
0.1
0.2
β_IV

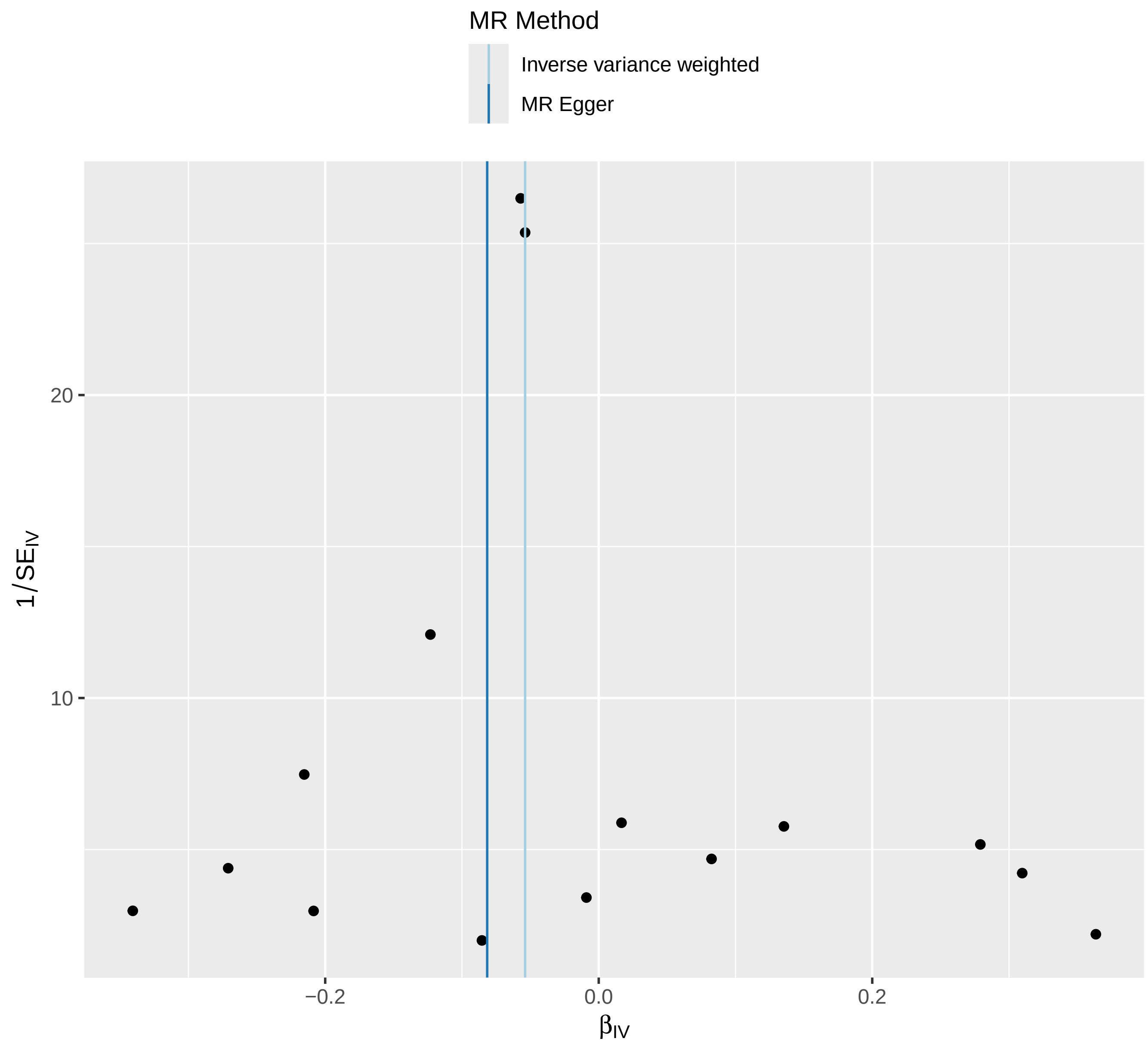

MR Method
Inverse variance weighted
MR Egger
20
10
1/SE$_{IV}$
−0.2
0.0
0.2
β$_{IV}$

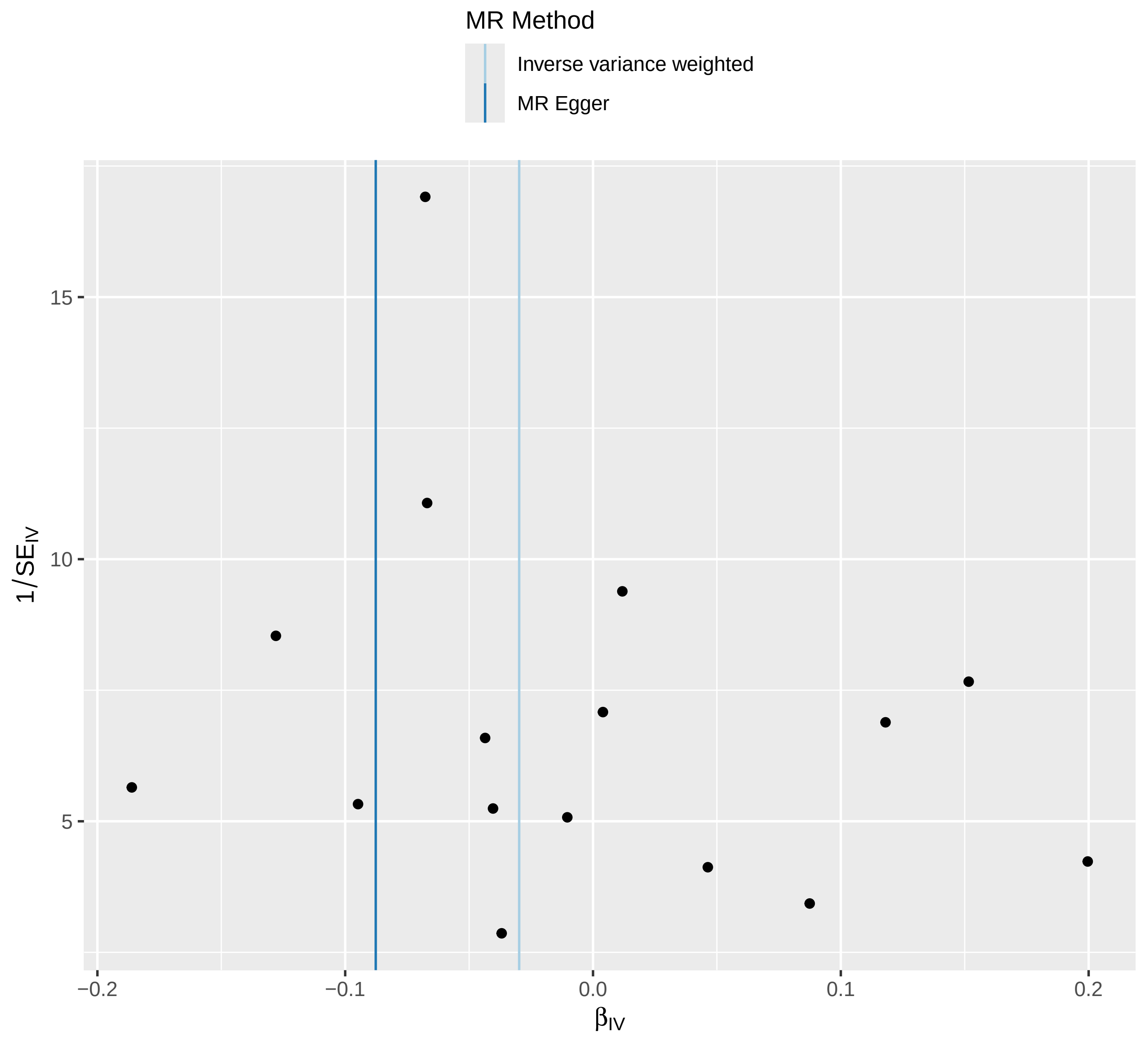

MR Method
Inverse variance weighted
MR Egger
15
10
5
$1/SE_{IV}$
−0.2
−0.1
0.0
0.1
0.2
$\beta_{IV}$

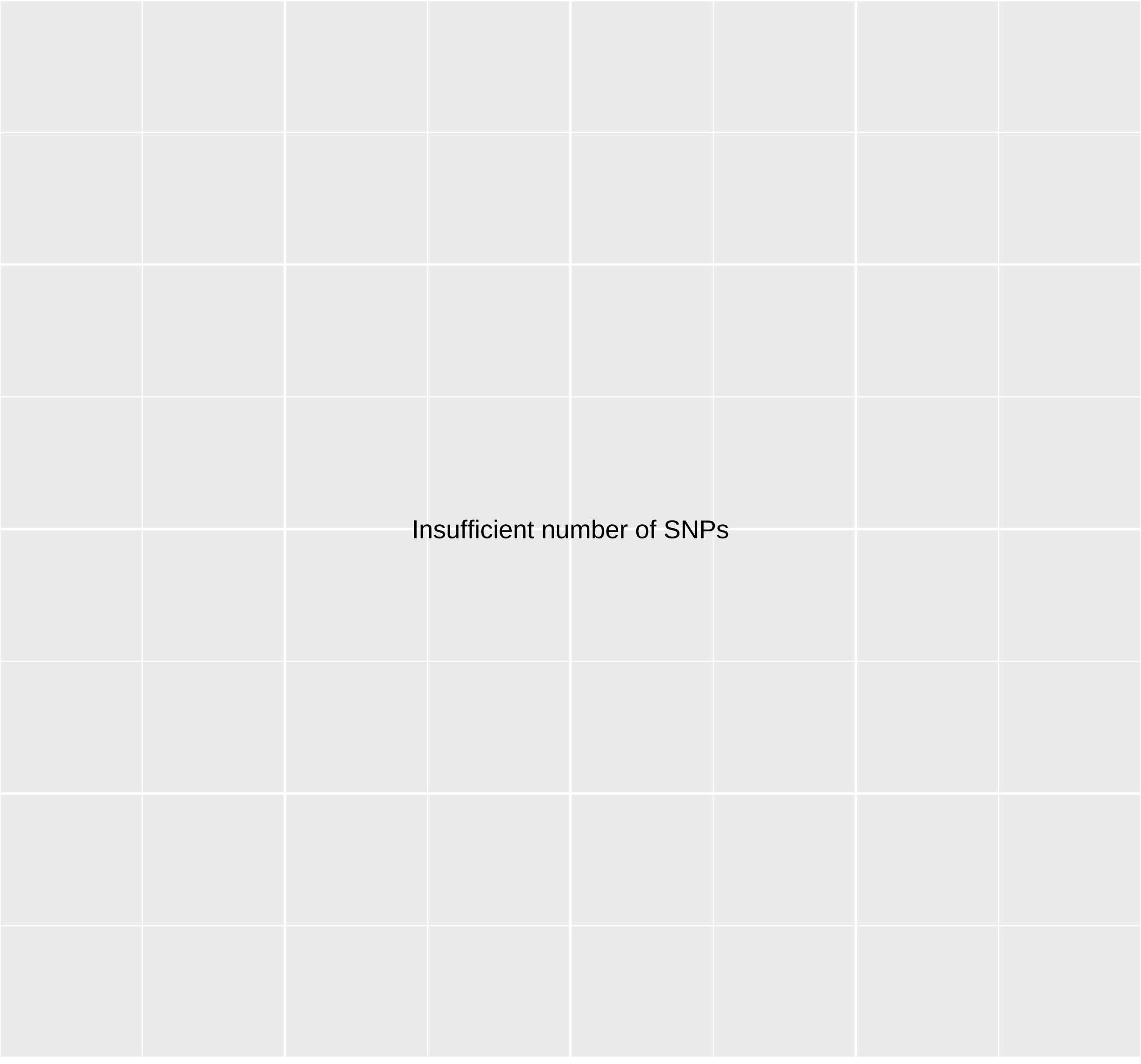
Insufficient number of SNPs

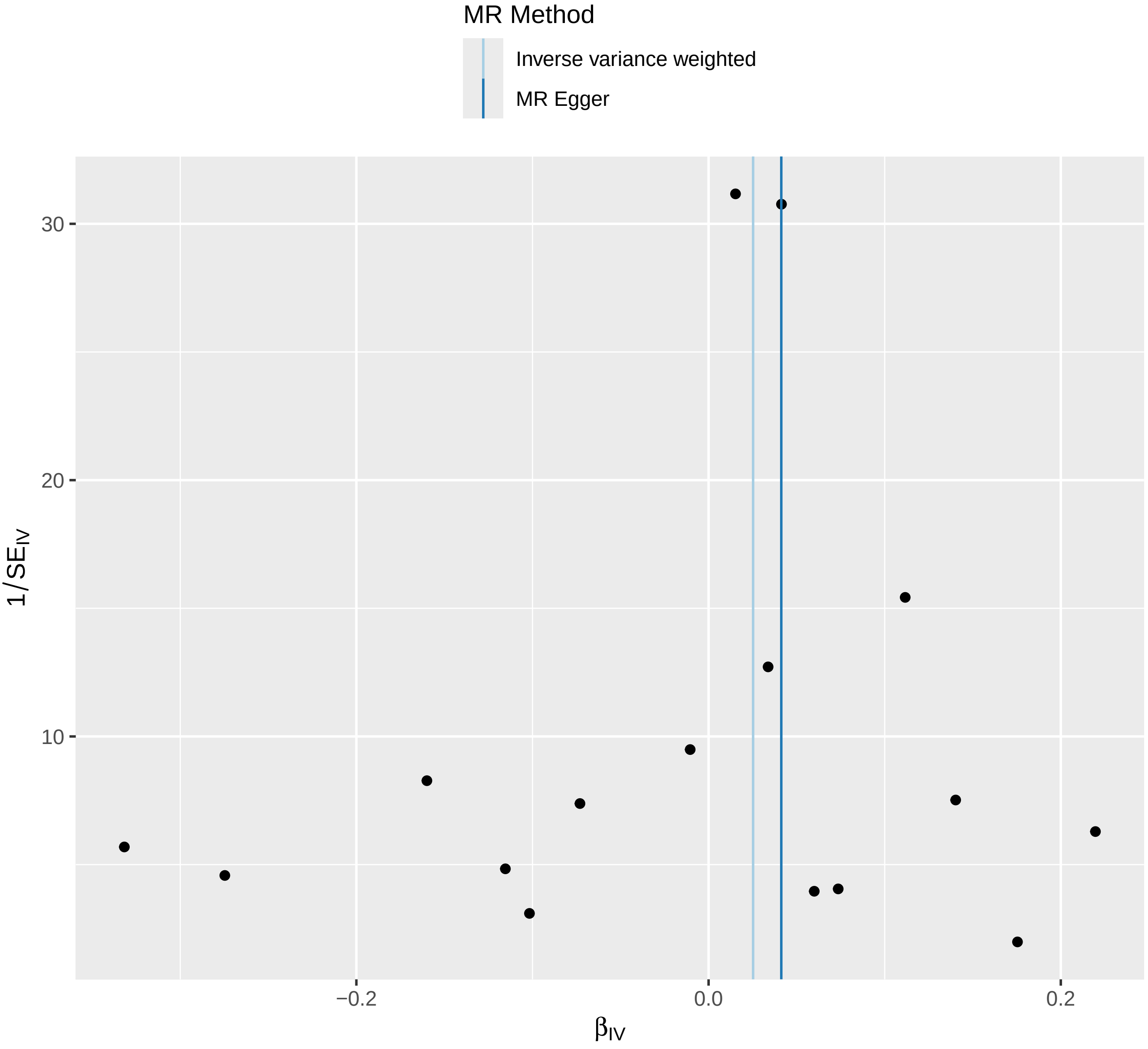
MR Method
Inverse variance weighted
MR Egger
30
20
10
$1/SE_{IV}$
−0.2
0.0
0.2
$\beta_{IV}$

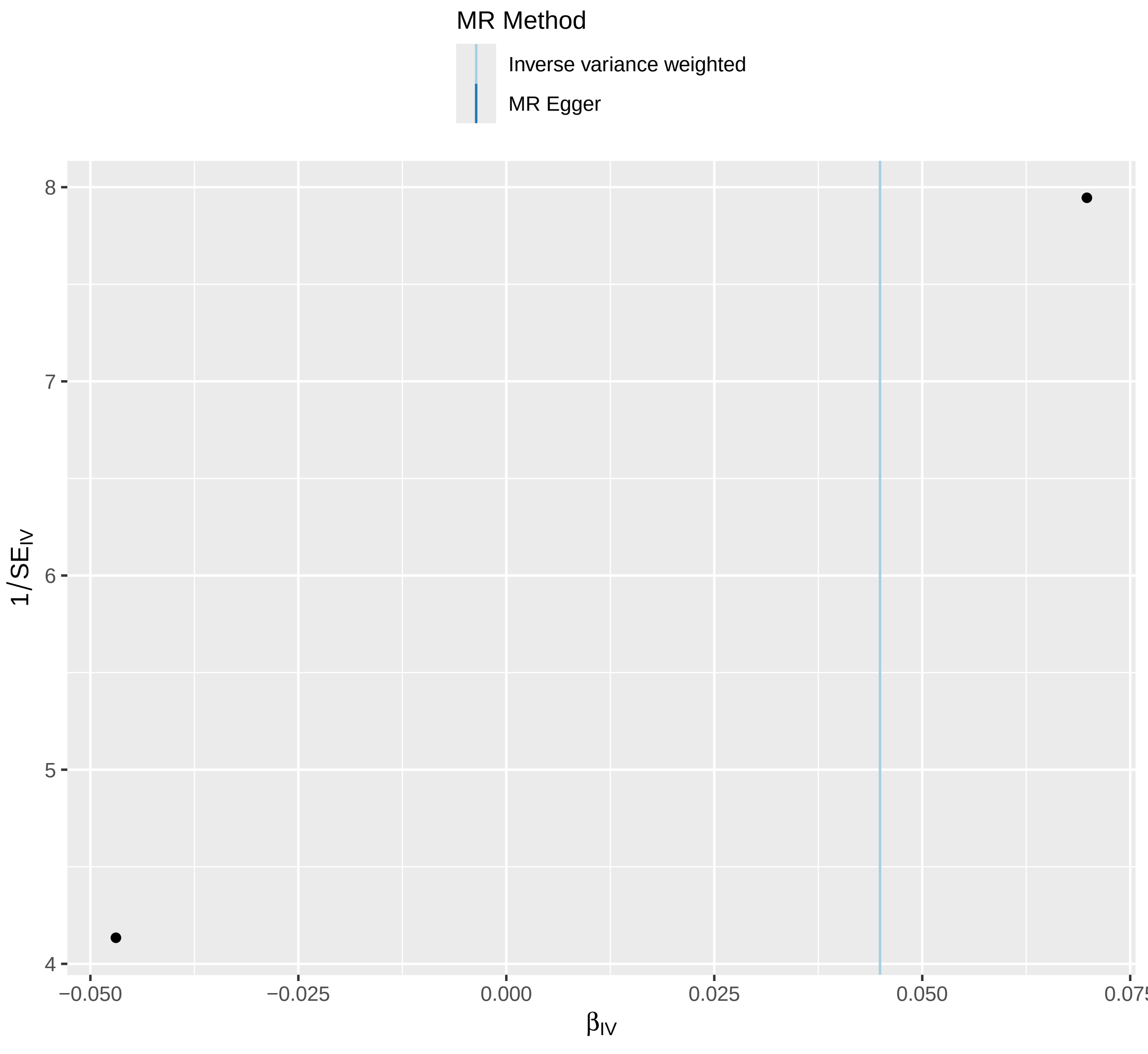

MR Method
Inverse variance weighted
MR Egger
8
7
6
5
4
1/SE$_{IV}$
−0.050
−0.025
0.000
0.025
0.050
0.075
β$_{IV}$

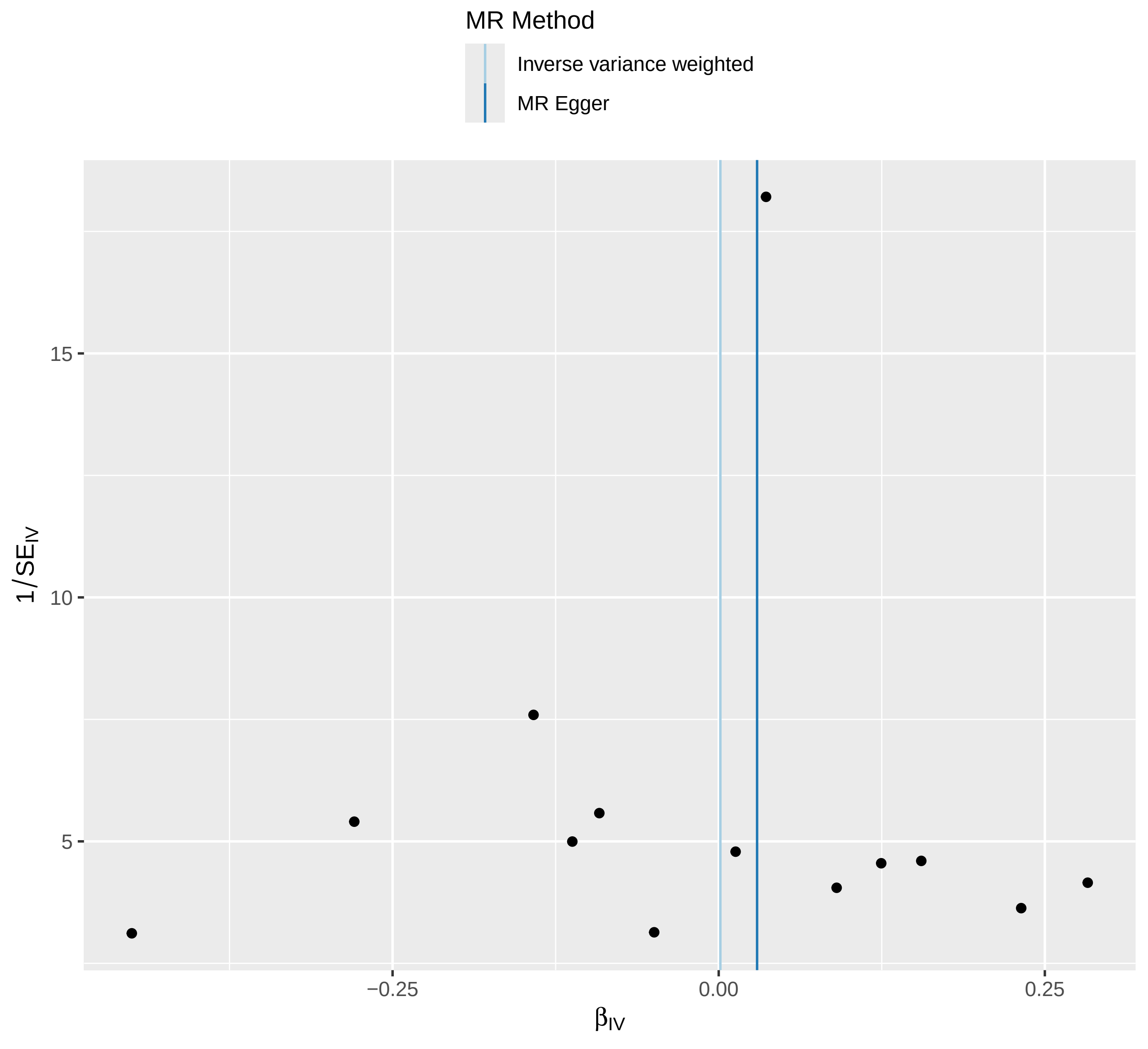

MR Method
Inverse variance weighted
MR Egger
15
10
5
−0.25
0.00
0.25
$1/SE_{IV}$
$\beta_{IV}$

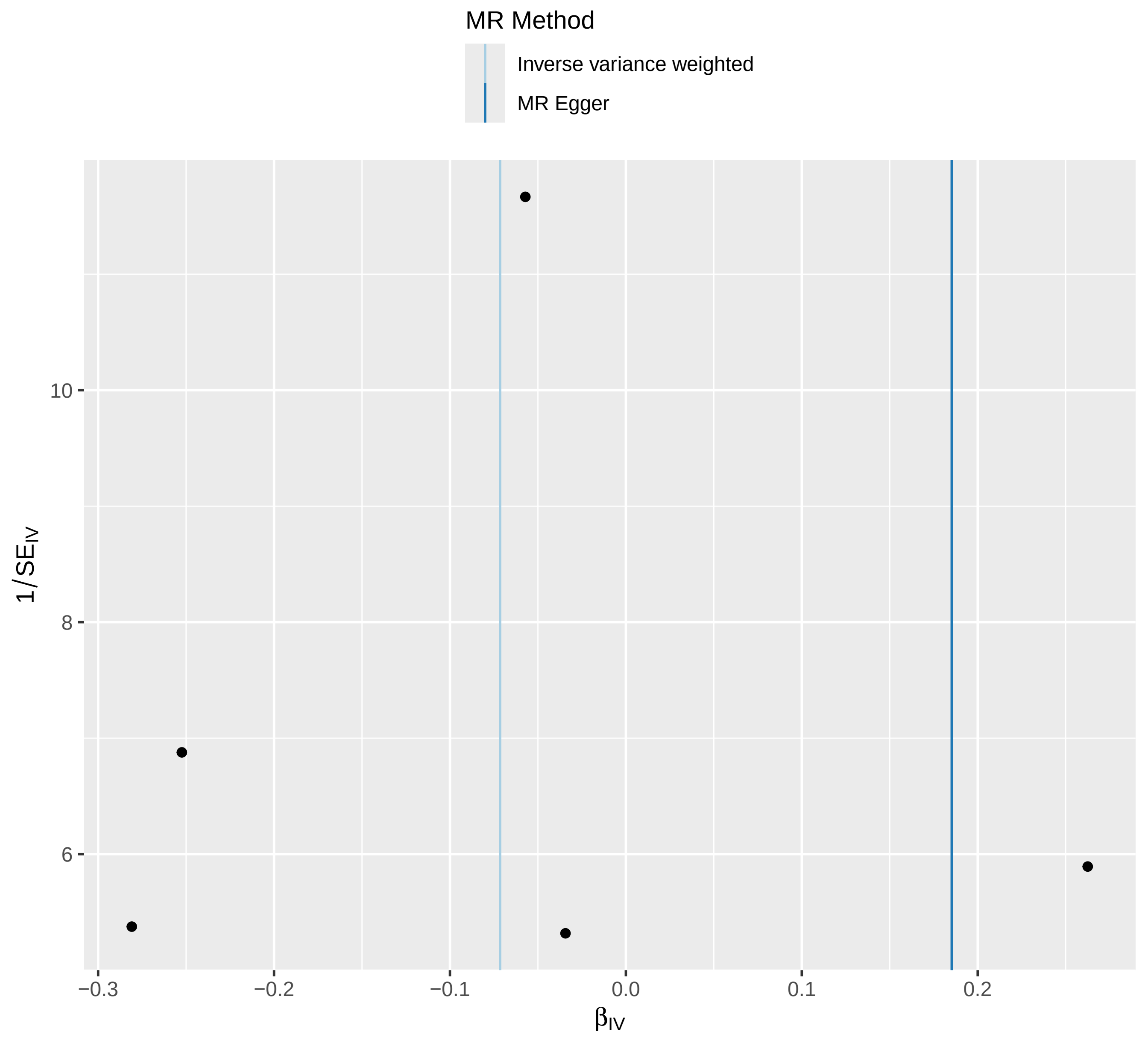
MR Method
Inverse variance weighted
MR Egger
1/SE_IV
10
8
6
−0.3
−0.2
−0.1
0.0
0.1
0.2
β_IV

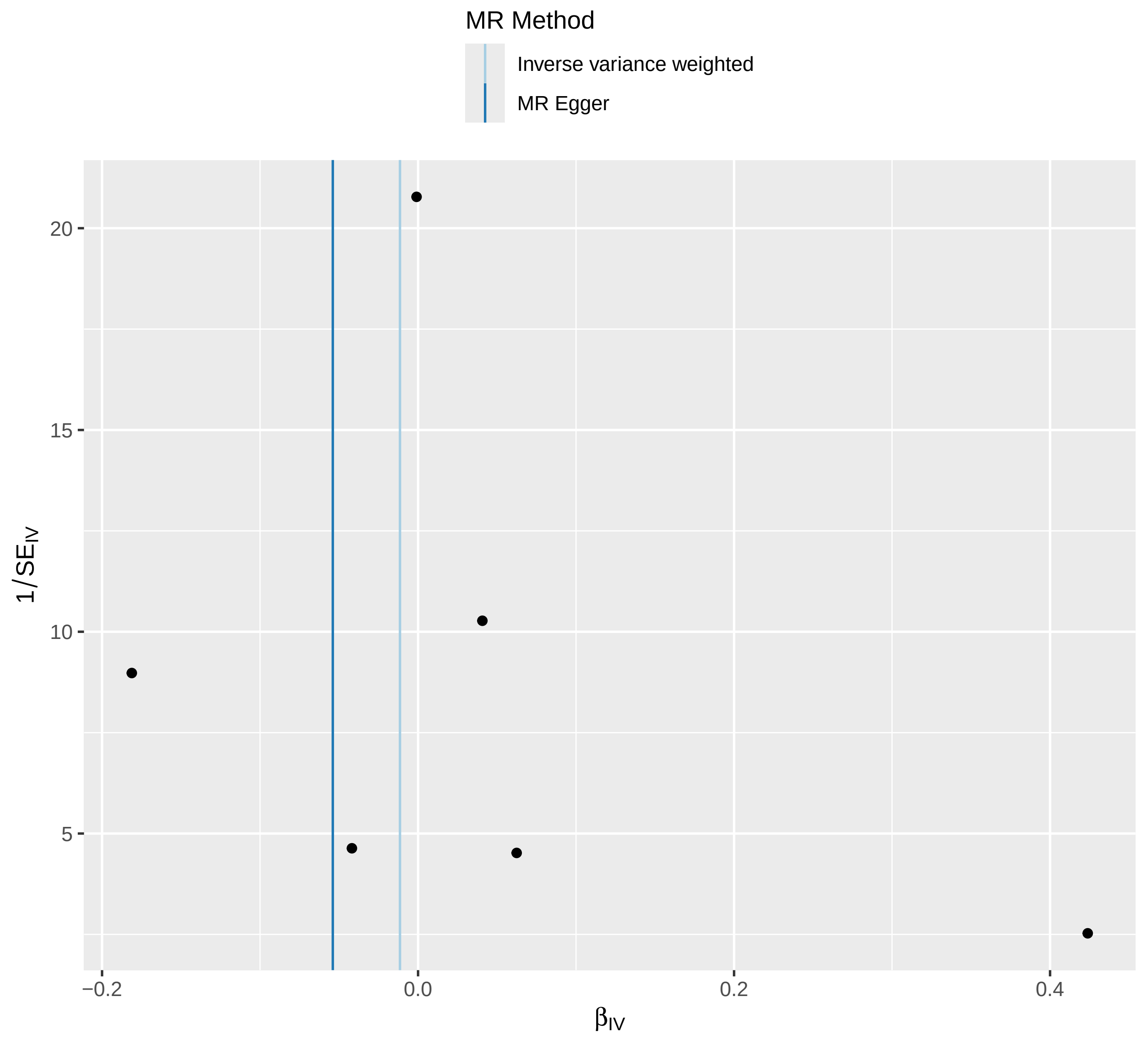
MR Method
Inverse variance weighted
MR Egger
20
15
10
5
1/SE_IV
−0.2
0.0
0.2
0.4
β_IV

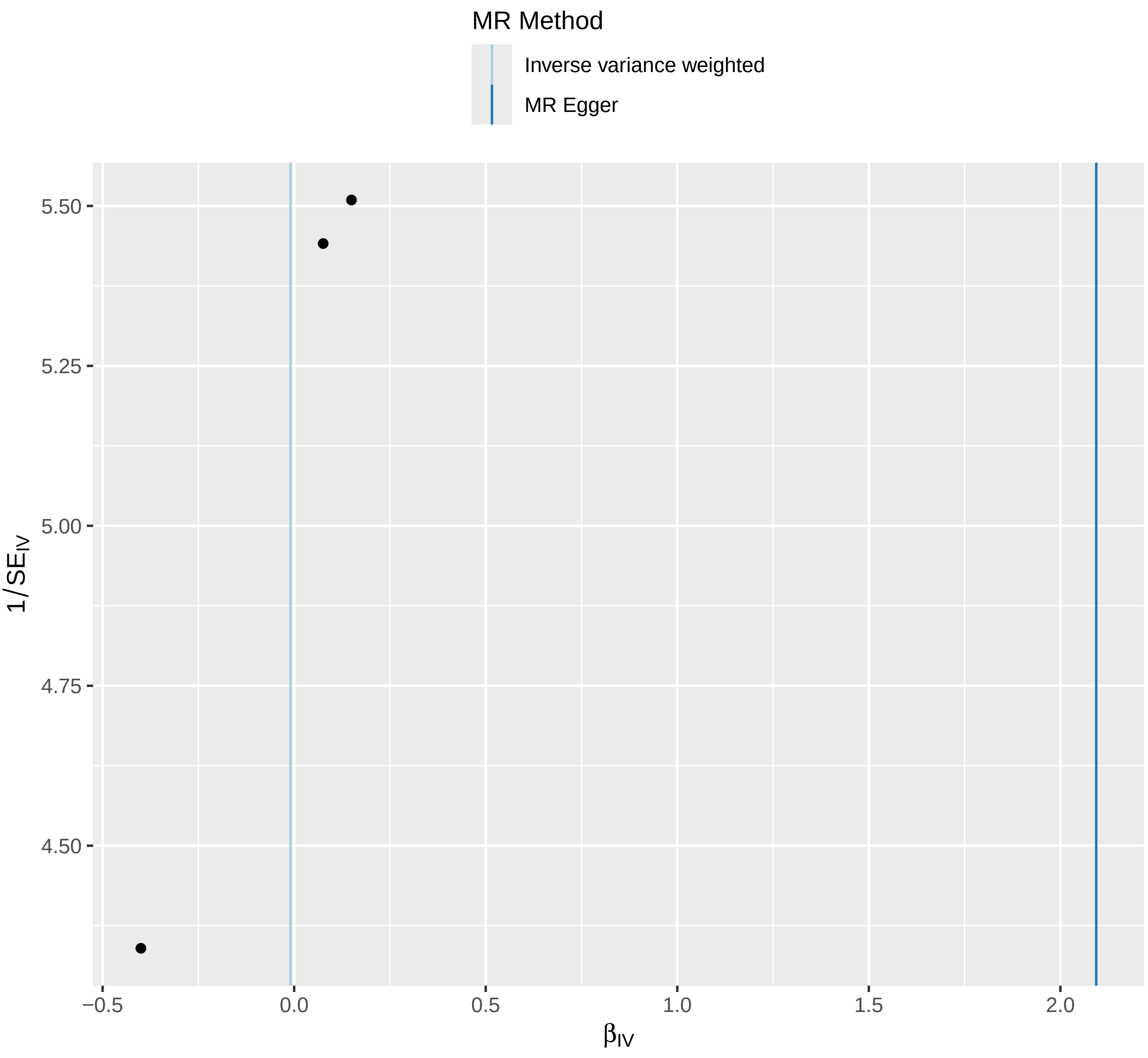
MR Method
Inverse variance weighted
MR Egger
5.50
5.25
5.00
4.75
4.50
$1/SE_{IV}$
−0.5
0.0
0.5
1.0
1.5
2.0
$\beta_{IV}$

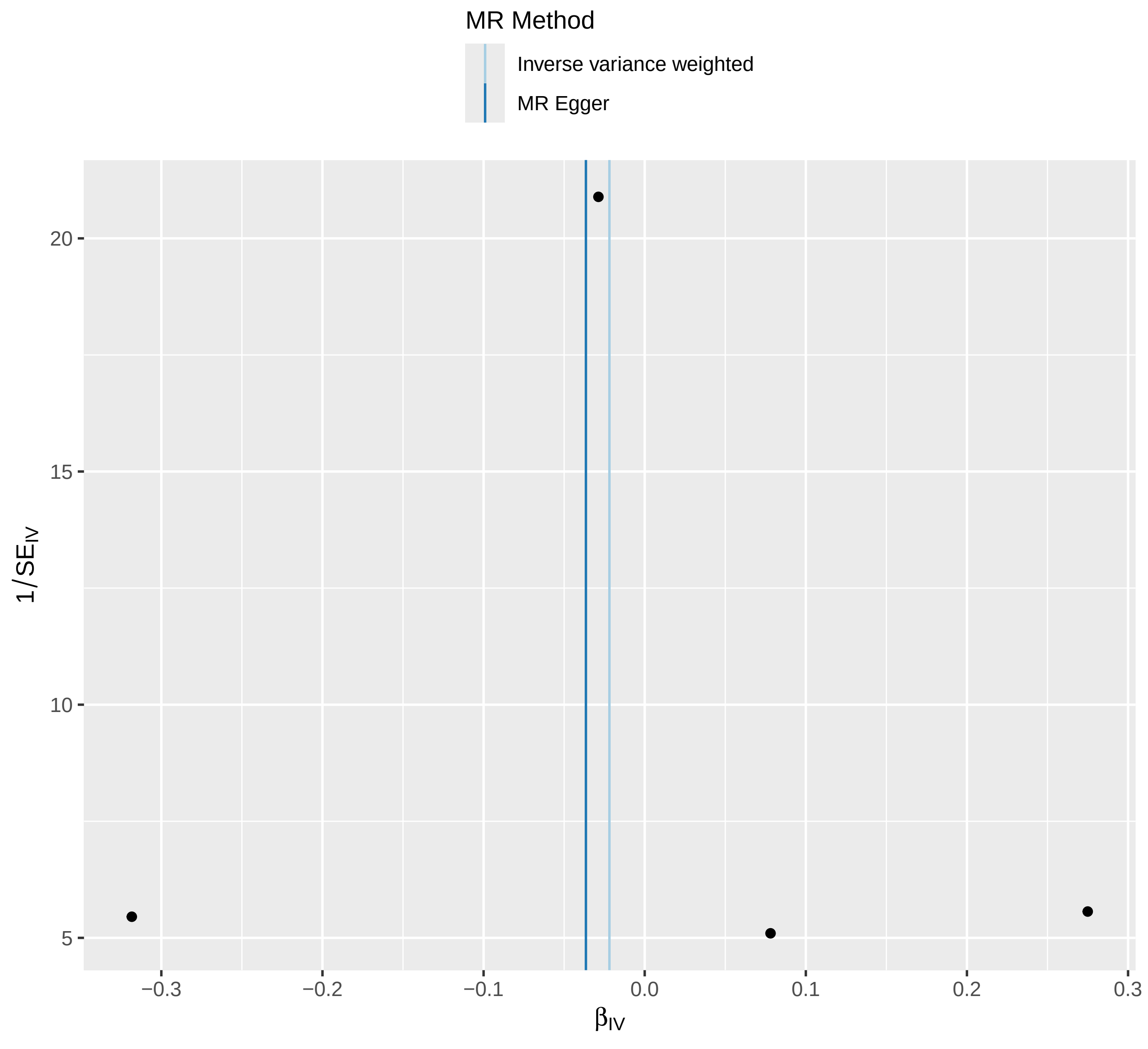

MR Method
Inverse variance weighted
MR Egger
20
15
10
5
1/SE$_{IV}$
−0.3
−0.2
−0.1
0.0
0.1
0.2
0.3
β$_{IV}$

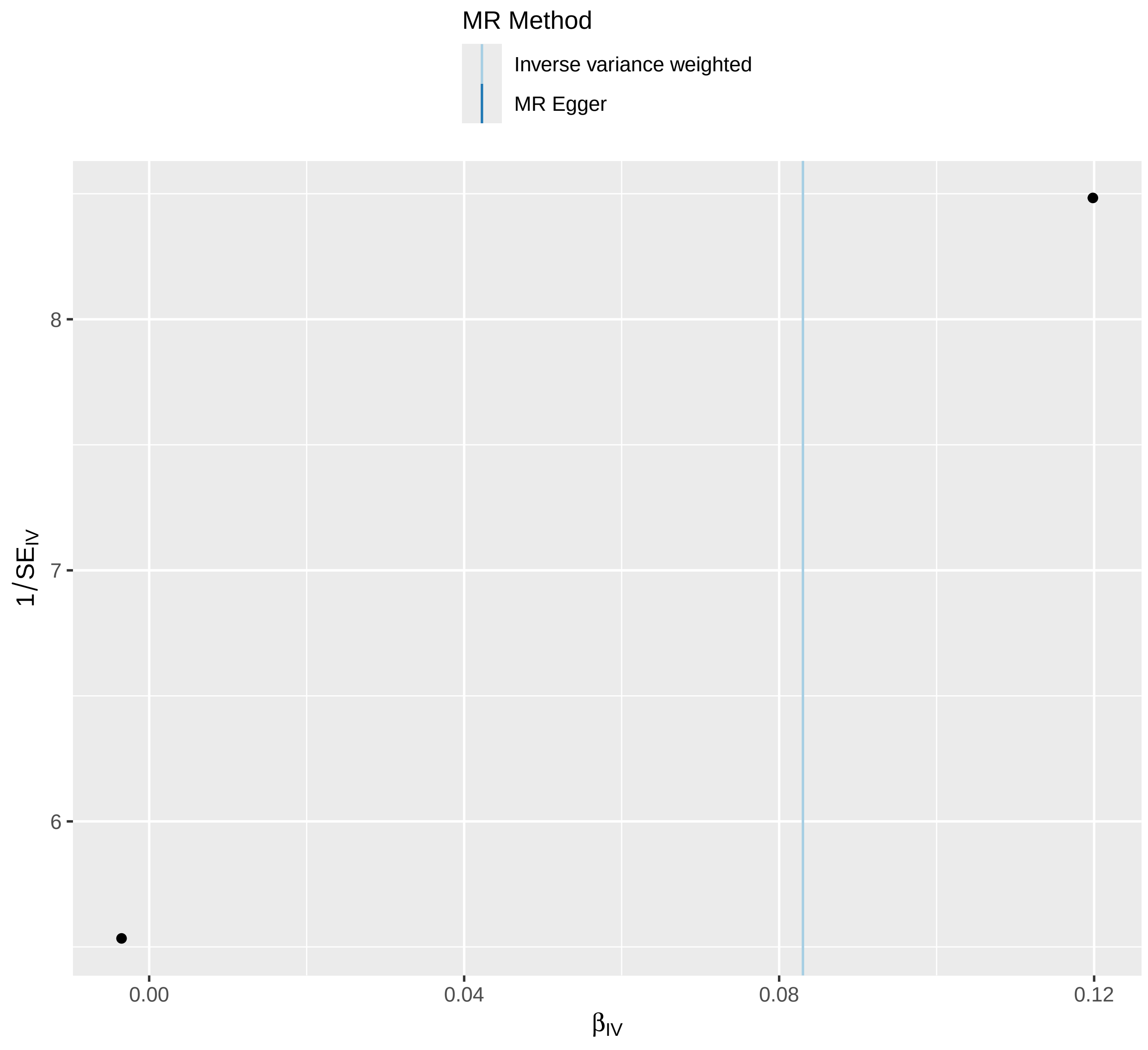
MR Method
Inverse variance weighted
MR Egger
8
7
6
0.00
0.04
0.08
0.12
$1/SE_{IV}$
$\beta_{IV}$

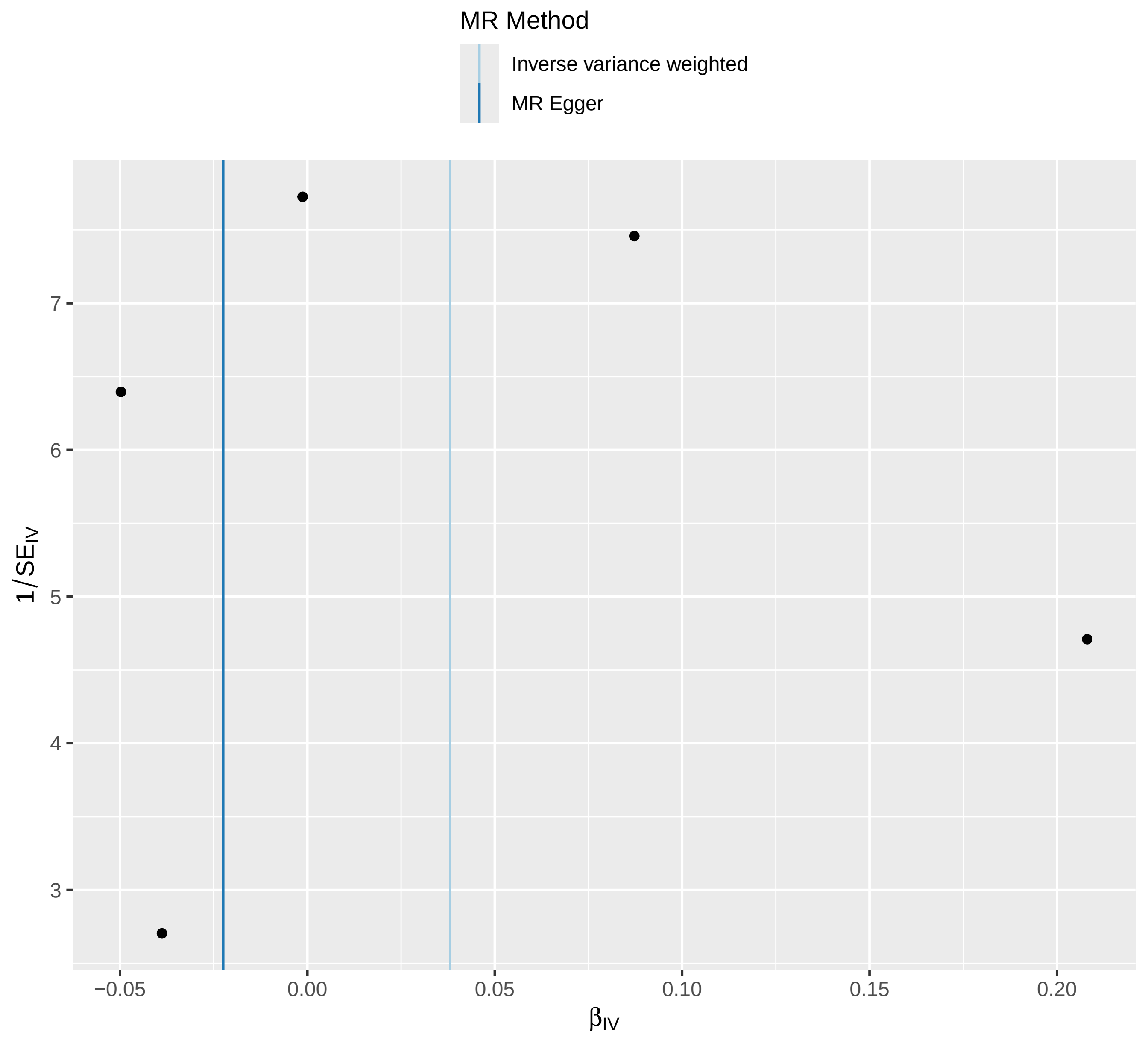

MR Method
Inverse variance weighted
MR Egger
7
6
5
4
3
1/SE_IV
−0.05
0.00
0.05
0.10
0.15
0.20
β_IV

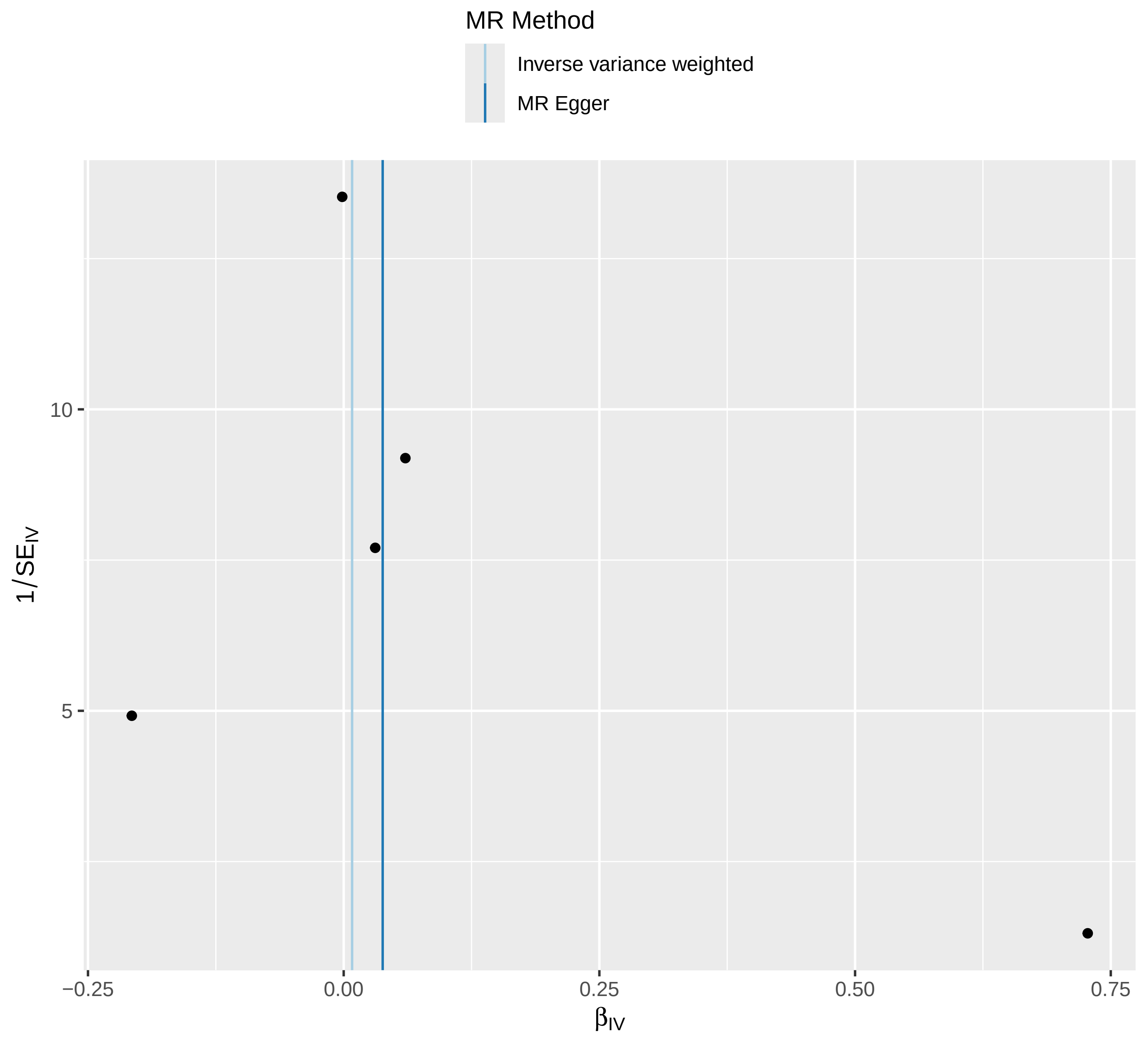

MR Method
Inverse variance weighted
MR Egger
10
5
1/SE$_{IV}$
−0.25
0.00
0.25
0.50
0.75
β$_{IV}$

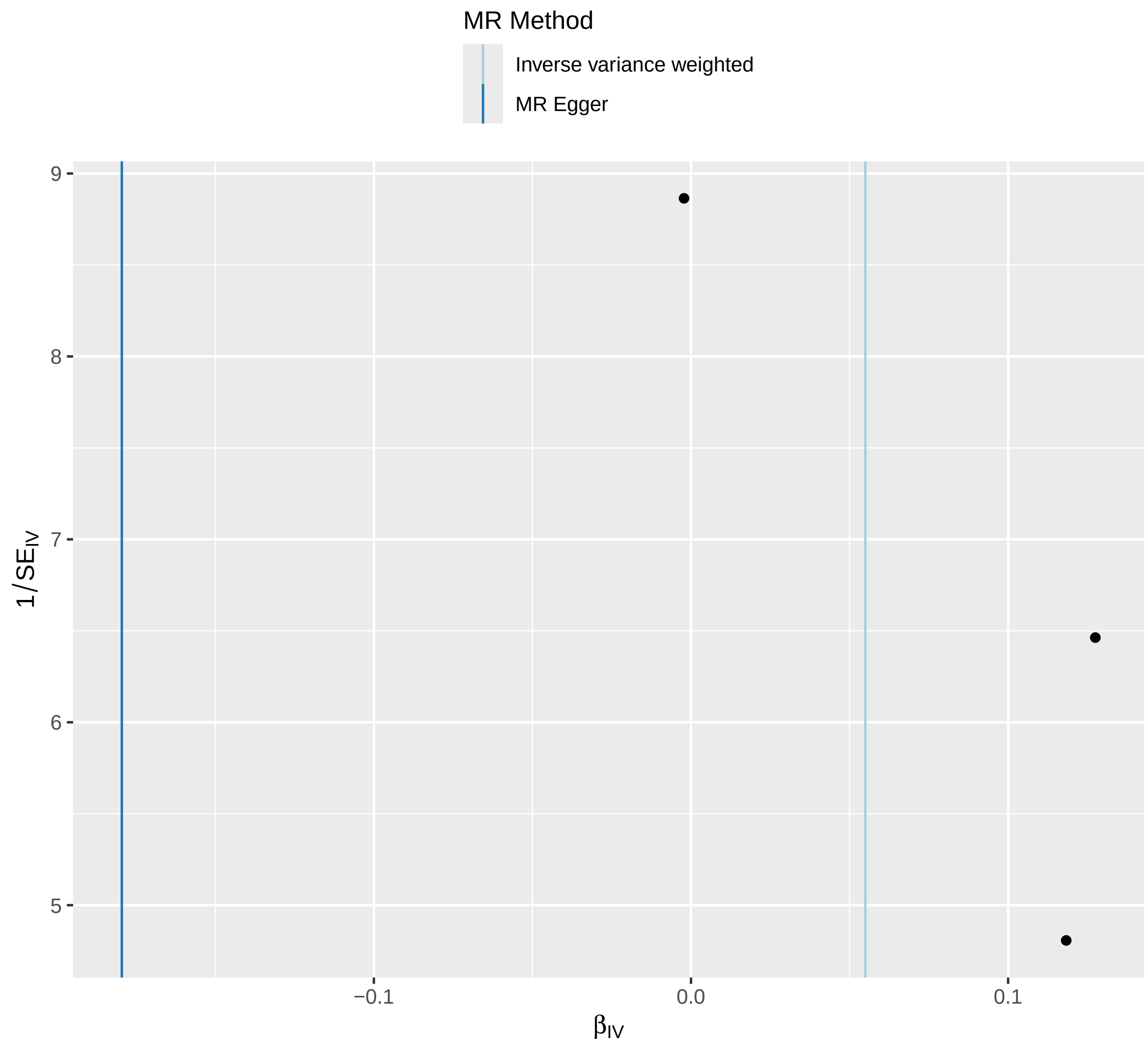

MR Method
Inverse variance weighted
MR Egger
9
8
7
6
5
1/SE$_{IV}$
−0.1
0.0
0.1
β$_{IV}$

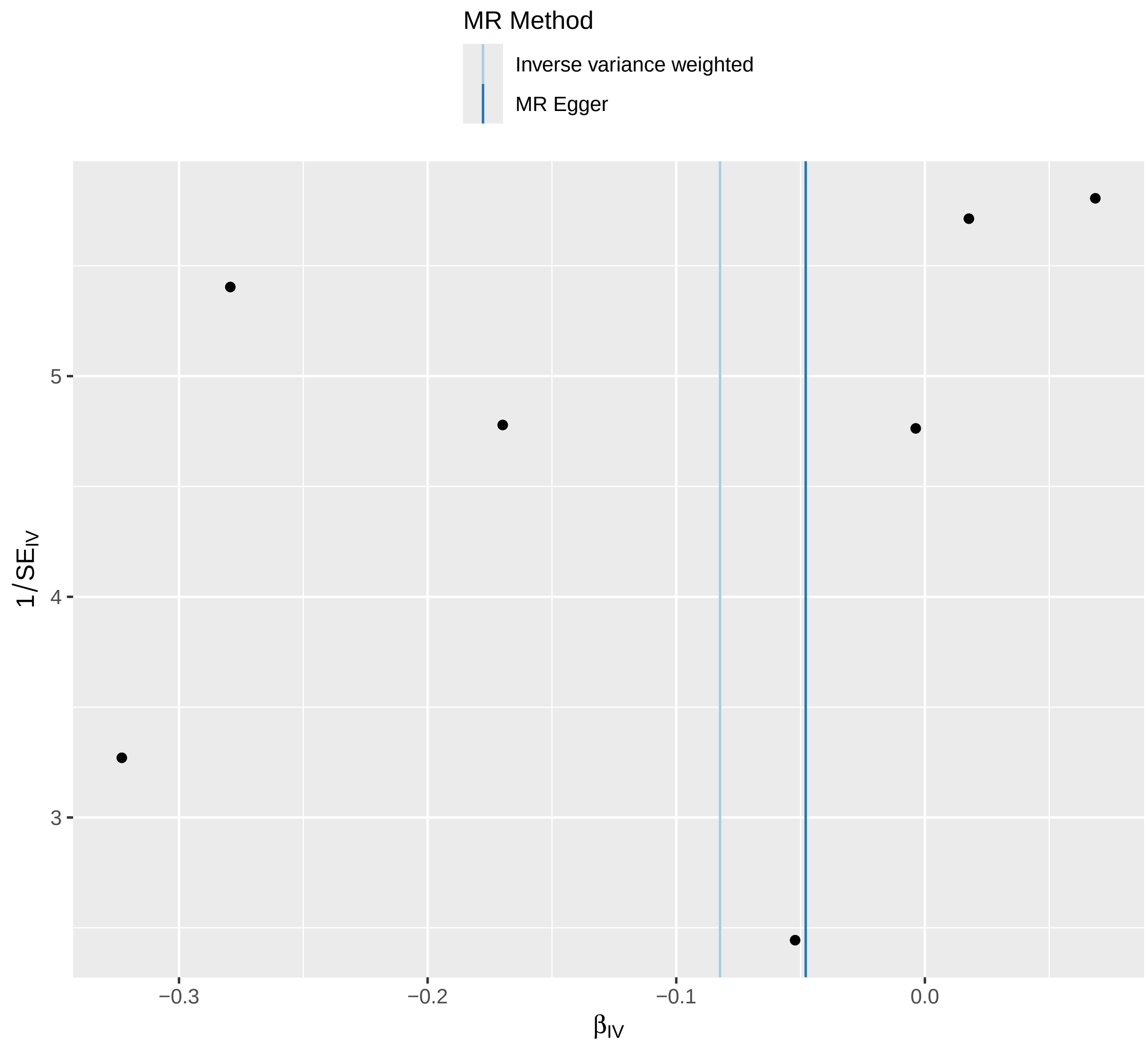
MR Method
Inverse variance weighted
MR Egger
5
4
3
1/SE$_{IV}$
−0.3
−0.2
−0.1
0.0
$\beta_{IV}$

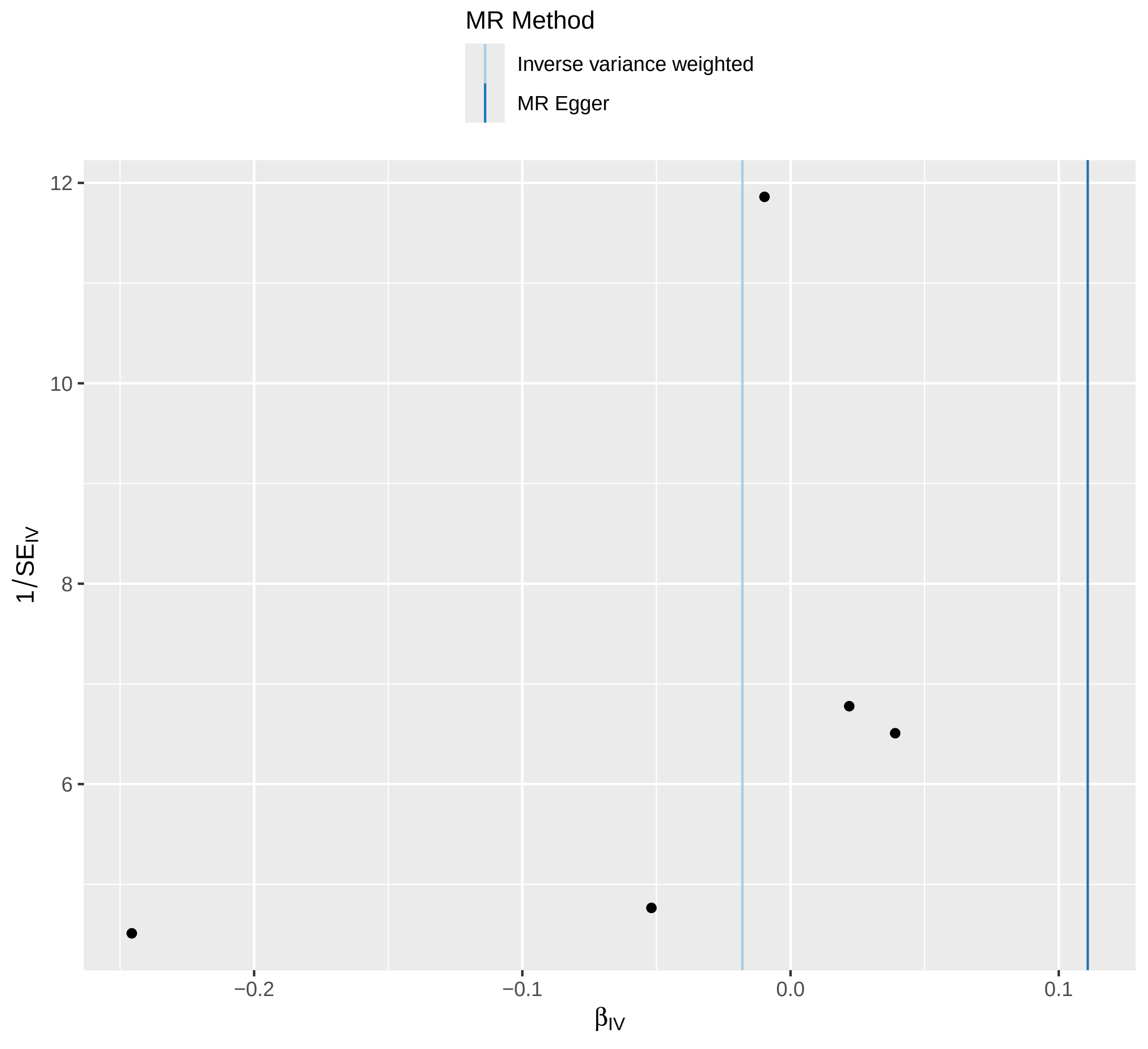
MR Method
Inverse variance weighted
MR Egger
12
10
8
6
$1/SE_{IV}$
−0.2
−0.1
0.0
0.1
$\beta_{IV}$

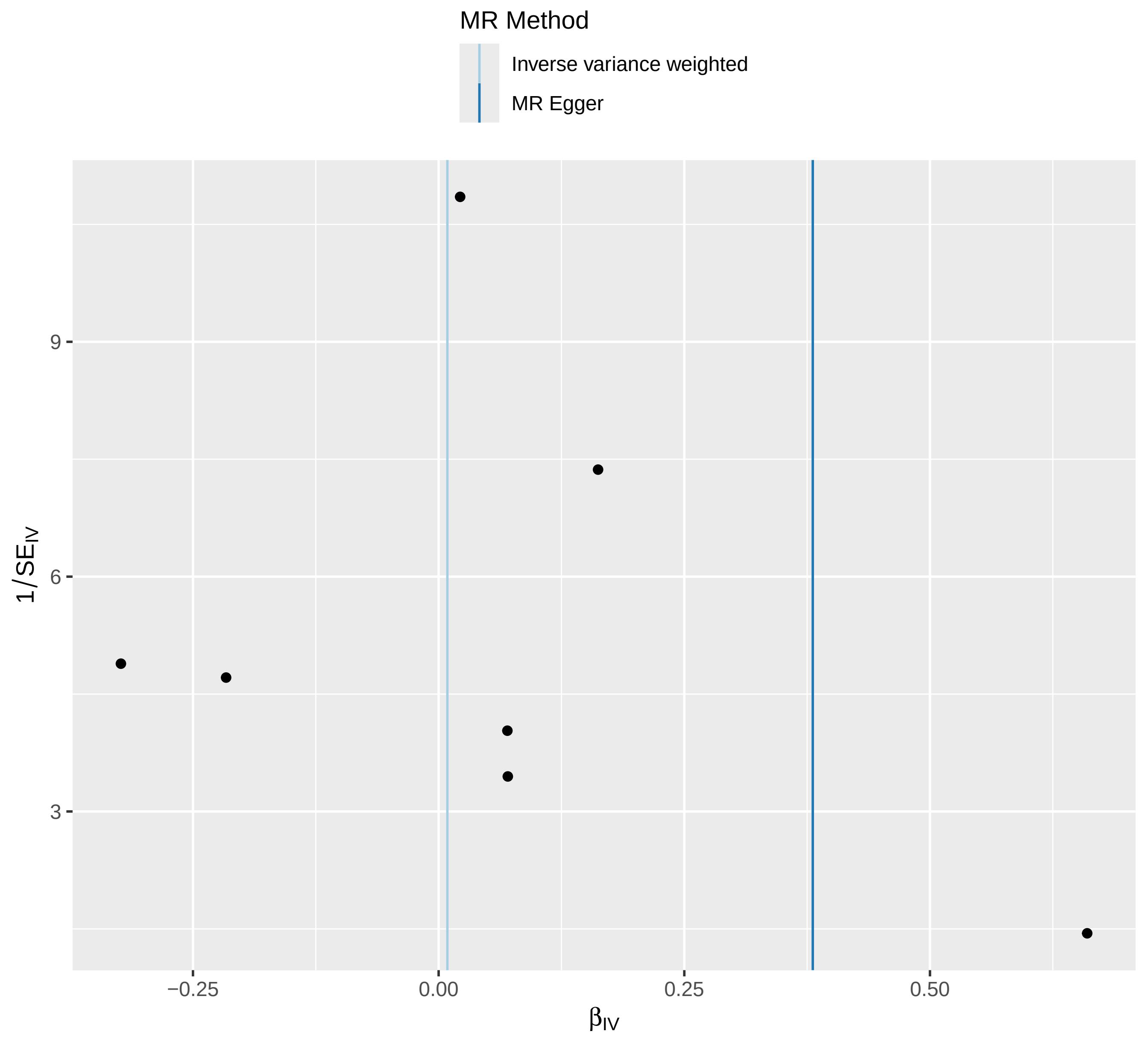
MR Method
Inverse variance weighted
MR Egger
9
6
3
$1/SE_{IV}$
−0.25
0.00
0.25
0.50
$\beta_{IV}$

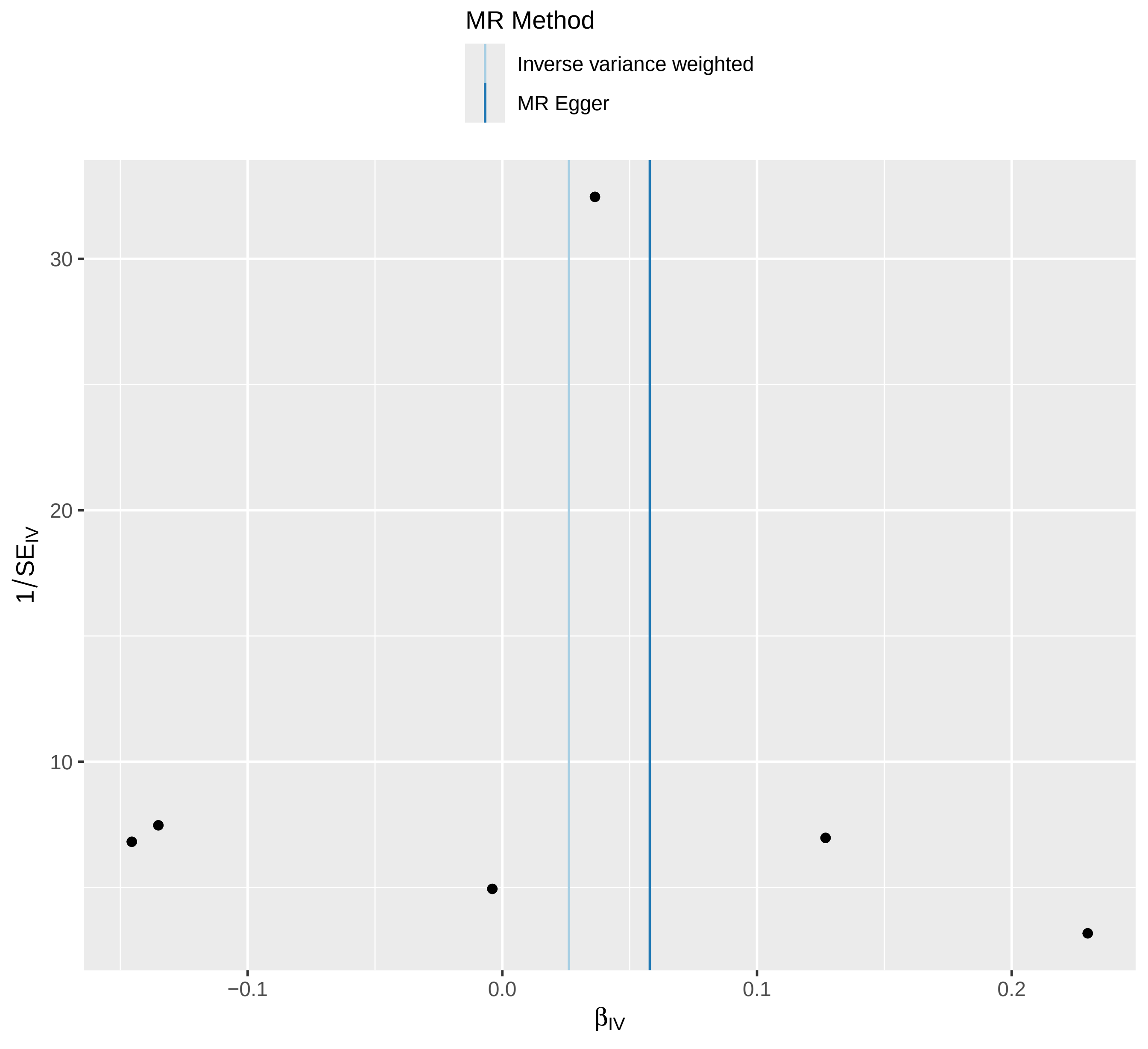
MR Method
Inverse variance weighted
MR Egger
30
20
10
1/SE$_{IV}$
−0.1
0.0
0.1
0.2
β$_{IV}$

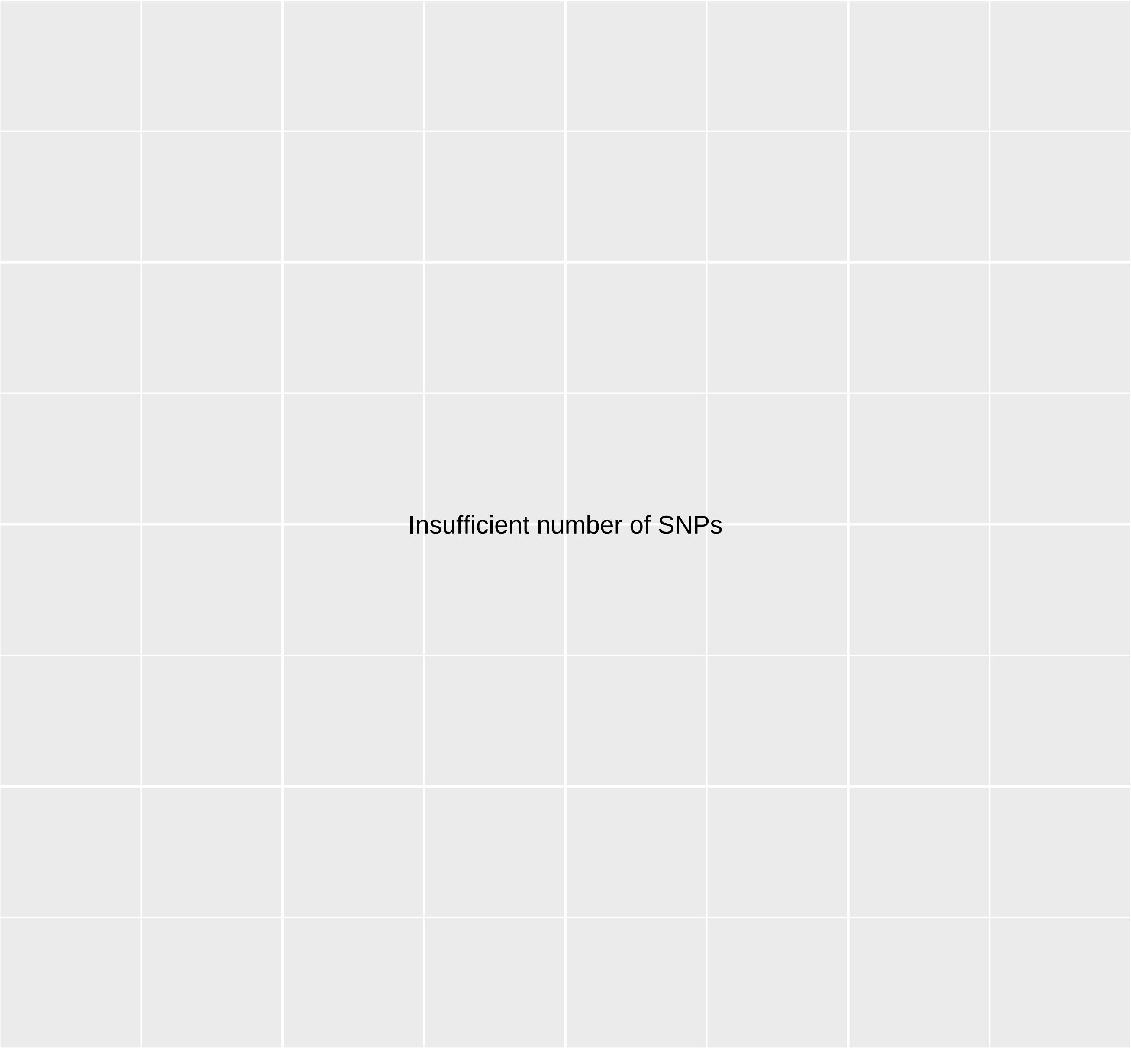
Insufficient number of SNPs

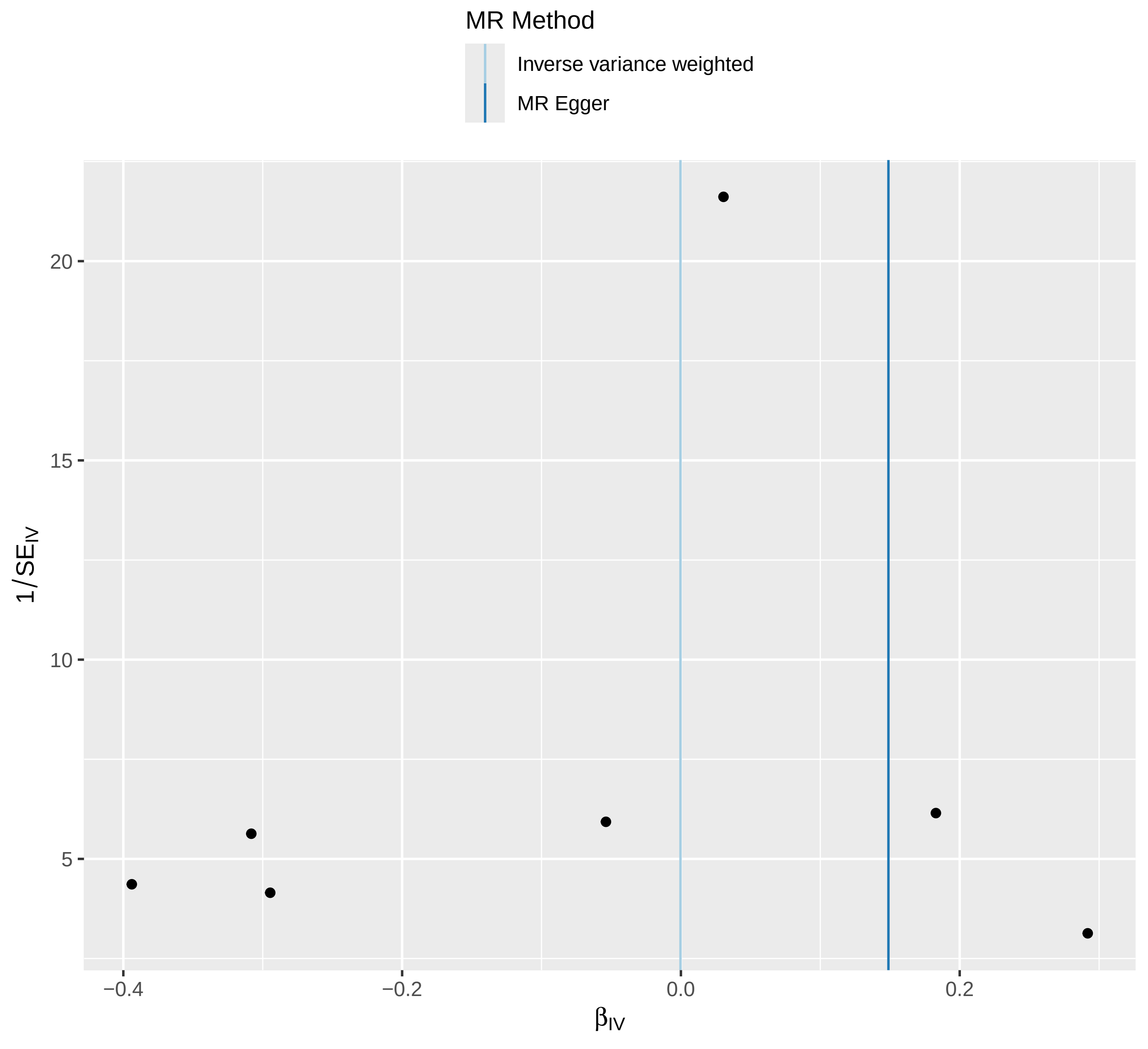

MR Method
Inverse variance weighted
MR Egger
20
15
10
5
1/SE_IV
−0.4
−0.2
0.0
0.2
β_IV

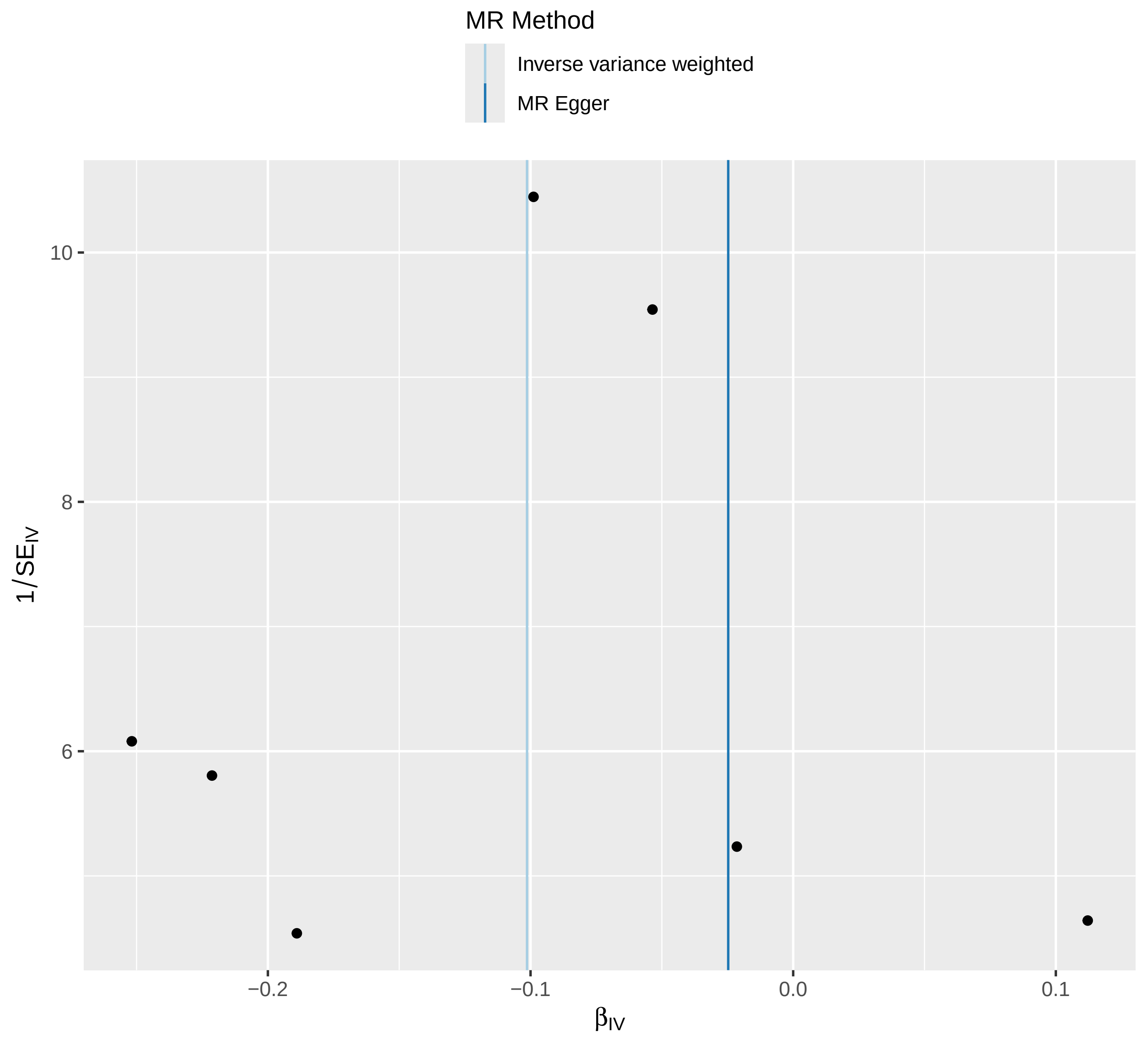

MR Method
Inverse variance weighted
MR Egger
10
8
6
−0.2
−0.1
0.0
0.1
$1/SE_{IV}$
$\beta_{IV}$

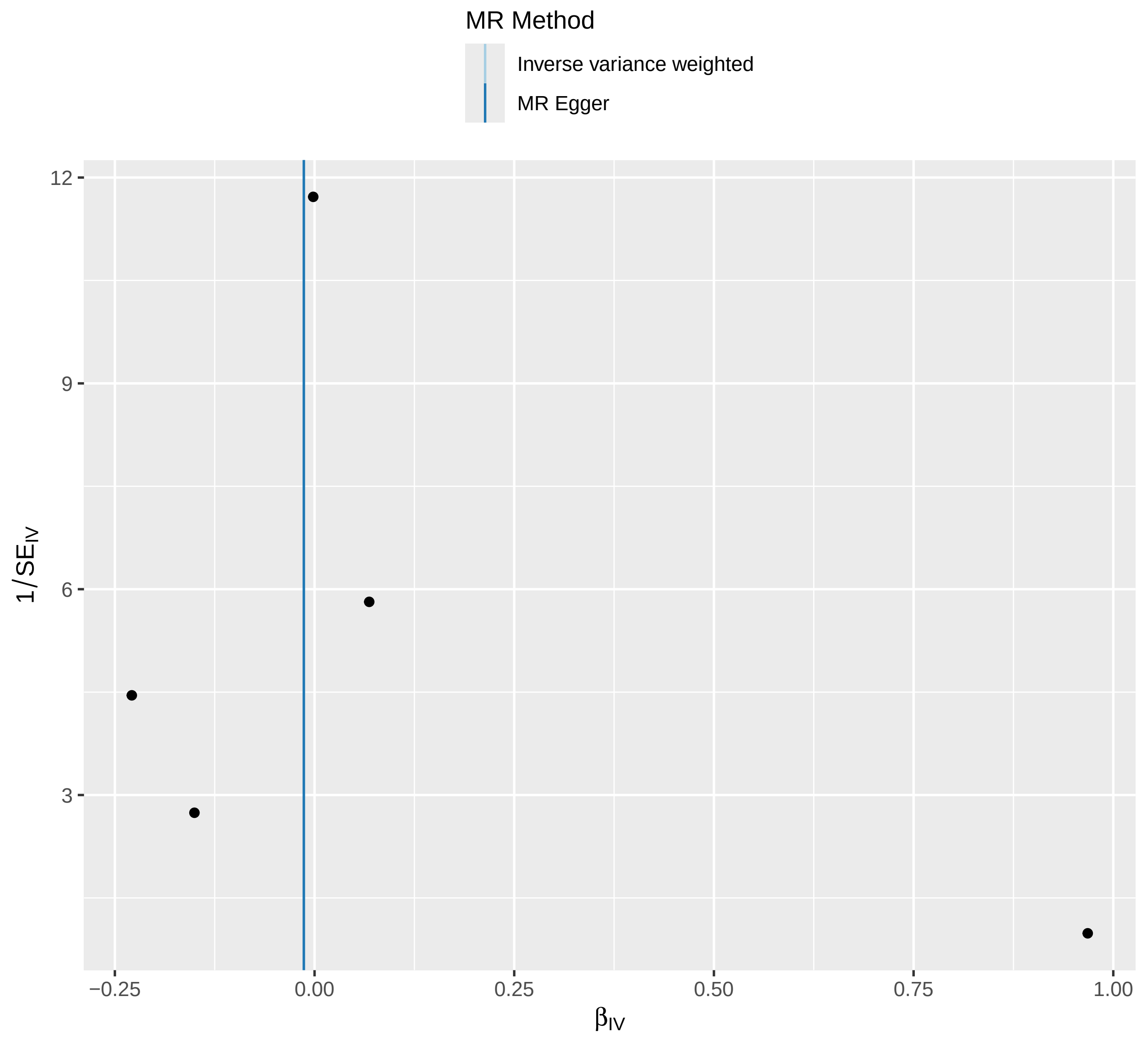

MR Method
Inverse variance weighted
MR Egger
12
9
6
3
$1/SE_{IV}$
−0.25
0.00
0.25
0.50
0.75
1.00
$\beta_{IV}$

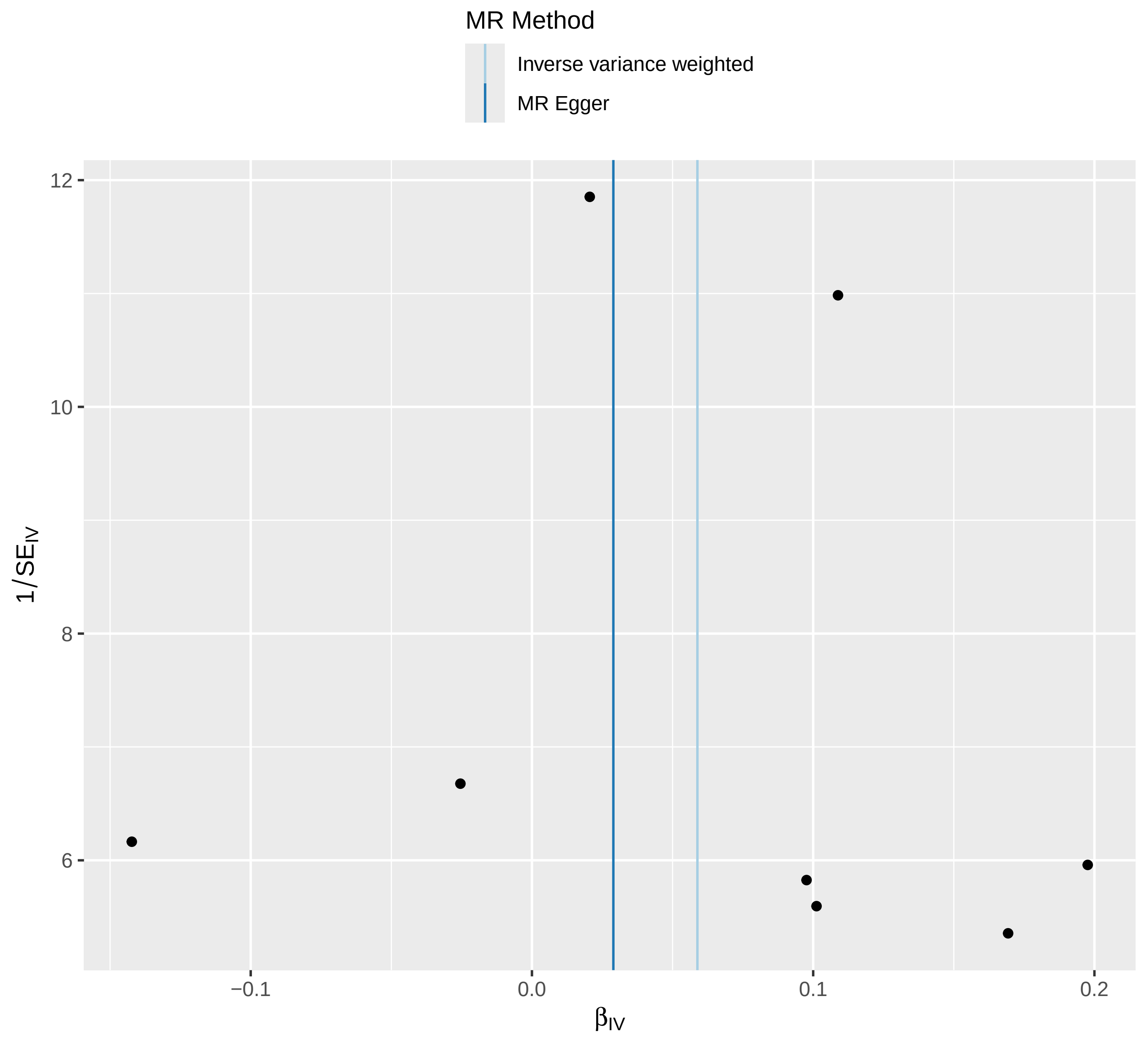
MR Method
Inverse variance weighted
MR Egger
12
10
8
6
1/SE$_{IV}$
−0.1
0.0
0.1
0.2
β$_{IV}$

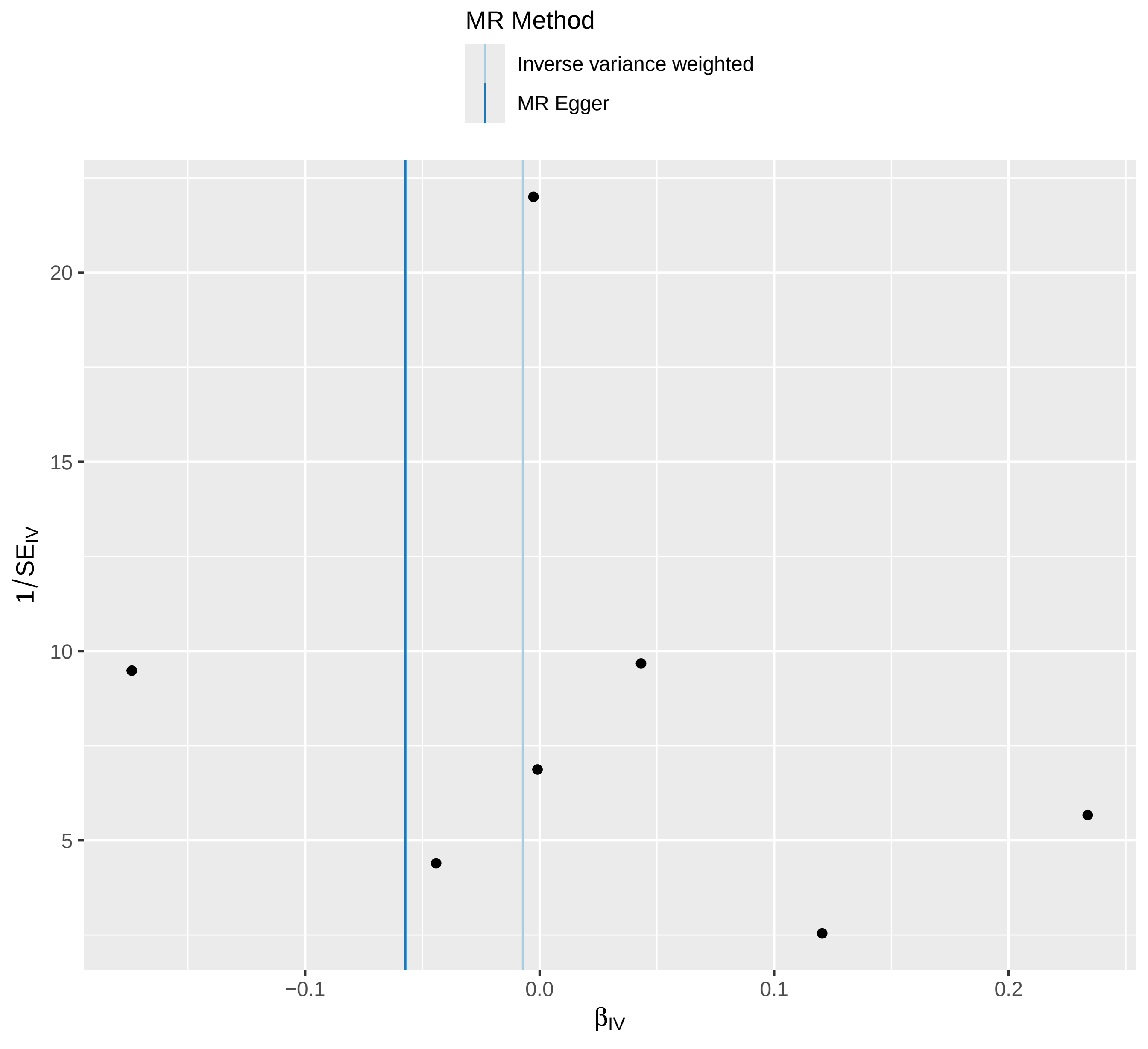
MR Method
Inverse variance weighted
MR Egger
20
15
10
5
1/SE$_{IV}$
−0.1
0.0
0.1
0.2
β$_{IV}$

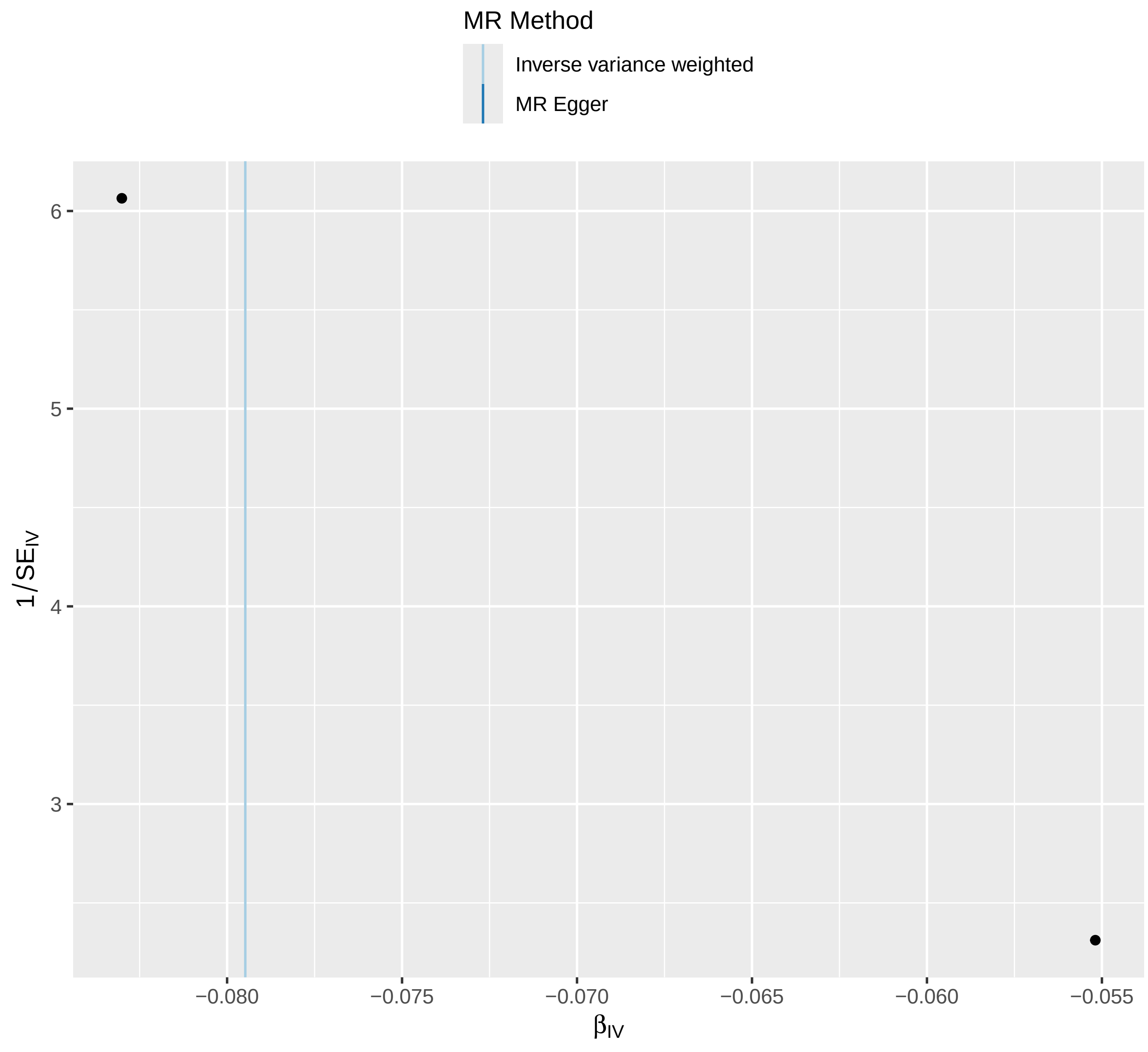

MR Method
Inverse variance weighted
MR Egger
6
5
4
3
$1/SE_{IV}$
−0.080
−0.075
−0.070
−0.065
−0.060
−0.055
$\beta_{IV}$

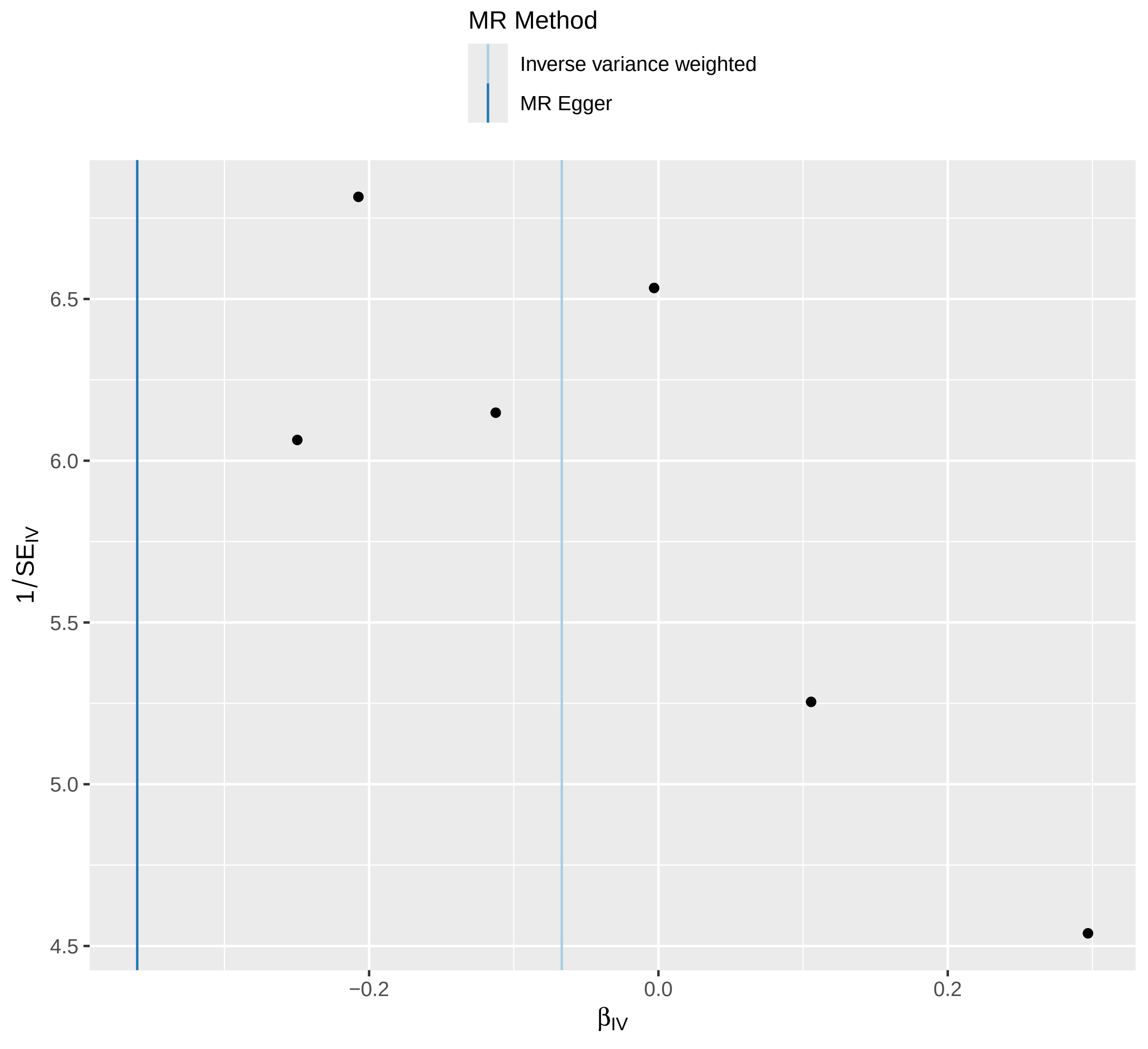
MR Method
Inverse variance weighted
MR Egger
6.5
6.0
5.5
5.0
4.5
1/SE_IV
−0.2
0.0
0.2
β_IV

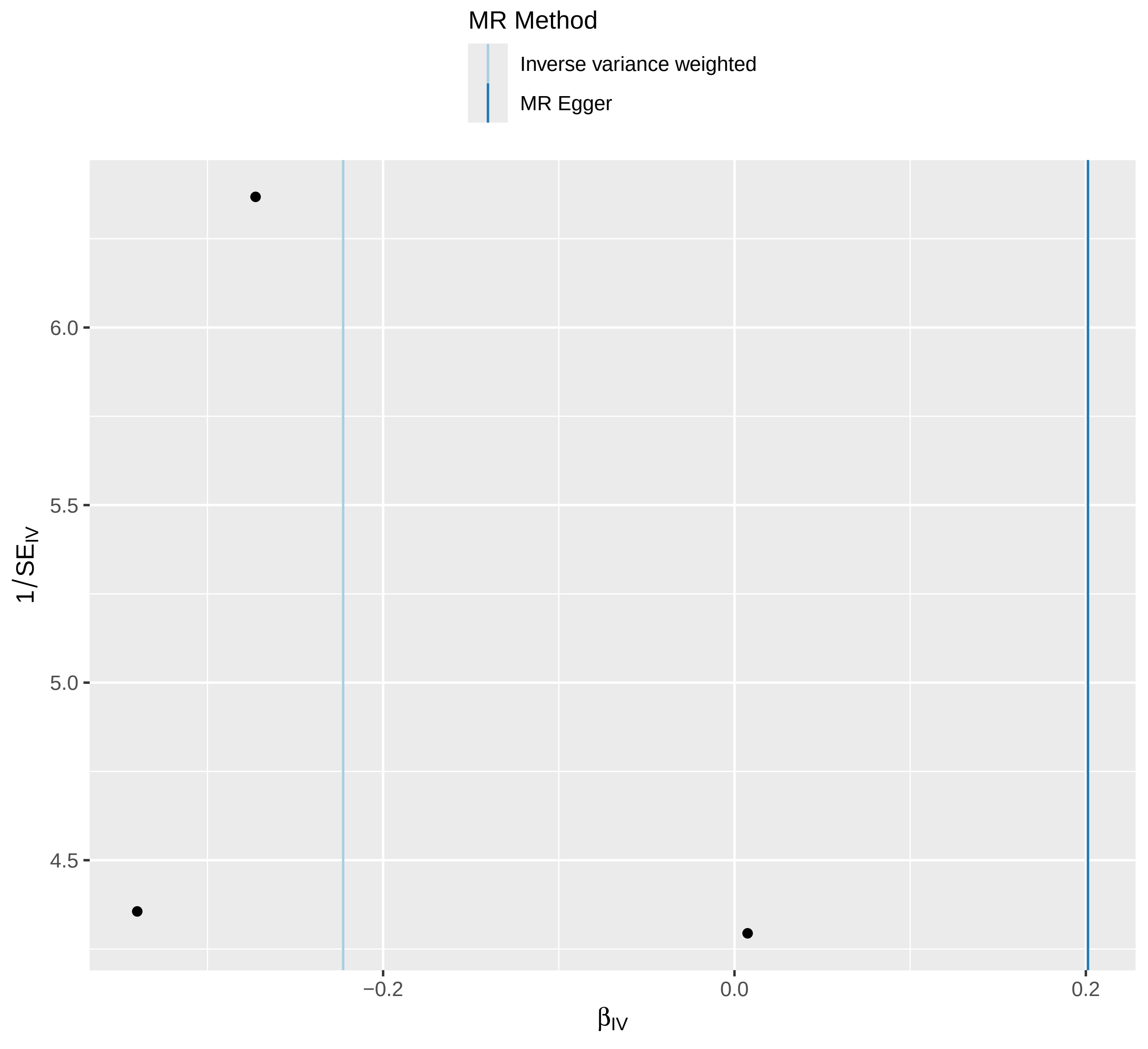

MR Method
Inverse variance weighted
MR Egger
6.0
5.5
5.0
4.5
$1/SE_{IV}$
−0.2
0.0
0.2
$\beta_{IV}$

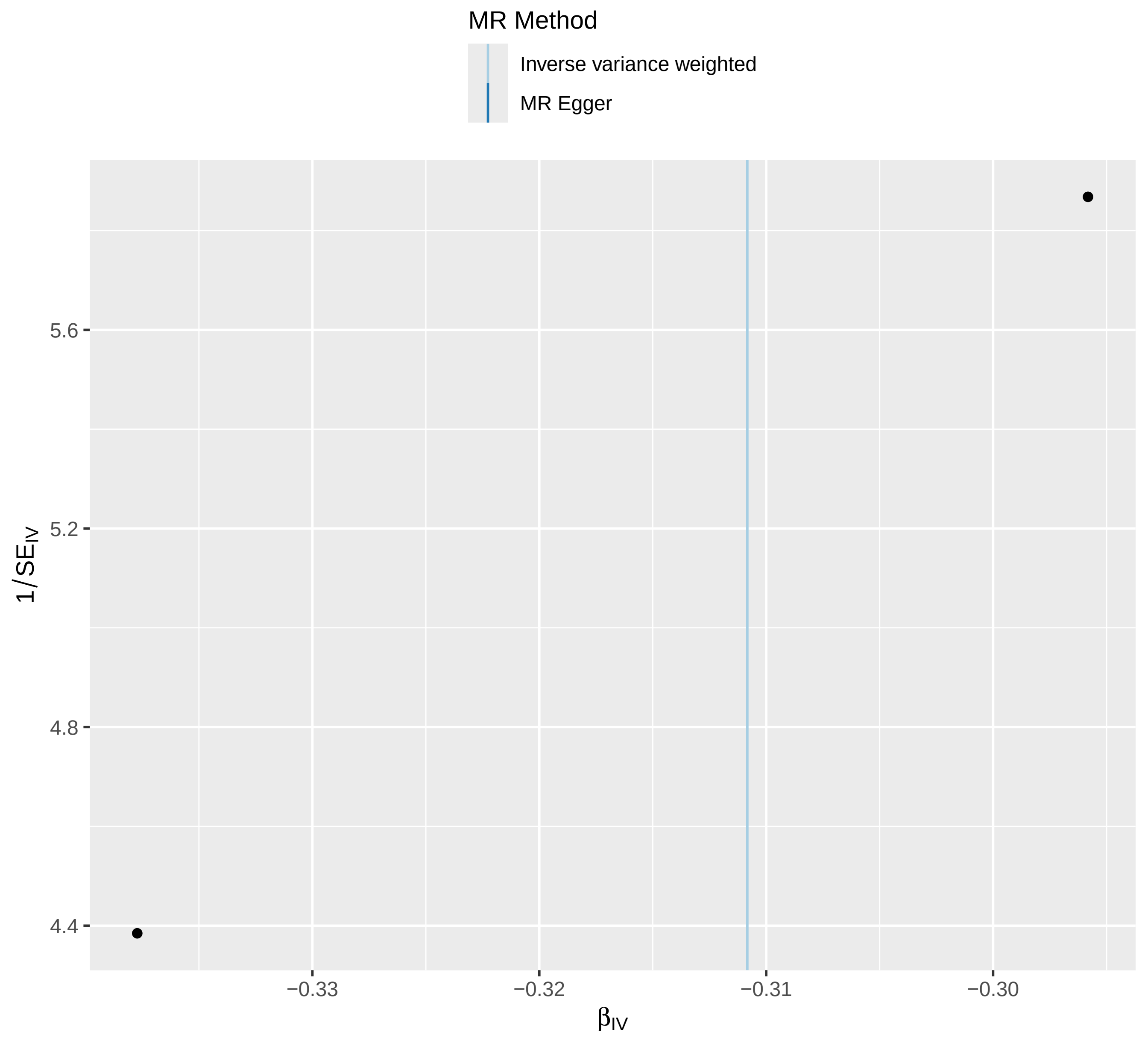

MR Method
Inverse variance weighted
MR Egger
5.6
5.2
4.8
4.4
$1/SE_{IV}$
−0.33
−0.32
−0.31
−0.30
$\beta_{IV}$

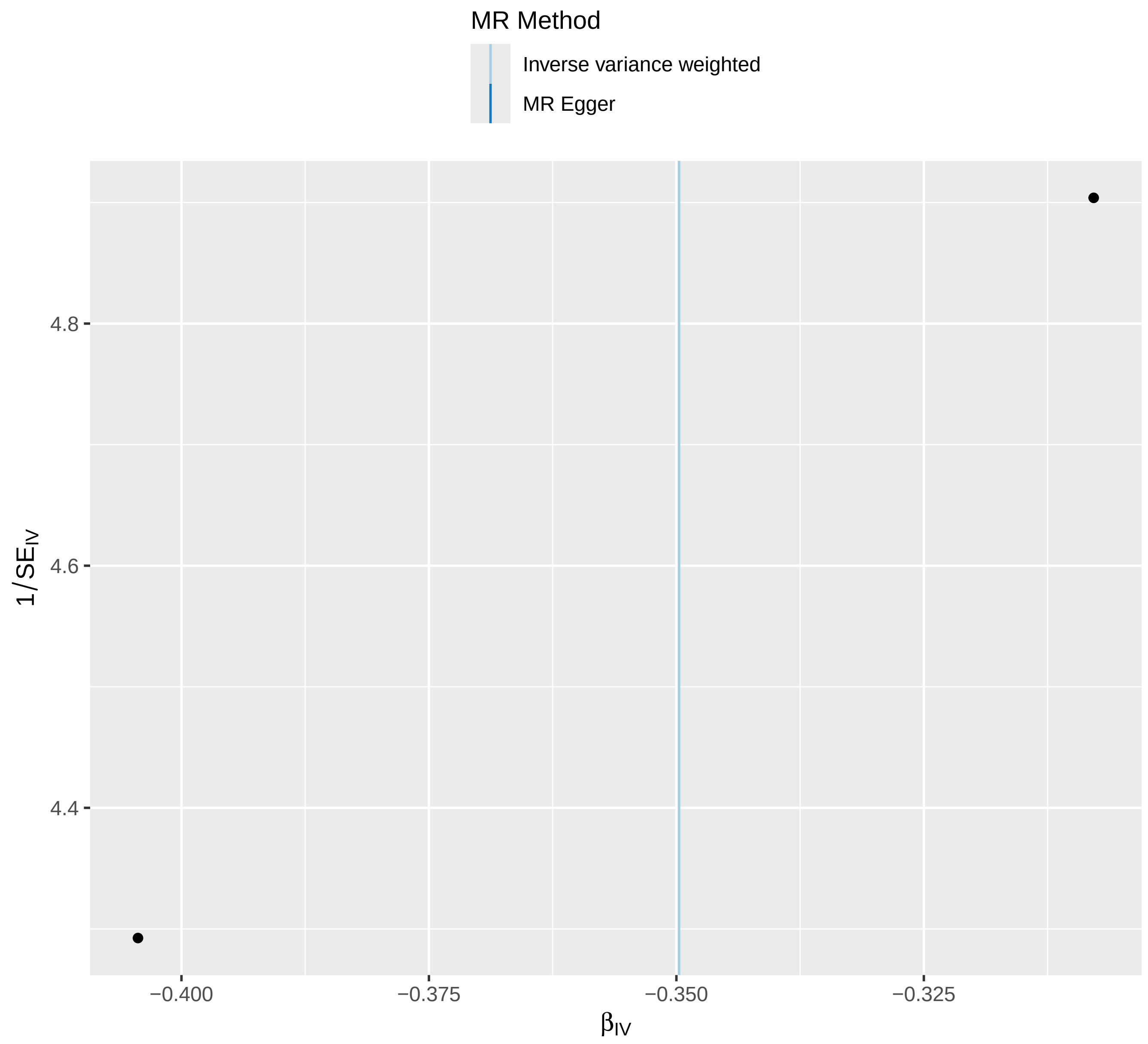

MR Method
Inverse variance weighted
MR Egger
4.8
4.6
4.4
−0.400
−0.375
−0.350
−0.325
$1/SE_{IV}$
$\beta_{IV}$

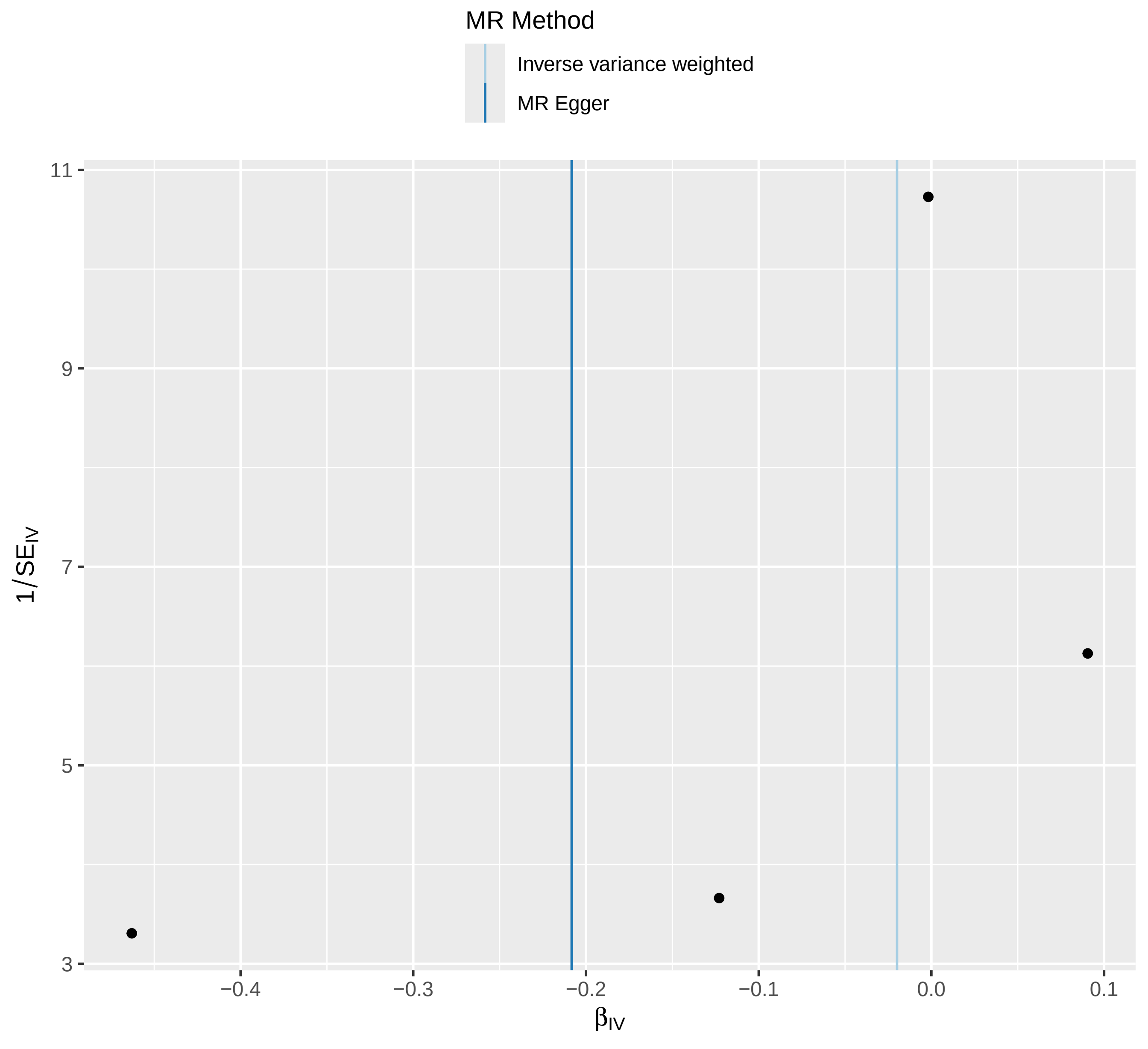
MR Method
Inverse variance weighted
MR Egger
11
9
7
5
3
1/SE_IV
−0.4
−0.3
−0.2
−0.1
0.0
0.1
β_IV

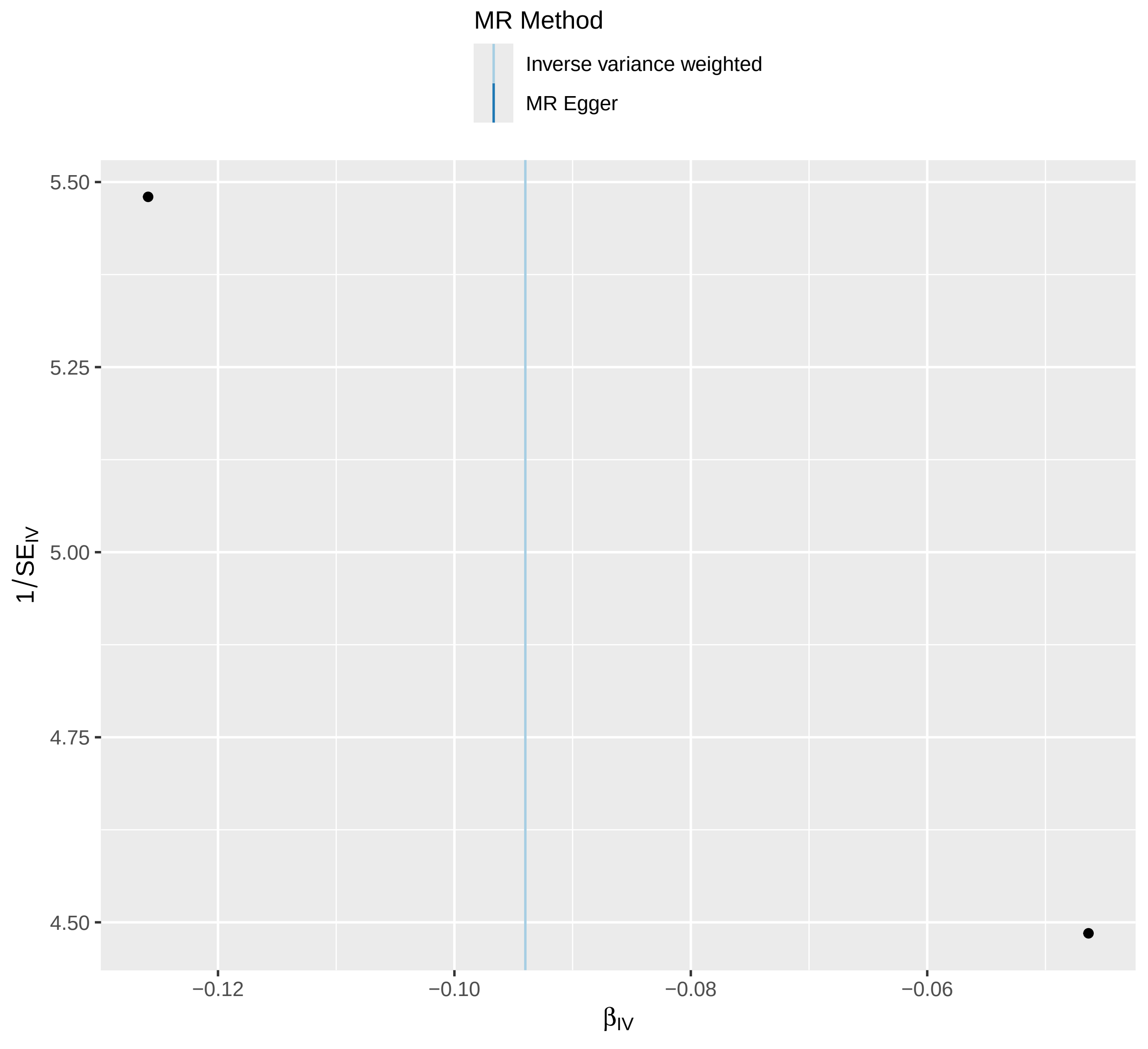

MR Method
Inverse variance weighted
MR Egger
5.50
5.25
5.00
4.75
4.50
1/SE$_{IV}$
−0.12
−0.10
−0.08
−0.06
β$_{IV}$

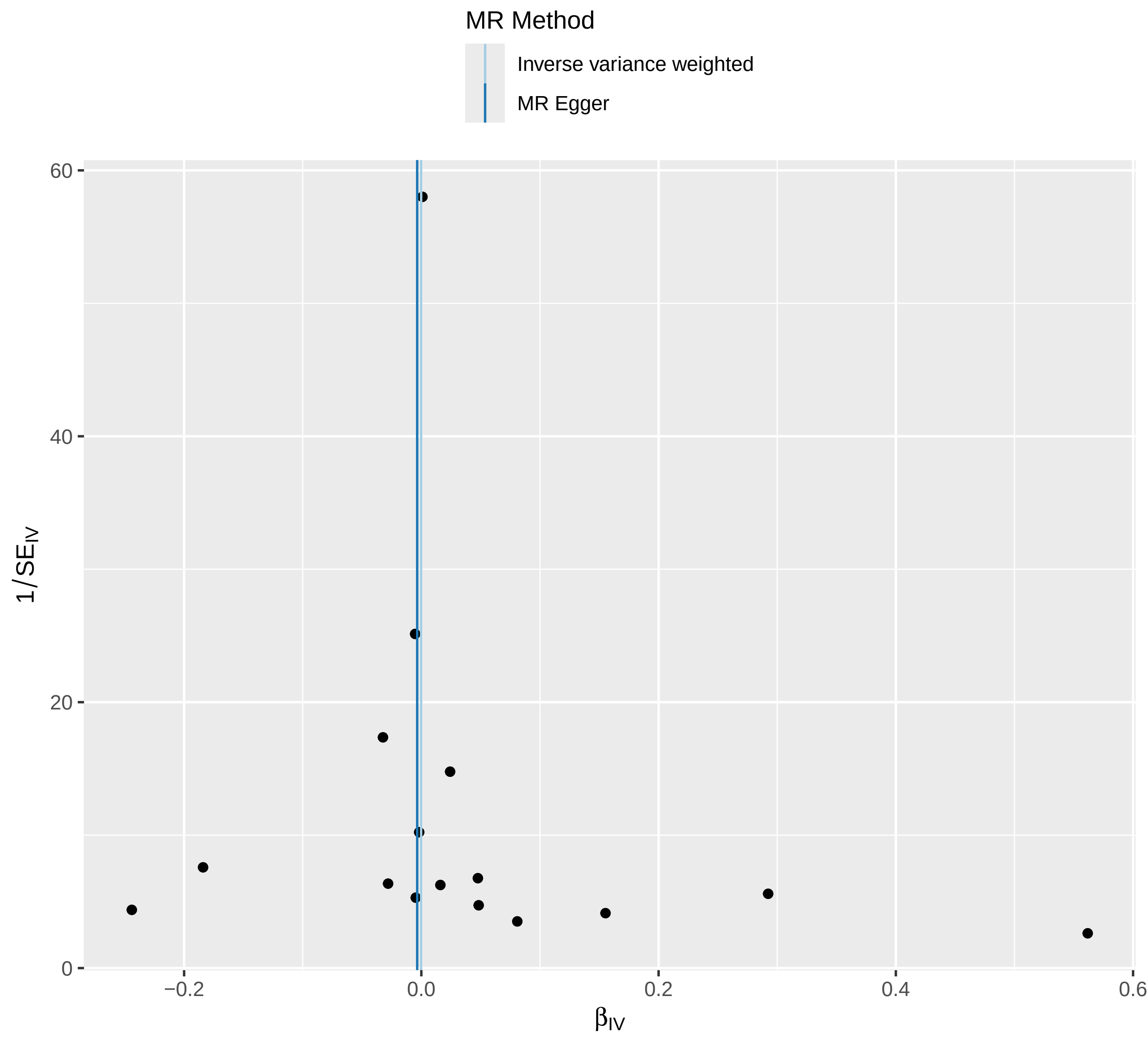

MR Method
Inverse variance weighted
MR Egger
60
40
20
0
1/SE$_{IV}$
−0.2
0.0
0.2
0.4
0.6
β$_{IV}$

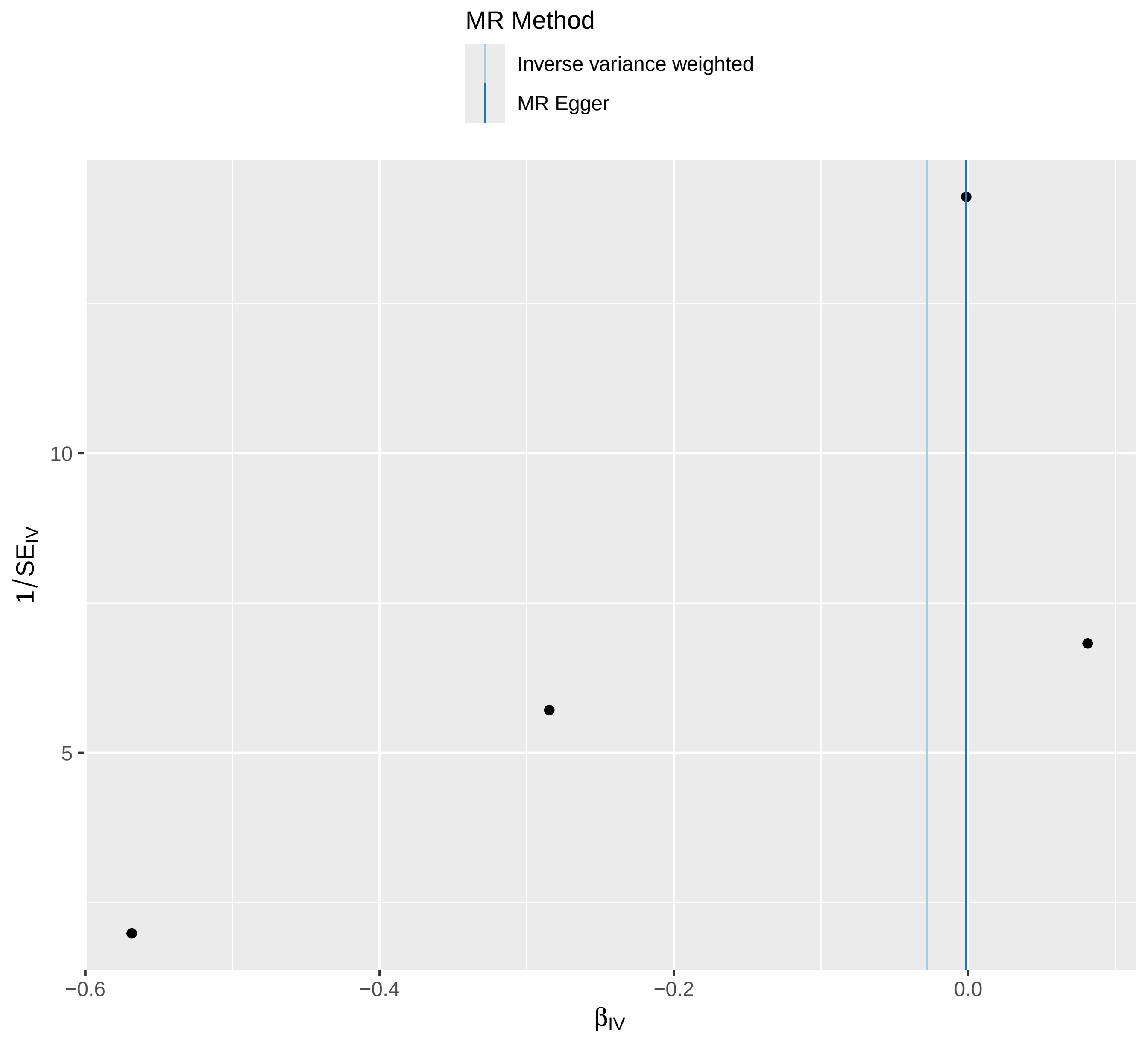

MR Method
Inverse variance weighted
MR Egger
1/SE$_{IV}$
10
5
−0.6
−0.4
−0.2
0.0
β$_{IV}$

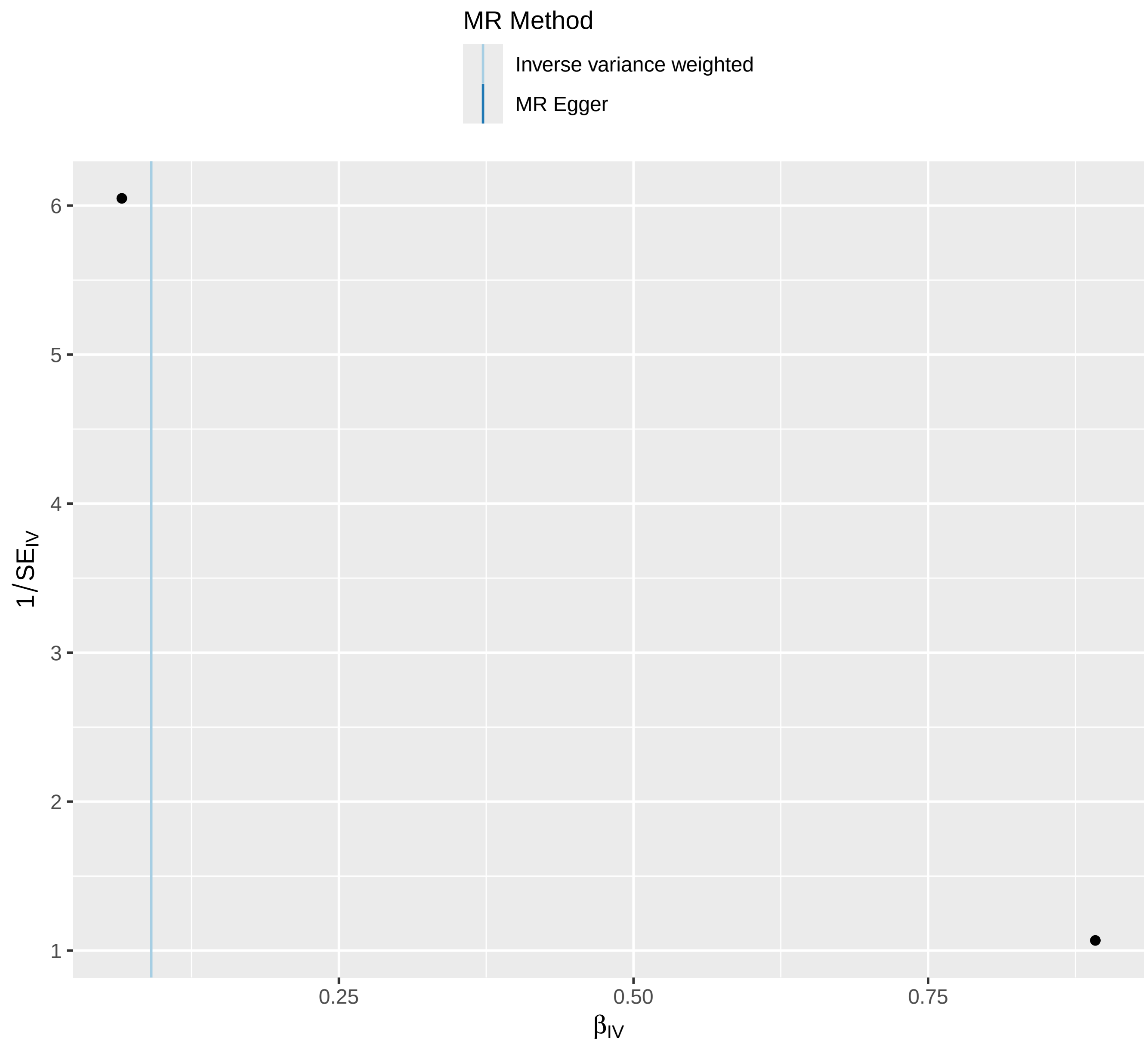
MR Method
Inverse variance weighted
MR Egger
1/SE_IV
β_IV
0.25
0.50
0.75
1
2
3
4
5
6

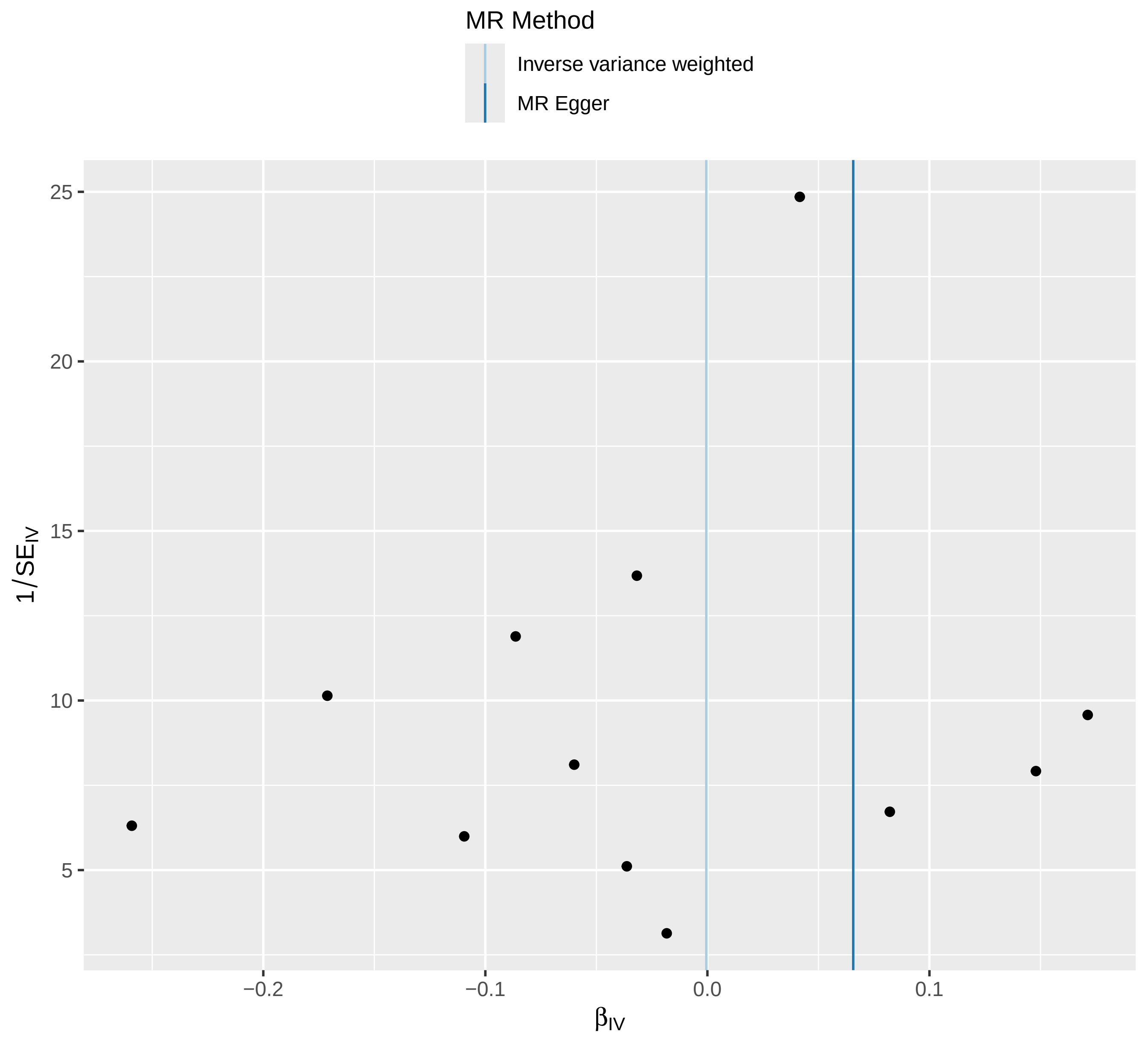
MR Method
Inverse variance weighted
MR Egger
25
20
15
10
5
$1/SE_{IV}$
−0.2
−0.1
0.0
0.1
$\beta_{IV}$

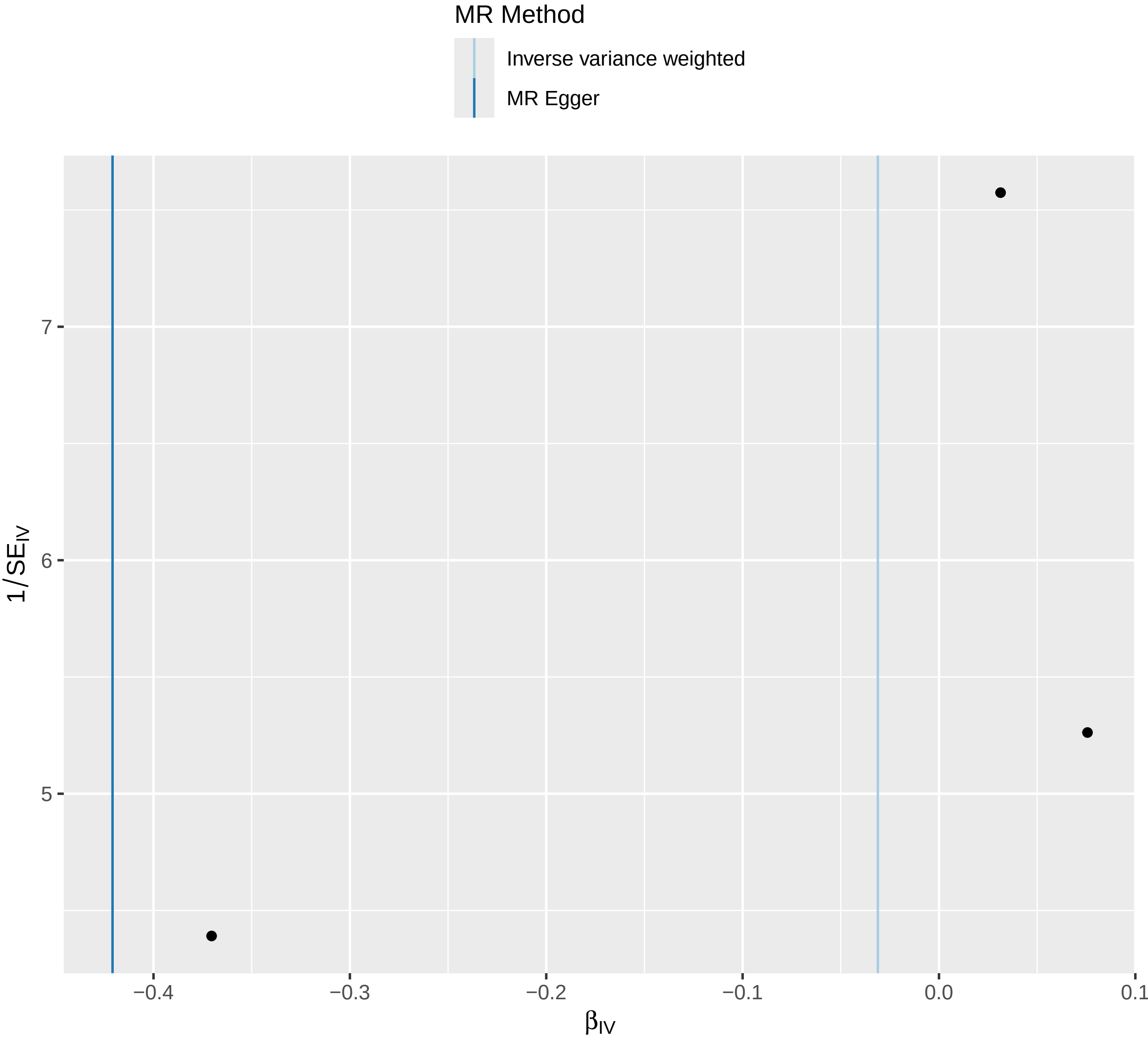

MR Method
Inverse variance weighted
MR Egger
7
6
5
1/SE_IV
−0.4
−0.3
−0.2
−0.1
0.0
0.1
β_IV

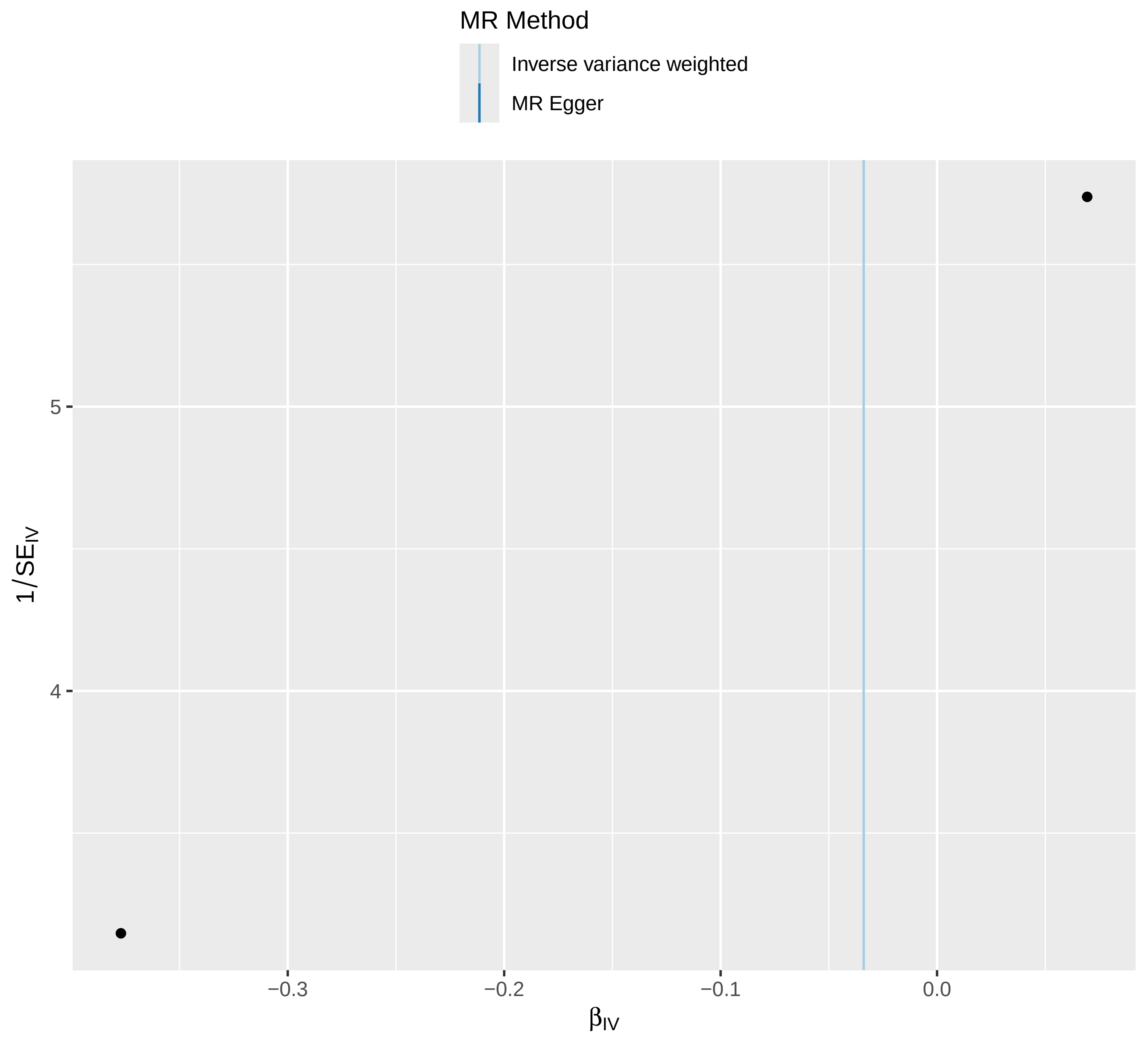
MR Method
Inverse variance weighted
MR Egger
5
4
−0.3
−0.2
−0.1
0.0
$1/SE_{IV}$
$\beta_{IV}$

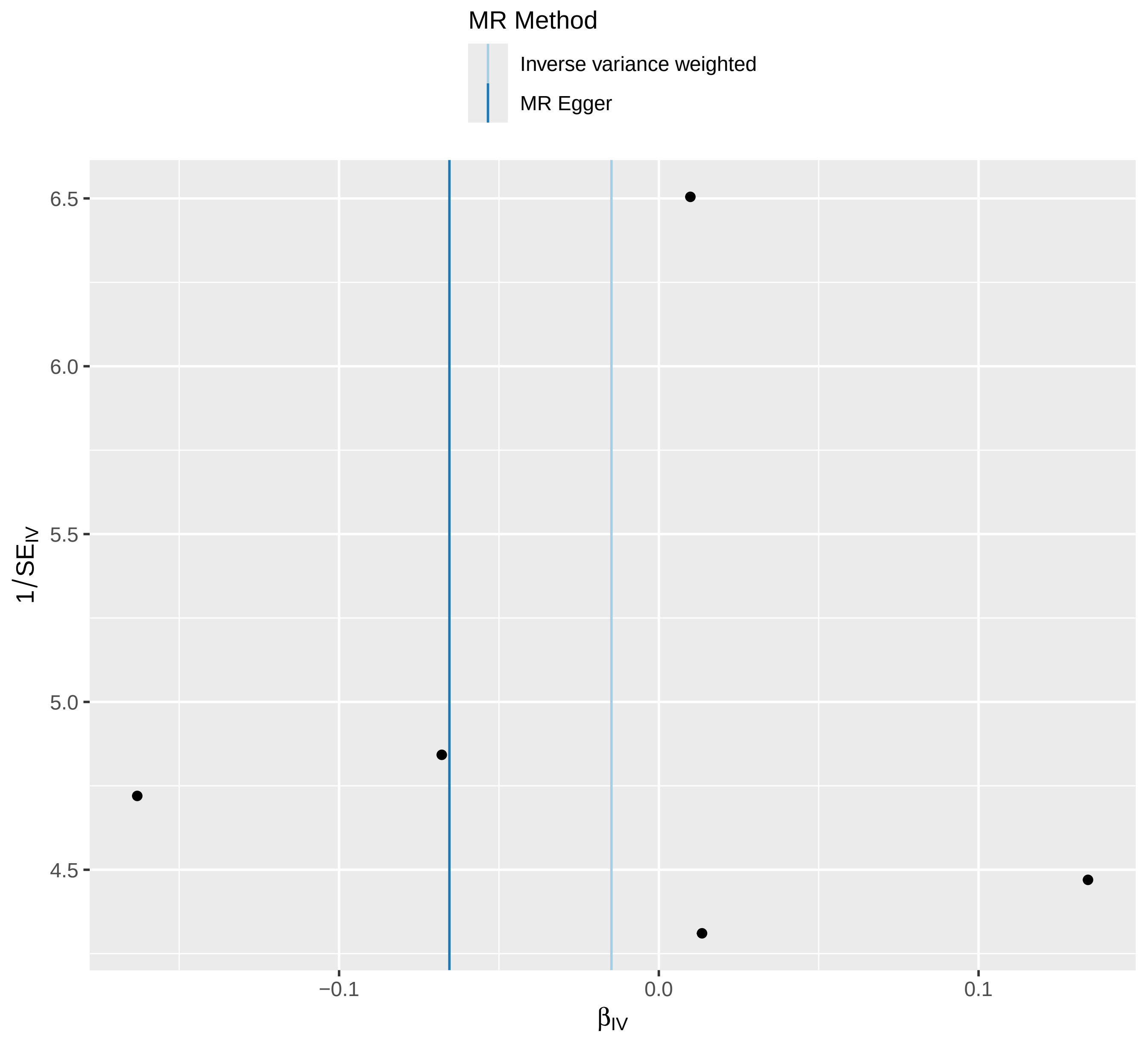
MR Method
Inverse variance weighted
MR Egger
6.5
6.0
5.5
5.0
4.5
1/SE_IV
−0.1
0.0
0.1
β_IV

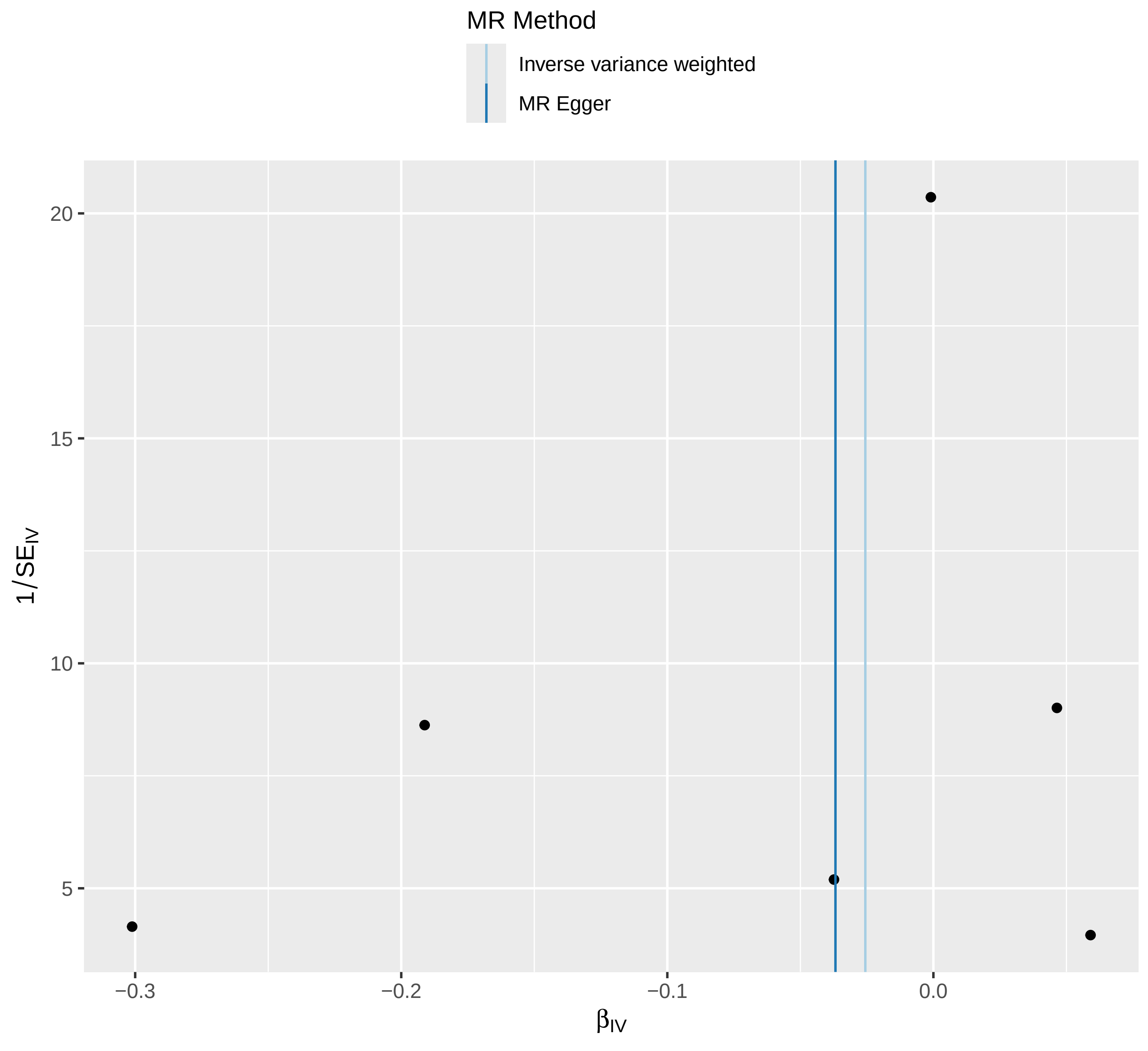

MR Method
Inverse variance weighted
MR Egger
20
15
10
5
1/SE$_{IV}$
−0.3
−0.2
−0.1
0.0
β$_{IV}$

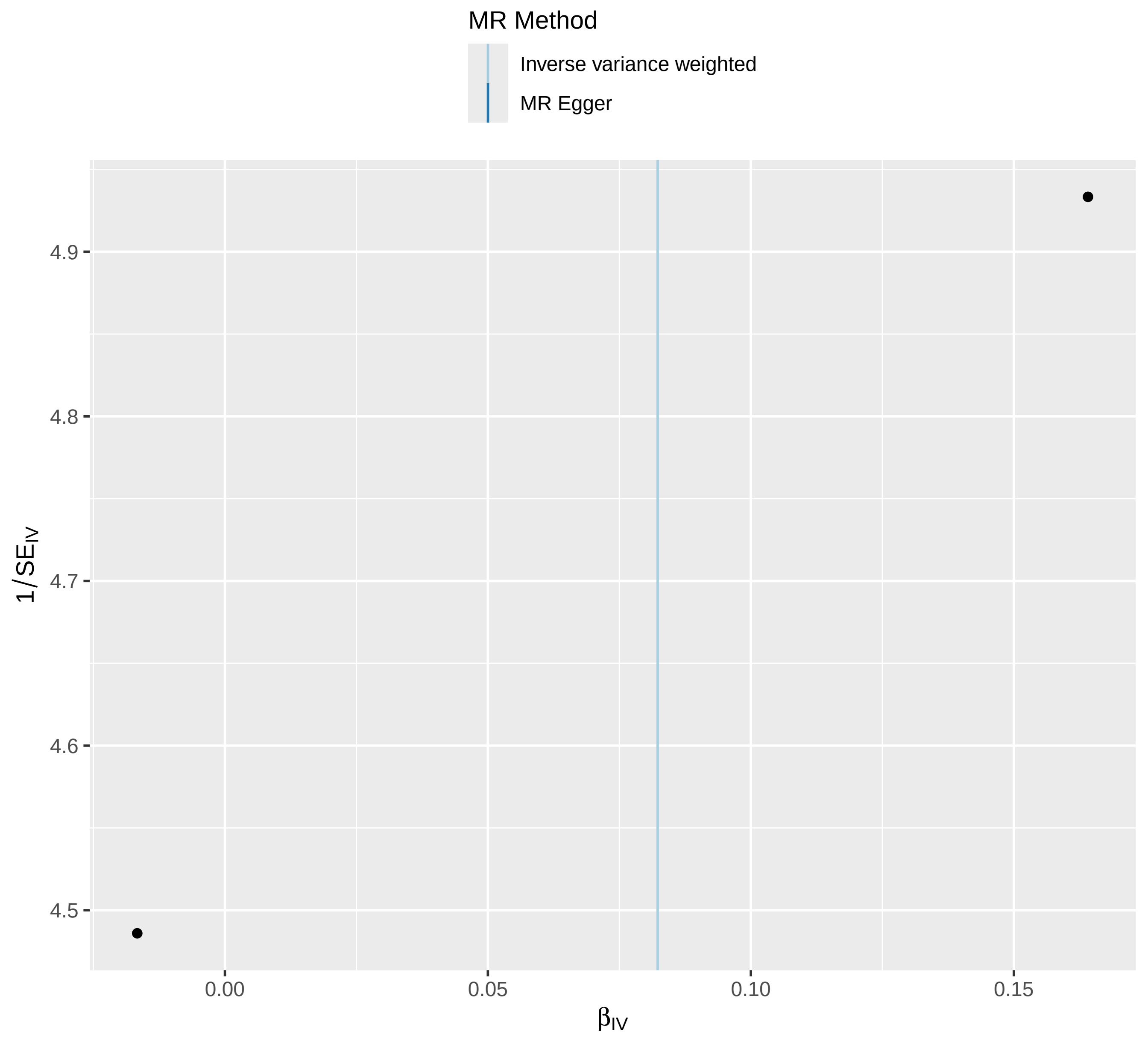

MR Method
Inverse variance weighted
MR Egger
4.9
4.8
4.7
4.6
4.5
1/SE$_{IV}$
0.00
0.05
0.10
0.15
β$_{IV}$

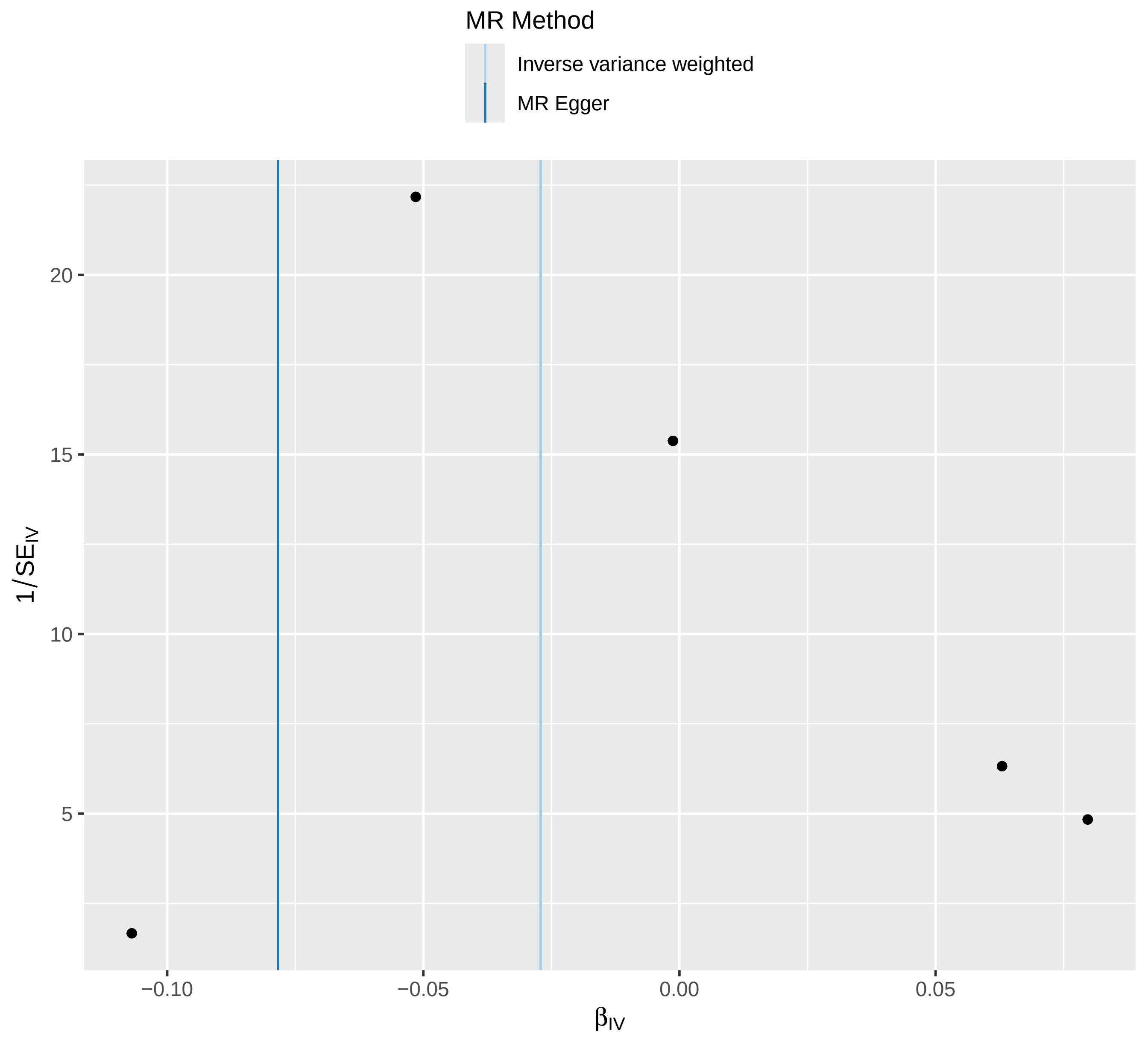
MR Method
Inverse variance weighted
MR Egger
20
15
10
5
1/SE_IV
−0.10
−0.05
0.00
0.05
β_IV

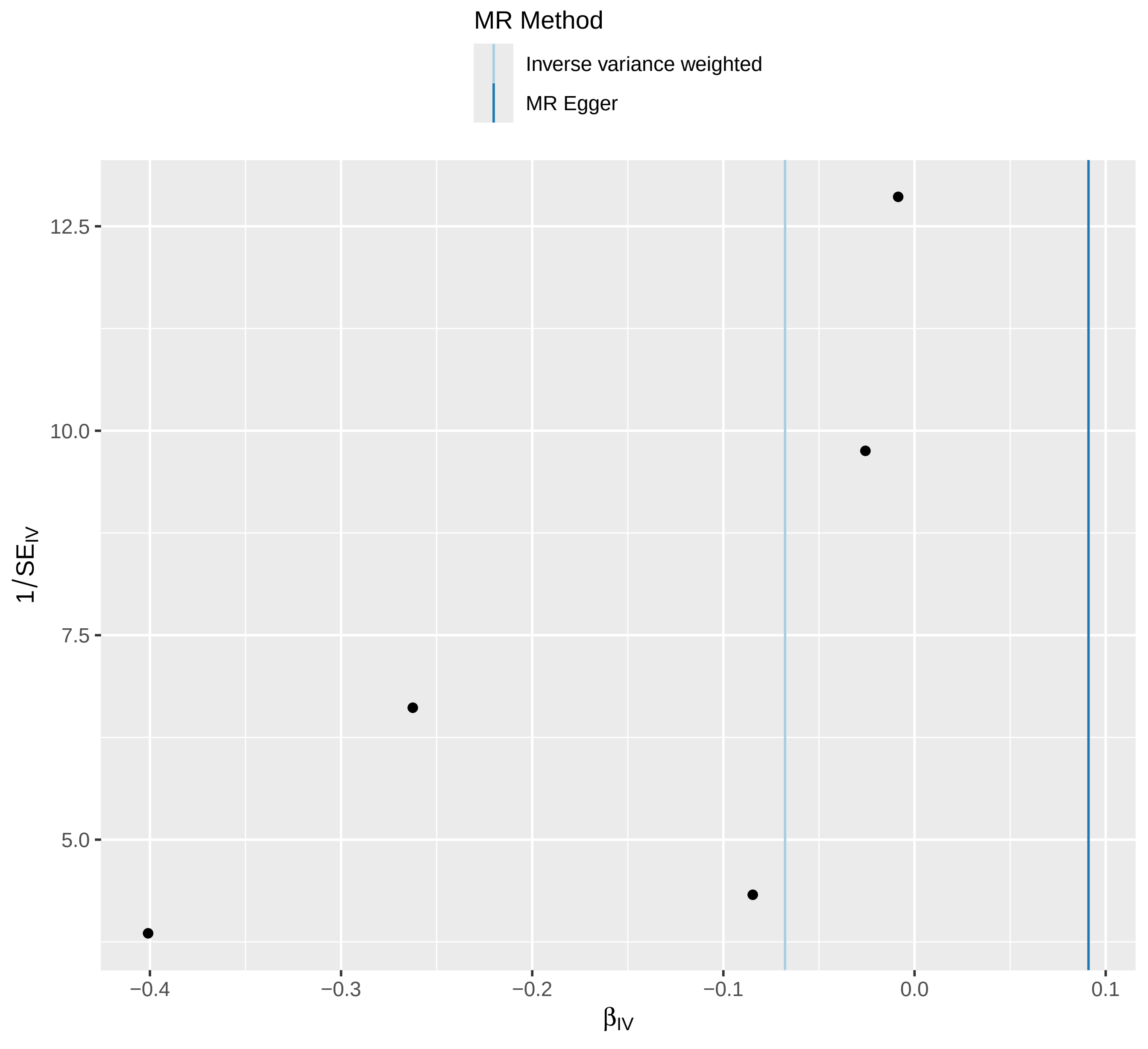

MR Method
Inverse variance weighted
MR Egger
12.5
10.0
7.5
5.0
1/SE$_{IV}$
−0.4
−0.3
−0.2
−0.1
0.0
0.1
β$_{IV}$

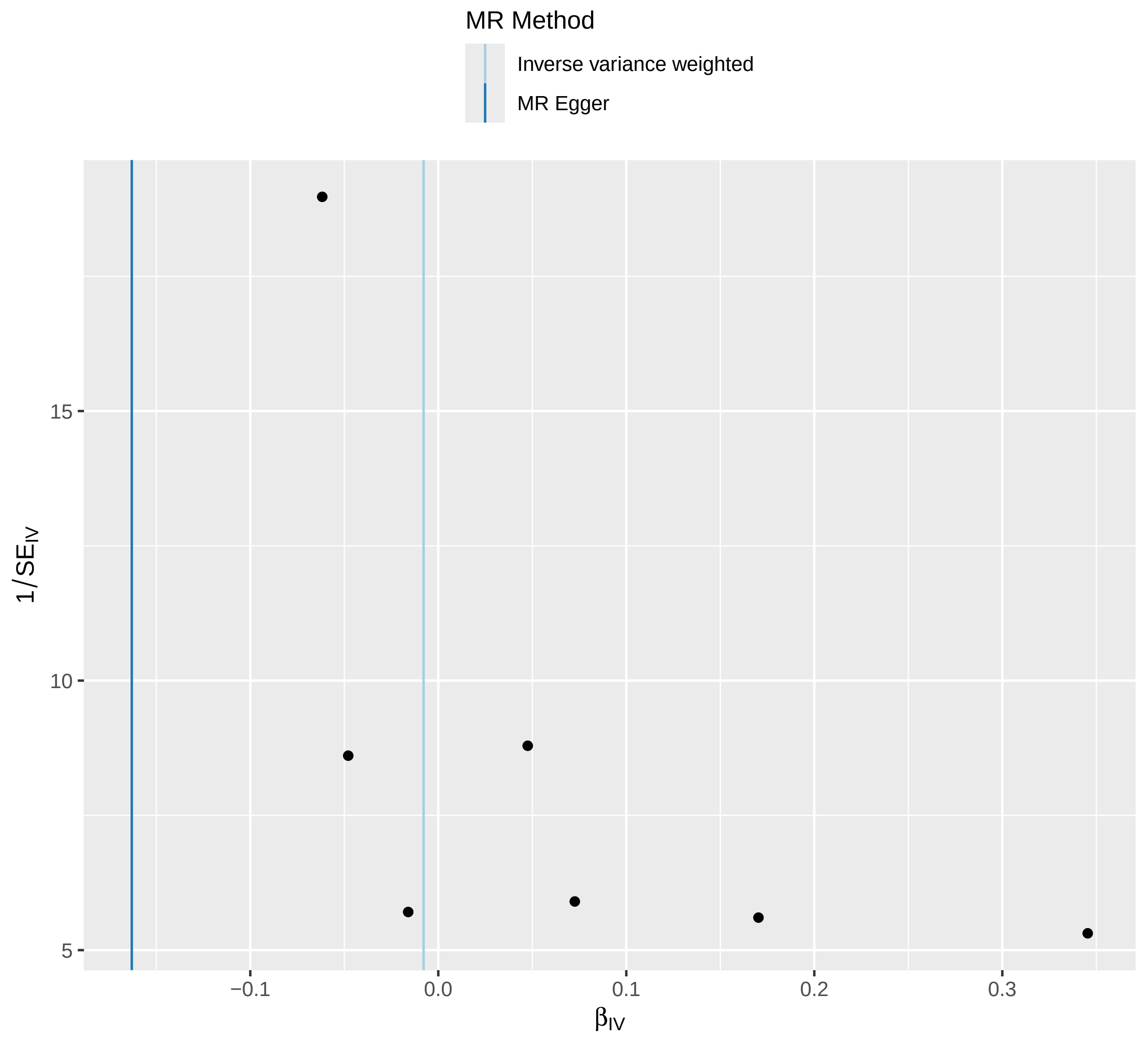
MR Method
Inverse variance weighted
MR Egger
1/SE$_{IV}$
15
10
5
−0.1
0.0
0.1
0.2
0.3
β$_{IV}$

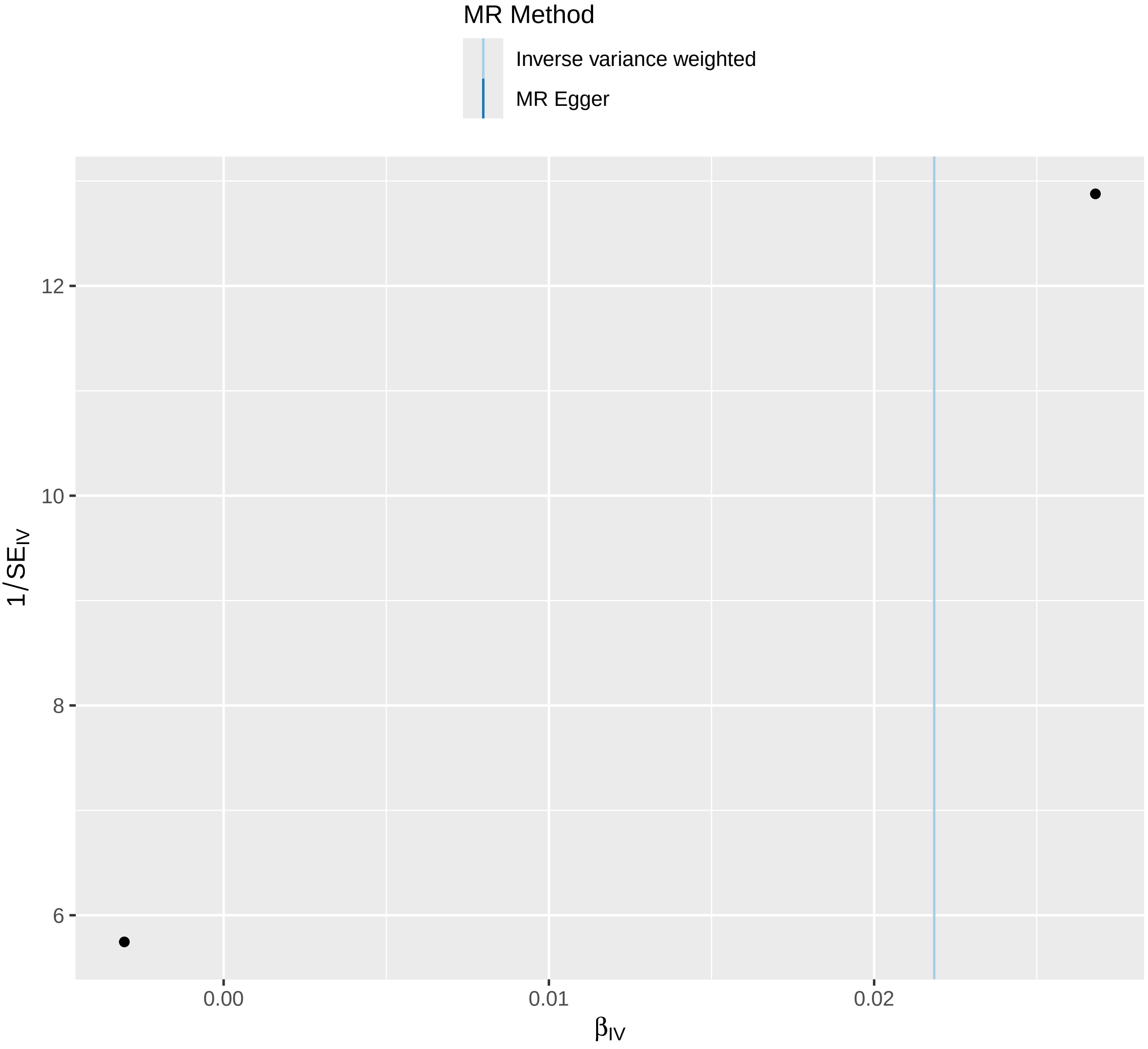

MR Method
Inverse variance weighted
MR Egger
12
10
8
6
1/SE_IV
0.00
0.01
0.02
β_IV

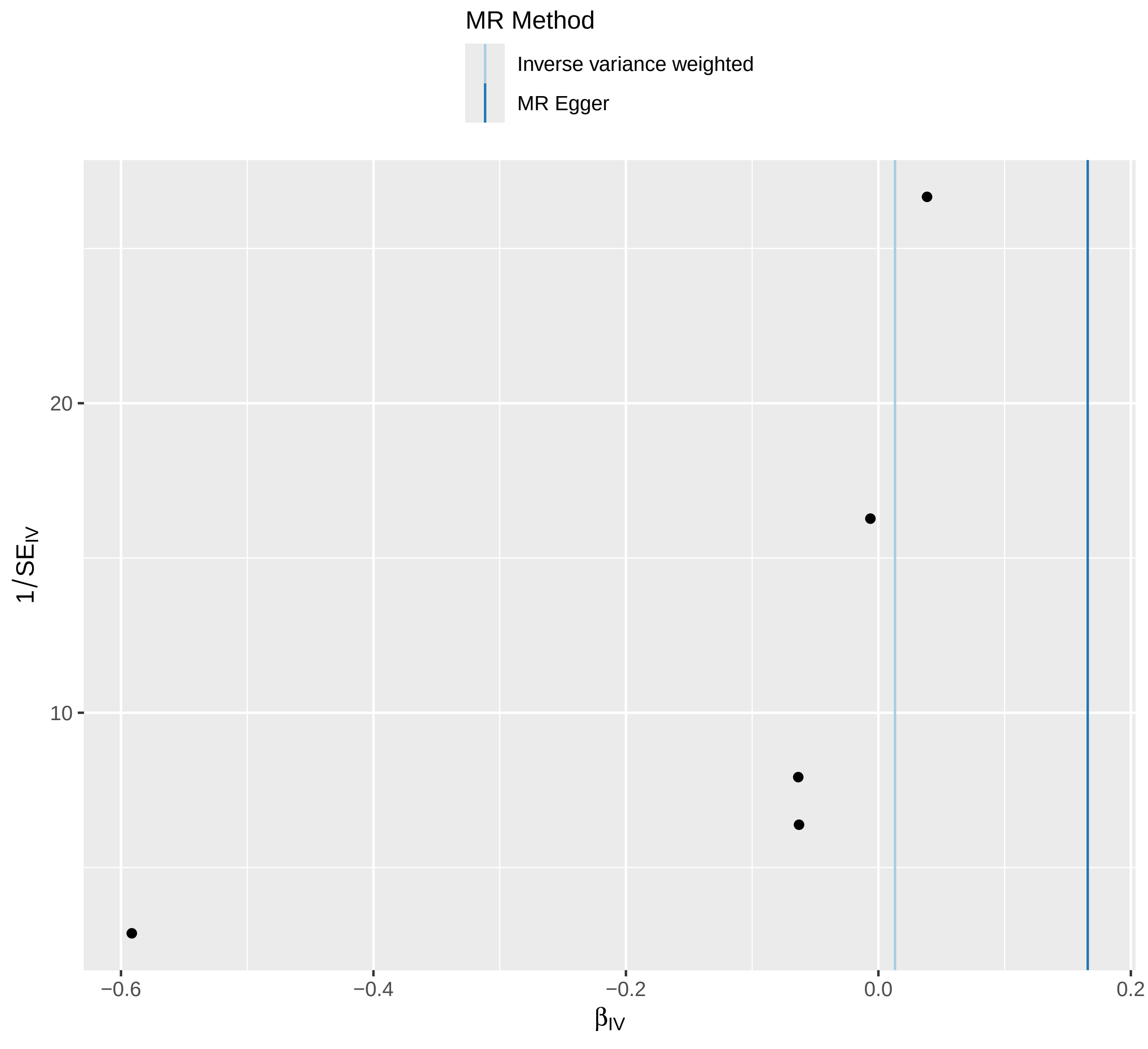
MR Method
Inverse variance weighted
MR Egger
20
10
1/SE$_{IV}$
−0.6
−0.4
−0.2
0.0
0.2
β$_{IV}$

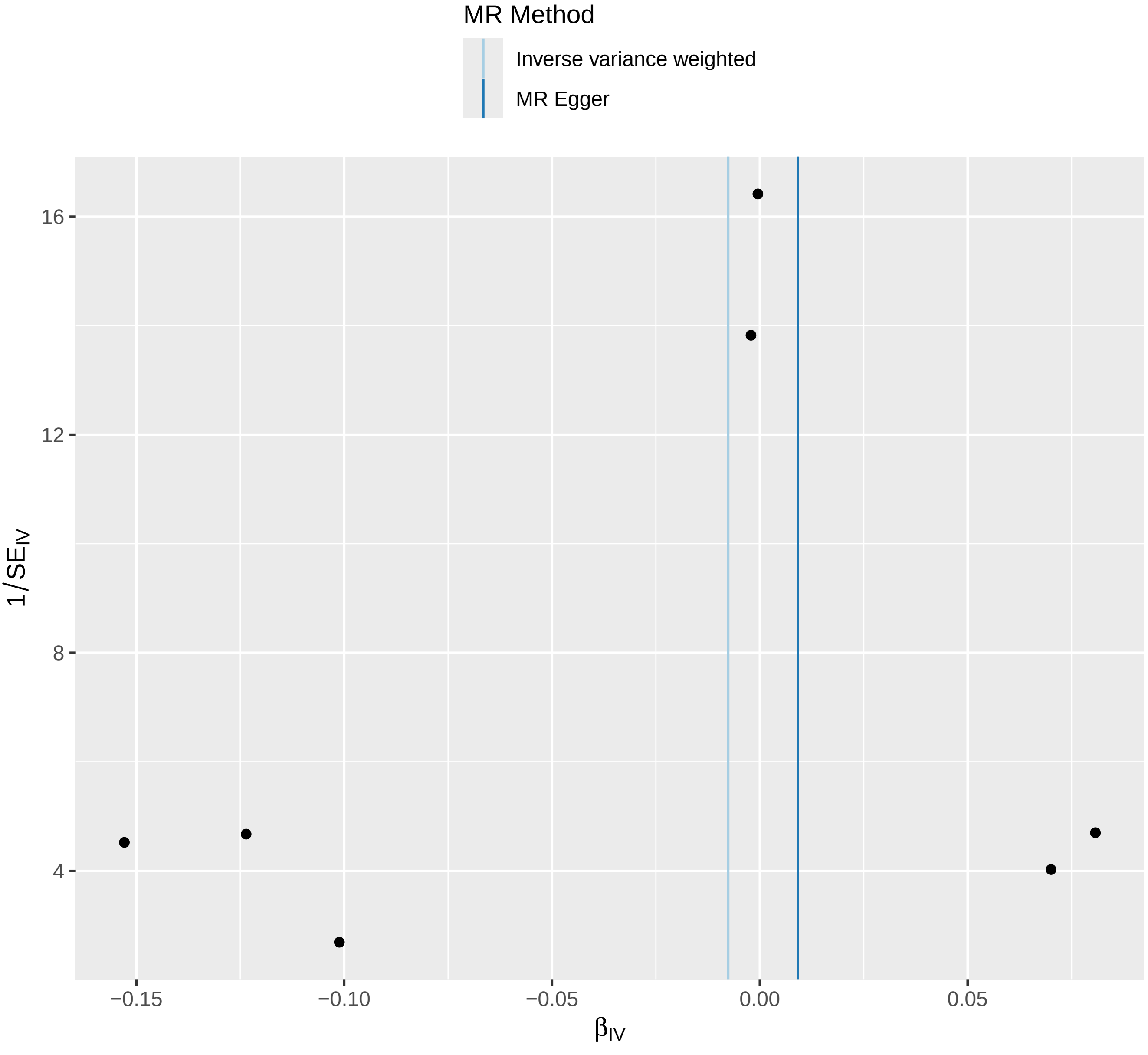
MR Method
Inverse variance weighted
MR Egger
16
12
8
4
1/SE$_{IV}$
−0.15
−0.10
−0.05
0.00
0.05
β$_{IV}$

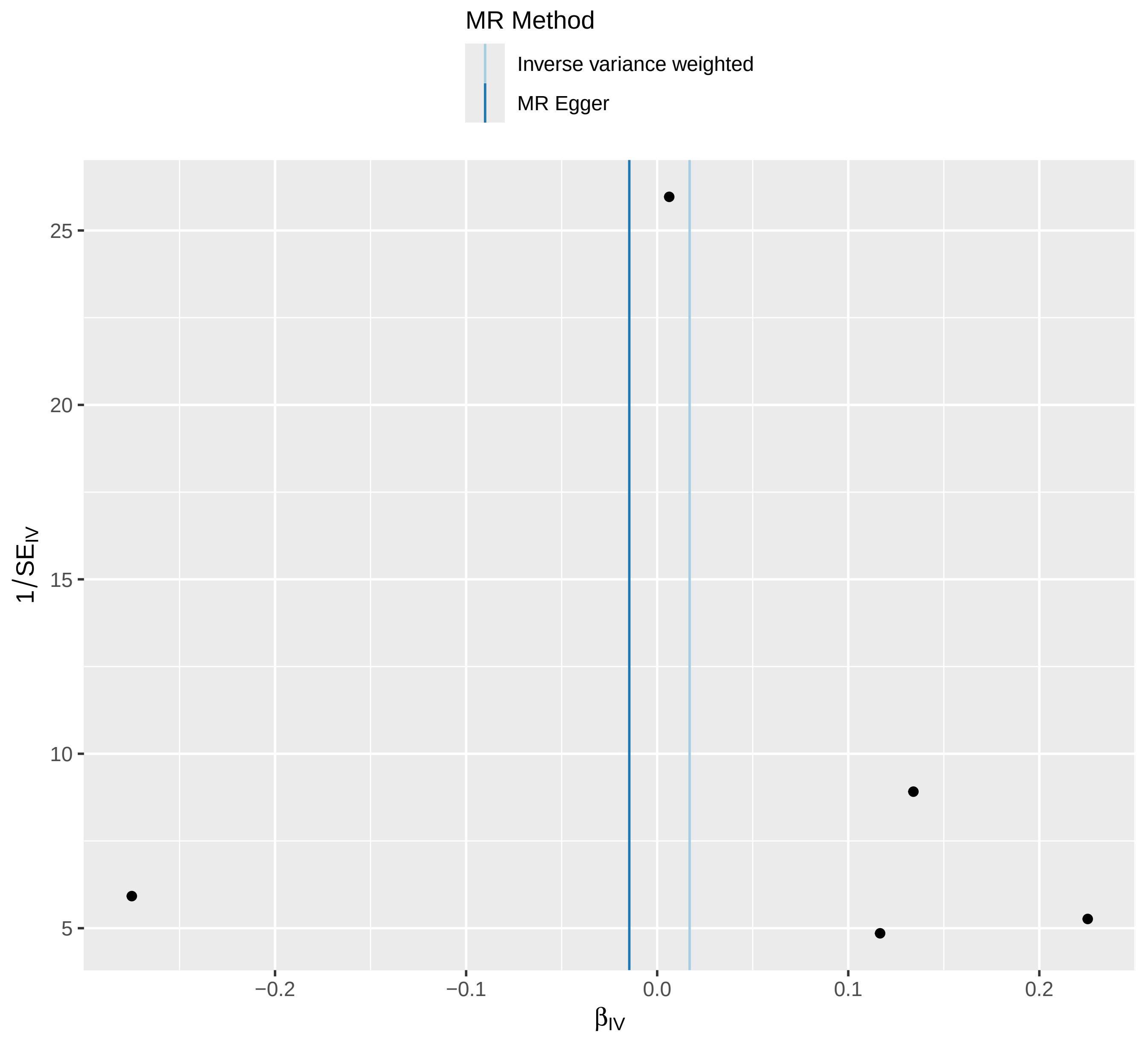

MR Method
Inverse variance weighted
MR Egger
25
20
15
10
5
$1/SE_{IV}$
−0.2
−0.1
0.0
0.1
0.2
$\beta_{IV}$

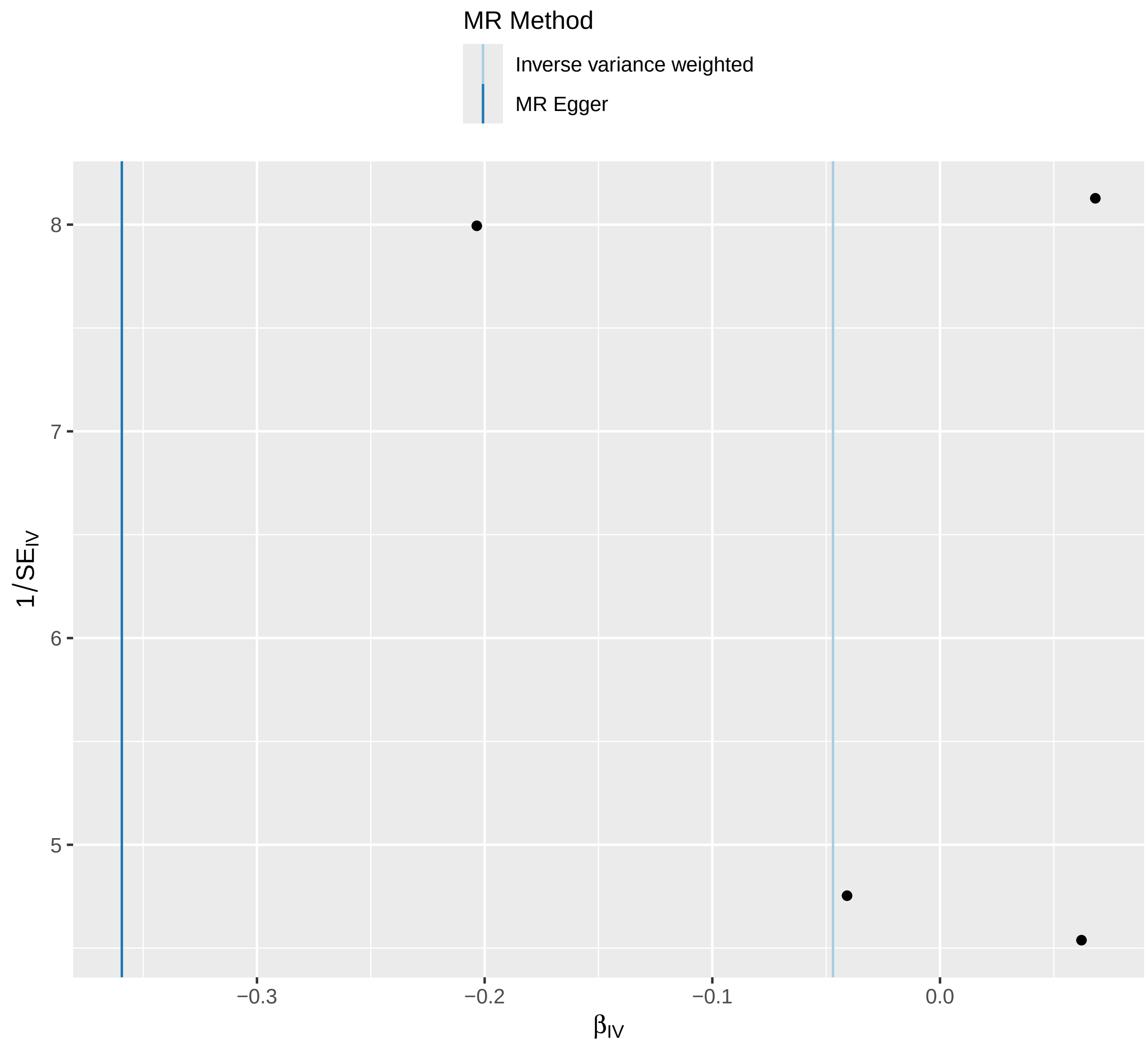

MR Method
Inverse variance weighted
MR Egger
8
7
6
5
$1/SE_{IV}$
−0.3
−0.2
−0.1
0.0
$\beta_{IV}$

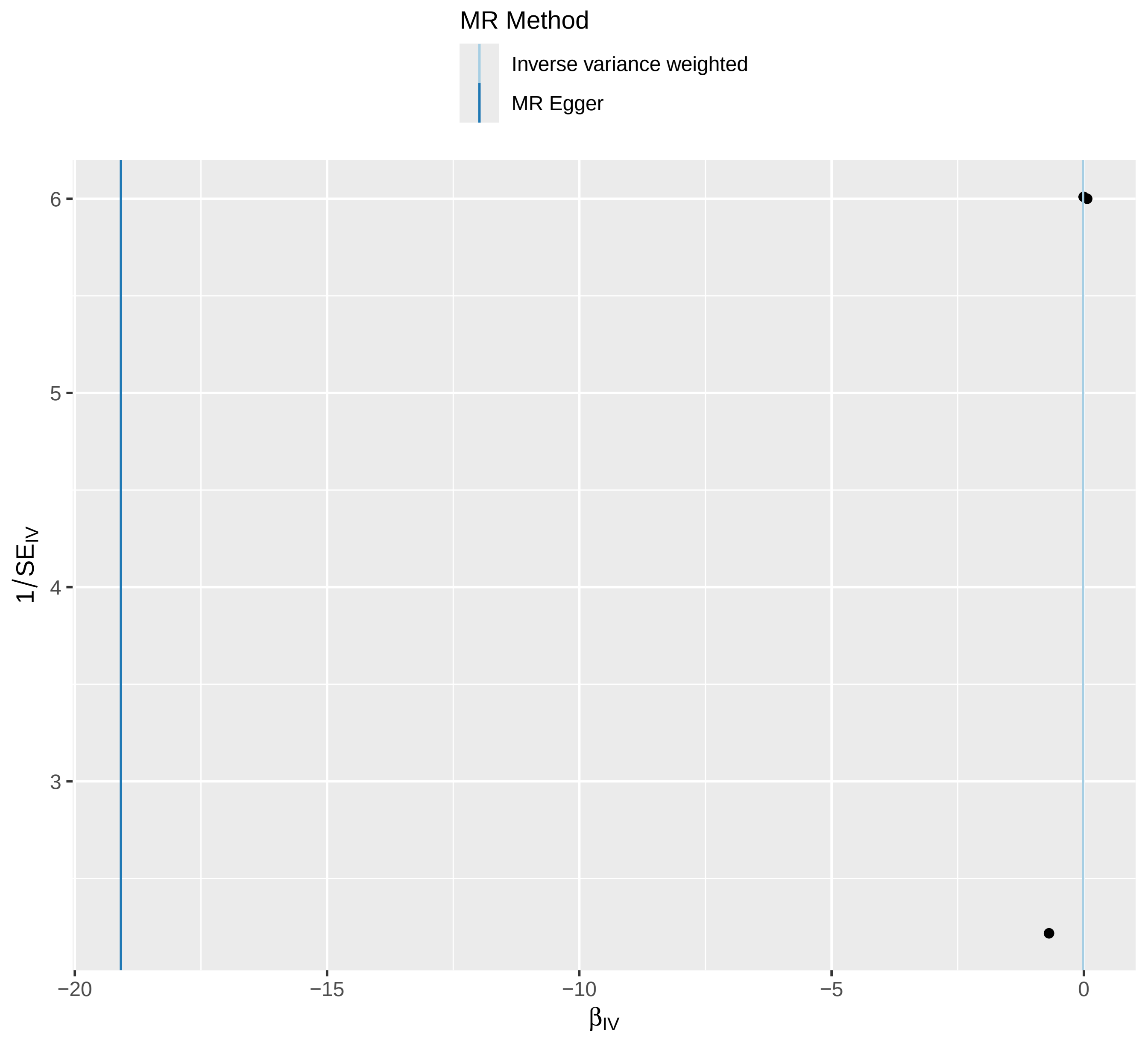

MR Method
Inverse variance weighted
MR Egger
1/SE_IV
β_IV
6
5
4
3
−20
−15
−10
−5
0

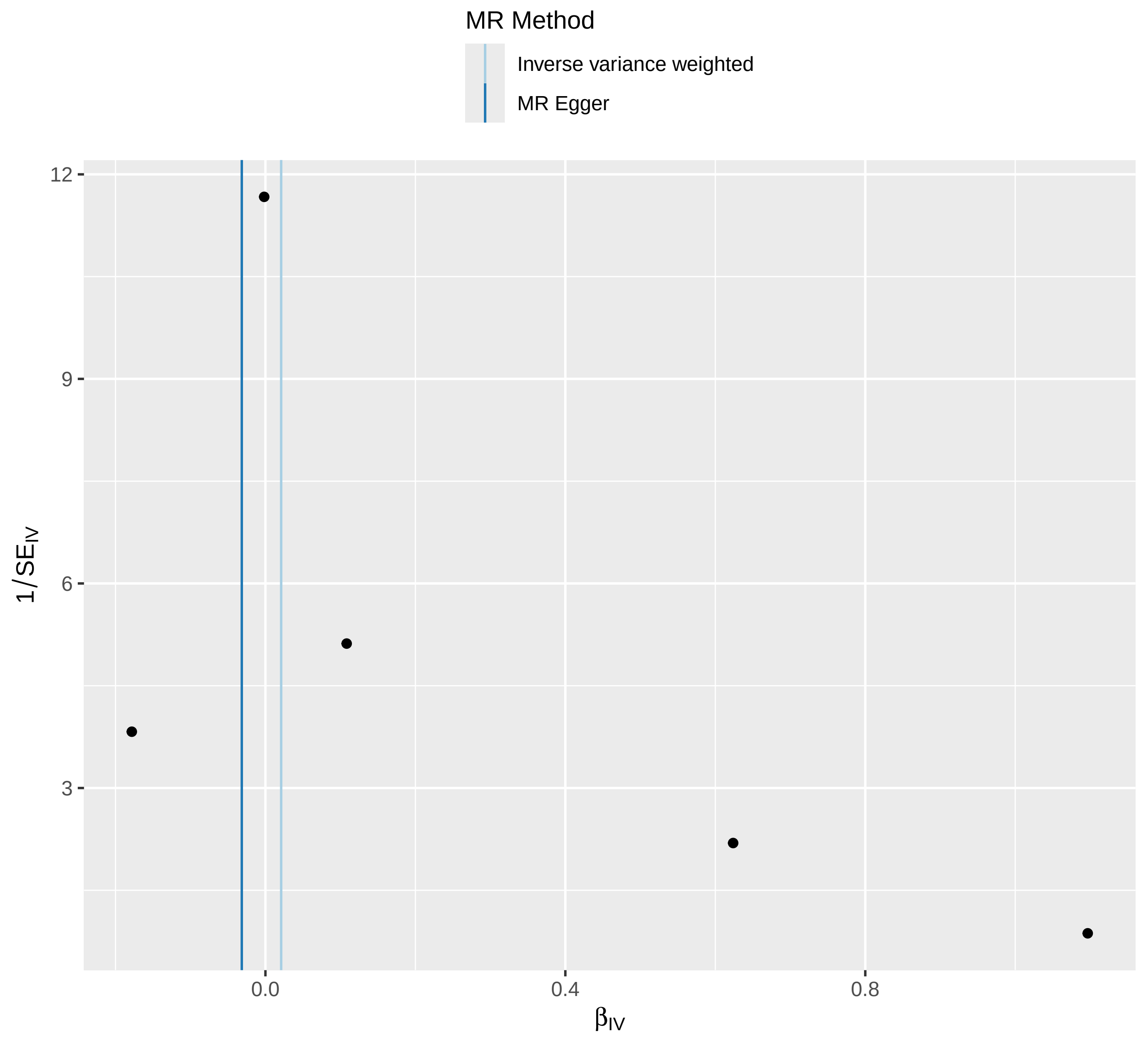
MR Method
Inverse variance weighted
MR Egger
12
9
6
3
1/SE_IV
0.0
0.4
0.8
β_IV

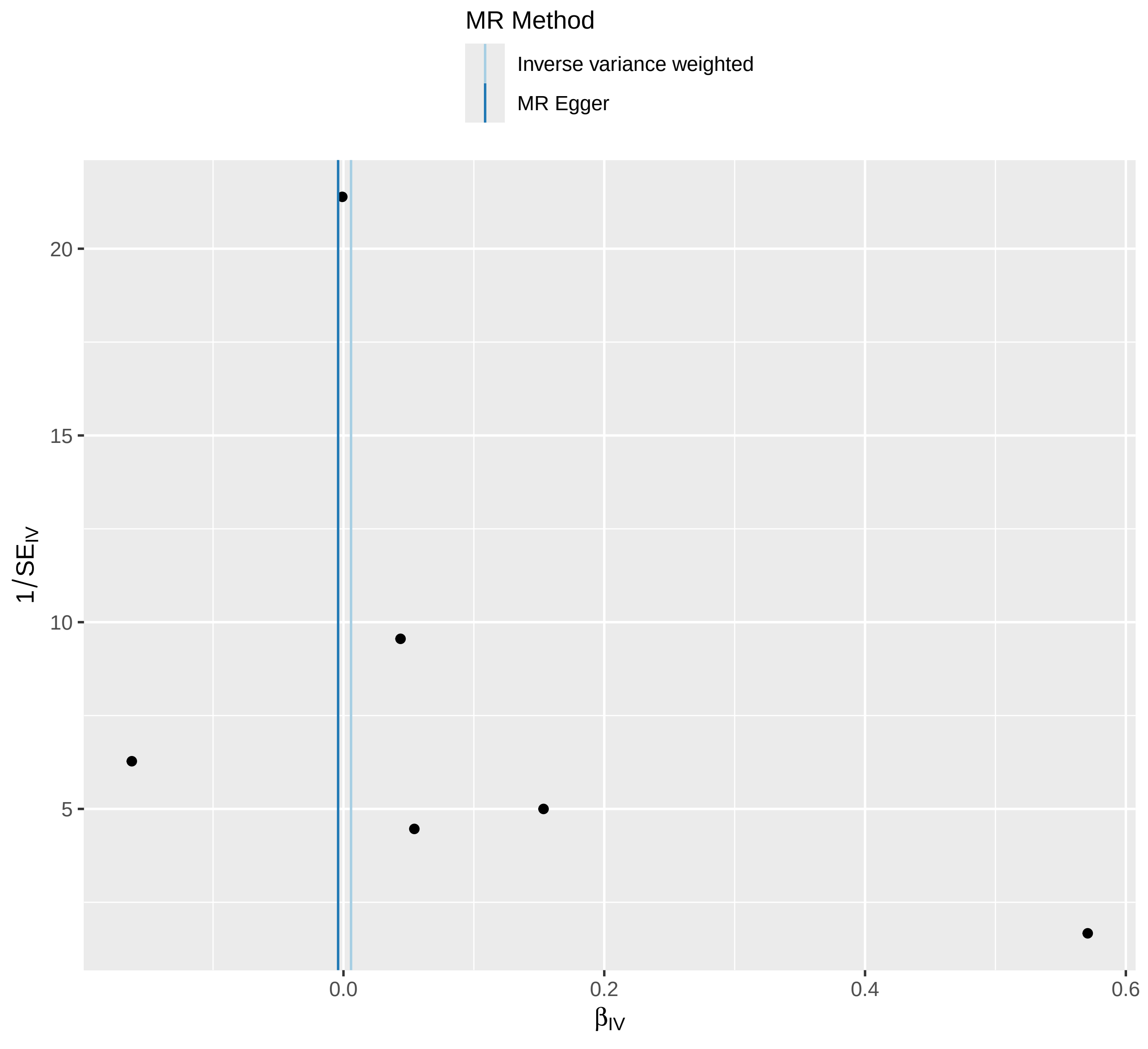
MR Method
Inverse variance weighted
MR Egger
20
15
10
5
1/SE$_{IV}$
0.0
0.2
0.4
0.6
β$_{IV}$

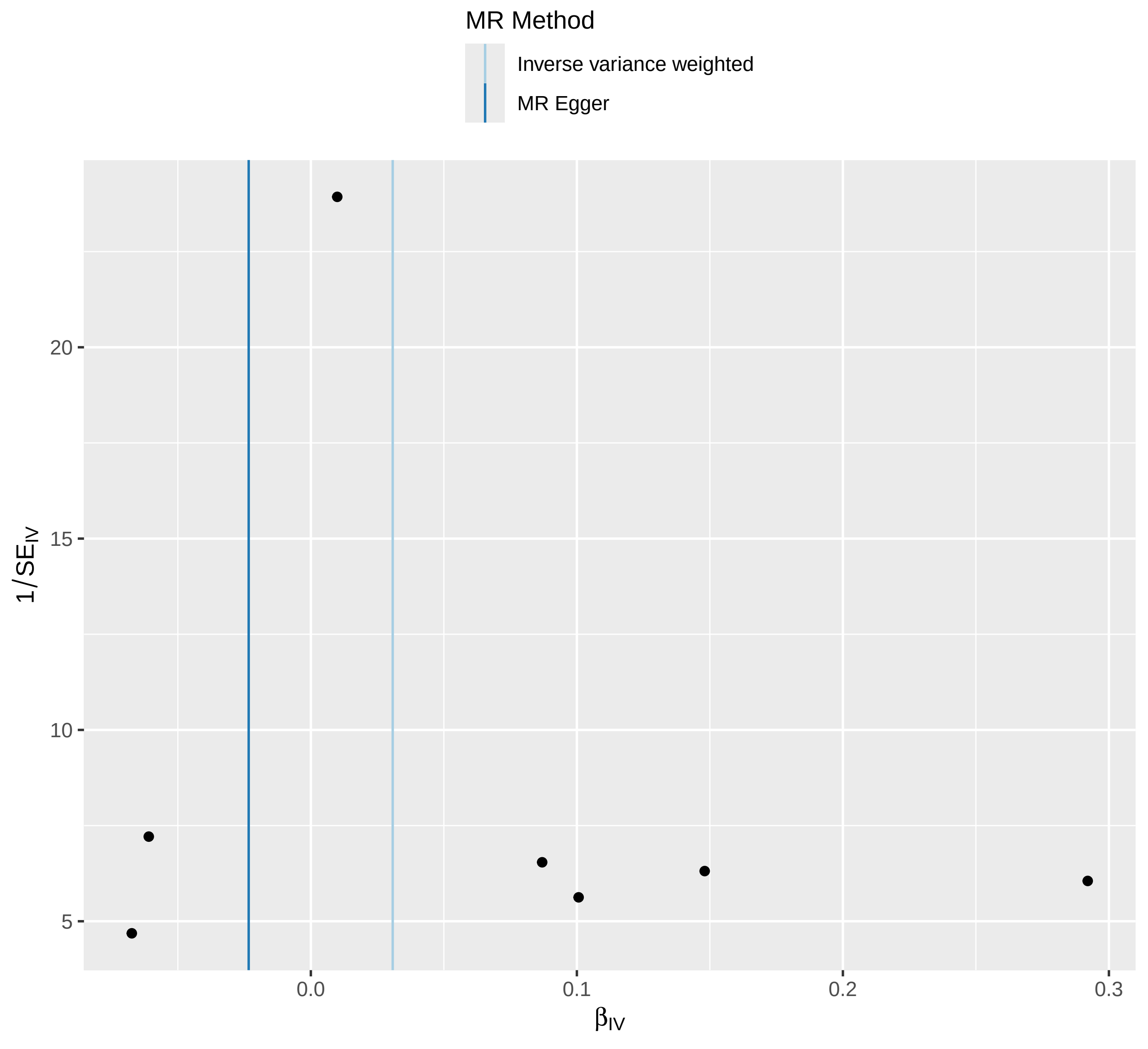
MR Method
Inverse variance weighted
MR Egger
1/SE_IV
20
15
10
5
0.0
0.1
0.2
0.3
β_IV

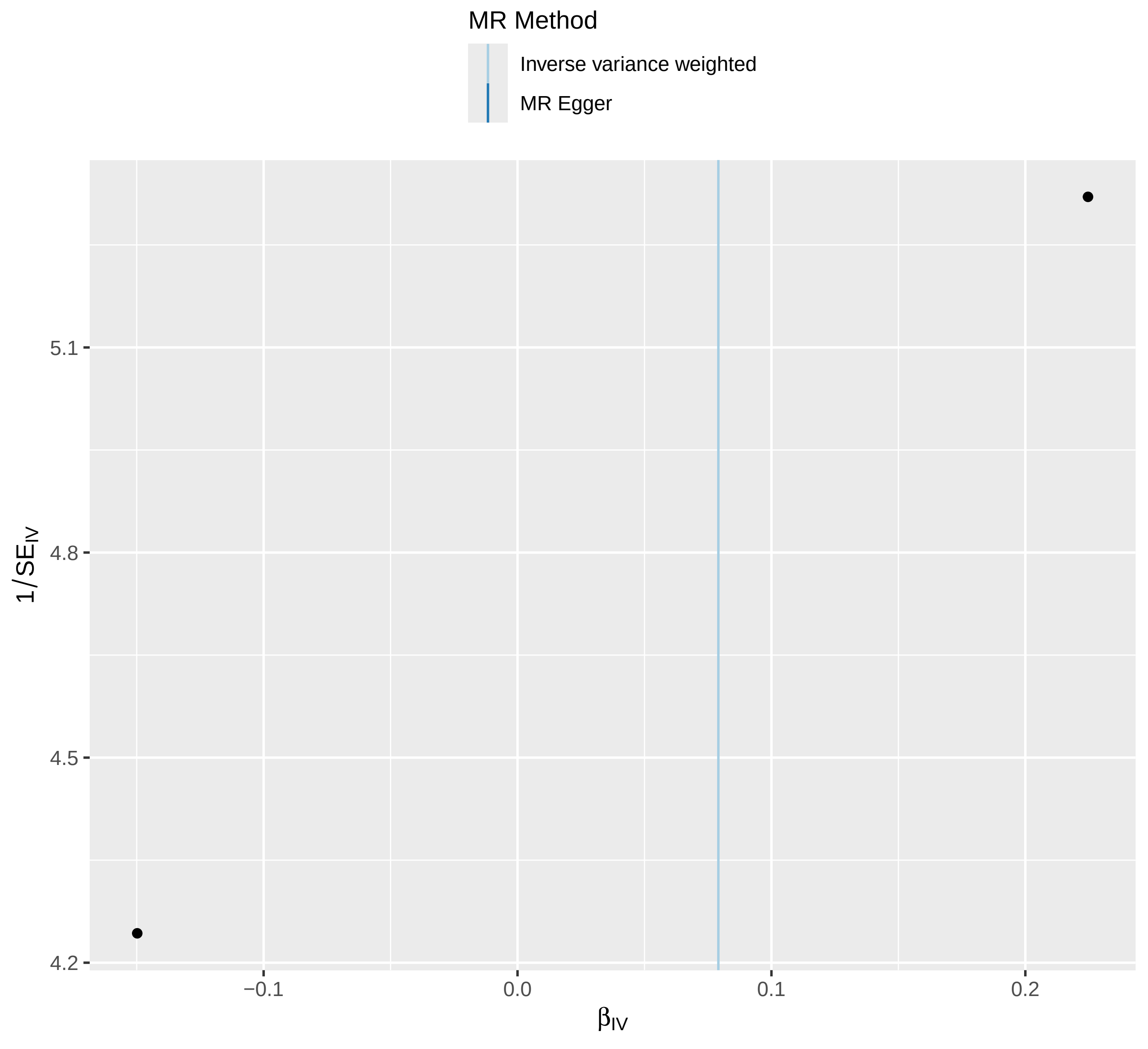
MR Method
Inverse variance weighted
MR Egger
5.1
4.8
4.5
4.2
1/SE_IV
−0.1
0.0
0.1
0.2
β_IV

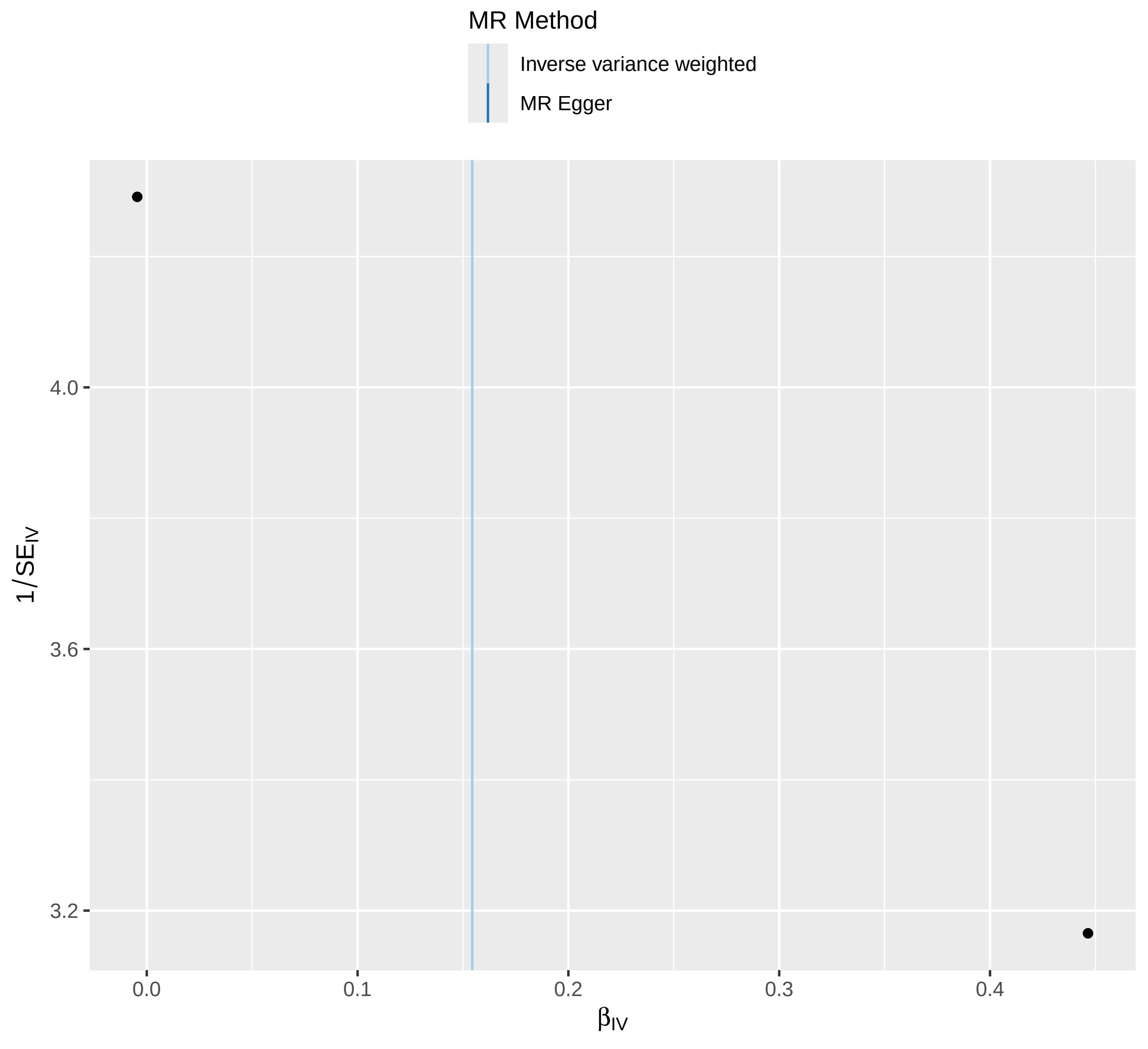
MR Method
Inverse variance weighted
MR Egger
4.0
3.6
3.2
0.0
0.1
0.2
0.3
0.4
$1/SE_{IV}$
$\beta_{IV}$

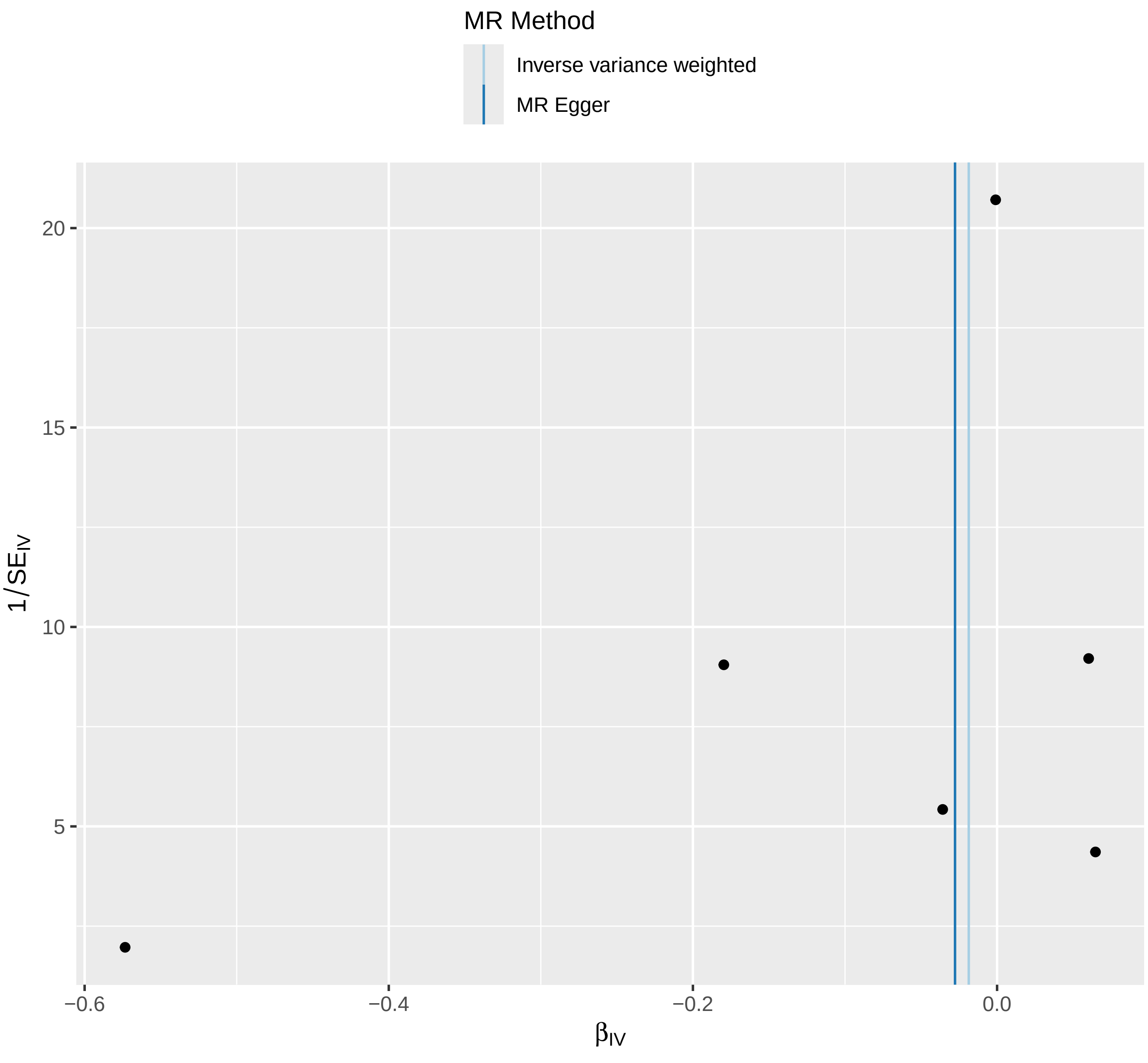
MR Method
Inverse variance weighted
MR Egger
20
15
10
5
1/SE_IV
−0.6
−0.4
−0.2
0.0
β_IV

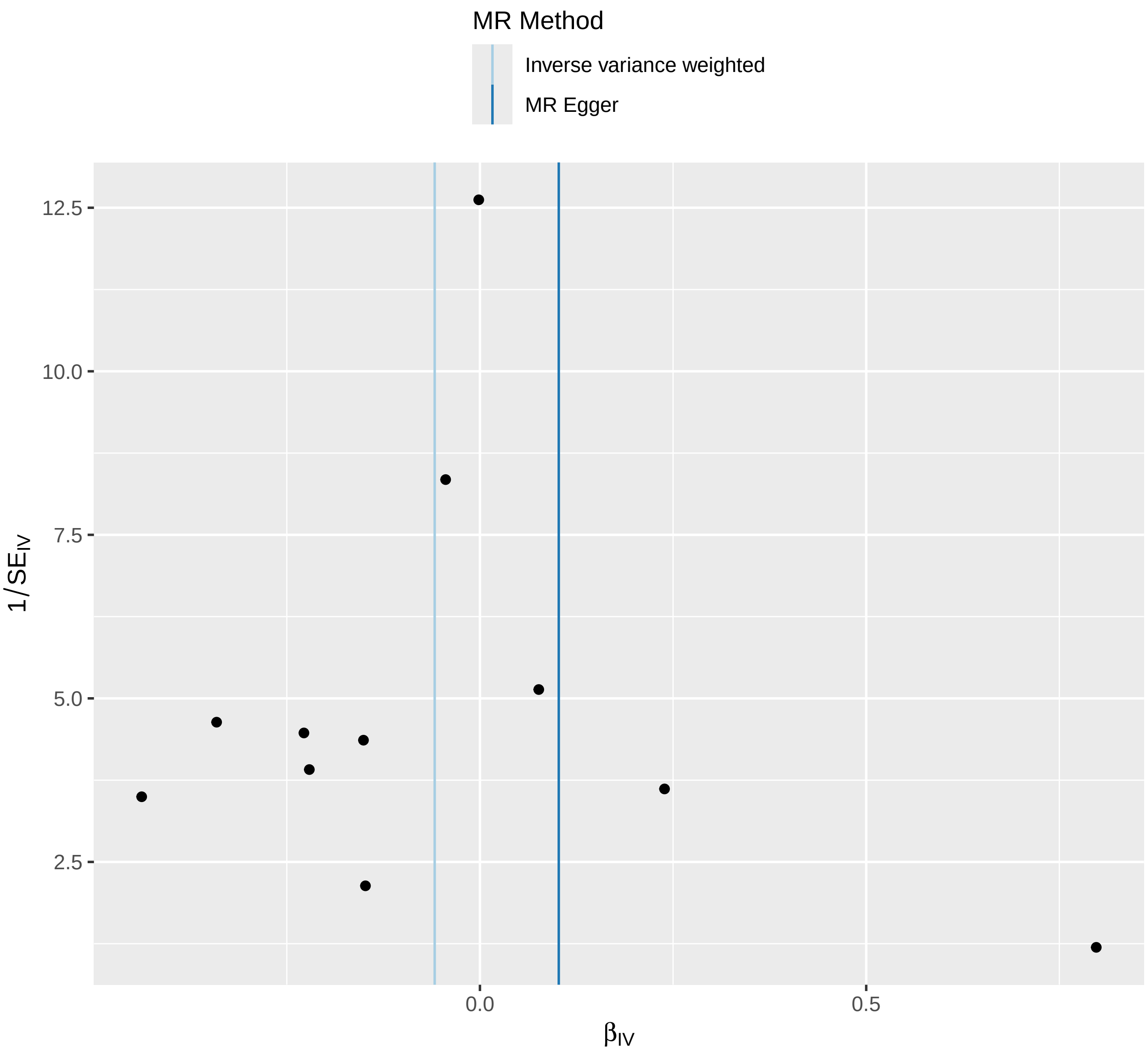

MR Method
Inverse variance weighted
MR Egger
12.5
10.0
7.5
5.0
2.5
1/SE_IV
0.0
0.5
β_IV

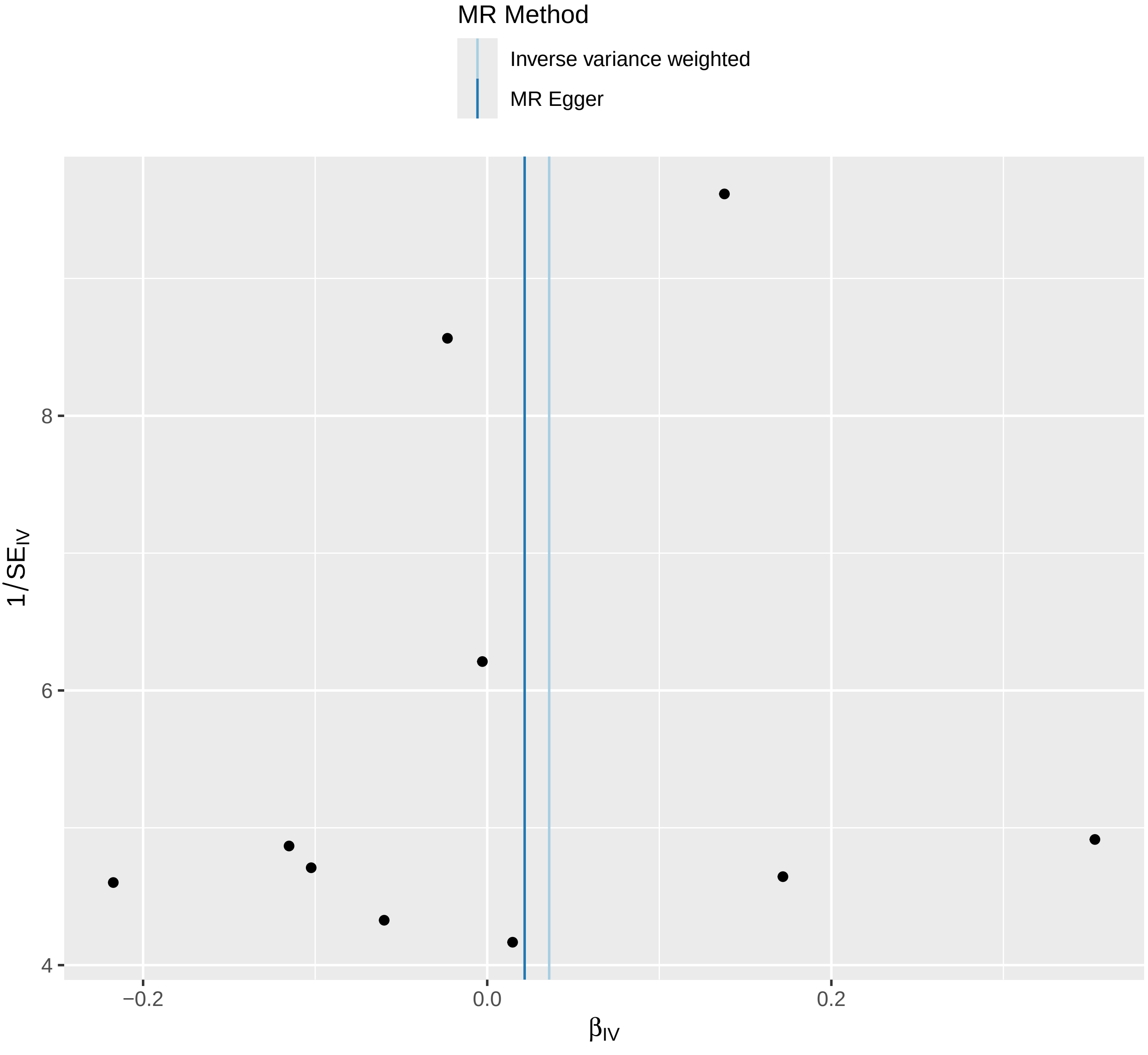
MR Method
Inverse variance weighted
MR Egger
1/$SE_{IV}$
8
6
4
−0.2
0.0
0.2
$\beta_{IV}$

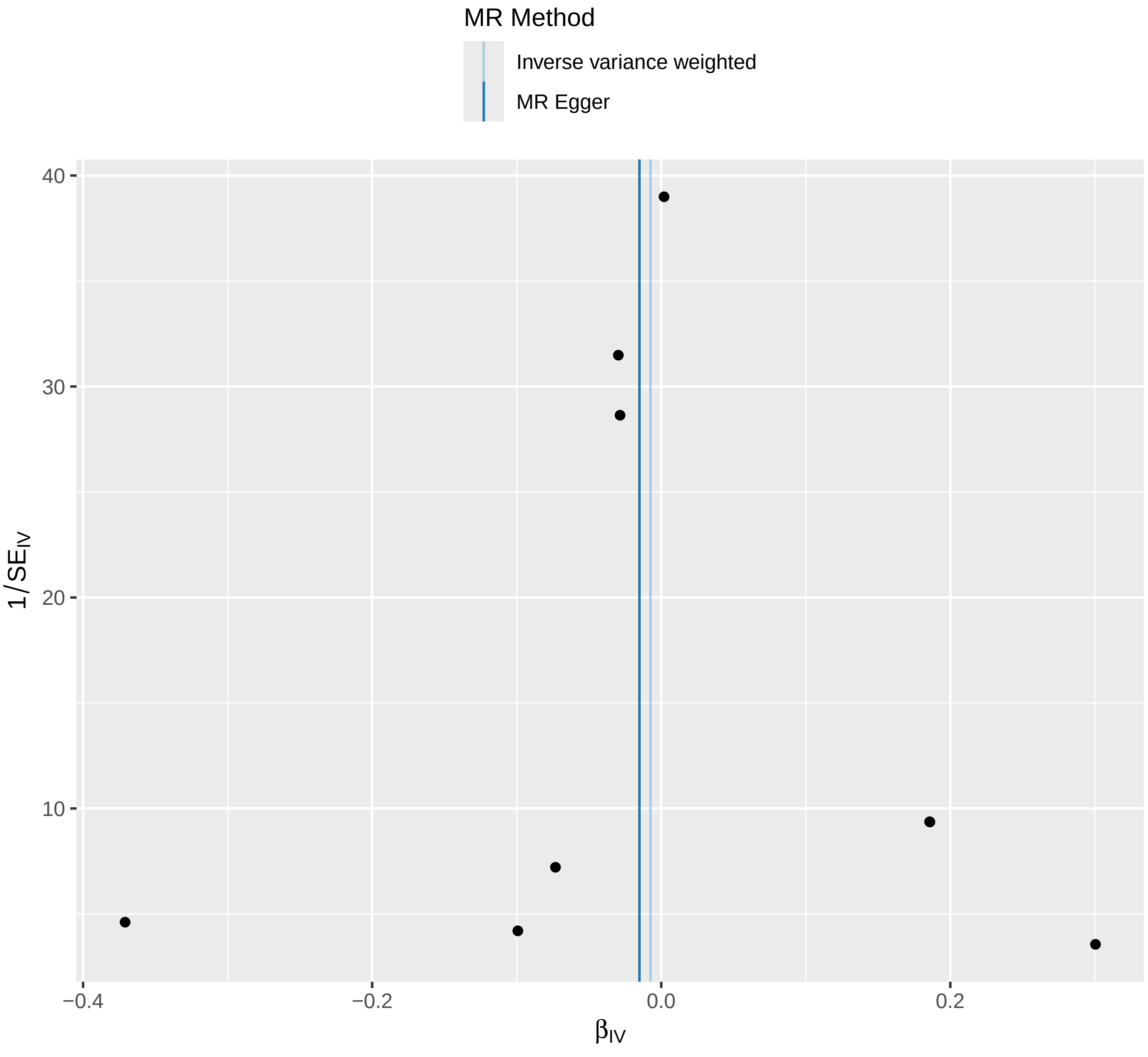
MR Method
Inverse variance weighted
MR Egger
40
30
20
10
−0.4
−0.2
0.0
0.2
$1/SE_{IV}$
$\beta_{IV}$

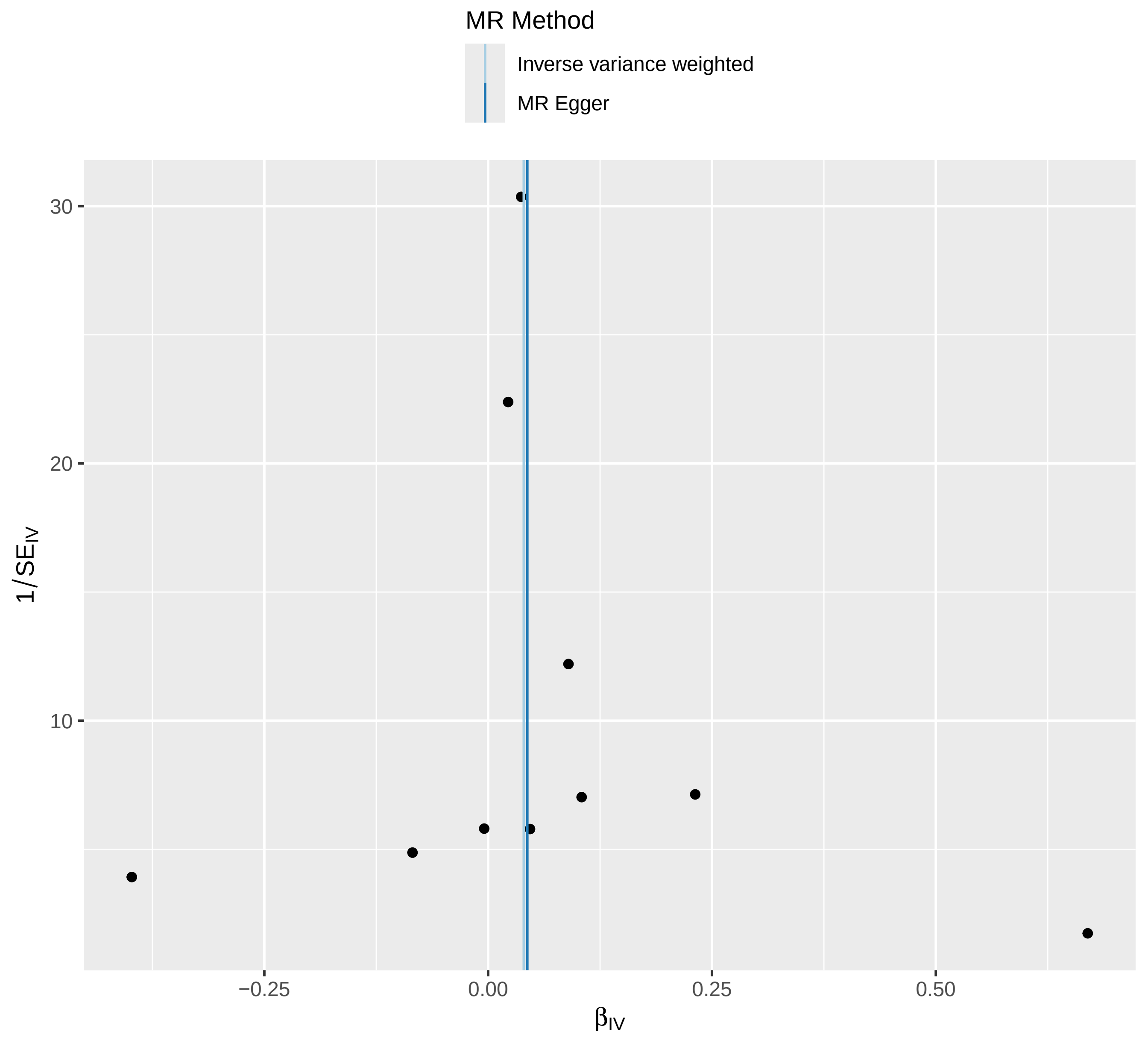

MR Method
Inverse variance weighted
MR Egger
30
20
10
1/SE_IV
−0.25
0.00
0.25
0.50
β_IV

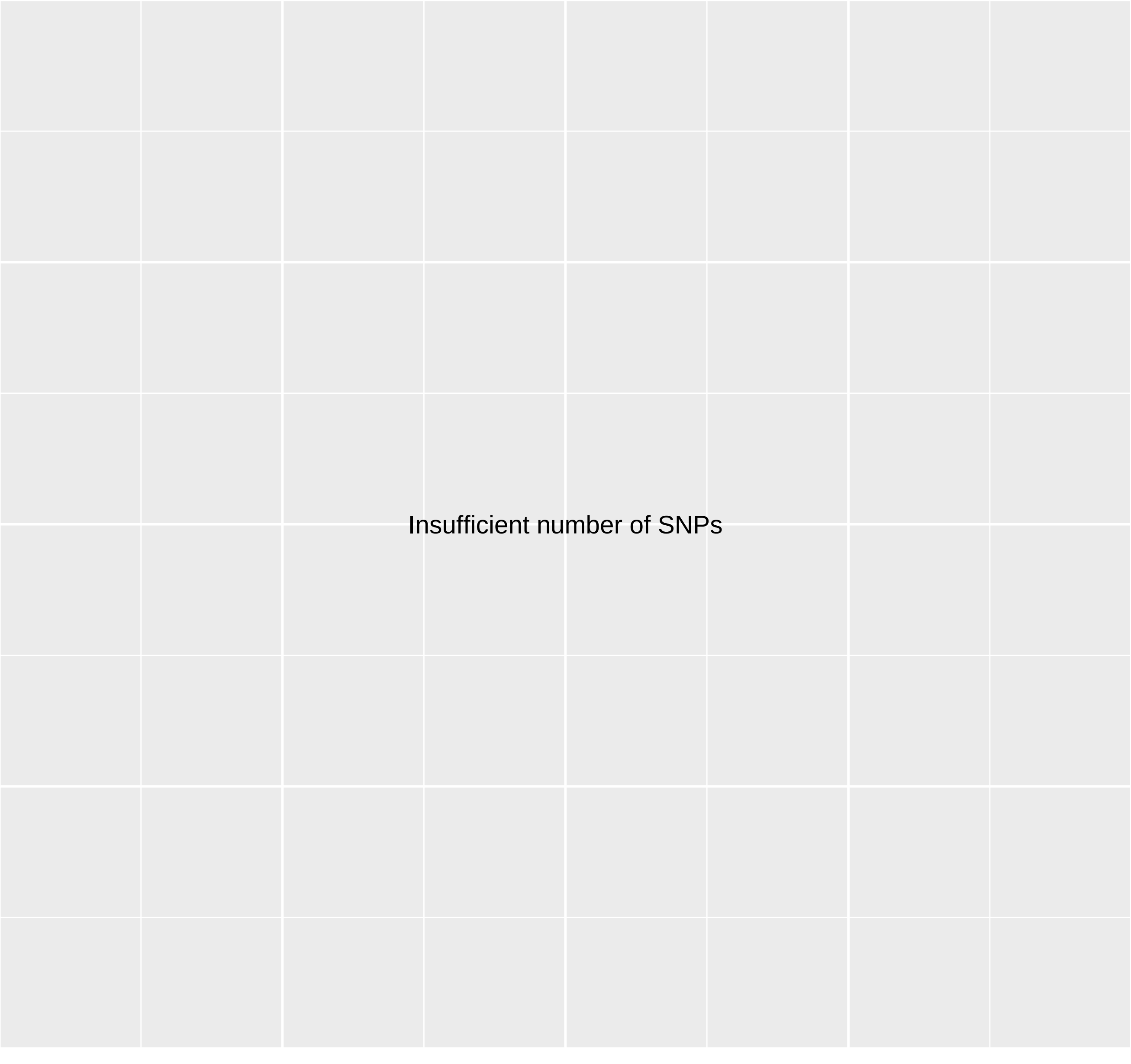
Insufficient number of SNPs

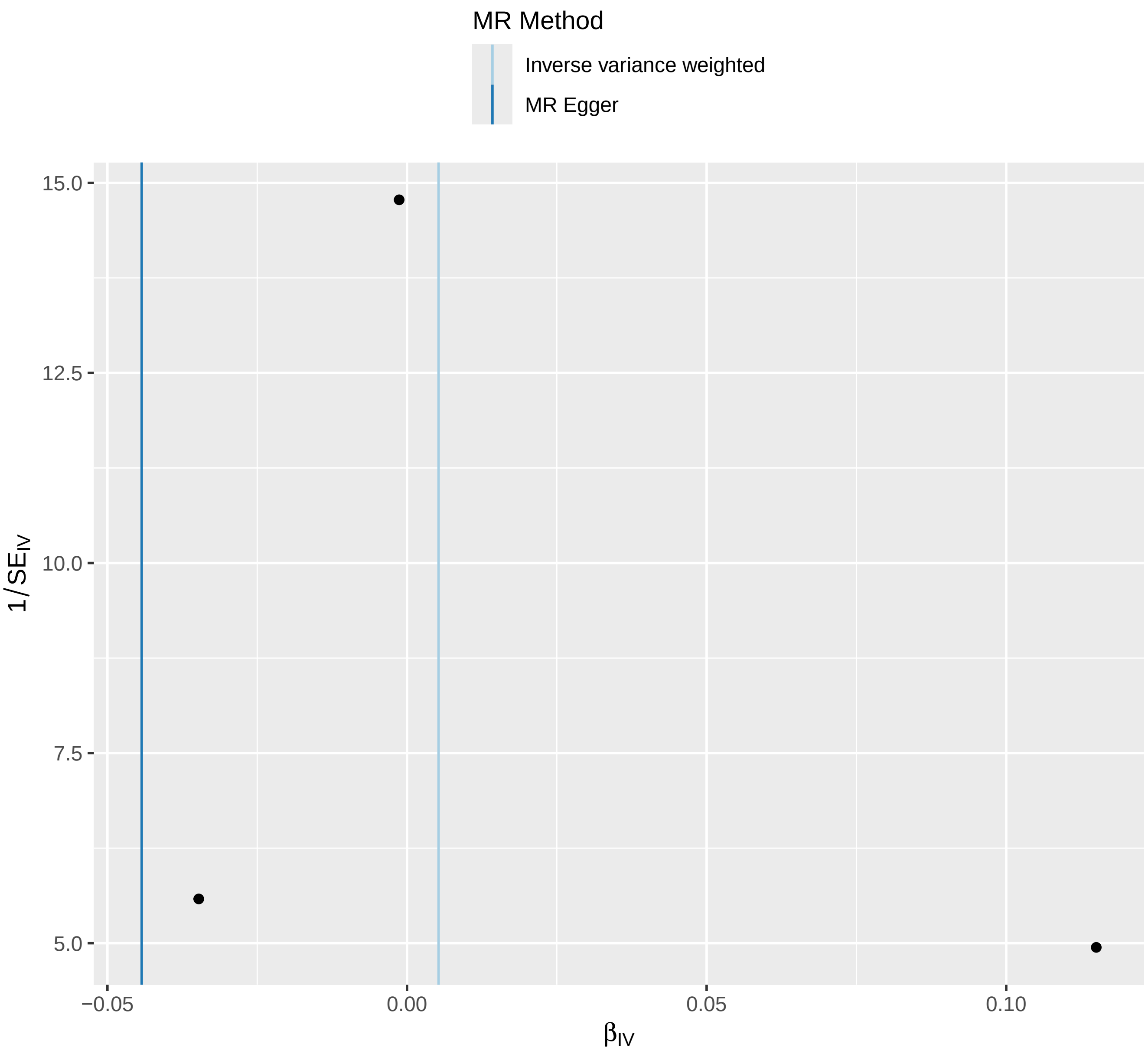
MR Method
Inverse variance weighted
MR Egger
15.0
12.5
10.0
7.5
5.0
1/SE_IV
−0.05
0.00
0.05
0.10
β_IV

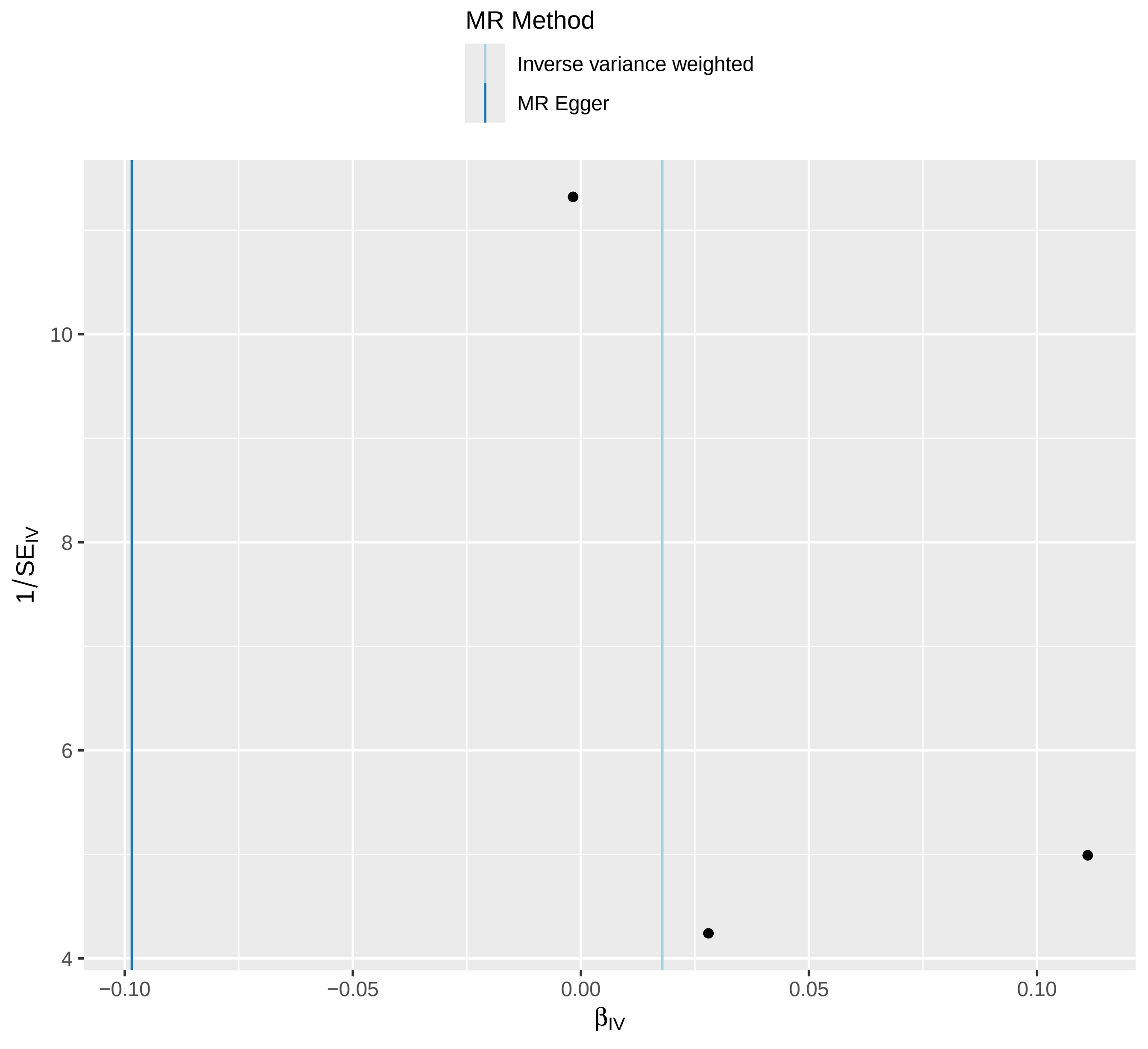

MR Method
Inverse variance weighted
MR Egger
10
8
6
4
1/SE$_{IV}$
−0.10
−0.05
0.00
0.05
0.10
β$_{IV}$

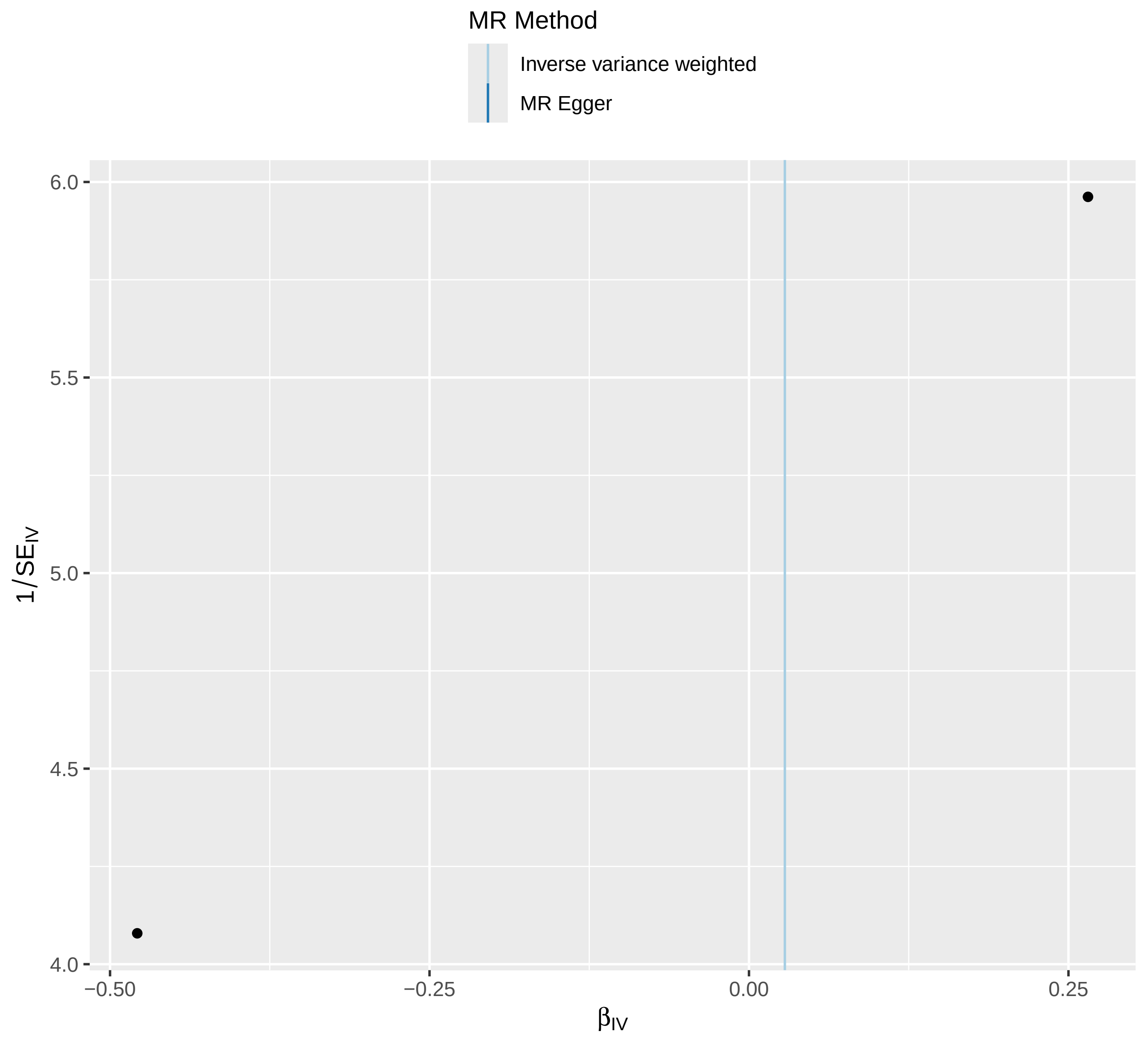
MR Method
Inverse variance weighted
MR Egger
6.0
5.5
5.0
4.5
4.0
$1/SE_{IV}$
−0.50
−0.25
0.00
0.25
$\beta_{IV}$

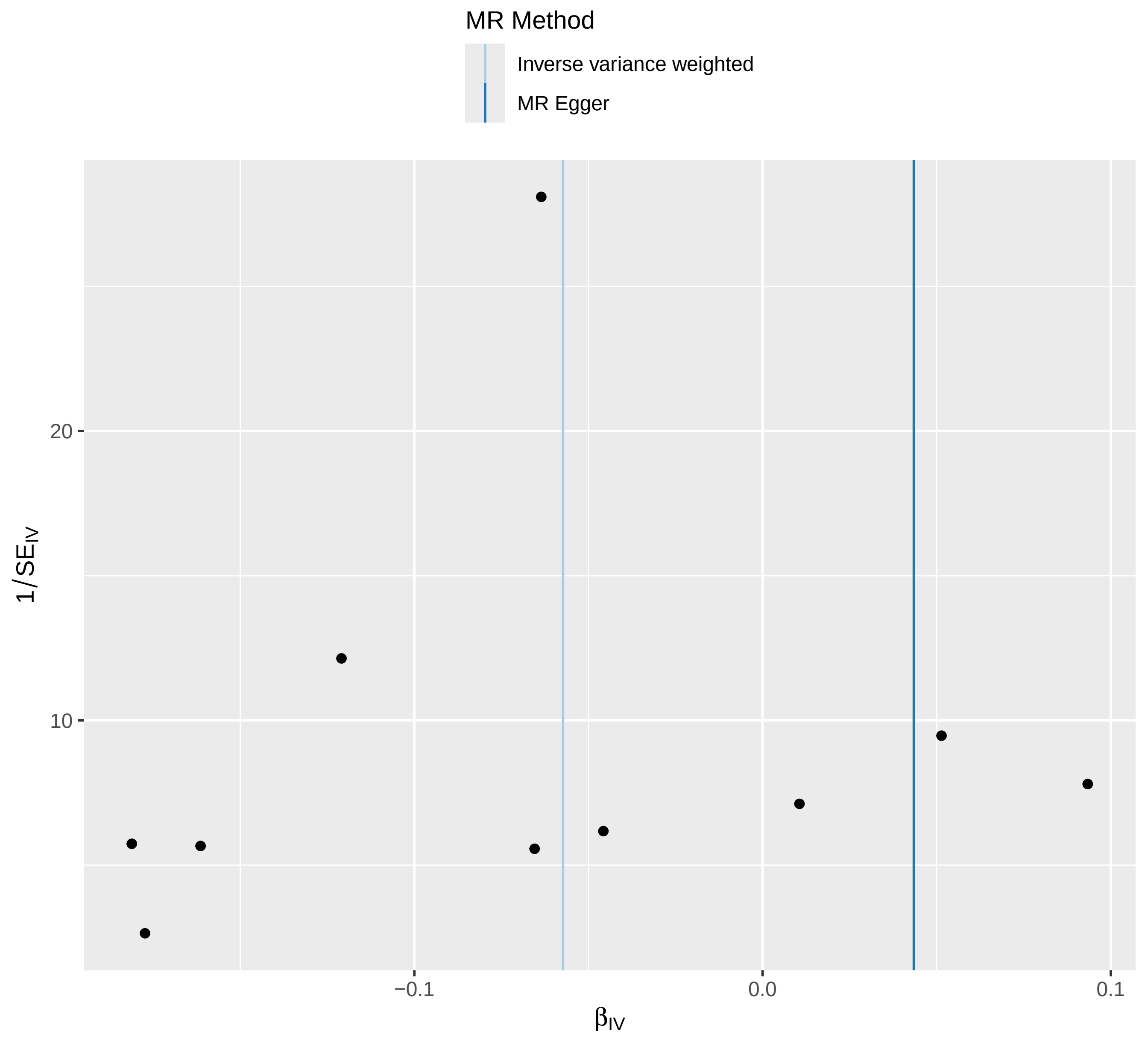

MR Method
Inverse variance weighted
MR Egger
20
10
1/SE_IV
−0.1
0.0
0.1
β_IV

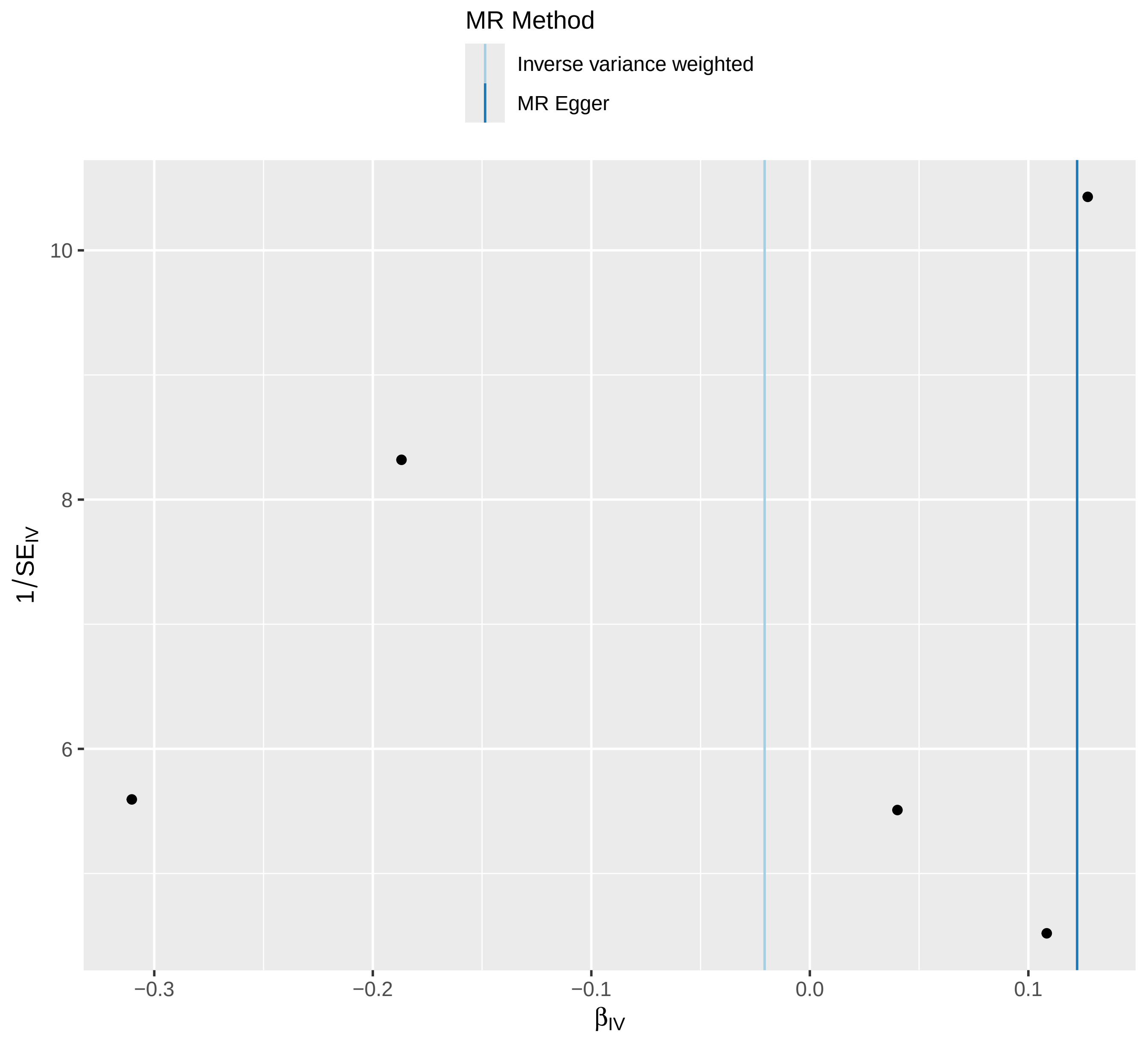
MR Method
Inverse variance weighted
MR Egger
10
8
6
$1/SE_{IV}$
−0.3
−0.2
−0.1
0.0
0.1
$\beta_{IV}$

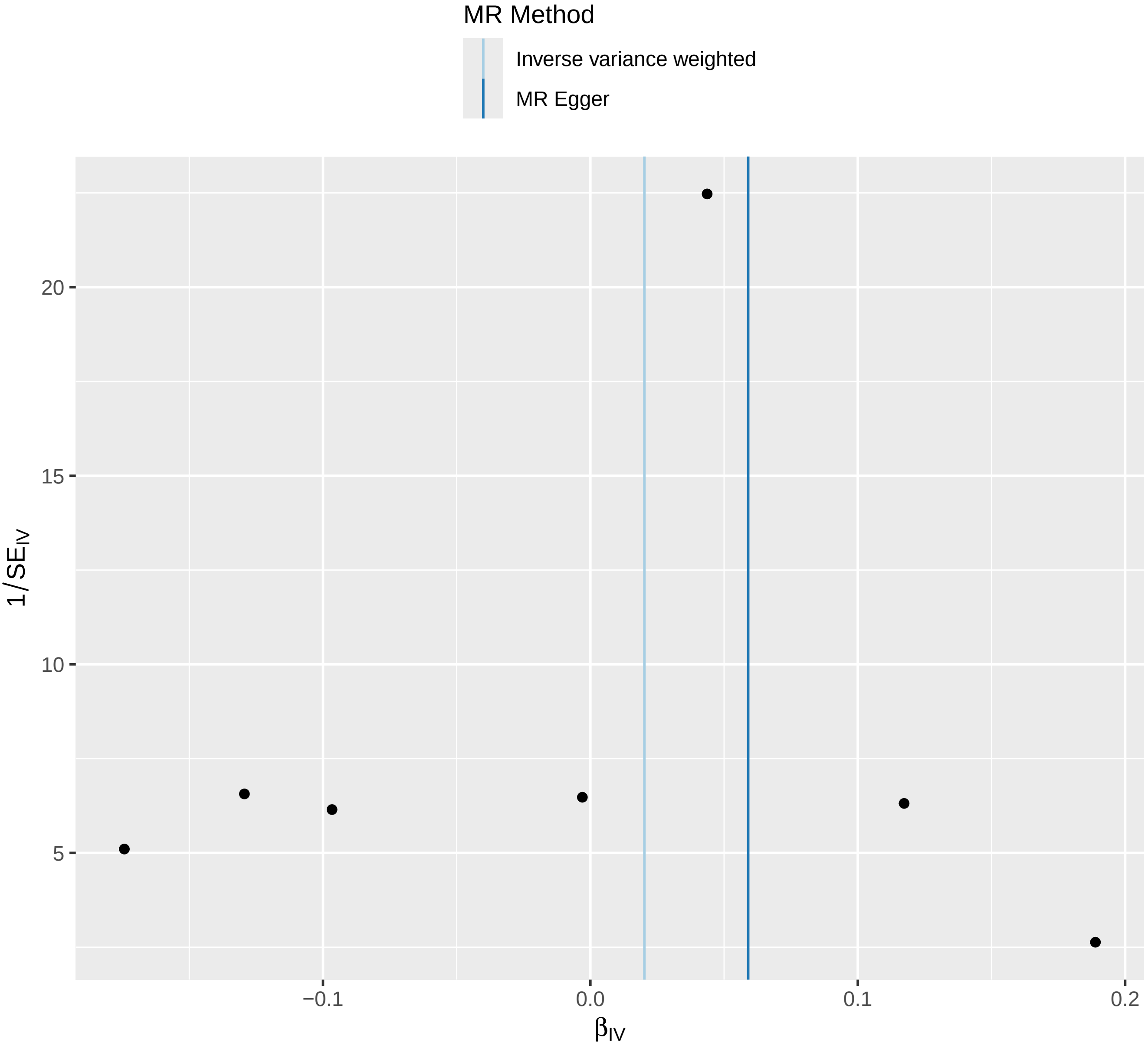
MR Method
Inverse variance weighted
MR Egger
20
15
10
5
−0.1
0.0
0.1
0.2
$1/SE_{IV}$
$\beta_{IV}$

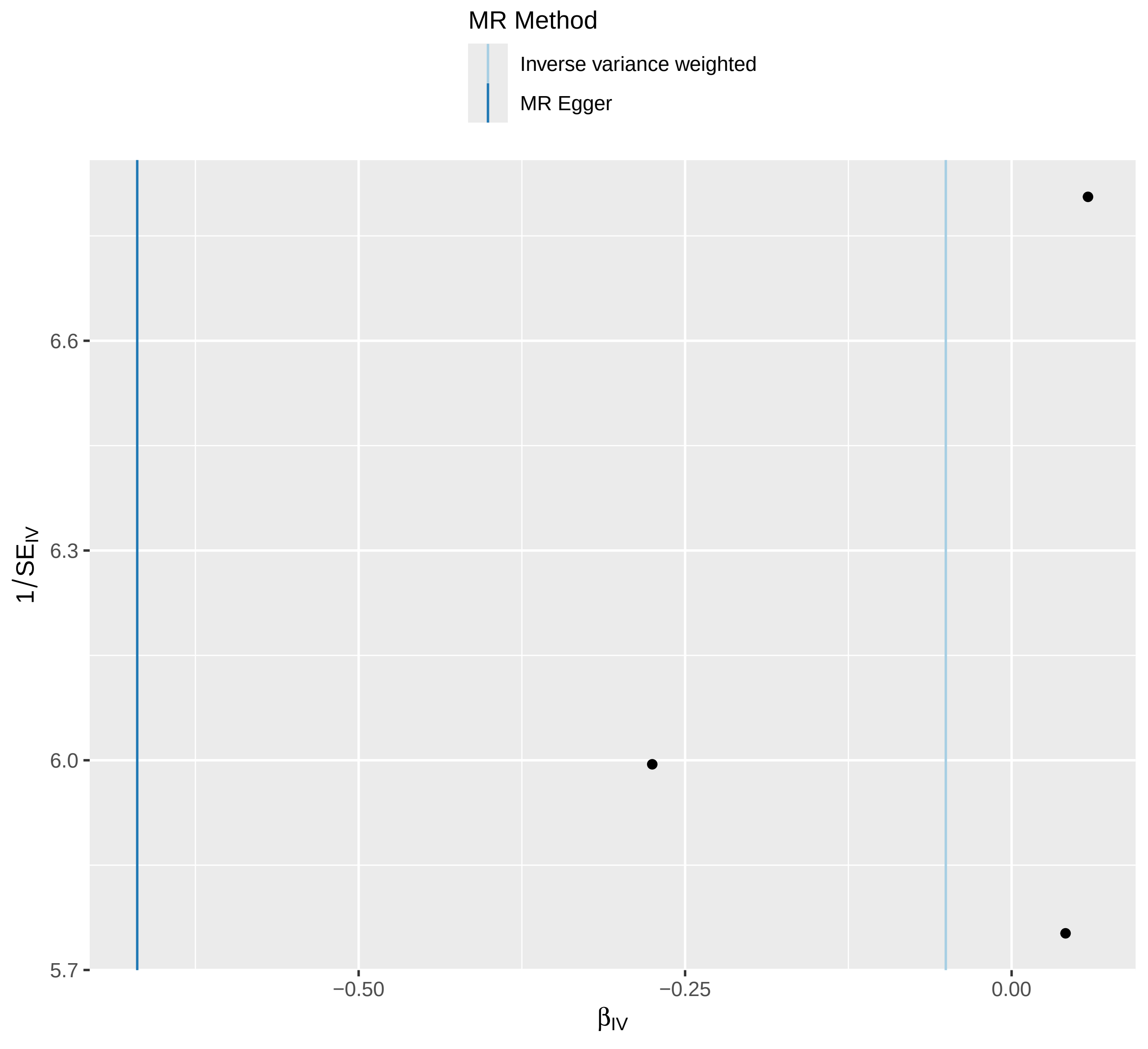

MR Method
Inverse variance weighted
MR Egger
6.6
6.3
6.0
5.7
$1/SE_{IV}$
−0.50
−0.25
0.00
$\beta_{IV}$

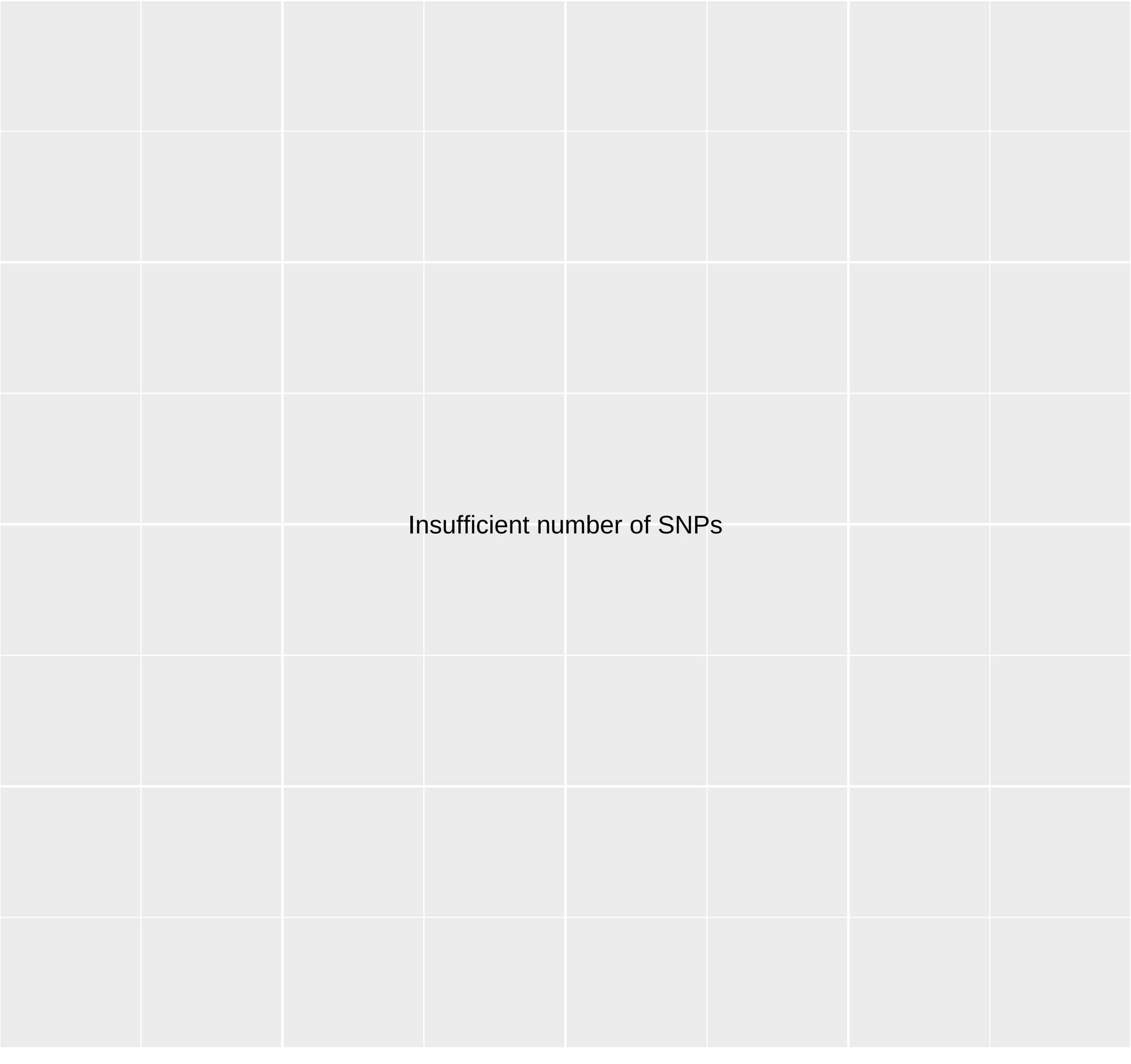
Insufficient number of SNPs

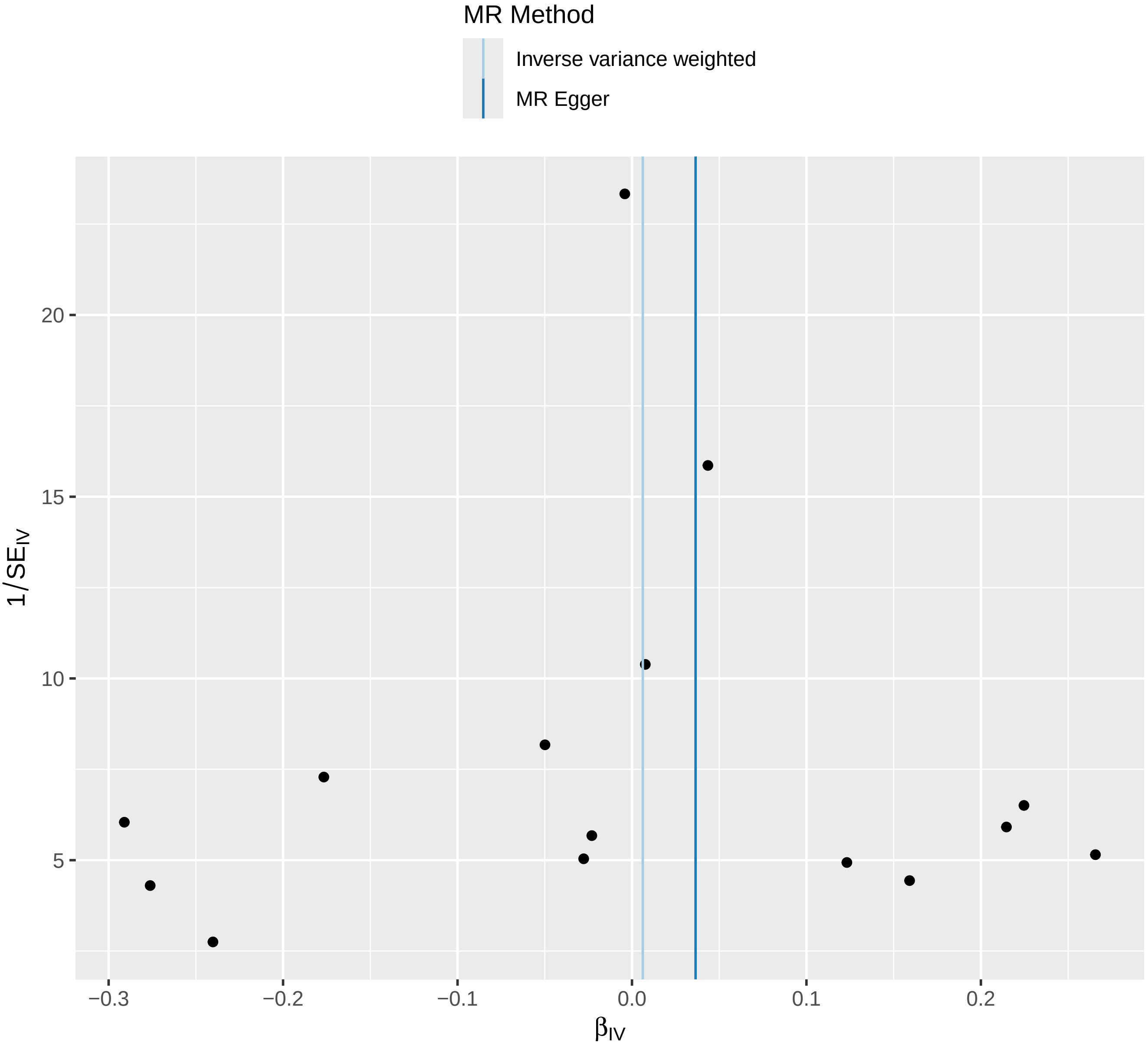

MR Method
Inverse variance weighted
MR Egger
20
15
10
5
1/SE$_{IV}$
−0.3
−0.2
−0.1
0.0
0.1
0.2
β$_{IV}$

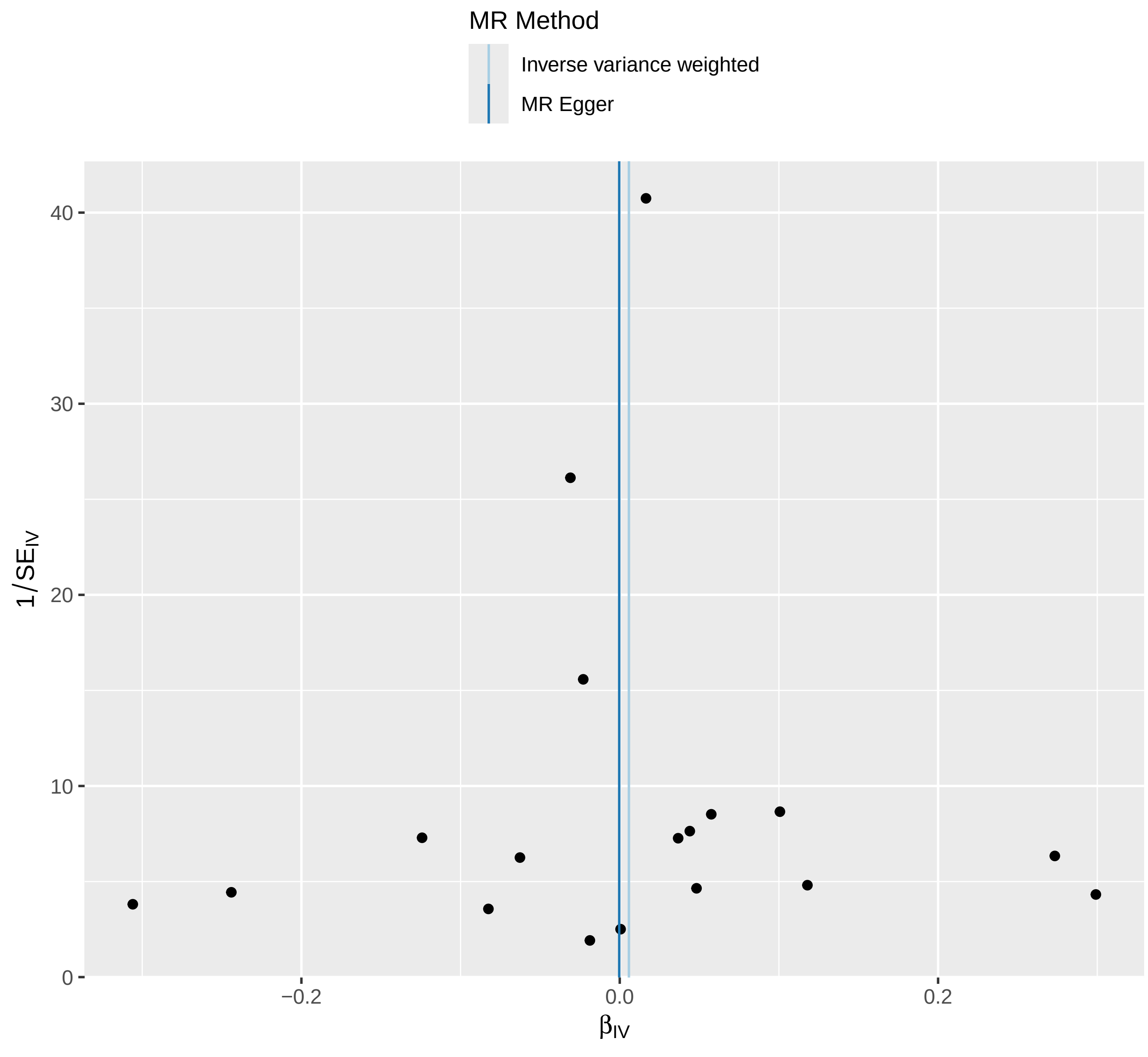

MR Method
Inverse variance weighted
MR Egger
40
30
20
10
0
1/SE_IV
−0.2
0.0
0.2
β_IV

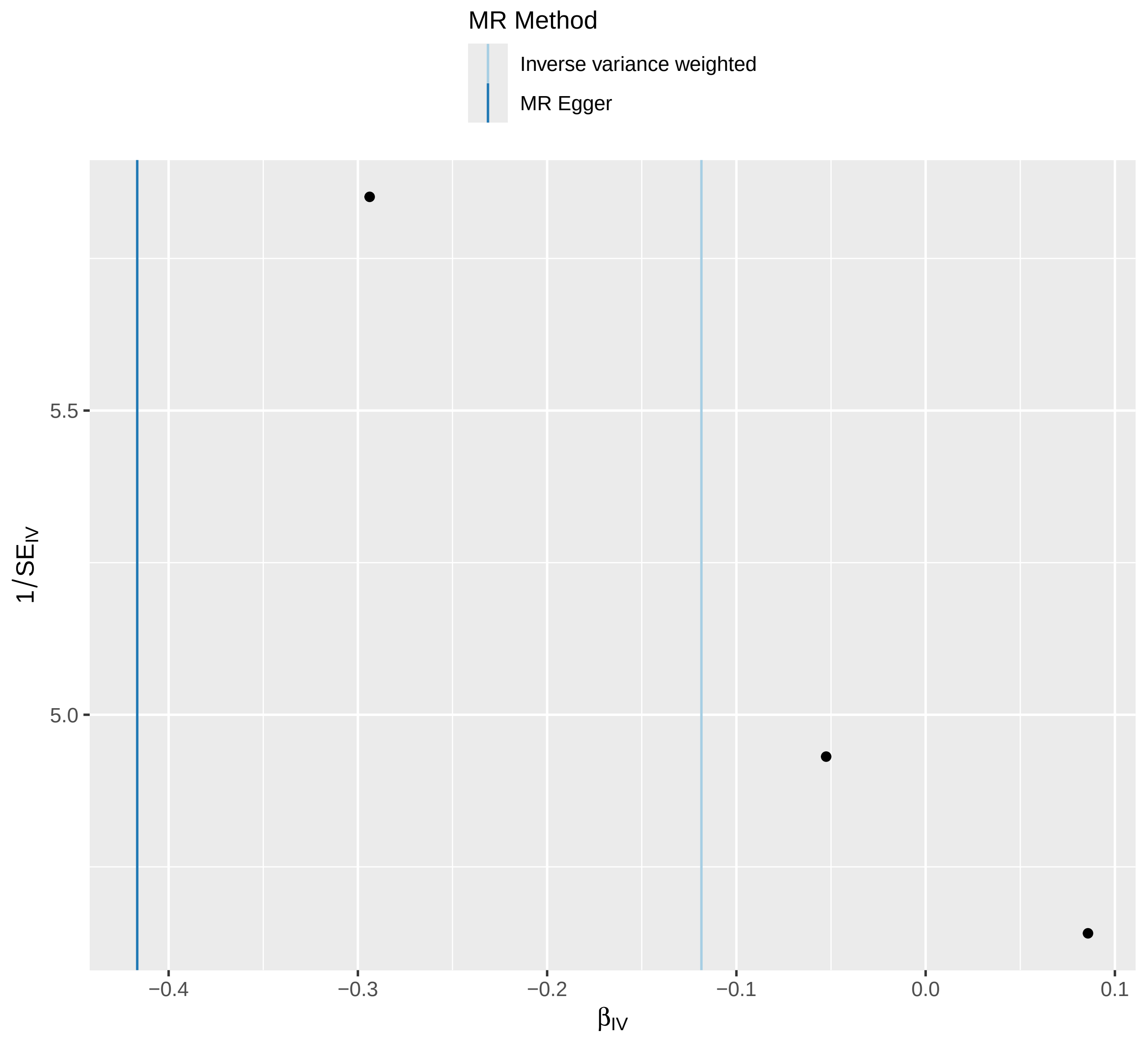
MR Method
Inverse variance weighted
MR Egger
5.5
5.0
1/SE_IV
−0.4
−0.3
−0.2
−0.1
0.0
0.1
β_IV

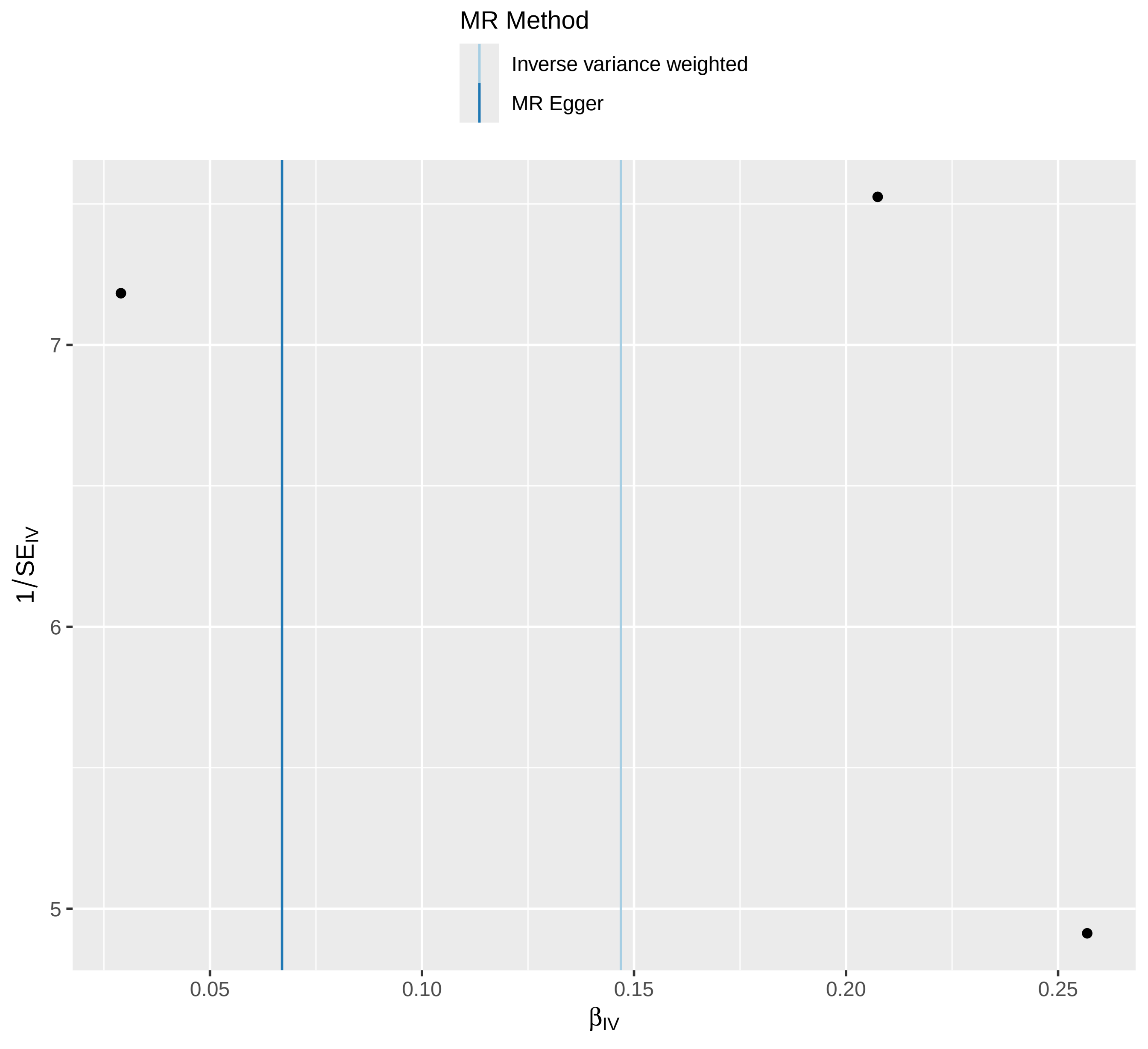

MR Method
Inverse variance weighted
MR Egger
1/SE_IV
7
6
5
0.05
0.10
0.15
0.20
0.25
β_IV

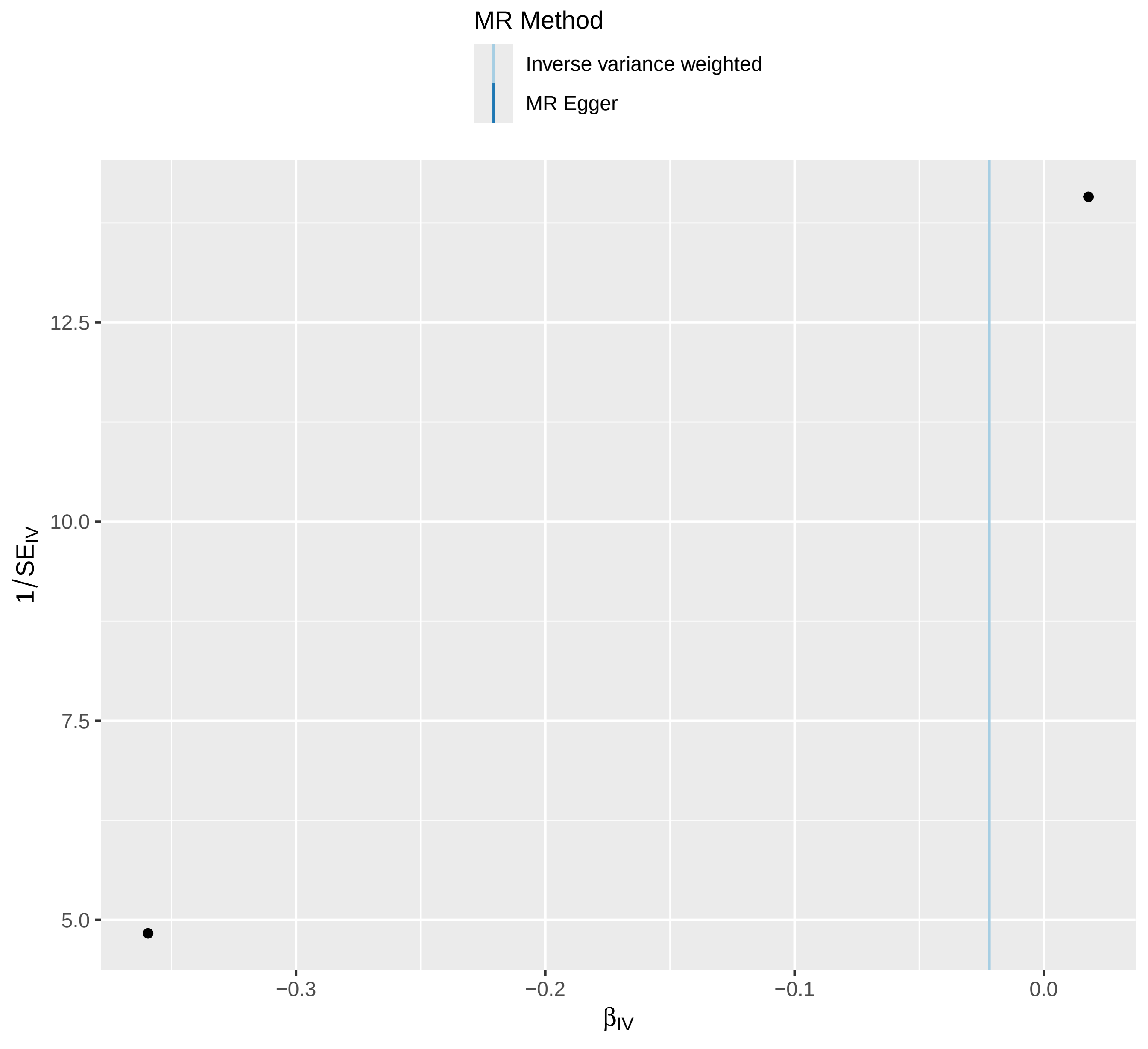
MR Method
Inverse variance weighted
MR Egger
12.5
10.0
7.5
5.0
1/SE_IV
−0.3
−0.2
−0.1
0.0
β_IV

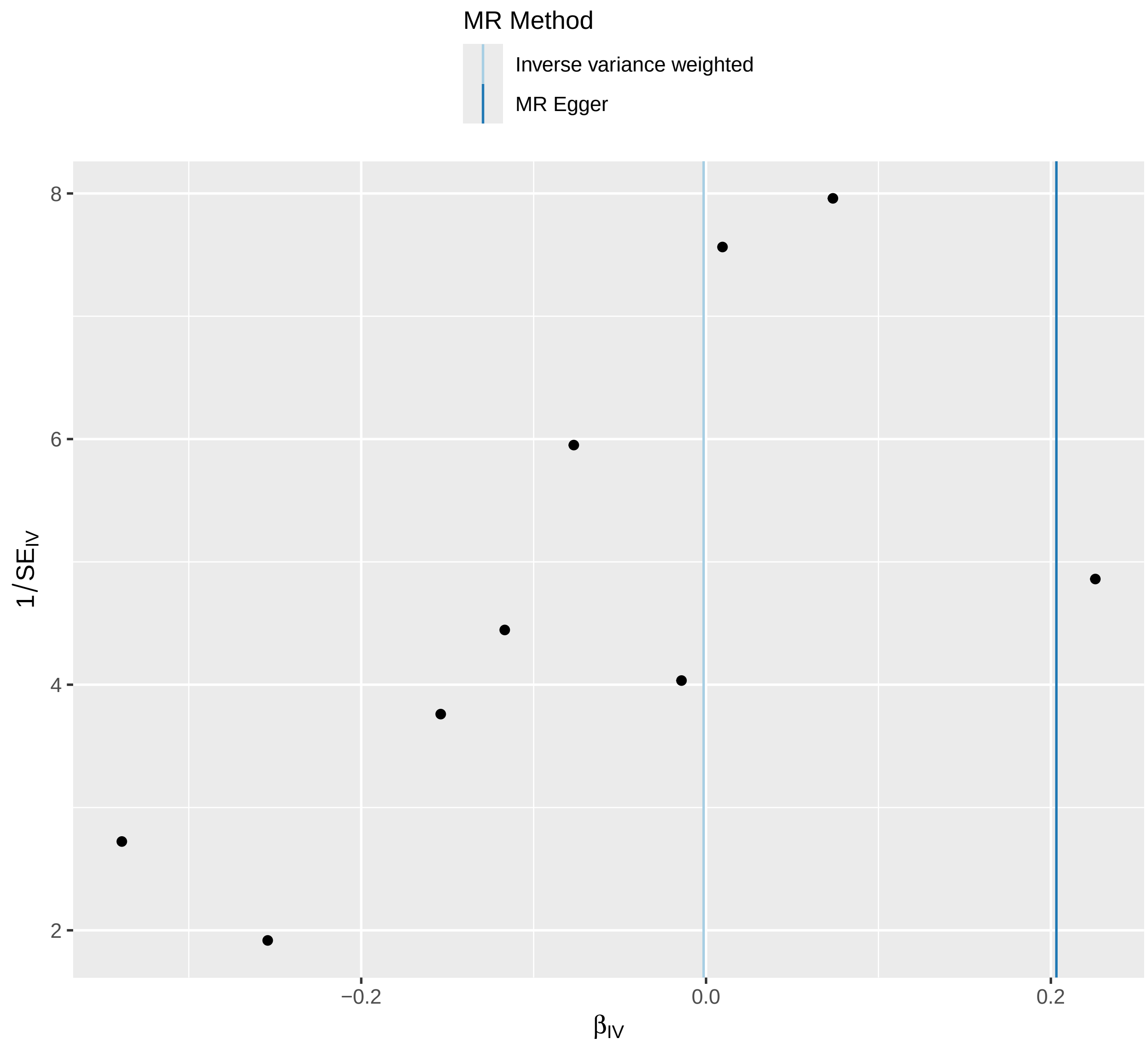

MR Method
Inverse variance weighted
MR Egger
8
6
4
2
−0.2
0.0
0.2
$1/SE_{IV}$
$\beta_{IV}$

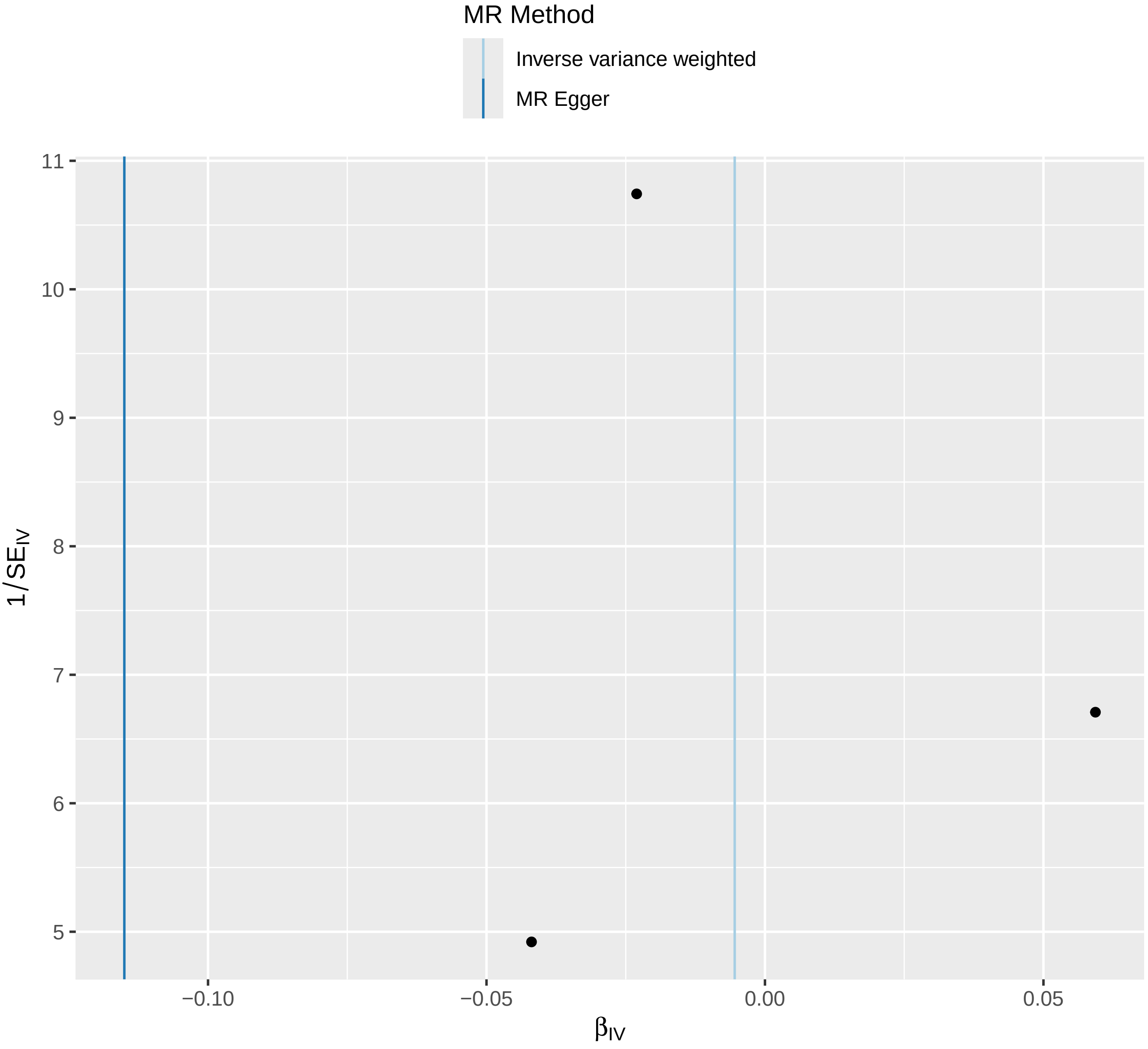
MR Method
Inverse variance weighted
MR Egger
11
10
9
8
7
6
5
1/SE$_{IV}$
−0.10
−0.05
0.00
0.05
$\beta_{IV}$

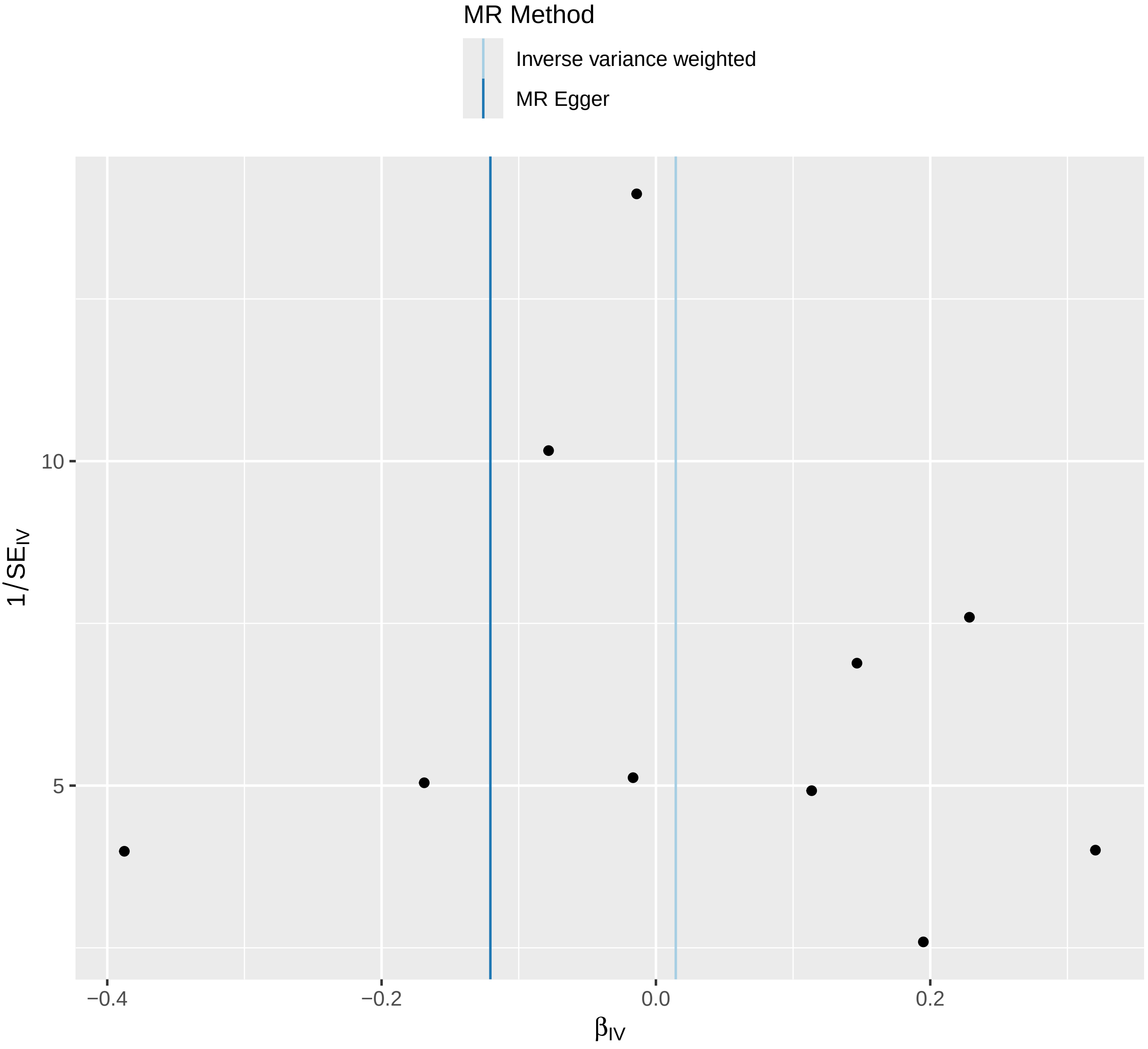

MR Method
Inverse variance weighted
MR Egger
10
5
1/SE$_{IV}$
−0.4
−0.2
0.0
0.2
β$_{IV}$

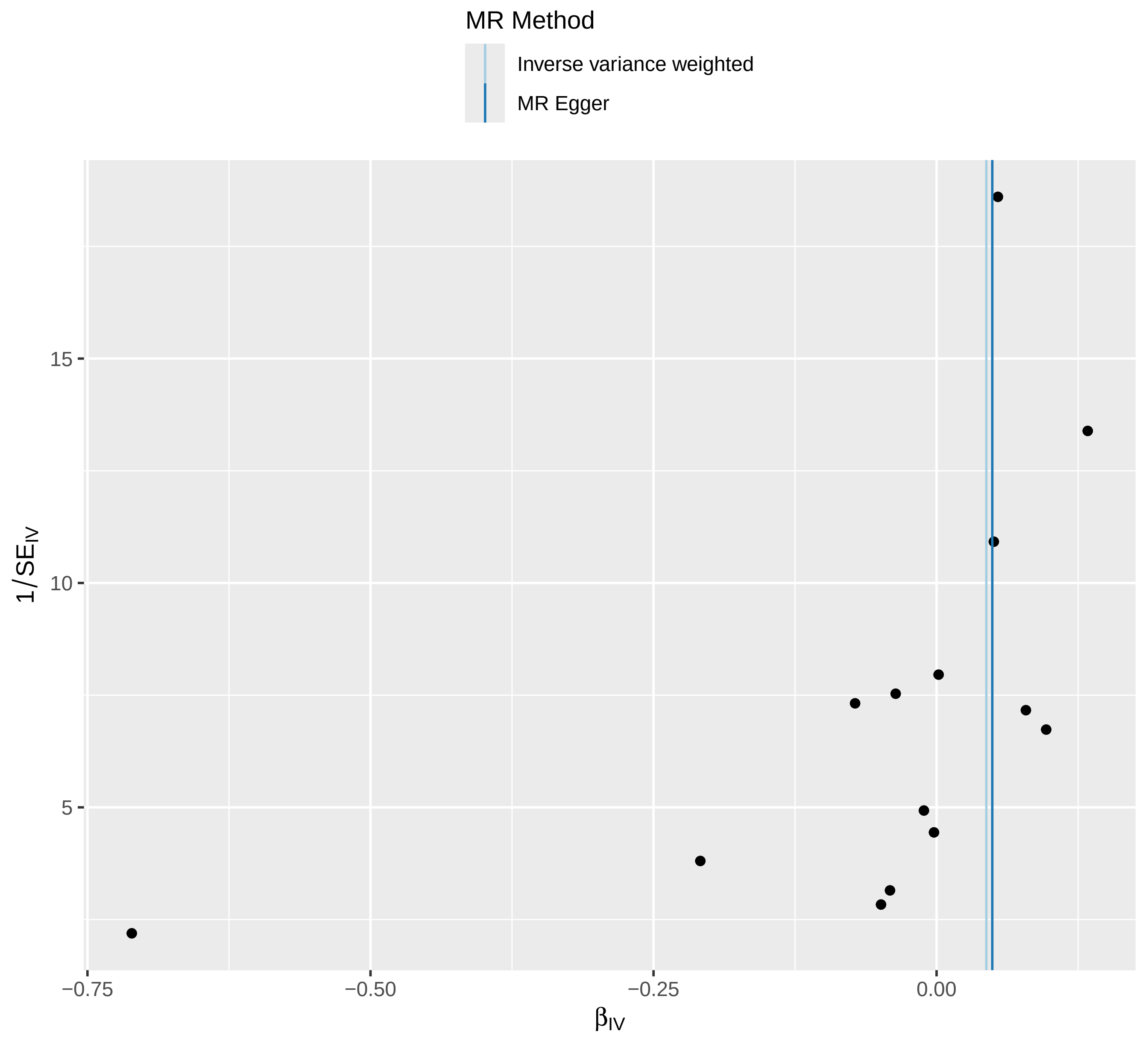
MR Method
Inverse variance weighted
MR Egger
1/SE_IV
15
10
5
β_IV
−0.75
−0.50
−0.25
0.00

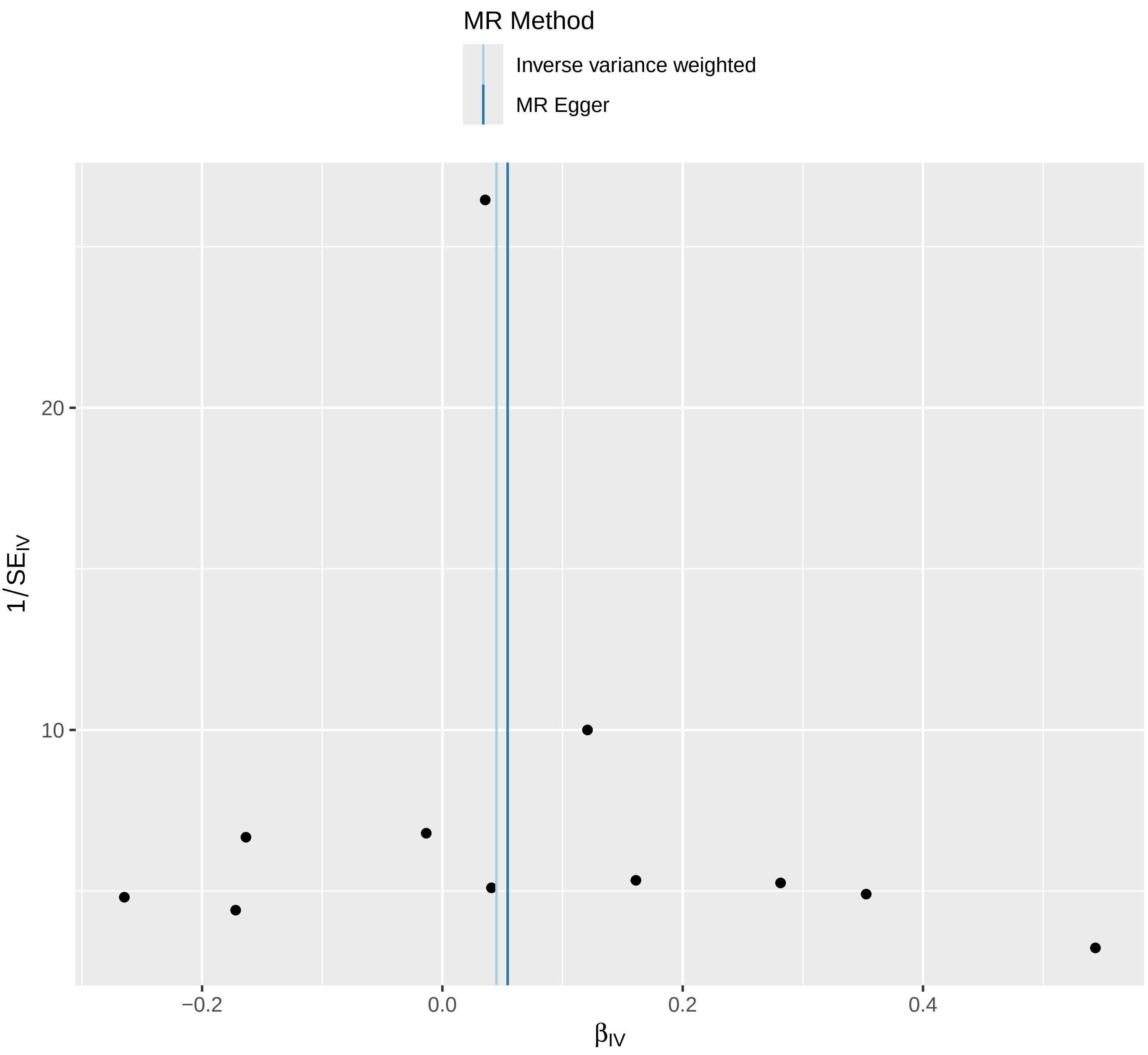

MR Method
Inverse variance weighted
MR Egger
20
10
1/SE$_{IV}$
−0.2
0.0
0.2
0.4
β$_{IV}$

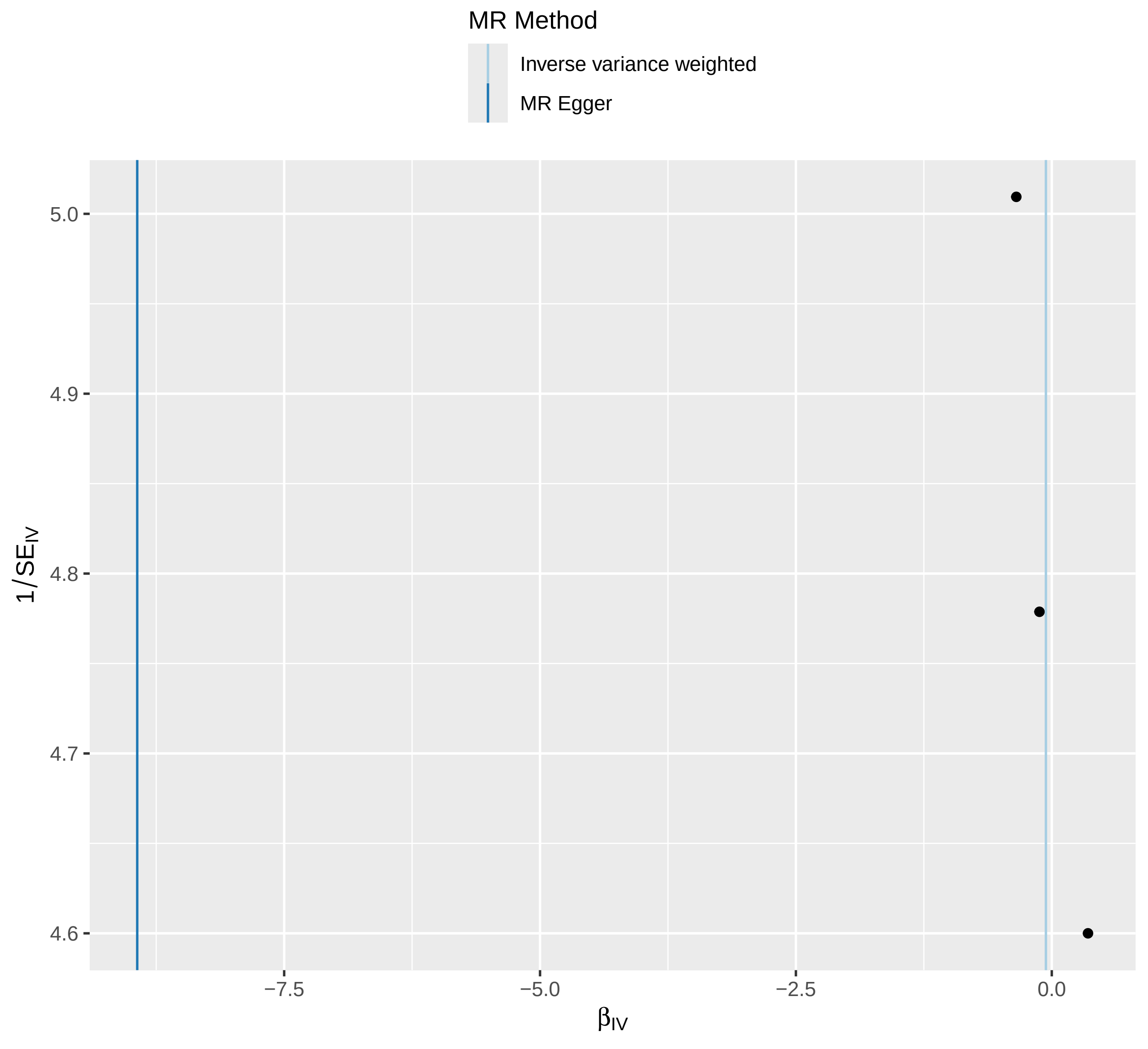
MR Method
Inverse variance weighted
MR Egger
1/SE$_{IV}$
5.0
4.9
4.8
4.7
4.6
−7.5
−5.0
−2.5
0.0
β$_{IV}$

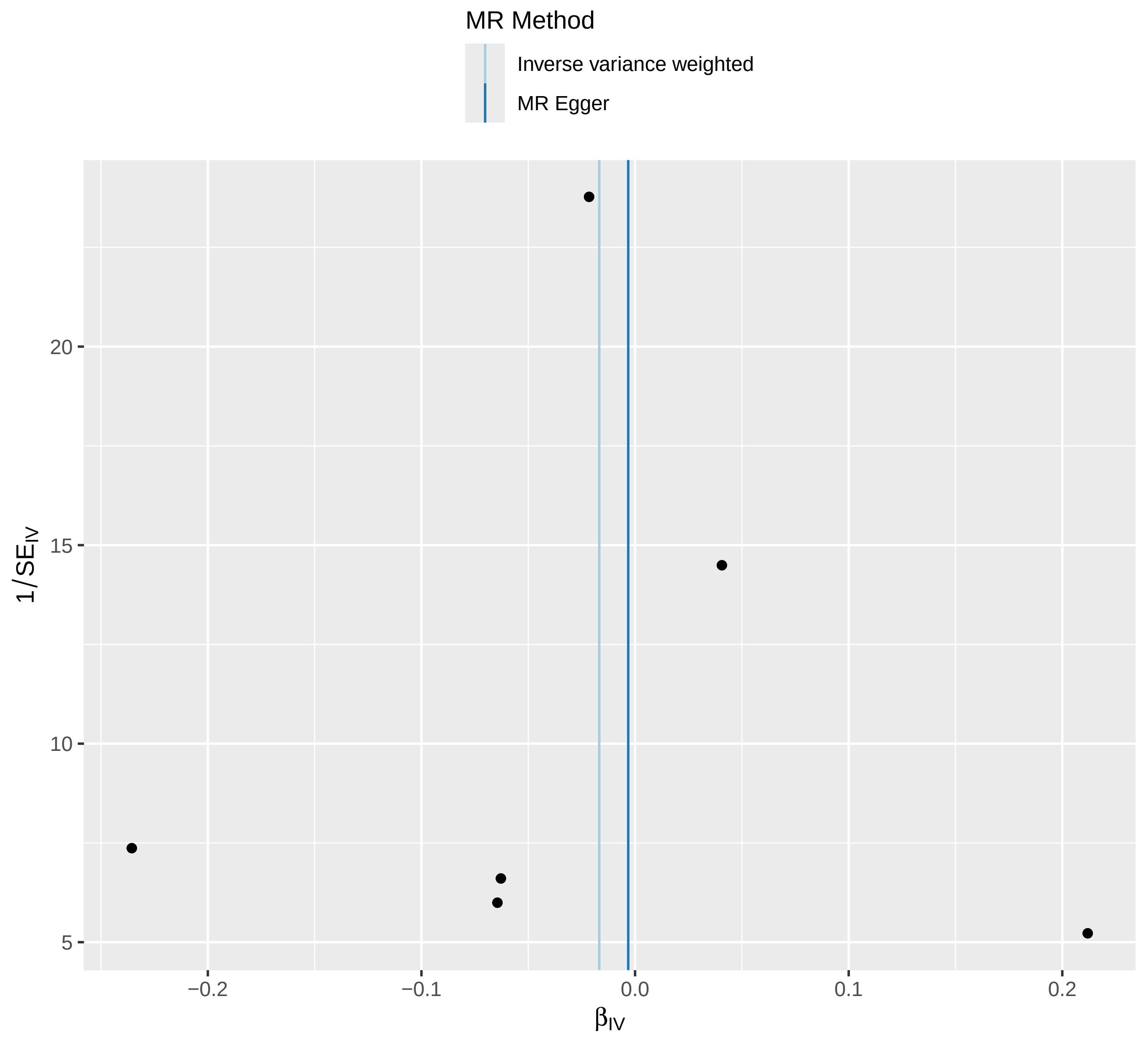

MR Method
Inverse variance weighted
MR Egger
20
15
10
5
1/SE$_{IV}$
−0.2
−0.1
0.0
0.1
0.2
β$_{IV}$

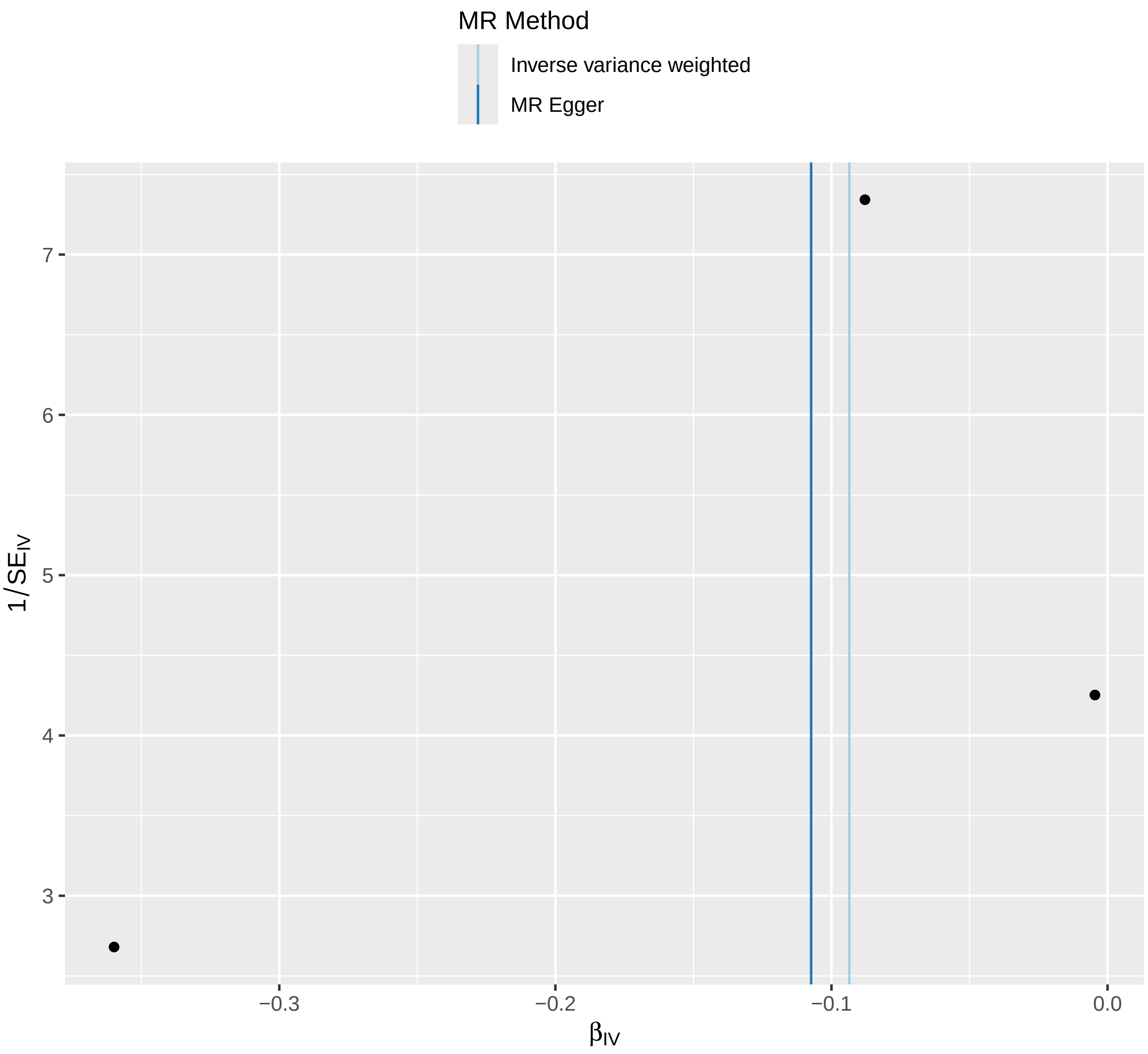
MR Method
Inverse variance weighted
MR Egger
1/SE_IV
7
6
5
4
3
−0.3
−0.2
−0.1
0.0
β_IV

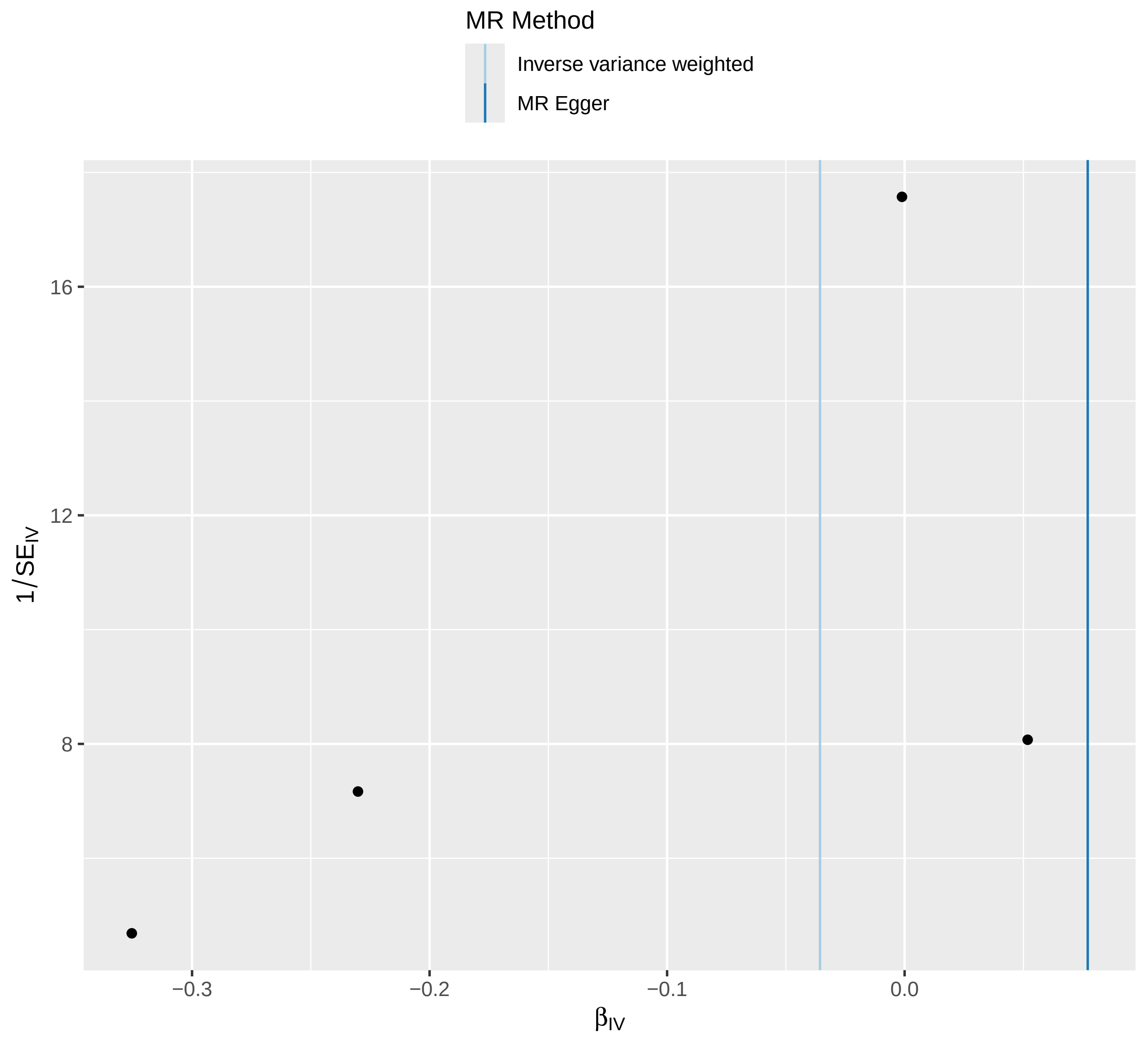

MR Method
Inverse variance weighted
MR Egger
16
12
8
1/SE$_{IV}$
−0.3
−0.2
−0.1
0.0
β$_{IV}$

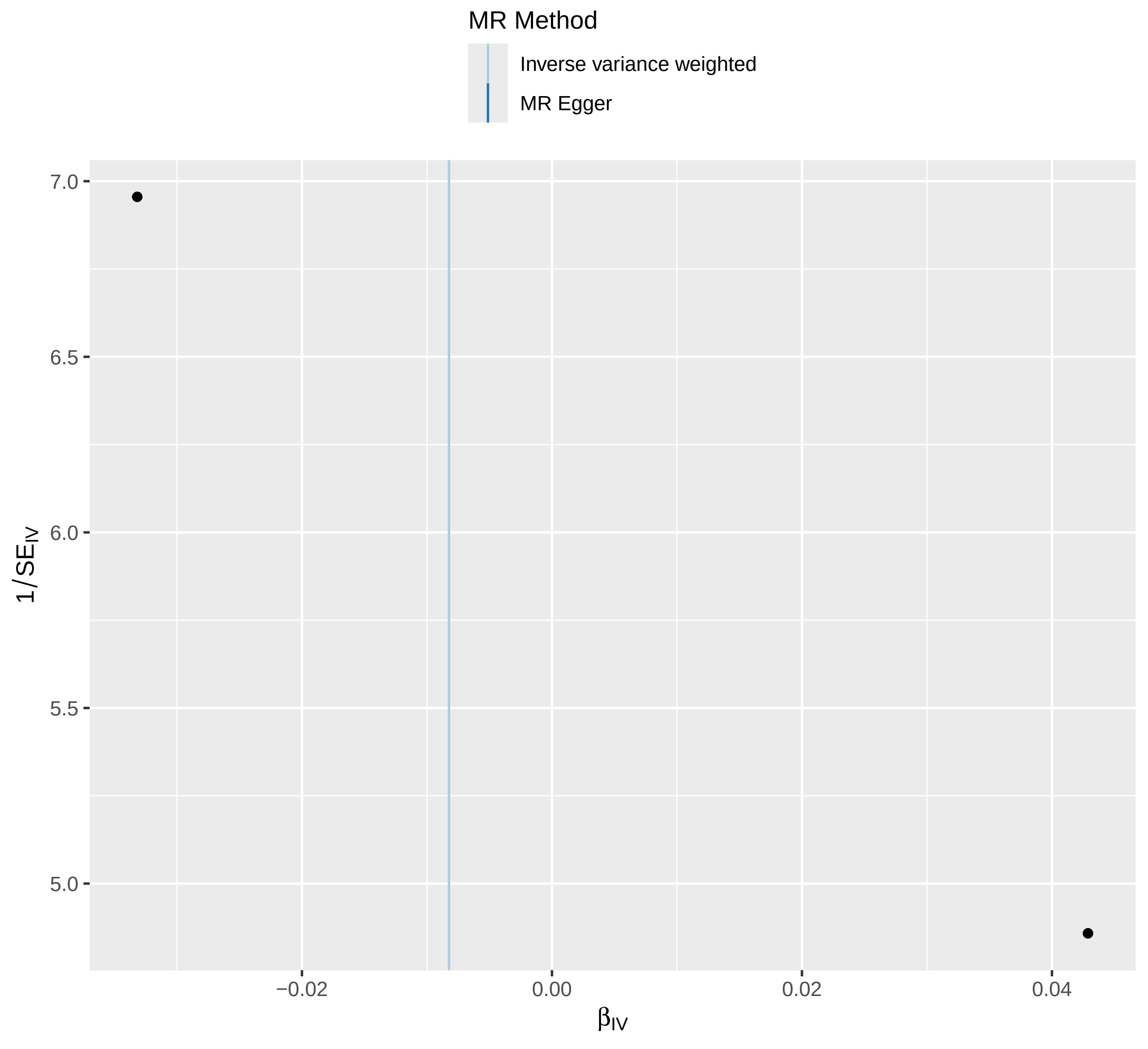

MR Method
Inverse variance weighted
MR Egger
7.0
6.5
6.0
5.5
5.0
1/SE_IV
−0.02
0.00
0.02
0.04
β_IV

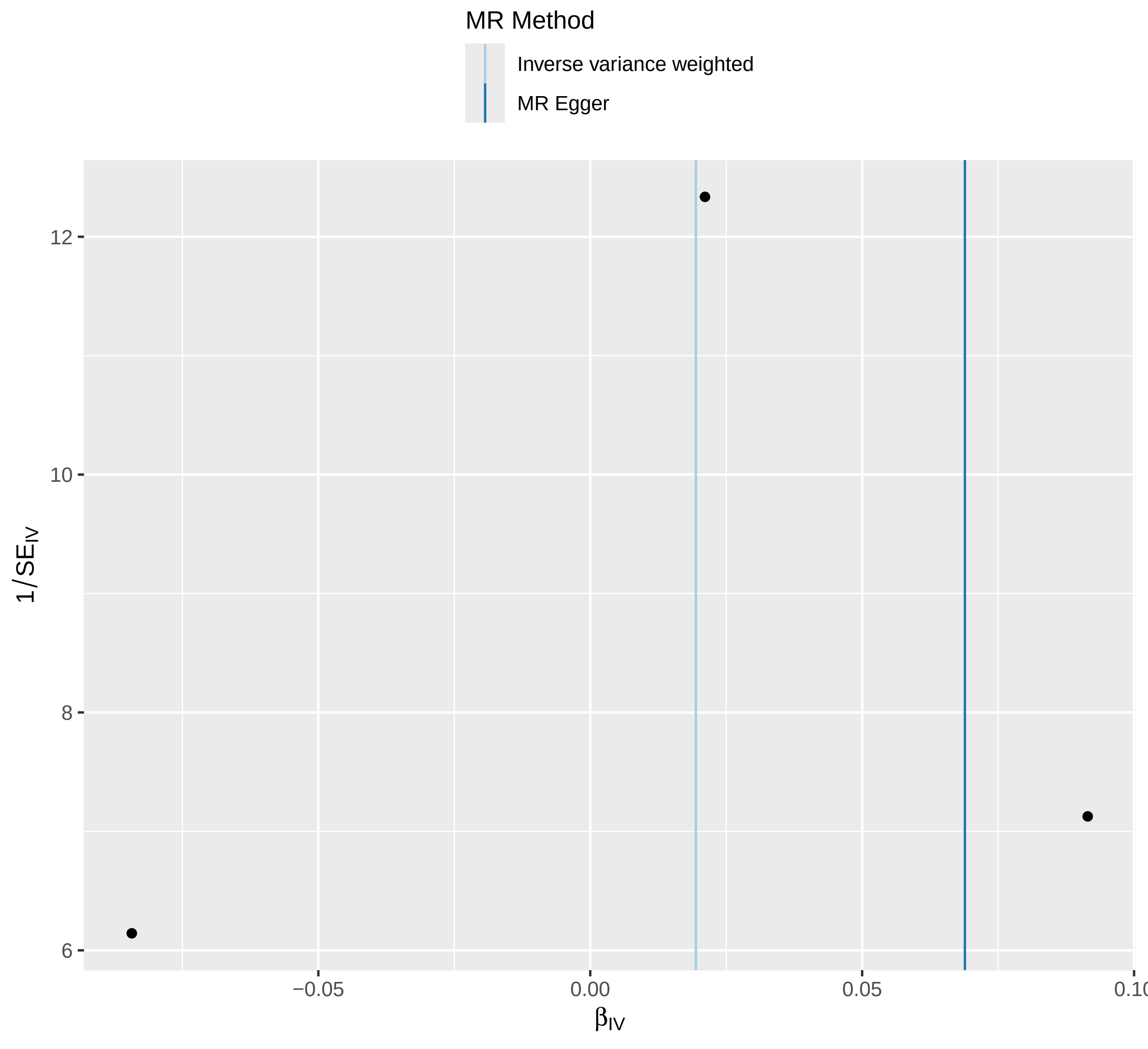

MR Method
Inverse variance weighted
MR Egger
12
10
8
6
1/SE$_{IV}$
−0.05
0.00
0.05
0.10
β$_{IV}$

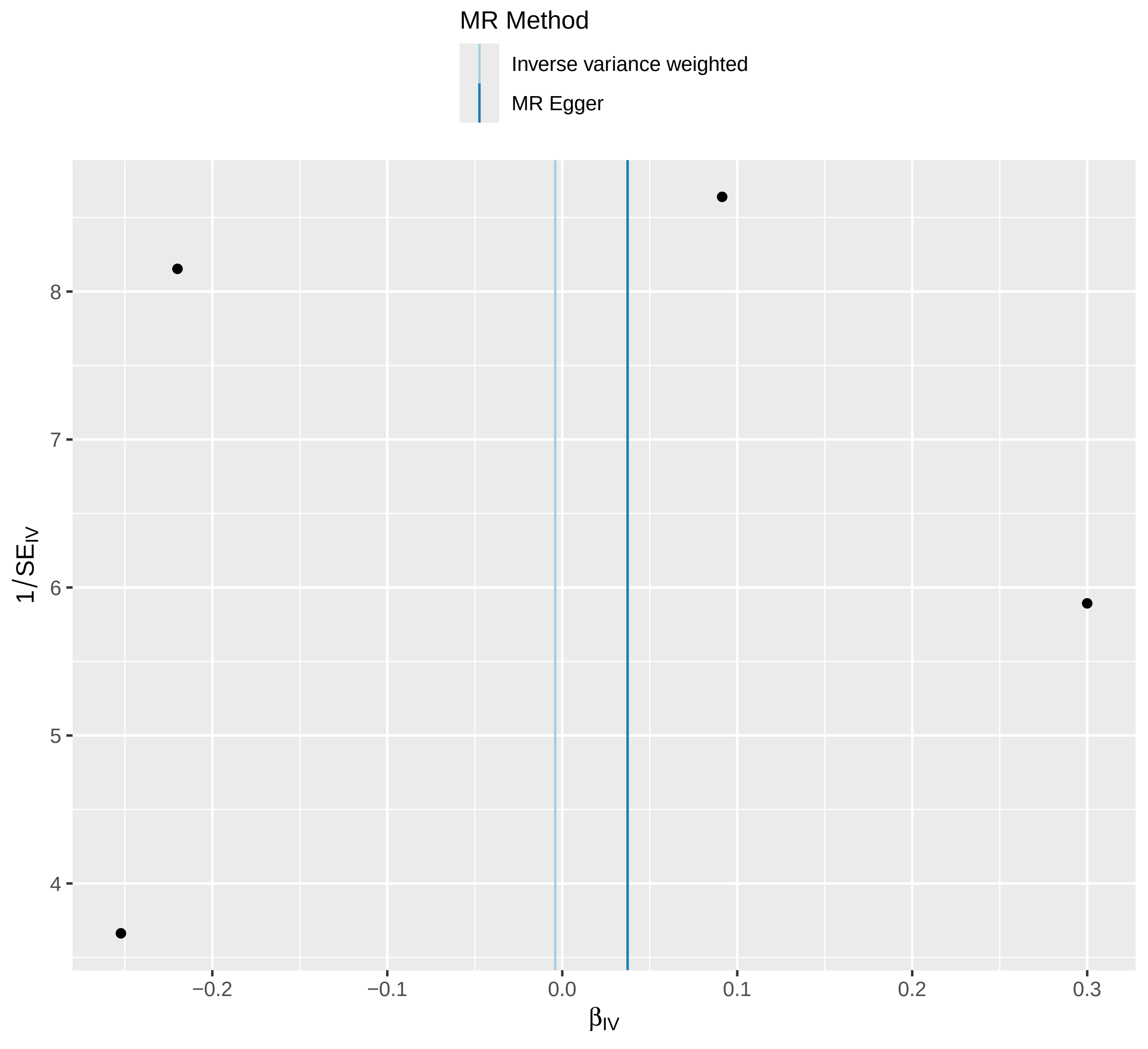

MR Method
Inverse variance weighted
MR Egger
8
7
6
5
4
−0.2
−0.1
0.0
0.1
0.2
0.3
$1/SE_{IV}$
$\beta_{IV}$

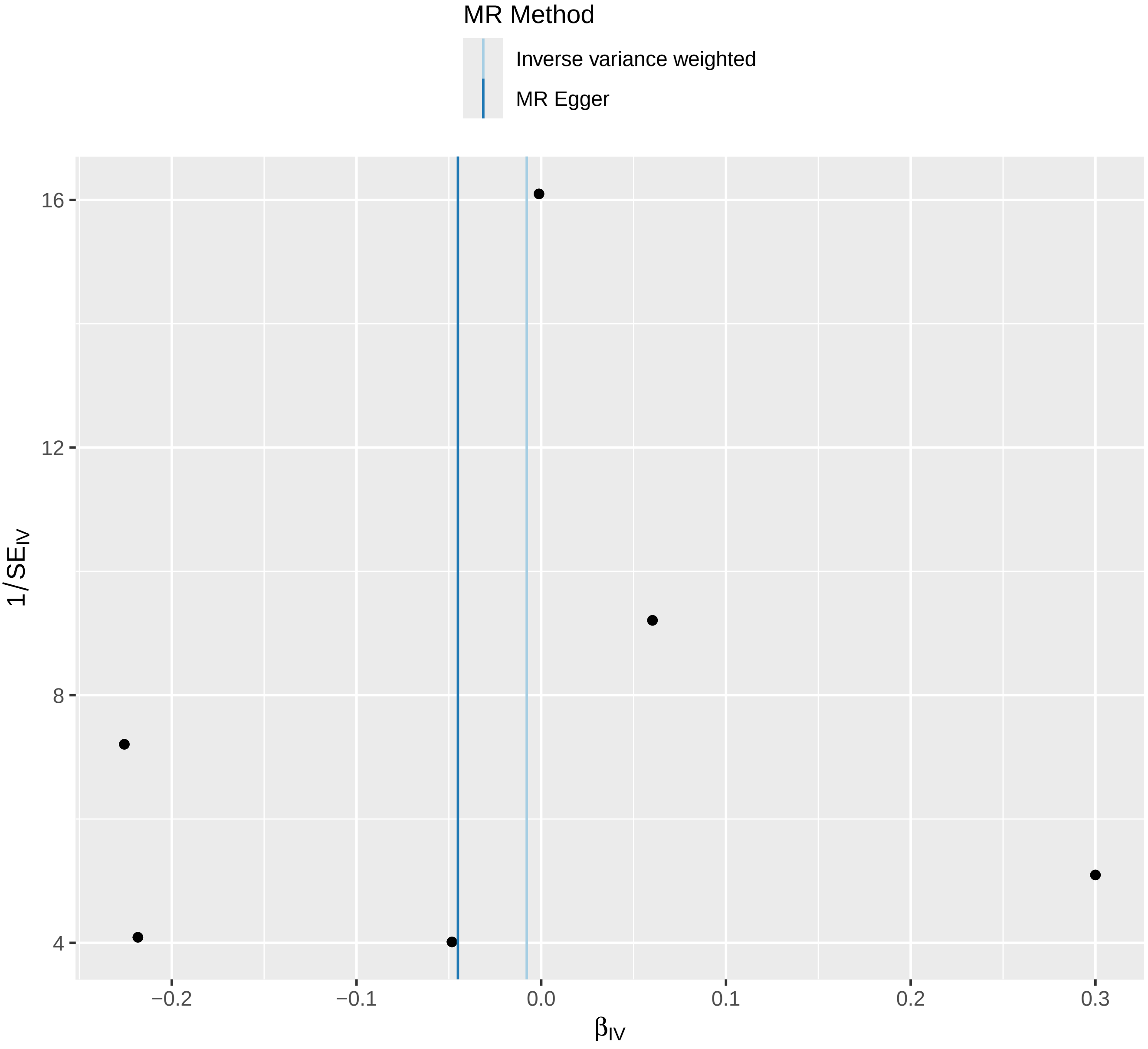

MR Method
Inverse variance weighted
MR Egger
16
12
8
4
−0.2
−0.1
0.0
0.1
0.2
0.3
$1/SE_{IV}$
$\beta_{IV}$

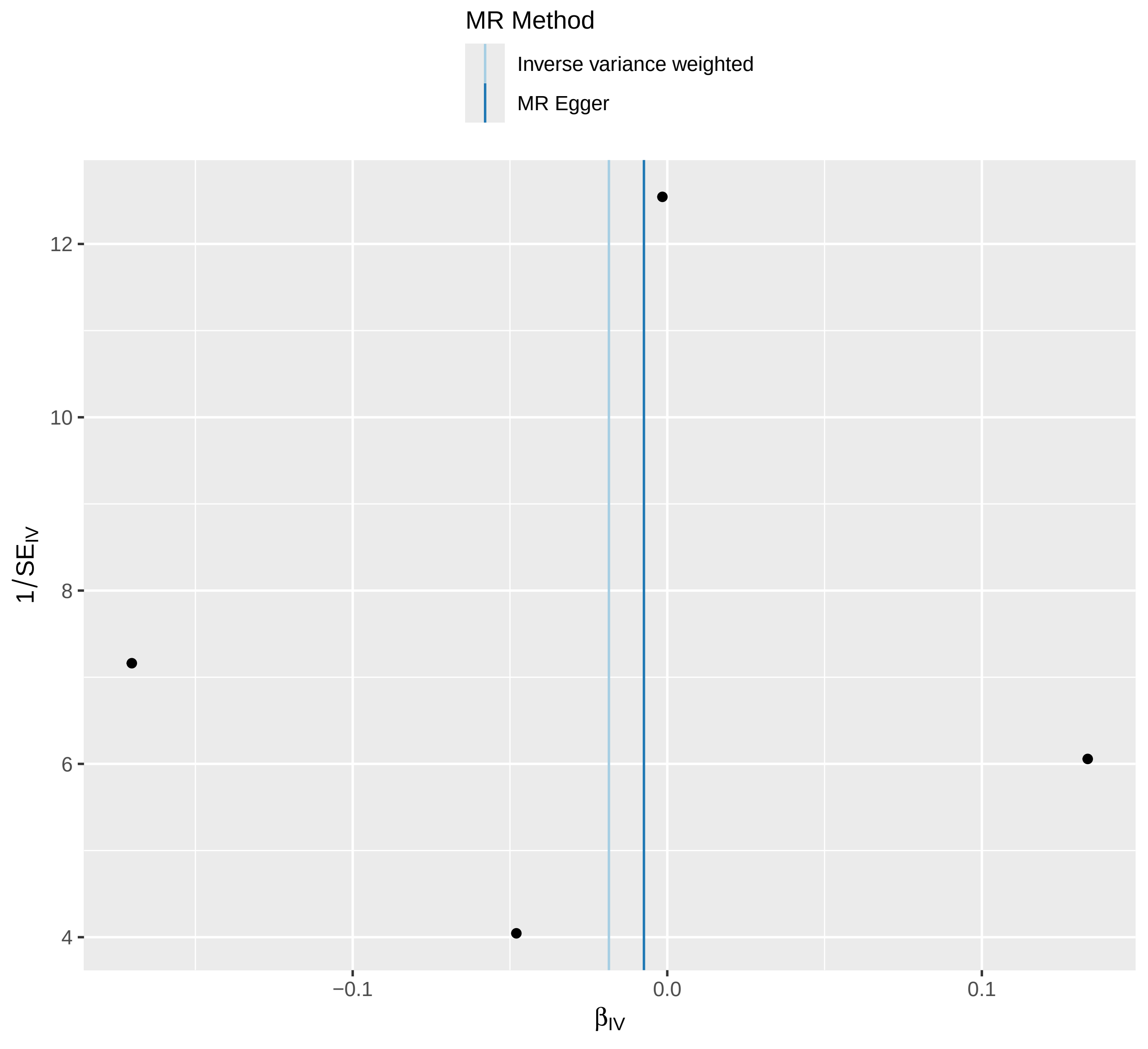
MR Method
Inverse variance weighted
MR Egger
12
10
8
6
4
1/SE_IV
−0.1
0.0
0.1
β_IV

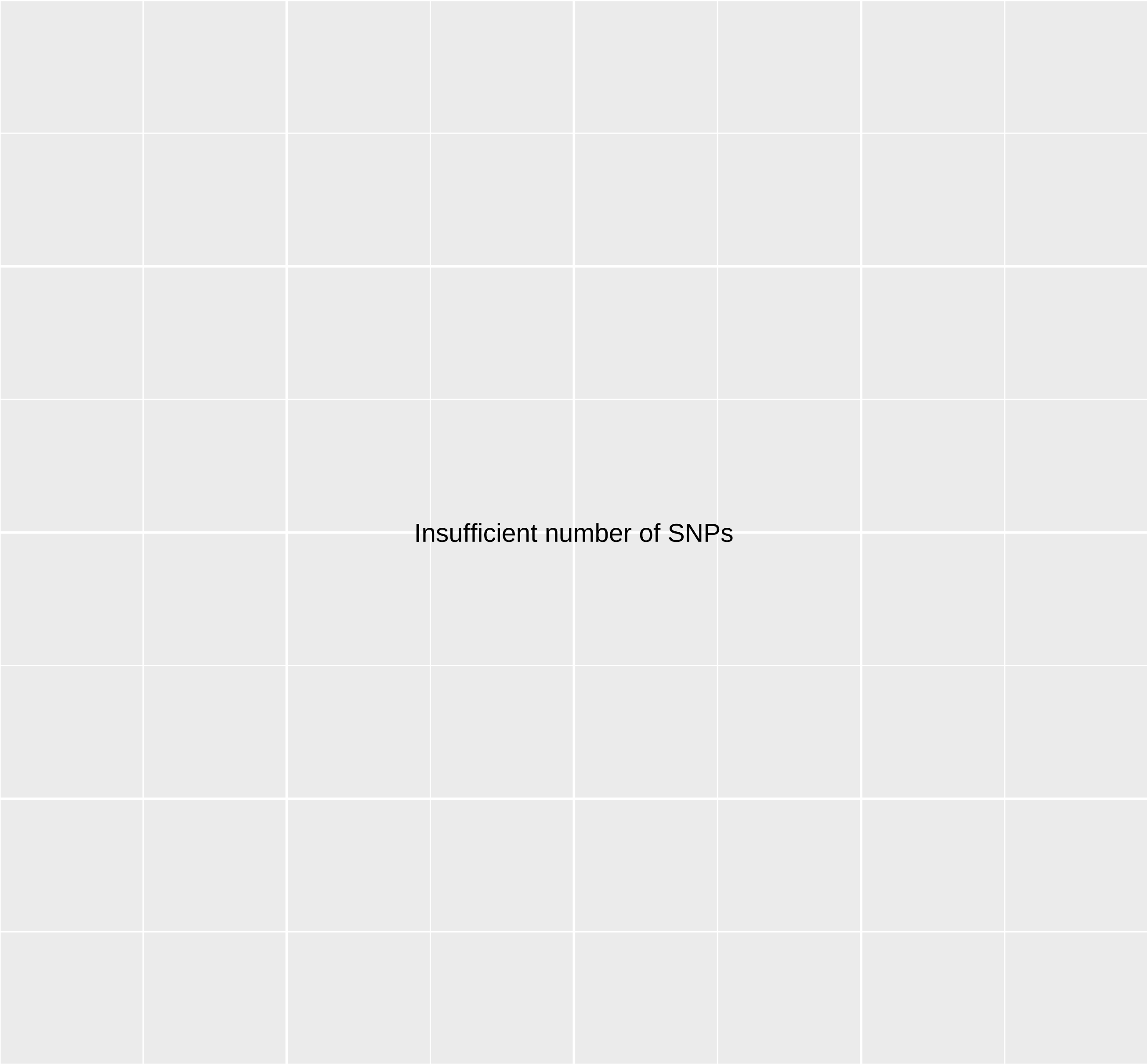
Insufficient number of SNPs

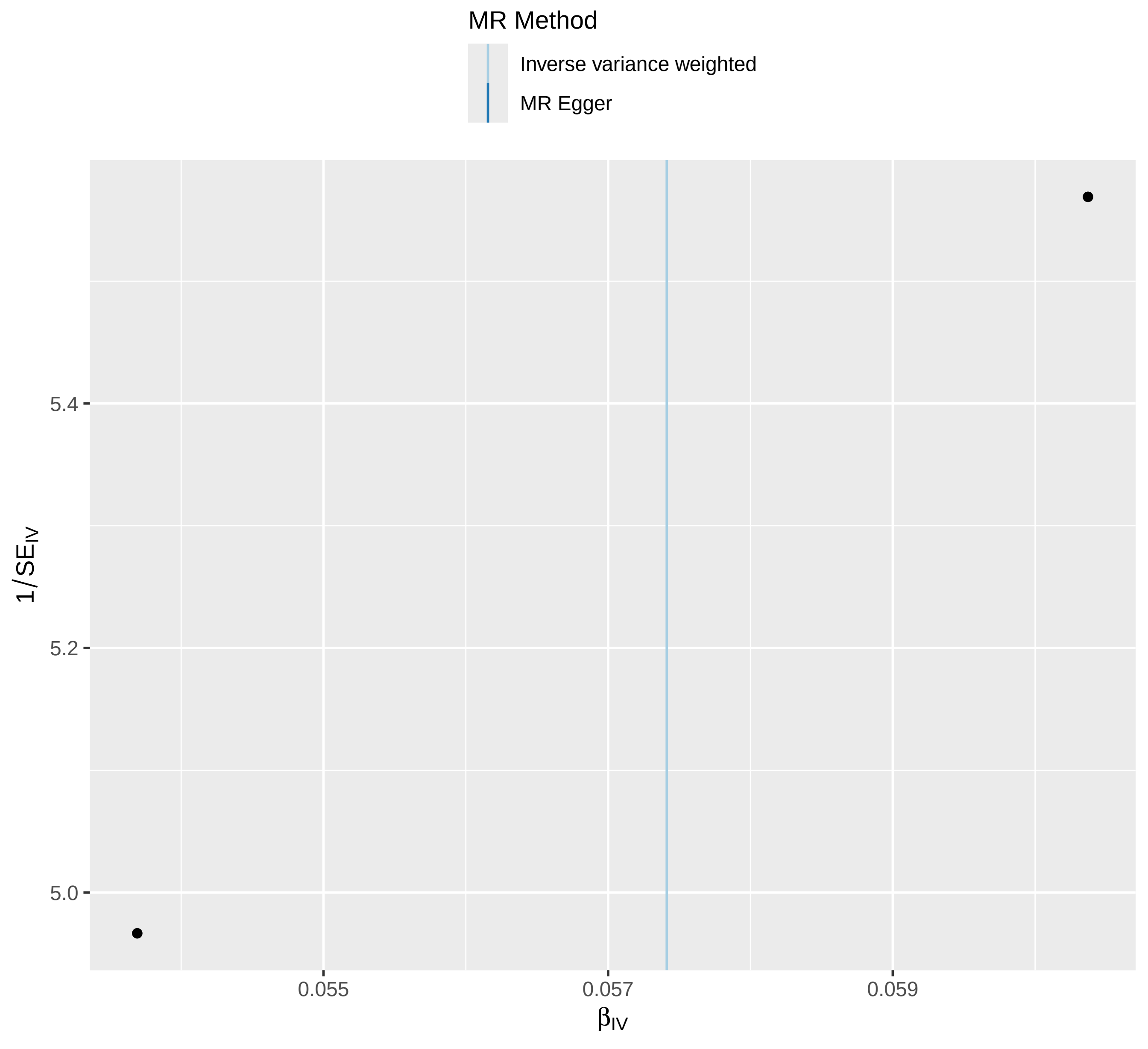

MR Method
Inverse variance weighted
MR Egger
5.4
5.2
5.0
1/SE_IV
0.055
0.057
0.059
β_IV

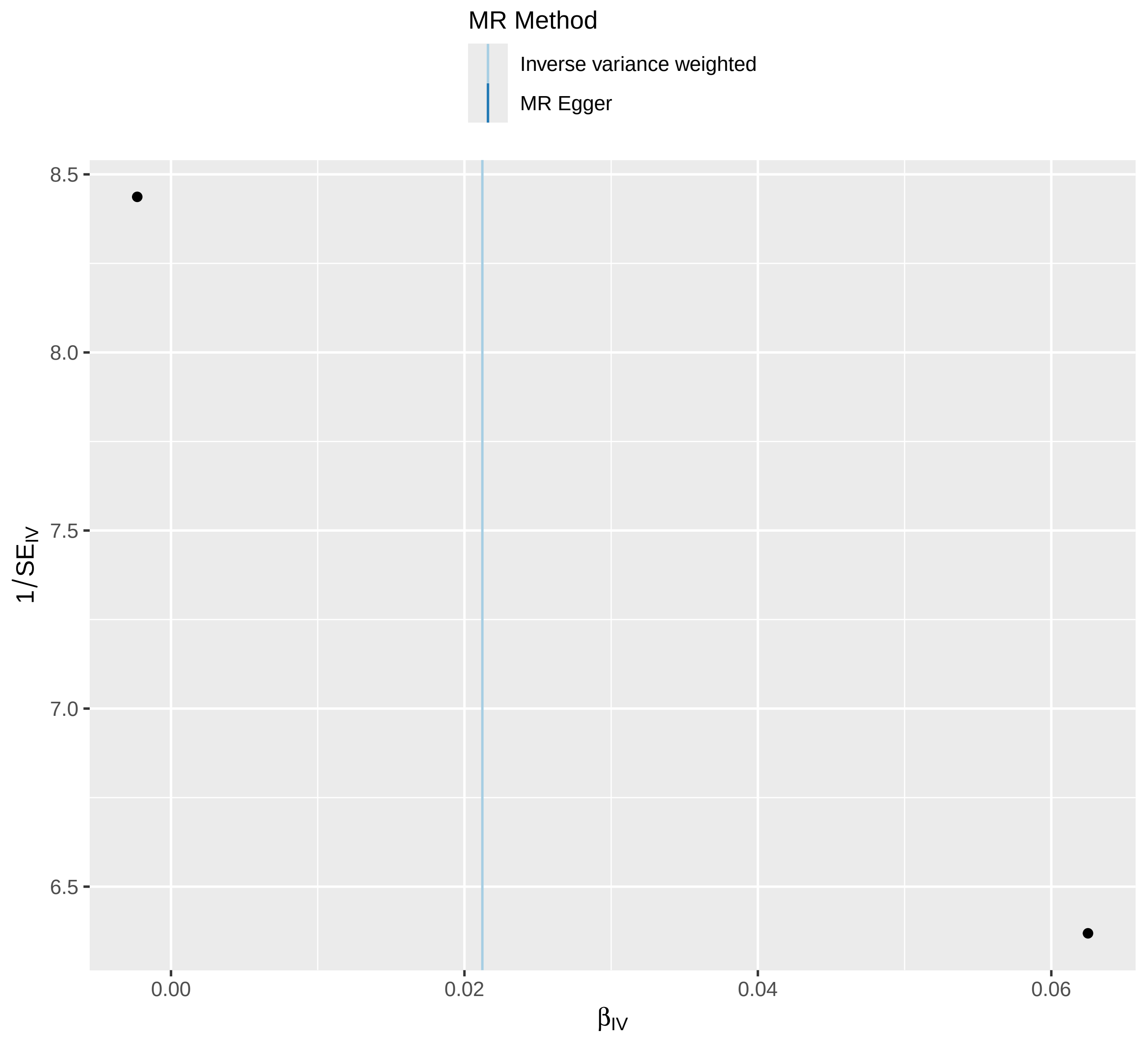

MR Method
Inverse variance weighted
MR Egger
8.5
8.0
7.5
7.0
6.5
1/SE_IV
0.00
0.02
0.04
0.06
β_IV

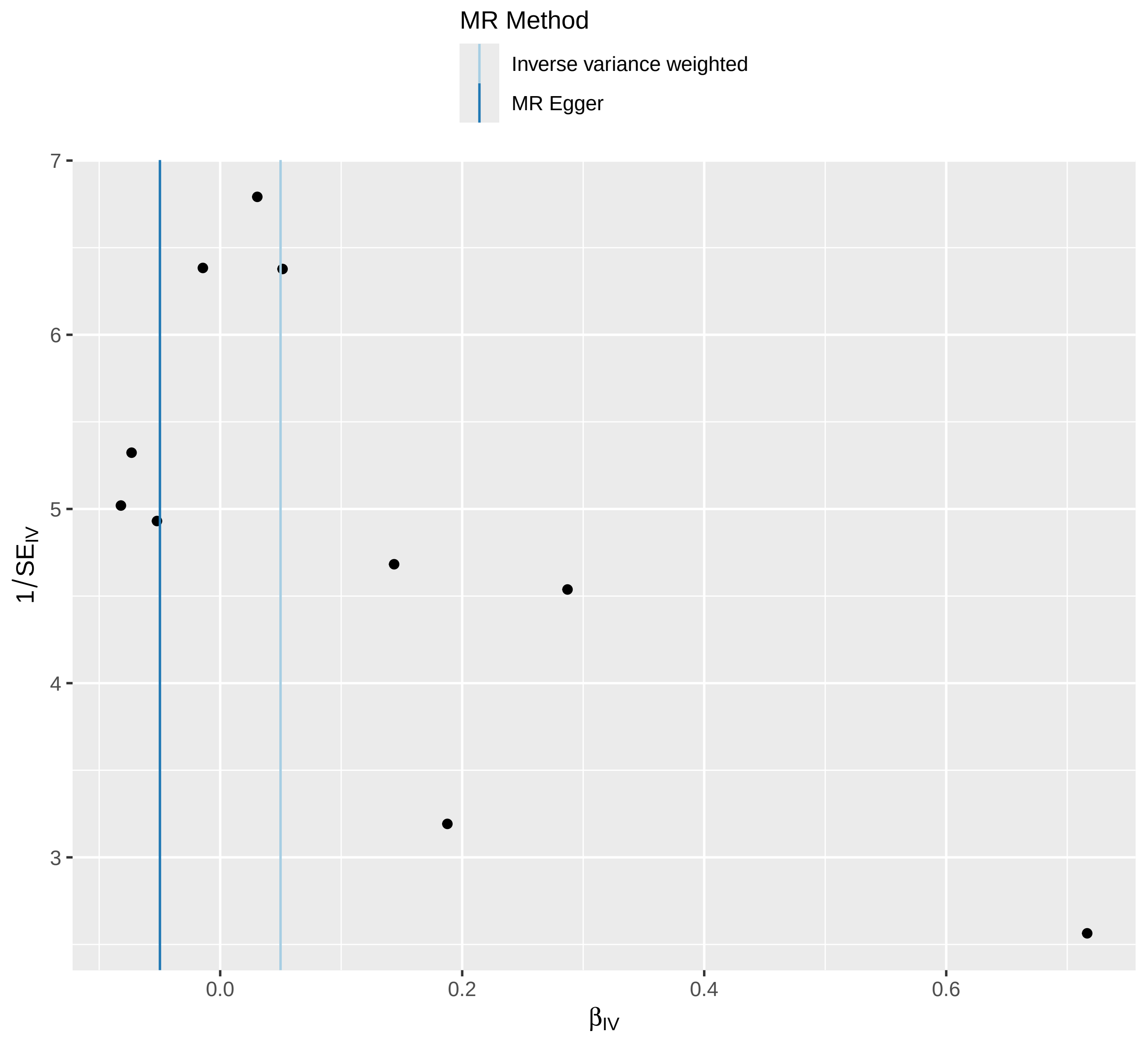

MR Method
Inverse variance weighted
MR Egger
7
6
5
4
3
1/SE_IV
0.0
0.2
0.4
0.6
β_IV

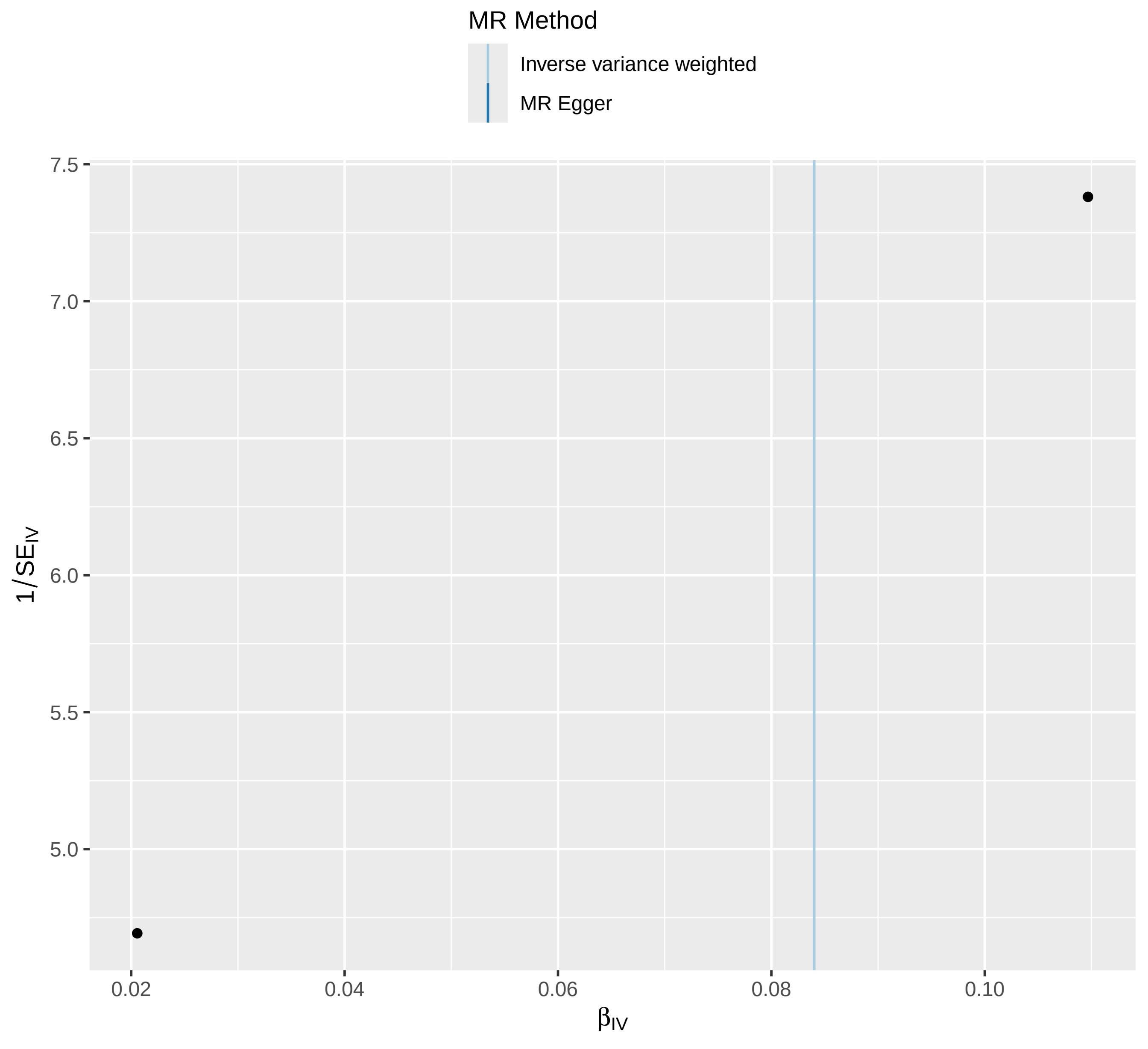
MR Method
Inverse variance weighted
MR Egger
7.5
7.0
6.5
6.0
5.5
5.0
$1/SE_{IV}$
0.02
0.04
0.06
0.08
0.10
$\beta_{IV}$

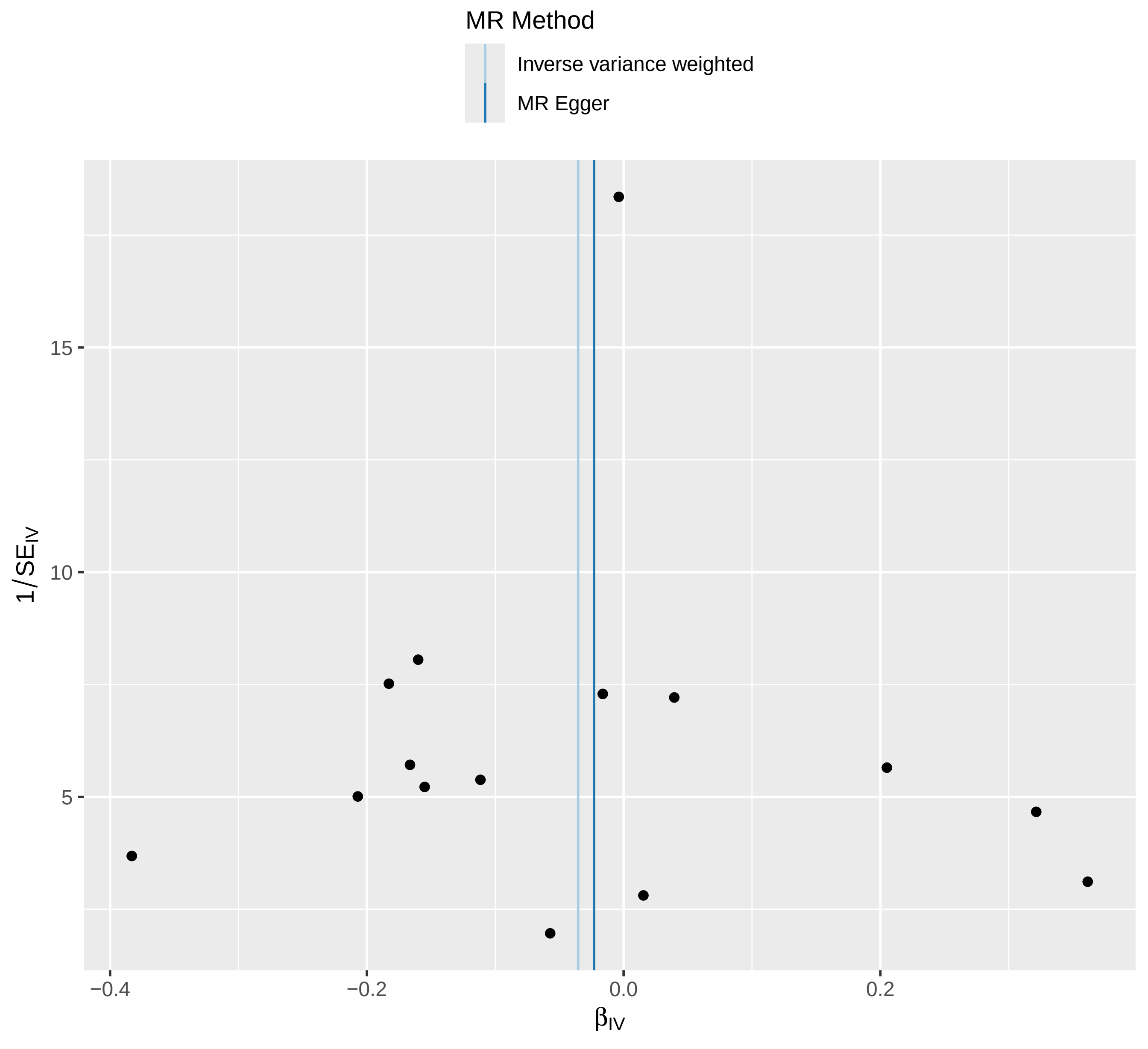

MR Method
Inverse variance weighted
MR Egger
15
10
5
1/SE_IV
−0.4
−0.2
0.0
0.2
β_IV

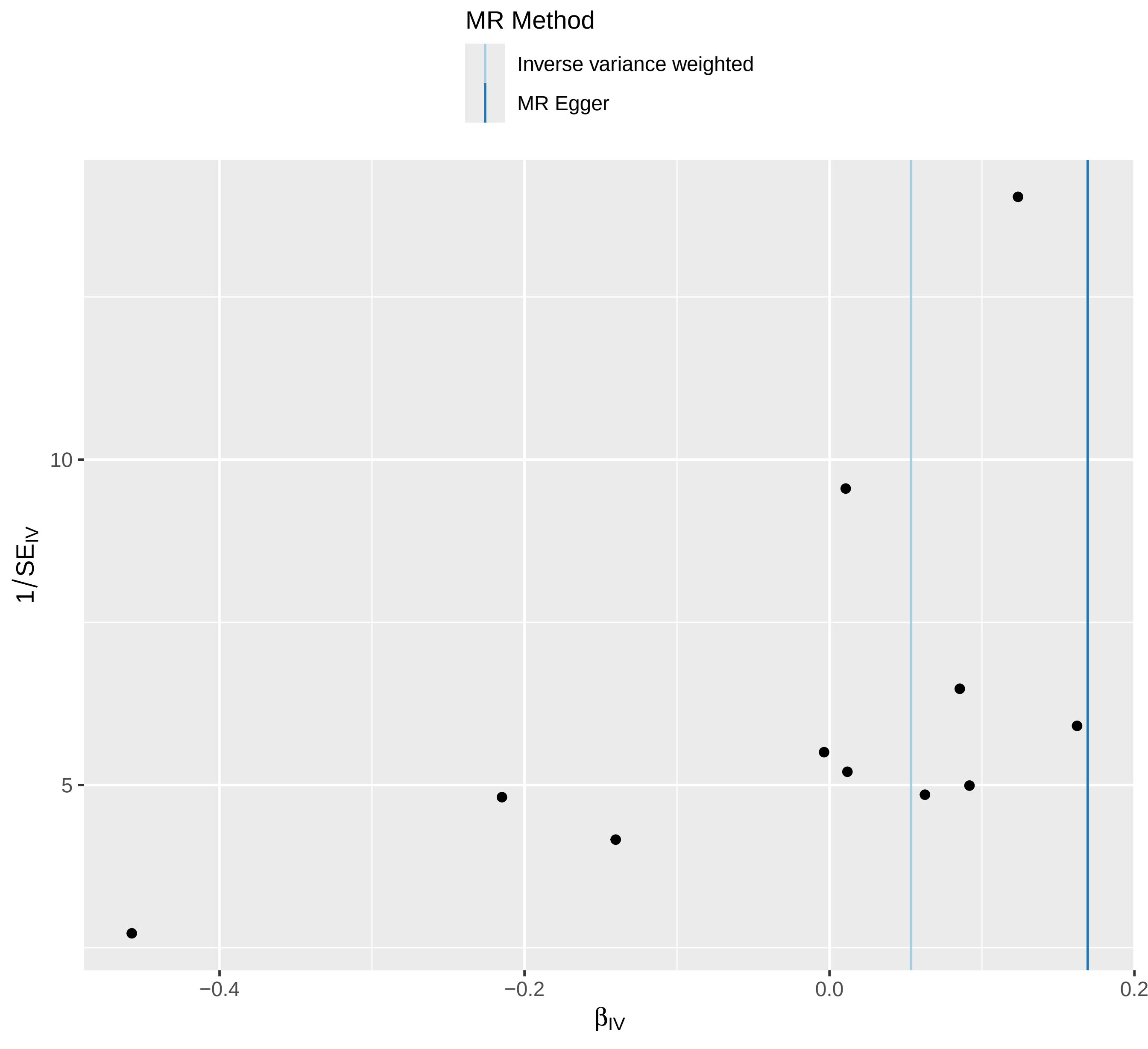
MR Method
Inverse variance weighted
MR Egger
1/SE_IV
10
5
−0.4
−0.2
0.0
0.2
β_IV

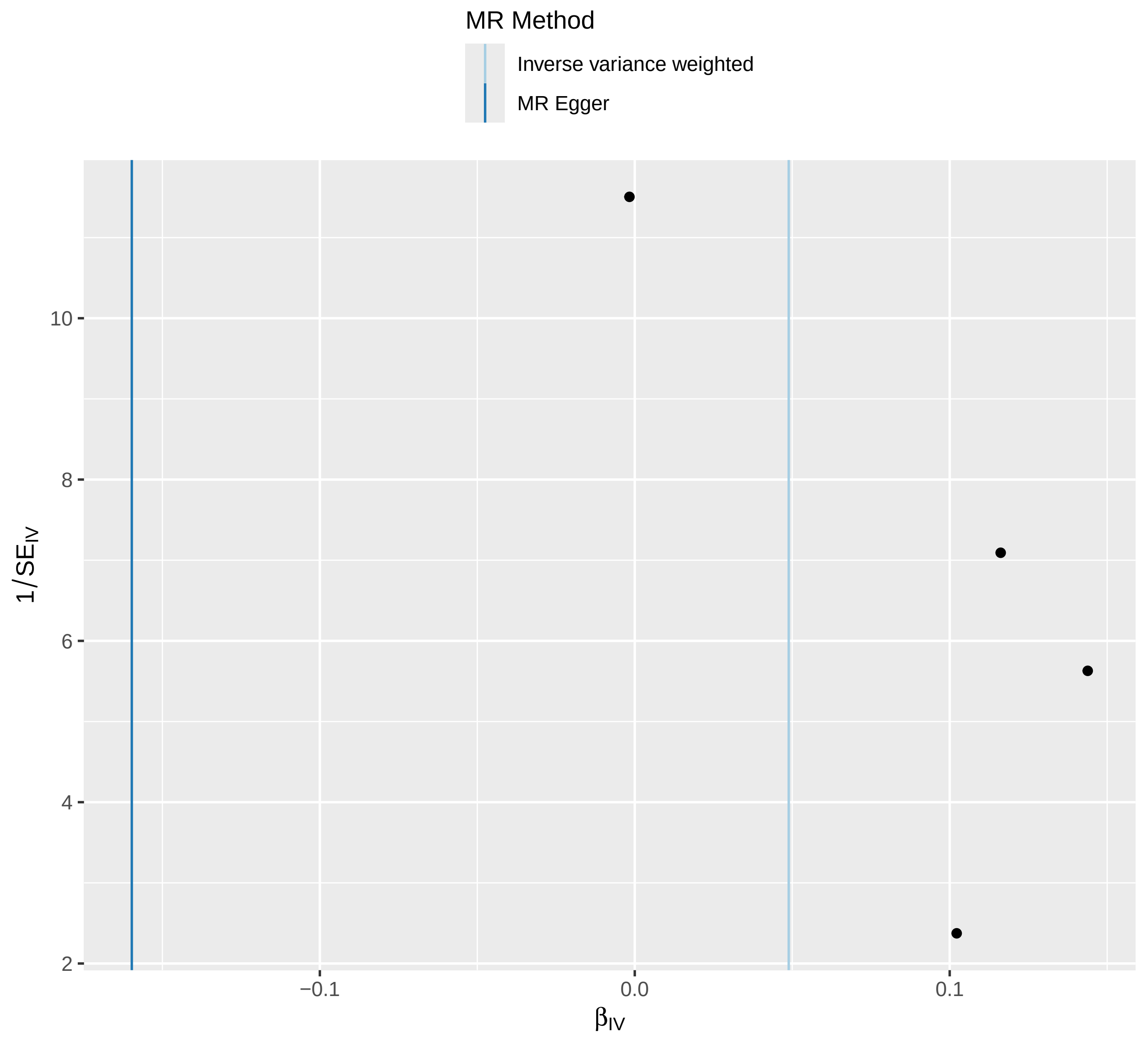

MR Method
Inverse variance weighted
MR Egger
1/SE_IV
10
8
6
4
2
−0.1
0.0
0.1
β_IV

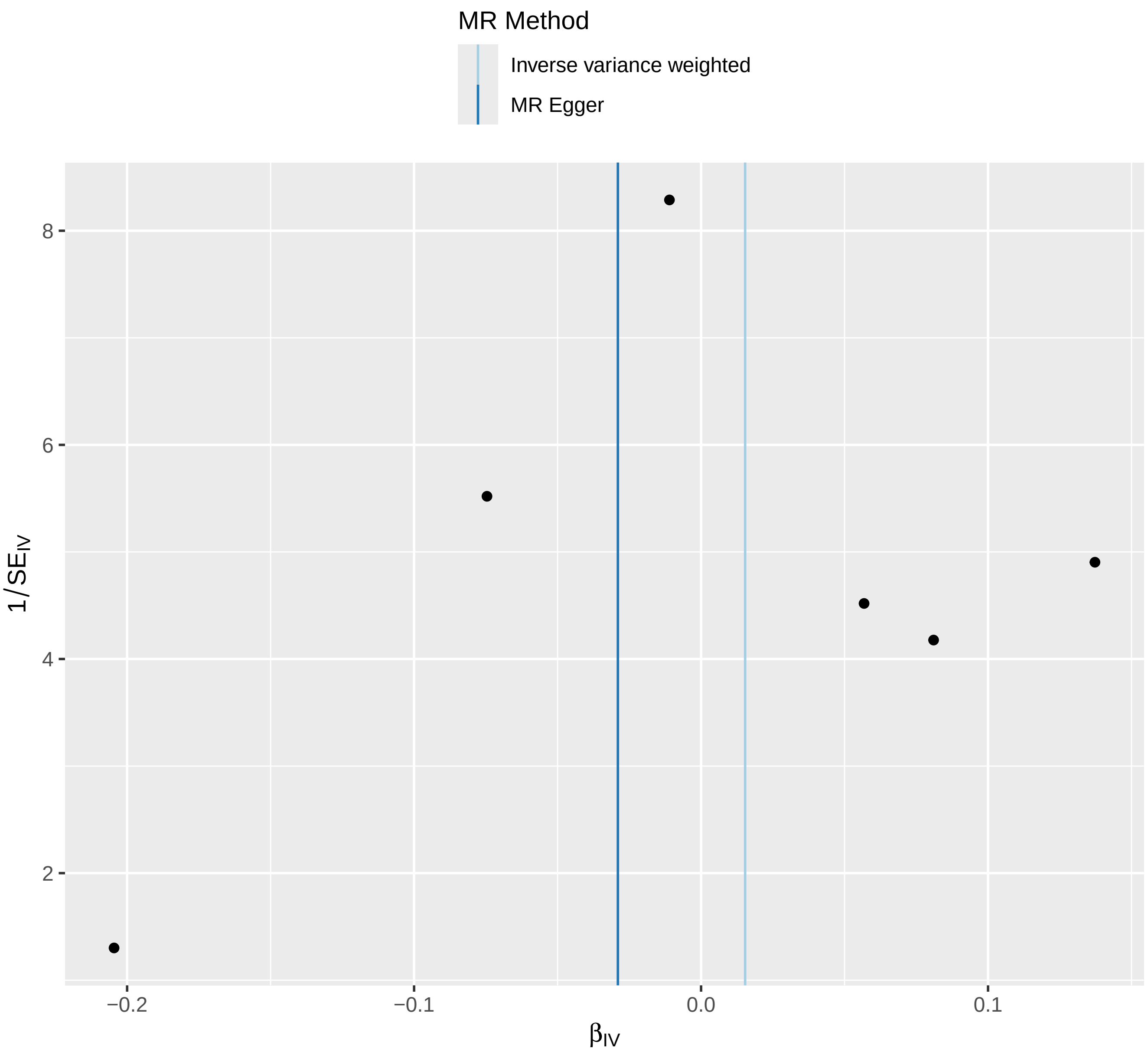

MR Method
Inverse variance weighted
MR Egger
8
6
4
2
1/SE_IV
−0.2
−0.1
0.0
0.1
β_IV

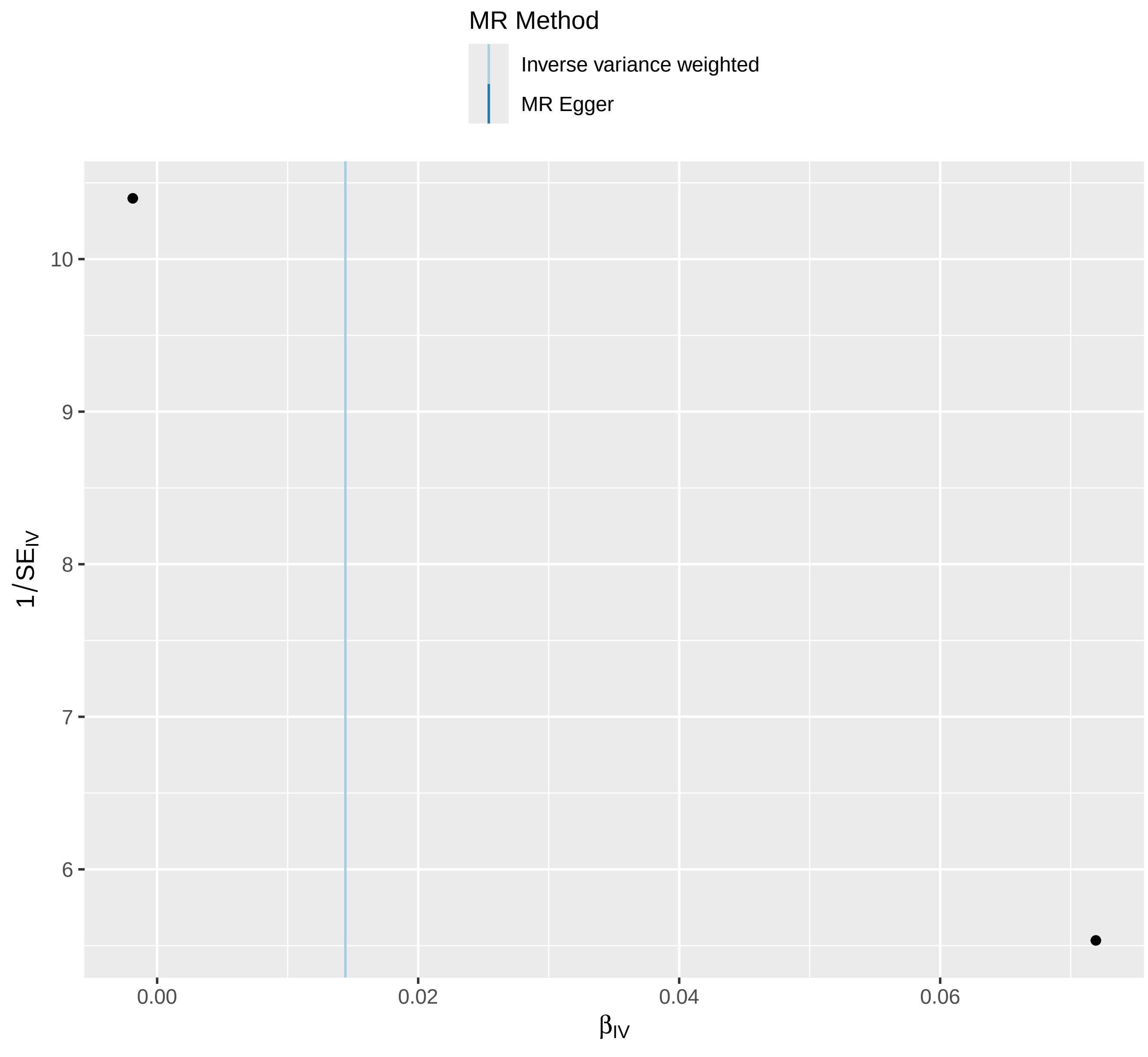
MR Method
Inverse variance weighted
MR Egger
10
9
8
7
6
1/SE_IV
0.00
0.02
0.04
0.06
β_IV

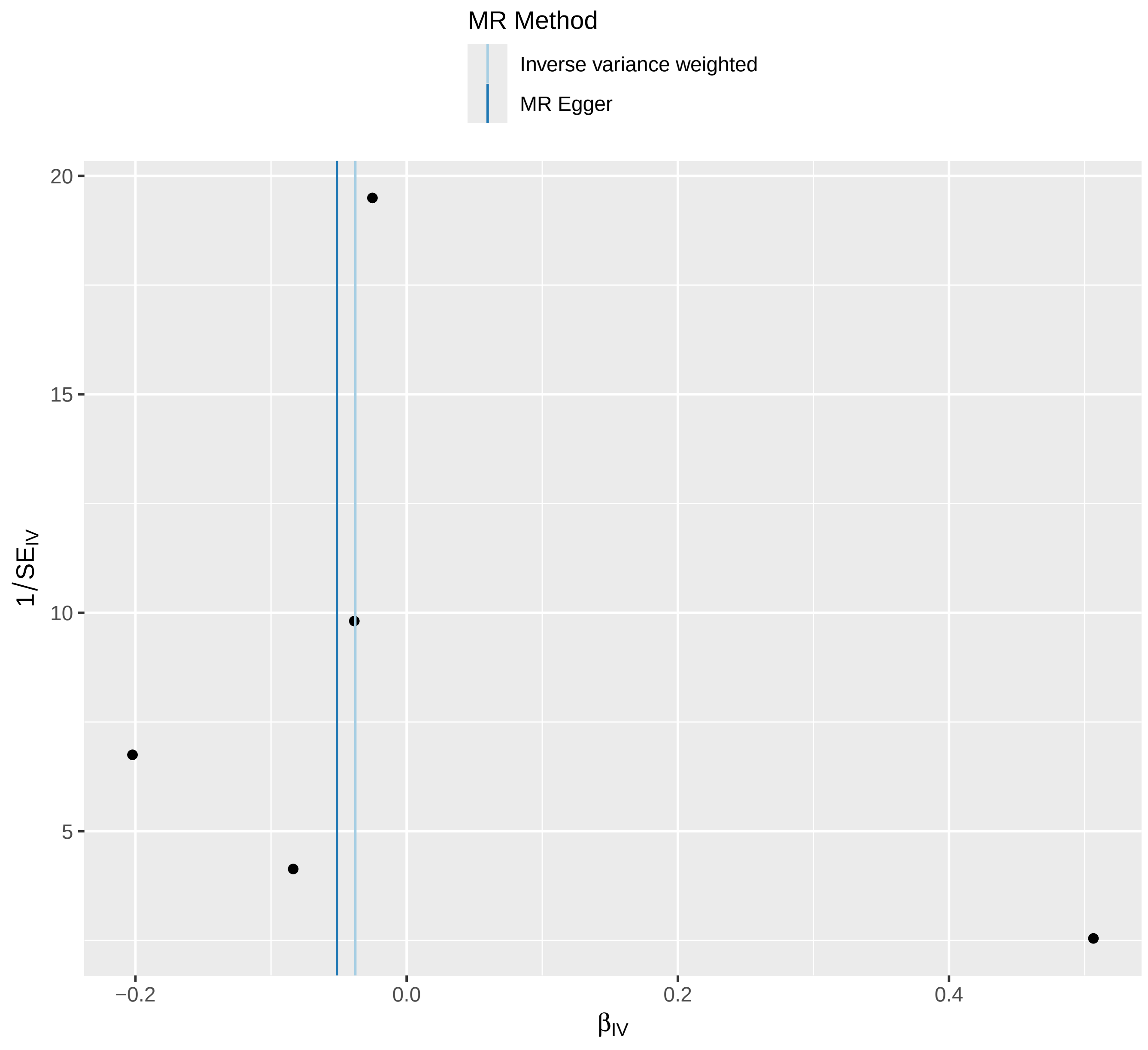
MR Method
Inverse variance weighted
MR Egger
20
15
10
5
−0.2
0.0
0.2
0.4
$1/SE_{IV}$
$\beta_{IV}$

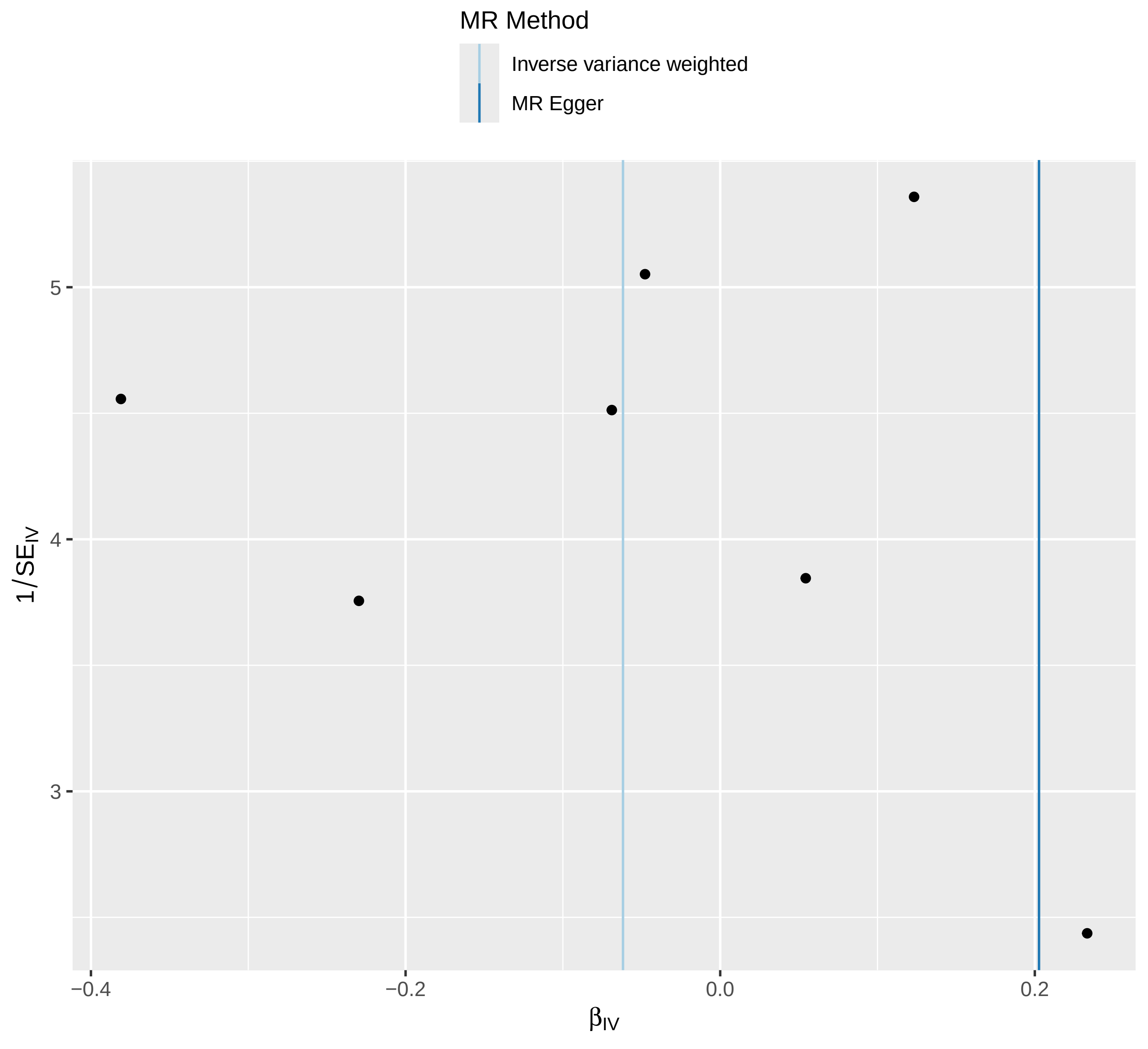
MR Method
Inverse variance weighted
MR Egger
5
4
3
1/SE_IV
−0.4
−0.2
0.0
0.2
β_IV

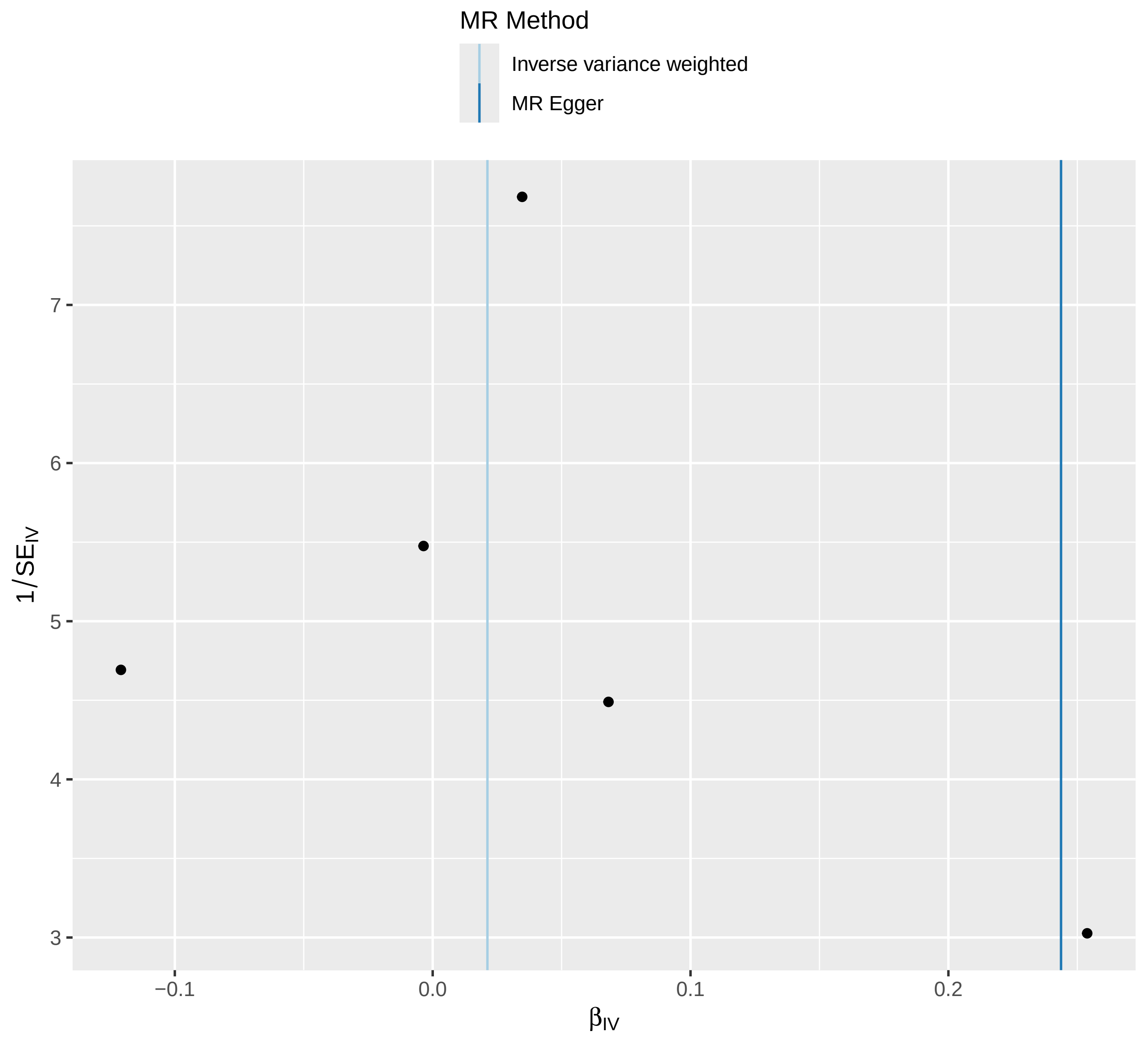

MR Method
Inverse variance weighted
MR Egger
7
6
5
4
3
1/SE$_{IV}$
−0.1
0.0
0.1
0.2
β$_{IV}$

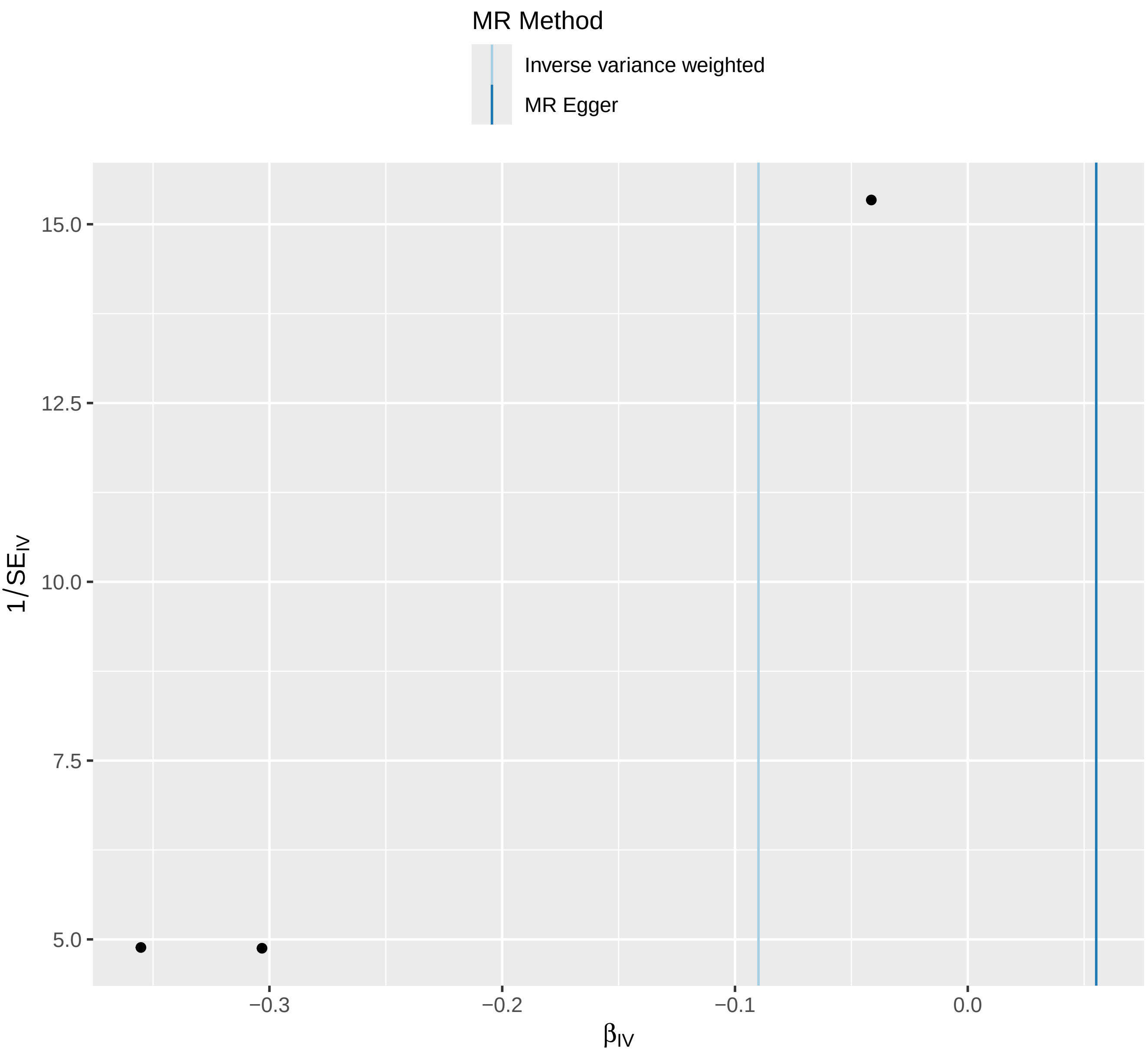
MR Method
Inverse variance weighted
MR Egger
15.0
12.5
10.0
7.5
5.0
−0.3
−0.2
−0.1
0.0
$1/SE_{IV}$
$\beta_{IV}$

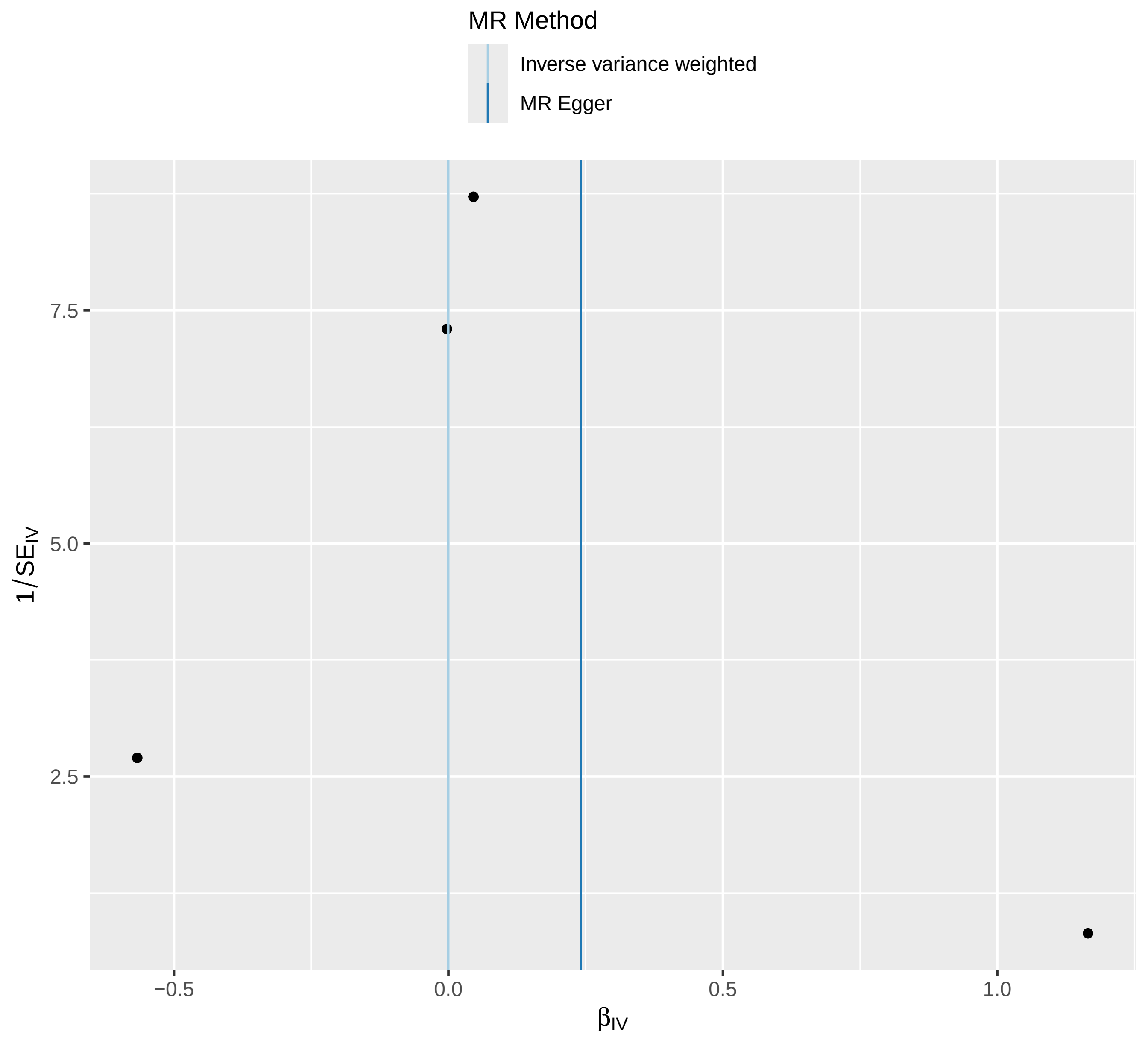
MR Method
Inverse variance weighted
MR Egger
7.5
5.0
2.5
1/SE_IV
−0.5
0.0
0.5
1.0
β_IV

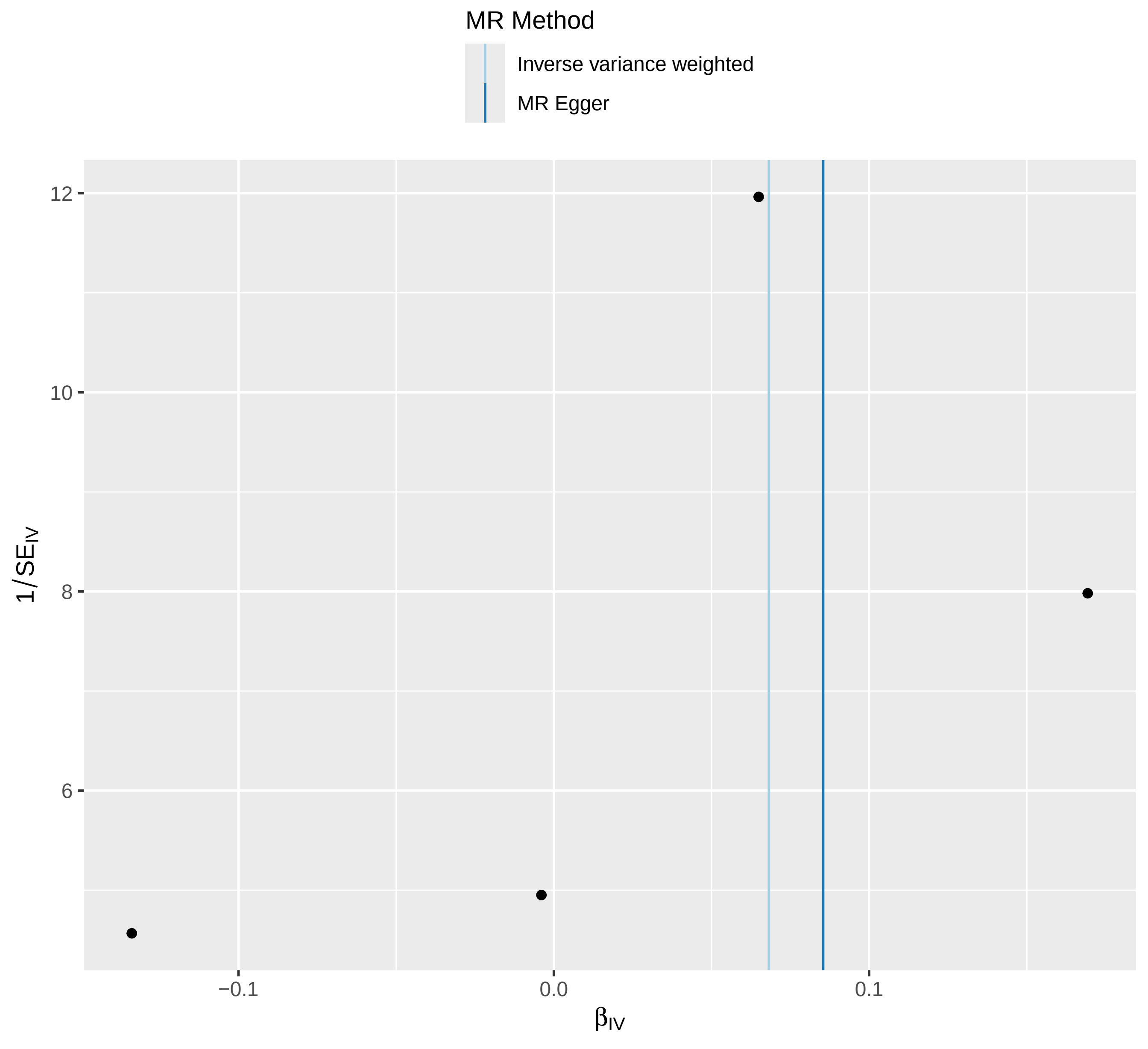

MR Method
Inverse variance weighted
MR Egger
12
10
8
6
$1/SE_{IV}$
−0.1
0.0
0.1
$\beta_{IV}$

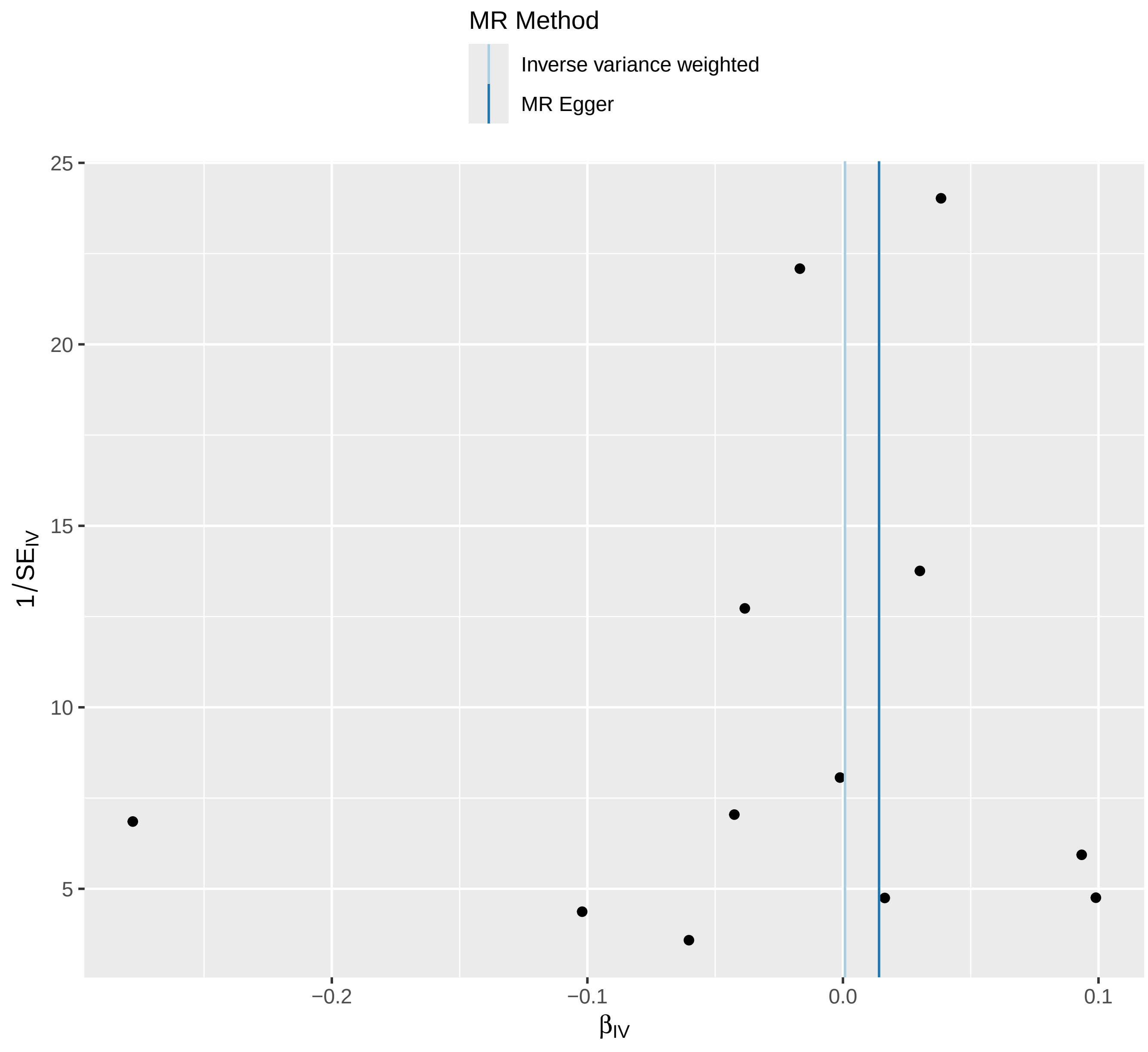
MR Method
Inverse variance weighted
MR Egger
25
20
15
10
5
1/SE_IV
−0.2
−0.1
0.0
0.1
β_IV

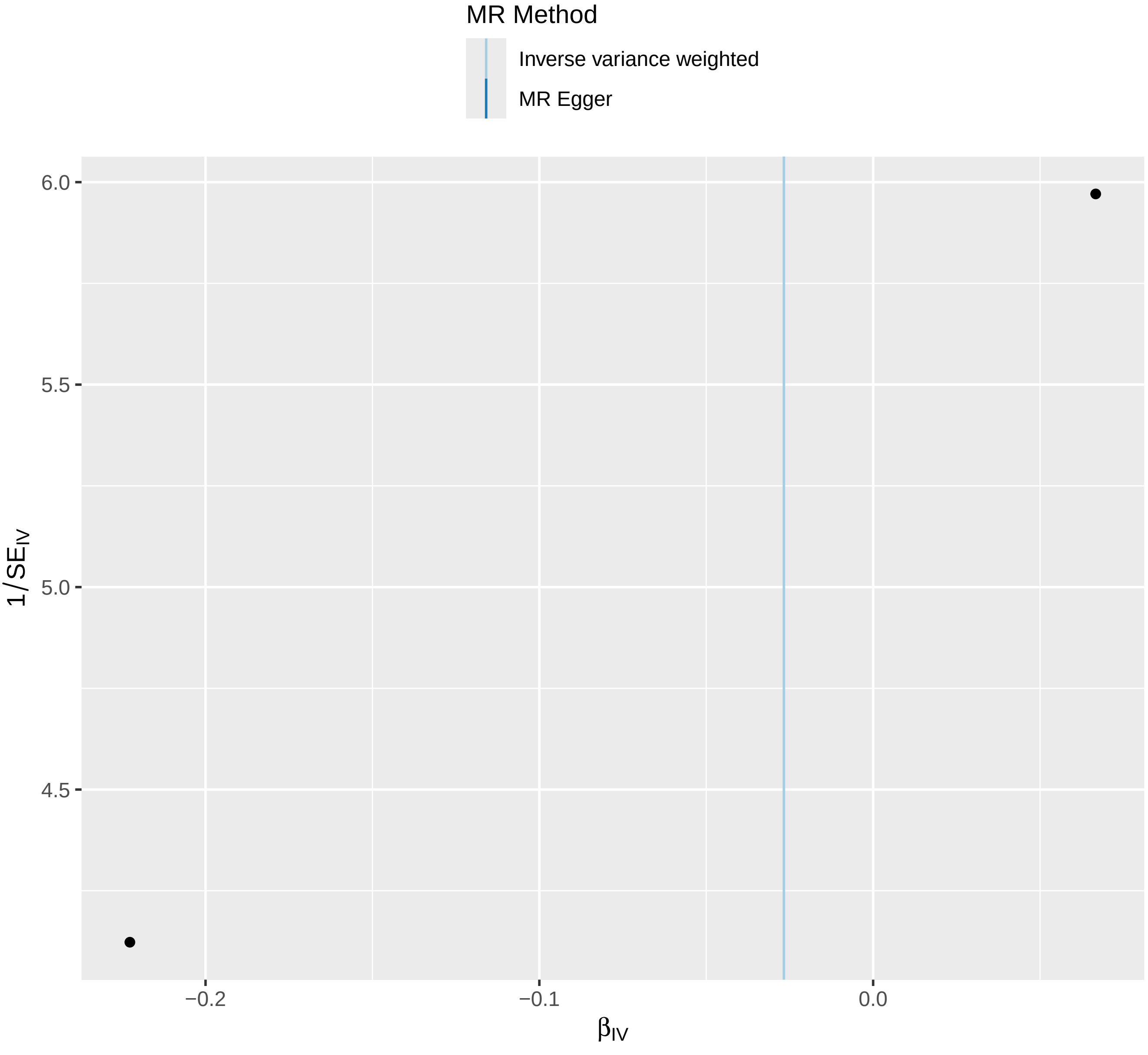
MR Method
Inverse variance weighted
MR Egger
6.0
5.5
5.0
4.5
1/SE$_{IV}$
−0.2
−0.1
0.0
β$_{IV}$

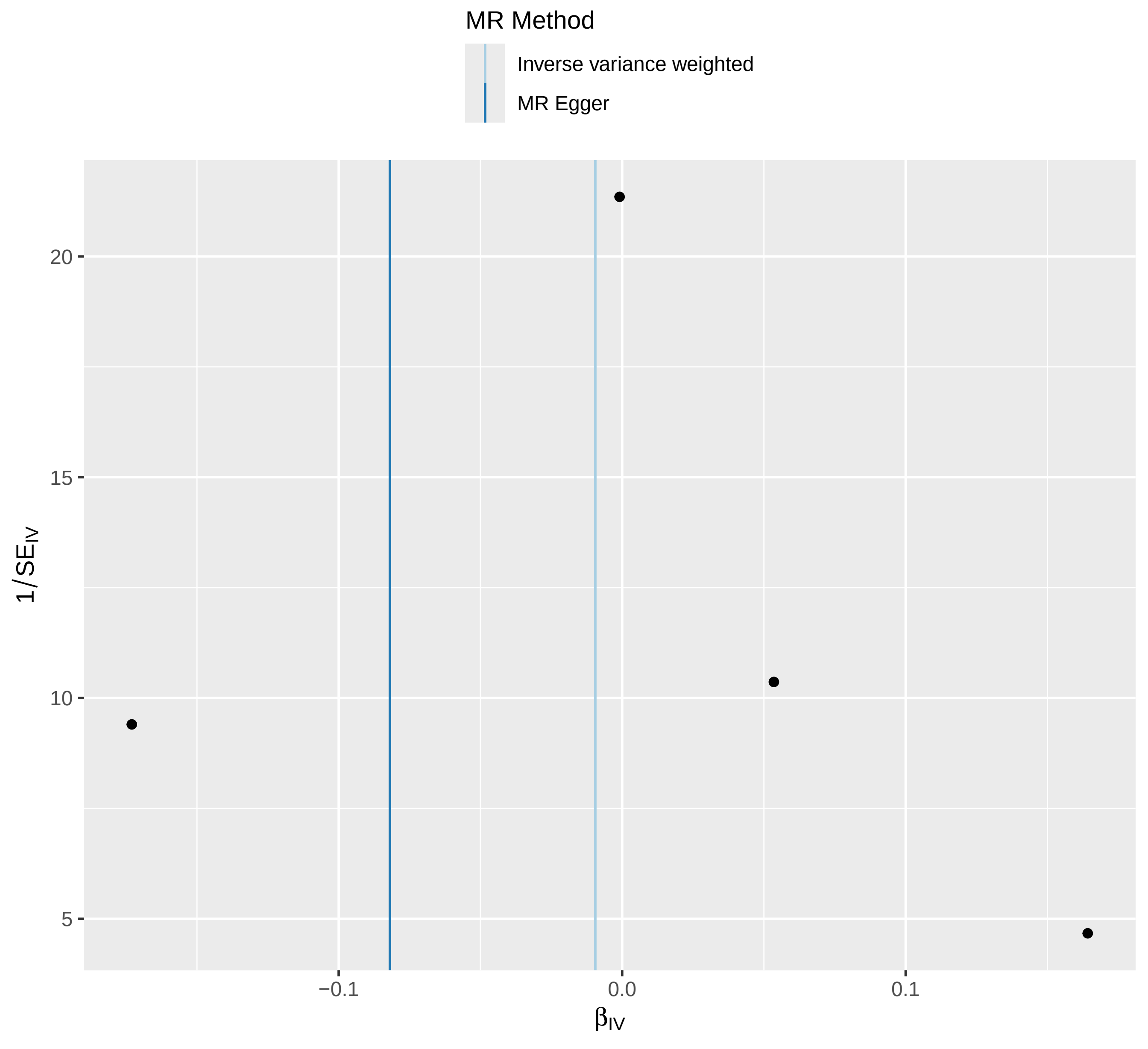

MR Method
Inverse variance weighted
MR Egger
20
15
10
5
1/SE$_{IV}$
−0.1
0.0
0.1
β$_{IV}$

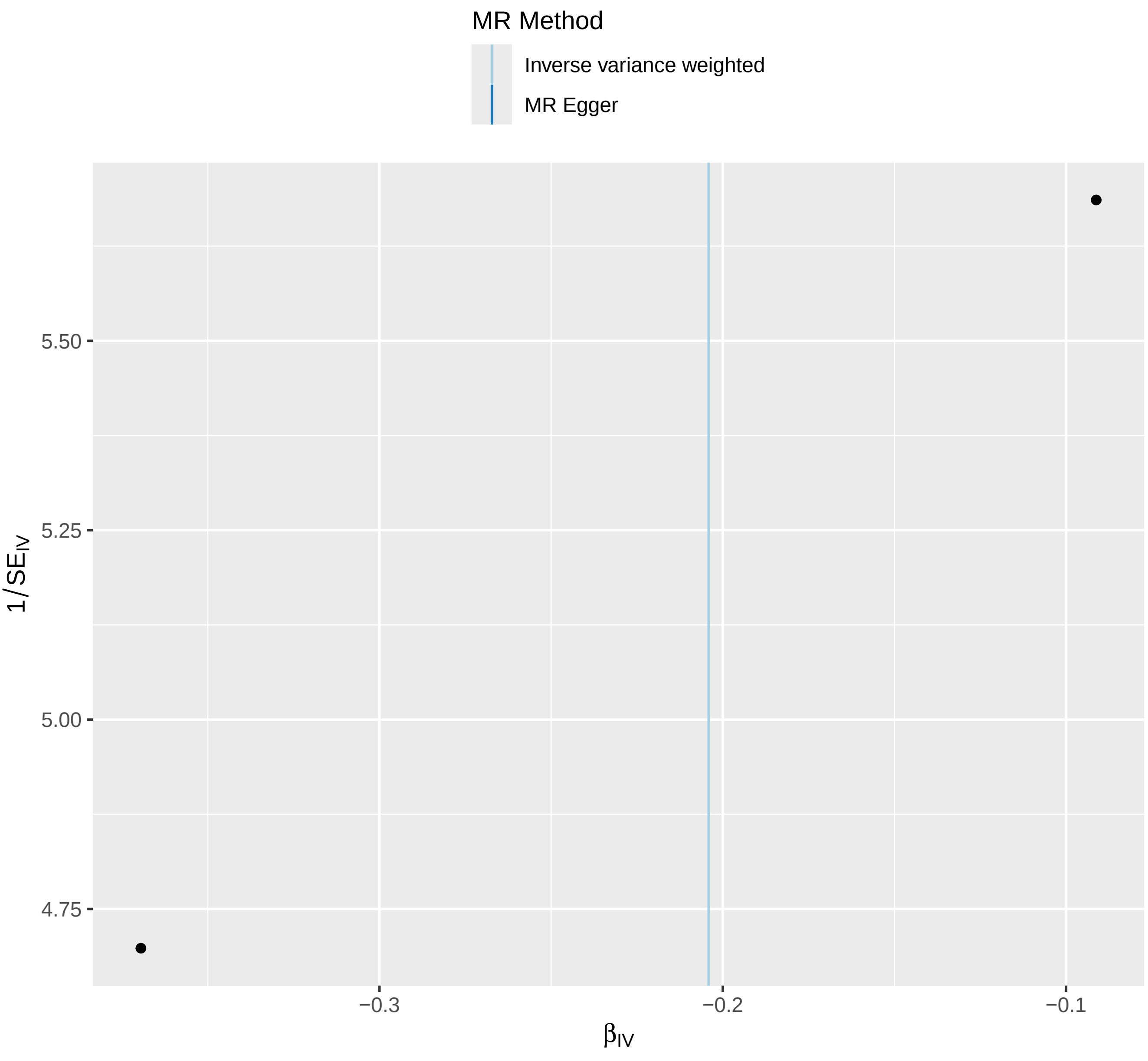

MR Method
Inverse variance weighted
MR Egger
5.50
5.25
5.00
4.75
1/SE$_{IV}$
−0.3
−0.2
−0.1
β$_{IV}$

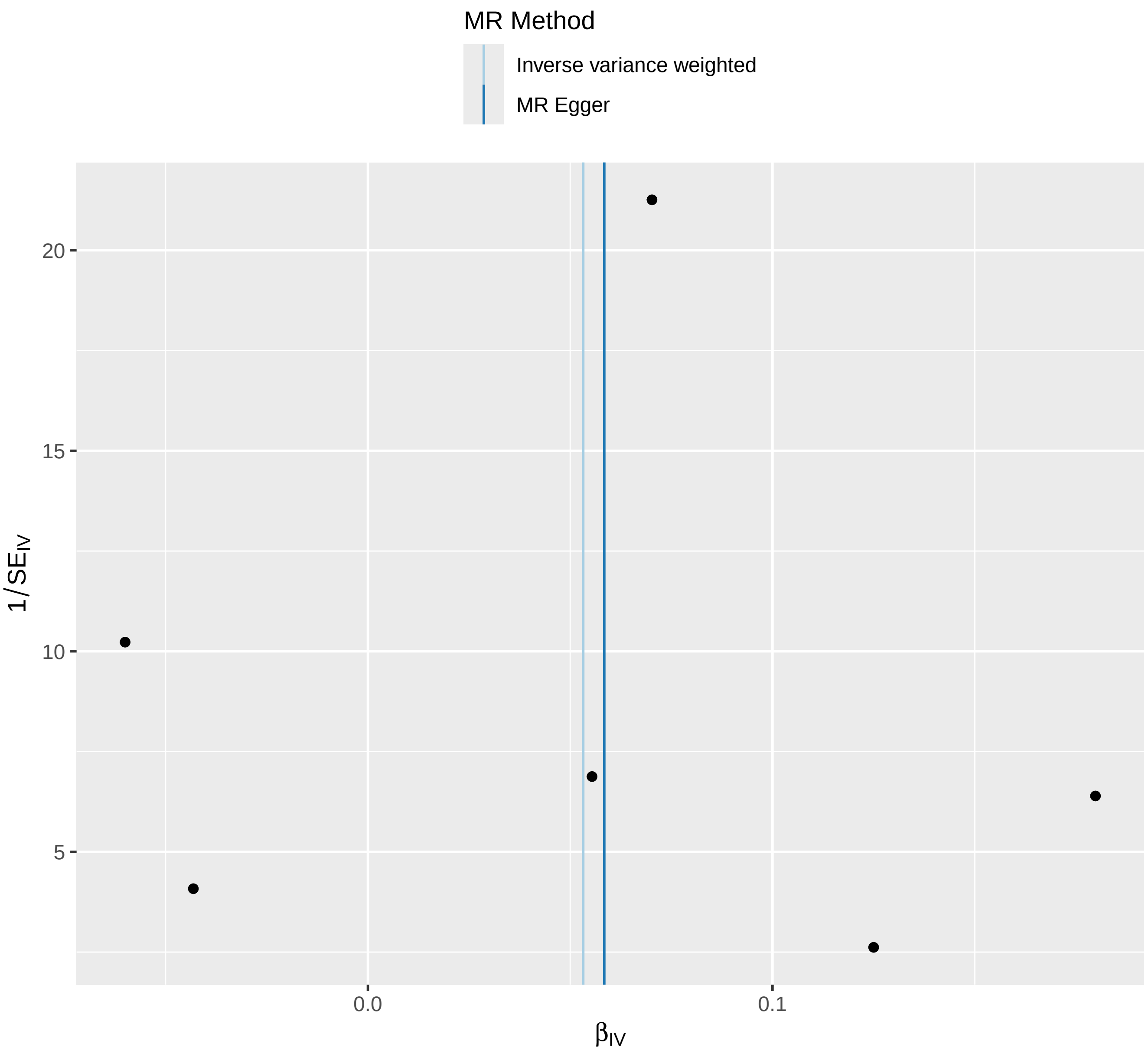

MR Method
Inverse variance weighted
MR Egger
20
15
10
5
1/SE$_{IV}$
0.0
0.1
$\beta_{IV}$

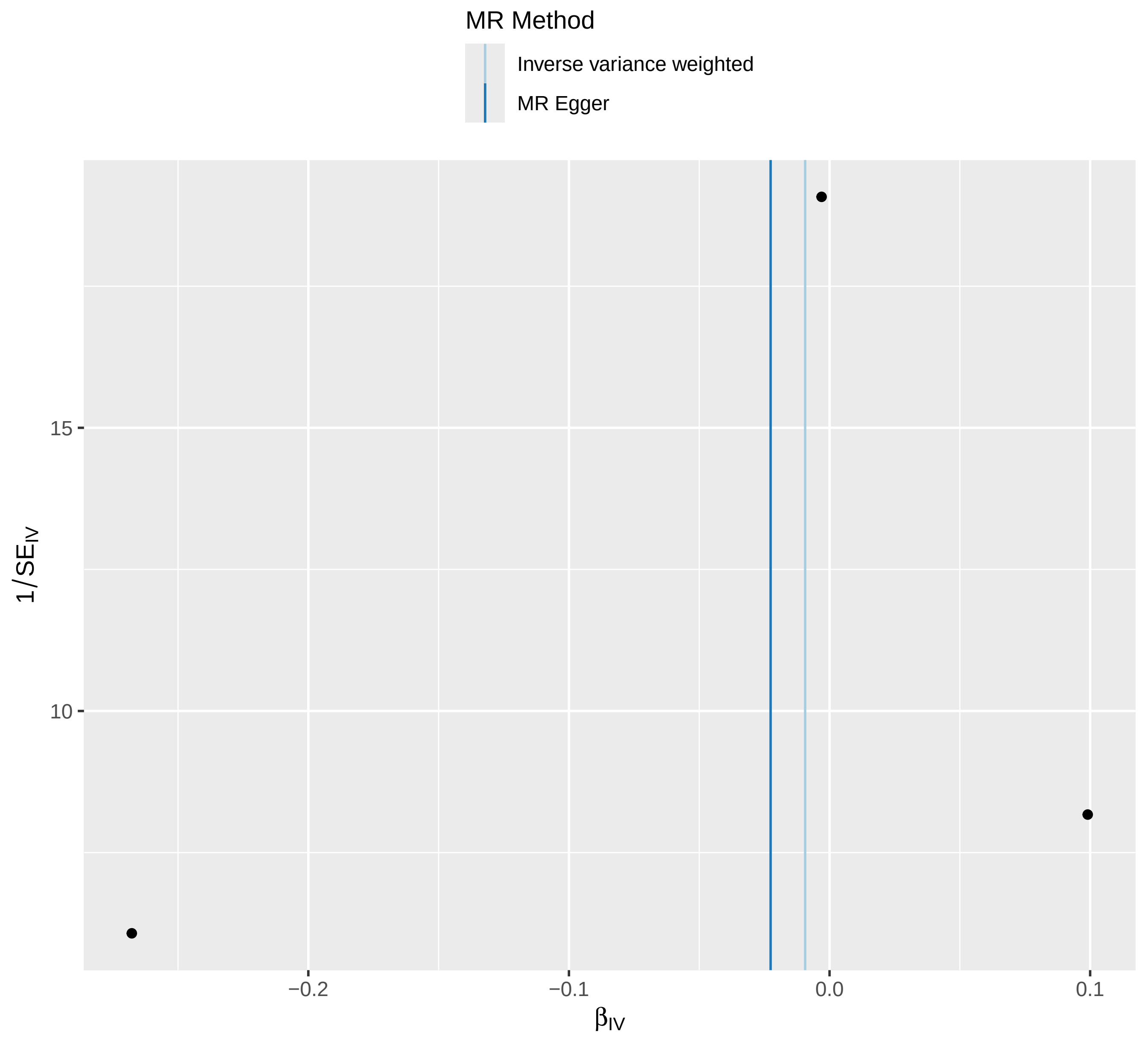
MR Method
Inverse variance weighted
MR Egger
15
10
1/SE_IV
−0.2
−0.1
0.0
0.1
β_IV

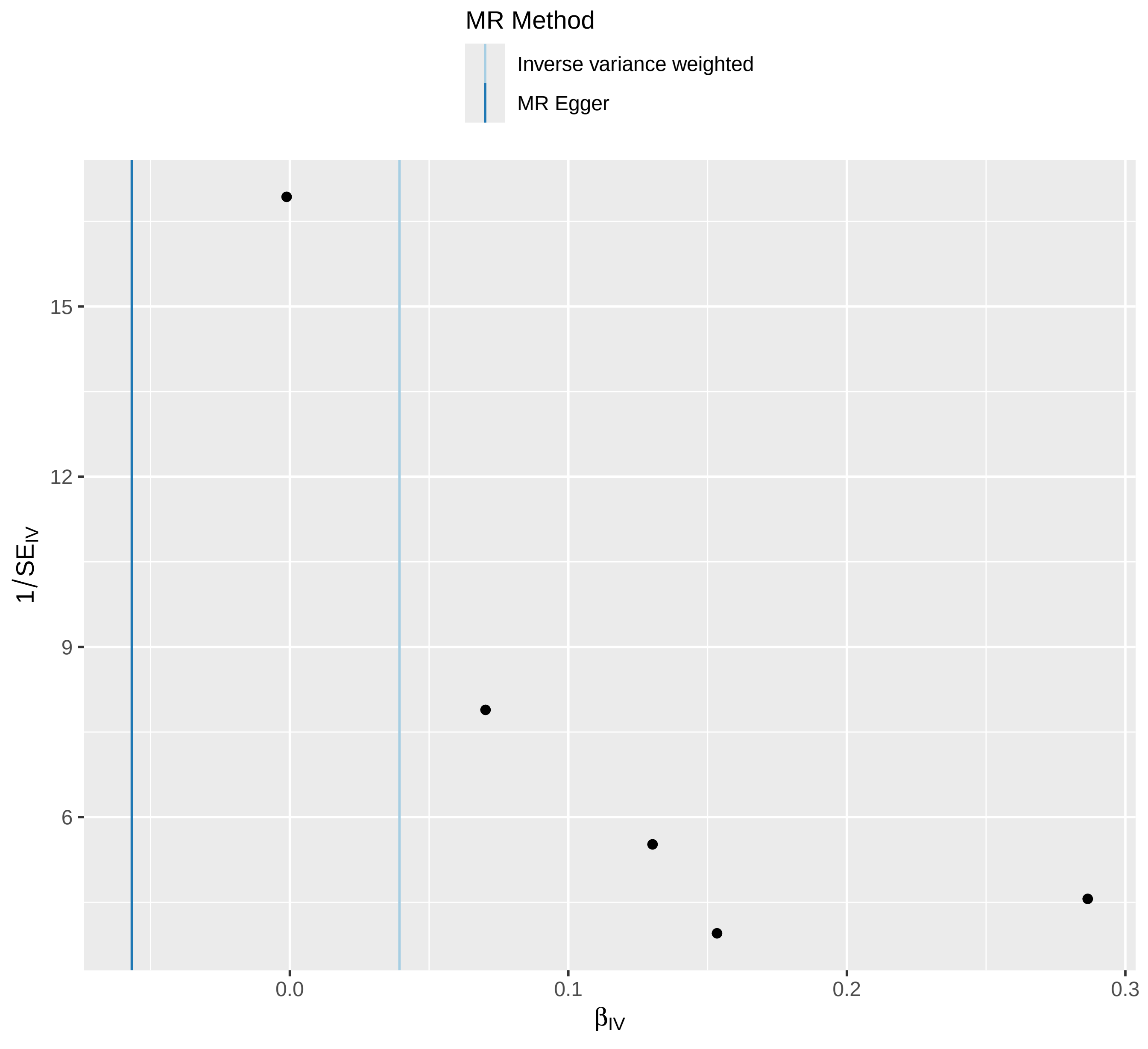

MR Method
Inverse variance weighted
MR Egger
15
12
9
6
0.0
0.1
0.2
0.3
$1/SE_{IV}$
$\beta_{IV}$

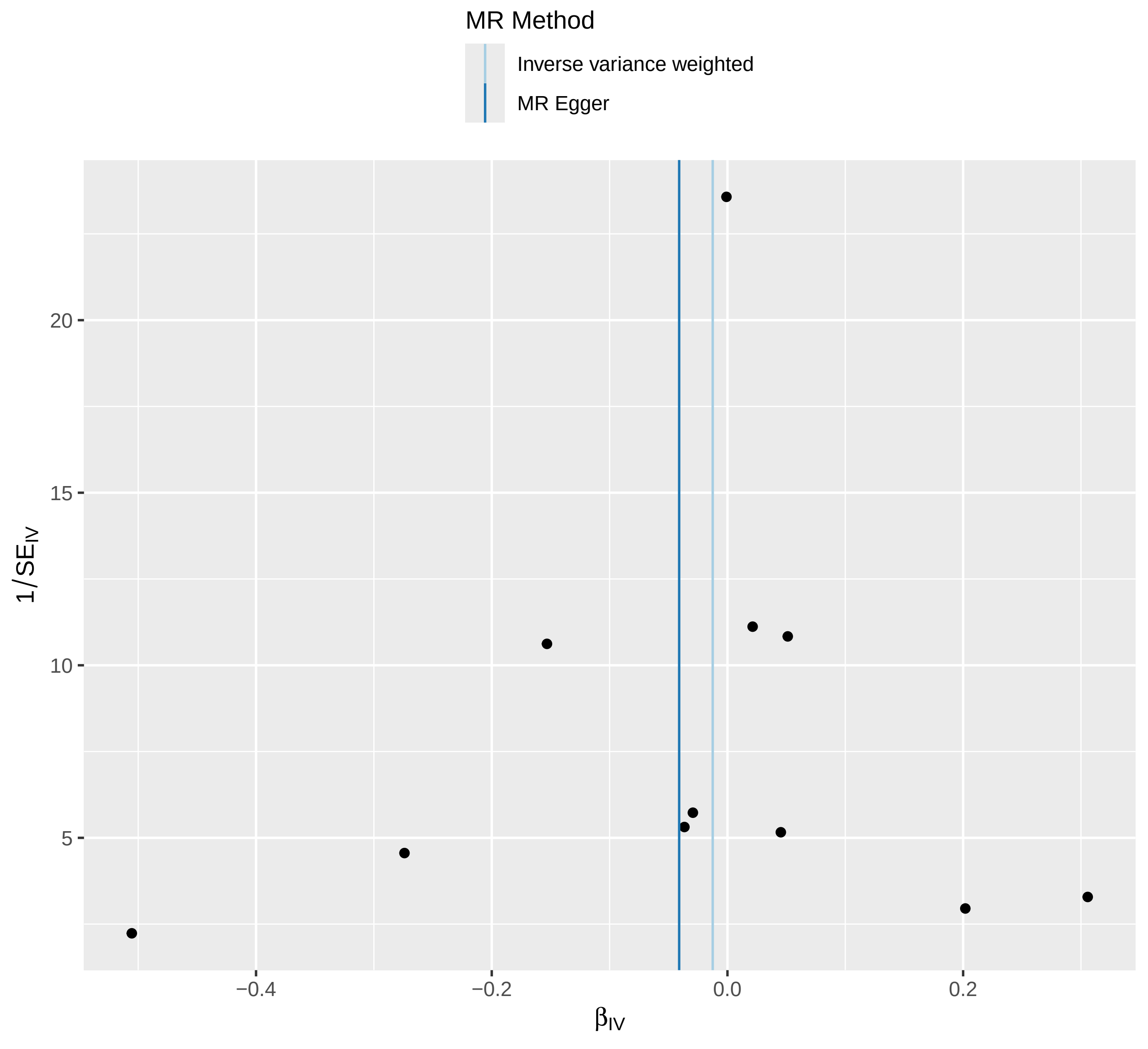
MR Method
Inverse variance weighted
MR Egger
20
15
10
5
−0.4
−0.2
0.0
0.2
$1/SE_{IV}$
$\beta_{IV}$

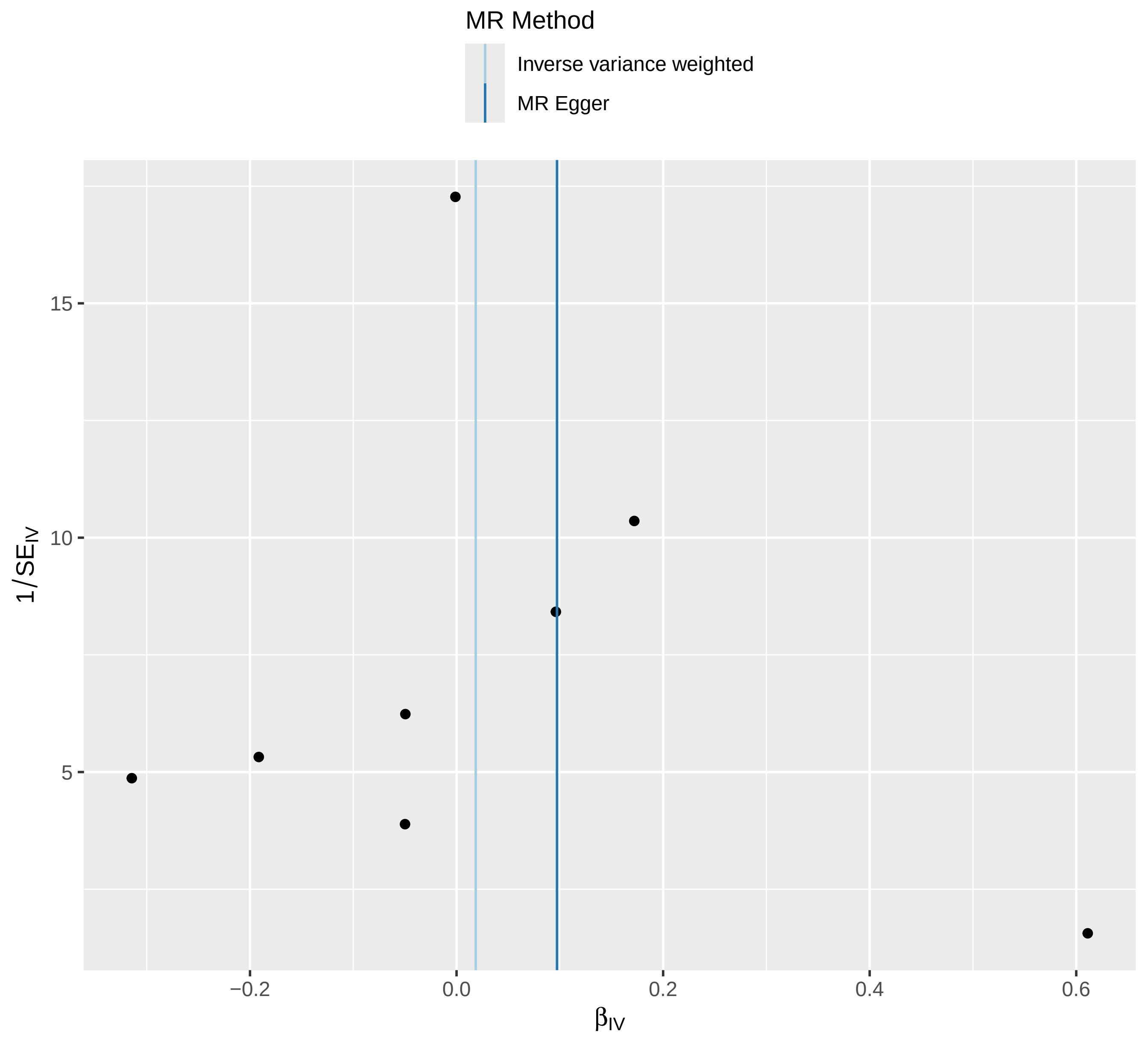

MR Method
Inverse variance weighted
MR Egger
15
10
5
−0.2
0.0
0.2
0.4
0.6
$1/SE_{IV}$
$\beta_{IV}$

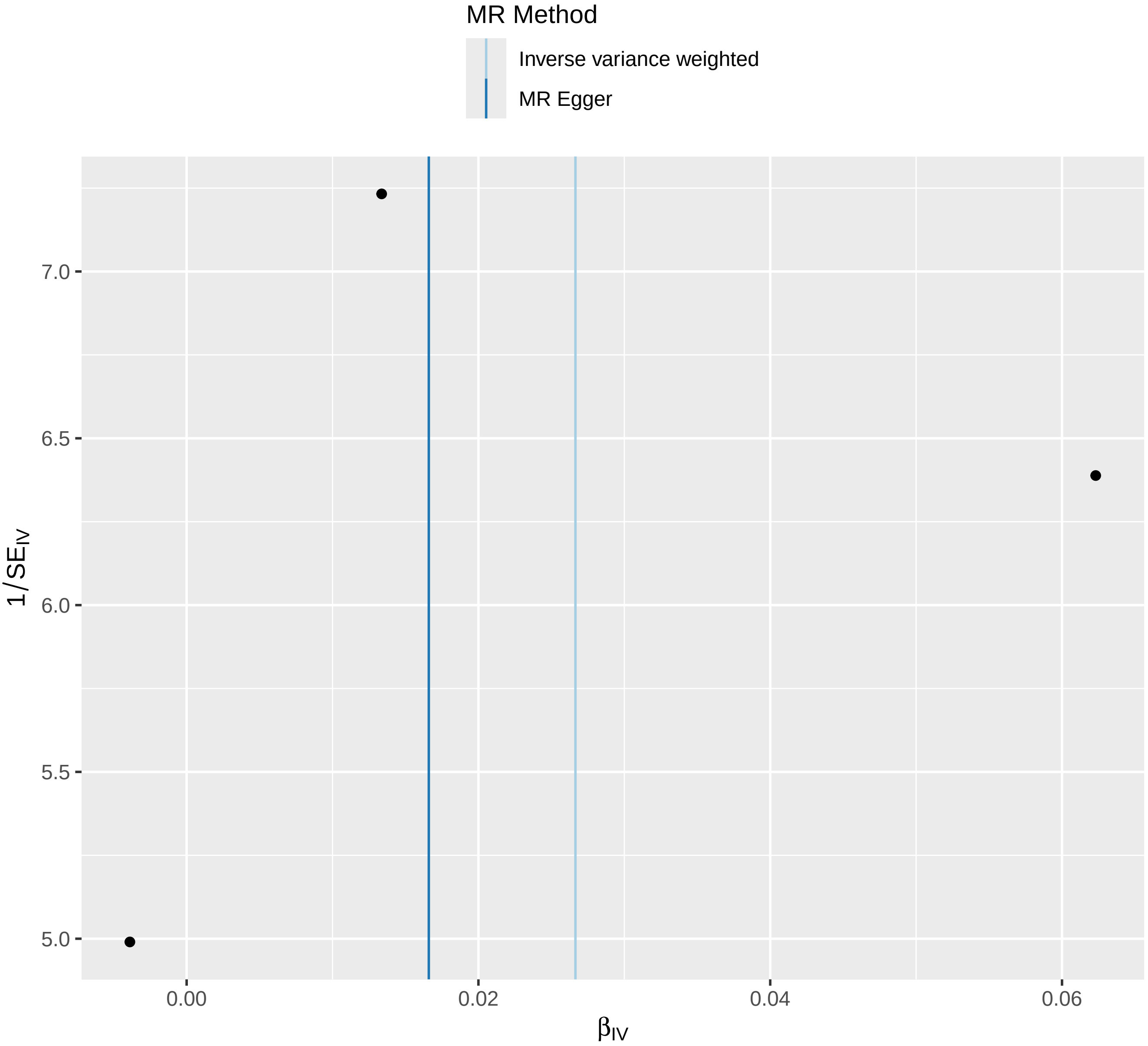
MR Method
Inverse variance weighted
MR Egger
7.0
6.5
6.0
5.5
5.0
1/SE$_{IV}$
0.00
0.02
0.04
0.06
β$_{IV}$

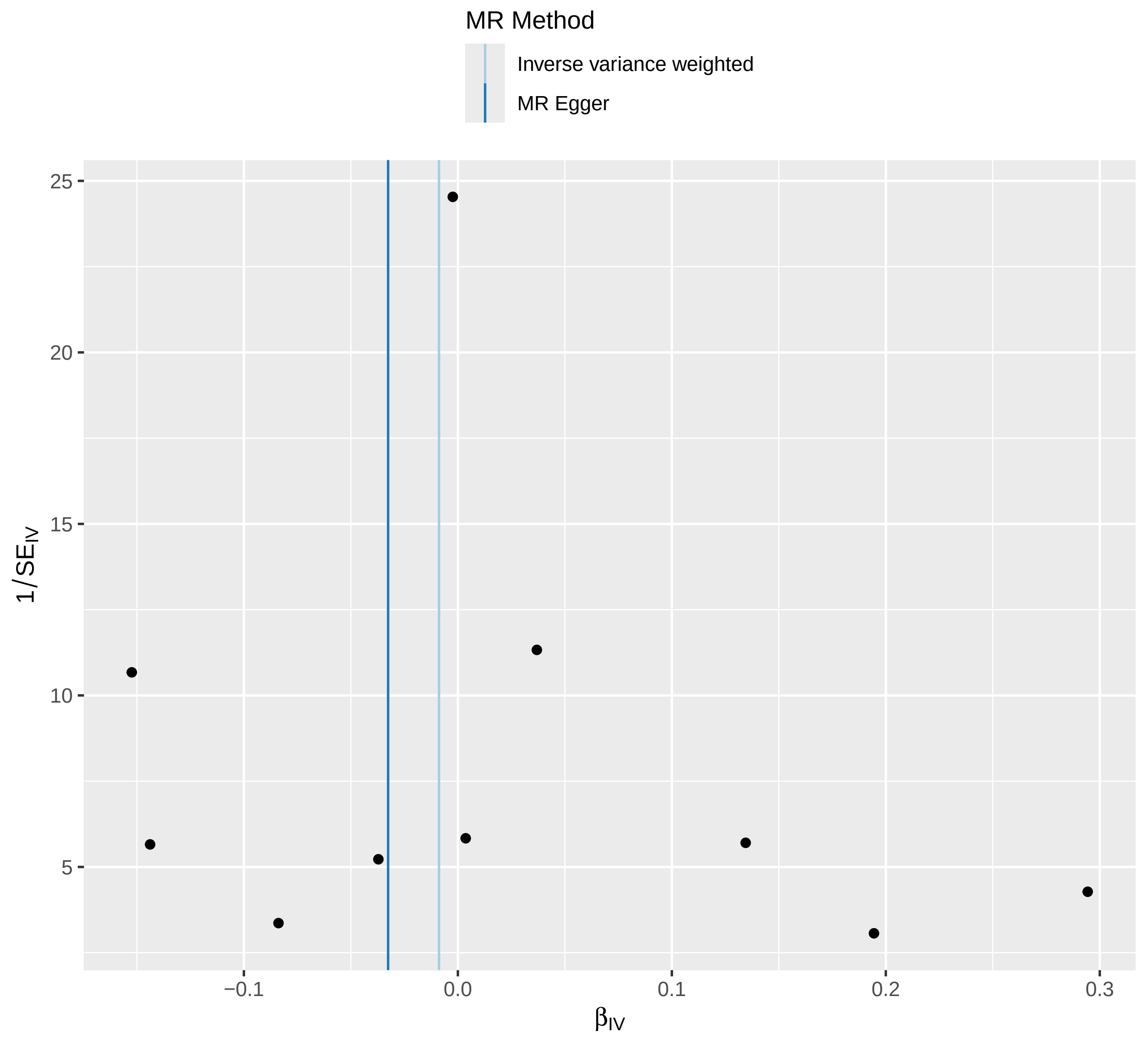

MR Method
Inverse variance weighted
MR Egger
25
20
15
10
5
1/SE$_{IV}$
−0.1
0.0
0.1
0.2
0.3
β$_{IV}$

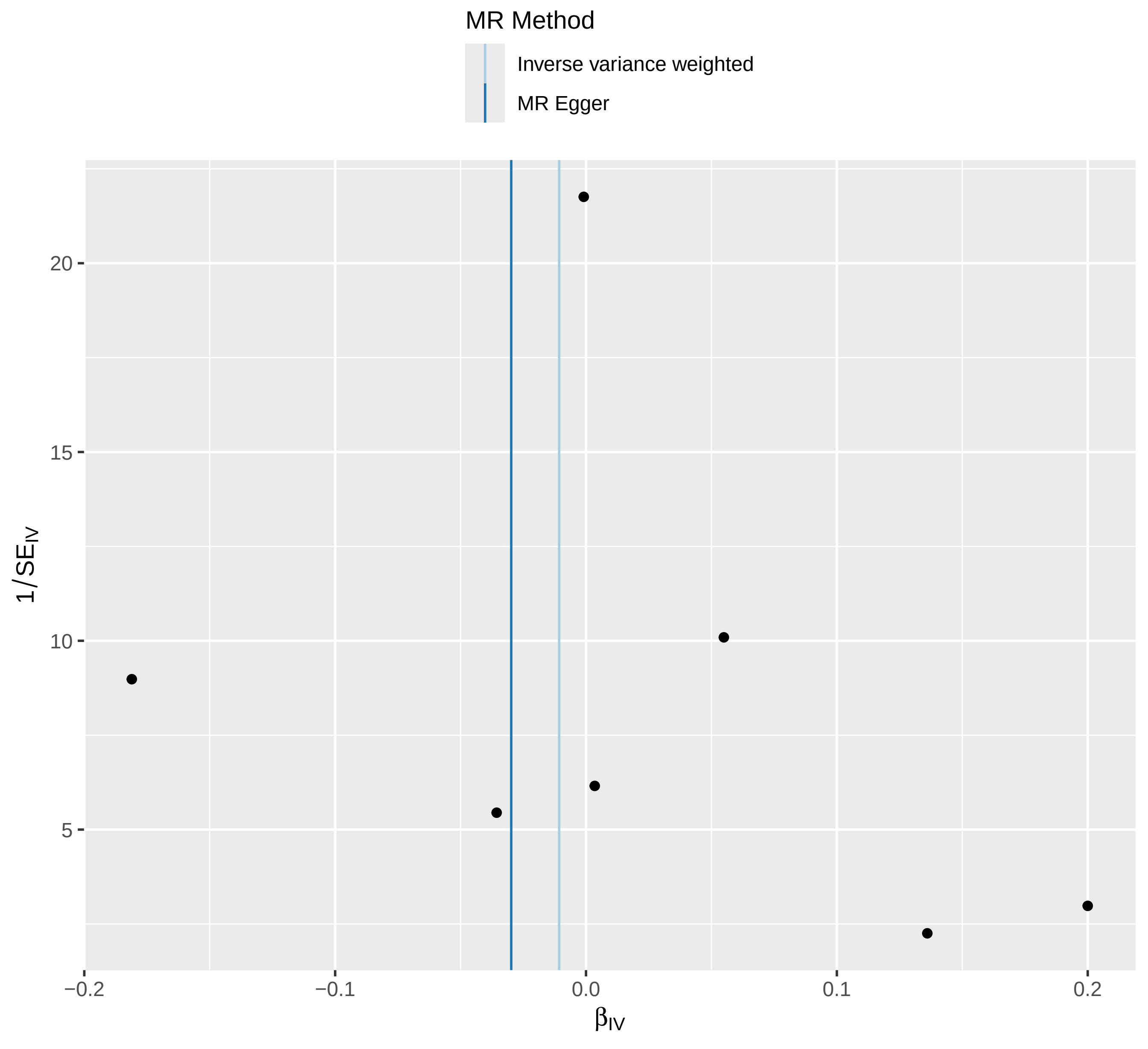

MR Method
Inverse variance weighted
MR Egger
20
15
10
5
$1/SE_{IV}$
−0.2
−0.1
0.0
0.1
0.2
$\beta_{IV}$

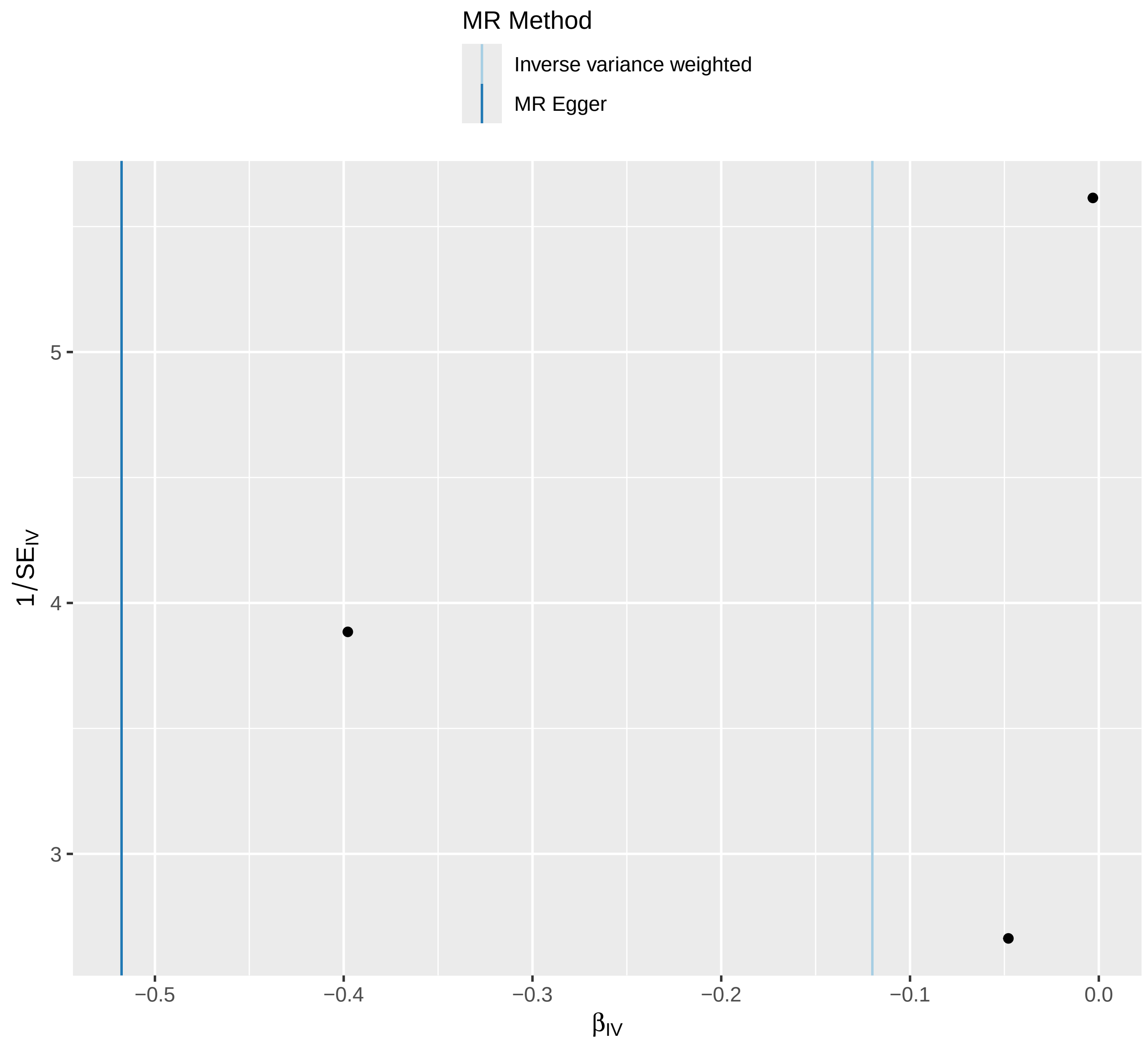

MR Method
Inverse variance weighted
MR Egger
5
4
3
1/SE$_{IV}$
−0.5
−0.4
−0.3
−0.2
−0.1
0.0
β$_{IV}$

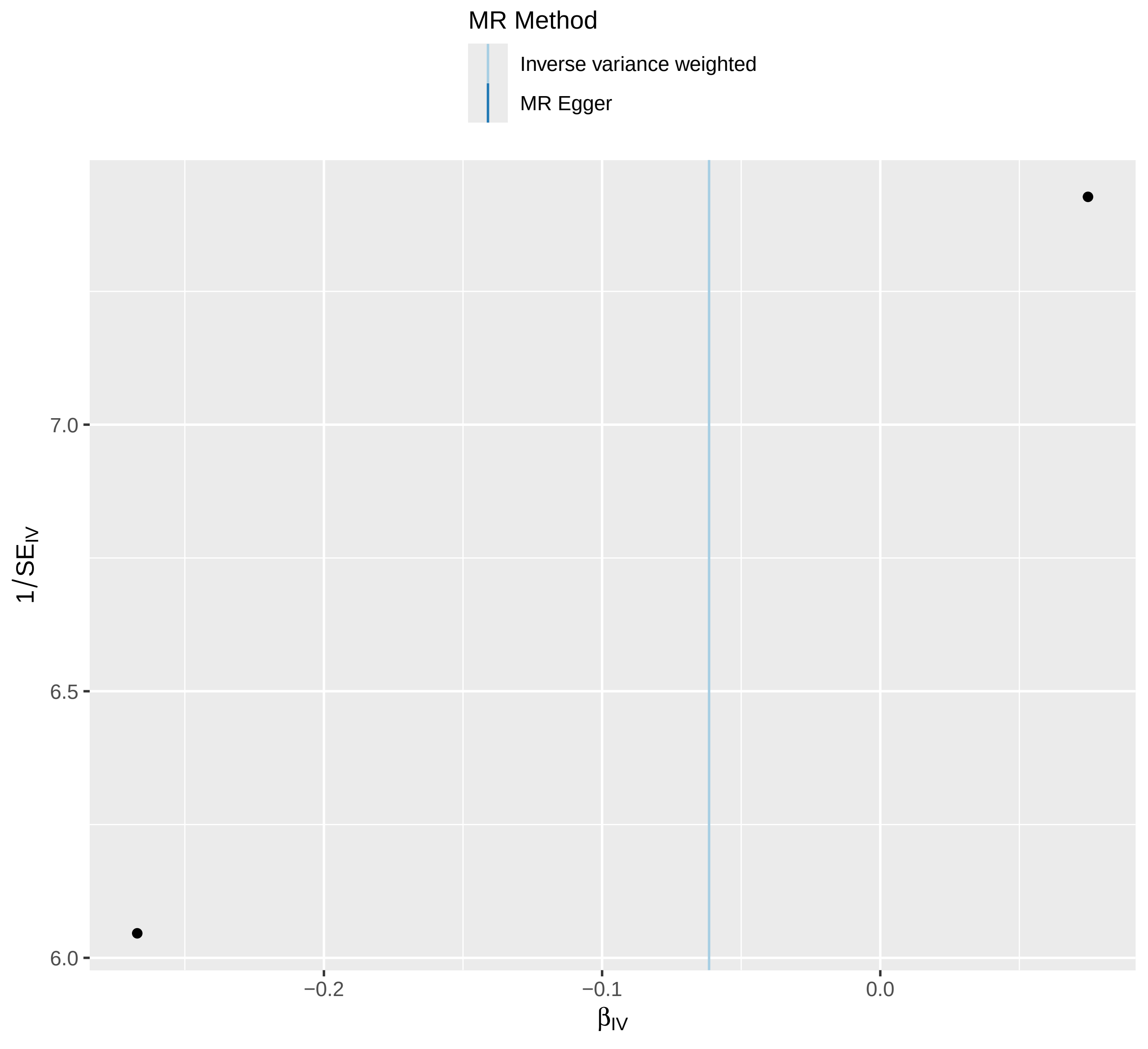
MR Method
Inverse variance weighted
MR Egger
7.0
6.5
6.0
1/SE$_{IV}$
−0.2
−0.1
0.0
β$_{IV}$

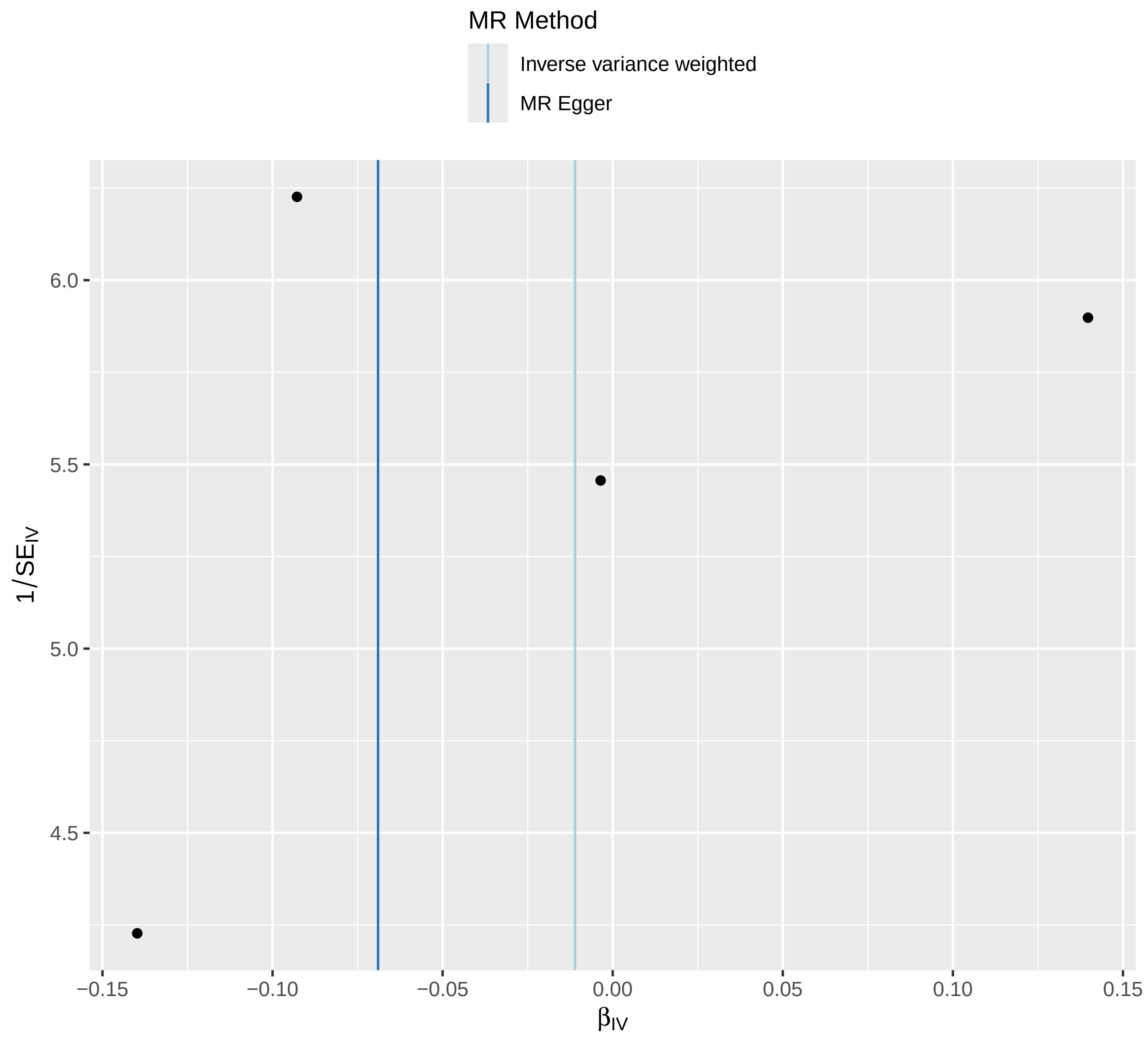

MR Method
Inverse variance weighted
MR Egger
6.0
5.5
5.0
4.5
1/SE$_{IV}$
−0.15
−0.10
−0.05
0.00
0.05
0.10
0.15
β$_{IV}$

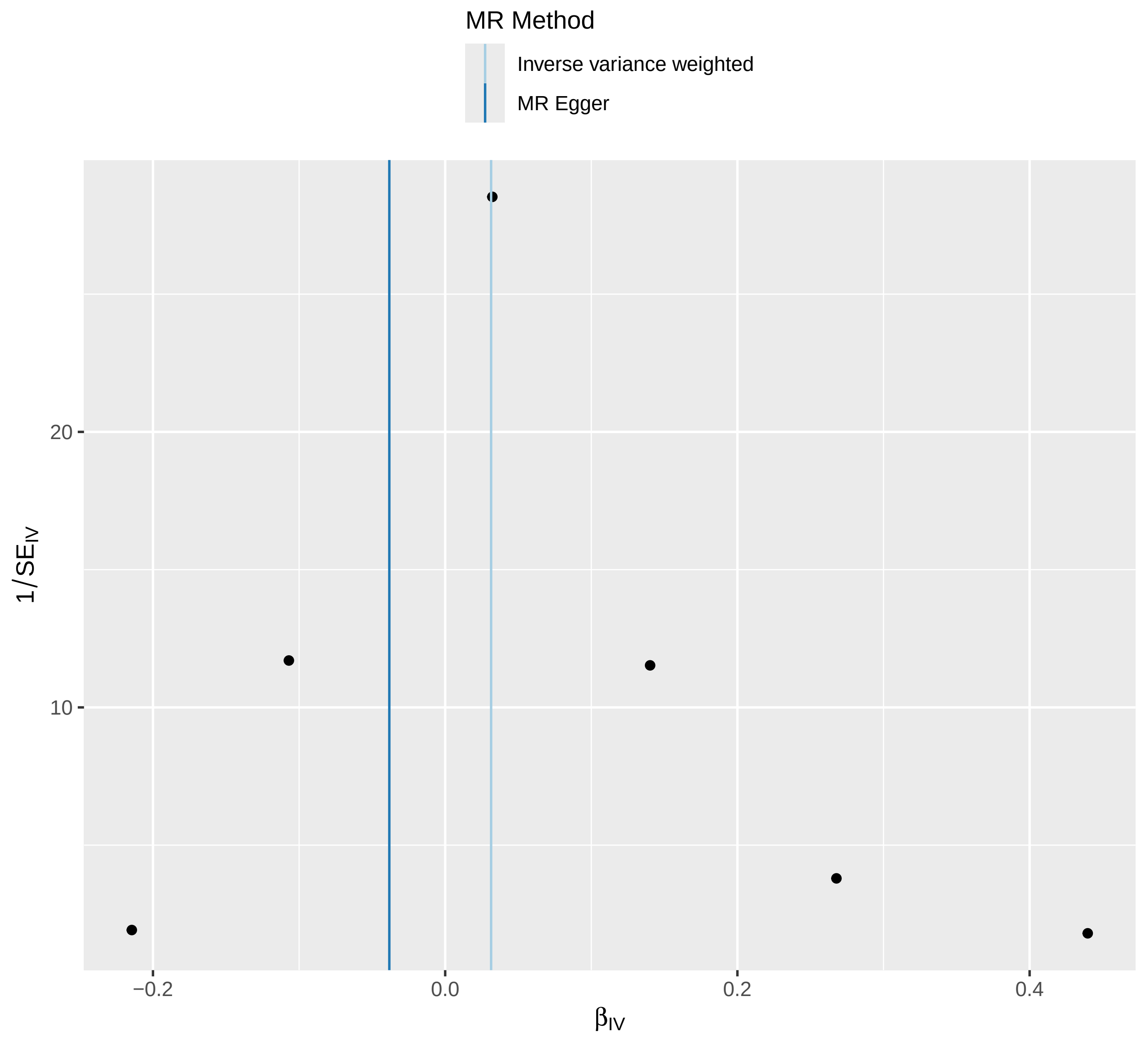
MR Method
Inverse variance weighted
MR Egger
20
10
1/SE_IV
−0.2
0.0
0.2
0.4
β_IV

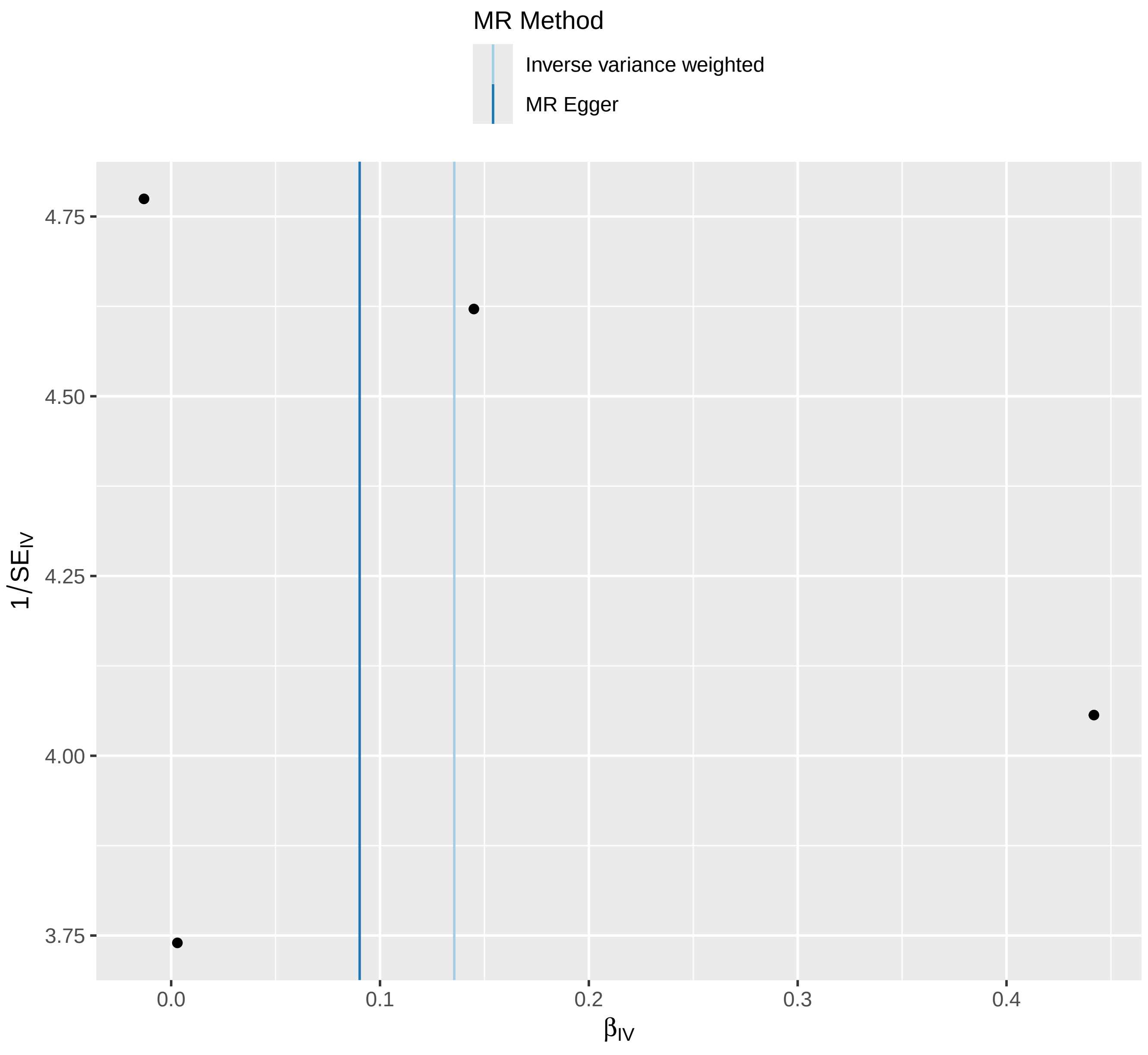
MR Method
Inverse variance weighted
MR Egger
4.75
4.50
4.25
4.00
3.75
1/SE_IV
0.0
0.1
0.2
0.3
0.4
β_IV

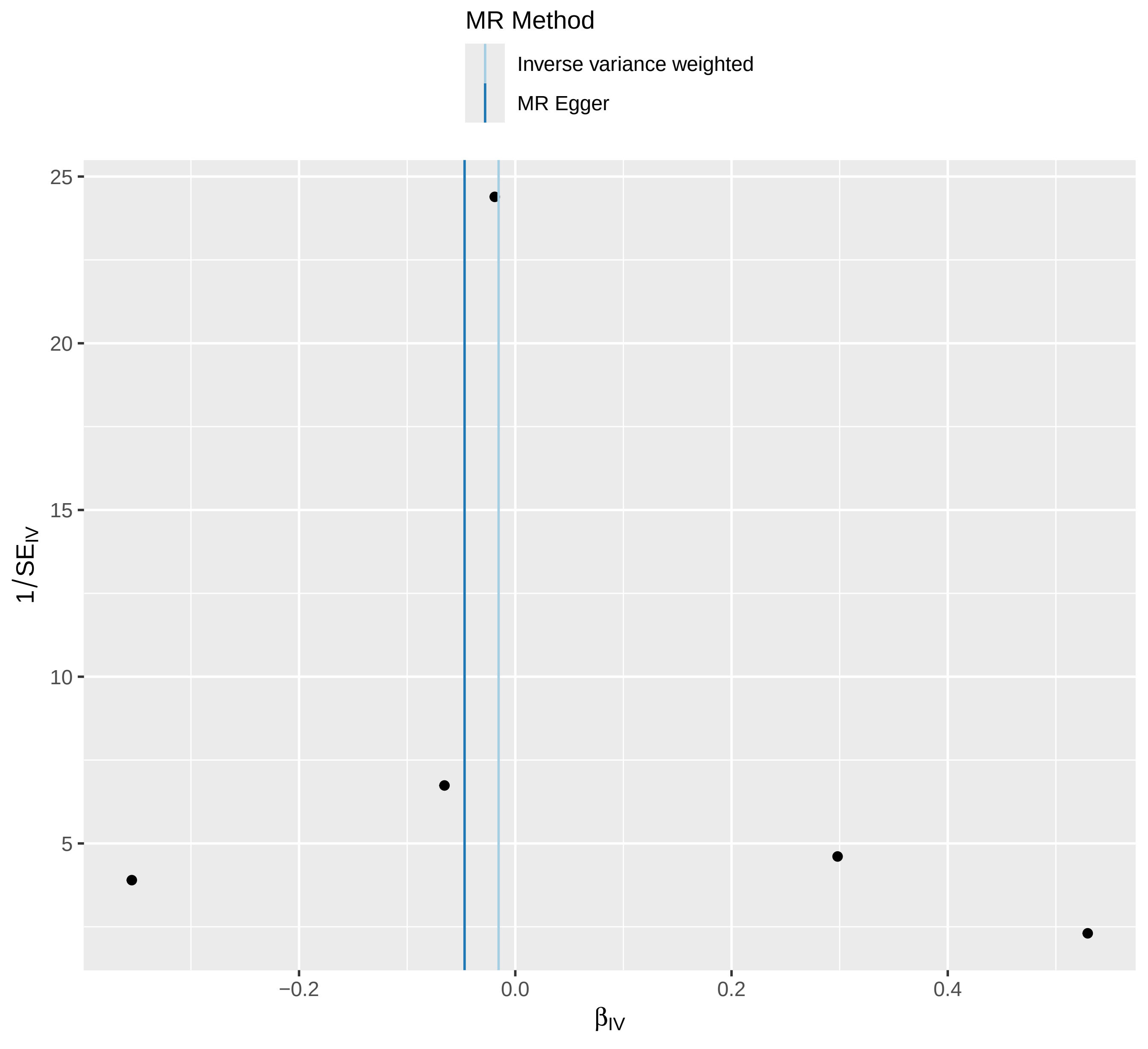

MR Method
Inverse variance weighted
MR Egger
25
20
15
10
5
1/SE$_{IV}$
−0.2
0.0
0.2
0.4
β$_{IV}$

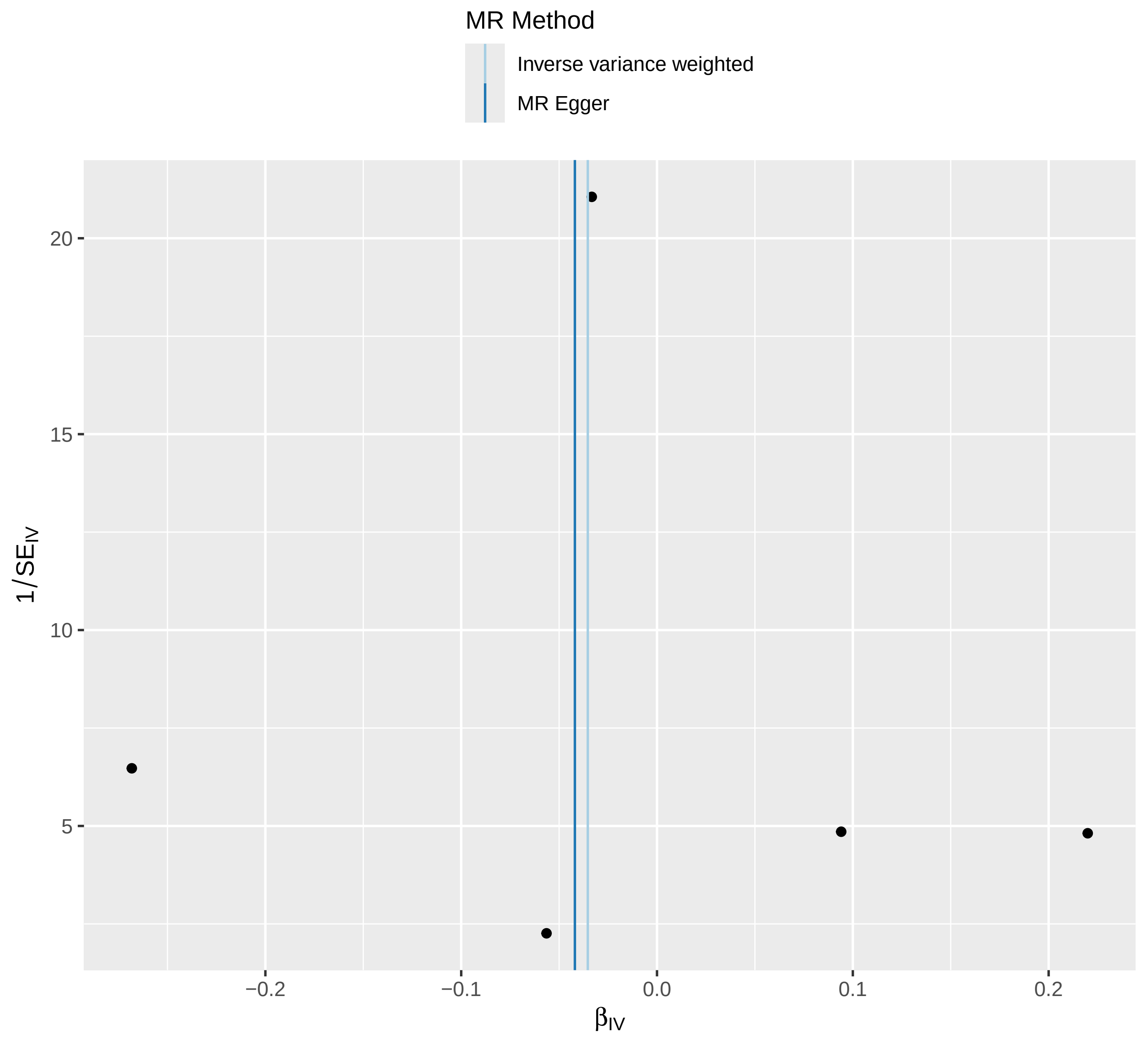
MR Method
Inverse variance weighted
MR Egger
20
15
10
5
1/SE_IV
−0.2
−0.1
0.0
0.1
0.2
β_IV

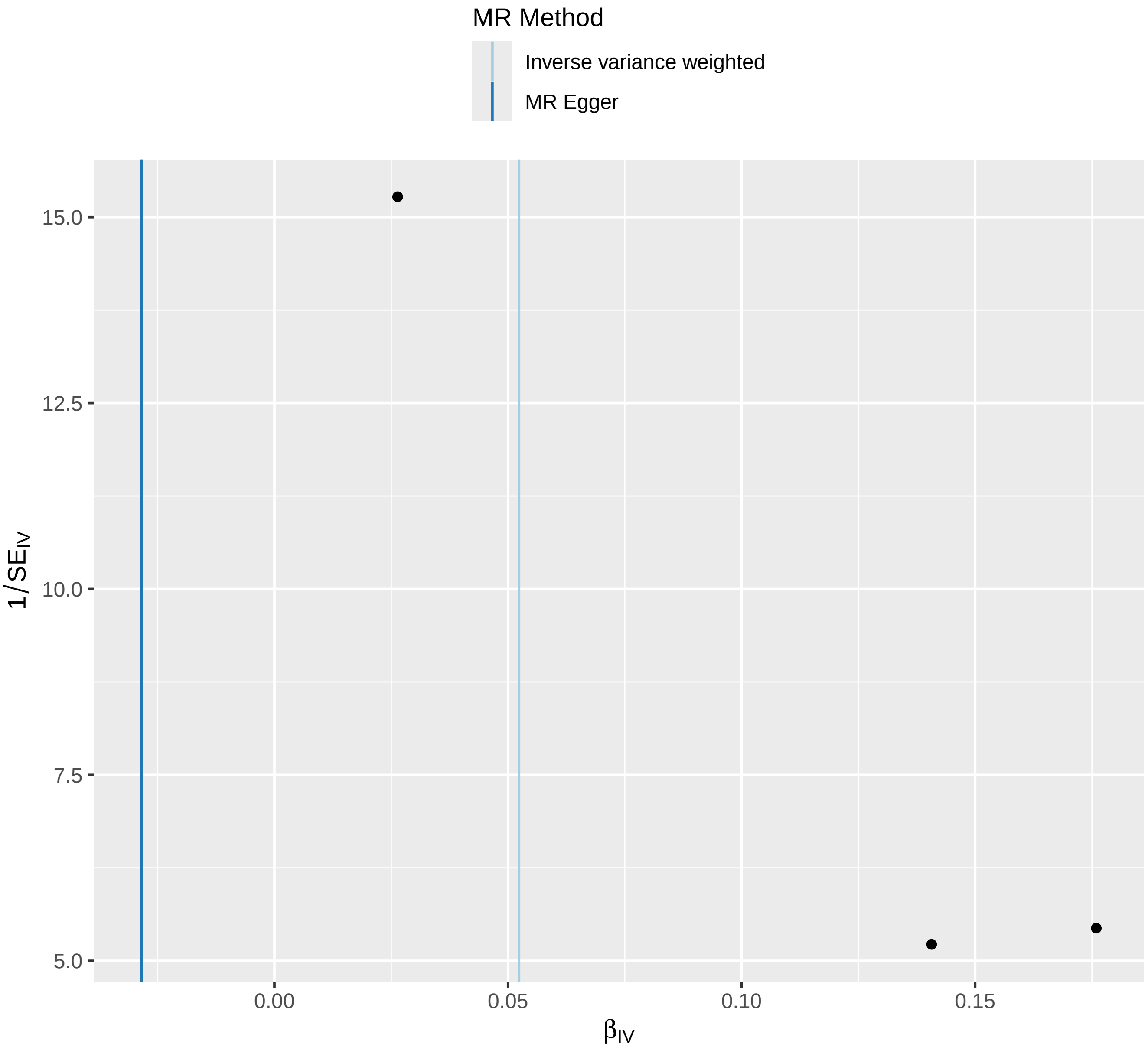
MR Method
Inverse variance weighted
MR Egger
15.0
12.5
10.0
7.5
5.0
$1/SE_{IV}$
0.00
0.05
0.10
0.15
$\beta_{IV}$

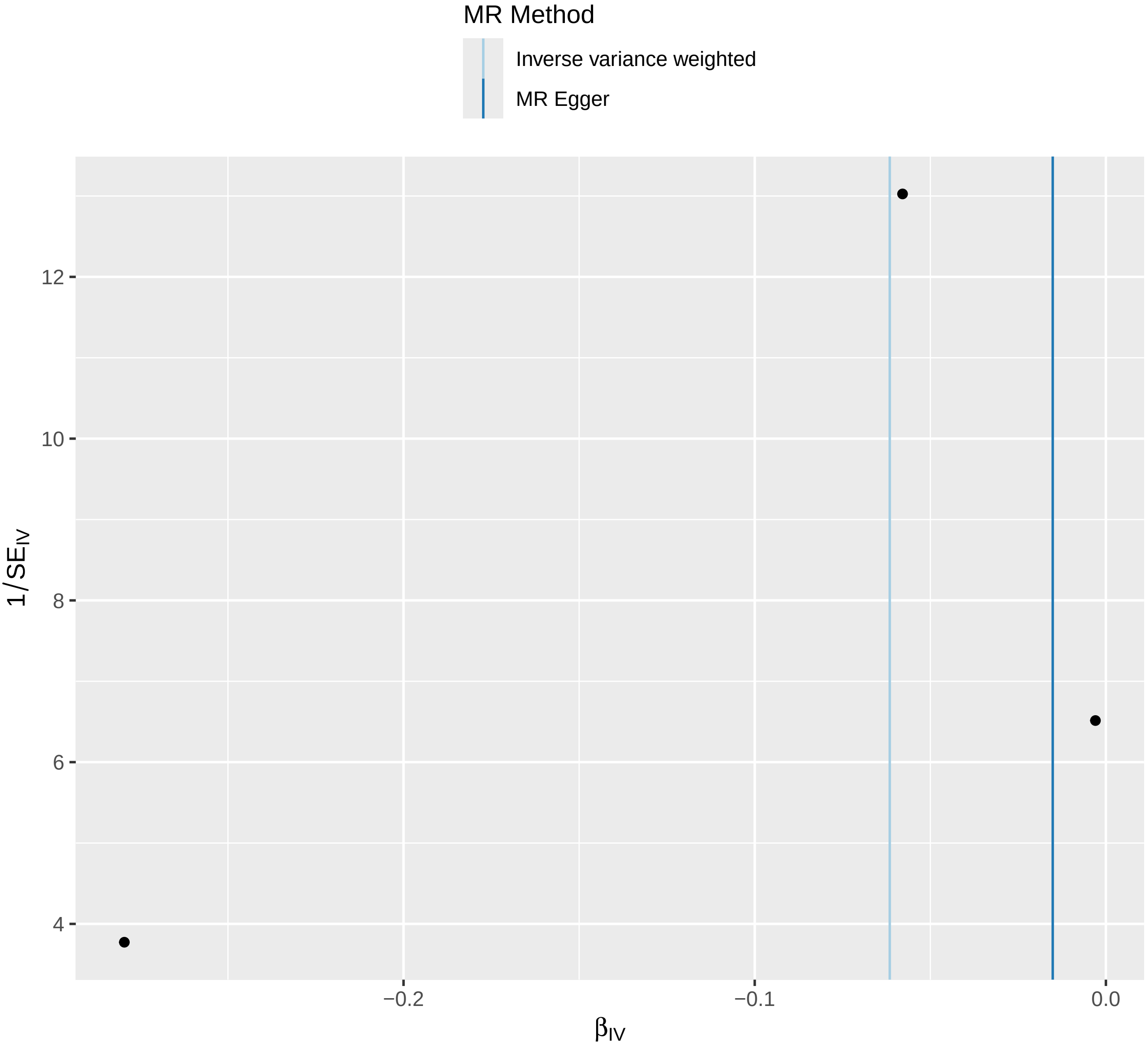

MR Method
Inverse variance weighted
MR Egger
12
10
8
6
4
1/SE_IV
−0.2
−0.1
0.0
β_IV

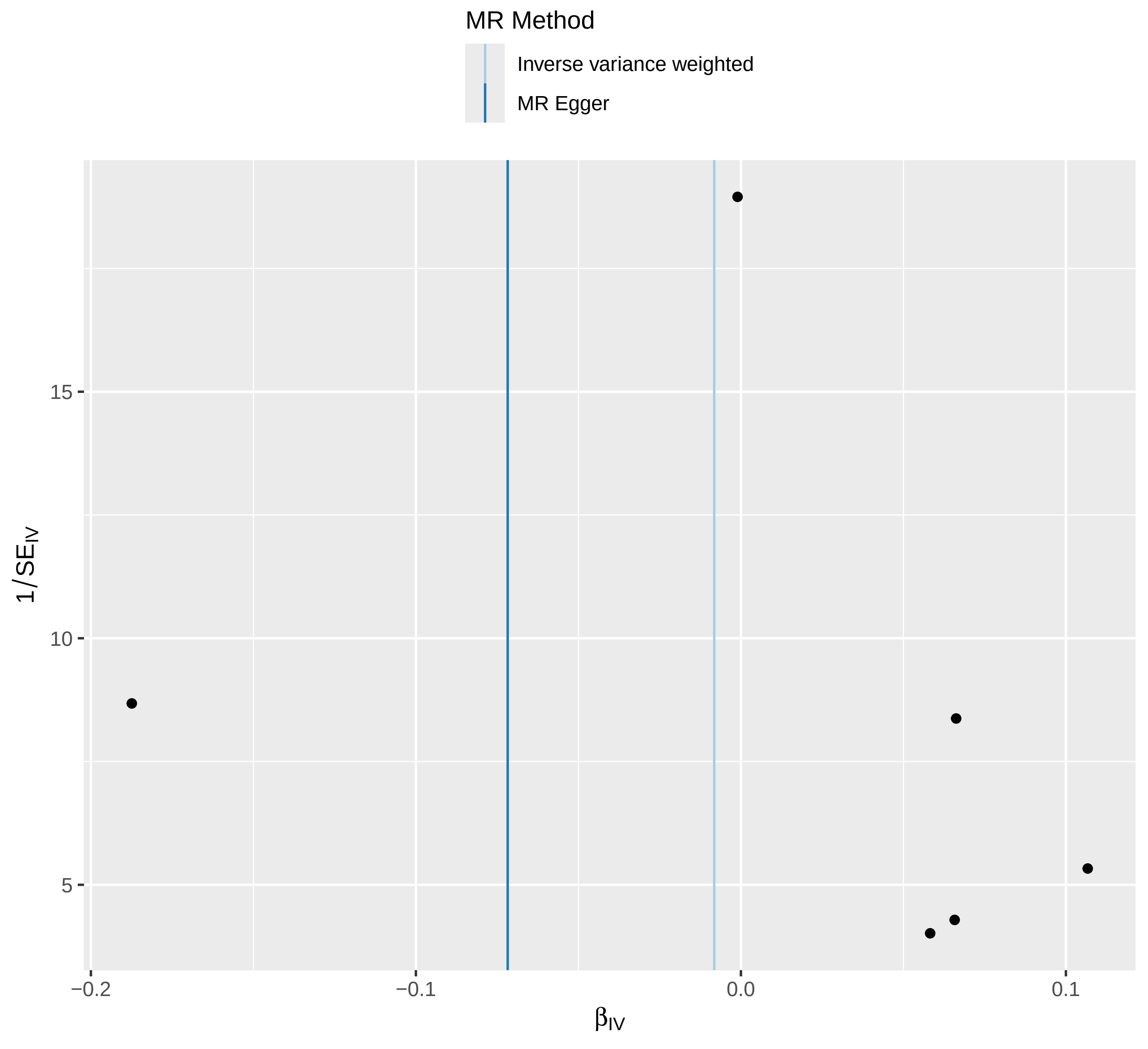
MR Method
Inverse variance weighted
MR Egger
15
10
5
1/SE_IV
−0.2
−0.1
0.0
0.1
β_IV

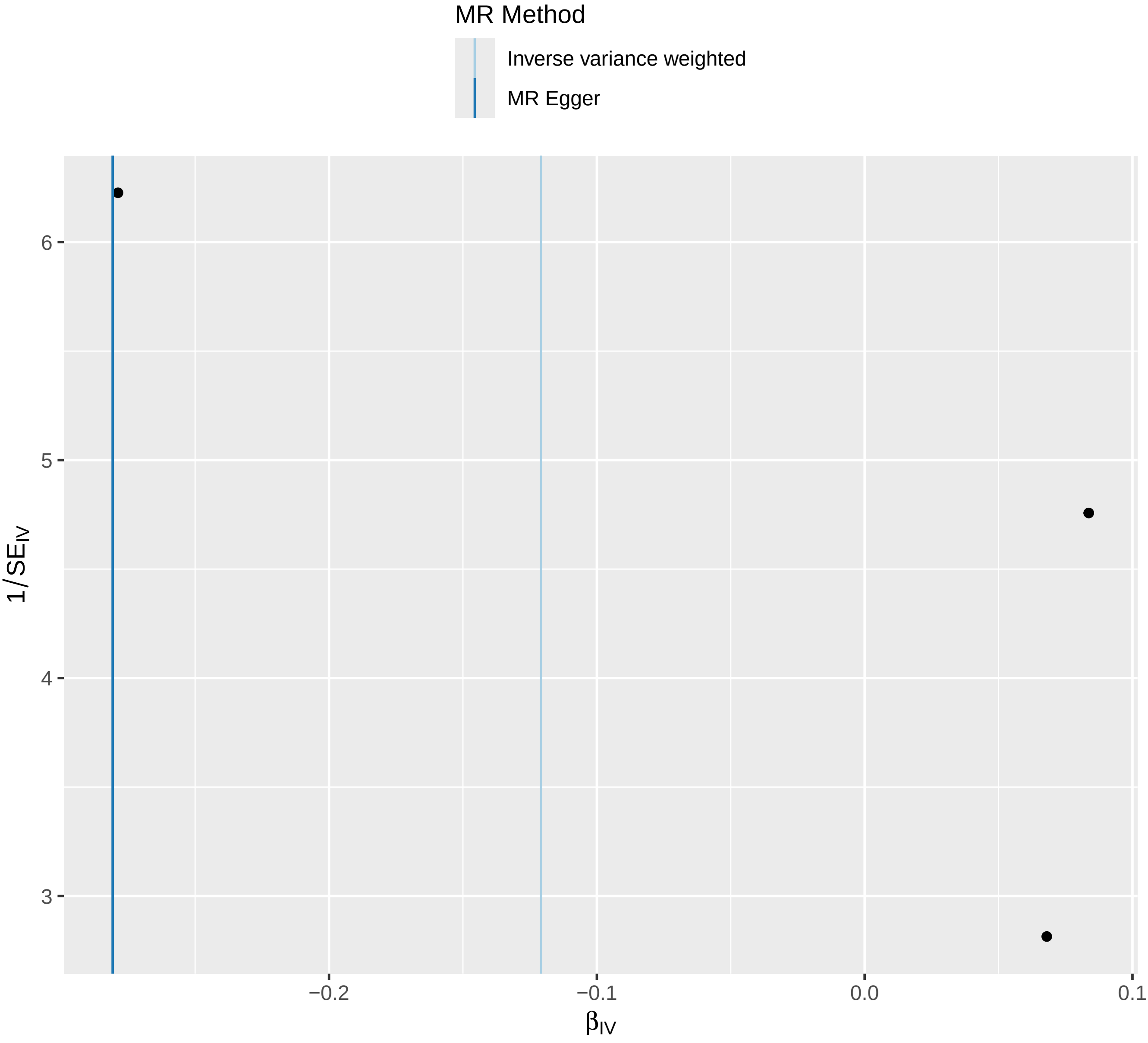

MR Method
Inverse variance weighted
MR Egger
6
5
4
3
−0.2
−0.1
0.0
0.1
$1/SE_{IV}$
$\beta_{IV}$

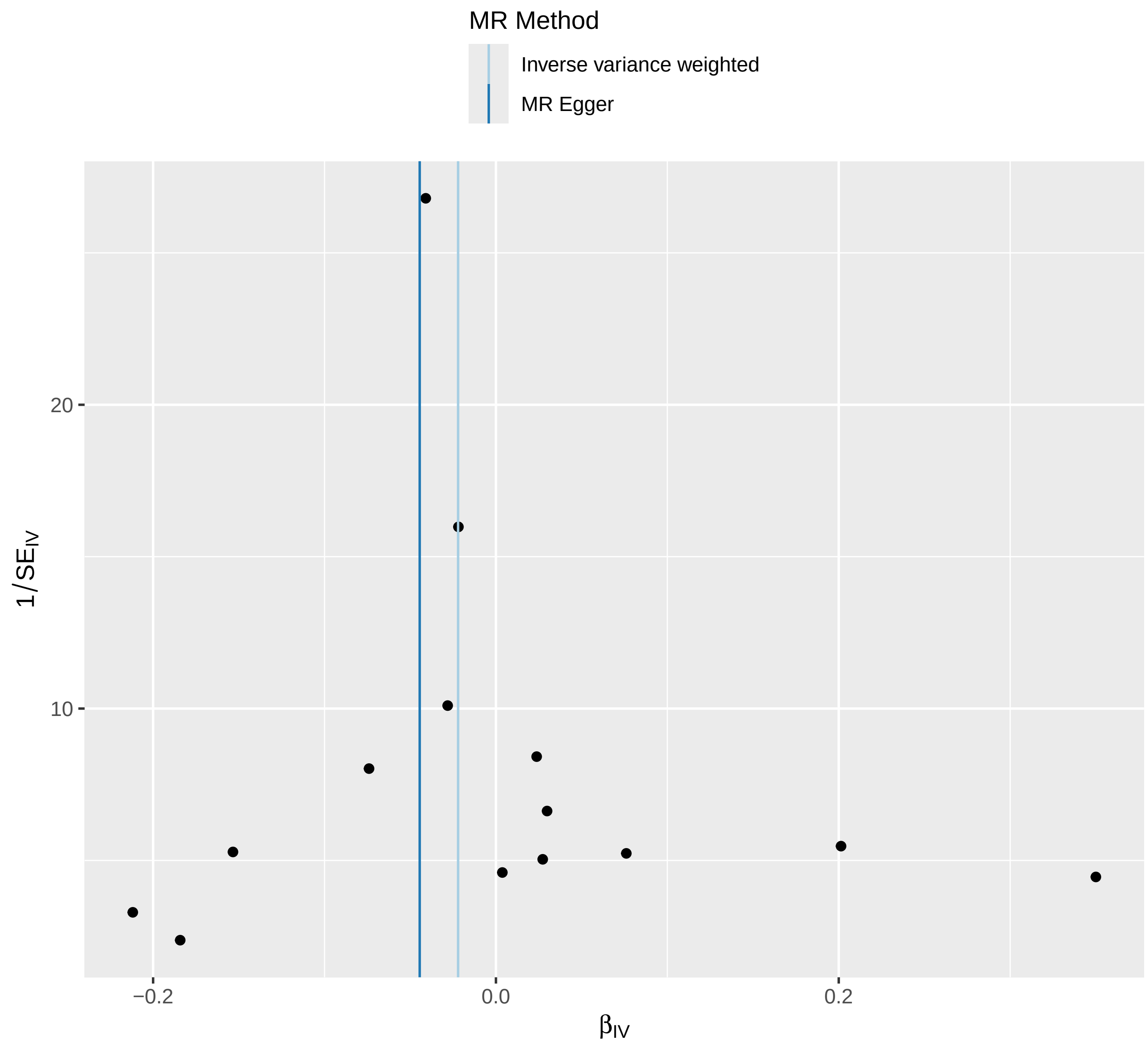
MR Method
Inverse variance weighted
MR Egger
20
10
1/SE$_{IV}$
−0.2
0.0
0.2
β$_{IV}$

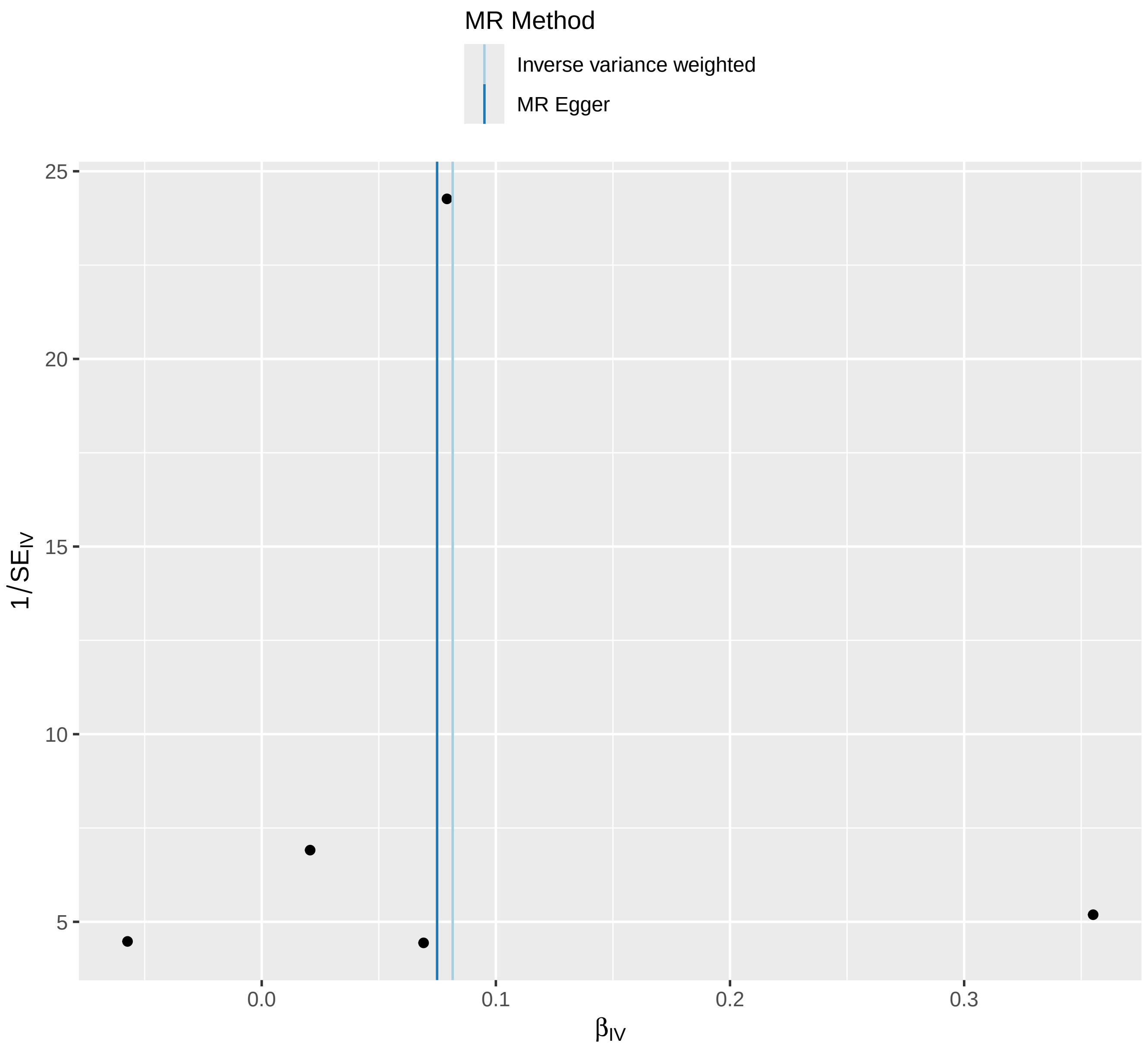
MR Method
Inverse variance weighted
MR Egger
25
20
15
10
5
1/SE$_{IV}$
0.0
0.1
0.2
0.3
β$_{IV}$

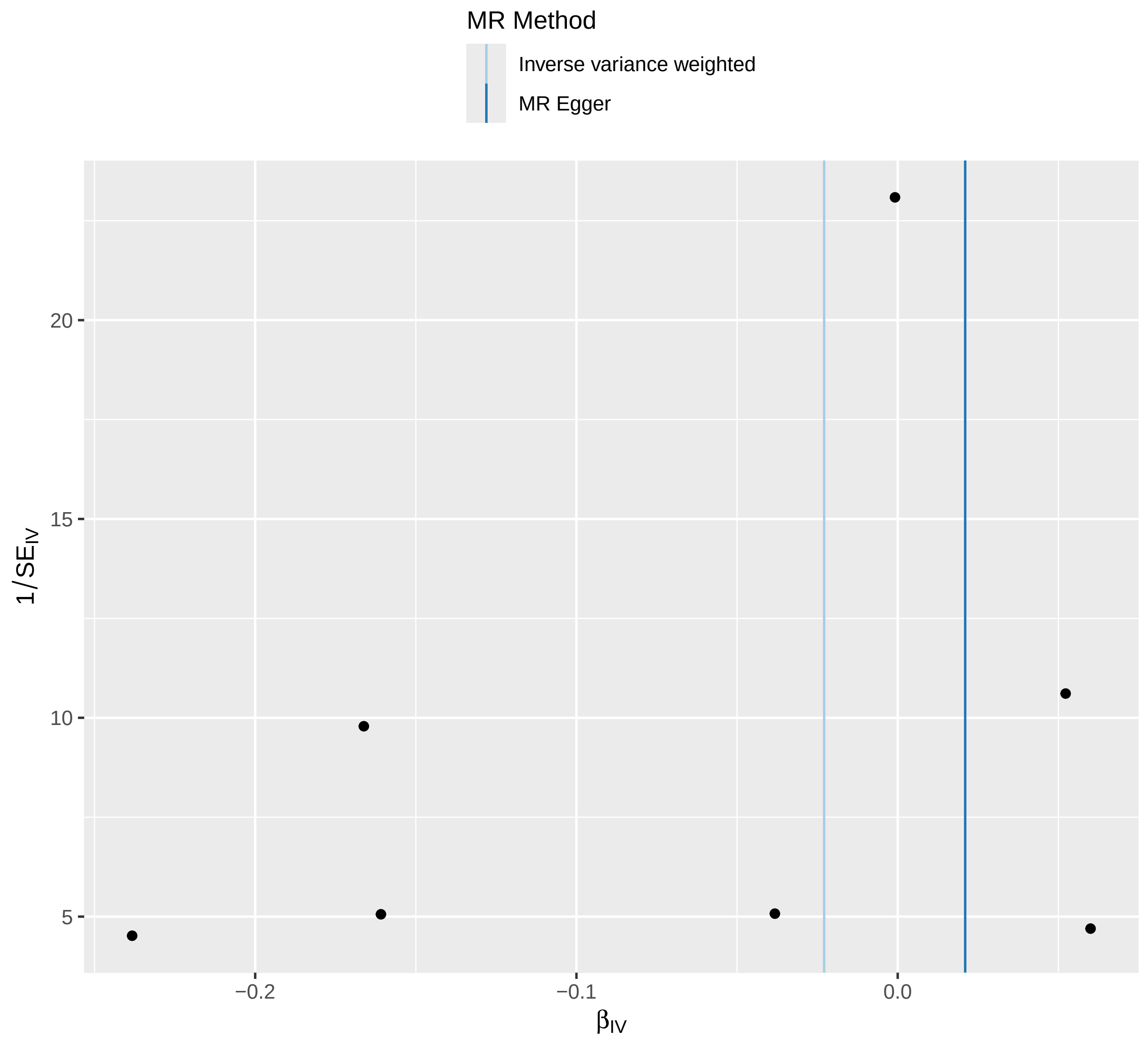
MR Method
Inverse variance weighted
MR Egger
20
15
10
5
$1/SE_{IV}$
−0.2
−0.1
0.0
$\beta_{IV}$

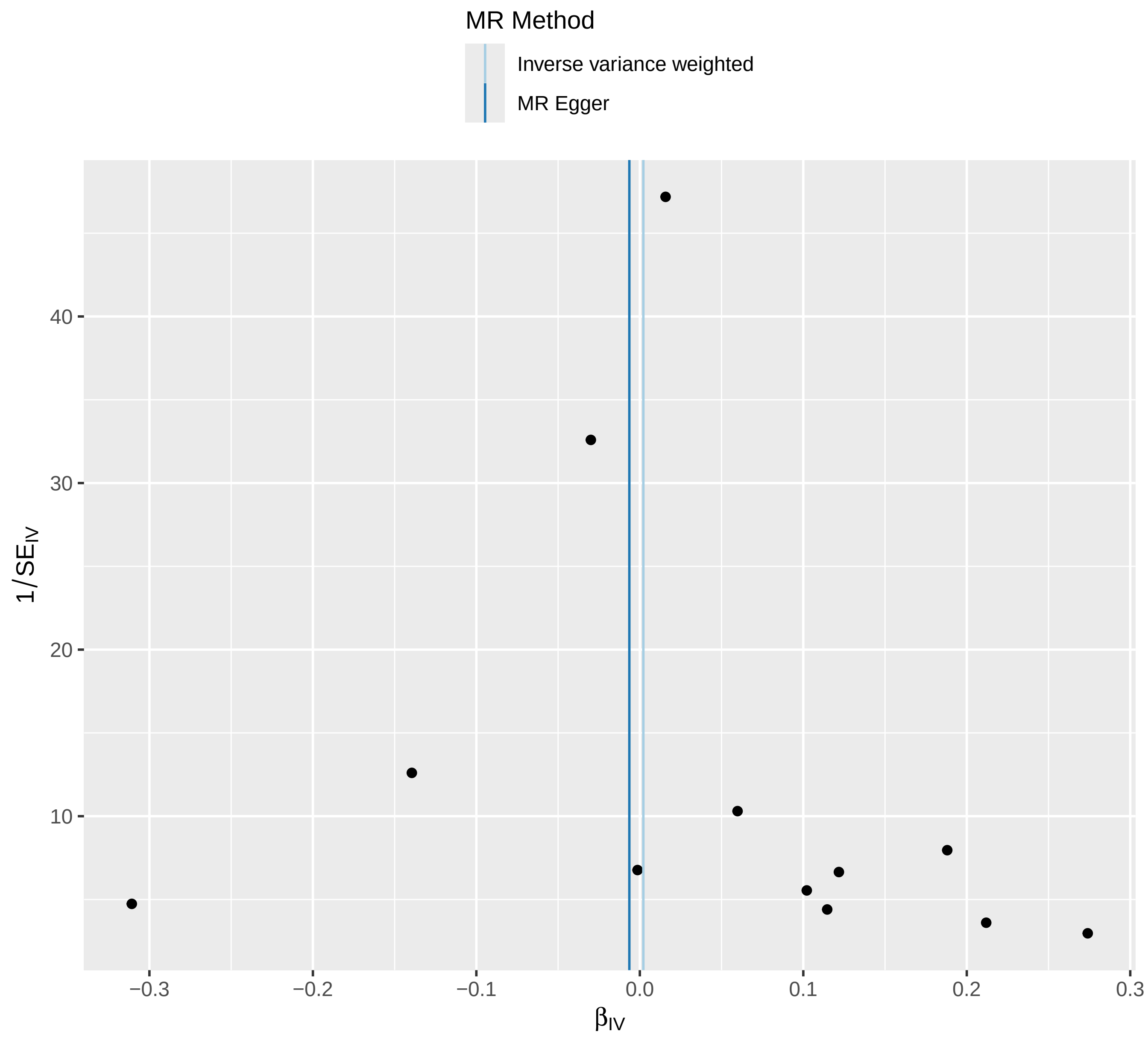

MR Method
Inverse variance weighted
MR Egger
40
30
20
10
1/SE$_{IV}$
−0.3
−0.2
−0.1
0.0
0.1
0.2
0.3
β$_{IV}$

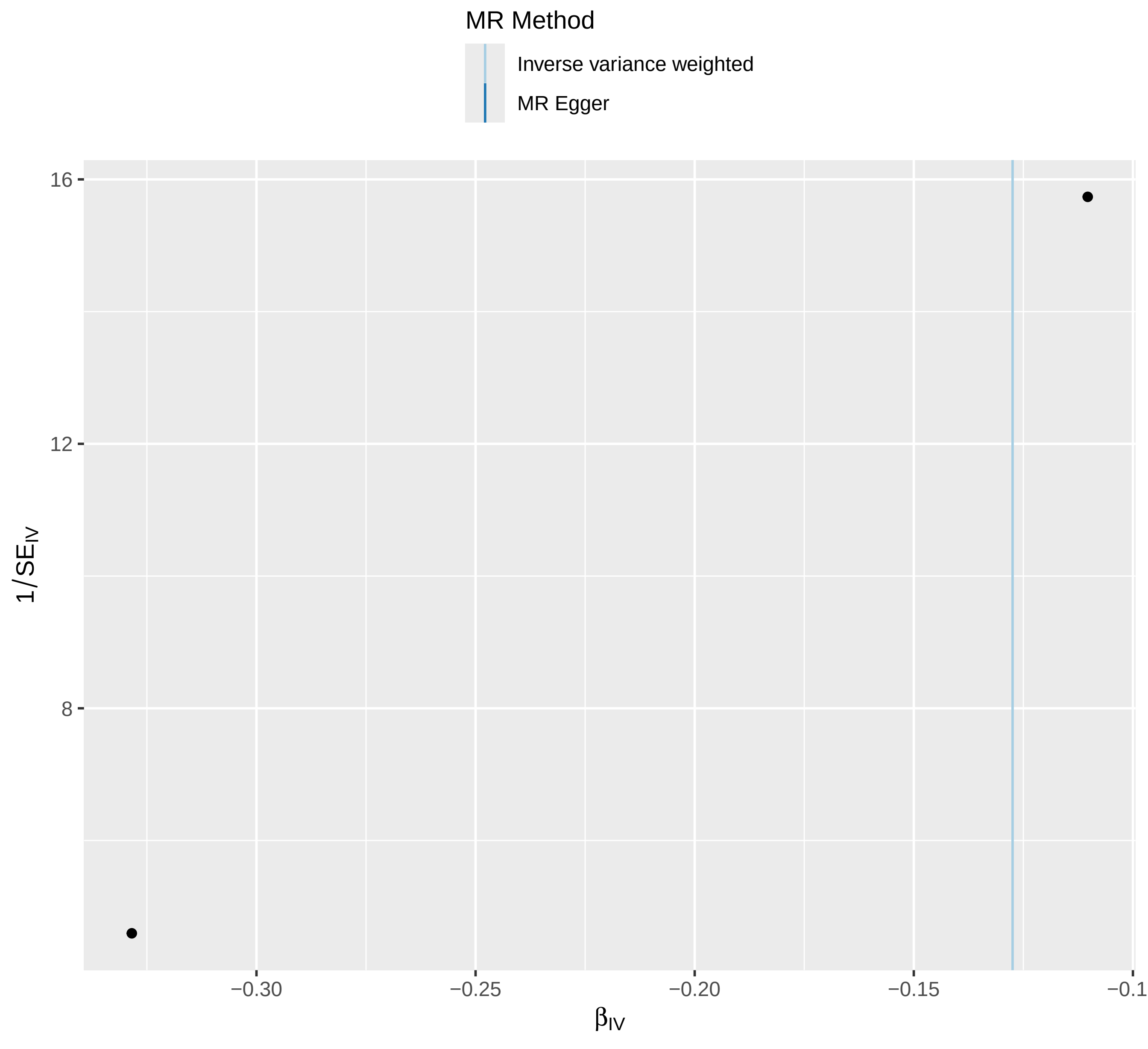

MR Method
Inverse variance weighted
MR Egger
16
12
8
1/SE$_{IV}$
−0.30
−0.25
−0.20
−0.15
−0.1
β$_{IV}$

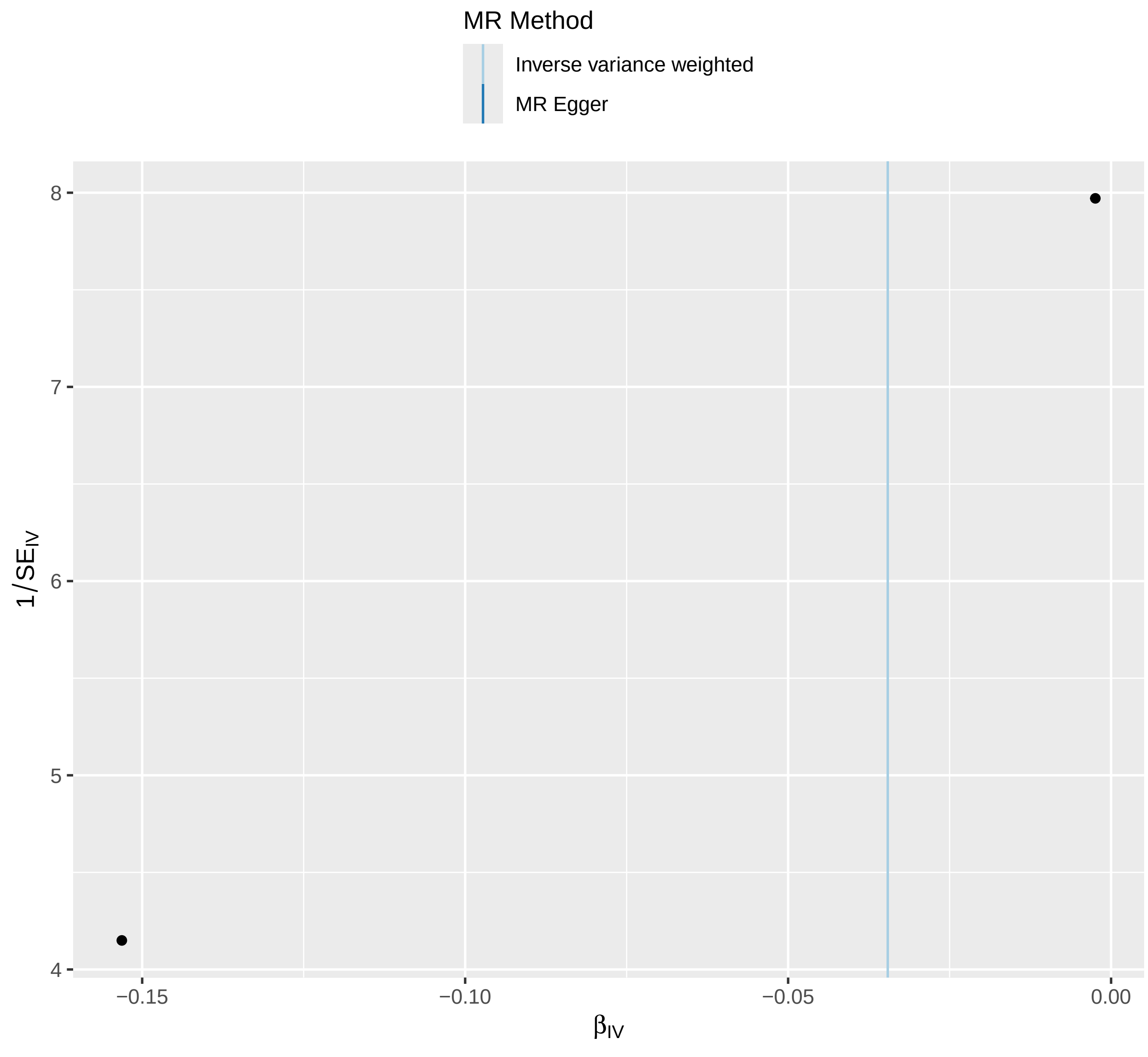
MR Method
Inverse variance weighted
MR Egger
8
7
6
5
4
1/SE$_{IV}$
−0.15
−0.10
−0.05
0.00
β$_{IV}$

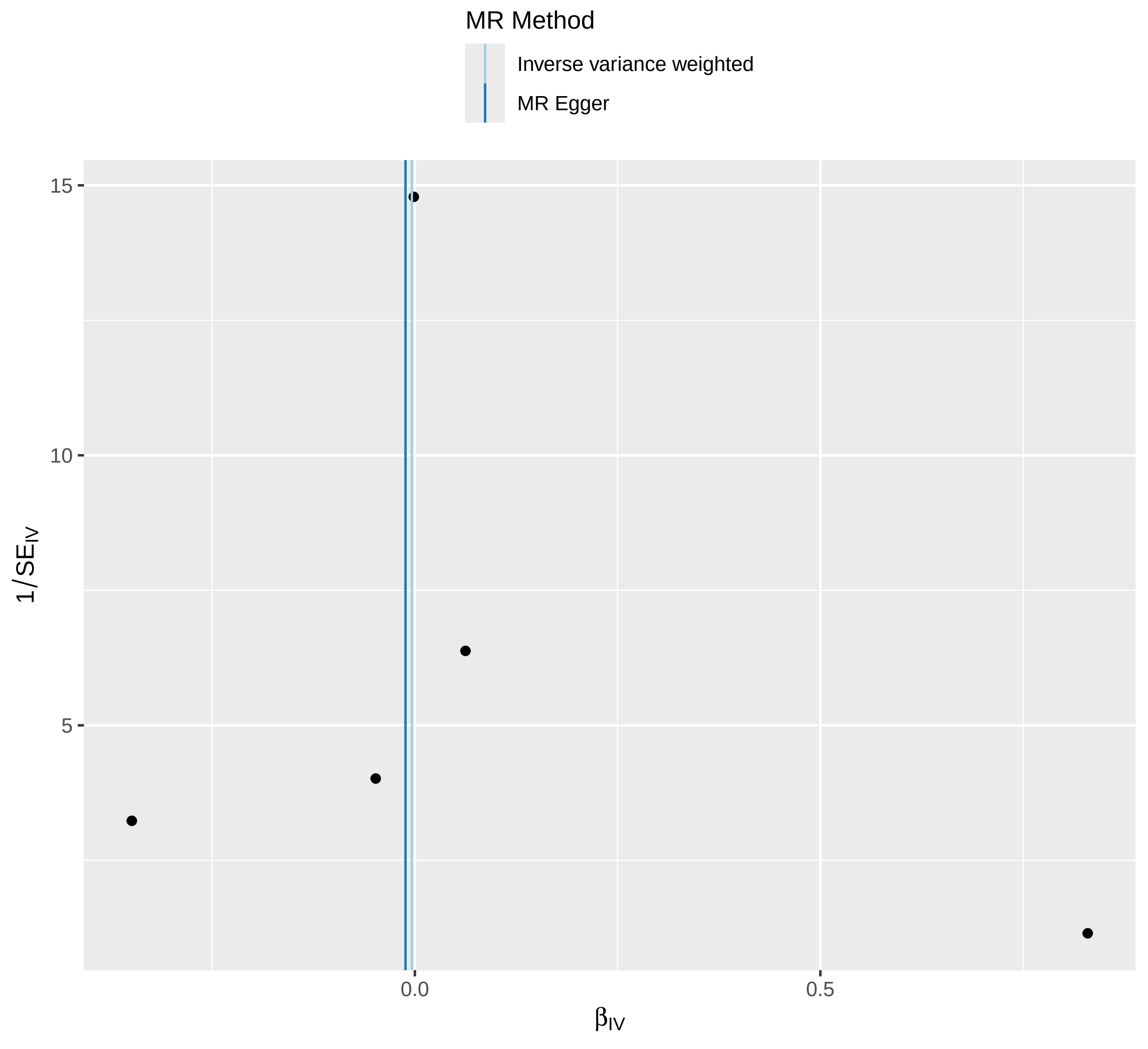

MR Method
Inverse variance weighted
MR Egger
15
10
5
0.0
0.5
1/SE_IV
β_IV

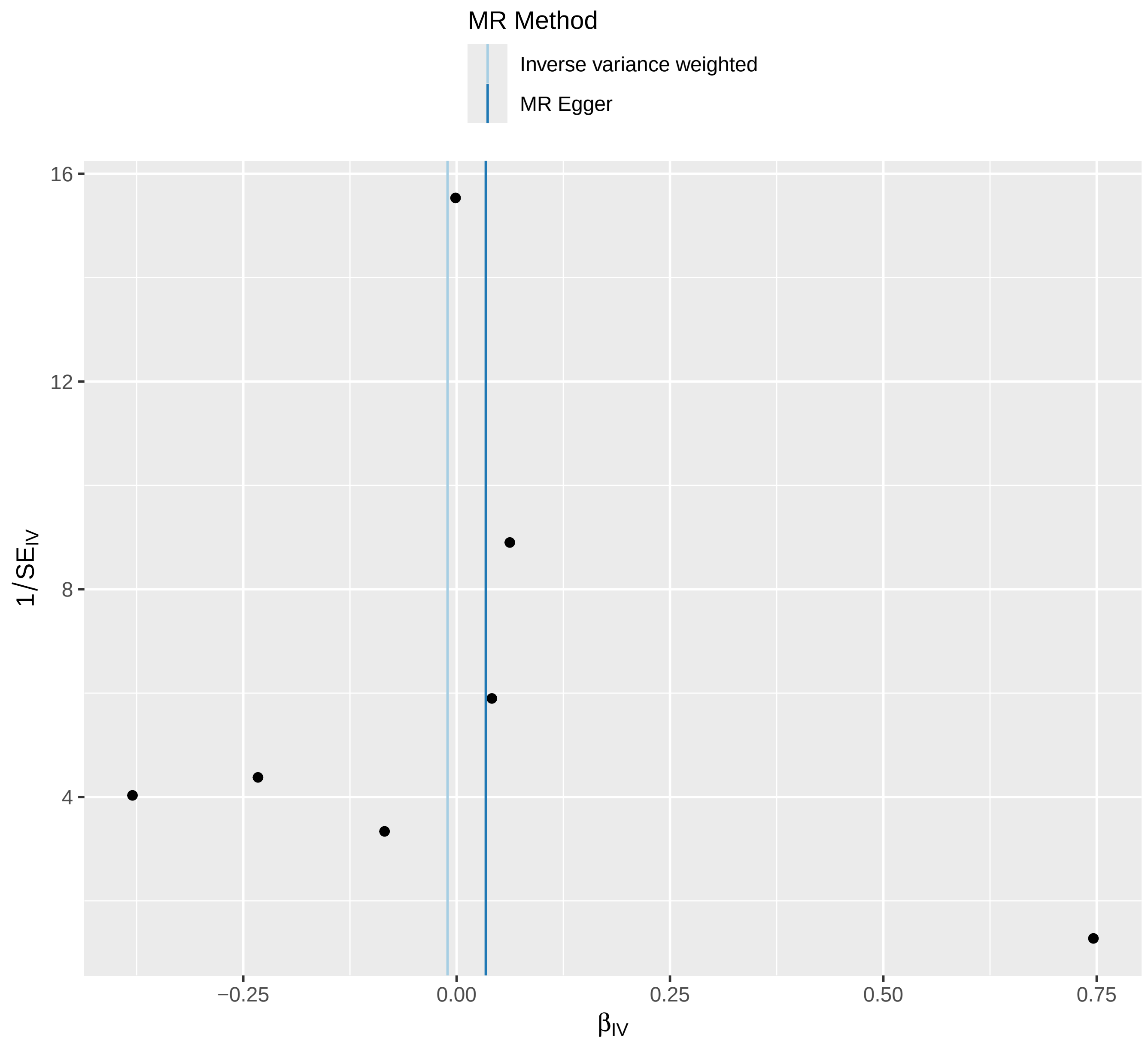

MR Method
Inverse variance weighted
MR Egger
16
12
8
4
1/SE$_{IV}$
−0.25
0.00
0.25
0.50
0.75
β$_{IV}$

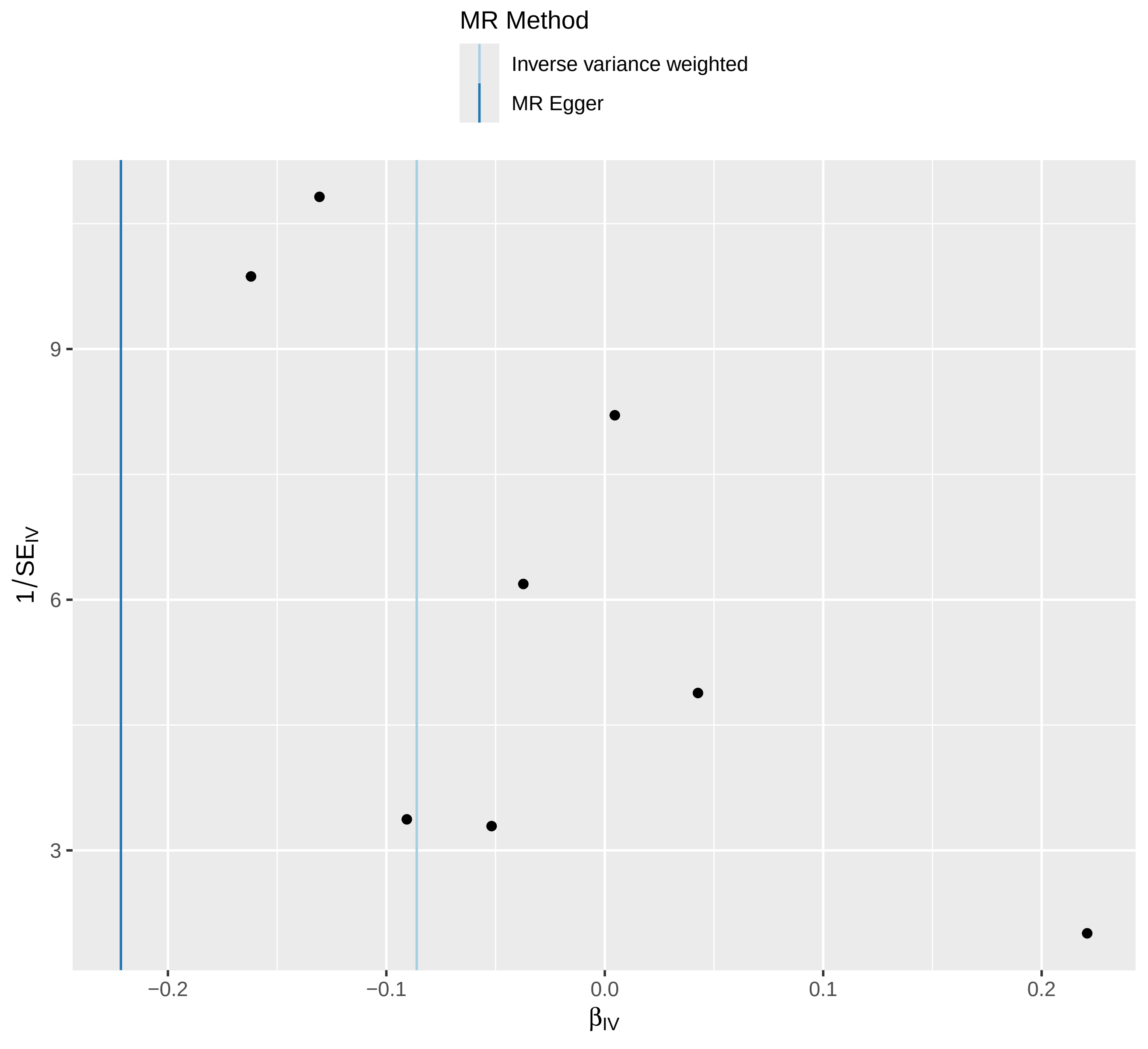
MR Method
Inverse variance weighted
MR Egger
9
6
3
1/SE$_{IV}$
−0.2
−0.1
0.0
0.1
0.2
β$_{IV}$

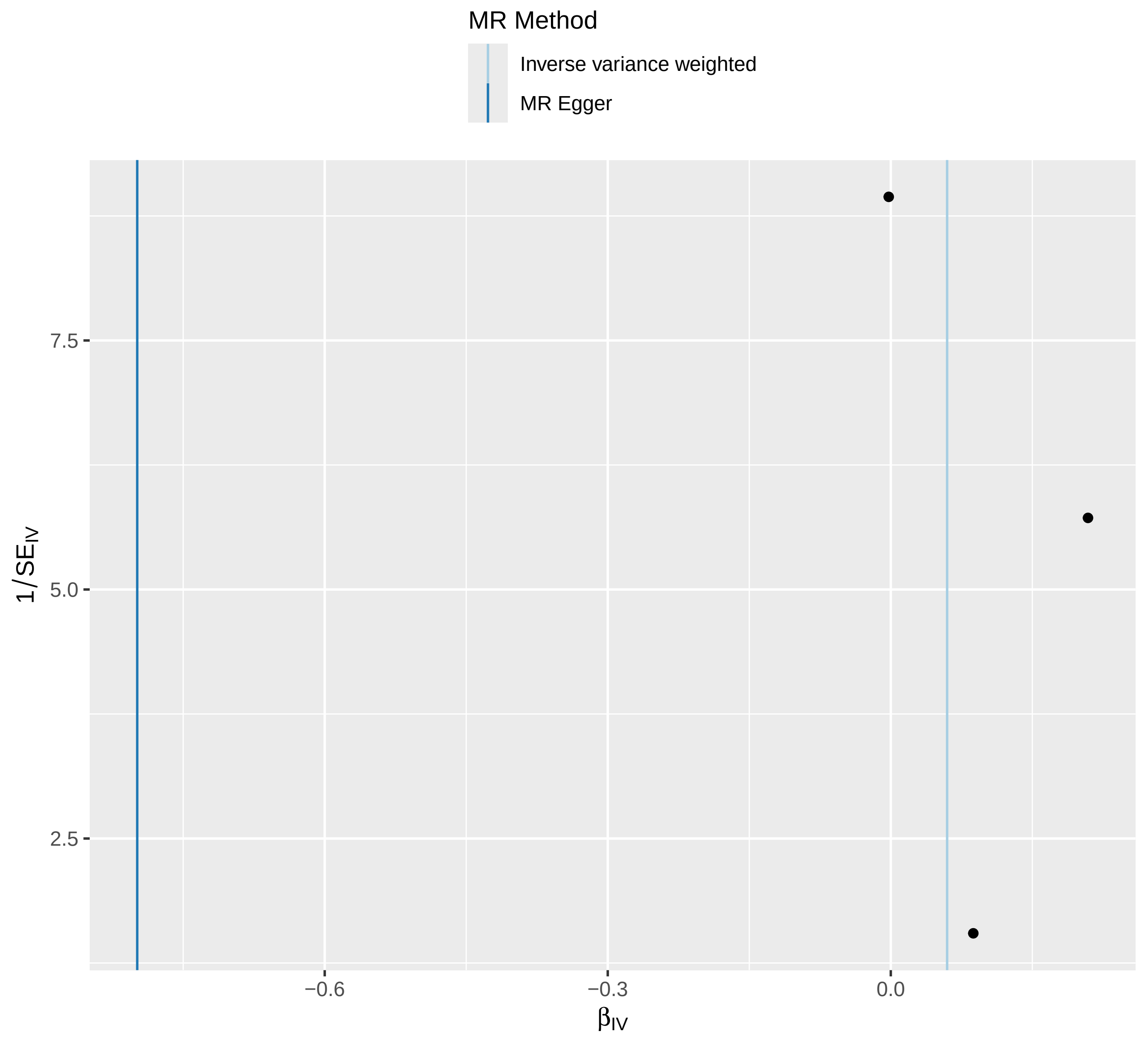

MR Method
Inverse variance weighted
MR Egger
7.5
5.0
2.5
1/SE_IV
−0.6
−0.3
0.0
β_IV

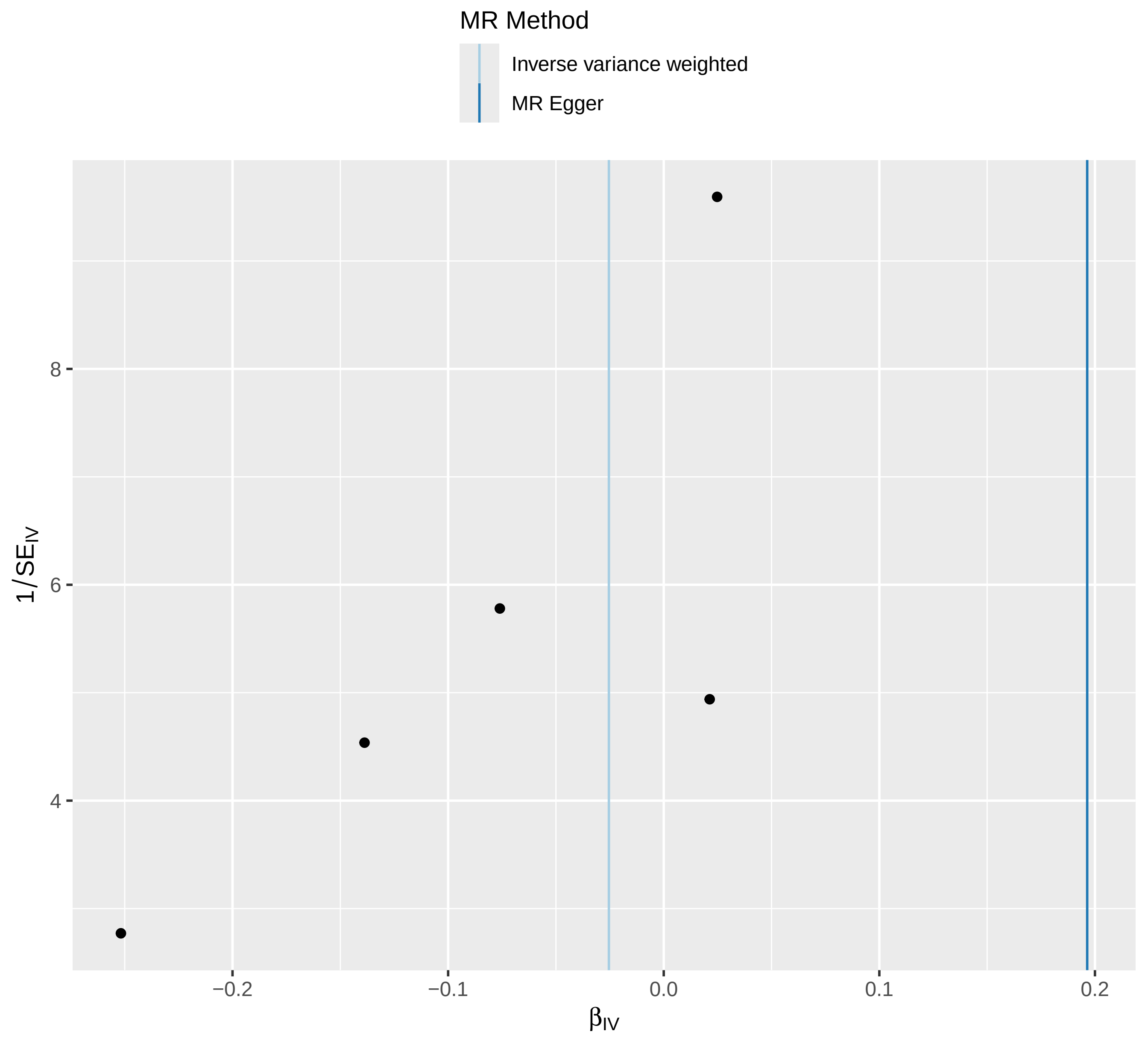
MR Method
Inverse variance weighted
MR Egger
8
6
4
−0.2
−0.1
0.0
0.1
0.2
1/SE_IV
β_IV

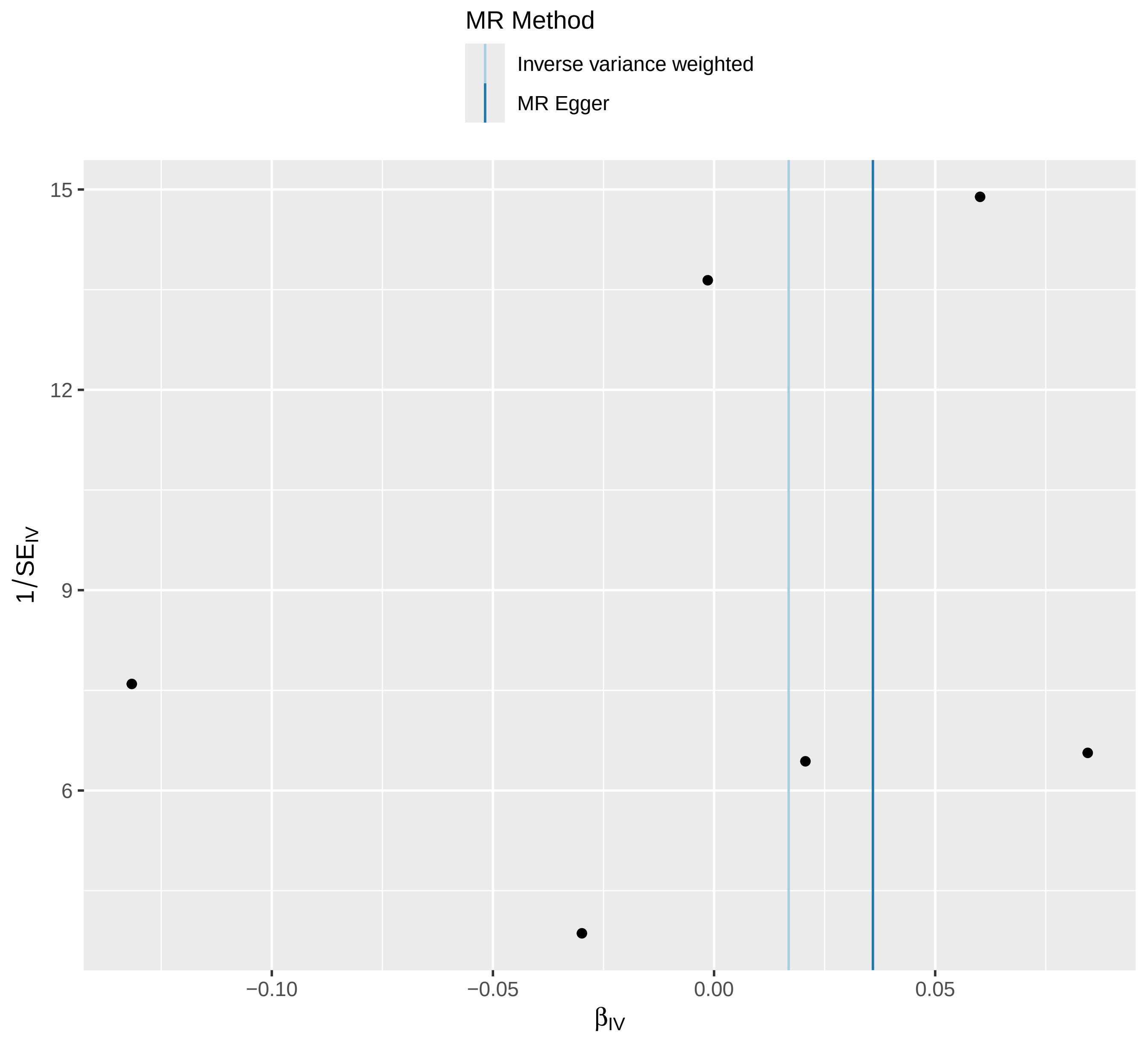
MR Method
Inverse variance weighted
MR Egger
15
12
9
6
1/SE$_{IV}$
−0.10
−0.05
0.00
0.05
β$_{IV}$

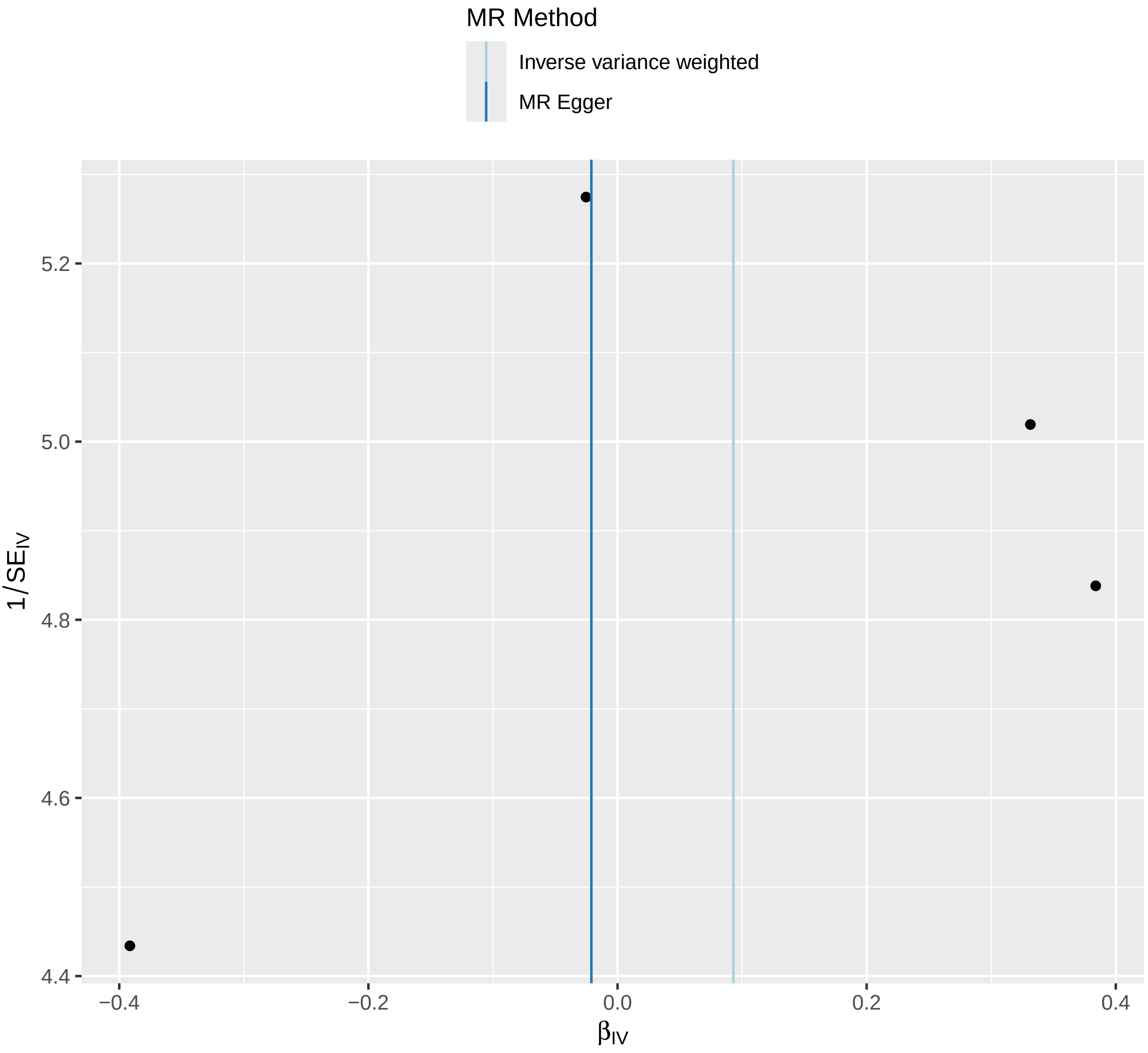

MR Method
Inverse variance weighted
MR Egger
5.2
5.0
4.8
4.6
4.4
1/SE_IV
−0.4
−0.2
0.0
0.2
0.4
β_IV

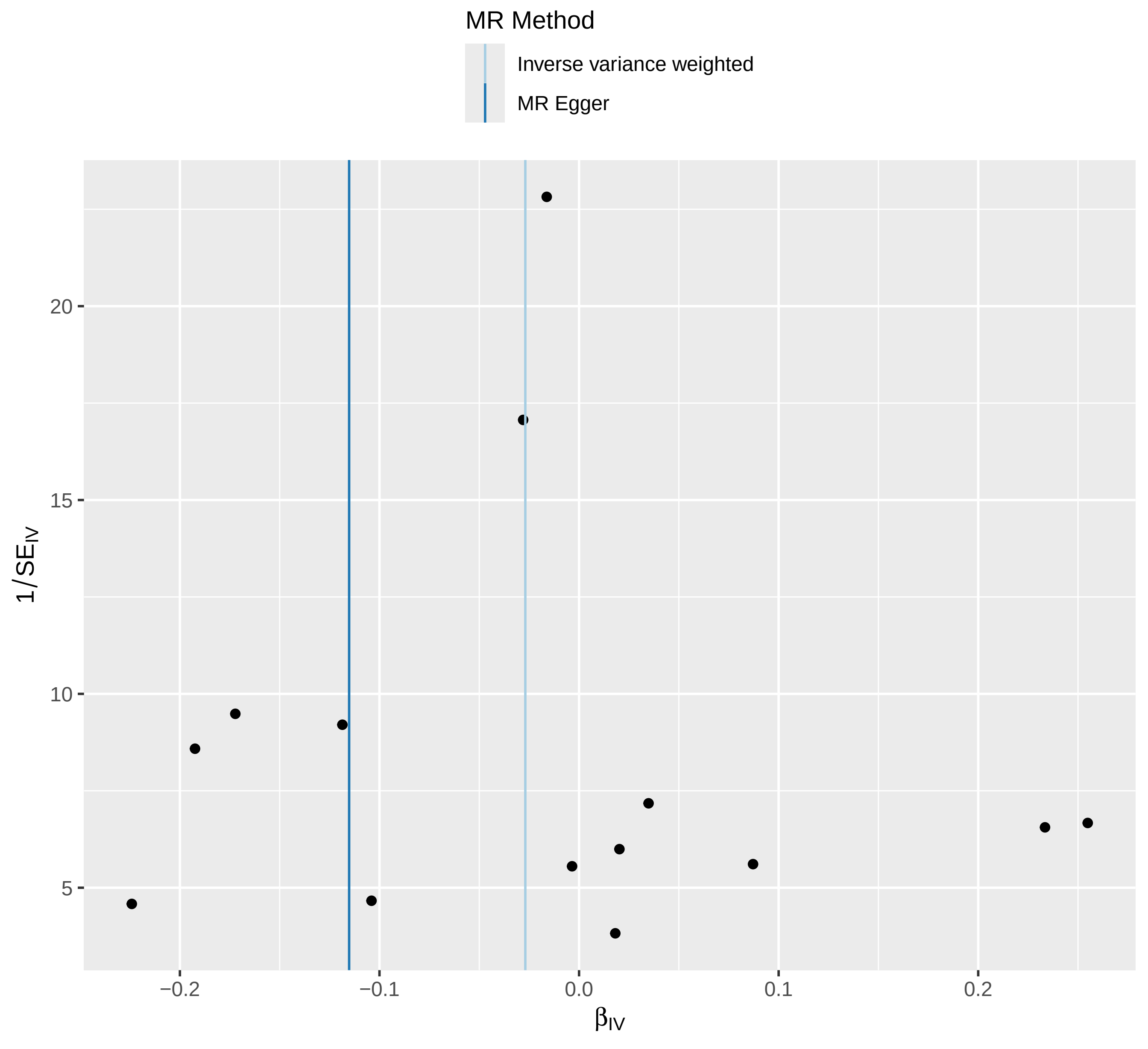
MR Method
Inverse variance weighted
MR Egger
20
15
10
5
−0.2
−0.1
0.0
0.1
0.2
$1/SE_{IV}$
$\beta_{IV}$

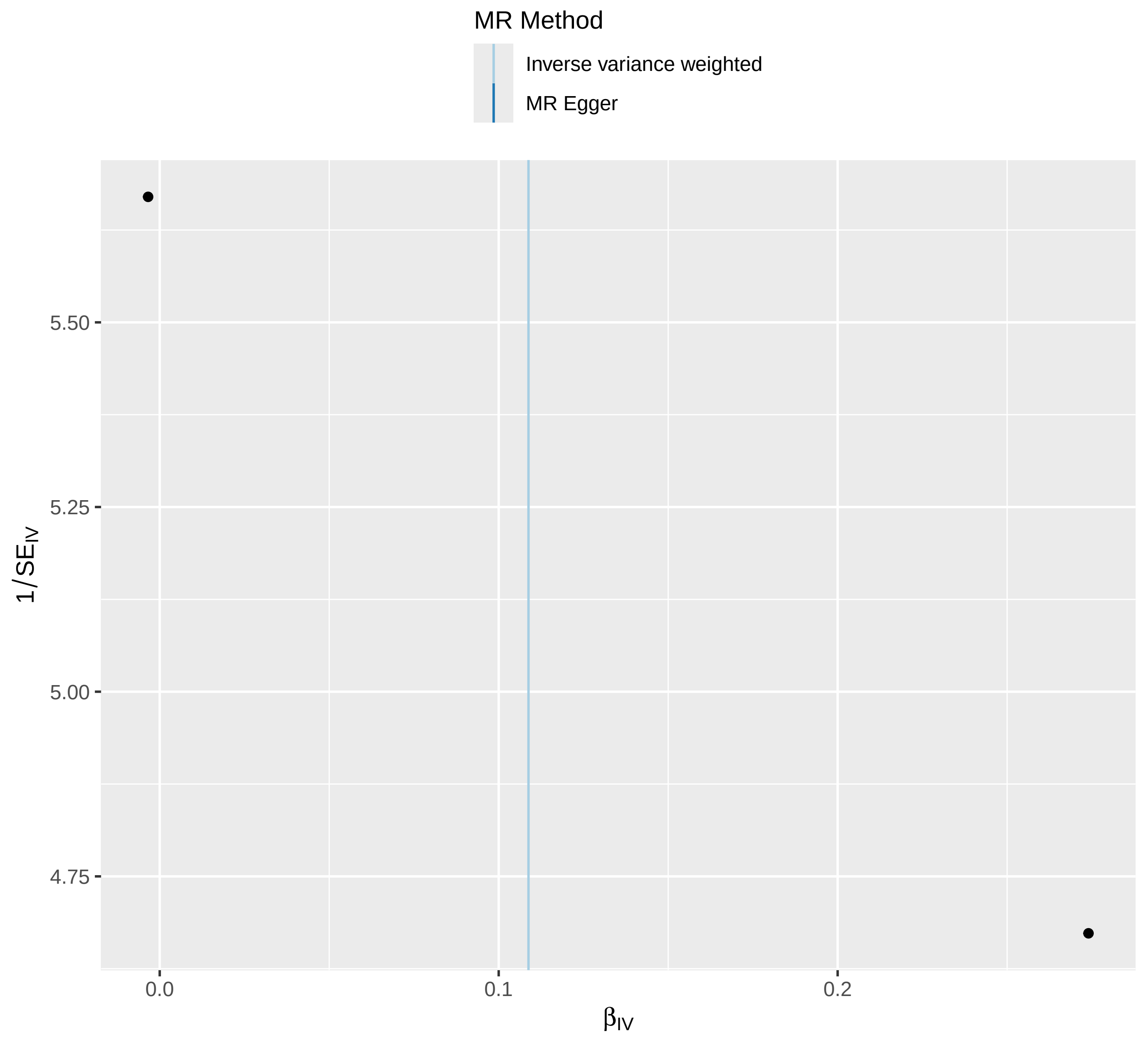
MR Method
Inverse variance weighted
MR Egger
5.50
5.25
5.00
4.75
1/SE_IV
0.0
0.1
0.2
β_IV

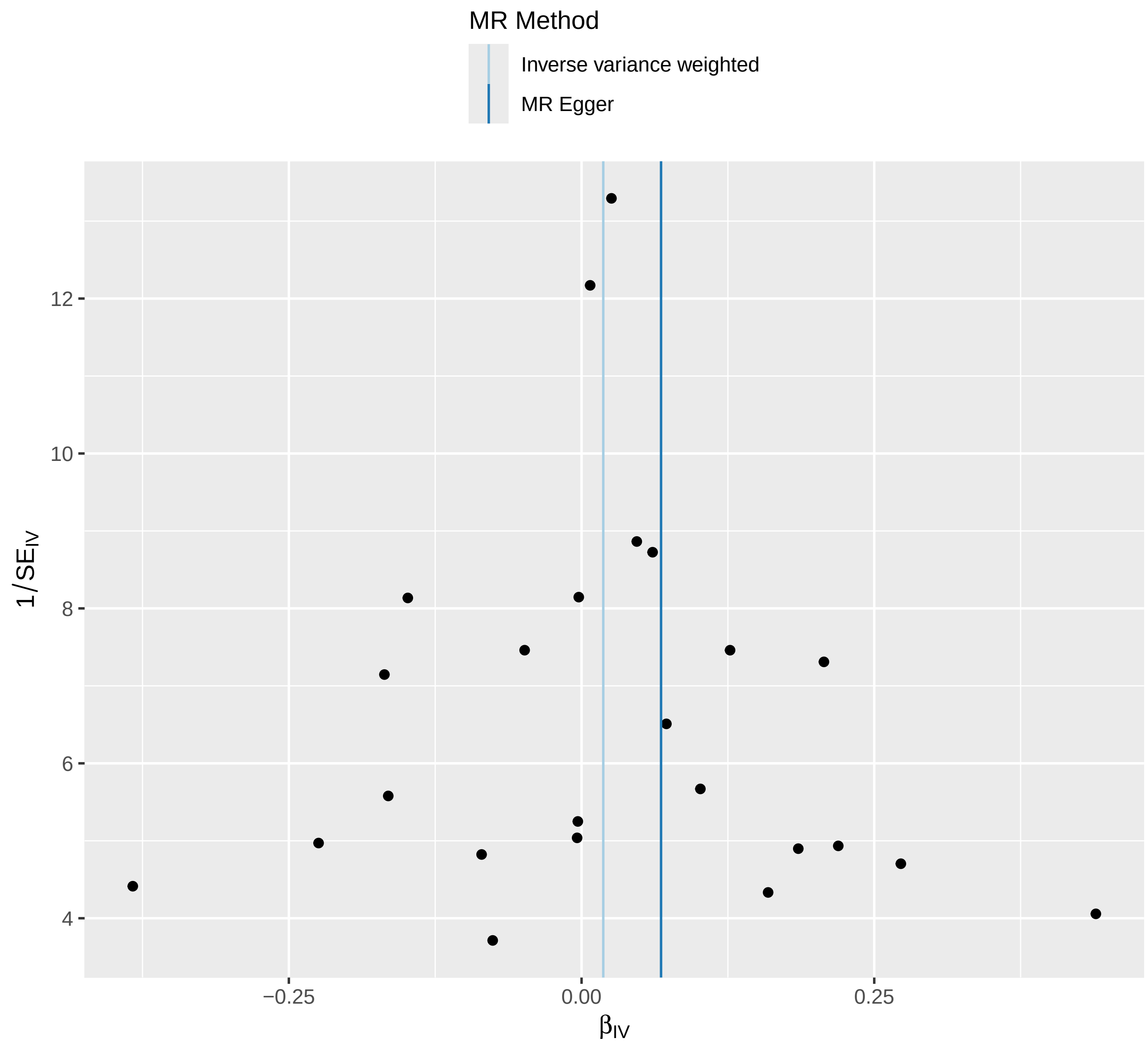
MR Method
Inverse variance weighted
MR Egger
12
10
8
6
4
1/SE$_{IV}$
−0.25
0.00
0.25
β$_{IV}$

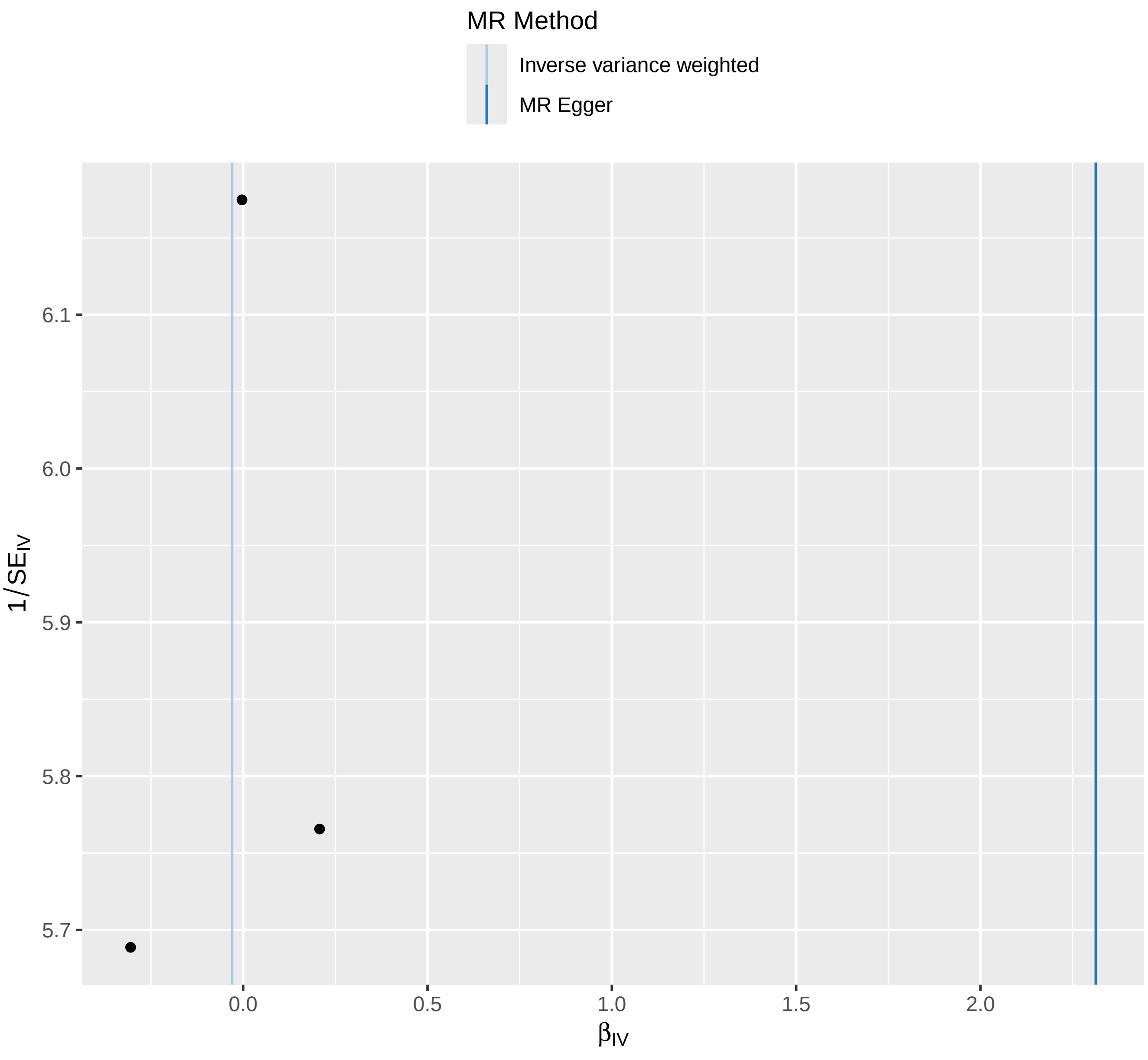

MR Method
Inverse variance weighted
MR Egger
6.1
6.0
5.9
5.8
5.7
$1/SE_{IV}$
0.0
0.5
1.0
1.5
2.0
$\beta_{IV}$

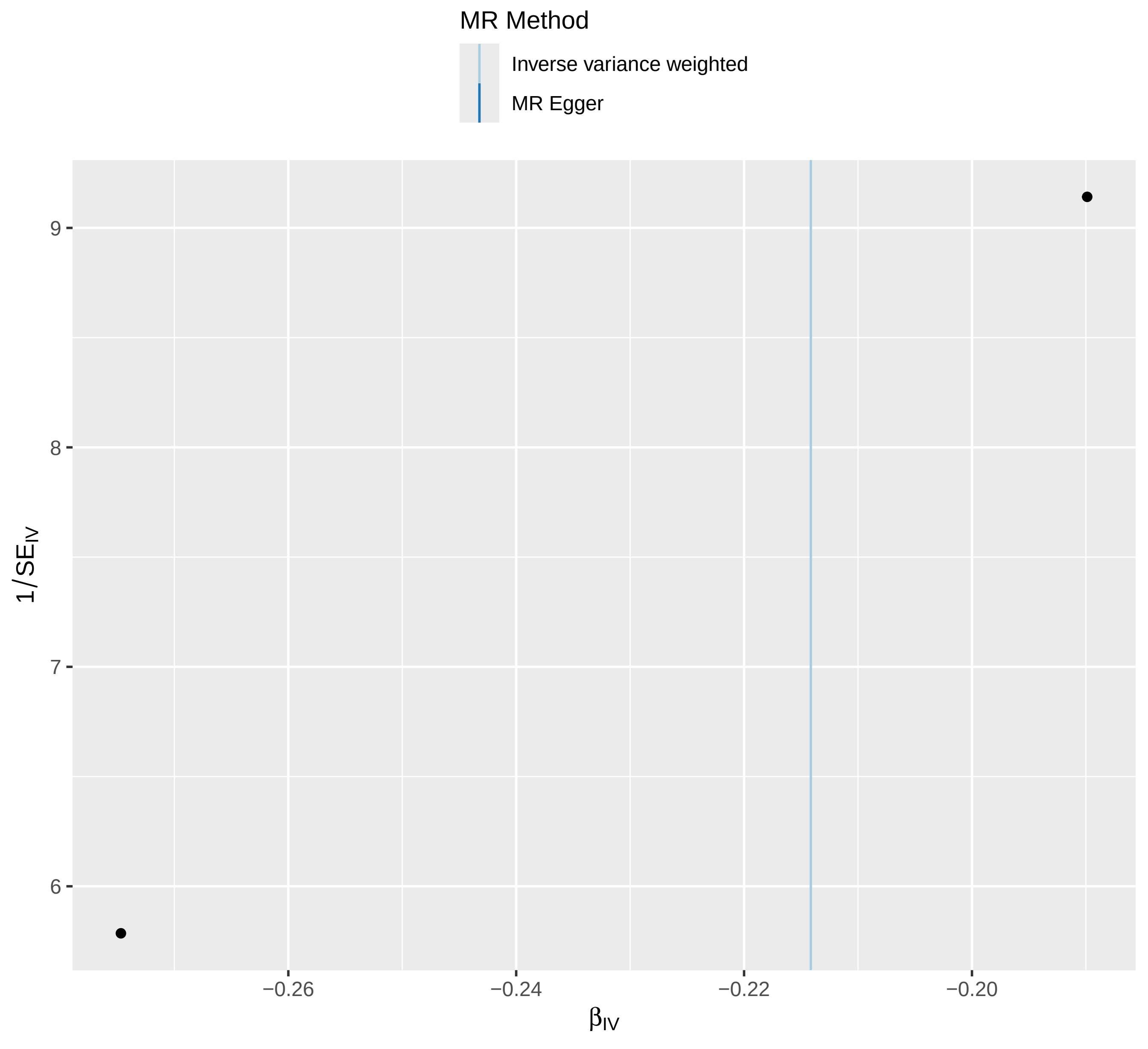
MR Method
Inverse variance weighted
MR Egger
9
8
7
6
−0.26
−0.24
−0.22
−0.20
$\beta_{IV}$
$1/SE_{IV}$

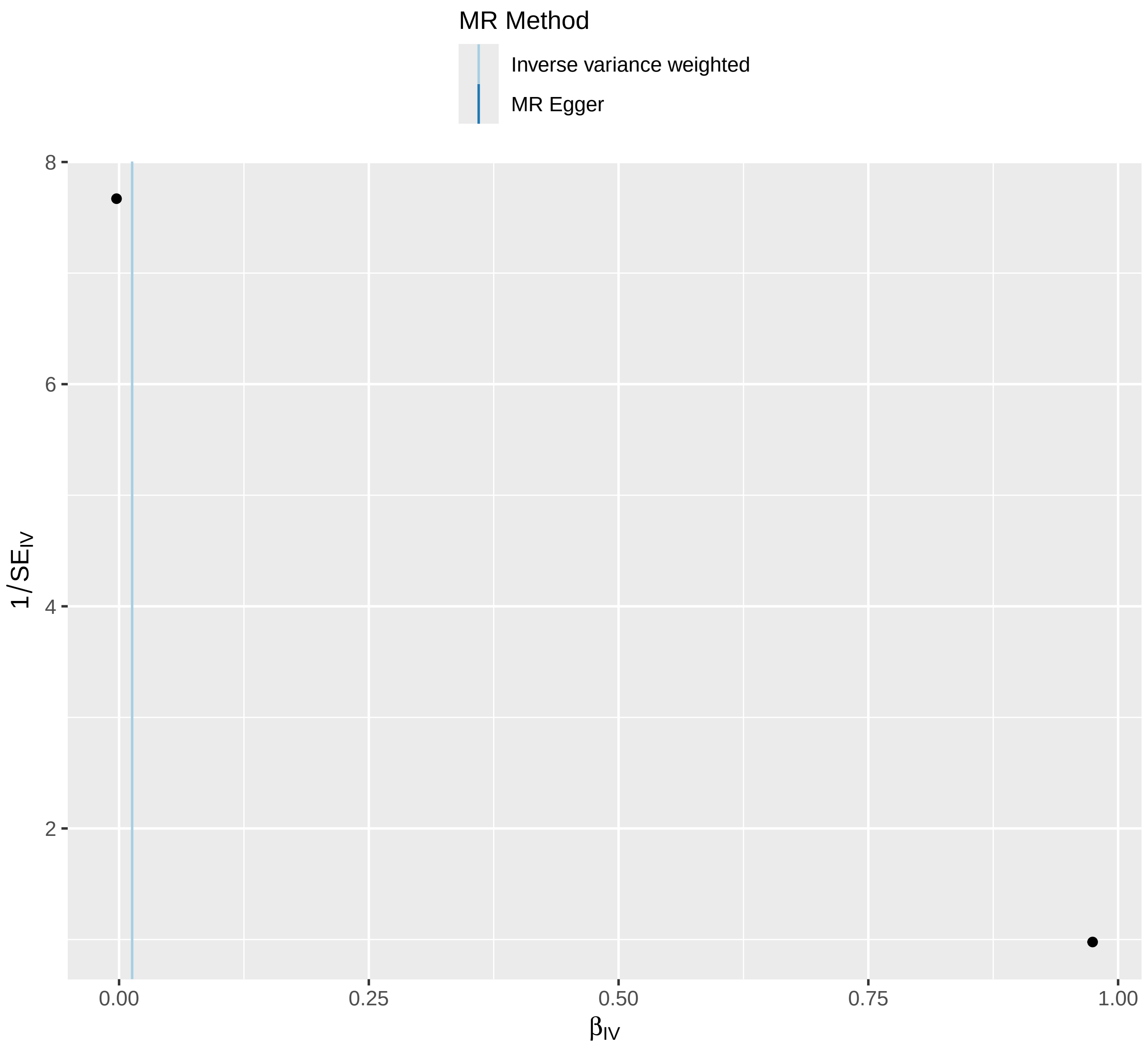
MR Method
Inverse variance weighted
MR Egger
8
6
4
2
1/SE$_{IV}$
0.00
0.25
0.50
0.75
1.00
β$_{IV}$

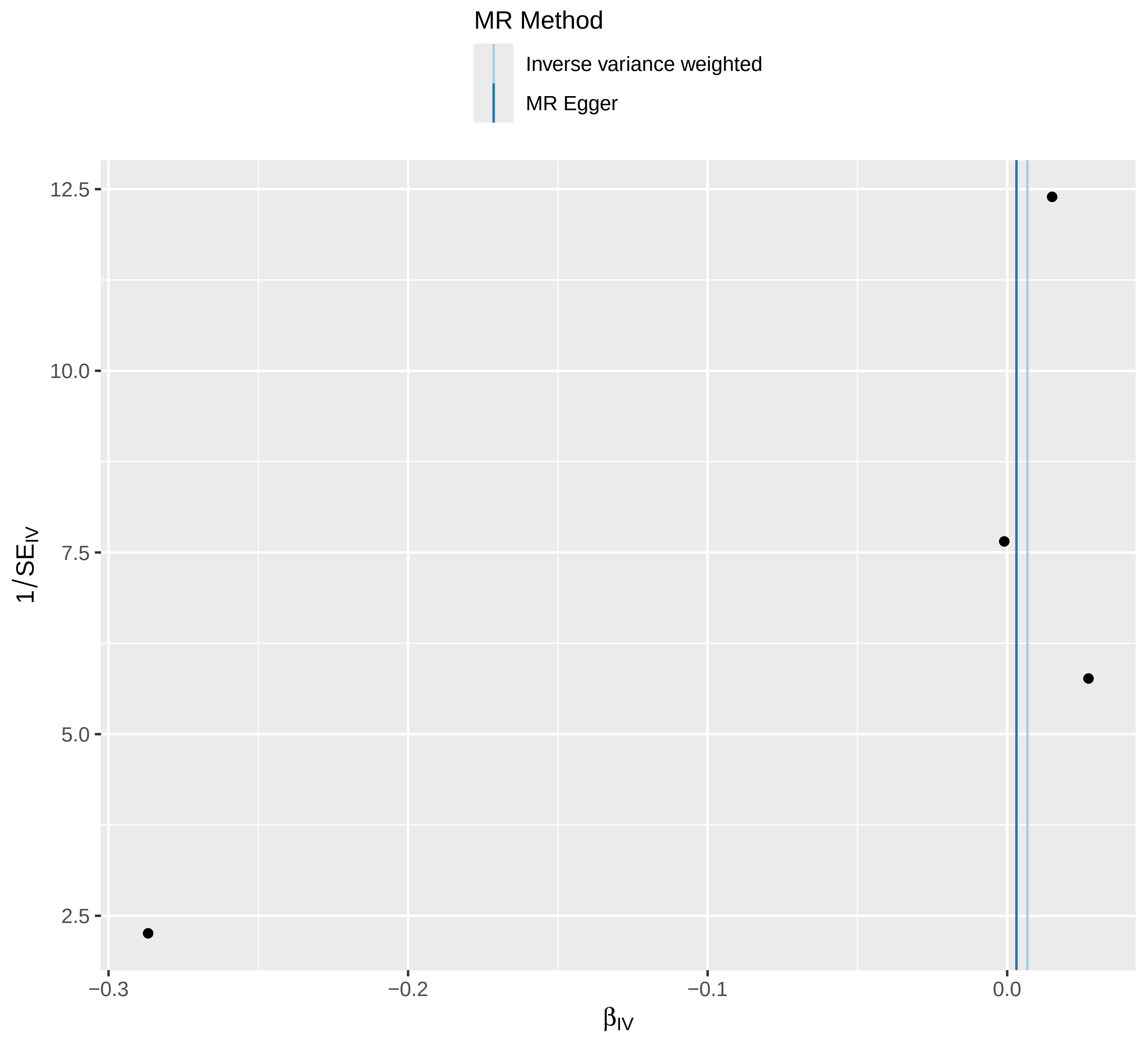

MR Method
Inverse variance weighted
MR Egger
12.5
10.0
7.5
5.0
2.5
1/SE_IV
−0.3
−0.2
−0.1
0.0
β_IV

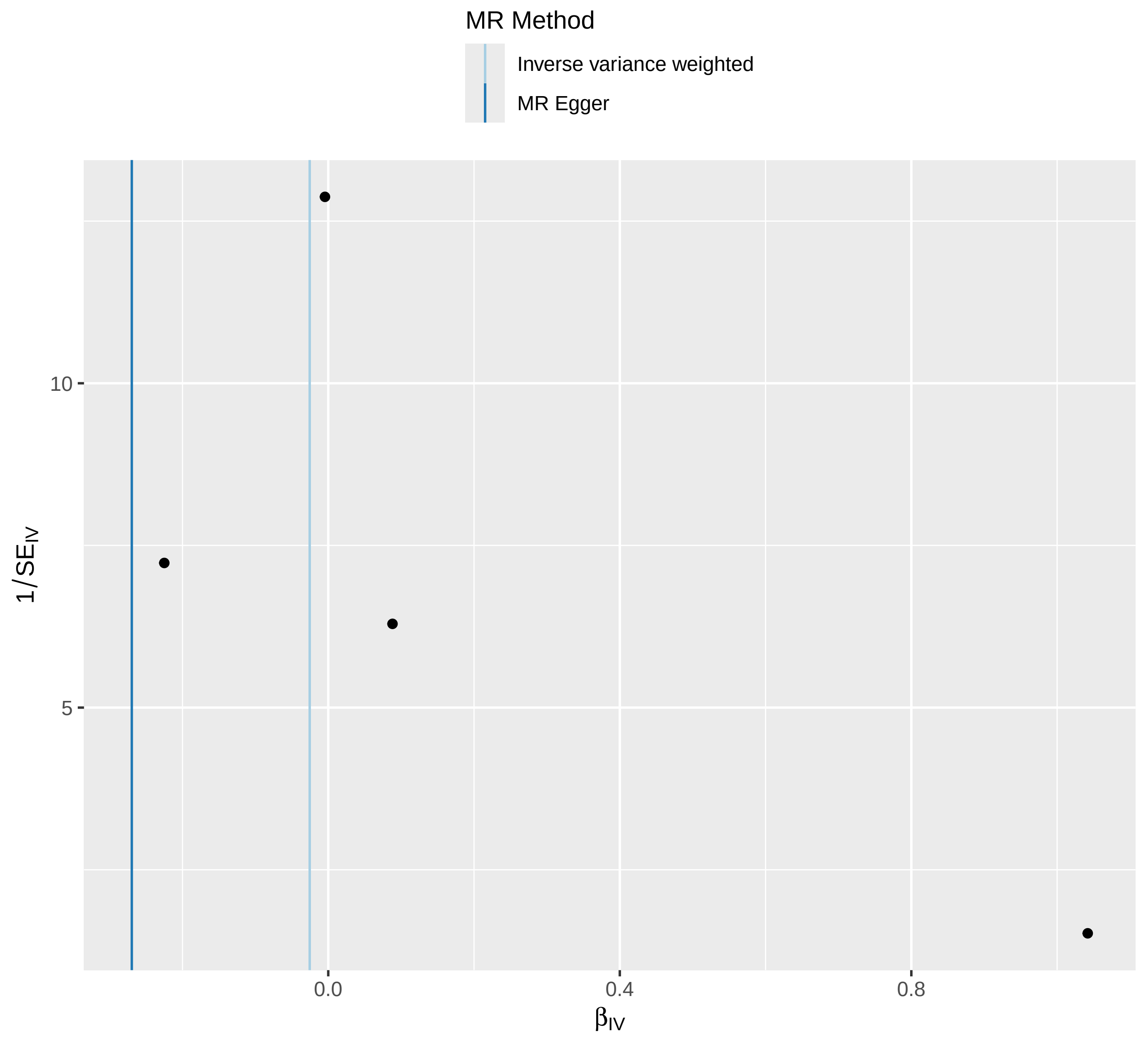
MR Method
Inverse variance weighted
MR Egger
10
5
1/SE_IV
0.0
0.4
0.8
β_IV

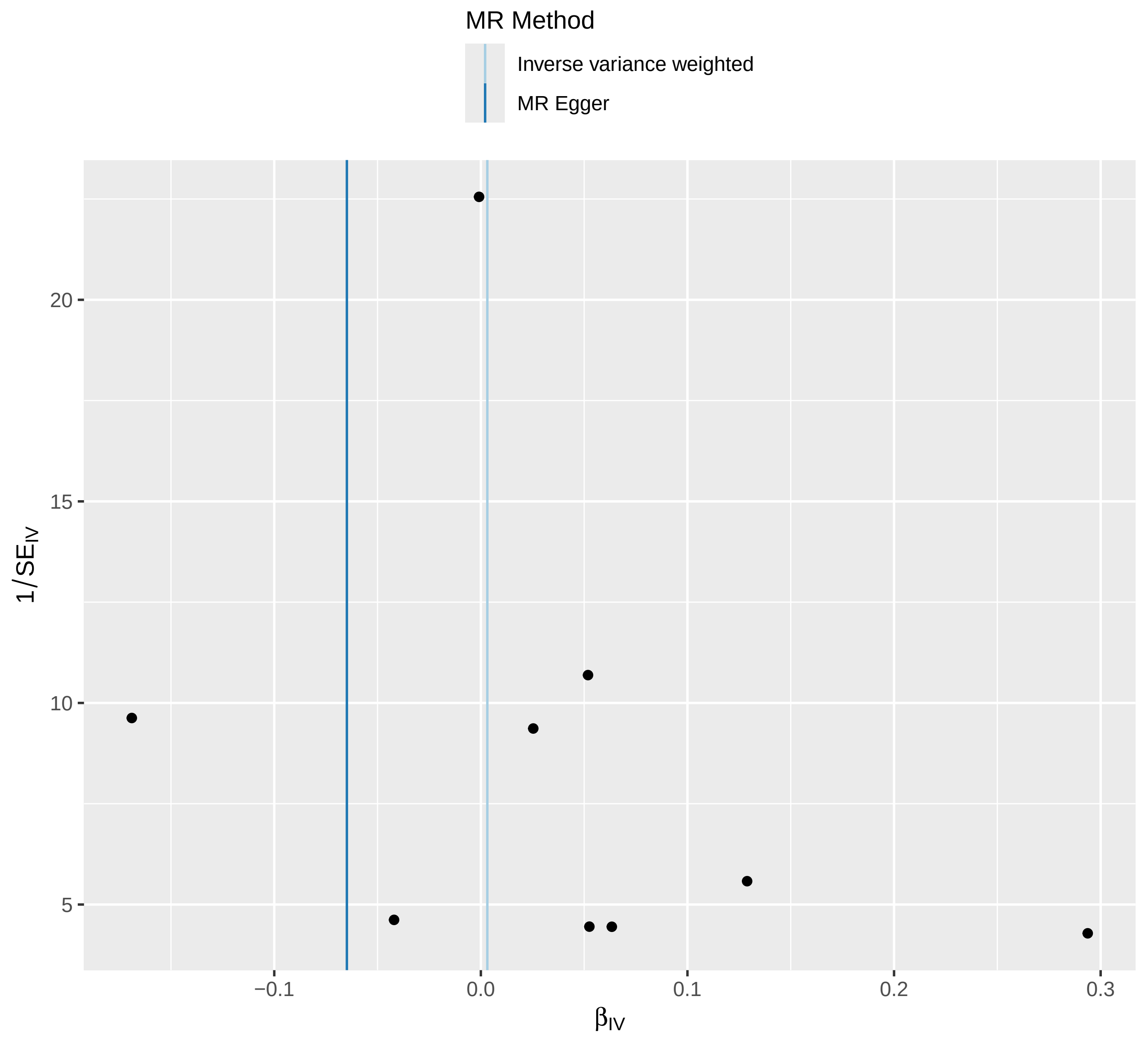
MR Method
Inverse variance weighted
MR Egger
20
15
10
5
1/SE$_{IV}$
−0.1
0.0
0.1
0.2
0.3
β$_{IV}$

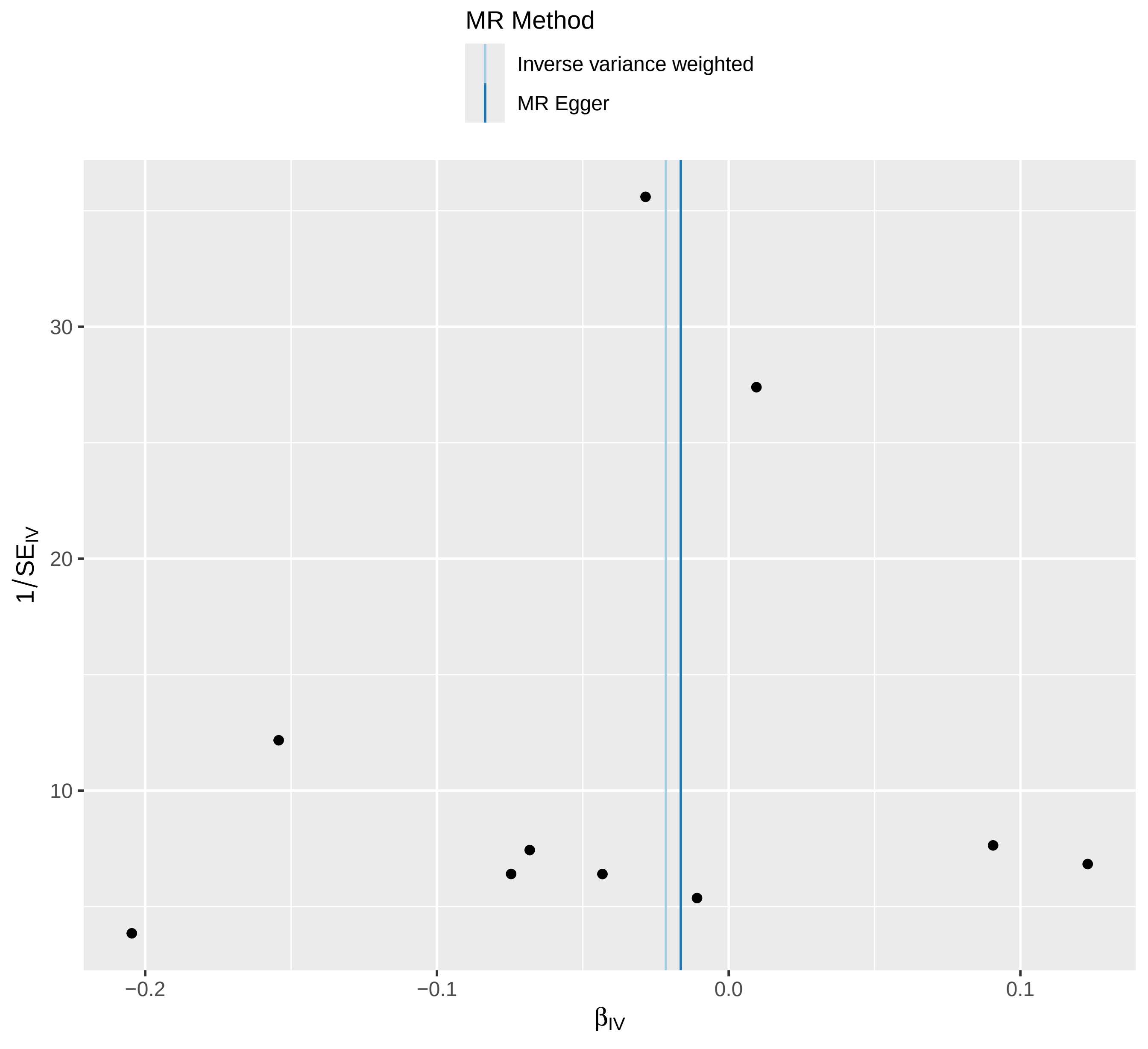

MR Method
Inverse variance weighted
MR Egger
30
20
10
$1/SE_{IV}$
−0.2
−0.1
0.0
0.1
$\beta_{IV}$

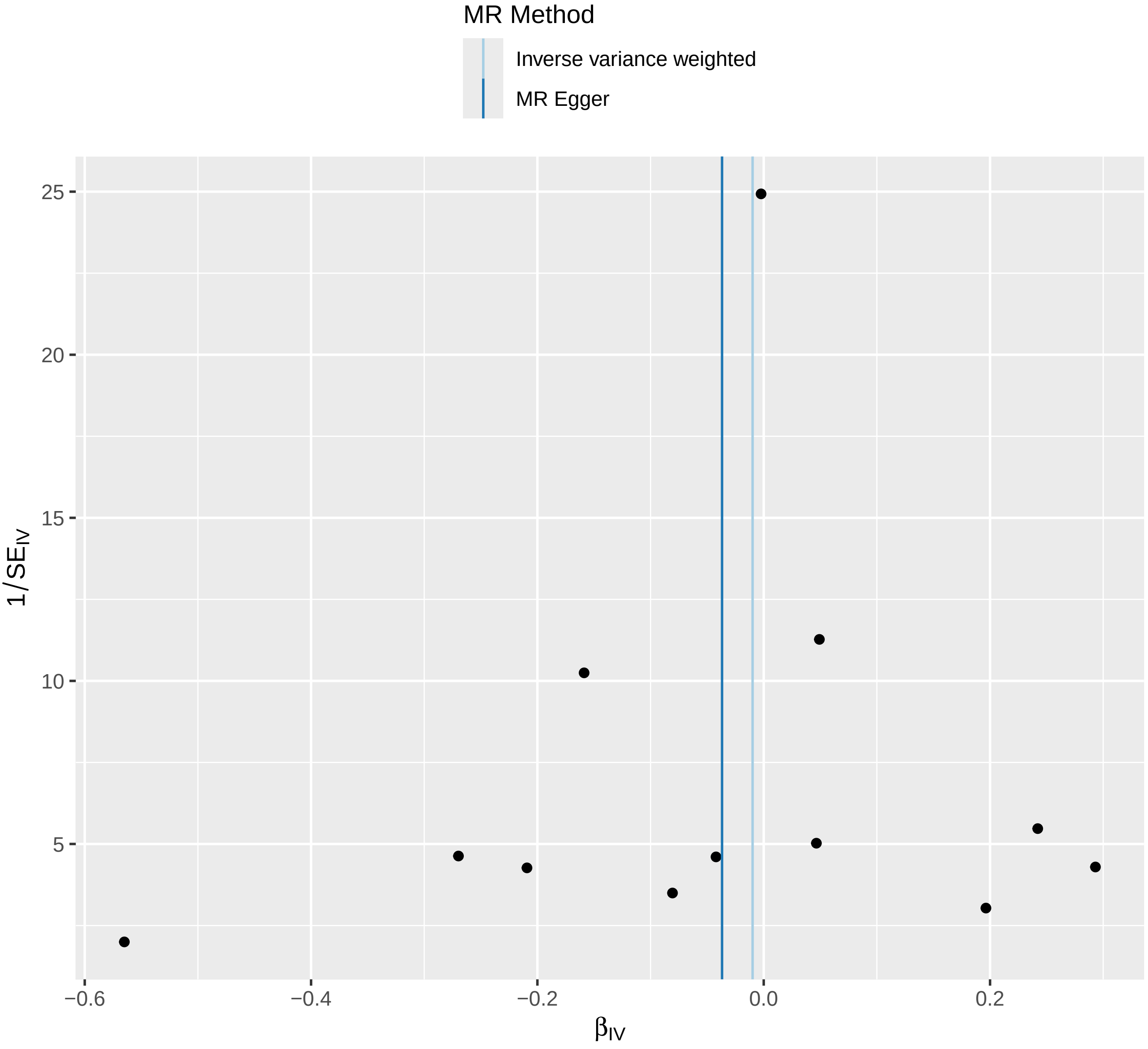

MR Method
Inverse variance weighted
MR Egger
25
20
15
10
5
1/SE$_{IV}$
−0.6
−0.4
−0.2
0.0
0.2
β$_{IV}$

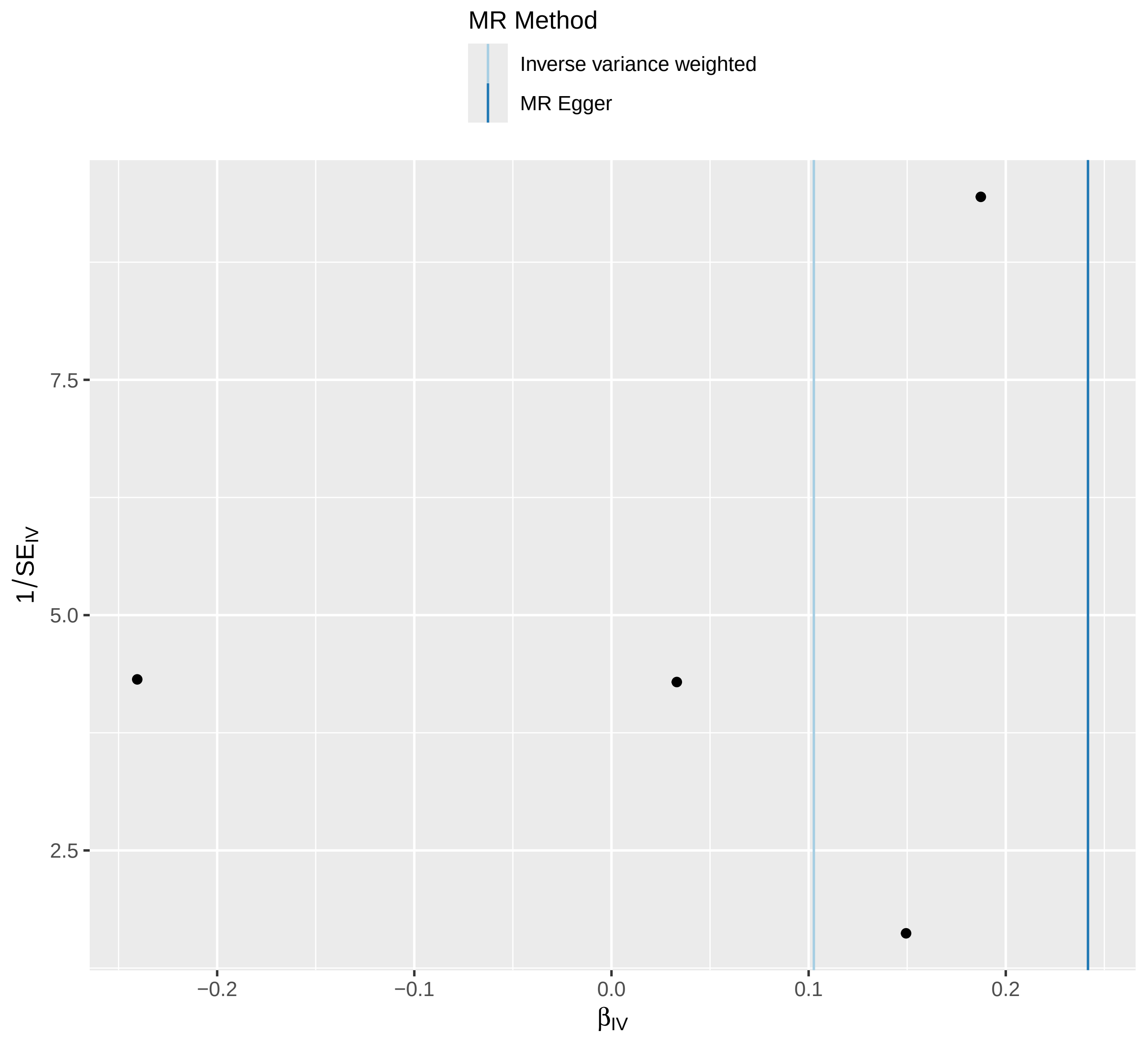

MR Method
Inverse variance weighted
MR Egger
7.5
5.0
2.5
1/SE$_{IV}$
−0.2
−0.1
0.0
0.1
0.2
β$_{IV}$

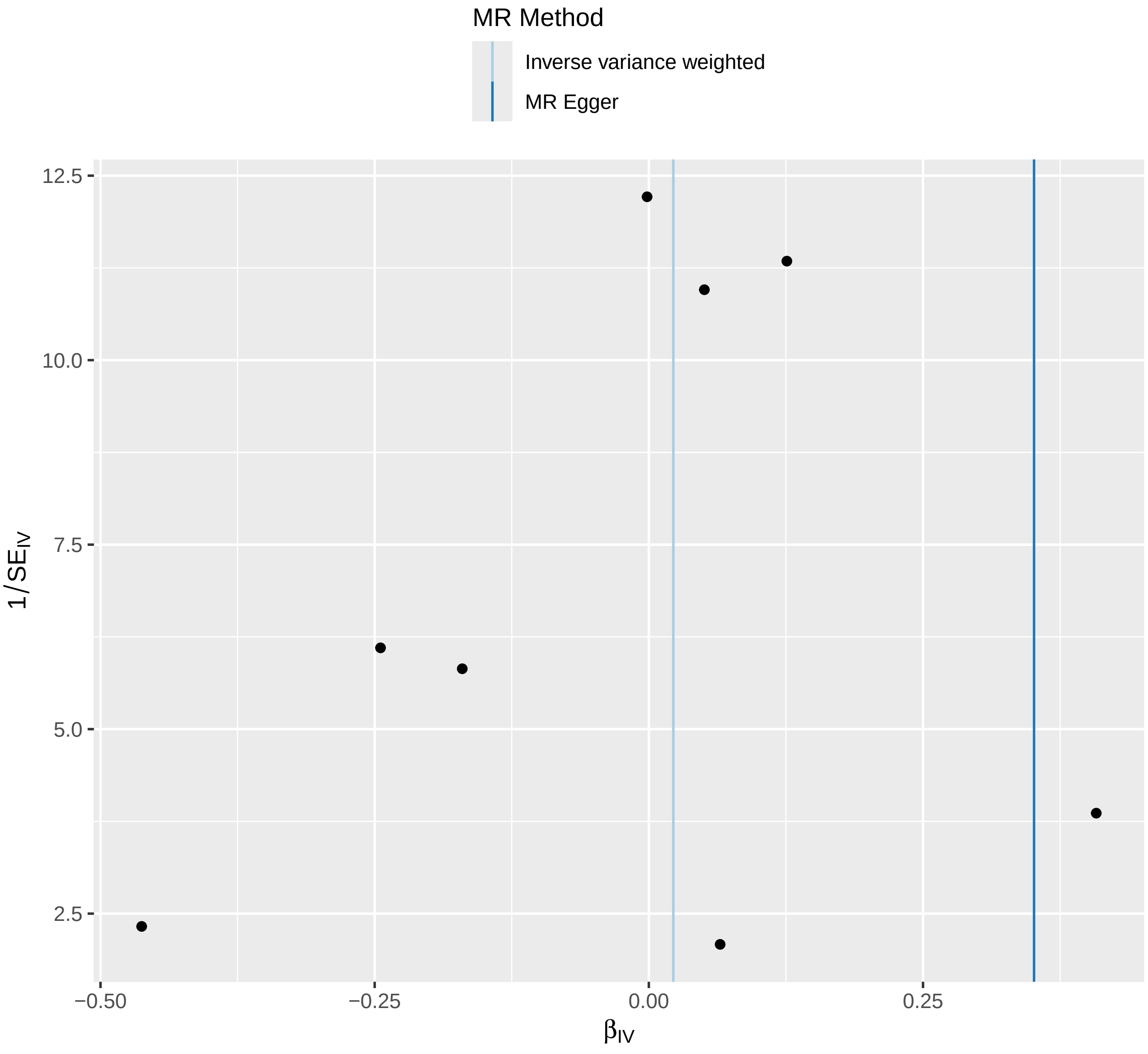

MR Method
Inverse variance weighted
MR Egger
12.5
10.0
7.5
5.0
2.5
1/SE_IV
−0.50
−0.25
0.00
0.25
β_IV

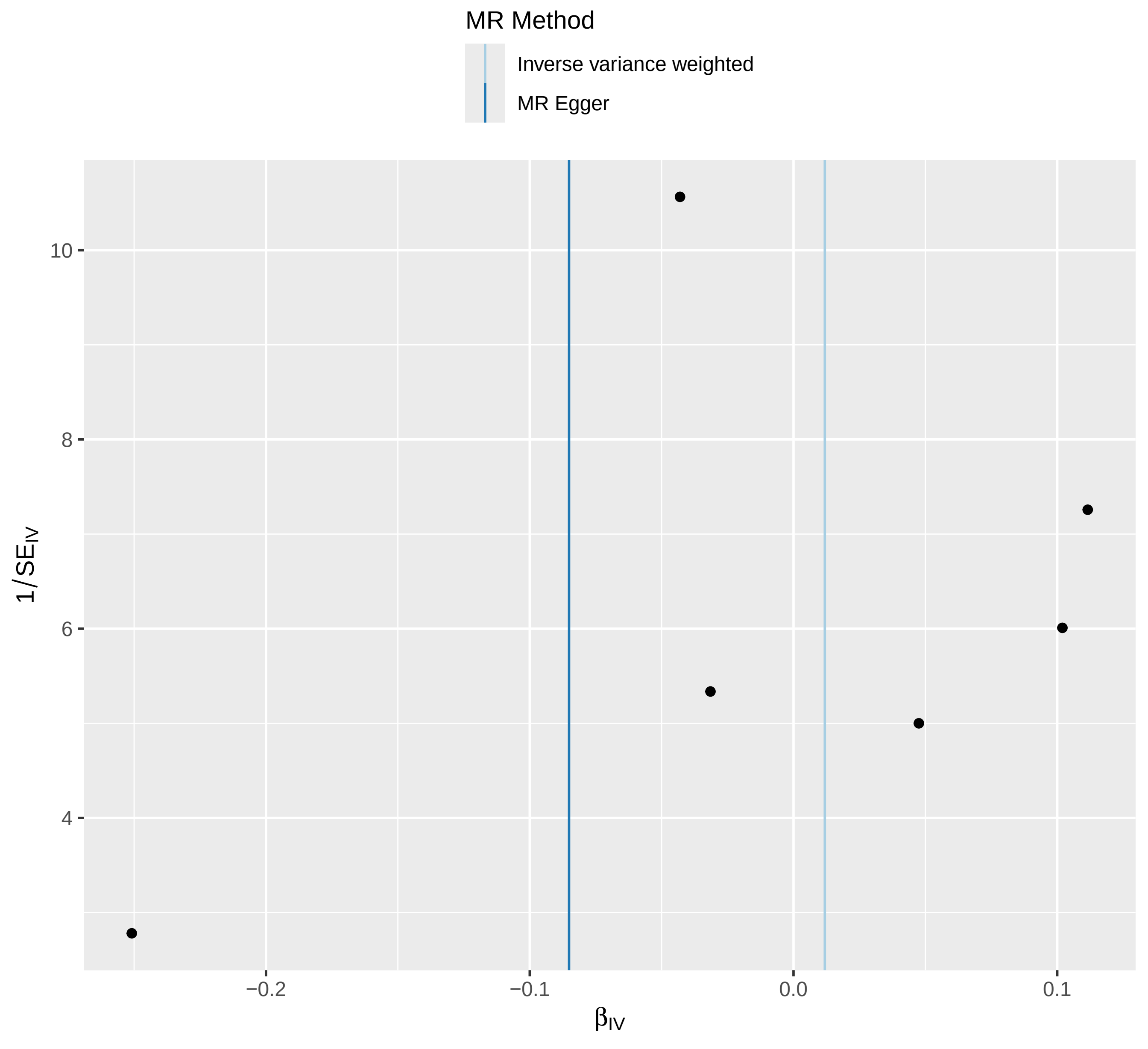
MR Method
Inverse variance weighted
MR Egger
10
8
6
4
1/SE$_{IV}$
−0.2
−0.1
0.0
0.1
β$_{IV}$

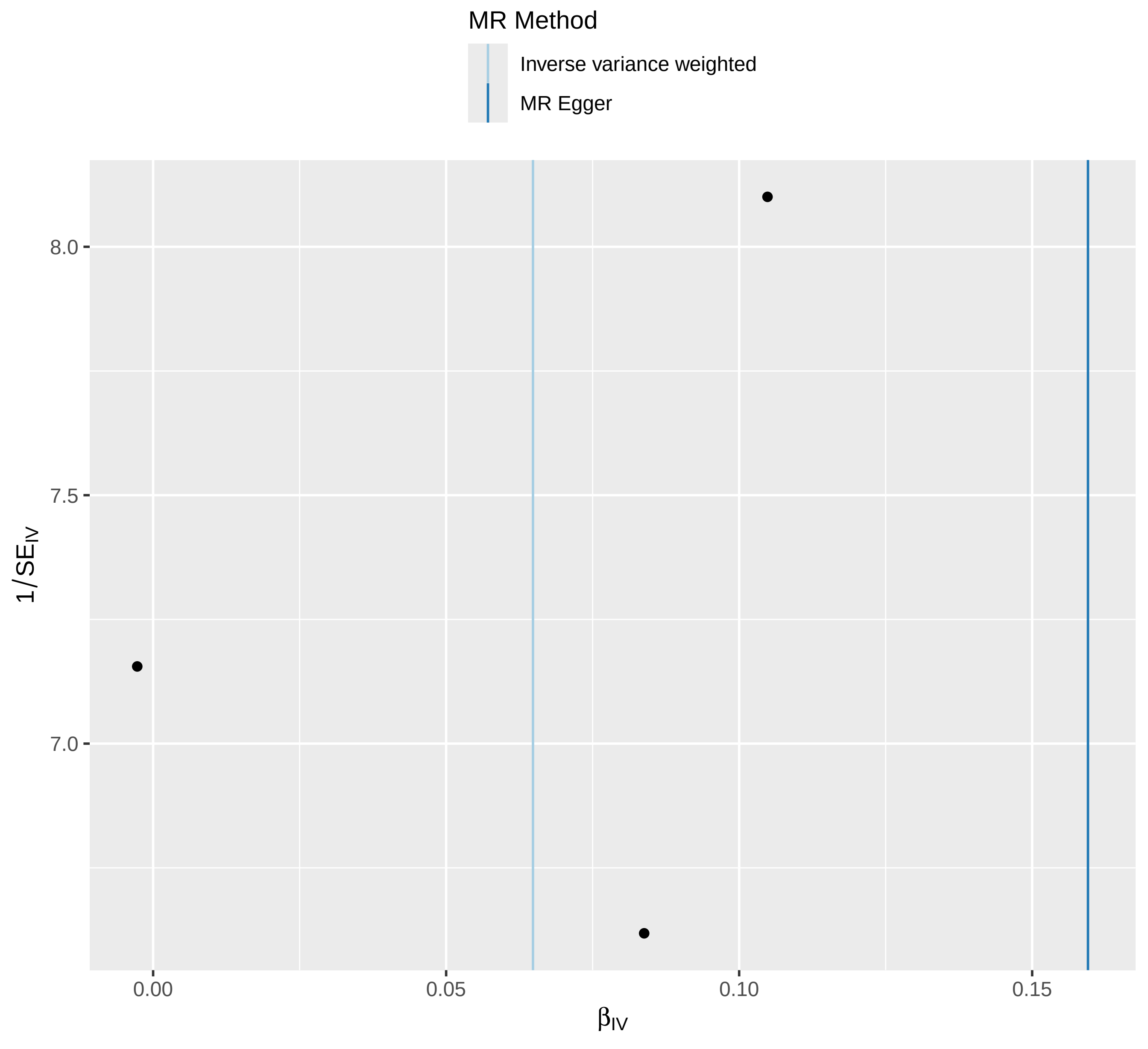
MR Method
Inverse variance weighted
MR Egger
8.0
7.5
7.0
0.00
0.05
0.10
0.15
1/SE_IV
β_IV

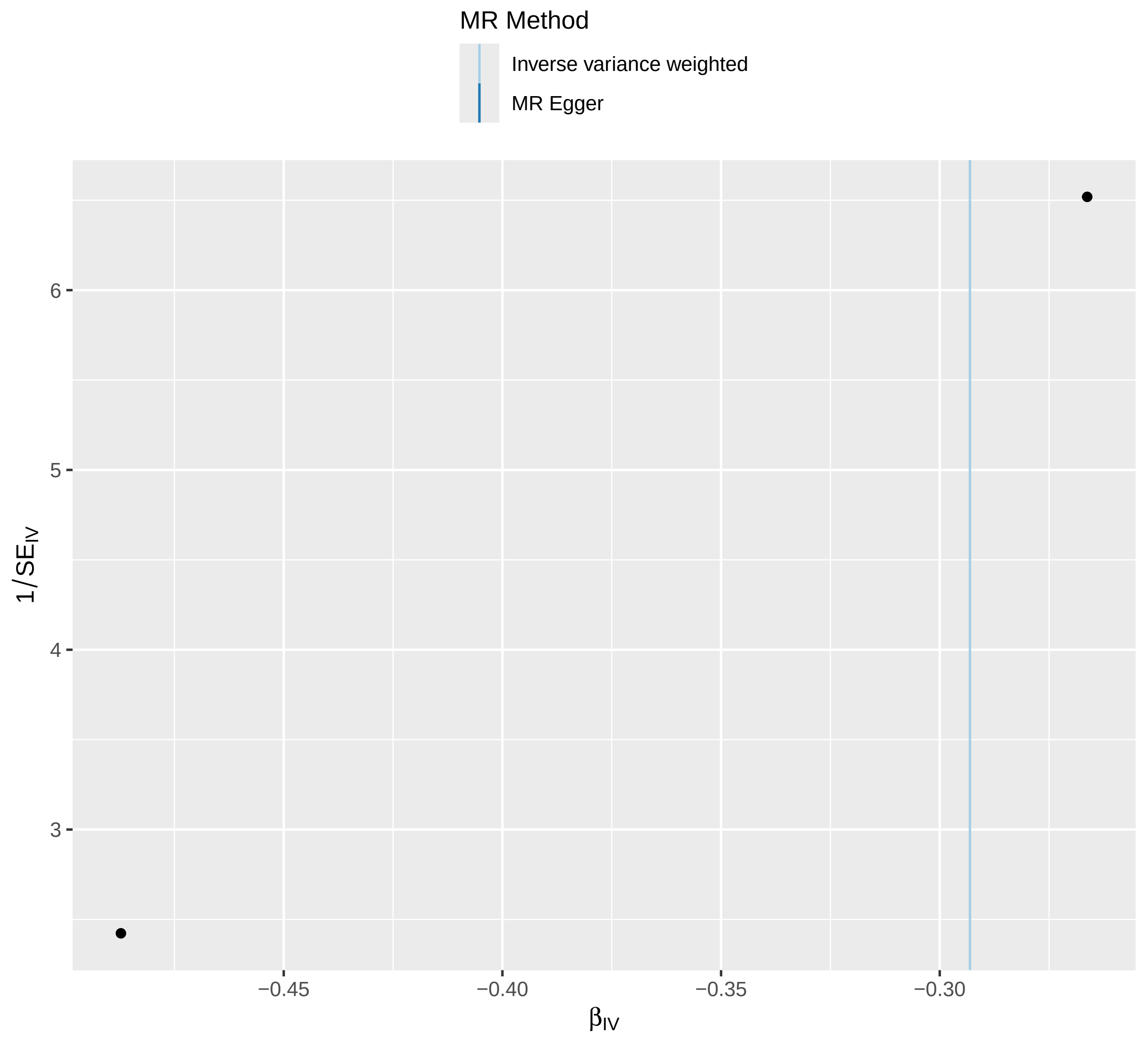
MR Method
Inverse variance weighted
MR Egger
6
5
4
3
1/SE$_{IV}$
−0.45
−0.40
−0.35
−0.30
β$_{IV}$

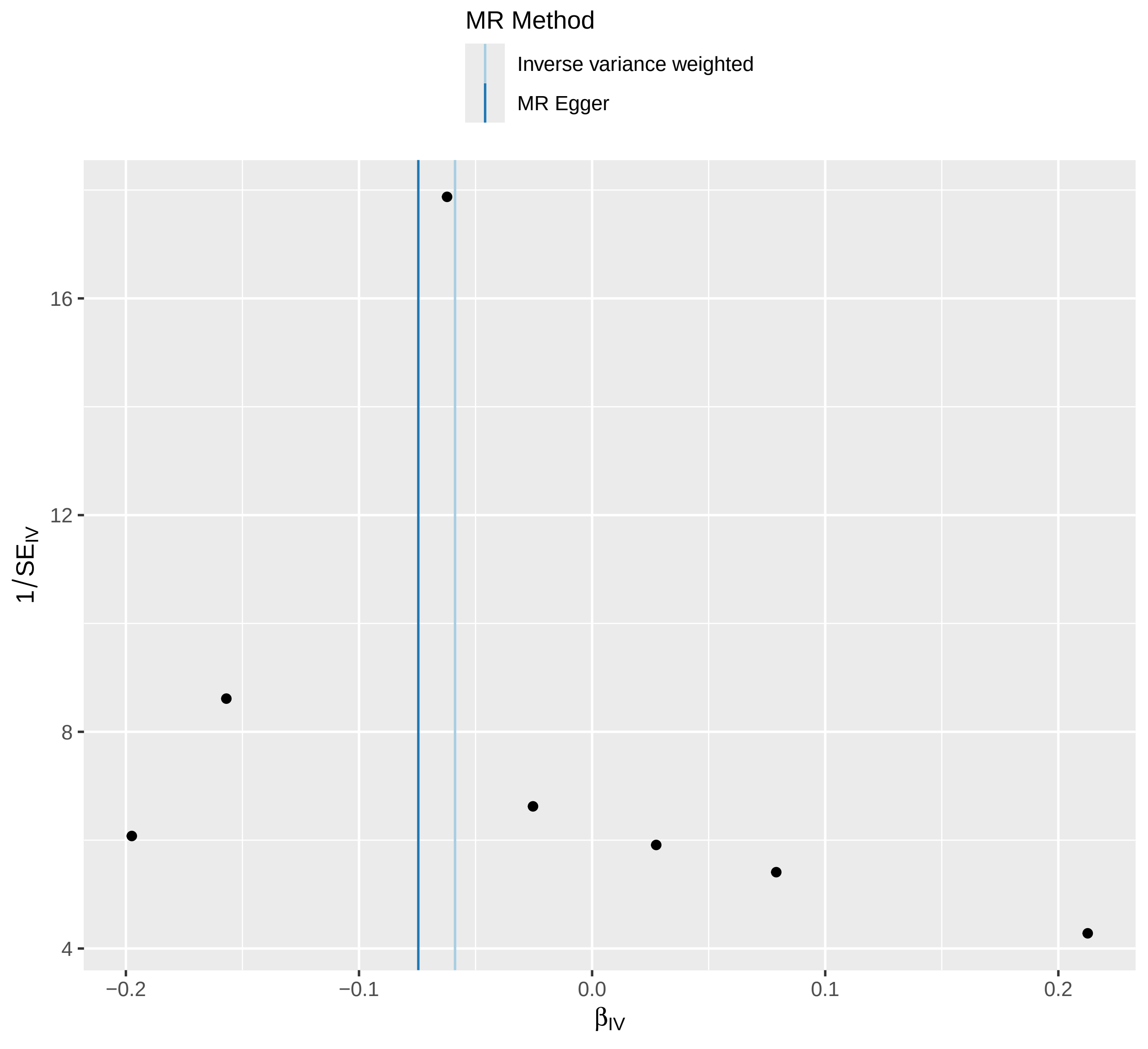

MR Method
Inverse variance weighted
MR Egger
16
12
8
4
$1/SE_{IV}$
−0.2
−0.1
0.0
0.1
0.2
$\beta_{IV}$

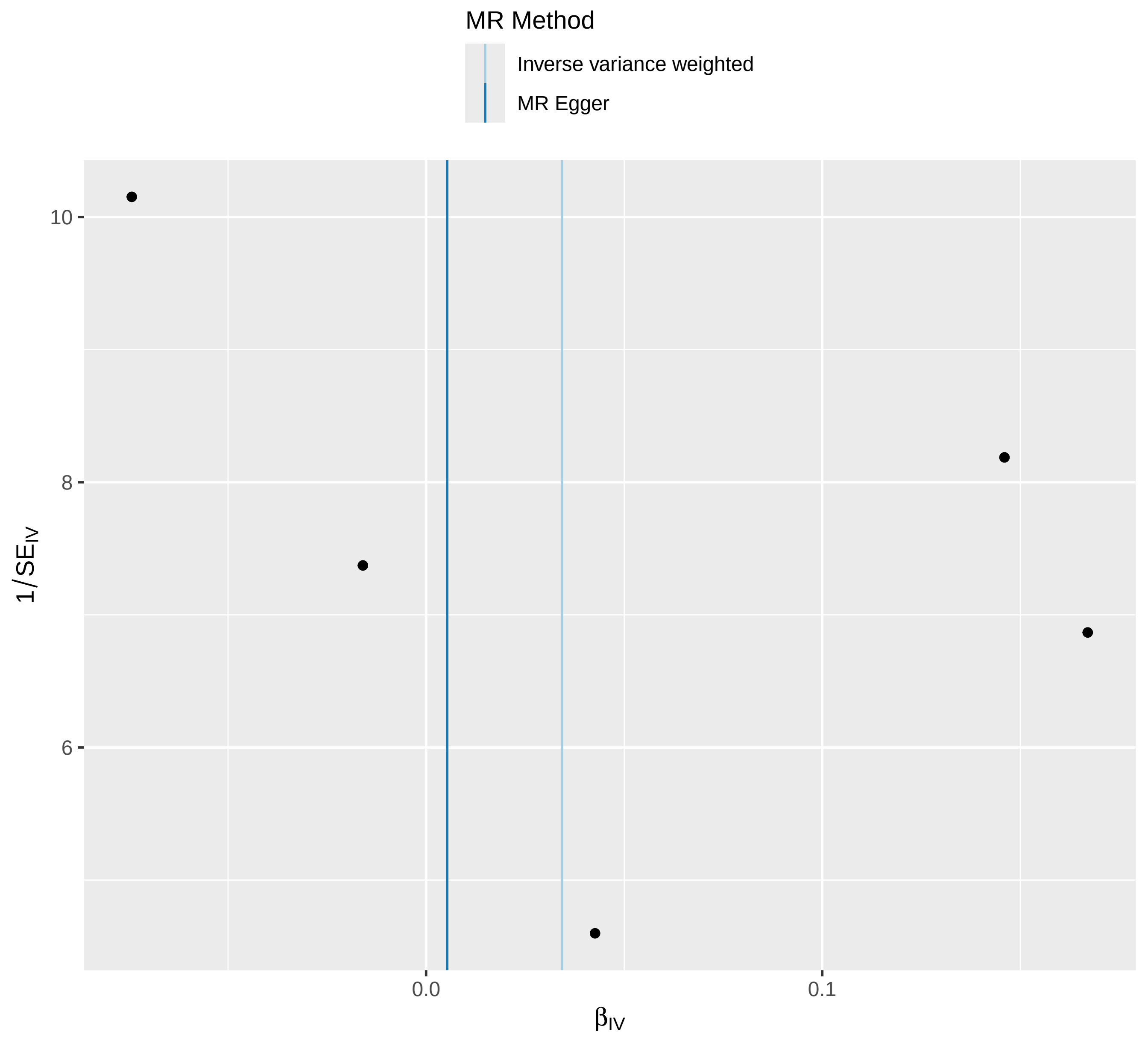
MR Method
Inverse variance weighted
MR Egger
10
8
6
1/SE_IV
0.0
0.1
β_IV

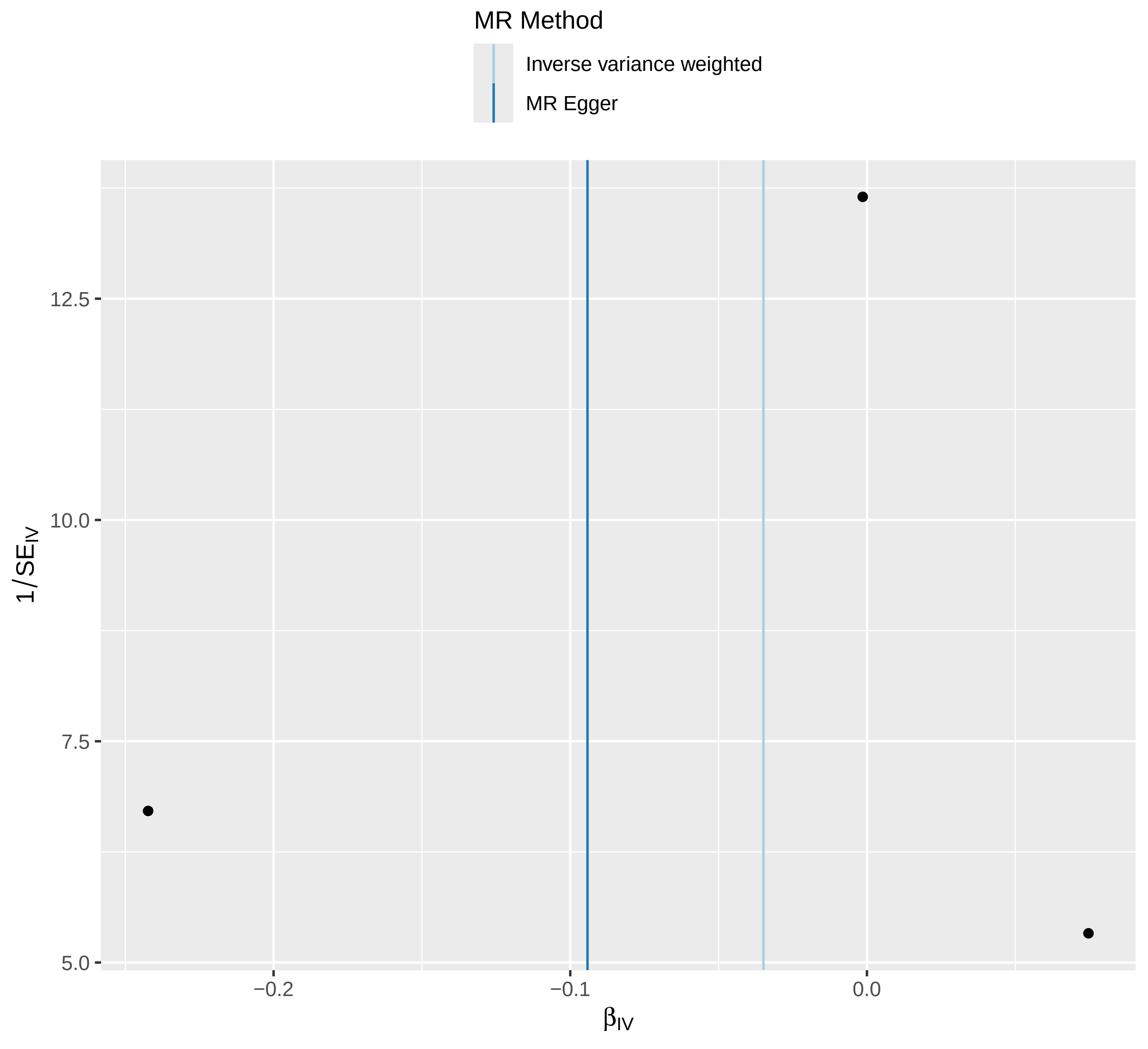

MR Method
Inverse variance weighted
MR Egger
12.5
10.0
7.5
5.0
1/SE_IV
−0.2
−0.1
0.0
β_IV

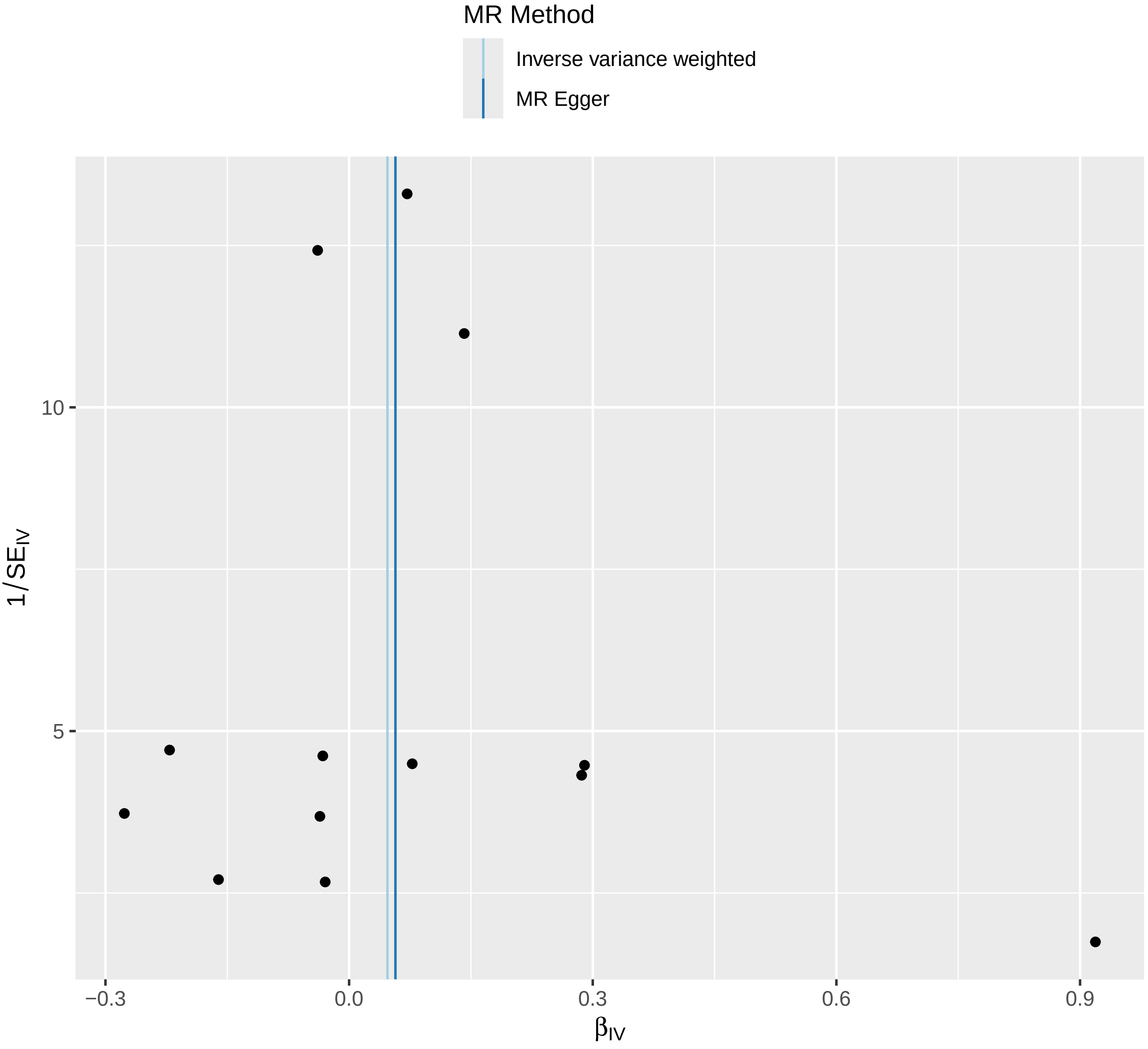

MR Method
Inverse variance weighted
MR Egger
10
5
$1/SE_{IV}$
−0.3
0.0
0.3
0.6
0.9
$\beta_{IV}$

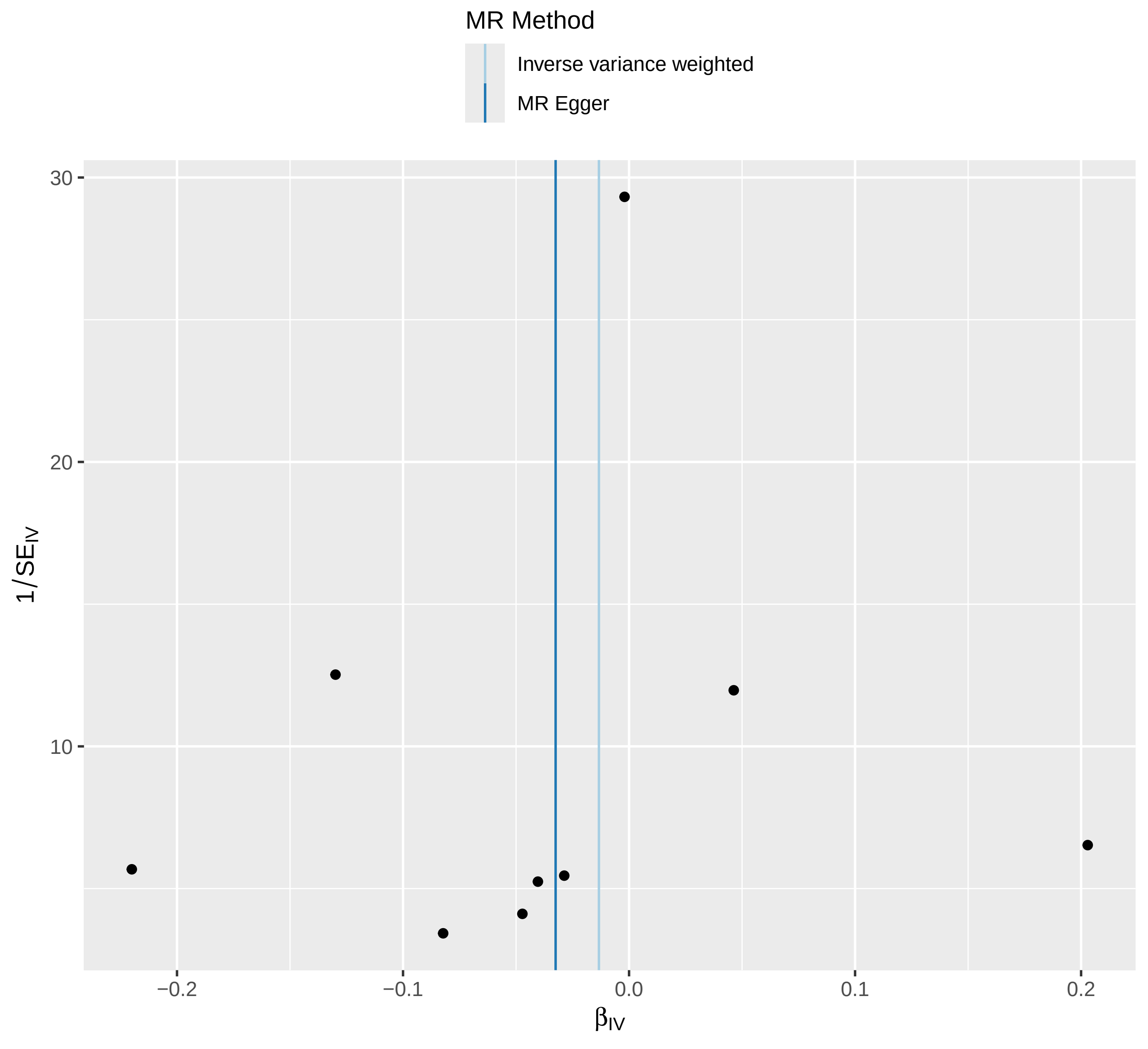
MR Method
Inverse variance weighted
MR Egger
30
20
10
$1/SE_{IV}$
−0.2
−0.1
0.0
0.1
0.2
$\beta_{IV}$

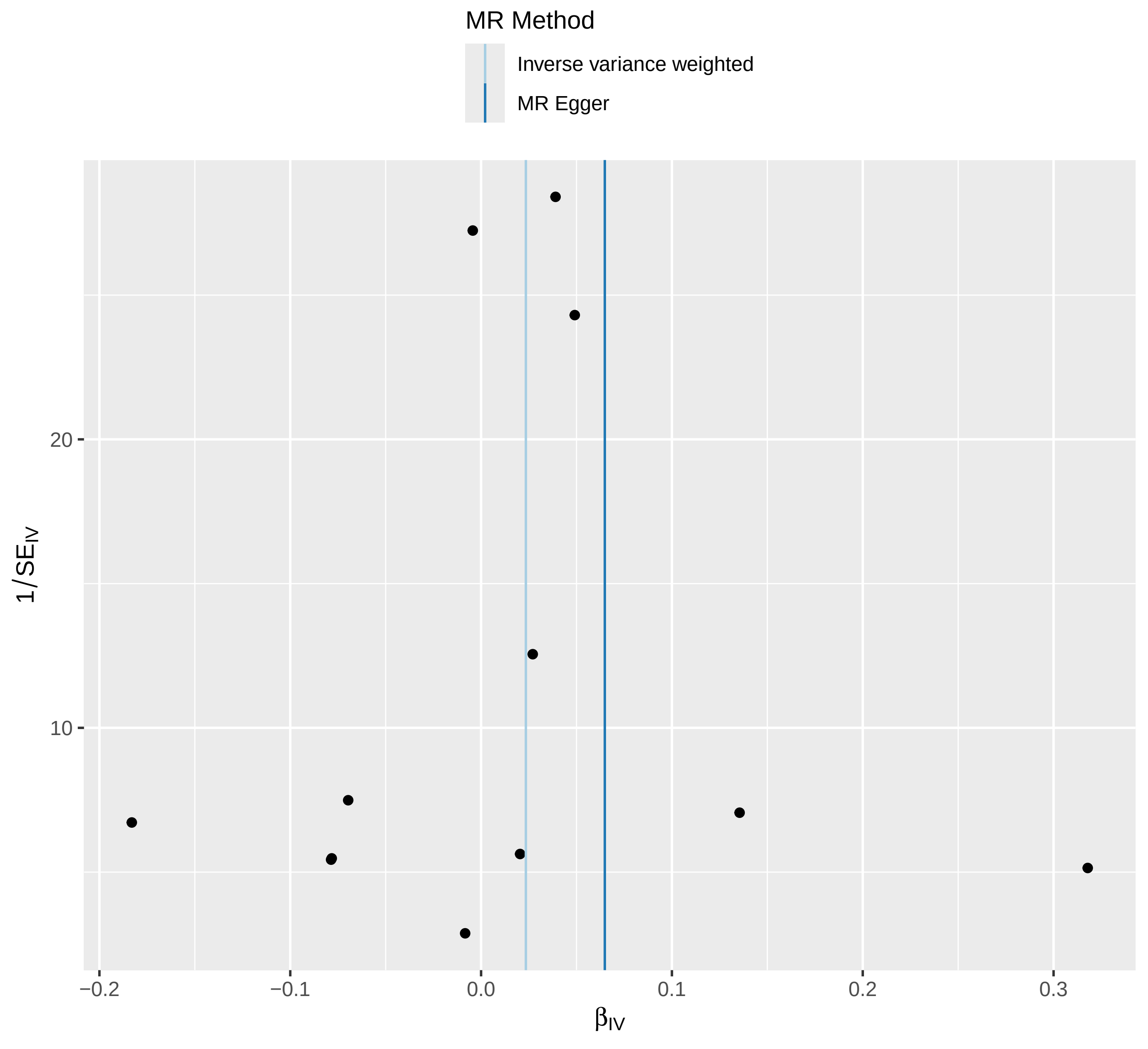

MR Method
Inverse variance weighted
MR Egger
20
10
$1/SE_{IV}$
−0.2
−0.1
0.0
0.1
0.2
0.3
$\beta_{IV}$

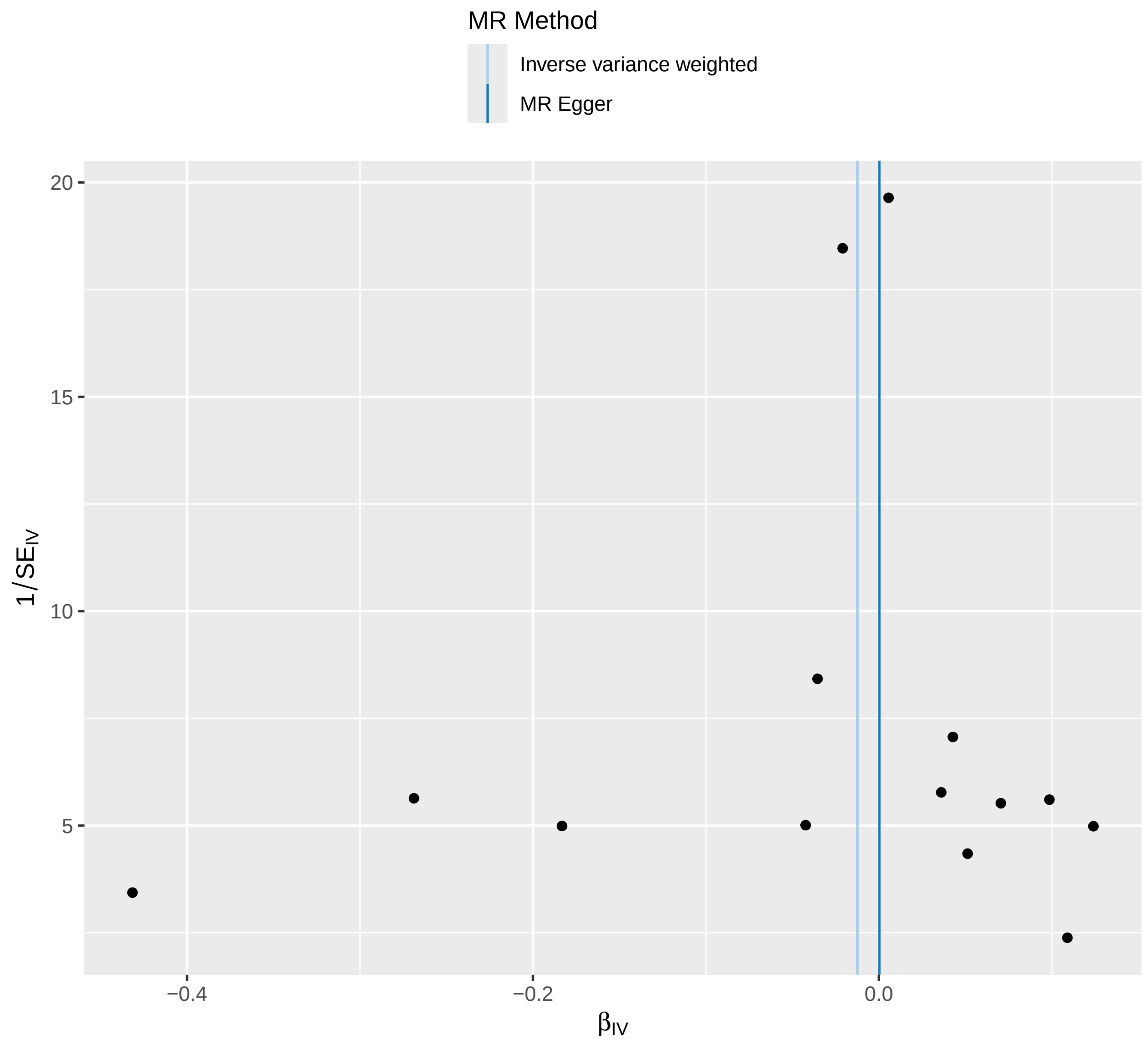

MR Method
Inverse variance weighted
MR Egger
20
15
10
5
−0.4
−0.2
0.0
$1/SE_{IV}$
$\beta_{IV}$

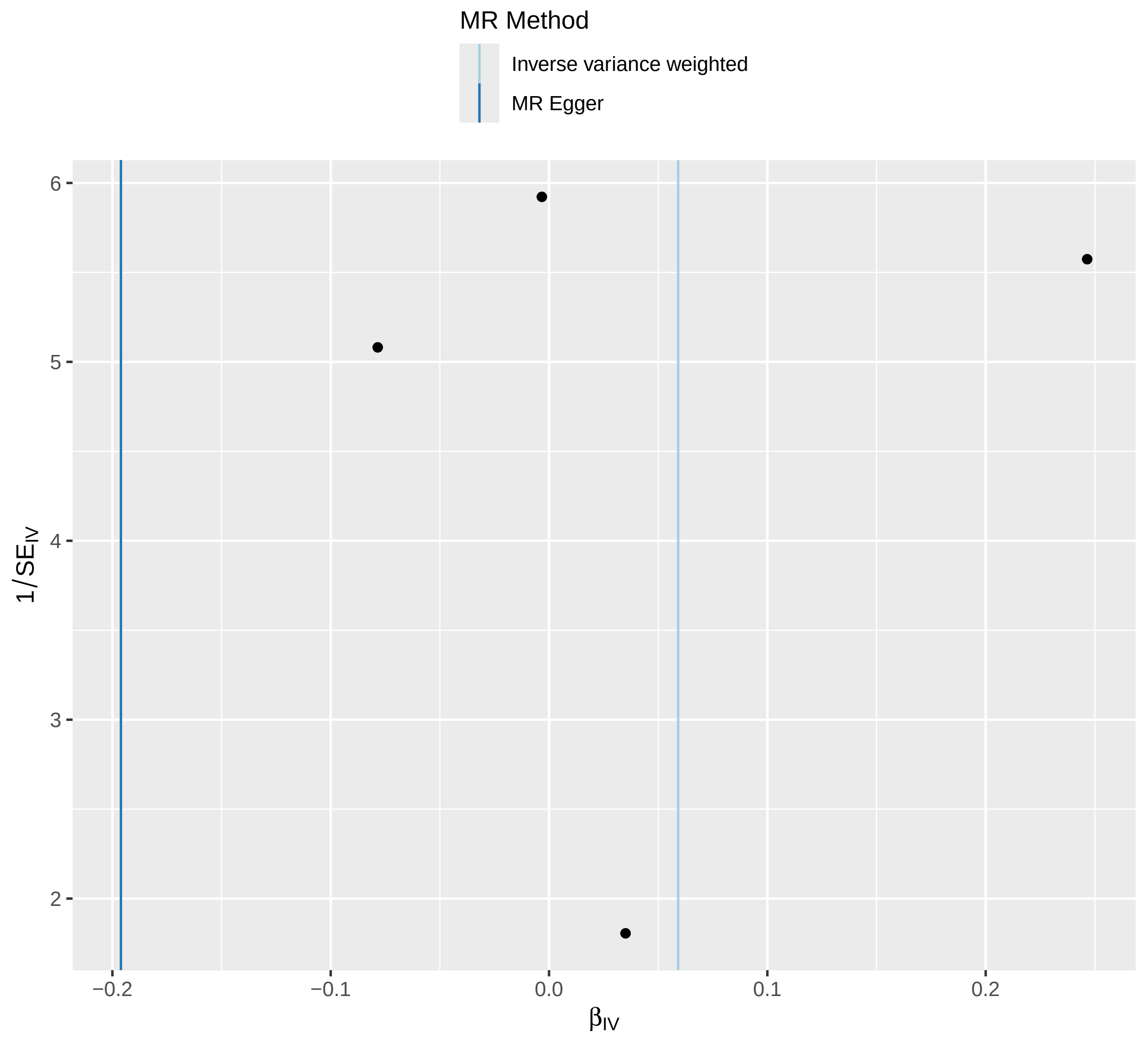
MR Method
Inverse variance weighted
MR Egger
1/SE$_{IV}$
6
5
4
3
2
−0.2
−0.1
0.0
0.1
0.2
β$_{IV}$

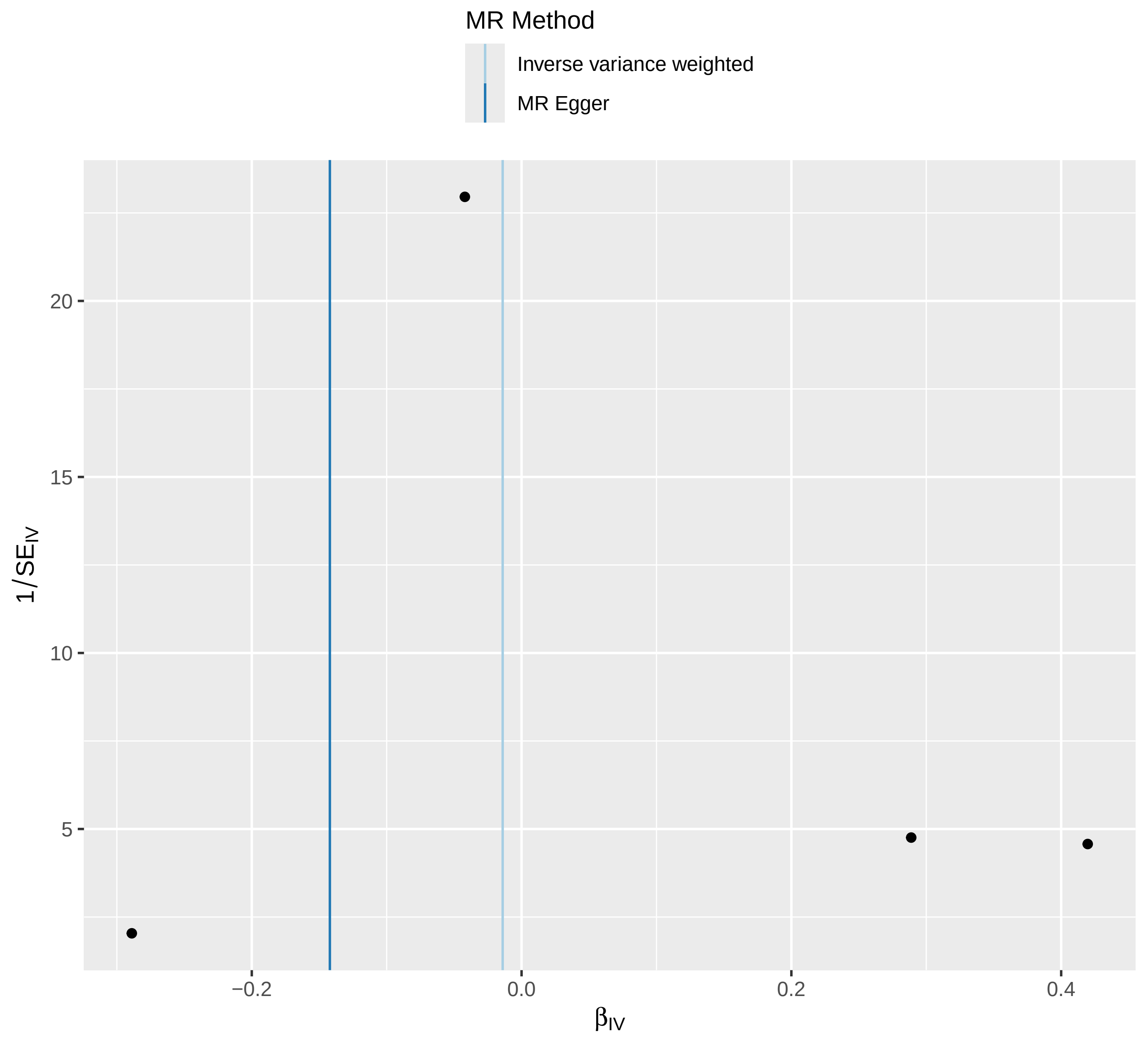

MR Method
Inverse variance weighted
MR Egger
20
15
10
5
1/SE_IV
−0.2
0.0
0.2
0.4
β_IV

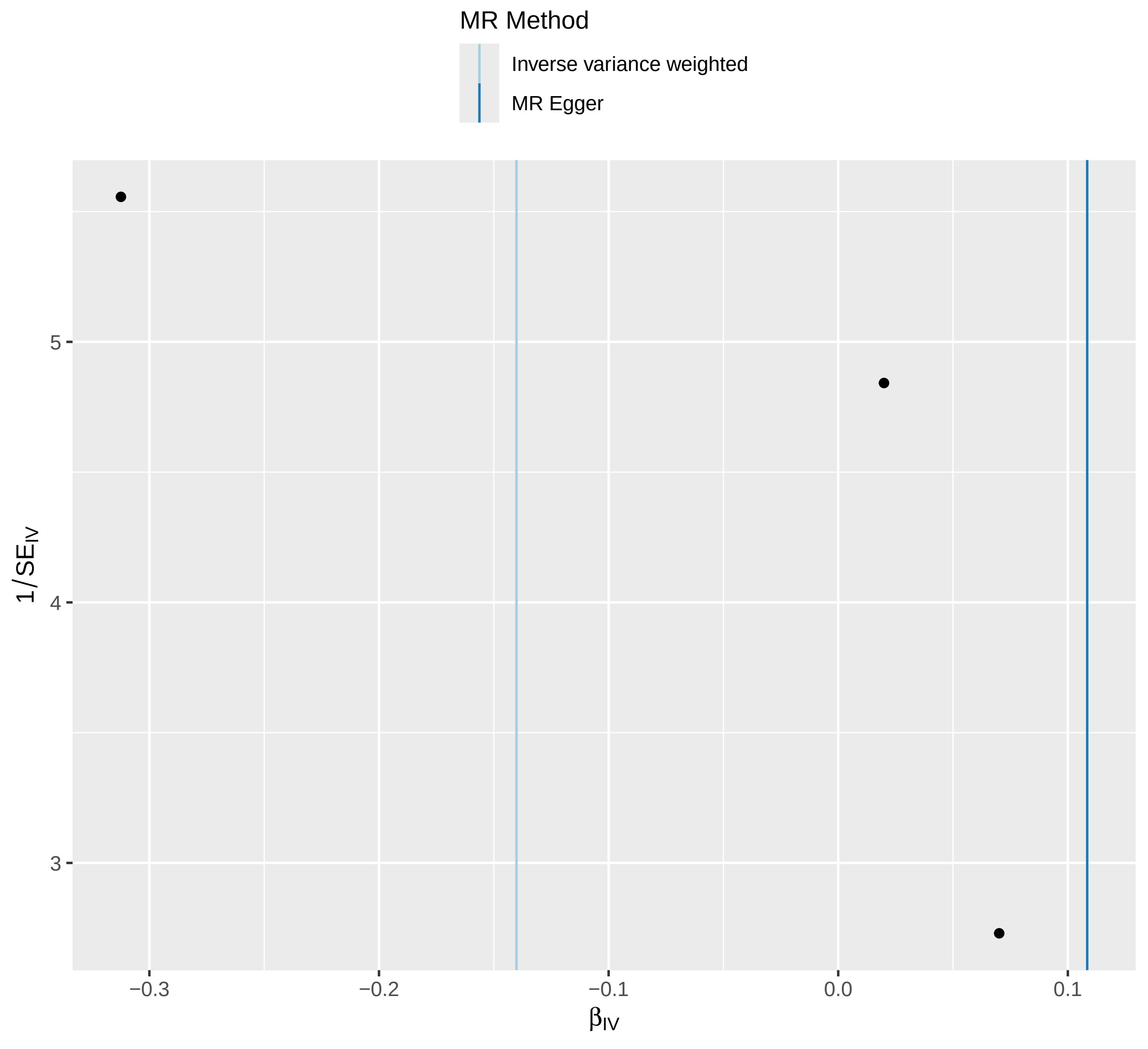

MR Method
Inverse variance weighted
MR Egger
5
4
3
1/SE_IV
−0.3
−0.2
−0.1
0.0
0.1
β_IV

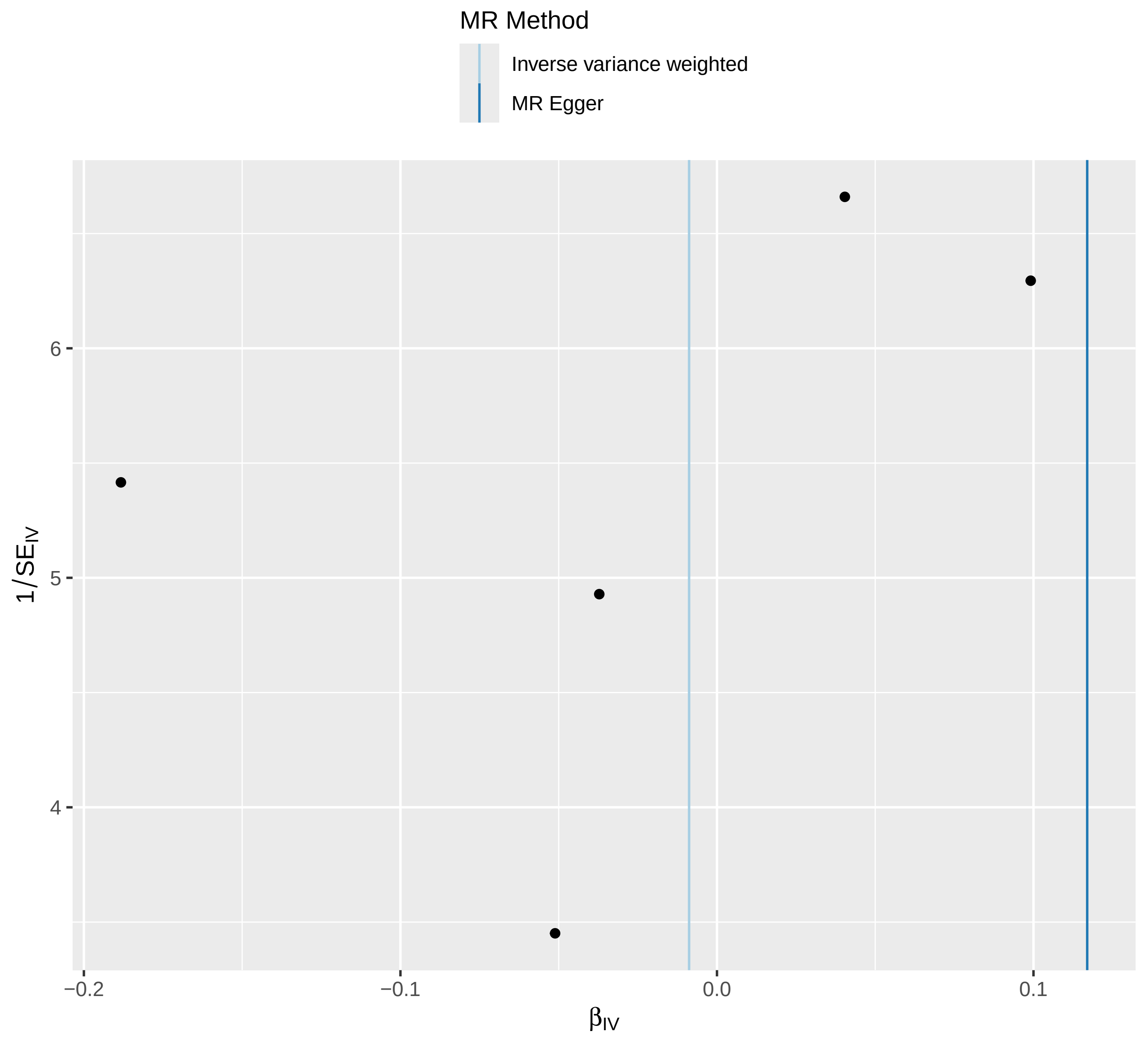
MR Method
Inverse variance weighted
MR Egger
6
5
4
1/SE$_{IV}$
−0.2
−0.1
0.0
0.1
β$_{IV}$

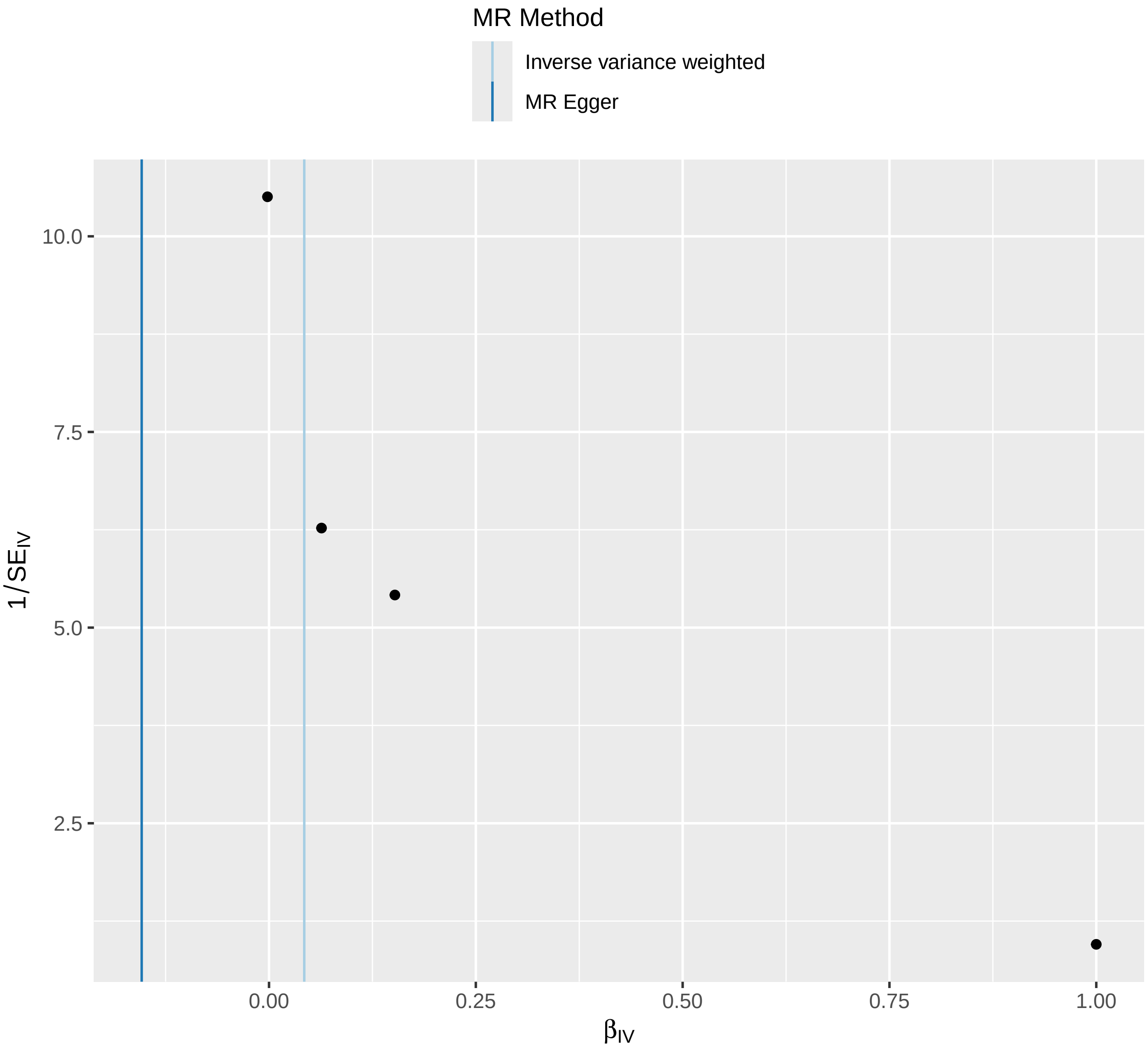

MR Method
Inverse variance weighted
MR Egger
10.0
7.5
5.0
2.5
1/SE_IV
0.00
0.25
0.50
0.75
1.00
β_IV

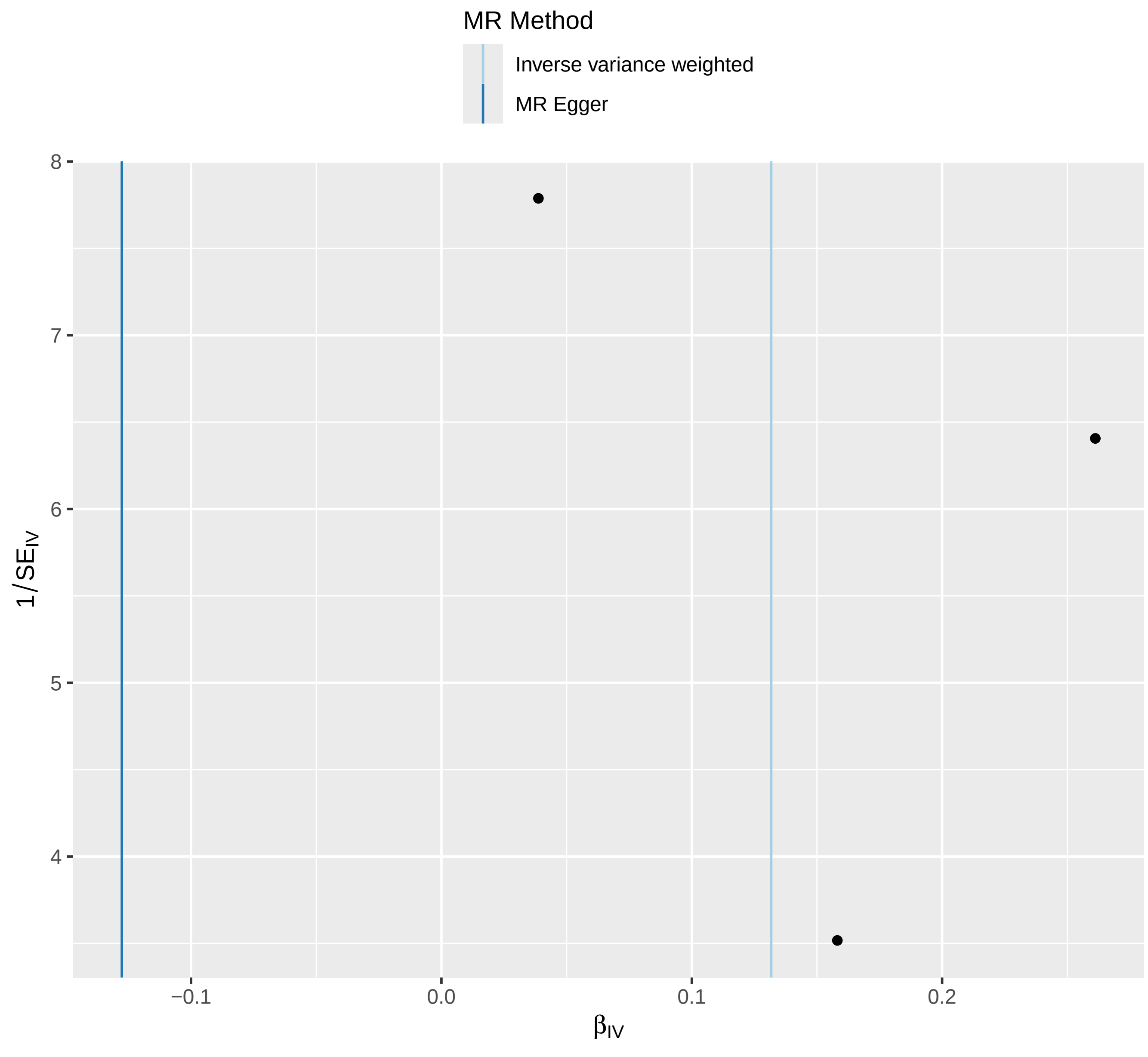

MR Method
Inverse variance weighted
MR Egger
8
7
6
5
4
1/SE_IV
−0.1
0.0
0.1
0.2
β_IV

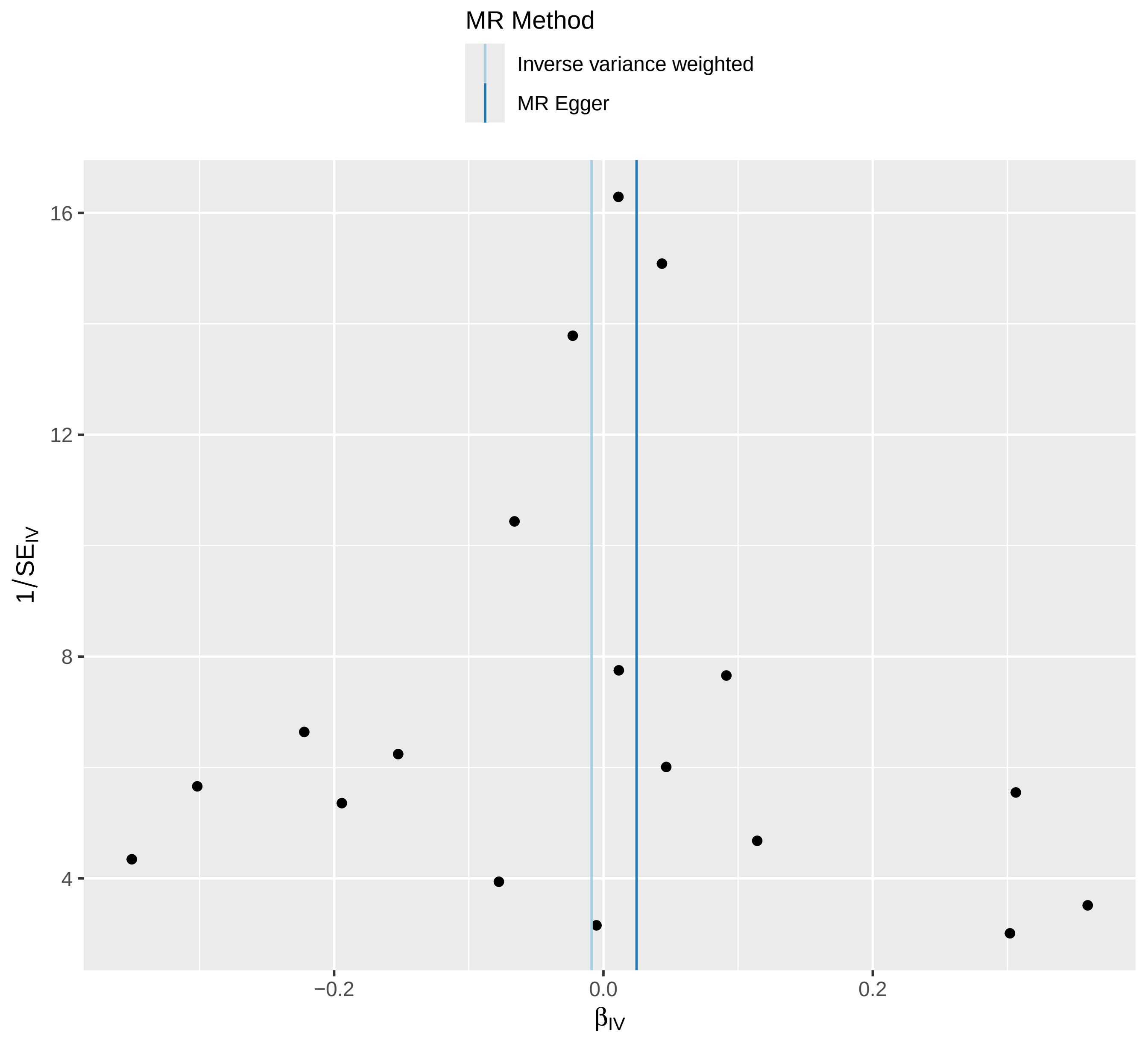

MR Method
Inverse variance weighted
MR Egger
16
12
8
4
1/SE$_{IV}$
−0.2
0.0
0.2
β$_{IV}$

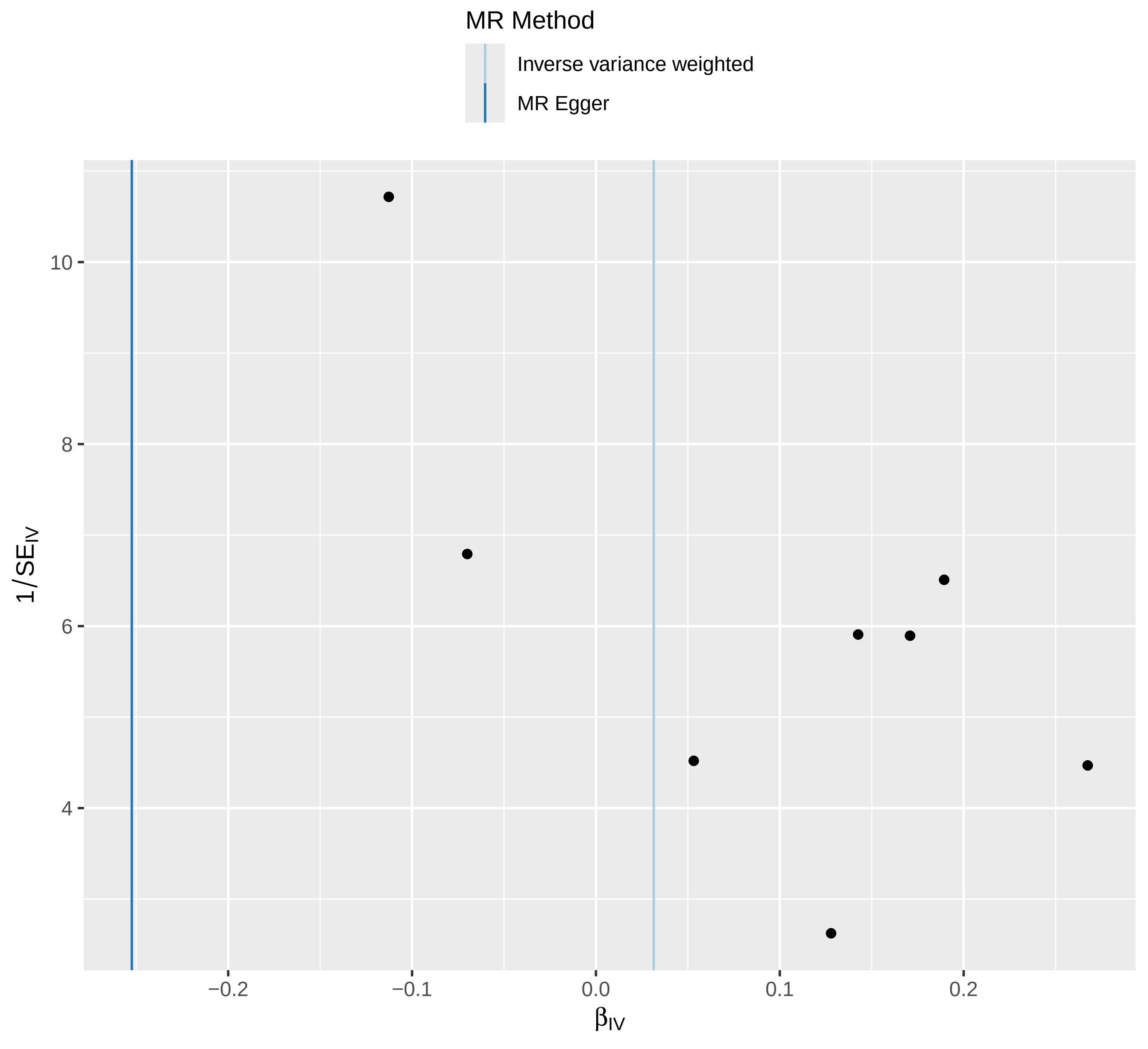
MR Method
Inverse variance weighted
MR Egger
1/SE_IV
10
8
6
4
−0.2
−0.1
0.0
0.1
0.2
β_IV

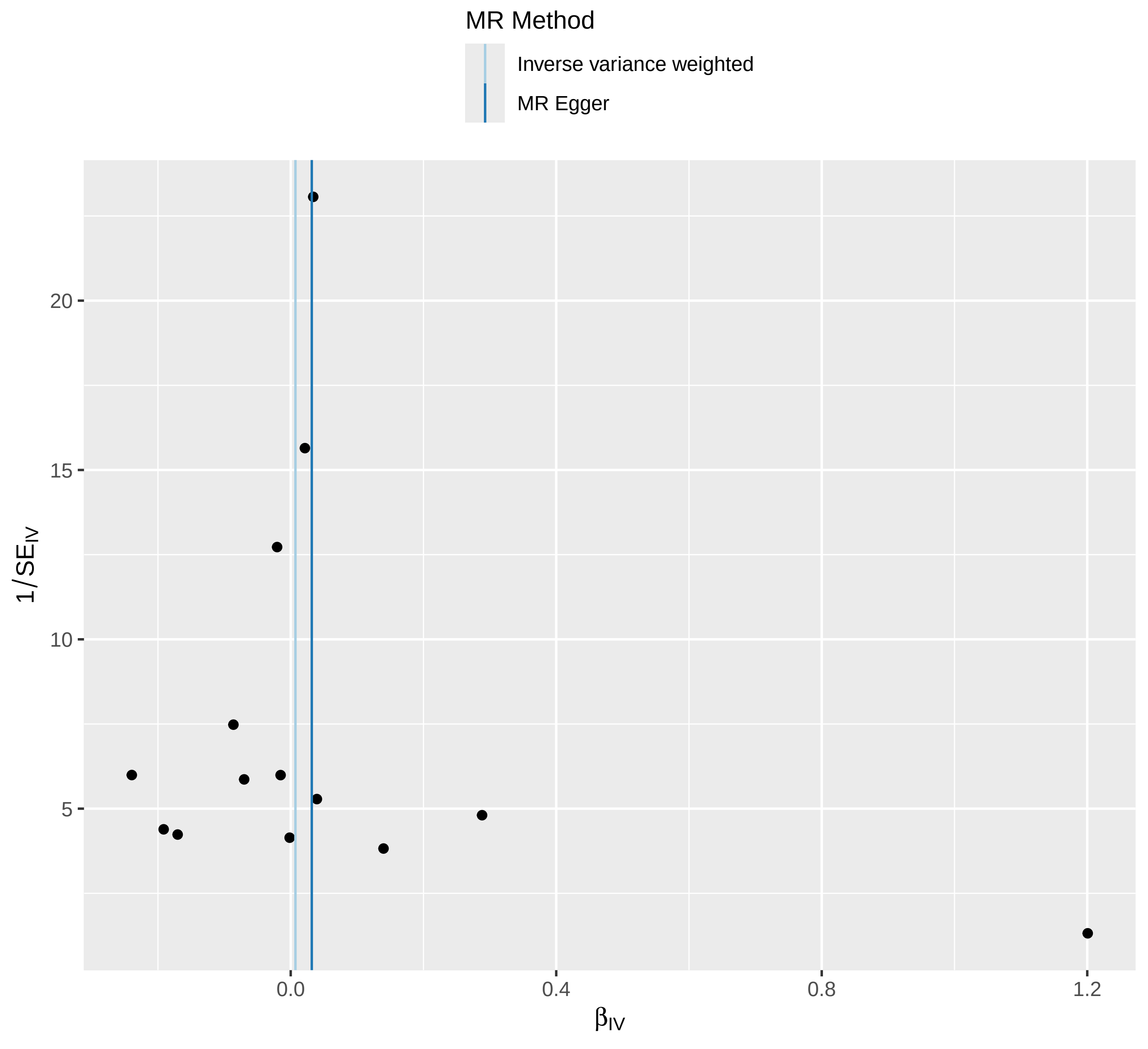

MR Method
Inverse variance weighted
MR Egger
20
15
10
5
1/SE_IV
0.0
0.4
0.8
1.2
β_IV

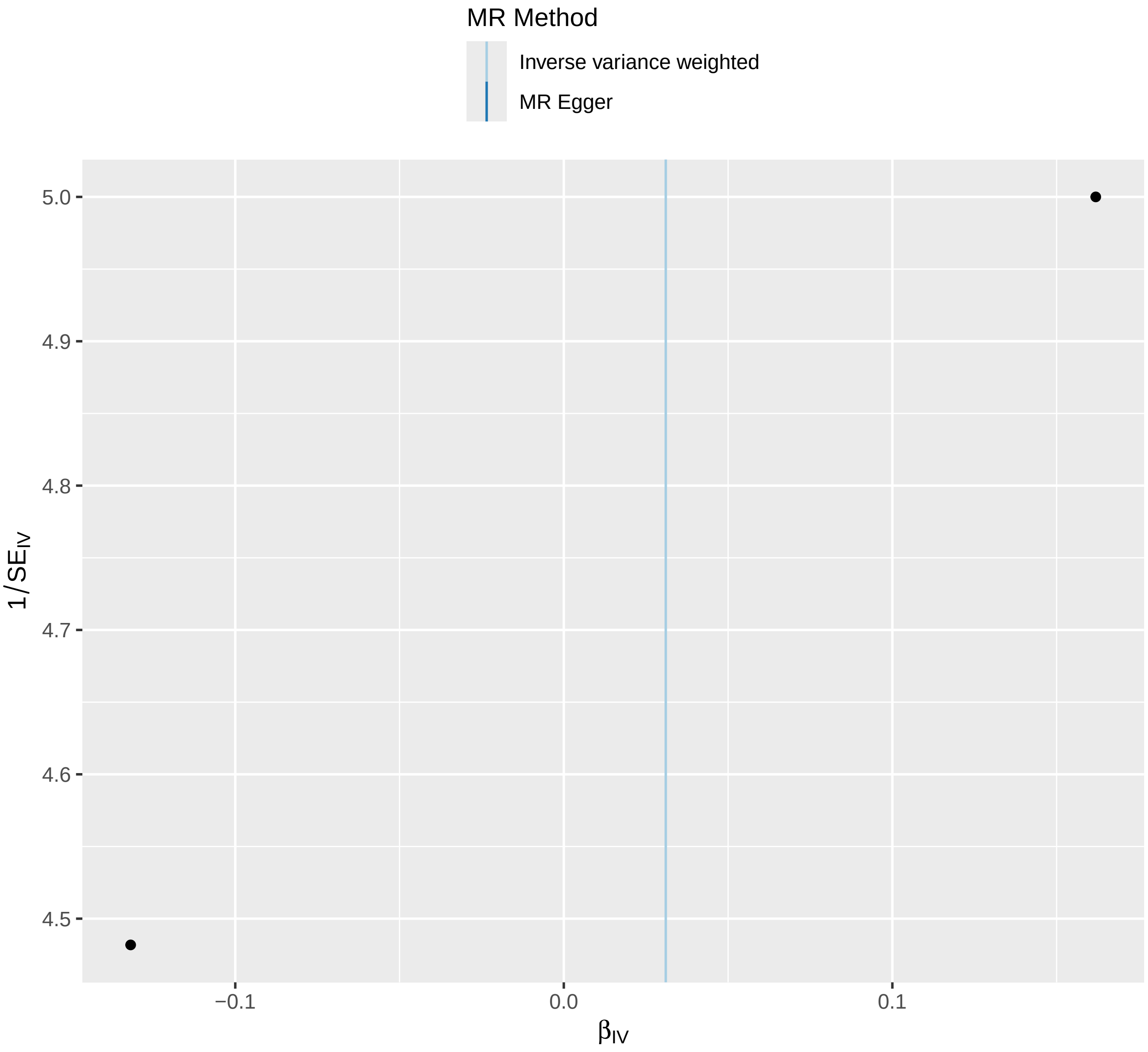
MR Method
Inverse variance weighted
MR Egger
5.0
4.9
4.8
4.7
4.6
4.5
1/SE_IV
−0.1
0.0
0.1
β_IV

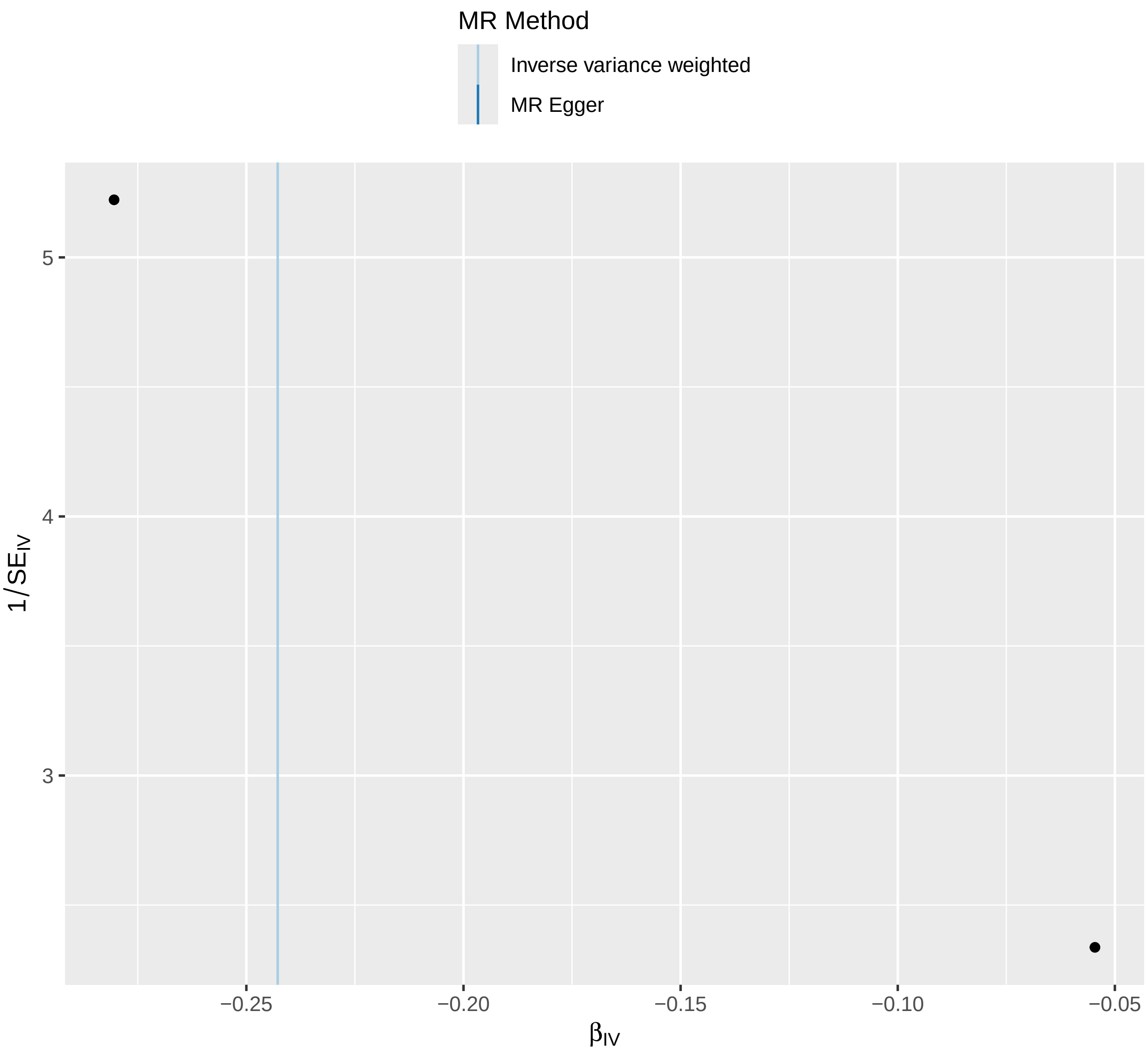
MR Method
Inverse variance weighted
MR Egger
5
4
3
1/SE_IV
−0.25
−0.20
−0.15
−0.10
−0.05
β_IV

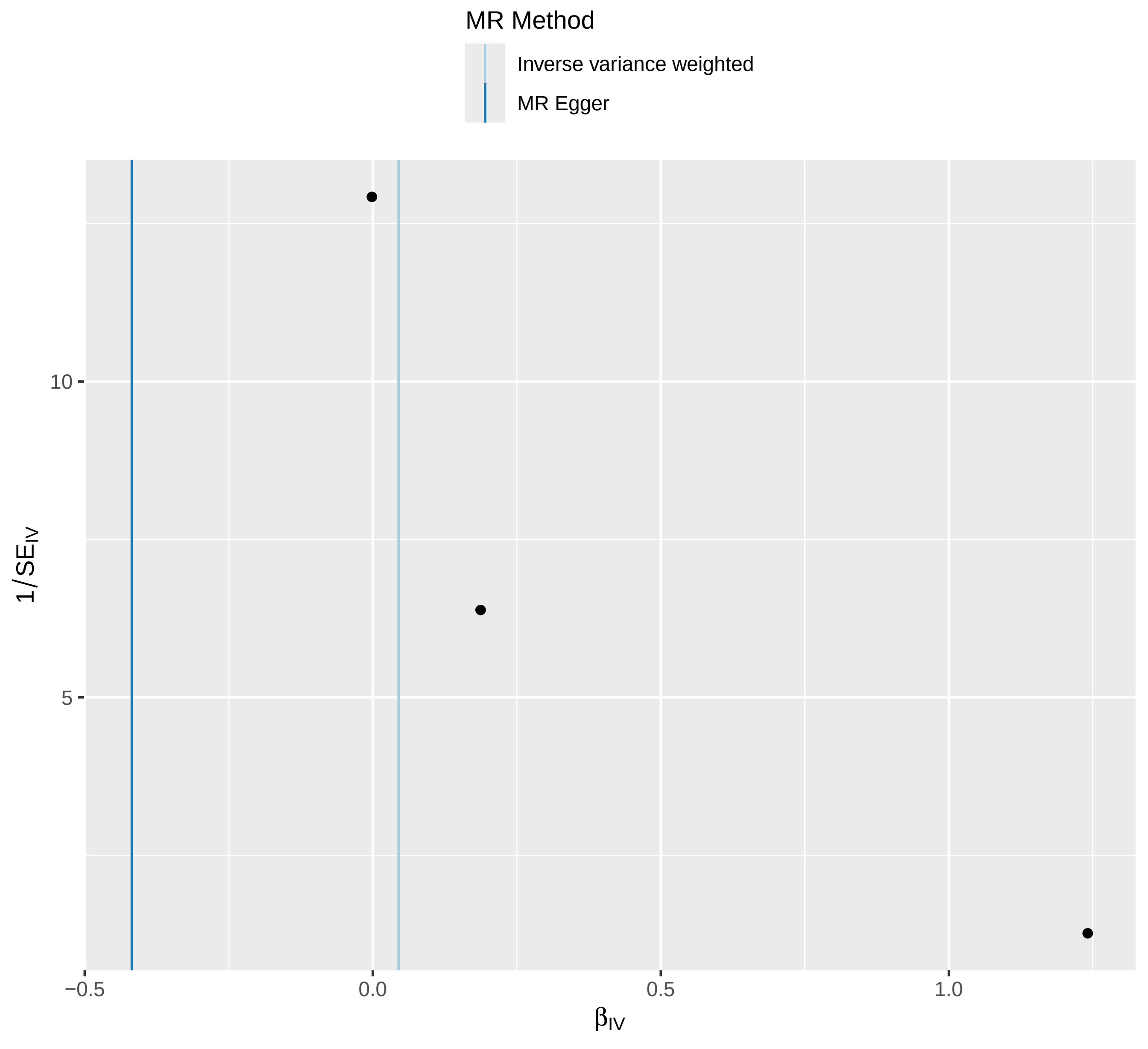
MR Method
Inverse variance weighted
MR Egger
10
5
1/SE_IV
−0.5
0.0
0.5
1.0
β_IV

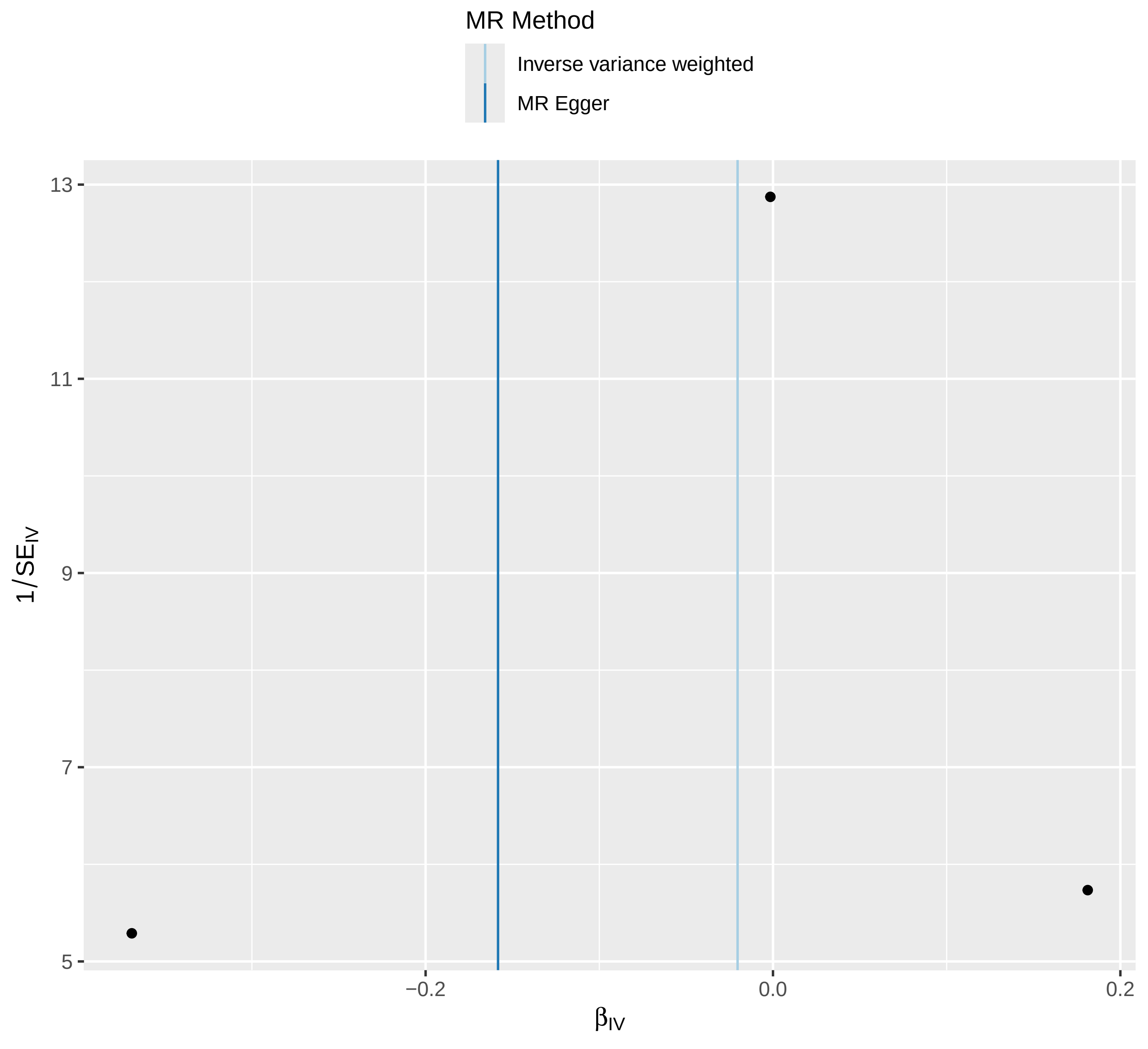
MR Method
Inverse variance weighted
MR Egger
13
11
9
7
5
1/SE_IV
−0.2
0.0
0.2
β_IV

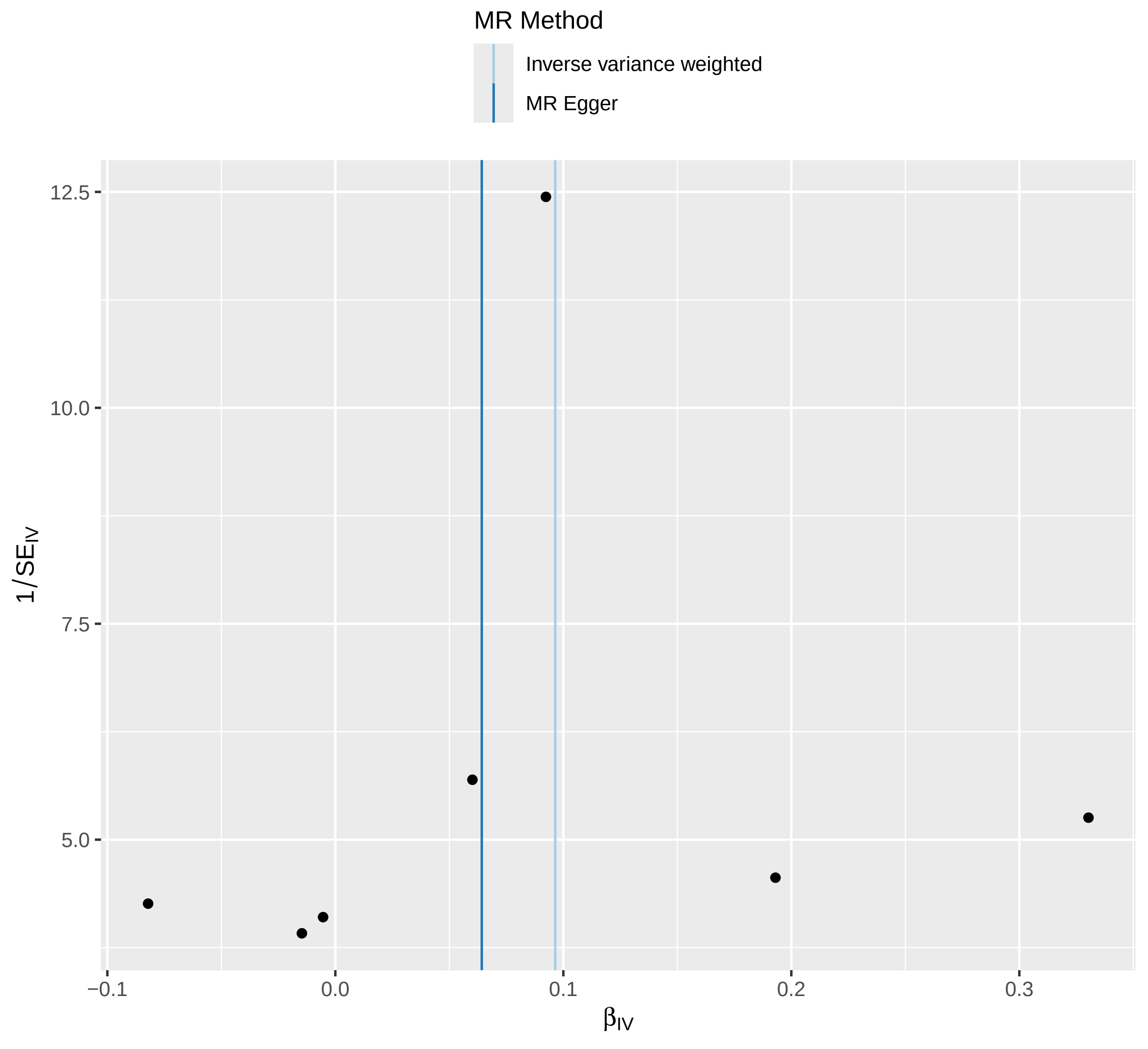
MR Method
Inverse variance weighted
MR Egger
12.5
10.0
7.5
5.0
1/SE_IV
−0.1
0.0
0.1
0.2
0.3
β_IV

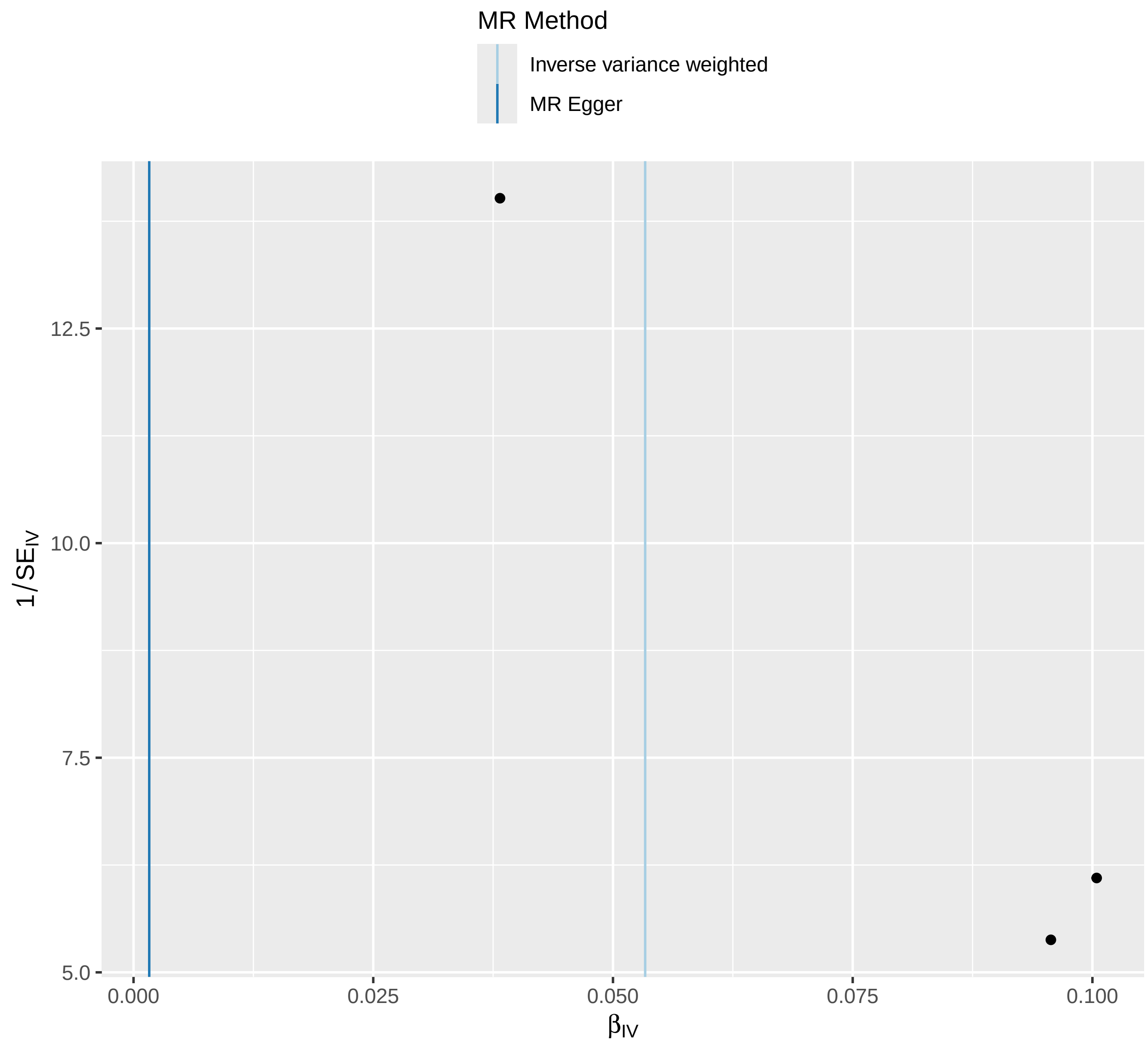

MR Method
Inverse variance weighted
MR Egger
12.5
10.0
7.5
5.0
1/SE_IV
0.000
0.025
0.050
0.075
0.100
β_IV

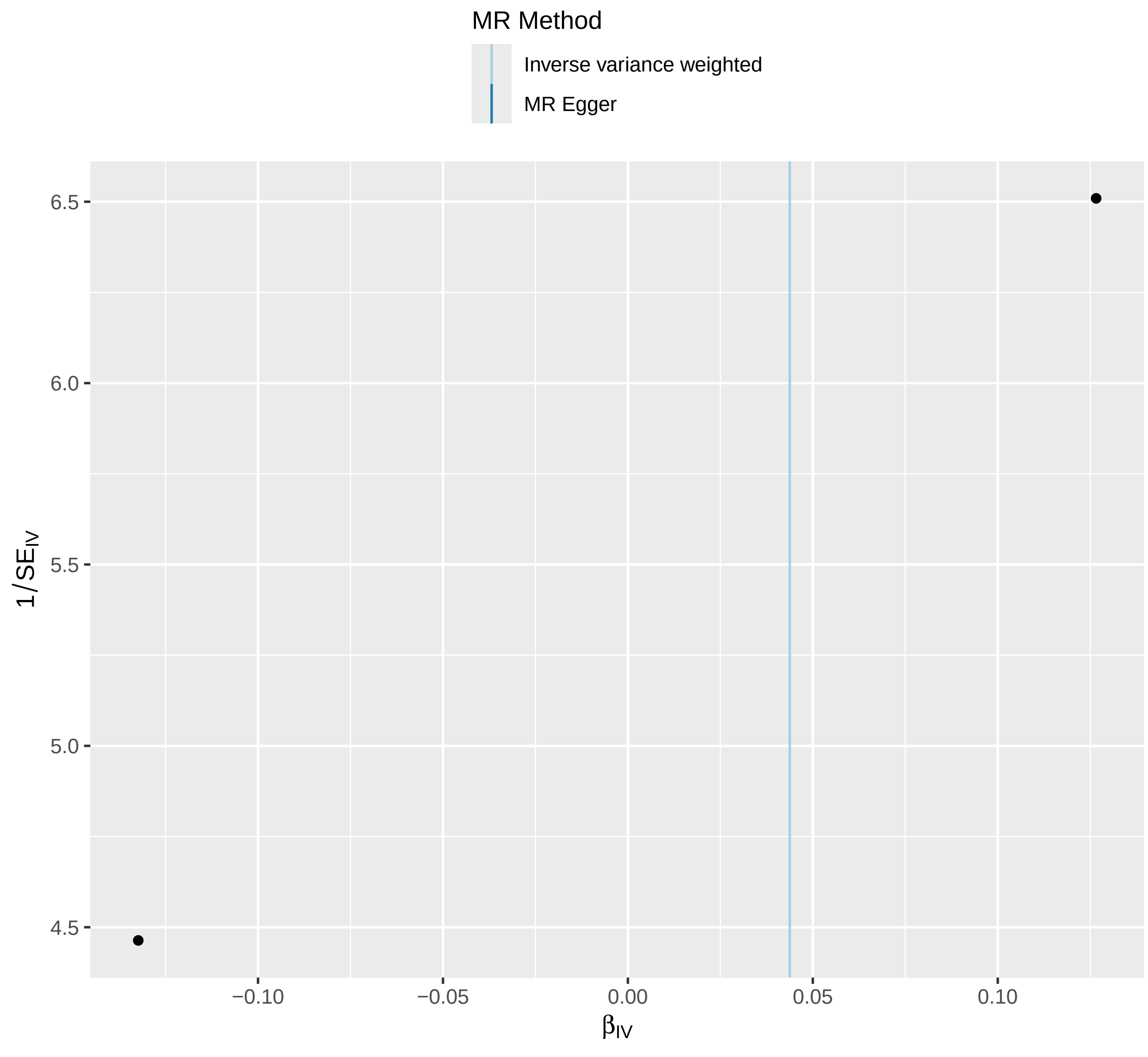

MR Method
Inverse variance weighted
MR Egger
6.5
6.0
5.5
5.0
4.5
$1/SE_{IV}$
−0.10
−0.05
0.00
0.05
0.10
$\beta_{IV}$

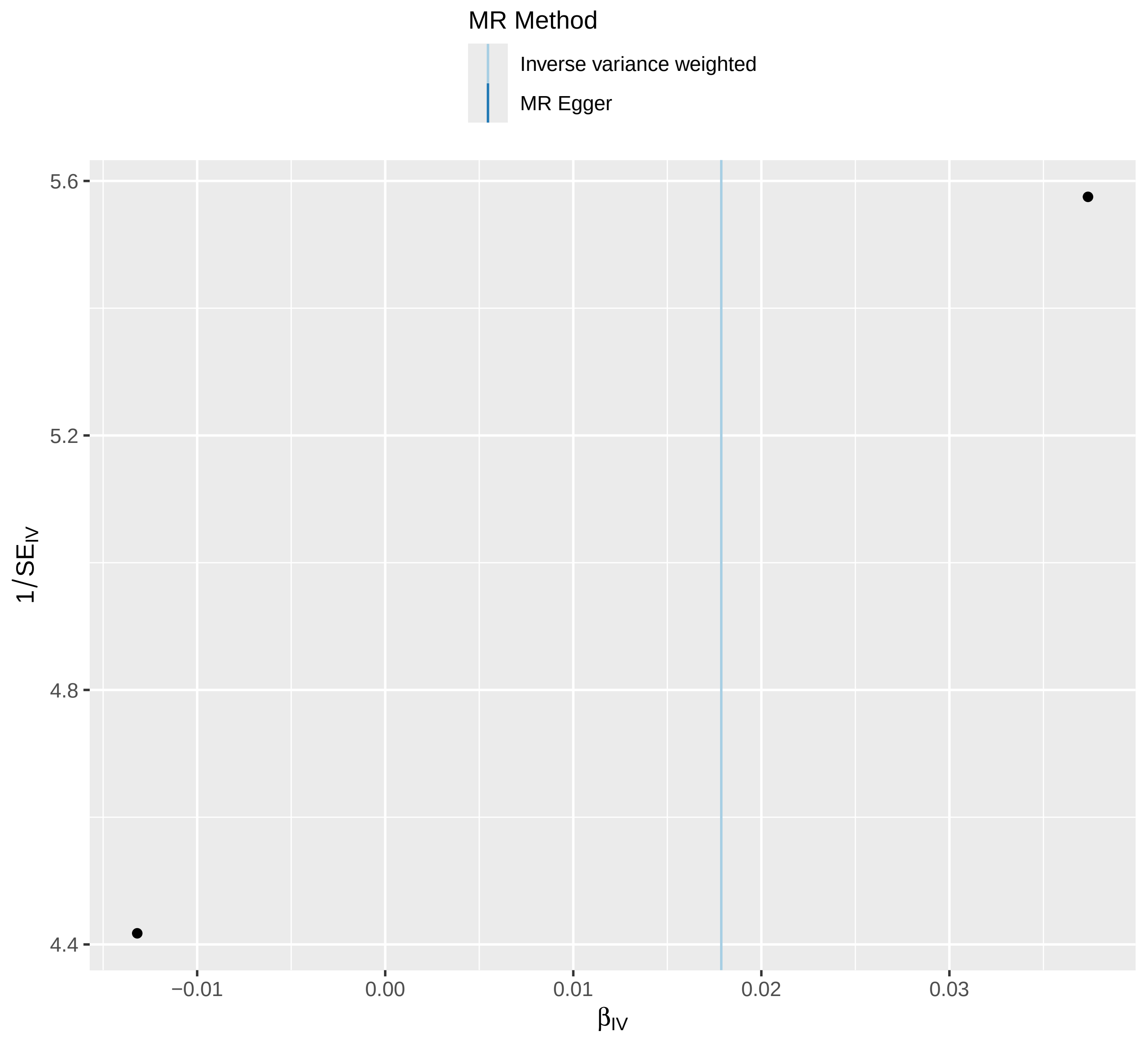
MR Method
Inverse variance weighted
MR Egger
5.6
5.2
4.8
4.4
1/SE$_{IV}$
−0.01
0.00
0.01
0.02
0.03
β$_{IV}$

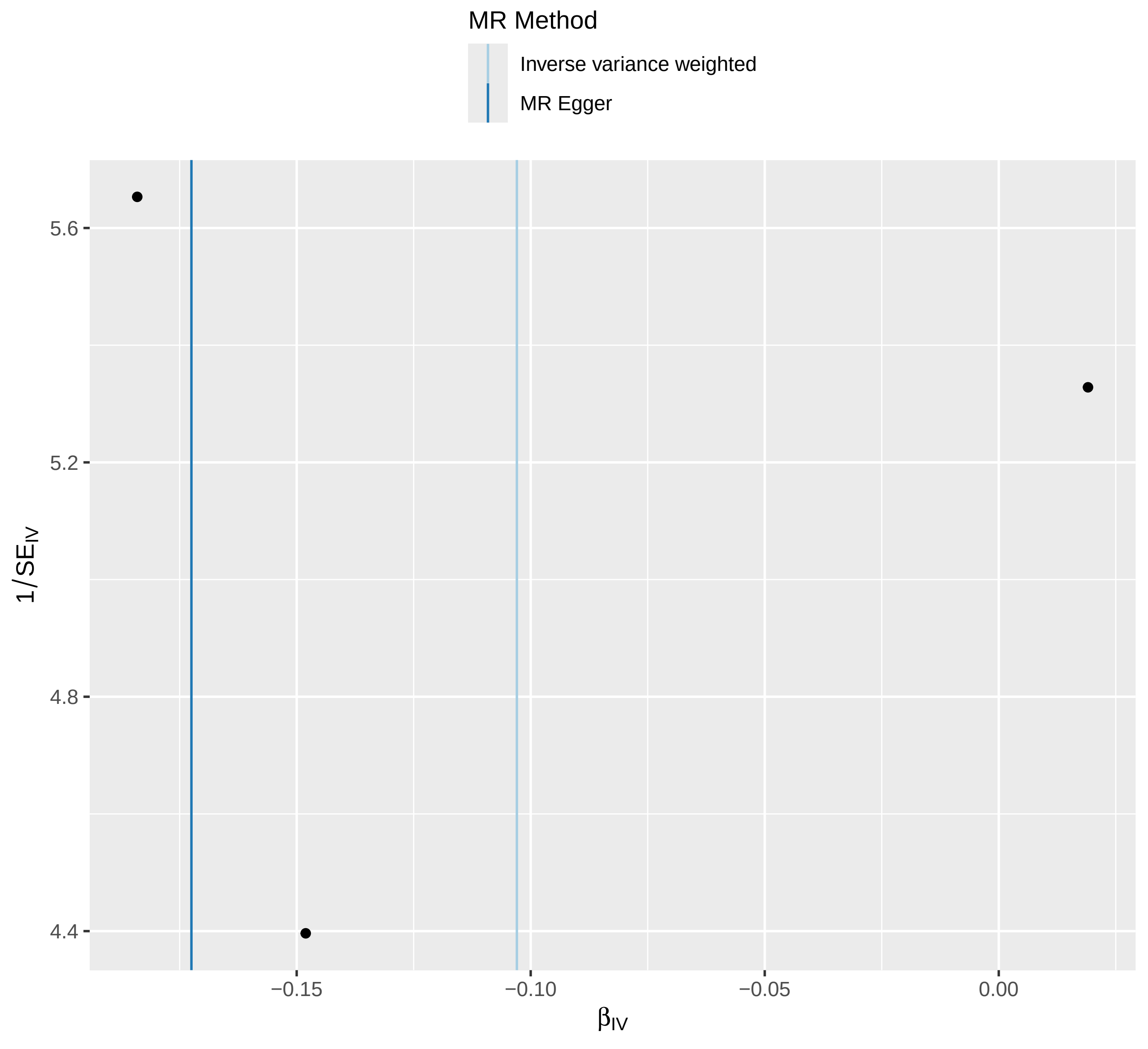
MR Method
Inverse variance weighted
MR Egger
5.6
5.2
4.8
4.4
1/SE_IV
−0.15
−0.10
−0.05
0.00
β_IV

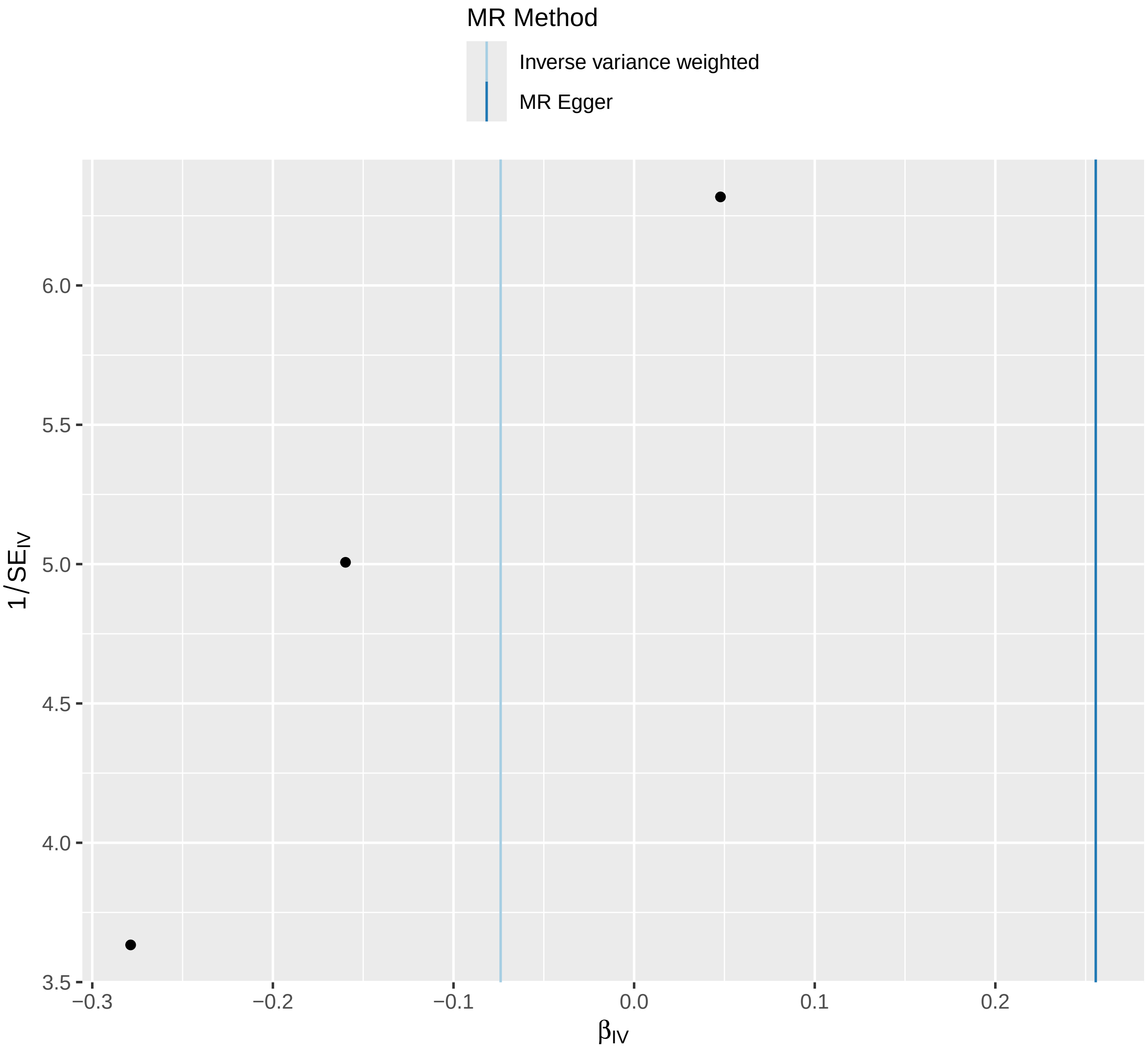

MR Method
Inverse variance weighted
MR Egger
6.0
5.5
5.0
4.5
4.0
3.5
1/SE_IV
−0.3
−0.2
−0.1
0.0
0.1
0.2
β_IV

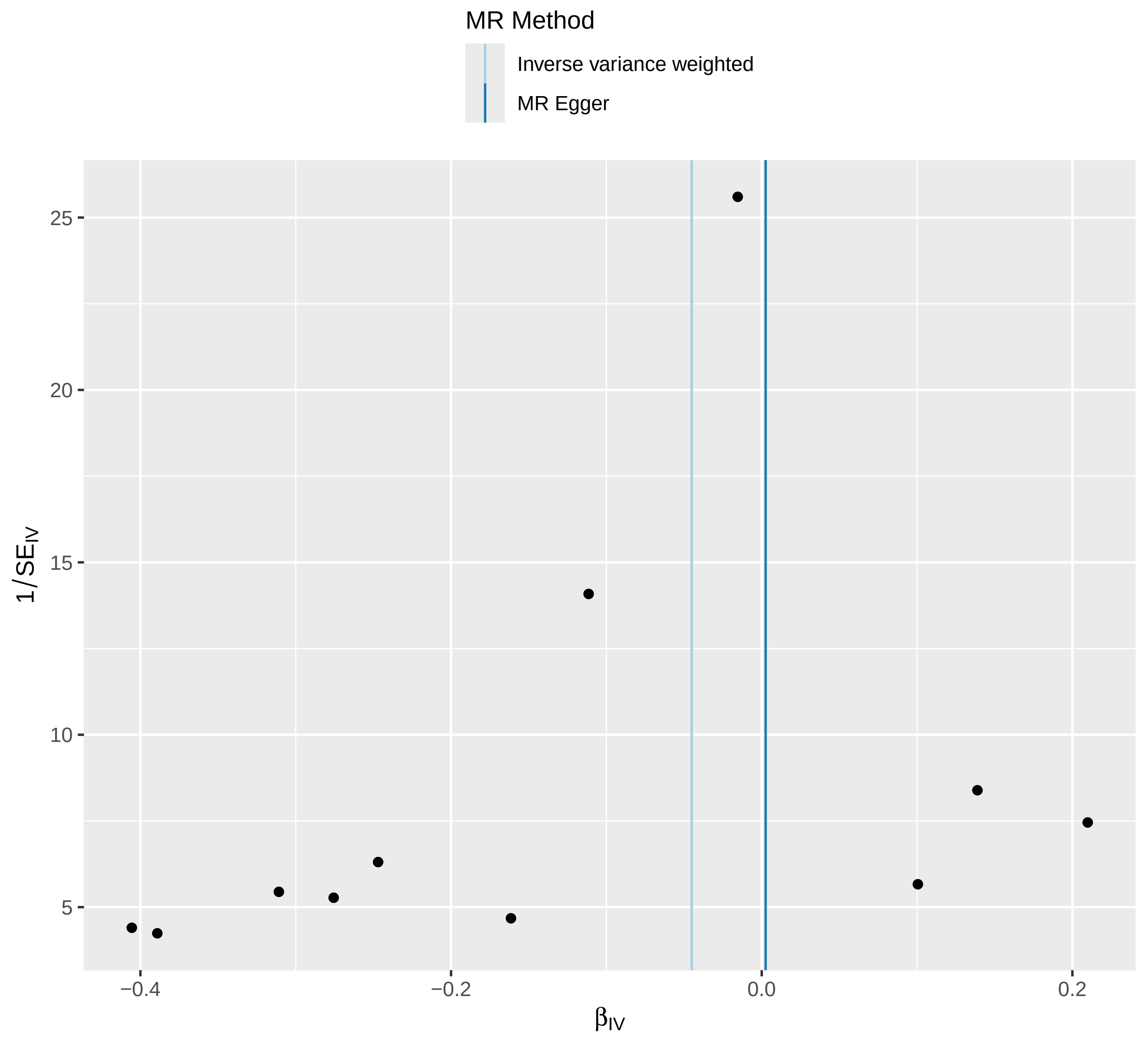
MR Method
Inverse variance weighted
MR Egger
25
20
15
10
5
1/SE_IV
−0.4
−0.2
0.0
0.2
β_IV

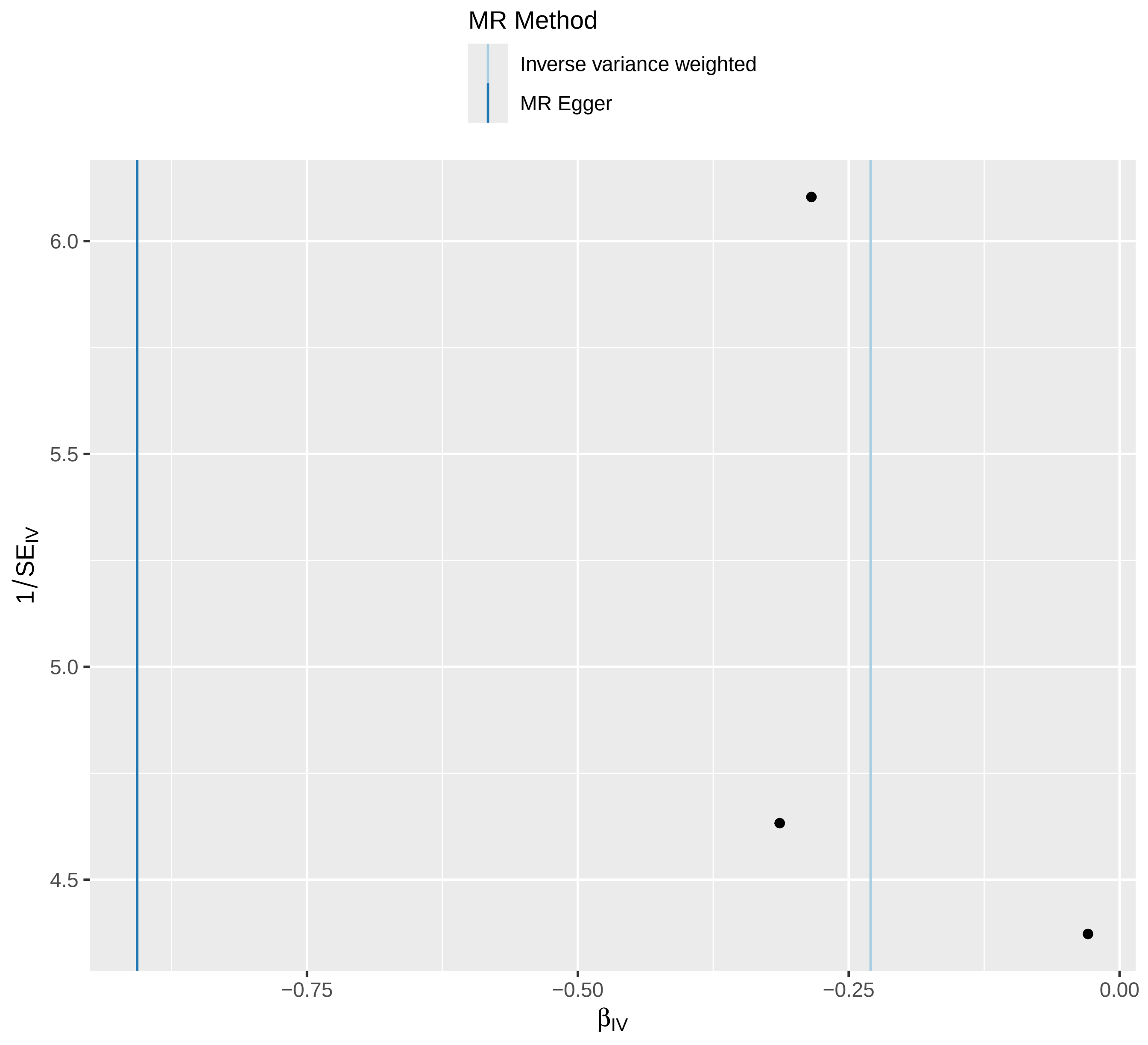

MR Method
Inverse variance weighted
MR Egger
6.0
5.5
5.0
4.5
1/SE_IV
−0.75
−0.50
−0.25
0.00
β_IV

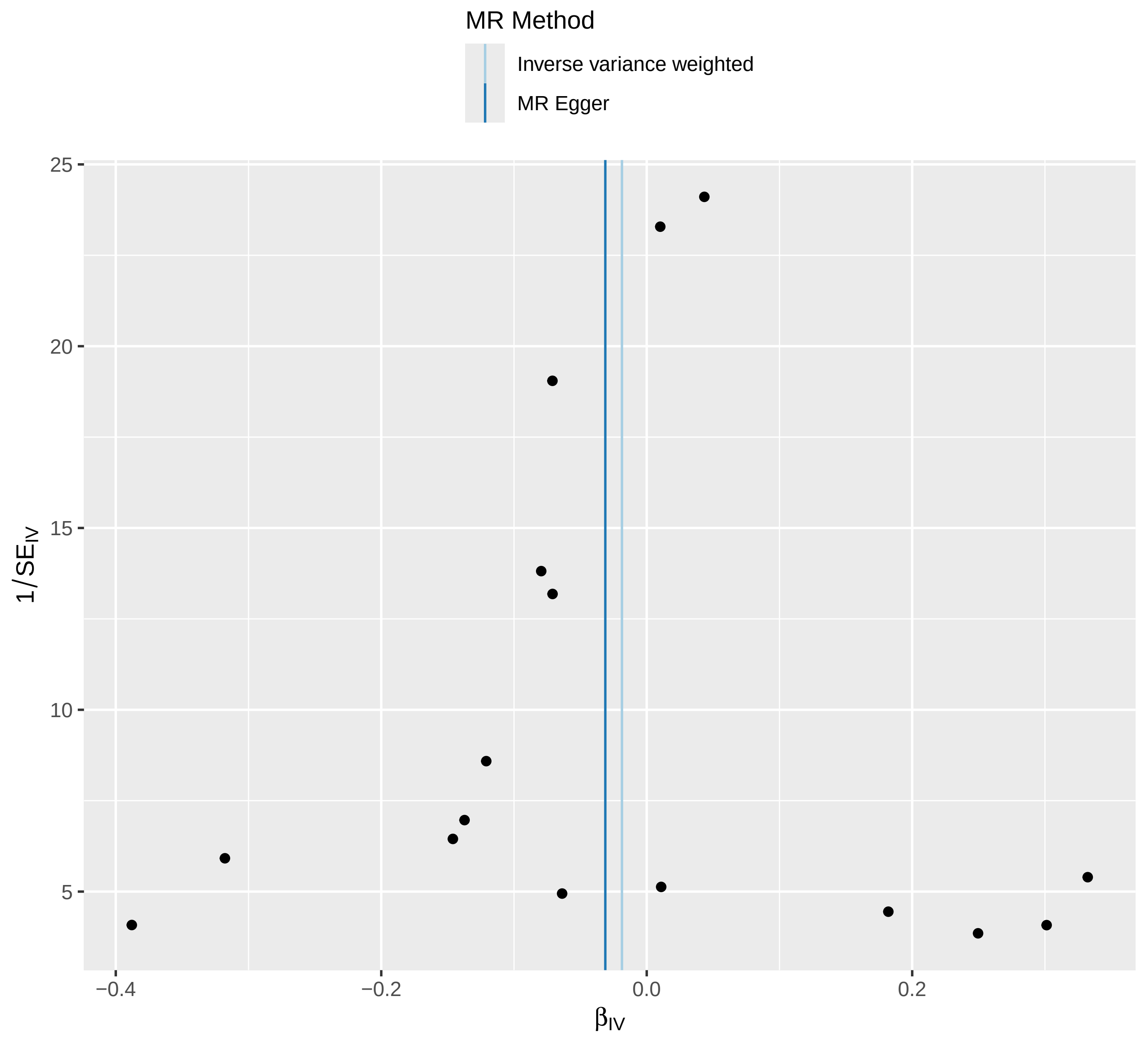

MR Method
Inverse variance weighted
MR Egger
25
20
15
10
5
1/SE_IV
−0.4
−0.2
0.0
0.2
β_IV

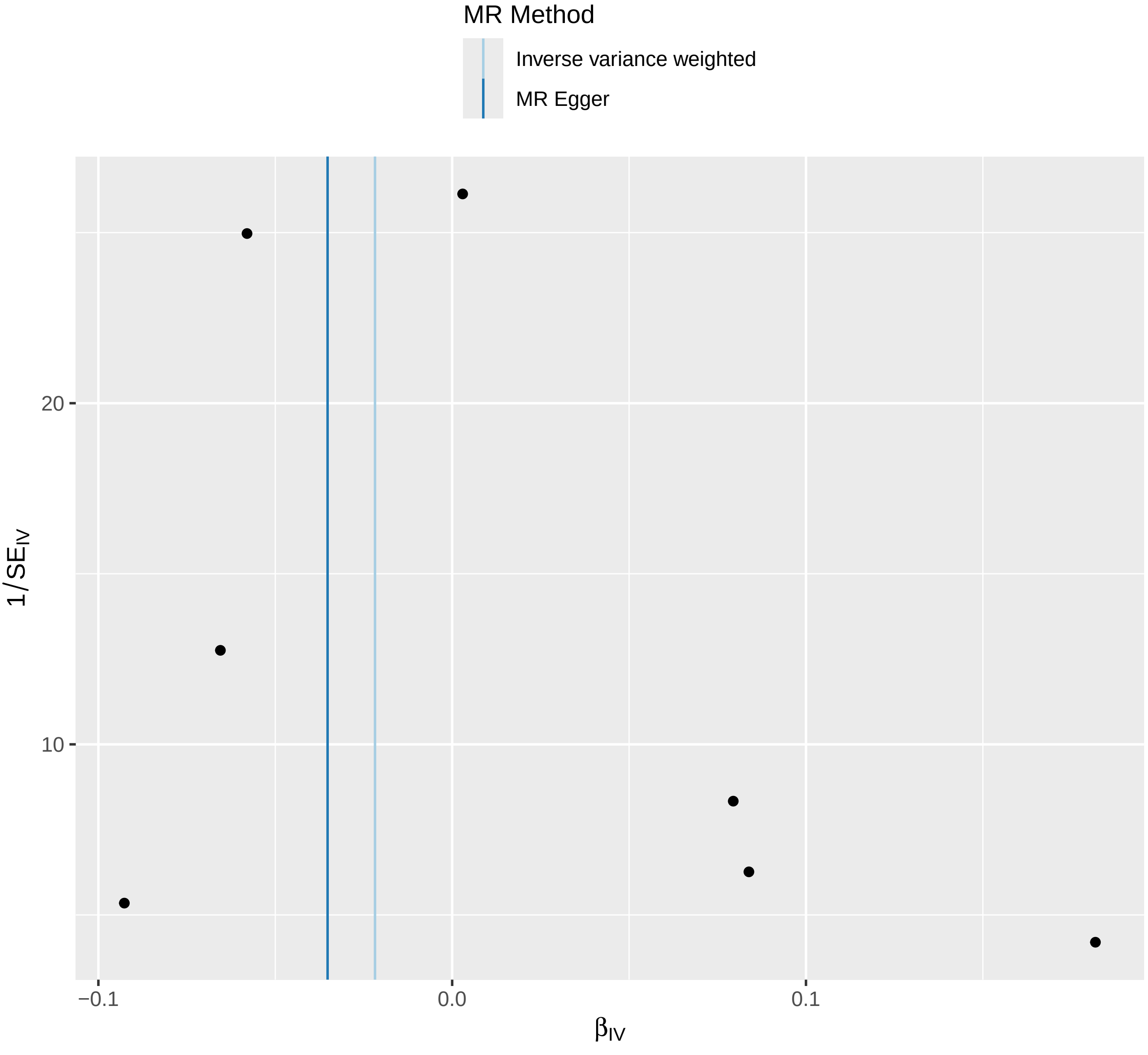

MR Method
Inverse variance weighted
MR Egger
20
10
1/SE$_{IV}$
−0.1
0.0
0.1
β$_{IV}$

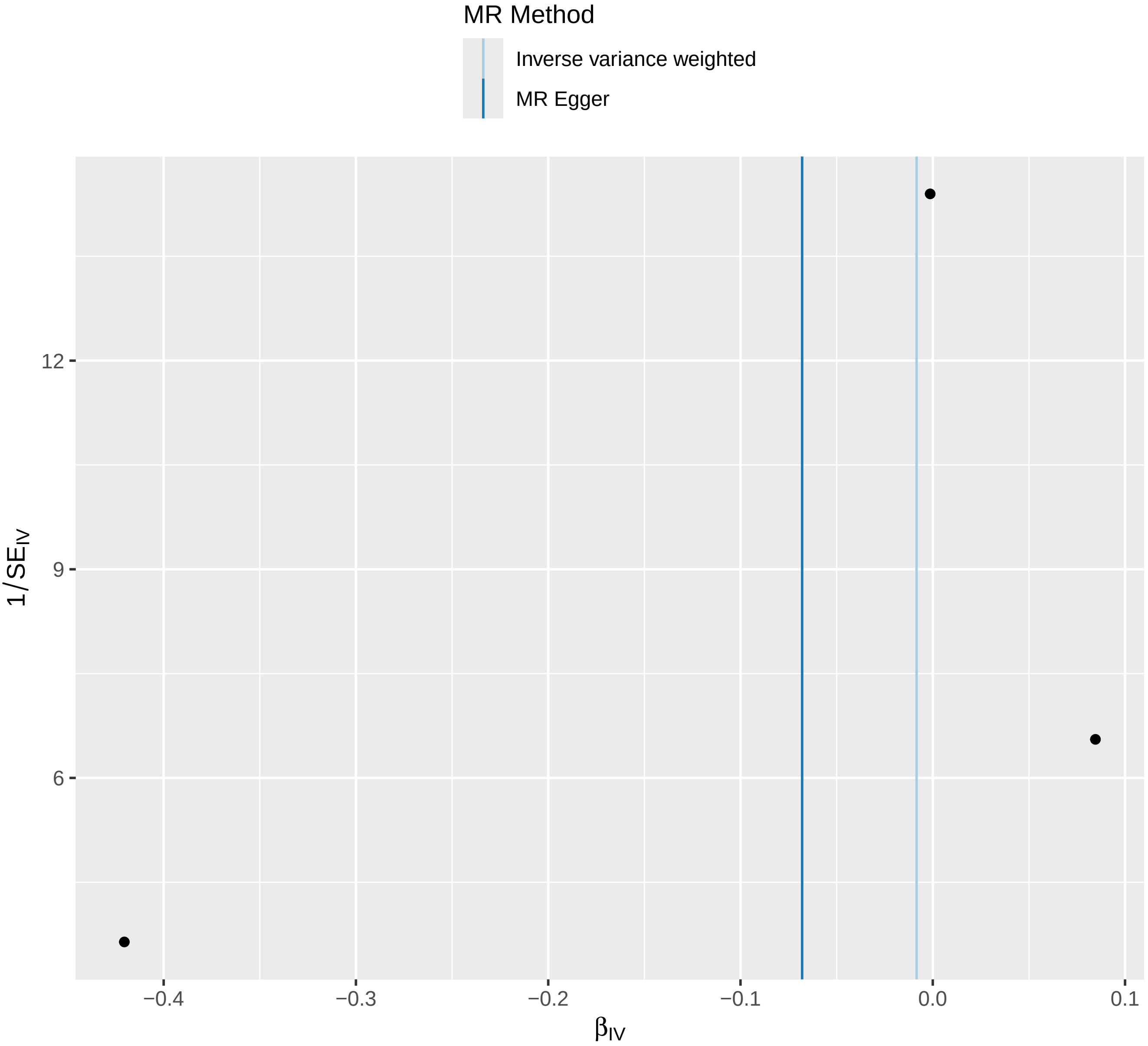

MR Method
Inverse variance weighted
MR Egger
12
9
6
$1/SE_{IV}$
−0.4
−0.3
−0.2
−0.1
0.0
0.1
$\beta_{IV}$

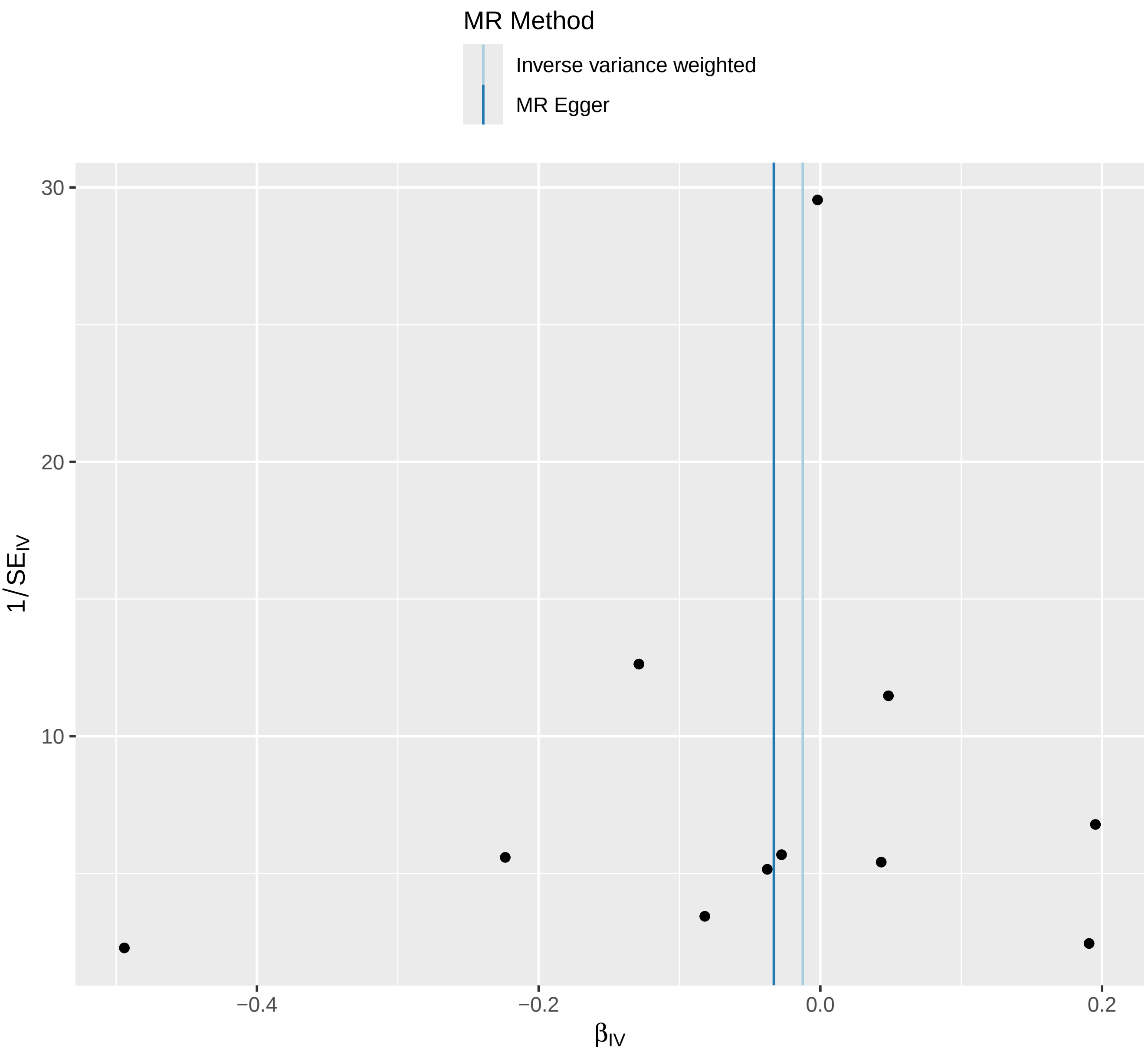
MR Method
Inverse variance weighted
MR Egger
30
20
10
1/$SE_{IV}$
−0.4
−0.2
0.0
0.2
$\beta_{IV}$

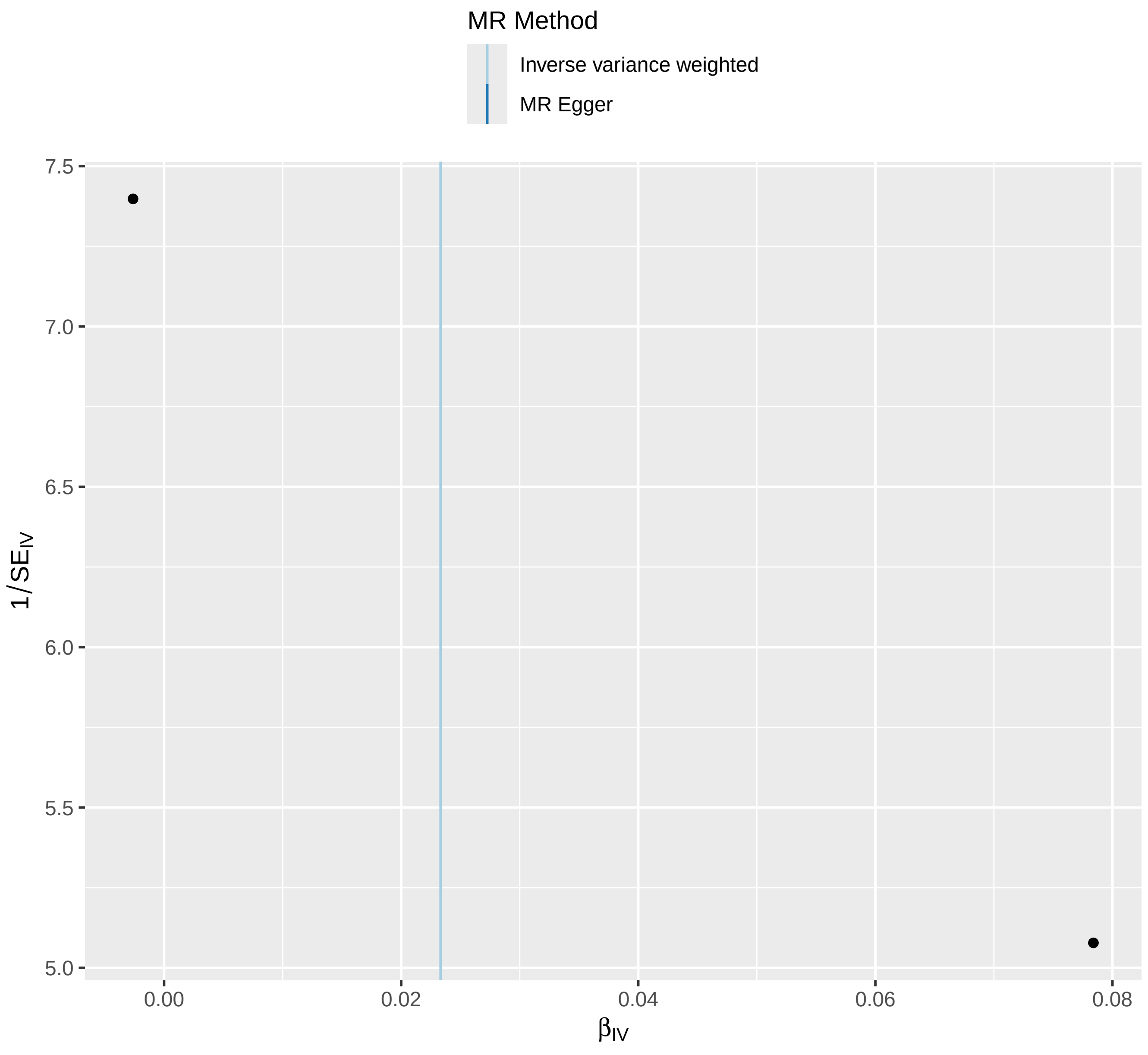

MR Method
Inverse variance weighted
MR Egger
7.5
7.0
6.5
6.0
5.5
5.0
0.00
0.02
0.04
0.06
0.08
$1/SE_{IV}$
$\beta_{IV}$

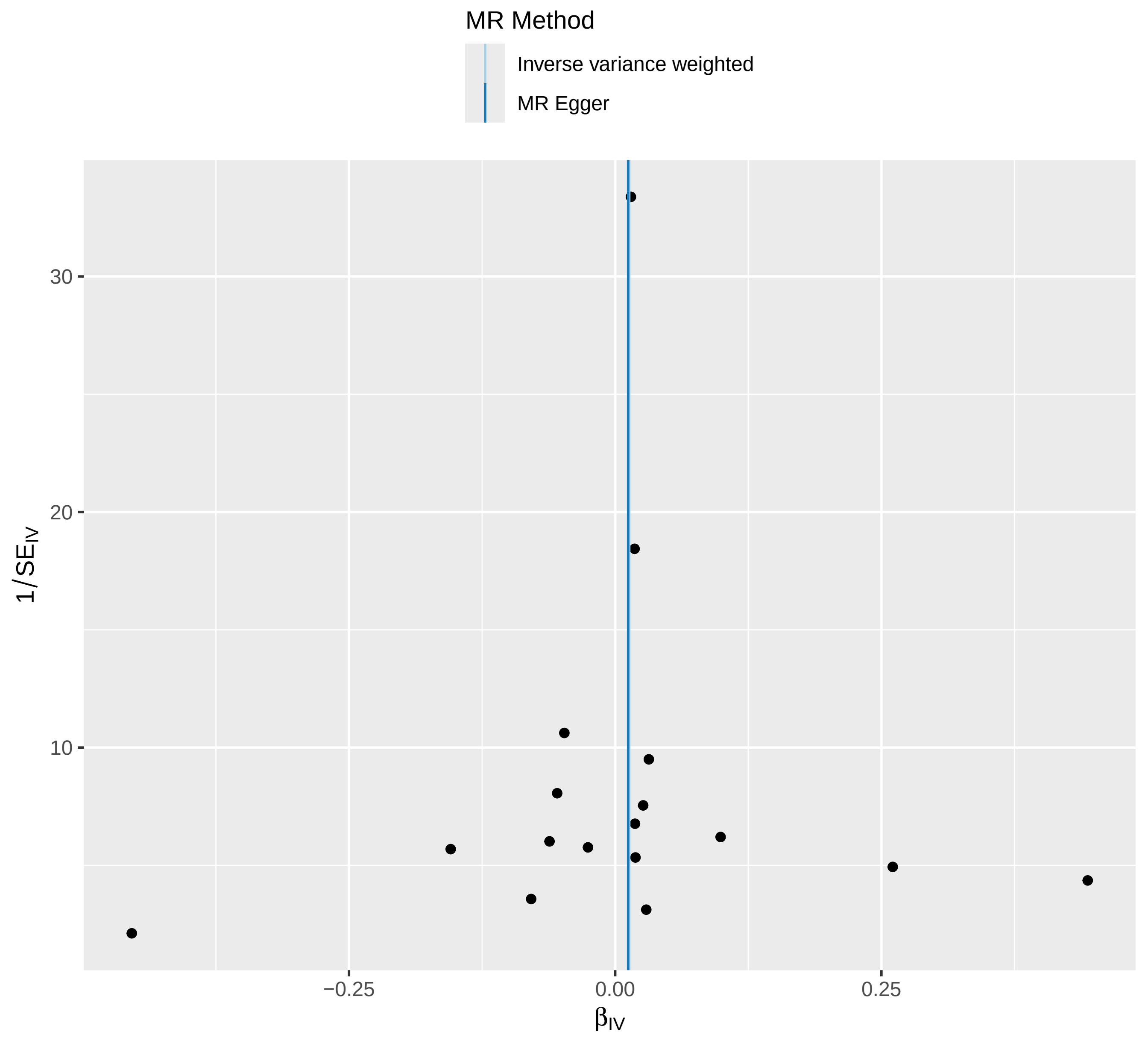
MR Method
Inverse variance weighted
MR Egger
30
20
10
1/SE_IV
−0.25
0.00
0.25
β_IV

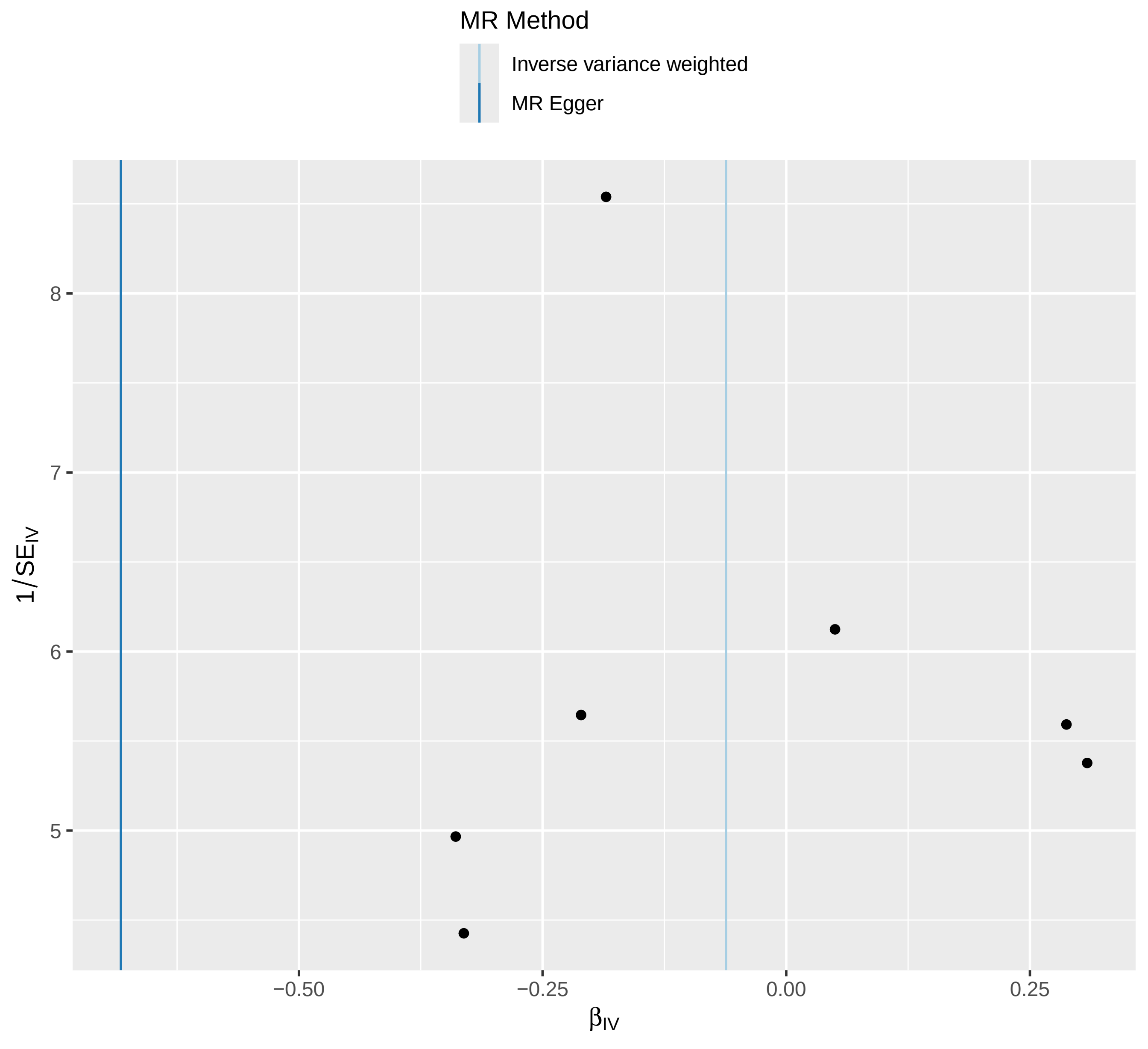
MR Method
Inverse variance weighted
MR Egger
1/SE$_{IV}$
8
7
6
5
−0.50
−0.25
0.00
0.25
β$_{IV}$

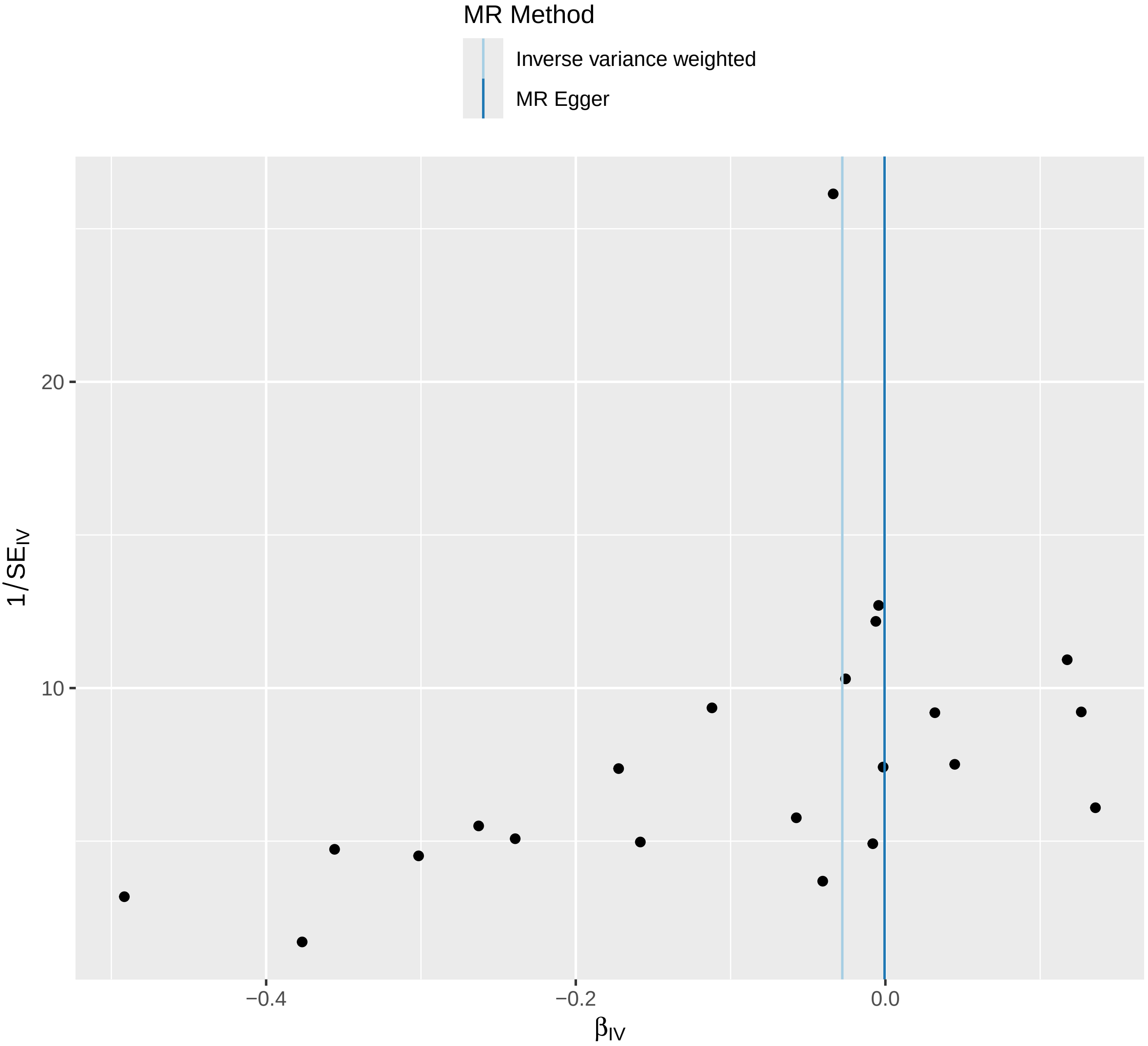

MR Method
Inverse variance weighted
MR Egger
20
10
1/SE$_{IV}$
−0.4
−0.2
0.0
β$_{IV}$

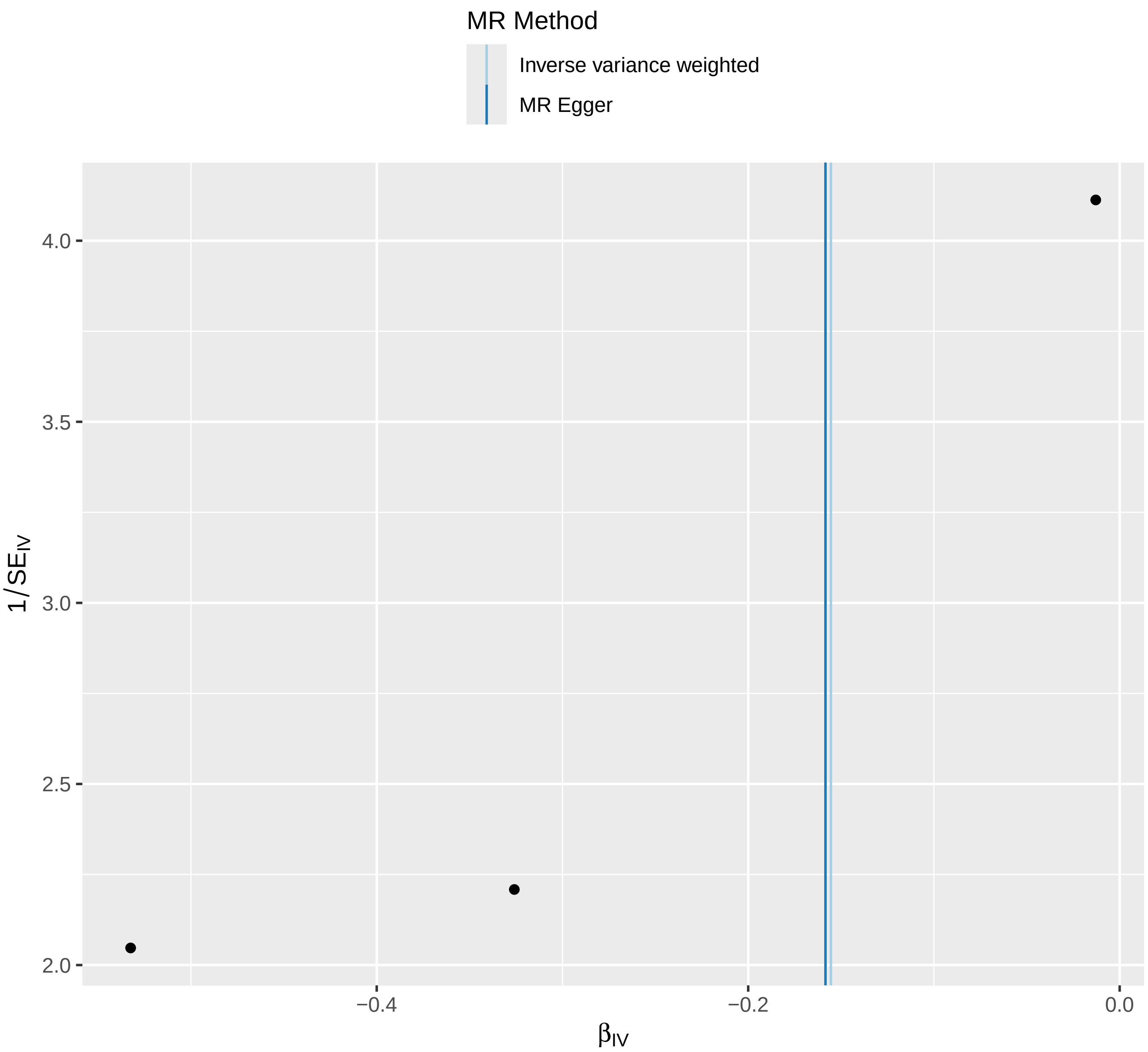
MR Method
Inverse variance weighted
MR Egger
4.0
3.5
3.0
2.5
2.0
1/SE$_{IV}$
−0.4
−0.2
0.0
β$_{IV}$

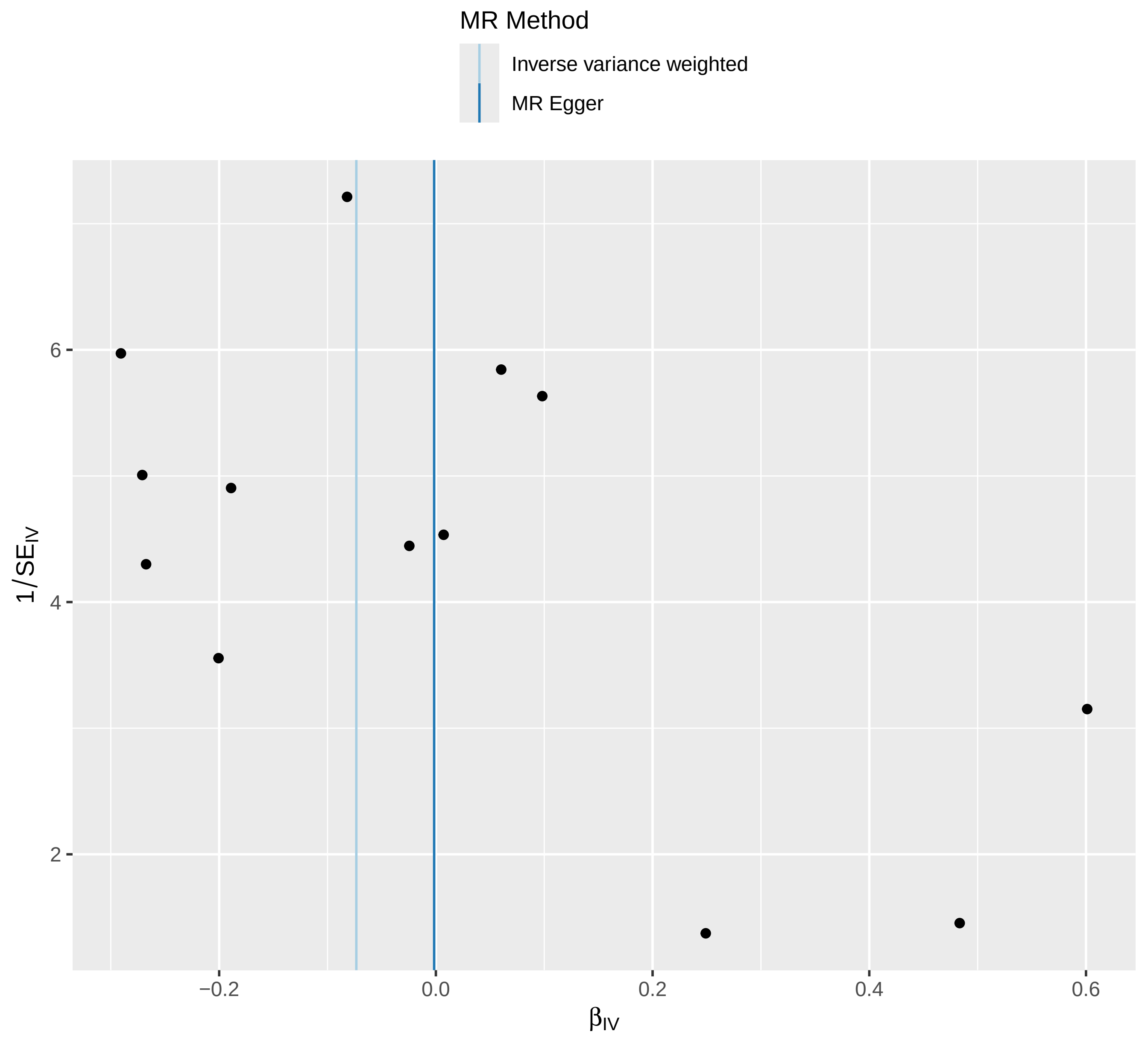

MR Method
Inverse variance weighted
MR Egger
6
4
2
1/SE$_{IV}$
−0.2
0.0
0.2
0.4
0.6
β$_{IV}$

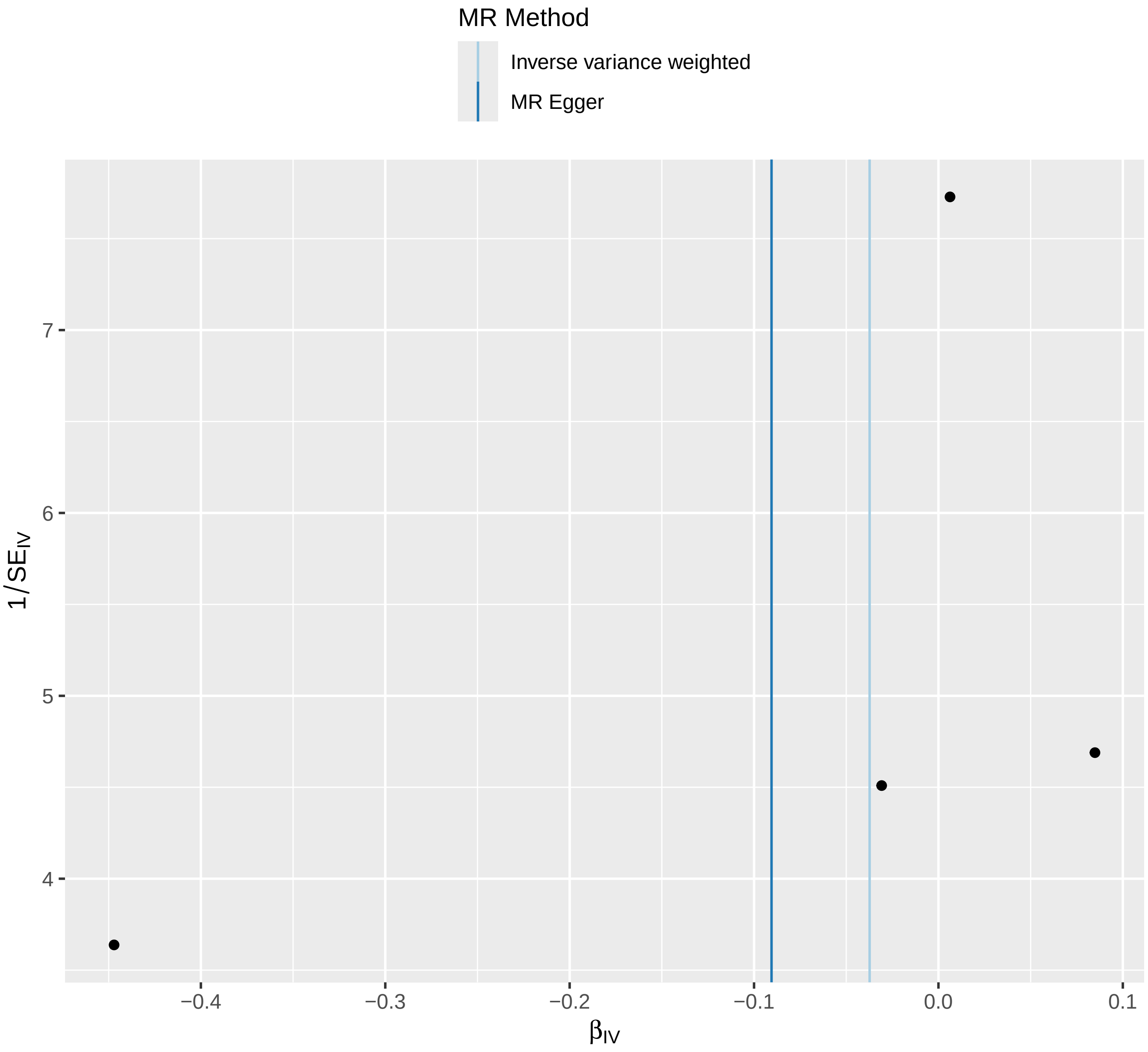

MR Method
Inverse variance weighted
MR Egger
7
6
5
4
1/SE$_{IV}$
−0.4
−0.3
−0.2
−0.1
0.0
0.1
β$_{IV}$

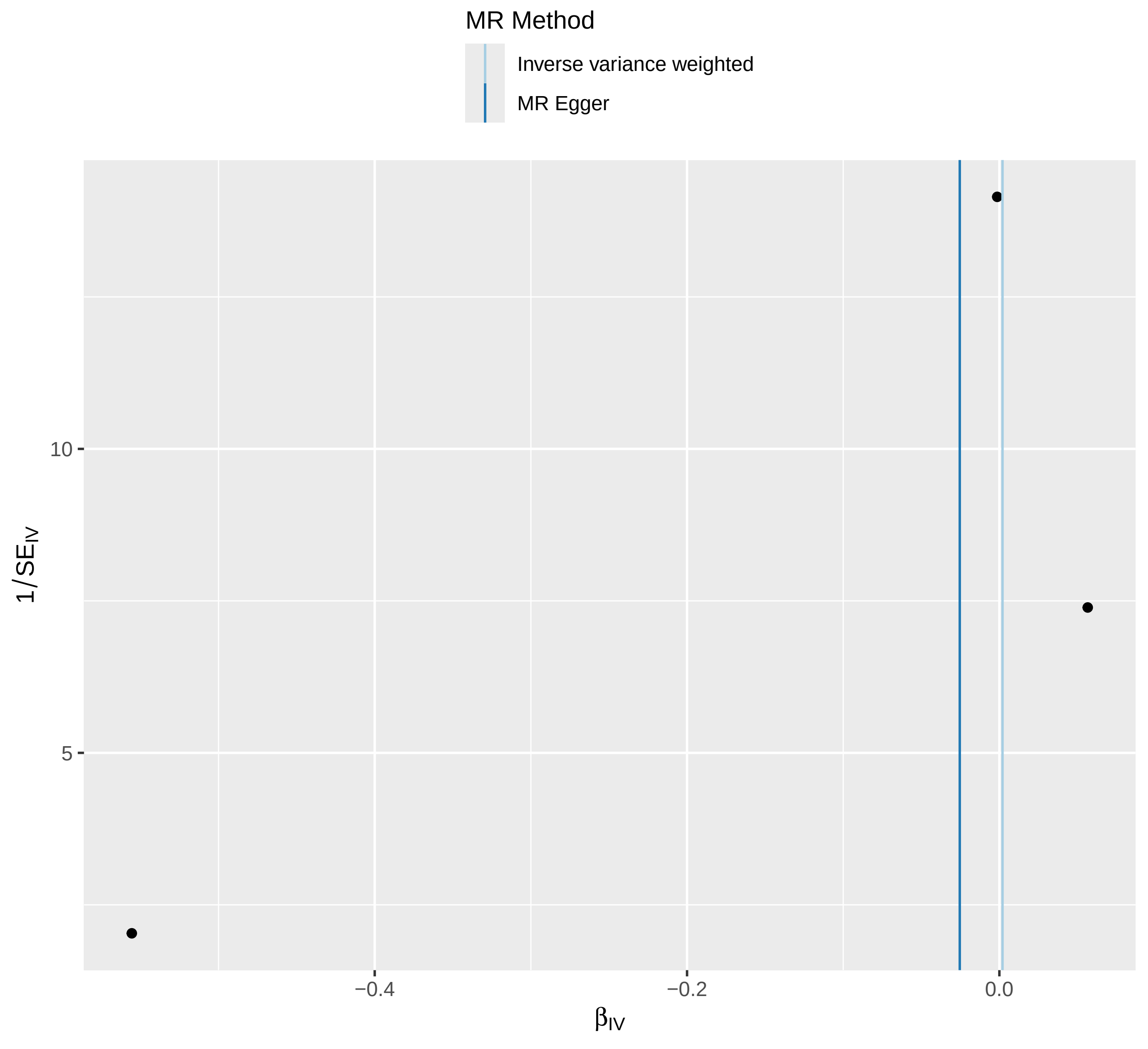

MR Method
Inverse variance weighted
MR Egger
10
5
1/SE_IV
−0.4
−0.2
0.0
β_IV

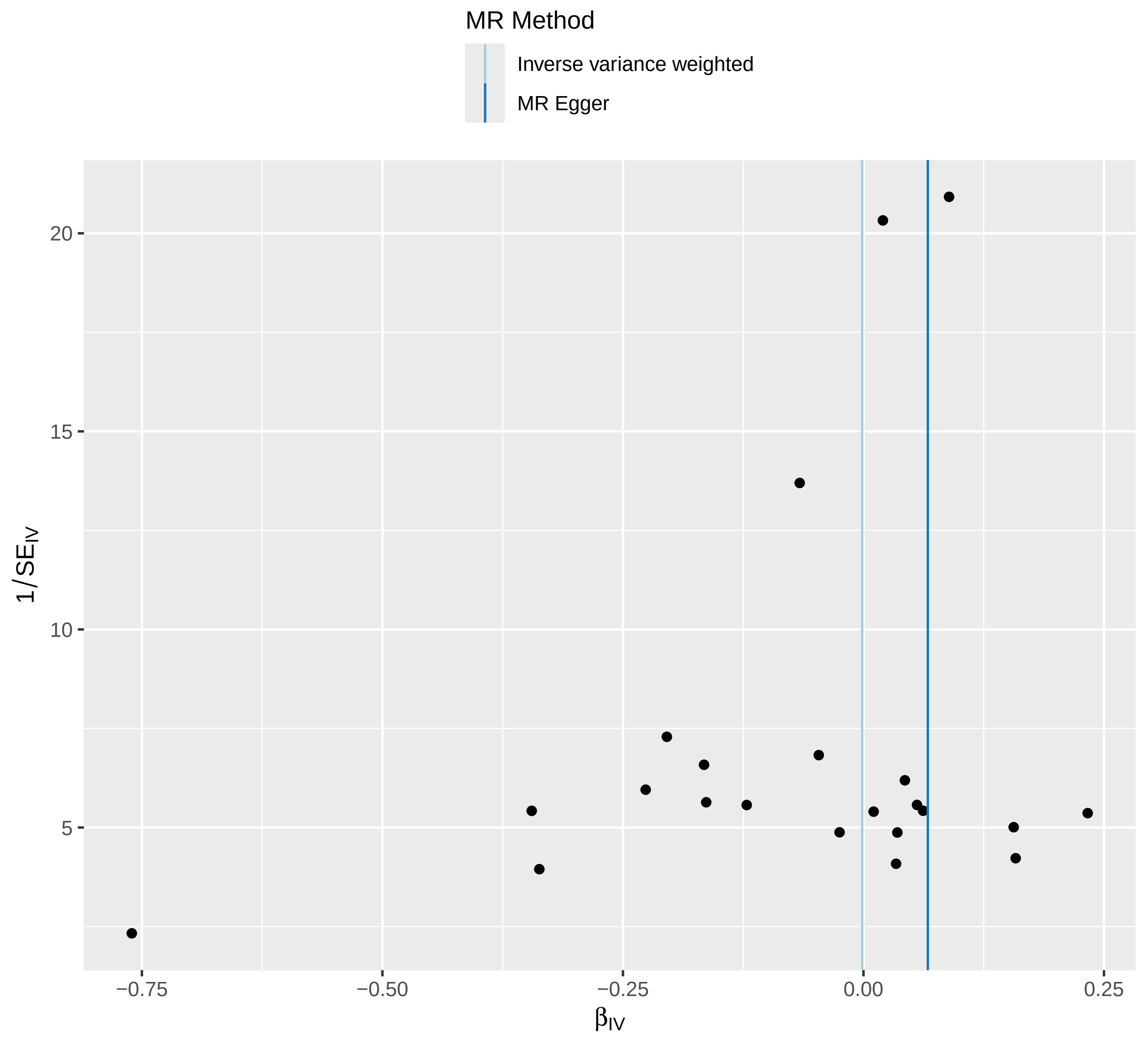
MR Method
Inverse variance weighted
MR Egger
1/SE_IV
β_IV
20
15
10
5
−0.75
−0.50
−0.25
0.00
0.25

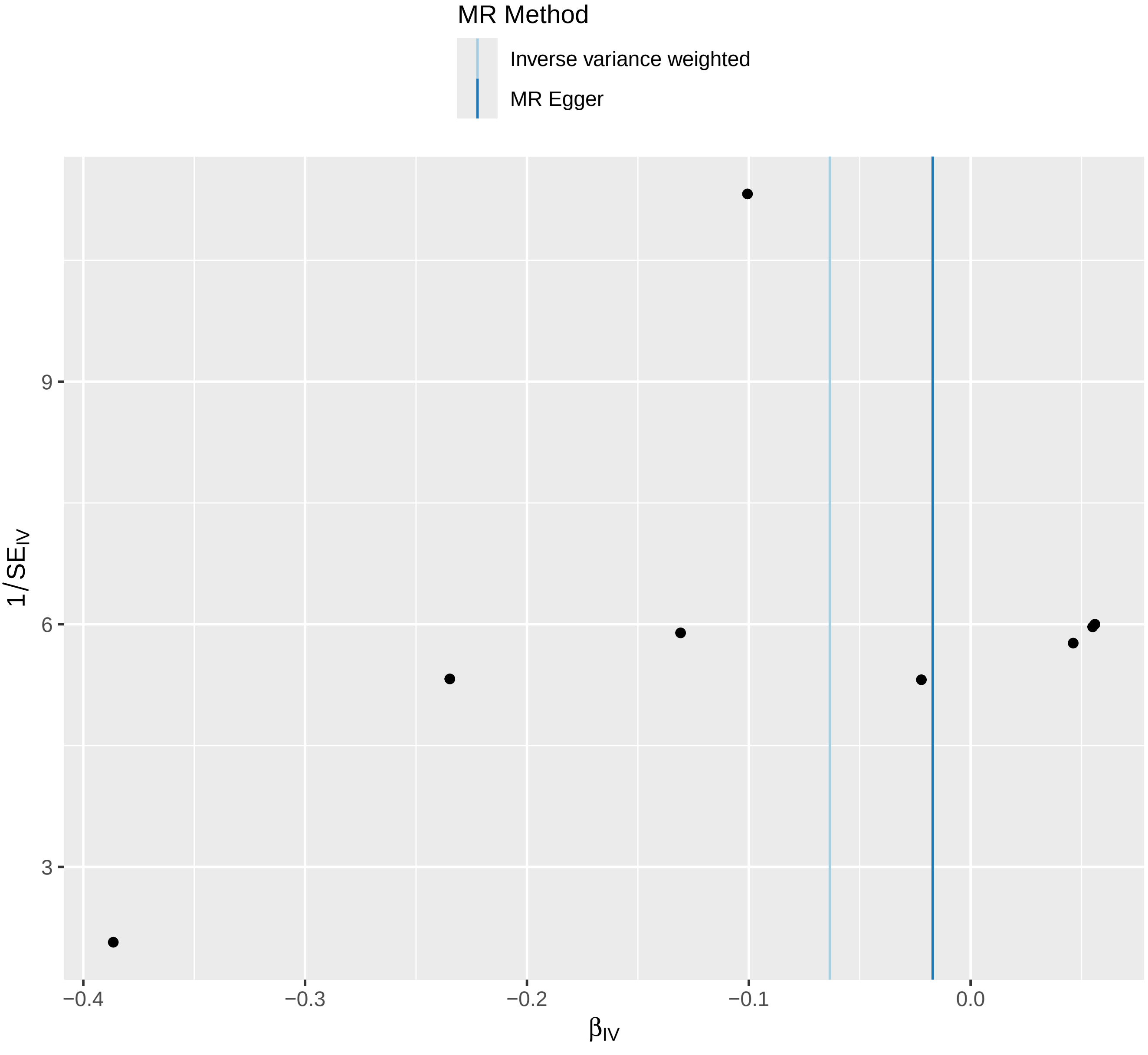
MR Method
Inverse variance weighted
MR Egger
9
6
3
$1/SE_{IV}$
−0.4
−0.3
−0.2
−0.1
0.0
$\beta_{IV}$

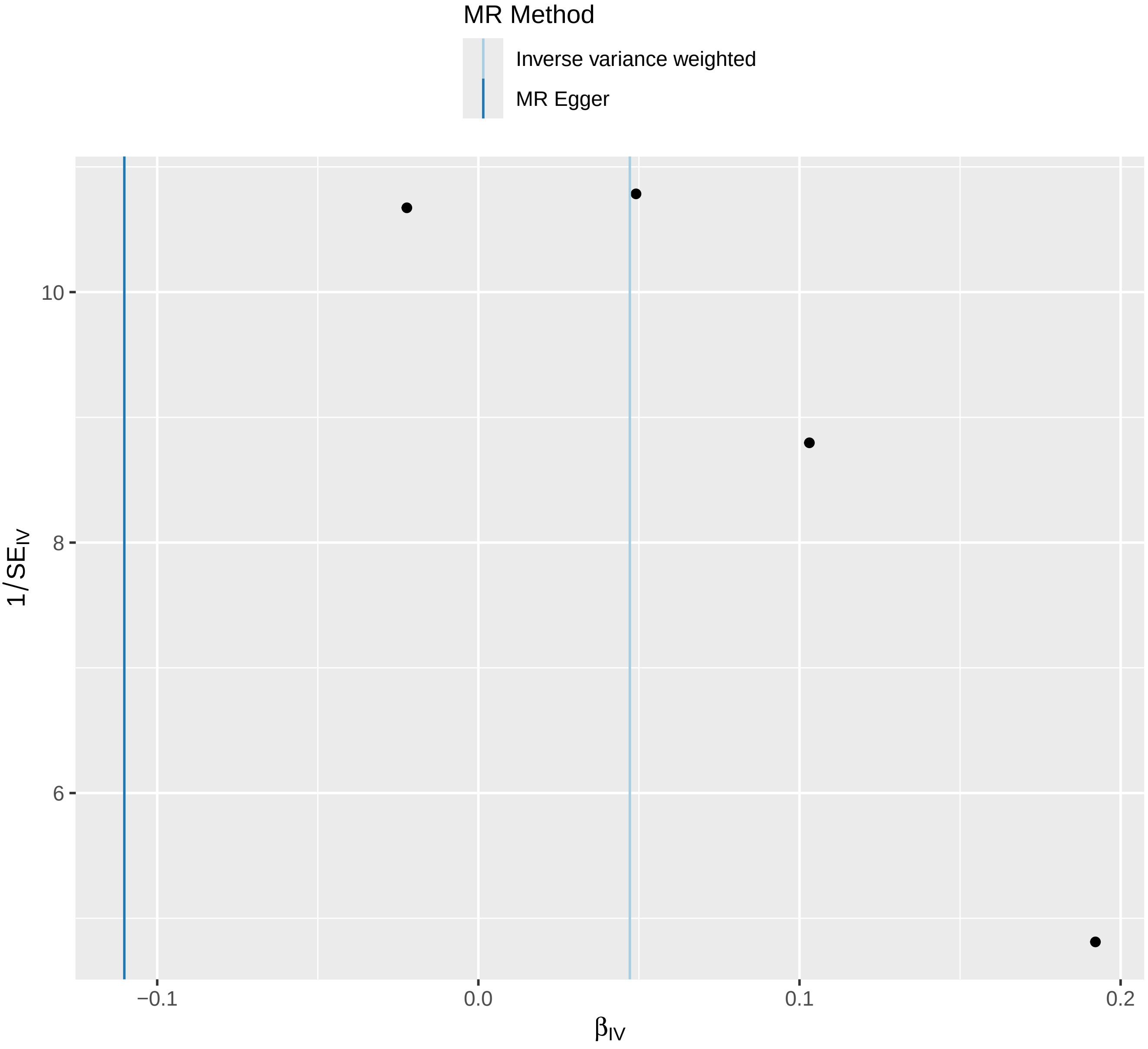

MR Method
Inverse variance weighted
MR Egger
10
8
6
1/SE$_{IV}$
−0.1
0.0
0.1
0.2
β$_{IV}$

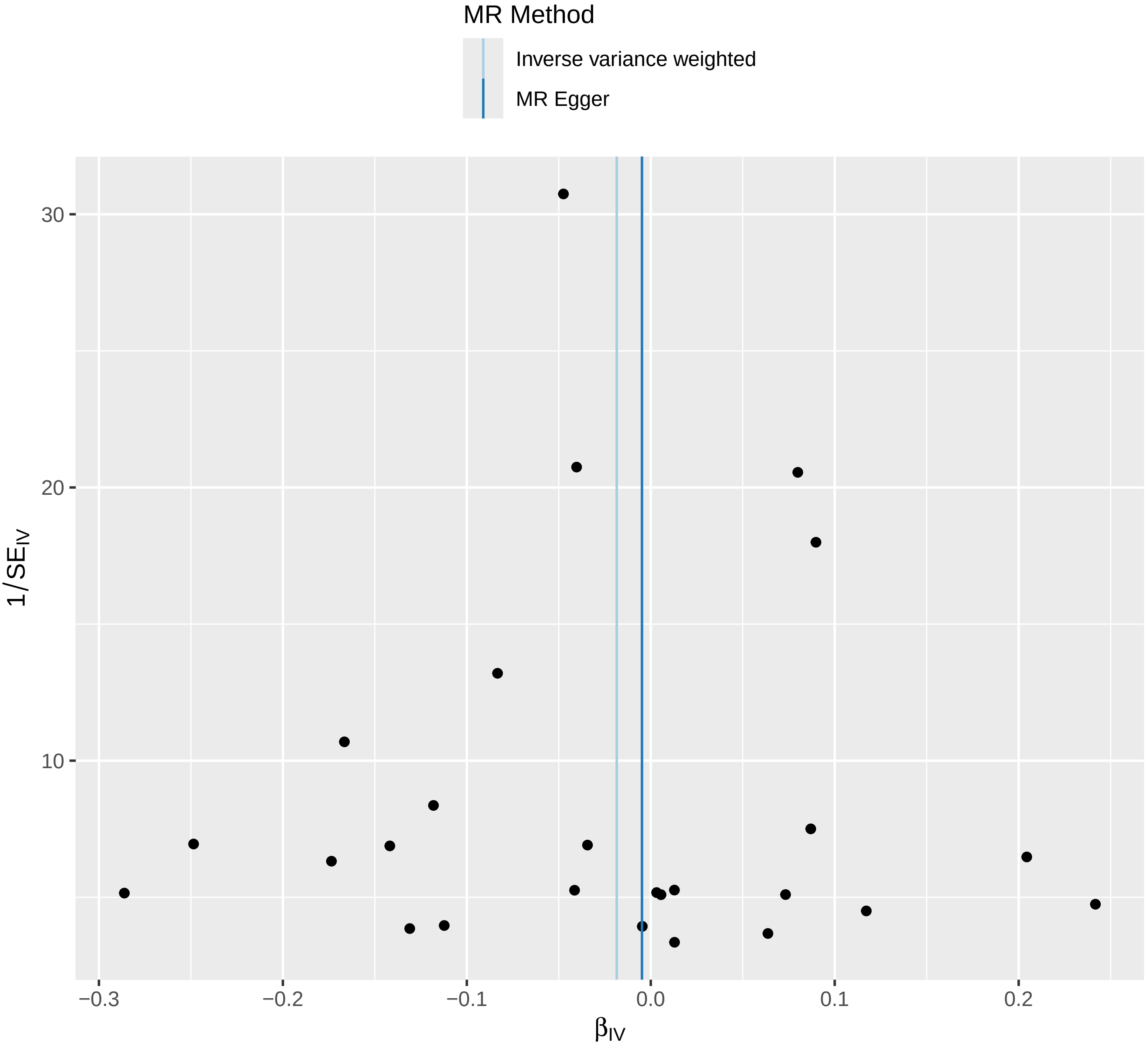
MR Method
Inverse variance weighted
MR Egger
30
20
10
$1/SE_{IV}$
−0.3
−0.2
−0.1
0.0
0.1
0.2
$\beta_{IV}$

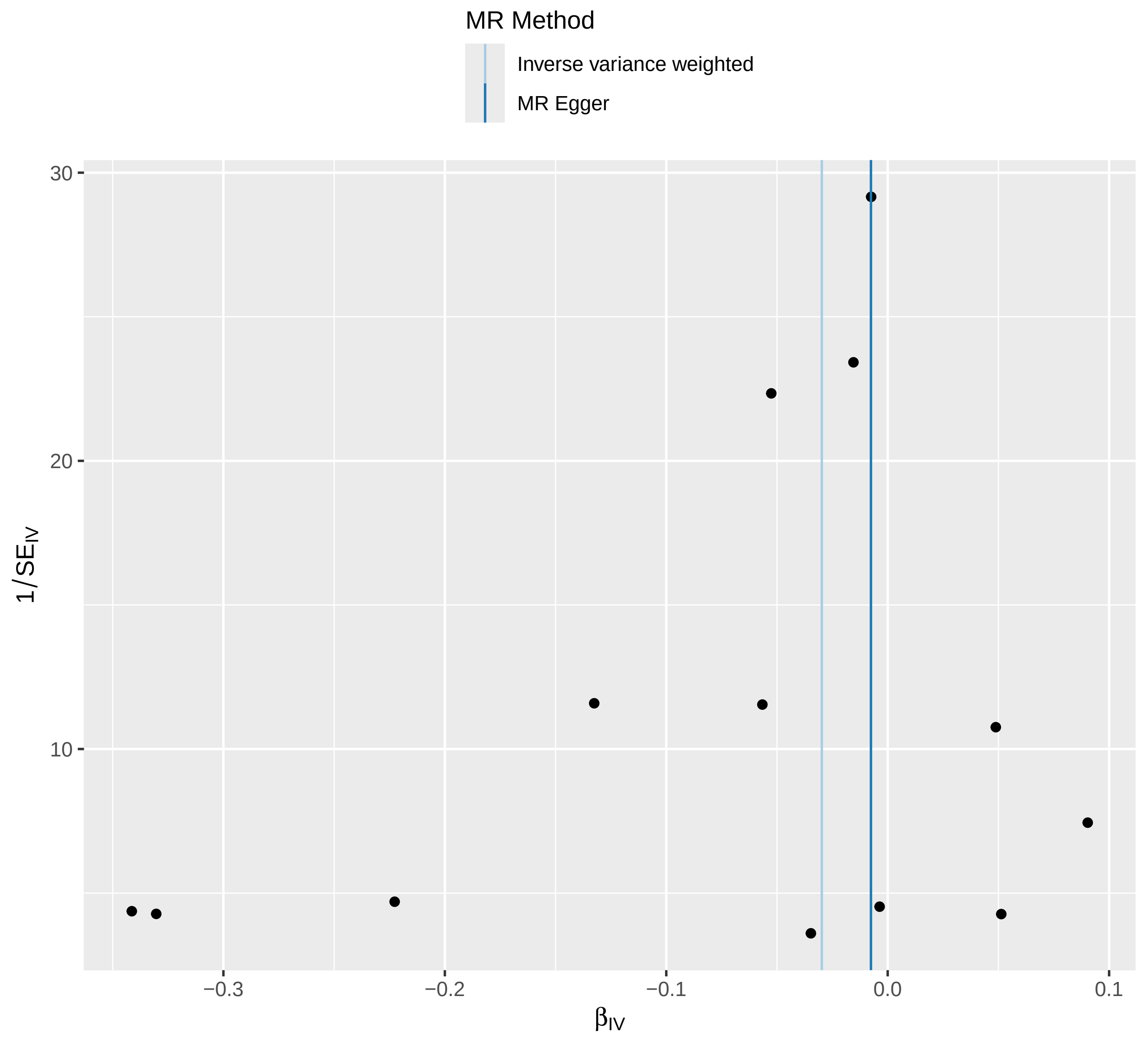
MR Method
Inverse variance weighted
MR Egger
30
20
10
1/SE$_{IV}$
−0.3
−0.2
−0.1
0.0
0.1
β$_{IV}$

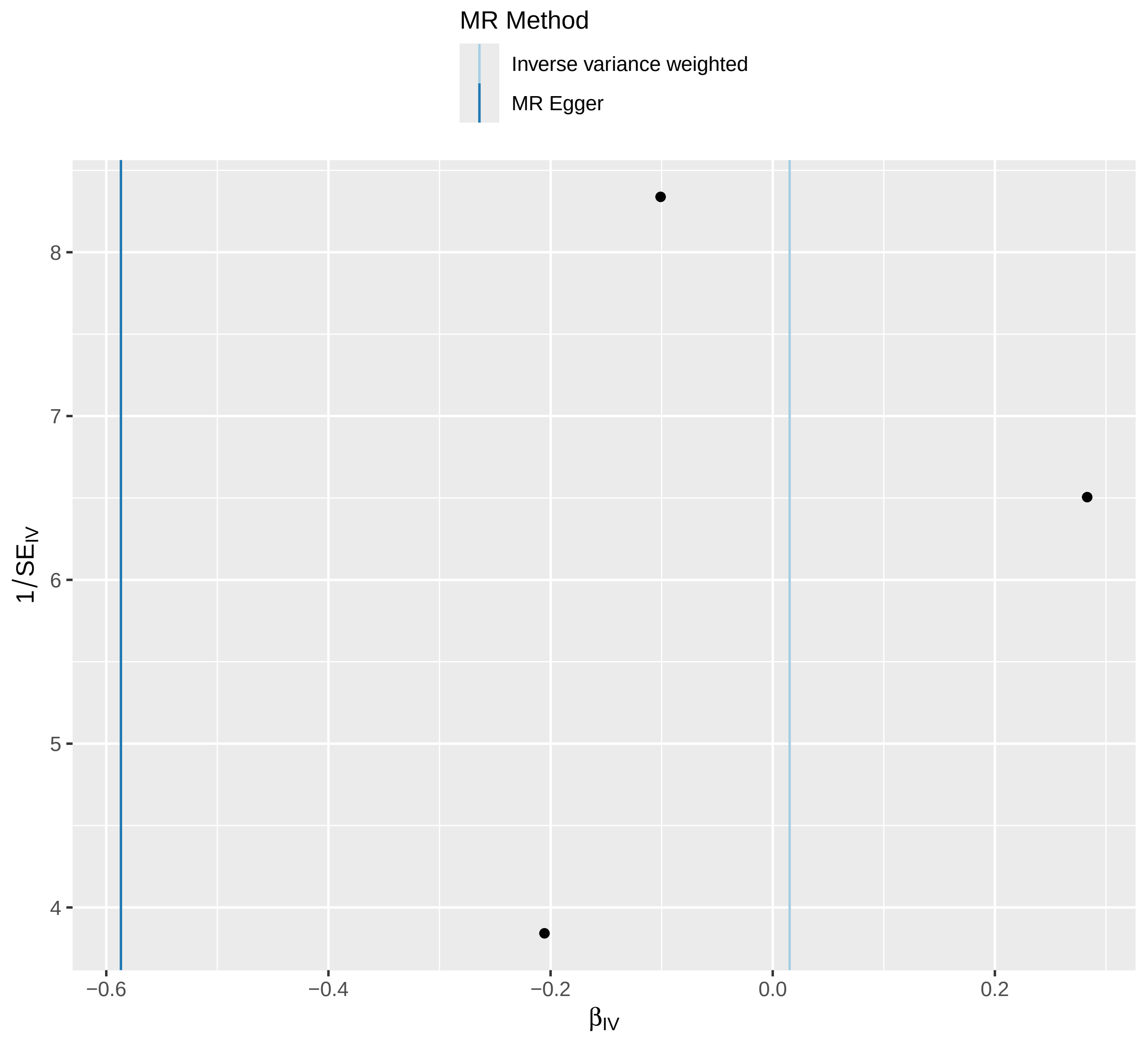

MR Method
Inverse variance weighted
MR Egger
8
7
6
5
4
1/SE$_{IV}$
−0.6
−0.4
−0.2
0.0
0.2
β$_{IV}$

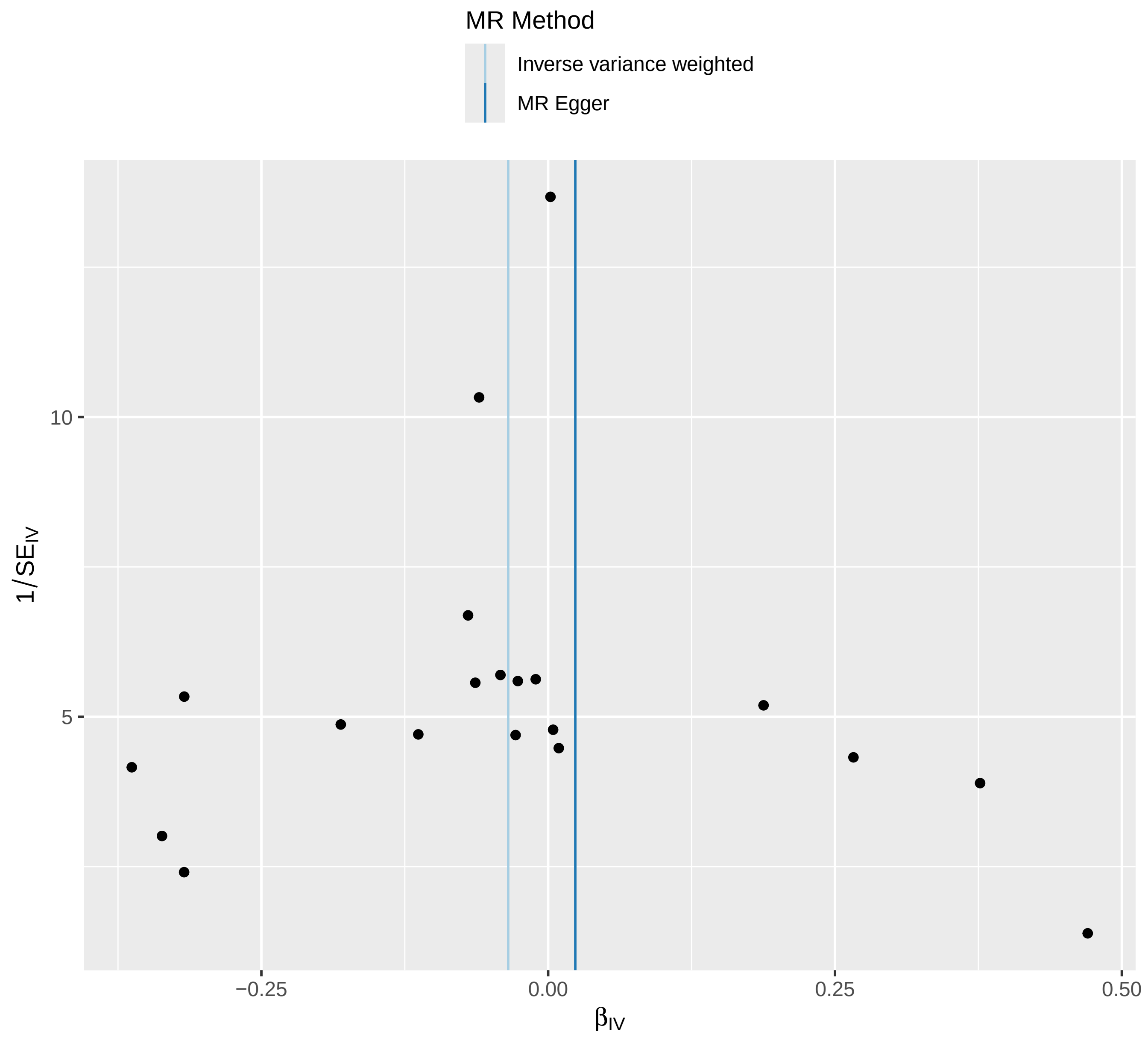
MR Method
Inverse variance weighted
MR Egger
10
5
1/SE_IV
−0.25
0.00
0.25
0.50
β_IV

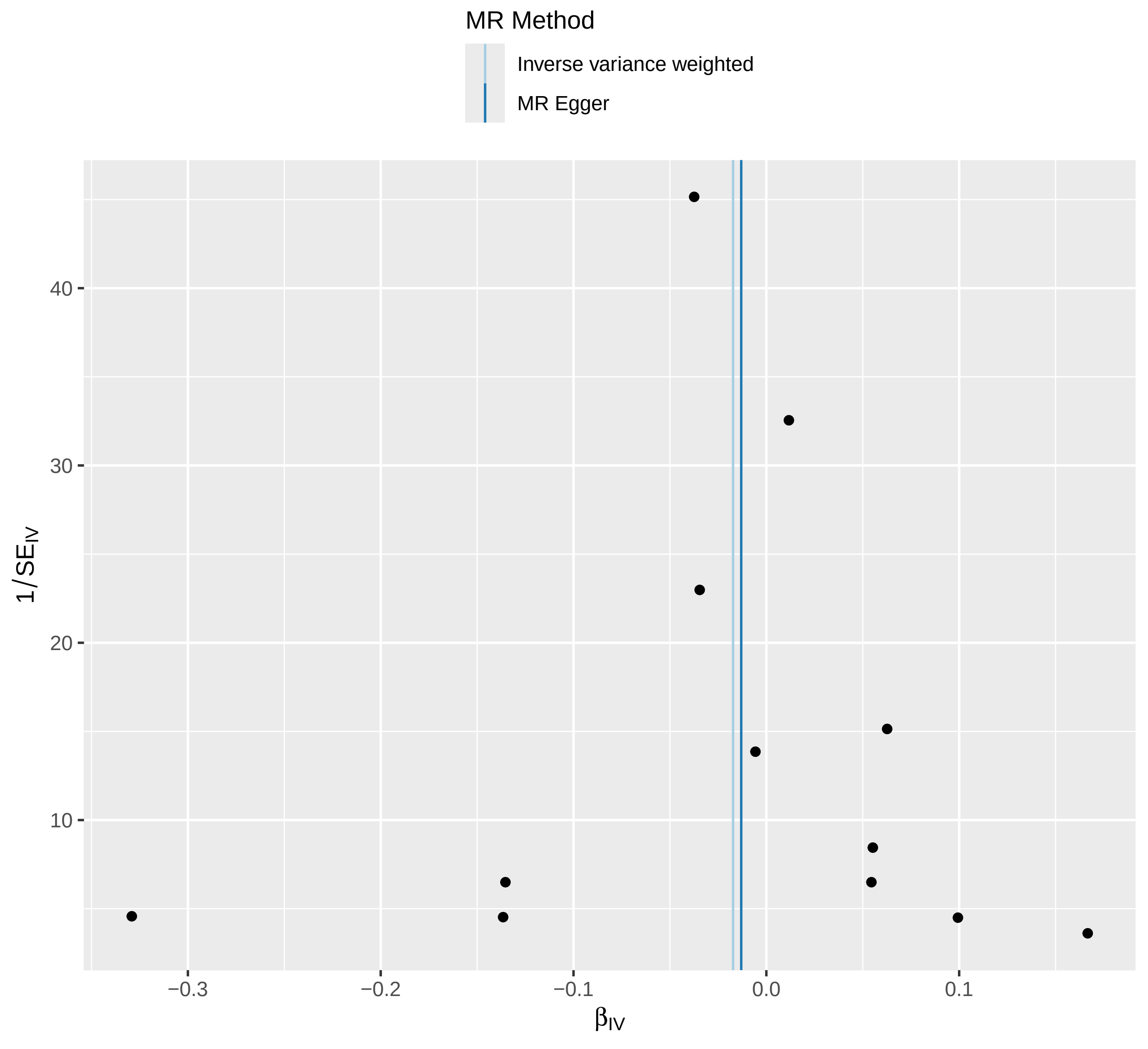

MR Method
Inverse variance weighted
MR Egger
1/SE_IV
40
30
20
10
−0.3
−0.2
−0.1
0.0
0.1
β_IV

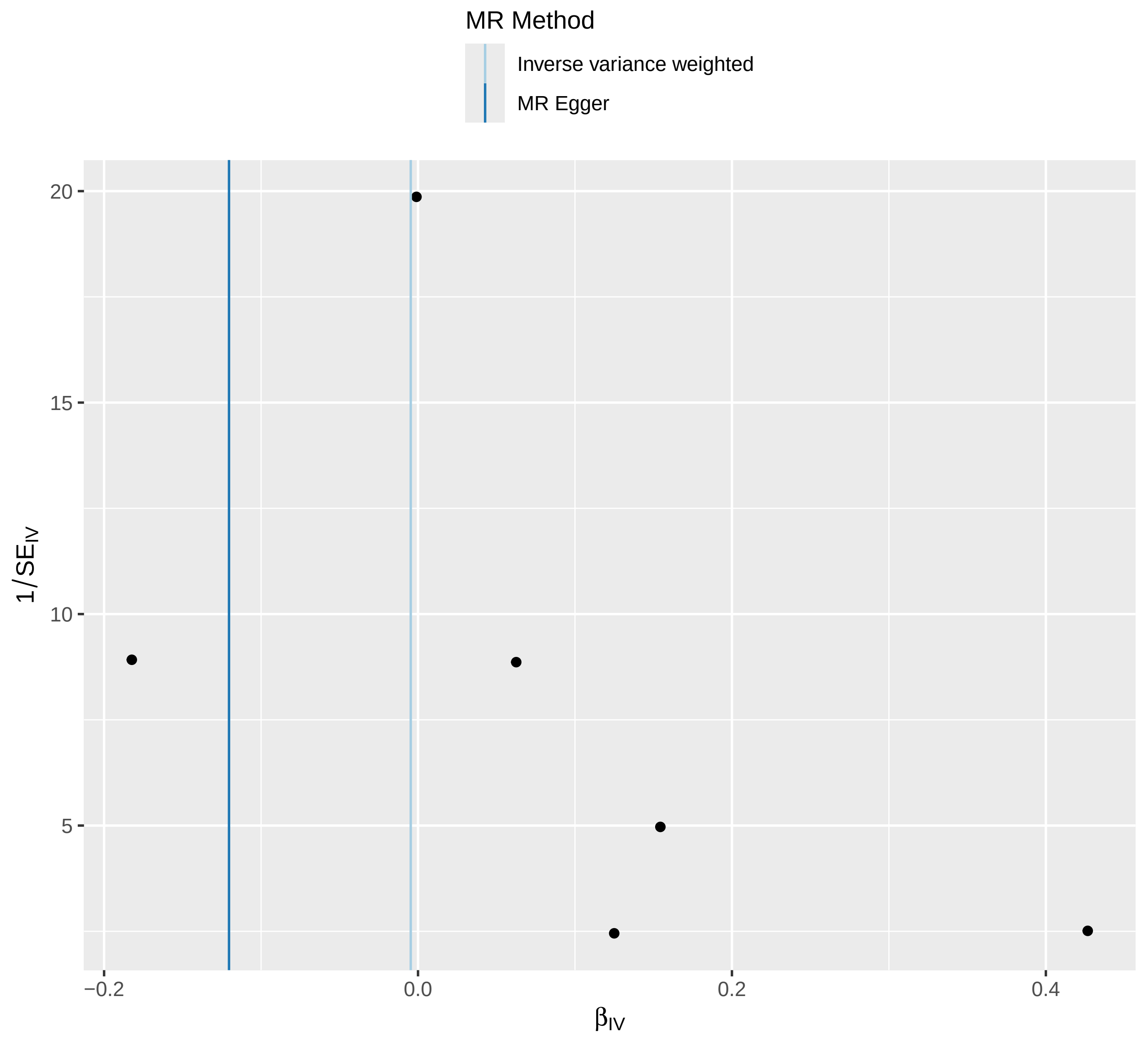
MR Method
Inverse variance weighted
MR Egger
20
15
10
5
$1/SE_{IV}$
−0.2
0.0
0.2
0.4
$\beta_{IV}$

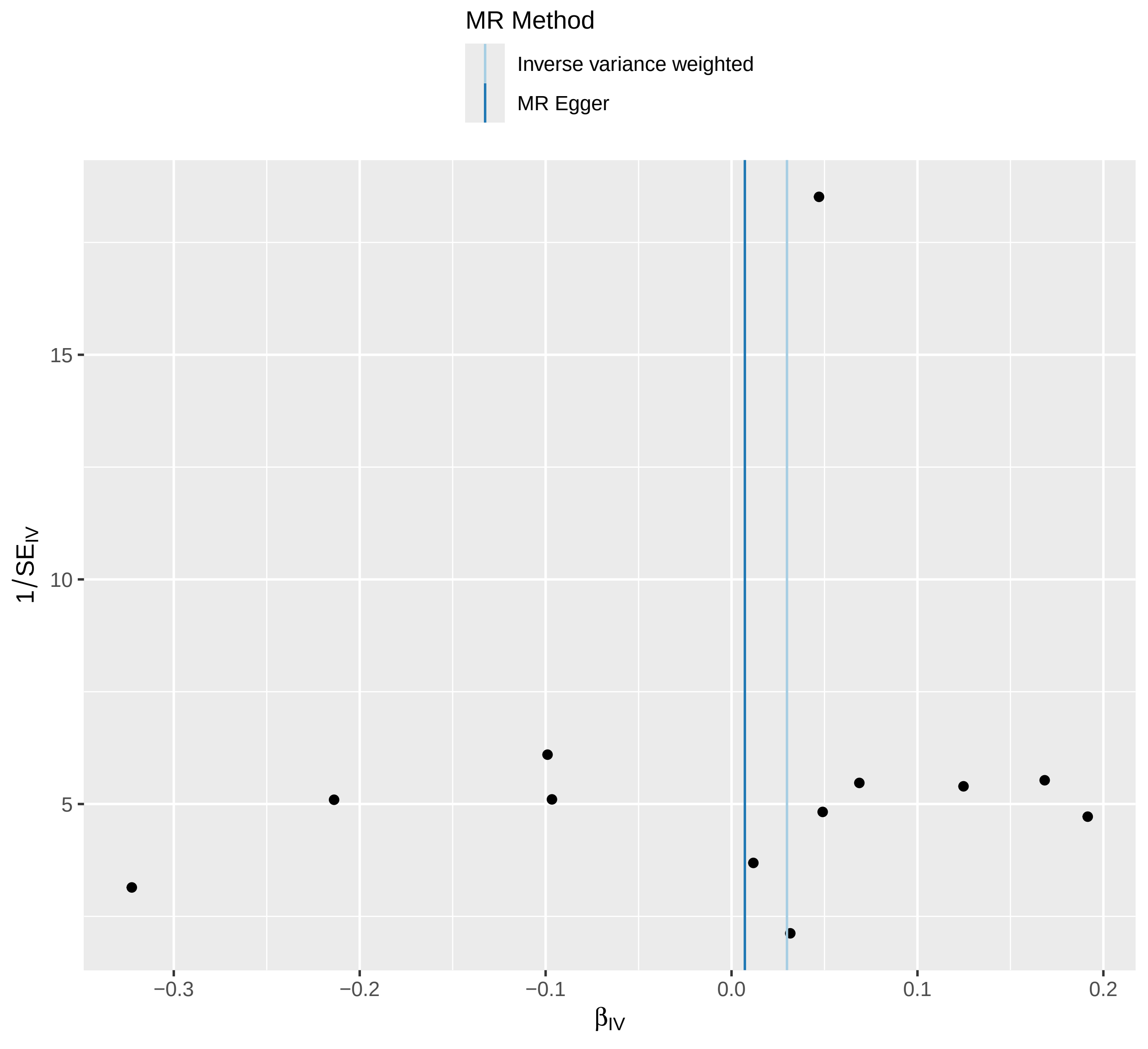
MR Method
Inverse variance weighted
MR Egger
15
10
5
1/$SE_{IV}$
−0.3
−0.2
−0.1
0.0
0.1
0.2
$\beta_{IV}$

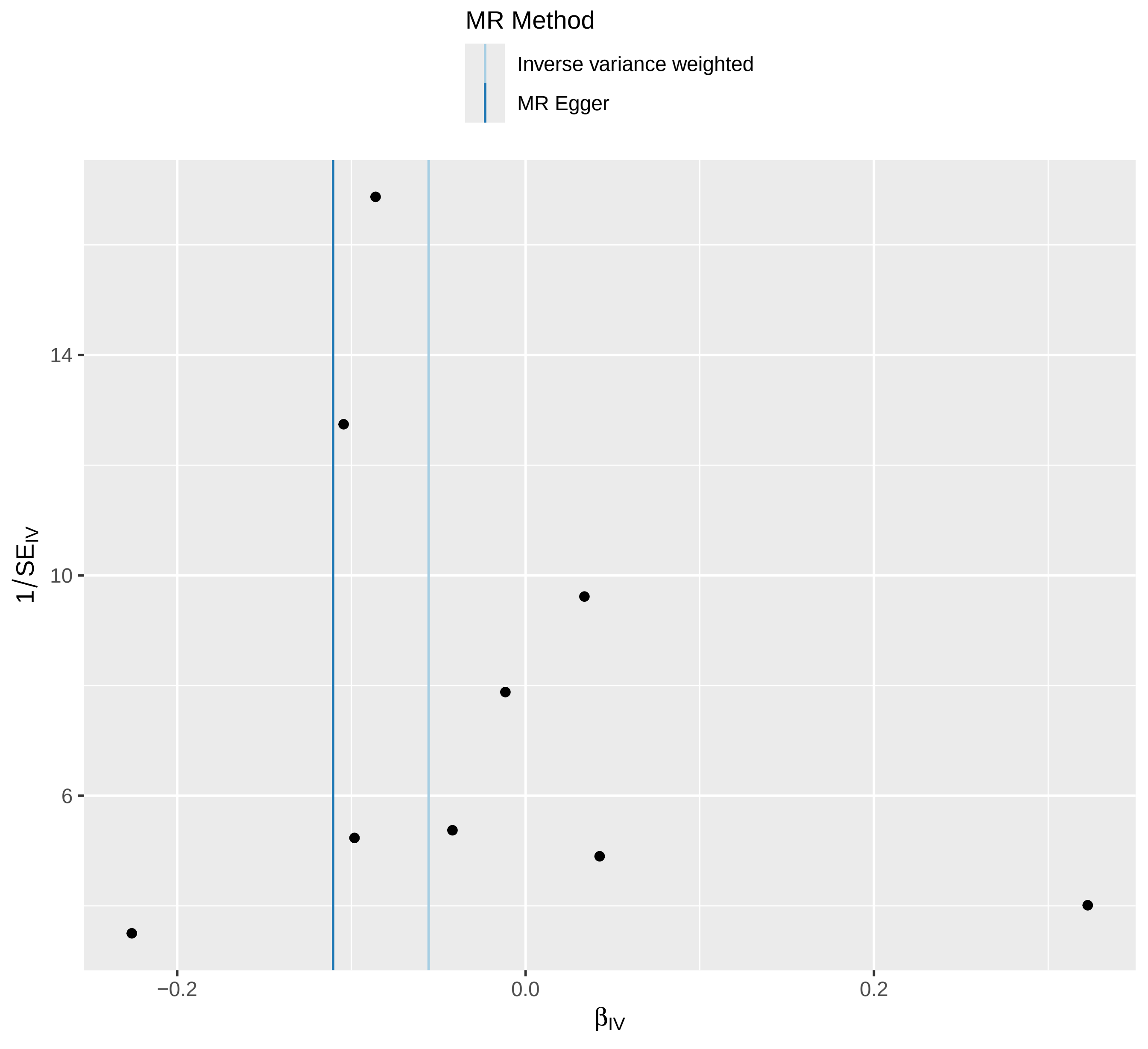

MR Method
Inverse variance weighted
MR Egger
14
10
6
$1/SE_{IV}$
−0.2
0.0
0.2
$\beta_{IV}$

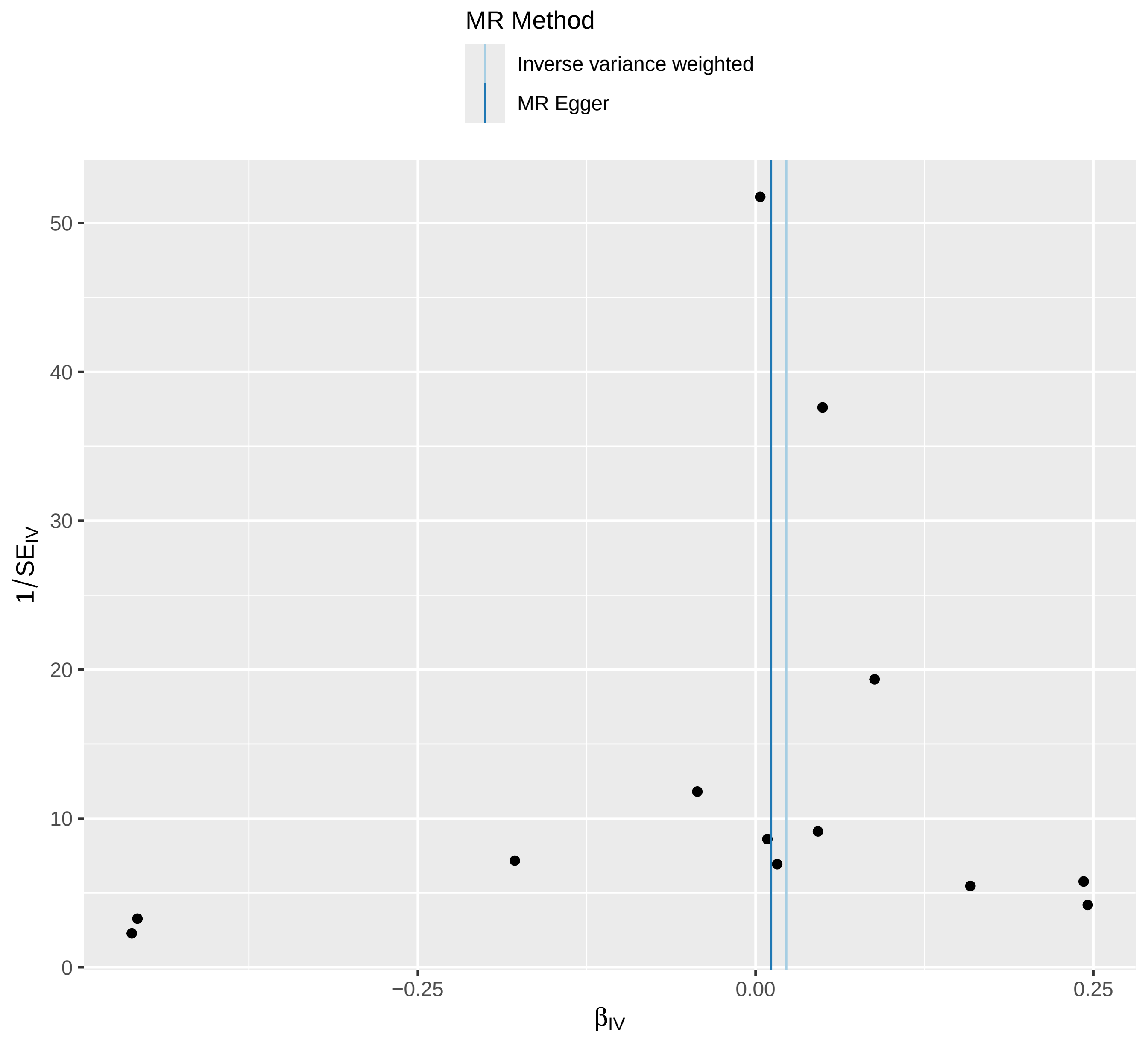
MR Method
Inverse variance weighted
MR Egger
1/SE$_{IV}$
β$_{IV}$
0
10
20
30
40
50
−0.25
0.00
0.25

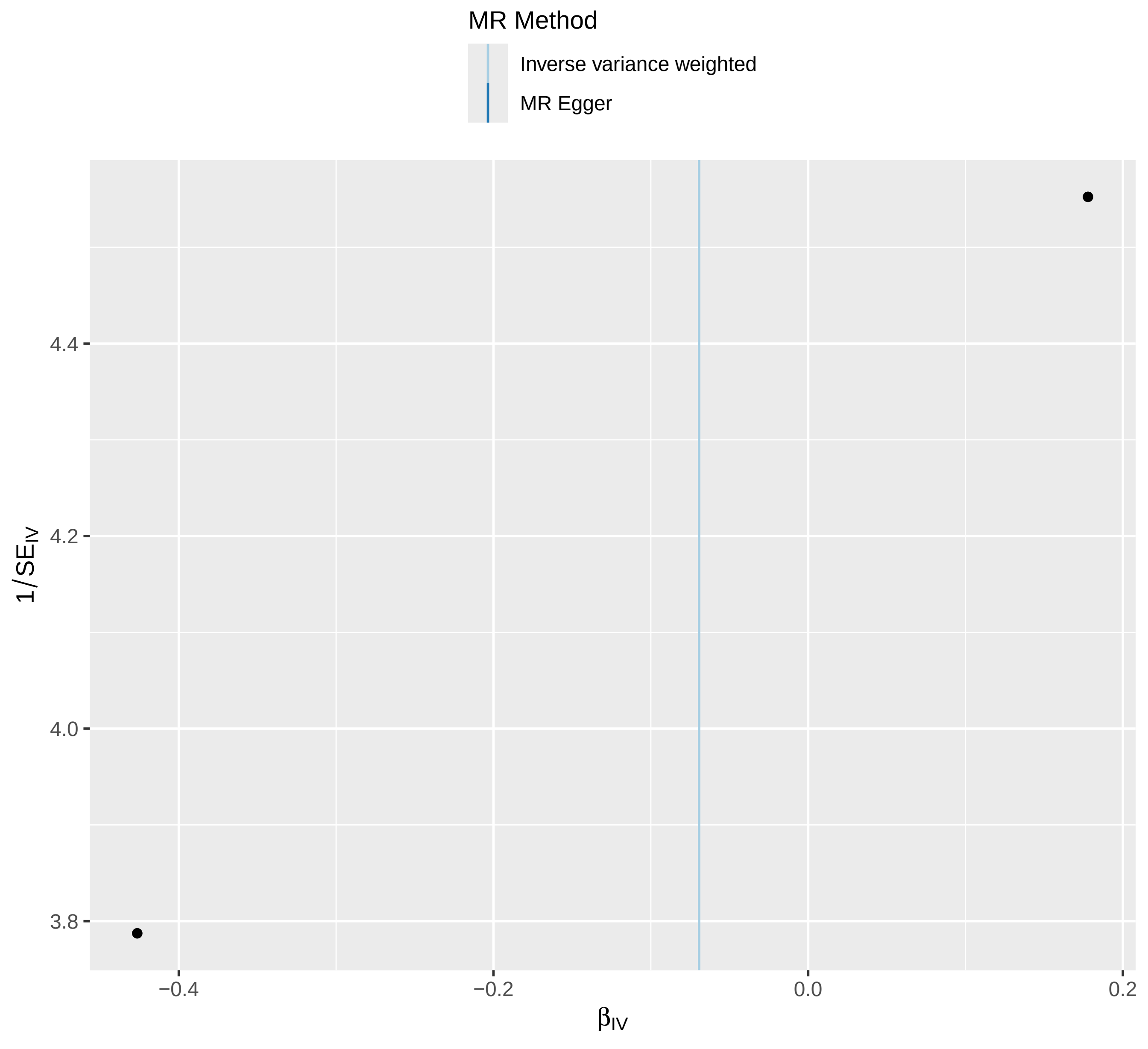
MR Method
Inverse variance weighted
MR Egger
4.4
4.2
4.0
3.8
1/SE_IV
−0.4
−0.2
0.0
0.2
β_IV

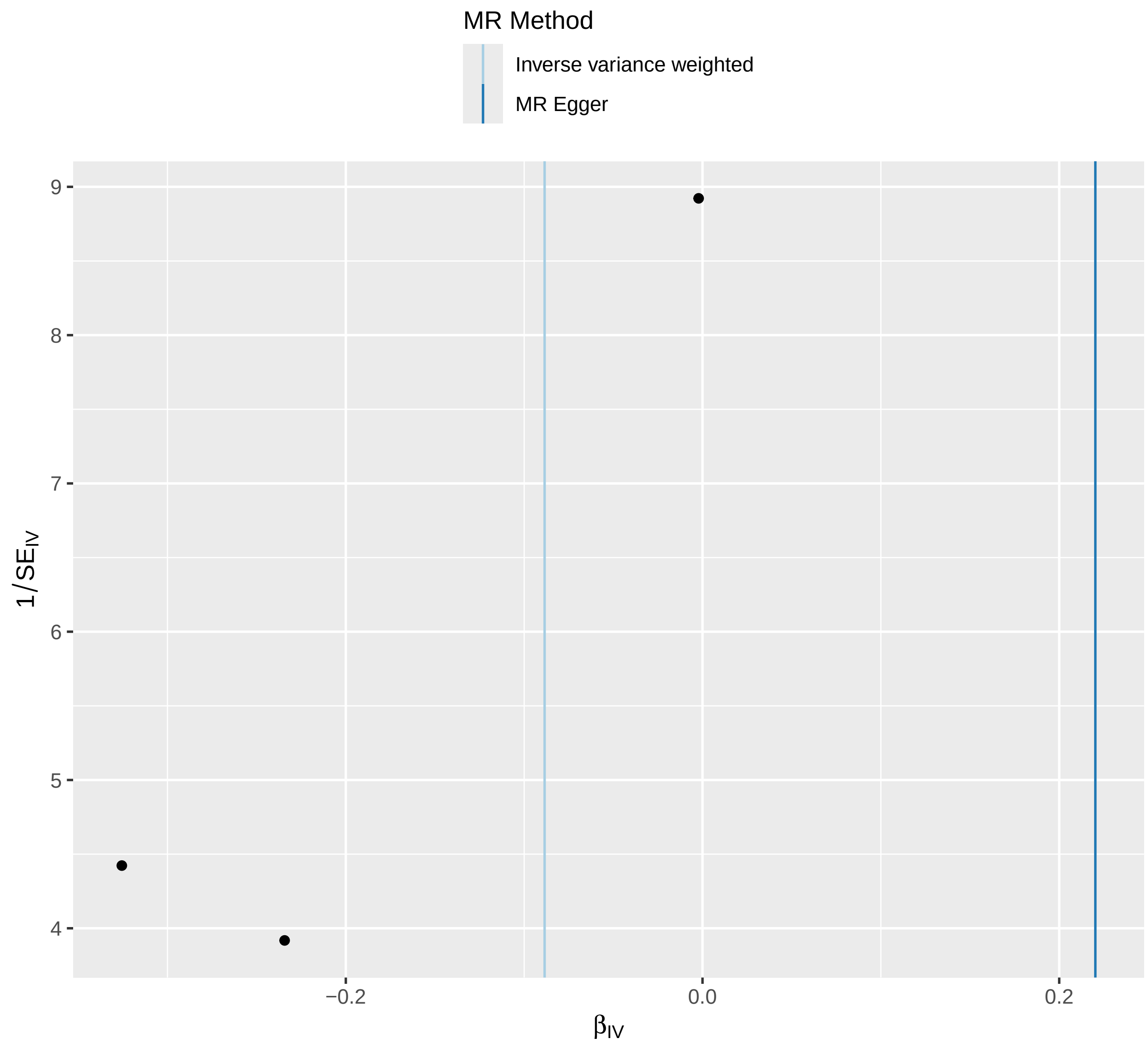
MR Method
Inverse variance weighted
MR Egger
9
8
7
6
5
4
1/SE_IV
−0.2
0.0
0.2
β_IV

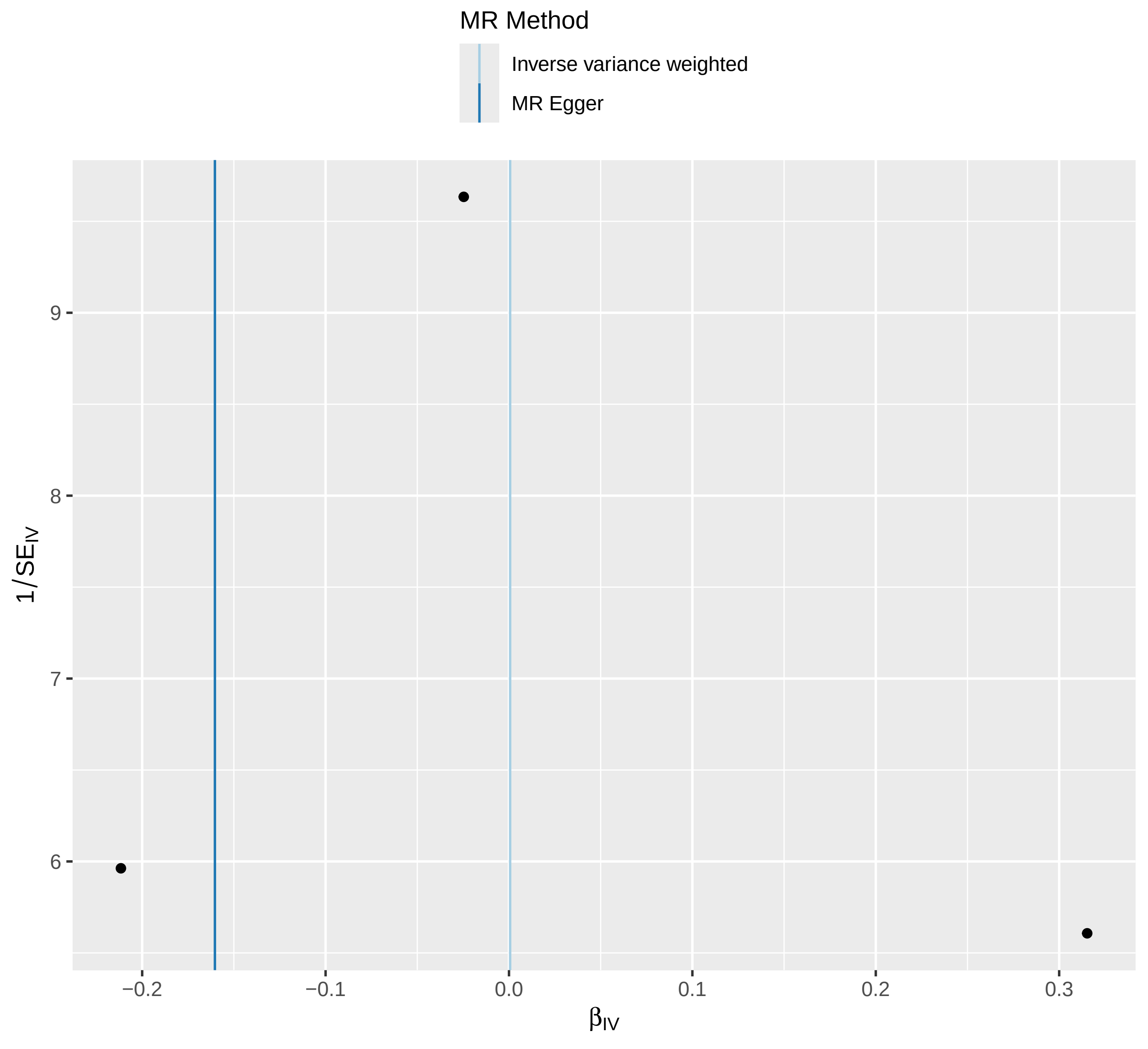
MR Method
Inverse variance weighted
MR Egger
1/SE_IV
9
8
7
6
−0.2
−0.1
0.0
0.1
0.2
0.3
β_IV

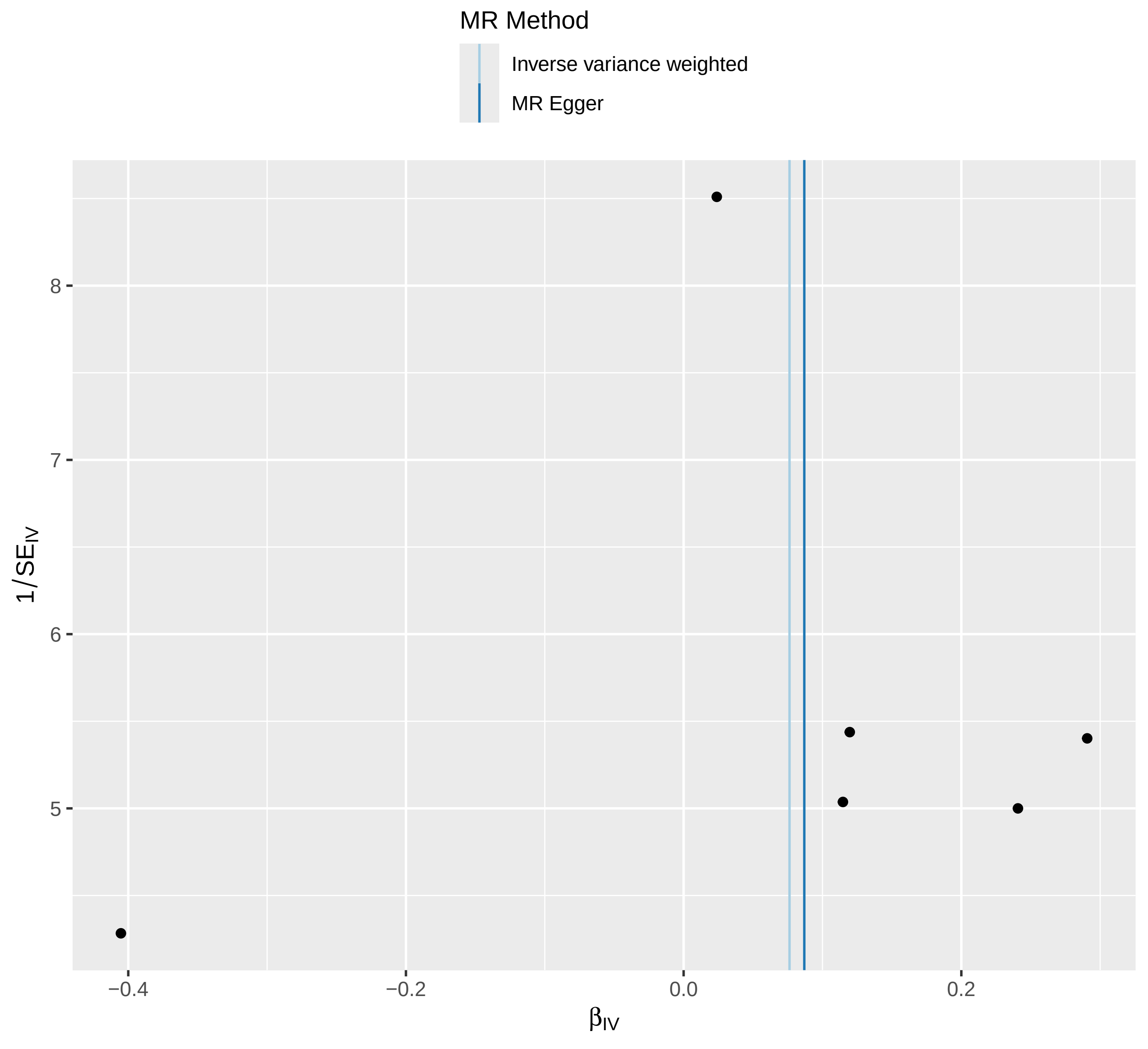

MR Method
Inverse variance weighted
MR Egger
8
7
6
5
1/SE_IV
−0.4
−0.2
0.0
0.2
β_IV

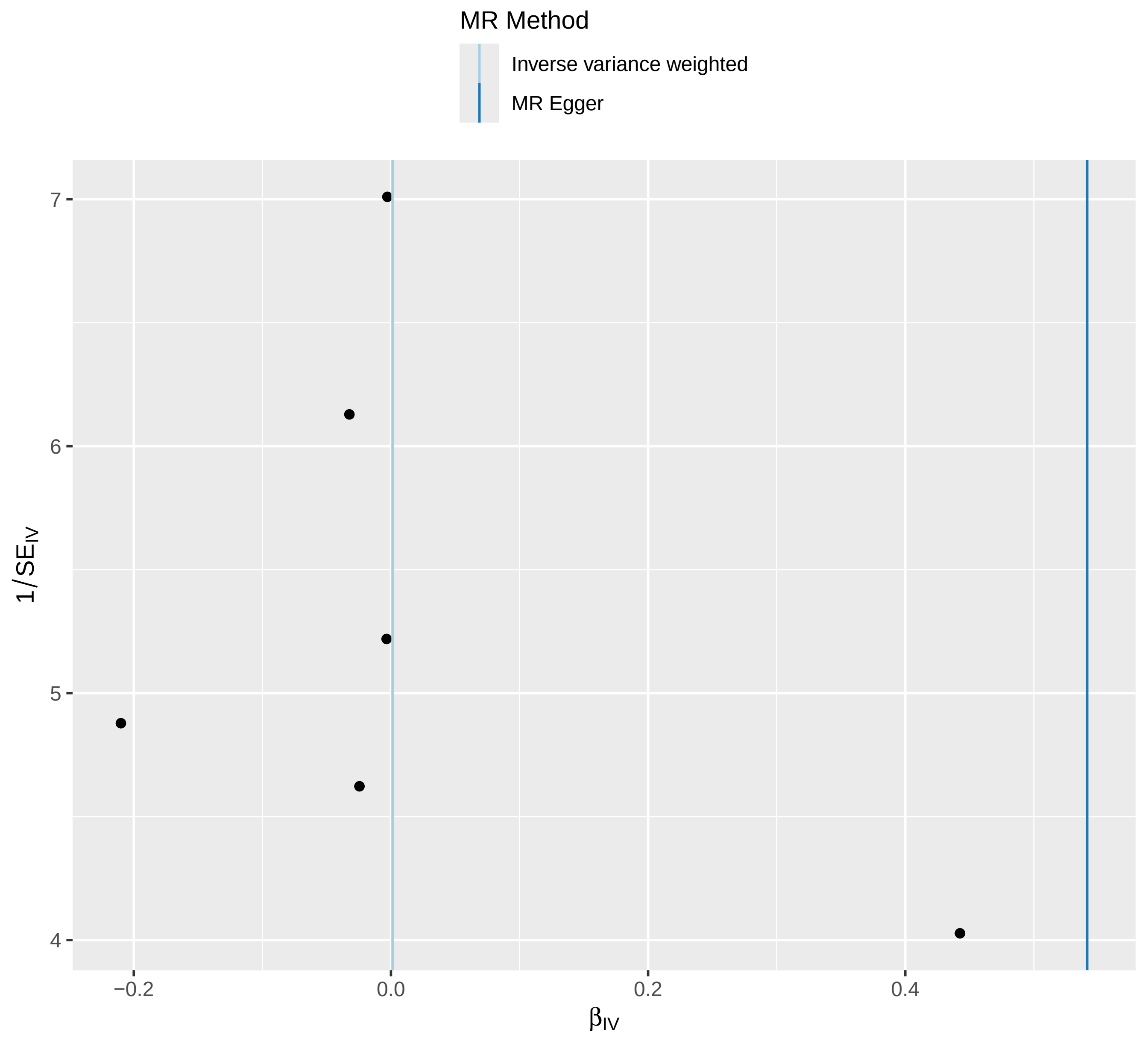

MR Method
Inverse variance weighted
MR Egger
7
6
5
4
−0.2
0.0
0.2
0.4
1/SE$_{IV}$
β$_{IV}$

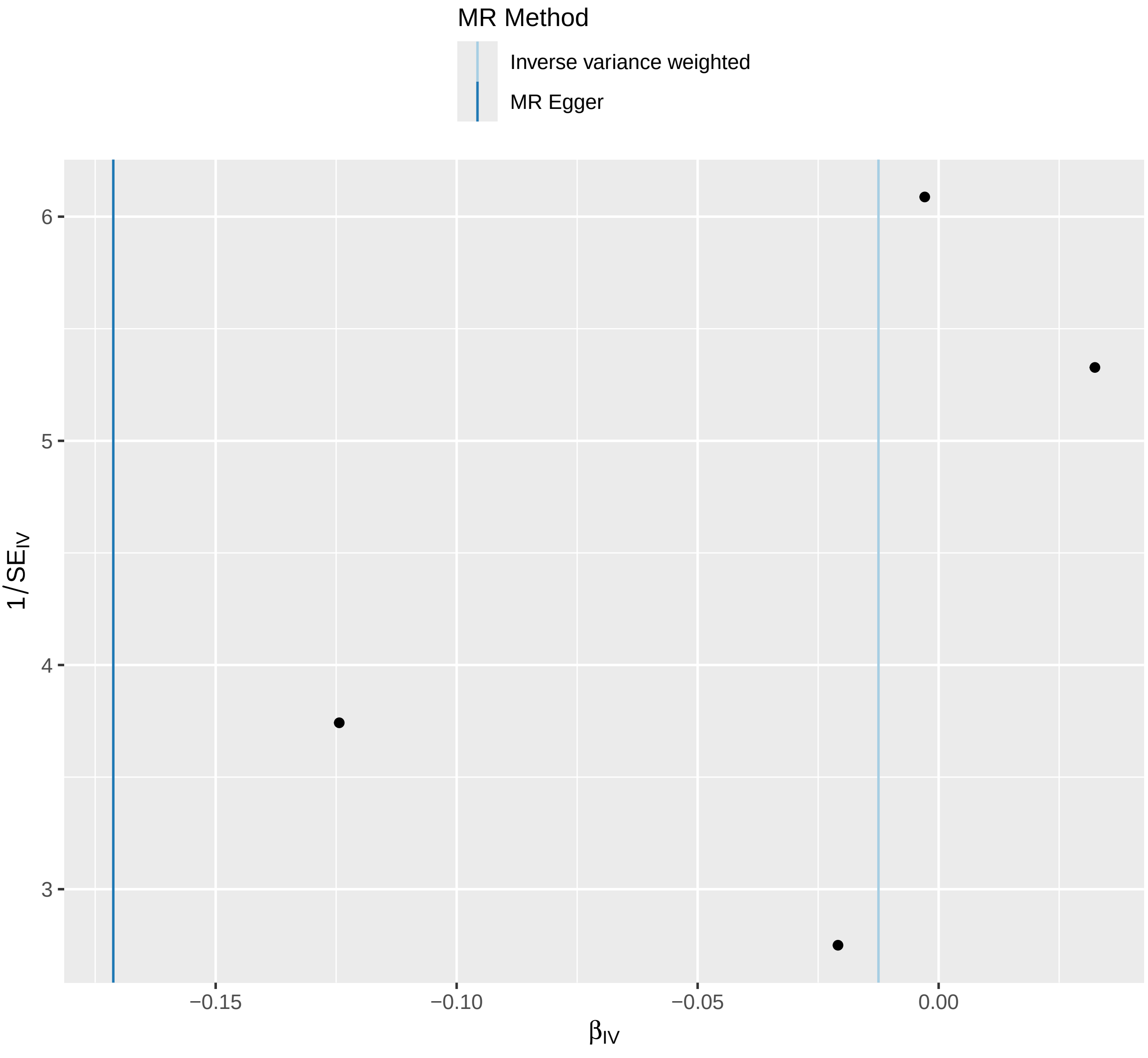
MR Method
Inverse variance weighted
MR Egger
6
5
4
3
1/SE$_{IV}$
−0.15
−0.10
−0.05
0.00
β$_{IV}$

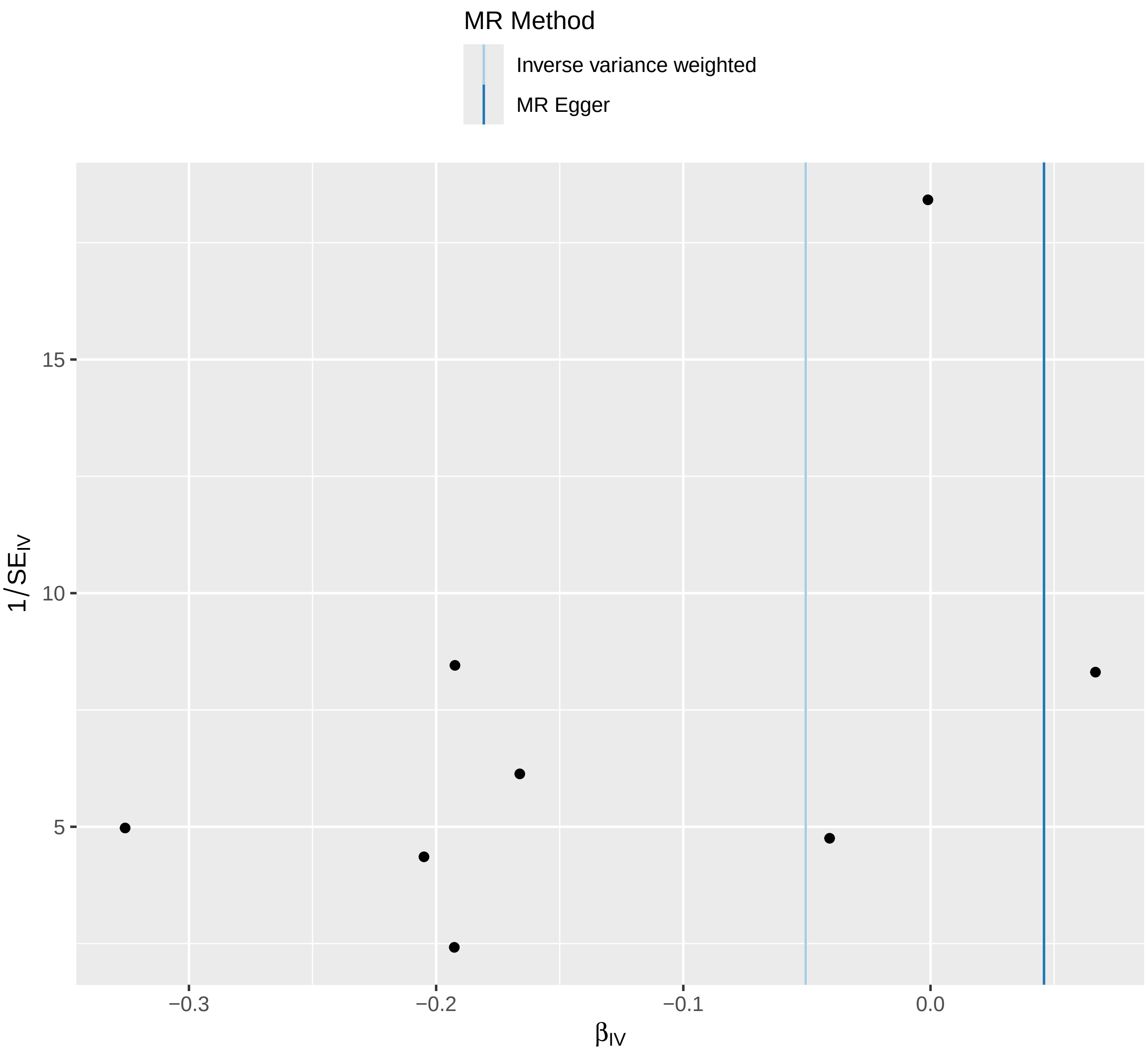
MR Method
Inverse variance weighted
MR Egger
15
10
5
1/SE$_{IV}$
−0.3
−0.2
−0.1
0.0
β$_{IV}$

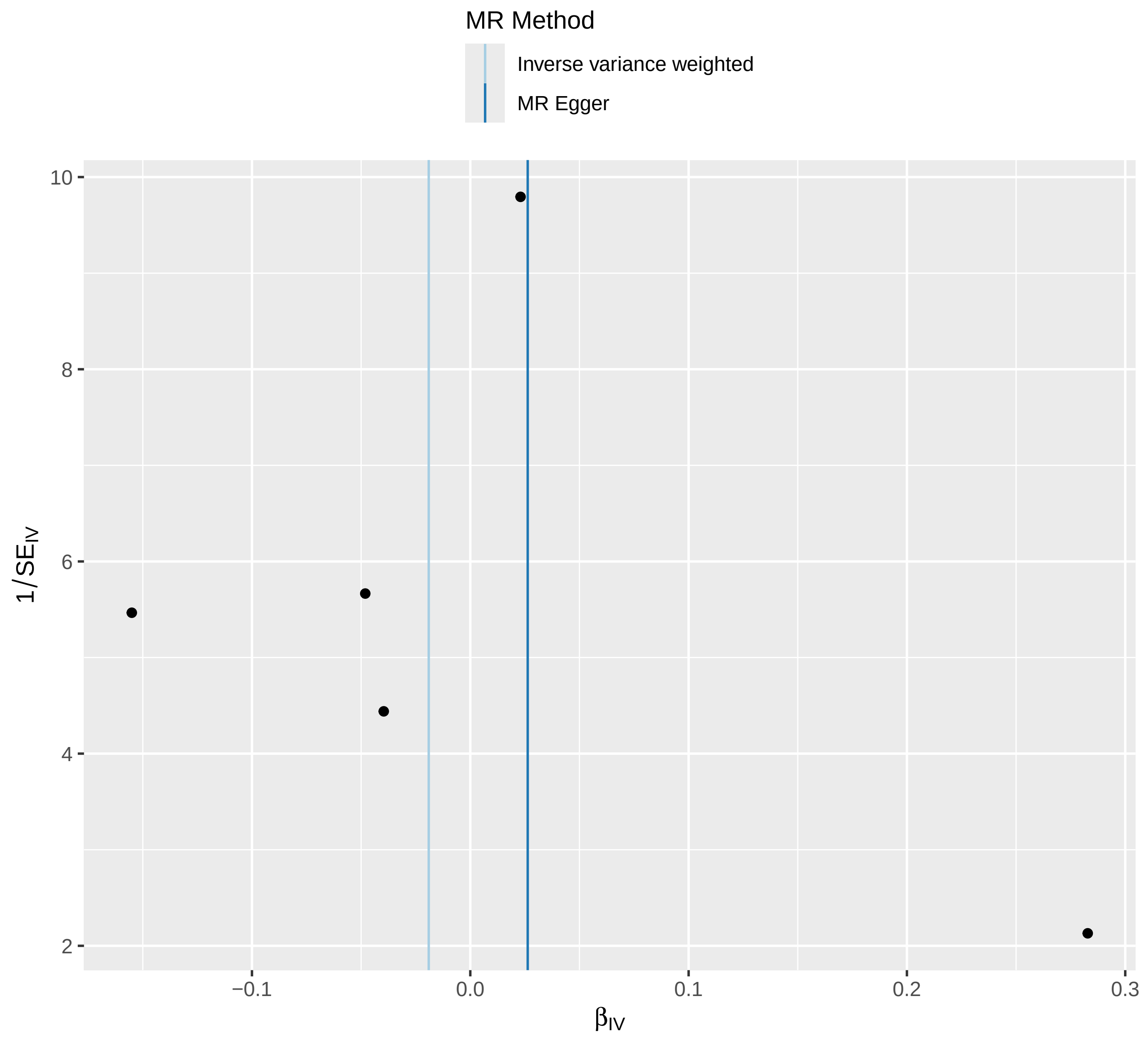
MR Method
Inverse variance weighted
MR Egger
1/SE_IV
β_IV
10
8
6
4
2
−0.1
0.0
0.1
0.2
0.3

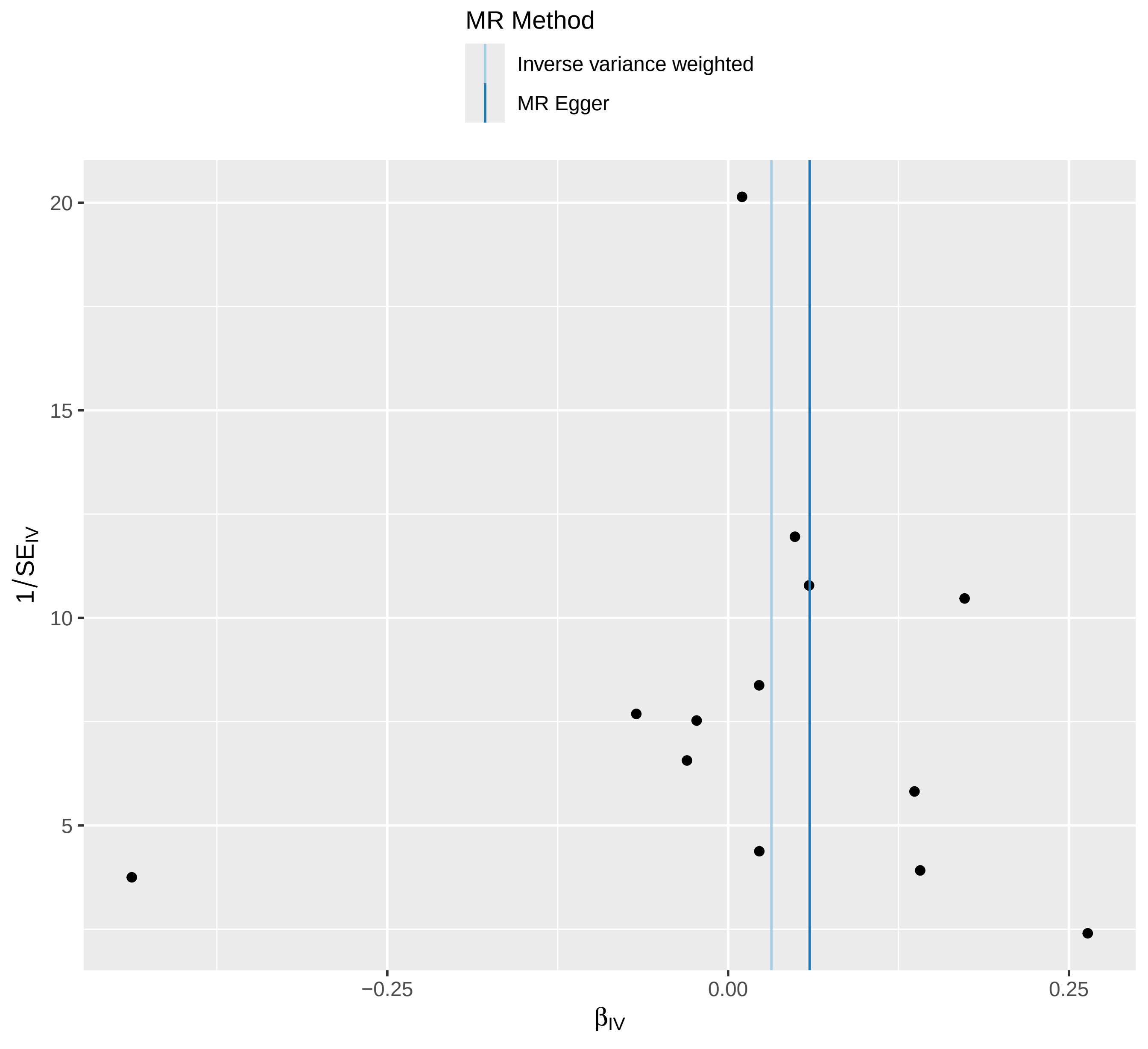
MR Method
Inverse variance weighted
MR Egger
20
15
10
5
1/SE_IV
−0.25
0.00
0.25
β_IV

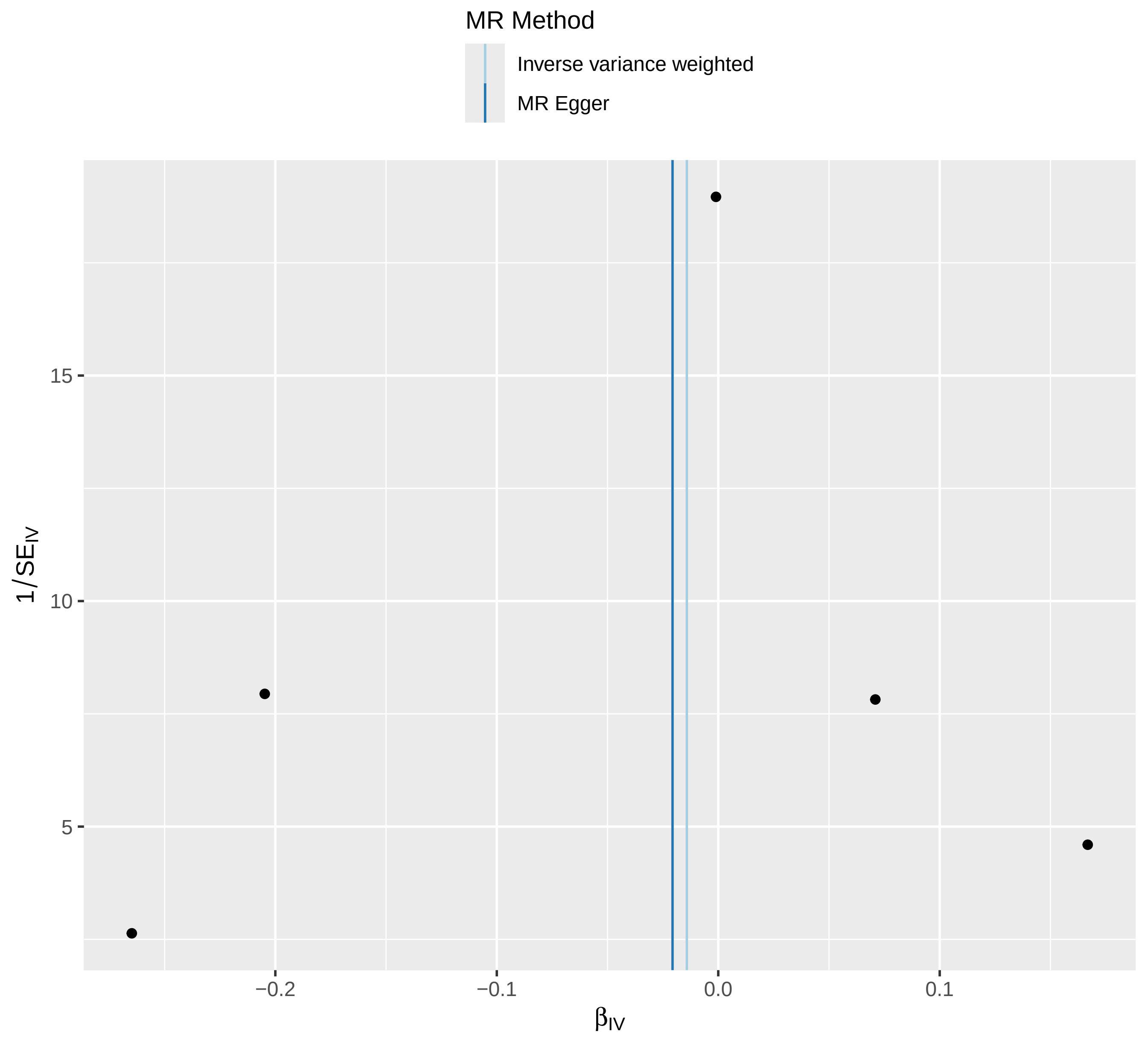

MR Method
Inverse variance weighted
MR Egger
1/SE_IV
15
10
5
−0.2
−0.1
0.0
0.1
β_IV

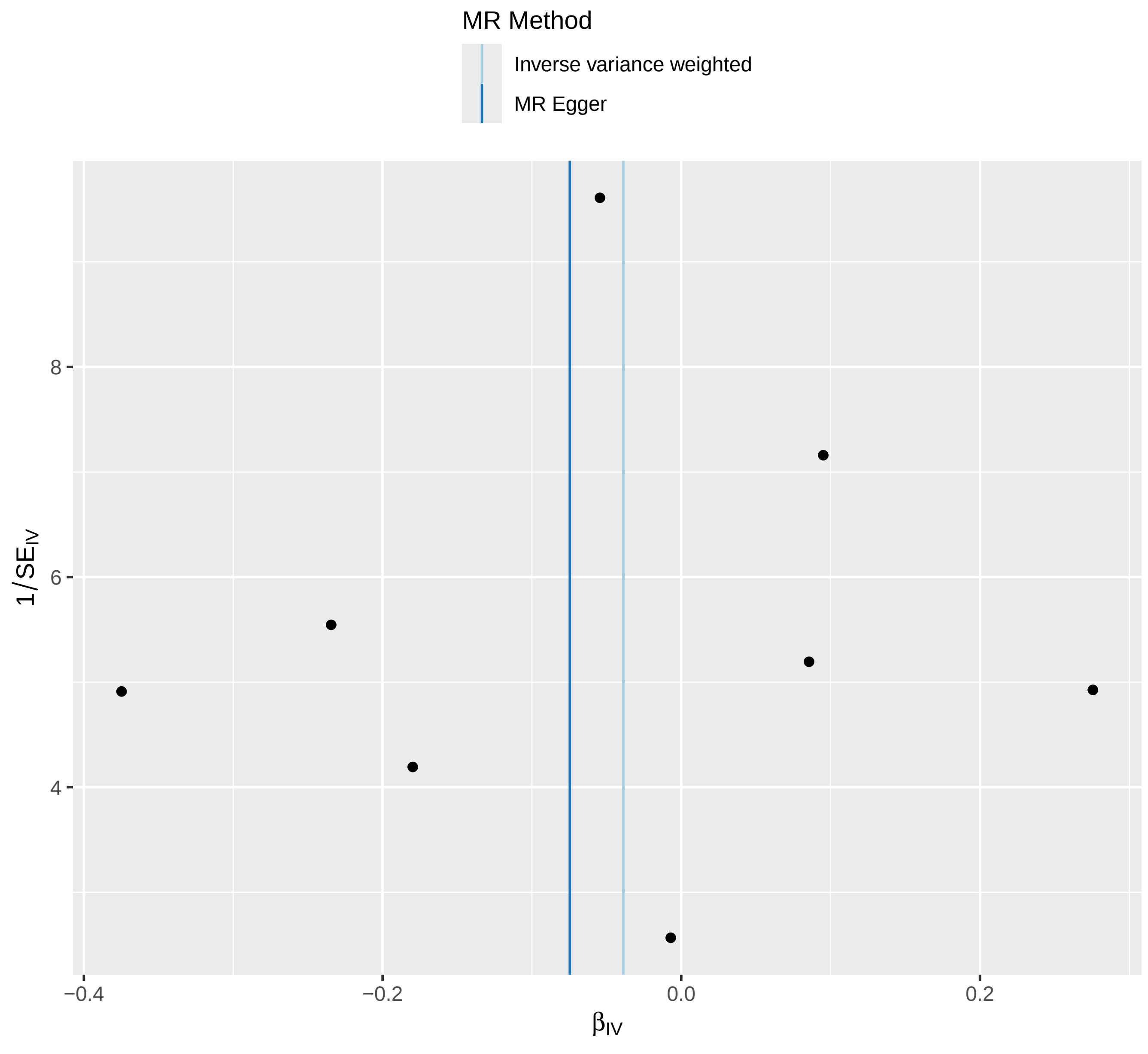

MR Method
Inverse variance weighted
MR Egger
1/SE$_{IV}$
8
6
4
−0.4
−0.2
0.0
0.2
β$_{IV}$

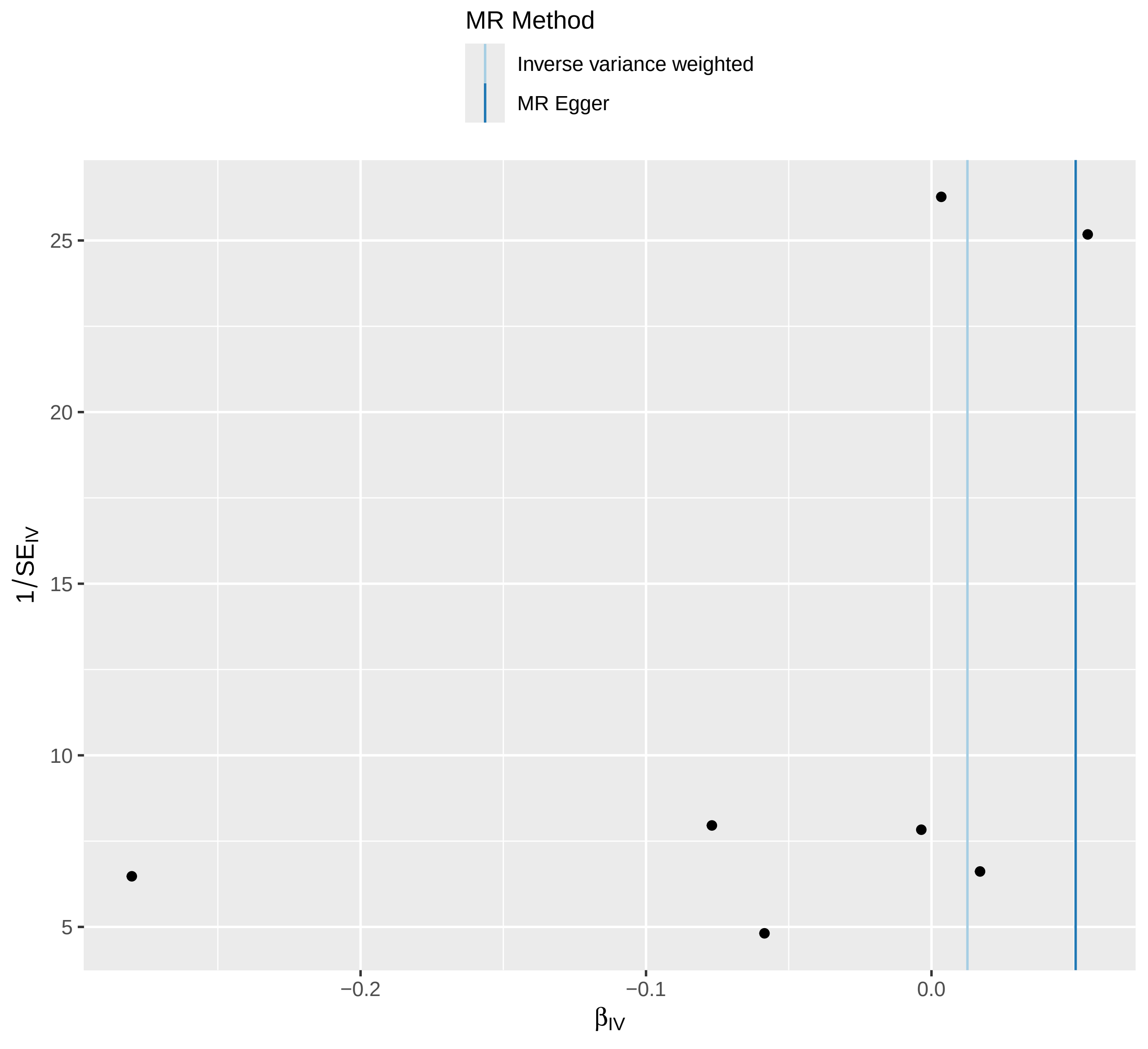

MR Method
Inverse variance weighted
MR Egger
25
20
15
10
5
1/SE_IV
−0.2
−0.1
0.0
β_IV

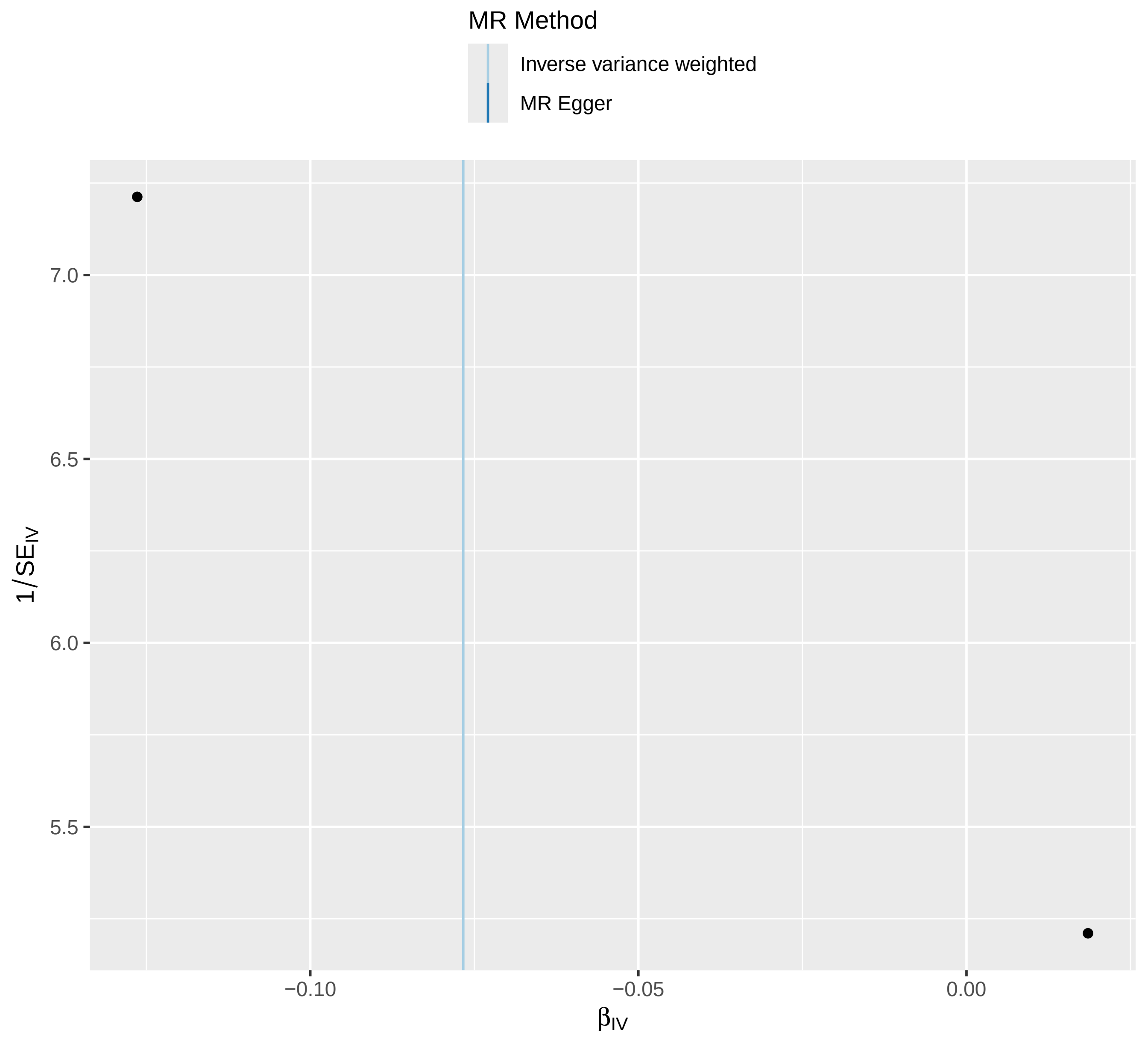

MR Method
Inverse variance weighted
MR Egger
7.0
6.5
6.0
5.5
1/SE_IV
−0.10
−0.05
0.00
β_IV

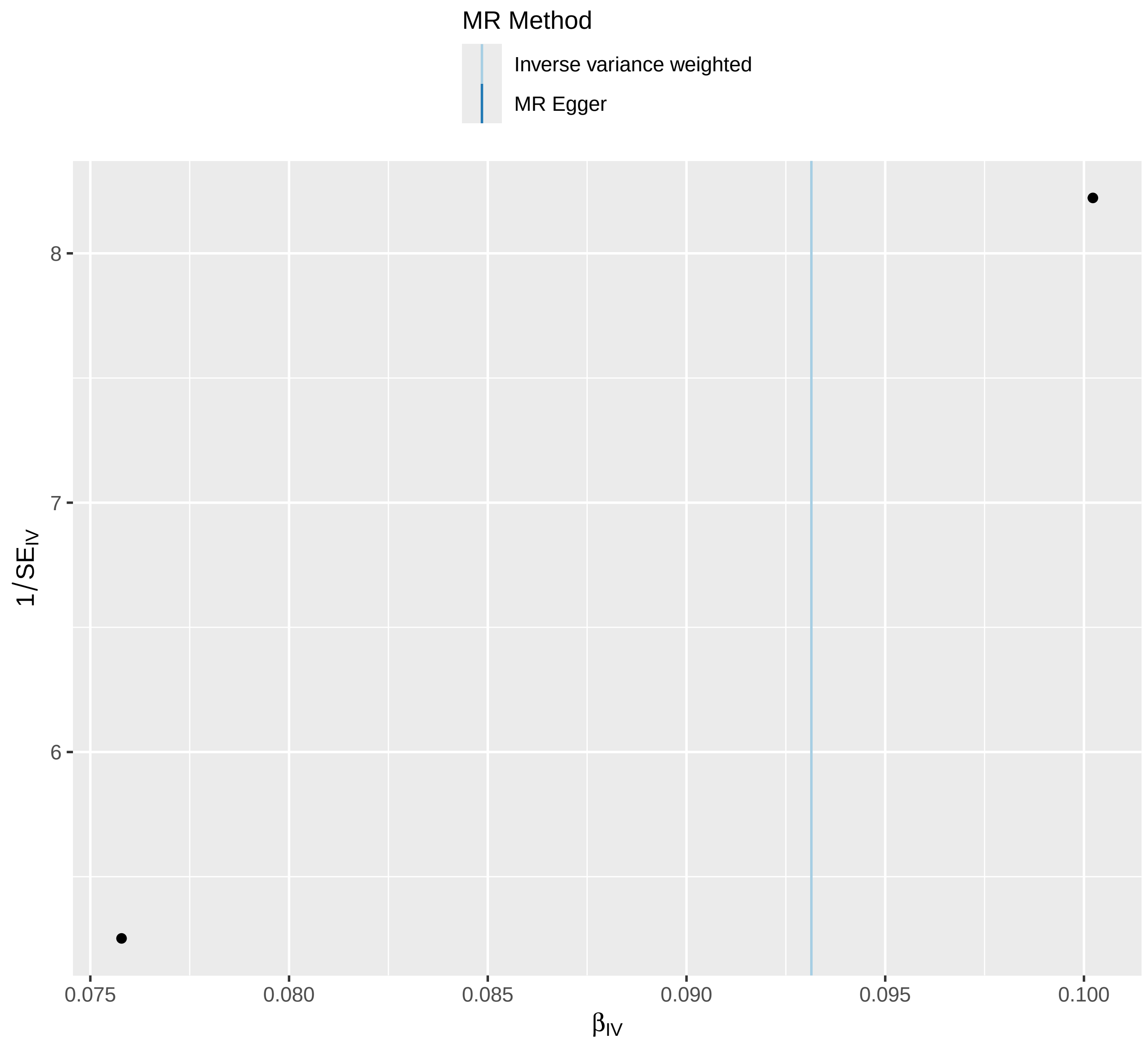

MR Method
Inverse variance weighted
MR Egger
8
7
6
1/SE$_{IV}$
0.075
0.080
0.085
0.090
0.095
0.100
β$_{IV}$

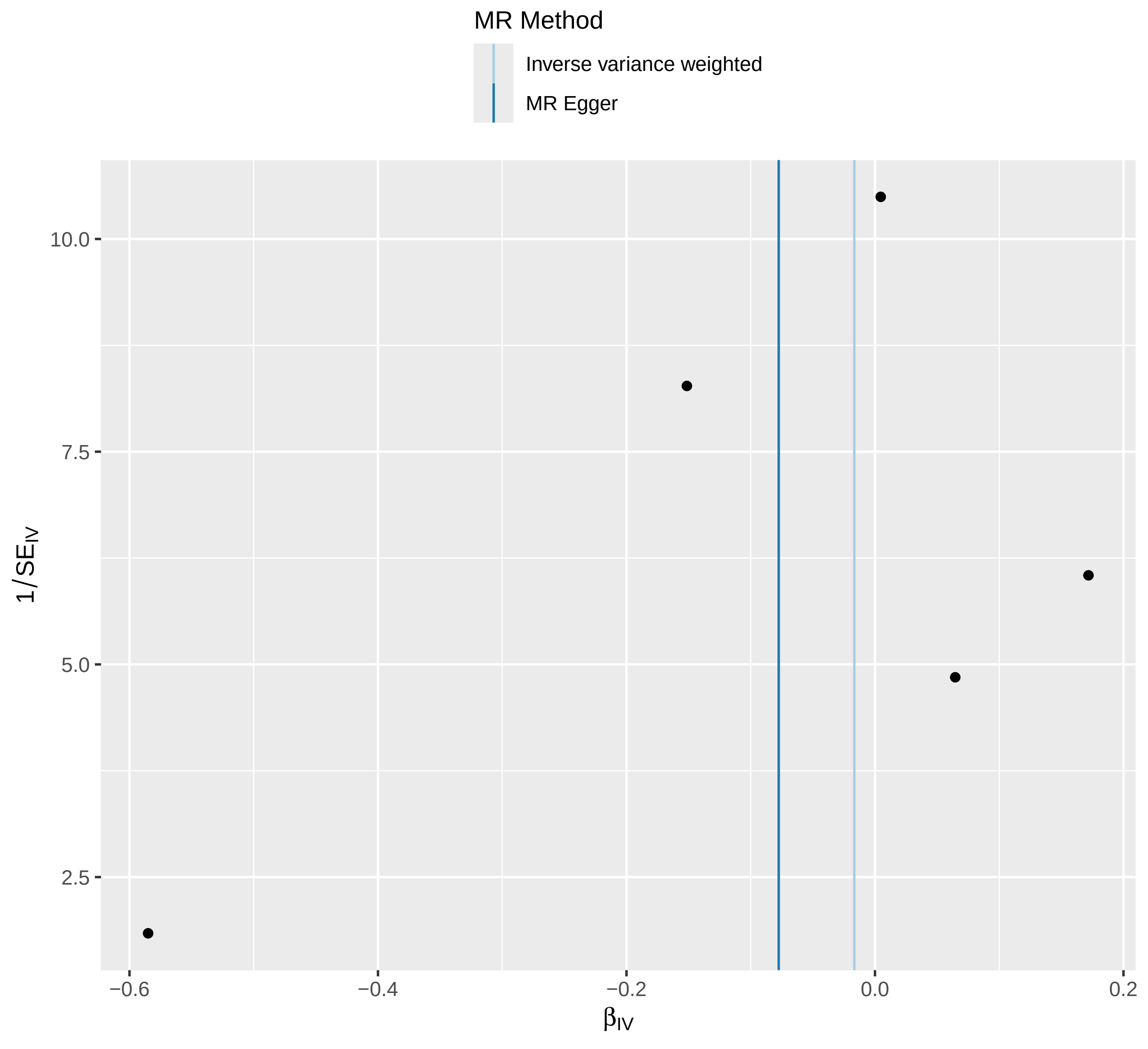

MR Method
Inverse variance weighted
MR Egger
10.0
7.5
5.0
2.5
1/SE_IV
−0.6
−0.4
−0.2
0.0
0.2
β_IV

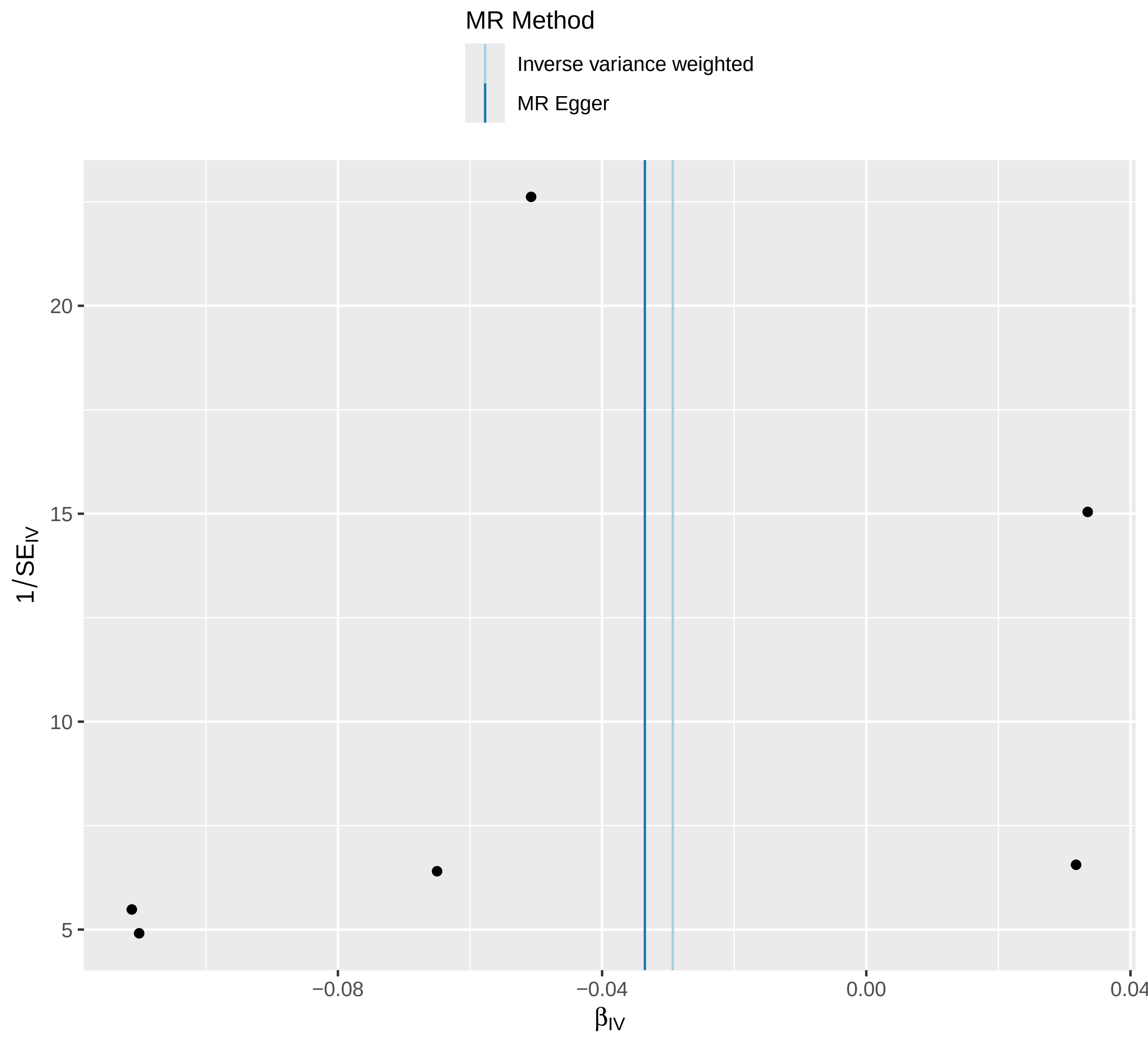
MR Method
Inverse variance weighted
MR Egger
20
15
10
5
1/SE$_{IV}$
−0.08
−0.04
0.00
0.04
β$_{IV}$

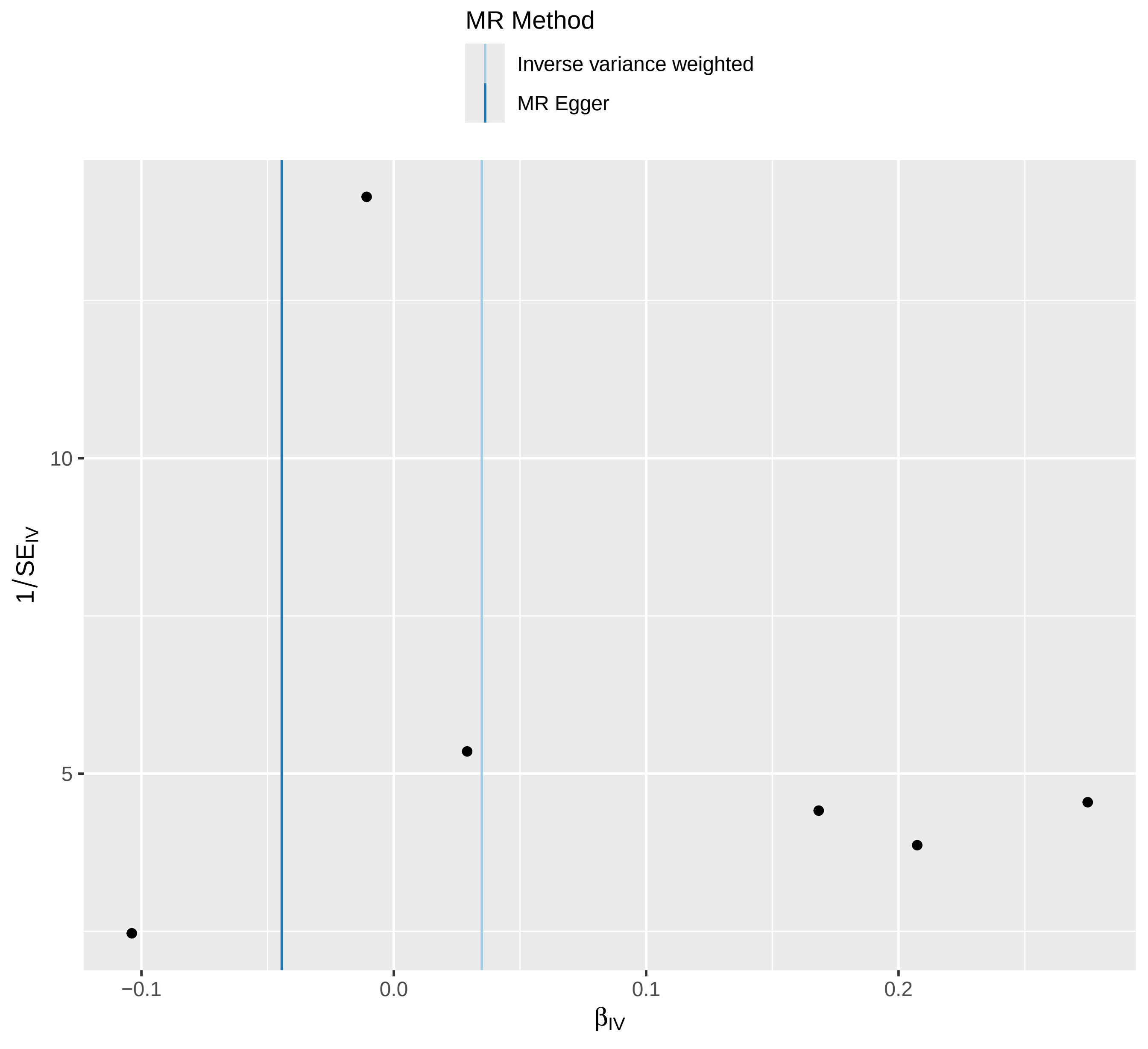
MR Method
Inverse variance weighted
MR Egger
10
5
1/SE_IV
−0.1
0.0
0.1
0.2
β_IV

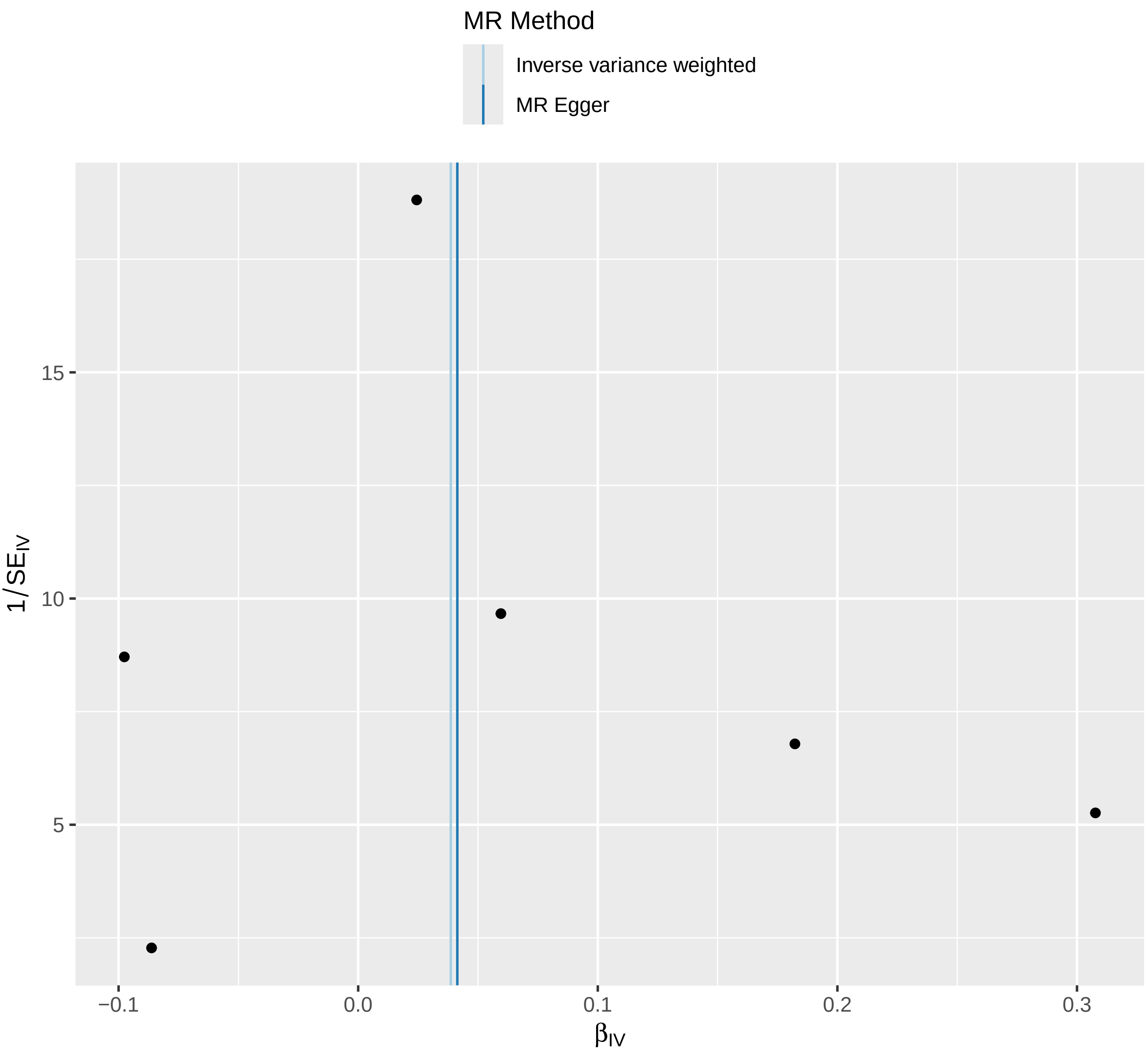
MR Method
Inverse variance weighted
MR Egger
15
10
5
1/SE$_{IV}$
−0.1
0.0
0.1
0.2
0.3
β$_{IV}$

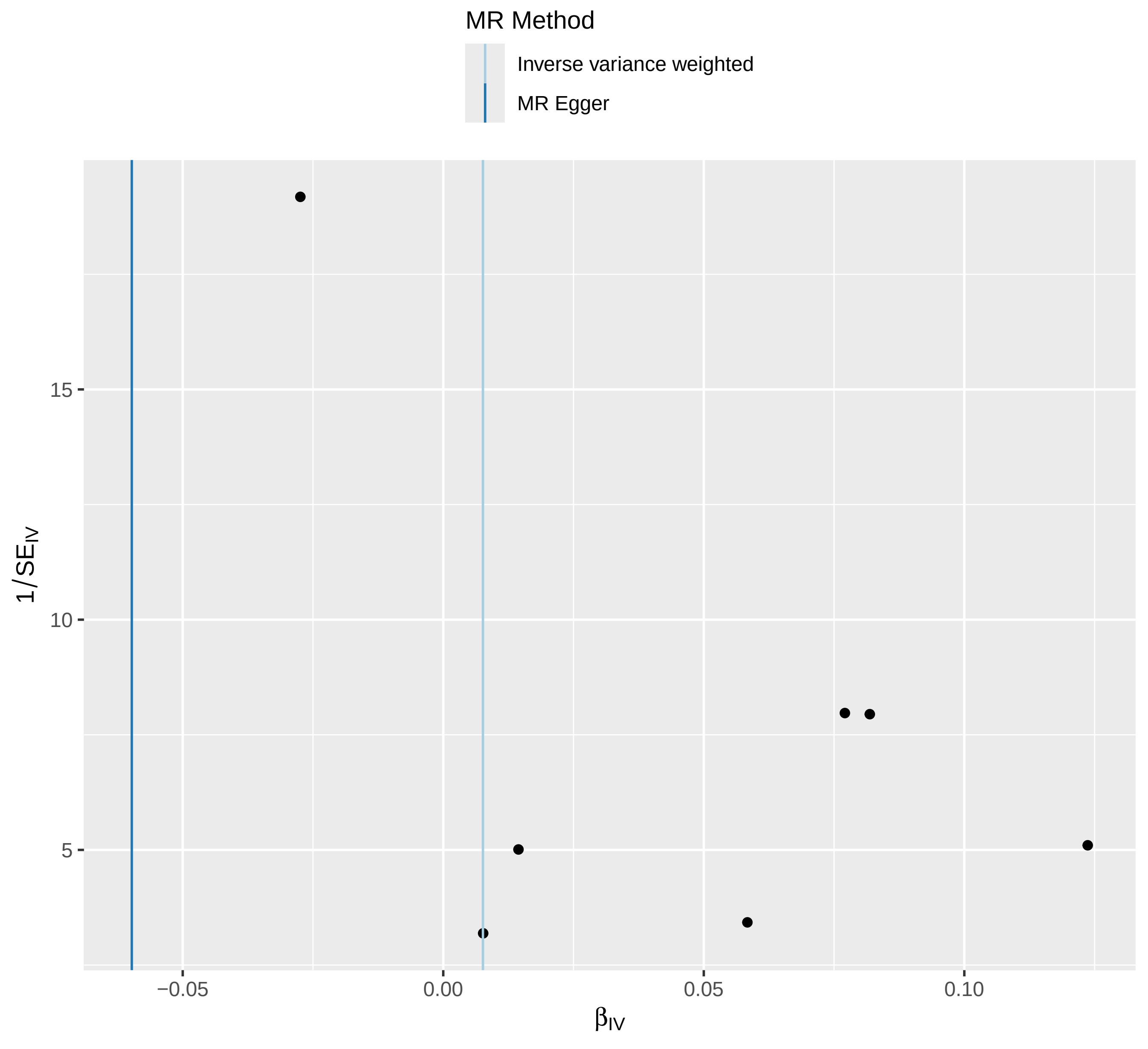

MR Method
Inverse variance weighted
MR Egger
15
10
5
1/SE$_{IV}$
−0.05
0.00
0.05
0.10
β$_{IV}$

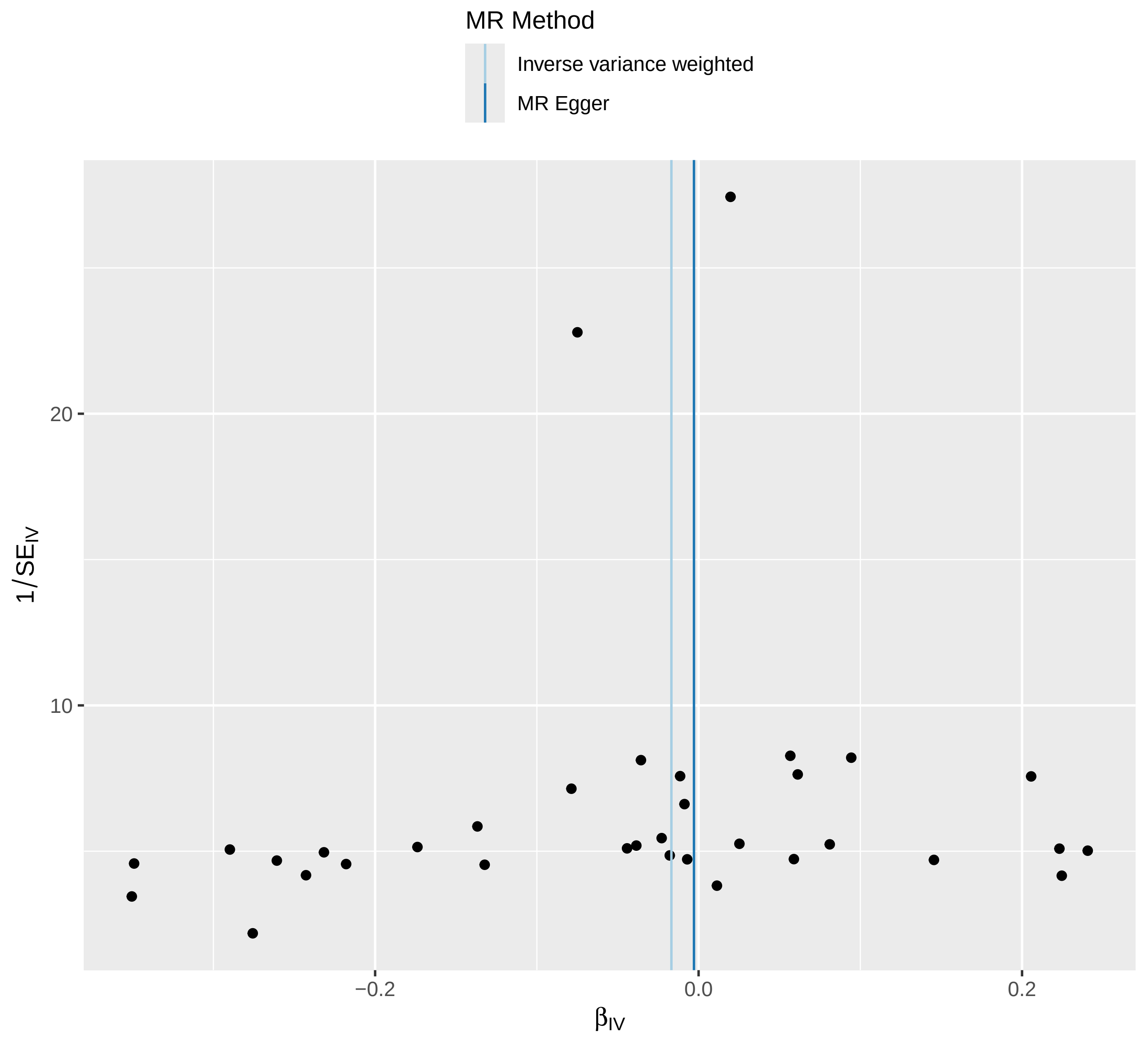
MR Method
Inverse variance weighted
MR Egger
20
10
$1/SE_{IV}$
−0.2
0.0
0.2
$\beta_{IV}$

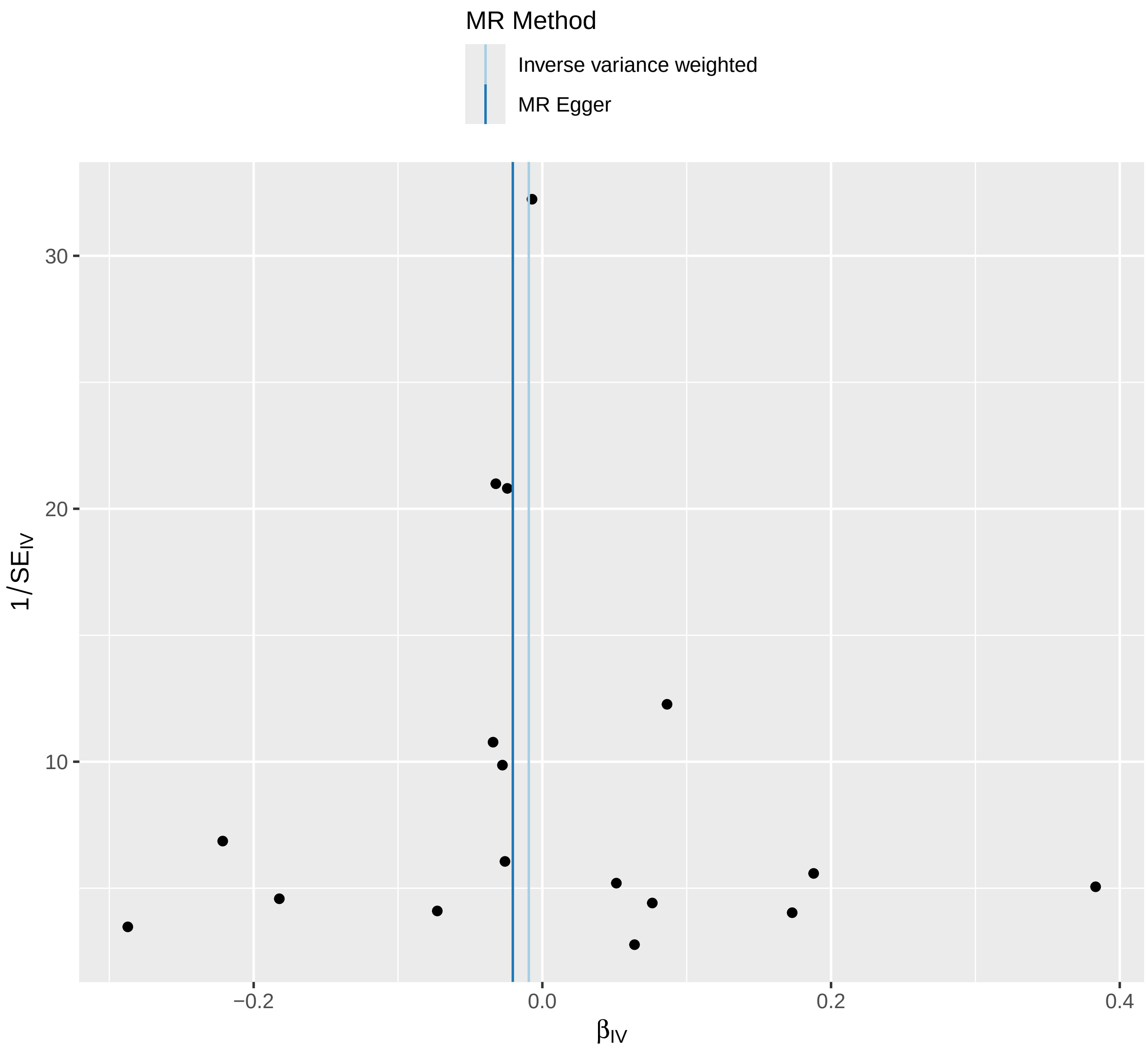

MR Method
Inverse variance weighted
MR Egger
30
20
10
1/SE_IV
−0.2
0.0
0.2
0.4
β_IV

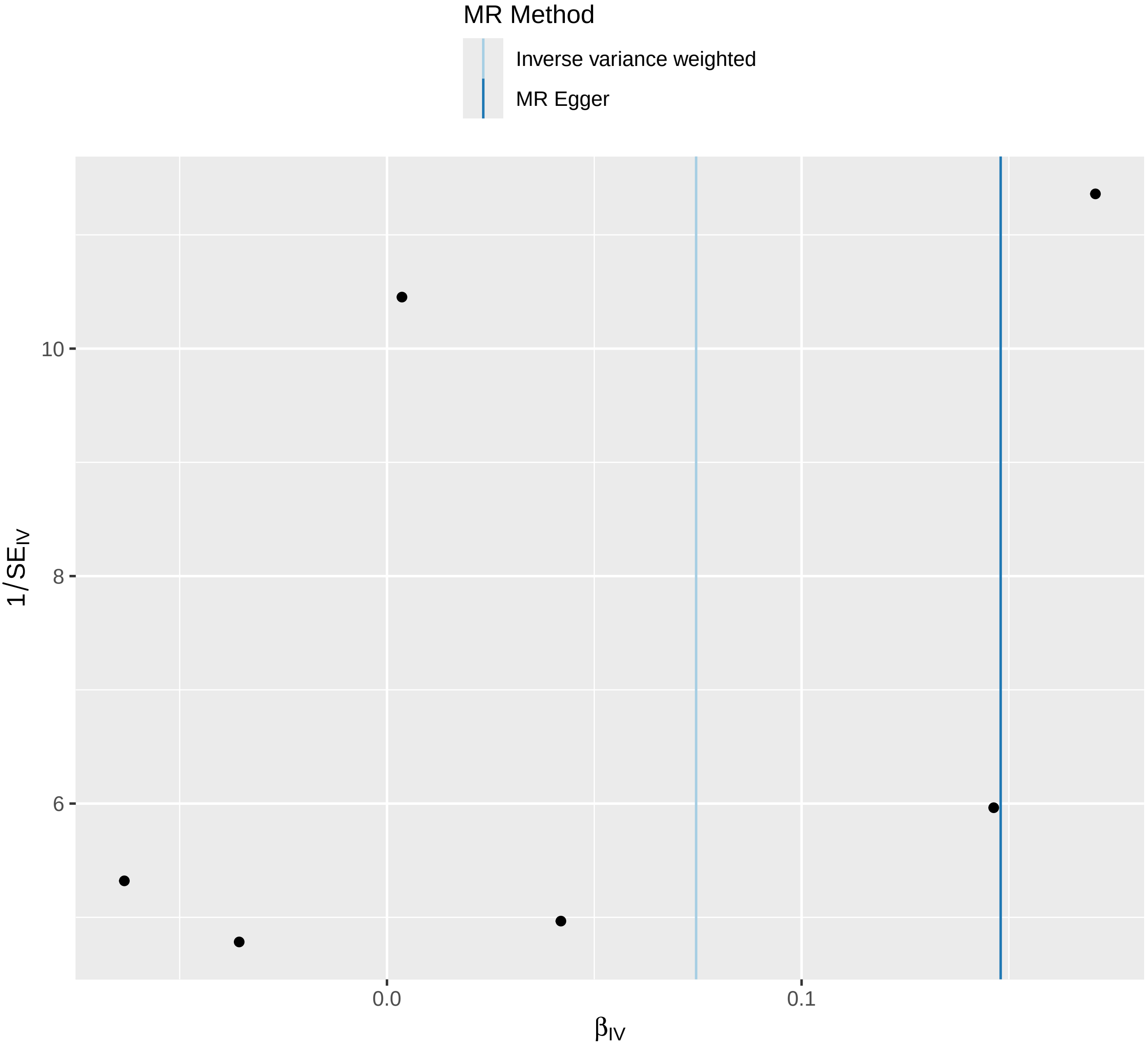
MR Method
Inverse variance weighted
MR Egger
10
8
6
1/SE_IV
0.0
0.1
β_IV

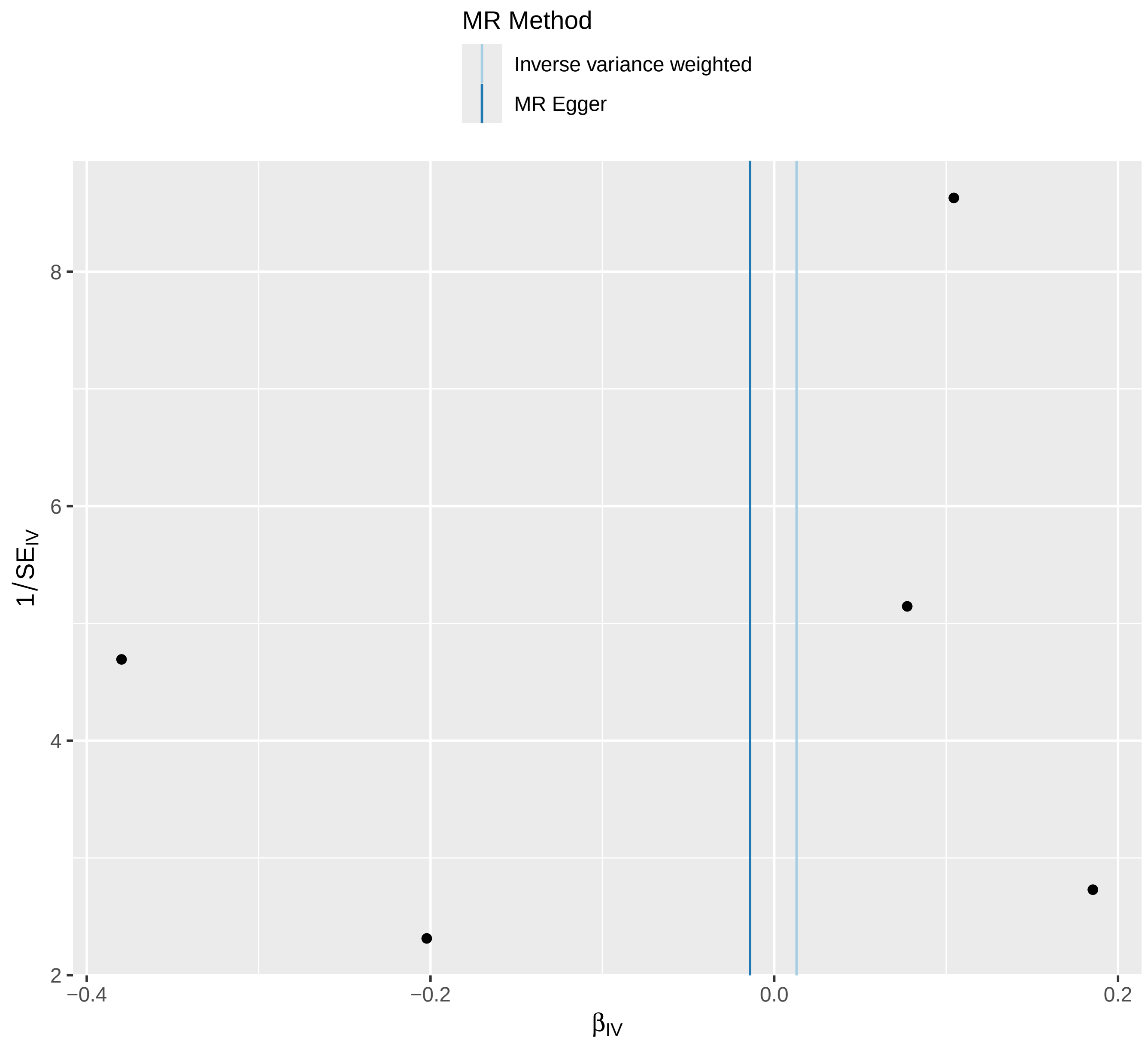
MR Method
Inverse variance weighted
MR Egger
1/SE_IV
8
6
4
2
−0.4
−0.2
0.0
0.2
β_IV

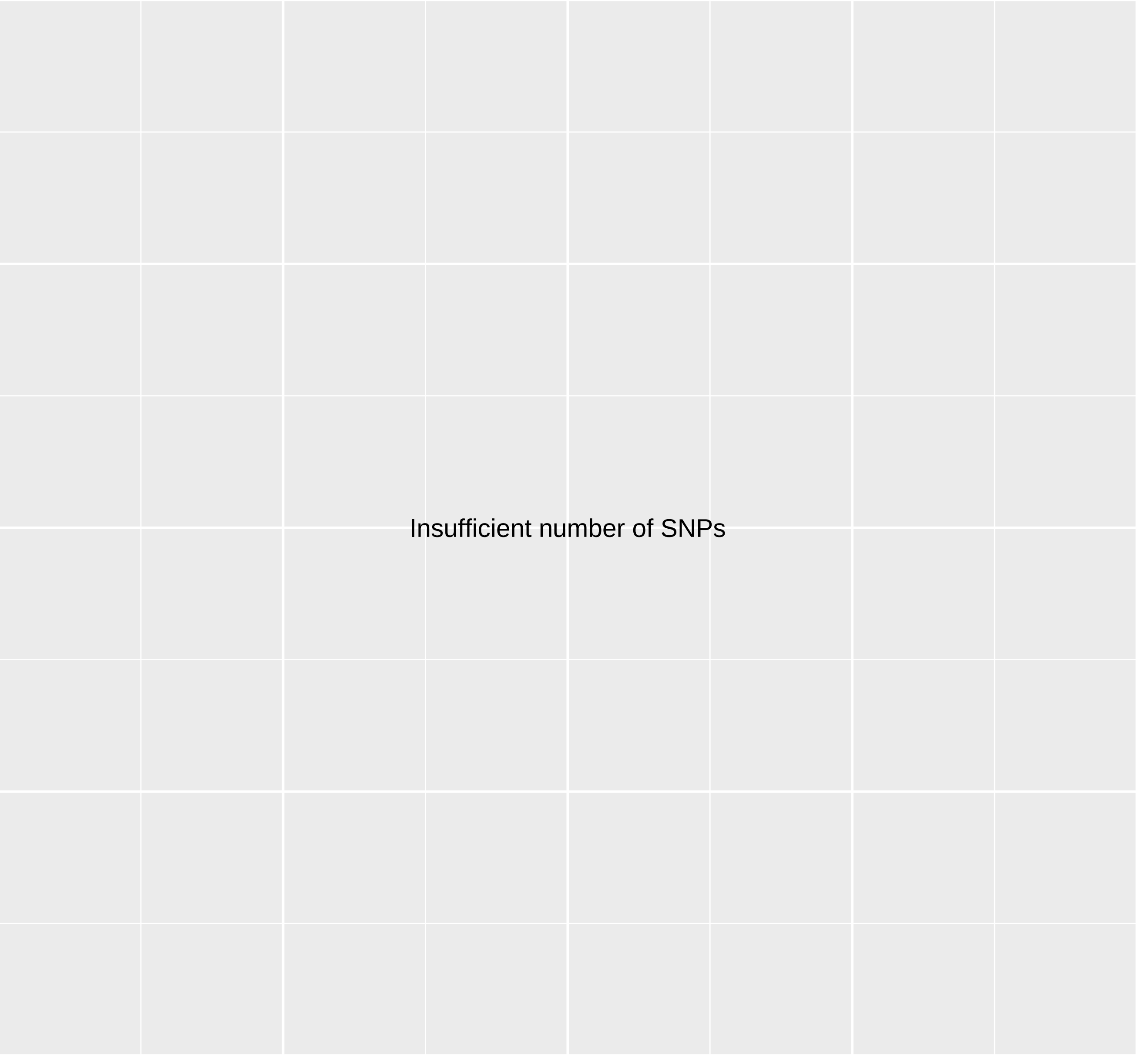
Insufficient number of SNPs

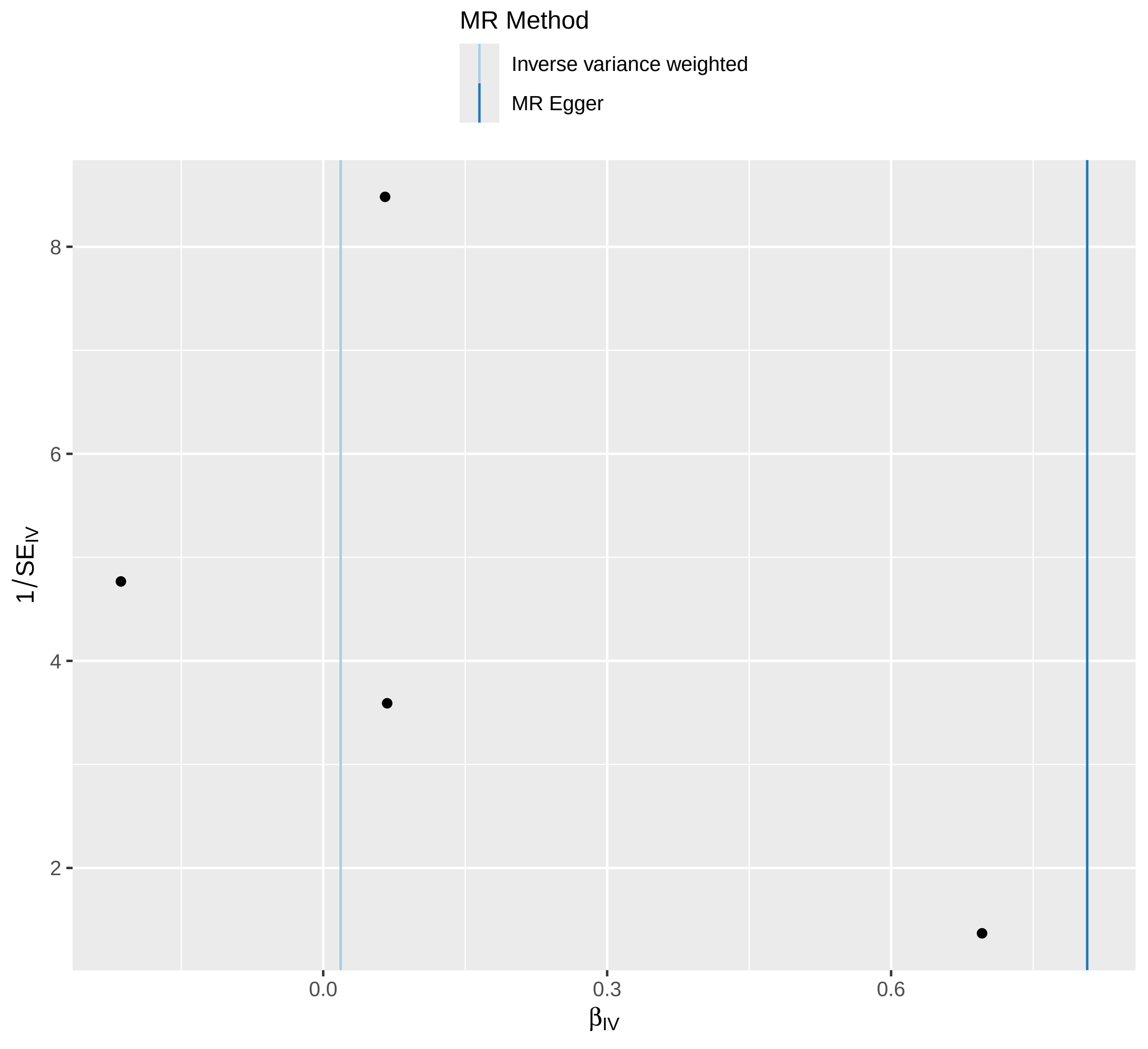

MR Method
Inverse variance weighted
MR Egger
8
6
4
2
1/SE$_{IV}$
0.0
0.3
0.6
β$_{IV}$

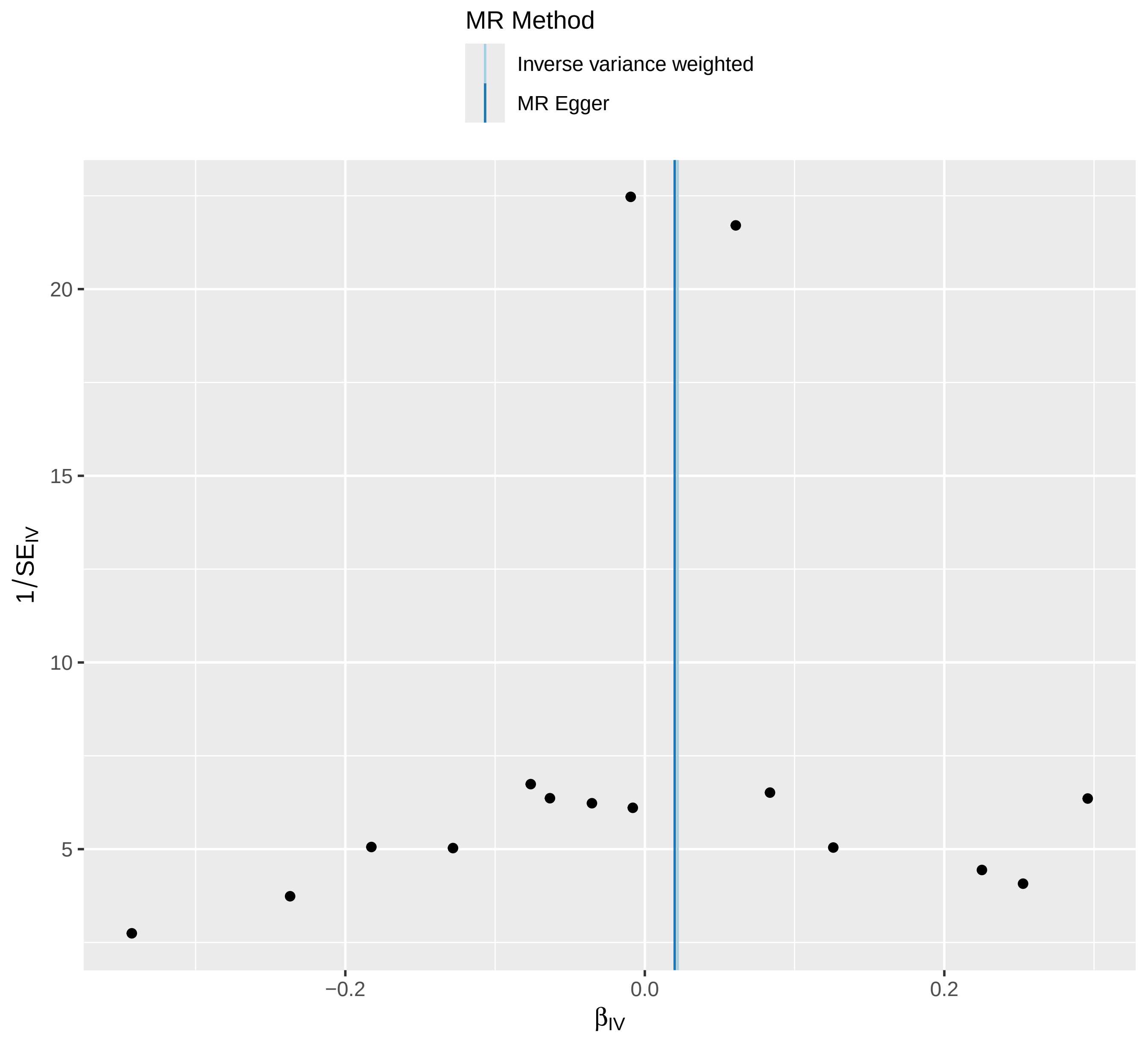
MR Method
Inverse variance weighted
MR Egger
20
15
10
5
1/SE_IV
−0.2
0.0
0.2
β_IV

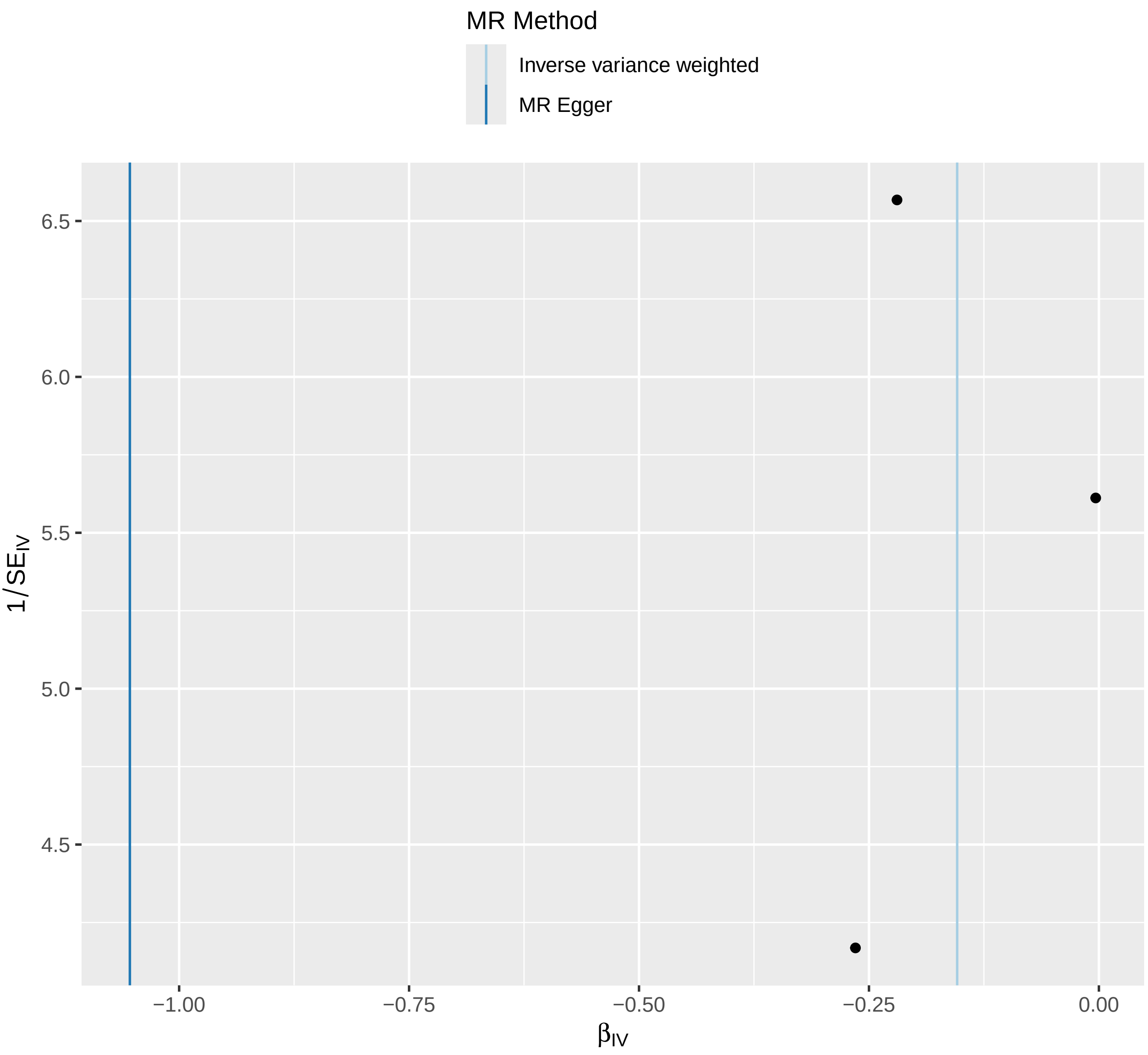

MR Method
Inverse variance weighted
MR Egger
6.5
6.0
5.5
5.0
4.5
$1/SE_{IV}$
−1.00
−0.75
−0.50
−0.25
0.00
$\beta_{IV}$

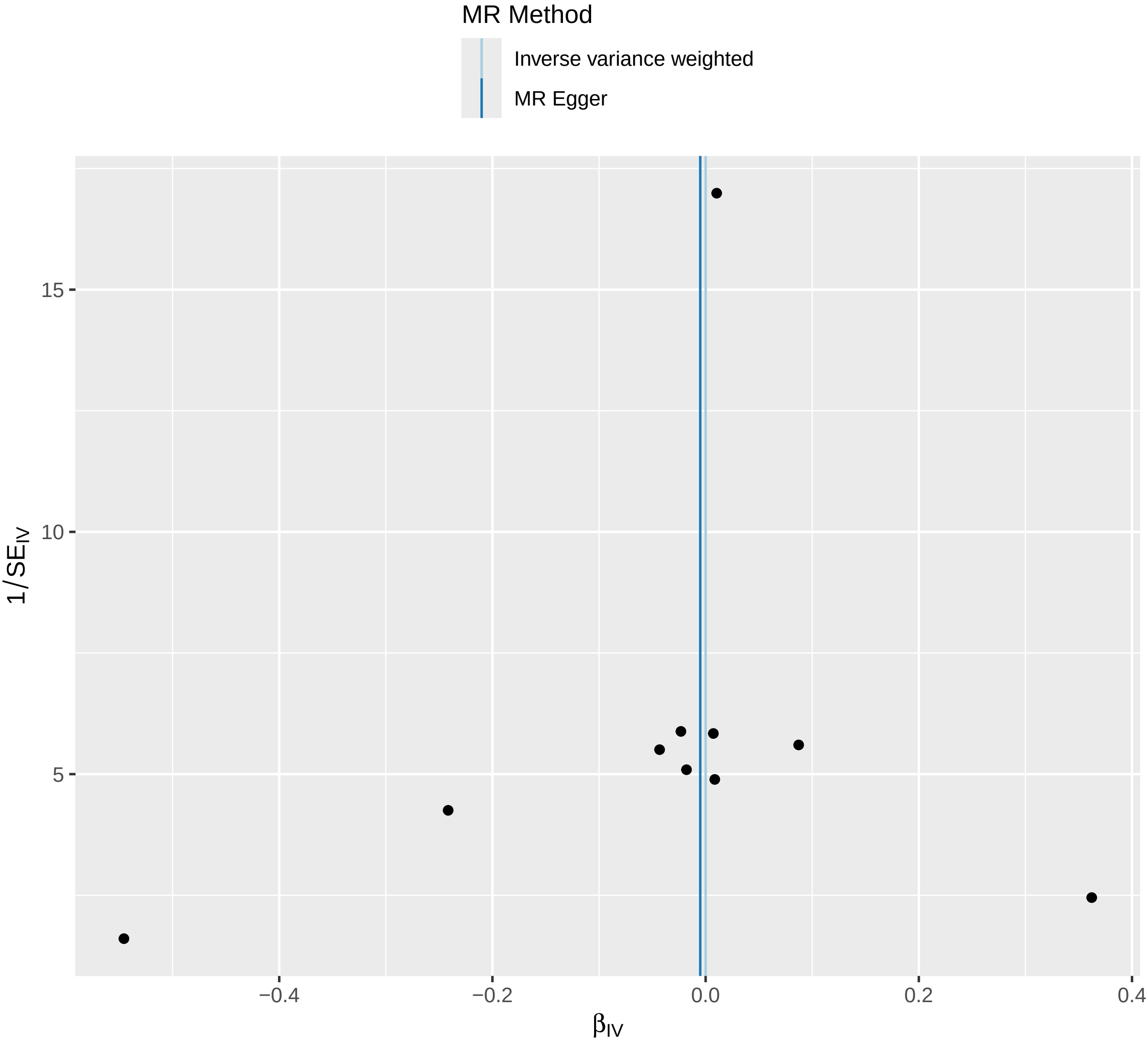
MR Method
Inverse variance weighted
MR Egger
15
10
5
1/$SE_{IV}$
−0.4
−0.2
0.0
0.2
0.4
$\beta_{IV}$

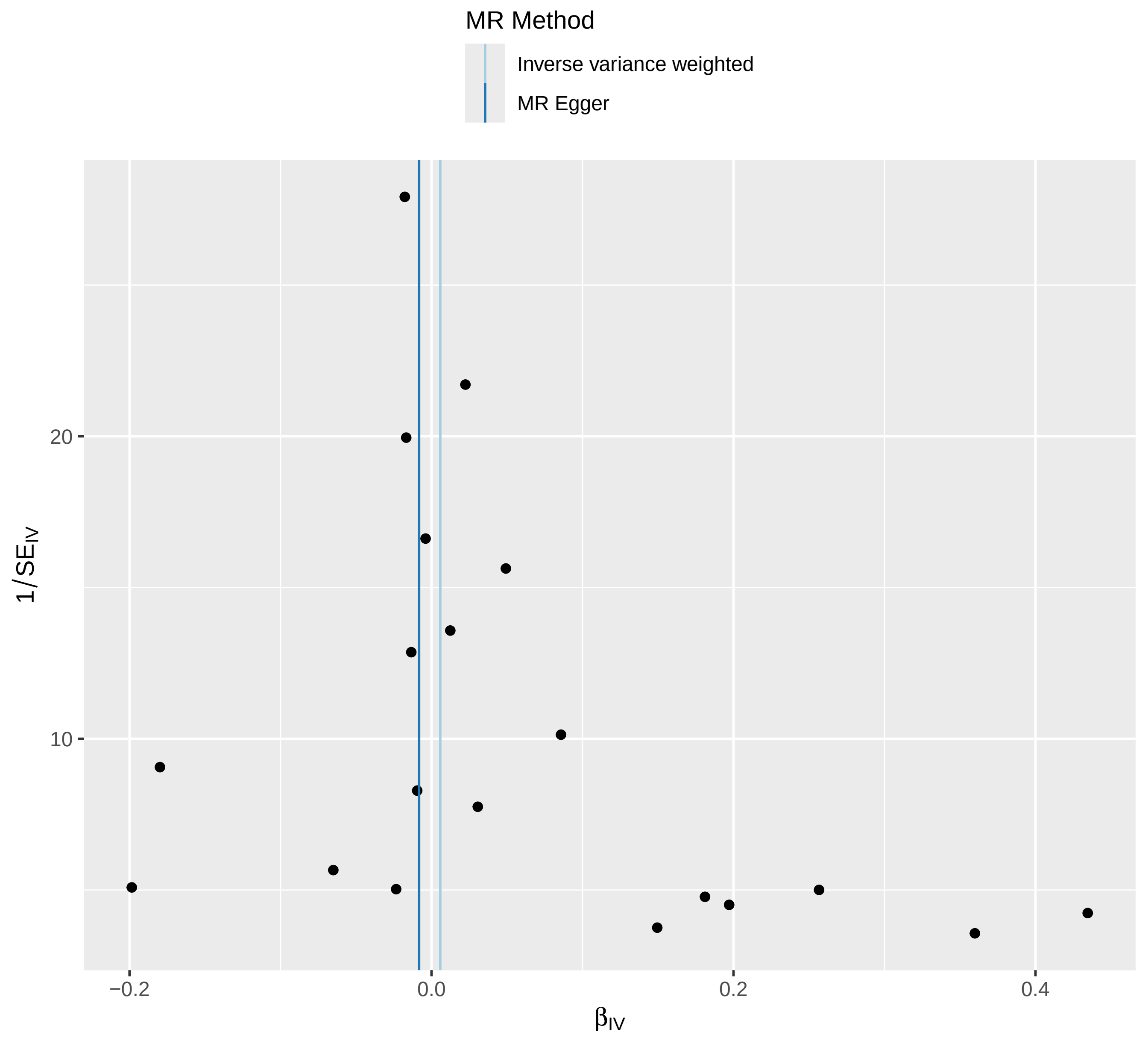

MR Method
Inverse variance weighted
MR Egger
20
10
1/SE_IV
−0.2
0.0
0.2
0.4
β_IV

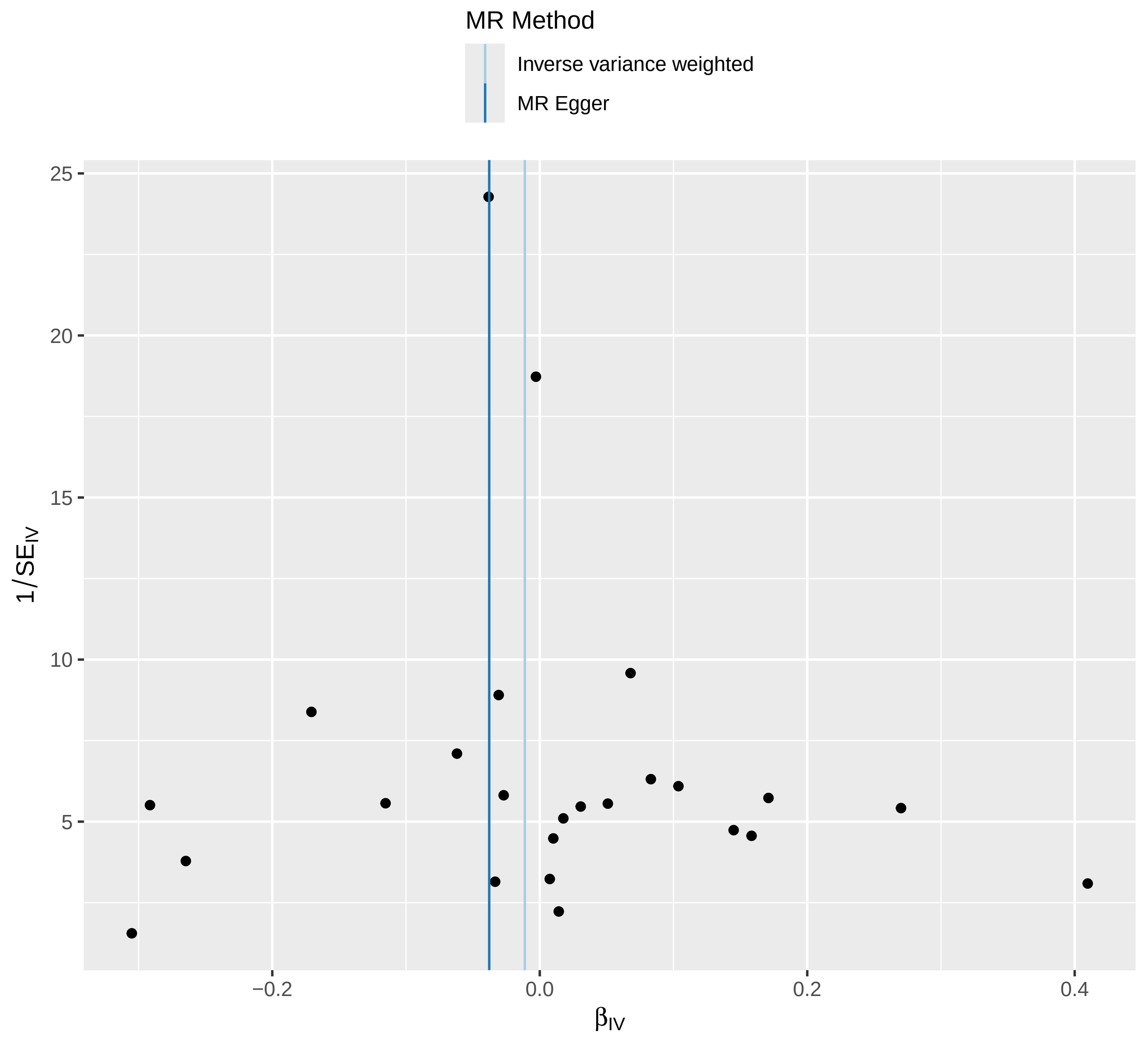

MR Method
Inverse variance weighted
MR Egger
25
20
15
10
5
1/SE$_{IV}$
−0.2
0.0
0.2
0.4
β$_{IV}$

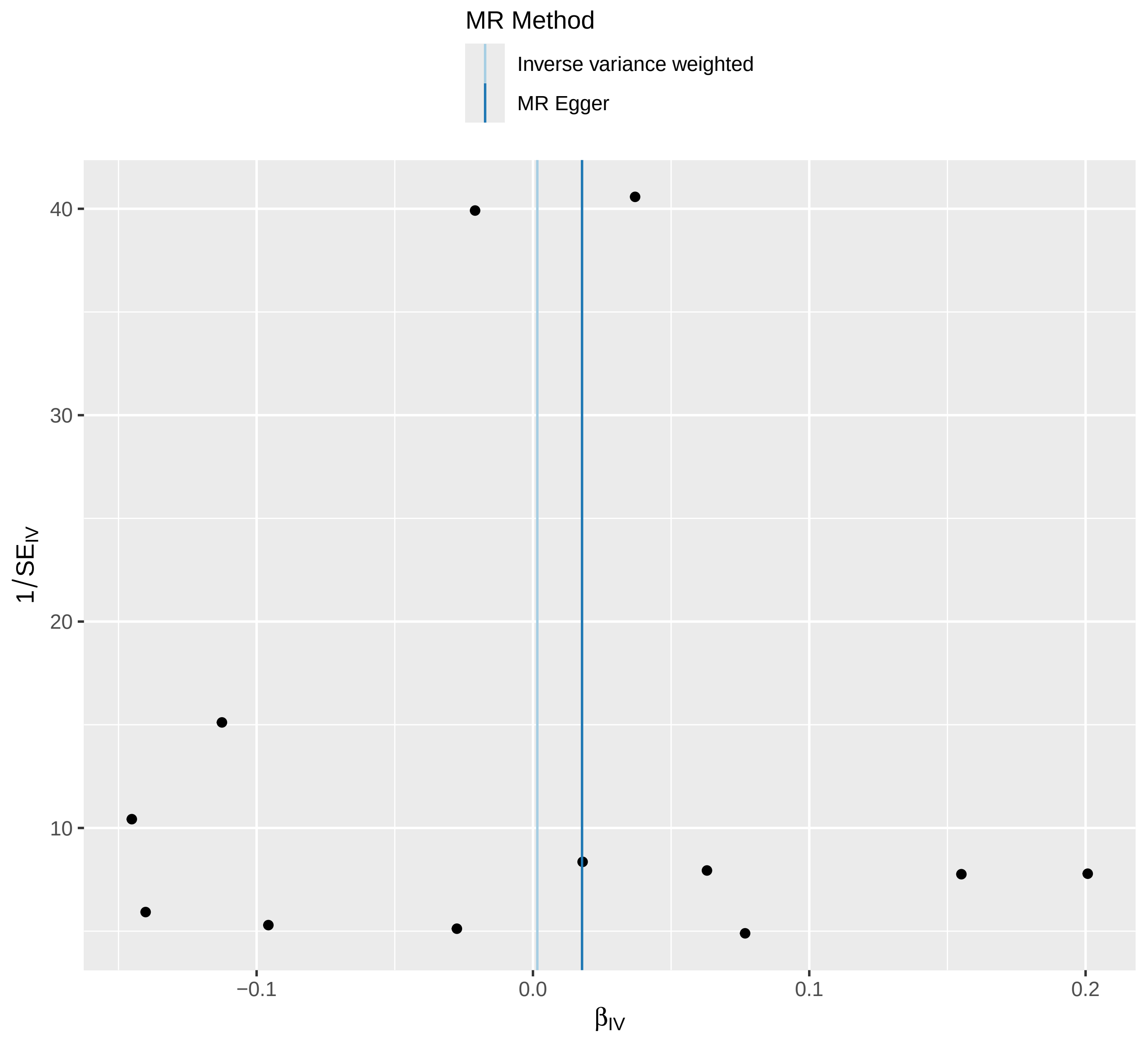

MR Method
Inverse variance weighted
MR Egger
40
30
20
10
$1/SE_{IV}$
−0.1
0.0
0.1
0.2
$\beta_{IV}$

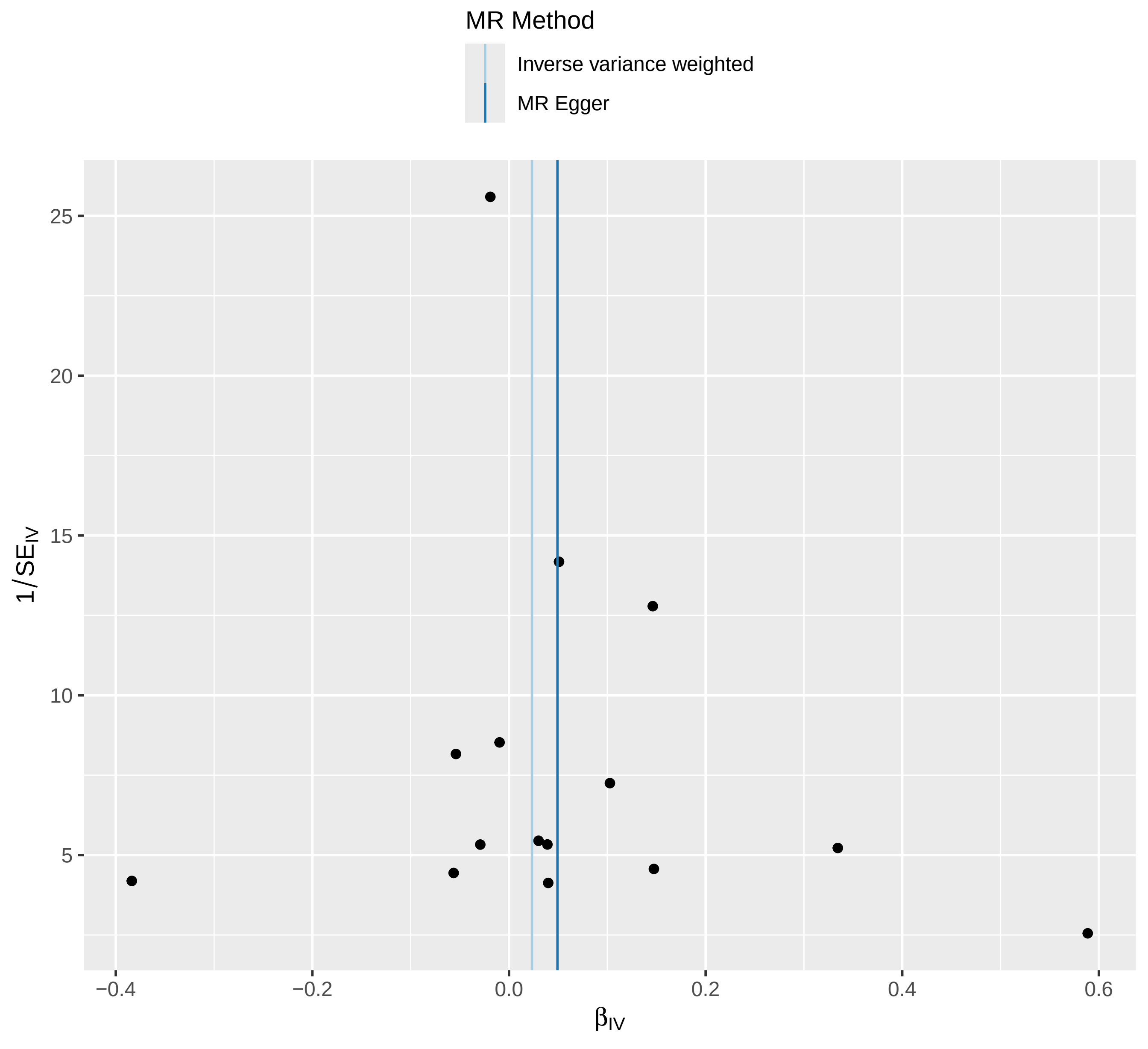
MR Method
Inverse variance weighted
MR Egger
25
20
15
10
5
1/SE$_{IV}$
−0.4
−0.2
0.0
0.2
0.4
0.6
β$_{IV}$

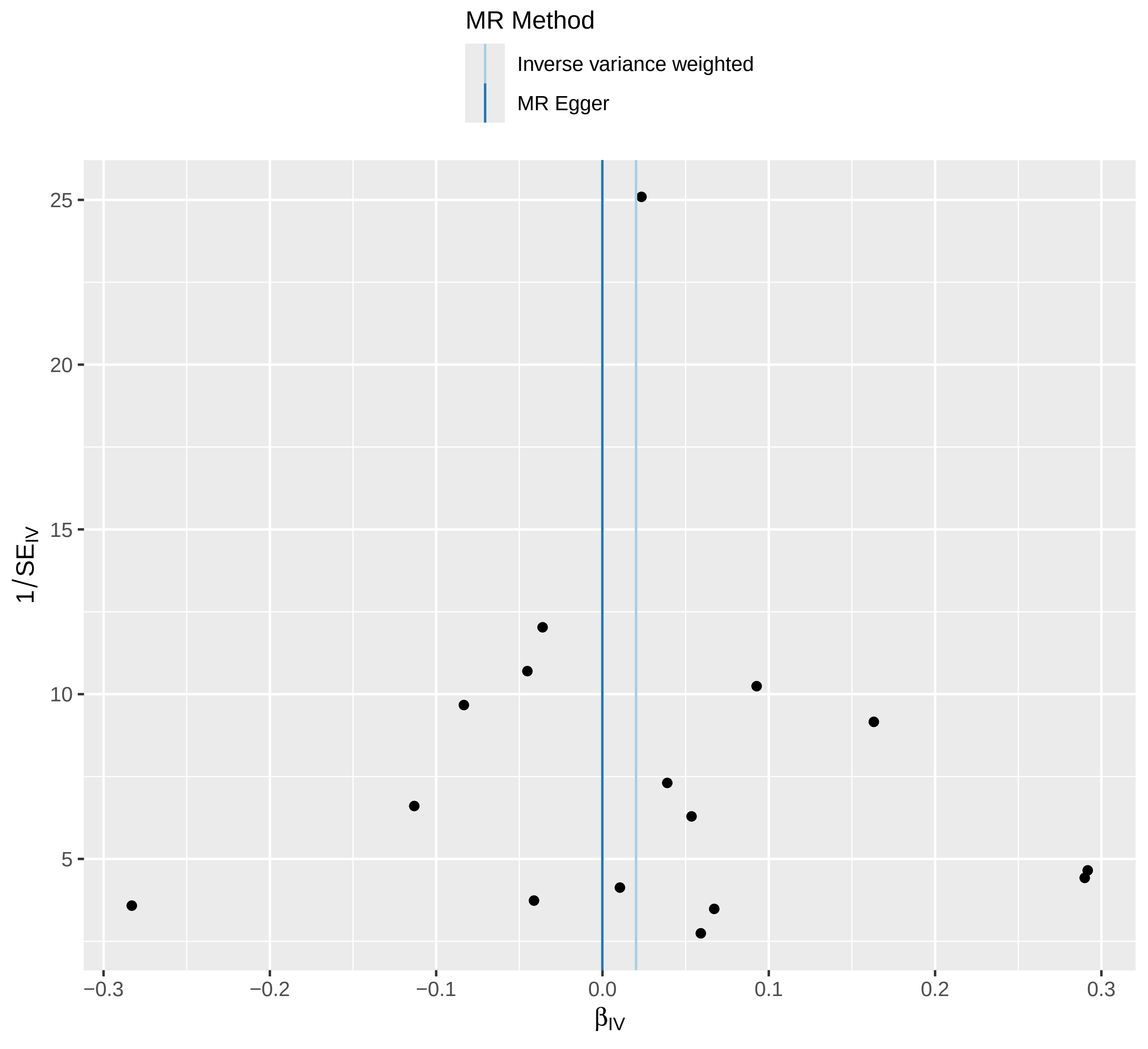

MR Method
Inverse variance weighted
MR Egger
25
20
15
10
5
$1/SE_{IV}$
−0.3
−0.2
−0.1
0.0
0.1
0.2
0.3
$\beta_{IV}$

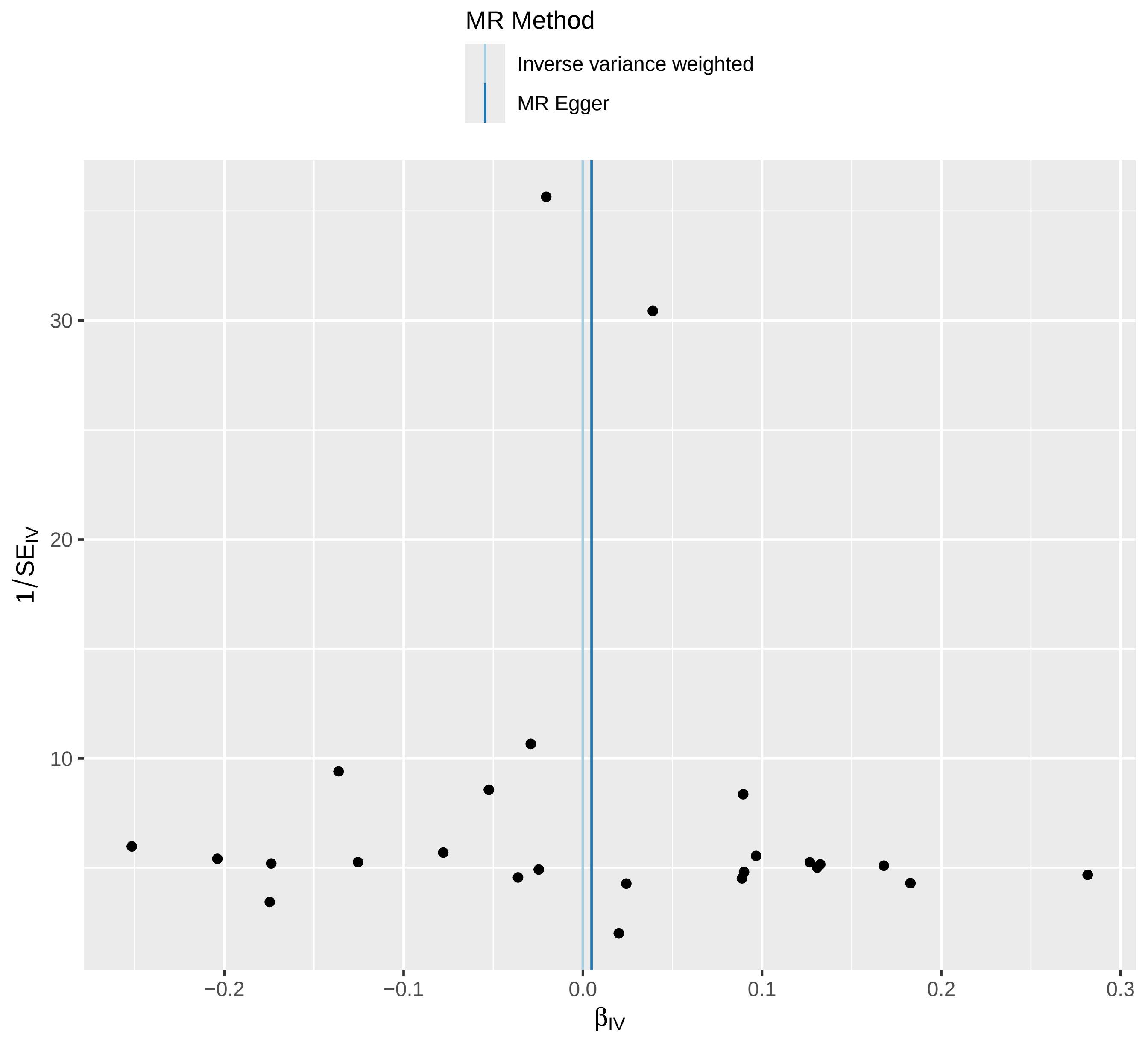
MR Method
Inverse variance weighted
MR Egger
30
20
10
−0.2
−0.1
0.0
0.1
0.2
0.3
1/SE_IV
β_IV

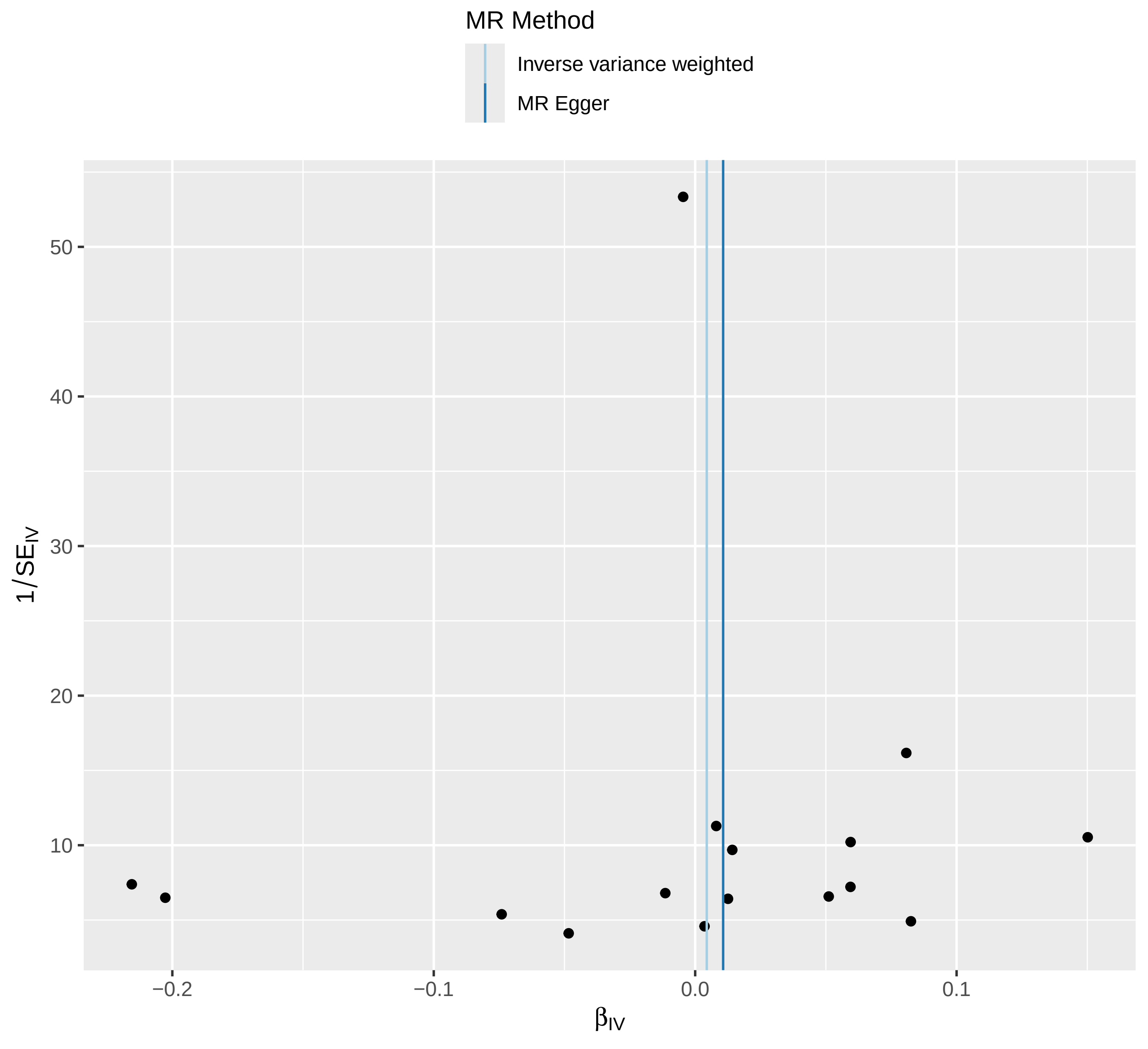

MR Method
Inverse variance weighted
MR Egger
50
40
30
20
10
1/SE_IV
−0.2
−0.1
0.0
0.1
β_IV

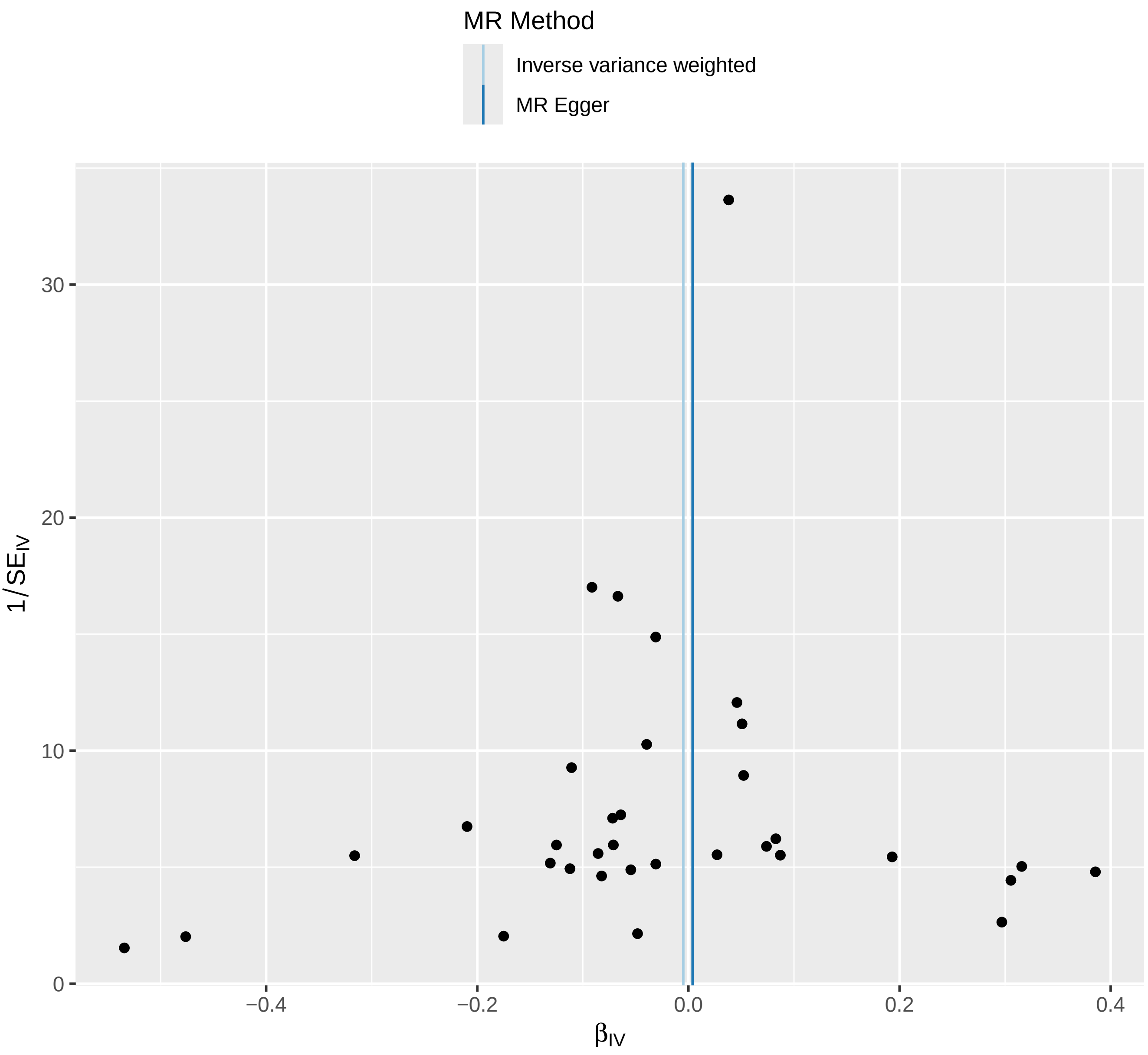

MR Method
Inverse variance weighted
MR Egger
30
20
10
0
$1/SE_{IV}$
−0.4
−0.2
0.0
0.2
0.4
$\beta_{IV}$

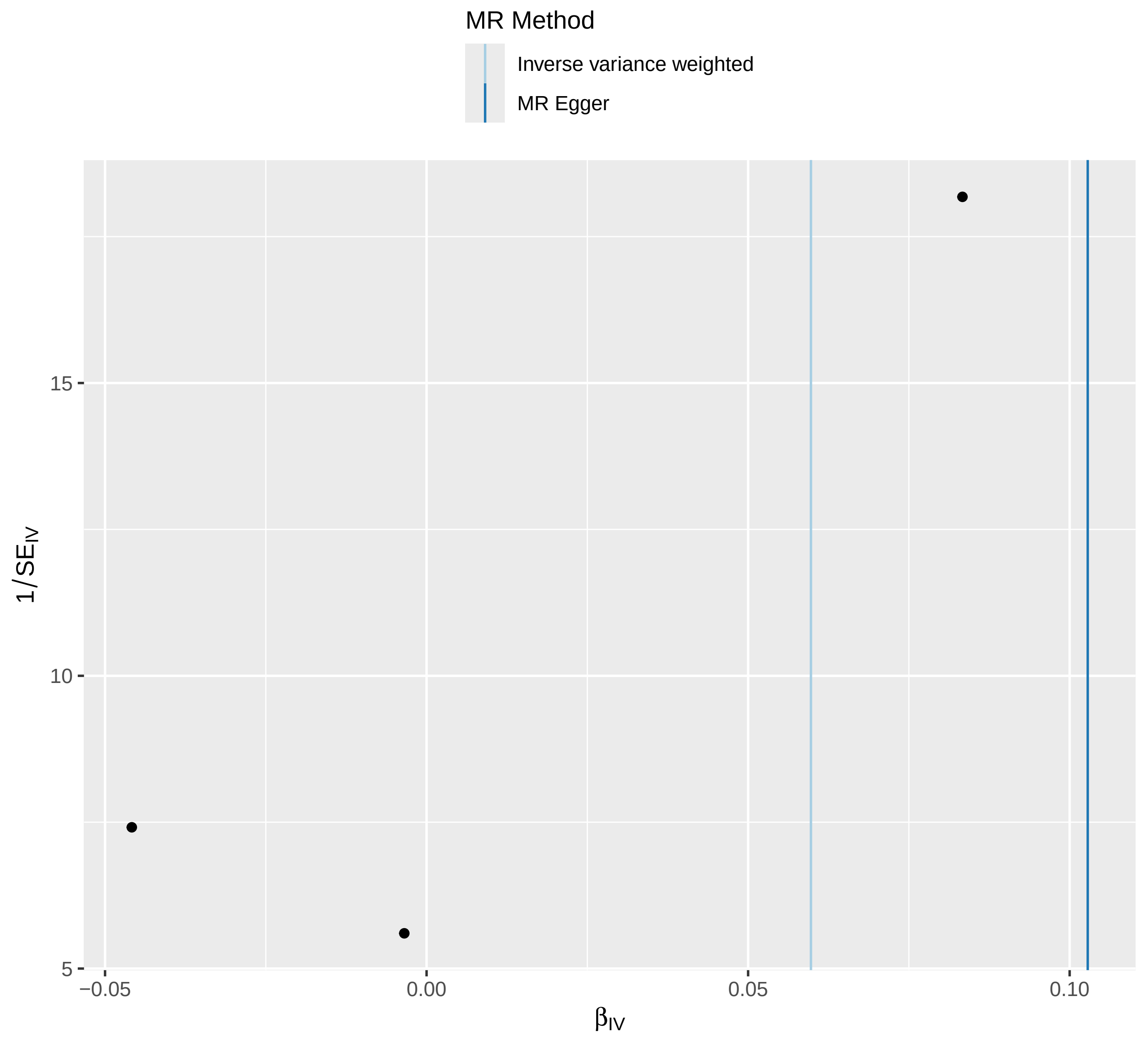

MR Method
Inverse variance weighted
MR Egger
15
10
5
1/SE$_{IV}$
−0.05
0.00
0.05
0.10
β$_{IV}$

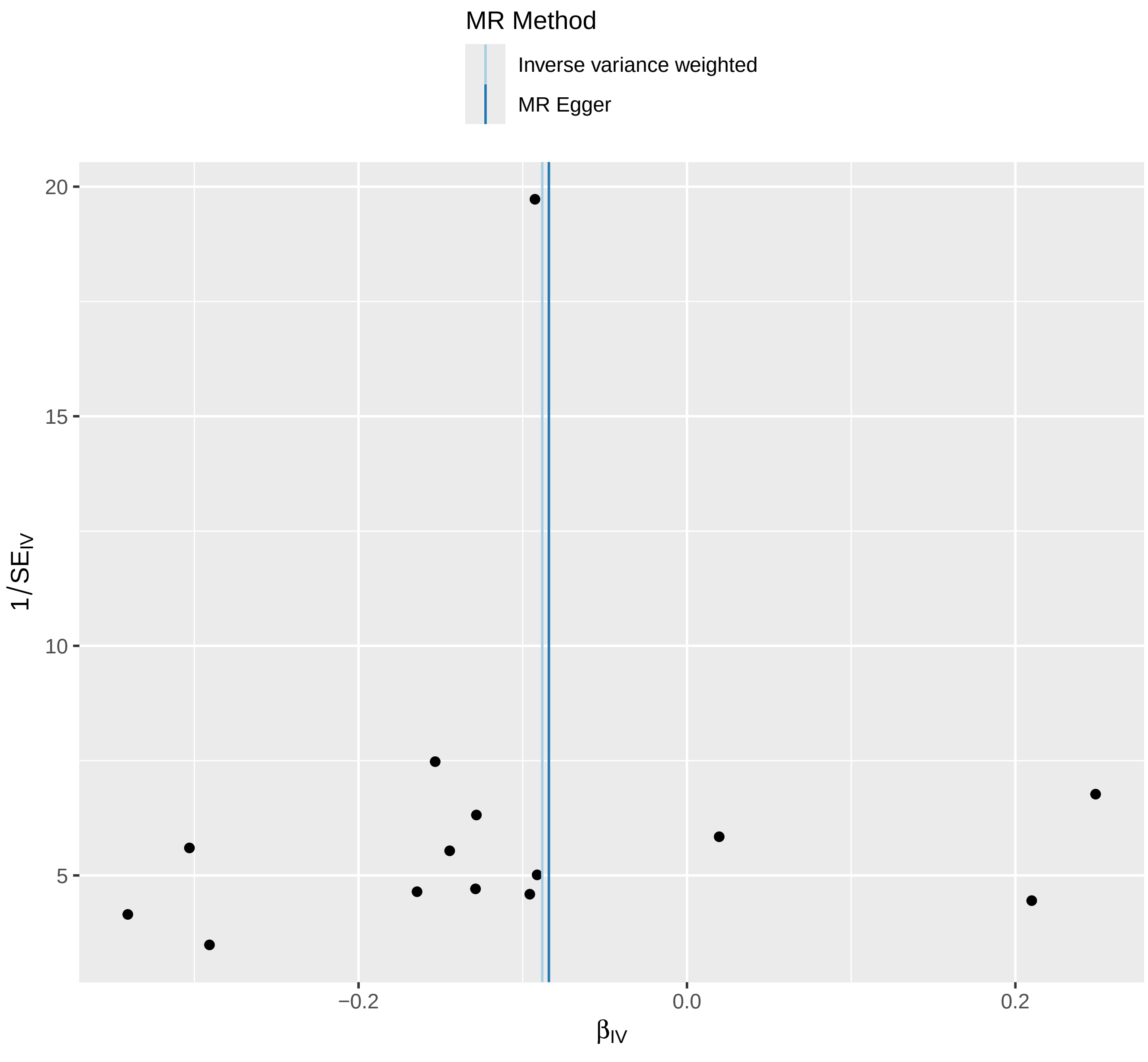

MR Method
Inverse variance weighted
MR Egger
20
15
10
5
1/SE$_{IV}$
−0.2
0.0
0.2
β$_{IV}$

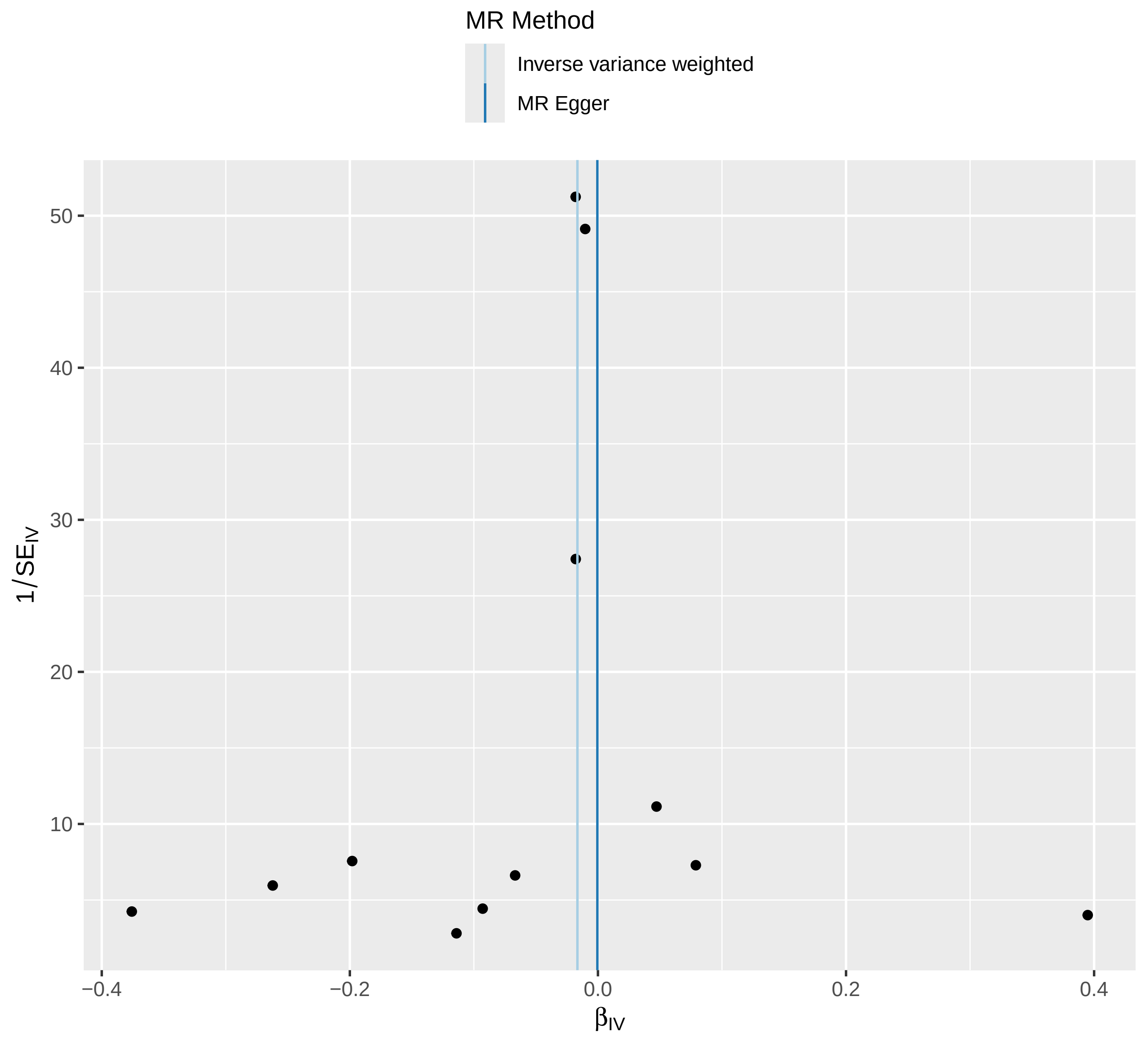

MR Method
Inverse variance weighted
MR Egger
1/SE_IV
50
40
30
20
10
−0.4
−0.2
0.0
0.2
0.4
β_IV

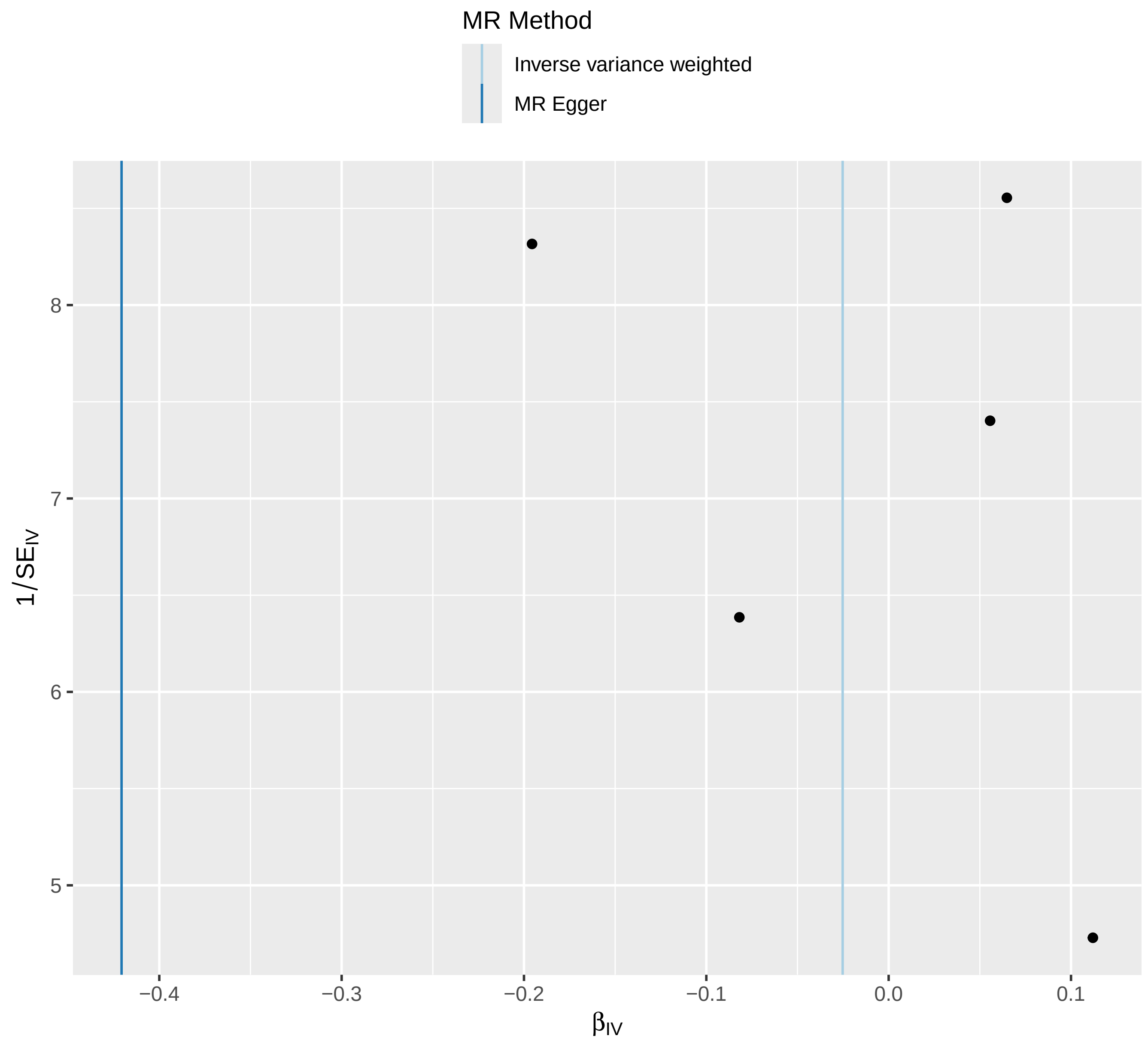

MR Method
Inverse variance weighted
MR Egger
8
7
6
5
1/SE$_{IV}$
−0.4
−0.3
−0.2
−0.1
0.0
0.1
β$_{IV}$

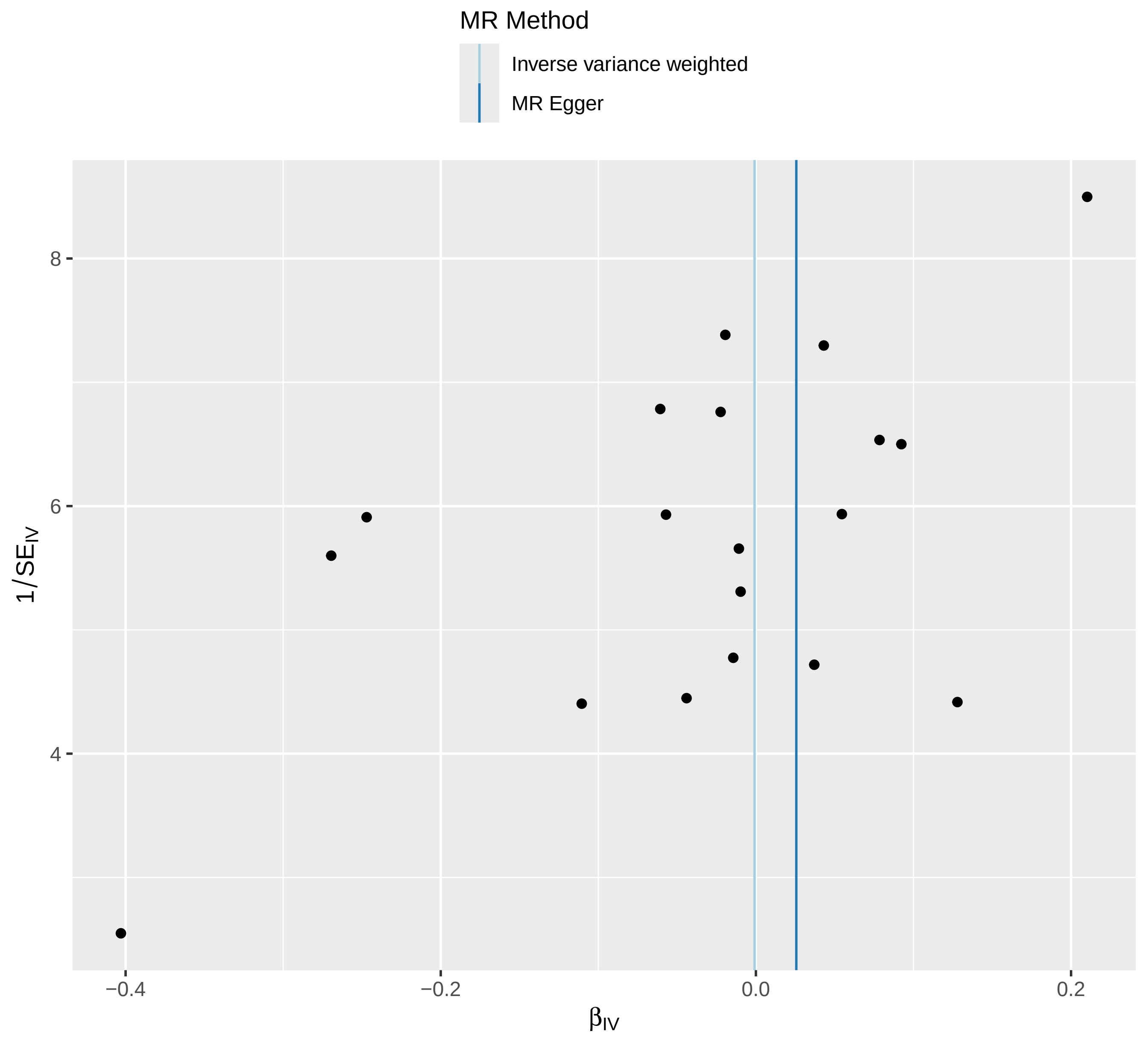
MR Method
Inverse variance weighted
MR Egger
8
6
4
$1/SE_{IV}$
−0.4
−0.2
0.0
0.2
$\beta_{IV}$

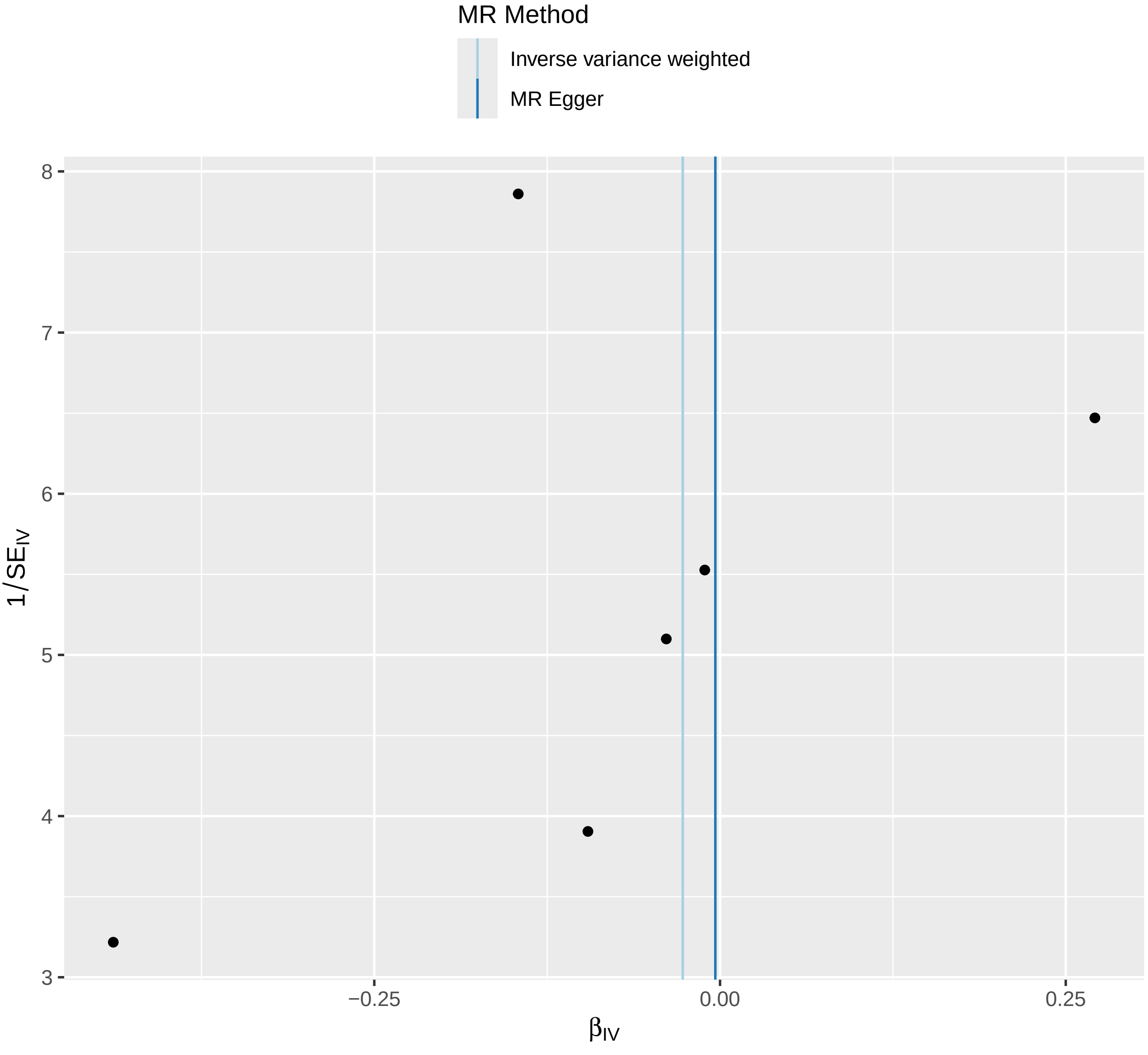
MR Method
Inverse variance weighted
MR Egger
8
7
6
5
4
3
1/SE$_{IV}$
−0.25
0.00
0.25
β$_{IV}$

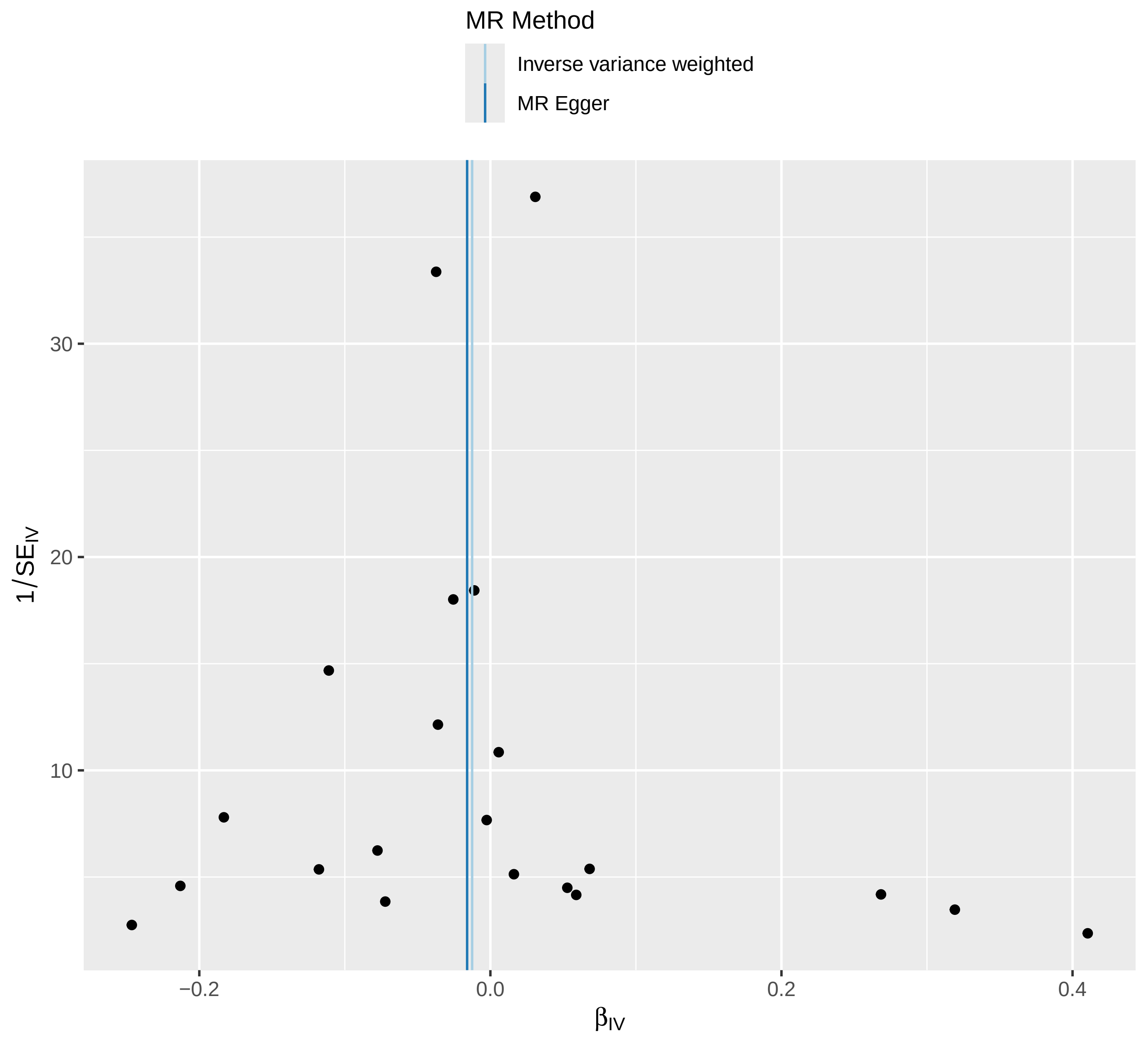
MR Method
Inverse variance weighted
MR Egger
30
20
10
$1/SE_{IV}$
−0.2
0.0
0.2
0.4
$\beta_{IV}$

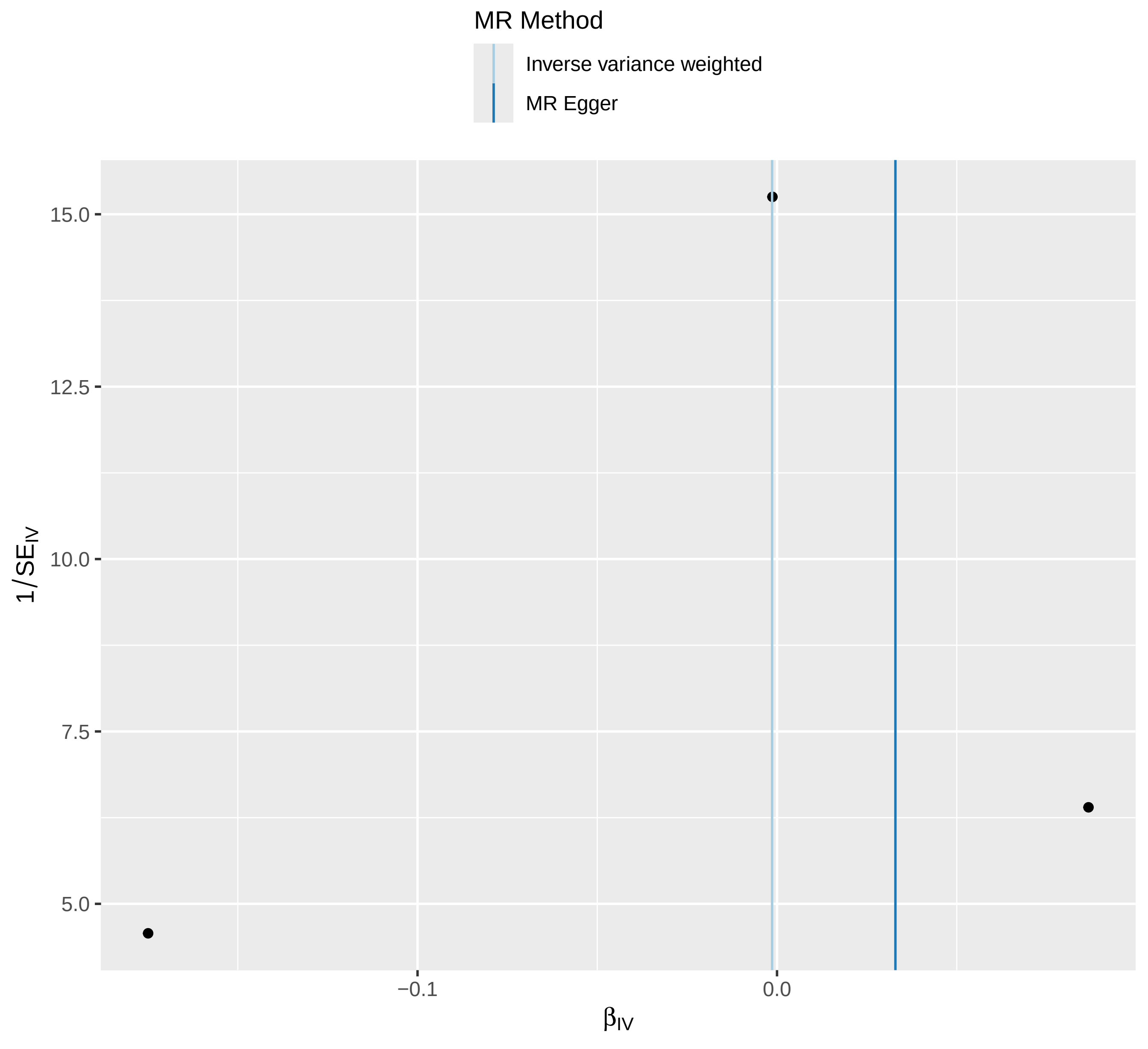
MR Method
Inverse variance weighted
MR Egger
15.0
12.5
10.0
7.5
5.0
1/SE$_{IV}$
−0.1
0.0
β$_{IV}$

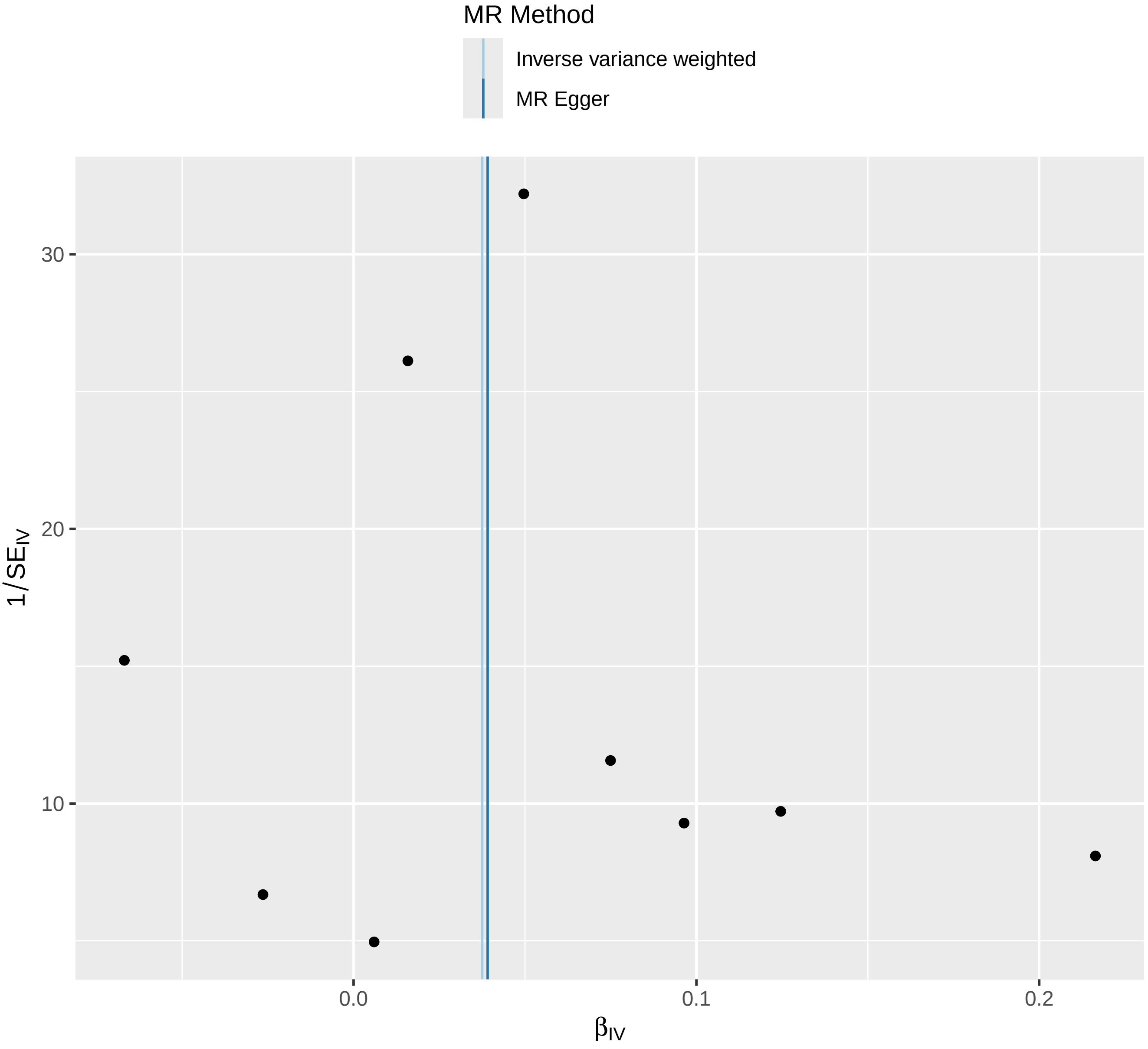

MR Method
Inverse variance weighted
MR Egger
30
20
10
1/SE_IV
0.0
0.1
0.2
β_IV

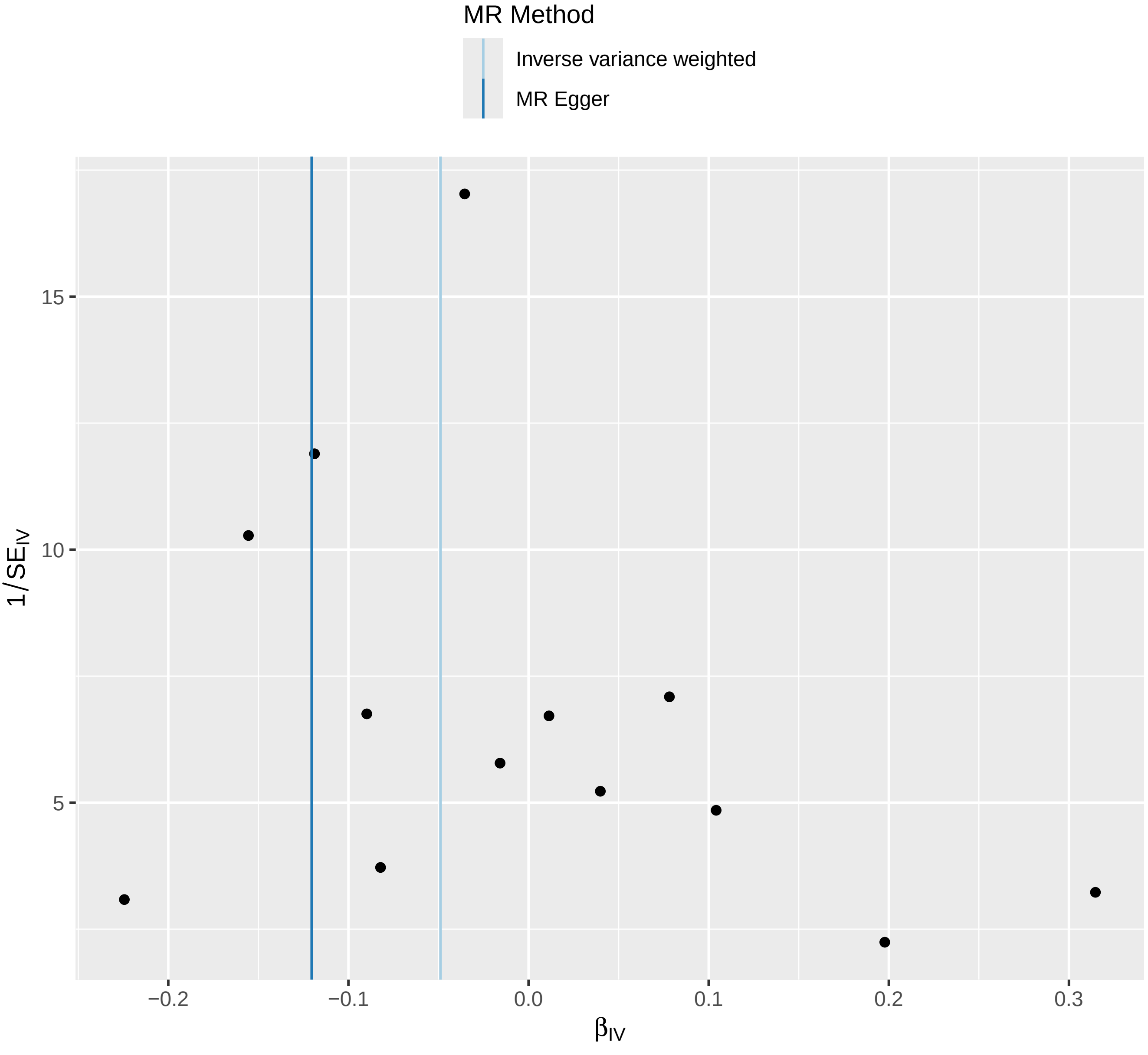

MR Method
Inverse variance weighted
MR Egger
15
10
5
1/SE$_{IV}$
−0.2
−0.1
0.0
0.1
0.2
0.3
β$_{IV}$

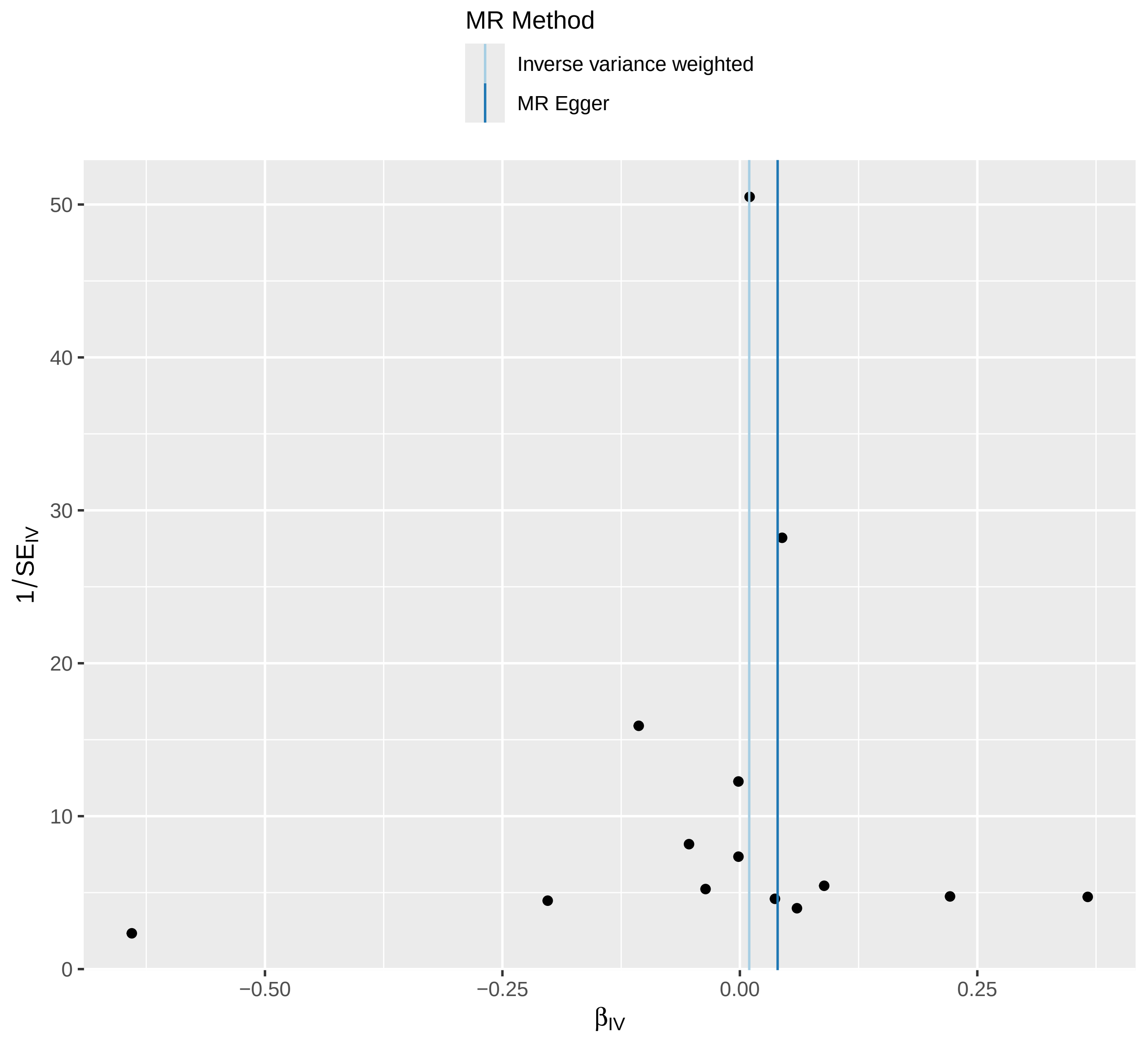

MR Method
Inverse variance weighted
MR Egger
50
40
30
20
10
0
$1/SE_{IV}$
−0.50
−0.25
0.00
0.25
$\beta_{IV}$

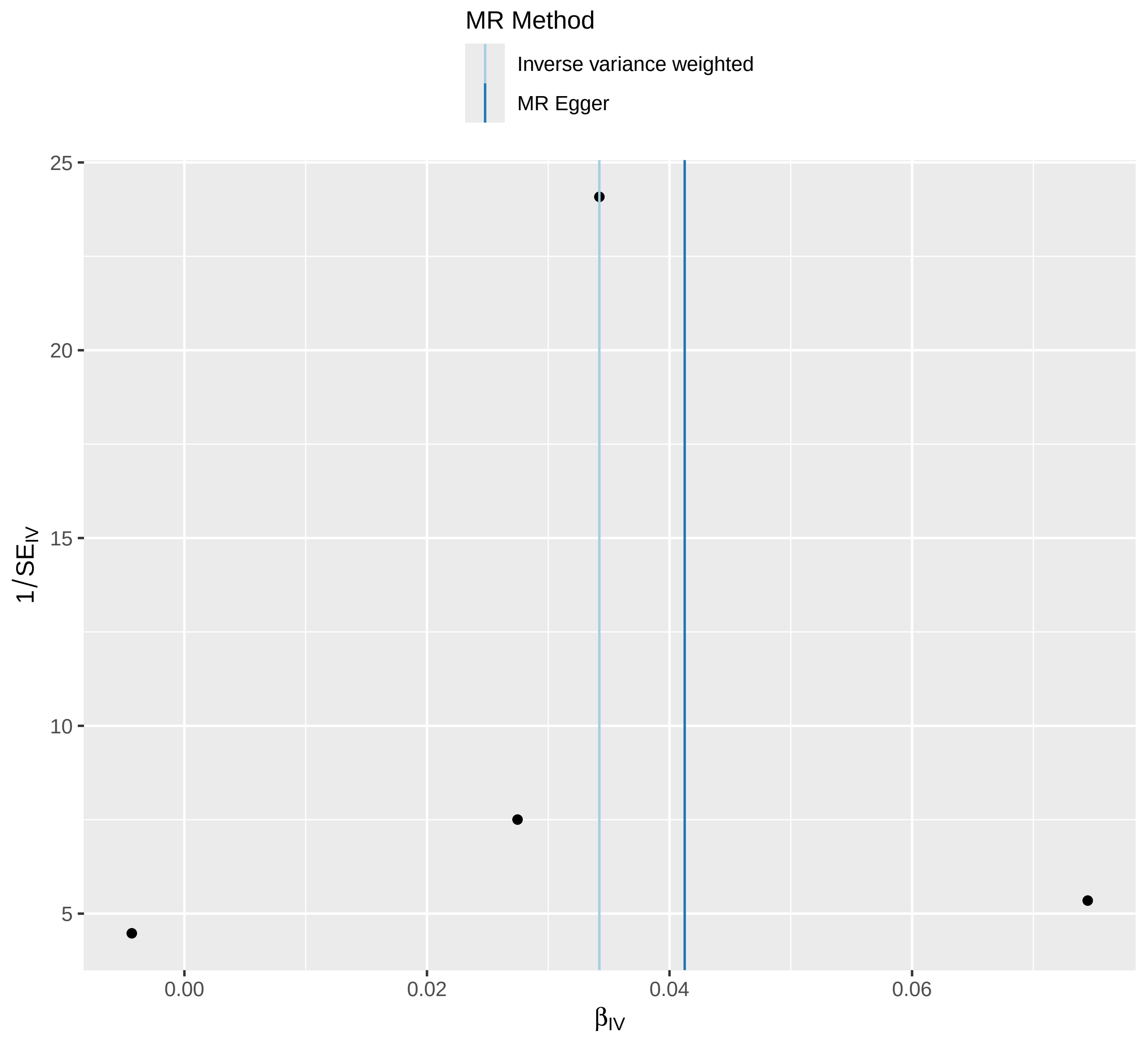
MR Method
Inverse variance weighted
MR Egger
25
20
15
10
5
0.00
0.02
0.04
0.06
1/SE_IV
β_IV

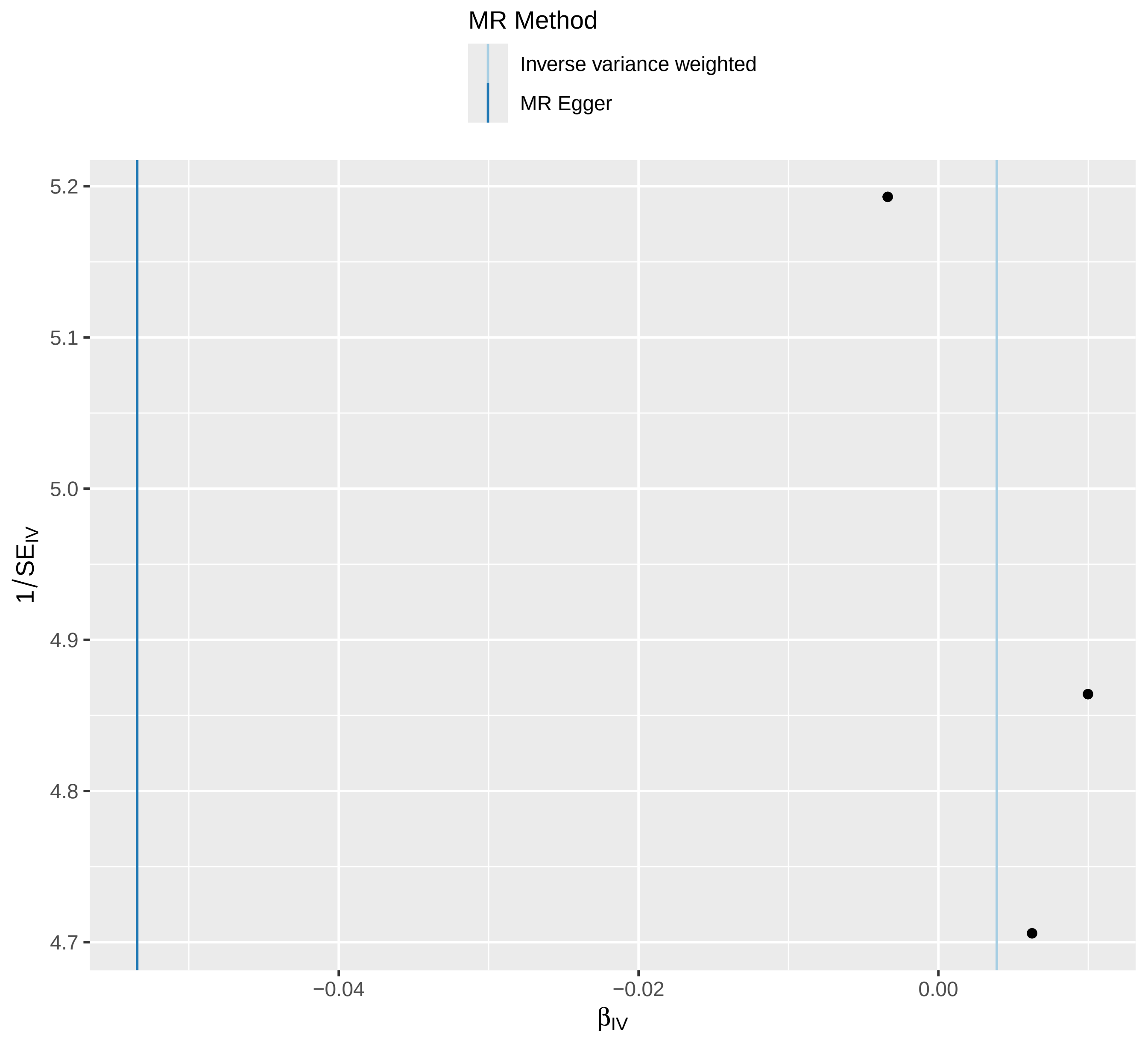
MR Method
Inverse variance weighted
MR Egger
5.2
5.1
5.0
4.9
4.8
4.7
1/SE$_{IV}$
−0.04
−0.02
0.00
β$_{IV}$

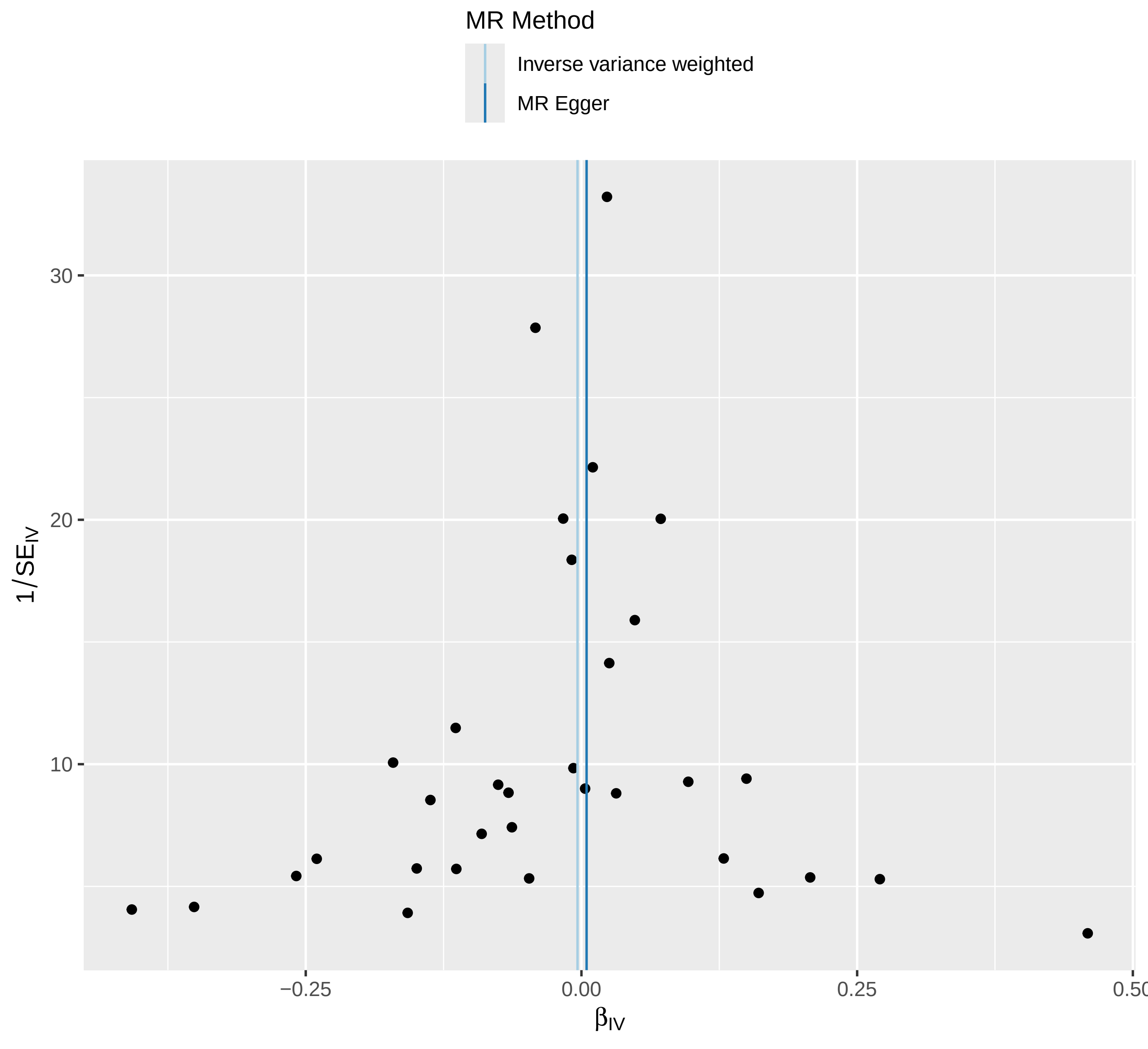
MR Method
Inverse variance weighted
MR Egger
30
20
10
$1/SE_{IV}$
−0.25
0.00
0.25
0.50
$\beta_{IV}$

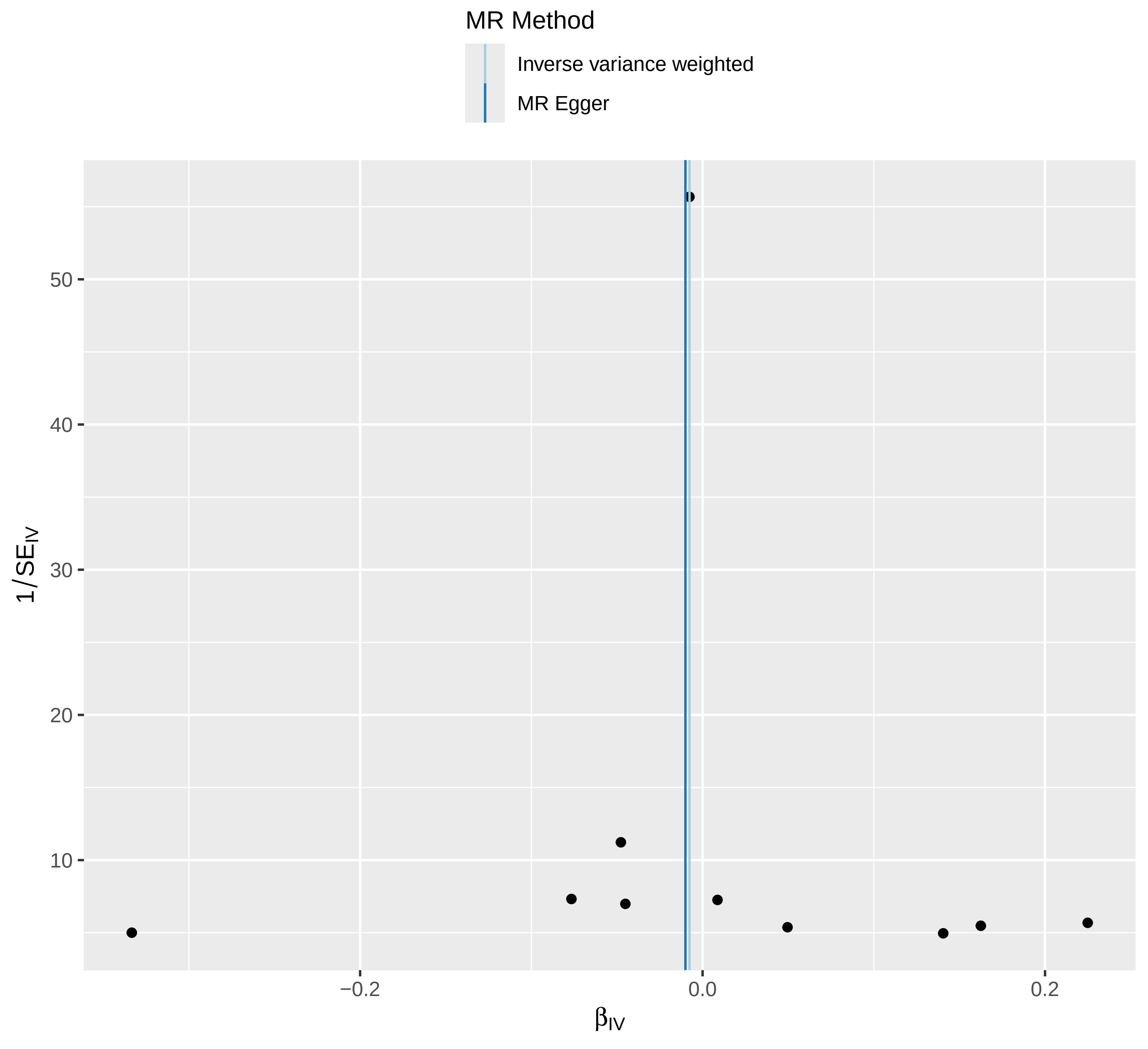
MR Method
Inverse variance weighted
MR Egger
50
40
30
20
10
1/SE_IV
−0.2
0.0
0.2
β_IV

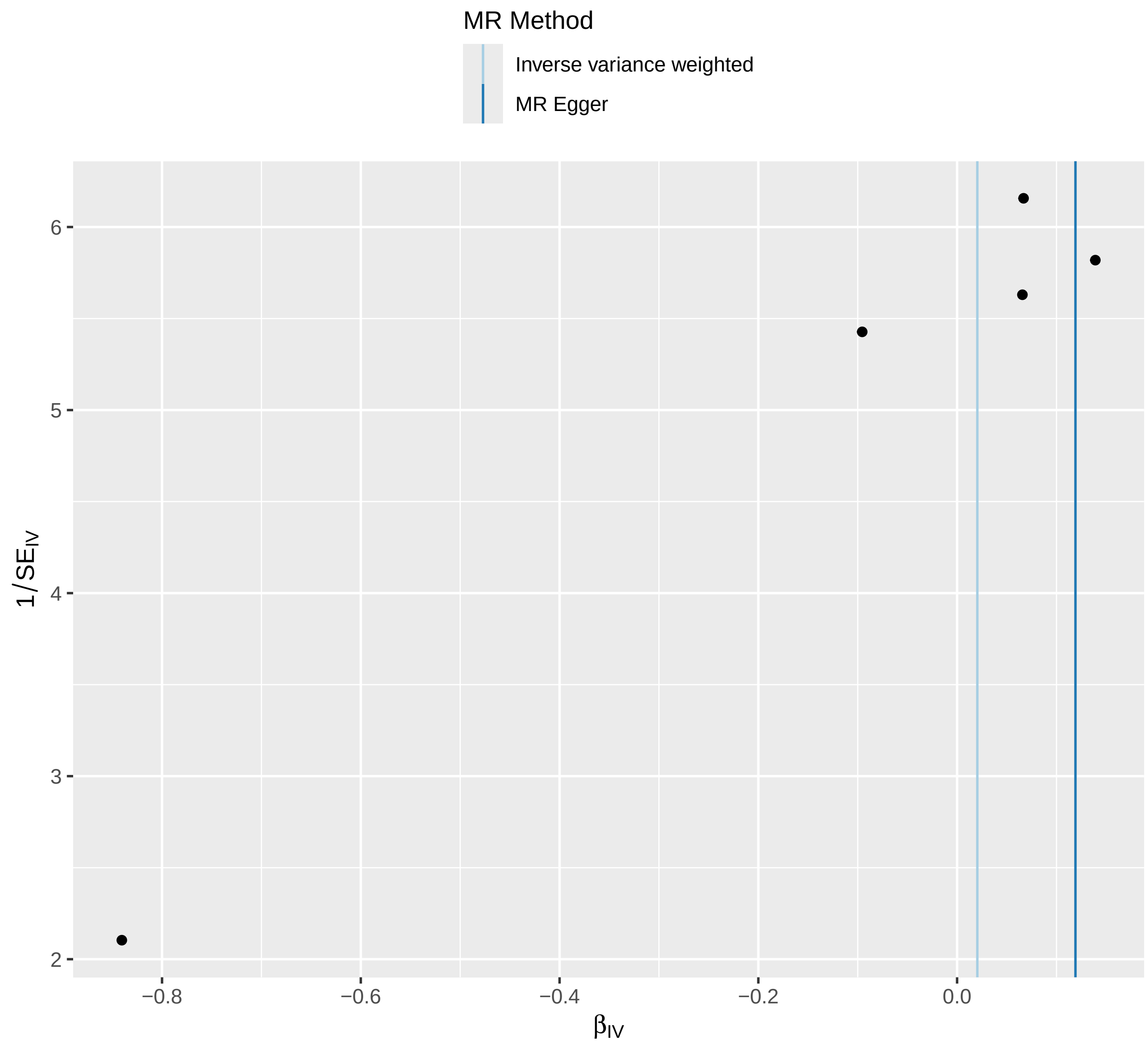
MR Method
Inverse variance weighted
MR Egger
6
5
4
3
2
1/SE$_{IV}$
−0.8
−0.6
−0.4
−0.2
0.0
β$_{IV}$

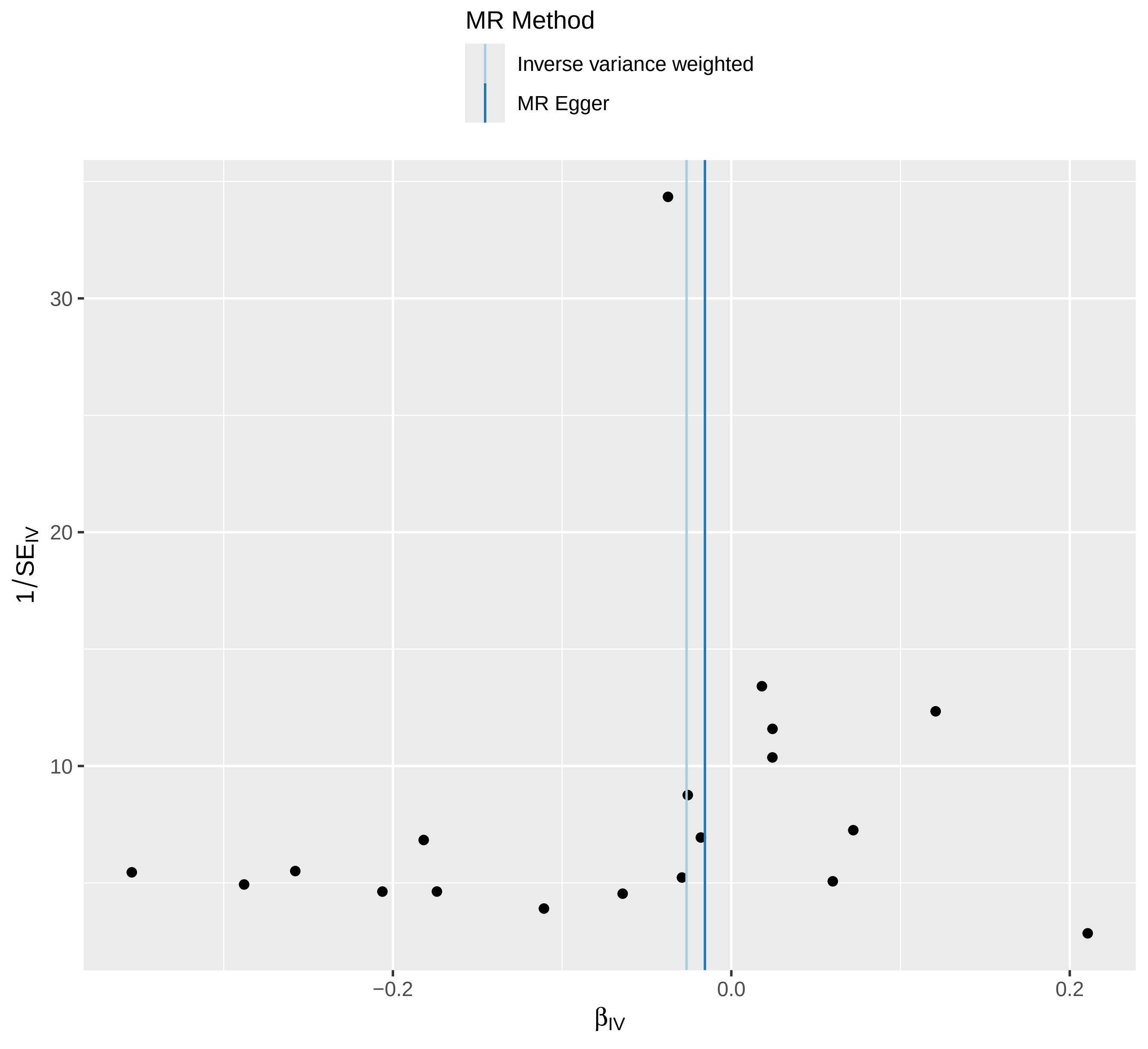

MR Method
Inverse variance weighted
MR Egger
30
20
10
1/SE_IV
−0.2
0.0
0.2
β_IV

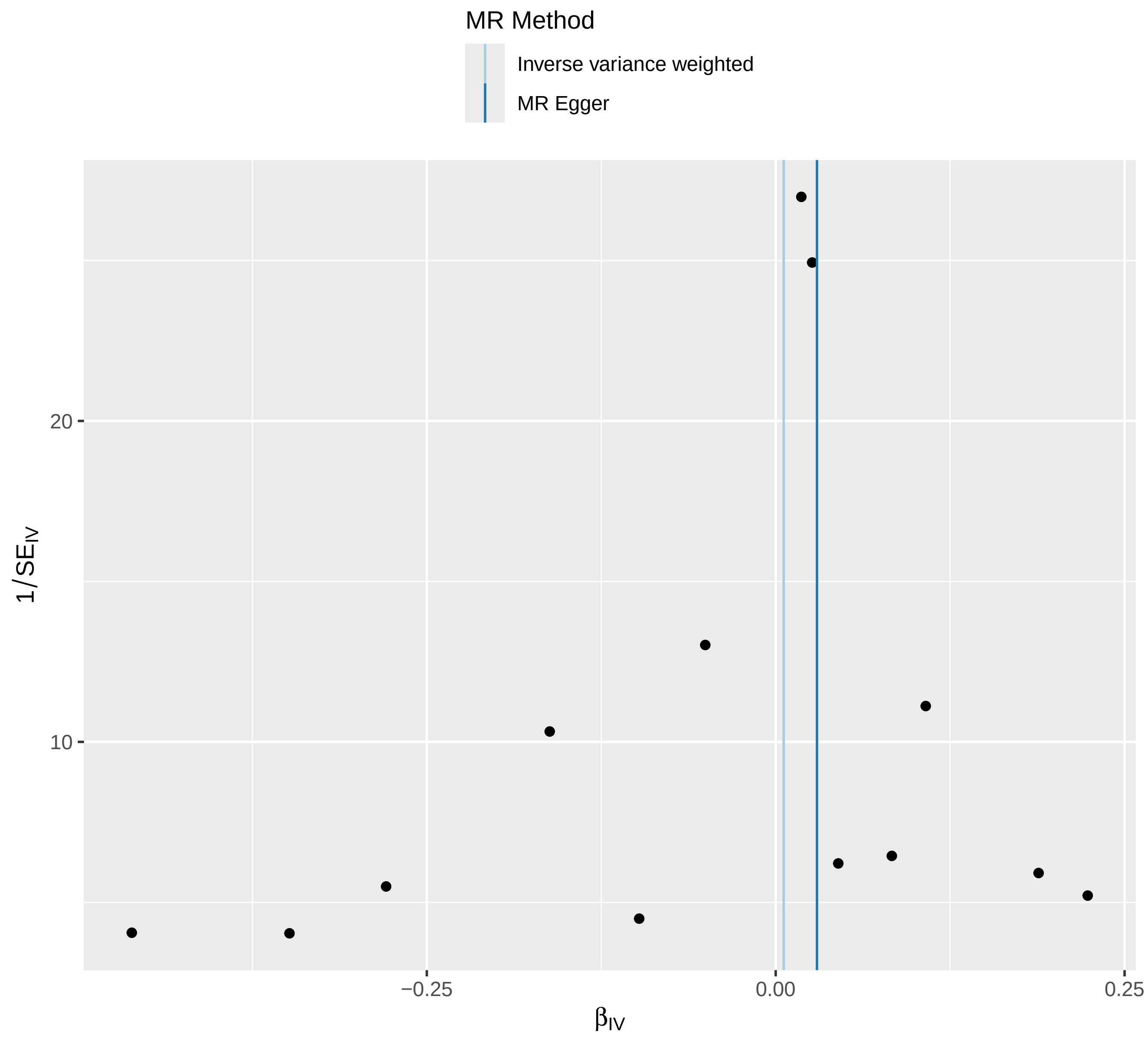
MR Method
Inverse variance weighted
MR Egger
20
10
$1/SE_{IV}$
−0.25
0.00
0.25
$\beta_{IV}$

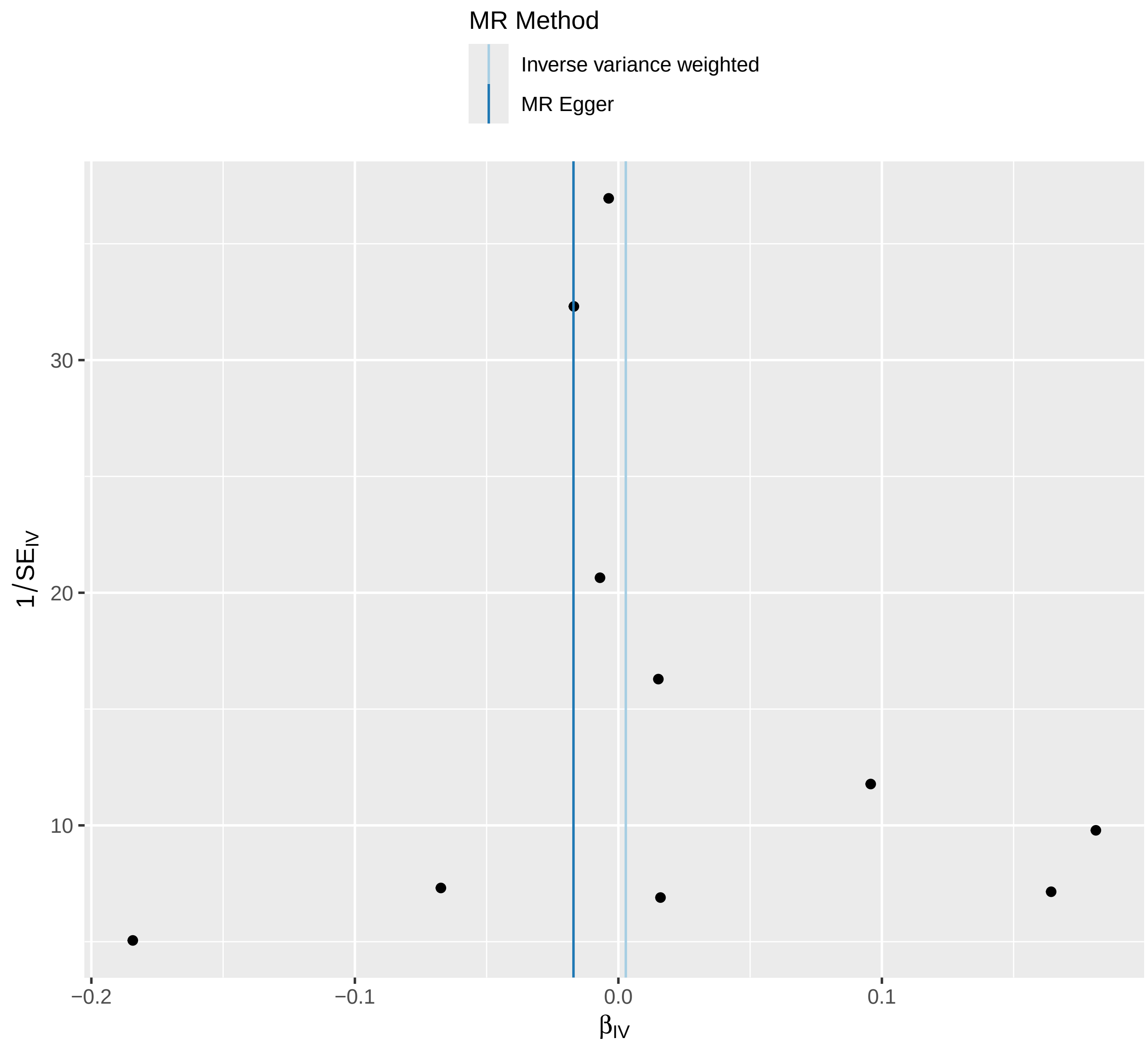
MR Method
Inverse variance weighted
MR Egger
30
20
10
1/SE$_{IV}$
−0.2
−0.1
0.0
0.1
β$_{IV}$

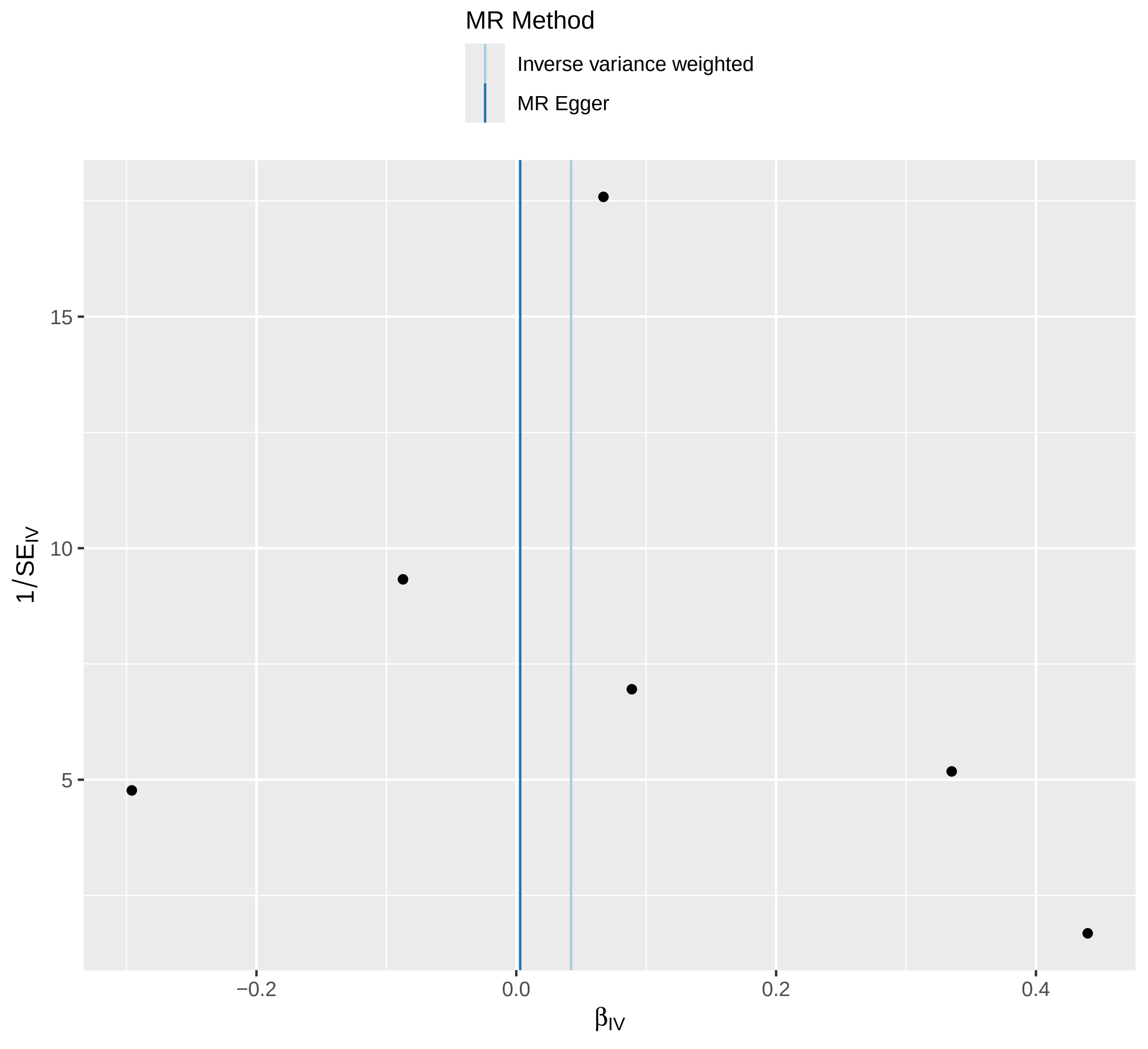
MR Method
Inverse variance weighted
MR Egger
15
10
5
$1/SE_{IV}$
−0.2
0.0
0.2
0.4
$\beta_{IV}$

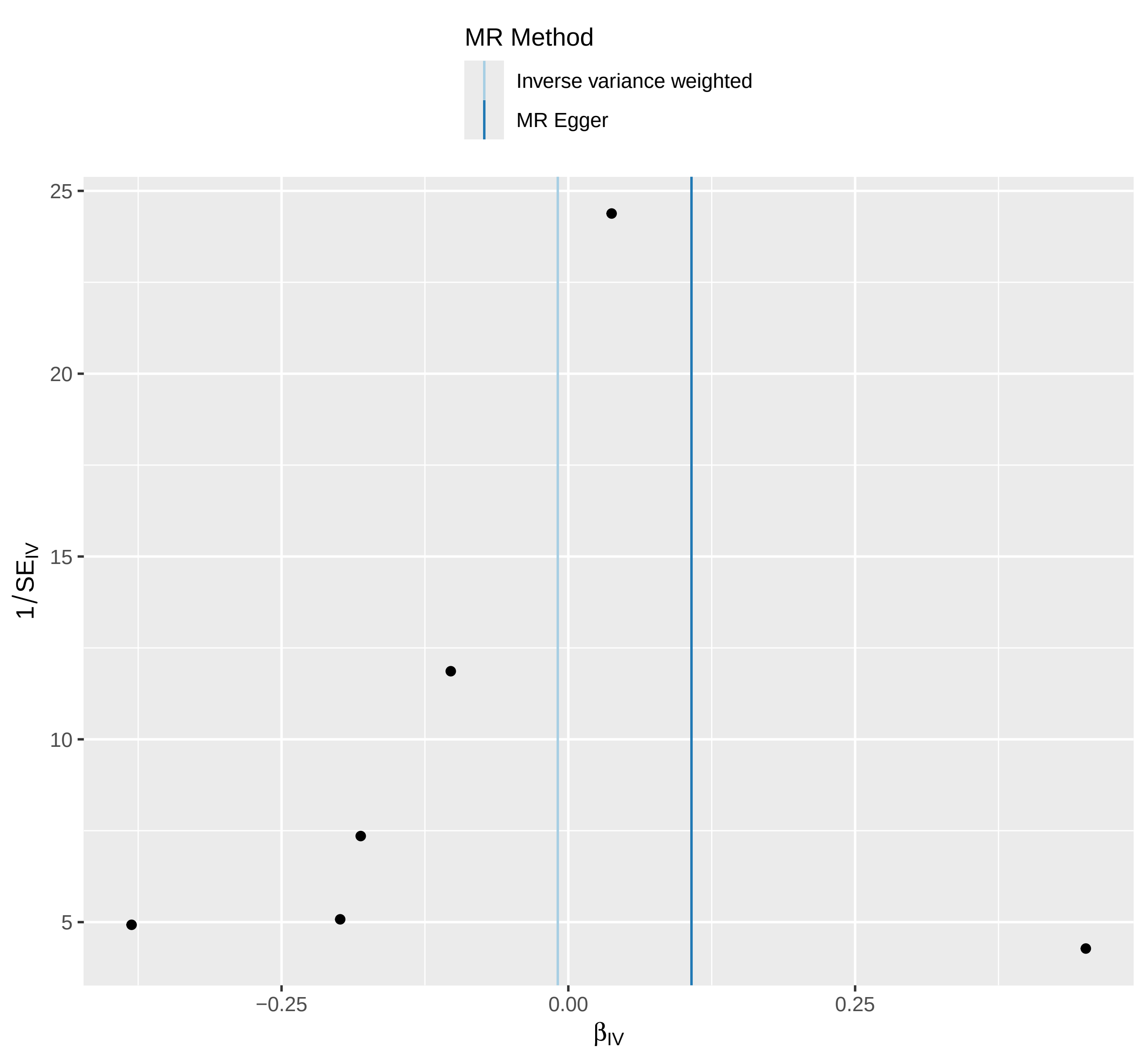
MR Method
Inverse variance weighted
MR Egger
25
20
15
10
5
−0.25
0.00
0.25
1/SE_IV
β_IV

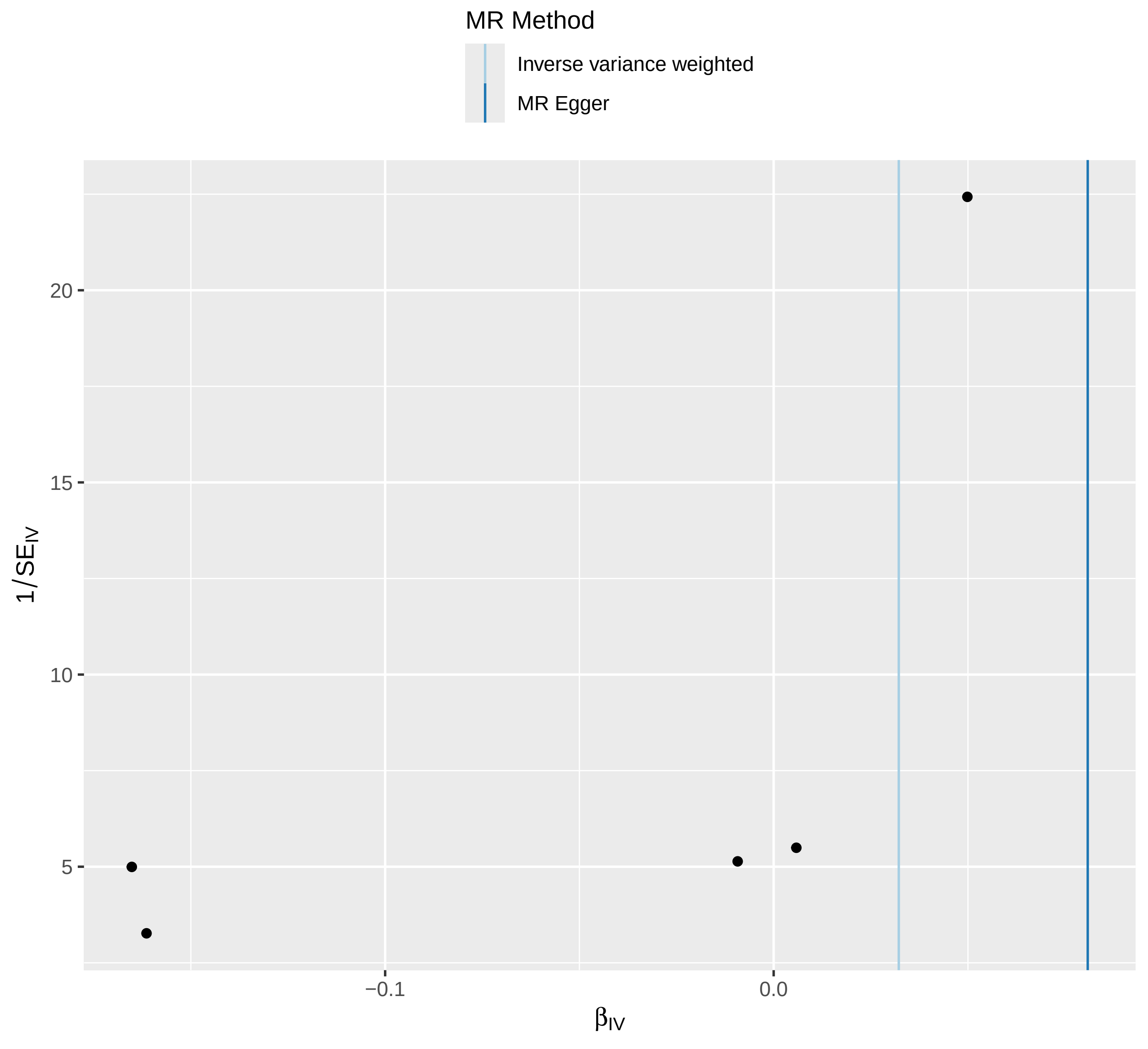

MR Method
Inverse variance weighted
MR Egger
20
15
10
5
1/SE$_{IV}$
−0.1
0.0
β$_{IV}$

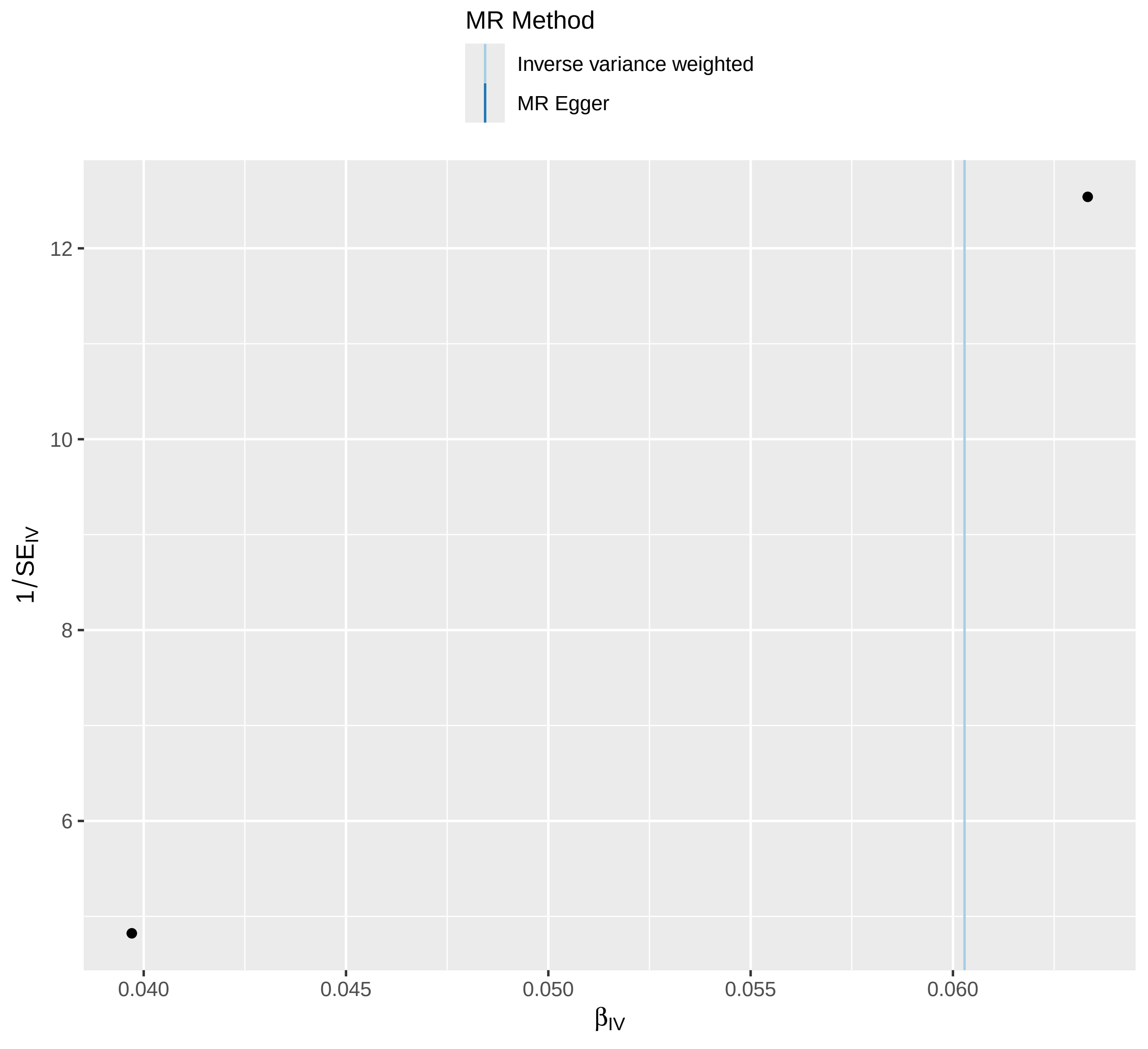
MR Method
Inverse variance weighted
MR Egger
$1/SE_{IV}$
12
10
8
6
0.040
0.045
0.050
0.055
0.060
$\beta_{IV}$

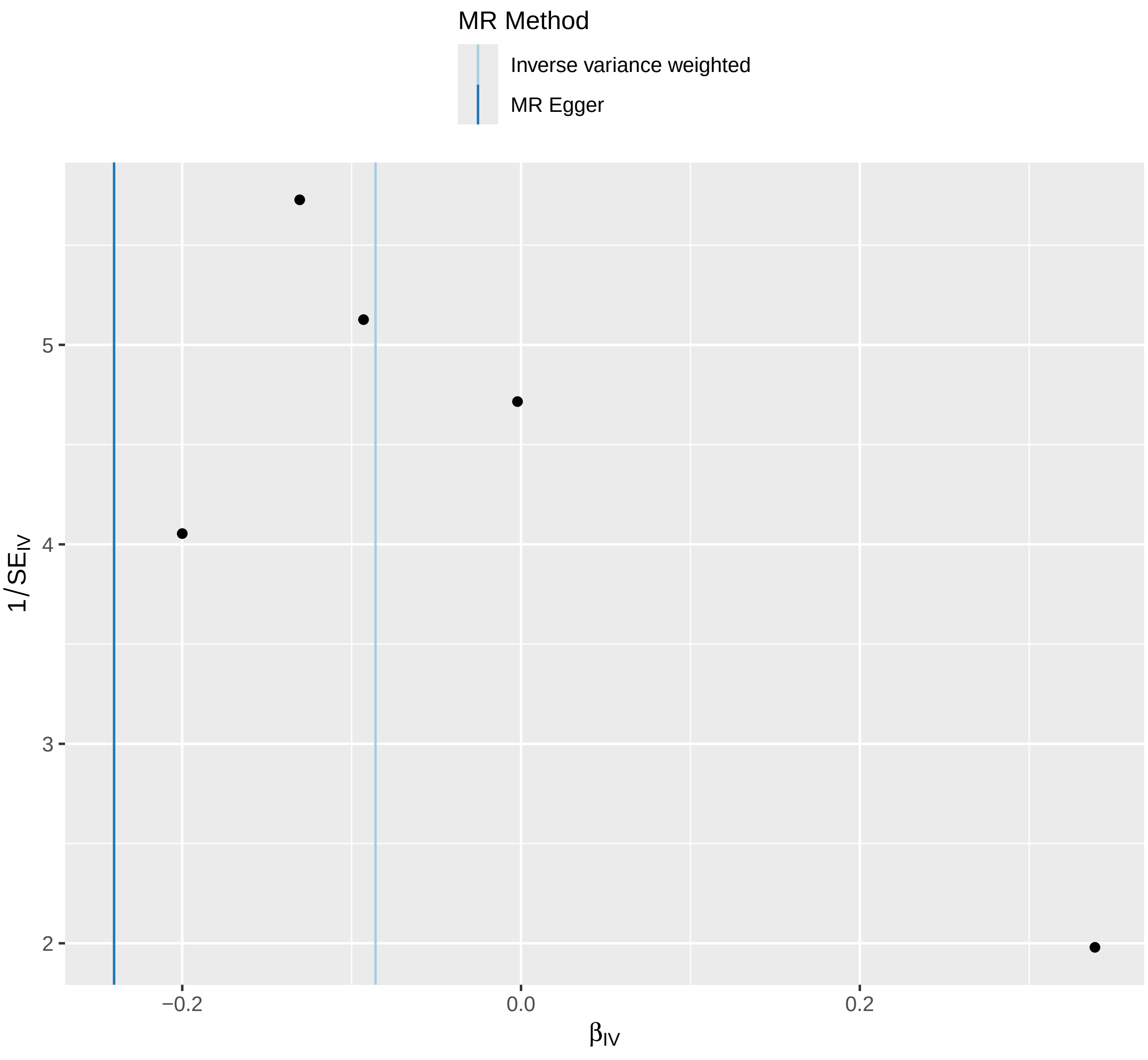

MR Method
Inverse variance weighted
MR Egger
5
4
3
2
1/SE$_{IV}$
−0.2
0.0
0.2
β$_{IV}$

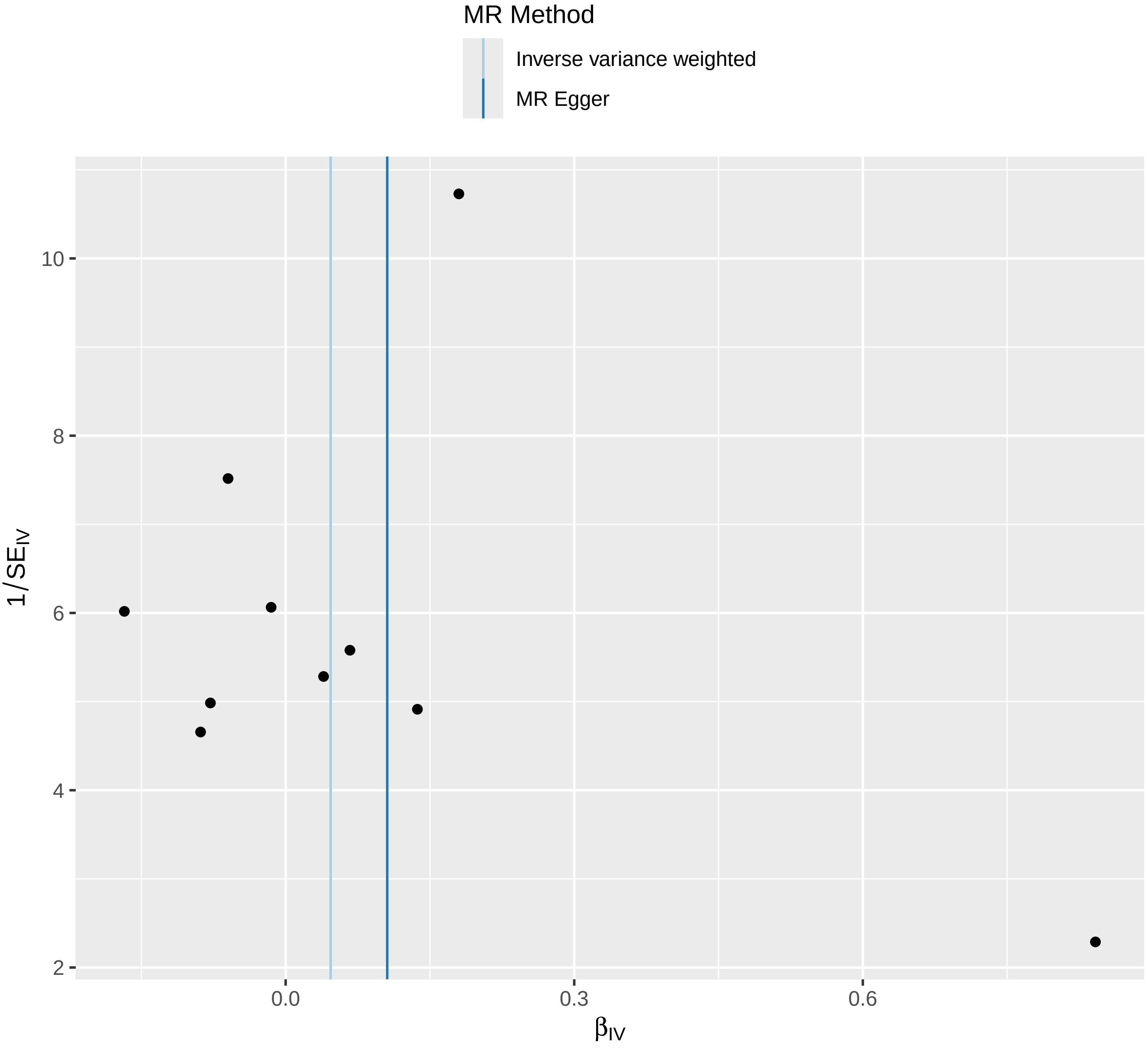
MR Method
Inverse variance weighted
MR Egger
10
8
6
4
2
0.0
0.3
0.6
$1/SE_{IV}$
$\beta_{IV}$

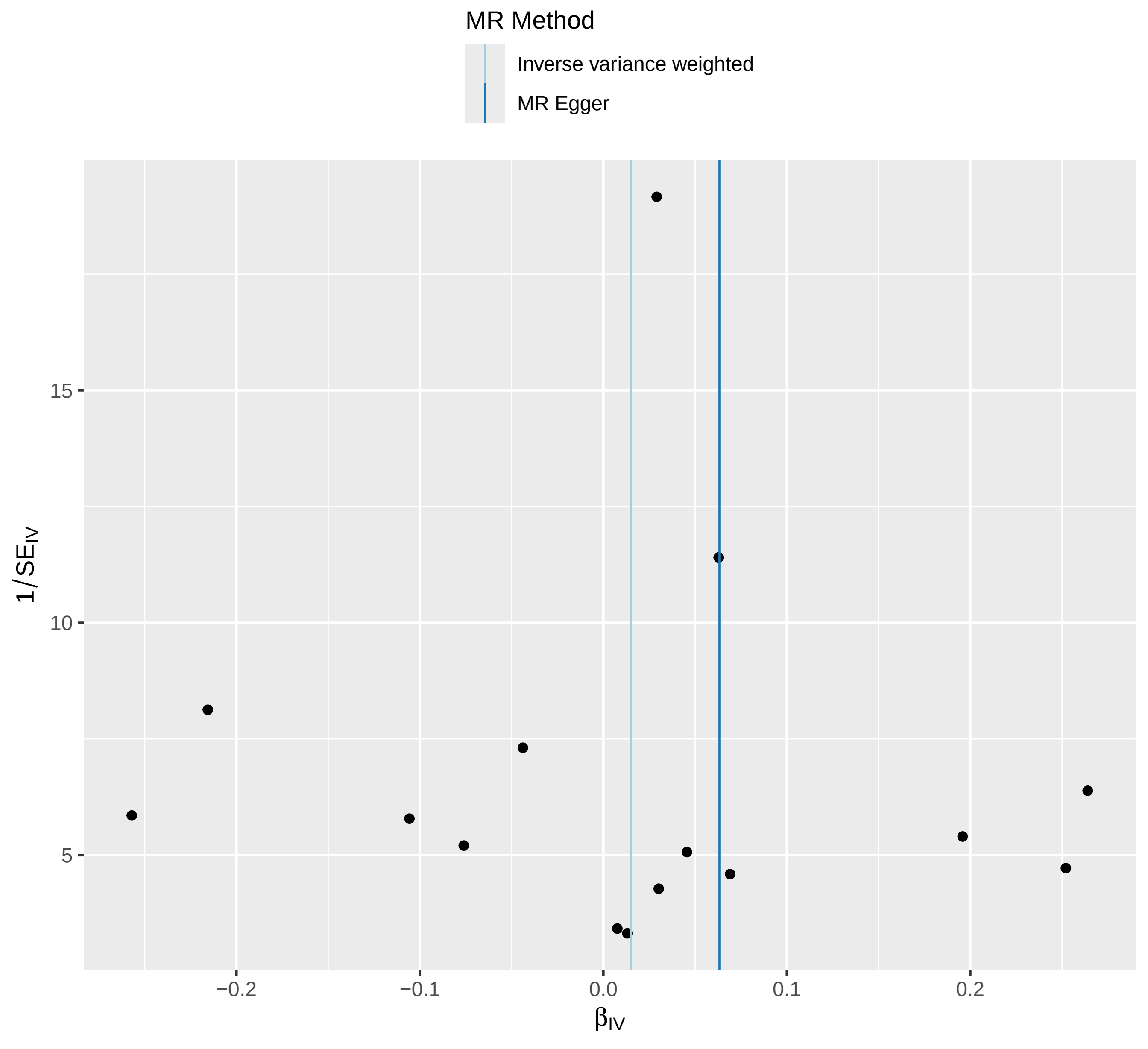
MR Method
Inverse variance weighted
MR Egger
1/SE_IV
β_IV
15
10
5
−0.2
−0.1
0.0
0.1
0.2

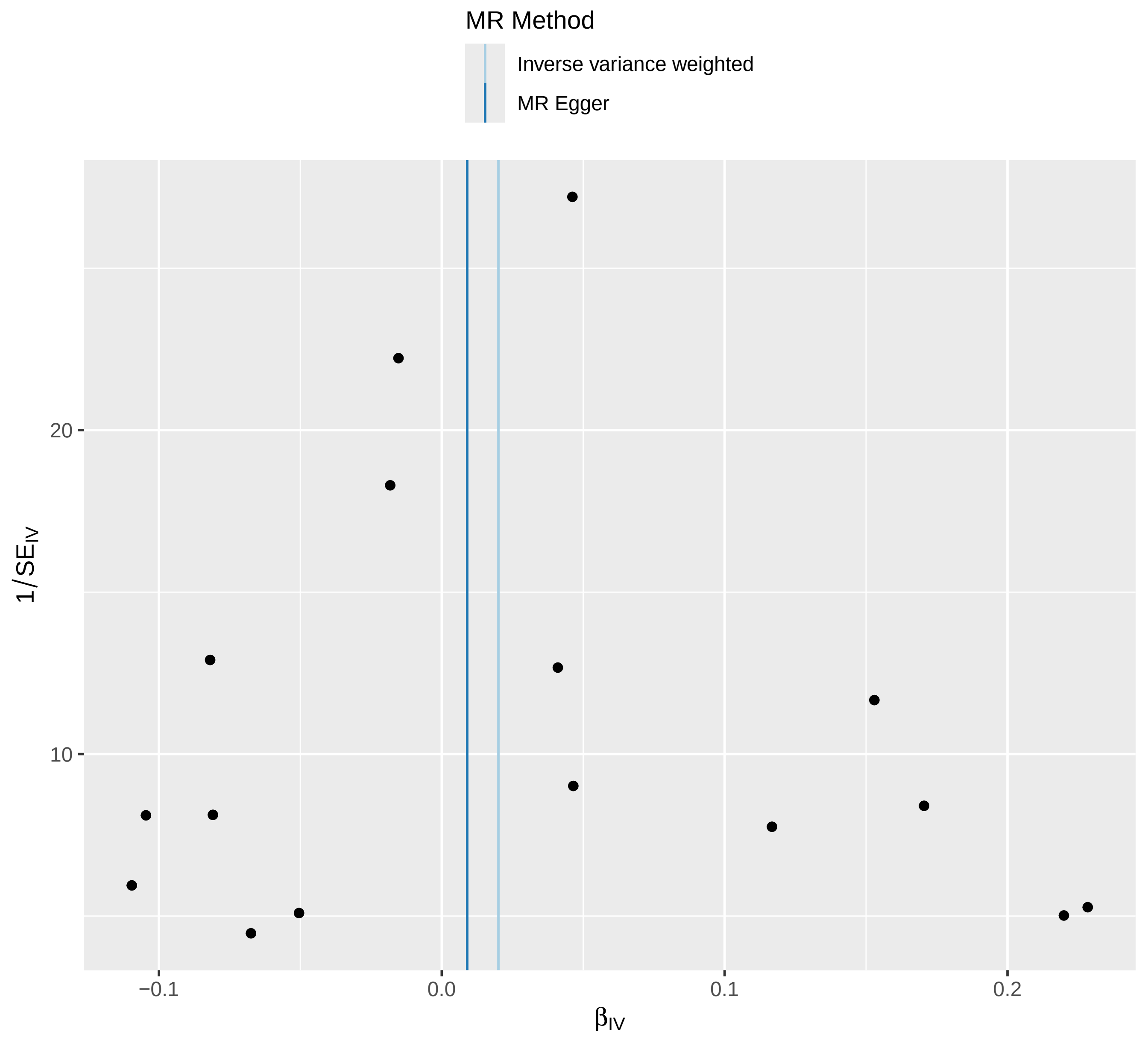

MR Method
Inverse variance weighted
MR Egger
20
10
$1/SE_{IV}$
−0.1
0.0
0.1
0.2
$\beta_{IV}$

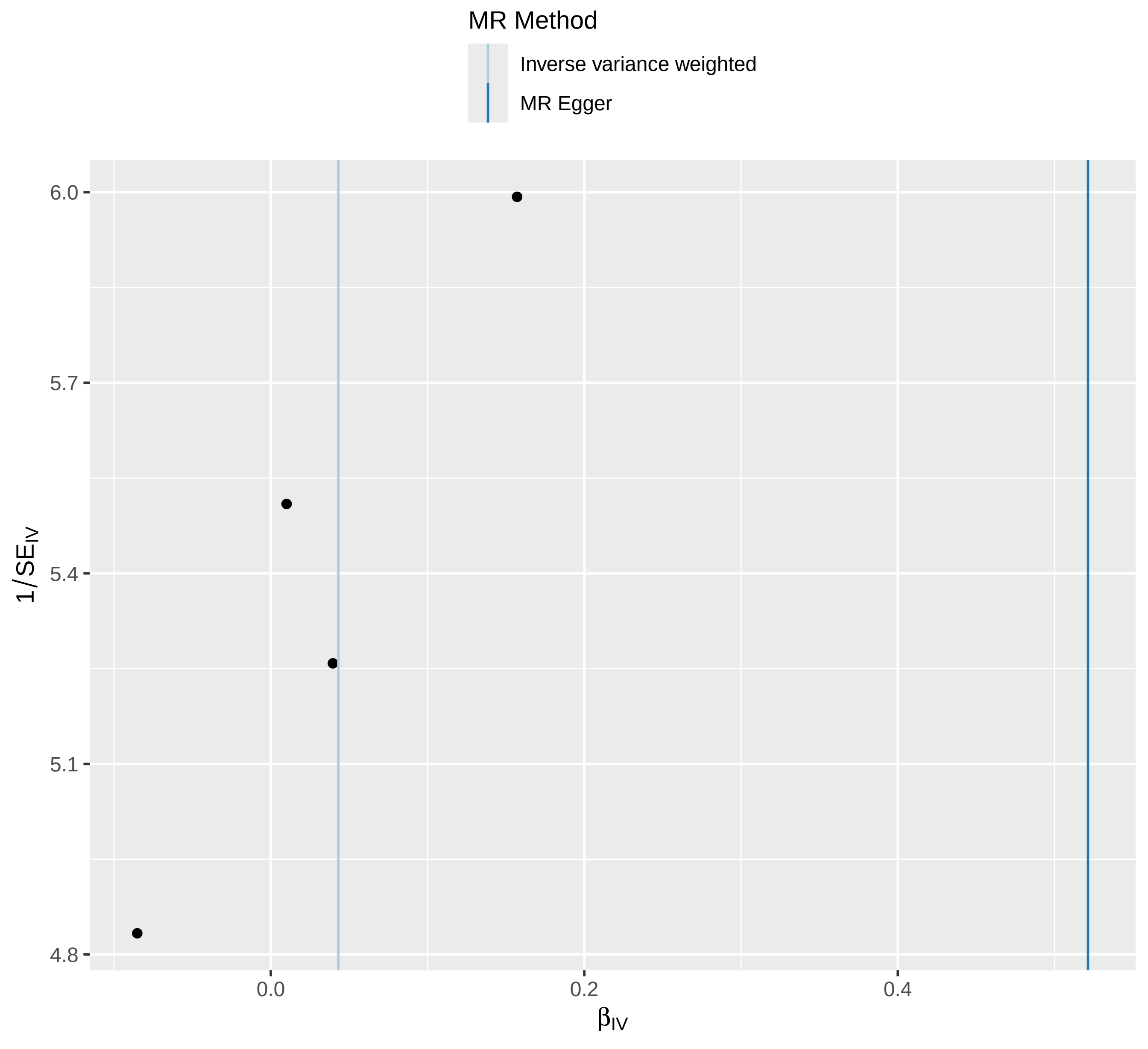

MR Method
Inverse variance weighted
MR Egger
6.0
5.7
5.4
5.1
4.8
$1/SE_{IV}$
0.0
0.2
0.4
$\beta_{IV}$

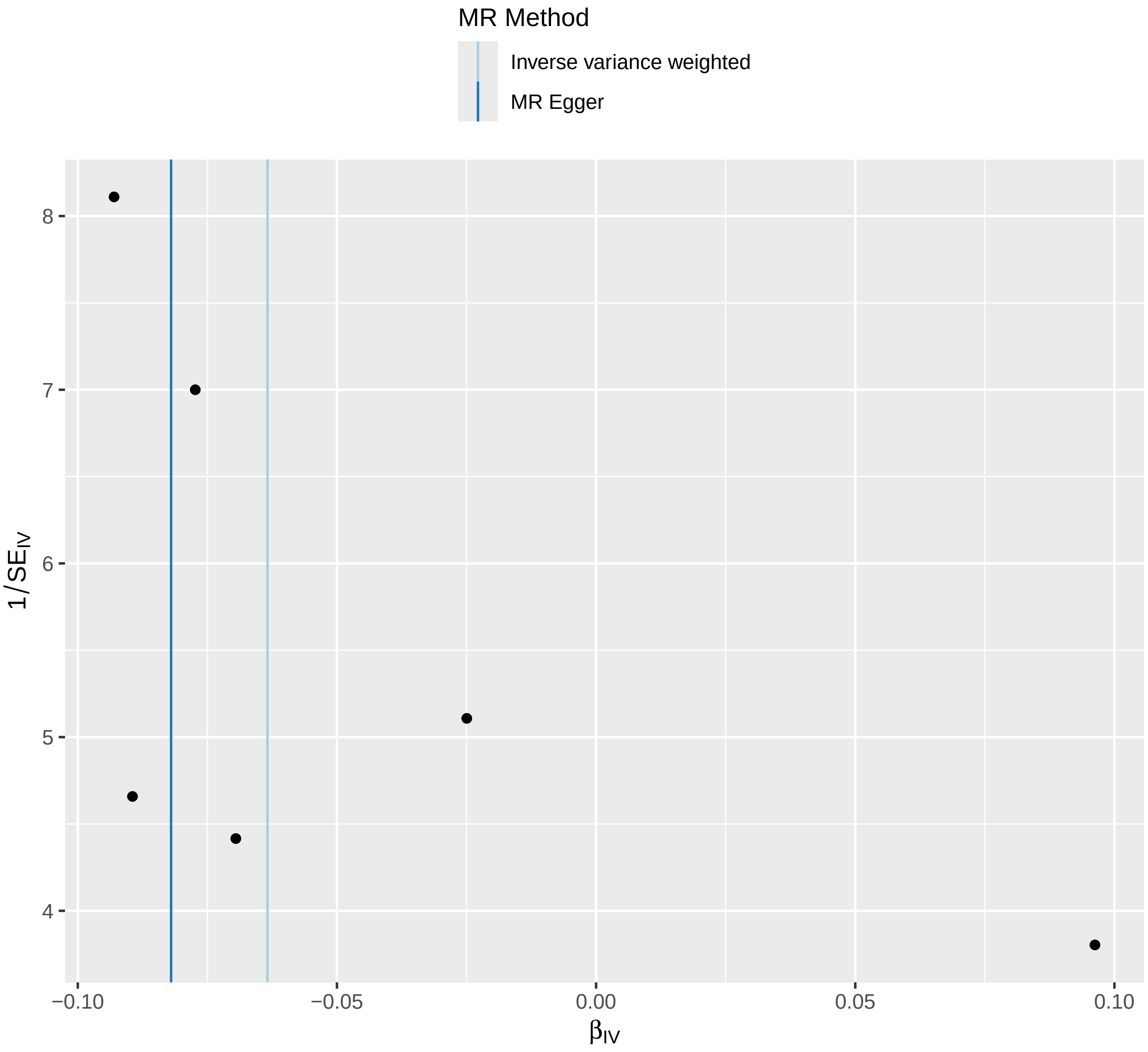
MR Method
Inverse variance weighted
MR Egger
8
7
6
5
4
$1/SE_{IV}$
−0.10
−0.05
0.00
0.05
0.10
$\beta_{IV}$

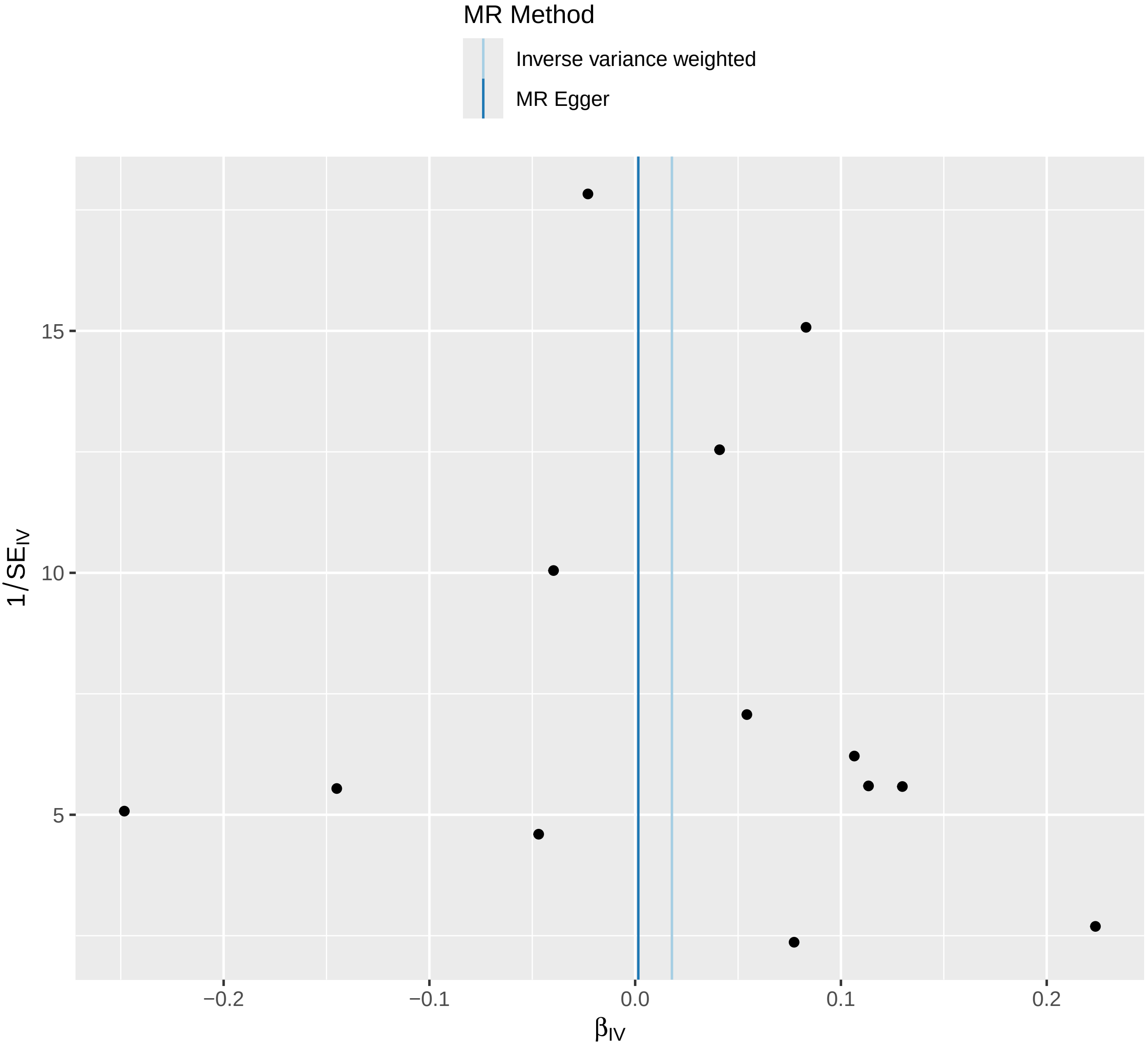
MR Method
Inverse variance weighted
MR Egger
15
10
5
1/SE$_{IV}$
−0.2
−0.1
0.0
0.1
0.2
β$_{IV}$

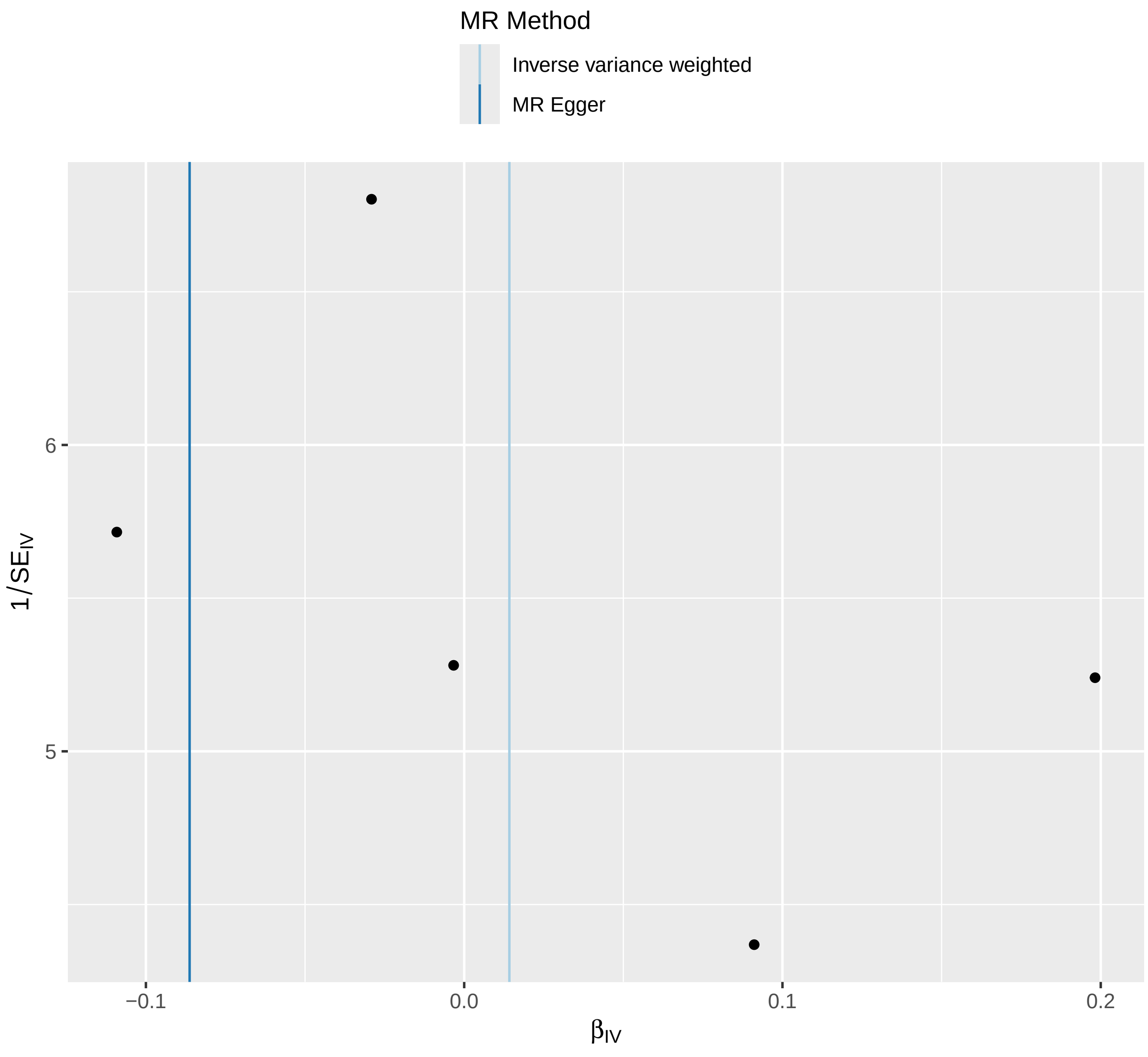

MR Method
Inverse variance weighted
MR Egger
6
5
1/SE$_{IV}$
−0.1
0.0
0.1
0.2
β$_{IV}$

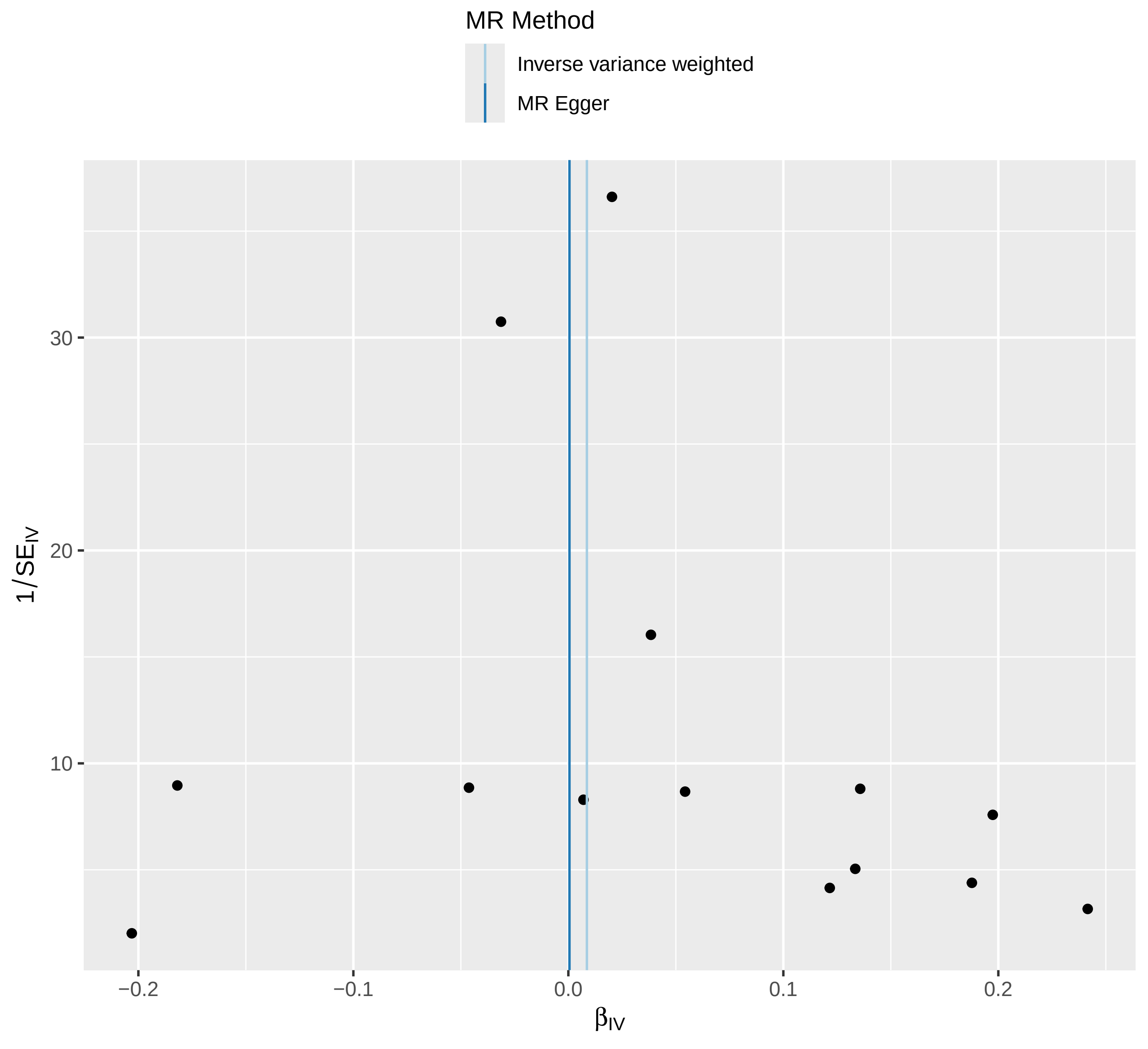
MR Method
Inverse variance weighted
MR Egger
30
20
10
1/SE_IV
−0.2
−0.1
0.0
0.1
0.2
β_IV

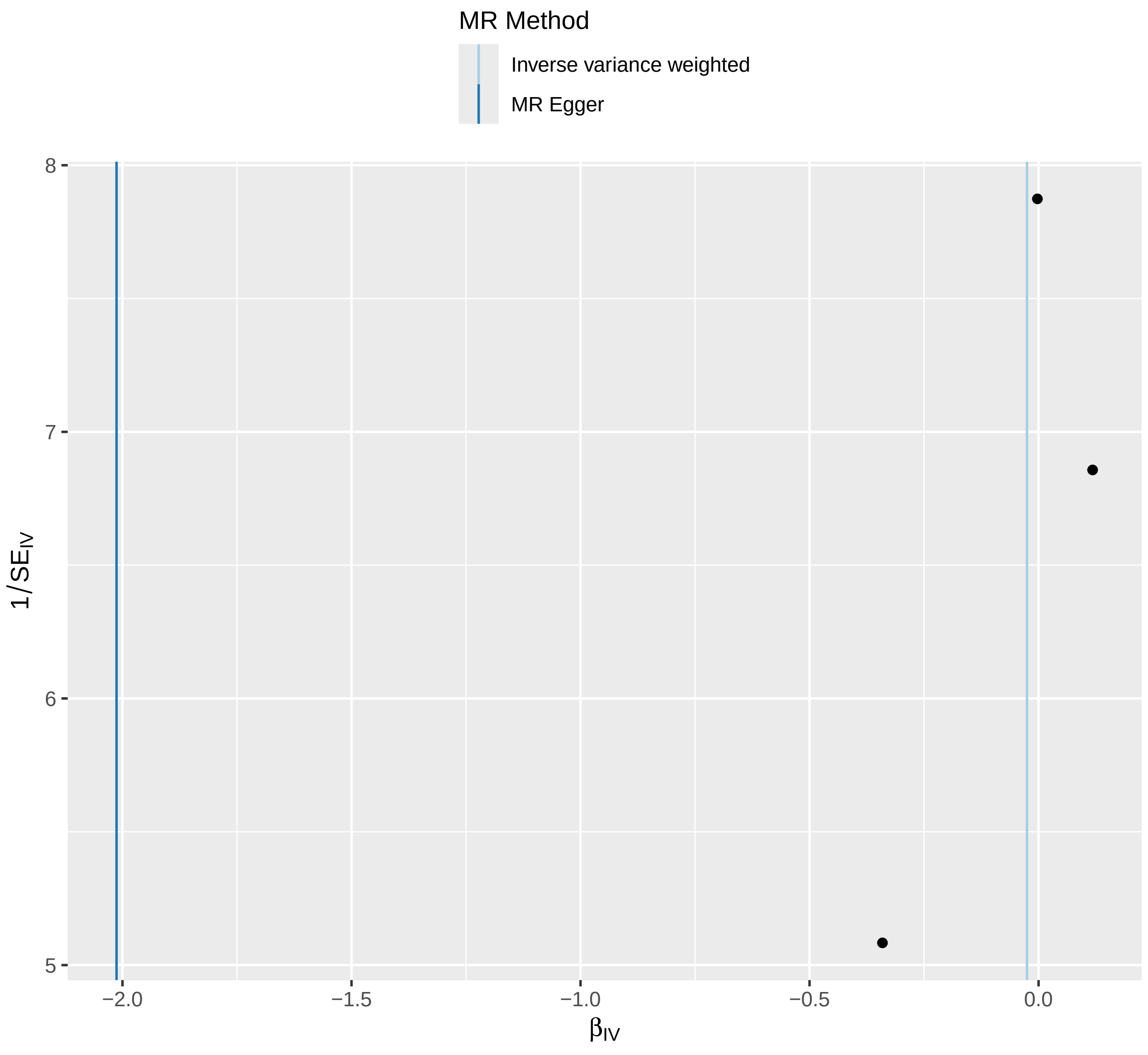
MR Method
Inverse variance weighted
MR Egger
8
7
6
5
1/SE_IV
−2.0
−1.5
−1.0
−0.5
0.0
β_IV

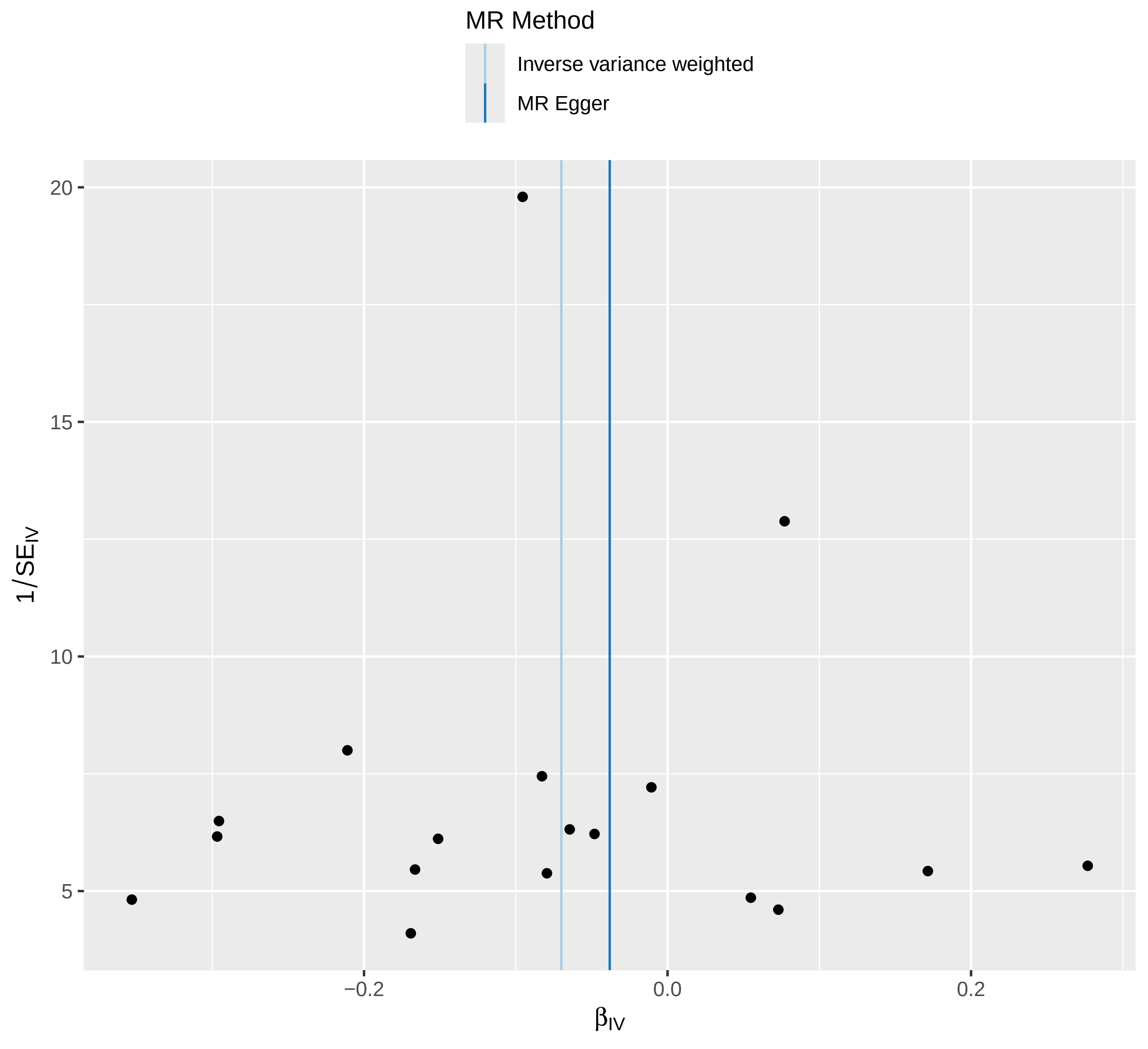
MR Method
Inverse variance weighted
MR Egger
20
15
10
5
1/SE_IV
−0.2
0.0
0.2
β_IV

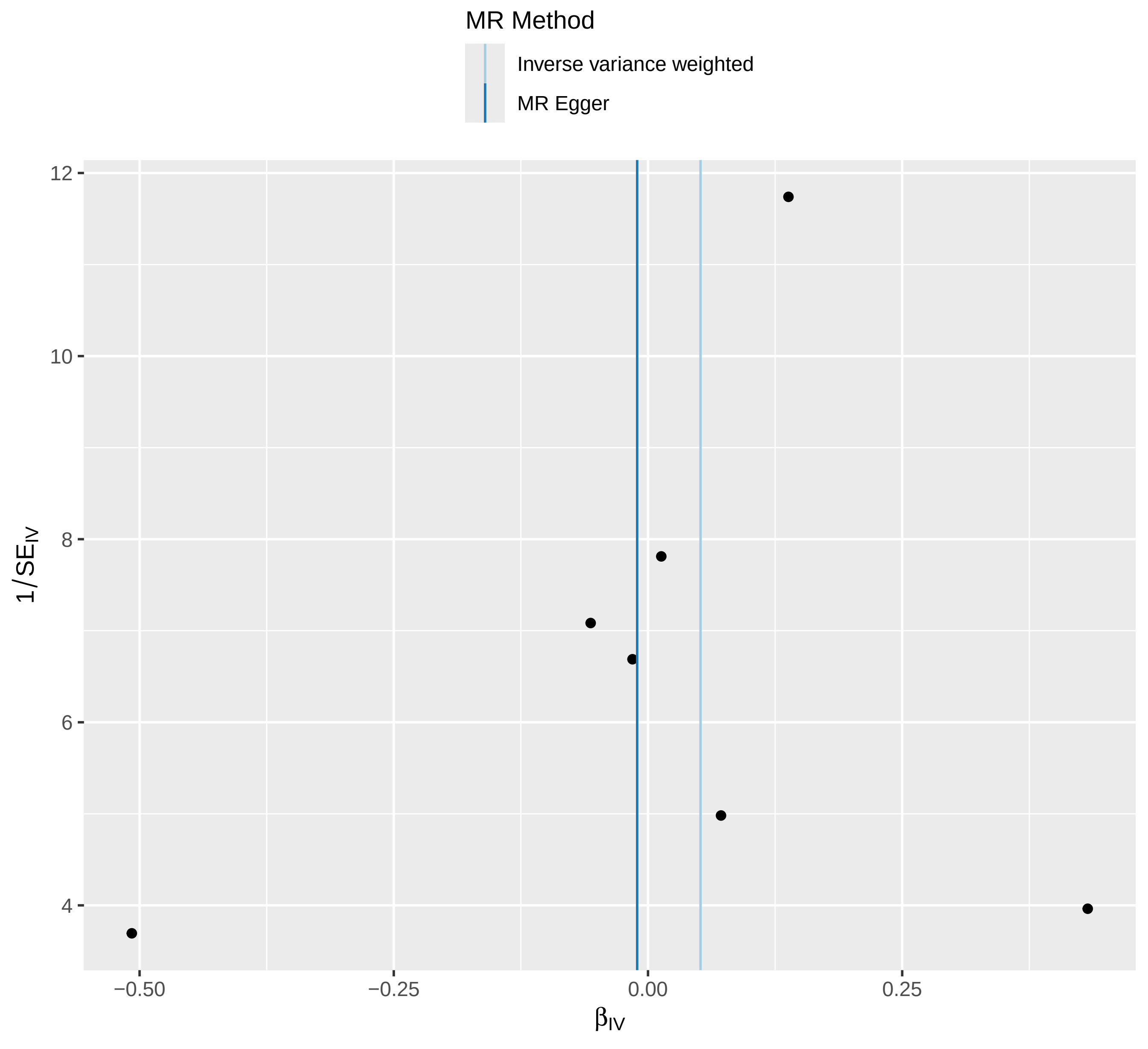
MR Method
Inverse variance weighted
MR Egger
12
10
8
6
4
1/SE_IV
−0.50
−0.25
0.00
0.25
β_IV

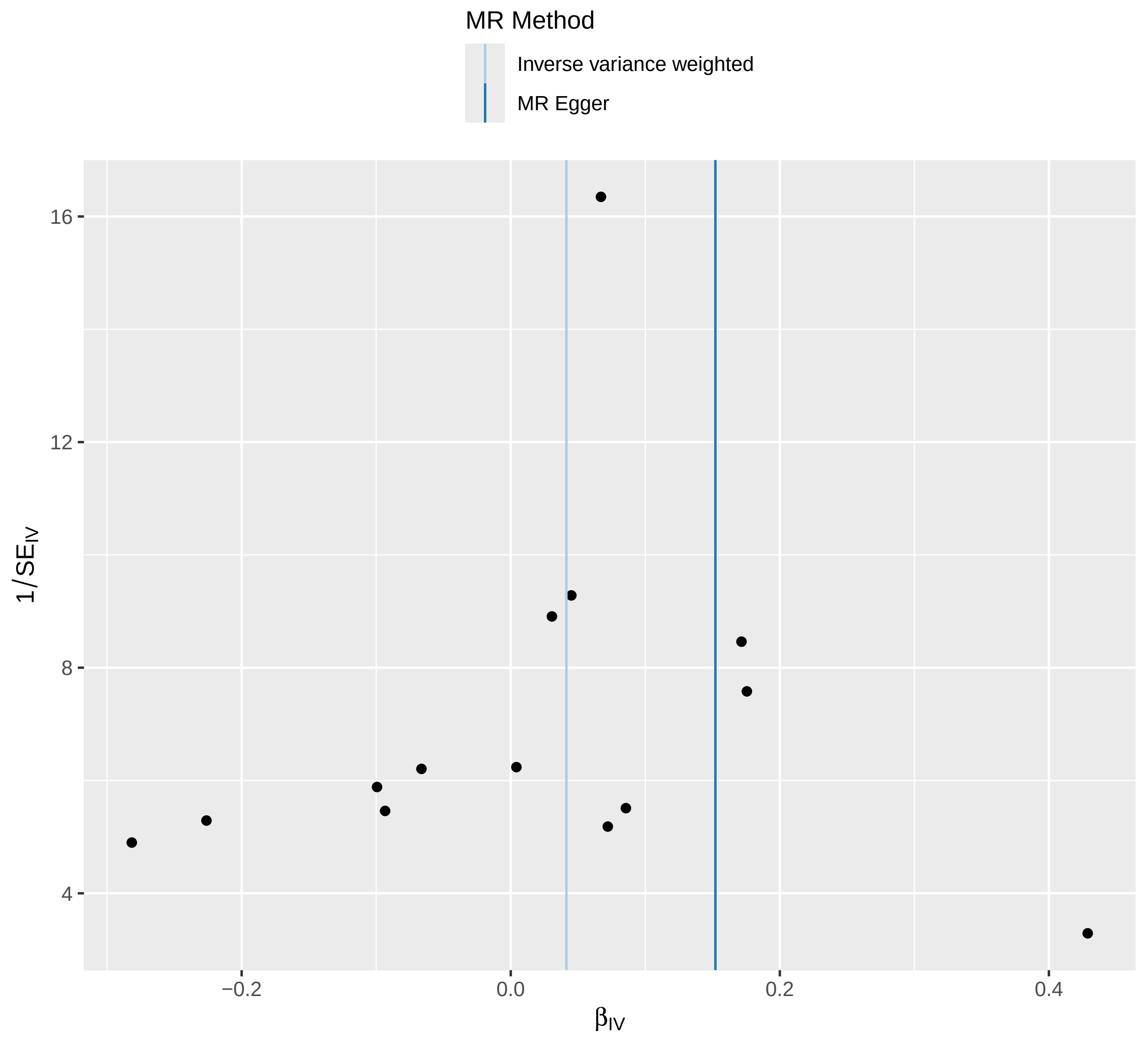

MR Method
Inverse variance weighted
MR Egger
16
12
8
4
1/SE_IV
−0.2
0.0
0.2
0.4
β_IV

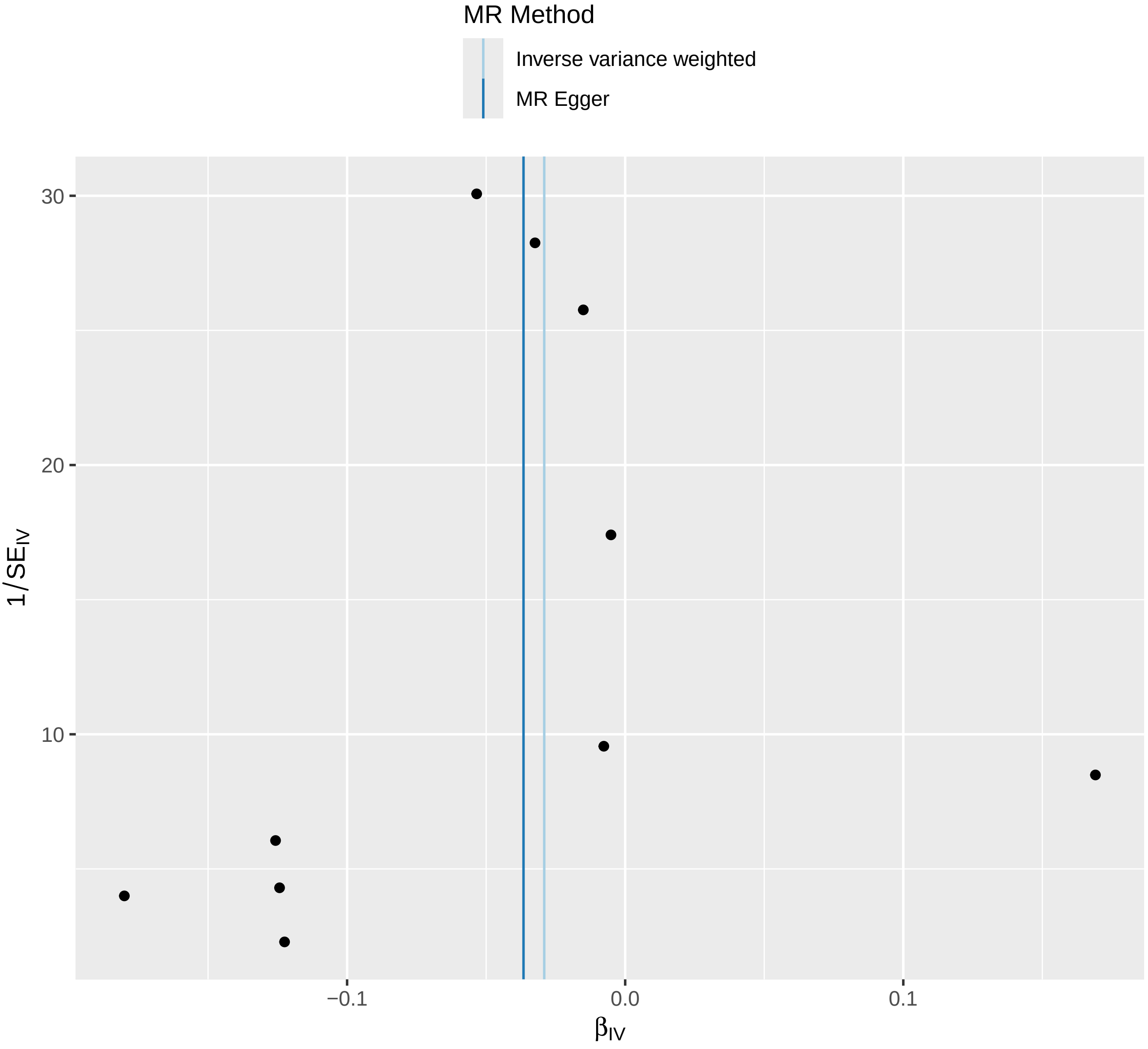
MR Method
Inverse variance weighted
MR Egger
30
20
10
1/SE$_{IV}$
−0.1
0.0
0.1
β$_{IV}$

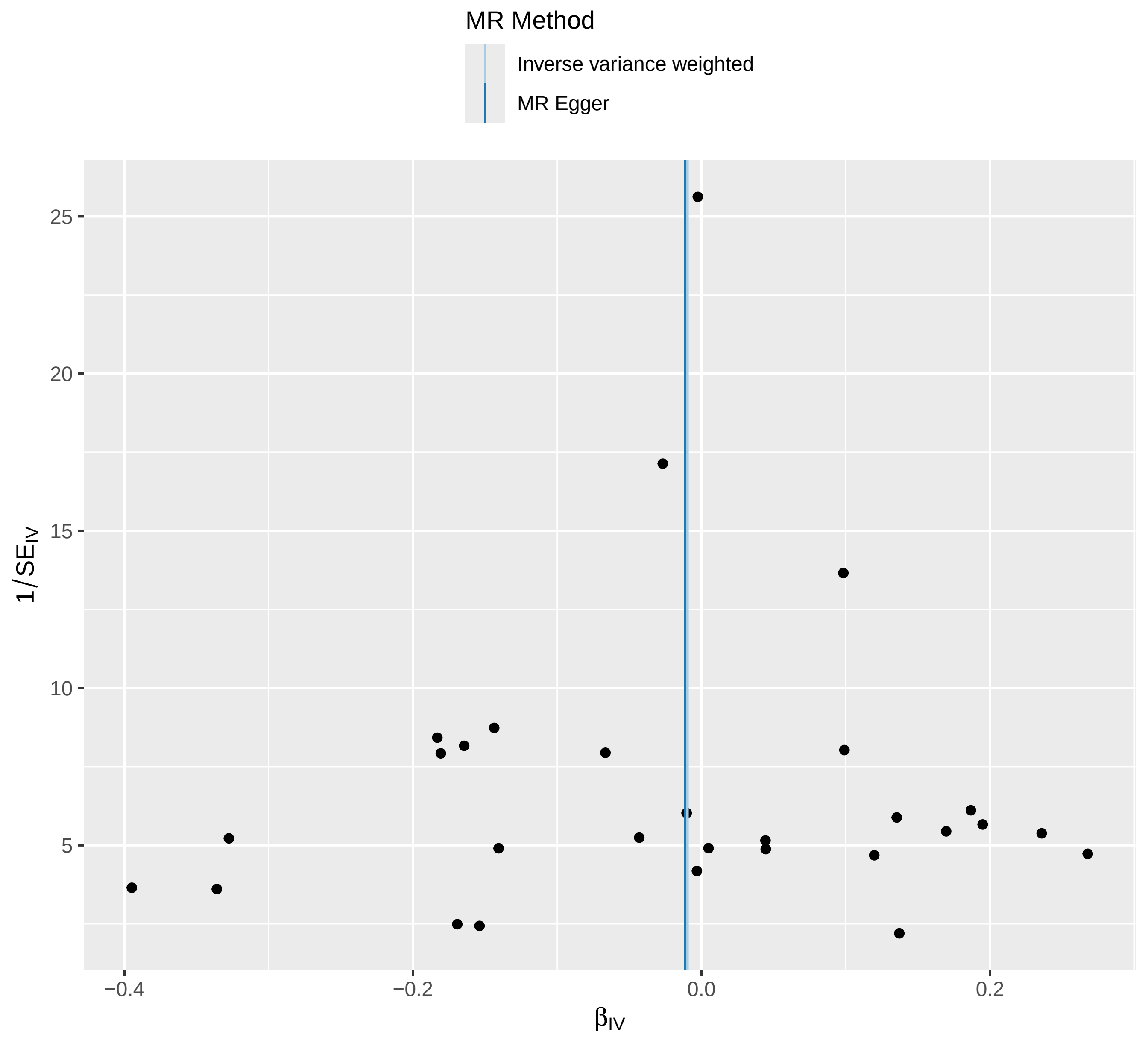

MR Method
Inverse variance weighted
MR Egger
25
20
15
10
5
1/SE$_{IV}$
−0.4
−0.2
0.0
0.2
β$_{IV}$

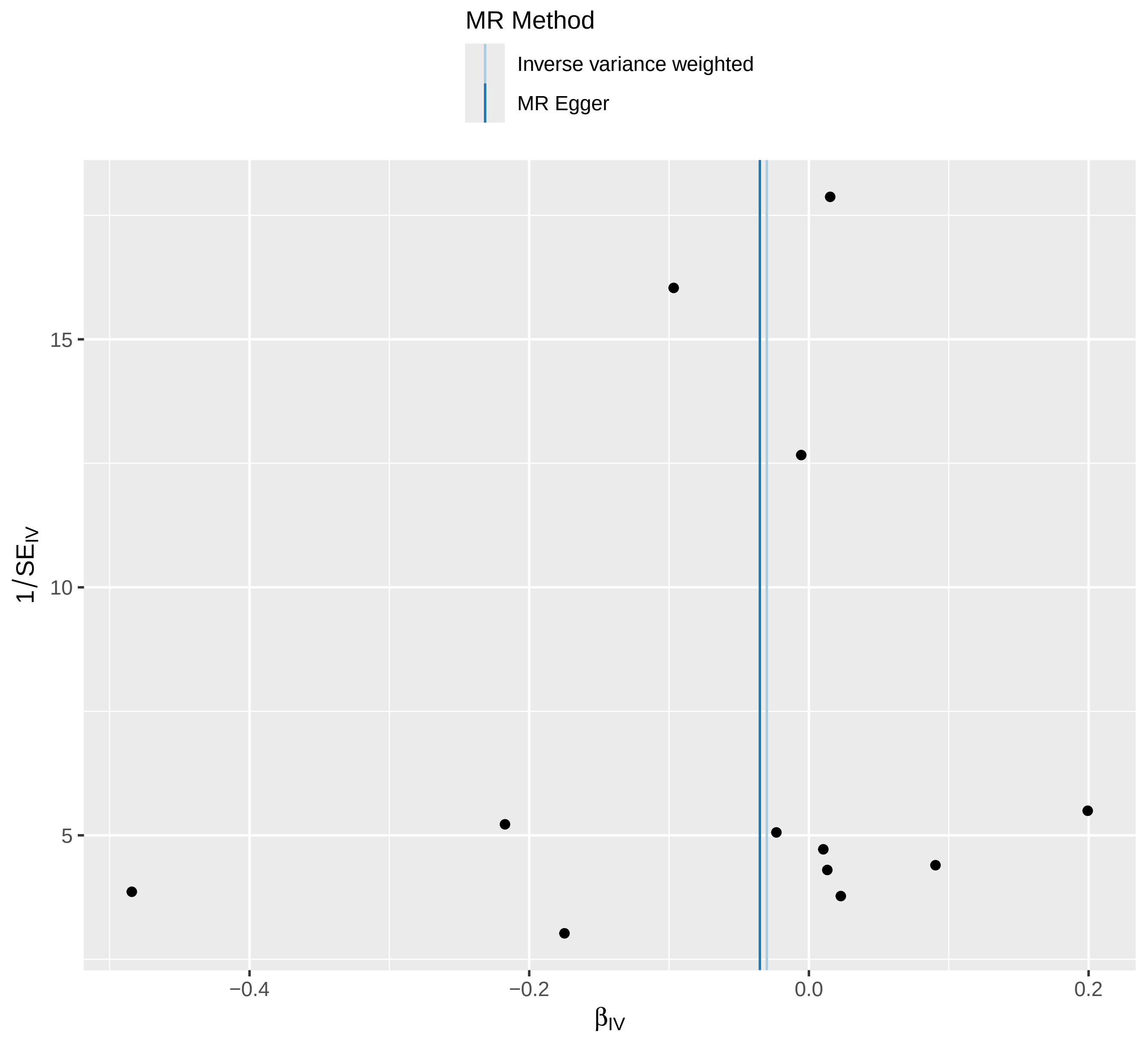

MR Method
Inverse variance weighted
MR Egger
15
10
5
1/SE$_{IV}$
−0.4
−0.2
0.0
0.2
β$_{IV}$

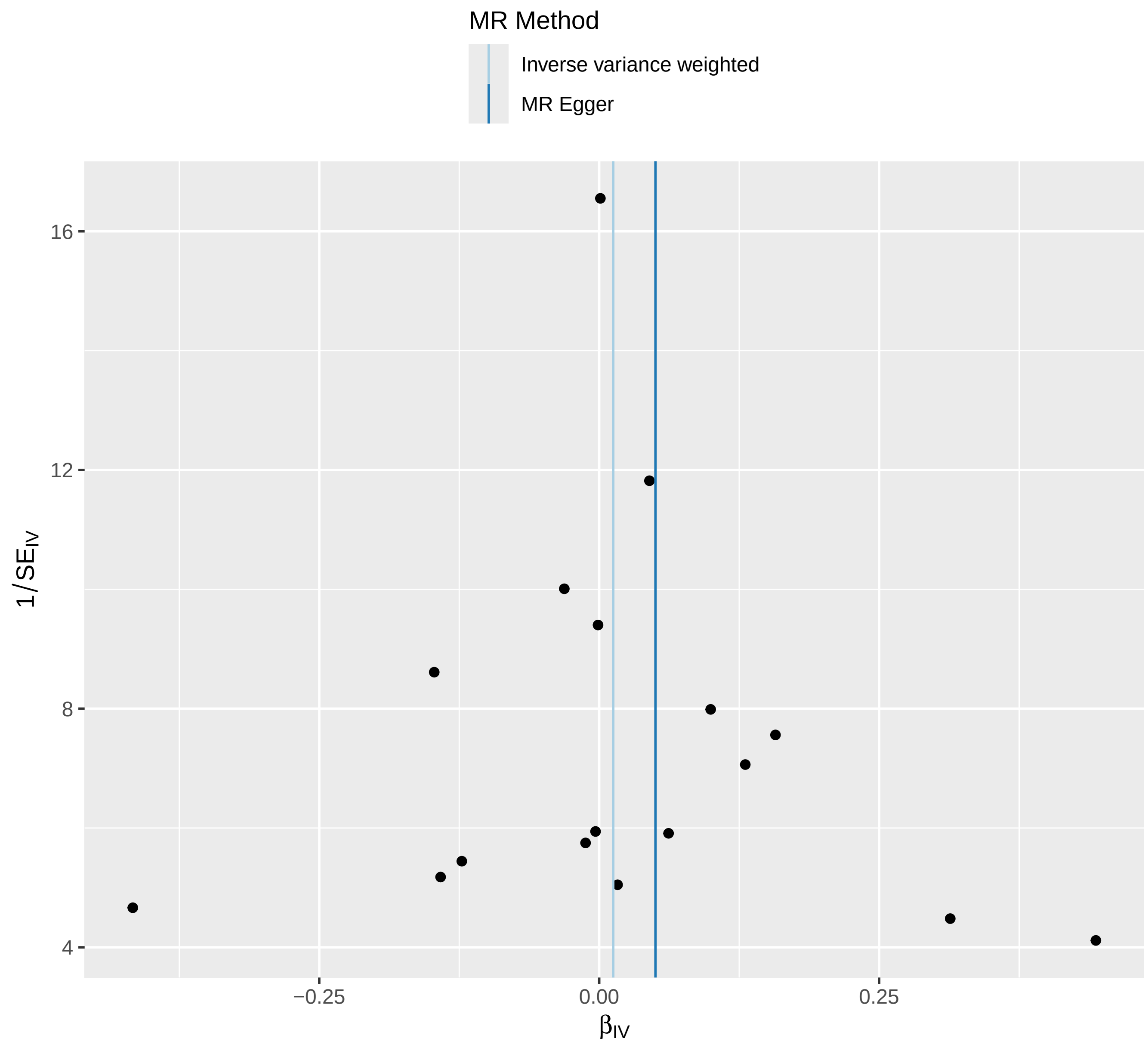
MR Method
Inverse variance weighted
MR Egger
16
12
8
4
1/SE$_{IV}$
−0.25
0.00
0.25
β$_{IV}$

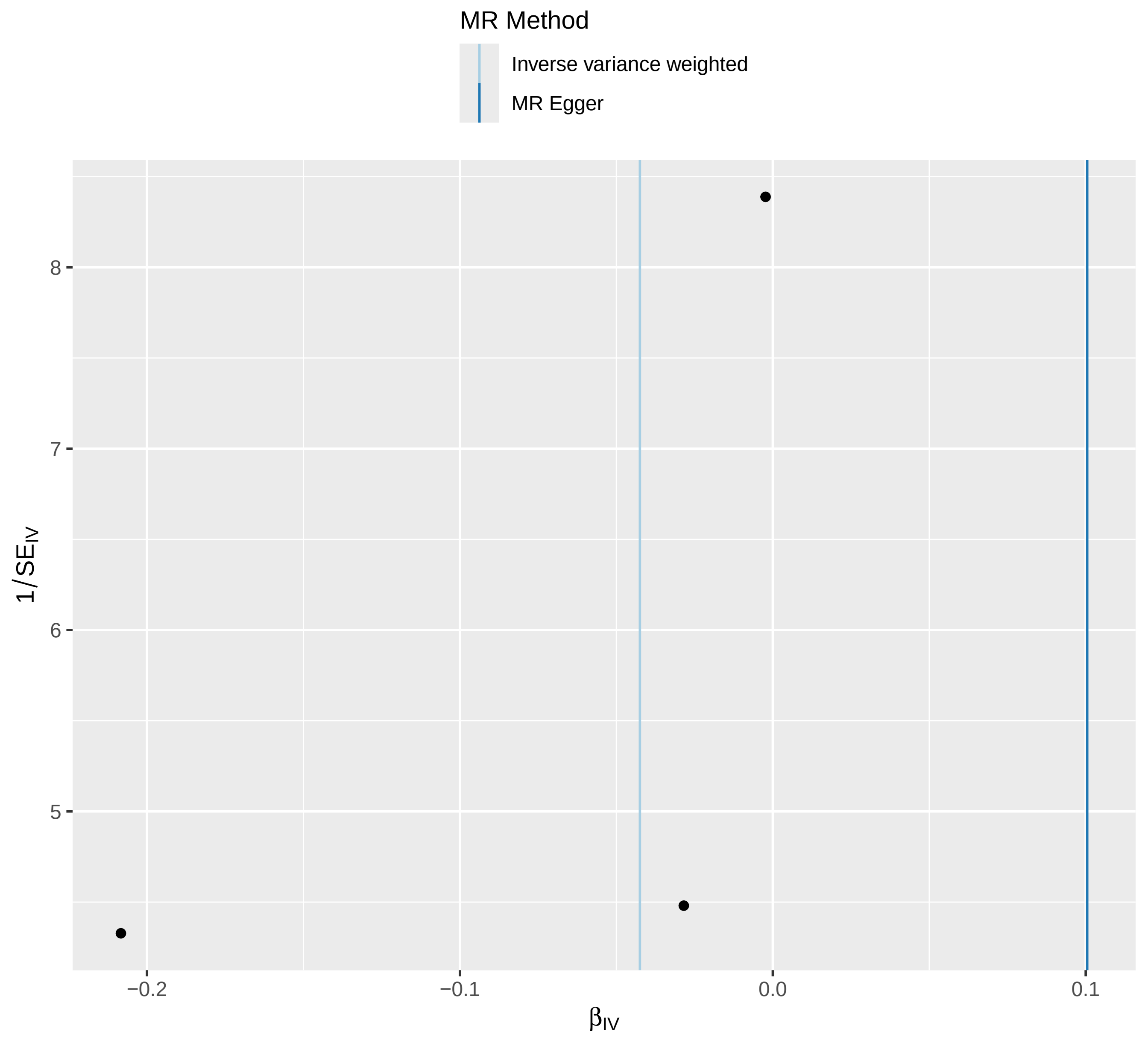
MR Method
Inverse variance weighted
MR Egger
8
7
6
5
1/SE_IV
−0.2
−0.1
0.0
0.1
β_IV

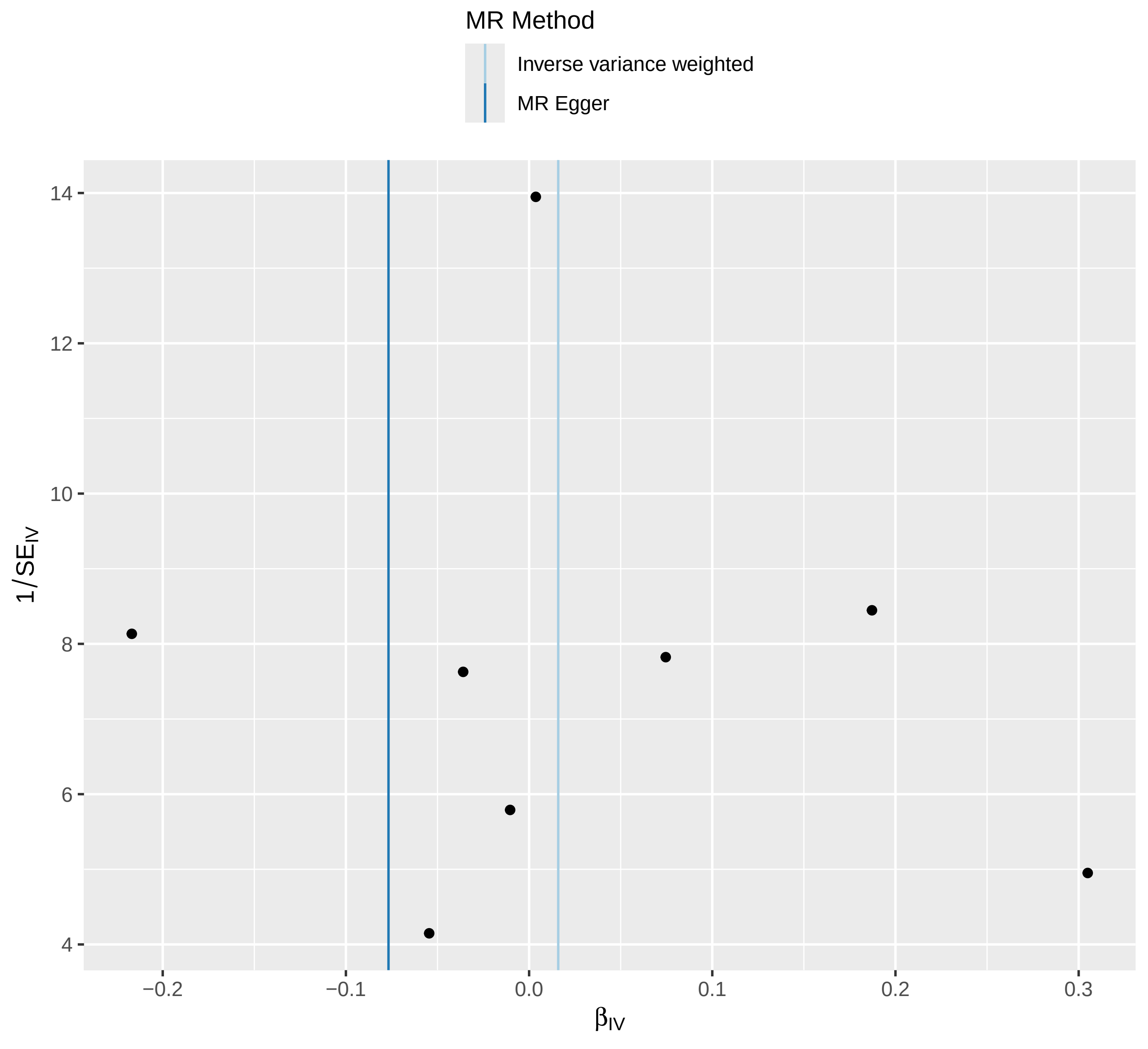

MR Method
Inverse variance weighted
MR Egger
14
12
10
8
6
4
1/SE$_{IV}$
−0.2
−0.1
0.0
0.1
0.2
0.3
β$_{IV}$

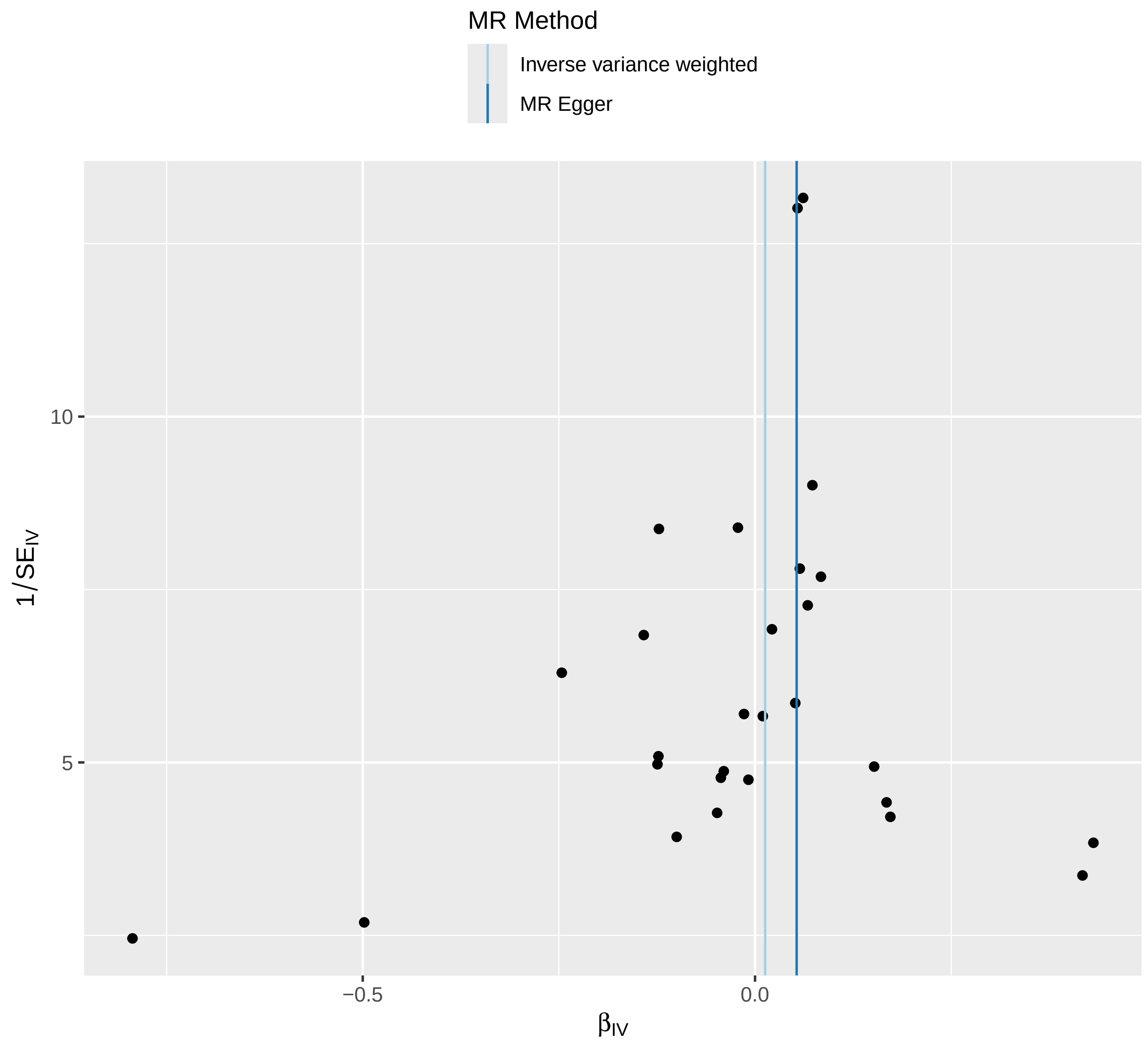

MR Method
Inverse variance weighted
MR Egger
10
5
1/SE$_{IV}$
−0.5
0.0
β$_{IV}$

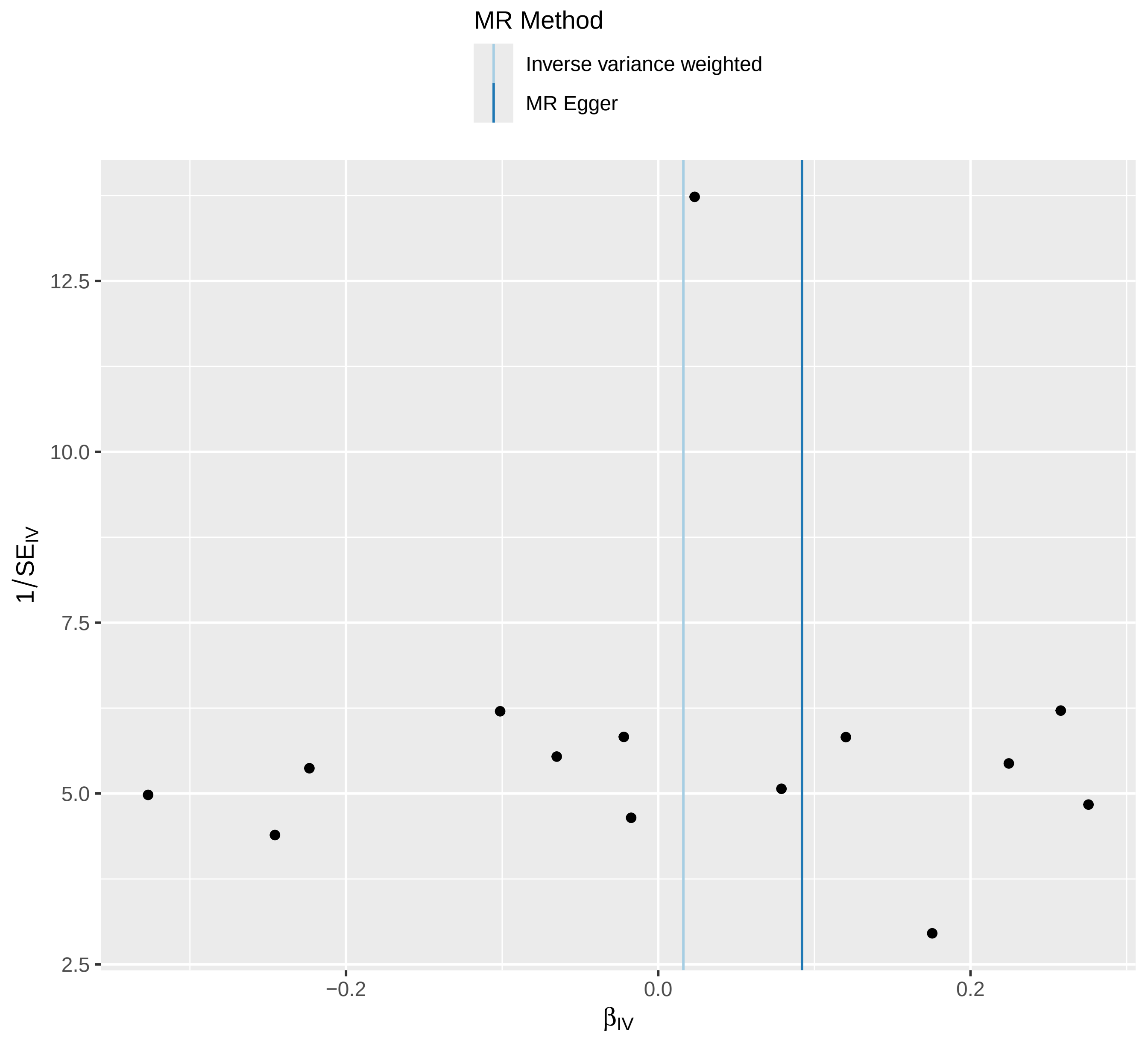

MR Method
Inverse variance weighted
MR Egger
1/SE_IV
β_IV
12.5
10.0
7.5
5.0
2.5
−0.2
0.0
0.2

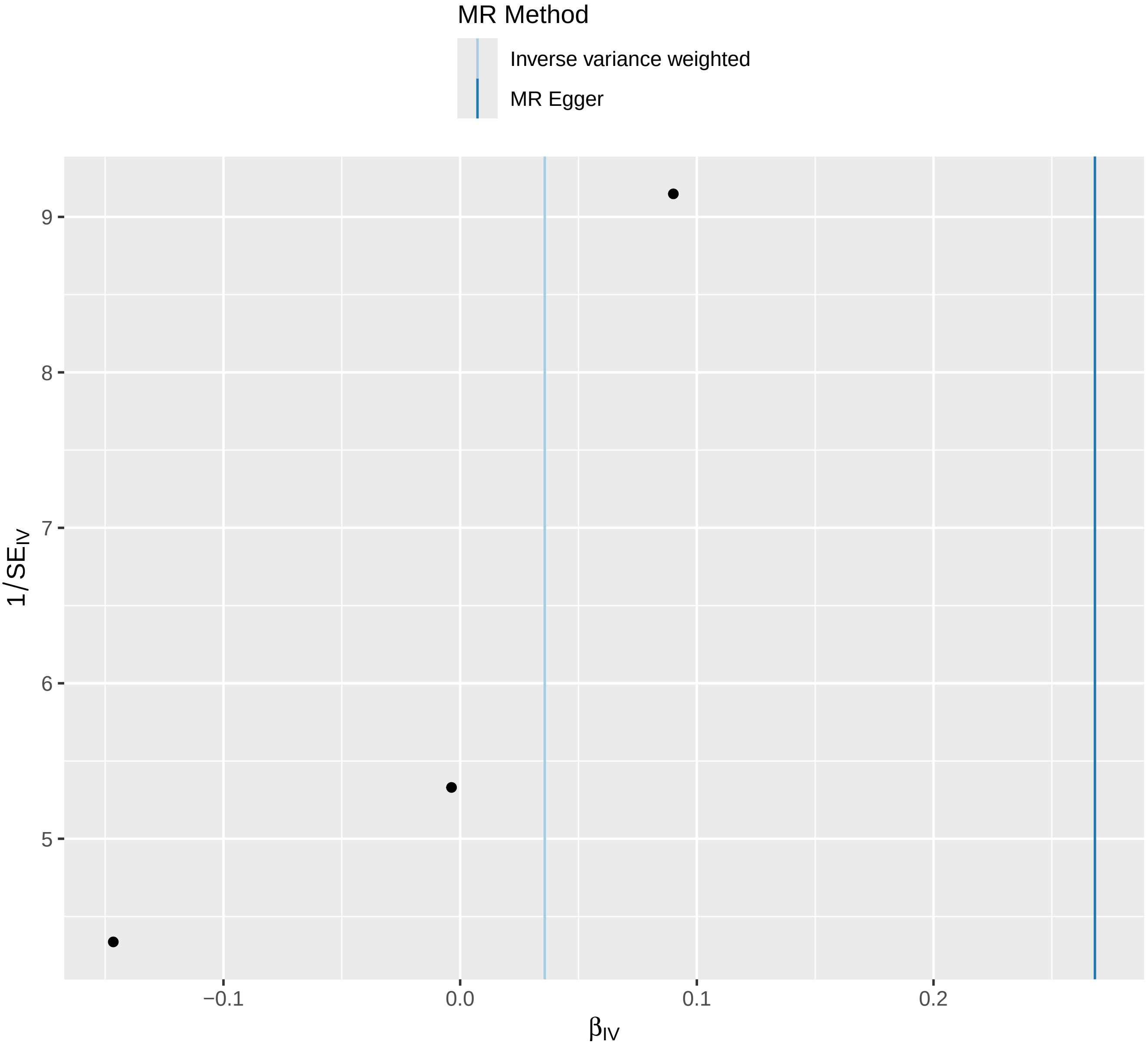

MR Method
Inverse variance weighted
MR Egger
9
8
7
6
5
1/SE_IV
−0.1
0.0
0.1
0.2
β_IV

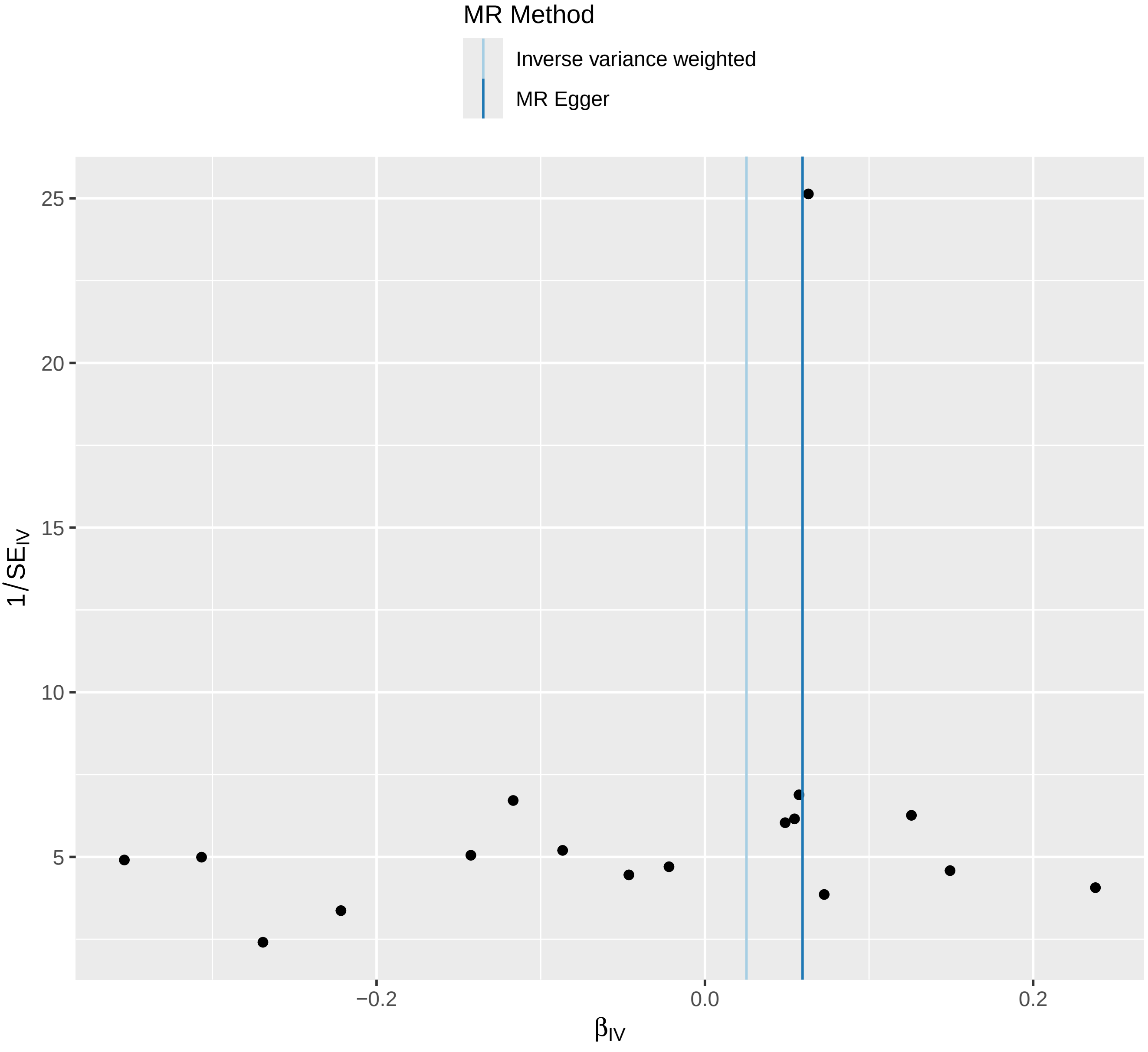
MR Method
Inverse variance weighted
MR Egger
25
20
15
10
5
$1/SE_{IV}$
−0.2
0.0
0.2
$\beta_{IV}$

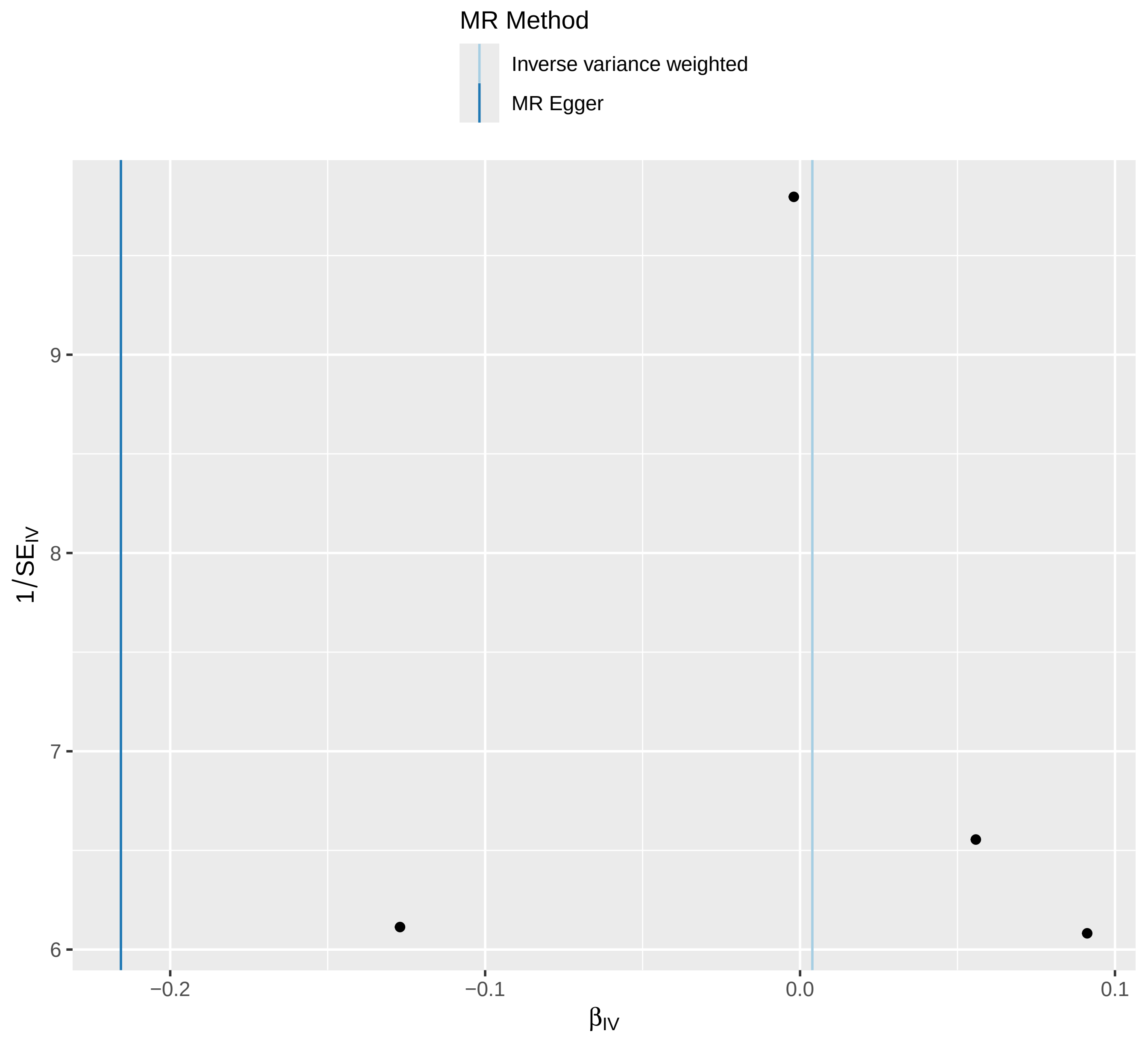

MR Method
Inverse variance weighted
MR Egger
9
8
7
6
$1/SE_{IV}$
−0.2
−0.1
0.0
0.1
$\beta_{IV}$

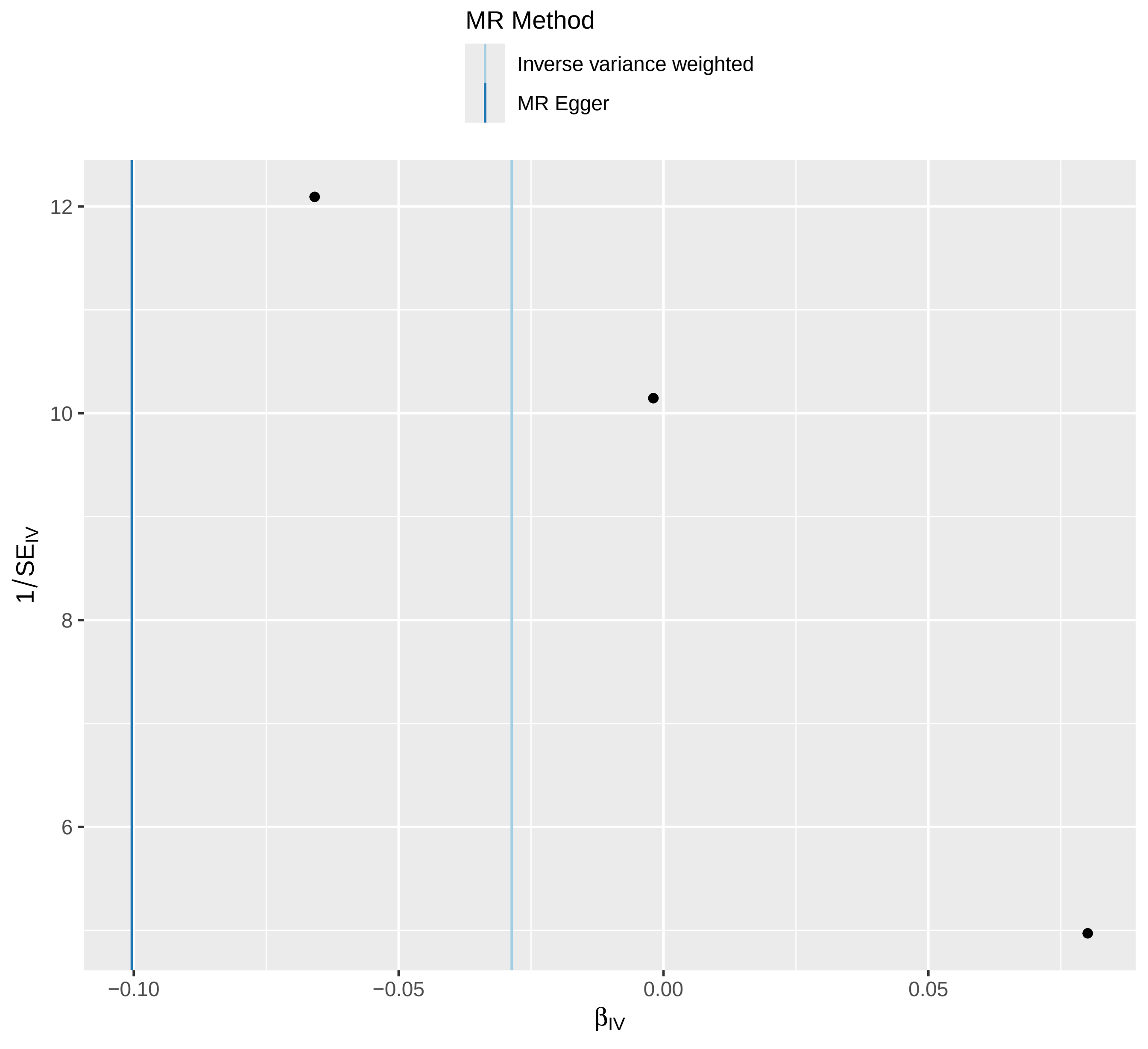
MR Method
Inverse variance weighted
MR Egger
12
10
8
6
1/SE_IV
−0.10
−0.05
0.00
0.05
β_IV

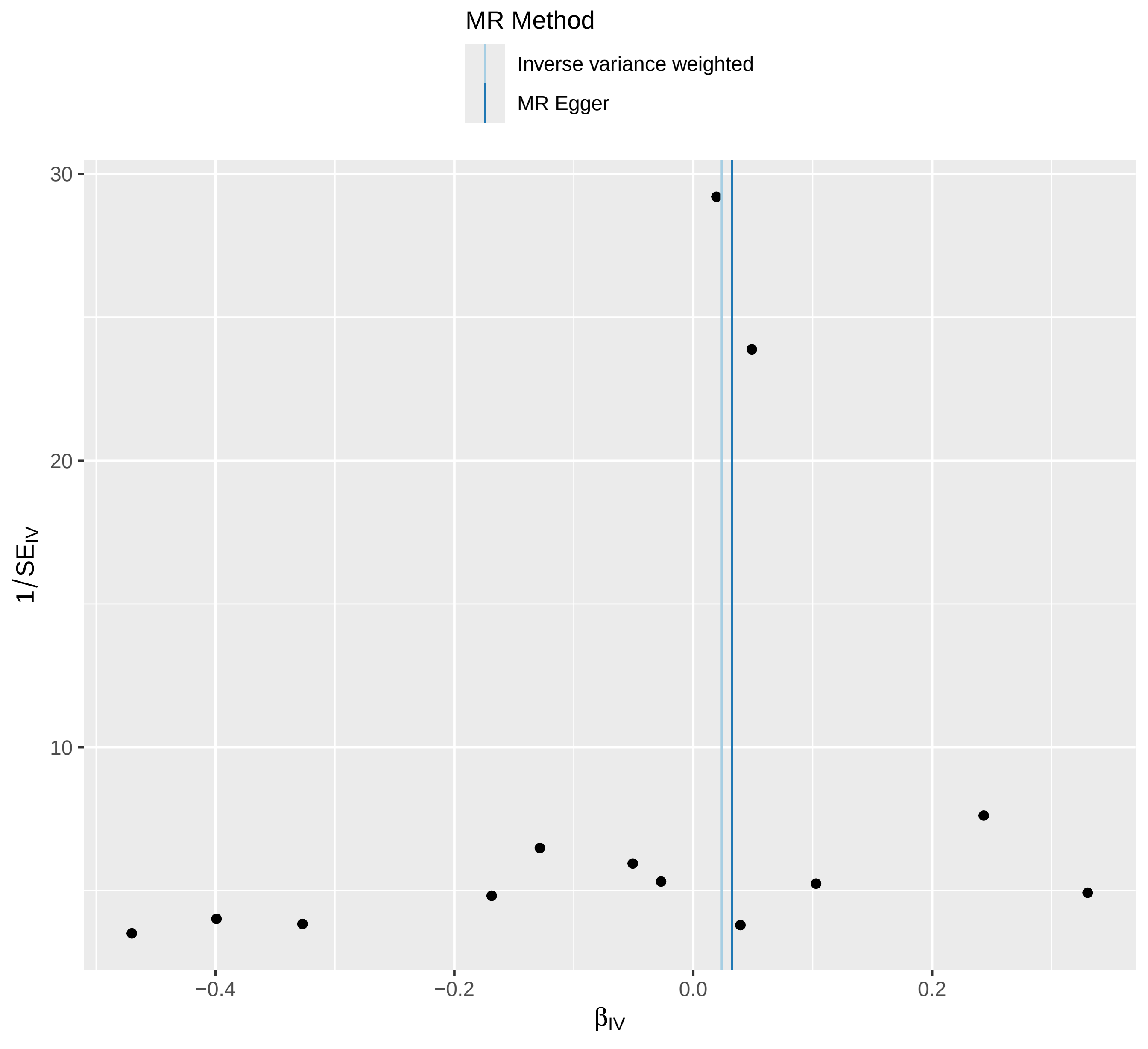
MR Method
Inverse variance weighted
MR Egger
30
20
10
$1/SE_{IV}$
−0.4
−0.2
0.0
0.2
$\beta_{IV}$

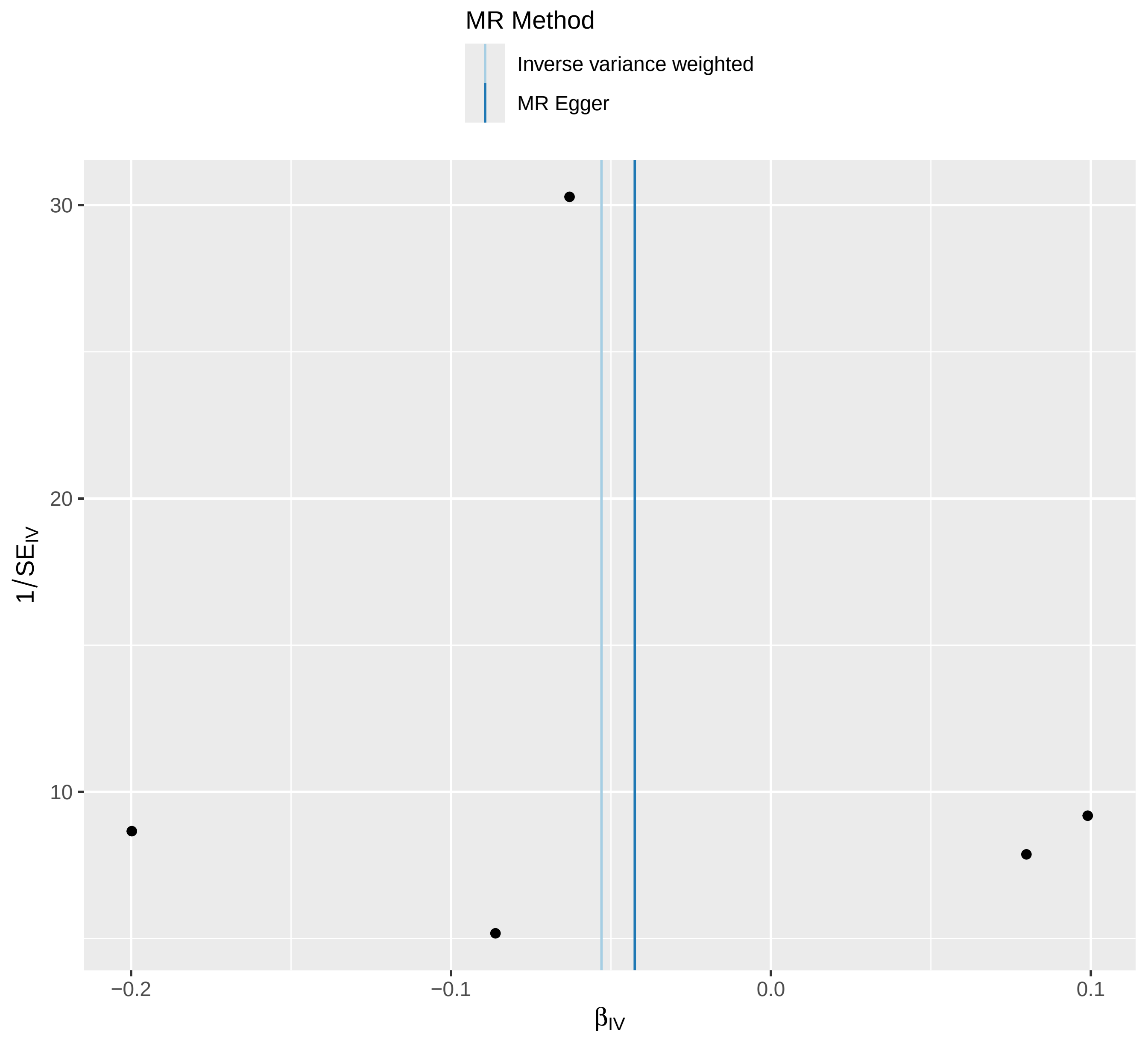
MR Method
Inverse variance weighted
MR Egger
30
20
10
1/SE_IV
−0.2
−0.1
0.0
0.1
β_IV

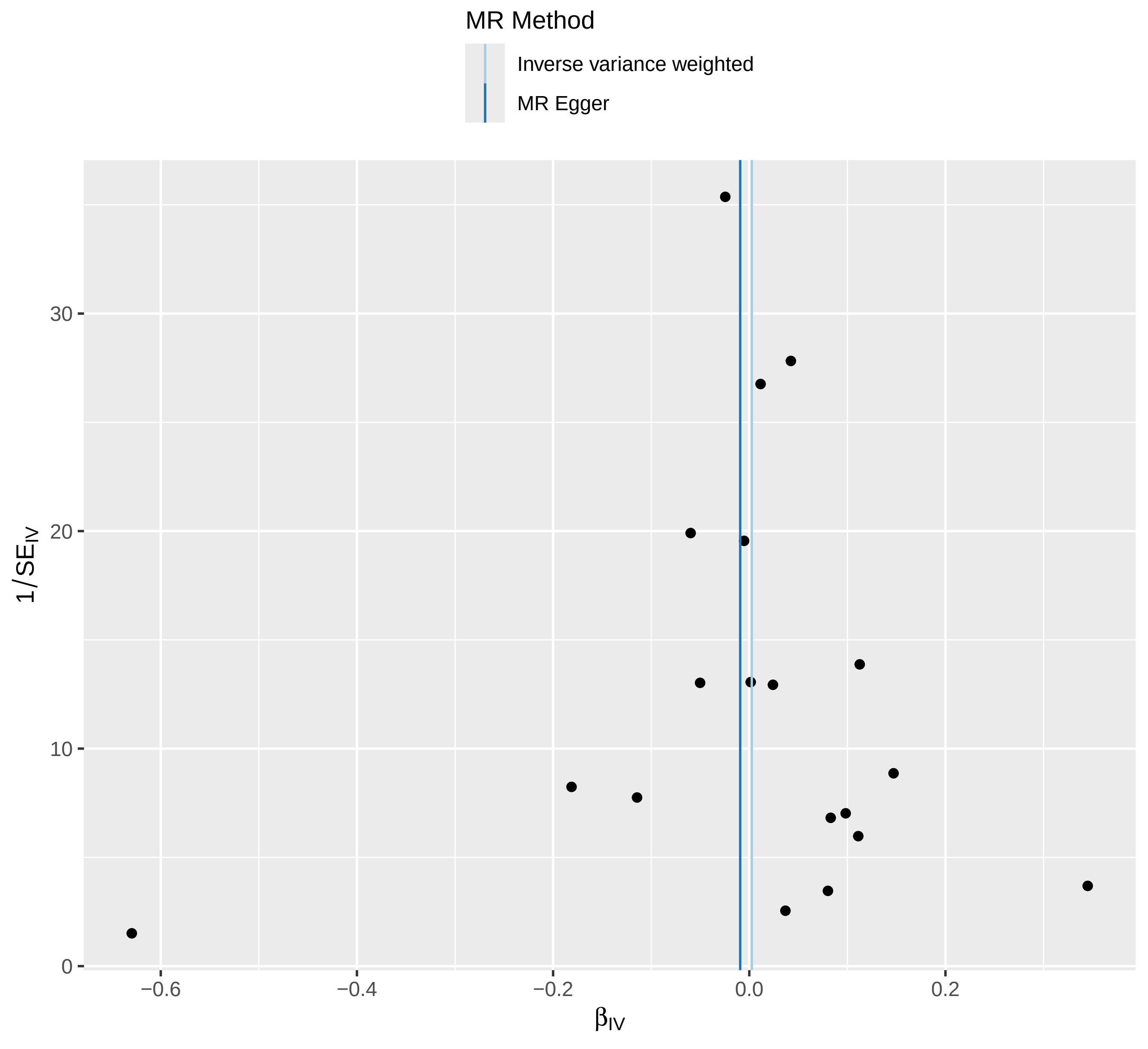
MR Method
Inverse variance weighted
MR Egger
30
20
10
0
1/SE_IV
−0.6
−0.4
−0.2
0.0
0.2
β_IV

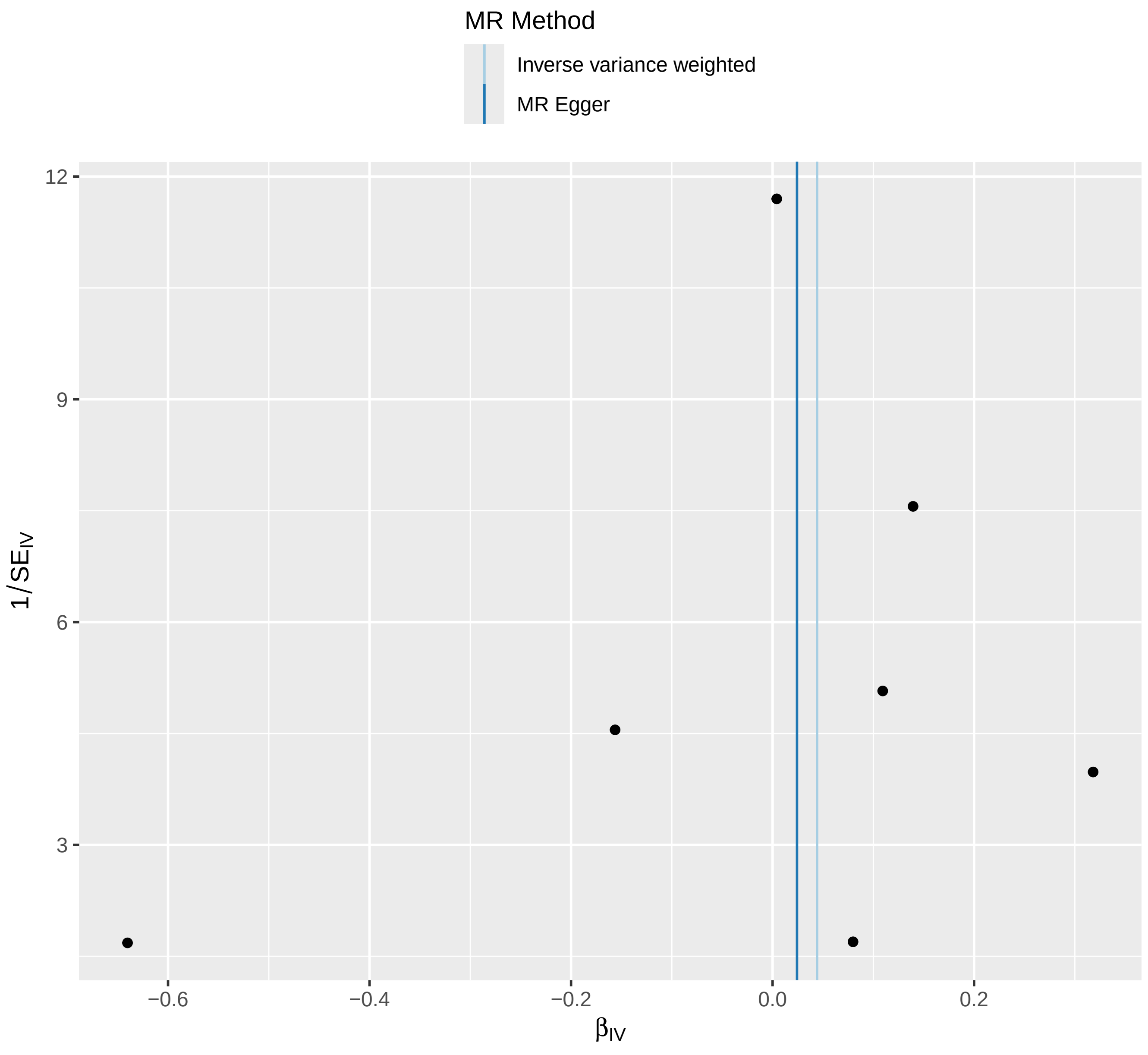
MR Method
Inverse variance weighted
MR Egger
12
9
6
3
1/SE_IV
−0.6
−0.4
−0.2
0.0
0.2
β_IV

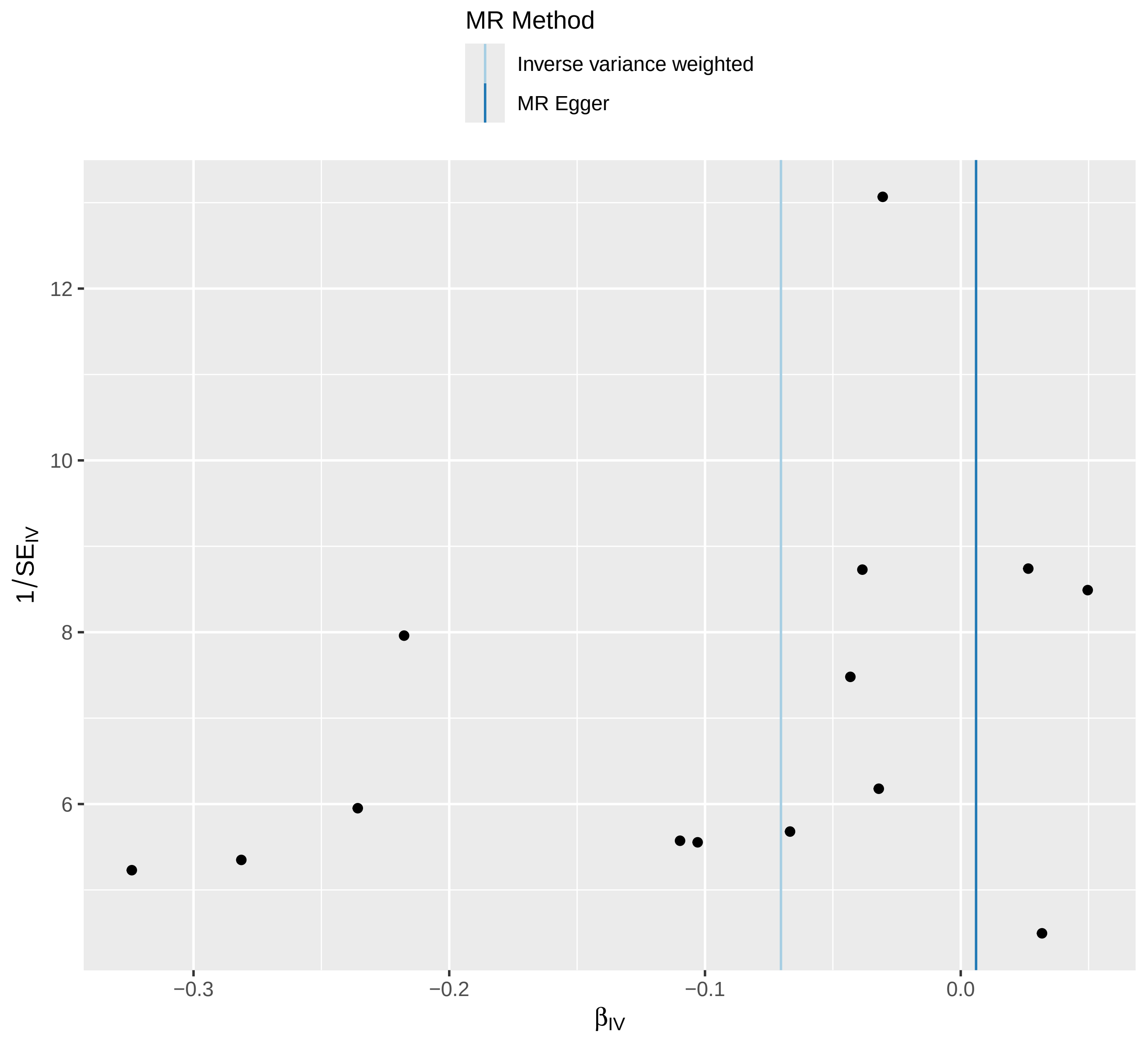
MR Method
Inverse variance weighted
MR Egger
12
10
8
6
1/SE$_{IV}$
−0.3
−0.2
−0.1
0.0
β$_{IV}$

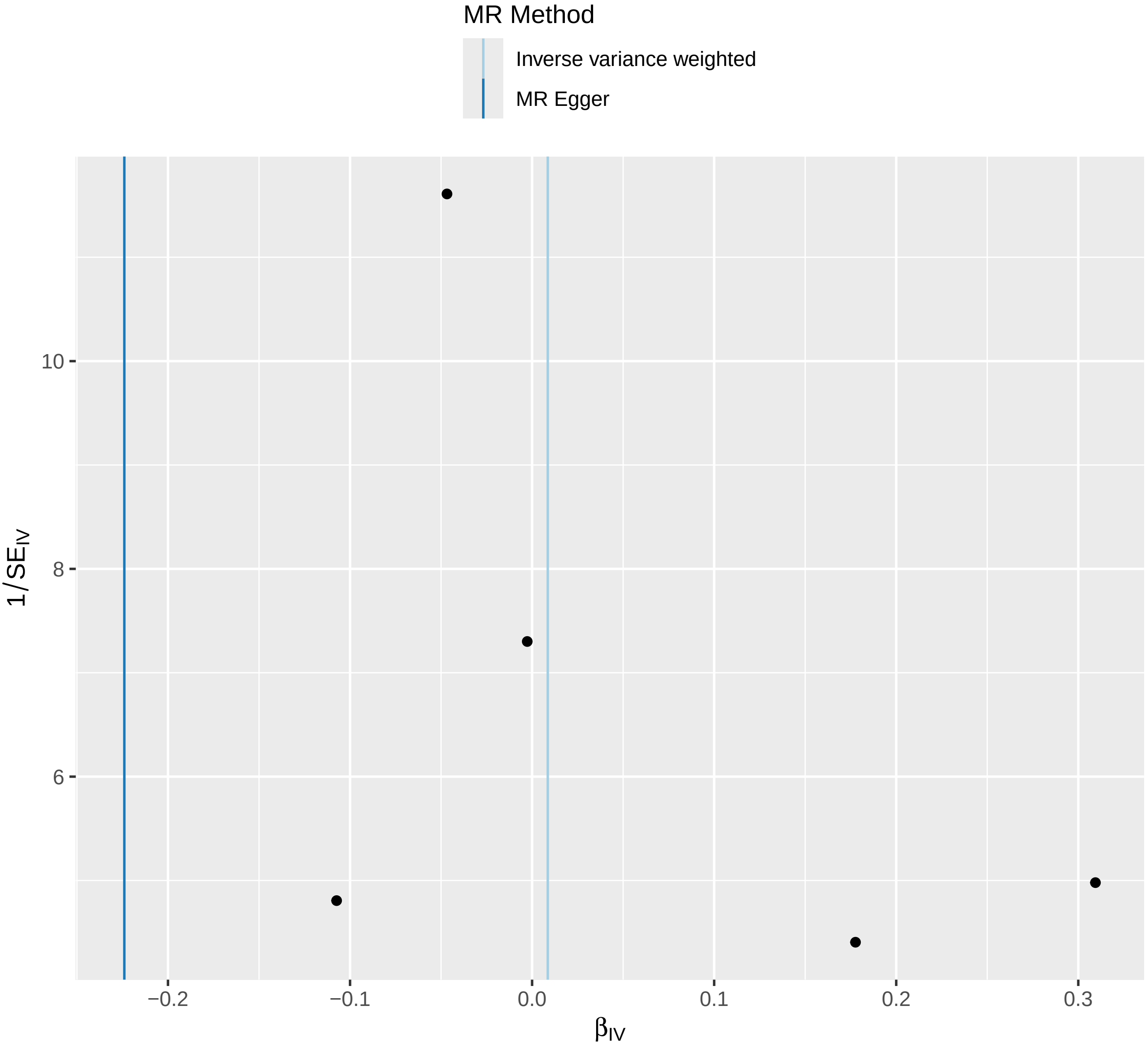

MR Method
Inverse variance weighted
MR Egger
10
8
6
−0.2
−0.1
0.0
0.1
0.2
0.3
$\beta_{IV}$
$1/SE_{IV}$

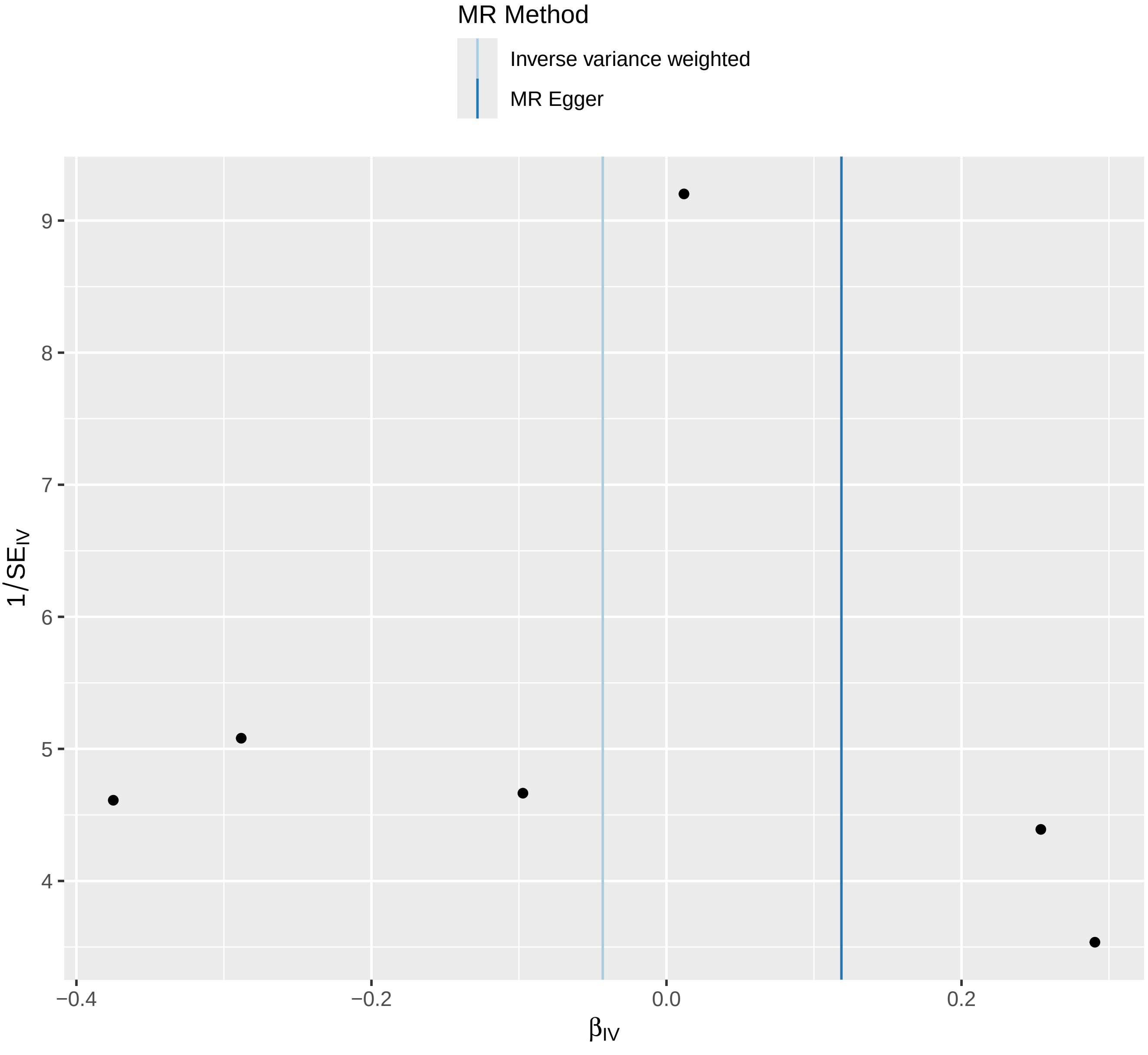
MR Method
Inverse variance weighted
MR Egger
1/SE_IV
β_IV
9
8
7
6
5
4
−0.4
−0.2
0.0
0.2

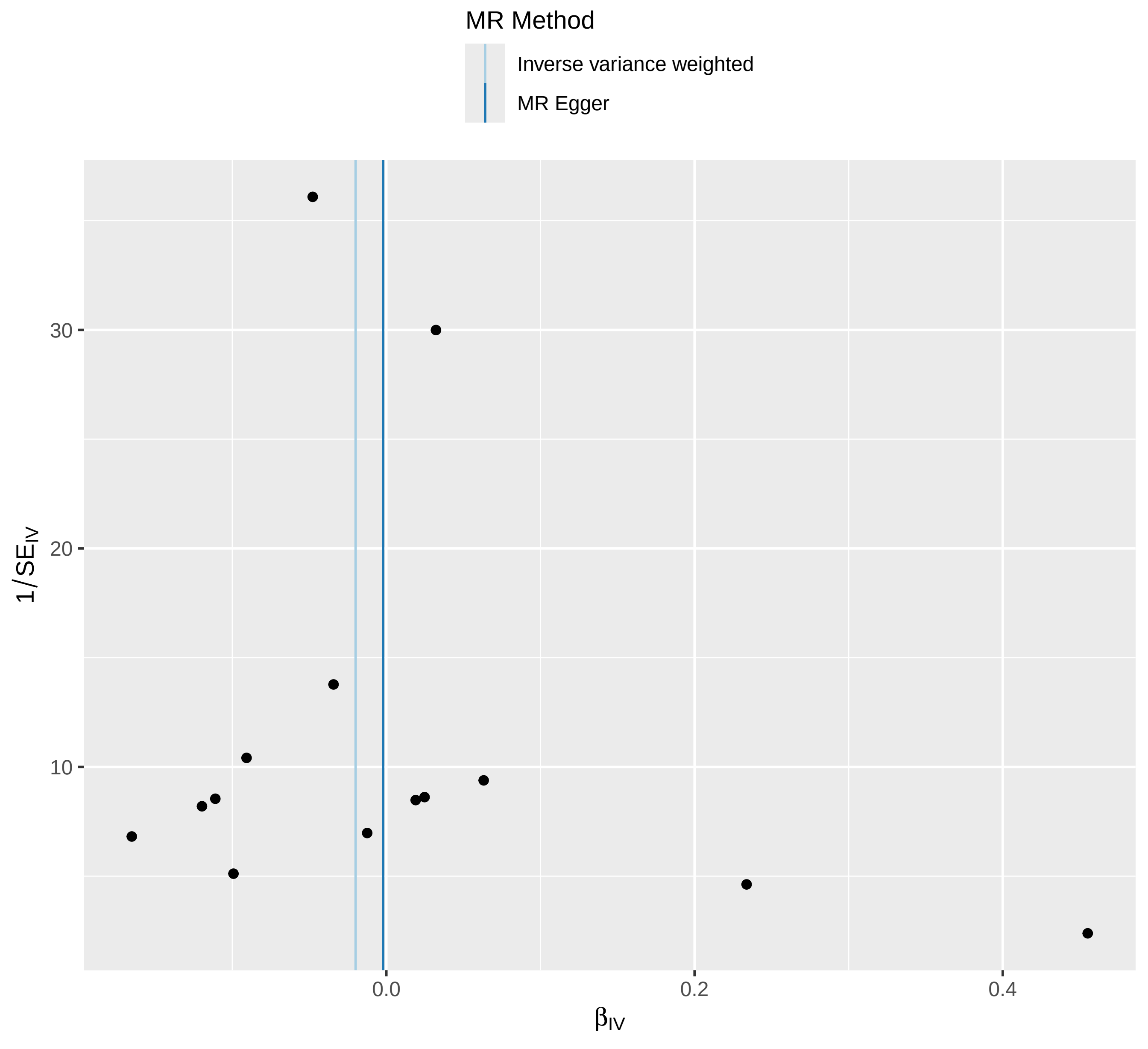
MR Method
Inverse variance weighted
MR Egger
30
20
10
0.0
0.2
0.4
$1/SE_{IV}$
$\beta_{IV}$

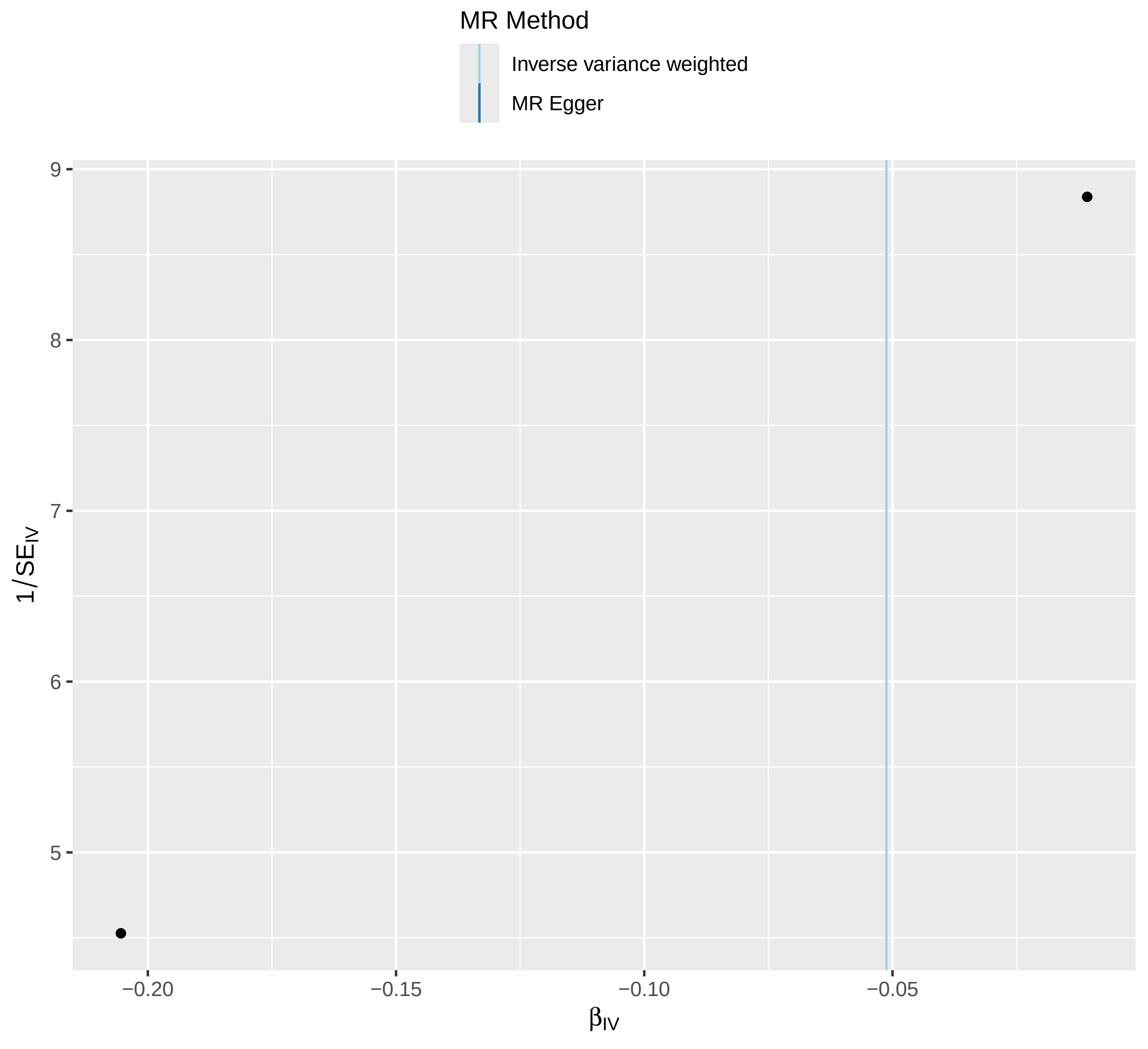
MR Method
Inverse variance weighted
MR Egger
9
8
7
6
5
1/SE$_{IV}$
−0.20
−0.15
−0.10
−0.05
β$_{IV}$

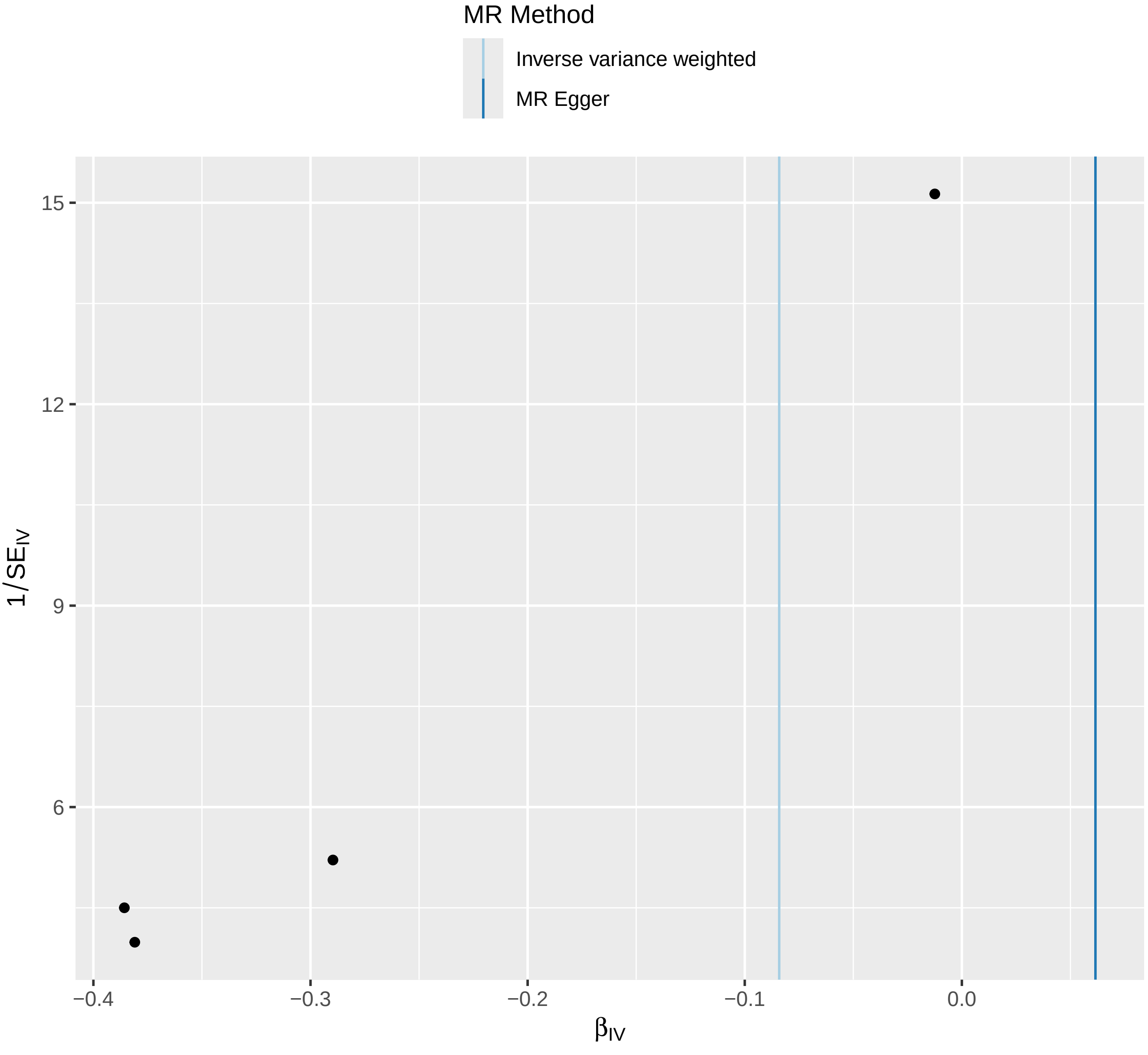
MR Method
Inverse variance weighted
MR Egger
15
12
9
6
1/SE_IV
−0.4
−0.3
−0.2
−0.1
0.0
β_IV

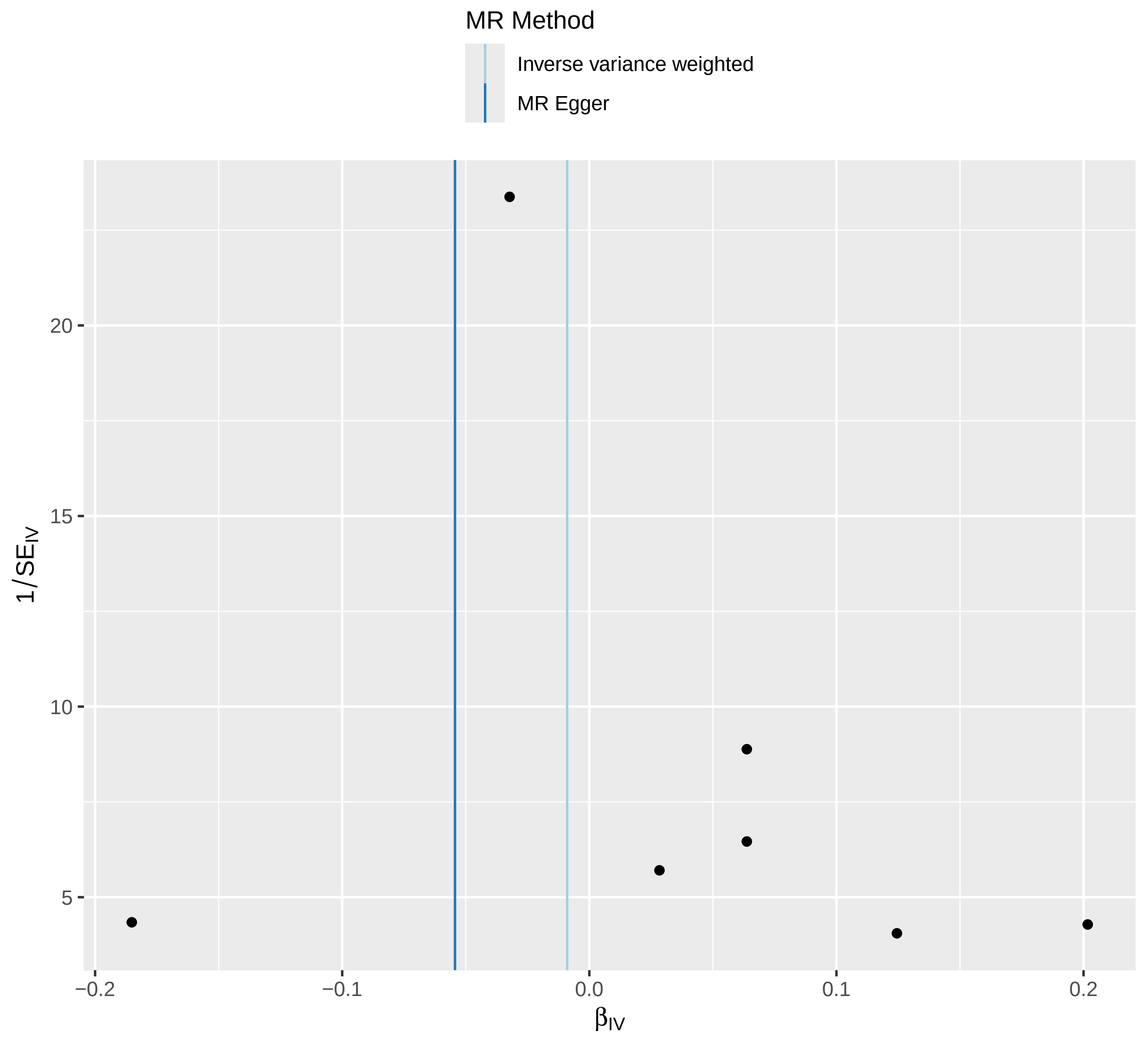

MR Method
Inverse variance weighted
MR Egger
20
15
10
5
1/SE_IV
−0.2
−0.1
0.0
0.1
0.2
β_IV

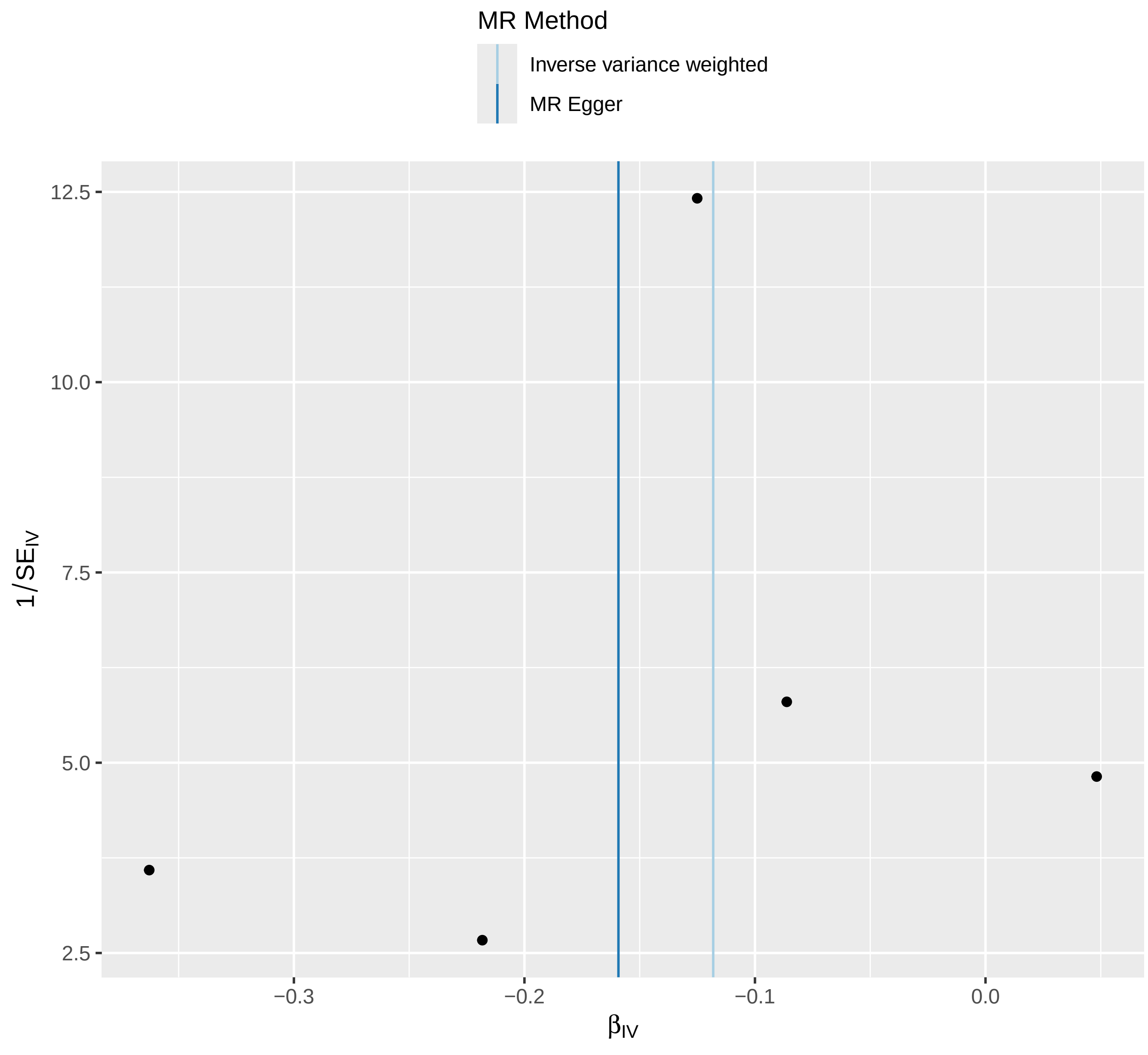
MR Method
Inverse variance weighted
MR Egger
12.5
10.0
7.5
5.0
2.5
1/SE$_{IV}$
−0.3
−0.2
−0.1
0.0
β$_{IV}$

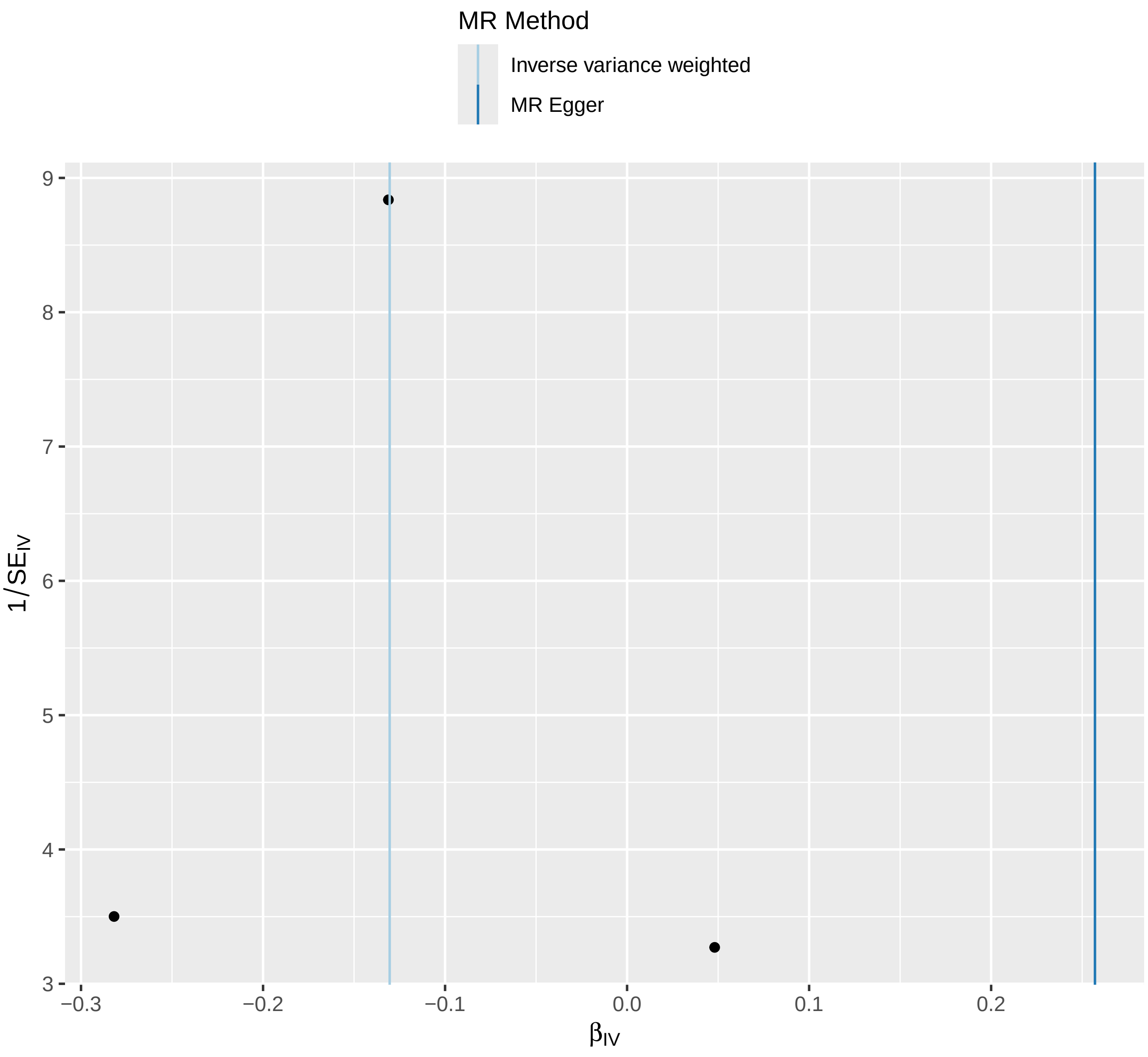
MR Method
Inverse variance weighted
MR Egger
1/SE_IV
β_IV
9
8
7
6
5
4
3
−0.3
−0.2
−0.1
0.0
0.1
0.2

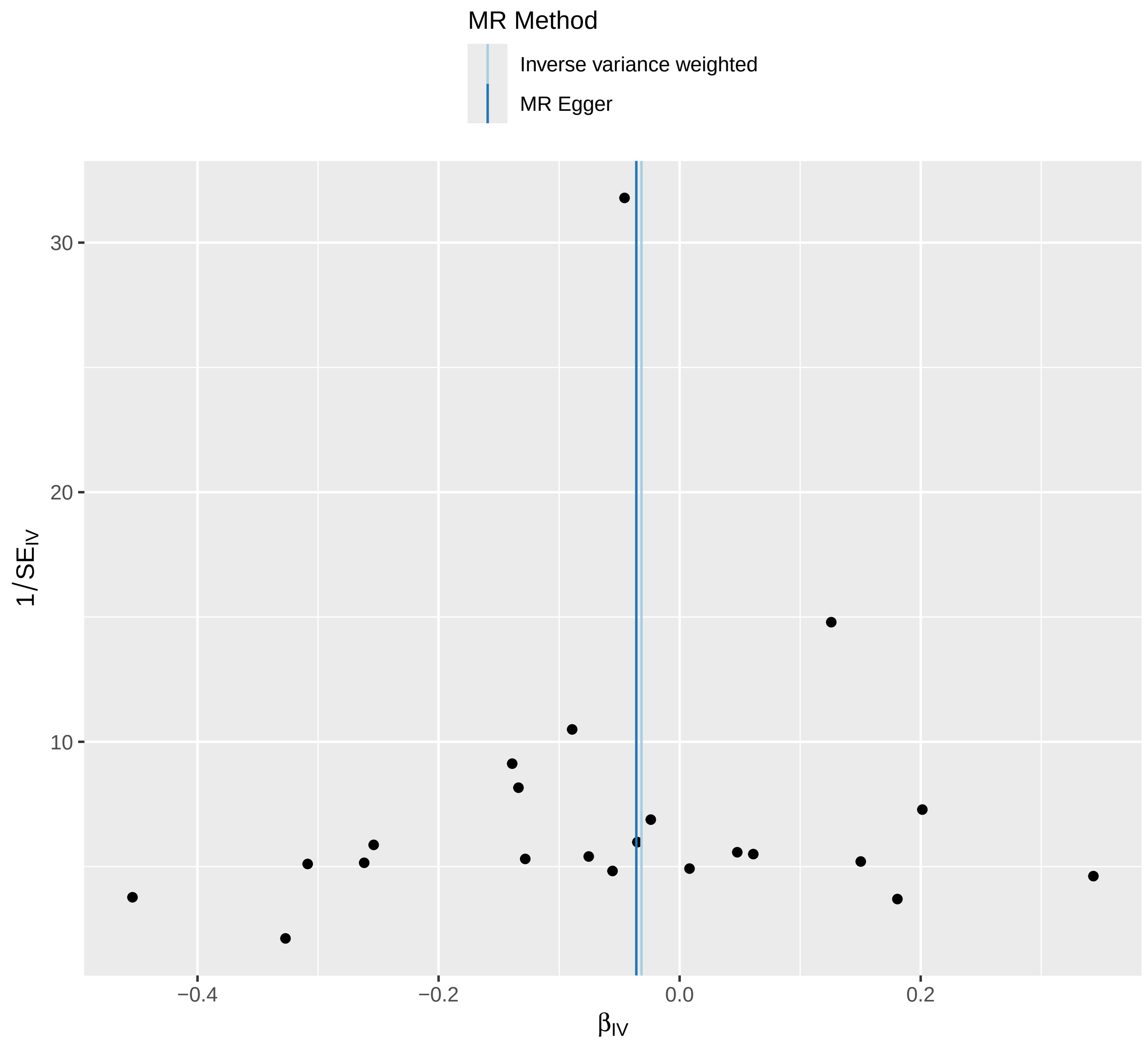

MR Method
Inverse variance weighted
MR Egger
30
20
10
$1/SE_{IV}$
−0.4
−0.2
0.0
0.2
$\beta_{IV}$

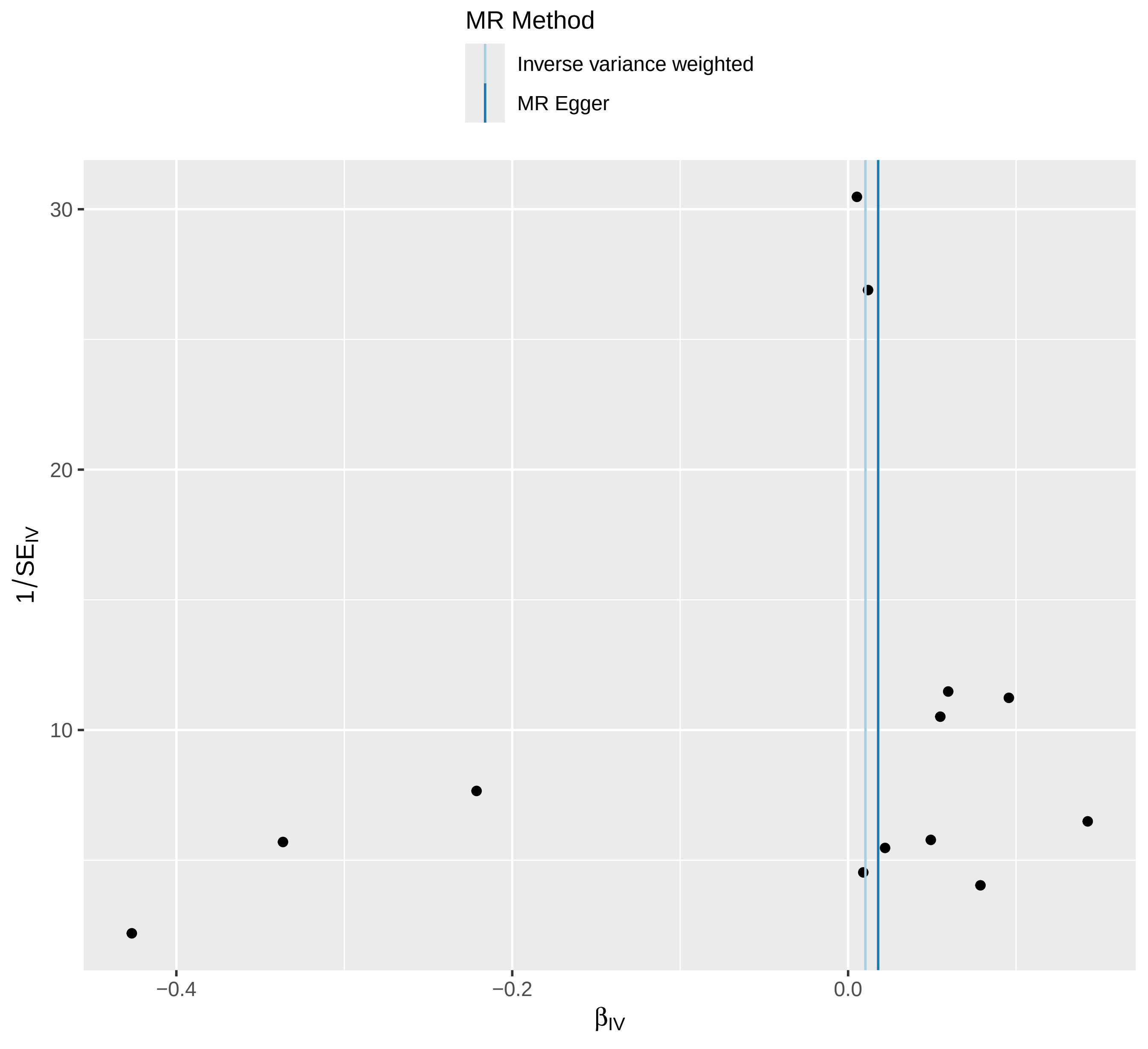
MR Method
Inverse variance weighted
MR Egger
30
20
10
$1/SE_{IV}$
−0.4
−0.2
0.0
$\beta_{IV}$

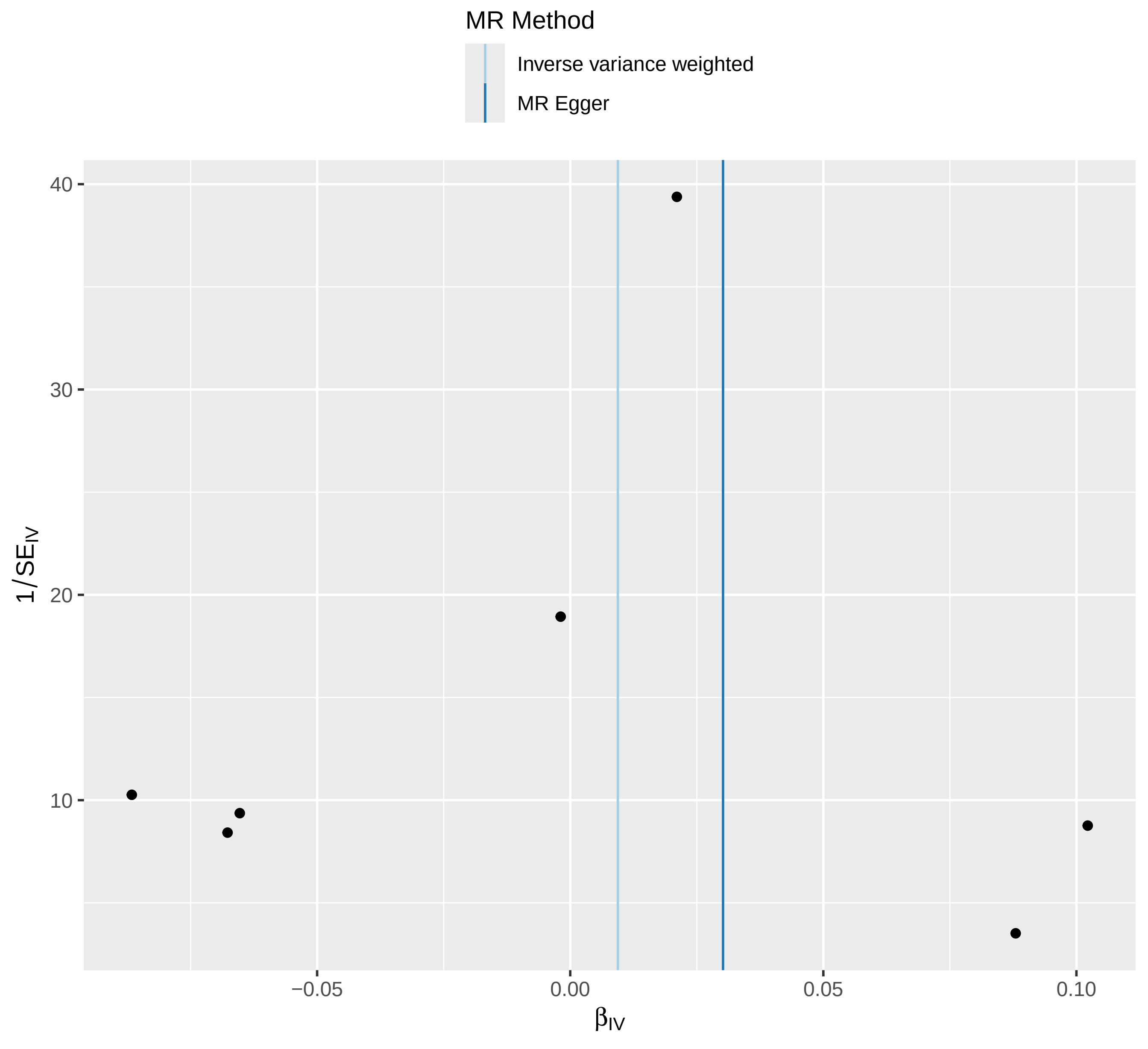
MR Method
Inverse variance weighted
MR Egger
40
30
20
10
1/SE_IV
−0.05
0.00
0.05
0.10
β_IV

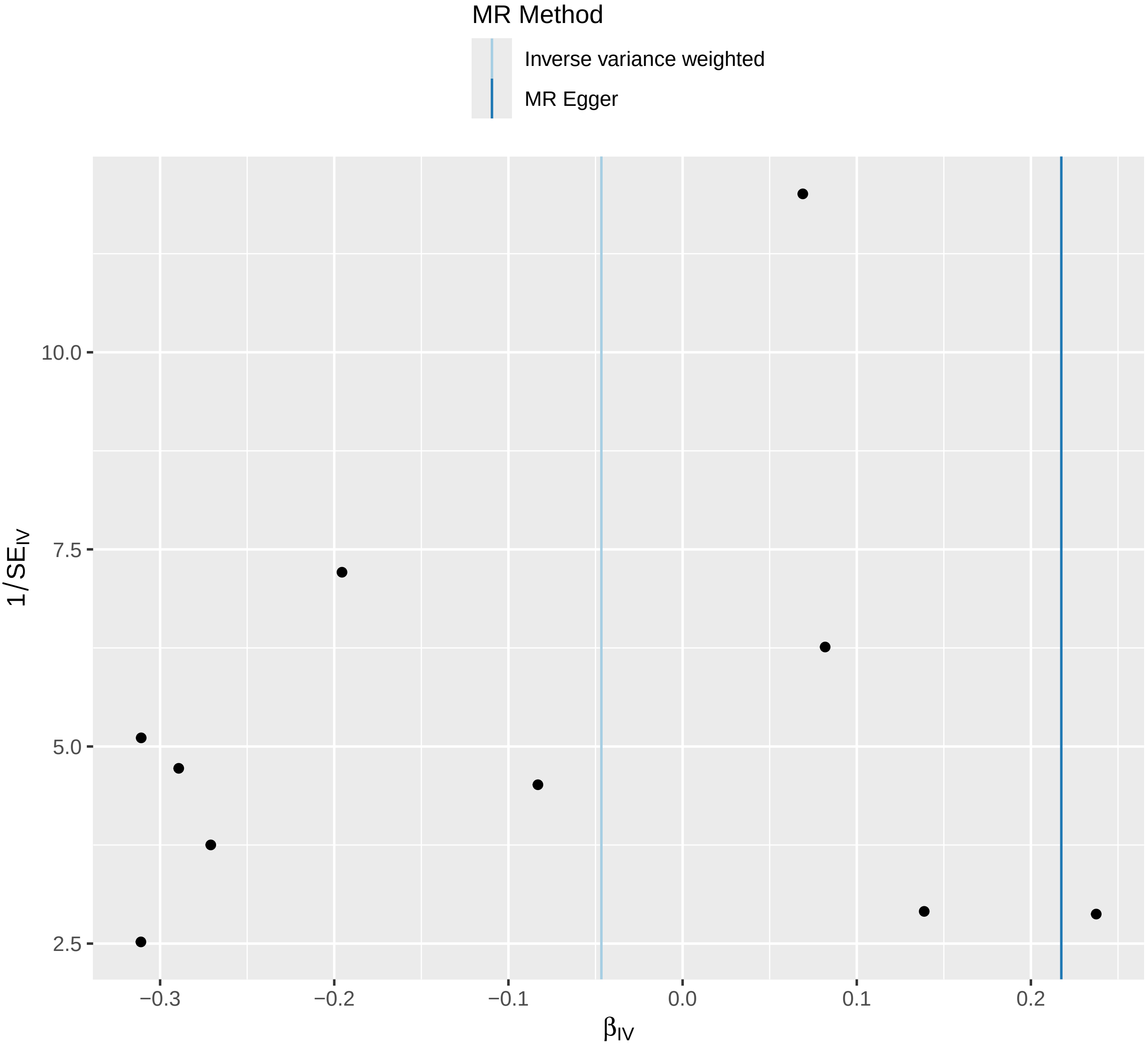

MR Method
Inverse variance weighted
MR Egger
10.0
7.5
5.0
2.5
1/SE$_{IV}$
−0.3
−0.2
−0.1
0.0
0.1
0.2
β$_{IV}$

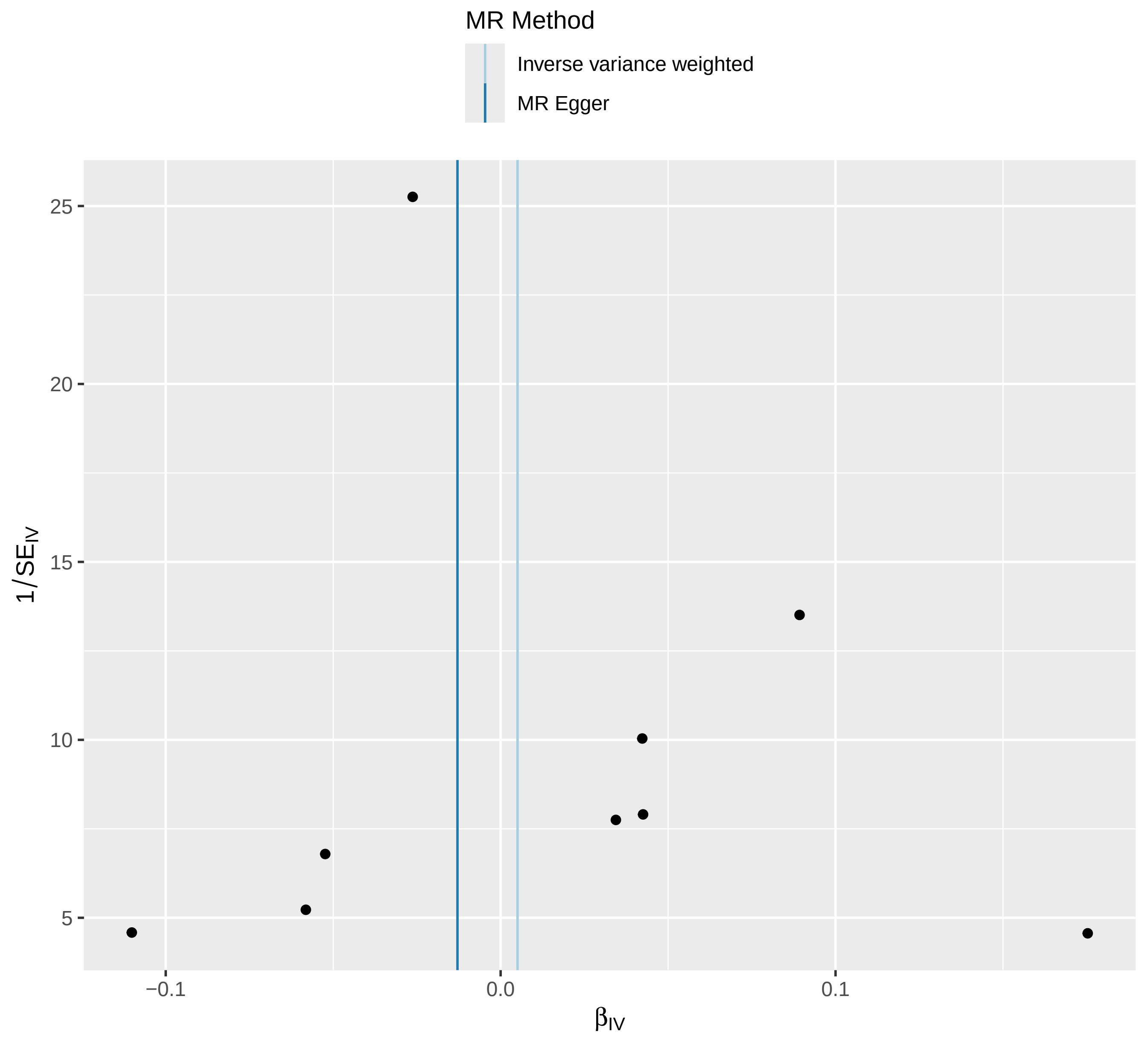

MR Method
Inverse variance weighted
MR Egger
25
20
15
10
5
1/SE$_{IV}$
−0.1
0.0
0.1
β$_{IV}$

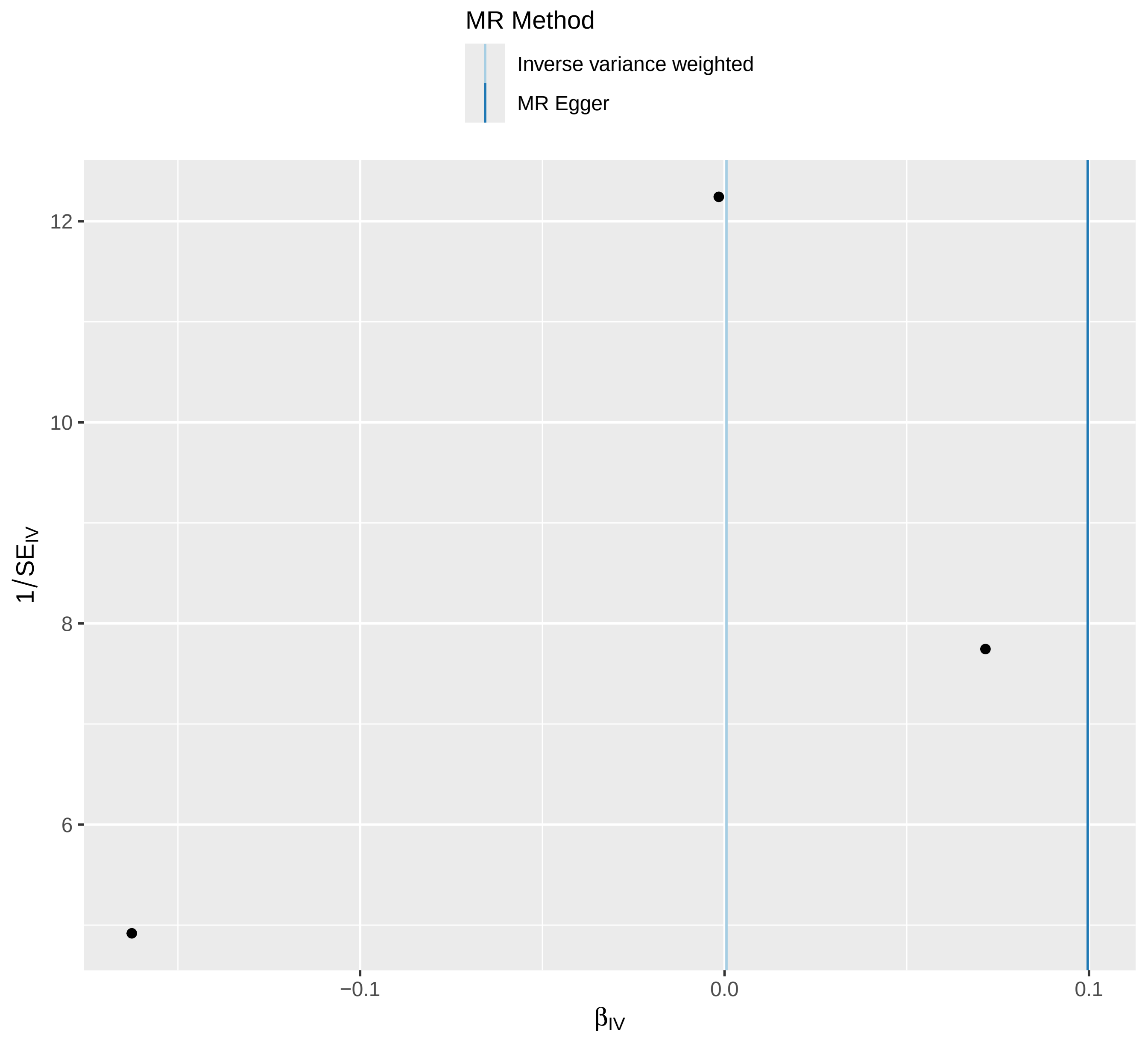
MR Method
Inverse variance weighted
MR Egger
1/SE$_{IV}$
12
10
8
6
−0.1
0.0
0.1
β$_{IV}$

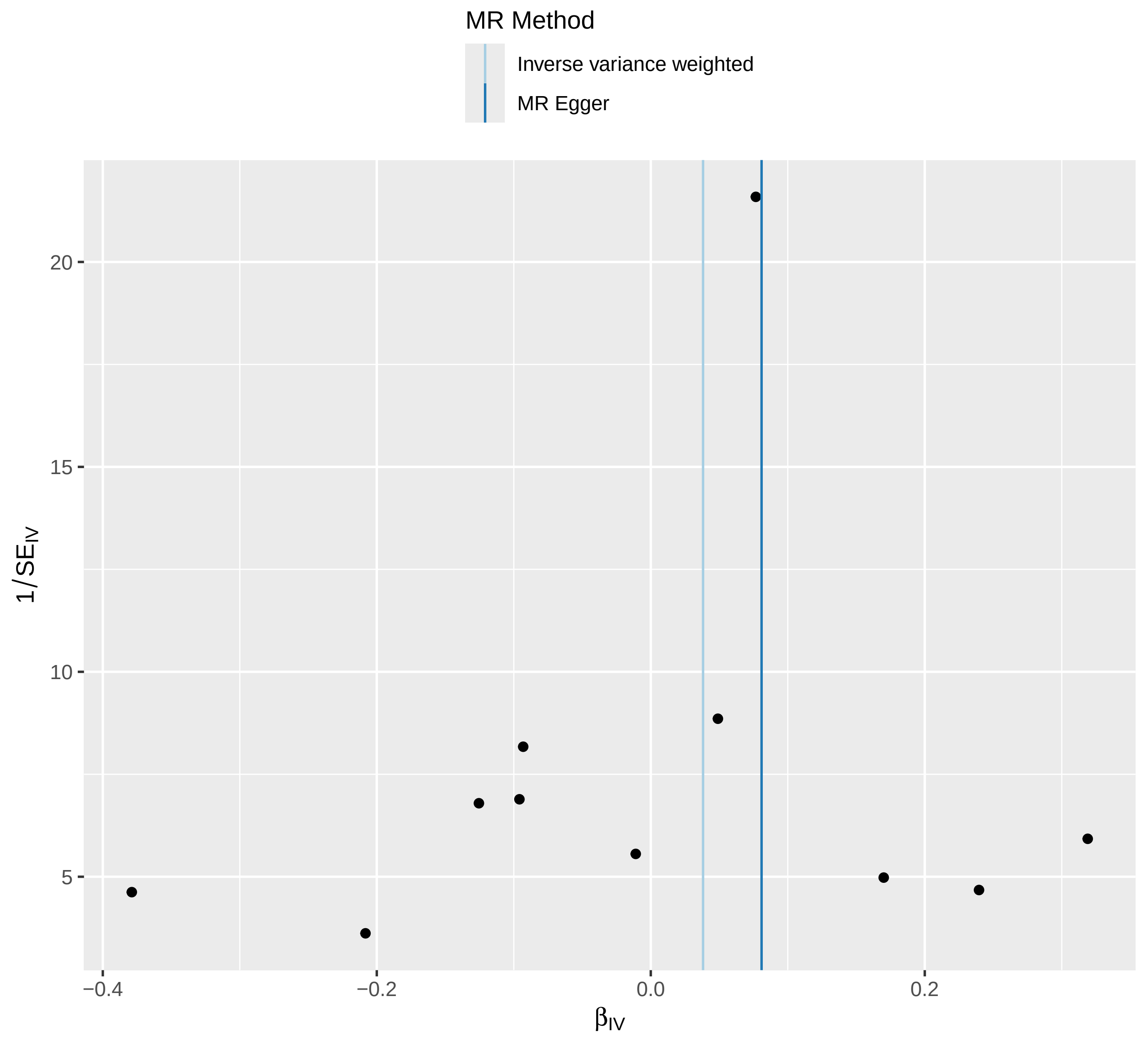

MR Method
Inverse variance weighted
MR Egger
20
15
10
5
1/SE$_{IV}$
−0.4
−0.2
0.0
0.2
β$_{IV}$

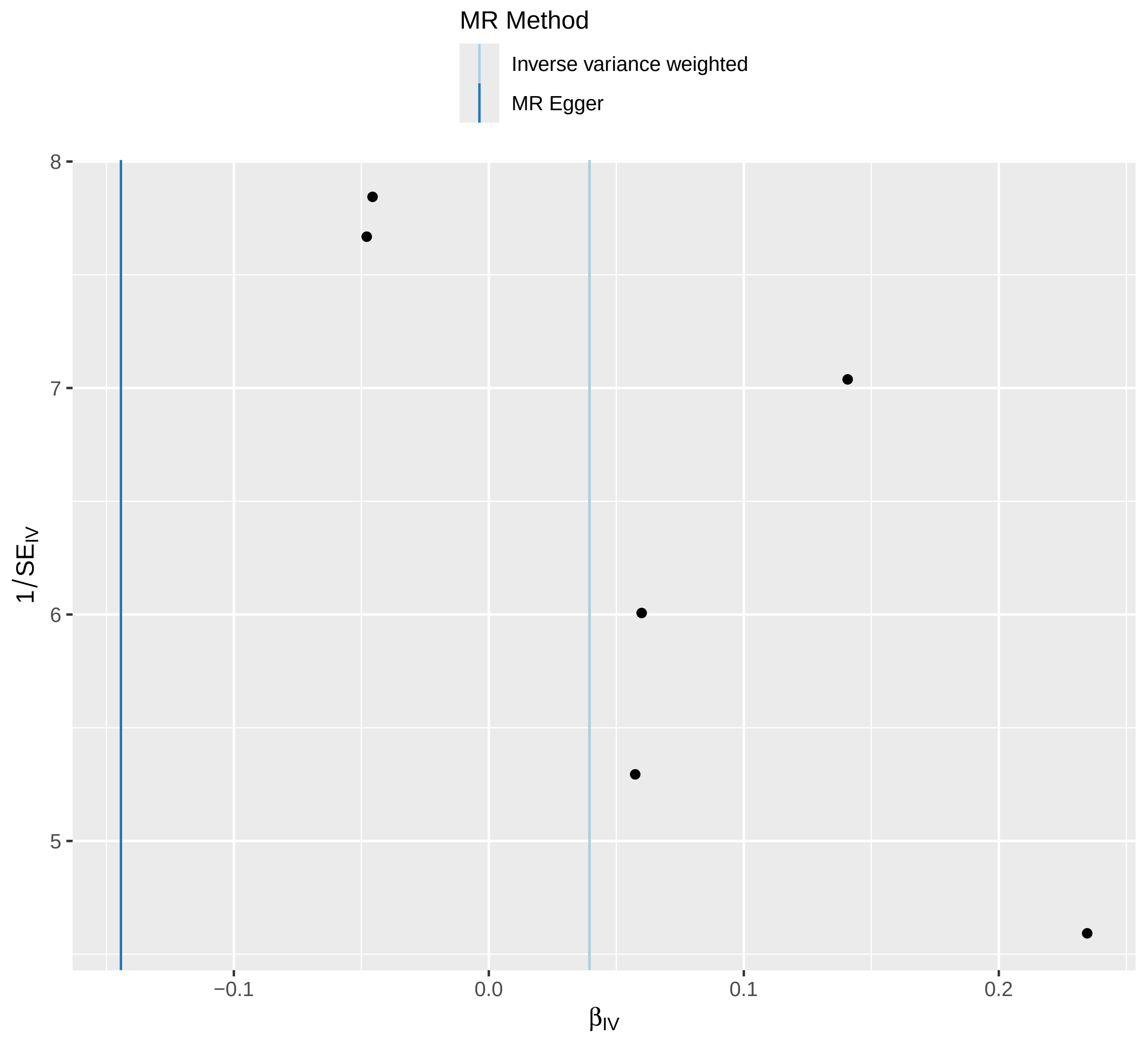
MR Method
Inverse variance weighted
MR Egger
8
7
6
5
1/SE$_{IV}$
−0.1
0.0
0.1
0.2
β$_{IV}$

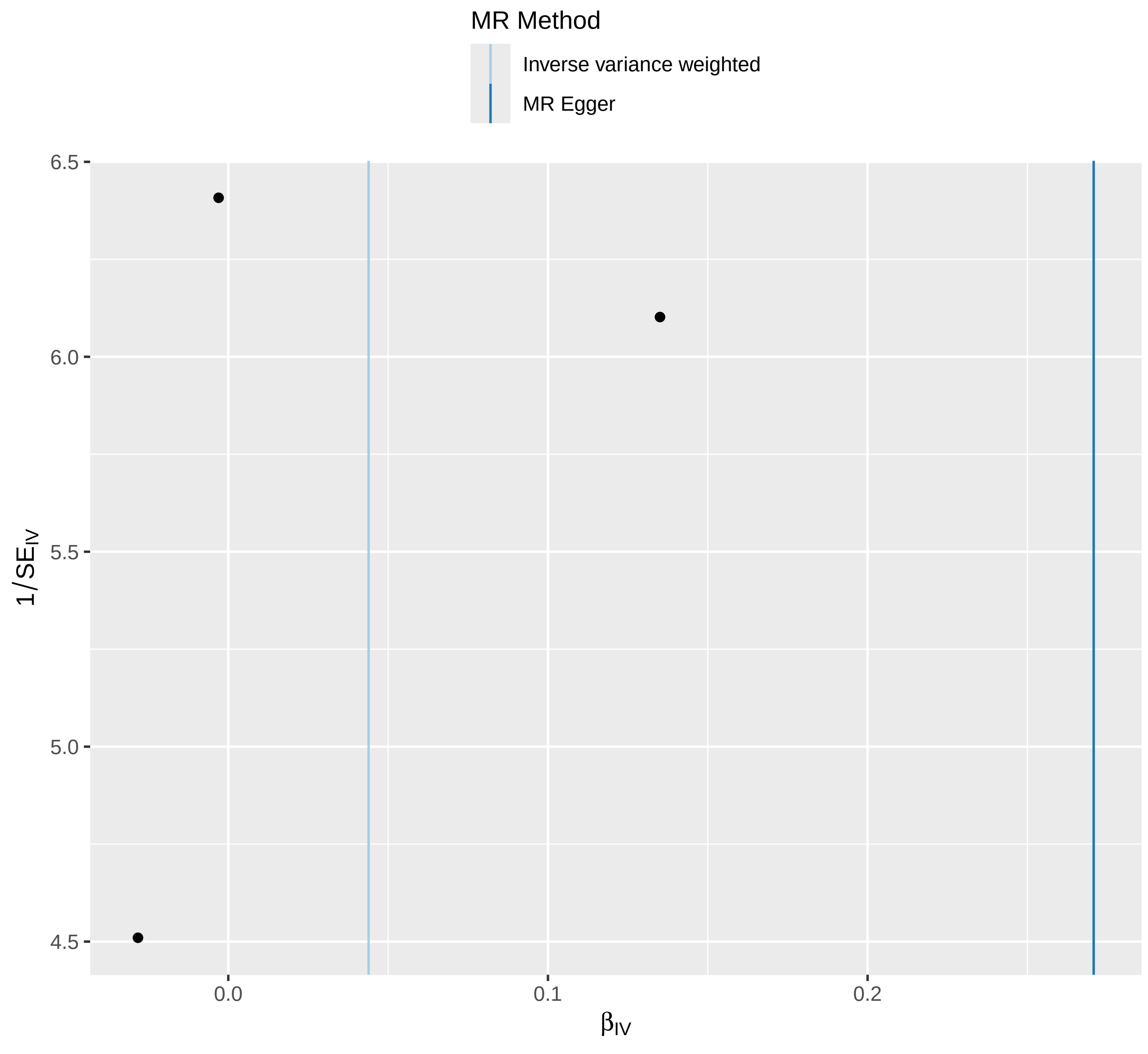
MR Method
Inverse variance weighted
MR Egger
6.5
6.0
5.5
5.0
4.5
$1/SE_{IV}$
0.0
0.1
0.2
$\beta_{IV}$

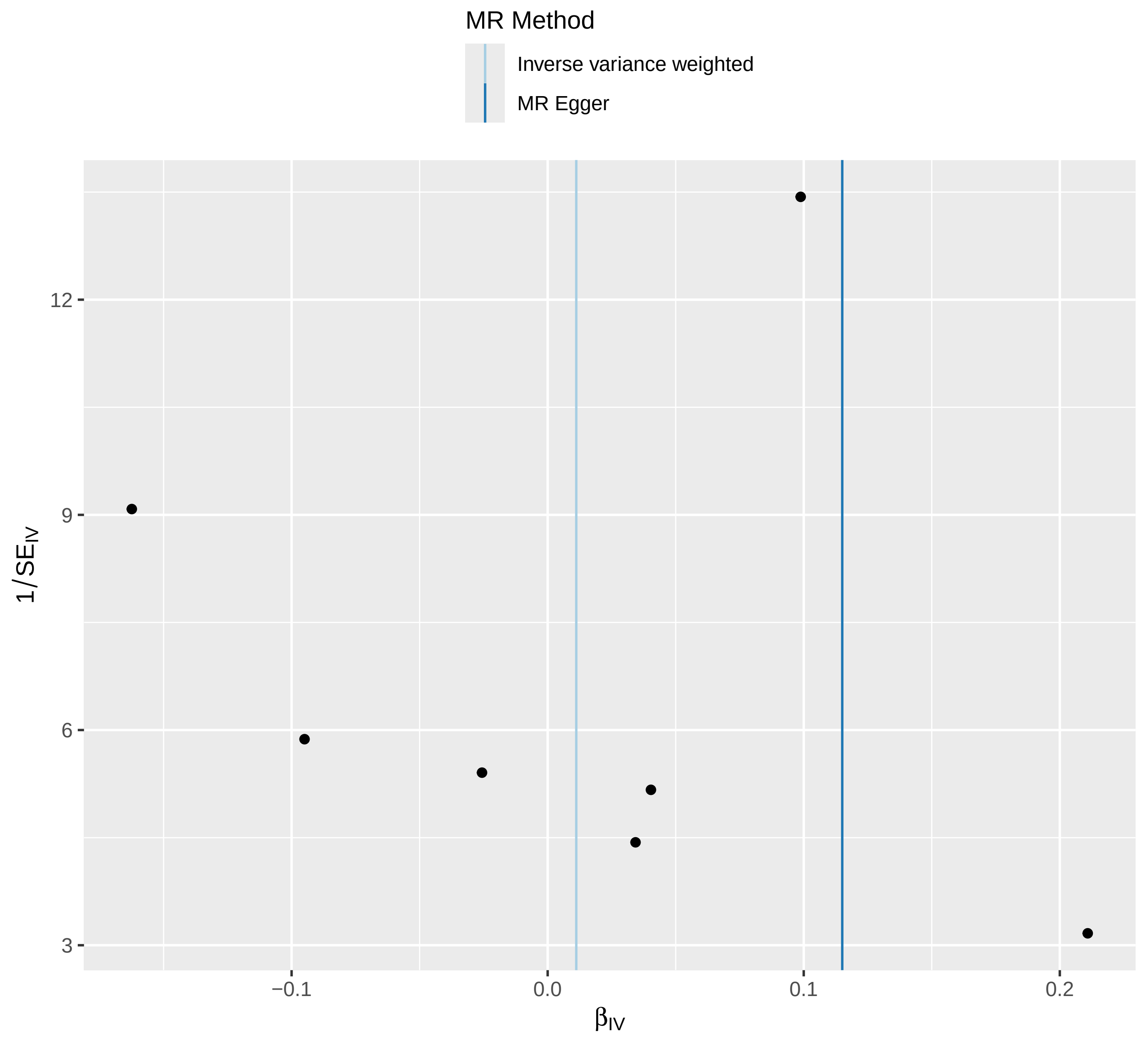
MR Method
Inverse variance weighted
MR Egger
12
9
6
3
1/SE_IV
−0.1
0.0
0.1
0.2
β_IV

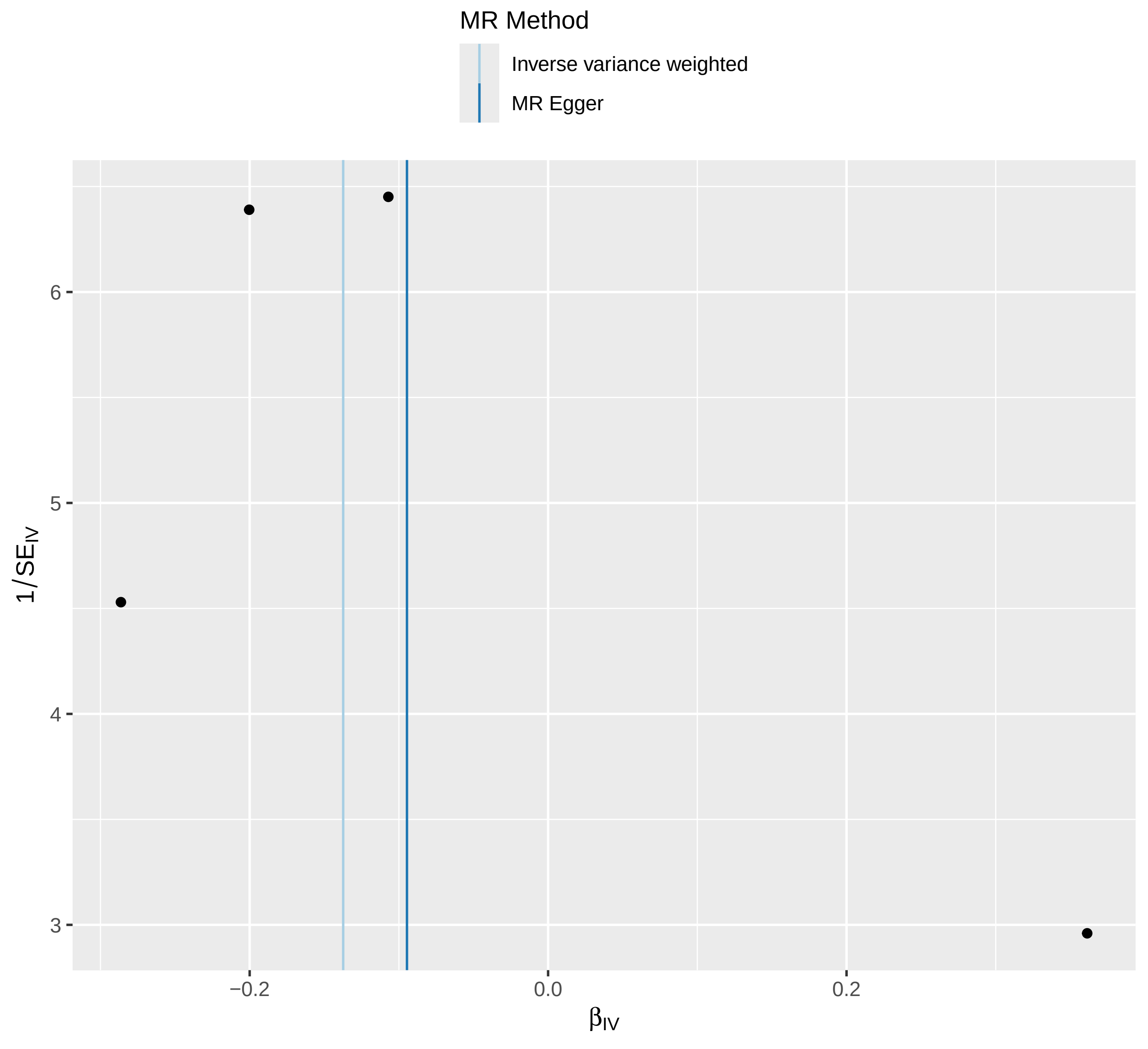

MR Method
Inverse variance weighted
MR Egger
6
5
4
3
1/SE$_{IV}$
−0.2
0.0
0.2
$\beta_{IV}$

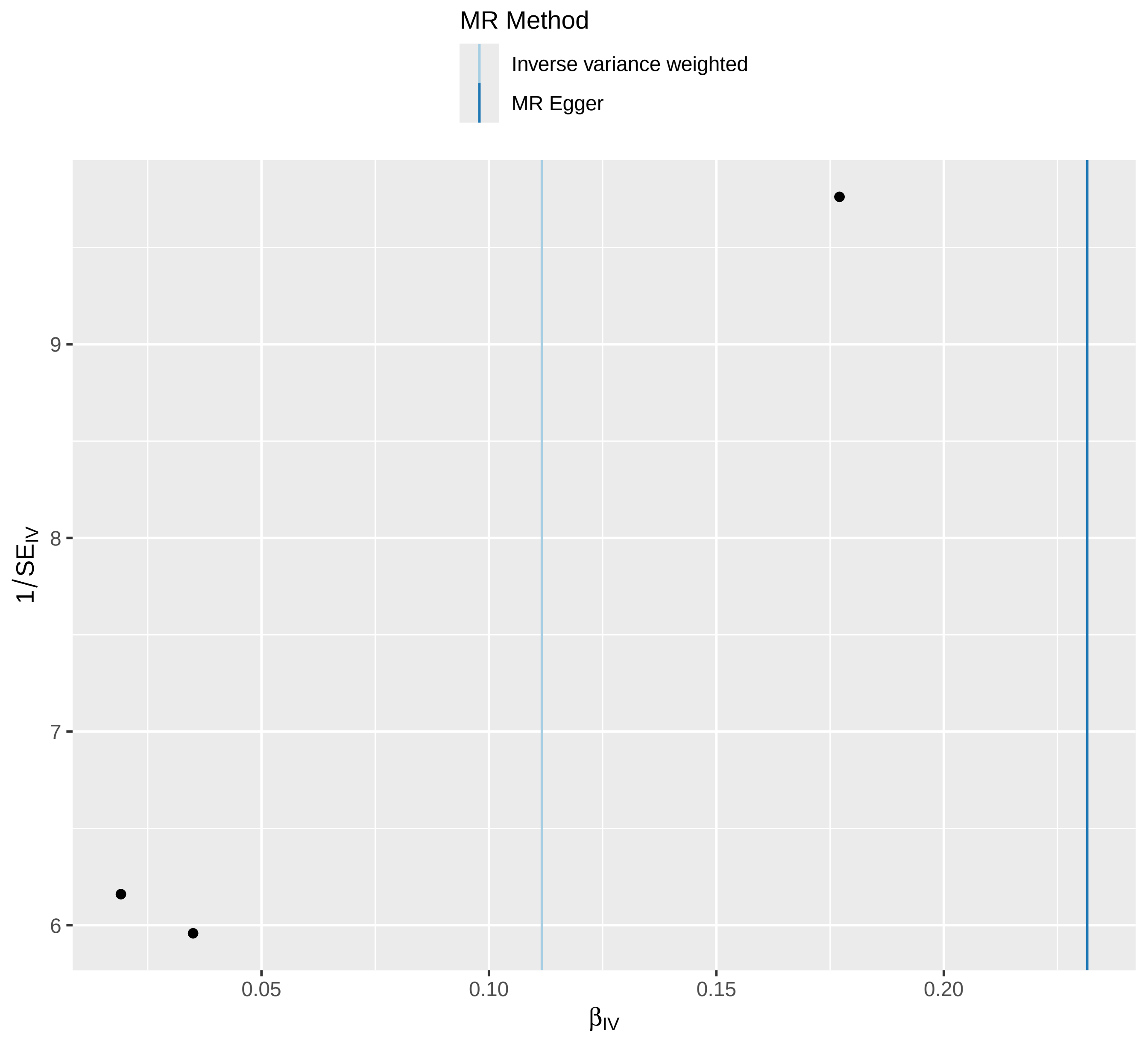
MR Method
Inverse variance weighted
MR Egger
9
8
7
6
1/SE_IV
0.05
0.10
0.15
0.20
β_IV

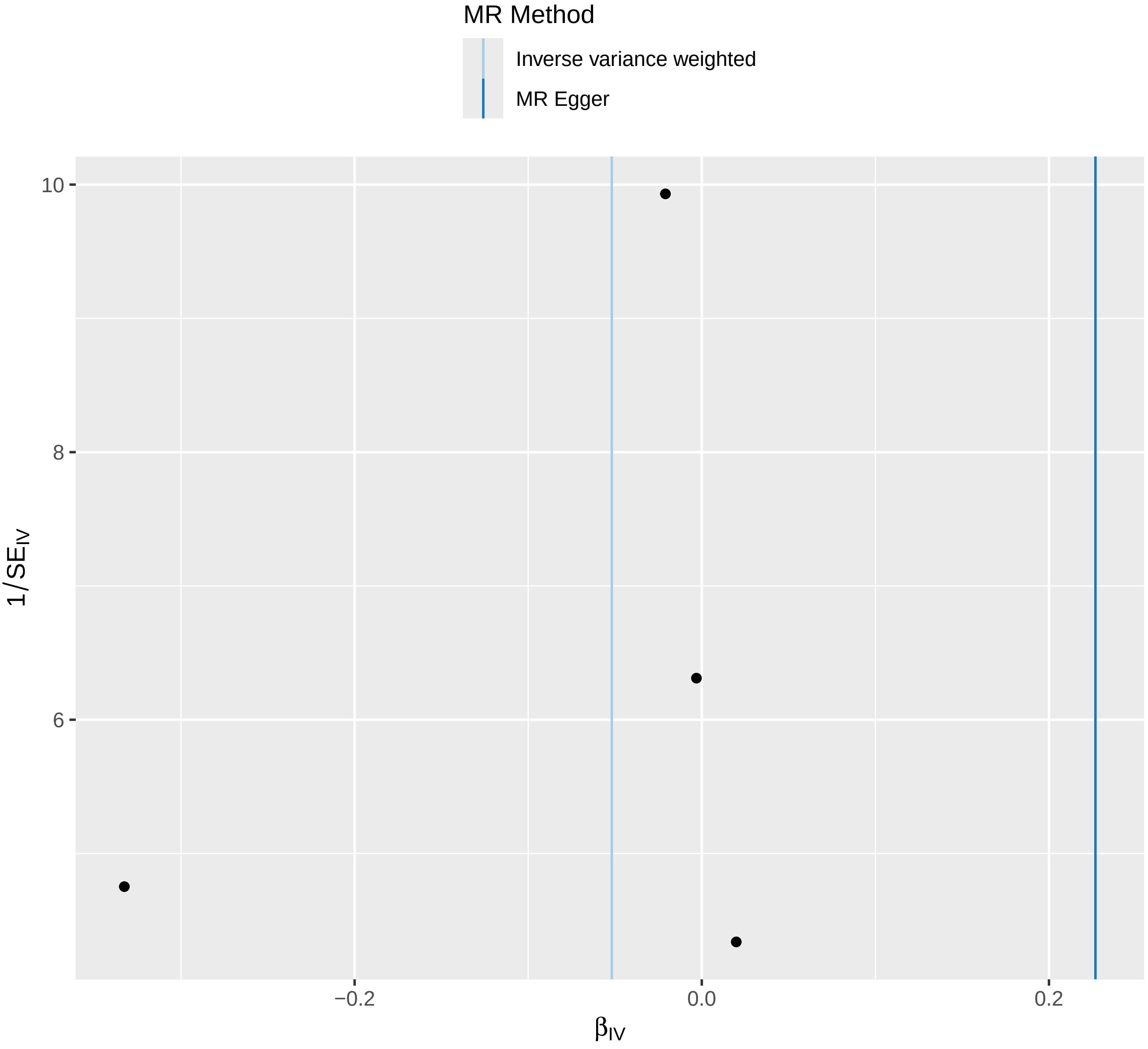
MR Method
Inverse variance weighted
MR Egger
10
8
6
1/SE_IV
−0.2
0.0
0.2
β_IV

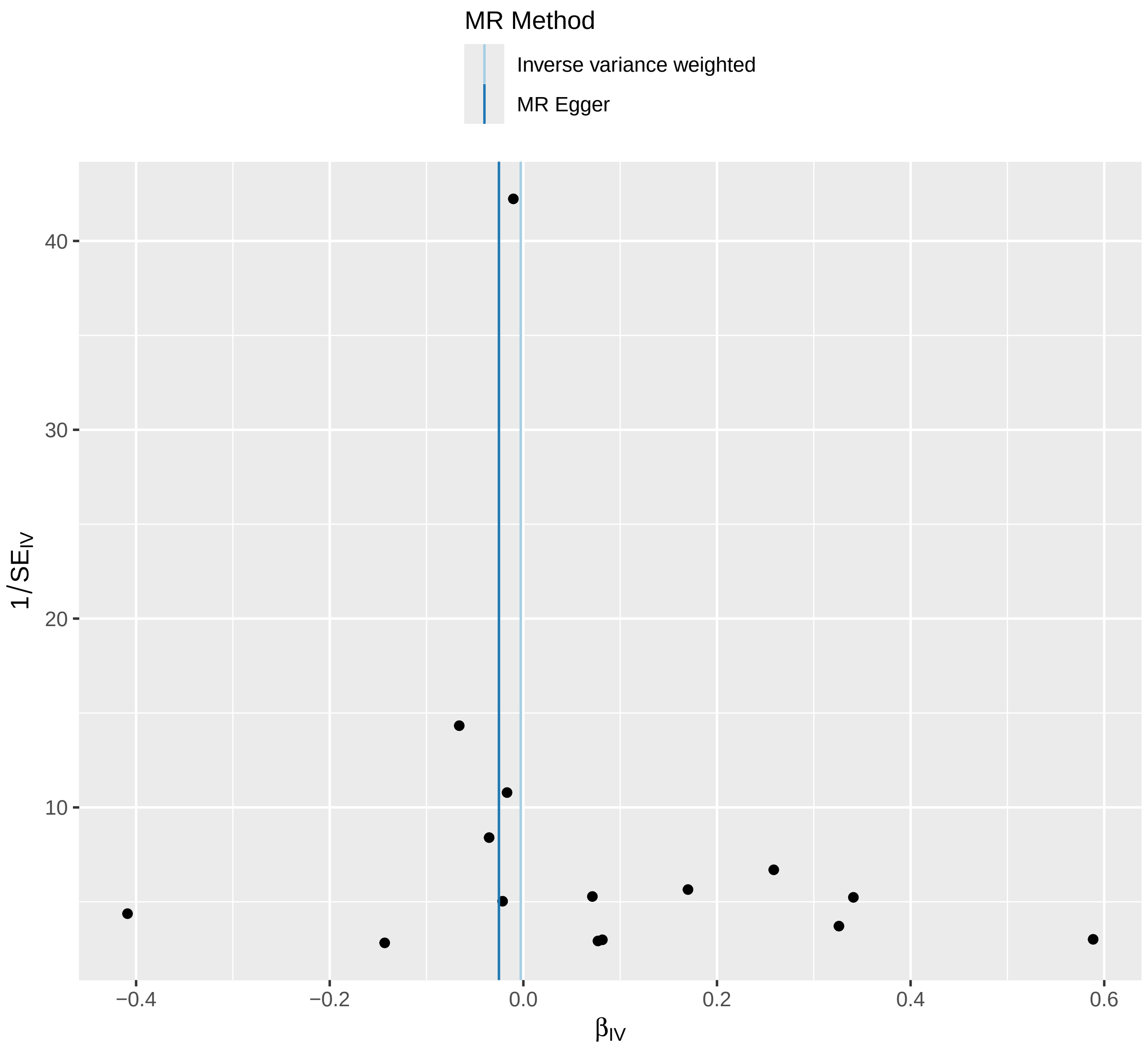
MR Method
Inverse variance weighted
MR Egger
40
30
20
10
1/SE$_{IV}$
−0.4
−0.2
0.0
0.2
0.4
0.6
β$_{IV}$

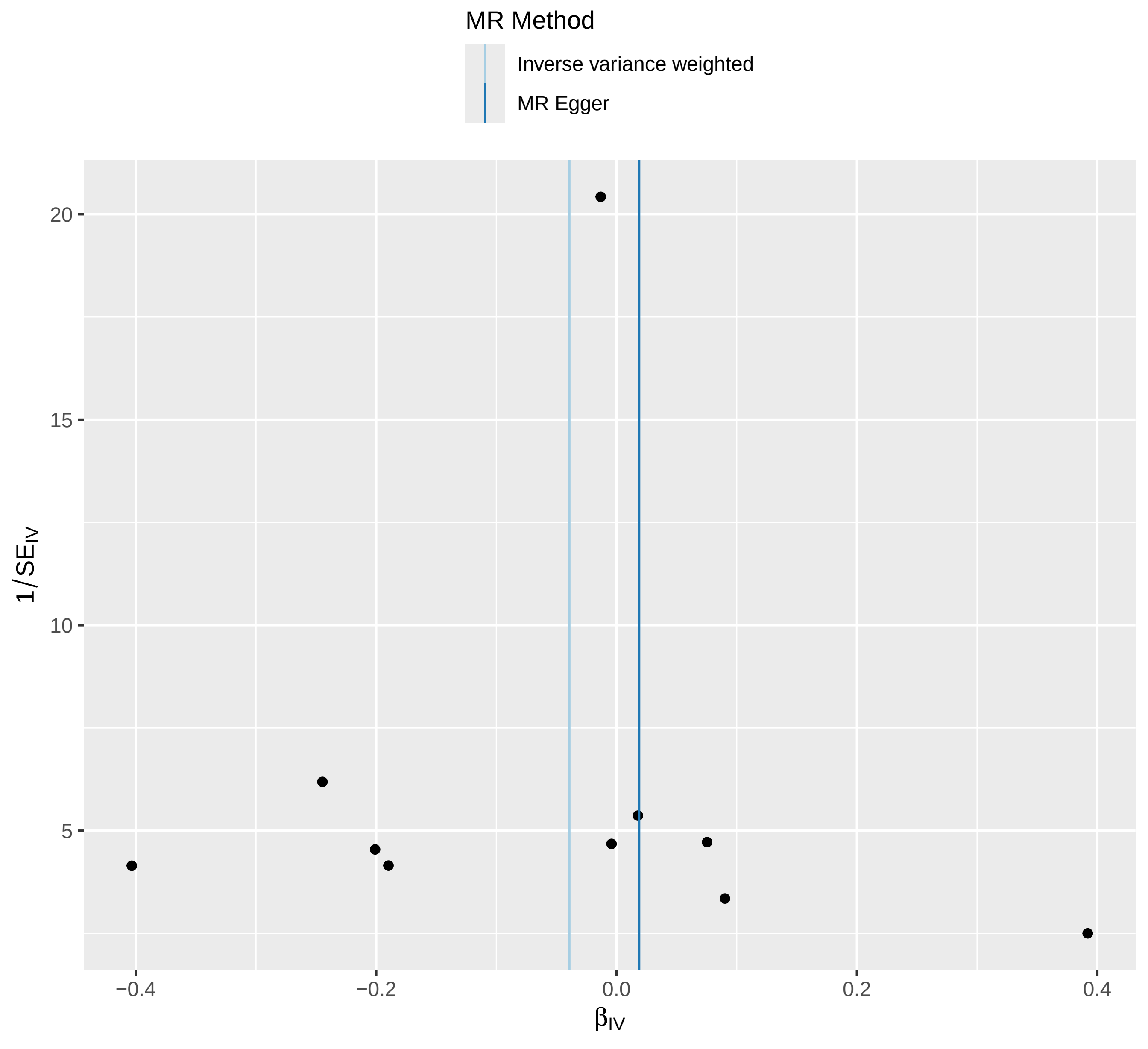
MR Method
Inverse variance weighted
MR Egger
1/SE_IV
20
15
10
5
−0.4
−0.2
0.0
0.2
0.4
β_IV

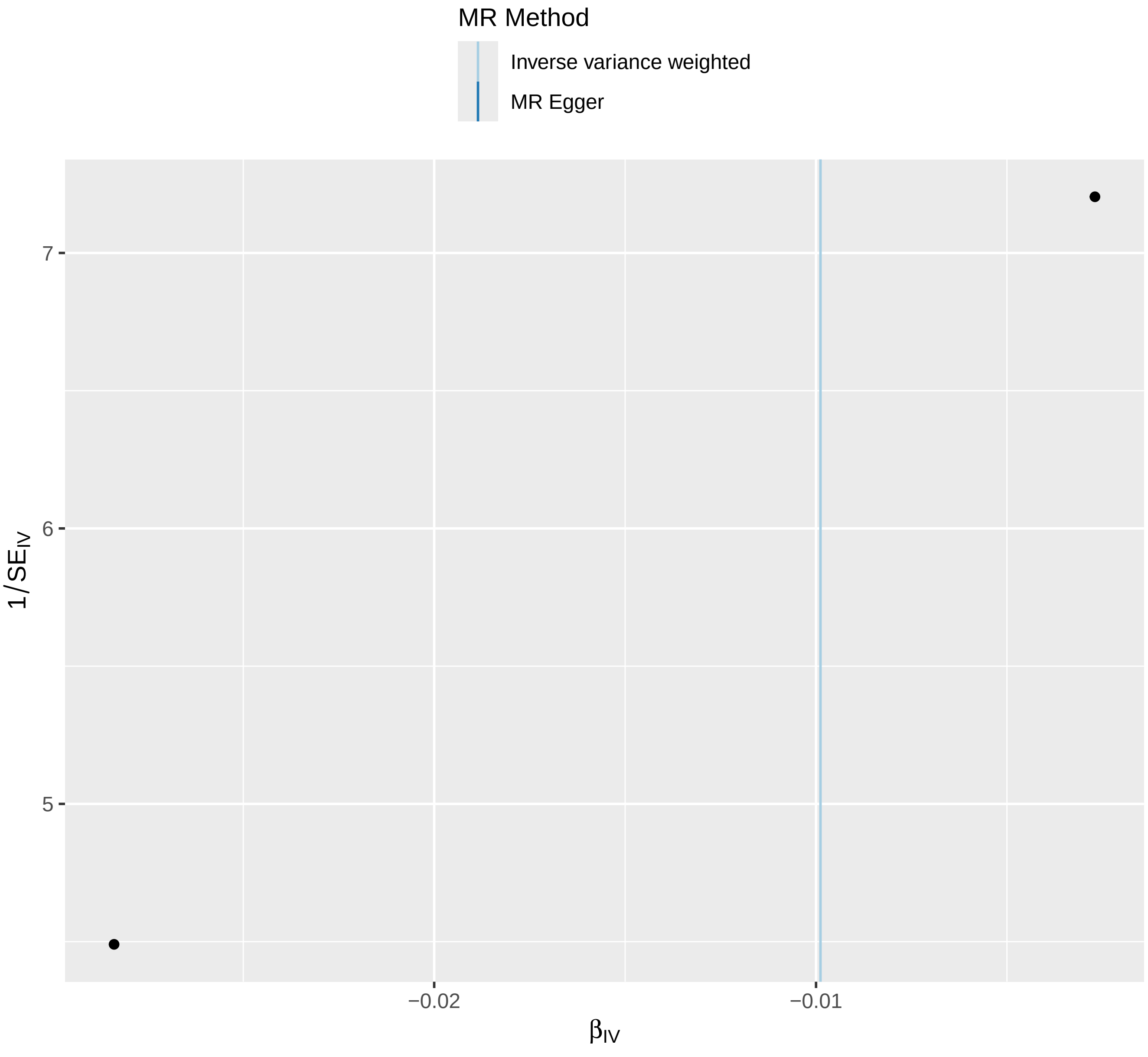
MR Method
Inverse variance weighted
MR Egger
1/SE$_{IV}$
7
6
5
−0.02
−0.01
β$_{IV}$

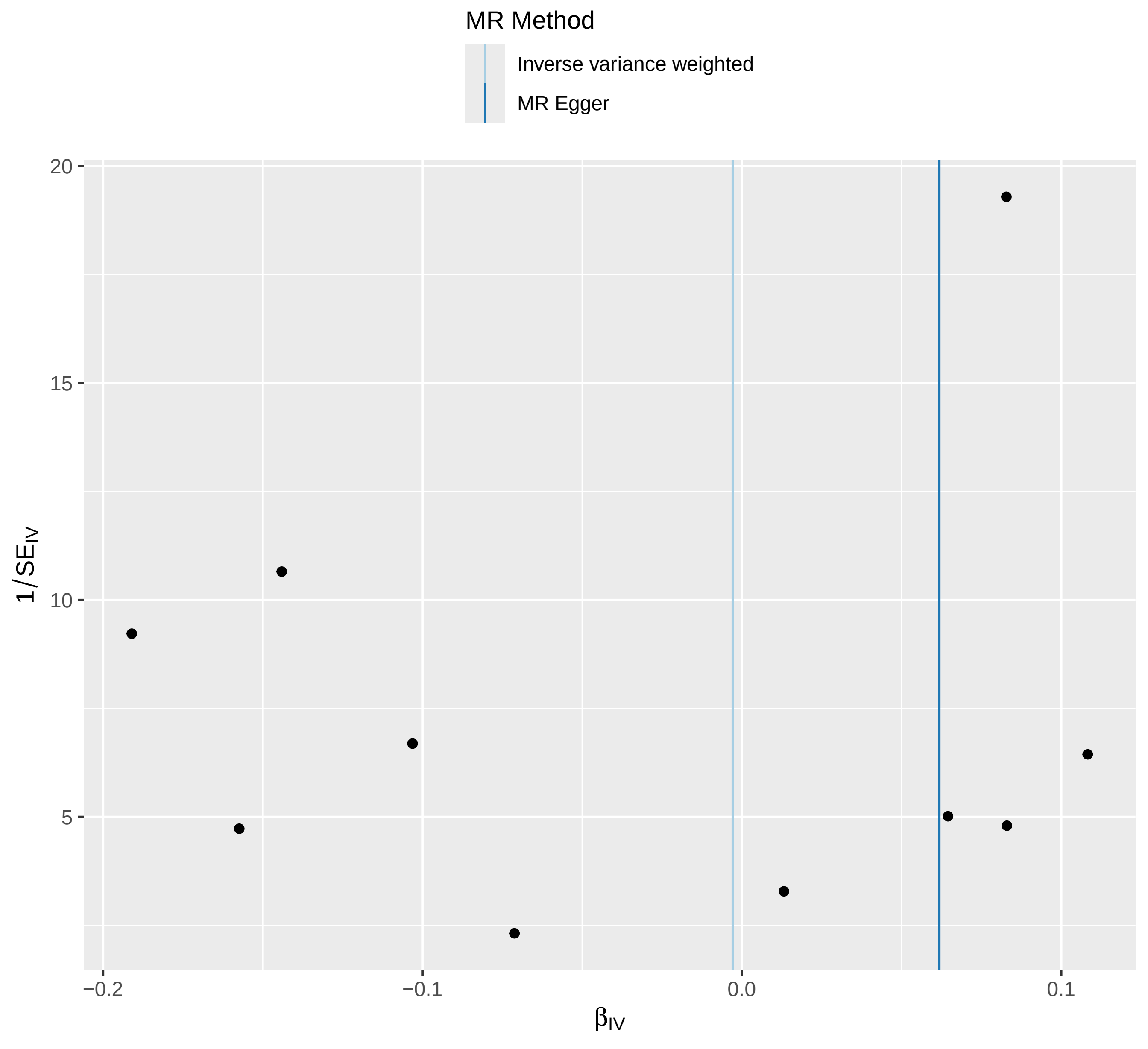

MR Method
Inverse variance weighted
MR Egger
20
15
10
5
$1/SE_{IV}$
−0.2
−0.1
0.0
0.1
$\beta_{IV}$

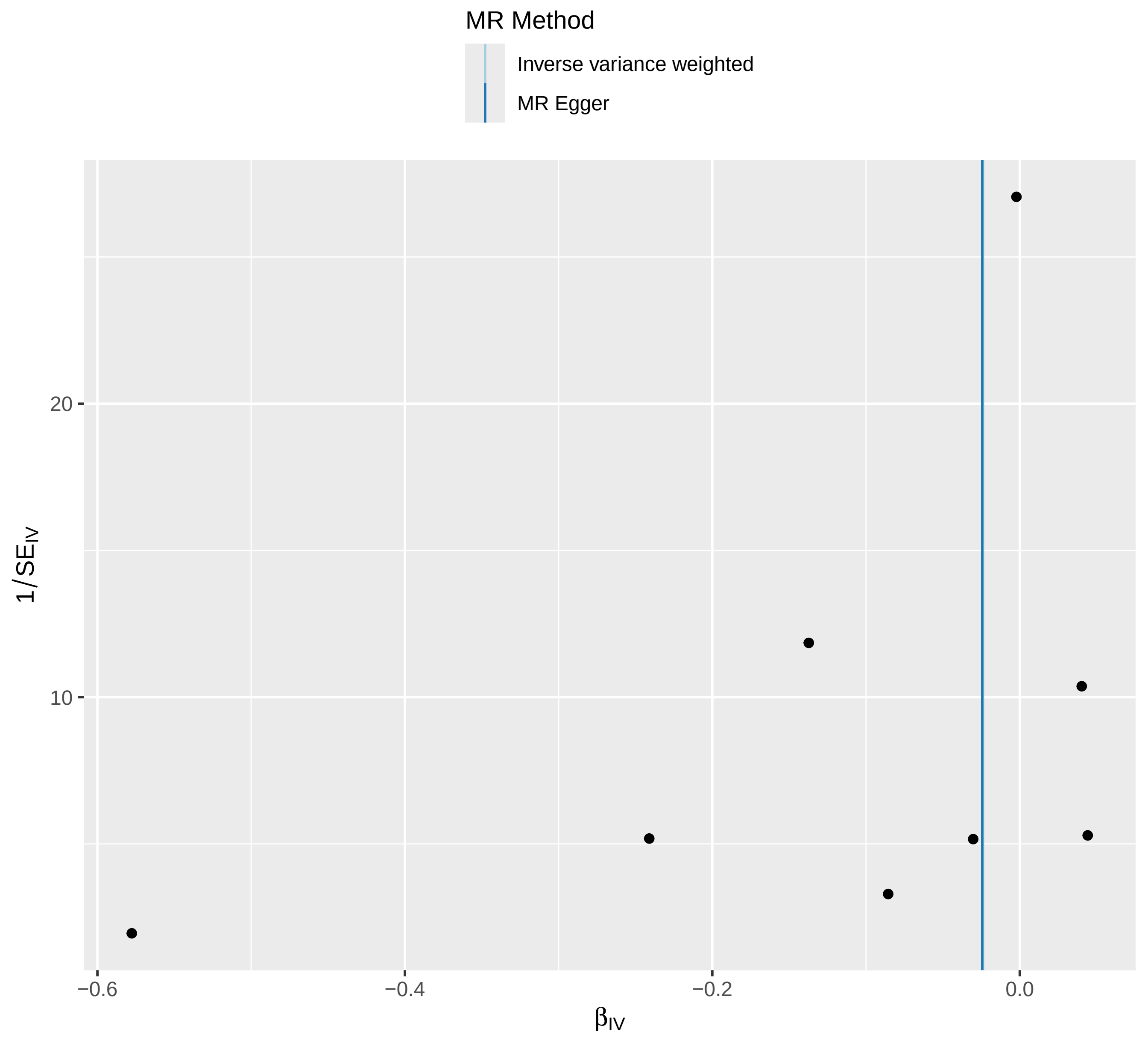

MR Method
Inverse variance weighted
MR Egger
20
10
1/SE$_{IV}$
−0.6
−0.4
−0.2
0.0
β$_{IV}$

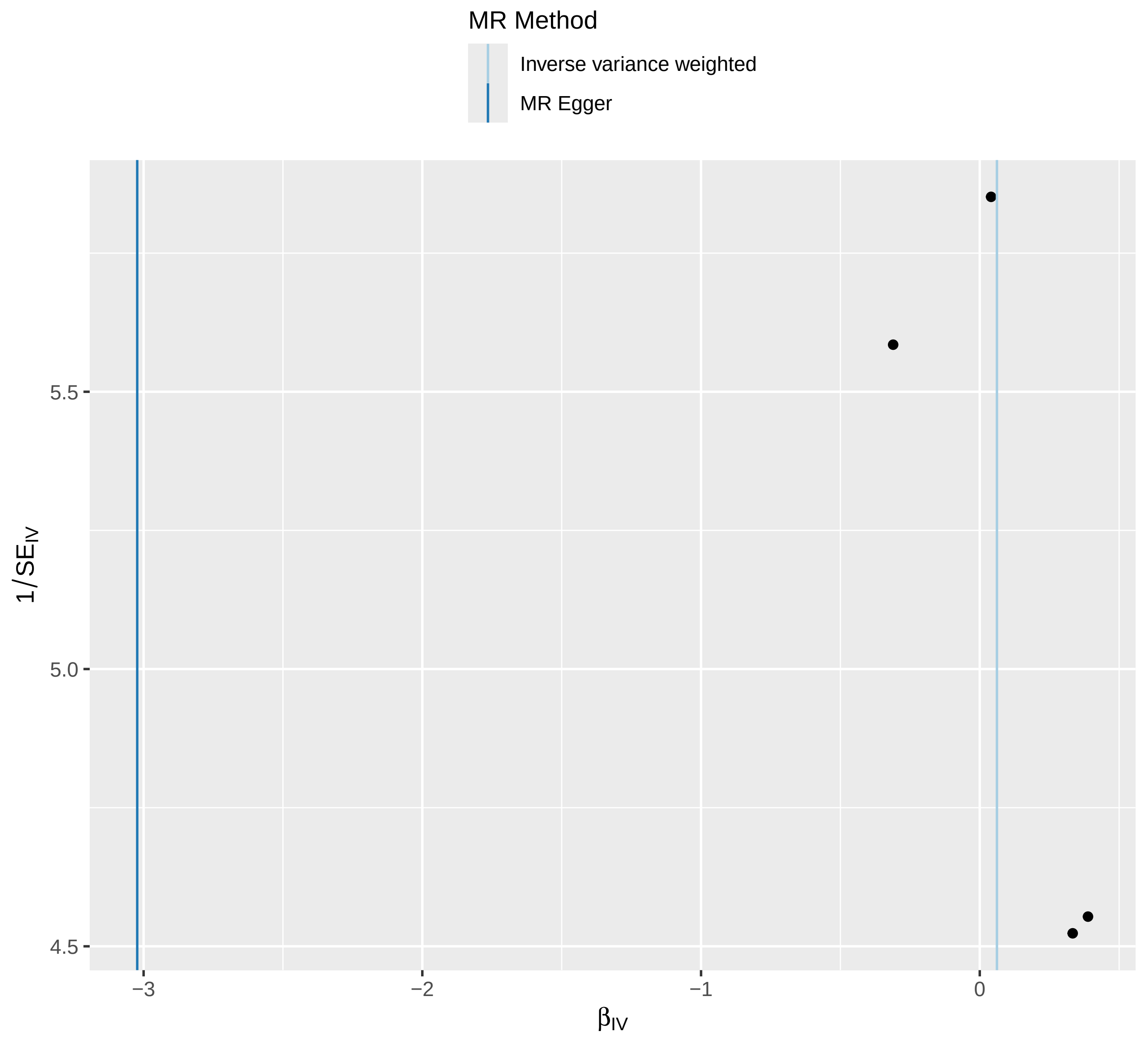

MR Method
Inverse variance weighted
MR Egger
5.5
5.0
4.5
1/SE_IV
−3
−2
−1
0
β_IV

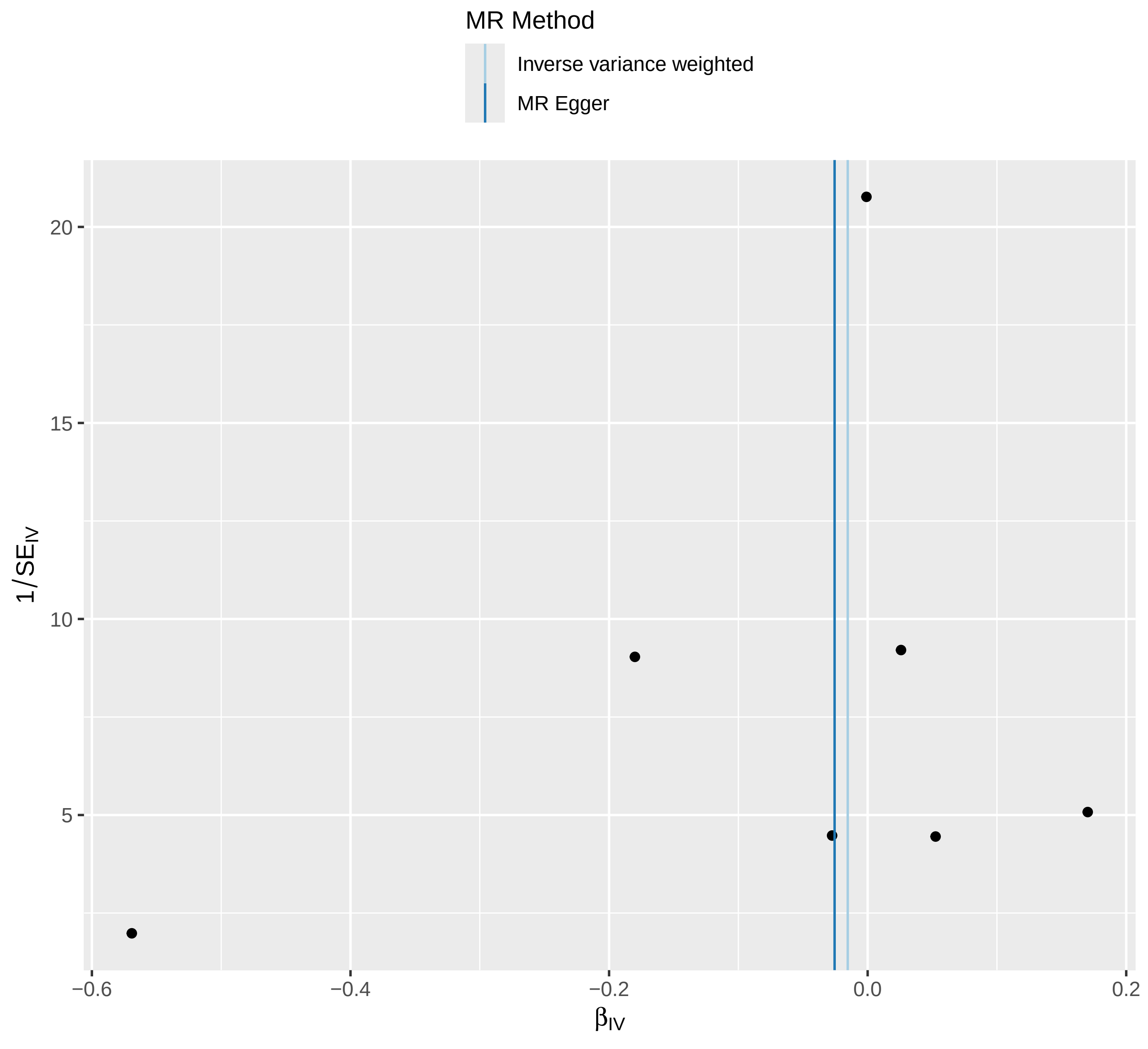
MR Method
Inverse variance weighted
MR Egger
20
15
10
5
1/SE$_{IV}$
−0.6
−0.4
−0.2
0.0
0.2
β$_{IV}$

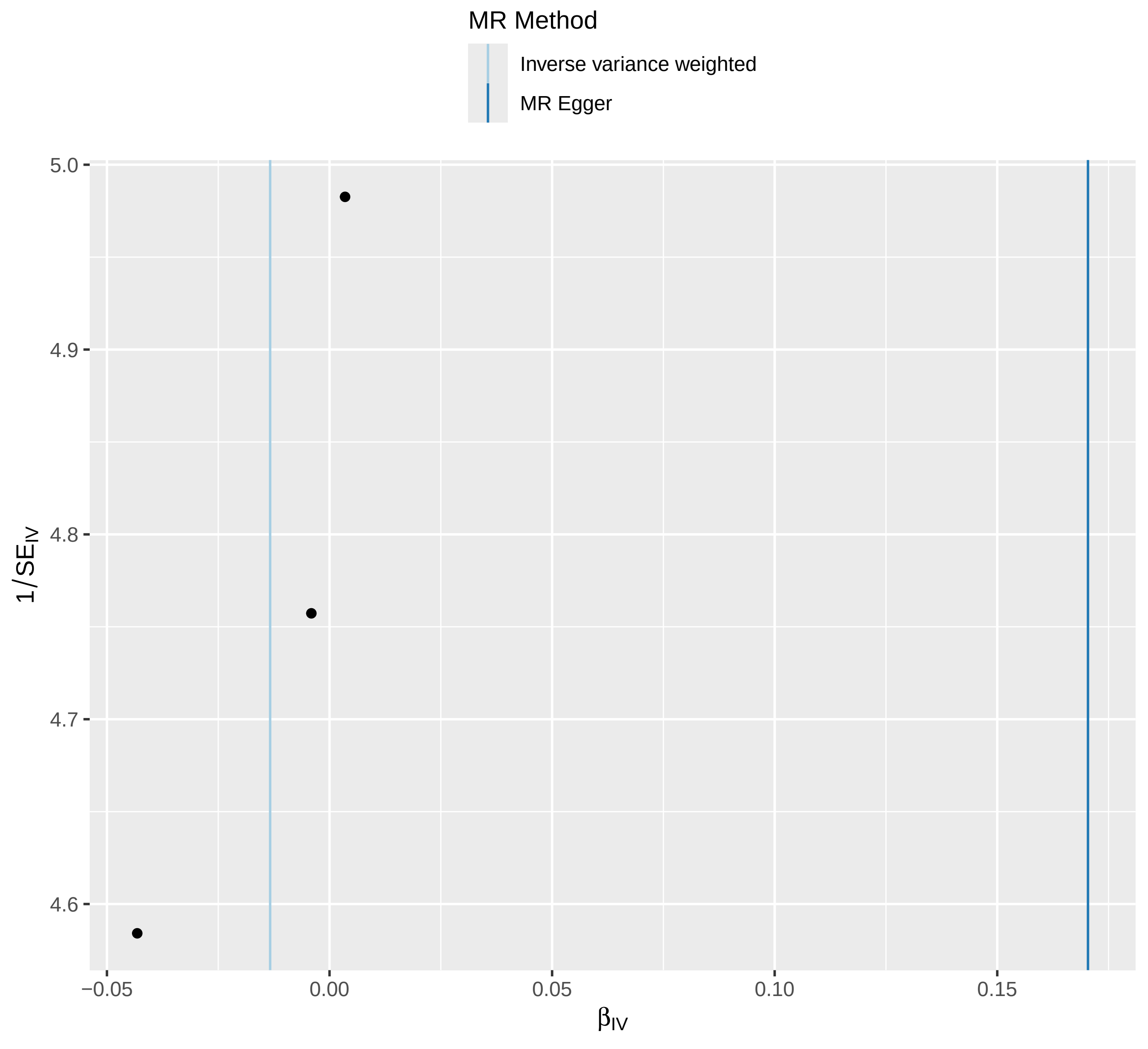

MR Method
Inverse variance weighted
MR Egger
5.0
4.9
4.8
4.7
4.6
1/SE$_{IV}$
−0.05
0.00
0.05
0.10
0.15
β$_{IV}$

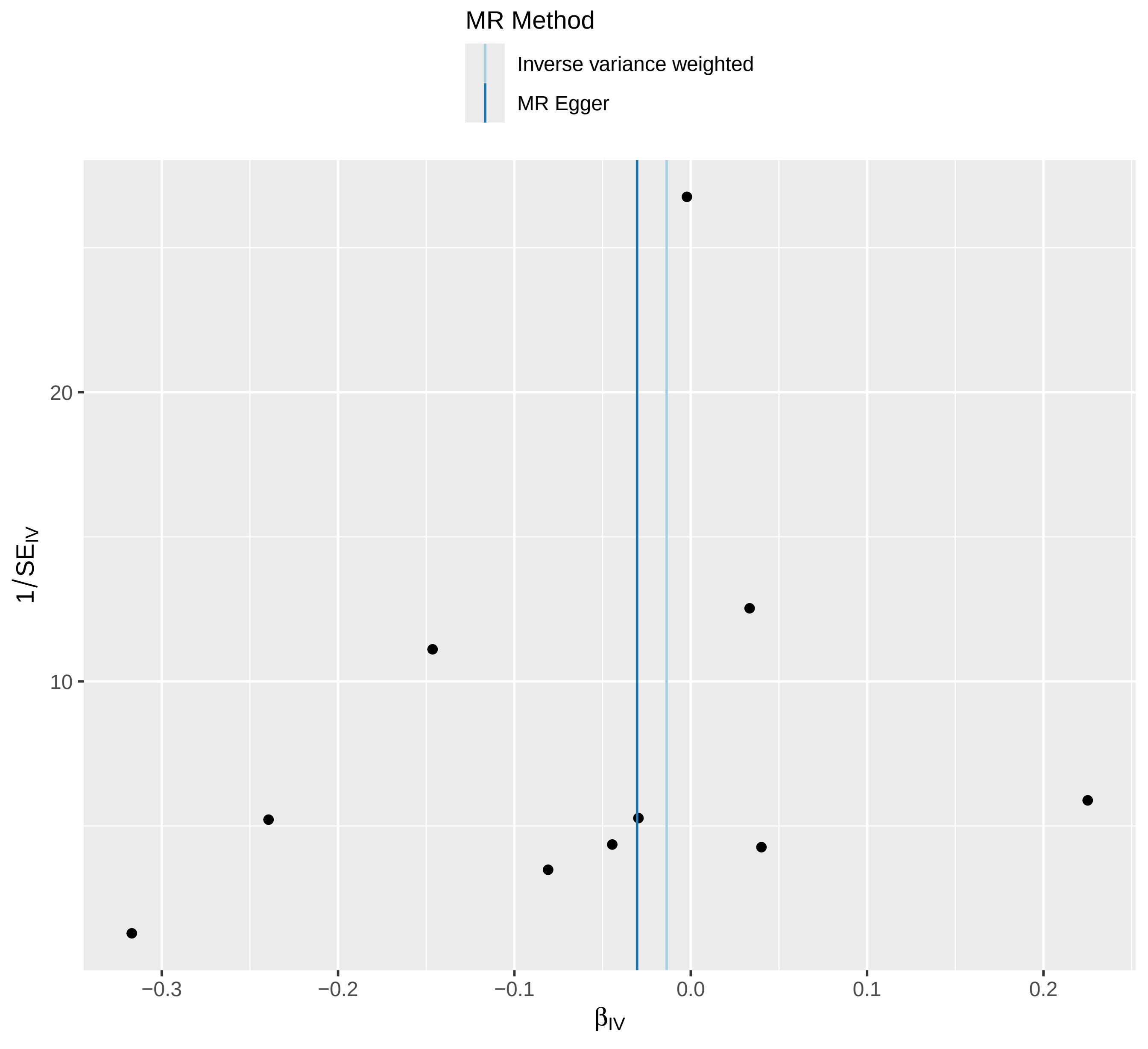
MR Method
Inverse variance weighted
MR Egger
20
10
$1/SE_{IV}$
−0.3
−0.2
−0.1
0.0
0.1
0.2
$\beta_{IV}$

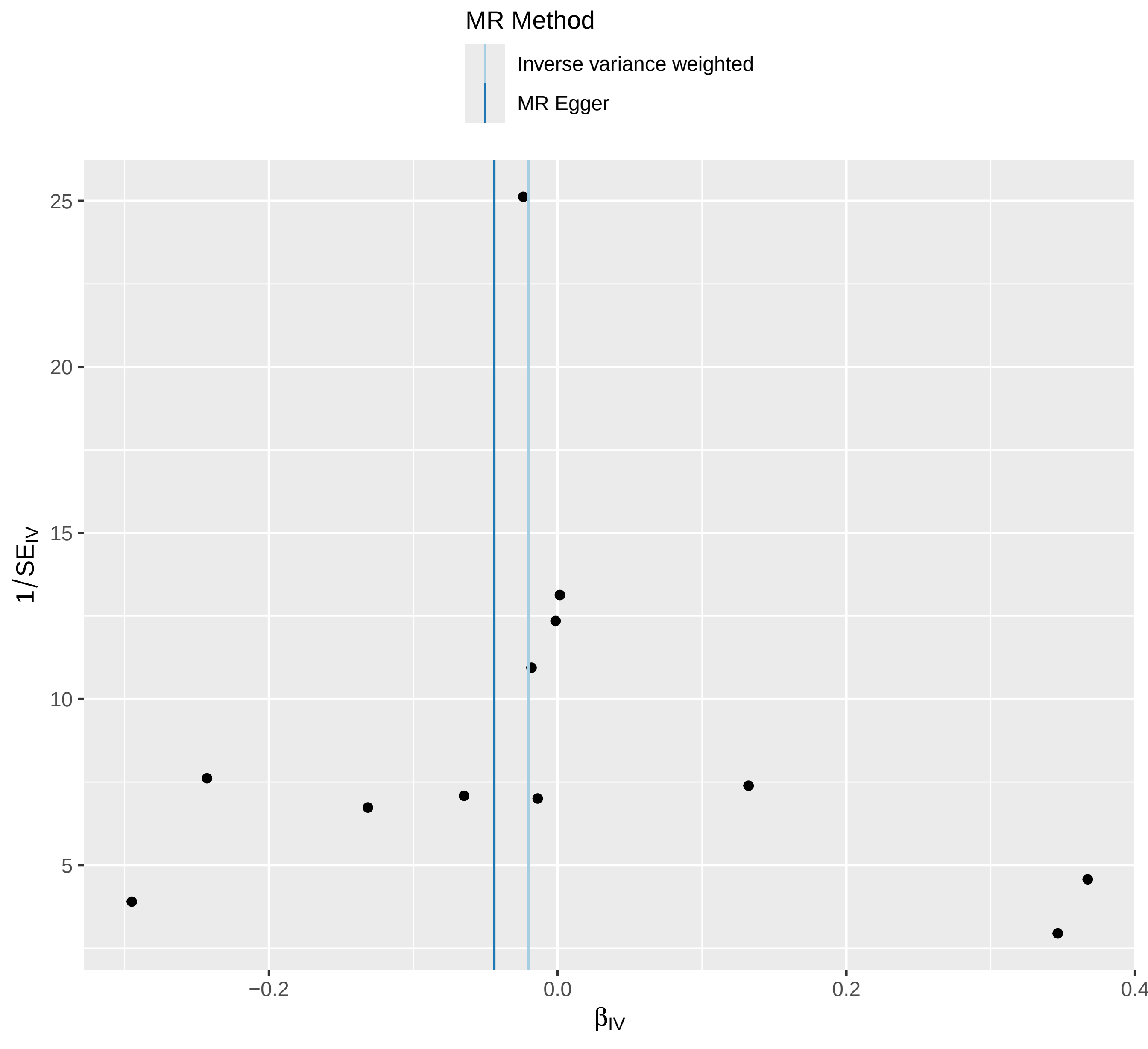
MR Method
Inverse variance weighted
MR Egger
25
20
15
10
5
$1/SE_{IV}$
−0.2
0.0
0.2
0.4
$\beta_{IV}$

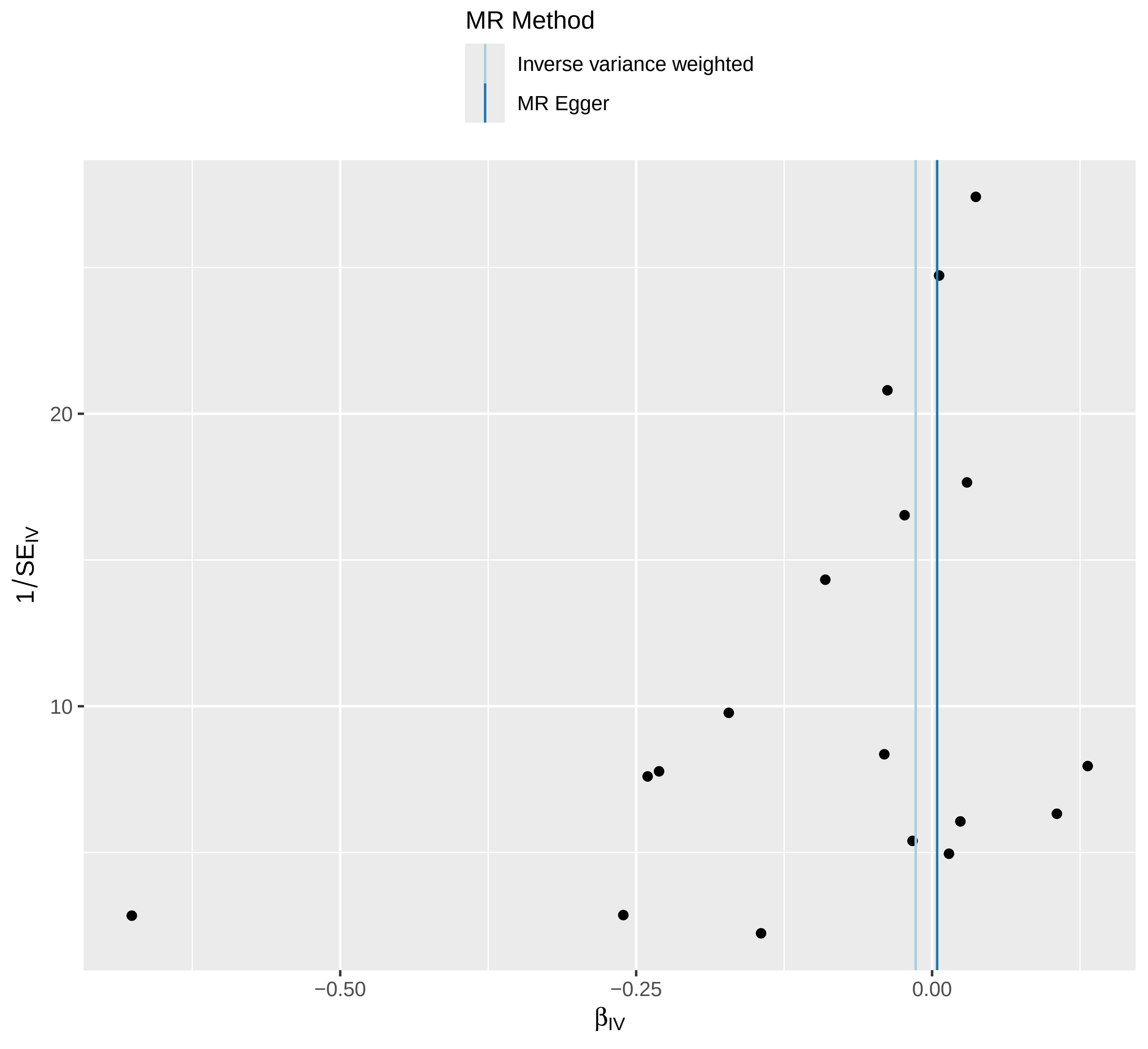
MR Method
Inverse variance weighted
MR Egger
20
10
1/SE$_{IV}$
−0.50
−0.25
0.00
β$_{IV}$

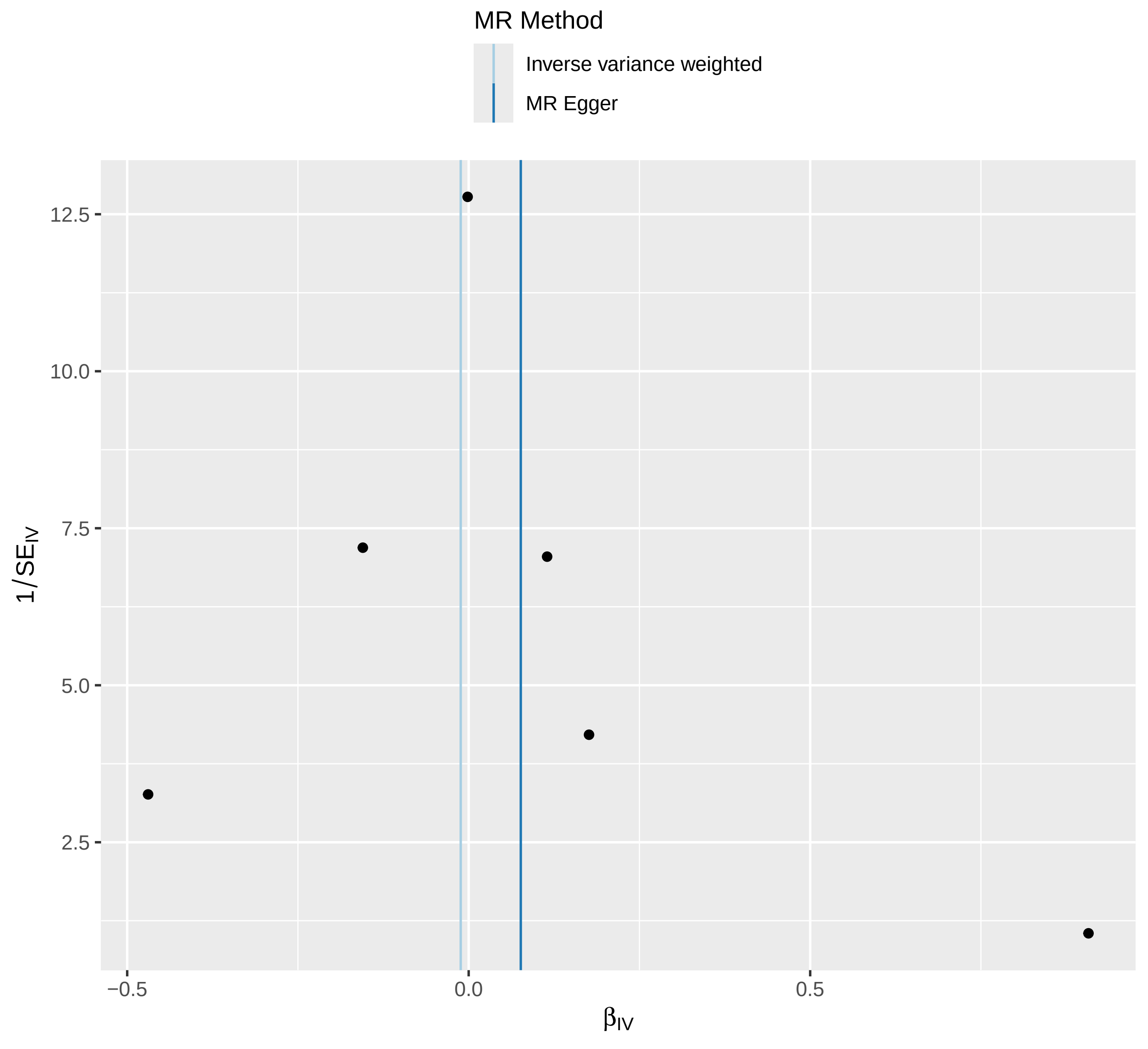
MR Method
Inverse variance weighted
MR Egger
12.5
10.0
7.5
5.0
2.5
1/SE_IV
−0.5
0.0
0.5
β_IV

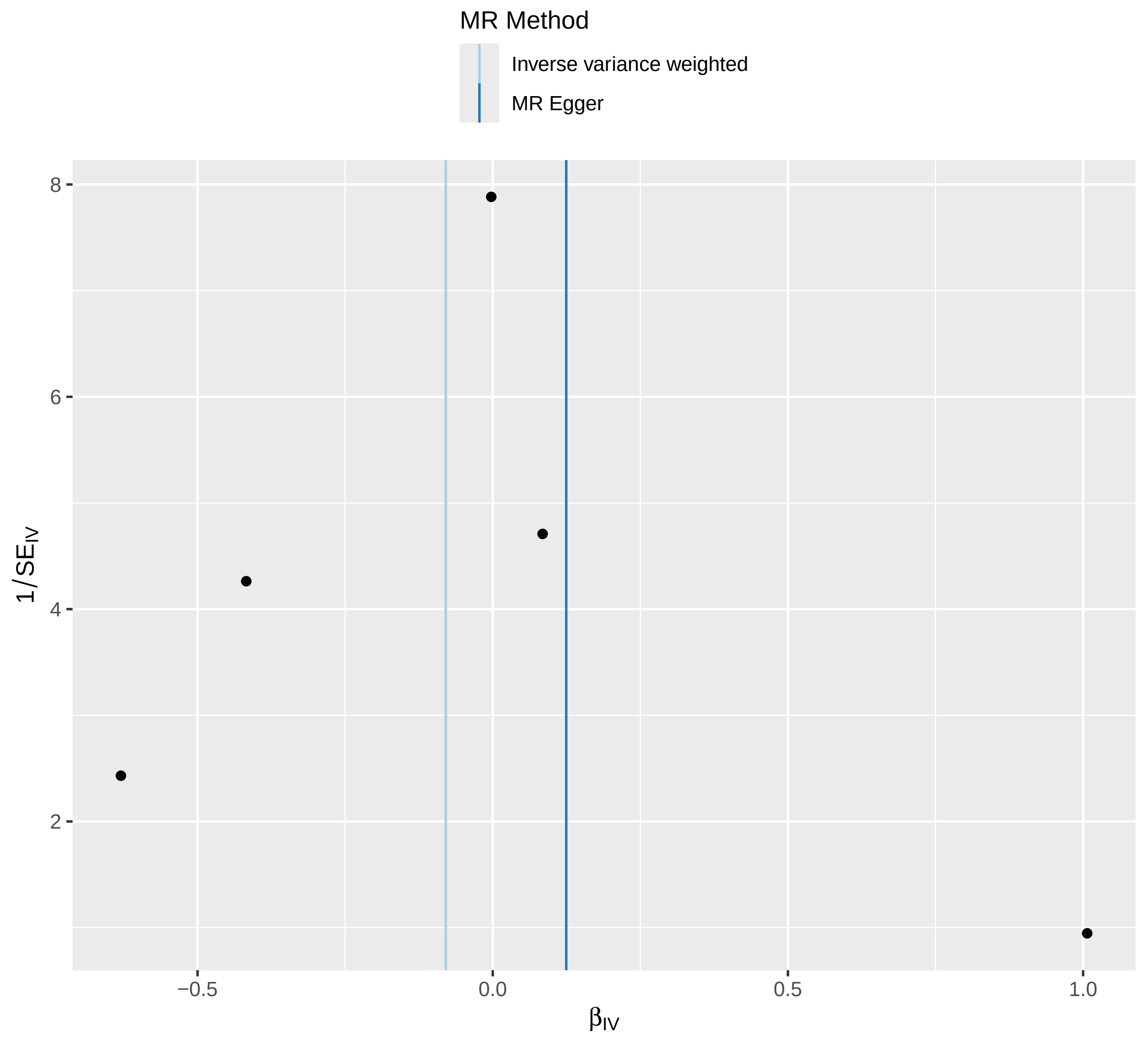
MR Method
Inverse variance weighted
MR Egger
8
6
4
2
$1/SE_{IV}$
−0.5
0.0
0.5
1.0
$\beta_{IV}$

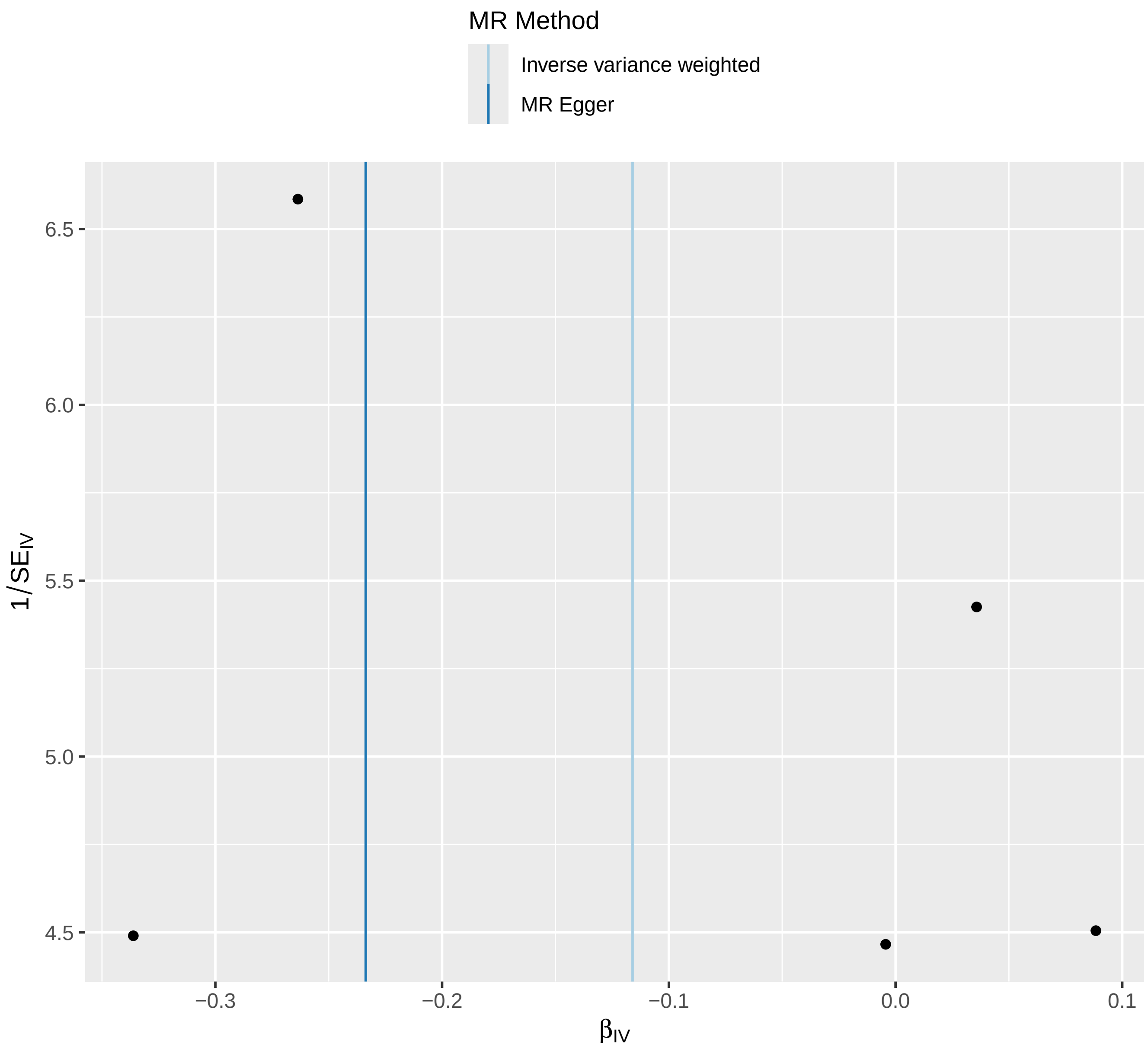
MR Method
Inverse variance weighted
MR Egger
6.5
6.0
5.5
5.0
4.5
1/SE_IV
−0.3
−0.2
−0.1
0.0
0.1
β_IV

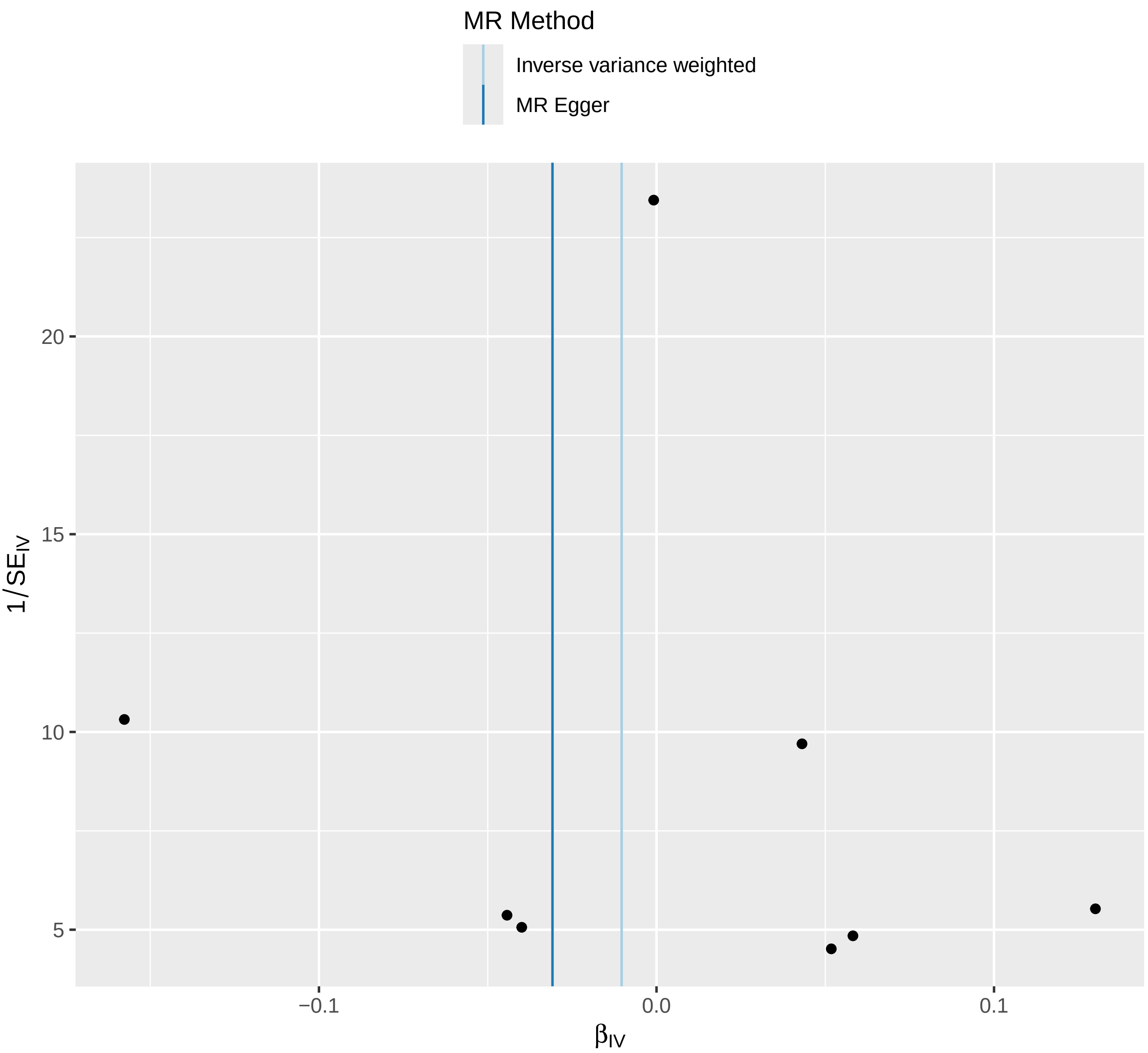
MR Method
Inverse variance weighted
MR Egger
20
15
10
5
1/SE_IV
−0.1
0.0
0.1
β_IV

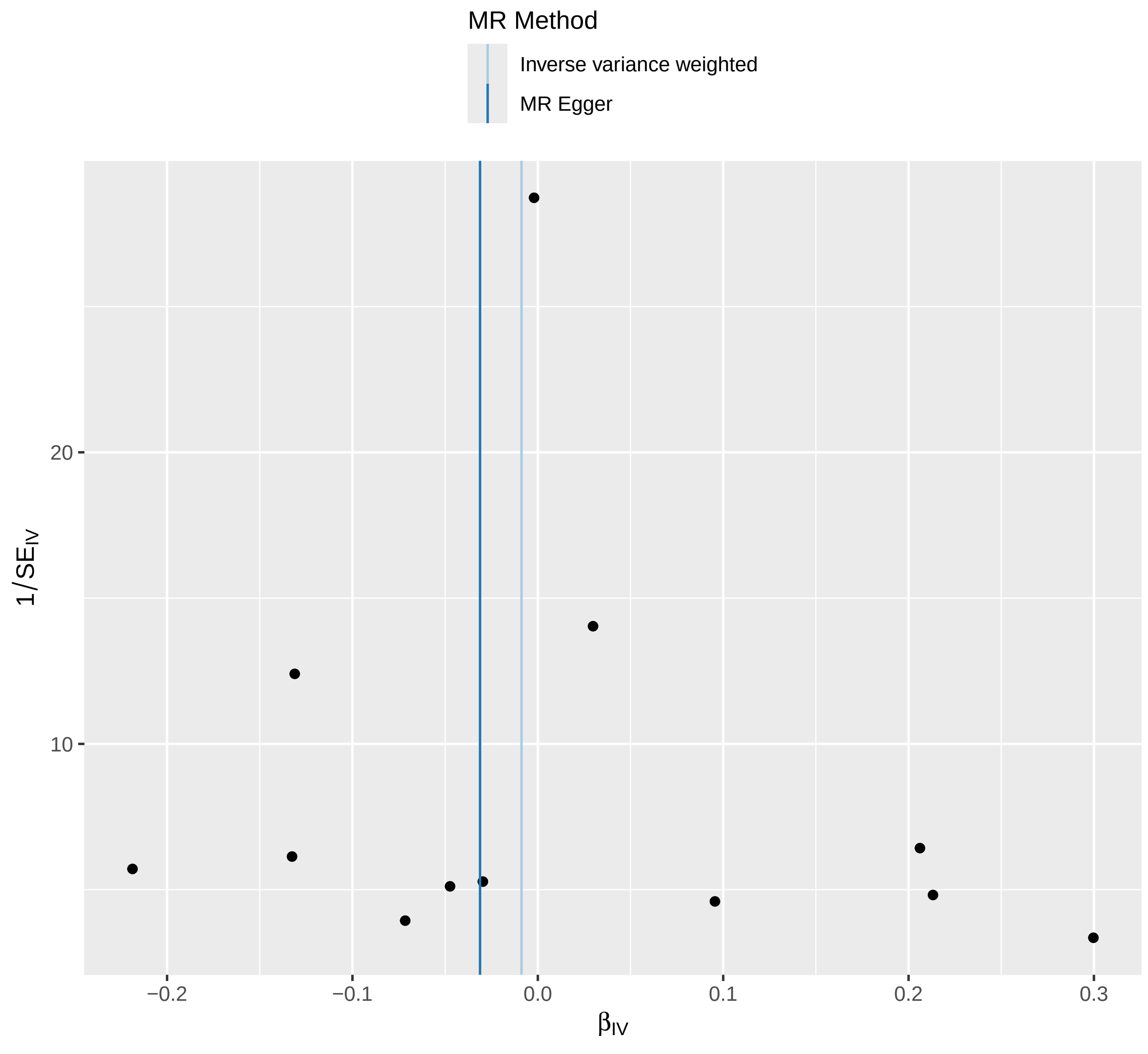
MR Method
Inverse variance weighted
MR Egger
20
10
1/SE_IV
−0.2
−0.1
0.0
0.1
0.2
0.3
β_IV

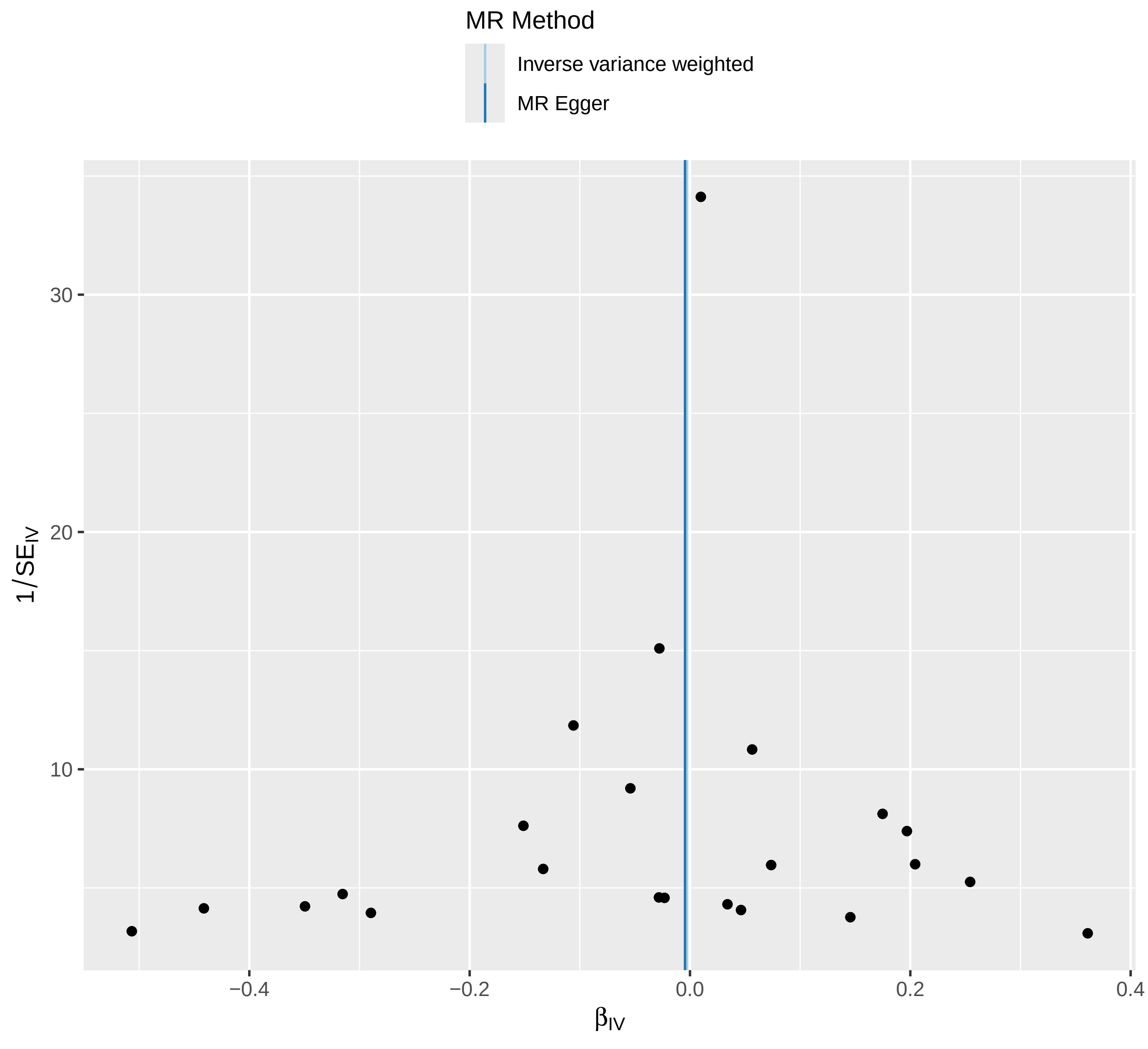

MR Method
Inverse variance weighted
MR Egger
30
20
10
1/SE$_{IV}$
−0.4
−0.2
0.0
0.2
0.4
β$_{IV}$

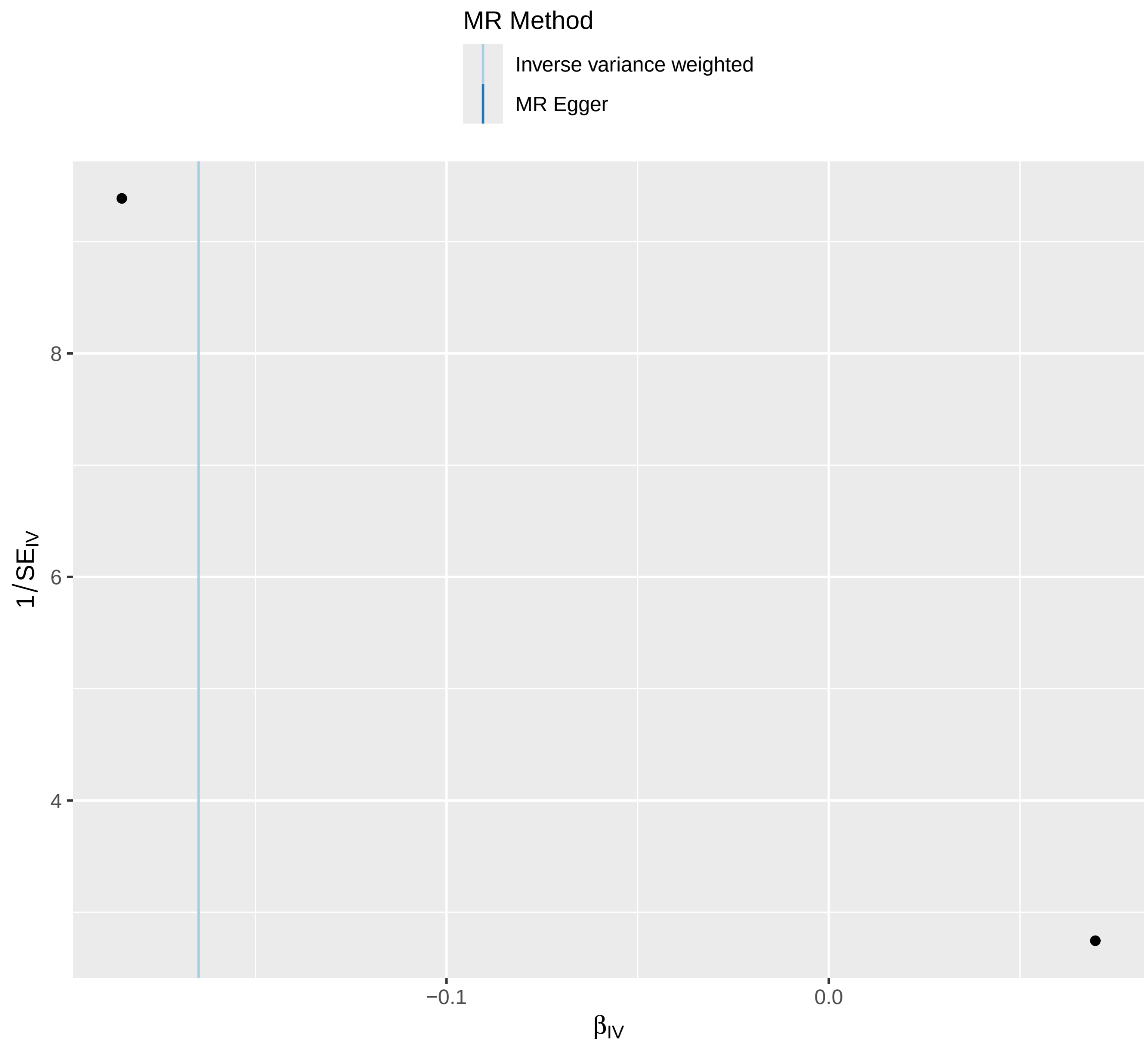

MR Method
Inverse variance weighted
MR Egger
8
6
4
1/SE_IV
−0.1
0.0
β_IV

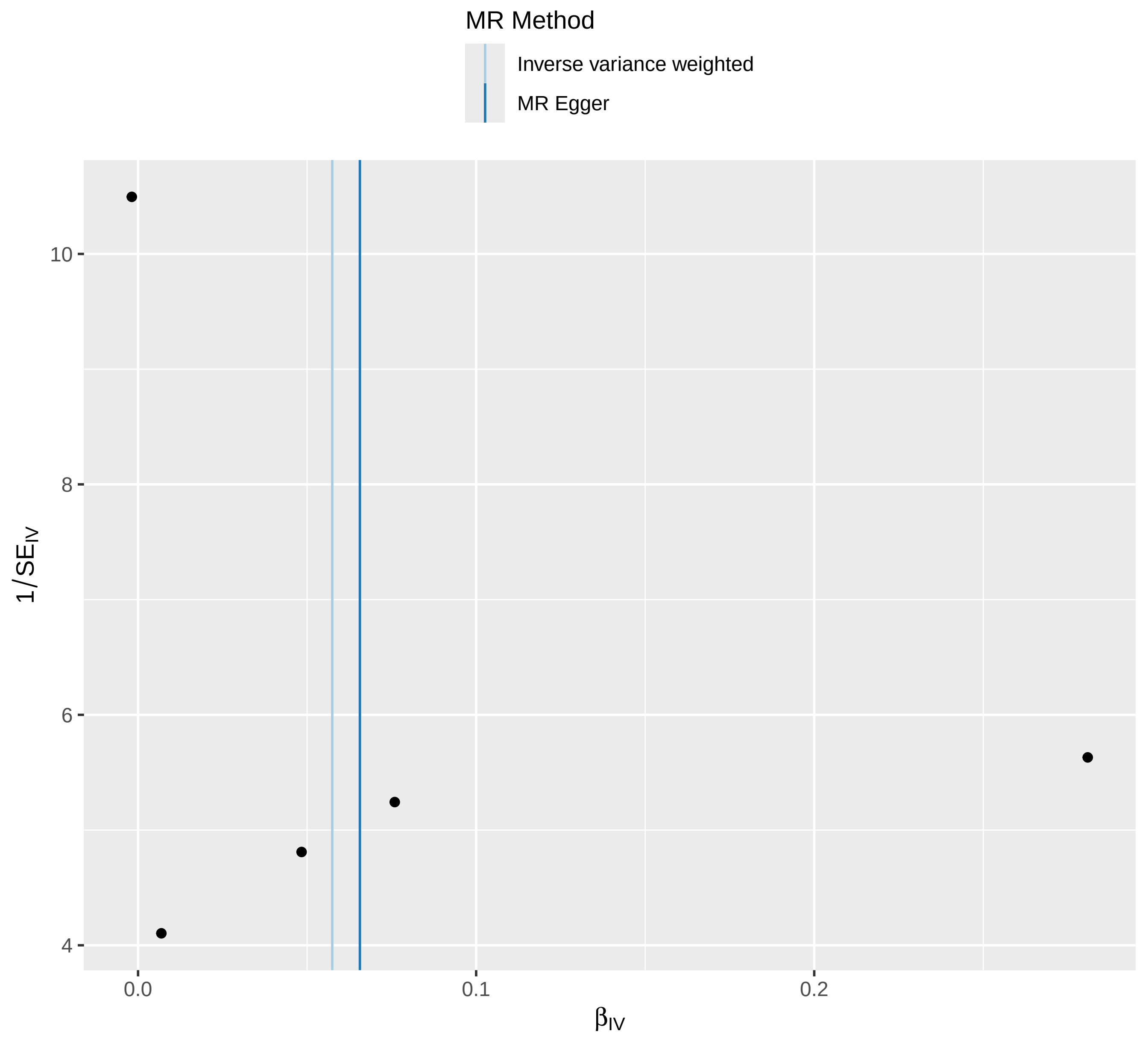

MR Method
Inverse variance weighted
MR Egger
10
8
6
4
$1/SE_{IV}$
0.0
0.1
0.2
$\beta_{IV}$

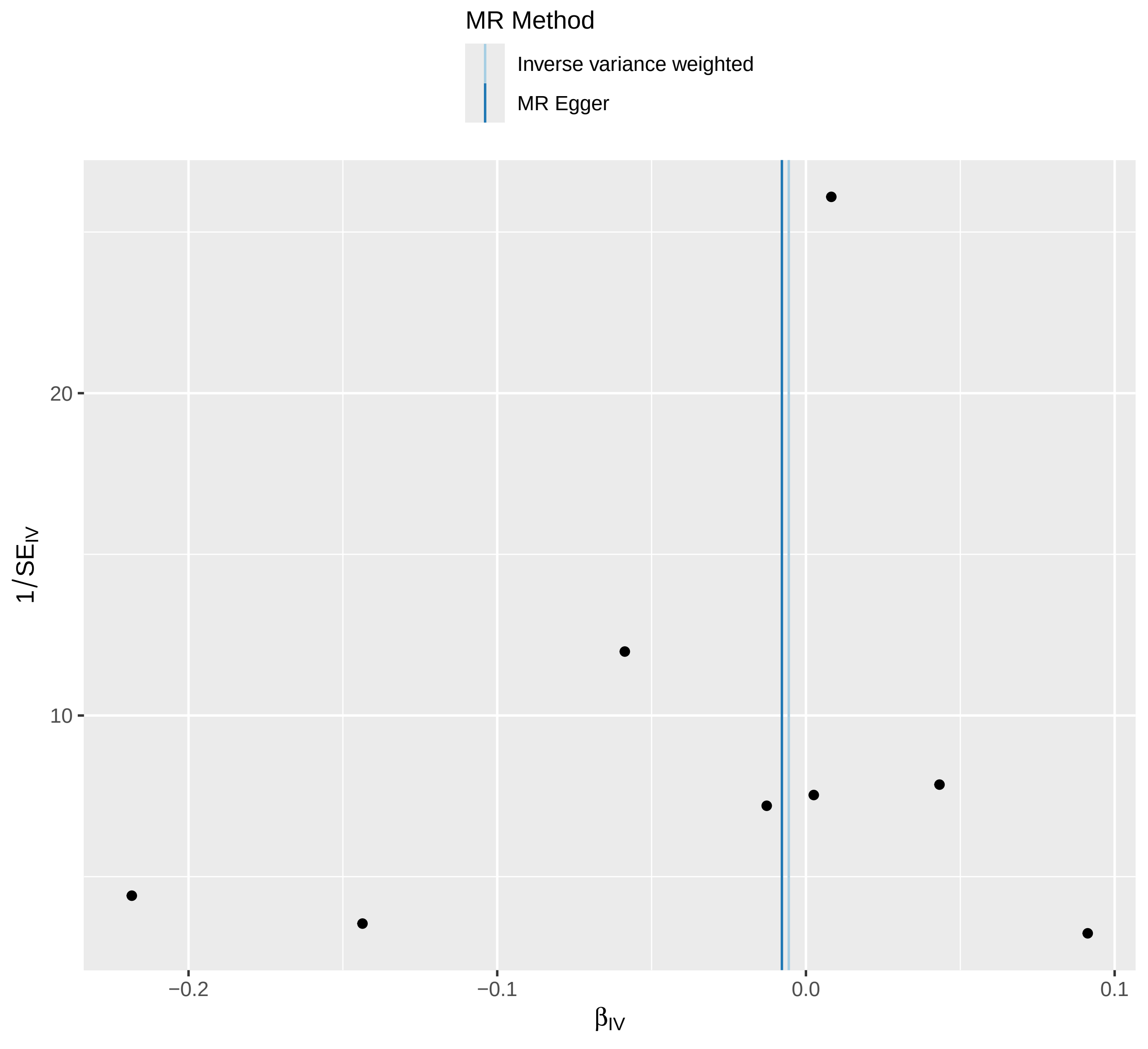

MR Method
Inverse variance weighted
MR Egger
20
10
1/SE$_{IV}$
−0.2
−0.1
0.0
0.1
β$_{IV}$

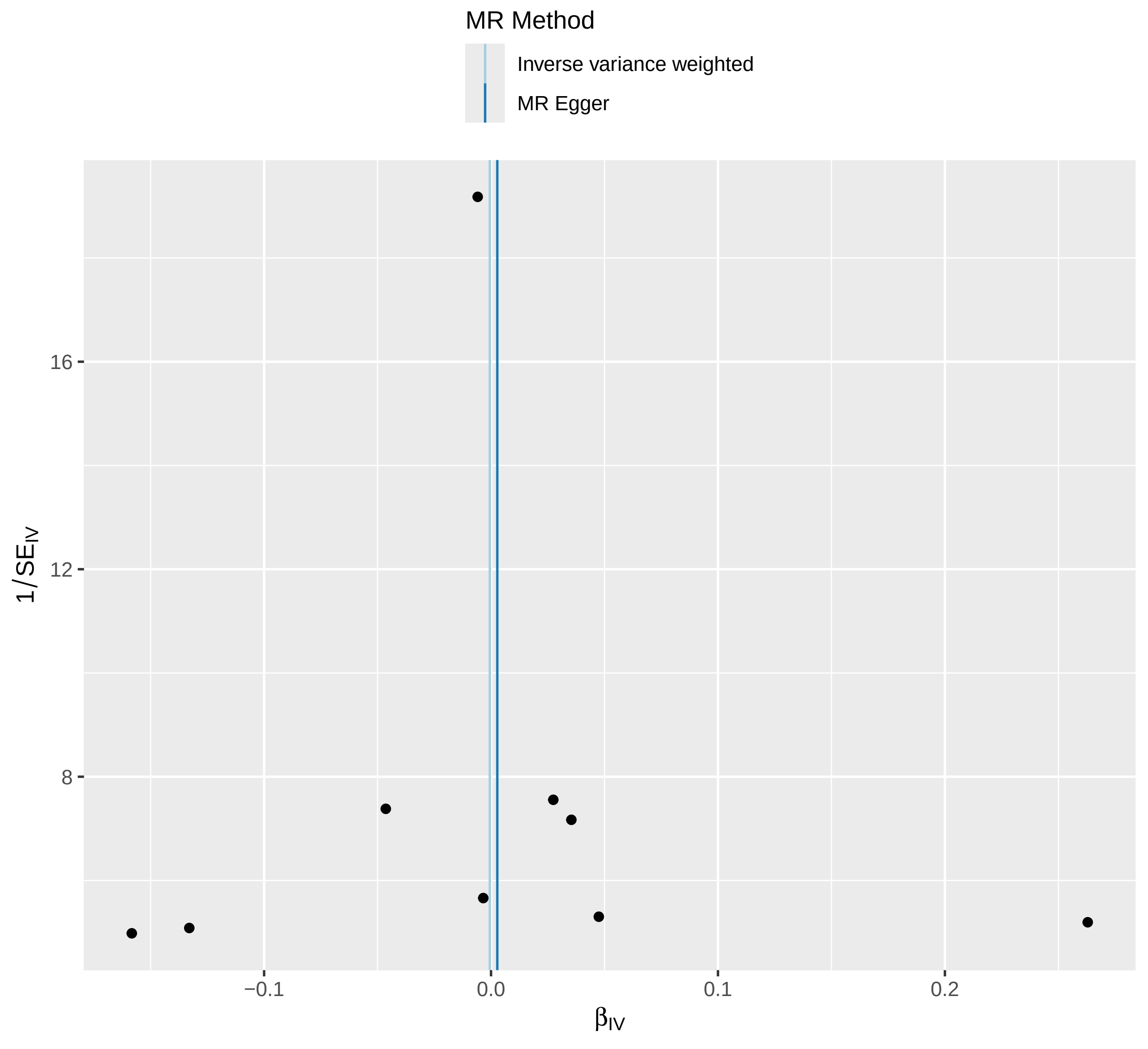
MR Method
Inverse variance weighted
MR Egger
16
12
8
1/SE$_{IV}$
−0.1
0.0
0.1
0.2
β$_{IV}$

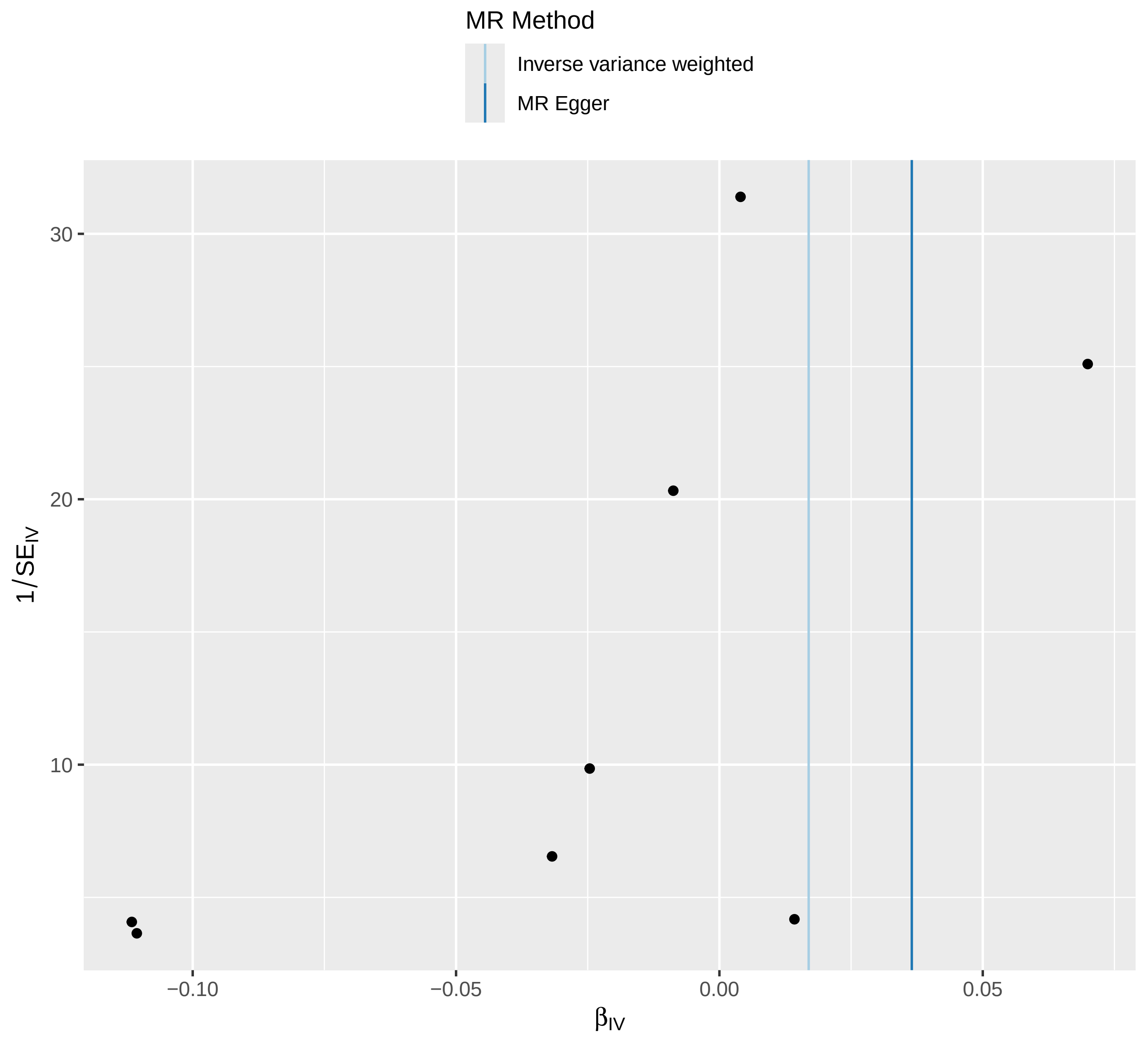
MR Method
Inverse variance weighted
MR Egger
30
20
10
$1/SE_{IV}$
−0.10
−0.05
0.00
0.05
$\beta_{IV}$

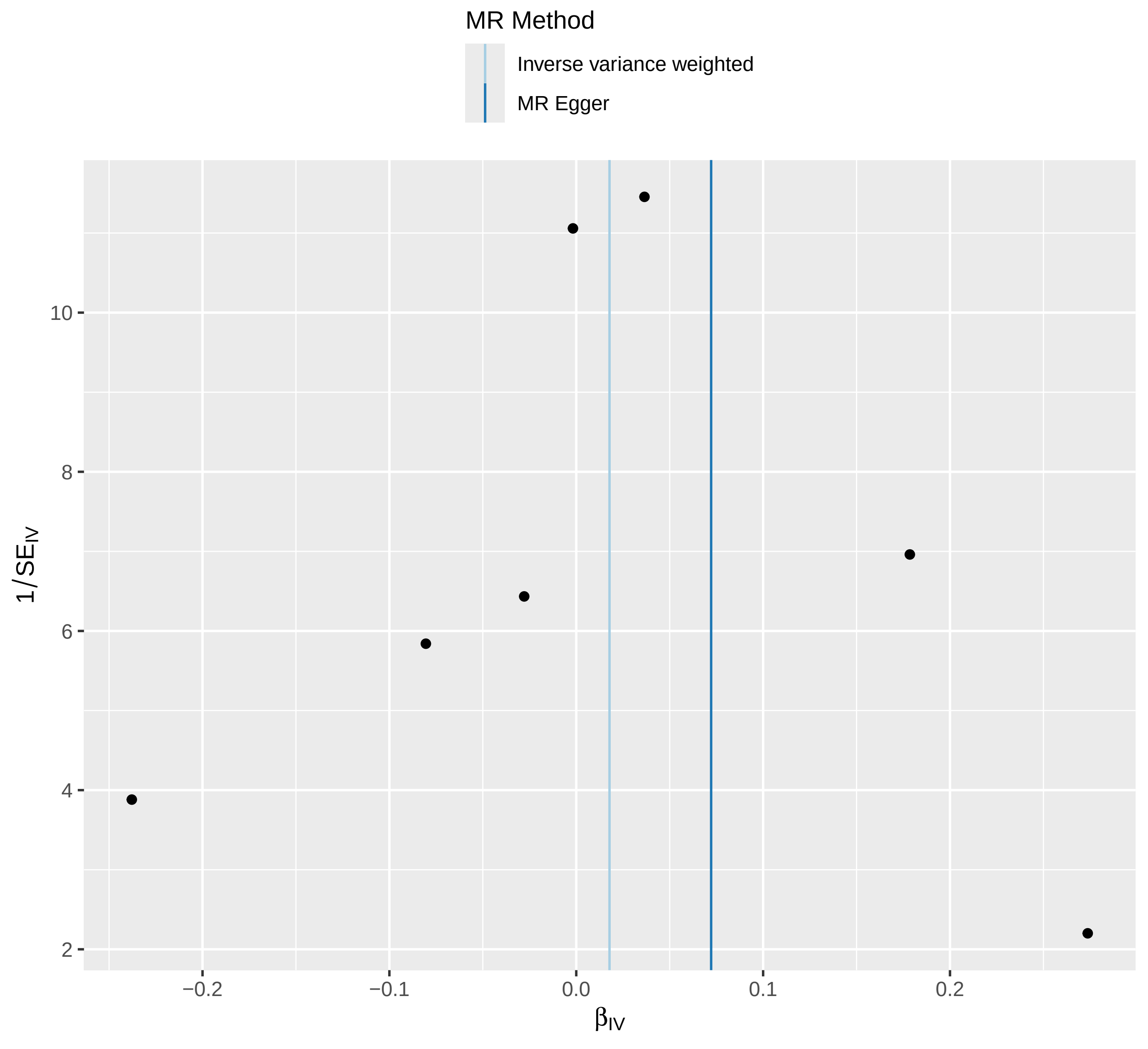

MR Method
Inverse variance weighted
MR Egger
1/SE_IV
10
8
6
4
2
−0.2
−0.1
0.0
0.1
0.2
β_IV

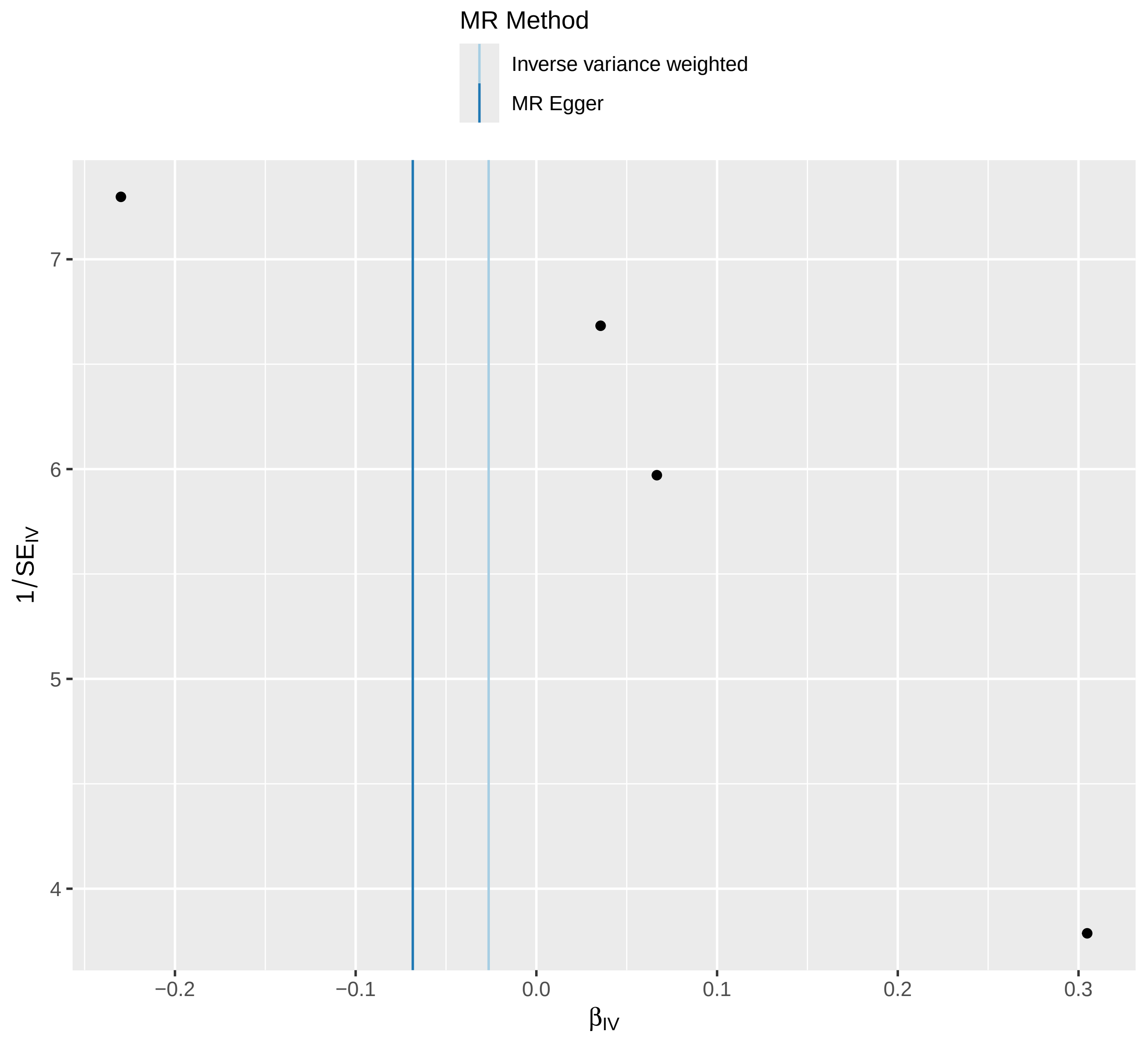

MR Method
Inverse variance weighted
MR Egger
1/SE_IV
7
6
5
4
−0.2
−0.1
0.0
0.1
0.2
0.3
β_IV

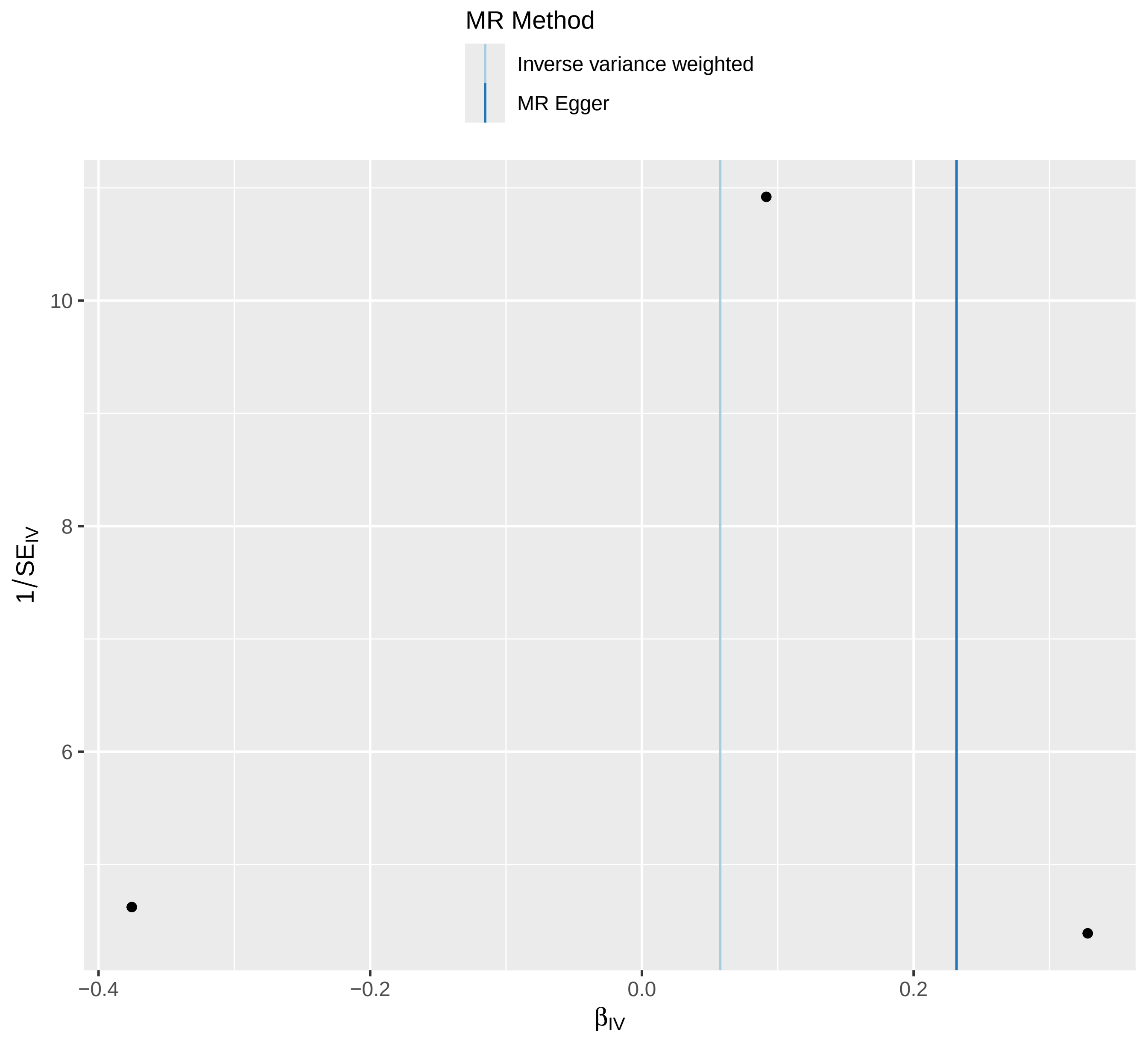

MR Method
Inverse variance weighted
MR Egger
10
8
6
$1/SE_{IV}$
−0.4
−0.2
0.0
0.2
$\beta_{IV}$

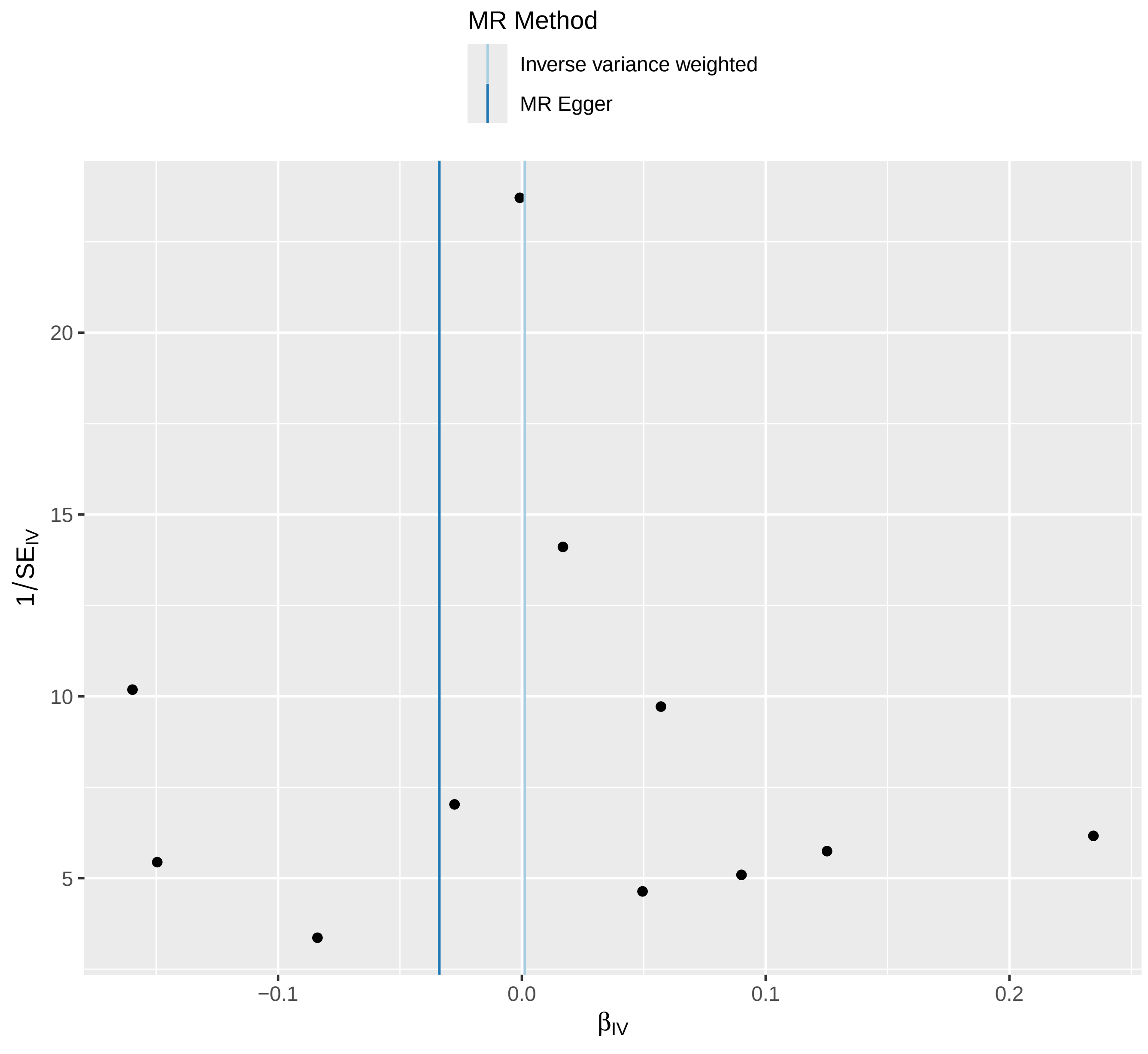
MR Method
Inverse variance weighted
MR Egger
20
15
10
5
1/SE_IV
−0.1
0.0
0.1
0.2
β_IV

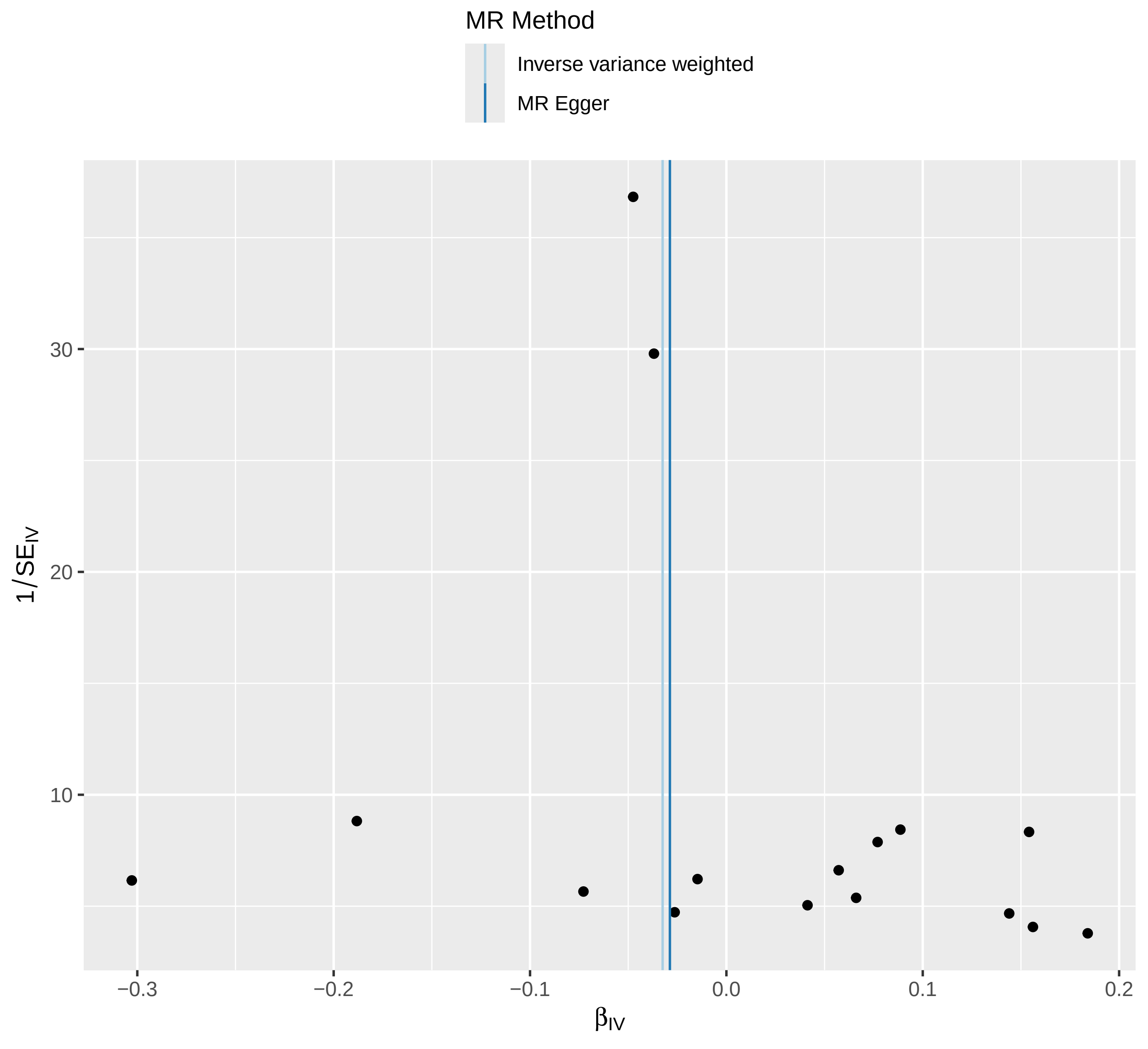
MR Method
Inverse variance weighted
MR Egger
1/SE$_{IV}$
30
20
10
−0.3
−0.2
−0.1
0.0
0.1
0.2
β$_{IV}$

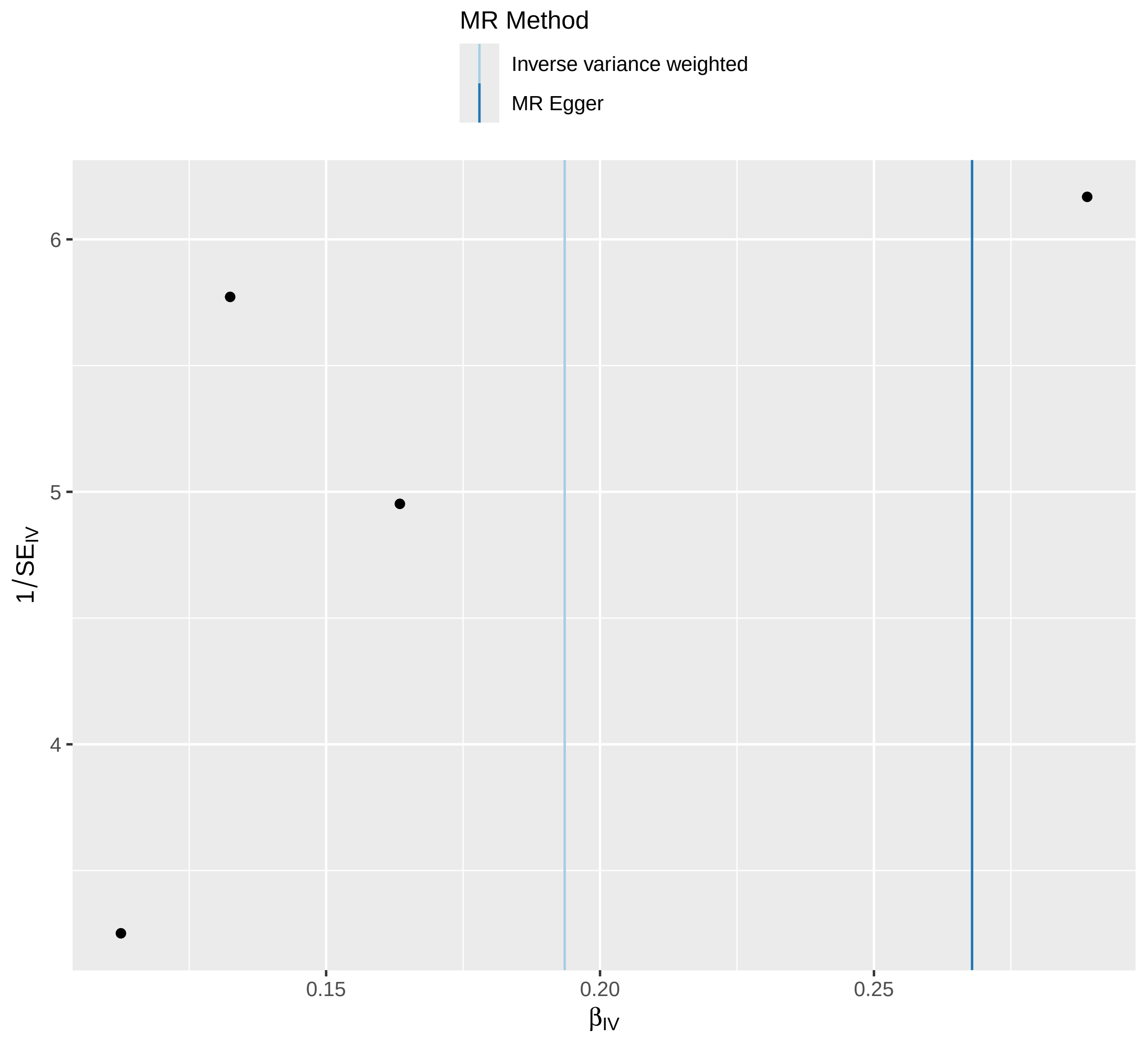

MR Method
Inverse variance weighted
MR Egger
6
5
4
0.15
0.20
0.25
1/SE_IV
β_IV

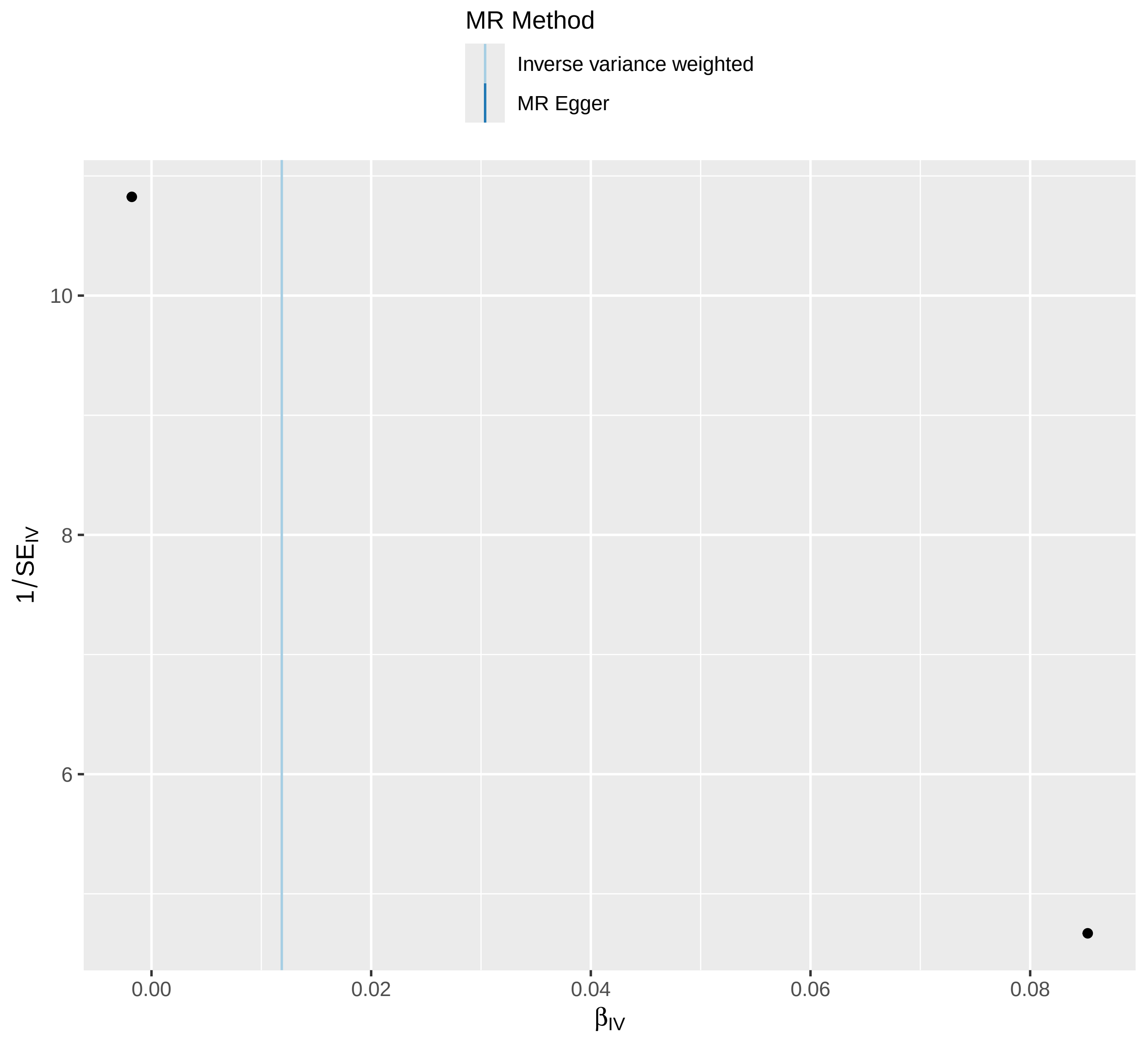

MR Method
Inverse variance weighted
MR Egger
10
8
6
1/SE_IV
0.00
0.02
0.04
0.06
0.08
β_IV

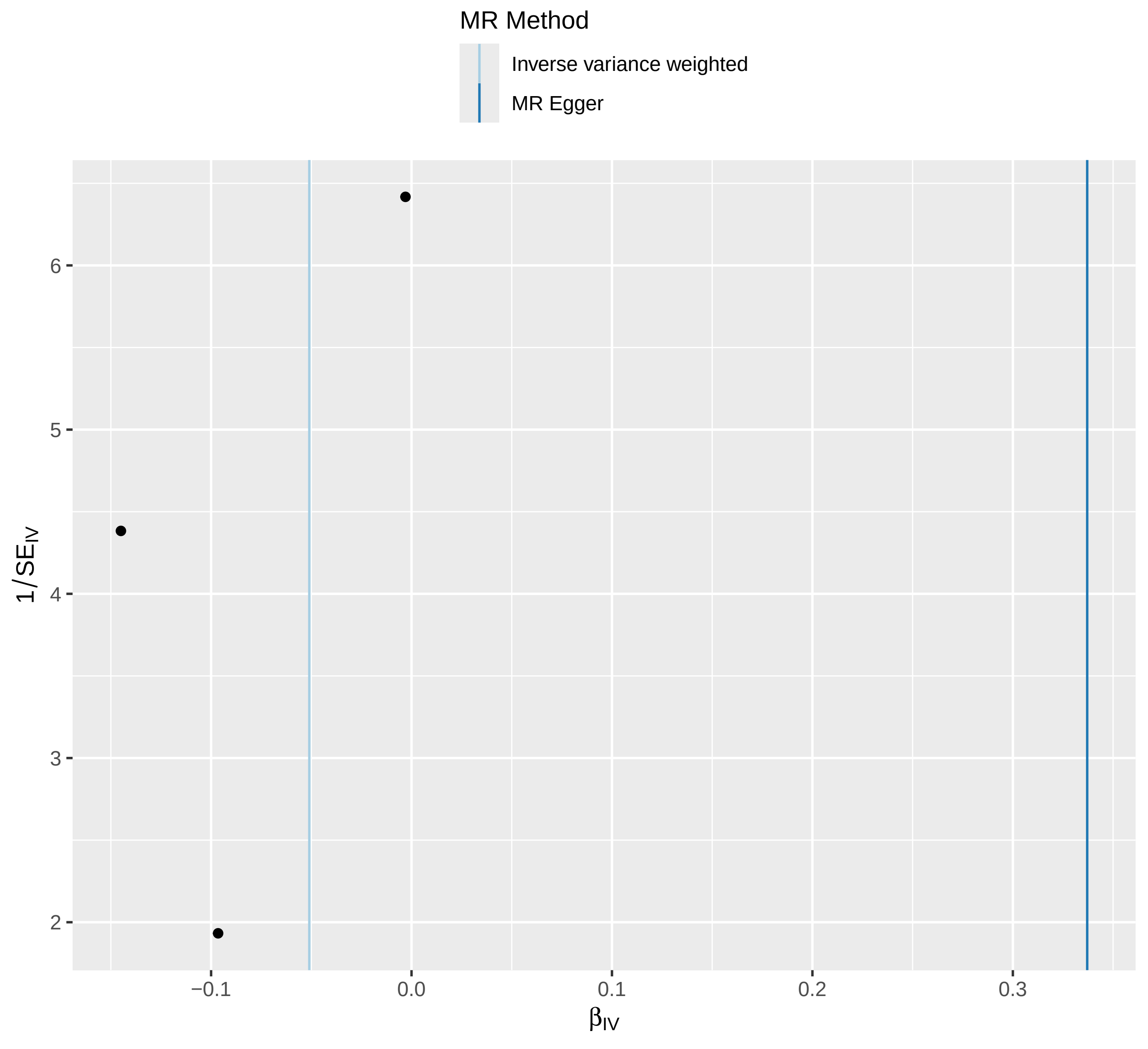

MR Method
Inverse variance weighted
MR Egger
6
5
4
3
2
1/SE_IV
−0.1
0.0
0.1
0.2
0.3
β_IV

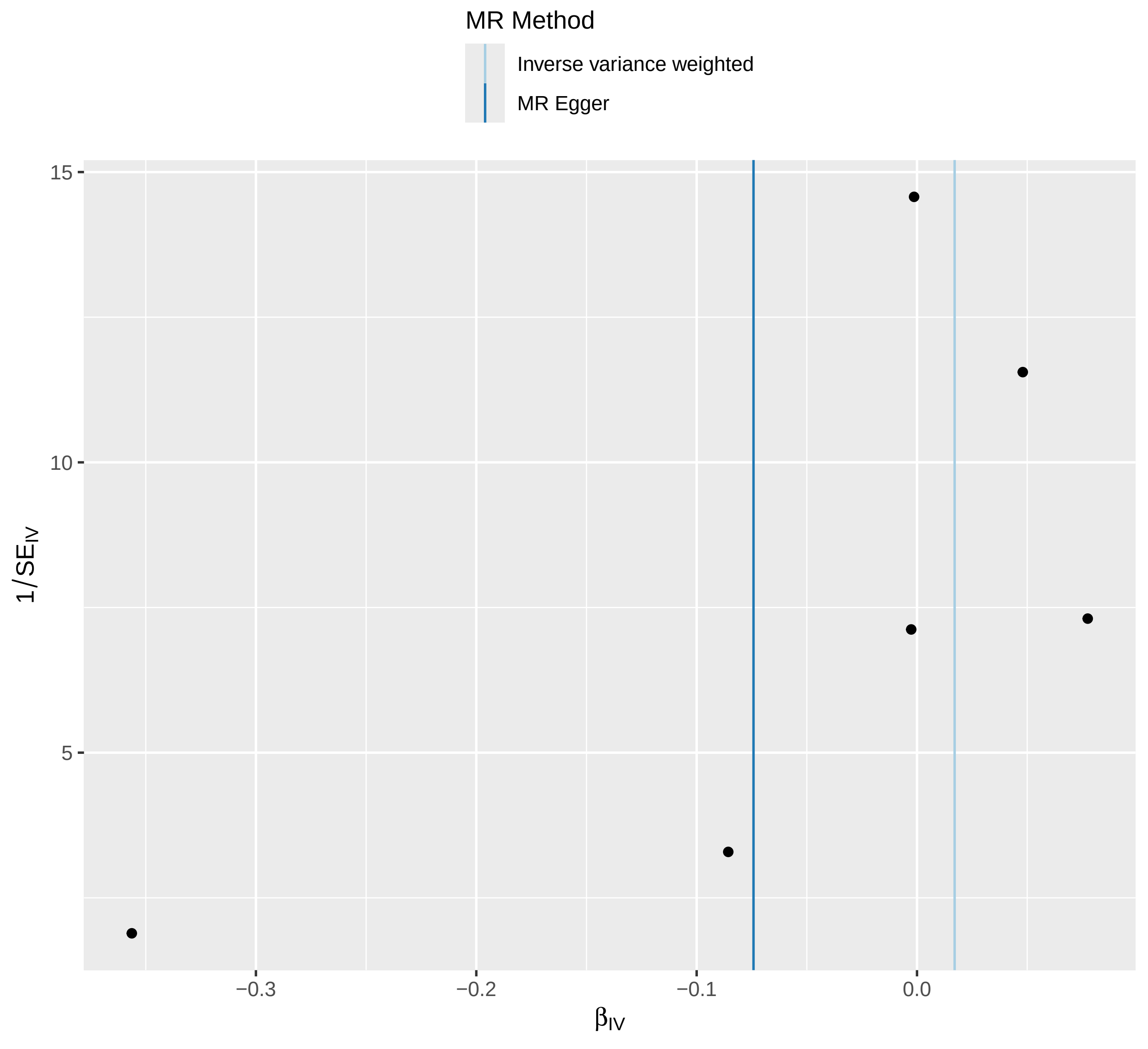
MR Method
Inverse variance weighted
MR Egger
15
10
5
1/SE_IV
−0.3
−0.2
−0.1
0.0
β_IV

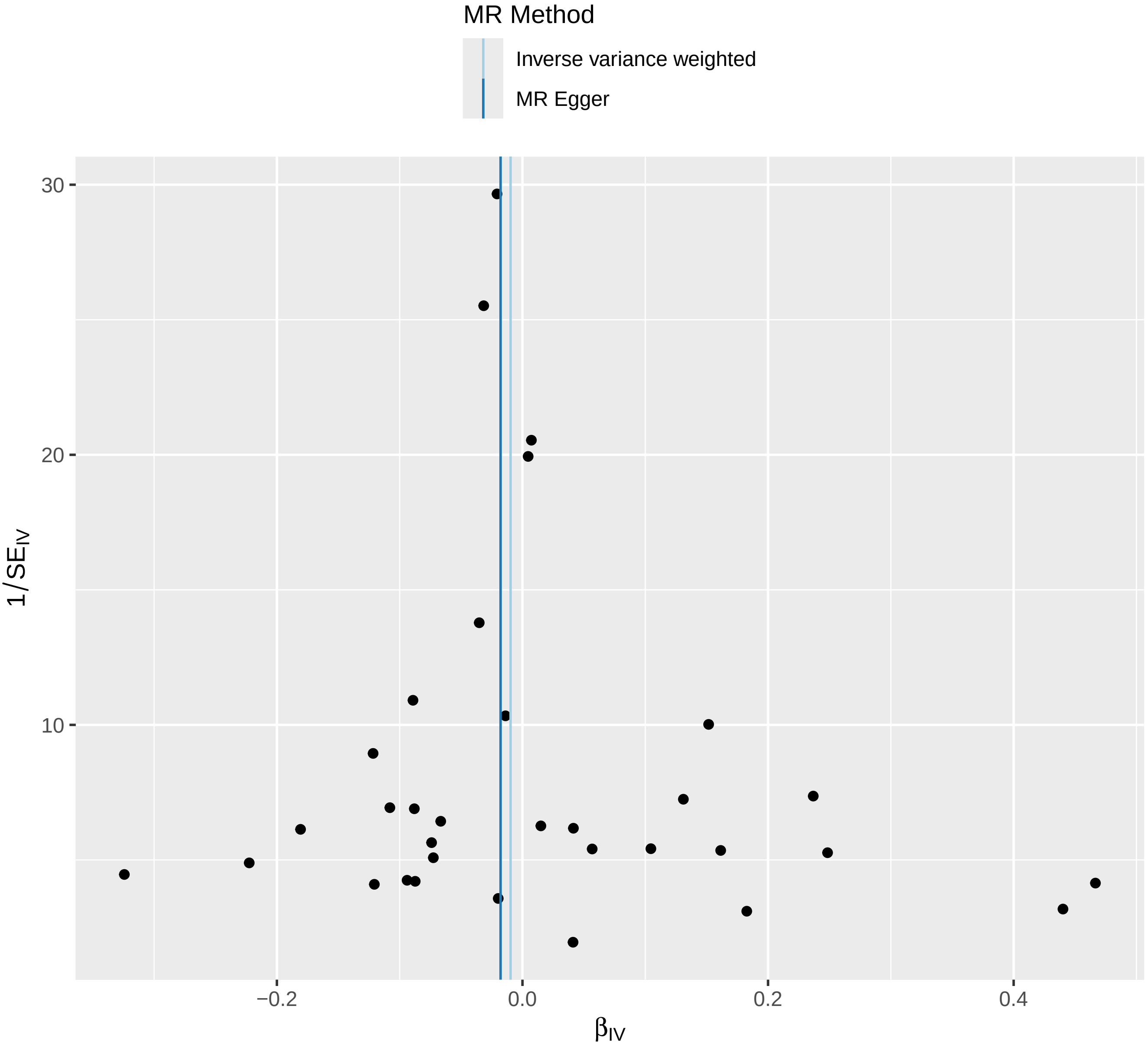
MR Method
Inverse variance weighted
MR Egger
30
20
10
1/SE$_{IV}$
−0.2
0.0
0.2
0.4
β$_{IV}$

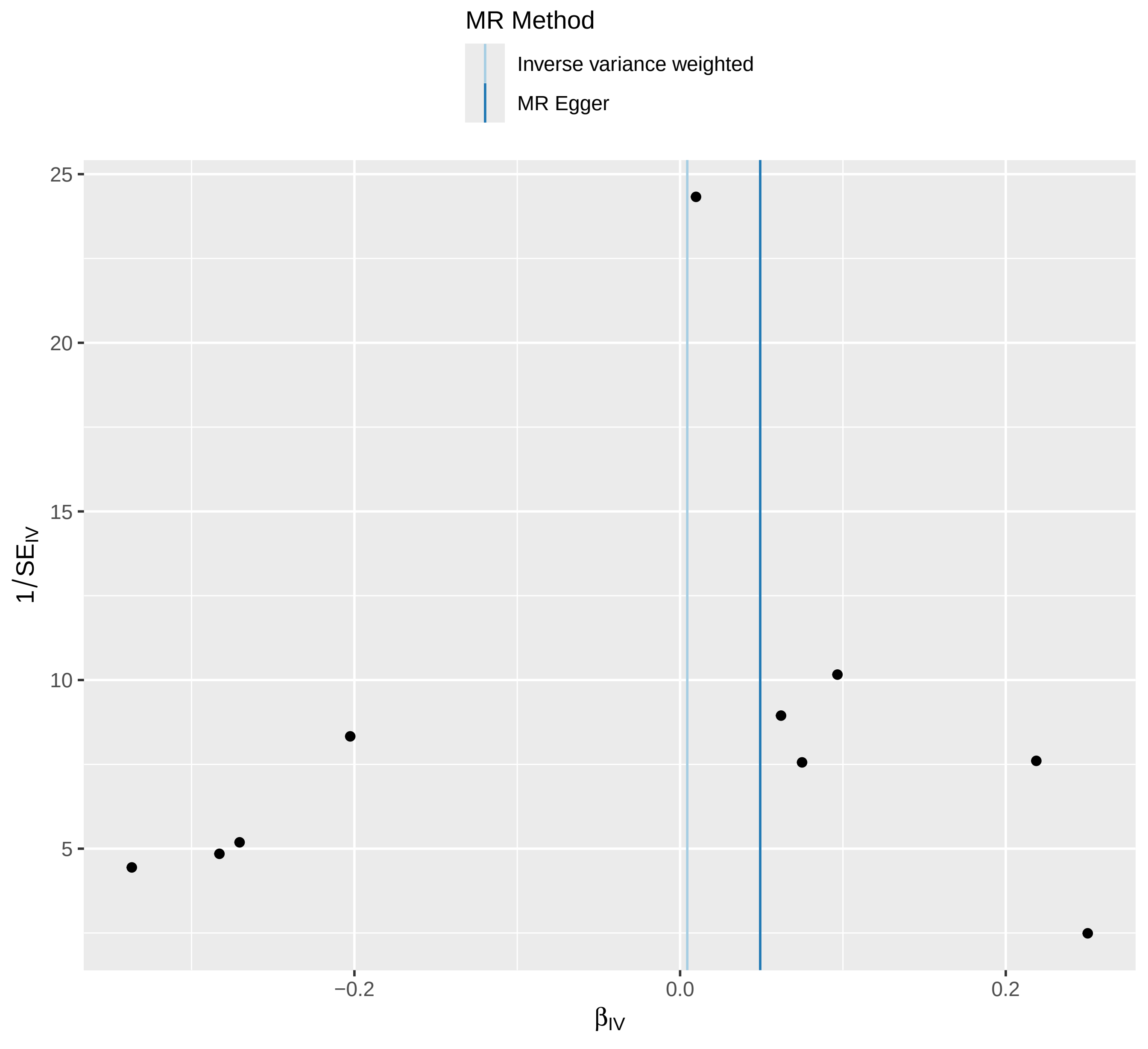
MR Method
Inverse variance weighted
MR Egger
25
20
15
10
5
1/SE$_{IV}$
−0.2
0.0
0.2
β$_{IV}$

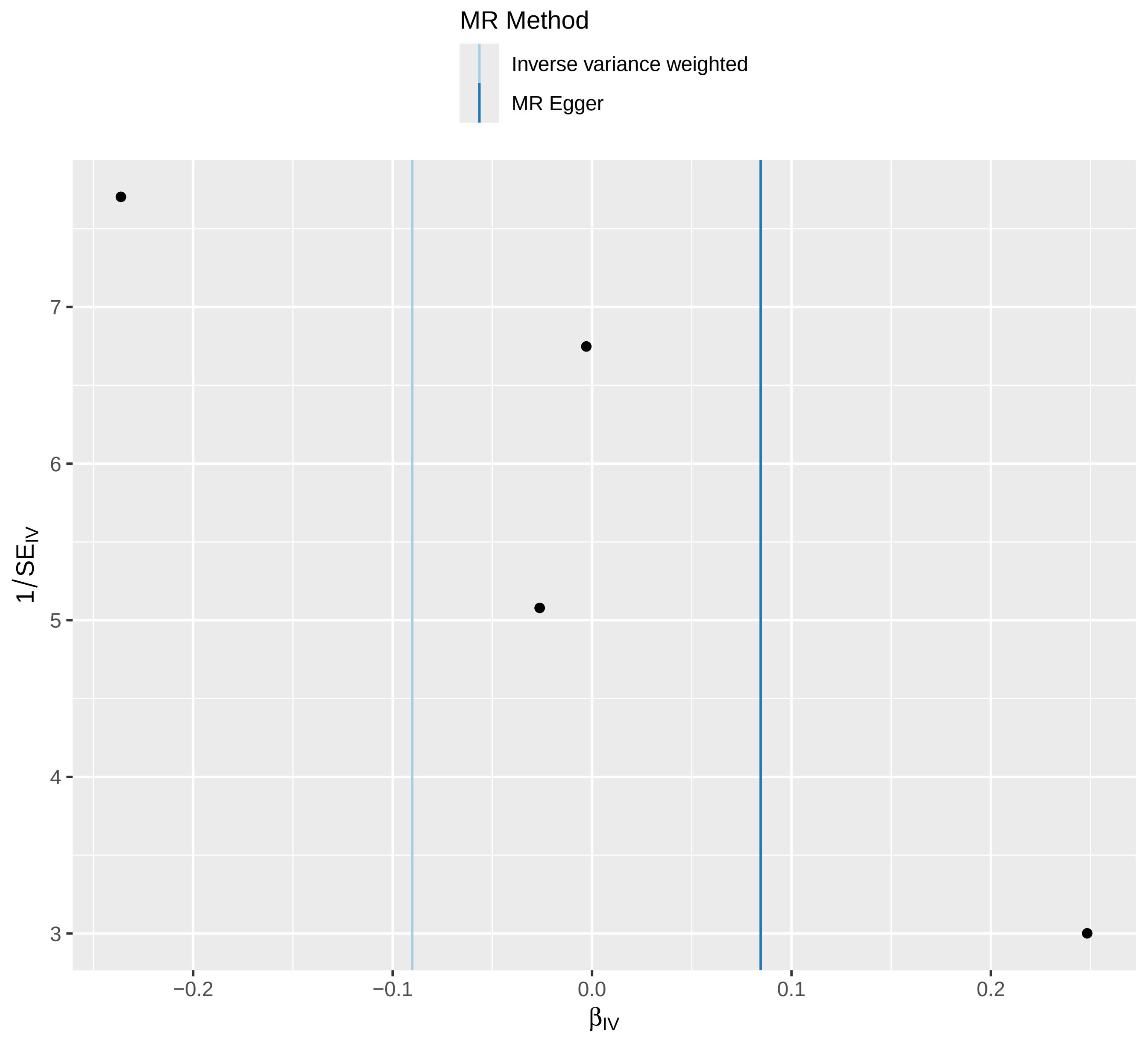
MR Method
Inverse variance weighted
MR Egger
7
6
5
4
3
1/SE$_{IV}$
−0.2
−0.1
0.0
0.1
0.2
$\beta_{IV}$

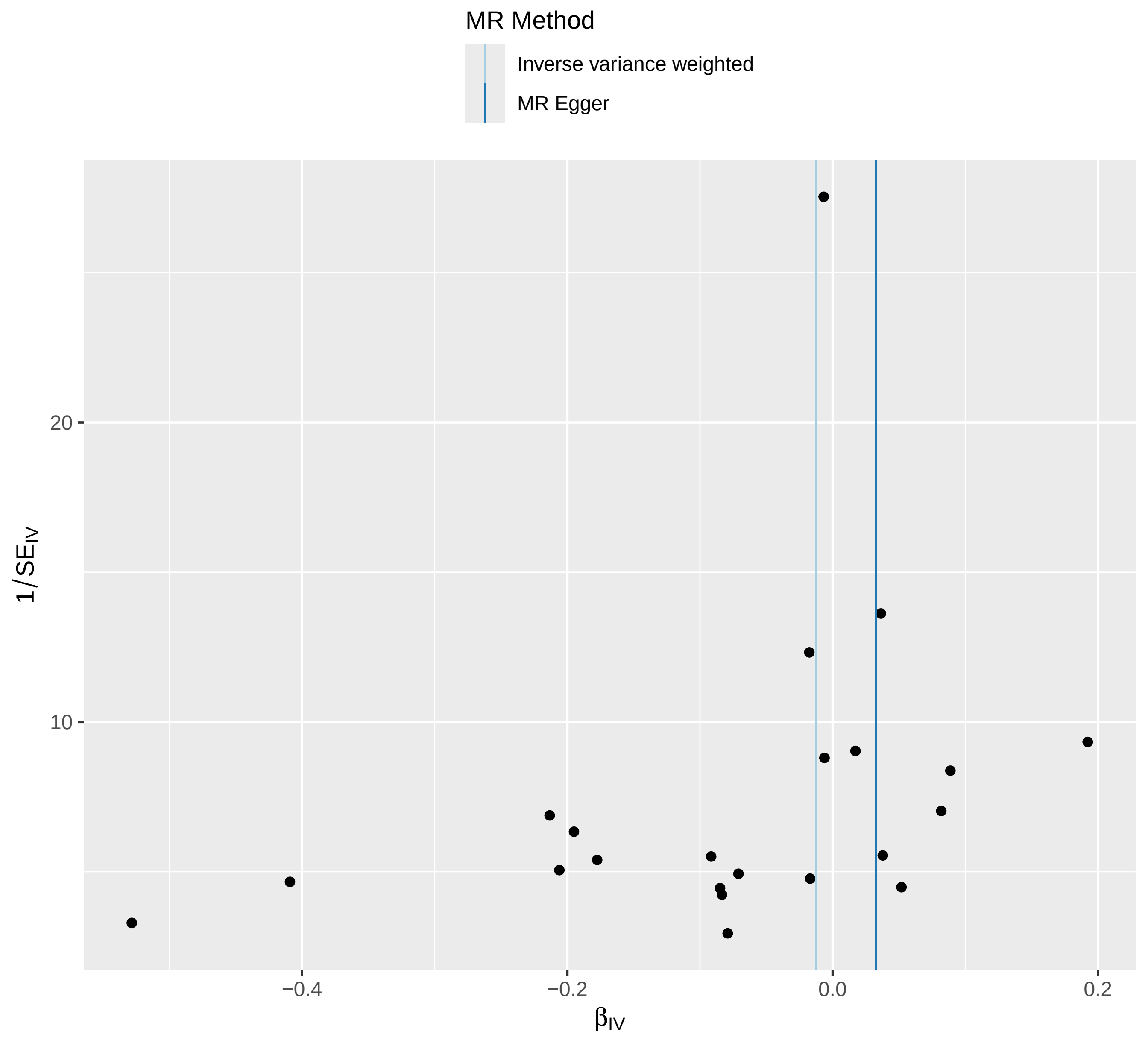
MR Method
Inverse variance weighted
MR Egger
20
10
1/SE_IV
−0.4
−0.2
0.0
0.2
β_IV

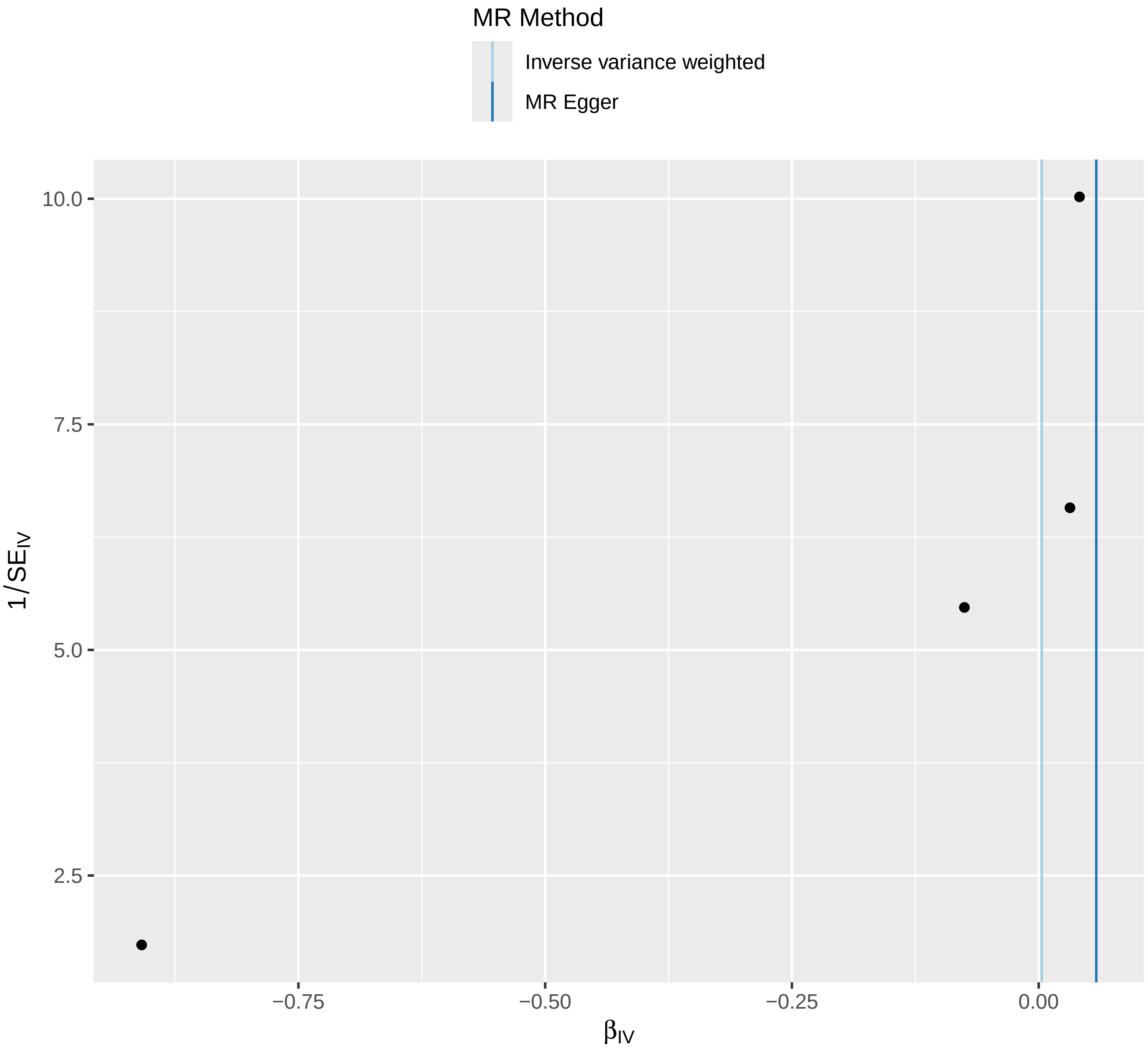

MR Method
Inverse variance weighted
MR Egger
10.0
7.5
5.0
2.5
1/SE$_{IV}$
−0.75
−0.50
−0.25
0.00
β$_{IV}$

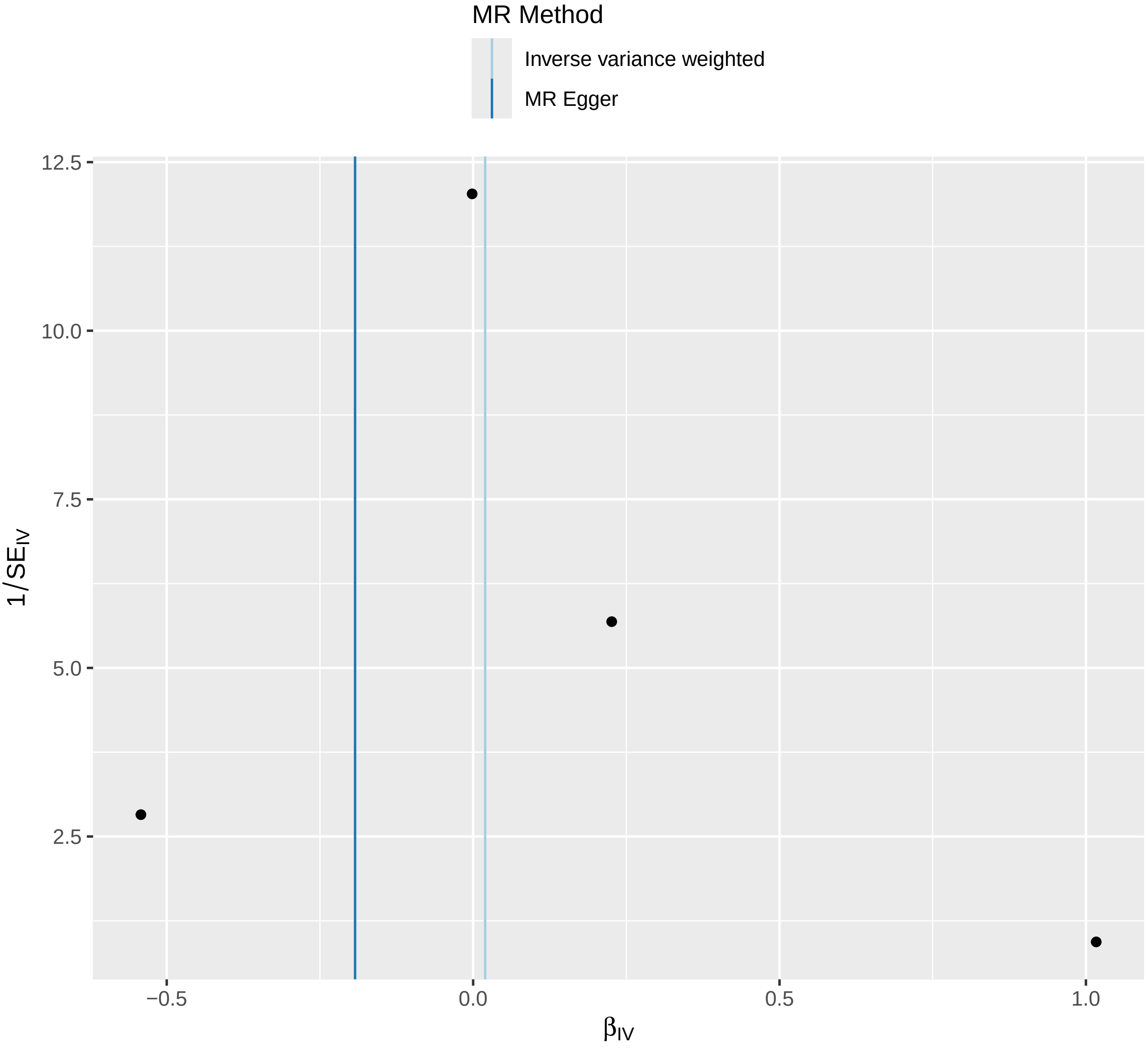

MR Method
Inverse variance weighted
MR Egger
12.5
10.0
7.5
5.0
2.5
$1/SE_{IV}$
−0.5
0.0
0.5
1.0
$\beta_{IV}$

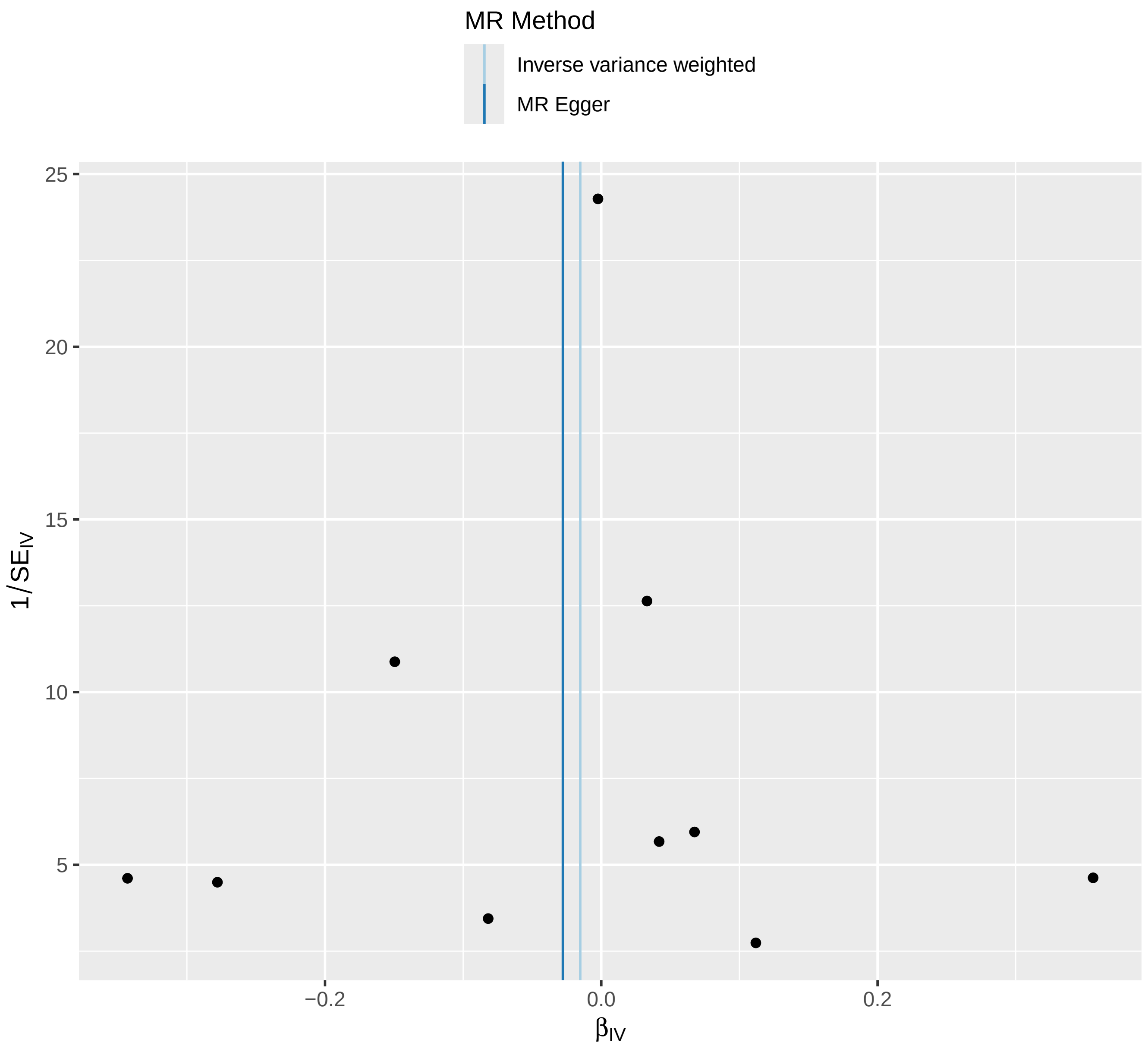
MR Method
Inverse variance weighted
MR Egger
25
20
15
10
5
1/SE$_{IV}$
−0.2
0.0
0.2
β$_{IV}$

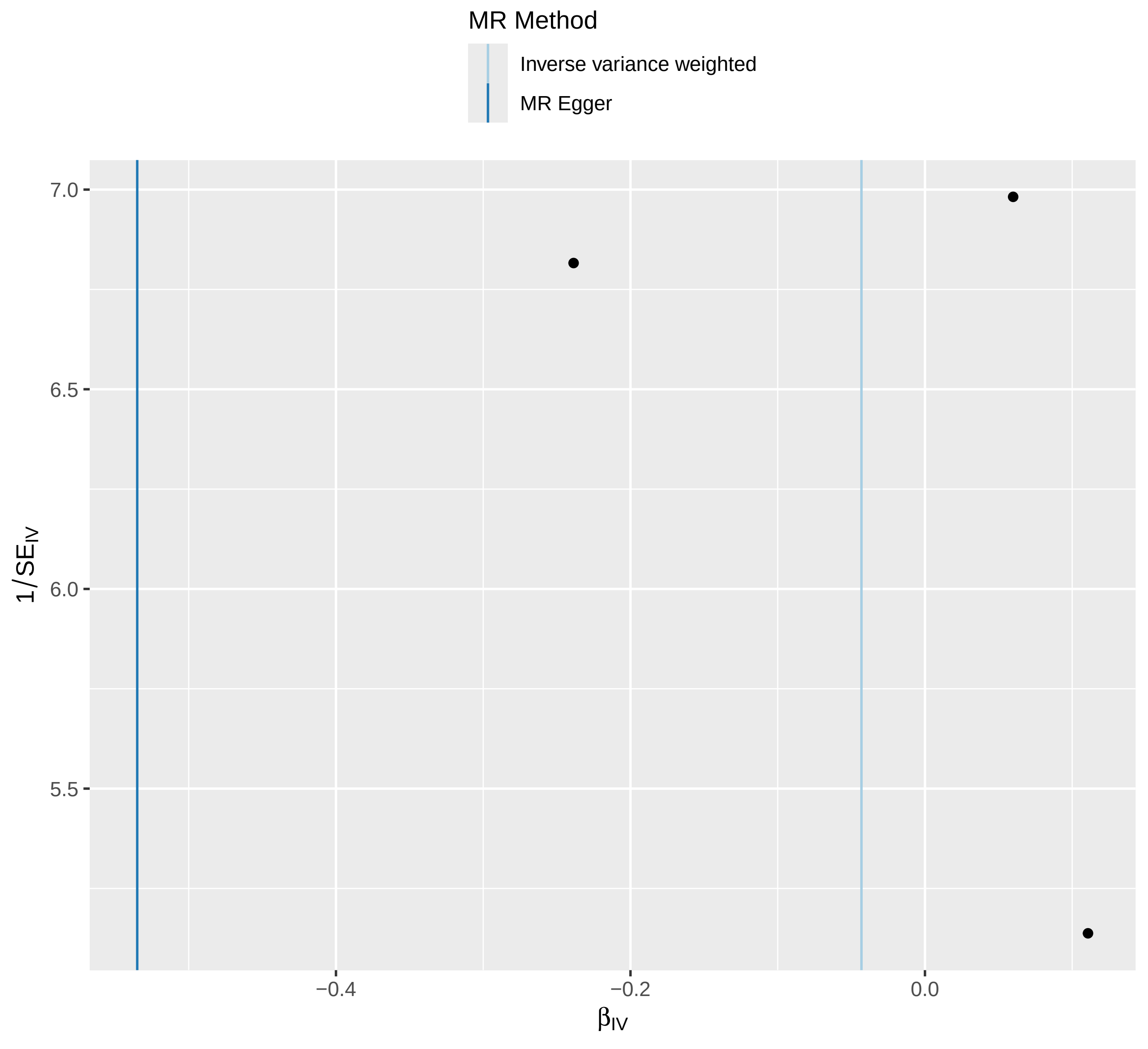

MR Method
Inverse variance weighted
MR Egger
7.0
6.5
6.0
5.5
1/SE_IV
−0.4
−0.2
0.0
β_IV

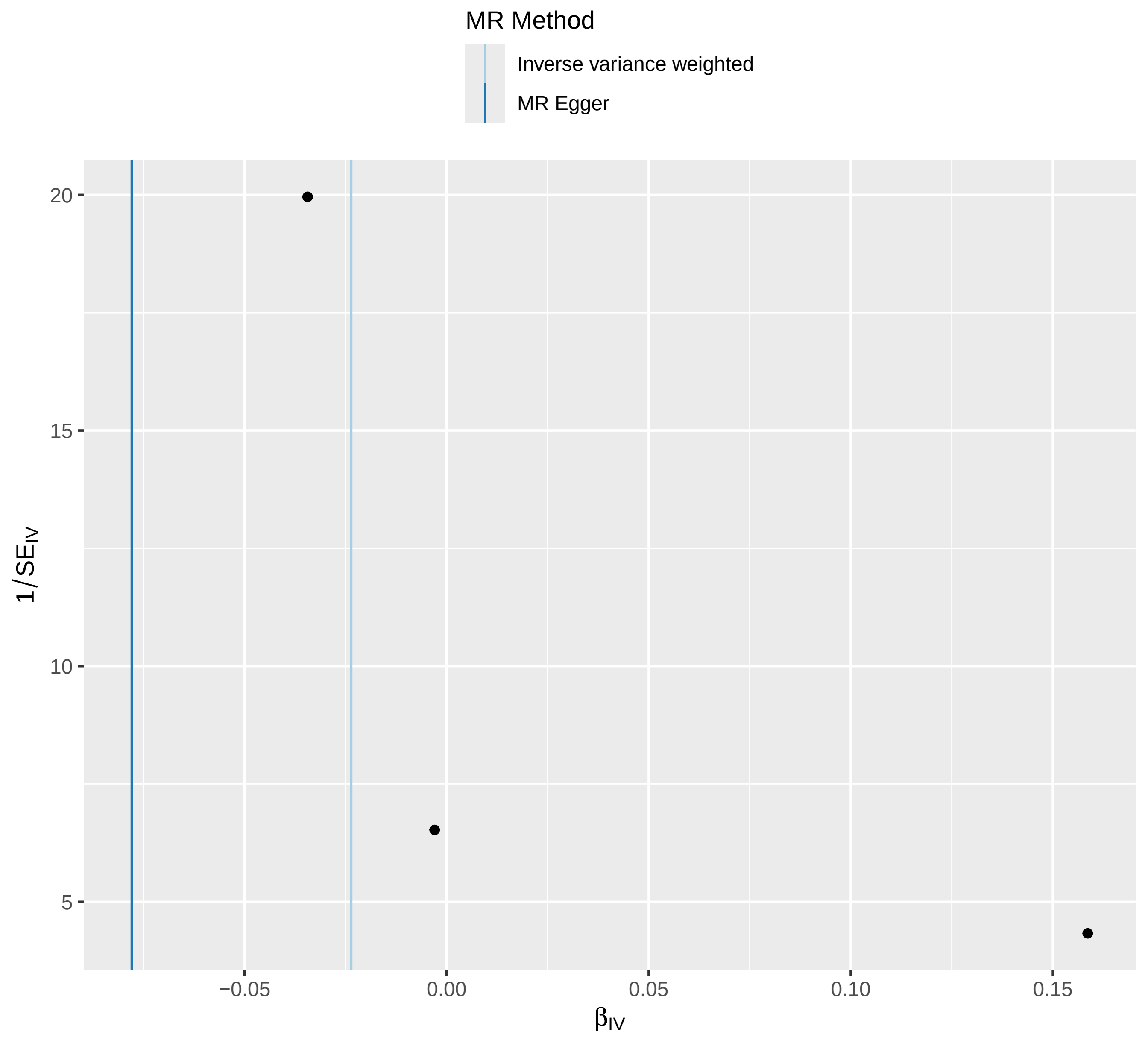

MR Method
Inverse variance weighted
MR Egger
1/SE_IV
20
15
10
5
−0.05
0.00
0.05
0.10
0.15
β_IV

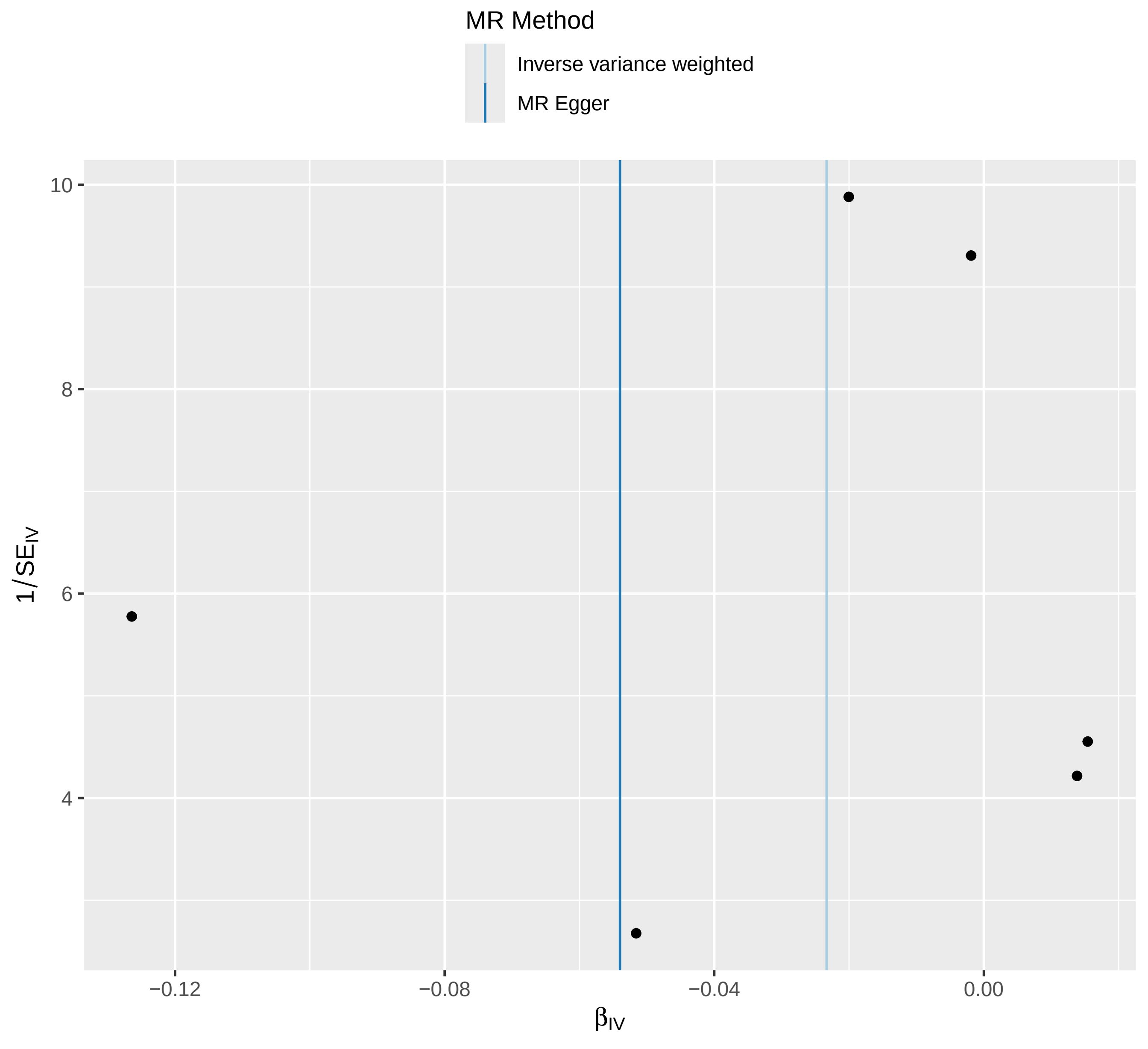
MR Method
Inverse variance weighted
MR Egger
10
8
6
4
$1/SE_{IV}$
−0.12
−0.08
−0.04
0.00
$\beta_{IV}$

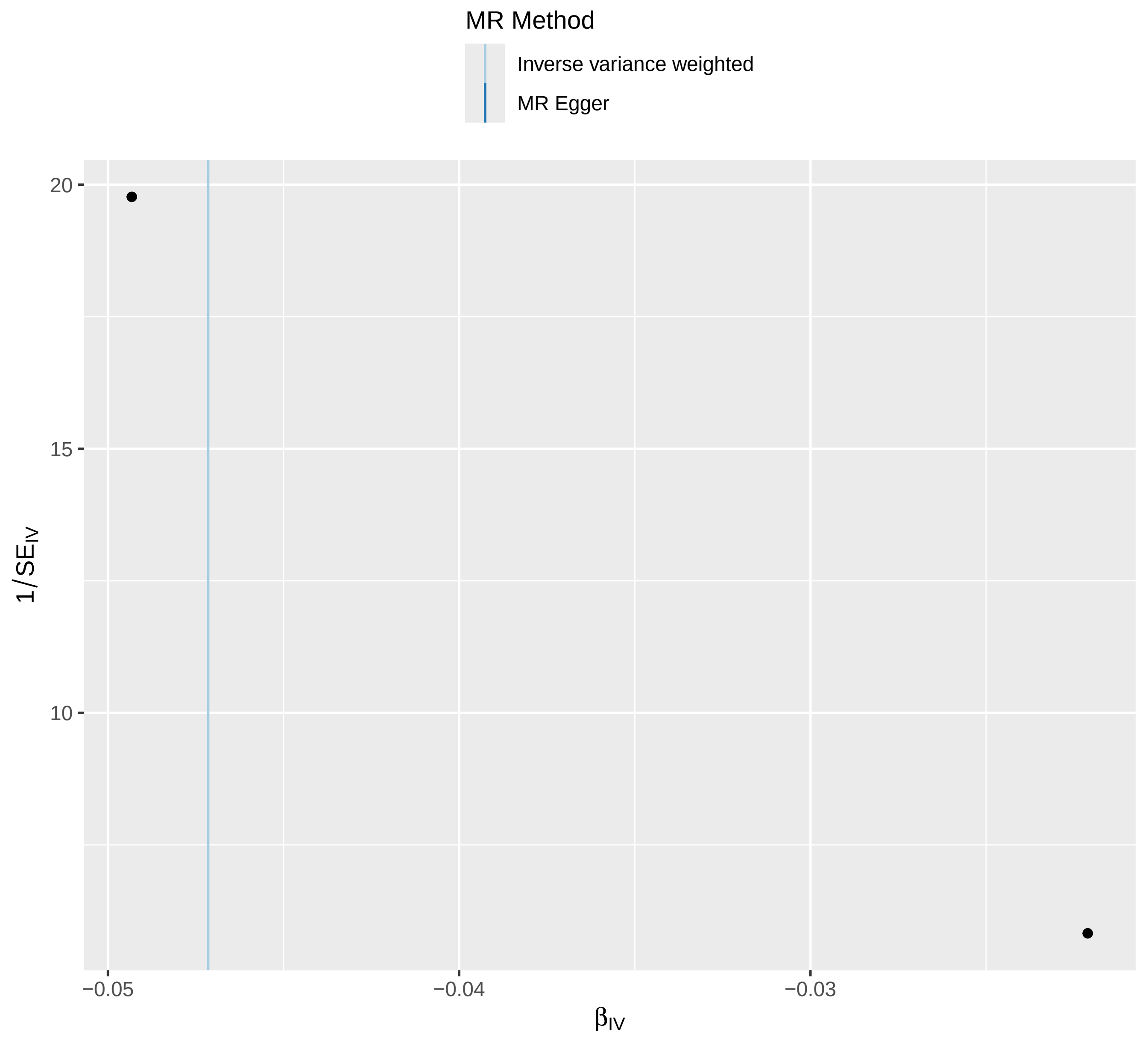
MR Method
Inverse variance weighted
MR Egger
20
15
10
1/SE$_{IV}$
−0.05
−0.04
−0.03
β$_{IV}$

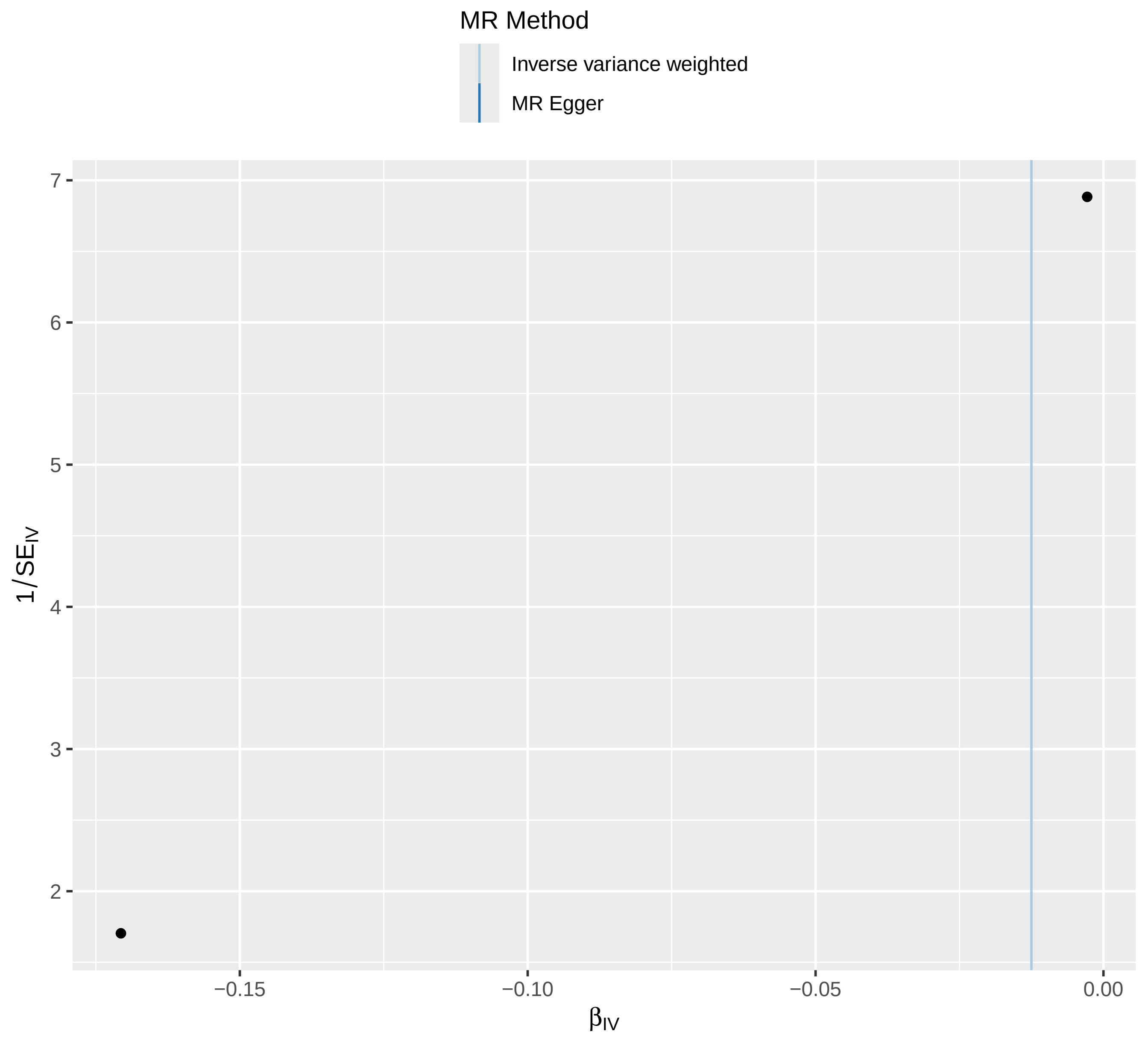
MR Method
Inverse variance weighted
MR Egger
7
6
5
4
3
2
$1/SE_{IV}$
−0.15
−0.10
−0.05
0.00
$\beta_{IV}$

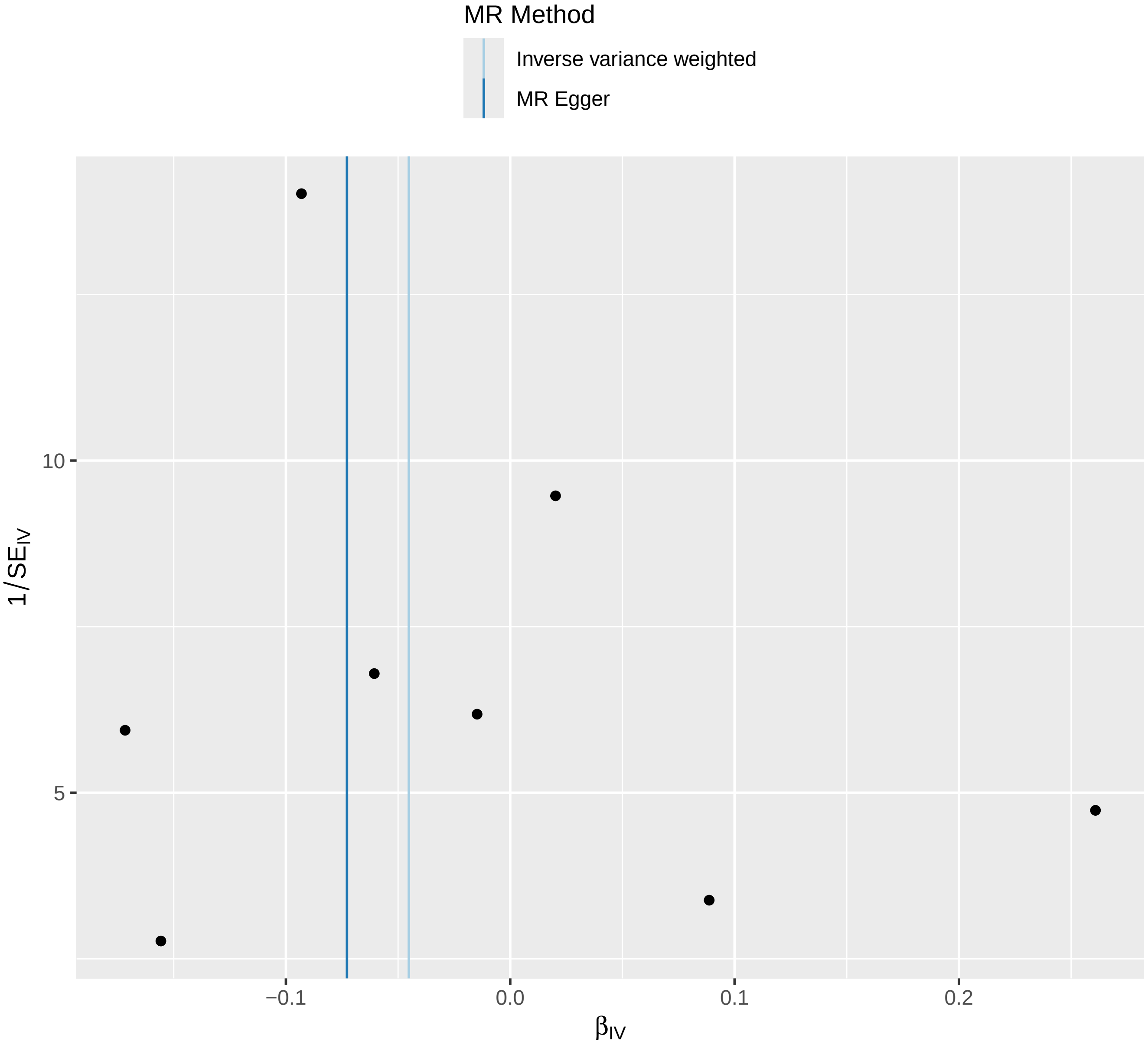

MR Method
Inverse variance weighted
MR Egger
10
5
1/SE$_{IV}$
−0.1
0.0
0.1
0.2
β$_{IV}$

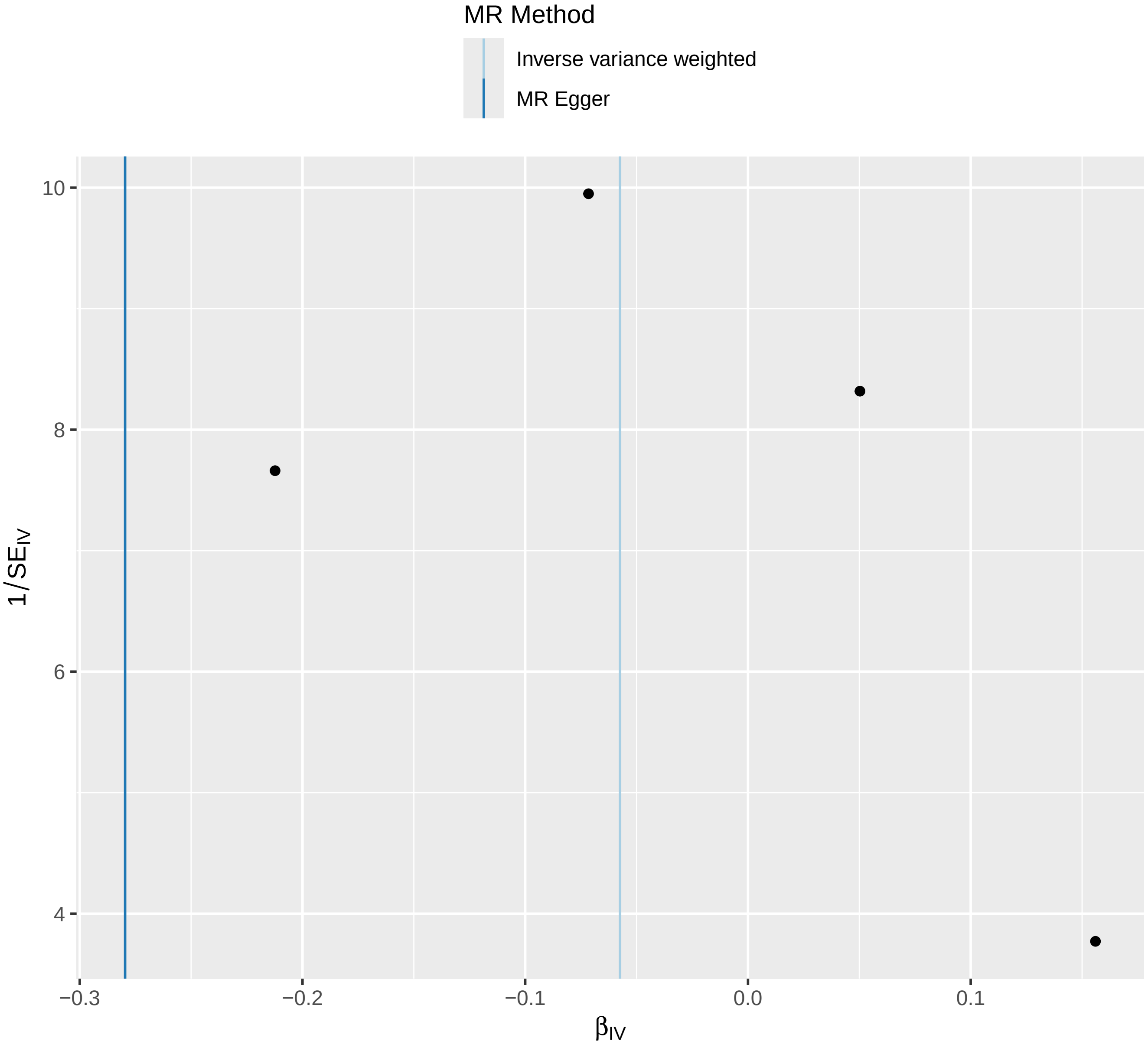
MR Method
Inverse variance weighted
MR Egger
10
8
6
4
1/SE$_{IV}$
−0.3
−0.2
−0.1
0.0
0.1
β$_{IV}$

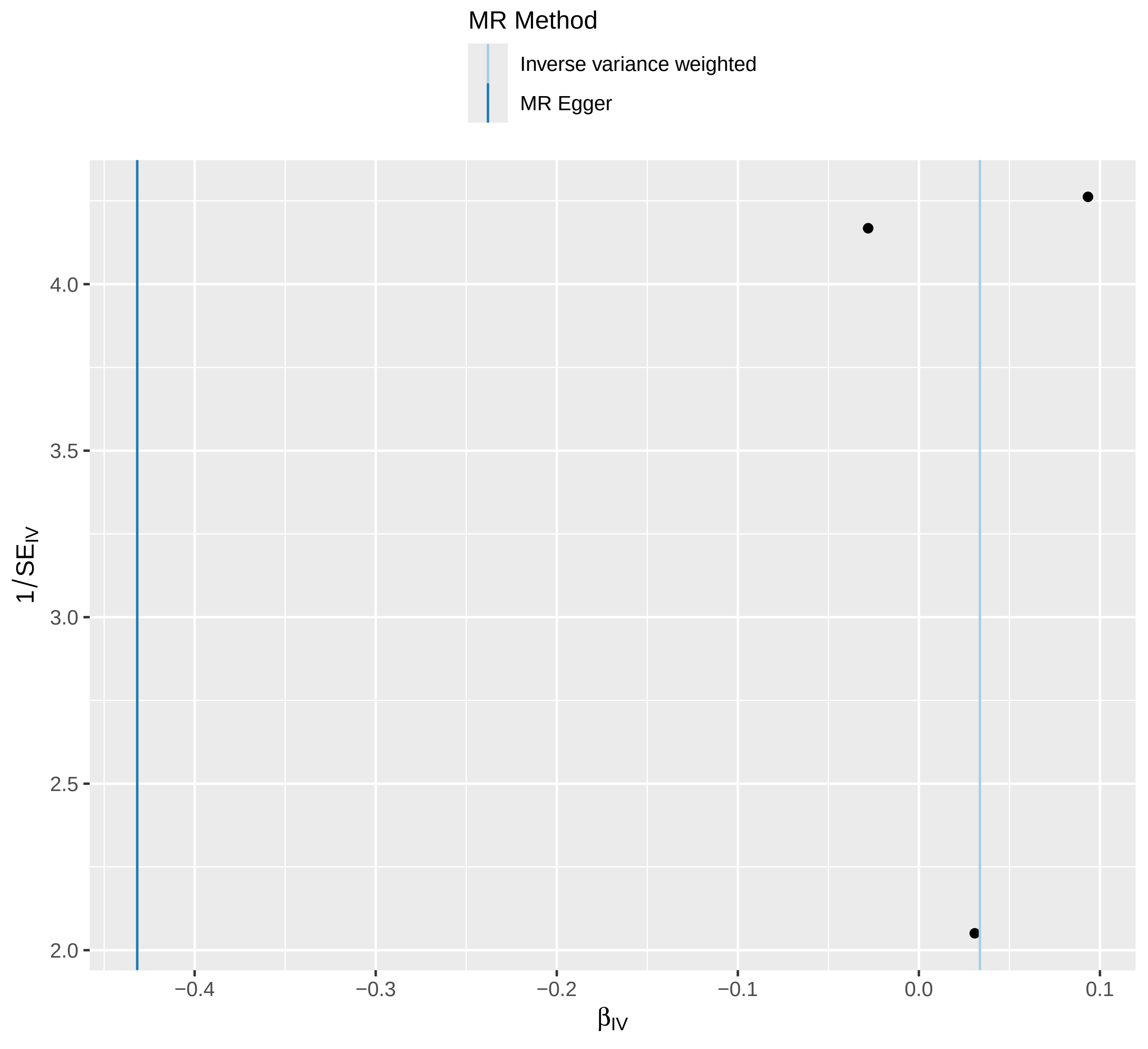

MR Method
Inverse variance weighted
MR Egger
4.0
3.5
3.0
2.5
2.0
1/SE_IV
−0.4
−0.3
−0.2
−0.1
0.0
0.1
β_IV

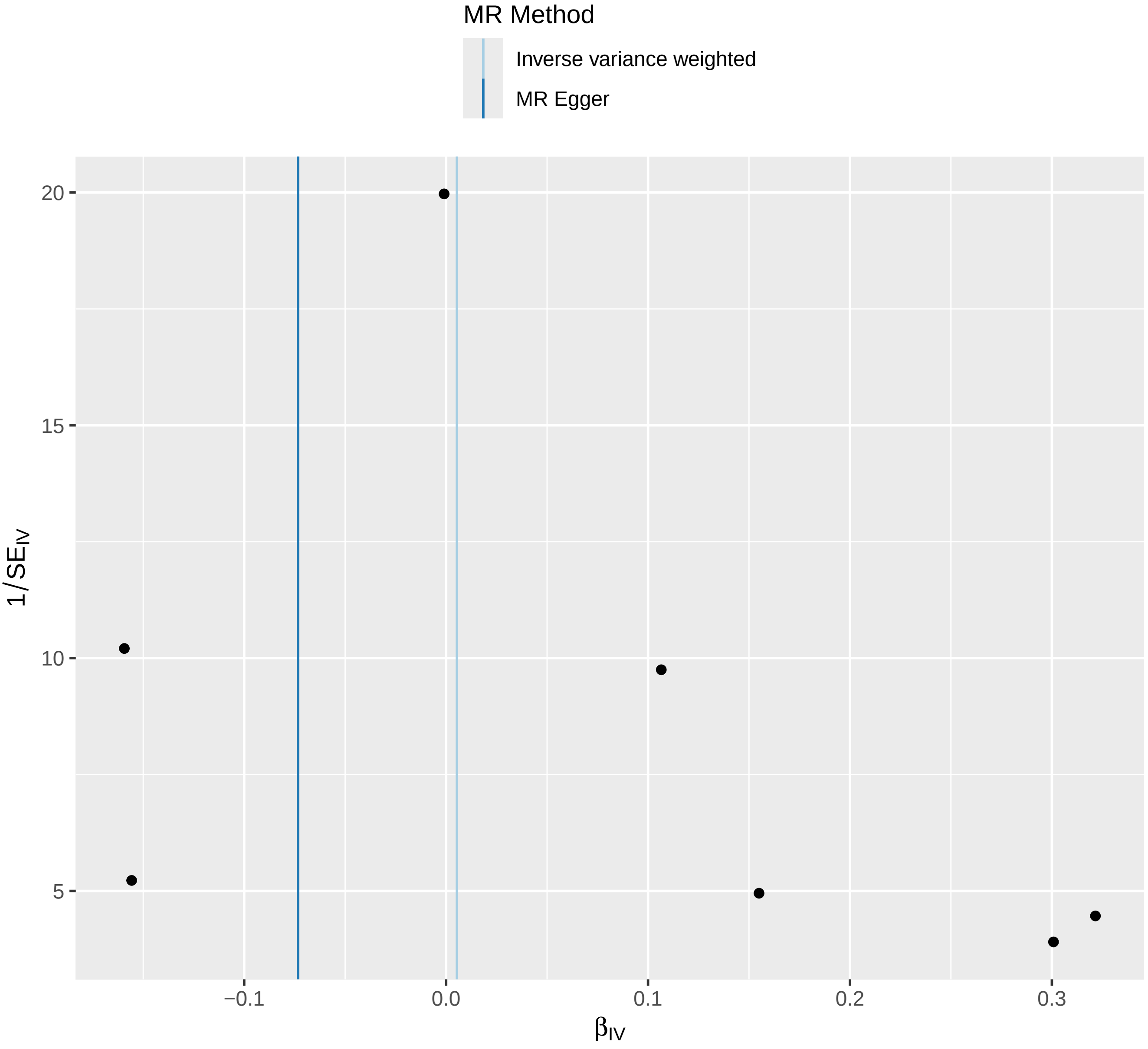
MR Method
Inverse variance weighted
MR Egger
20
15
10
5
$1/SE_{IV}$
−0.1
0.0
0.1
0.2
0.3
$\beta_{IV}$

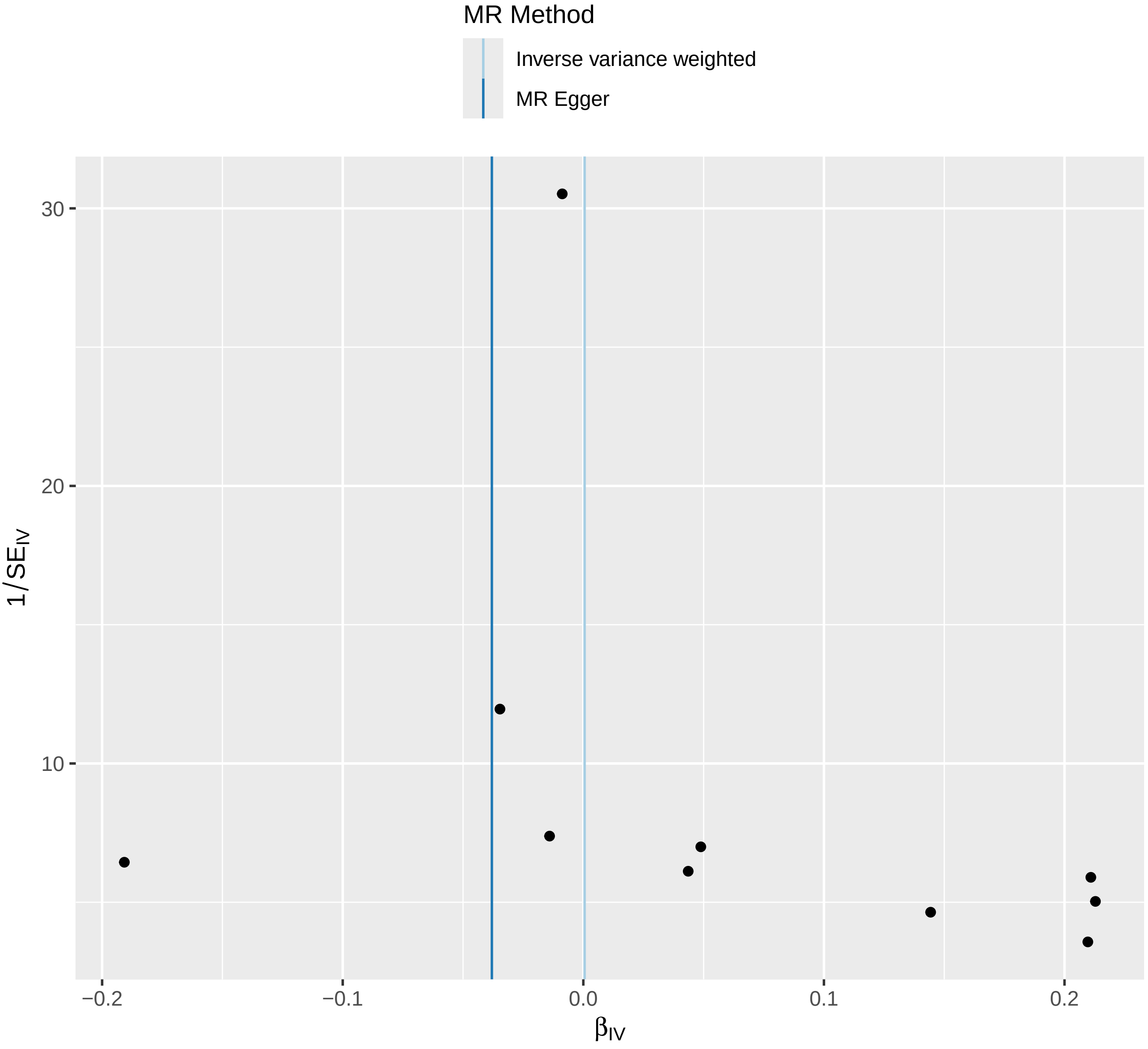

MR Method
Inverse variance weighted
MR Egger
30
20
10
1/SE_IV
−0.2
−0.1
0.0
0.1
0.2
β_IV

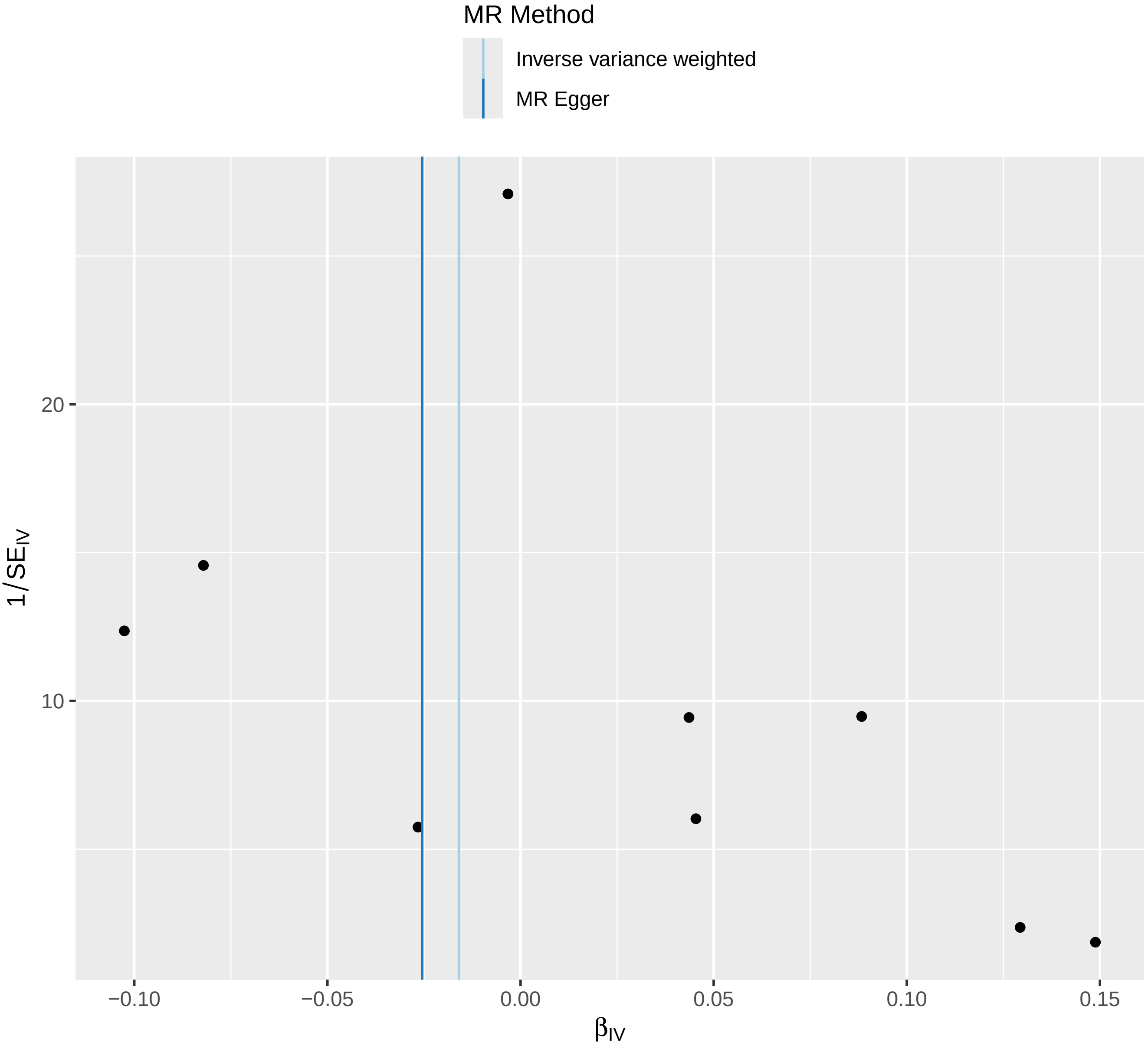
MR Method
Inverse variance weighted
MR Egger
20
10
1/SE$_{IV}$
−0.10
−0.05
0.00
0.05
0.10
0.15
β$_{IV}$

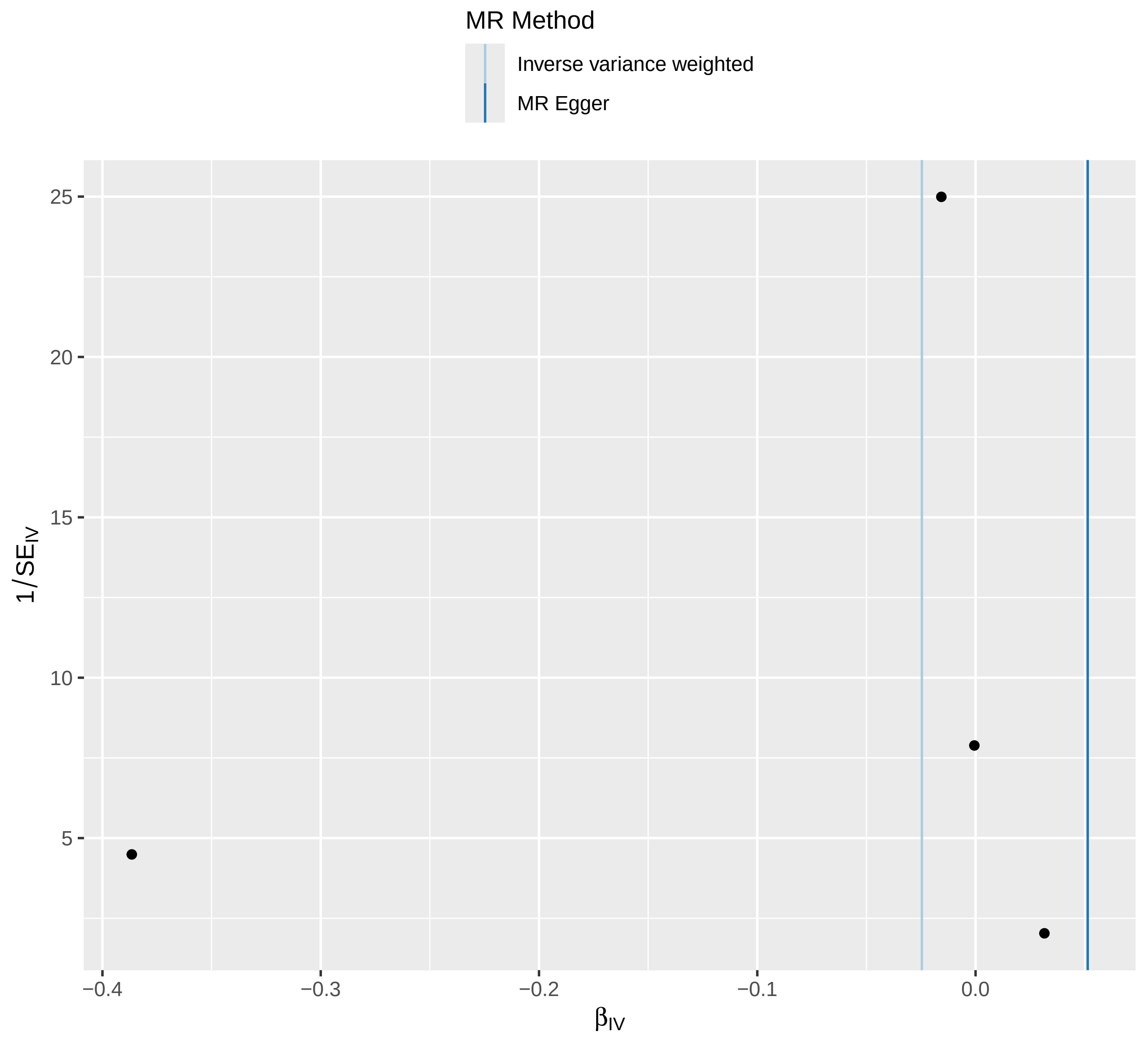
MR Method
Inverse variance weighted
MR Egger
25
20
15
10
5
1/SE_IV
−0.4
−0.3
−0.2
−0.1
0.0
β_IV

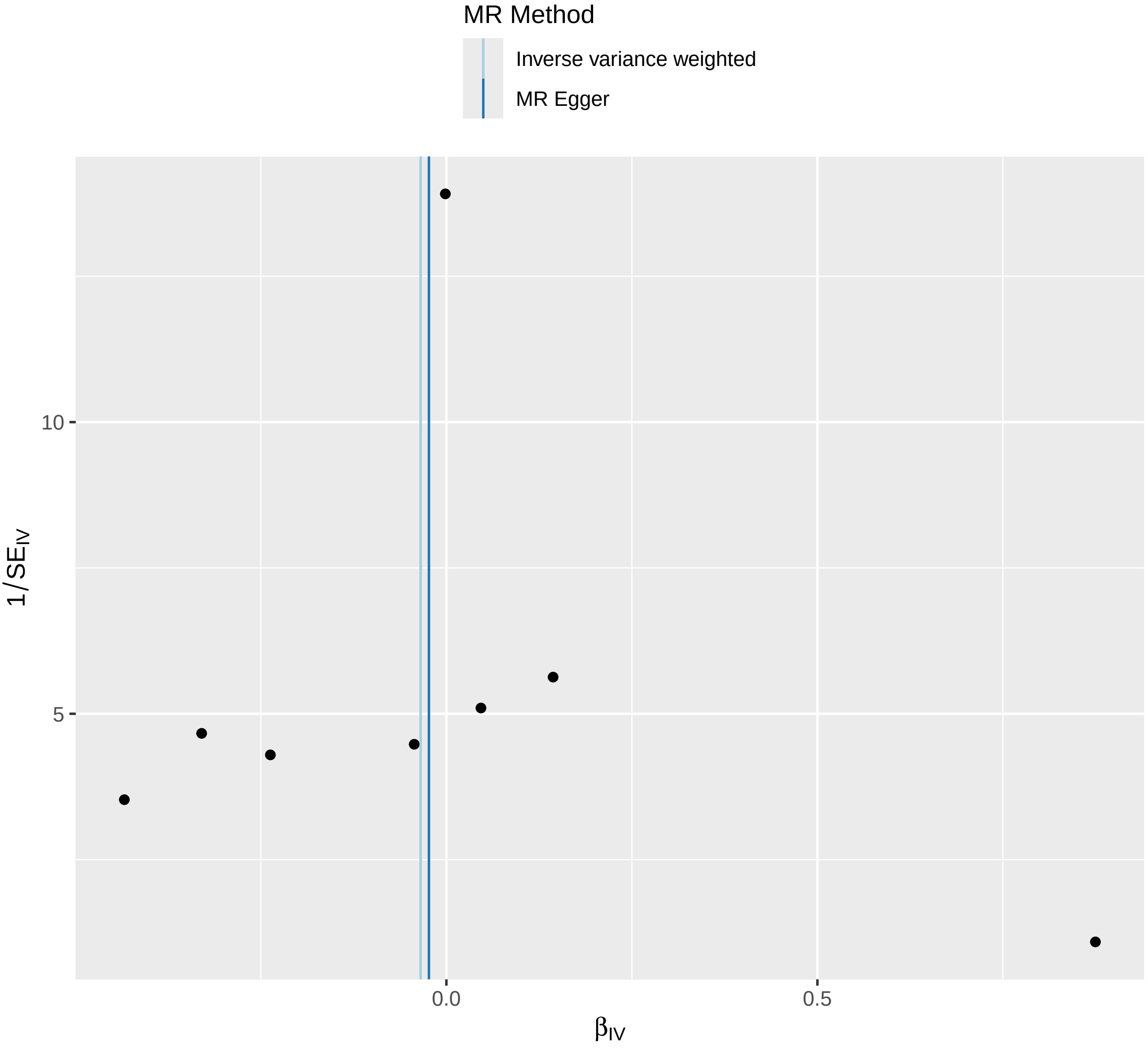
MR Method
Inverse variance weighted
MR Egger
10
5
0.0
0.5
1/SE$_{IV}$
β$_{IV}$

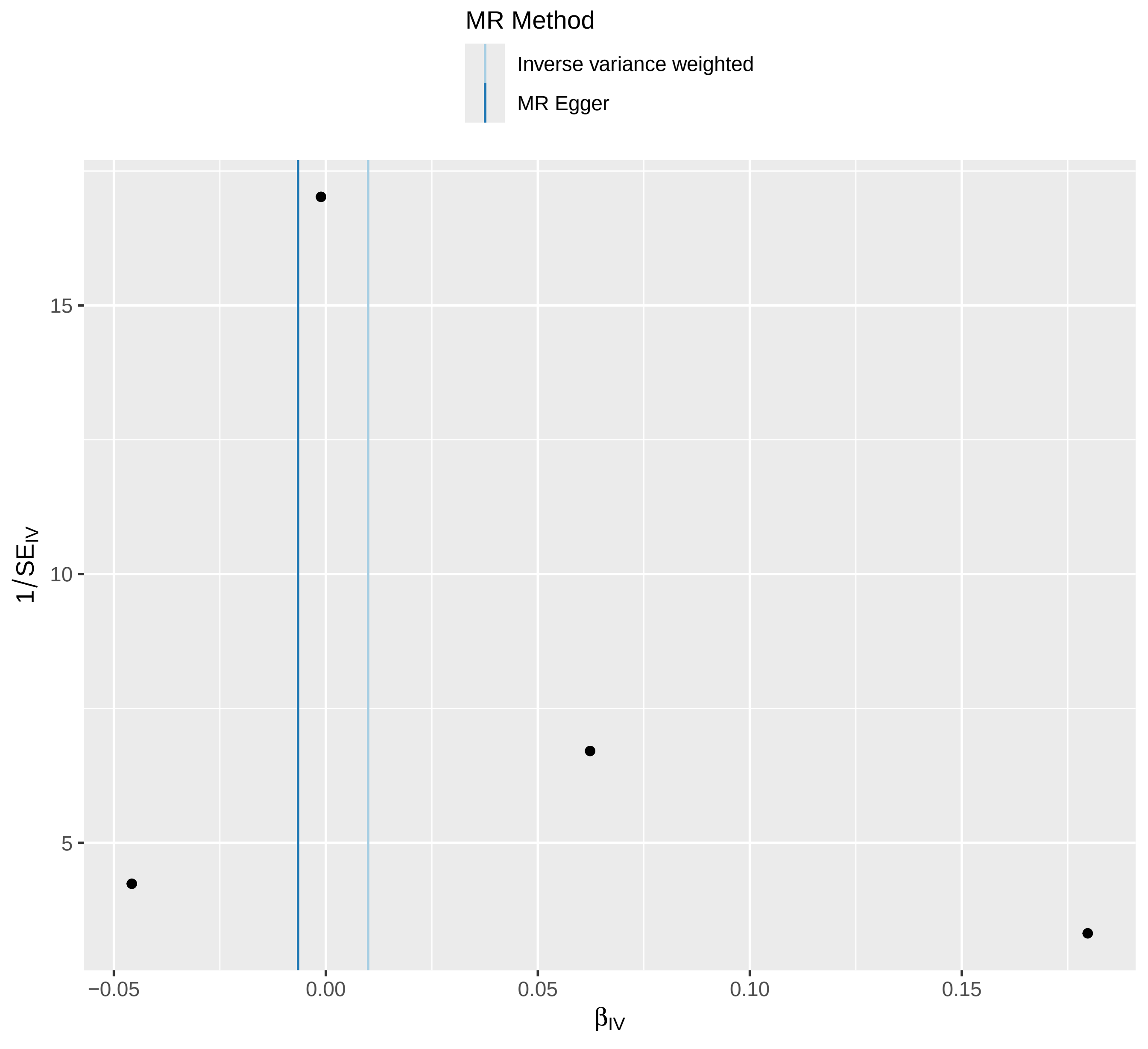

MR Method
Inverse variance weighted
MR Egger
15
10
5
$1/SE_{IV}$
−0.05
0.00
0.05
0.10
0.15
$\beta_{IV}$

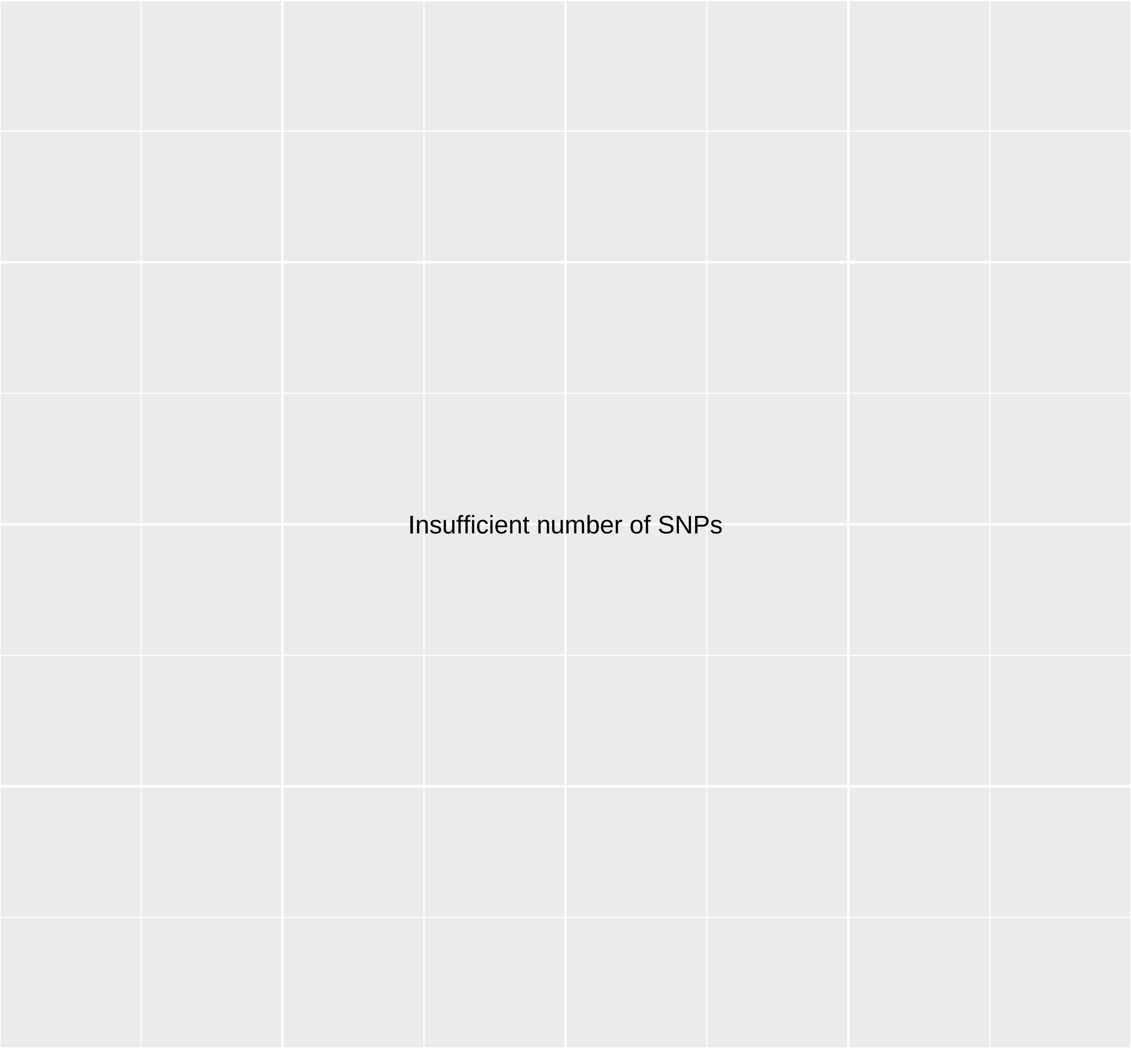
Insufficient number of SNPs

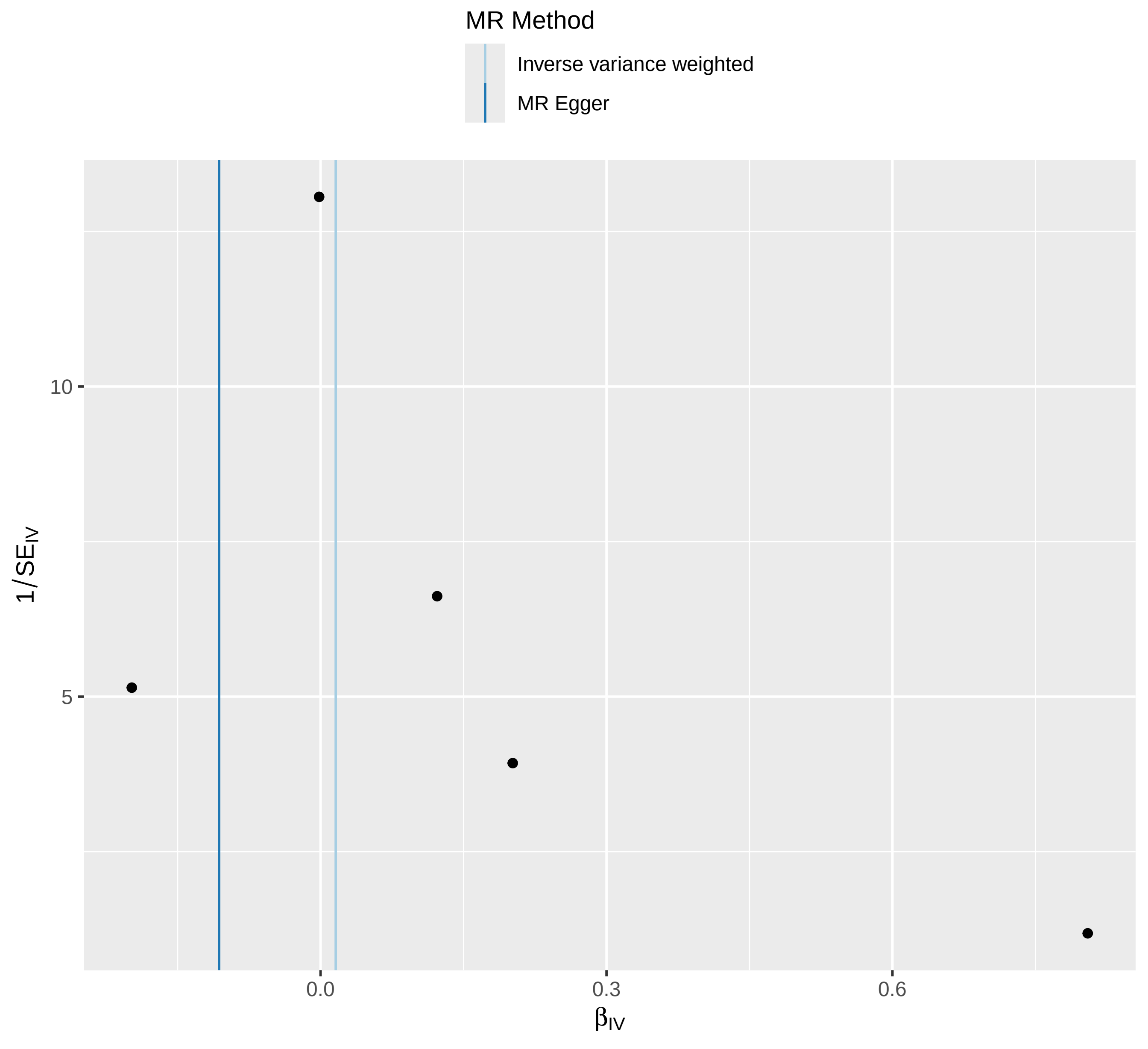
MR Method
Inverse variance weighted
MR Egger
10
5
$1/SE_{IV}$
0.0
0.3
0.6
$\beta_{IV}$

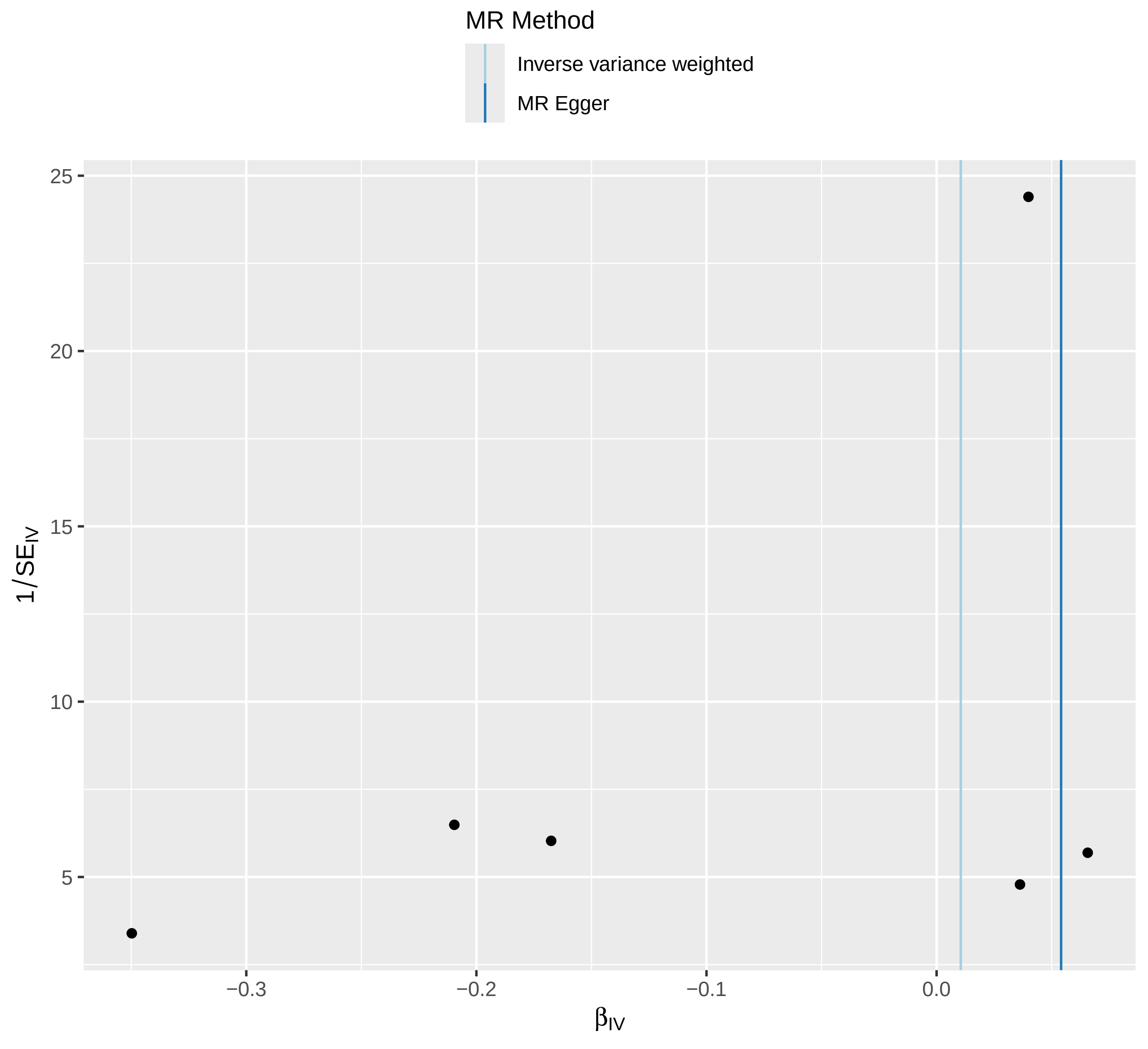

MR Method
Inverse variance weighted
MR Egger
25
20
15
10
5
1/SE$_{IV}$
−0.3
−0.2
−0.1
0.0
β$_{IV}$

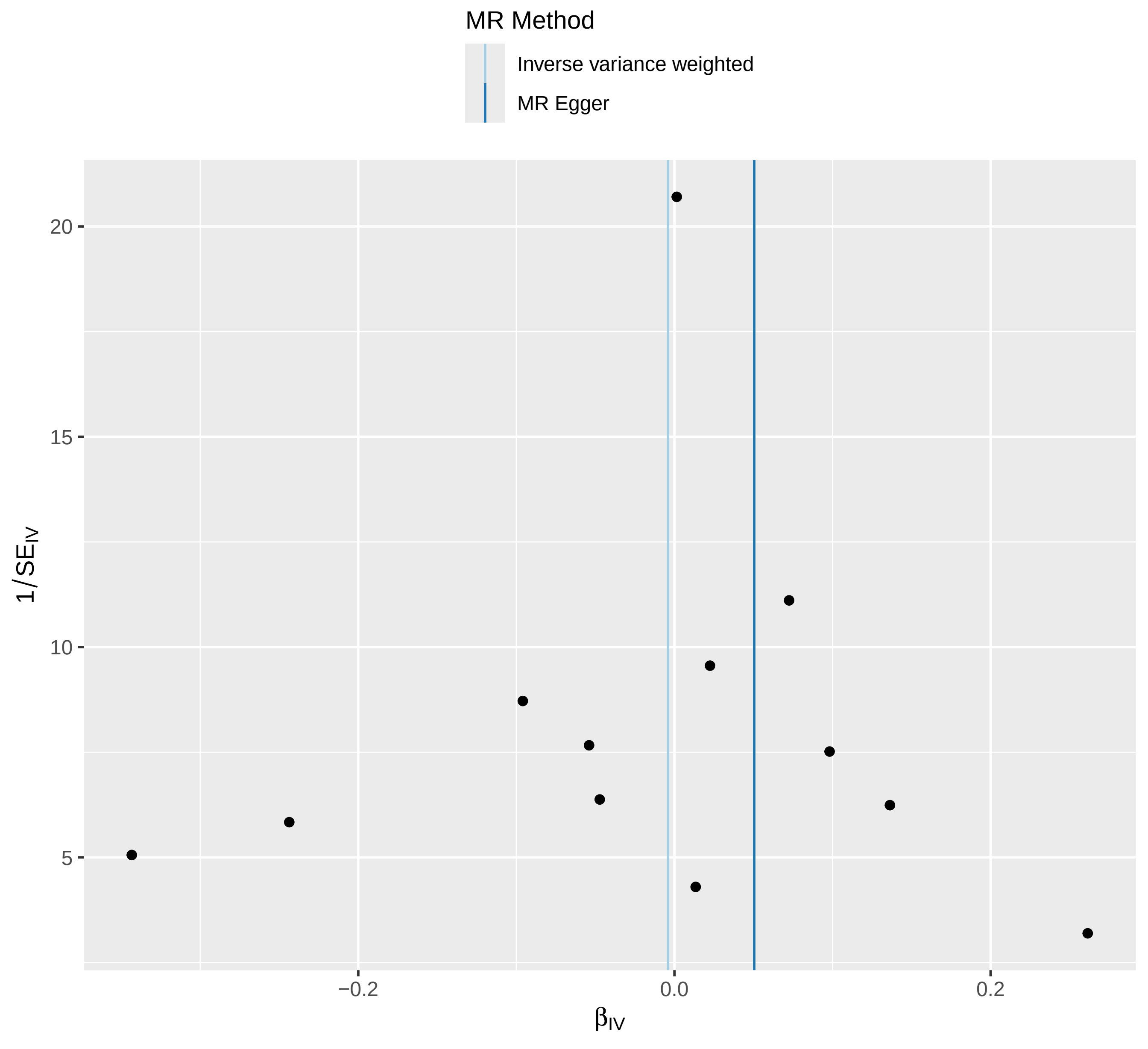

MR Method
Inverse variance weighted
MR Egger
20
15
10
5
1/SE$_{IV}$
−0.2
0.0
0.2
β$_{IV}$

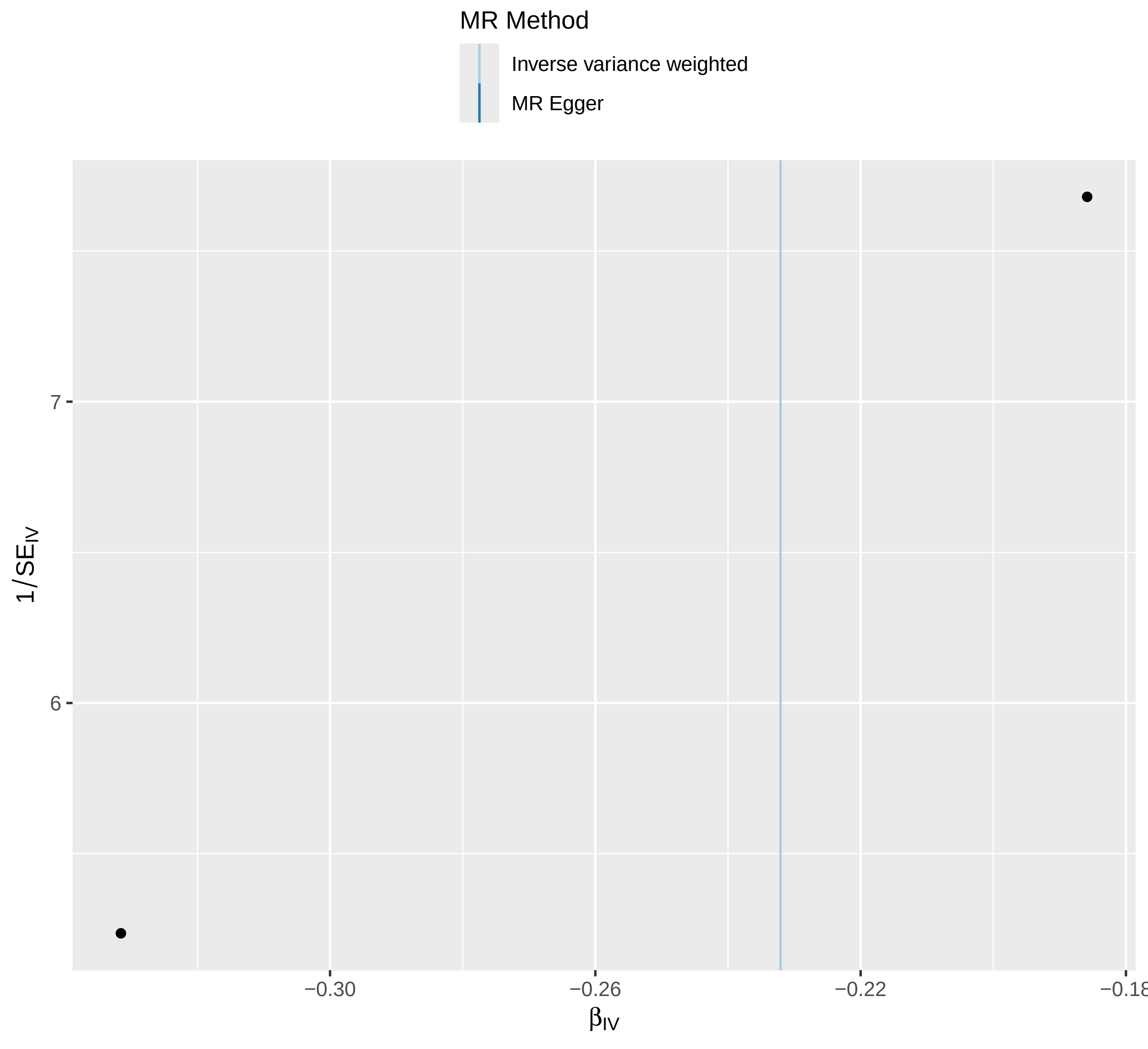

MR Method
Inverse variance weighted
MR Egger
7
6
−0.30
−0.26
−0.22
−0.18
$1/SE_{IV}$
$\beta_{IV}$

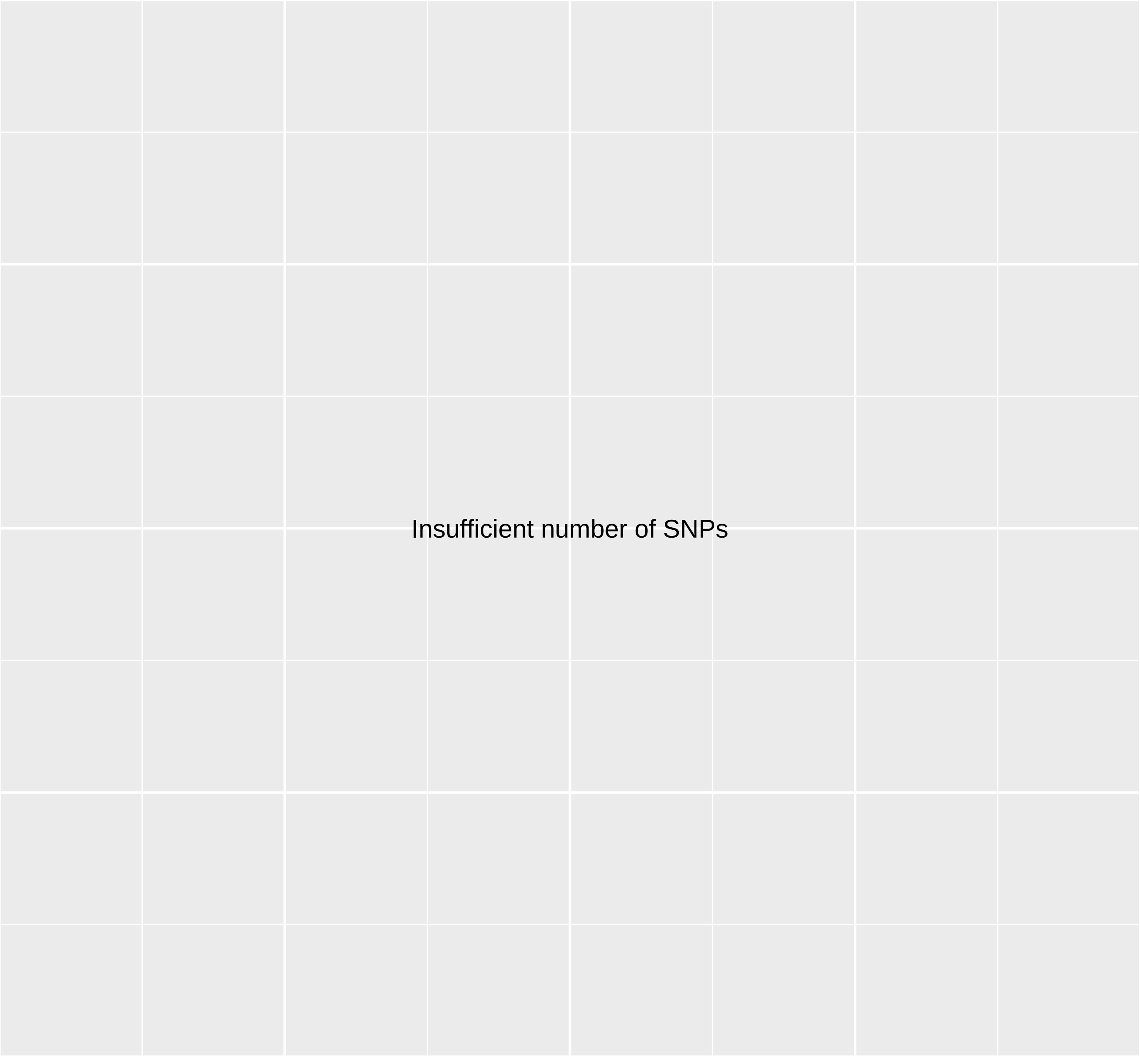
Insufficient number of SNPs

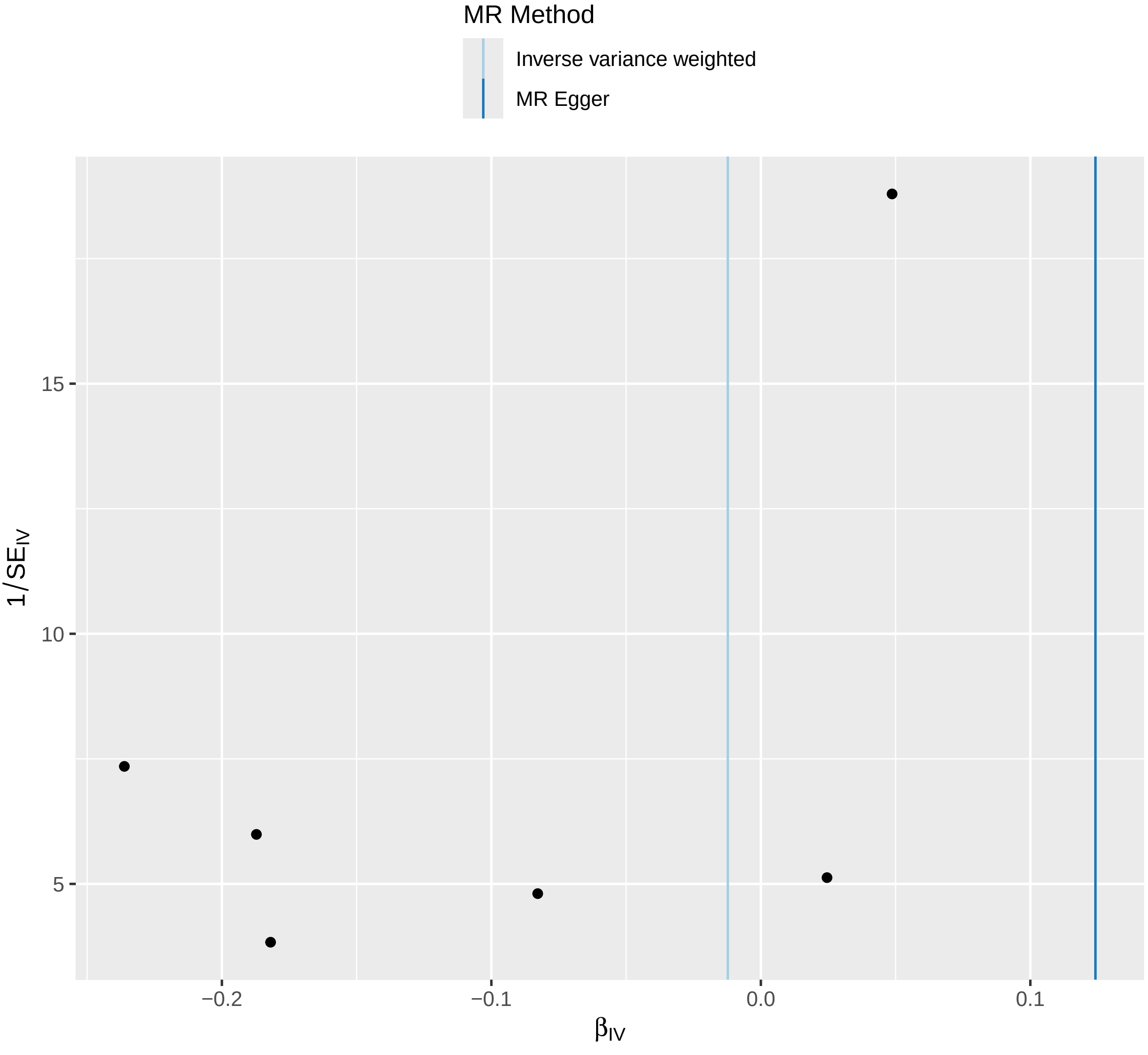
MR Method
Inverse variance weighted
MR Egger
15
10
5
$1/SE_{IV}$
−0.2
−0.1
0.0
0.1
$\beta_{IV}$

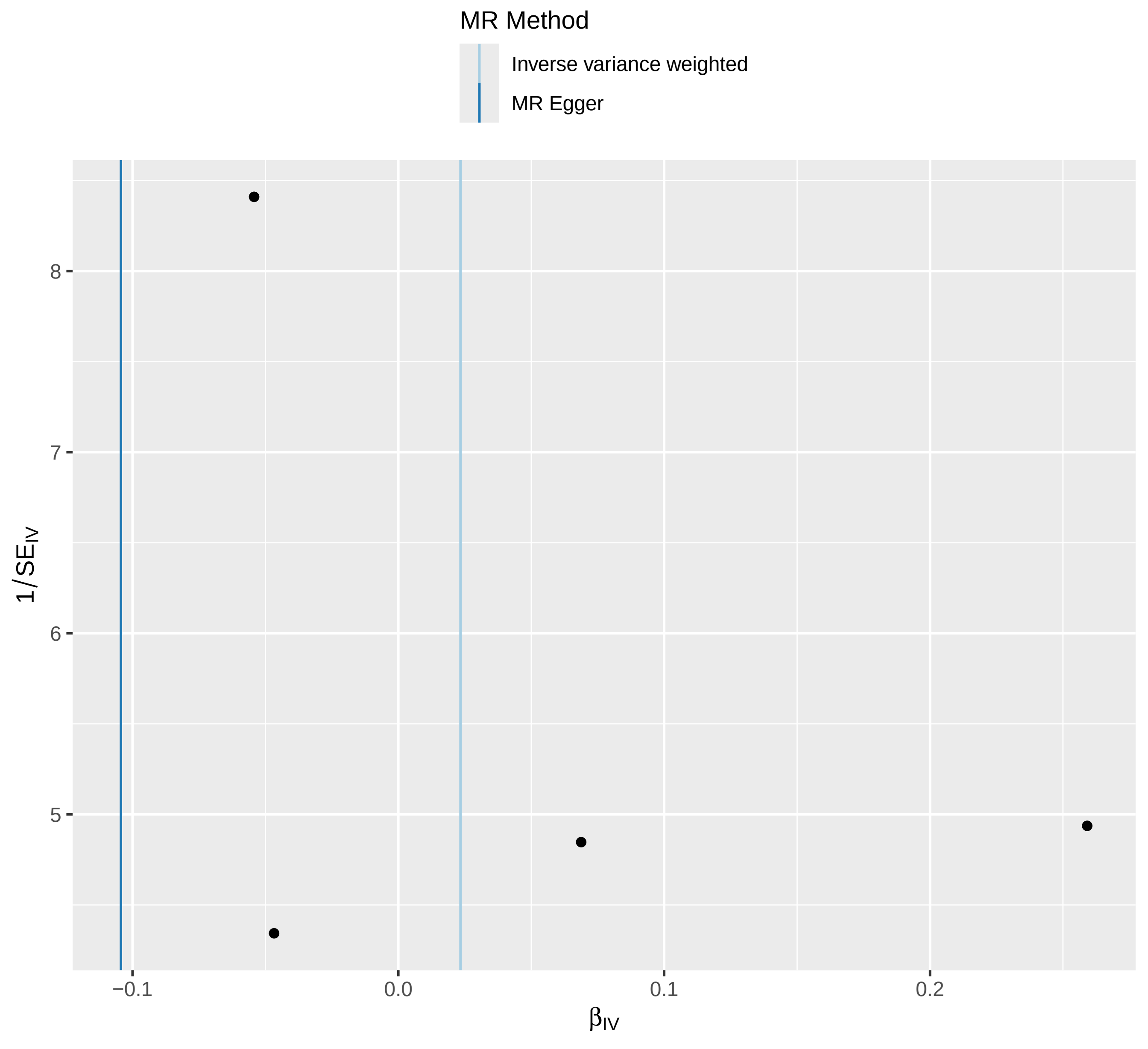

MR Method
Inverse variance weighted
MR Egger
8
7
6
5
$1/SE_{IV}$
−0.1
0.0
0.1
0.2
$\beta_{IV}$

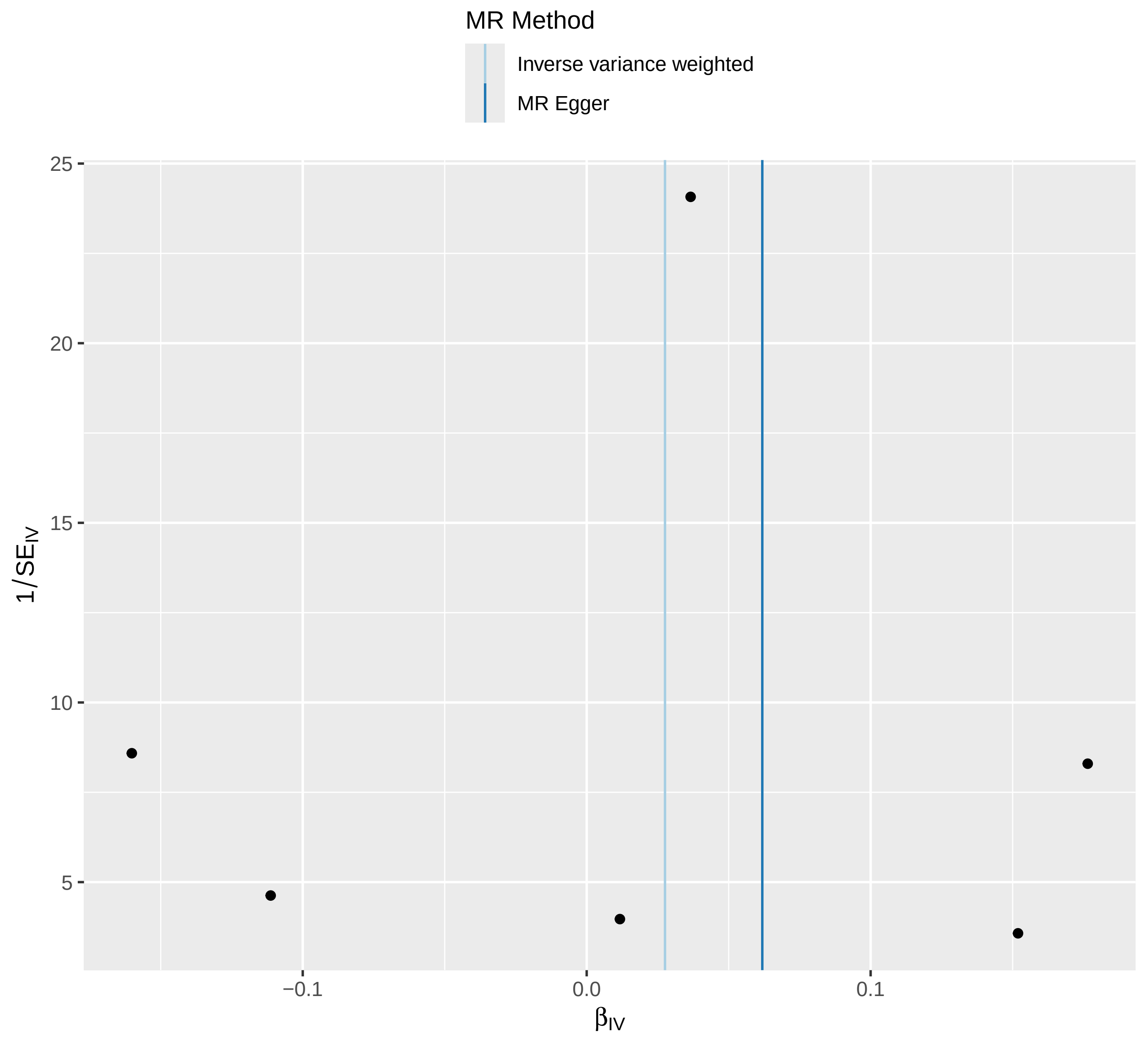

MR Method
Inverse variance weighted
MR Egger
25
20
15
10
5
$1/SE_{IV}$
−0.1
0.0
0.1
$\beta_{IV}$

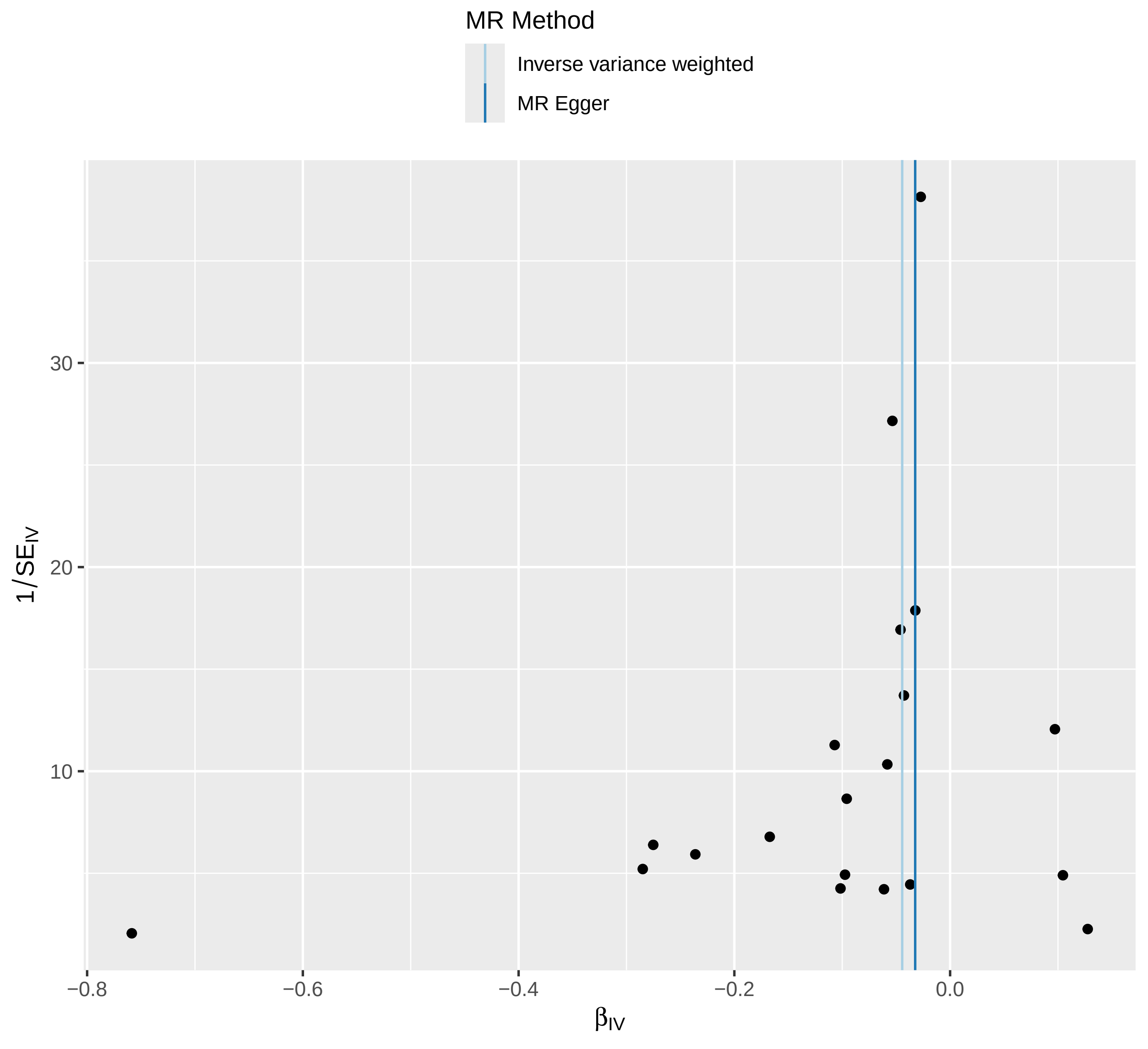
MR Method
Inverse variance weighted
MR Egger
30
20
10
1/SE$_{IV}$
−0.8
−0.6
−0.4
−0.2
0.0
β$_{IV}$

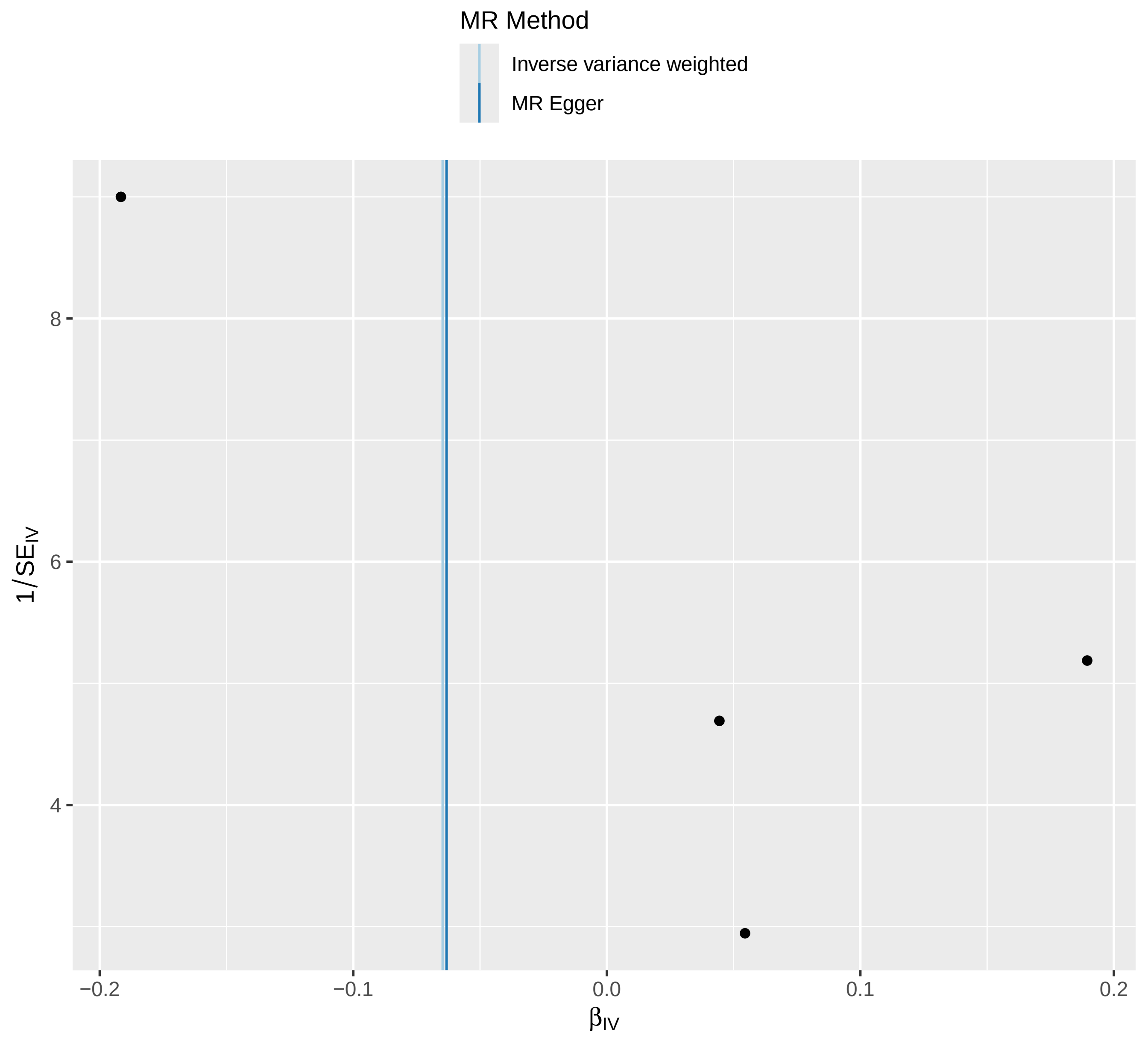

MR Method
Inverse variance weighted
MR Egger
8
6
4
1/SE_IV
−0.2
−0.1
0.0
0.1
0.2
β_IV

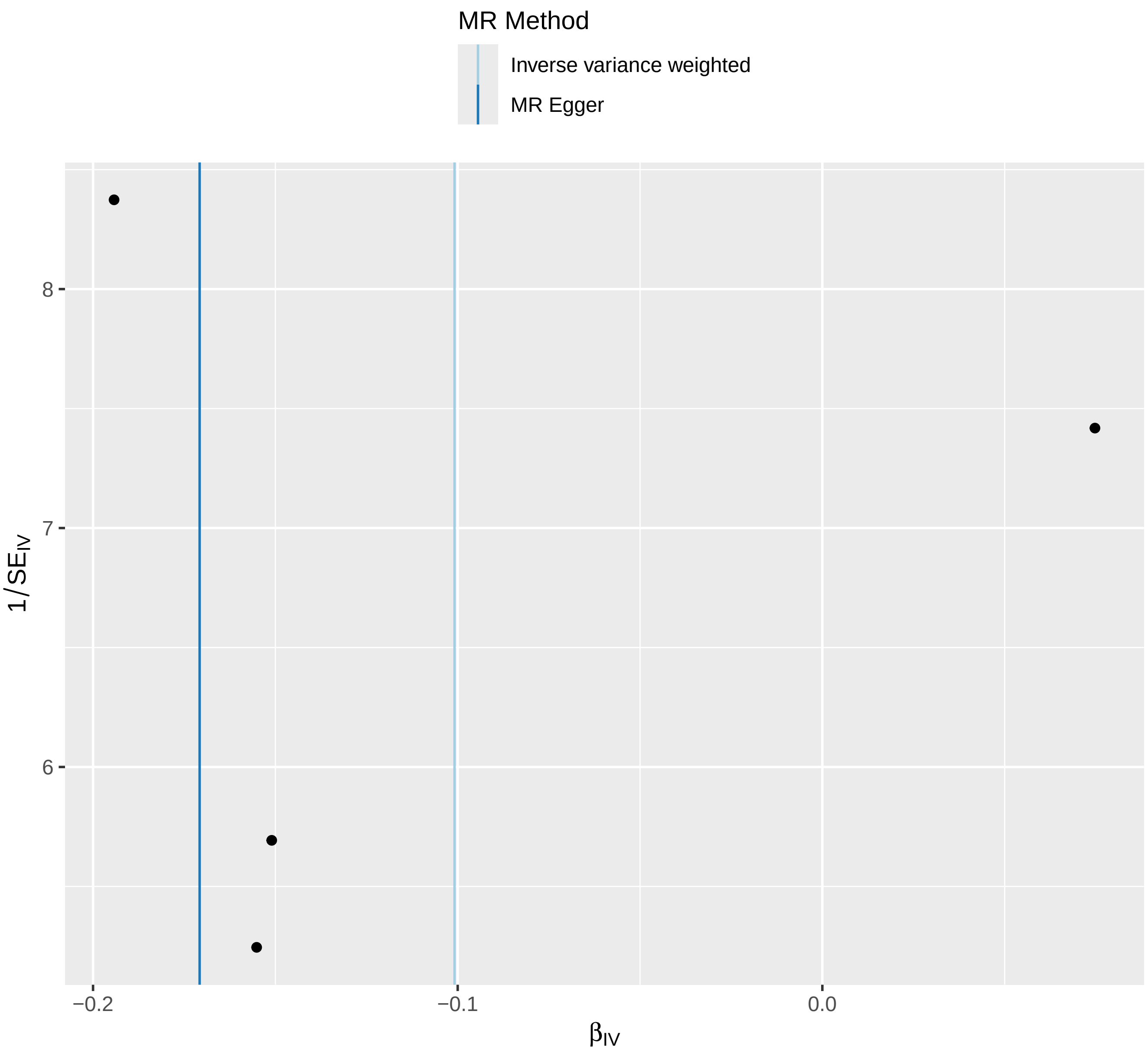
MR Method
Inverse variance weighted
MR Egger
8
7
6
1/SE$_{IV}$
−0.2
−0.1
0.0
β$_{IV}$

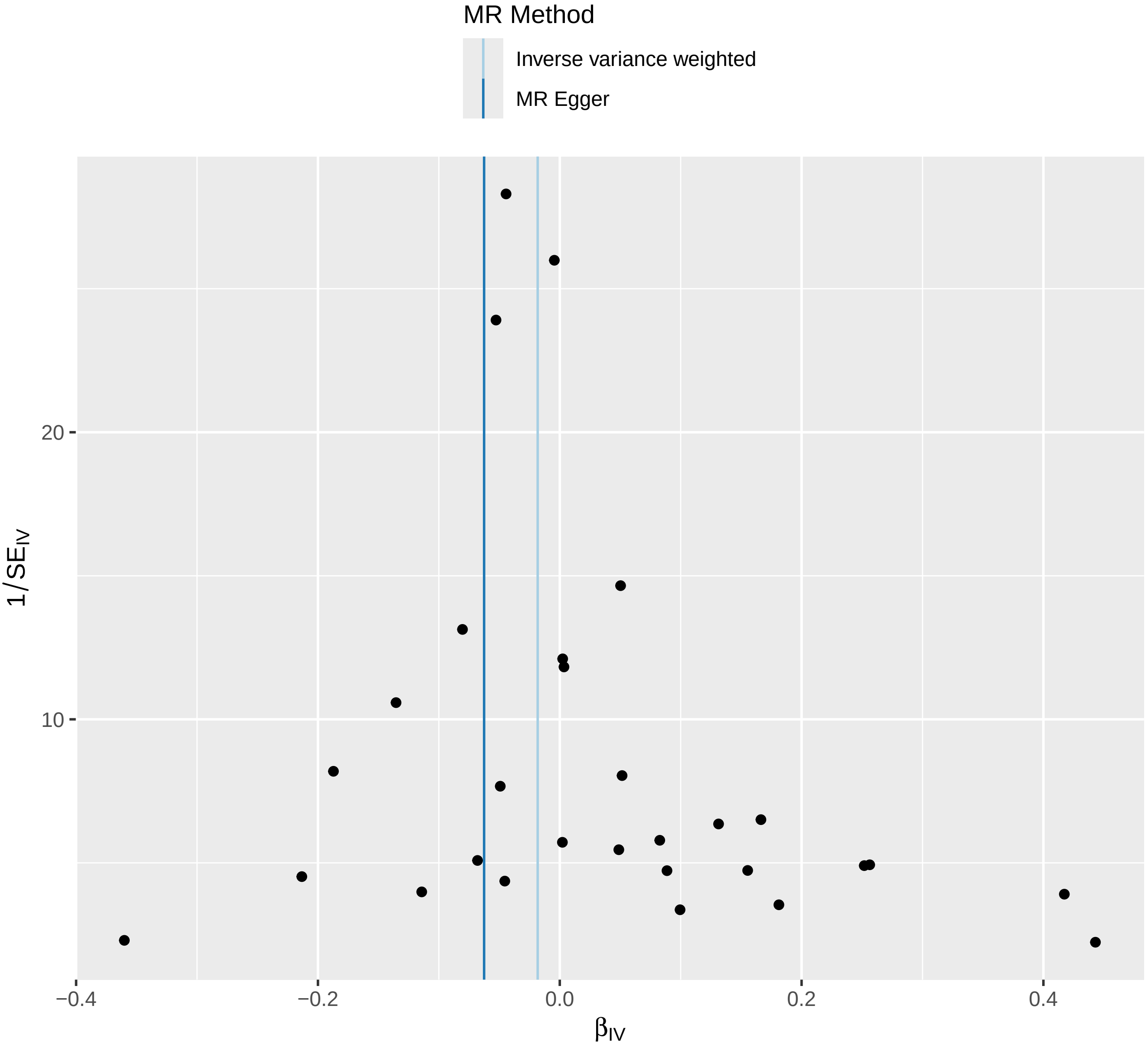
MR Method
Inverse variance weighted
MR Egger
20
10
1/SE$_{IV}$
−0.4
−0.2
0.0
0.2
0.4
β$_{IV}$

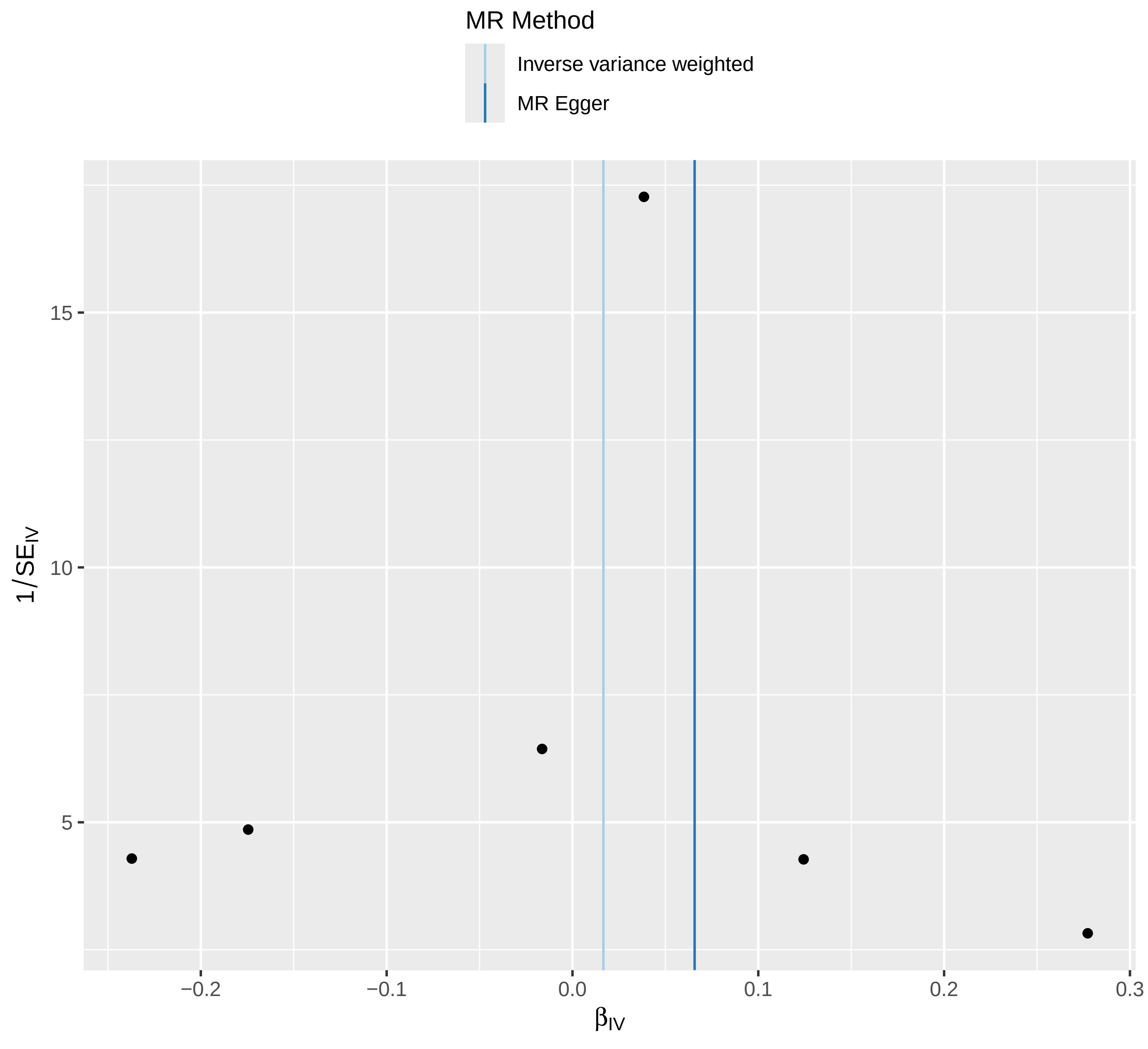
MR Method
Inverse variance weighted
MR Egger
15
10
5
1/SE_IV
−0.2
−0.1
0.0
0.1
0.2
0.3
β_IV

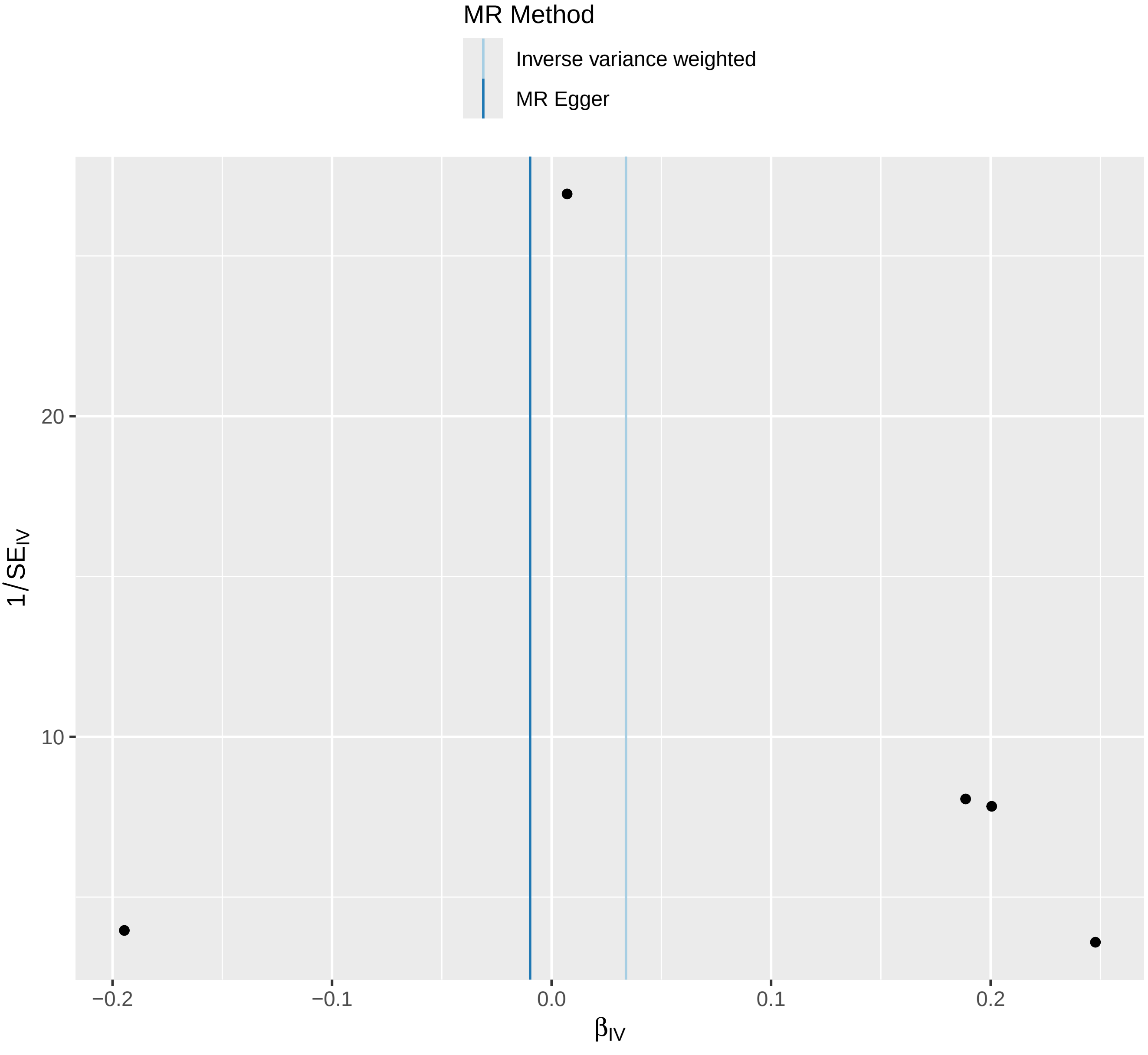

MR Method
Inverse variance weighted
MR Egger
20
10
1/SE_IV
−0.2
−0.1
0.0
0.1
0.2
β_IV

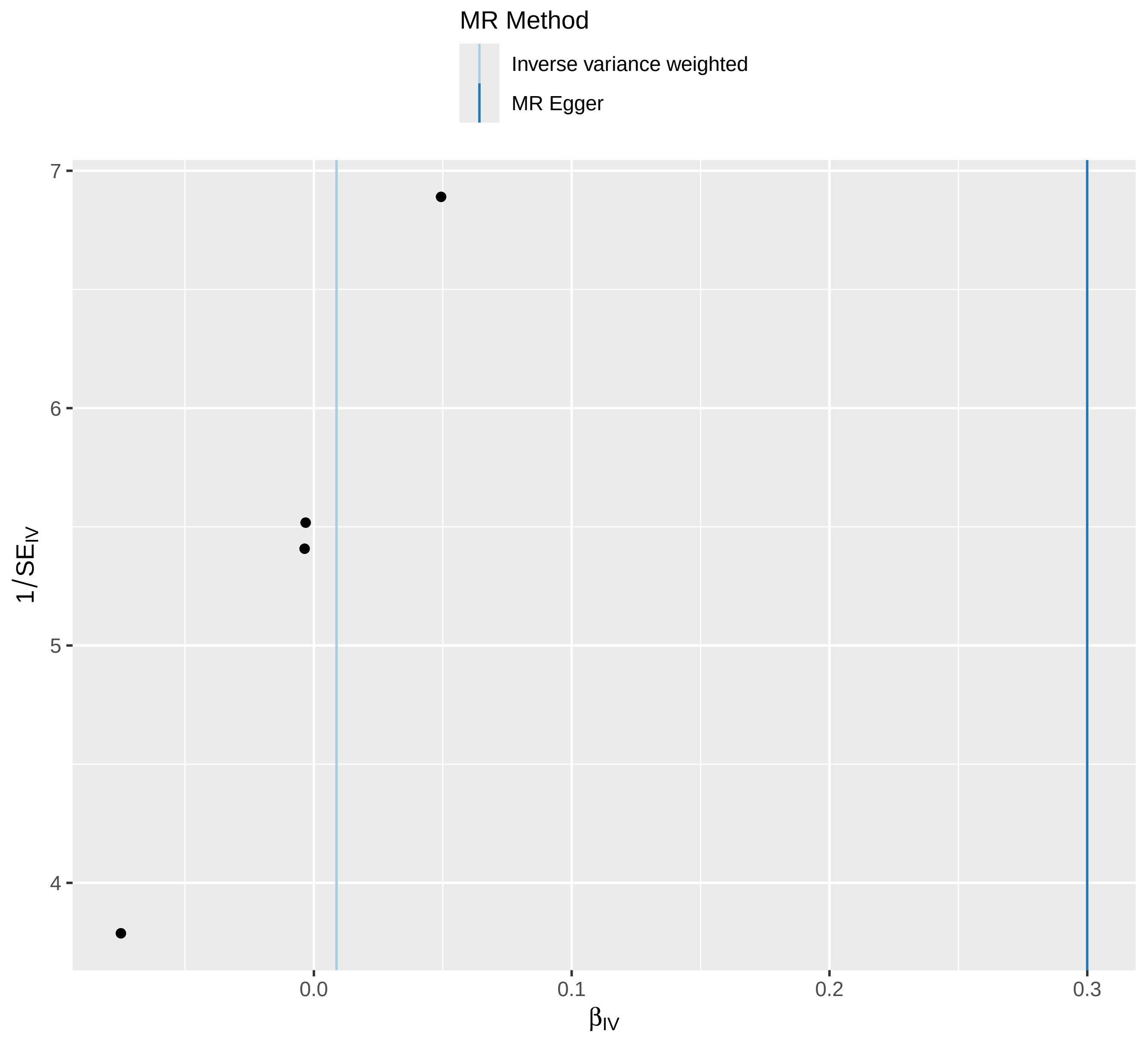
MR Method
Inverse variance weighted
MR Egger
7
6
5
4
0.0
0.1
0.2
0.3
1/SE_IV
β_IV

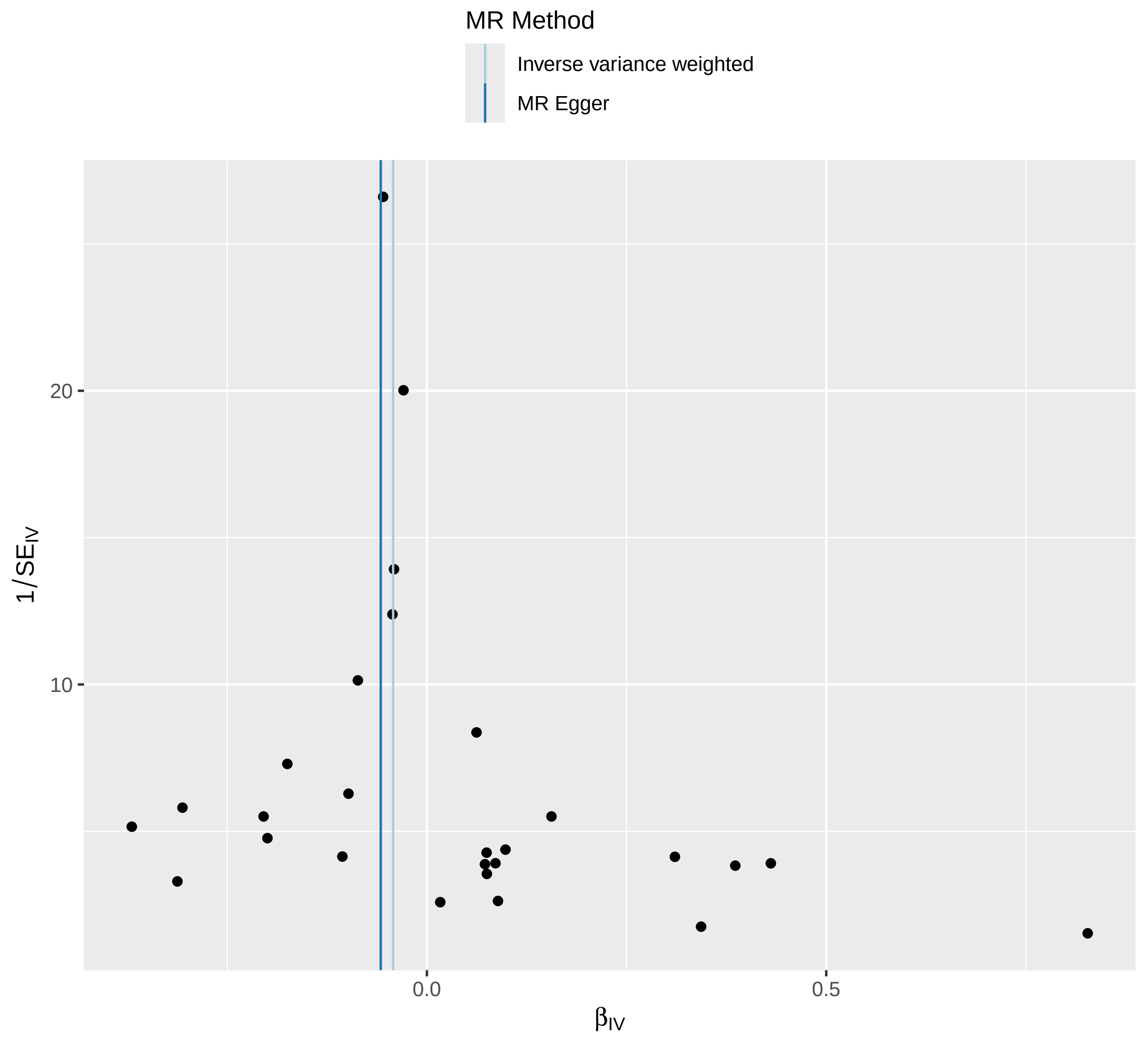

MR Method
Inverse variance weighted
MR Egger
20
10
1/SE$_{IV}$
0.0
0.5
β$_{IV}$

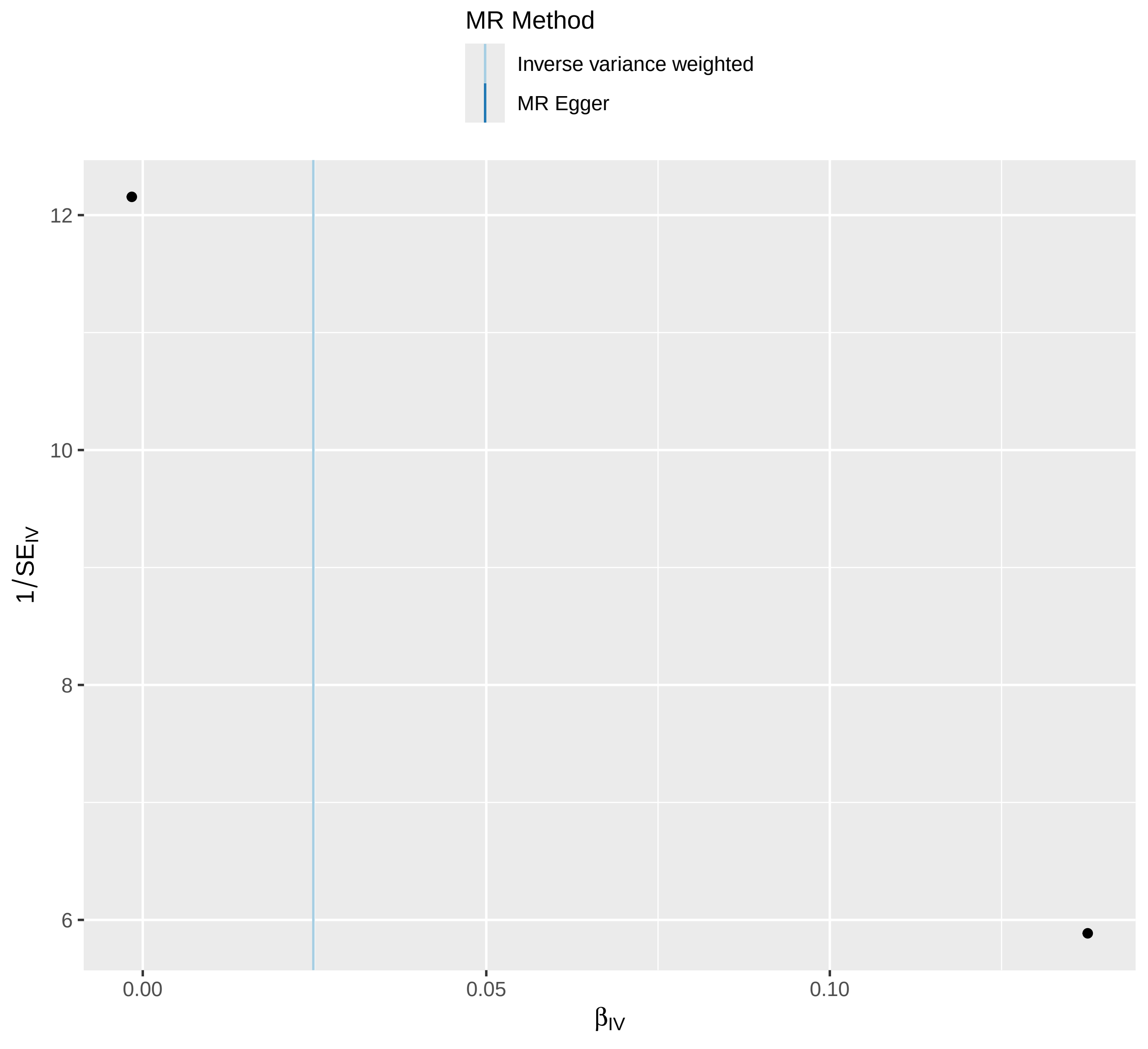

MR Method
Inverse variance weighted
MR Egger
12
10
8
6
1/SE_IV
0.00
0.05
0.10
β_IV

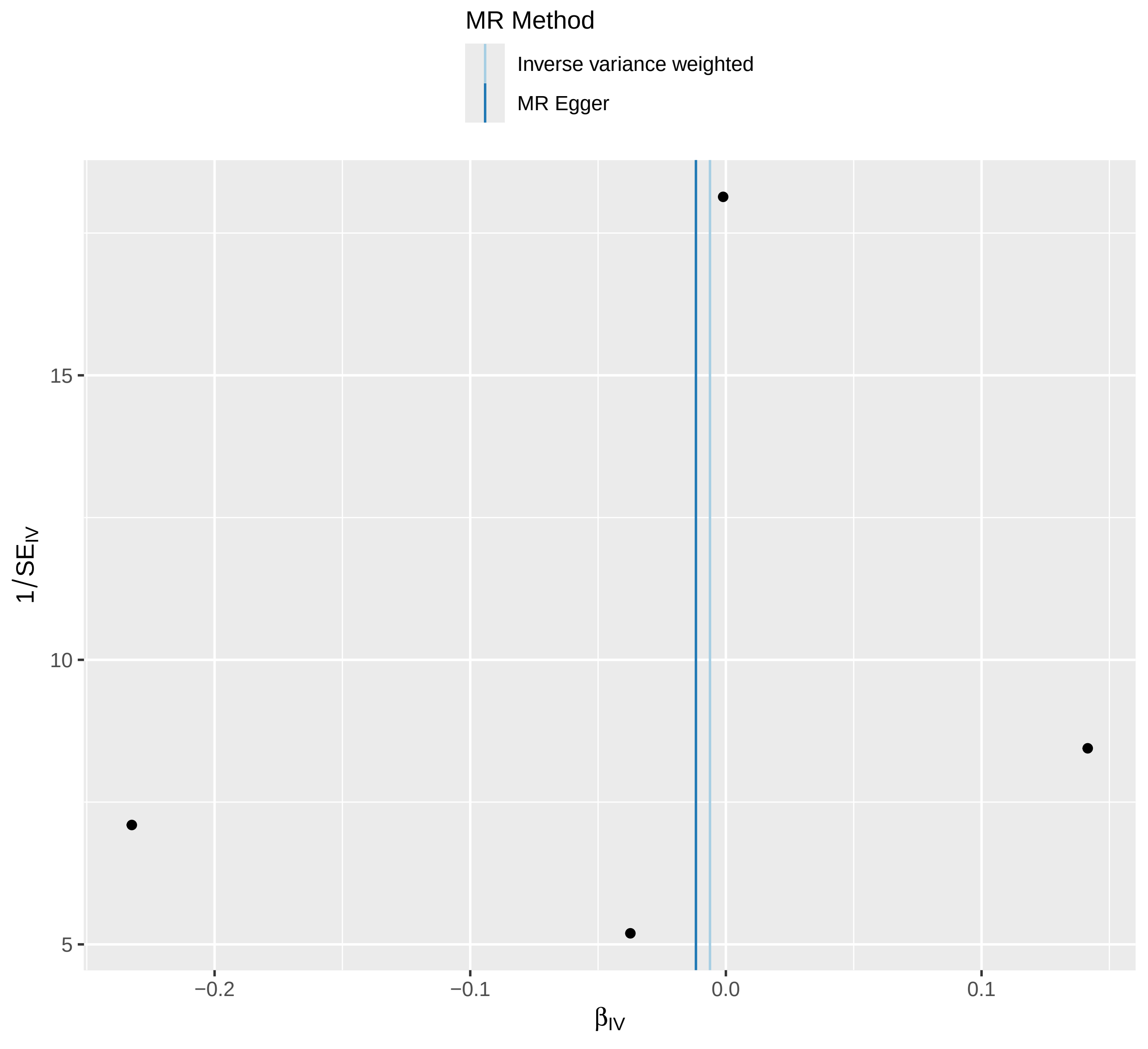
MR Method
Inverse variance weighted
MR Egger
15
10
5
1/SE_IV
−0.2
−0.1
0.0
0.1
β_IV

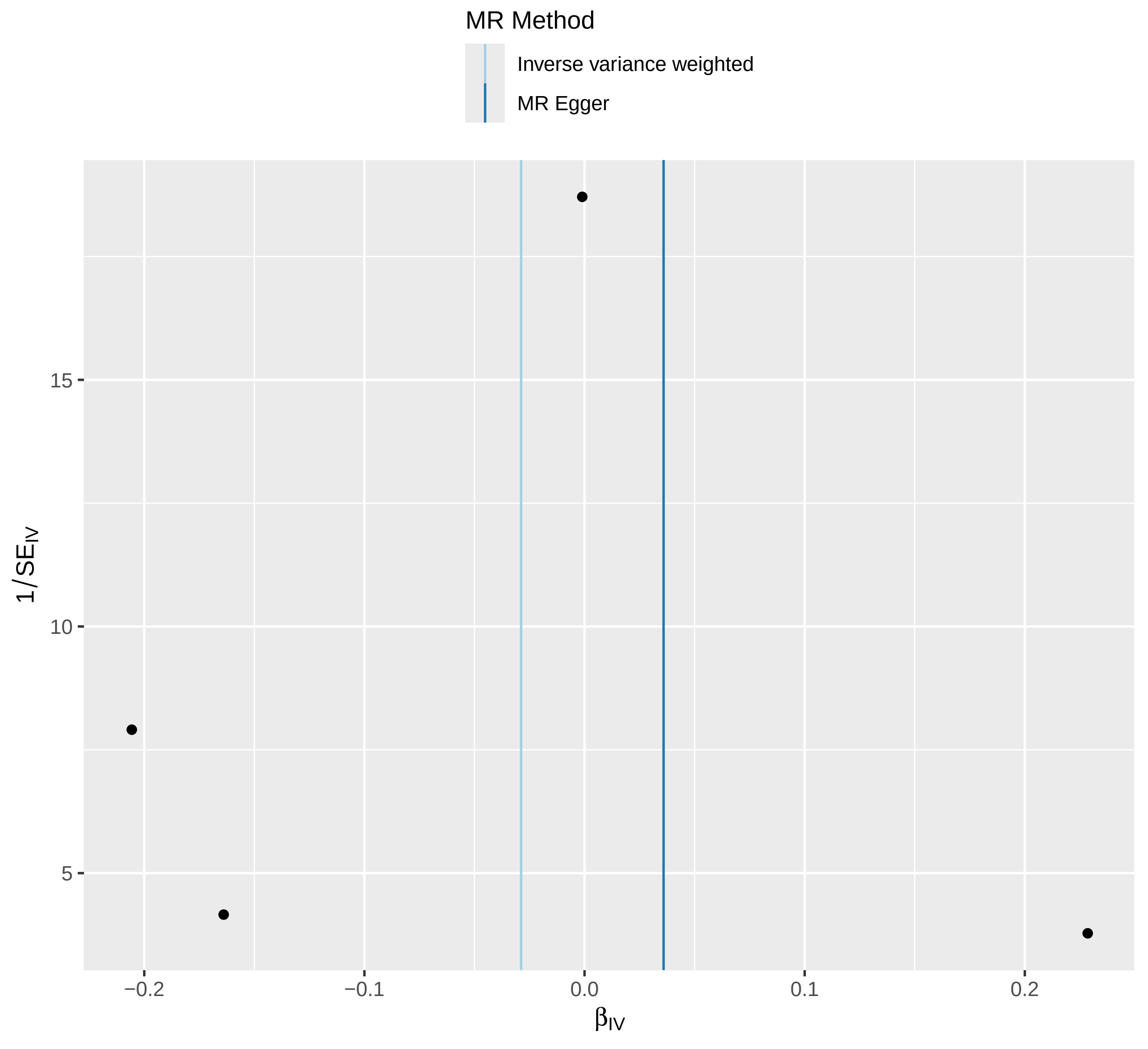

MR Method
Inverse variance weighted
MR Egger
15
10
5
$1/SE_{IV}$
−0.2
−0.1
0.0
0.1
0.2
$\beta_{IV}$

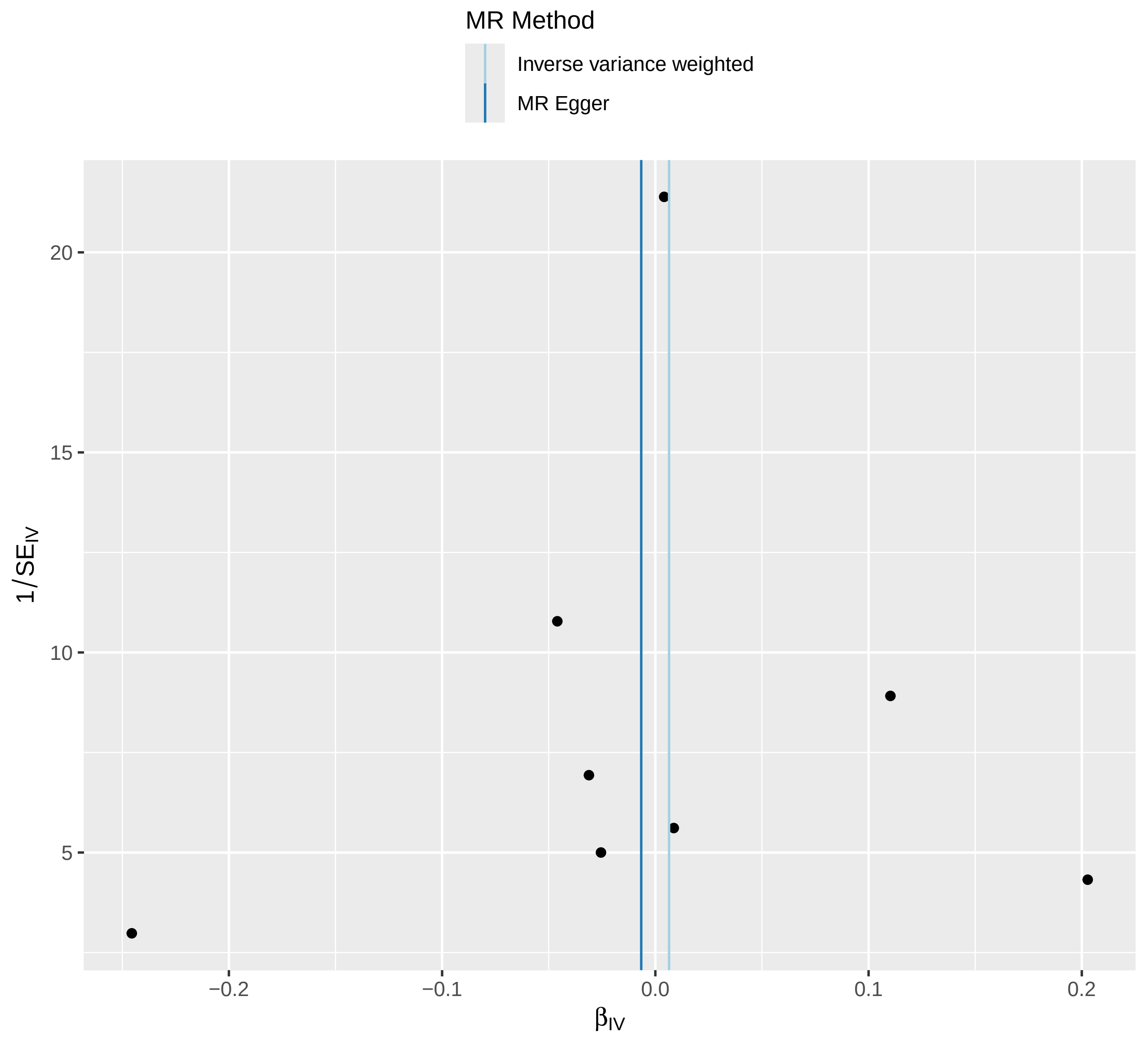

MR Method
Inverse variance weighted
MR Egger
20
15
10
5
1/SE_IV
−0.2
−0.1
0.0
0.1
0.2
β_IV

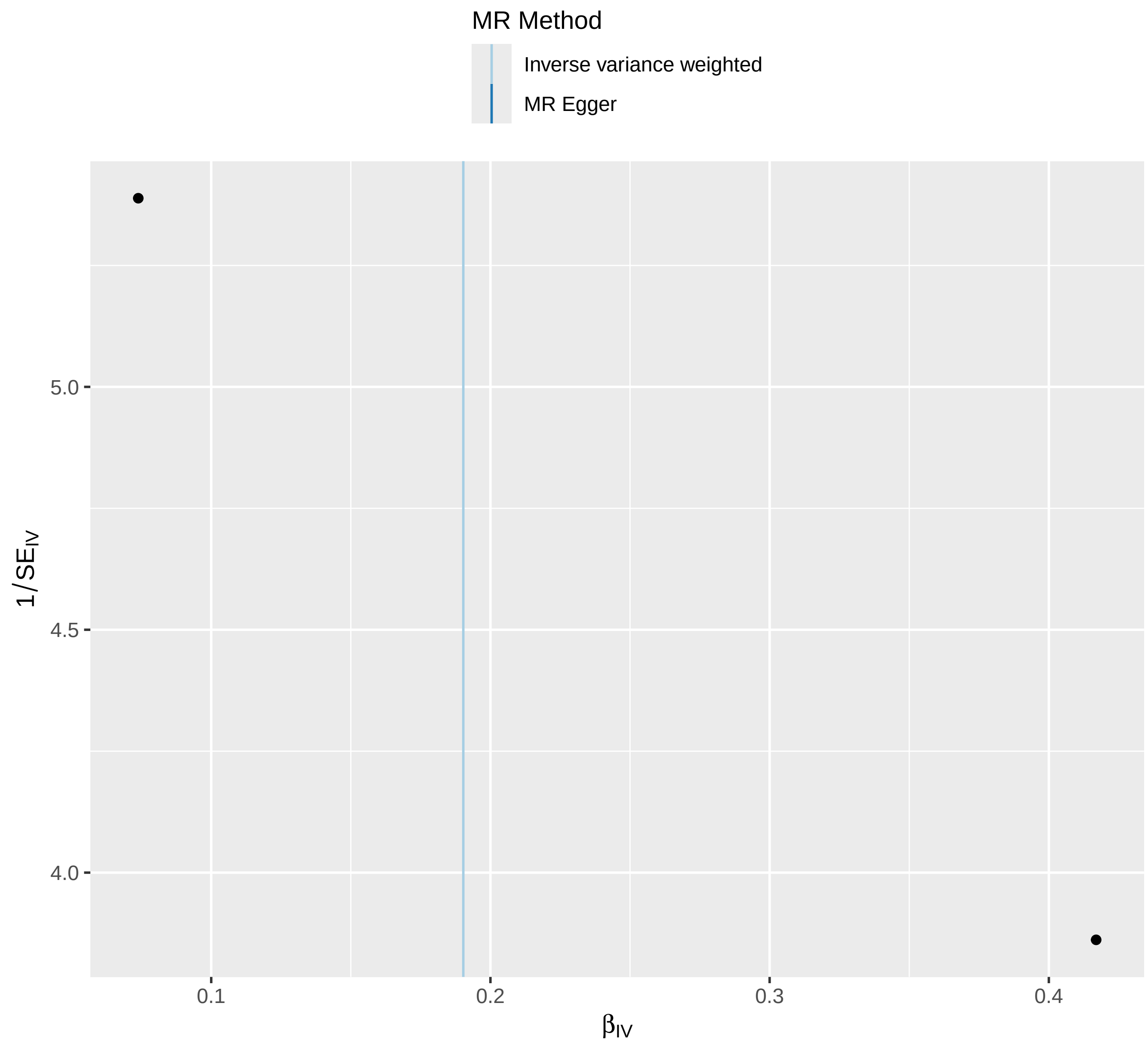
MR Method
Inverse variance weighted
MR Egger
5.0
4.5
4.0
1/$SE_{IV}$
0.1
0.2
0.3
0.4
$\beta_{IV}$

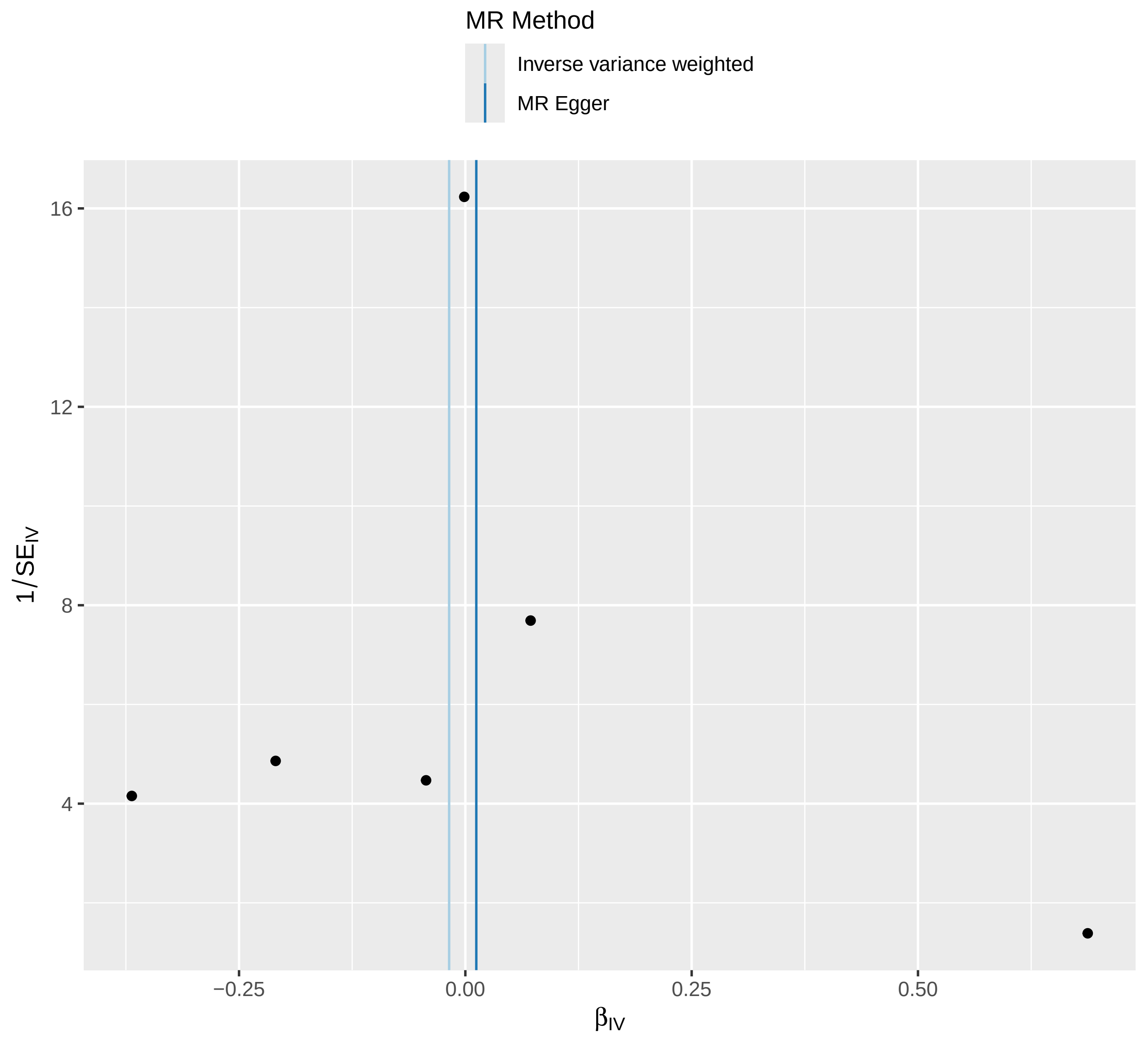

MR Method
Inverse variance weighted
MR Egger
16
12
8
4
1/SE_IV
−0.25
0.00
0.25
0.50
β_IV

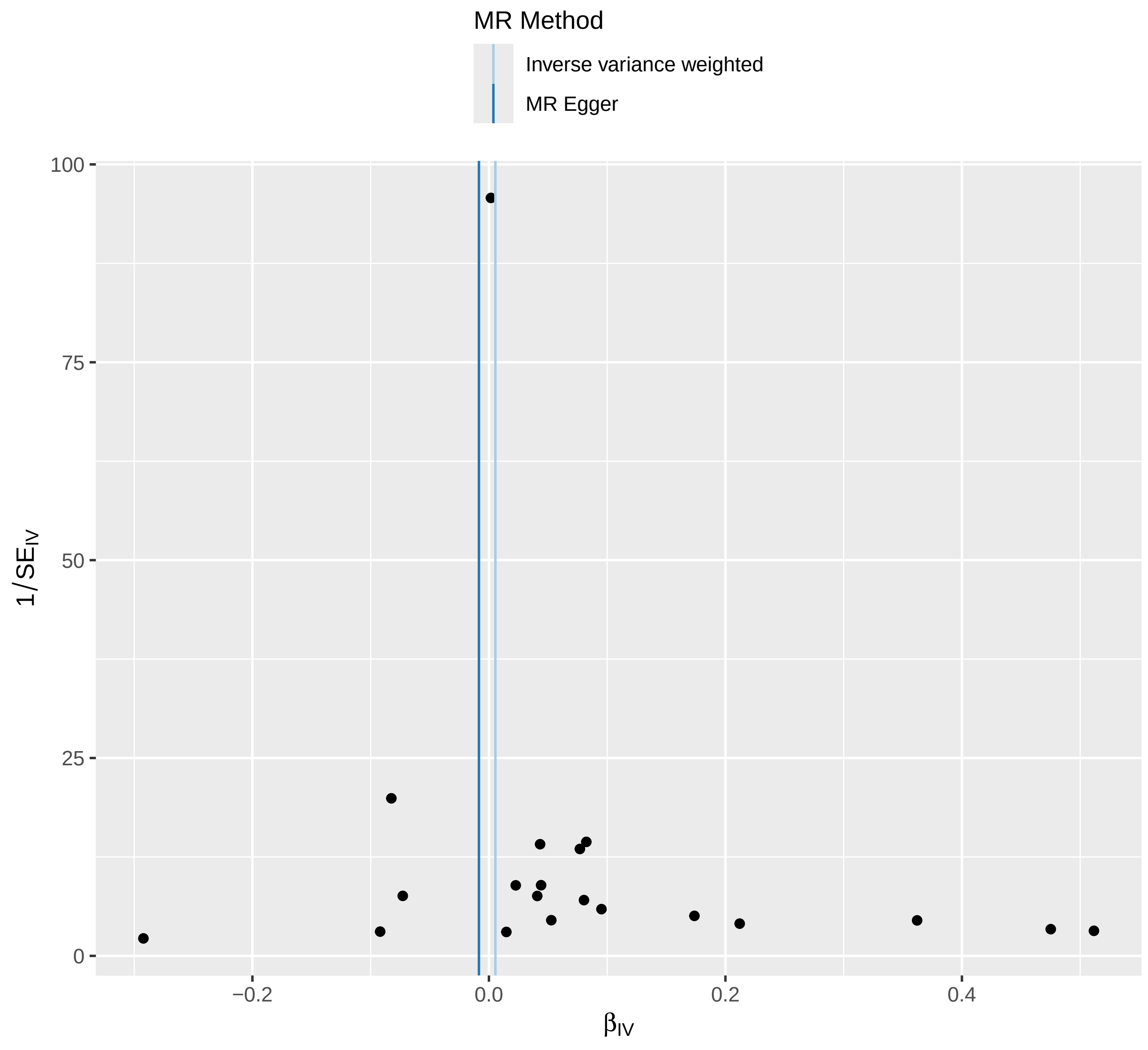

MR Method
Inverse variance weighted
MR Egger
100
75
50
25
0
1/SE_IV
−0.2
0.0
0.2
0.4
β_IV

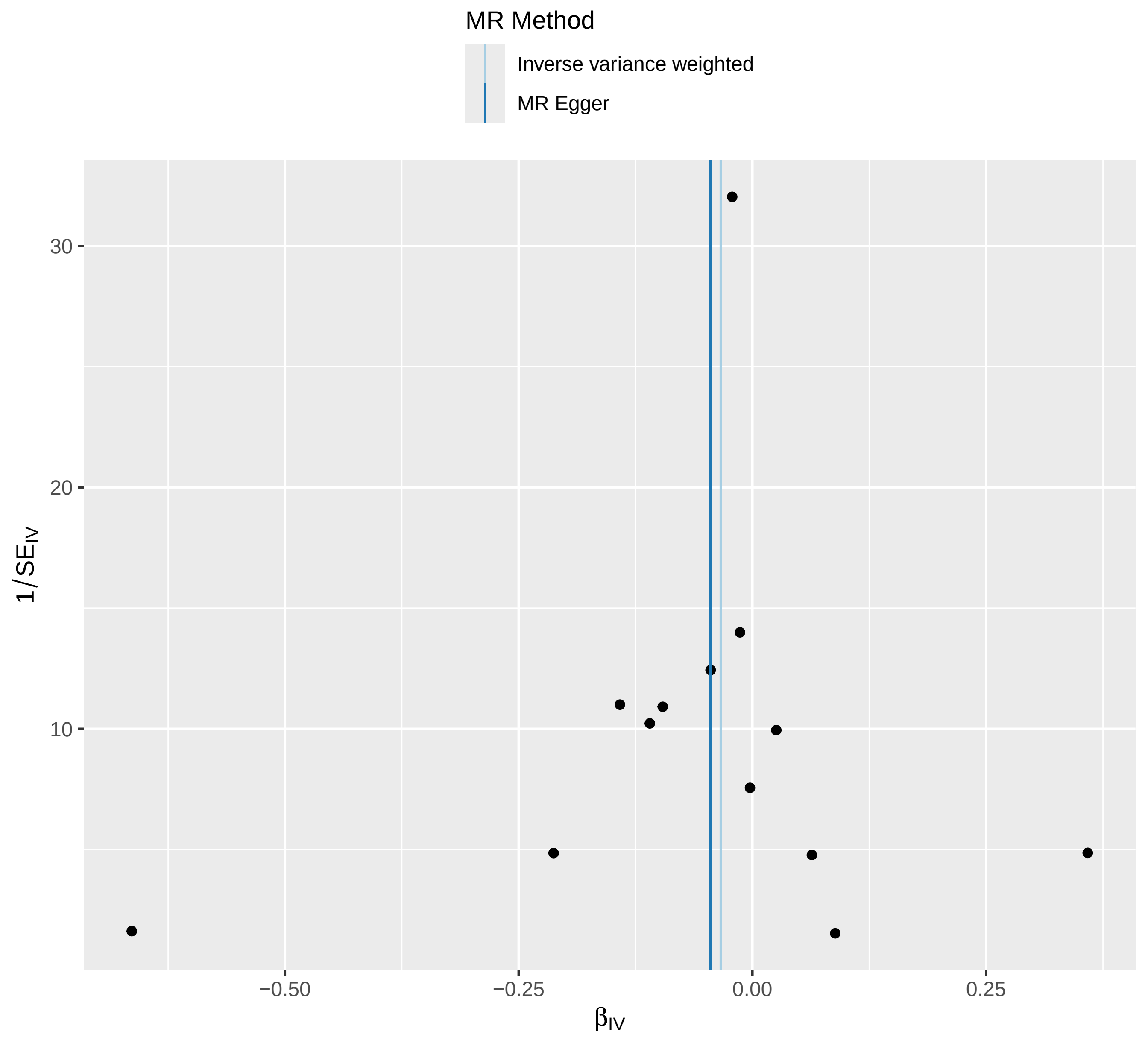
MR Method
Inverse variance weighted
MR Egger
30
20
10
1/SE_IV
−0.50
−0.25
0.00
0.25
β_IV

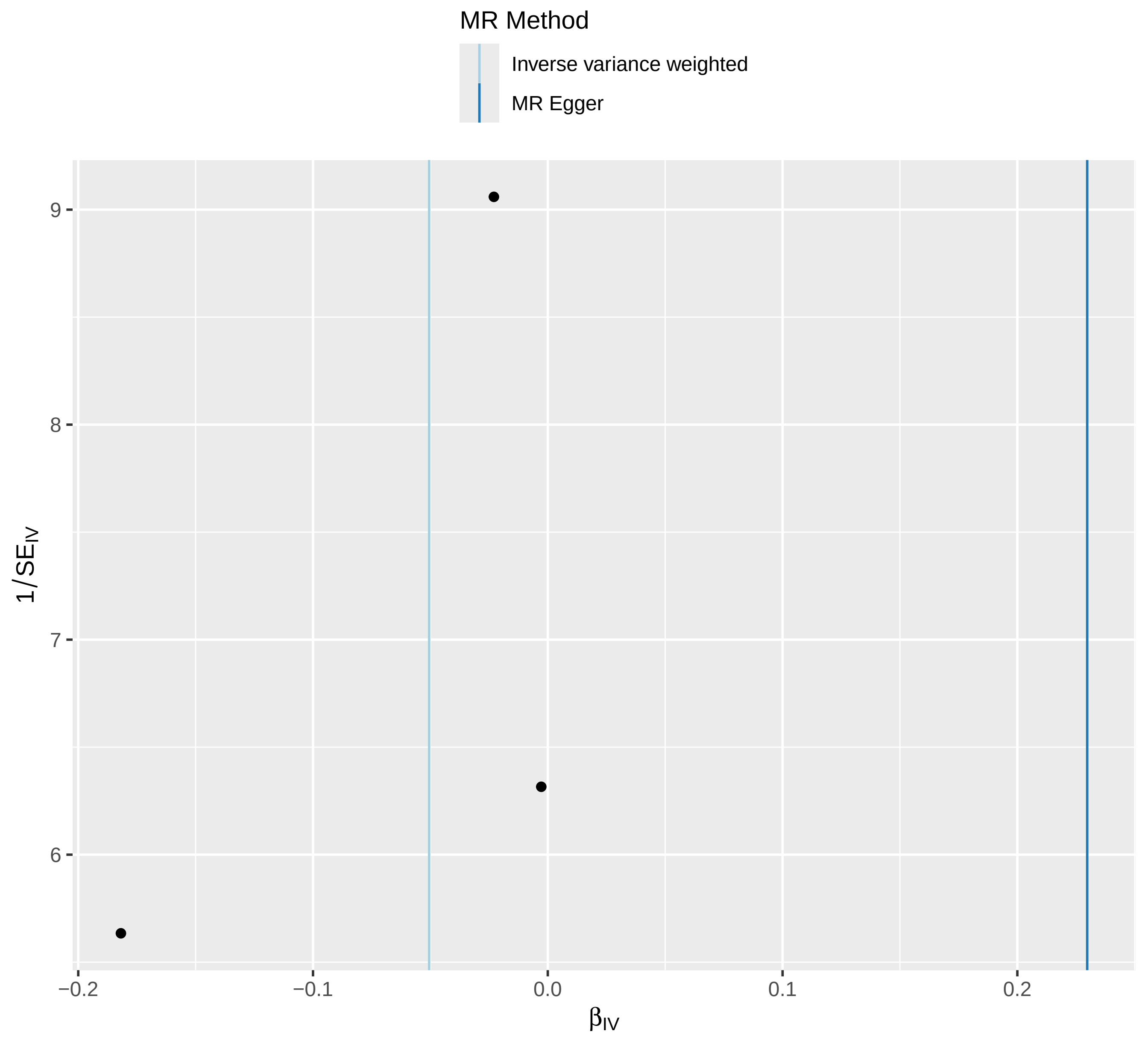
MR Method
Inverse variance weighted
MR Egger
9
8
7
6
1/SE_IV
−0.2
−0.1
0.0
0.1
0.2
β_IV

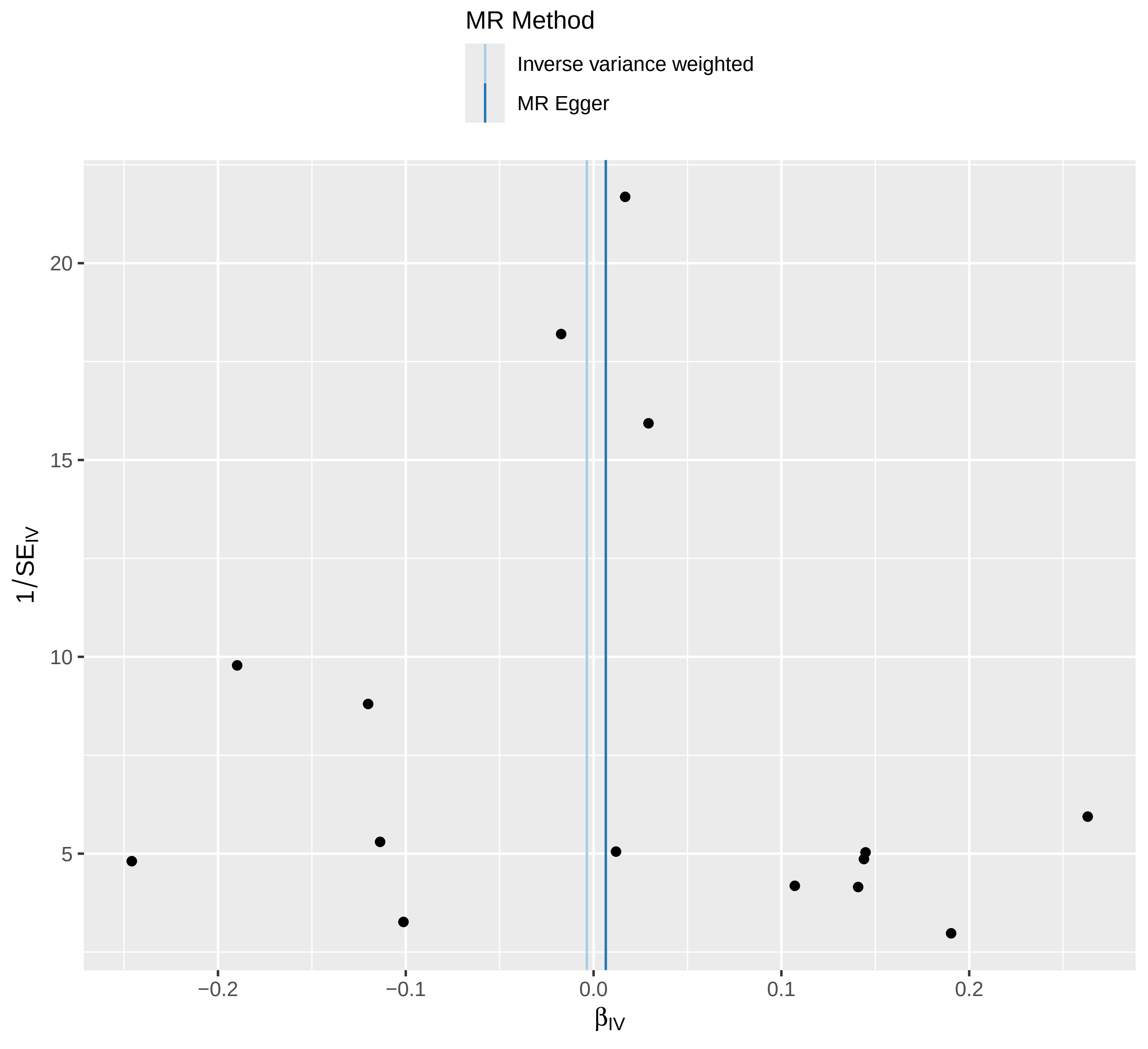
MR Method
Inverse variance weighted
MR Egger
20
15
10
5
1/SE_IV
−0.2
−0.1
0.0
0.1
0.2
β_IV

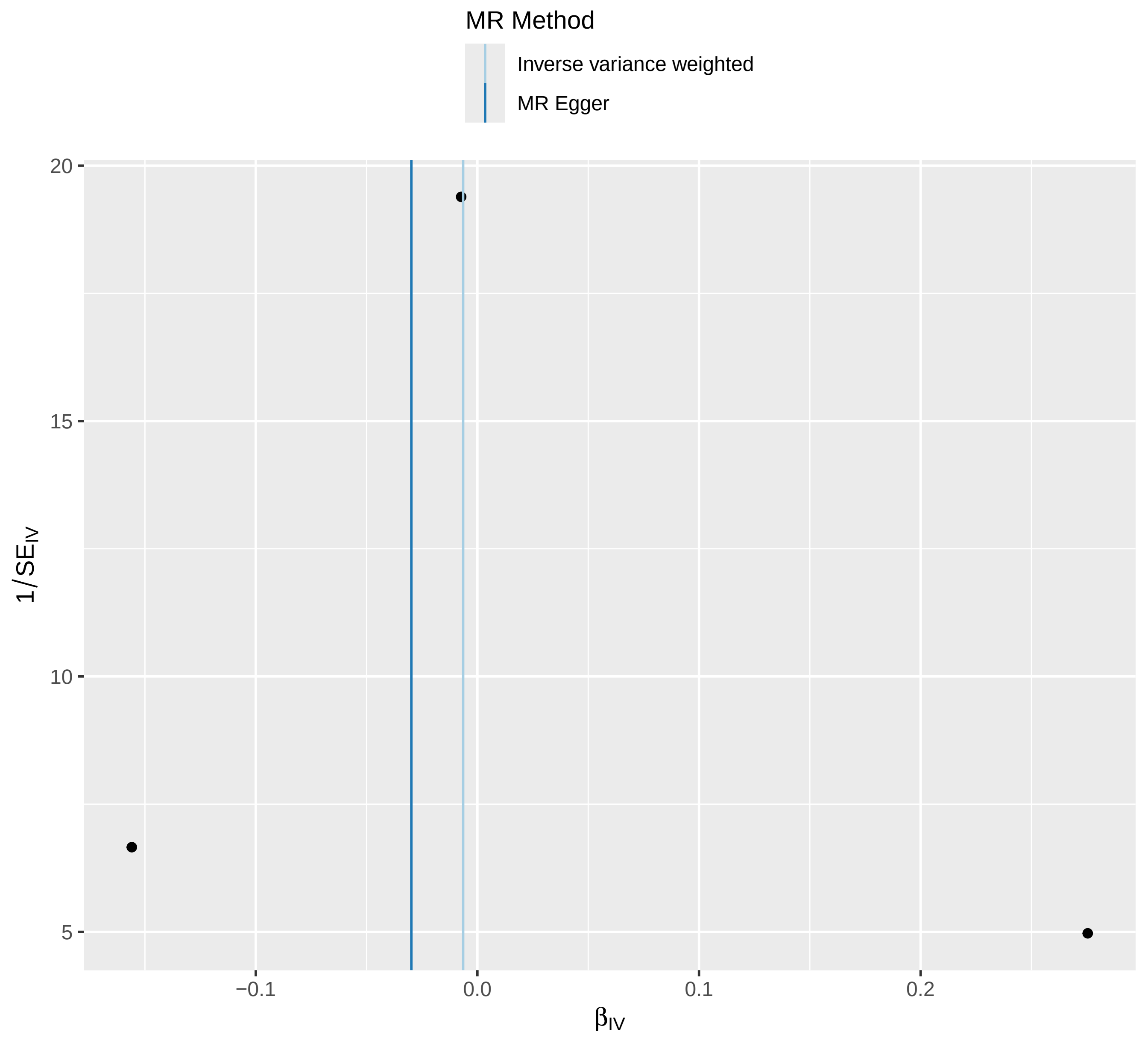

MR Method
Inverse variance weighted
MR Egger
20
15
10
5
1/SE_IV
−0.1
0.0
0.1
0.2
β_IV

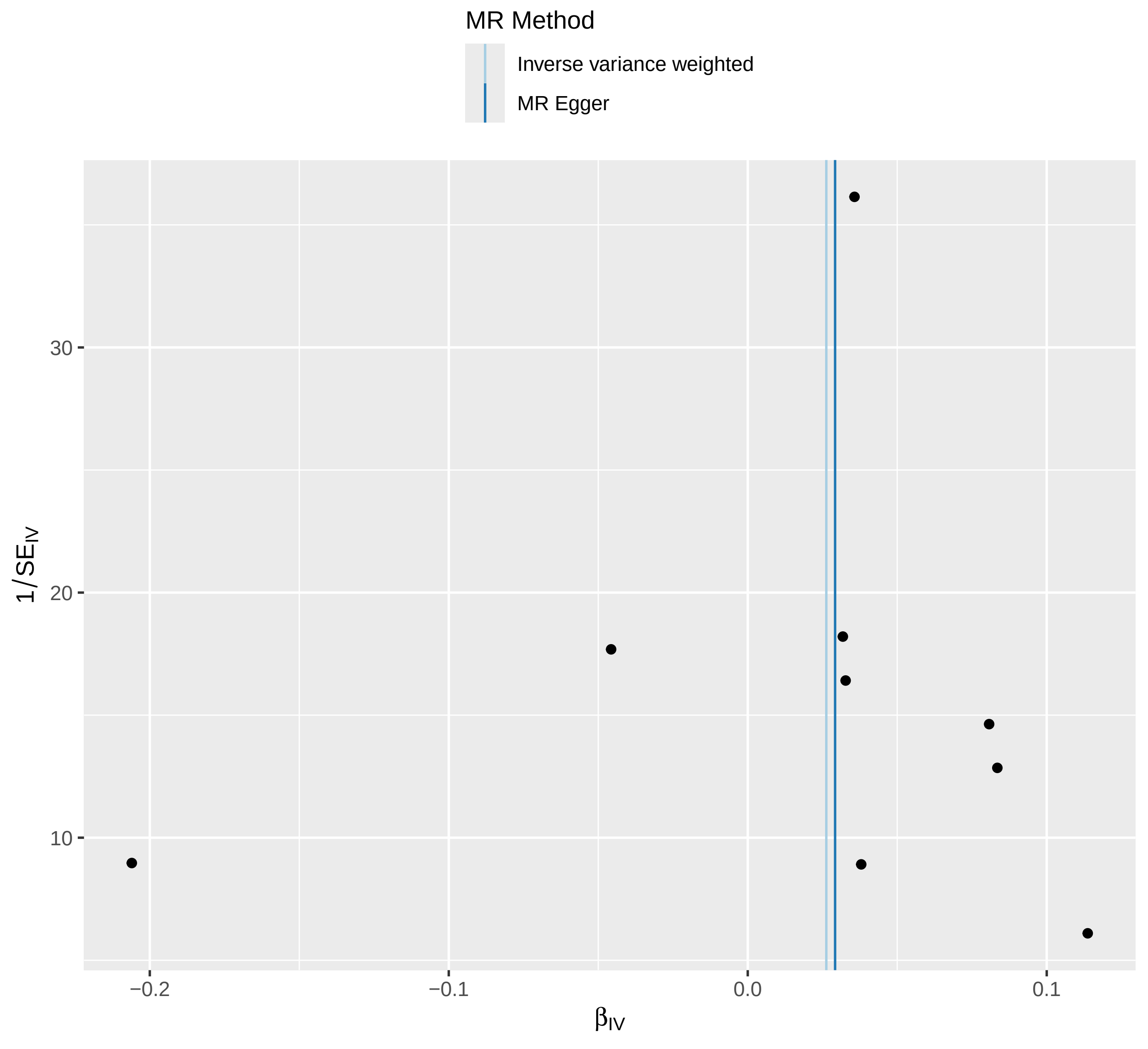
MR Method
Inverse variance weighted
MR Egger
1/$SE_{IV}$
30
20
10
−0.2
−0.1
0.0
0.1
$\beta_{IV}$

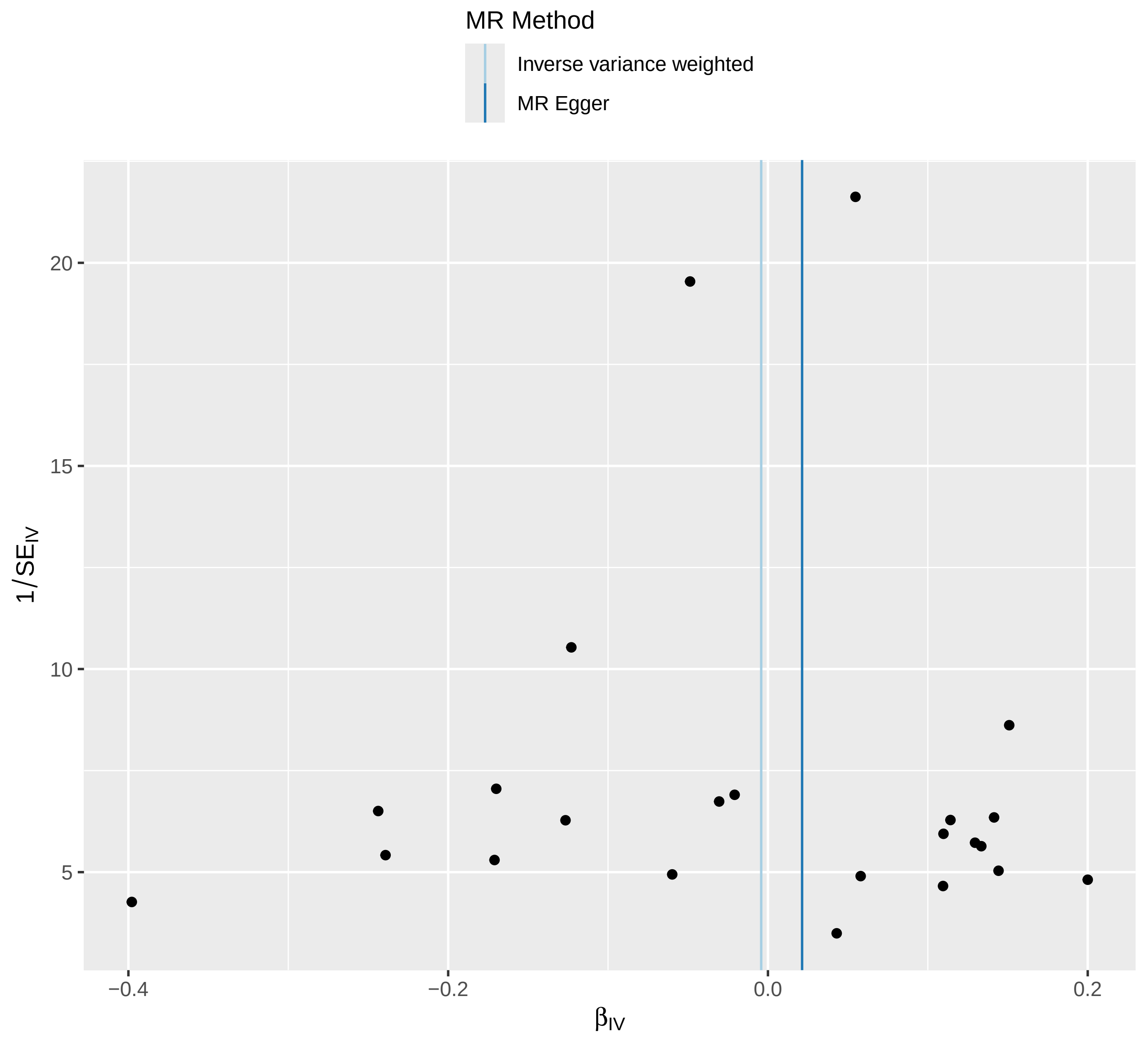
MR Method
Inverse variance weighted
MR Egger
20
15
10
5
1/SE_IV
−0.4
−0.2
0.0
0.2
β_IV

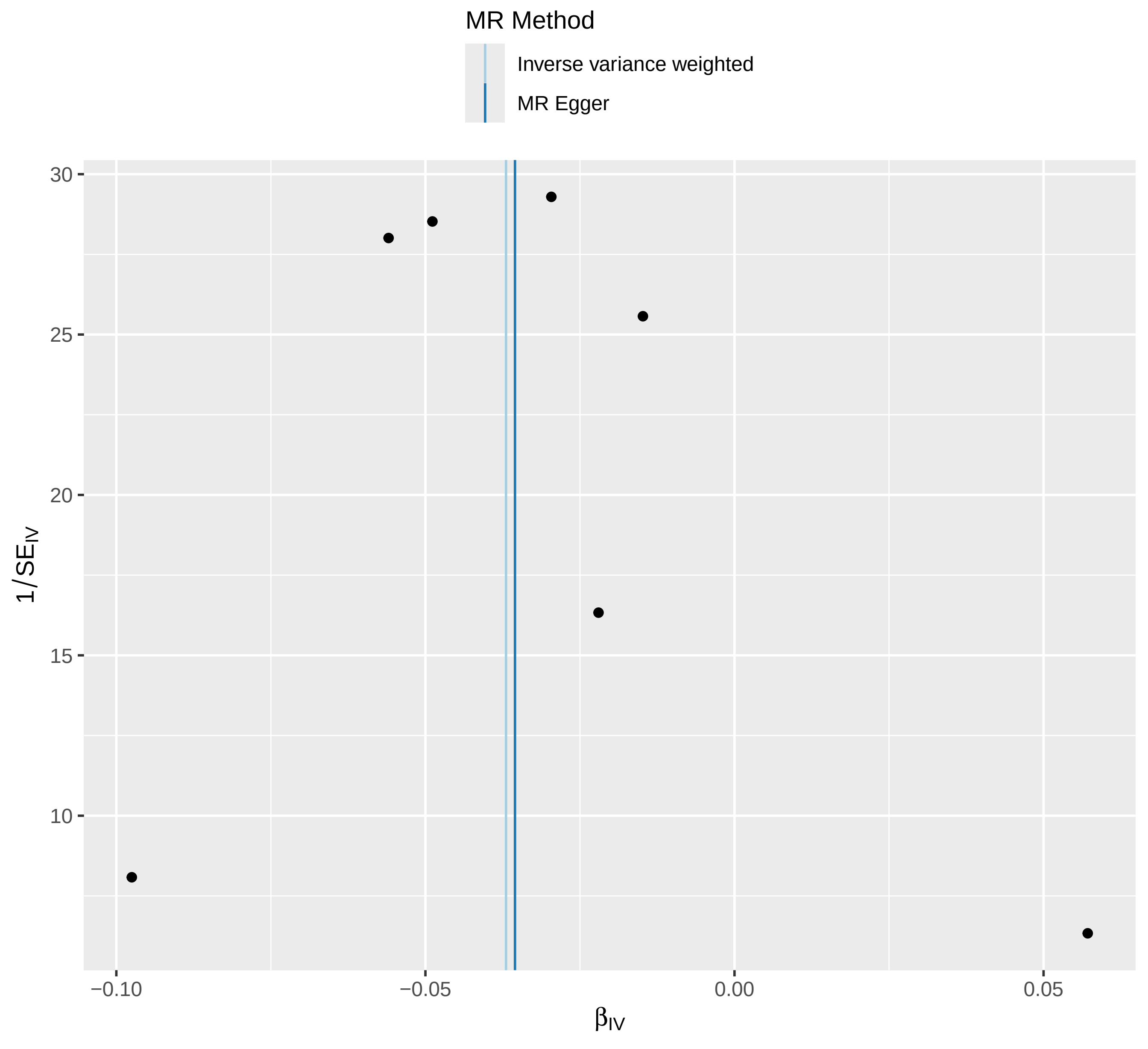
MR Method
Inverse variance weighted
MR Egger
30
25
20
15
10
1/SE$_{IV}$
−0.10
−0.05
0.00
0.05
β$_{IV}$

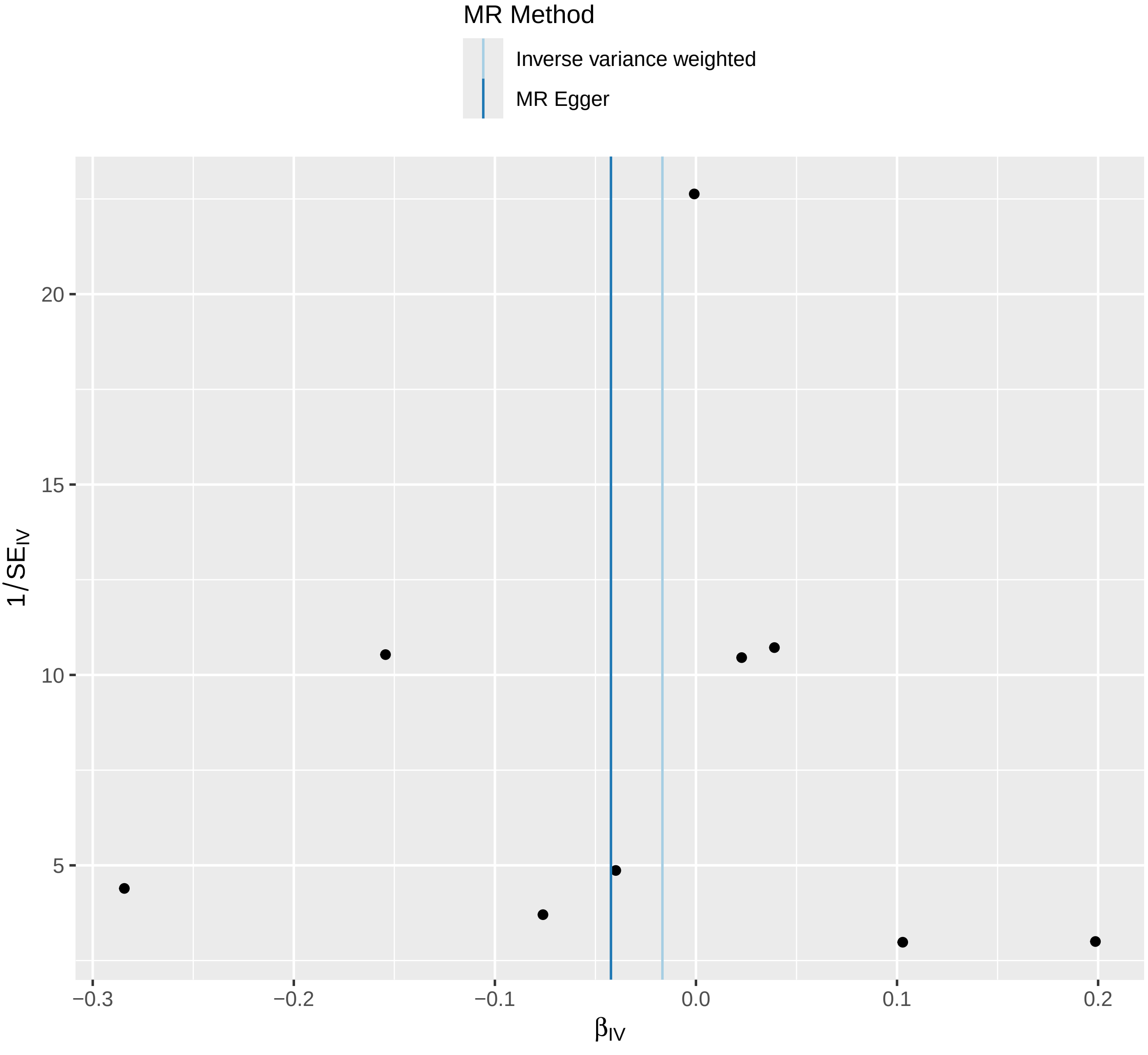
MR Method
Inverse variance weighted
MR Egger
1/SE$_{IV}$
20
15
10
5
−0.3
−0.2
−0.1
0.0
0.1
0.2
β$_{IV}$

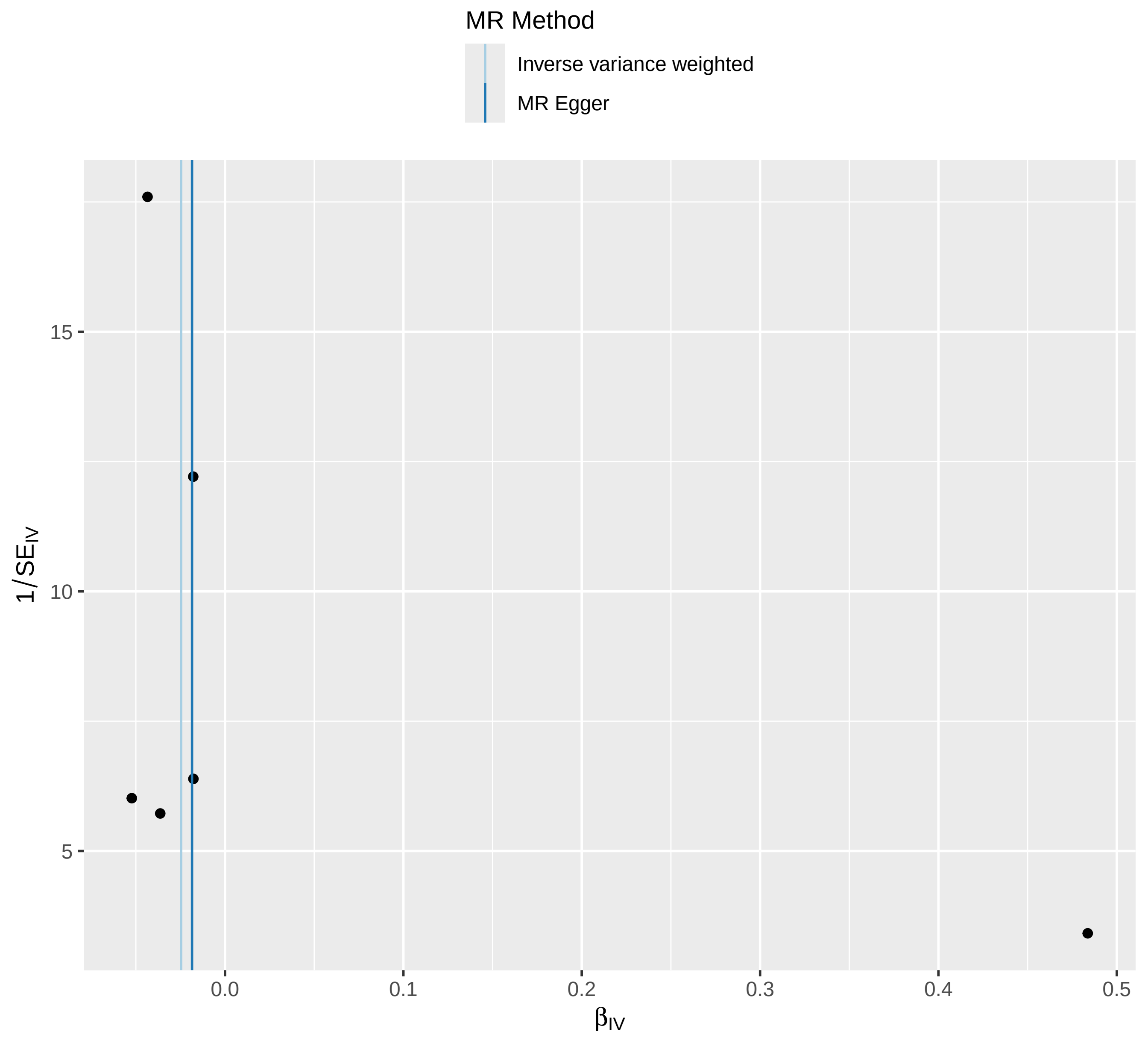
MR Method
Inverse variance weighted
MR Egger
15
10
5
1/SE_IV
0.0
0.1
0.2
0.3
0.4
0.5
β_IV

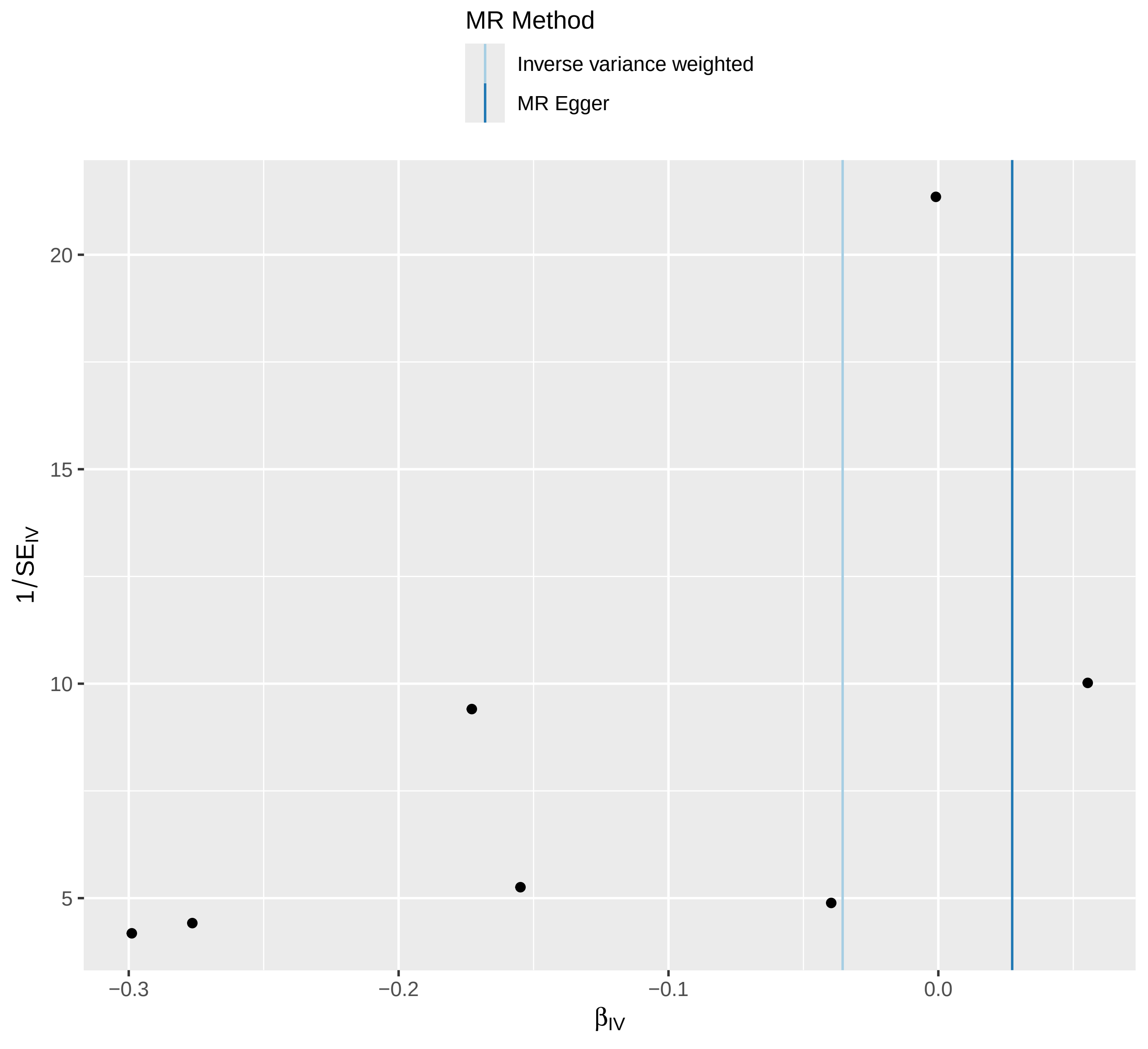

MR Method
Inverse variance weighted
MR Egger
20
15
10
5
$1/SE_{IV}$
−0.3
−0.2
−0.1
0.0
$\beta_{IV}$

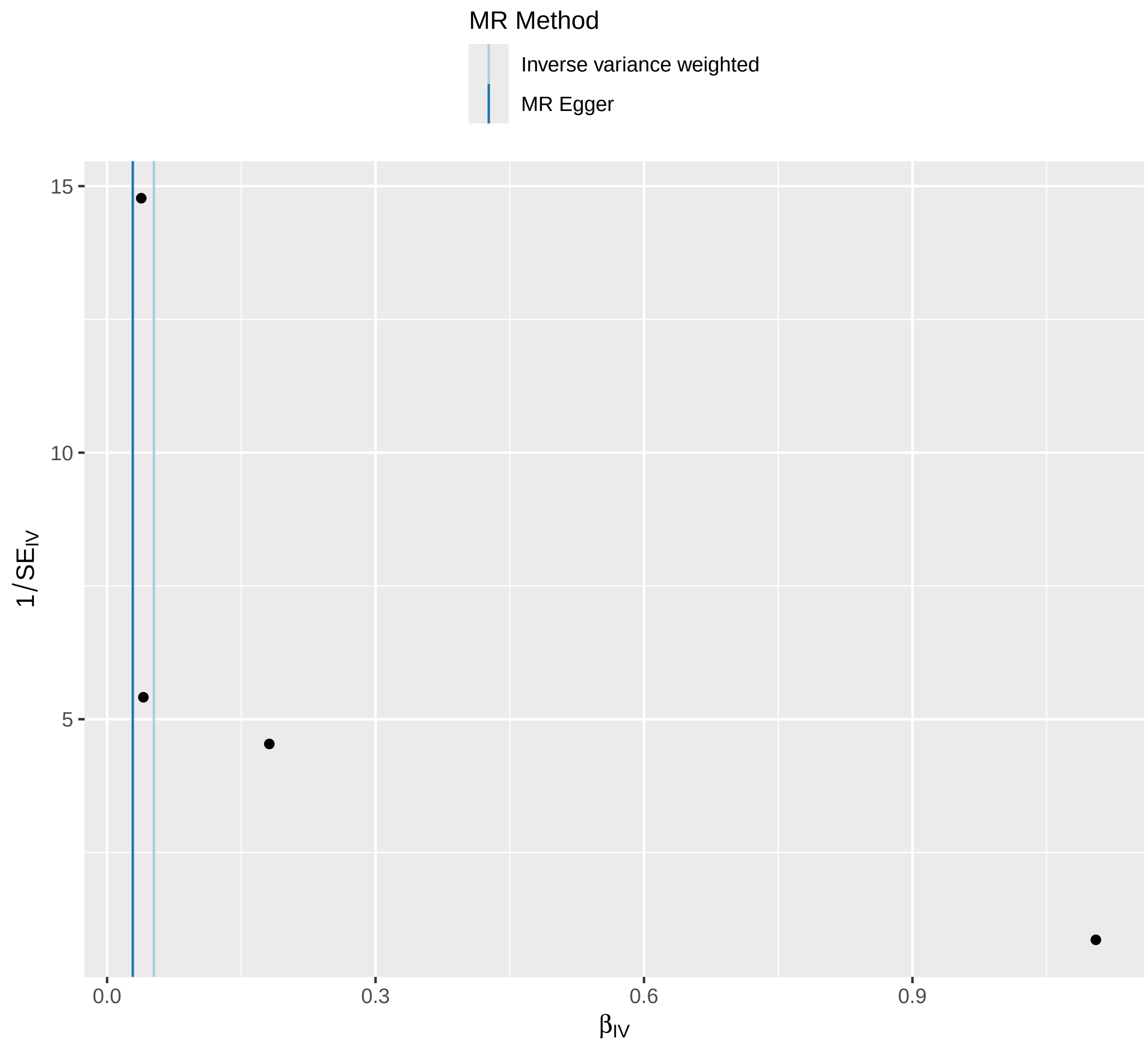
MR Method
Inverse variance weighted
MR Egger
15
10
5
$1/SE_{IV}$
0.0
0.3
0.6
0.9
$\beta_{IV}$

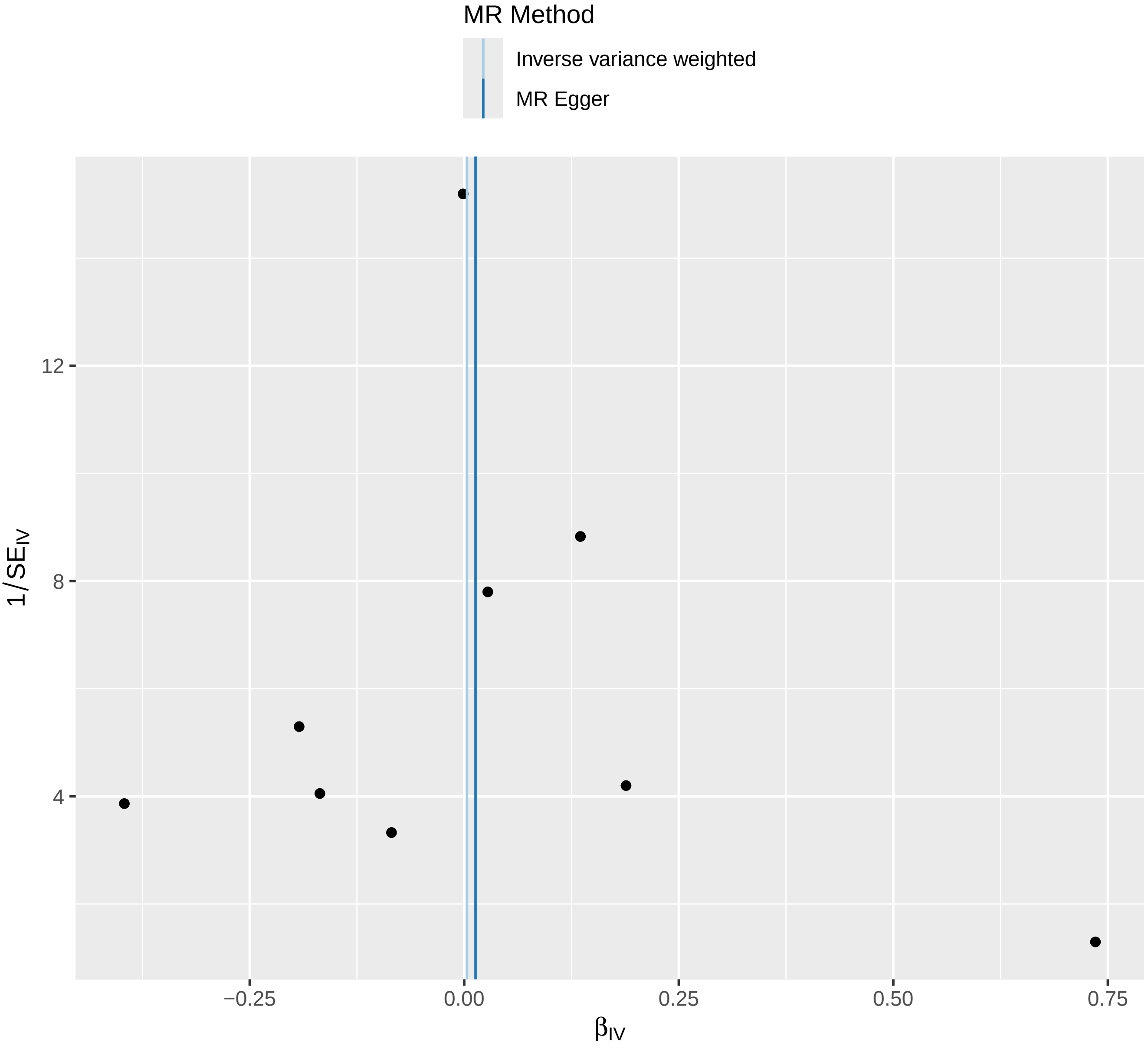
MR Method
Inverse variance weighted
MR Egger
12
8
4
1/SE_IV
−0.25
0.00
0.25
0.50
0.75
β_IV

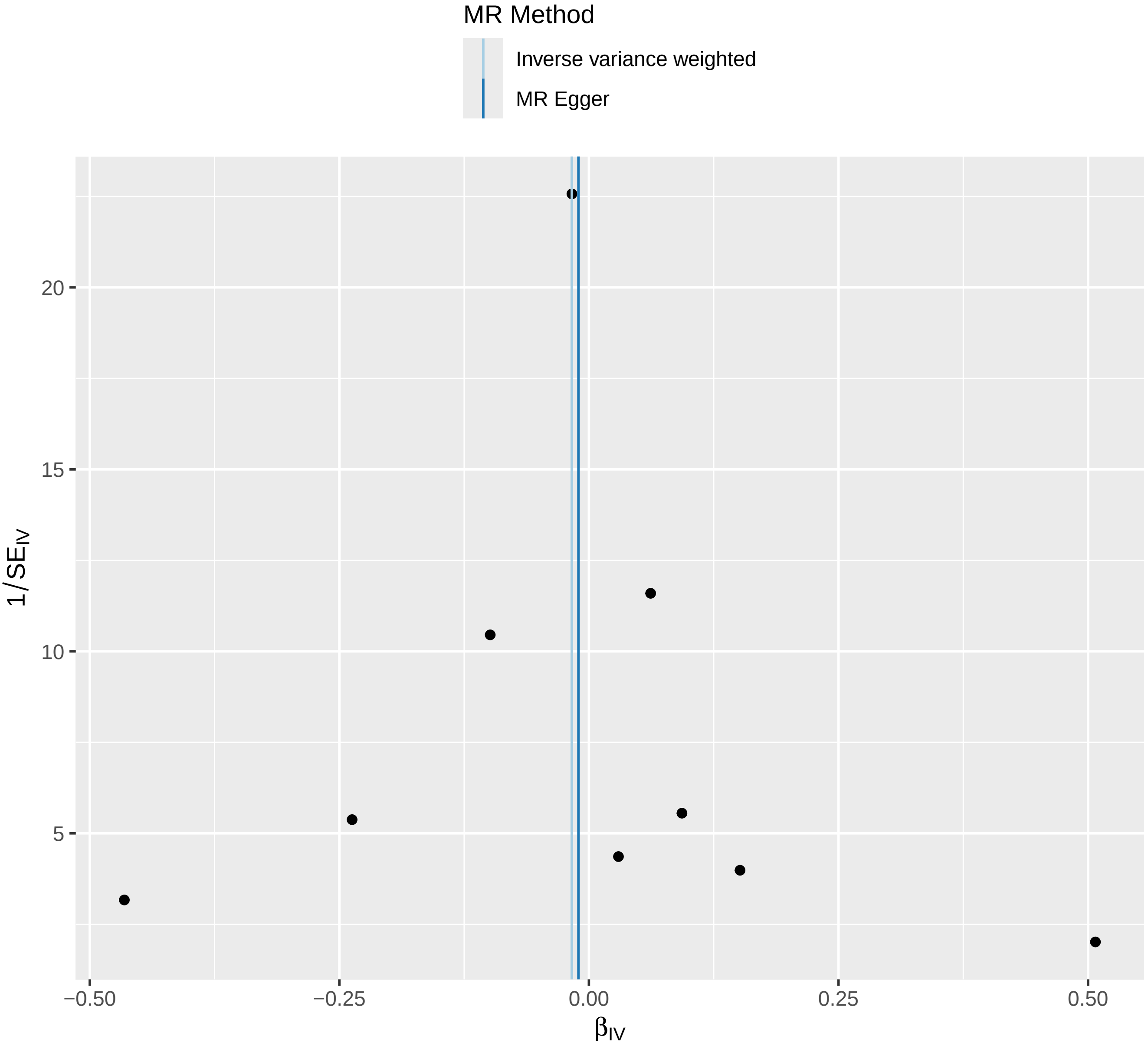

MR Method
Inverse variance weighted
MR Egger
20
15
10
5
$1/SE_{IV}$
−0.50
−0.25
0.00
0.25
0.50
$\beta_{IV}$

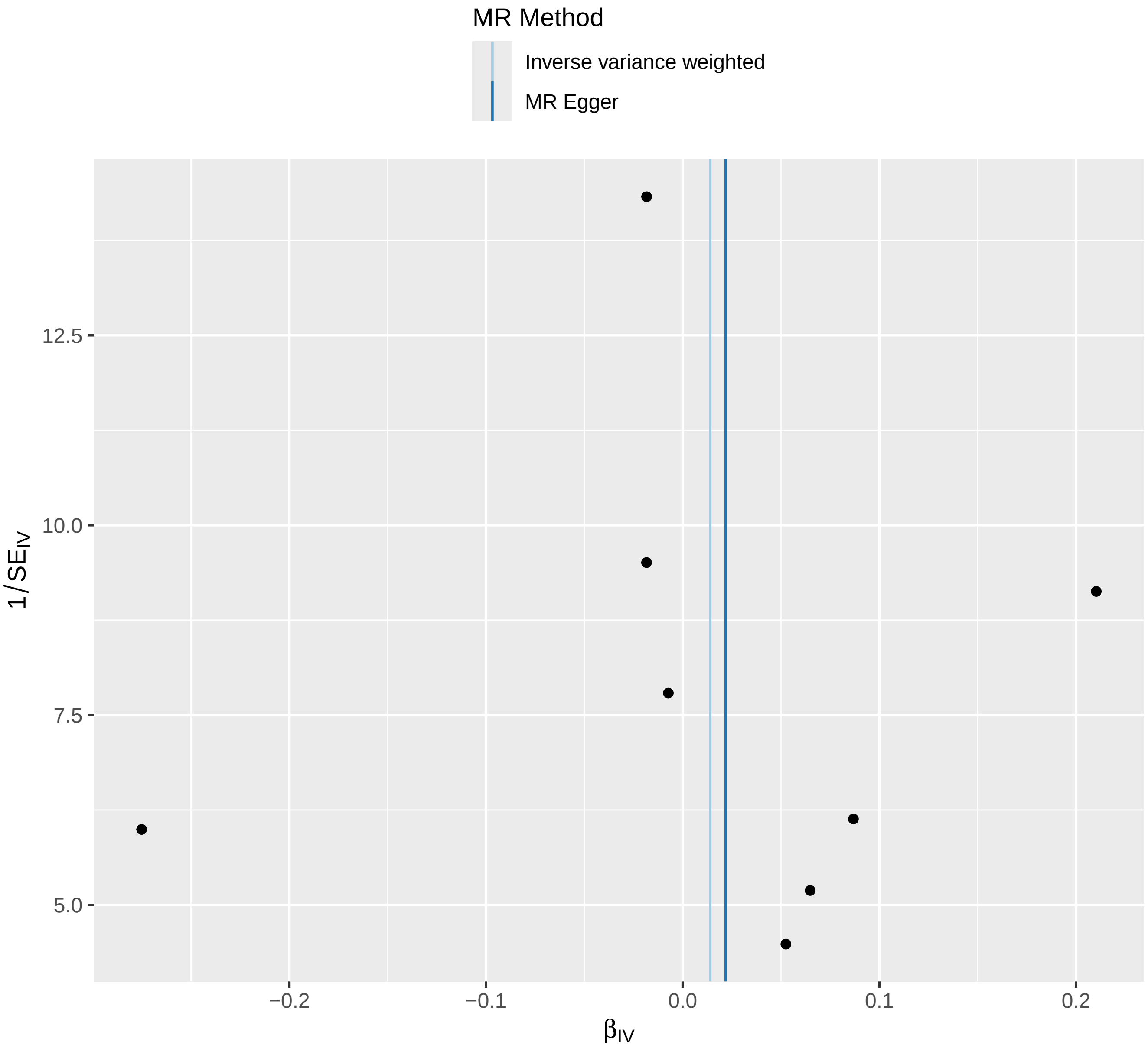

MR Method
Inverse variance weighted
MR Egger
12.5
10.0
7.5
5.0
1/SE_IV
−0.2
−0.1
0.0
0.1
0.2
β_IV

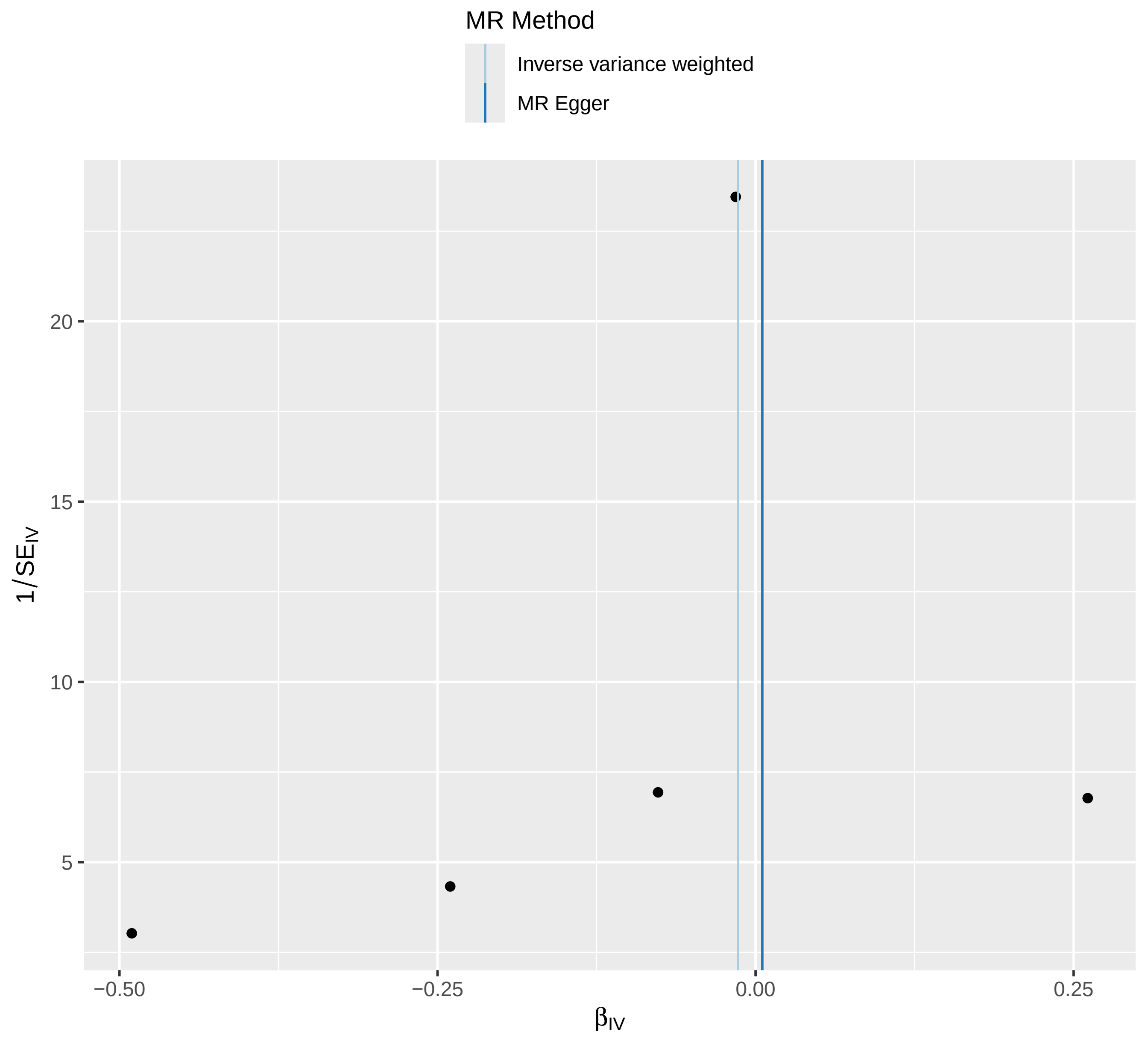
MR Method
Inverse variance weighted
MR Egger
20
15
10
5
1/SE_IV
−0.50
−0.25
0.00
0.25
β_IV

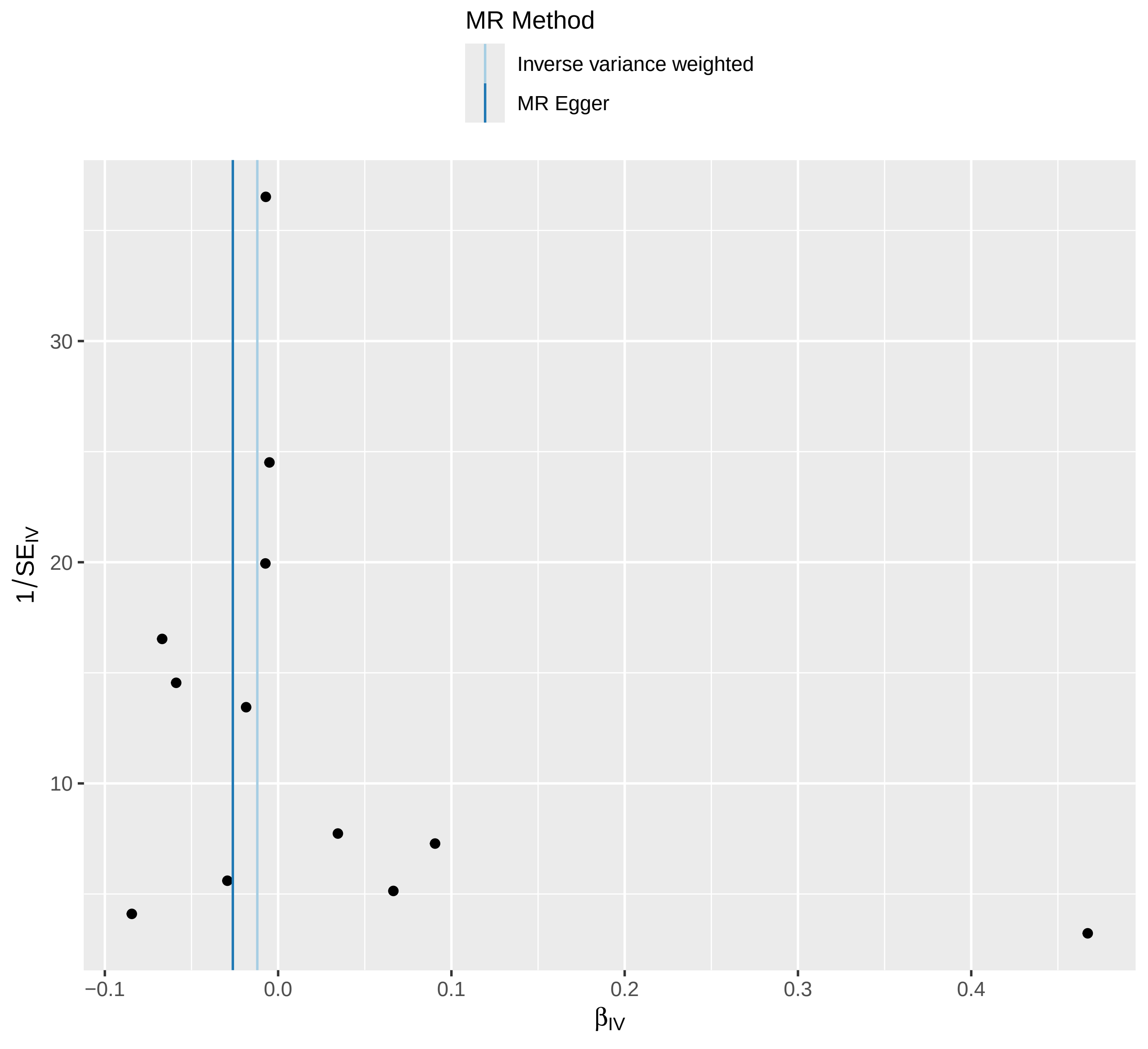

MR Method
Inverse variance weighted
MR Egger
30
20
10
1/SE_IV
−0.1
0.0
0.1
0.2
0.3
0.4
β_IV

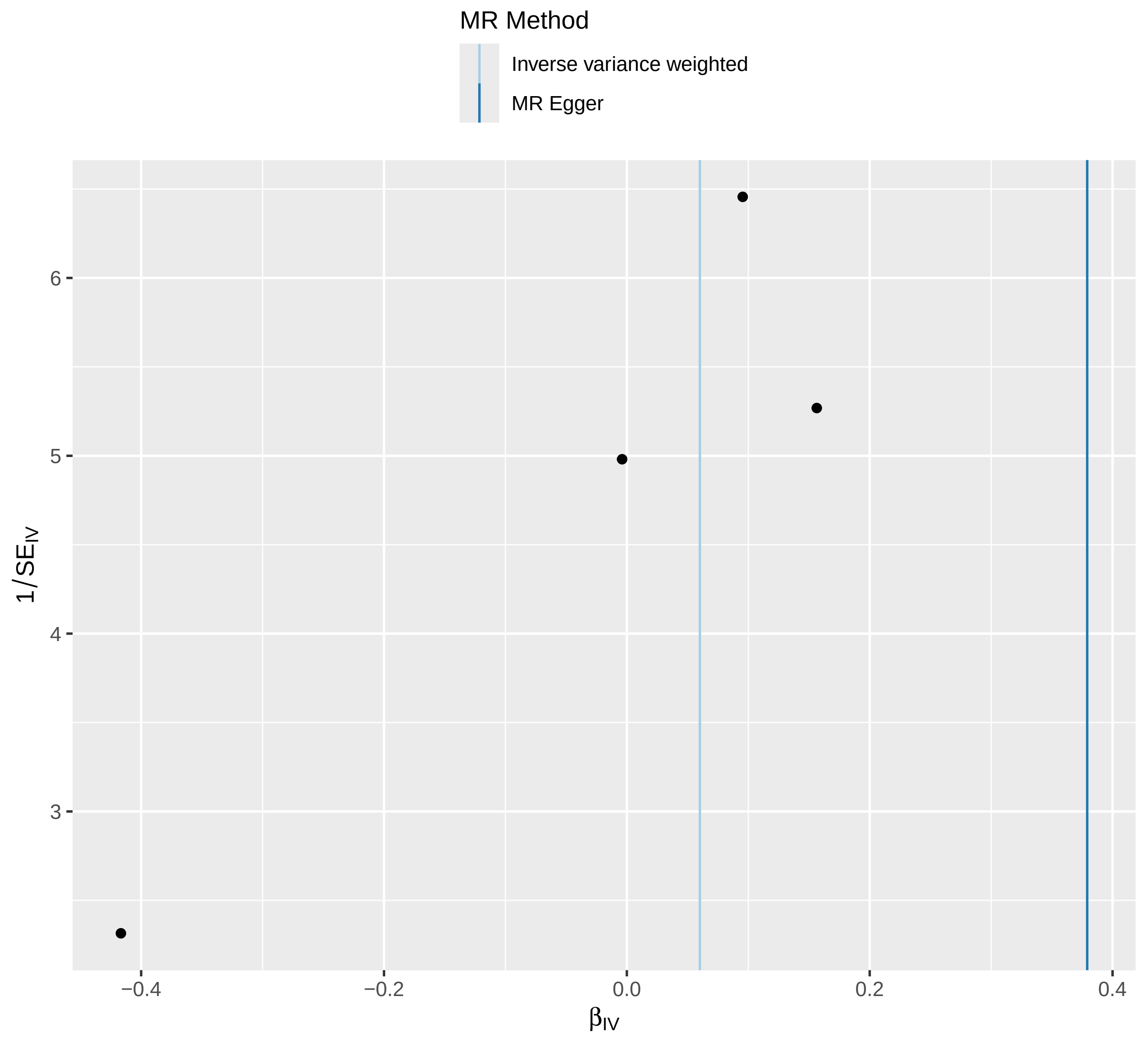
MR Method
Inverse variance weighted
MR Egger
6
5
4
3
1/SE$_{IV}$
−0.4
−0.2
0.0
0.2
0.4
β$_{IV}$

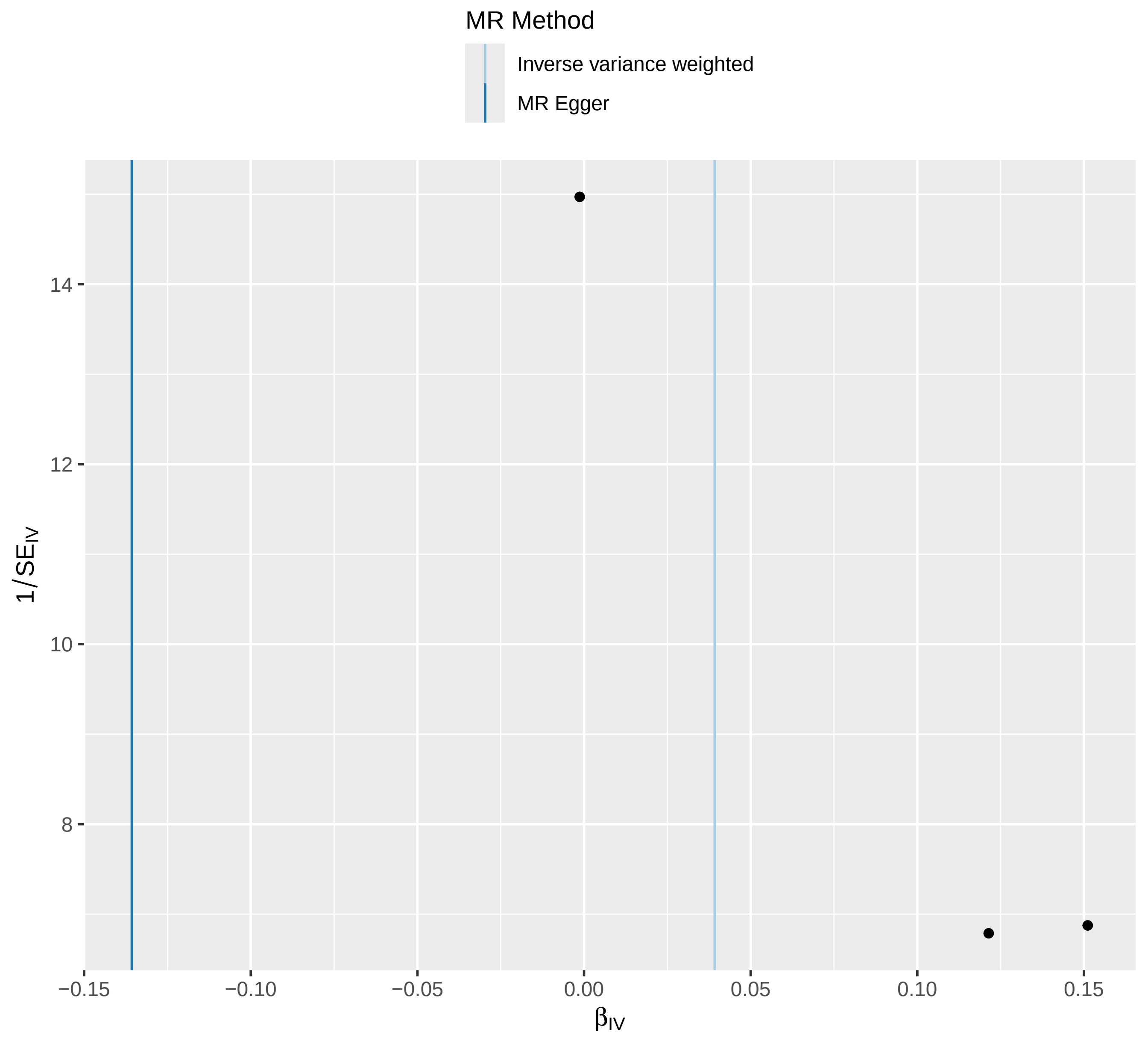

MR Method
Inverse variance weighted
MR Egger
14
12
10
8
$1/SE_{IV}$
−0.15
−0.10
−0.05
0.00
0.05
0.10
0.15
$\beta_{IV}$

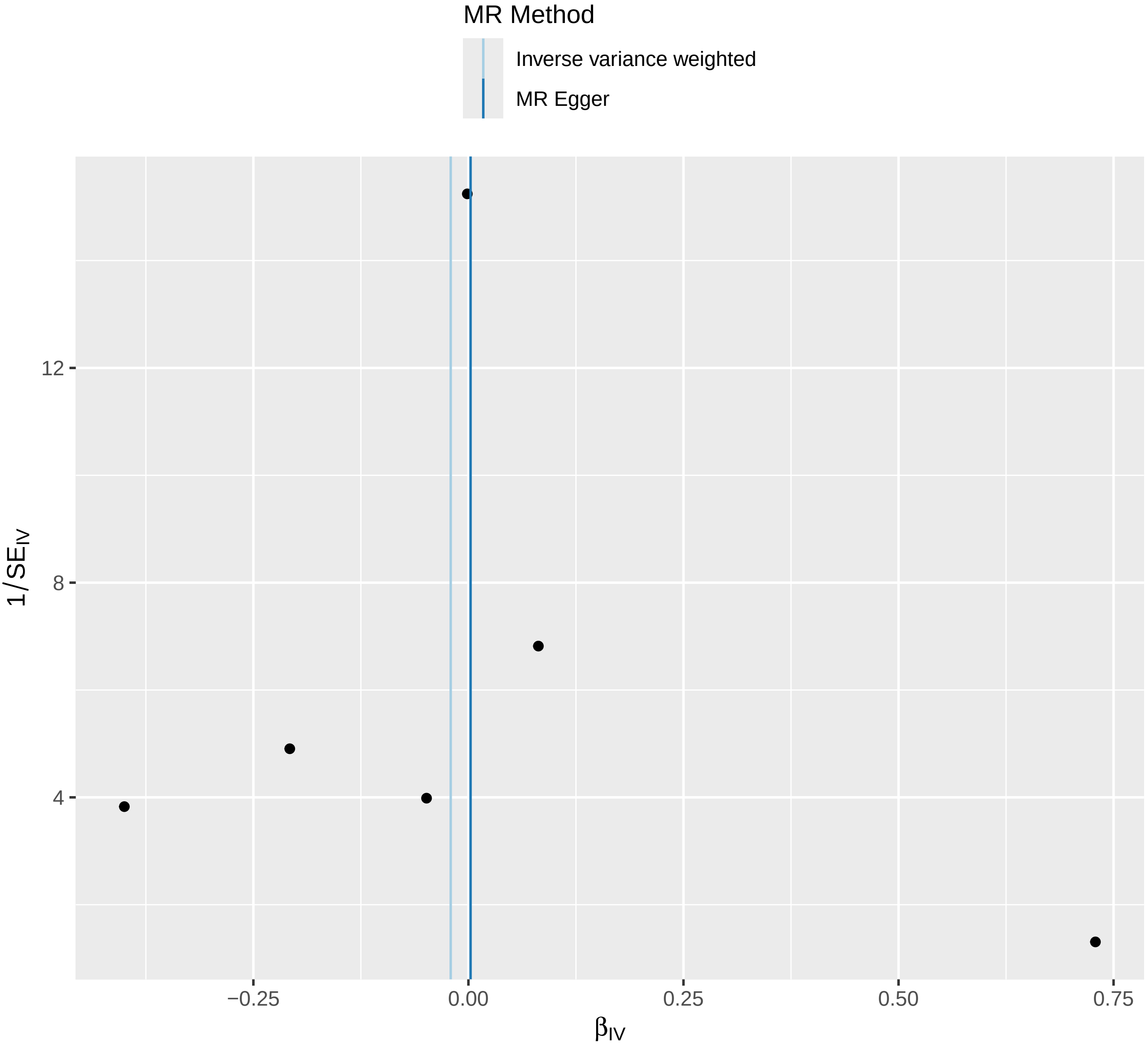

MR Method
Inverse variance weighted
MR Egger
12
8
4
1/SE$_{IV}$
−0.25
0.00
0.25
0.50
0.75
β$_{IV}$

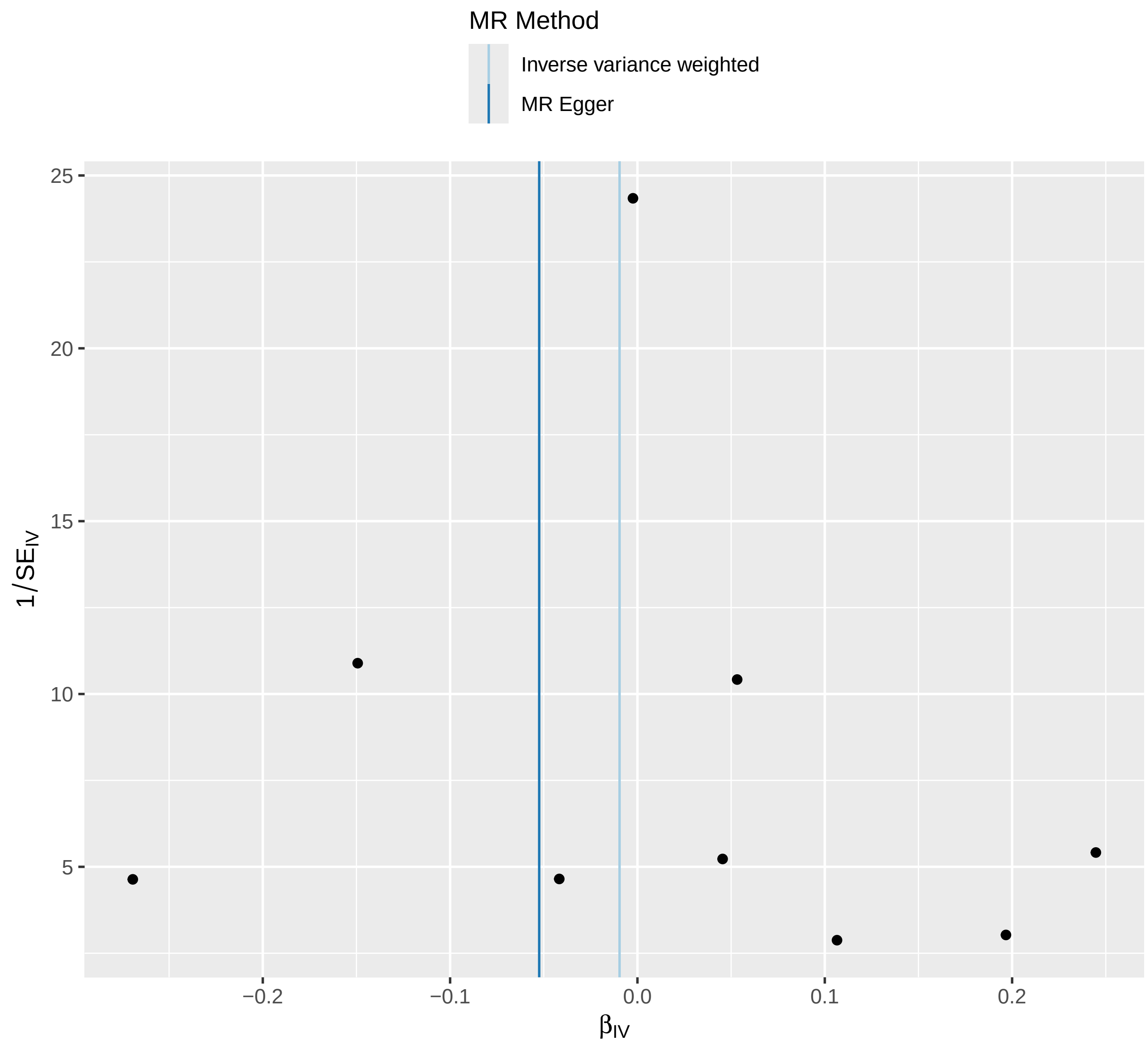
MR Method
Inverse variance weighted
MR Egger
25
20
15
10
5
1/SE$_{IV}$
−0.2
−0.1
0.0
0.1
0.2
β$_{IV}$

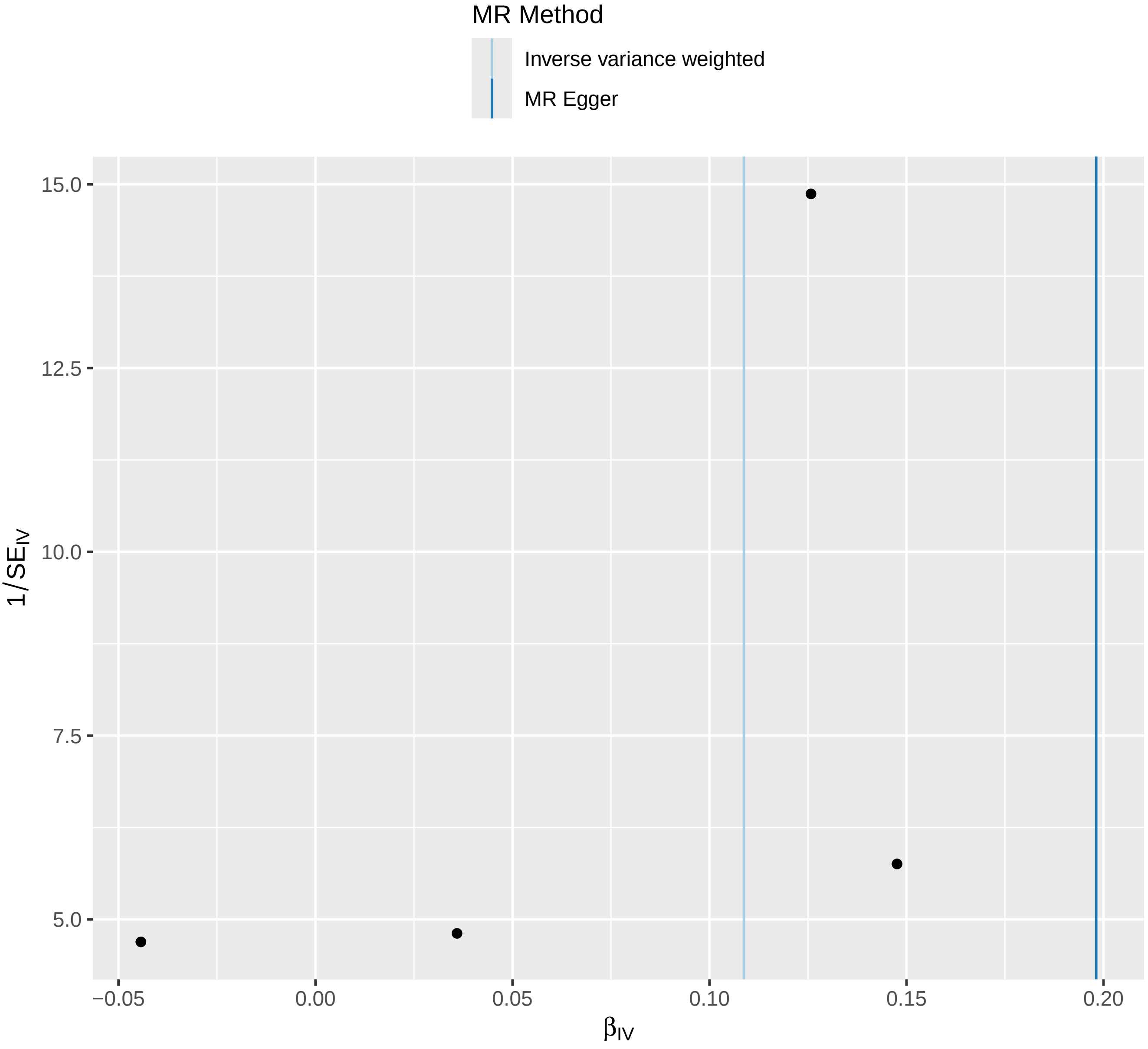
MR Method
Inverse variance weighted
MR Egger
15.0
12.5
10.0
7.5
5.0
$1/SE_{IV}$
−0.05
0.00
0.05
0.10
0.15
0.20
$\beta_{IV}$

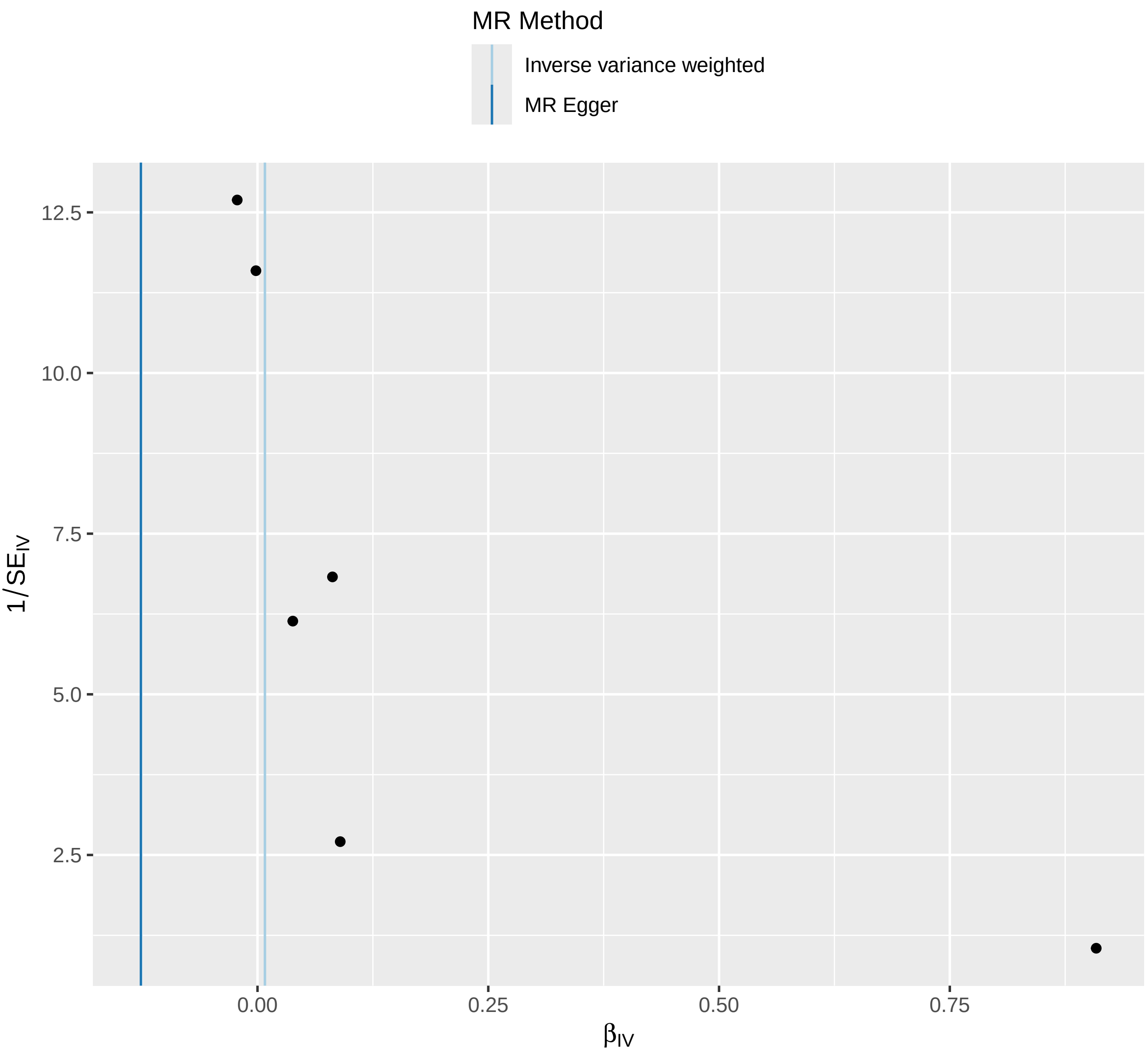
MR Method
Inverse variance weighted
MR Egger
12.5
10.0
7.5
5.0
2.5
0.00
0.25
0.50
0.75
1/SE_IV
β_IV

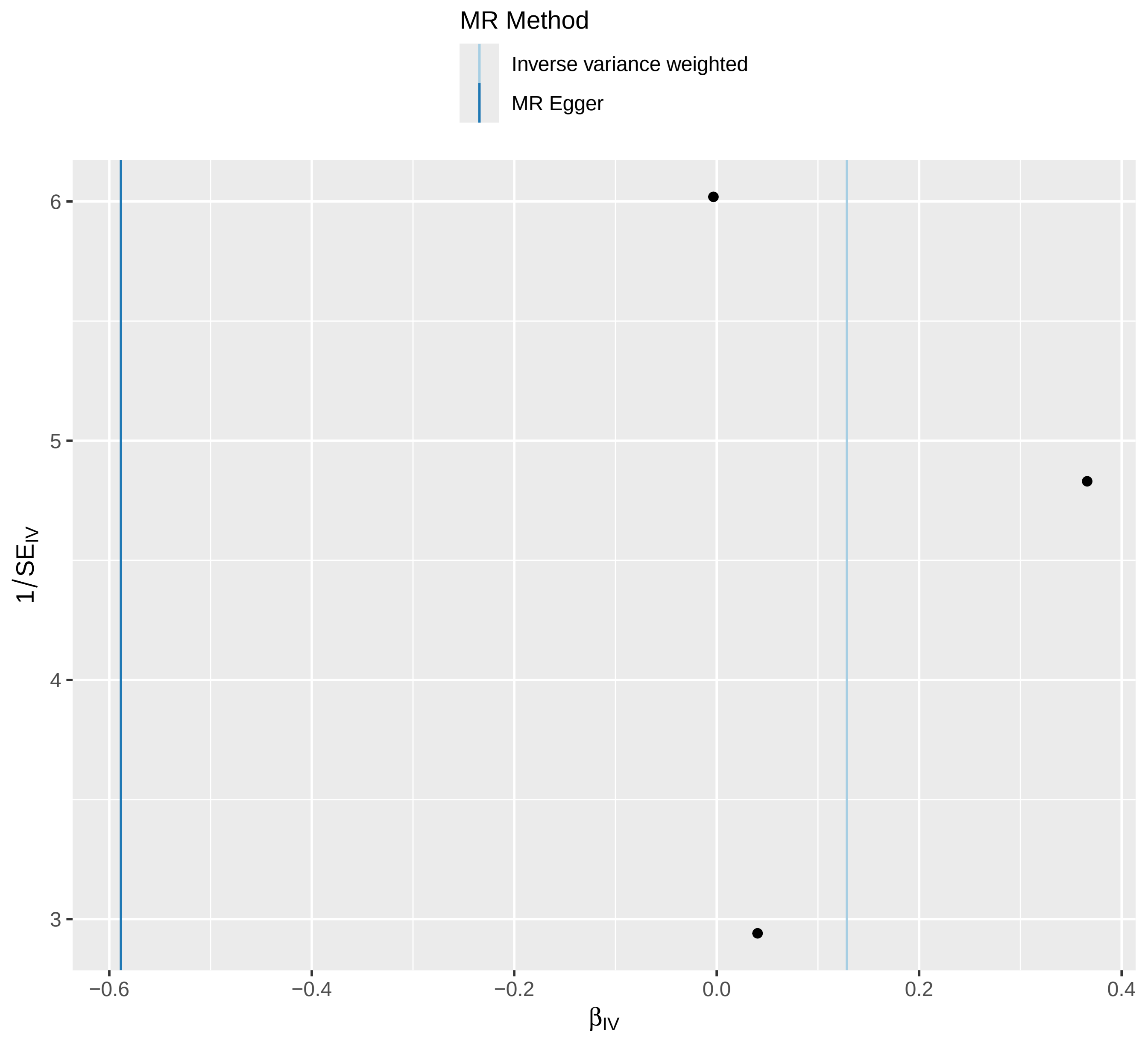

MR Method
Inverse variance weighted
MR Egger
6
5
4
3
1/SE$_{IV}$
−0.6
−0.4
−0.2
0.0
0.2
0.4
β$_{IV}$

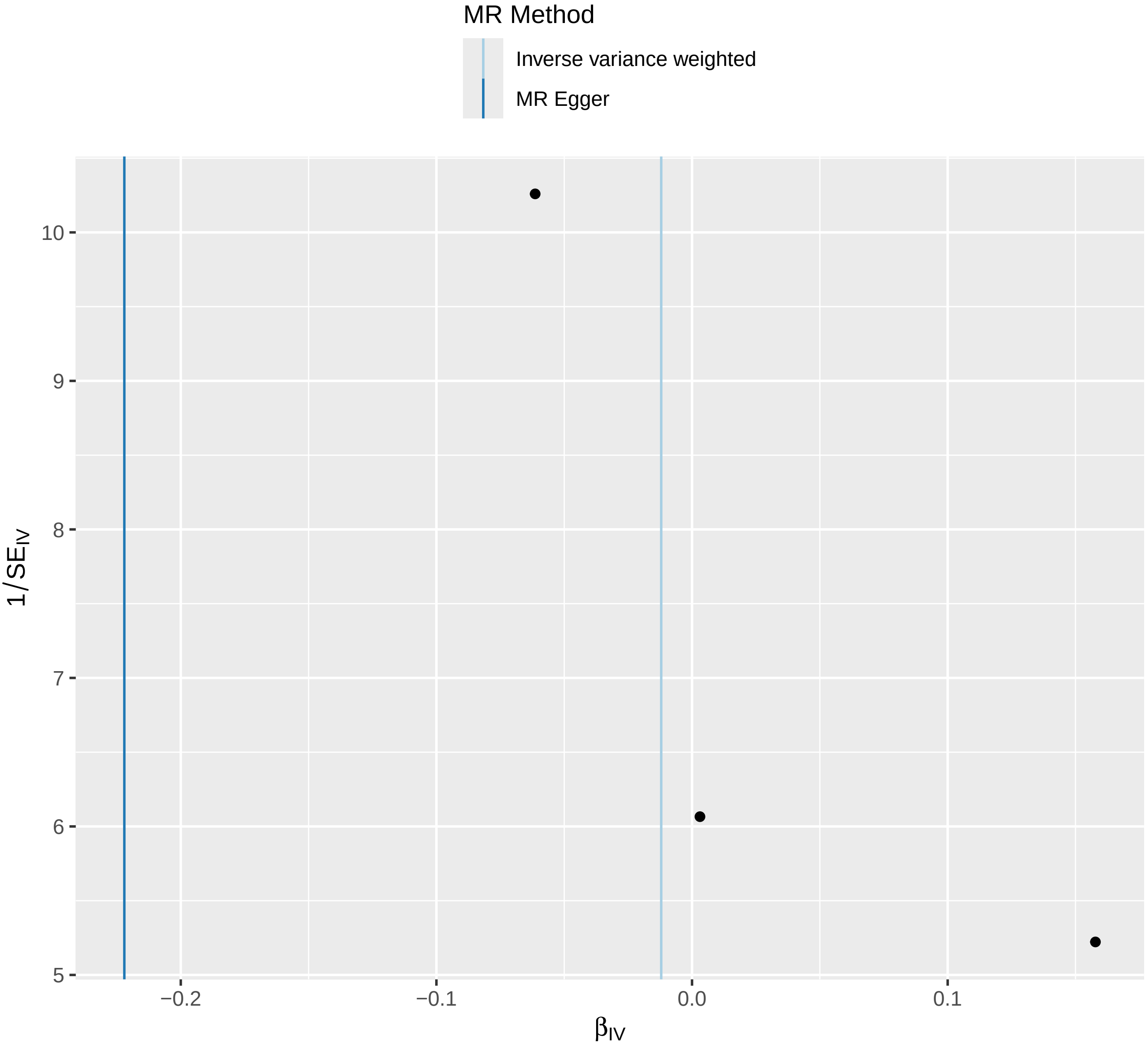

MR Method
Inverse variance weighted
MR Egger
10
9
8
7
6
5
1/SE$_{IV}$
−0.2
−0.1
0.0
0.1
β$_{IV}$

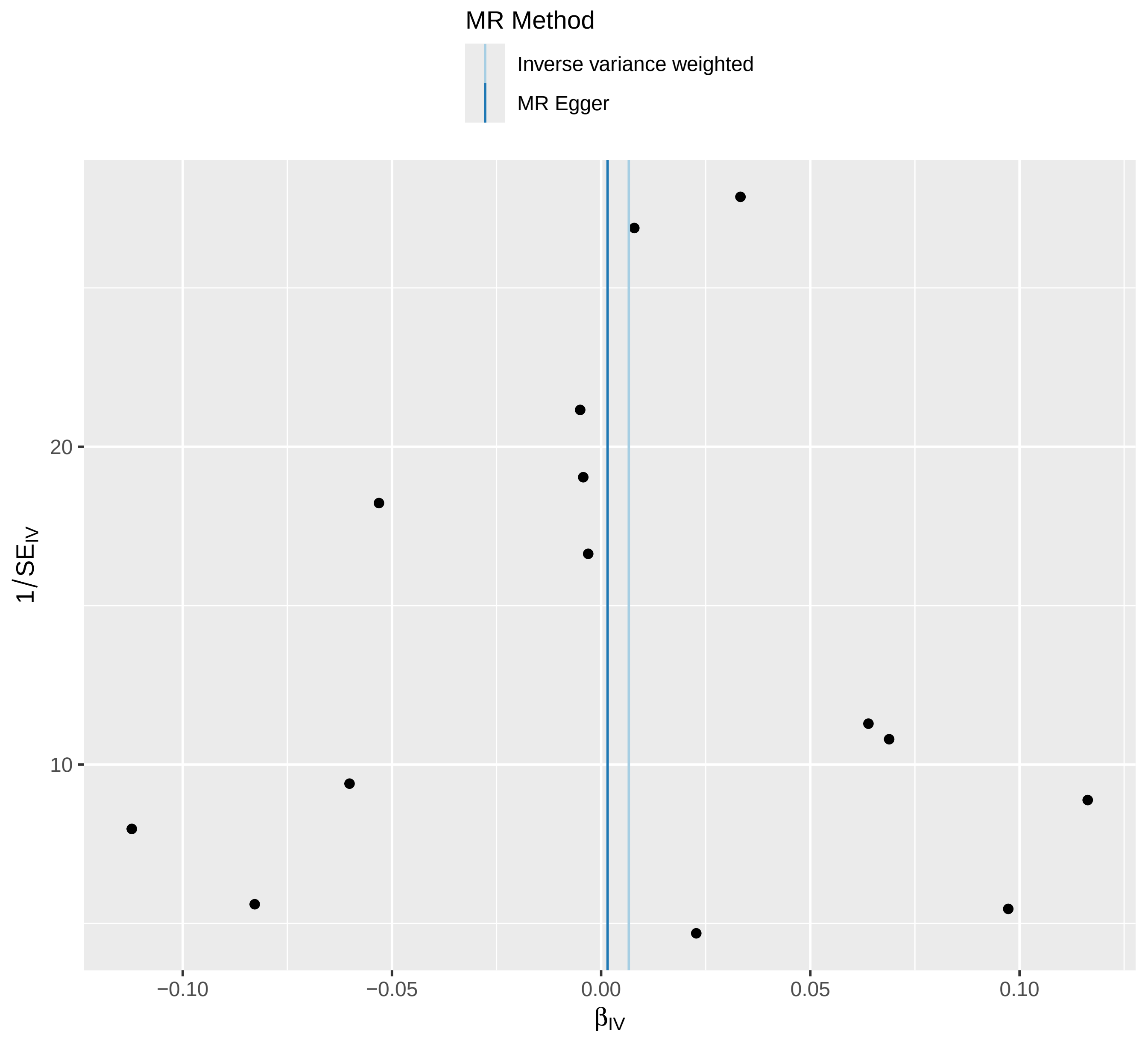
MR Method
Inverse variance weighted
MR Egger
1/SE_IV
20
10
−0.10
−0.05
0.00
0.05
0.10
β_IV

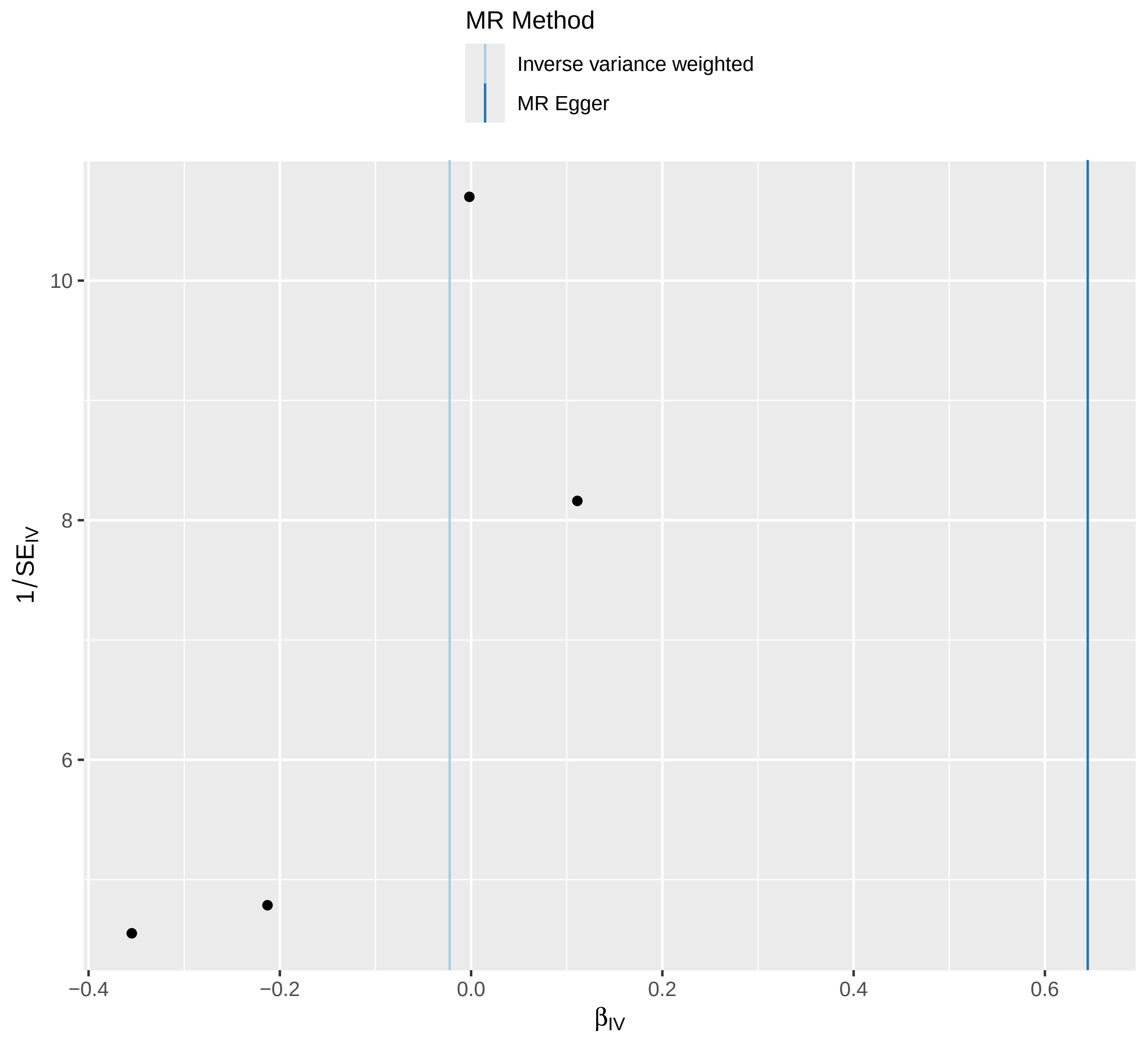

MR Method
Inverse variance weighted
MR Egger
10
8
6
1/SE_IV
−0.4
−0.2
0.0
0.2
0.4
0.6
β_IV

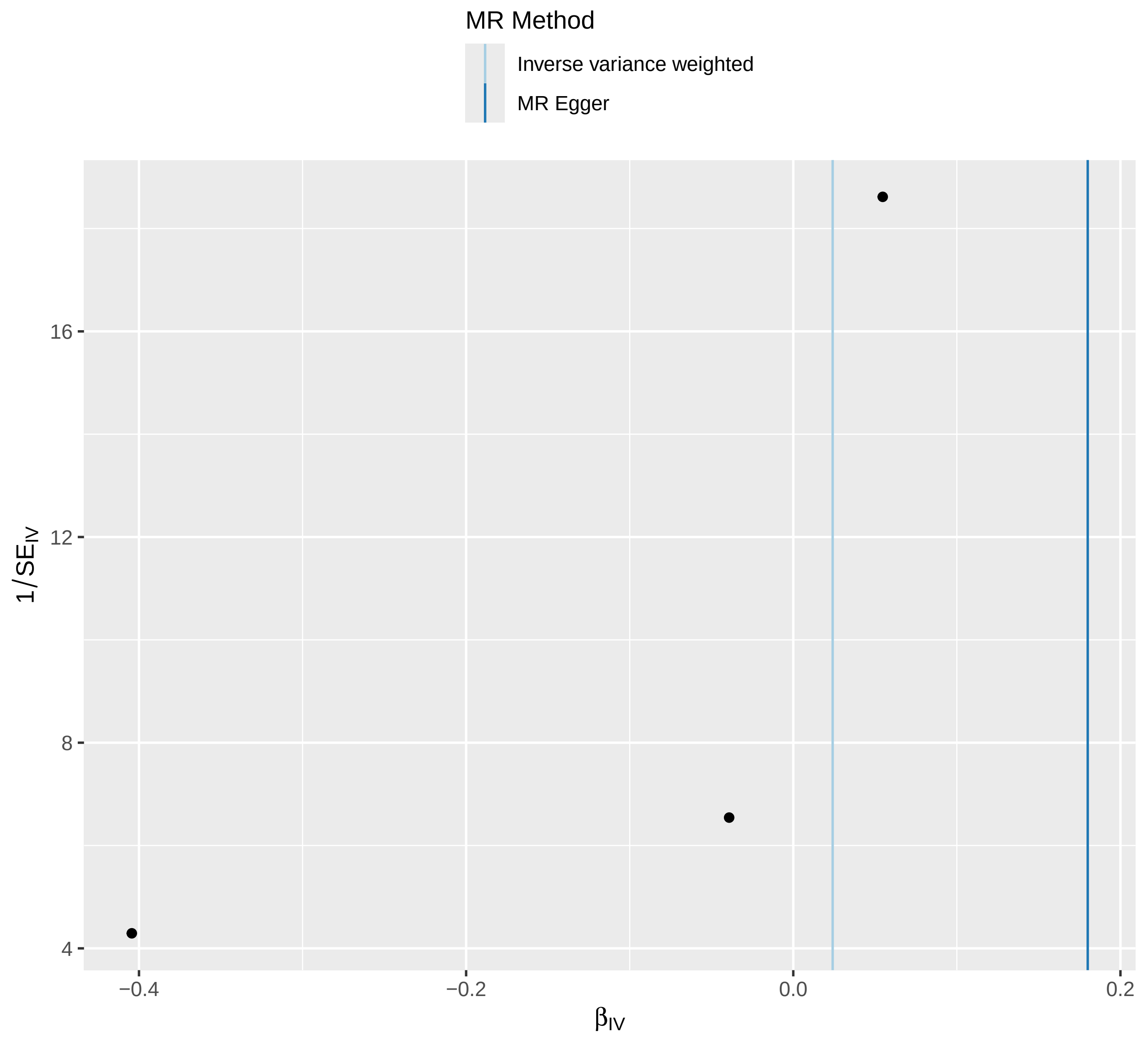
MR Method
Inverse variance weighted
MR Egger
16
12
8
4
1/SE$_{IV}$
−0.4
−0.2
0.0
0.2
β$_{IV}$

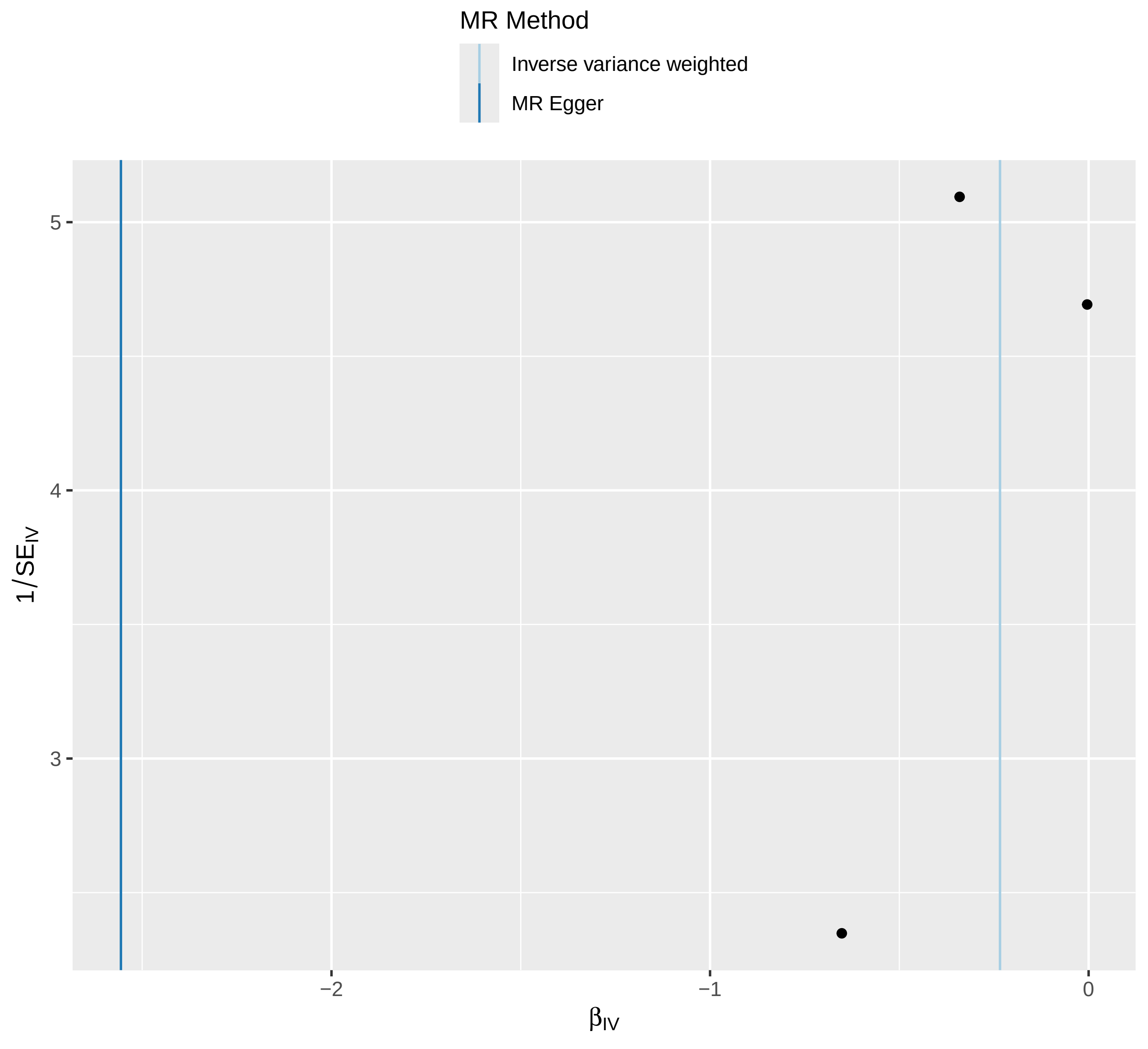
MR Method
Inverse variance weighted
MR Egger
5
4
3
1/SE$_{IV}$
−2
−1
0
$\beta_{IV}$

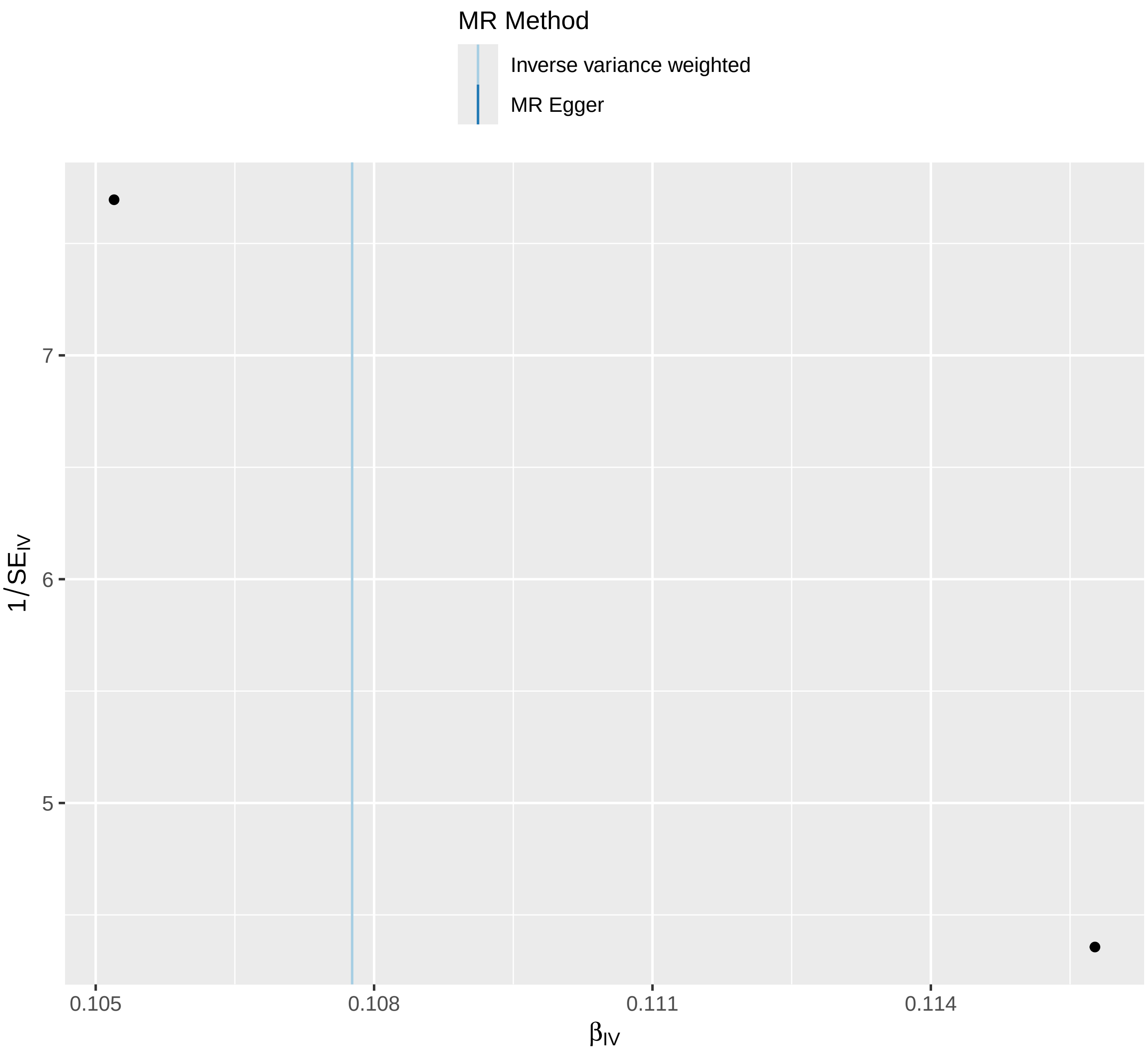
MR Method
Inverse variance weighted
MR Egger
1/SE_IV
7
6
5
0.105
0.108
0.111
0.114
β_IV

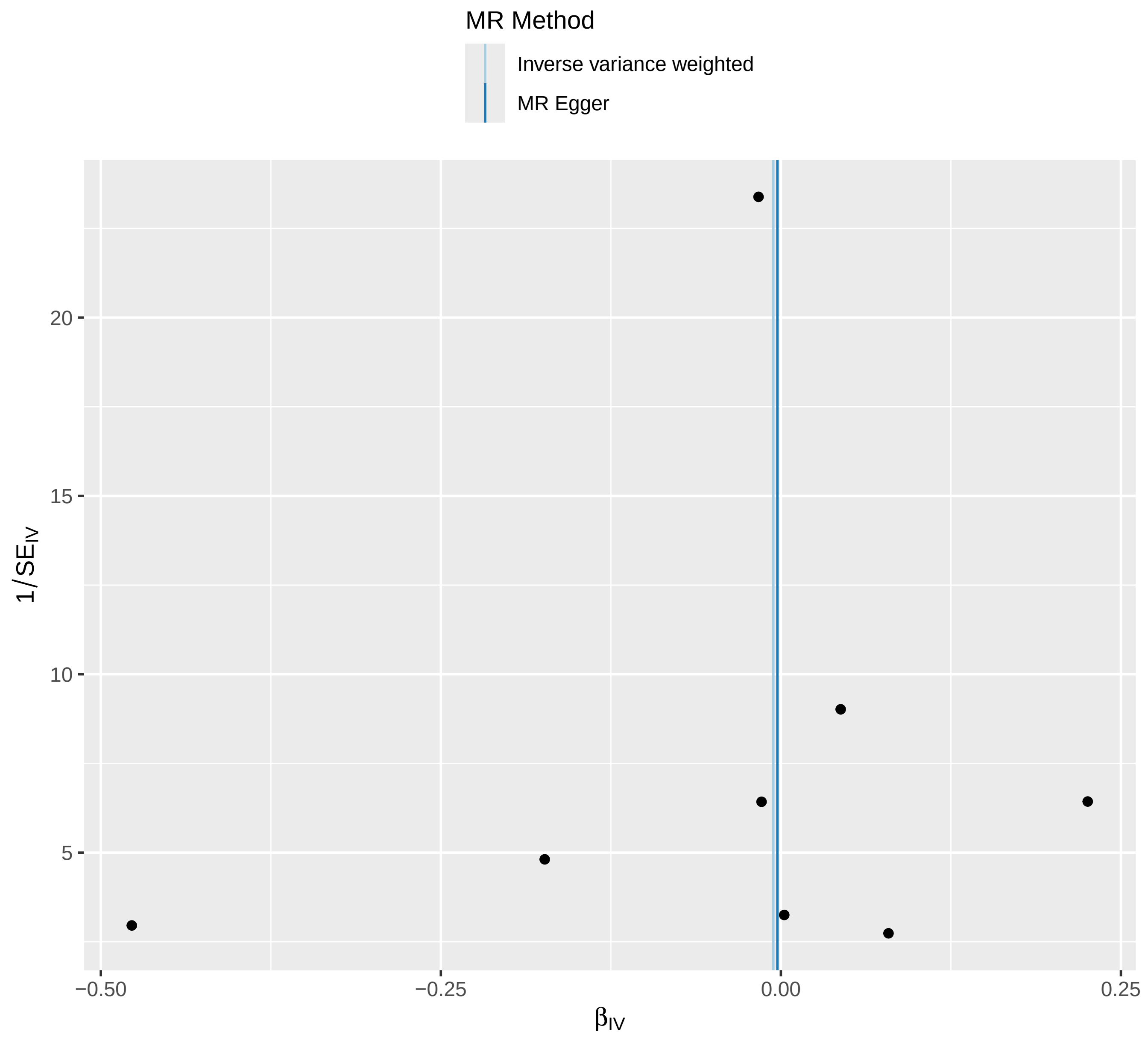

MR Method
Inverse variance weighted
MR Egger
20
15
10
5
1/SE_IV
−0.50
−0.25
0.00
0.25
β_IV

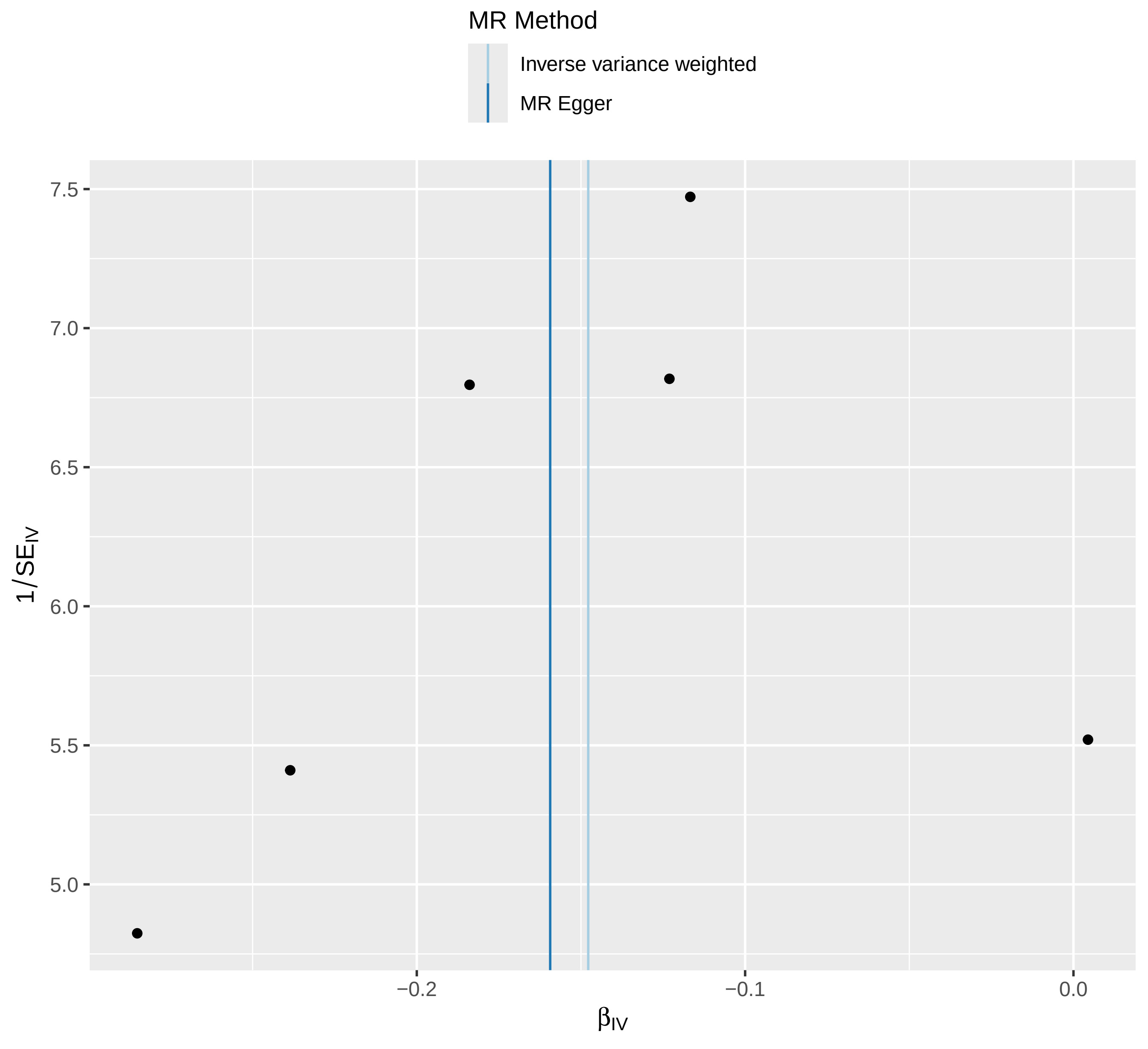

MR Method
Inverse variance weighted
MR Egger
7.5
7.0
6.5
6.0
5.5
5.0
1/SE$_{IV}$
−0.2
−0.1
0.0
β$_{IV}$

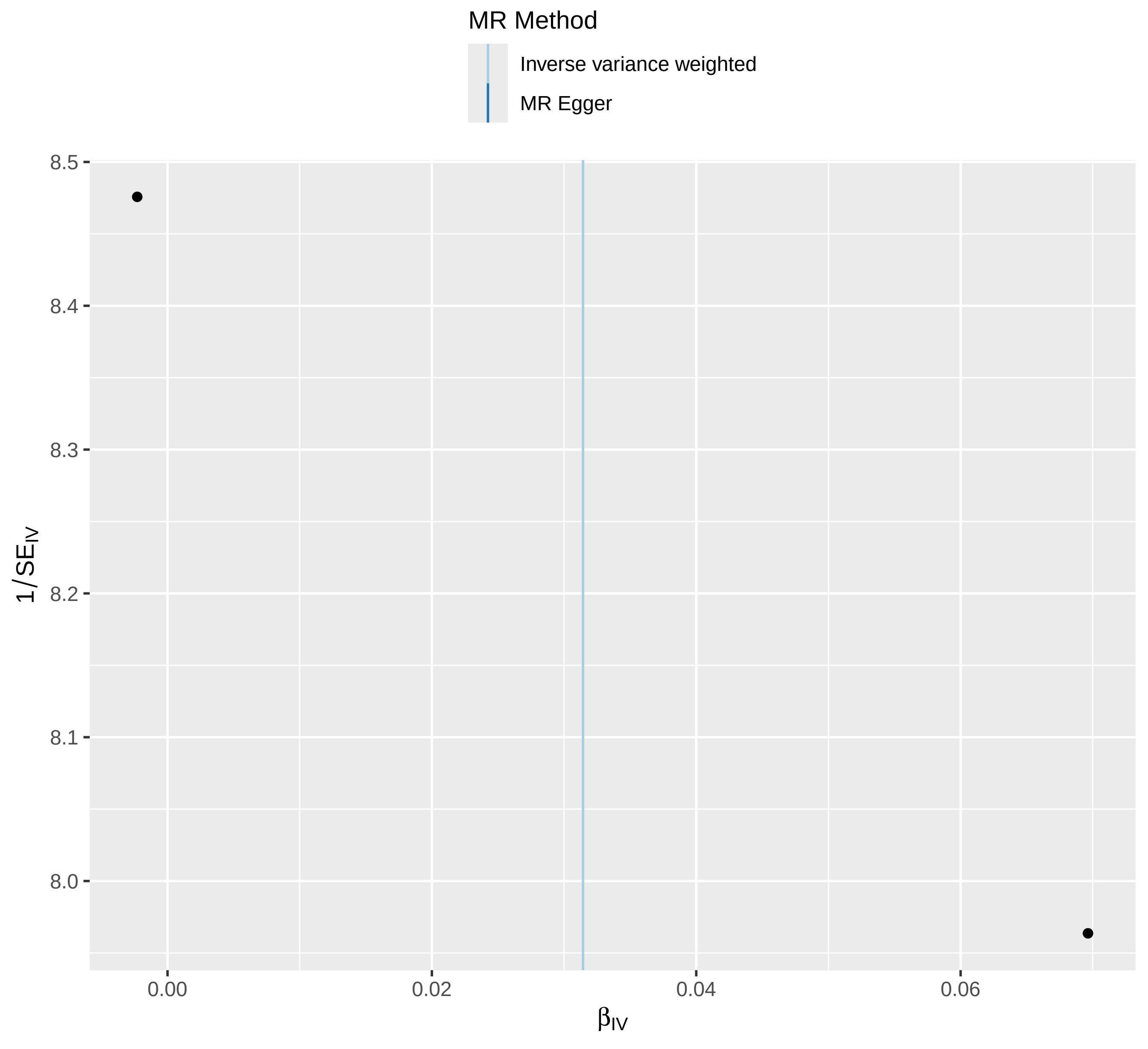
MR Method
Inverse variance weighted
MR Egger
8.5
8.4
8.3
8.2
8.1
8.0
1/SE$_{IV}$
0.00
0.02
0.04
0.06
β$_{IV}$

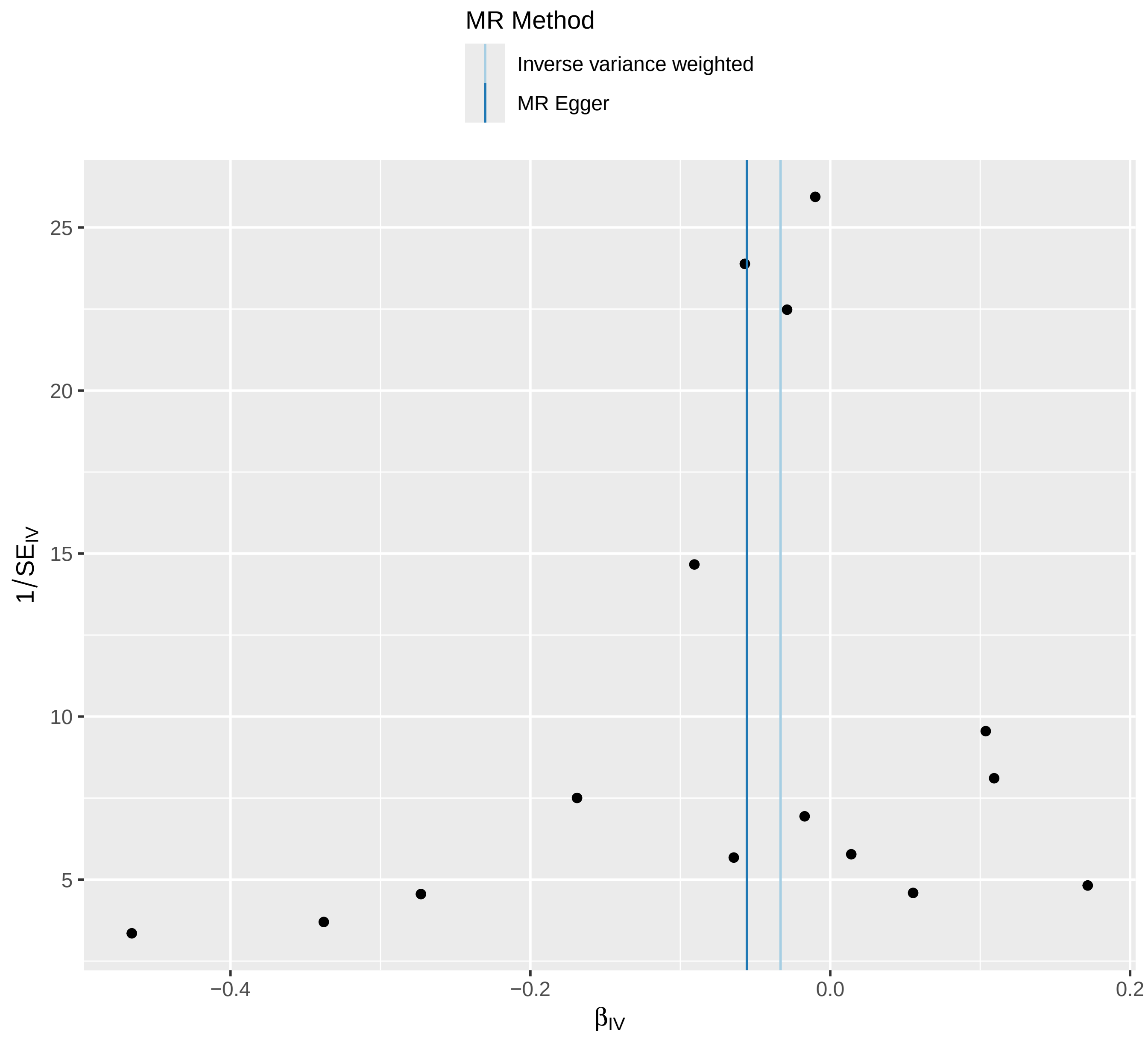
MR Method
Inverse variance weighted
MR Egger
25
20
15
10
5
1/SE_IV
−0.4
−0.2
0.0
0.2
β_IV

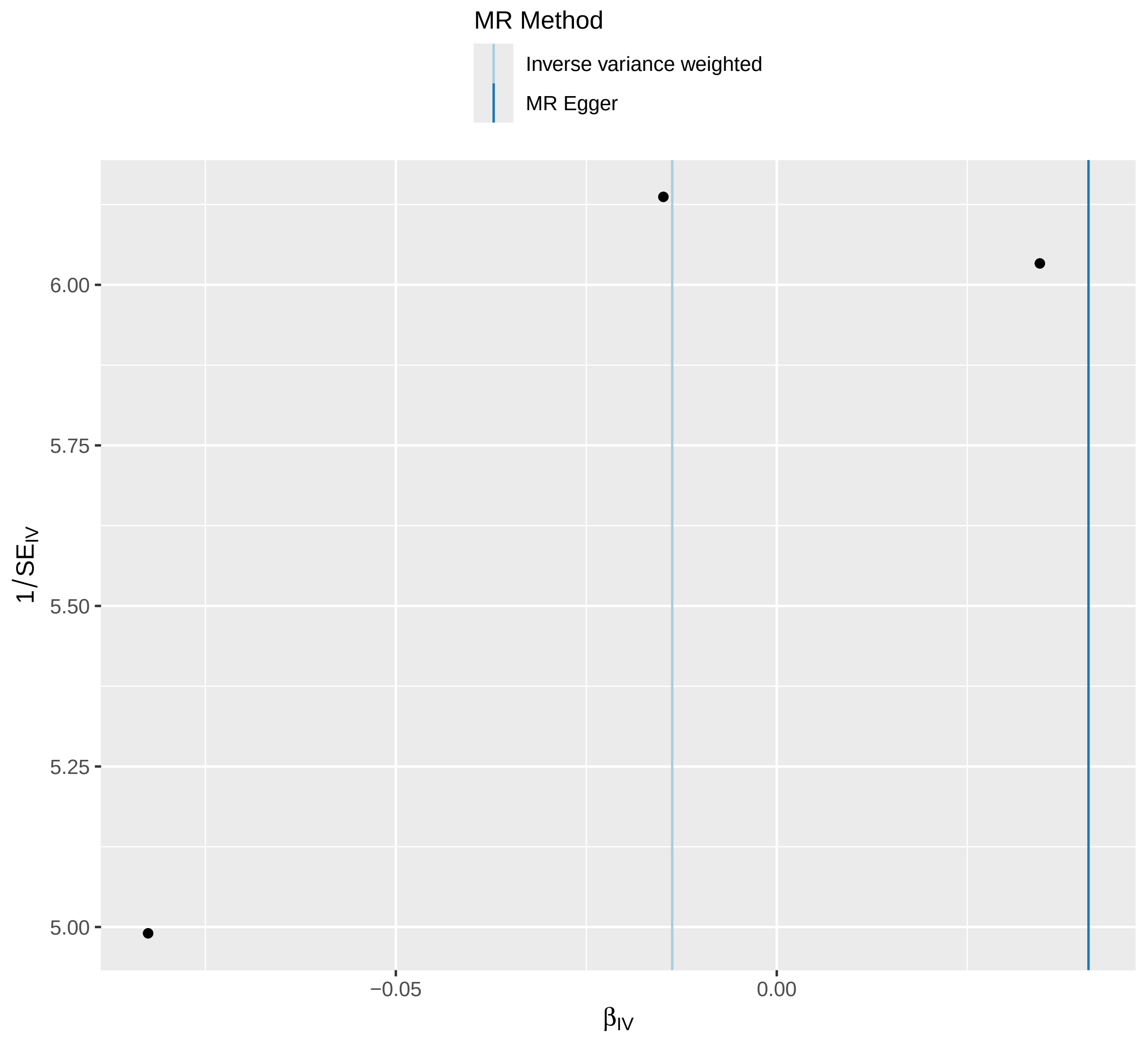
MR Method
Inverse variance weighted
MR Egger
6.00
5.75
5.50
5.25
5.00
−0.05
0.00
1/SE_IV
β_IV

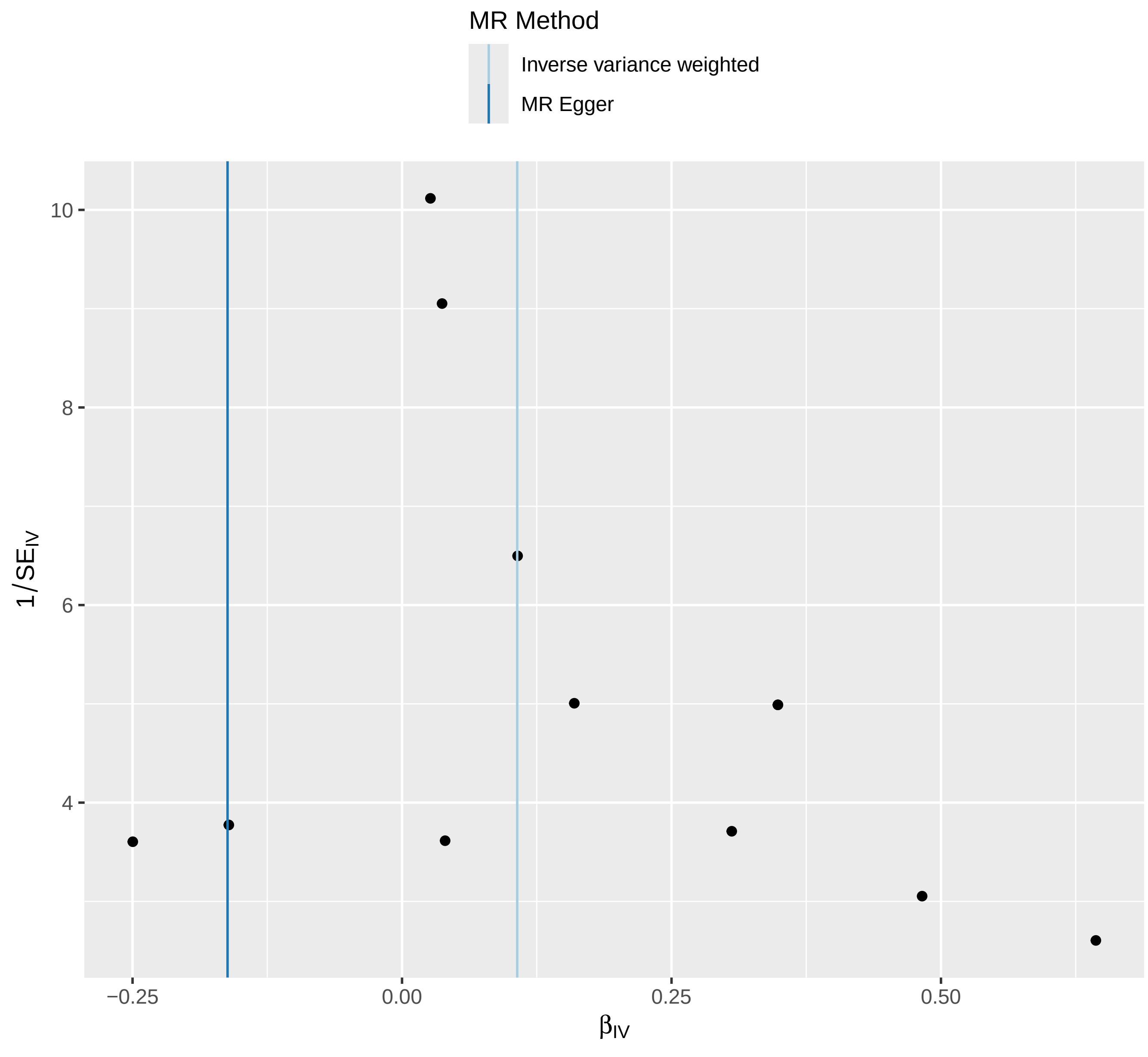
MR Method
Inverse variance weighted
MR Egger
10
8
6
4
1/SE_IV
−0.25
0.00
0.25
0.50
β_IV

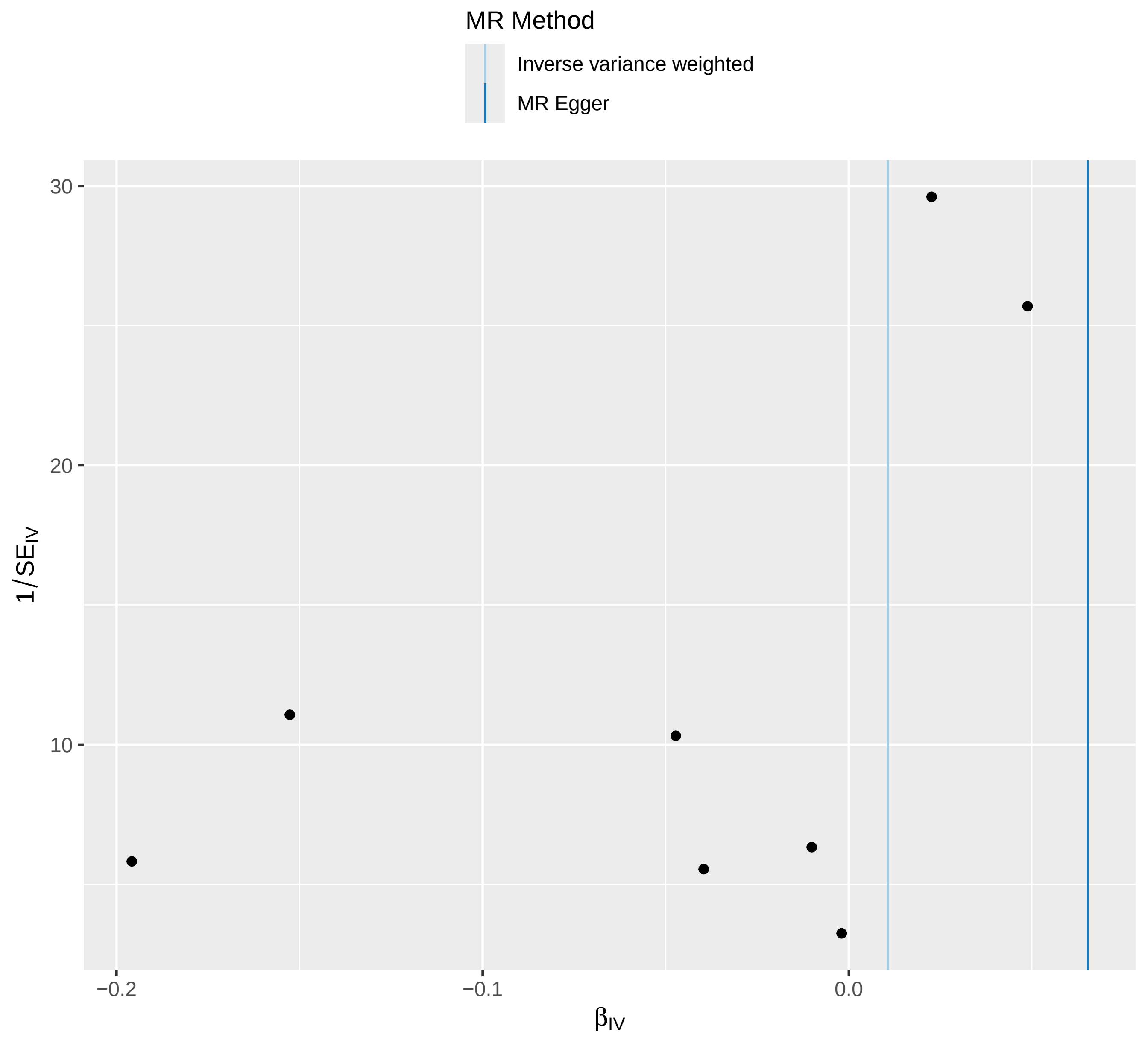
MR Method
Inverse variance weighted
MR Egger
30
20
10
1/SE_IV
−0.2
−0.1
0.0
β_IV

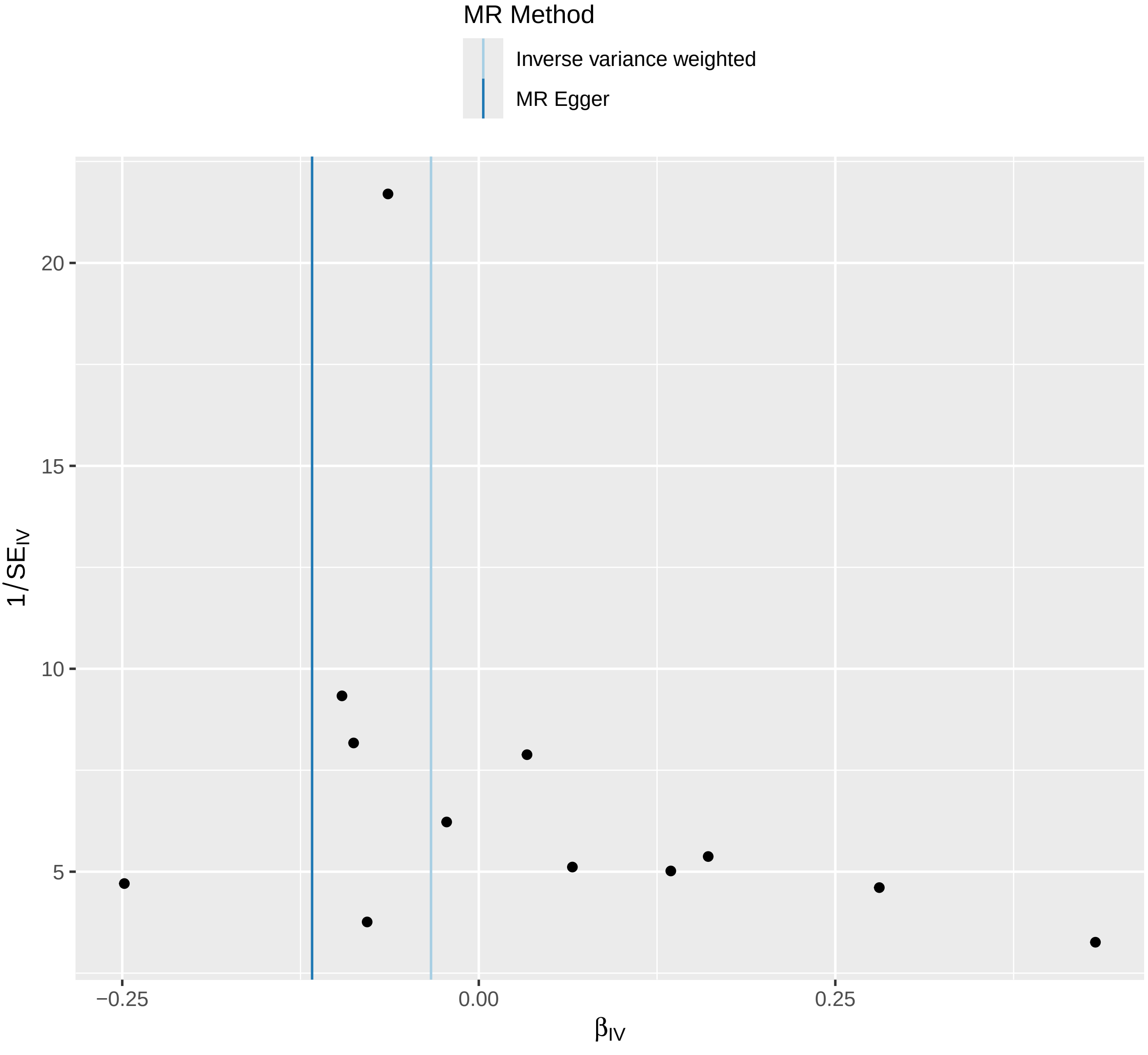
MR Method
Inverse variance weighted
MR Egger
20
15
10
5
1/SE_IV
−0.25
0.00
0.25
β_IV

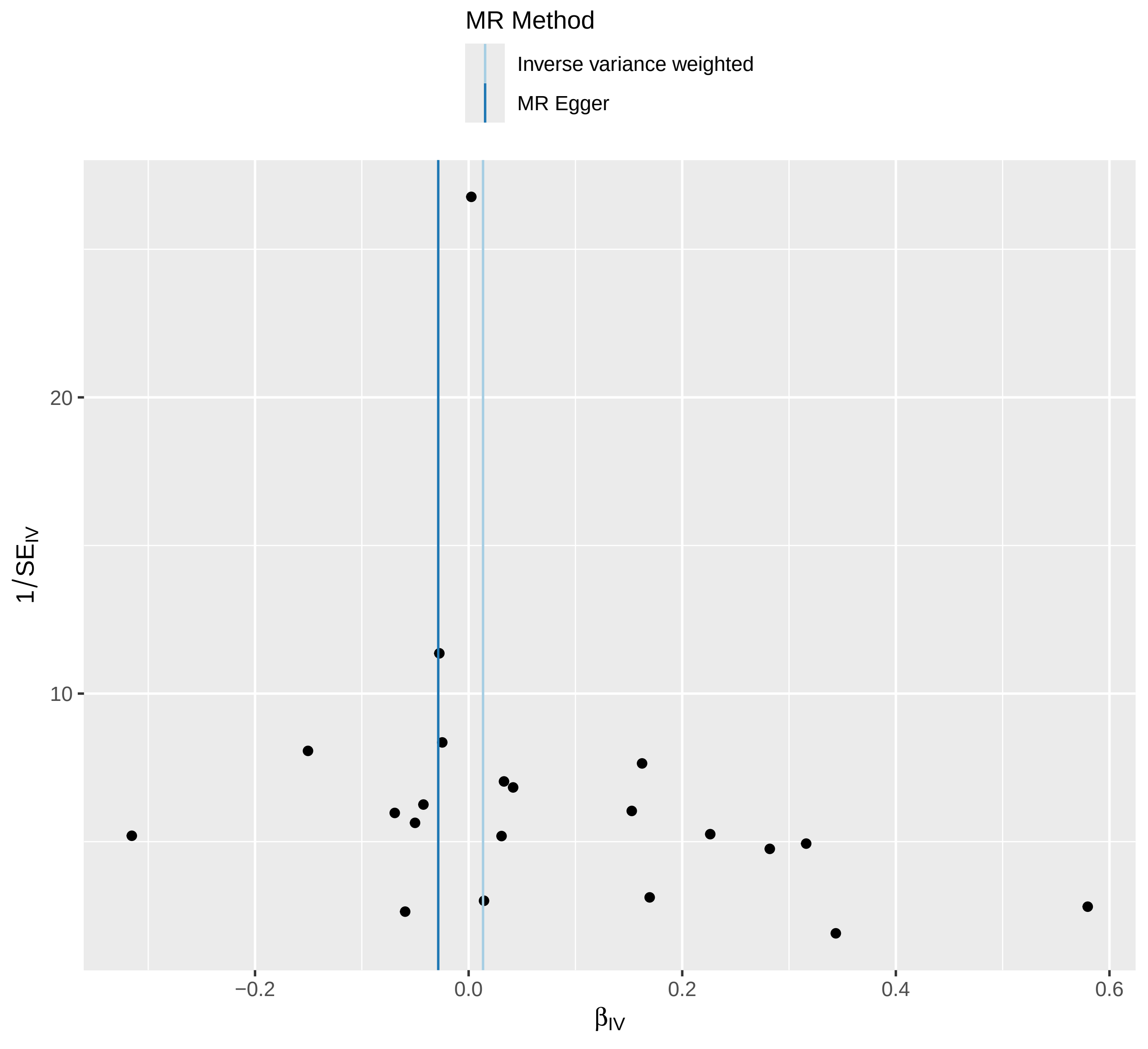
MR Method
Inverse variance weighted
MR Egger
20
10
1/SE$_{IV}$
−0.2
0.0
0.2
0.4
0.6
β$_{IV}$

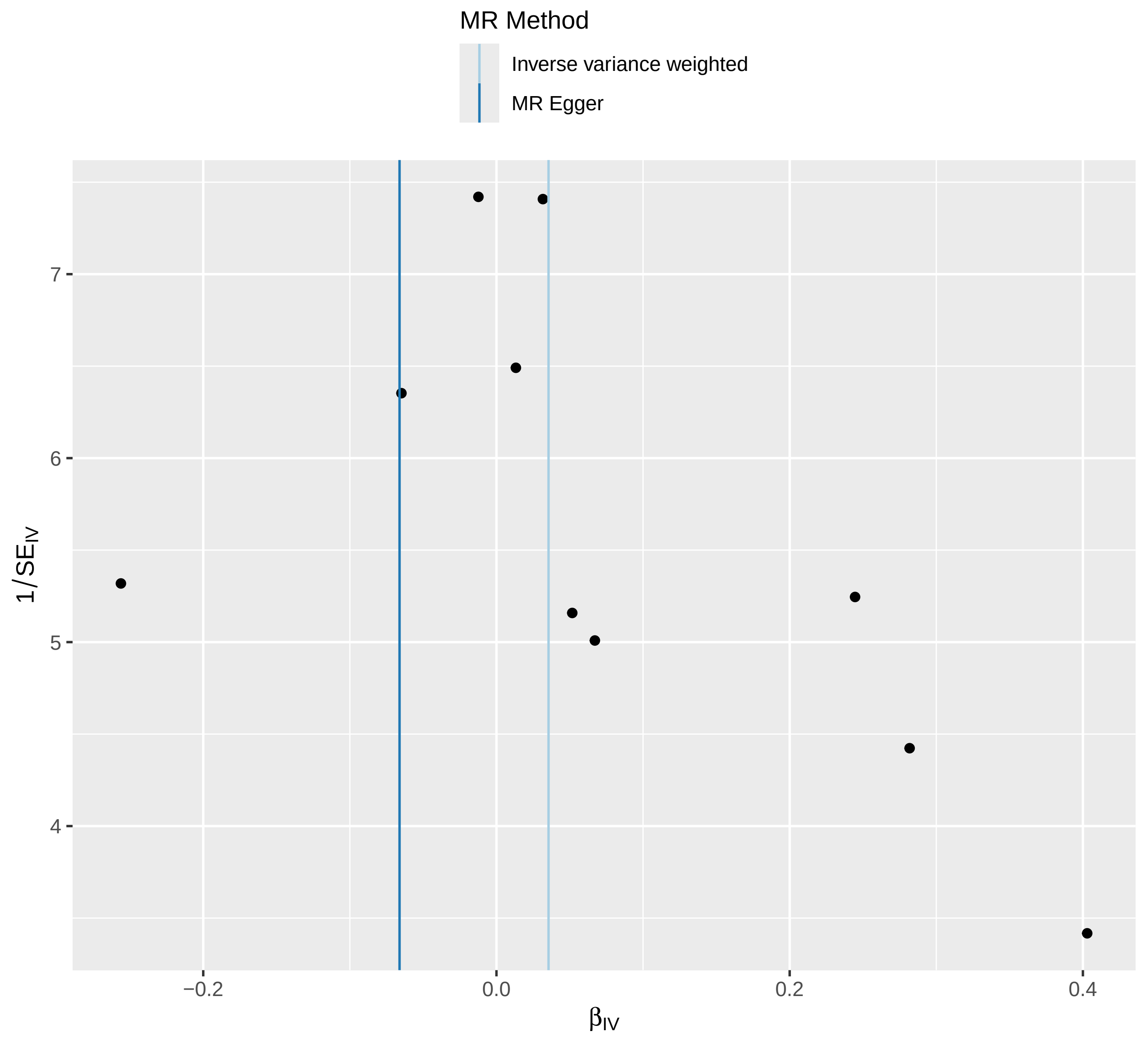
MR Method
Inverse variance weighted
MR Egger
1/SE$_{IV}$
7
6
5
4
−0.2
0.0
0.2
0.4
β$_{IV}$

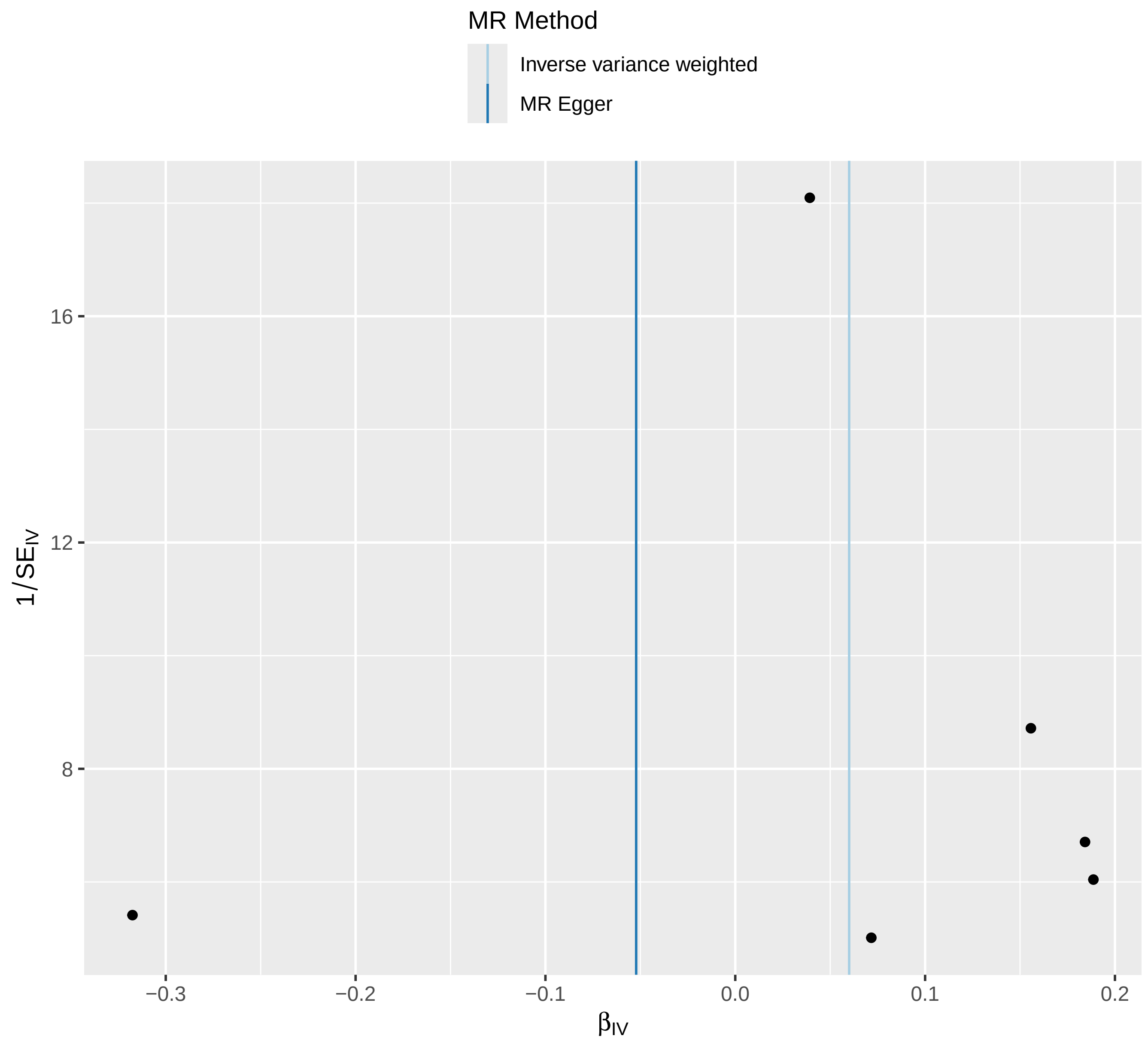
MR Method
Inverse variance weighted
MR Egger
16
12
8
$1/SE_{IV}$
−0.3
−0.2
−0.1
0.0
0.1
0.2
$\beta_{IV}$

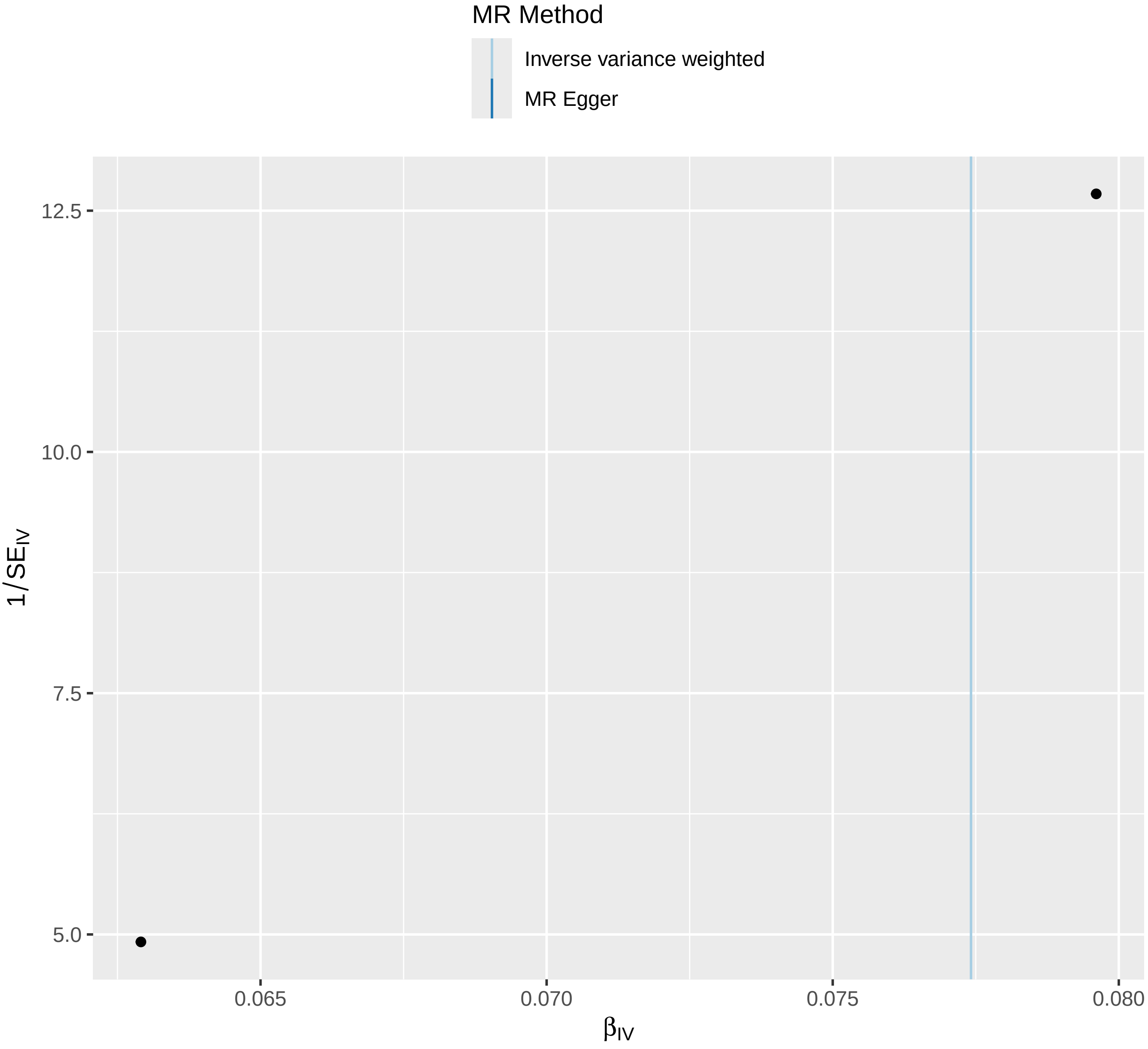
MR Method
Inverse variance weighted
MR Egger
12.5
10.0
7.5
5.0
1/SE$_{IV}$
0.065
0.070
0.075
0.080
β$_{IV}$

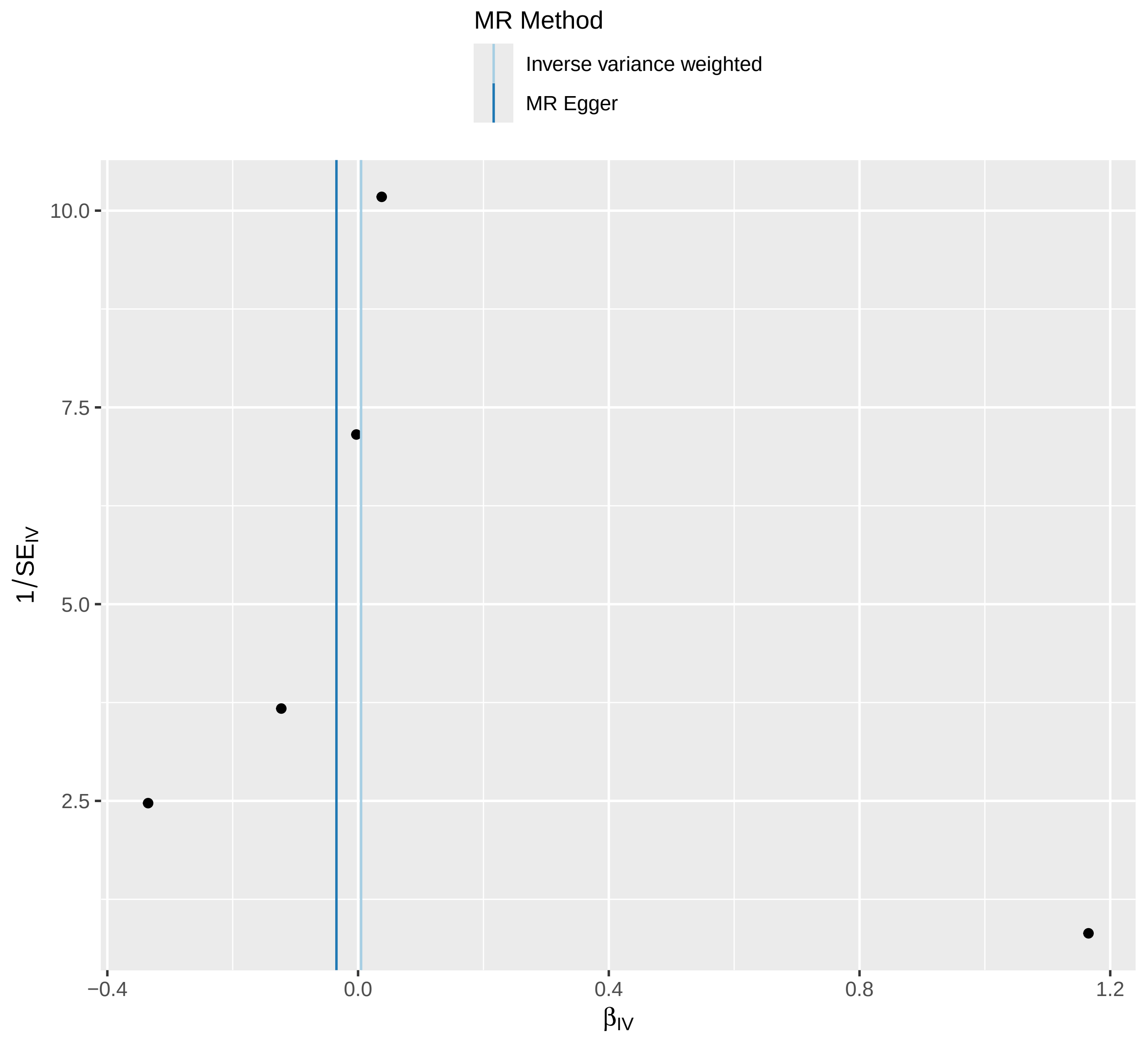
MR Method
Inverse variance weighted
MR Egger
10.0
7.5
5.0
2.5
$1/SE_{IV}$
−0.4
0.0
0.4
0.8
1.2
$\beta_{IV}$

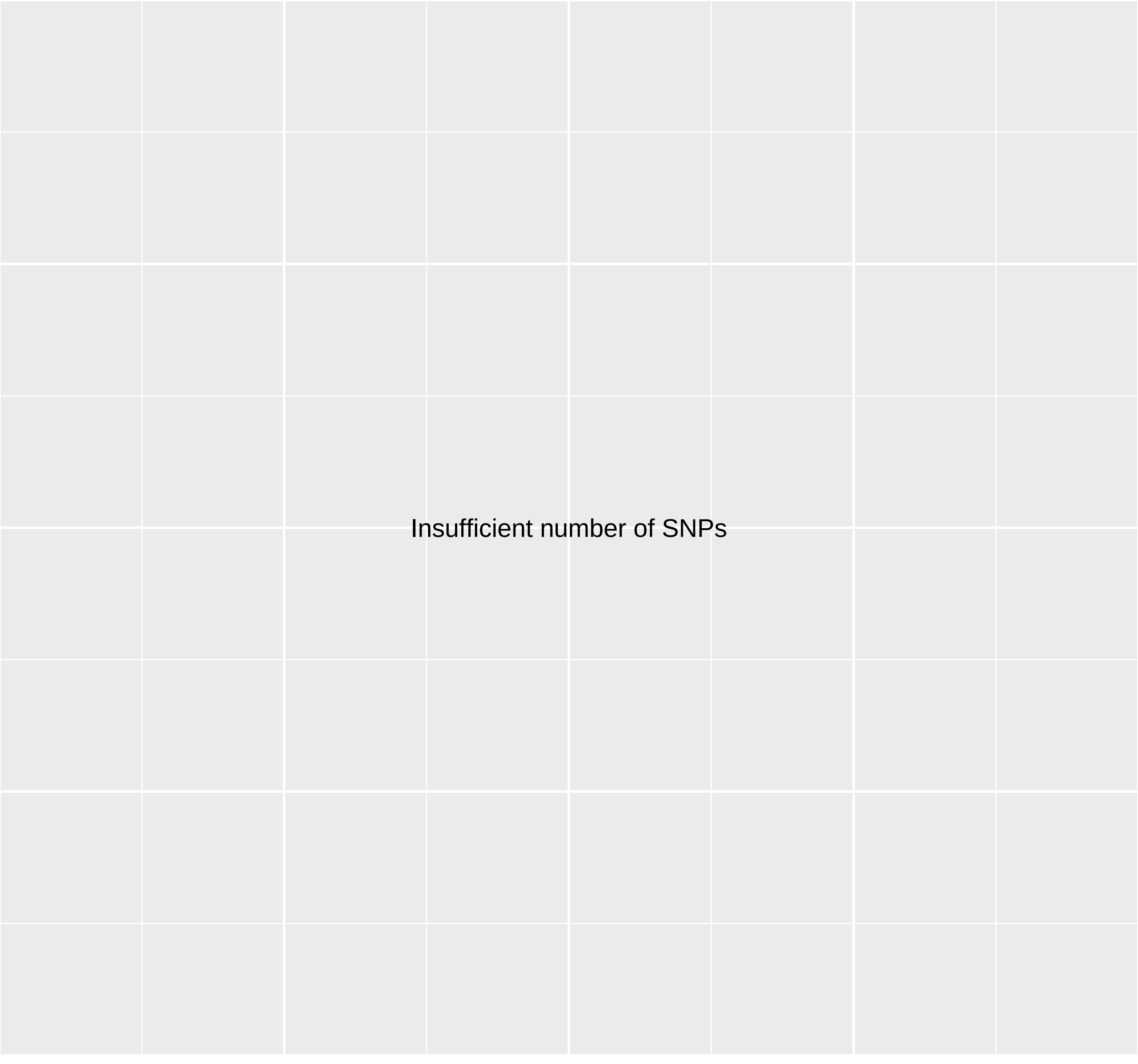
Insufficient number of SNPs

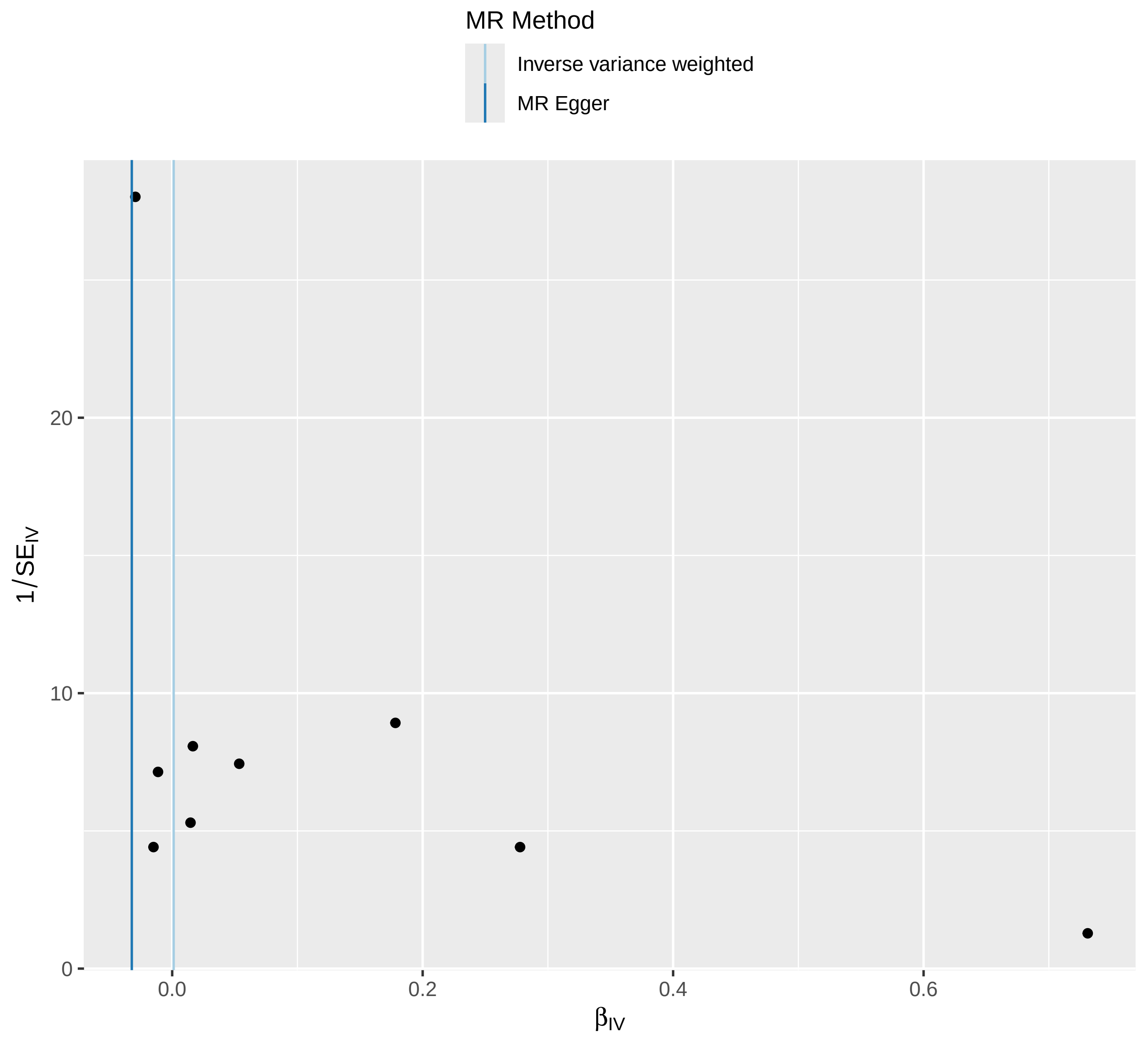
MR Method
Inverse variance weighted
MR Egger
20
10
0
0.0
0.2
0.4
0.6
$1/SE_{IV}$
$\beta_{IV}$

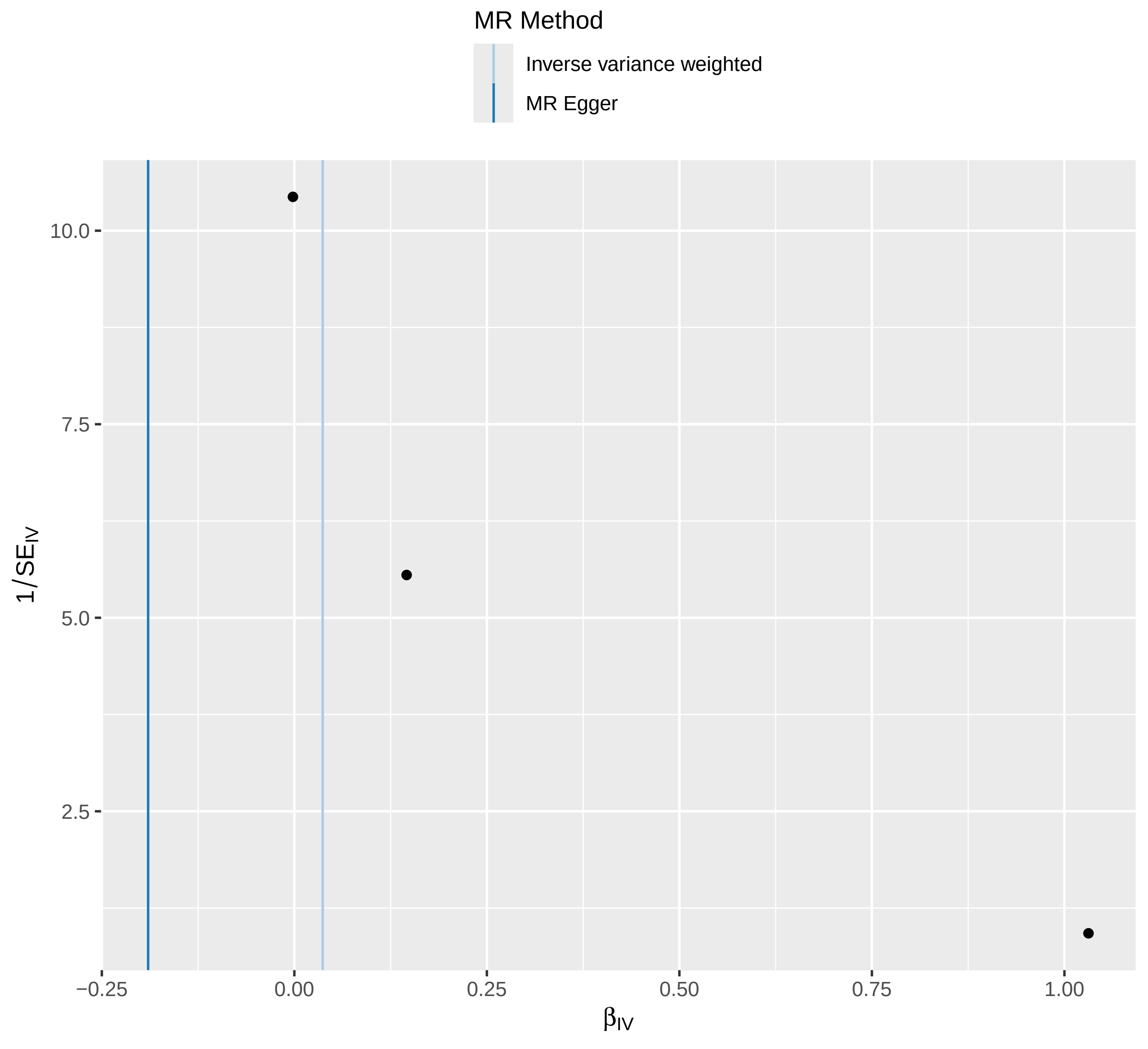
MR Method
Inverse variance weighted
MR Egger
10.0
7.5
5.0
2.5
$1/SE_{IV}$
−0.25
0.00
0.25
0.50
0.75
1.00
$\beta_{IV}$

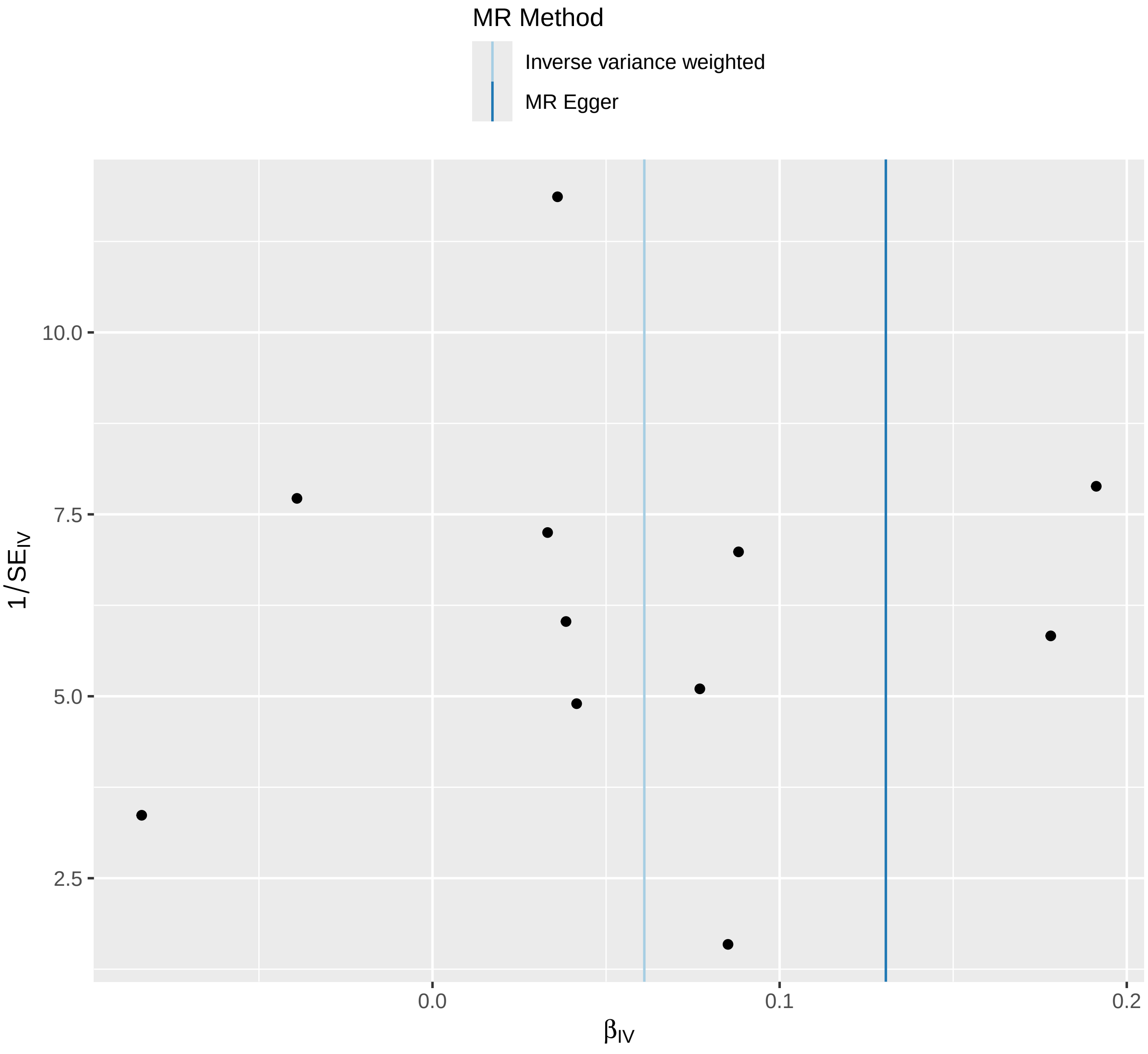
MR Method
Inverse variance weighted
MR Egger
10.0
7.5
5.0
2.5
$1/SE_{IV}$
0.0
0.1
0.2
$\beta_{IV}$

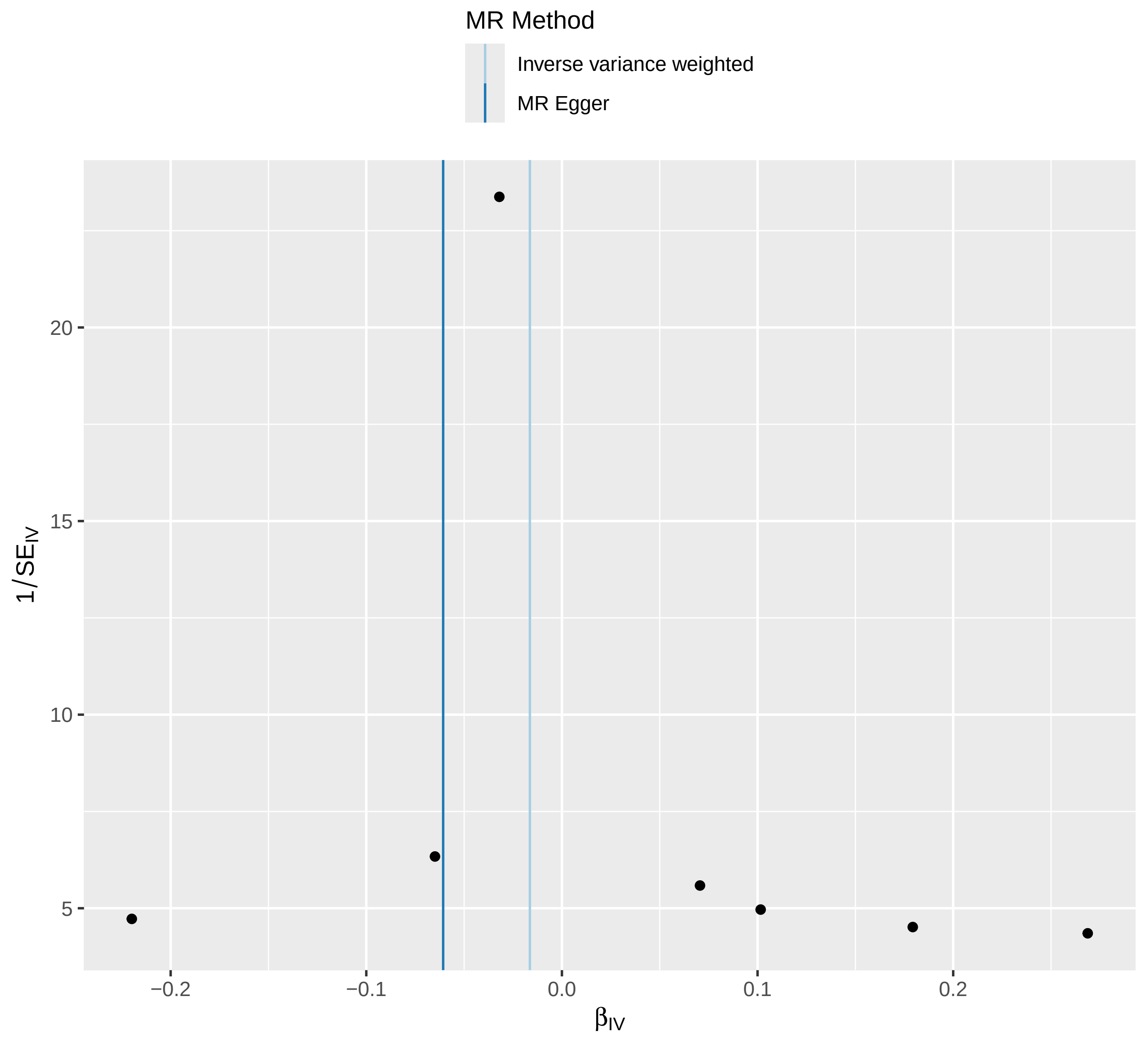
MR Method
Inverse variance weighted
MR Egger
20
15
10
5
1/SE$_{IV}$
−0.2
−0.1
0.0
0.1
0.2
β$_{IV}$

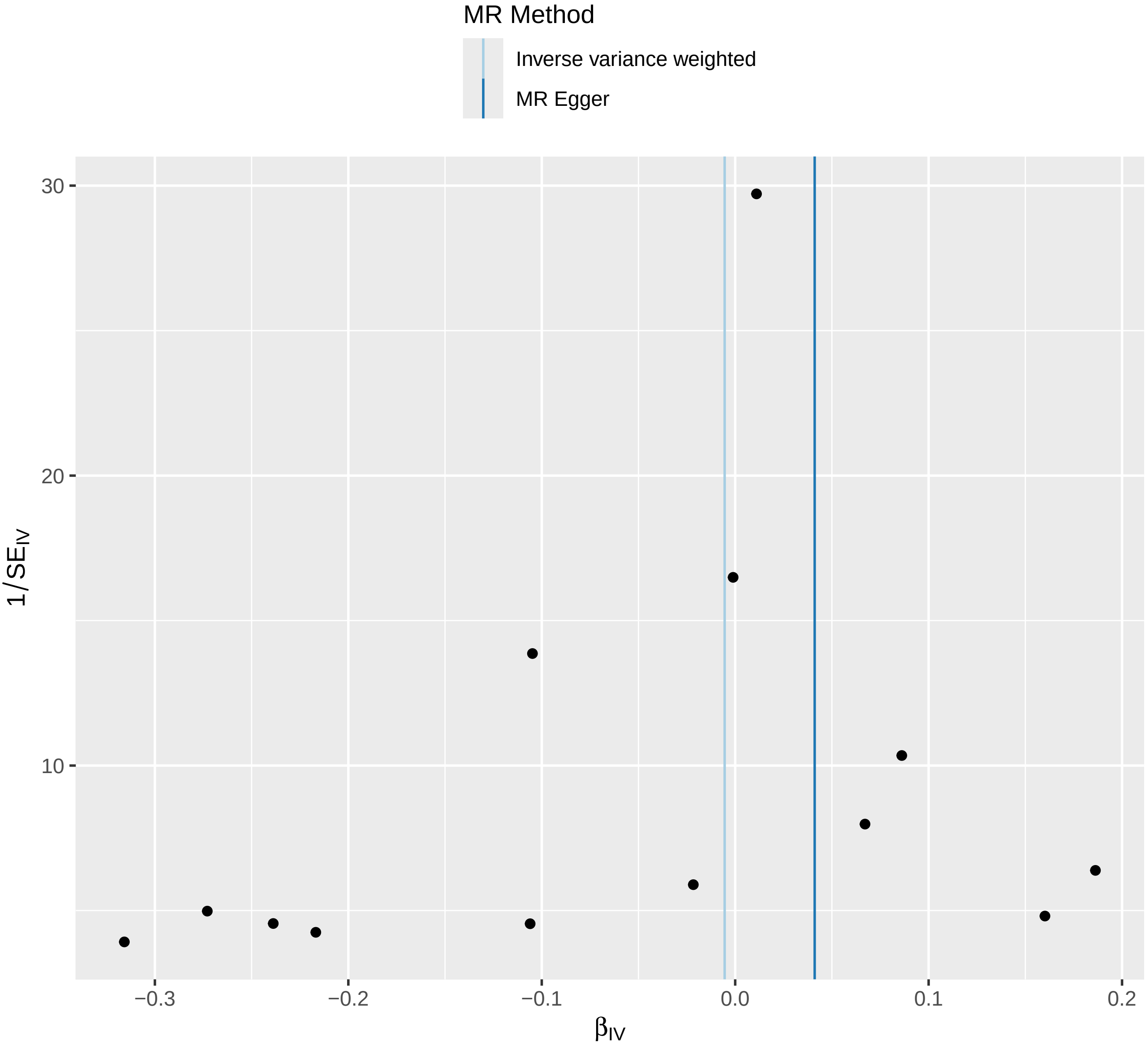
MR Method
Inverse variance weighted
MR Egger
30
20
10
1/SE$_{IV}$
−0.3
−0.2
−0.1
0.0
0.1
0.2
β$_{IV}$

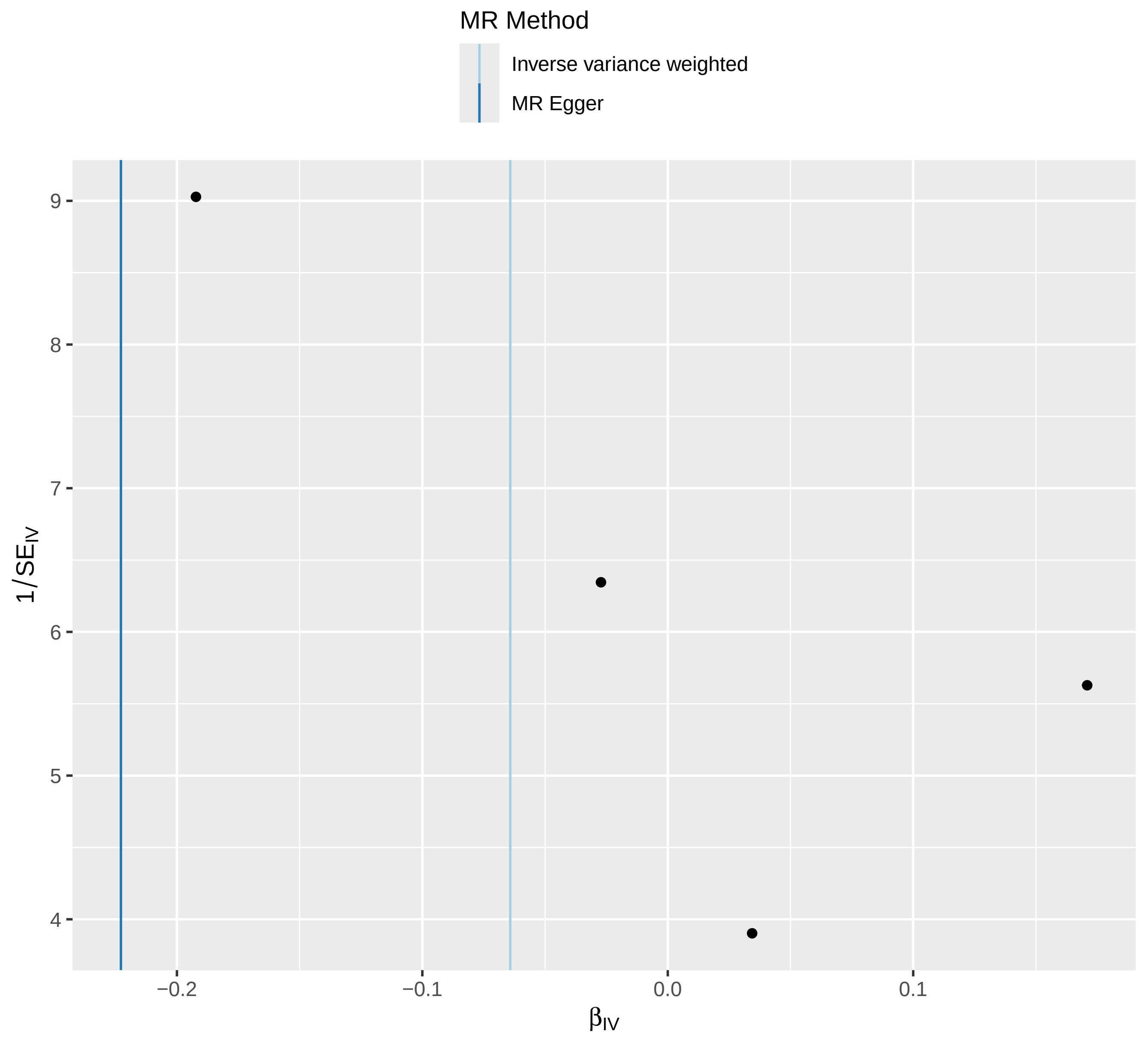
MR Method
Inverse variance weighted
MR Egger
1/SE_IV
9
8
7
6
5
4
−0.2
−0.1
0.0
0.1
β_IV

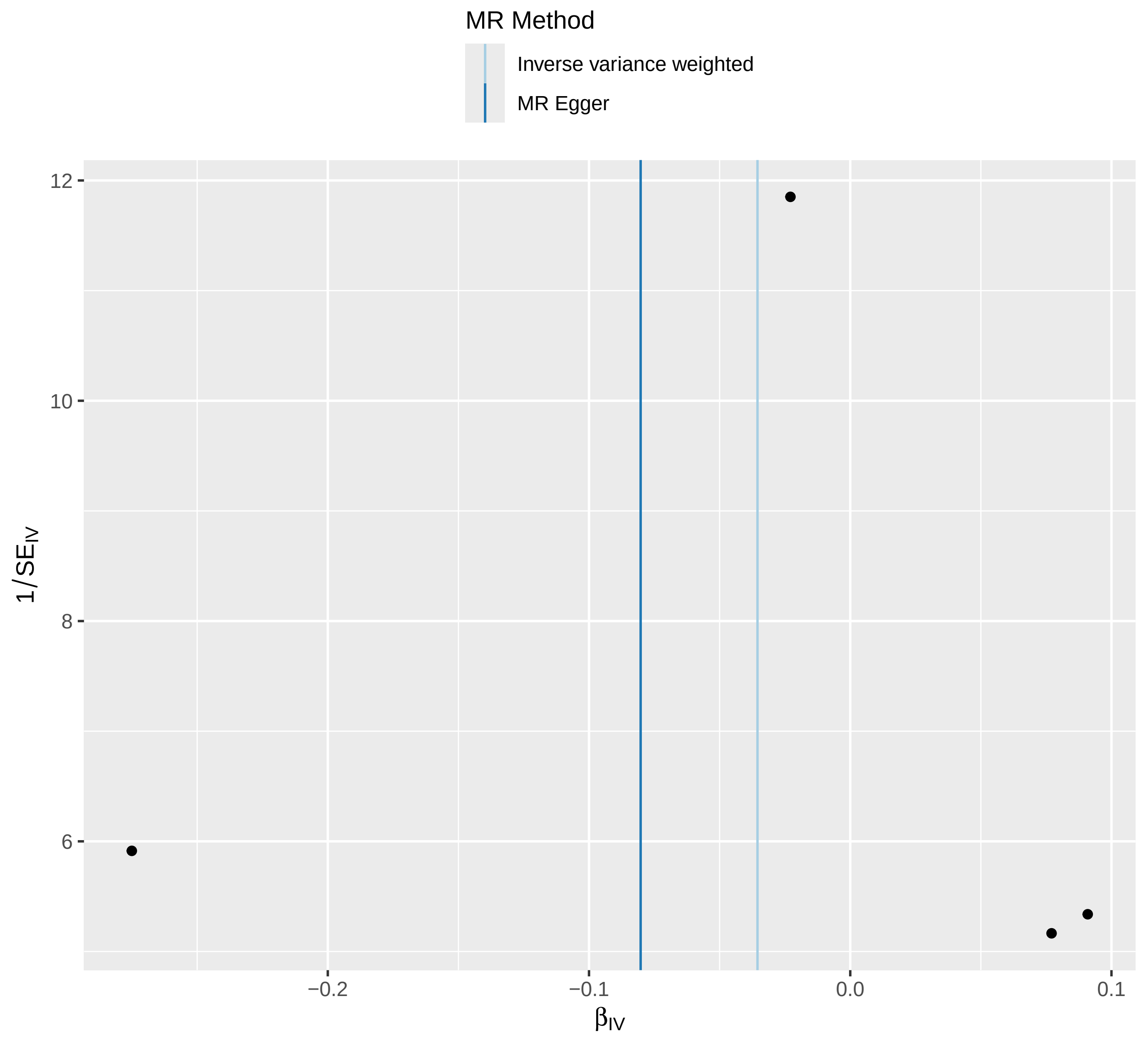
MR Method
Inverse variance weighted
MR Egger
12
10
8
6
1/SE$_{IV}$
−0.2
−0.1
0.0
0.1
$\beta_{IV}$

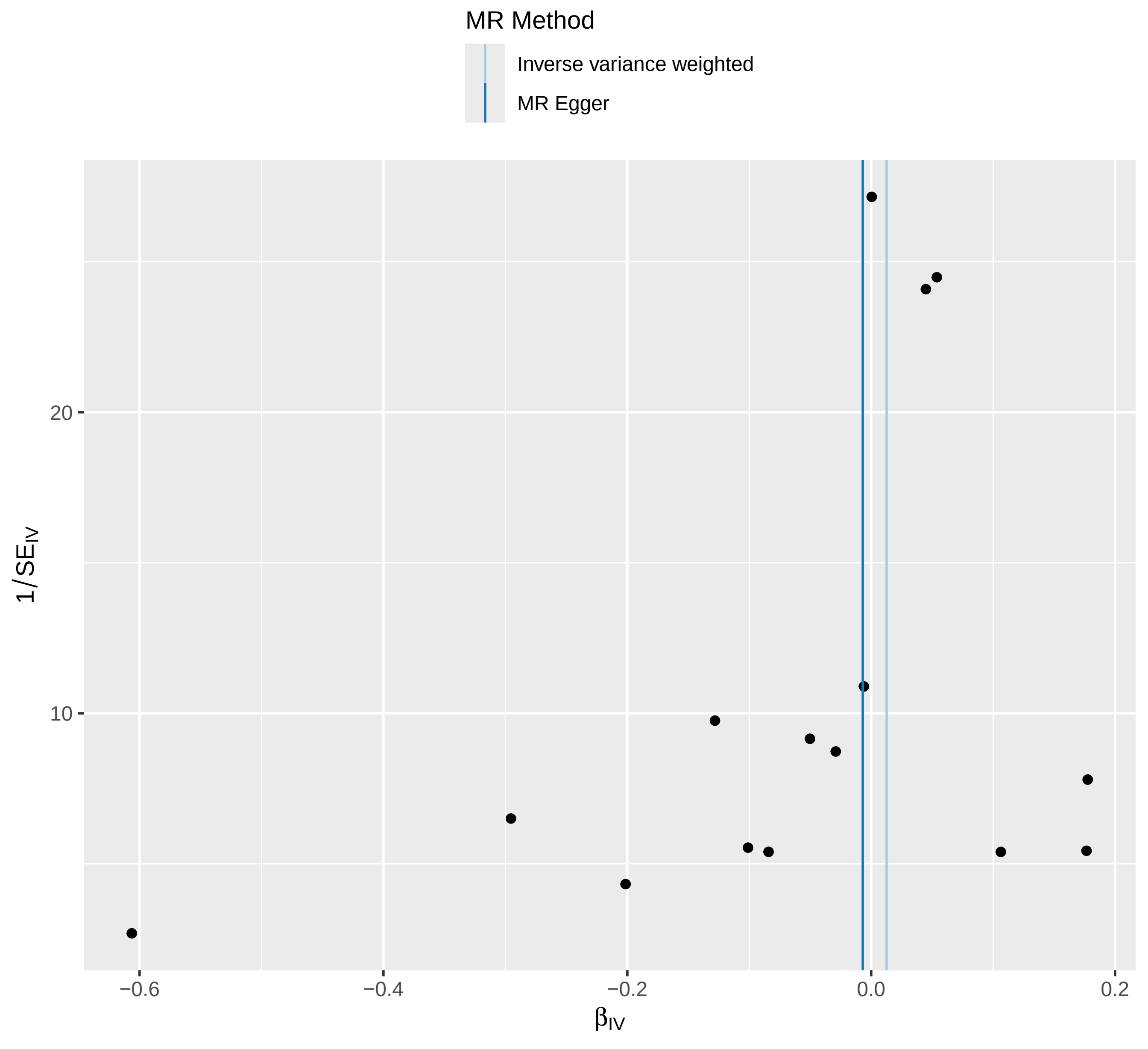
MR Method
Inverse variance weighted
MR Egger
20
10
−0.6
−0.4
−0.2
0.0
0.2
$1/SE_{IV}$
$\beta_{IV}$

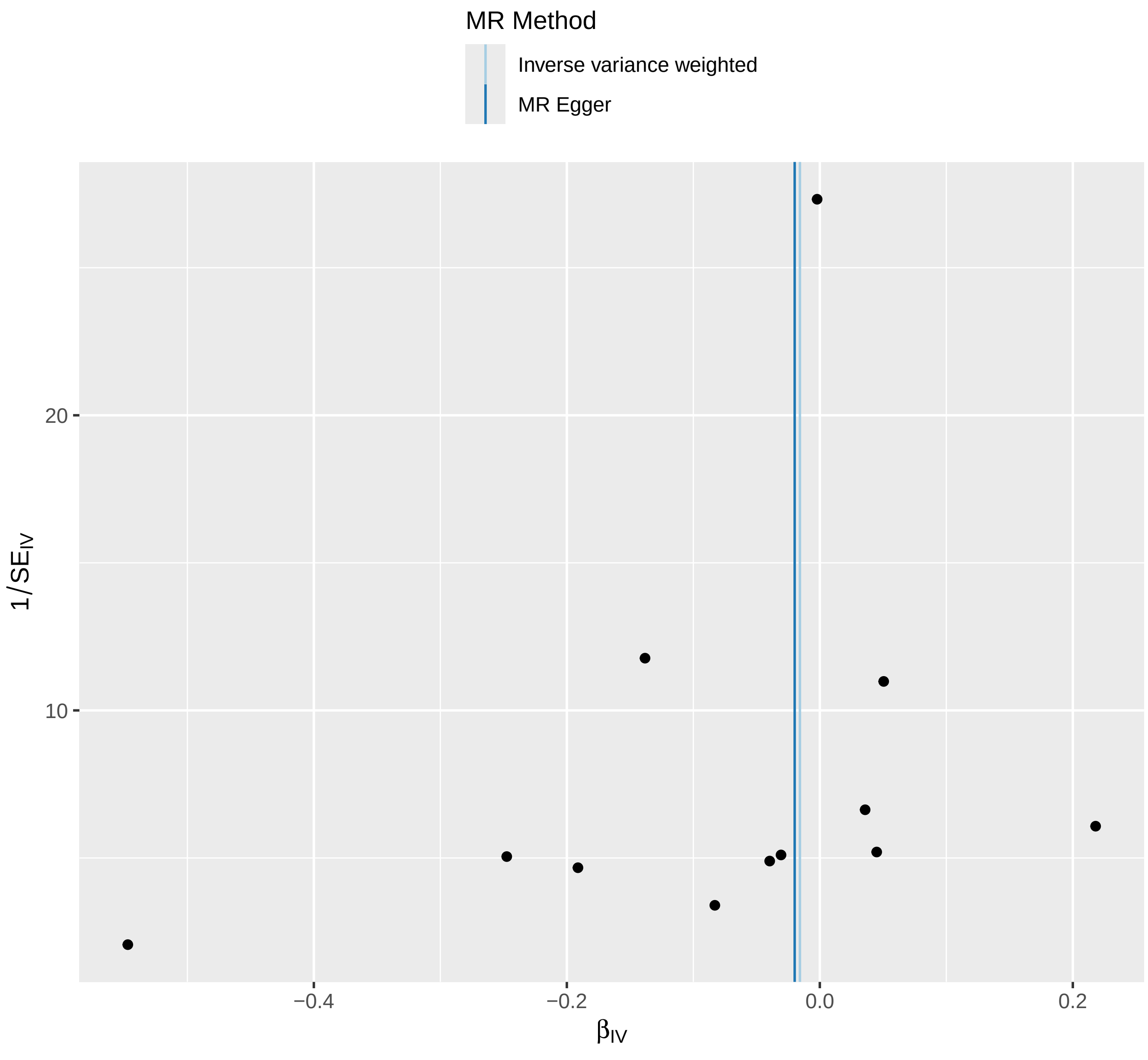
MR Method
Inverse variance weighted
MR Egger
20
10
1/SE$_{IV}$
−0.4
−0.2
0.0
0.2
β$_{IV}$

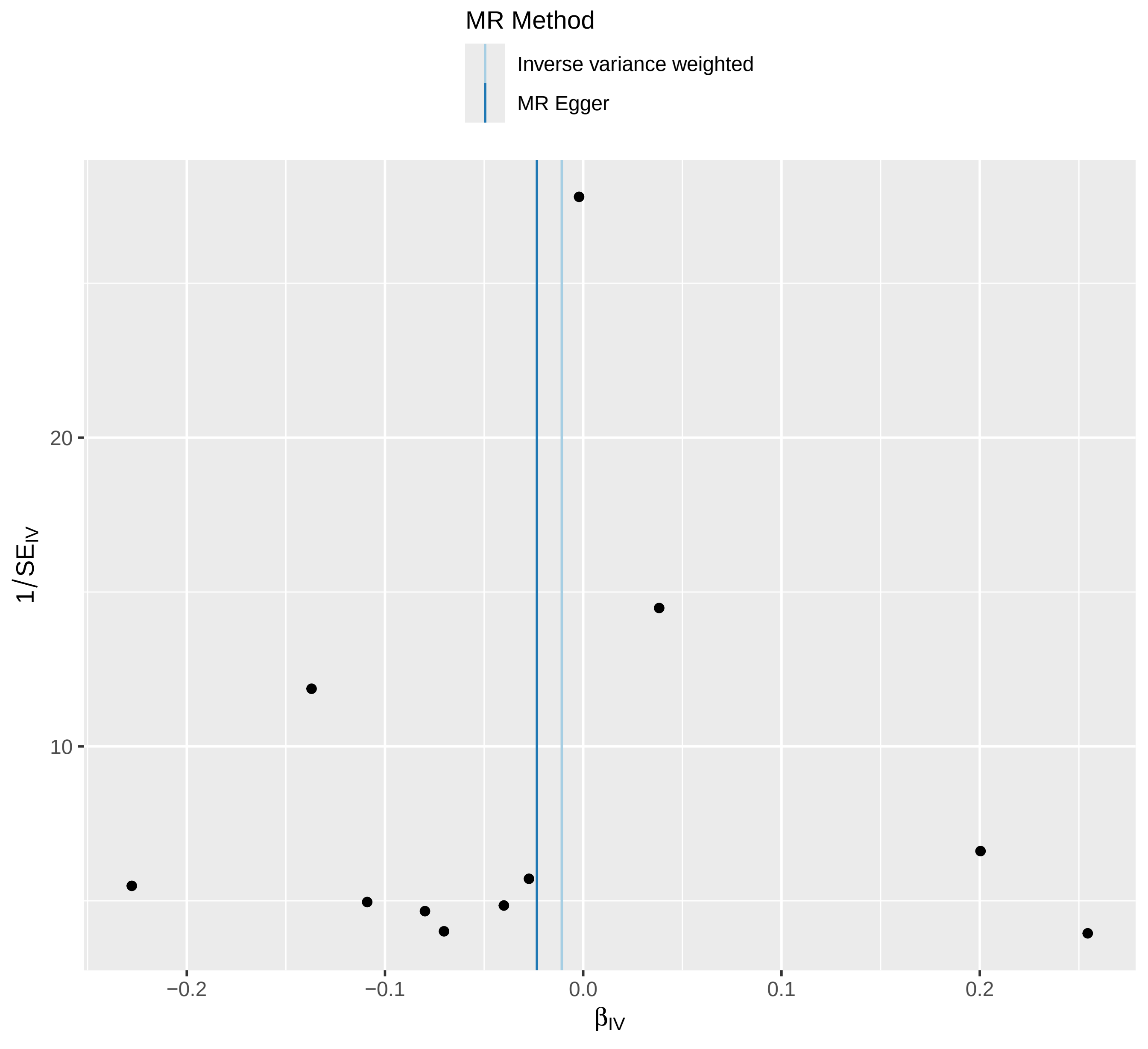

MR Method
Inverse variance weighted
MR Egger
1/SE_IV
20
10
β_IV
−0.2
−0.1
0.0
0.1
0.2

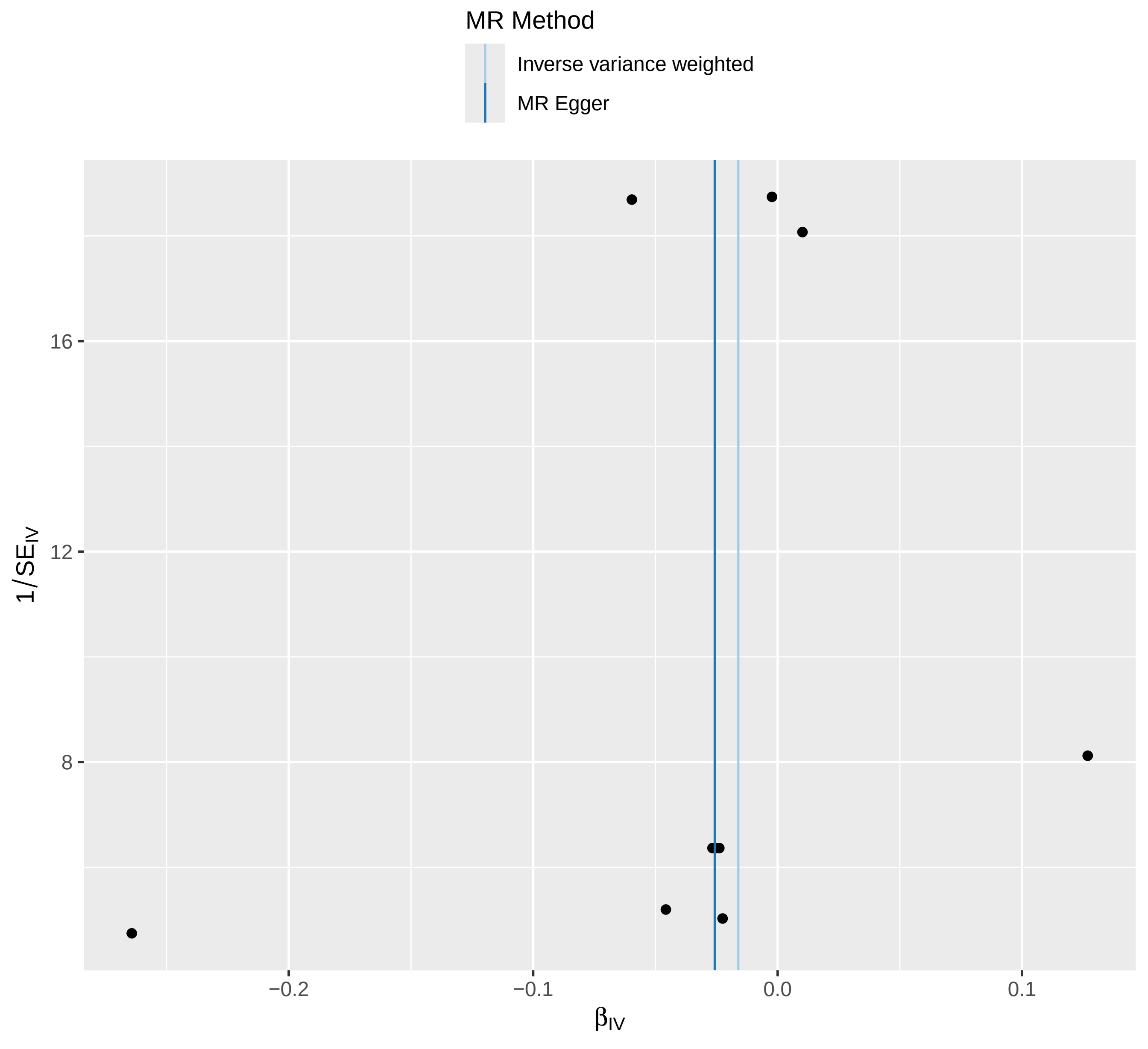
MR Method
Inverse variance weighted
MR Egger
16
12
8
1/SE$_{IV}$
−0.2
−0.1
0.0
0.1
β$_{IV}$

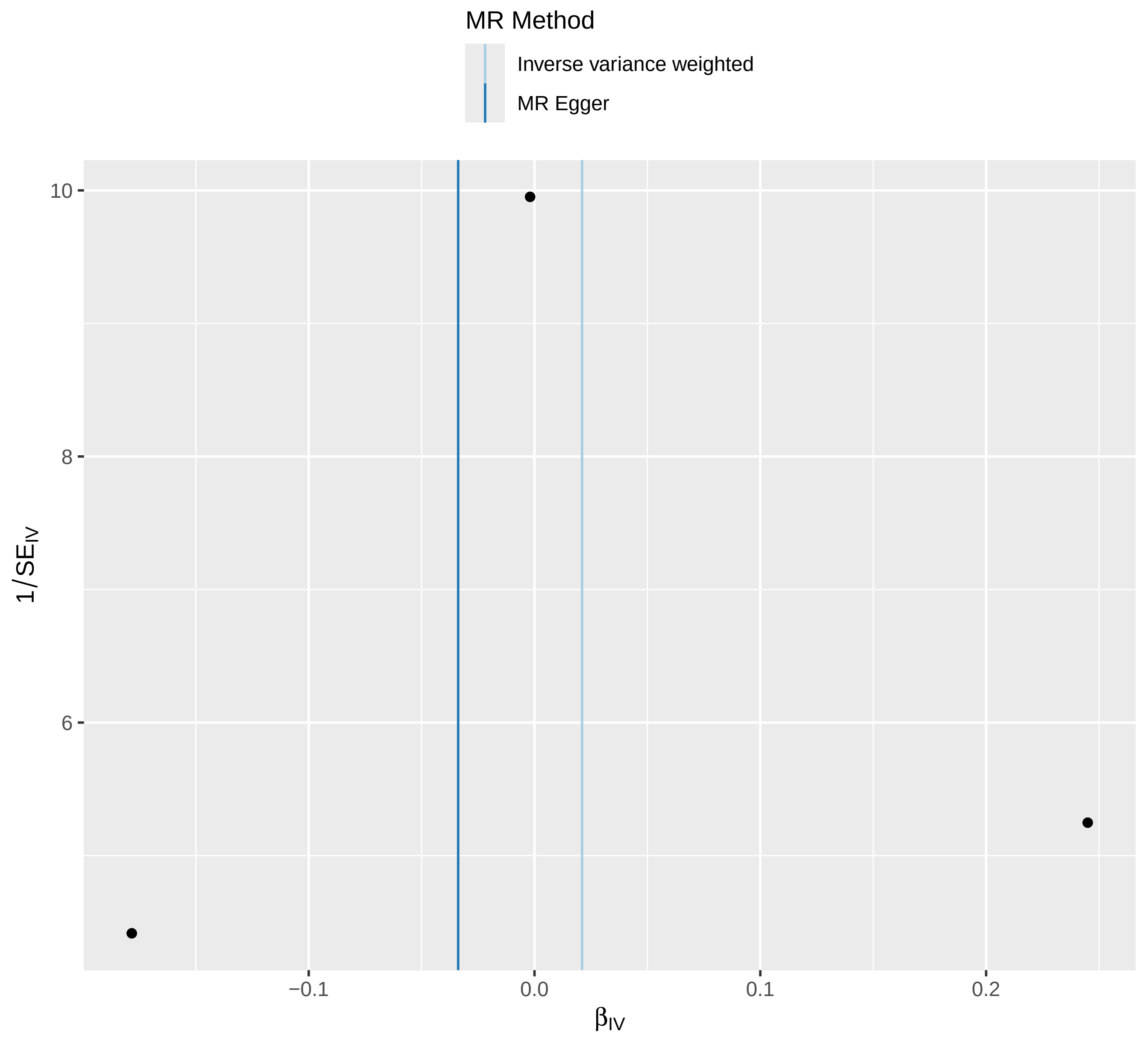
MR Method
Inverse variance weighted
MR Egger
10
8
6
$1/SE_{IV}$
−0.1
0.0
0.1
0.2
$\beta_{IV}$

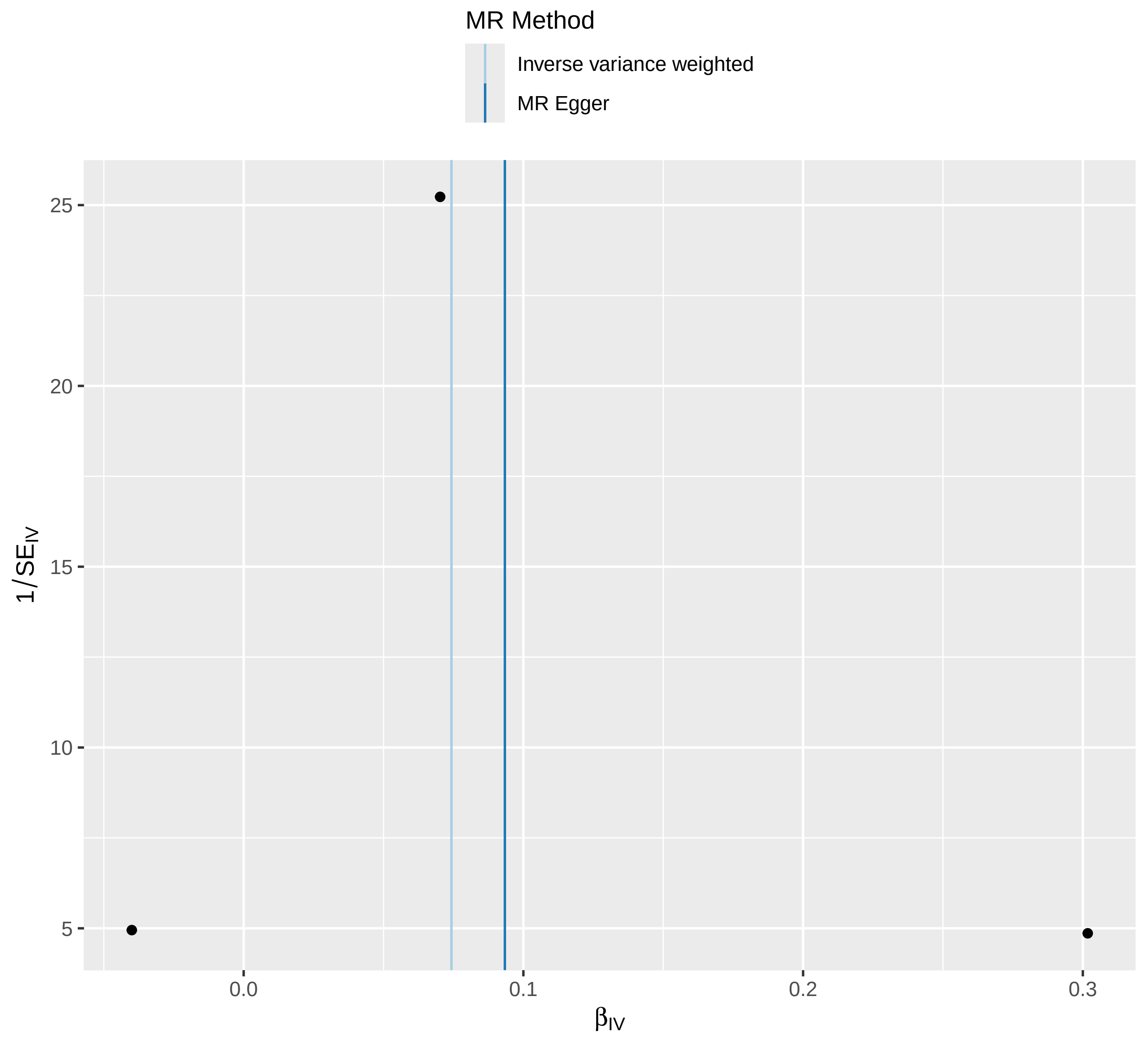

MR Method
Inverse variance weighted
MR Egger
25
20
15
10
5
0.0
0.1
0.2
0.3
$1/SE_{IV}$
$\beta_{IV}$

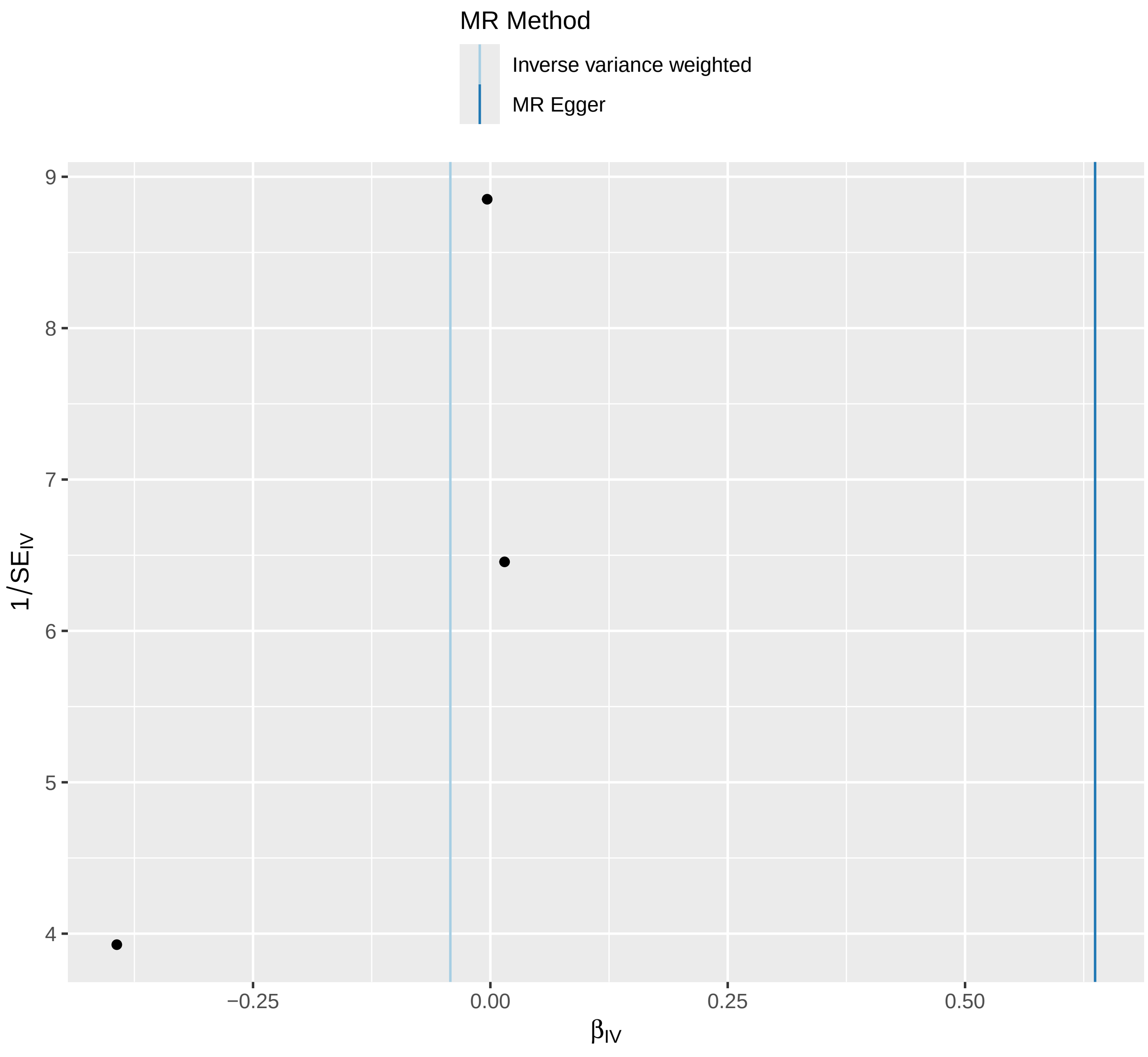

MR Method
Inverse variance weighted
MR Egger
9
8
7
6
5
4
1/SE$_{IV}$
−0.25
0.00
0.25
0.50
β$_{IV}$

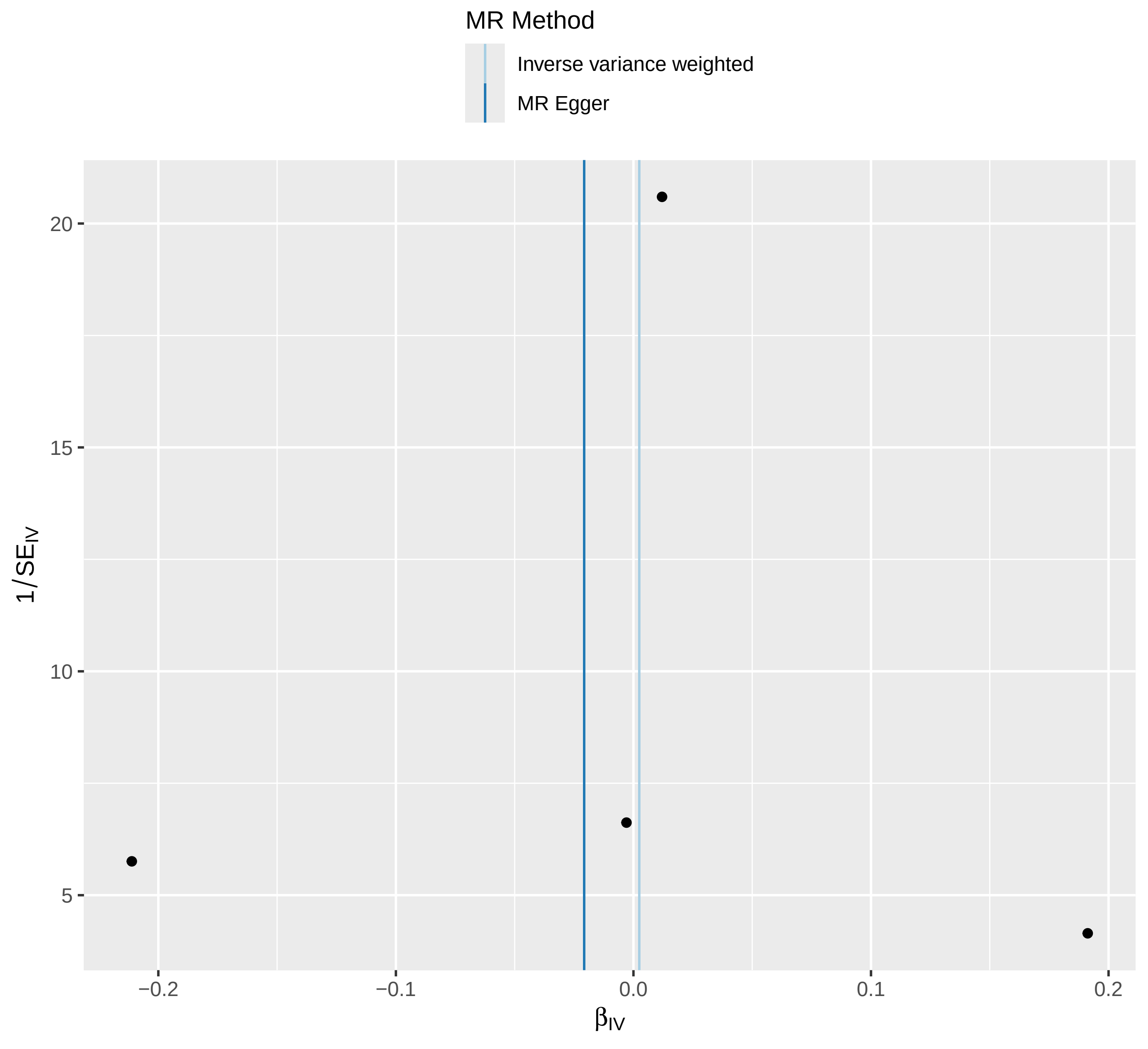

MR Method
Inverse variance weighted
MR Egger
20
15
10
5
1/SE$_{IV}$
−0.2
−0.1
0.0
0.1
0.2
β$_{IV}$

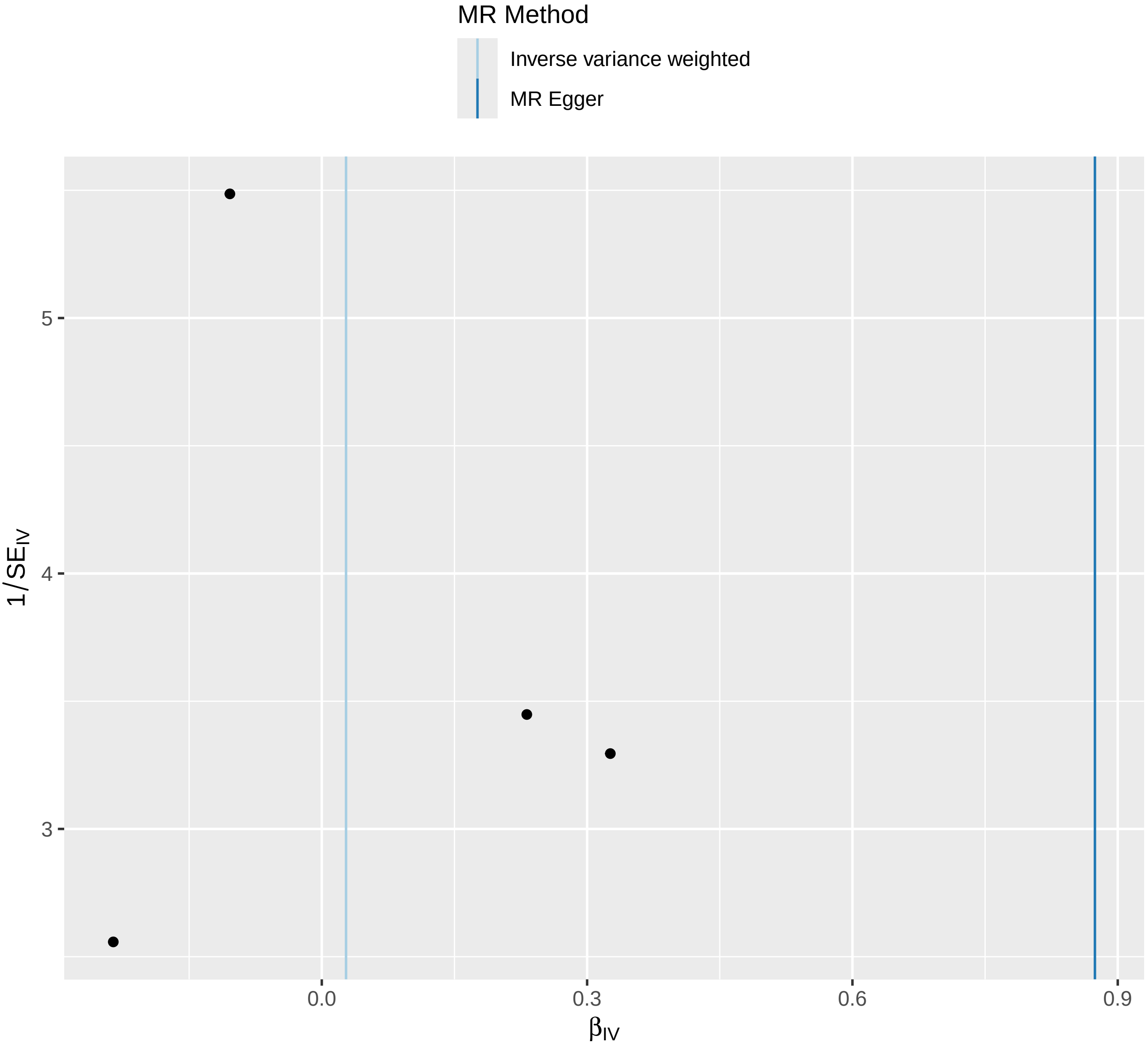
MR Method
Inverse variance weighted
MR Egger
5
4
3
1/$SE_{IV}$
0.0
0.3
0.6
0.9
$\beta_{IV}$

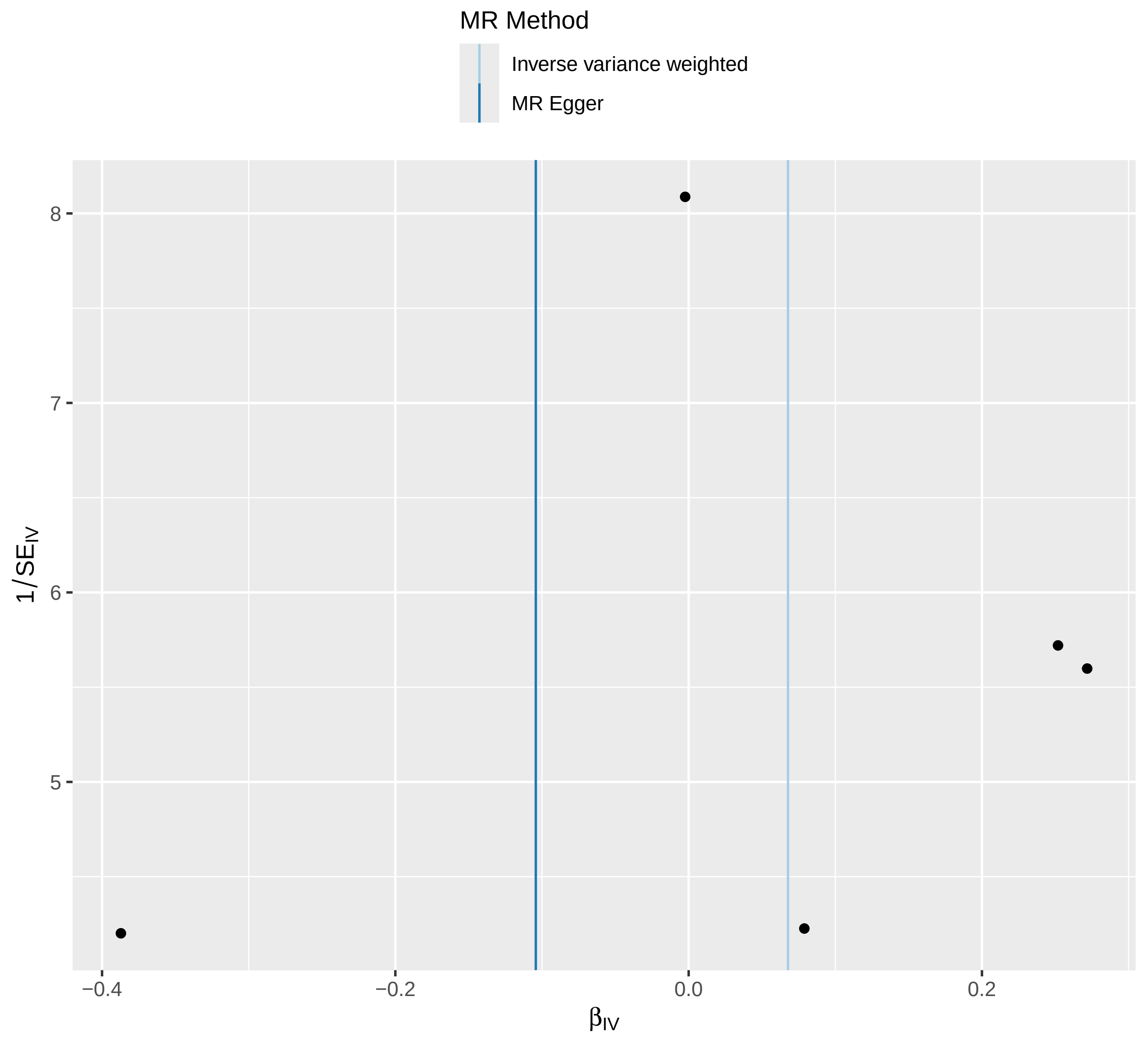

MR Method
Inverse variance weighted
MR Egger
8
7
6
5
1/SE$_{IV}$
−0.4
−0.2
0.0
0.2
β$_{IV}$

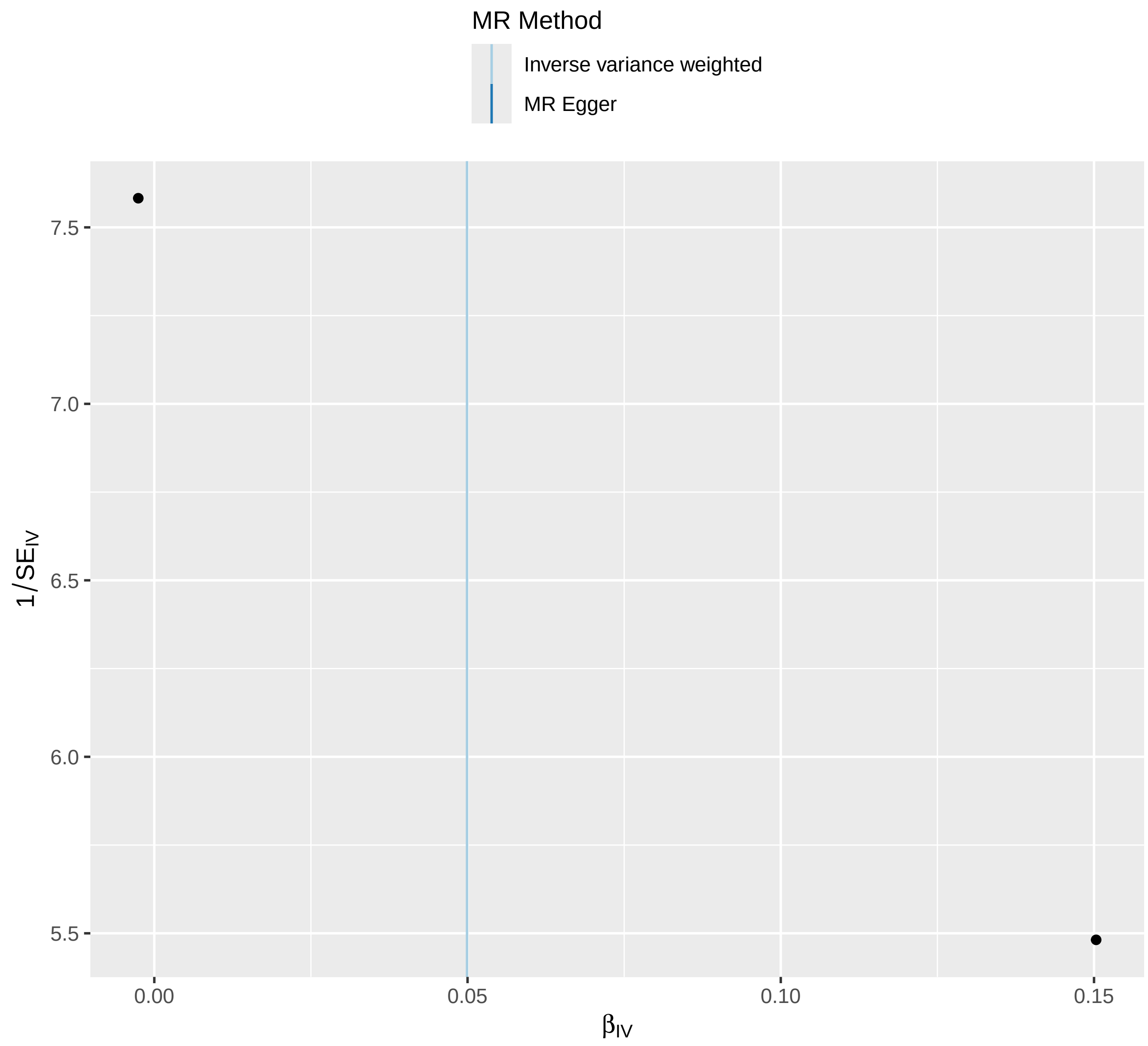
MR Method
Inverse variance weighted
MR Egger
7.5
7.0
6.5
6.0
5.5
$1/SE_{IV}$
0.00
0.05
0.10
0.15
$\beta_{IV}$

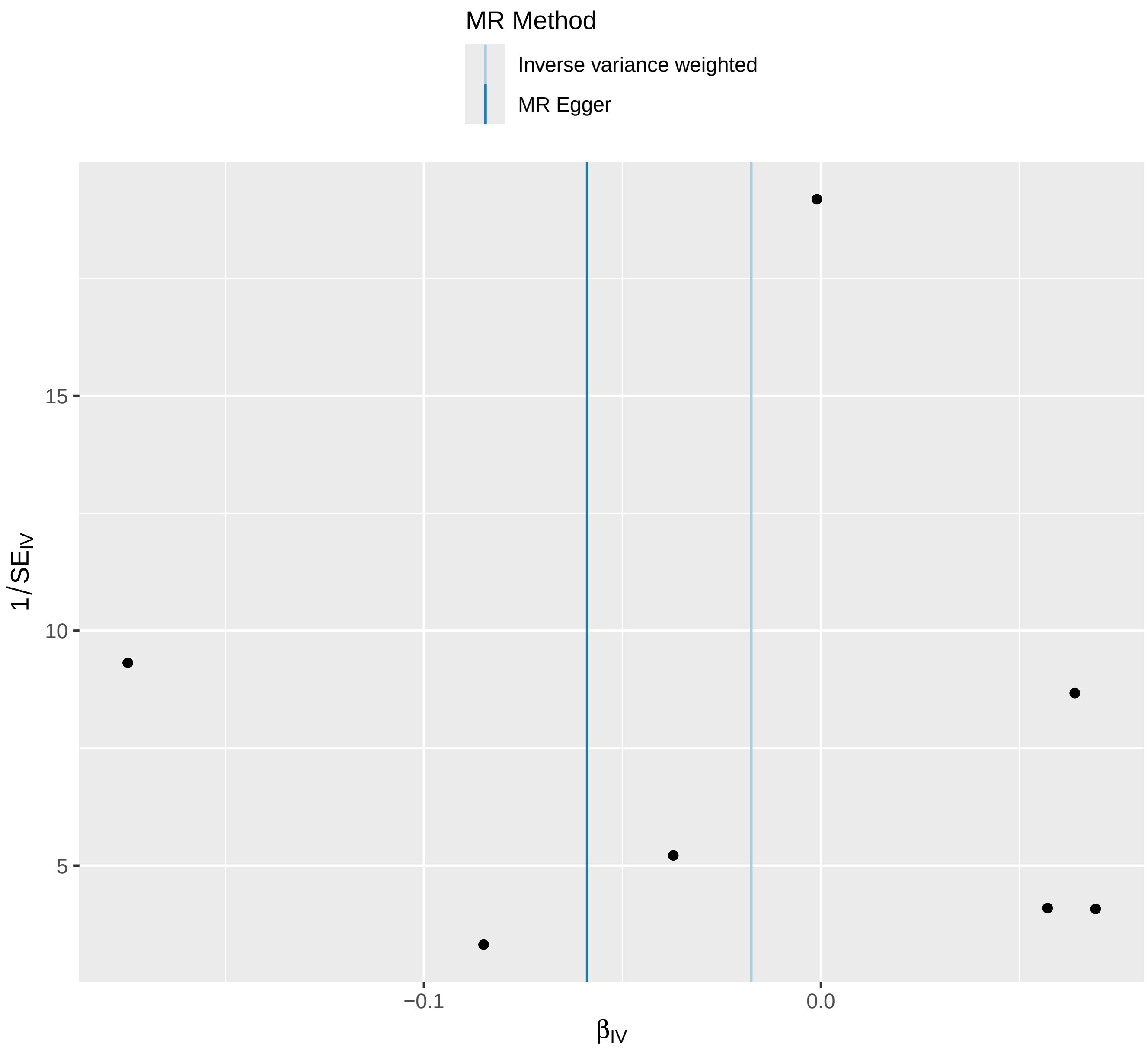

MR Method
Inverse variance weighted
MR Egger
1/SE_IV
15
10
5
−0.1
0.0
β_IV

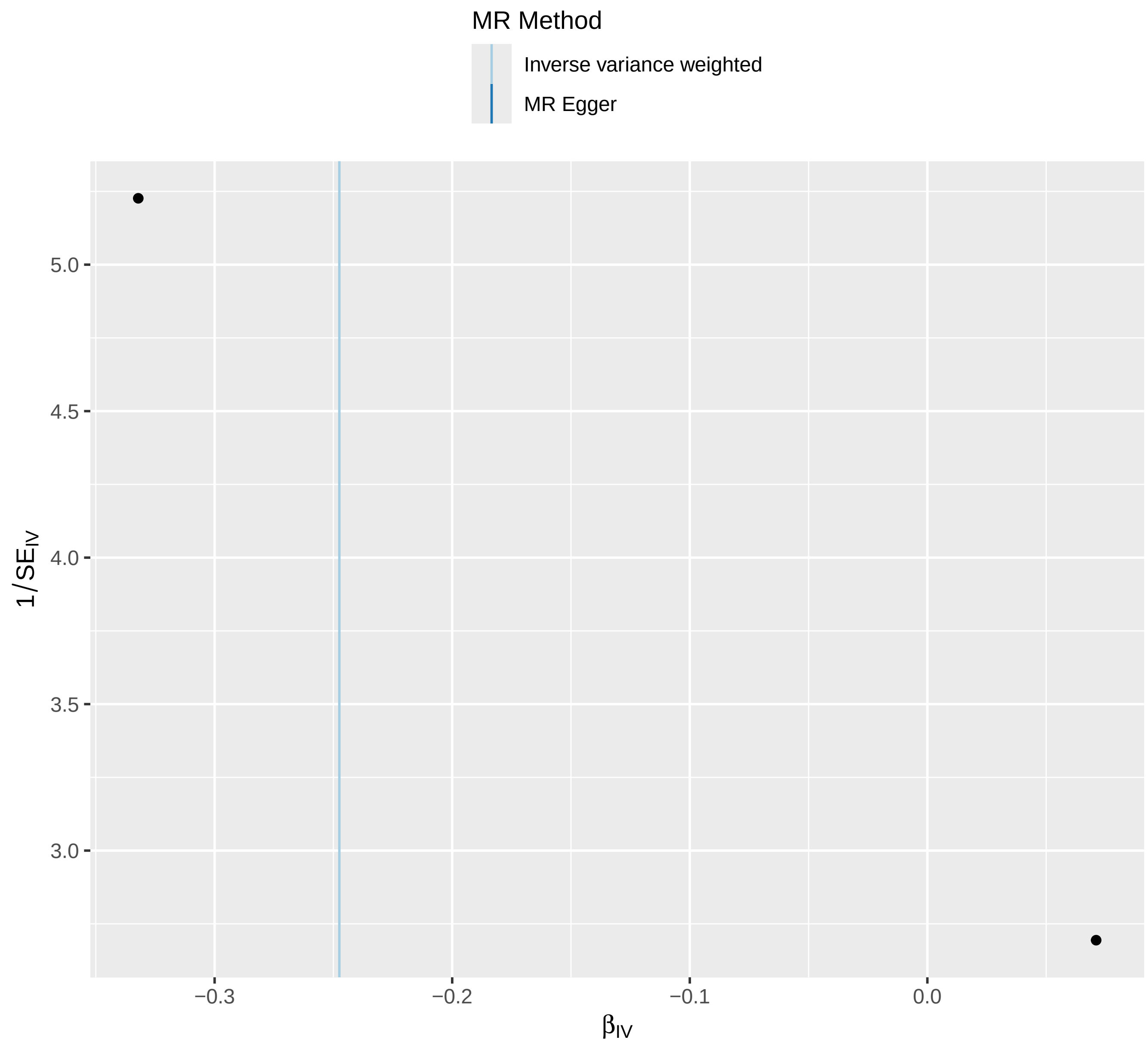

MR Method
Inverse variance weighted
MR Egger
5.0
4.5
4.0
3.5
3.0
1/SE$_{IV}$
−0.3
−0.2
−0.1
0.0
β$_{IV}$

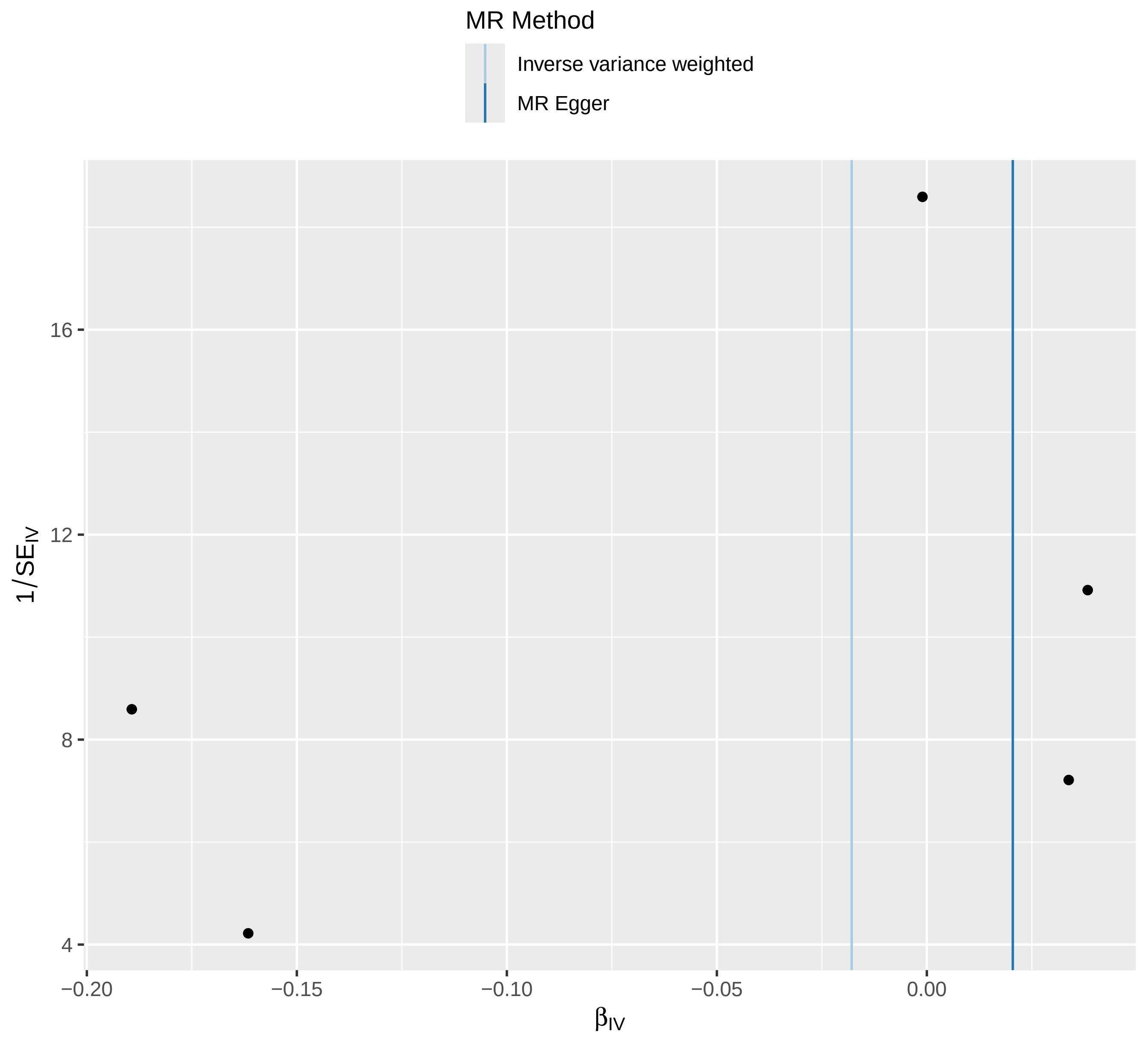

MR Method
Inverse variance weighted
MR Egger
16
12
8
4
1/SE_IV
−0.20
−0.15
−0.10
−0.05
0.00
β_IV

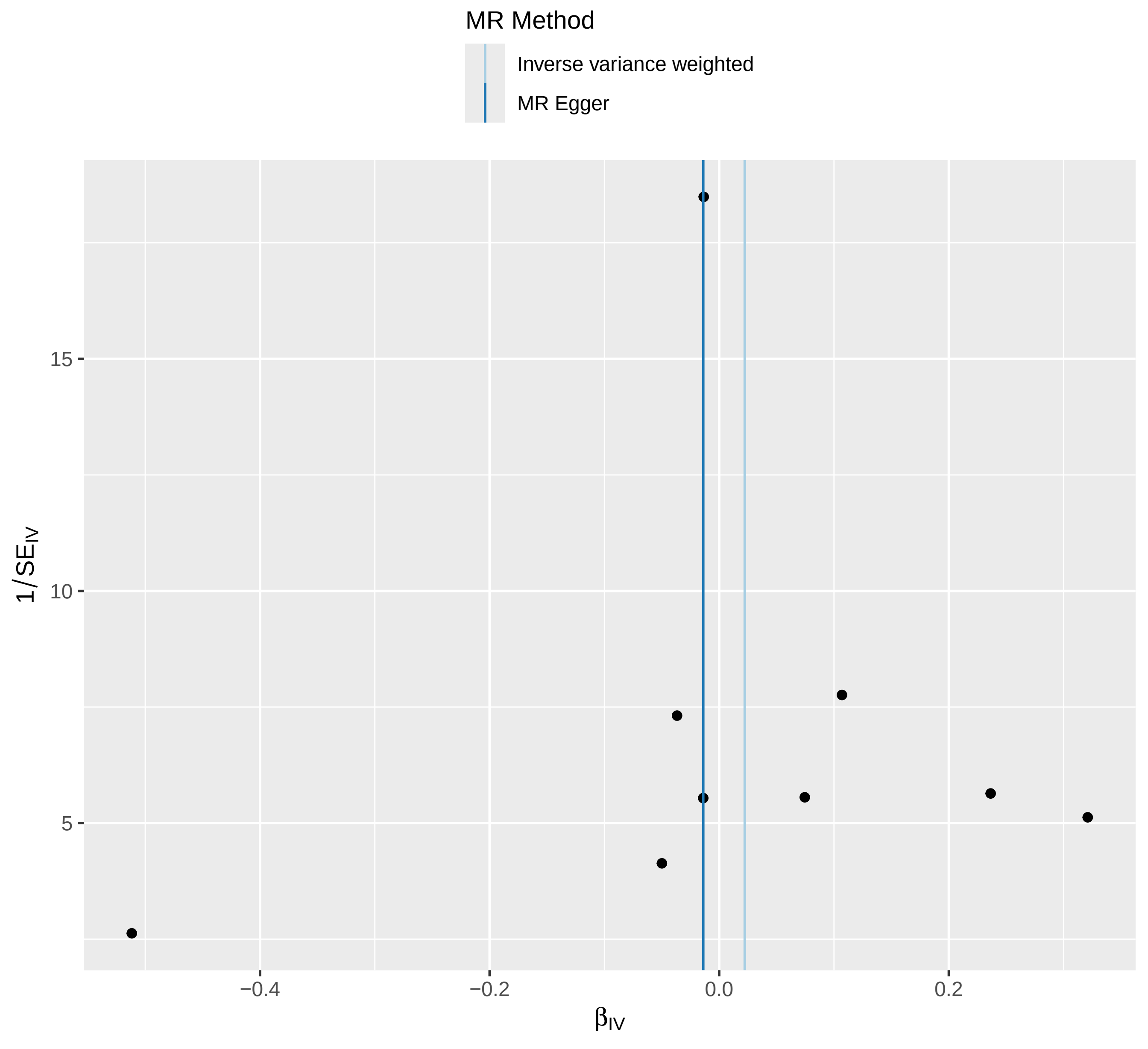

MR Method
Inverse variance weighted
MR Egger
15
10
5
1/SE$_{IV}$
−0.4
−0.2
0.0
0.2
β$_{IV}$

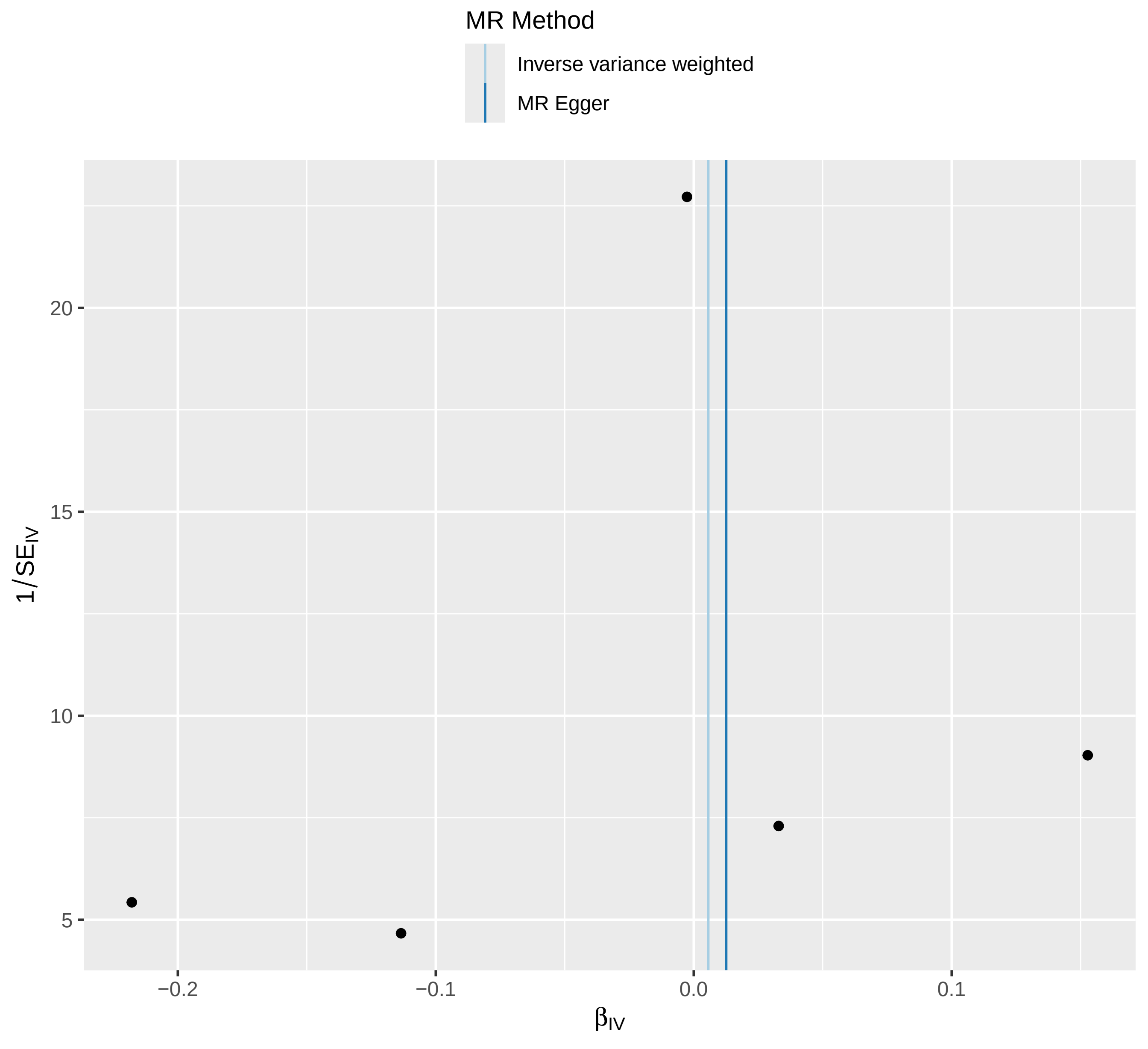
MR Method
Inverse variance weighted
MR Egger
1/SE_IV
β_IV
20
15
10
5
−0.2
−0.1
0.0
0.1

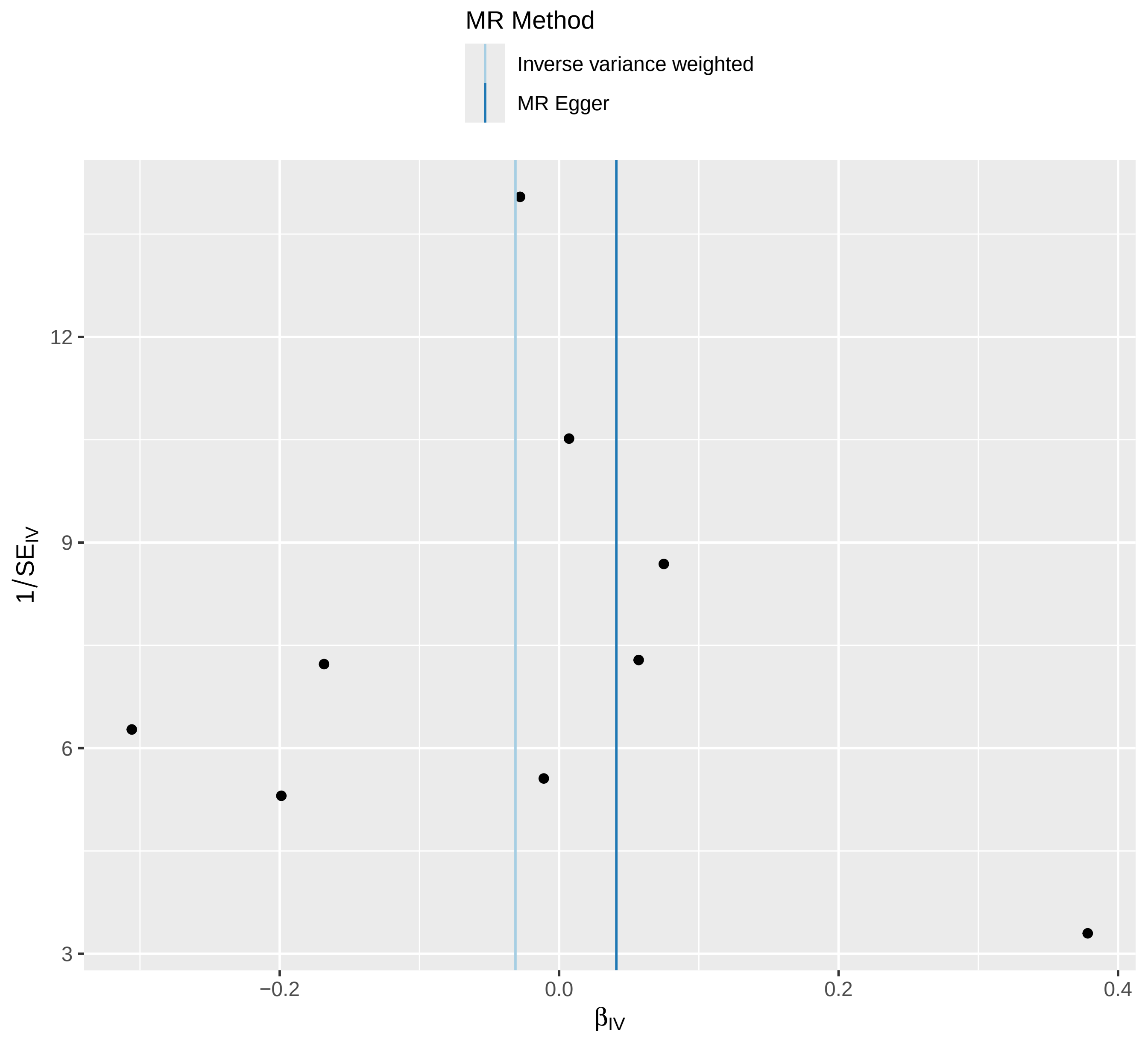

MR Method
Inverse variance weighted
MR Egger
12
9
6
3
1/SE_IV
−0.2
0.0
0.2
0.4
β_IV

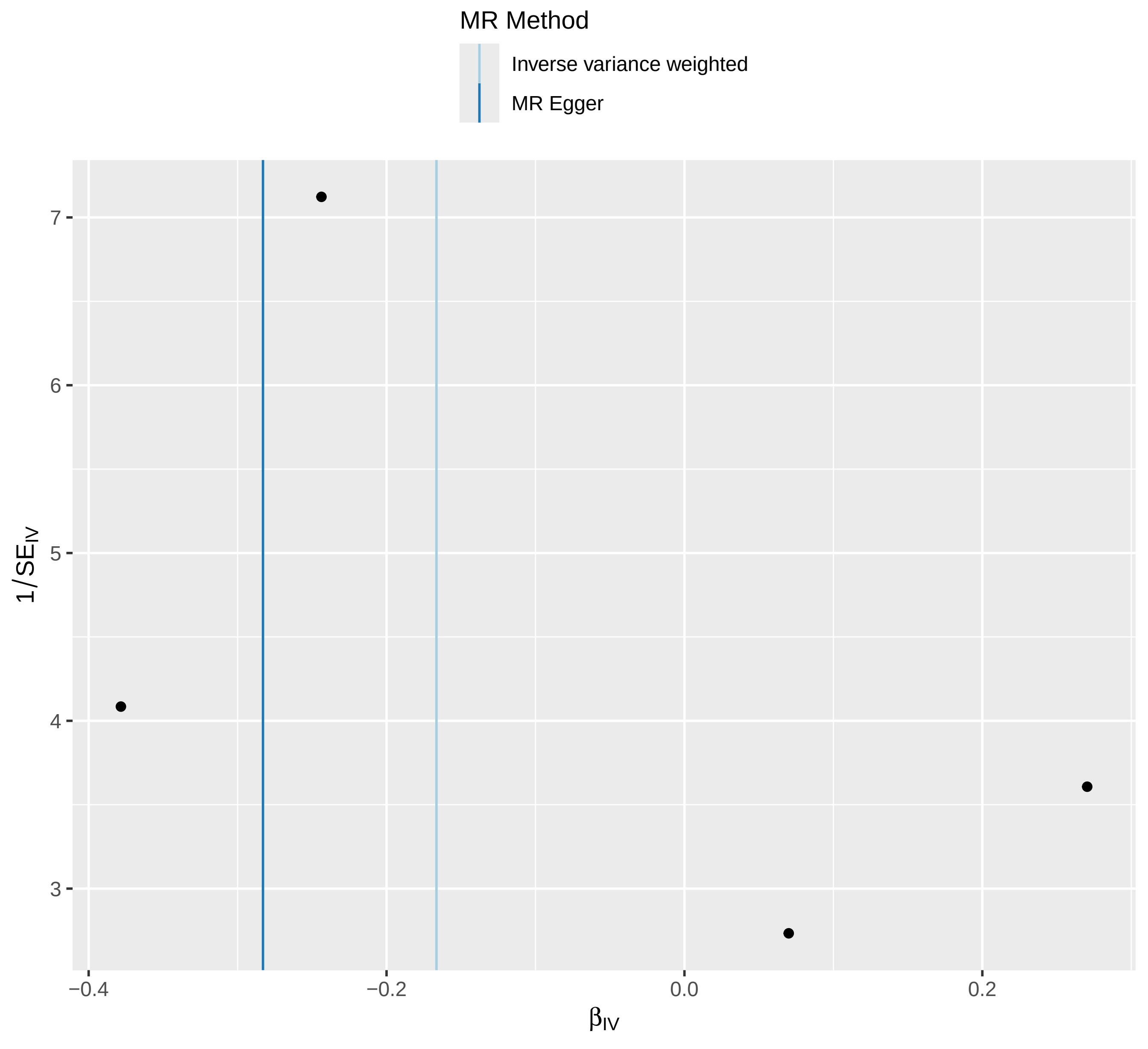

MR Method
Inverse variance weighted
MR Egger
7
6
5
4
3
1/SE_IV
−0.4
−0.2
0.0
0.2
β_IV

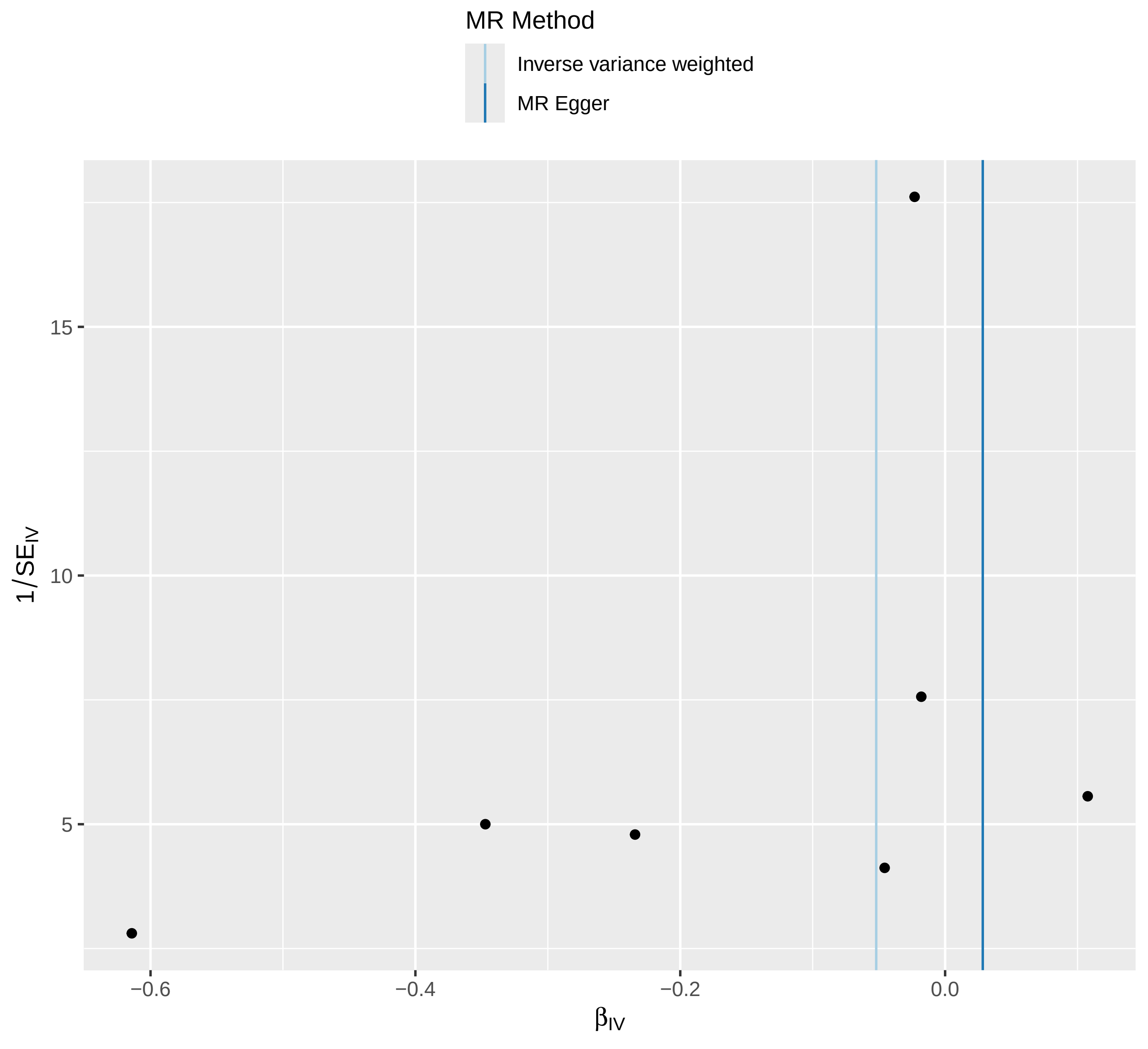
MR Method
Inverse variance weighted
MR Egger
15
10
5
$1/SE_{IV}$
−0.6
−0.4
−0.2
0.0
$\beta_{IV}$

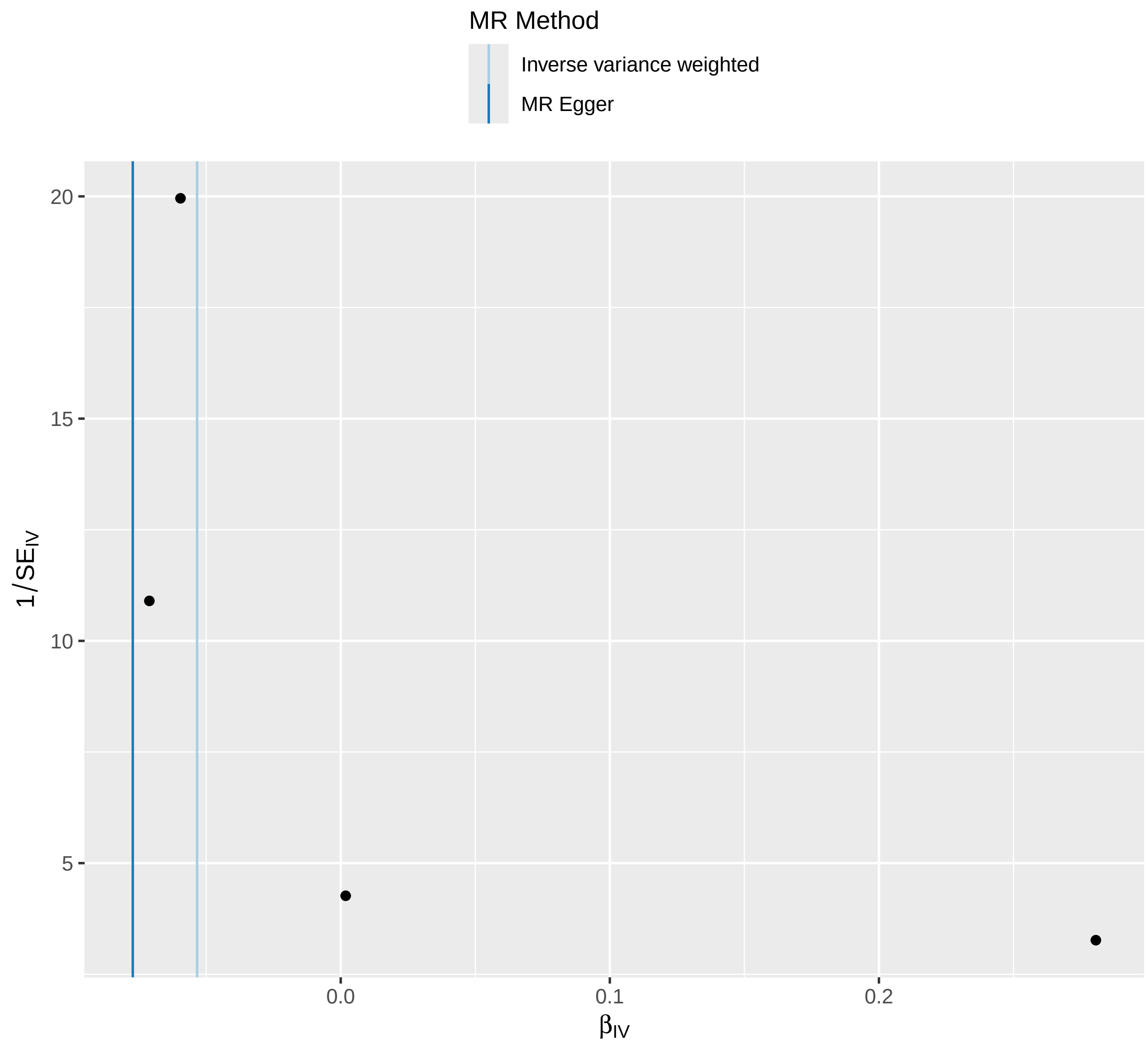
MR Method
Inverse variance weighted
MR Egger
20
15
10
5
$1/SE_{IV}$
0.0
0.1
0.2
$\beta_{IV}$

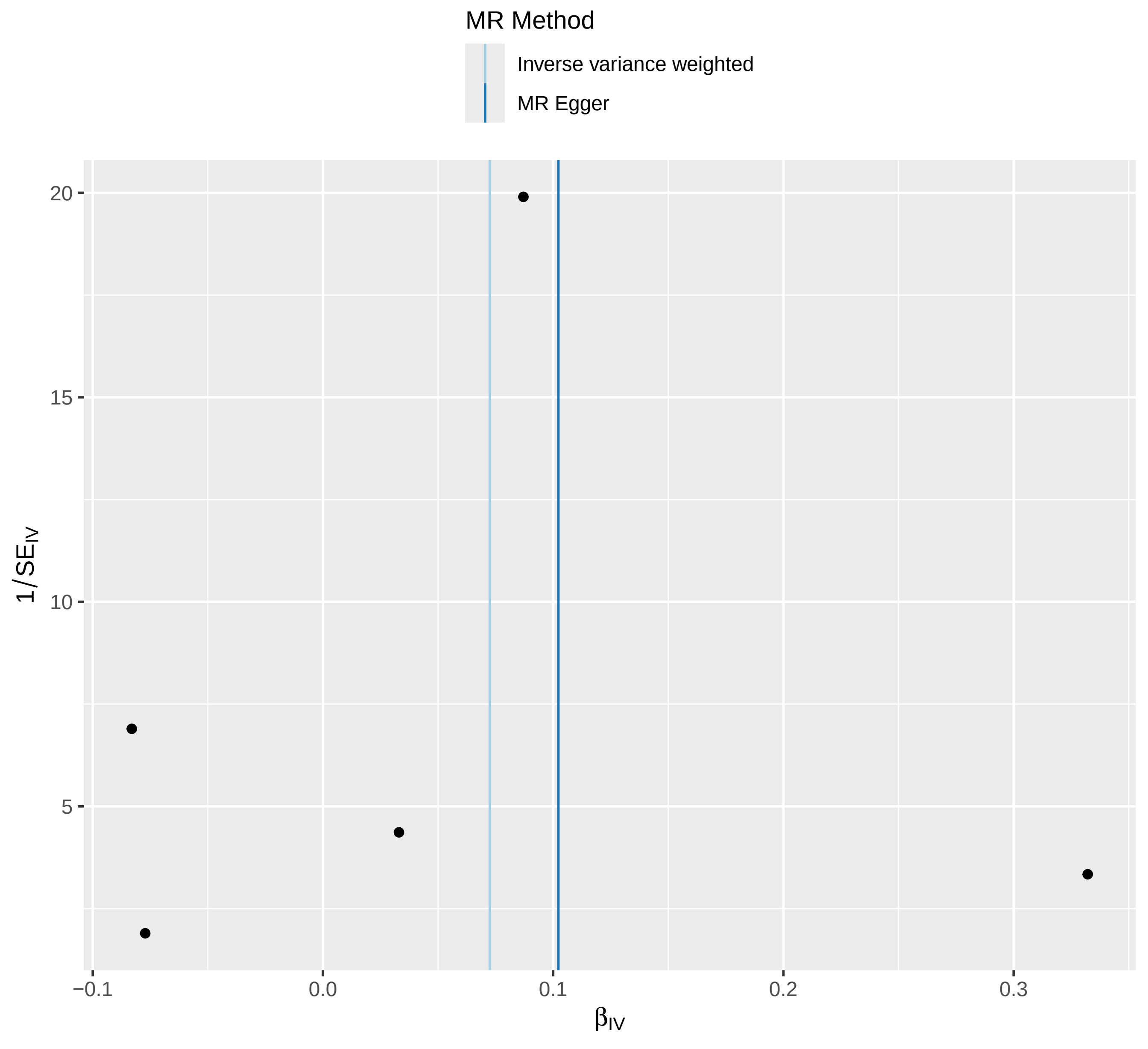

MR Method
Inverse variance weighted
MR Egger
20
15
10
5
1/SE_IV
−0.1
0.0
0.1
0.2
0.3
β_IV

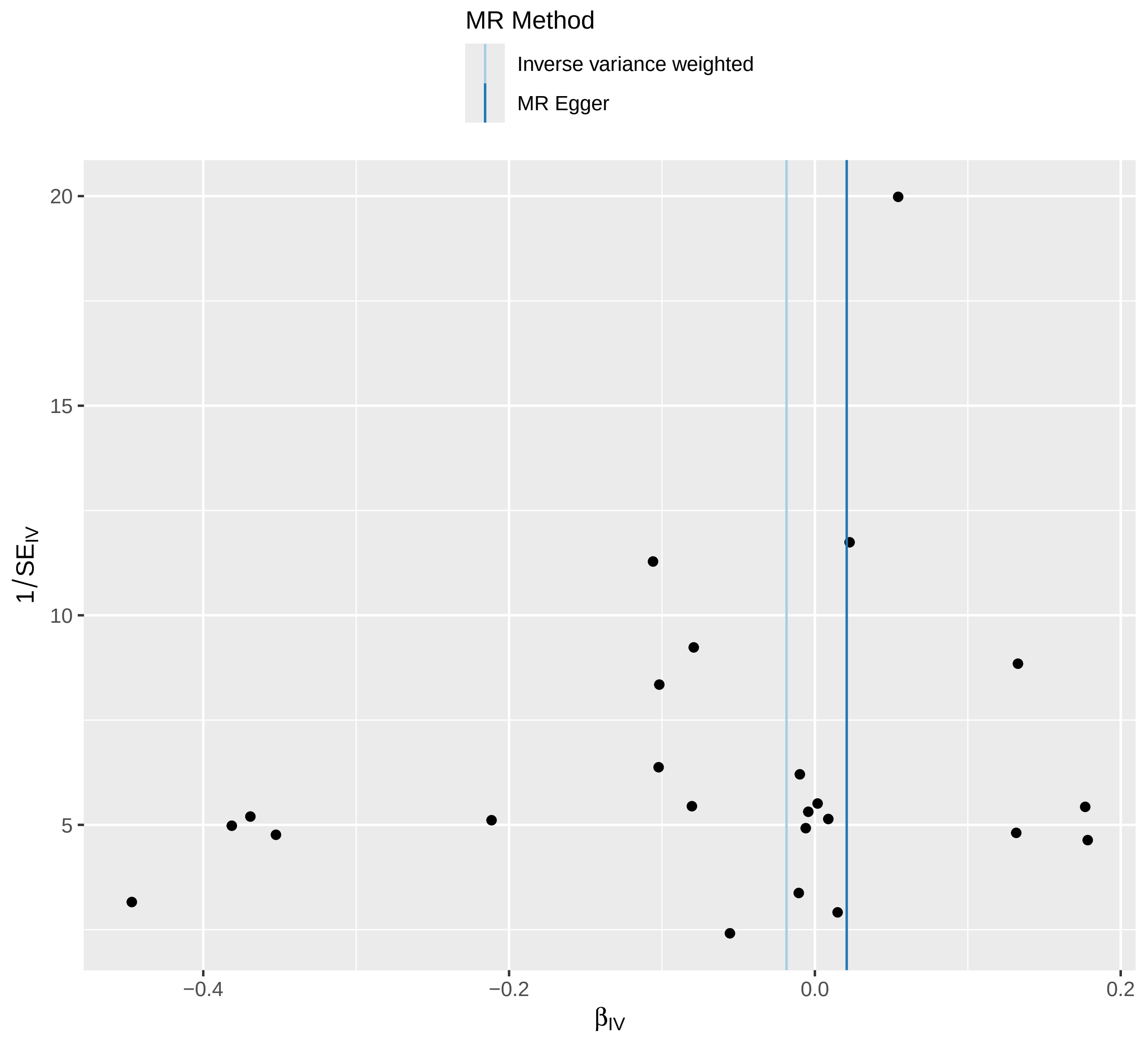

MR Method
Inverse variance weighted
MR Egger
20
15
10
5
1/SE$_{IV}$
−0.4
−0.2
0.0
0.2
β$_{IV}$

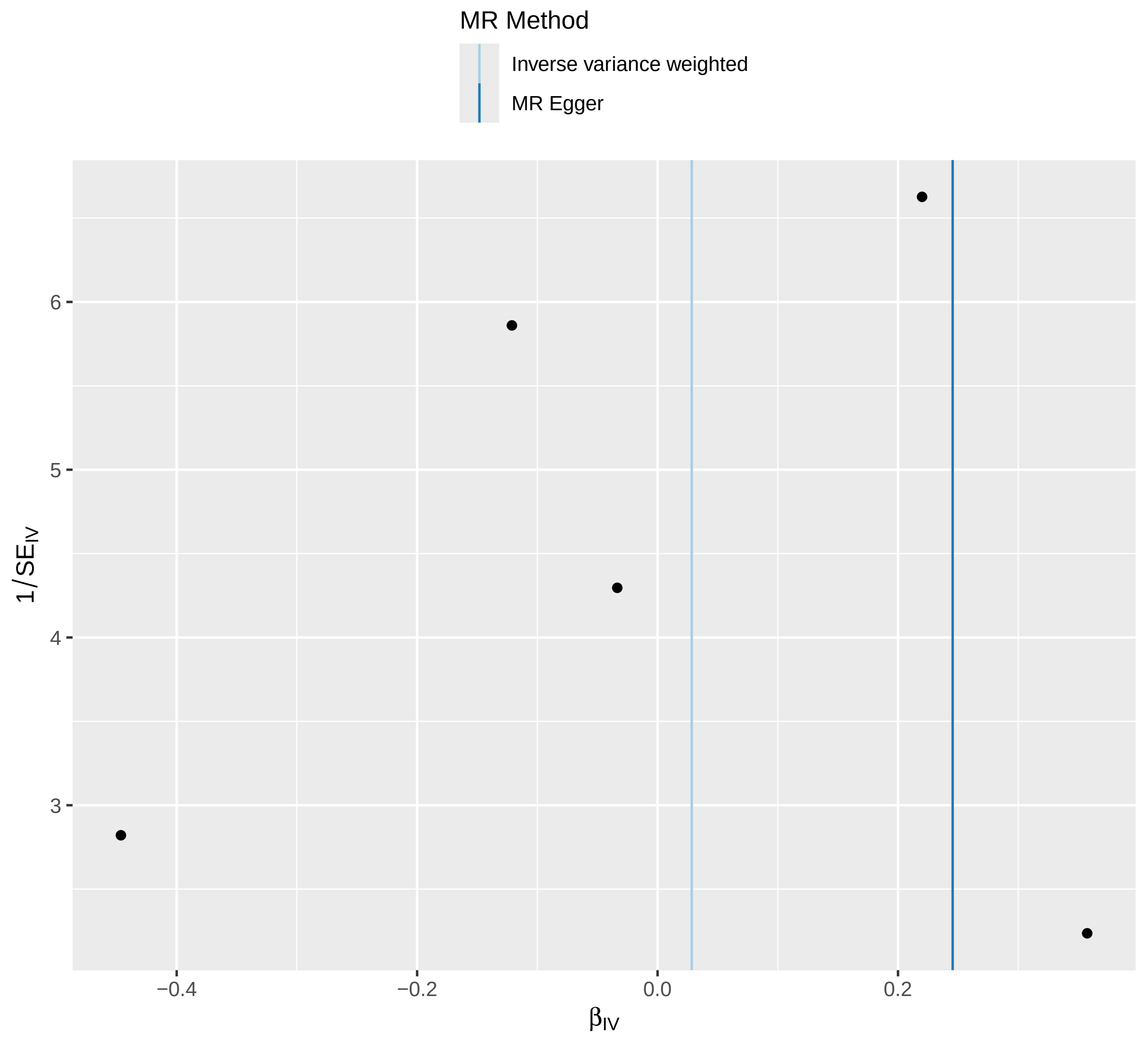

MR Method
Inverse variance weighted
MR Egger
6
5
4
3
1/SE$_{IV}$
−0.4
−0.2
0.0
0.2
β$_{IV}$

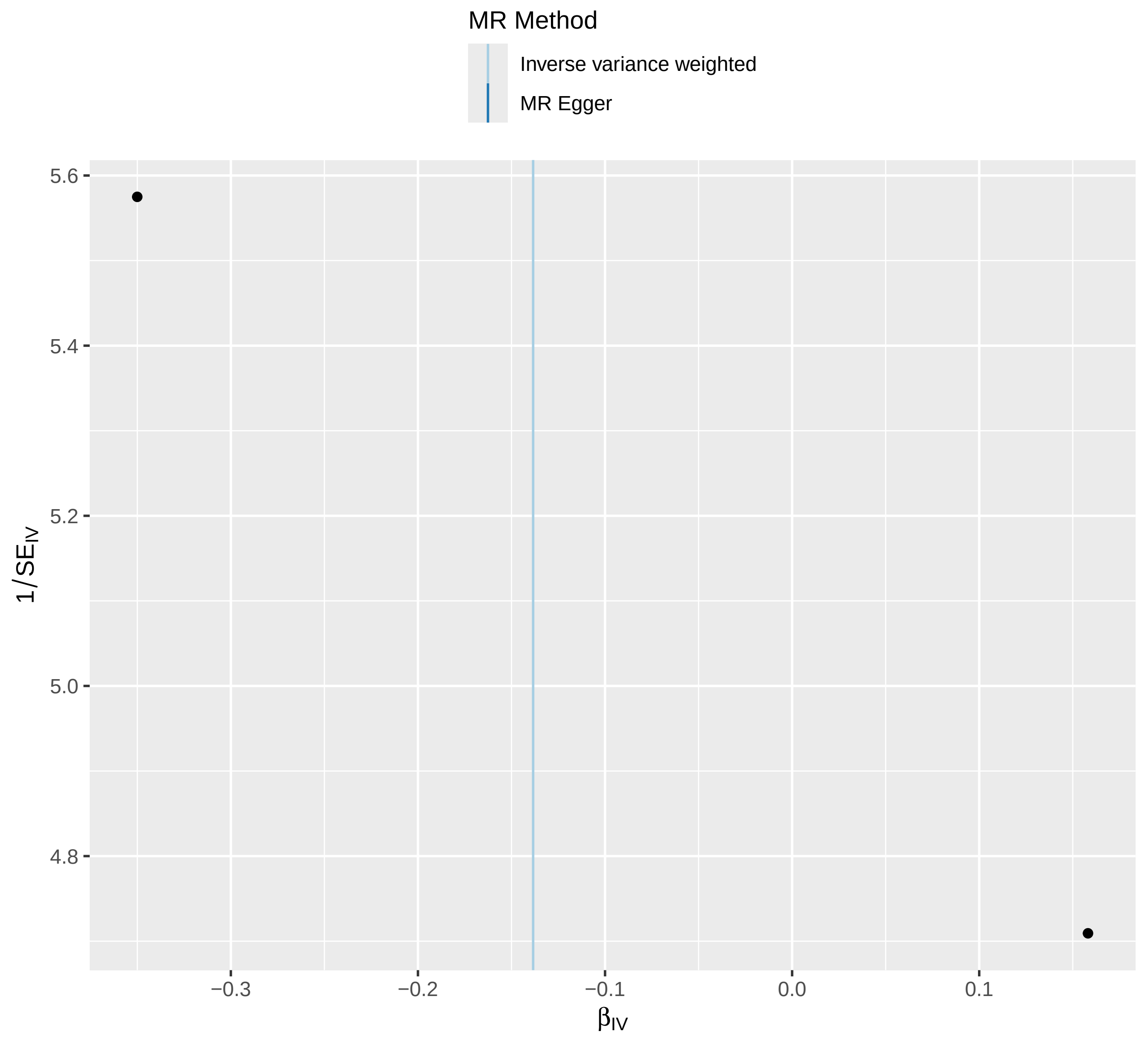

MR Method
Inverse variance weighted
MR Egger
5.6
5.4
5.2
5.0
4.8
$1/SE_{IV}$
−0.3
−0.2
−0.1
0.0
0.1
$\beta_{IV}$

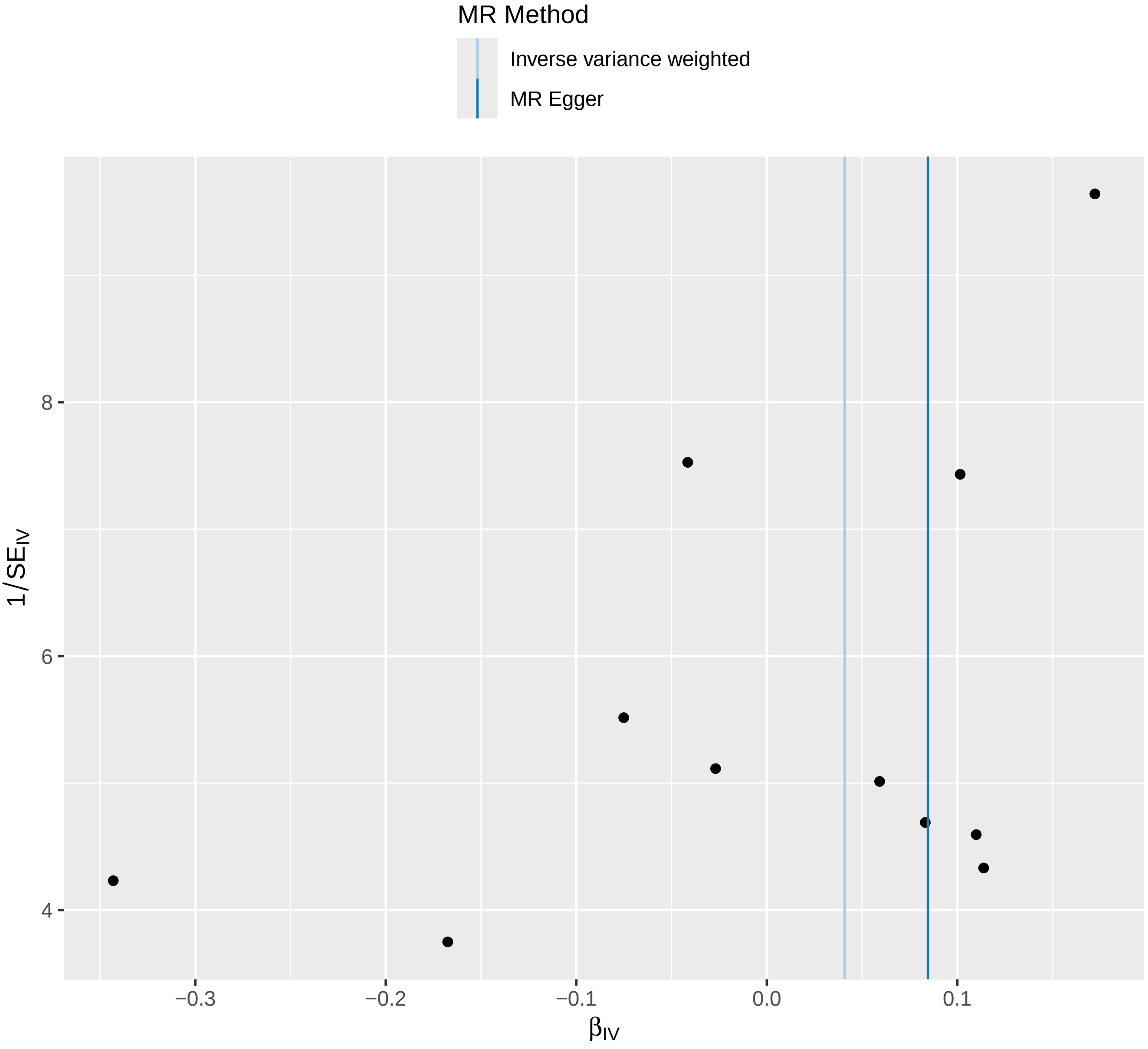
MR Method
Inverse variance weighted
MR Egger
1/SE$_{IV}$
8
6
4
−0.3
−0.2
−0.1
0.0
0.1
β$_{IV}$

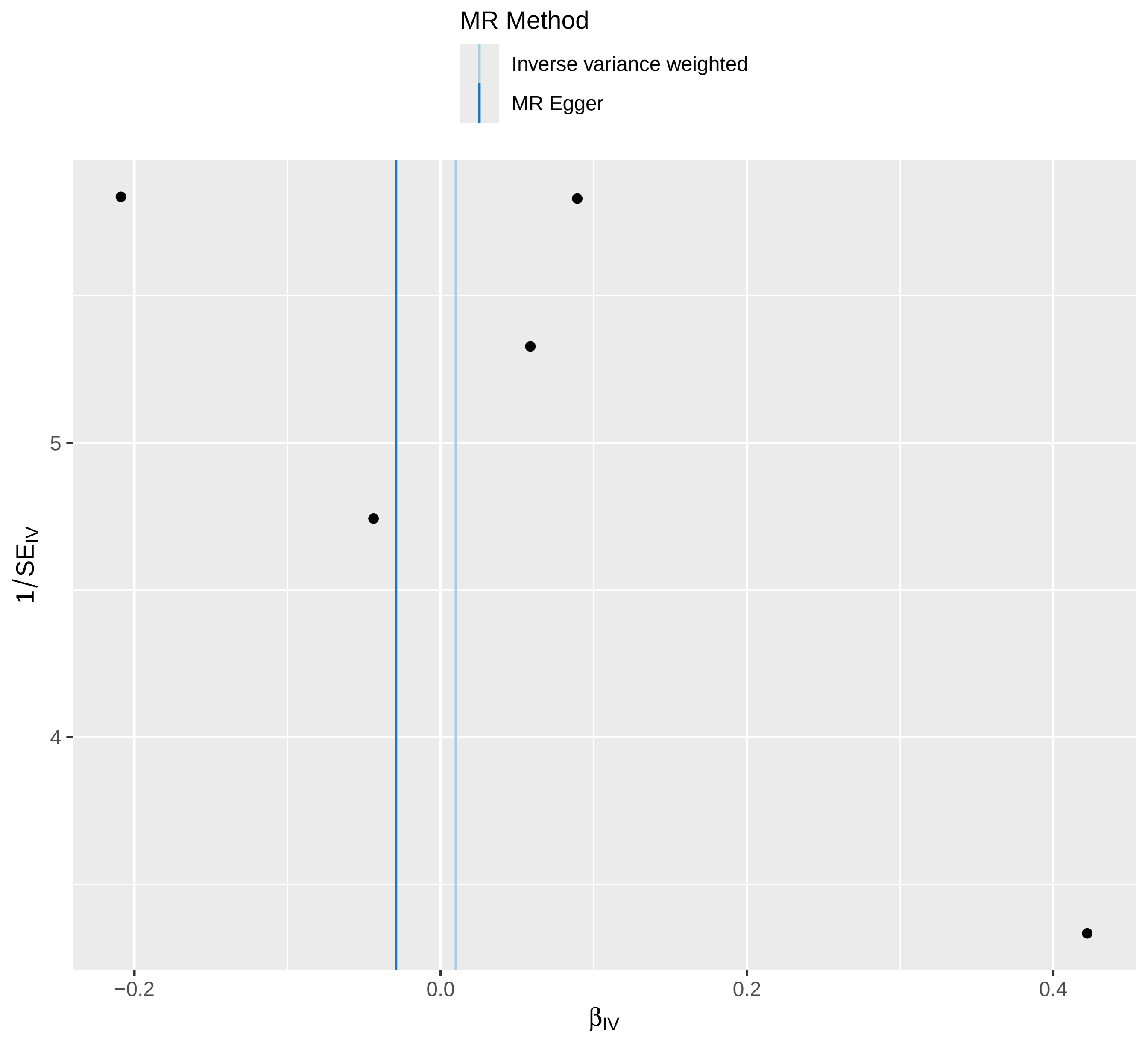

MR Method
Inverse variance weighted
MR Egger
5
4
$1/SE_{IV}$
−0.2
0.0
0.2
0.4
$\beta_{IV}$

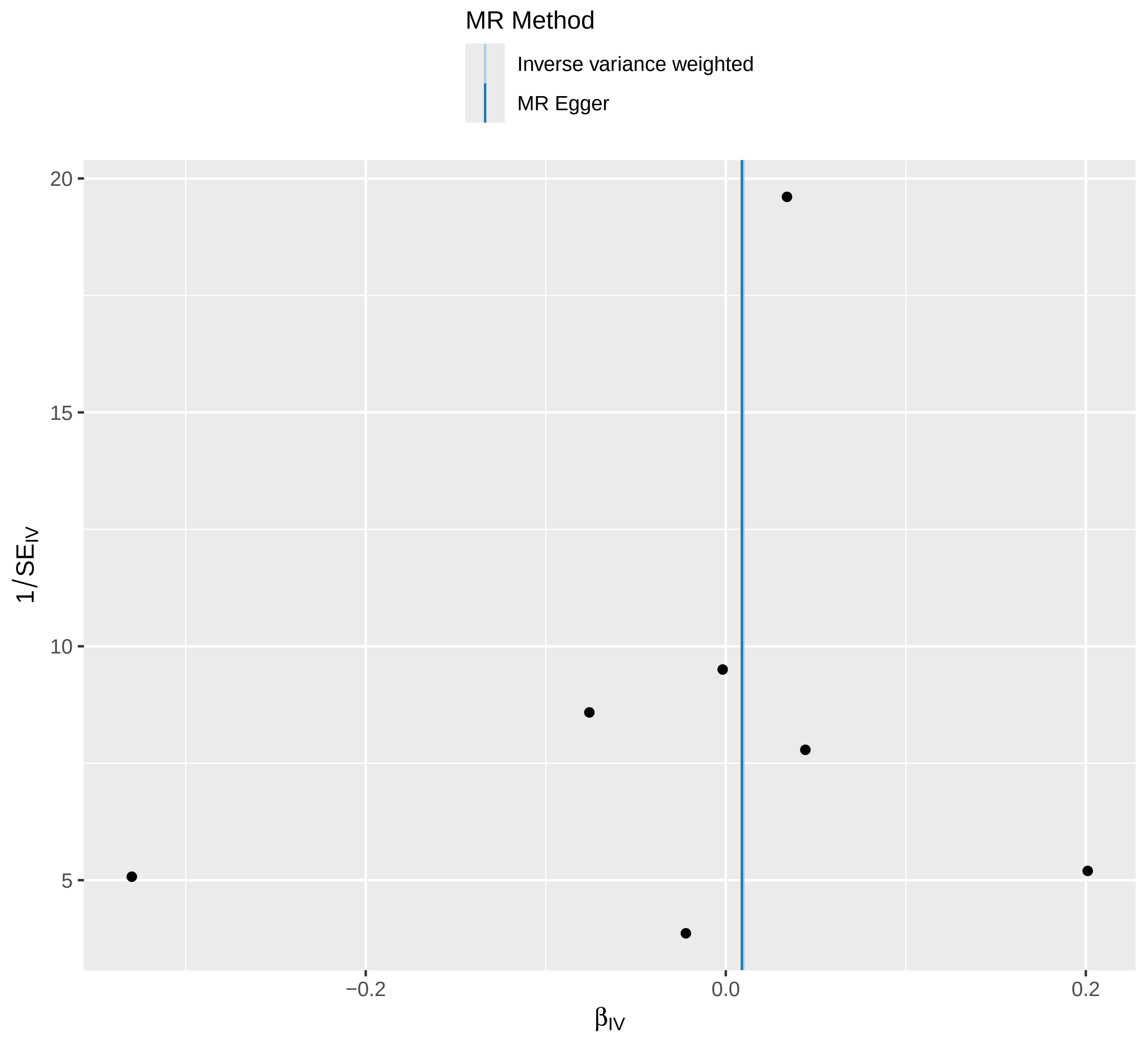

MR Method
Inverse variance weighted
MR Egger
20
15
10
5
−0.2
0.0
0.2
1/SE$_{IV}$
β$_{IV}$

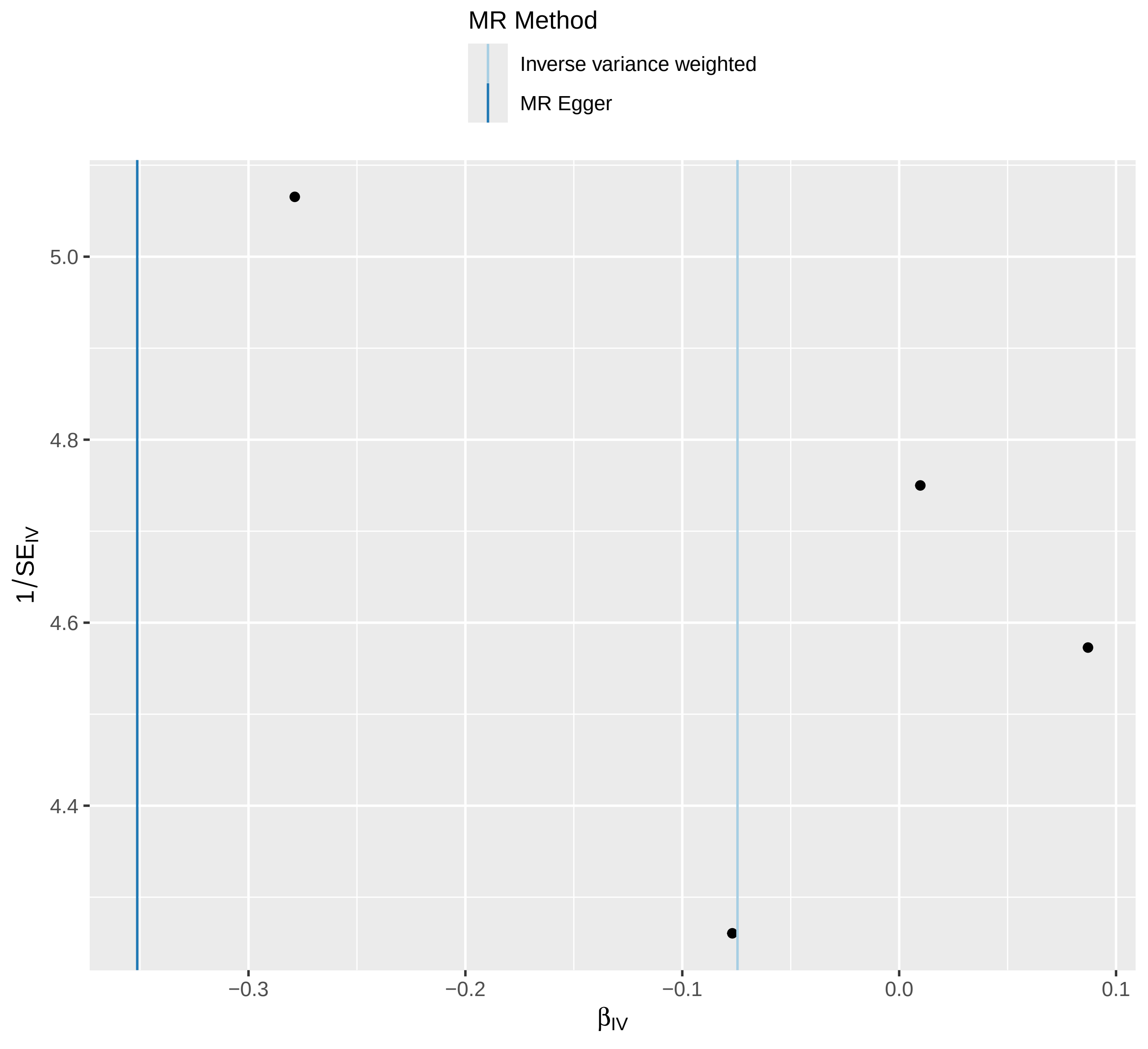

MR Method
Inverse variance weighted
MR Egger
5.0
4.8
4.6
4.4
1/SE$_{IV}$
−0.3
−0.2
−0.1
0.0
0.1
β$_{IV}$

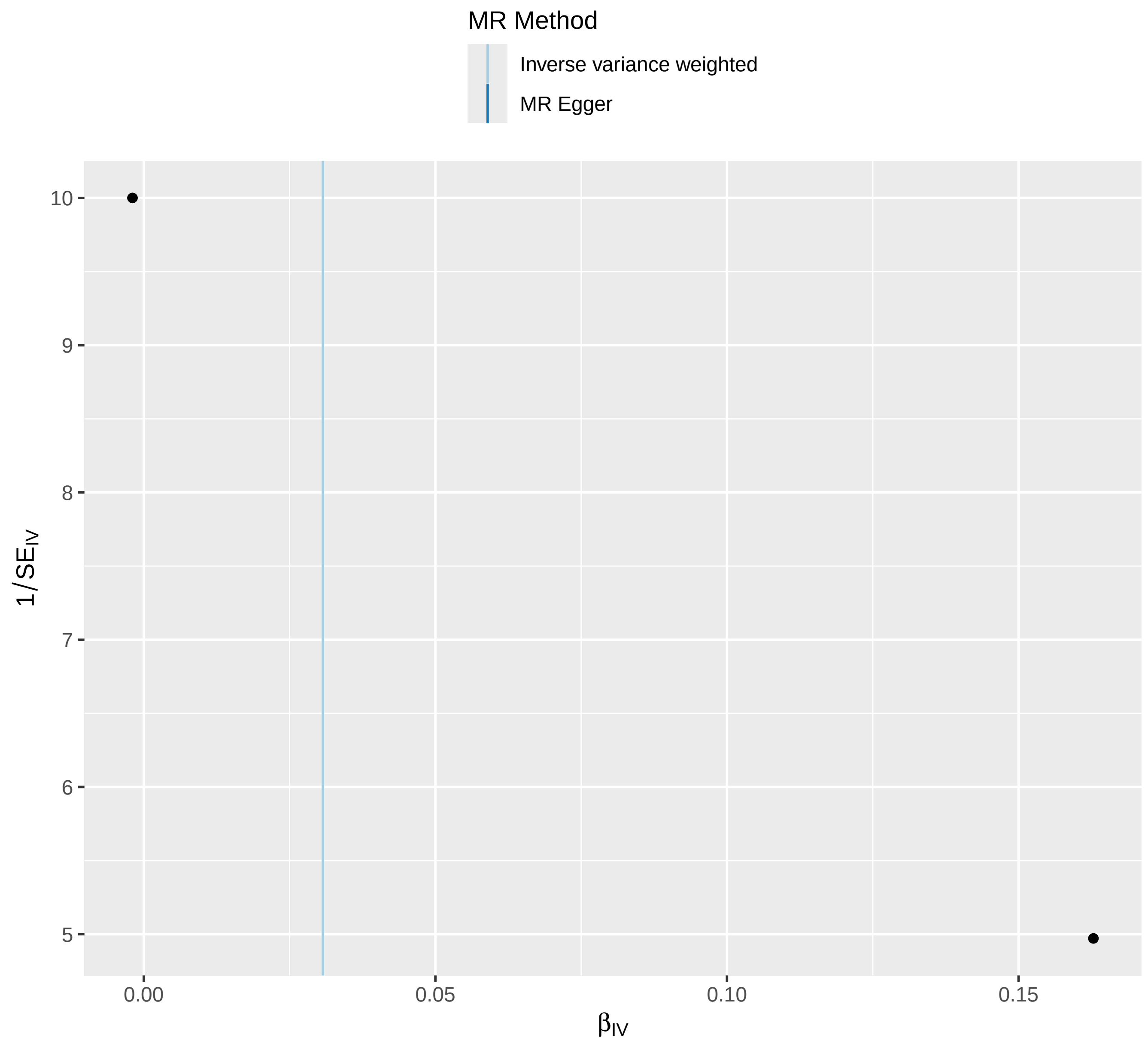

MR Method
Inverse variance weighted
MR Egger
10
9
8
7
6
5
1/SE_IV
0.00
0.05
0.10
0.15
β_IV

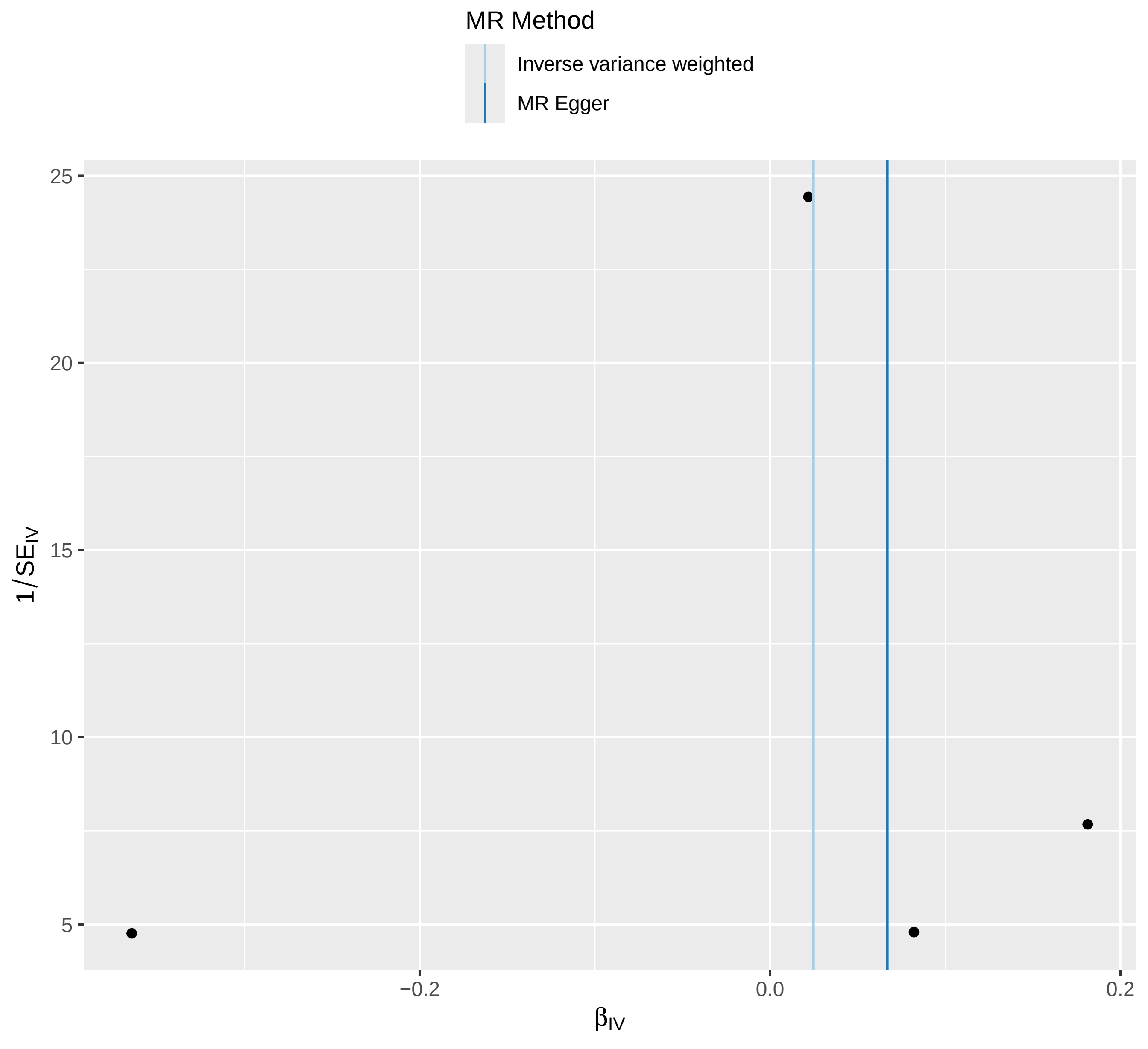
MR Method
Inverse variance weighted
MR Egger
25
20
15
10
5
1/SE$_{IV}$
−0.2
0.0
0.2
β$_{IV}$

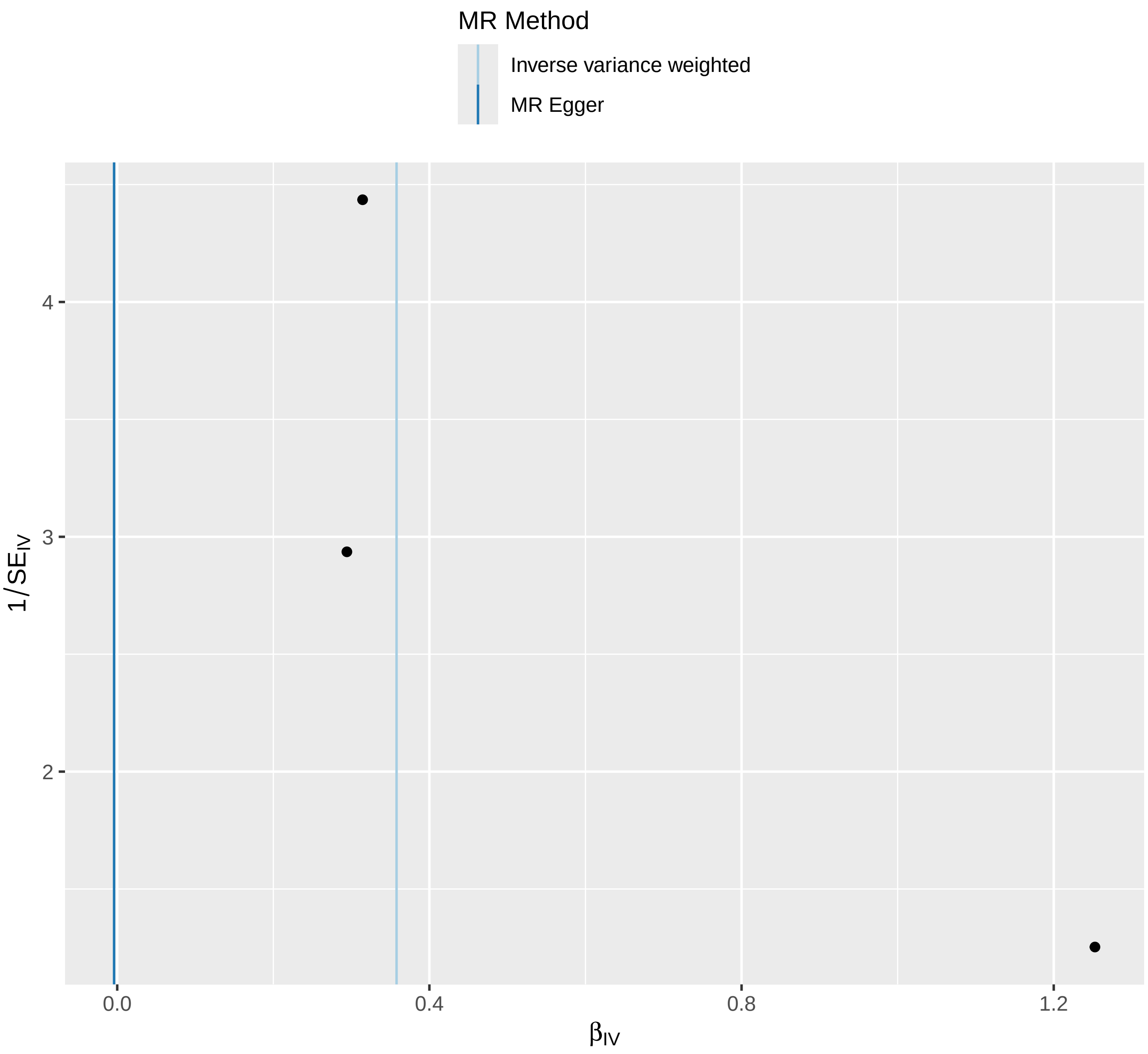
MR Method
Inverse variance weighted
MR Egger
4
3
2
$1/SE_{IV}$
0.0
0.4
0.8
1.2
$\beta_{IV}$

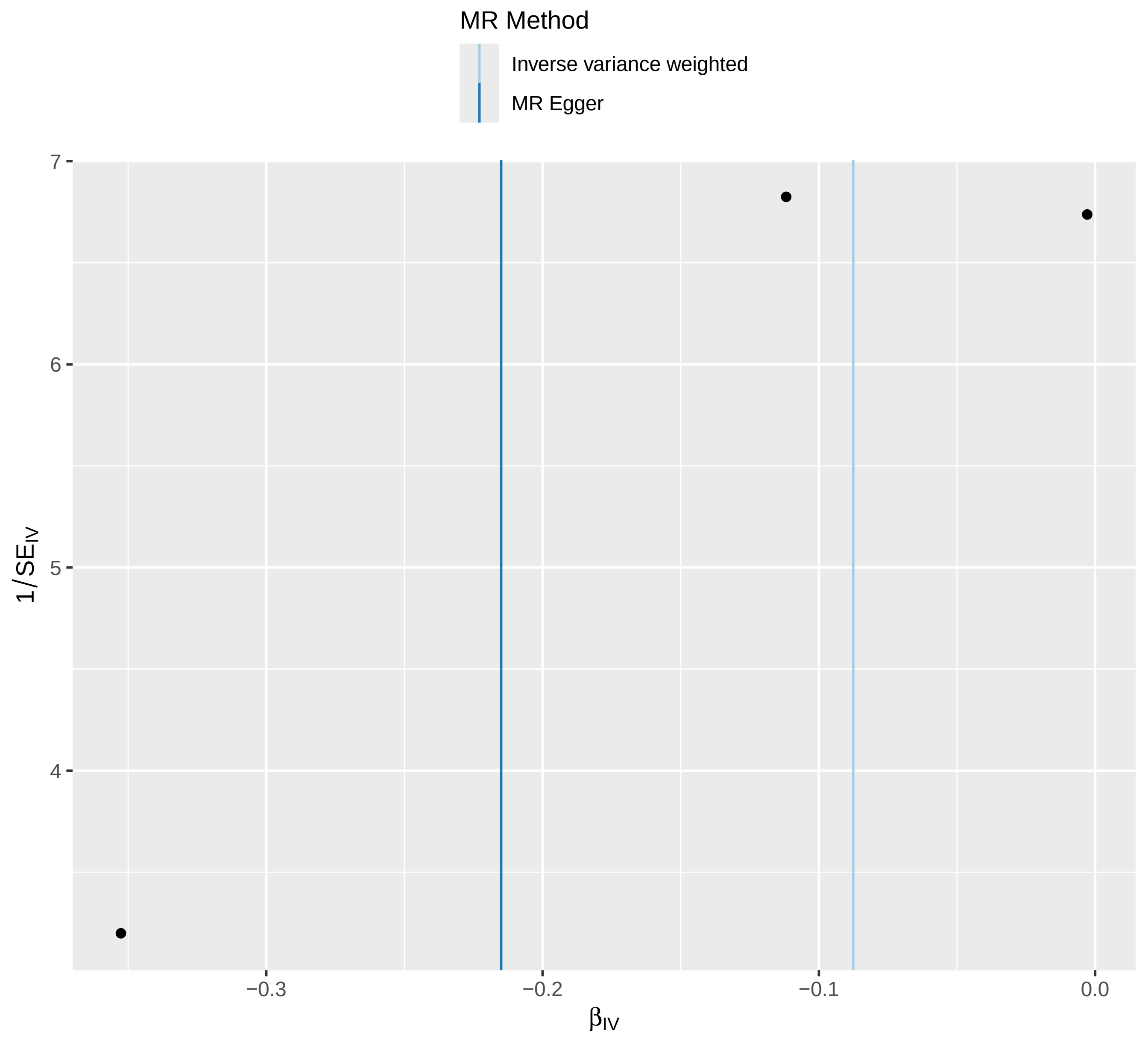

MR Method
Inverse variance weighted
MR Egger
7
6
5
4
1/SE_IV
−0.3
−0.2
−0.1
0.0
β_IV

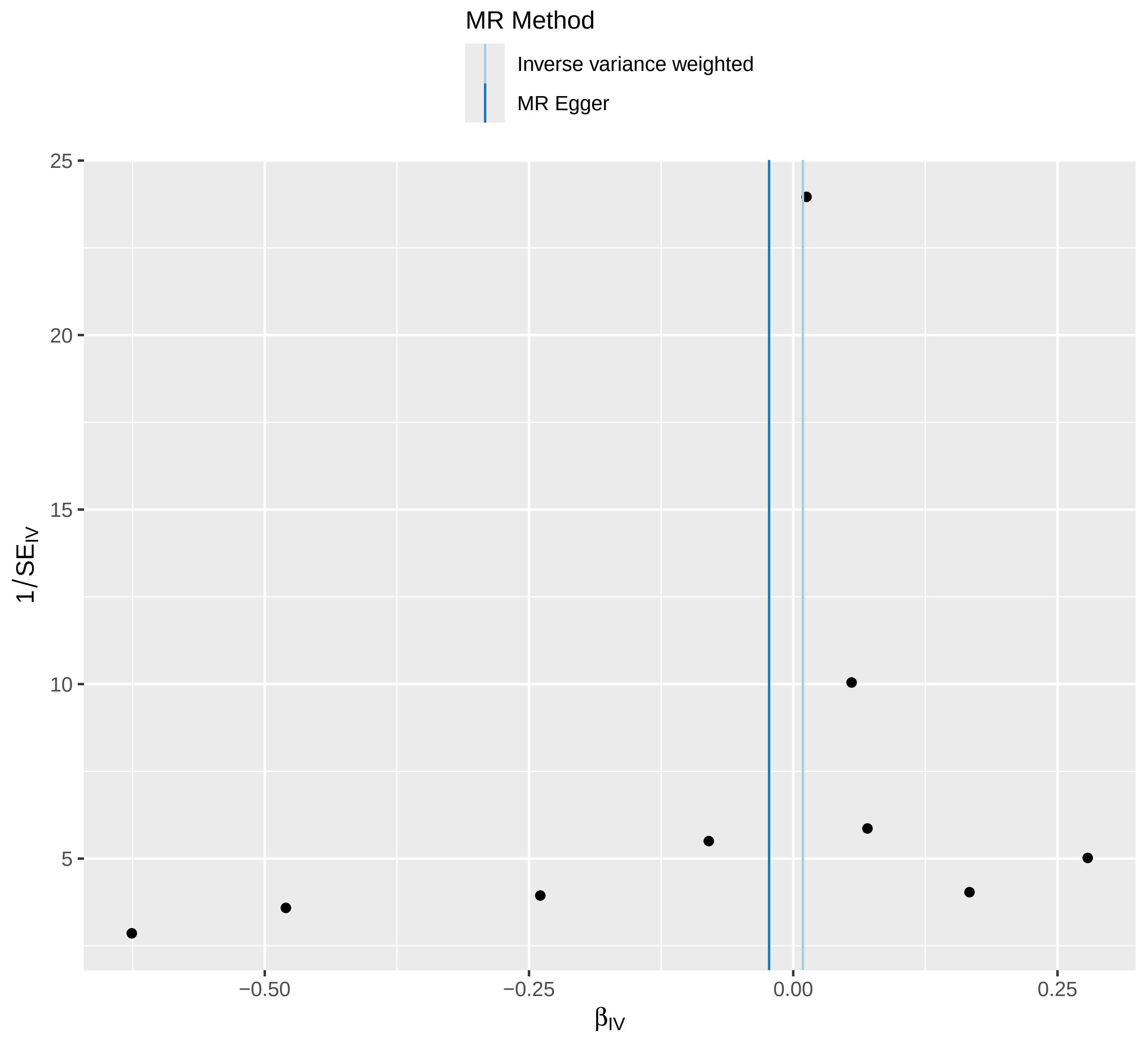
MR Method
Inverse variance weighted
MR Egger
25
20
15
10
5
1/SE_IV
−0.50
−0.25
0.00
0.25
β_IV

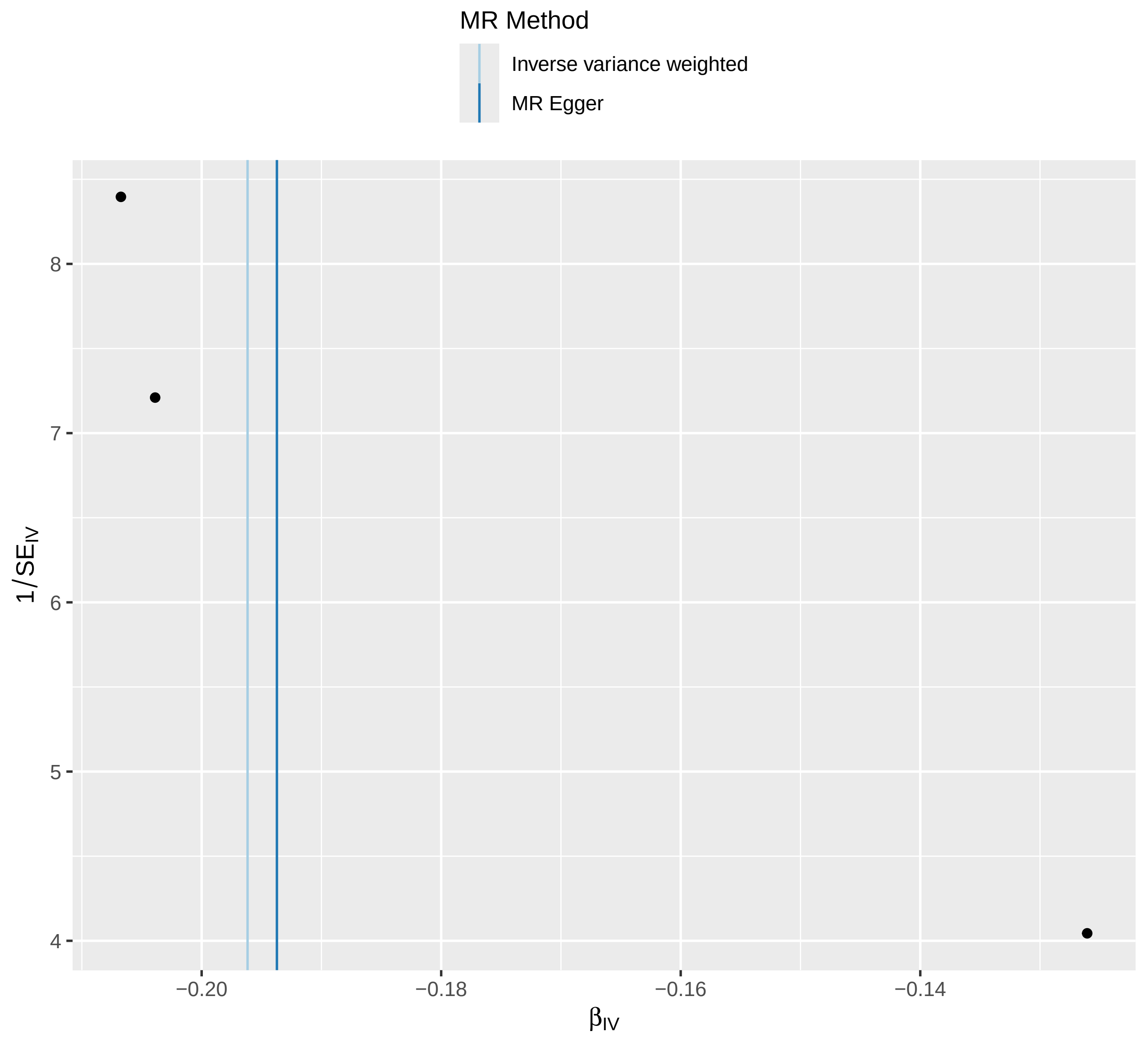
MR Method
Inverse variance weighted
MR Egger
1/SE_IV
8
7
6
5
4
β_IV
−0.20
−0.18
−0.16
−0.14

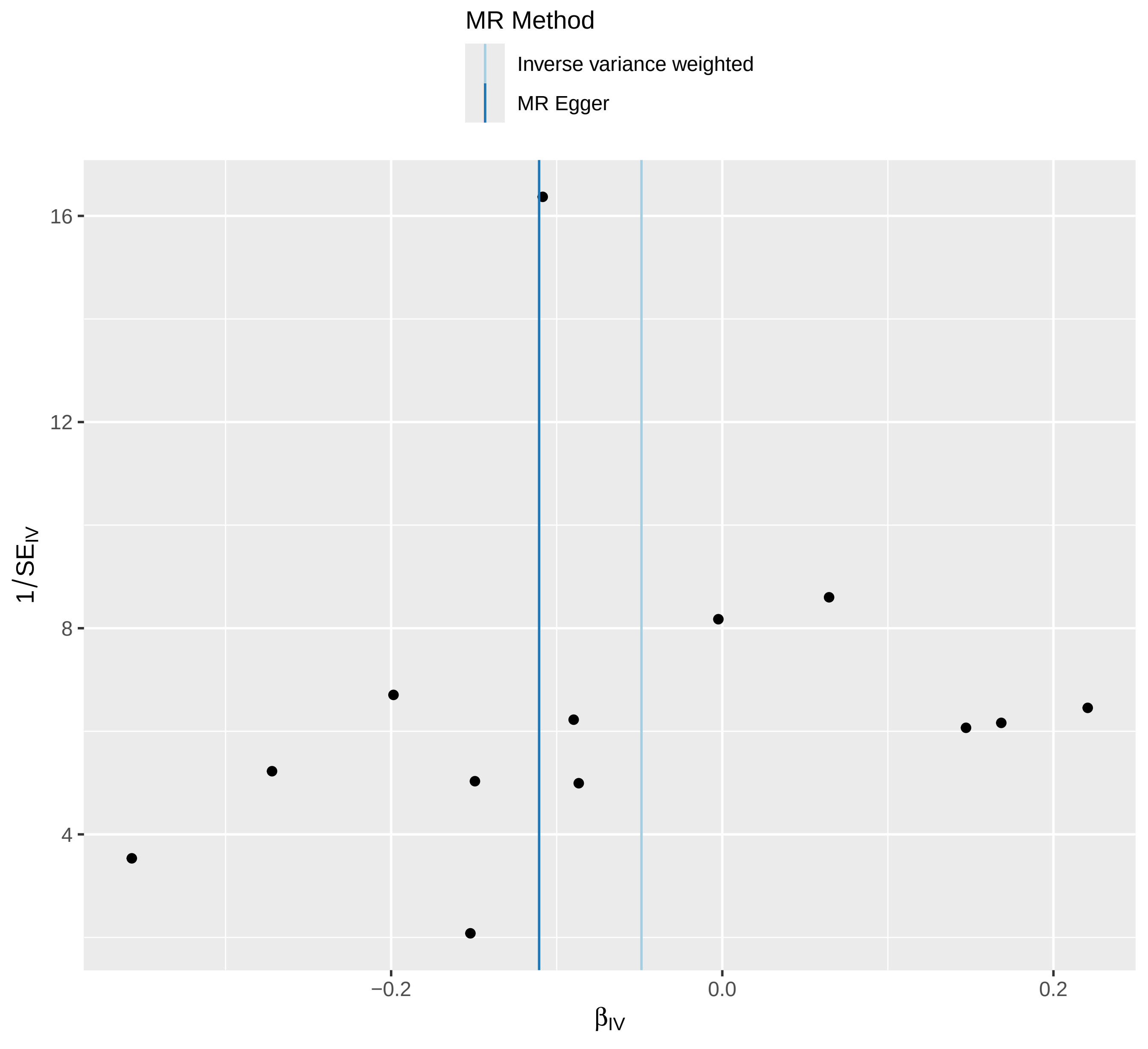
MR Method
Inverse variance weighted
MR Egger
16
12
8
4
1/SE_IV
−0.2
0.0
0.2
β_IV

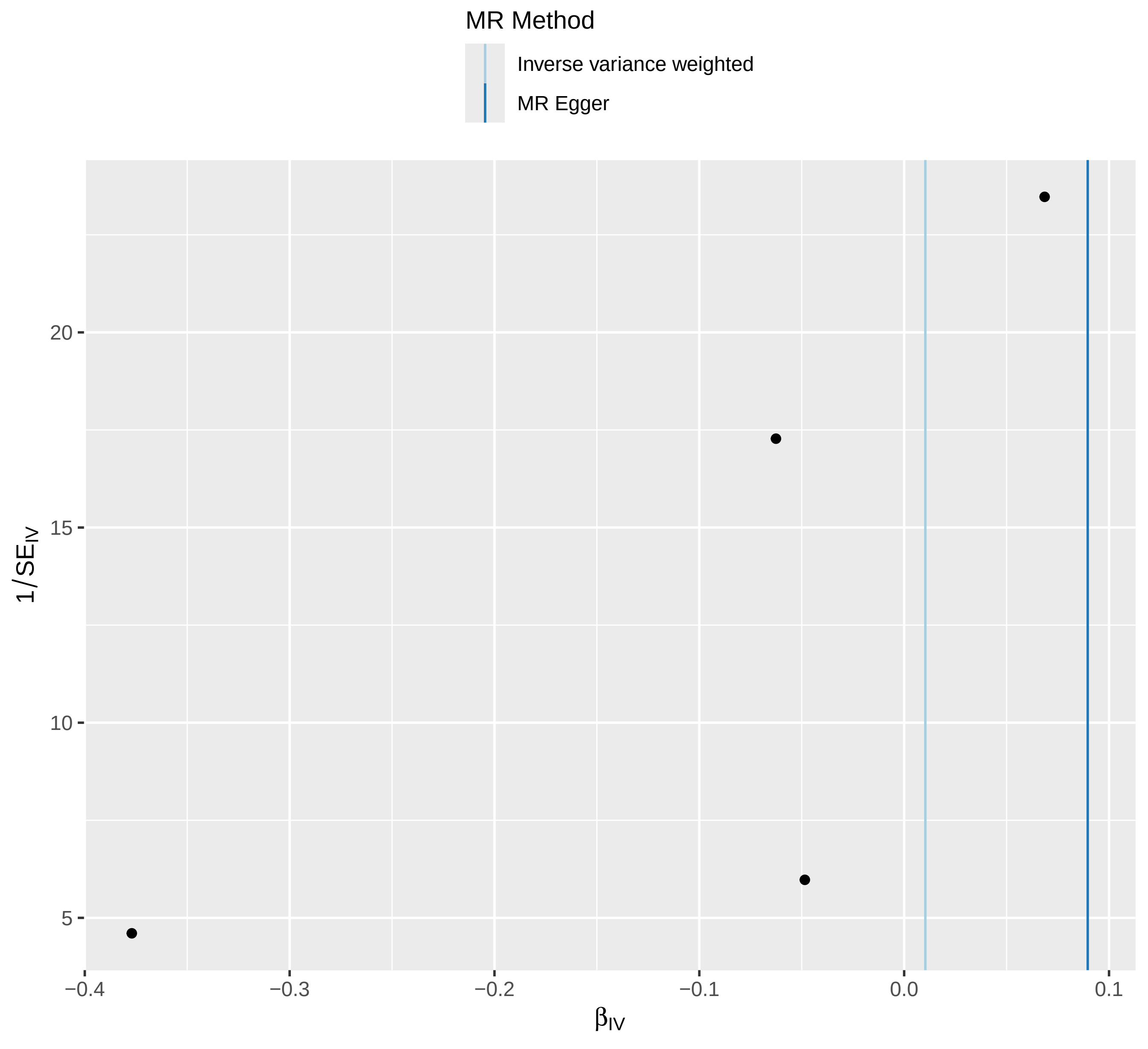
MR Method
Inverse variance weighted
MR Egger
20
15
10
5
1/SE_IV
−0.4
−0.3
−0.2
−0.1
0.0
0.1
β_IV

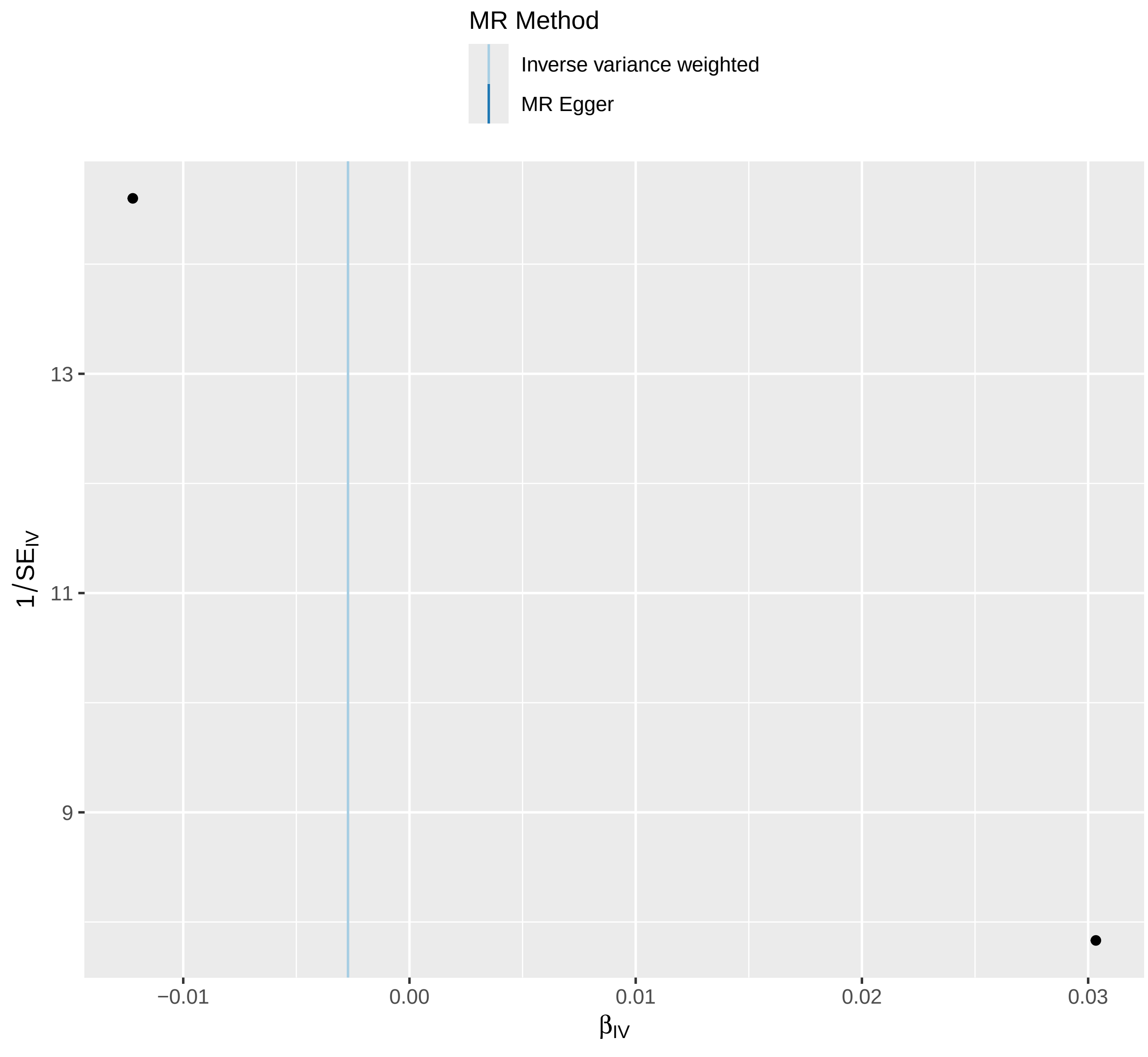
MR Method
Inverse variance weighted
MR Egger
13
11
9
$1/SE_{IV}$
−0.01
0.00
0.01
0.02
0.03
$\beta_{IV}$

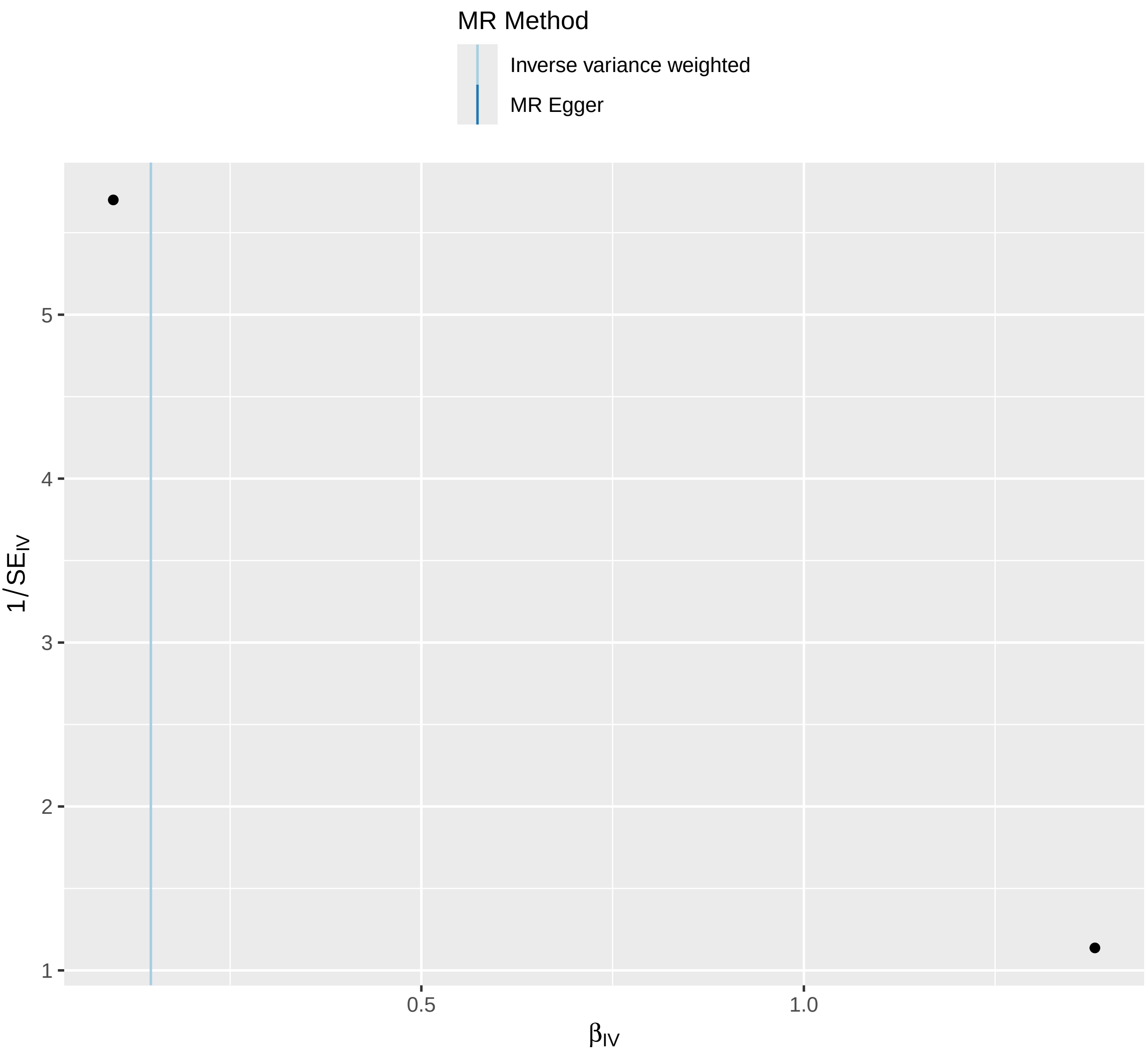

MR Method
Inverse variance weighted
MR Egger
5
4
3
2
1
$1/SE_{IV}$
0.5
1.0
$\beta_{IV}$

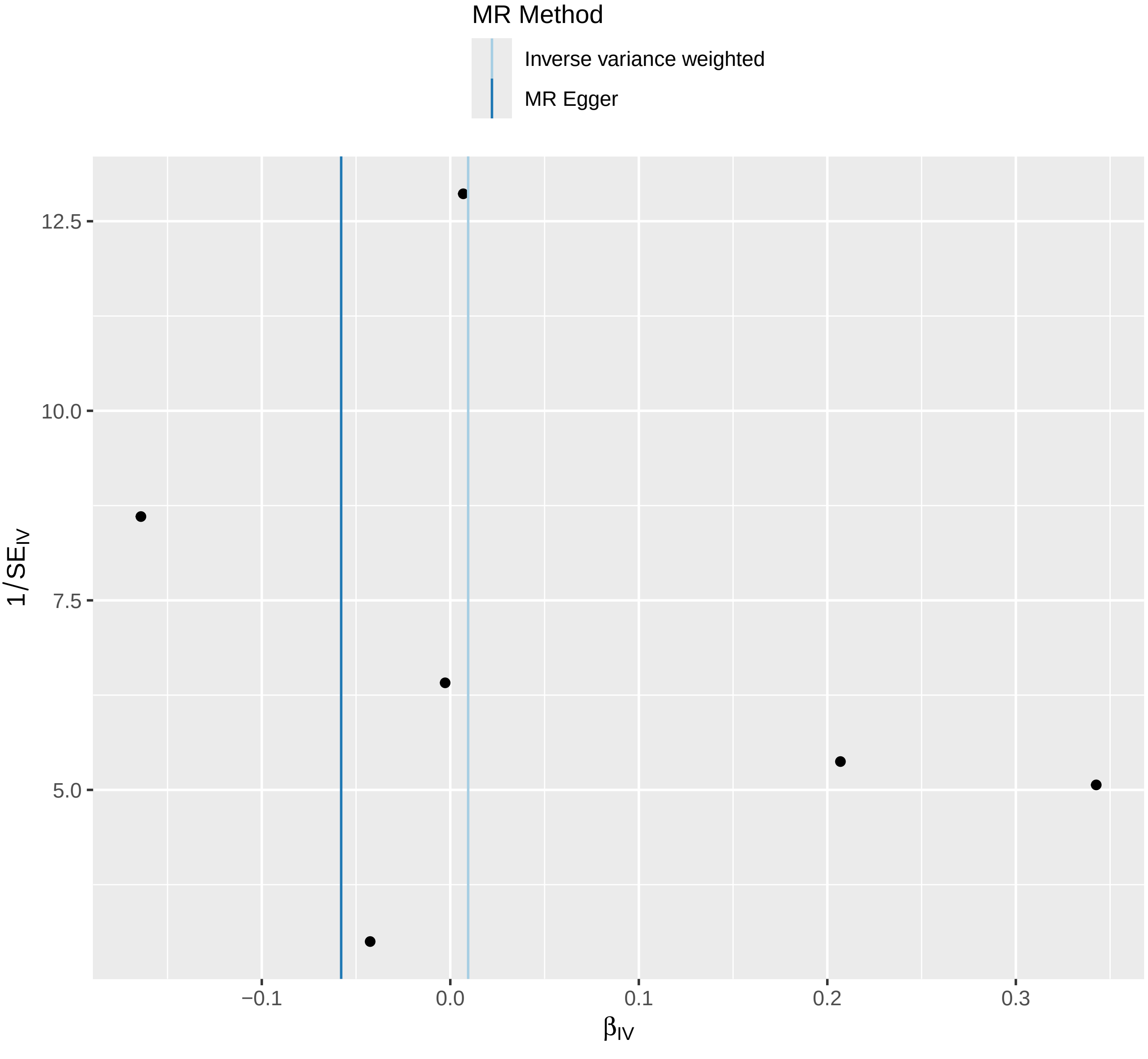

MR Method
Inverse variance weighted
MR Egger
12.5
10.0
7.5
5.0
1/SE_IV
−0.1
0.0
0.1
0.2
0.3
β_IV

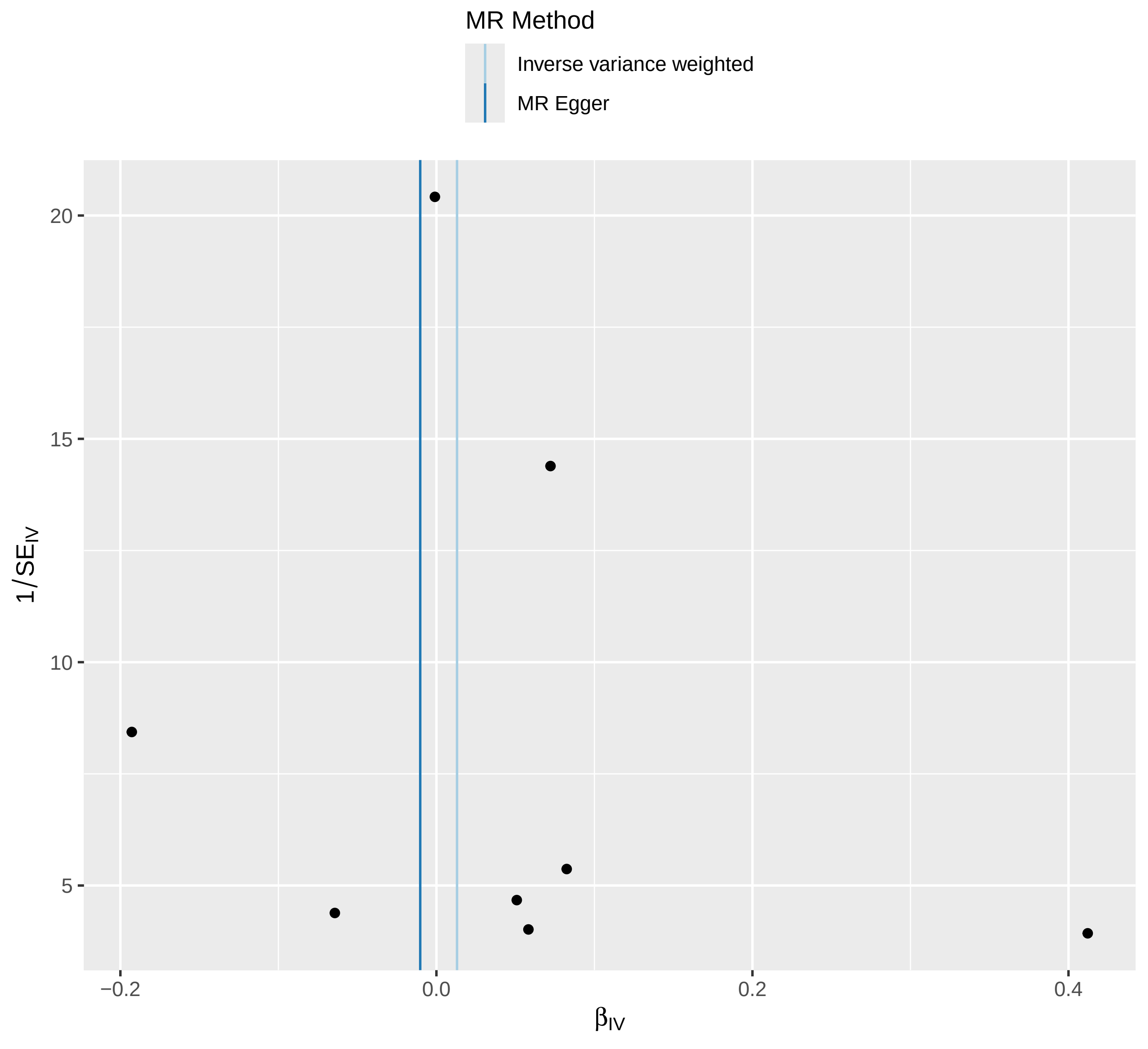

MR Method
Inverse variance weighted
MR Egger
20
15
10
5
1/SE$_{IV}$
−0.2
0.0
0.2
0.4
β$_{IV}$

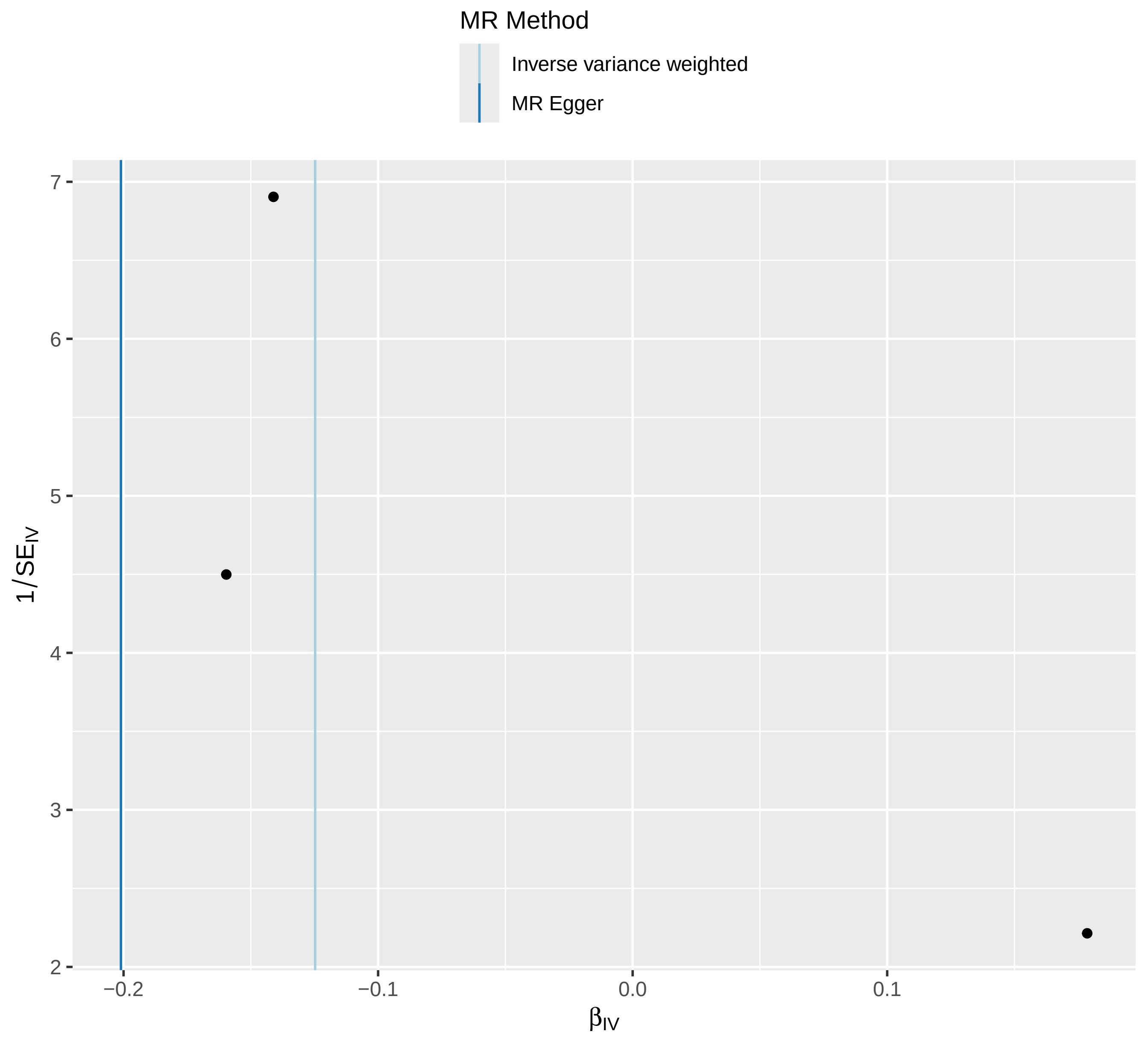
MR Method
Inverse variance weighted
MR Egger
1/SE_IV
β_IV
7
6
5
4
3
2
−0.2
−0.1
0.0
0.1

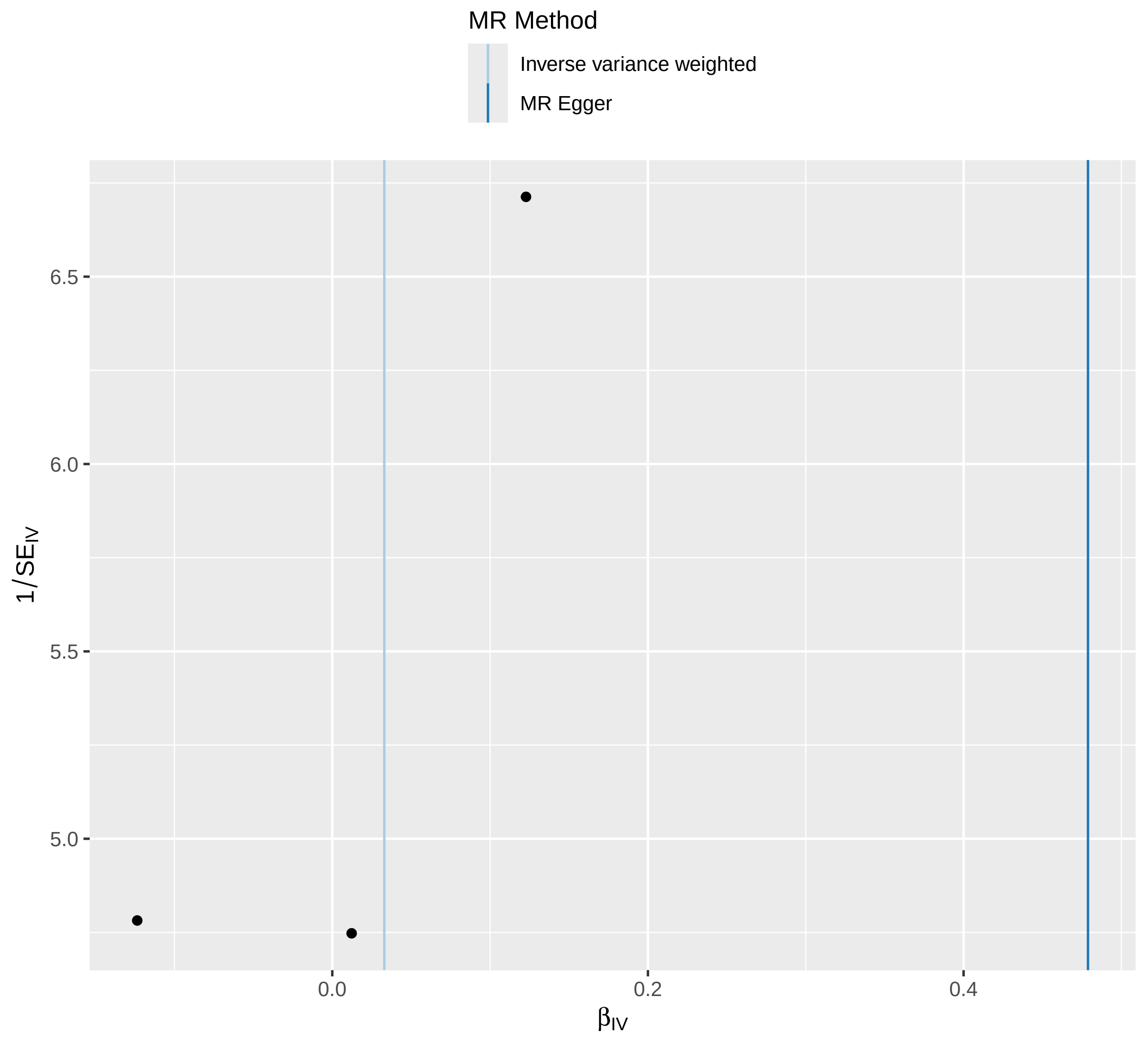

MR Method
Inverse variance weighted
MR Egger
6.5
6.0
5.5
5.0
1/SE$_{IV}$
0.0
0.2
0.4
β$_{IV}$

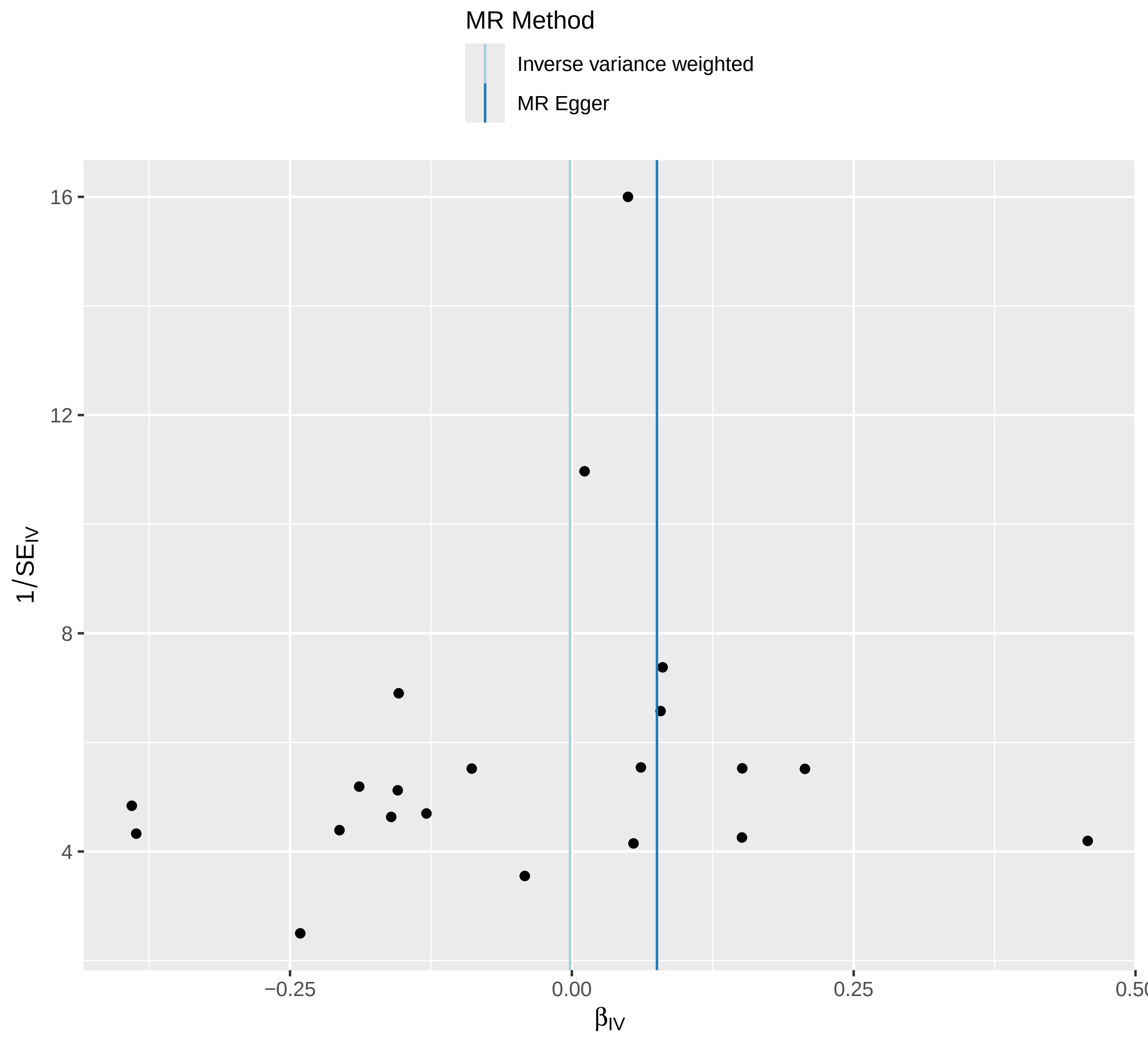
MR Method
Inverse variance weighted
MR Egger
16
12
8
4
1/SE$_{IV}$
−0.25
0.00
0.25
0.50
β$_{IV}$

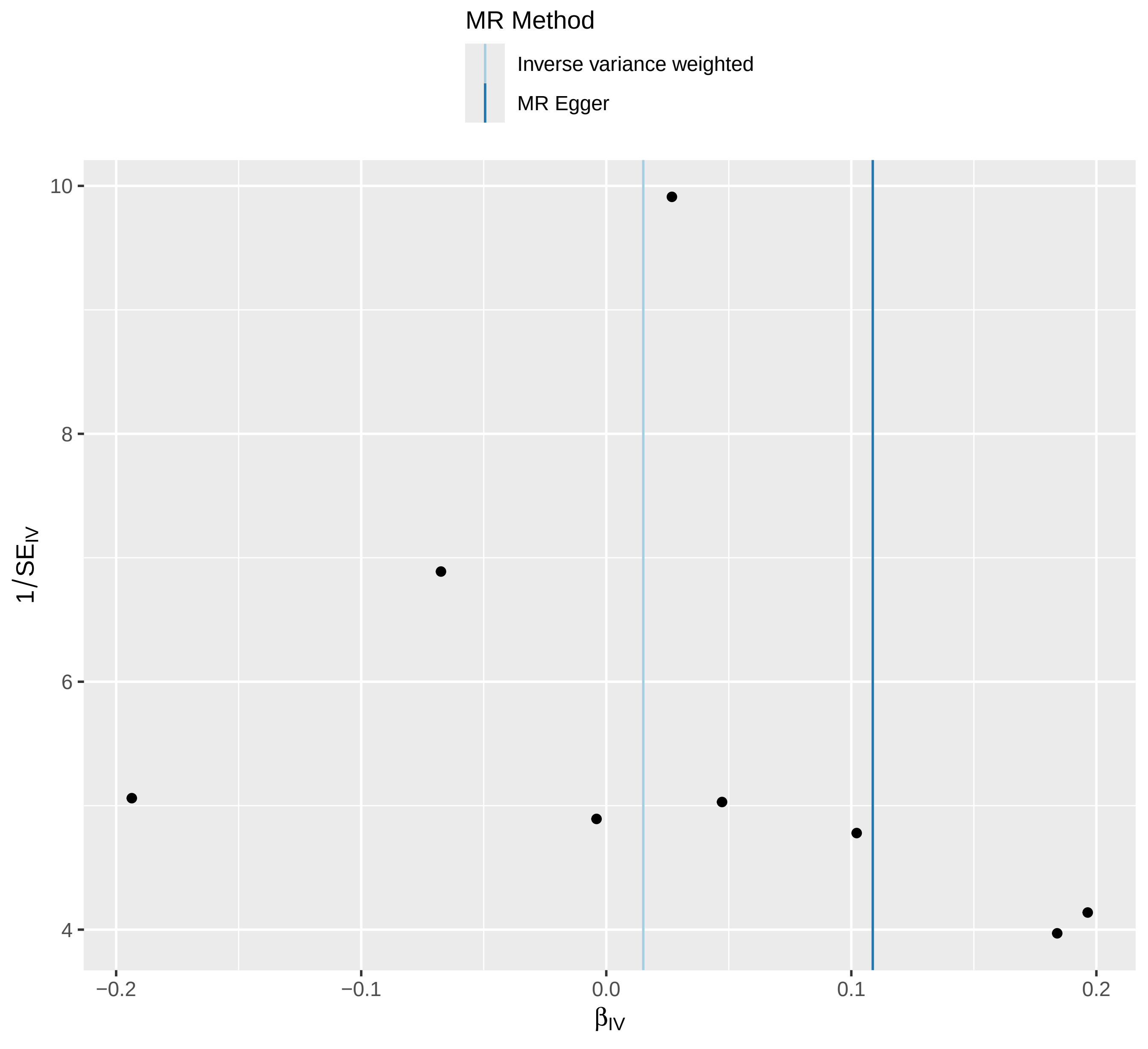
MR Method
Inverse variance weighted
MR Egger
10
8
6
4
1/SE$_{IV}$
−0.2
−0.1
0.0
0.1
0.2
β$_{IV}$

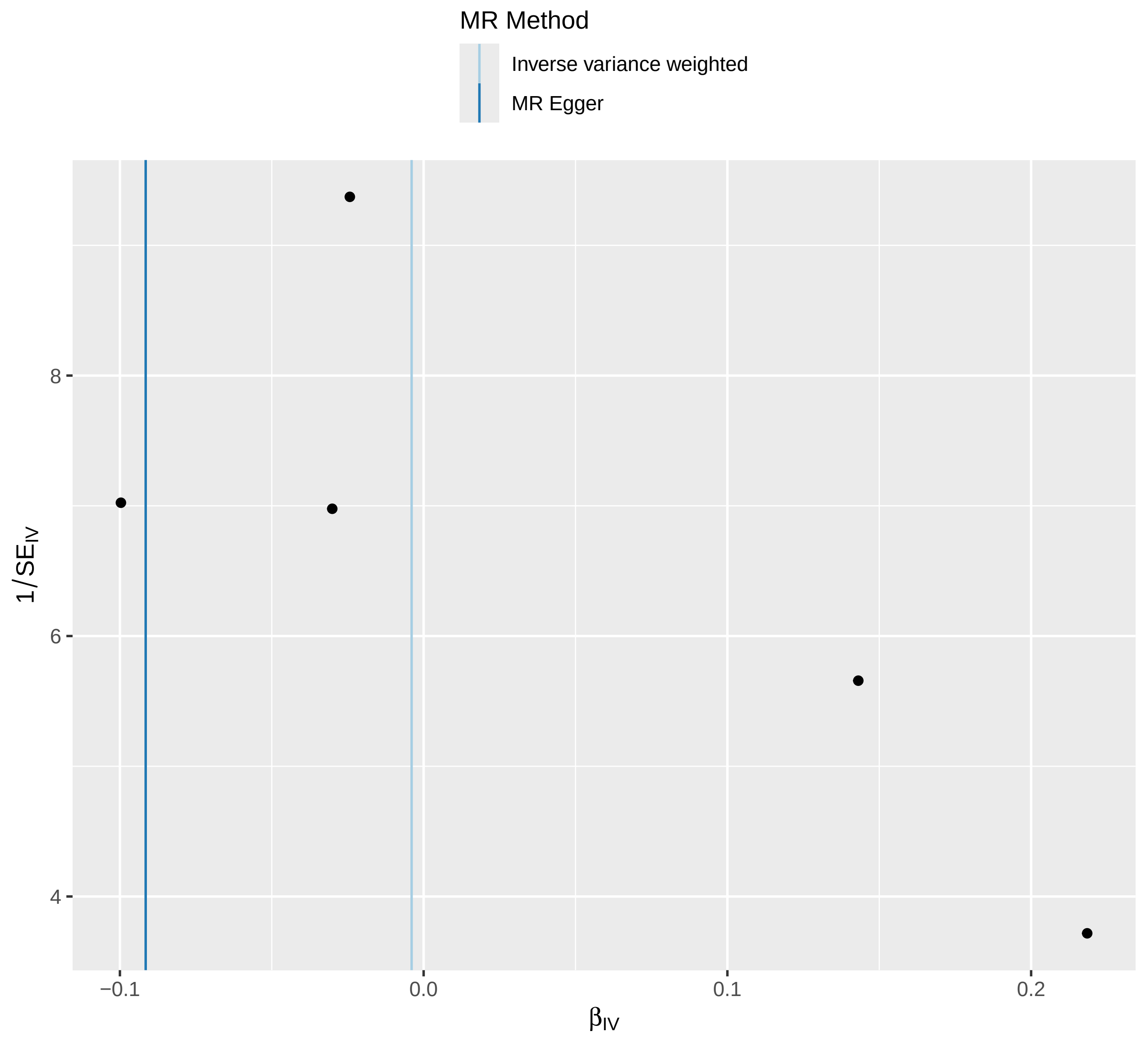

MR Method
Inverse variance weighted
MR Egger
8
6
4
1/SE$_{IV}$
−0.1
0.0
0.1
0.2
$\beta_{IV}$

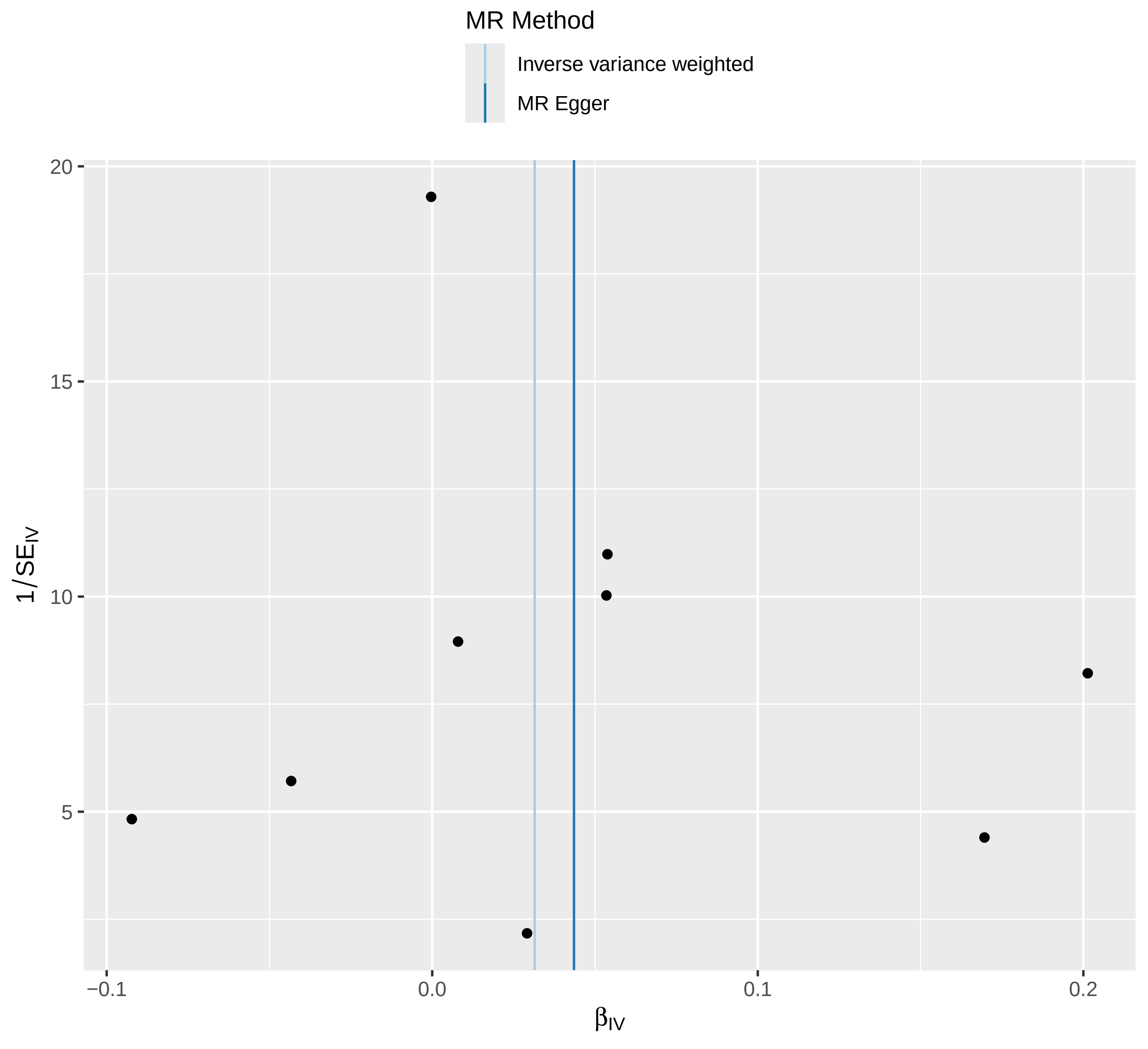

MR Method
Inverse variance weighted
MR Egger
20
15
10
5
$1/SE_{IV}$
−0.1
0.0
0.1
0.2
$\beta_{IV}$

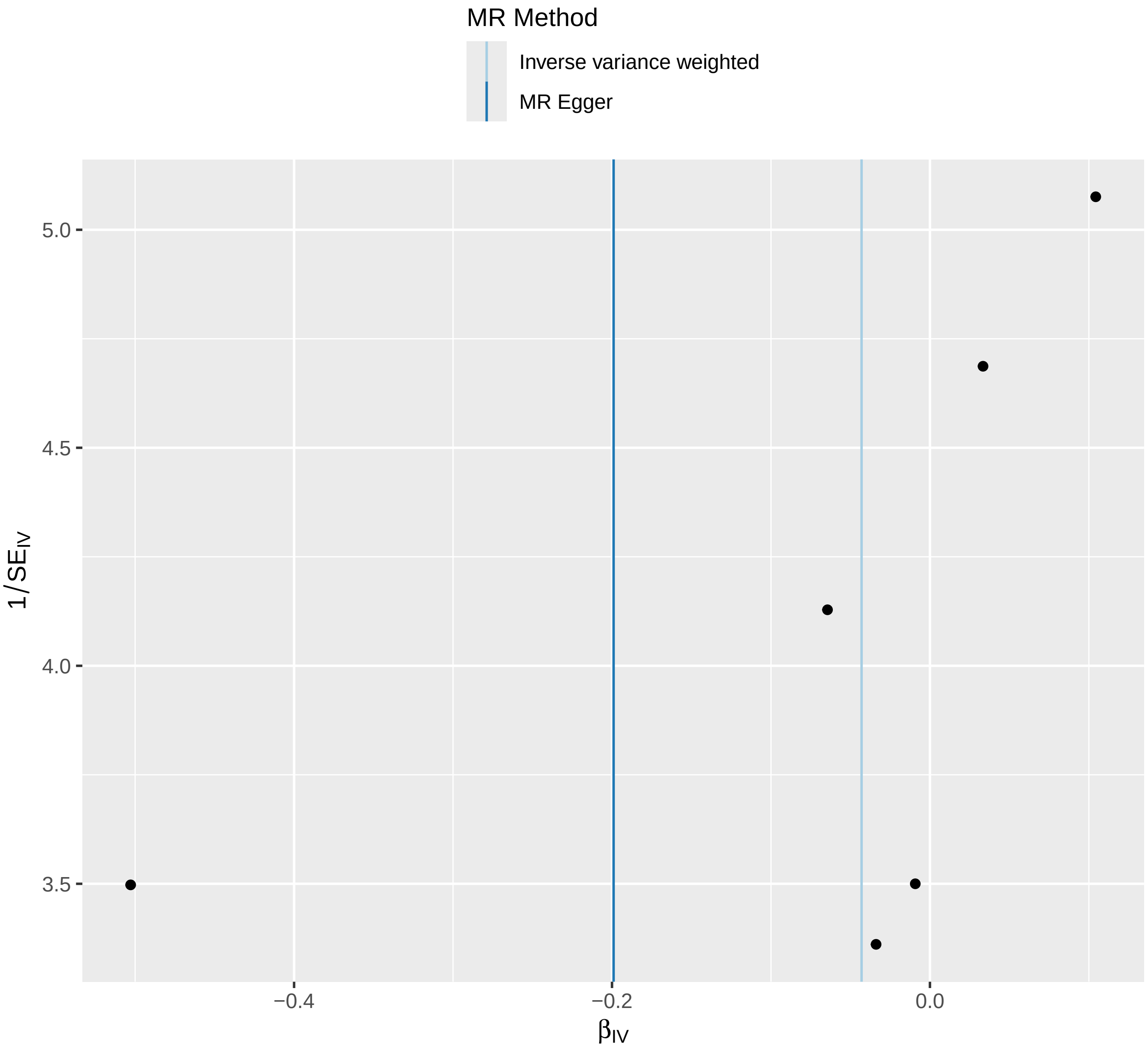

MR Method
Inverse variance weighted
MR Egger
5.0
4.5
4.0
3.5
1/SE$_{IV}$
−0.4
−0.2
0.0
β$_{IV}$

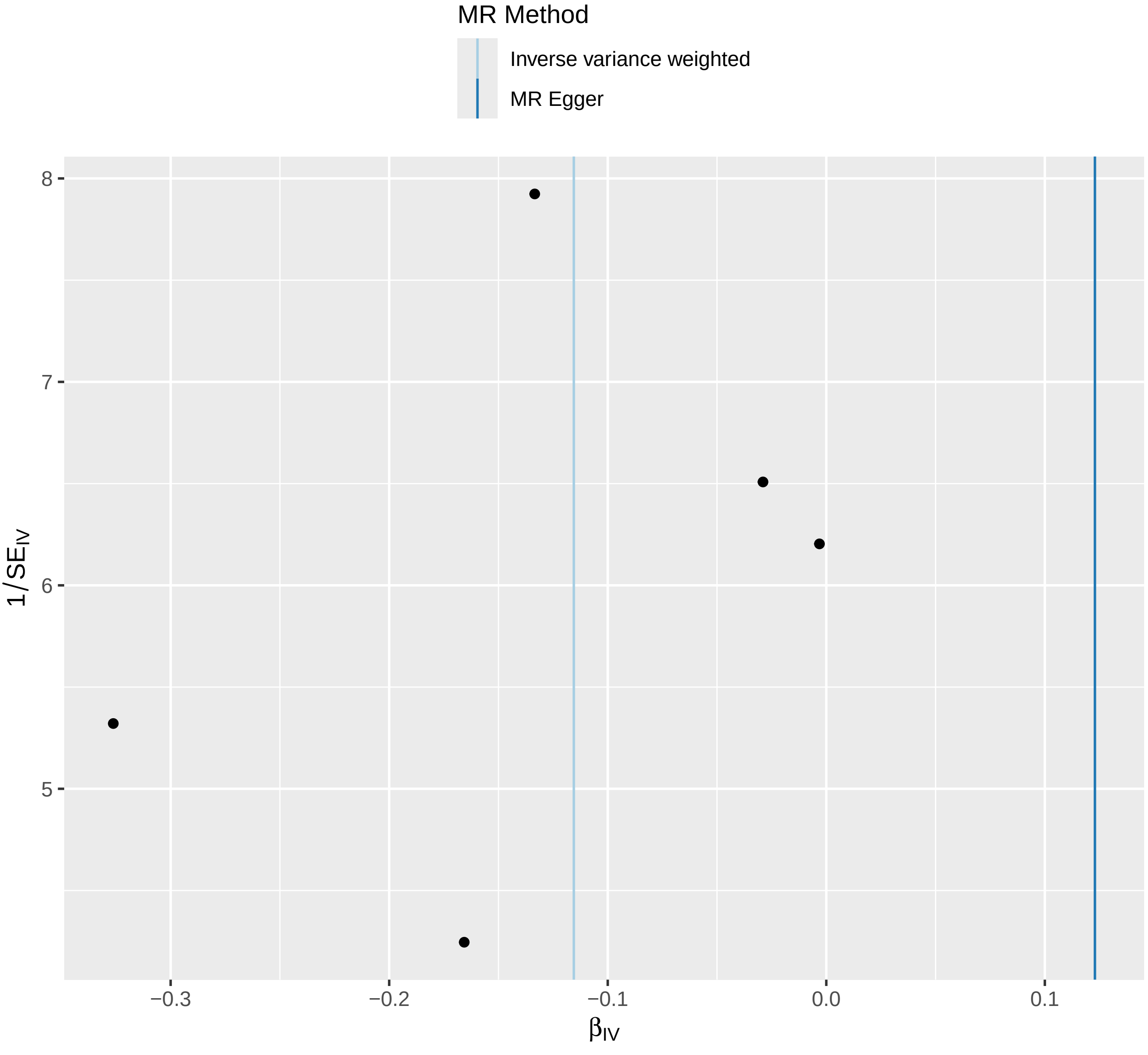
MR Method
Inverse variance weighted
MR Egger
8
7
6
5
1/SE_IV
−0.3
−0.2
−0.1
0.0
0.1
β_IV

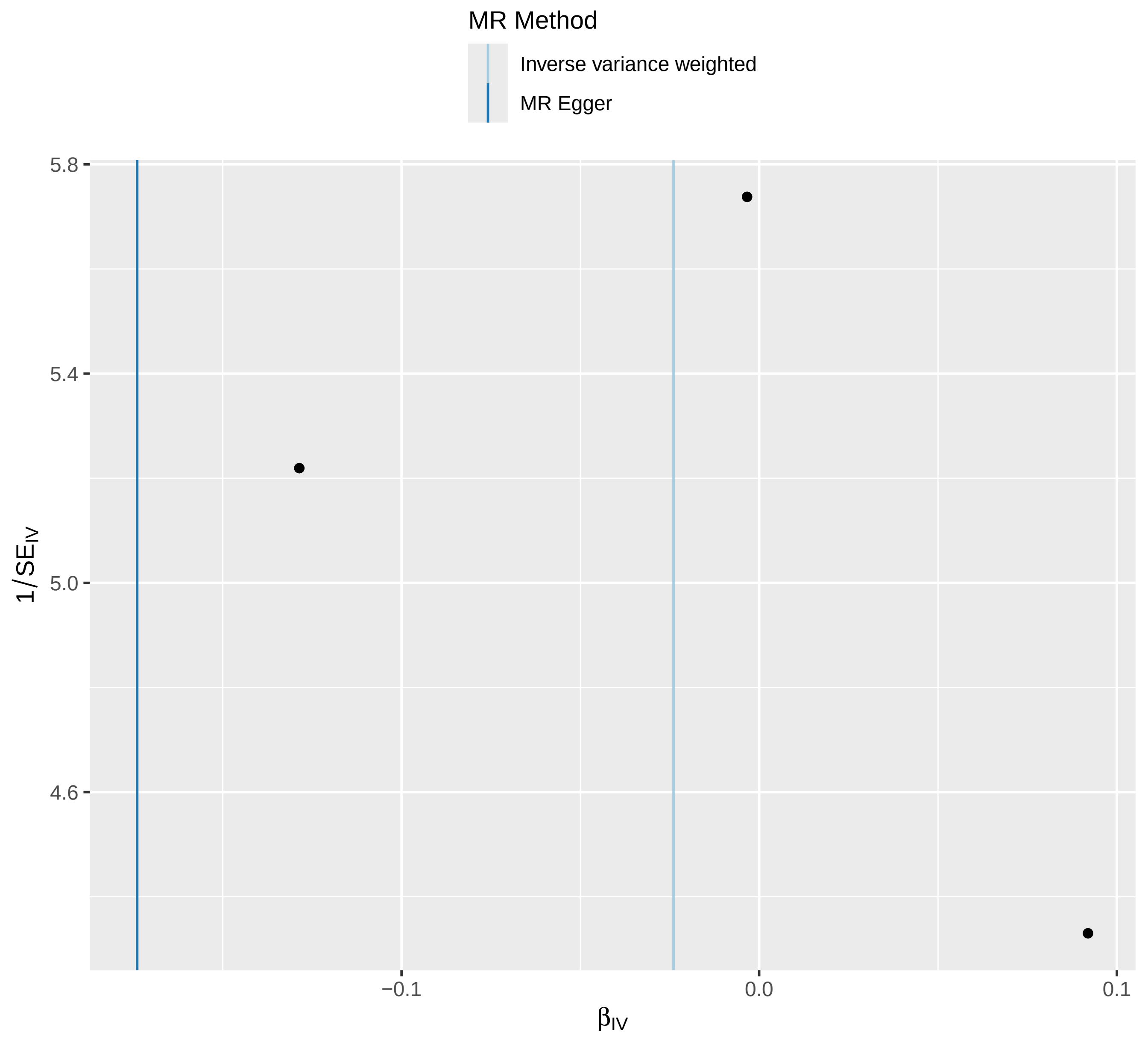
MR Method
Inverse variance weighted
MR Egger
5.8
5.4
5.0
4.6
1/SE_IV
−0.1
0.0
0.1
β_IV

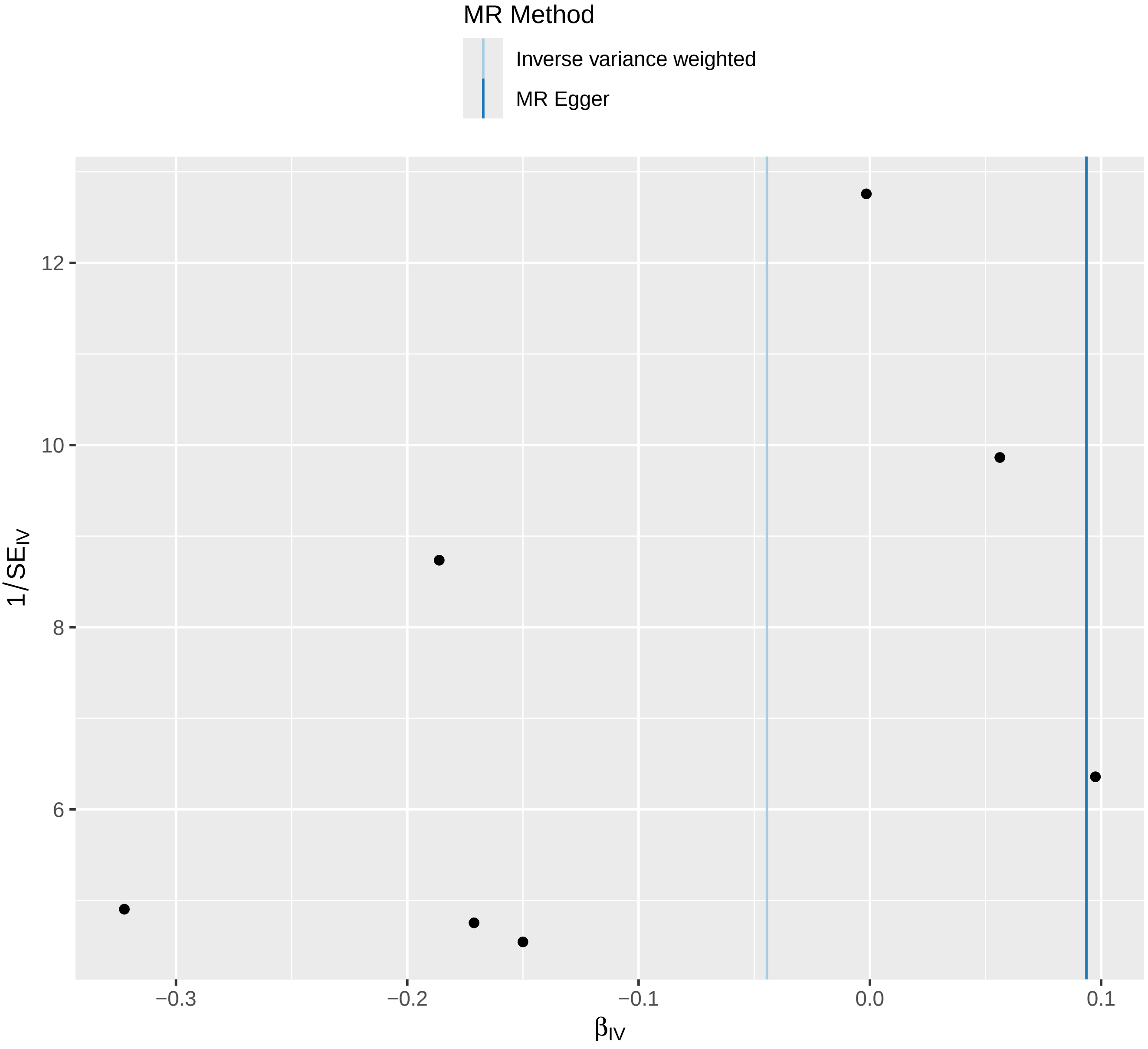
MR Method
Inverse variance weighted
MR Egger
12
10
8
6
1/SE$_{IV}$
−0.3
−0.2
−0.1
0.0
0.1
β$_{IV}$

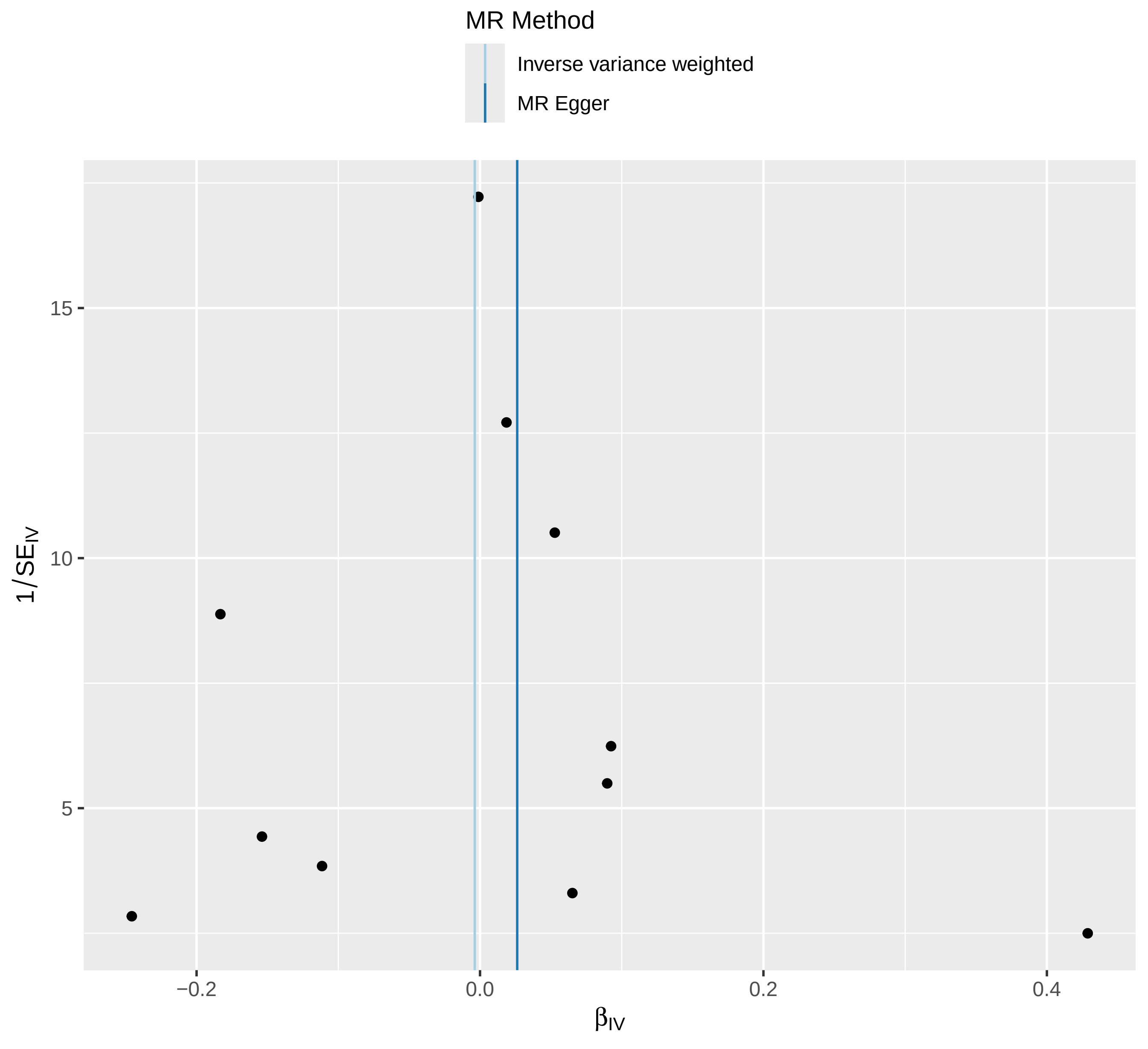
MR Method
Inverse variance weighted
MR Egger
15
10
5
1/SE$_{IV}$
−0.2
0.0
0.2
0.4
β$_{IV}$

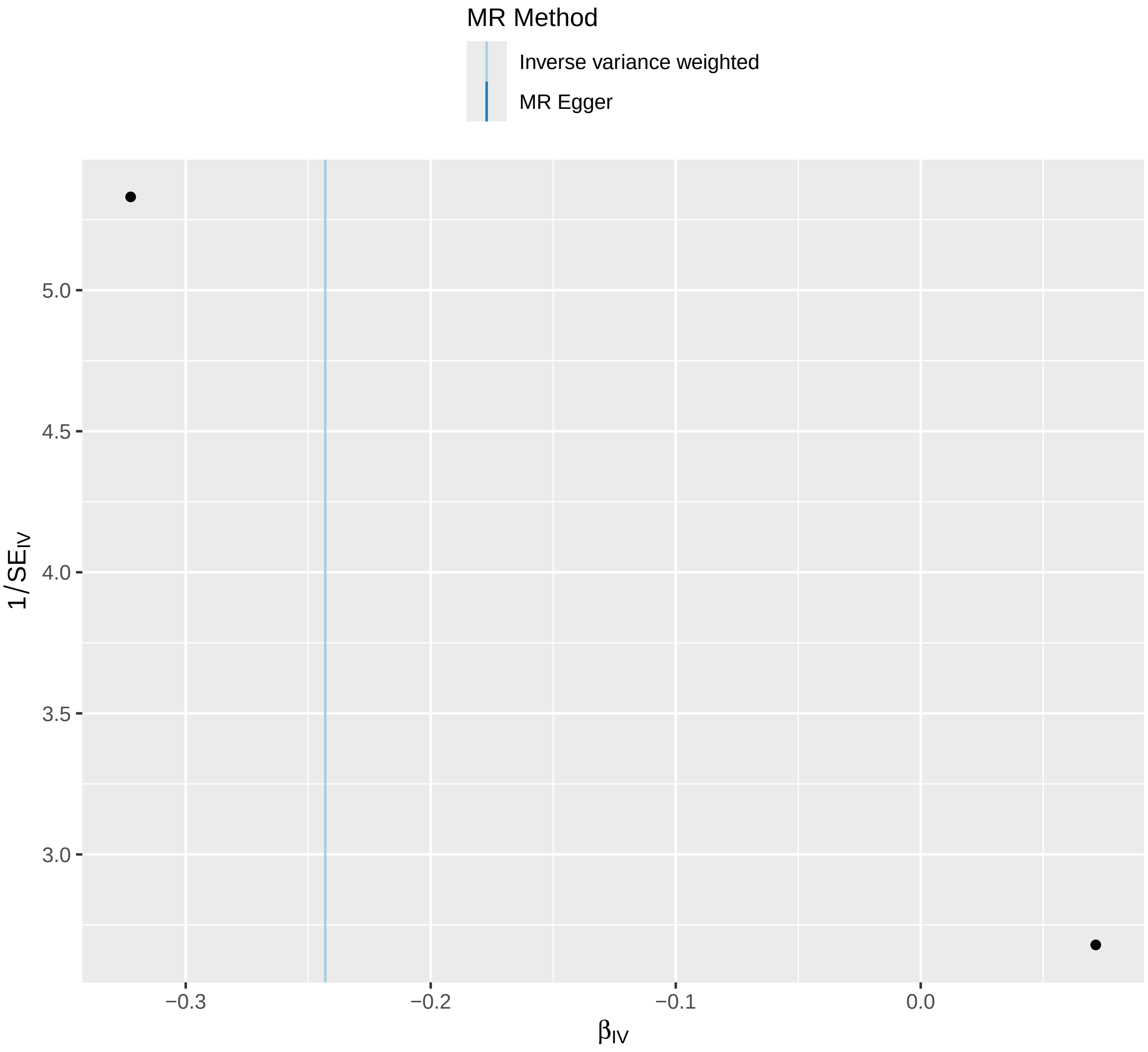

MR Method
Inverse variance weighted
MR Egger
5.0
4.5
4.0
3.5
3.0
$1/SE_{IV}$
−0.3
−0.2
−0.1
0.0
$\beta_{IV}$

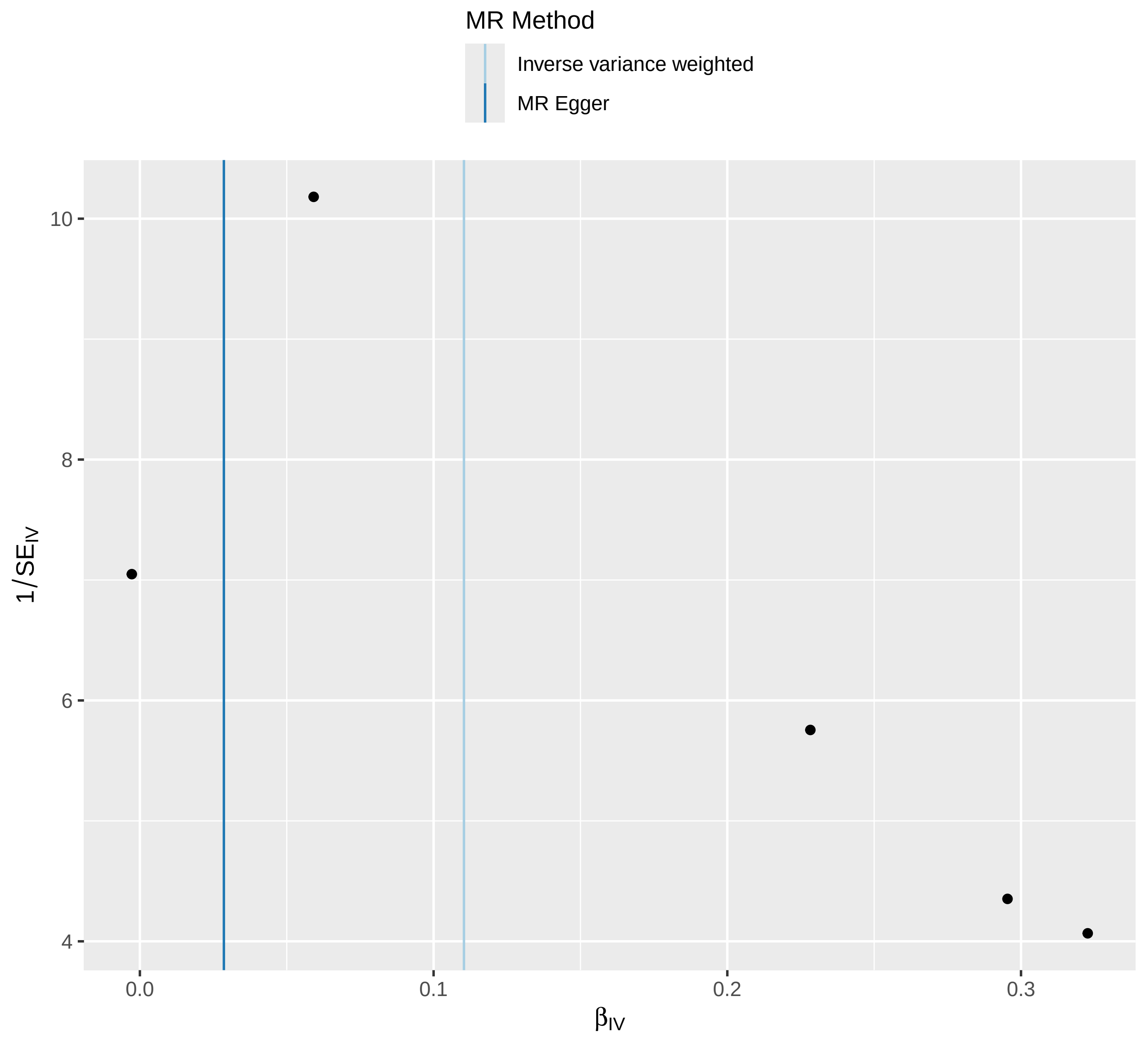
MR Method
Inverse variance weighted
MR Egger
10
8
6
4
0.0
0.1
0.2
0.3
$\beta_{IV}$
$1/SE_{IV}$

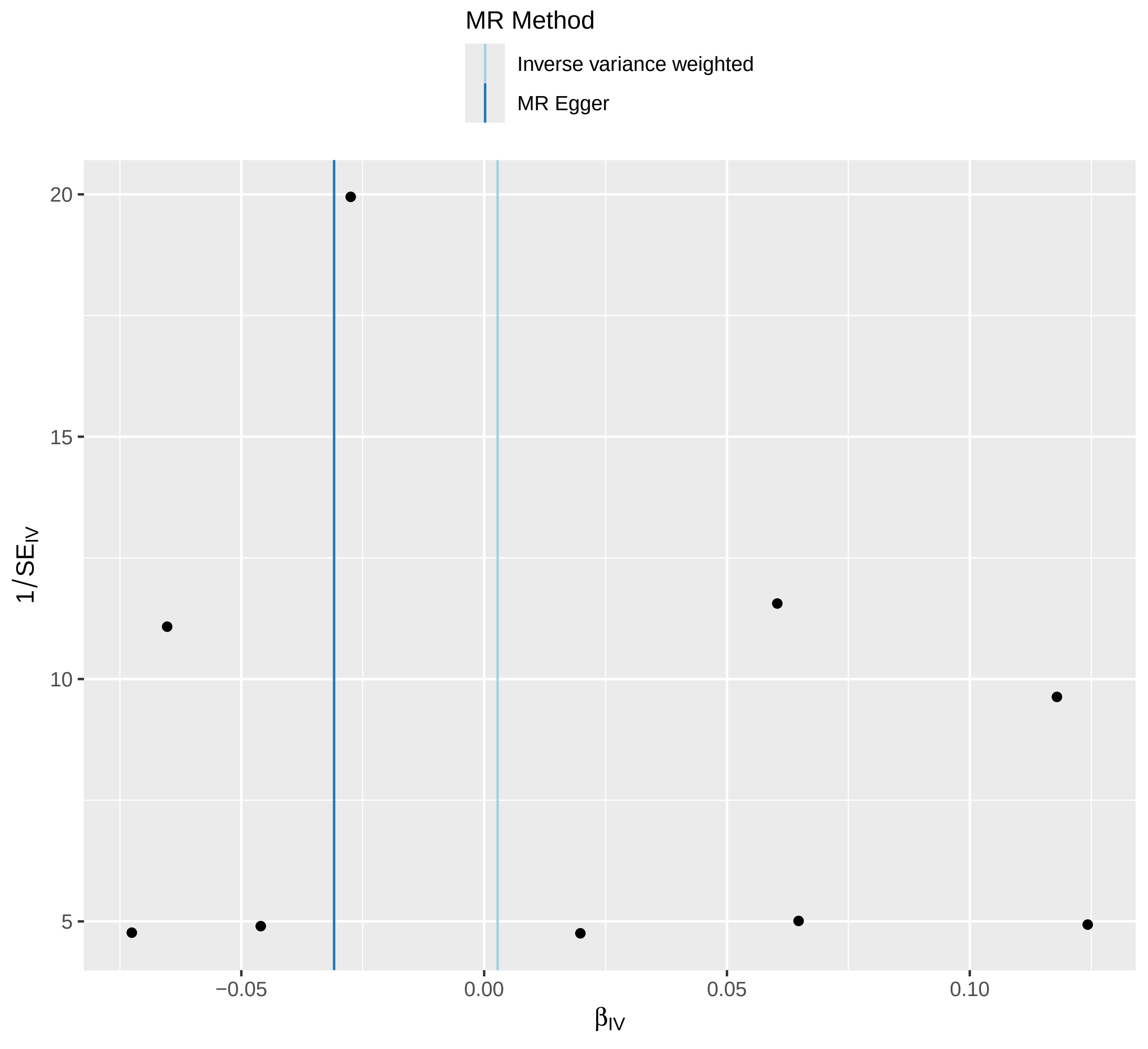
MR Method
Inverse variance weighted
MR Egger
20
15
10
5
1/SE$_{IV}$
−0.05
0.00
0.05
0.10
β$_{IV}$

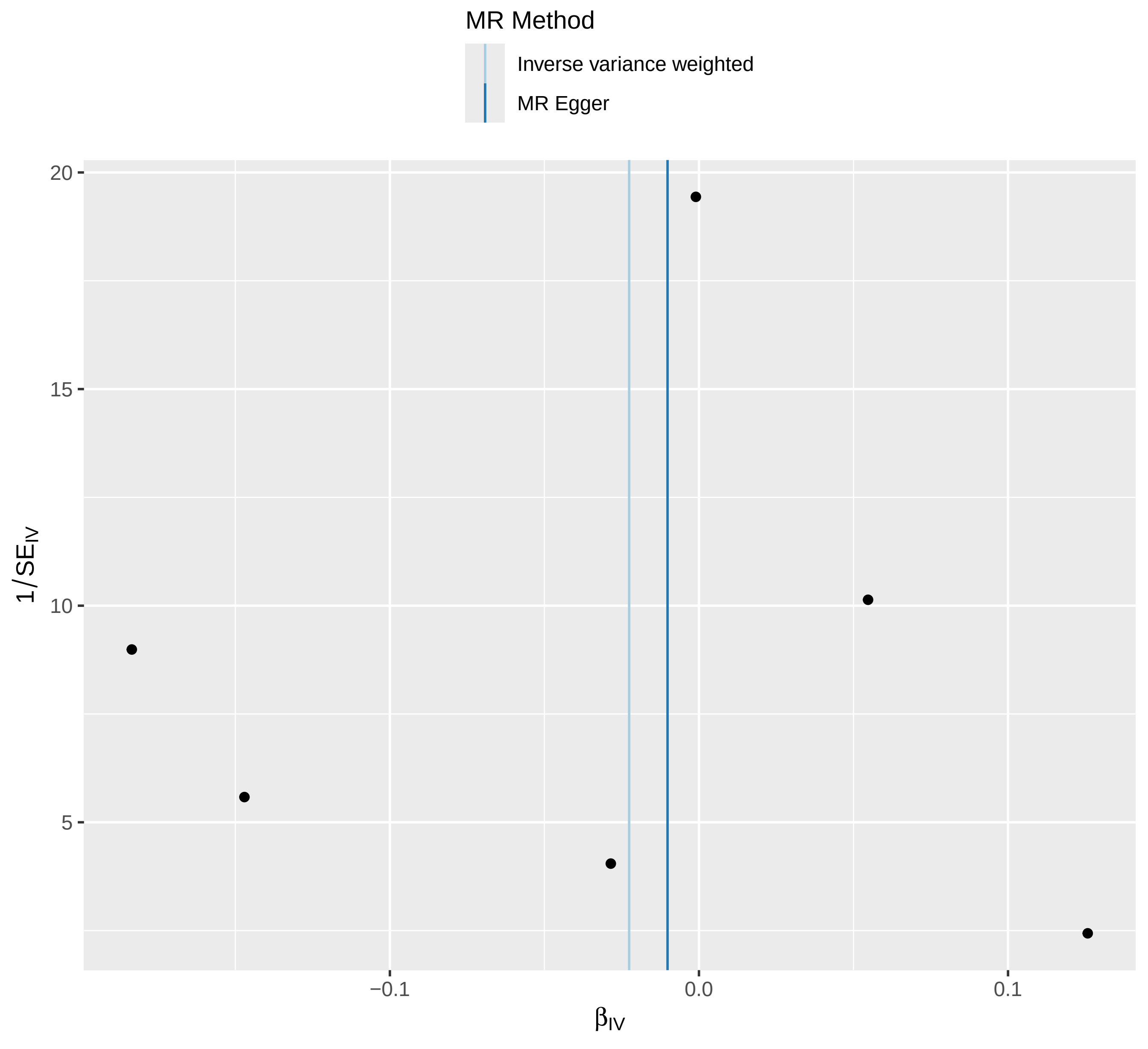

MR Method
Inverse variance weighted
MR Egger
20
15
10
5
1/SE_IV
−0.1
0.0
0.1
β_IV

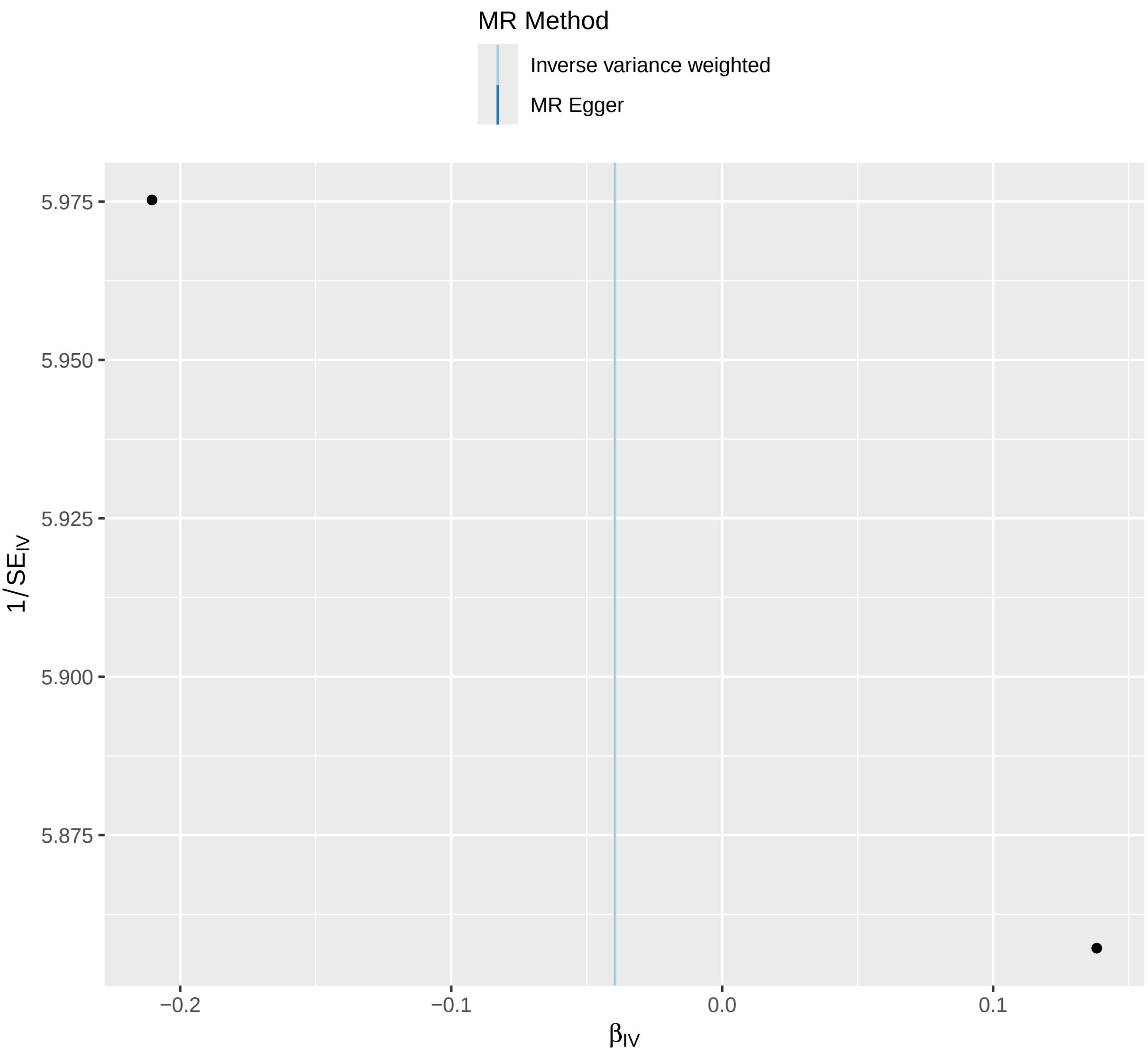
MR Method
Inverse variance weighted
MR Egger
5.975
5.950
5.925
5.900
5.875
1/SE$_{IV}$
−0.2
−0.1
0.0
0.1
β$_{IV}$

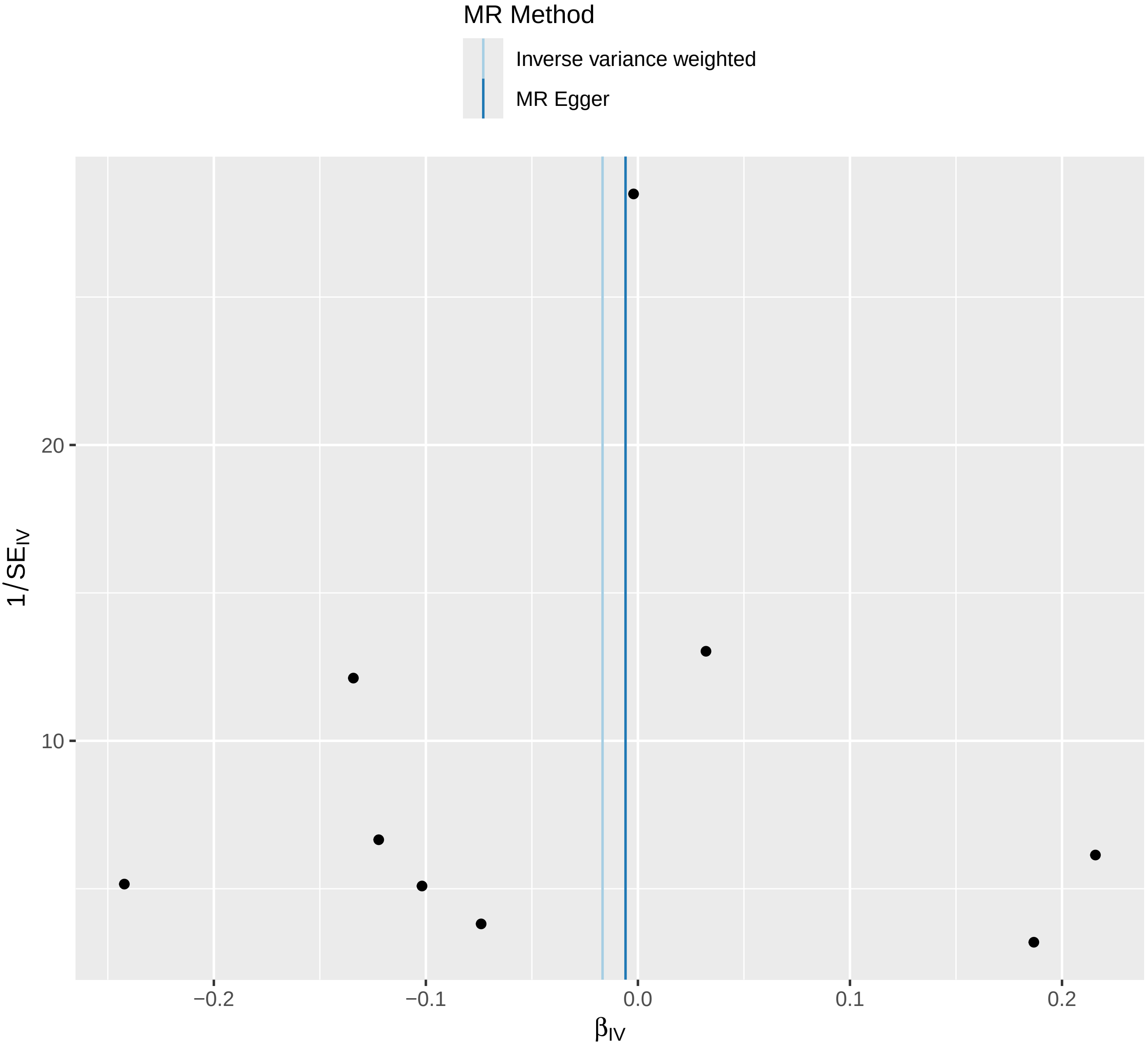
MR Method
Inverse variance weighted
MR Egger
20
10
1/SE_IV
−0.2
−0.1
0.0
0.1
0.2
β_IV

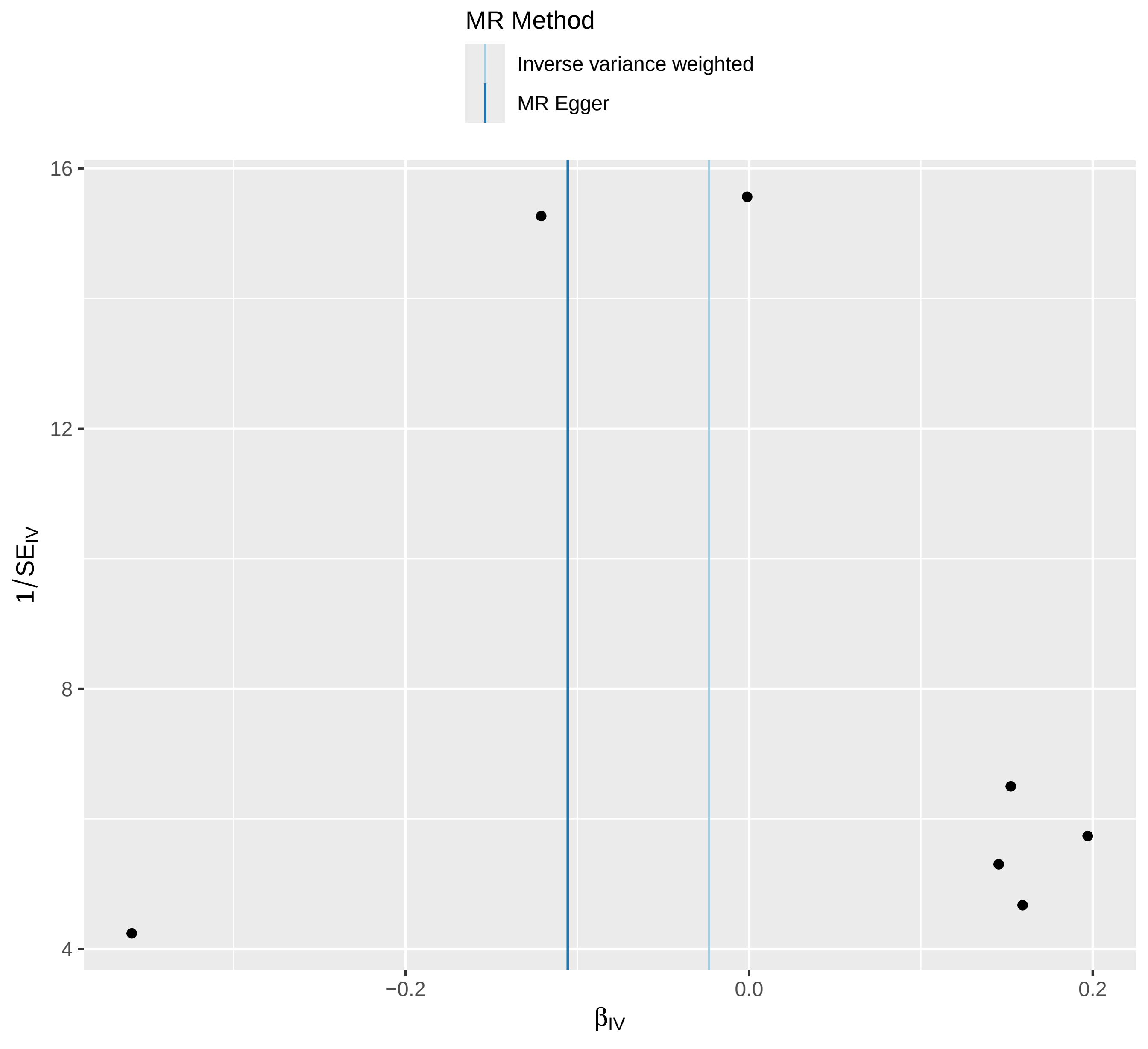

MR Method
Inverse variance weighted
MR Egger
16
12
8
4
1/SE_IV
−0.2
0.0
0.2
β_IV

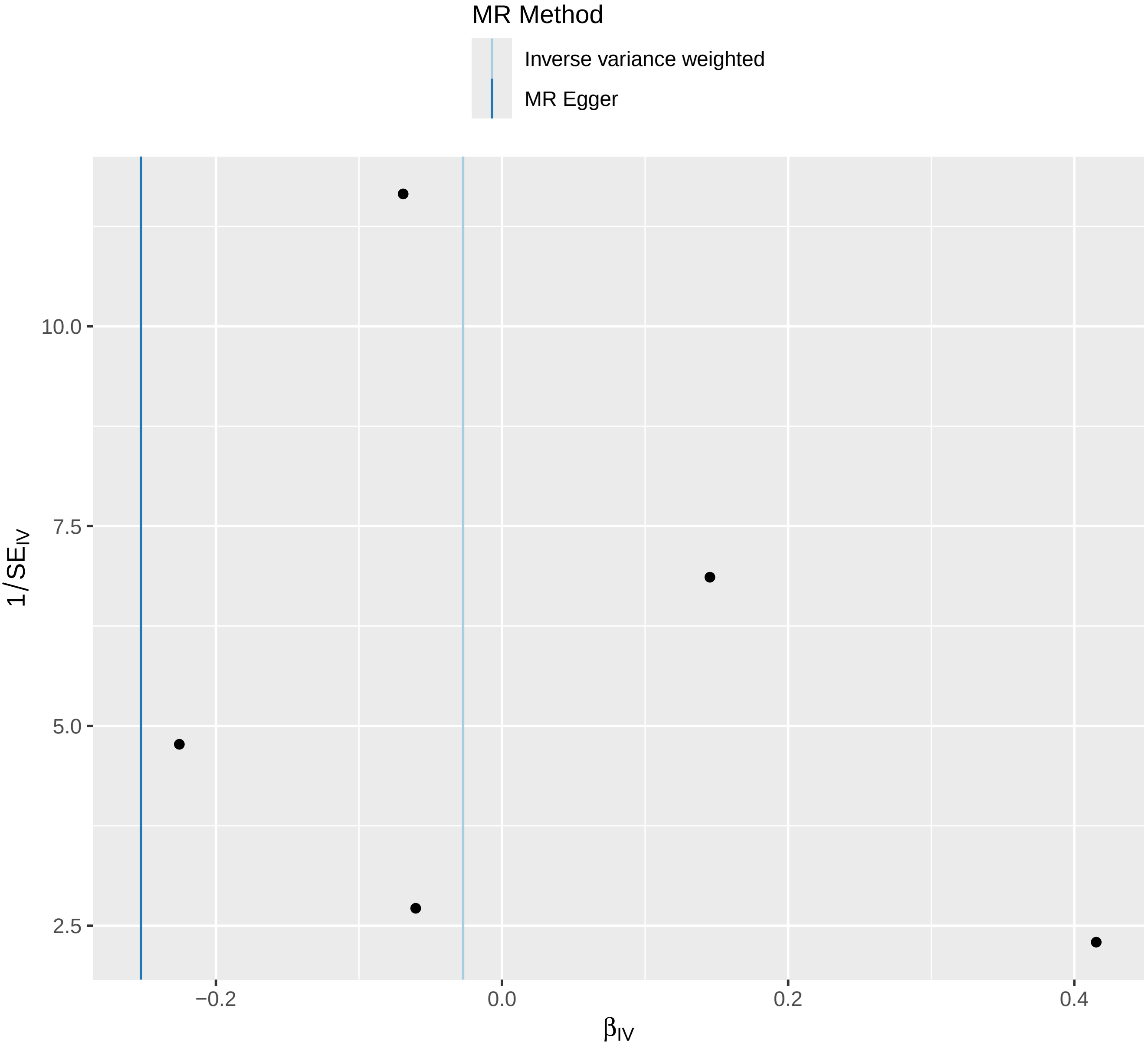
MR Method
Inverse variance weighted
MR Egger
10.0
7.5
5.0
2.5
1/SE$_{IV}$
−0.2
0.0
0.2
0.4
β$_{IV}$

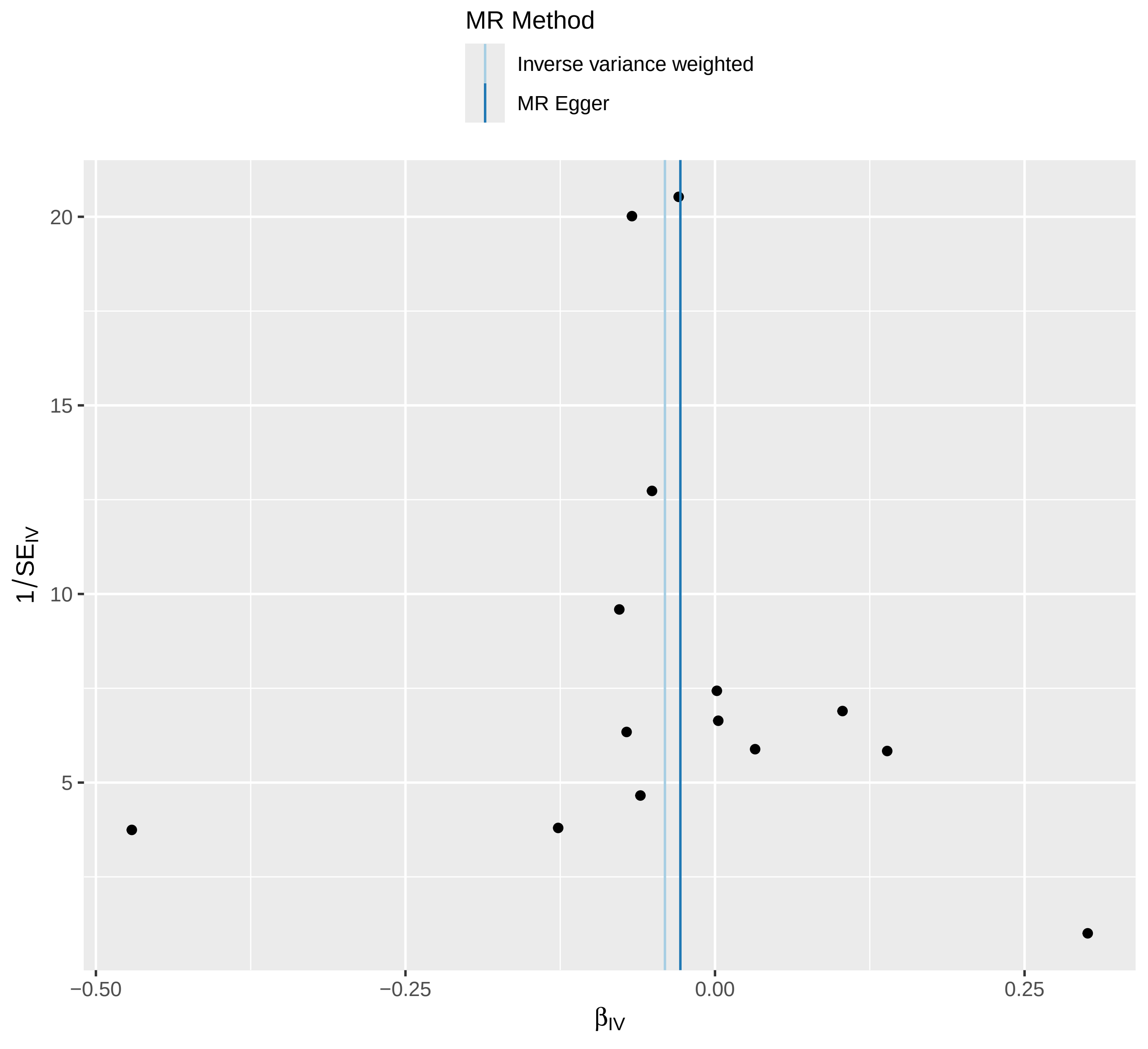
MR Method
Inverse variance weighted
MR Egger
20
15
10
5
$1/SE_{IV}$
−0.50
−0.25
0.00
0.25
$\beta_{IV}$

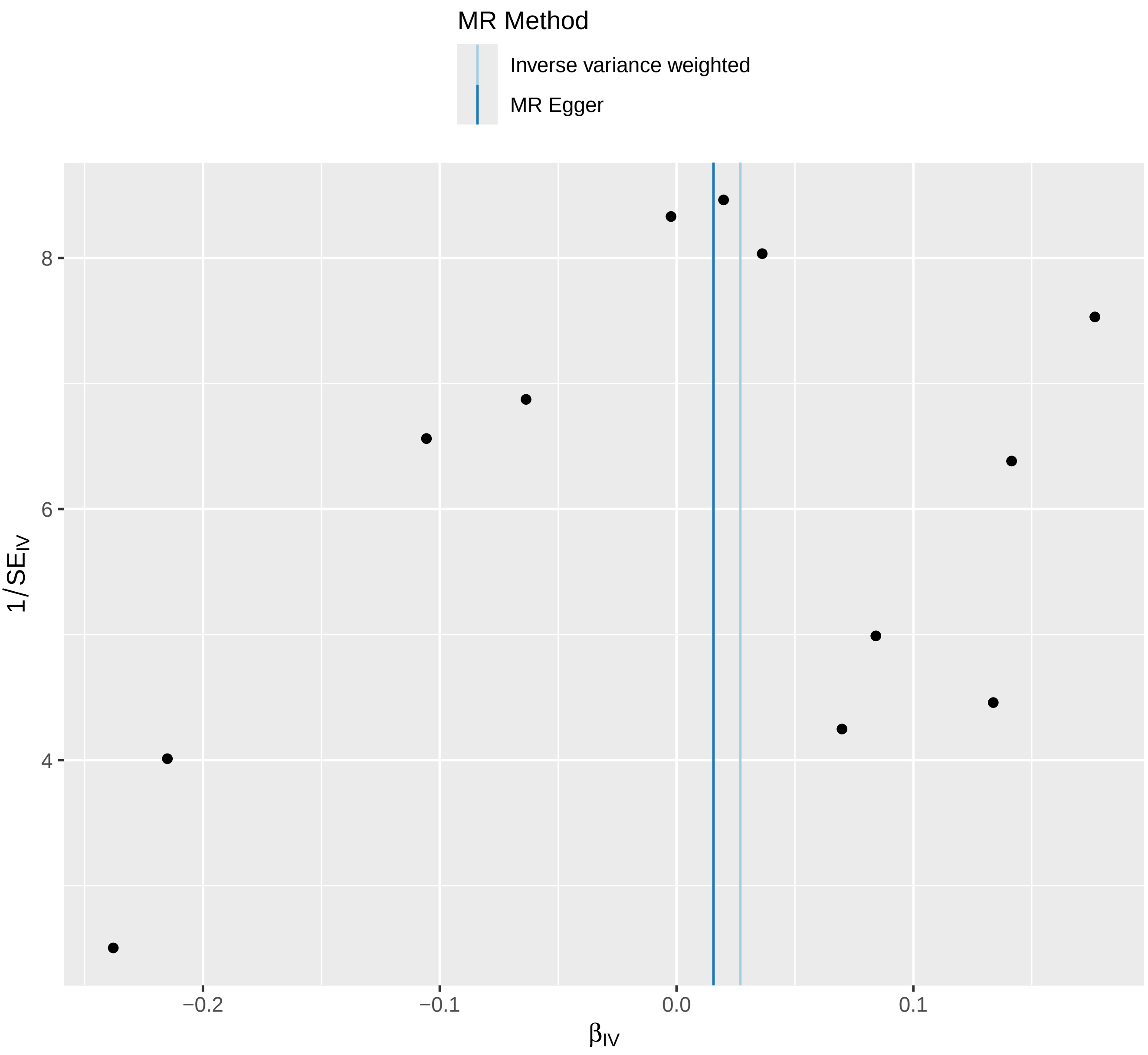

MR Method
Inverse variance weighted
MR Egger
8
6
4
1/SE_IV
−0.2
−0.1
0.0
0.1
β_IV

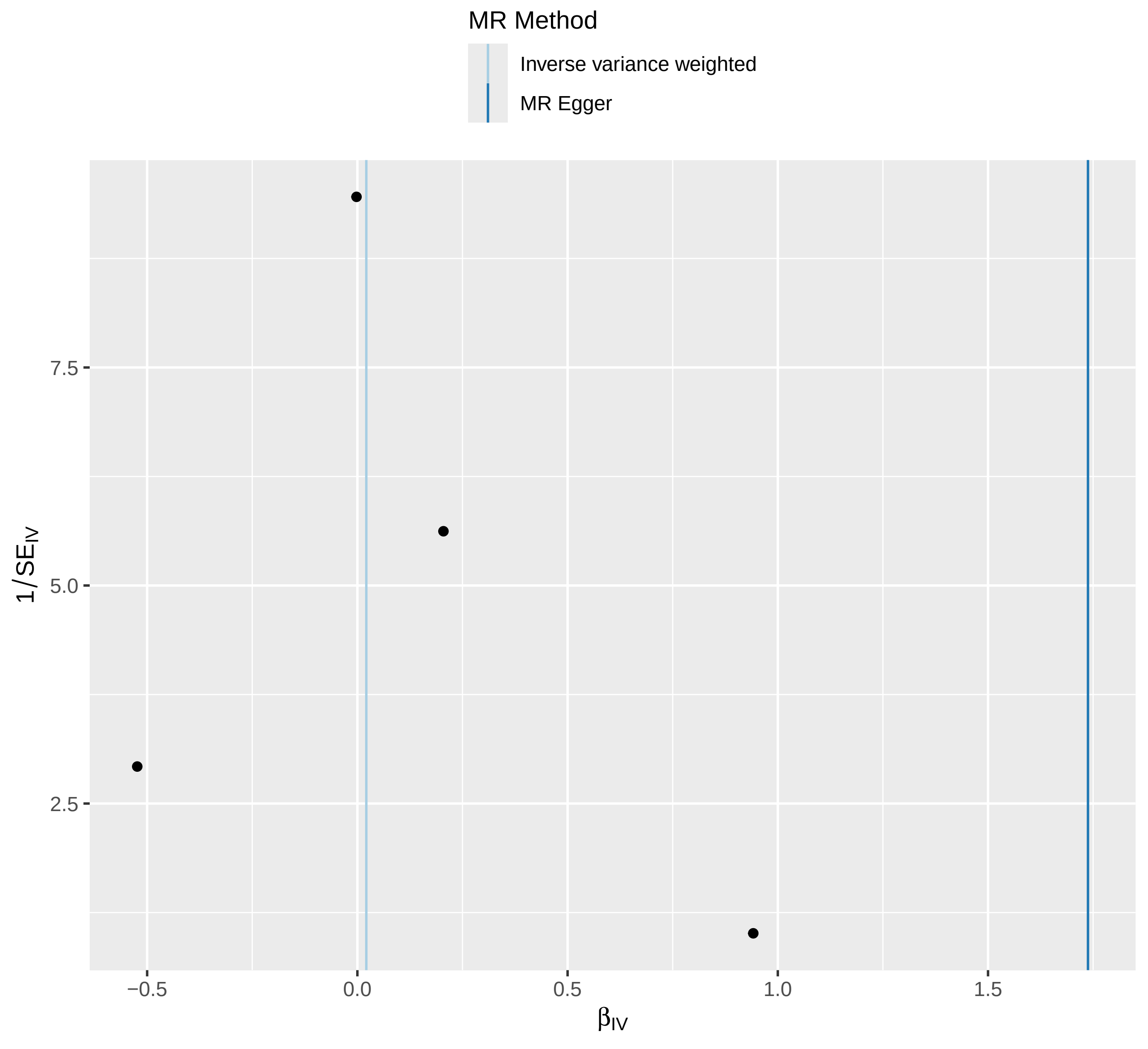
MR Method
Inverse variance weighted
MR Egger
7.5
5.0
2.5
1/SE$_{IV}$
−0.5
0.0
0.5
1.0
1.5
β$_{IV}$

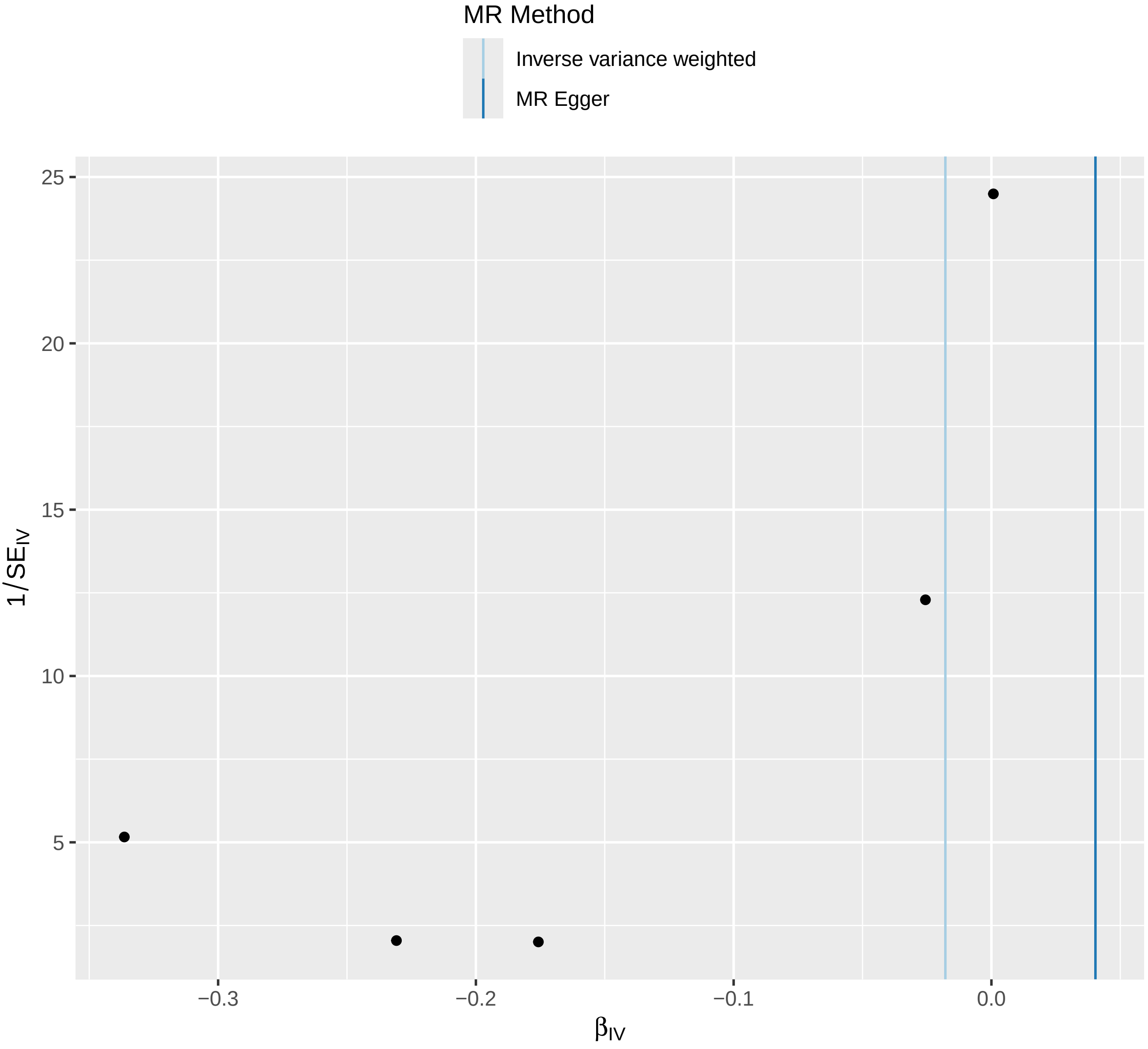
MR Method
Inverse variance weighted
MR Egger
25
20
15
10
5
1/SE_IV
−0.3
−0.2
−0.1
0.0
β_IV

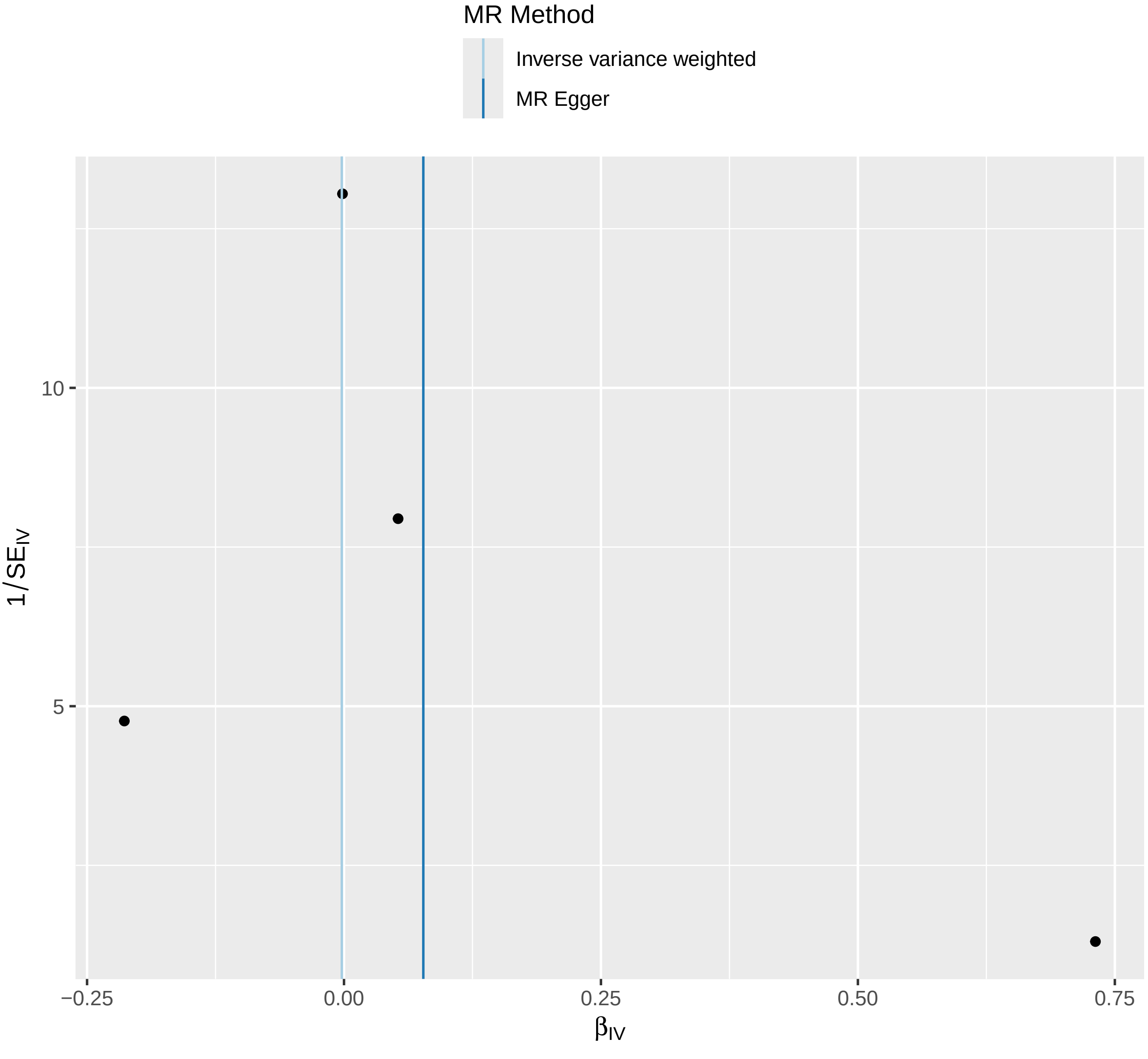
MR Method
Inverse variance weighted
MR Egger
10
5
1/SE_IV
−0.25
0.00
0.25
0.50
0.75
β_IV

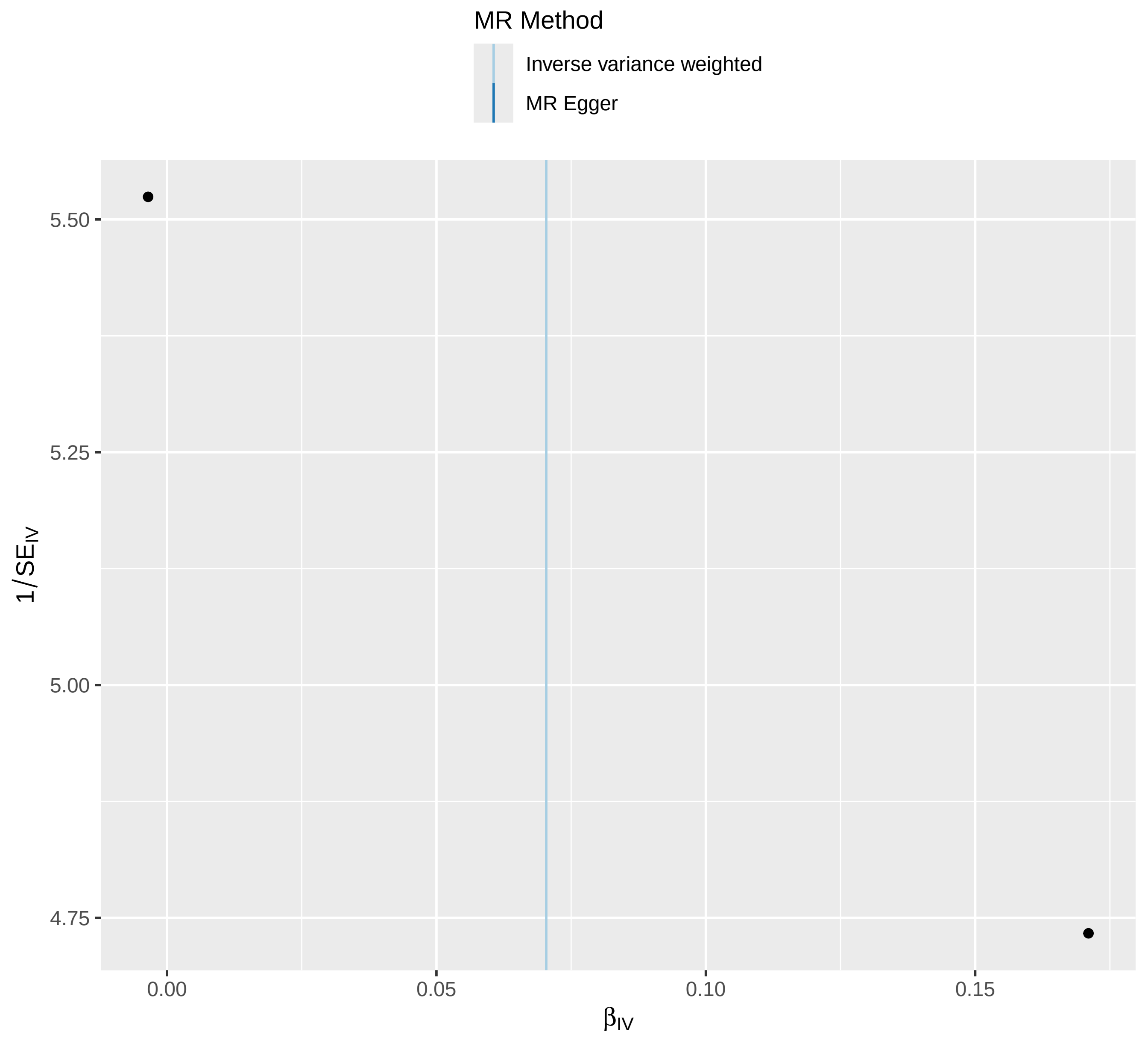
MR Method
Inverse variance weighted
MR Egger
5.50
5.25
5.00
4.75
1/SE_IV
0.00
0.05
0.10
0.15
β_IV

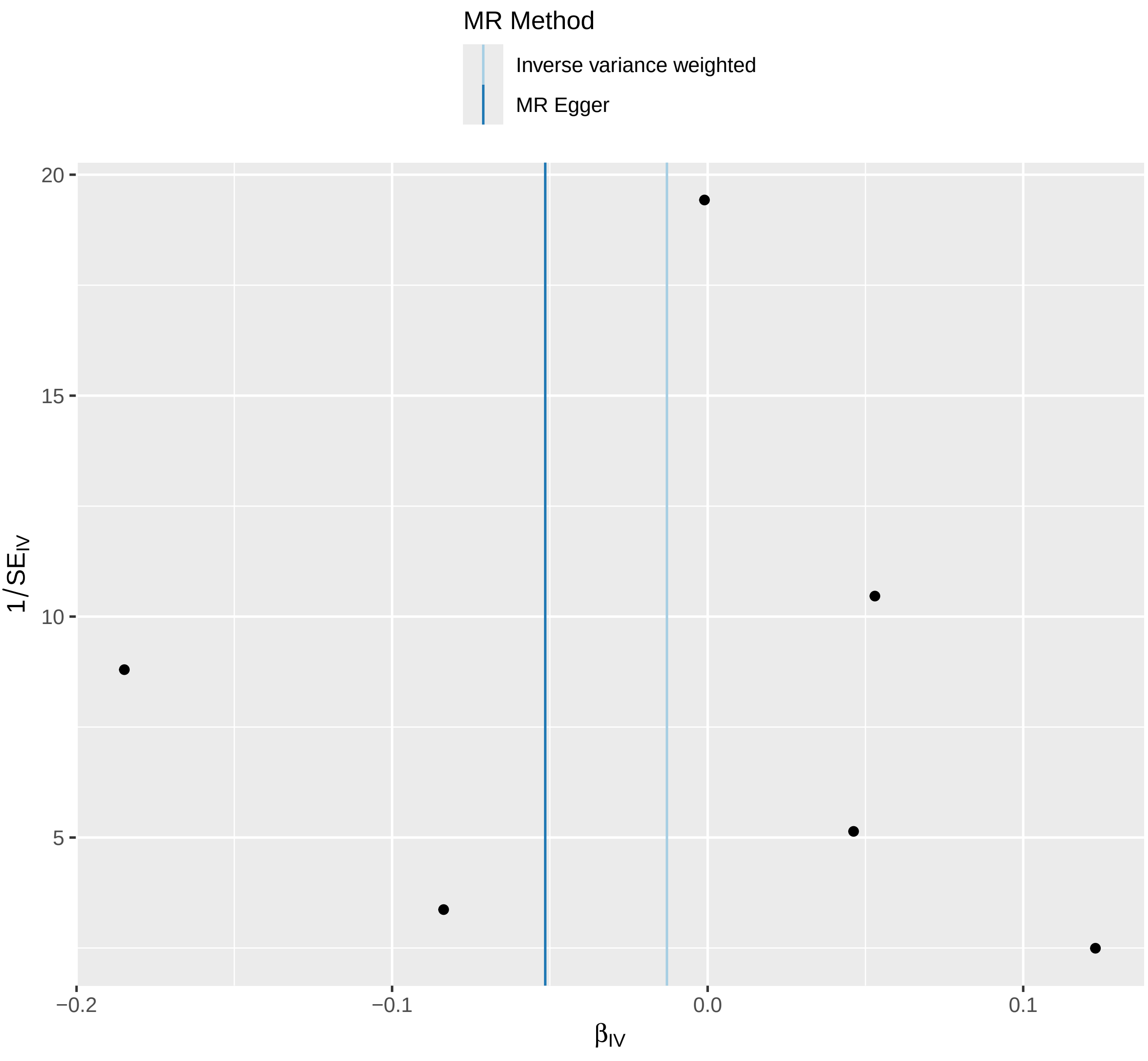
MR Method
Inverse variance weighted
MR Egger
20
15
10
5
1/SE_IV
−0.2
−0.1
0.0
0.1
β_IV

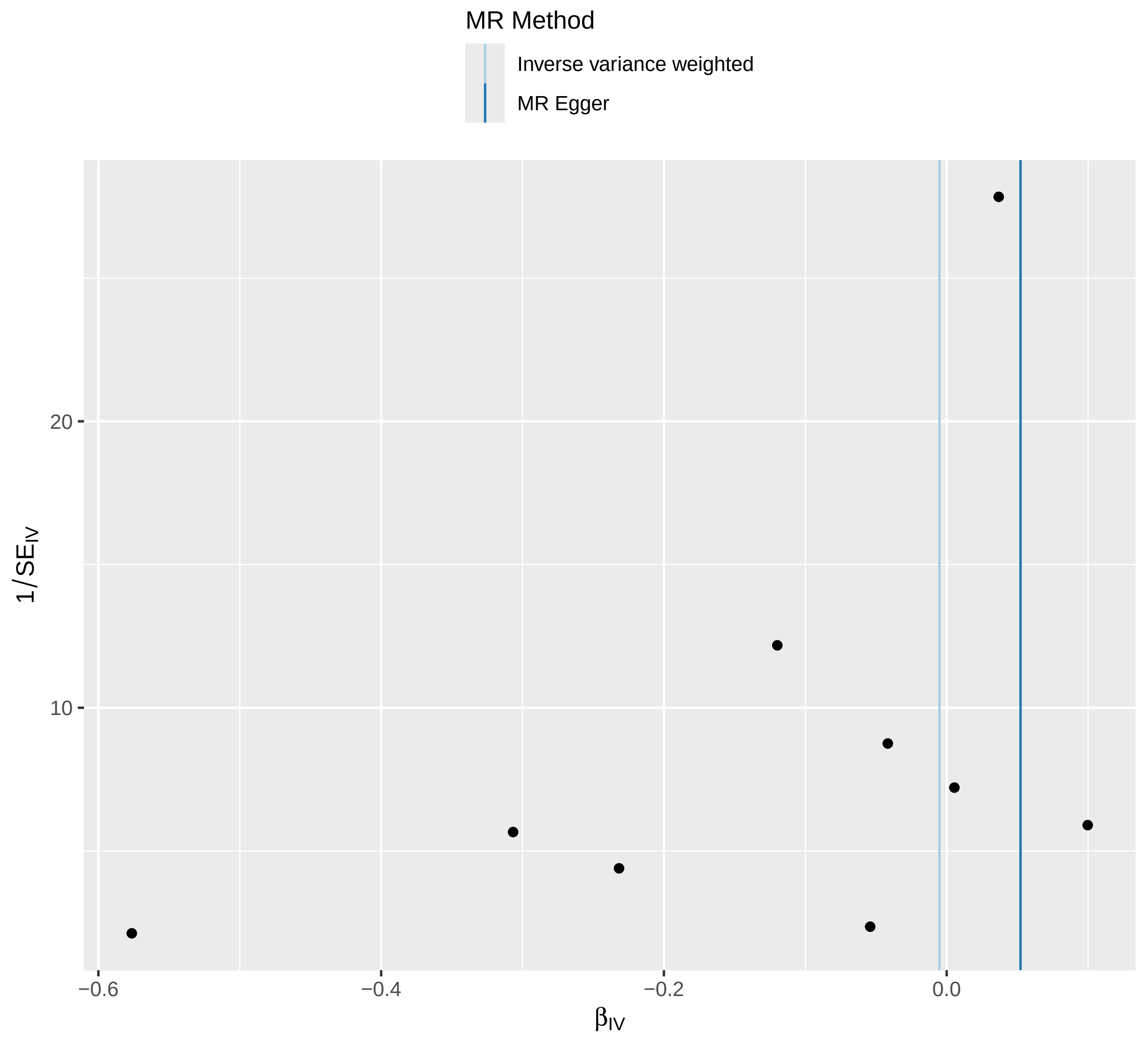
MR Method
Inverse variance weighted
MR Egger
20
10
$1/SE_{IV}$
−0.6
−0.4
−0.2
0.0
$\beta_{IV}$

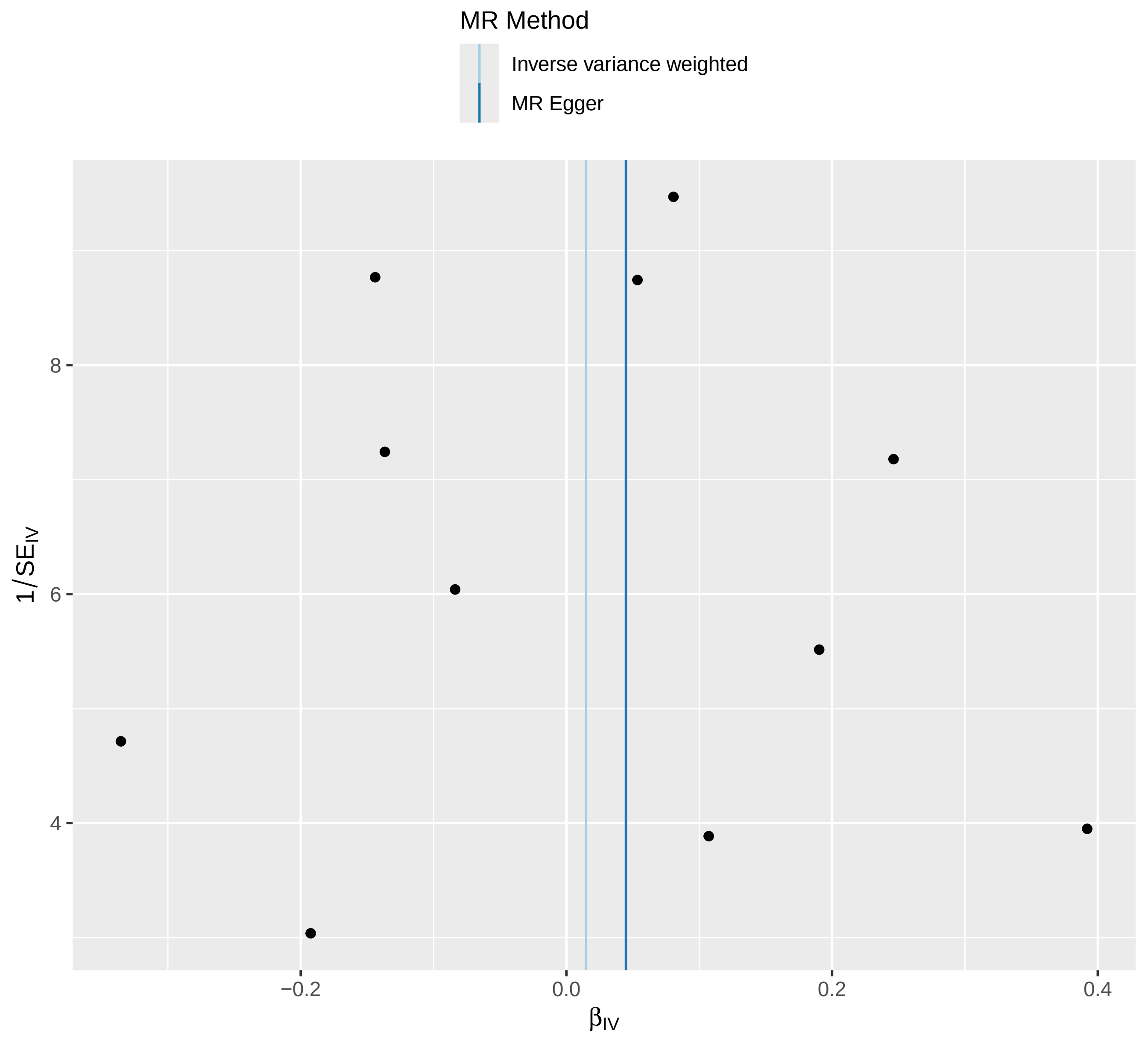
MR Method
Inverse variance weighted
MR Egger
8
6
4
1/SE_IV
−0.2
0.0
0.2
0.4
β_IV

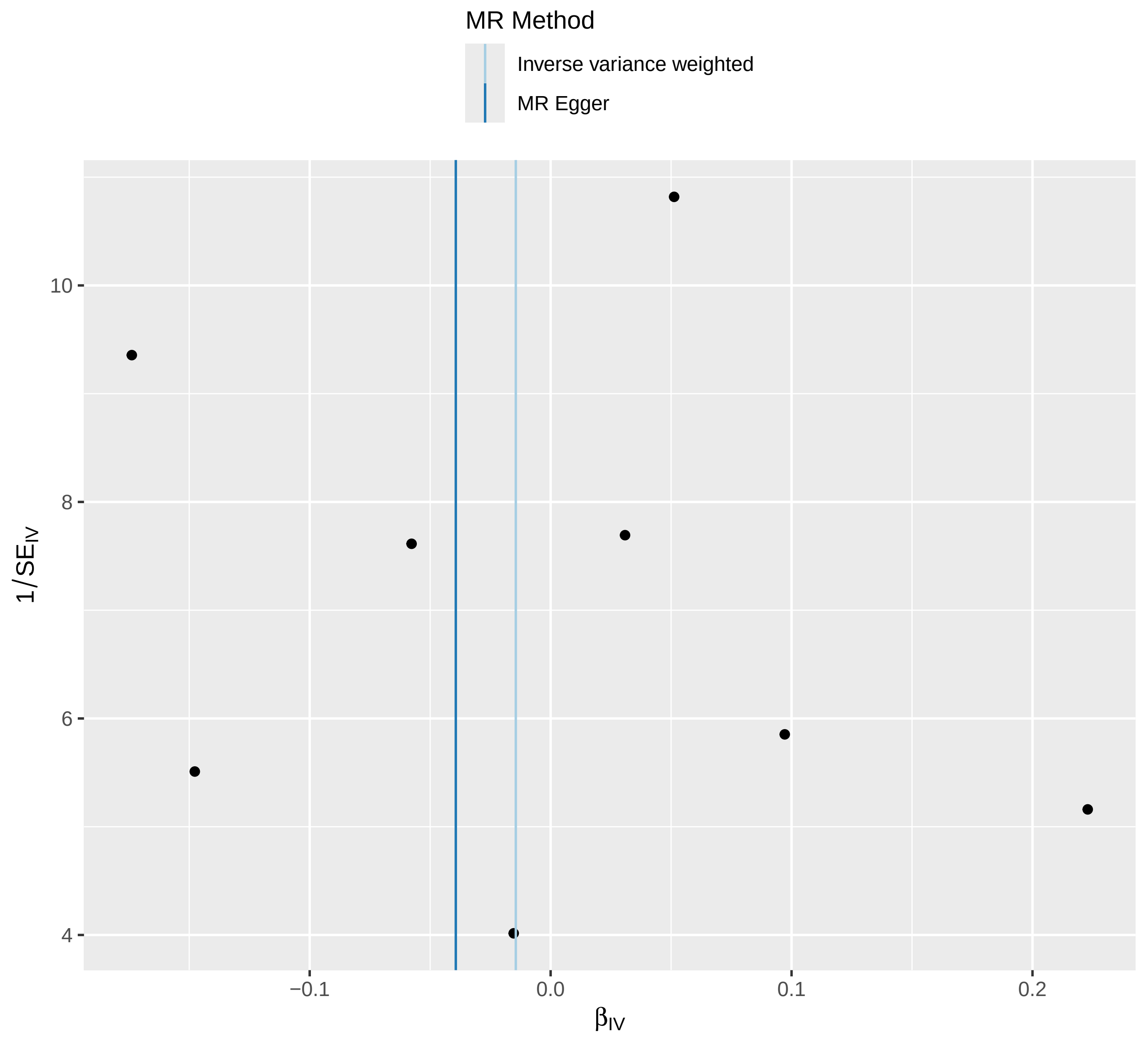
MR Method
Inverse variance weighted
MR Egger
10
8
6
4
$1/SE_{IV}$
−0.1
0.0
0.1
0.2
$\beta_{IV}$

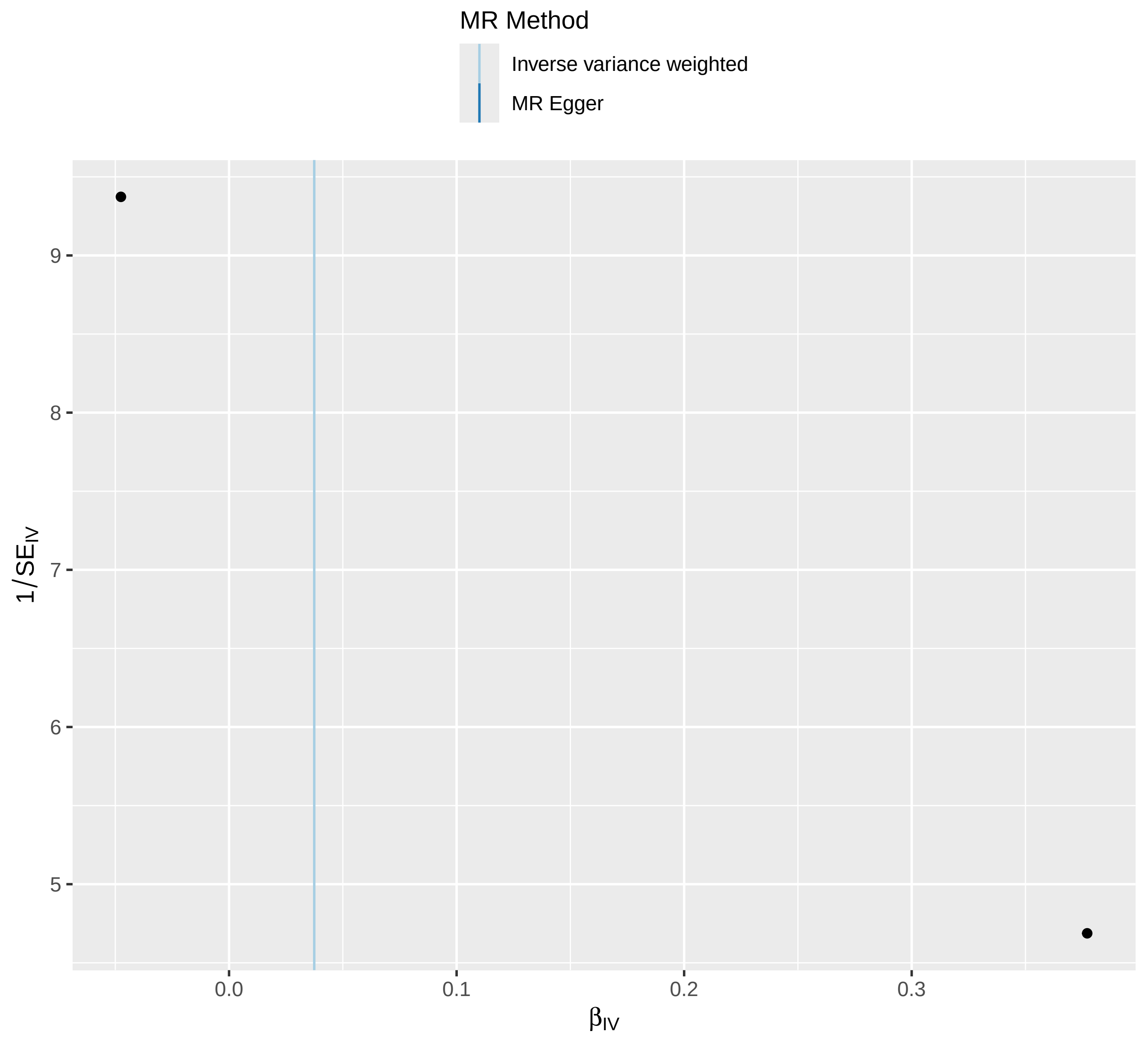
MR Method
Inverse variance weighted
MR Egger
9
8
7
6
5
1/SE_IV
0.0
0.1
0.2
0.3
β_IV

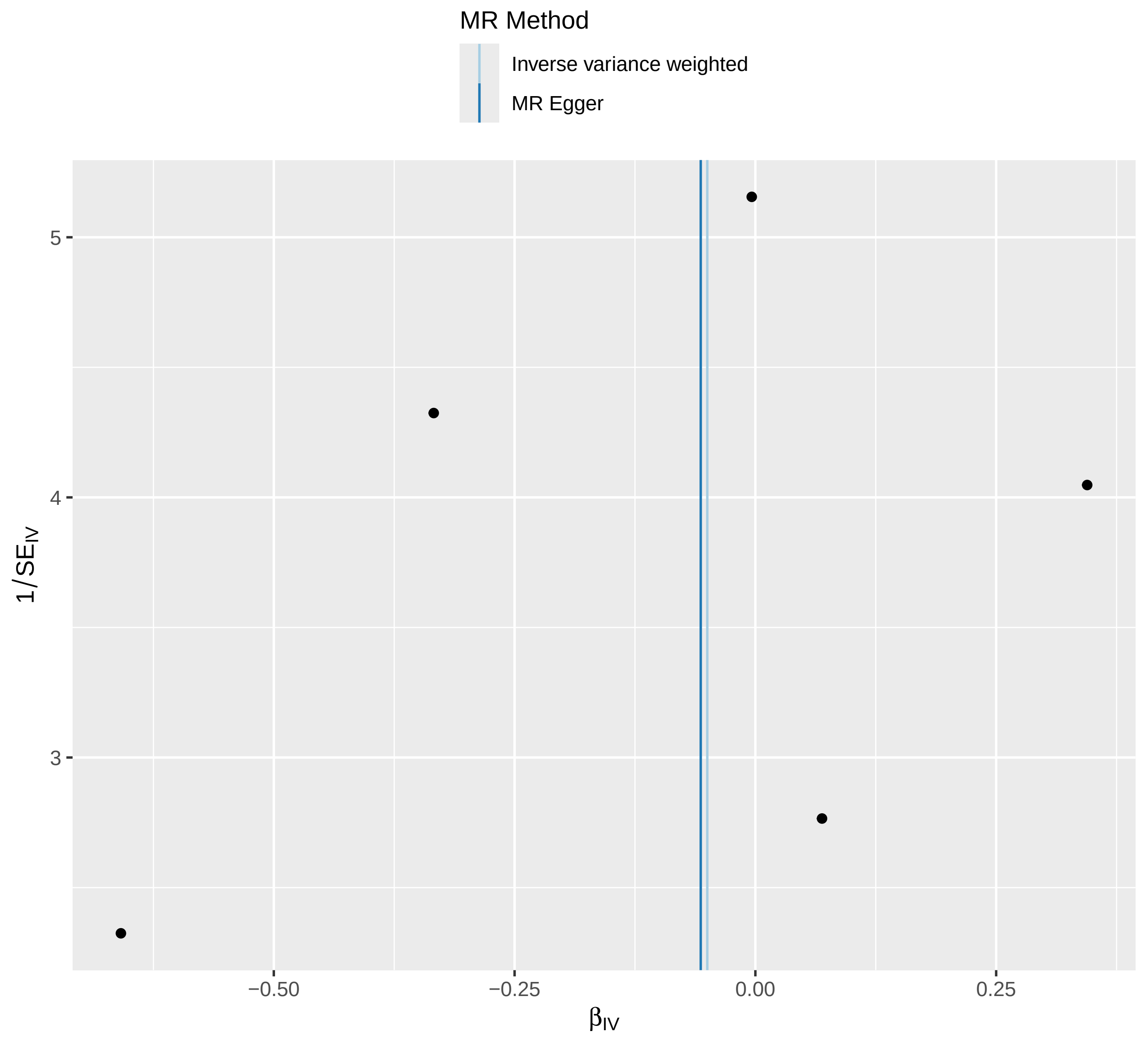
MR Method
Inverse variance weighted
MR Egger
5
4
3
$1/SE_{IV}$
−0.50
−0.25
0.00
0.25
$\beta_{IV}$

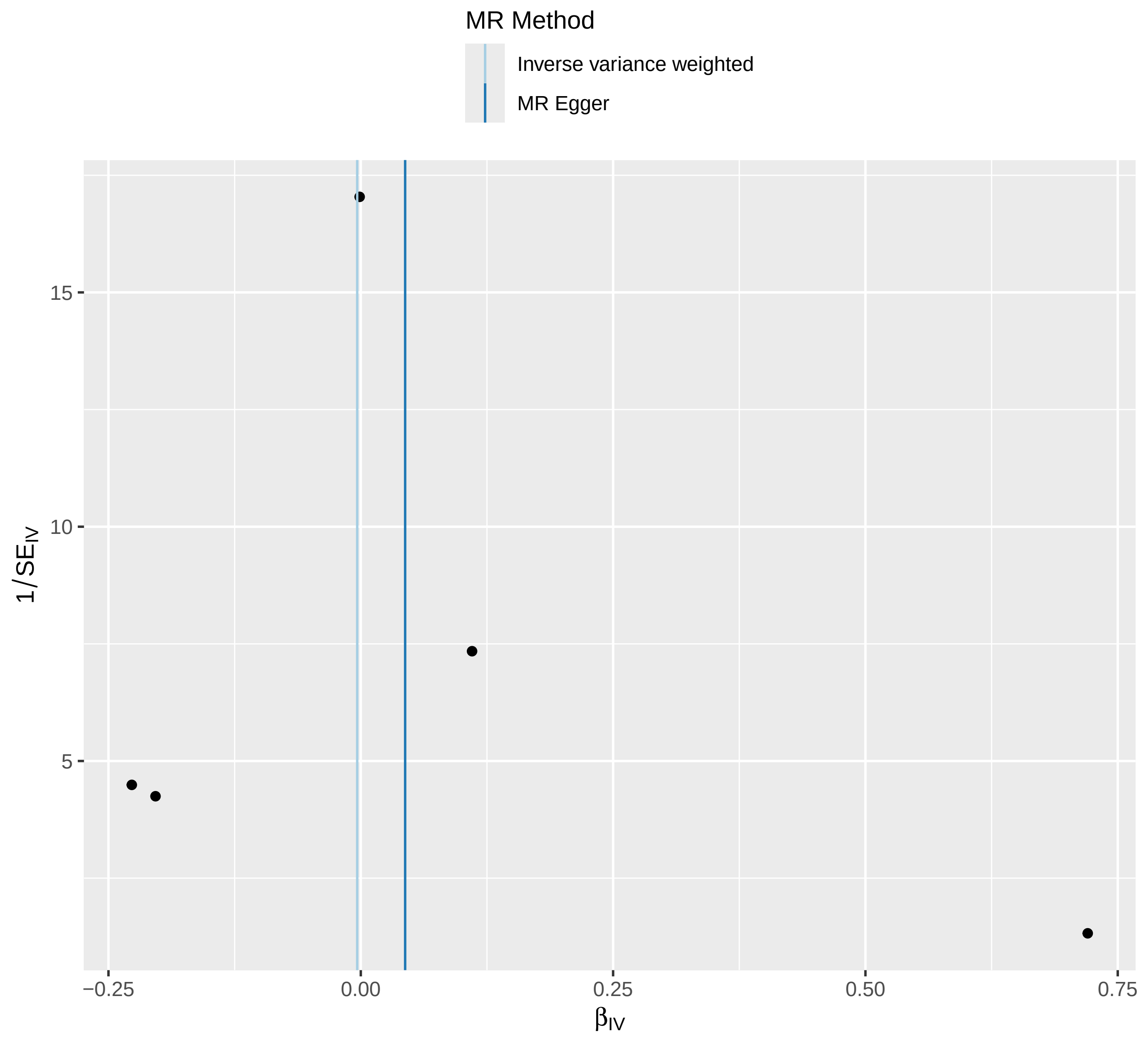
MR Method
Inverse variance weighted
MR Egger
15
10
5
1/SE_IV
−0.25
0.00
0.25
0.50
0.75
β_IV

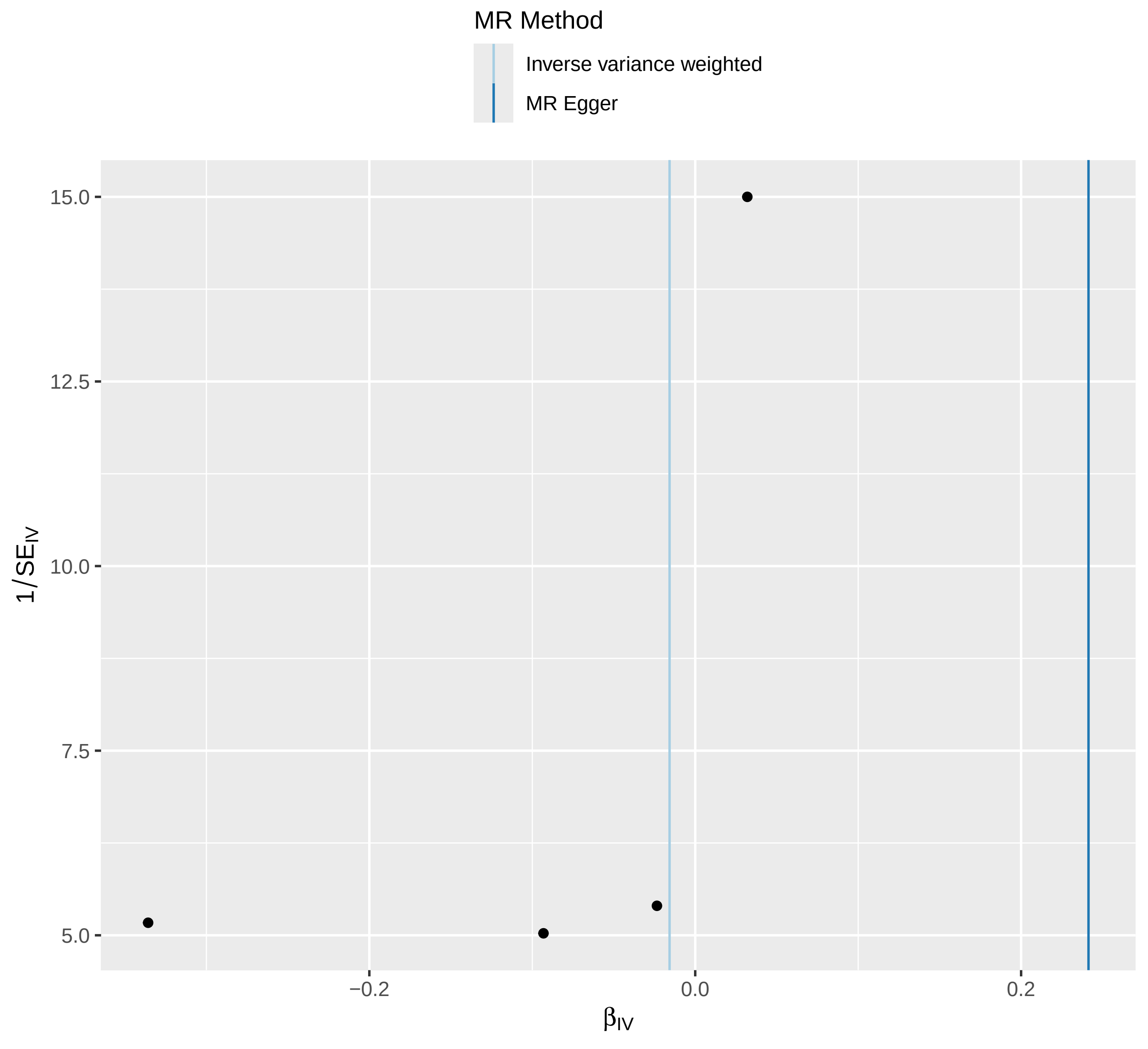
MR Method
Inverse variance weighted
MR Egger
15.0
12.5
10.0
7.5
5.0
1/SE_IV
−0.2
0.0
0.2
β_IV

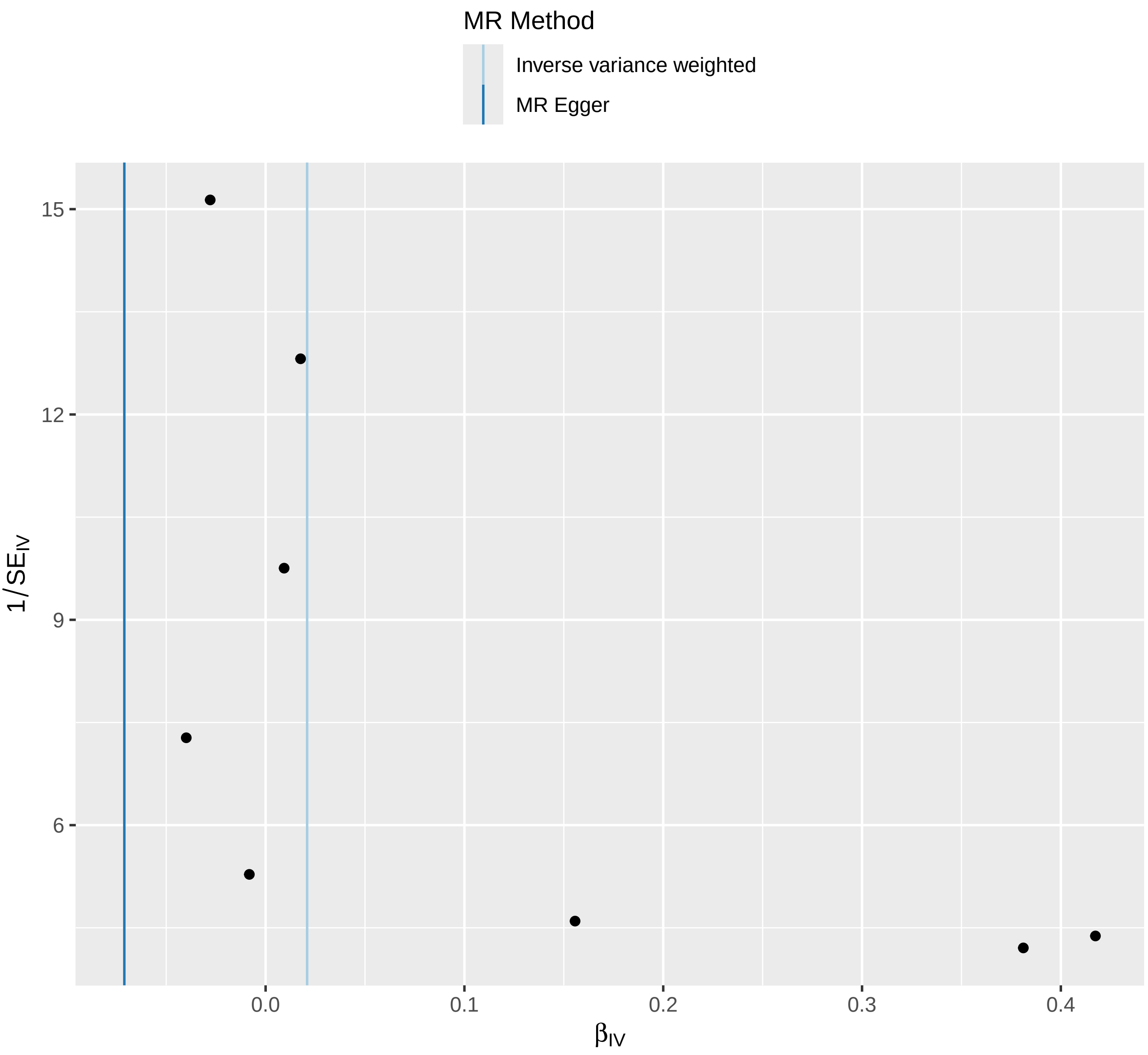
MR Method
Inverse variance weighted
MR Egger
15
12
9
6
1/SE$_{IV}$
0.0
0.1
0.2
0.3
0.4
β$_{IV}$

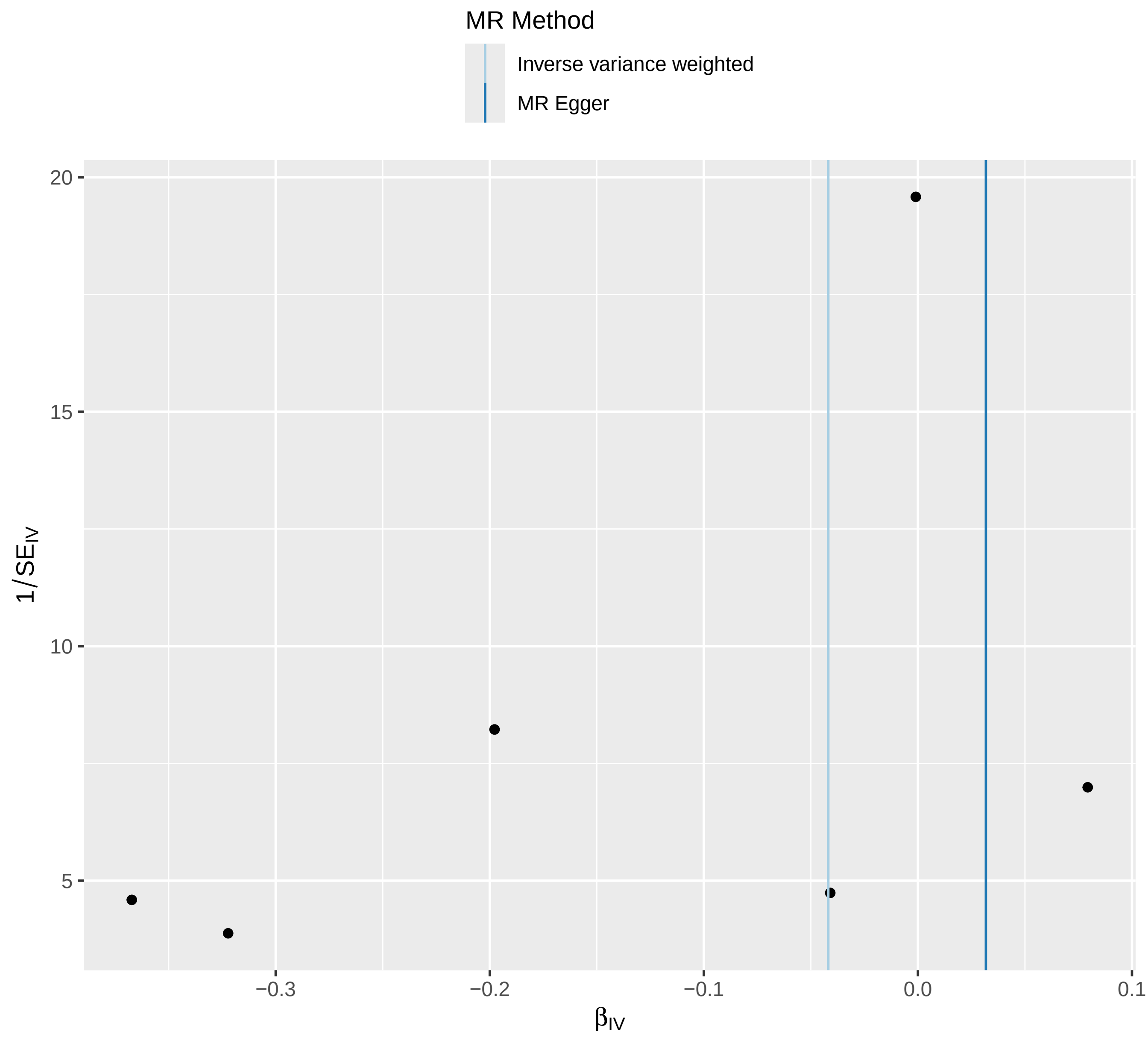

MR Method
Inverse variance weighted
MR Egger
20
15
10
5
1/SE$_{IV}$
−0.3
−0.2
−0.1
0.0
0.1
β$_{IV}$

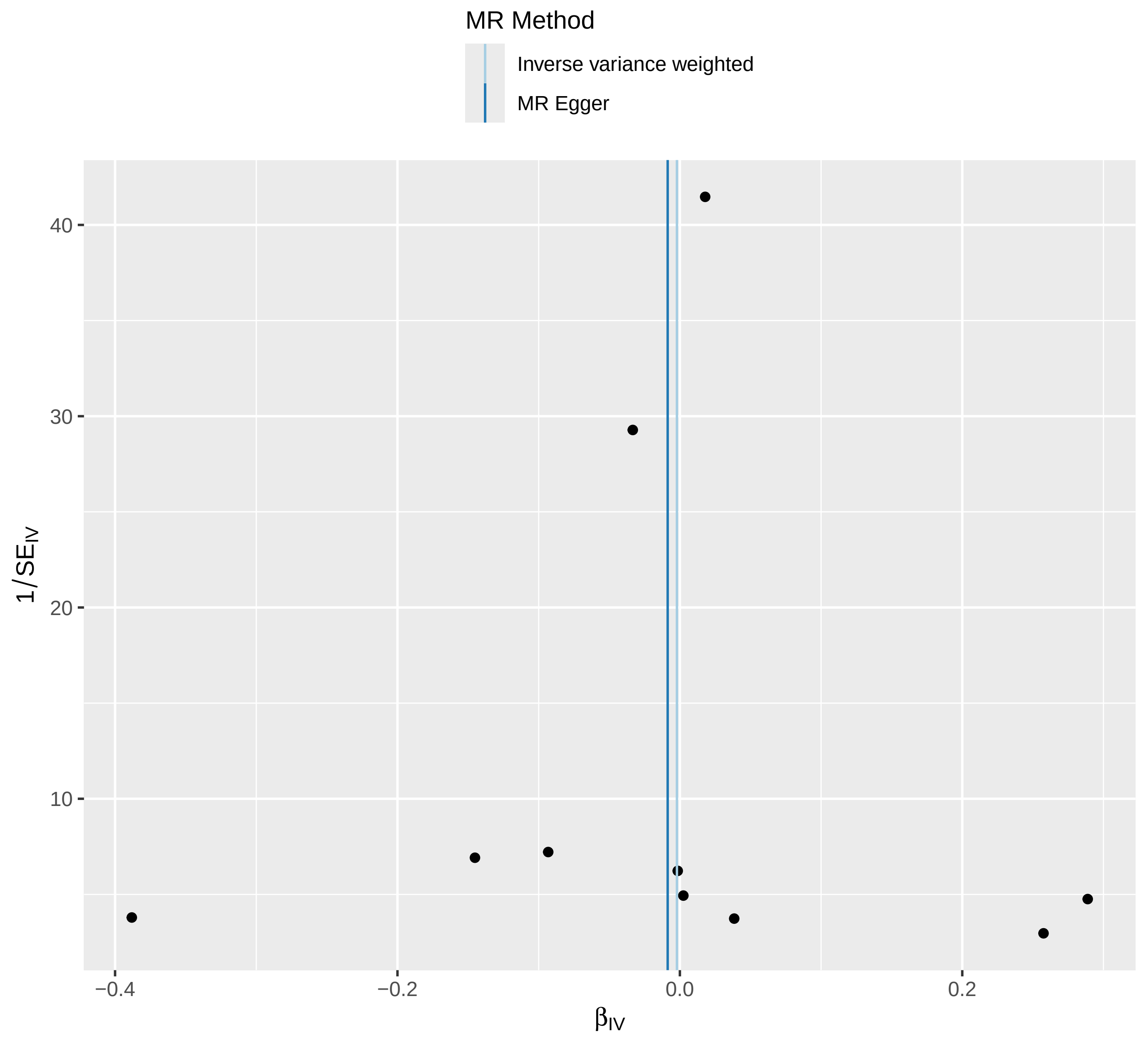
MR Method
Inverse variance weighted
MR Egger
1/SE$_{IV}$
40
30
20
10
−0.4
−0.2
0.0
0.2
β$_{IV}$

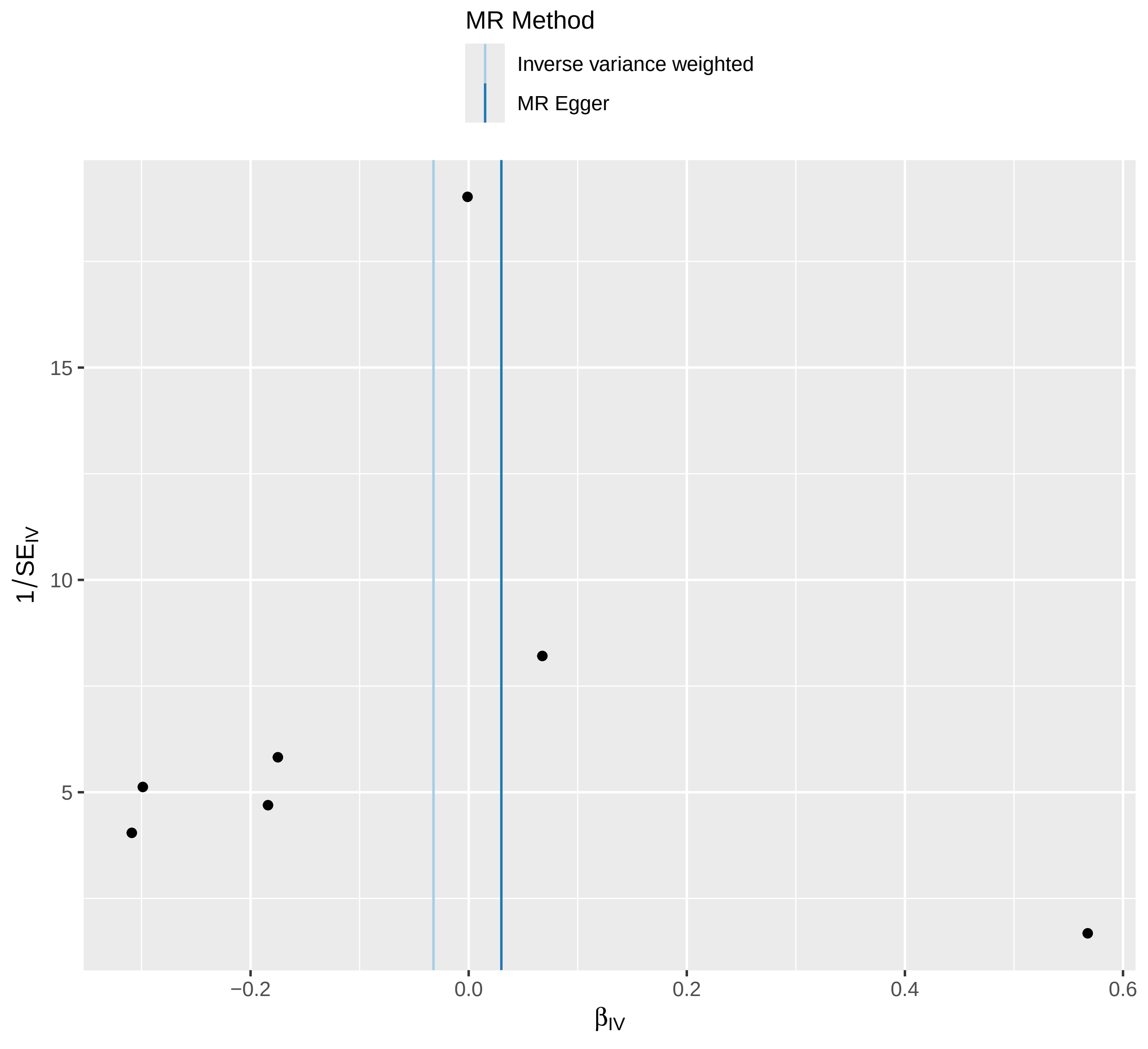
MR Method
Inverse variance weighted
MR Egger
15
10
5
1/SE$_{IV}$
−0.2
0.0
0.2
0.4
0.6
β$_{IV}$

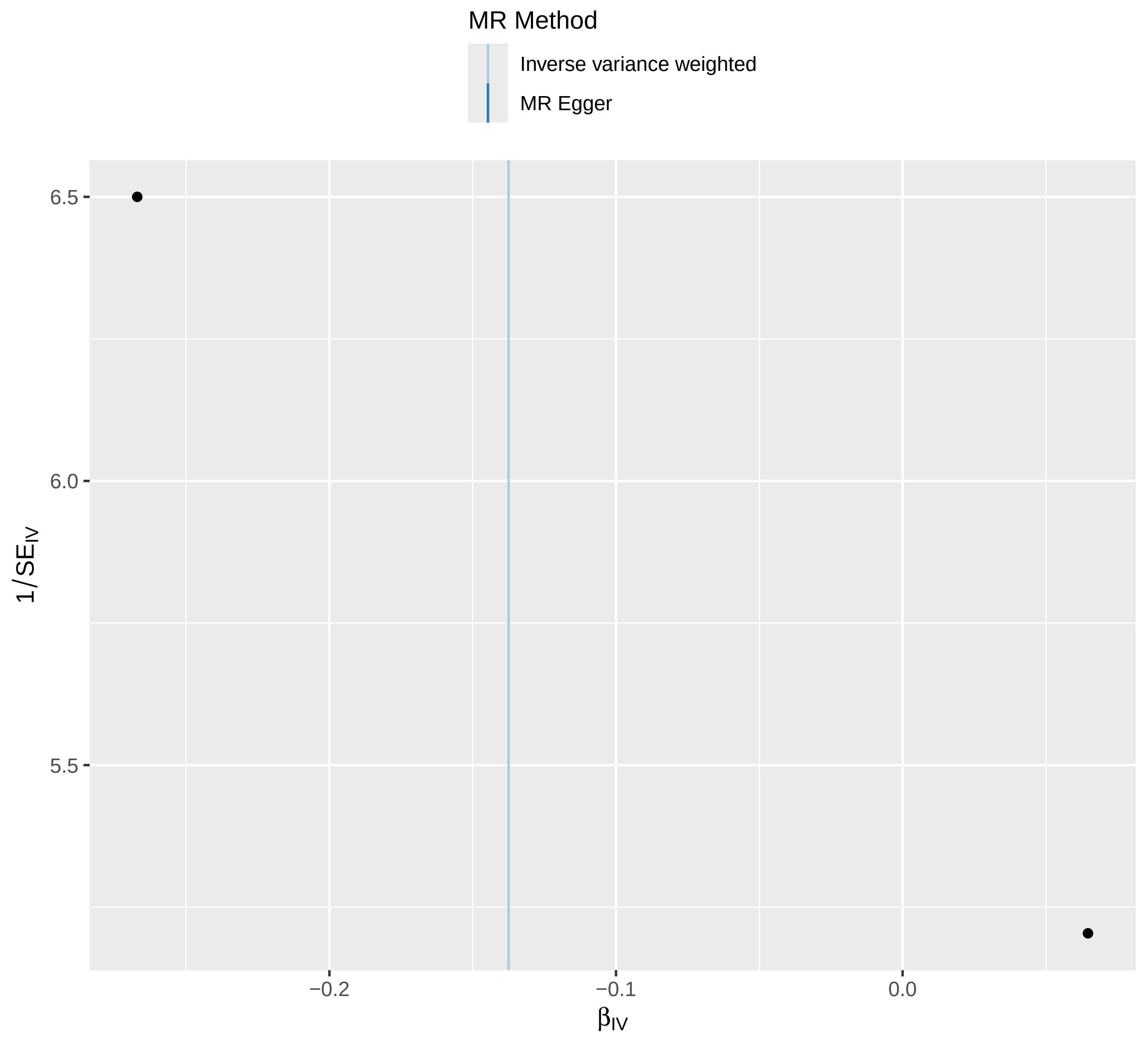
MR Method
Inverse variance weighted
MR Egger
6.5
6.0
5.5
$1/SE_{IV}$
−0.2
−0.1
0.0
$\beta_{IV}$

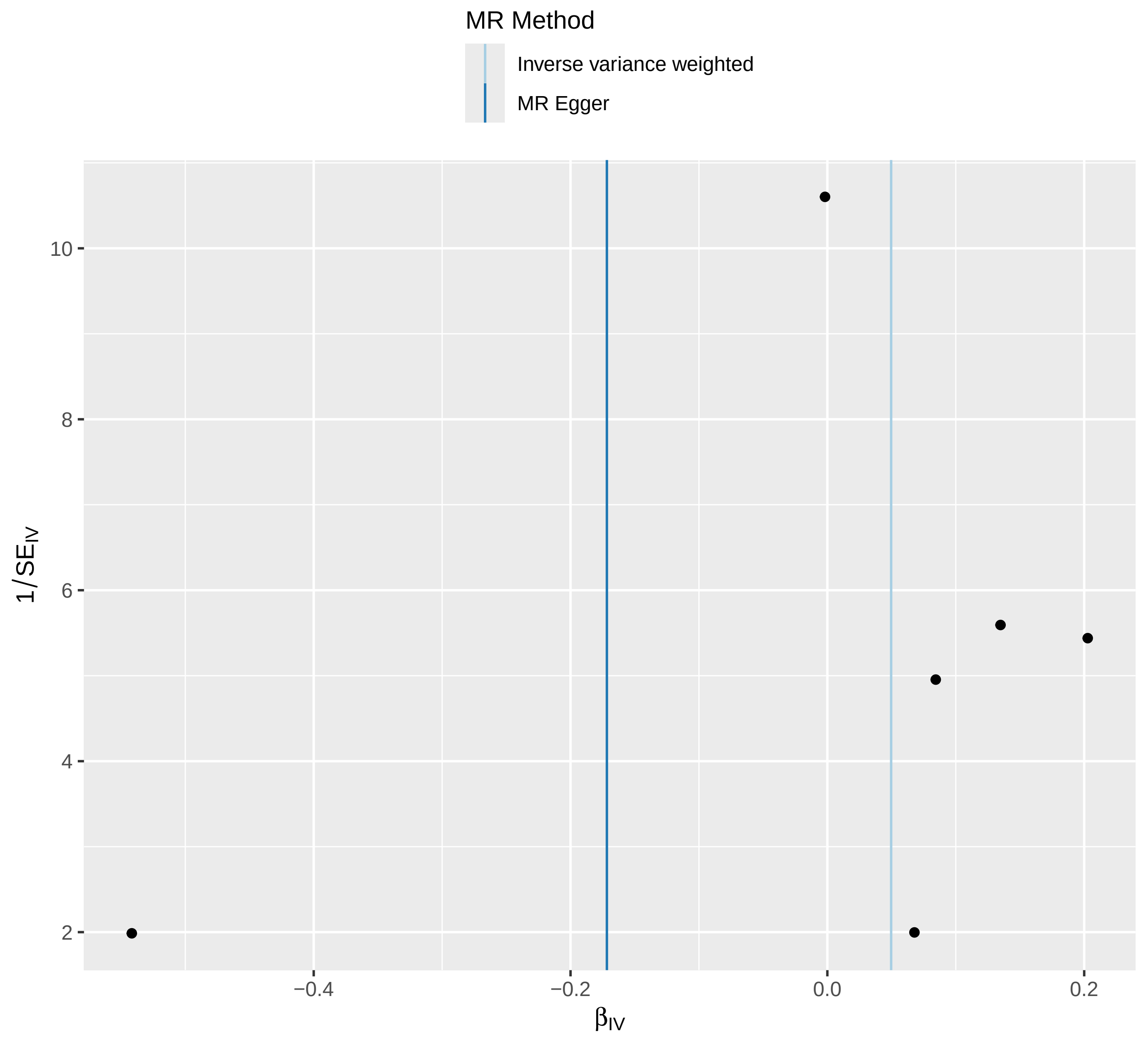

MR Method
Inverse variance weighted
MR Egger
10
8
6
4
2
1/SE_IV
−0.4
−0.2
0.0
0.2
β_IV

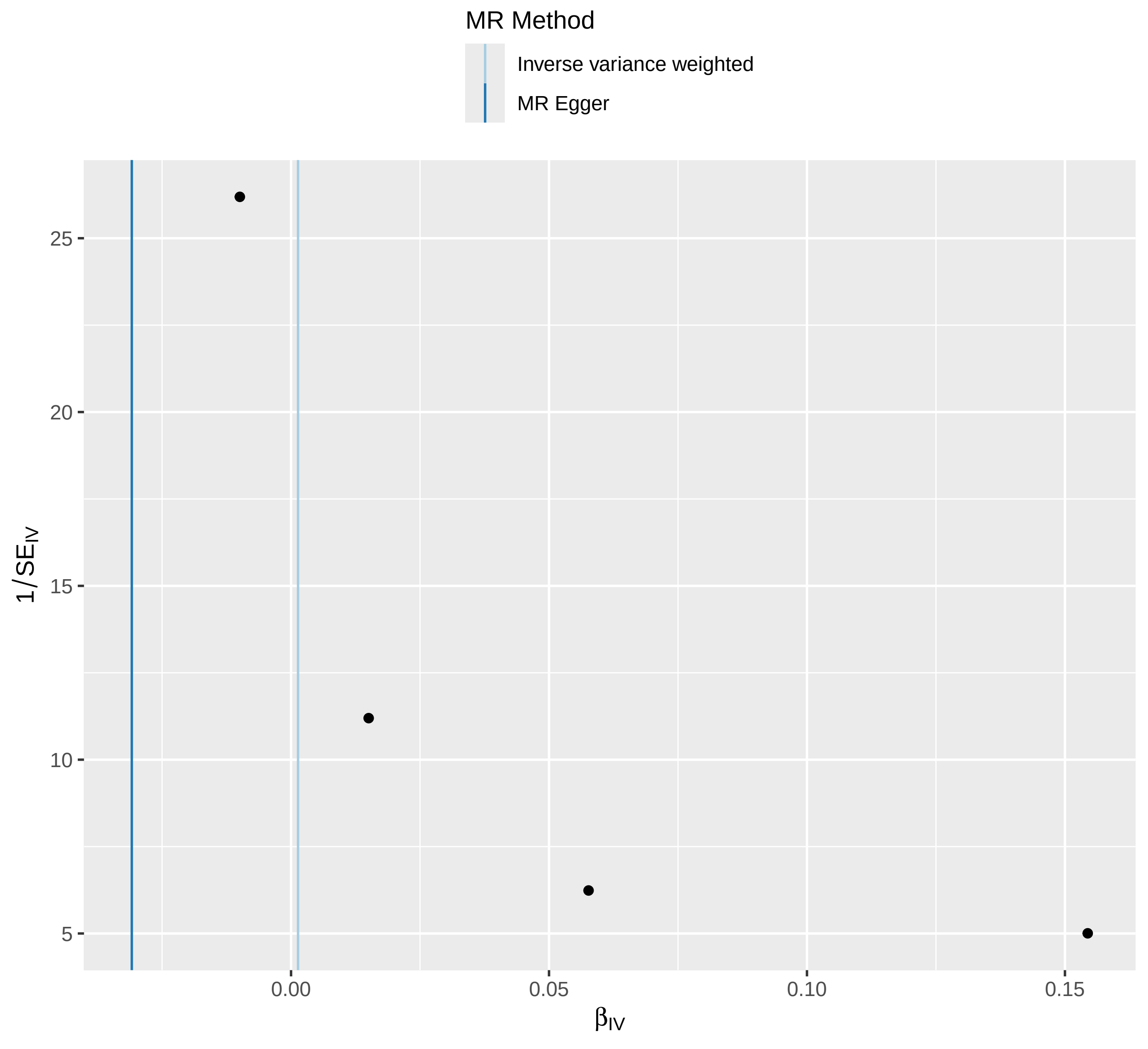

MR Method
Inverse variance weighted
MR Egger
25
20
15
10
5
1/SE_IV
0.00
0.05
0.10
0.15
β_IV

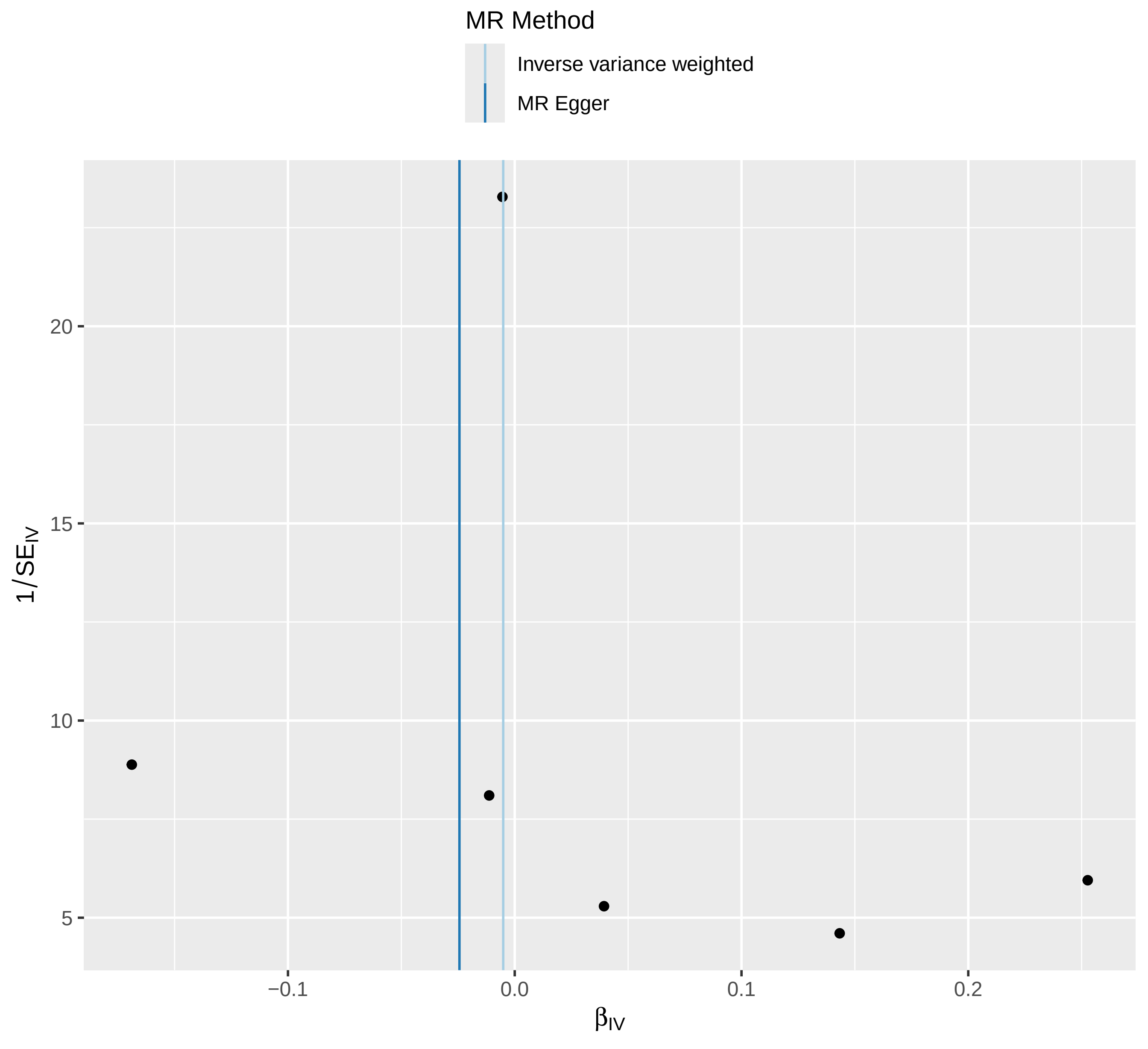
MR Method
Inverse variance weighted
MR Egger
20
15
10
5
1/SE_IV
−0.1
0.0
0.1
0.2
β_IV

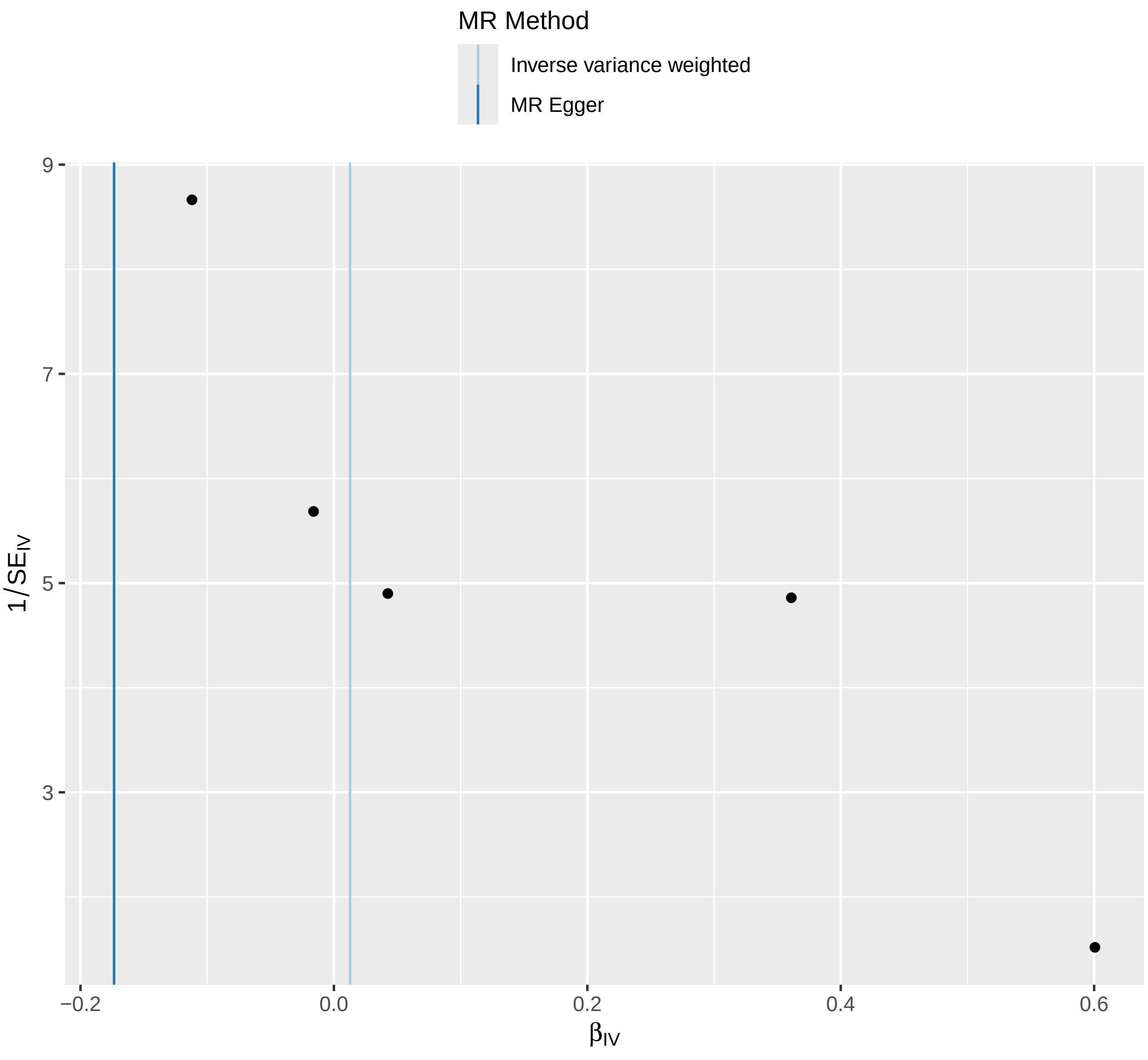

MR Method
Inverse variance weighted
MR Egger
9
7
5
3
1/SE_IV
−0.2
0.0
0.2
0.4
0.6
β_IV

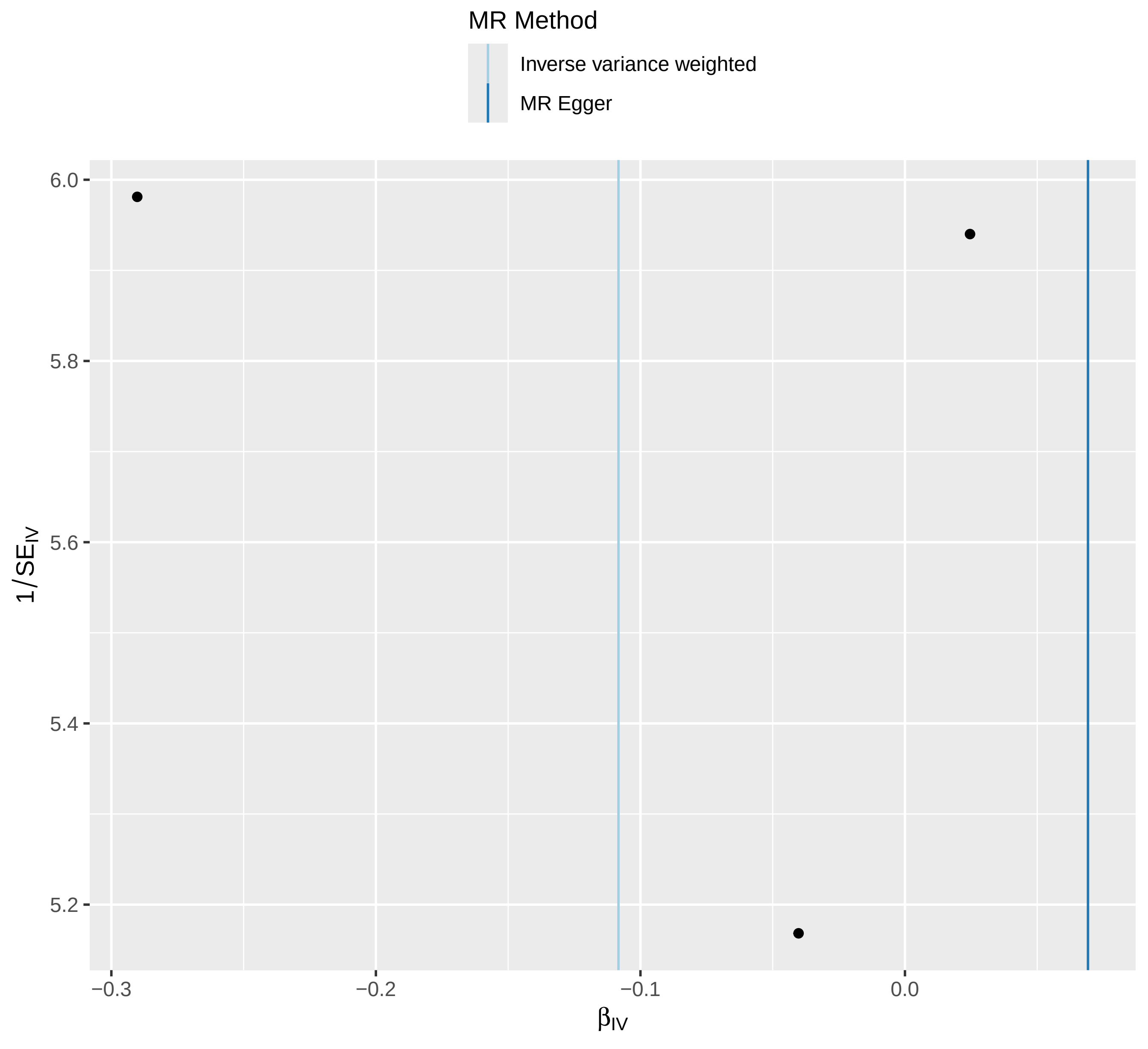

MR Method
Inverse variance weighted
MR Egger
6.0
5.8
5.6
5.4
5.2
1/SE_IV
−0.3
−0.2
−0.1
0.0
β_IV

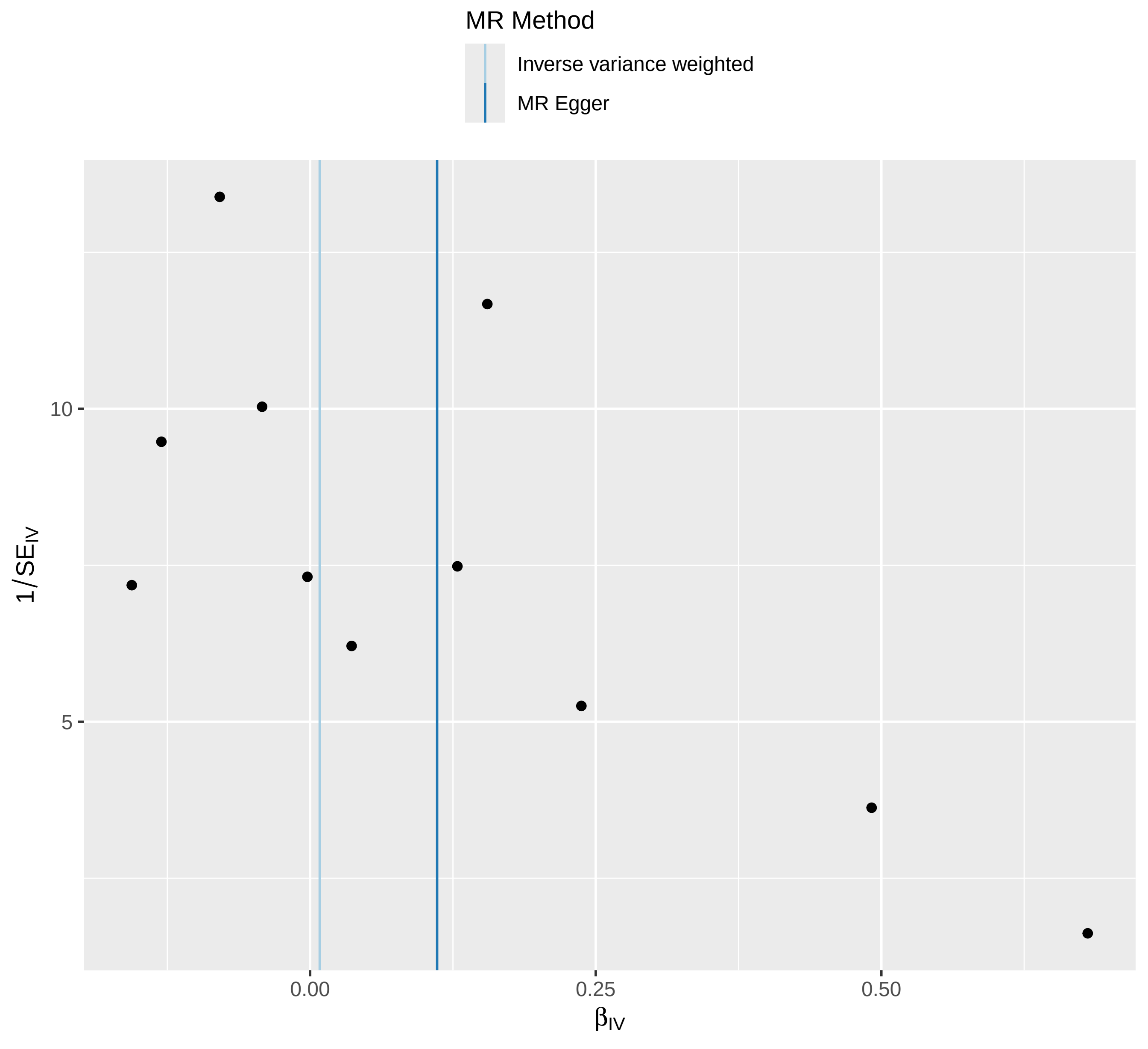
MR Method
Inverse variance weighted
MR Egger
10
5
1/SE_IV
0.00
0.25
0.50
β_IV

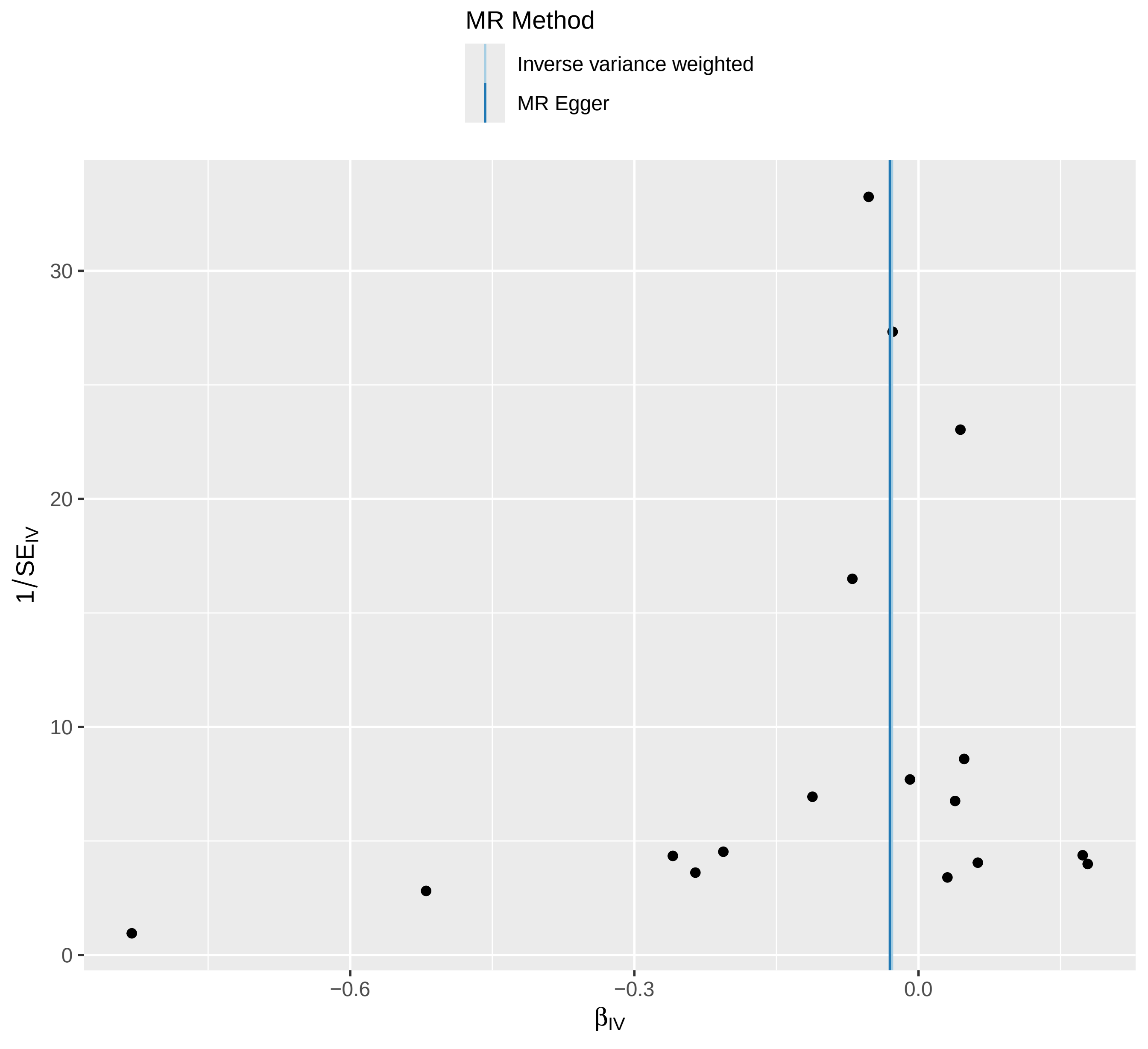

MR Method
Inverse variance weighted
MR Egger
30
20
10
0
1/SE_IV
−0.6
−0.3
0.0
β_IV

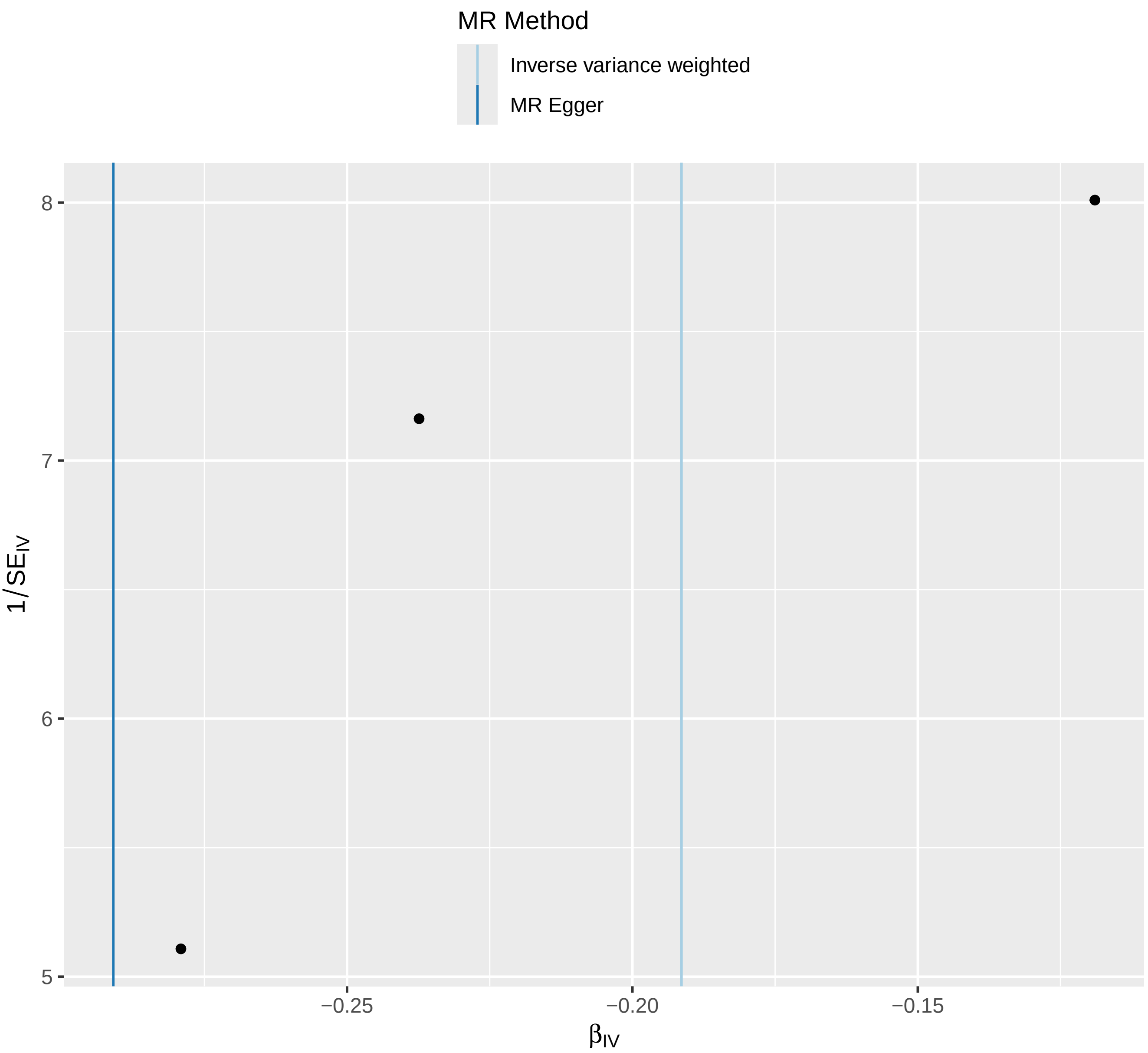
MR Method
Inverse variance weighted
MR Egger
8
7
6
5
1/SE_IV
−0.25
−0.20
−0.15
β_IV

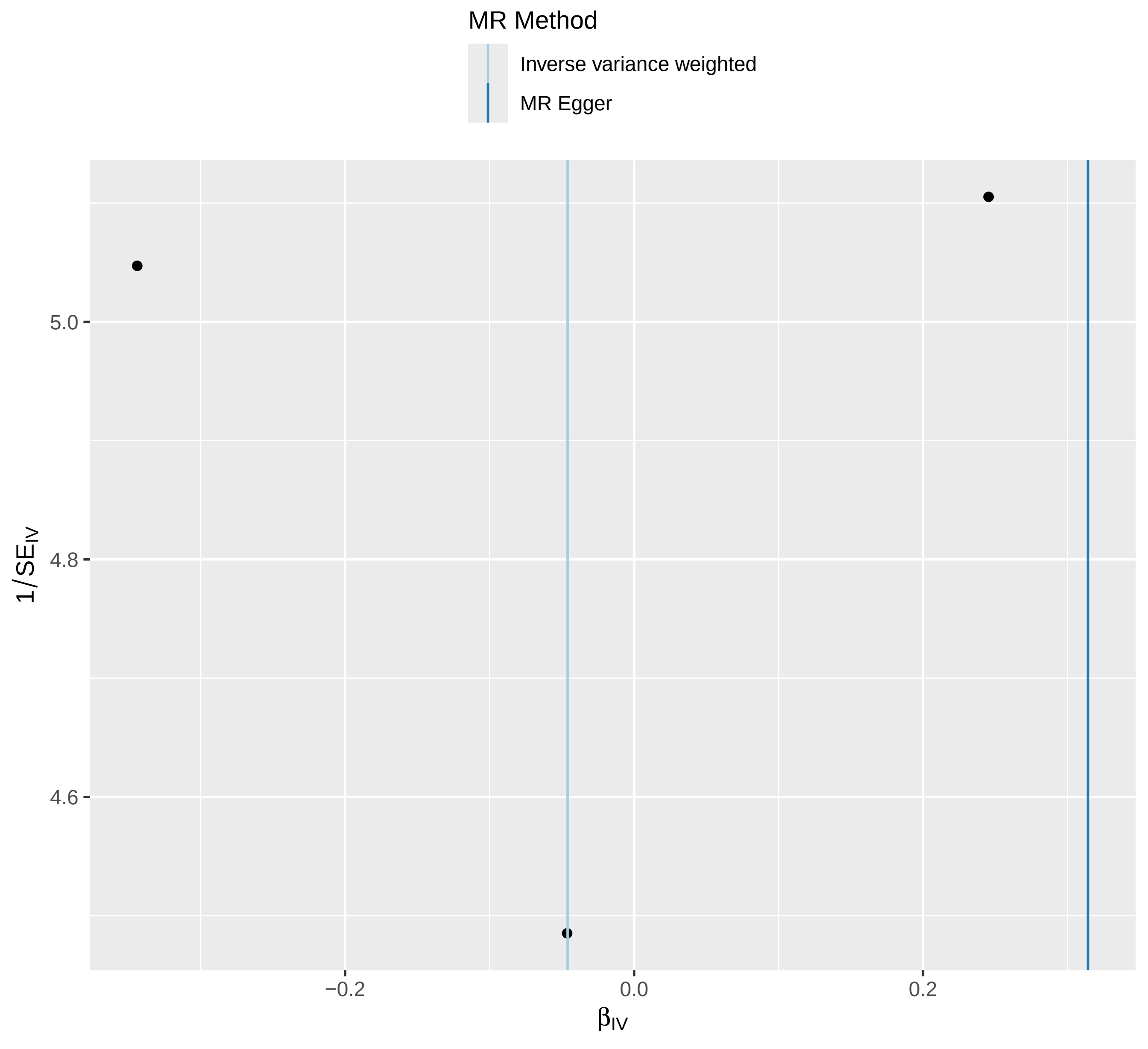
MR Method
Inverse variance weighted
MR Egger
5.0
4.8
4.6
1/SE_IV
−0.2
0.0
0.2
β_IV

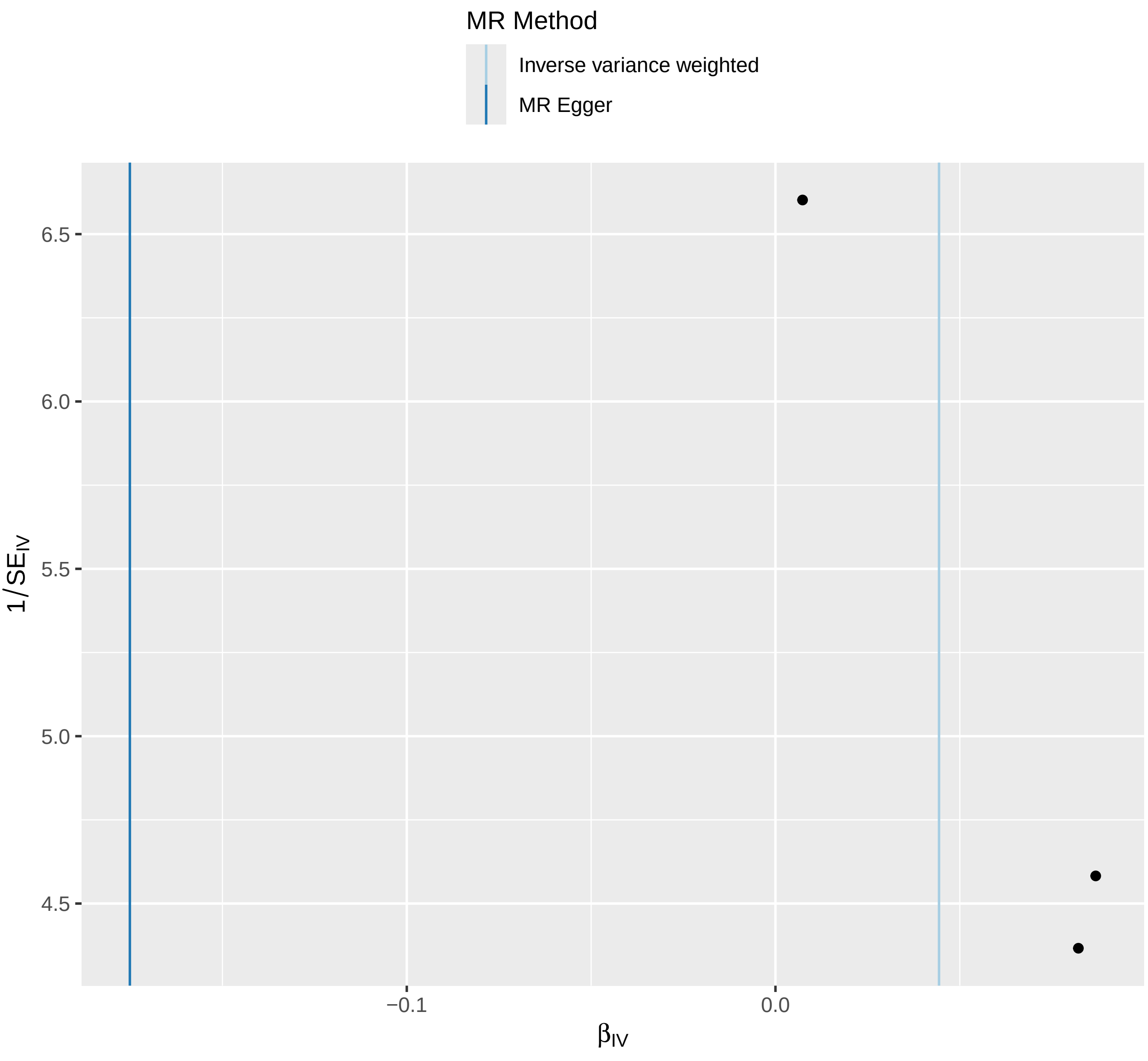
MR Method
Inverse variance weighted
MR Egger
6.5
6.0
5.5
5.0
4.5
1/SE$_{IV}$
−0.1
0.0
β$_{IV}$

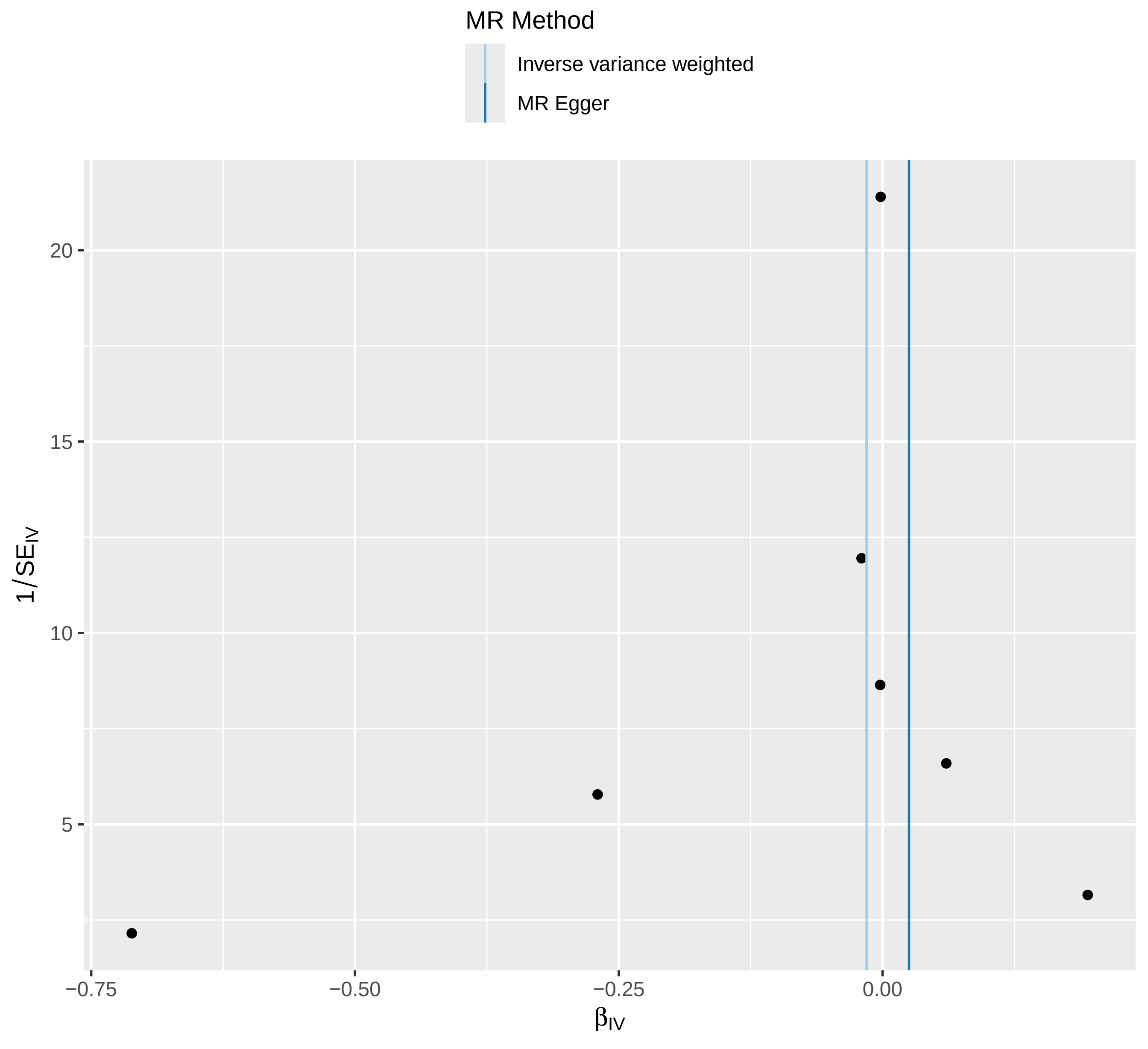
MR Method
Inverse variance weighted
MR Egger
1/SE$_{IV}$
20
15
10
5
−0.75
−0.50
−0.25
0.00
β$_{IV}$

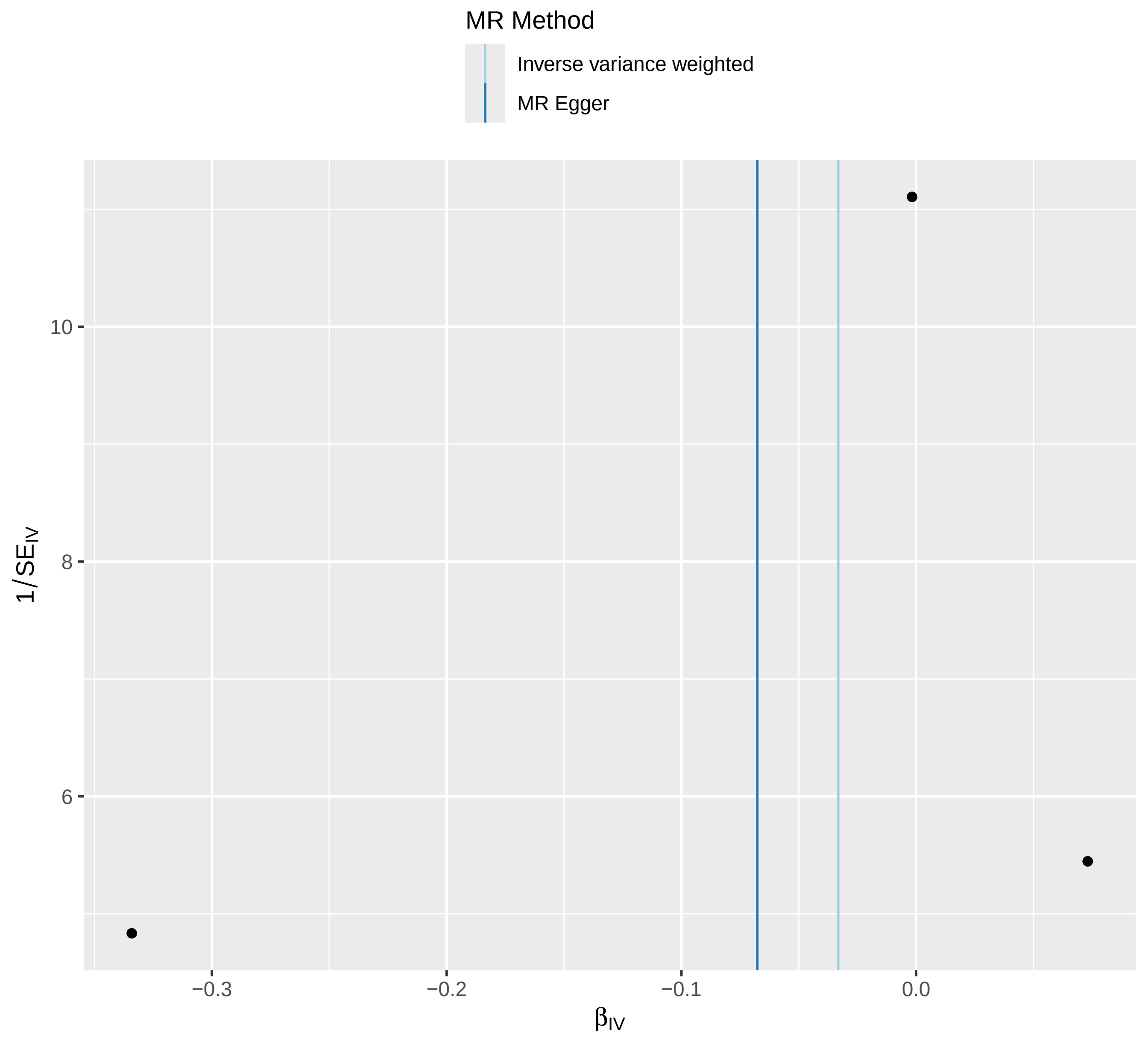
MR Method
Inverse variance weighted
MR Egger
10
8
6
1/SE$_{IV}$
−0.3
−0.2
−0.1
0.0
β$_{IV}$

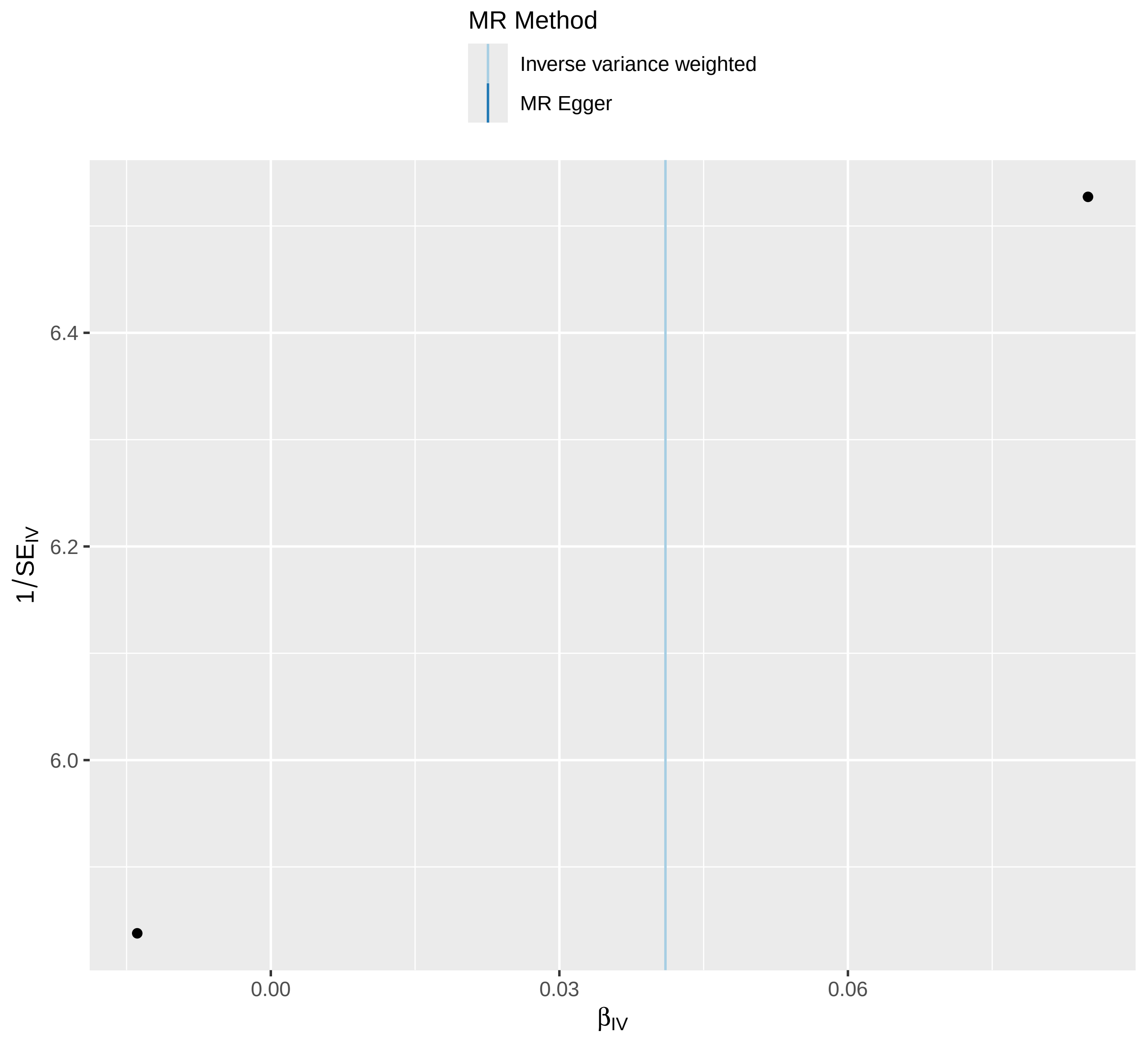

MR Method
Inverse variance weighted
MR Egger
6.4
6.2
6.0
1/SE$_{IV}$
0.00
0.03
0.06
$\beta_{IV}$

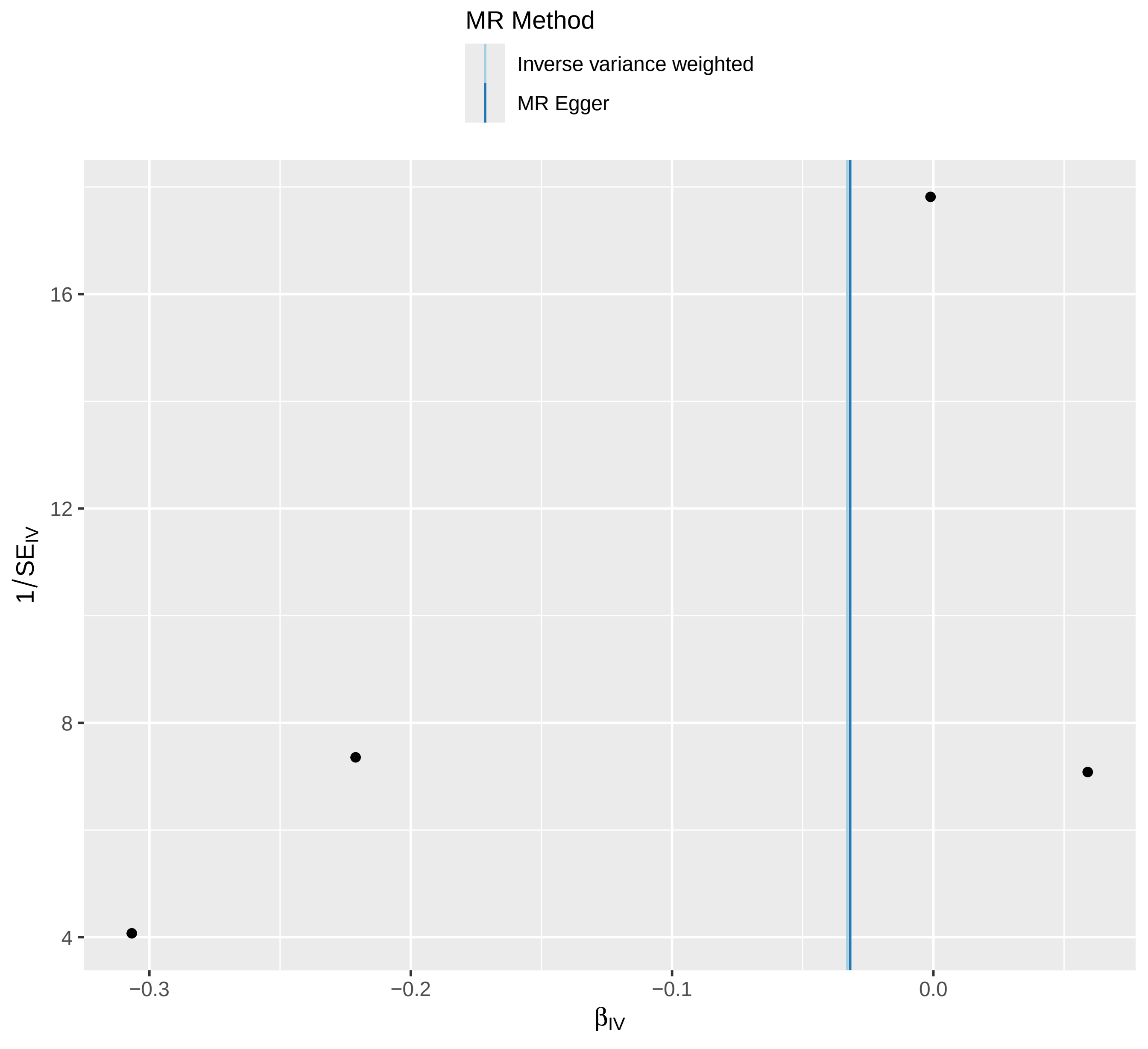
MR Method
Inverse variance weighted
MR Egger
16
12
8
4
1/SE_IV
−0.3
−0.2
−0.1
0.0
β_IV

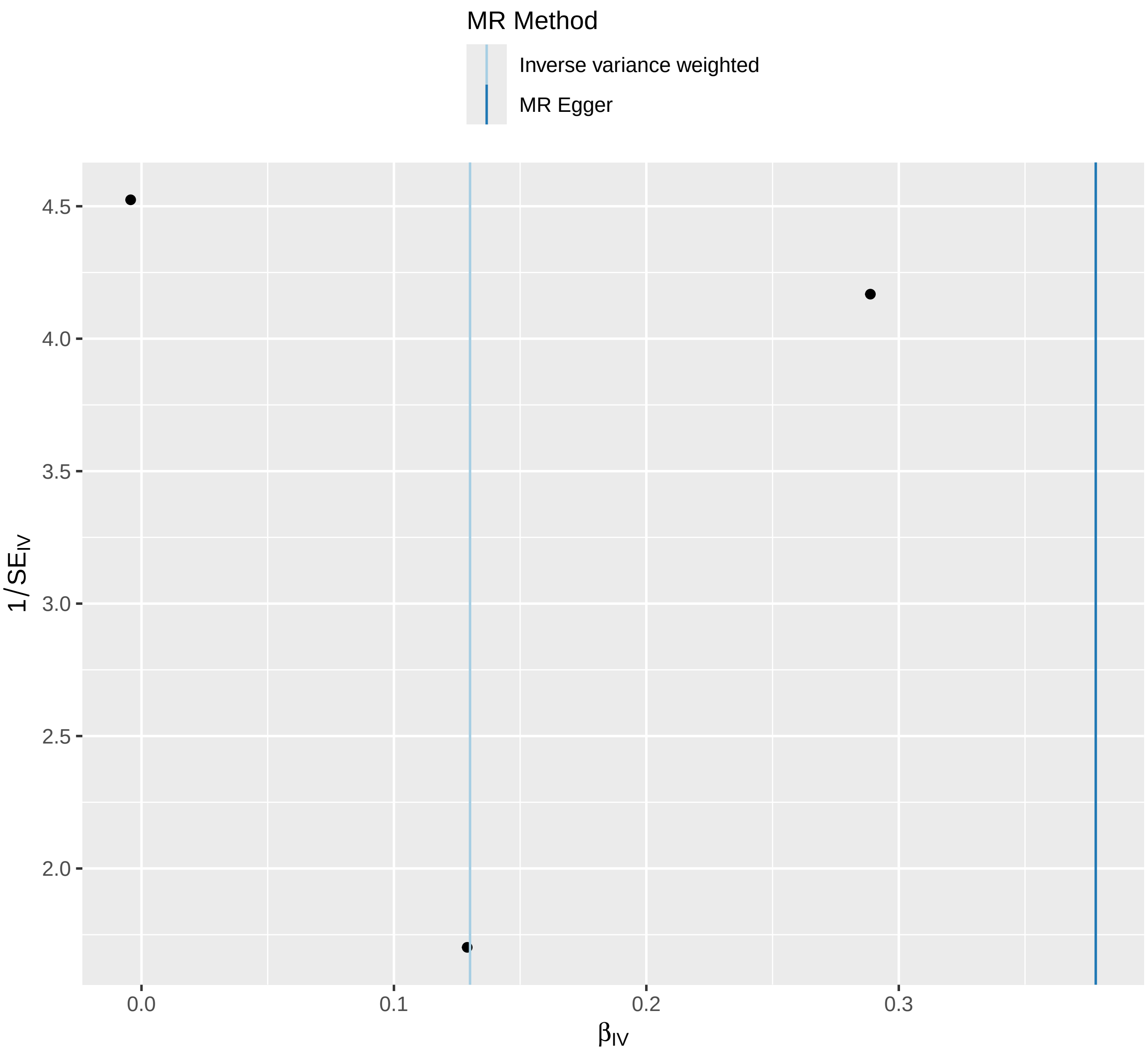
MR Method
Inverse variance weighted
MR Egger
4.5
4.0
3.5
3.0
2.5
2.0
1/SE_IV
0.0
0.1
0.2
0.3
β_IV

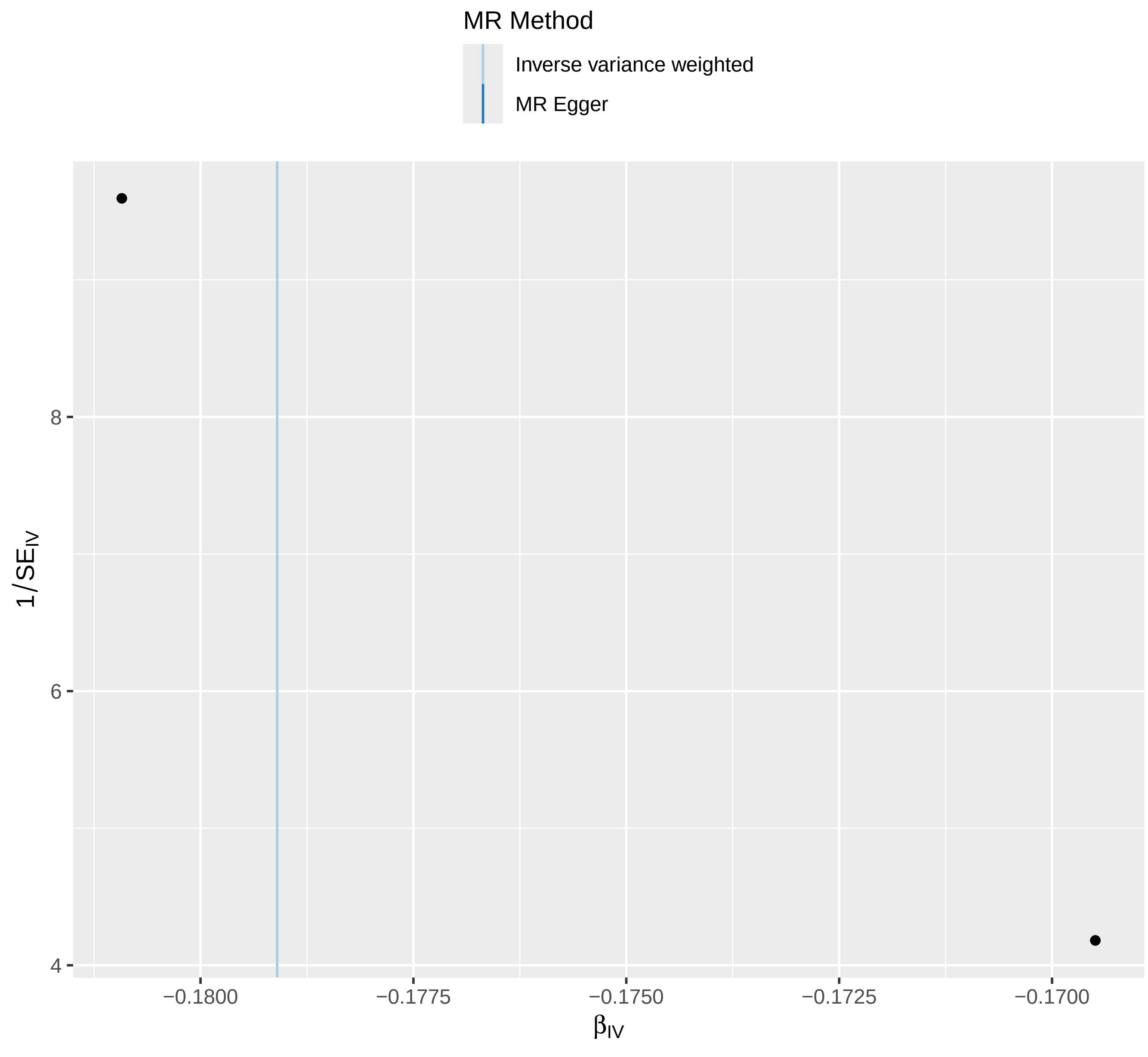
MR Method
Inverse variance weighted
MR Egger
8
6
4
$1/SE_{IV}$
−0.1800
−0.1775
−0.1750
−0.1725
−0.1700
$\beta_{IV}$

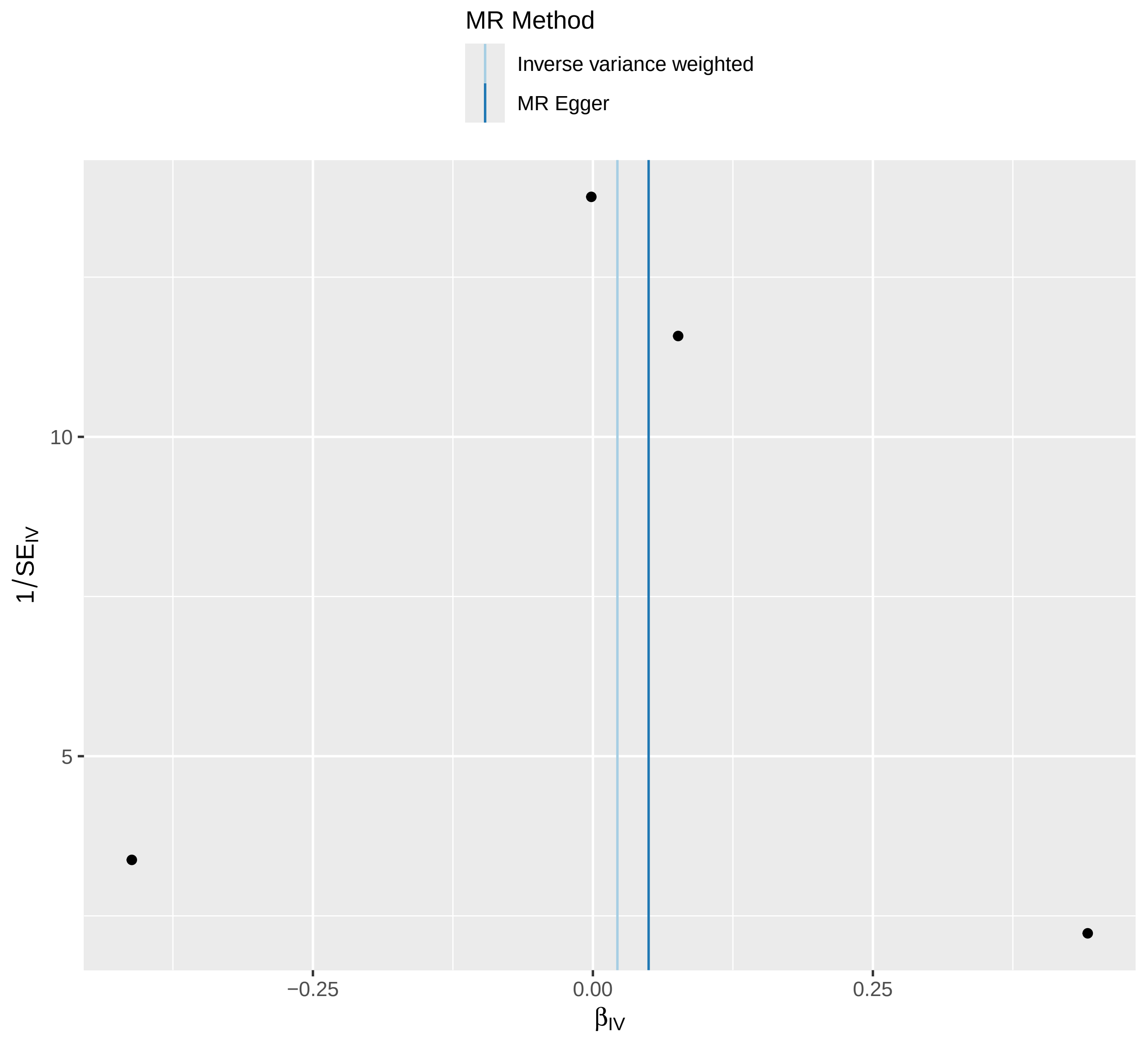

MR Method
Inverse variance weighted
MR Egger
10
5
1/SE$_{IV}$
−0.25
0.00
0.25
β$_{IV}$

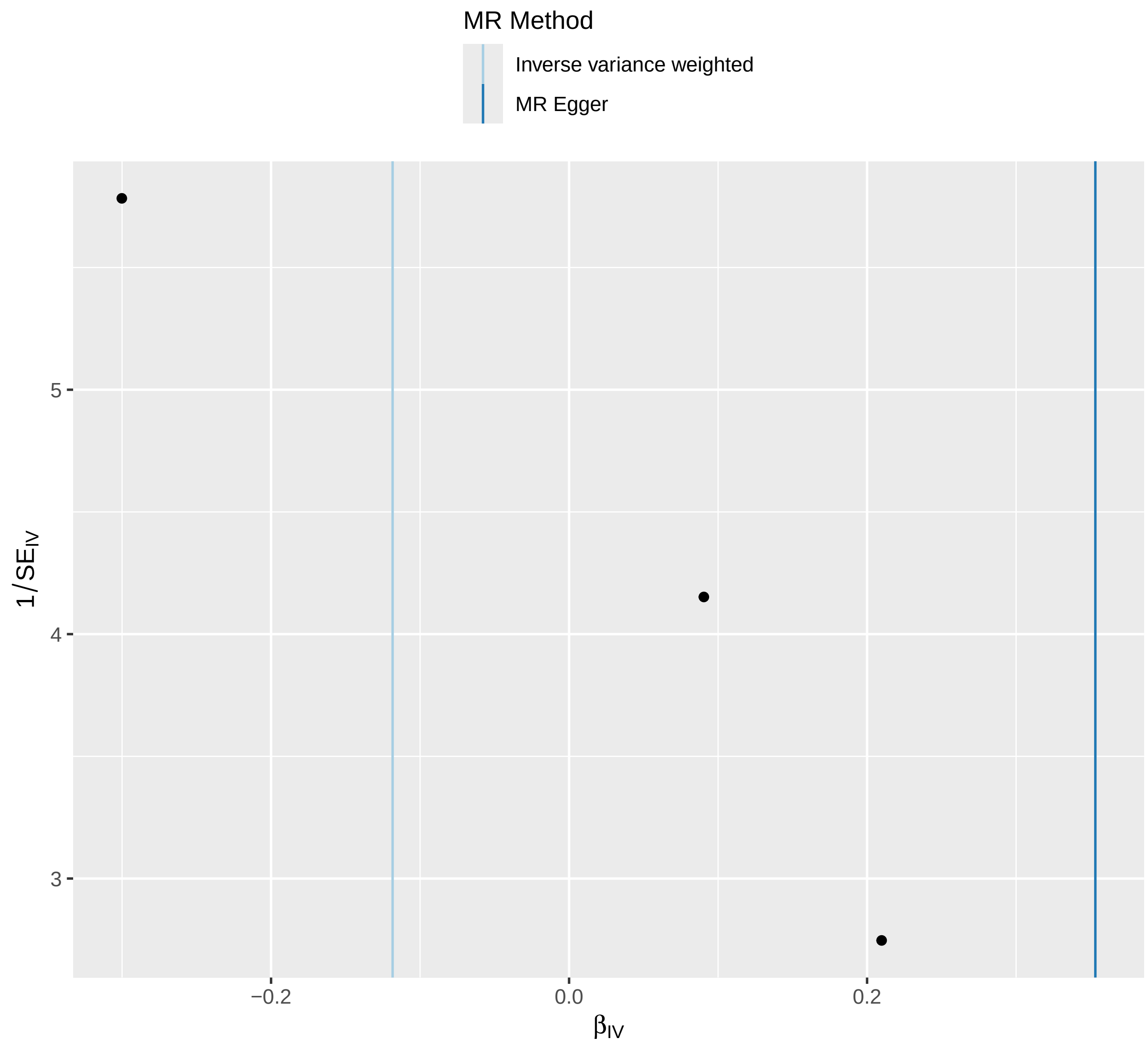
MR Method
Inverse variance weighted
MR Egger
1/SE_IV
5
4
3
−0.2
0.0
0.2
β_IV

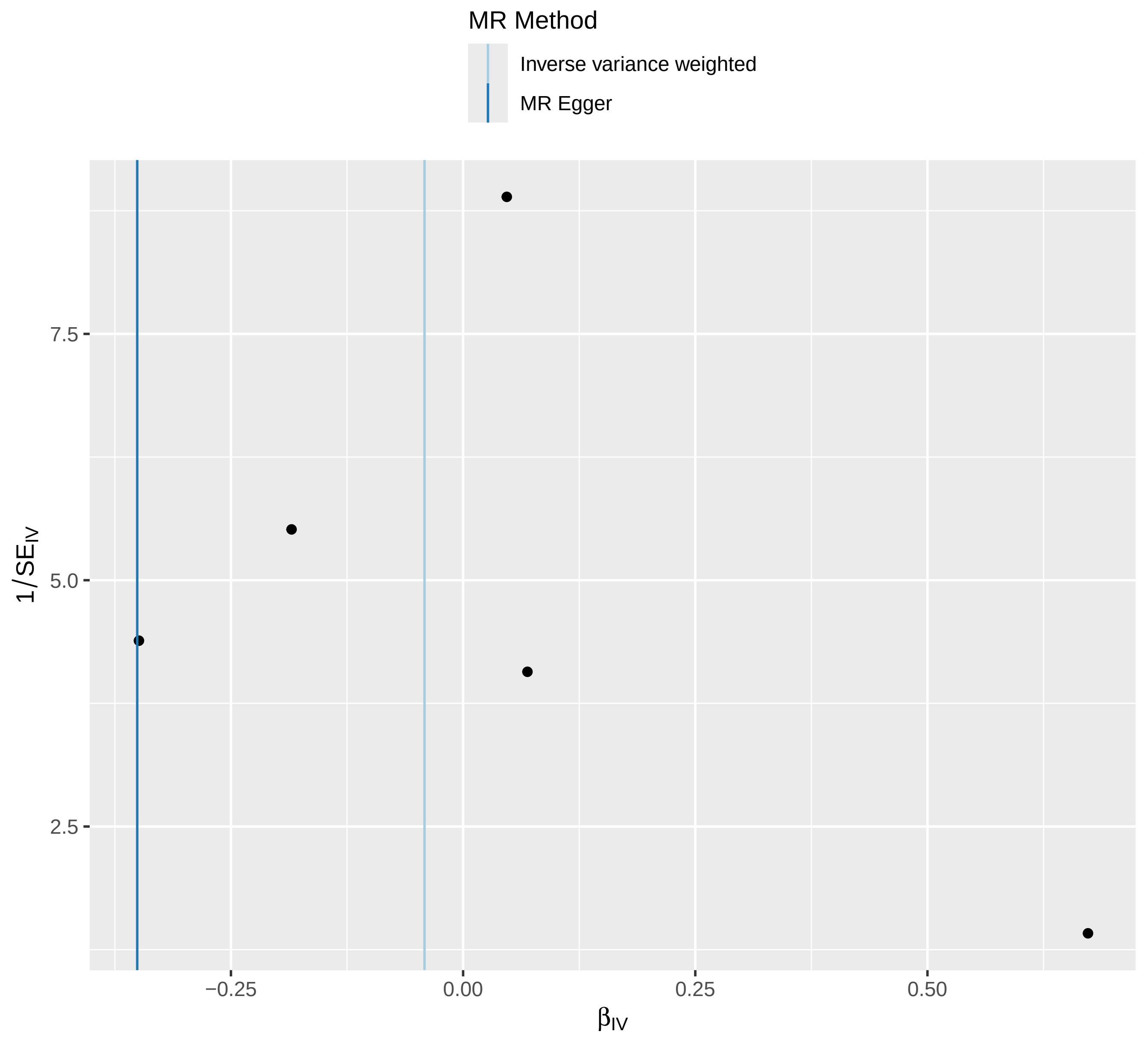

MR Method
Inverse variance weighted
MR Egger
7.5
5.0
2.5
1/SE_IV
−0.25
0.00
0.25
0.50
β_IV

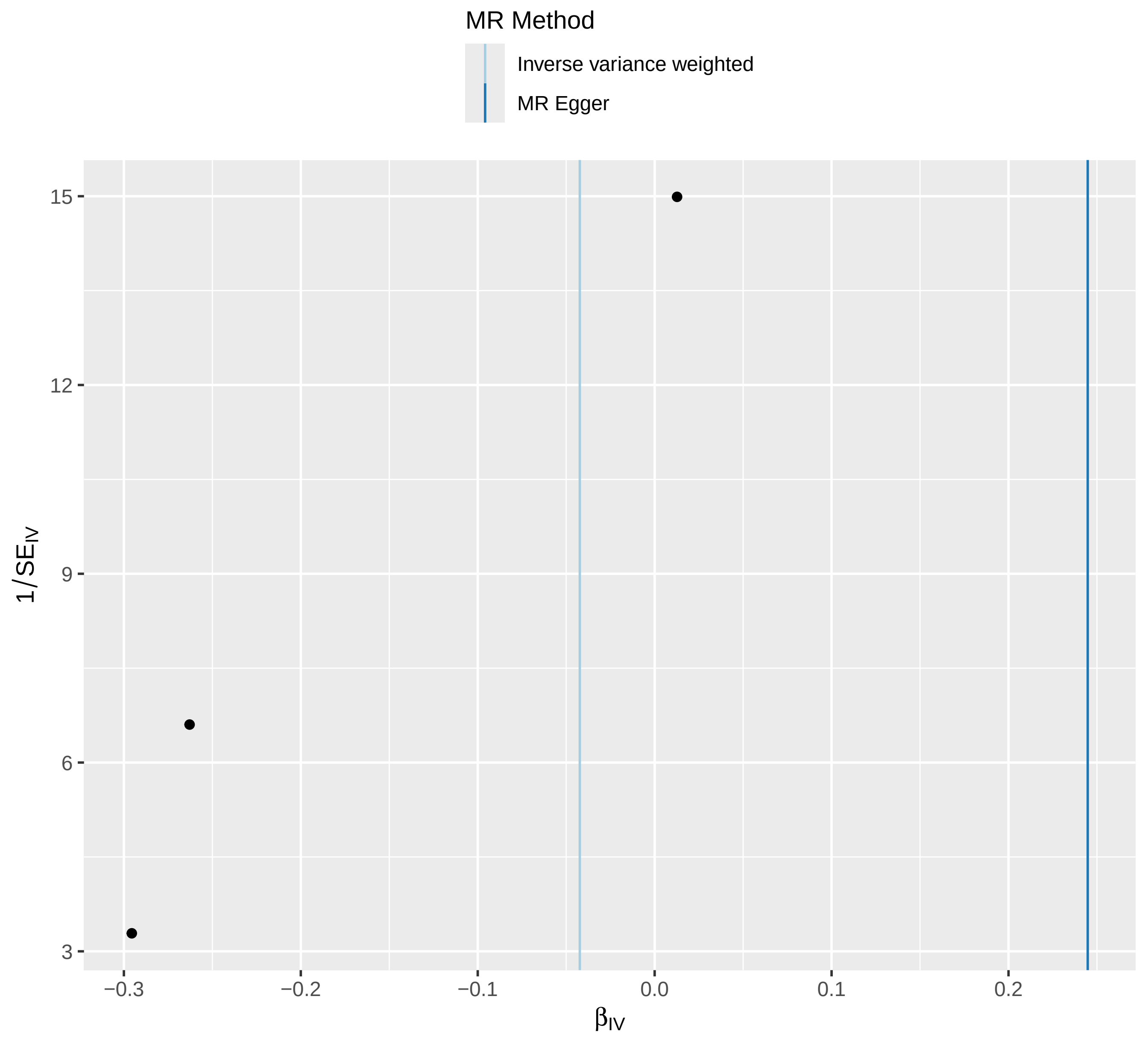
MR Method
Inverse variance weighted
MR Egger
15
12
9
6
3
1/SE$_{IV}$
−0.3
−0.2
−0.1
0.0
0.1
0.2
β$_{IV}$

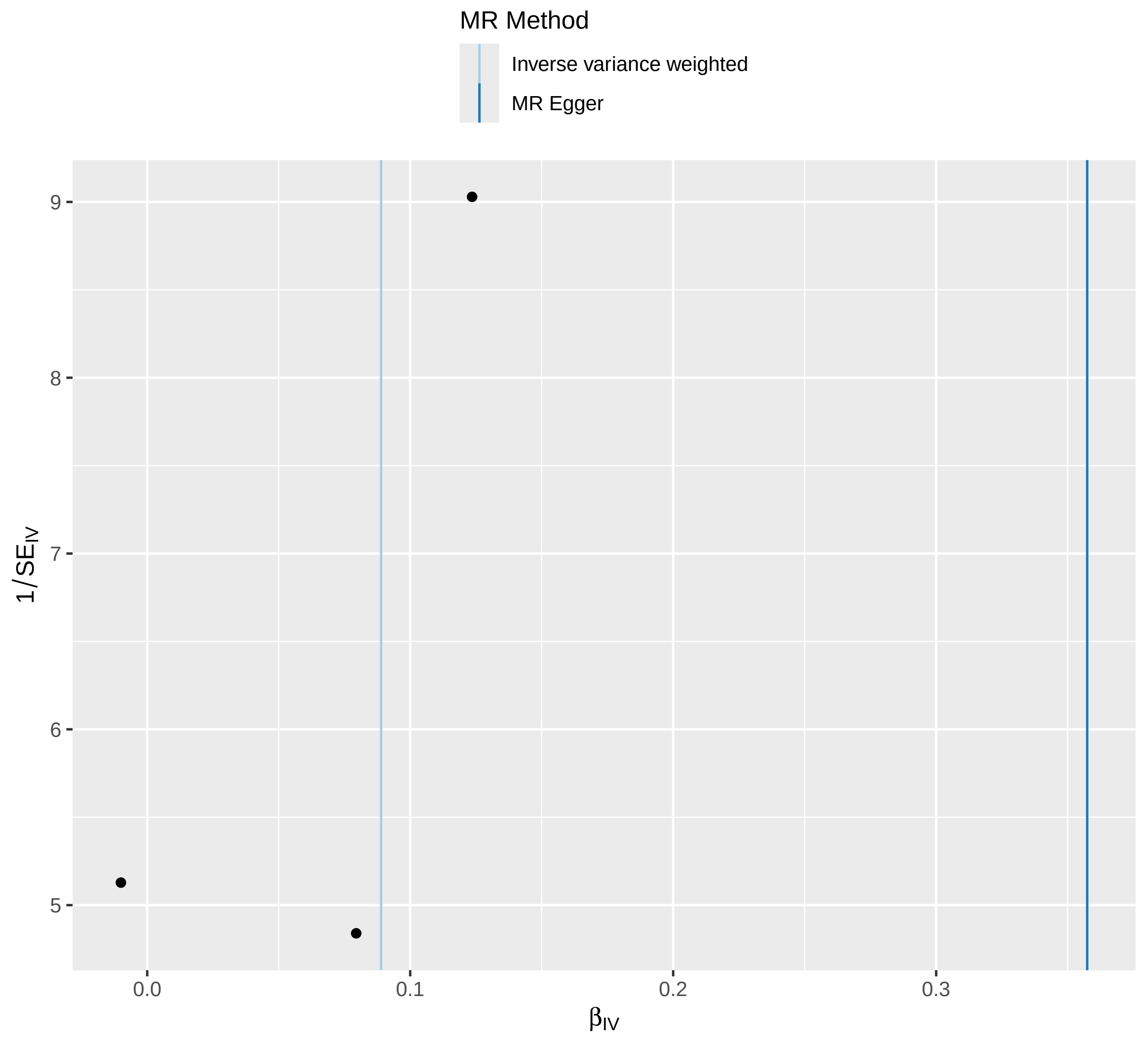
MR Method
Inverse variance weighted
MR Egger
9
8
7
6
5
1/SE$_{IV}$
0.0
0.1
0.2
0.3
β$_{IV}$

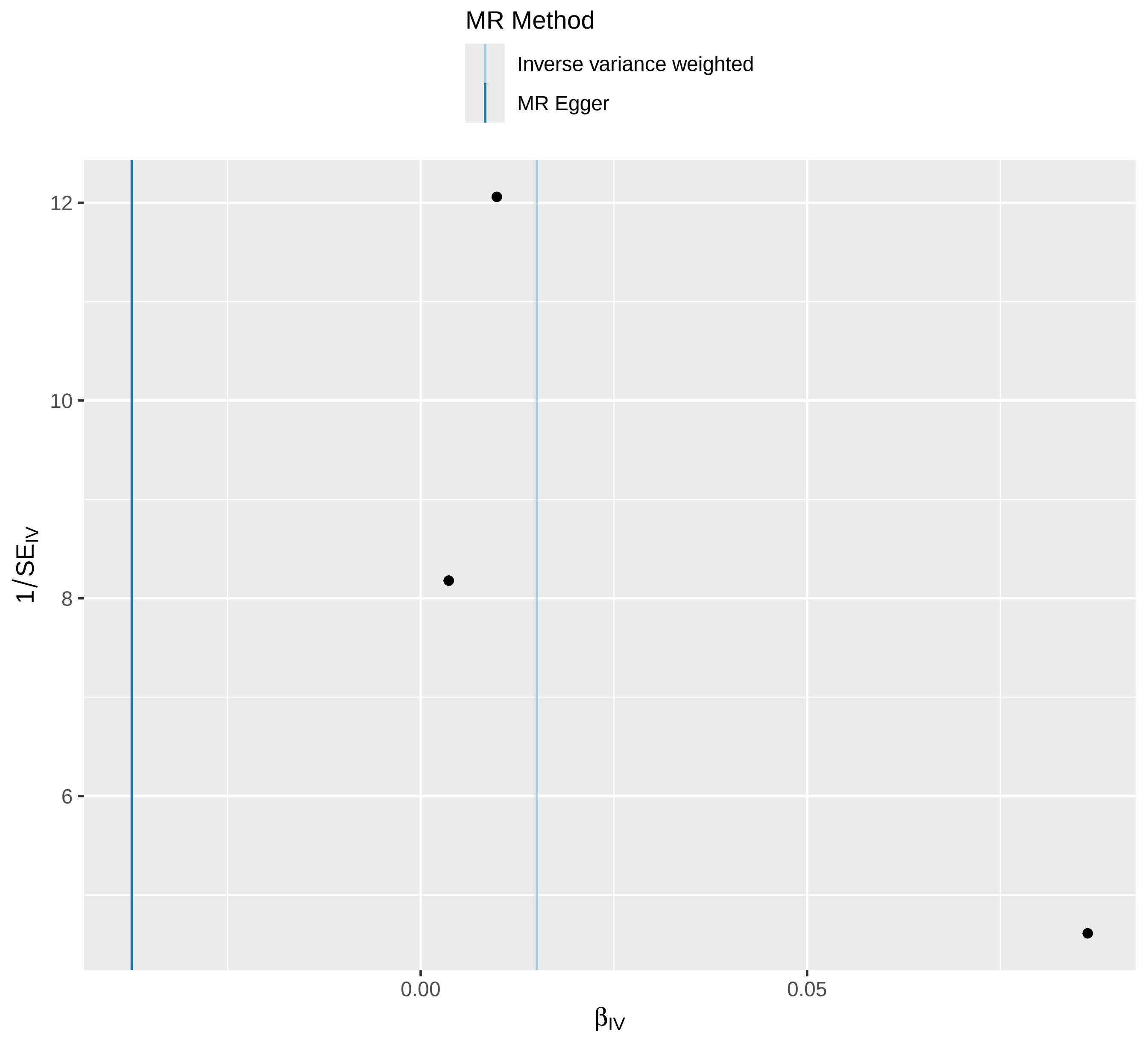

MR Method
Inverse variance weighted
MR Egger
12
10
8
6
1/SE_IV
0.00
0.05
β_IV

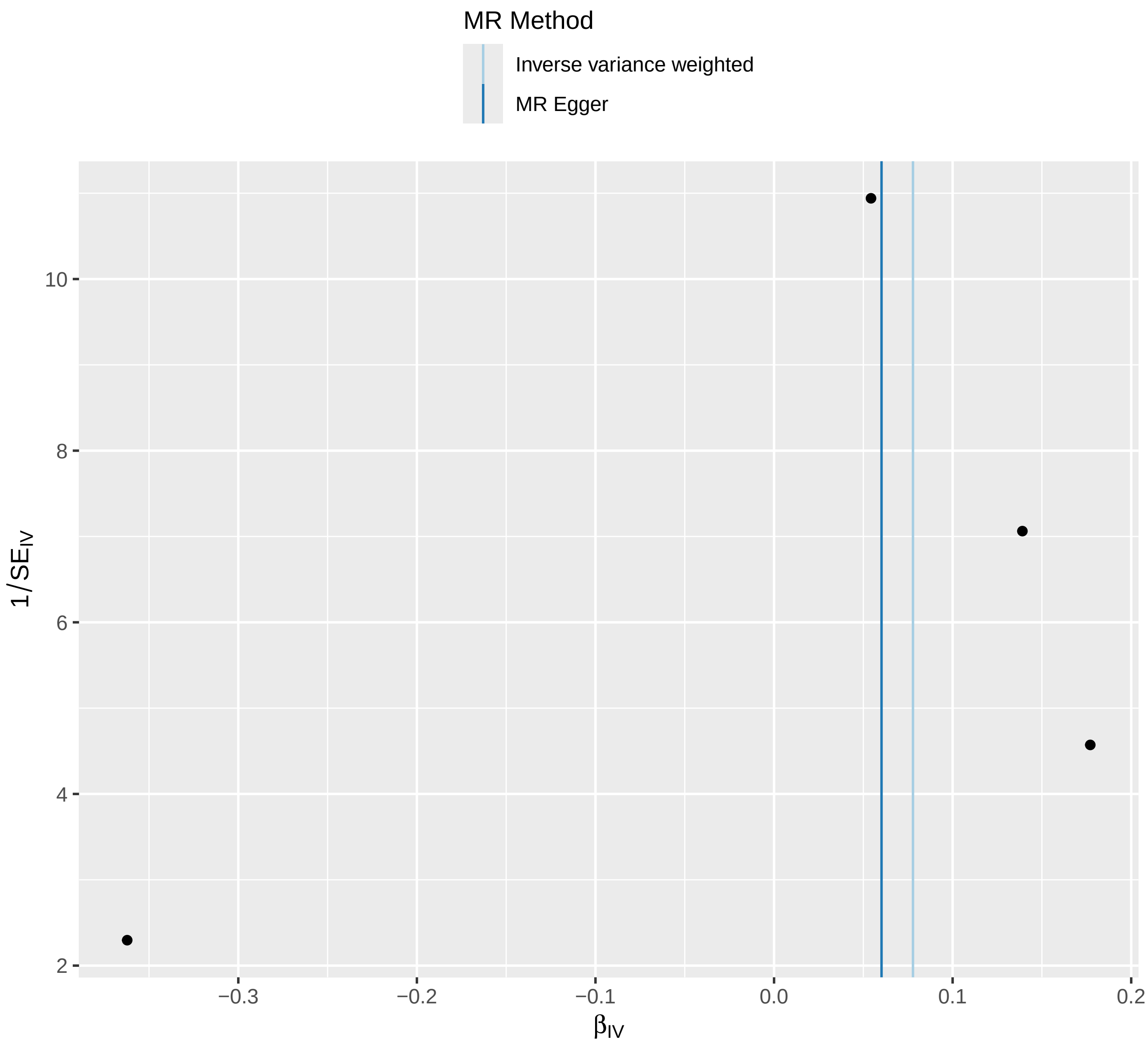
MR Method
Inverse variance weighted
MR Egger
1/SE_IV
10
8
6
4
2
β_IV
−0.3
−0.2
−0.1
0.0
0.1
0.2

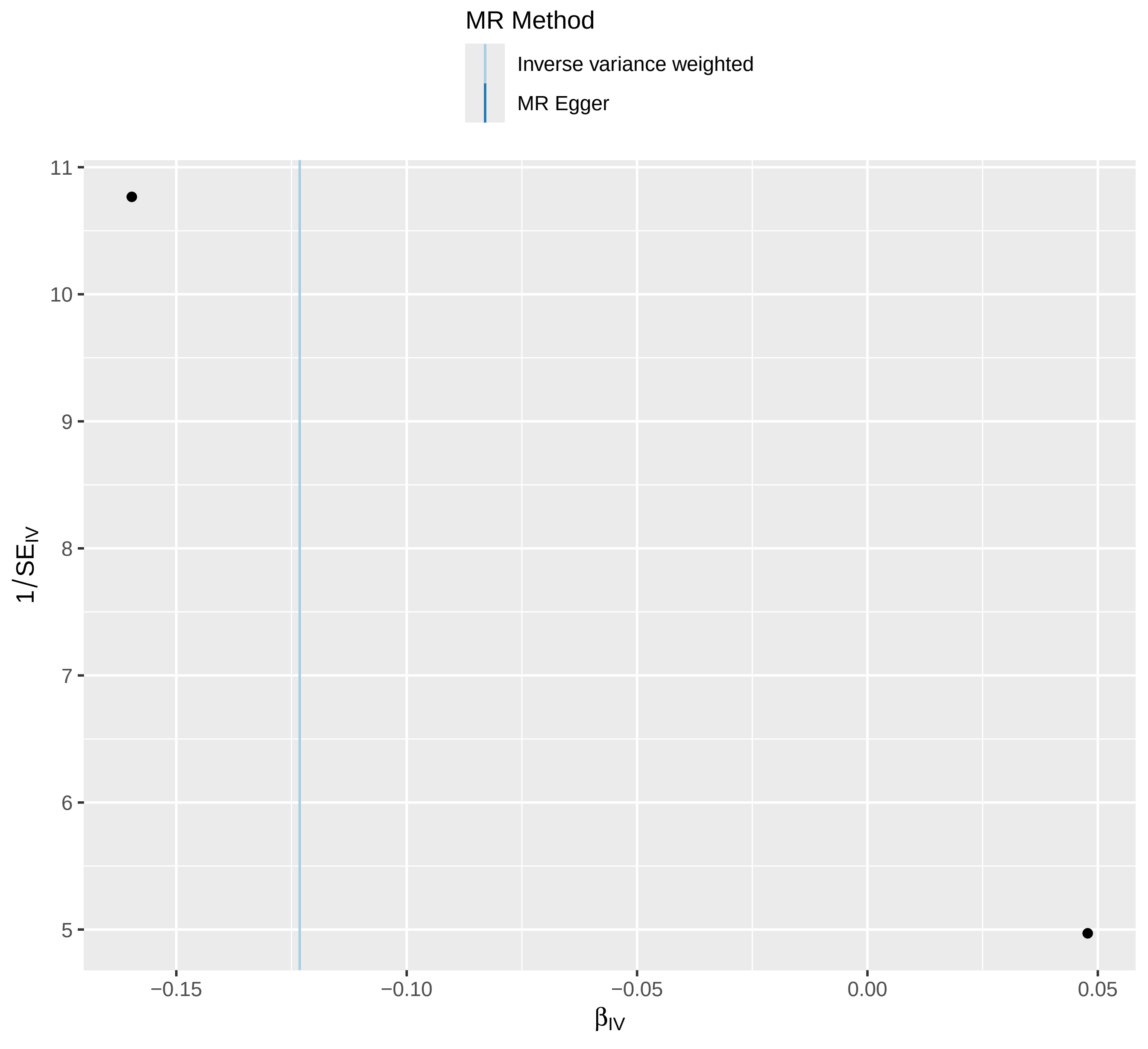
MR Method
Inverse variance weighted
MR Egger
11
10
9
8
7
6
5
1/SE$_{IV}$
−0.15
−0.10
−0.05
0.00
0.05
β$_{IV}$

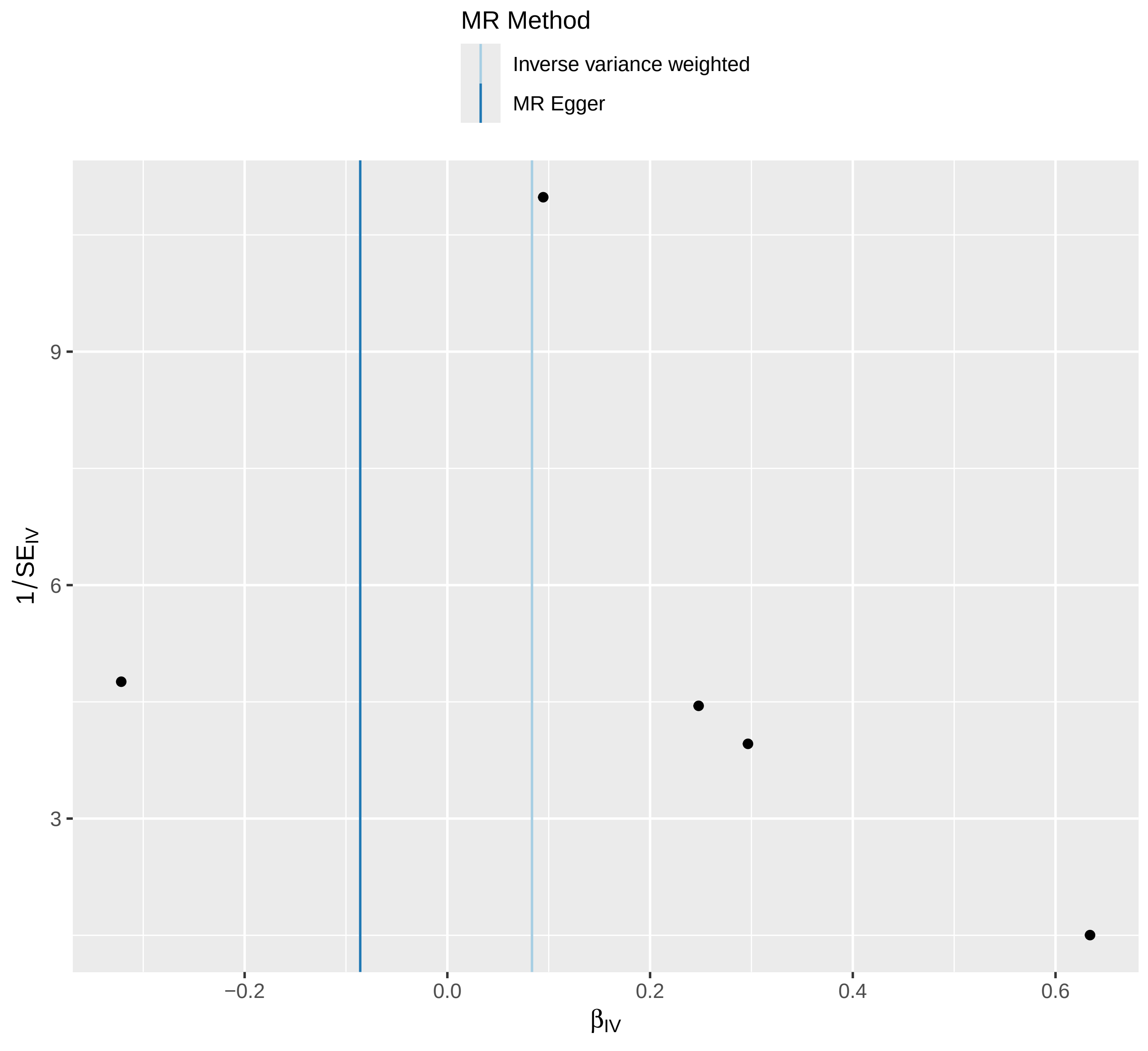

MR Method
Inverse variance weighted
MR Egger
9
6
3
1/SE_IV
−0.2
0.0
0.2
0.4
0.6
β_IV

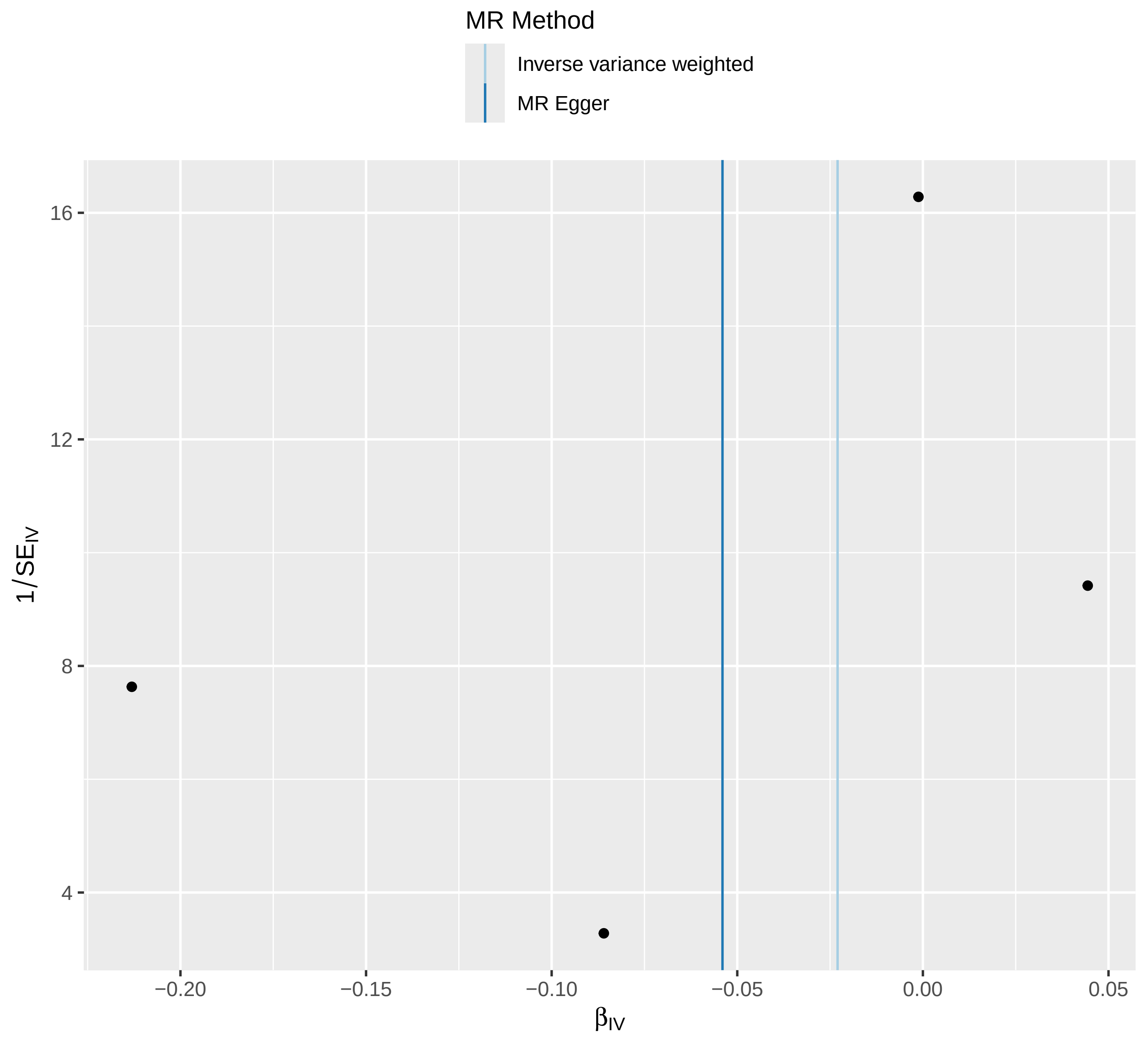
MR Method
Inverse variance weighted
MR Egger
16
12
8
4
1/SE$_{IV}$
−0.20
−0.15
−0.10
−0.05
0.00
0.05
β$_{IV}$

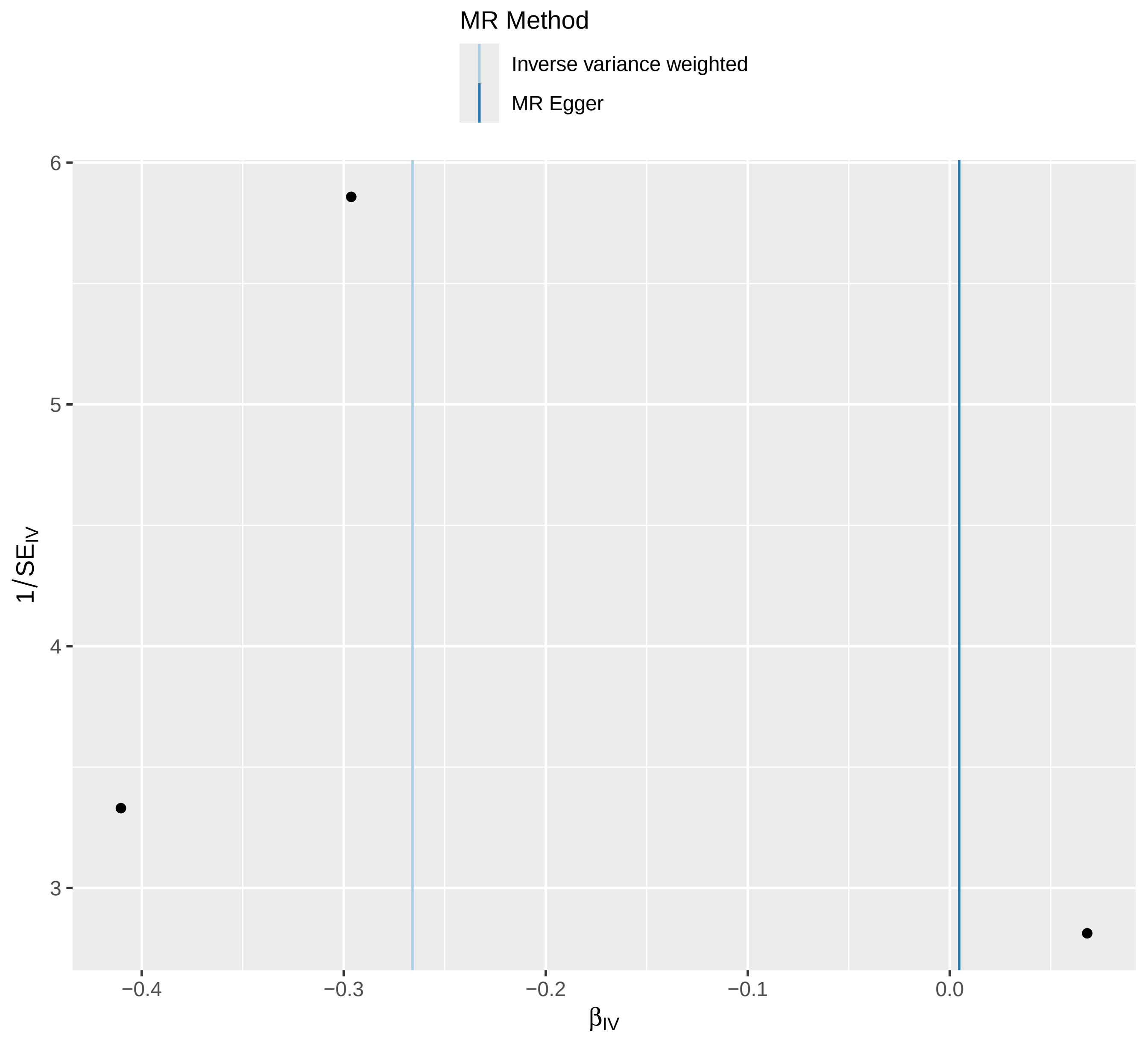
MR Method
Inverse variance weighted
MR Egger
6
5
4
3
1/SE_IV
−0.4
−0.3
−0.2
−0.1
0.0
β_IV

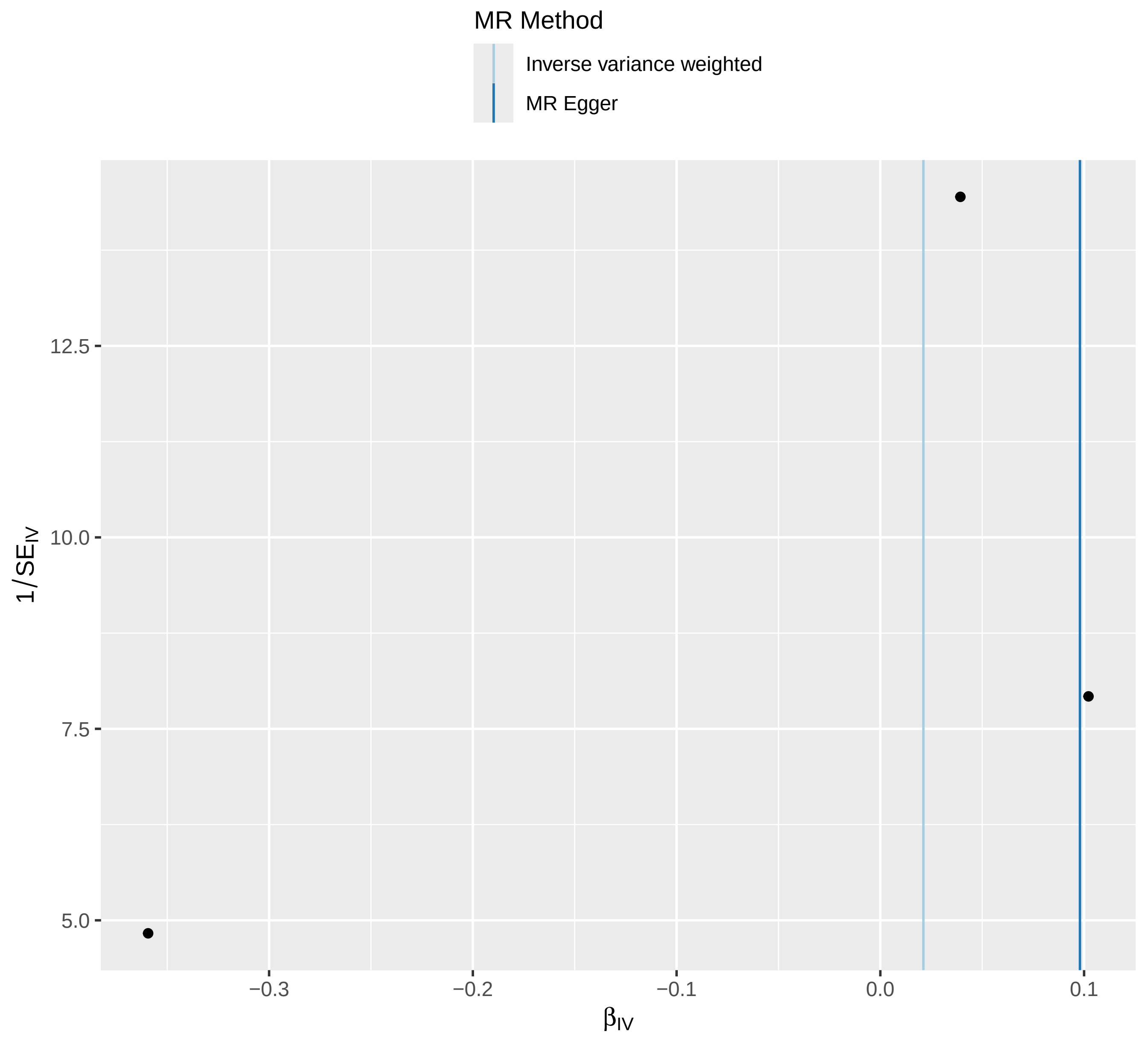

MR Method
Inverse variance weighted
MR Egger
12.5
10.0
7.5
5.0
1/SE_IV
−0.3
−0.2
−0.1
0.0
0.1
β_IV

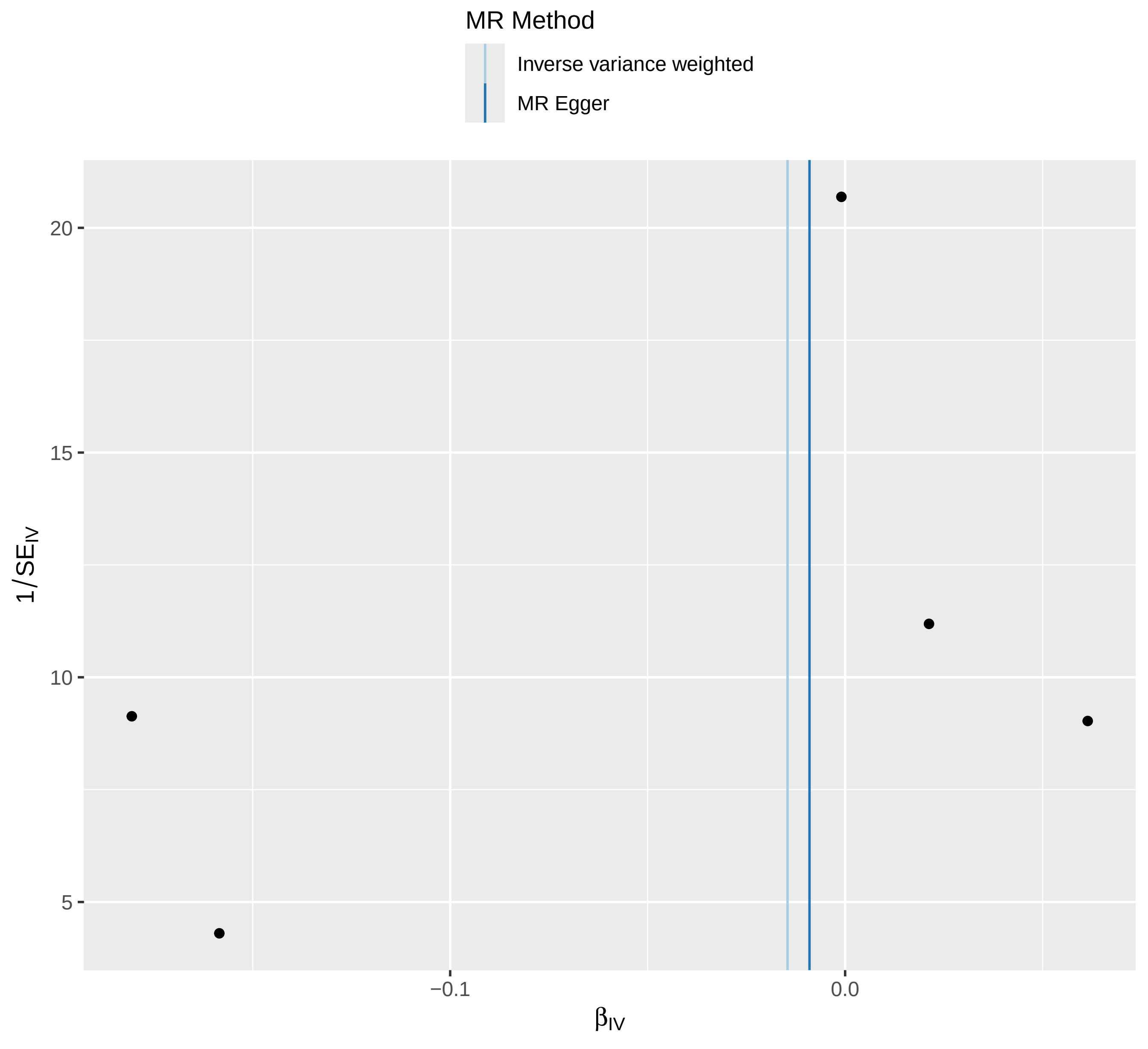

MR Method
Inverse variance weighted
MR Egger
20
15
10
5
1/SE$_{IV}$
−0.1
0.0
β$_{IV}$

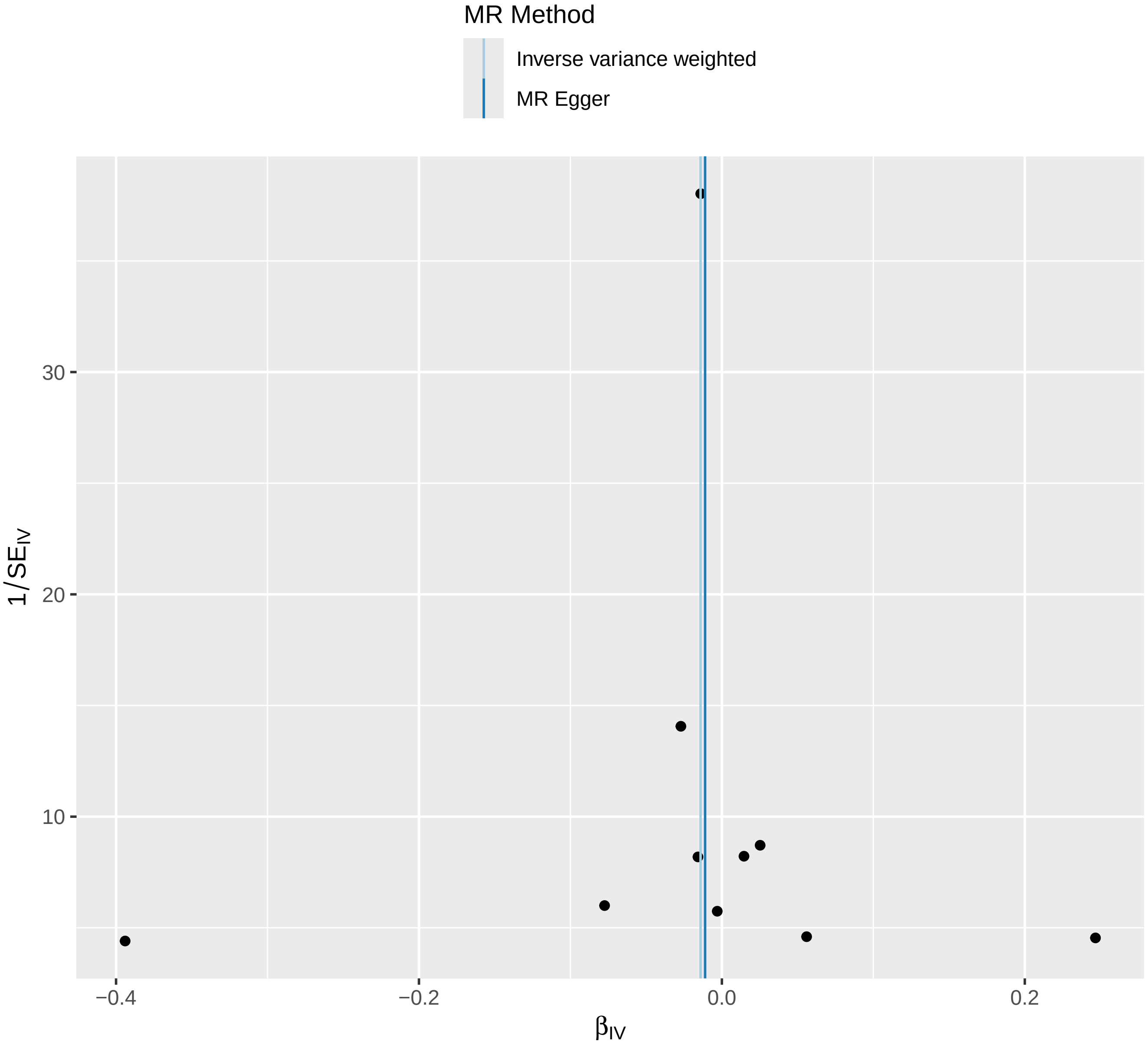
MR Method
Inverse variance weighted
MR Egger
30
20
10
1/SE$_{IV}$
−0.4
−0.2
0.0
0.2
β$_{IV}$

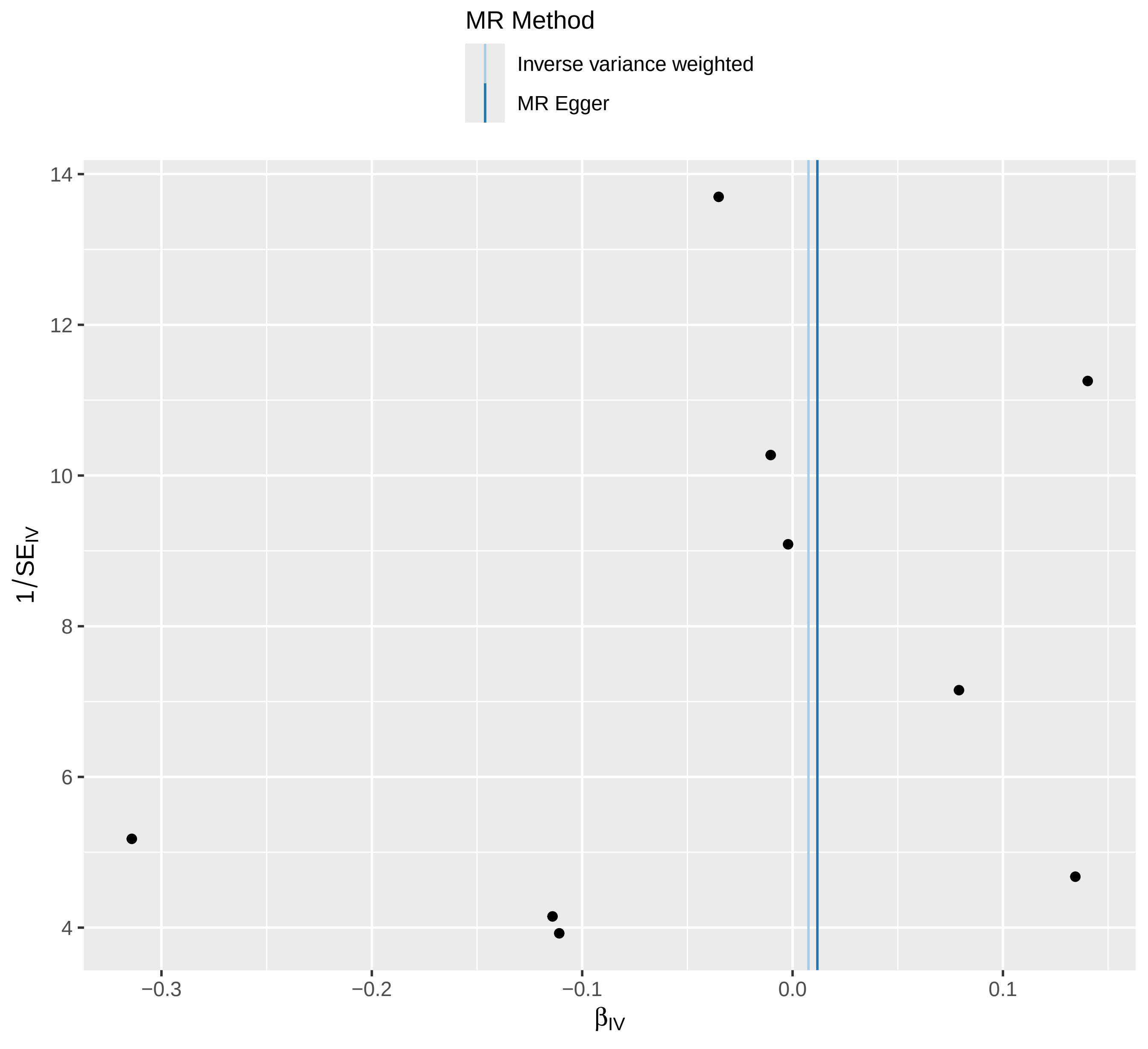
MR Method
Inverse variance weighted
MR Egger
14
12
10
8
6
4
−0.3
−0.2
−0.1
0.0
0.1
$1/SE_{IV}$
$\beta_{IV}$

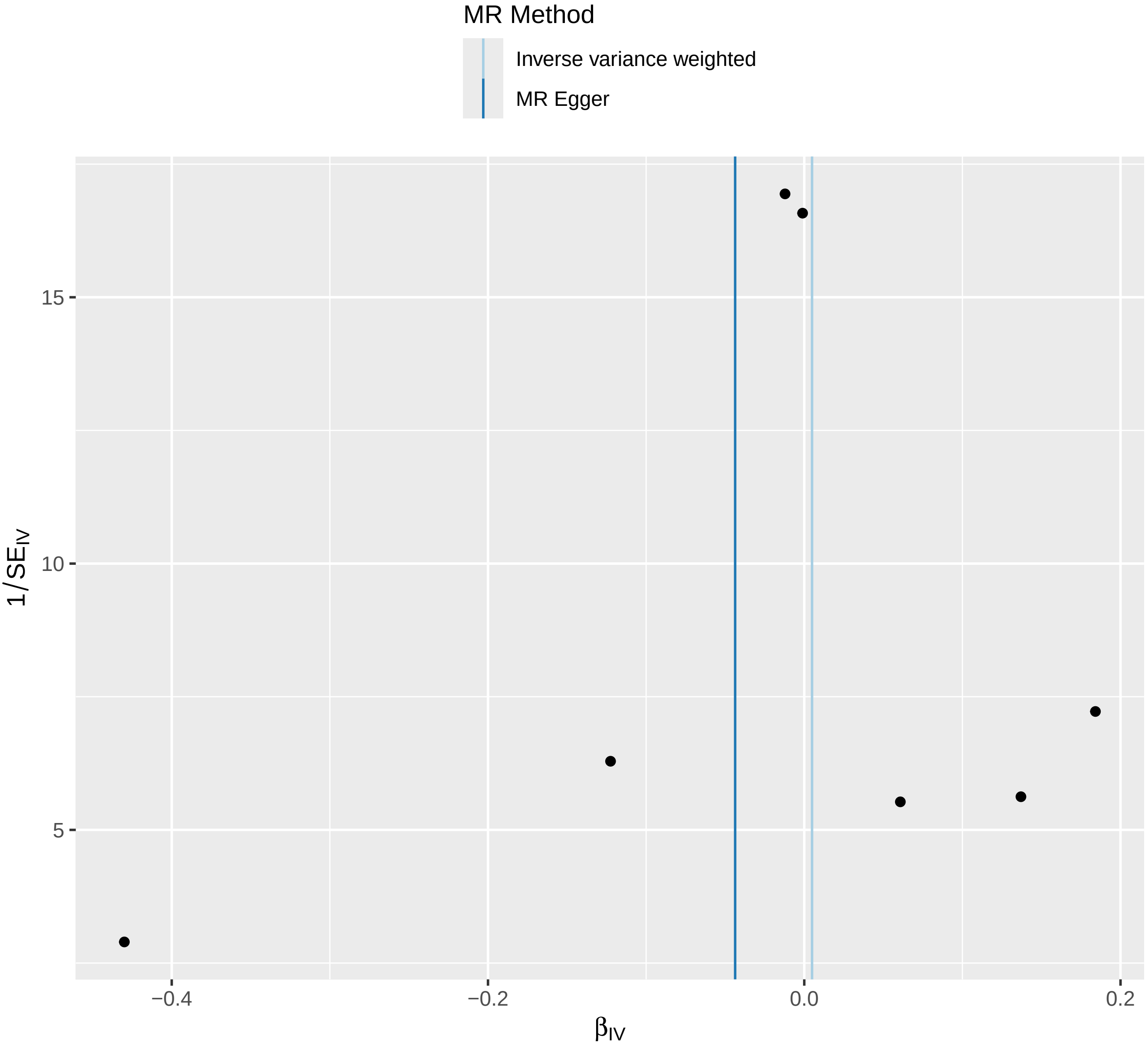

MR Method
Inverse variance weighted
MR Egger
15
10
5
1/SE_IV
−0.4
−0.2
0.0
0.2
β_IV

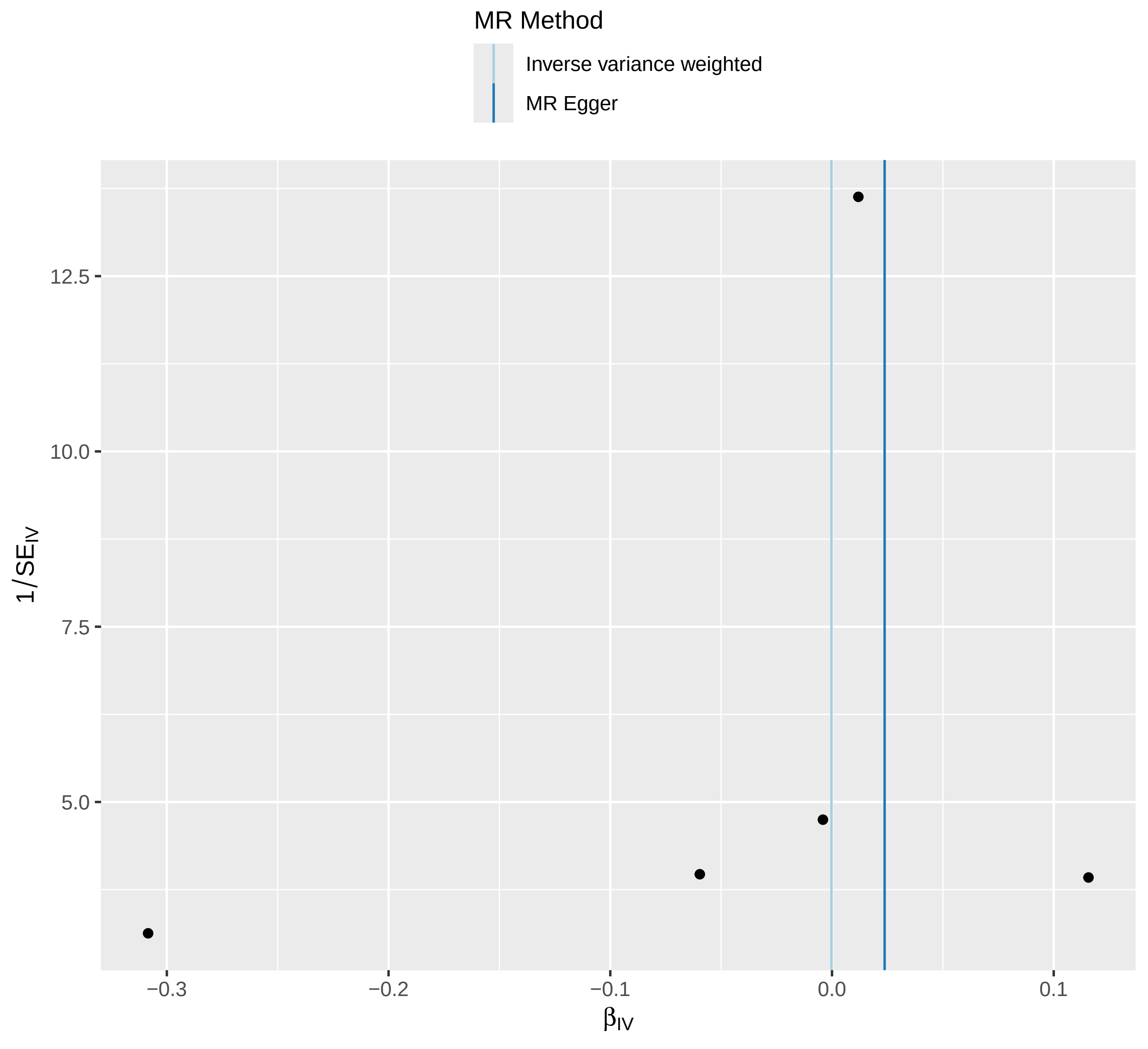
MR Method
Inverse variance weighted
MR Egger
12.5
10.0
7.5
5.0
−0.3
−0.2
−0.1
0.0
0.1
1/SE_IV
β_IV

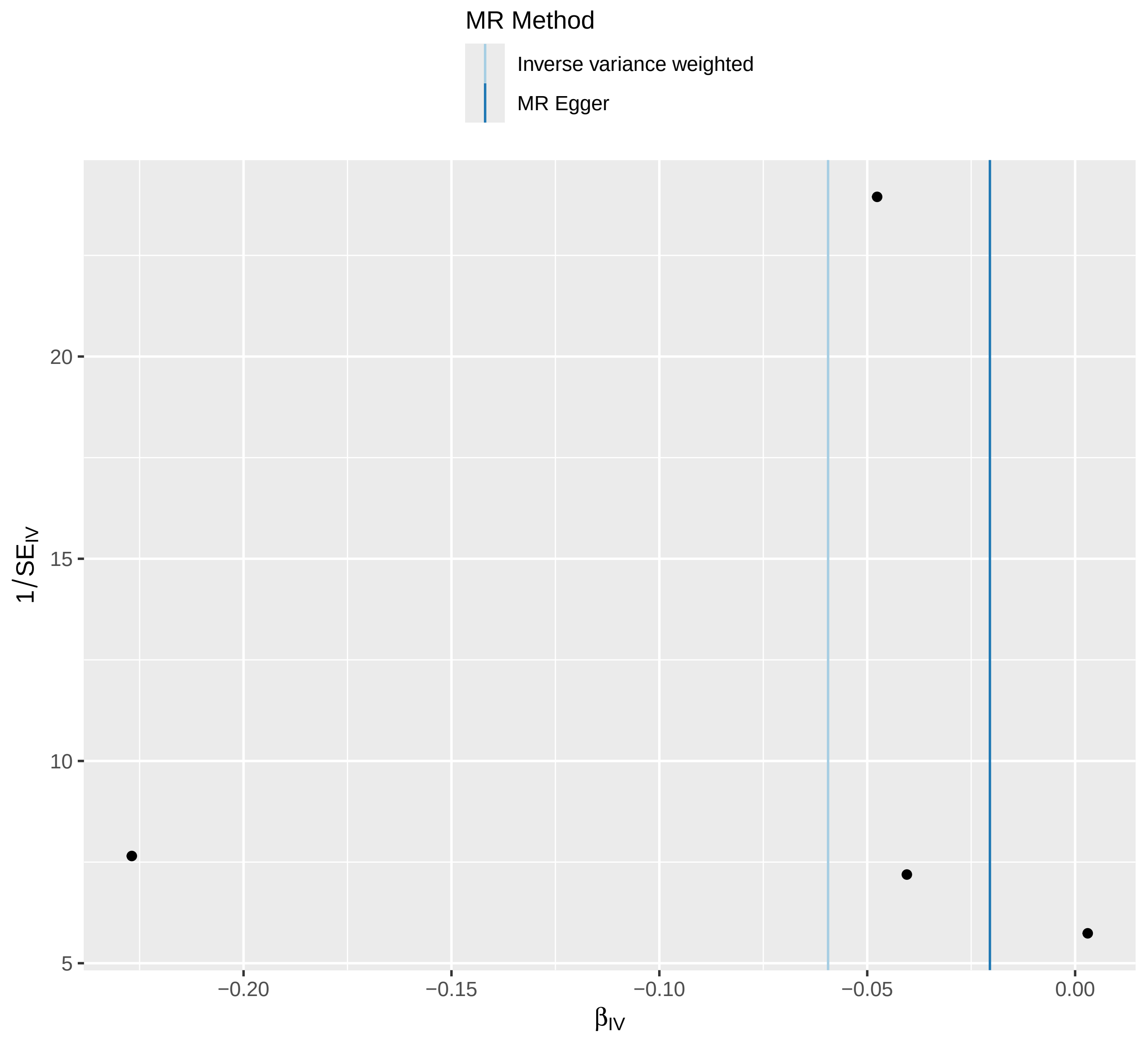
MR Method
Inverse variance weighted
MR Egger
20
15
10
5
1/SE$_{IV}$
−0.20
−0.15
−0.10
−0.05
0.00
β$_{IV}$

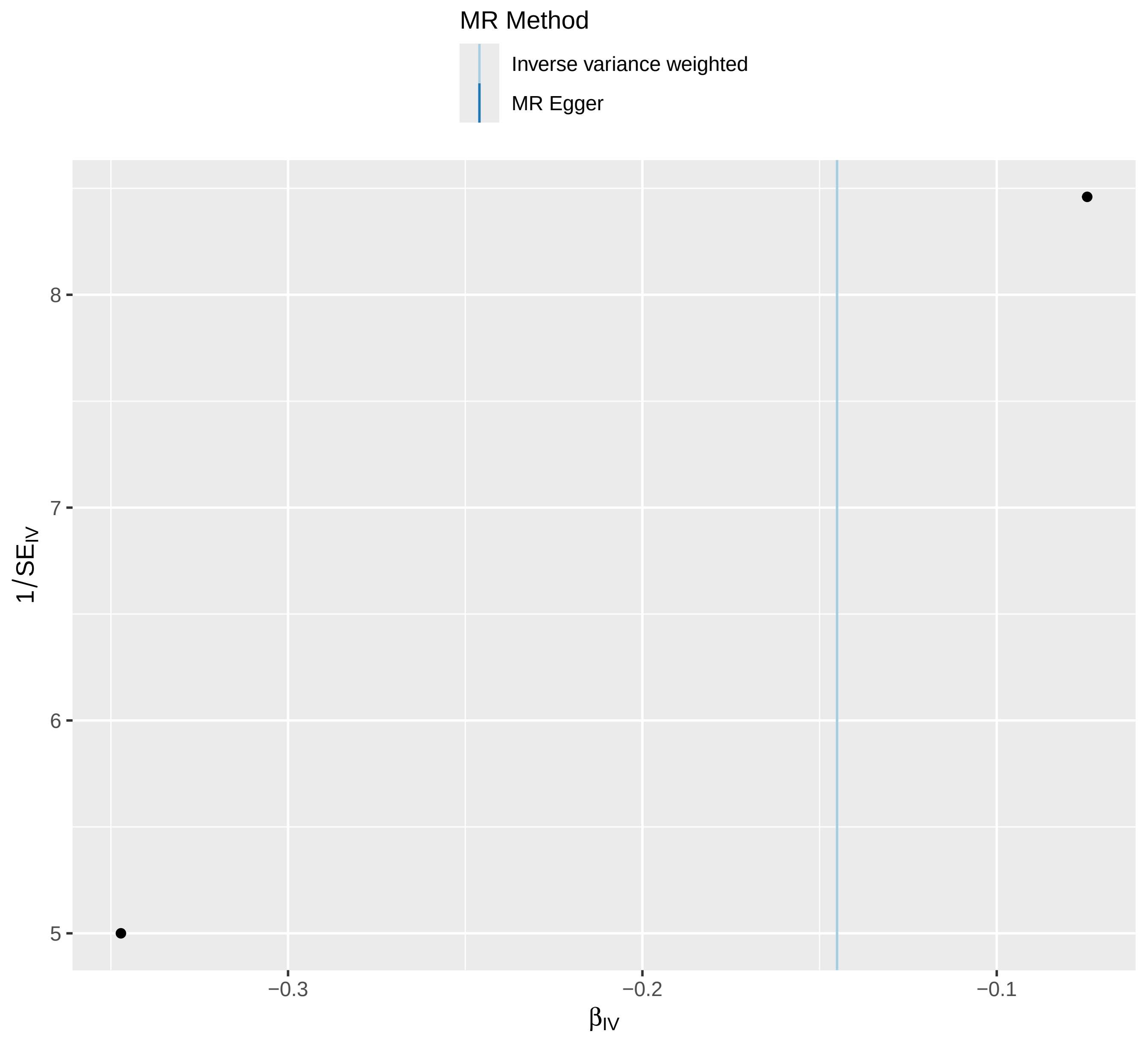
MR Method
Inverse variance weighted
MR Egger
1/SE_IV
β_IV
8
7
6
5
−0.3
−0.2
−0.1

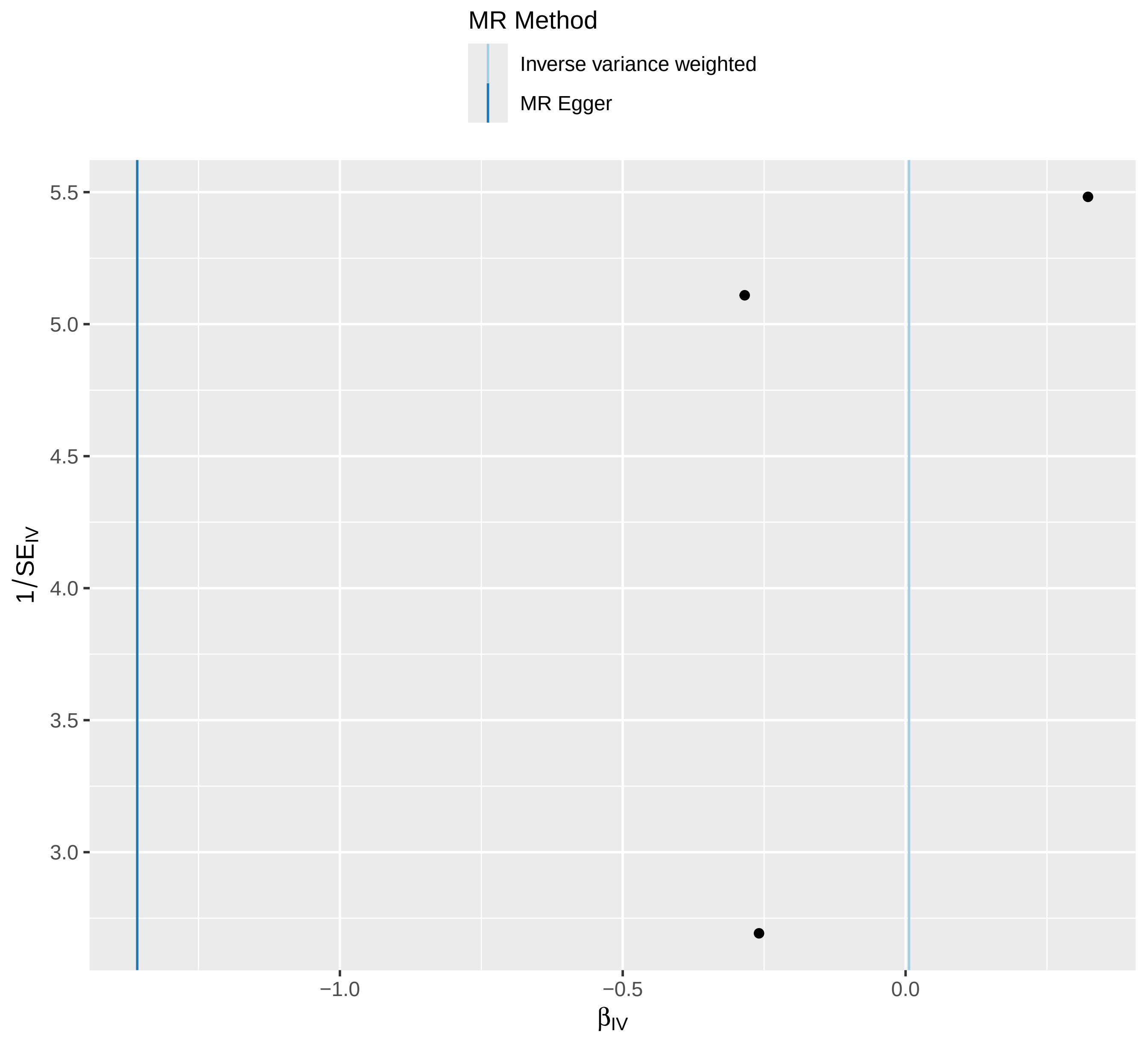

MR Method
Inverse variance weighted
MR Egger
5.5
5.0
4.5
4.0
3.5
3.0
$1/SE_{IV}$
−1.0
−0.5
0.0
$\beta_{IV}$

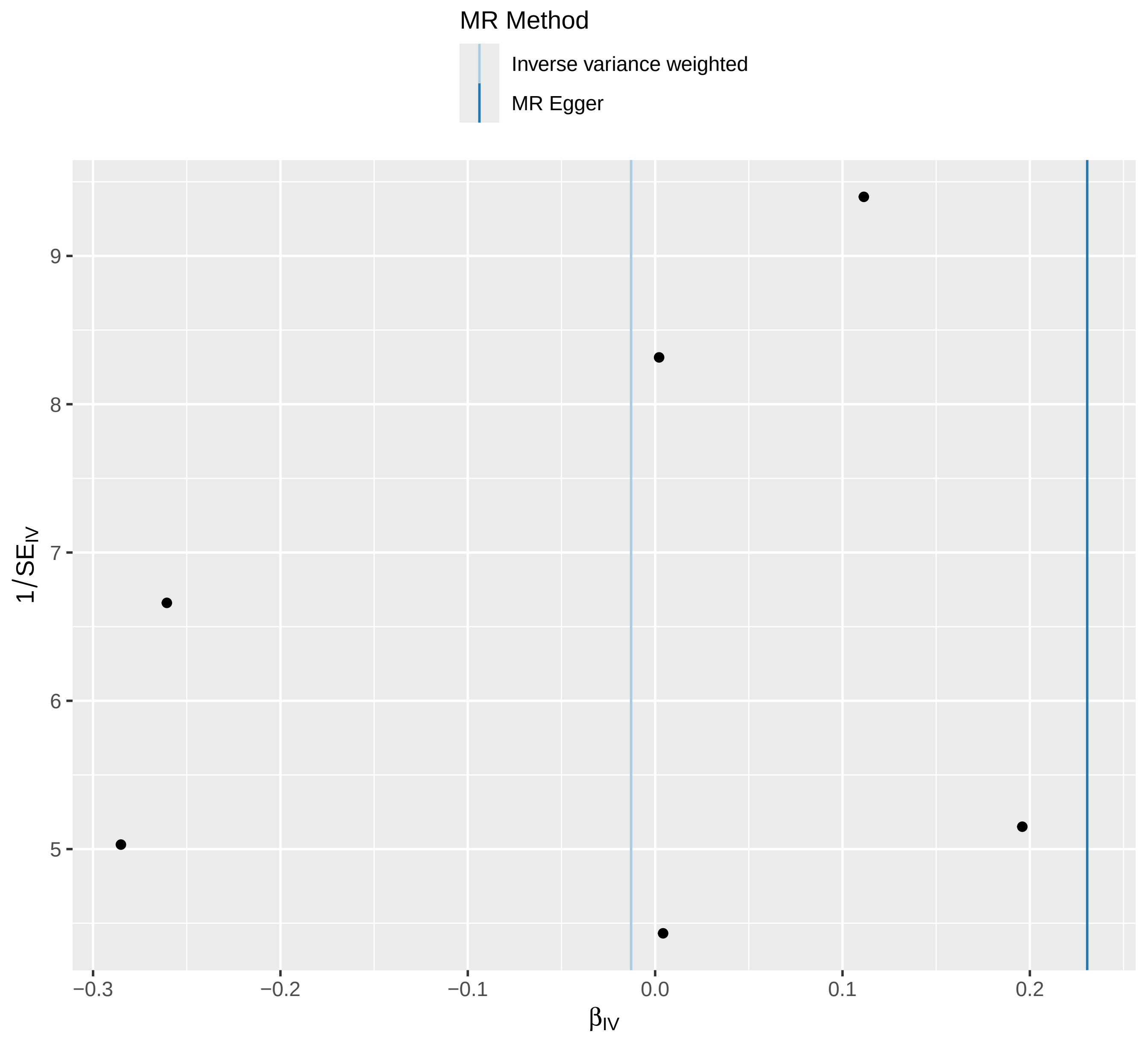
MR Method
Inverse variance weighted
MR Egger
9
8
7
6
5
1/SE$_{IV}$
−0.3
−0.2
−0.1
0.0
0.1
0.2
β$_{IV}$

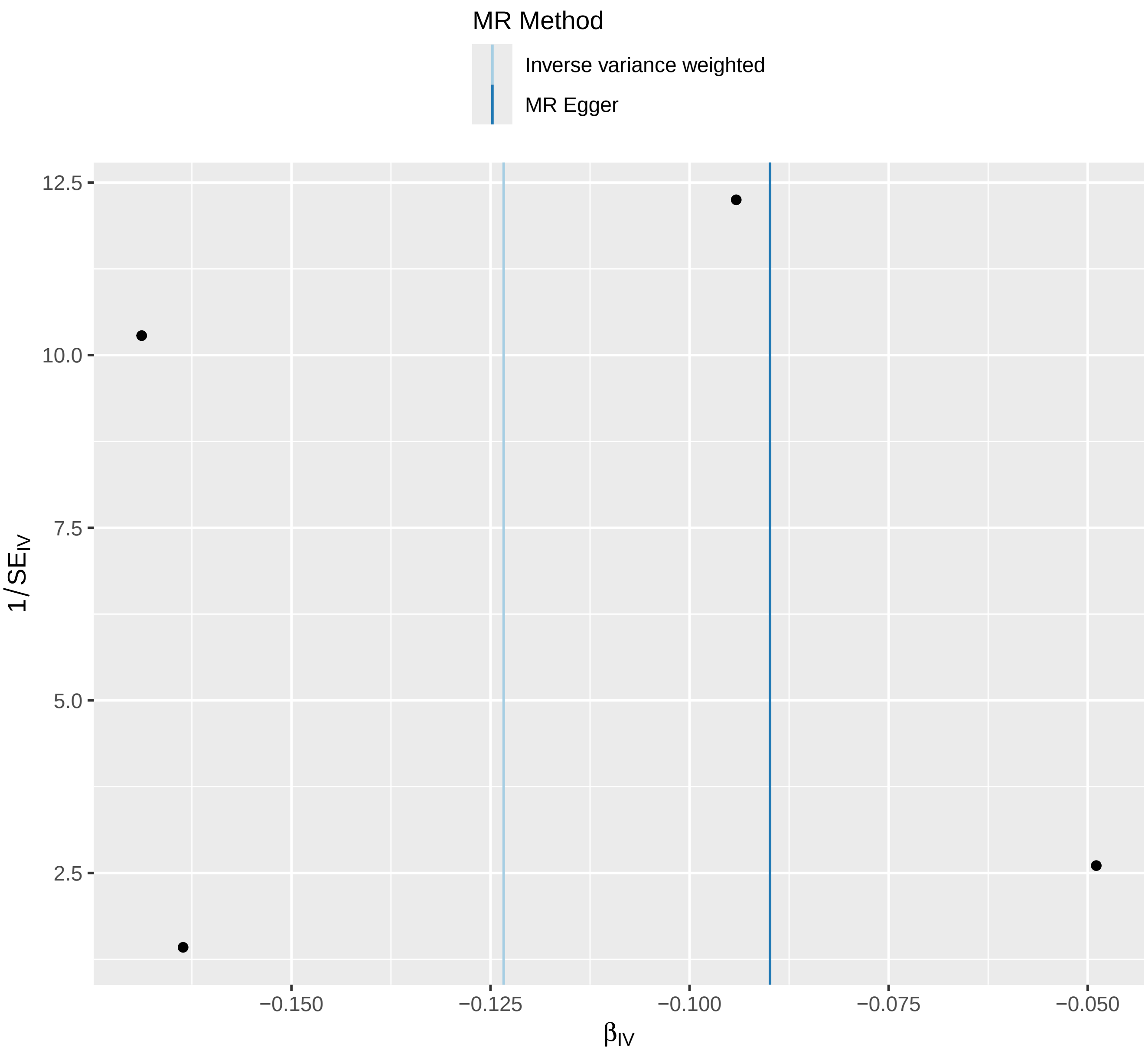

MR Method
Inverse variance weighted
MR Egger
12.5
10.0
7.5
5.0
2.5
1/SE$_{IV}$
−0.150
−0.125
−0.100
−0.075
−0.050
β$_{IV}$

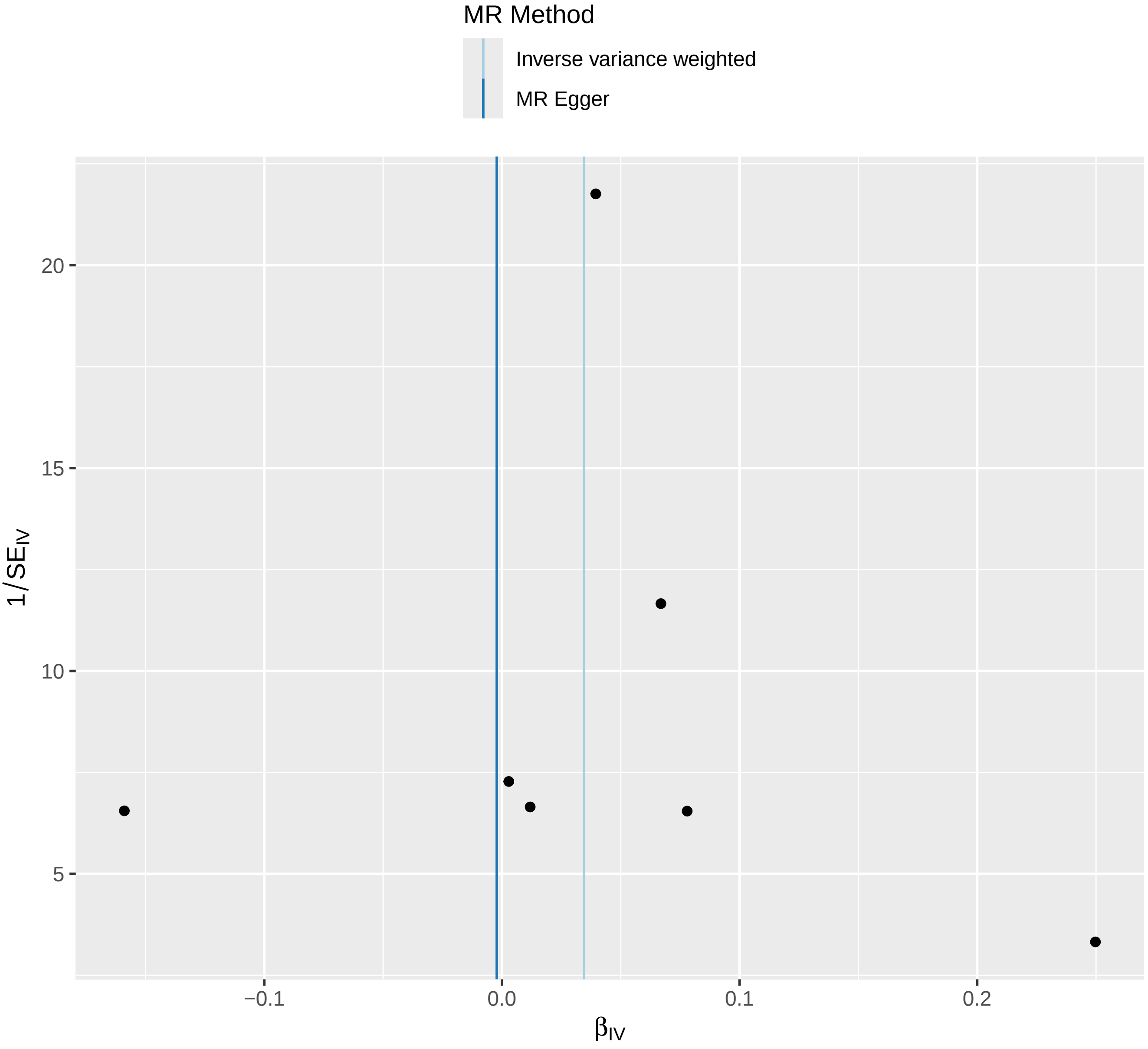
MR Method
Inverse variance weighted
MR Egger
20
15
10
5
$1/SE_{IV}$
−0.1
0.0
0.1
0.2
$\beta_{IV}$

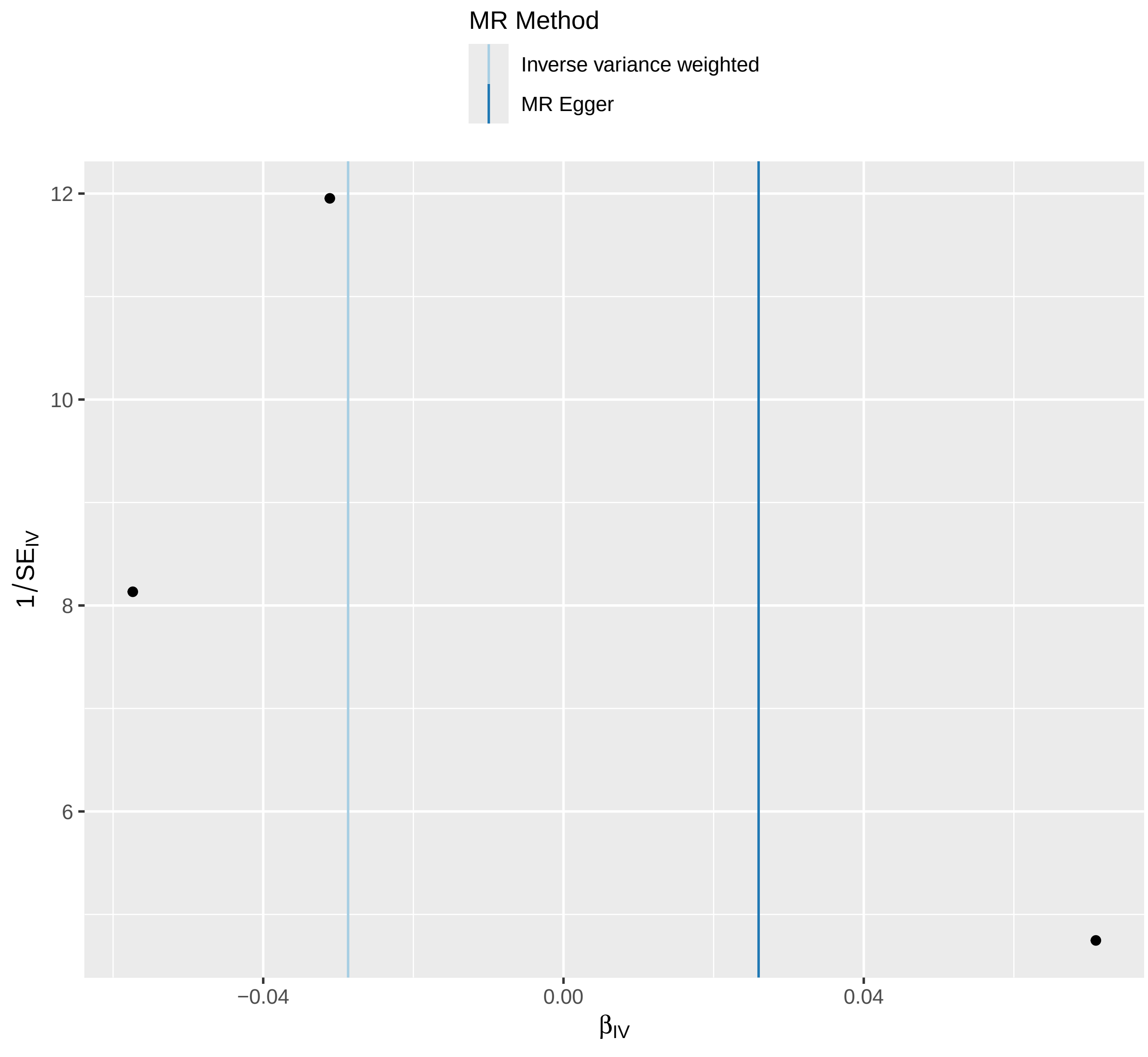
MR Method
Inverse variance weighted
MR Egger
12
10
8
6
1/$SE_{IV}$
−0.04
0.00
0.04
$\beta_{IV}$

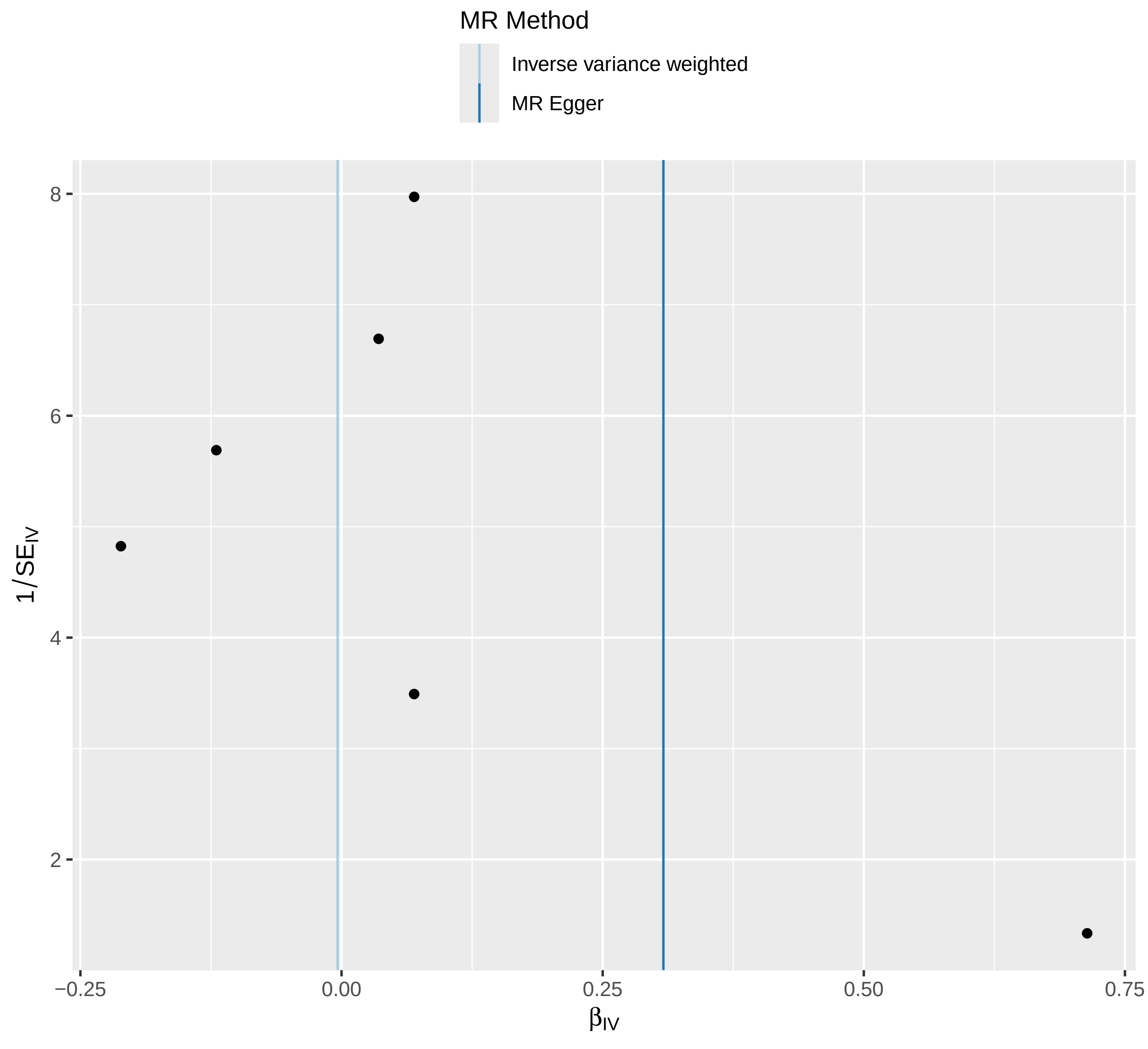
MR Method
Inverse variance weighted
MR Egger
8
6
4
2
$1/SE_{IV}$
−0.25
0.00
0.25
0.50
0.75
$\beta_{IV}$

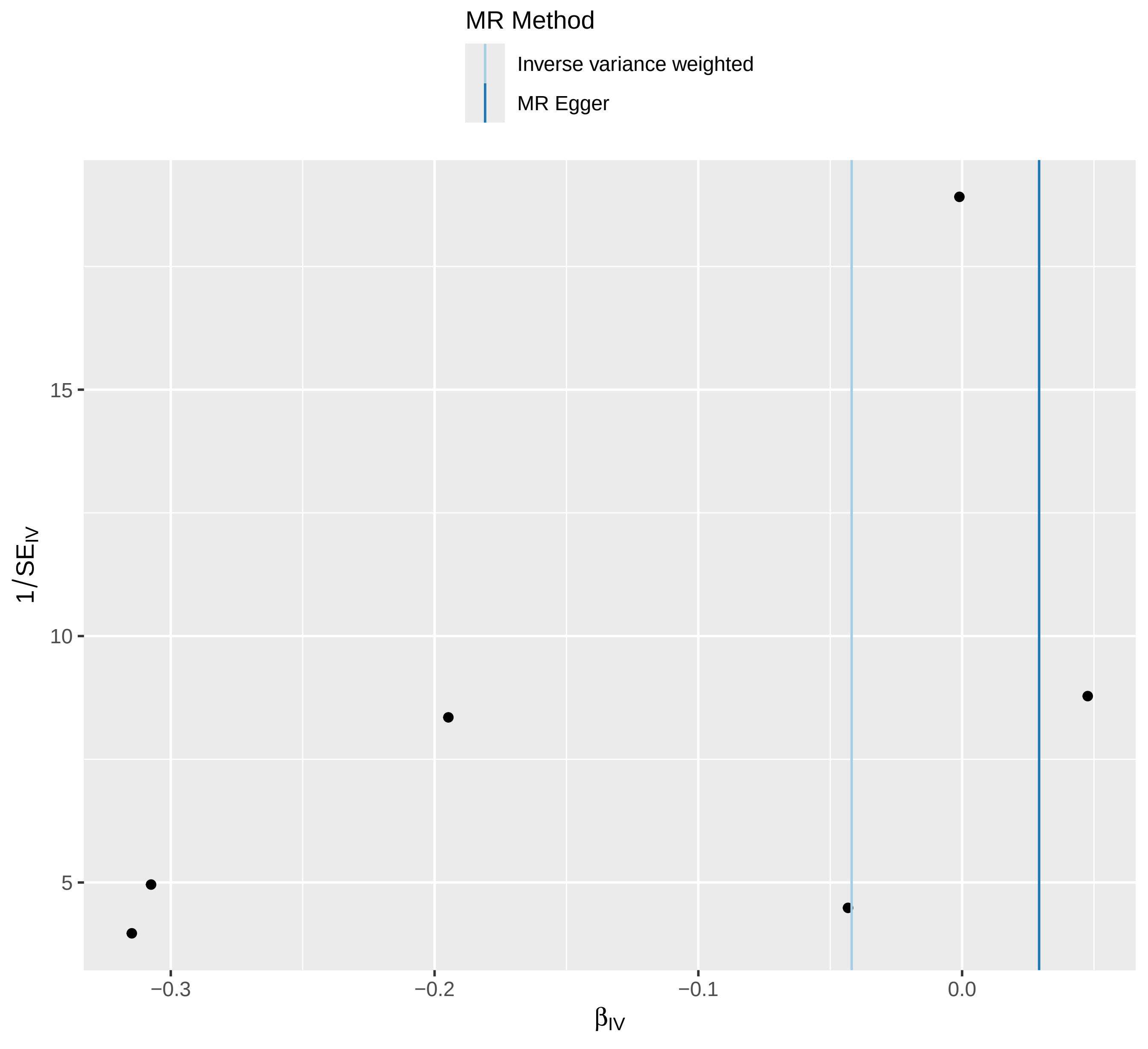

MR Method
Inverse variance weighted
MR Egger
15
10
5
1/SE$_{IV}$
−0.3
−0.2
−0.1
0.0
β$_{IV}$

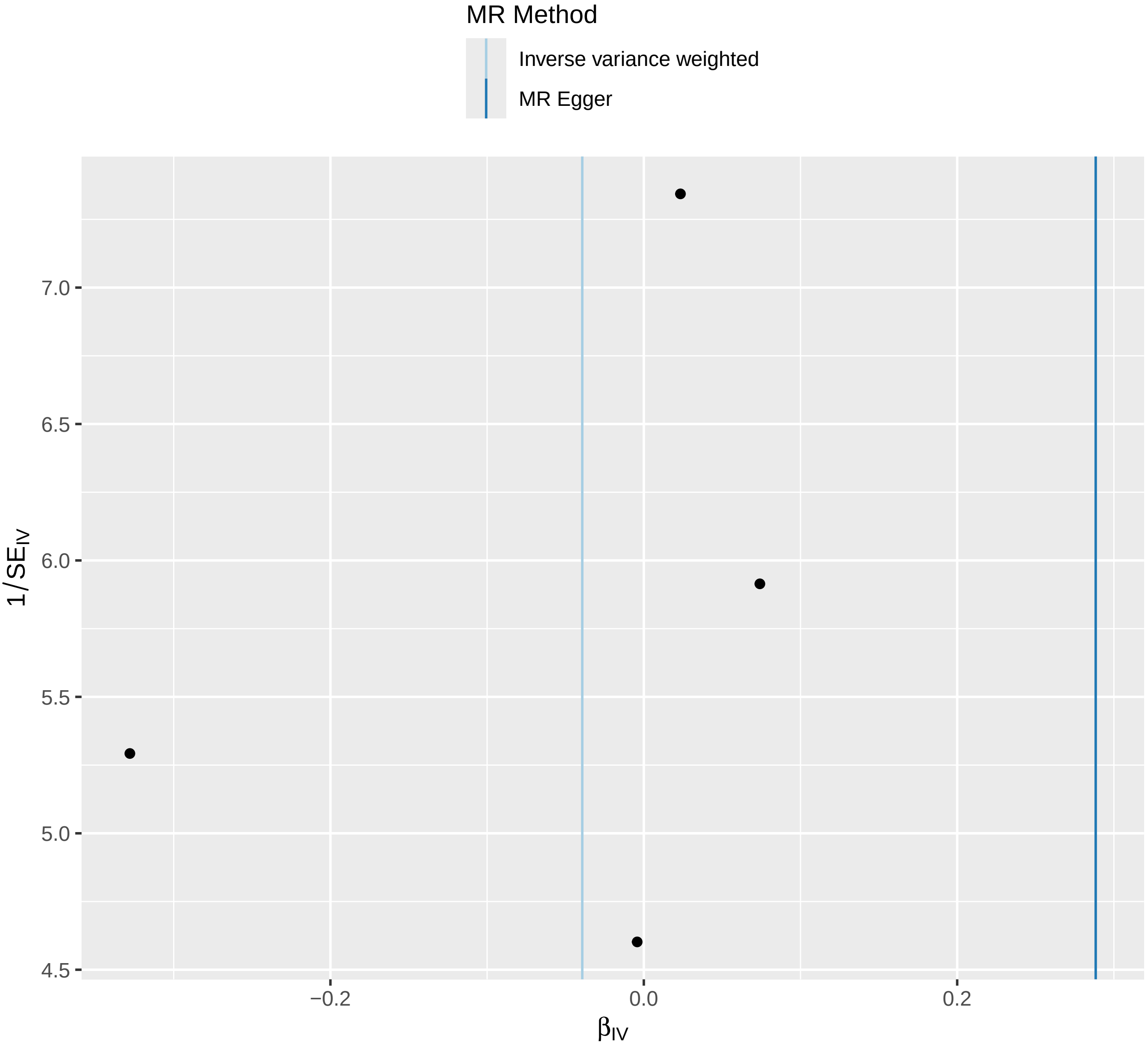
MR Method
Inverse variance weighted
MR Egger
1/SE_IV
7.0
6.5
6.0
5.5
5.0
4.5
−0.2
0.0
0.2
β_IV

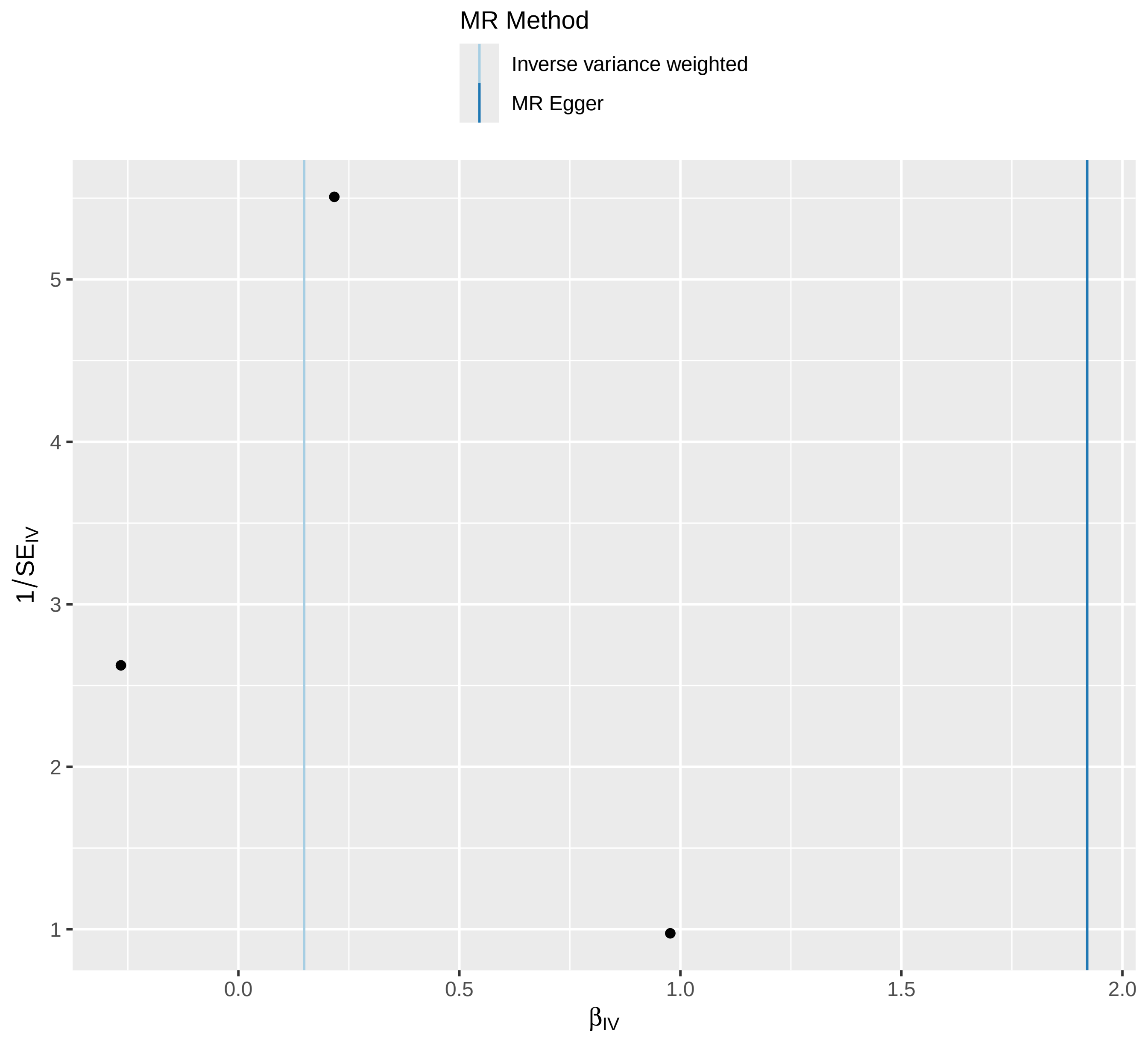

MR Method
Inverse variance weighted
MR Egger
5
4
3
2
1
1/SE$_{IV}$
0.0
0.5
1.0
1.5
2.0
β$_{IV}$

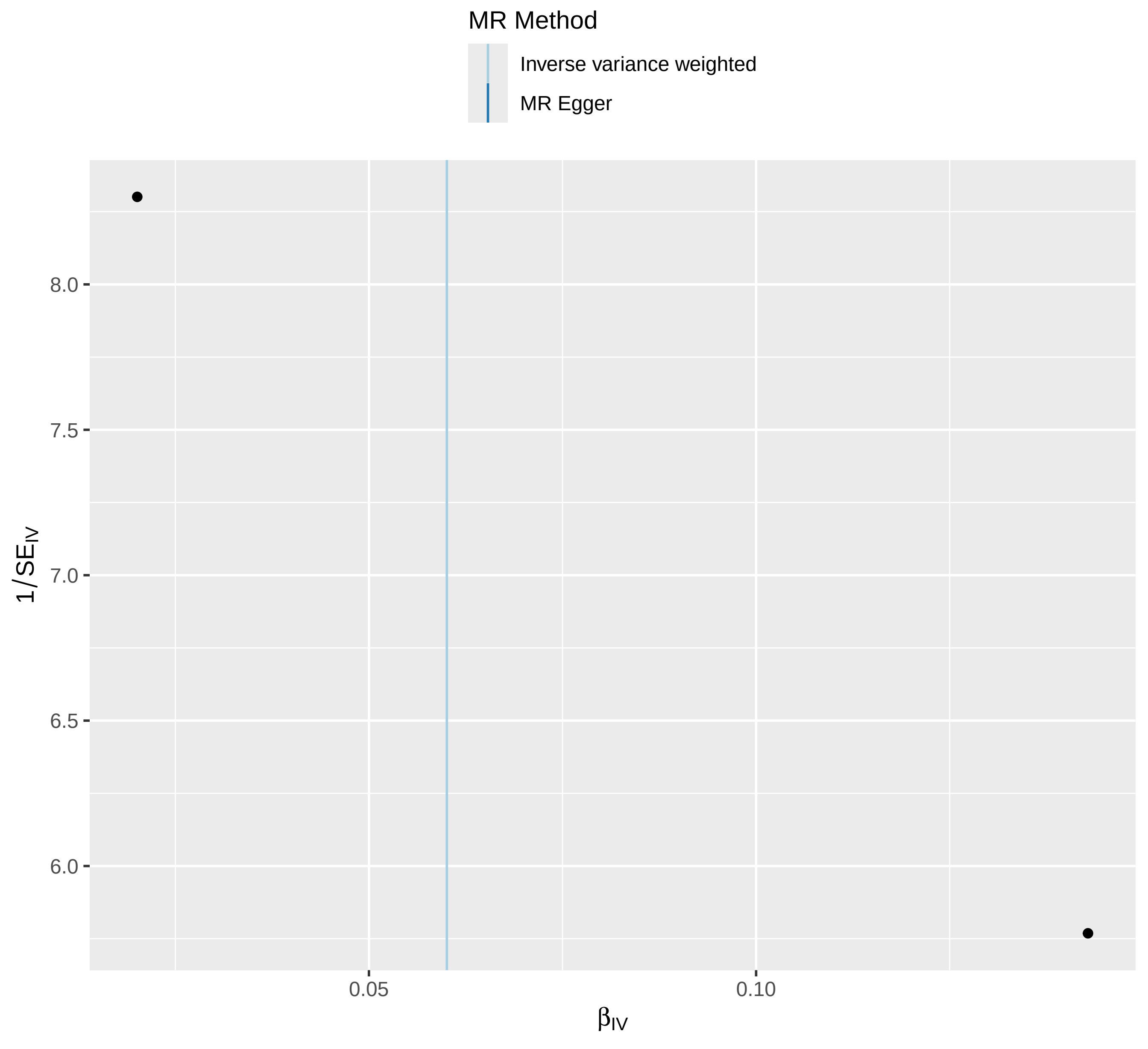
MR Method
Inverse variance weighted
MR Egger
8.0
7.5
7.0
6.5
6.0
1/SE_IV
0.05
0.10
β_IV

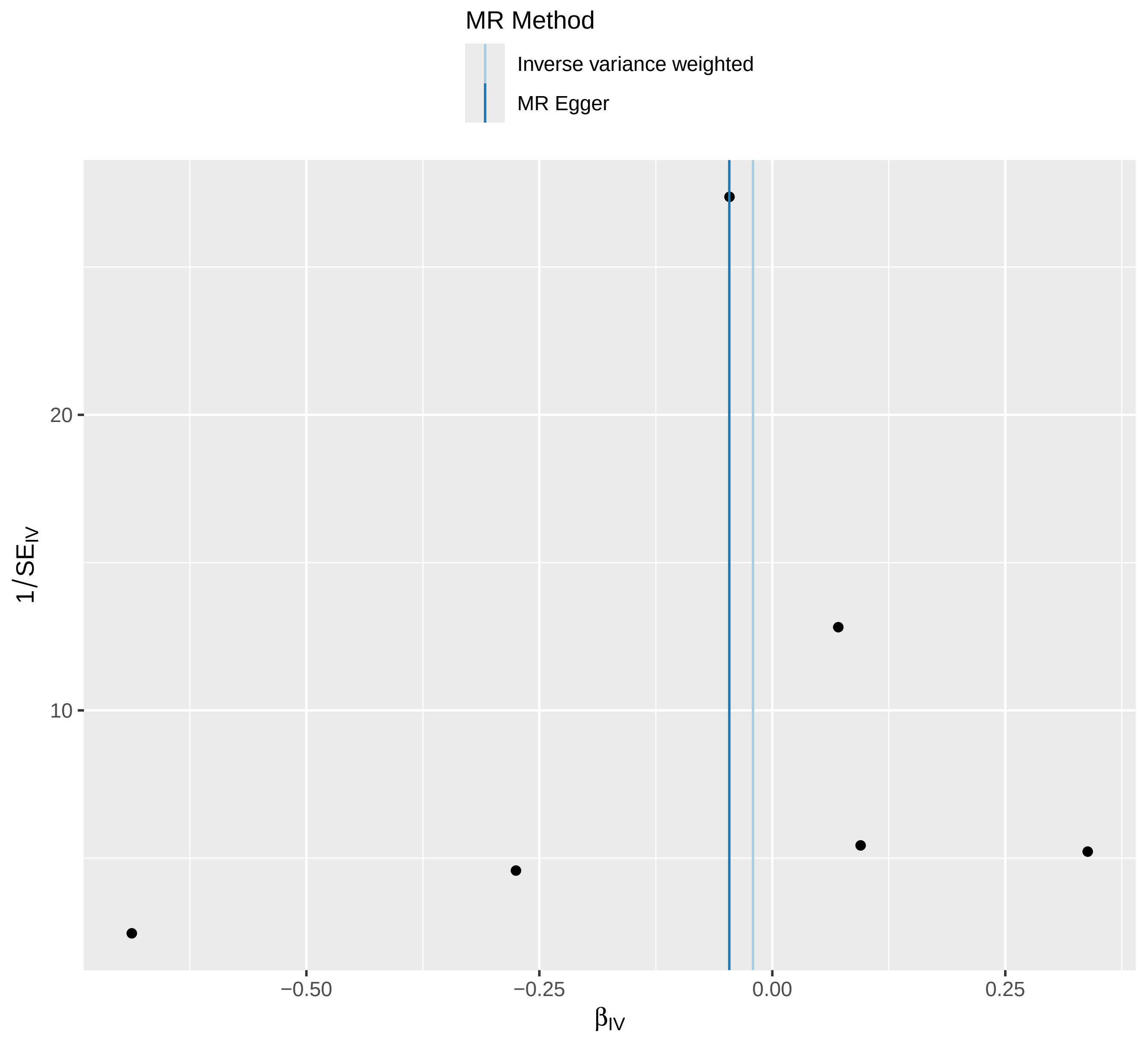

MR Method
Inverse variance weighted
MR Egger
20
10
1/SE$_{IV}$
−0.50
−0.25
0.00
0.25
β$_{IV}$

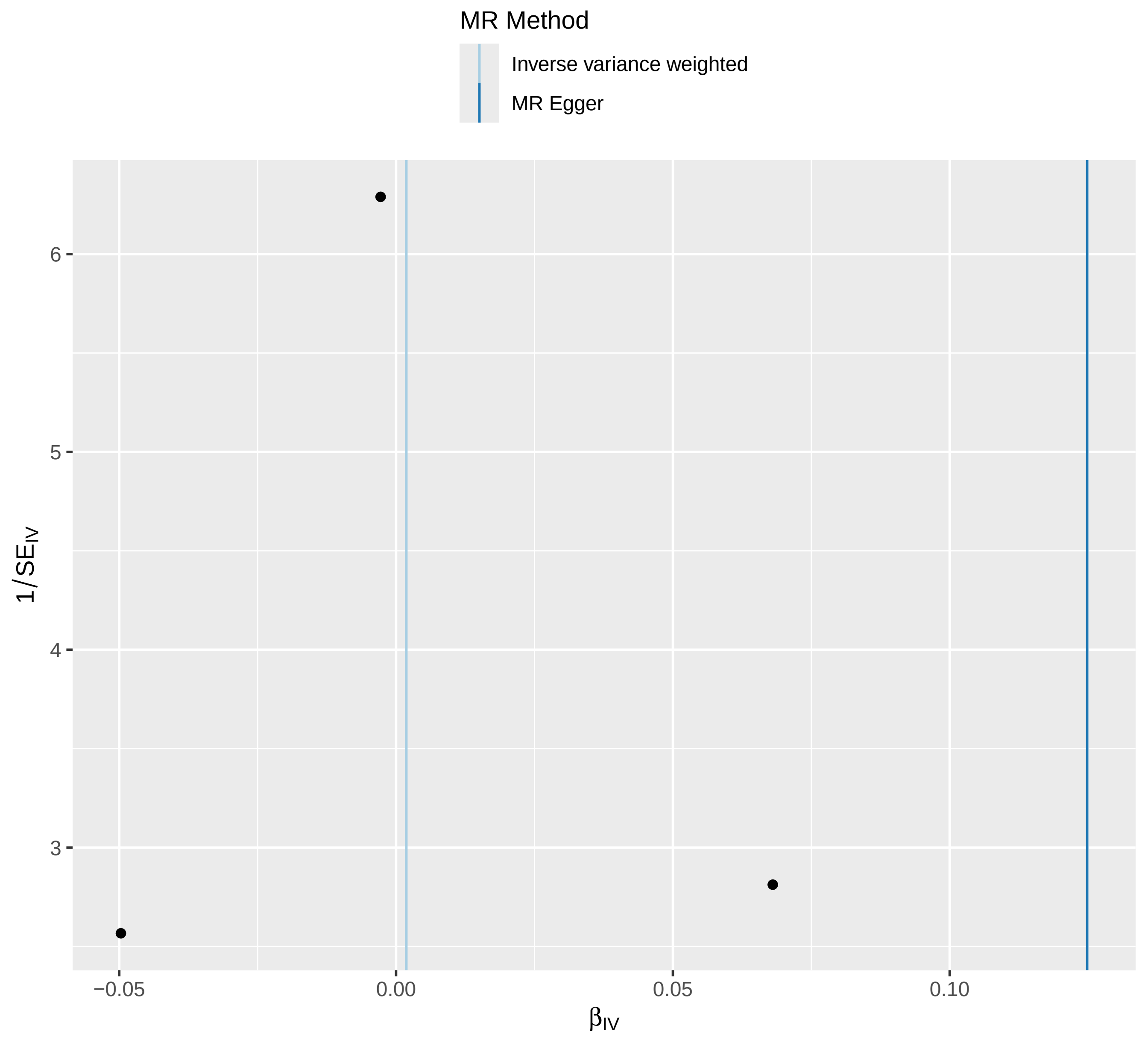

MR Method
Inverse variance weighted
MR Egger
6
5
4
3
1/SE$_{IV}$
−0.05
0.00
0.05
0.10
β$_{IV}$

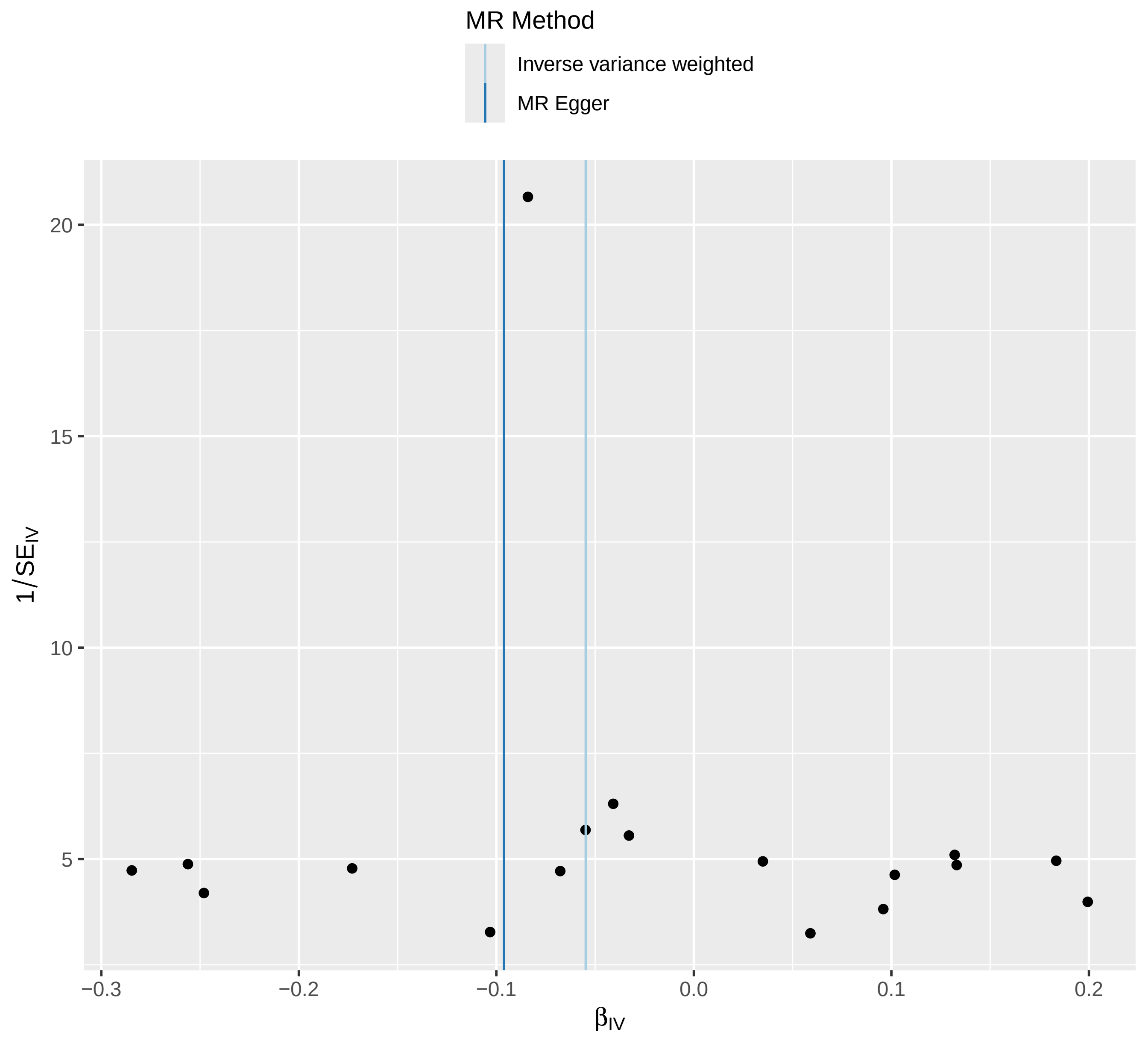
MR Method
Inverse variance weighted
MR Egger
20
15
10
5
1/SE_IV
−0.3
−0.2
−0.1
0.0
0.1
0.2
β_IV

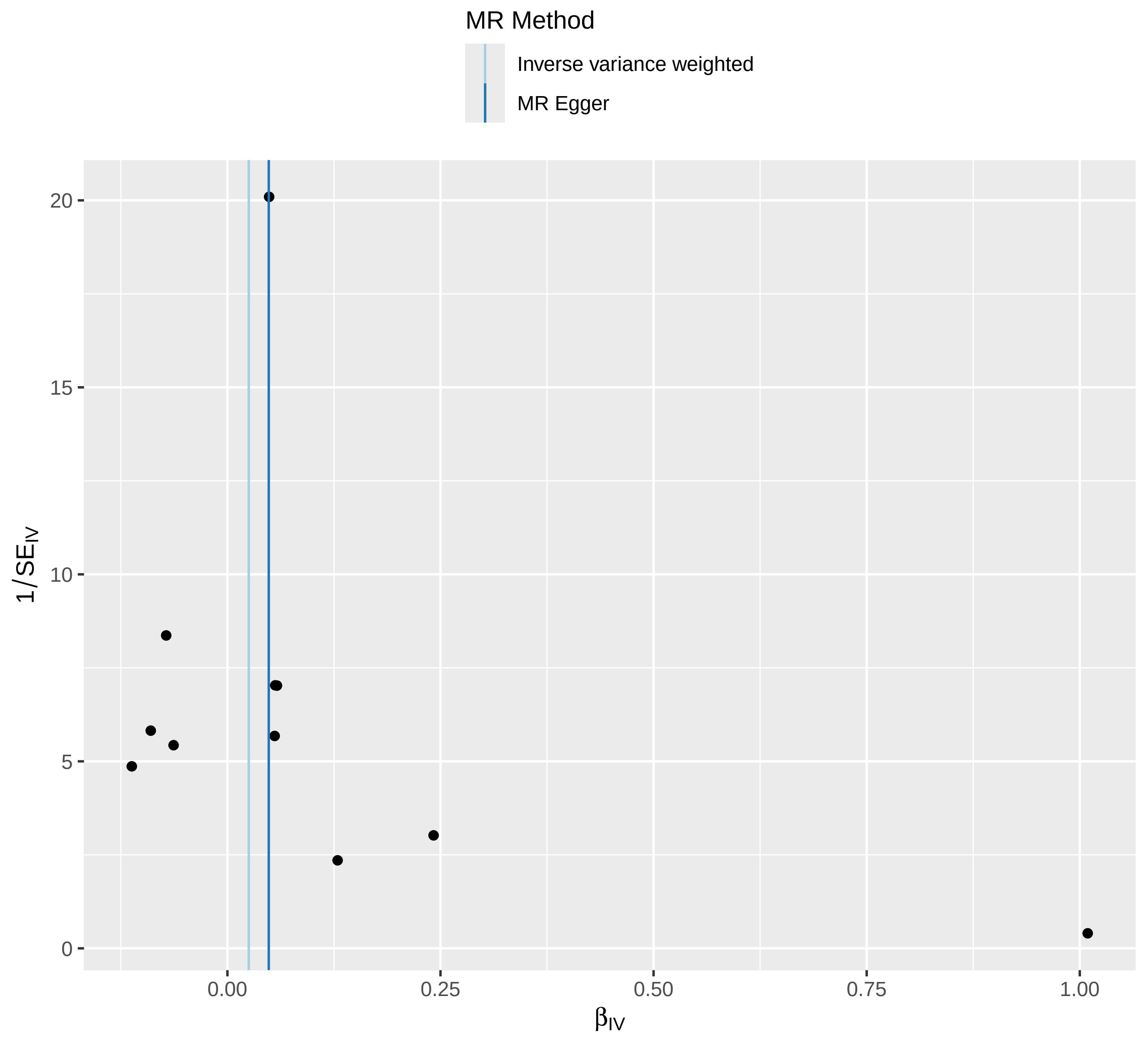
MR Method
Inverse variance weighted
MR Egger
20
15
10
5
0
1/SE_IV
0.00
0.25
0.50
0.75
1.00
β_IV

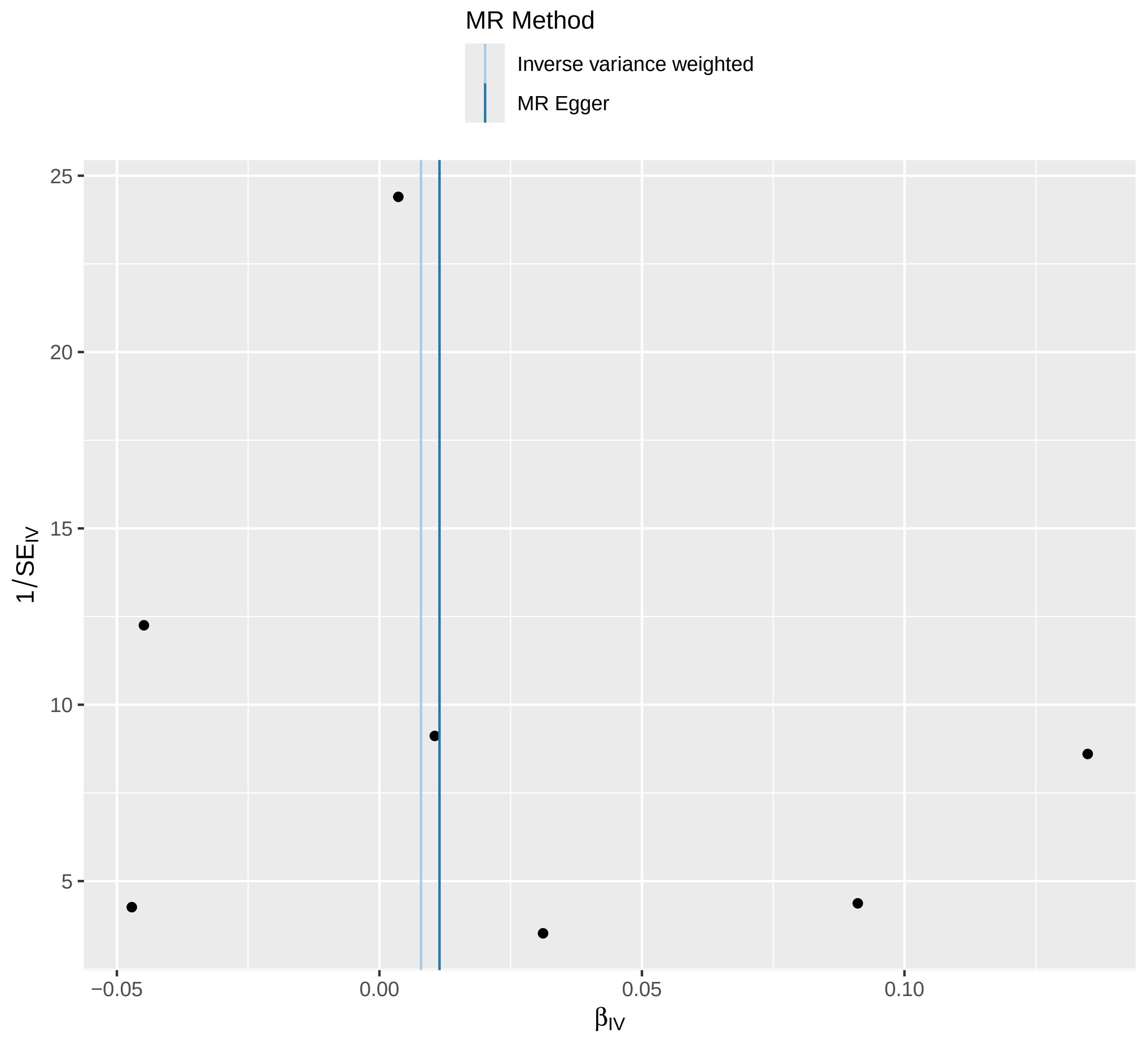
MR Method
Inverse variance weighted
MR Egger
25
20
15
10
5
1/SE$_{IV}$
−0.05
0.00
0.05
0.10
β$_{IV}$

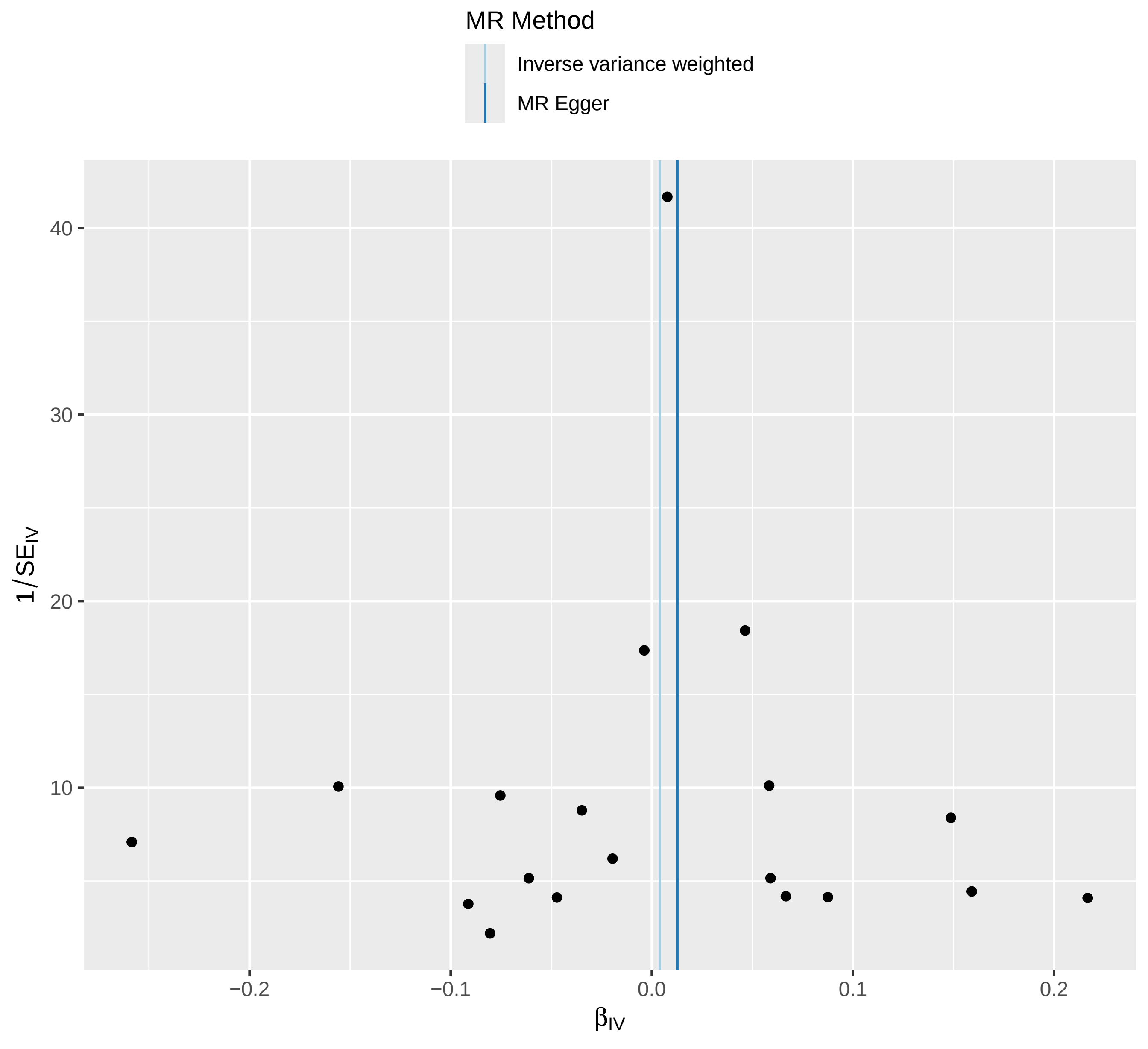
MR Method
Inverse variance weighted
MR Egger
40
30
20
10
1/SE$_{IV}$
−0.2
−0.1
0.0
0.1
0.2
β$_{IV}$

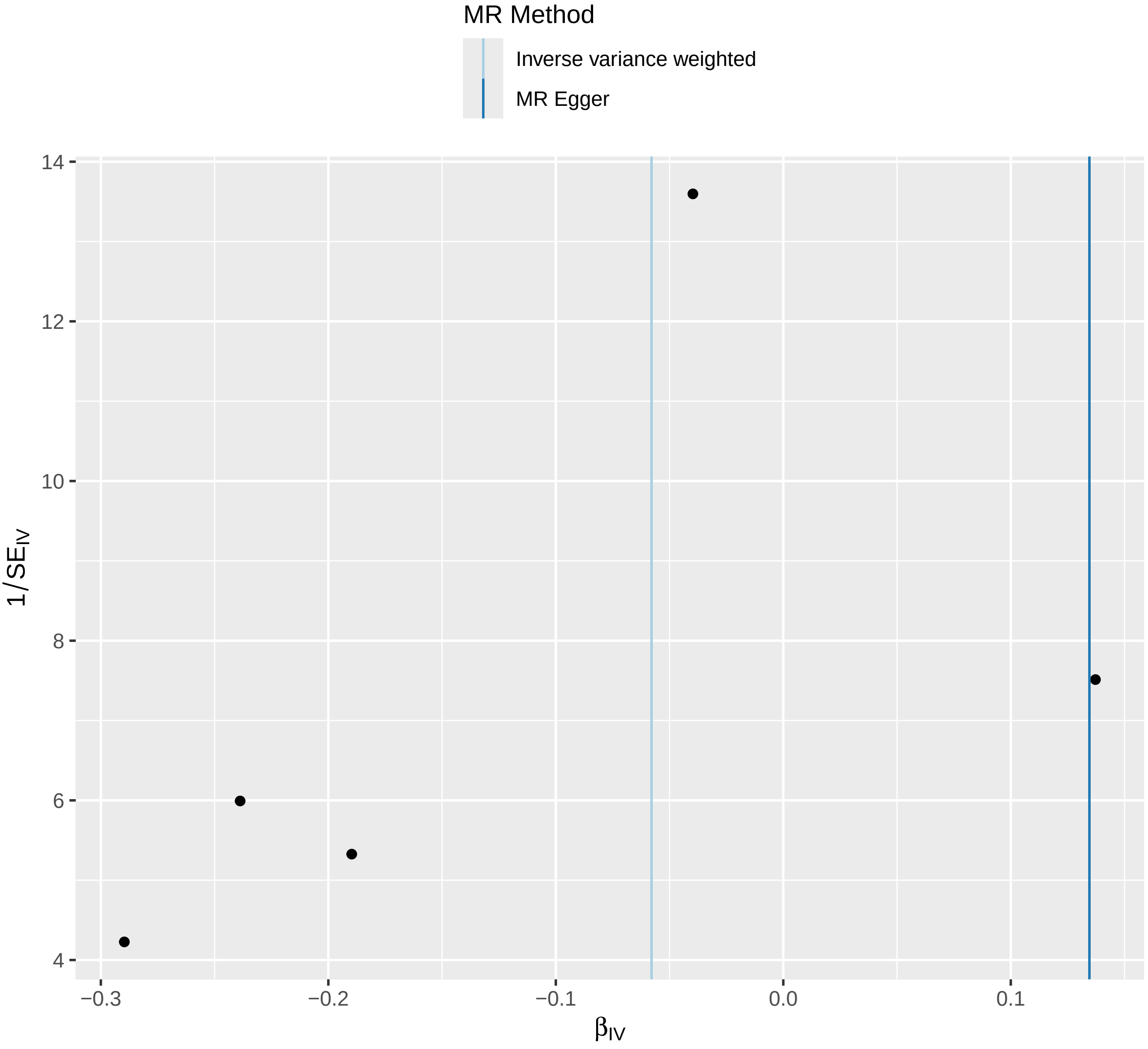
MR Method
Inverse variance weighted
MR Egger
14
12
10
8
6
4
$1/SE_{IV}$
−0.3
−0.2
−0.1
0.0
0.1
$\beta_{IV}$

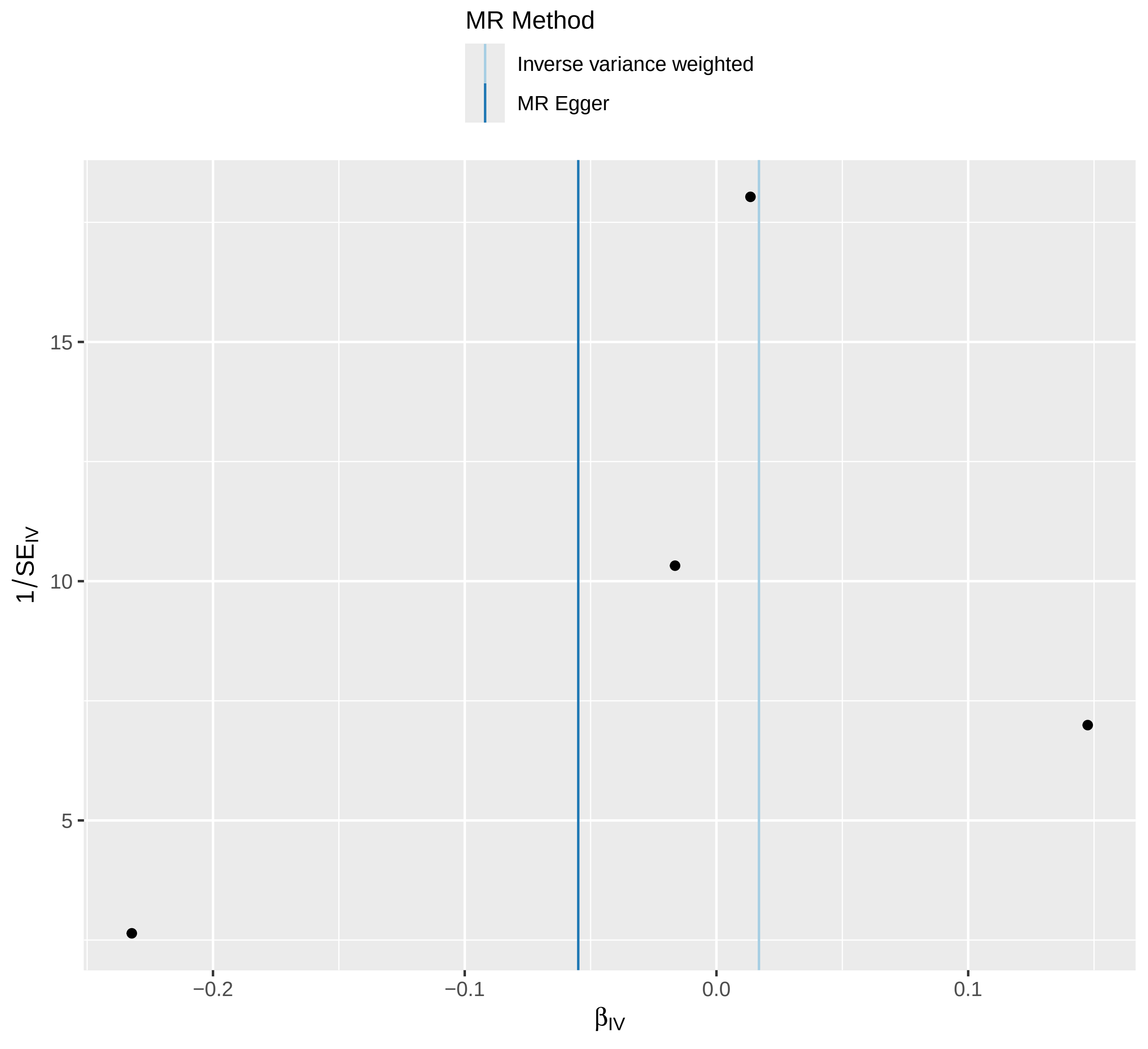

MR Method
Inverse variance weighted
MR Egger
15
10
5
1/SE$_{IV}$
−0.2
−0.1
0.0
0.1
β$_{IV}$

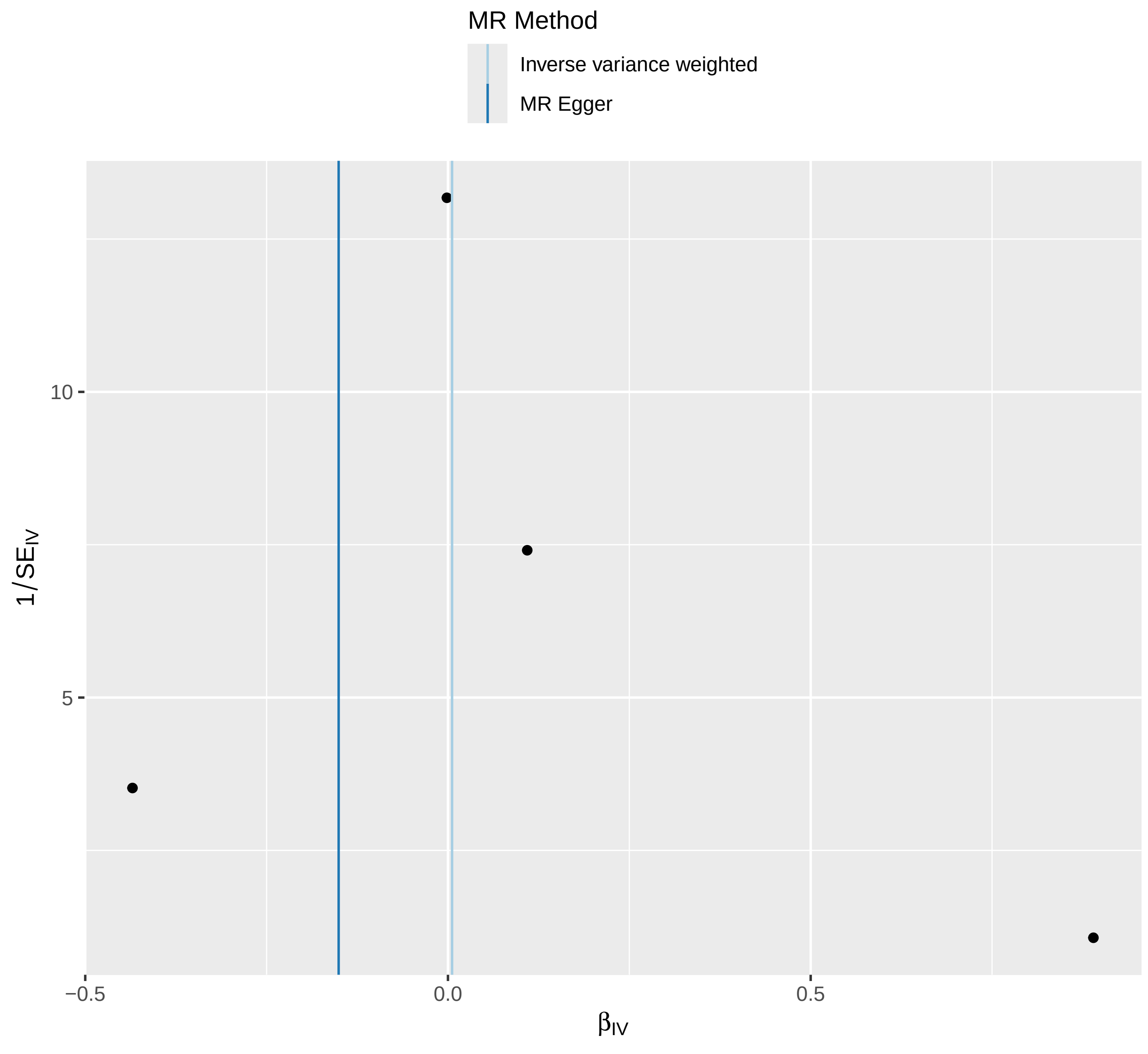
MR Method
Inverse variance weighted
MR Egger
10
5
1/SE_IV
−0.5
0.0
0.5
β_IV

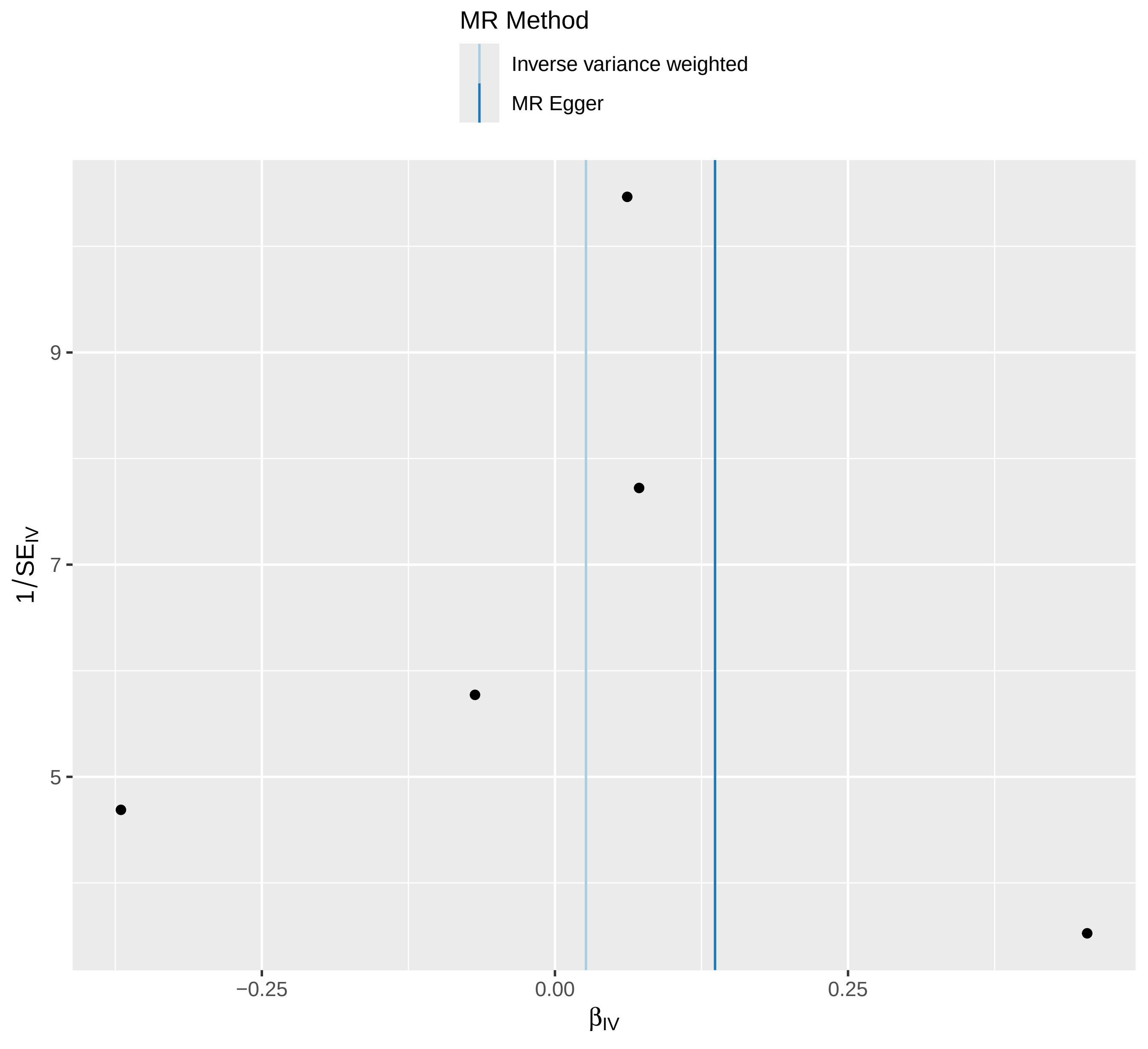
MR Method
Inverse variance weighted
MR Egger
9
7
5
1/SE_IV
−0.25
0.00
0.25
β_IV

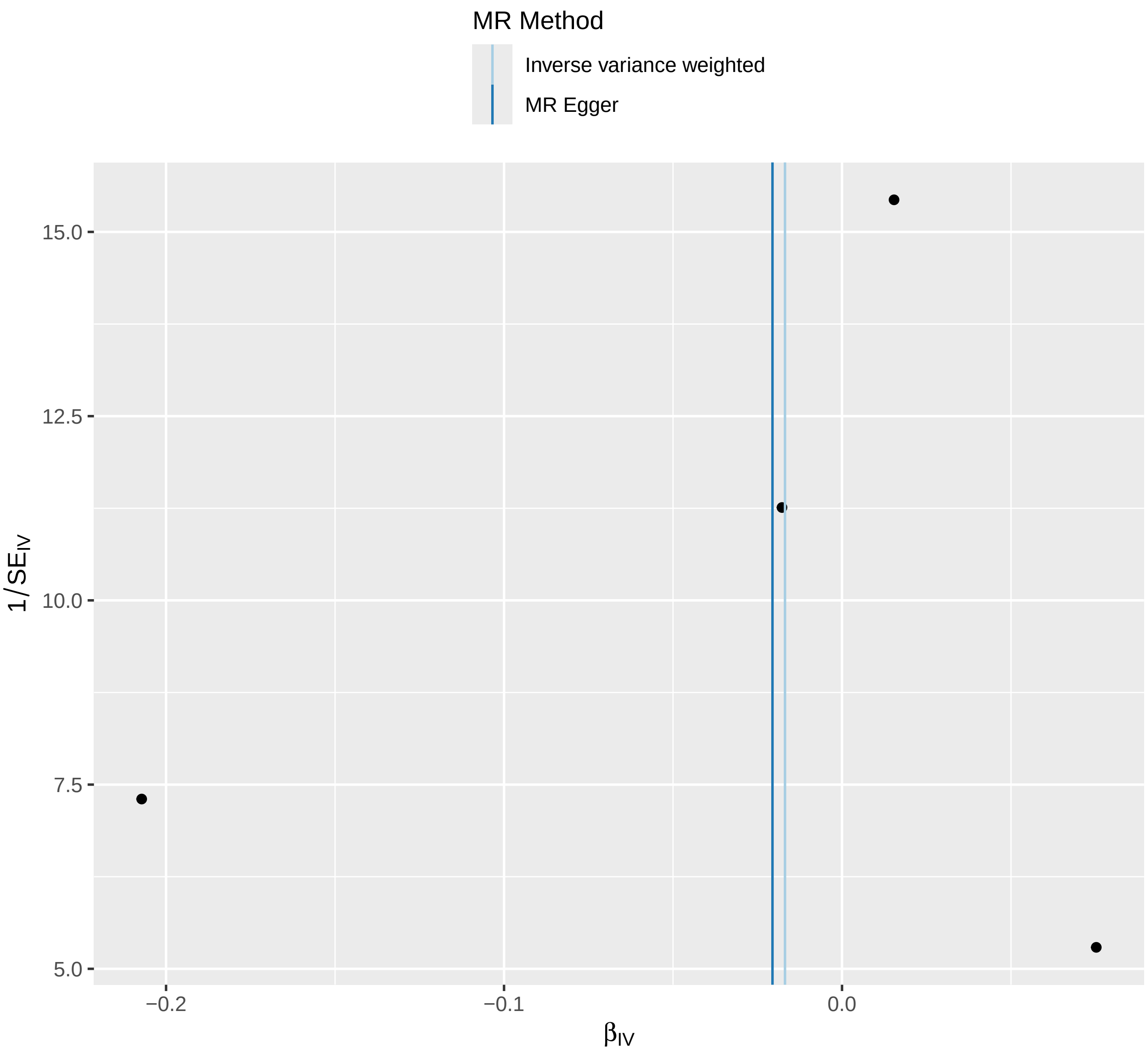
MR Method
Inverse variance weighted
MR Egger
15.0
12.5
10.0
7.5
5.0
$1/SE_{IV}$
−0.2
−0.1
0.0
$\beta_{IV}$

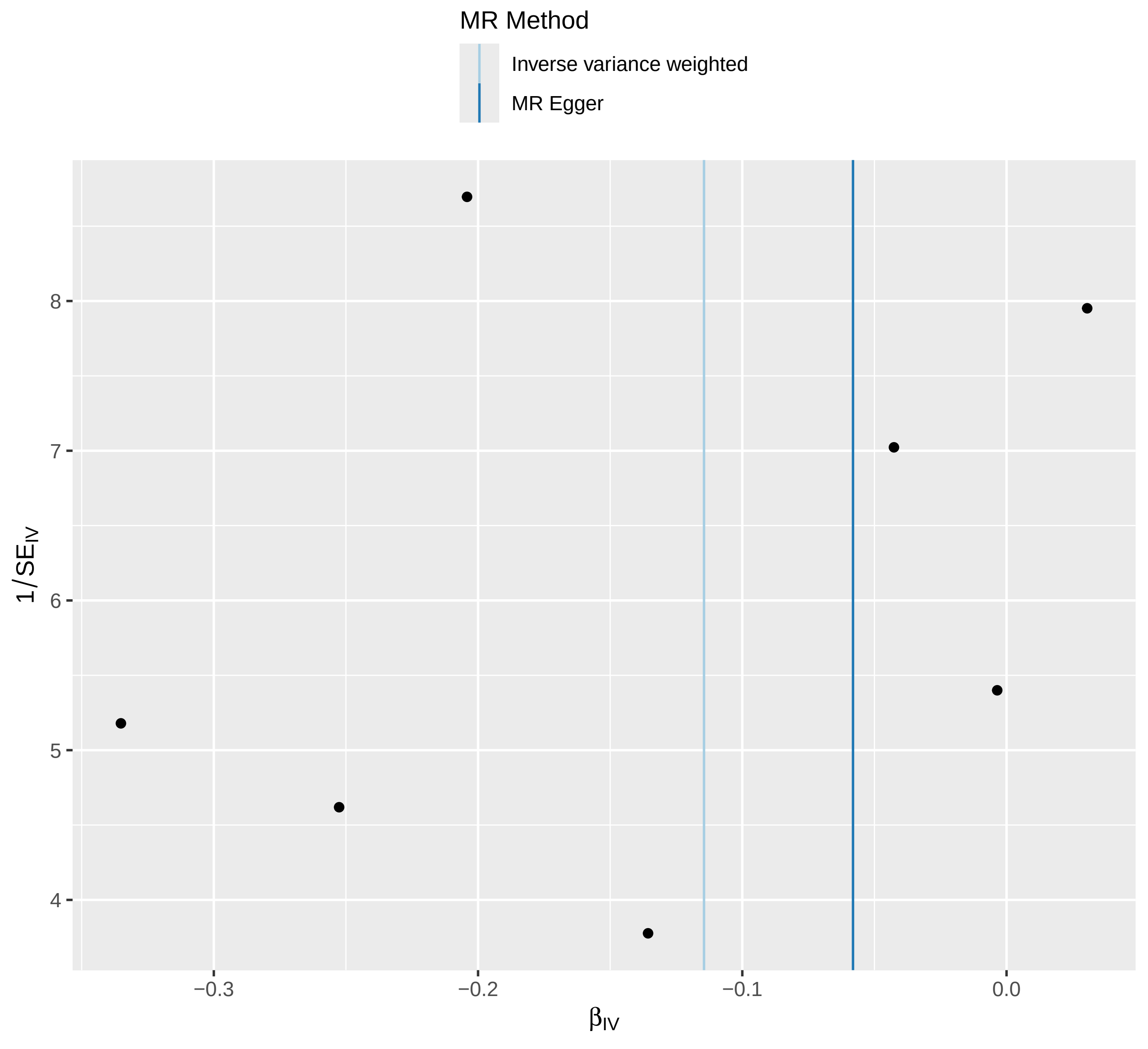

MR Method
Inverse variance weighted
MR Egger
8
7
6
5
4
1/SE_IV
−0.3
−0.2
−0.1
0.0
β_IV

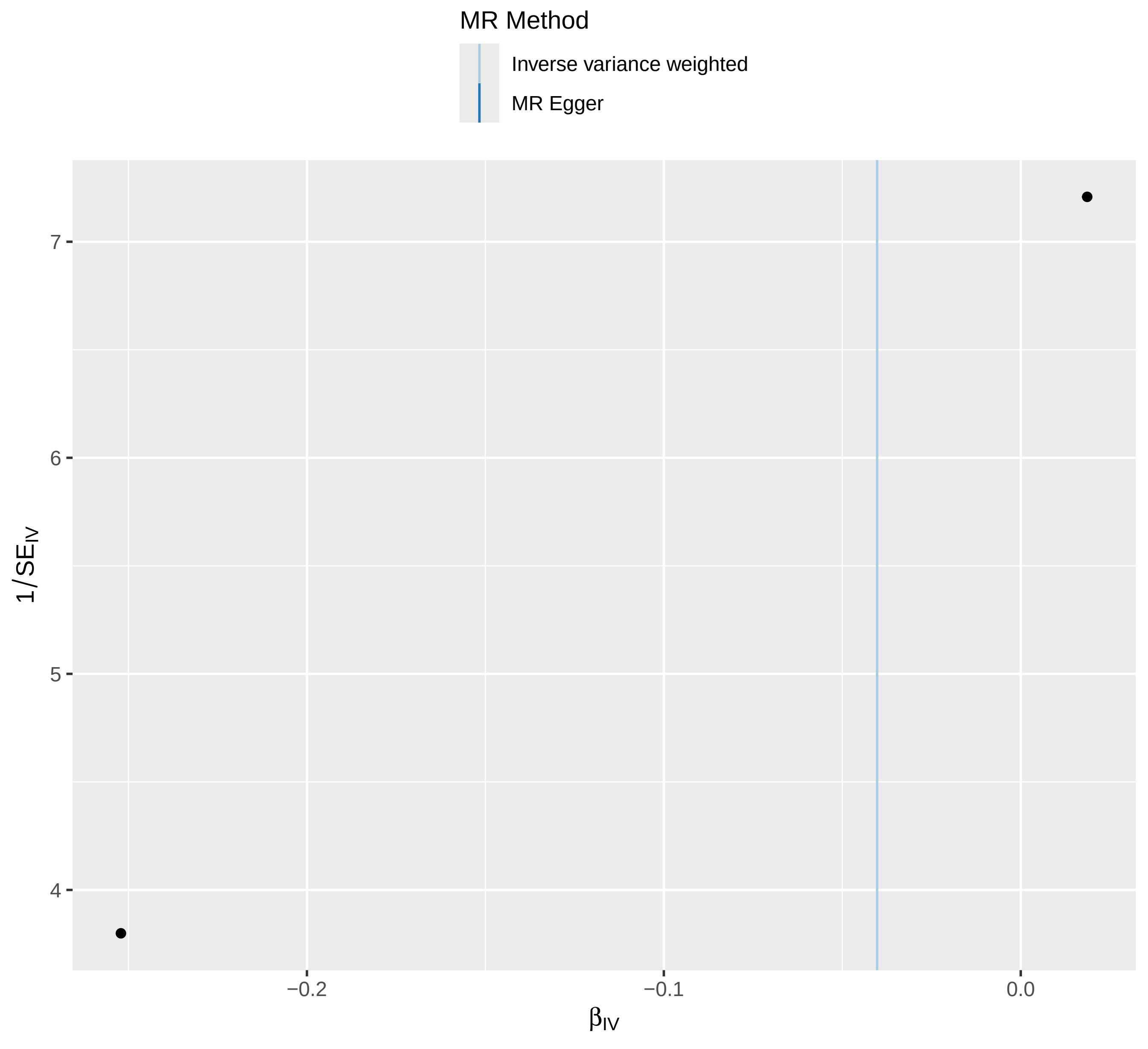

MR Method
Inverse variance weighted
MR Egger
7
6
5
4
1/SE_IV
−0.2
−0.1
0.0
β_IV

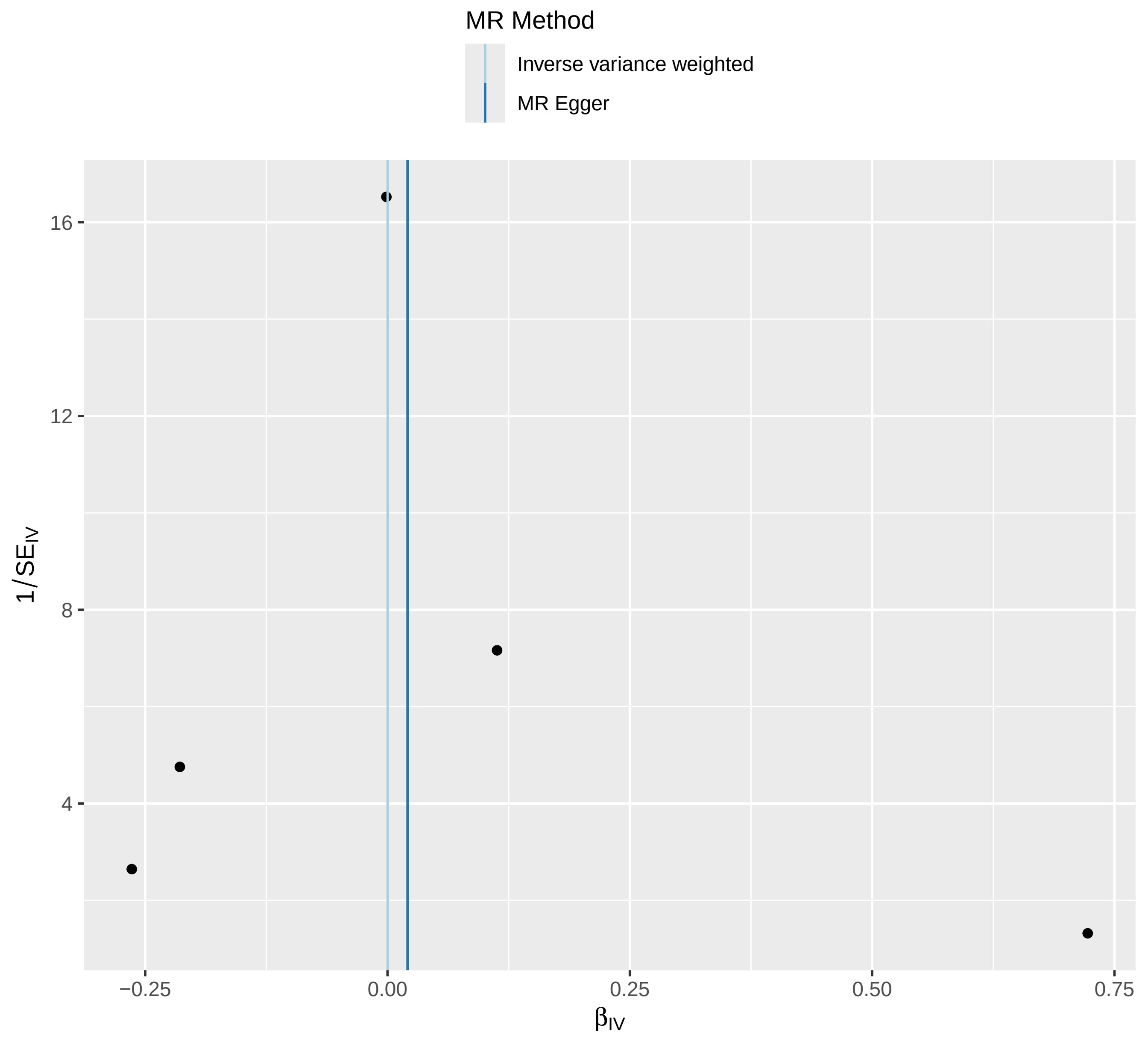

MR Method
Inverse variance weighted
MR Egger
16
12
8
4
1/SE_IV
−0.25
0.00
0.25
0.50
0.75
β_IV

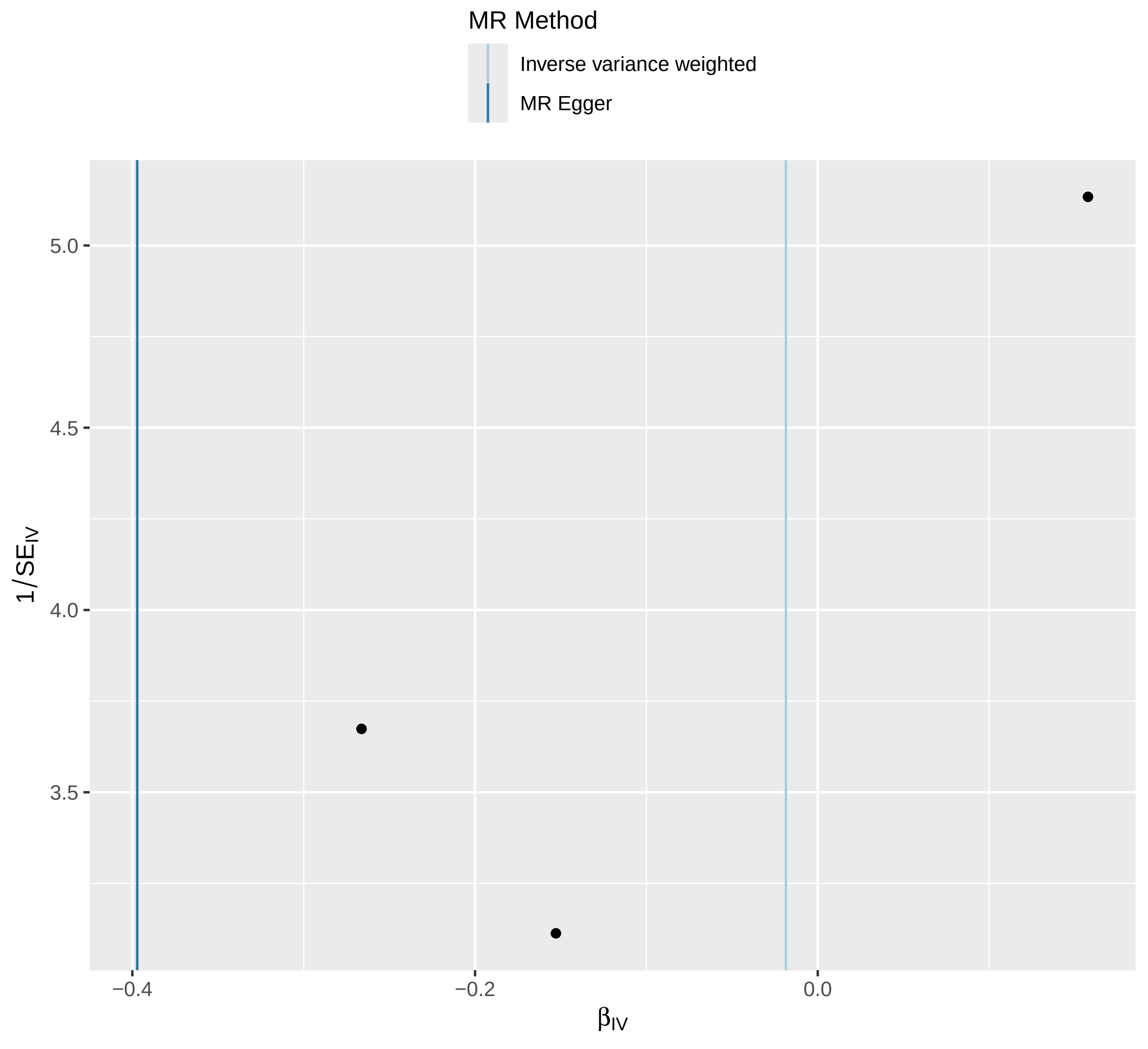

MR Method
Inverse variance weighted
MR Egger
5.0
4.5
4.0
3.5
1/SE_IV
−0.4
−0.2
0.0
β_IV

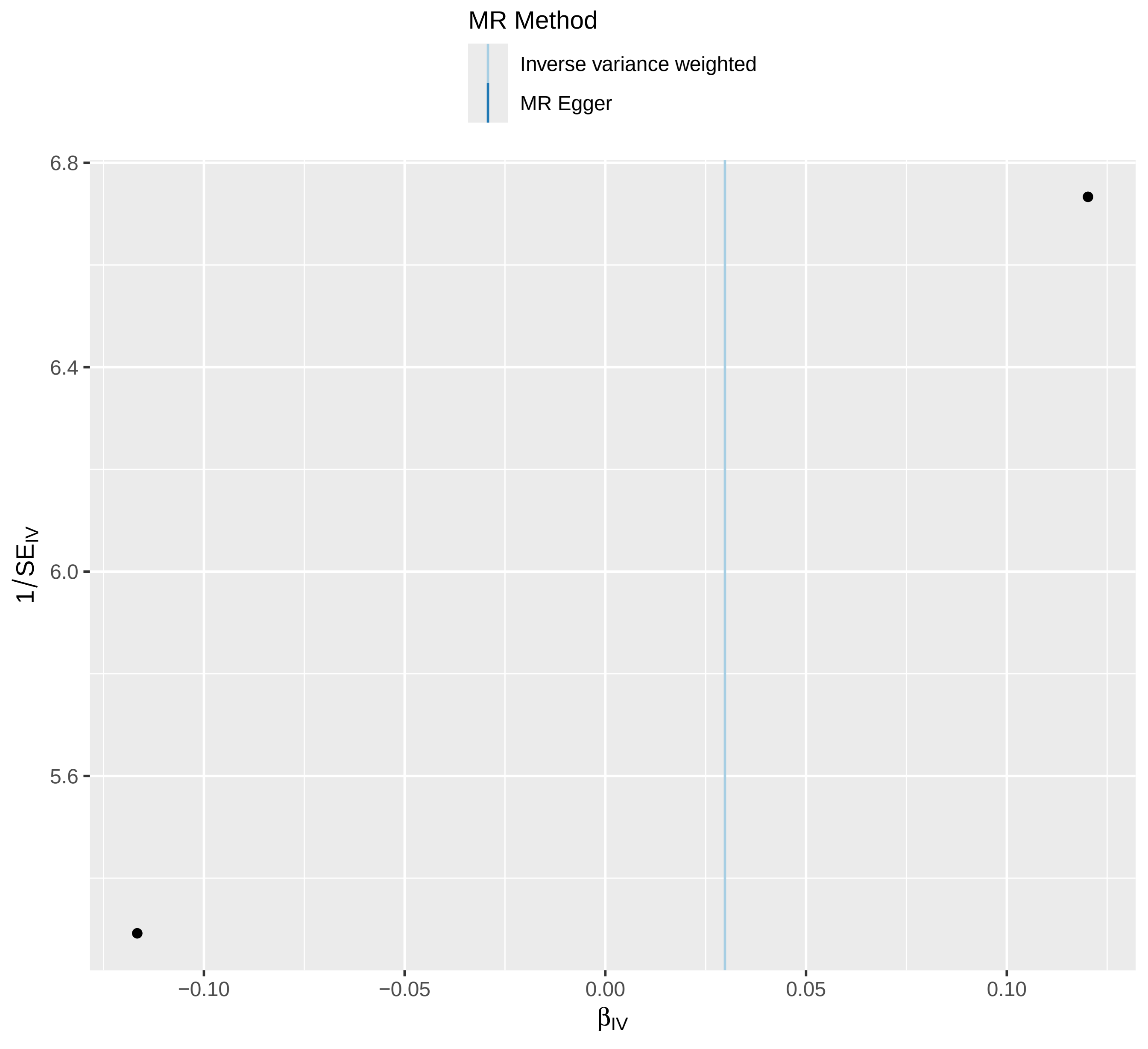
MR Method
Inverse variance weighted
MR Egger
6.8
6.4
6.0
5.6
$1/SE_{IV}$
−0.10
−0.05
0.00
0.05
0.10
$\beta_{IV}$

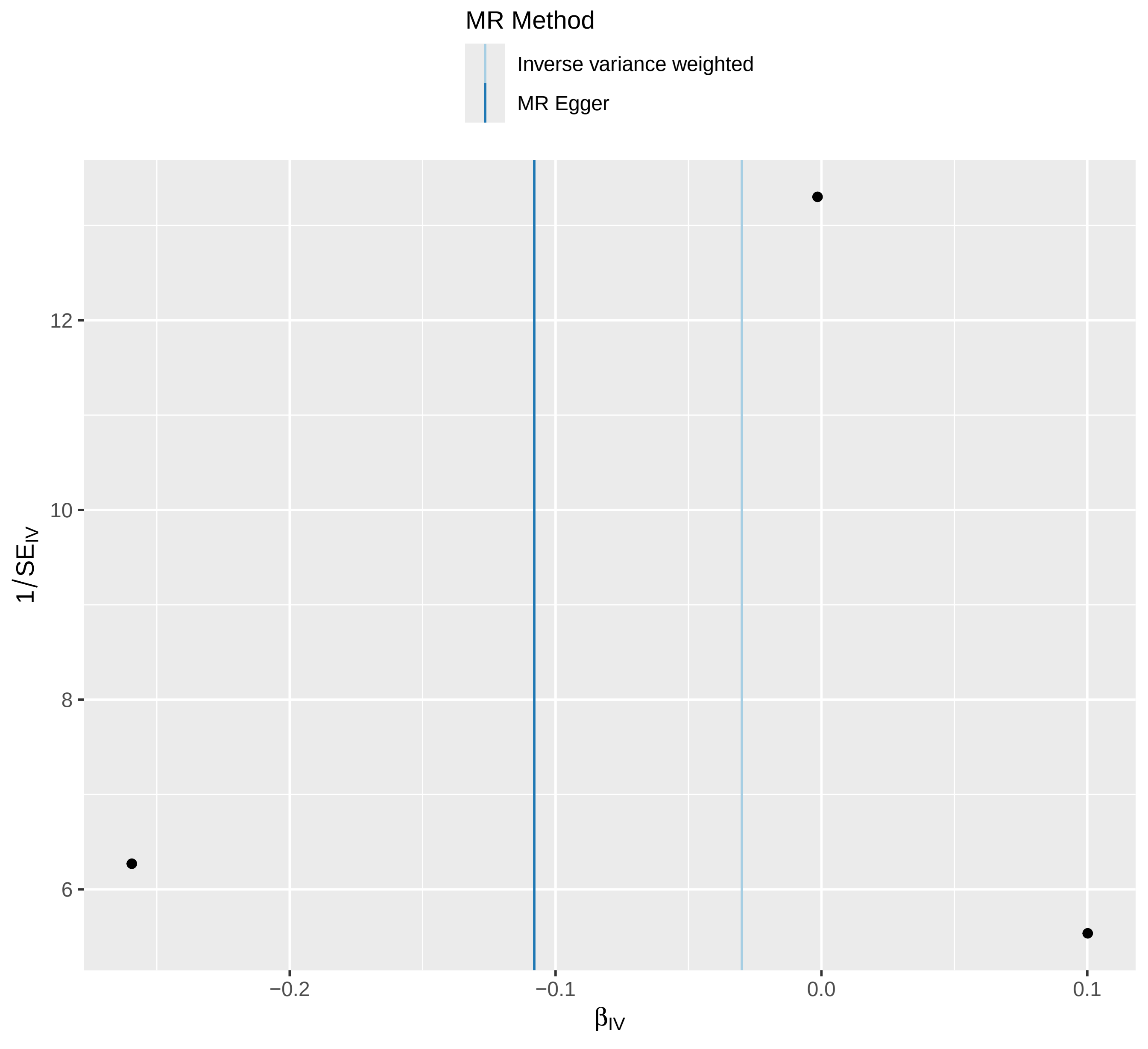
MR Method
Inverse variance weighted
MR Egger
12
10
8
6
−0.2
−0.1
0.0
0.1
β_IV
1/SE_IV

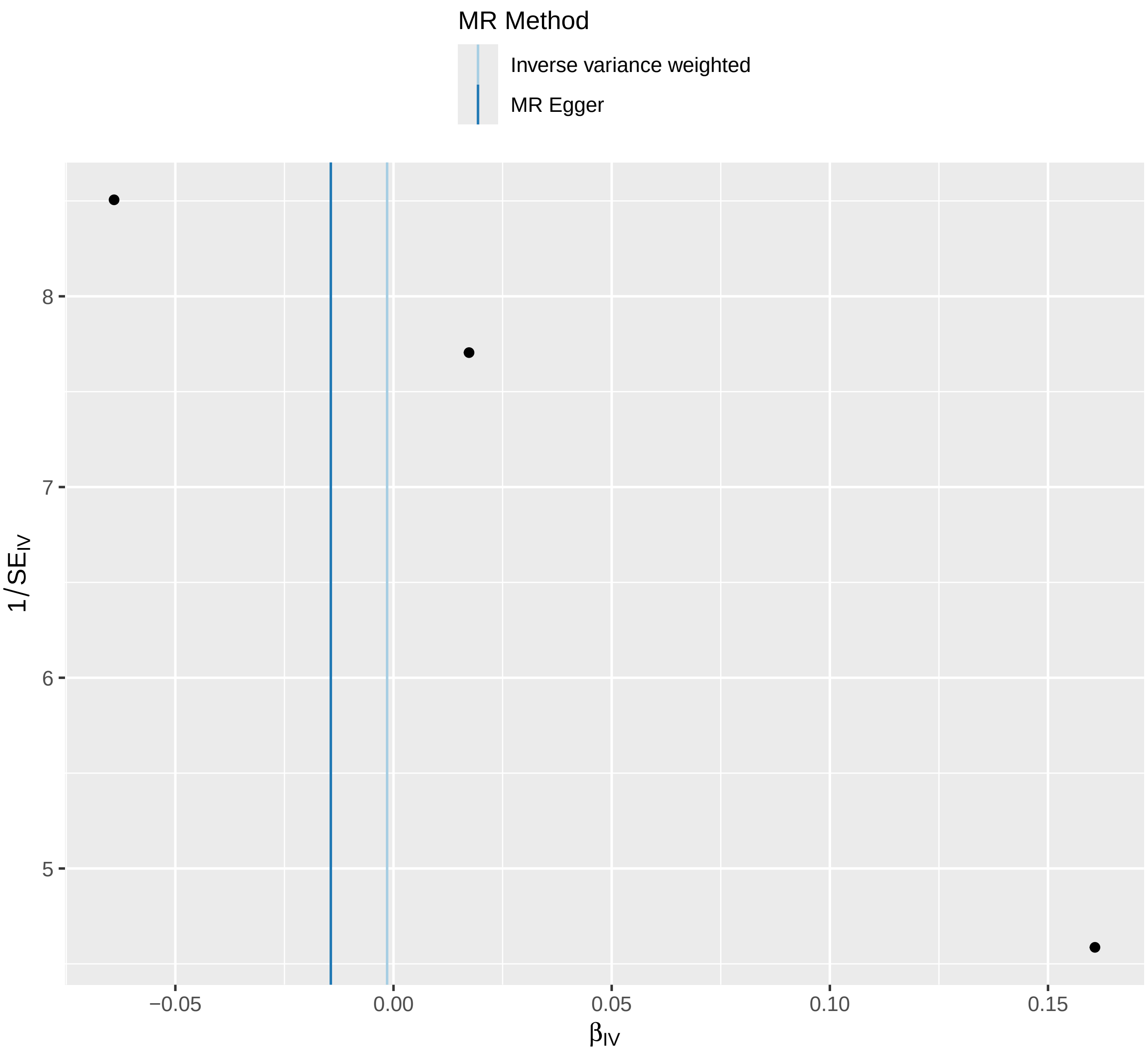

MR Method
Inverse variance weighted
MR Egger
1/SE_IV
8
7
6
5
−0.05
0.00
0.05
0.10
0.15
β_IV

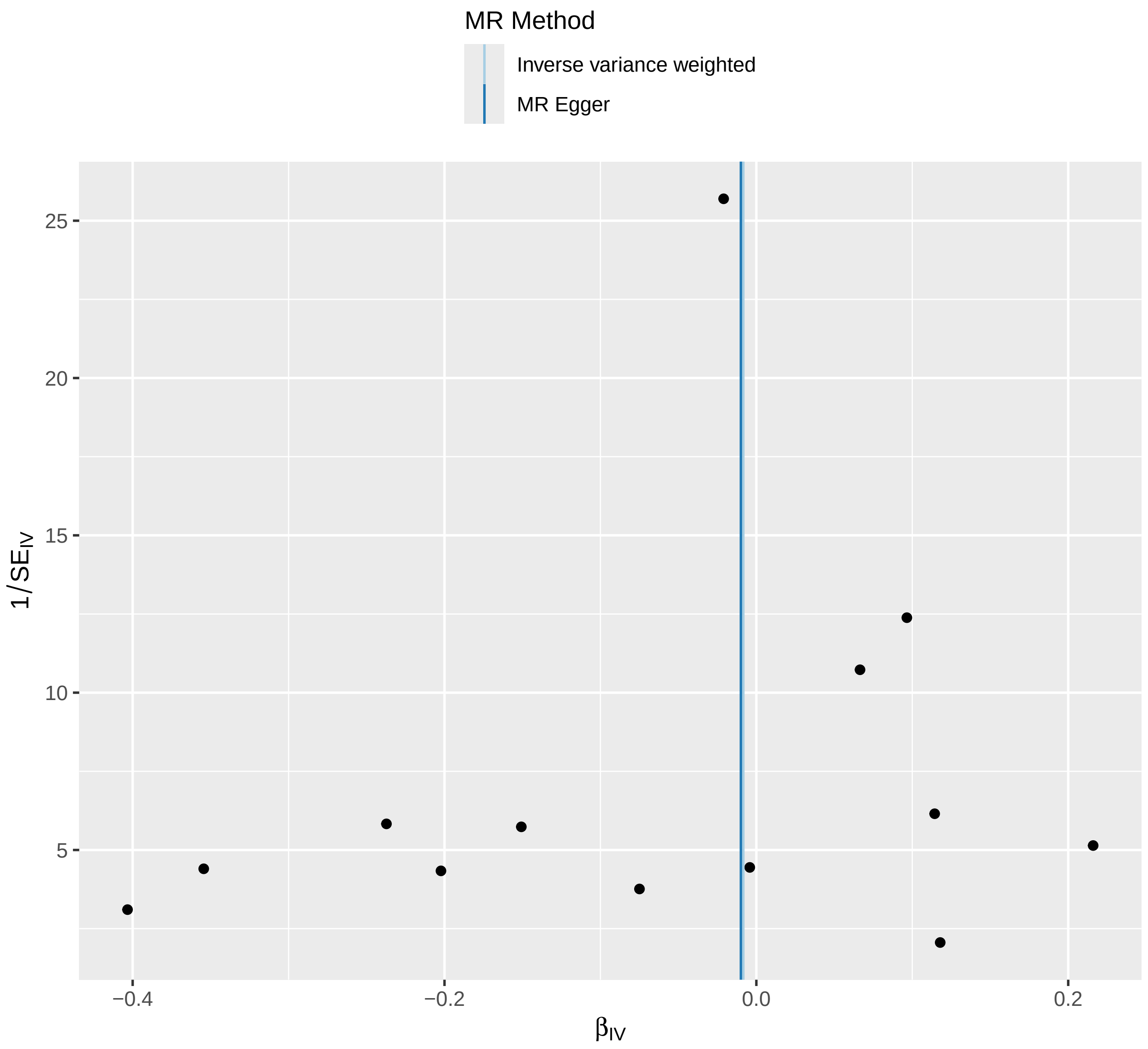

MR Method
Inverse variance weighted
MR Egger
25
20
15
10
5
1/SE$_{IV}$
−0.4
−0.2
0.0
0.2
β$_{IV}$

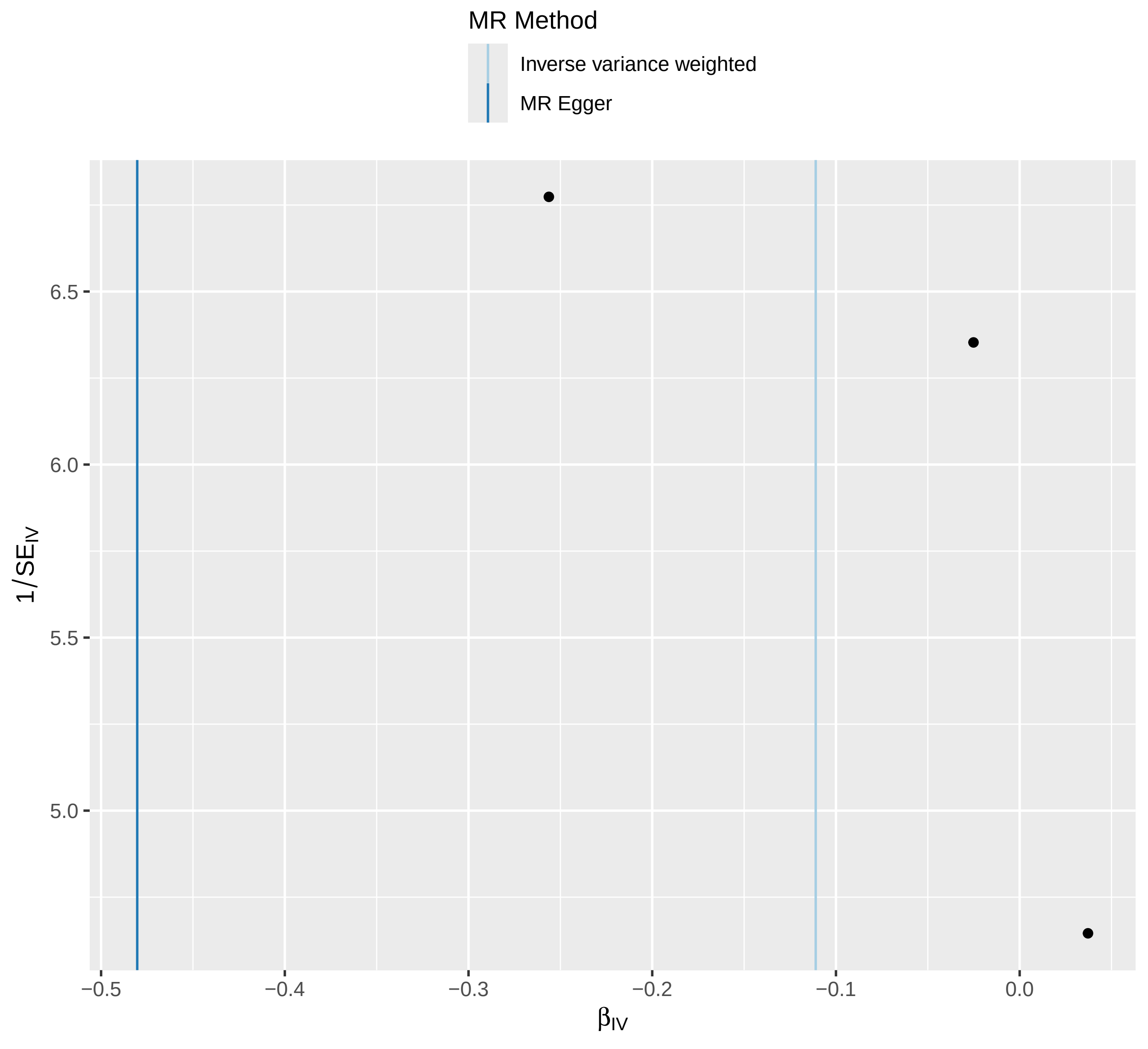

MR Method
Inverse variance weighted
MR Egger
6.5
6.0
5.5
5.0
1/SE_IV
−0.5
−0.4
−0.3
−0.2
−0.1
0.0
β_IV

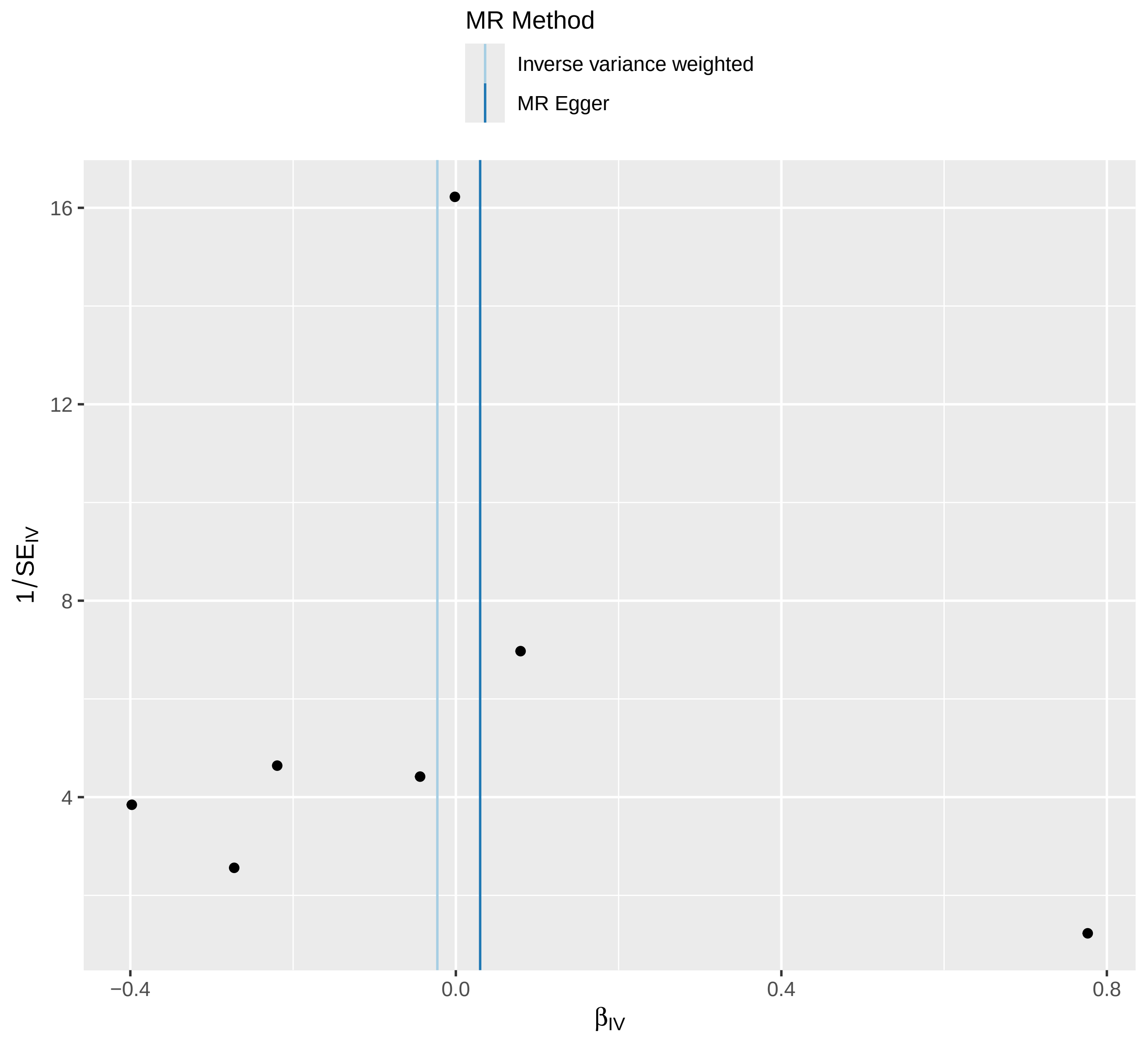
MR Method
Inverse variance weighted
MR Egger
16
12
8
4
$1/SE_{IV}$
−0.4
0.0
0.4
0.8
$\beta_{IV}$

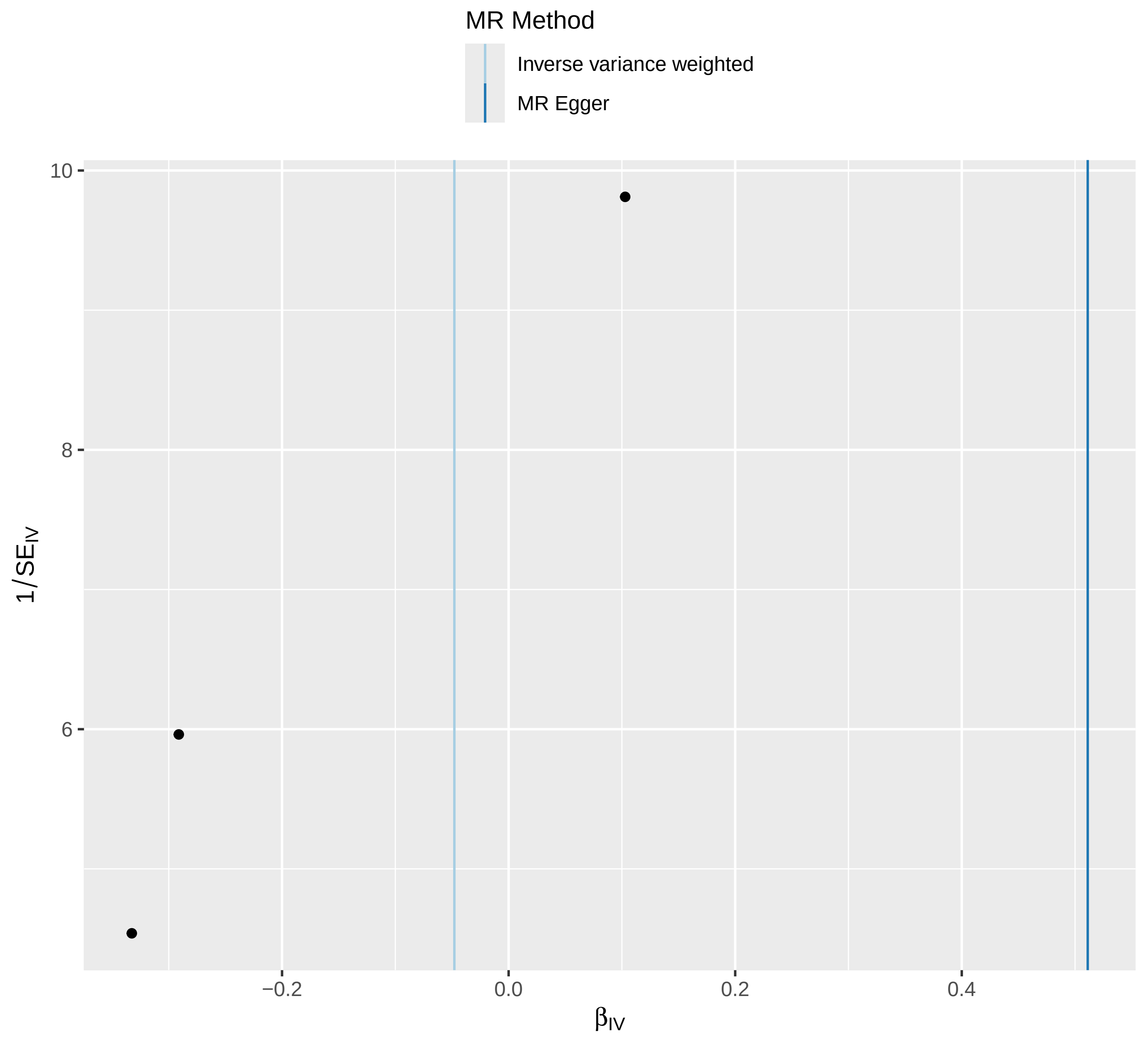
MR Method
Inverse variance weighted
MR Egger
10
8
6
1/SE_IV
−0.2
0.0
0.2
0.4
β_IV

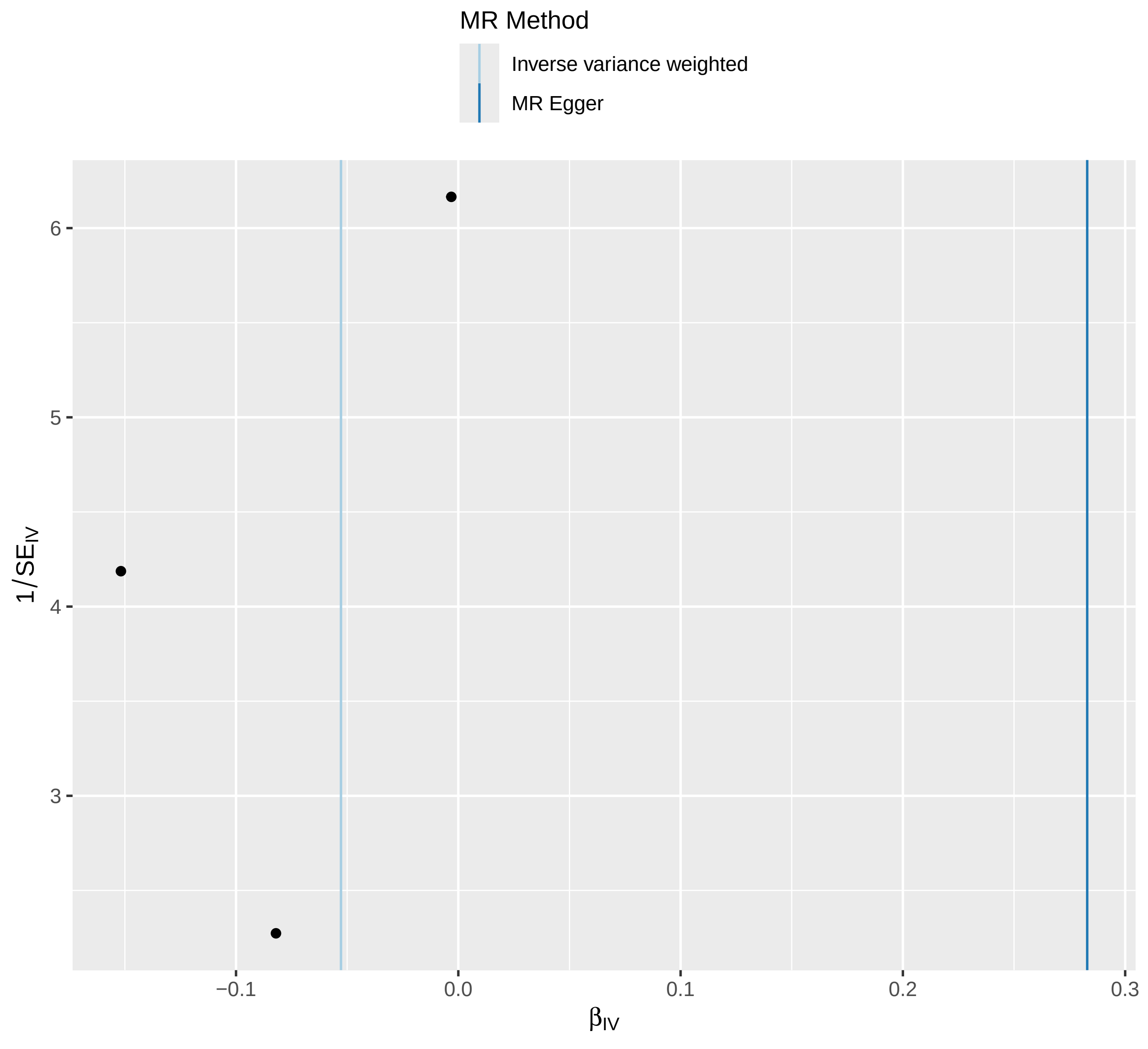
MR Method
Inverse variance weighted
MR Egger
1/SE_IV
6
5
4
3
−0.1
0.0
0.1
0.2
0.3
β_IV

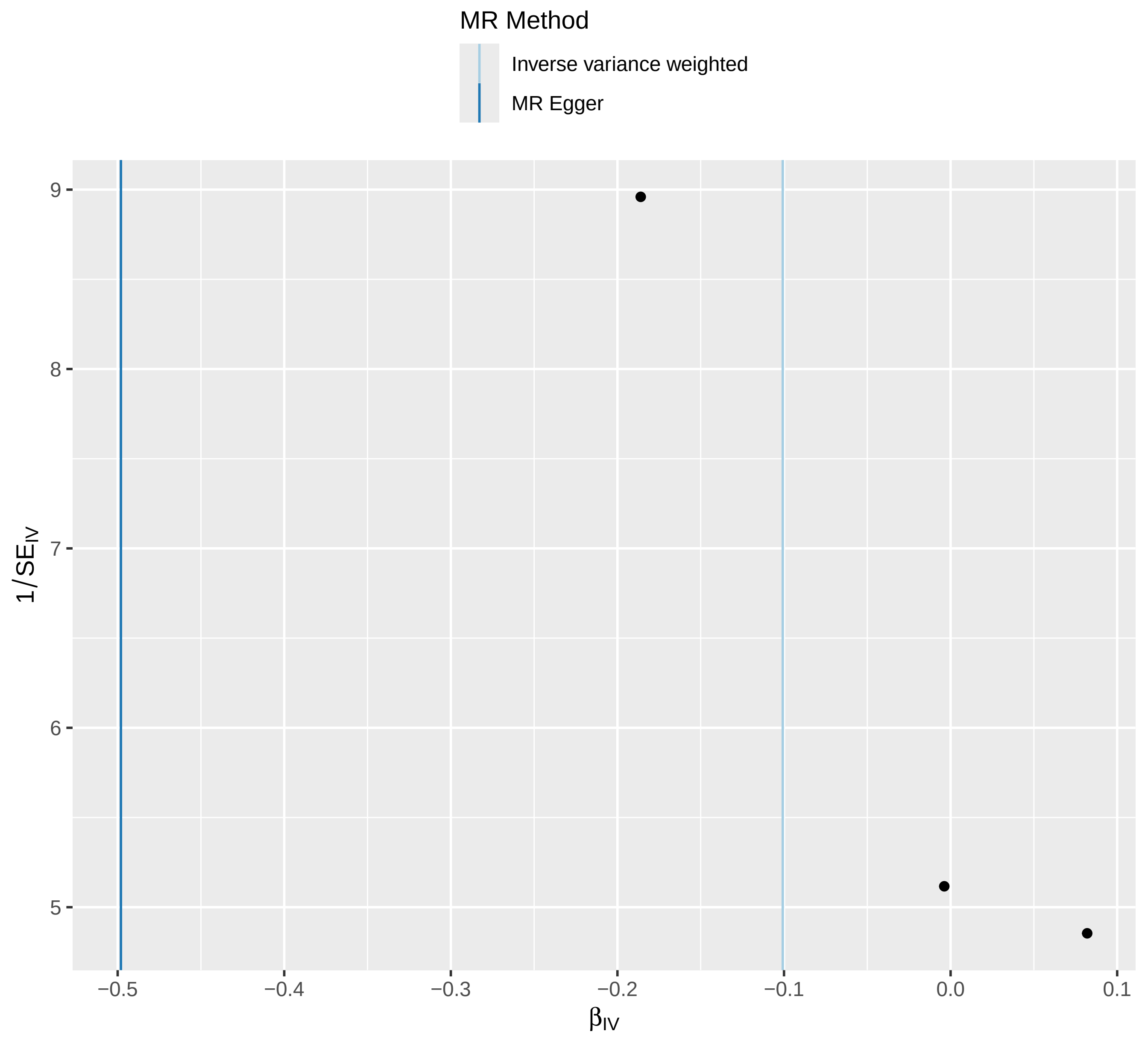
MR Method
Inverse variance weighted
MR Egger
9
8
7
6
5
−0.5
−0.4
−0.3
−0.2
−0.1
0.0
0.1
$1/SE_{IV}$
$\beta_{IV}$

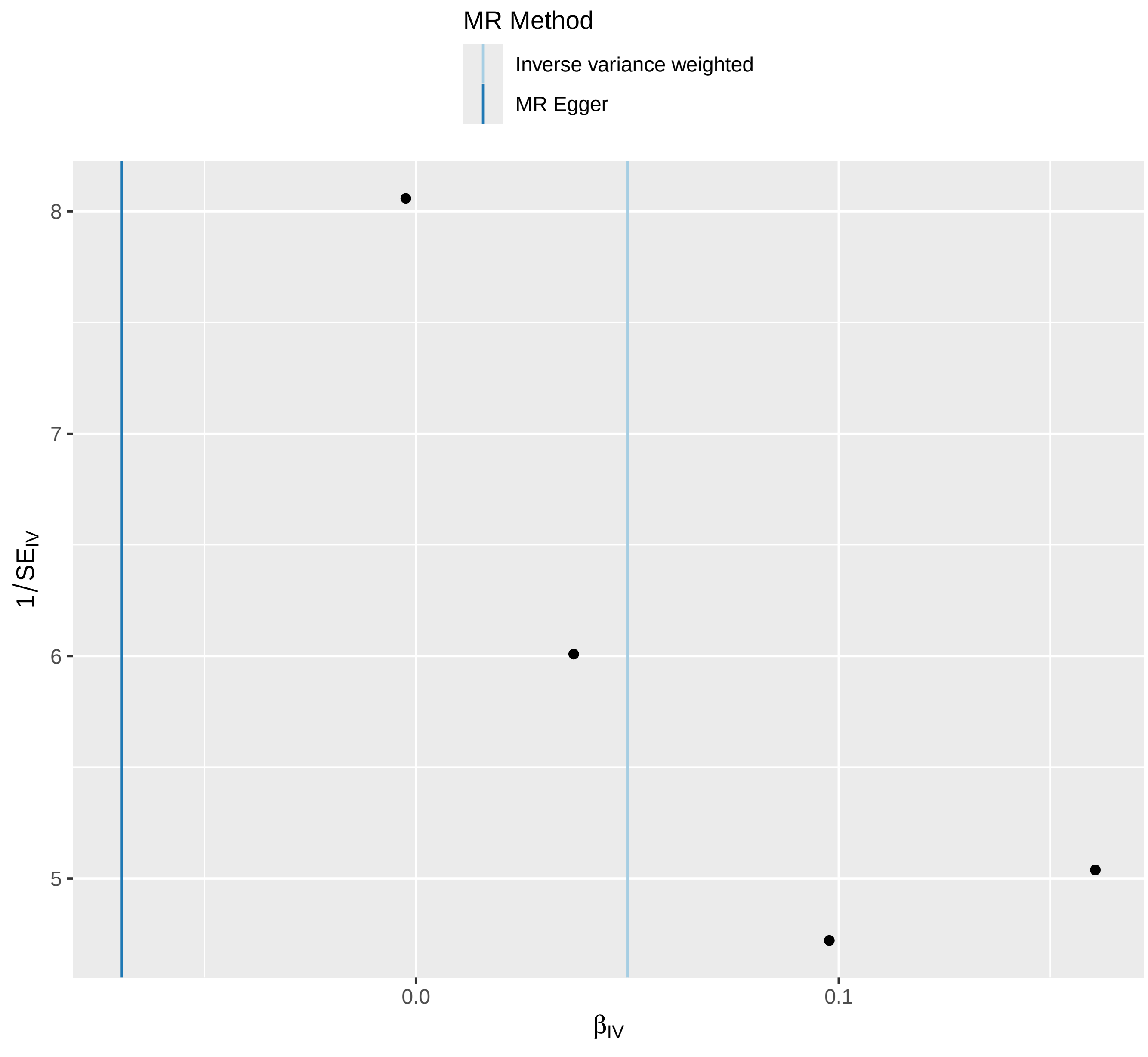
MR Method
Inverse variance weighted
MR Egger
8
7
6
5
1/SE$_{IV}$
0.0
0.1
$\beta_{IV}$

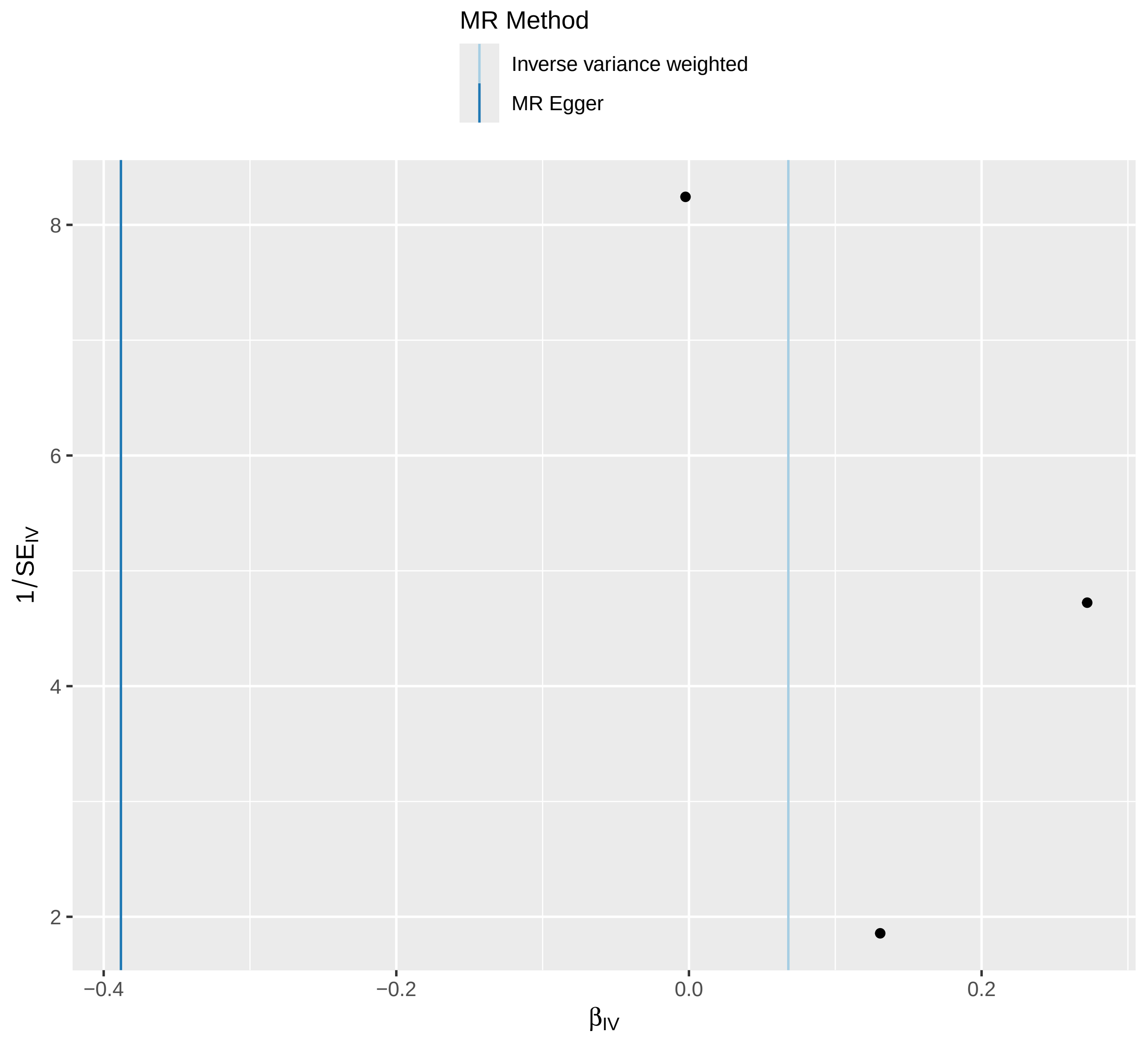

MR Method
Inverse variance weighted
MR Egger
8
6
4
2
1/SE$_{IV}$
−0.4
−0.2
0.0
0.2
β$_{IV}$

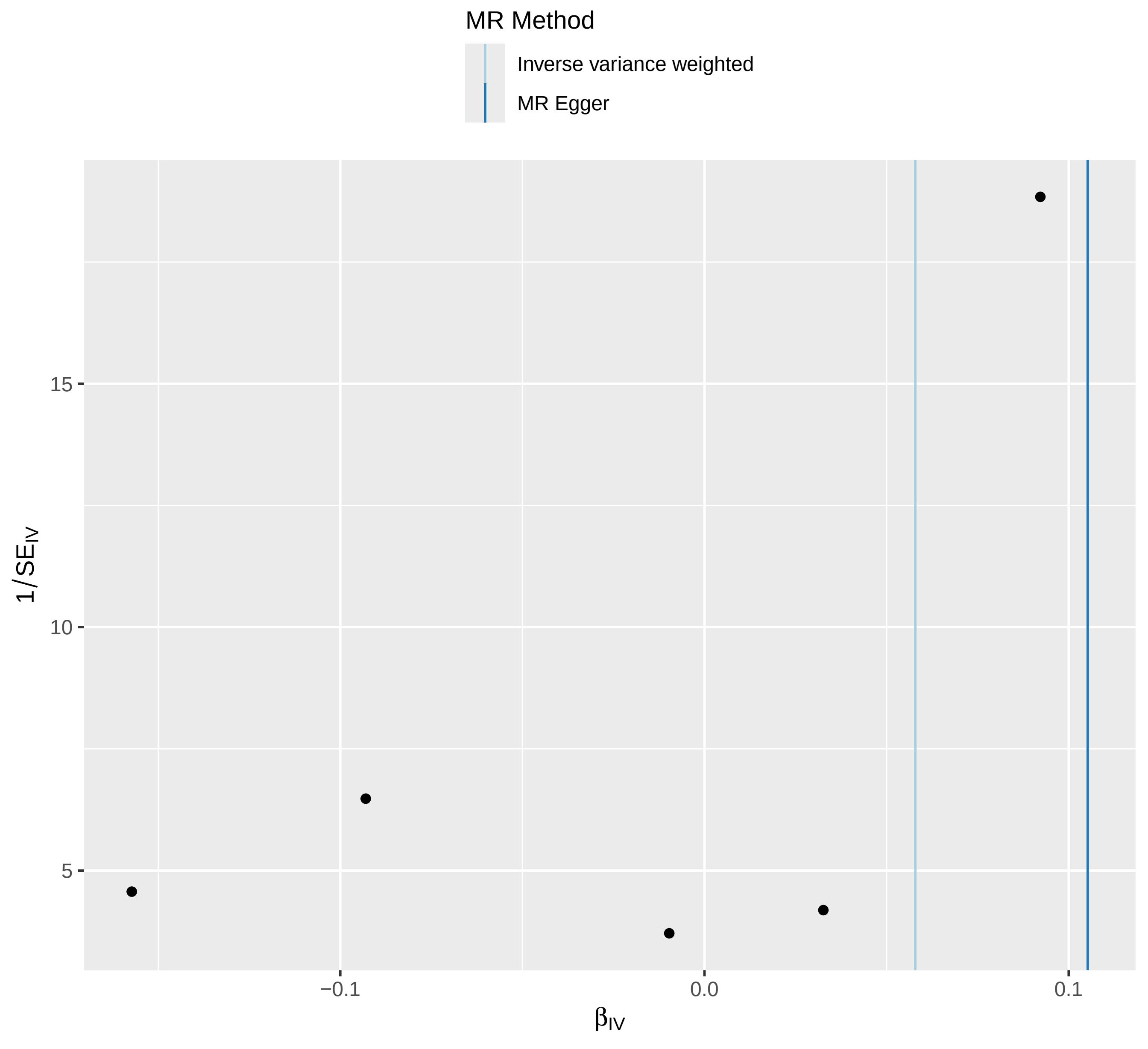

MR Method
Inverse variance weighted
MR Egger
15
10
5
1/SE$_{IV}$
−0.1
0.0
0.1
β$_{IV}$

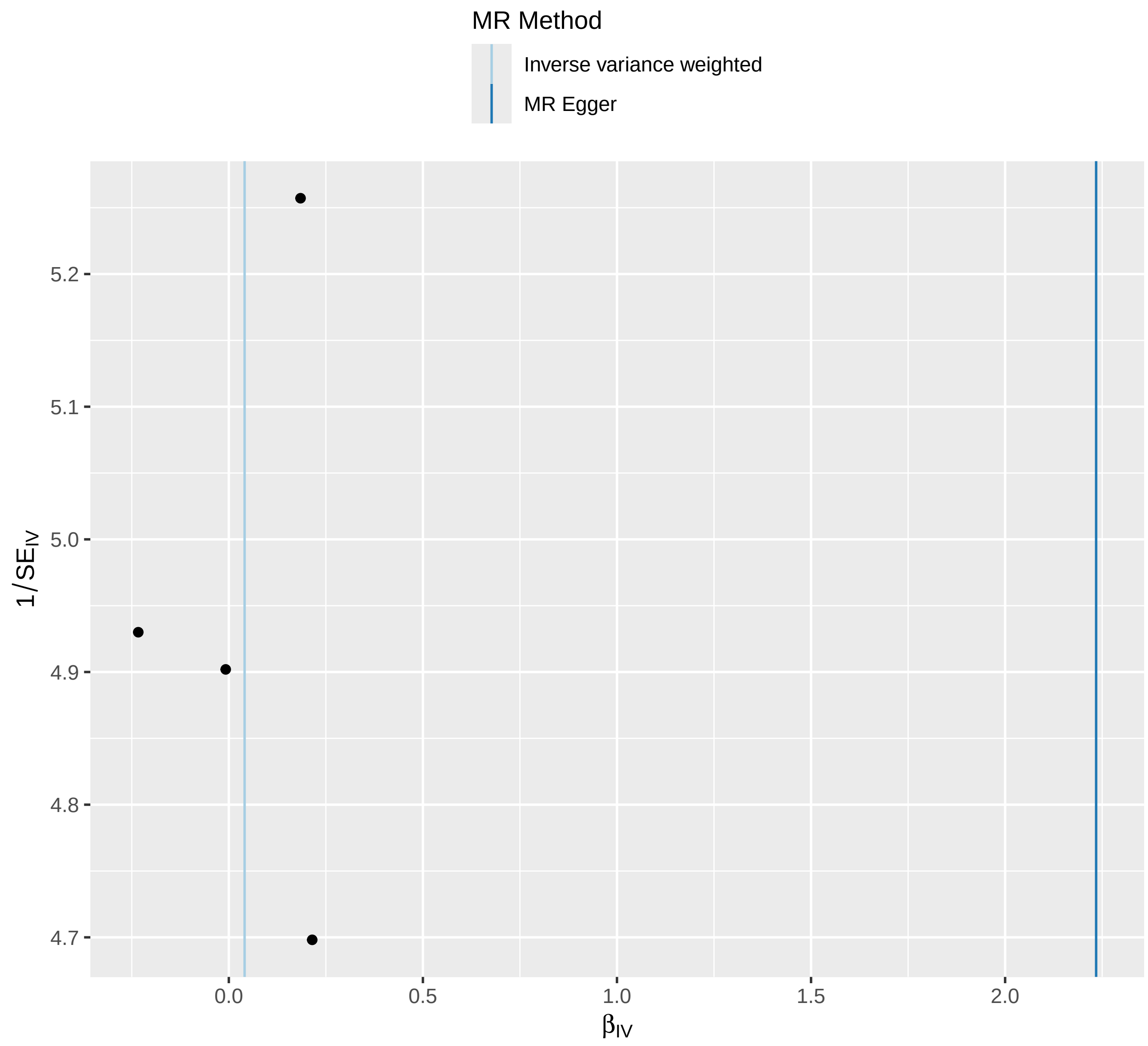

MR Method
Inverse variance weighted
MR Egger
5.2
5.1
5.0
4.9
4.8
4.7
1/SE$_{IV}$
0.0
0.5
1.0
1.5
2.0
β$_{IV}$

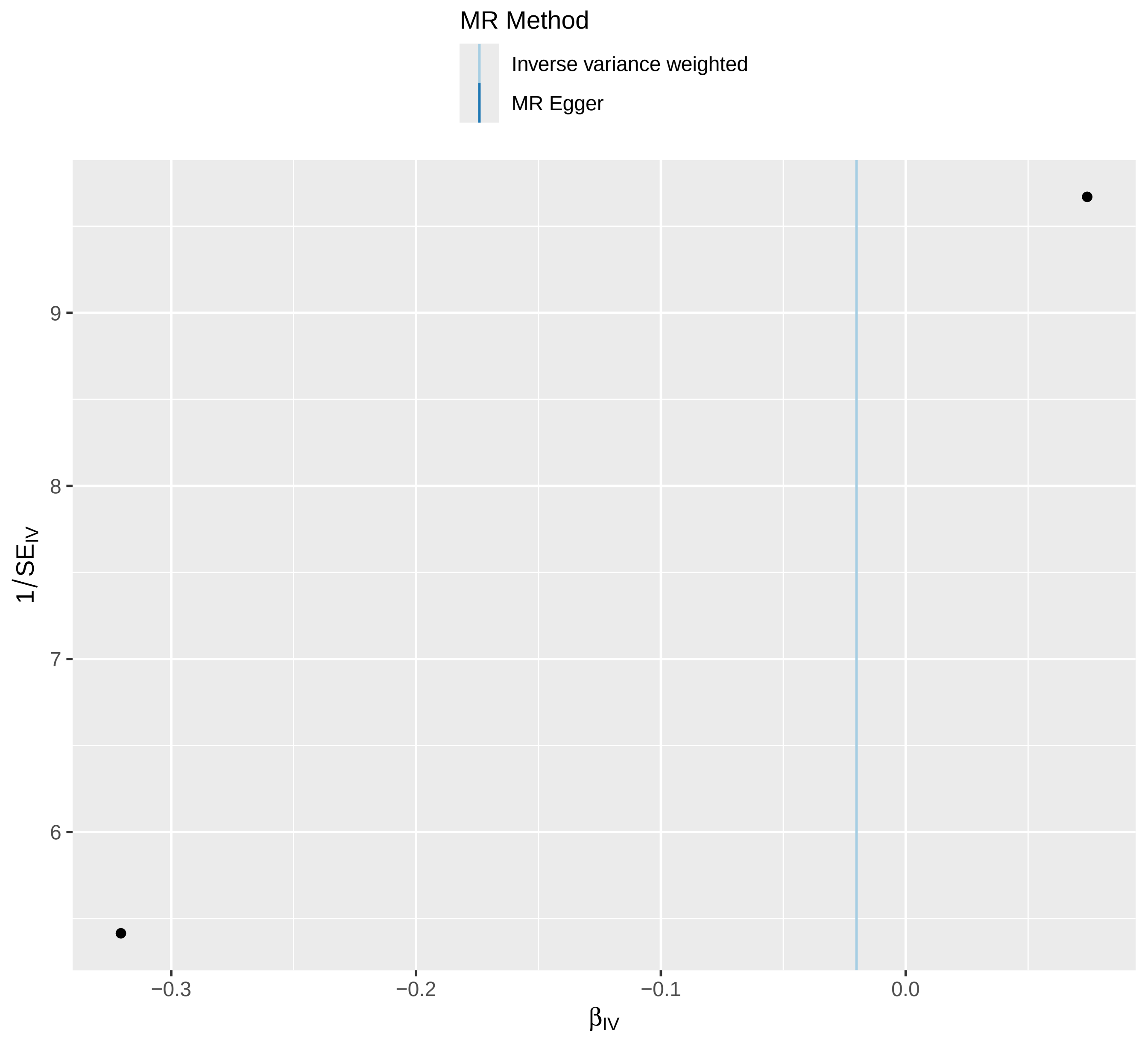

MR Method
Inverse variance weighted
MR Egger
9
8
7
6
1/SE_IV
−0.3
−0.2
−0.1
0.0
β_IV

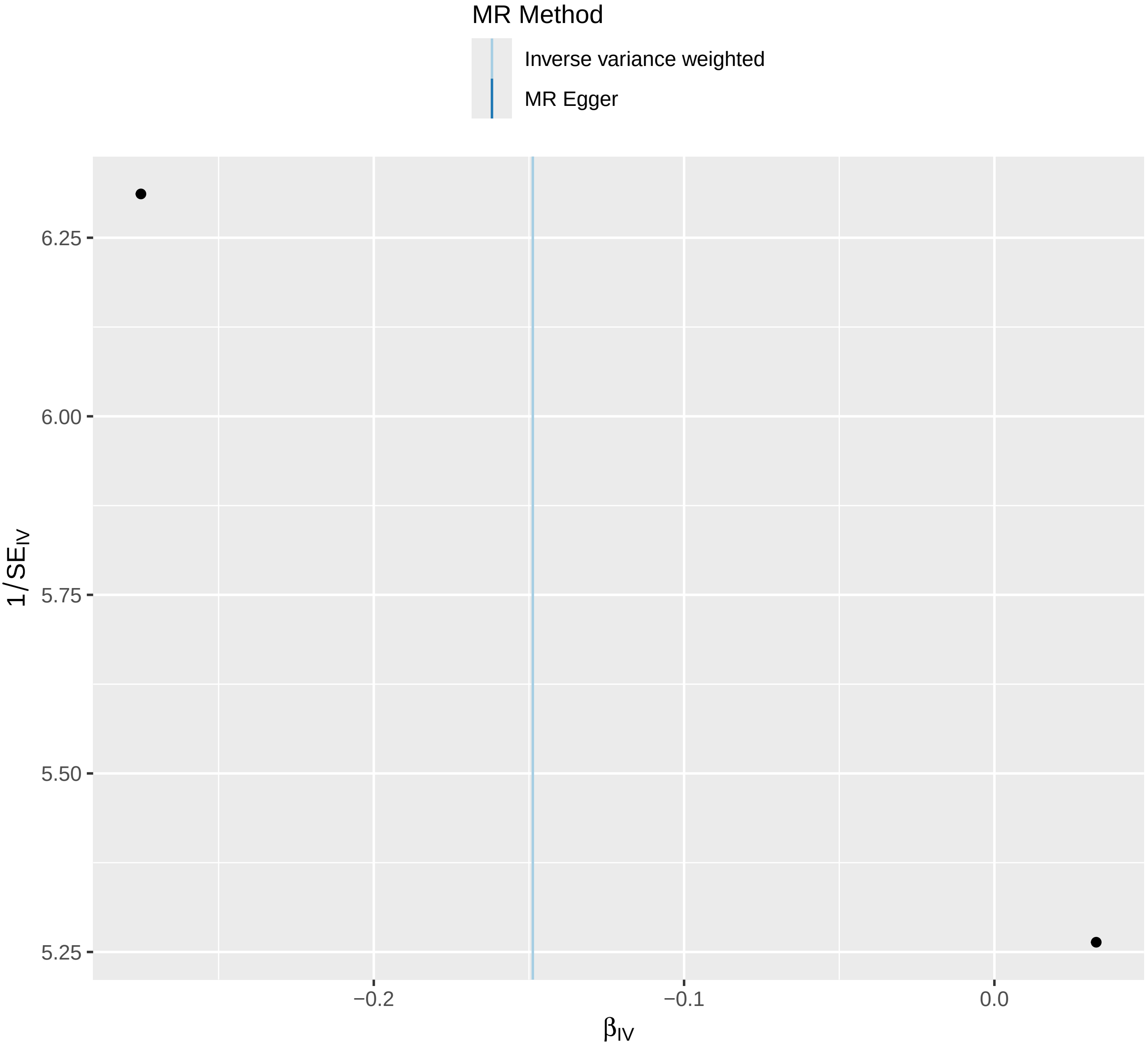
MR Method
Inverse variance weighted
MR Egger
6.25
6.00
5.75
5.50
5.25
−0.2
−0.1
0.0
1/SE_IV
β_IV

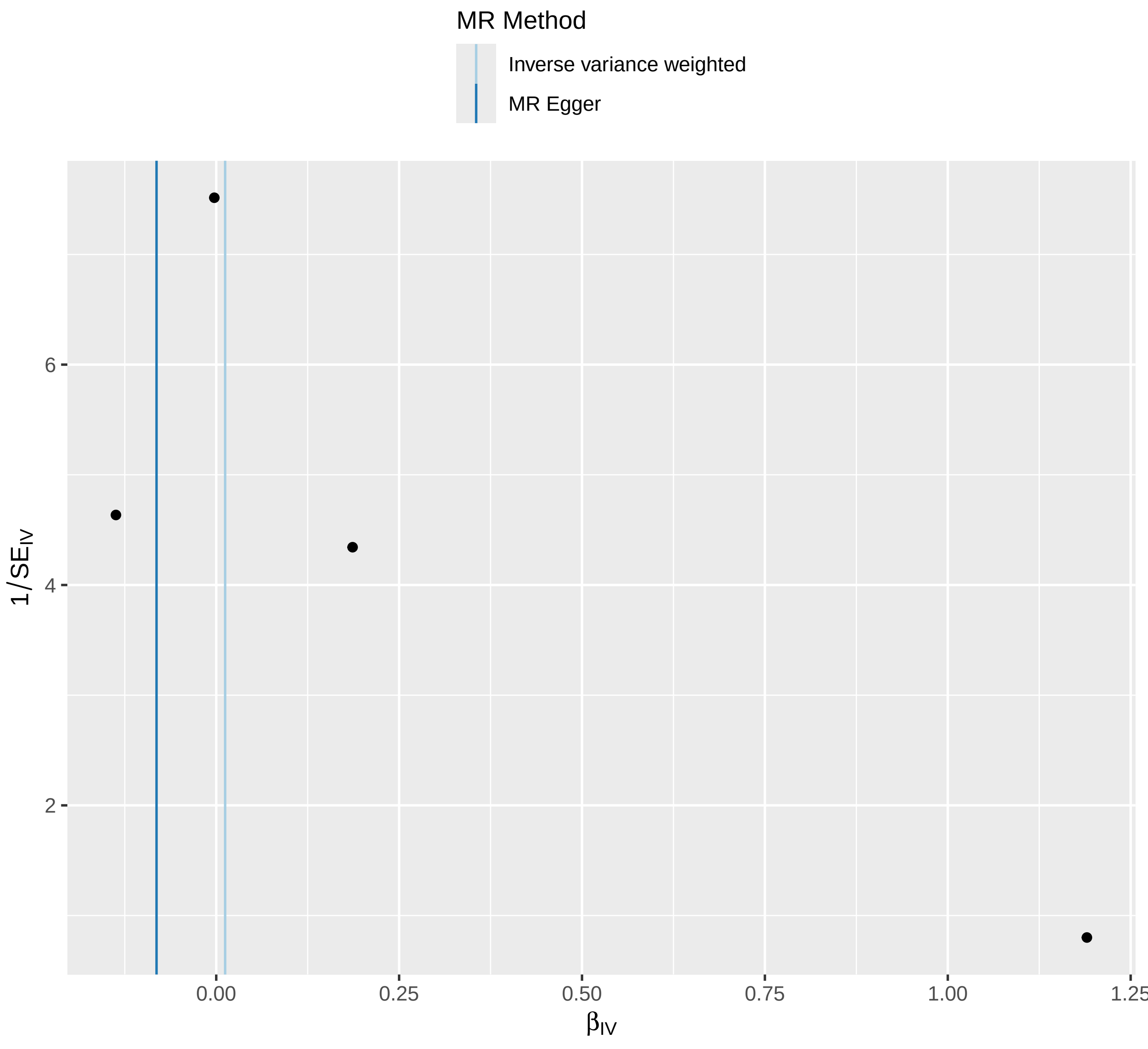

MR Method
Inverse variance weighted
MR Egger
6
4
2
$1/SE_{IV}$
0.00
0.25
0.50
0.75
1.00
1.25
$\beta_{IV}$

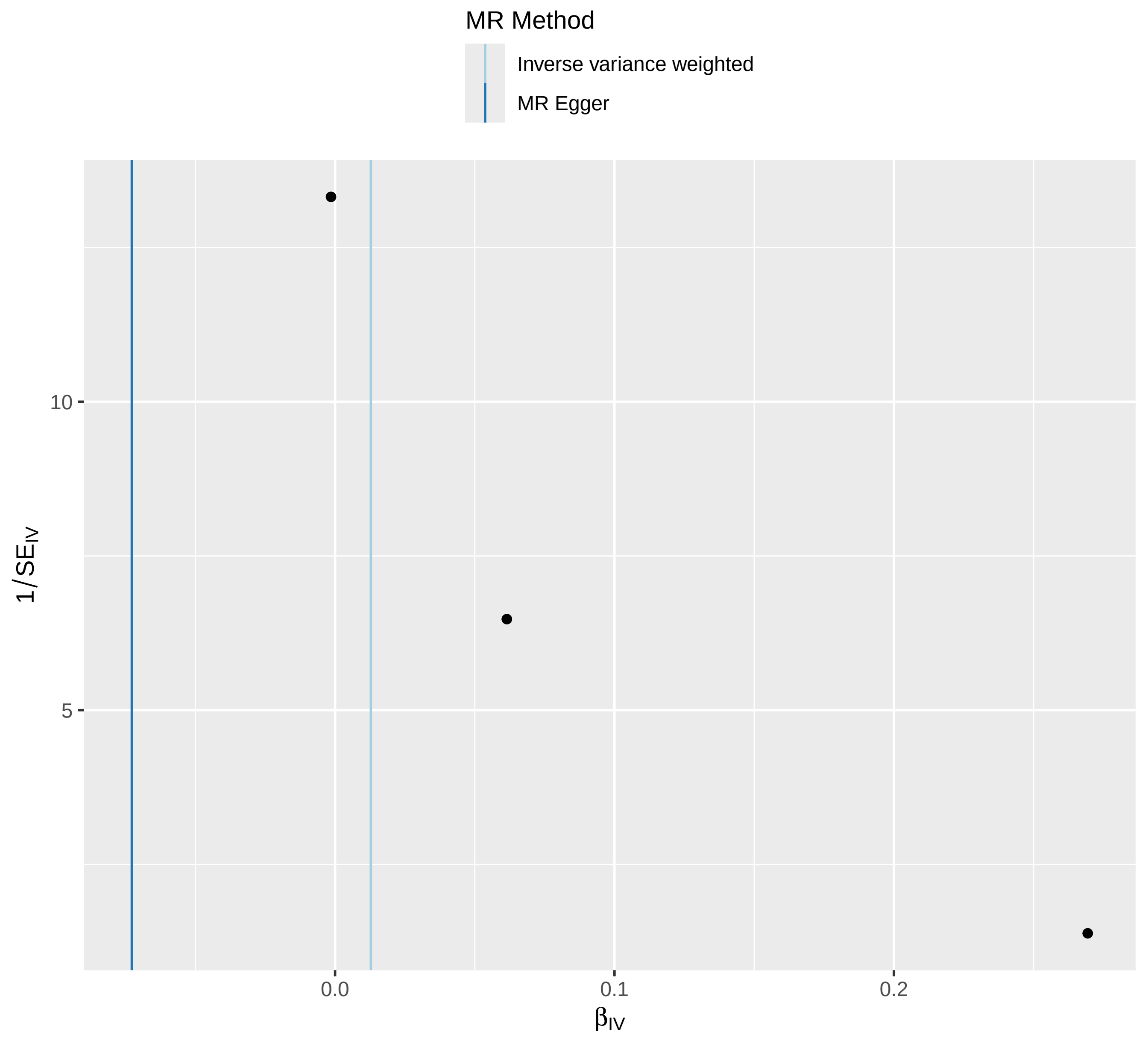
MR Method
Inverse variance weighted
MR Egger
10
5
$1/SE_{IV}$
0.0
0.1
0.2
$\beta_{IV}$

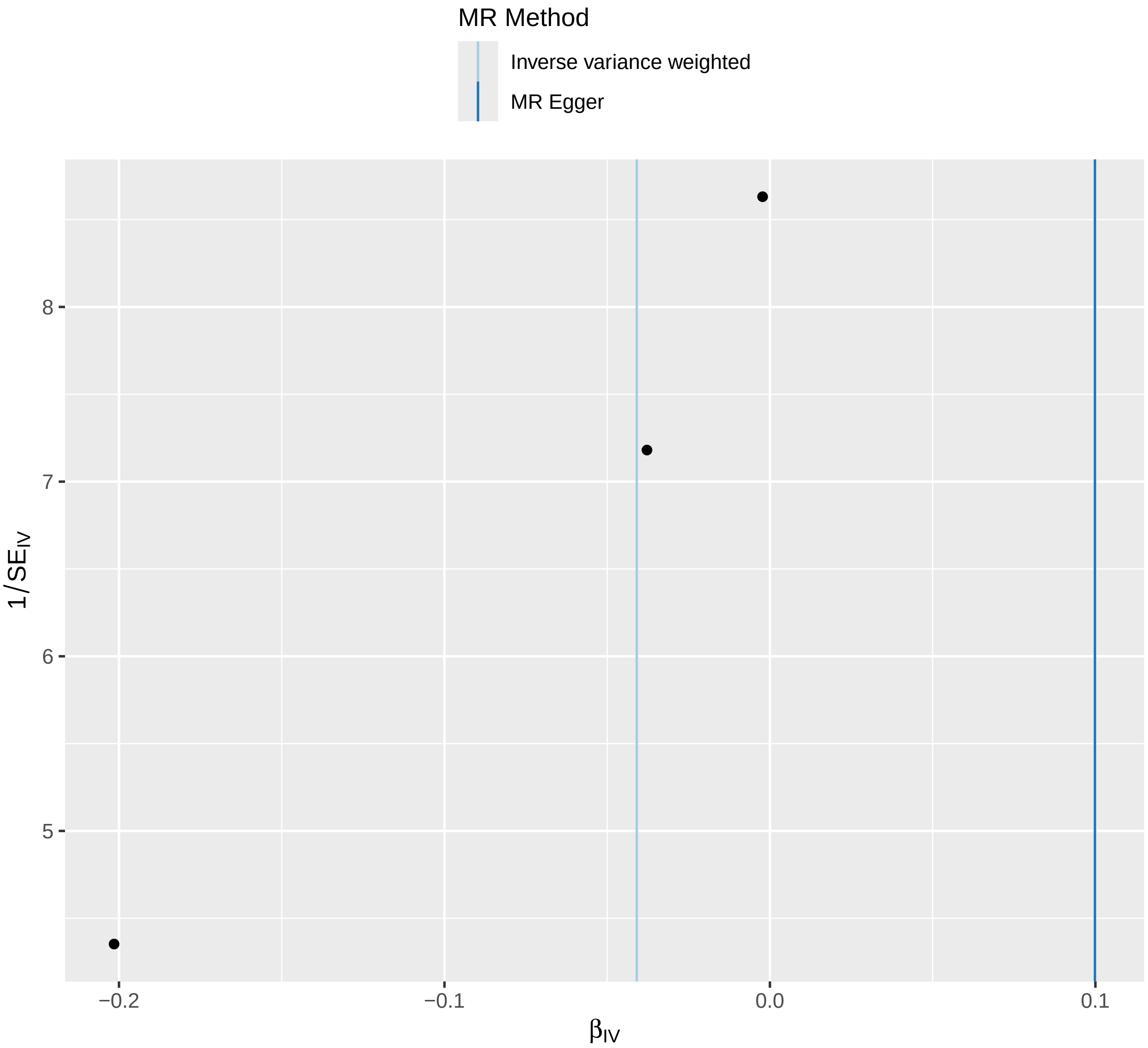

MR Method
Inverse variance weighted
MR Egger
8
7
6
5
$1/SE_{IV}$
−0.2
−0.1
0.0
0.1
$\beta_{IV}$

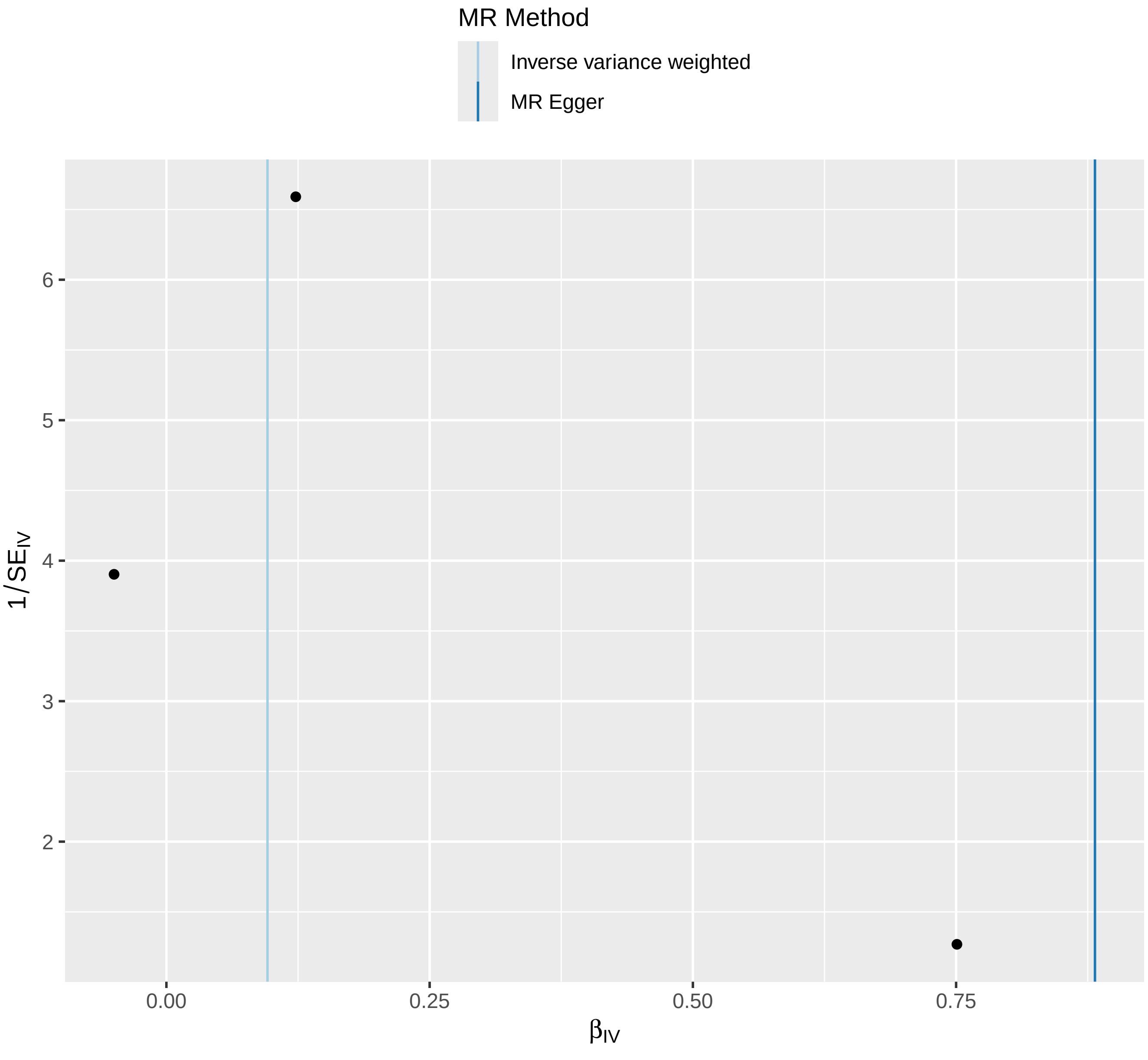
MR Method
Inverse variance weighted
MR Egger
6
5
4
3
2
1/SE_IV
0.00
0.25
0.50
0.75
β_IV

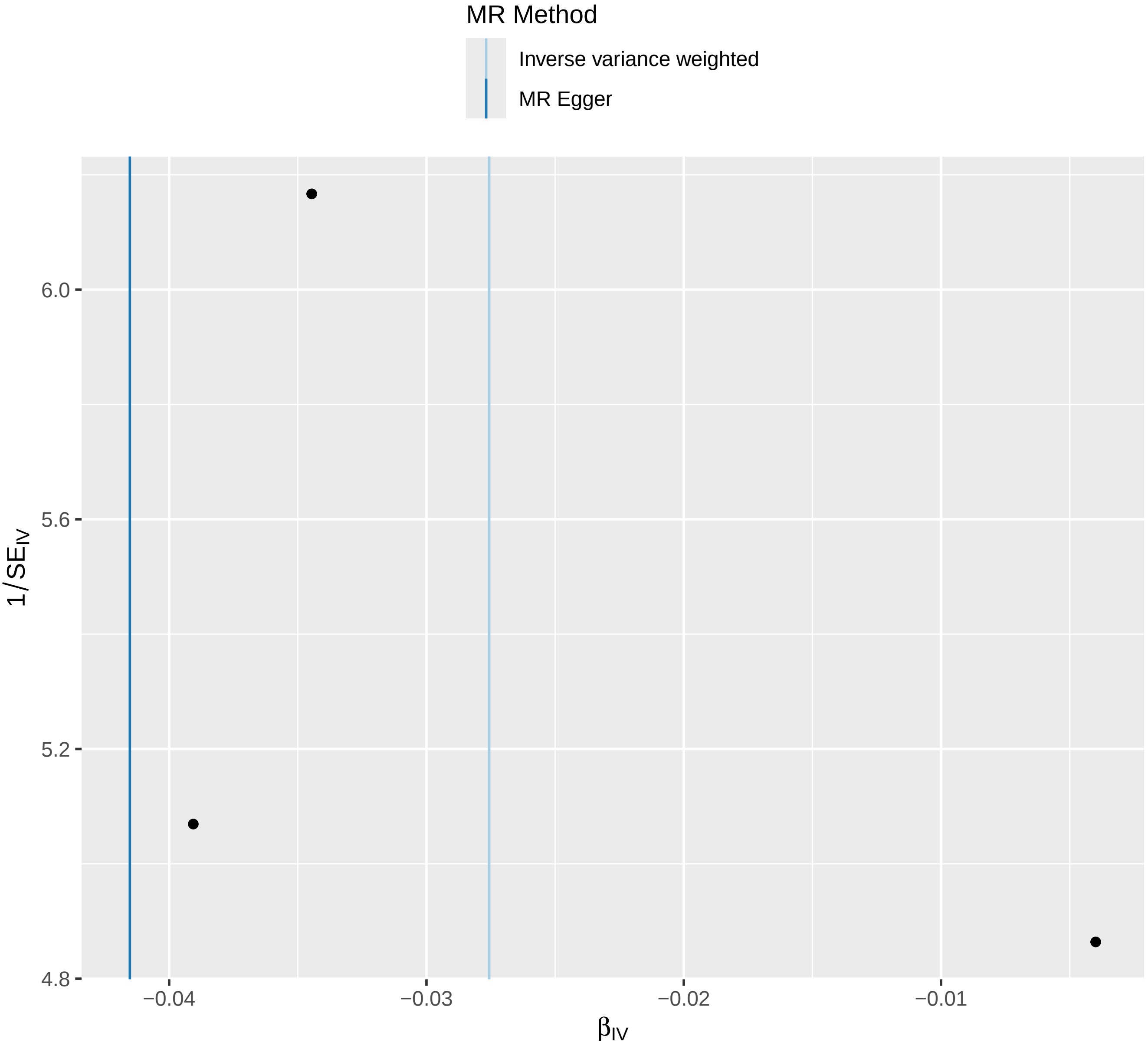
MR Method
Inverse variance weighted
MR Egger
6.0
5.6
5.2
4.8
1/SE$_{IV}$
−0.04
−0.03
−0.02
−0.01
β$_{IV}$

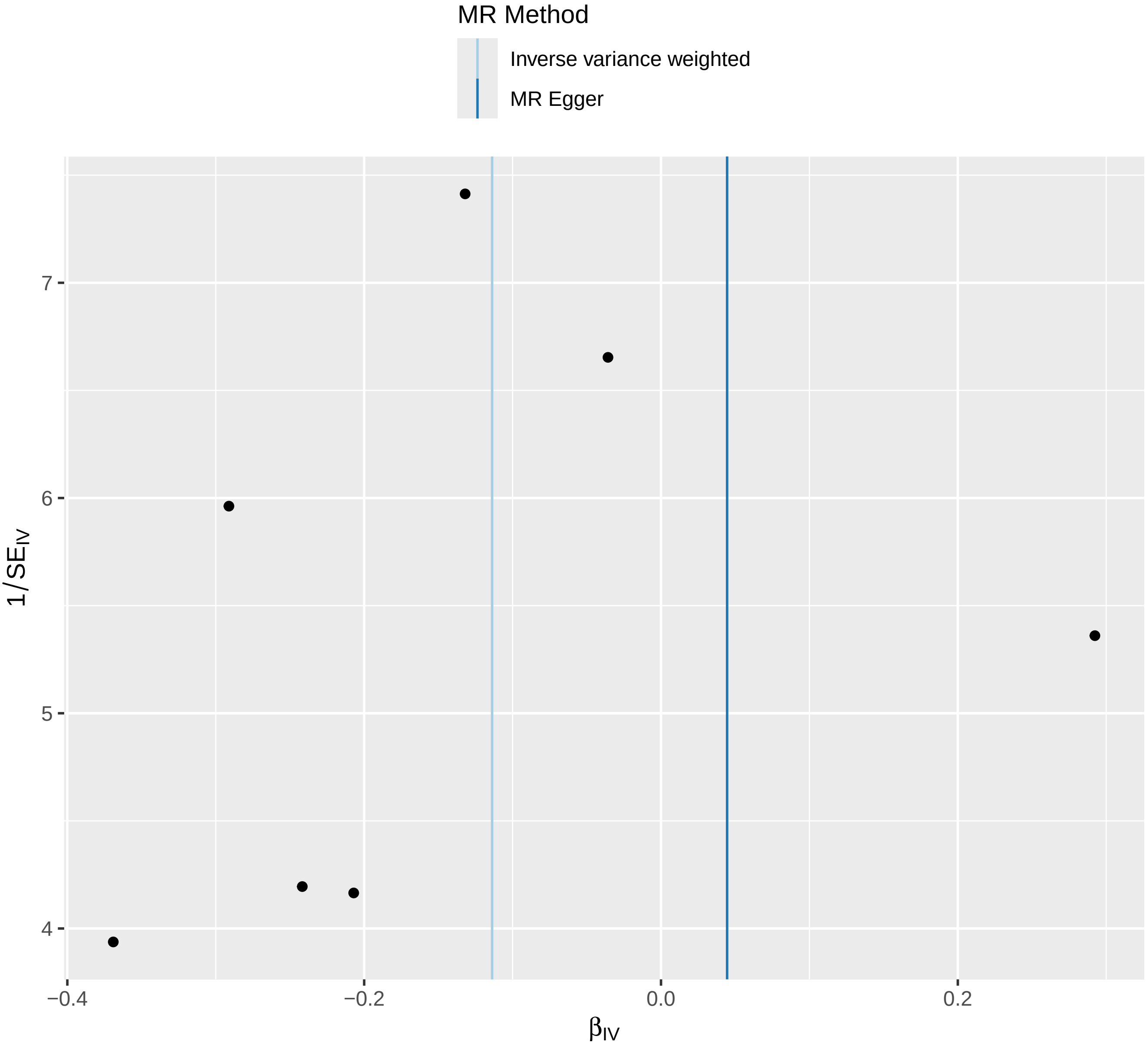

MR Method
Inverse variance weighted
MR Egger
7
6
5
4
1/SE$_{IV}$
−0.4
−0.2
0.0
0.2
β$_{IV}$

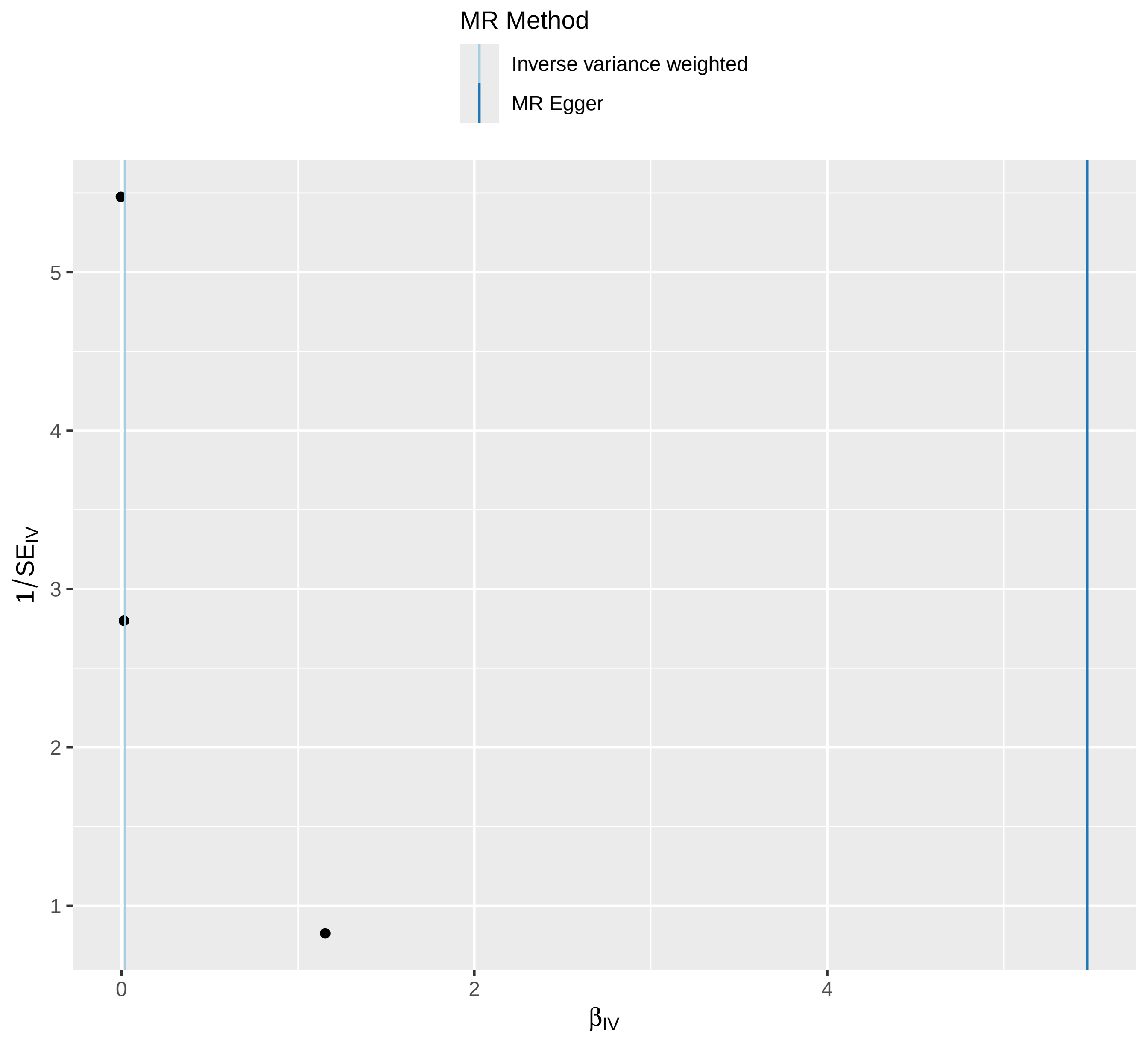
MR Method
Inverse variance weighted
MR Egger
1/SE_IV
5
4
3
2
1
0
2
4
β_IV

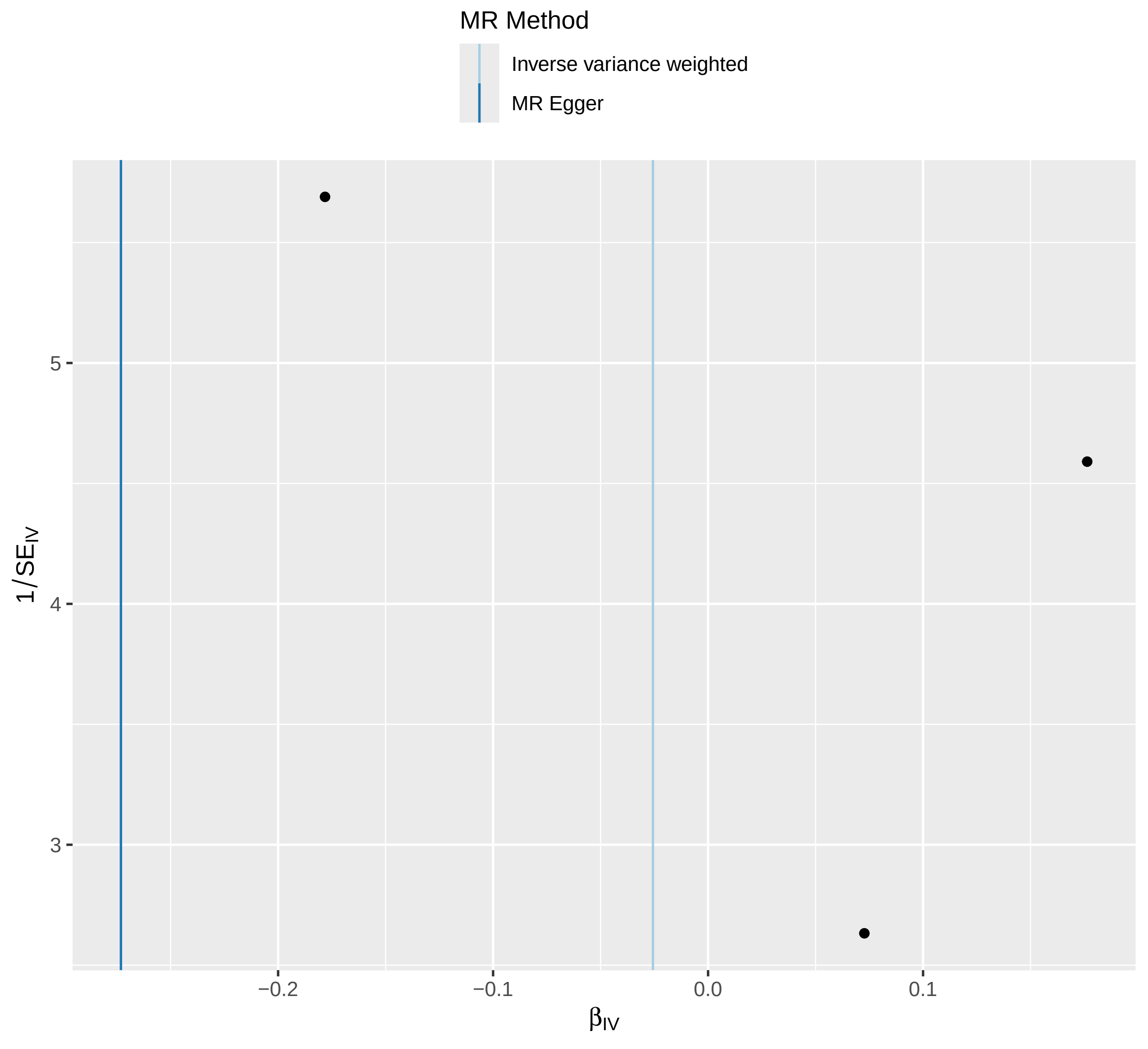

MR Method
Inverse variance weighted
MR Egger
5
4
3
1/SE$_{IV}$
−0.2
−0.1
0.0
0.1
β$_{IV}$

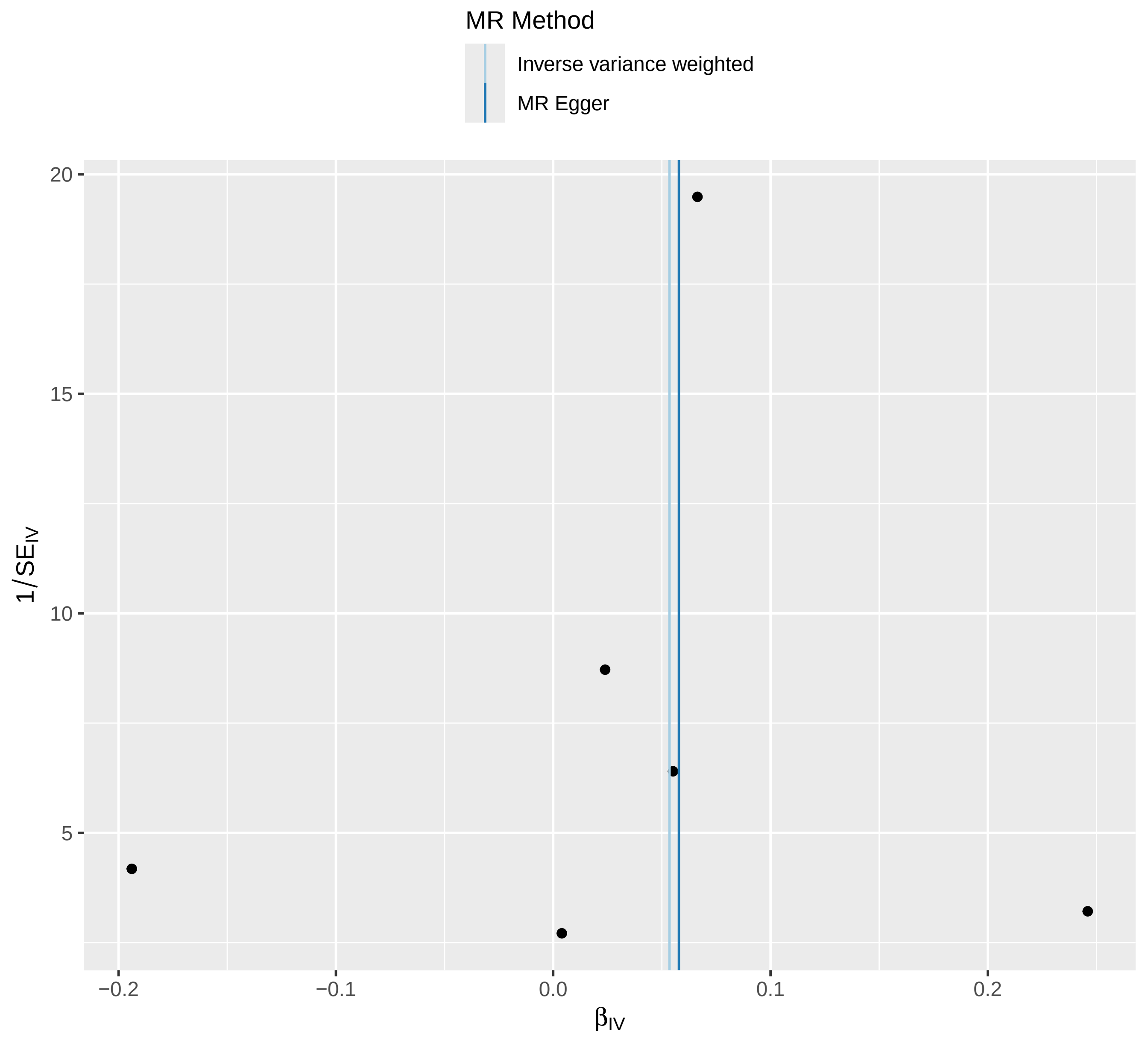
MR Method
Inverse variance weighted
MR Egger
20
15
10
5
$1/SE_{IV}$
−0.2
−0.1
0.0
0.1
0.2
$\beta_{IV}$

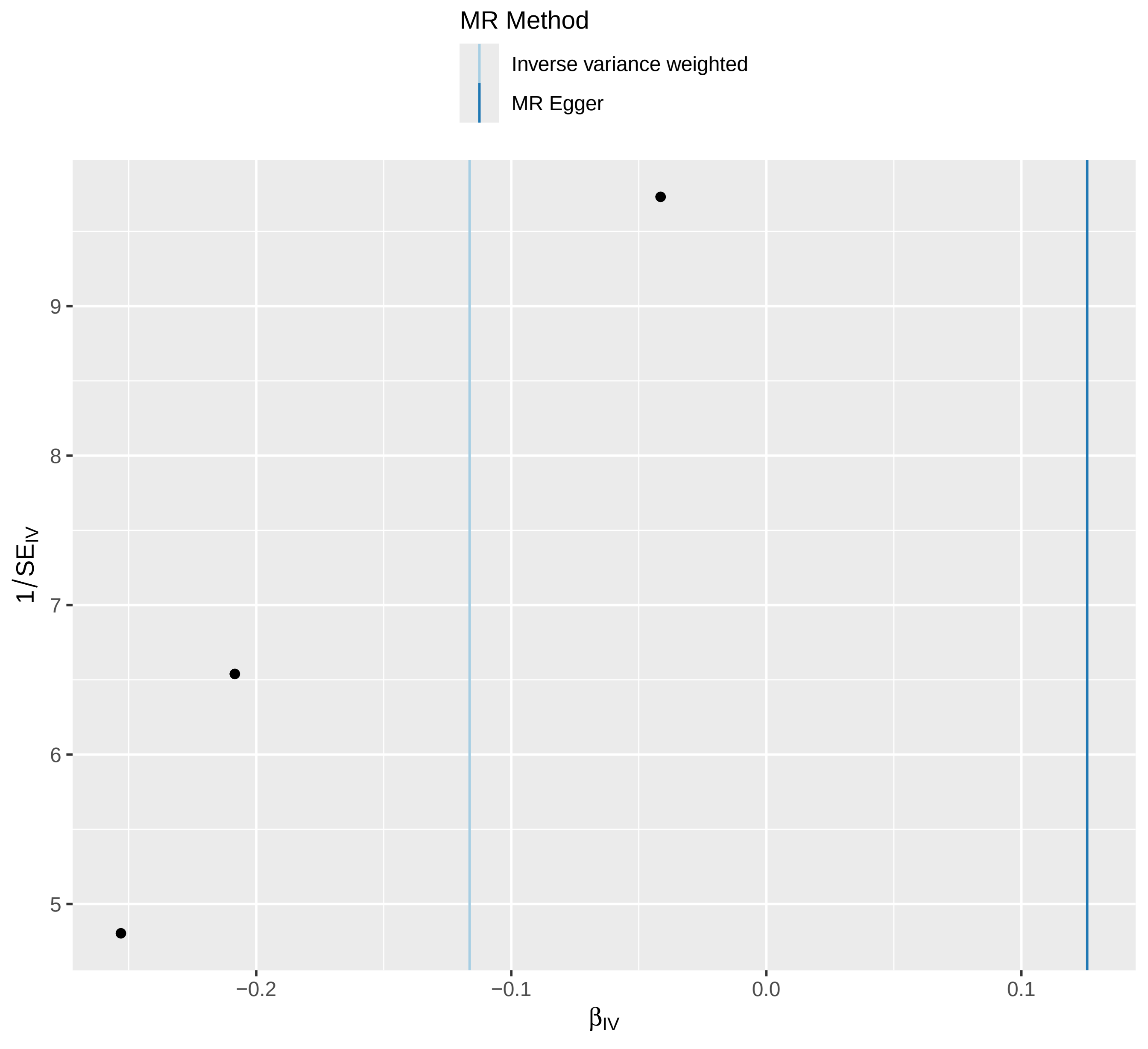
MR Method
Inverse variance weighted
MR Egger
1/SE_IV
9
8
7
6
5
−0.2
−0.1
0.0
0.1
β_IV

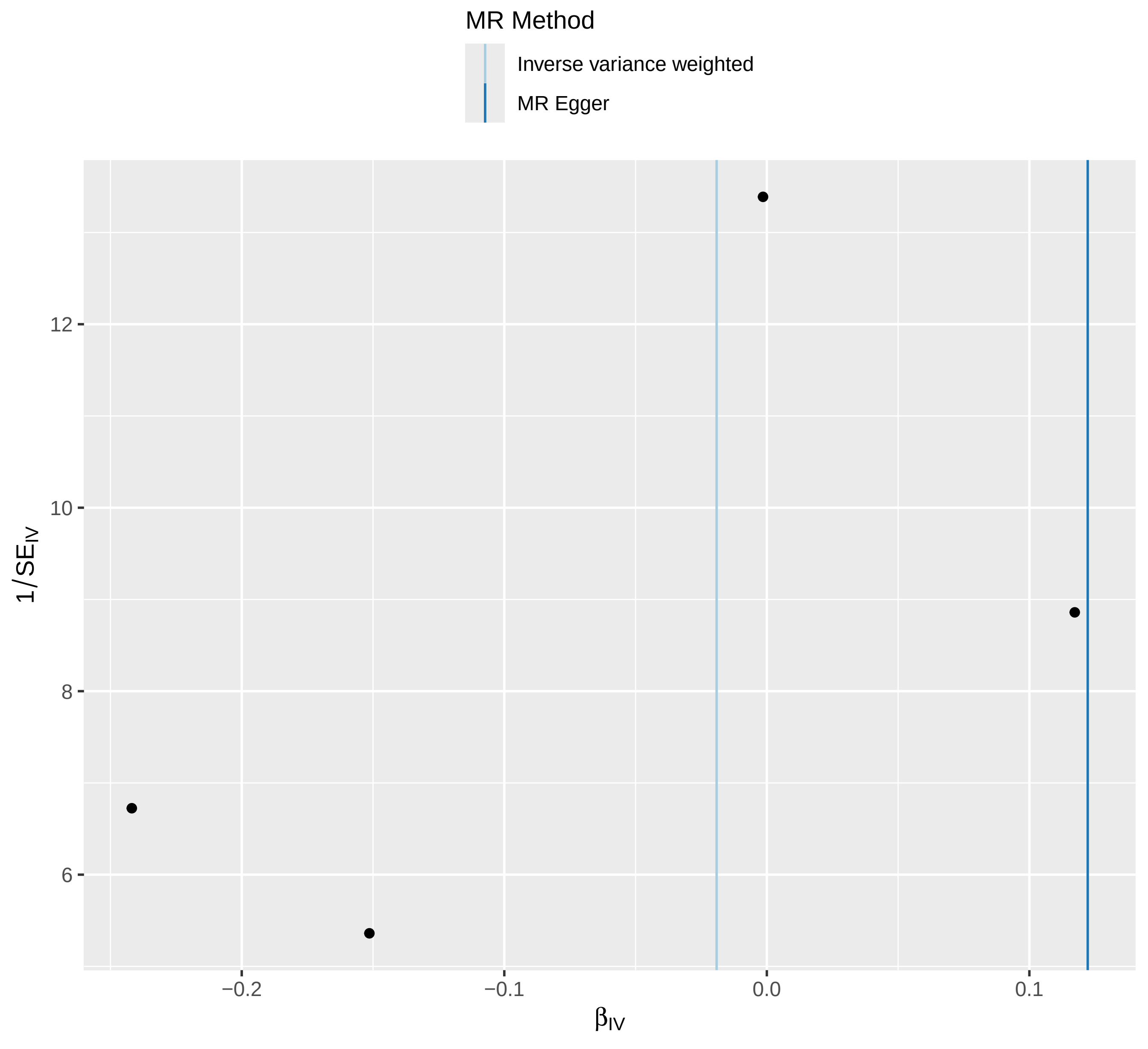
MR Method
Inverse variance weighted
MR Egger
1/SE_IV
12
10
8
6
−0.2
−0.1
0.0
0.1
β_IV

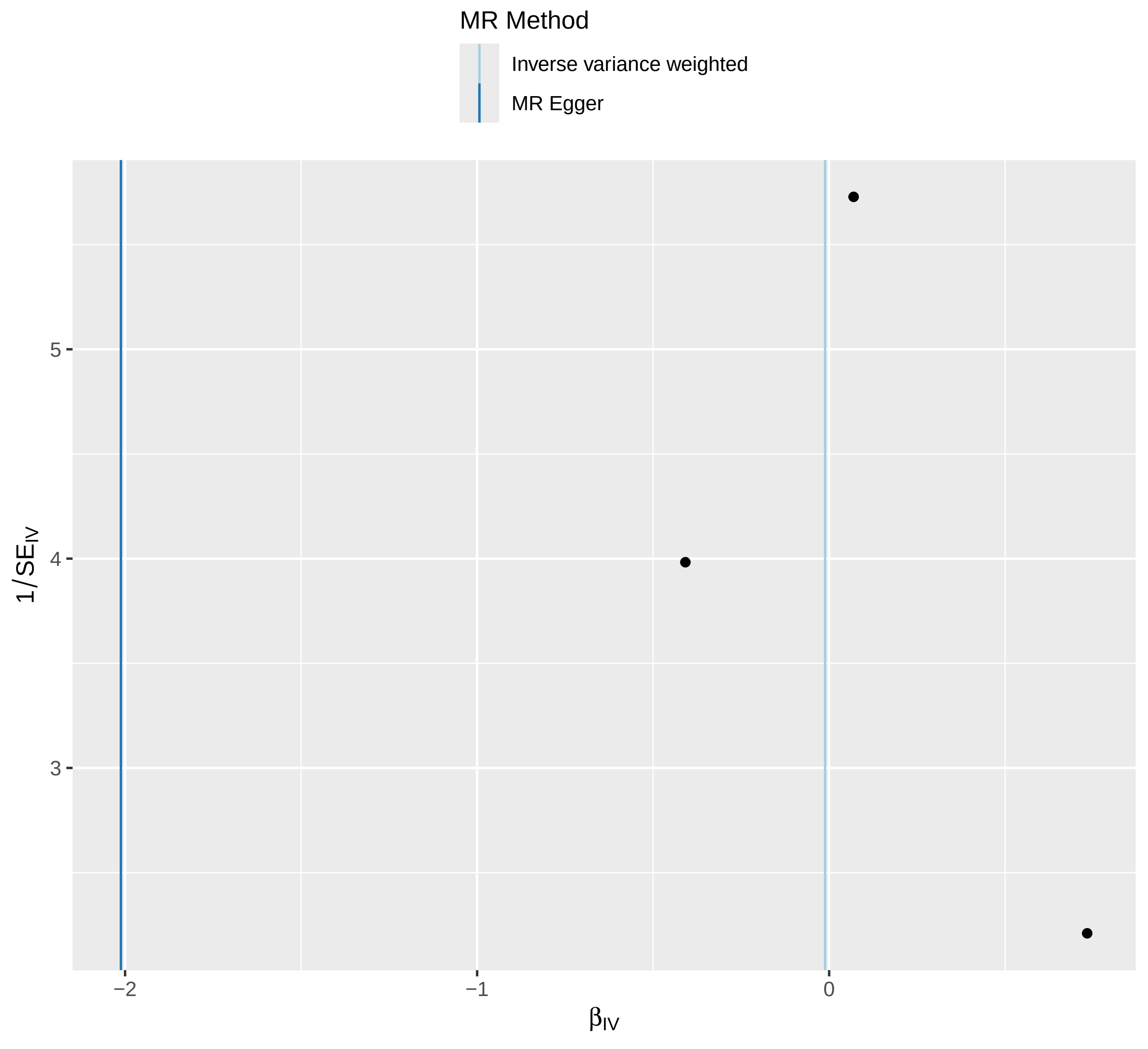
MR Method
Inverse variance weighted
MR Egger
5
4
3
1/SE_IV
−2
−1
0
β_IV

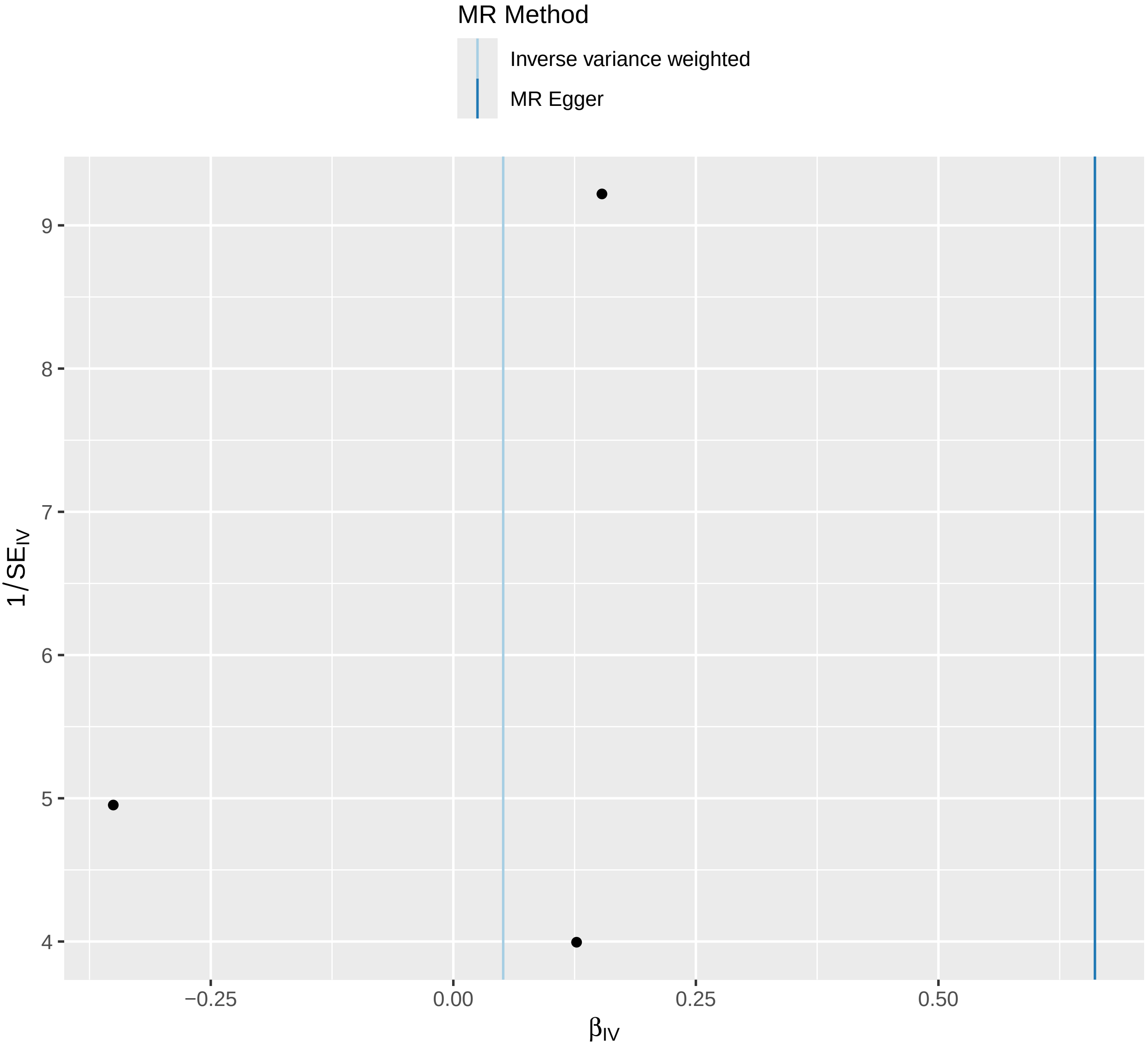

MR Method
Inverse variance weighted
MR Egger
9
8
7
6
5
4
1/SE$_{IV}$
−0.25
0.00
0.25
0.50
β$_{IV}$

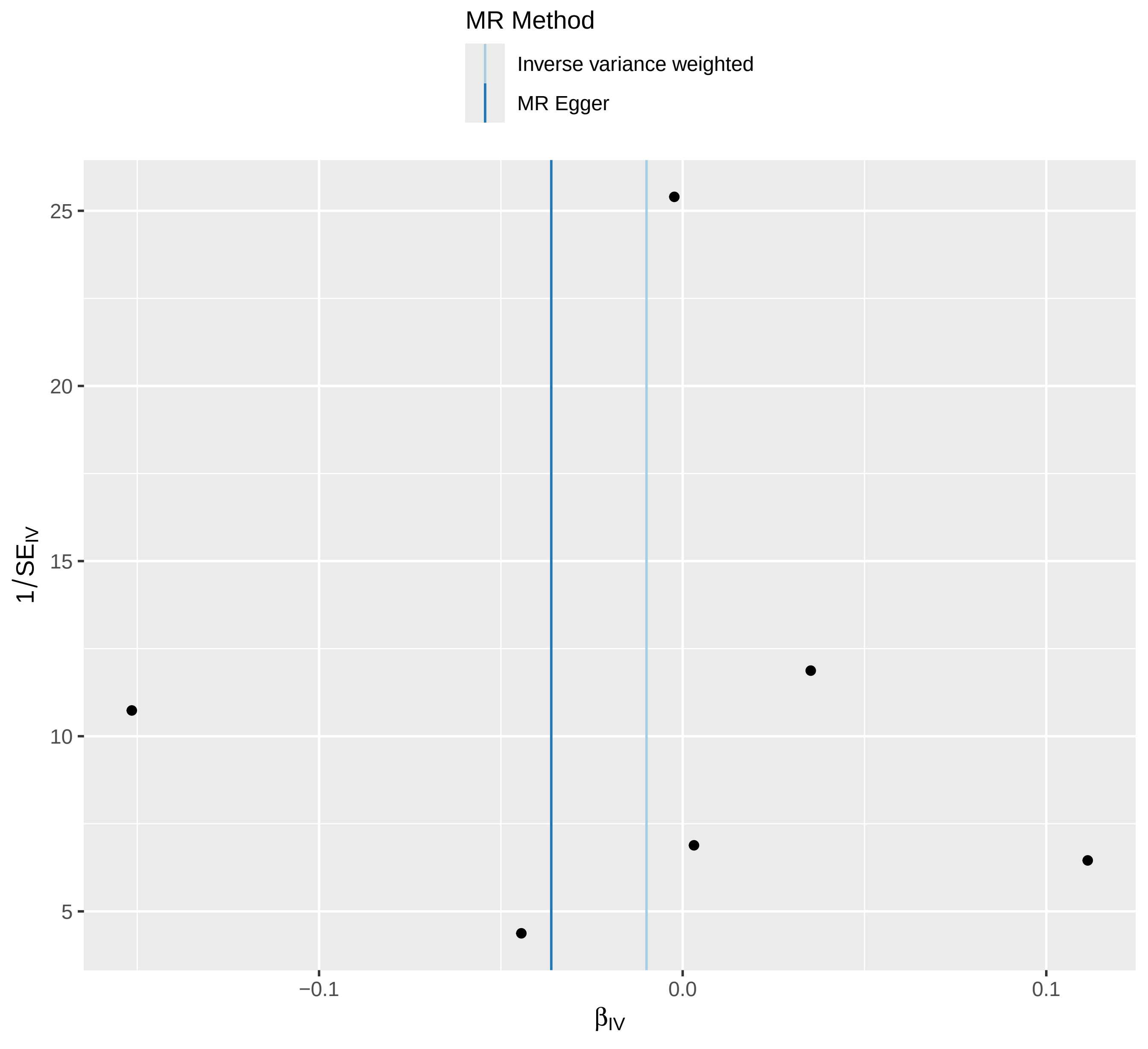

MR Method
Inverse variance weighted
MR Egger
25
20
15
10
5
1/SE$_{IV}$
−0.1
0.0
0.1
β$_{IV}$

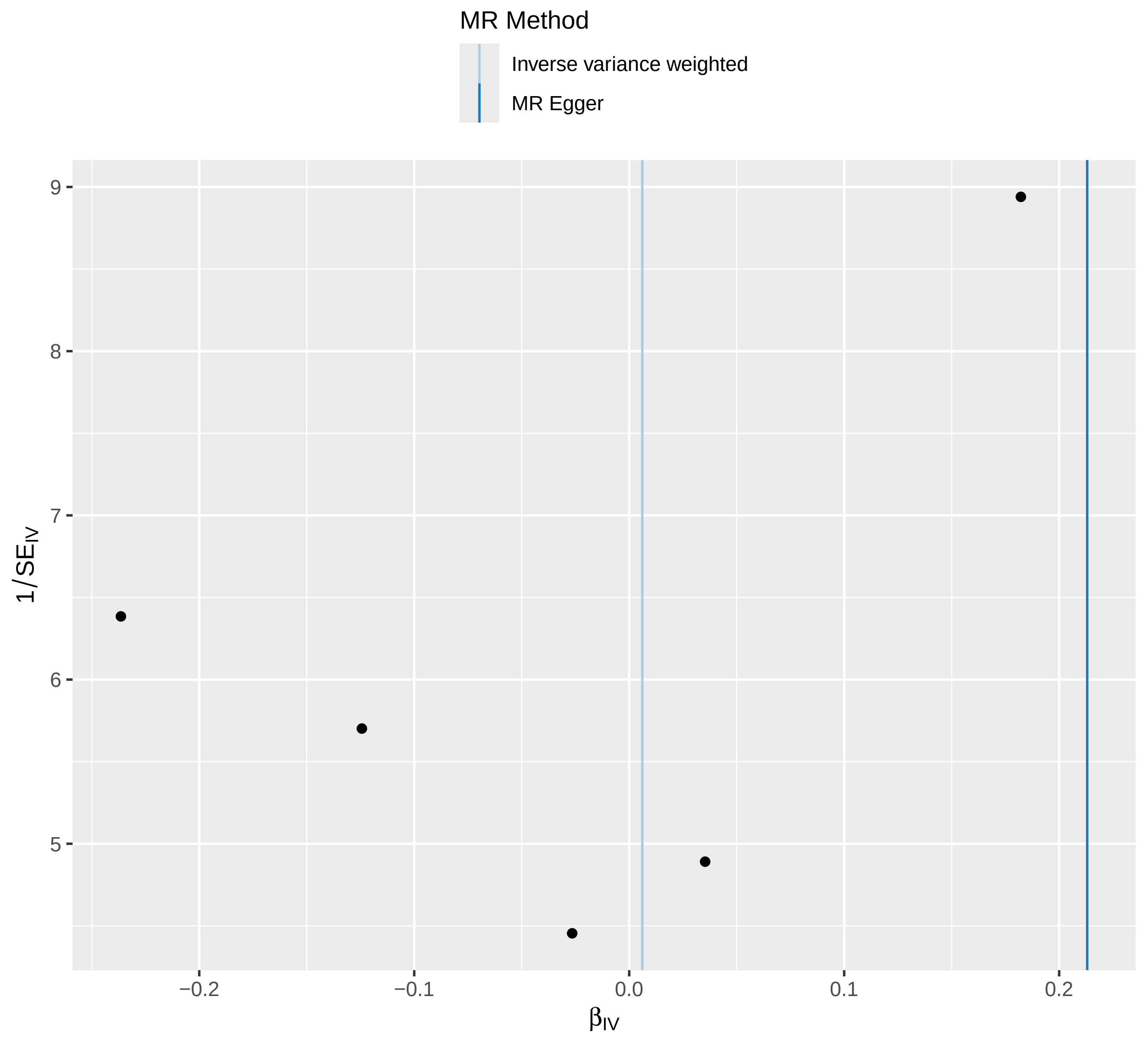
MR Method
Inverse variance weighted
MR Egger
9
8
7
6
5
1/SE_IV
−0.2
−0.1
0.0
0.1
0.2
β_IV

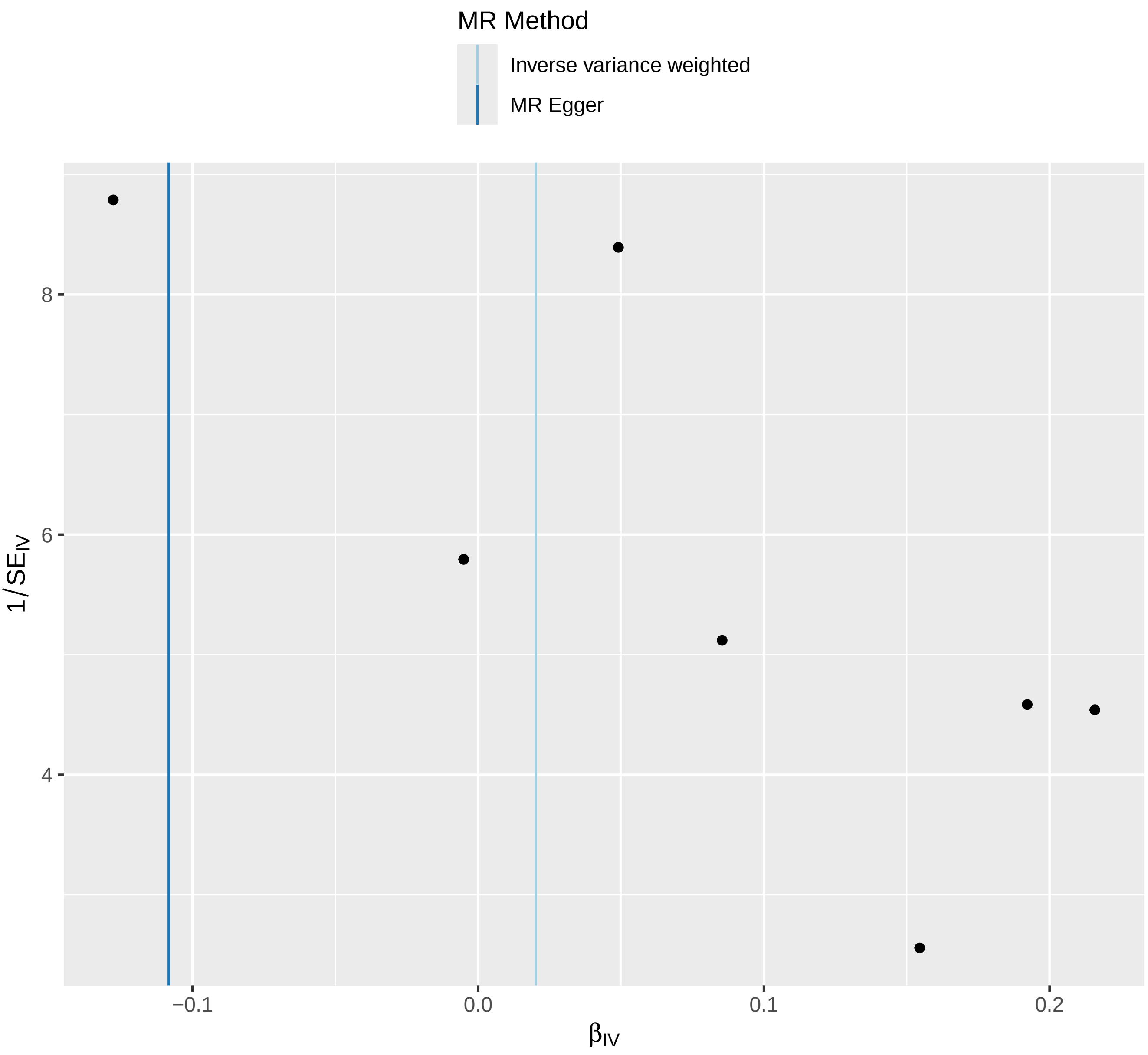

MR Method
Inverse variance weighted
MR Egger
8
6
4
1/SE_IV
−0.1
0.0
0.1
0.2
β_IV

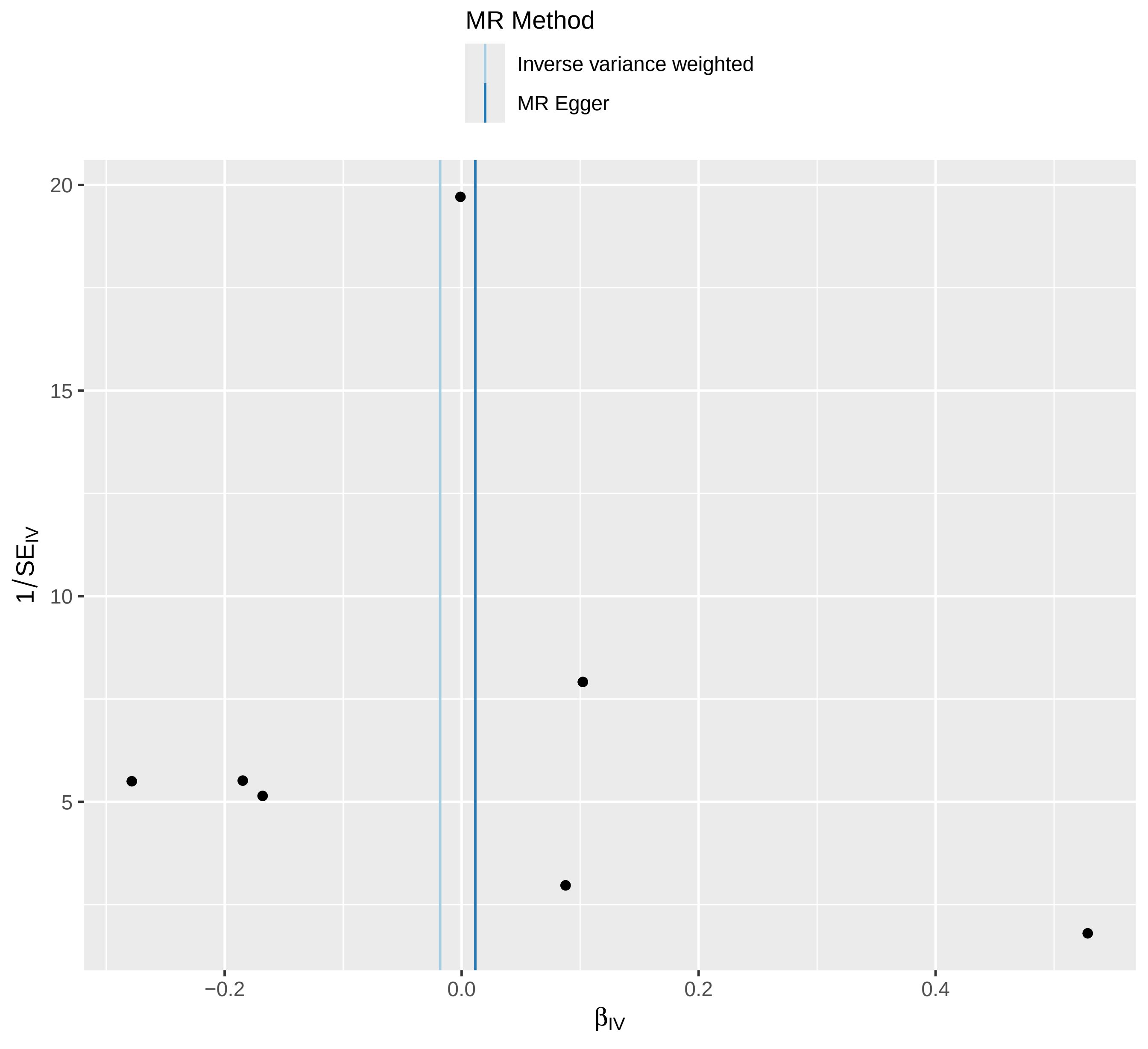

MR Method
Inverse variance weighted
MR Egger
20
15
10
5
1/SE$_{IV}$
−0.2
0.0
0.2
0.4
β$_{IV}$

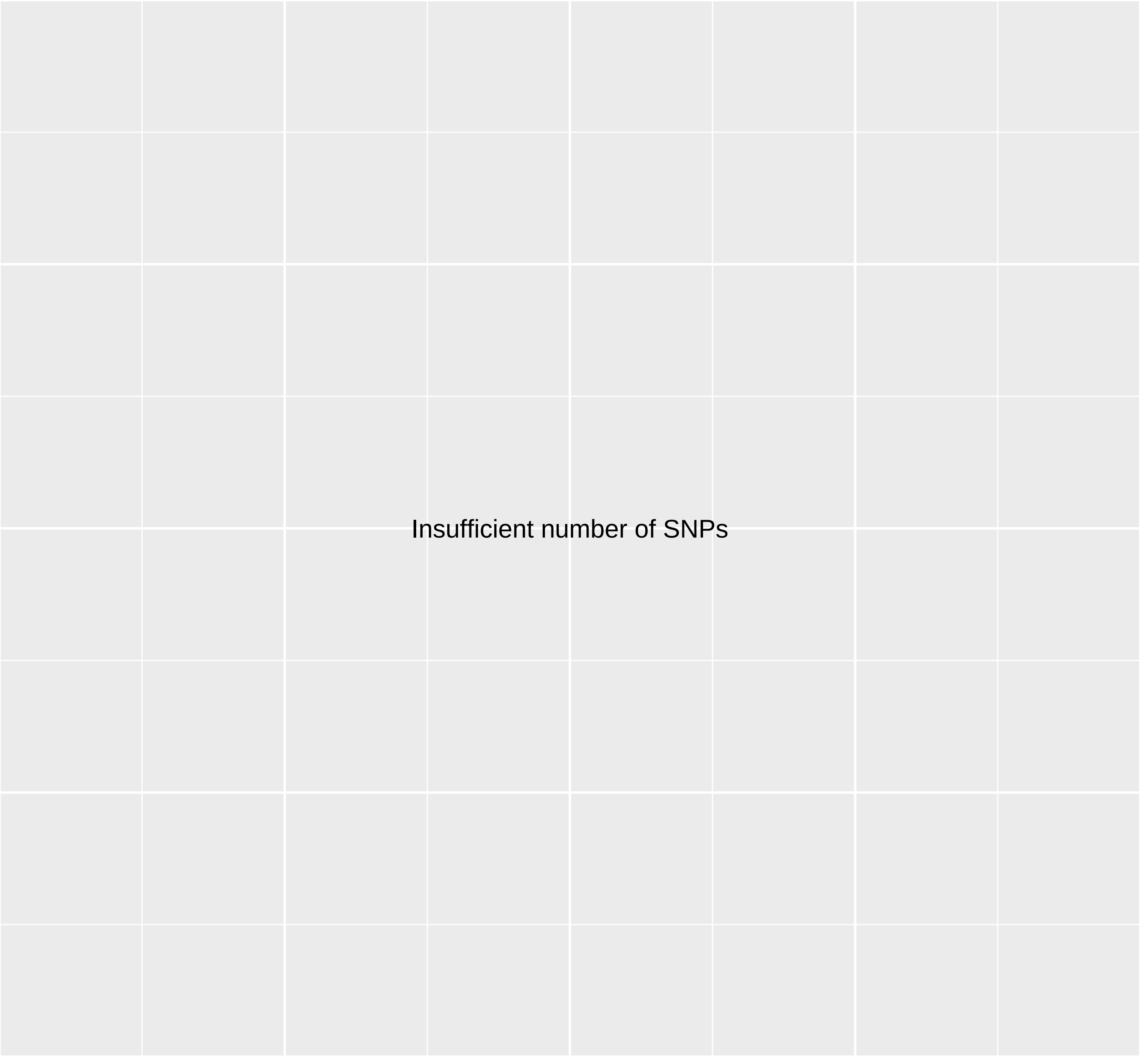
Insufficient number of SNPs

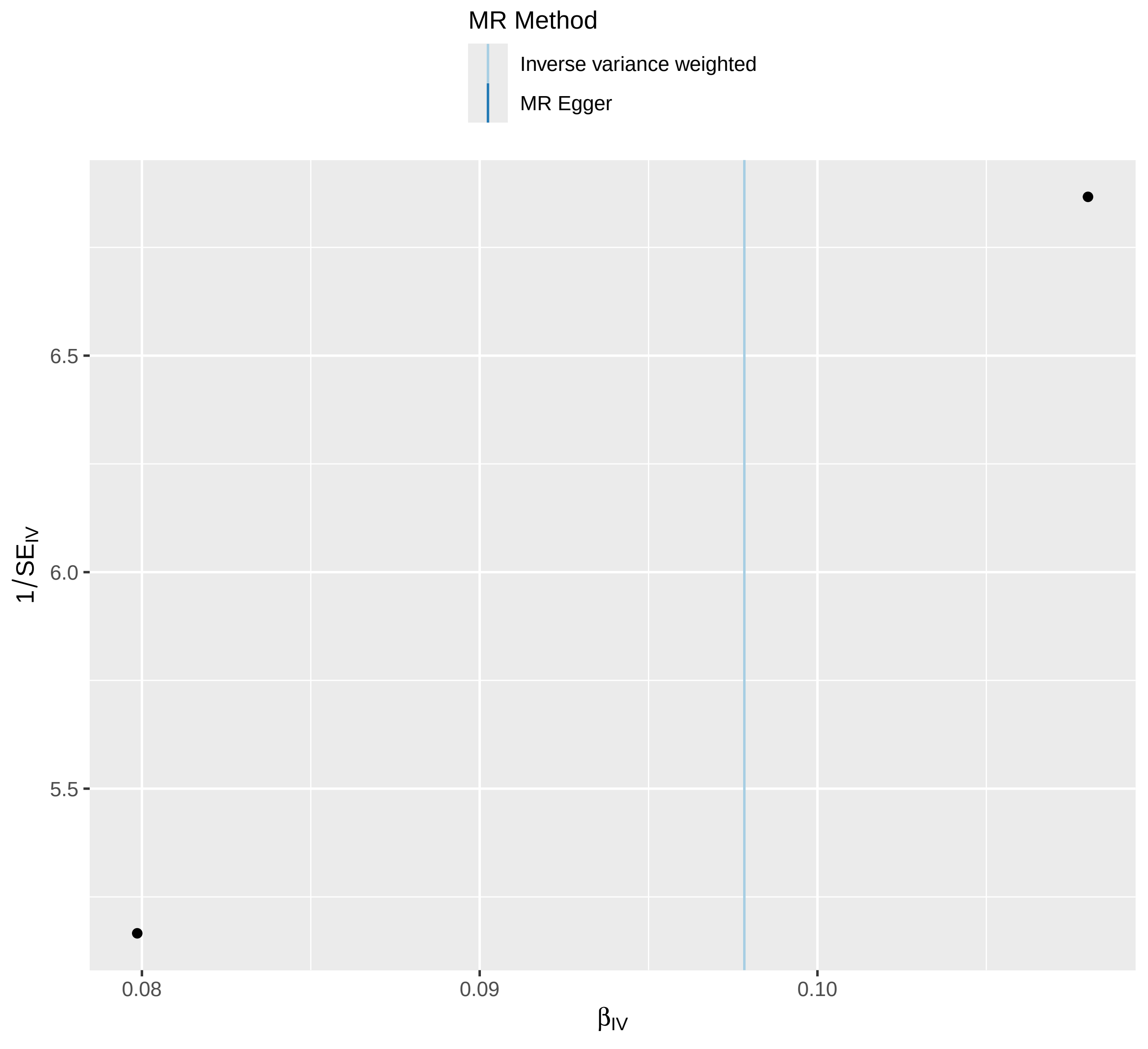
MR Method
Inverse variance weighted
MR Egger
6.5
6.0
5.5
1/SE_IV
0.08
0.09
0.10
β_IV

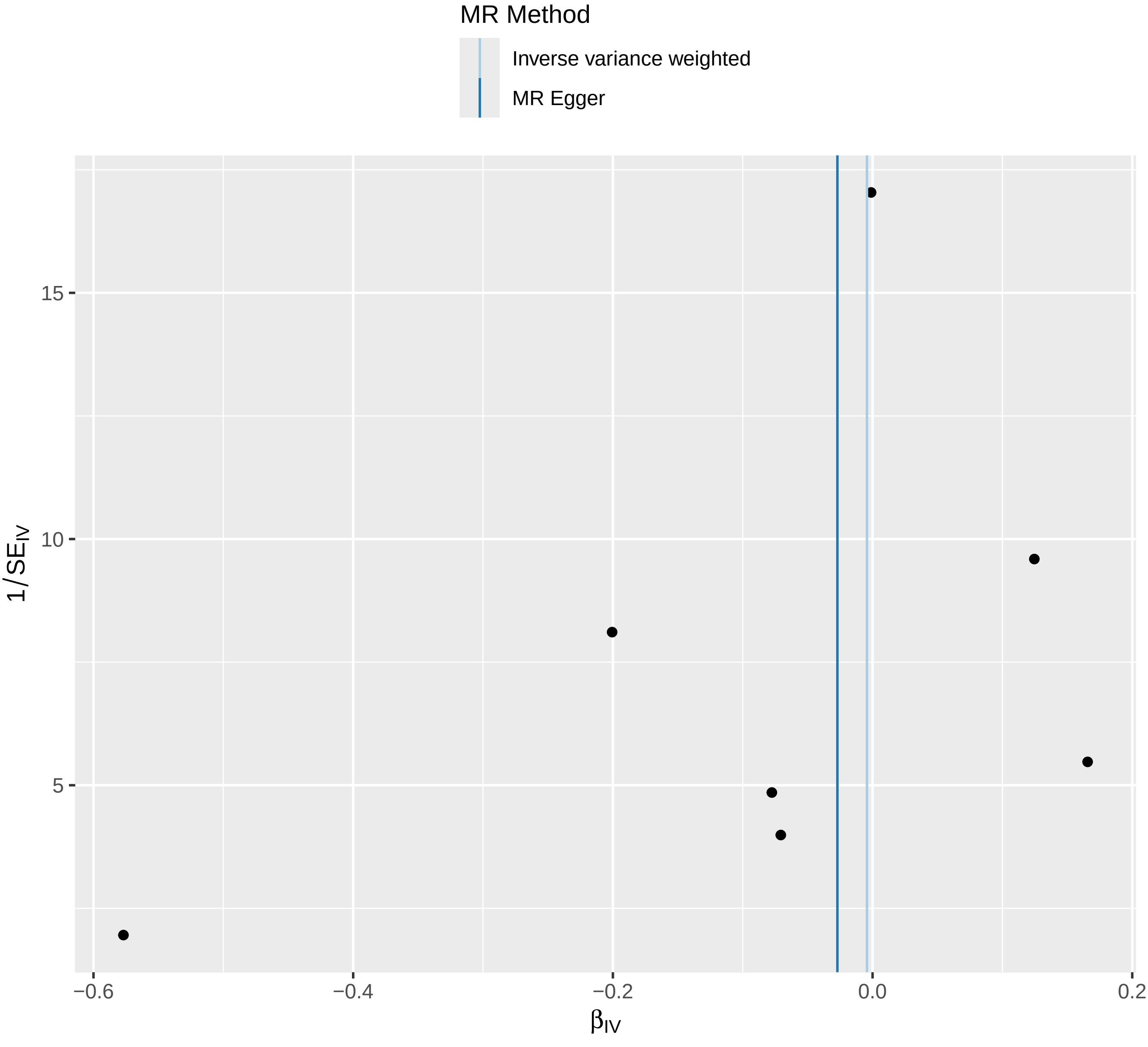

MR Method
Inverse variance weighted
MR Egger
15
10
5
$1/SE_{IV}$
−0.6
−0.4
−0.2
0.0
0.2
$\beta_{IV}$

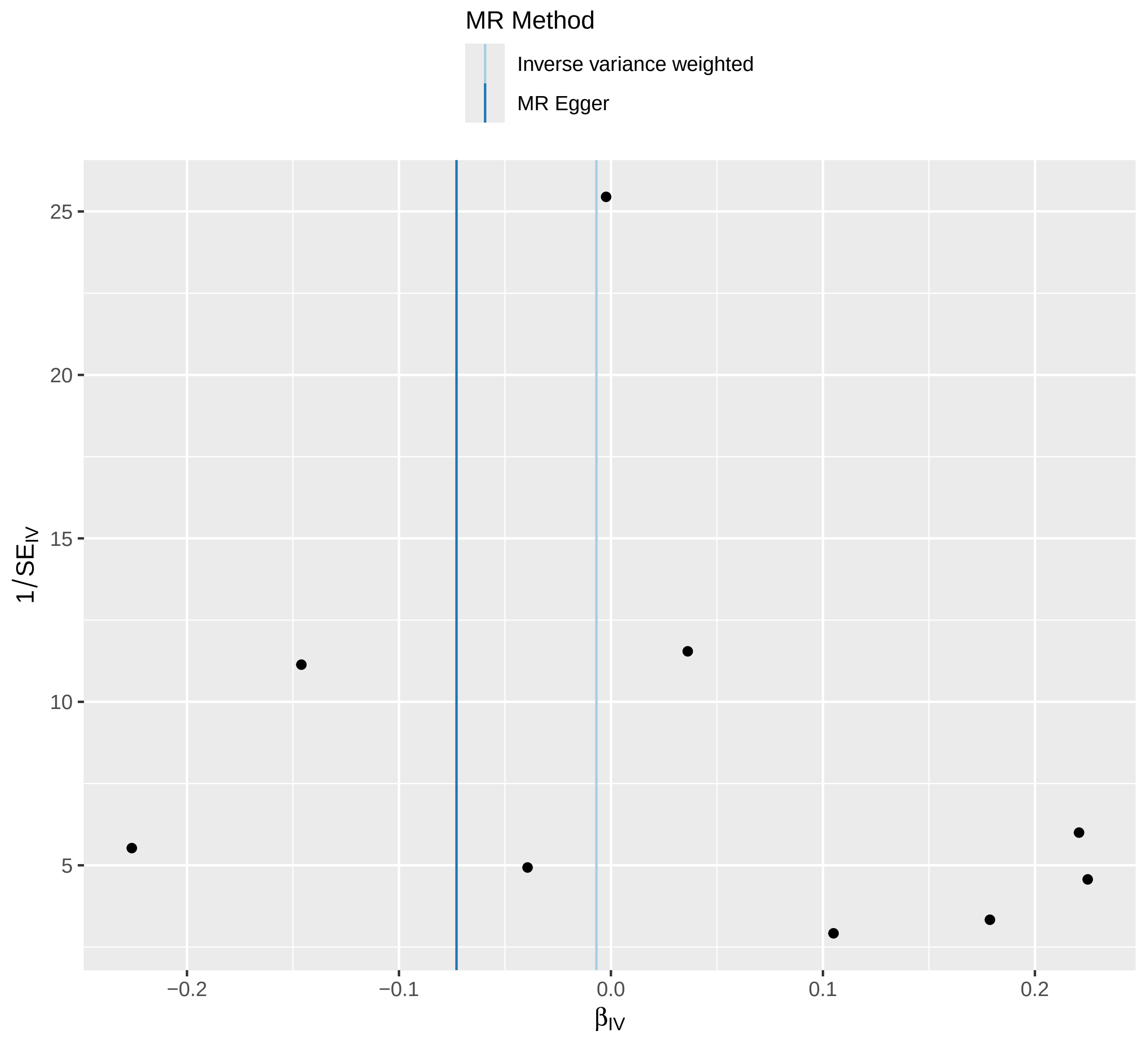
MR Method
Inverse variance weighted
MR Egger
25
20
15
10
5
1/SE_IV
−0.2
−0.1
0.0
0.1
0.2
β_IV

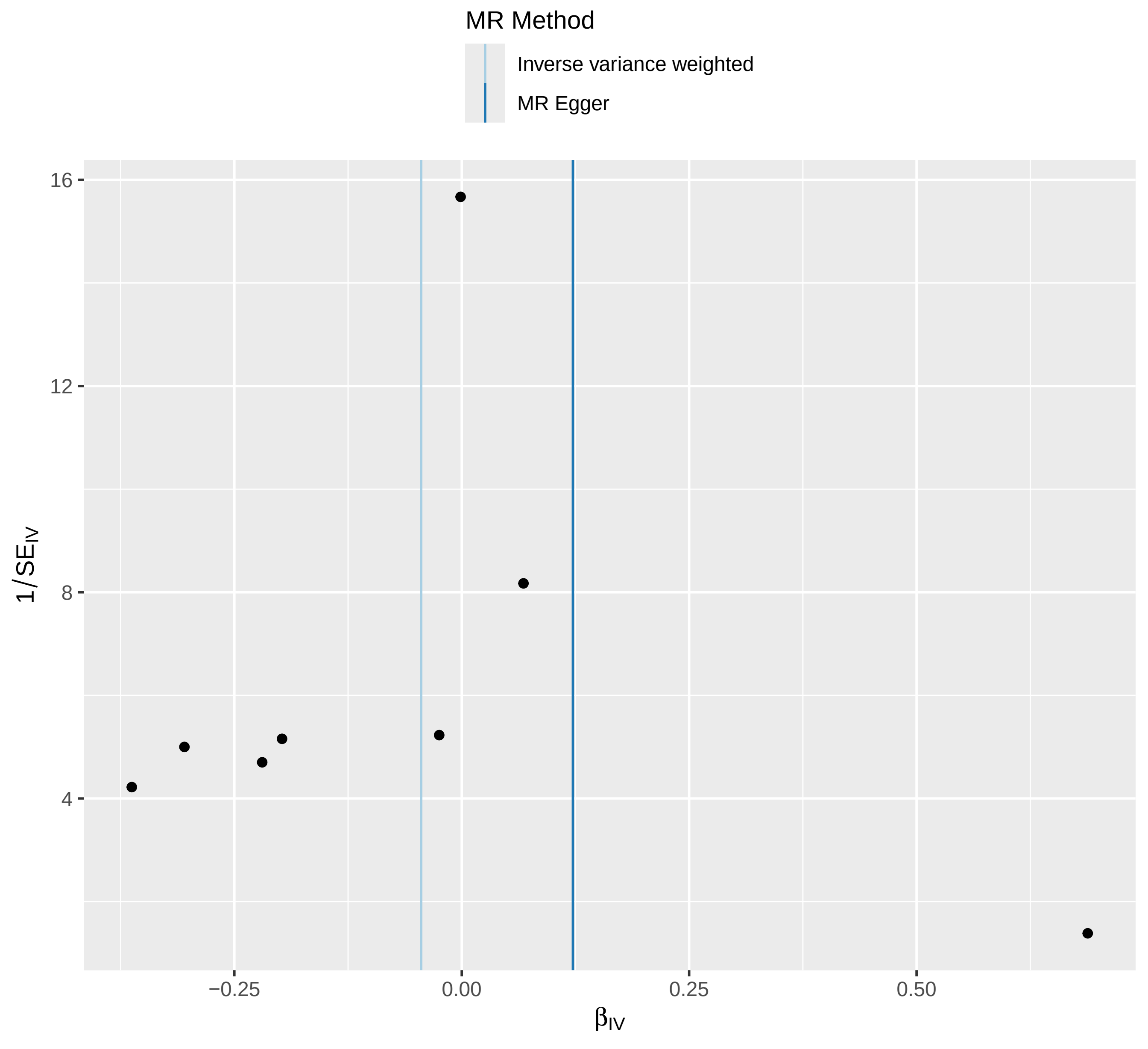

MR Method
Inverse variance weighted
MR Egger
16
12
8
4
1/SE_IV
−0.25
0.00
0.25
0.50
β_IV

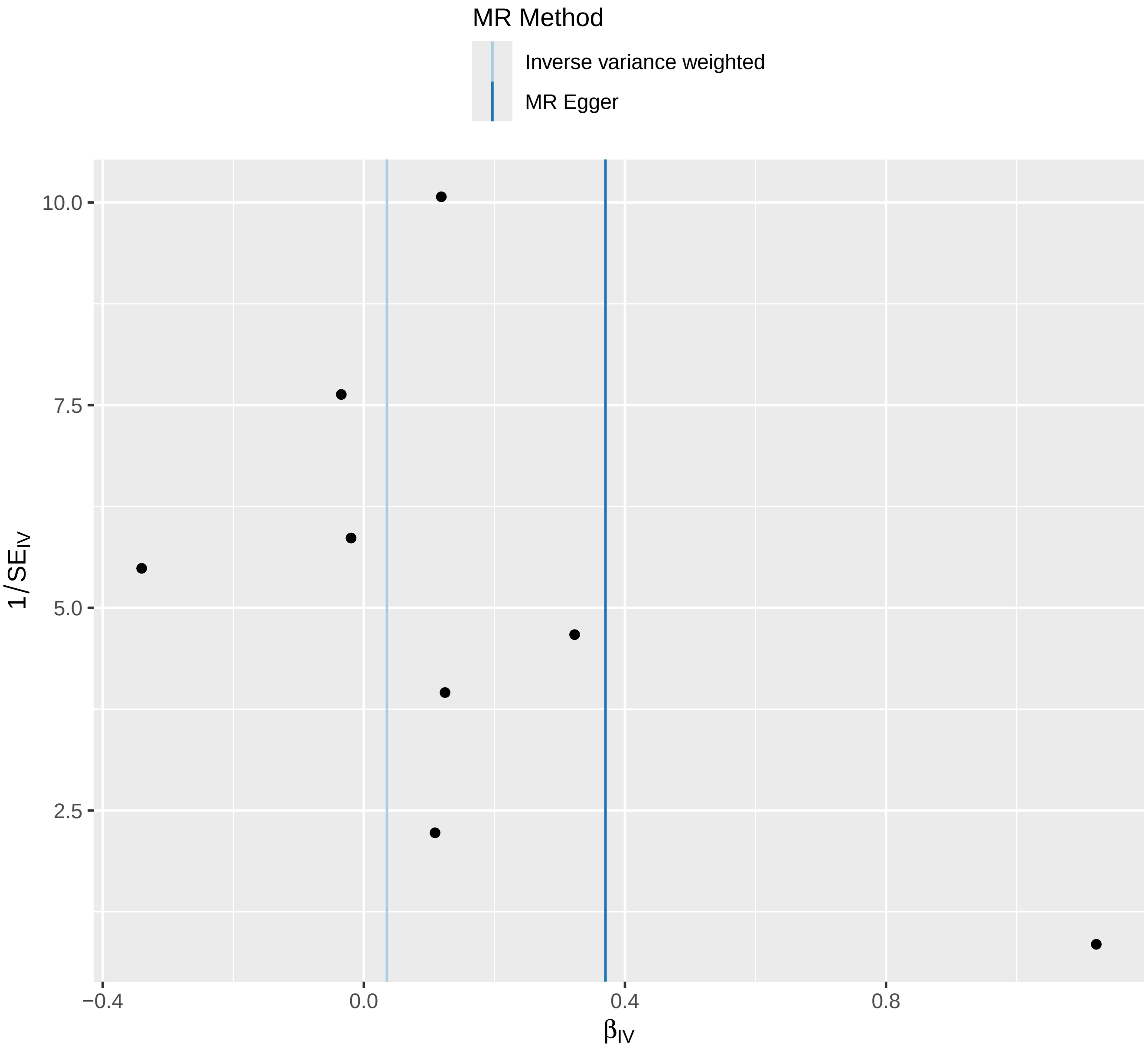
MR Method
Inverse variance weighted
MR Egger
10.0
7.5
5.0
2.5
$1/SE_{IV}$
−0.4
0.0
0.4
0.8
$\beta_{IV}$

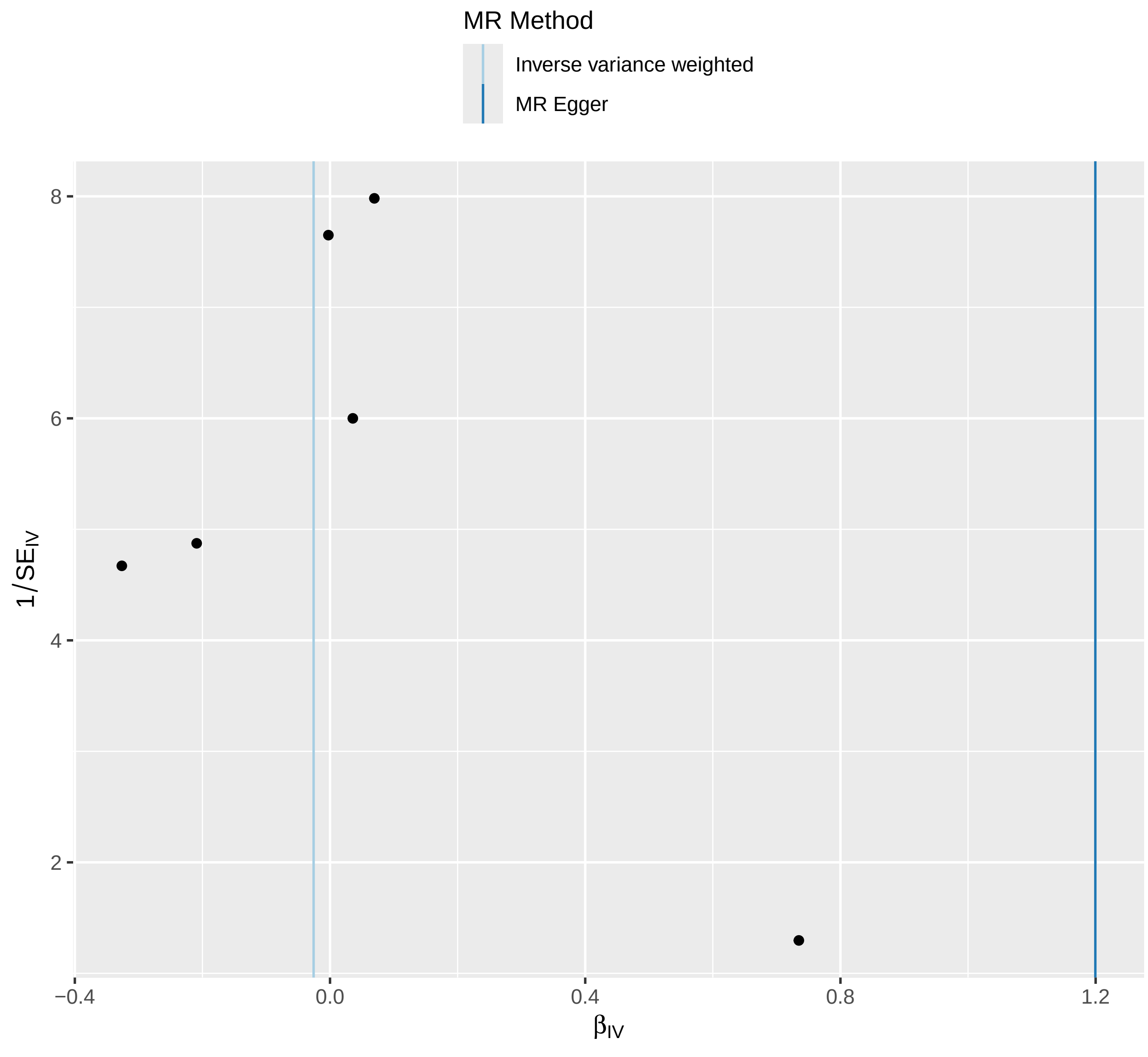
MR Method
Inverse variance weighted
MR Egger
8
6
4
2
1/SE_IV
−0.4
0.0
0.4
0.8
1.2
β_IV

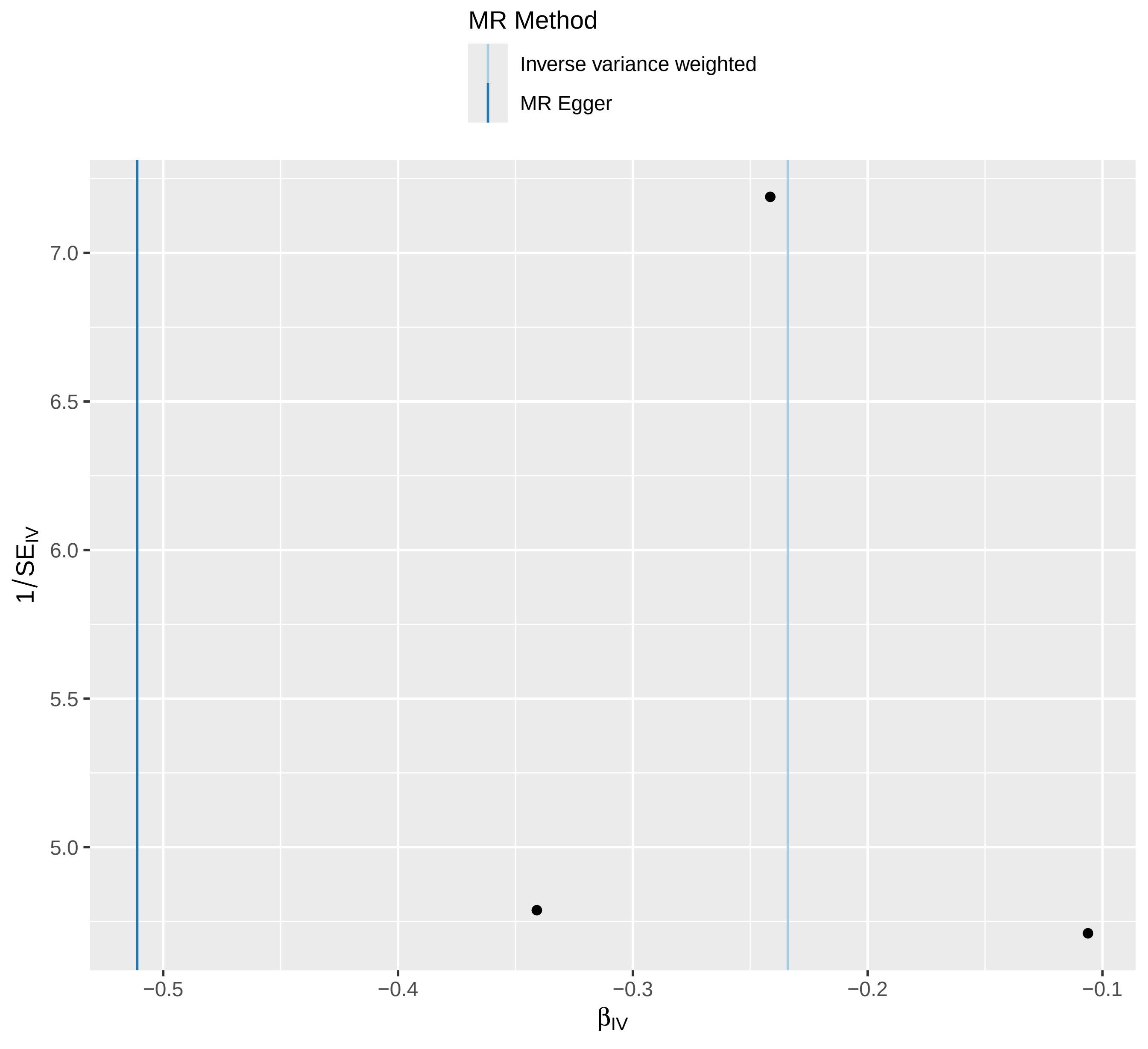

MR Method
Inverse variance weighted
MR Egger
7.0
6.5
6.0
5.5
5.0
1/SE$_{IV}$
−0.5
−0.4
−0.3
−0.2
−0.1
β$_{IV}$

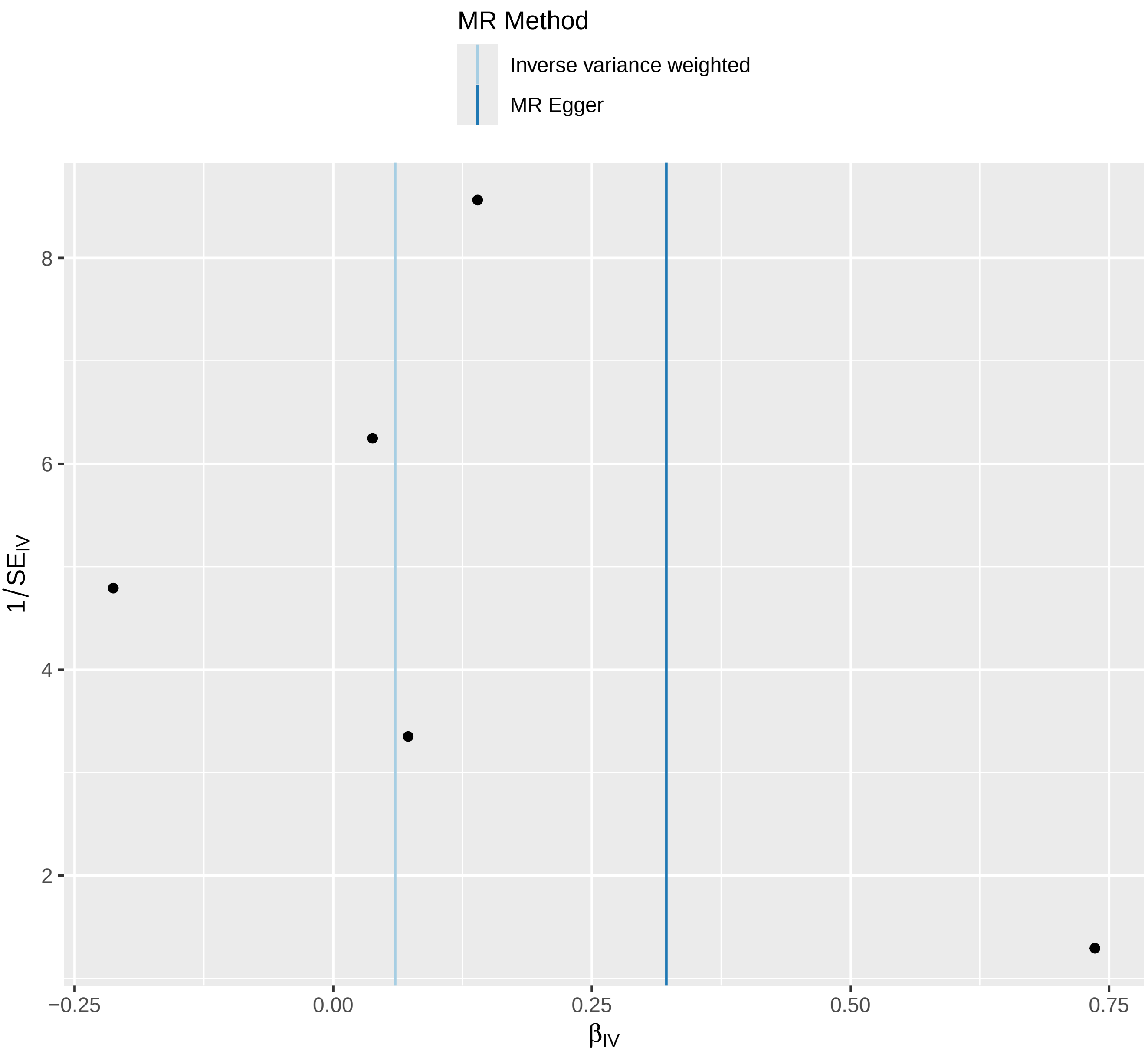
MR Method
Inverse variance weighted
MR Egger
8
6
4
2
1/SE_IV
−0.25
0.00
0.25
0.50
0.75
β_IV

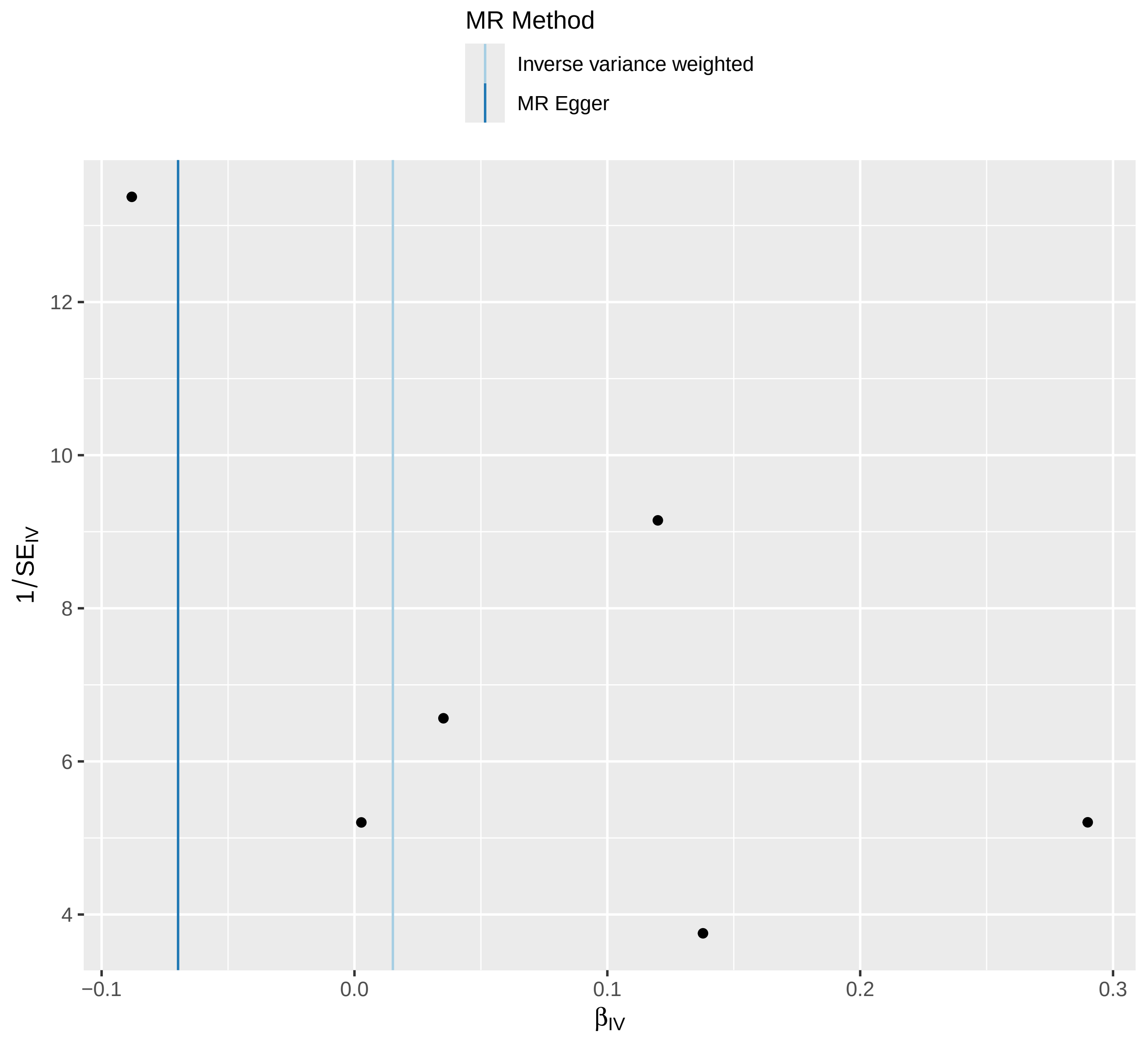
MR Method
Inverse variance weighted
MR Egger
1/SE$_{IV}$
12
10
8
6
4
−0.1
0.0
0.1
0.2
0.3
β$_{IV}$

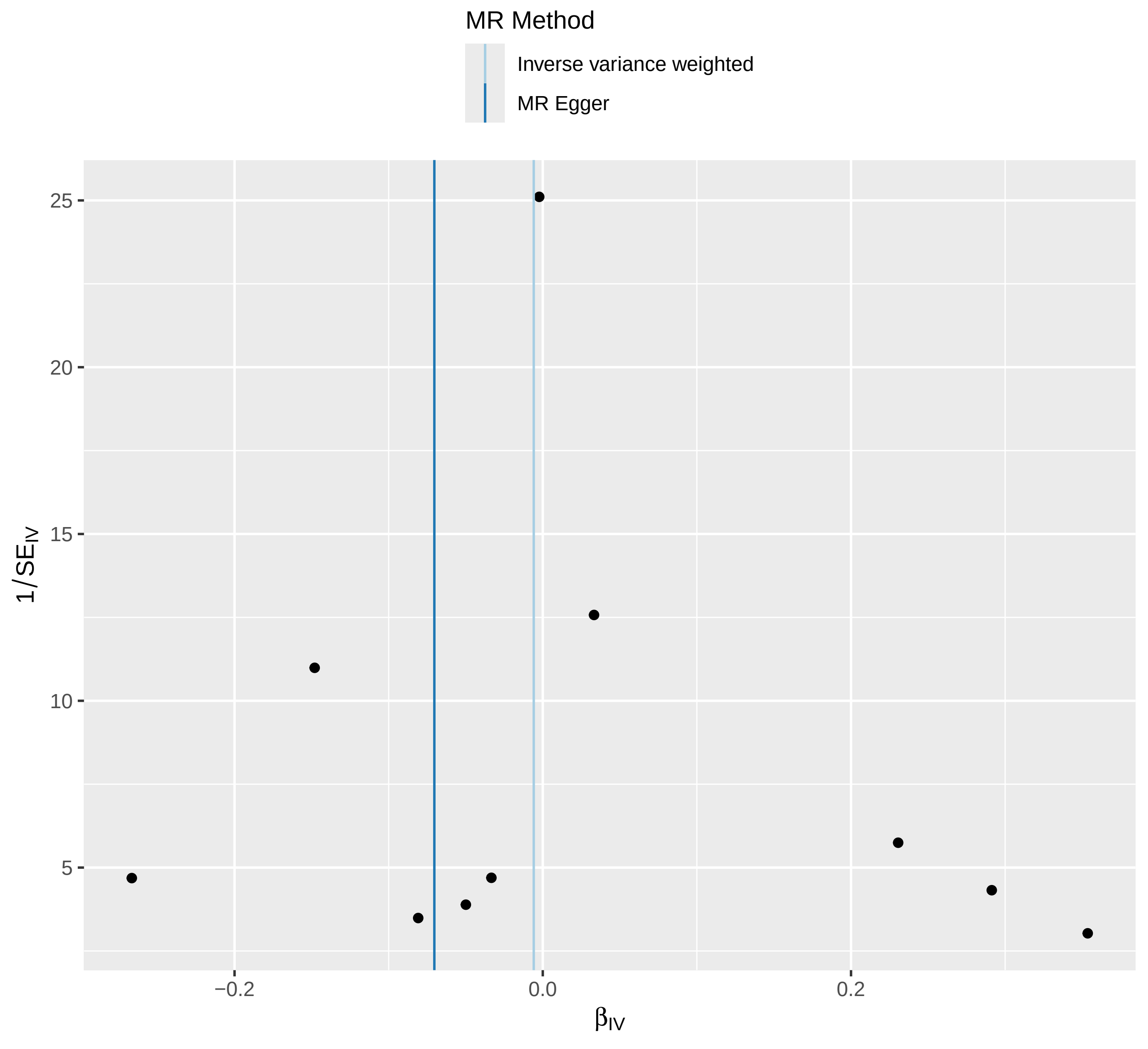
MR Method
Inverse variance weighted
MR Egger
25
20
15
10
5
−0.2
0.0
0.2
$1/SE_{IV}$
$\beta_{IV}$

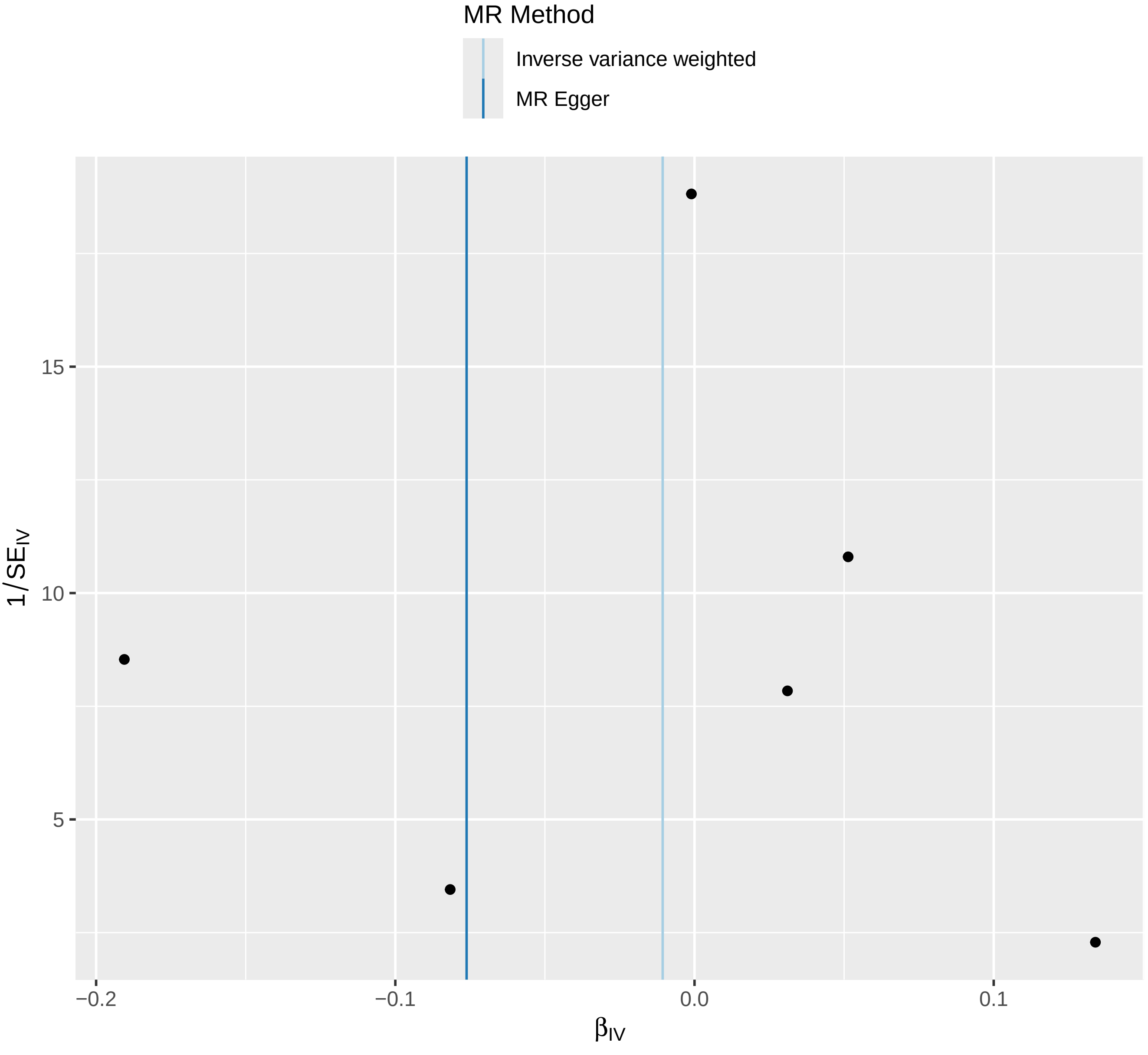
MR Method
Inverse variance weighted
MR Egger
15
10
5
1/SE_IV
−0.2
−0.1
0.0
0.1
β_IV

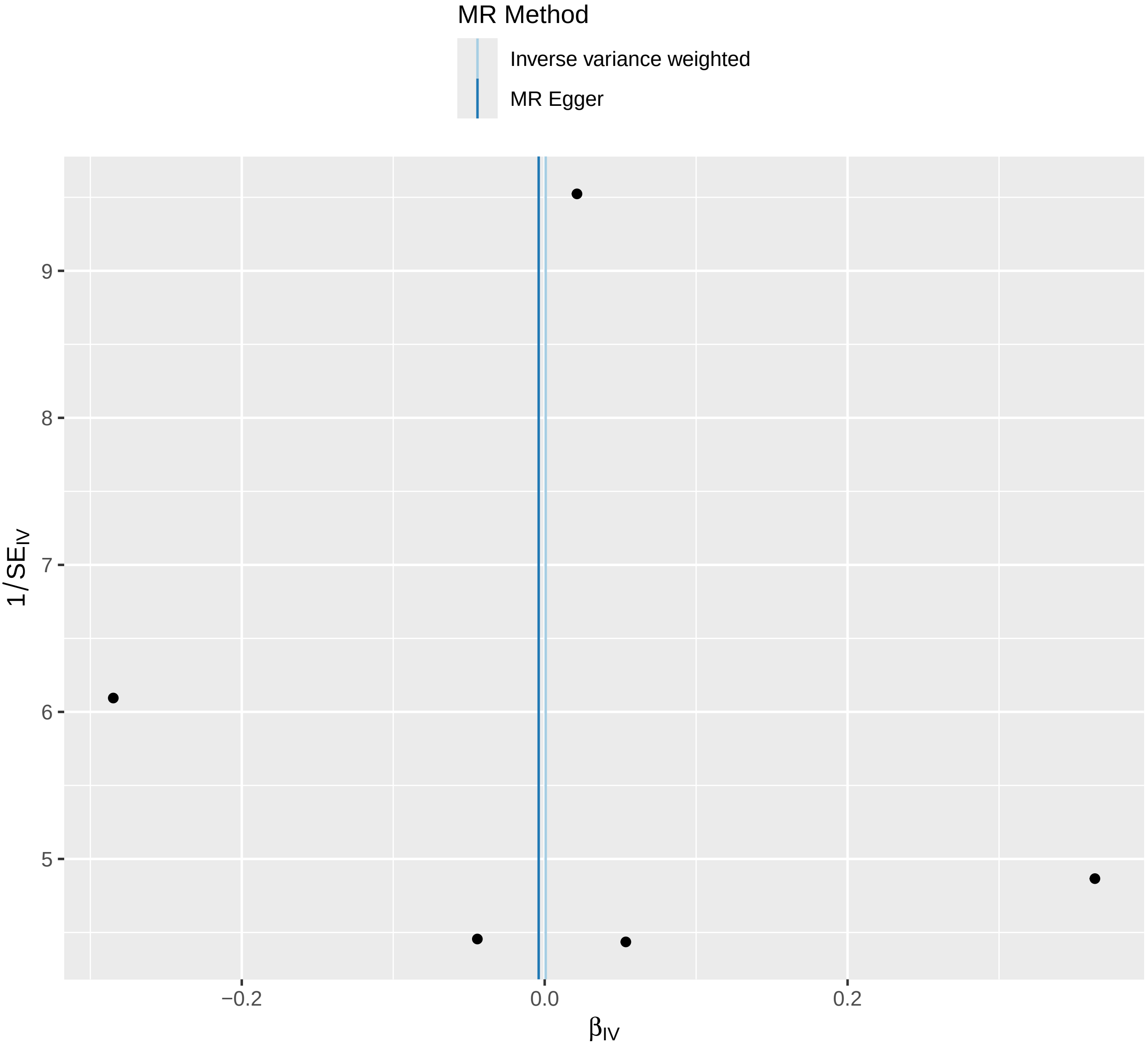
MR Method
Inverse variance weighted
MR Egger
9
8
7
6
5
1/SE$_{IV}$
−0.2
0.0
0.2
β$_{IV}$

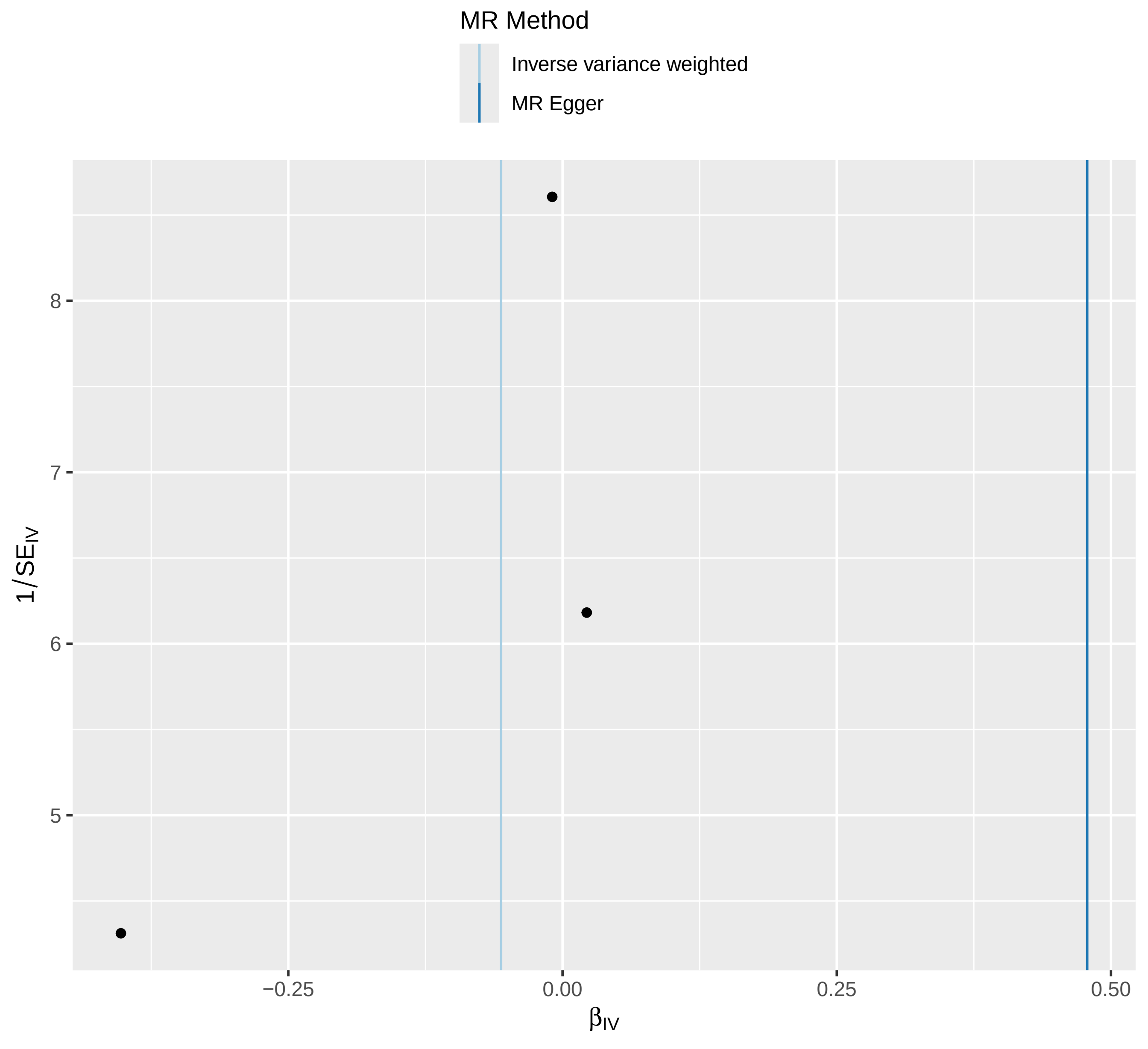

MR Method
Inverse variance weighted
MR Egger
8
7
6
5
1/SE$_{IV}$
−0.25
0.00
0.25
0.50
β$_{IV}$

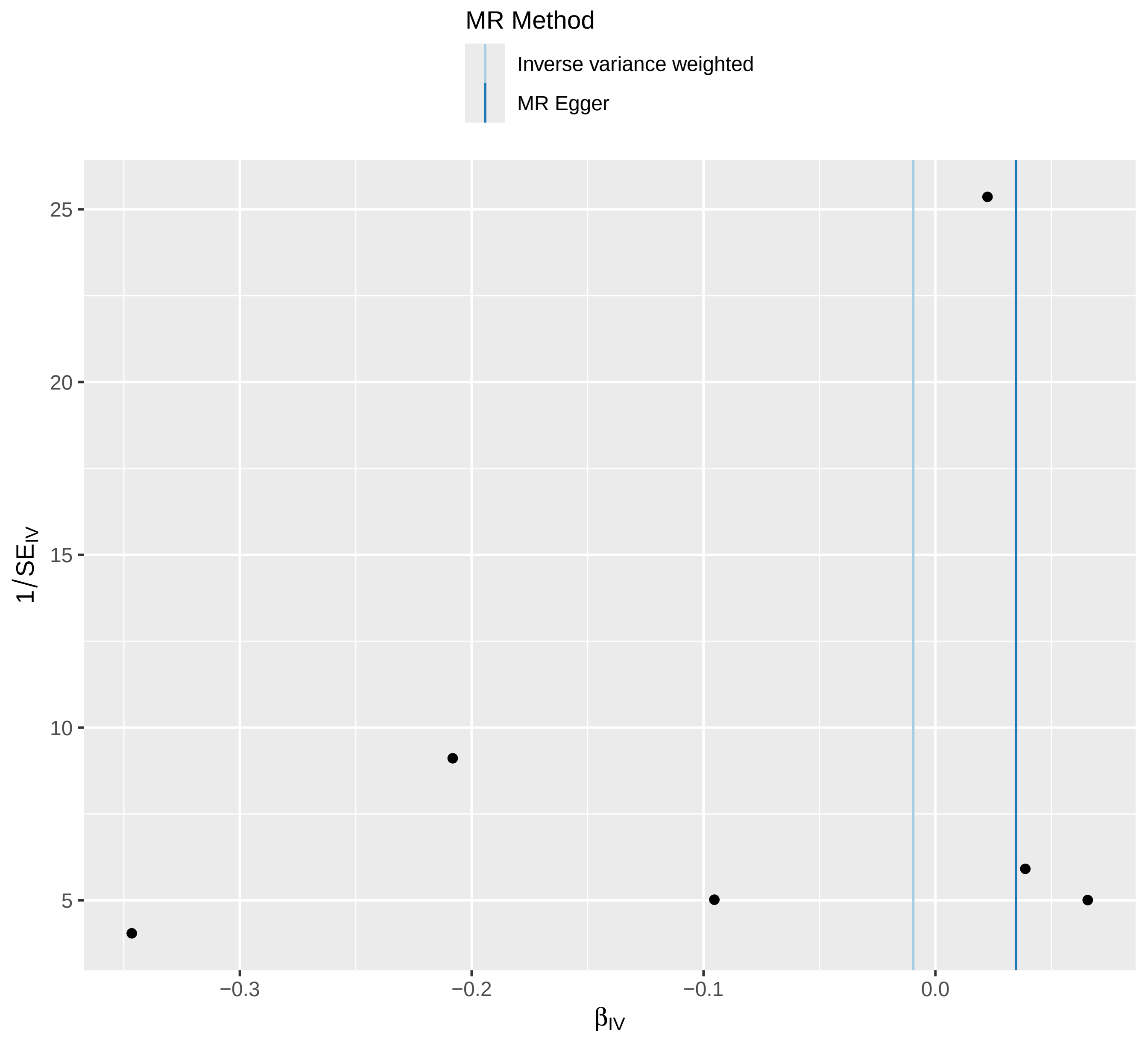

MR Method
Inverse variance weighted
MR Egger
1/SE_IV
25
20
15
10
5
−0.3
−0.2
−0.1
0.0
β_IV

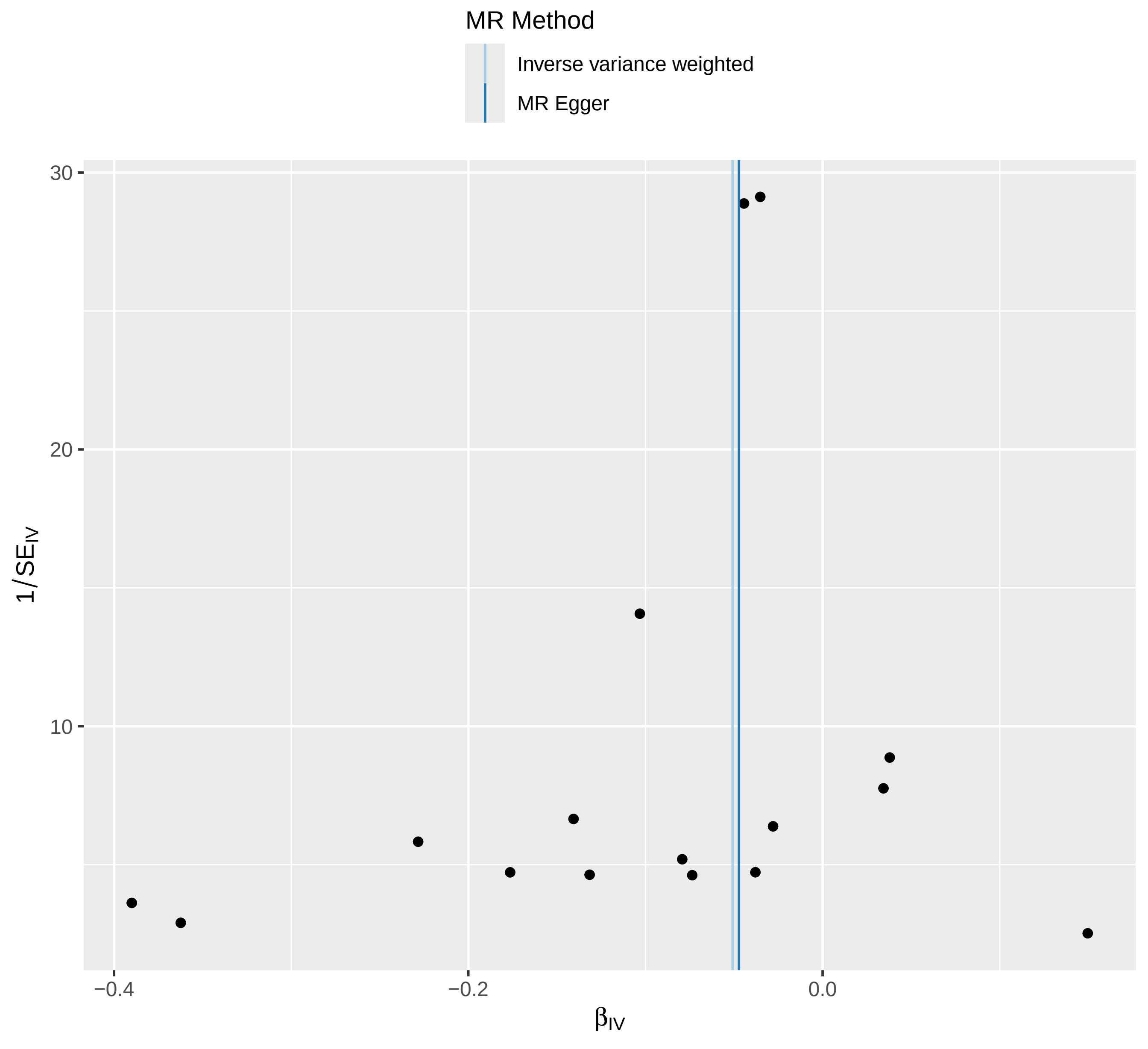
MR Method
Inverse variance weighted
MR Egger
30
20
10
1/SE$_{IV}$
−0.4
−0.2
0.0
β$_{IV}$

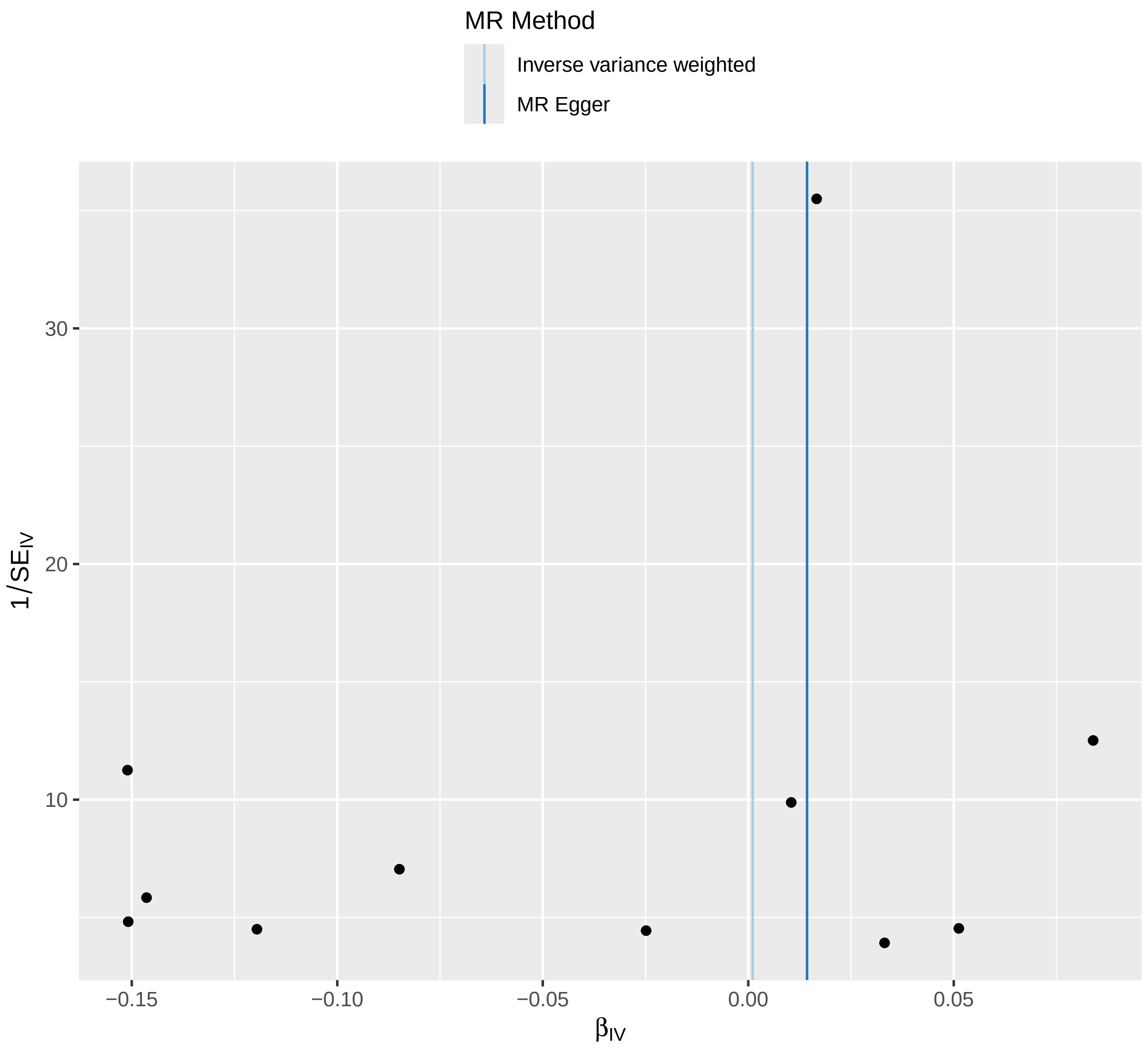

MR Method
Inverse variance weighted
MR Egger
30
20
10
1/SE$_{IV}$
−0.15
−0.10
−0.05
0.00
0.05
β$_{IV}$

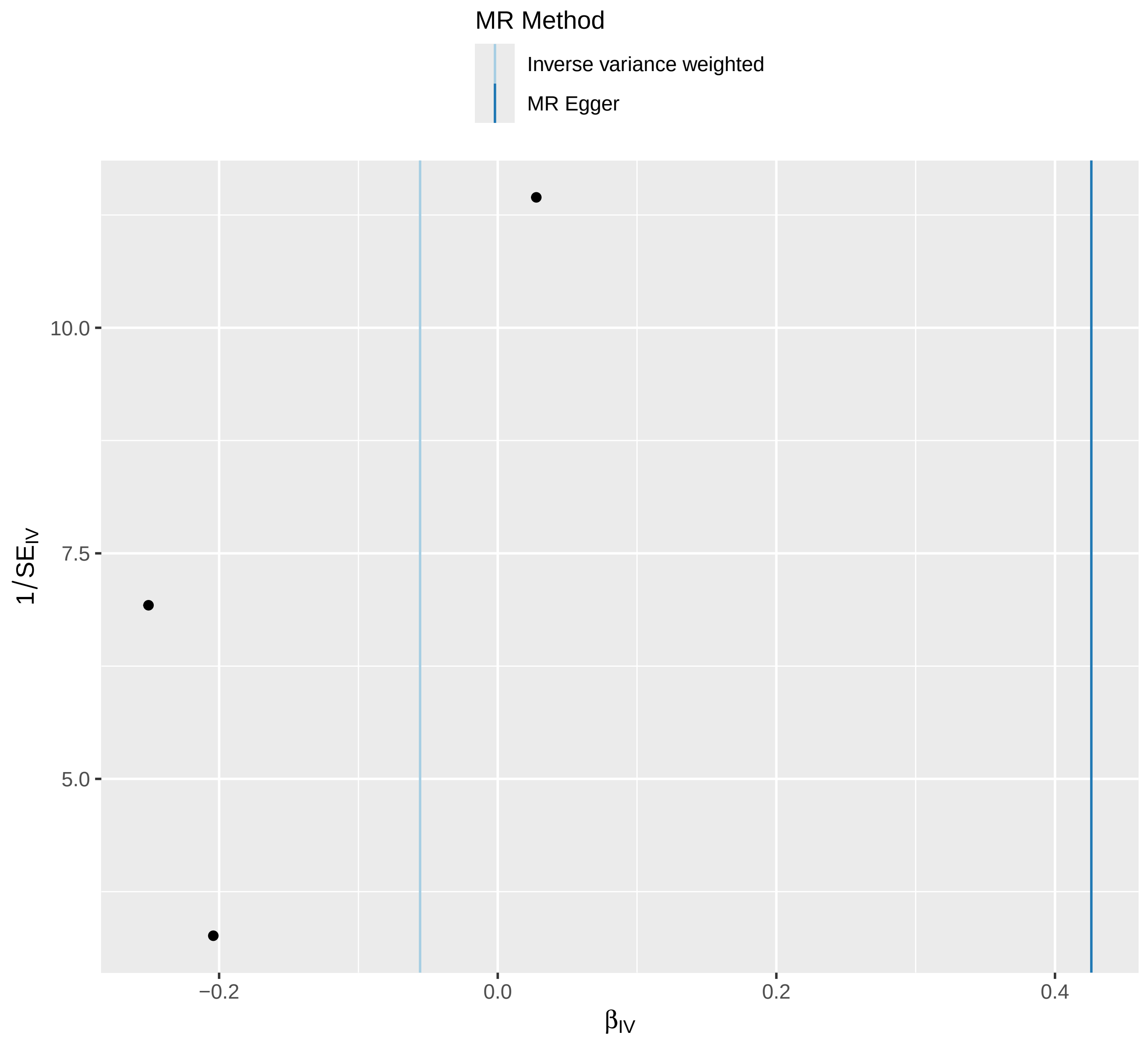
MR Method
Inverse variance weighted
MR Egger
10.0
7.5
5.0
1/SE_IV
−0.2
0.0
0.2
0.4
β_IV

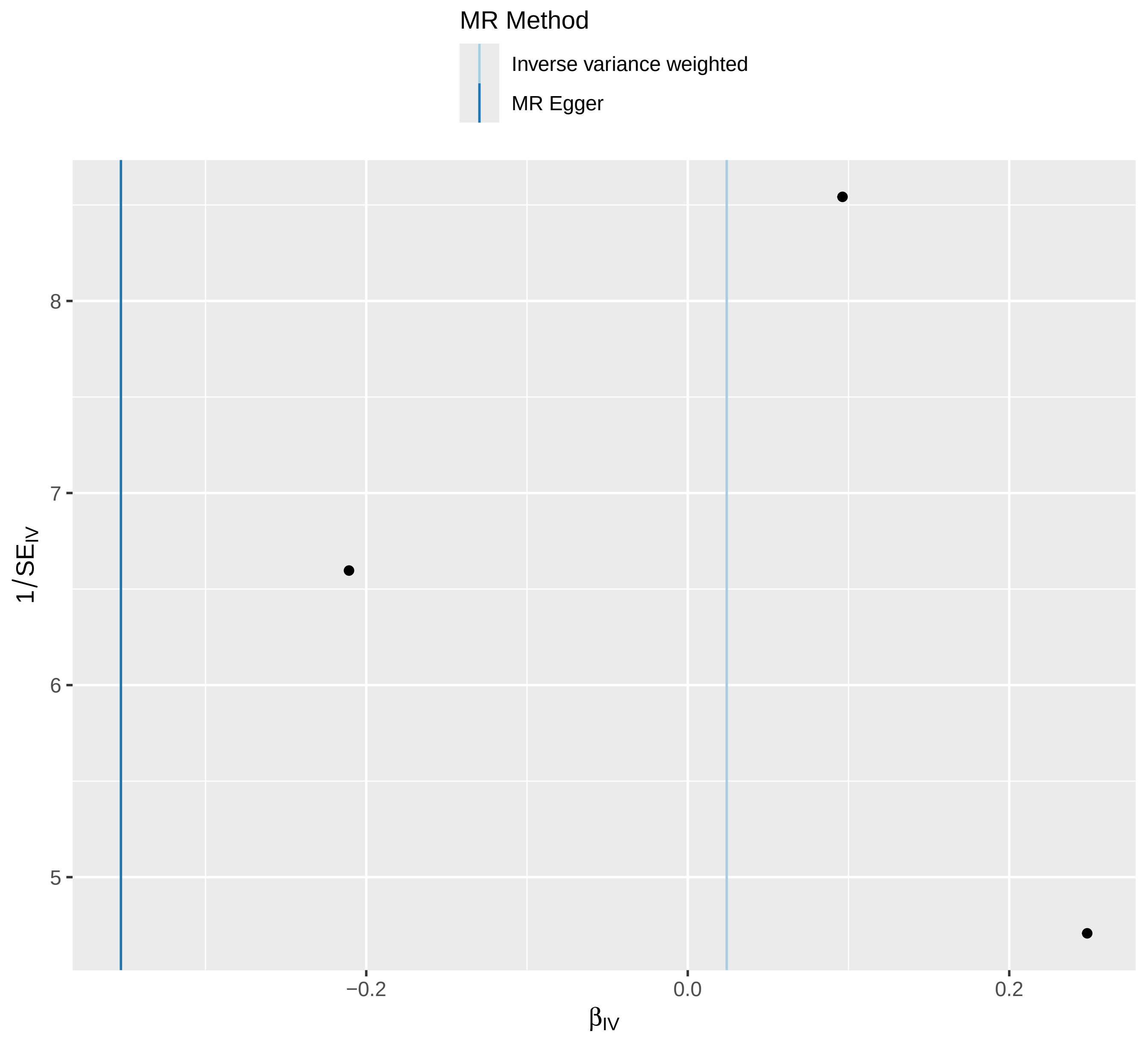
MR Method
Inverse variance weighted
MR Egger
8
7
6
5
1/SE_IV
−0.2
0.0
0.2
β_IV

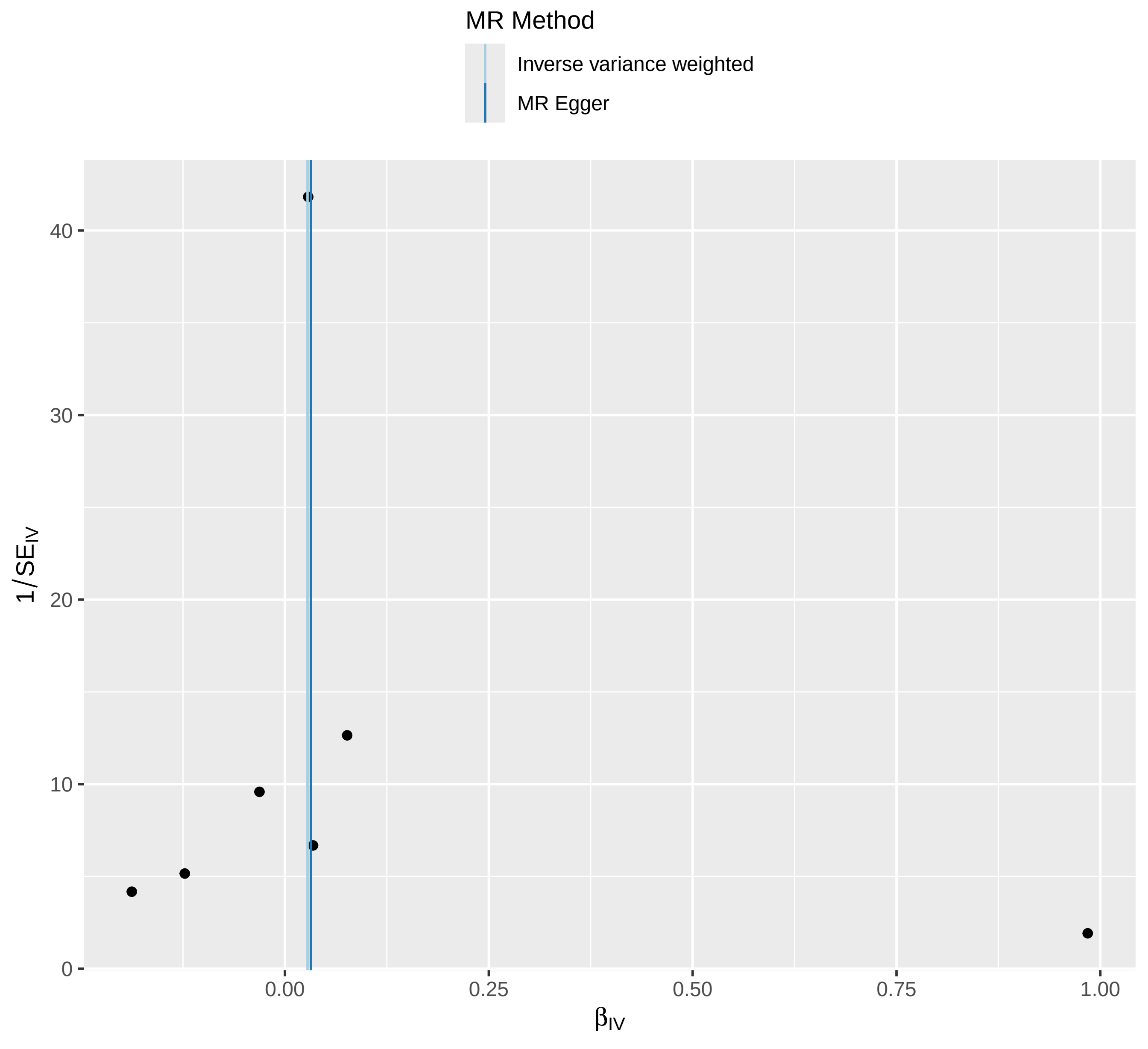

MR Method
Inverse variance weighted
MR Egger
40
30
20
10
0
1/SE$_{IV}$
0.00
0.25
0.50
0.75
1.00
β$_{IV}$

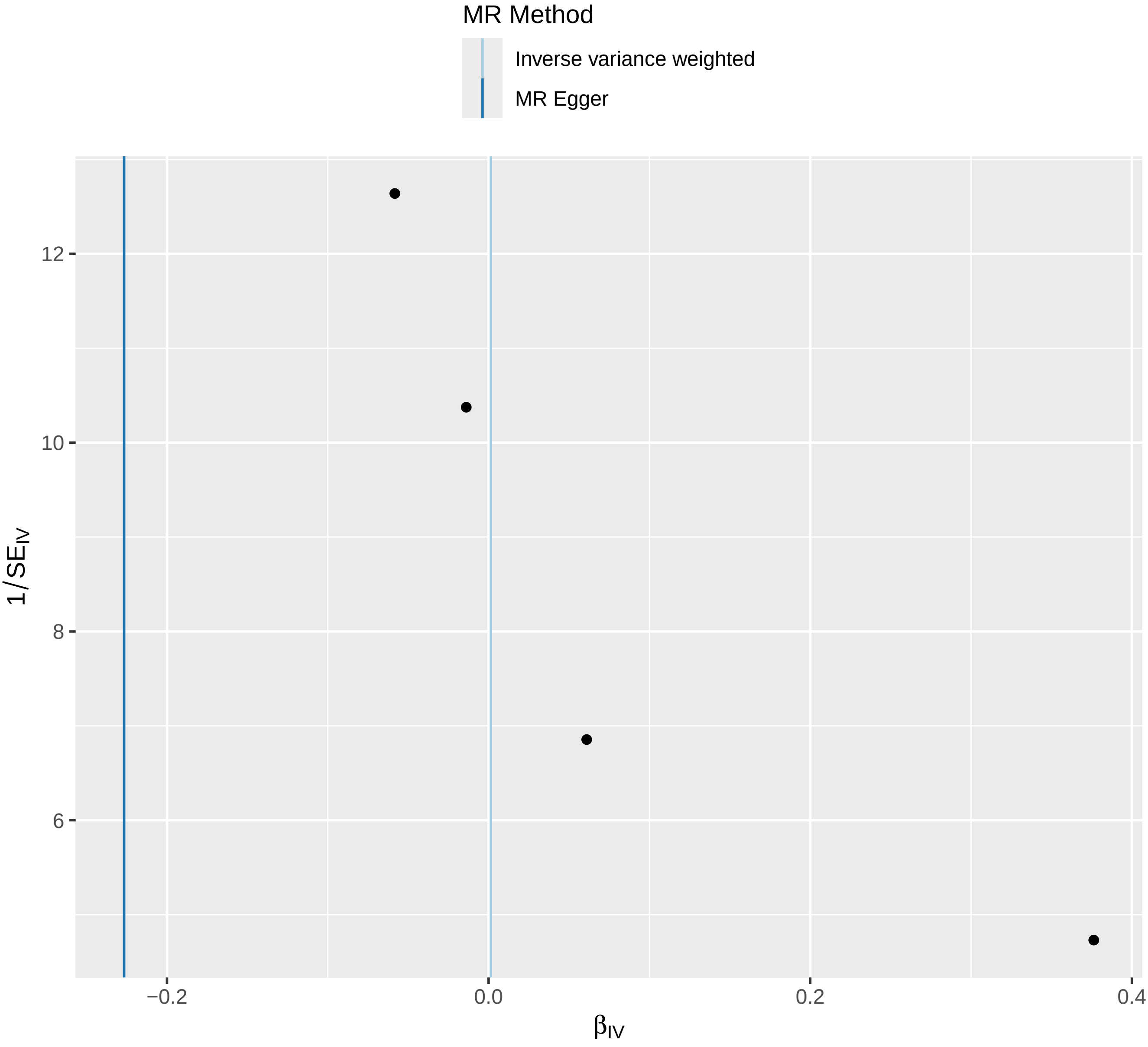
MR Method
Inverse variance weighted
MR Egger
12
10
8
6
1/SE$_{IV}$
−0.2
0.0
0.2
0.4
β$_{IV}$

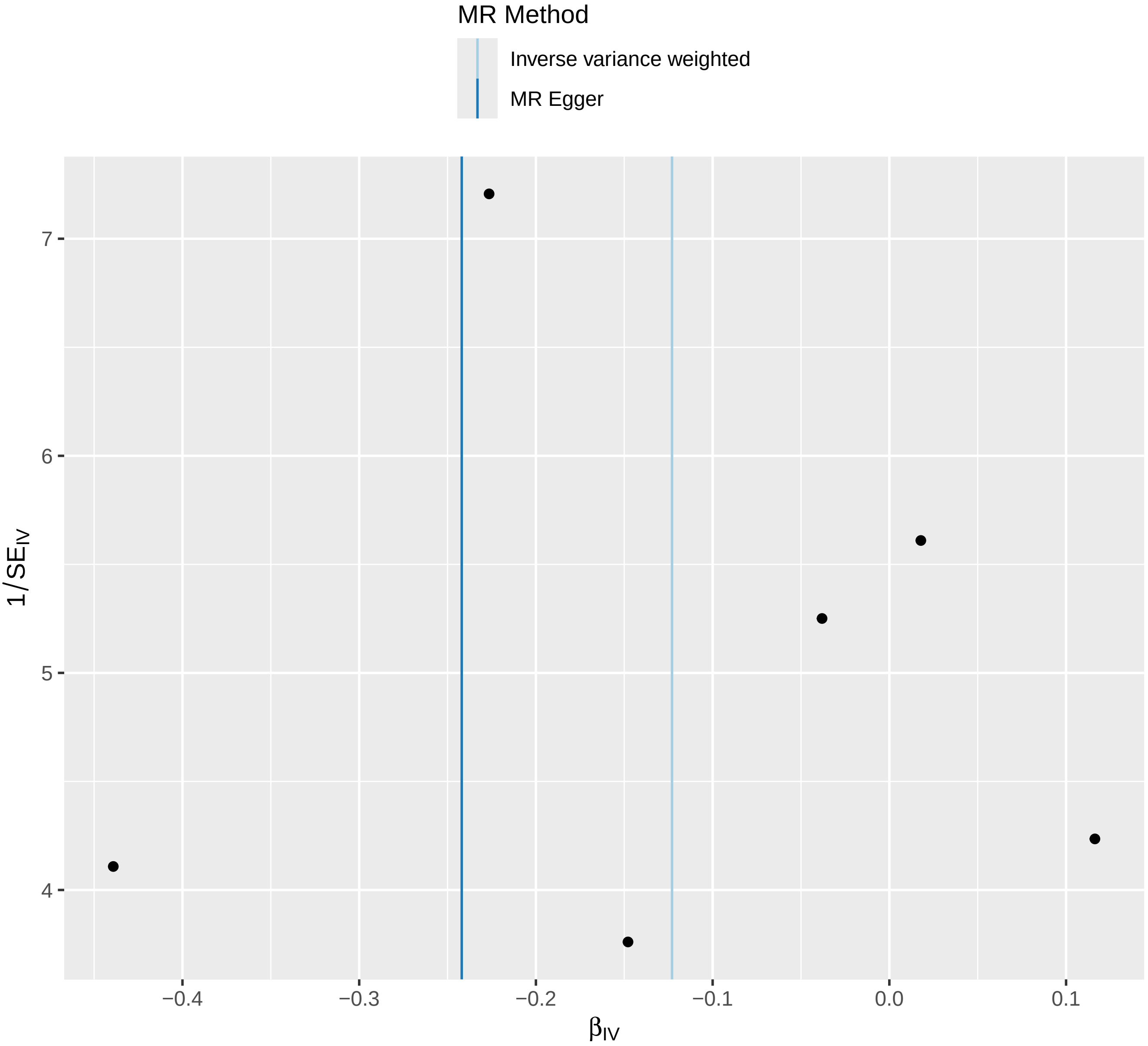
MR Method
Inverse variance weighted
MR Egger
7
6
5
4
$1/SE_{IV}$
−0.4
−0.3
−0.2
−0.1
0.0
0.1
$\beta_{IV}$

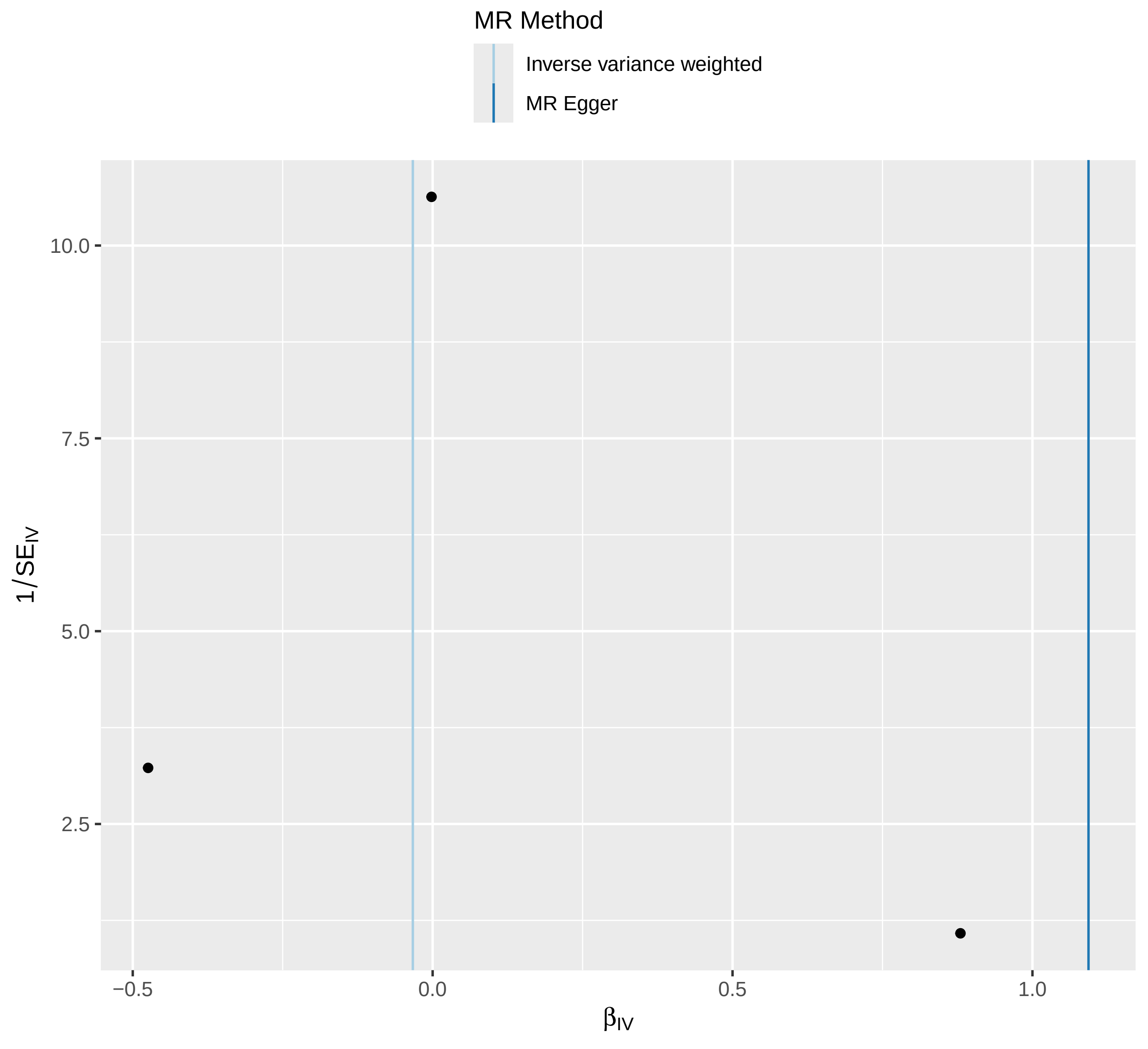

MR Method
Inverse variance weighted
MR Egger
10.0
7.5
5.0
2.5
1/SE$_{IV}$
−0.5
0.0
0.5
1.0
β$_{IV}$

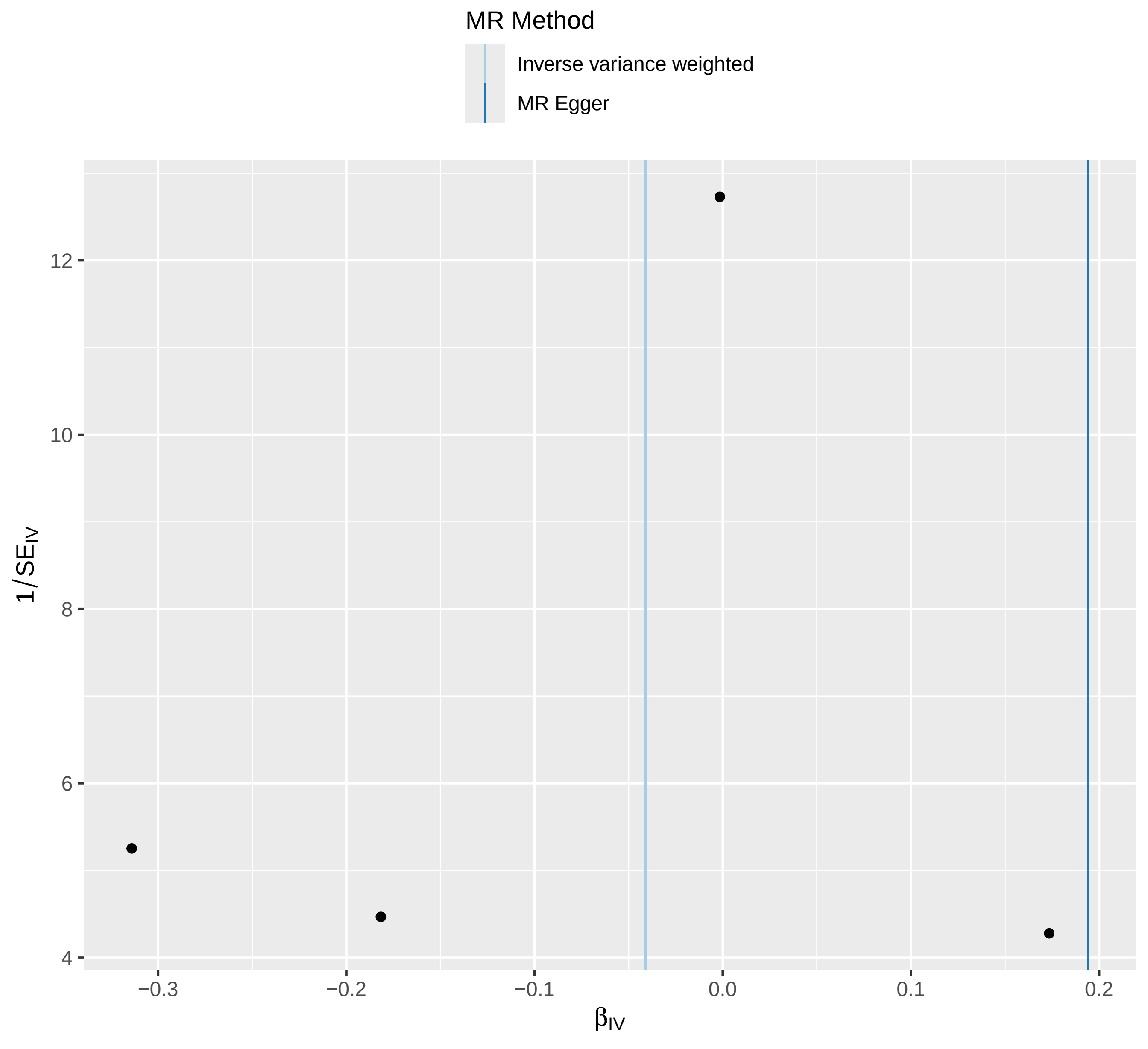

MR Method
Inverse variance weighted
MR Egger
12
10
8
6
4
−0.3
−0.2
−0.1
0.0
0.1
0.2
1/SE_IV
β_IV

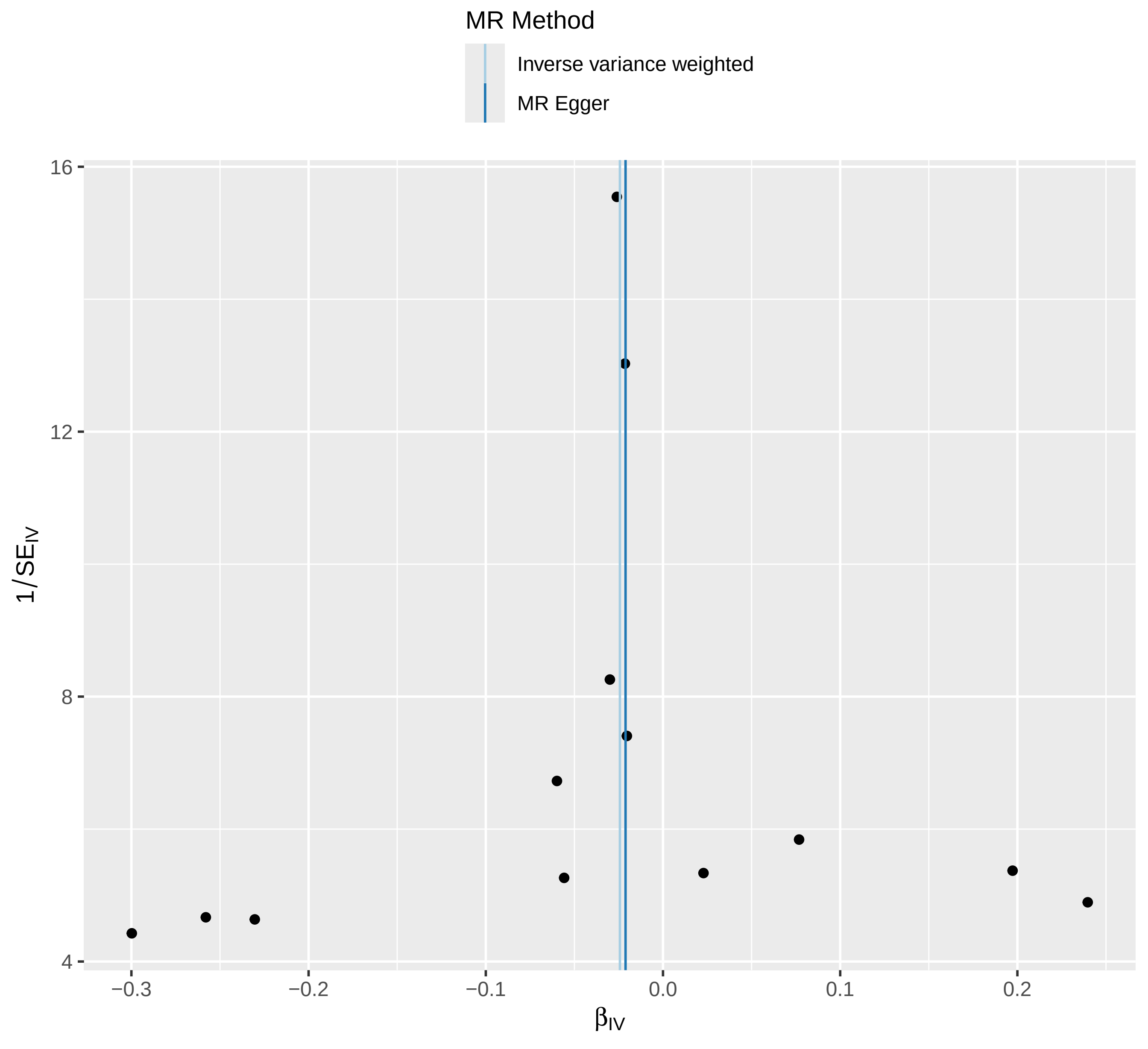

MR Method
Inverse variance weighted
MR Egger
16
12
8
4
1/SE$_{IV}$
−0.3
−0.2
−0.1
0.0
0.1
0.2
β$_{IV}$

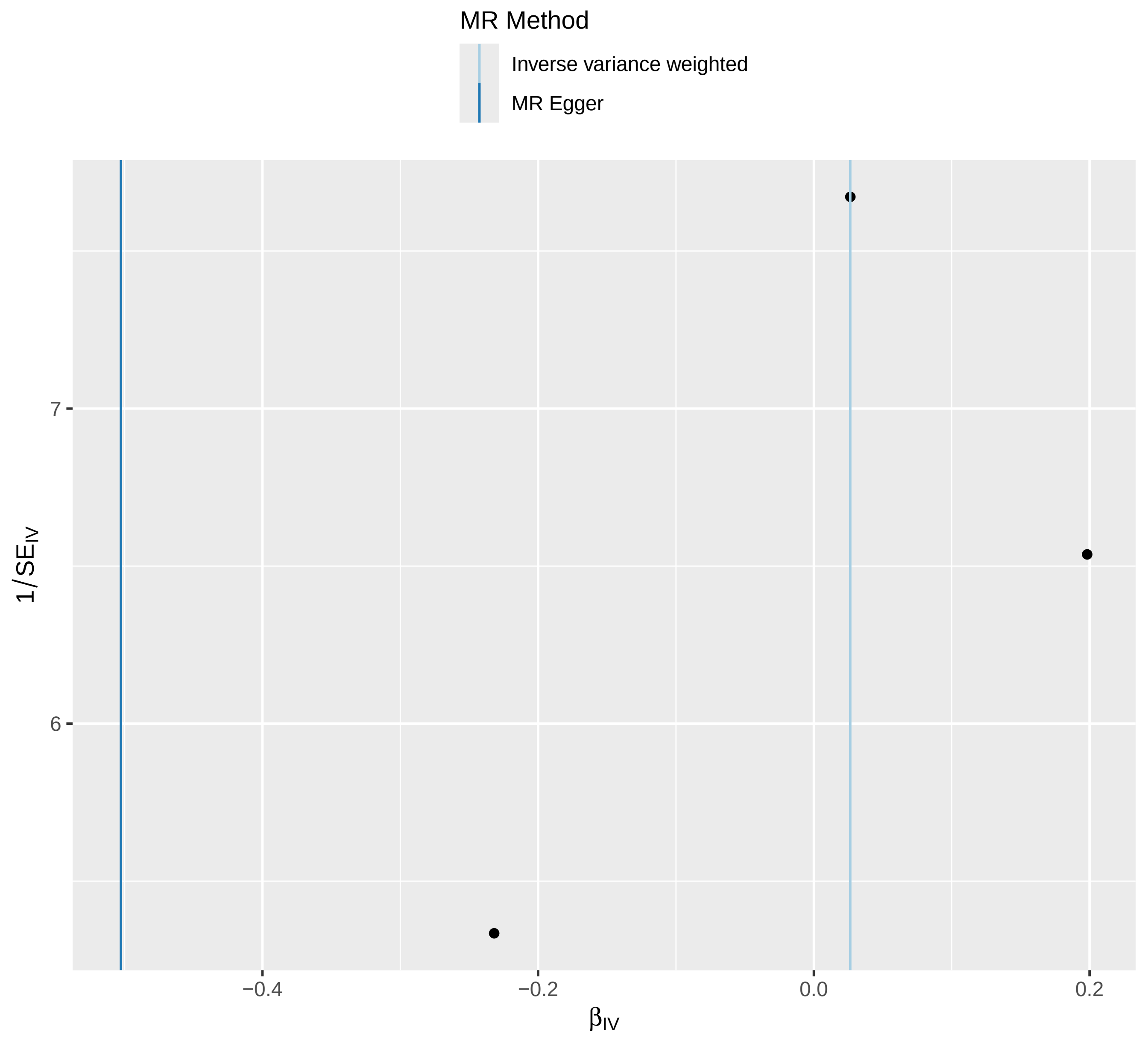
MR Method
Inverse variance weighted
MR Egger
7
6
1/SE_IV
−0.4
−0.2
0.0
0.2
β_IV

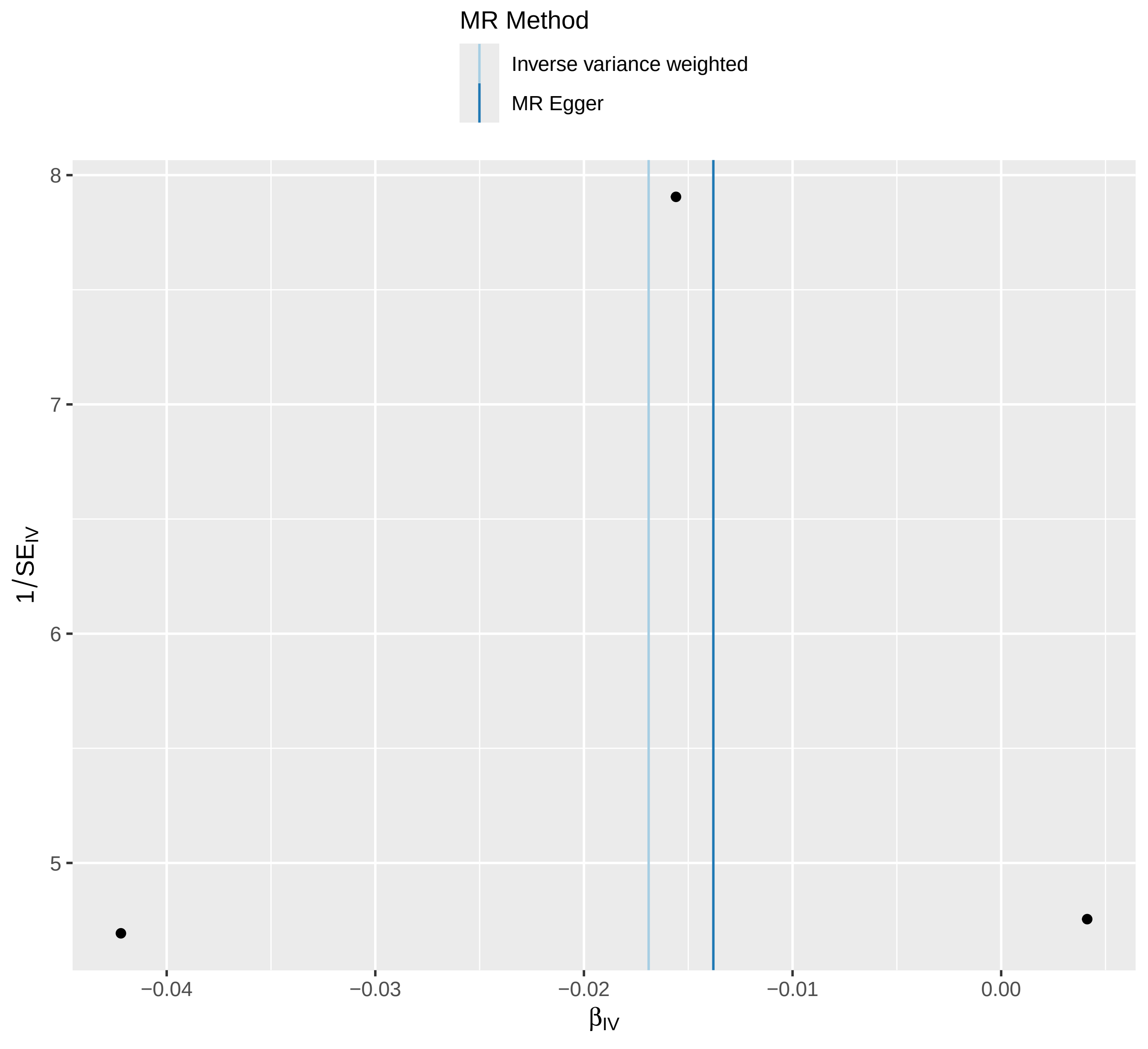
MR Method
Inverse variance weighted
MR Egger
8
7
6
5
1/SE_IV
−0.04
−0.03
−0.02
−0.01
0.00
β_IV

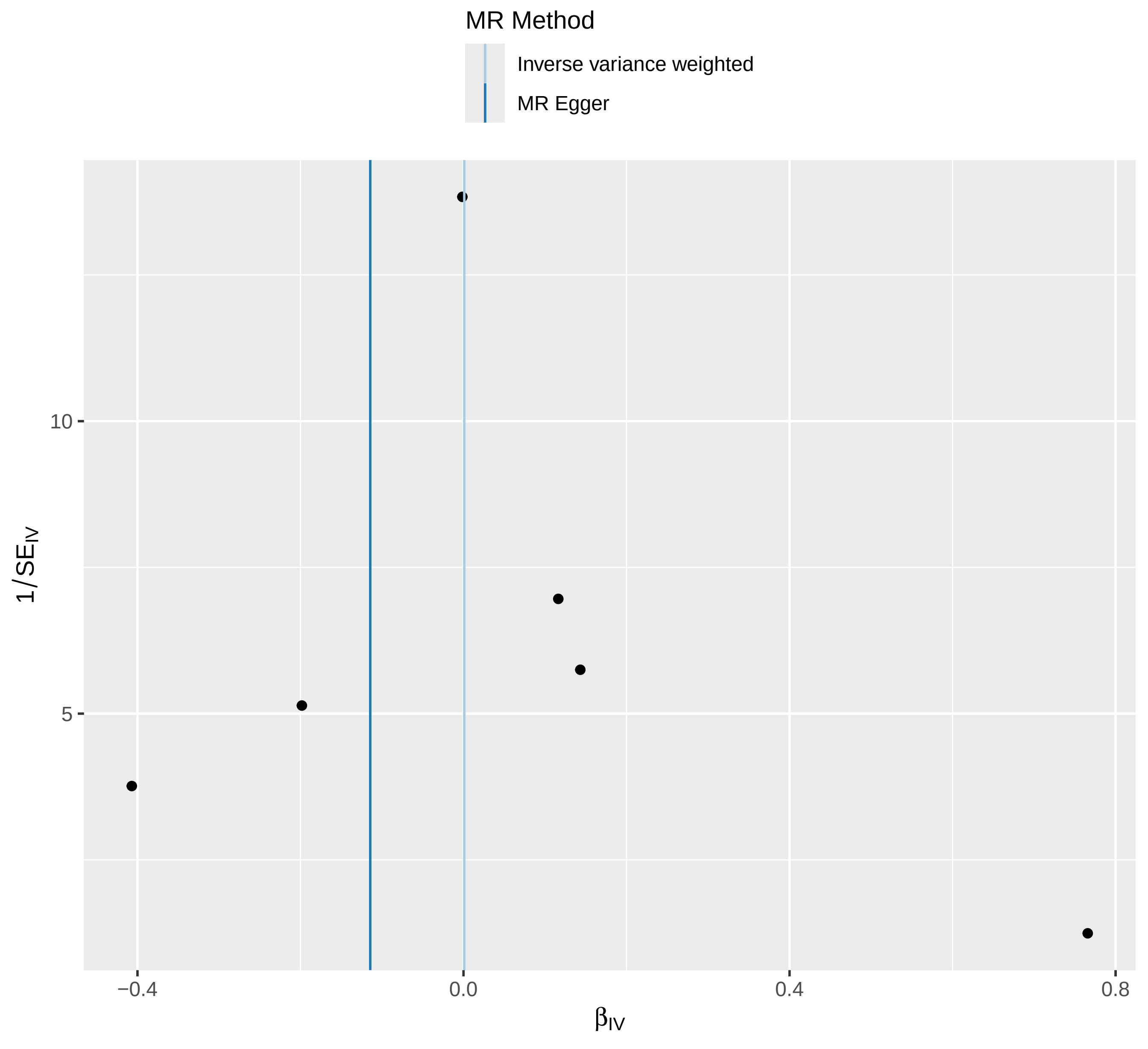
MR Method
Inverse variance weighted
MR Egger
1/SE_IV
10
5
−0.4
0.0
0.4
0.8
β_IV

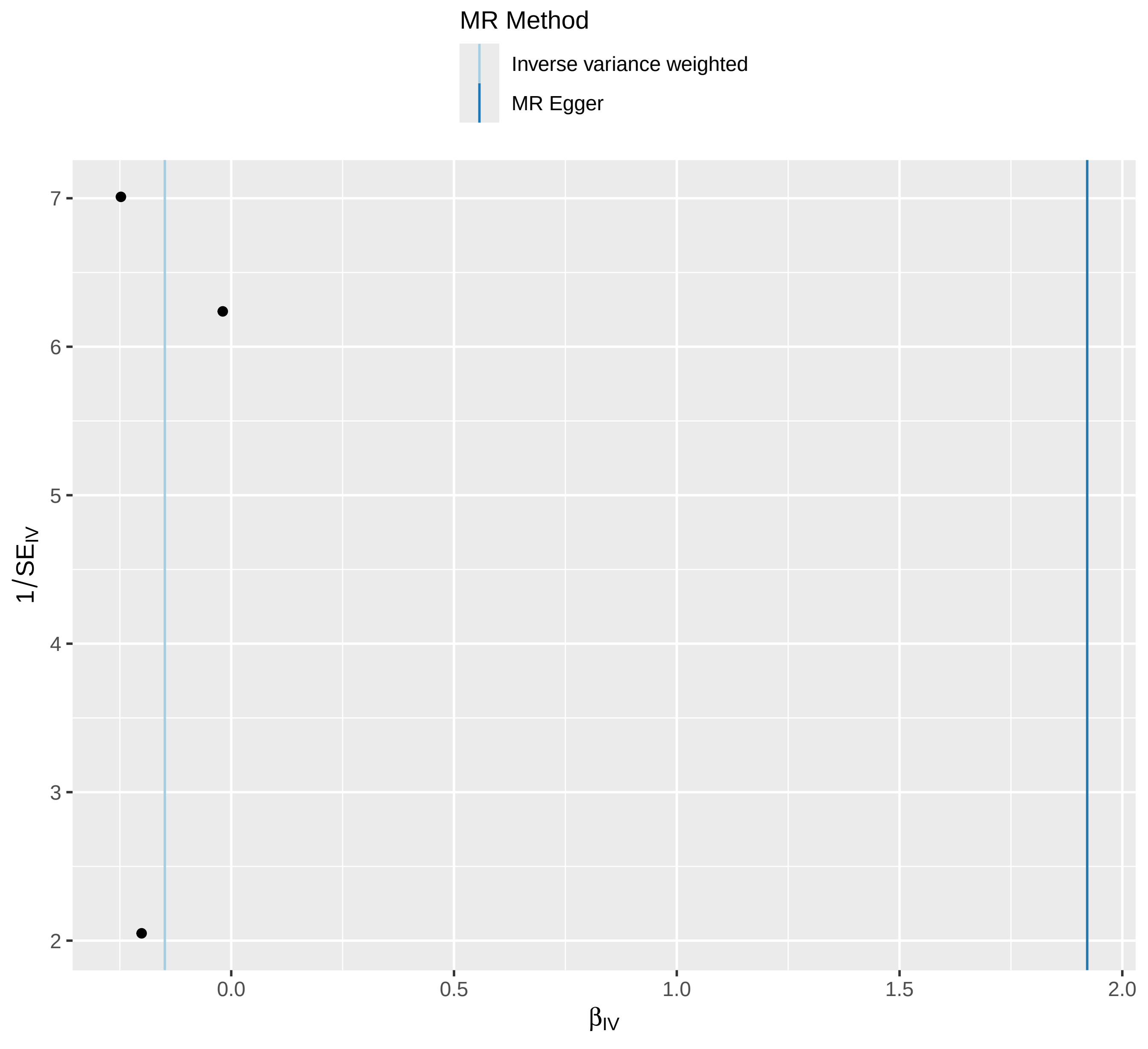

MR Method
Inverse variance weighted
MR Egger
7
6
5
4
3
2
$1/SE_{IV}$
0.0
0.5
1.0
1.5
2.0
$\beta_{IV}$

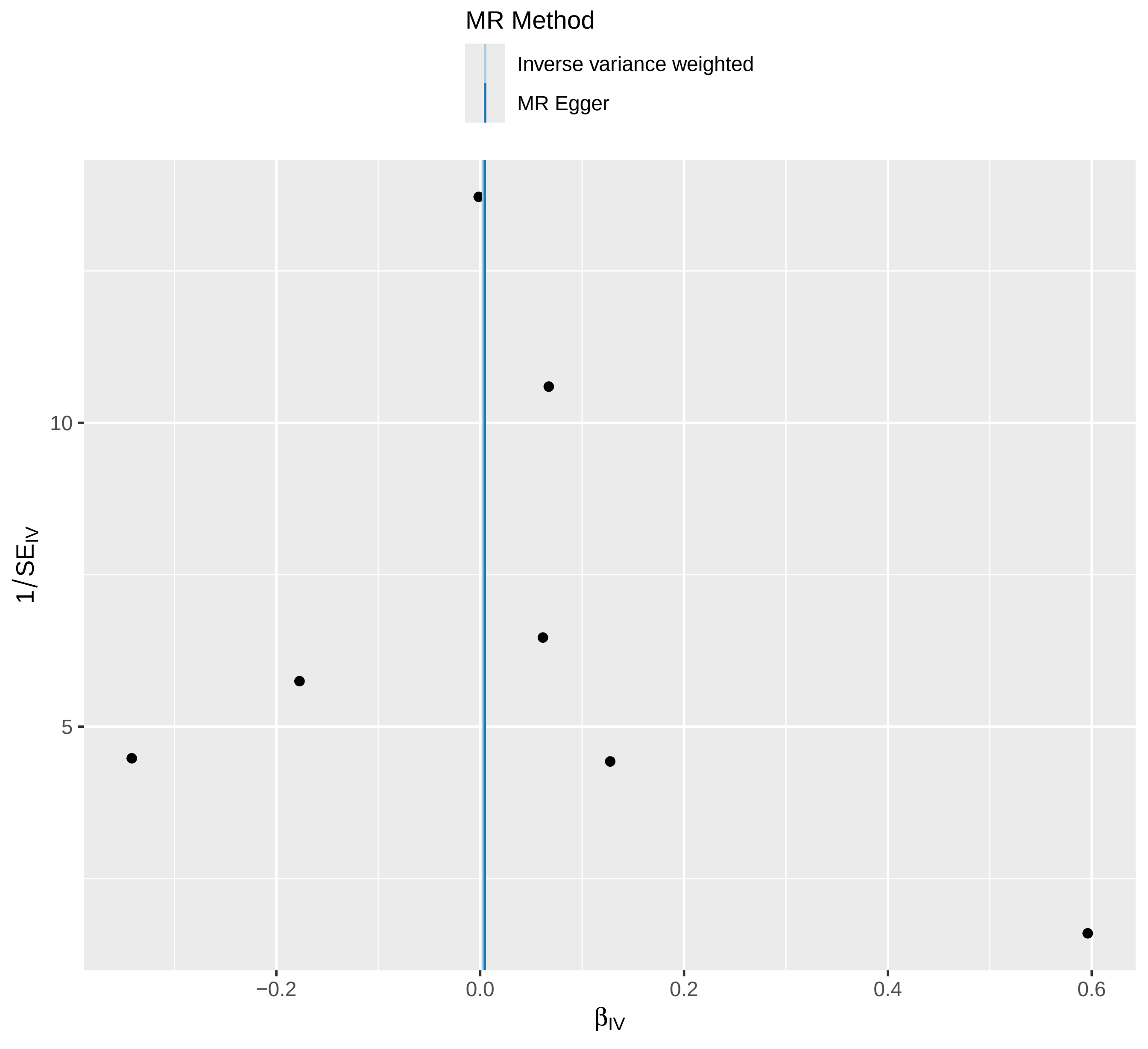

MR Method
Inverse variance weighted
MR Egger
10
5
$1/SE_{IV}$
−0.2
0.0
0.2
0.4
0.6
$\beta_{IV}$

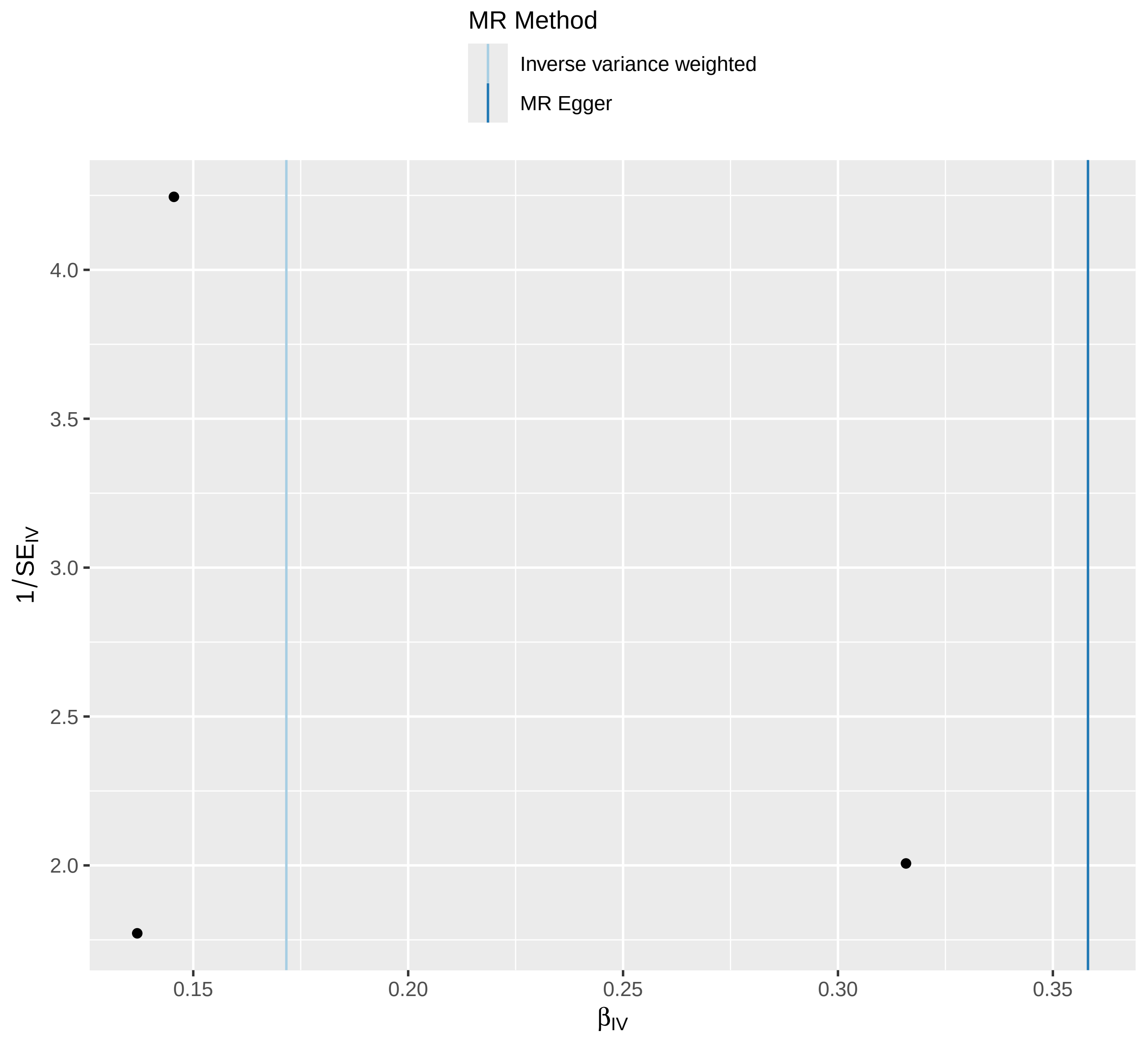
MR Method
Inverse variance weighted
MR Egger
4.0
3.5
3.0
2.5
2.0
1/SE$_{IV}$
0.15
0.20
0.25
0.30
0.35
β$_{IV}$

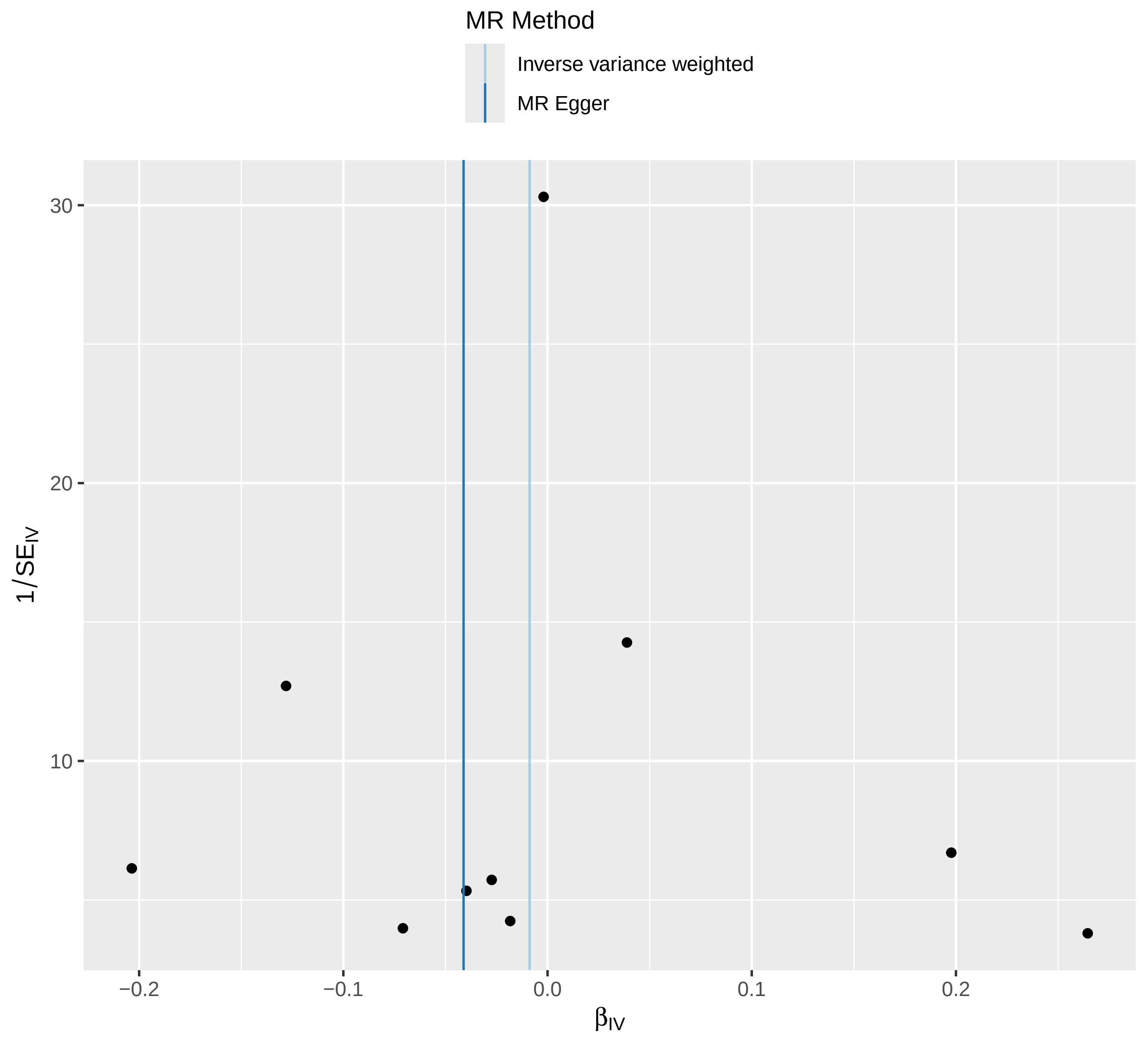
MR Method
Inverse variance weighted
MR Egger
30
20
10
1/SE_IV
−0.2
−0.1
0.0
0.1
0.2
β_IV

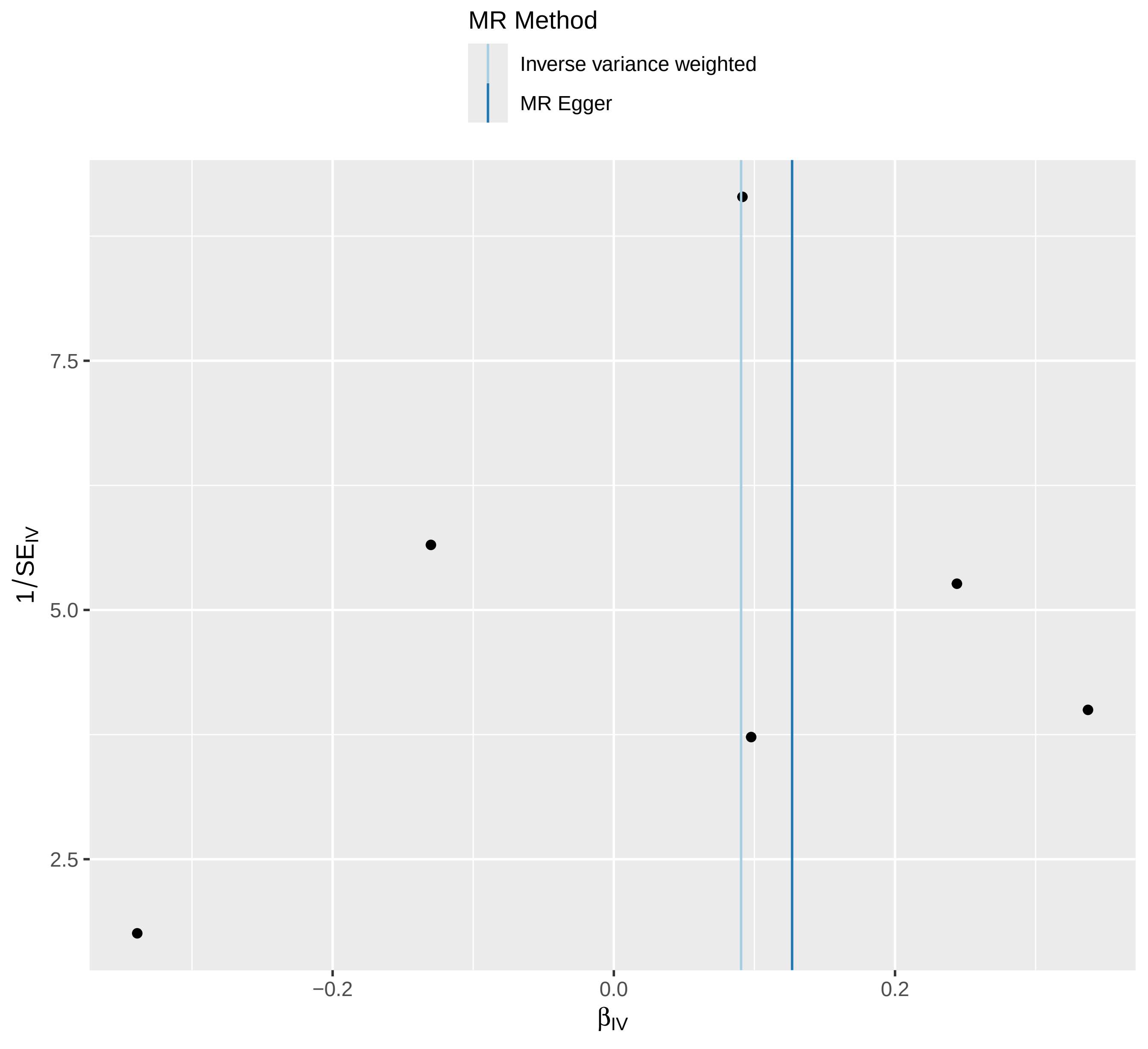
MR Method
Inverse variance weighted
MR Egger
7.5
5.0
2.5
1/SE$_{IV}$
−0.2
0.0
0.2
β$_{IV}$

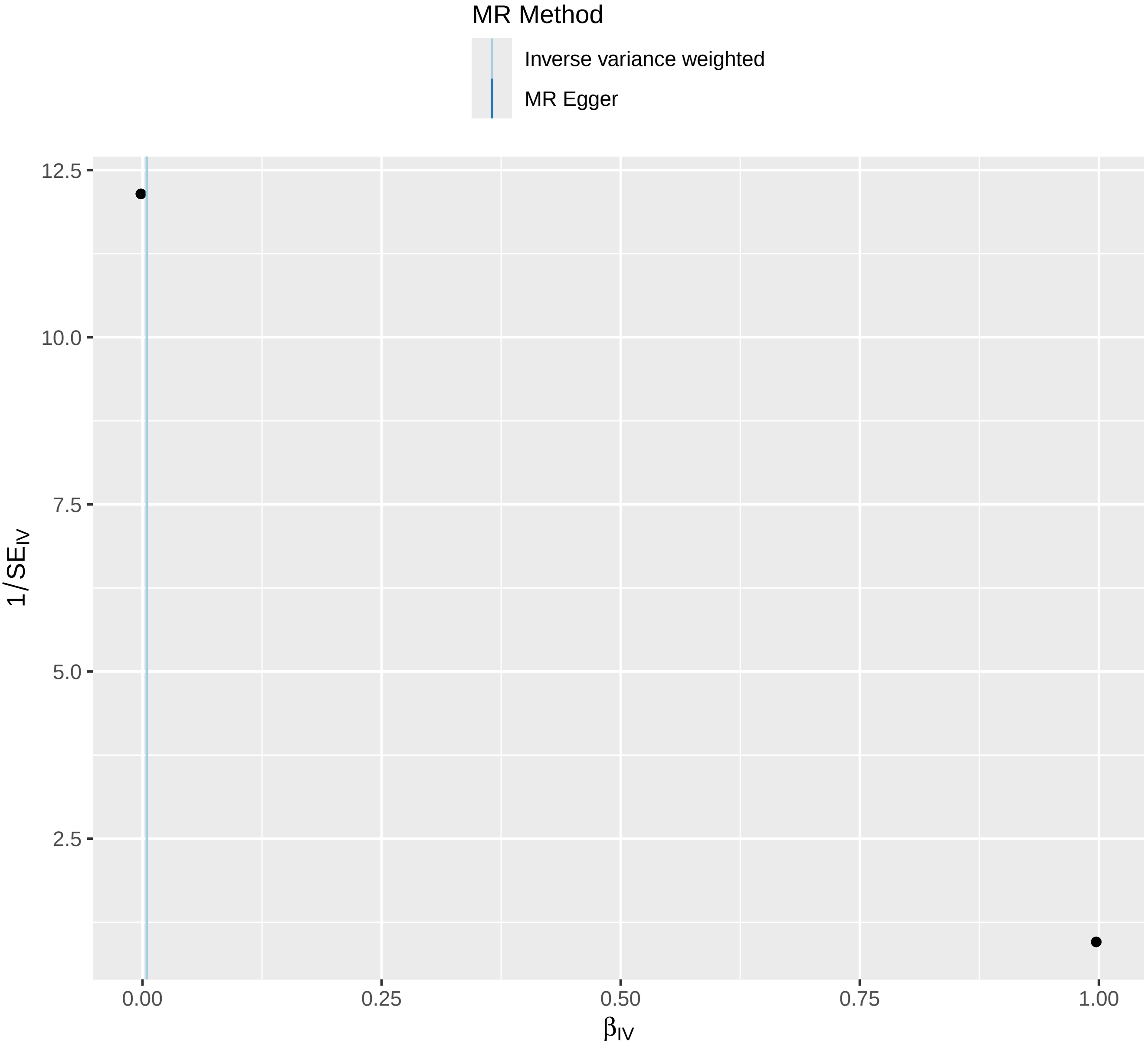
MR Method
Inverse variance weighted
MR Egger
12.5
10.0
7.5
5.0
2.5
1/SE_IV
0.00
0.25
0.50
0.75
1.00
β_IV

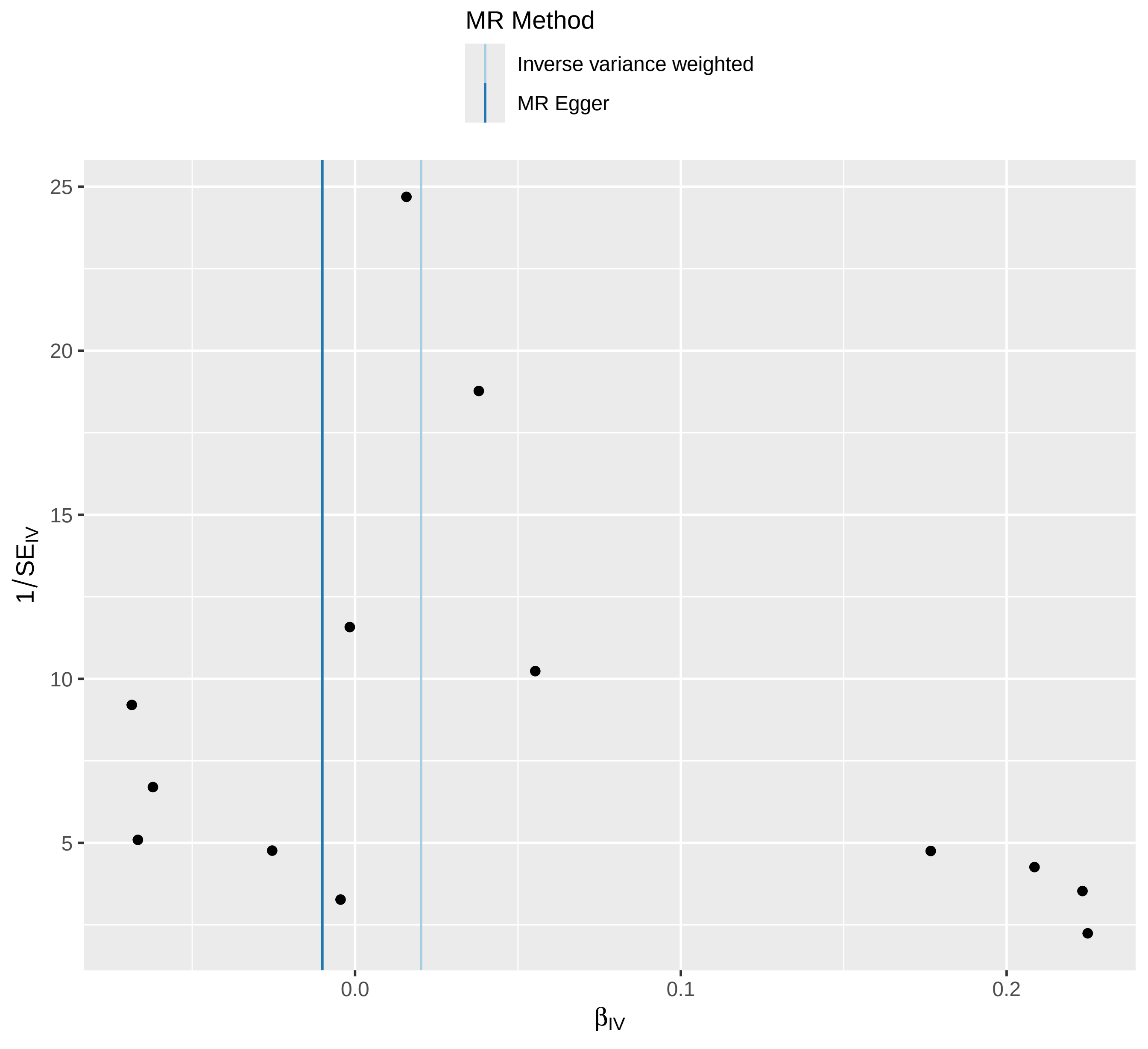

MR Method
Inverse variance weighted
MR Egger
25
20
15
10
5
1/SE_IV
0.0
0.1
0.2
β_IV

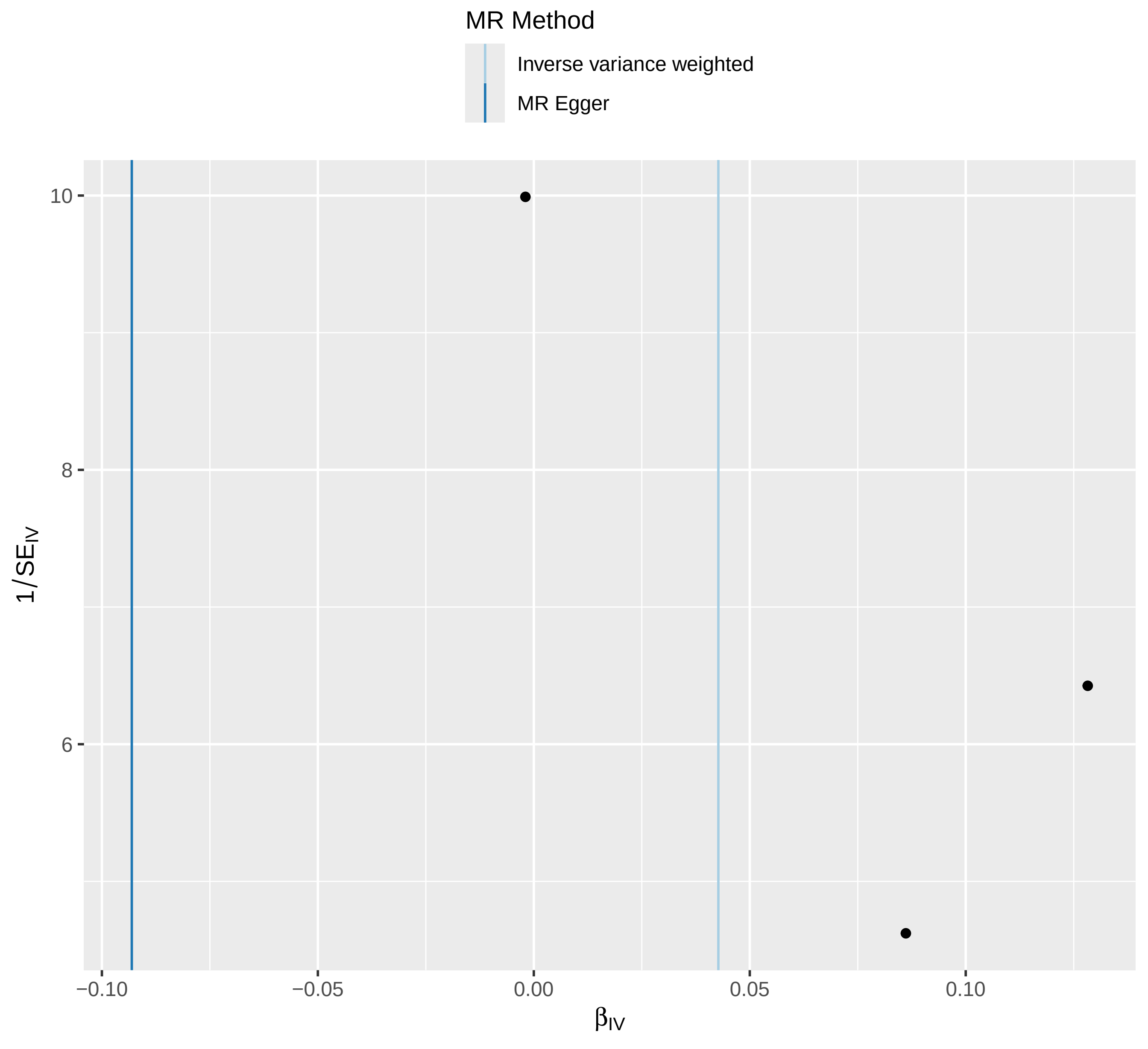

MR Method
Inverse variance weighted
MR Egger
10
8
6
1/SE$_{IV}$
−0.10
−0.05
0.00
0.05
0.10
β$_{IV}$

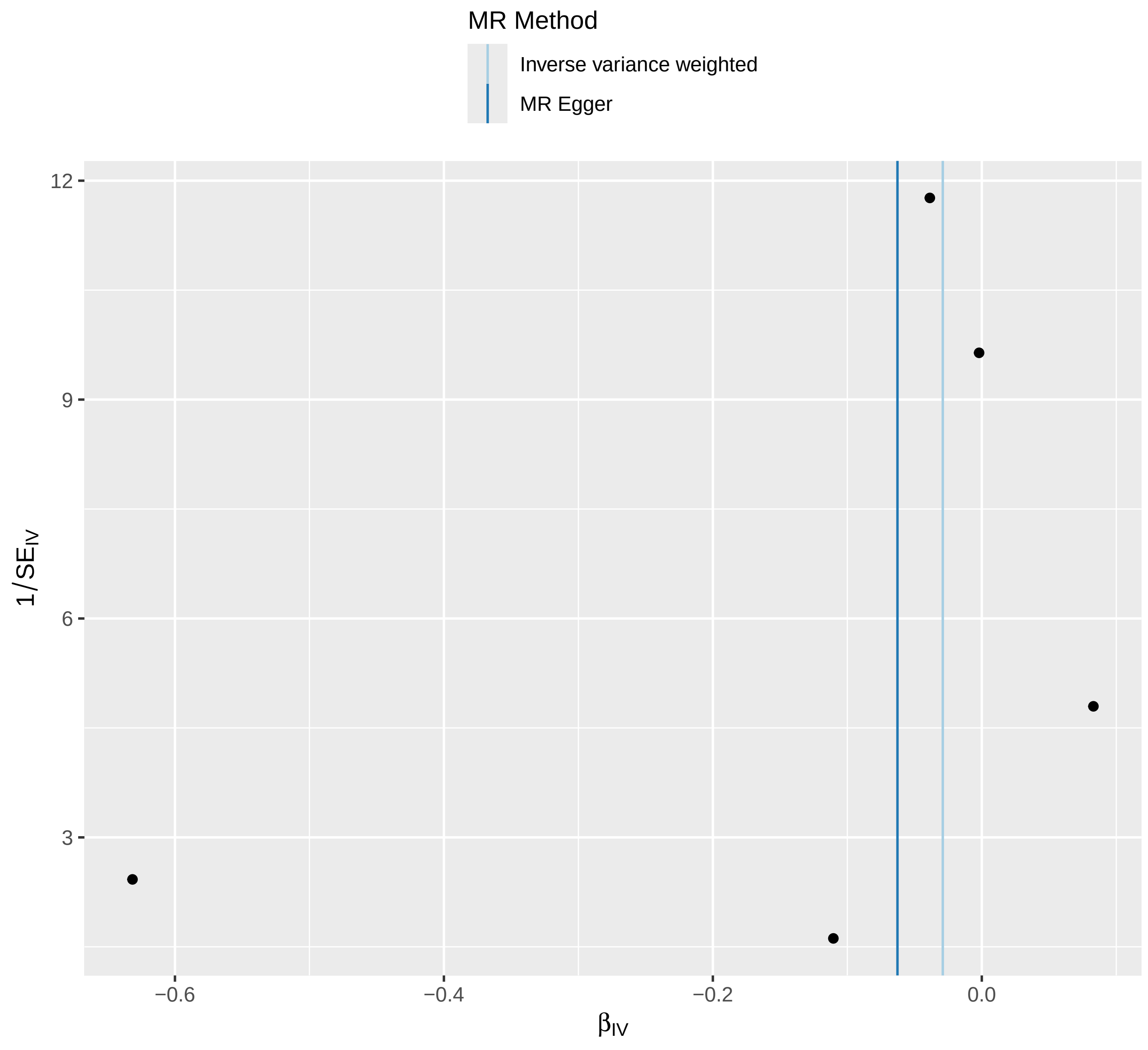

MR Method
Inverse variance weighted
MR Egger
12
9
6
3
1/SE_IV
−0.6
−0.4
−0.2
0.0
β_IV

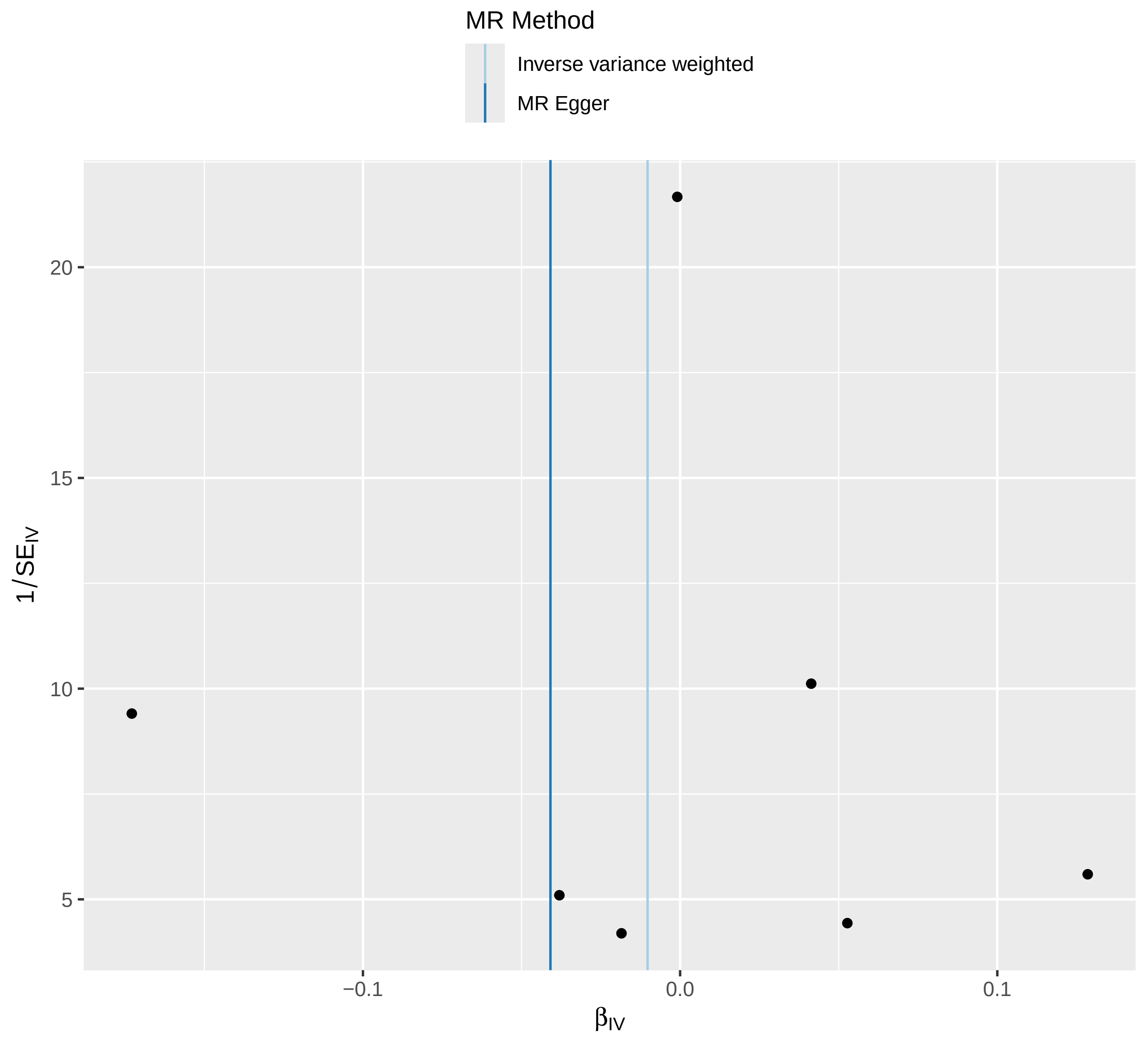
MR Method
Inverse variance weighted
MR Egger
20
15
10
5
1/SE_IV
−0.1
0.0
0.1
β_IV

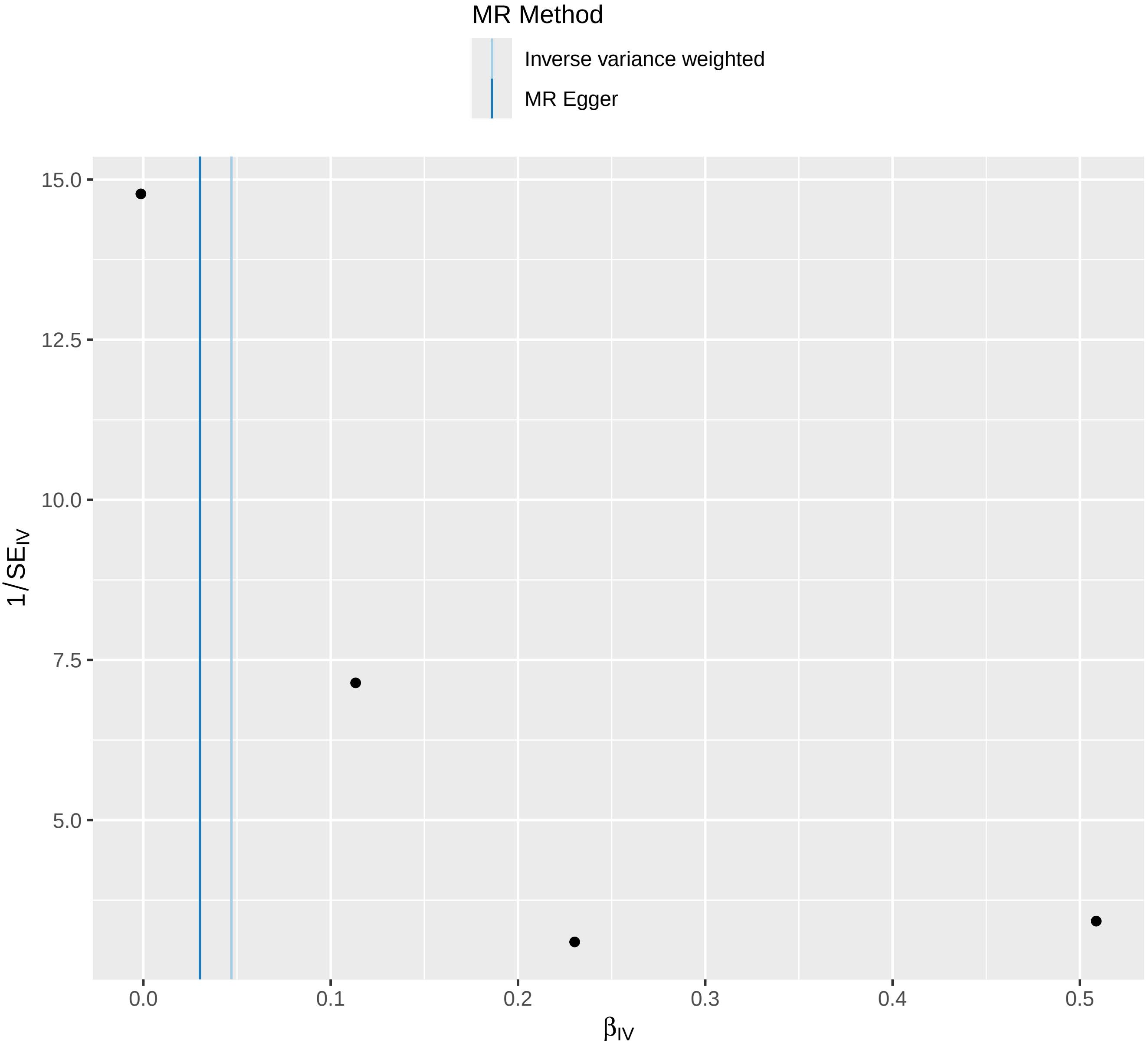
MR Method
Inverse variance weighted
MR Egger
15.0
12.5
10.0
7.5
5.0
1/SE_IV
0.0
0.1
0.2
0.3
0.4
0.5
β_IV

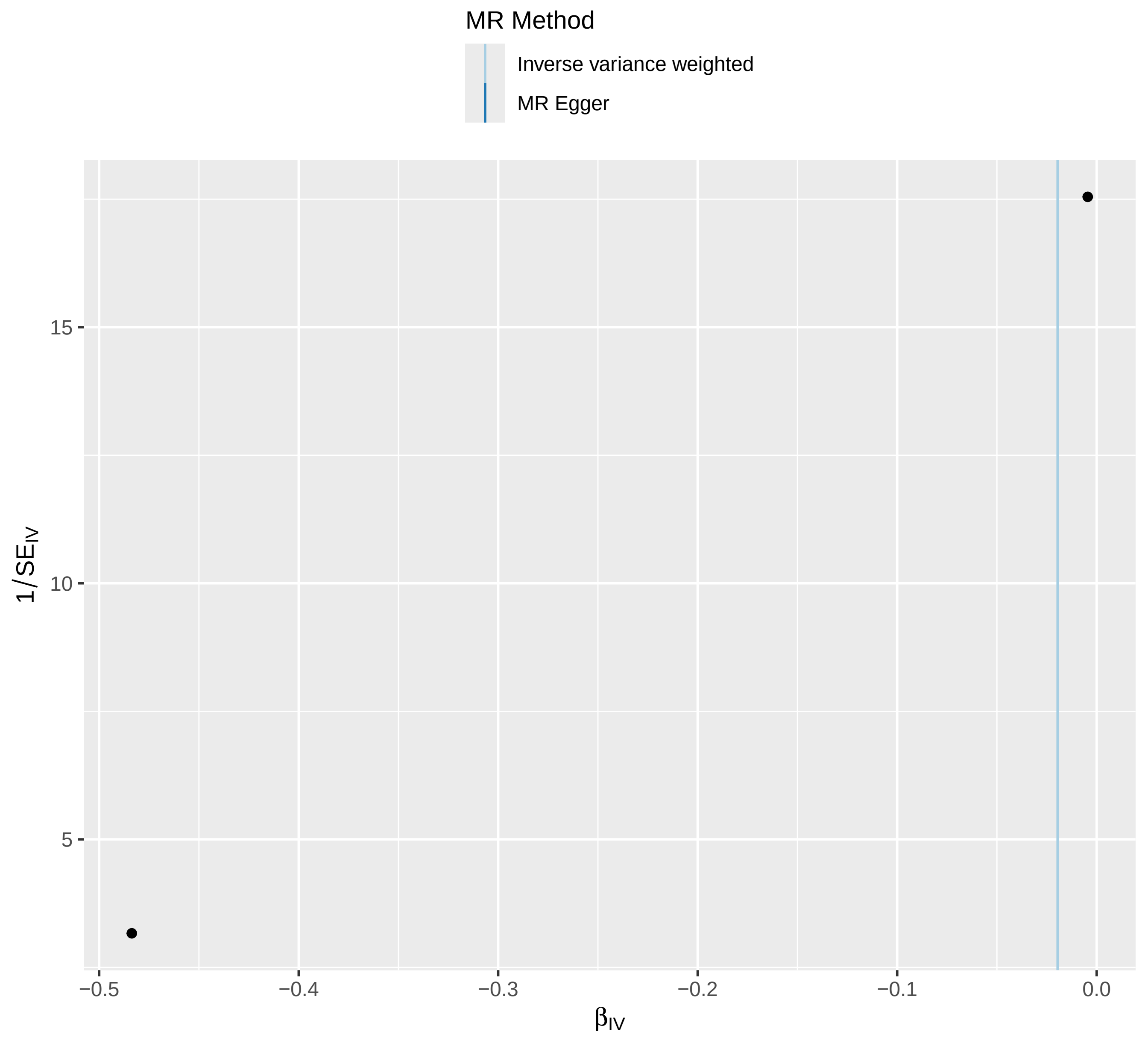

MR Method
Inverse variance weighted
MR Egger
1/SE_IV
15
10
5
−0.5
−0.4
−0.3
−0.2
−0.1
0.0
β_IV

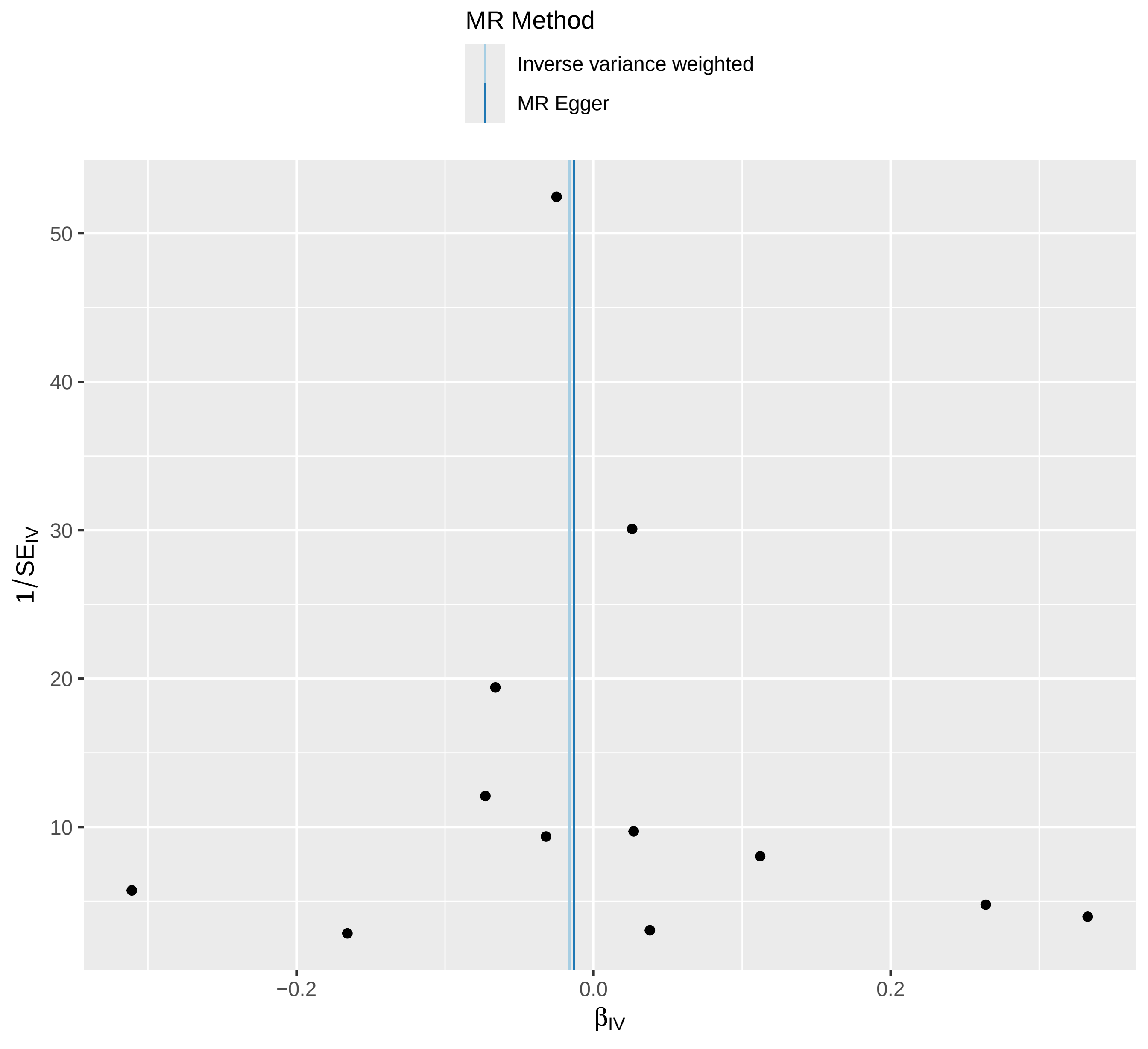

MR Method
Inverse variance weighted
MR Egger
50
40
30
20
10
$1/SE_{IV}$
−0.2
0.0
0.2
$\beta_{IV}$

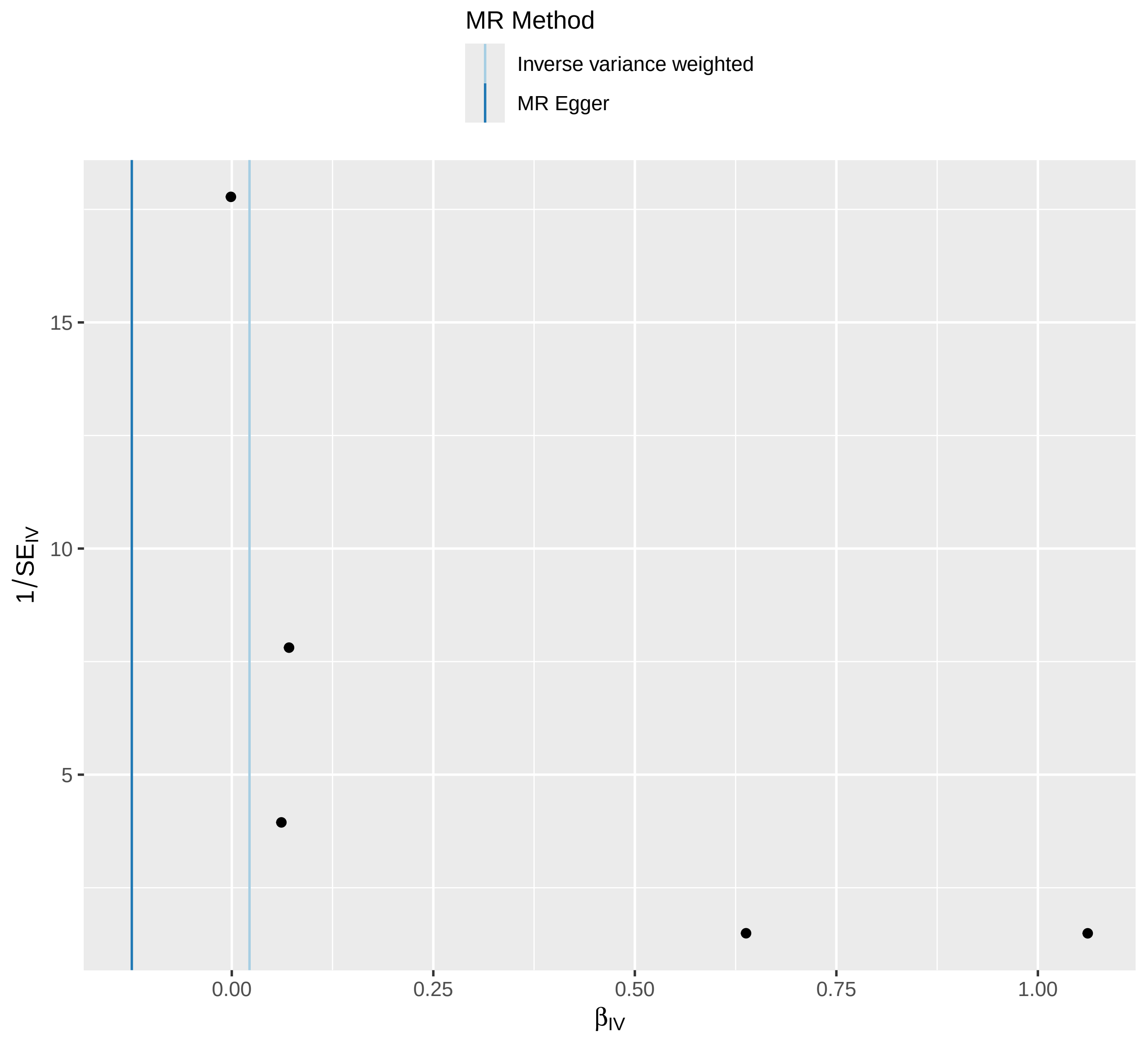
MR Method
Inverse variance weighted
MR Egger
15
10
5
$1/SE_{IV}$
0.00
0.25
0.50
0.75
1.00
$\beta_{IV}$

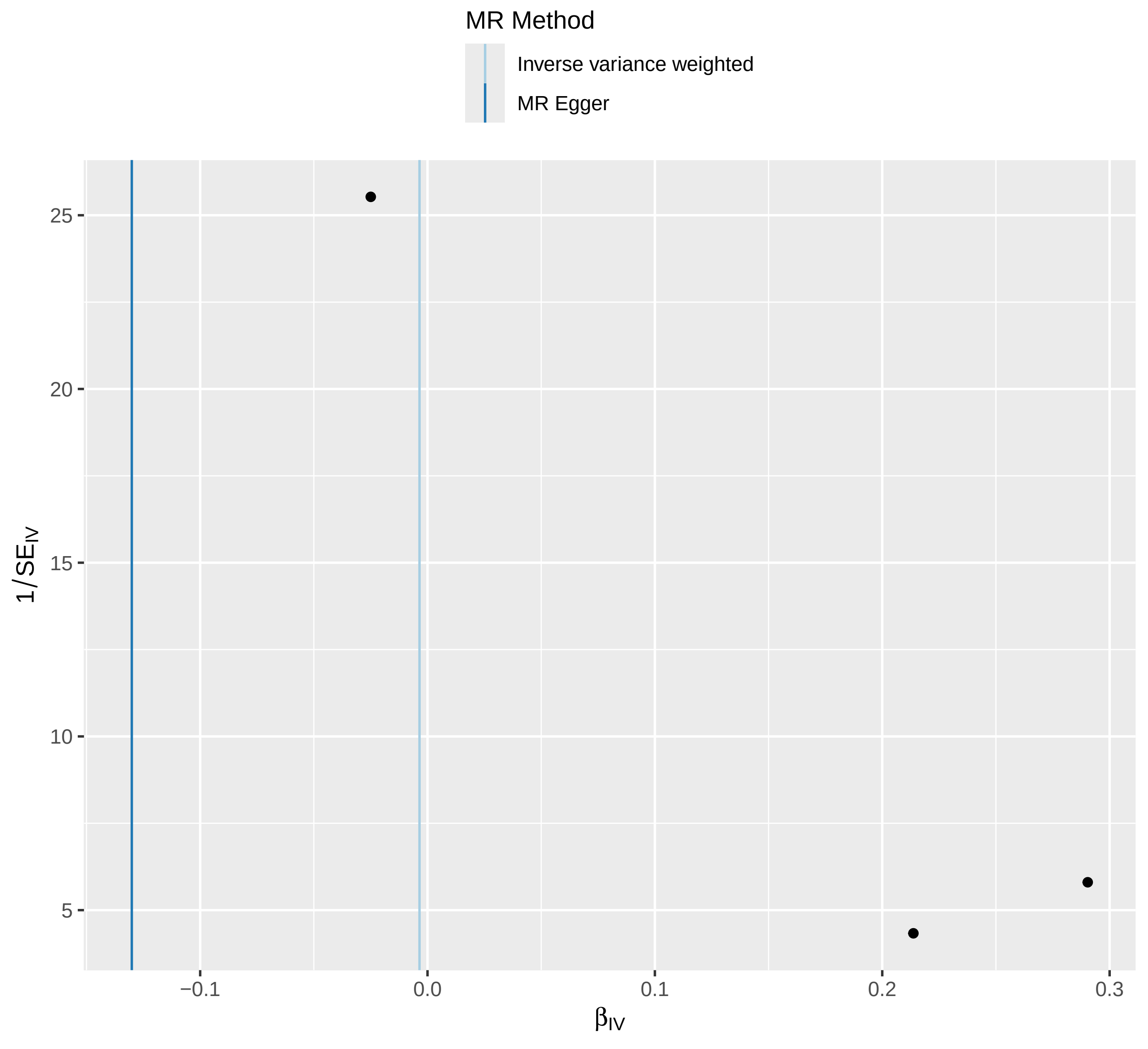
MR Method
Inverse variance weighted
MR Egger
25
20
15
10
5
−0.1
0.0
0.1
0.2
0.3
$1/SE_{IV}$
$\beta_{IV}$

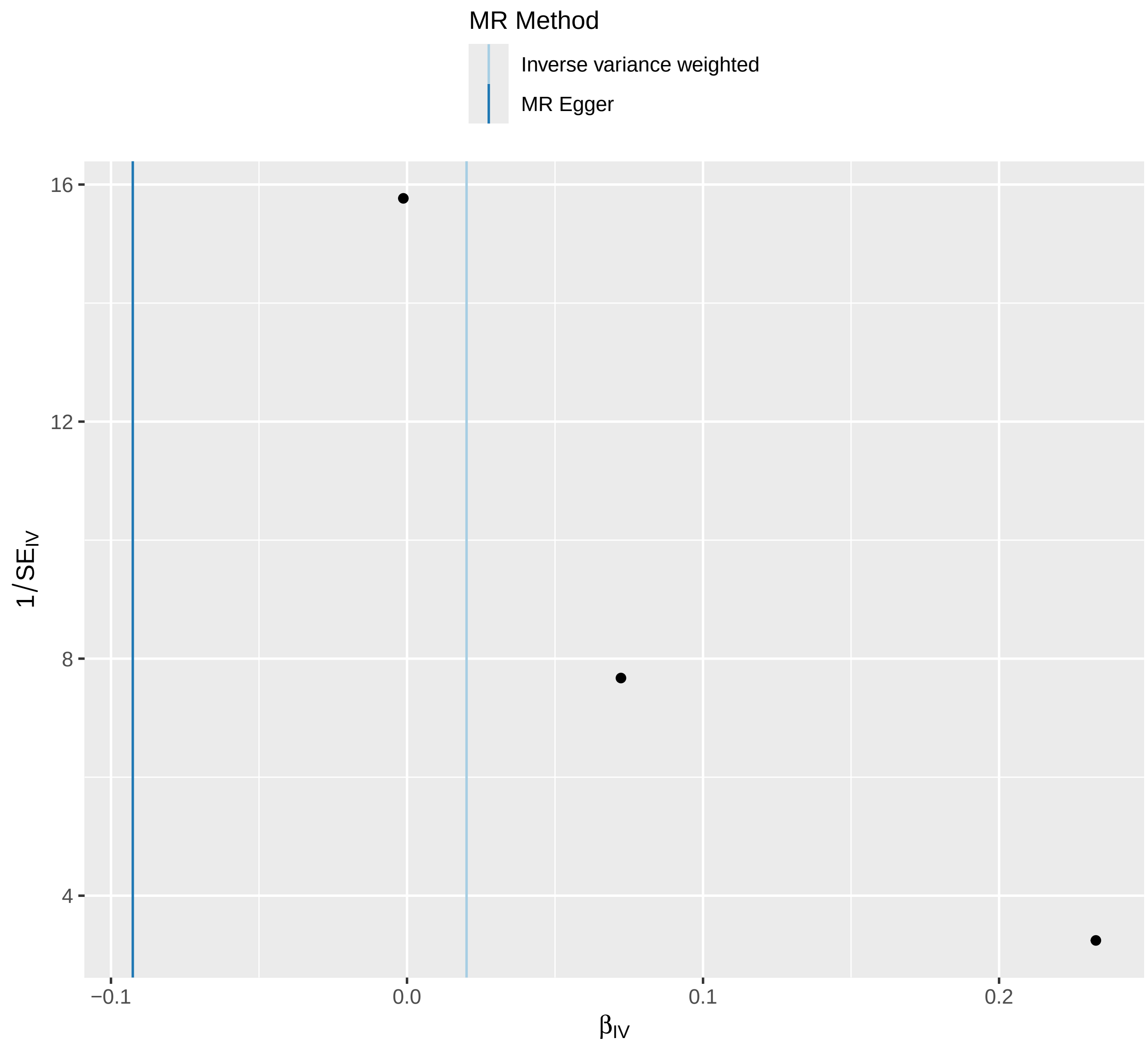

MR Method
Inverse variance weighted
MR Egger
16
12
8
4
1/SE$_{IV}$
−0.1
0.0
0.1
0.2
β$_{IV}$

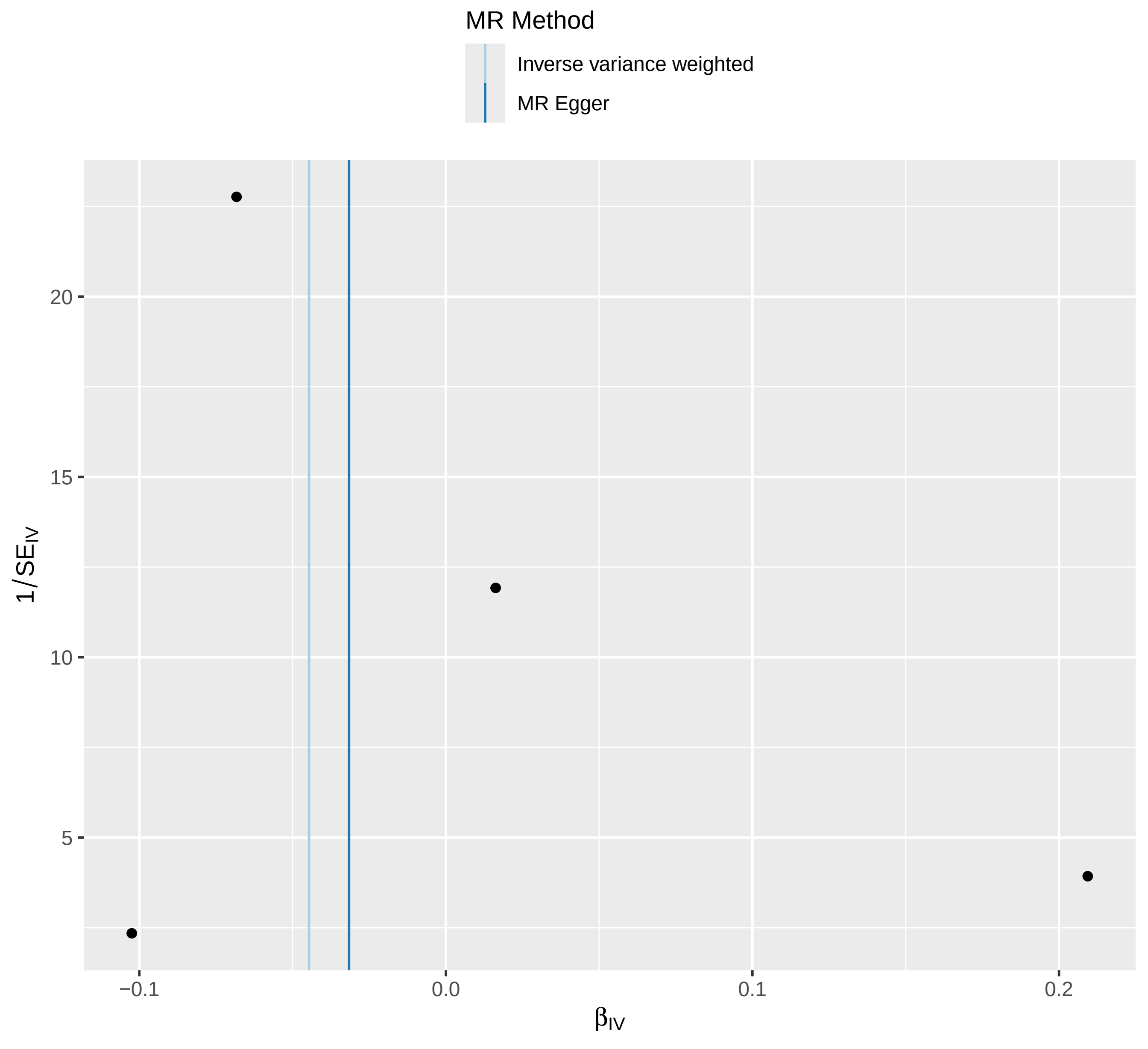

MR Method
Inverse variance weighted
MR Egger
1/SE_IV
20
15
10
5
−0.1
0.0
0.1
0.2
β_IV

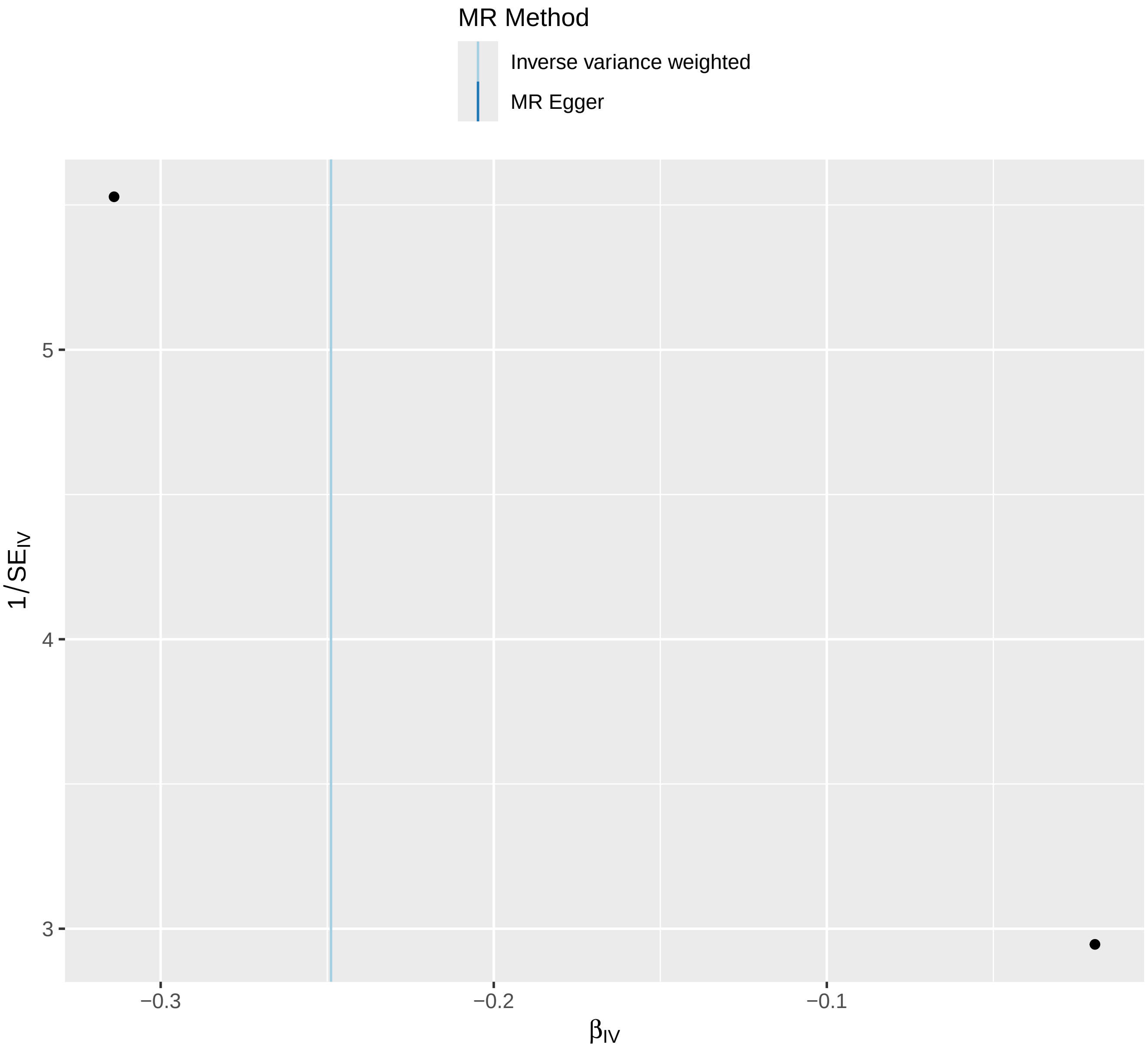
MR Method
Inverse variance weighted
MR Egger
5
4
3
$1/SE_{IV}$
−0.3
−0.2
−0.1
$\beta_{IV}$

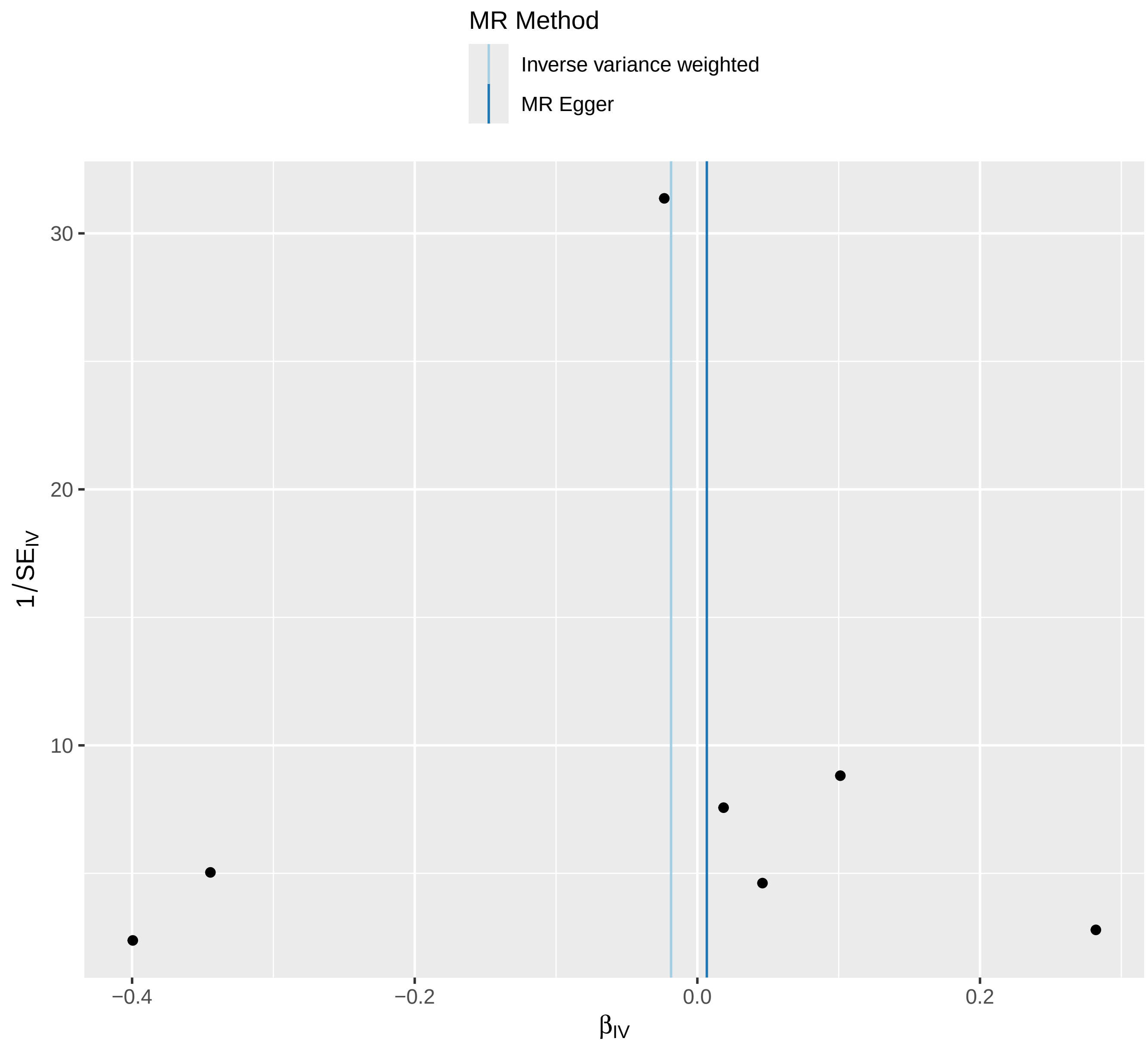
MR Method
Inverse variance weighted
MR Egger
30
20
10
1/SE_IV
−0.4
−0.2
0.0
0.2
β_IV

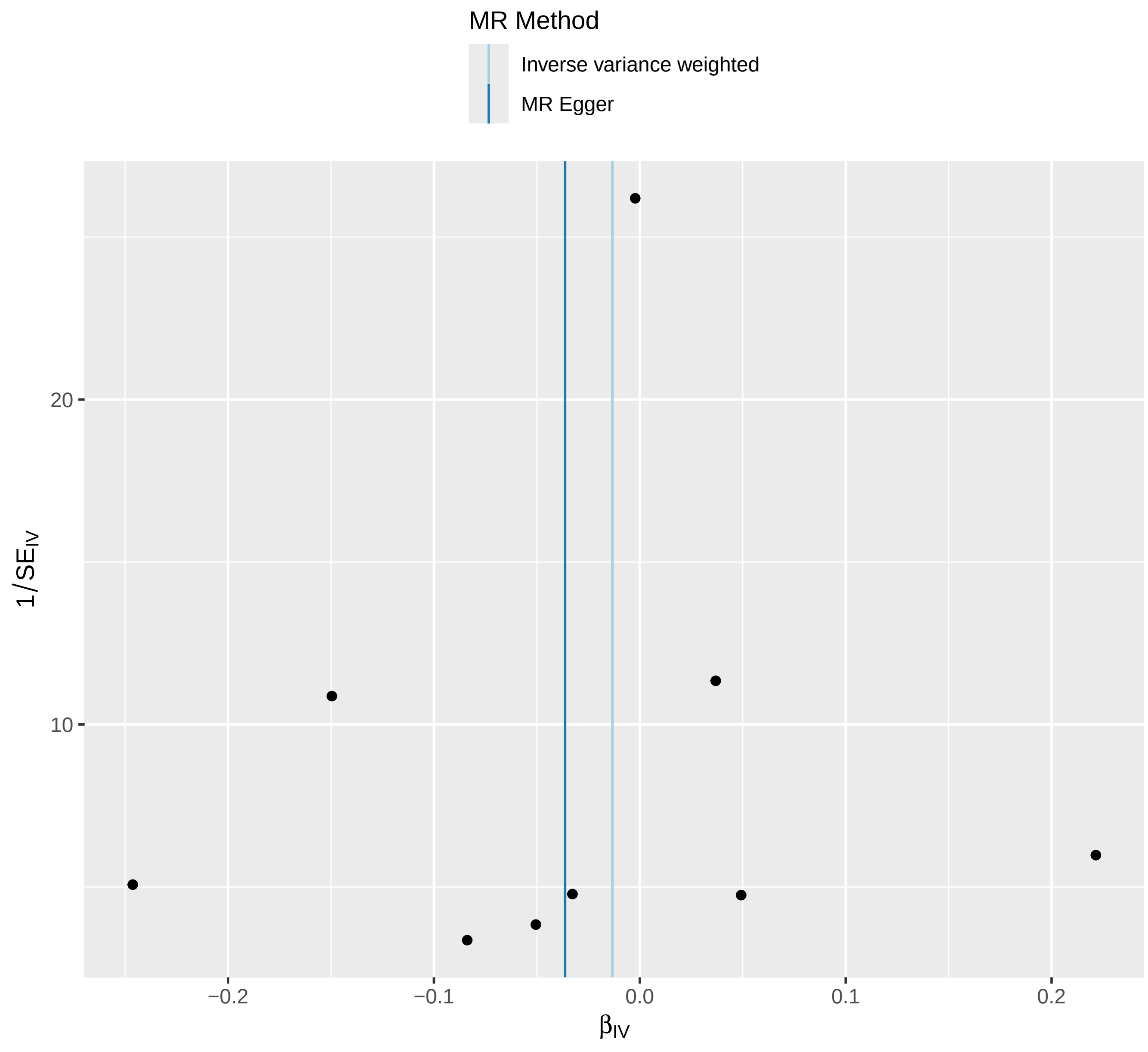

MR Method
Inverse variance weighted
MR Egger
20
10
$1/SE_{IV}$
−0.2
−0.1
0.0
0.1
0.2
$\beta_{IV}$

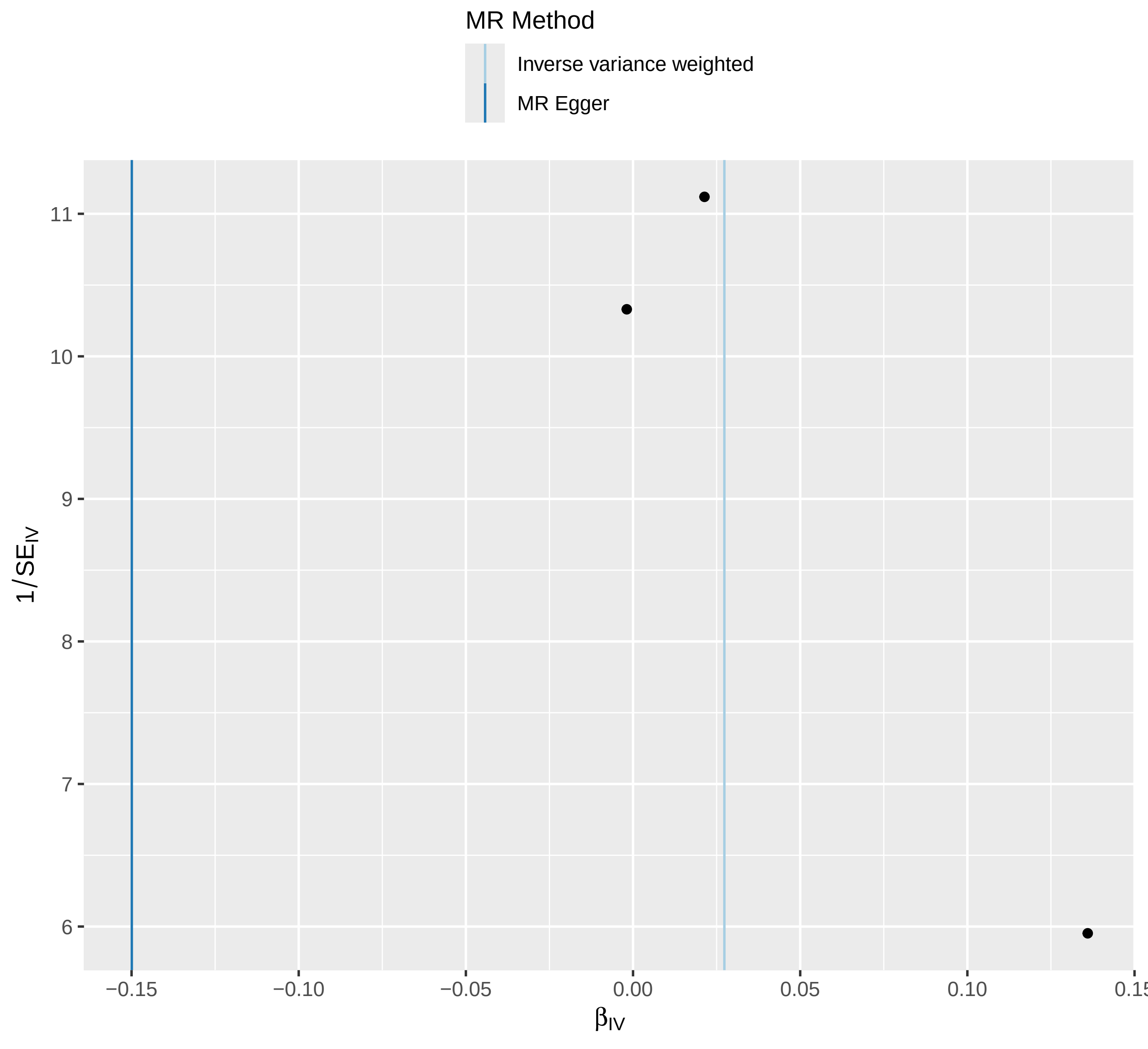
MR Method
Inverse variance weighted
MR Egger
11
10
9
8
7
6
1/SE_IV
−0.15
−0.10
−0.05
0.00
0.05
0.10
0.15
β_IV

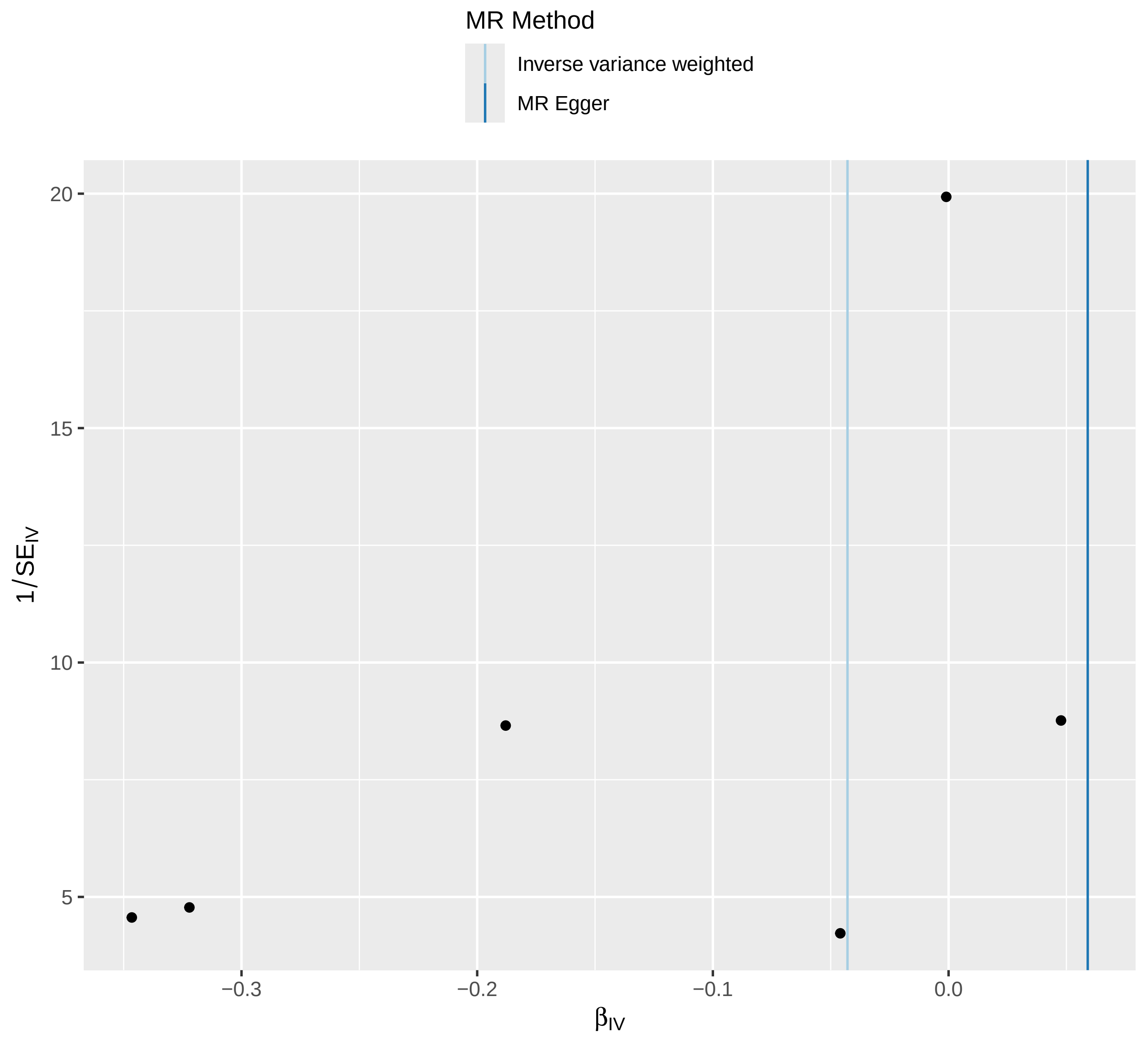
MR Method
Inverse variance weighted
MR Egger
20
15
10
5
1/SE_IV
−0.3
−0.2
−0.1
0.0
β_IV

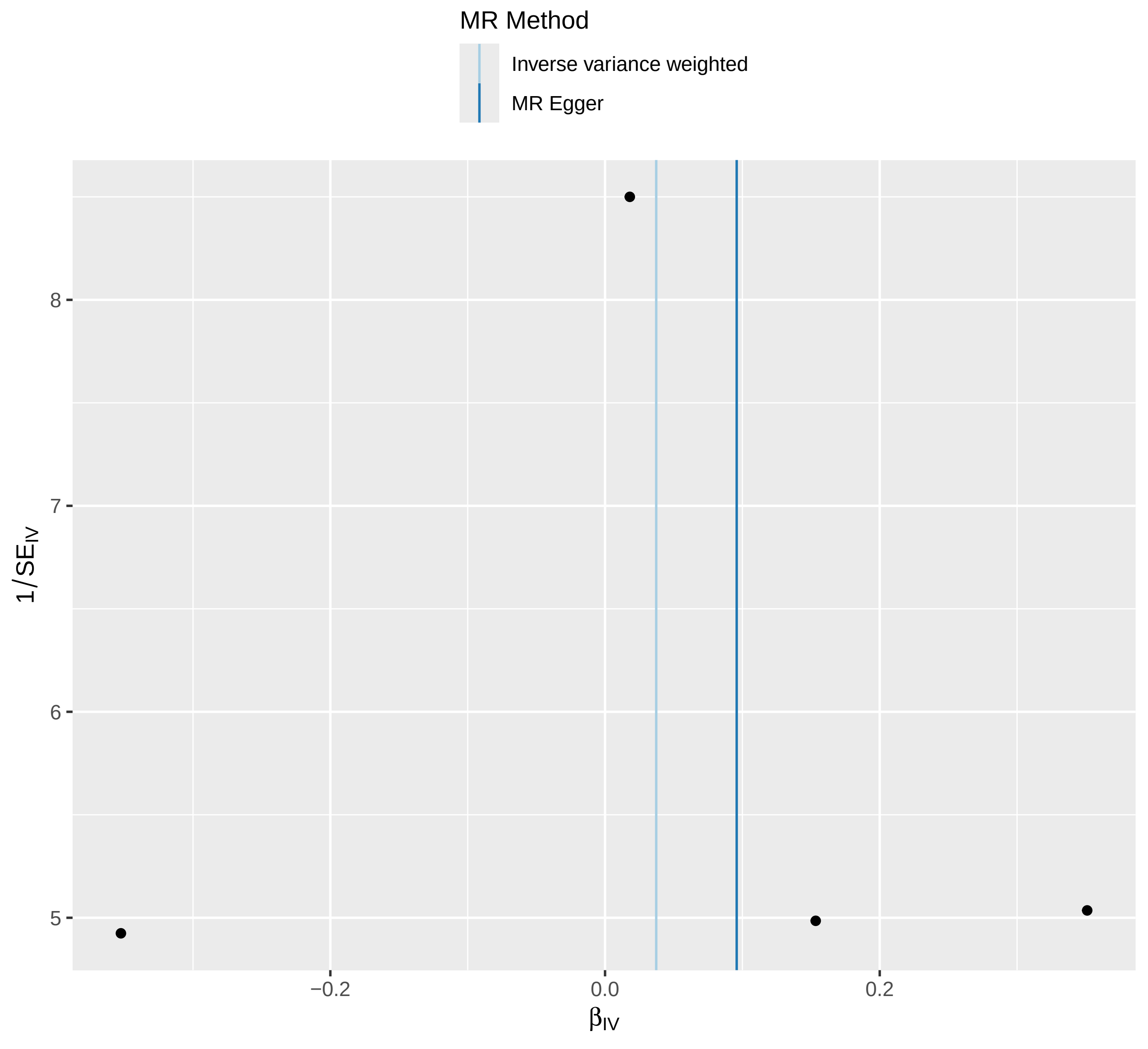

MR Method
Inverse variance weighted
MR Egger
1/SE_IV
8
7
6
5
−0.2
0.0
0.2
β_IV

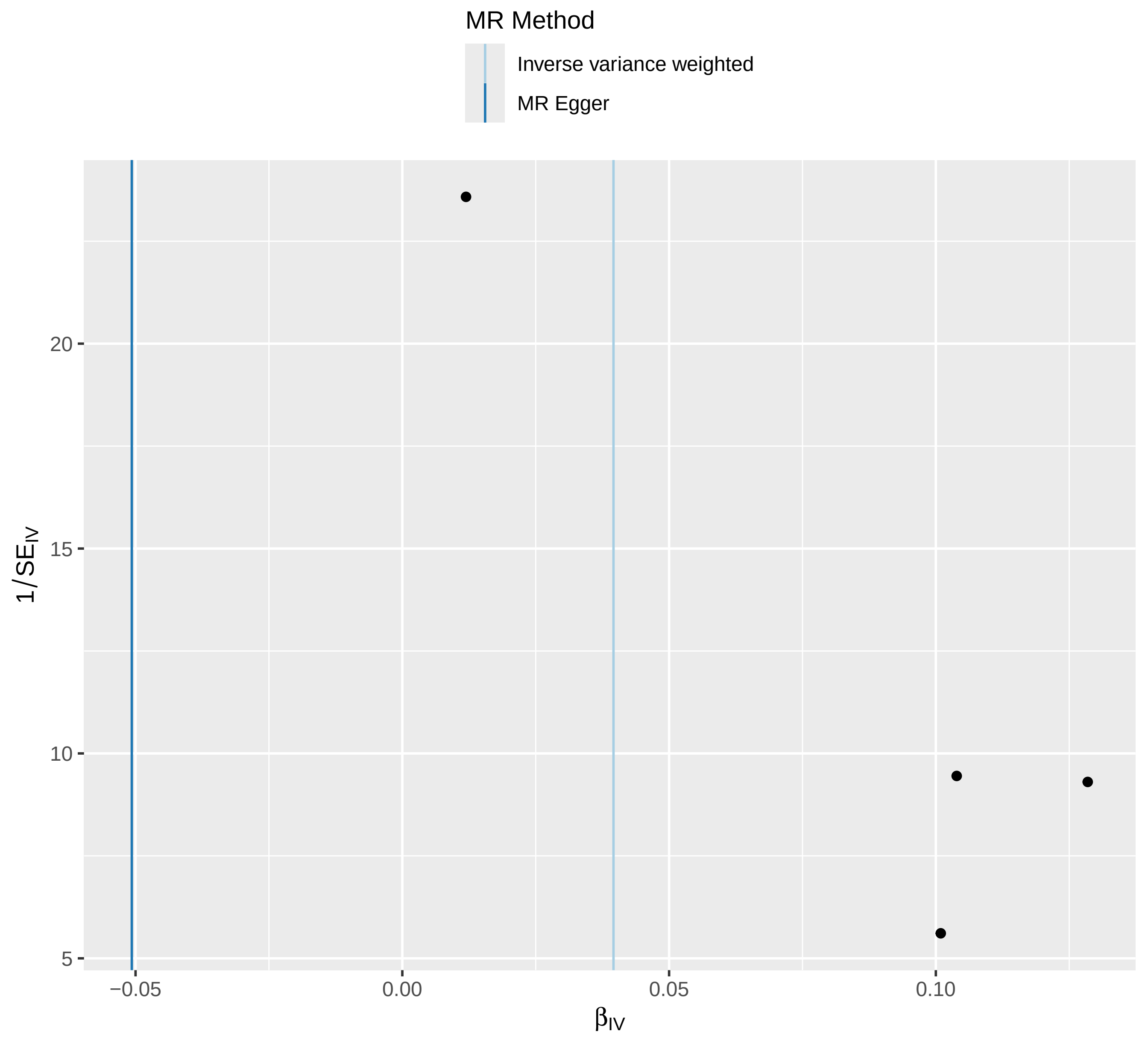
MR Method
Inverse variance weighted
MR Egger
20
15
10
5
1/SE$_{IV}$
−0.05
0.00
0.05
0.10
β$_{IV}$

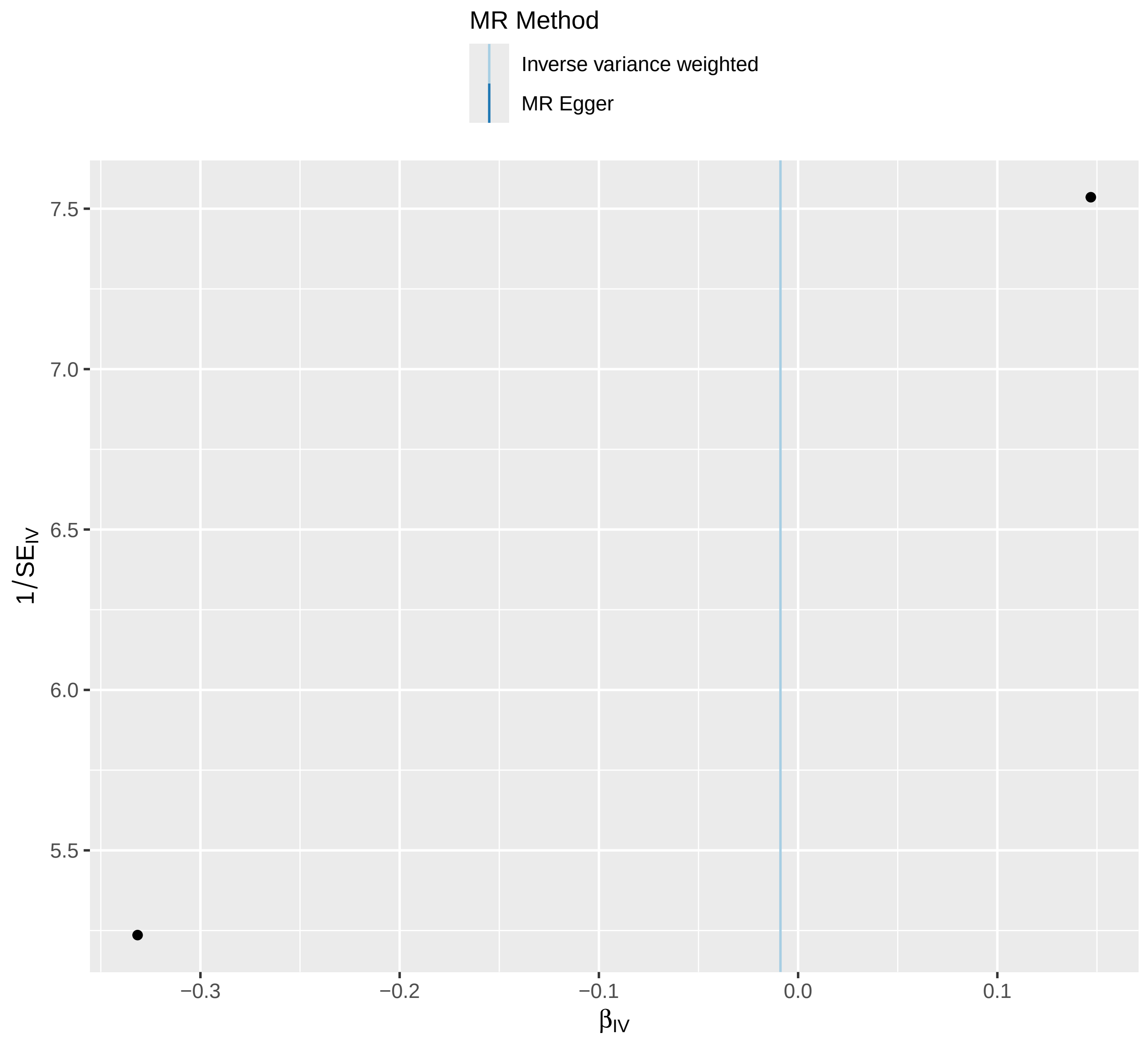
MR Method
Inverse variance weighted
MR Egger
7.5
7.0
6.5
6.0
5.5
1/SE_IV
−0.3
−0.2
−0.1
0.0
0.1
β_IV

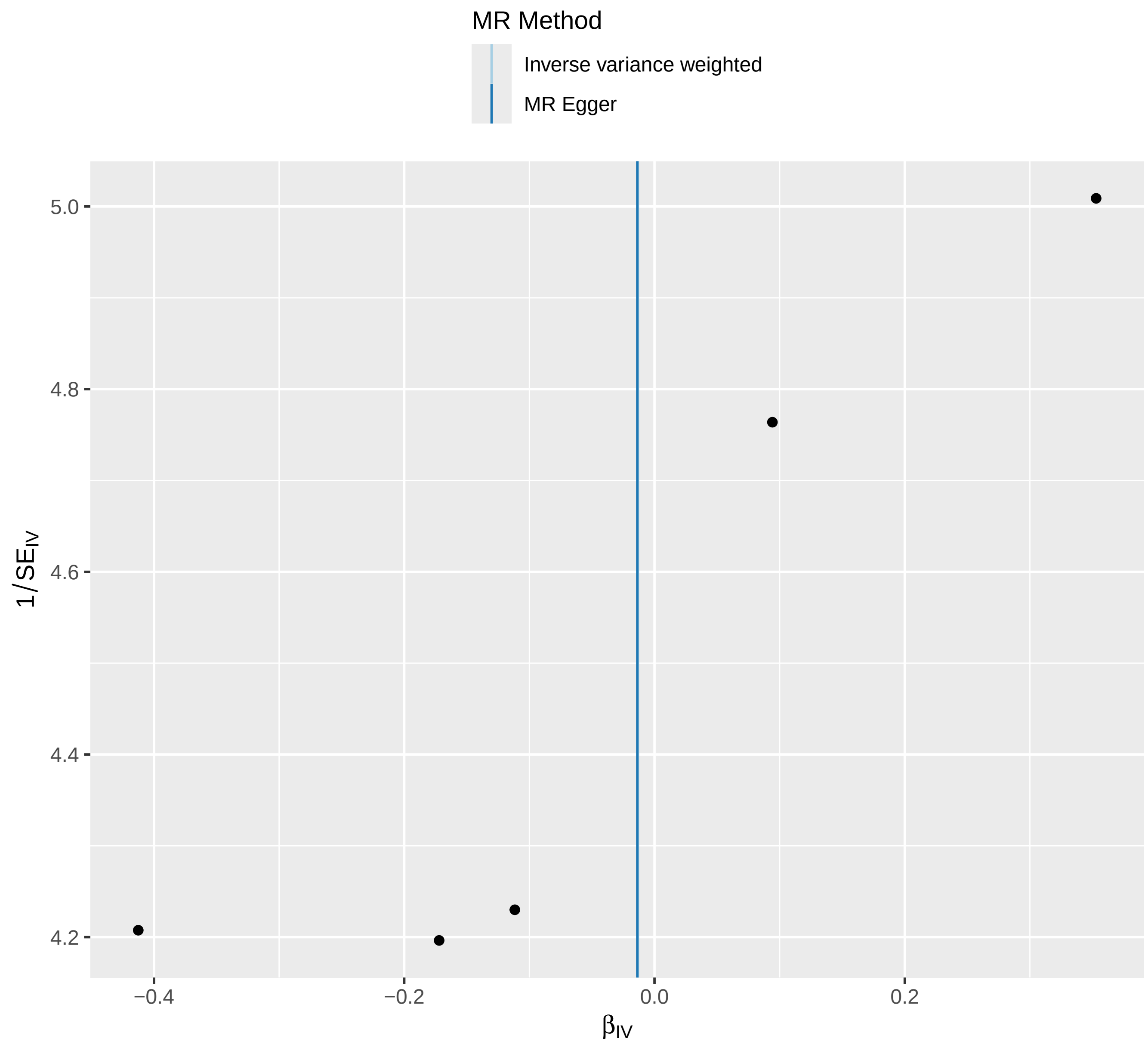
MR Method
Inverse variance weighted
MR Egger
5.0
4.8
4.6
4.4
4.2
$1/SE_{IV}$
−0.4
−0.2
0.0
0.2
$\beta_{IV}$

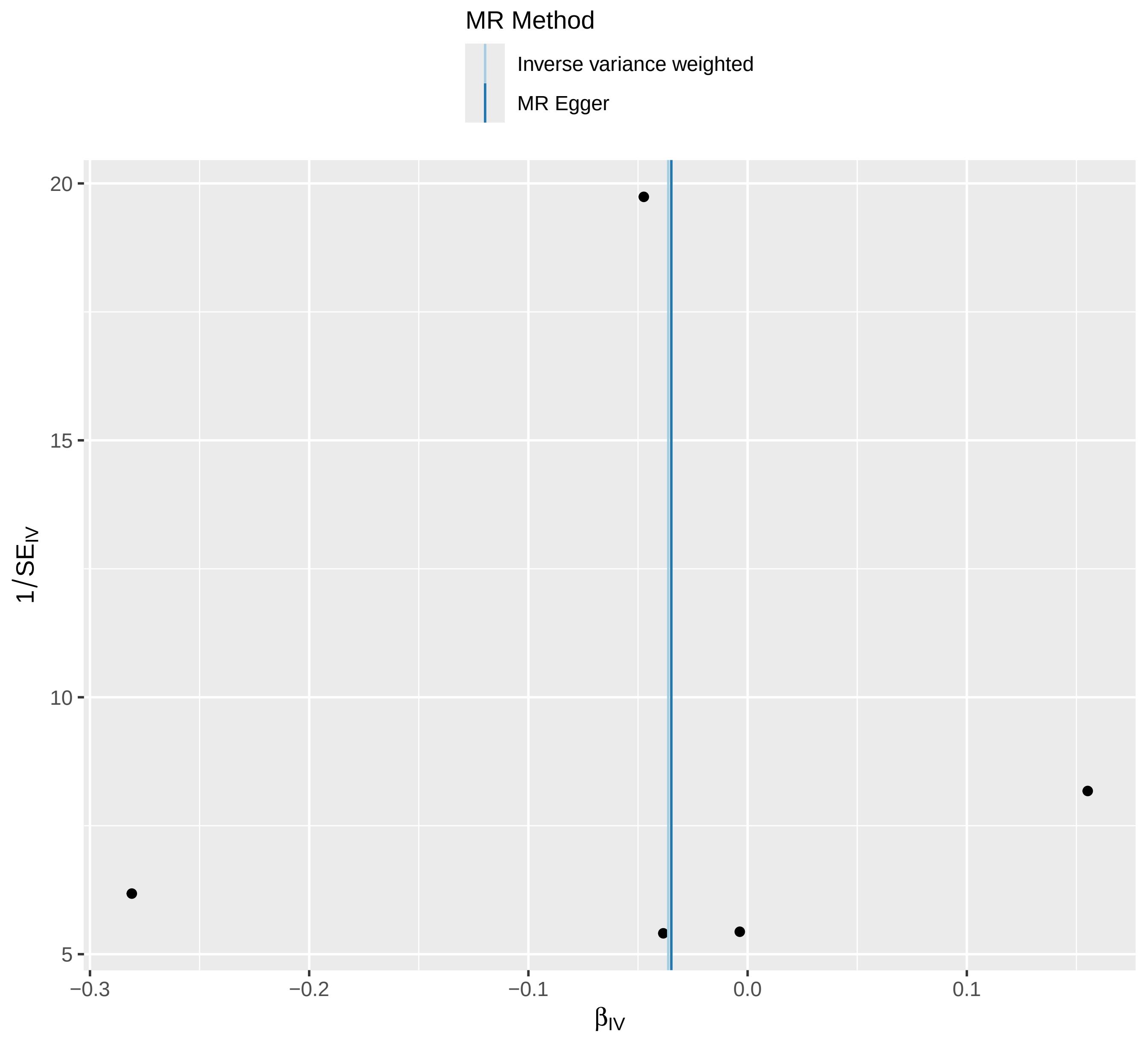
MR Method
Inverse variance weighted
MR Egger
20
15
10
5
1/SE_IV
−0.3
−0.2
−0.1
0.0
0.1
β_IV

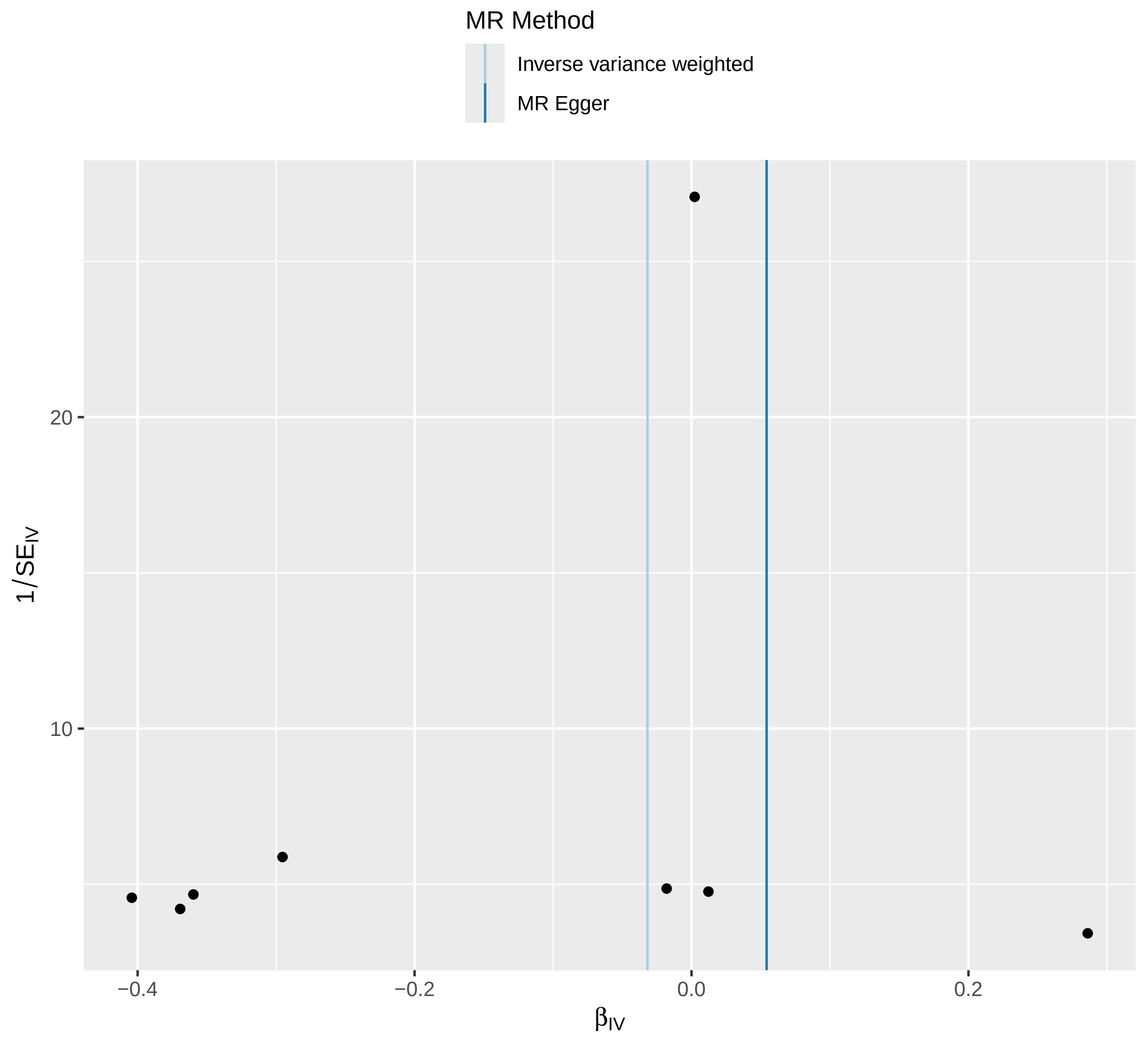
MR Method
Inverse variance weighted
MR Egger
20
10
$1/SE_{IV}$
−0.4
−0.2
0.0
0.2
$\beta_{IV}$

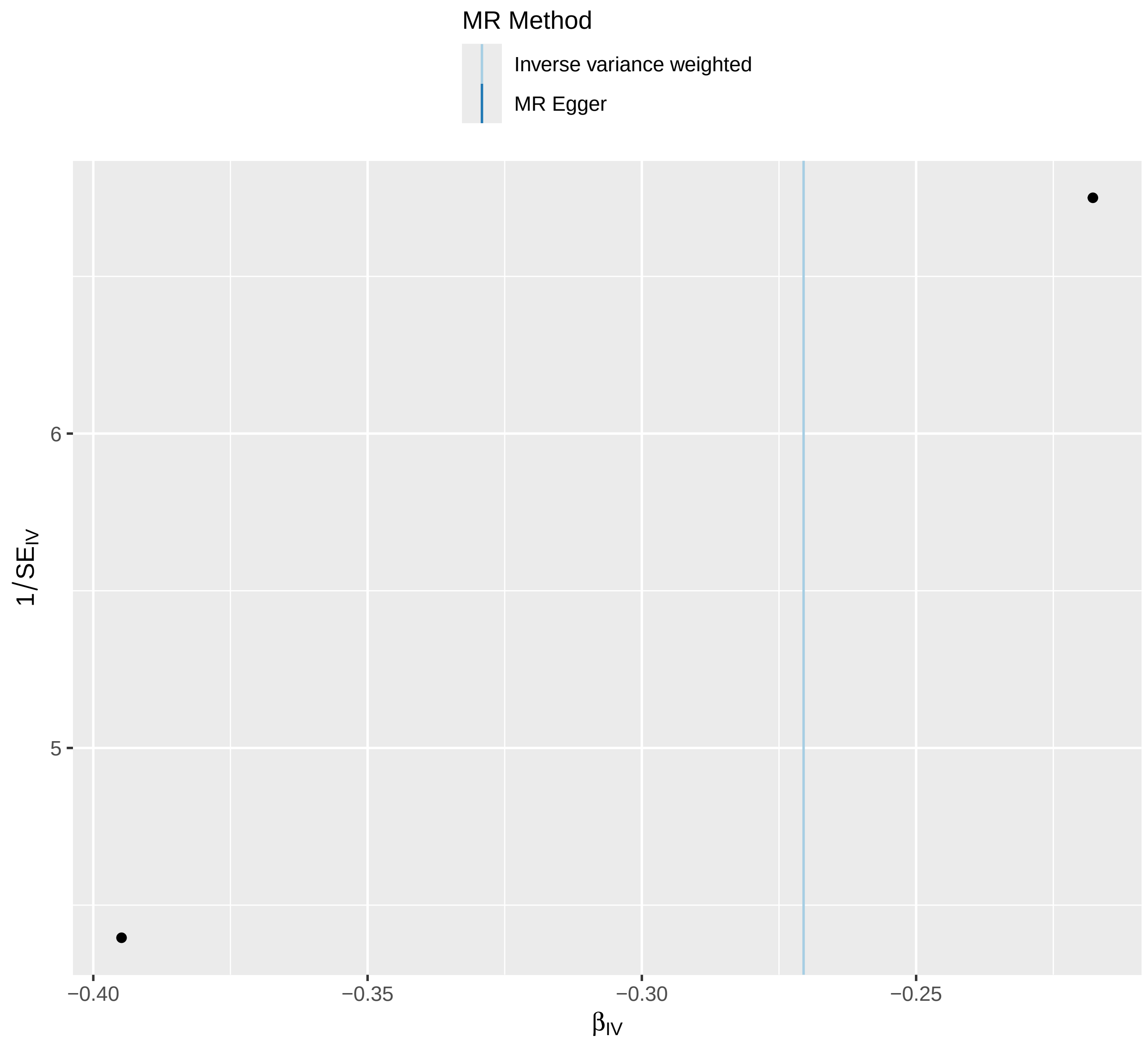
MR Method
Inverse variance weighted
MR Egger
1/SE$_{IV}$
6
5
−0.40
−0.35
−0.30
−0.25
β$_{IV}$

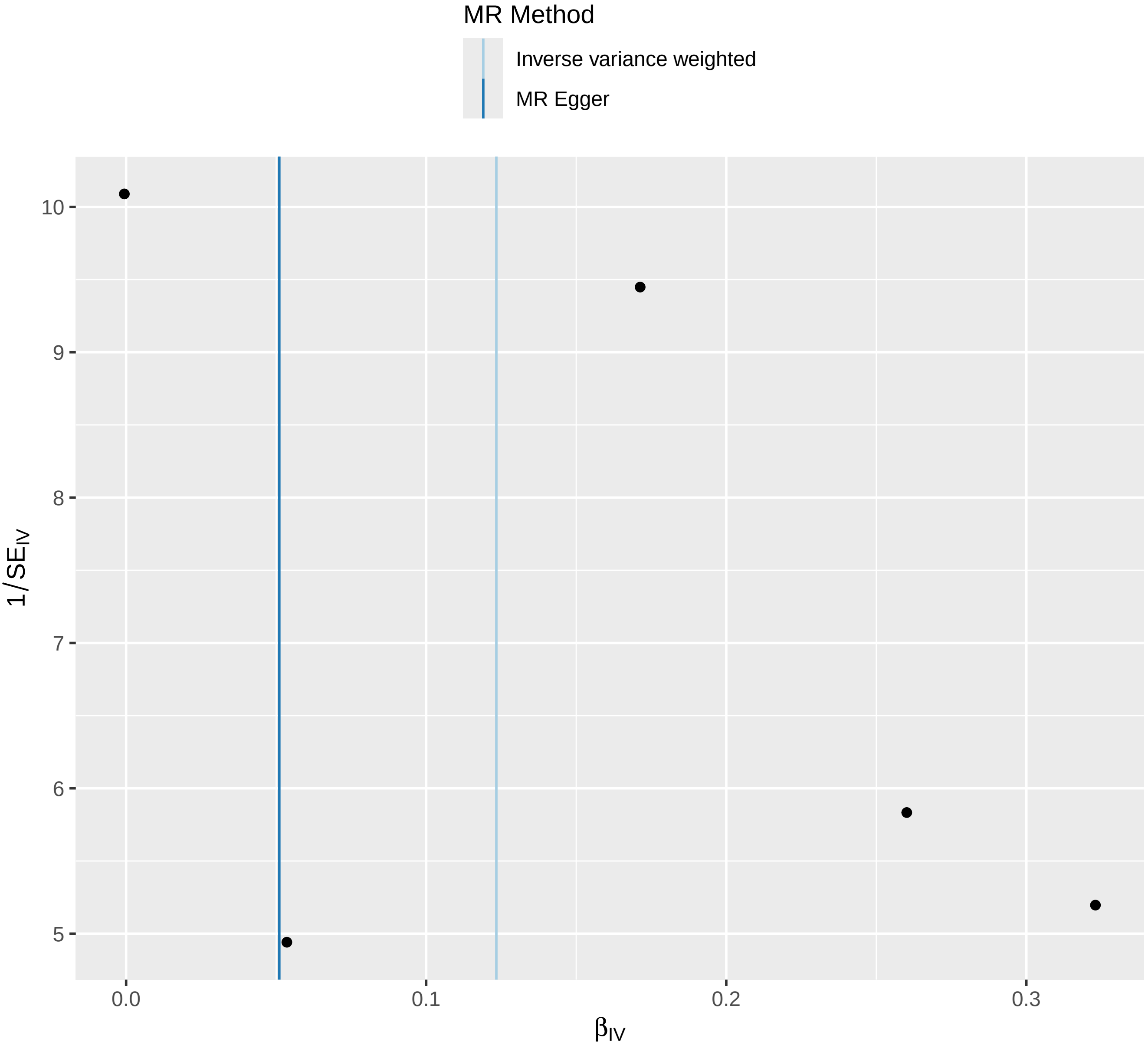

MR Method
Inverse variance weighted
MR Egger
10
9
8
7
6
5
1/SE_IV
0.0
0.1
0.2
0.3
β_IV

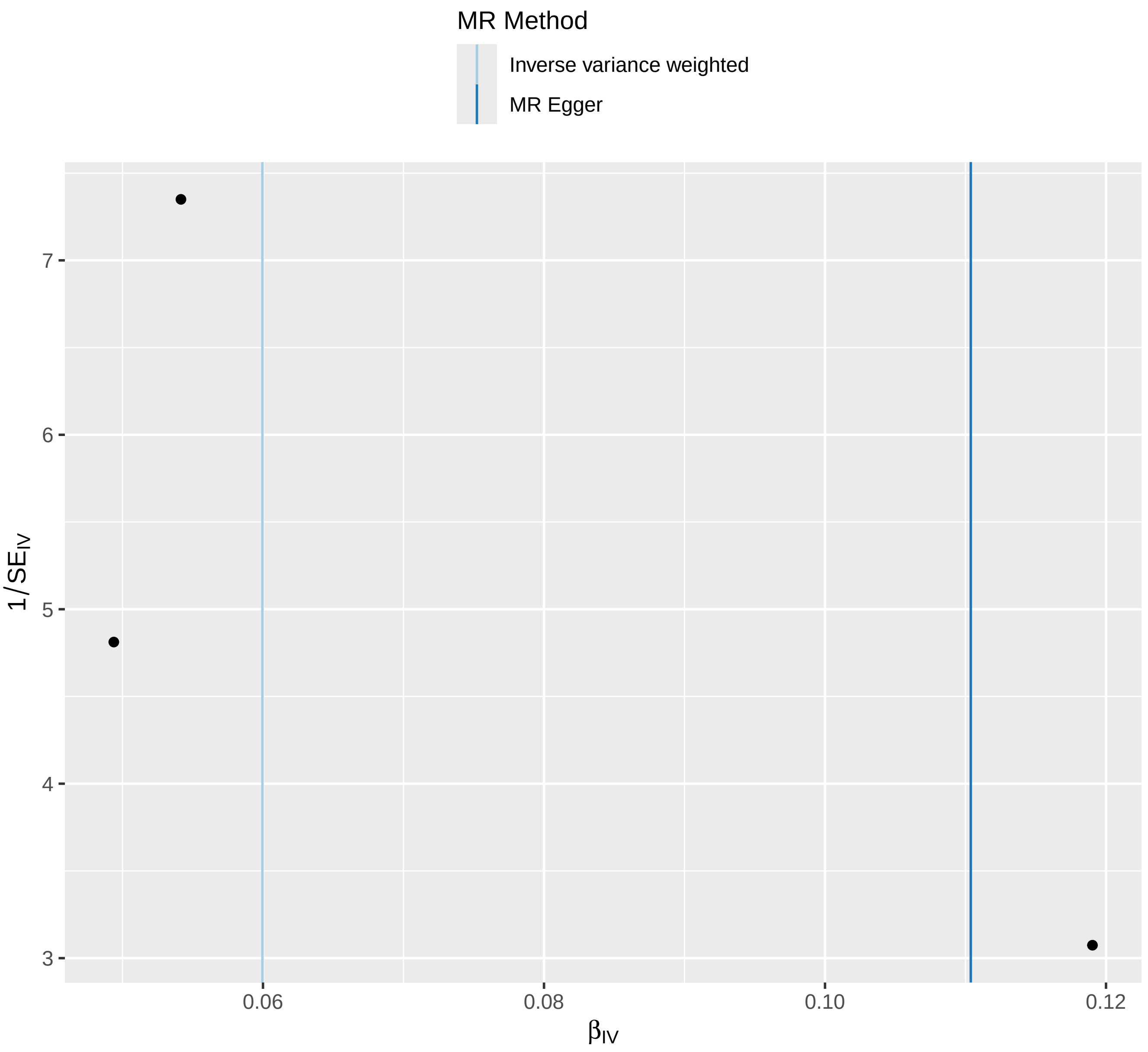
MR Method
Inverse variance weighted
MR Egger
1/SE_IV
β_IV
7
6
5
4
3
0.06
0.08
0.10
0.12

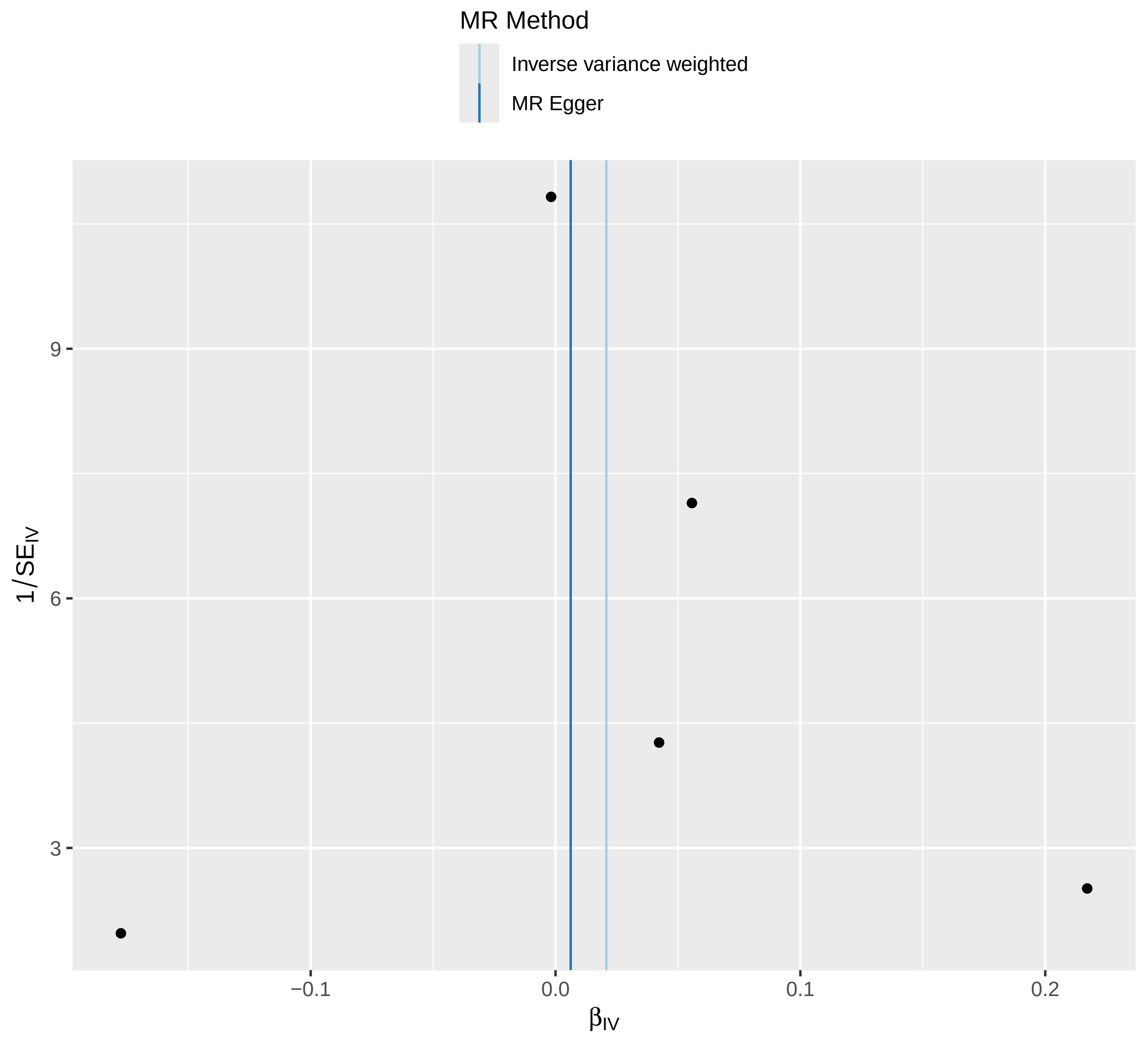
MR Method
Inverse variance weighted
MR Egger
9
6
3
1/SE_IV
−0.1
0.0
0.1
0.2
β_IV

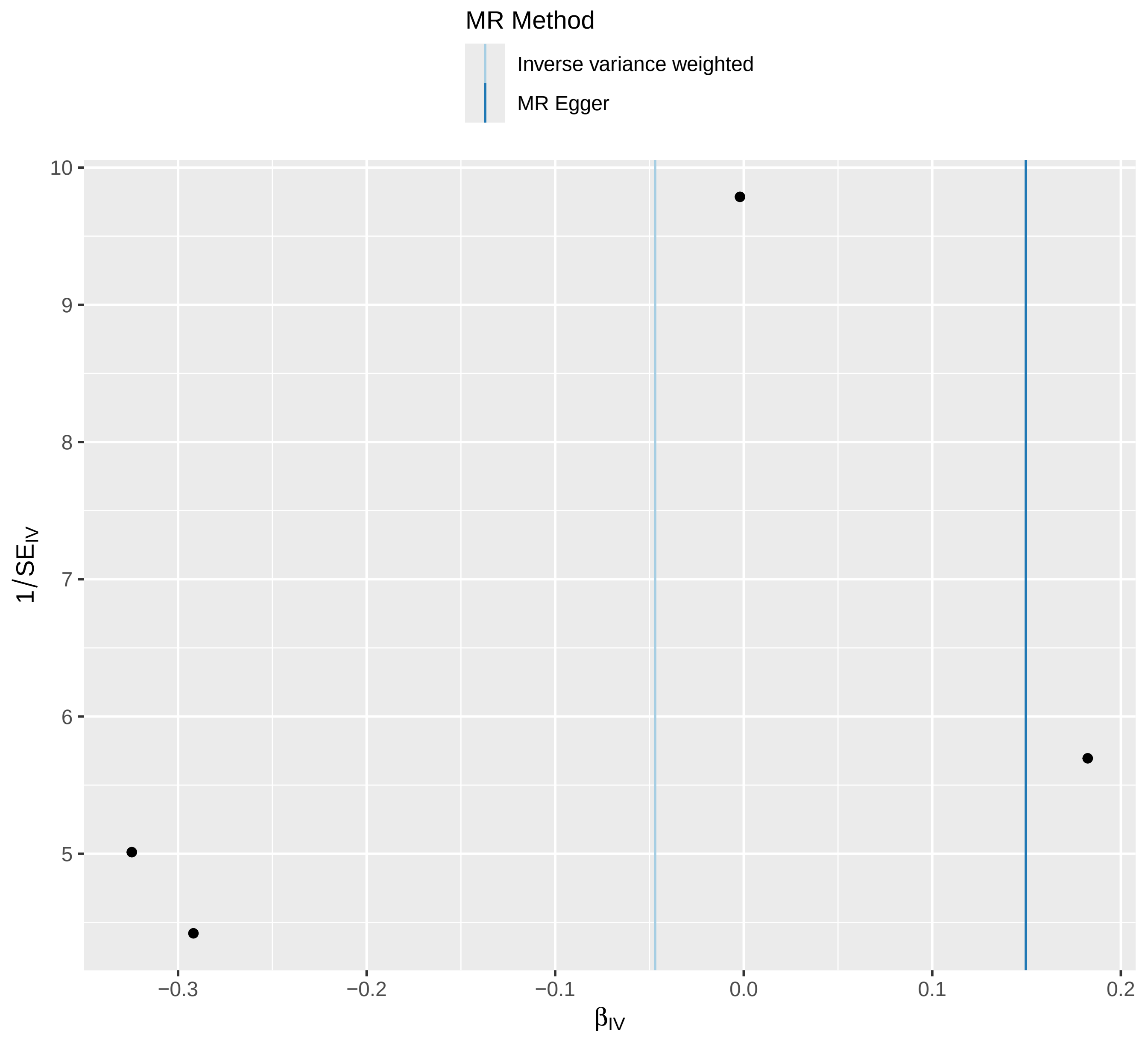
MR Method
Inverse variance weighted
MR Egger
10
9
8
7
6
5
1/SE$_{IV}$
−0.3
−0.2
−0.1
0.0
0.1
0.2
$\beta_{IV}$

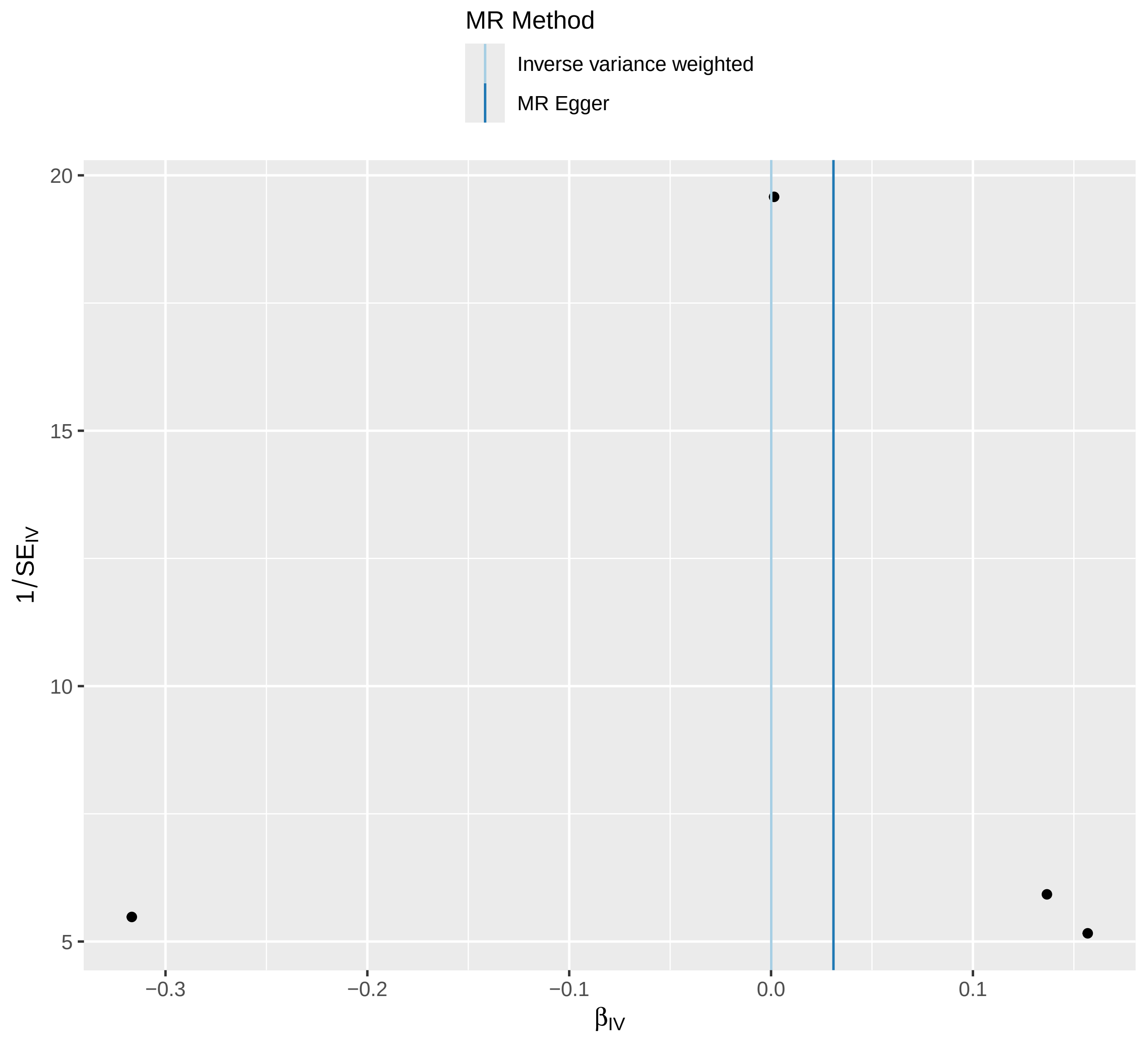
MR Method
Inverse variance weighted
MR Egger
20
15
10
5
1/SE$_{IV}$
−0.3
−0.2
−0.1
0.0
0.1
β$_{IV}$

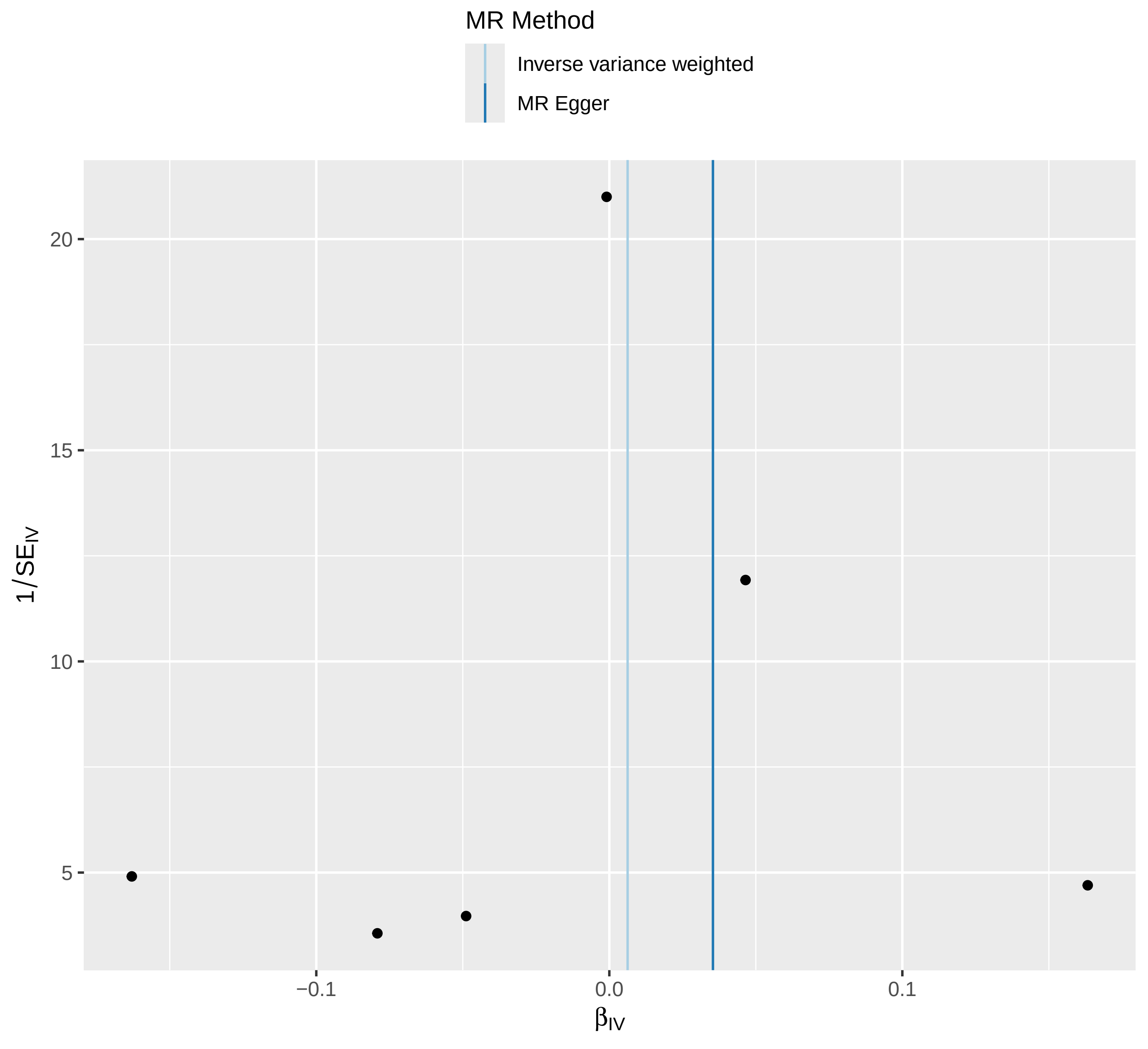
MR Method
Inverse variance weighted
MR Egger
20
15
10
5
$1/SE_{IV}$
−0.1
0.0
0.1
$\beta_{IV}$

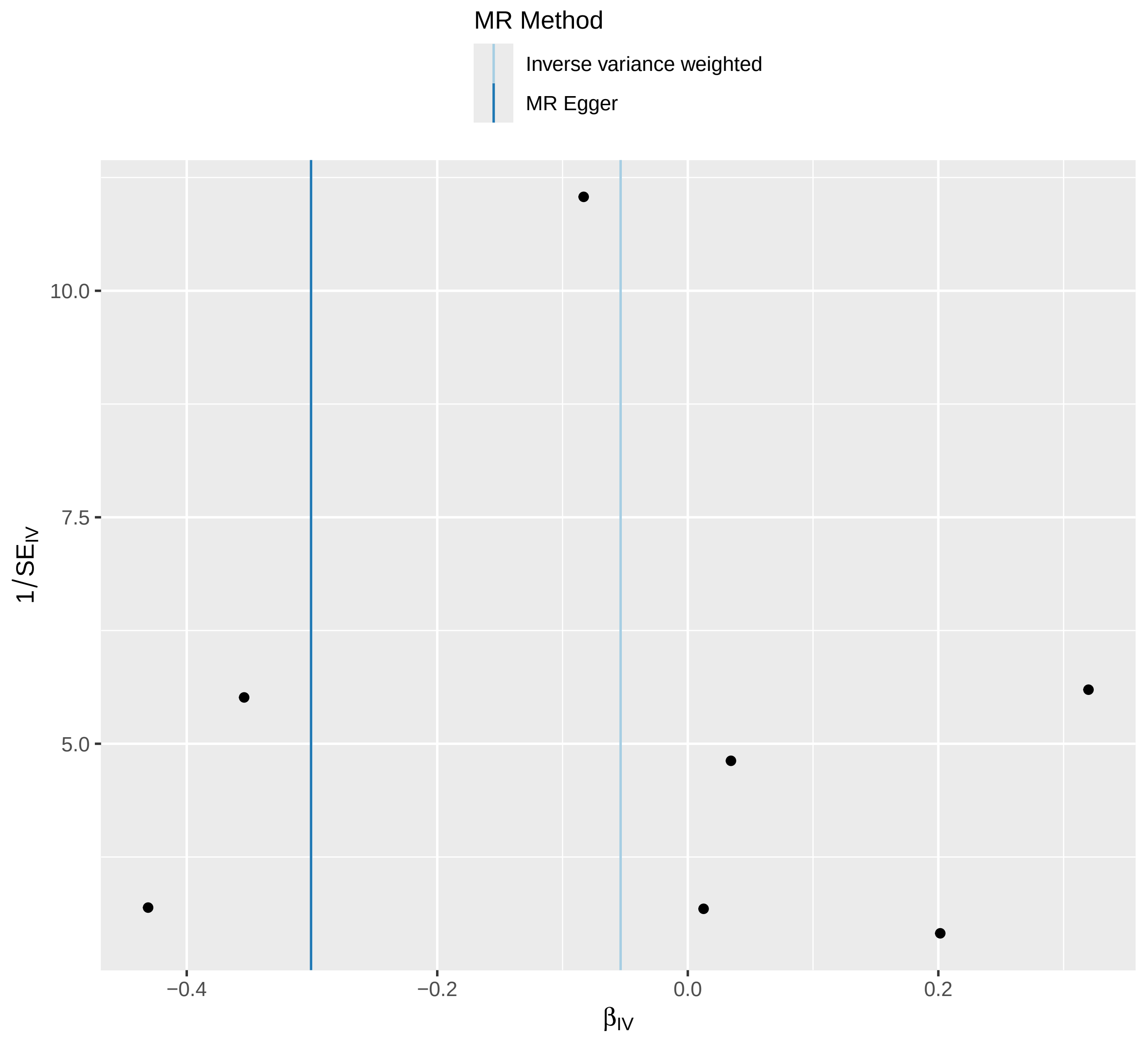

MR Method
Inverse variance weighted
MR Egger
10.0
7.5
5.0
1/SE_IV
−0.4
−0.2
0.0
0.2
β_IV

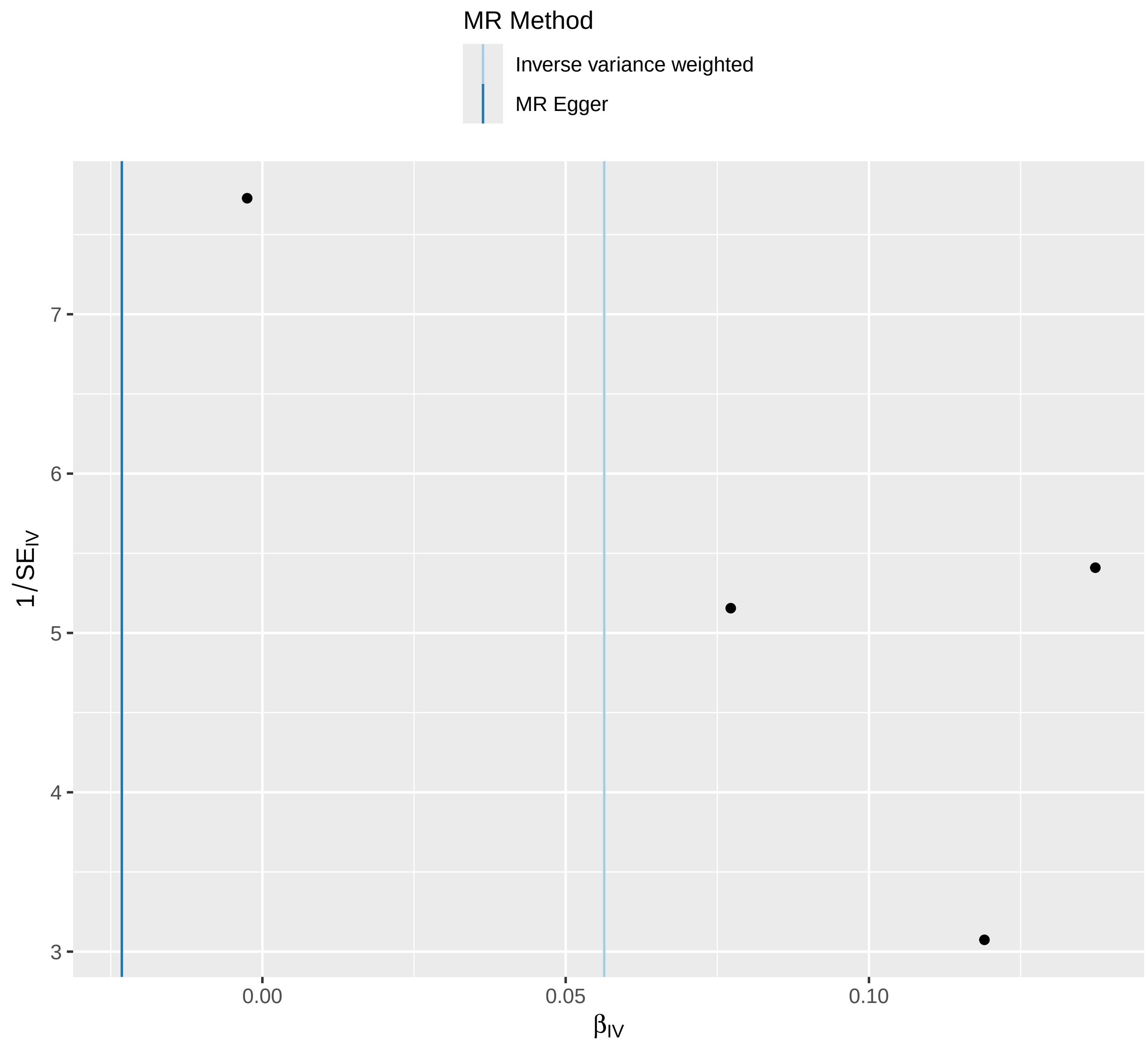

MR Method
Inverse variance weighted
MR Egger
7
6
5
4
3
1/SE_IV
0.00
0.05
0.10
β_IV

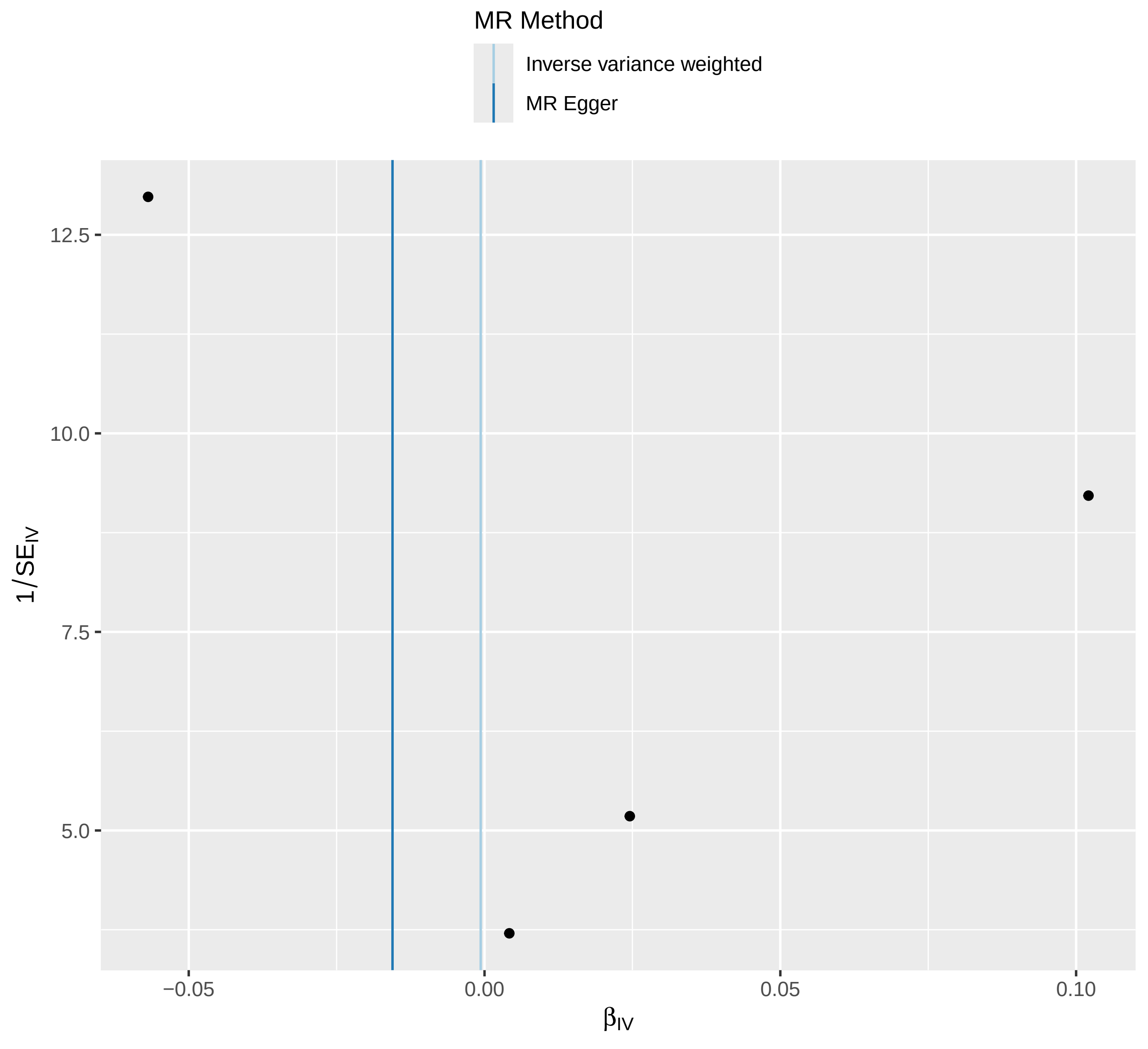

MR Method
Inverse variance weighted
MR Egger
12.5
10.0
7.5
5.0
1/SE$_{IV}$
−0.05
0.00
0.05
0.10
β$_{IV}$

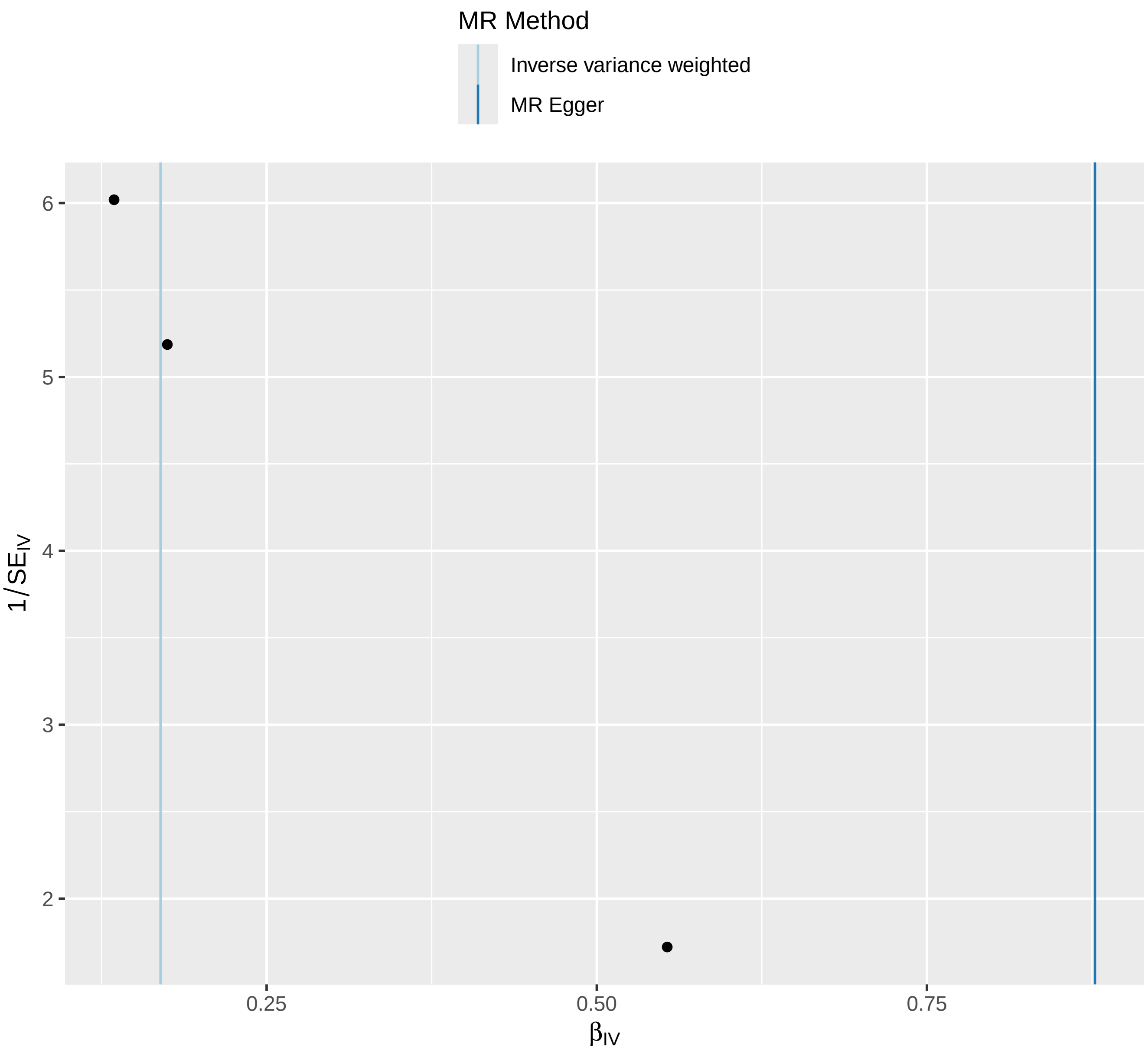

MR Method
Inverse variance weighted
MR Egger
6
5
4
3
2
1/SE$_{IV}$
0.25
0.50
0.75
β$_{IV}$

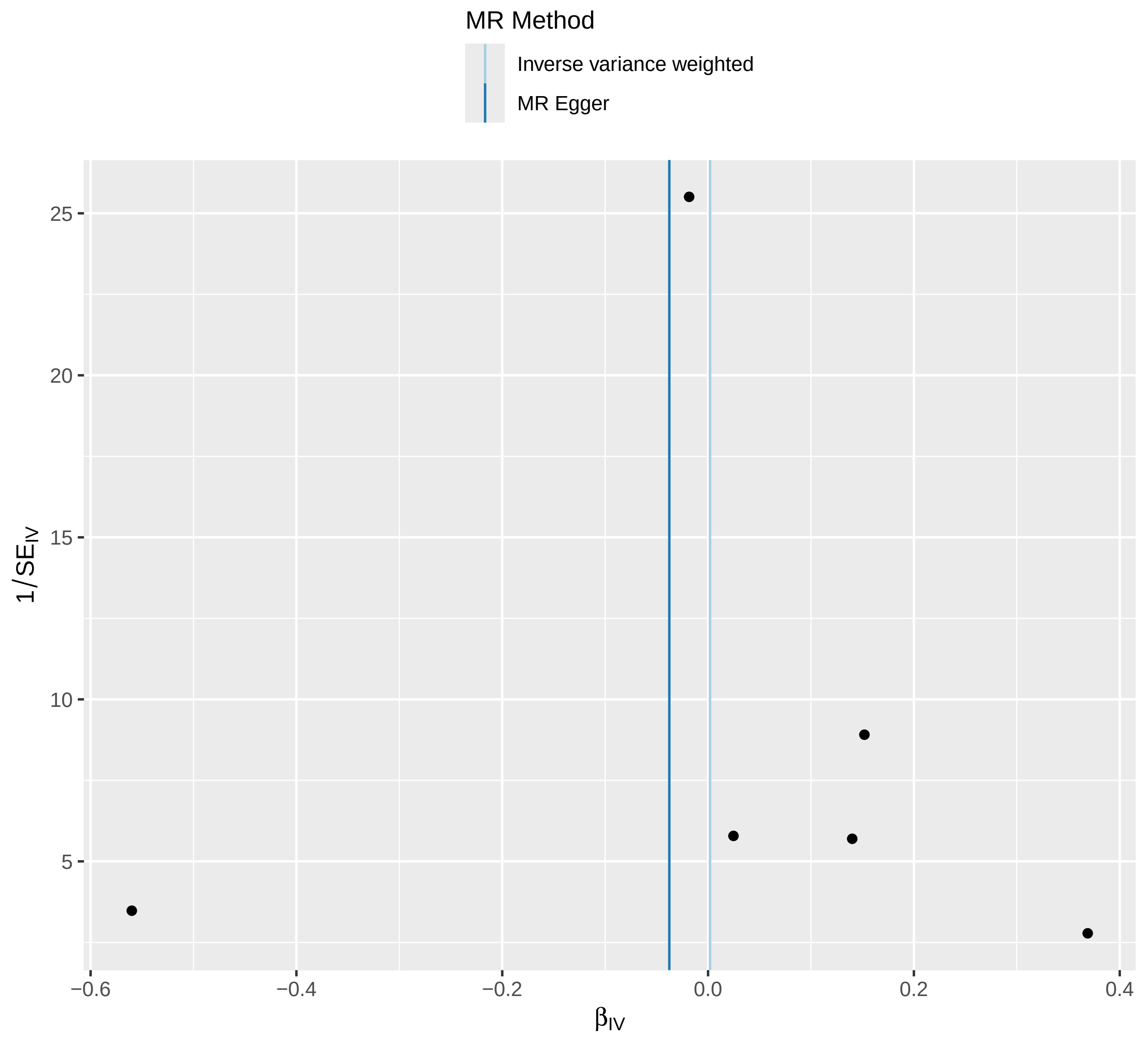
MR Method
Inverse variance weighted
MR Egger
25
20
15
10
5
1/SE$_{IV}$
−0.6
−0.4
−0.2
0.0
0.2
0.4
β$_{IV}$

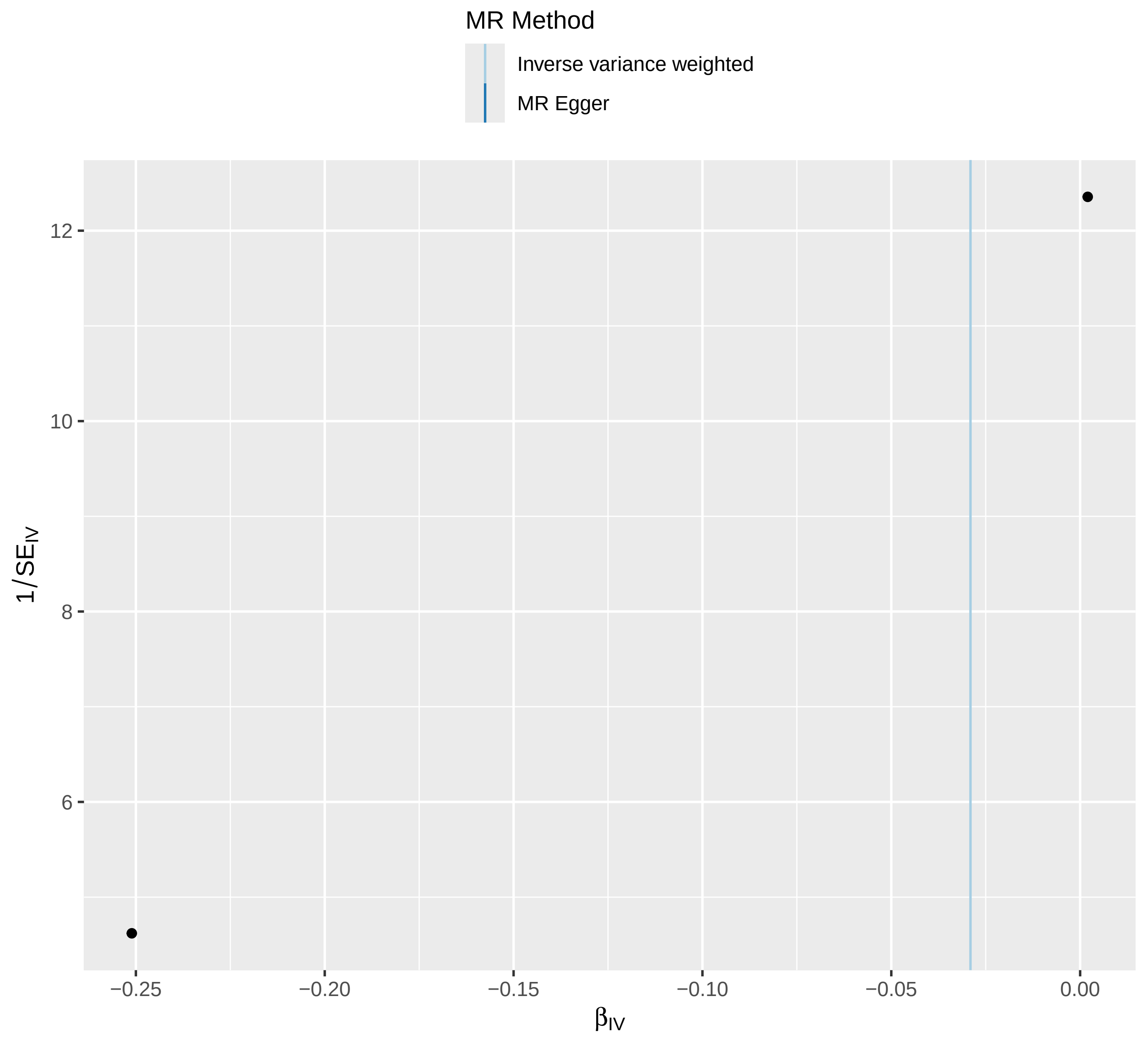
MR Method
Inverse variance weighted
MR Egger
12
10
8
6
−0.25
−0.20
−0.15
−0.10
−0.05
0.00
$1/SE_{IV}$
$\beta_{IV}$

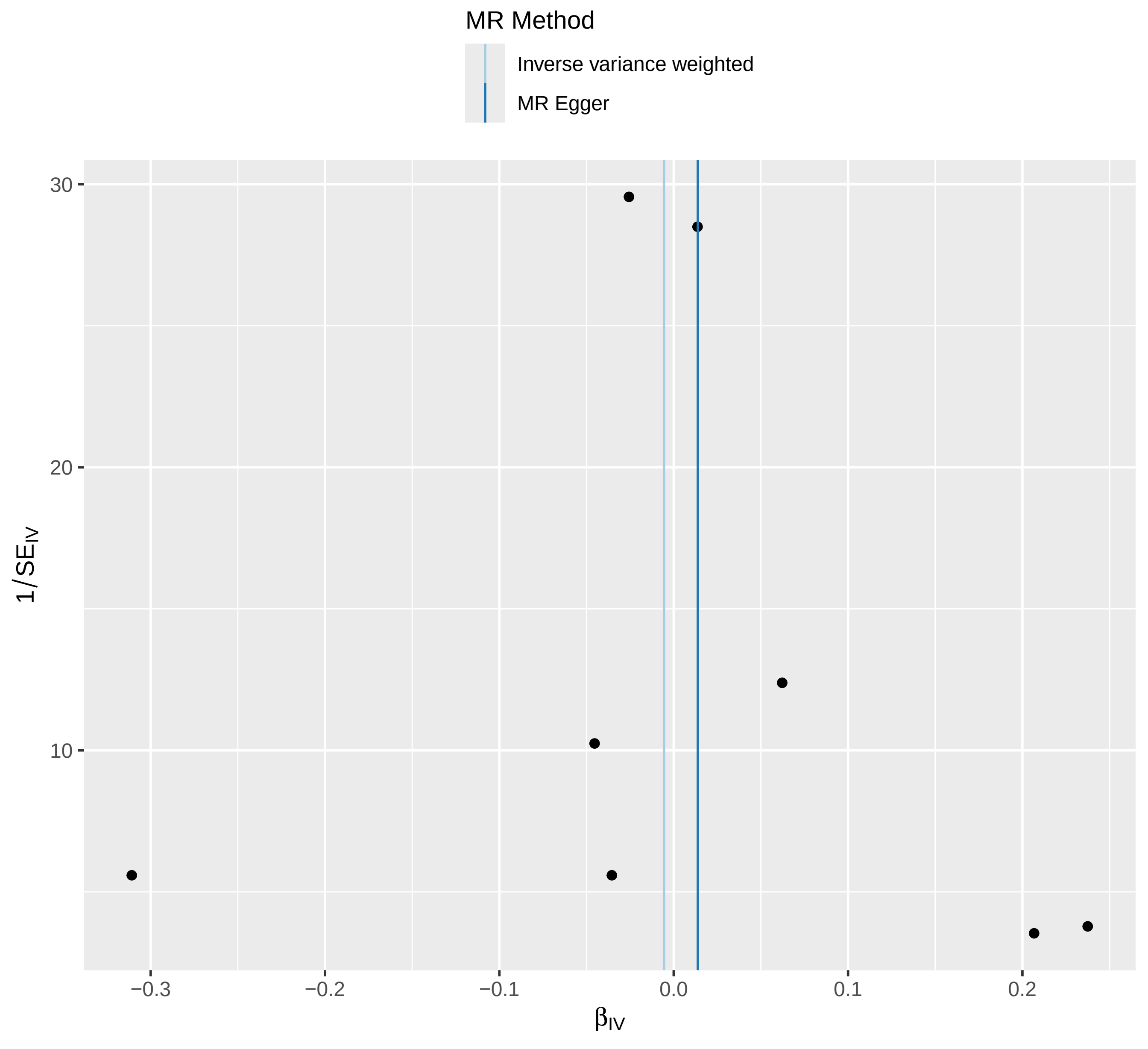

MR Method
Inverse variance weighted
MR Egger
30
20
10
1/SE$_{IV}$
−0.3
−0.2
−0.1
0.0
0.1
0.2
β$_{IV}$

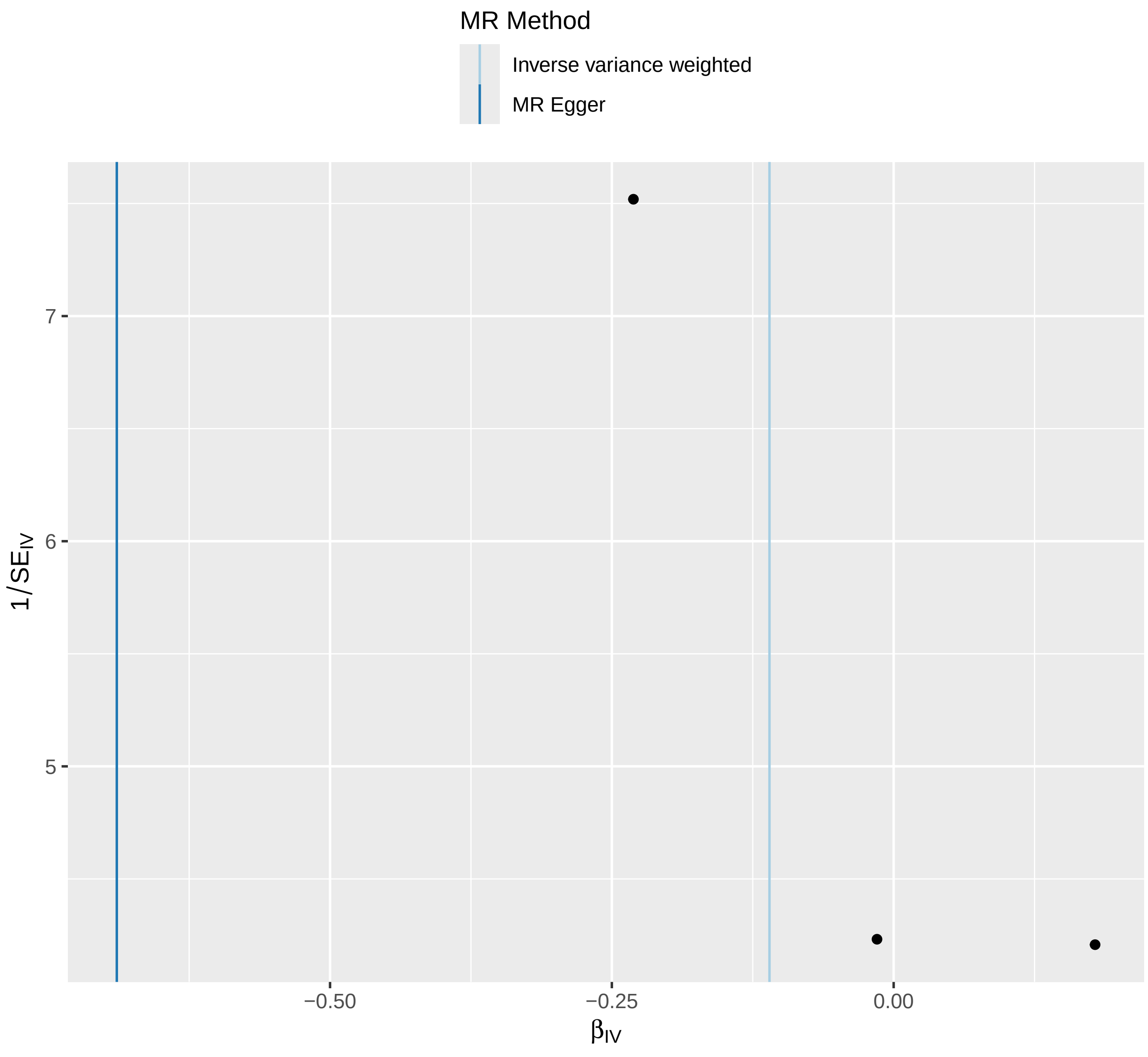
MR Method
Inverse variance weighted
MR Egger
7
6
5
$1/SE_{IV}$
−0.50
−0.25
0.00
$\beta_{IV}$

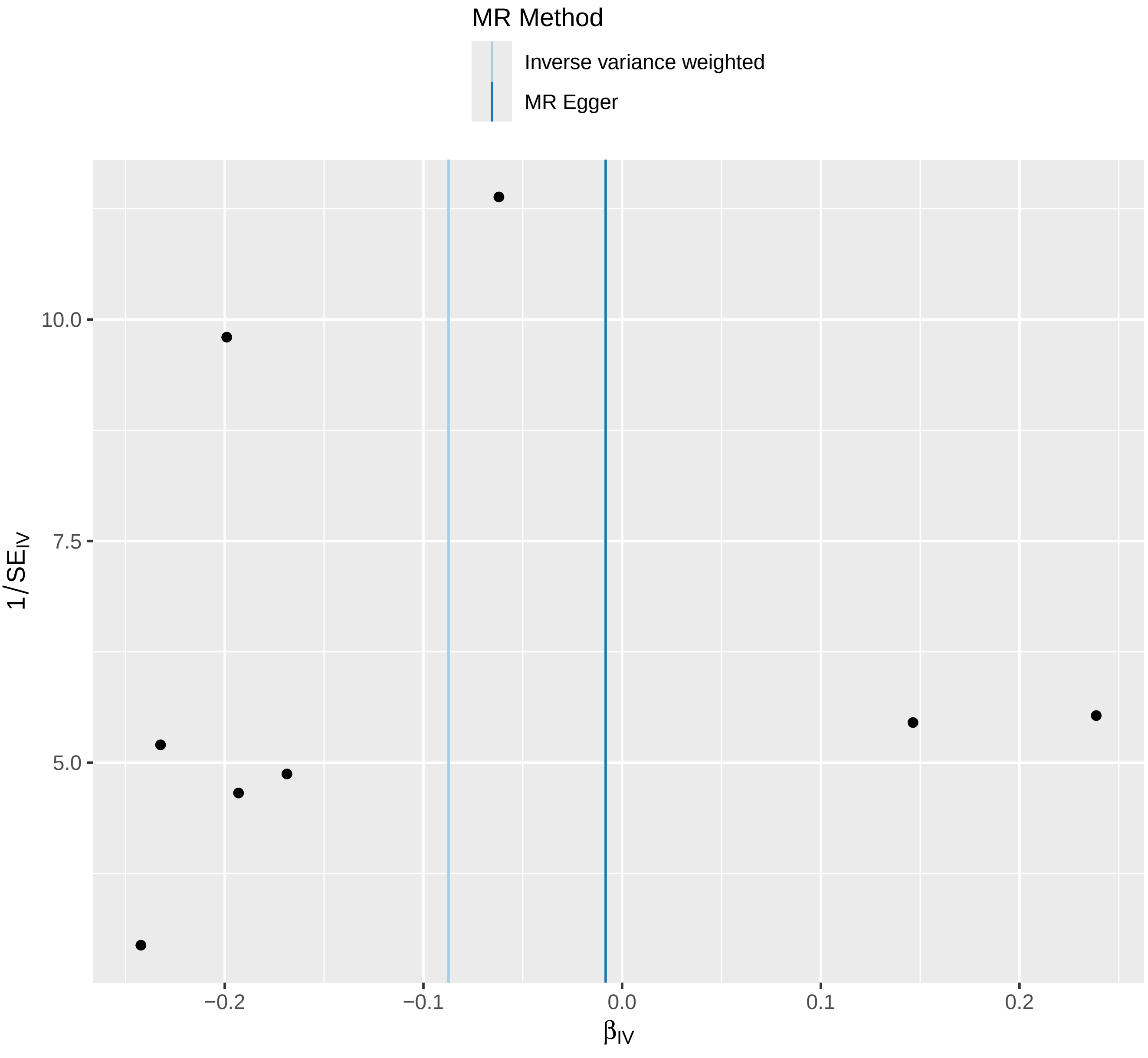
MR Method
Inverse variance weighted
MR Egger
10.0
7.5
5.0
1/SE_IV
−0.2
−0.1
0.0
0.1
0.2
β_IV

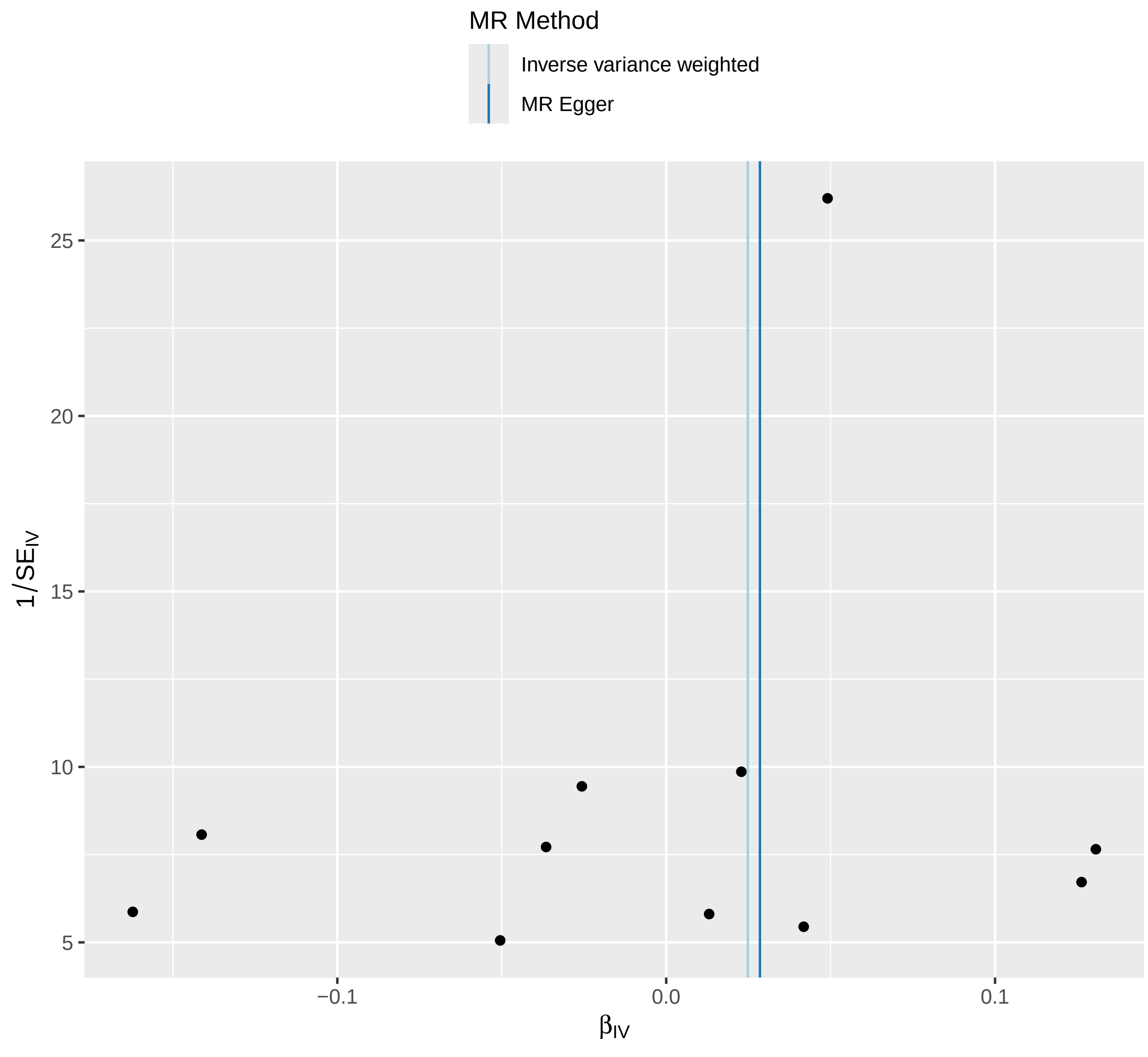

MR Method
Inverse variance weighted
MR Egger
25
20
15
10
5
1/SE$_{IV}$
−0.1
0.0
0.1
β$_{IV}$

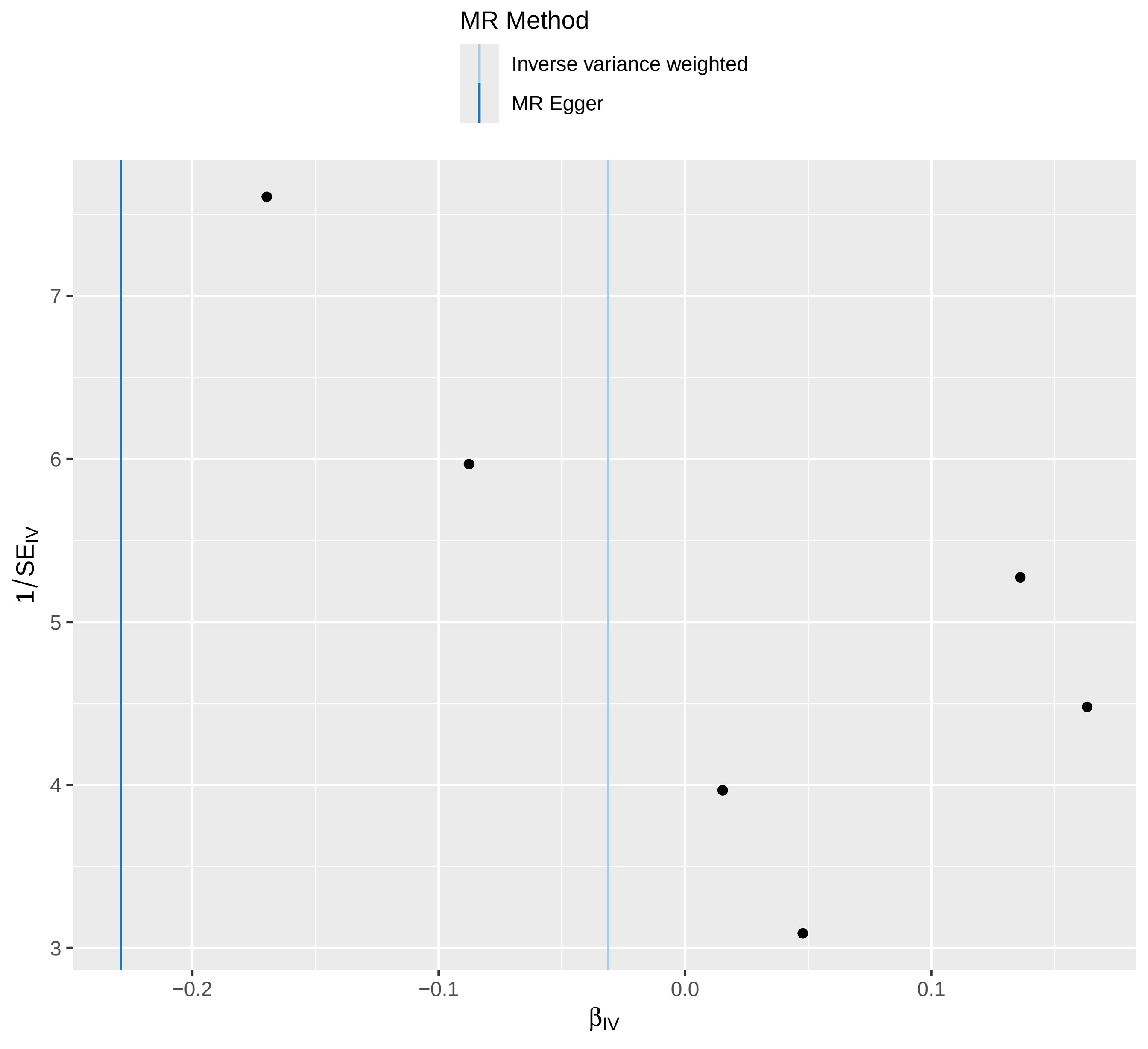
MR Method
Inverse variance weighted
MR Egger
1/SE_IV
7
6
5
4
3
β_IV
−0.2
−0.1
0.0
0.1

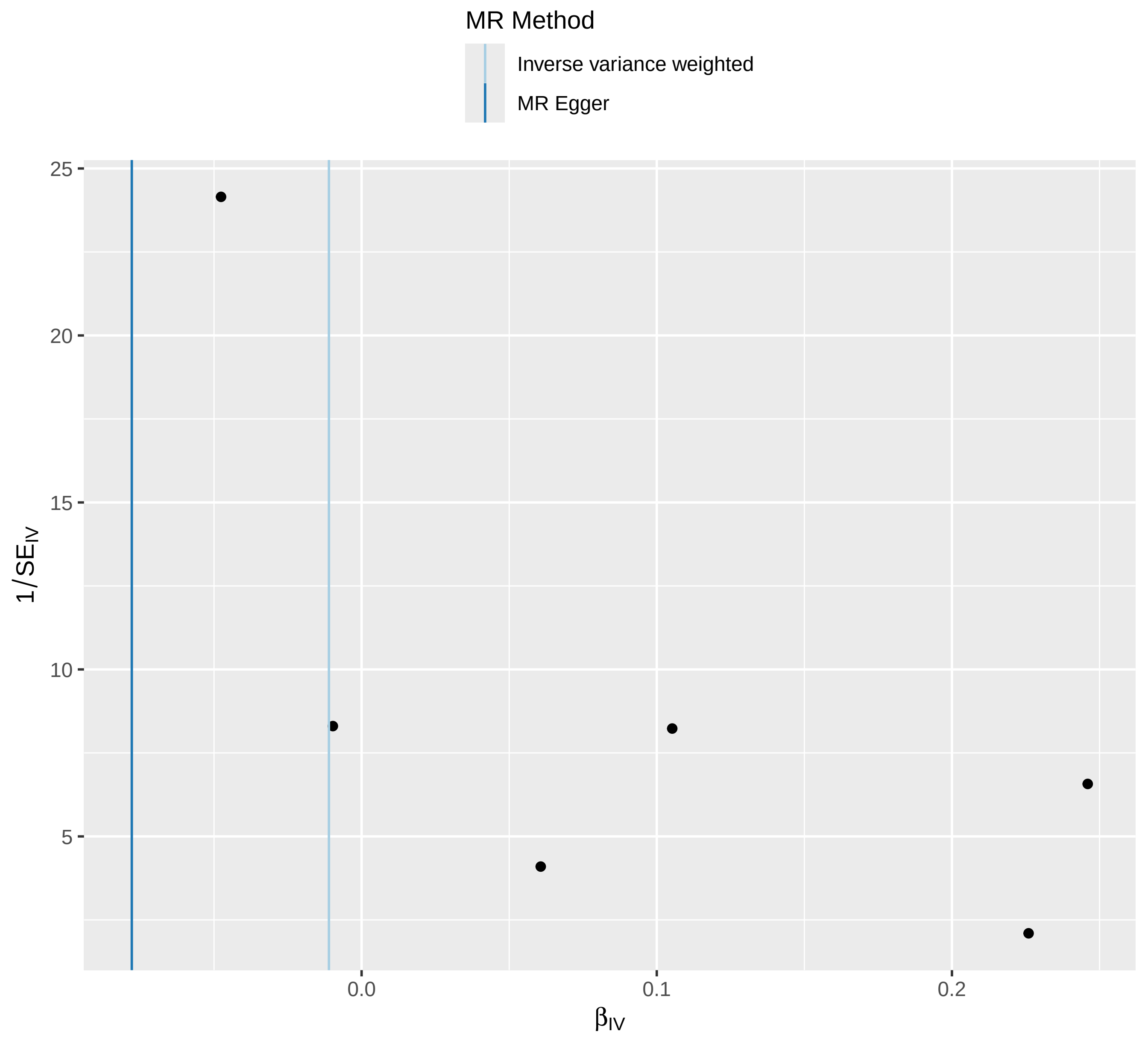

MR Method
Inverse variance weighted
MR Egger
25
20
15
10
5
1/SE$_{IV}$
0.0
0.1
0.2
β$_{IV}$

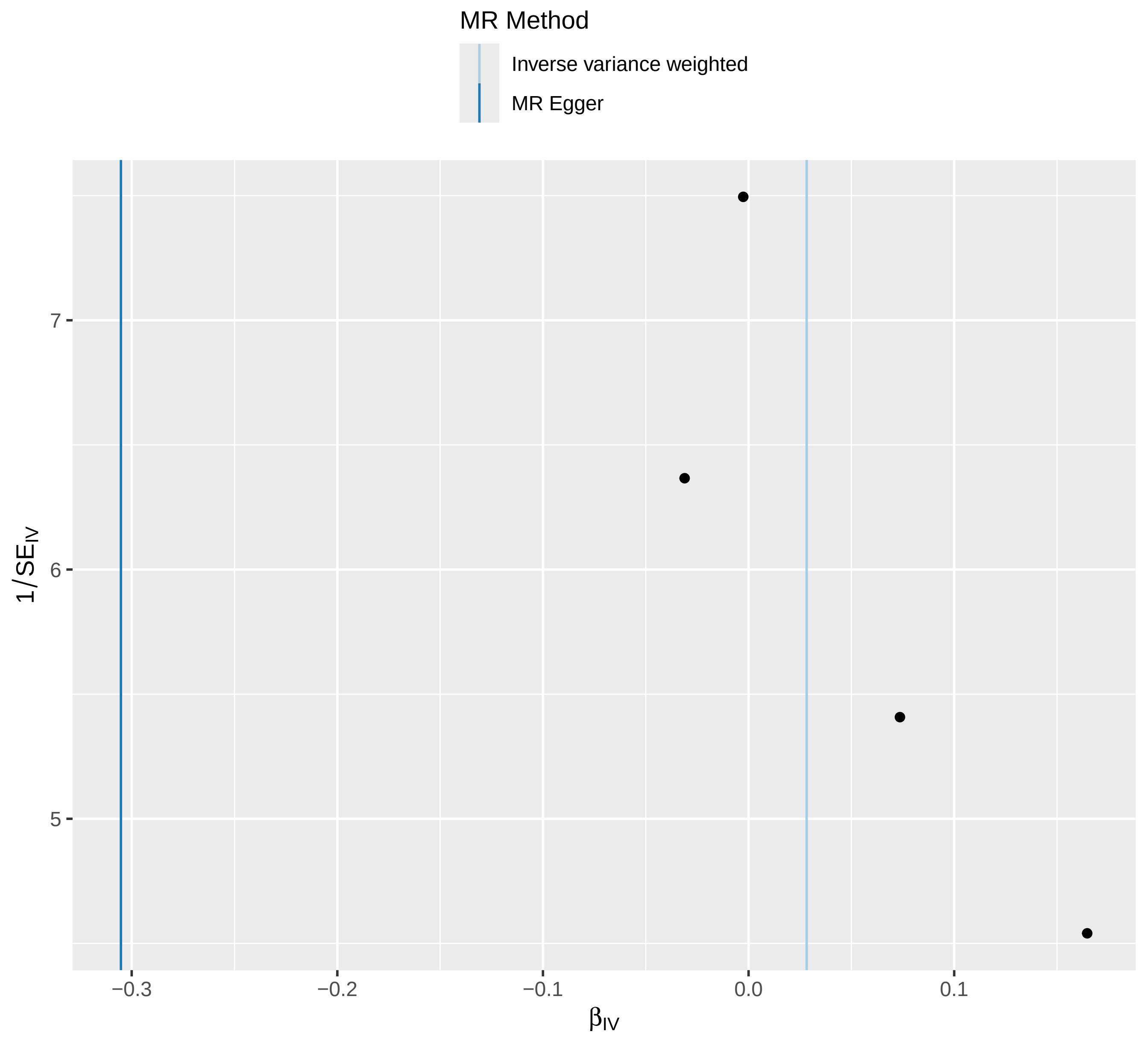

MR Method
Inverse variance weighted
MR Egger
7
6
5
$1/SE_{IV}$
−0.3
−0.2
−0.1
0.0
0.1
$\beta_{IV}$

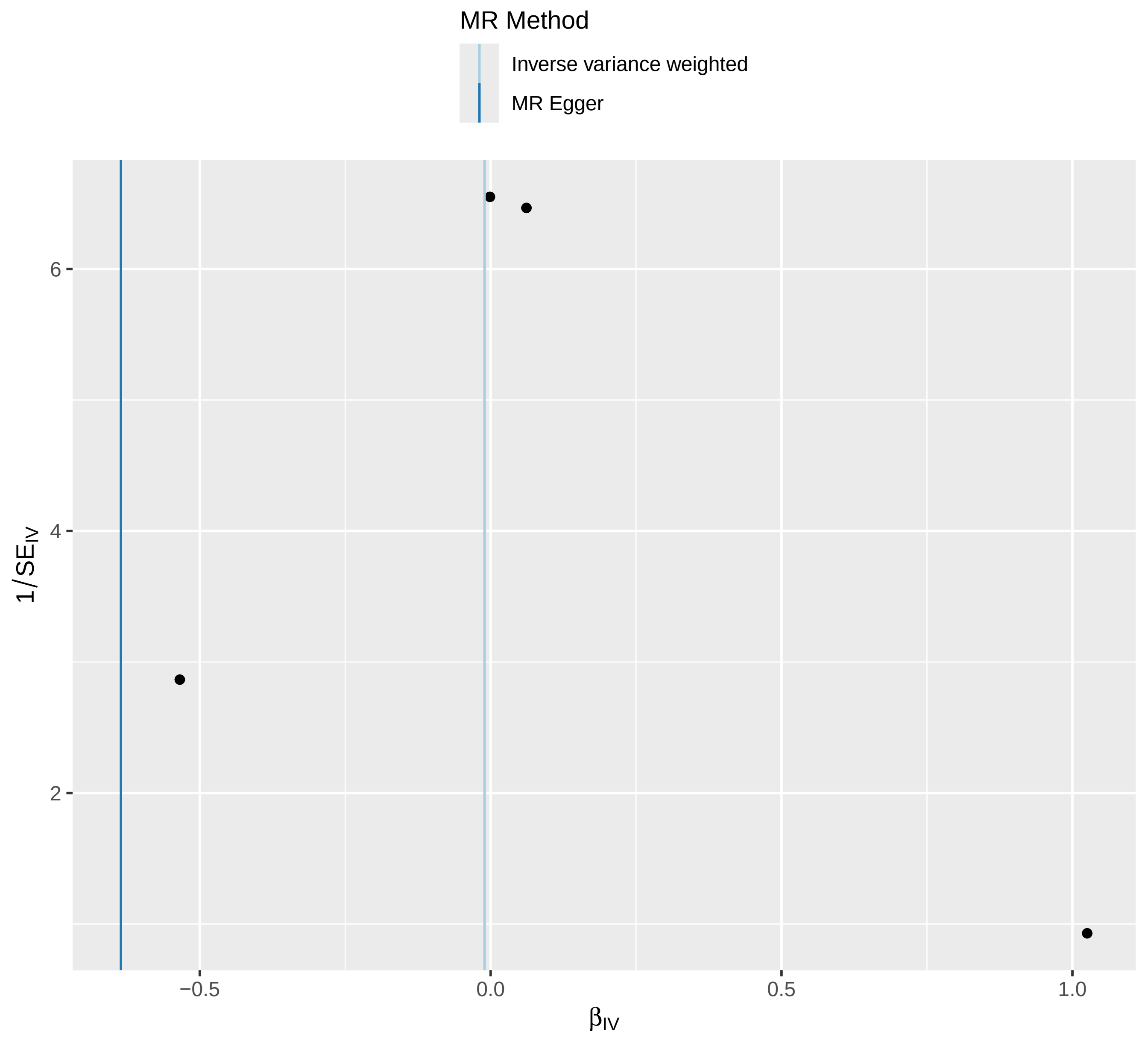

MR Method
Inverse variance weighted
MR Egger
6
4
2
1/SE_IV
−0.5
0.0
0.5
1.0
β_IV

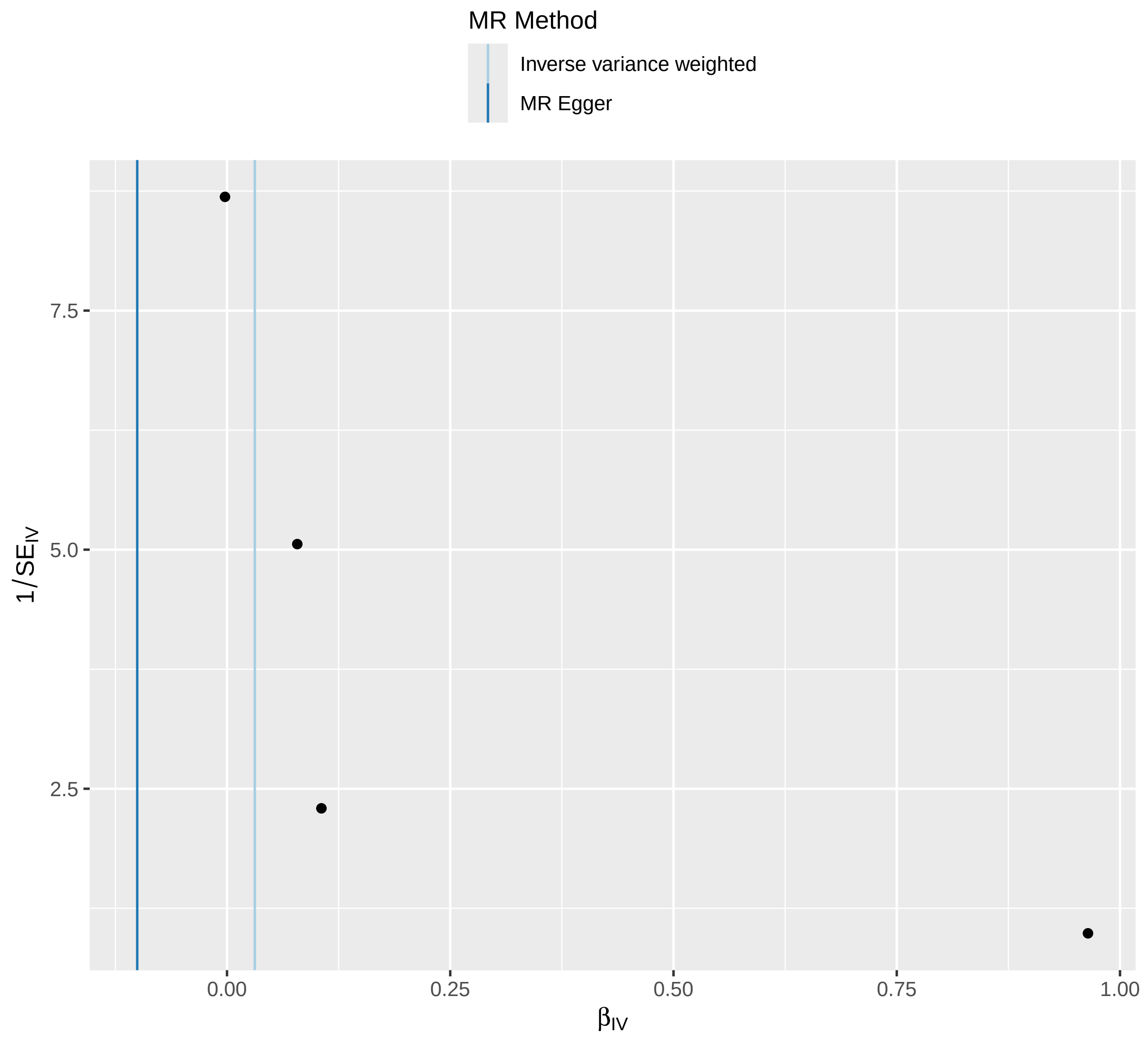
MR Method
Inverse variance weighted
MR Egger
7.5
5.0
2.5
$1/SE_{IV}$
0.00
0.25
0.50
0.75
1.00
$\beta_{IV}$

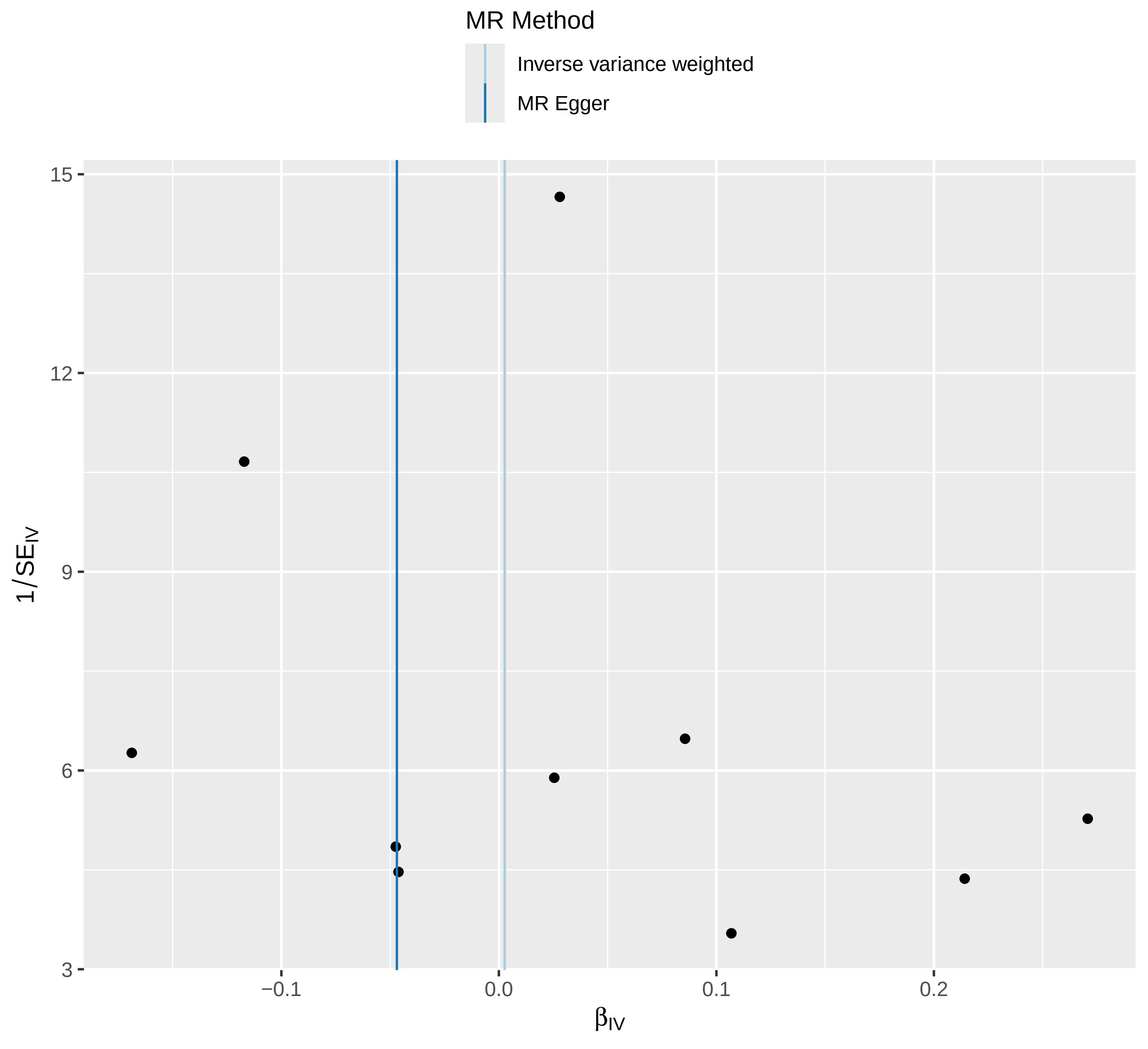

MR Method
Inverse variance weighted
MR Egger
15
12
9
6
3
1/SE_IV
−0.1
0.0
0.1
0.2
β_IV

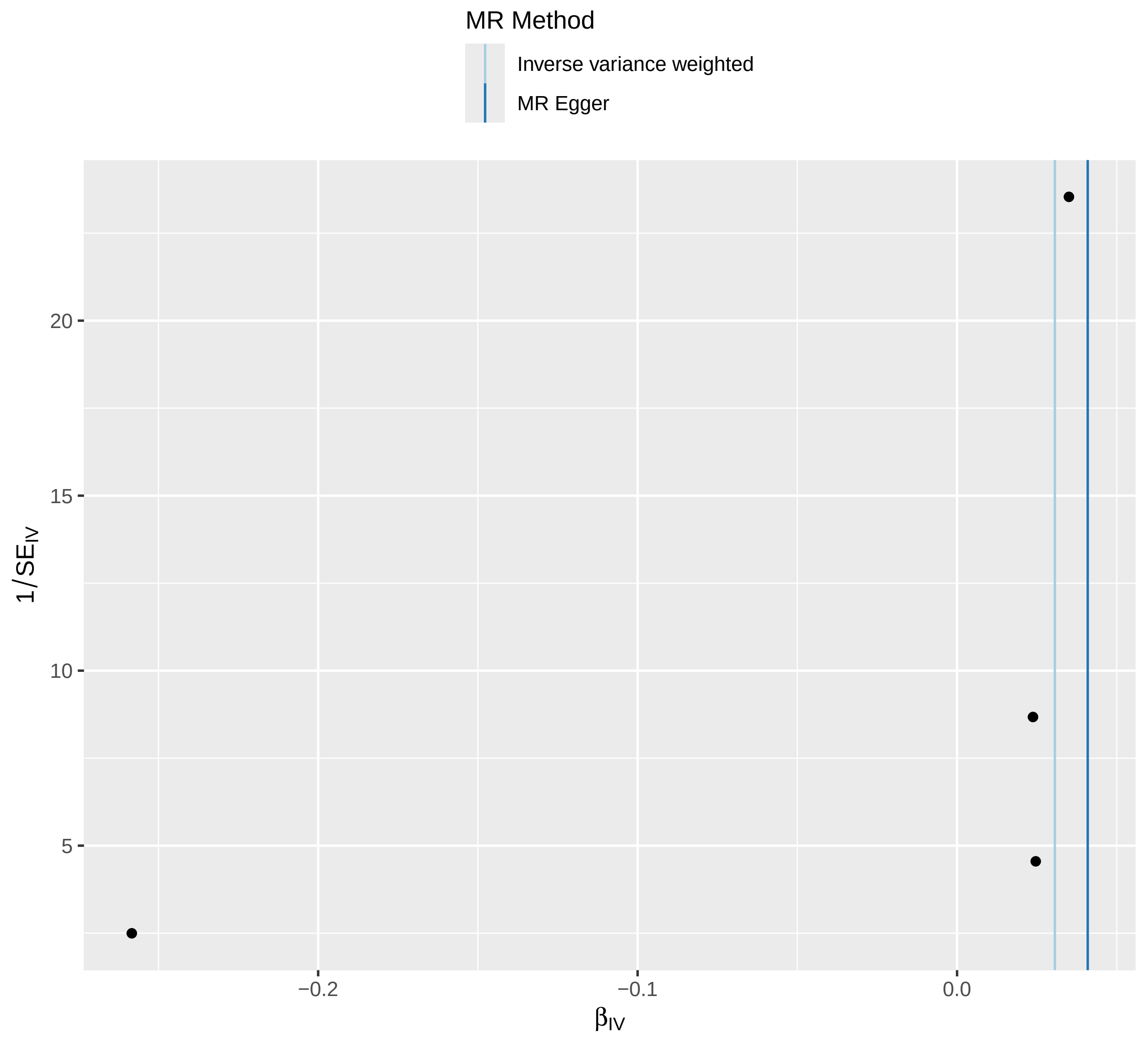

MR Method
Inverse variance weighted
MR Egger
20
15
10
5
1/SE_IV
−0.2
−0.1
0.0
β_IV

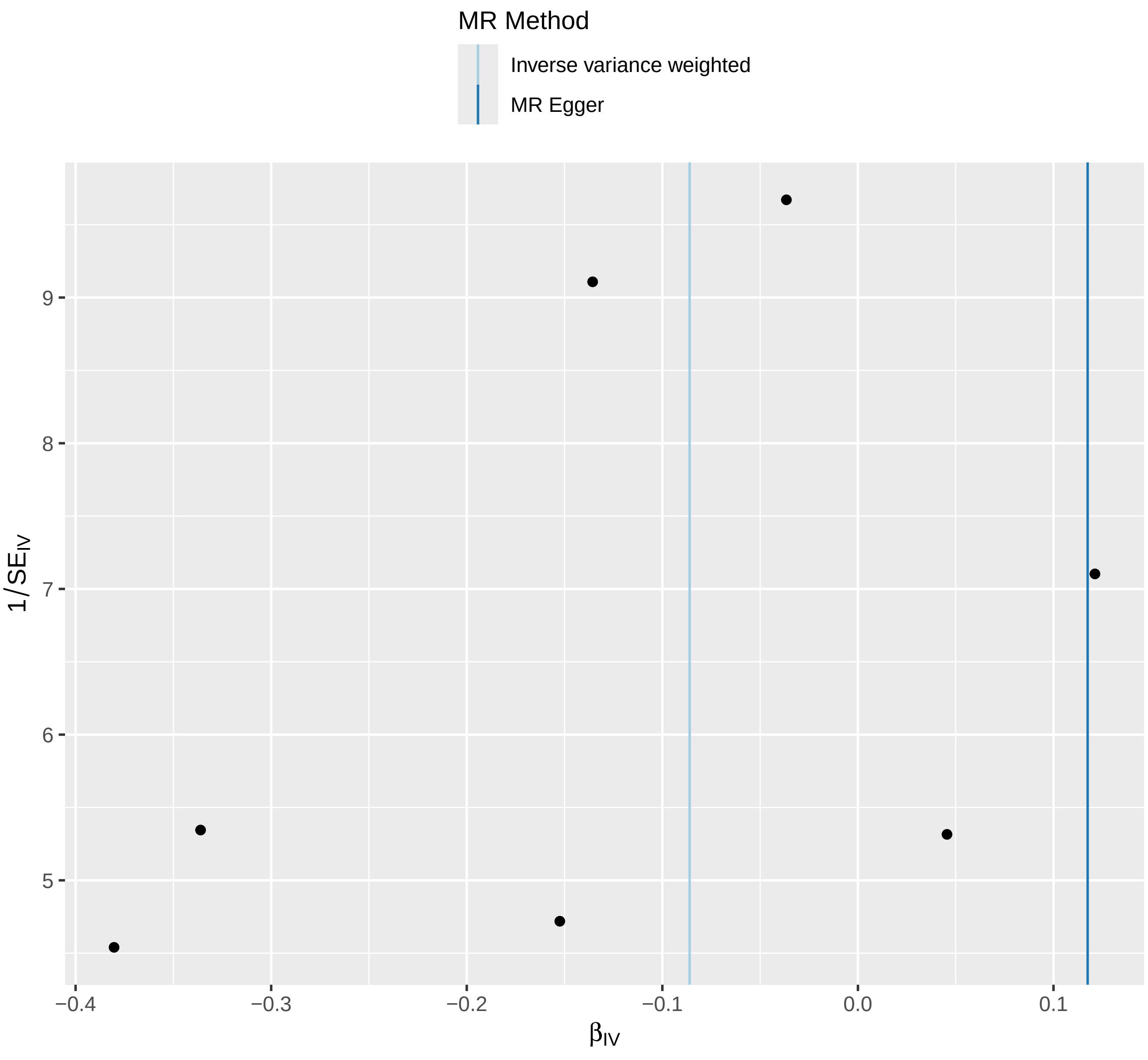
MR Method
Inverse variance weighted
MR Egger
1/SE$_{IV}$
9
8
7
6
5
−0.4
−0.3
−0.2
−0.1
0.0
0.1
β$_{IV}$

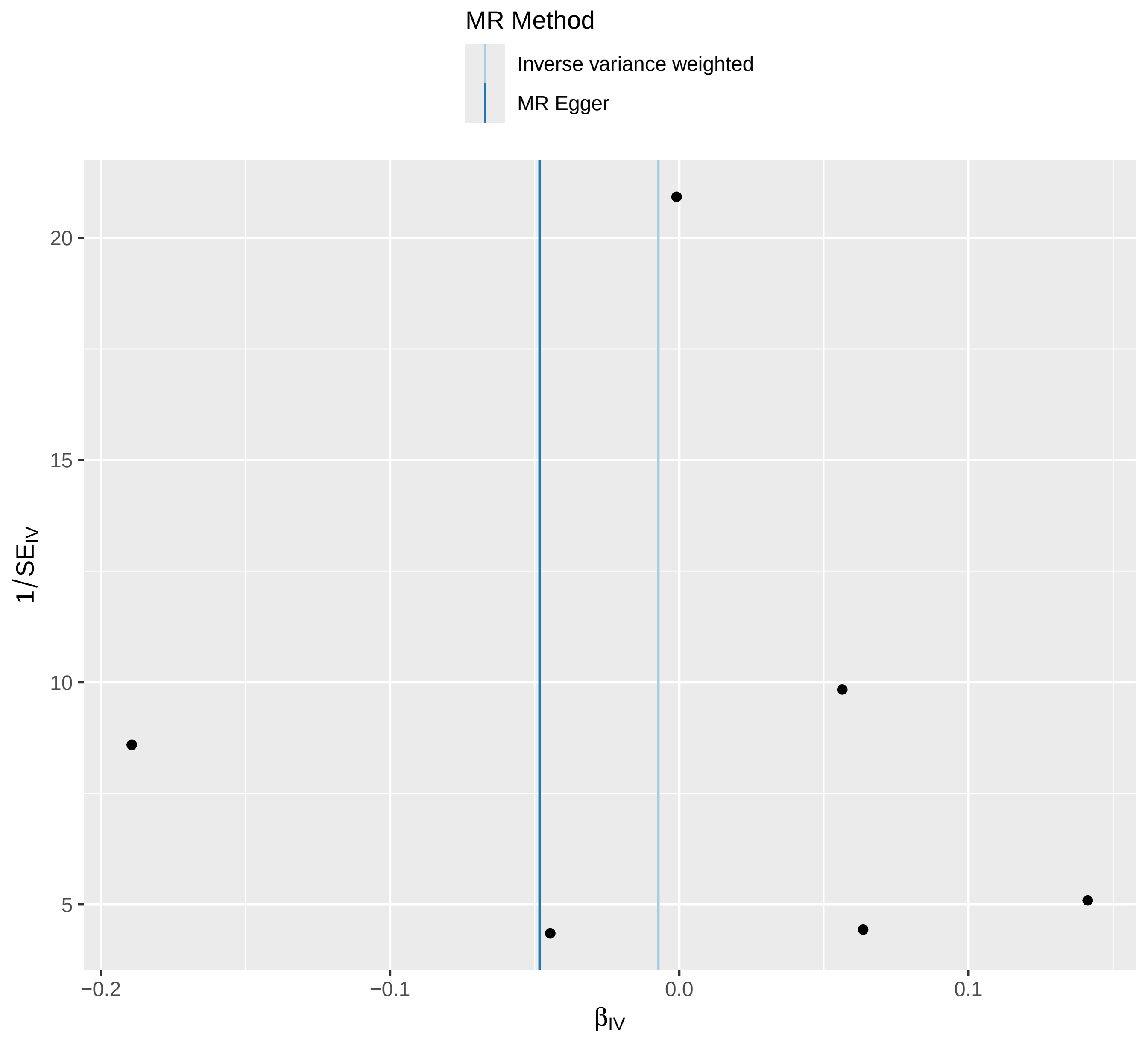
MR Method
Inverse variance weighted
MR Egger
20
15
10
5
1/SE_IV
−0.2
−0.1
0.0
0.1
β_IV

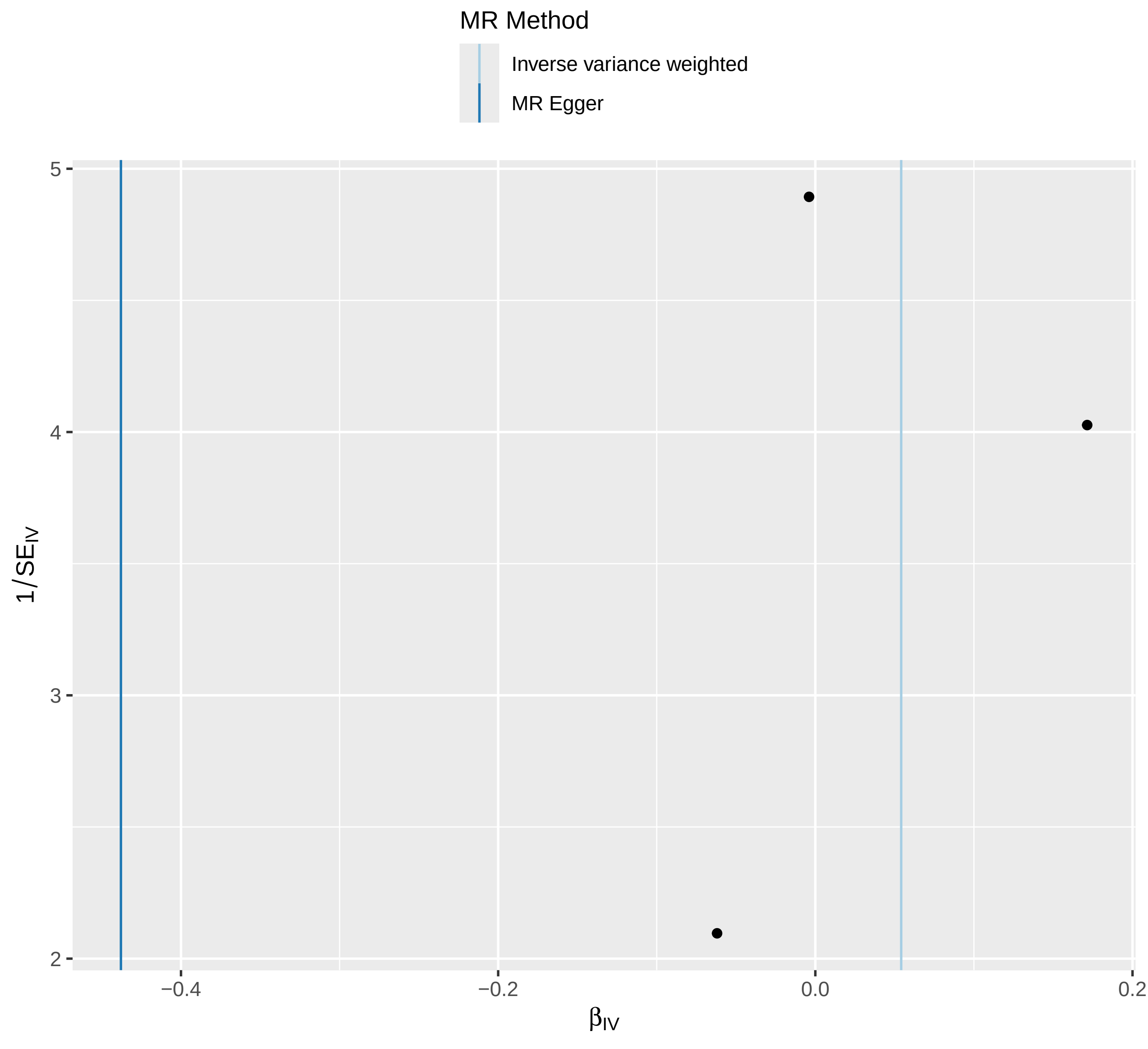
MR Method
Inverse variance weighted
MR Egger
1/SE$_{IV}$
5
4
3
2
−0.4
−0.2
0.0
0.2
β$_{IV}$

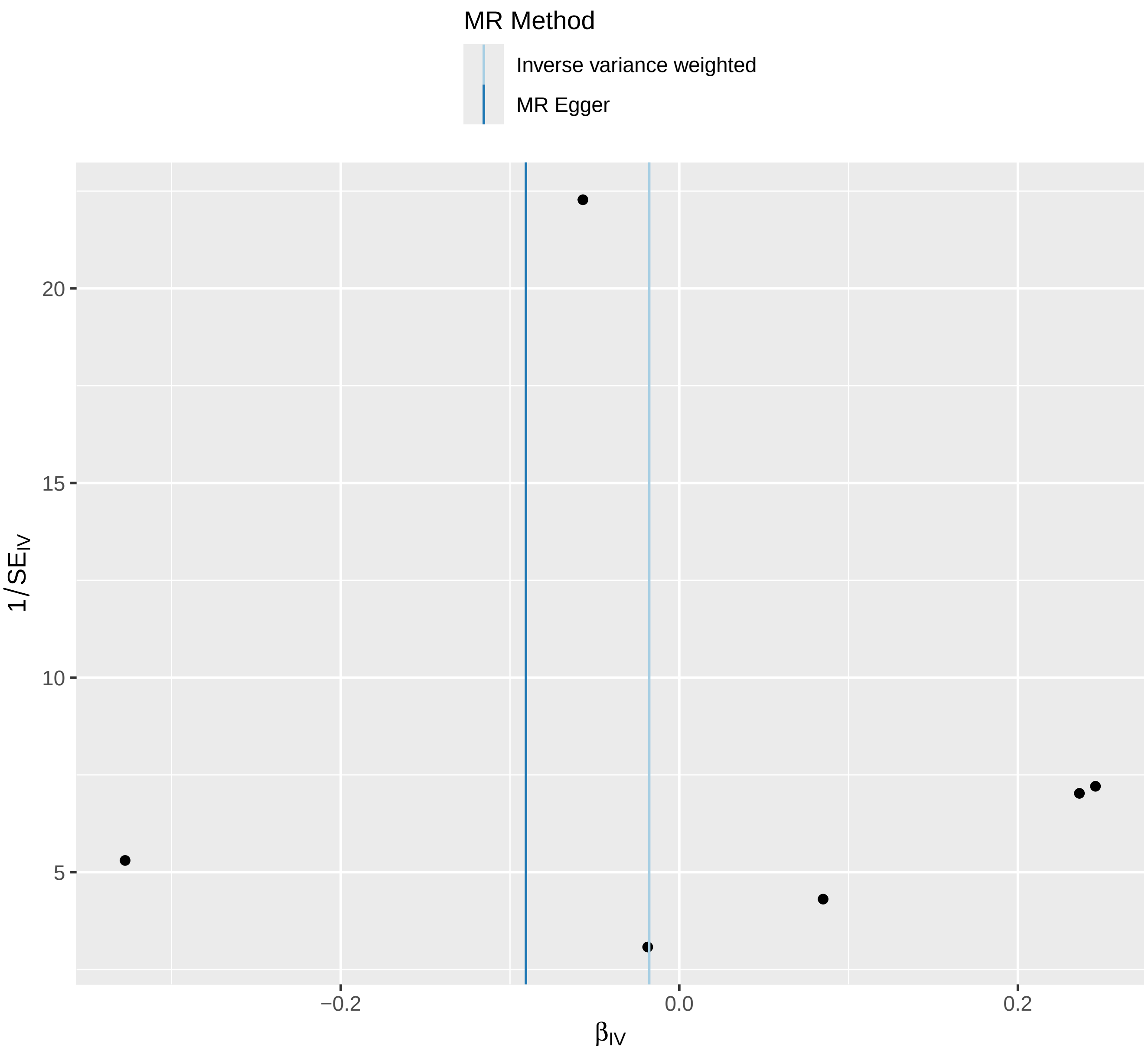
MR Method
Inverse variance weighted
MR Egger
20
15
10
5
1/SE_IV
−0.2
0.0
0.2
β_IV

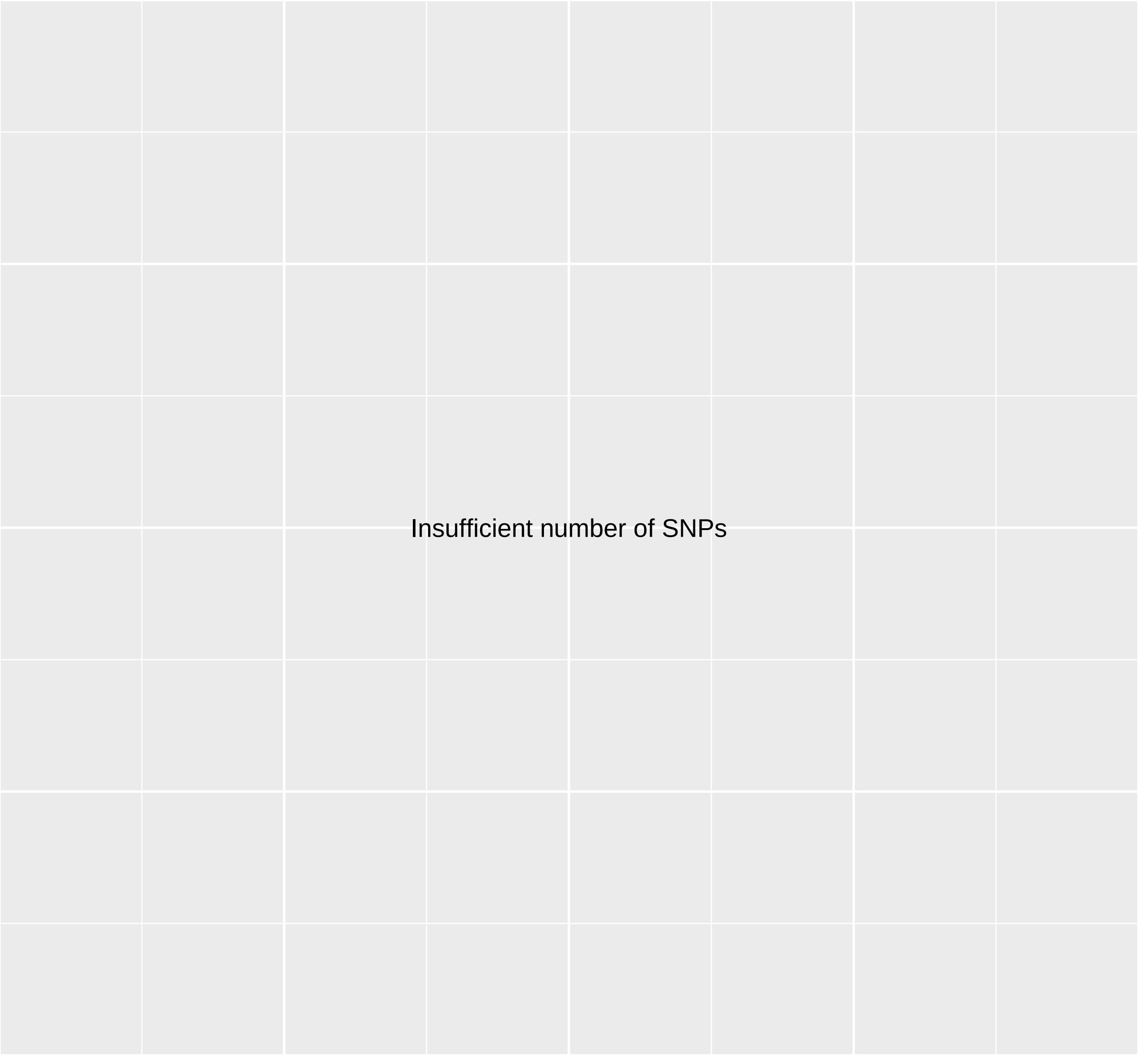
Insufficient number of SNPs

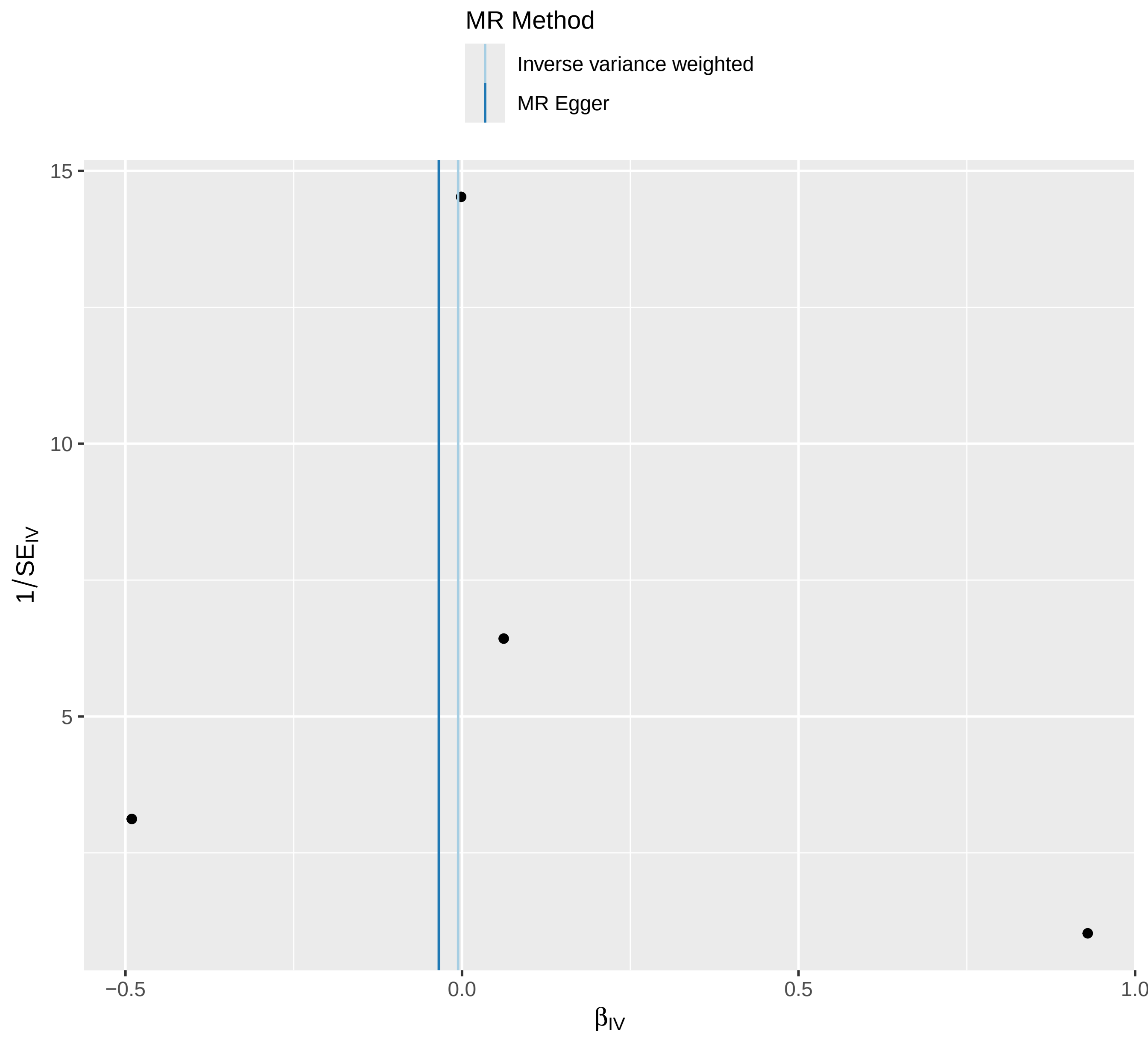
MR Method
Inverse variance weighted
MR Egger
15
10
5
1/SE$_{IV}$
−0.5
0.0
0.5
1.0
β$_{IV}$

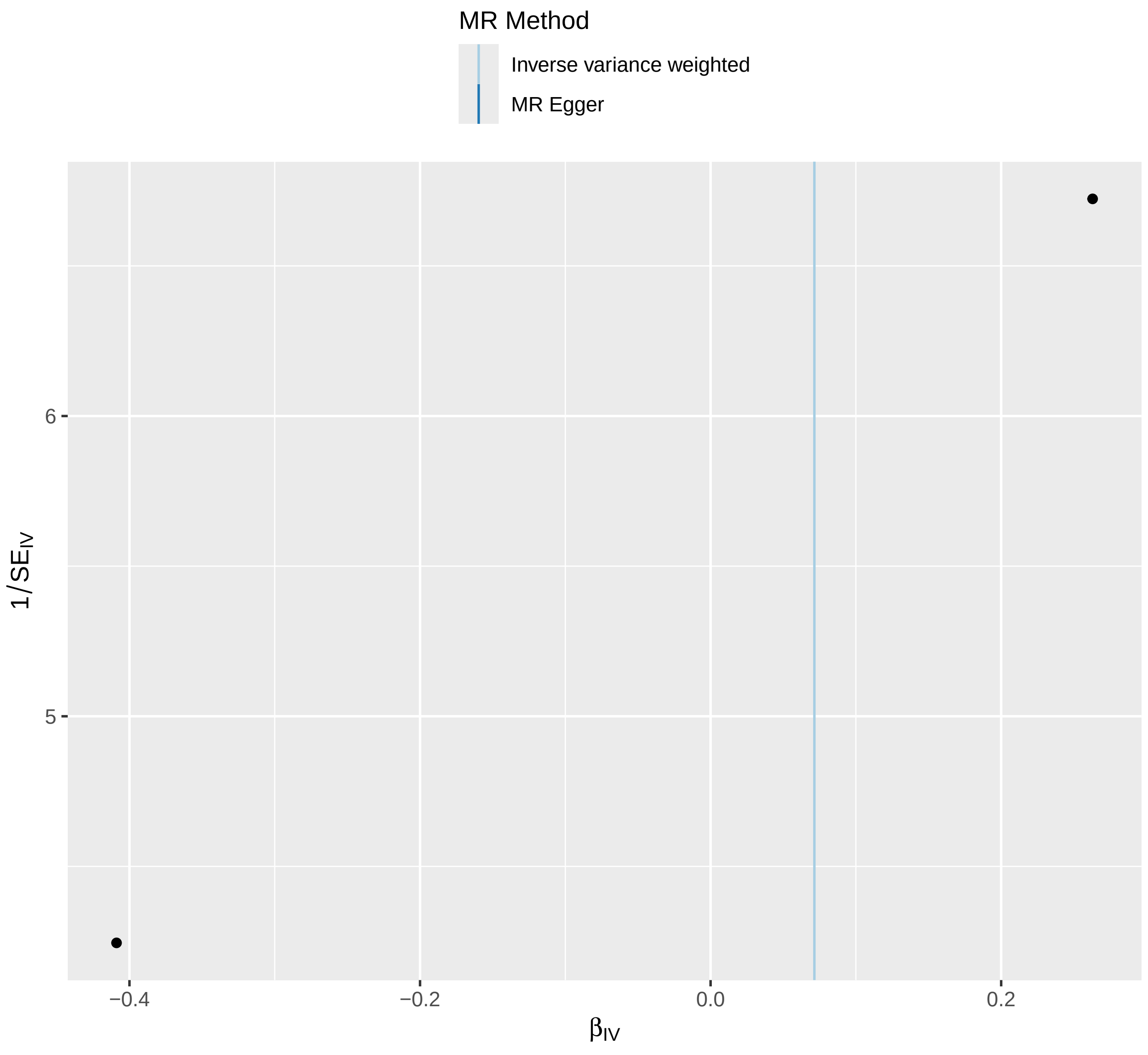

MR Method
Inverse variance weighted
MR Egger
6
5
1/SE$_{IV}$
−0.4
−0.2
0.0
0.2
β$_{IV}$

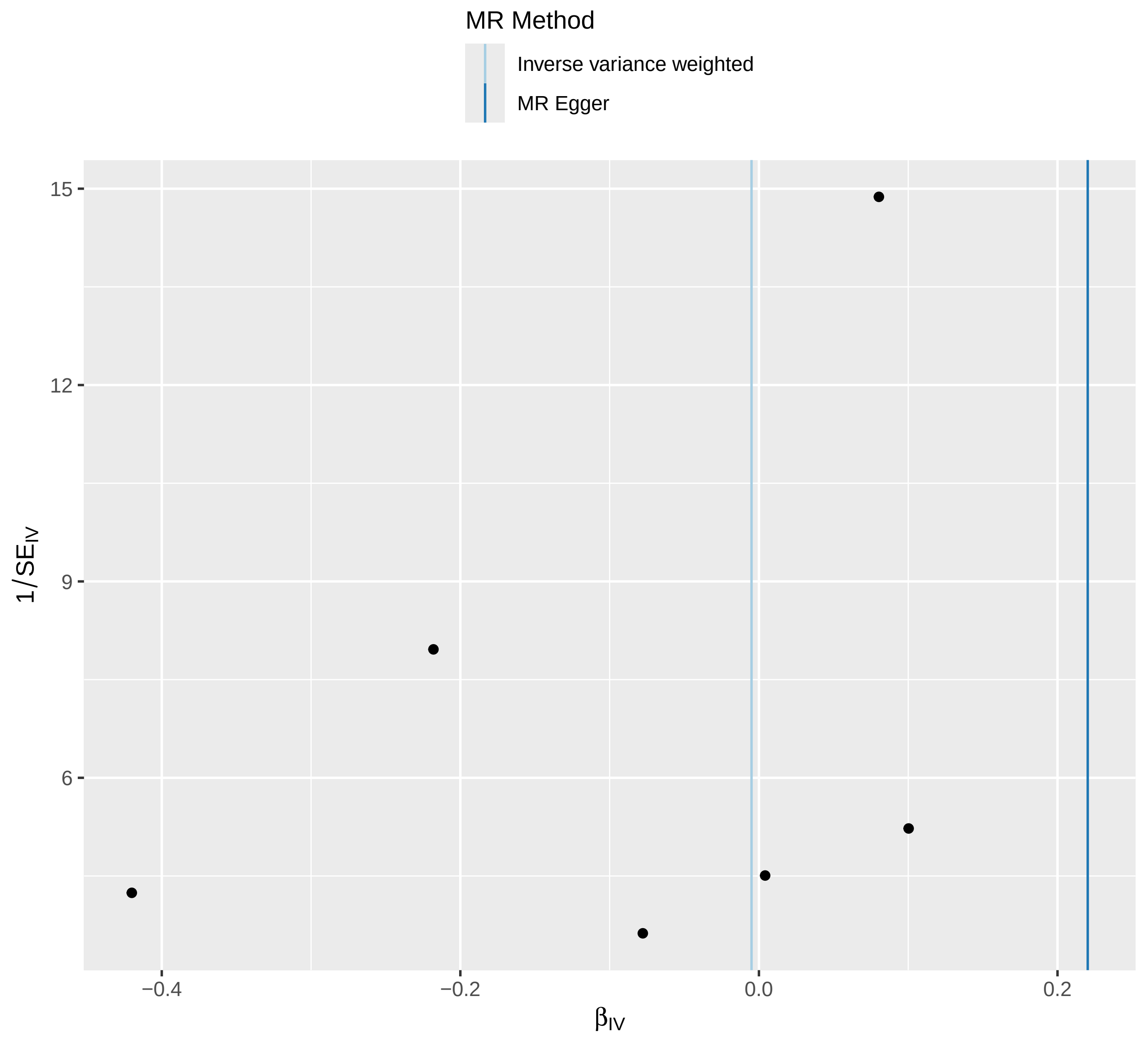
MR Method
Inverse variance weighted
MR Egger
15
12
9
6
1/SE_IV
−0.4
−0.2
0.0
0.2
β_IV

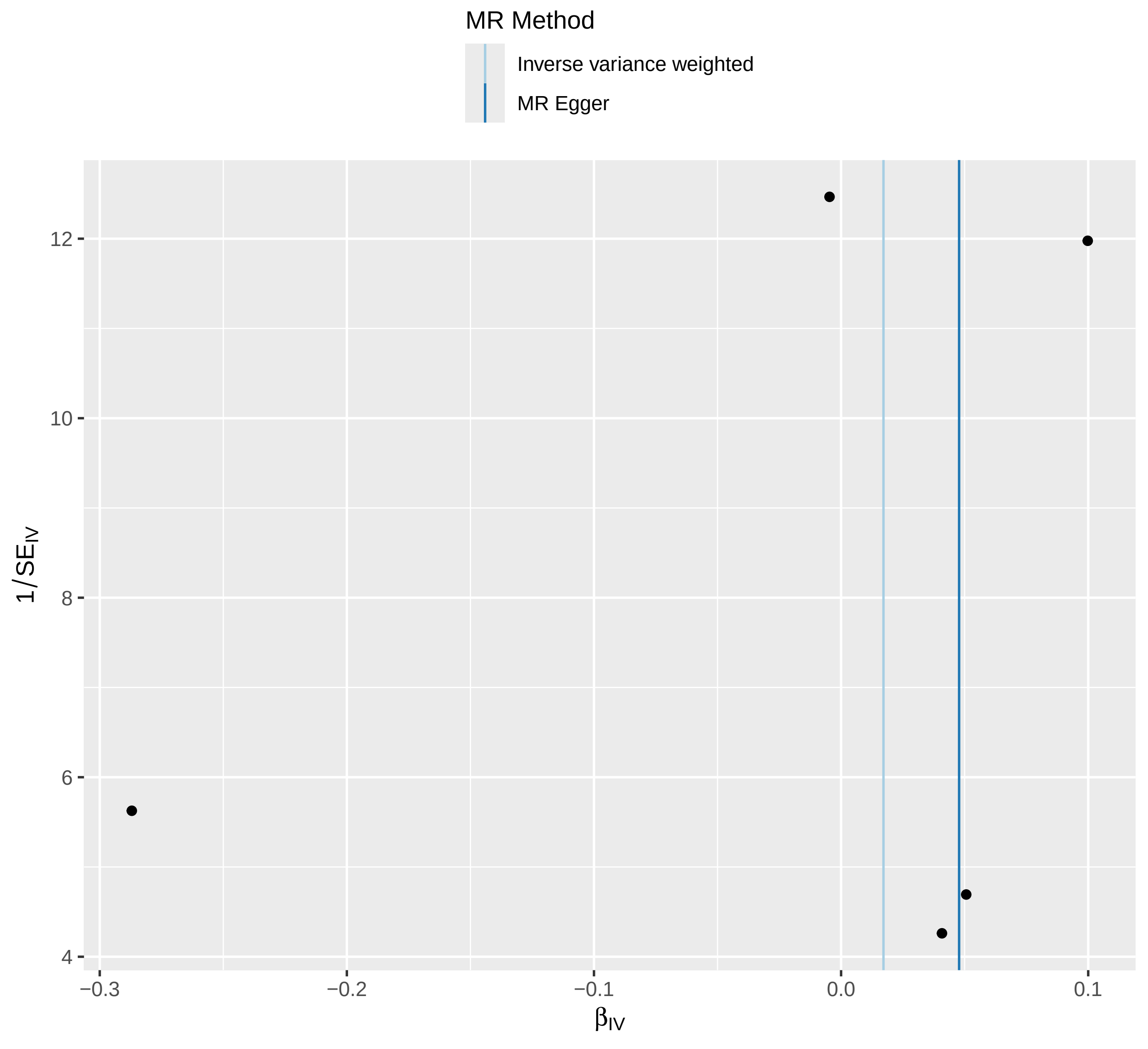

MR Method
Inverse variance weighted
MR Egger
12
10
8
6
4
1/SE_IV
−0.3
−0.2
−0.1
0.0
0.1
β_IV

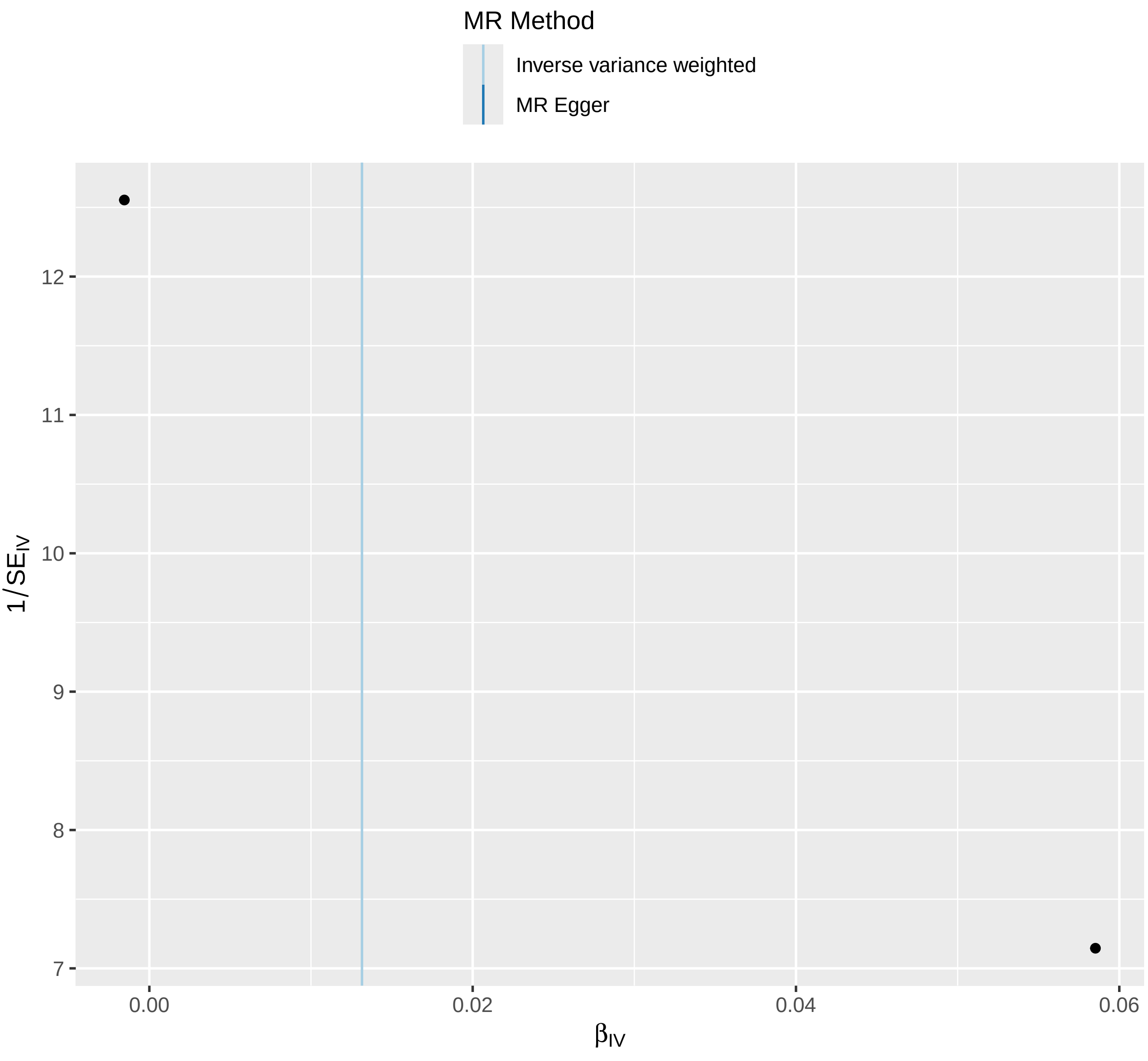
MR Method
Inverse variance weighted
MR Egger
12
11
10
9
8
7
1/SE_IV
0.00
0.02
0.04
0.06
β_IV

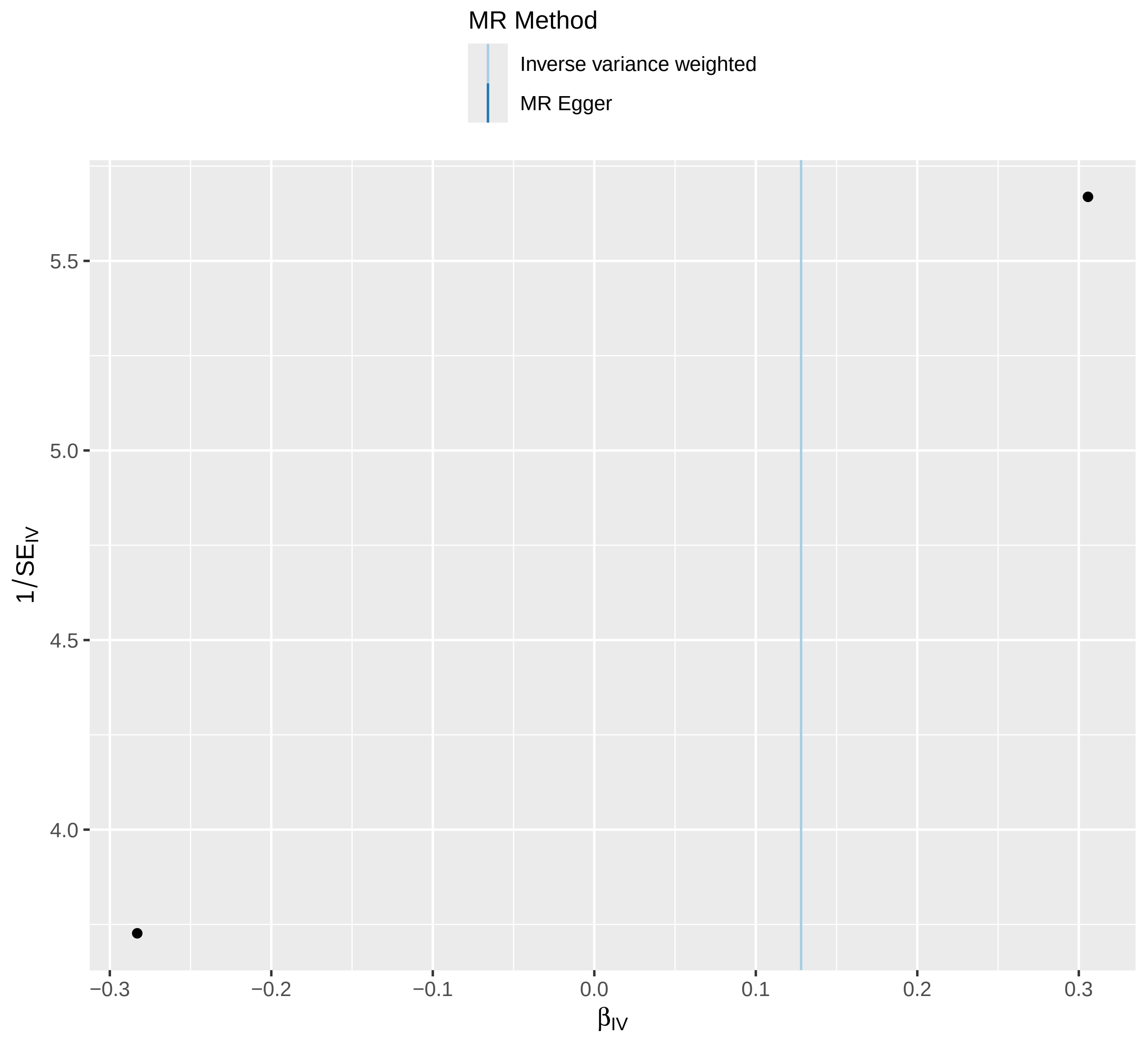
MR Method
Inverse variance weighted
MR Egger
5.5
5.0
4.5
4.0
$1/SE_{IV}$
−0.3
−0.2
−0.1
0.0
0.1
0.2
0.3
$\beta_{IV}$

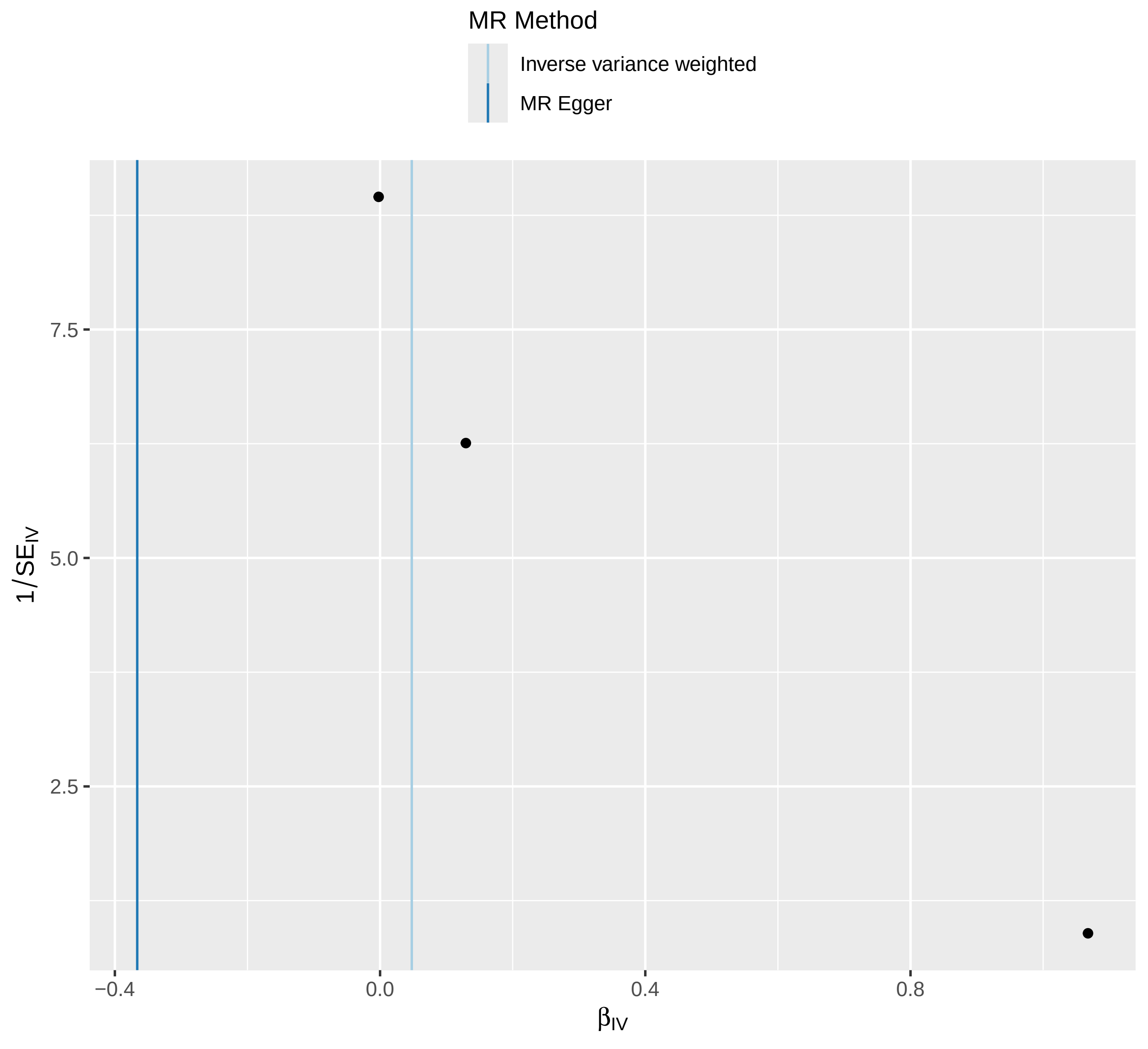
MR Method
Inverse variance weighted
MR Egger
7.5
5.0
2.5
1/SE$_{IV}$
−0.4
0.0
0.4
0.8
β$_{IV}$

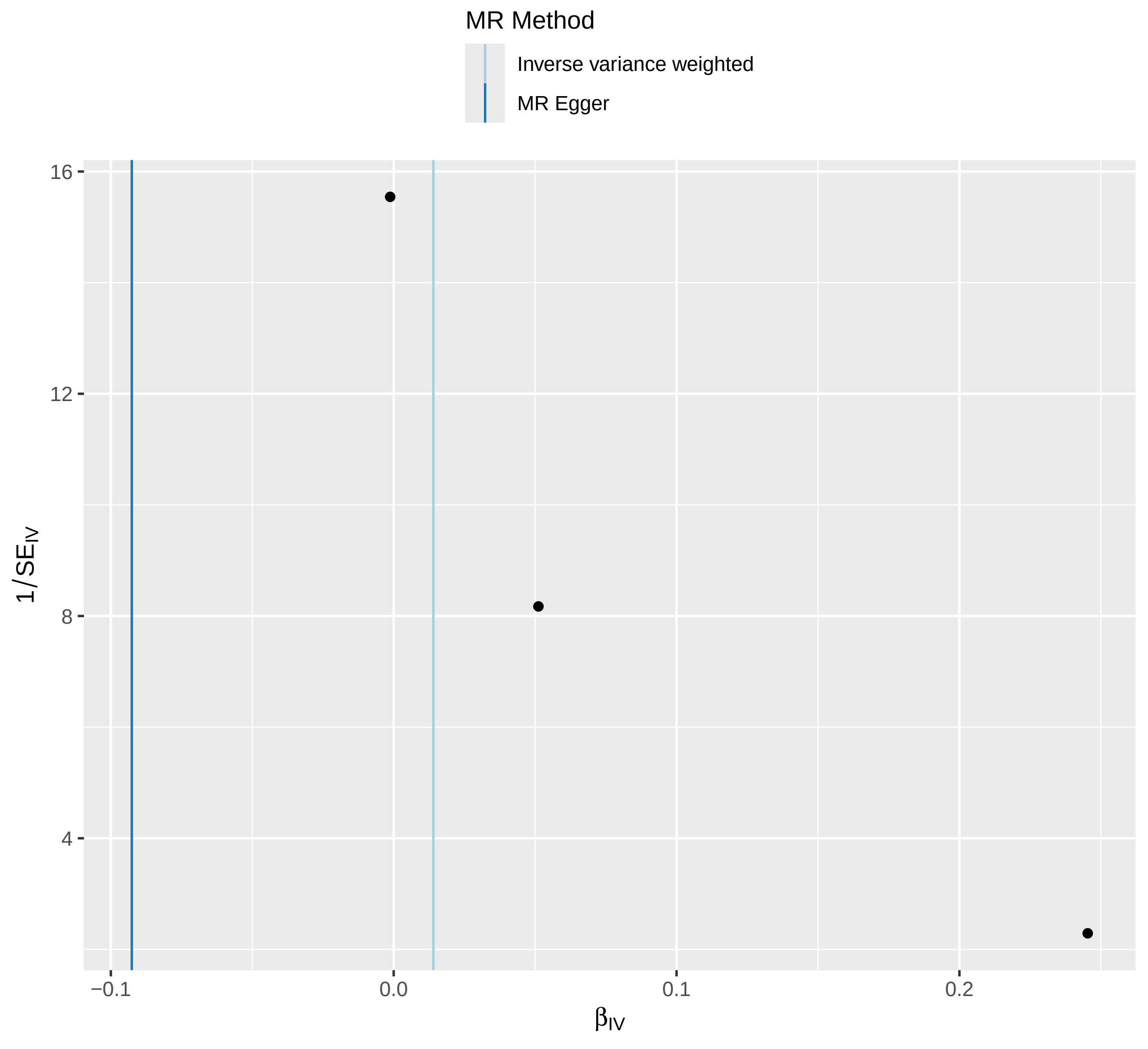
MR Method
Inverse variance weighted
MR Egger
16
12
8
4
1/SE_IV
−0.1
0.0
0.1
0.2
β_IV

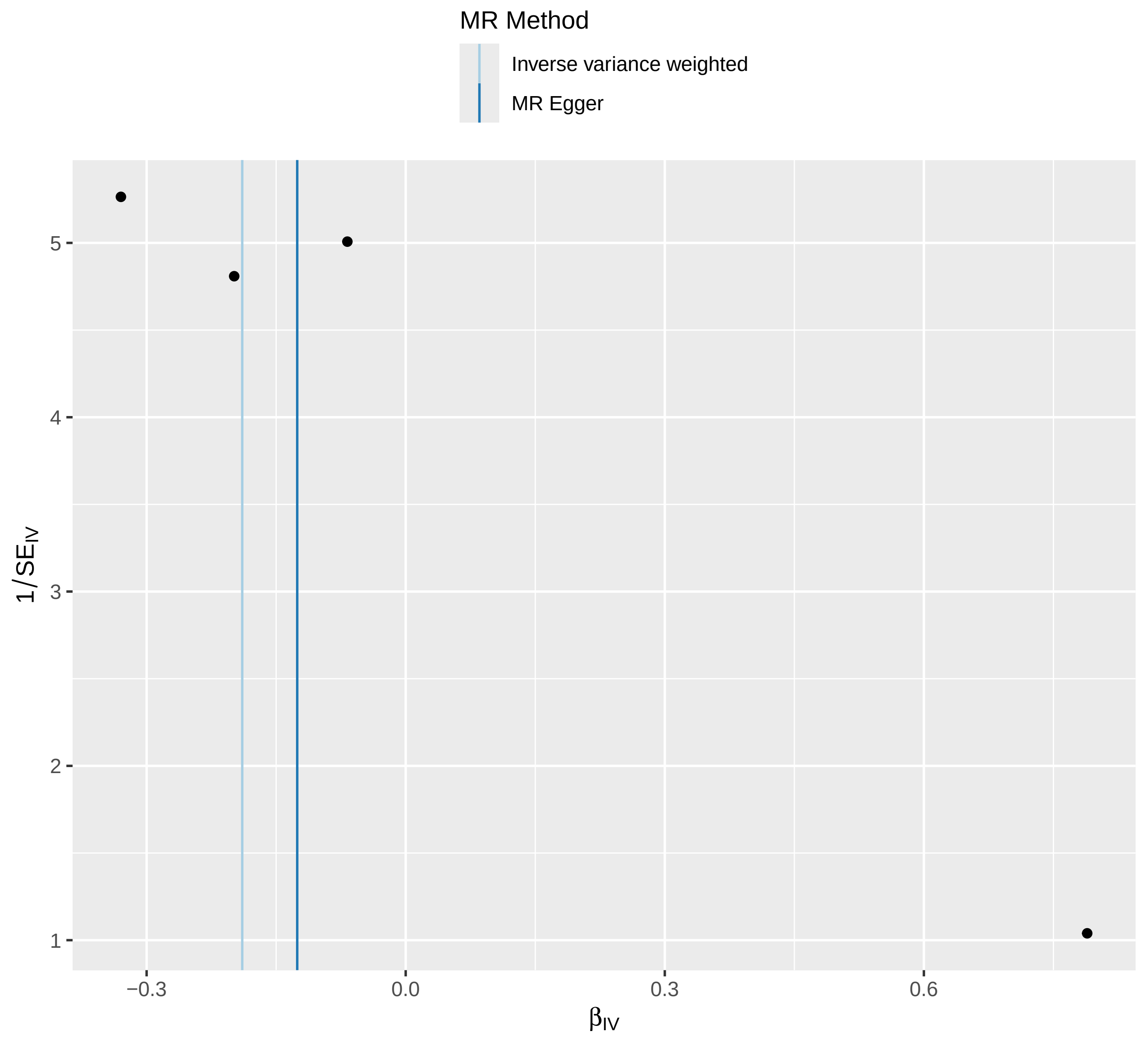
MR Method
Inverse variance weighted
MR Egger
1/SE$_{IV}$
5
4
3
2
1
−0.3
0.0
0.3
0.6
β$_{IV}$

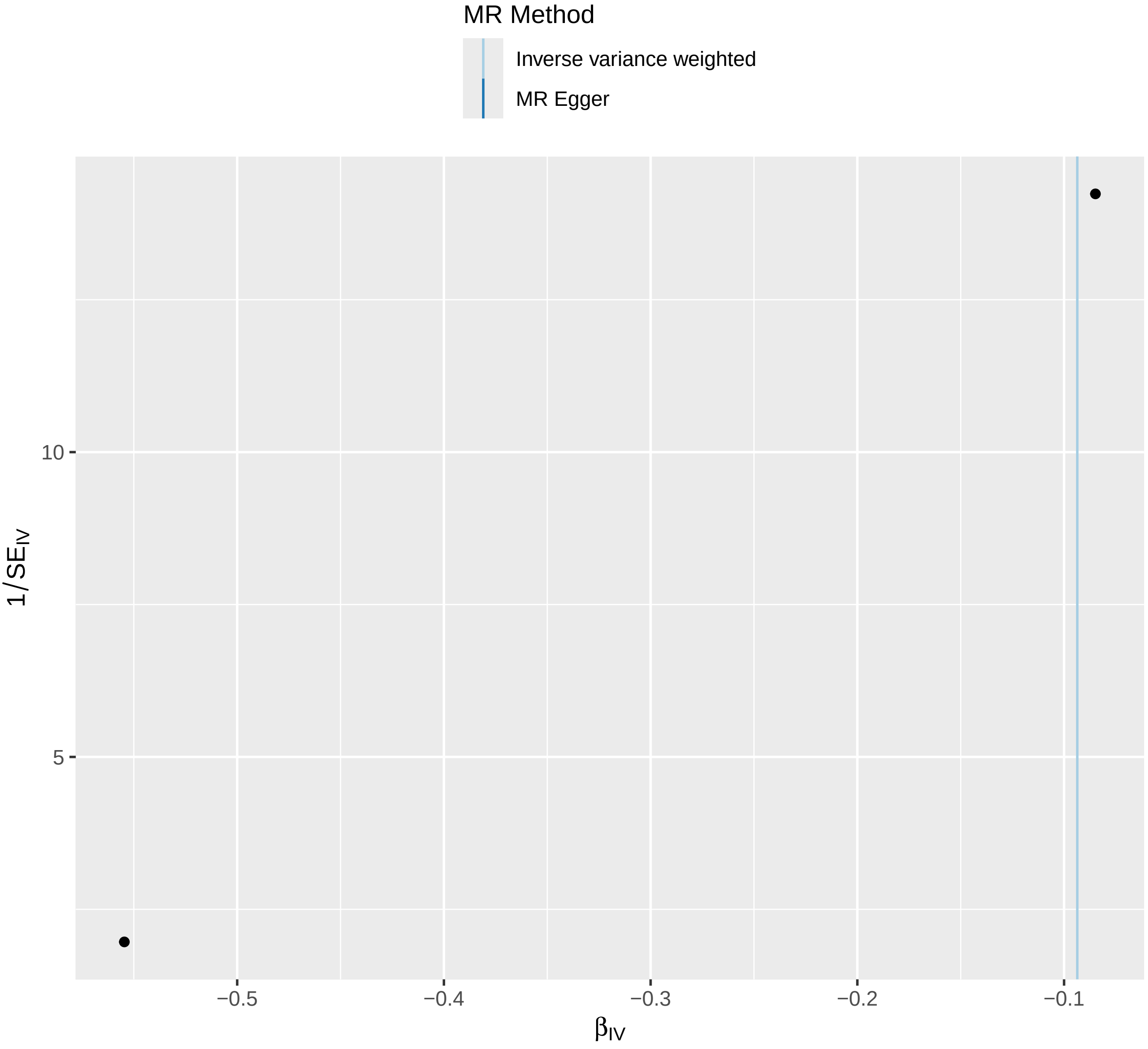

MR Method
Inverse variance weighted
MR Egger
10
5
1/SE$_{IV}$
−0.5
−0.4
−0.3
−0.2
−0.1
$\beta_{IV}$

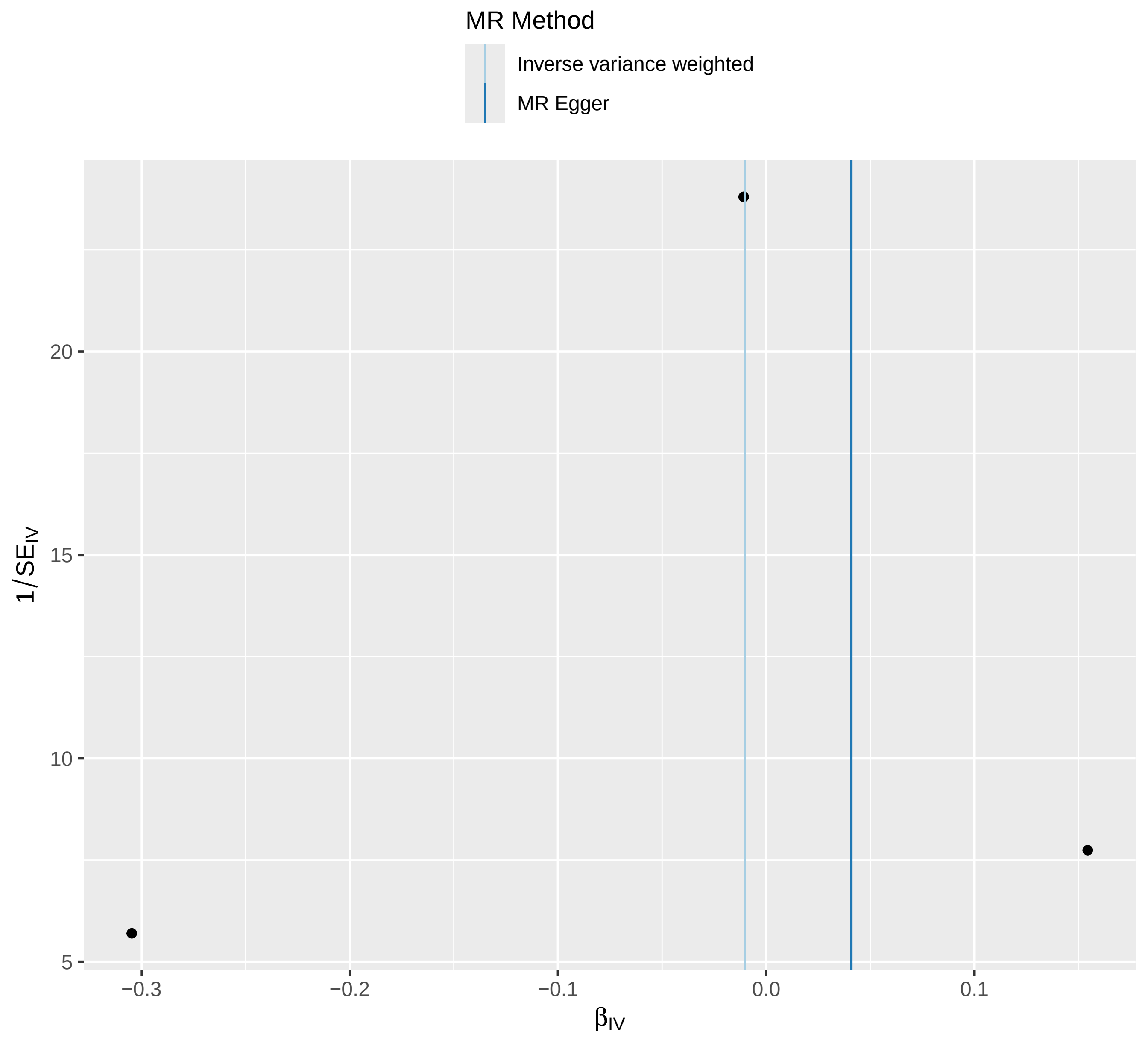

MR Method
Inverse variance weighted
MR Egger
20
15
10
5
1/SE$_{IV}$
−0.3
−0.2
−0.1
0.0
0.1
β$_{IV}$

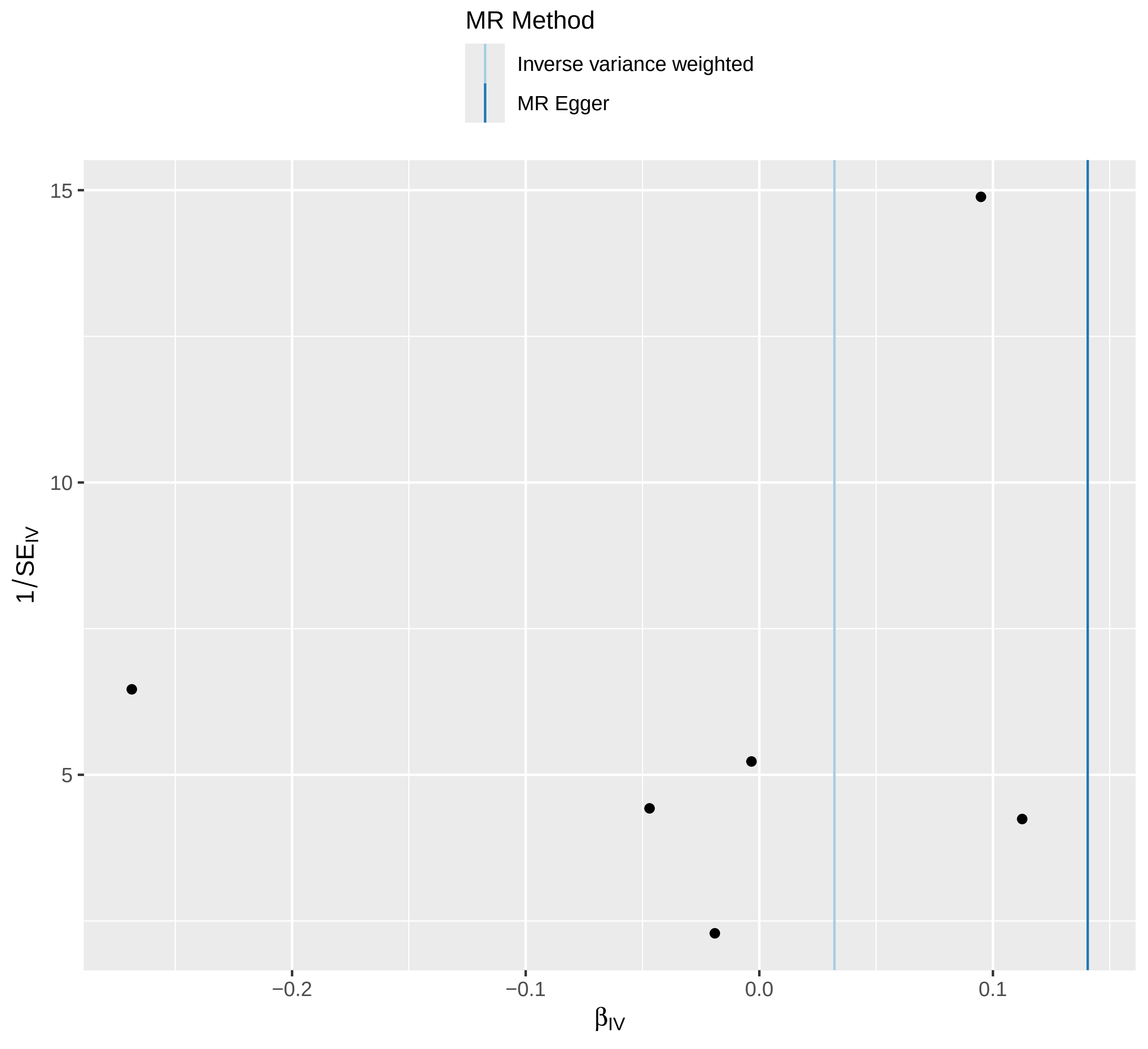
MR Method
Inverse variance weighted
MR Egger
15
10
5
1/SE_IV
−0.2
−0.1
0.0
0.1
β_IV

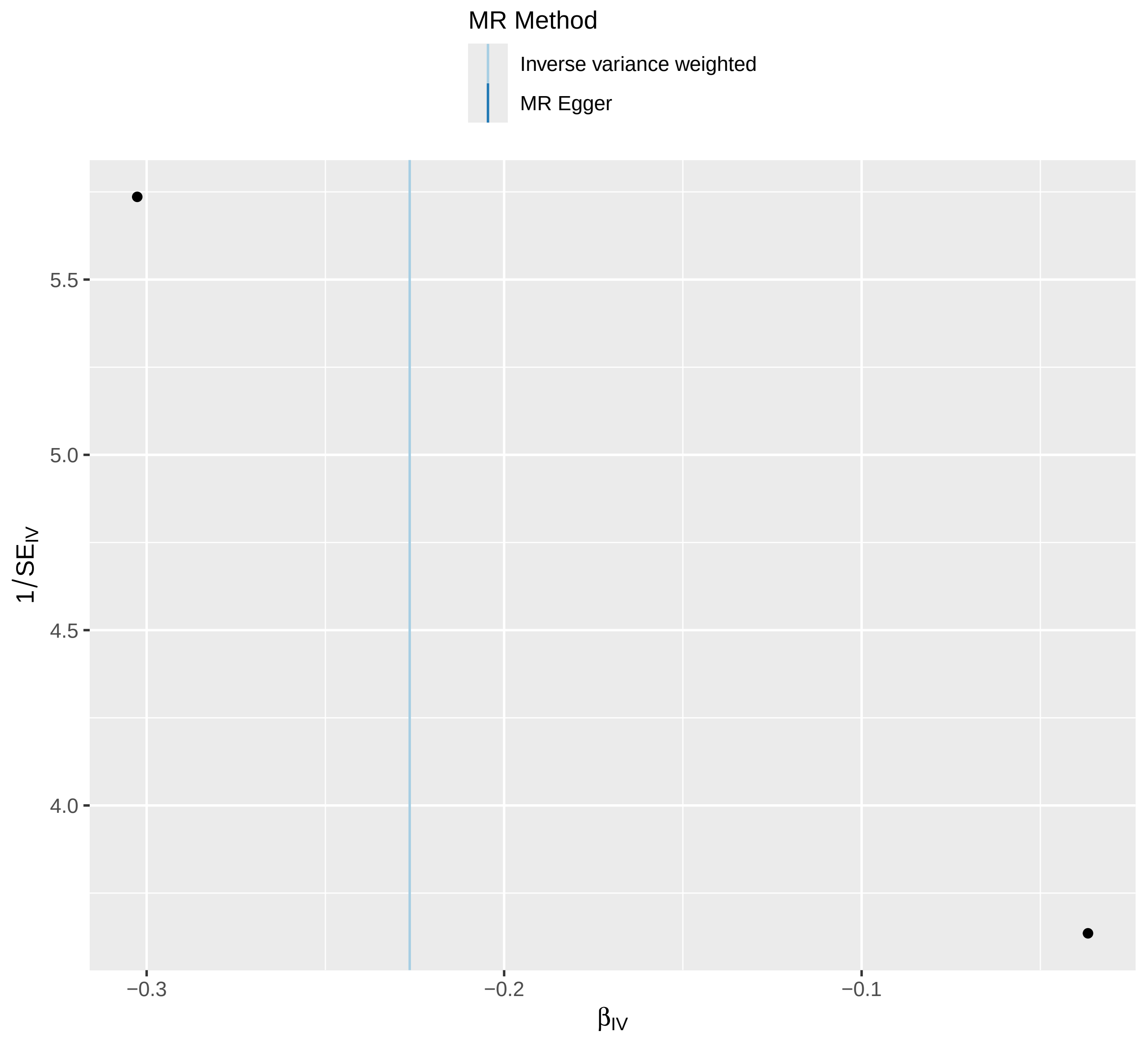

MR Method
Inverse variance weighted
MR Egger
5.5
5.0
4.5
4.0
1/SE_IV
−0.3
−0.2
−0.1
β_IV

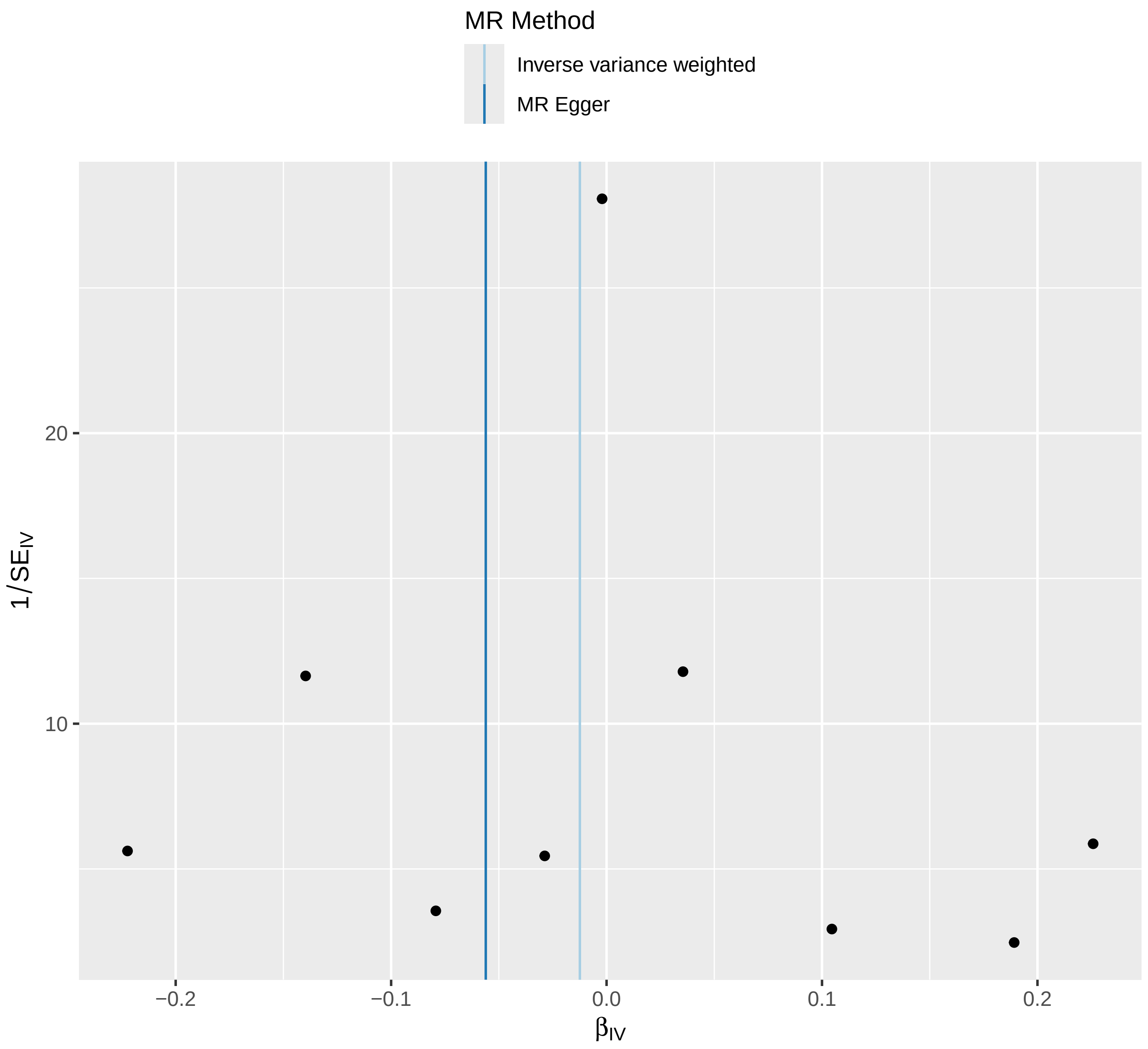
MR Method
Inverse variance weighted
MR Egger
20
10
1/SE$_{IV}$
−0.2
−0.1
0.0
0.1
0.2
β$_{IV}$

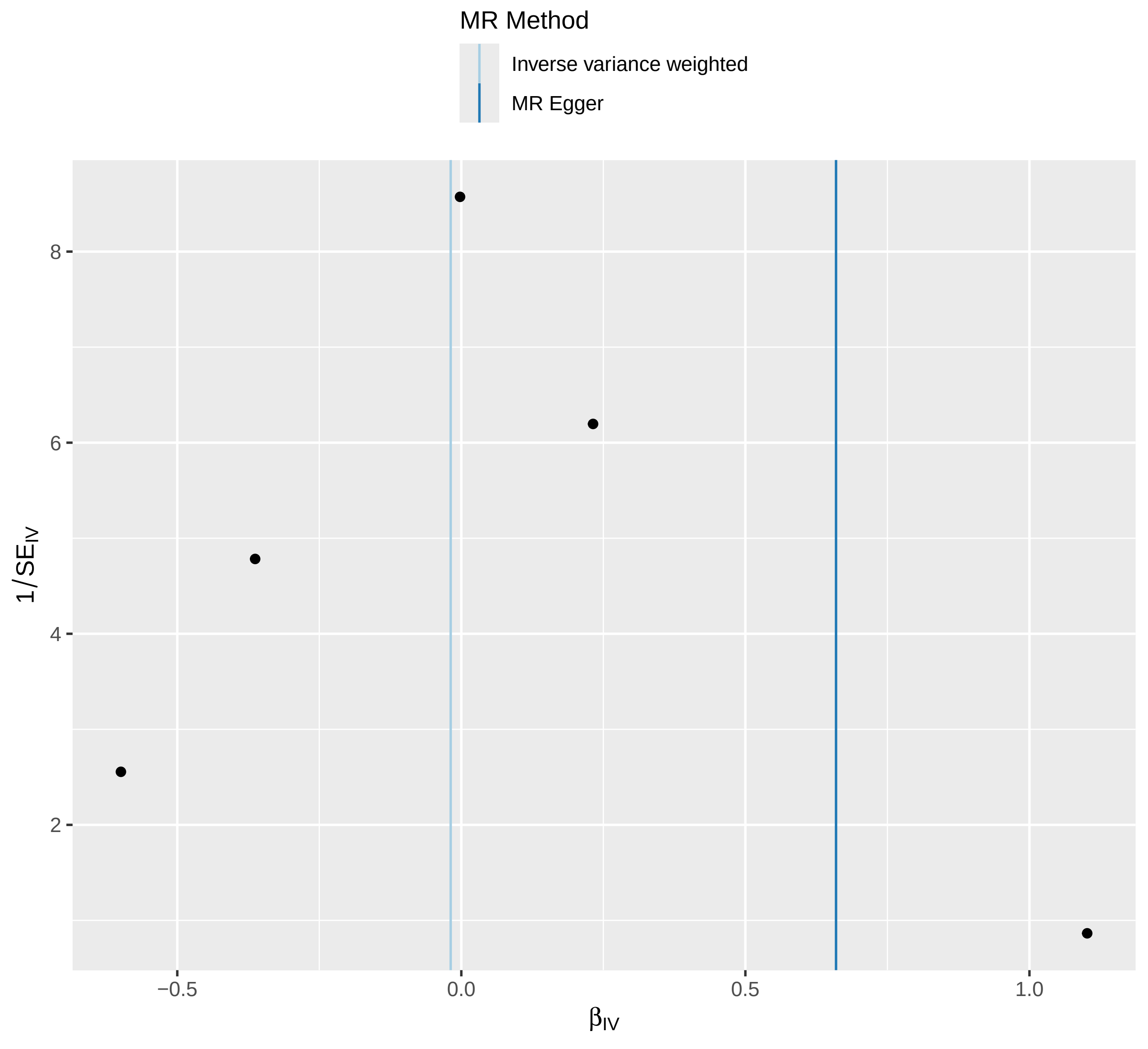

MR Method
Inverse variance weighted
MR Egger
8
6
4
2
$1/SE_{IV}$
−0.5
0.0
0.5
1.0
$\beta_{IV}$

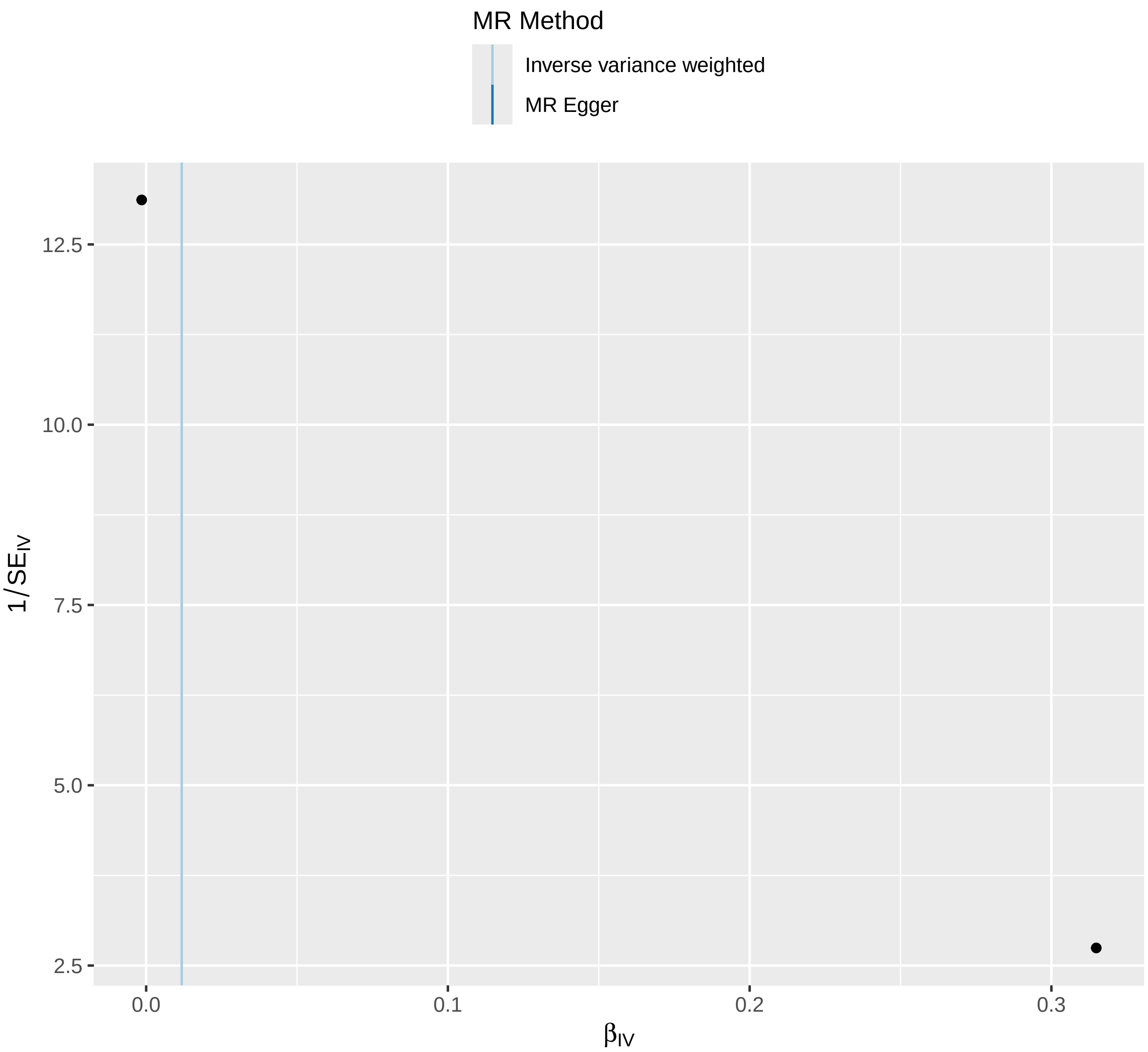

MR Method
Inverse variance weighted
MR Egger
12.5
10.0
7.5
5.0
2.5
1/SE_IV
0.0
0.1
0.2
0.3
β_IV

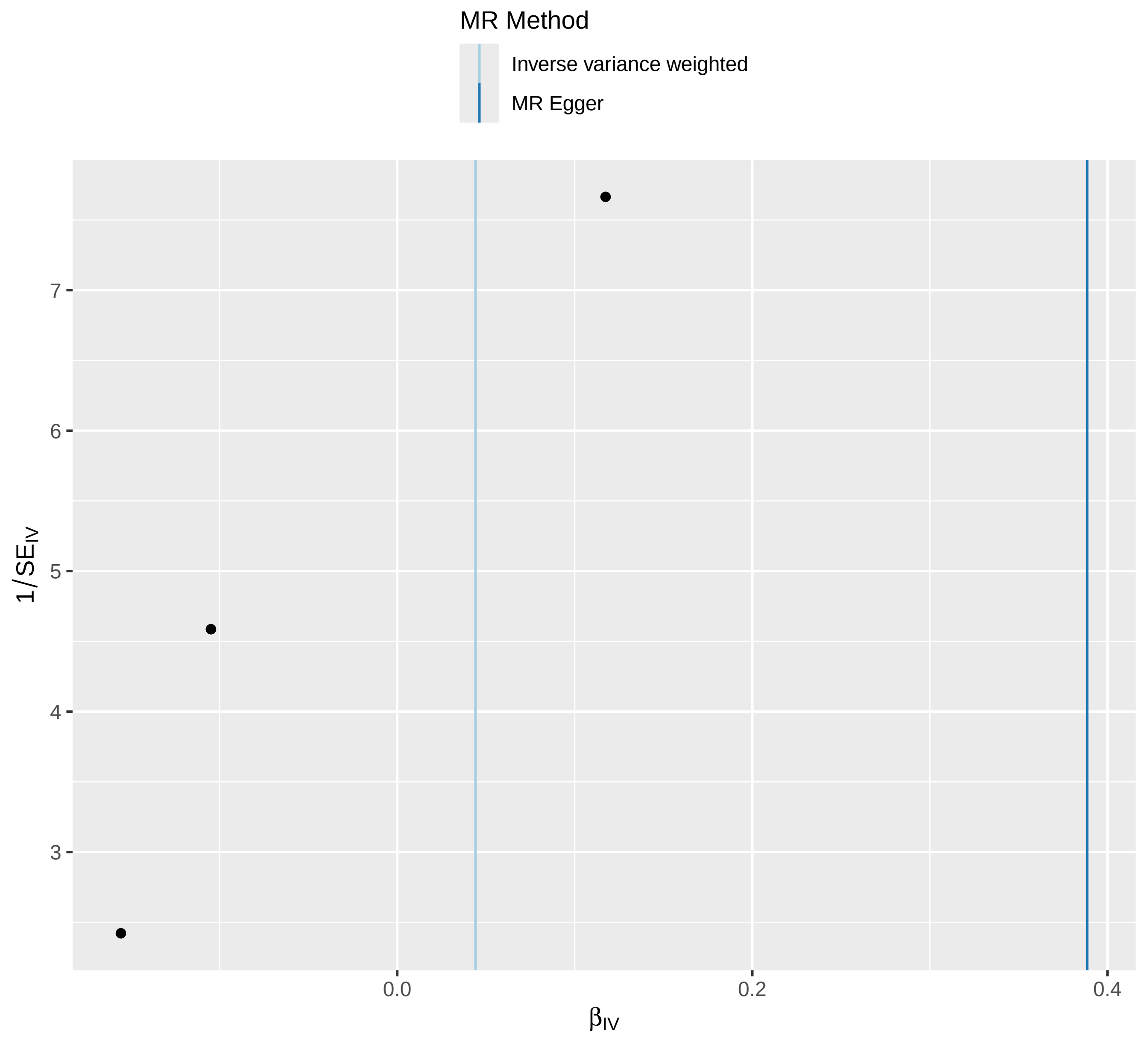
MR Method
Inverse variance weighted
MR Egger
7
6
5
4
3
1/SE_IV
0.0
0.2
0.4
β_IV

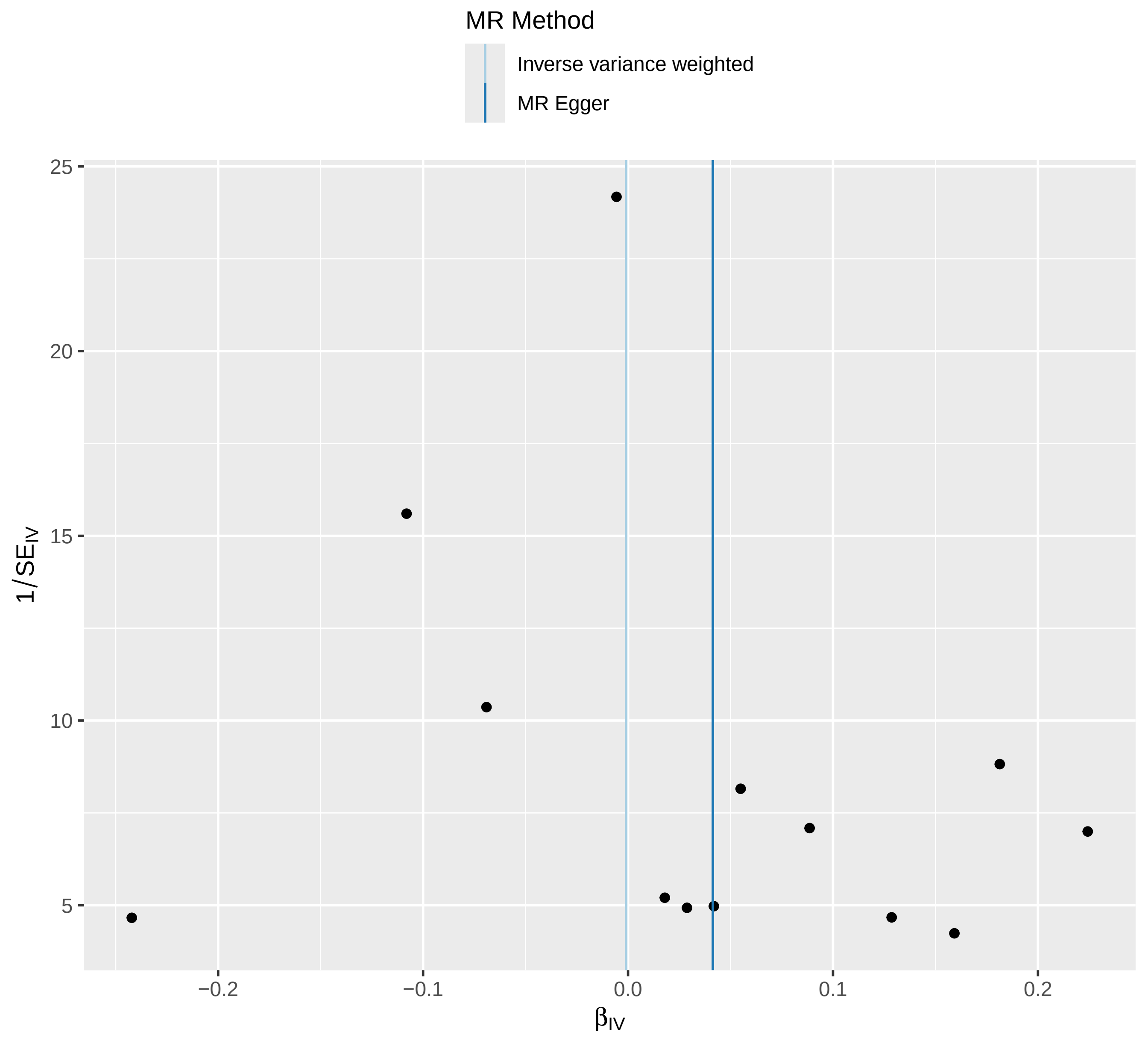
MR Method
Inverse variance weighted
MR Egger
25
20
15
10
5
$1/SE_{IV}$
−0.2
−0.1
0.0
0.1
0.2
$\beta_{IV}$

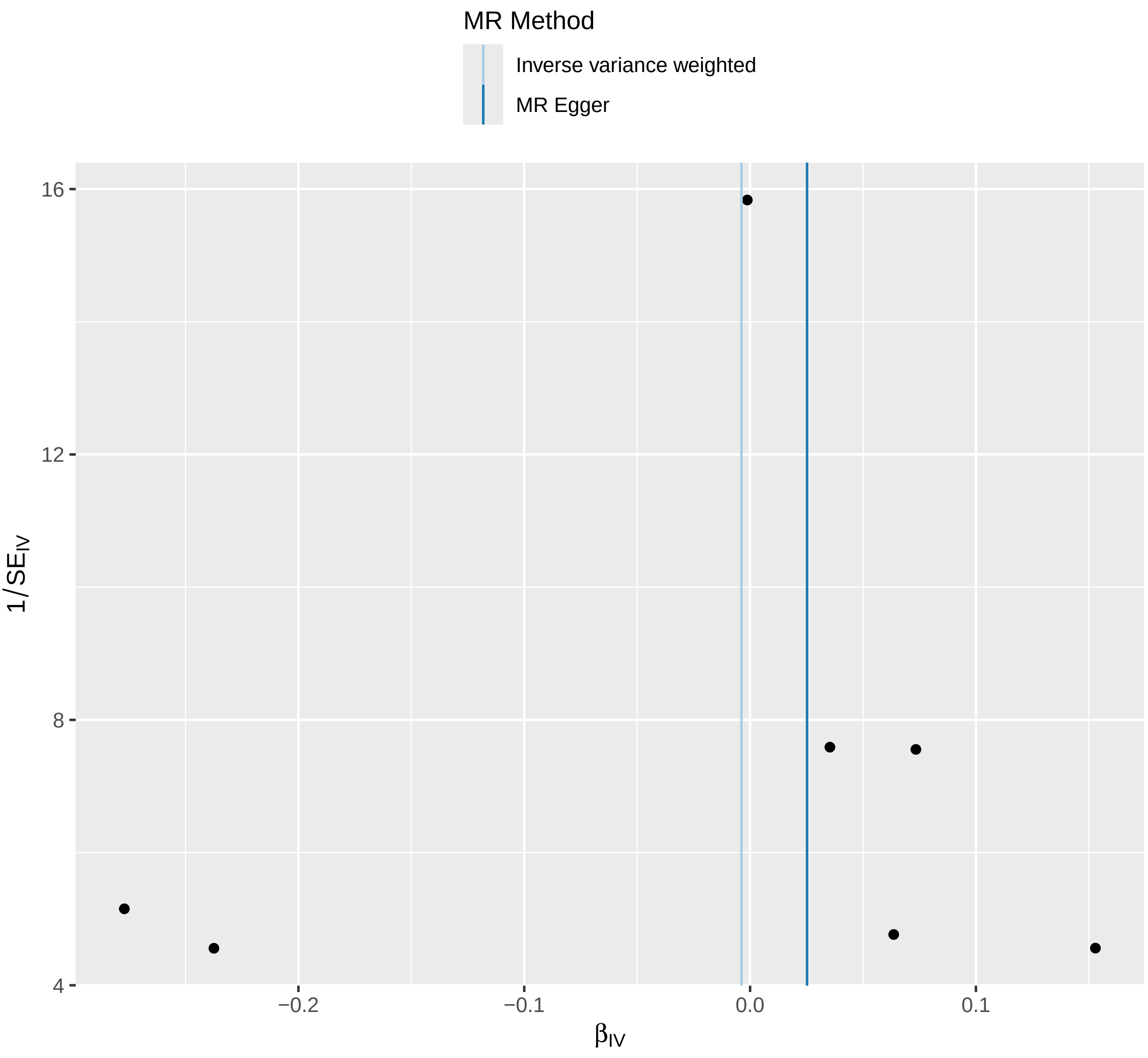
MR Method
Inverse variance weighted
MR Egger
16
12
8
4
$1/SE_{IV}$
−0.2
−0.1
0.0
0.1
$\beta_{IV}$

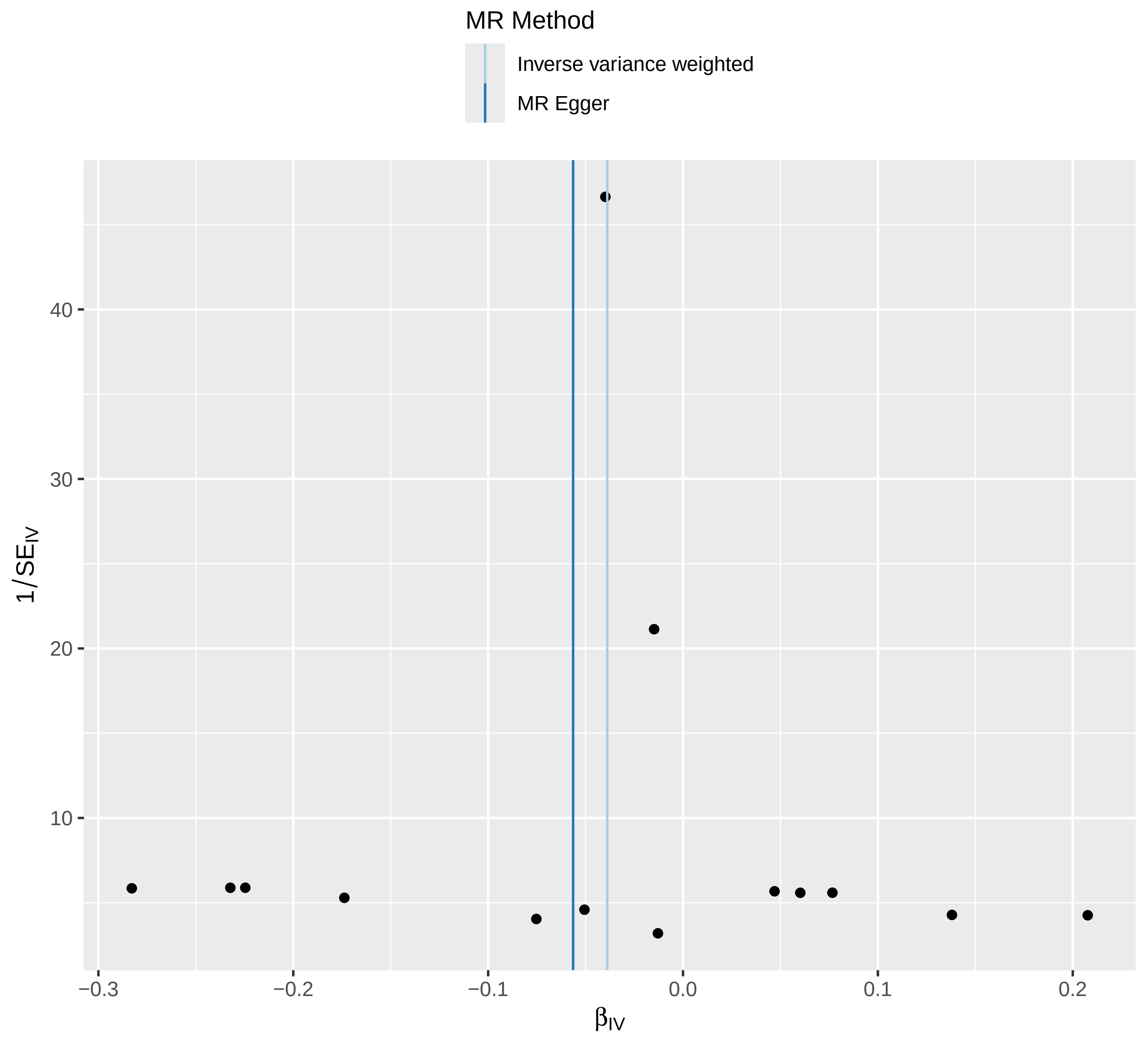

MR Method
Inverse variance weighted
MR Egger
40
30
20
10
$1/SE_{IV}$
−0.3
−0.2
−0.1
0.0
0.1
0.2
$\beta_{IV}$

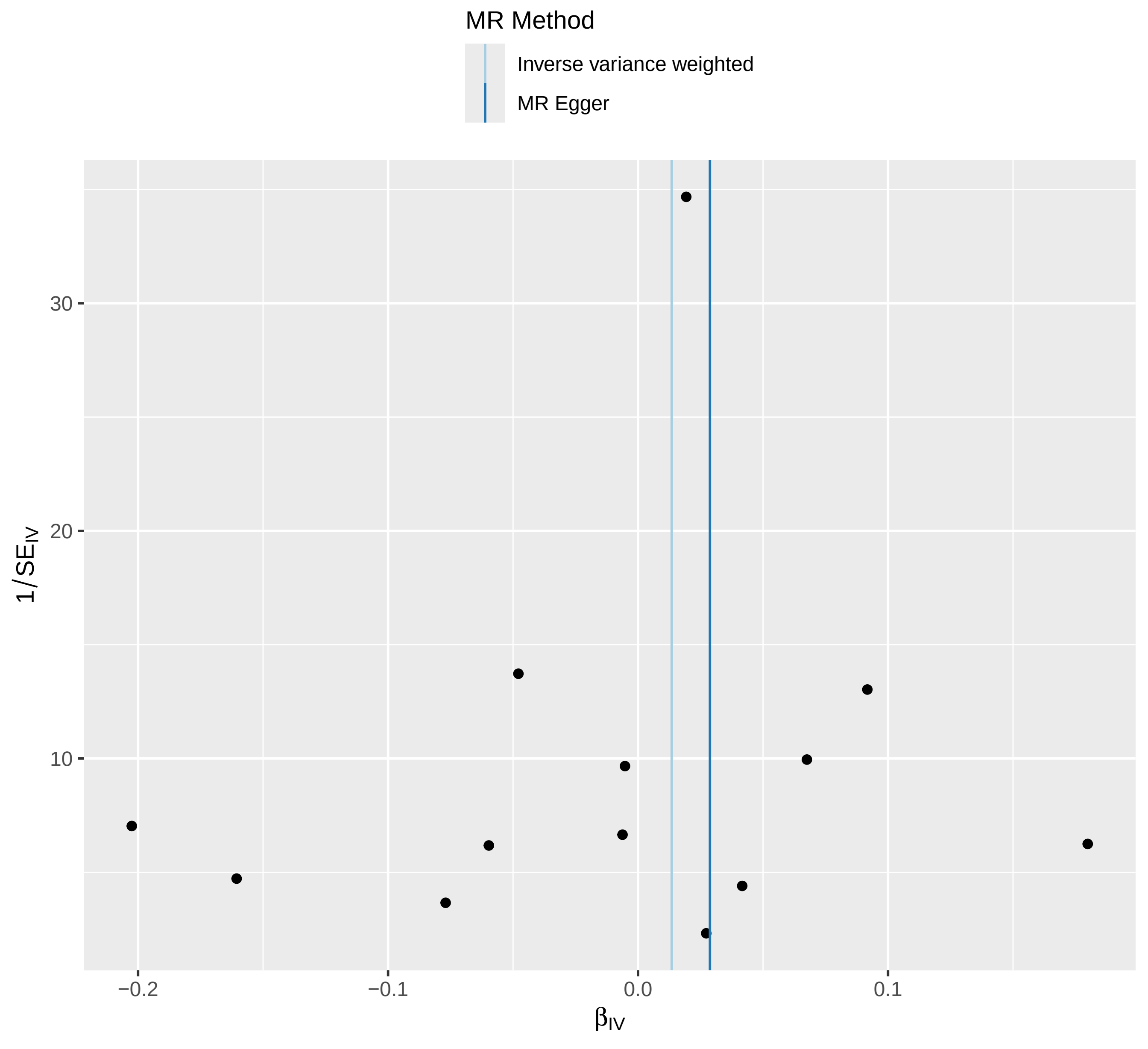

MR Method
Inverse variance weighted
MR Egger
30
20
10
1/SE_IV
−0.2
−0.1
0.0
0.1
β_IV

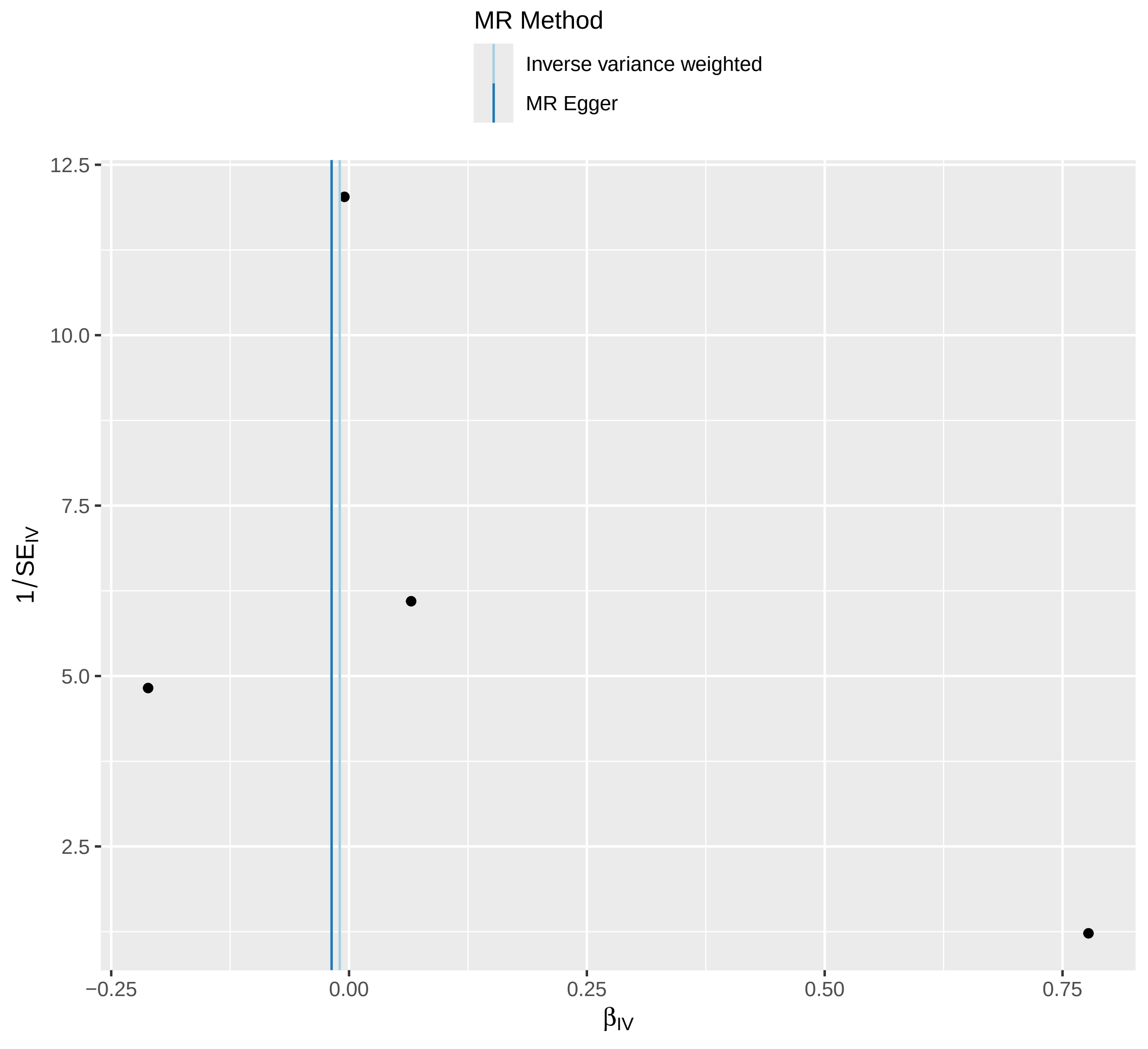
MR Method
Inverse variance weighted
MR Egger
12.5
10.0
7.5
5.0
2.5
1/SE_IV
−0.25
0.00
0.25
0.50
0.75
β_IV

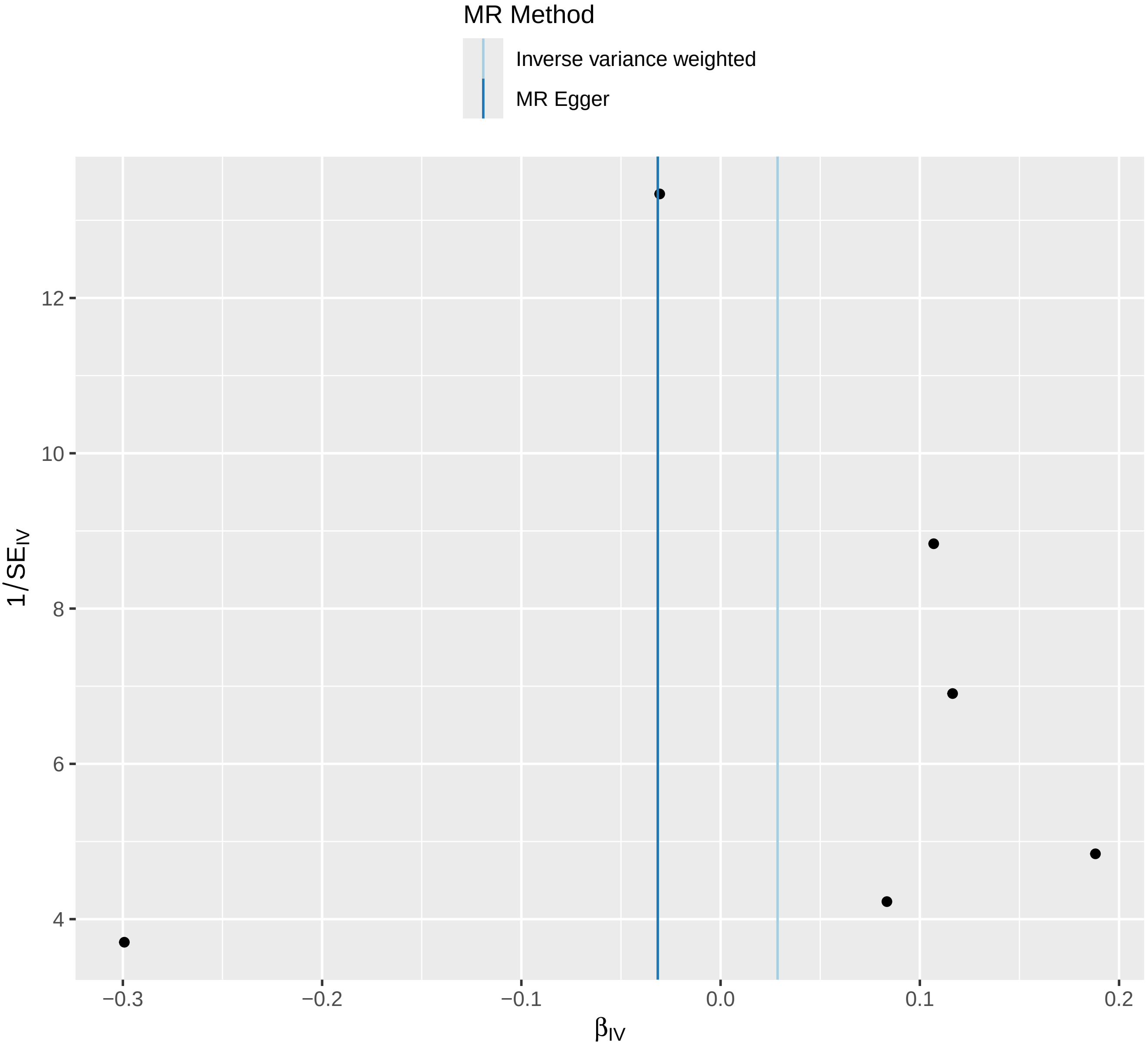
MR Method
Inverse variance weighted
MR Egger
12
10
8
6
4
1/SE_IV
−0.3
−0.2
−0.1
0.0
0.1
0.2
β_IV

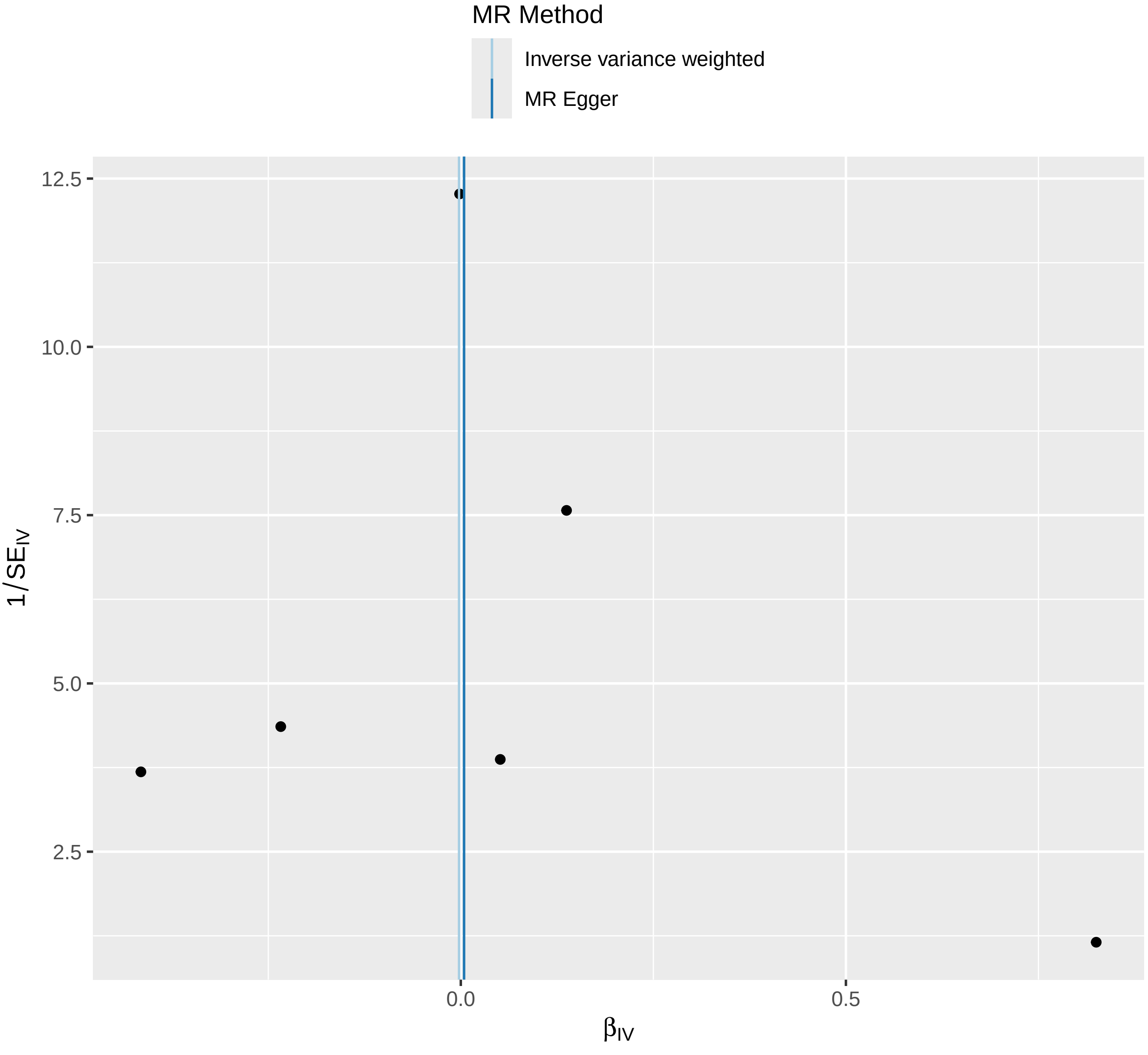
MR Method
Inverse variance weighted
MR Egger
12.5
10.0
7.5
5.0
2.5
$1/SE_{IV}$
0.0
0.5
$\beta_{IV}$

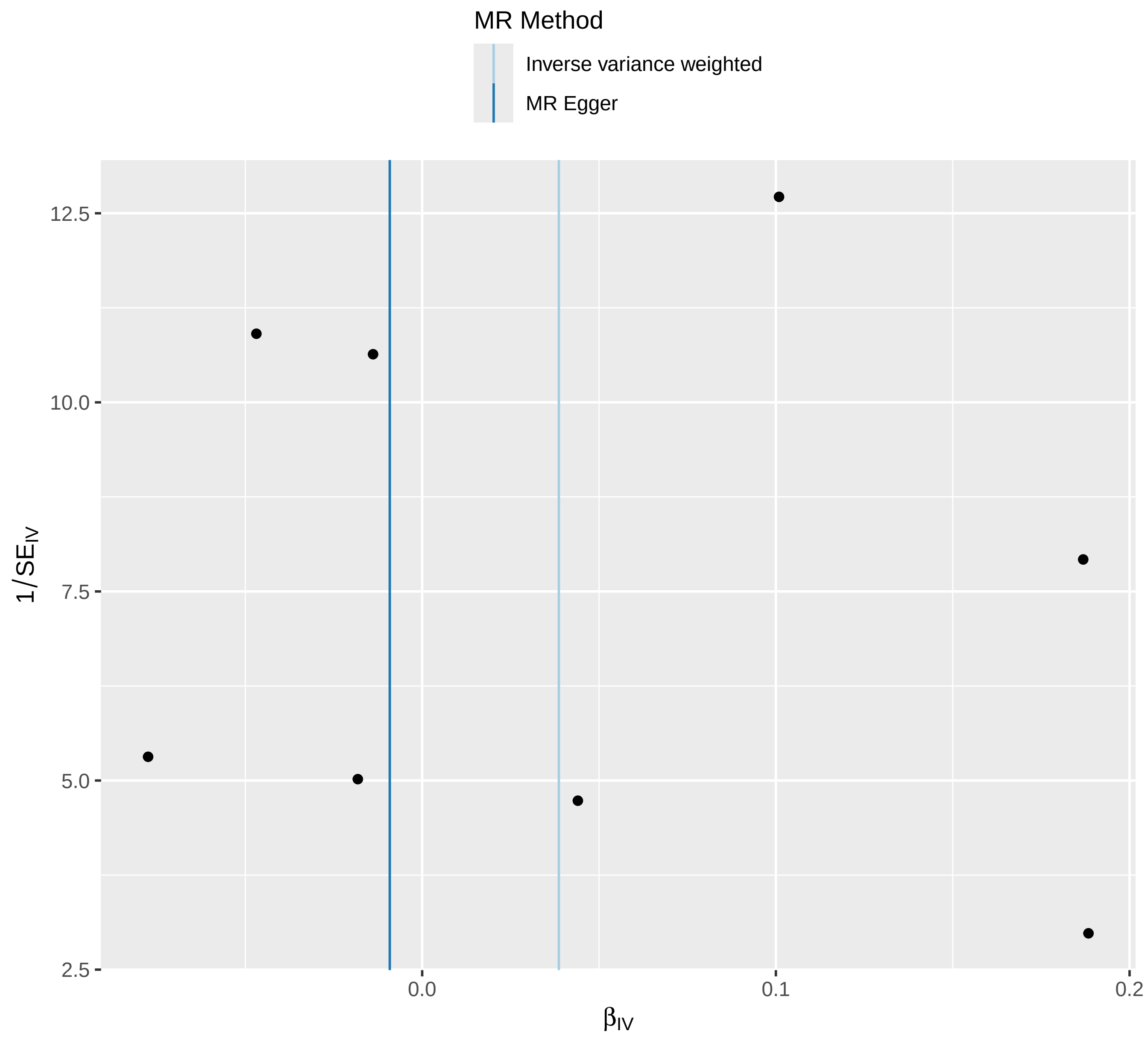

MR Method
Inverse variance weighted
MR Egger
12.5
10.0
7.5
5.0
2.5
1/SE_IV
0.0
0.1
0.2
β_IV

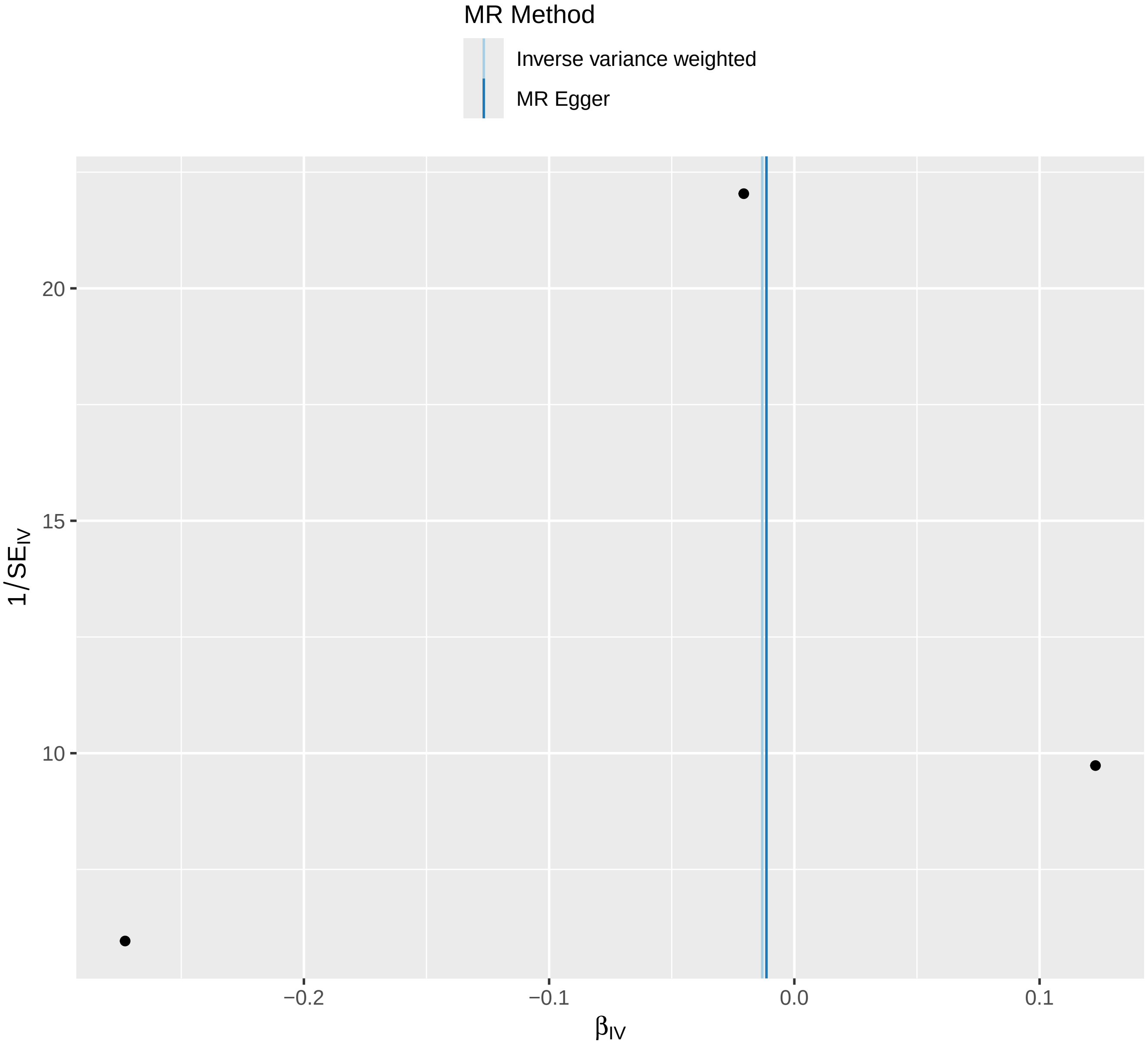

MR Method
Inverse variance weighted
MR Egger
20
15
10
−0.2
−0.1
0.0
0.1
$1/SE_{IV}$
$\beta_{IV}$

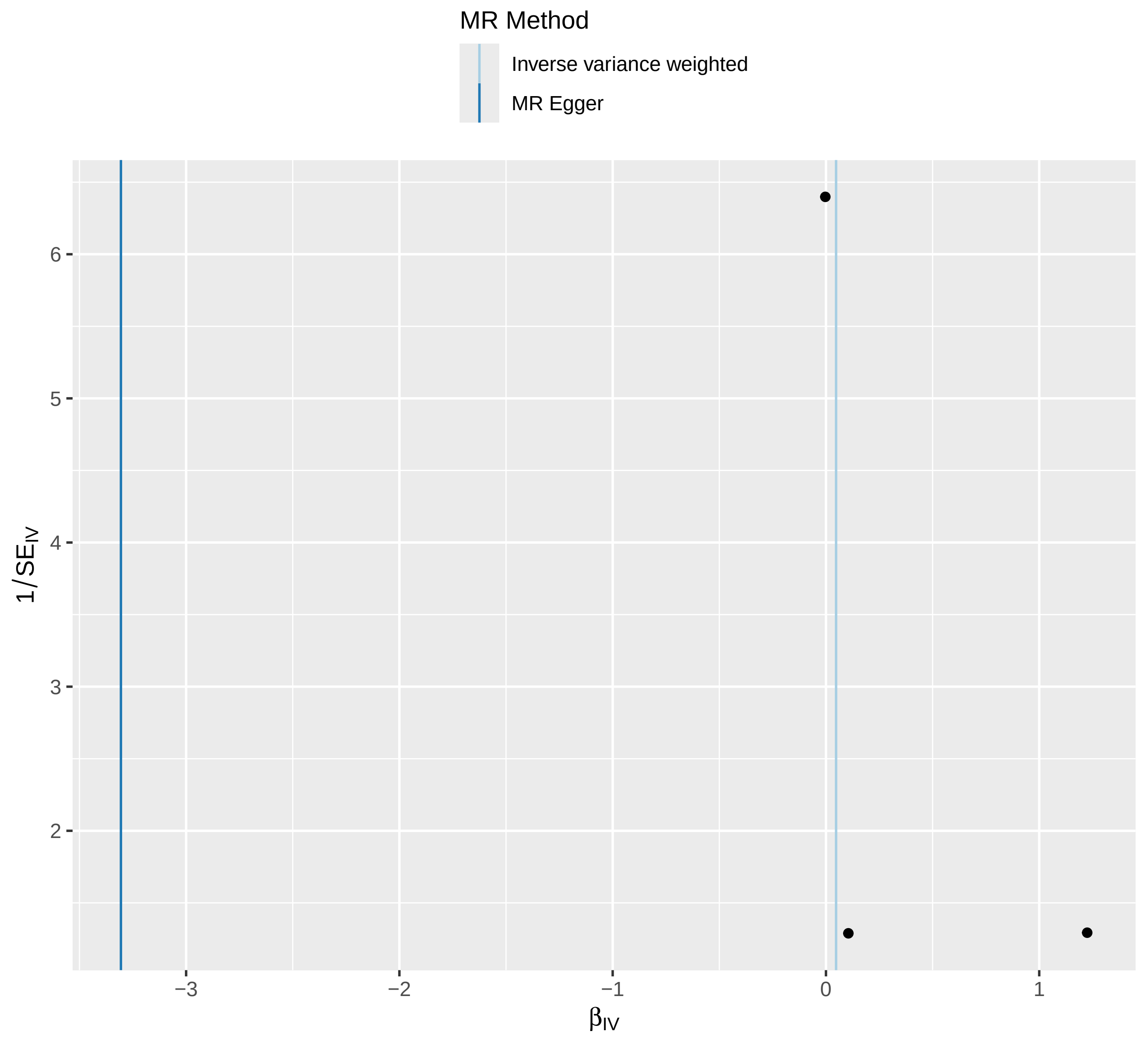

MR Method
Inverse variance weighted
MR Egger
6
5
4
3
2
1/SE_IV
−3
−2
−1
0
1
β_IV

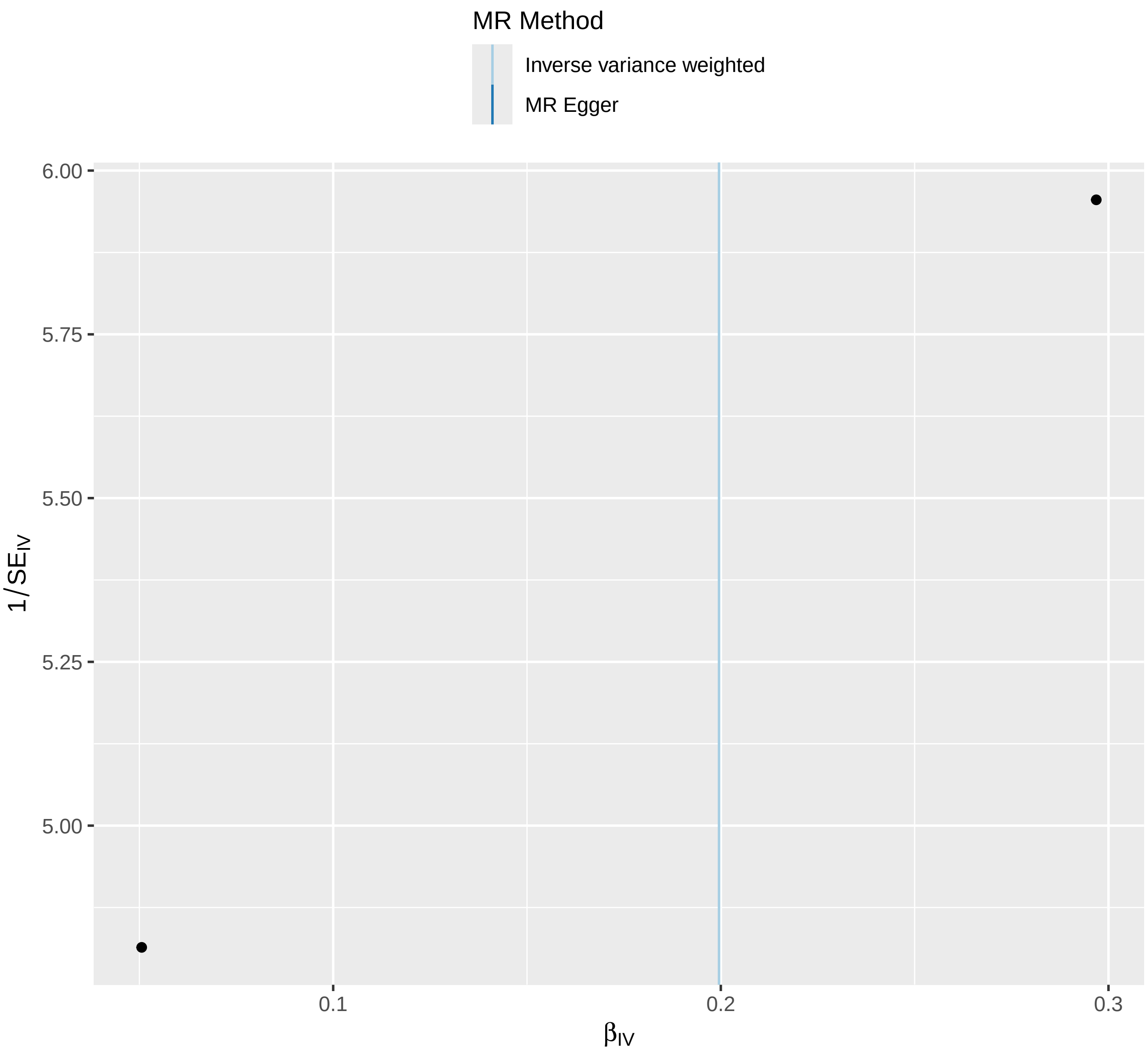

MR Method
Inverse variance weighted
MR Egger
6.00
5.75
5.50
5.25
5.00
1/SE$_{IV}$
0.1
0.2
0.3
β$_{IV}$

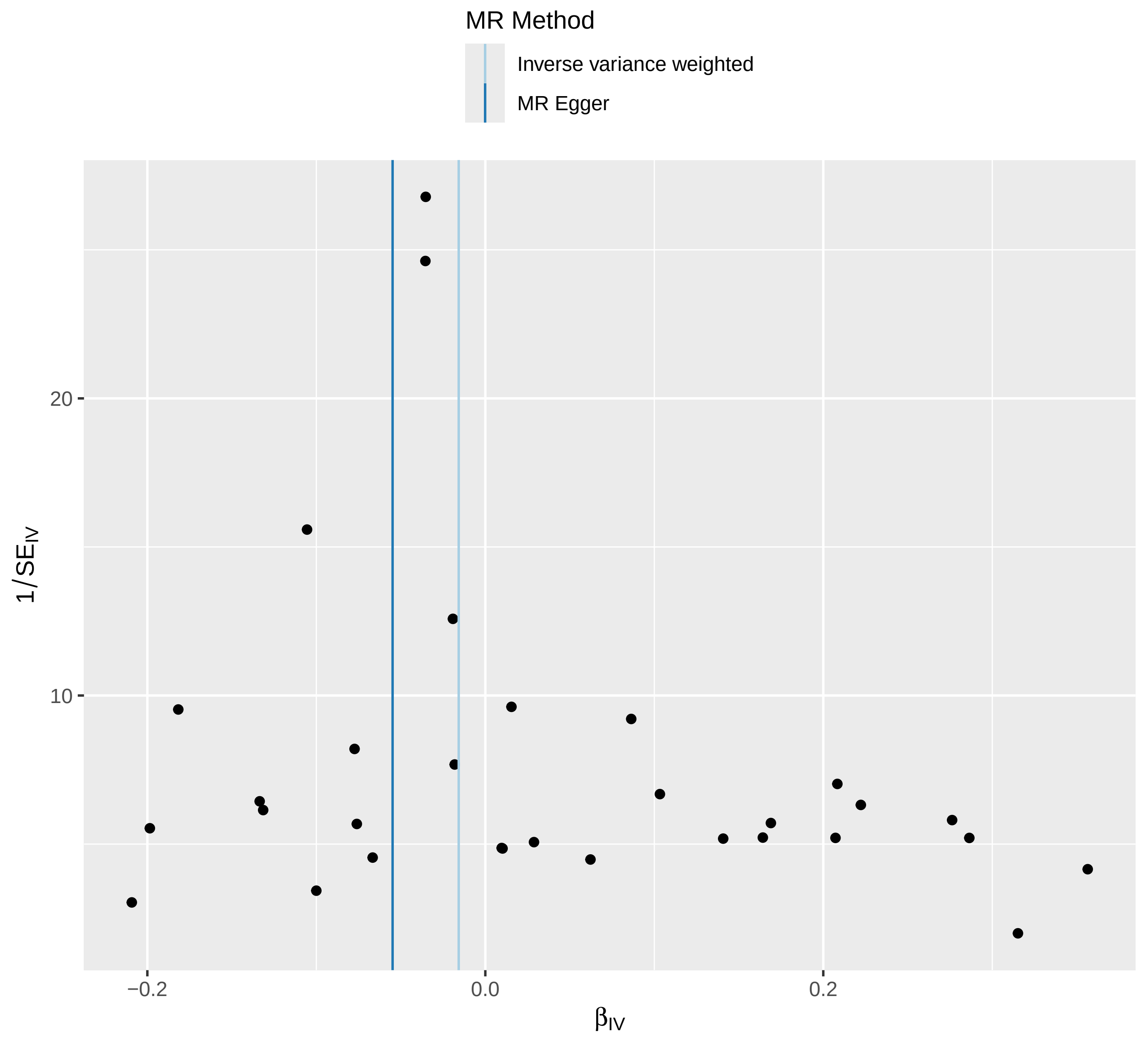
MR Method
Inverse variance weighted
MR Egger
20
10
1/SE$_{IV}$
−0.2
0.0
0.2
β$_{IV}$

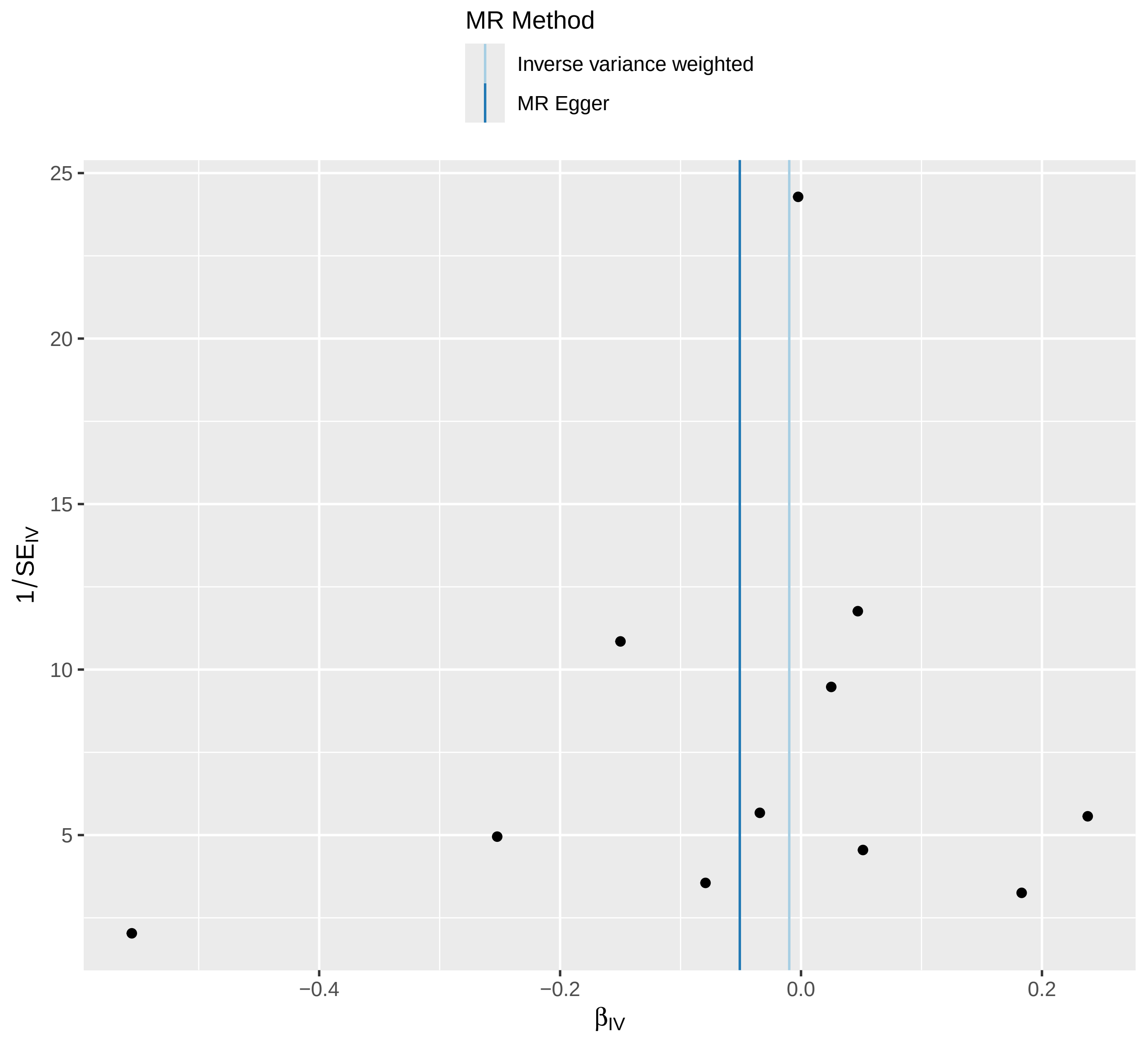
MR Method
Inverse variance weighted
MR Egger
25
20
15
10
5
1/SE$_{IV}$
−0.4
−0.2
0.0
0.2
β$_{IV}$

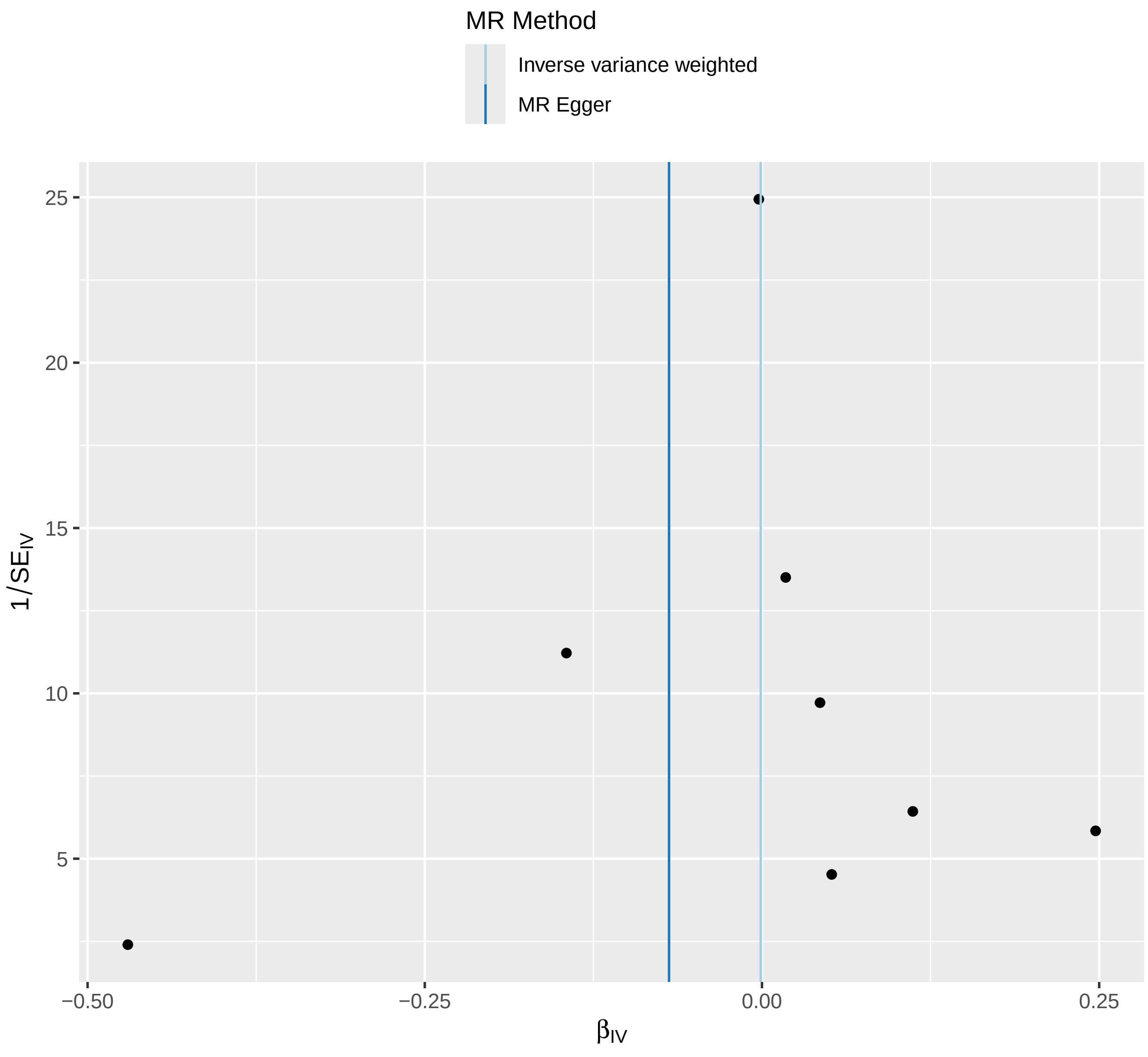

MR Method
Inverse variance weighted
MR Egger
25
20
15
10
5
1/SE$_{IV}$
−0.50
−0.25
0.00
0.25
β$_{IV}$

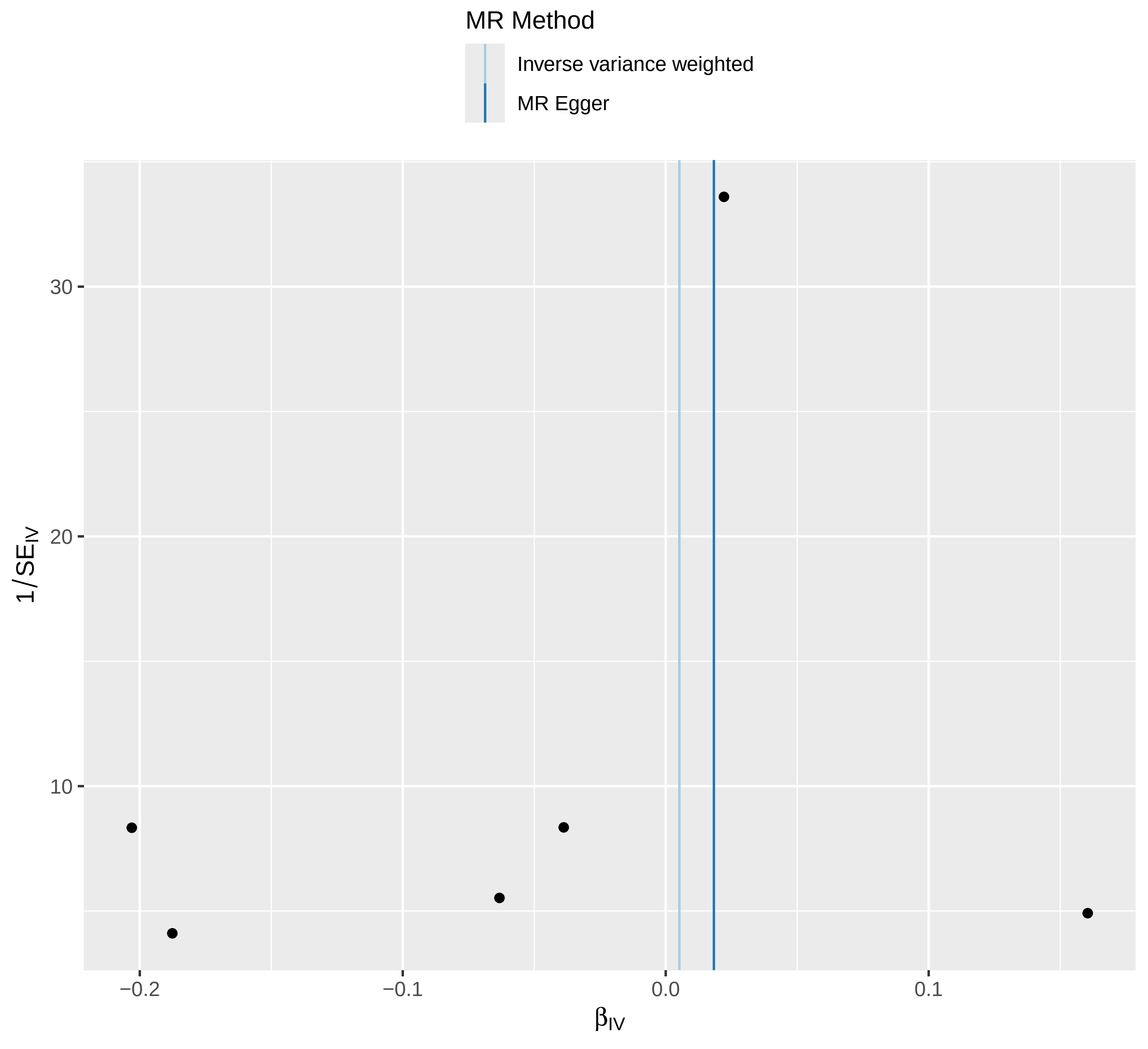
MR Method
Inverse variance weighted
MR Egger
30
20
10
1/SE$_{IV}$
−0.2
−0.1
0.0
0.1
β$_{IV}$

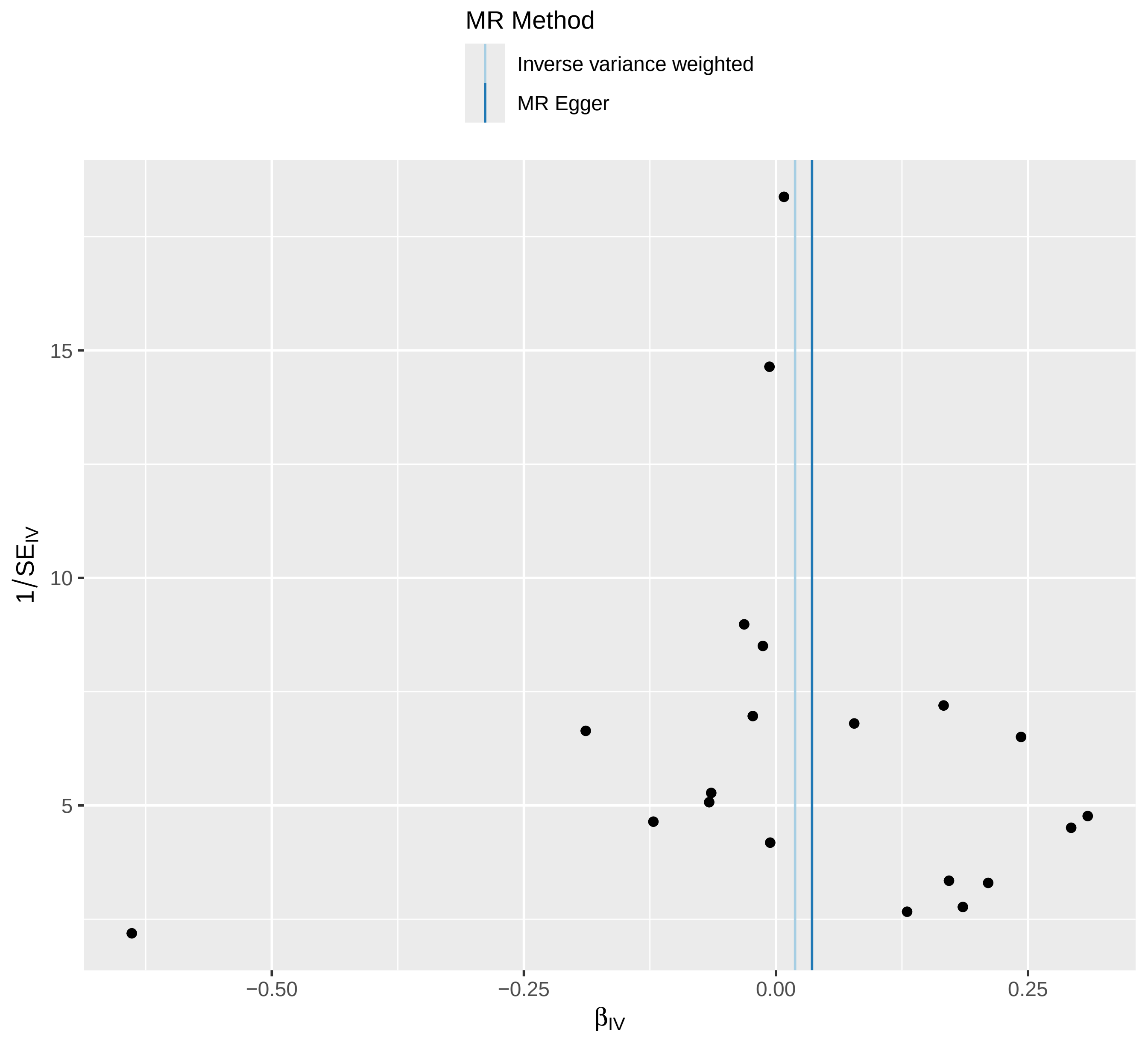

MR Method
Inverse variance weighted
MR Egger
15
10
5
$1/SE_{IV}$
−0.50
−0.25
0.00
0.25
$\beta_{IV}$

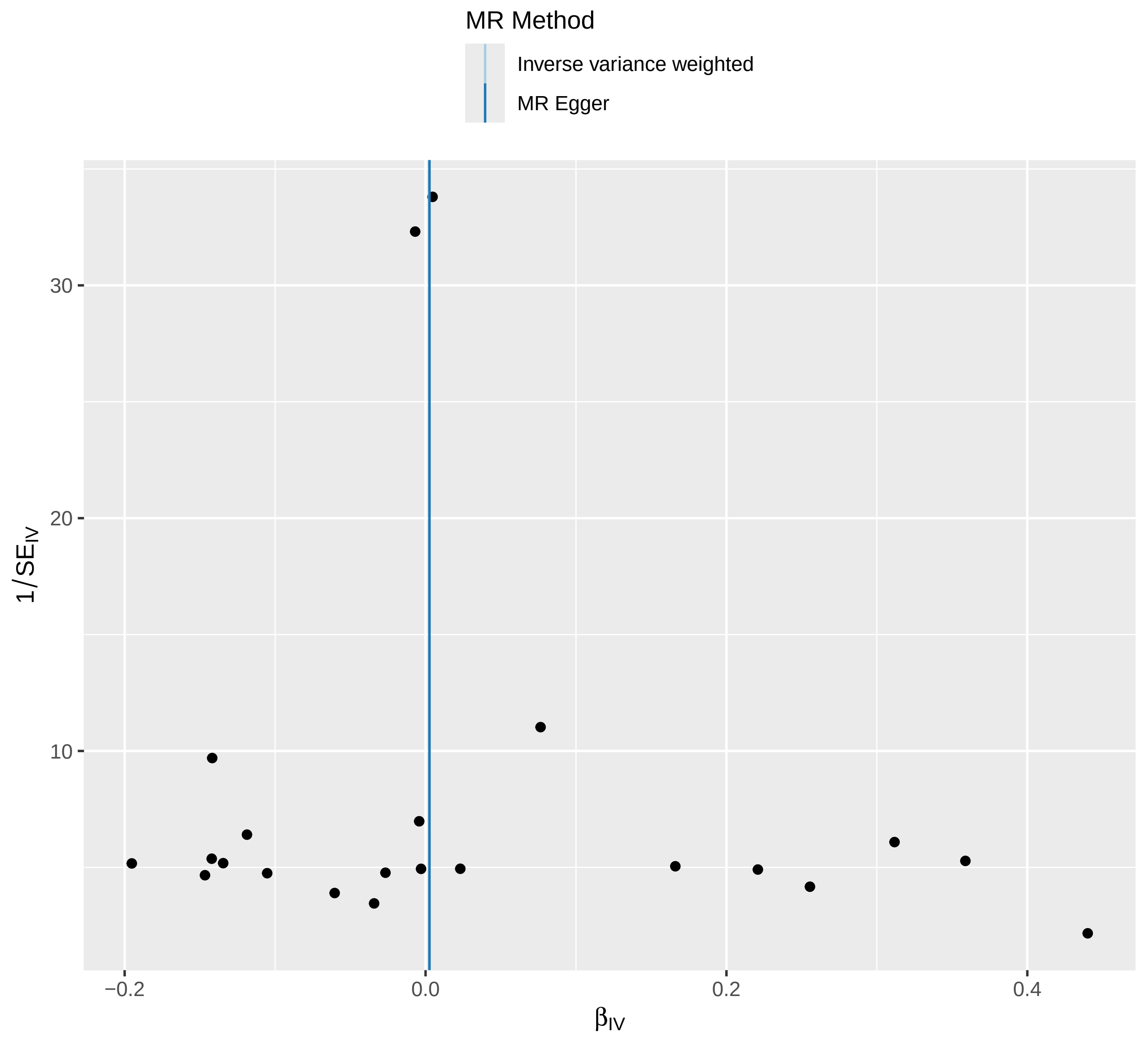
MR Method
Inverse variance weighted
MR Egger
30
20
10
1/SE$_{IV}$
−0.2
0.0
0.2
0.4
β$_{IV}$

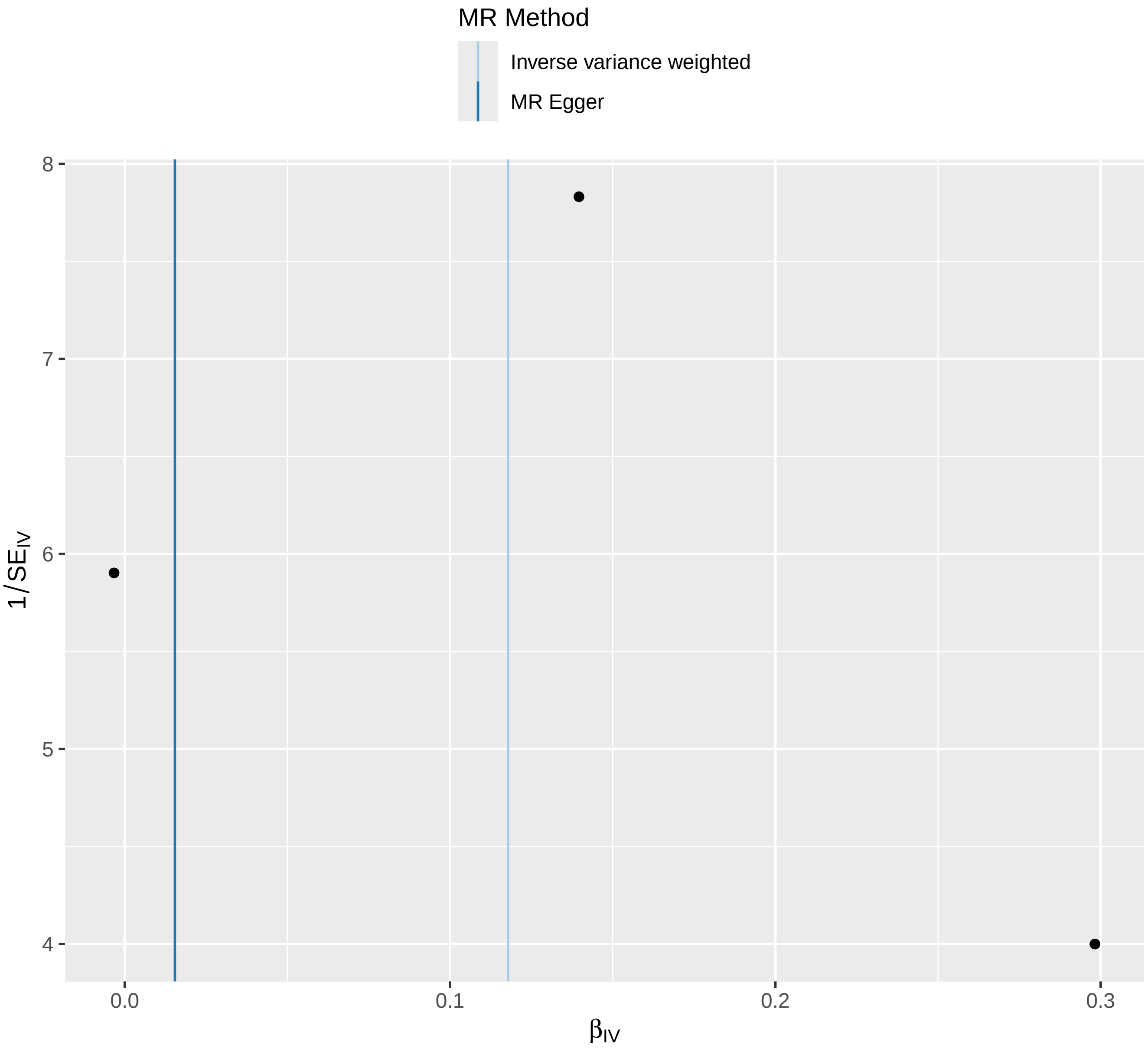

MR Method
Inverse variance weighted
MR Egger
8
7
6
5
4
1/SE$_{IV}$
0.0
0.1
0.2
0.3
$\beta_{IV}$

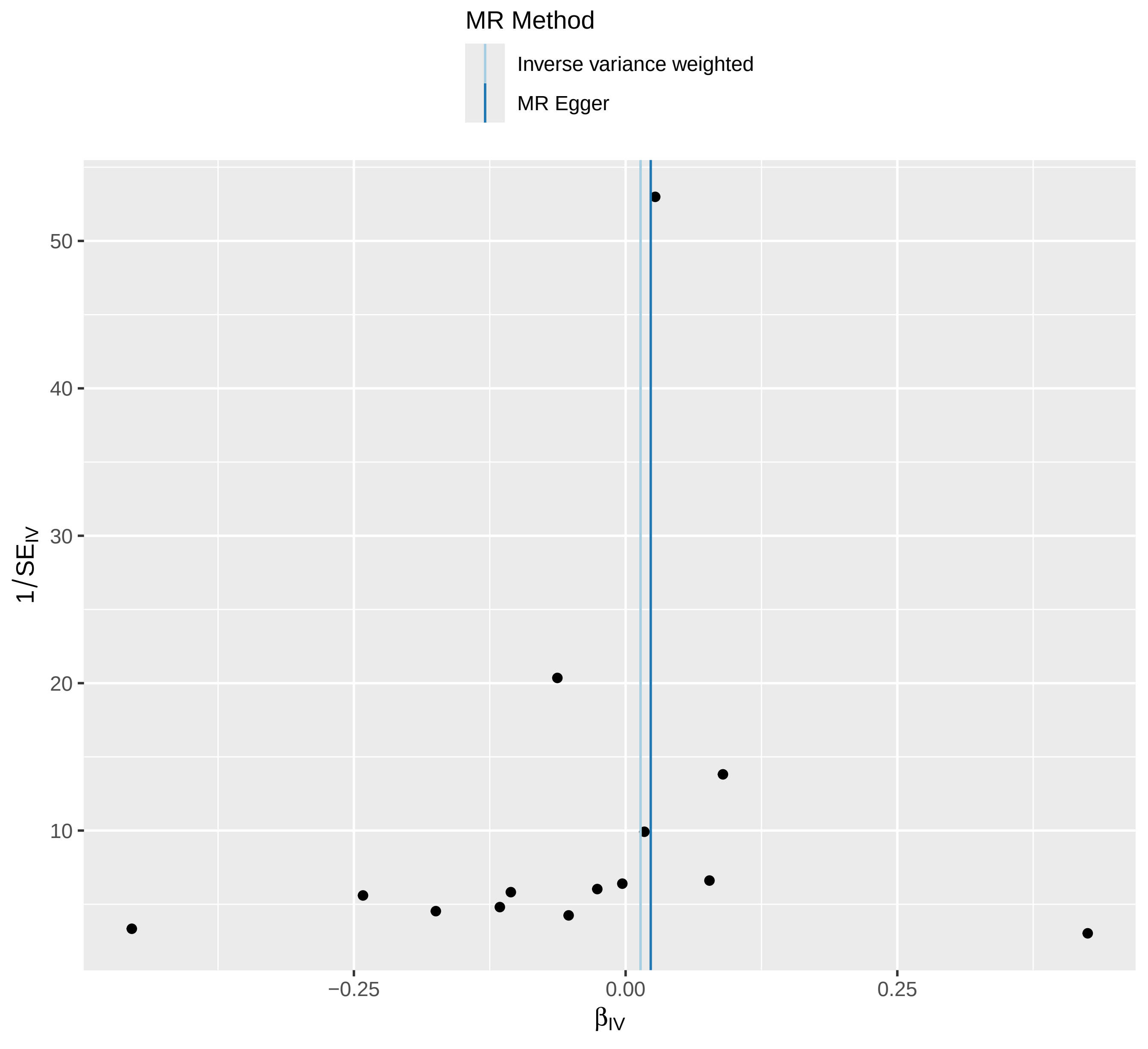

MR Method
Inverse variance weighted
MR Egger
50
40
30
20
10
1/SE_IV
−0.25
0.00
0.25
β_IV

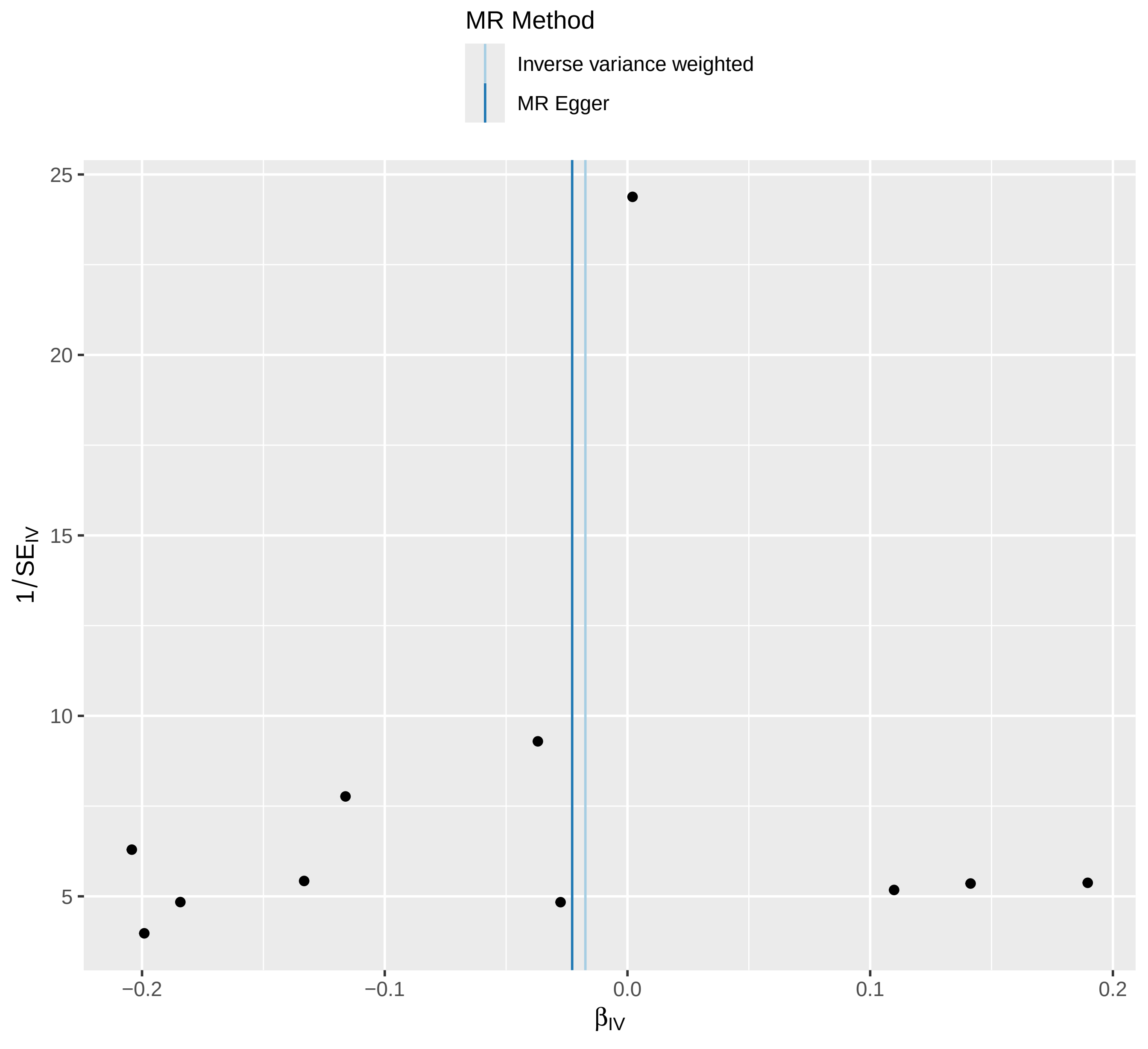
MR Method
Inverse variance weighted
MR Egger
25
20
15
10
5
1/SE_IV
−0.2
−0.1
0.0
0.1
0.2
β_IV

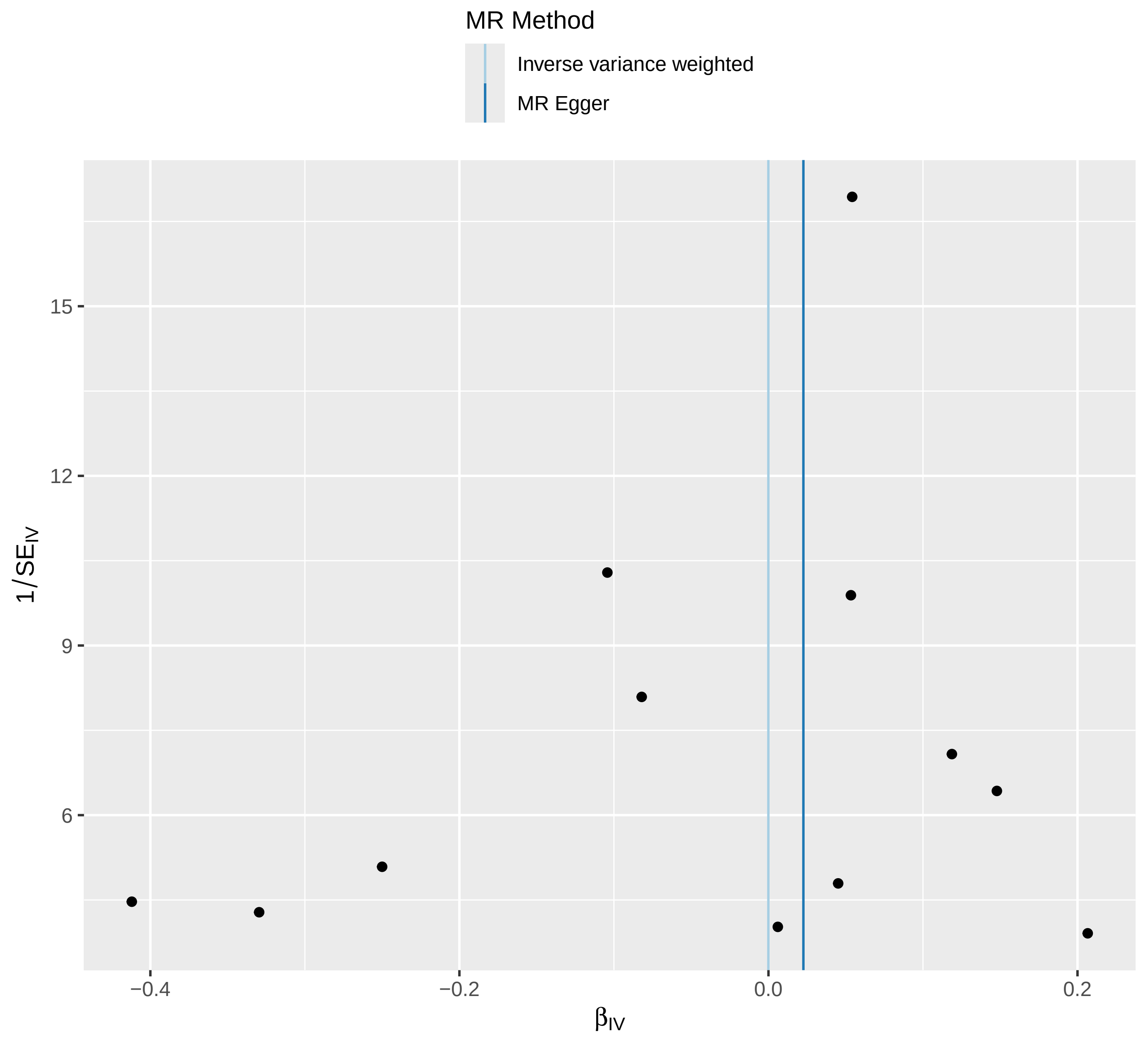
MR Method
Inverse variance weighted
MR Egger
1/SE_IV
15
12
9
6
−0.4
−0.2
0.0
0.2
β_IV

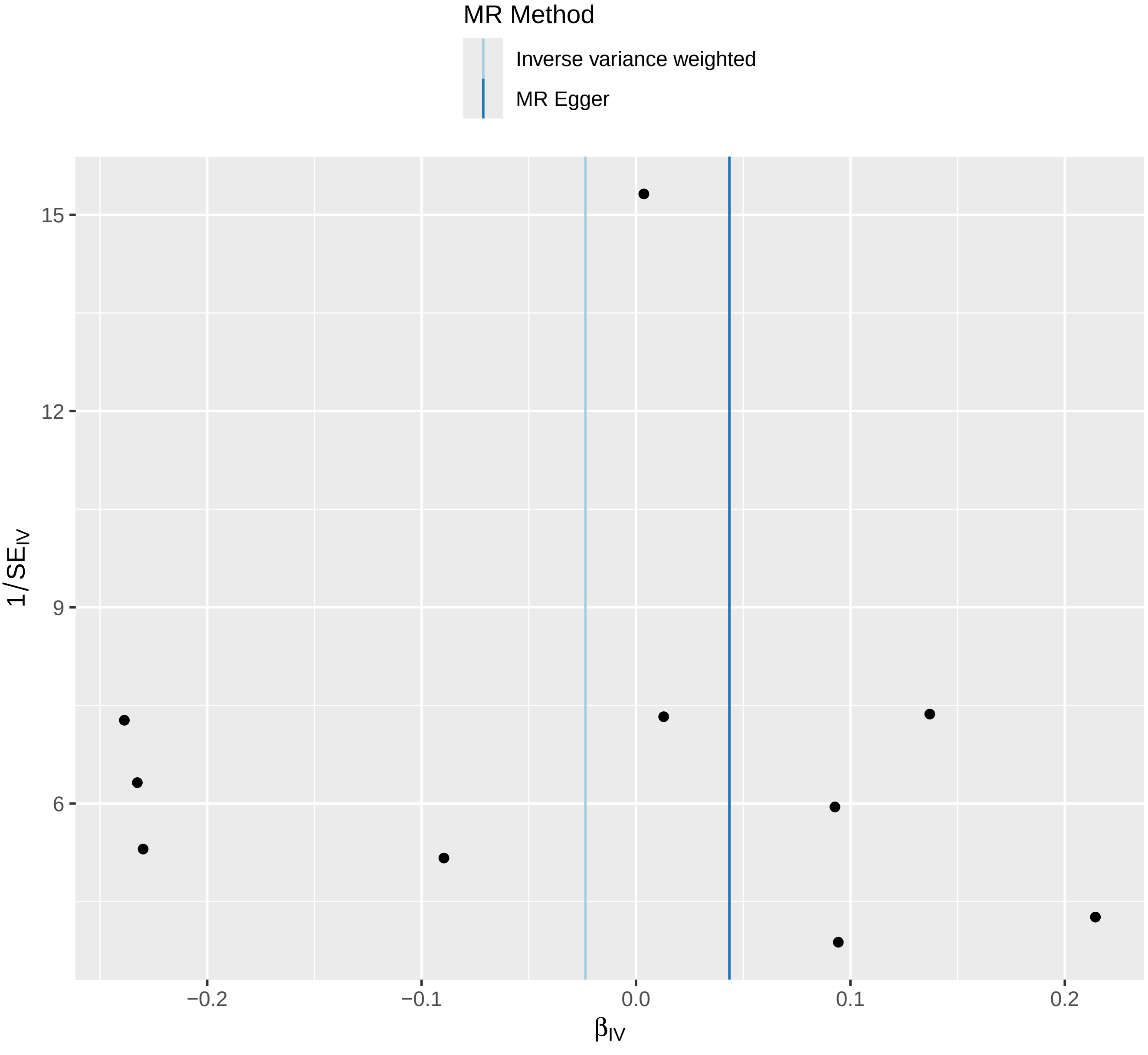
MR Method
Inverse variance weighted
MR Egger
15
12
9
6
1/SE$_{IV}$
−0.2
−0.1
0.0
0.1
0.2
β$_{IV}$

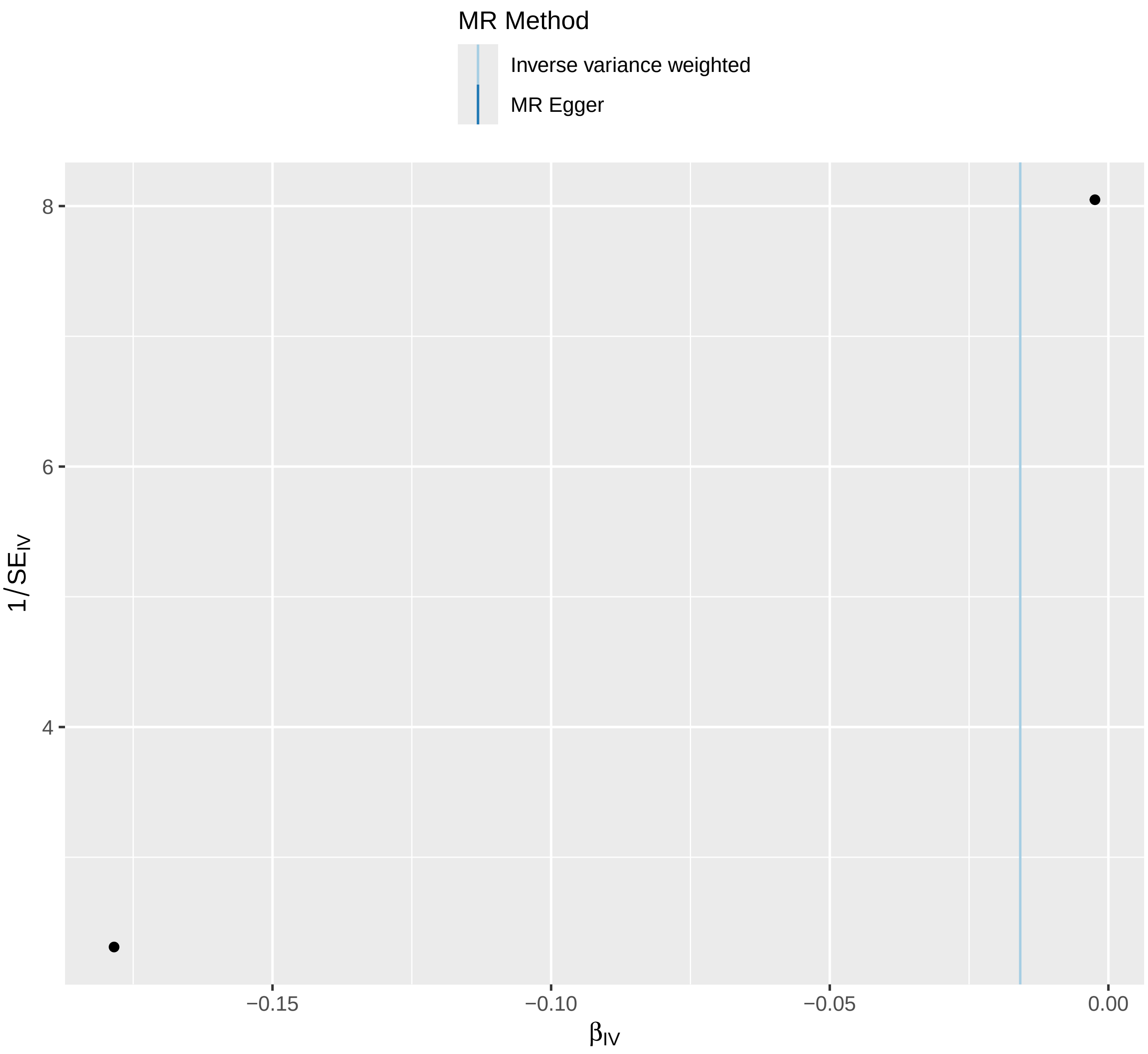
MR Method
Inverse variance weighted
MR Egger
8
6
4
1/SE_IV
−0.15
−0.10
−0.05
0.00
β_IV

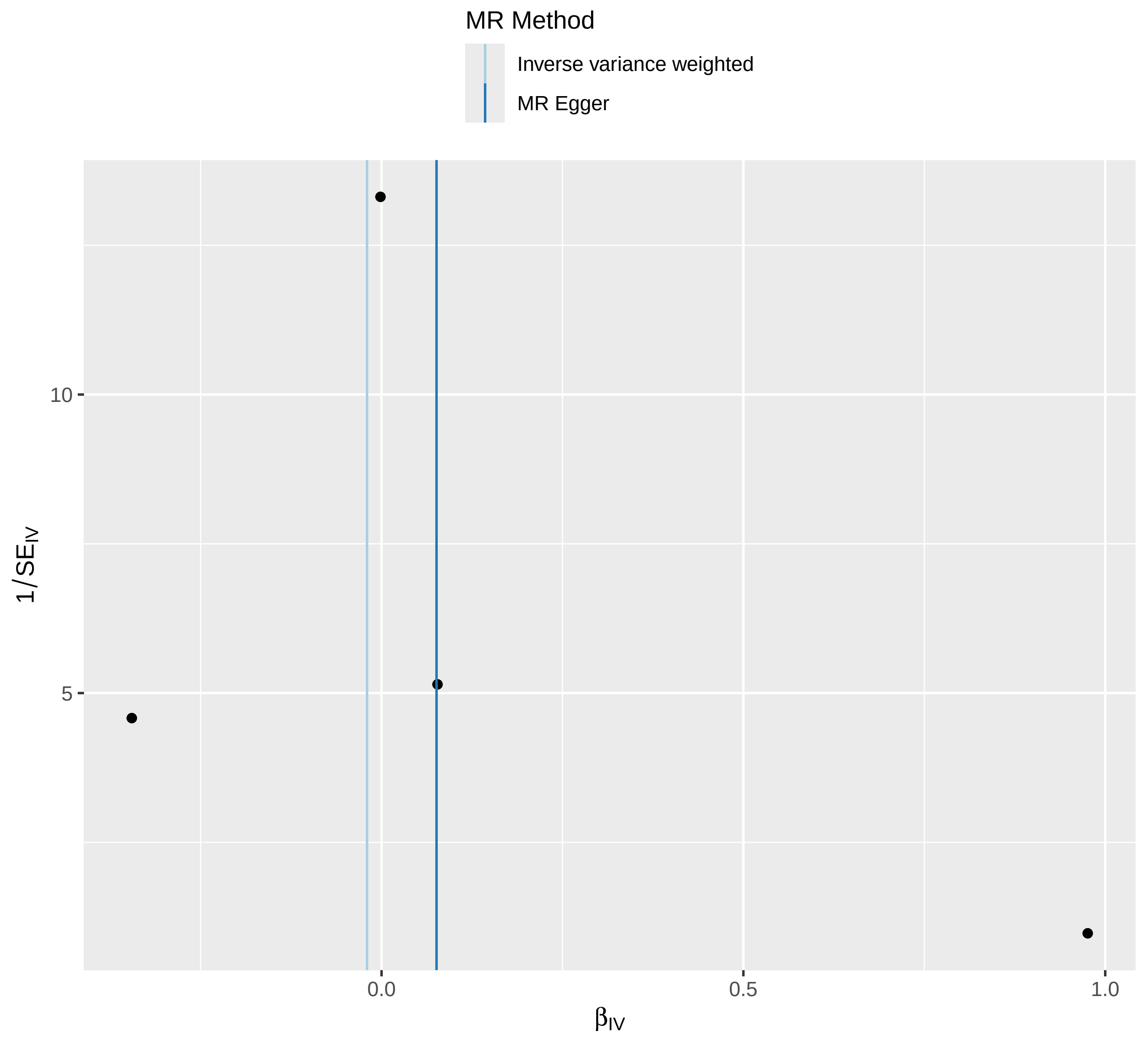
MR Method
Inverse variance weighted
MR Egger
10
5
0.0
0.5
1.0
1/SE_IV
β_IV

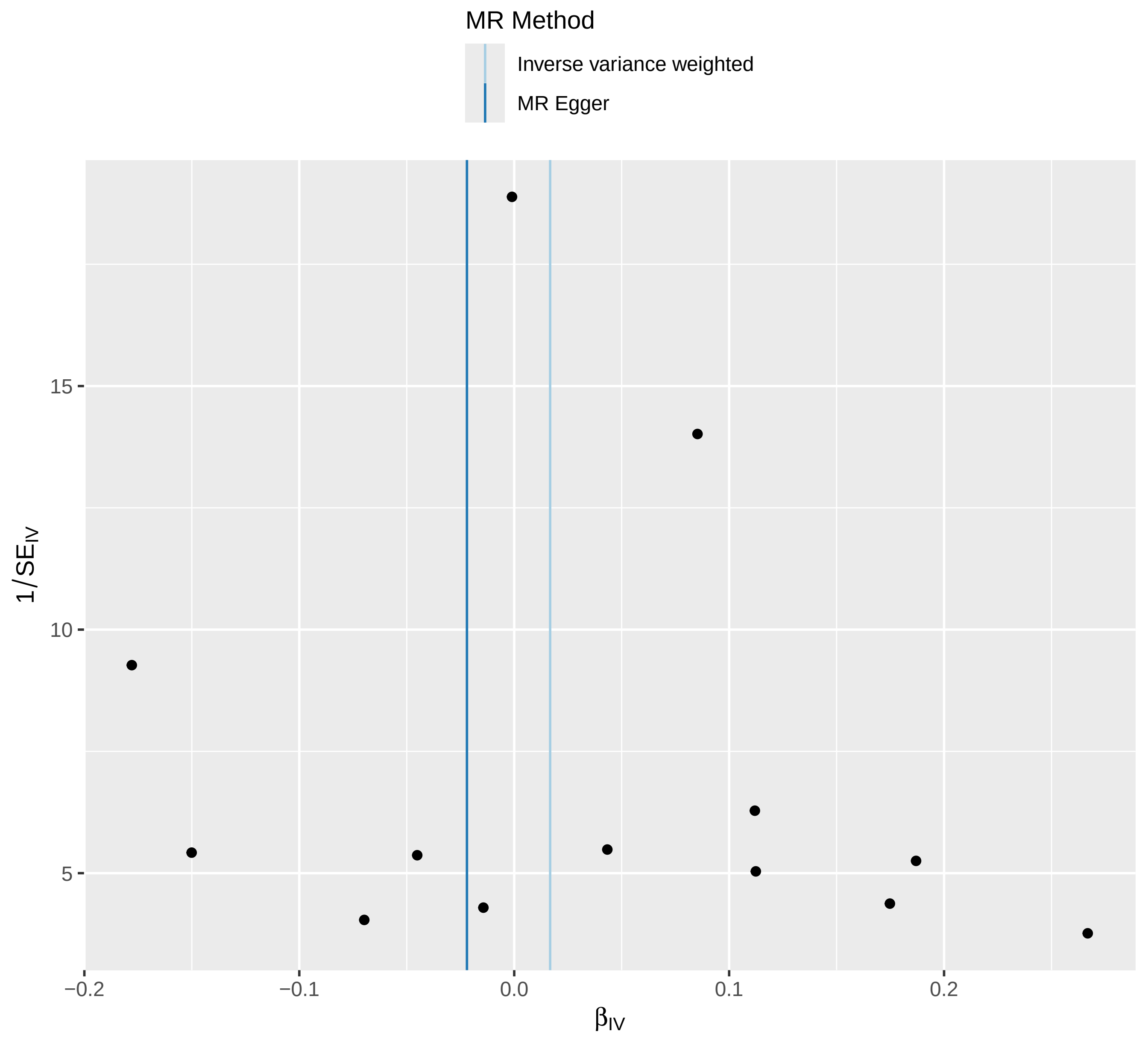
MR Method
Inverse variance weighted
MR Egger
15
10
5
1/SE$_{IV}$
−0.2
−0.1
0.0
0.1
0.2
β$_{IV}$

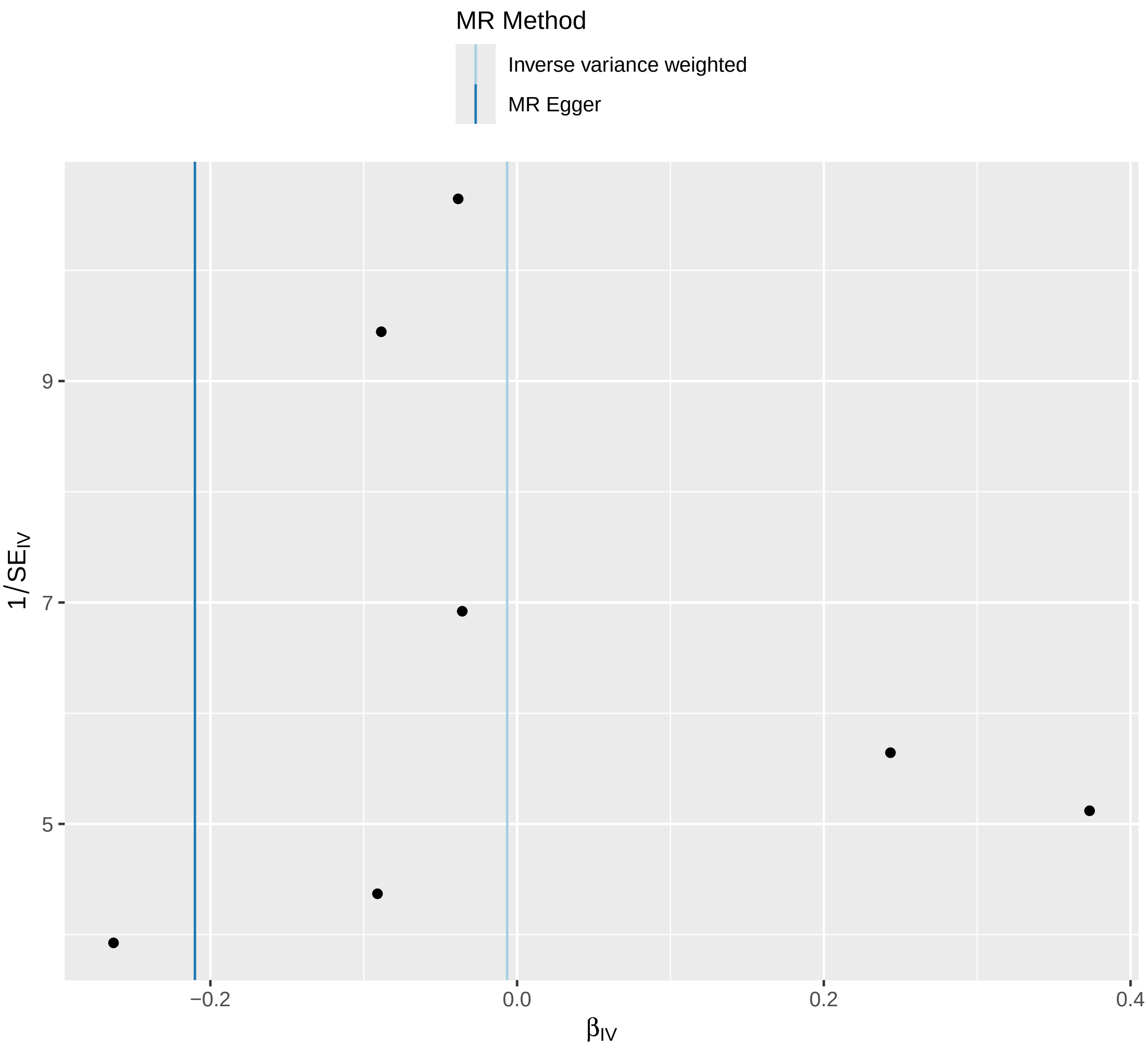

MR Method
Inverse variance weighted
MR Egger
9
7
5
1/SE_IV
−0.2
0.0
0.2
0.4
β_IV

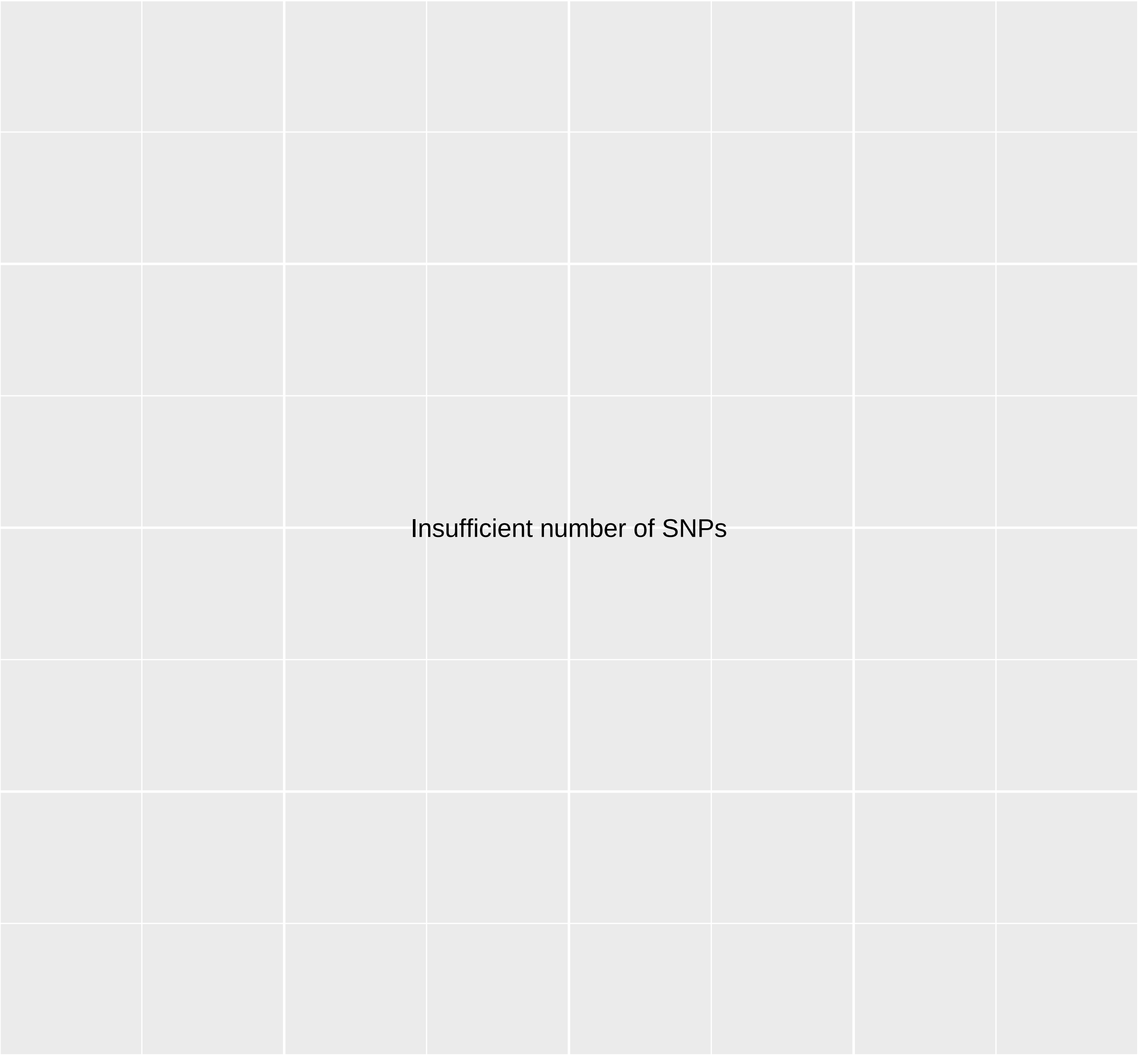
Insufficient number of SNPs

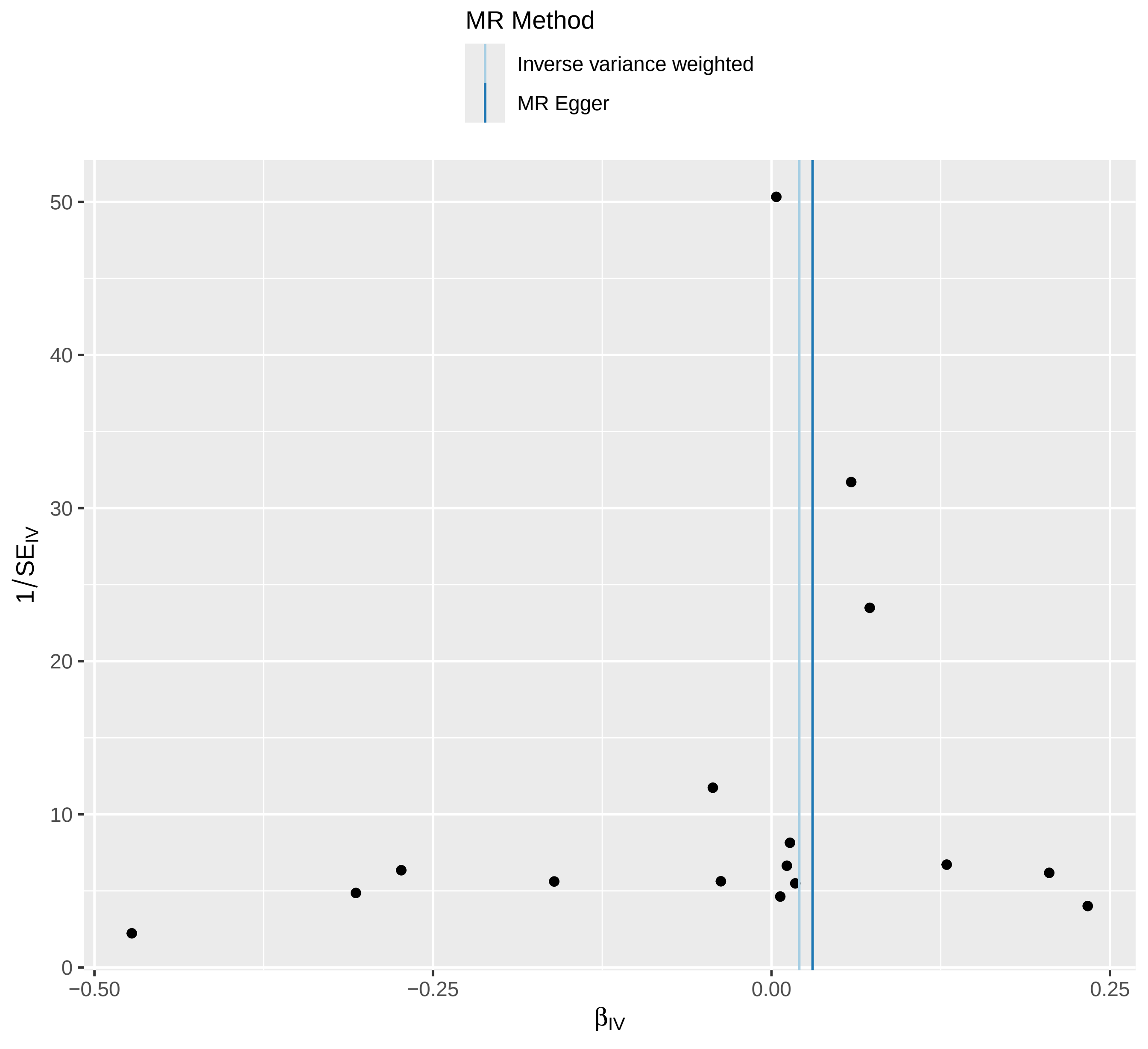

MR Method
Inverse variance weighted
MR Egger
50
40
30
20
10
0
1/SE$_{IV}$
−0.50
−0.25
0.00
0.25
β$_{IV}$

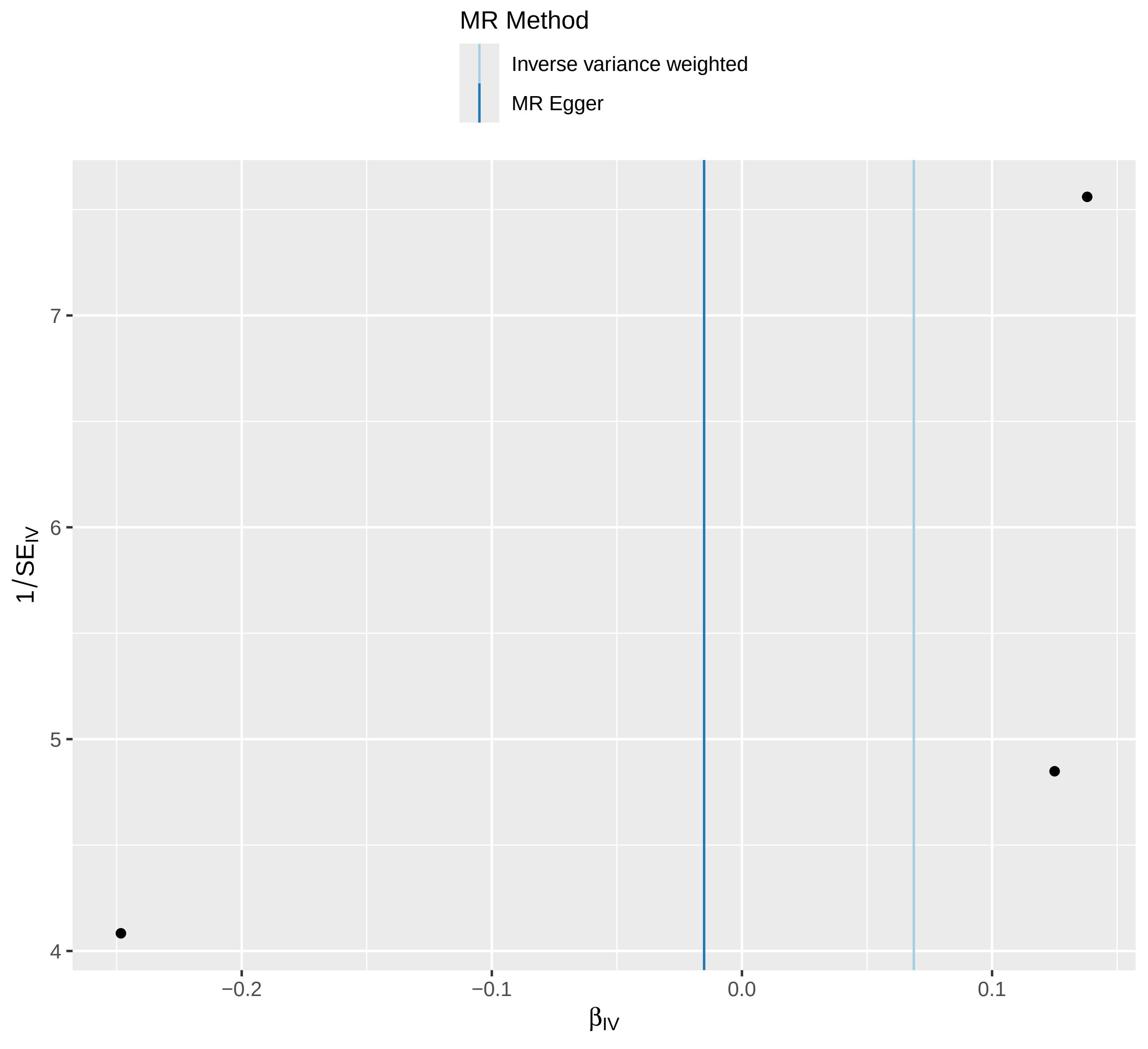
MR Method
Inverse variance weighted
MR Egger
7
6
5
4
1/SE_IV
−0.2
−0.1
0.0
0.1
β_IV

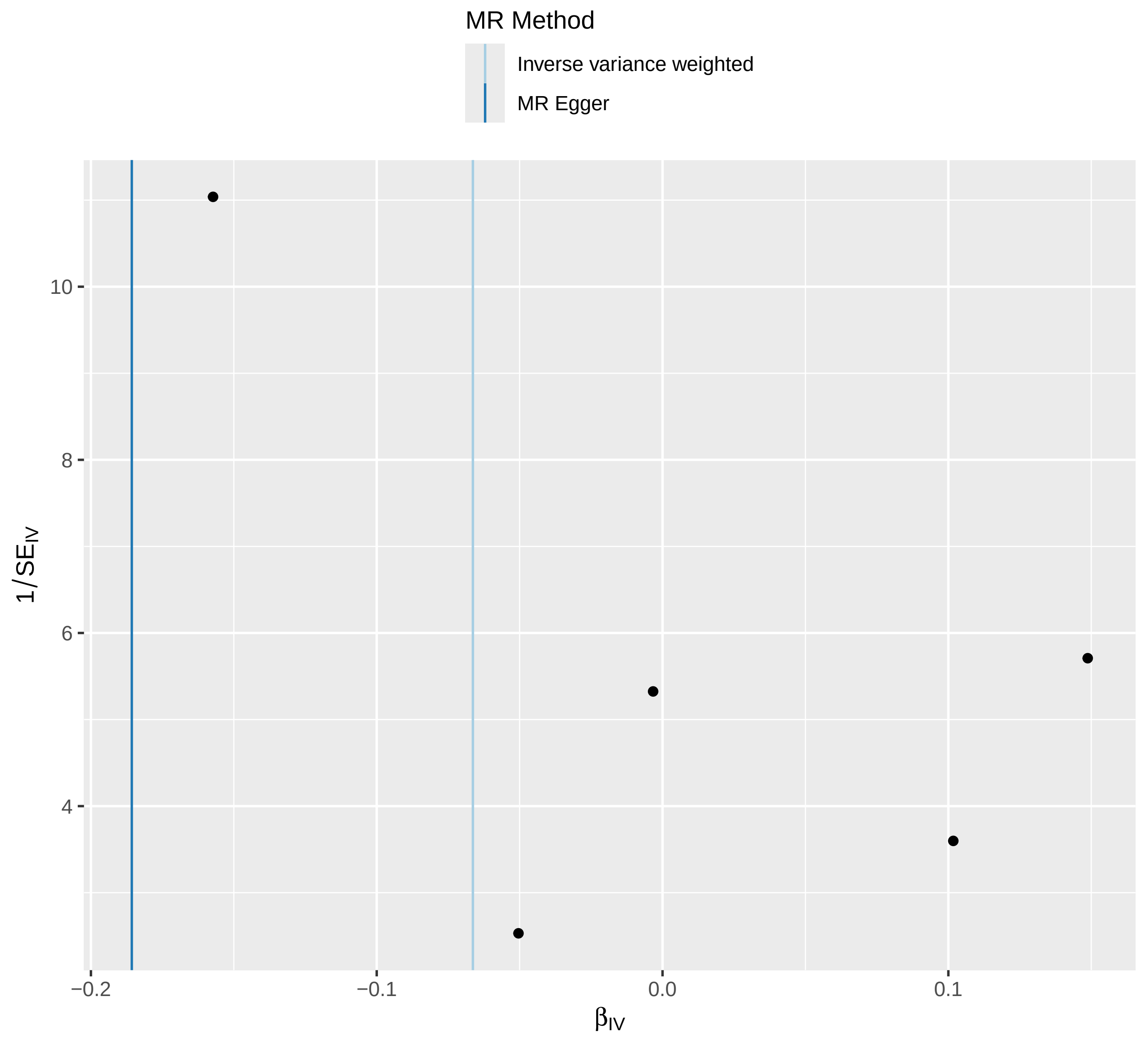

MR Method
Inverse variance weighted
MR Egger
1/SE$_{IV}$
10
8
6
4
−0.2
−0.1
0.0
0.1
β$_{IV}$

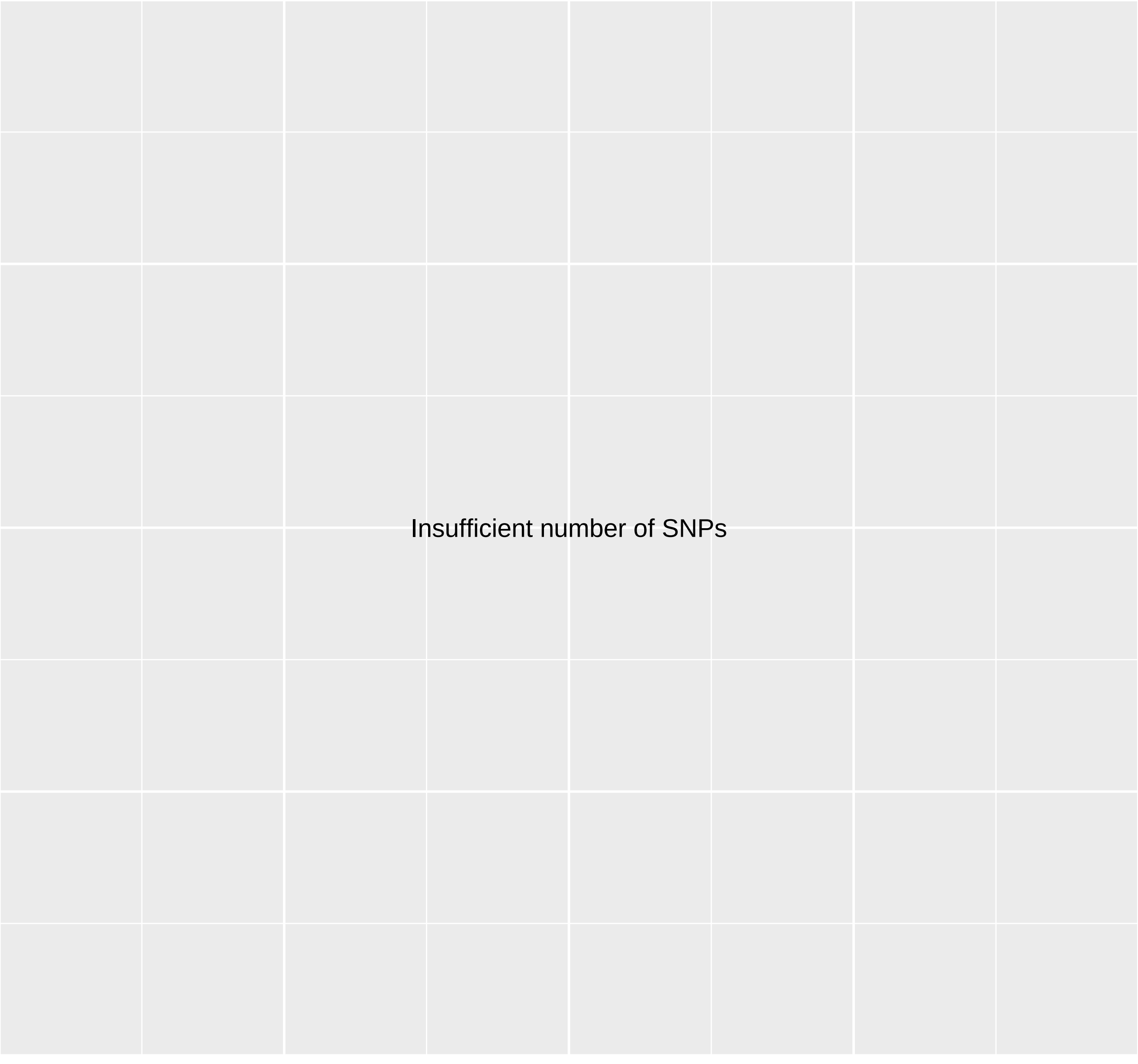
Insufficient number of SNPs

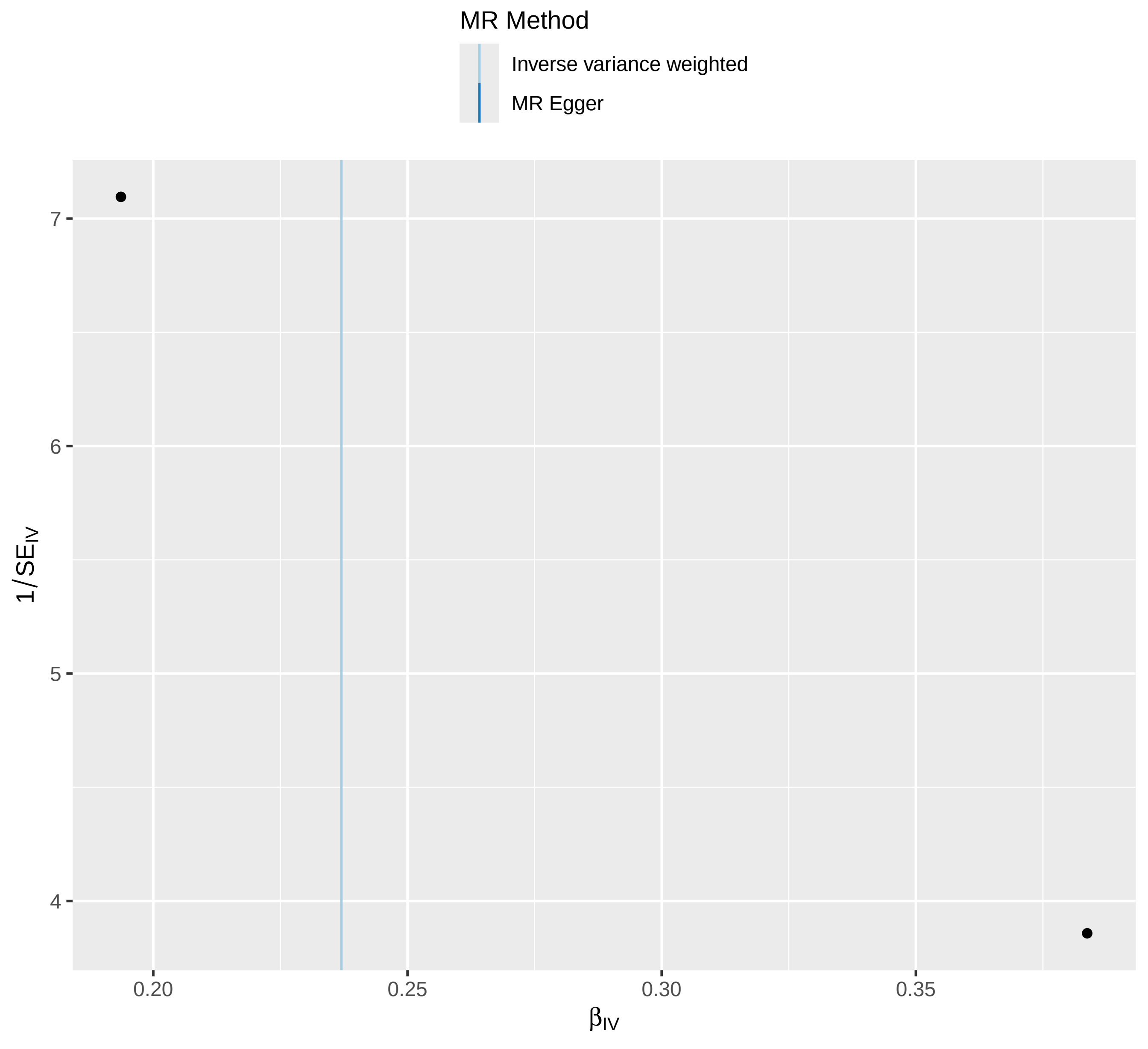
MR Method
Inverse variance weighted
MR Egger
7
6
5
4
$1/SE_{IV}$
0.20
0.25
0.30
0.35
$\beta_{IV}$

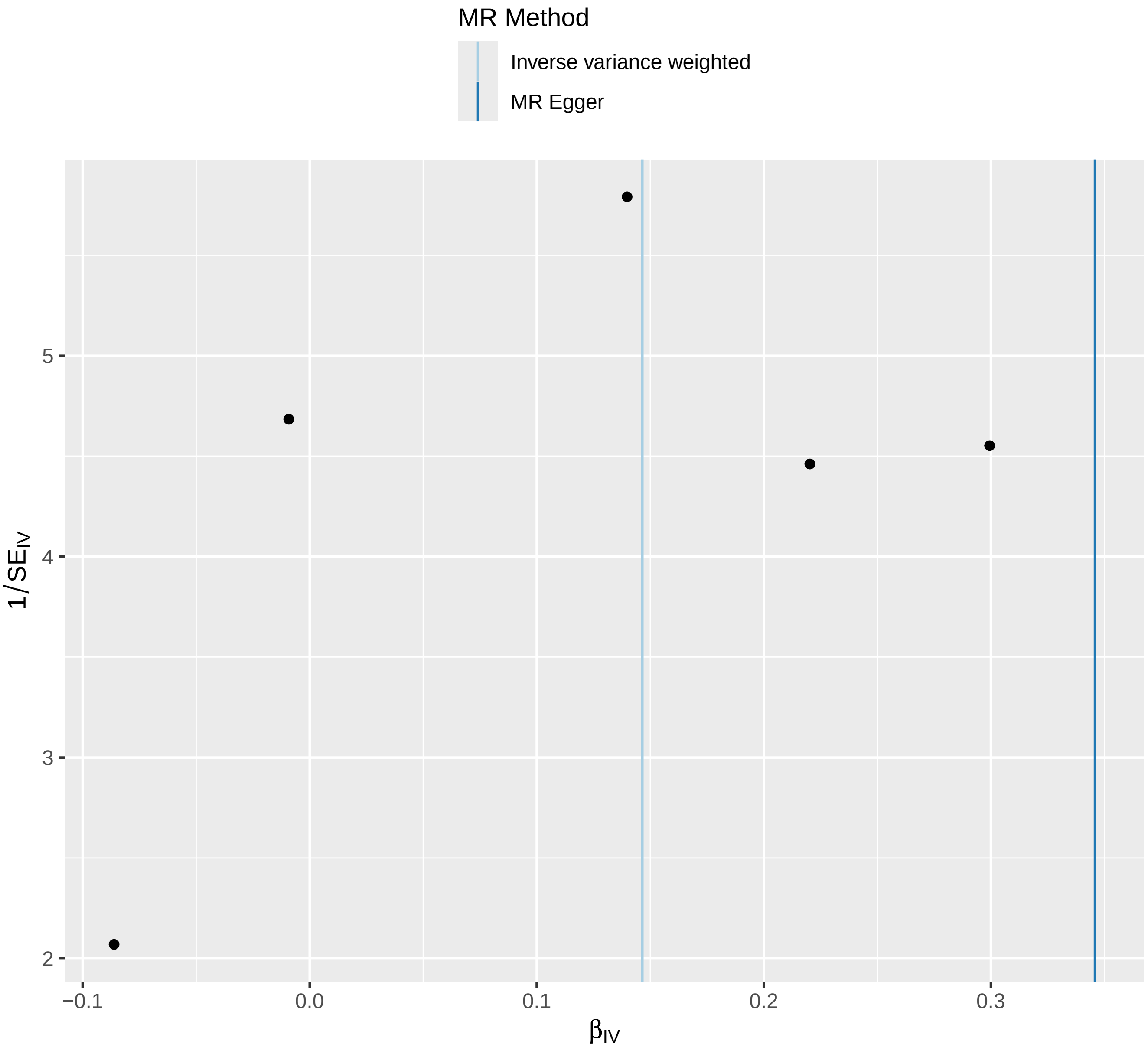
MR Method
Inverse variance weighted
MR Egger
1/SE_IV
5
4
3
2
−0.1
0.0
0.1
0.2
0.3
β_IV

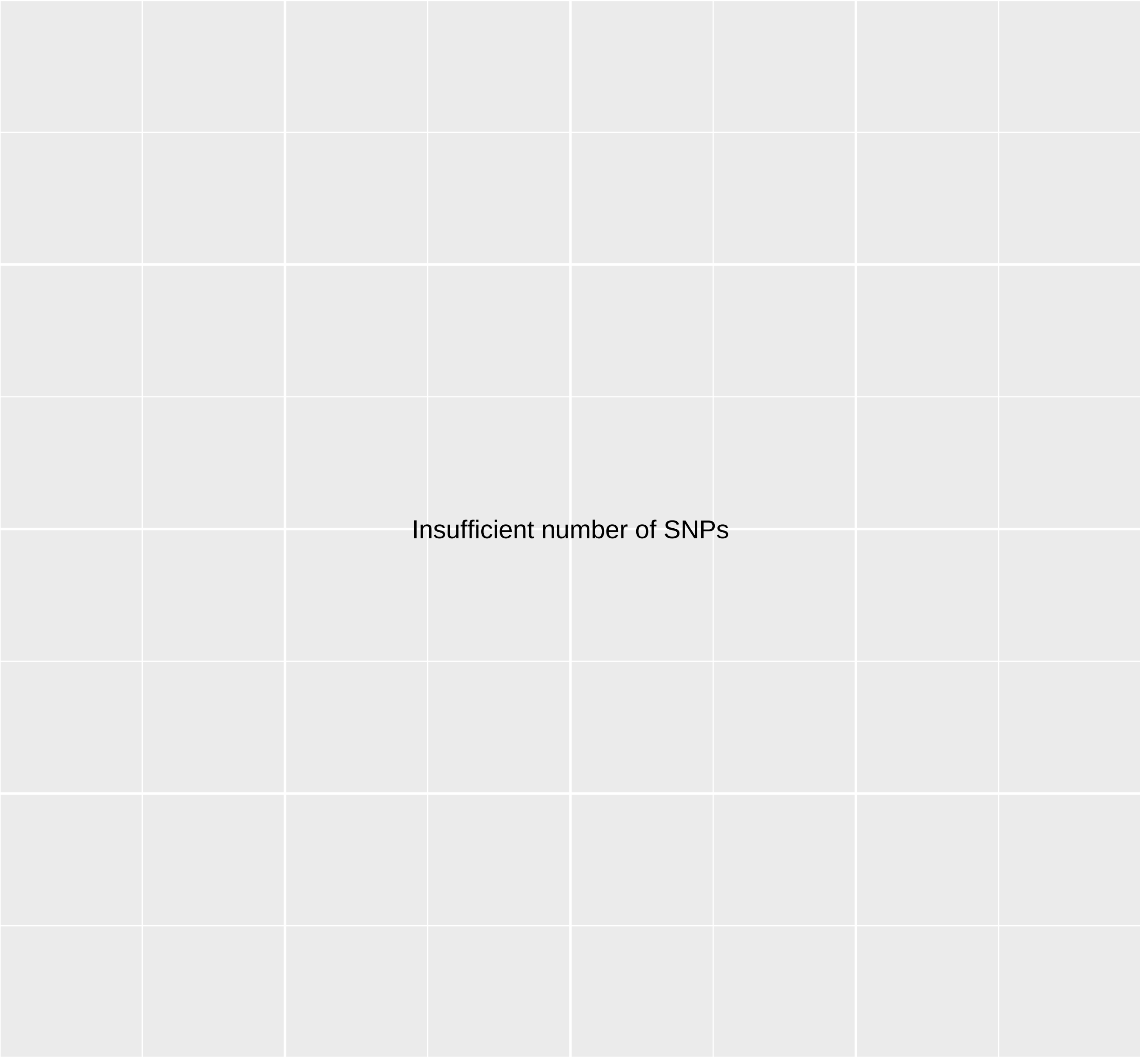
Insufficient number of SNPs

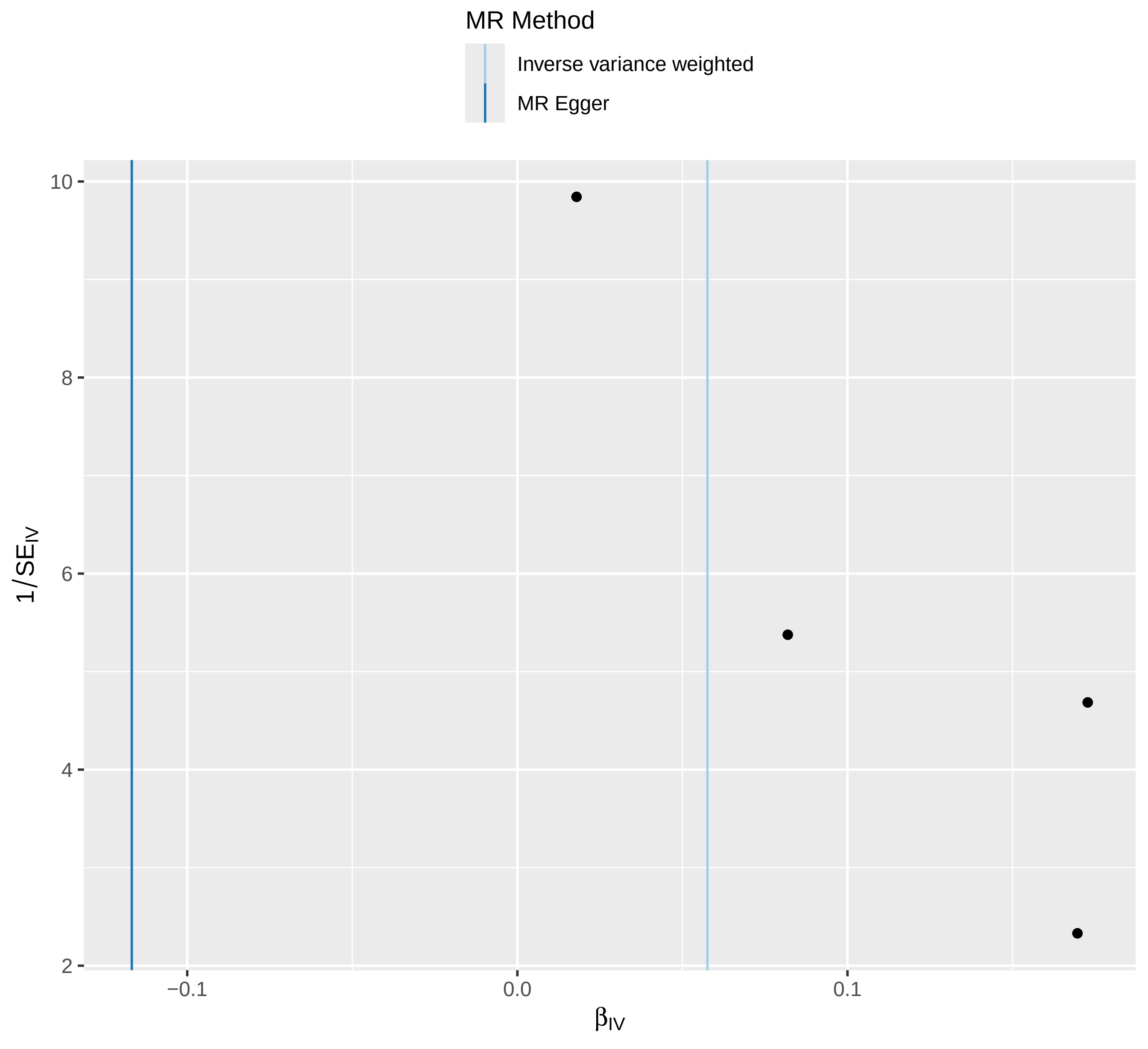
MR Method
Inverse variance weighted
MR Egger
10
8
6
4
2
−0.1
0.0
0.1
1/SE_IV
β_IV

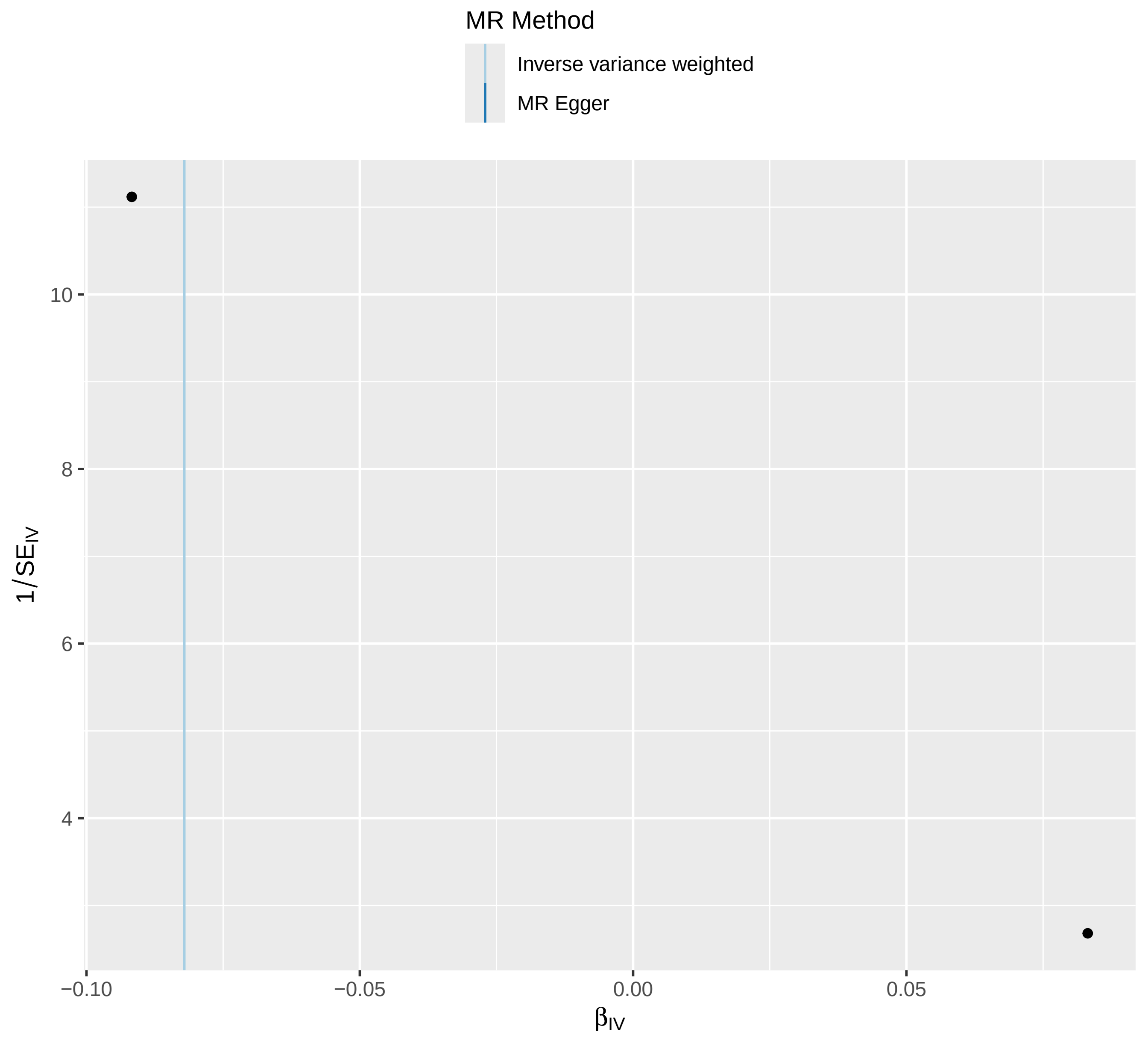
MR Method
Inverse variance weighted
MR Egger
10
8
6
4
$1/SE_{IV}$
−0.10
−0.05
0.00
0.05
$\beta_{IV}$

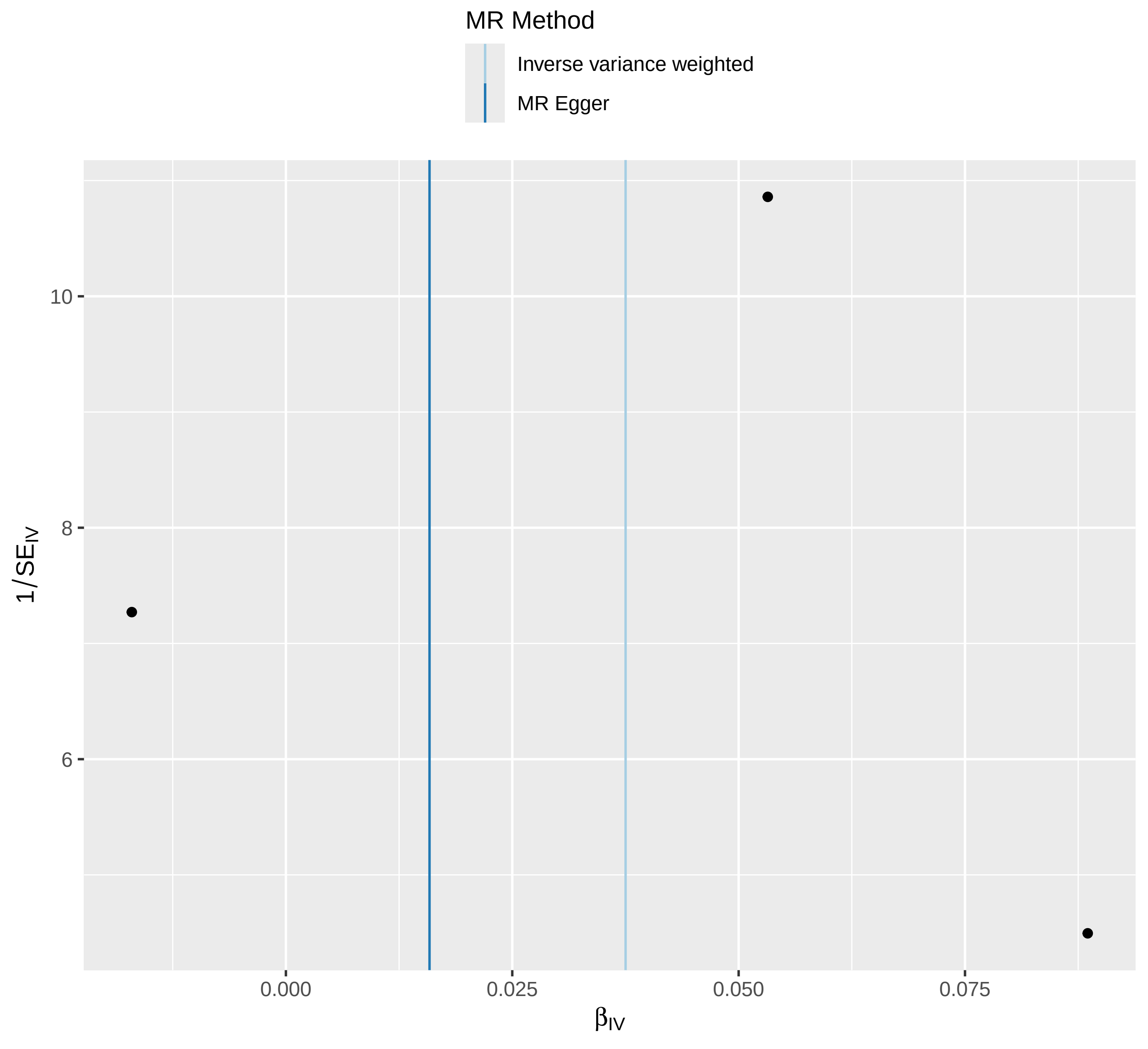
MR Method
Inverse variance weighted
MR Egger
10
8
6
1/SE_IV
0.000
0.025
0.050
0.075
β_IV

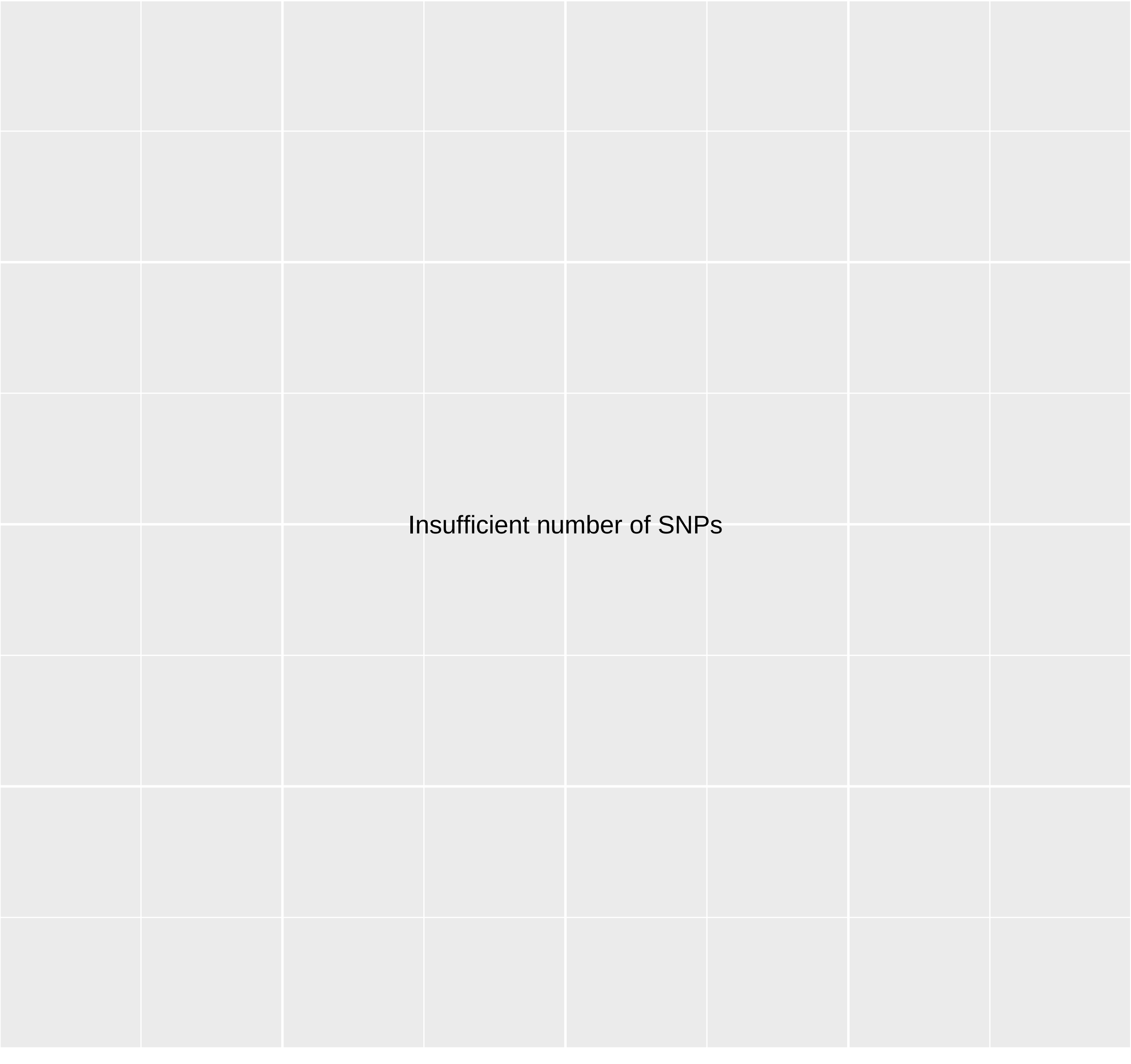
Insufficient number of SNPs

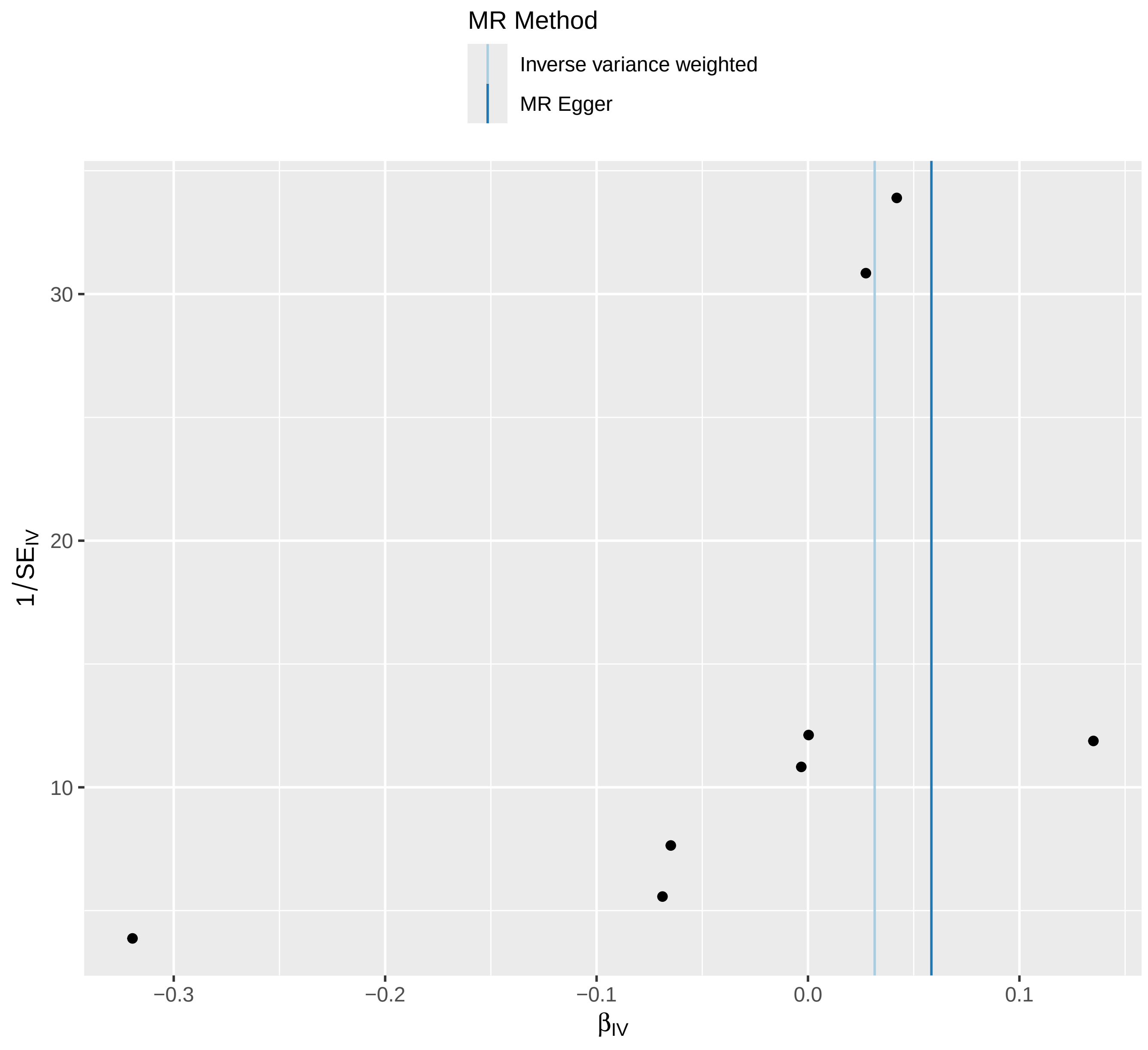
MR Method
Inverse variance weighted
MR Egger
30
20
10
1/$SE_{IV}$
−0.3
−0.2
−0.1
0.0
0.1
$\beta_{IV}$

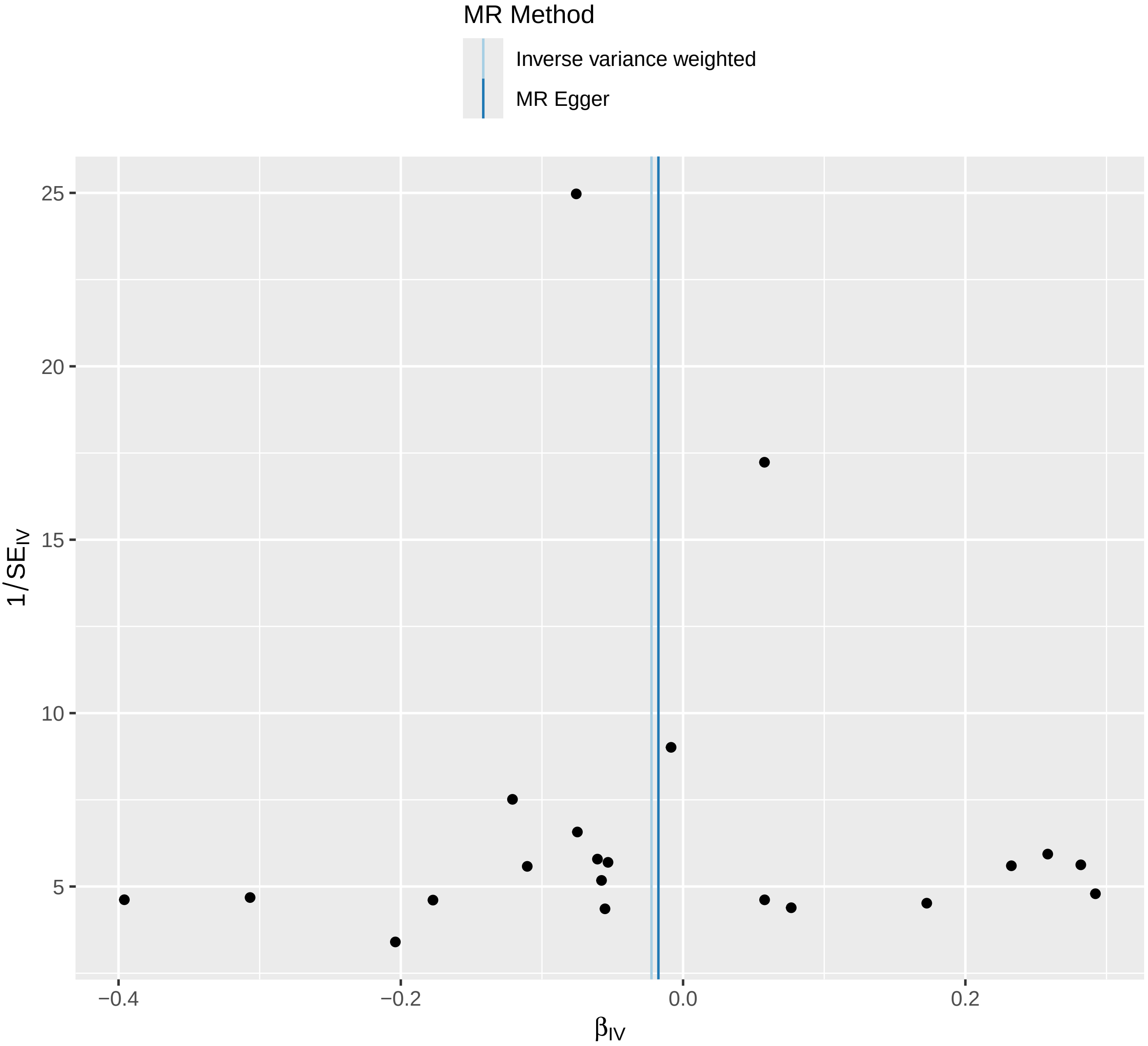
MR Method
Inverse variance weighted
MR Egger
25
20
15
10
5
1/SE_IV
−0.4
−0.2
0.0
0.2
β_IV

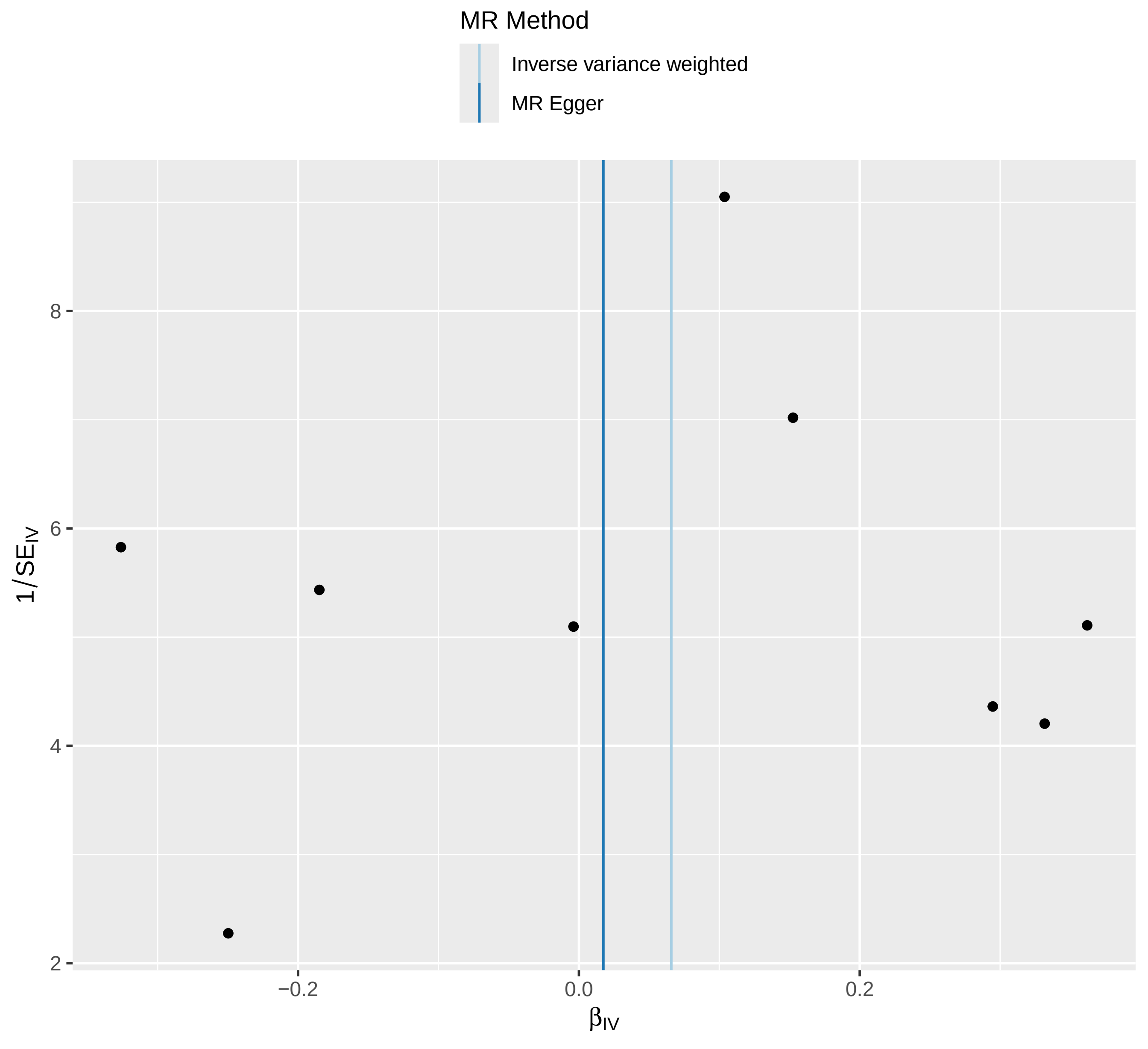
MR Method
Inverse variance weighted
MR Egger
8
6
4
2
1/SE$_{IV}$
−0.2
0.0
0.2
β$_{IV}$

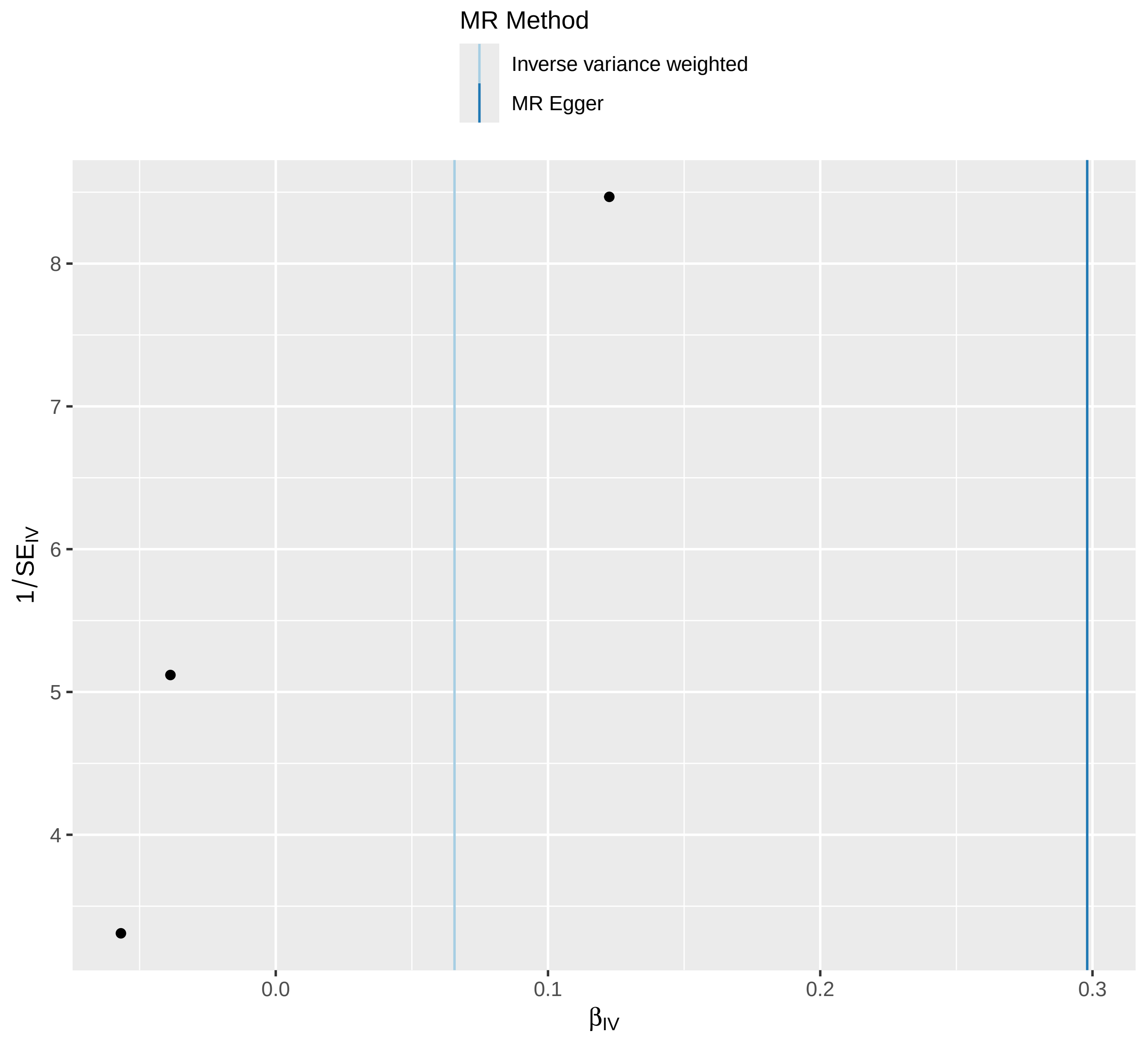
MR Method
Inverse variance weighted
MR Egger
1/SE$_{IV}$
8
7
6
5
4
0.0
0.1
0.2
0.3
$\beta_{IV}$

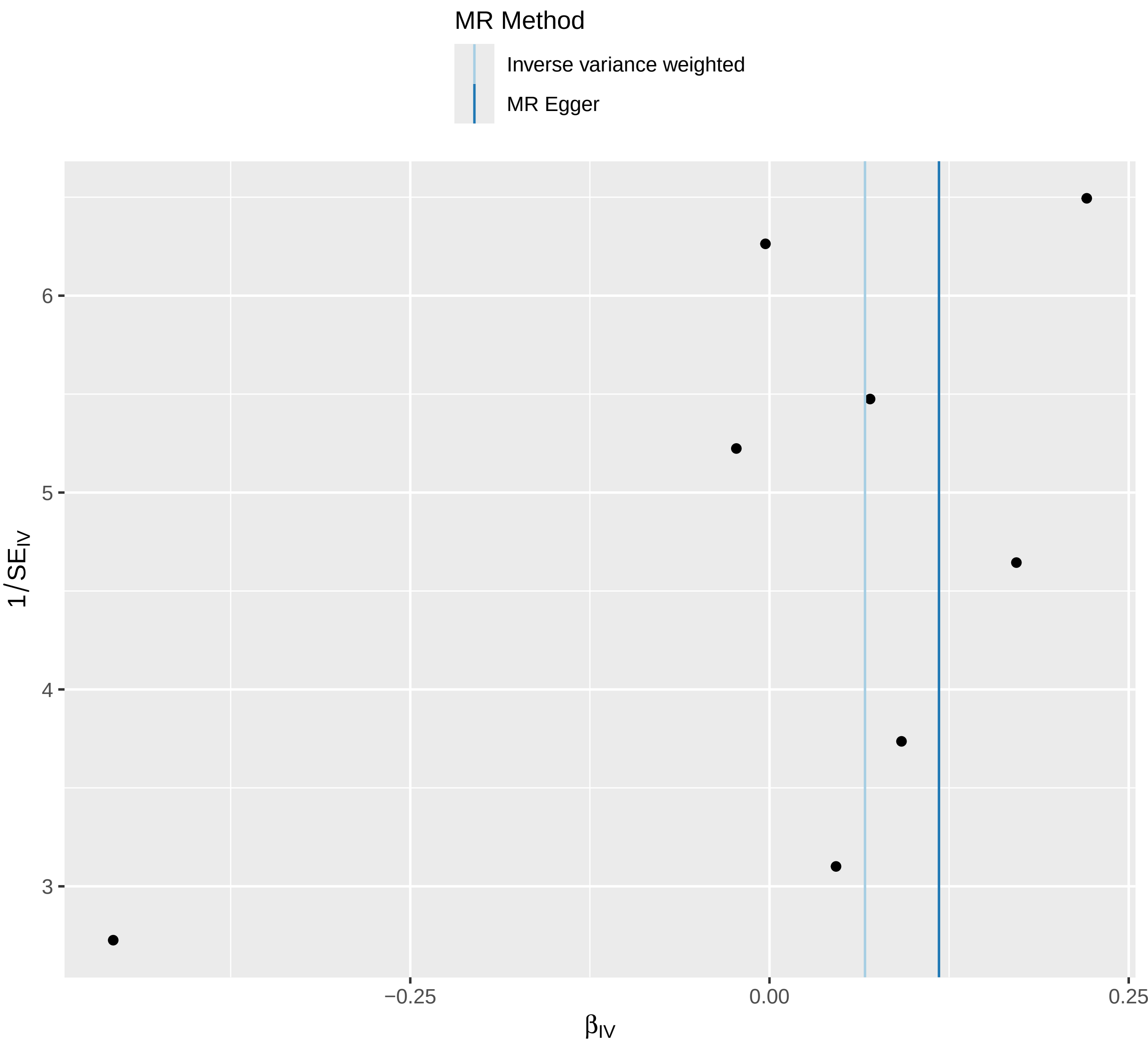
MR Method
Inverse variance weighted
MR Egger
6
5
4
3
−0.25
0.00
0.25
$\beta_{IV}$
$1/SE_{IV}$

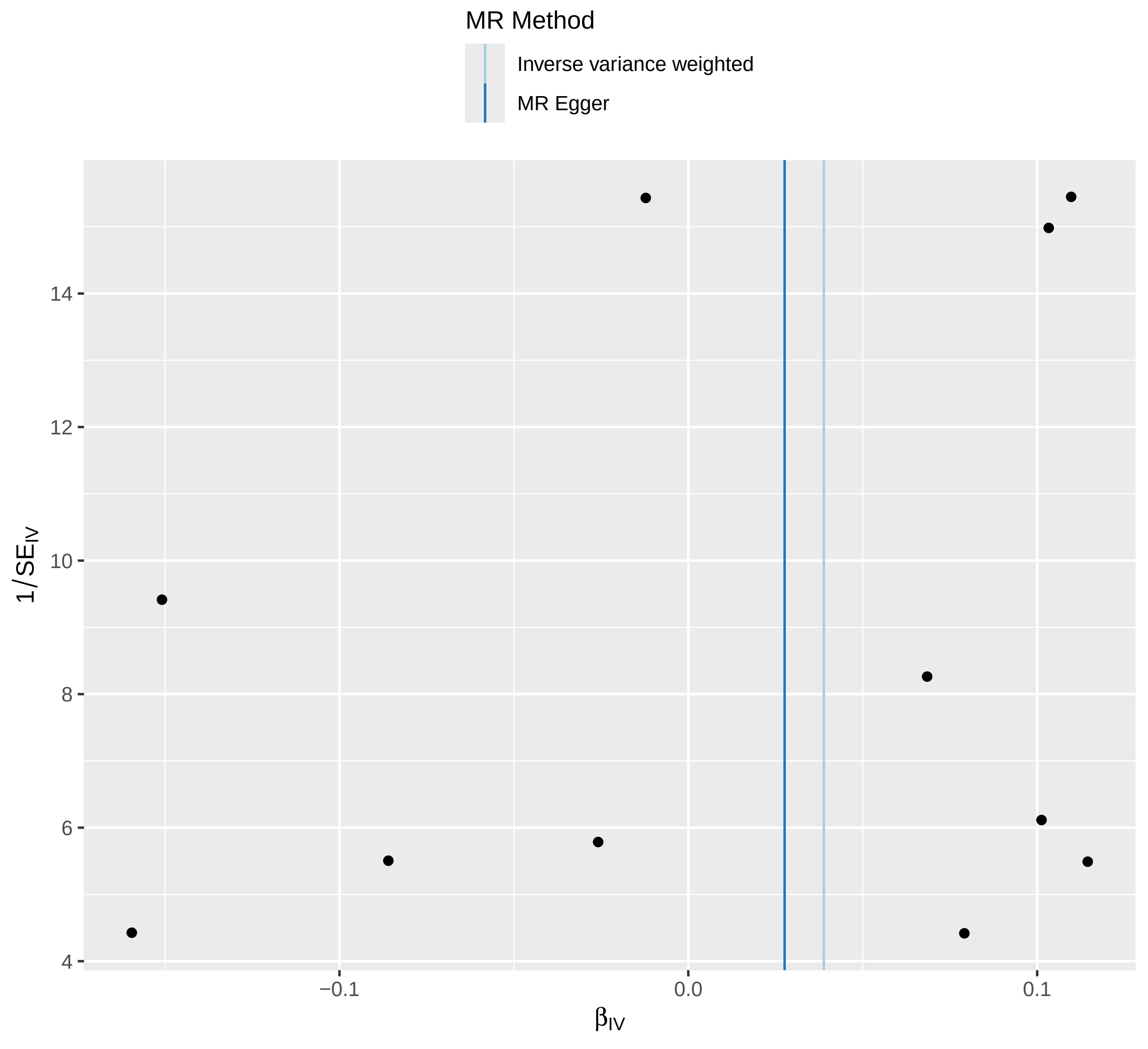
MR Method
Inverse variance weighted
MR Egger
14
12
10
8
6
4
1/SE_IV
−0.1
0.0
0.1
β_IV

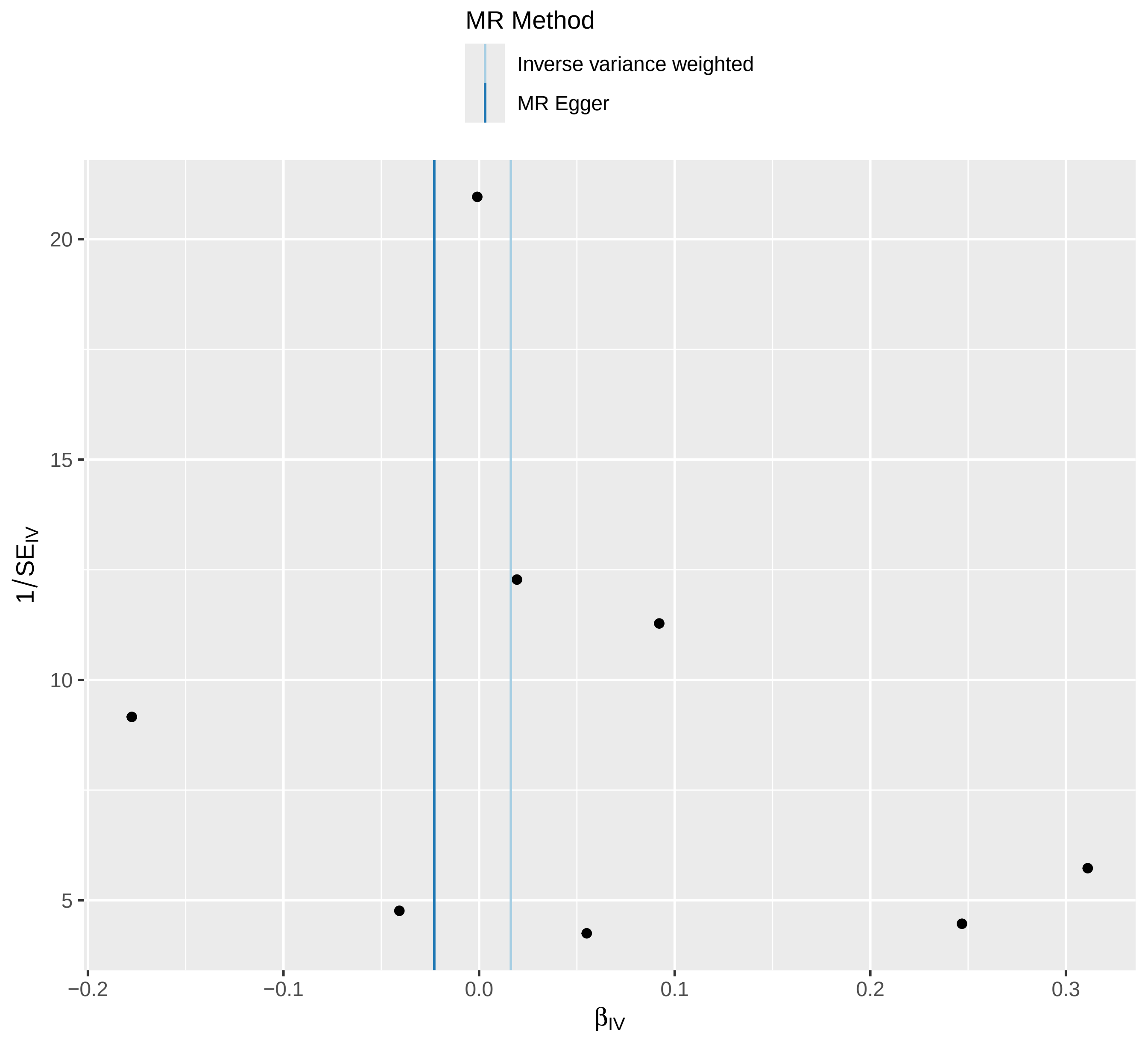

MR Method
Inverse variance weighted
MR Egger
20
15
10
5
1/SE$_{IV}$
−0.2
−0.1
0.0
0.1
0.2
0.3
β$_{IV}$

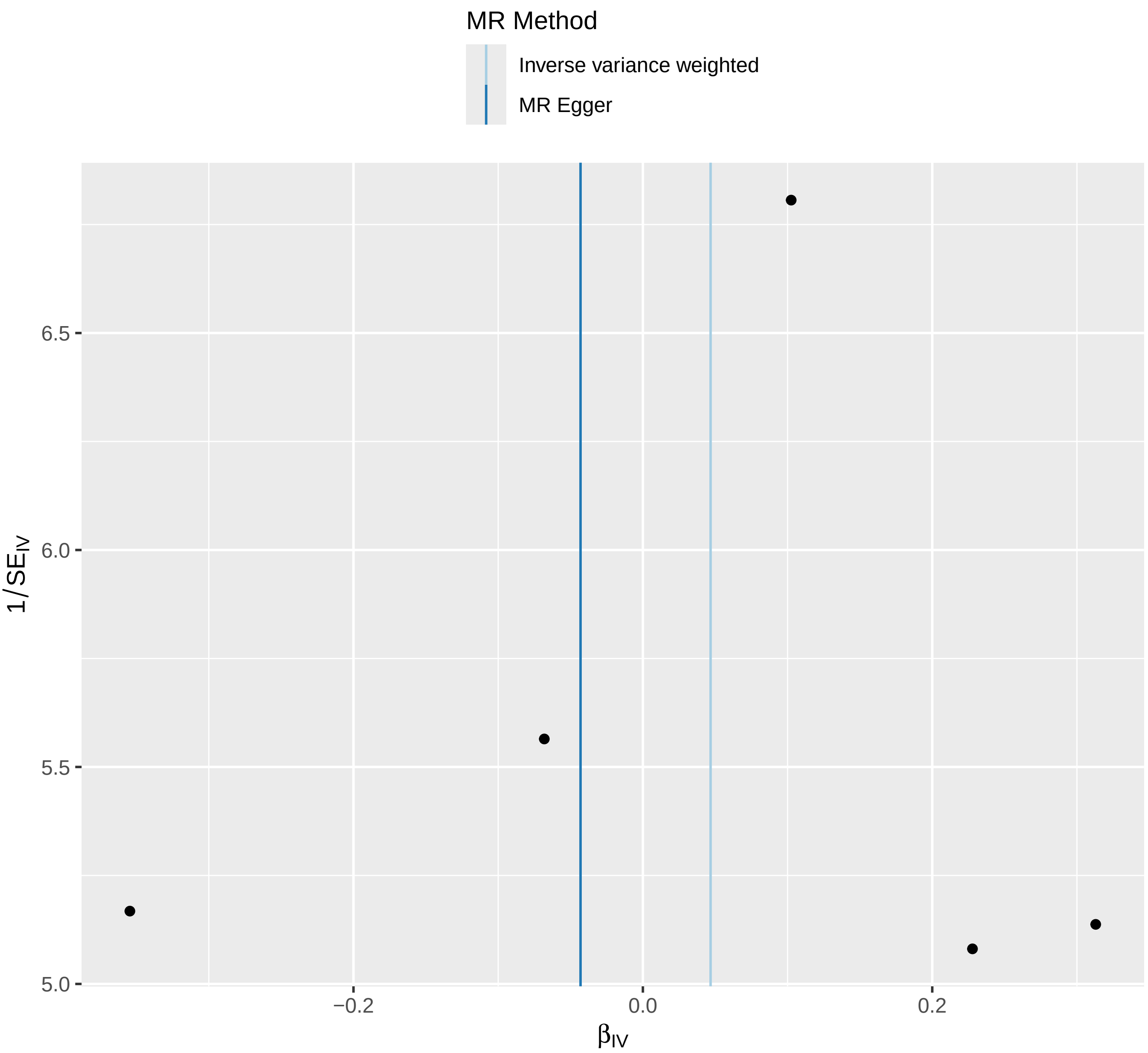
MR Method
Inverse variance weighted
MR Egger
6.5
6.0
5.5
5.0
1/SE$_{IV}$
−0.2
0.0
0.2
β$_{IV}$

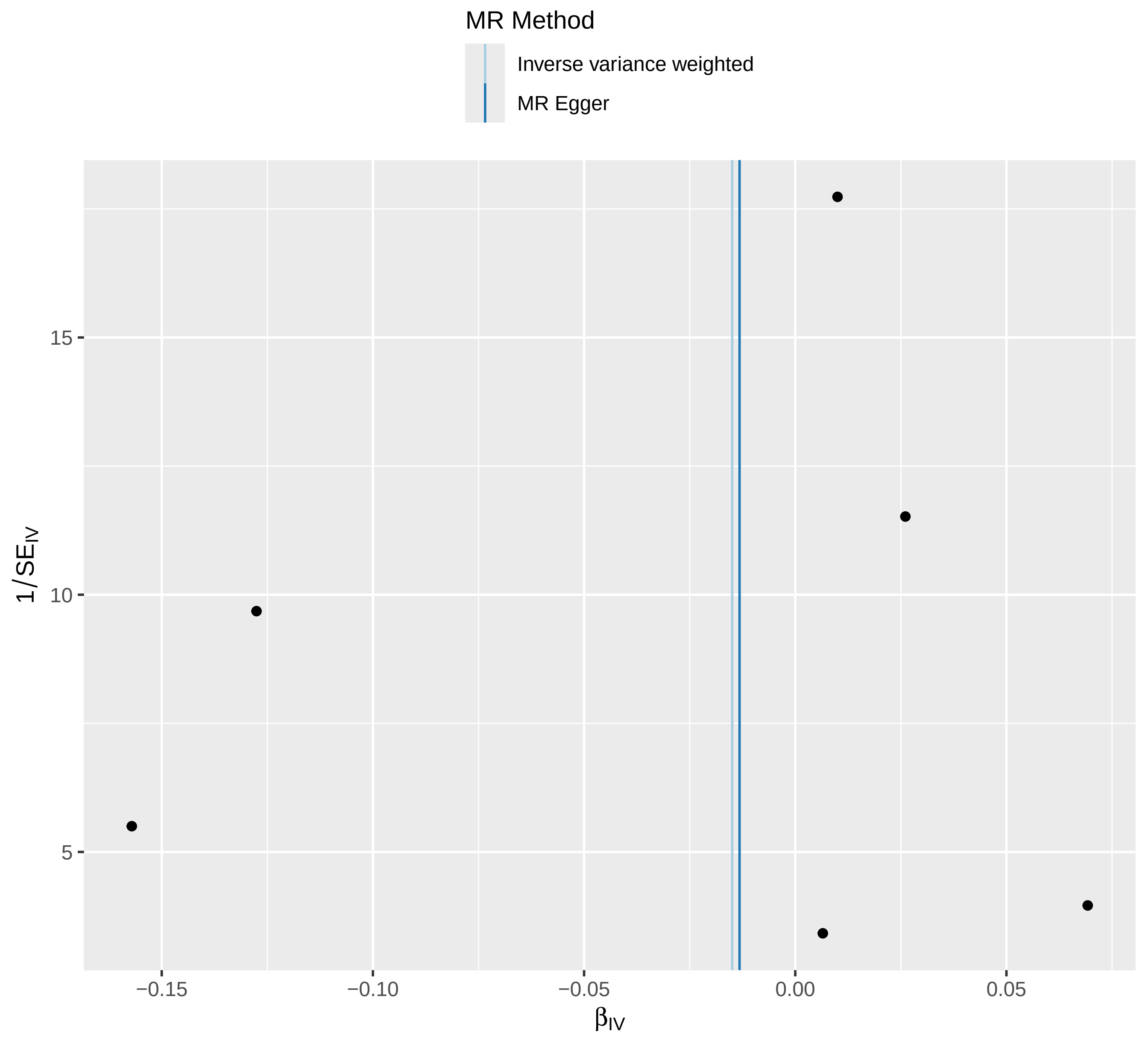

MR Method
Inverse variance weighted
MR Egger
15
10
5
1/SE_IV
−0.15
−0.10
−0.05
0.00
0.05
β_IV

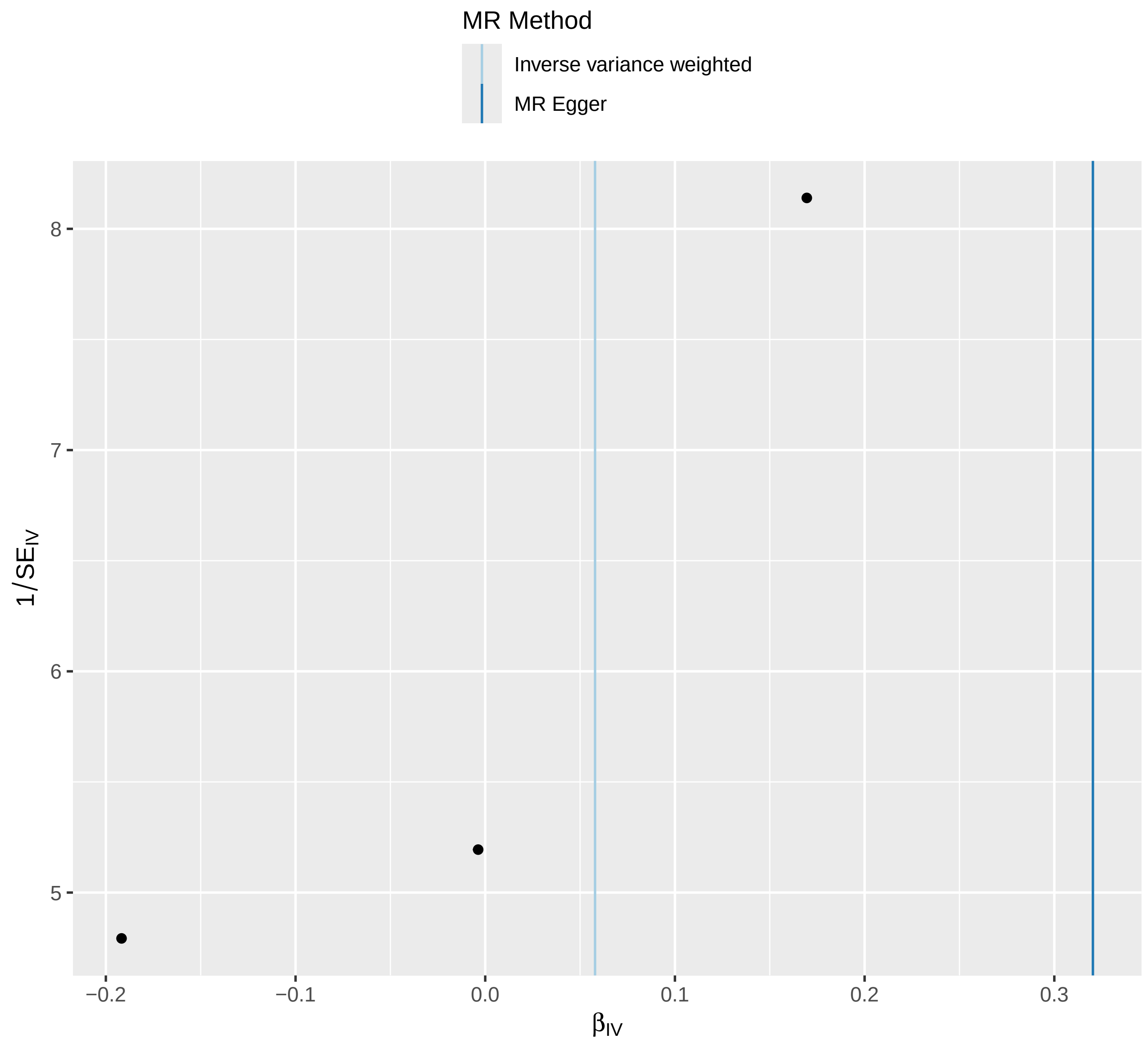

MR Method
Inverse variance weighted
MR Egger
8
7
6
5
$1/SE_{IV}$
−0.2
−0.1
0.0
0.1
0.2
0.3
$\beta_{IV}$

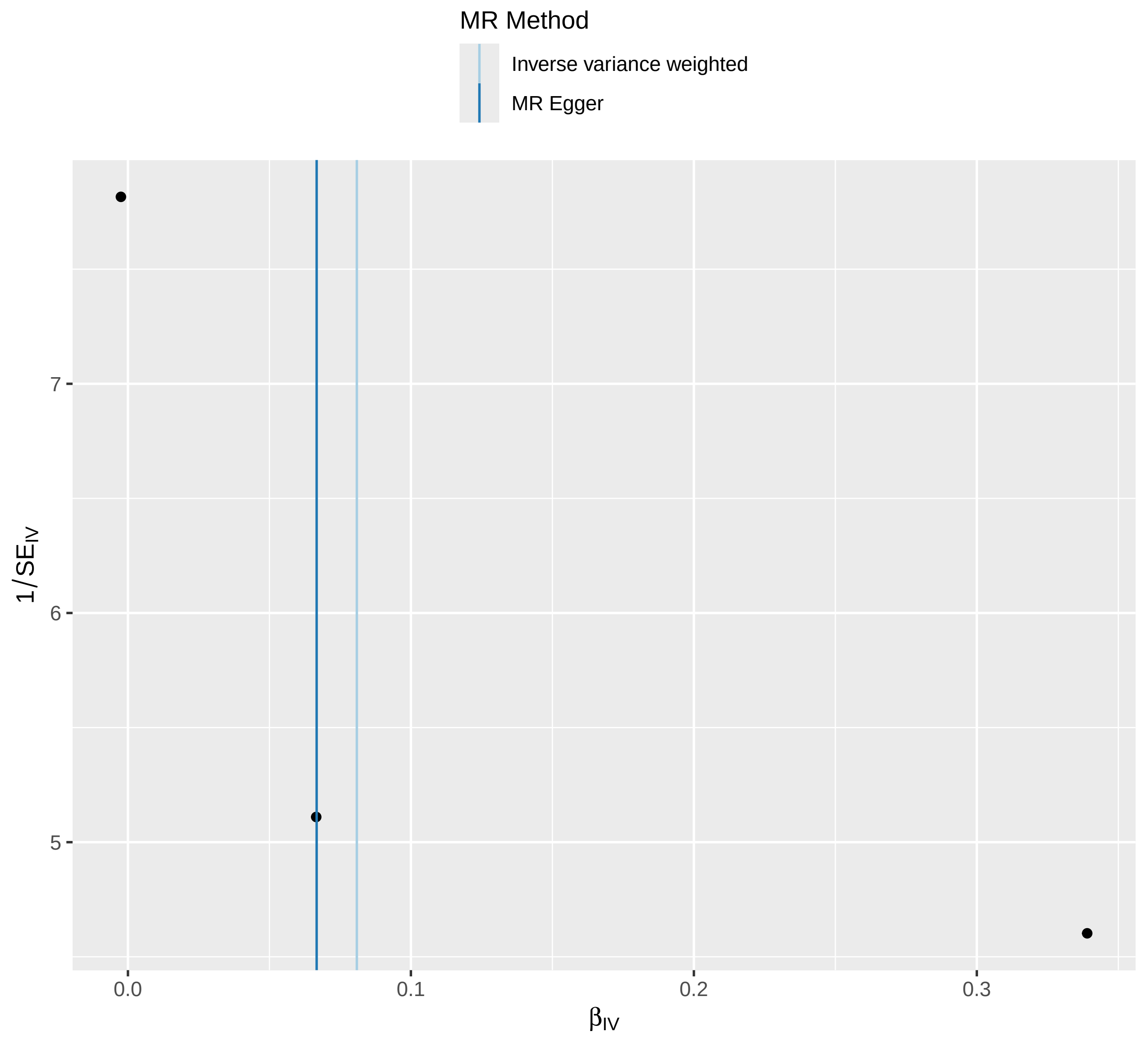
MR Method
Inverse variance weighted
MR Egger
7
6
5
1/SE$_{IV}$
0.0
0.1
0.2
0.3
β$_{IV}$

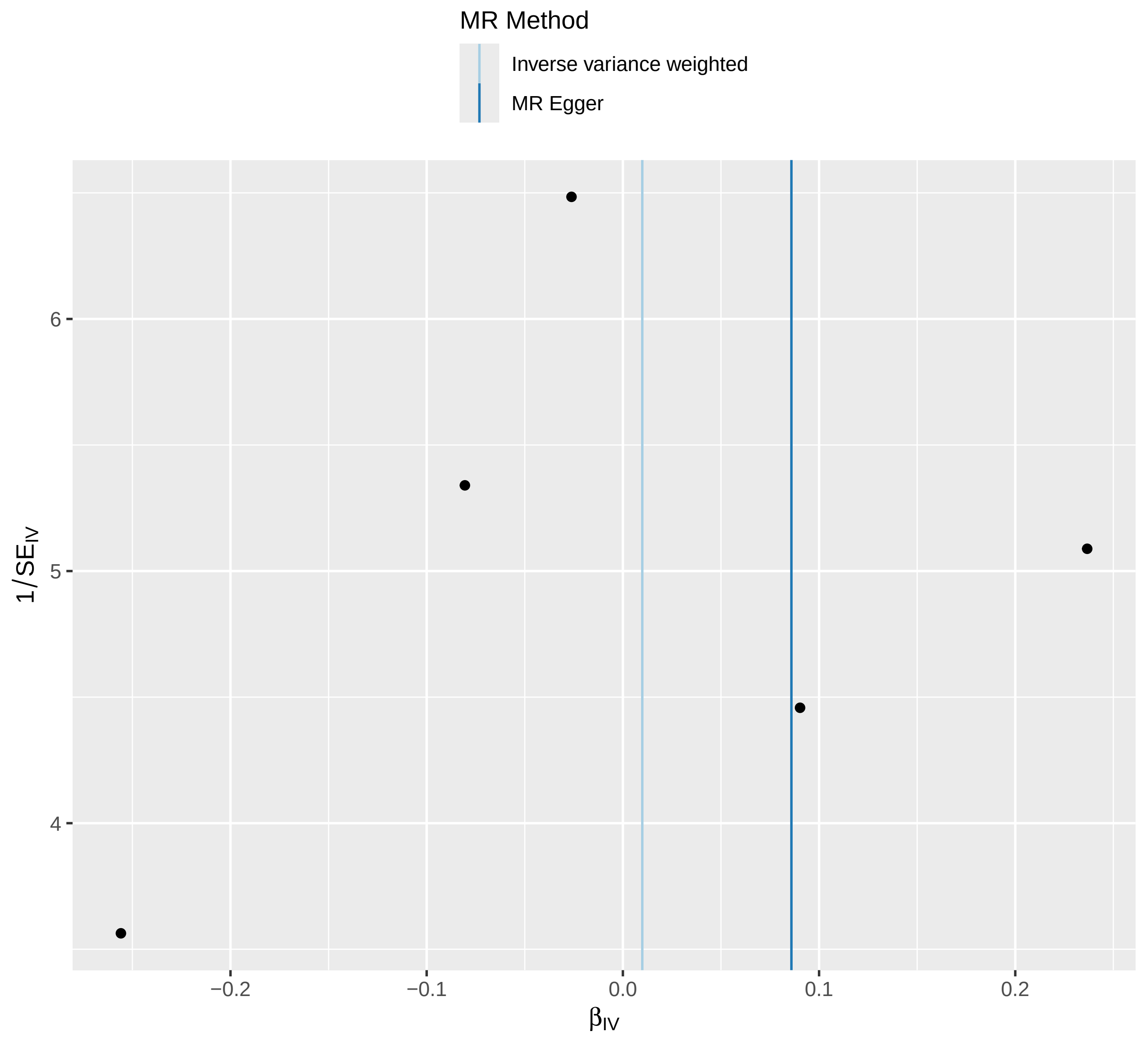

MR Method
Inverse variance weighted
MR Egger
6
5
4
1/SE$_{IV}$
−0.2
−0.1
0.0
0.1
0.2
β$_{IV}$

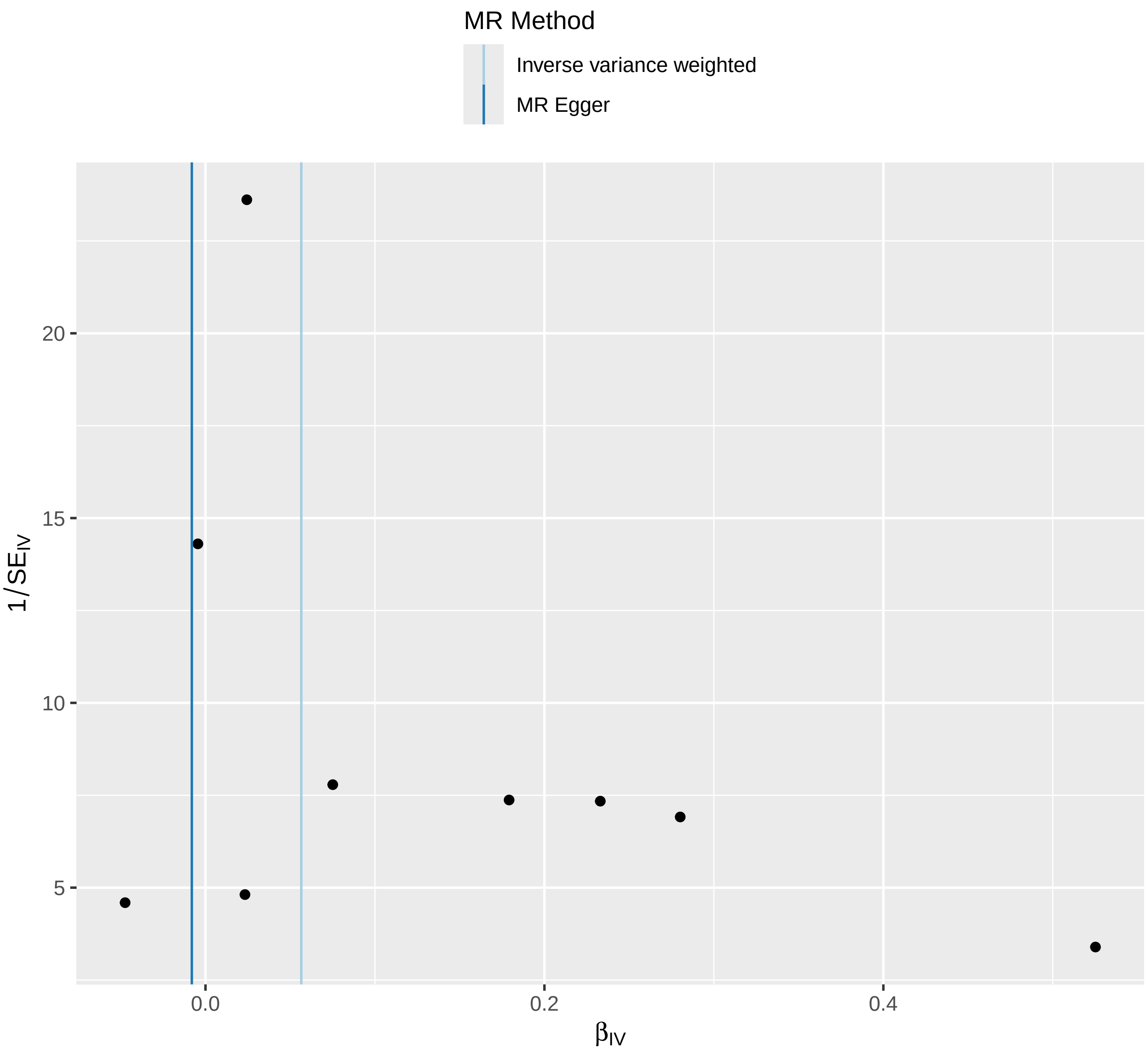

MR Method
Inverse variance weighted
MR Egger
20
15
10
5
0.0
0.2
0.4
1/SE$_{IV}$
β$_{IV}$

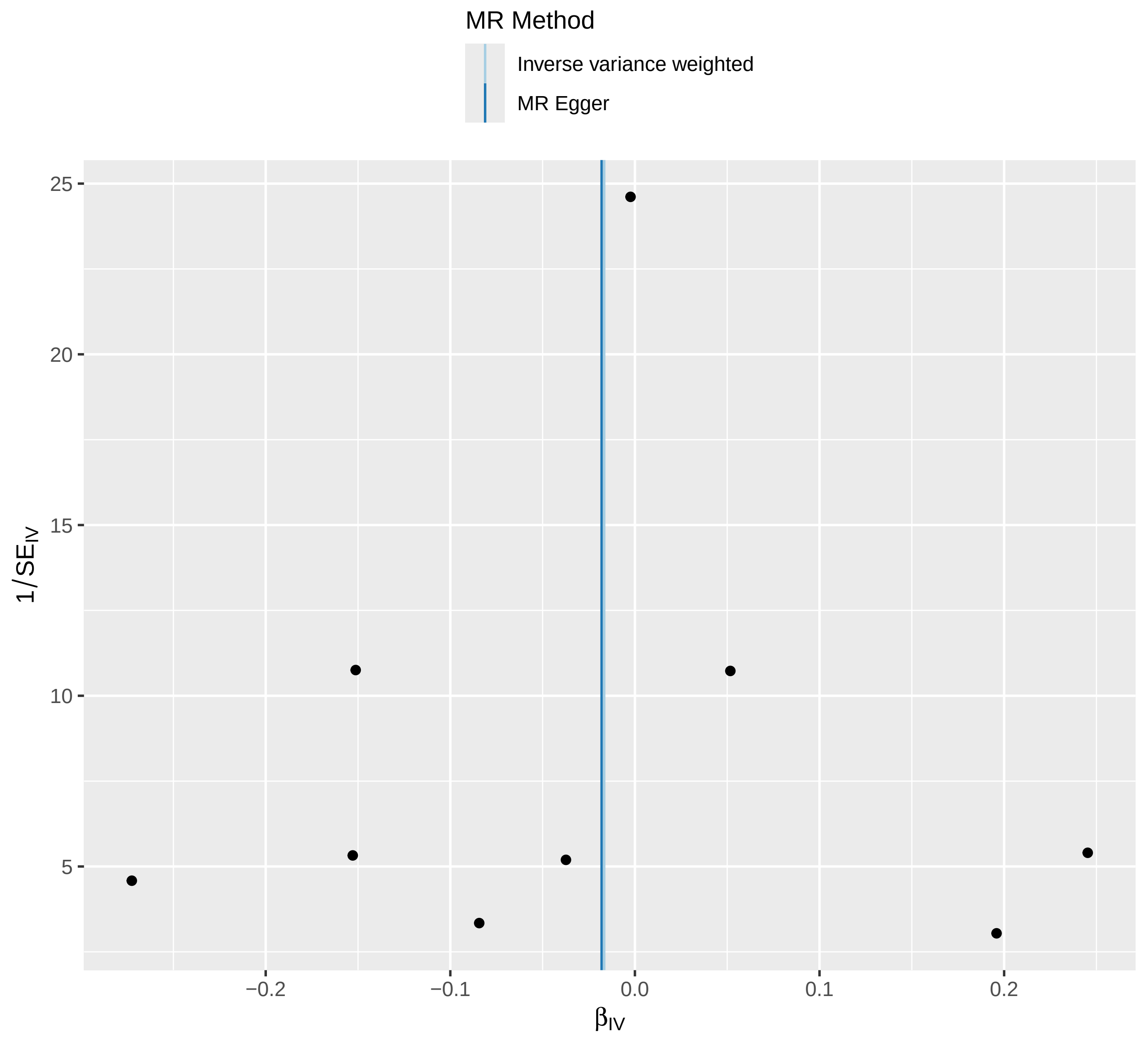

MR Method
Inverse variance weighted
MR Egger
25
20
15
10
5
1/SE$_{IV}$
−0.2
−0.1
0.0
0.1
0.2
β$_{IV}$

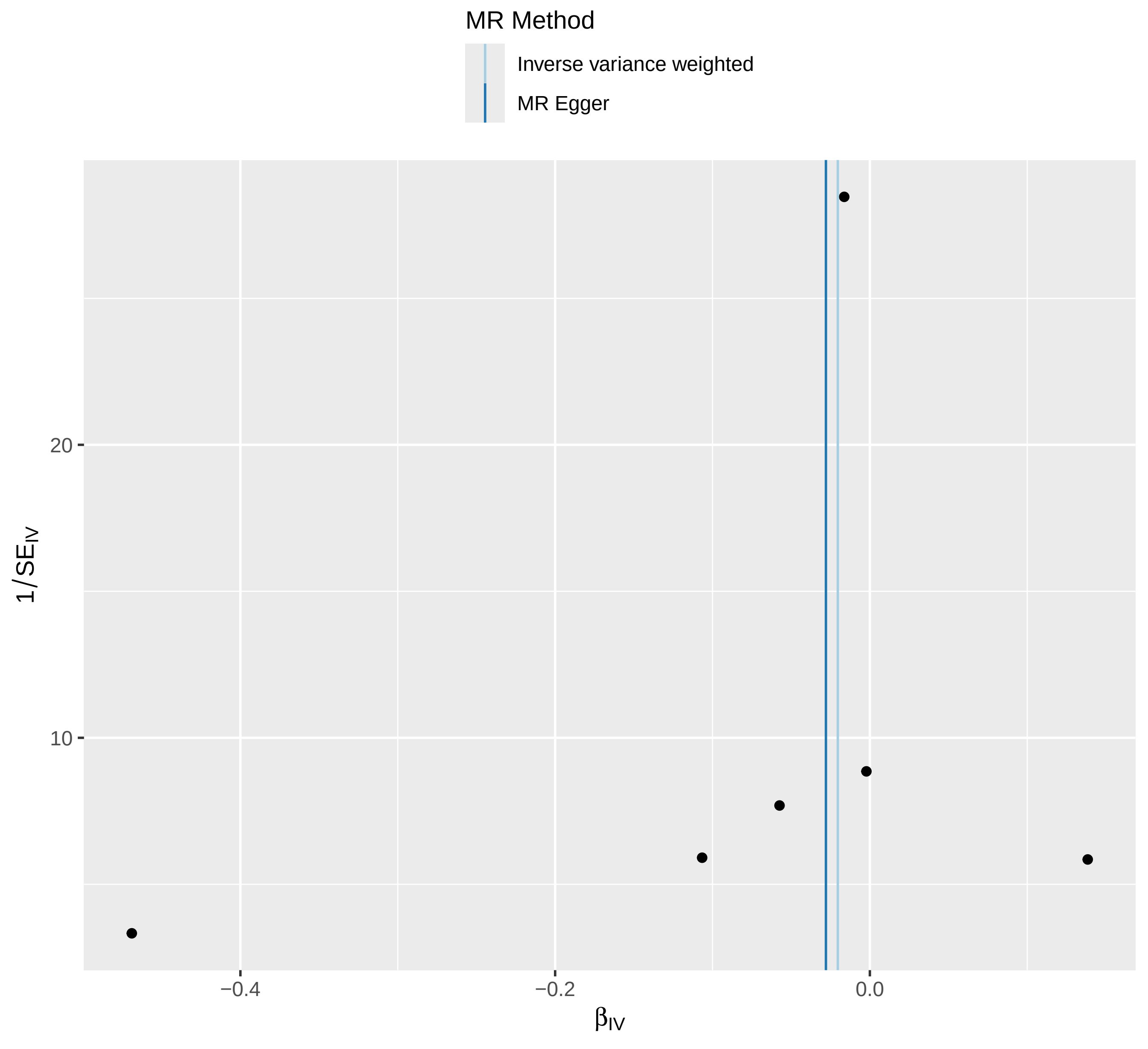
MR Method
Inverse variance weighted
MR Egger
20
10
1/SE$_{IV}$
−0.4
−0.2
0.0
β$_{IV}$

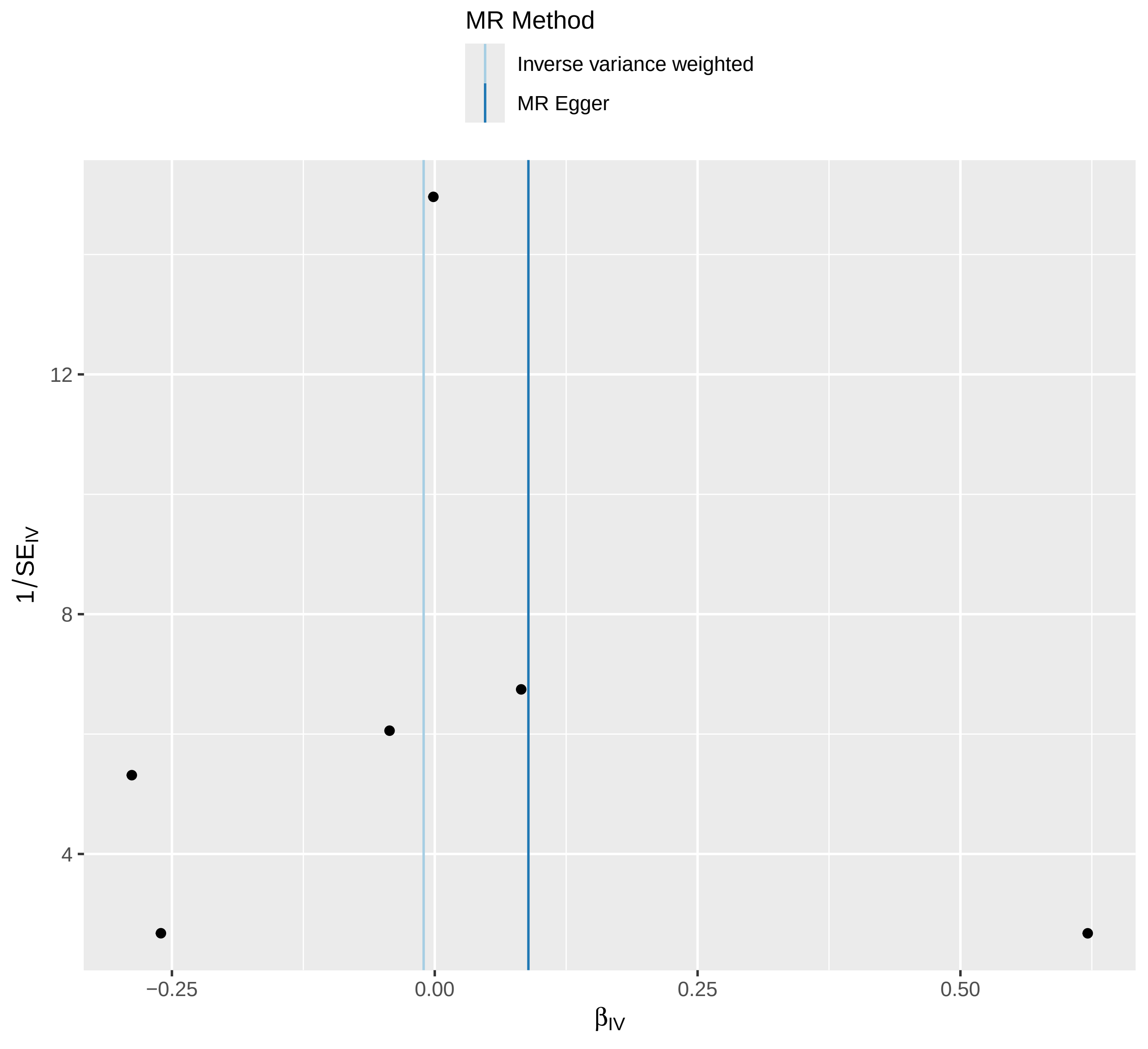
MR Method
Inverse variance weighted
MR Egger
$1/SE_{IV}$
12
8
4
−0.25
0.00
0.25
0.50
$\beta_{IV}$

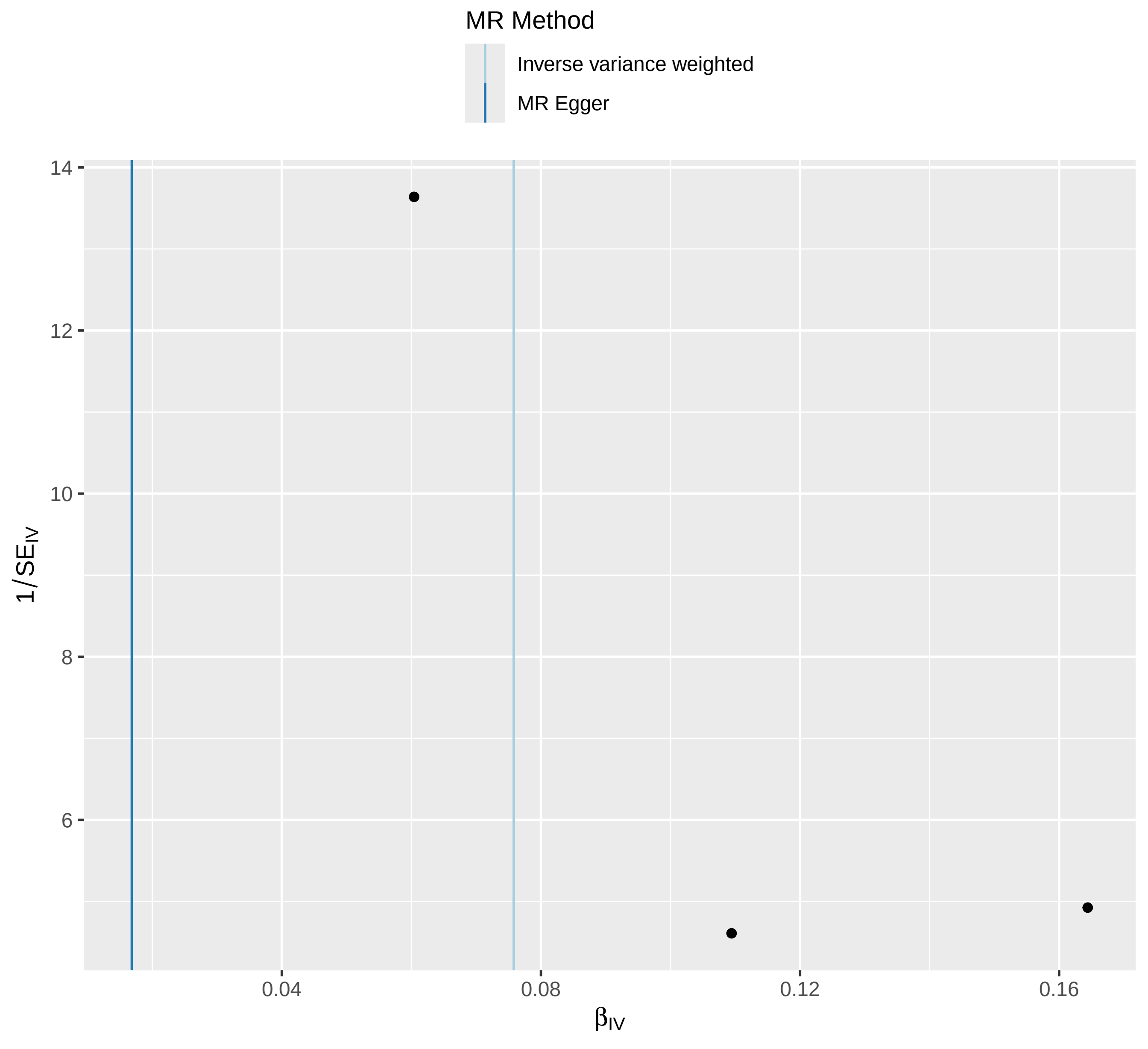
MR Method
Inverse variance weighted
MR Egger
14
12
10
8
6
1/SE_IV
0.04
0.08
0.12
0.16
β_IV

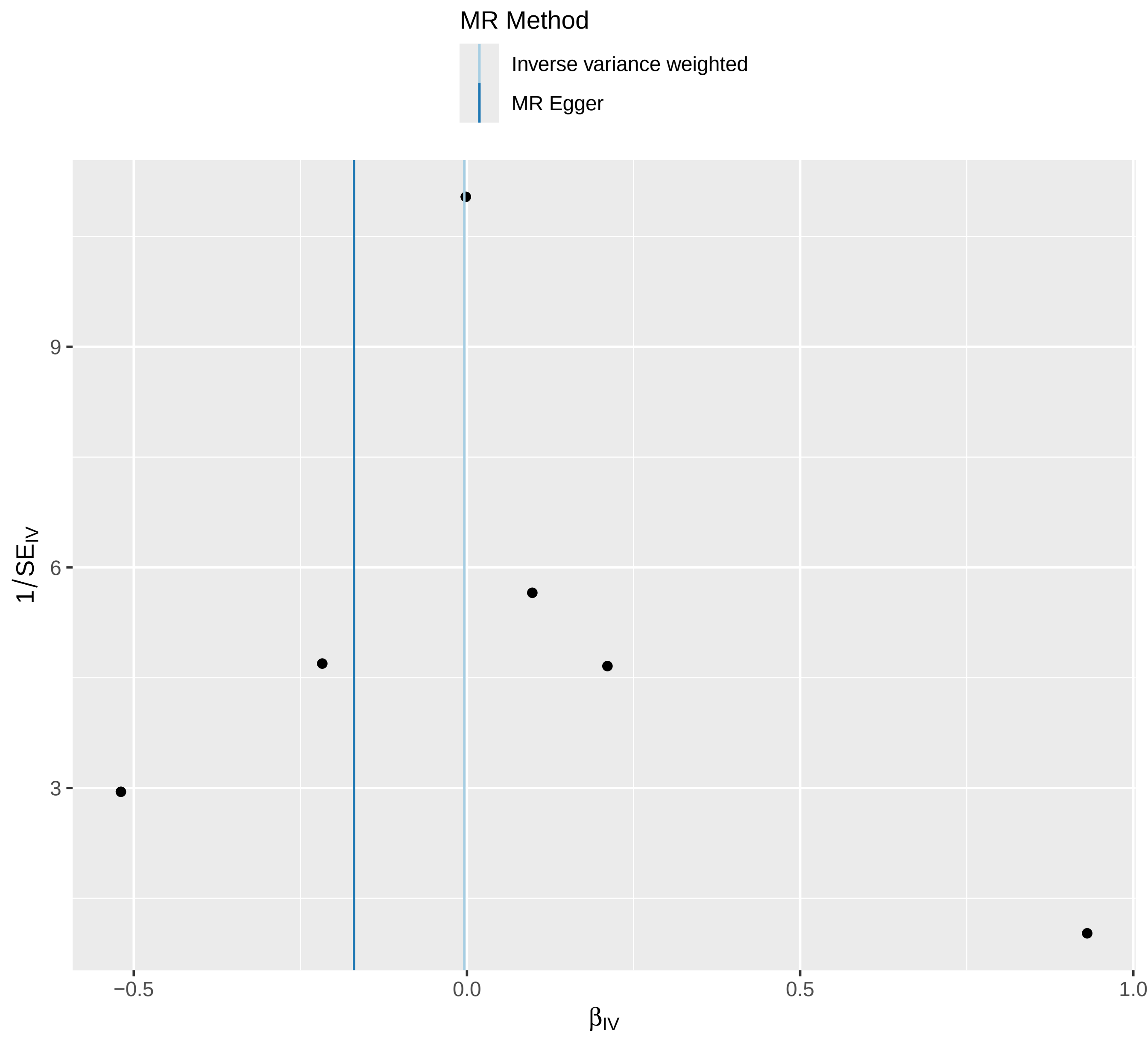

MR Method
Inverse variance weighted
MR Egger
1/SE_IV
9
6
3
−0.5
0.0
0.5
1.0
β_IV

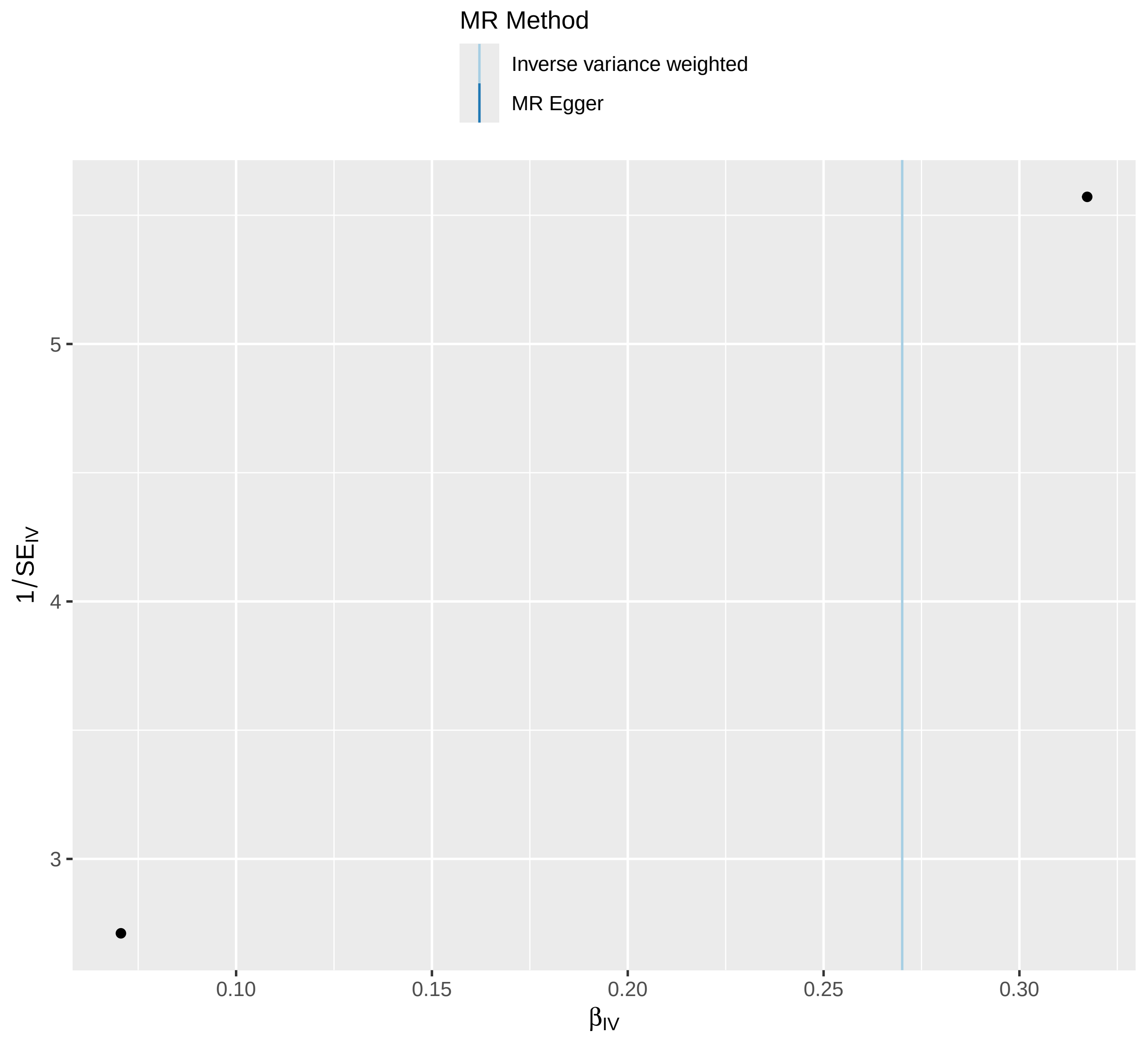

MR Method
Inverse variance weighted
MR Egger
5
4
3
1/SE_IV
0.10
0.15
0.20
0.25
0.30
β_IV

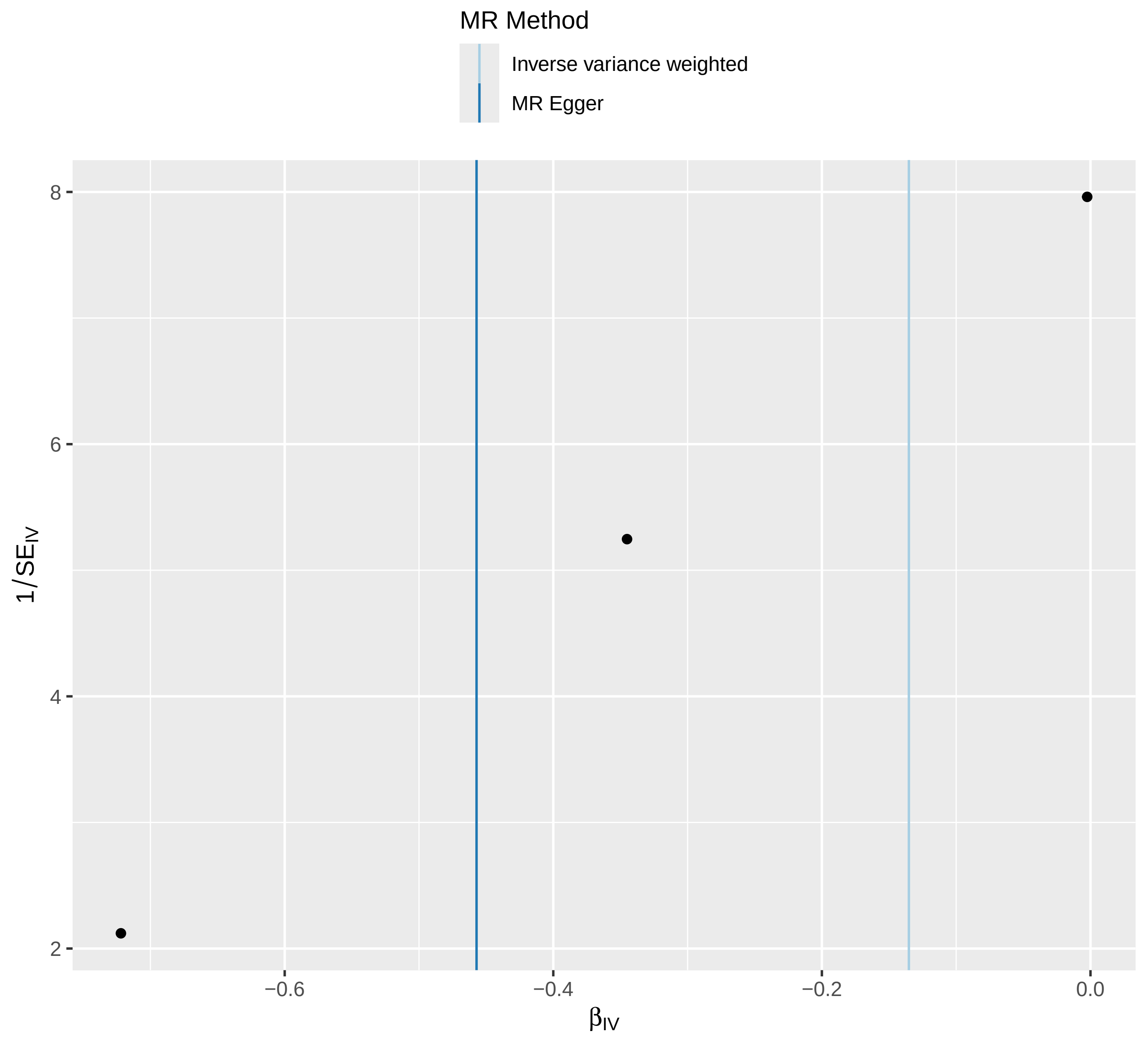
MR Method
Inverse variance weighted
MR Egger
8
6
4
2
1/SE_IV
−0.6
−0.4
−0.2
0.0
β_IV

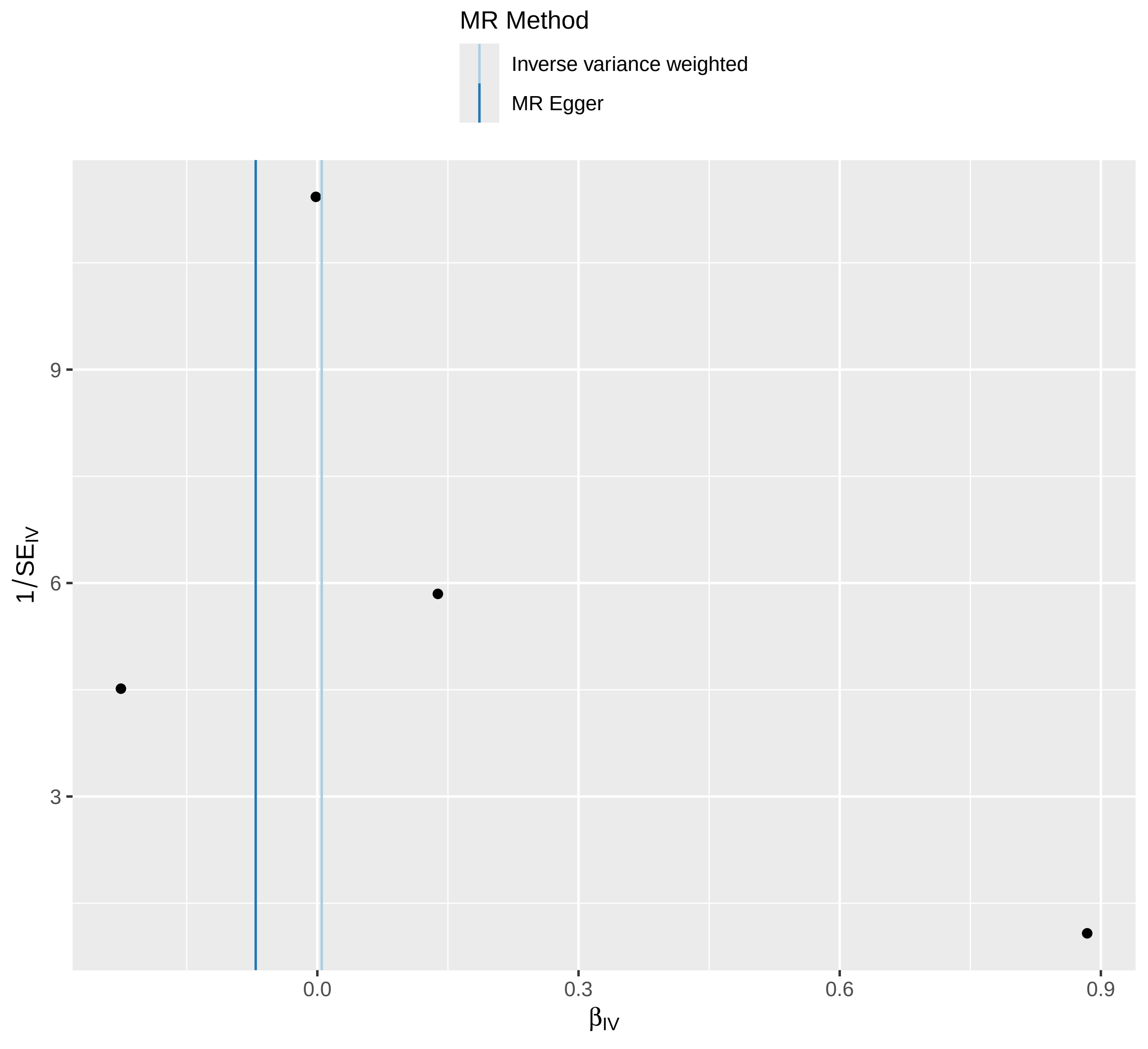
MR Method
Inverse variance weighted
MR Egger
9
6
3
1/SE_IV
0.0
0.3
0.6
0.9
β_IV

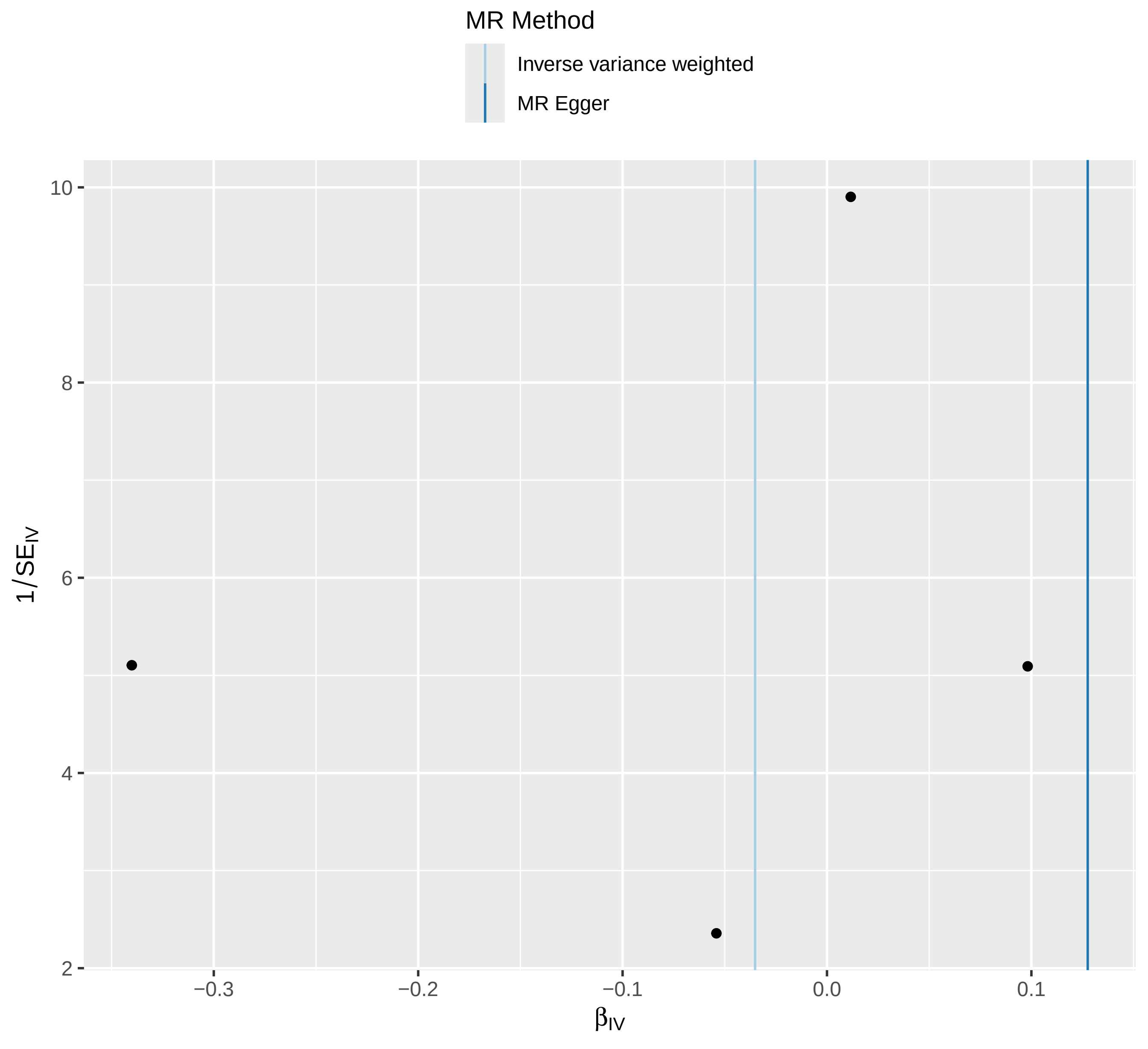

MR Method
Inverse variance weighted
MR Egger
10
8
6
4
2
1/SE$_{IV}$
−0.3
−0.2
−0.1
0.0
0.1
β$_{IV}$

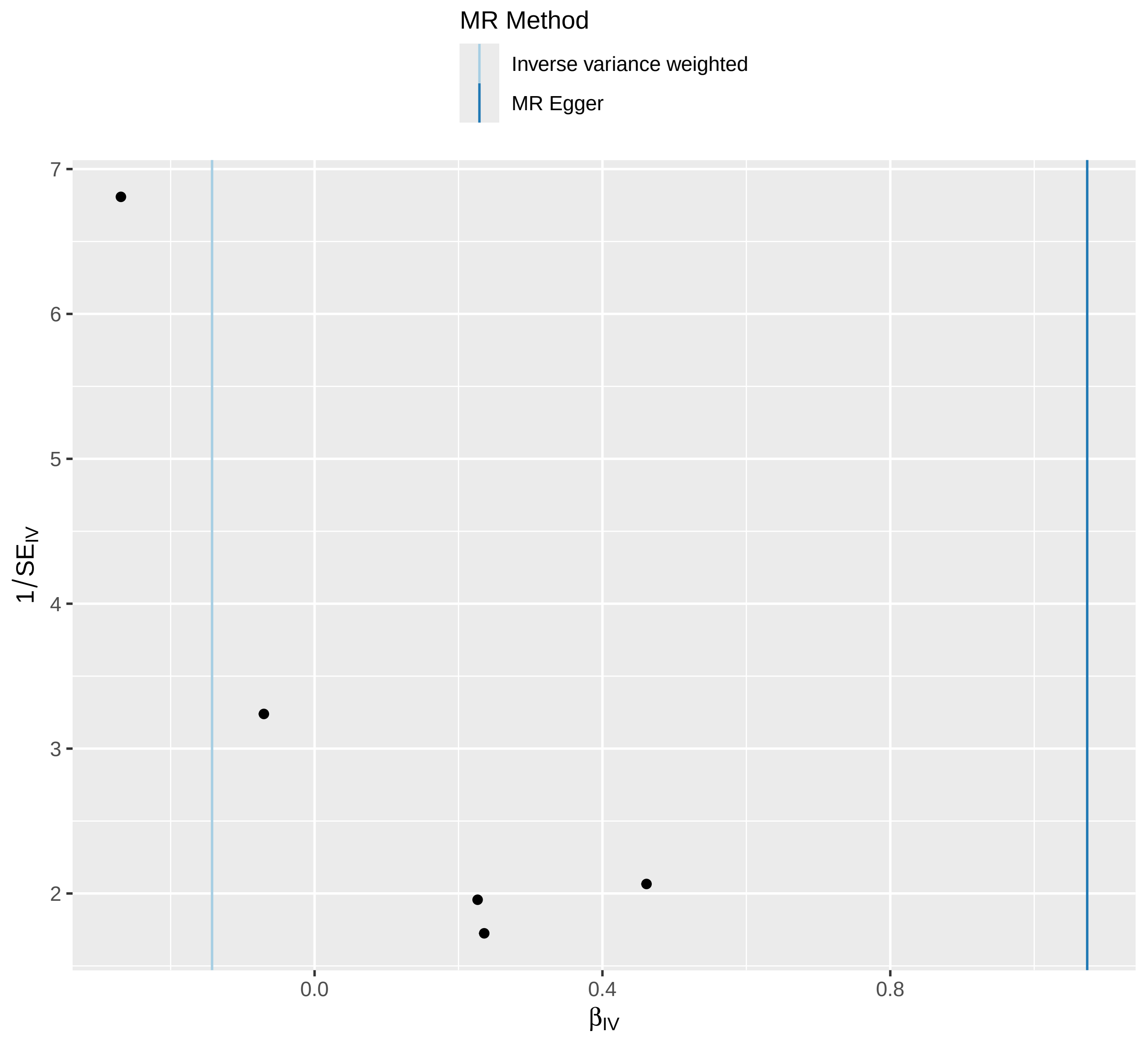
MR Method
Inverse variance weighted
MR Egger
7
6
5
4
3
2
1/SE$_{IV}$
0.0
0.4
0.8
β$_{IV}$

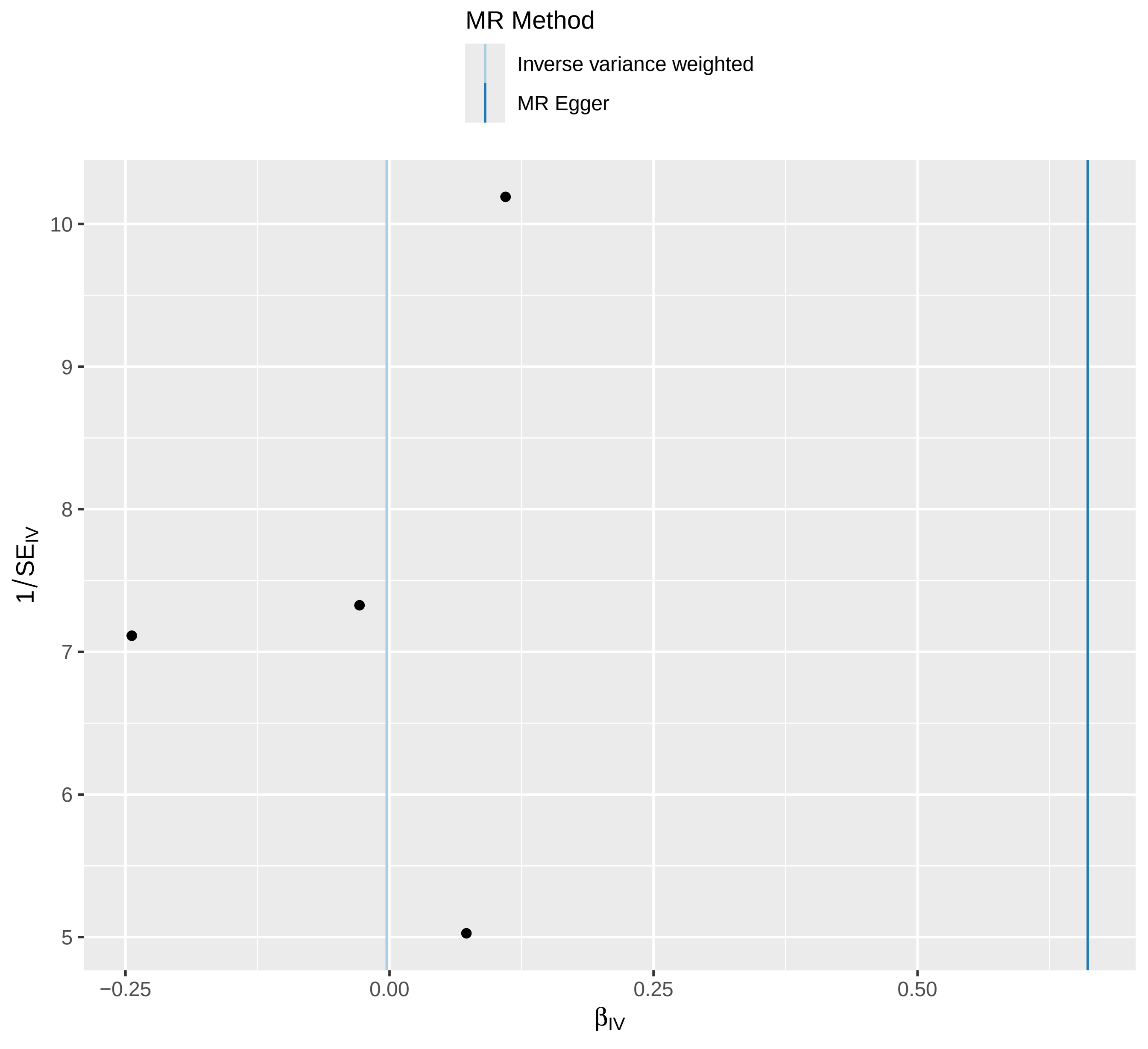

MR Method
Inverse variance weighted
MR Egger
1/SE_IV
β_IV
10
9
8
7
6
5
−0.25
0.00
0.25
0.50

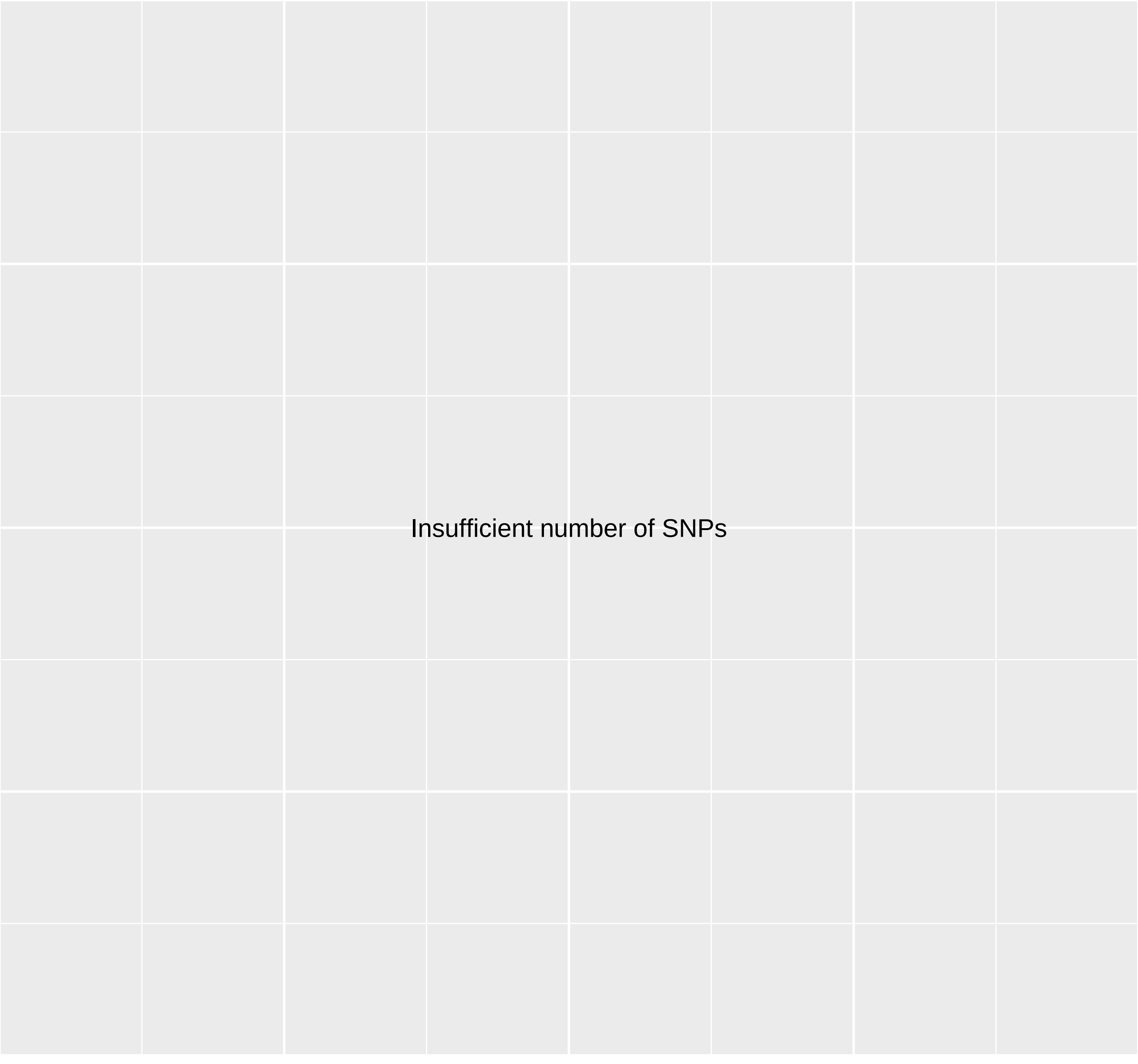
Insufficient number of SNPs

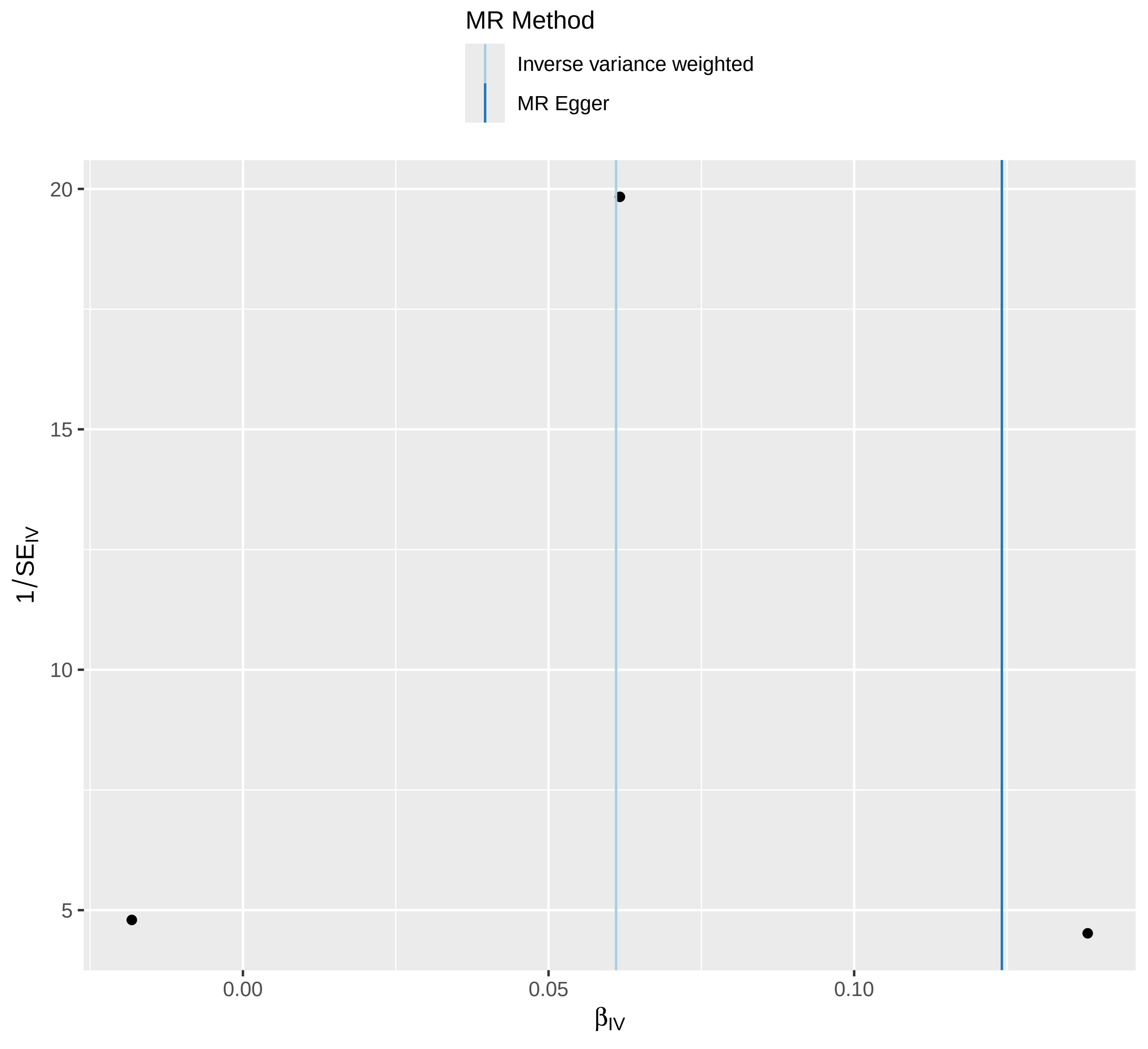
MR Method
Inverse variance weighted
MR Egger
20
15
10
5
0.00
0.05
0.10
1/SE_IV
β_IV

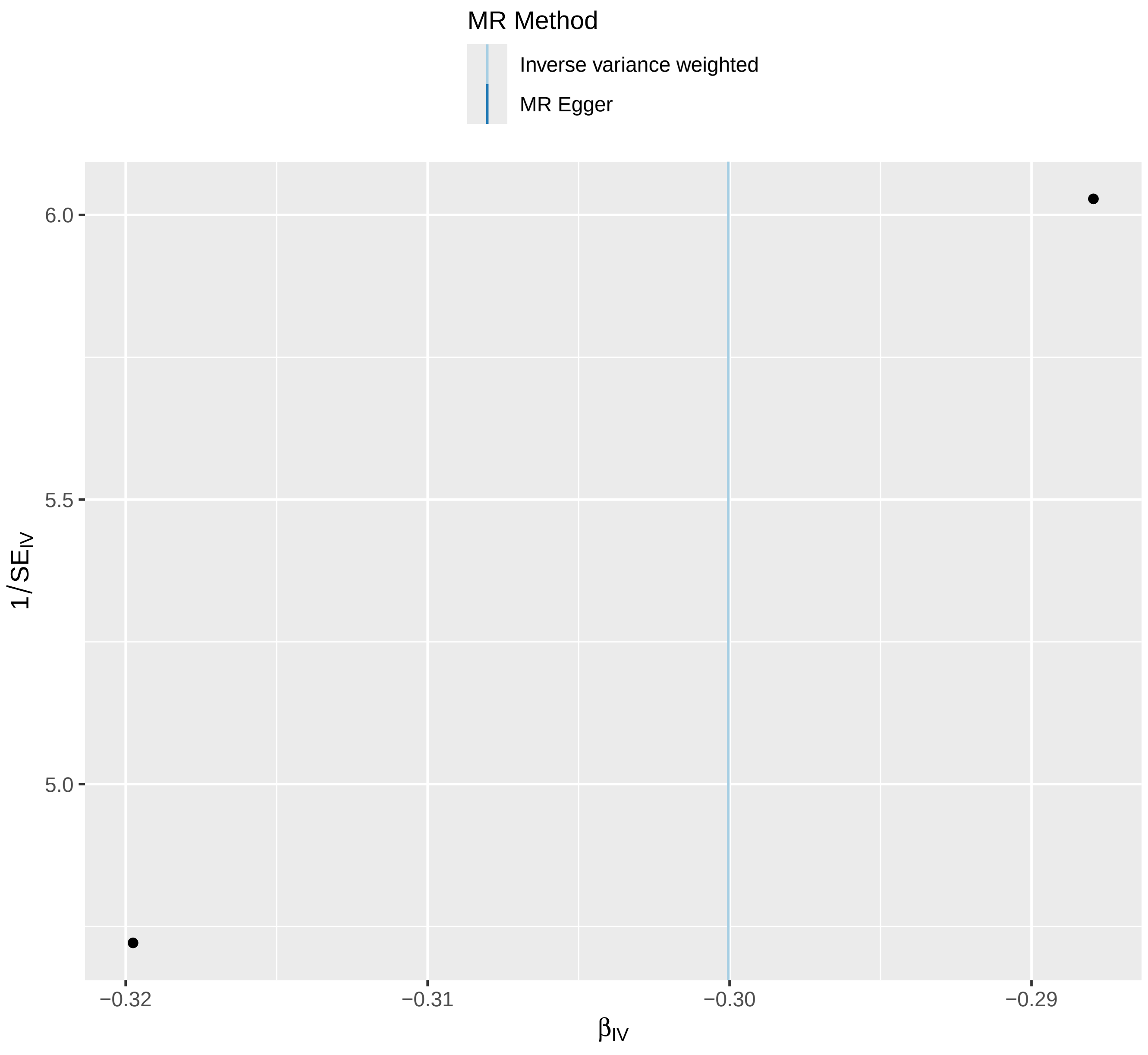
MR Method
Inverse variance weighted
MR Egger
6.0
5.5
5.0
1/SE_IV
−0.32
−0.31
−0.30
−0.29
β_IV

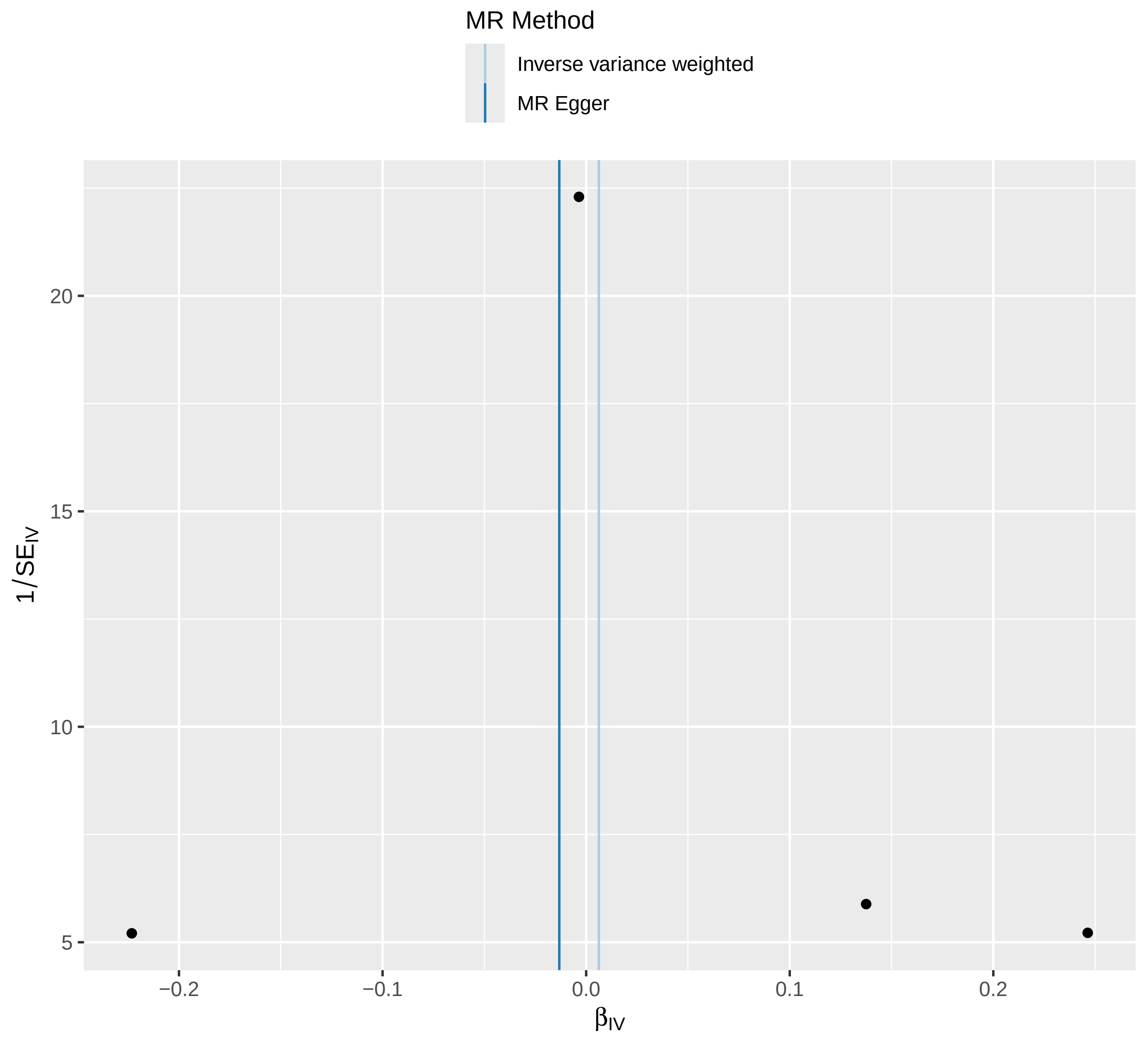

MR Method
Inverse variance weighted
MR Egger
20
15
10
5
1/SE_IV
−0.2
−0.1
0.0
0.1
0.2
β_IV

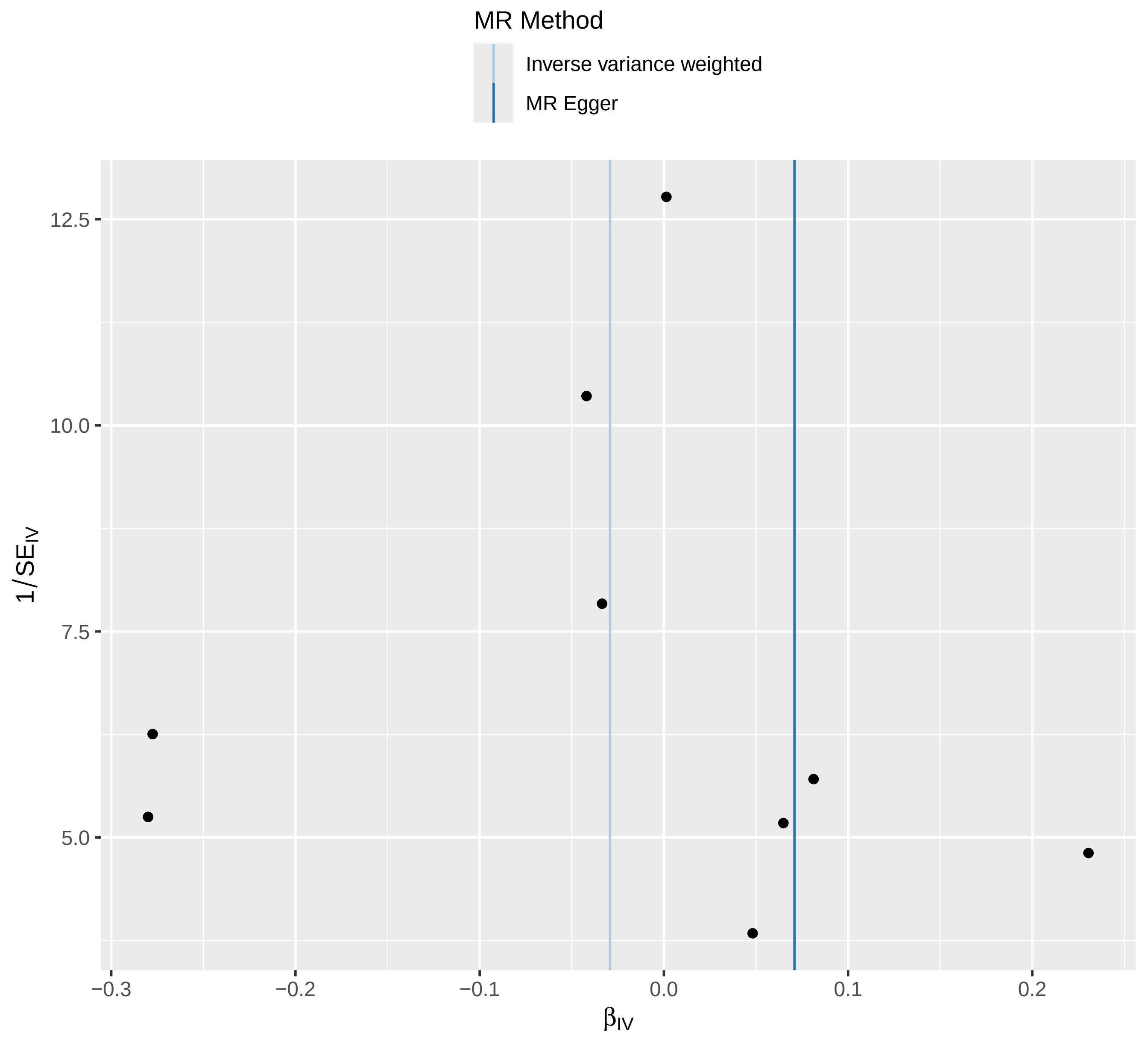
MR Method
Inverse variance weighted
MR Egger
12.5
10.0
7.5
5.0
1/SE$_{IV}$
−0.3
−0.2
−0.1
0.0
0.1
0.2
β$_{IV}$

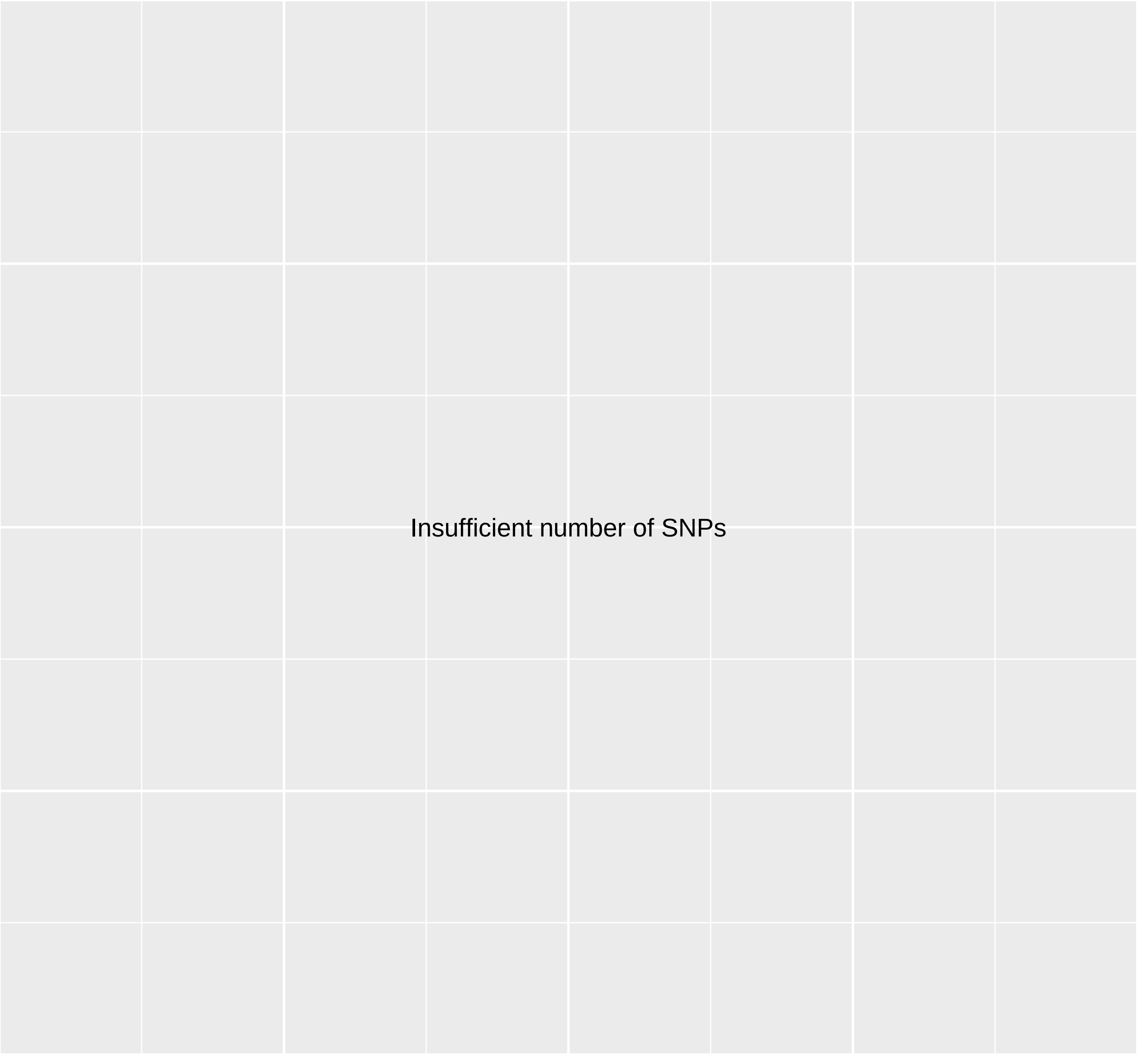
Insufficient number of SNPs

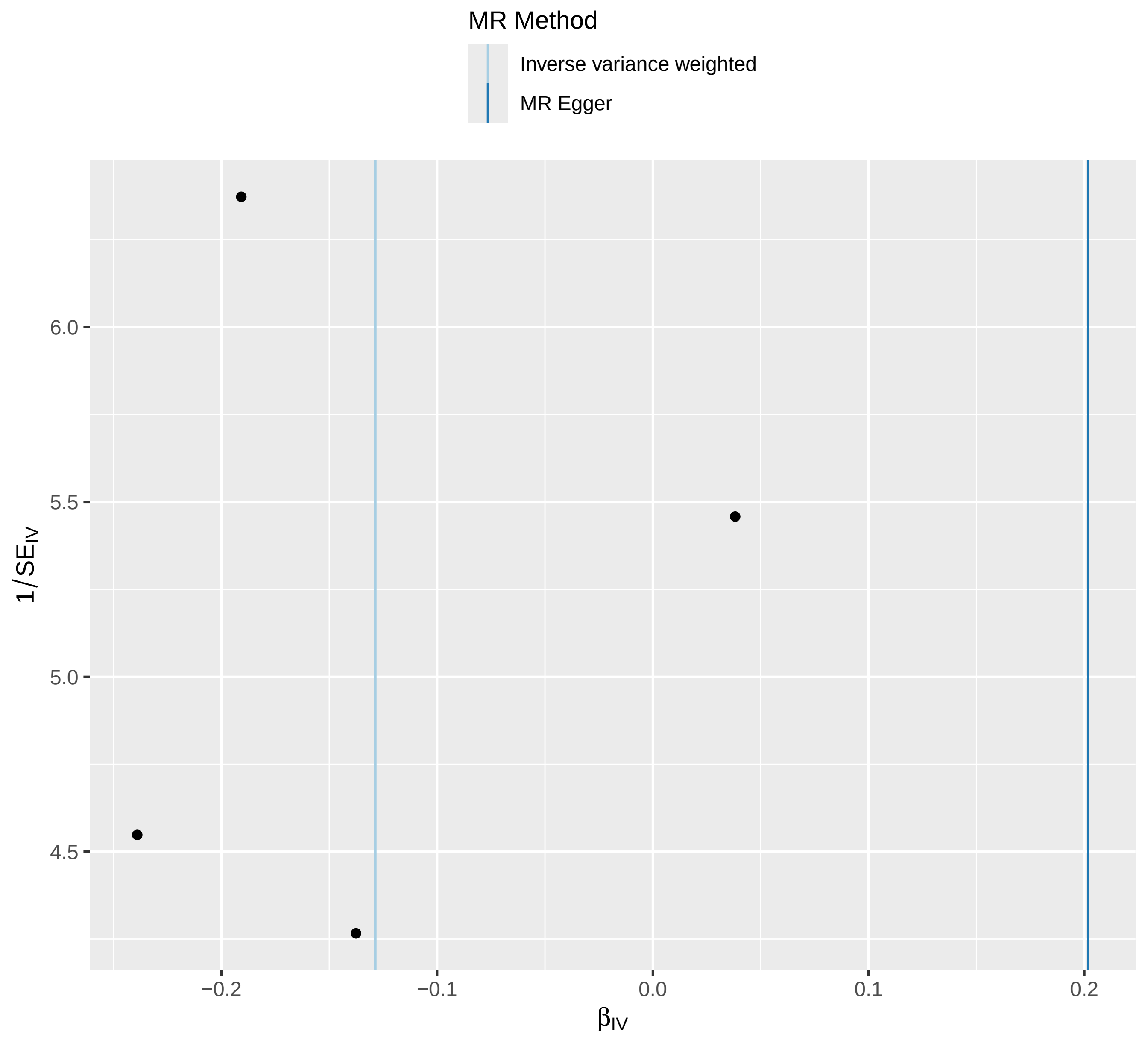
MR Method
Inverse variance weighted
MR Egger
6.0
5.5
5.0
4.5
1/SE$_{IV}$
−0.2
−0.1
0.0
0.1
0.2
β$_{IV}$

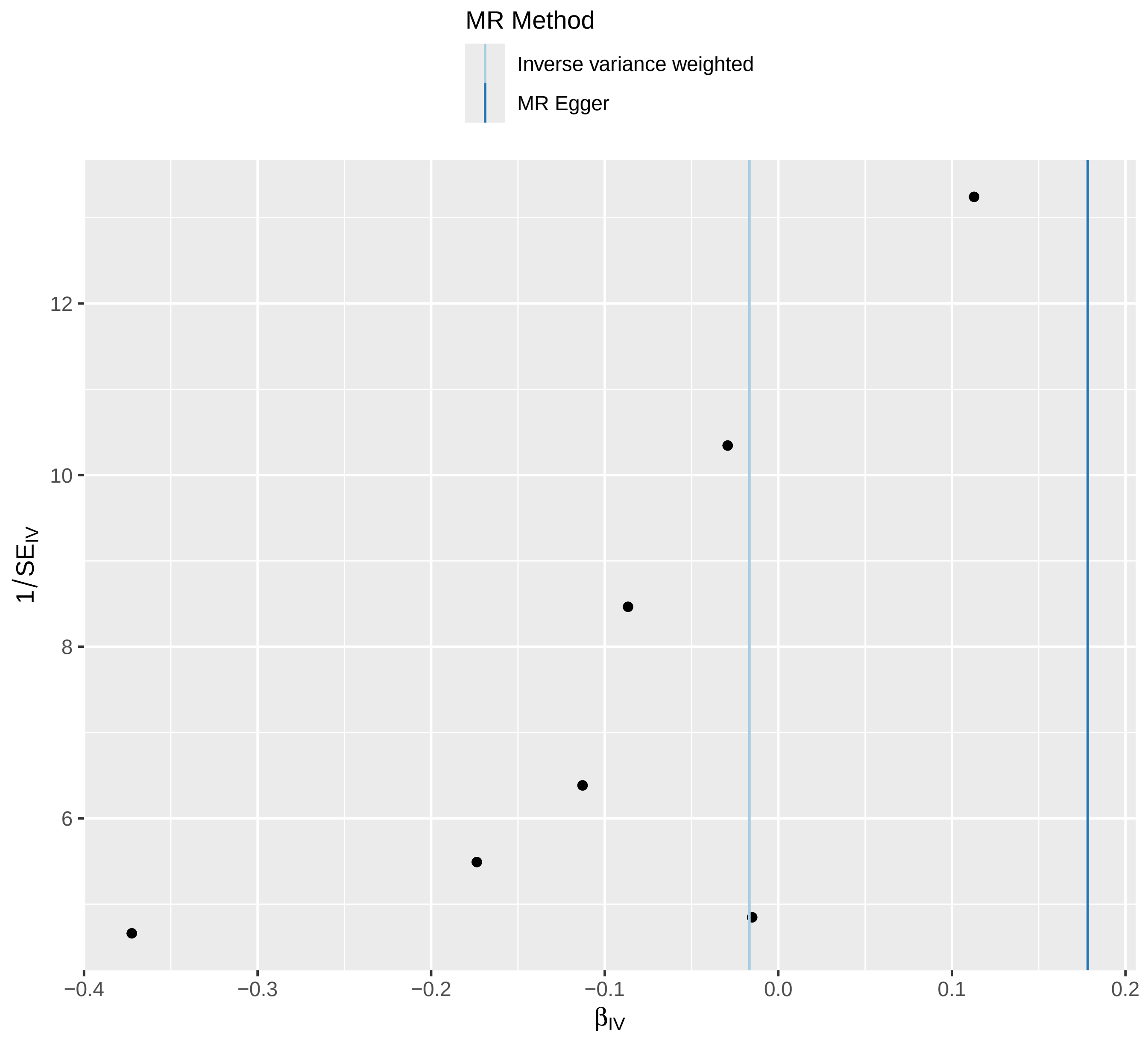
MR Method
Inverse variance weighted
MR Egger
1/SE$_{IV}$
12
10
8
6
−0.4
−0.3
−0.2
−0.1
0.0
0.1
0.2
β$_{IV}$

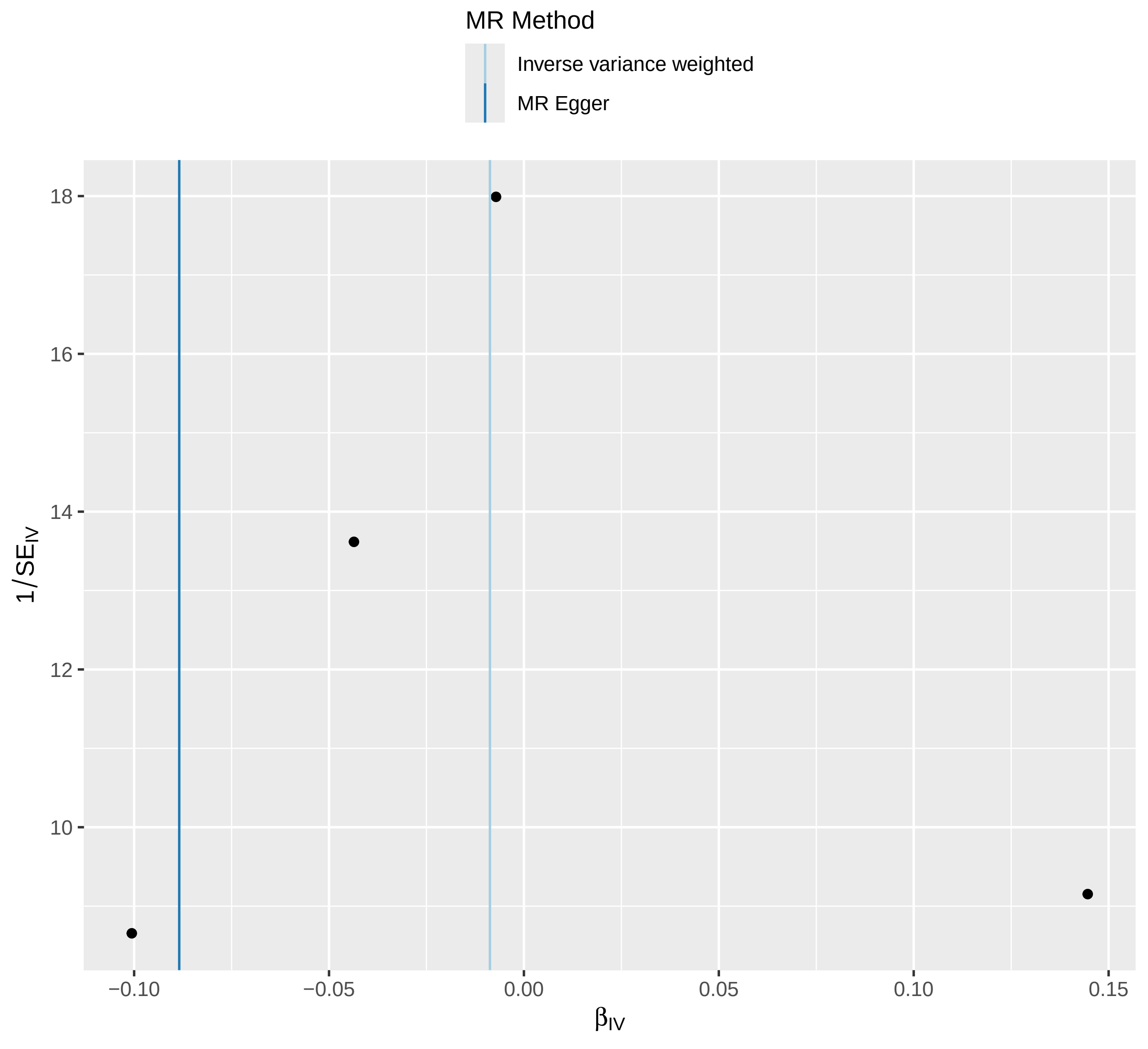
MR Method
Inverse variance weighted
MR Egger
18
16
14
12
10
1/SE_IV
−0.10
−0.05
0.00
0.05
0.10
0.15
β_IV

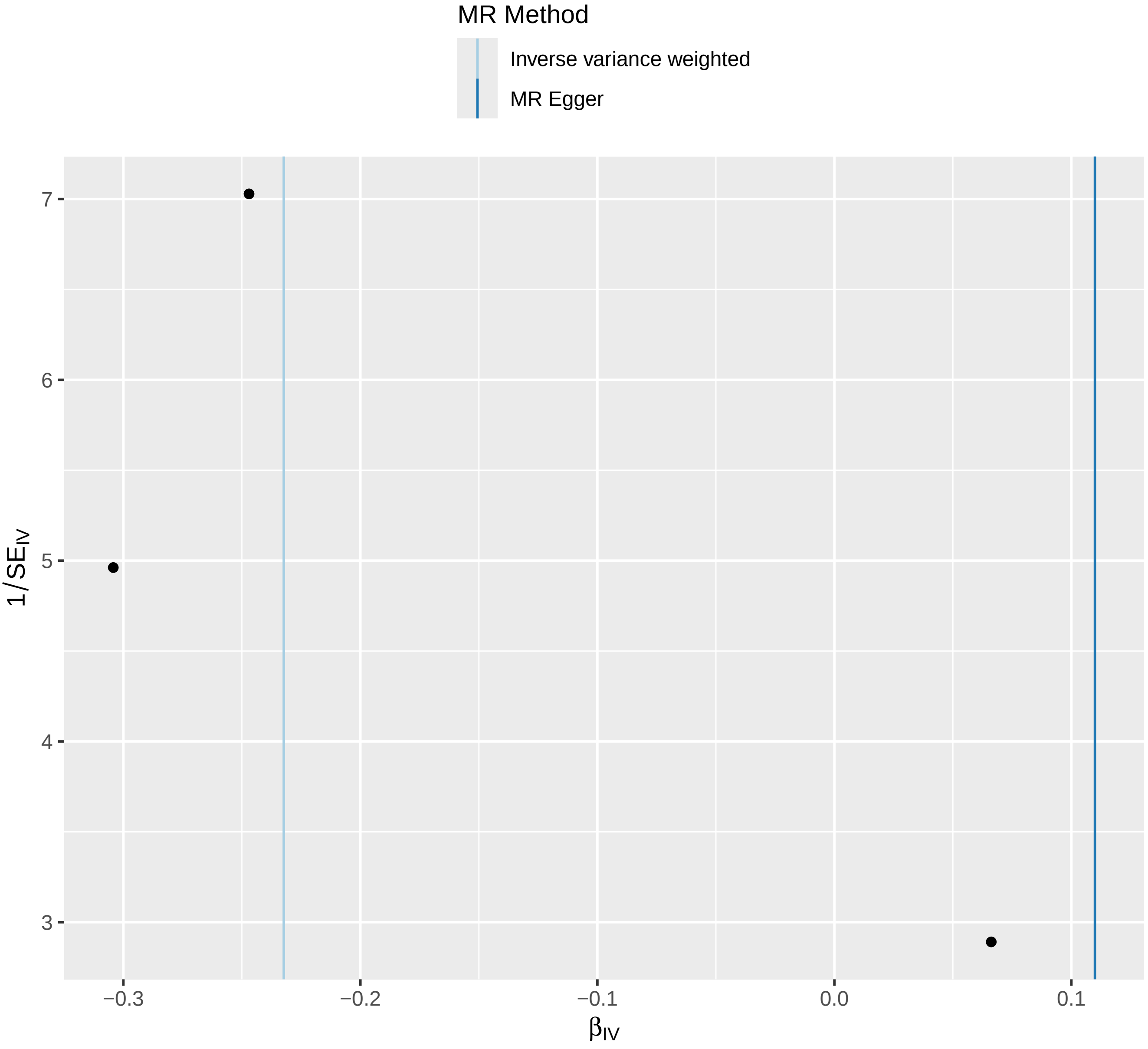

MR Method
Inverse variance weighted
MR Egger
7
6
5
4
3
1/SE$_{IV}$
−0.3
−0.2
−0.1
0.0
0.1
β$_{IV}$

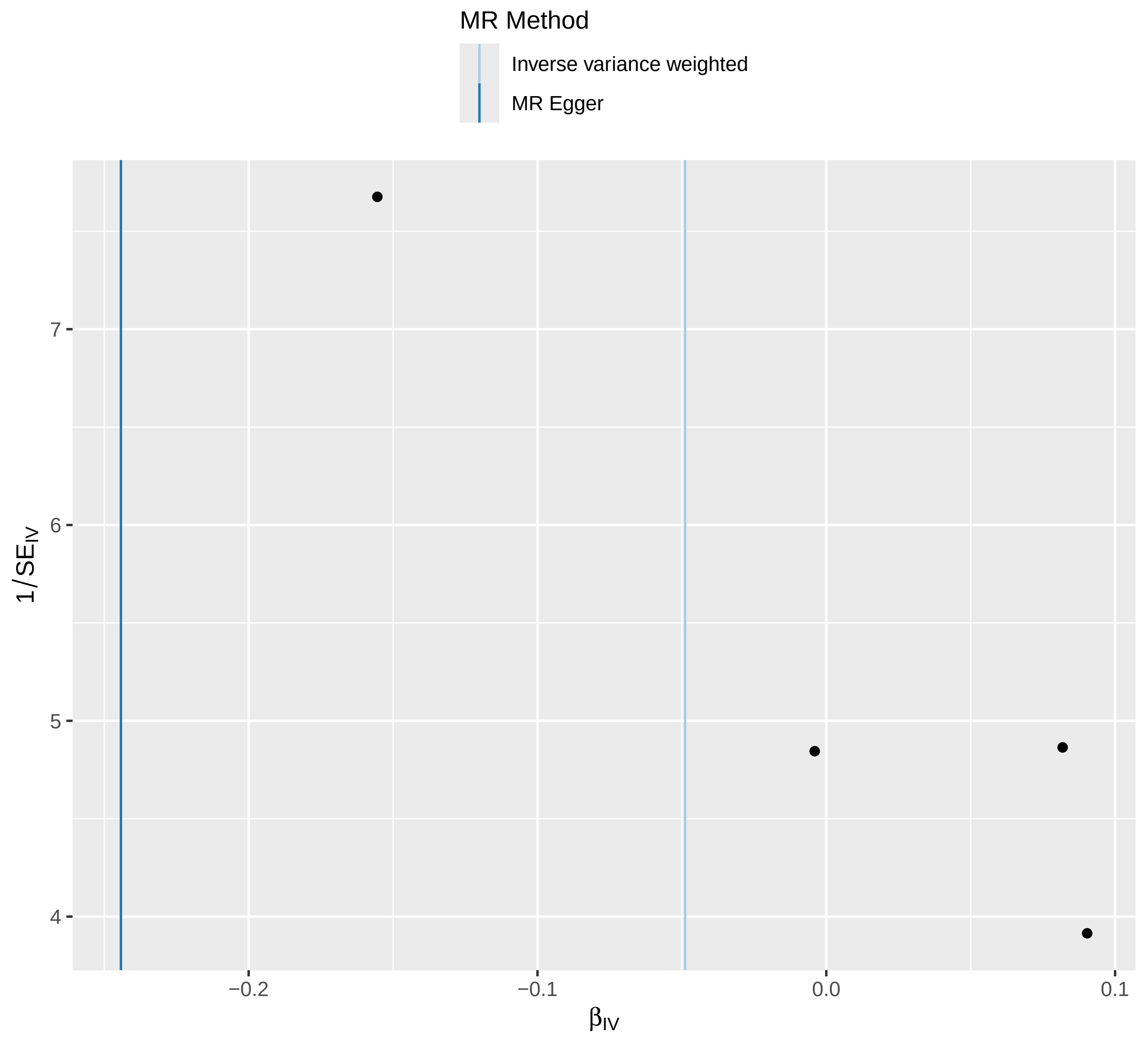
MR Method
Inverse variance weighted
MR Egger
7
6
5
4
1/SE_IV
−0.2
−0.1
0.0
0.1
β_IV

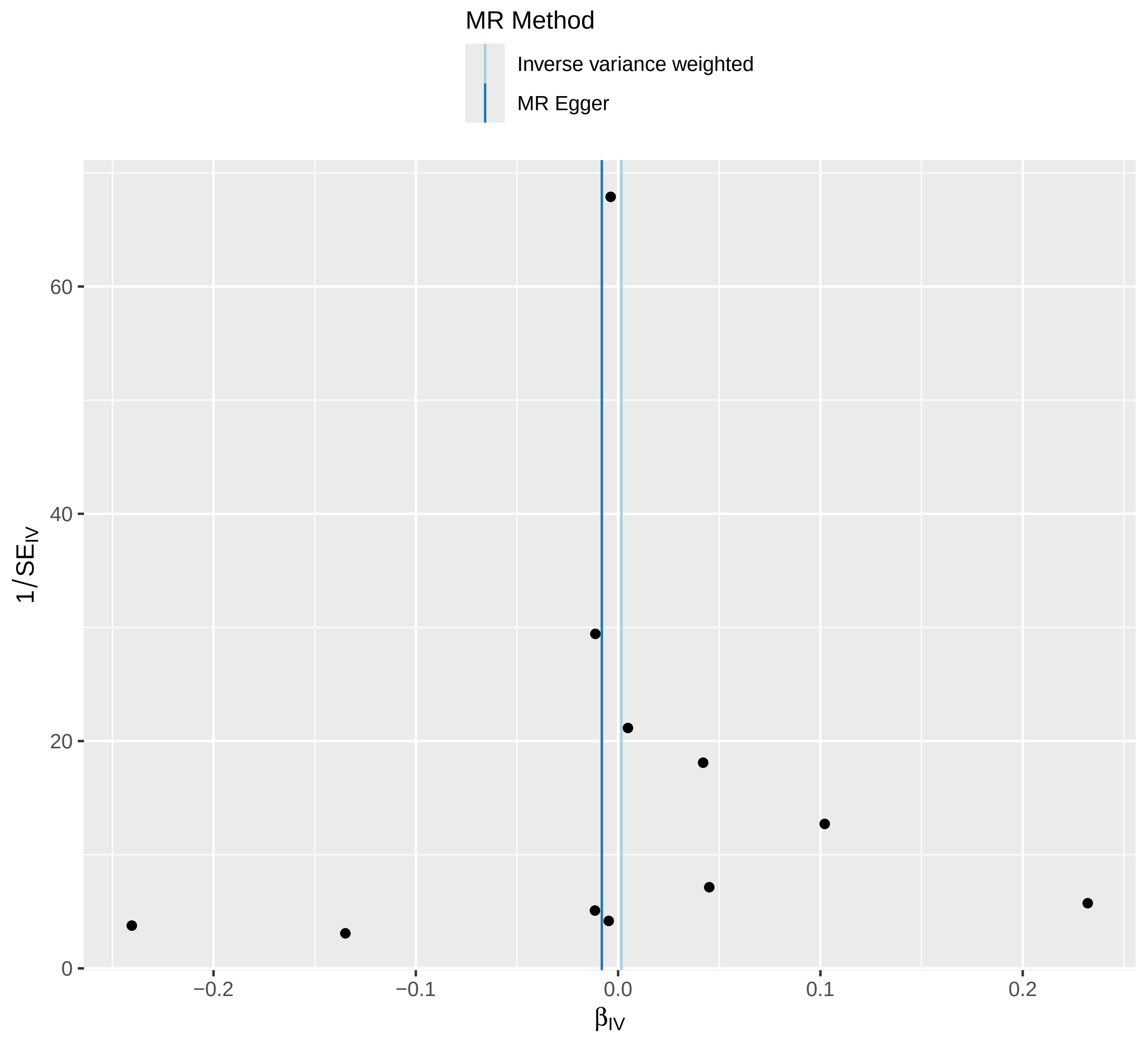

MR Method
Inverse variance weighted
MR Egger
60
40
20
0
1/SE_IV
−0.2
−0.1
0.0
0.1
0.2
β_IV

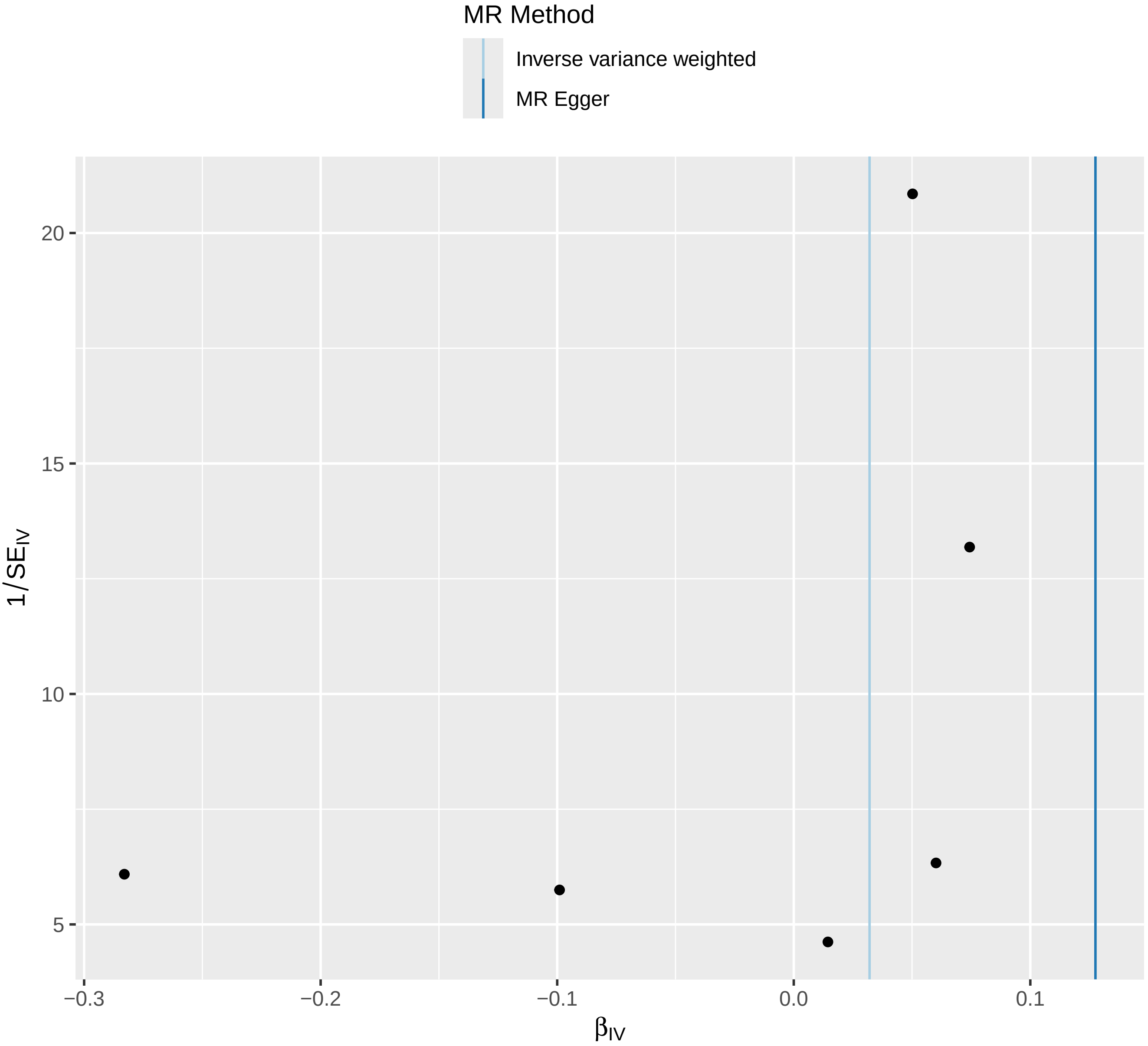

MR Method
Inverse variance weighted
MR Egger
20
15
10
5
1/SE$_{IV}$
−0.3
−0.2
−0.1
0.0
0.1
β$_{IV}$

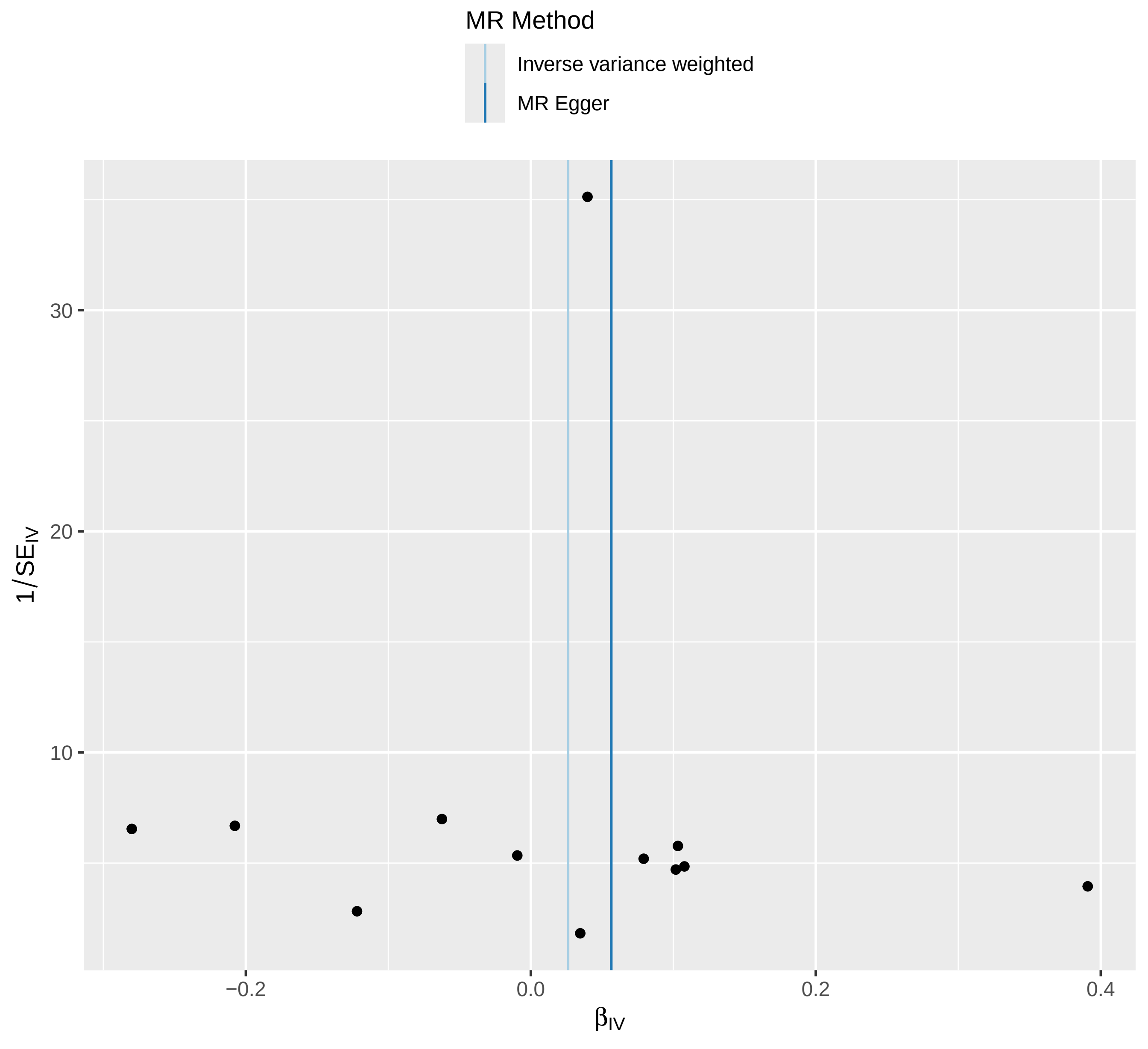
MR Method
Inverse variance weighted
MR Egger
30
20
10
1/SE$_{IV}$
−0.2
0.0
0.2
0.4
β$_{IV}$

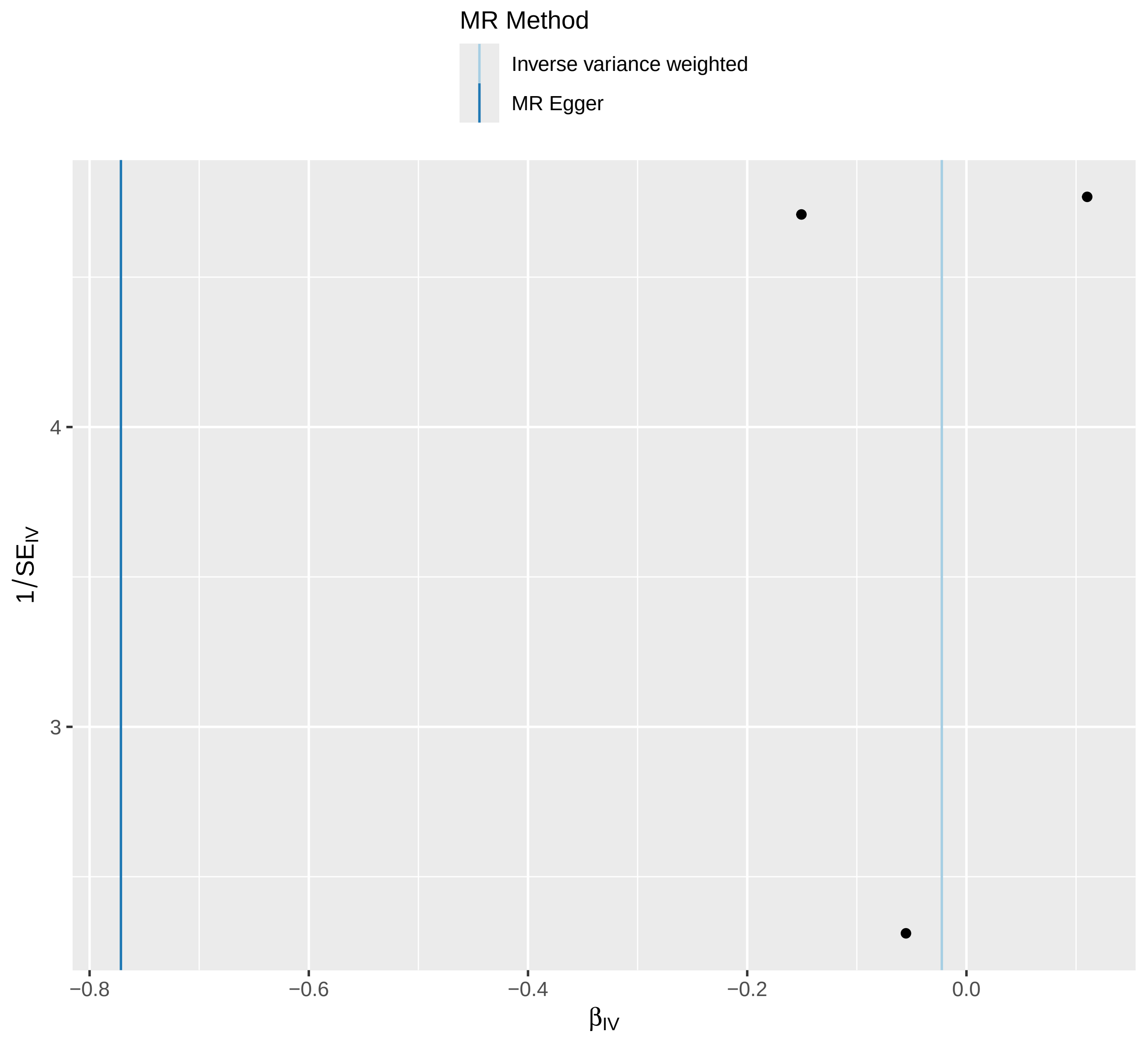
MR Method
Inverse variance weighted
MR Egger
4
3
1/SE_IV
−0.8
−0.6
−0.4
−0.2
0.0
β_IV

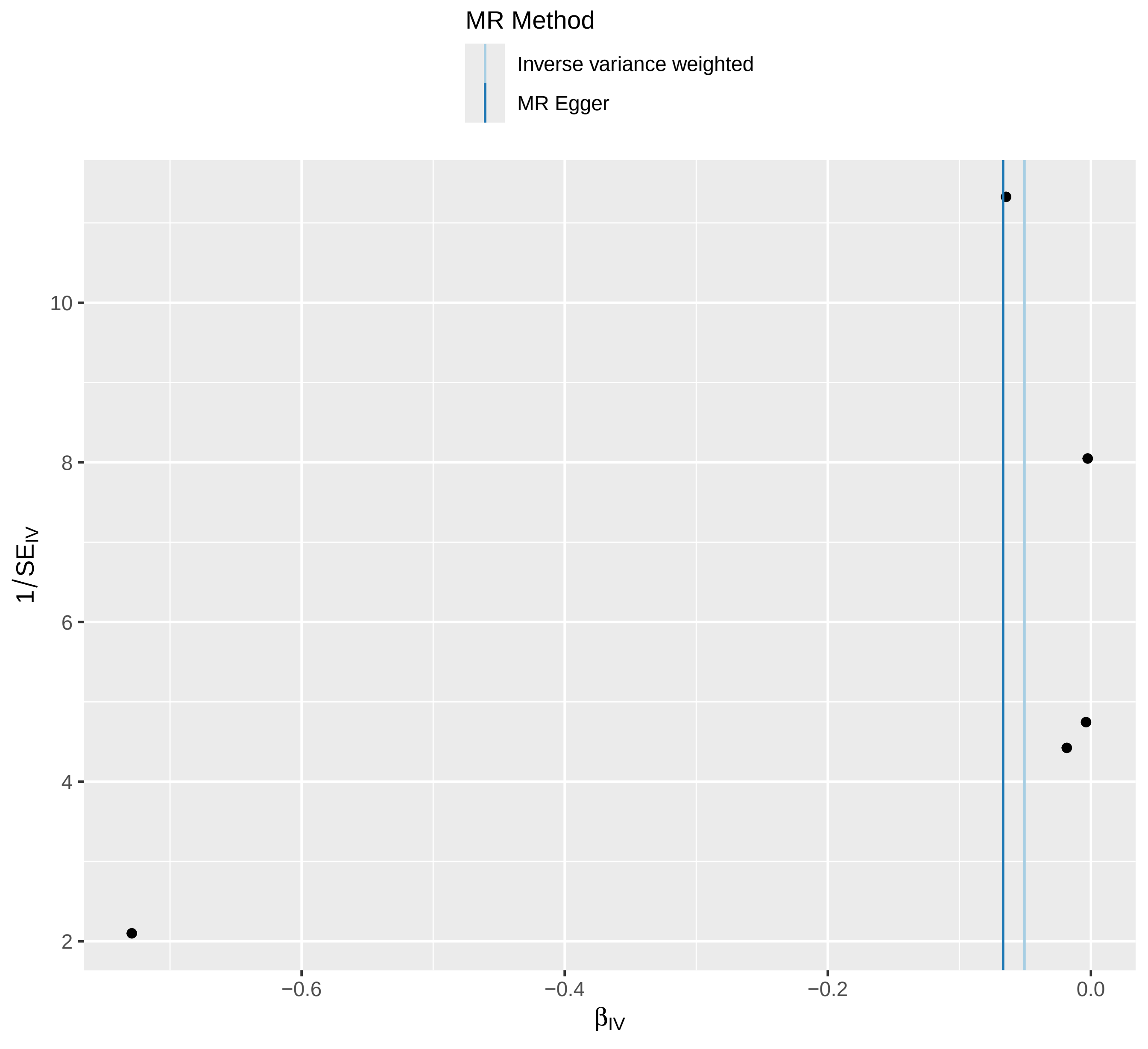
MR Method
Inverse variance weighted
MR Egger
10
8
6
4
2
1/SE$_{IV}$
−0.6
−0.4
−0.2
0.0
β$_{IV}$

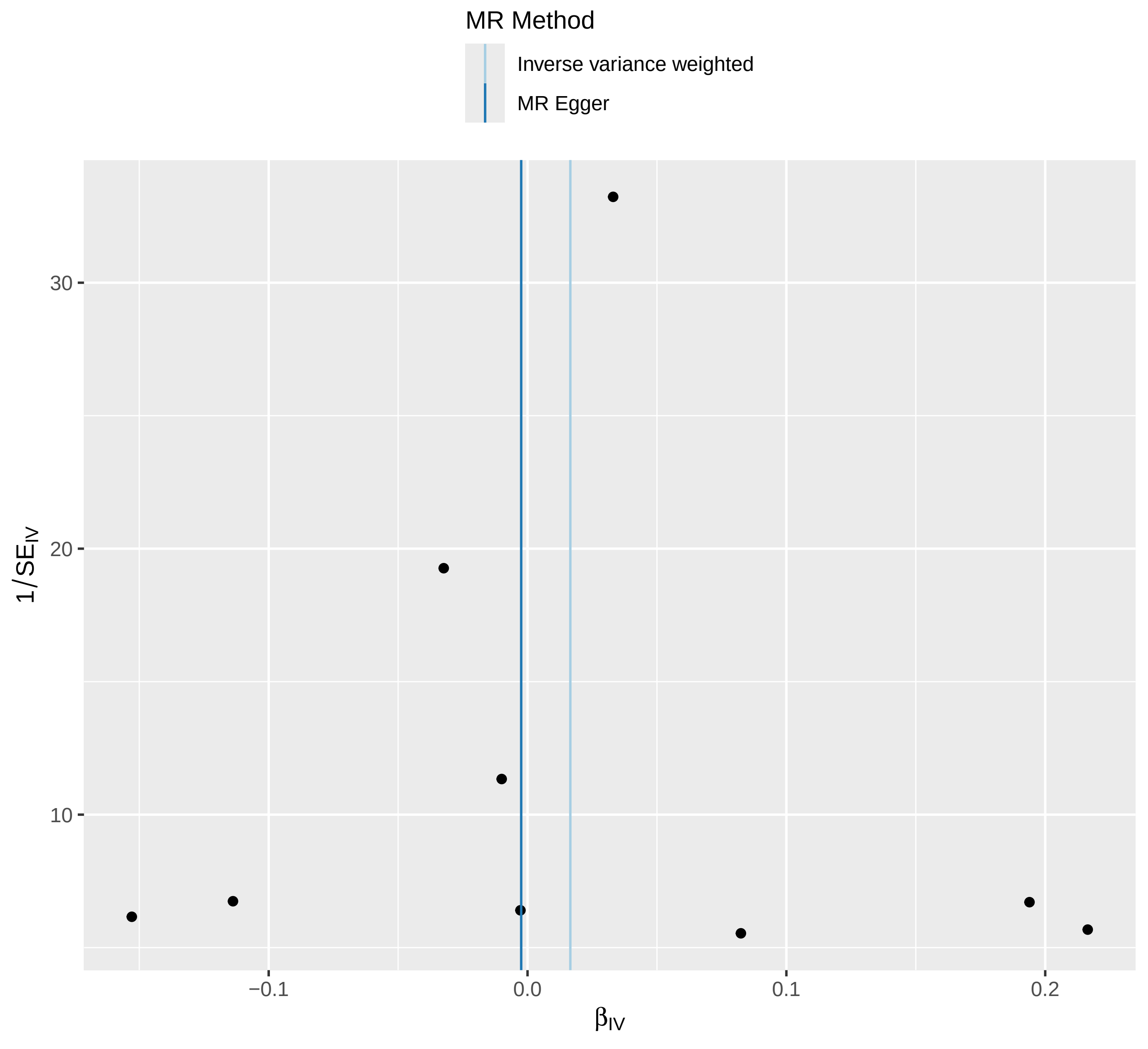

MR Method
Inverse variance weighted
MR Egger
30
20
10
$1/SE_{IV}$
−0.1
0.0
0.1
0.2
$\beta_{IV}$

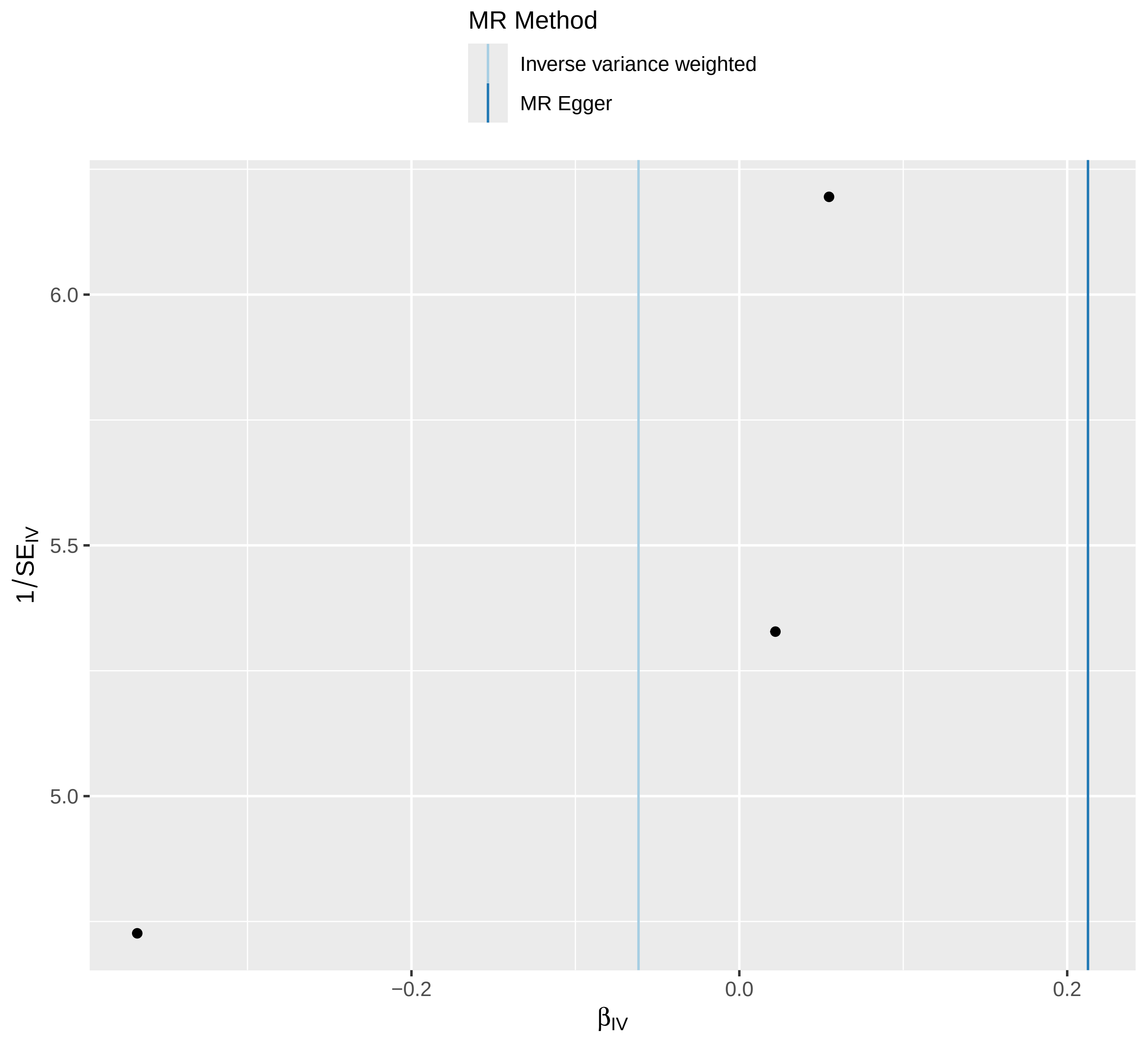
MR Method
Inverse variance weighted
MR Egger
6.0
5.5
5.0
$1/SE_{IV}$
−0.2
0.0
0.2
$\beta_{IV}$

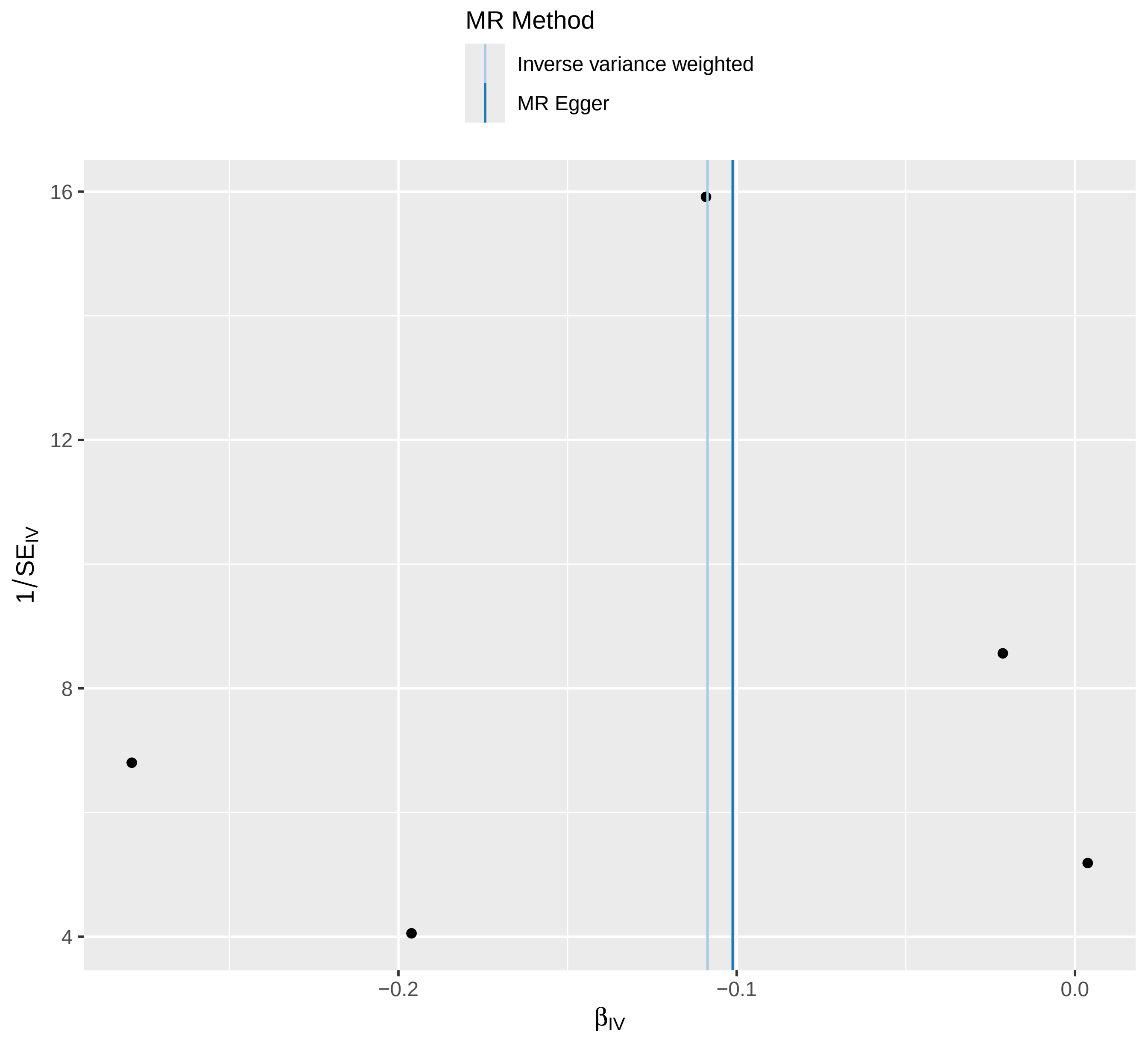

MR Method
Inverse variance weighted
MR Egger
16
12
8
4
1/SE$_{IV}$
−0.2
−0.1
0.0
β$_{IV}$

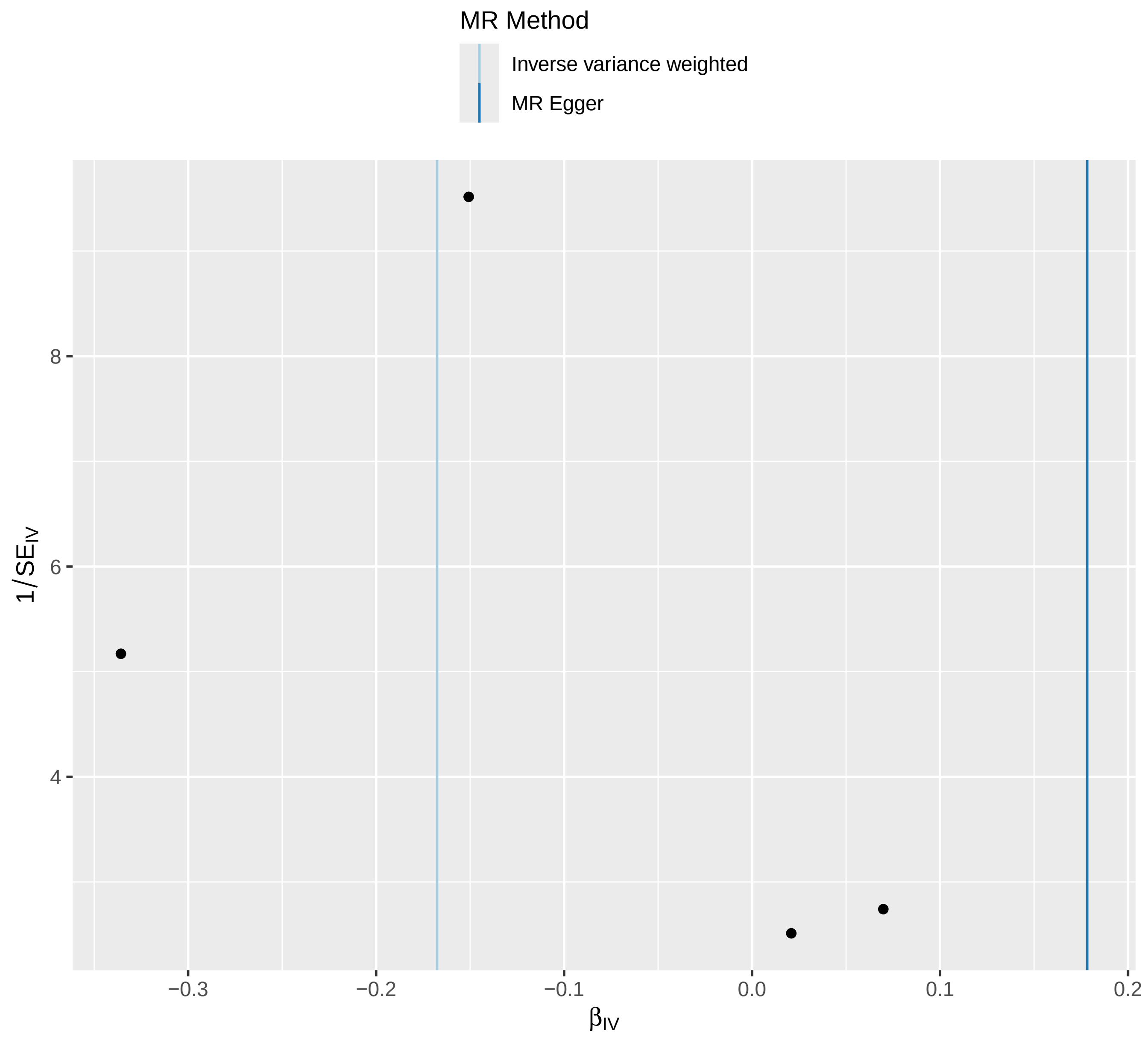
MR Method
Inverse variance weighted
MR Egger
8
6
4
1/SE_IV
−0.3
−0.2
−0.1
0.0
0.1
0.2
β_IV

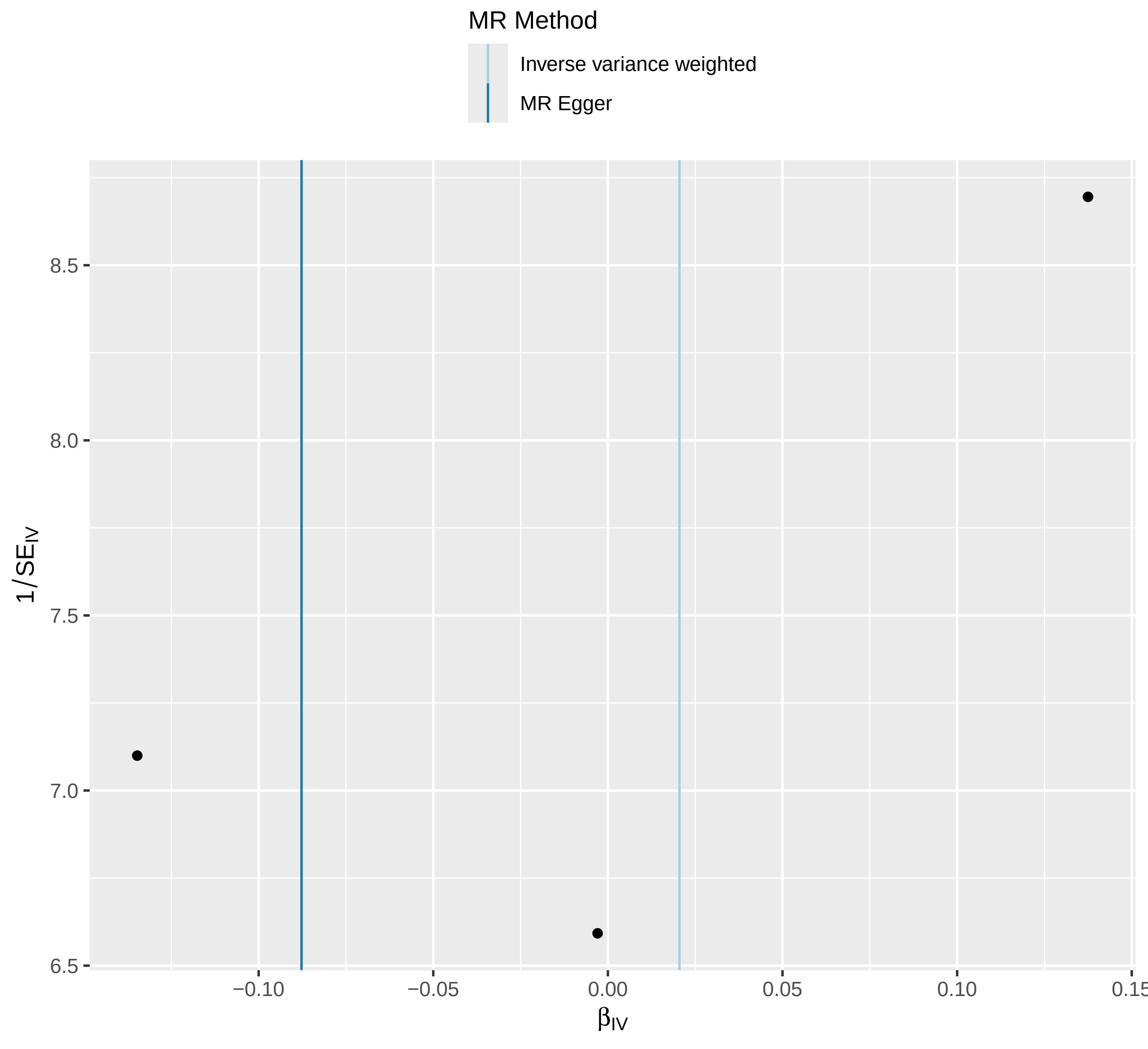
MR Method
Inverse variance weighted
MR Egger
8.5
8.0
7.5
7.0
6.5
1/SE_IV
−0.10
−0.05
0.00
0.05
0.10
0.15
β_IV

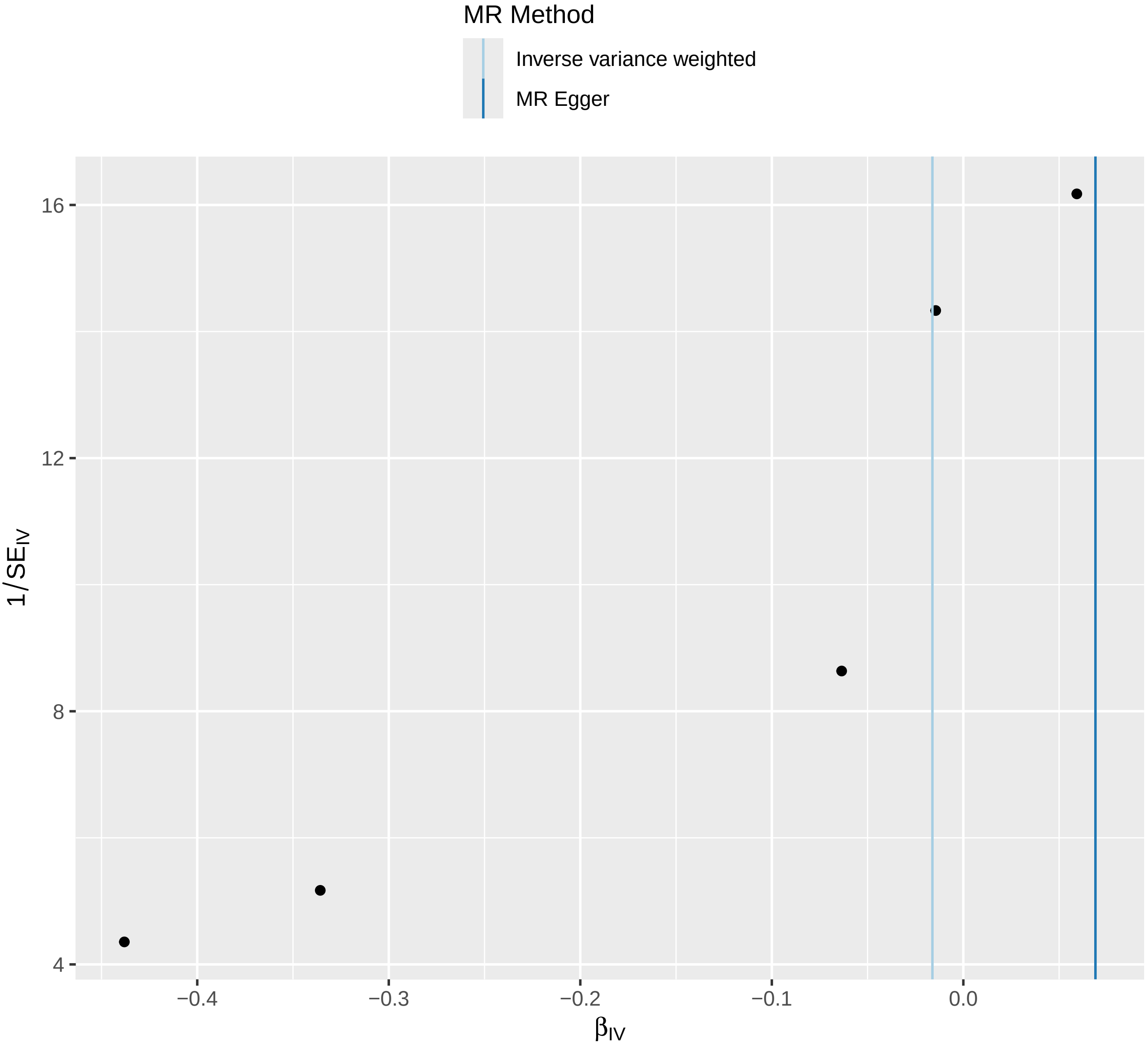

MR Method
Inverse variance weighted
MR Egger
16
12
8
4
1/SE$_{IV}$
−0.4
−0.3
−0.2
−0.1
0.0
β$_{IV}$

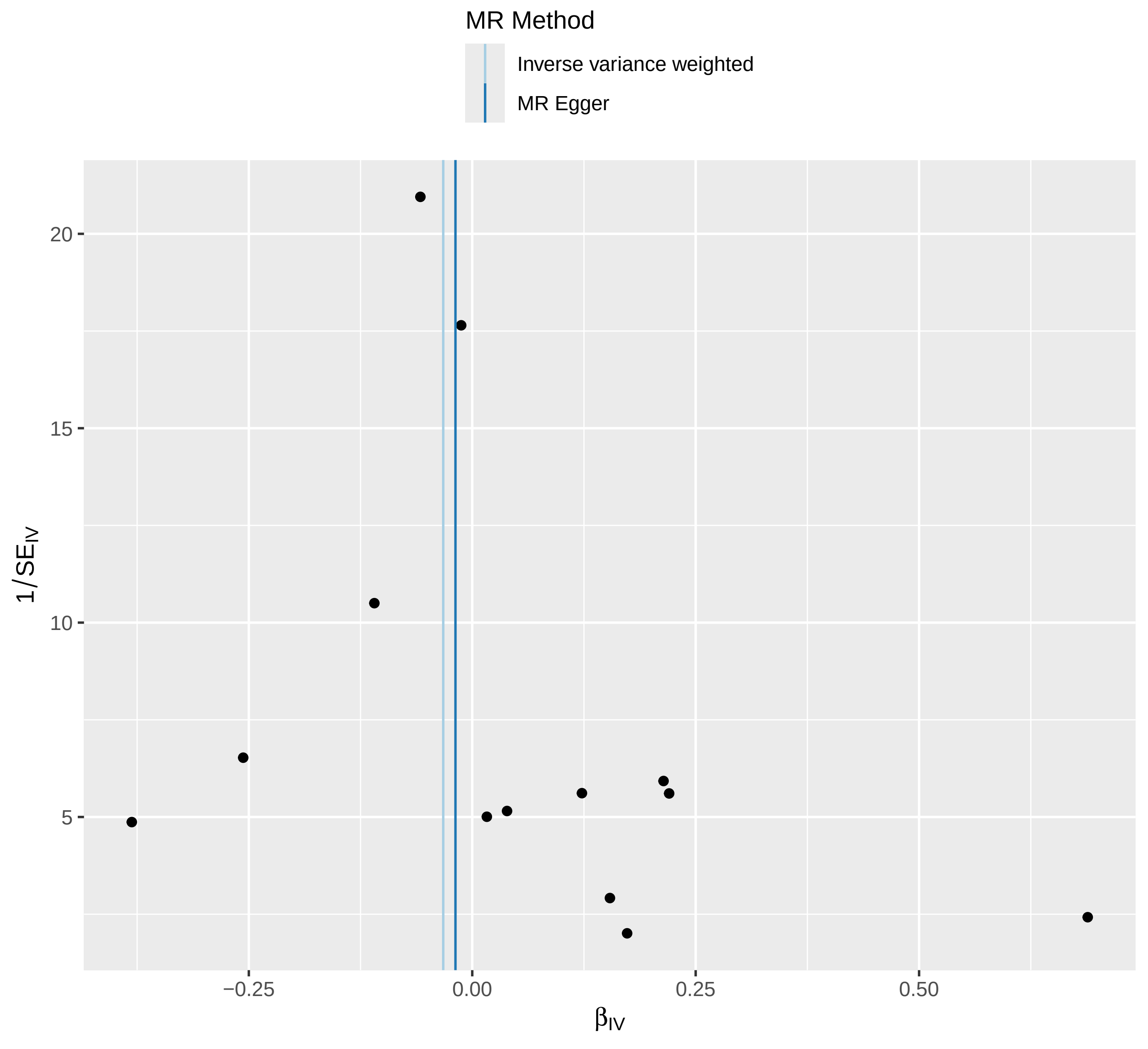

MR Method
Inverse variance weighted
MR Egger
20
15
10
5
1/SE_IV
−0.25
0.00
0.25
0.50
β_IV

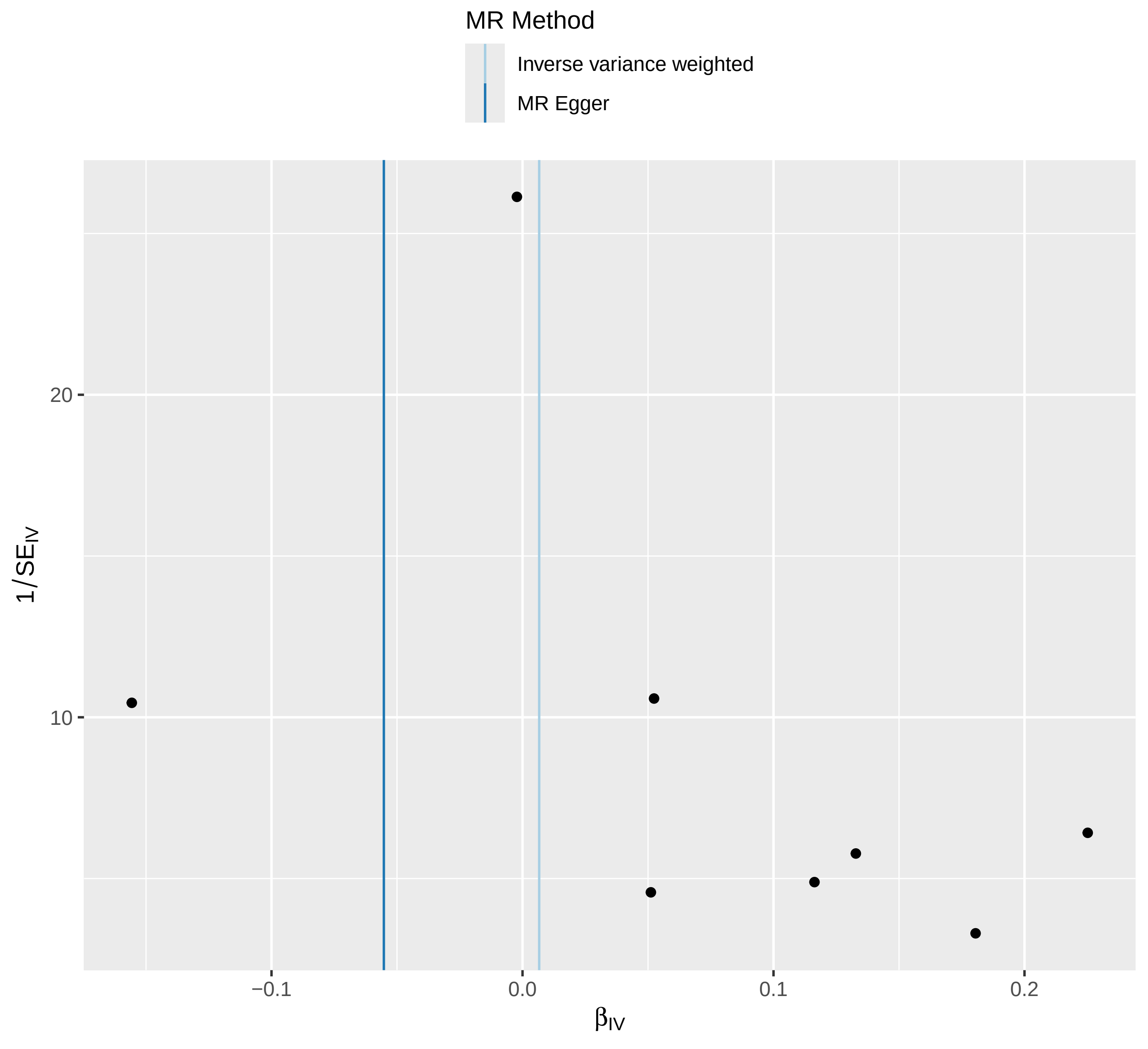

MR Method
Inverse variance weighted
MR Egger
20
10
1/SE_IV
−0.1
0.0
0.1
0.2
β_IV

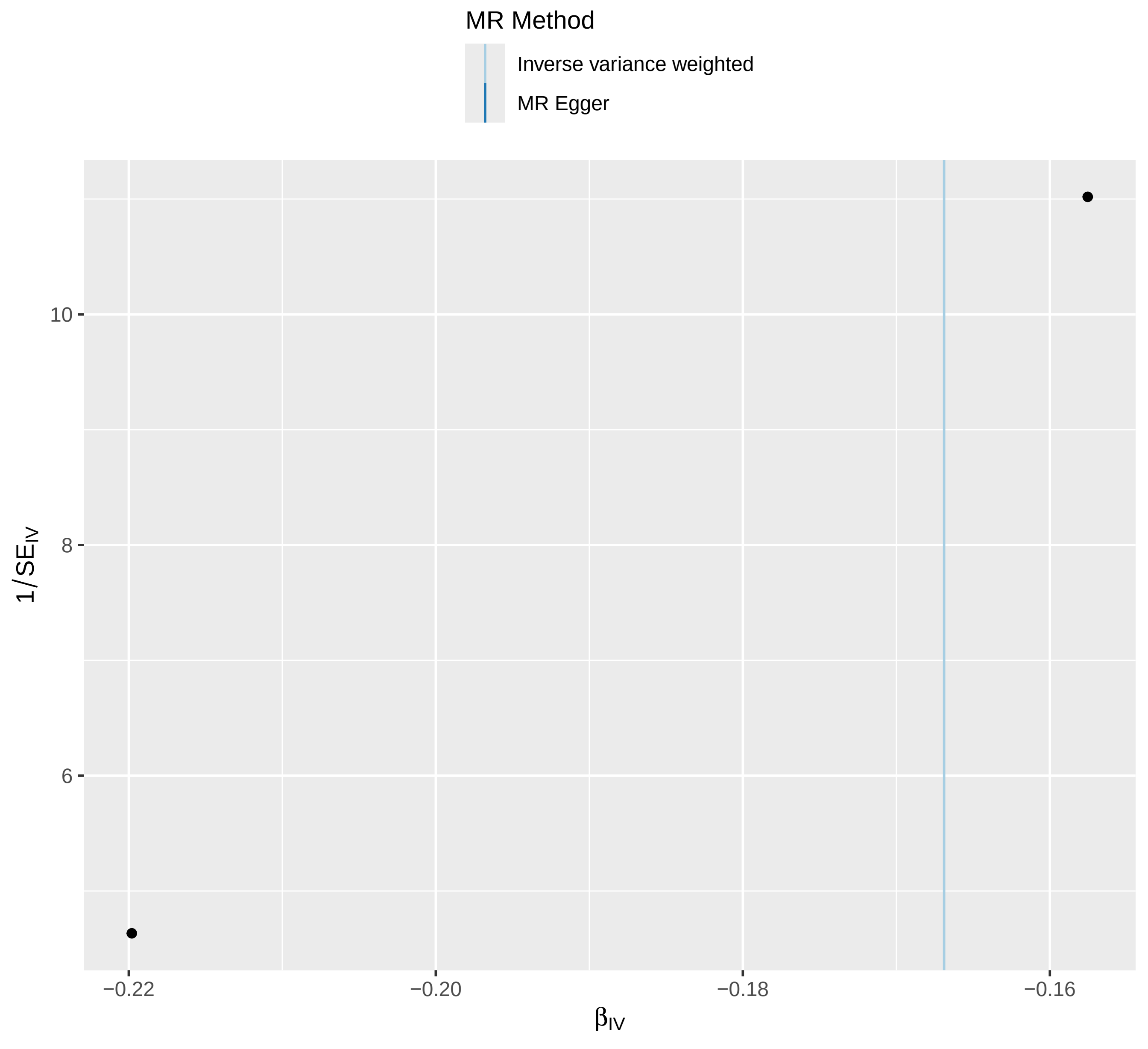

MR Method
Inverse variance weighted
MR Egger
10
8
6
$1/SE_{IV}$
−0.22
−0.20
−0.18
−0.16
$\beta_{IV}$

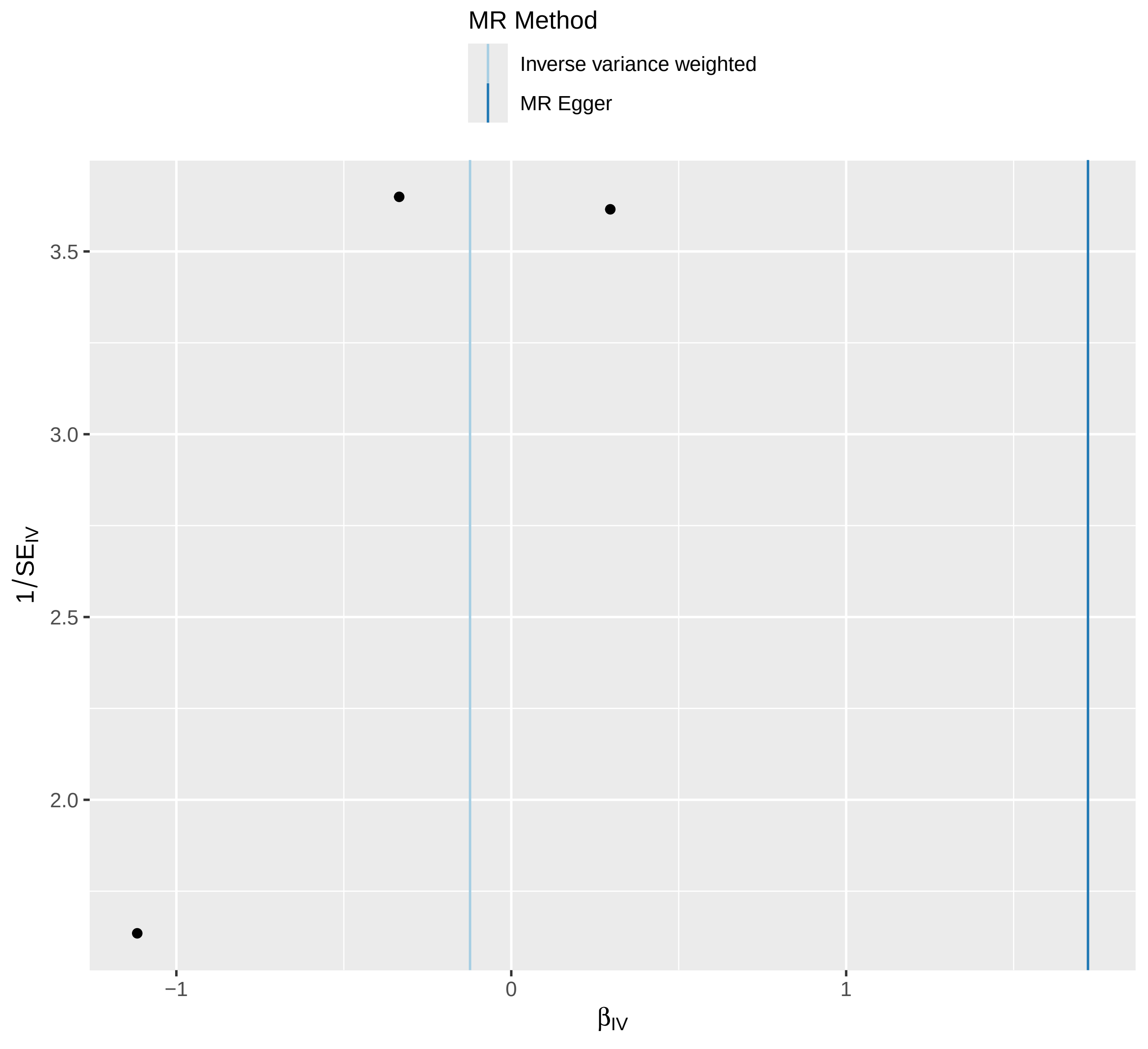
MR Method
Inverse variance weighted
MR Egger
3.5
3.0
2.5
2.0
1/SE$_{IV}$
−1
0
1
β$_{IV}$

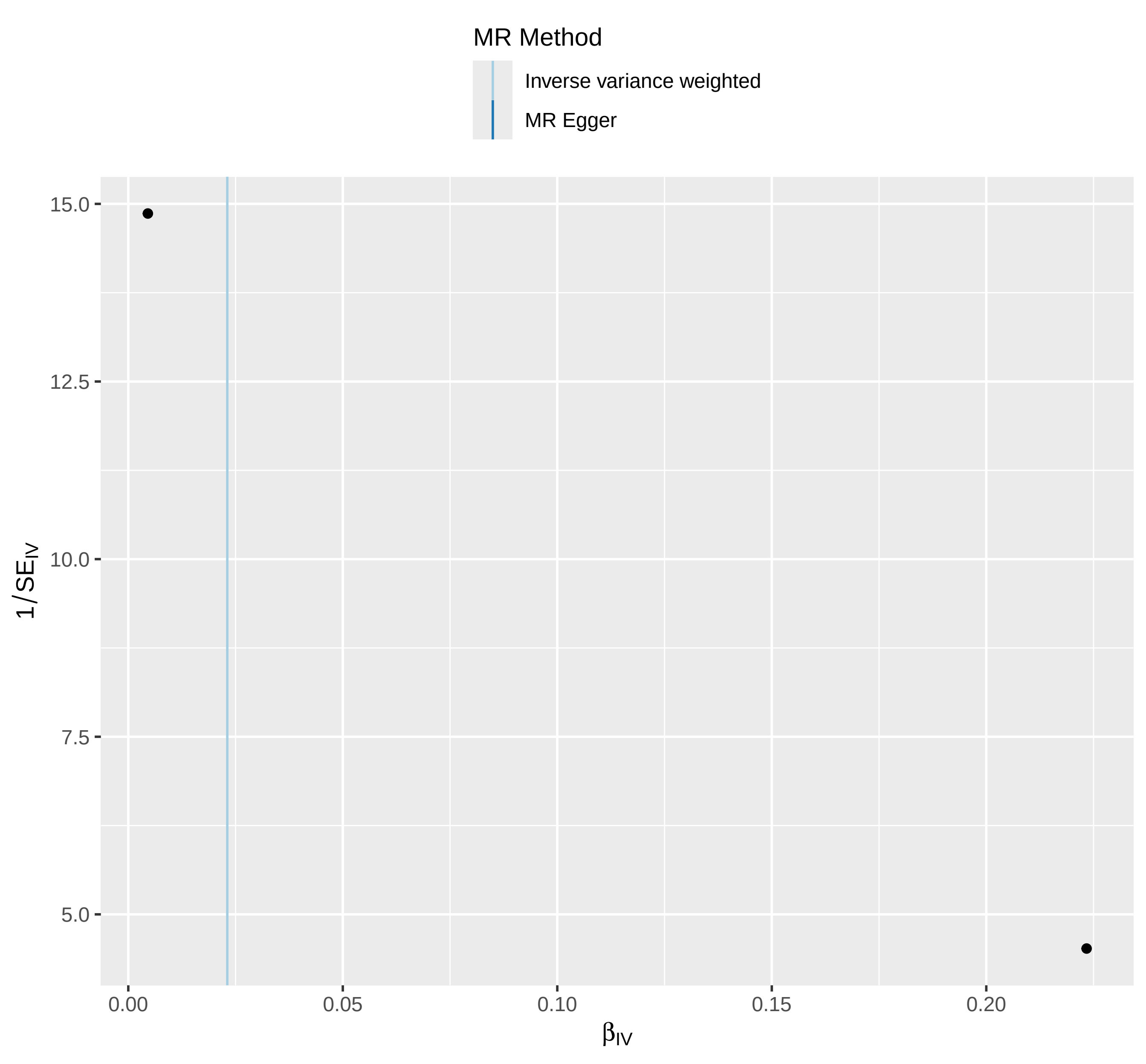
MR Method
Inverse variance weighted
MR Egger
15.0
12.5
10.0
7.5
5.0
1/SE_IV
0.00
0.05
0.10
0.15
0.20
β_IV

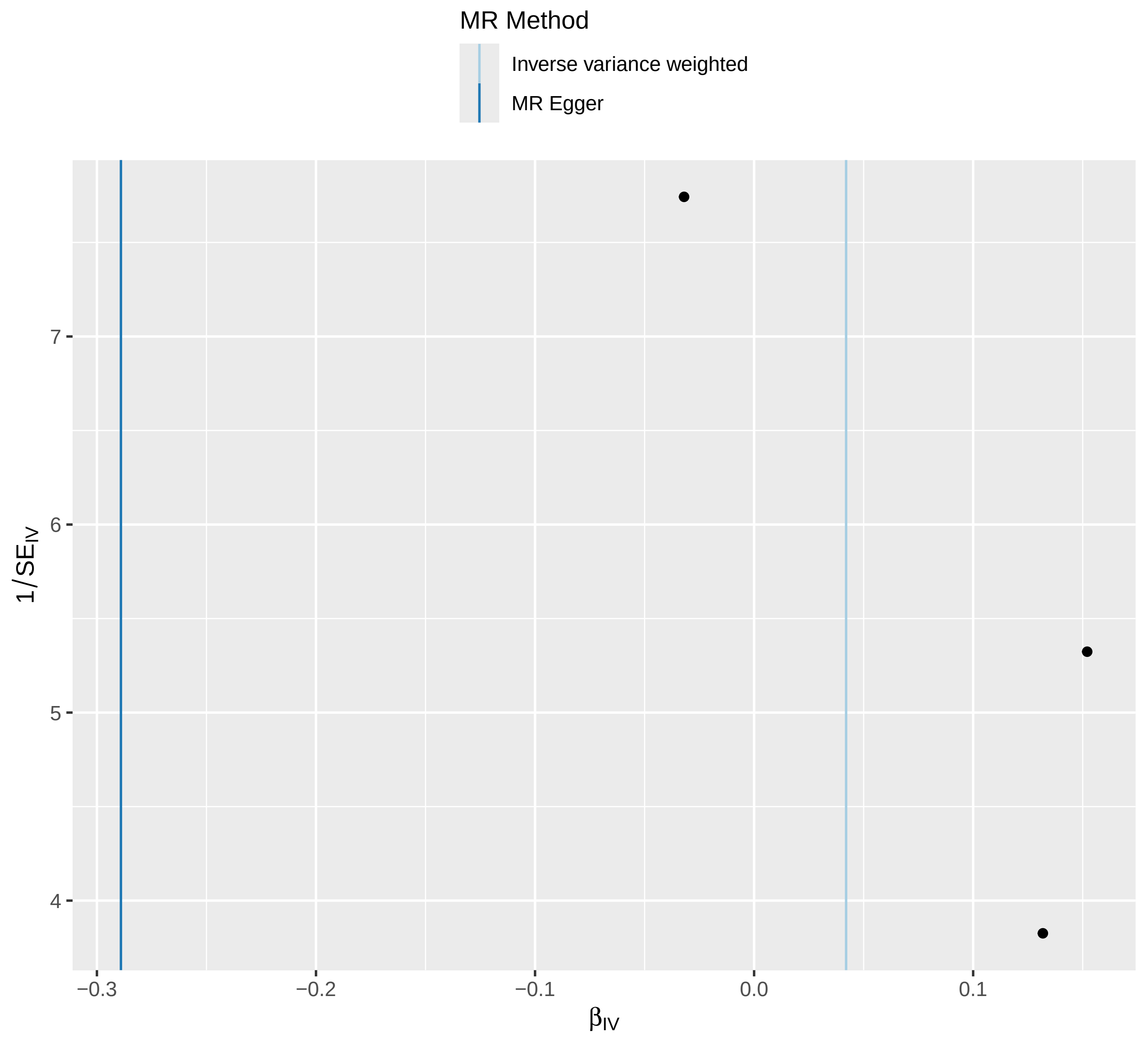

MR Method
Inverse variance weighted
MR Egger
7
6
5
4
1/SE_IV
−0.3
−0.2
−0.1
0.0
0.1
β_IV

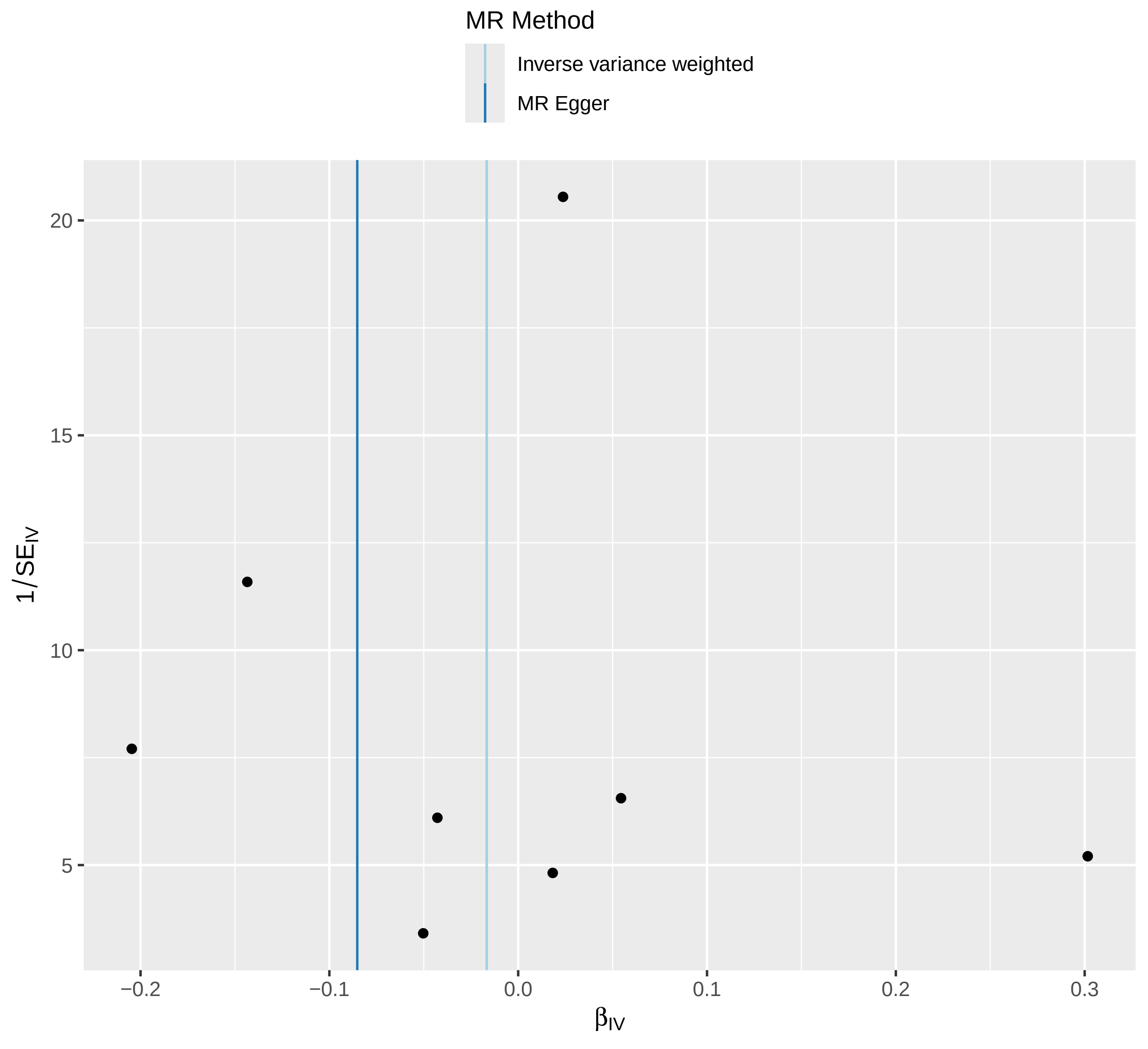
MR Method
Inverse variance weighted
MR Egger
1/SE_IV
20
15
10
5
β_IV
−0.2
−0.1
0.0
0.1
0.2
0.3

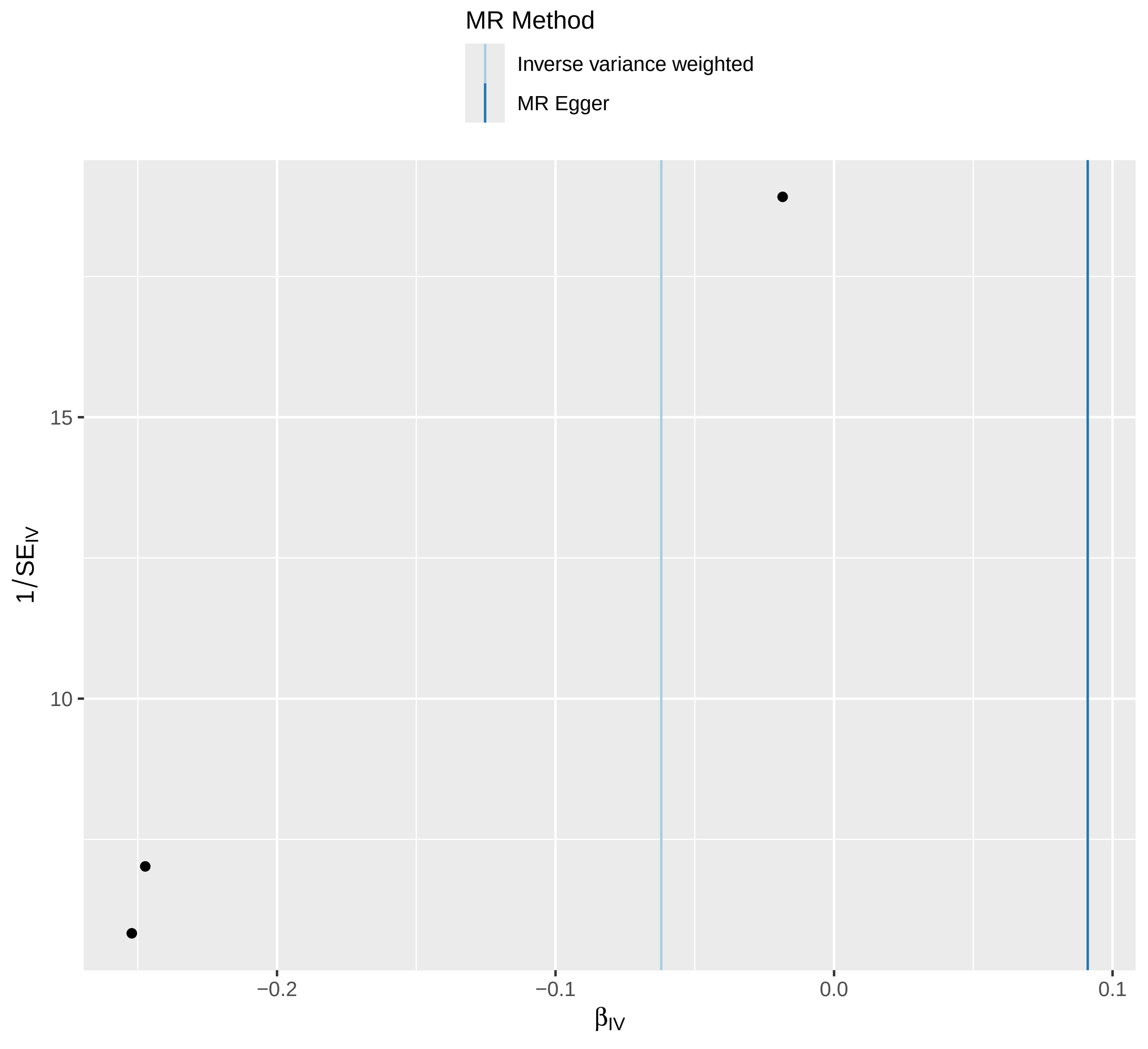

MR Method
Inverse variance weighted
MR Egger
15
10
$1/SE_{IV}$
−0.2
−0.1
0.0
0.1
$\beta_{IV}$

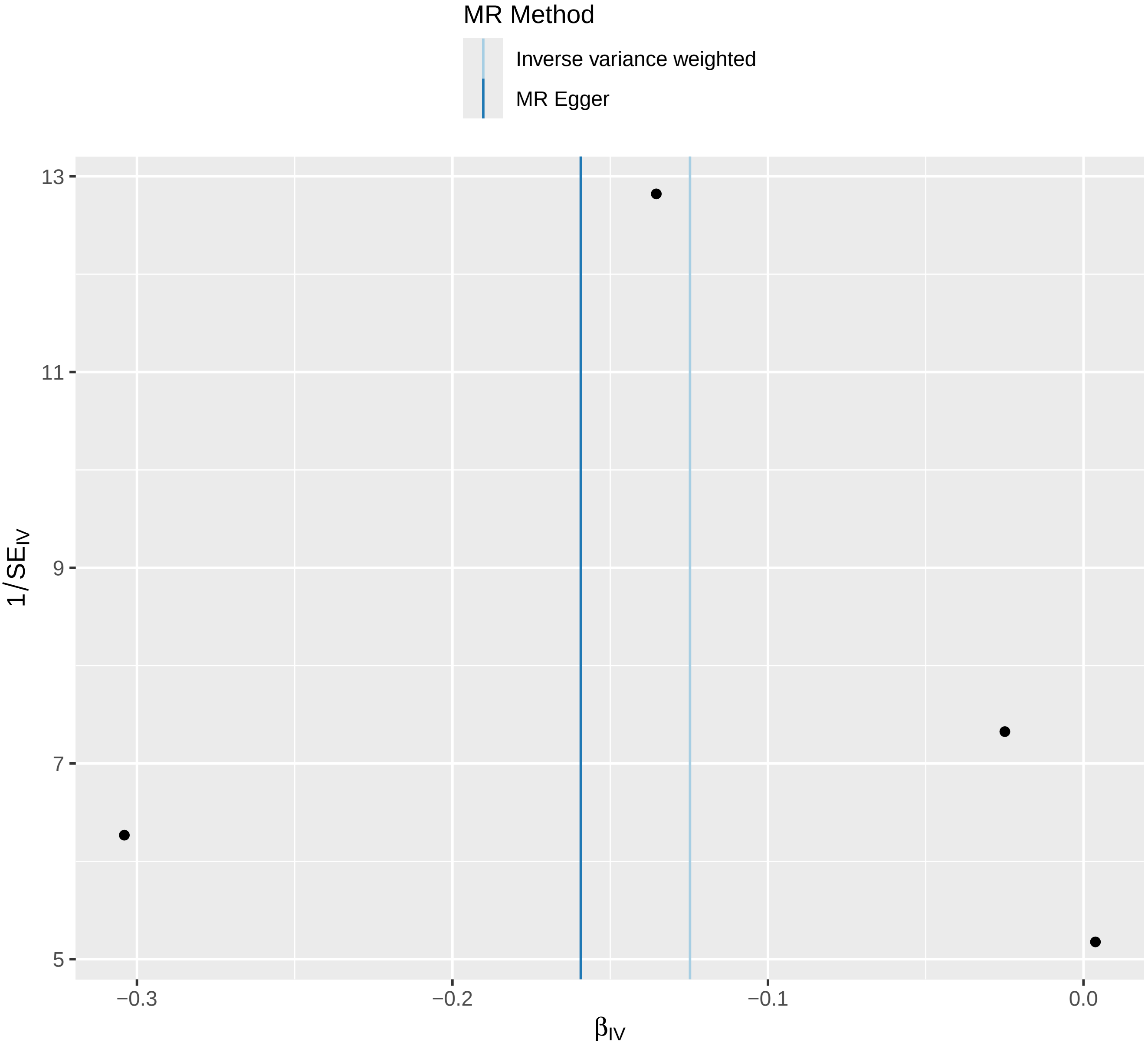

MR Method
Inverse variance weighted
MR Egger
13
11
9
7
5
1/SE_IV
−0.3
−0.2
−0.1
0.0
β_IV

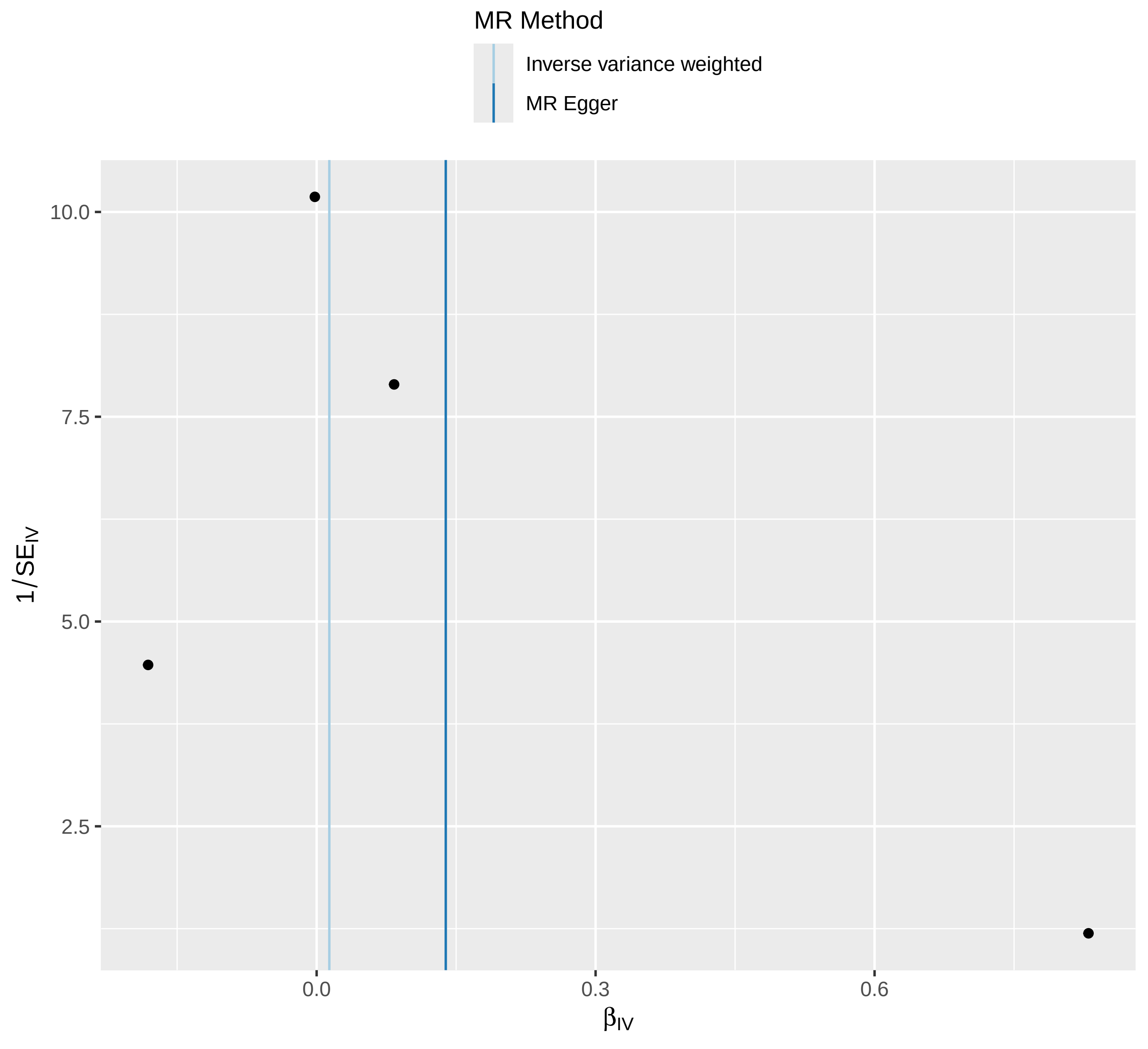
MR Method
Inverse variance weighted
MR Egger
10.0
7.5
5.0
2.5
$1/SE_{IV}$
0.0
0.3
0.6
$\beta_{IV}$

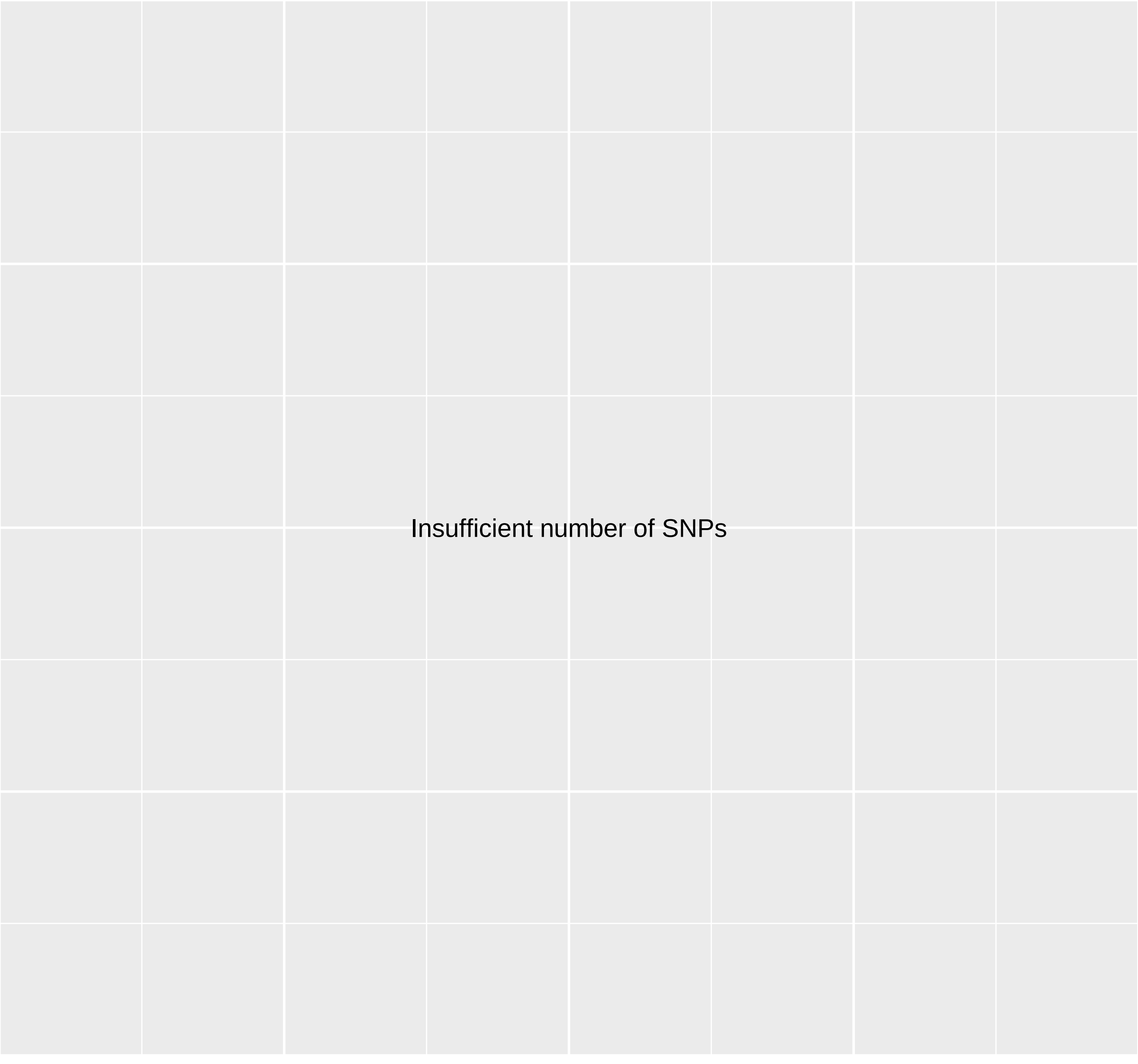
Insufficient number of SNPs

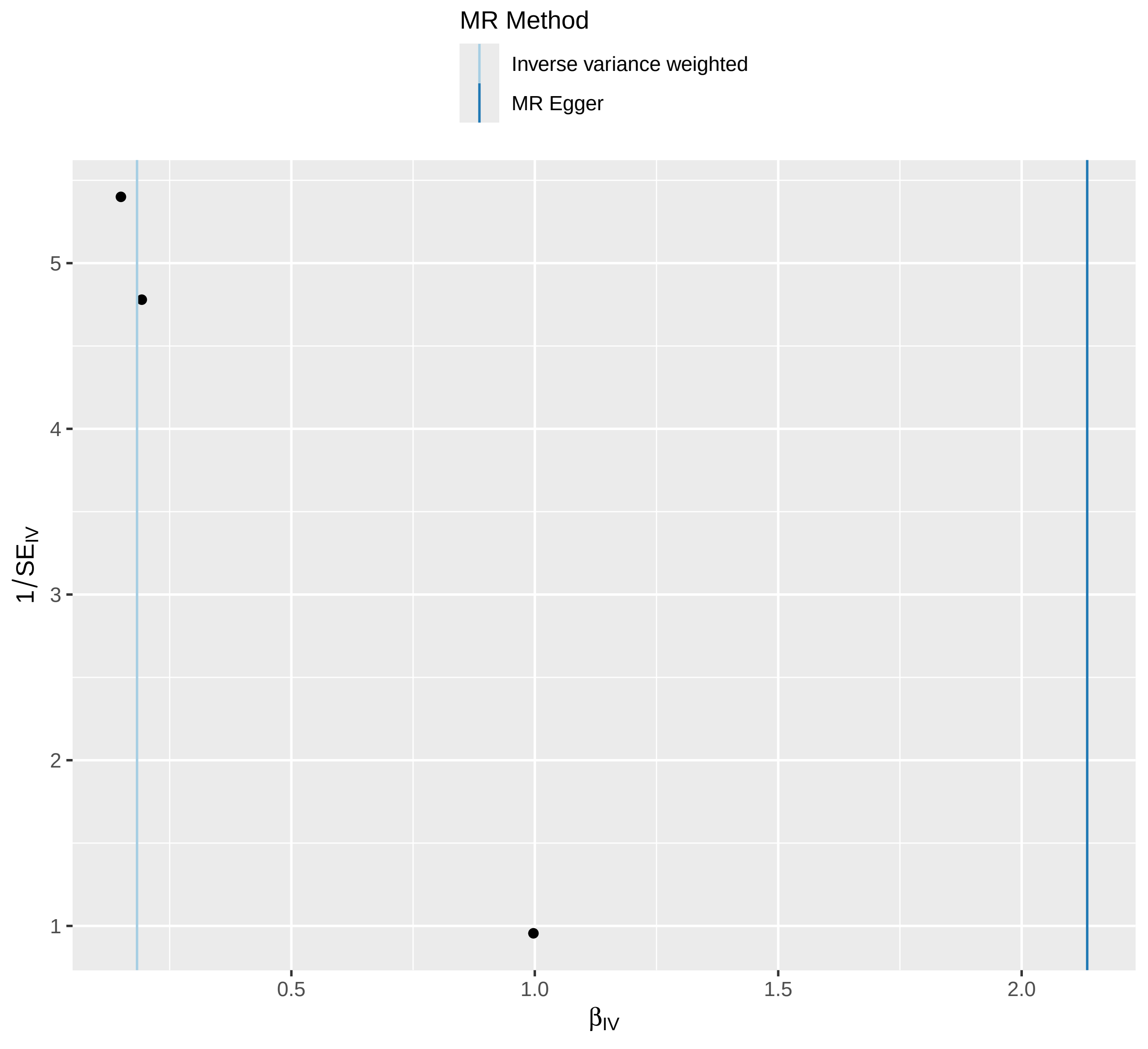
MR Method
Inverse variance weighted
MR Egger
1/SE_IV
5
4
3
2
1
0.5
1.0
1.5
2.0
β_IV

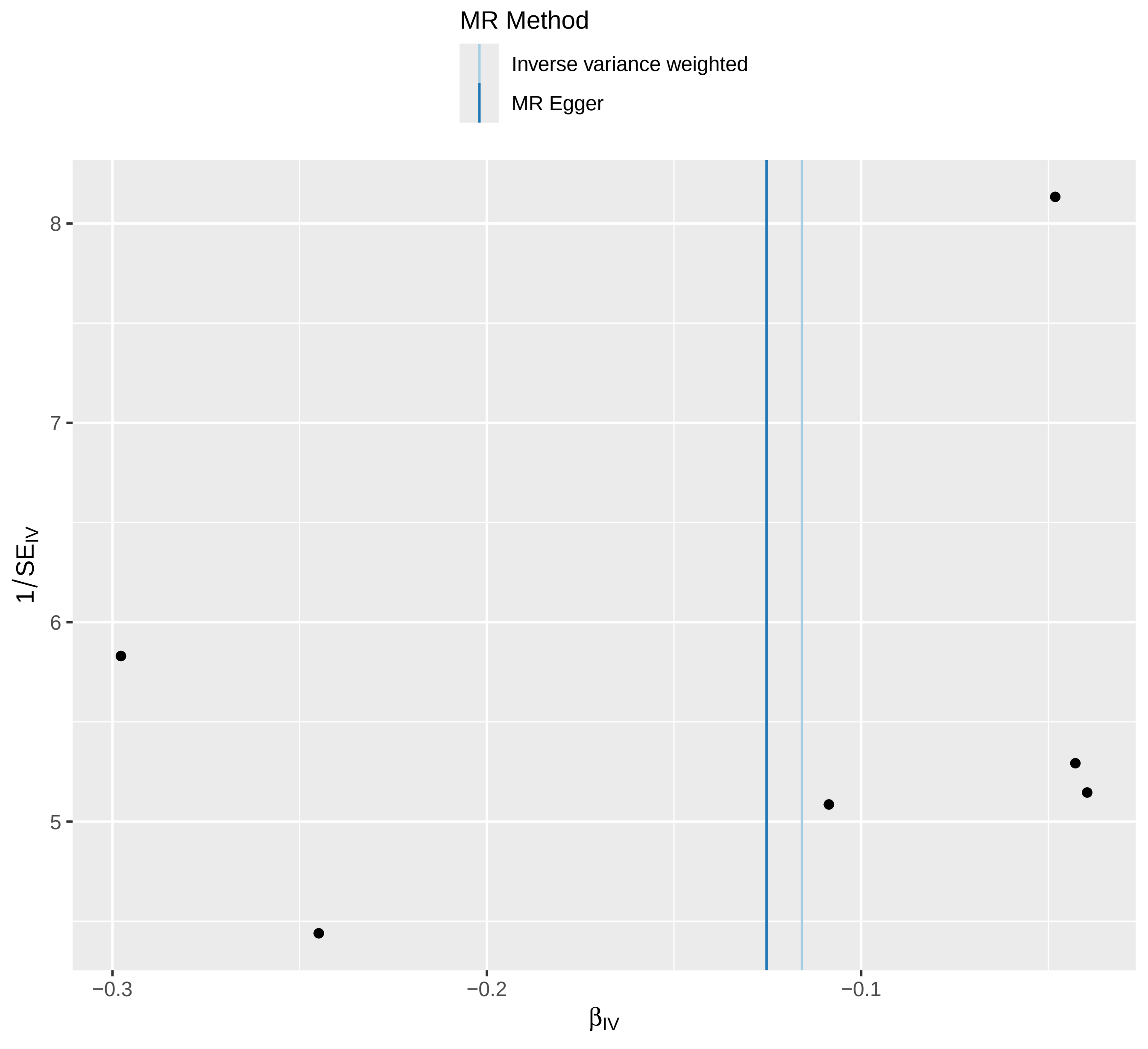
MR Method
Inverse variance weighted
MR Egger
8
7
6
5
1/SE_IV
−0.3
−0.2
−0.1
β_IV

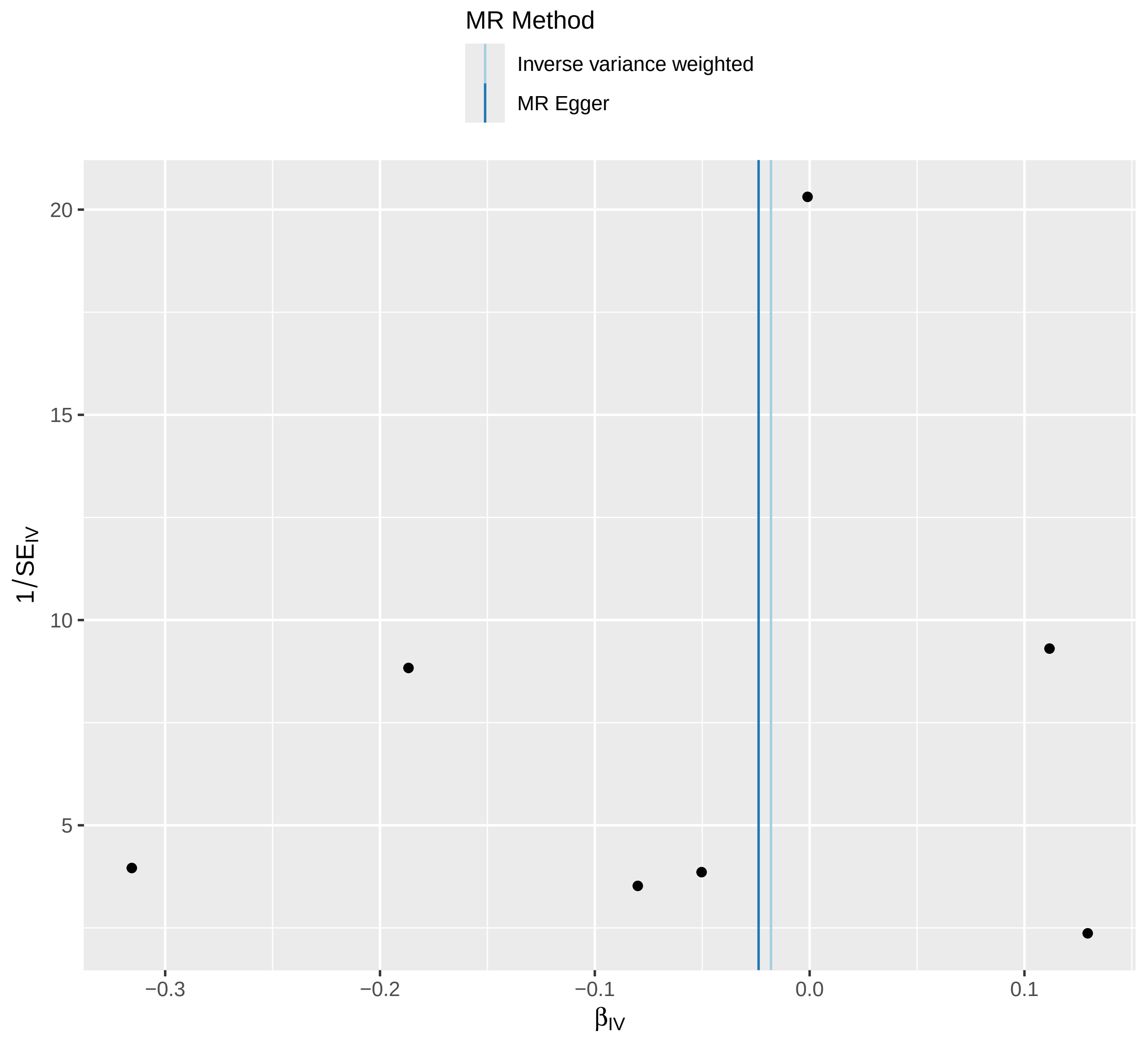

MR Method
Inverse variance weighted
MR Egger
20
15
10
5
1/SE$_{IV}$
−0.3
−0.2
−0.1
0.0
0.1
β$_{IV}$

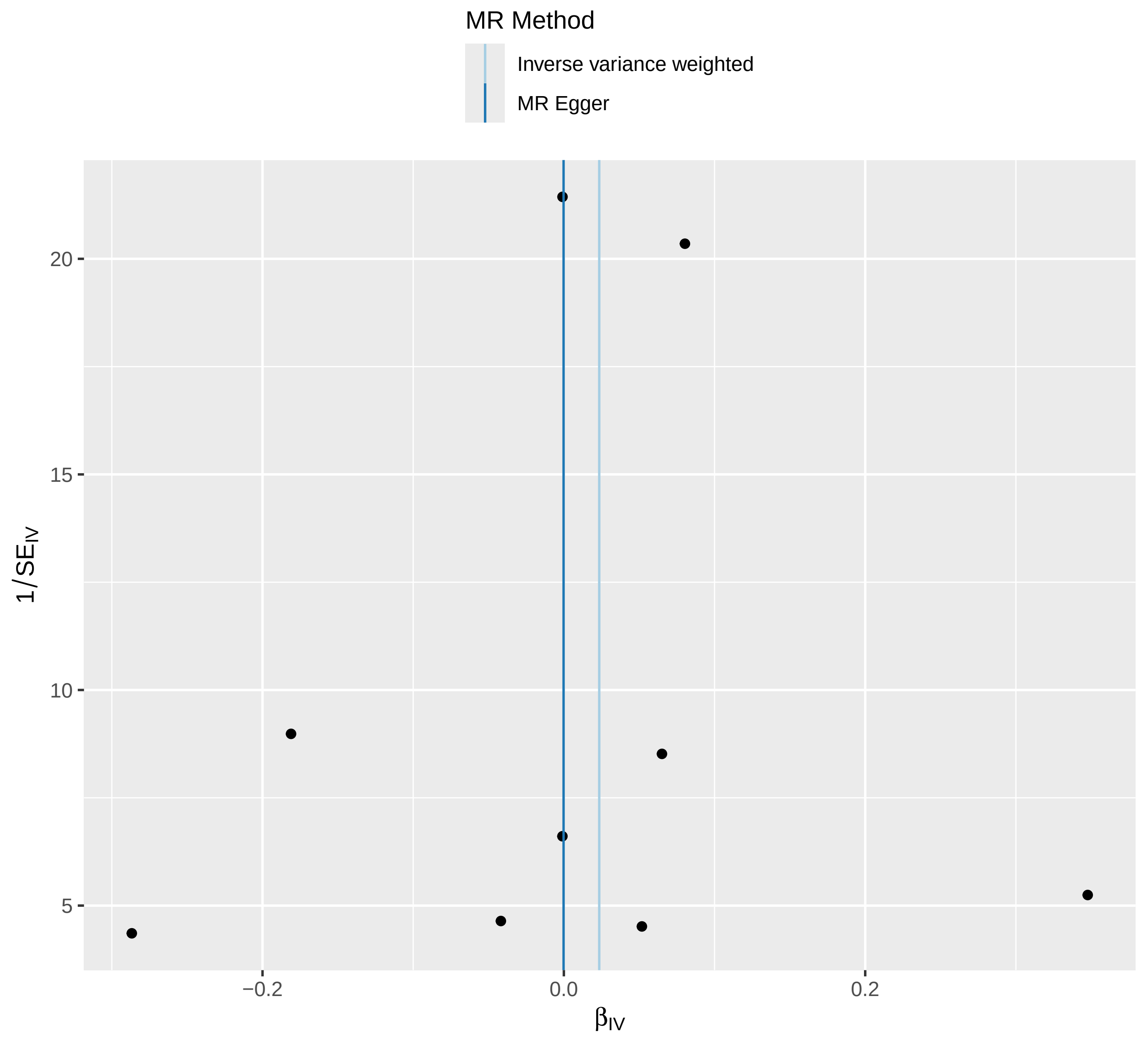
MR Method
Inverse variance weighted
MR Egger
20
15
10
5
−0.2
0.0
0.2
1/SE_IV
β_IV

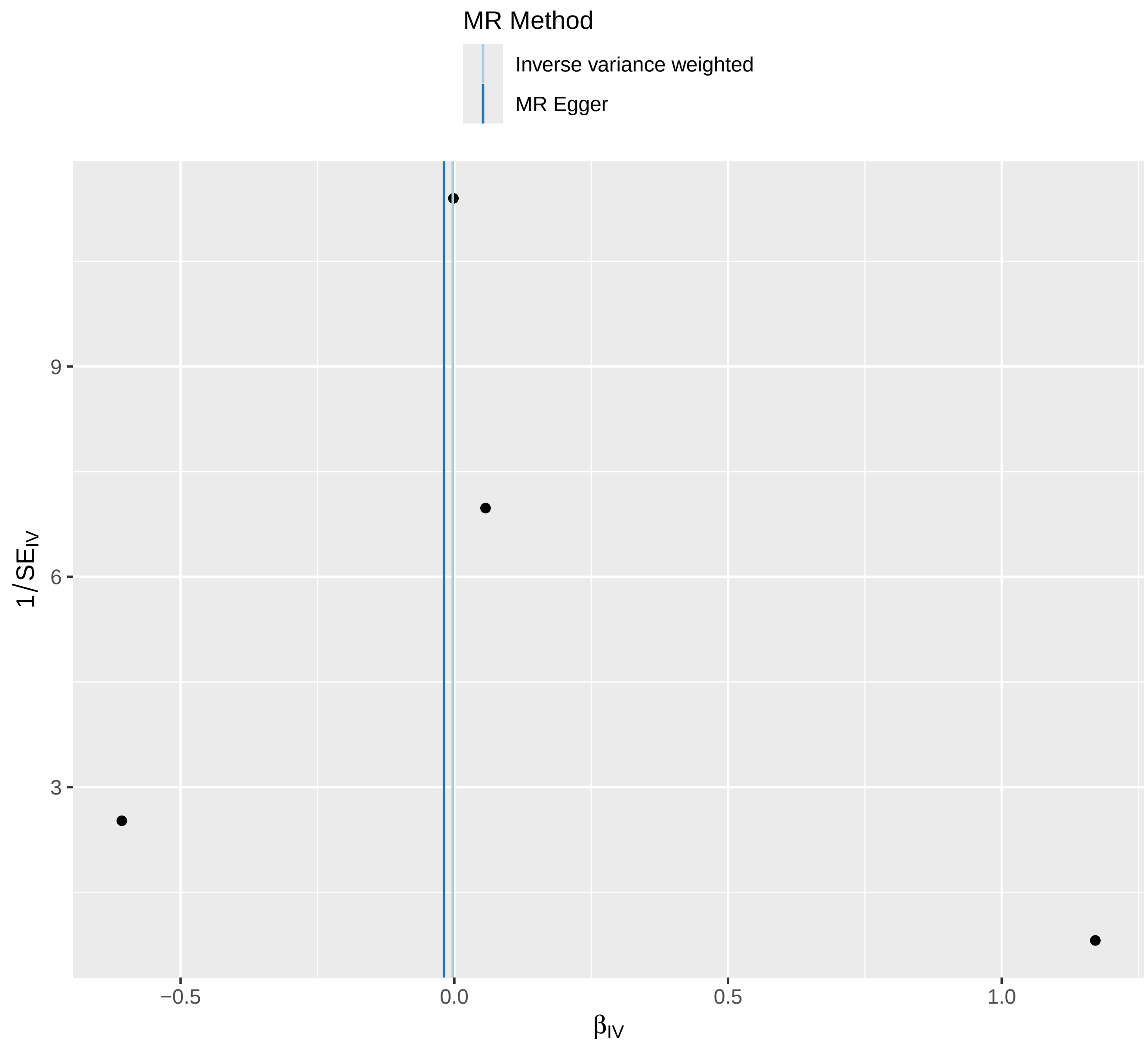
MR Method
Inverse variance weighted
MR Egger
9
6
3
1/SE_IV
−0.5
0.0
0.5
1.0
β_IV

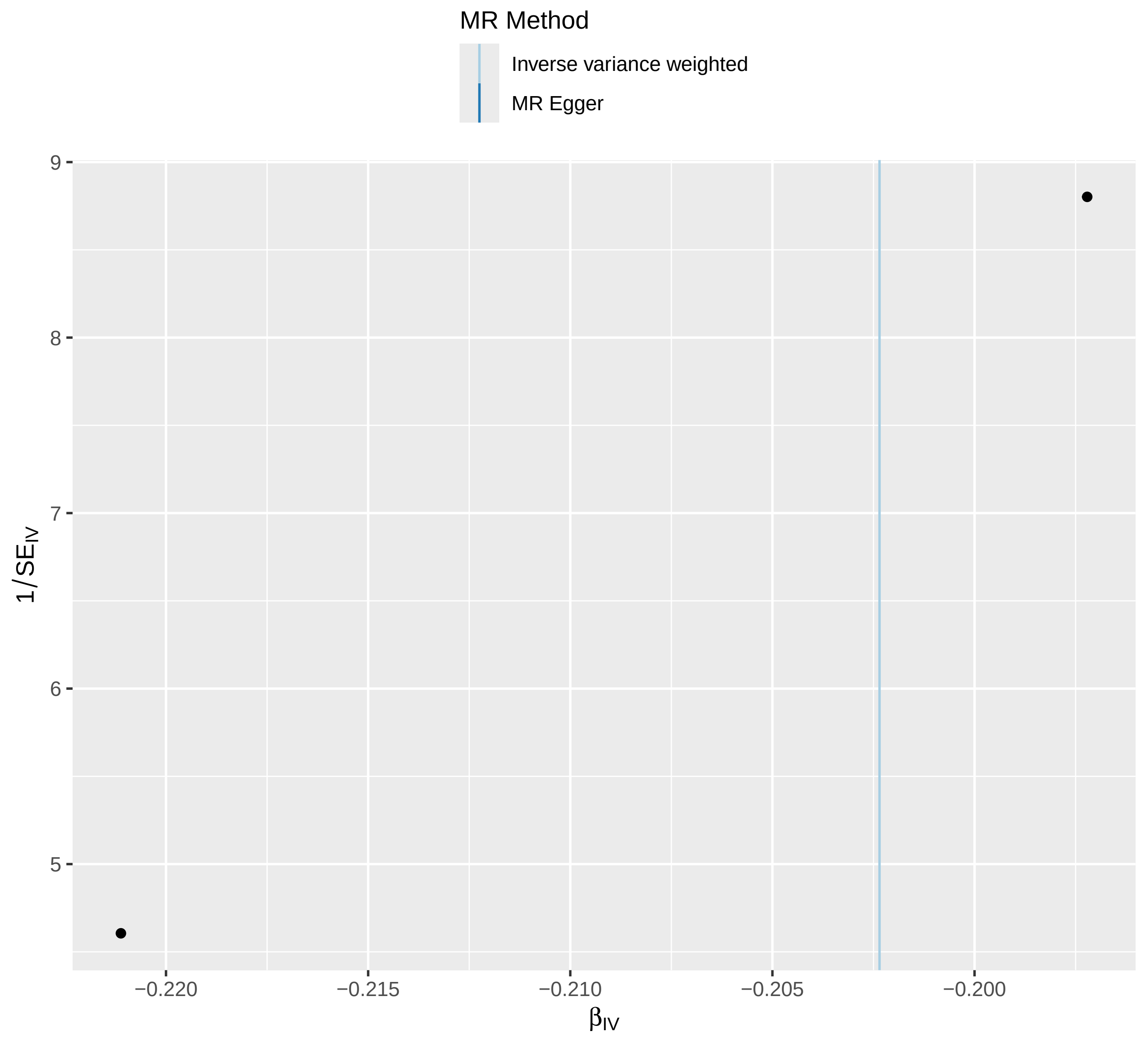
MR Method
Inverse variance weighted
MR Egger
9
8
7
6
5
1/SE_IV
−0.220
−0.215
−0.210
−0.205
−0.200
β_IV

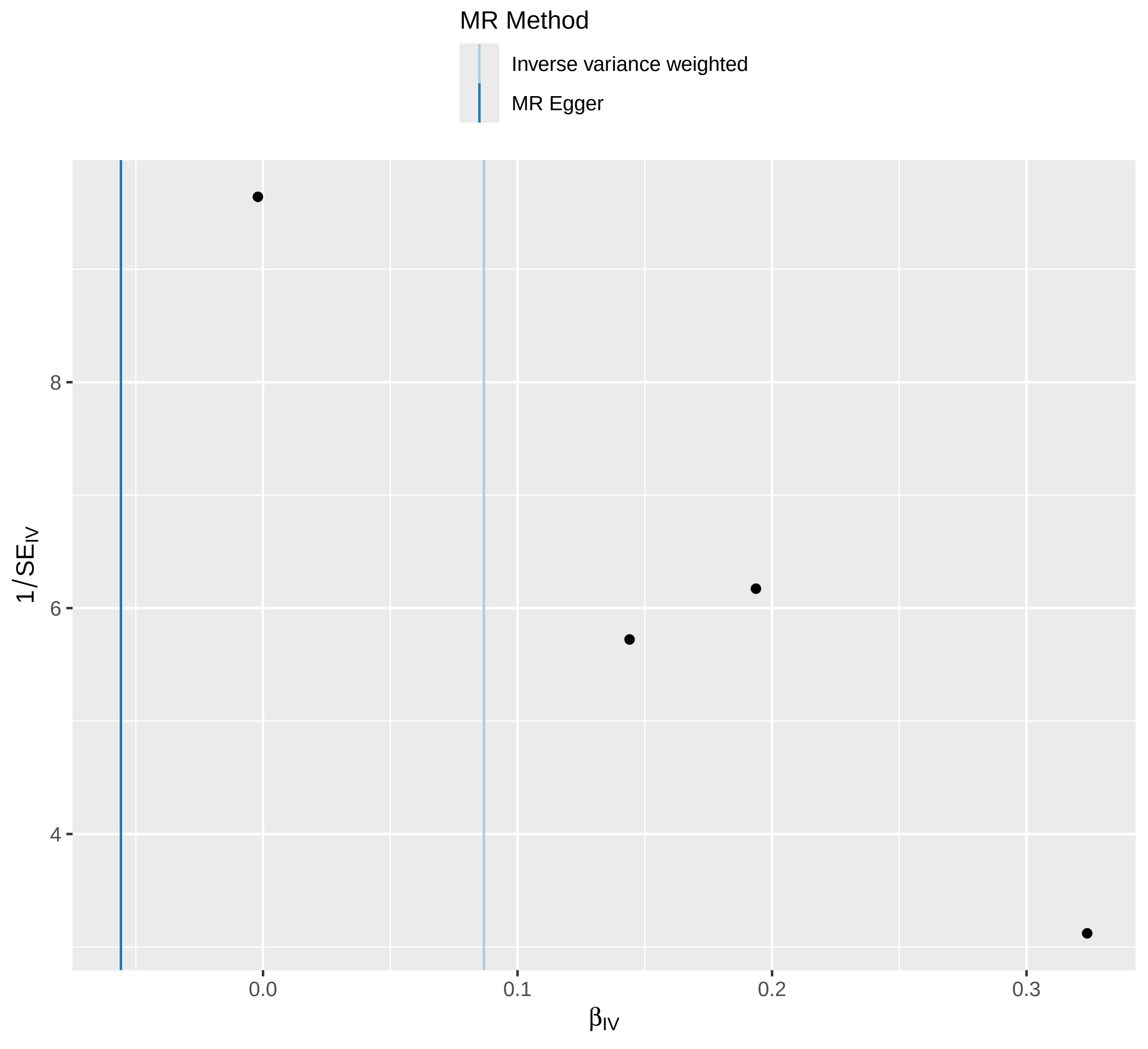
MR Method
Inverse variance weighted
MR Egger
8
6
4
1/SE_IV
0.0
0.1
0.2
0.3
β_IV

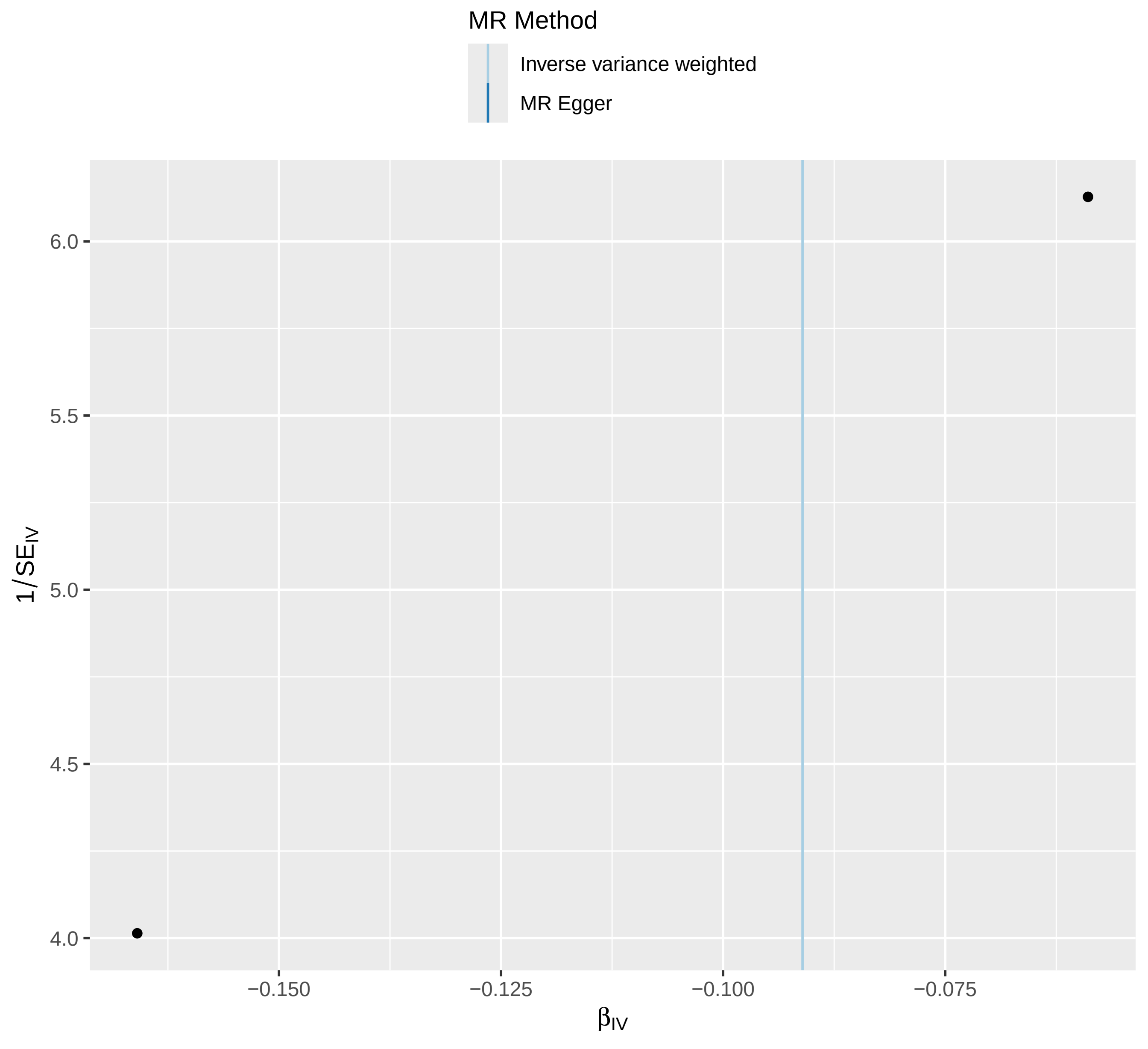

MR Method
Inverse variance weighted
MR Egger
6.0
5.5
5.0
4.5
4.0
1/SE$_{IV}$
−0.150
−0.125
−0.100
−0.075
β$_{IV}$

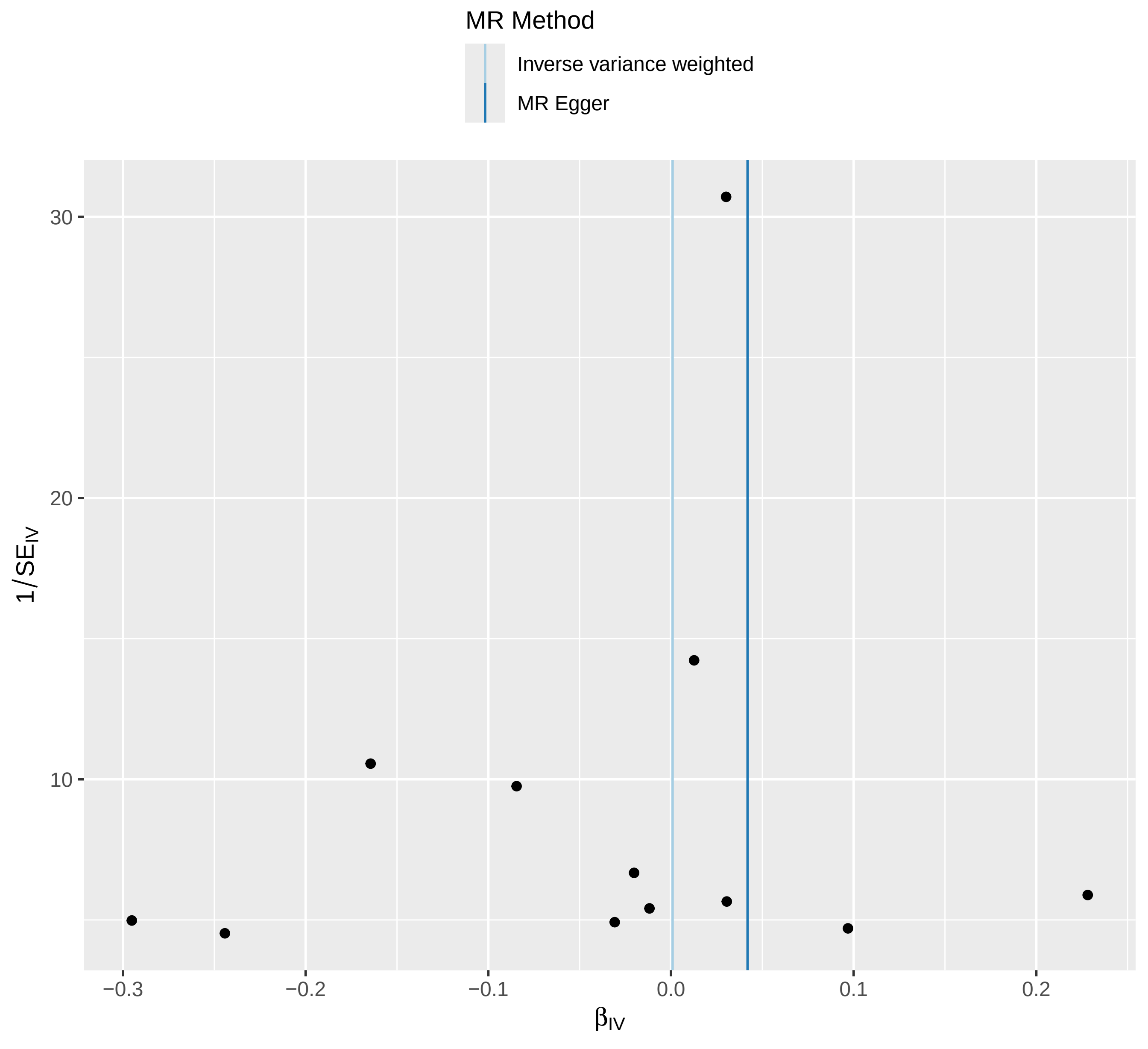
MR Method
Inverse variance weighted
MR Egger
30
20
10
1/SE_IV
−0.3
−0.2
−0.1
0.0
0.1
0.2
β_IV

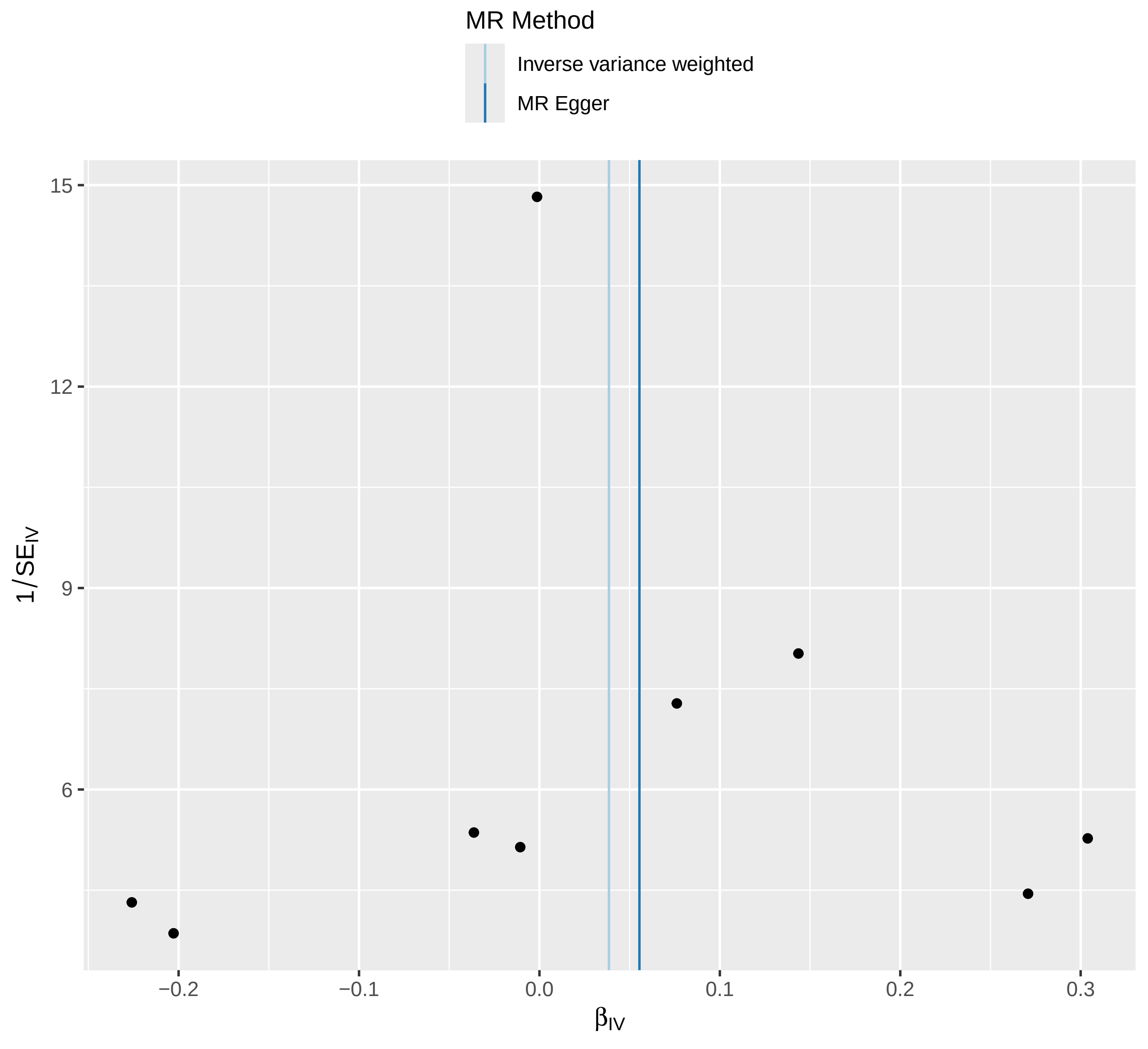
MR Method
Inverse variance weighted
MR Egger
15
12
9
6
1/SE$_{IV}$
−0.2
−0.1
0.0
0.1
0.2
0.3
β$_{IV}$

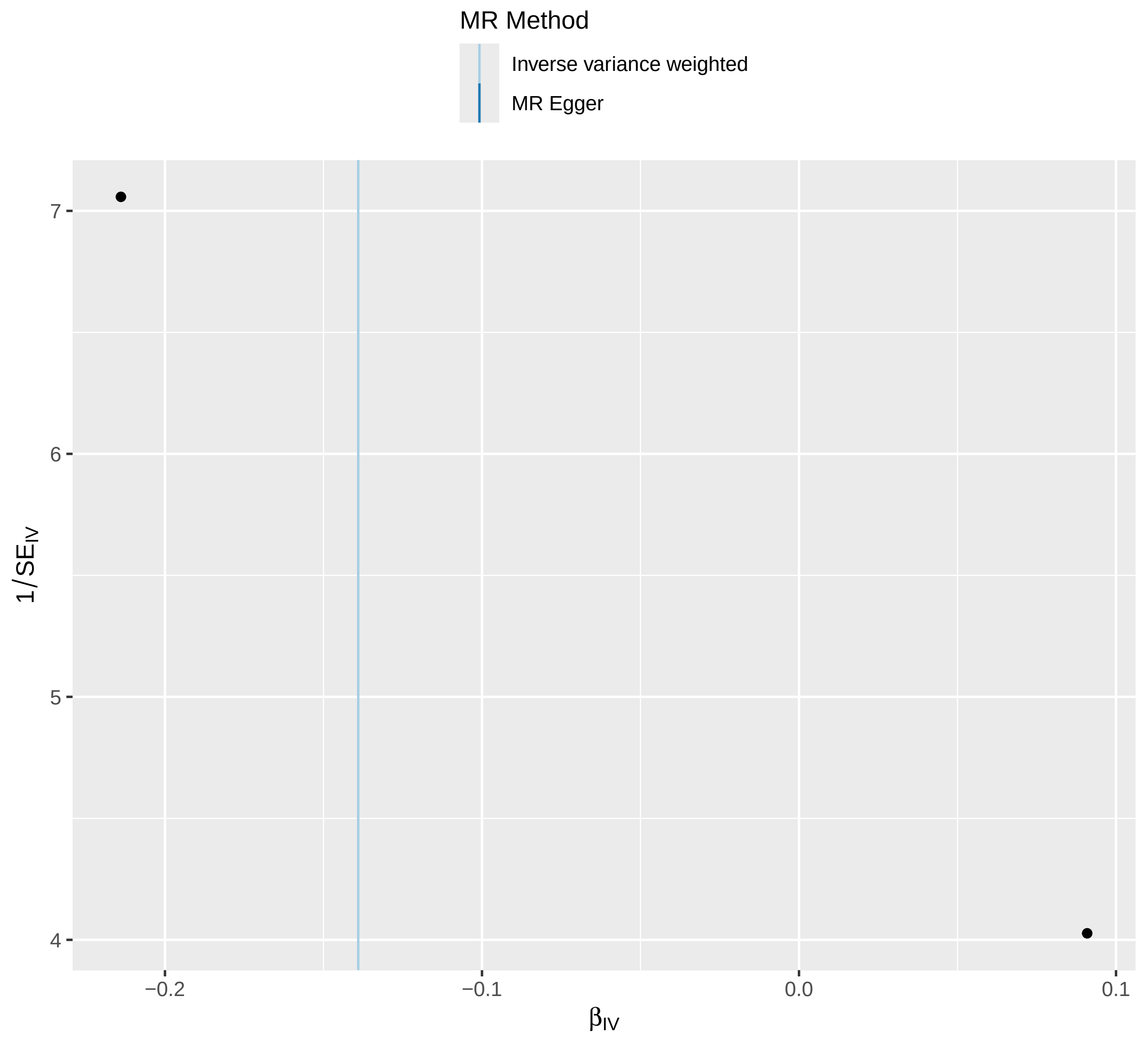
MR Method
Inverse variance weighted
MR Egger
1/SE$_{IV}$
7
6
5
4
−0.2
−0.1
0.0
0.1
β$_{IV}$

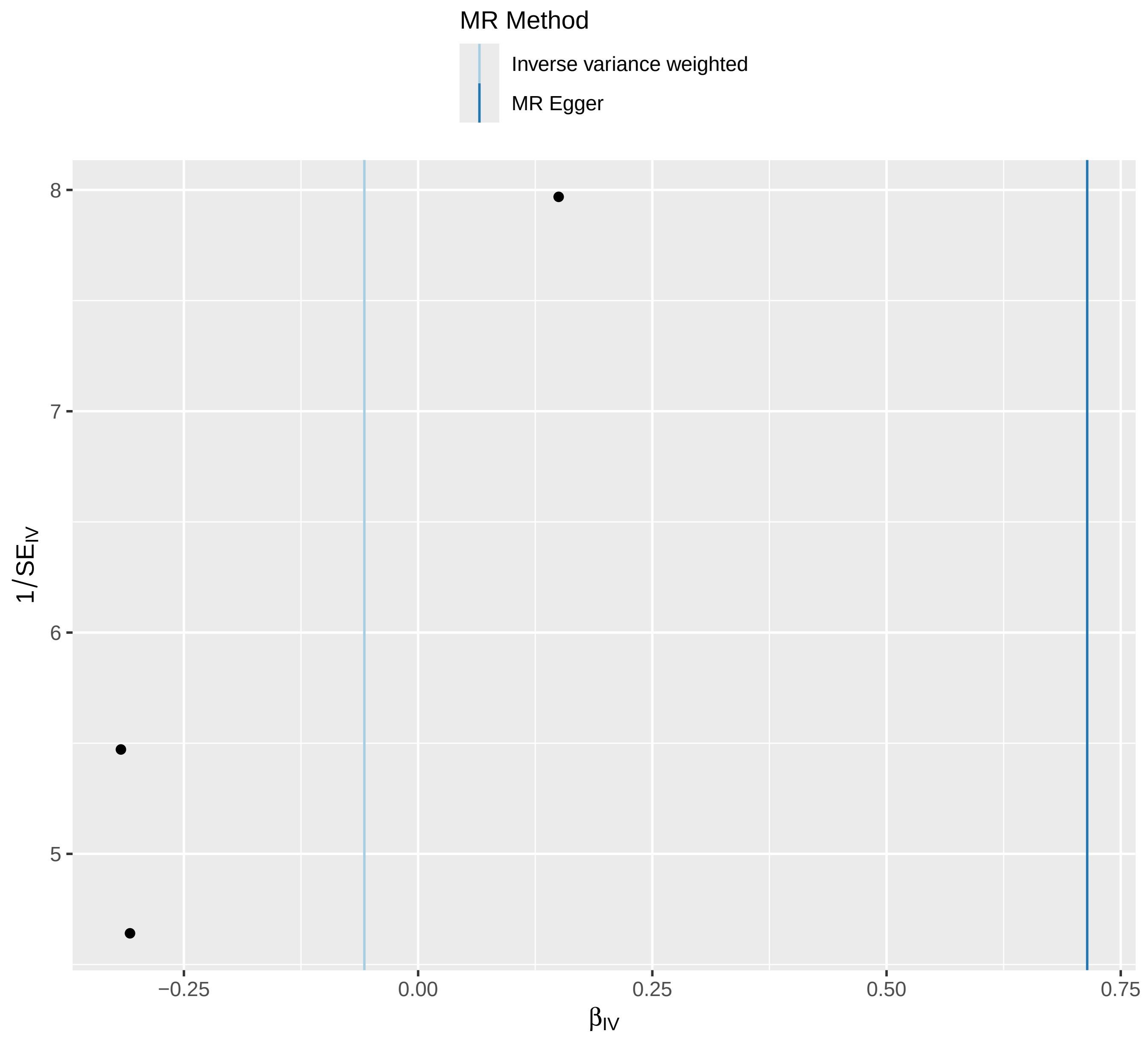
MR Method
Inverse variance weighted
MR Egger
8
7
6
5
1/SE_IV
−0.25
0.00
0.25
0.50
0.75
β_IV

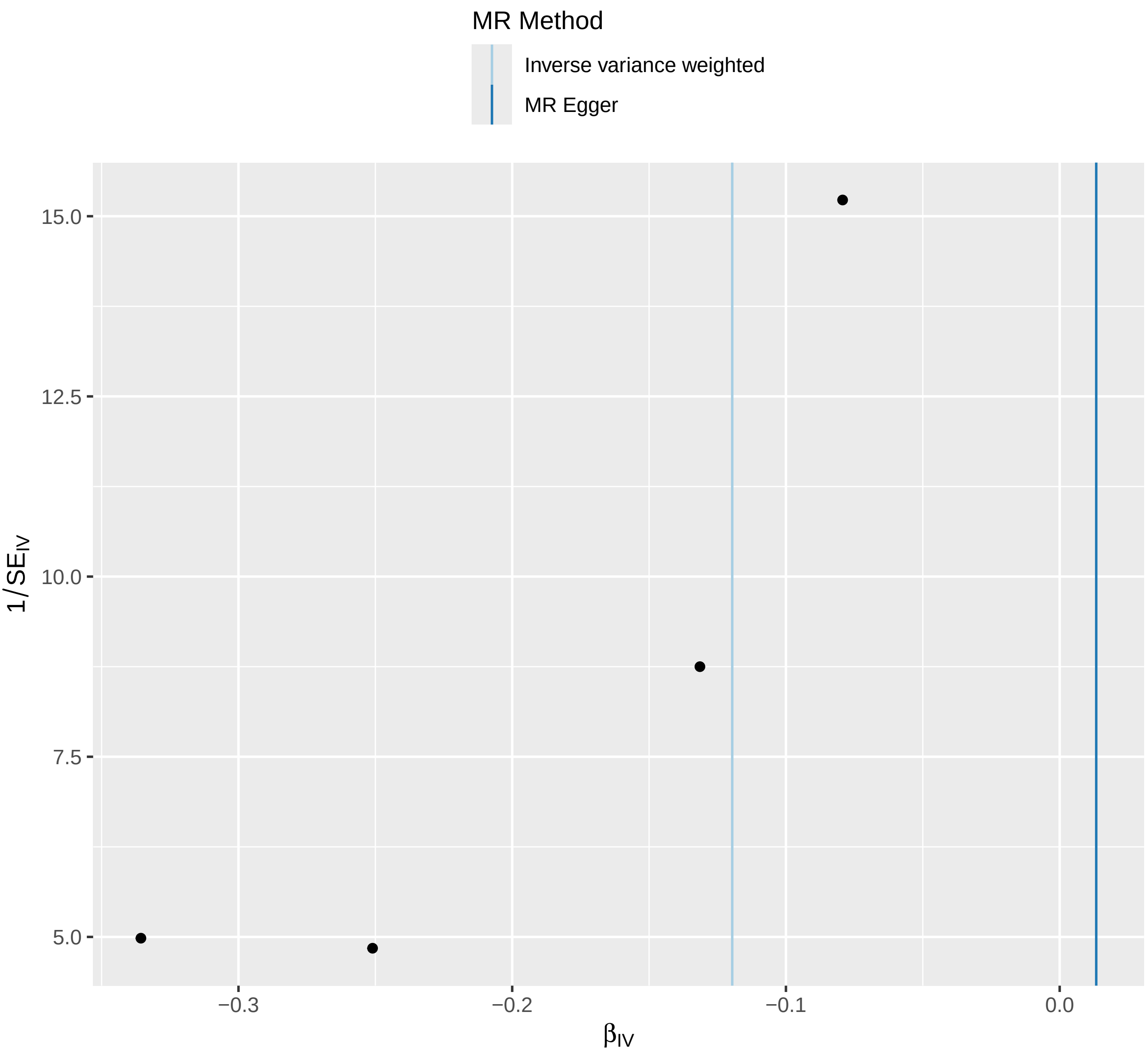

MR Method
Inverse variance weighted
MR Egger
15.0
12.5
10.0
7.5
5.0
$1/SE_{IV}$
−0.3
−0.2
−0.1
0.0
$\beta_{IV}$

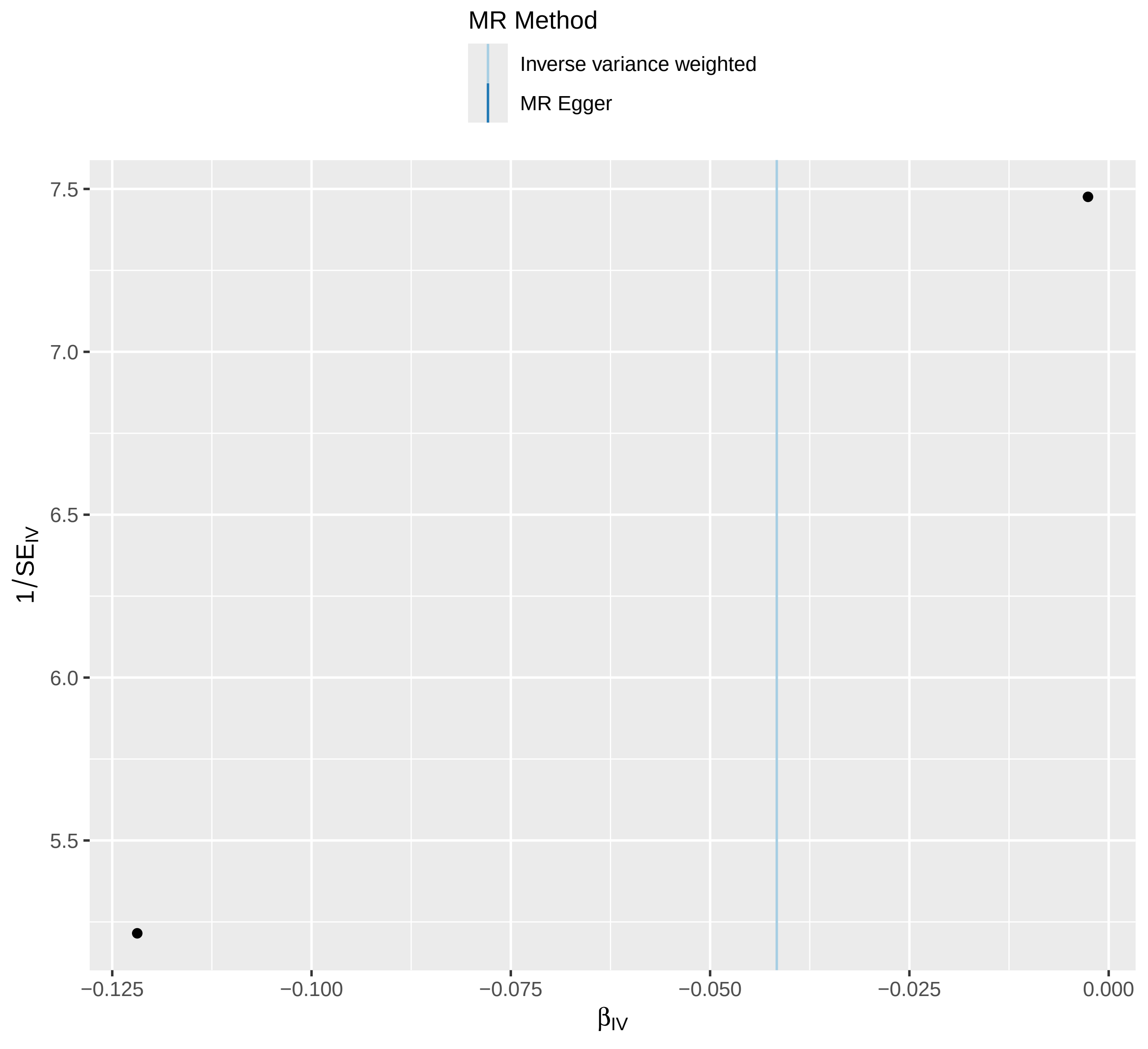

MR Method
Inverse variance weighted
MR Egger
7.5
7.0
6.5
6.0
5.5
1/SE_IV
−0.125
−0.100
−0.075
−0.050
−0.025
0.000
β_IV

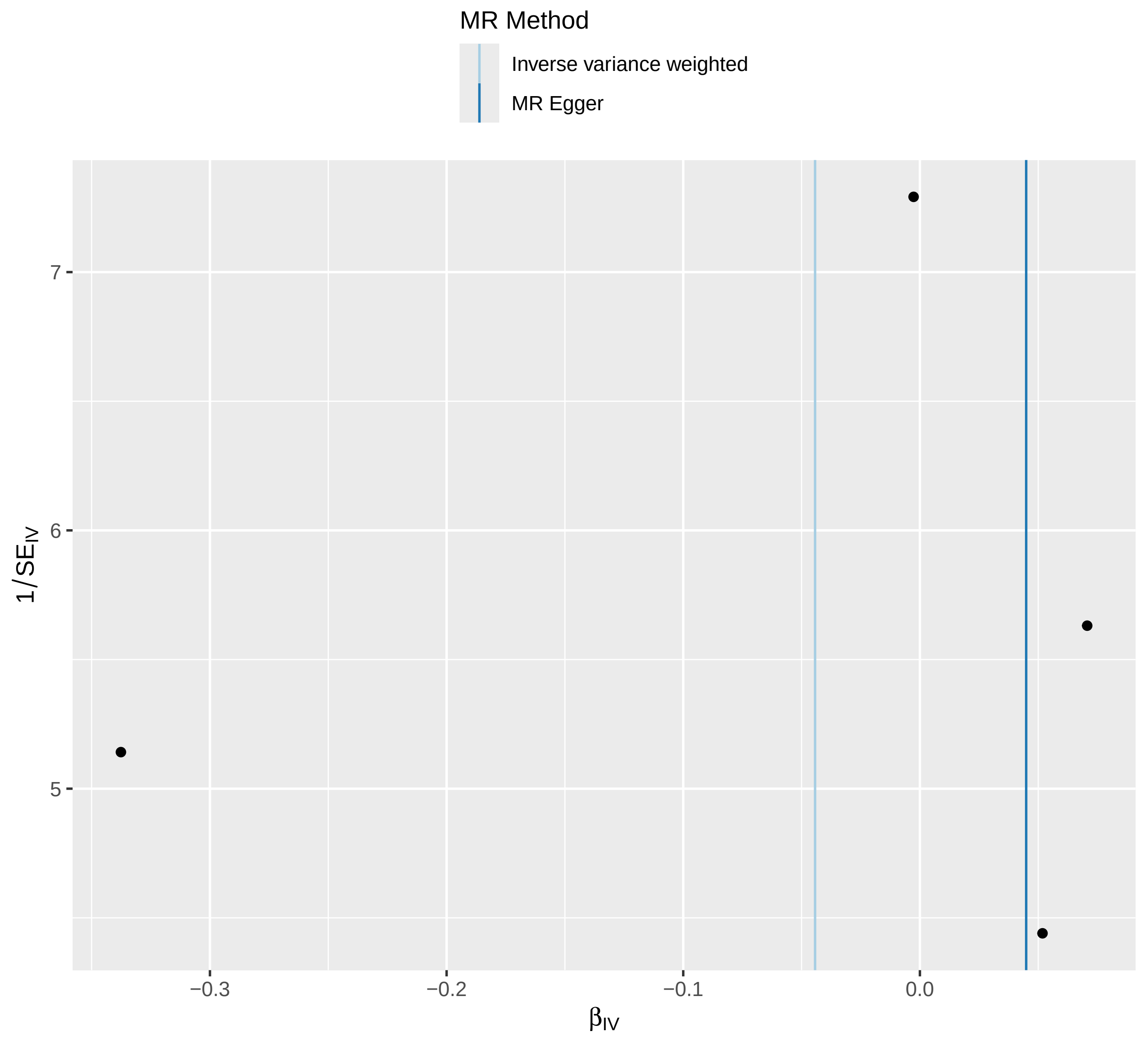
MR Method
Inverse variance weighted
MR Egger
7
6
5
1/SE$_{IV}$
−0.3
−0.2
−0.1
0.0
β$_{IV}$

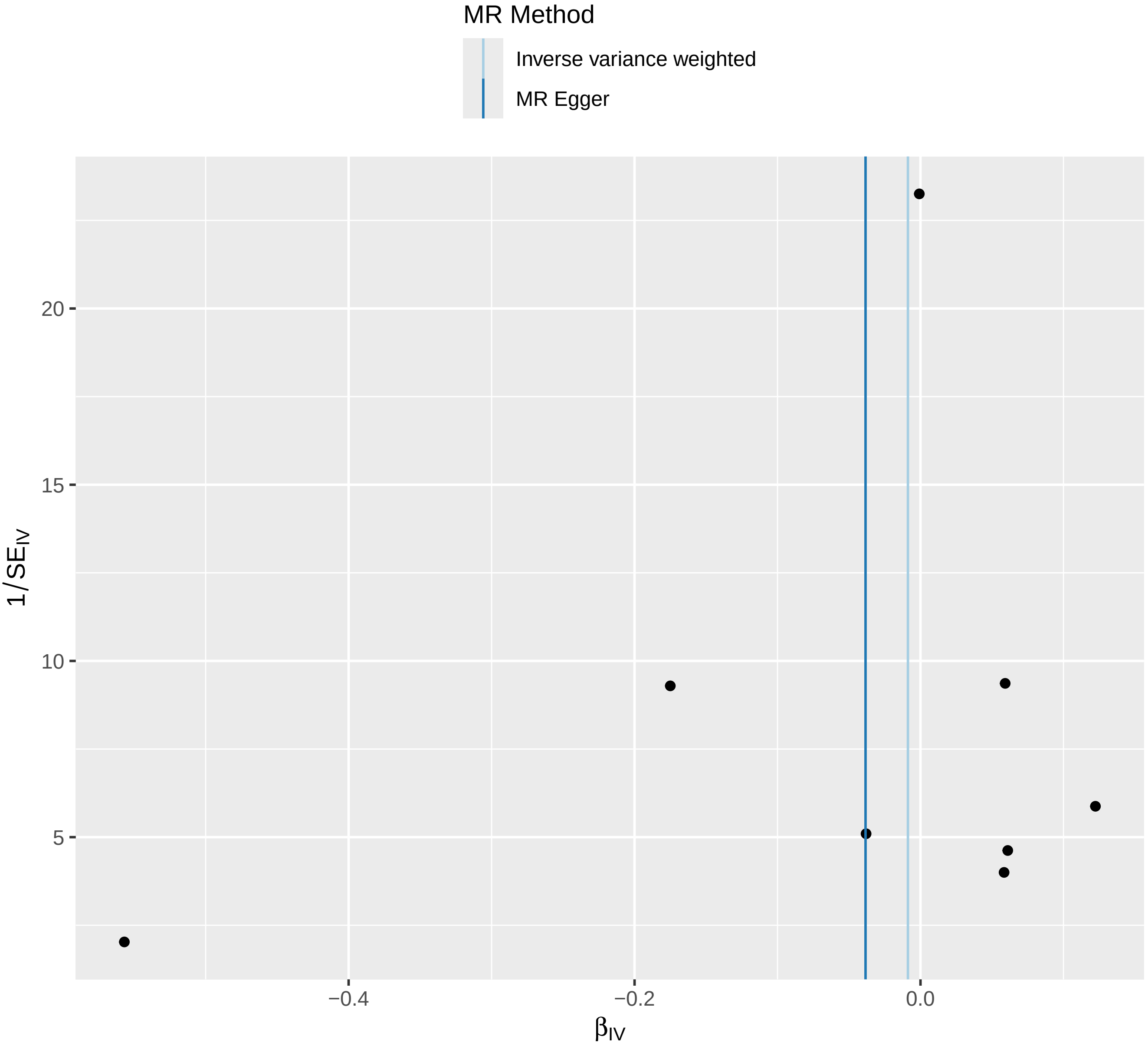

MR Method
Inverse variance weighted
MR Egger
20
15
10
5
1/SE_IV
−0.4
−0.2
0.0
β_IV

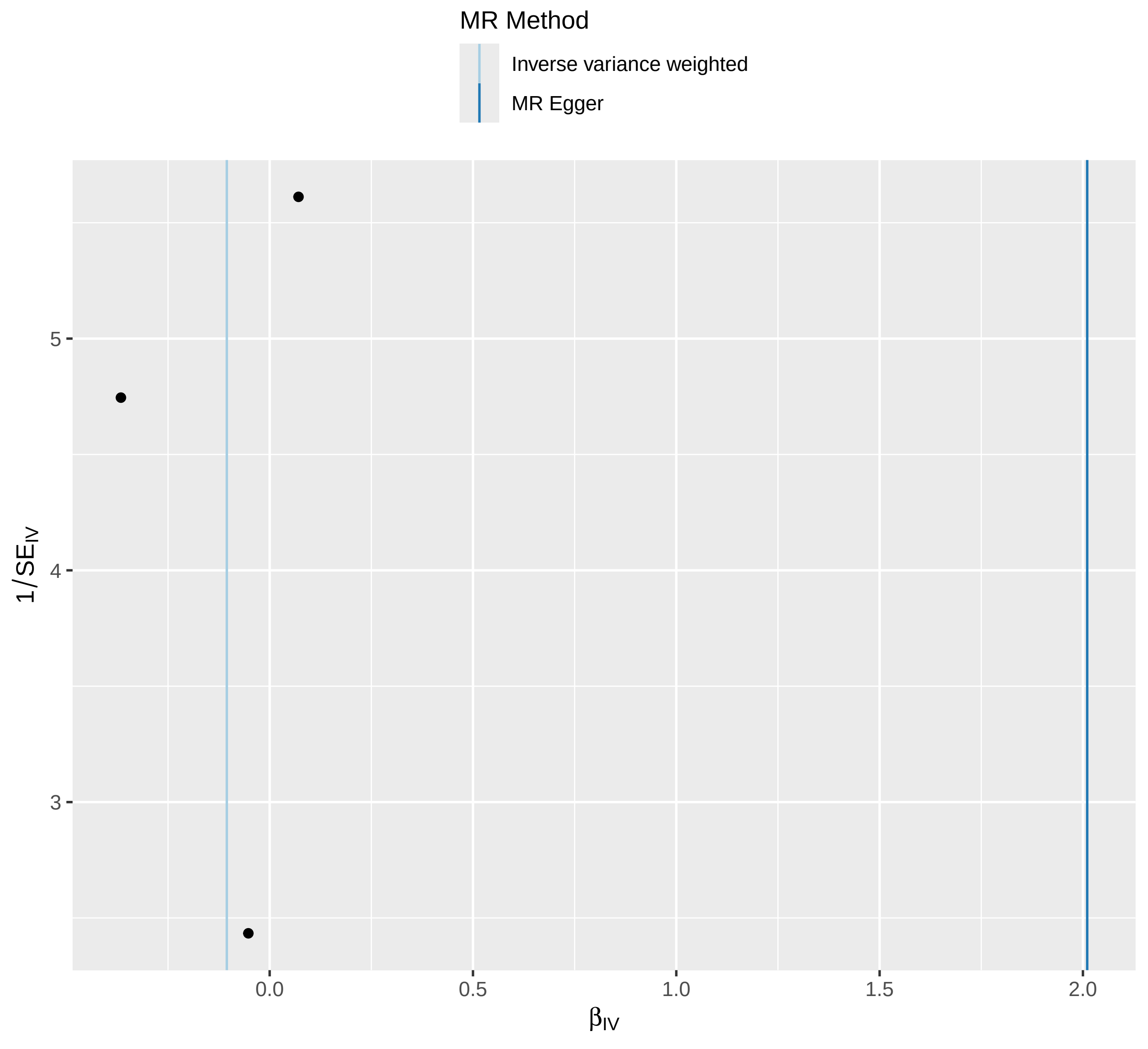
MR Method
Inverse variance weighted
MR Egger
5
4
3
1/$SE_{IV}$
0.0
0.5
1.0
1.5
2.0
$\beta_{IV}$

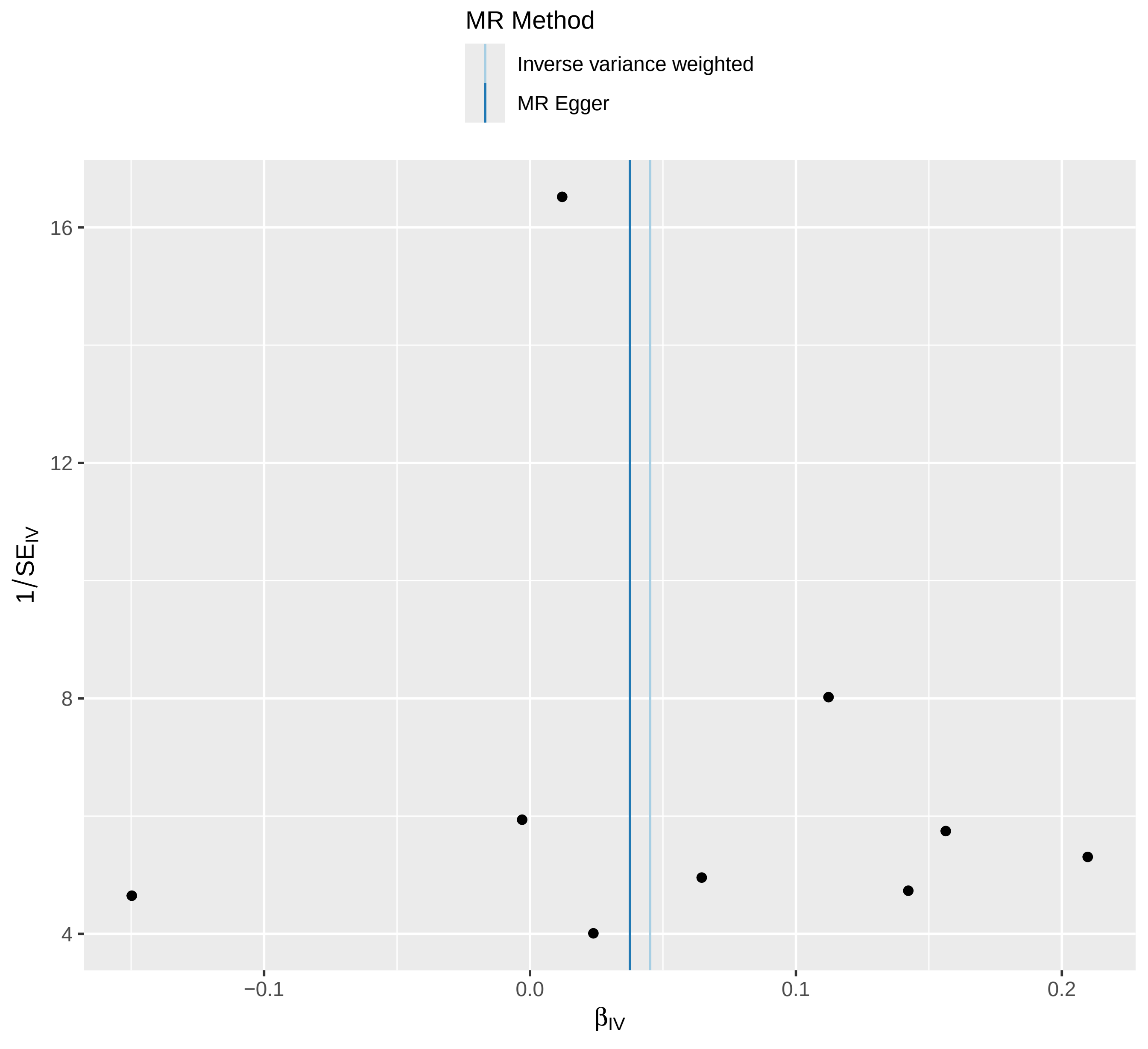

MR Method
Inverse variance weighted
MR Egger
16
12
8
4
1/SE_IV
−0.1
0.0
0.1
0.2
β_IV

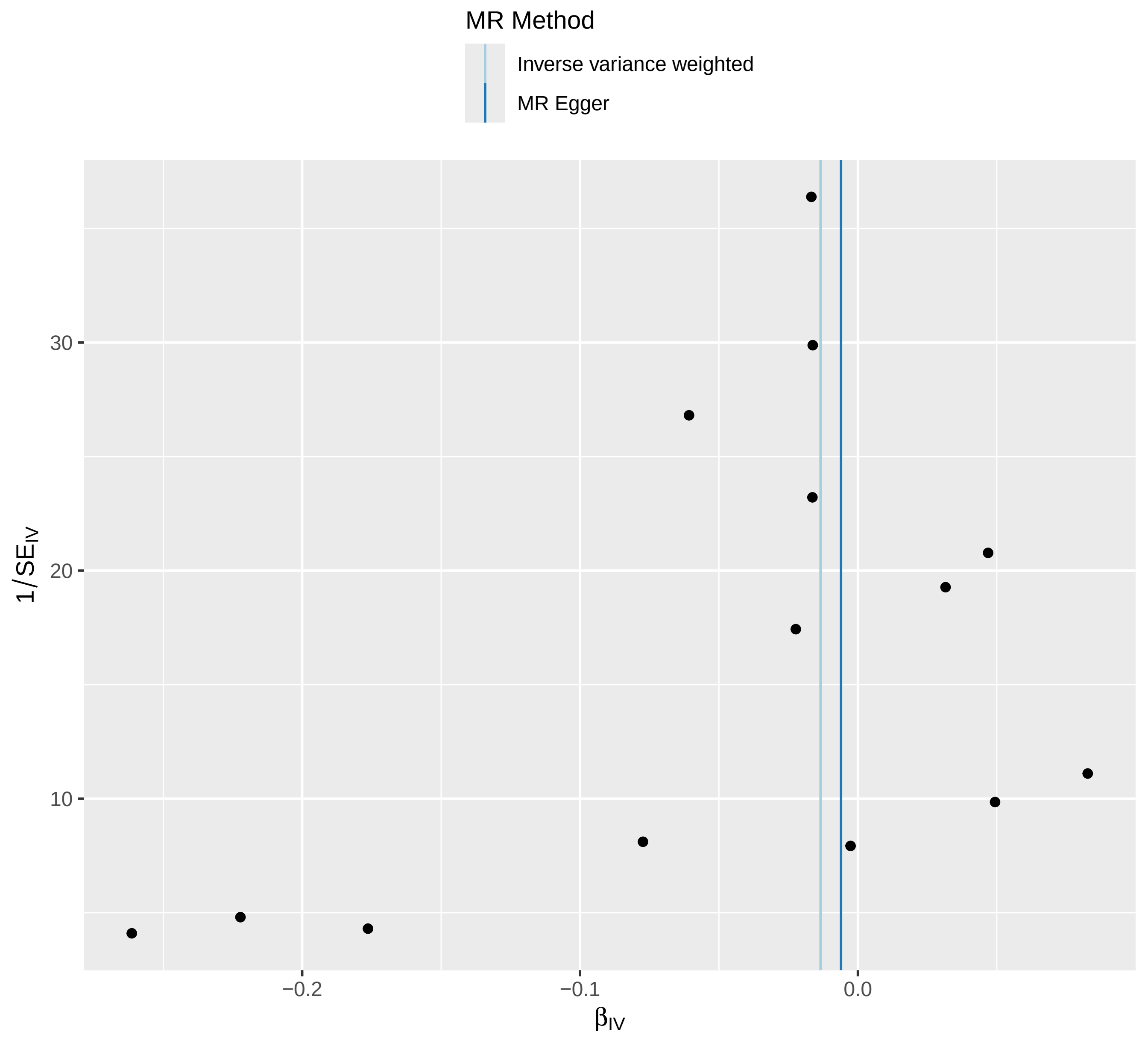
MR Method
Inverse variance weighted
MR Egger
30
20
10
1/SE_IV
−0.2
−0.1
0.0
β_IV

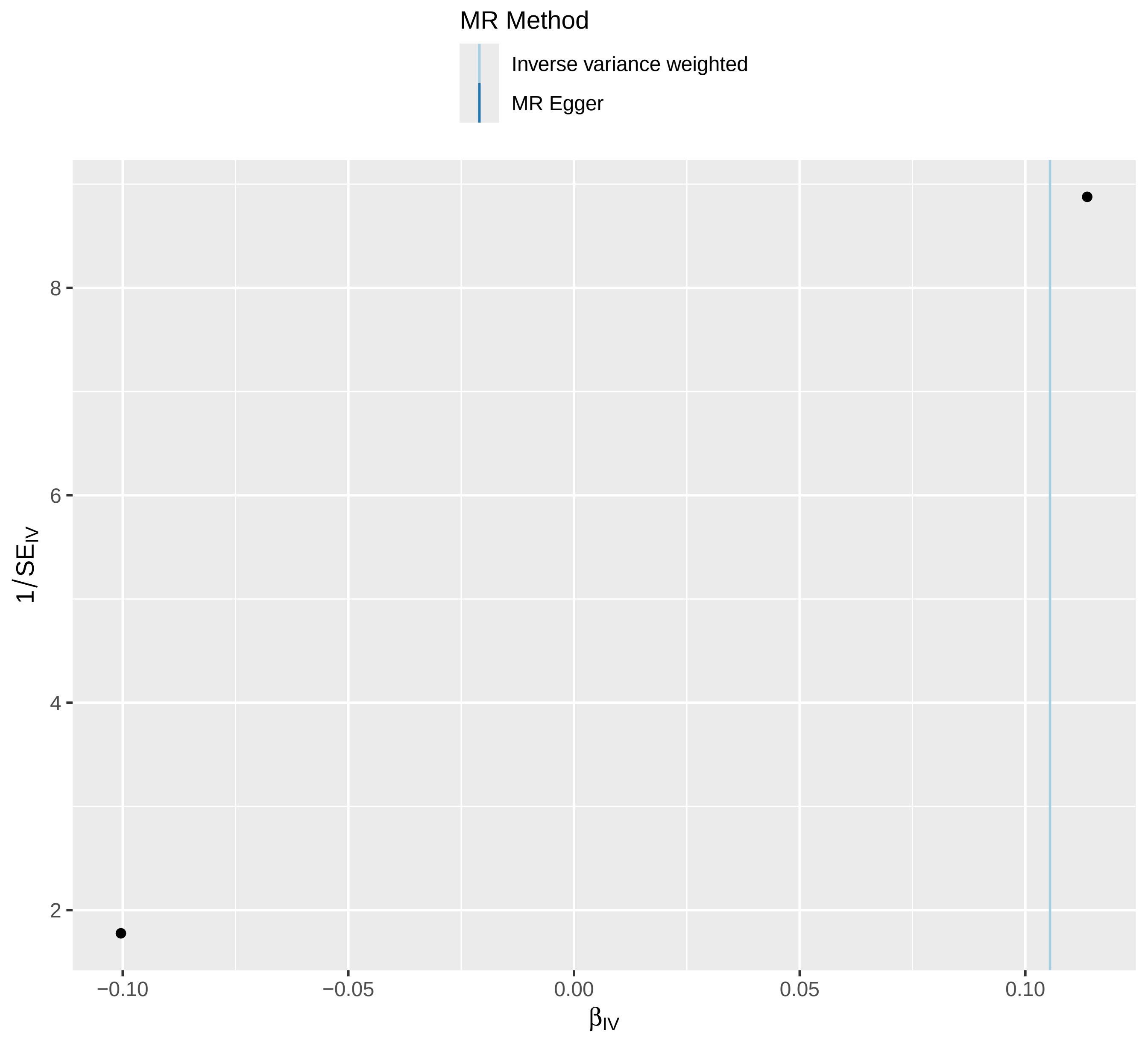
MR Method
Inverse variance weighted
MR Egger
8
6
4
2
1/SE_IV
−0.10
−0.05
0.00
0.05
0.10
β_IV

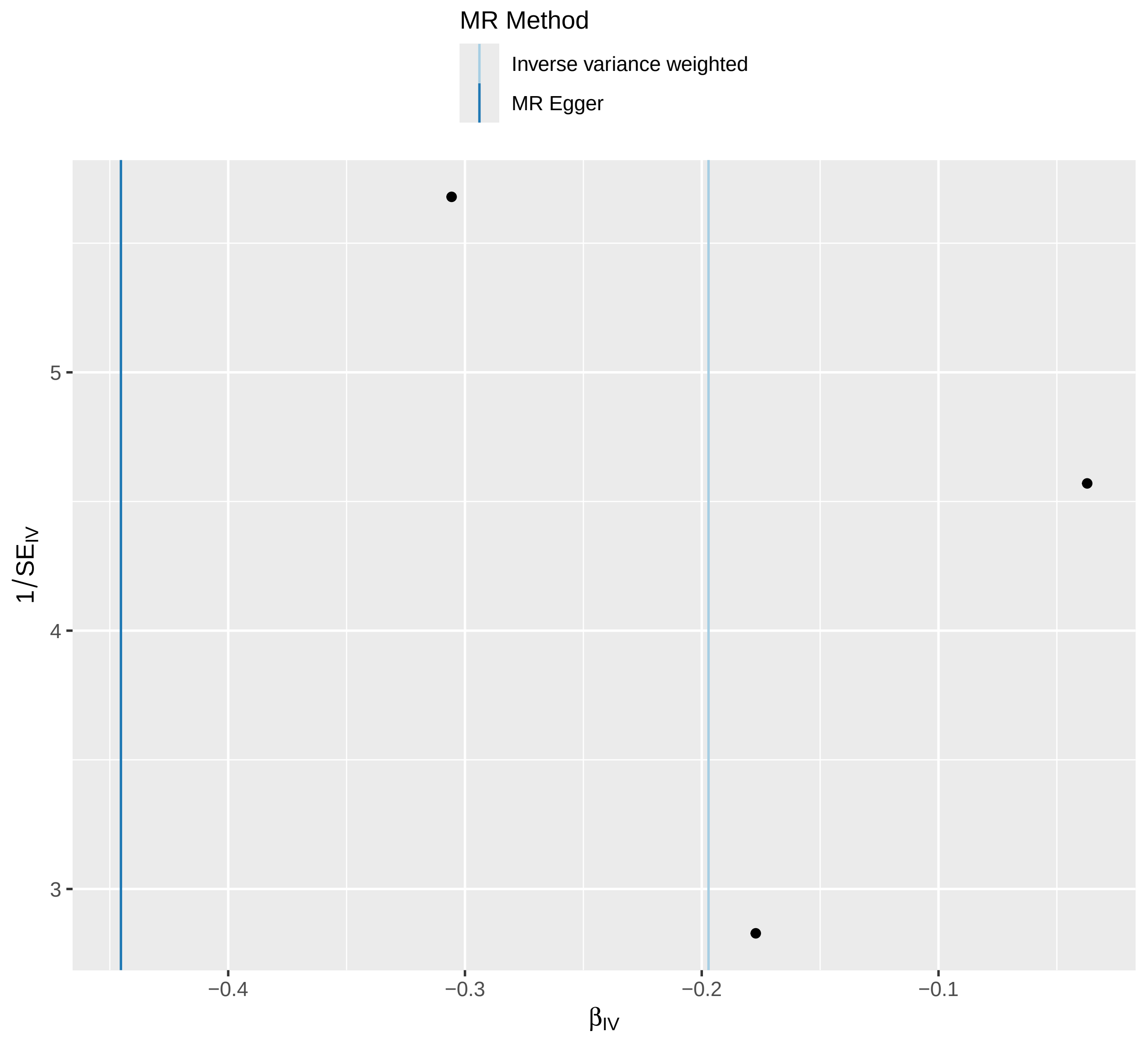

MR Method
Inverse variance weighted
MR Egger
5
4
3
1/SE$_{IV}$
−0.4
−0.3
−0.2
−0.1
β$_{IV}$

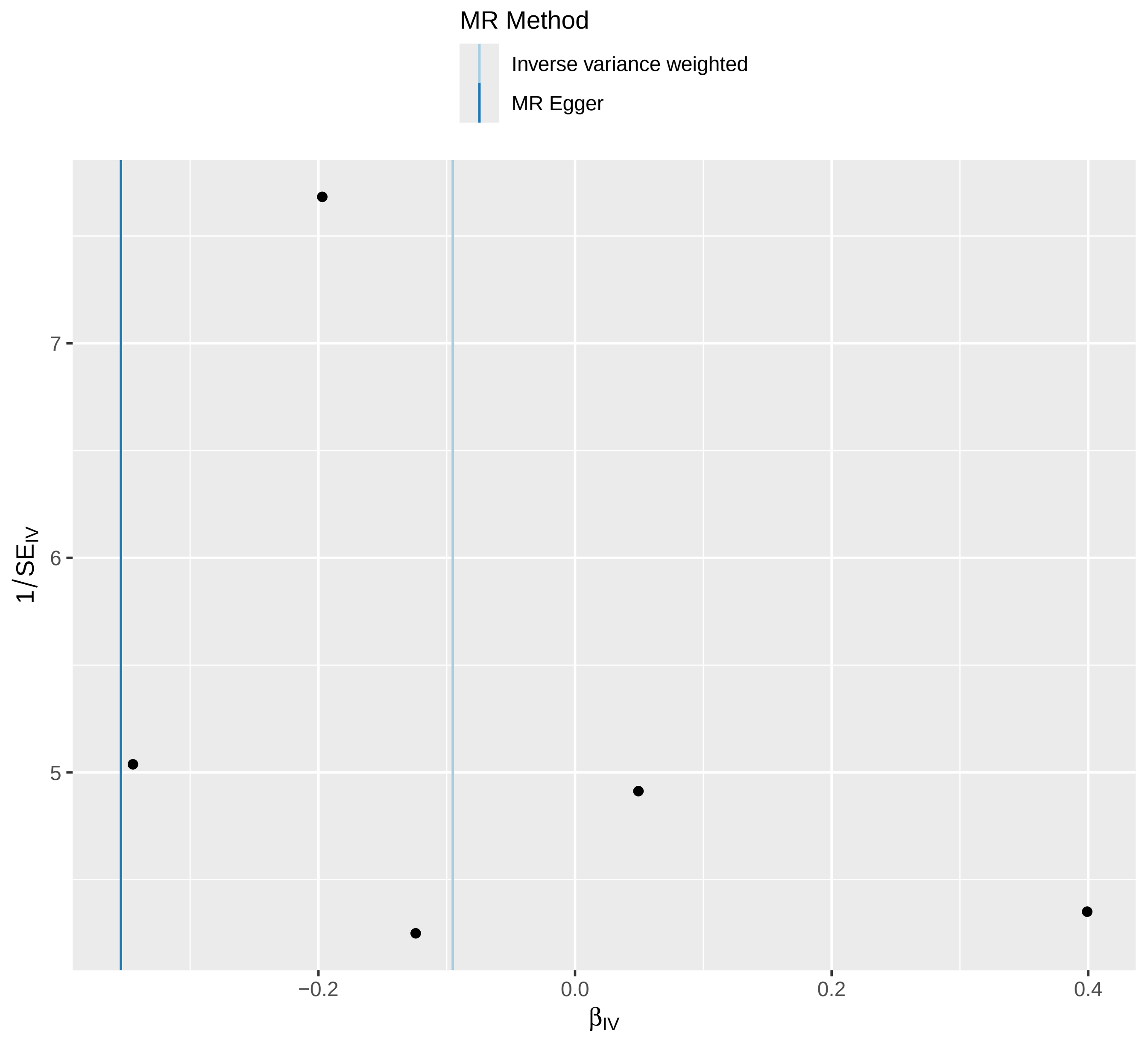
MR Method
Inverse variance weighted
MR Egger
1/SE_IV
7
6
5
−0.2
0.0
0.2
0.4
β_IV

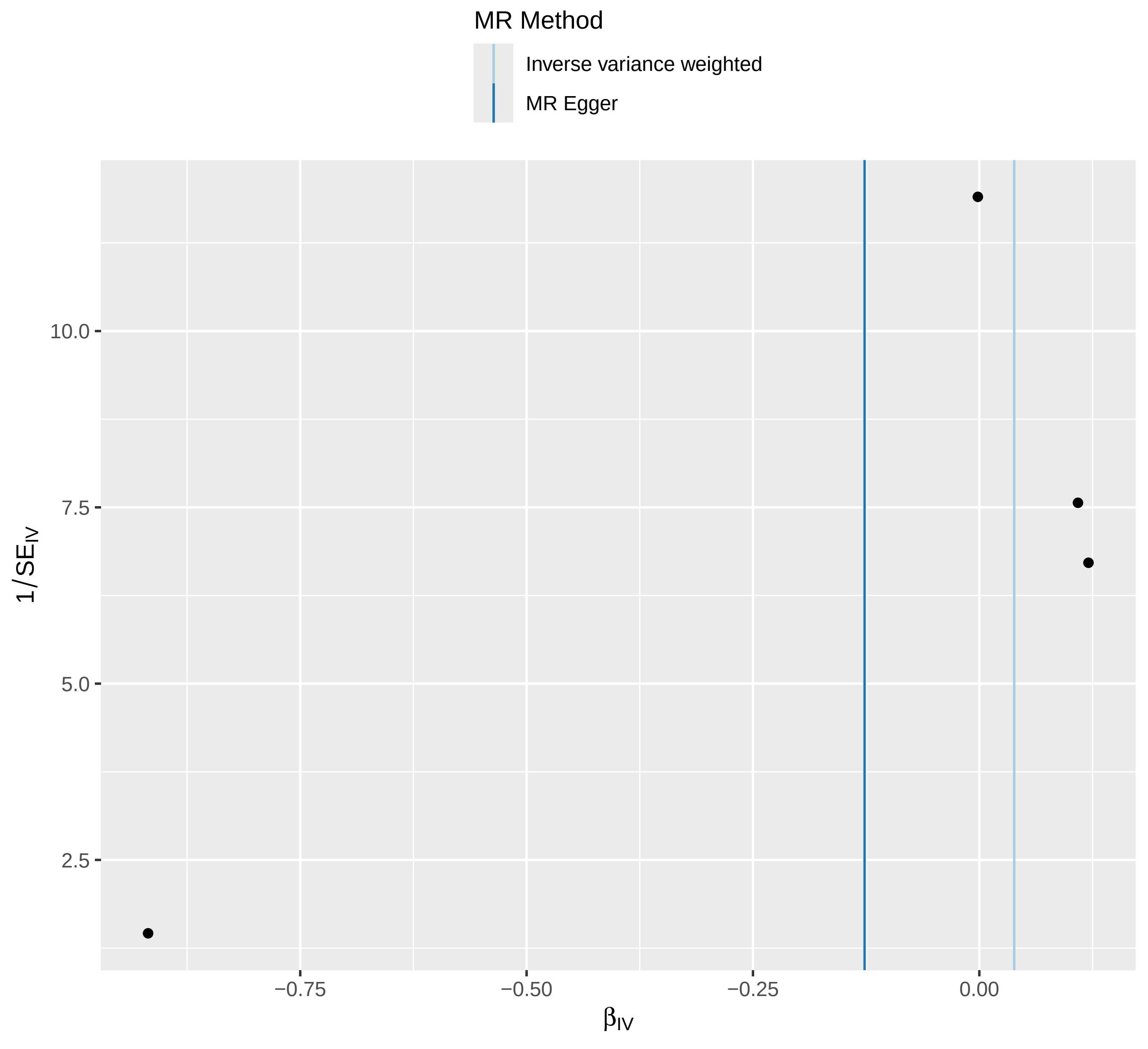
MR Method
Inverse variance weighted
MR Egger
1/SE$_{IV}$
10.0
7.5
5.0
2.5
−0.75
−0.50
−0.25
0.00
β$_{IV}$

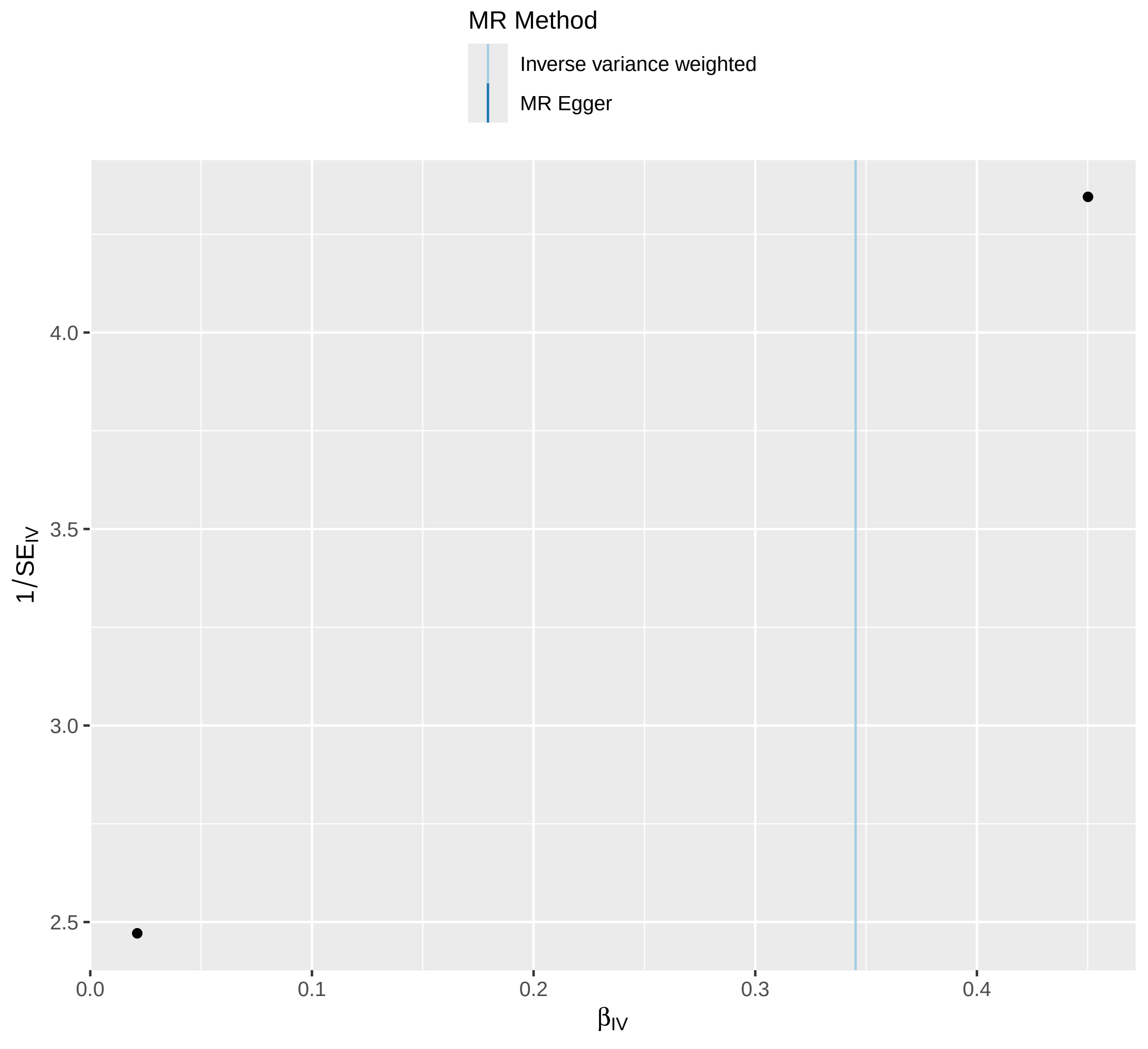
MR Method
Inverse variance weighted
MR Egger
4.0
3.5
3.0
2.5
1/SE$_{IV}$
0.0
0.1
0.2
0.3
0.4
β$_{IV}$

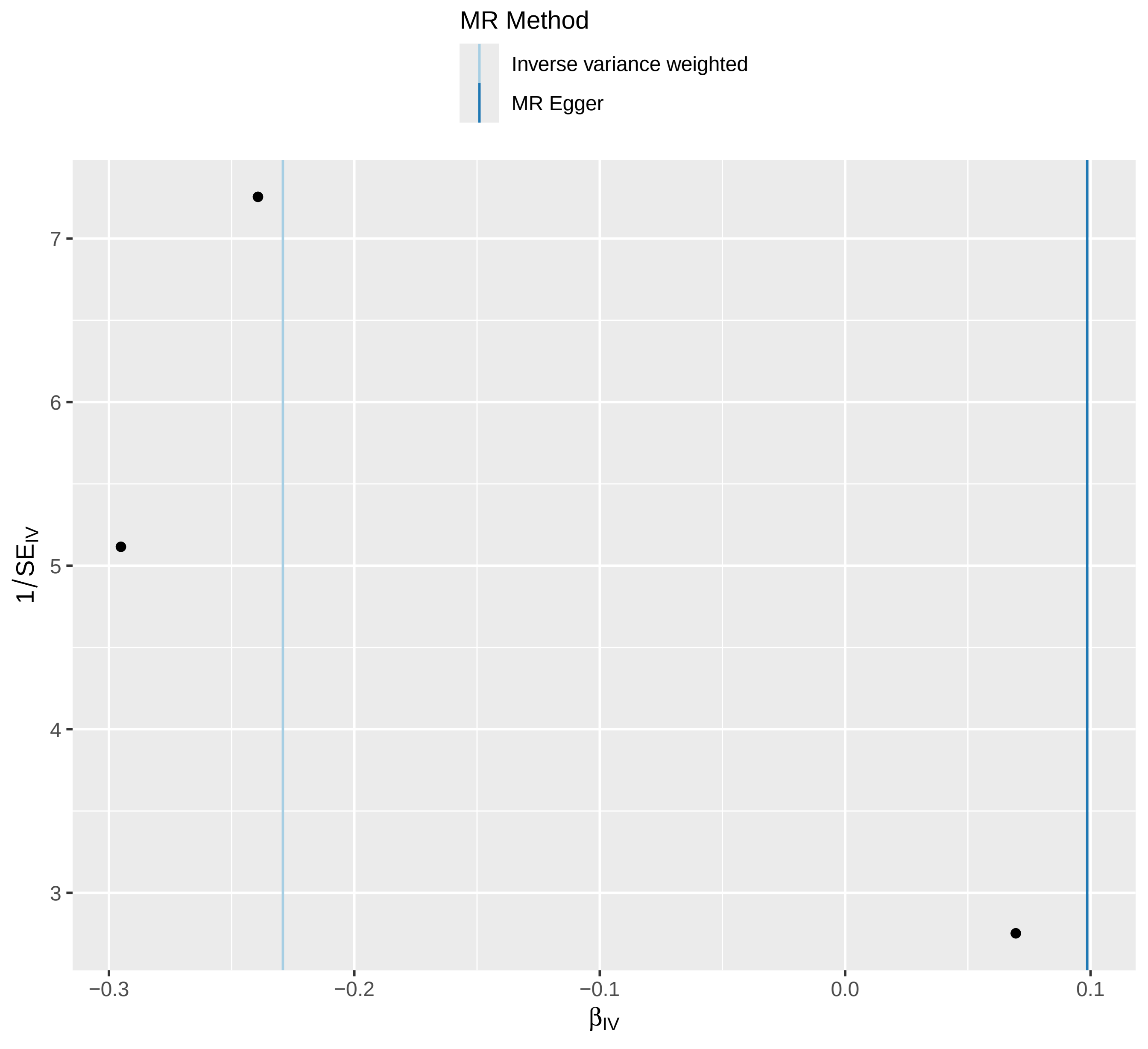
MR Method
Inverse variance weighted
MR Egger
7
6
5
4
3
1/SE_IV
−0.3
−0.2
−0.1
0.0
0.1
β_IV

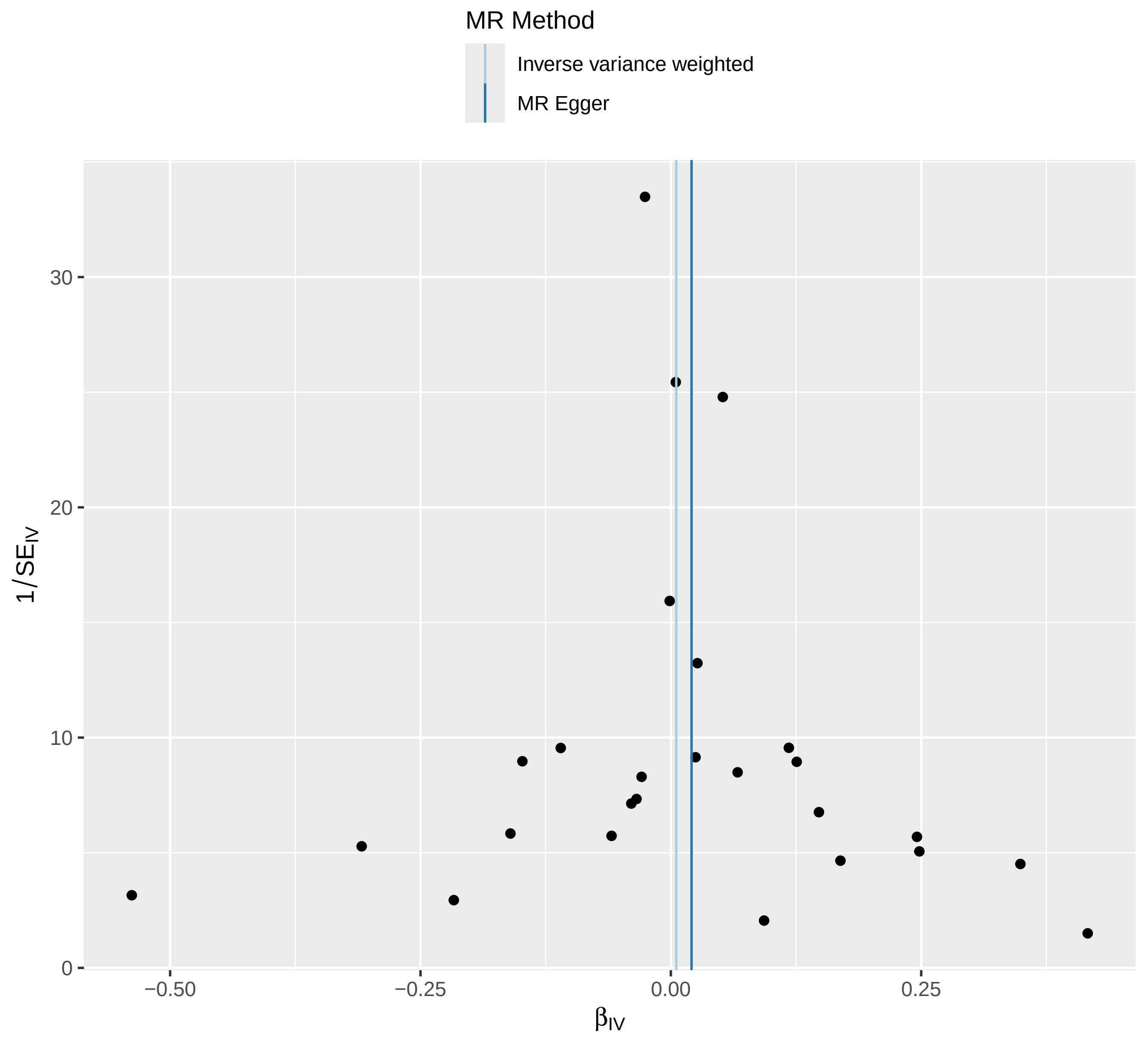
MR Method
Inverse variance weighted
MR Egger
30
20
10
0
1/SE$_{IV}$
−0.50
−0.25
0.00
0.25
β$_{IV}$

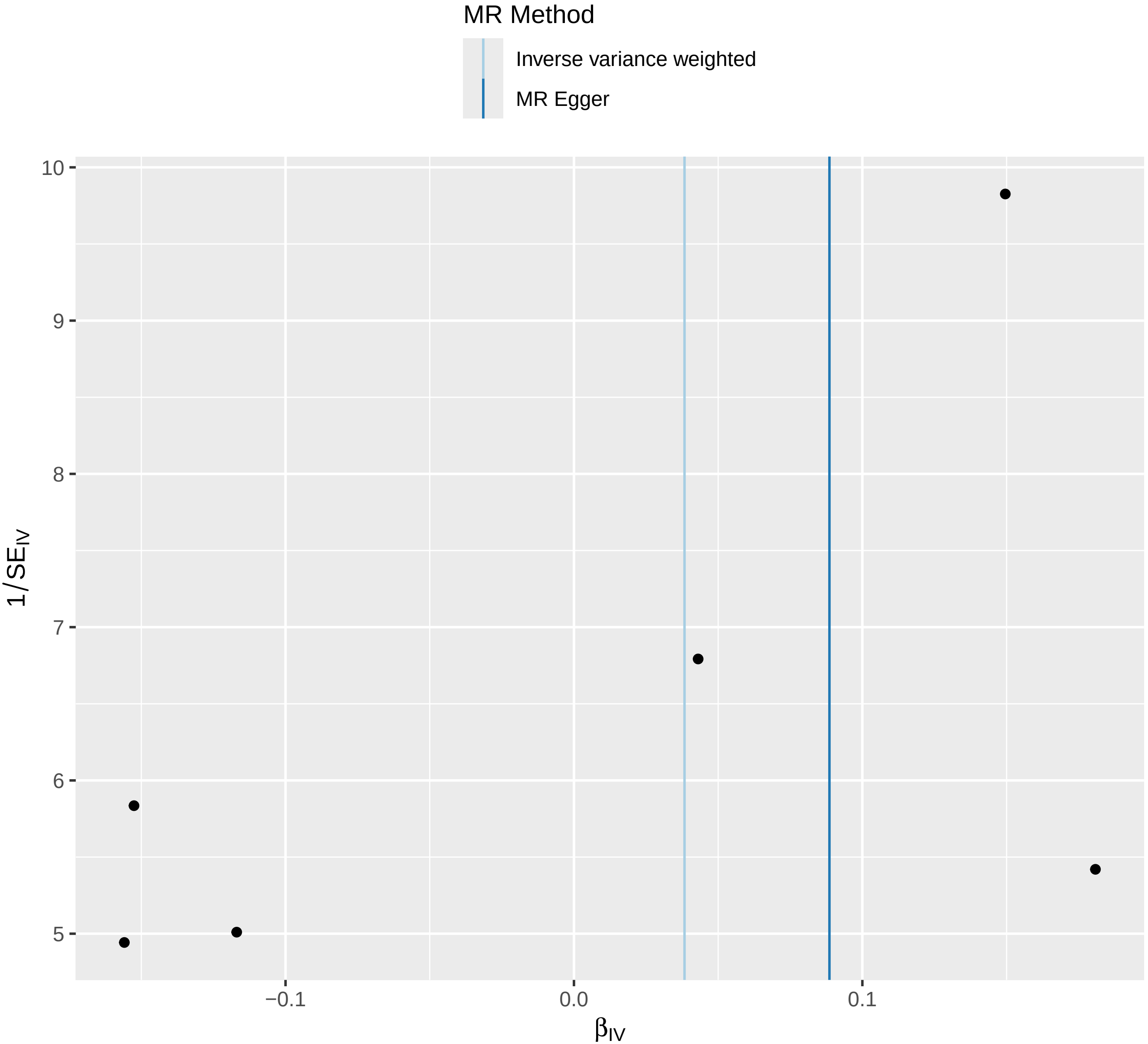
MR Method
Inverse variance weighted
MR Egger
10
9
8
7
6
5
1/SE$_{IV}$
−0.1
0.0
0.1
β$_{IV}$

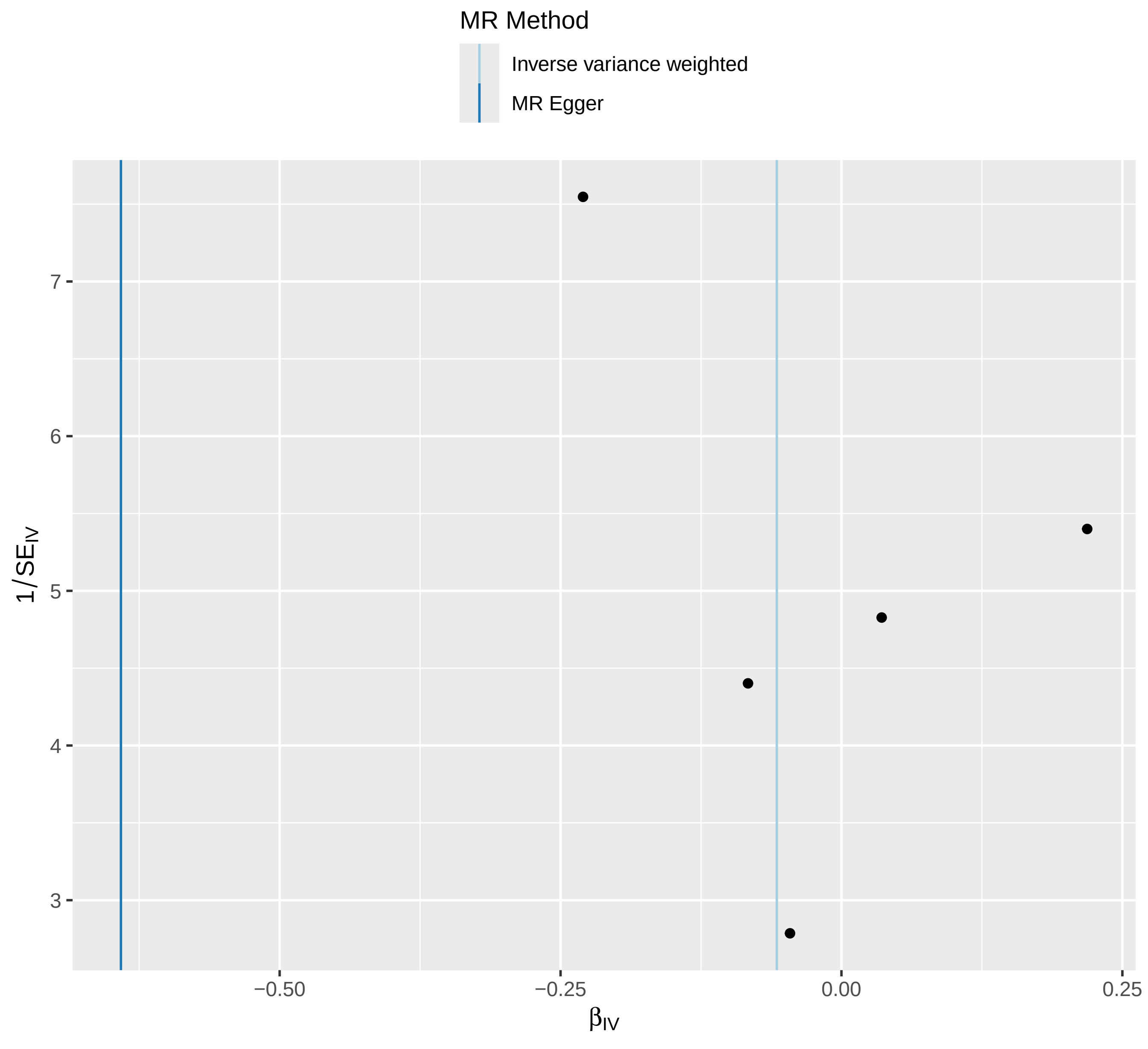
MR Method
Inverse variance weighted
MR Egger
1/SE$_{IV}$
β$_{IV}$
7
6
5
4
3
−0.50
−0.25
0.00
0.25

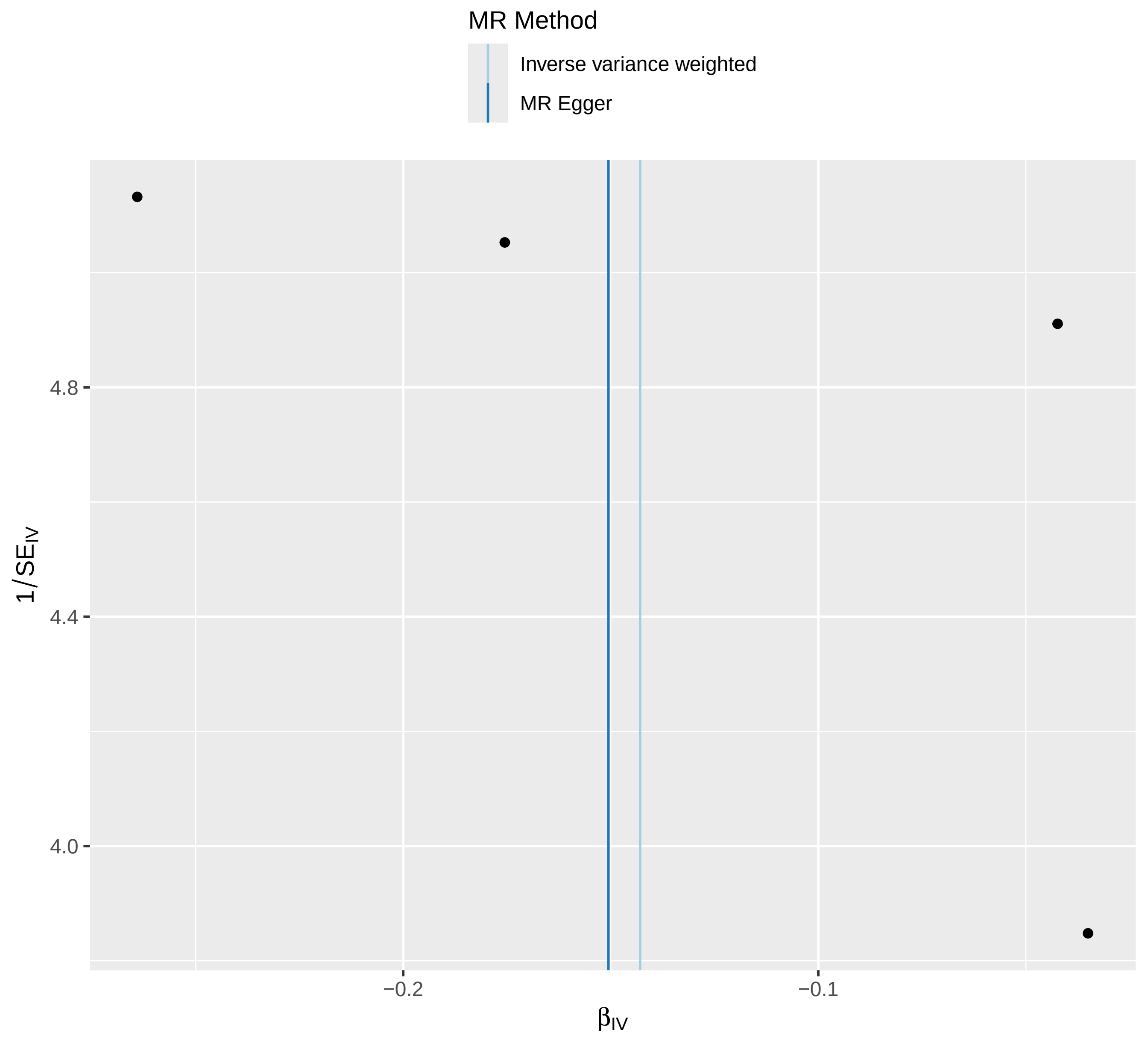

MR Method
Inverse variance weighted
MR Egger
4.8
4.4
4.0
1/SE_IV
−0.2
−0.1
β_IV

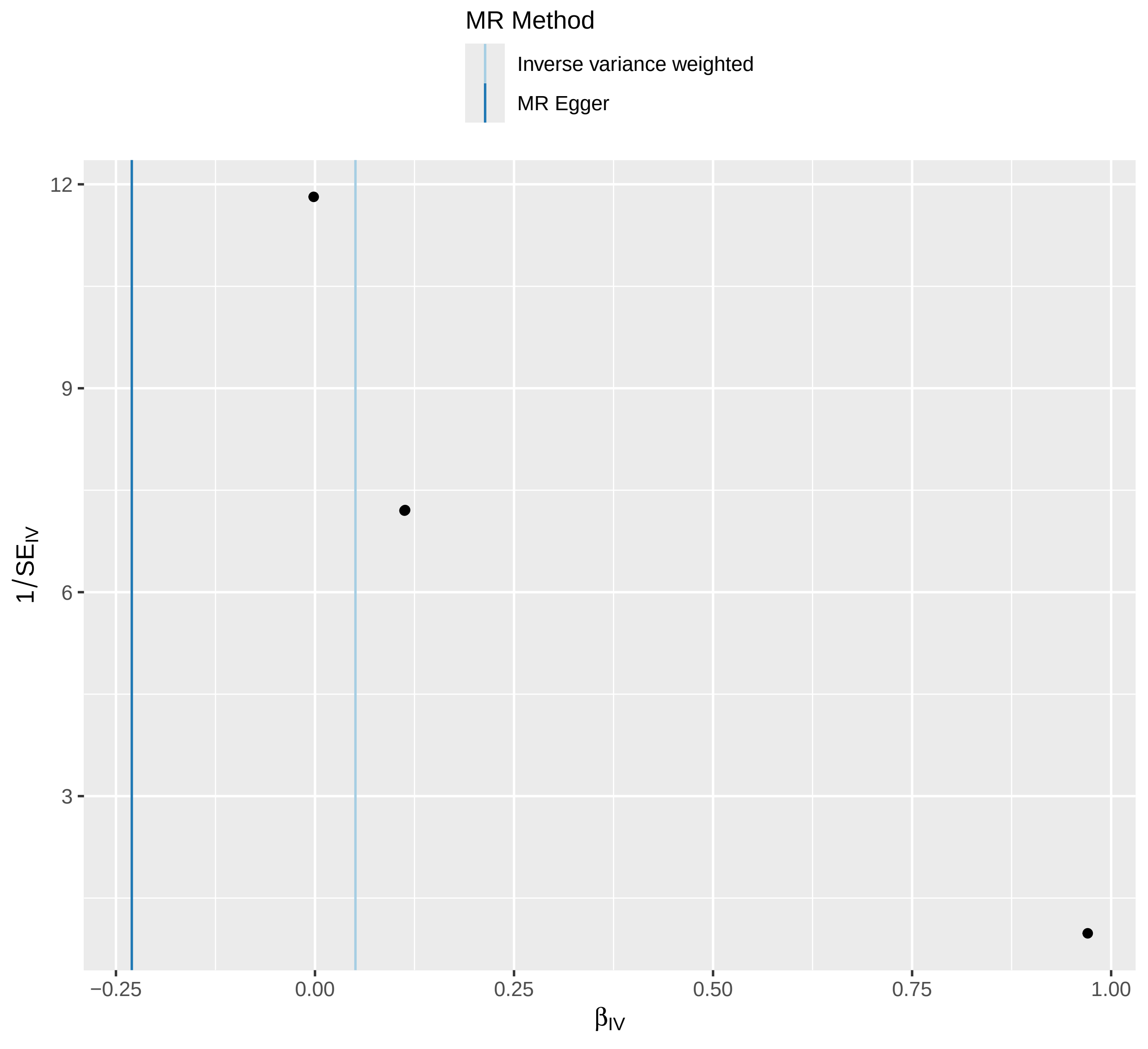
MR Method
Inverse variance weighted
MR Egger
12
9
6
3
$1/SE_{IV}$
−0.25
0.00
0.25
0.50
0.75
1.00
$\beta_{IV}$

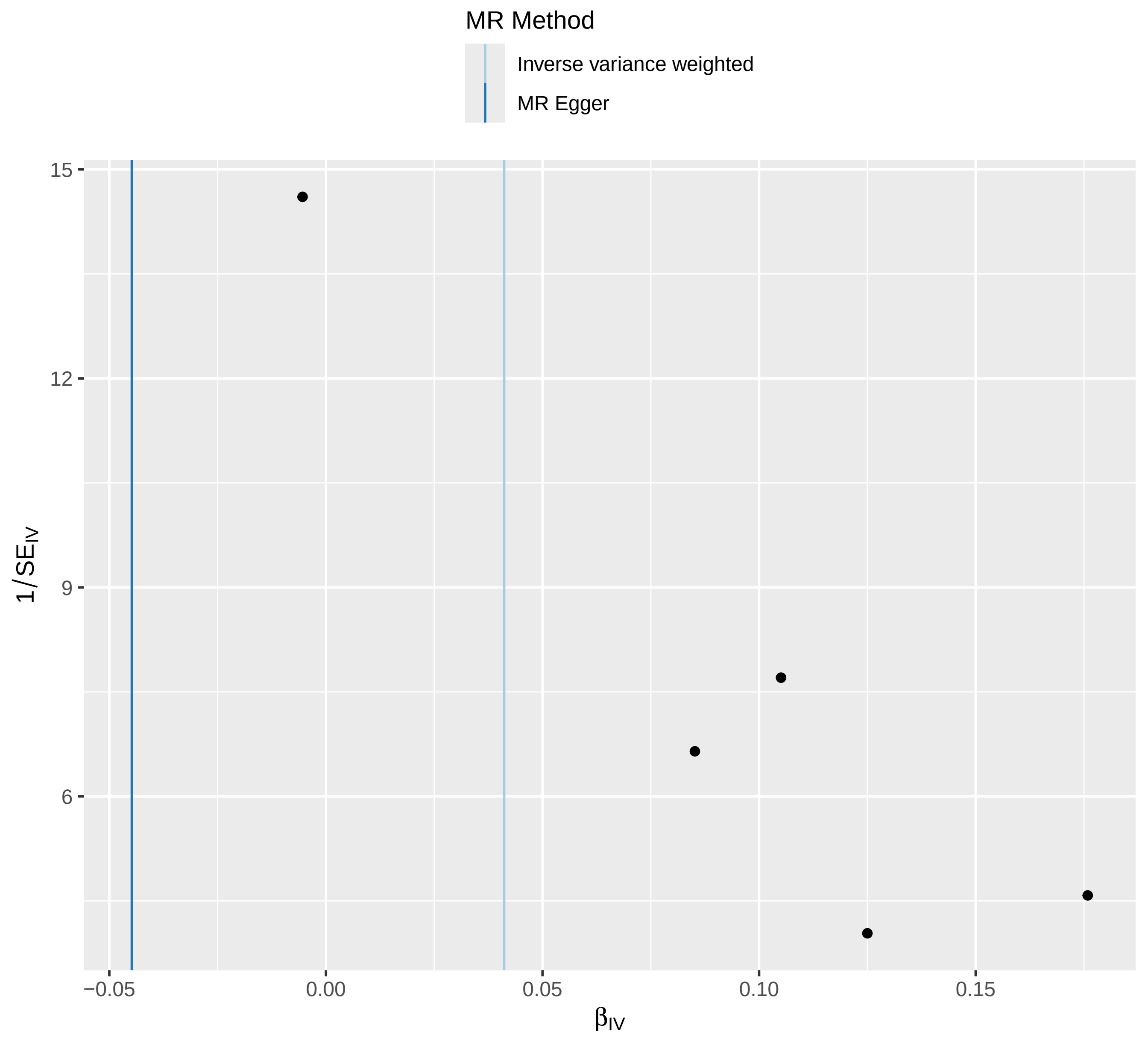
MR Method
Inverse variance weighted
MR Egger
15
12
9
6
1/SE_IV
−0.05
0.00
0.05
0.10
0.15
β_IV

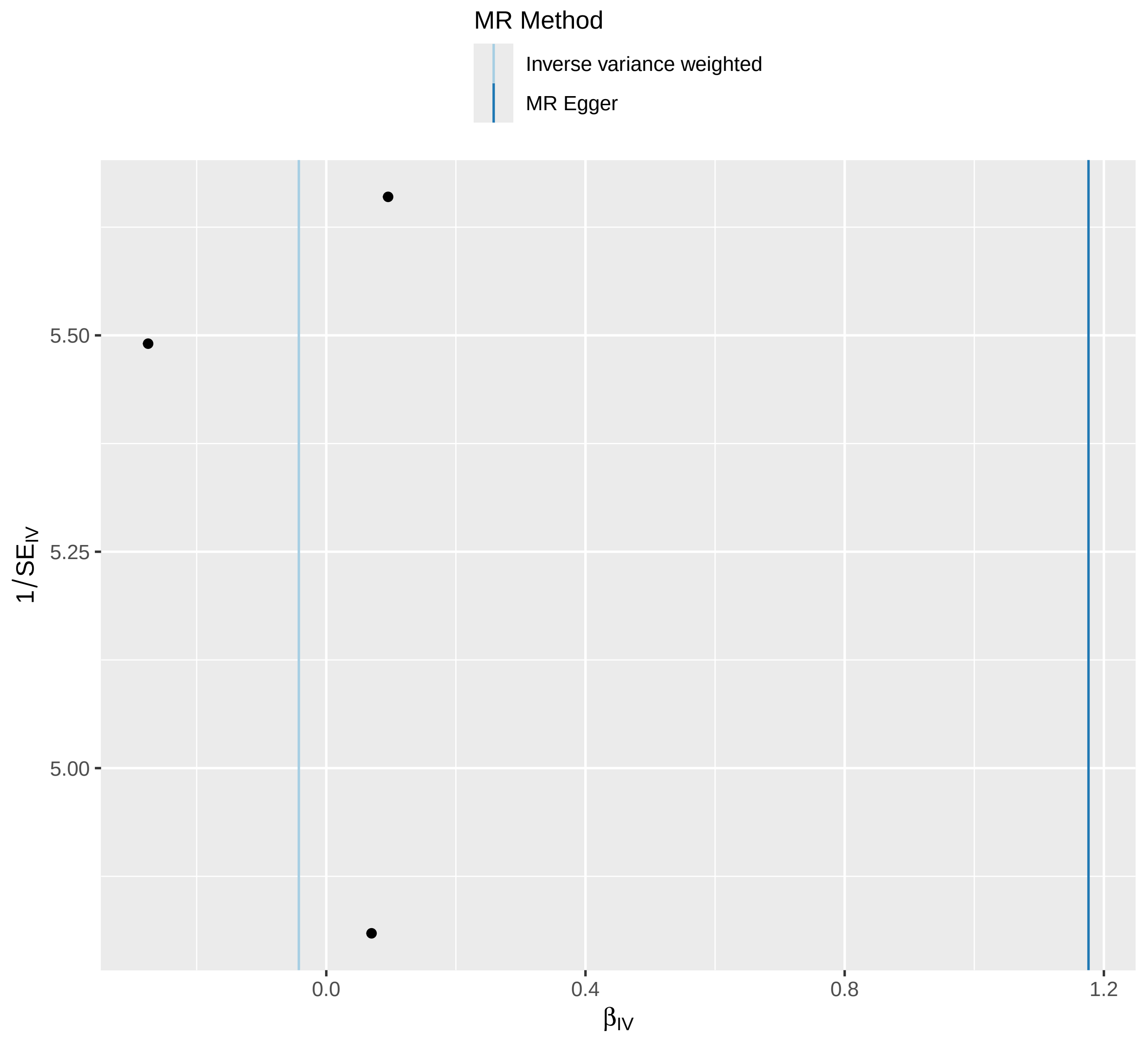
MR Method
Inverse variance weighted
MR Egger
5.50
5.25
5.00
1/SE_IV
0.0
0.4
0.8
1.2
β_IV

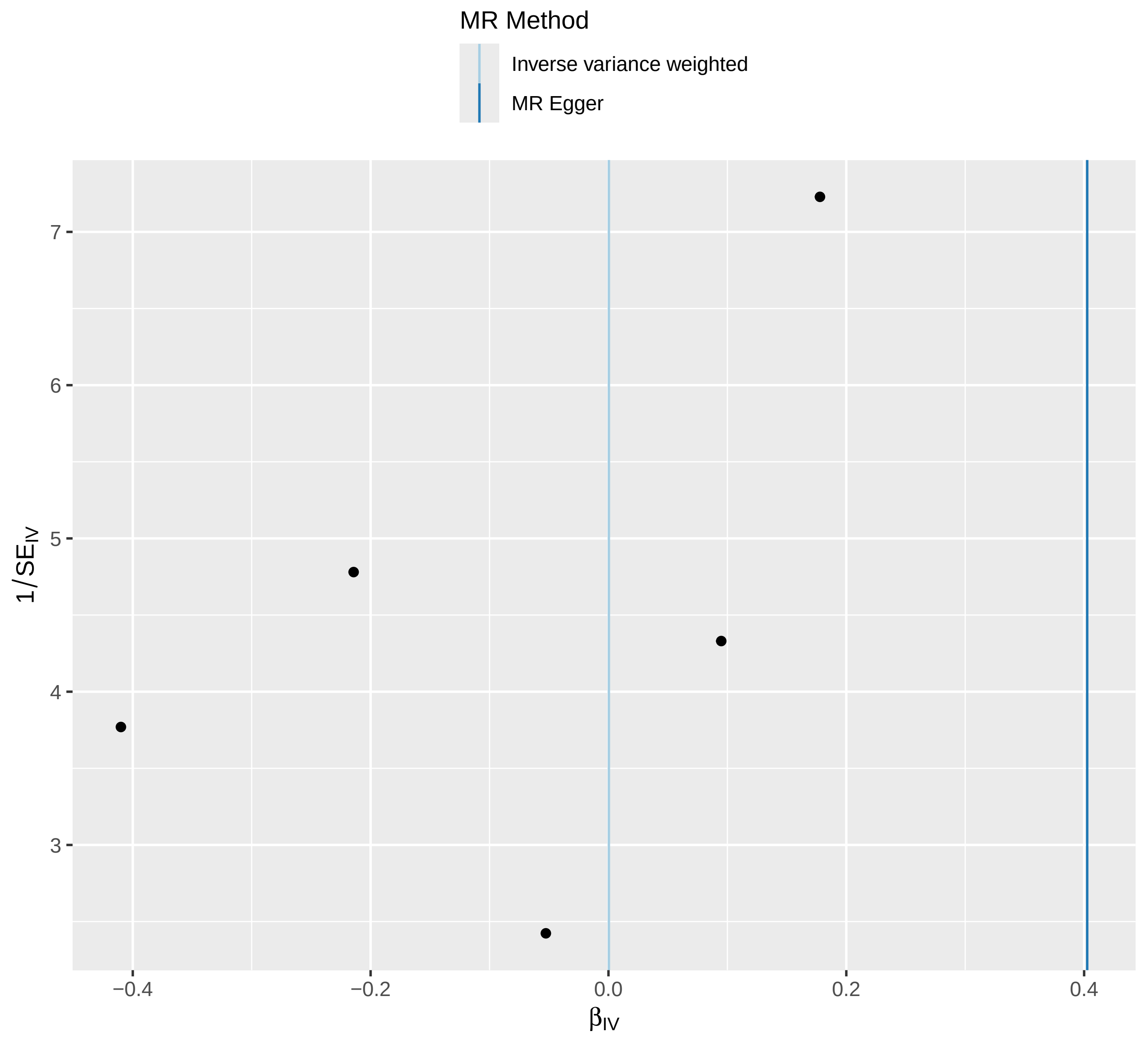
MR Method
Inverse variance weighted
MR Egger
1/SE_IV
β_IV
7
6
5
4
3
−0.4
−0.2
0.0
0.2
0.4

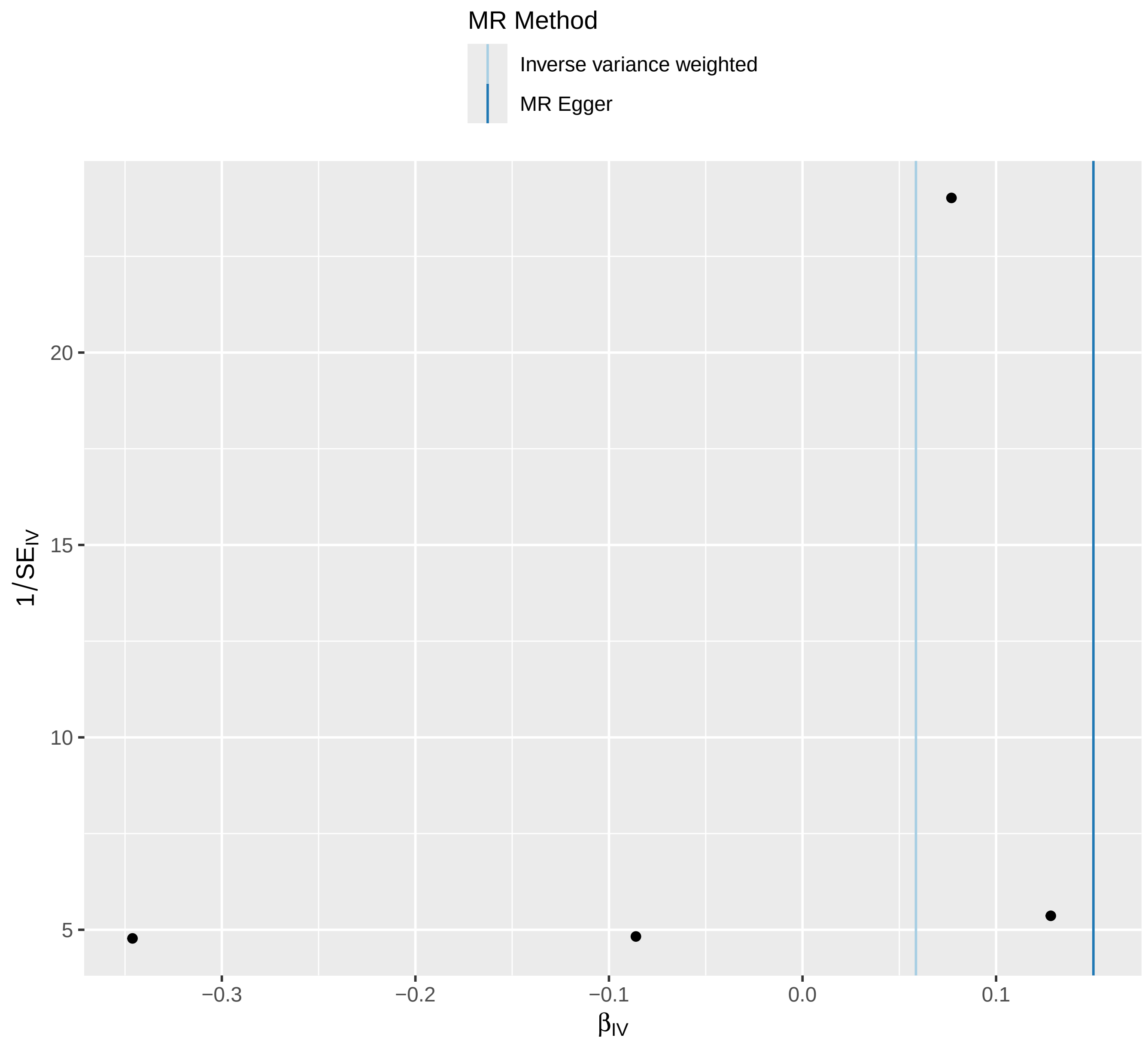

MR Method
Inverse variance weighted
MR Egger
20
15
10
5
1/SE_IV
−0.3
−0.2
−0.1
0.0
0.1
β_IV

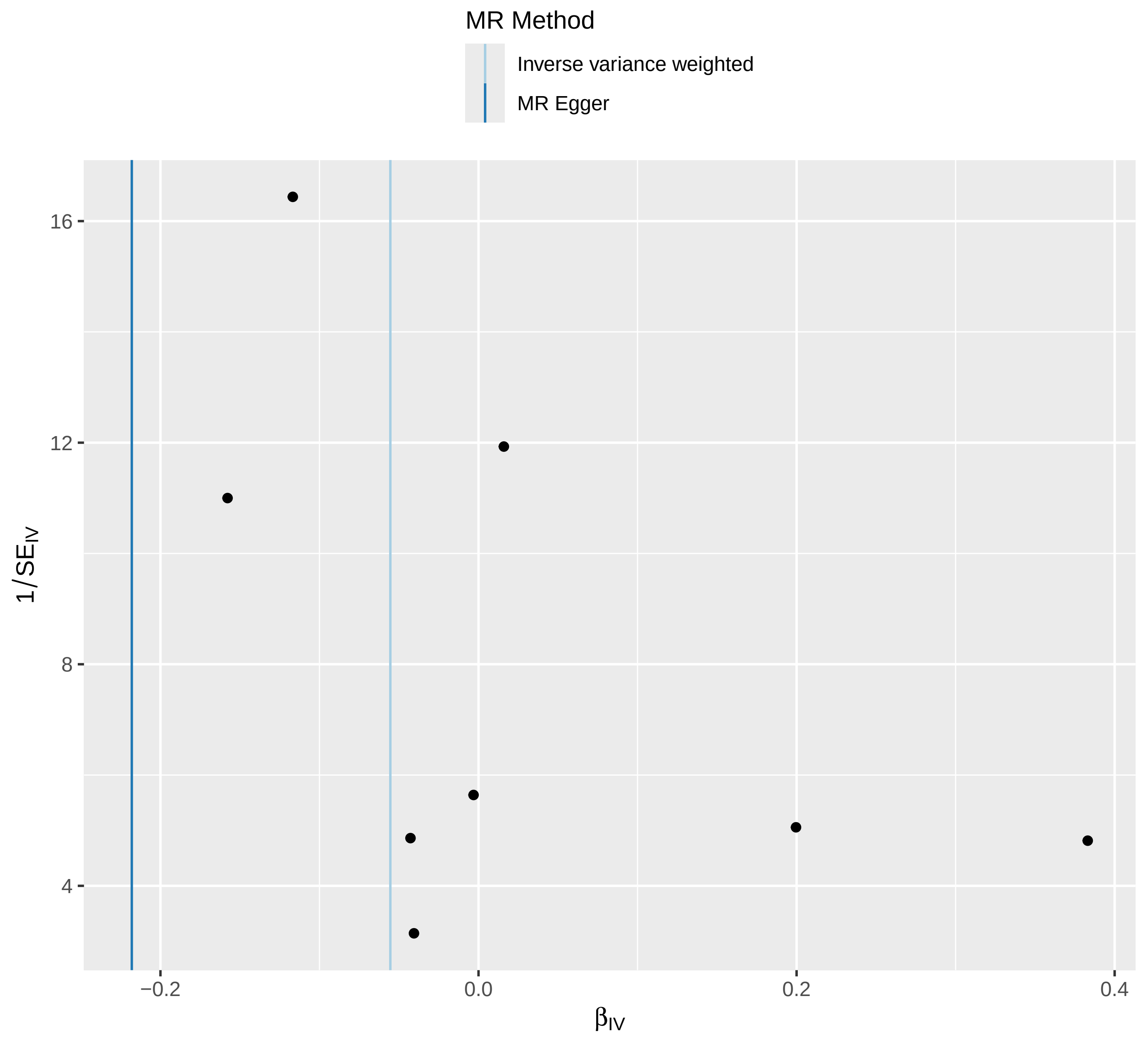

MR Method
Inverse variance weighted
MR Egger
16
12
8
4
1/SE_IV
−0.2
0.0
0.2
0.4
β_IV

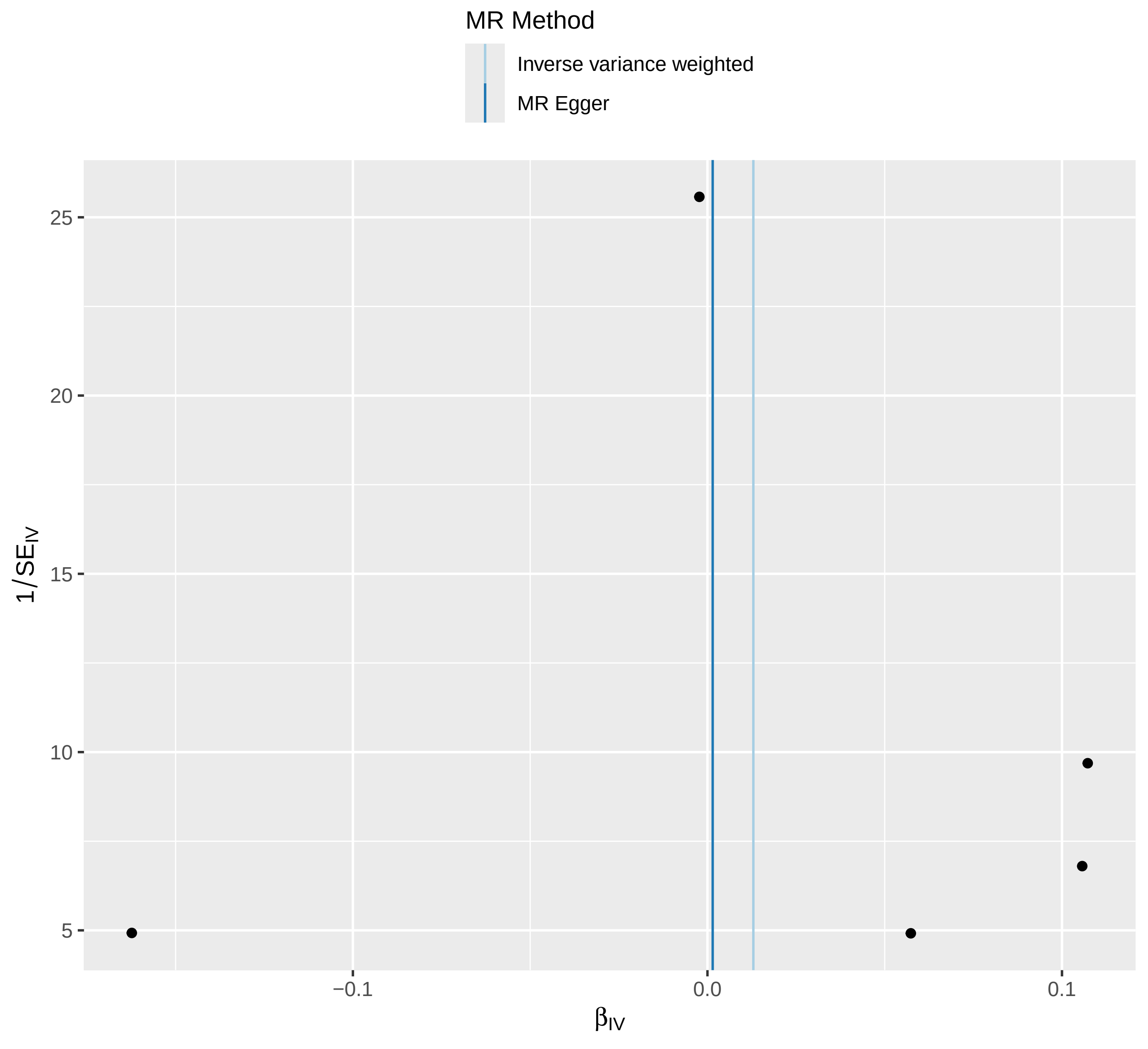
MR Method
Inverse variance weighted
MR Egger
25
20
15
10
5
1/SE_IV
−0.1
0.0
0.1
β_IV

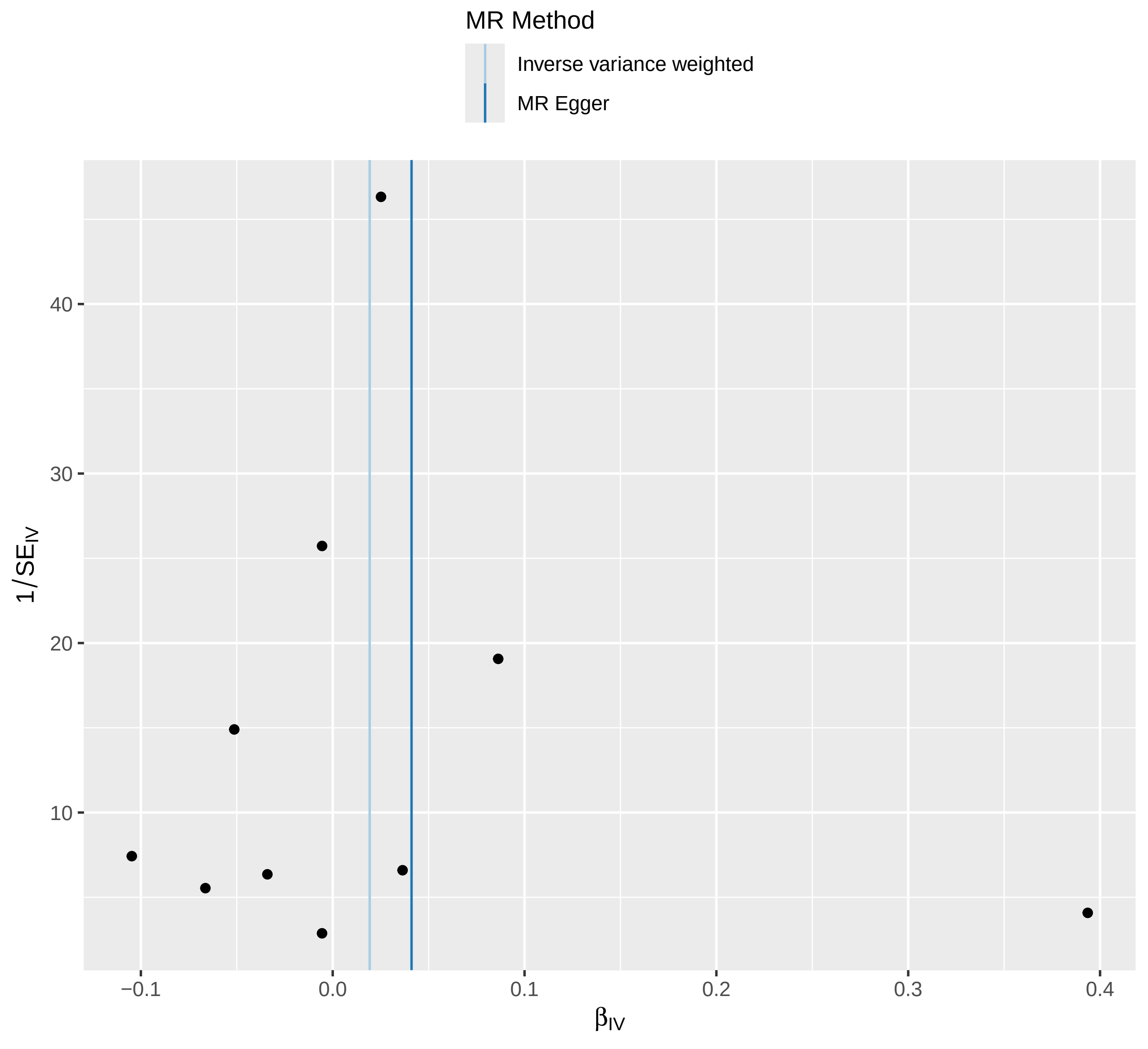
MR Method
Inverse variance weighted
MR Egger
40
30
20
10
$1/SE_{IV}$
−0.1
0.0
0.1
0.2
0.3
0.4
$\beta_{IV}$

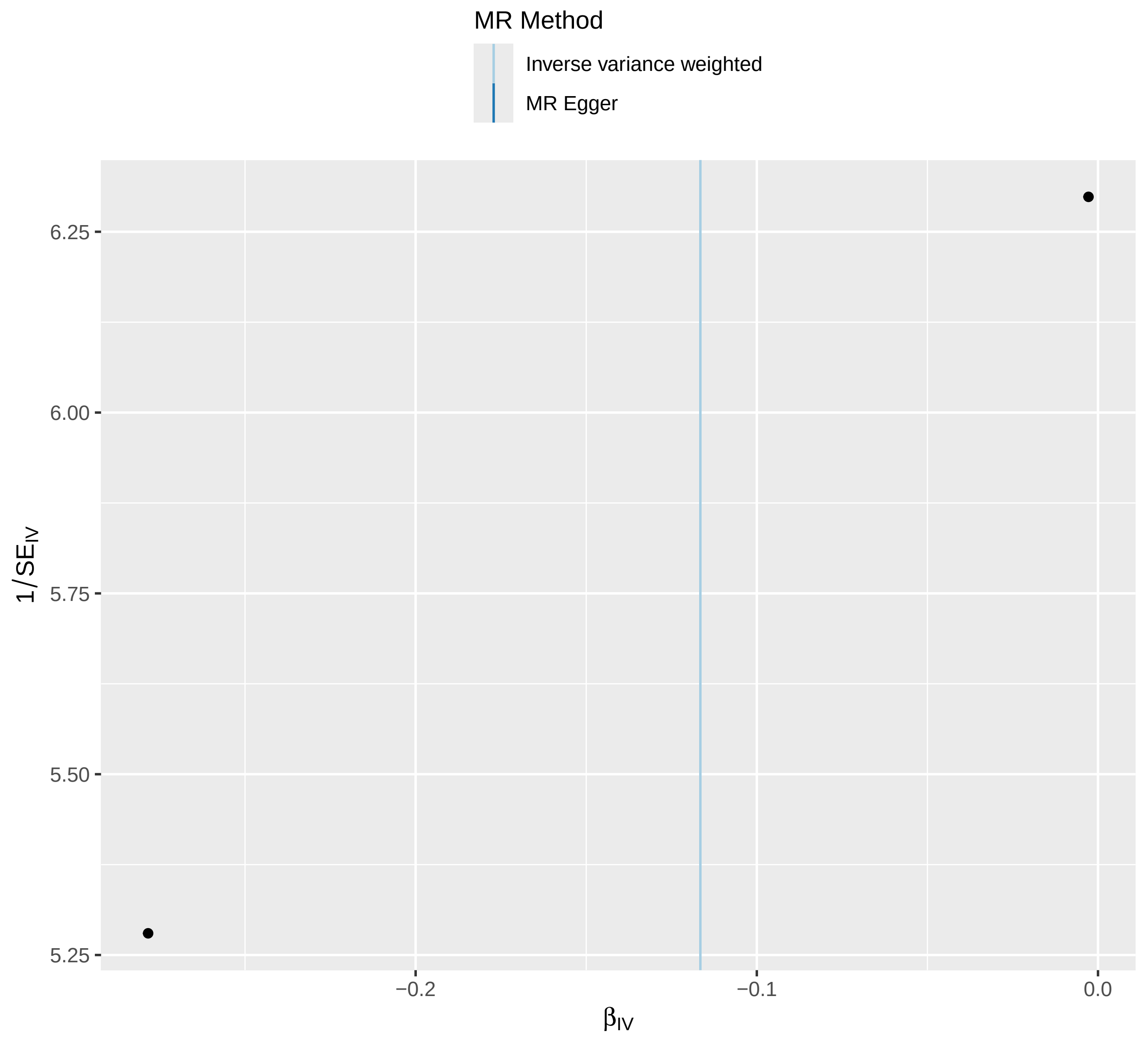

MR Method
Inverse variance weighted
MR Egger
6.25
6.00
5.75
5.50
5.25
$1/SE_{IV}$
−0.2
−0.1
0.0
$\beta_{IV}$

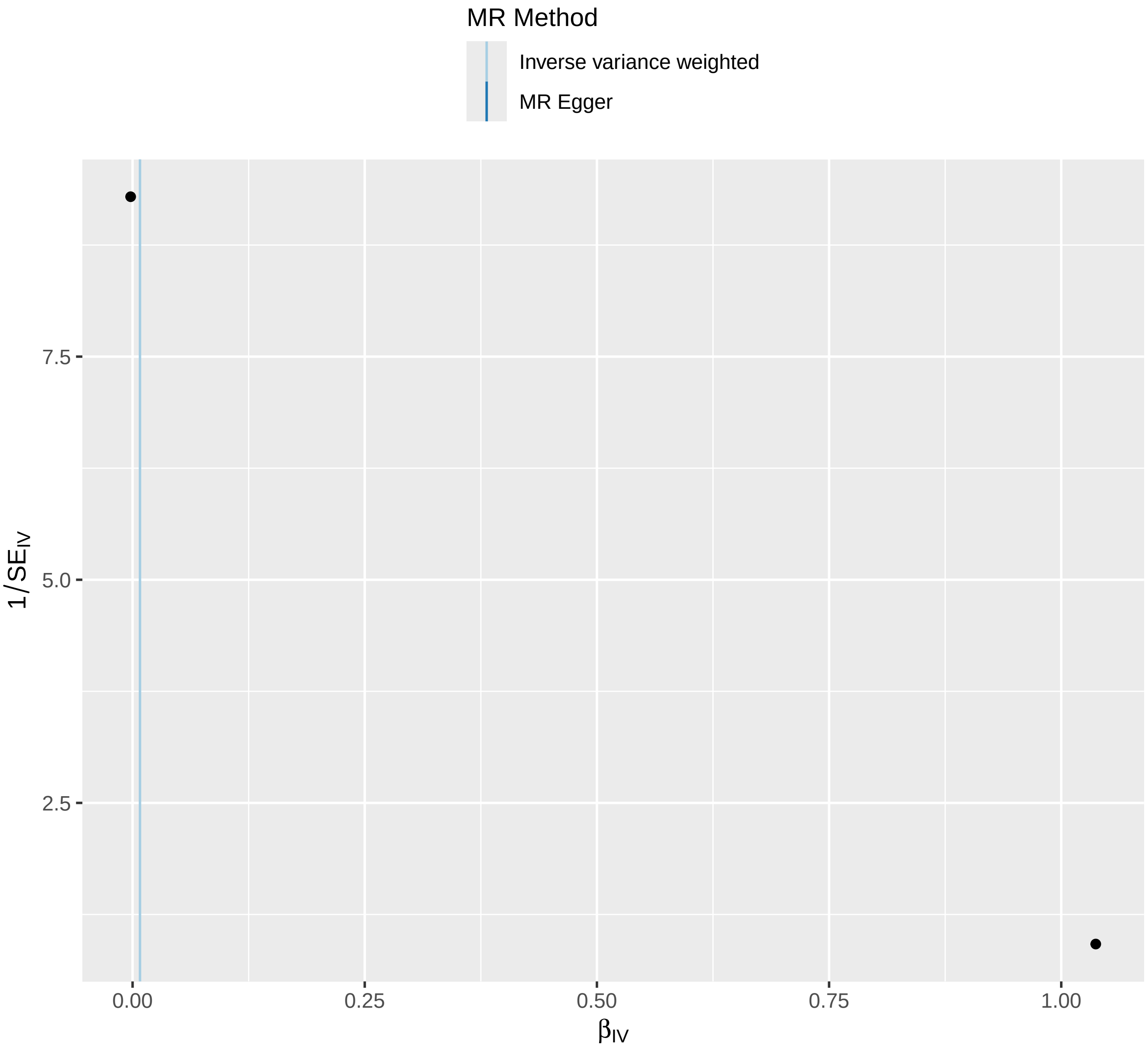

MR Method
Inverse variance weighted
MR Egger
7.5
5.0
2.5
1/$SE_{IV}$
0.00
0.25
0.50
0.75
1.00
$\beta_{IV}$

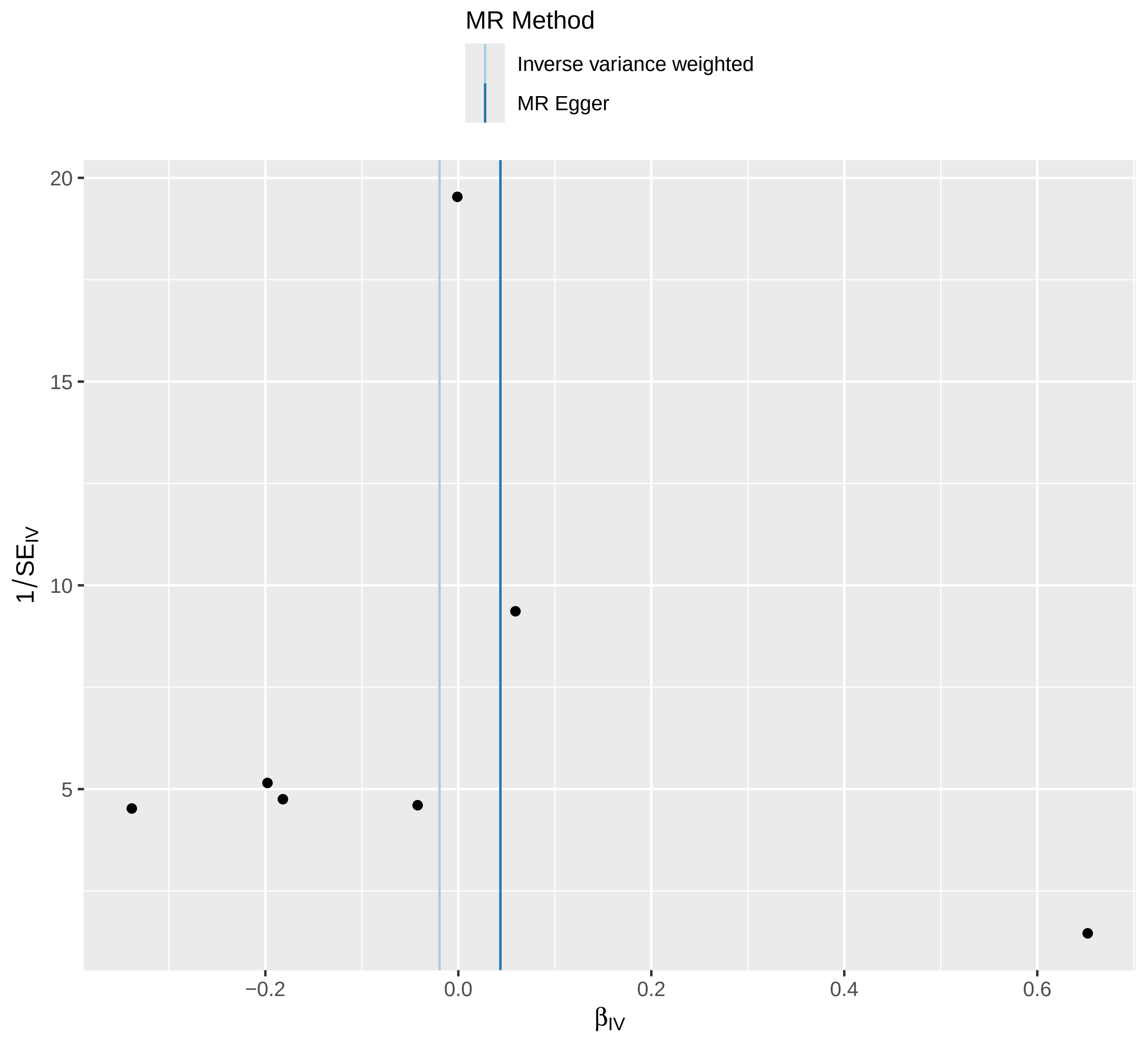
MR Method
Inverse variance weighted
MR Egger
20
15
10
5
1/SE$_{IV}$
−0.2
0.0
0.2
0.4
0.6
β$_{IV}$

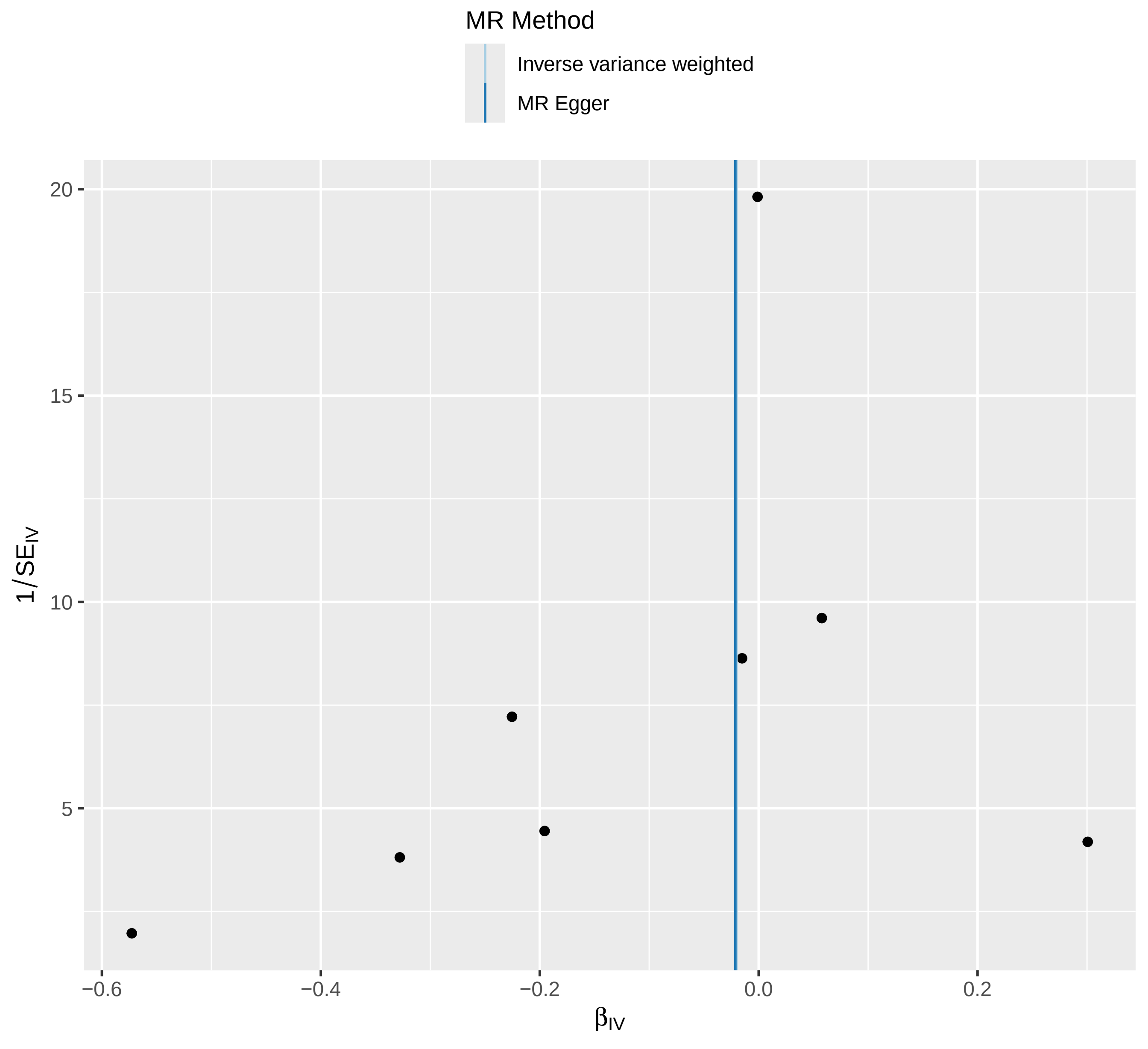

MR Method
Inverse variance weighted
MR Egger
20
15
10
5
1/SE_IV
−0.6
−0.4
−0.2
0.0
0.2
β_IV

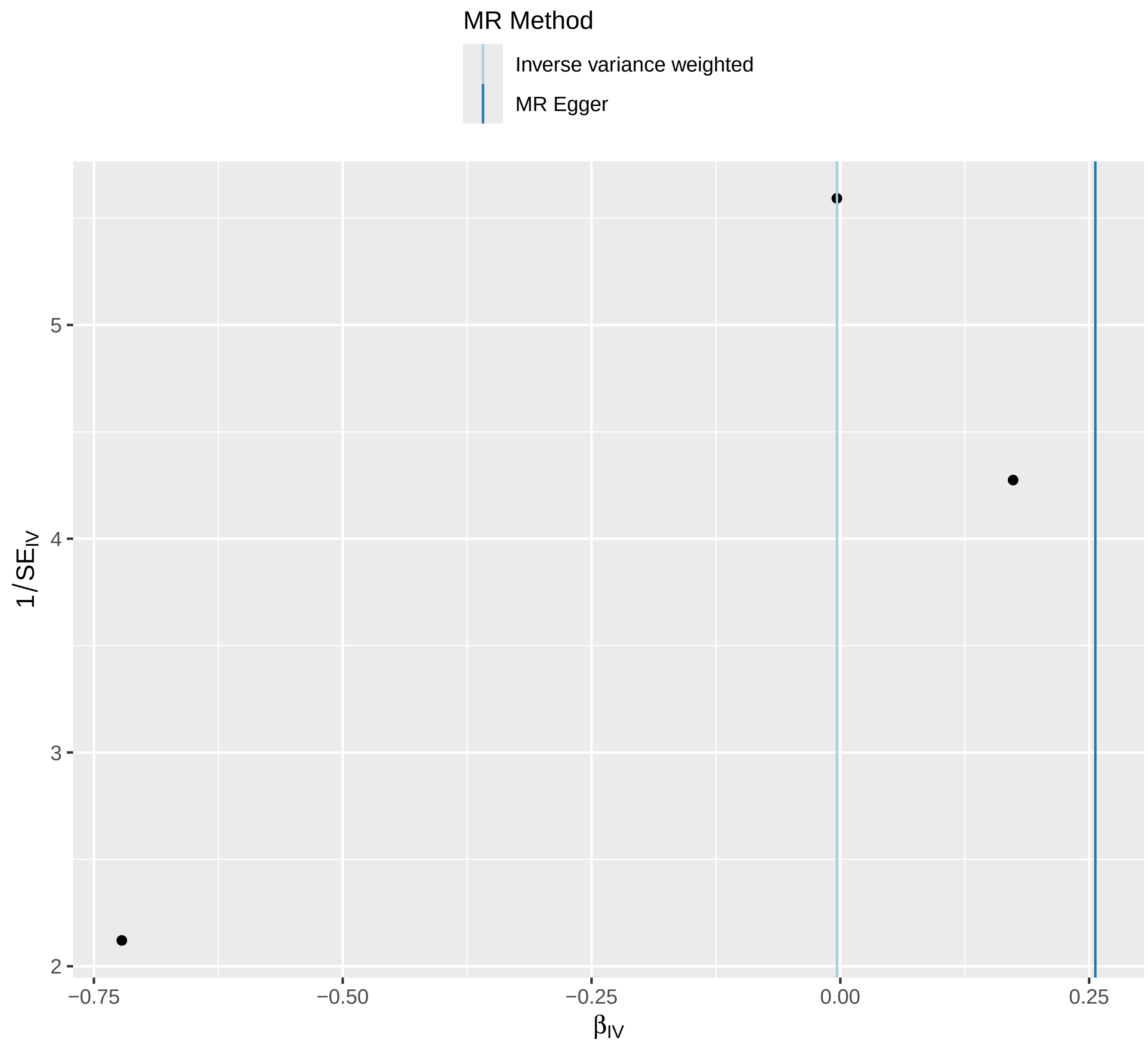
MR Method
Inverse variance weighted
MR Egger
5
4
3
2
1/SE$_{IV}$
−0.75
−0.50
−0.25
0.00
0.25
β$_{IV}$

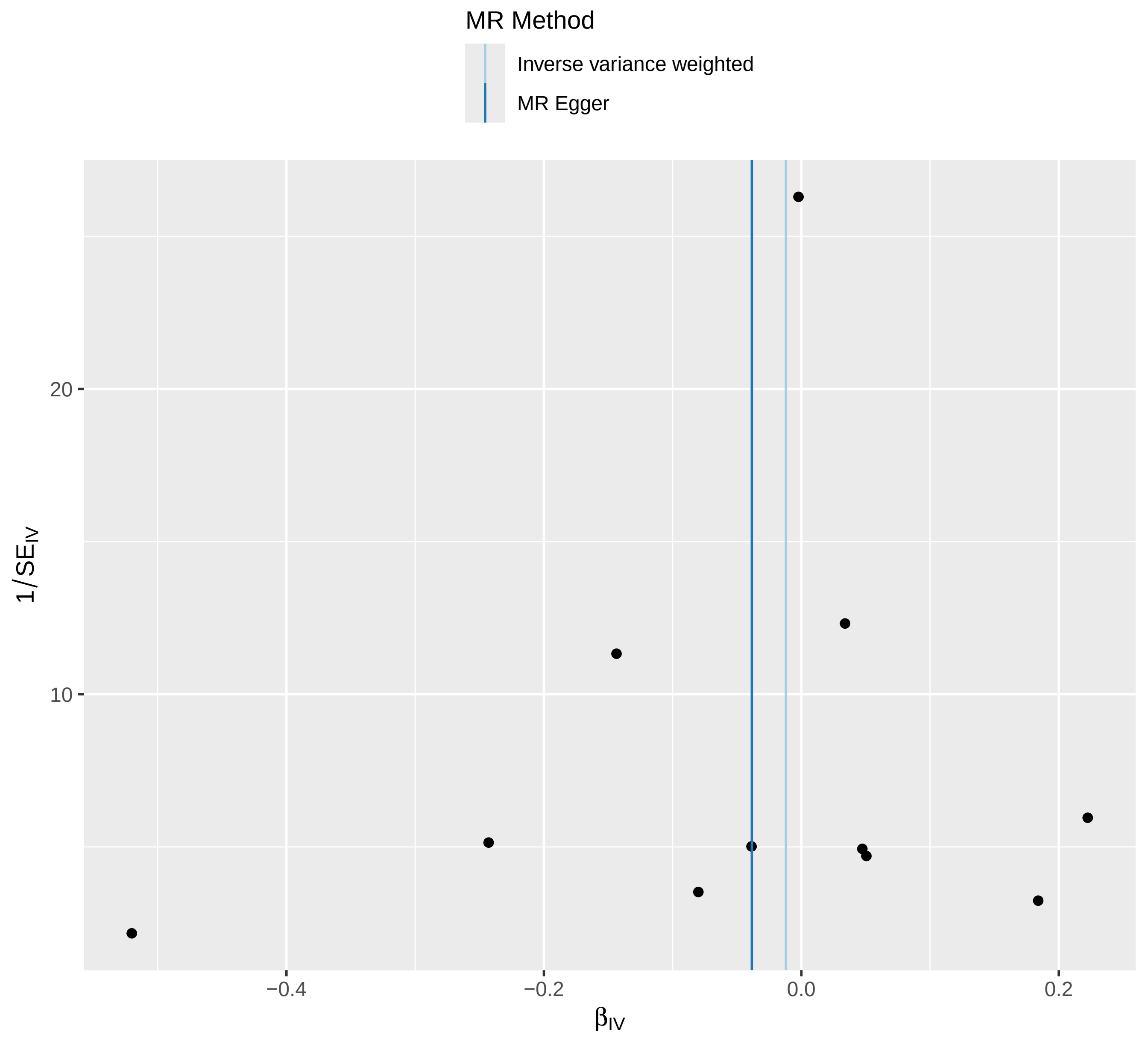
MR Method
Inverse variance weighted
MR Egger
20
10
$1/SE_{IV}$
−0.4
−0.2
0.0
0.2
$\beta_{IV}$

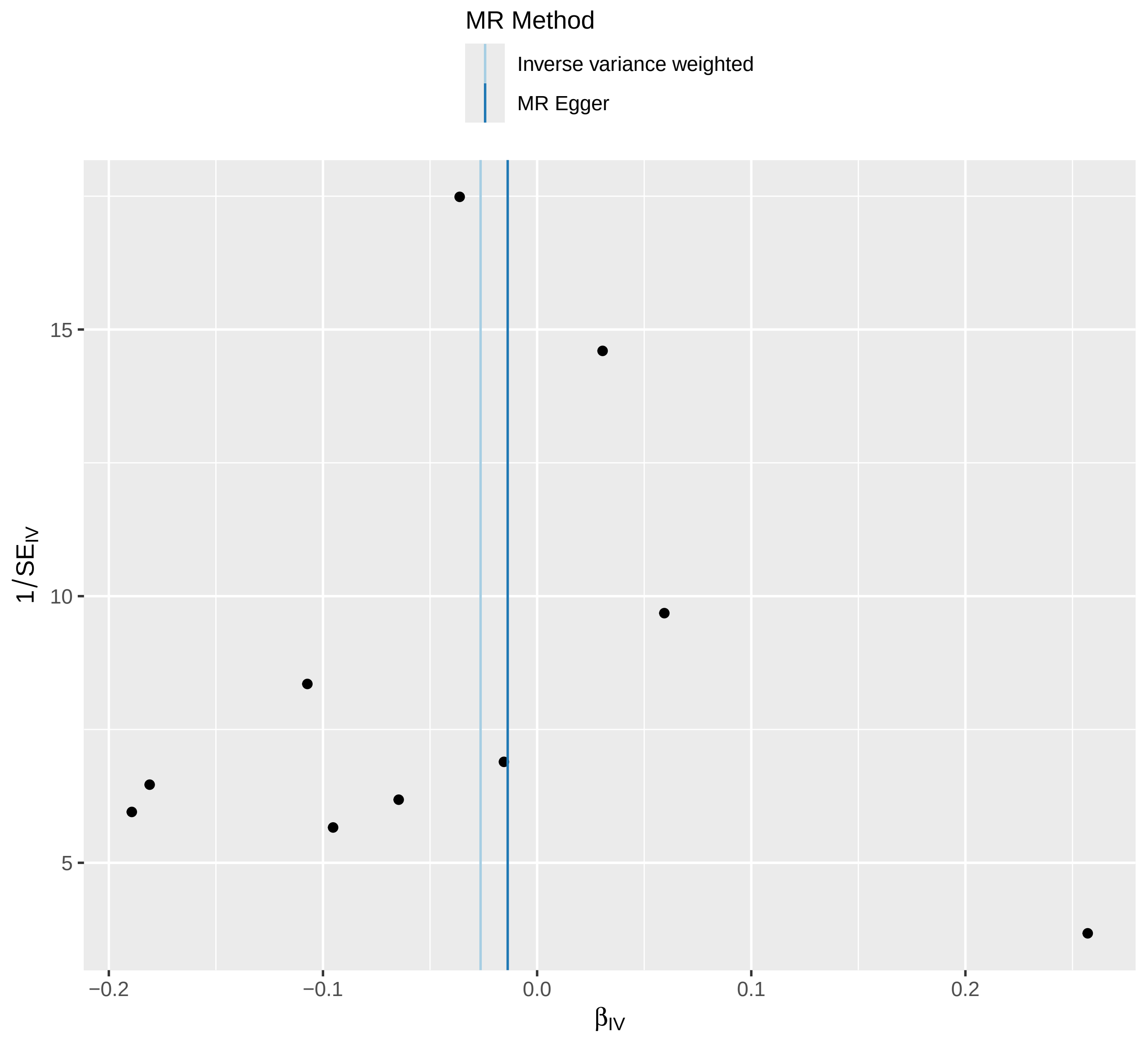

MR Method
Inverse variance weighted
MR Egger
1/SE_IV
15
10
5
−0.2
−0.1
0.0
0.1
0.2
β_IV

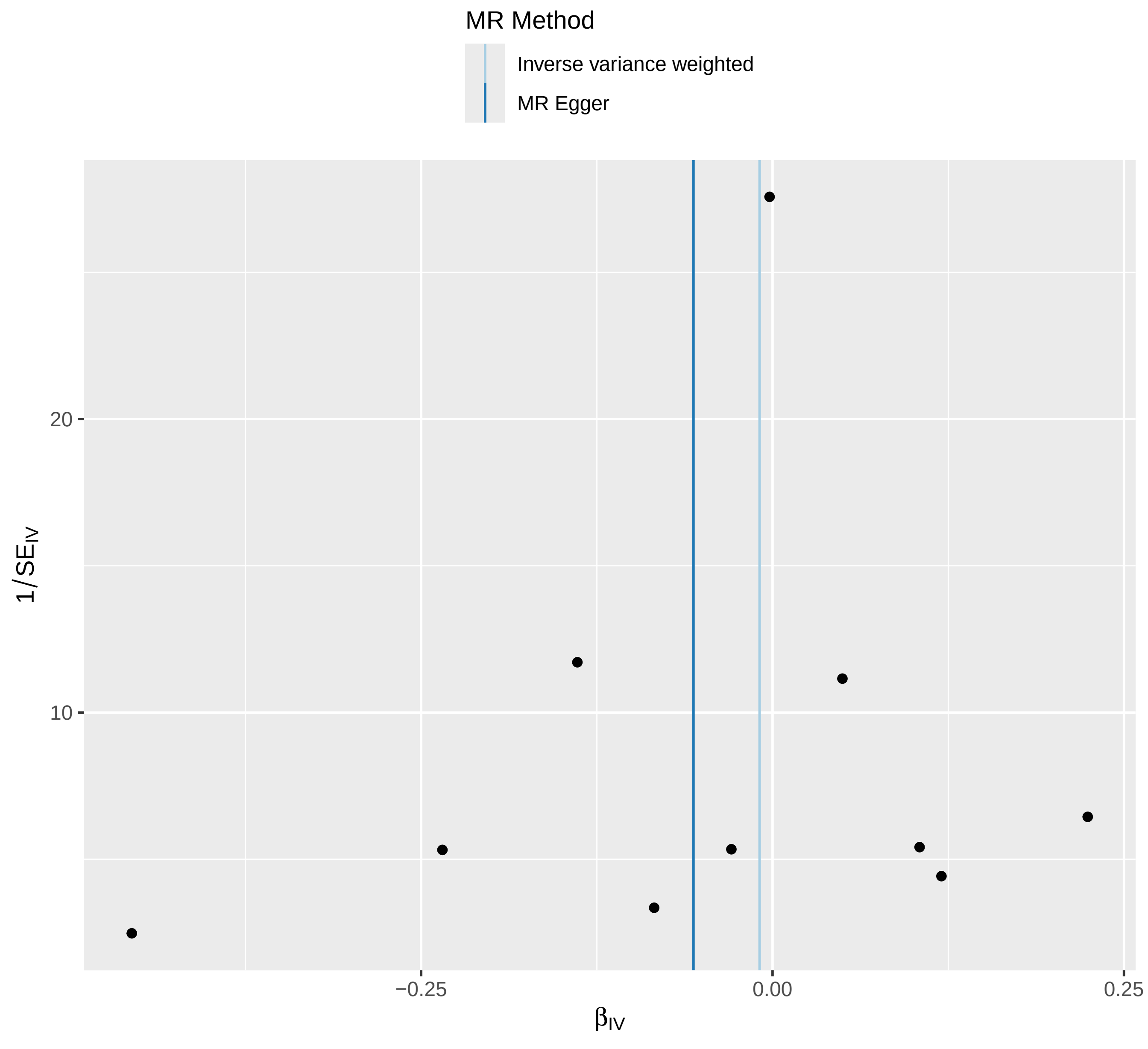

MR Method
Inverse variance weighted
MR Egger
20
10
1/SE_IV
−0.25
0.00
0.25
β_IV

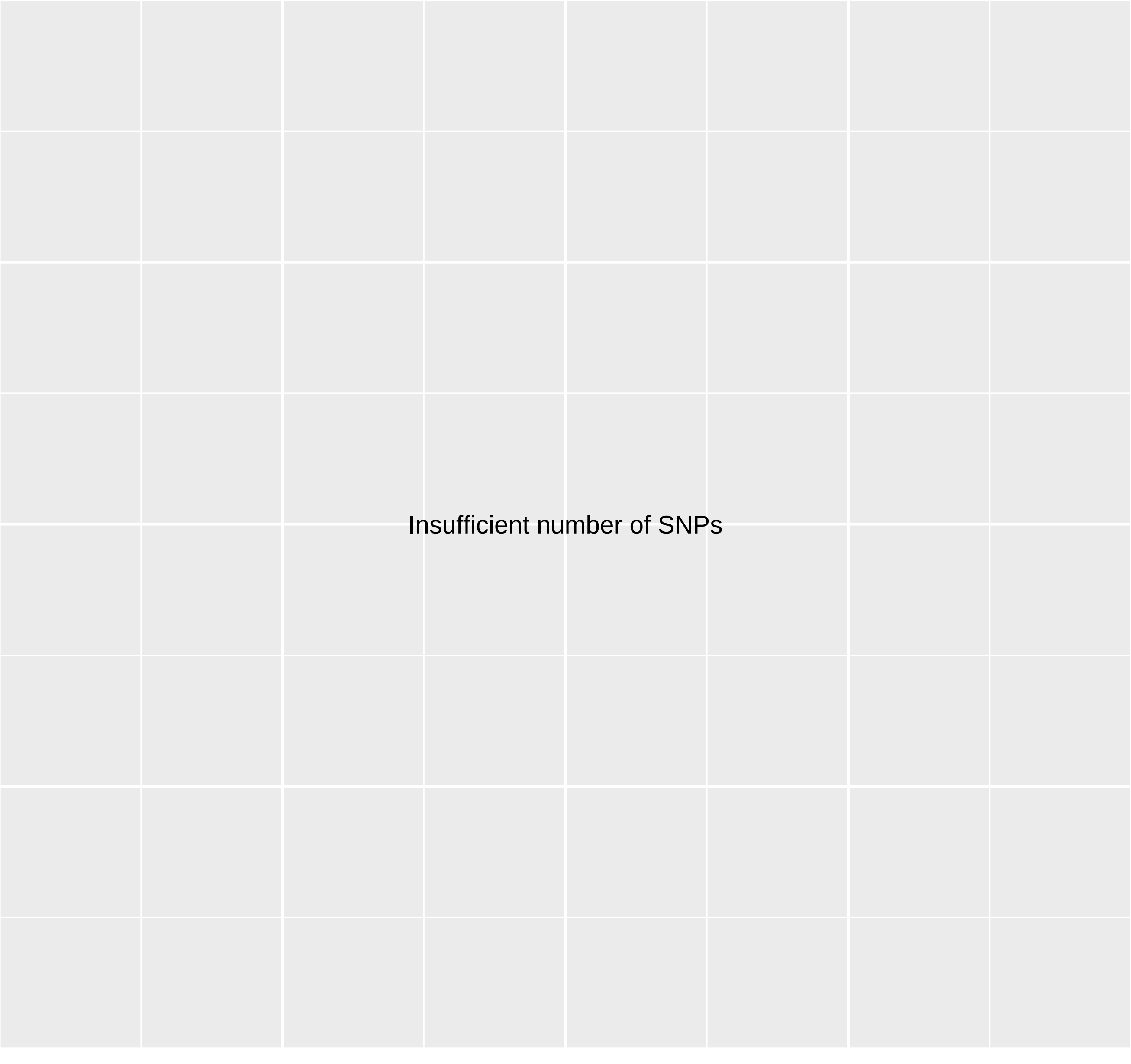
Insufficient number of SNPs

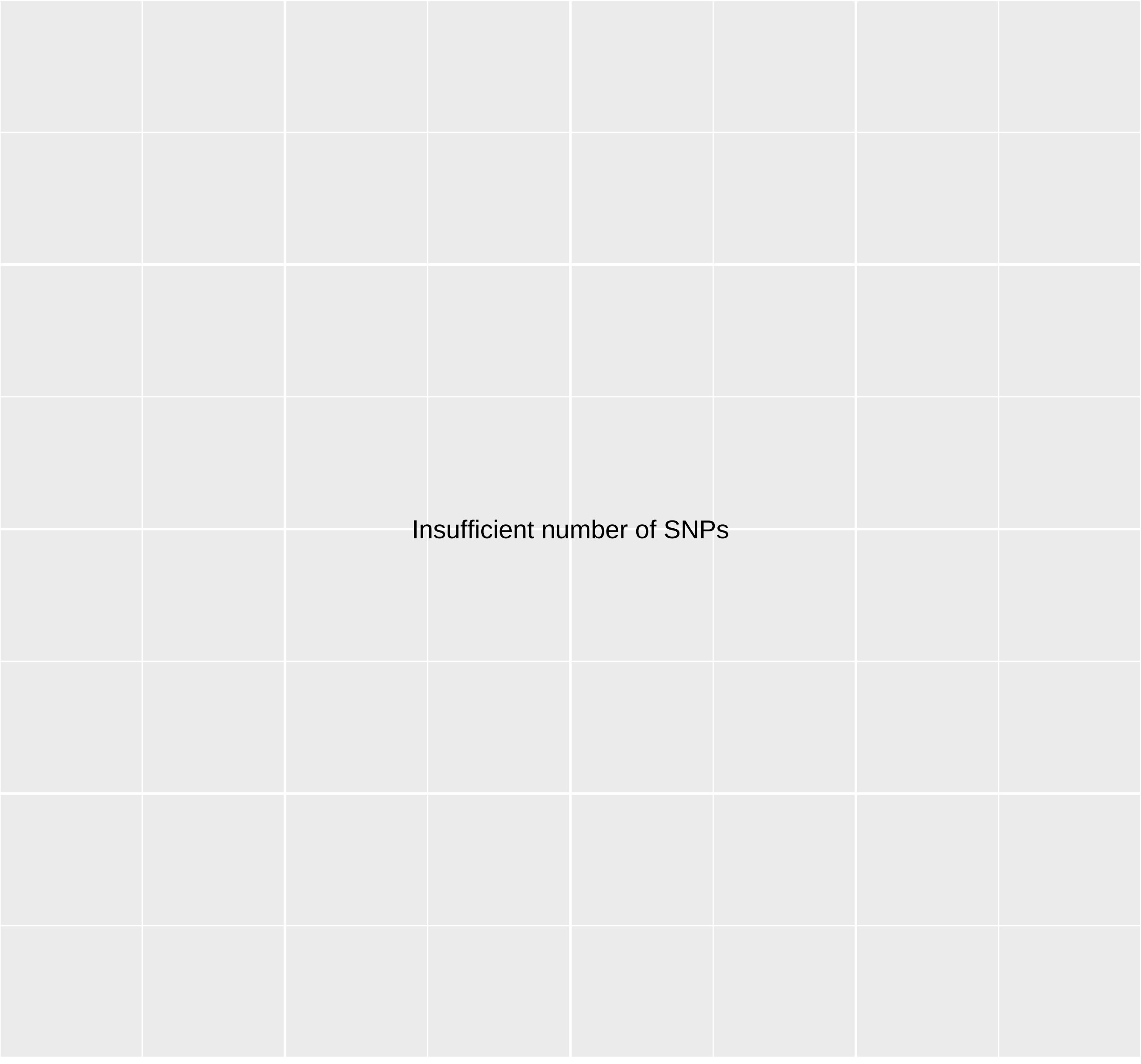
Insufficient number of SNPs

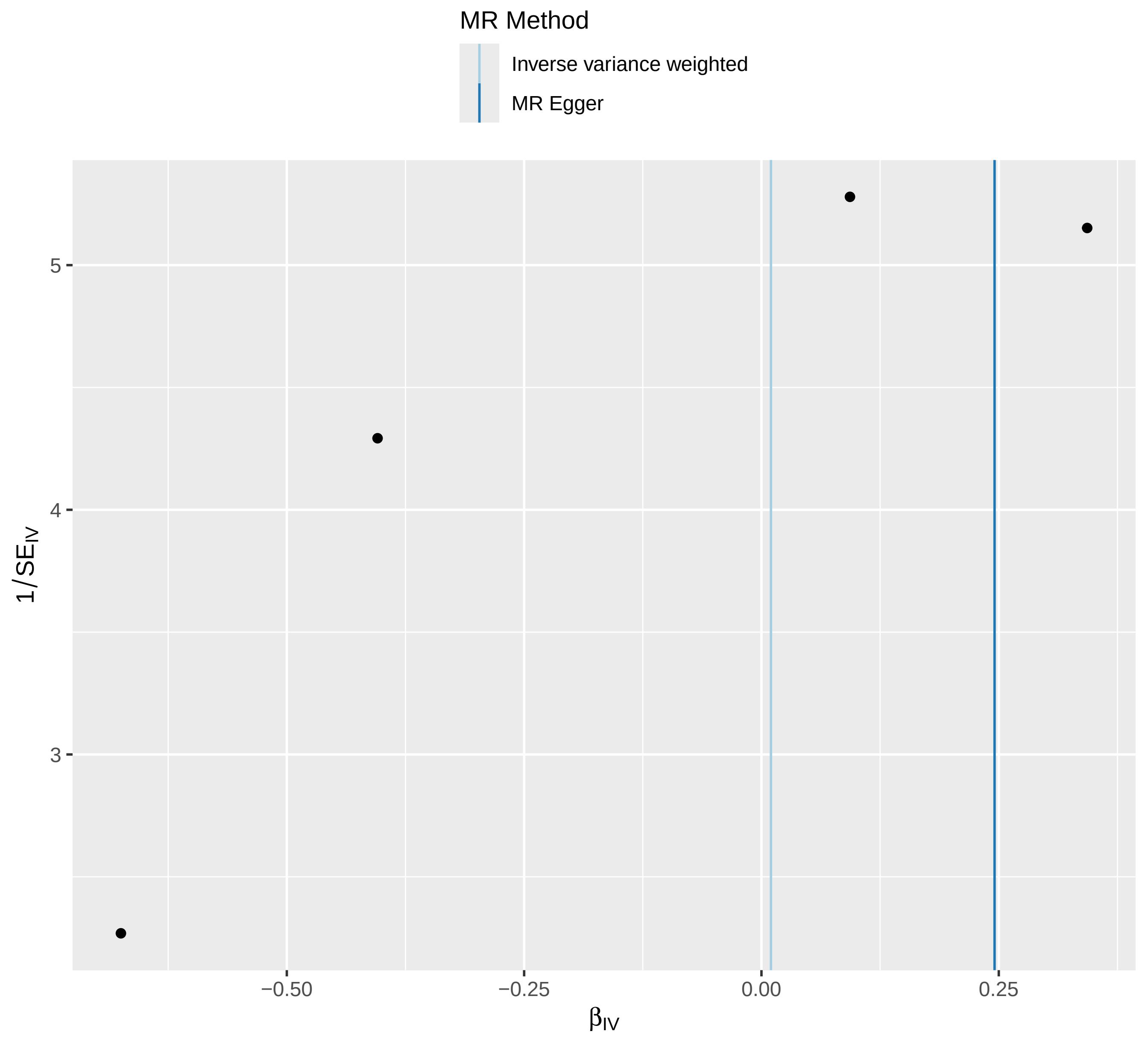
MR Method
Inverse variance weighted
MR Egger
5
4
3
1/SE$_{IV}$
−0.50
−0.25
0.00
0.25
β$_{IV}$

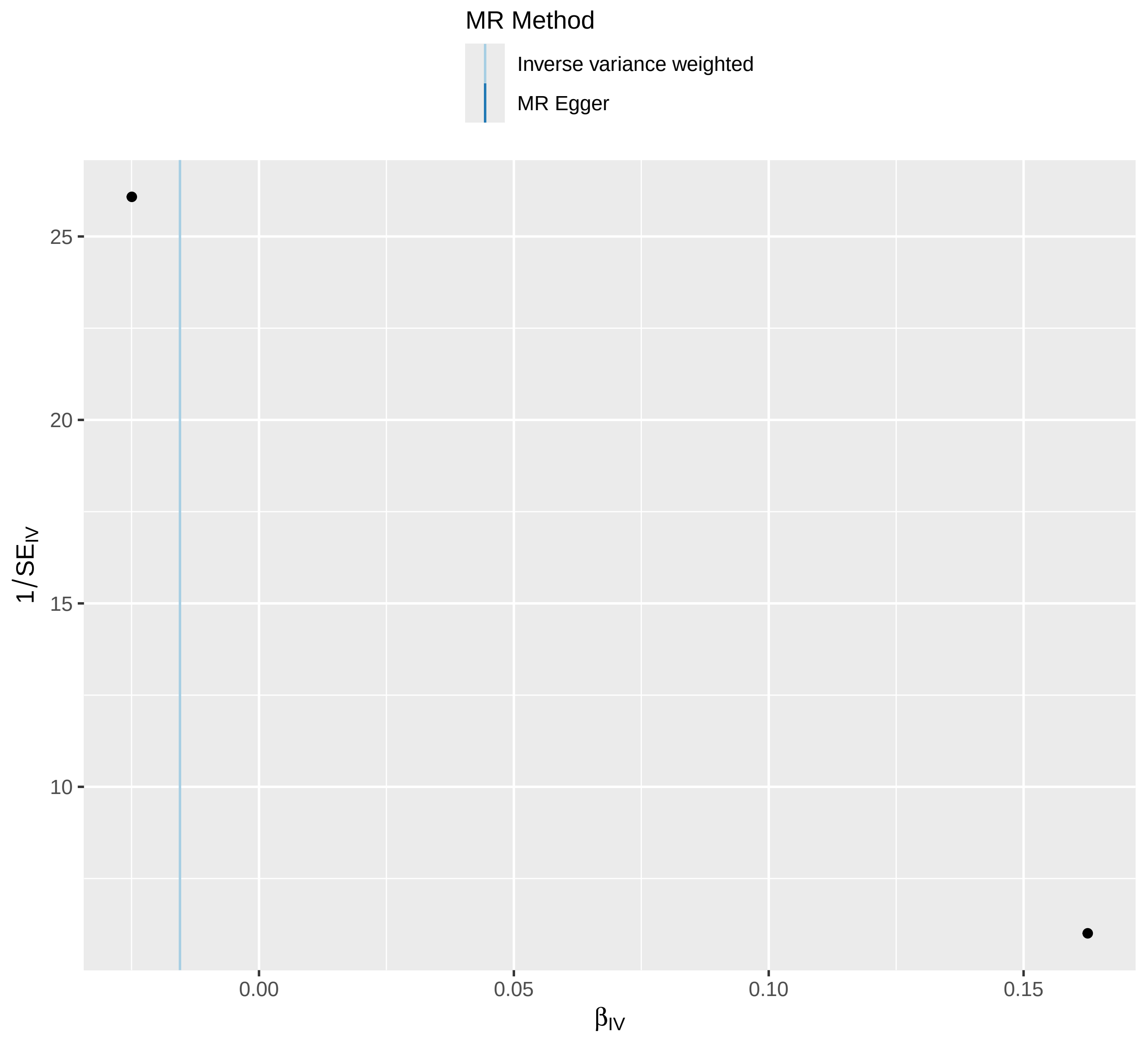
MR Method
Inverse variance weighted
MR Egger
25
20
15
10
0.00
0.05
0.10
0.15
$1/SE_{IV}$
$\beta_{IV}$

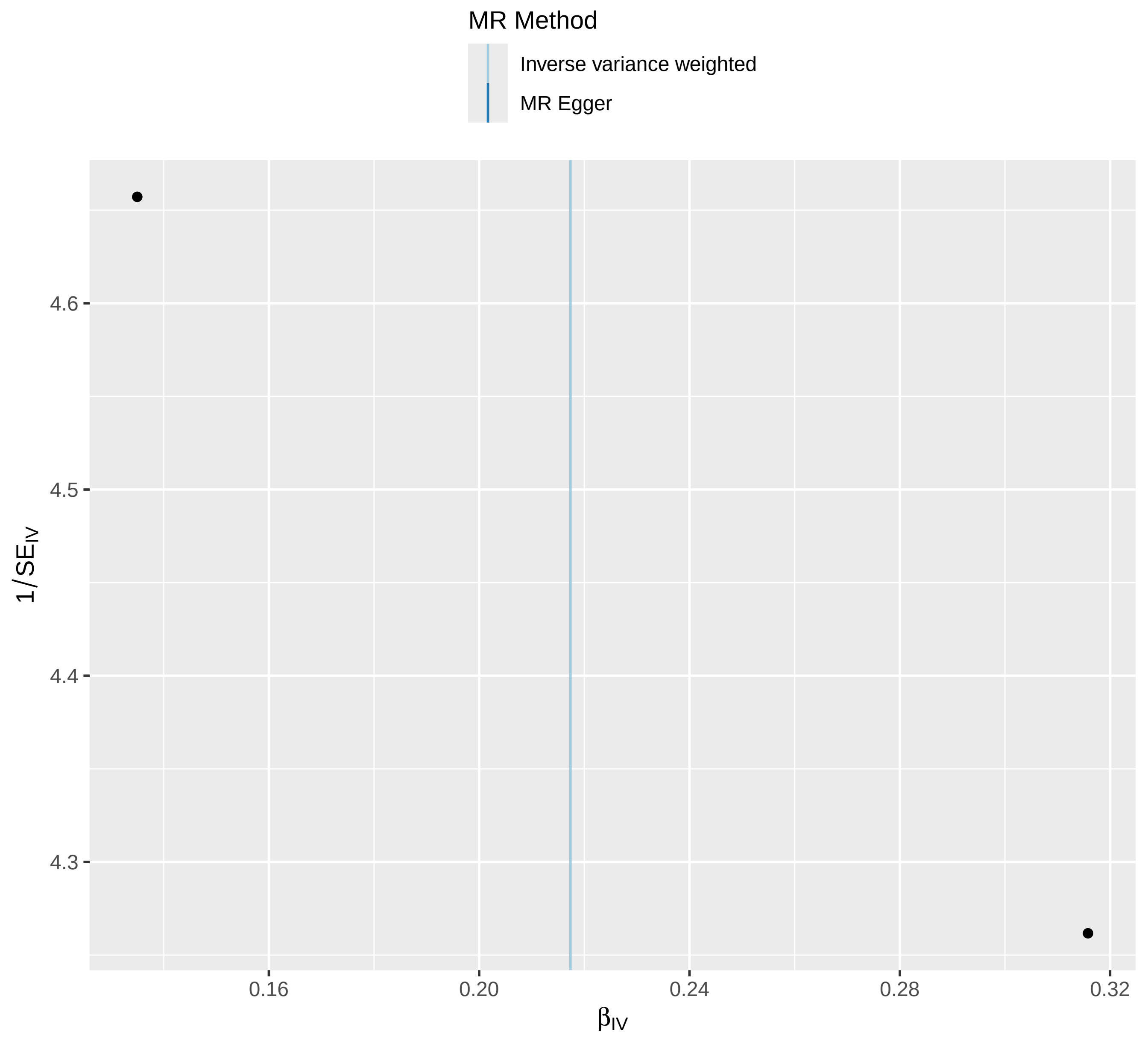

MR Method
Inverse variance weighted
MR Egger
4.6
4.5
4.4
4.3
1/SE$_{IV}$
0.16
0.20
0.24
0.28
0.32
β$_{IV}$

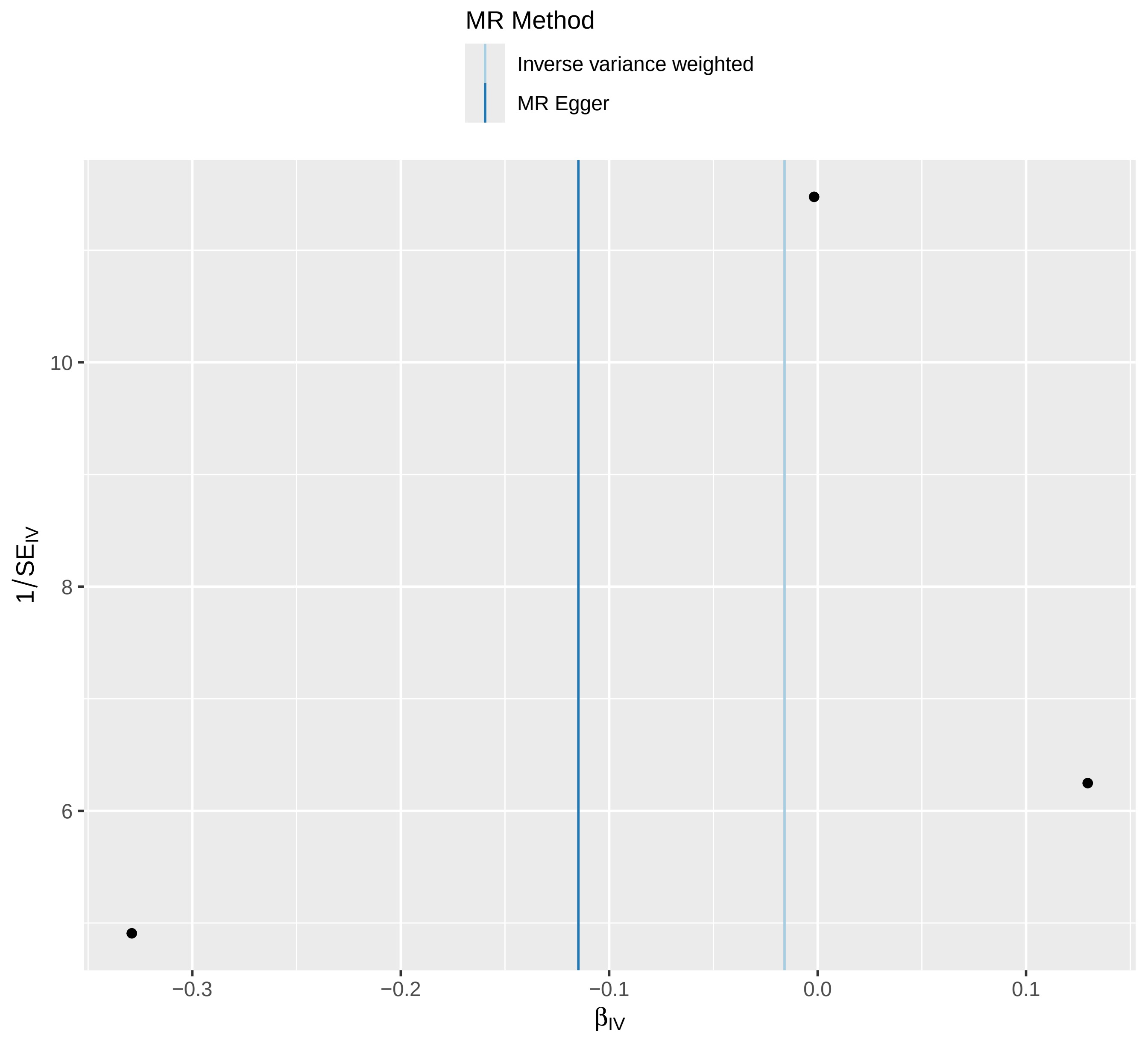

MR Method
Inverse variance weighted
MR Egger
10
8
6
1/SE_IV
−0.3
−0.2
−0.1
0.0
0.1
β_IV

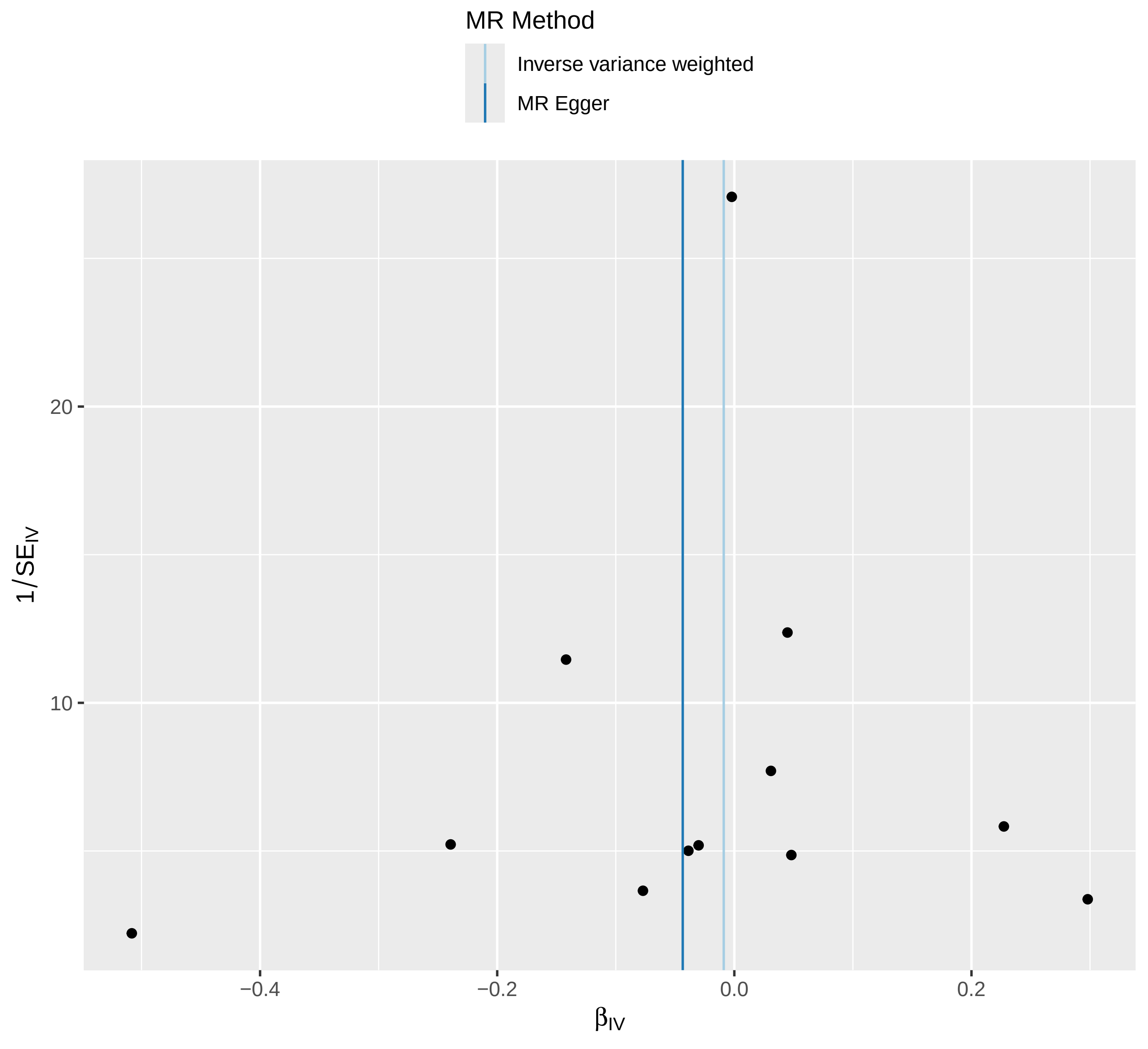

MR Method
Inverse variance weighted
MR Egger
20
10
1/SE_IV
−0.4
−0.2
0.0
0.2
β_IV

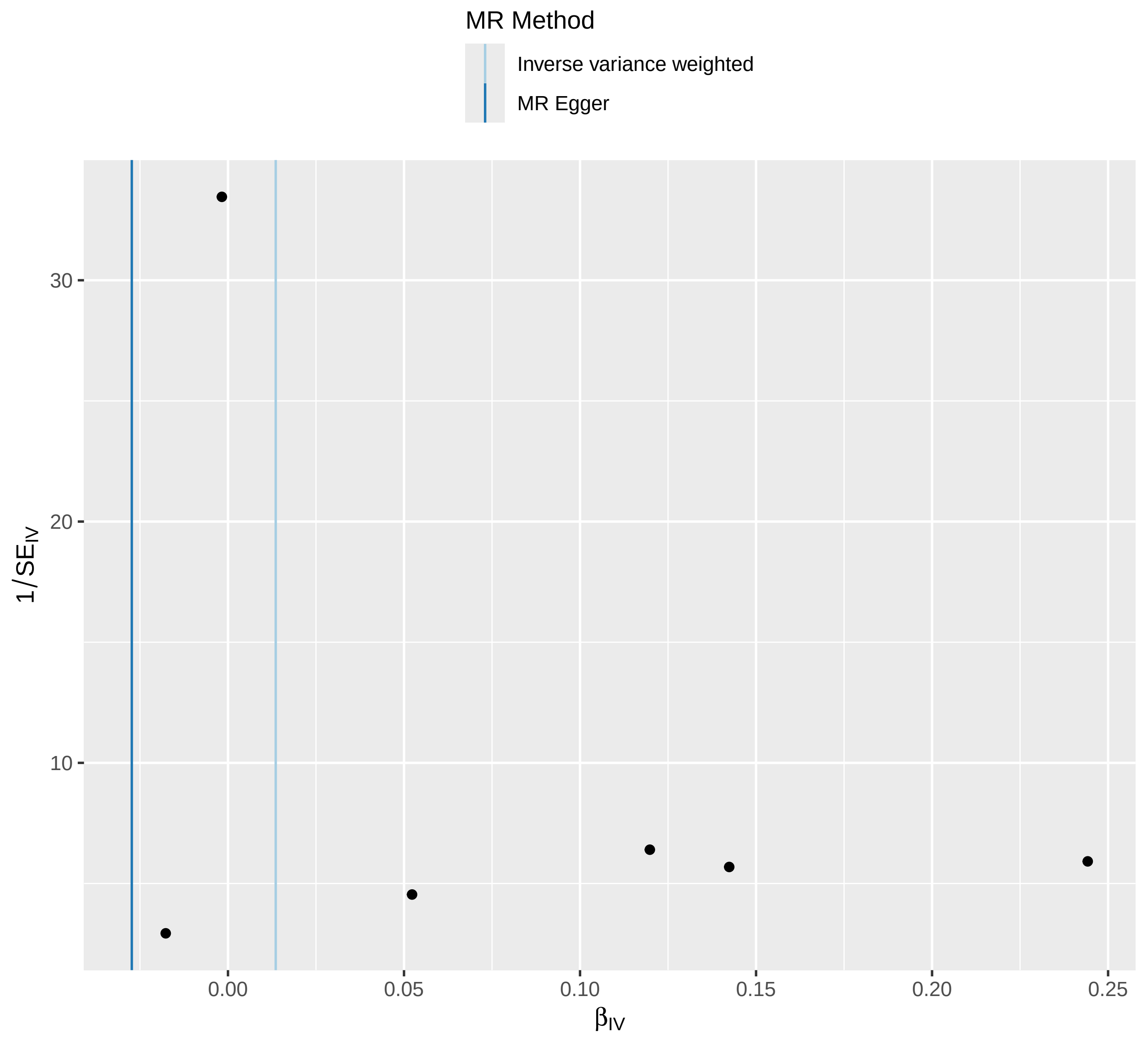

MR Method
Inverse variance weighted
MR Egger
30
20
10
1/SE_IV
0.00
0.05
0.10
0.15
0.20
0.25
β_IV

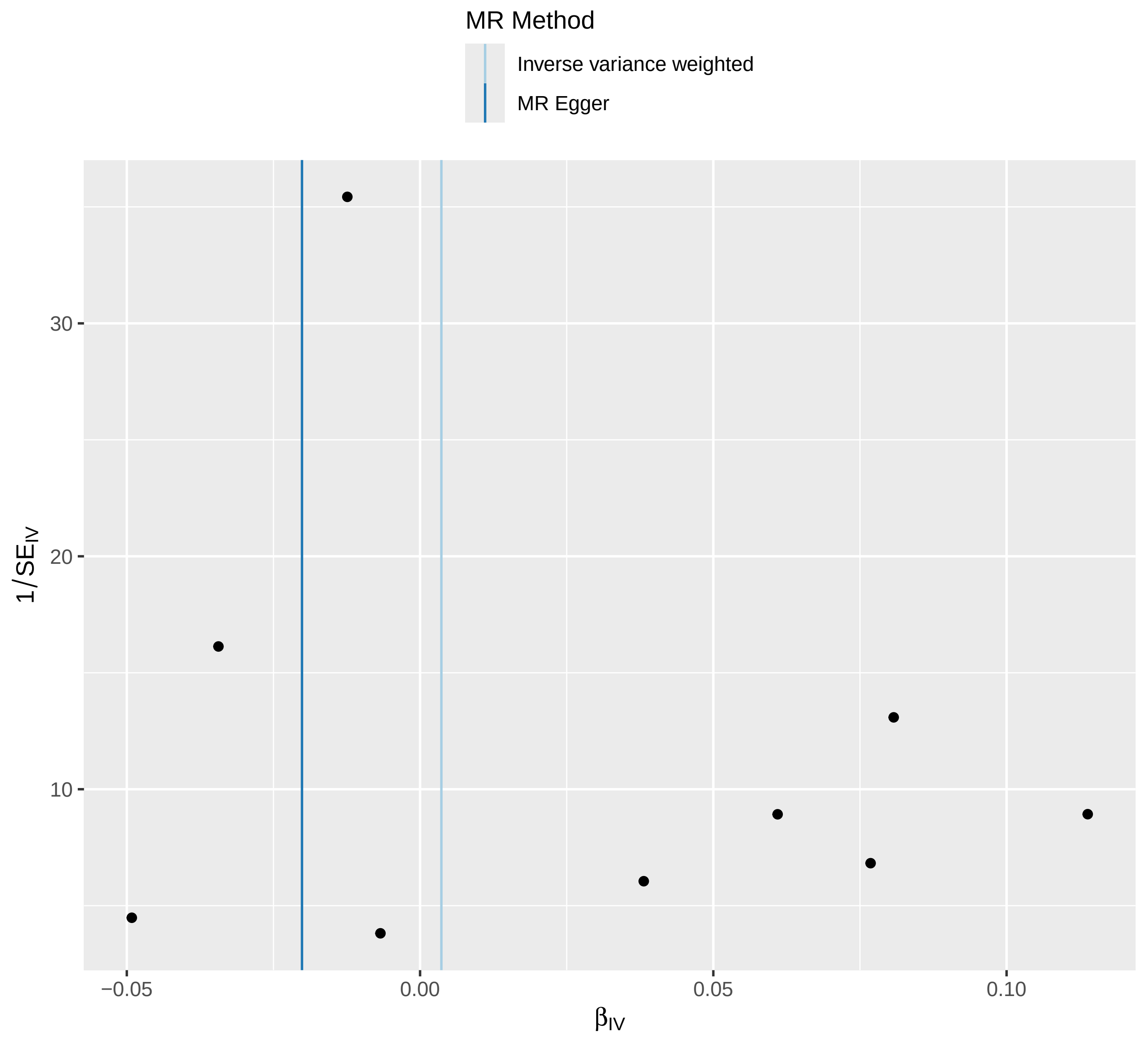
MR Method
Inverse variance weighted
MR Egger
30
20
10
−0.05
0.00
0.05
0.10
$1/SE_{IV}$
$\beta_{IV}$

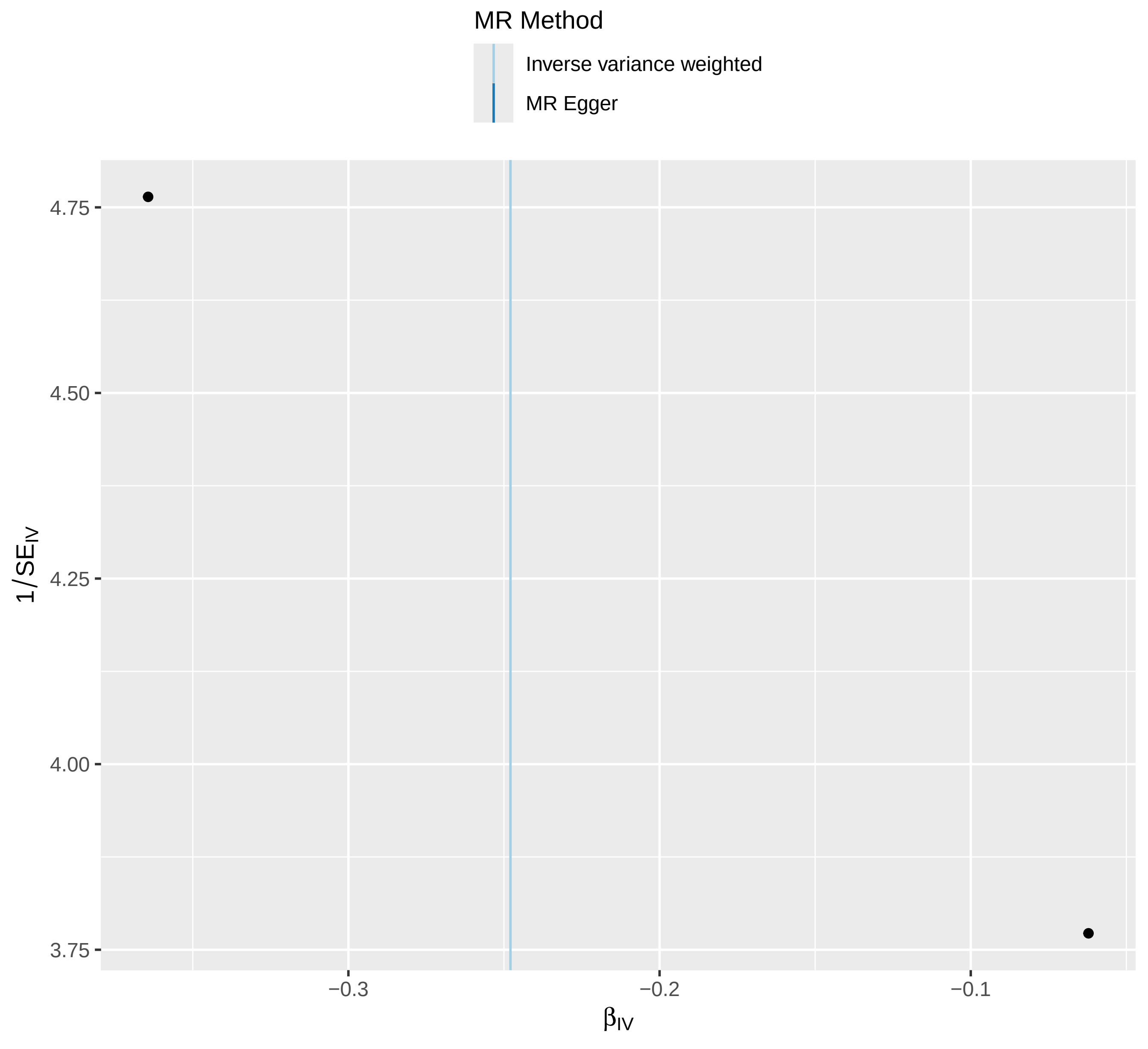
MR Method
Inverse variance weighted
MR Egger
4.75
4.50
4.25
4.00
3.75
1/SE_IV
−0.3
−0.2
−0.1
β_IV

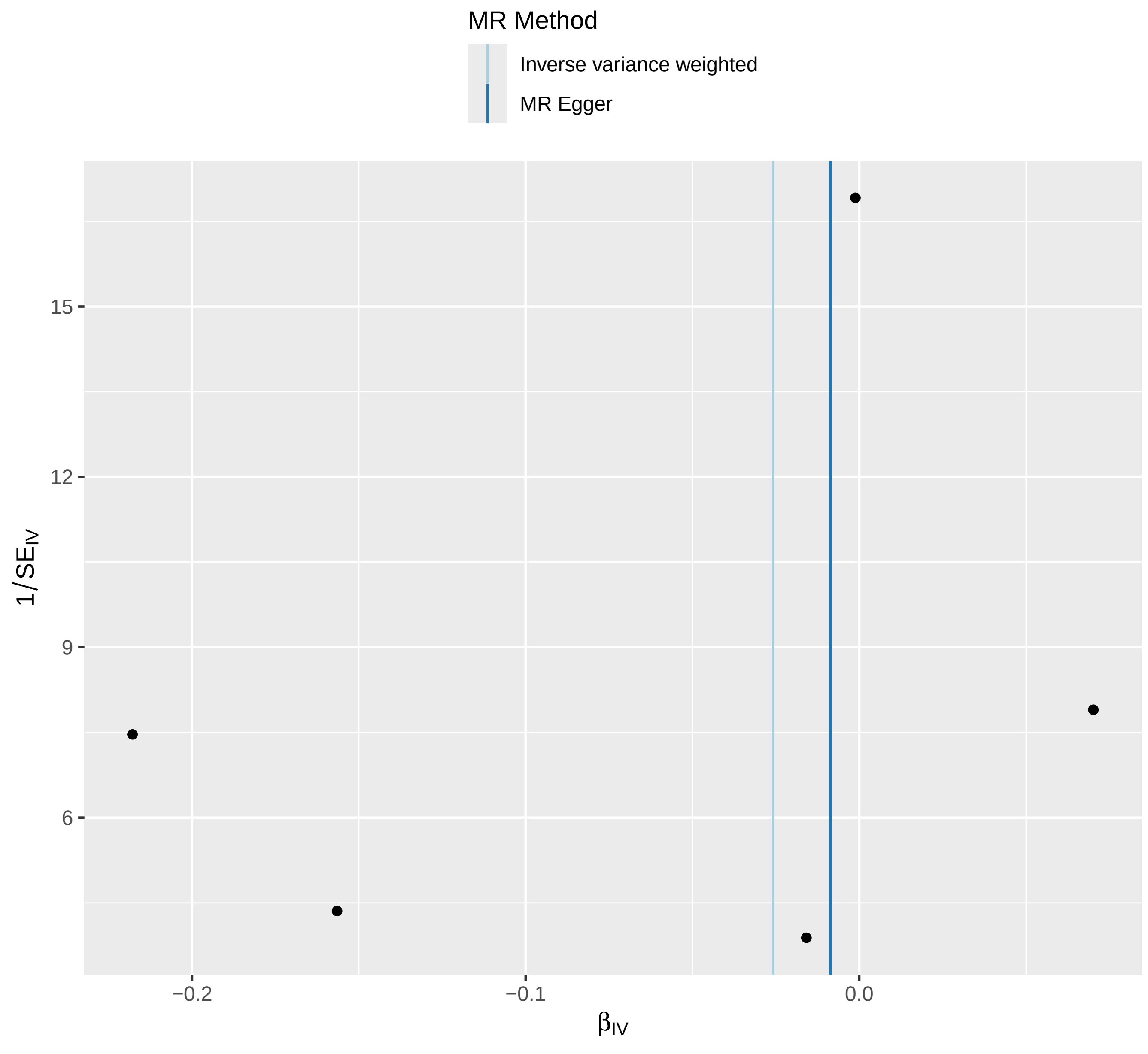

MR Method
Inverse variance weighted
MR Egger
1/SE$_{IV}$
15
12
9
6
−0.2
−0.1
0.0
β$_{IV}$

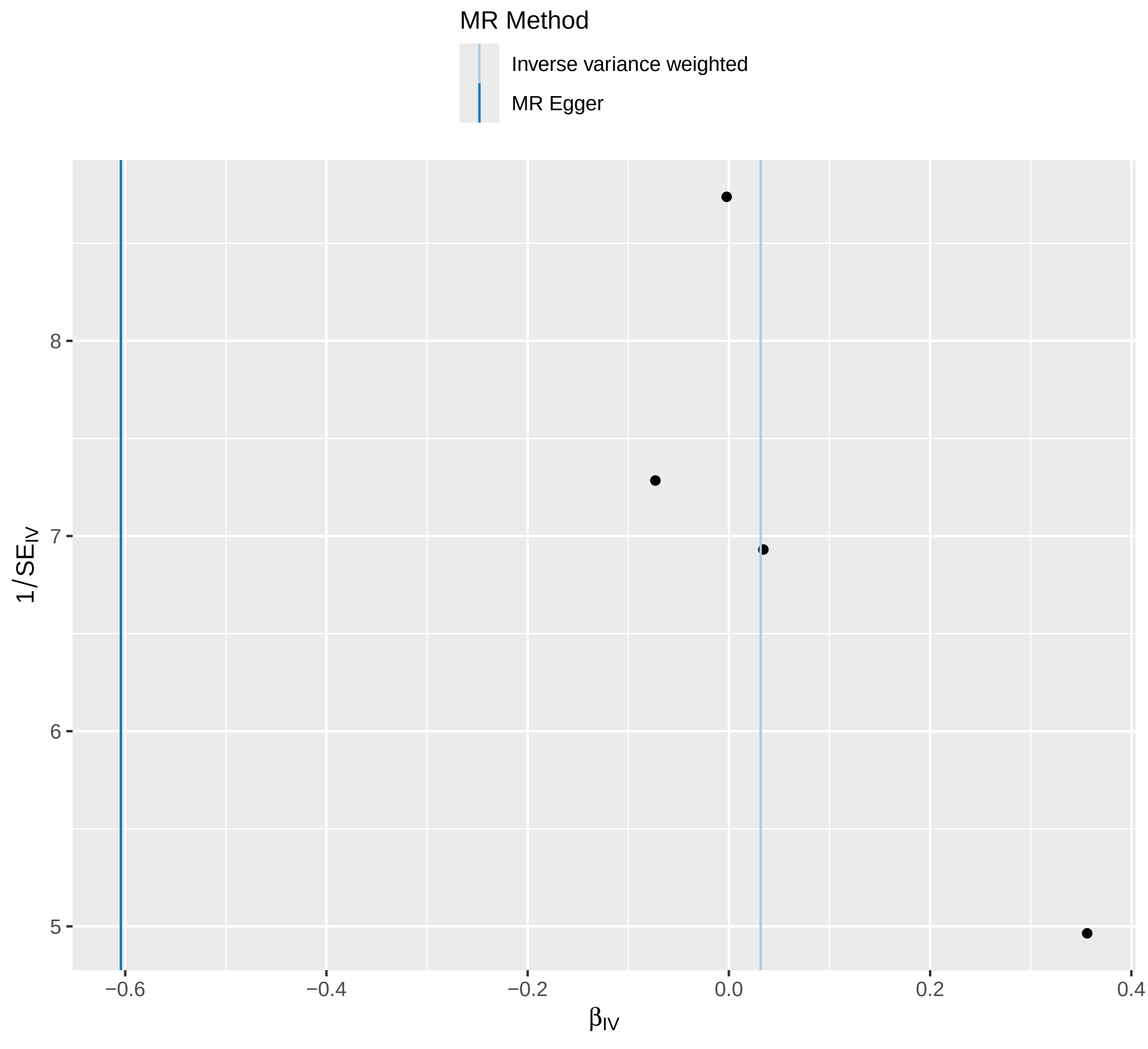
MR Method
Inverse variance weighted
MR Egger
8
7
6
5
1/SE$_{IV}$
−0.6
−0.4
−0.2
0.0
0.2
0.4
β$_{IV}$

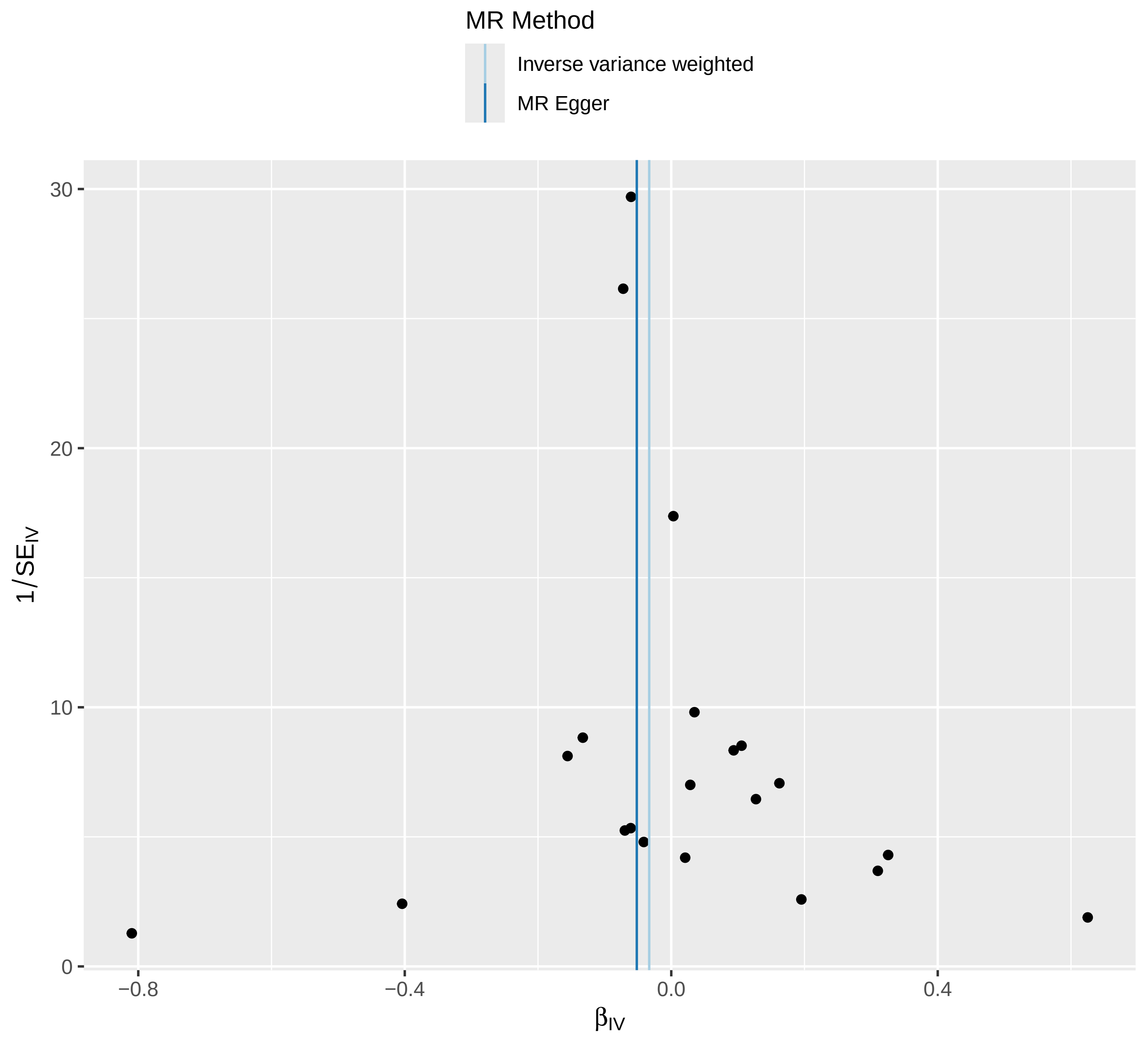

MR Method
Inverse variance weighted
MR Egger
30
20
10
0
1/SE$_{IV}$
−0.8
−0.4
0.0
0.4
β$_{IV}$

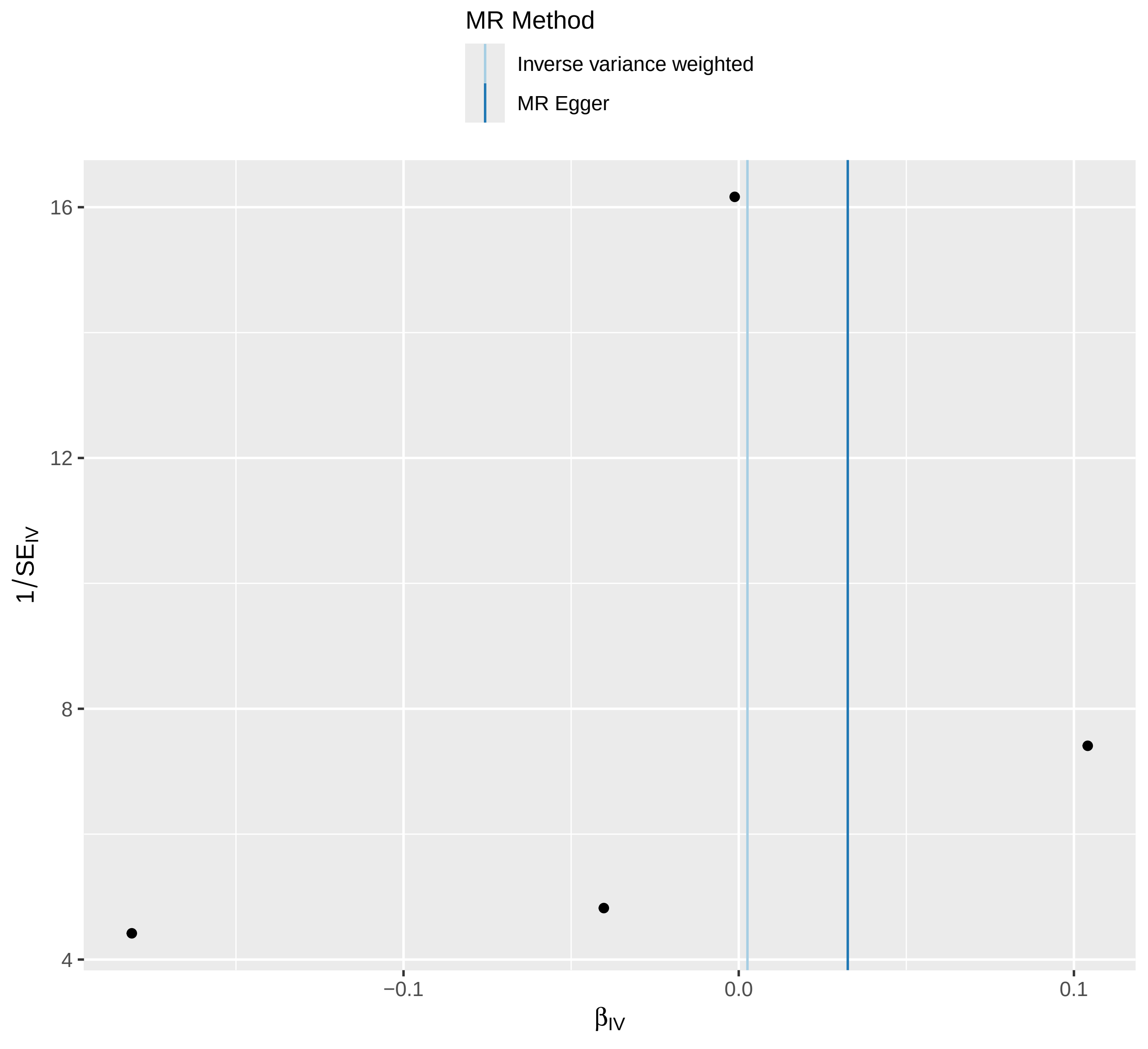
MR Method
Inverse variance weighted
MR Egger
16
12
8
4
−0.1
0.0
0.1
1/SE_IV
β_IV

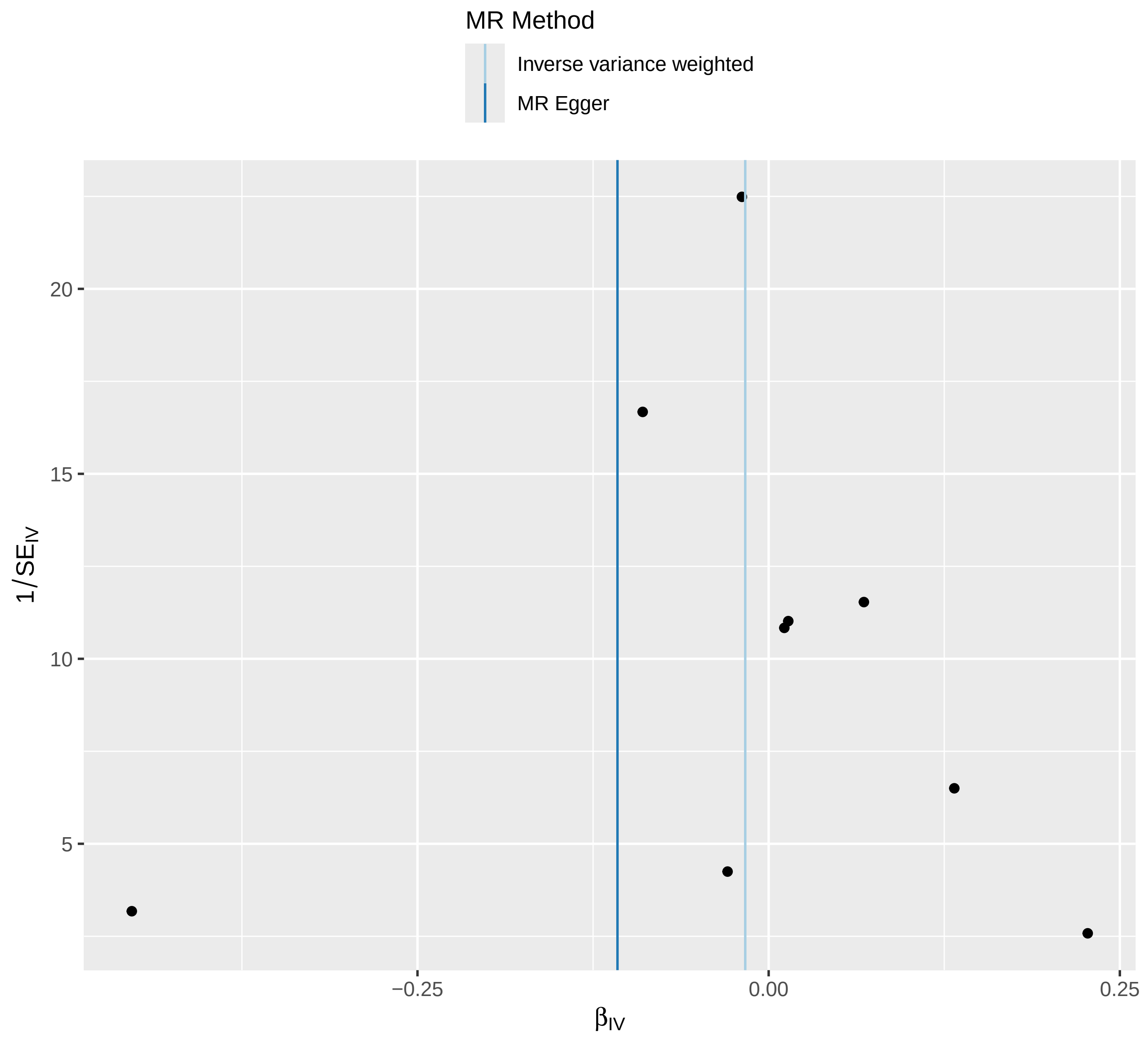
MR Method
Inverse variance weighted
MR Egger
20
15
10
5
1/SE_IV
−0.25
0.00
0.25
β_IV

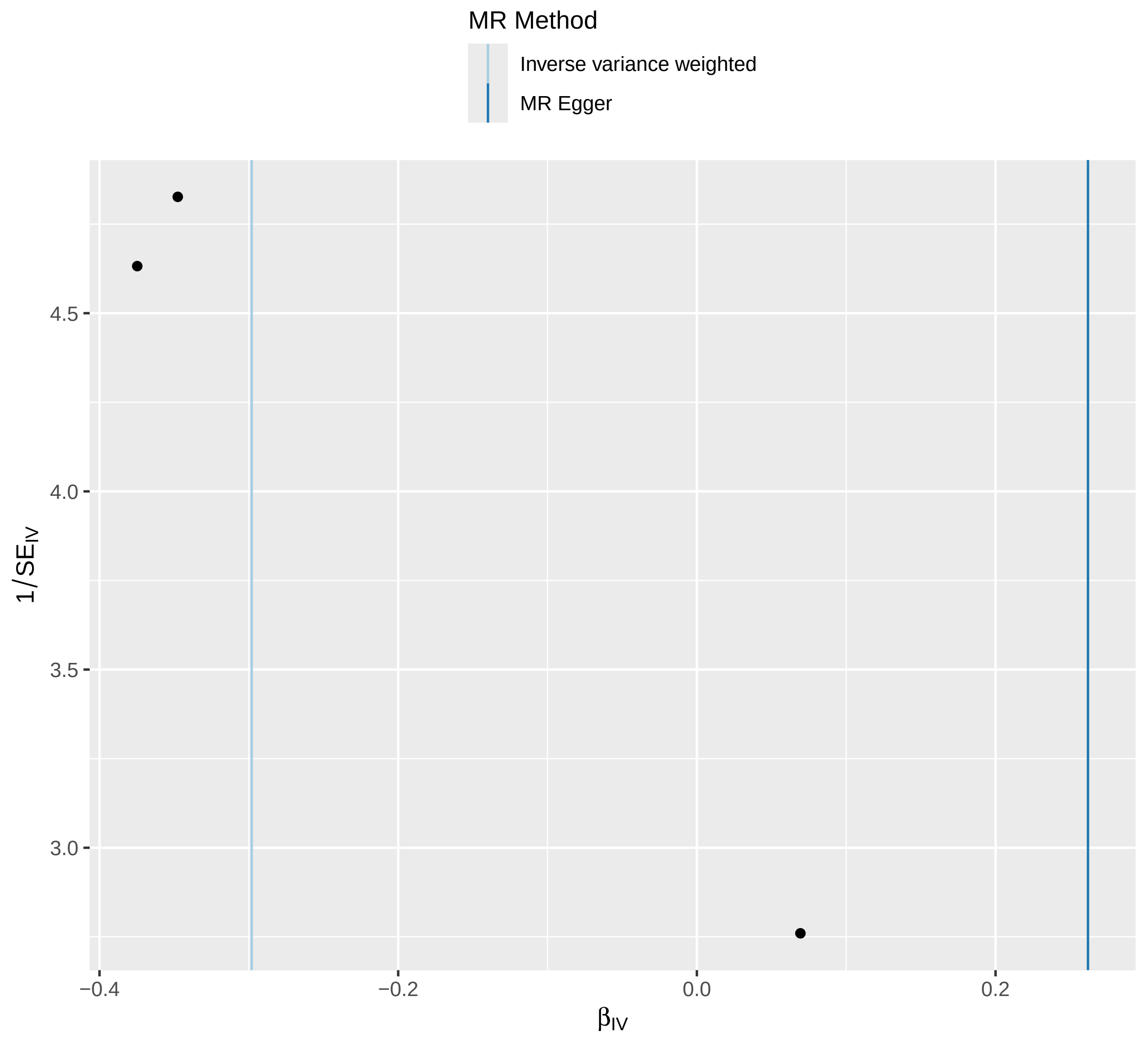
MR Method
Inverse variance weighted
MR Egger
4.5
4.0
3.5
3.0
1/SE$_{IV}$
−0.4
−0.2
0.0
0.2
β$_{IV}$

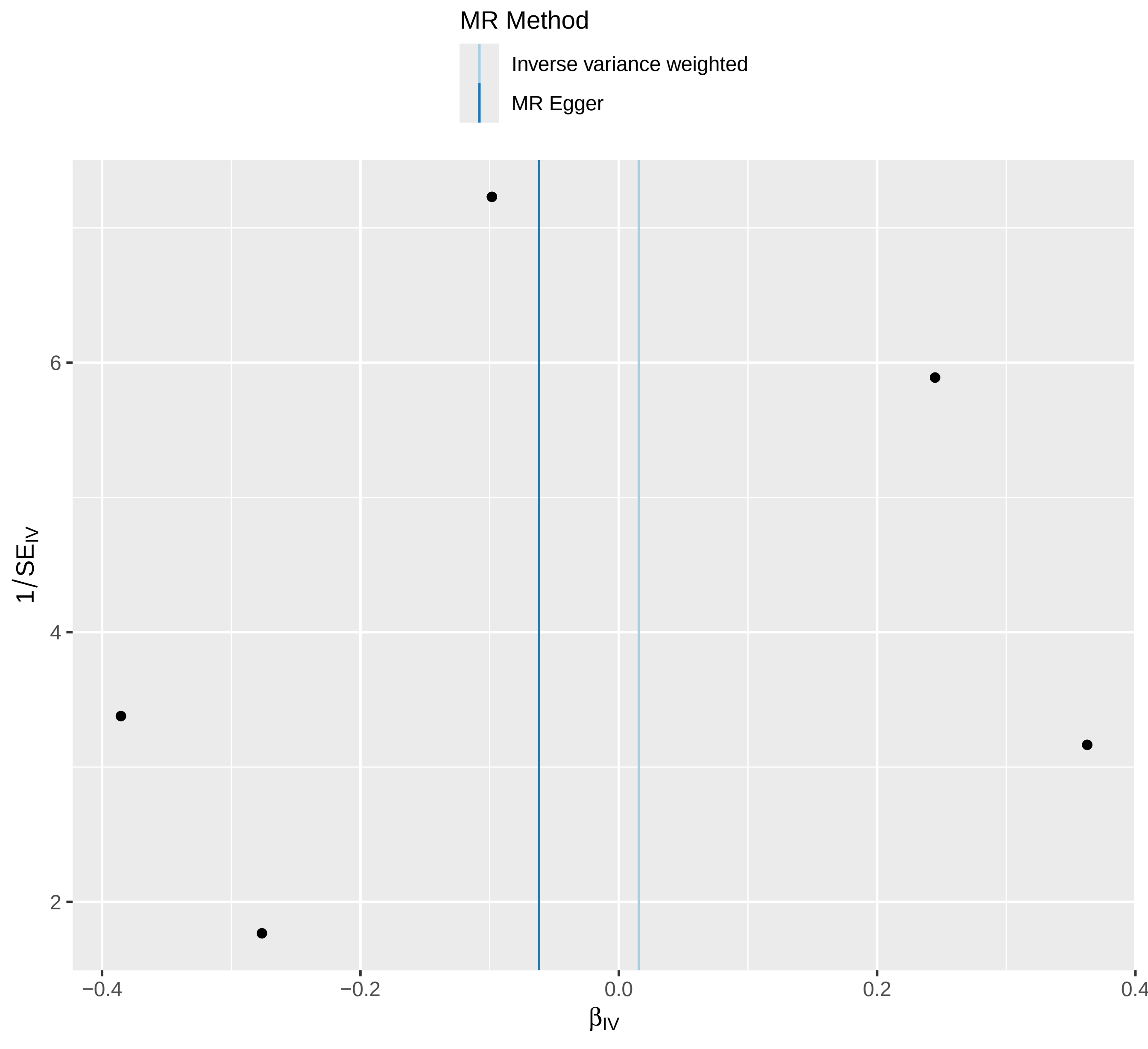
MR Method
Inverse variance weighted
MR Egger
1/SE_IV
6
4
2
−0.4
−0.2
0.0
0.2
0.4
β_IV

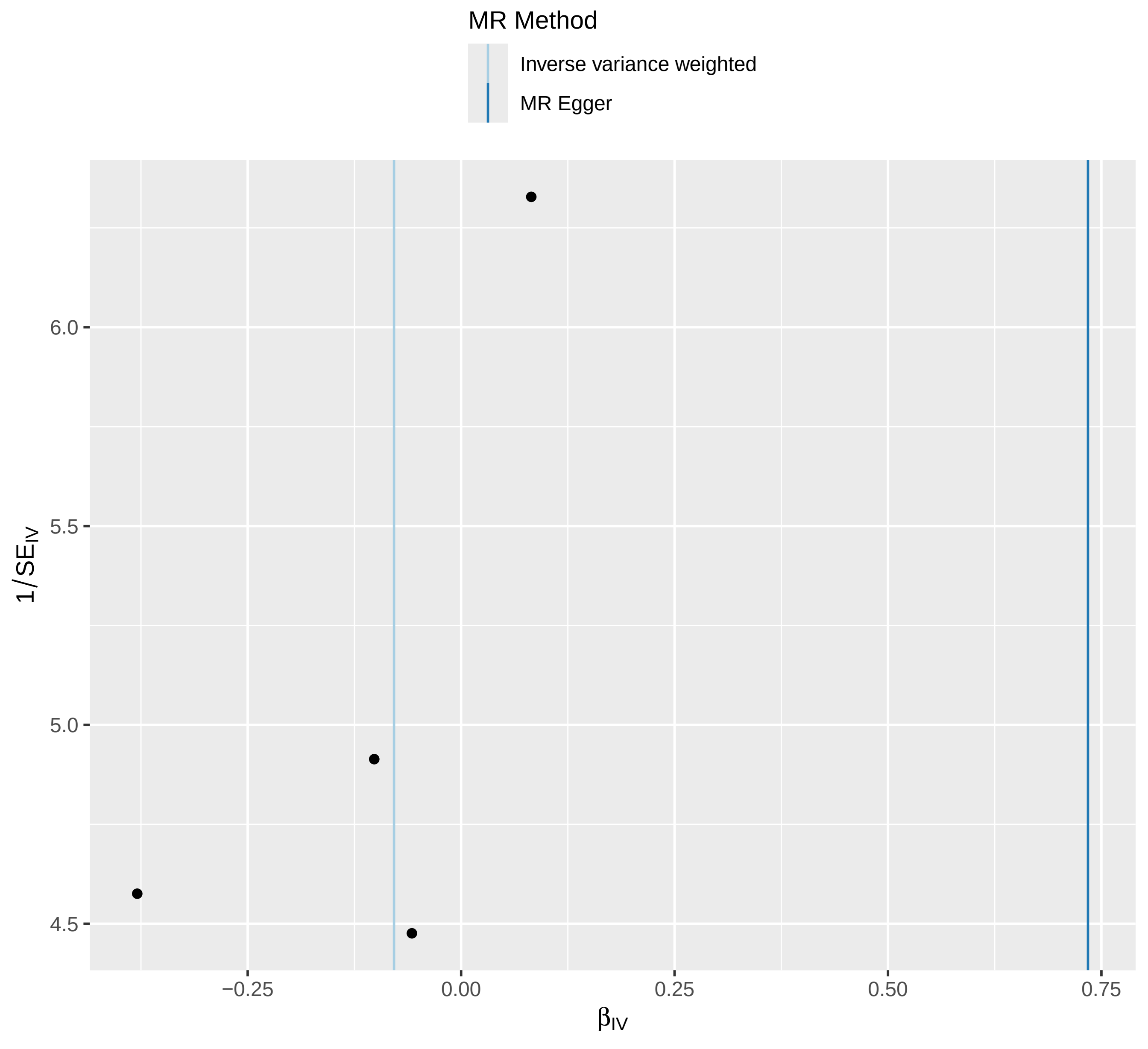
MR Method
Inverse variance weighted
MR Egger
6.0
5.5
5.0
4.5
$1/SE_{IV}$
−0.25
0.00
0.25
0.50
0.75
$\beta_{IV}$

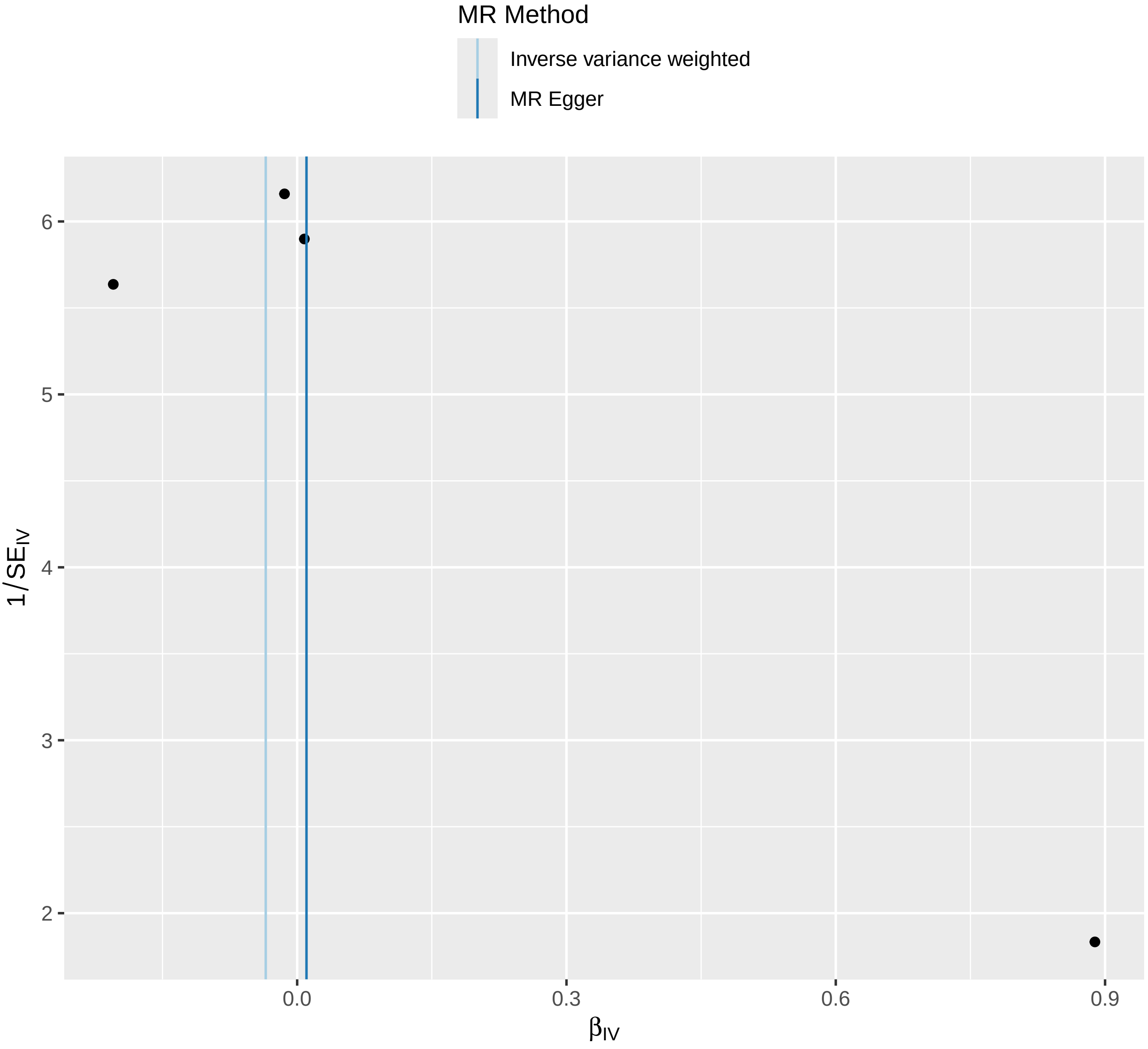

MR Method
Inverse variance weighted
MR Egger
6
5
4
3
2
1/SE$_{IV}$
0.0
0.3
0.6
0.9
β$_{IV}$

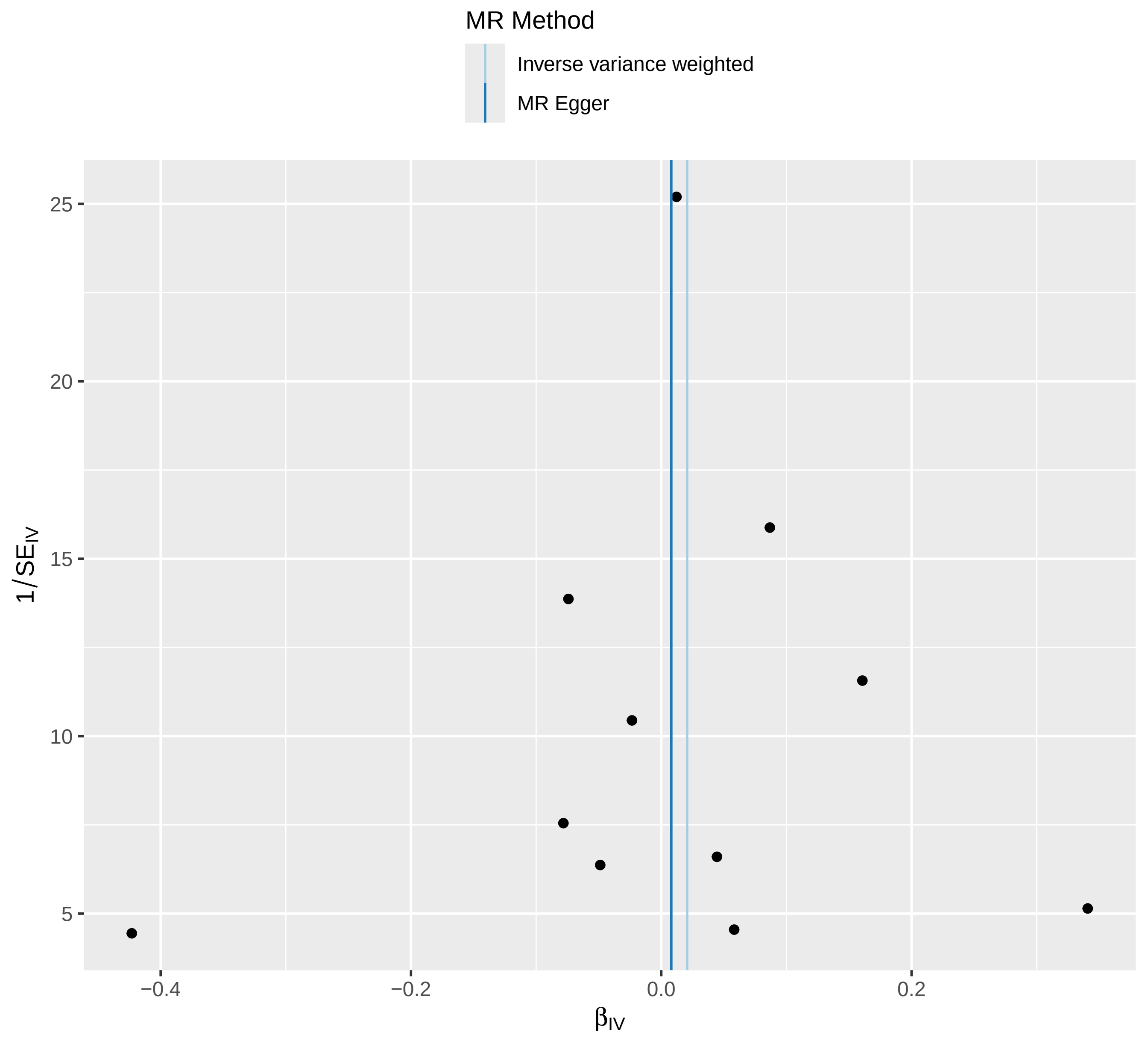

MR Method
Inverse variance weighted
MR Egger
25
20
15
10
5
1/SE_IV
−0.4
−0.2
0.0
0.2
β_IV

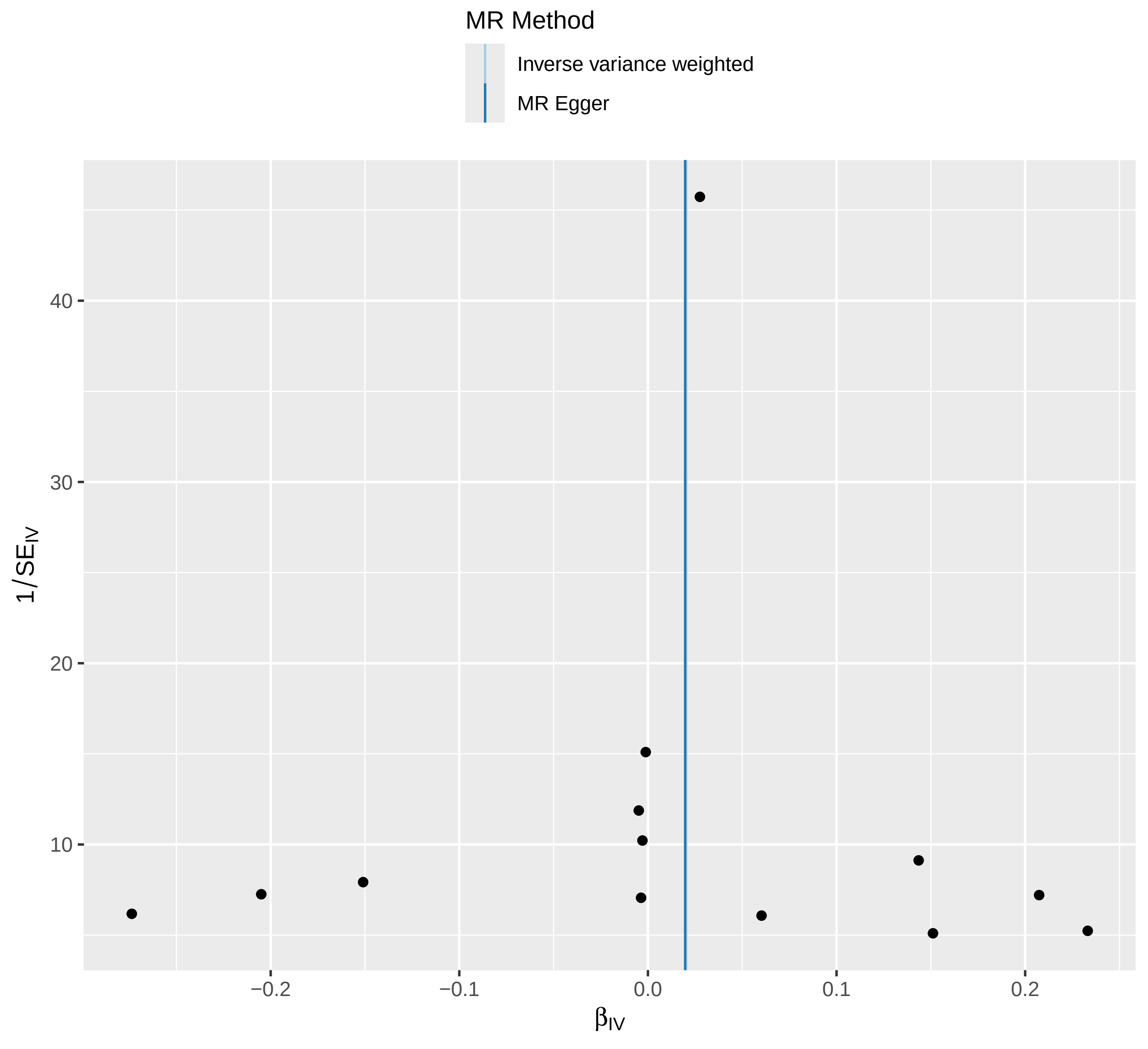
MR Method
Inverse variance weighted
MR Egger
40
30
20
10
1/$SE_{IV}$
−0.2
−0.1
0.0
0.1
0.2
$\beta_{IV}$

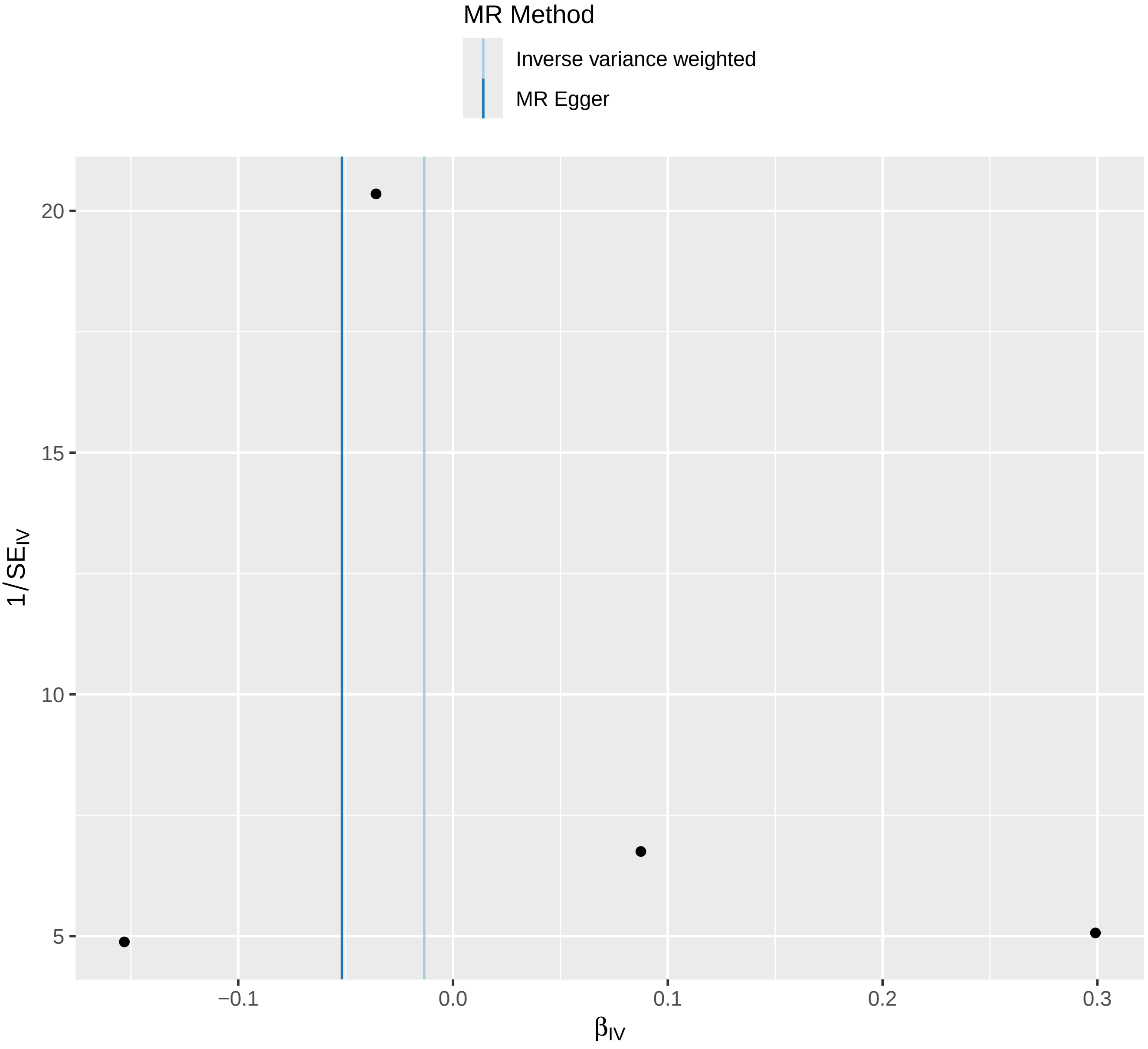
MR Method
Inverse variance weighted
MR Egger
20
15
10
5
1/SE$_{IV}$
−0.1
0.0
0.1
0.2
0.3
β$_{IV}$

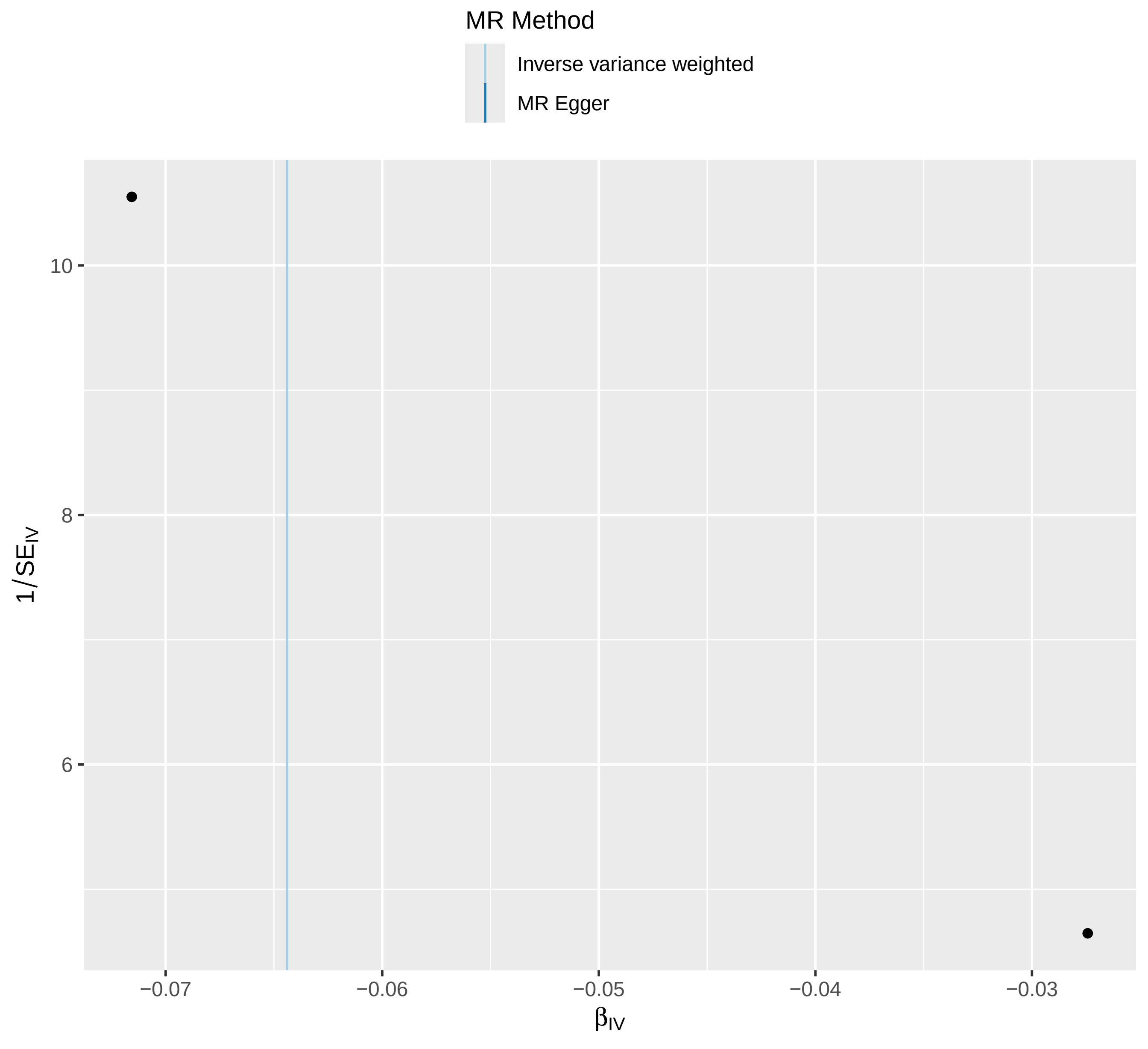
MR Method
Inverse variance weighted
MR Egger
10
8
6
1/SE$_{IV}$
−0.07
−0.06
−0.05
−0.04
−0.03
β$_{IV}$

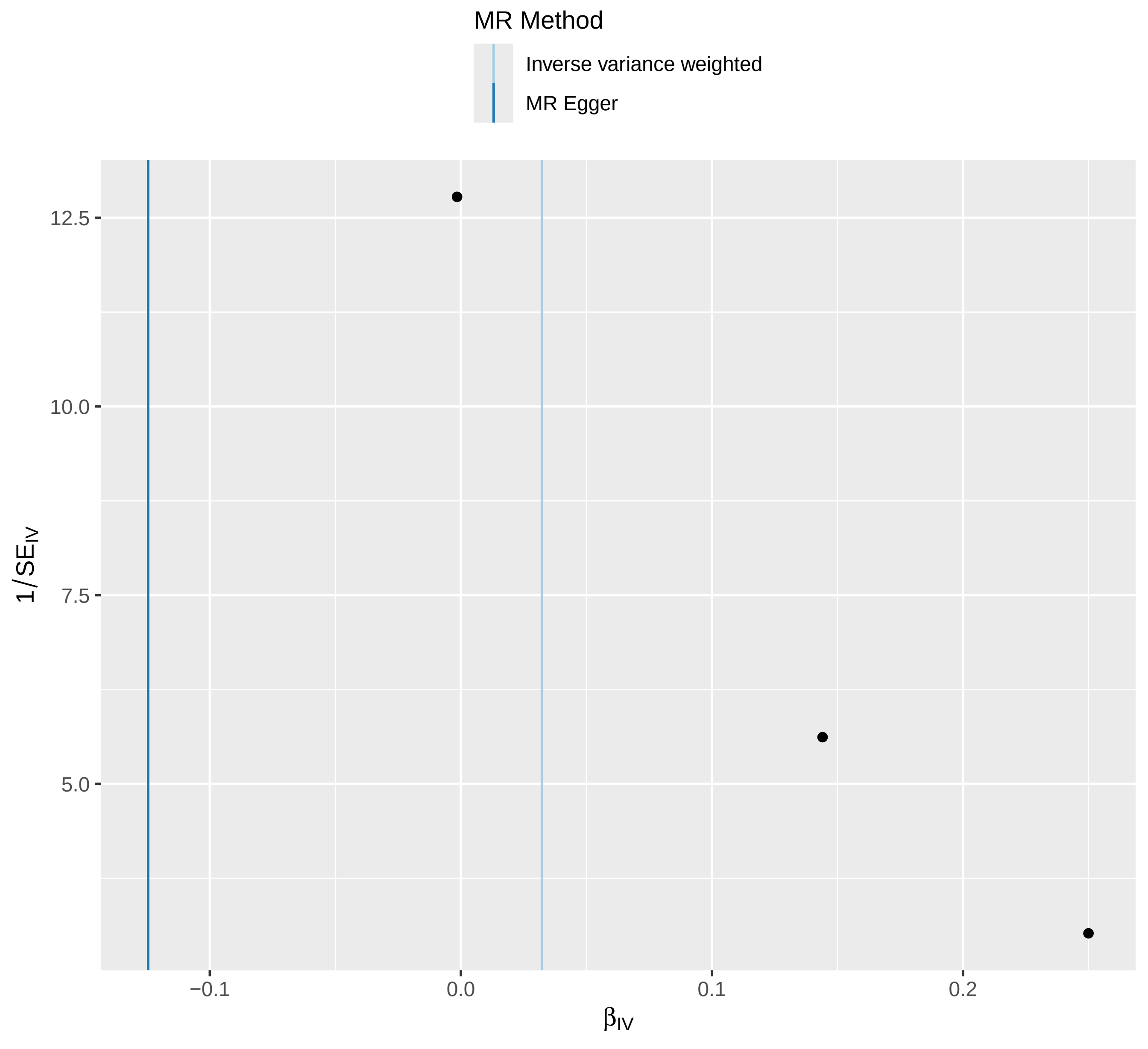
MR Method
Inverse variance weighted
MR Egger
12.5
10.0
7.5
5.0
$1/SE_{IV}$
−0.1
0.0
0.1
0.2
$\beta_{IV}$

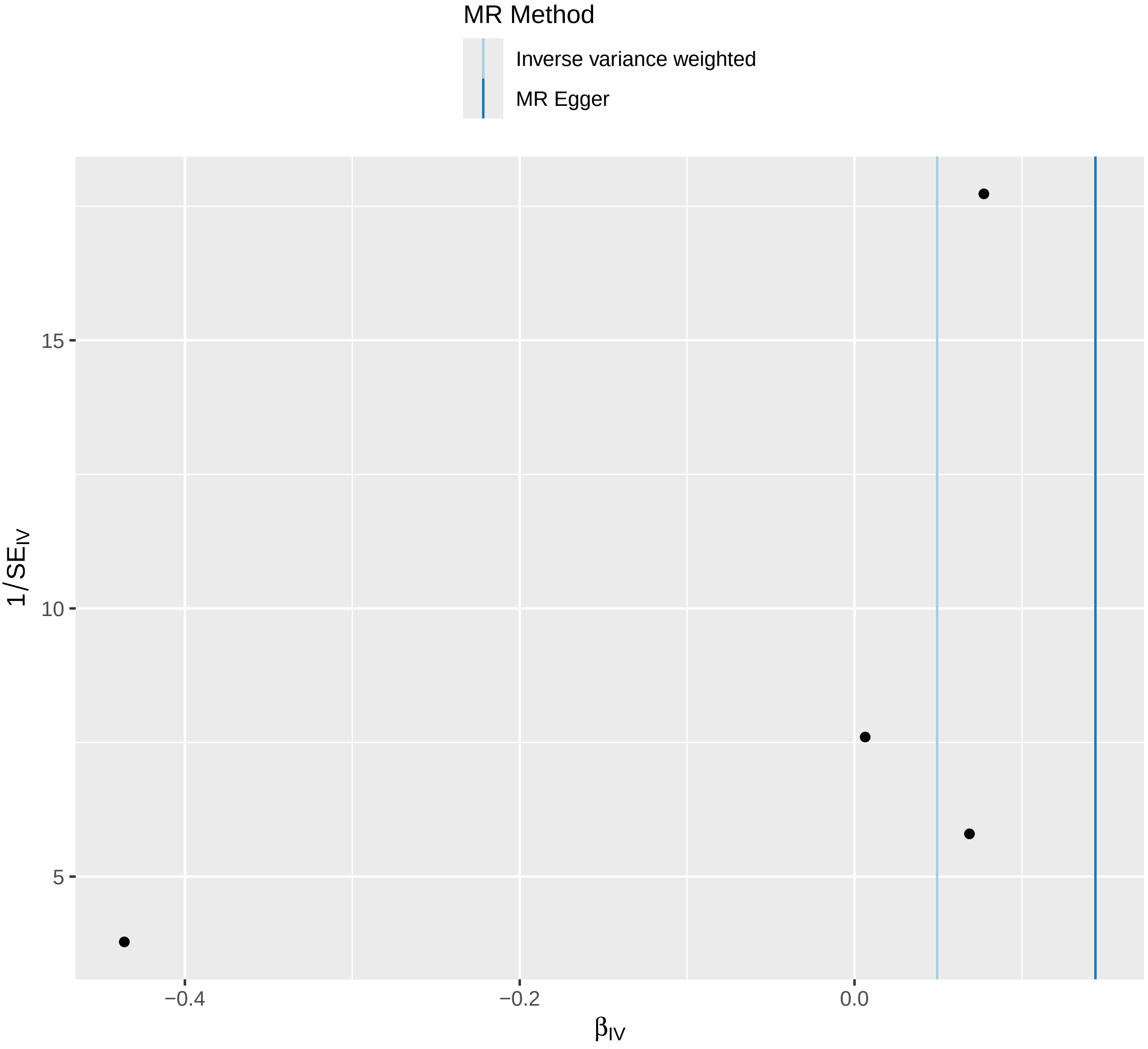
MR Method
Inverse variance weighted
MR Egger
15
10
5
1/SE$_{IV}$
−0.4
−0.2
0.0
β$_{IV}$

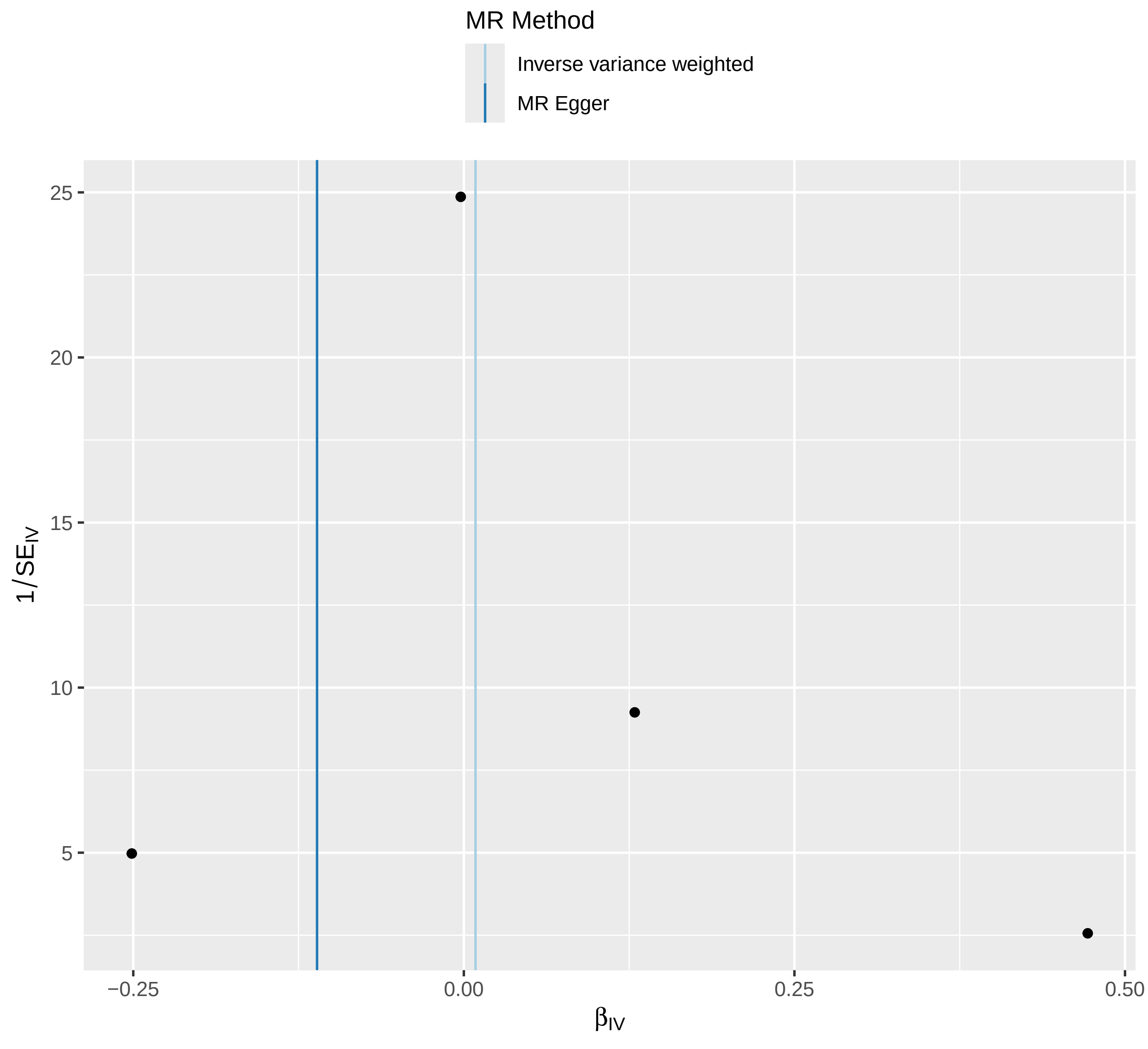

MR Method
Inverse variance weighted
MR Egger
25
20
15
10
5
1/SE_IV
−0.25
0.00
0.25
0.50
β_IV

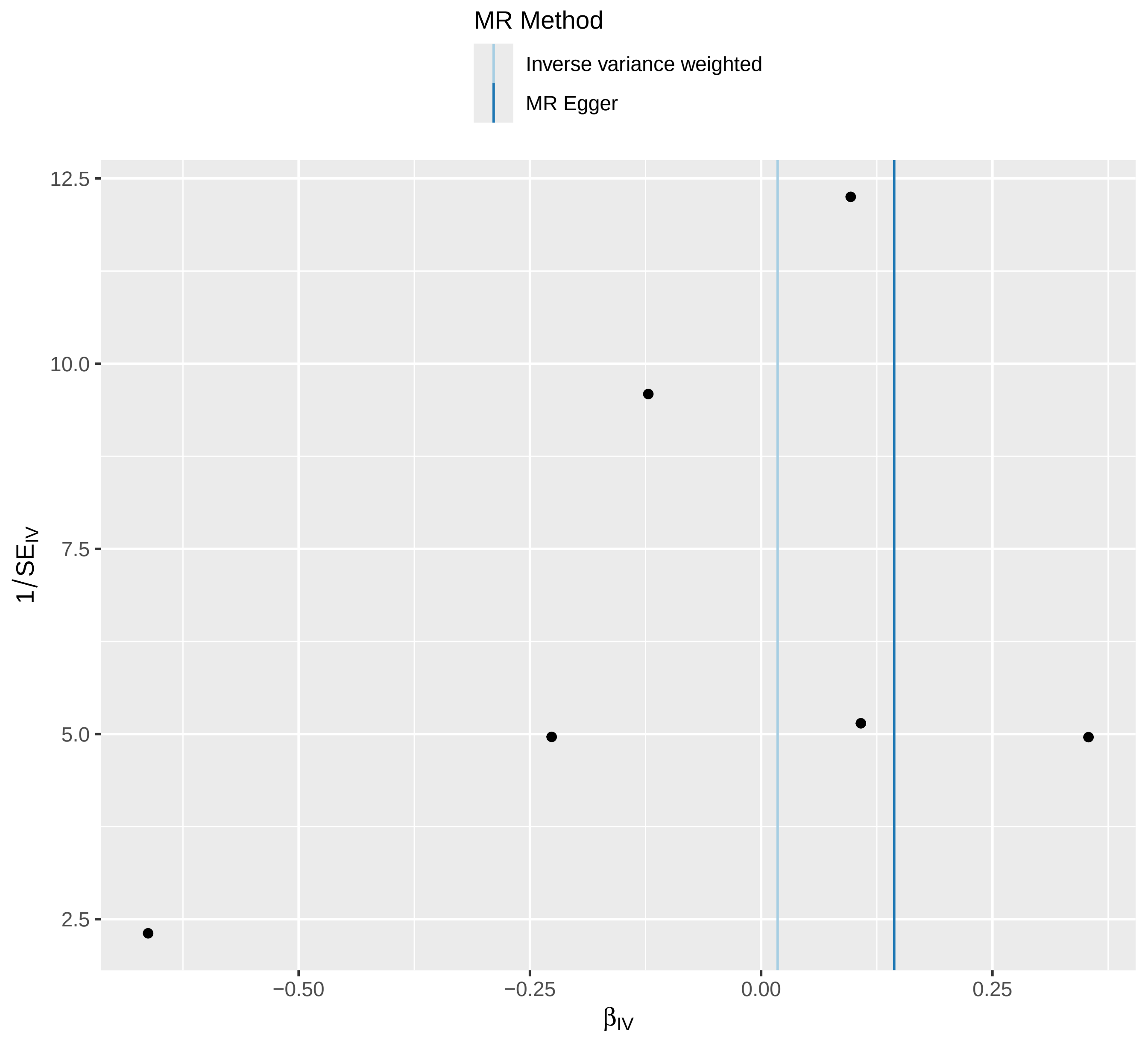

MR Method
Inverse variance weighted
MR Egger
12.5
10.0
7.5
5.0
2.5
$1/SE_{IV}$
−0.50
−0.25
0.00
0.25
$\beta_{IV}$

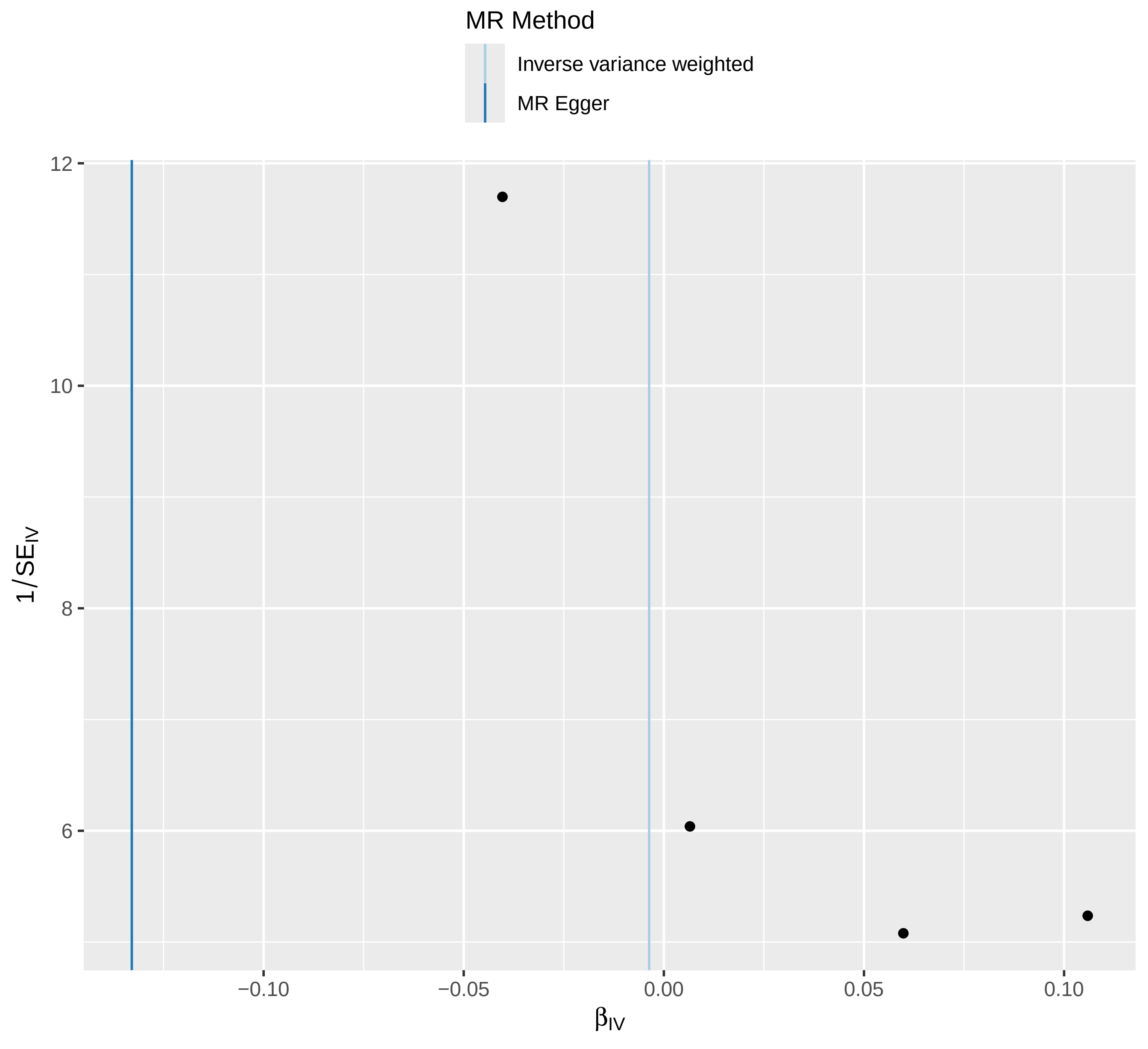

MR Method
Inverse variance weighted
MR Egger
12
10
8
6
1/SE$_{IV}$
−0.10
−0.05
0.00
0.05
0.10
β$_{IV}$

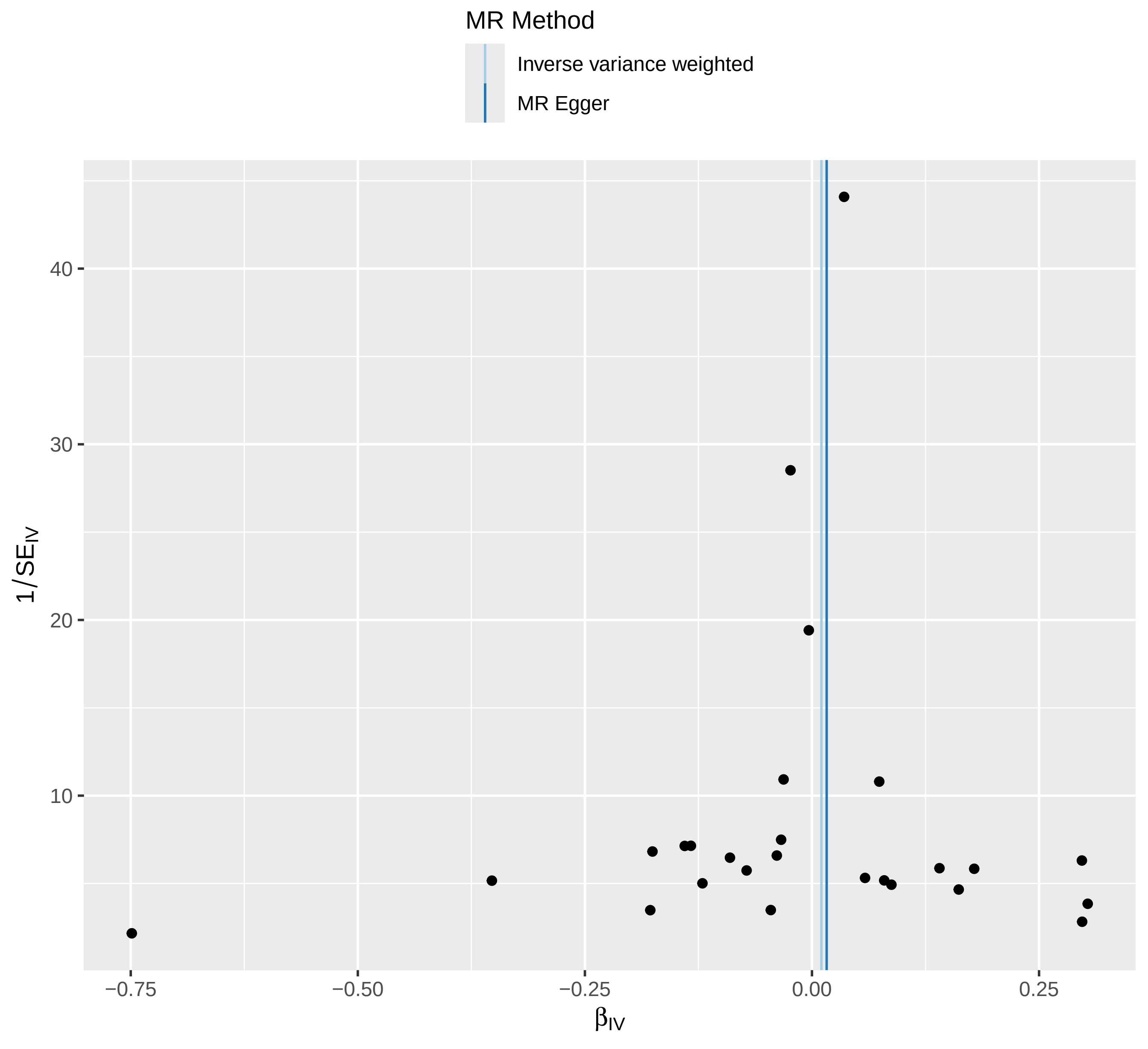

MR Method
Inverse variance weighted
MR Egger
40
30
20
10
1/SE_IV
−0.75
−0.50
−0.25
0.00
0.25
β_IV

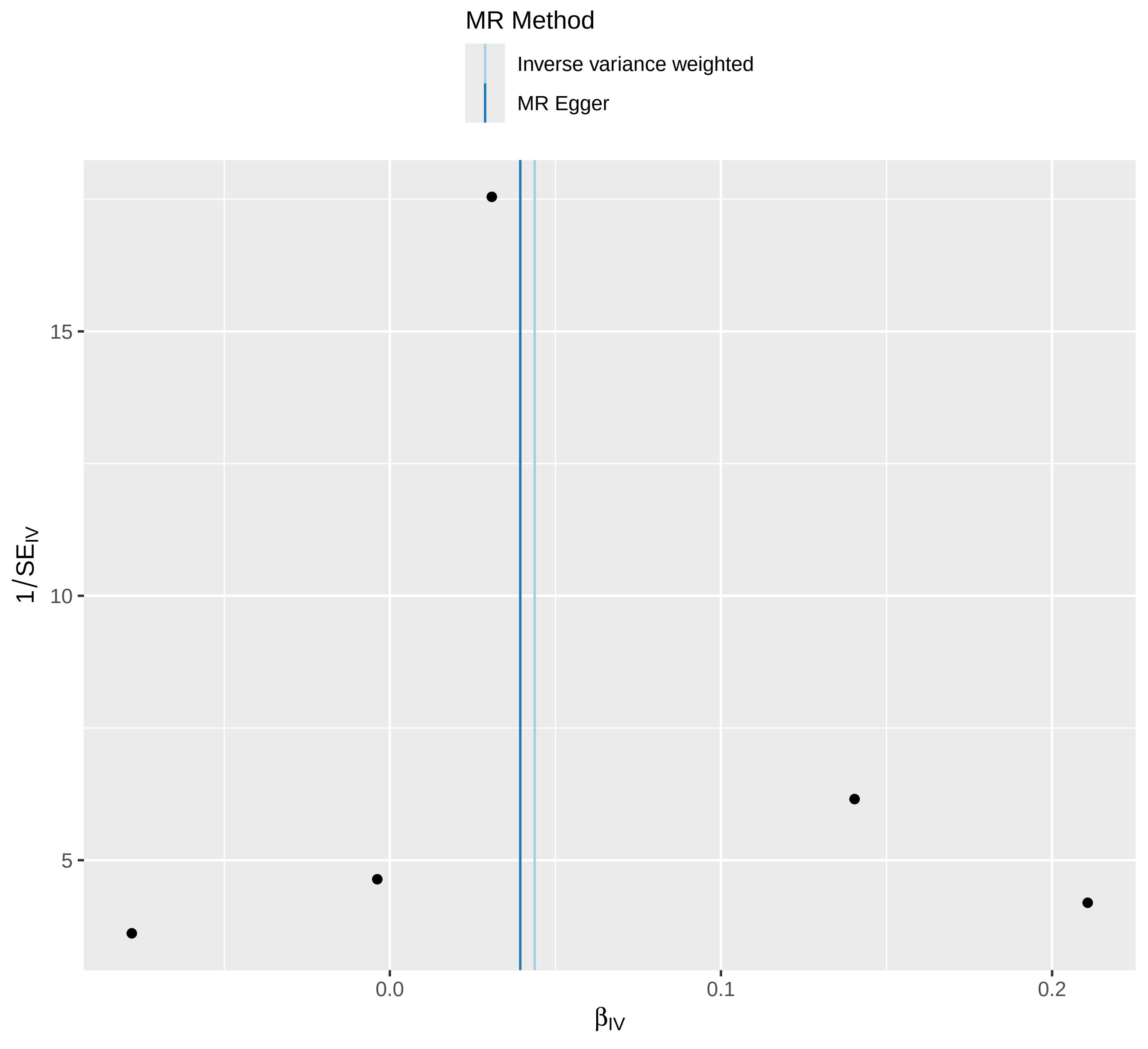
MR Method
Inverse variance weighted
MR Egger
15
10
5
1/SE_IV
0.0
0.1
0.2
β_IV

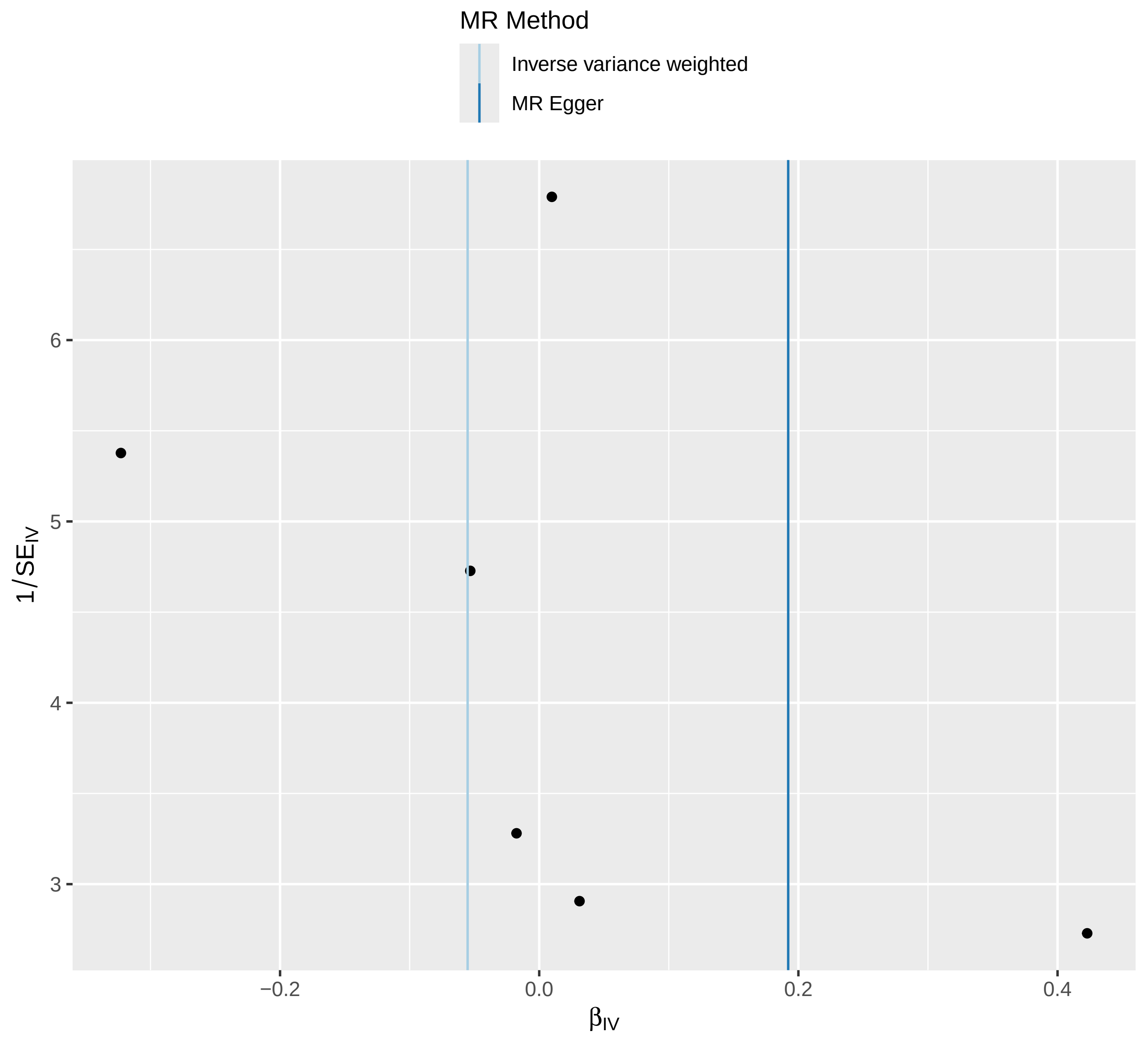
MR Method
Inverse variance weighted
MR Egger
6
5
4
3
1/SE_IV
−0.2
0.0
0.2
0.4
β_IV

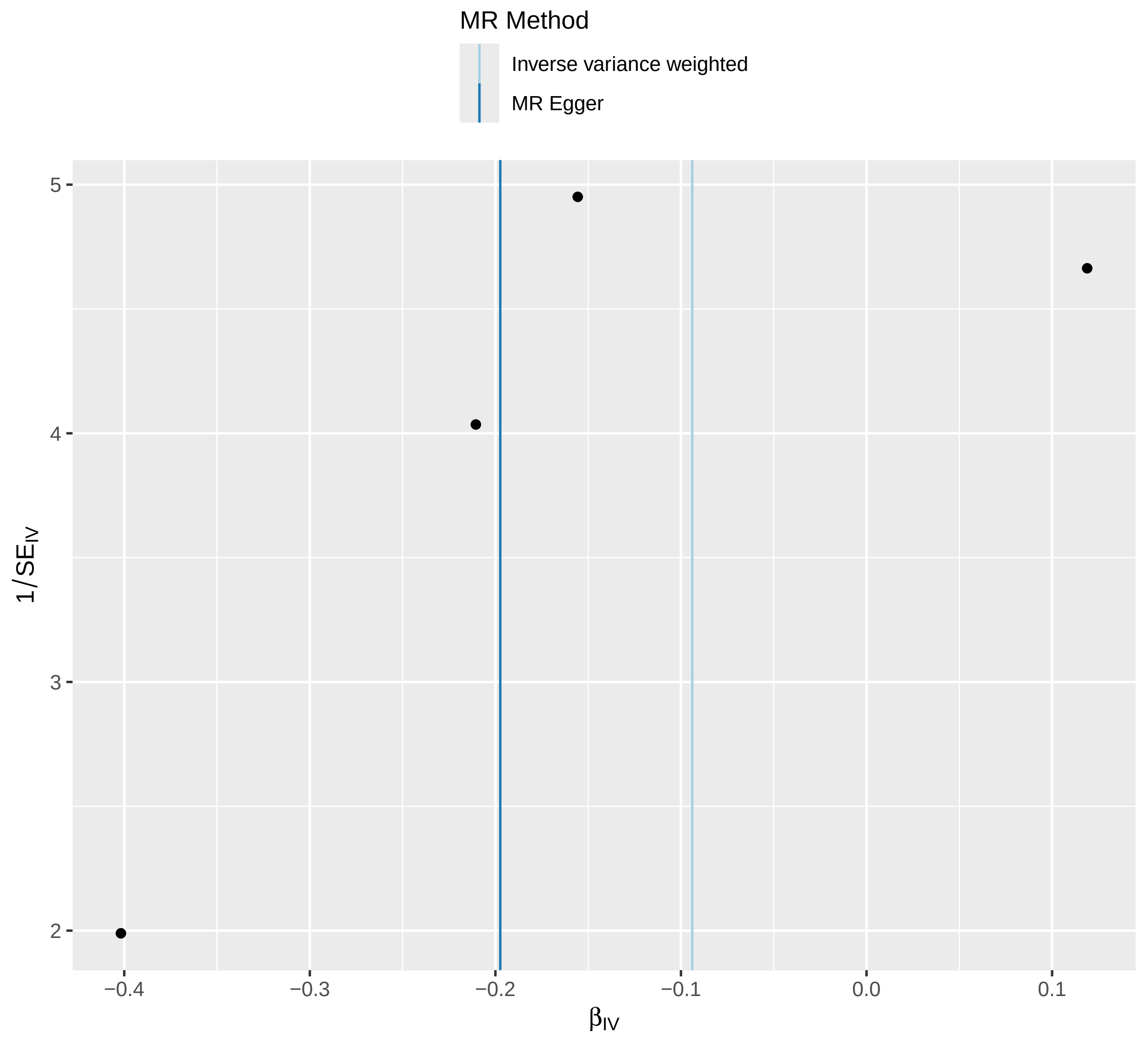
MR Method
Inverse variance weighted
MR Egger
5
4
3
2
1/SE_IV
−0.4
−0.3
−0.2
−0.1
0.0
0.1
β_IV

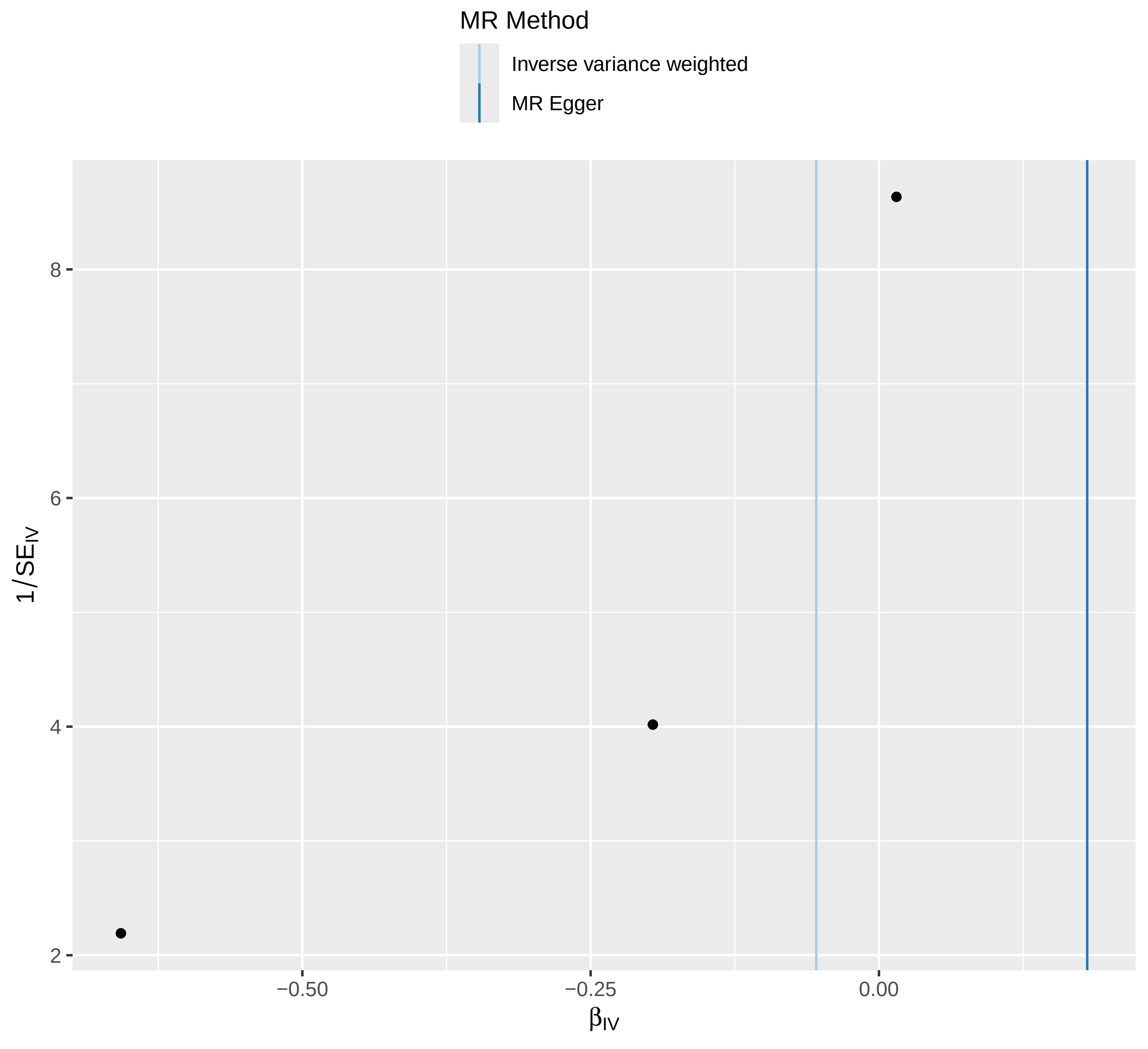
MR Method
Inverse variance weighted
MR Egger
8
6
4
2
1/SE$_{IV}$
−0.50
−0.25
0.00
β$_{IV}$

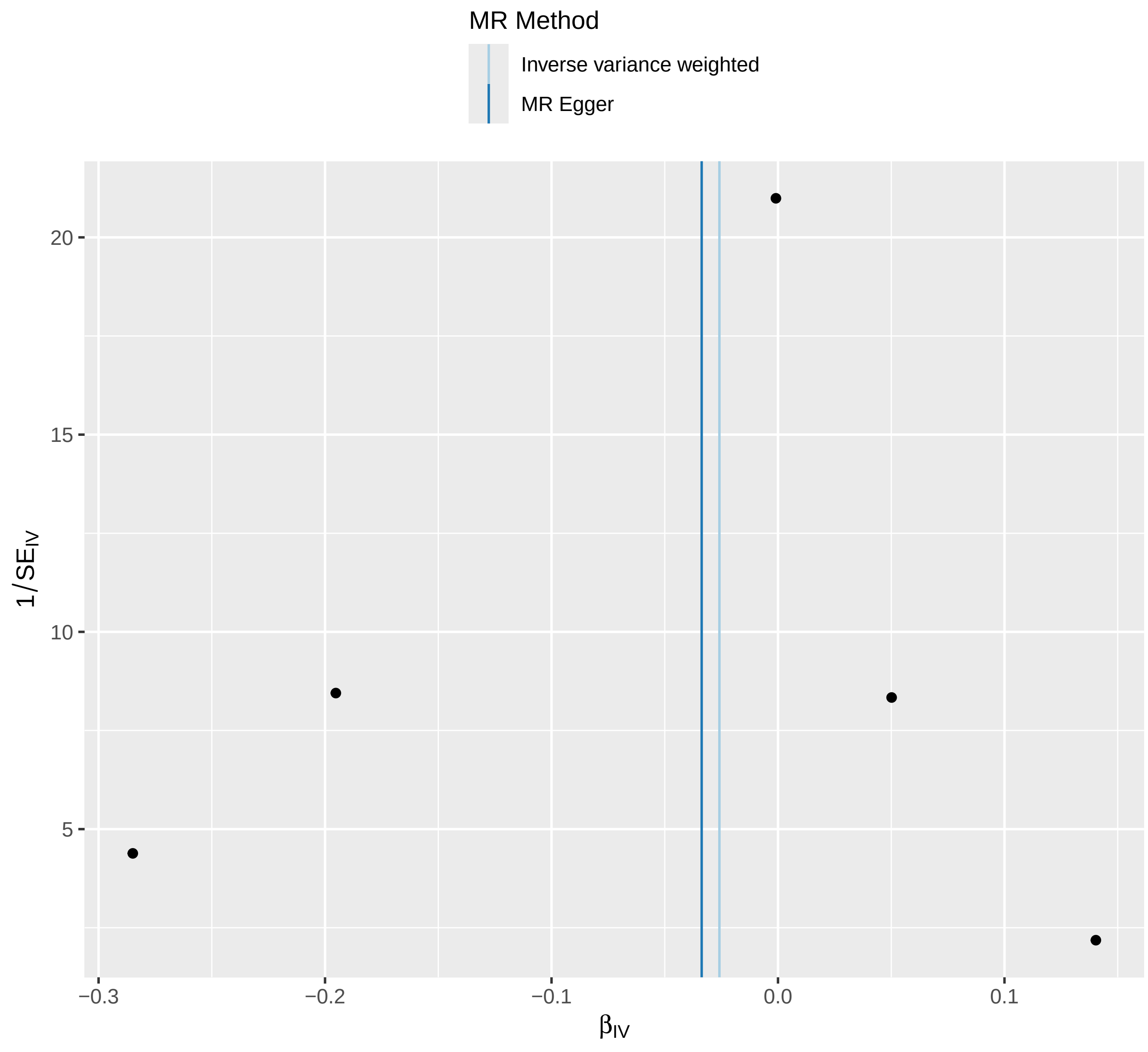

MR Method
Inverse variance weighted
MR Egger
20
15
10
5
1/SE_IV
−0.3
−0.2
−0.1
0.0
0.1
β_IV

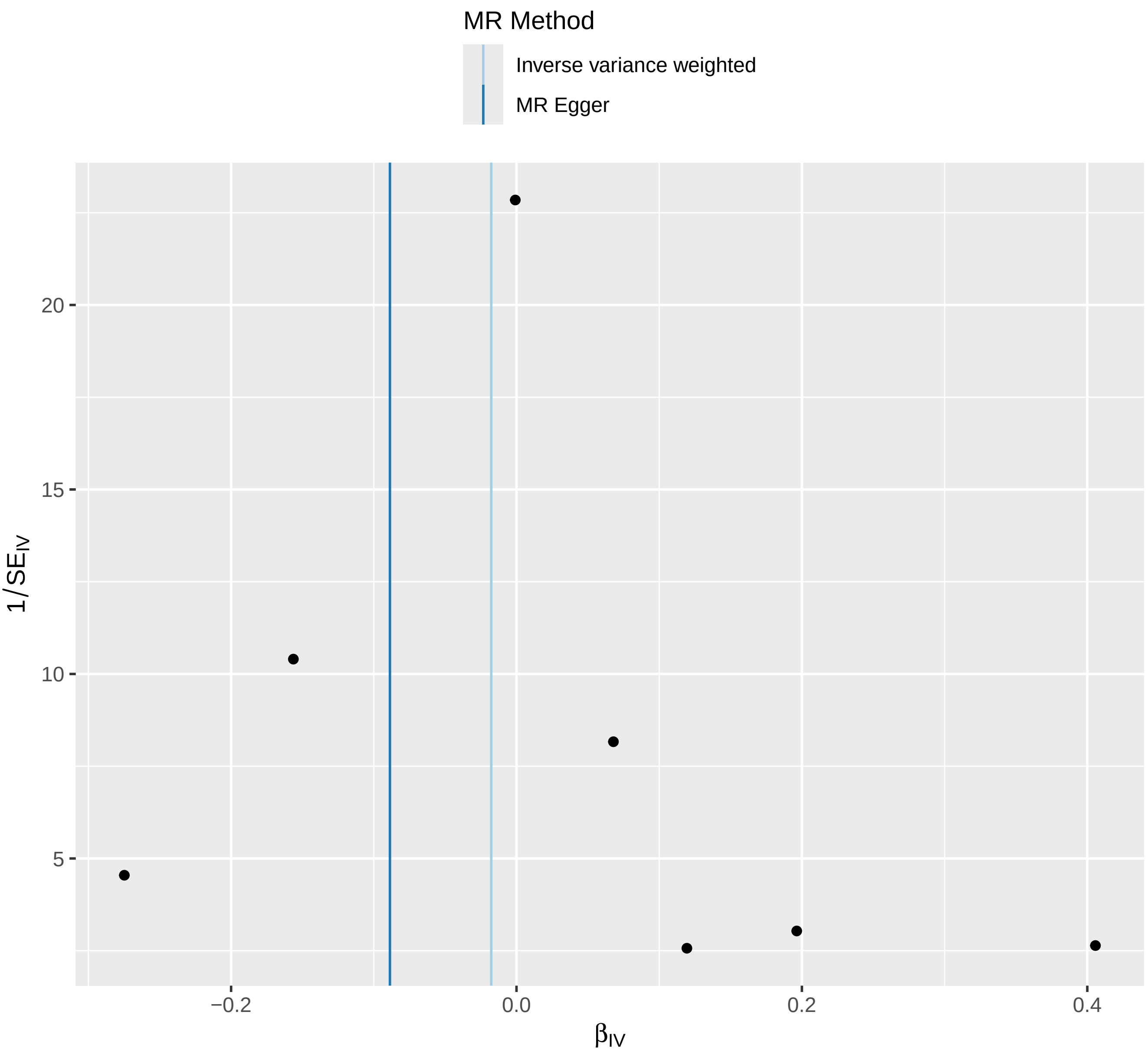
MR Method
Inverse variance weighted
MR Egger
20
15
10
5
1/SE$_{IV}$
−0.2
0.0
0.2
0.4
β$_{IV}$

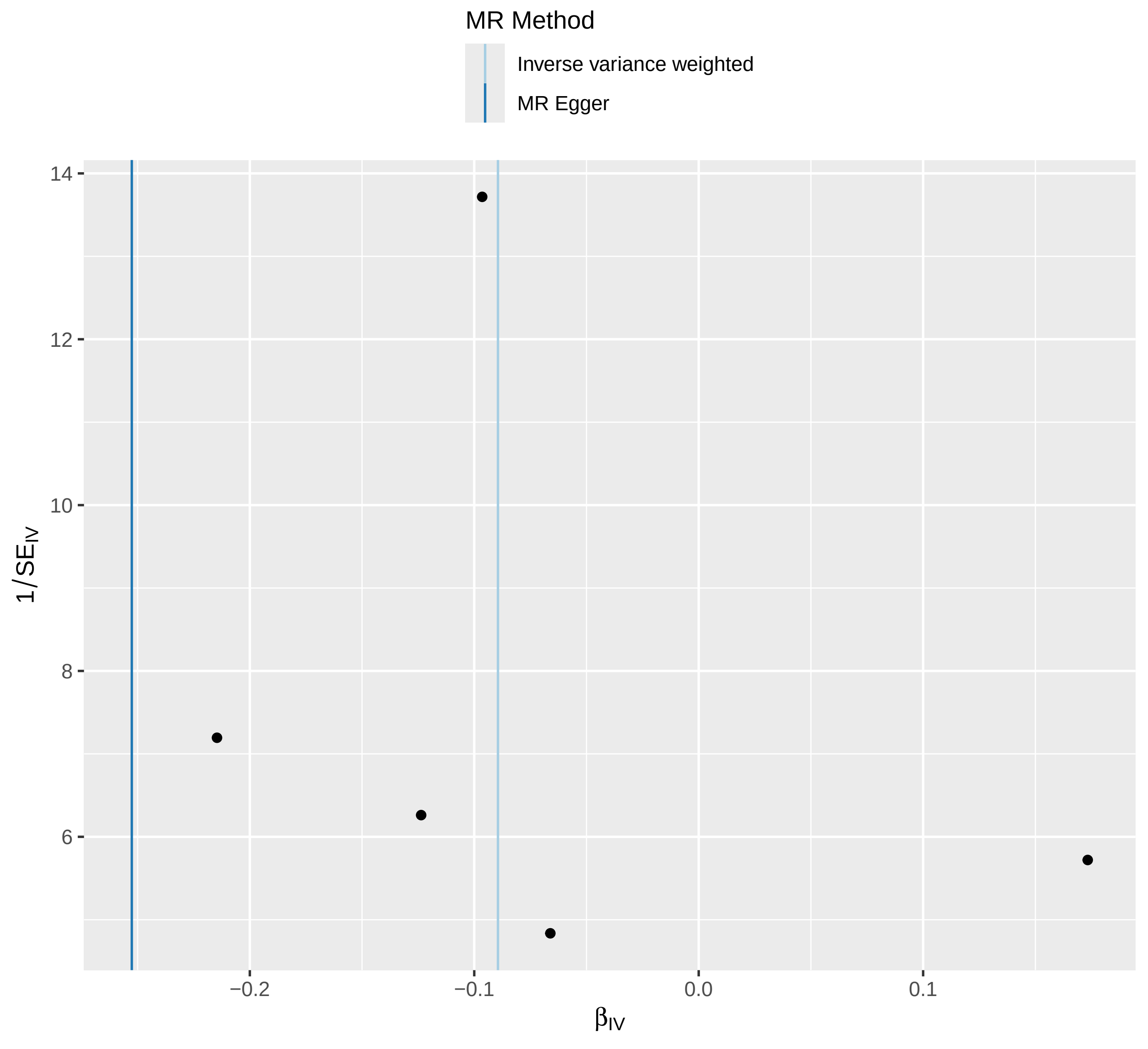
MR Method
Inverse variance weighted
MR Egger
14
12
10
8
6
1/SE$_{IV}$
−0.2
−0.1
0.0
0.1
β$_{IV}$

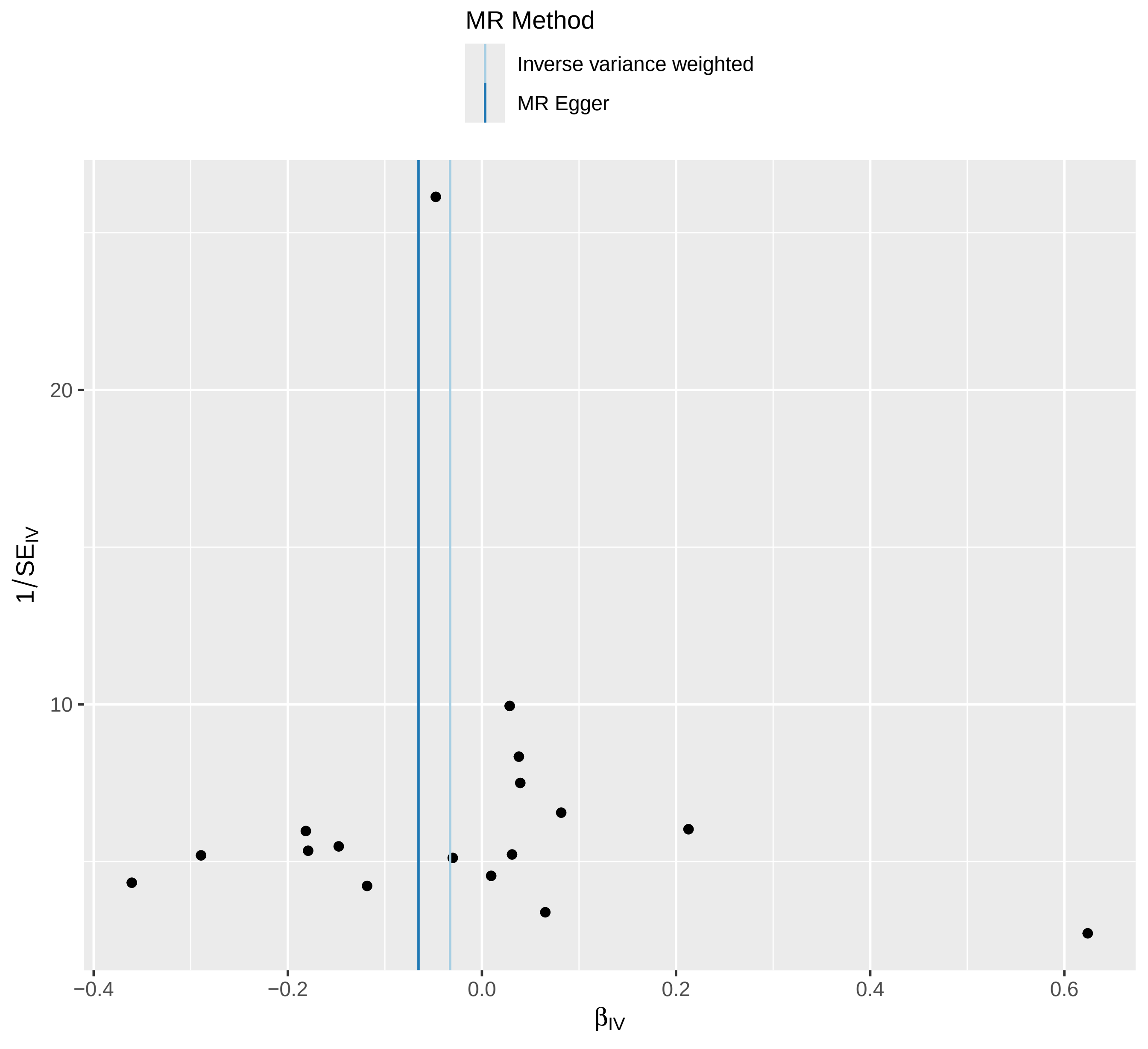

MR Method
Inverse variance weighted
MR Egger
20
10
1/SE$_{IV}$
−0.4
−0.2
0.0
0.2
0.4
0.6
β$_{IV}$

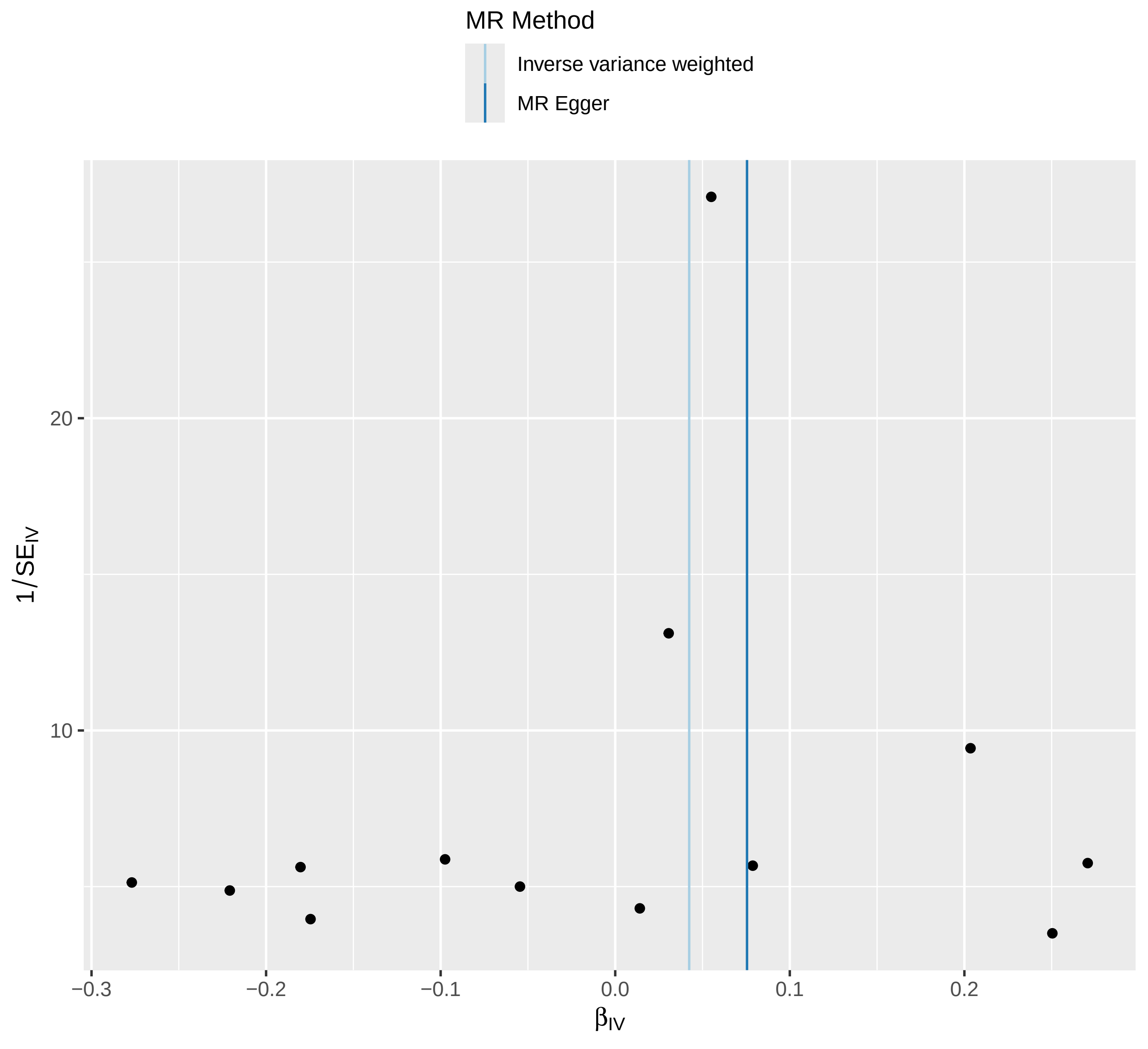
MR Method
Inverse variance weighted
MR Egger
20
10
$1/SE_{IV}$
−0.3
−0.2
−0.1
0.0
0.1
0.2
$\beta_{IV}$

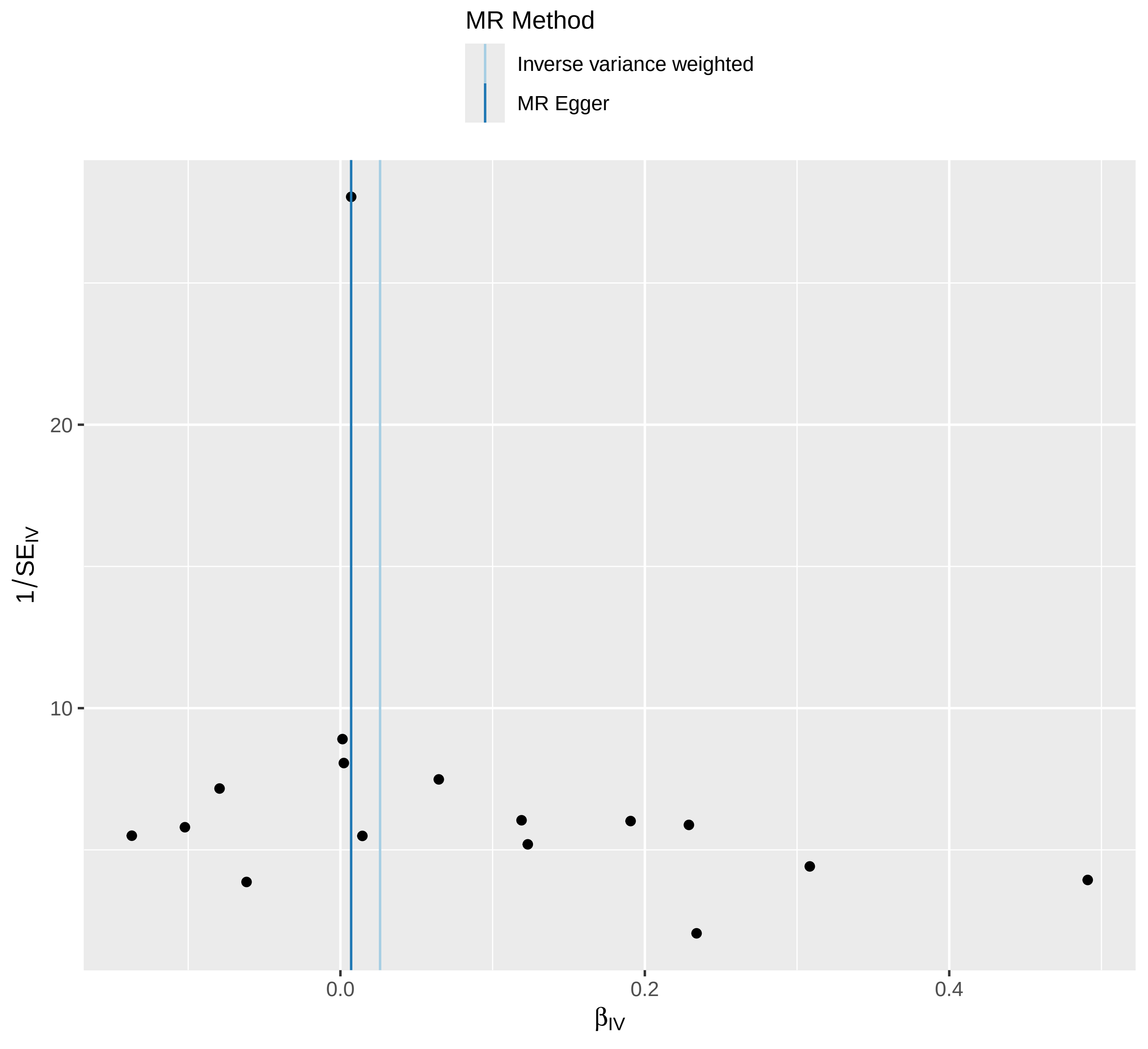
MR Method
Inverse variance weighted
MR Egger
20
10
$1/SE_{IV}$
0.0
0.2
0.4
$\beta_{IV}$

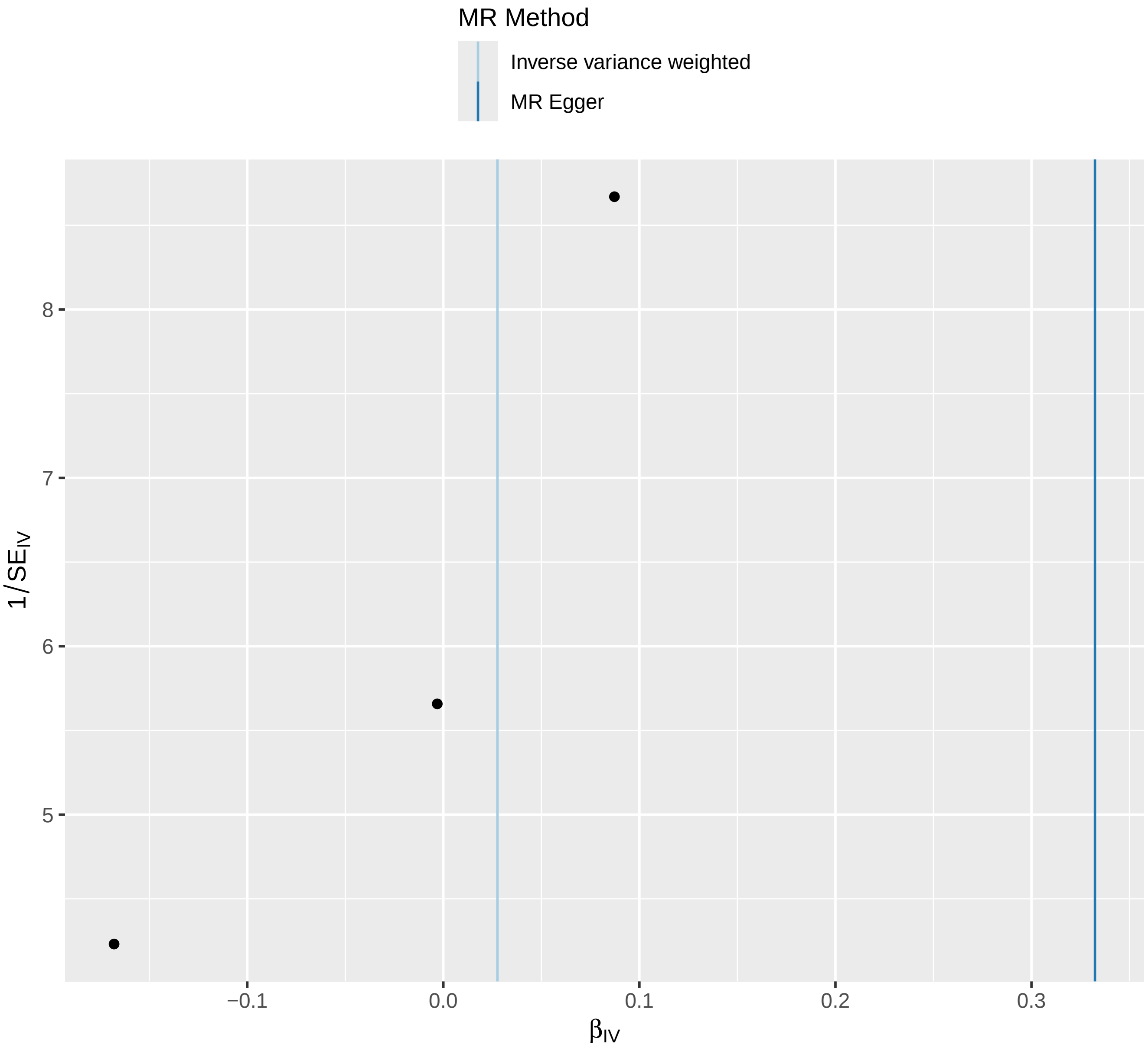

MR Method
Inverse variance weighted
MR Egger
8
7
6
5
1/SE$_{IV}$
−0.1
0.0
0.1
0.2
0.3
β$_{IV}$

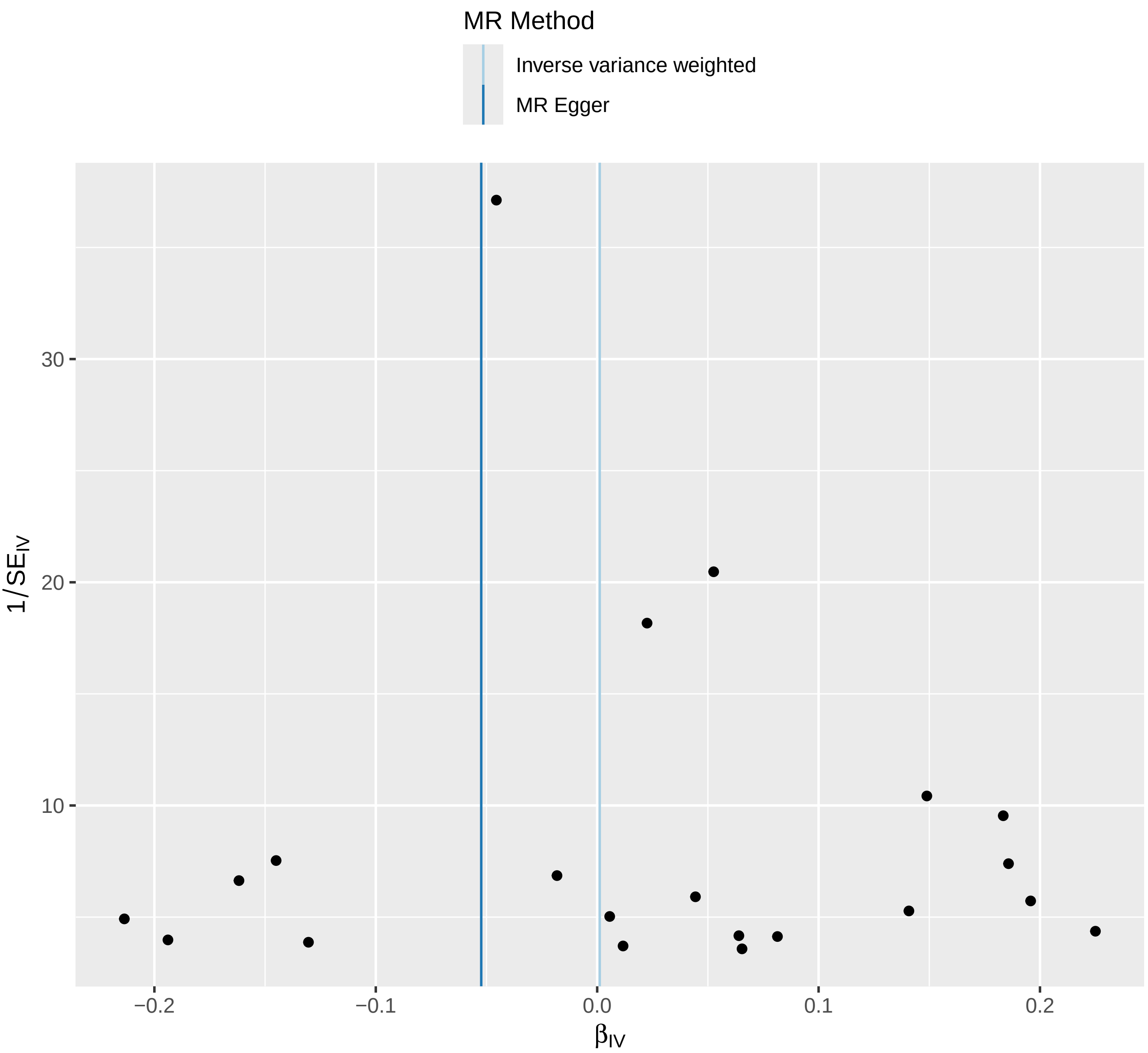

MR Method
Inverse variance weighted
MR Egger
1/SE_IV
30
20
10
−0.2
−0.1
0.0
0.1
0.2
β_IV

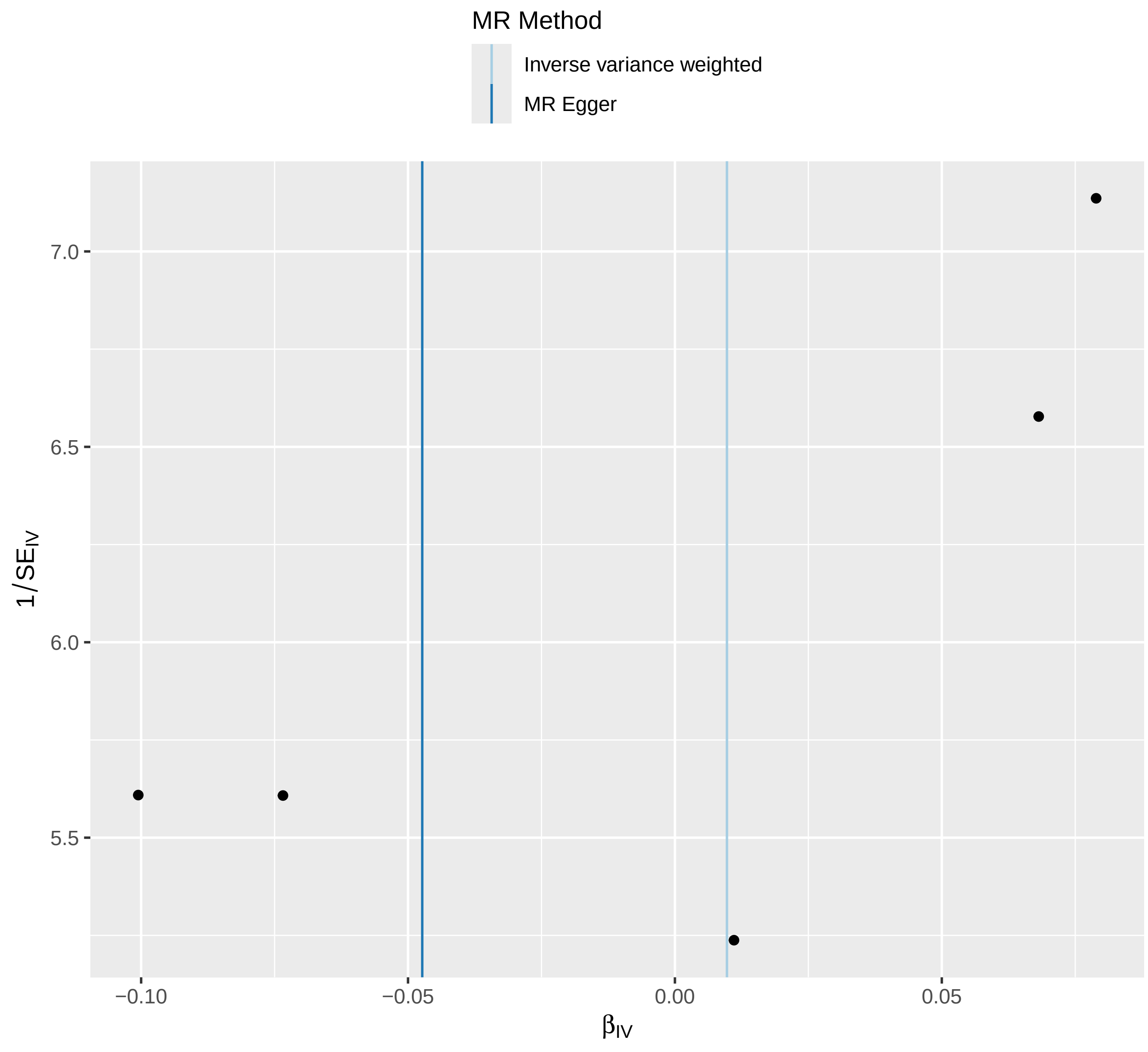
MR Method
Inverse variance weighted
MR Egger
7.0
6.5
6.0
5.5
1/SE$_{IV}$
−0.10
−0.05
0.00
0.05
β$_{IV}$

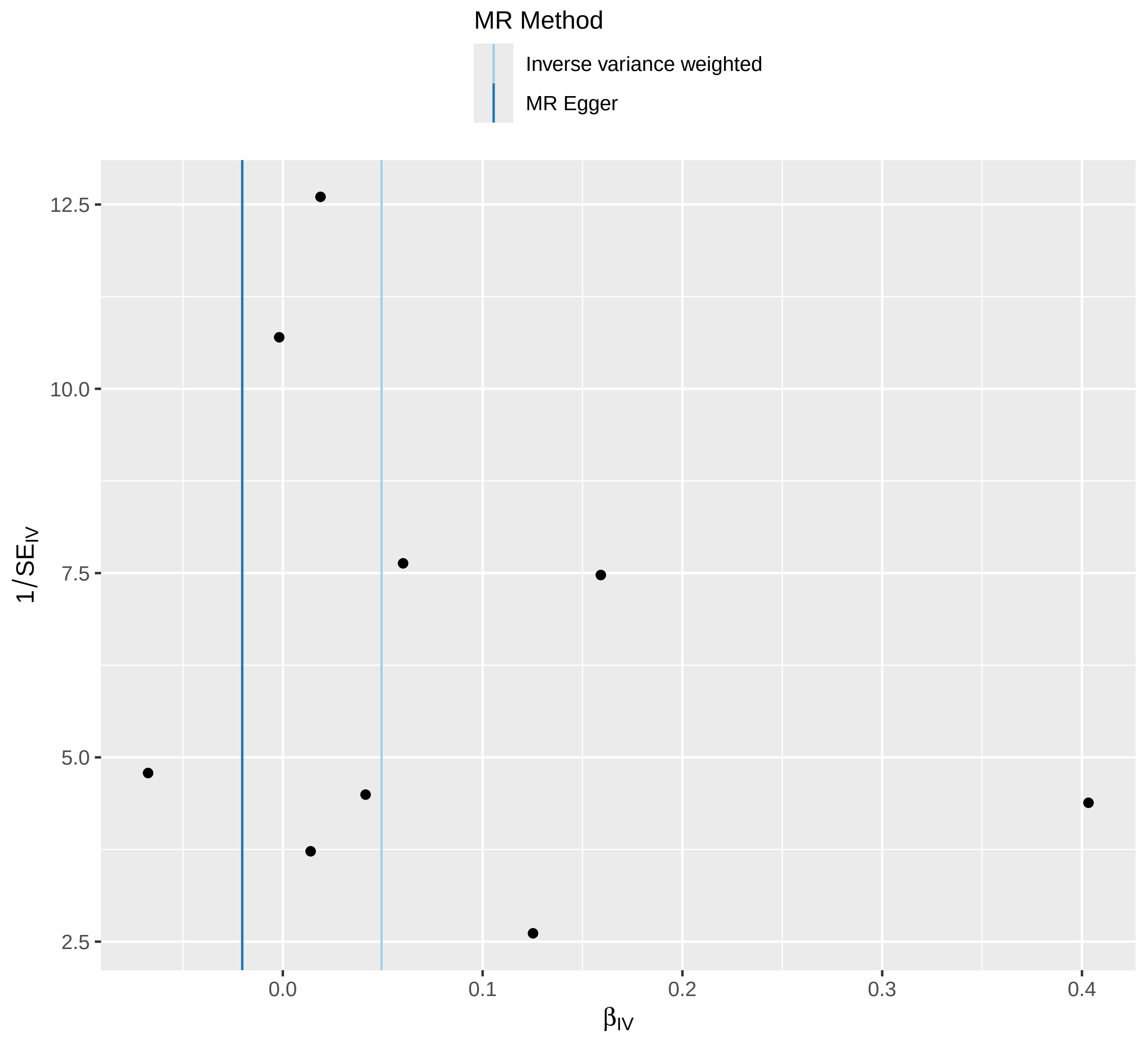

MR Method
Inverse variance weighted
MR Egger
12.5
10.0
7.5
5.0
2.5
1/SE_IV
0.0
0.1
0.2
0.3
0.4
β_IV

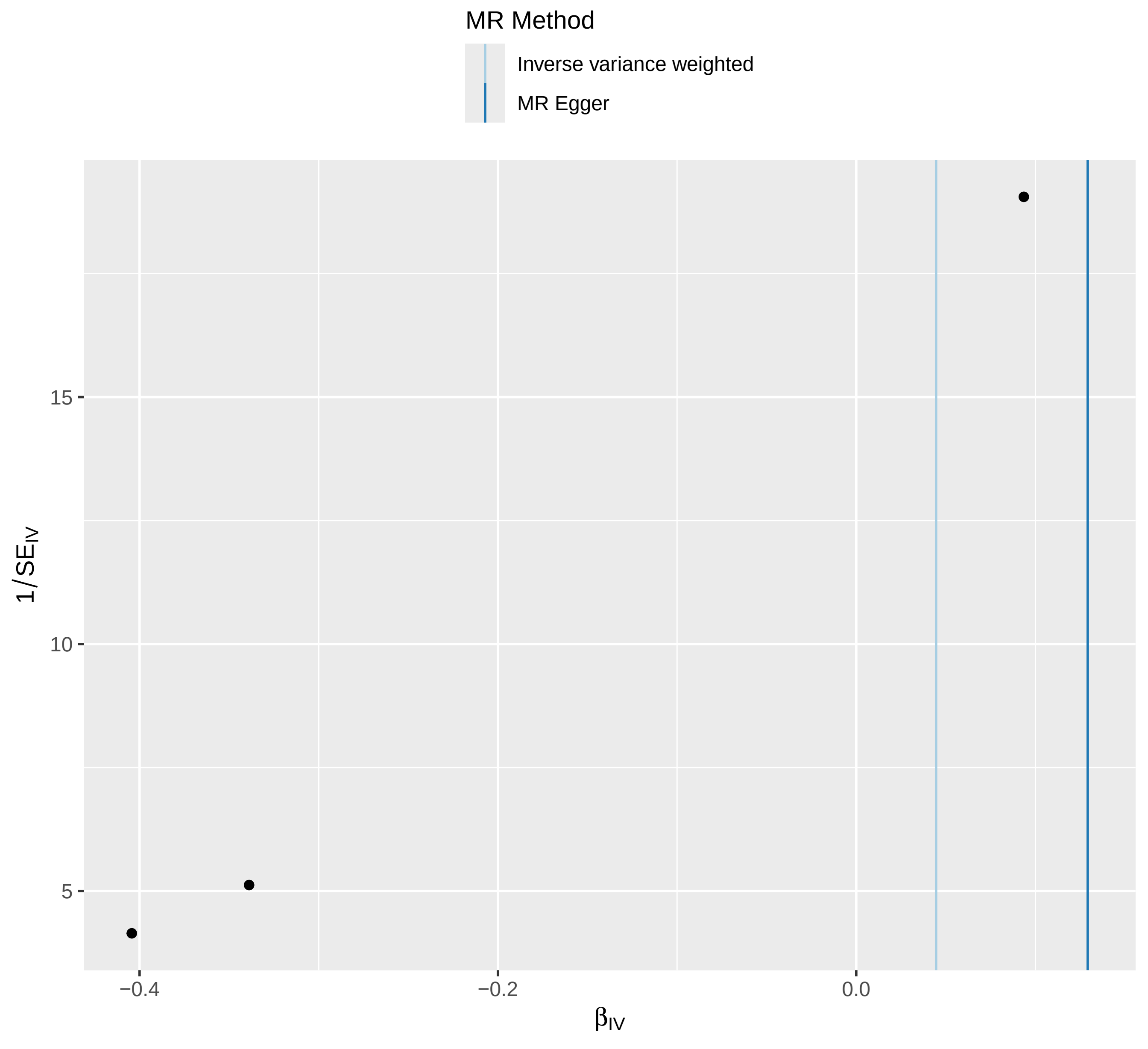
MR Method
Inverse variance weighted
MR Egger
1/SE$_{IV}$
15
10
5
−0.4
−0.2
0.0
β$_{IV}$

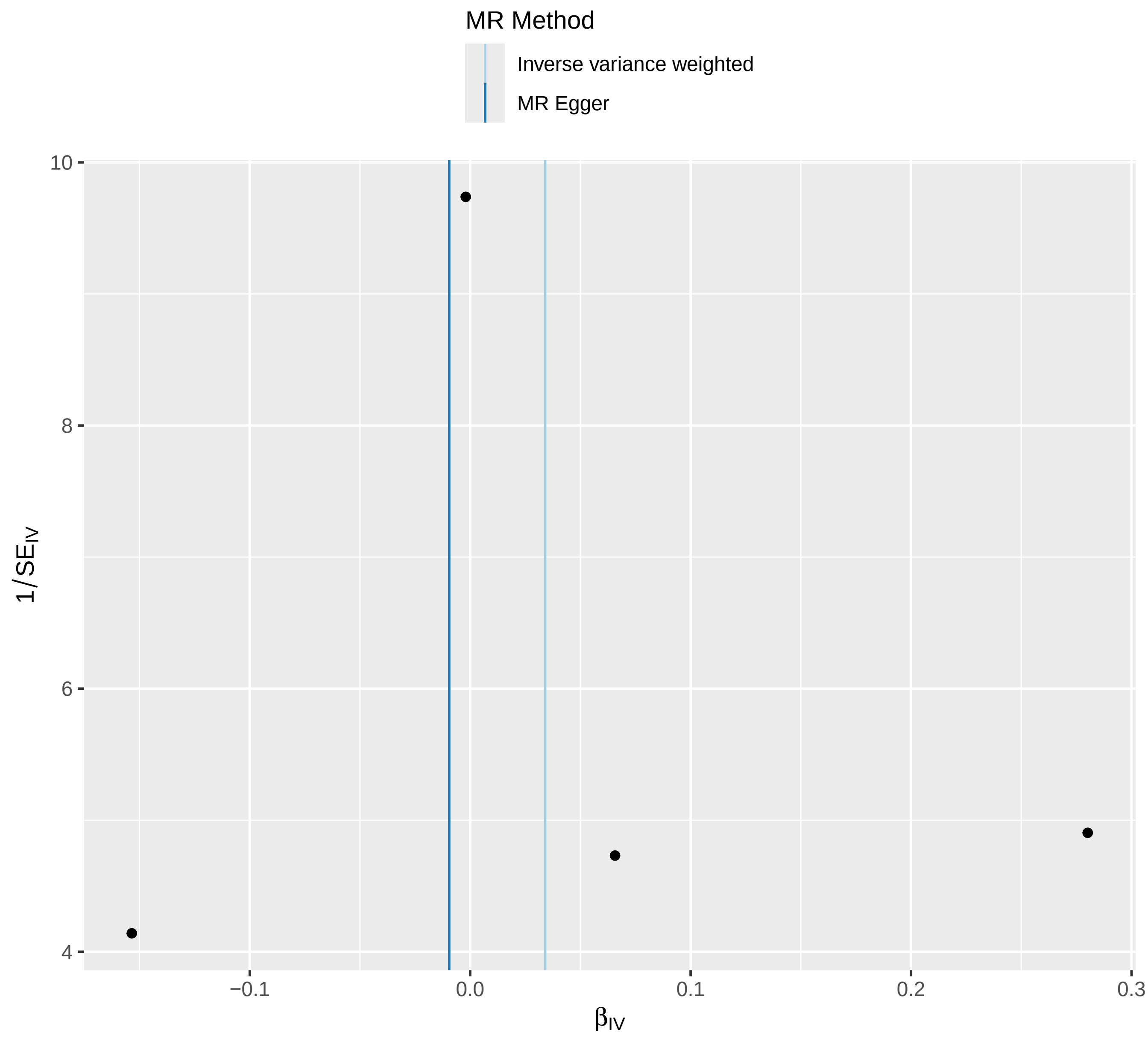
MR Method
Inverse variance weighted
MR Egger
10
8
6
4
1/SE$_{IV}$
−0.1
0.0
0.1
0.2
0.3
β$_{IV}$

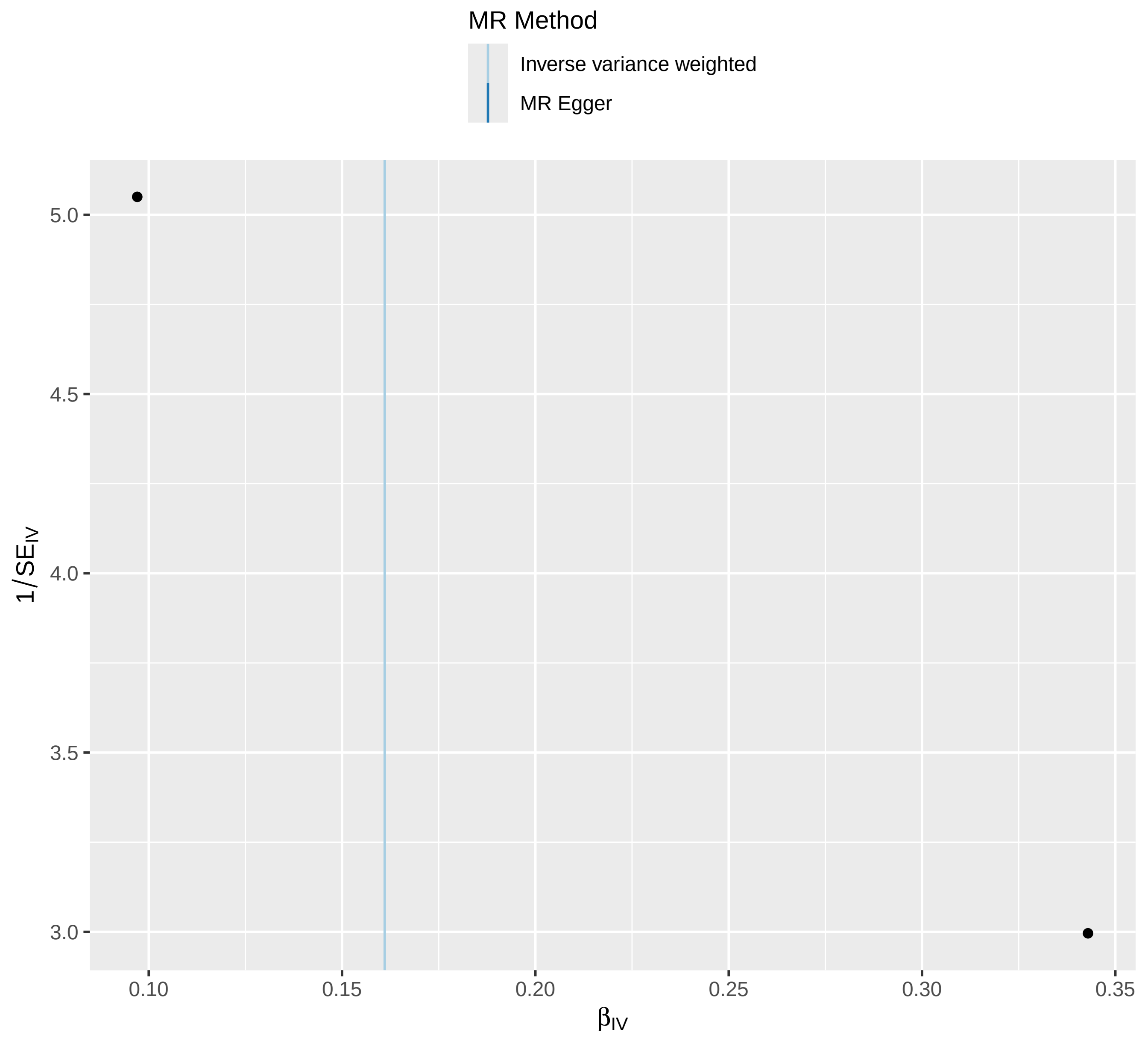

MR Method
Inverse variance weighted
MR Egger
5.0
4.5
4.0
3.5
3.0
1/SE_IV
0.10
0.15
0.20
0.25
0.30
0.35
β_IV

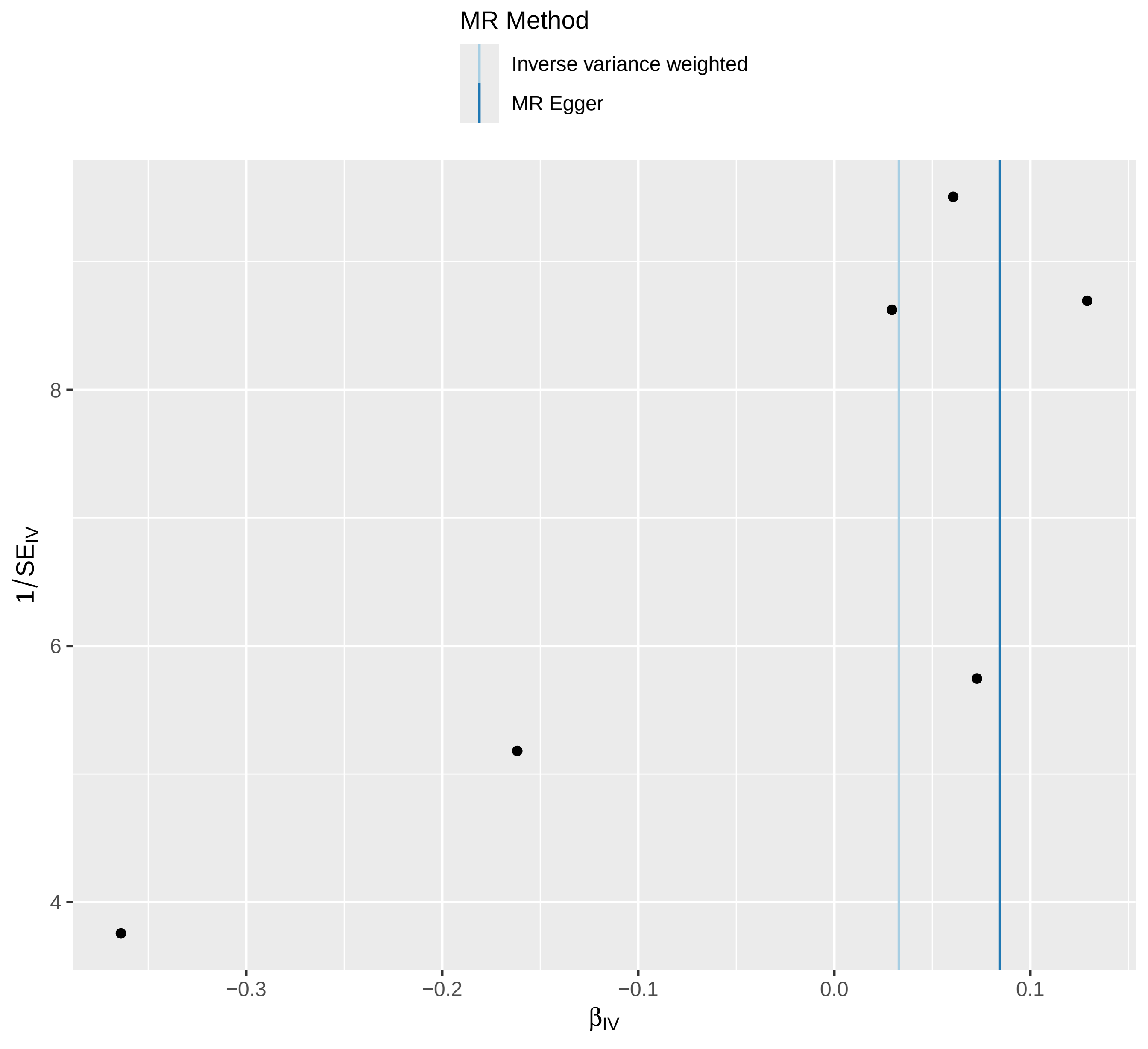

MR Method
Inverse variance weighted
MR Egger
8
6
4
$1/SE_{IV}$
−0.3
−0.2
−0.1
0.0
0.1
$\beta_{IV}$

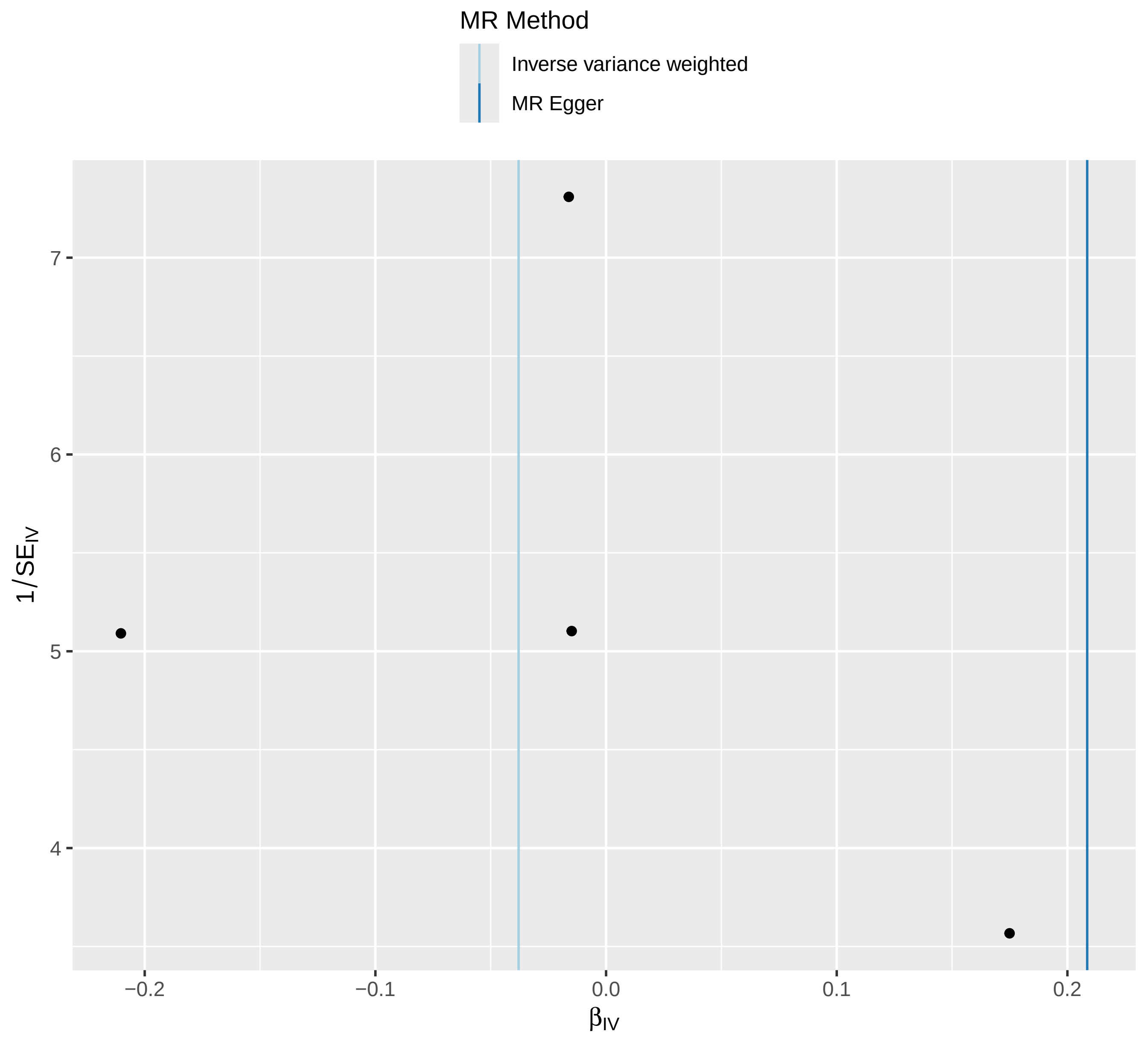

MR Method
Inverse variance weighted
MR Egger
7
6
5
4
1/SE$_{IV}$
−0.2
−0.1
0.0
0.1
0.2
β$_{IV}$

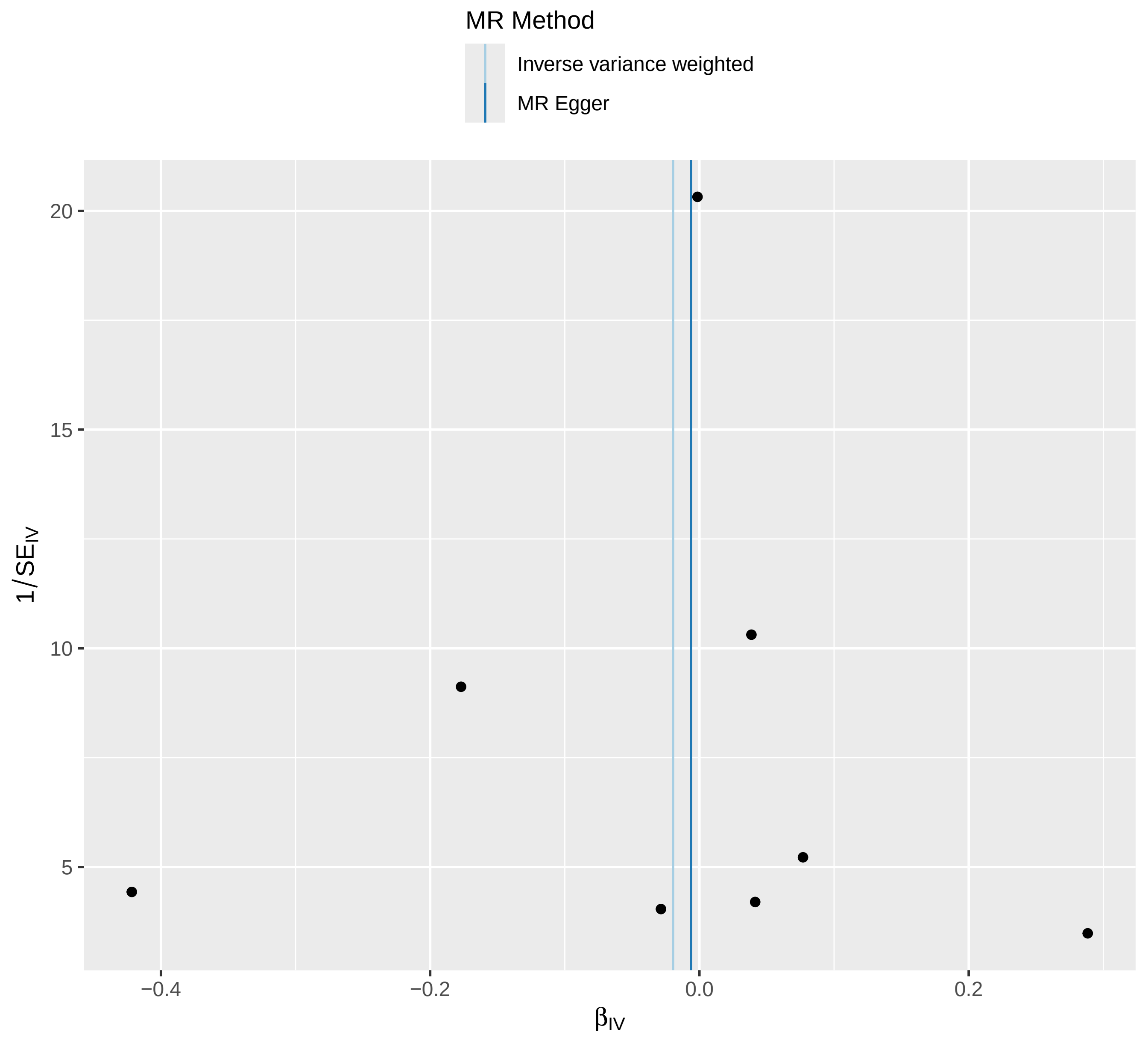
MR Method
Inverse variance weighted
MR Egger
20
15
10
5
1/SE$_{IV}$
−0.4
−0.2
0.0
0.2
β$_{IV}$

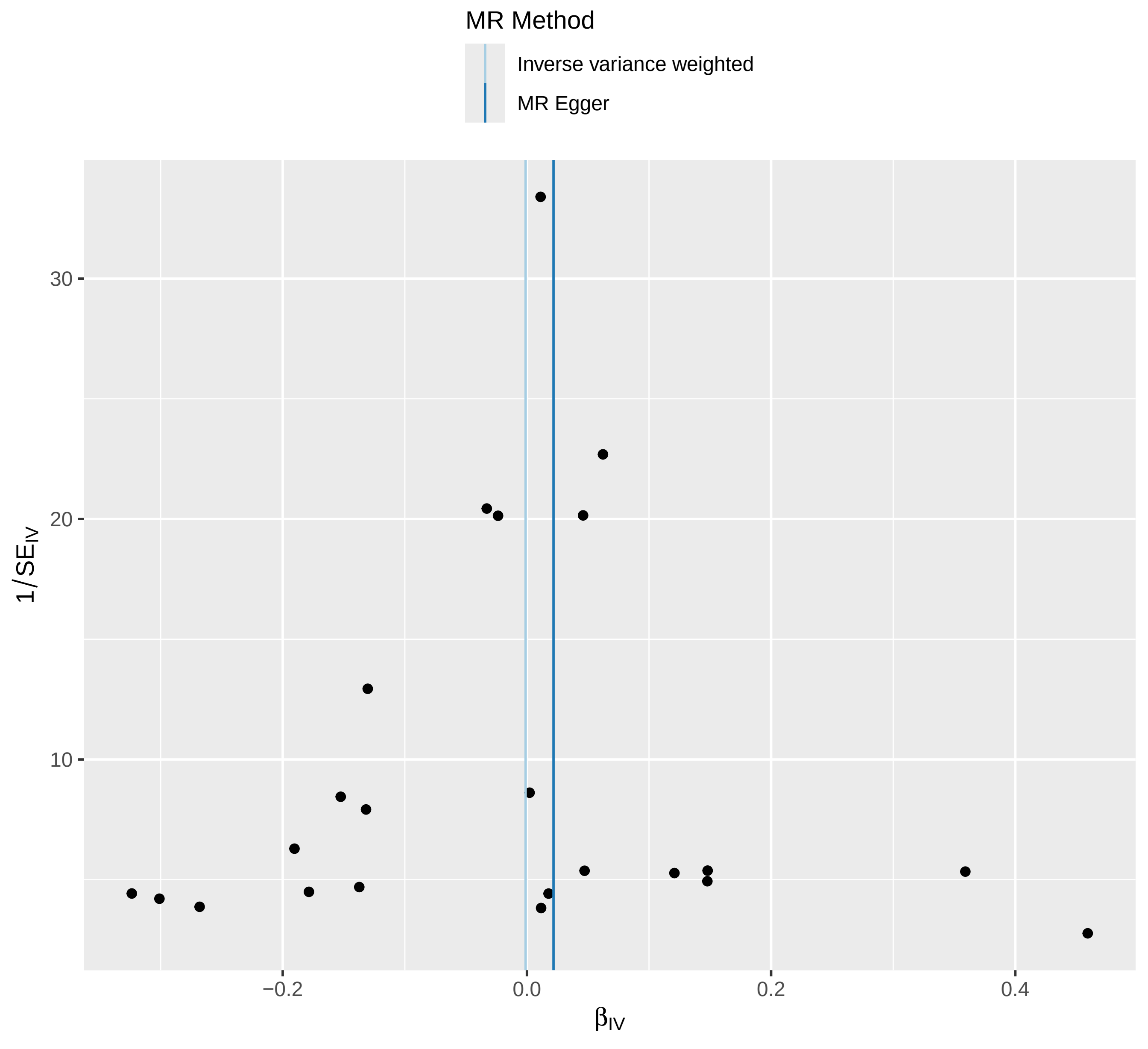
MR Method
Inverse variance weighted
MR Egger
30
20
10
1/SE_IV
−0.2
0.0
0.2
0.4
β_IV

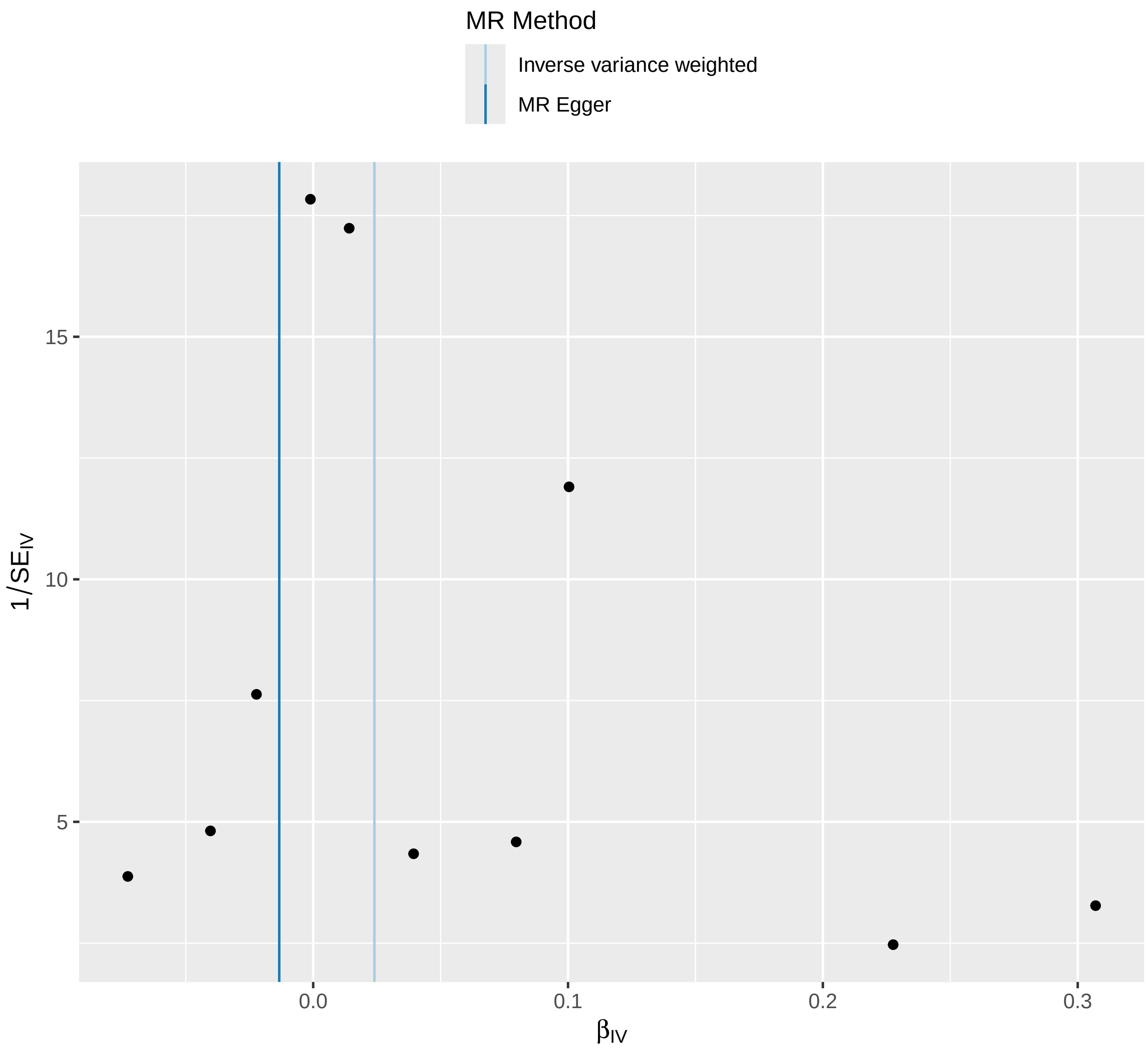
MR Method
Inverse variance weighted
MR Egger
15
10
5
$1/SE_{IV}$
0.0
0.1
0.2
0.3
$\beta_{IV}$

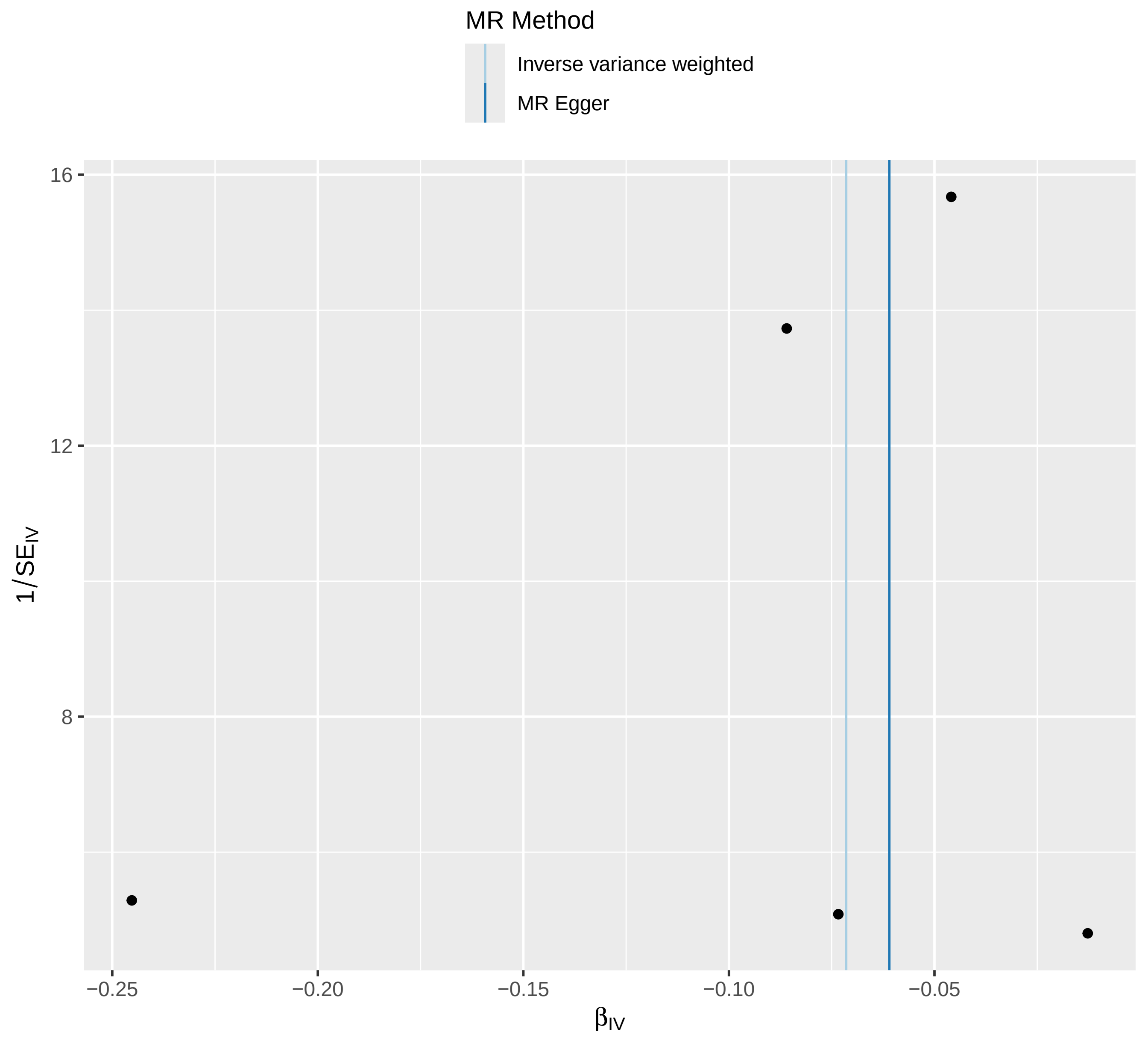

MR Method
Inverse variance weighted
MR Egger
16
12
8
1/SE_IV
−0.25
−0.20
−0.15
−0.10
−0.05
β_IV

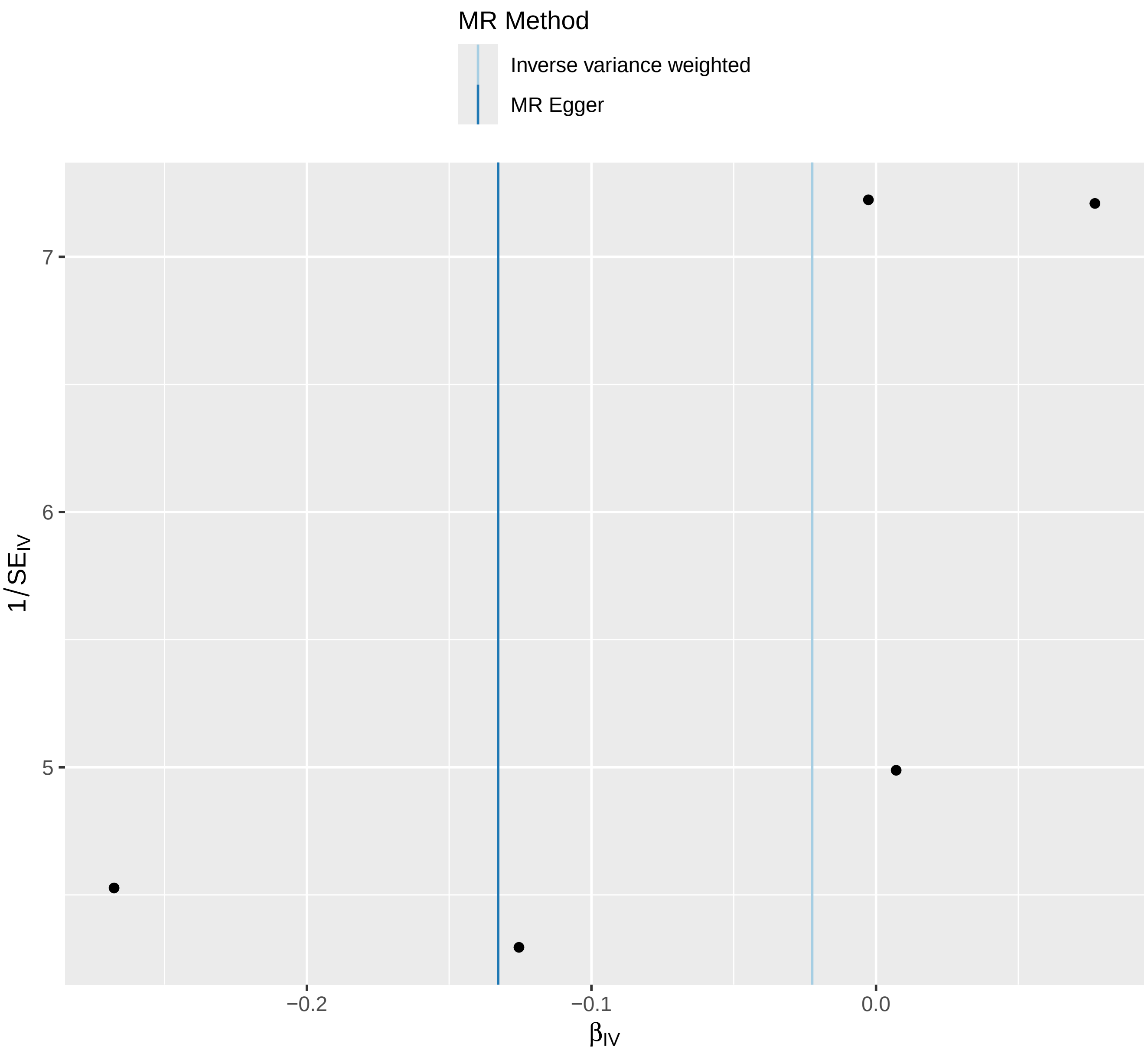

MR Method
Inverse variance weighted
MR Egger
7
6
5
$1/SE_{IV}$
−0.2
−0.1
0.0
$\beta_{IV}$

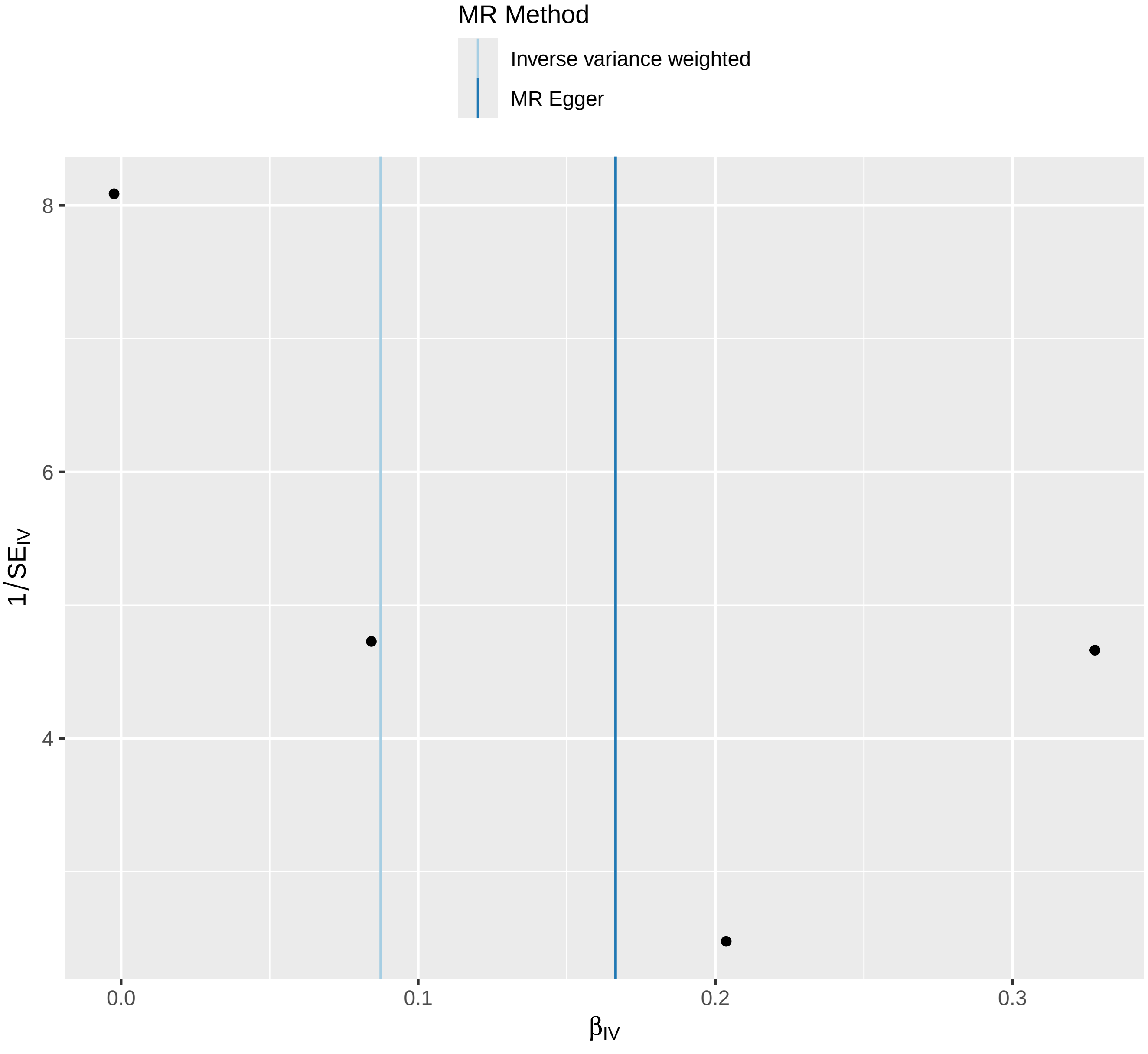

MR Method
Inverse variance weighted
MR Egger
8
6
4
$1/SE_{IV}$
0.0
0.1
0.2
0.3
$\beta_{IV}$

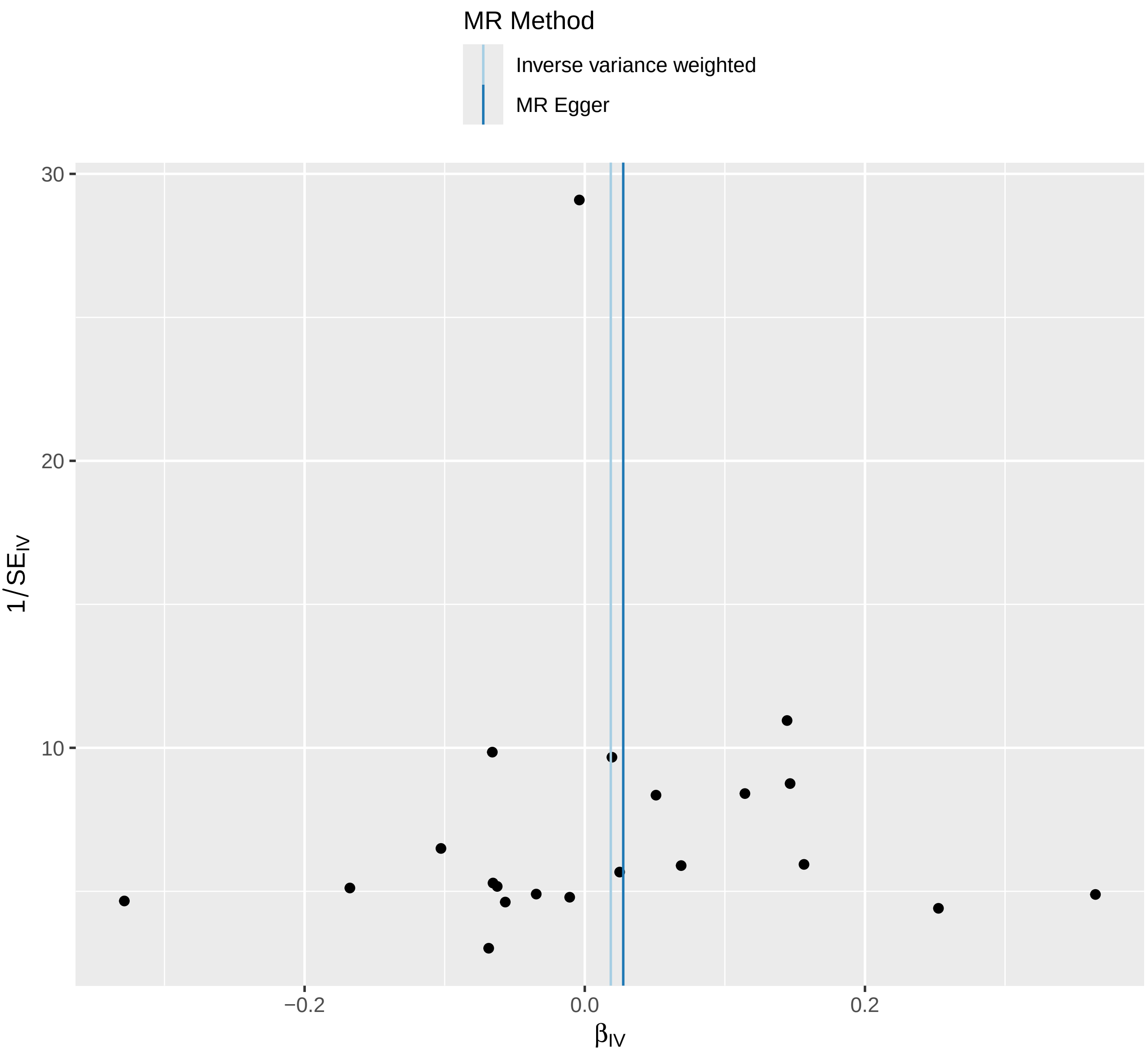

MR Method
Inverse variance weighted
MR Egger
30
20
10
1/SE_IV
−0.2
0.0
0.2
β_IV

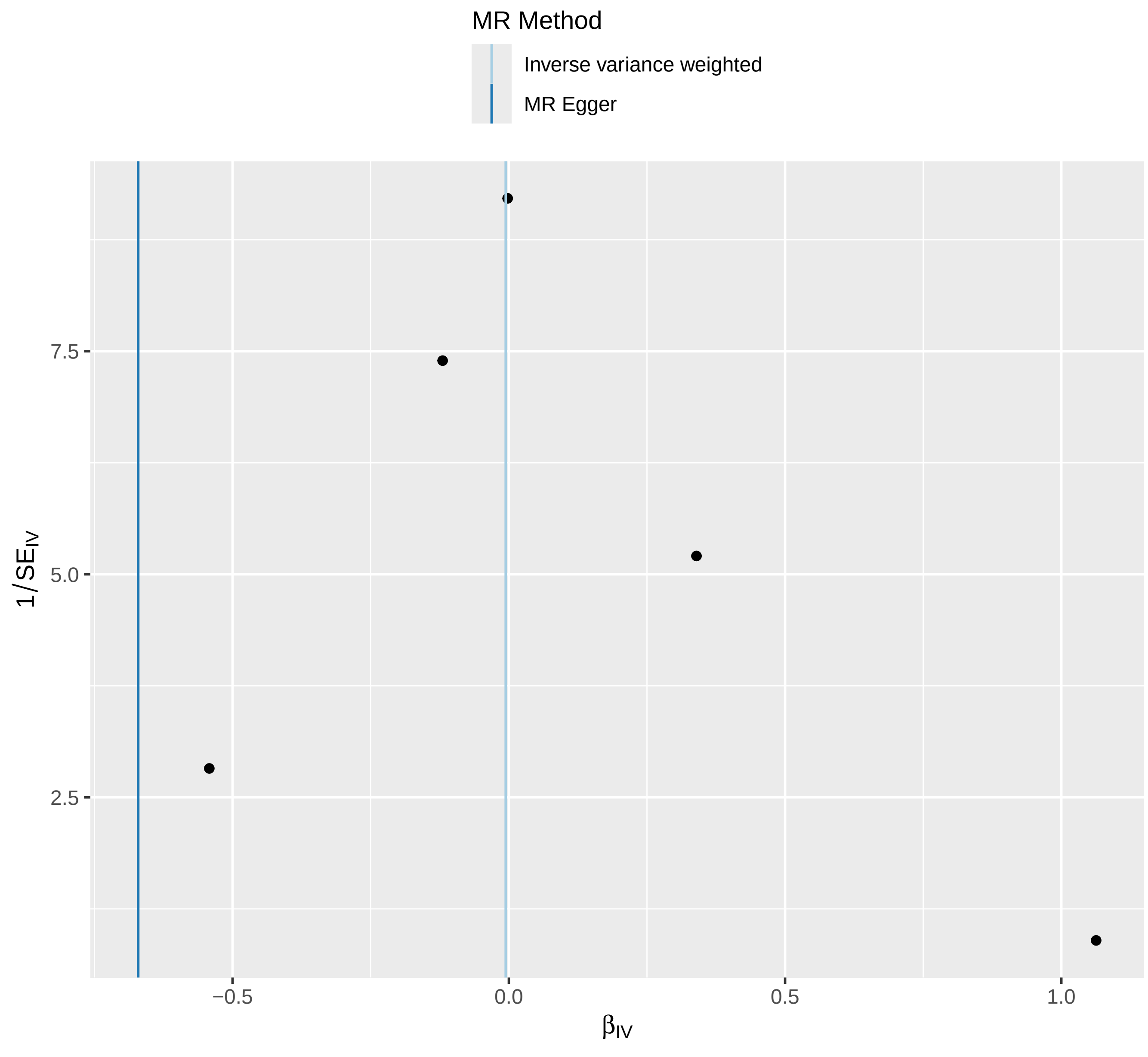

MR Method
Inverse variance weighted
MR Egger
7.5
5.0
2.5
$1/SE_{IV}$
−0.5
0.0
0.5
1.0
$\beta_{IV}$

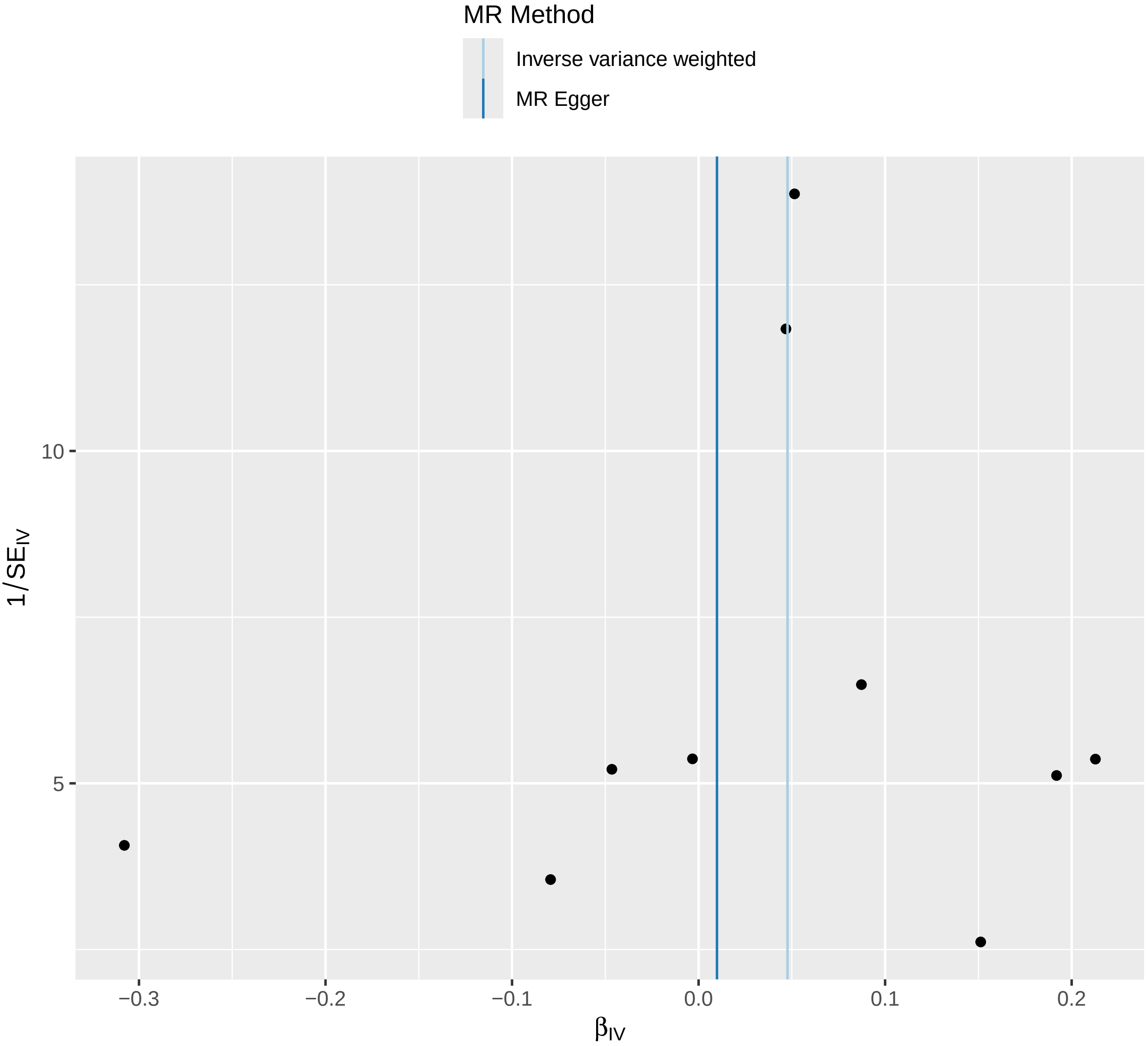

MR Method
Inverse variance weighted
MR Egger
10
5
1/SE$_{IV}$
−0.3
−0.2
−0.1
0.0
0.1
0.2
β$_{IV}$

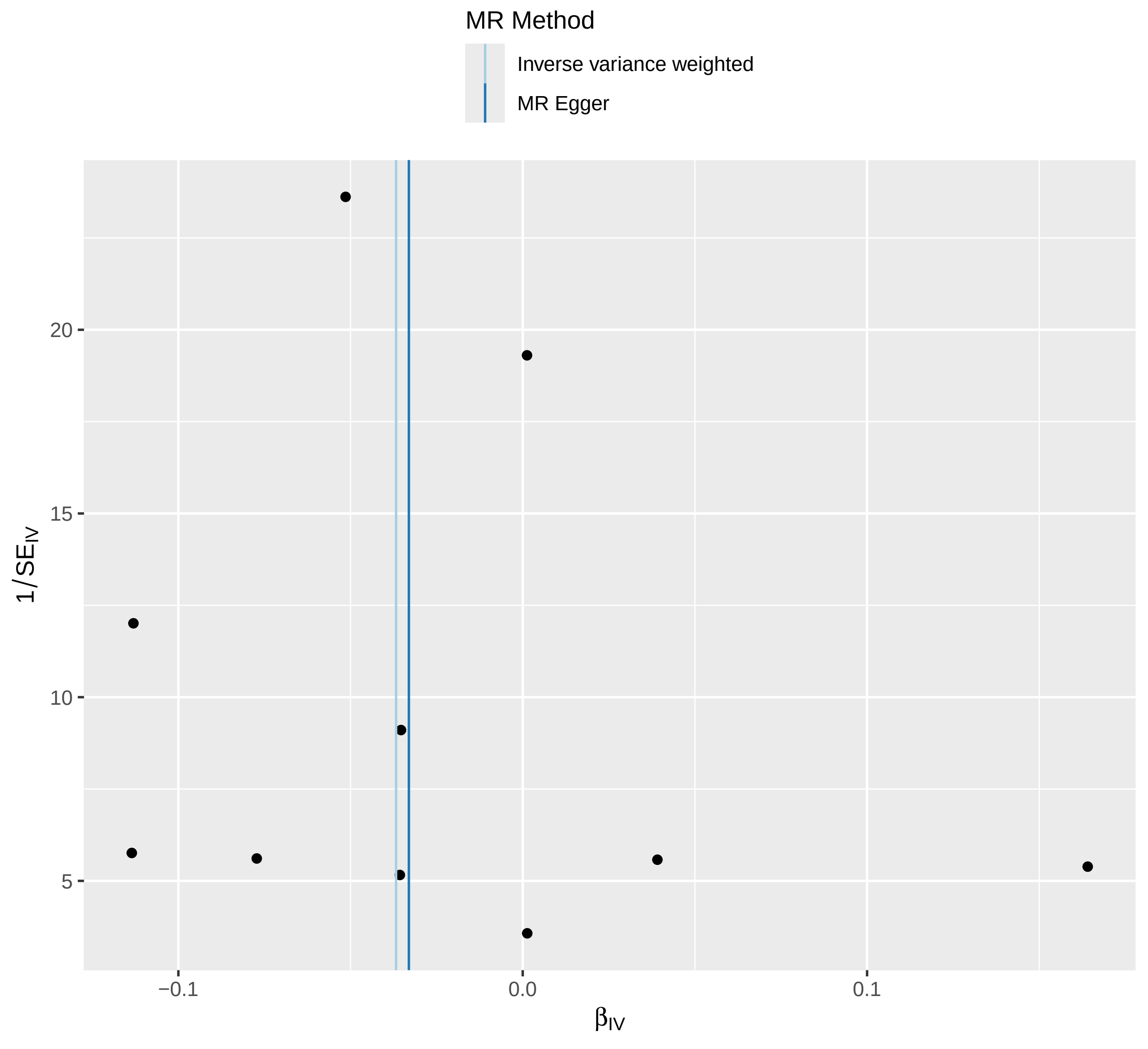
MR Method
Inverse variance weighted
MR Egger
20
15
10
5
1/SE_IV
−0.1
0.0
0.1
β_IV

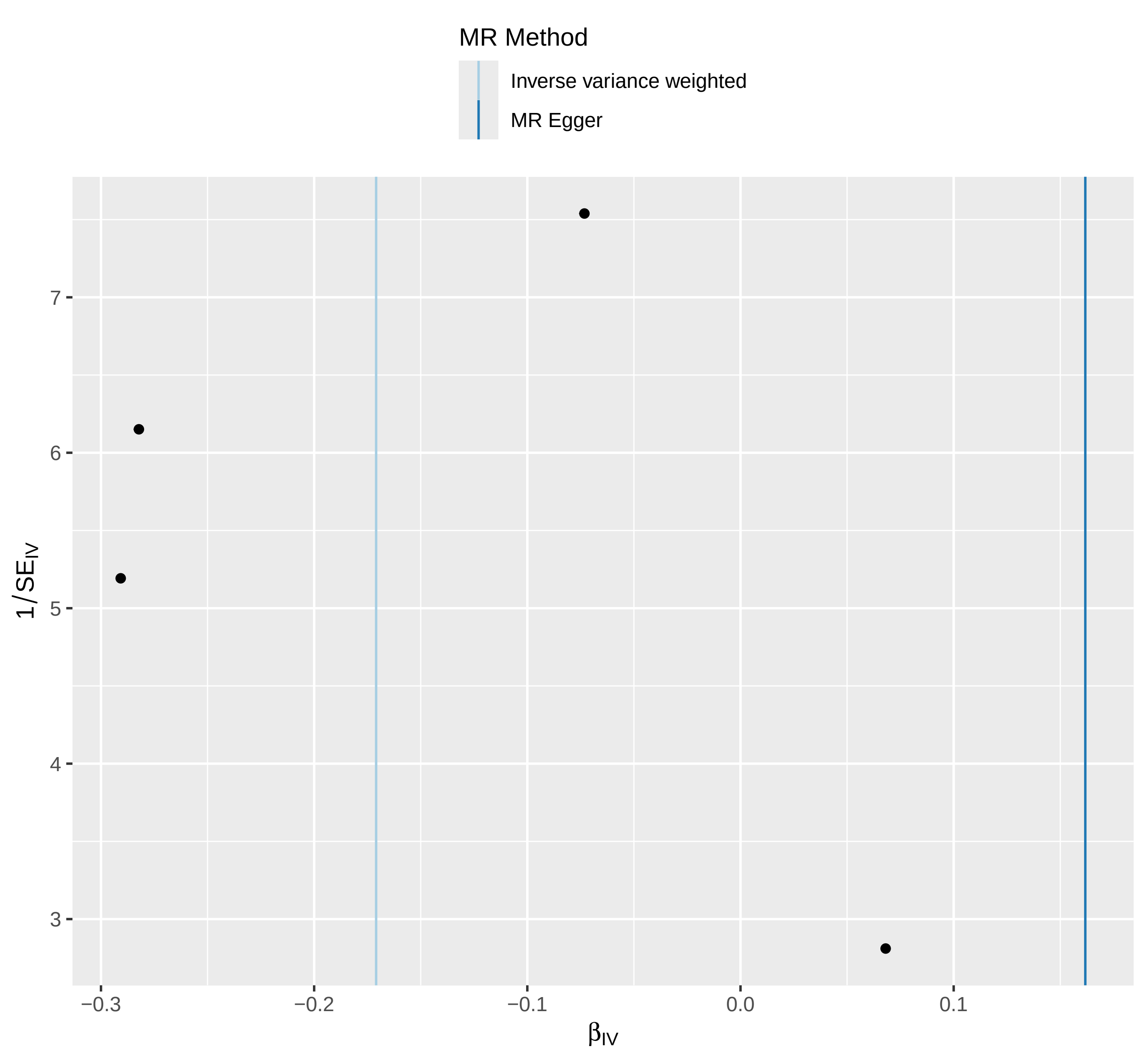

MR Method
Inverse variance weighted
MR Egger
7
6
5
4
3
1/SE_IV
−0.3
−0.2
−0.1
0.0
0.1
β_IV

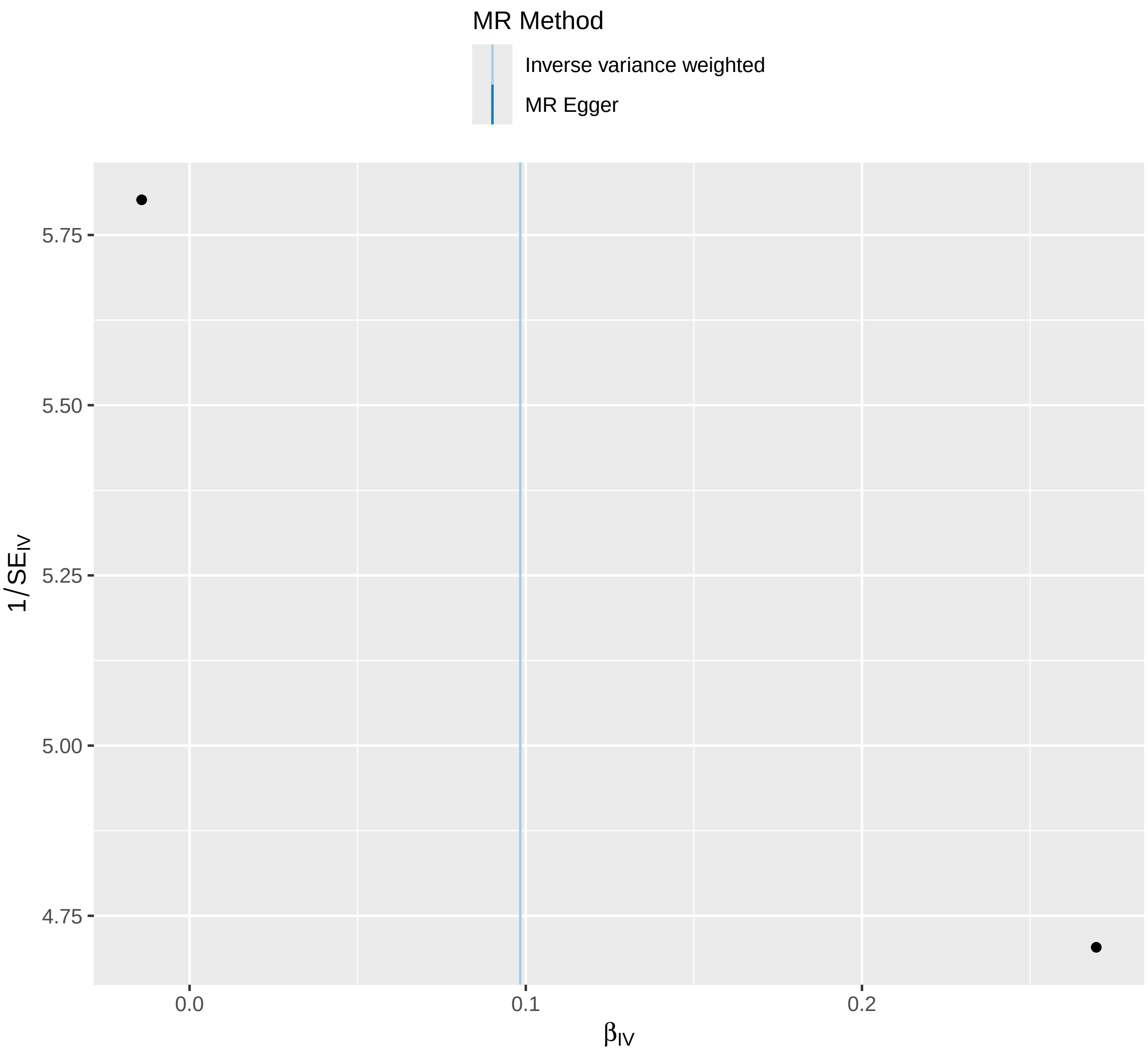
MR Method
Inverse variance weighted
MR Egger
5.75
5.50
5.25
5.00
4.75
1/SE_IV
0.0
0.1
0.2
β_IV

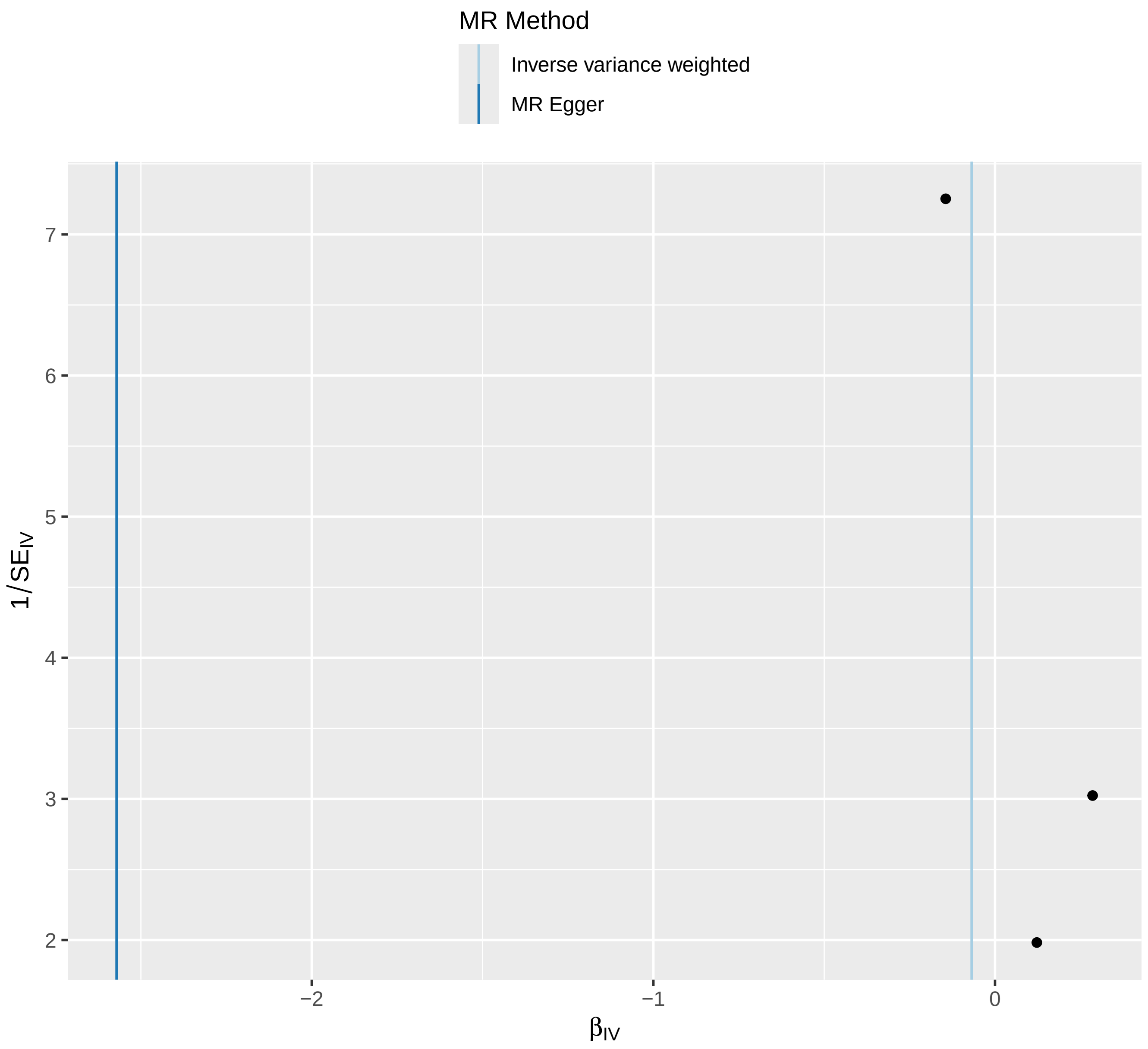

MR Method
Inverse variance weighted
MR Egger
7
6
5
4
3
2
$1/SE_{IV}$
−2
−1
0
$\beta_{IV}$

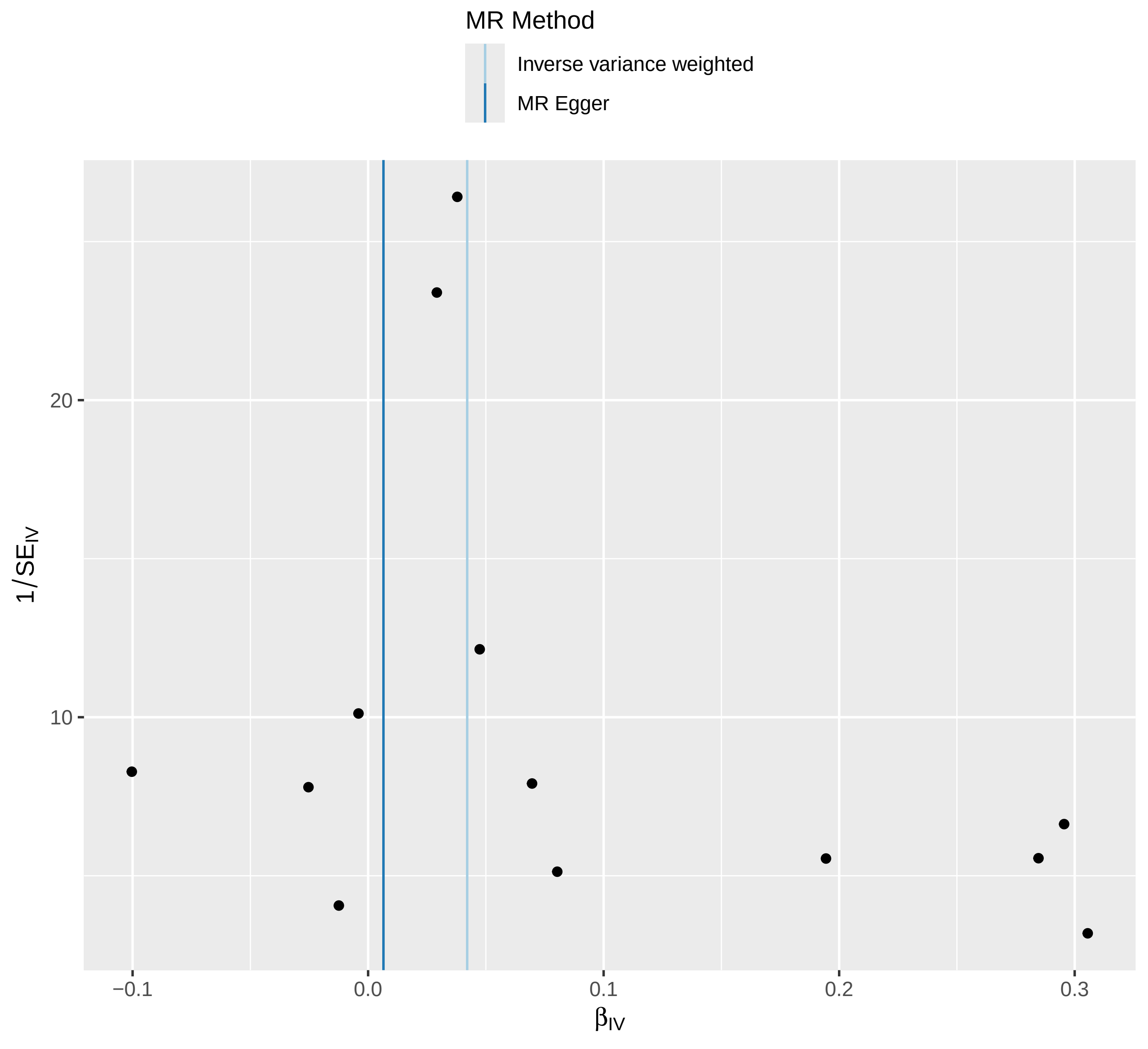
MR Method
Inverse variance weighted
MR Egger
20
10
$1/SE_{IV}$
−0.1
0.0
0.1
0.2
0.3
$\beta_{IV}$

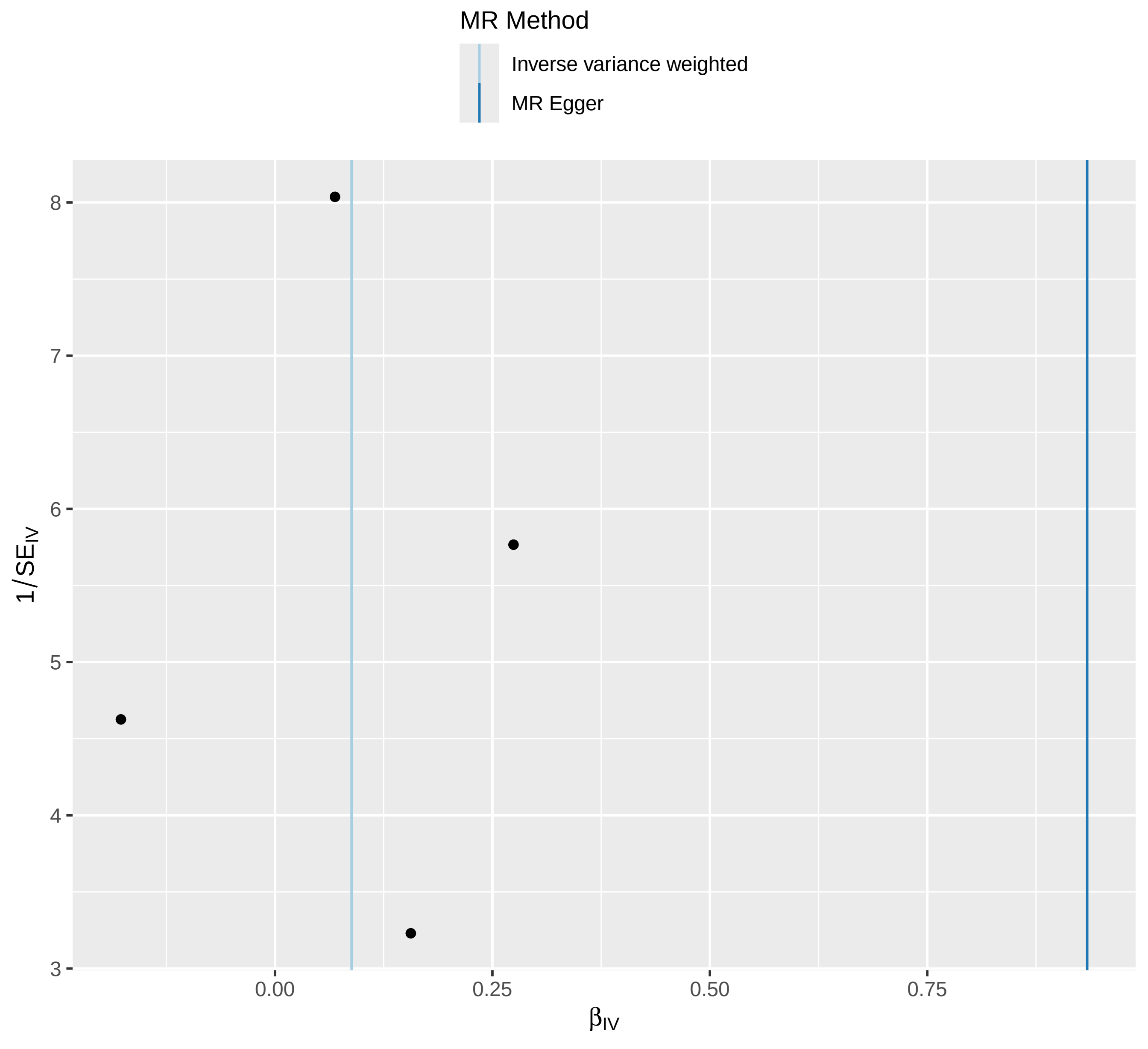
MR Method
Inverse variance weighted
MR Egger
8
7
6
5
4
3
1/SE_IV
0.00
0.25
0.50
0.75
β_IV

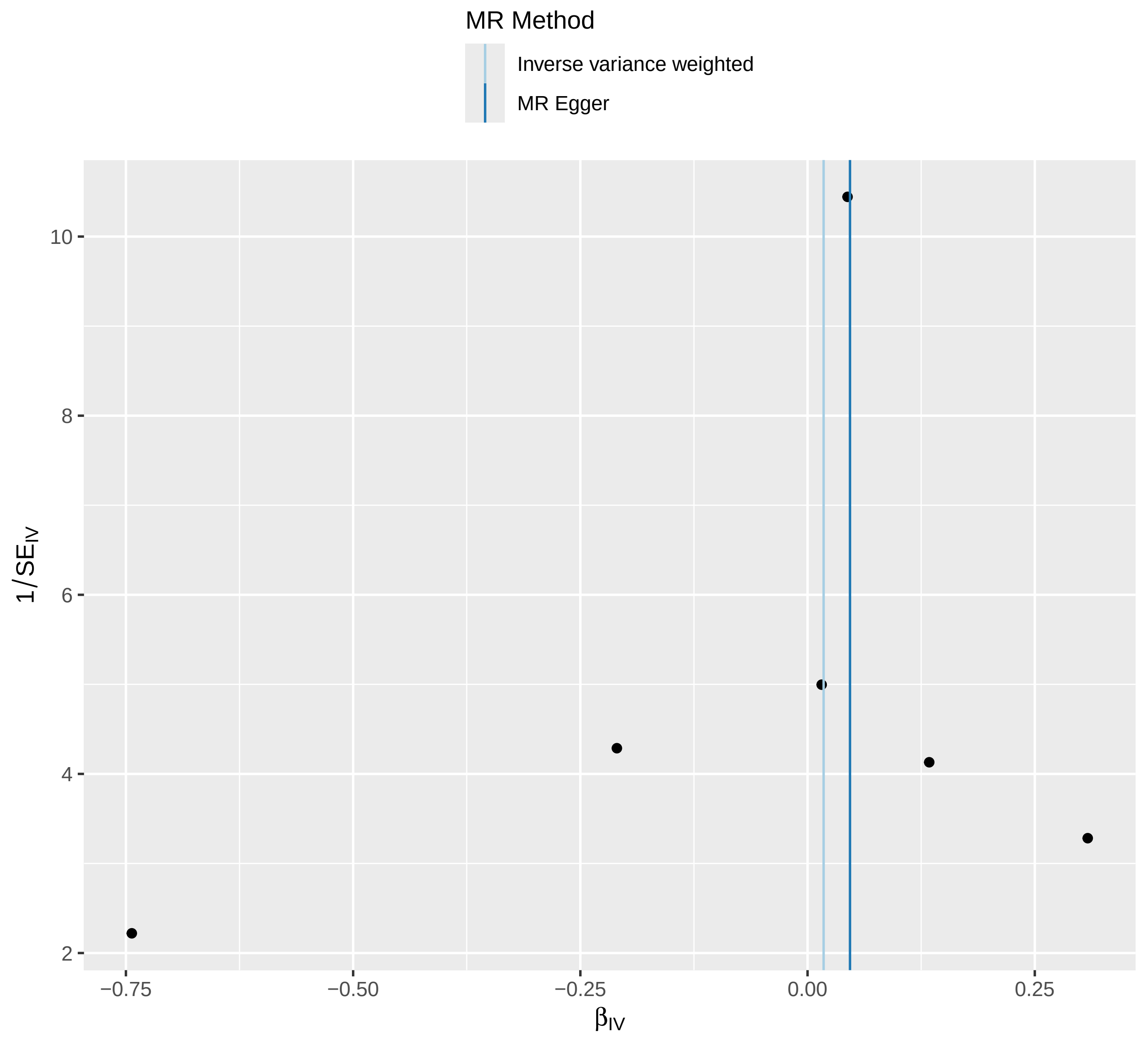
MR Method
Inverse variance weighted
MR Egger
1/SE$_{IV}$
β$_{IV}$
10
8
6
4
2
−0.75
−0.50
−0.25
0.00
0.25

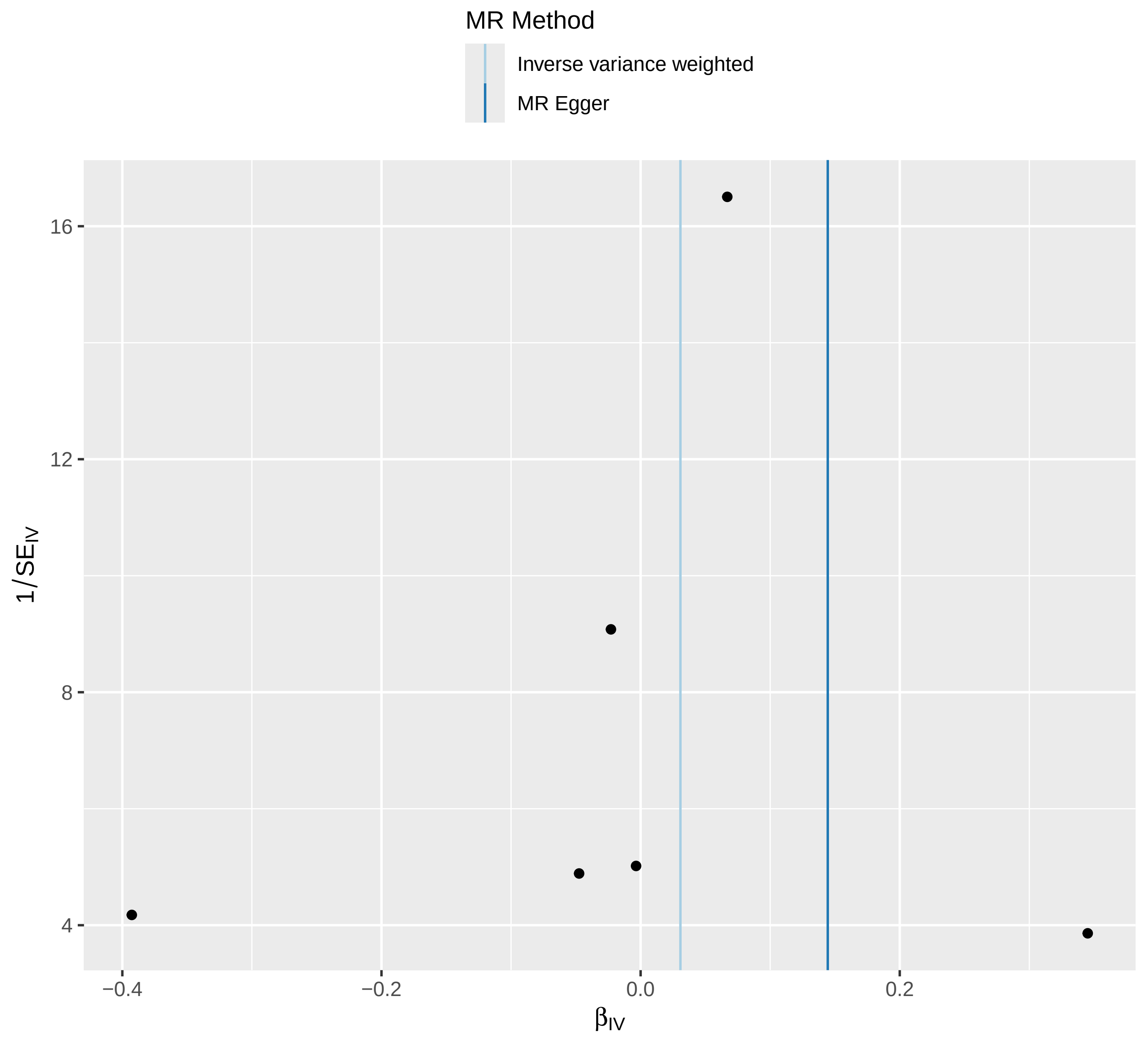

MR Method
Inverse variance weighted
MR Egger
16
12
8
4
1/SE_IV
−0.4
−0.2
0.0
0.2
β_IV

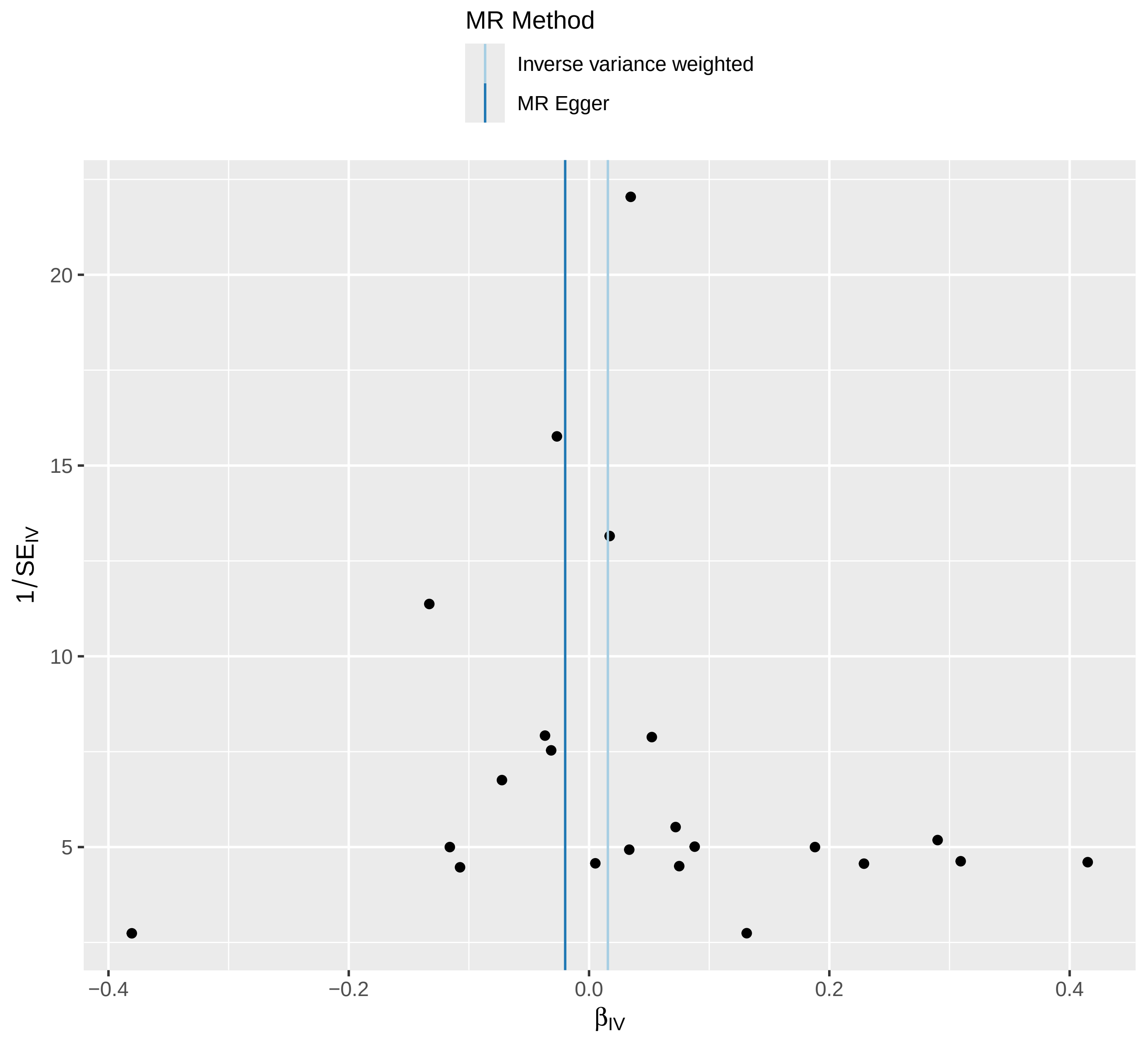

MR Method
Inverse variance weighted
MR Egger
20
15
10
5
1/SE$_{IV}$
−0.4
−0.2
0.0
0.2
0.4
β$_{IV}$

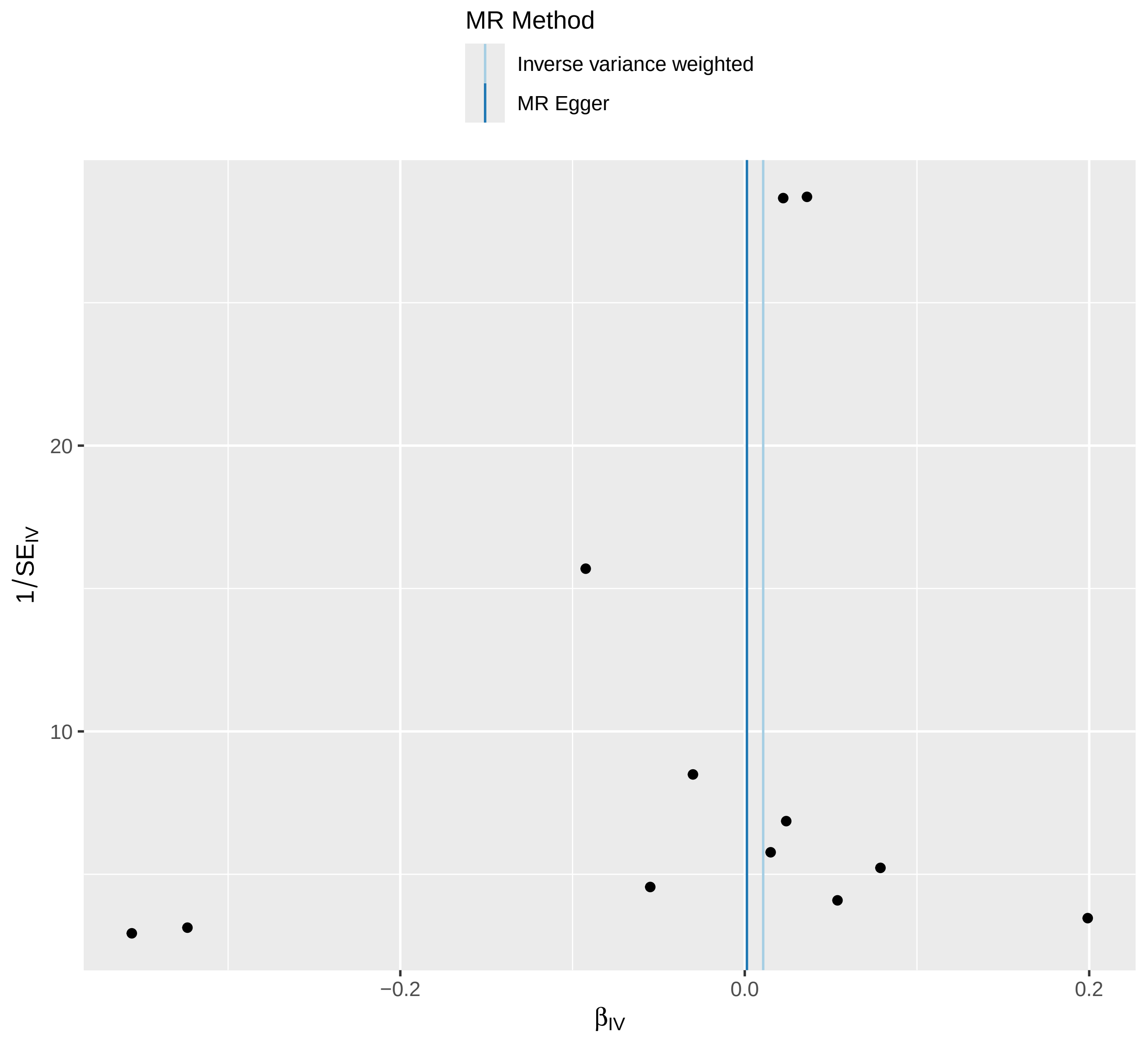

MR Method
Inverse variance weighted
MR Egger
20
10
1/SE_IV
−0.2
0.0
0.2
β_IV

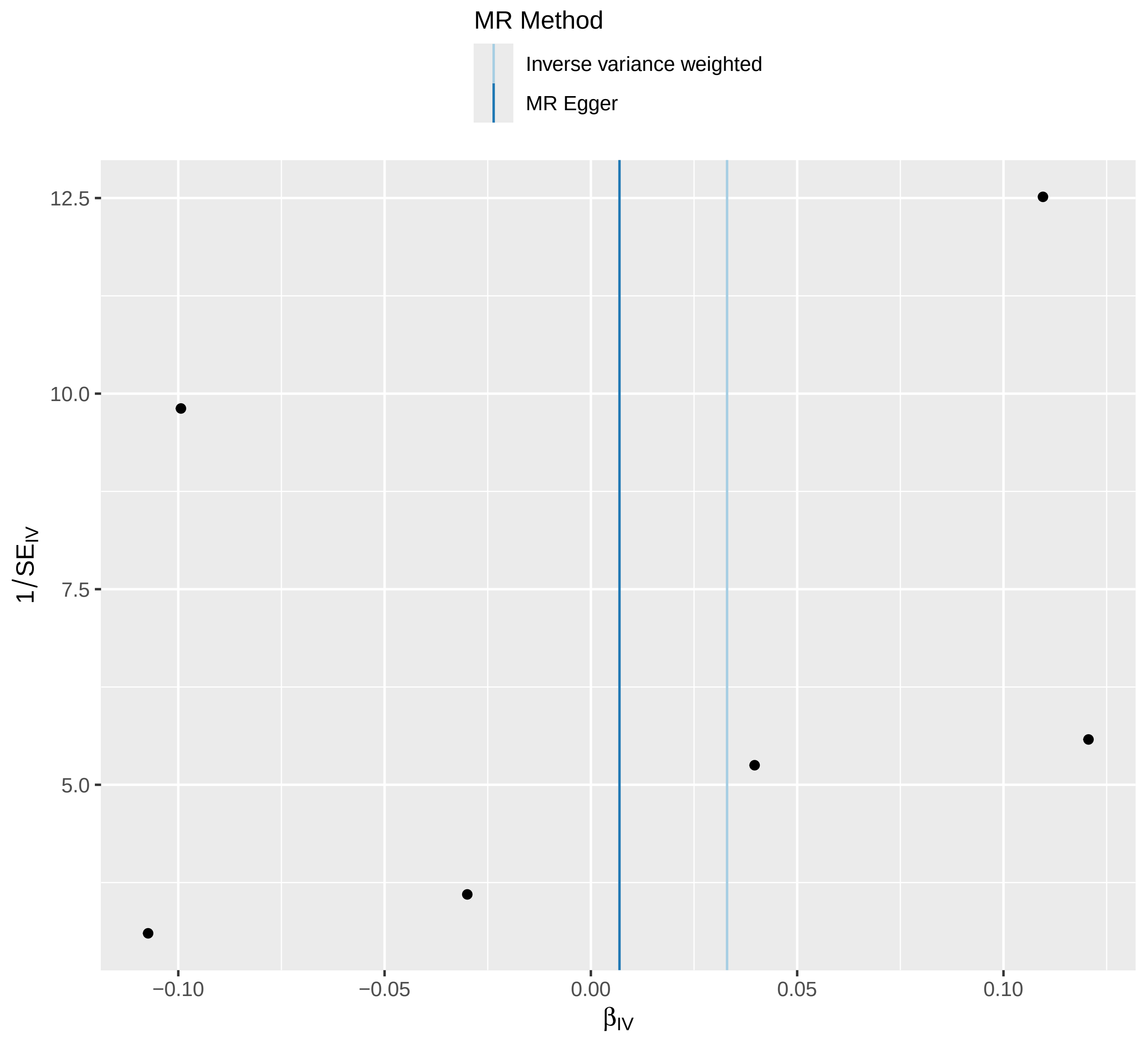
MR Method
Inverse variance weighted
MR Egger
12.5
10.0
7.5
5.0
1/SE$_{IV}$
−0.10
−0.05
0.00
0.05
0.10
β$_{IV}$

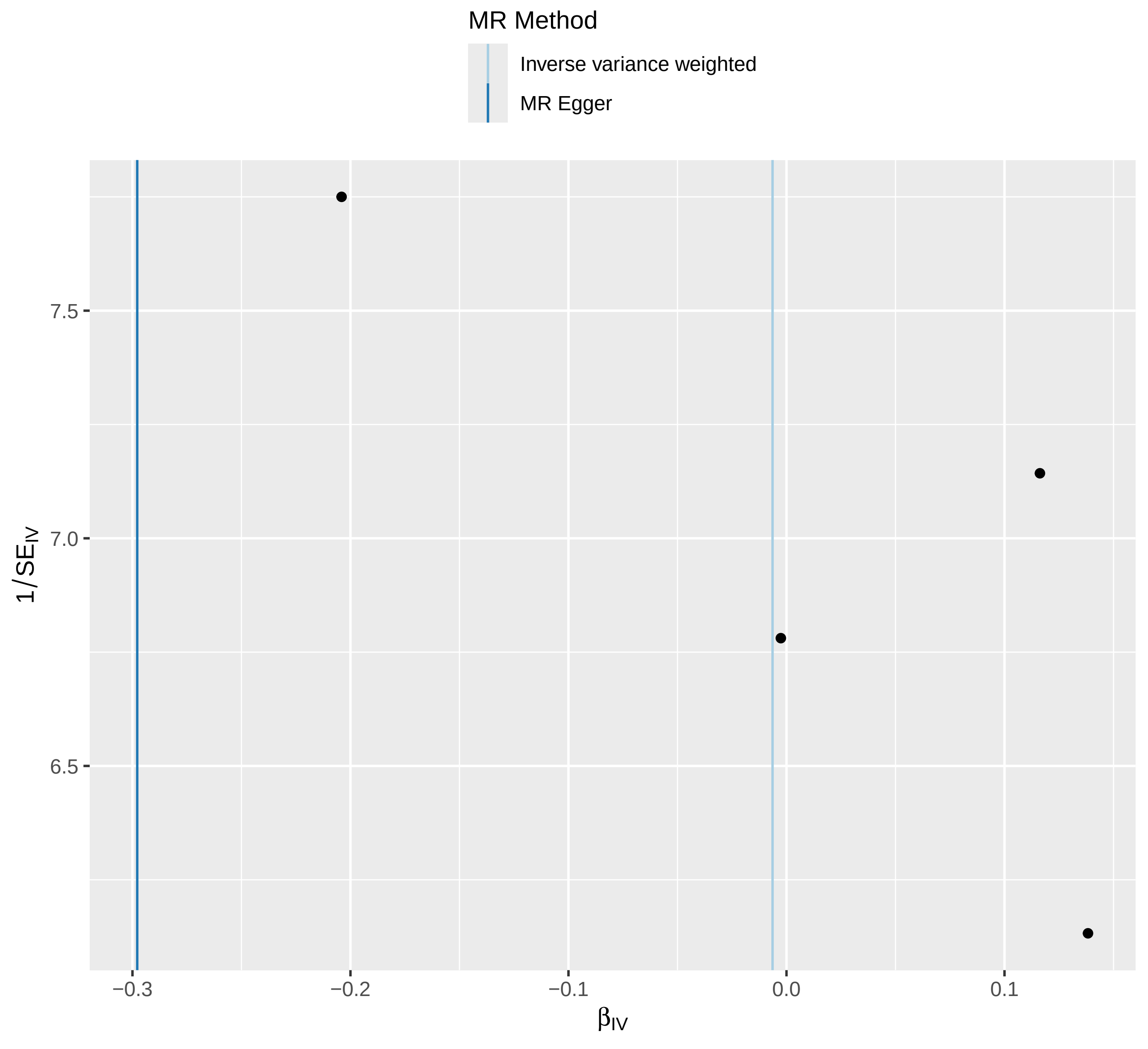

MR Method
Inverse variance weighted
MR Egger
7.5
7.0
6.5
1/SE_IV
−0.3
−0.2
−0.1
0.0
0.1
β_IV

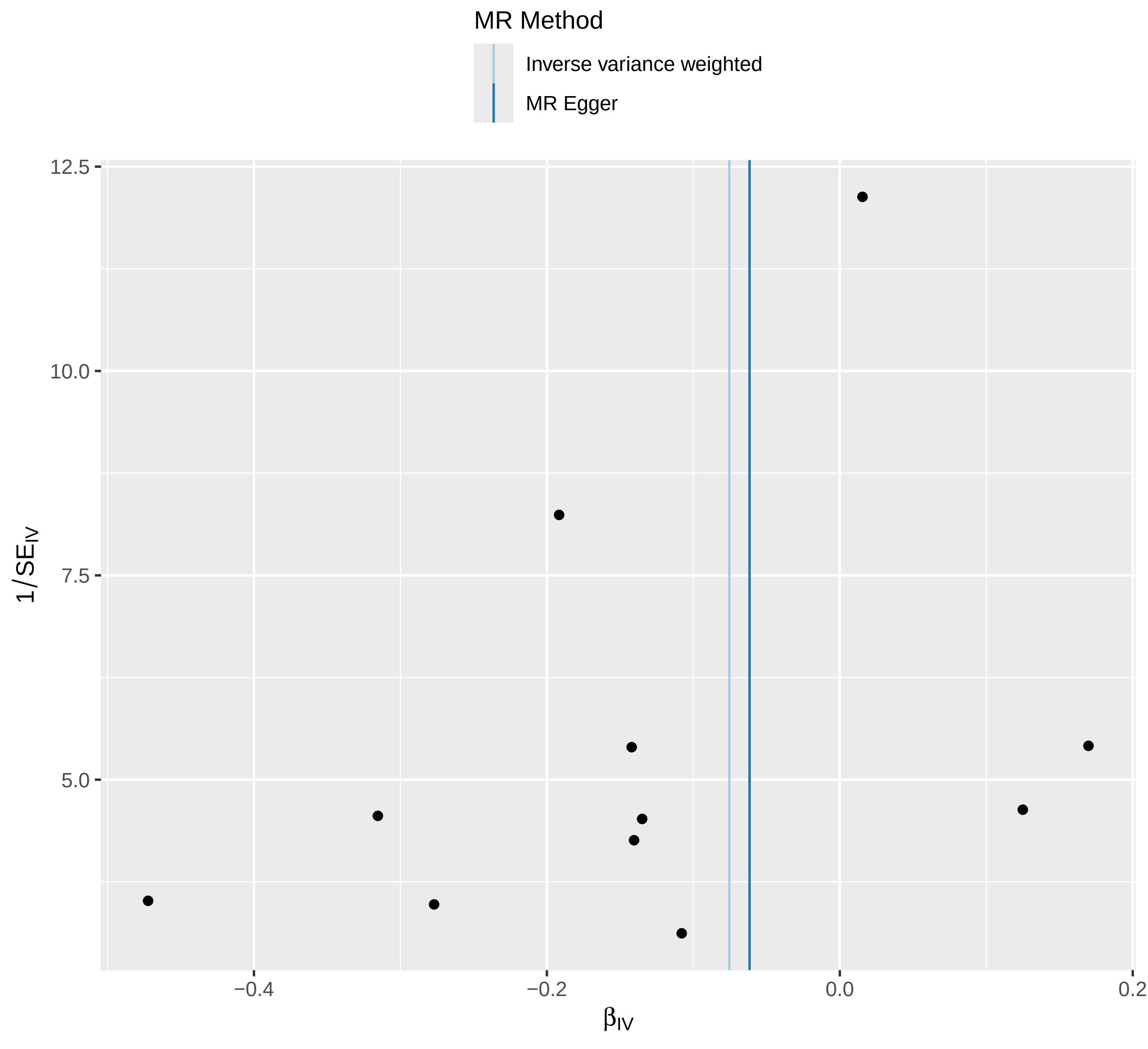

MR Method
Inverse variance weighted
MR Egger
12.5
10.0
7.5
5.0
1/SE_IV
−0.4
−0.2
0.0
0.2
β_IV

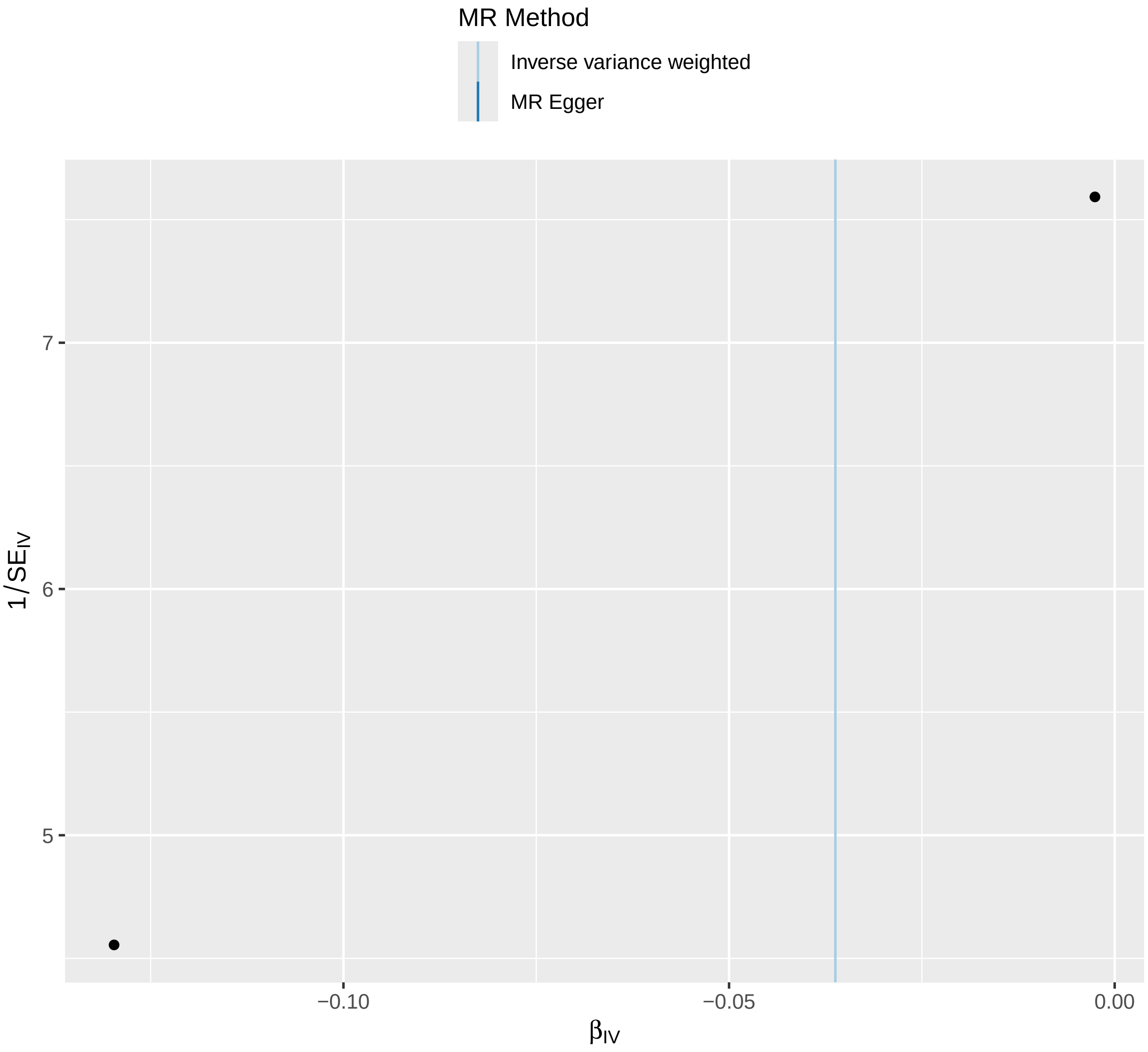
MR Method
Inverse variance weighted
MR Egger
7
6
5
1/SE$_{IV}$
−0.10
−0.05
0.00
β$_{IV}$

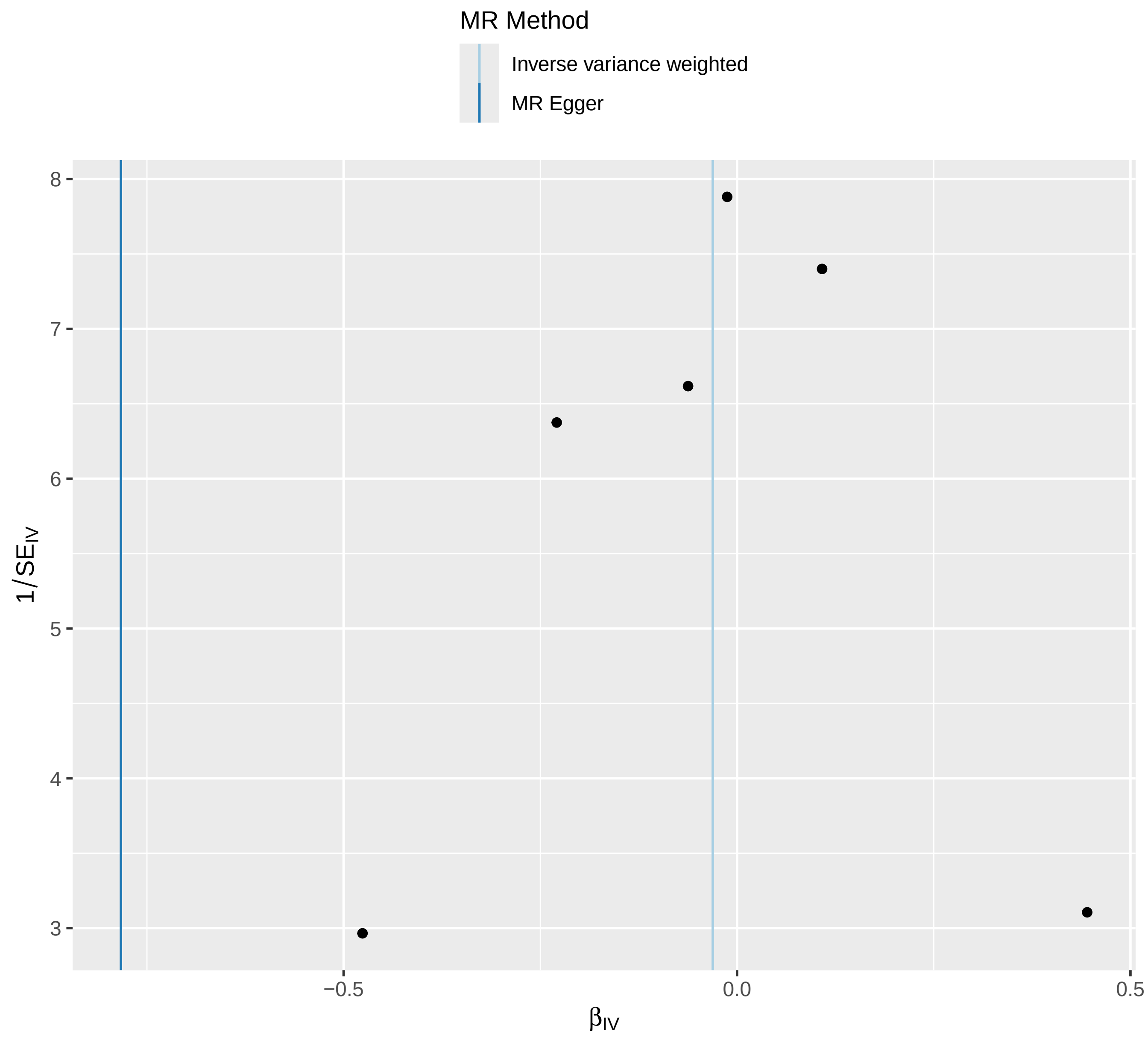
MR Method
Inverse variance weighted
MR Egger
8
7
6
5
4
3
1/SE_IV
−0.5
0.0
0.5
β_IV

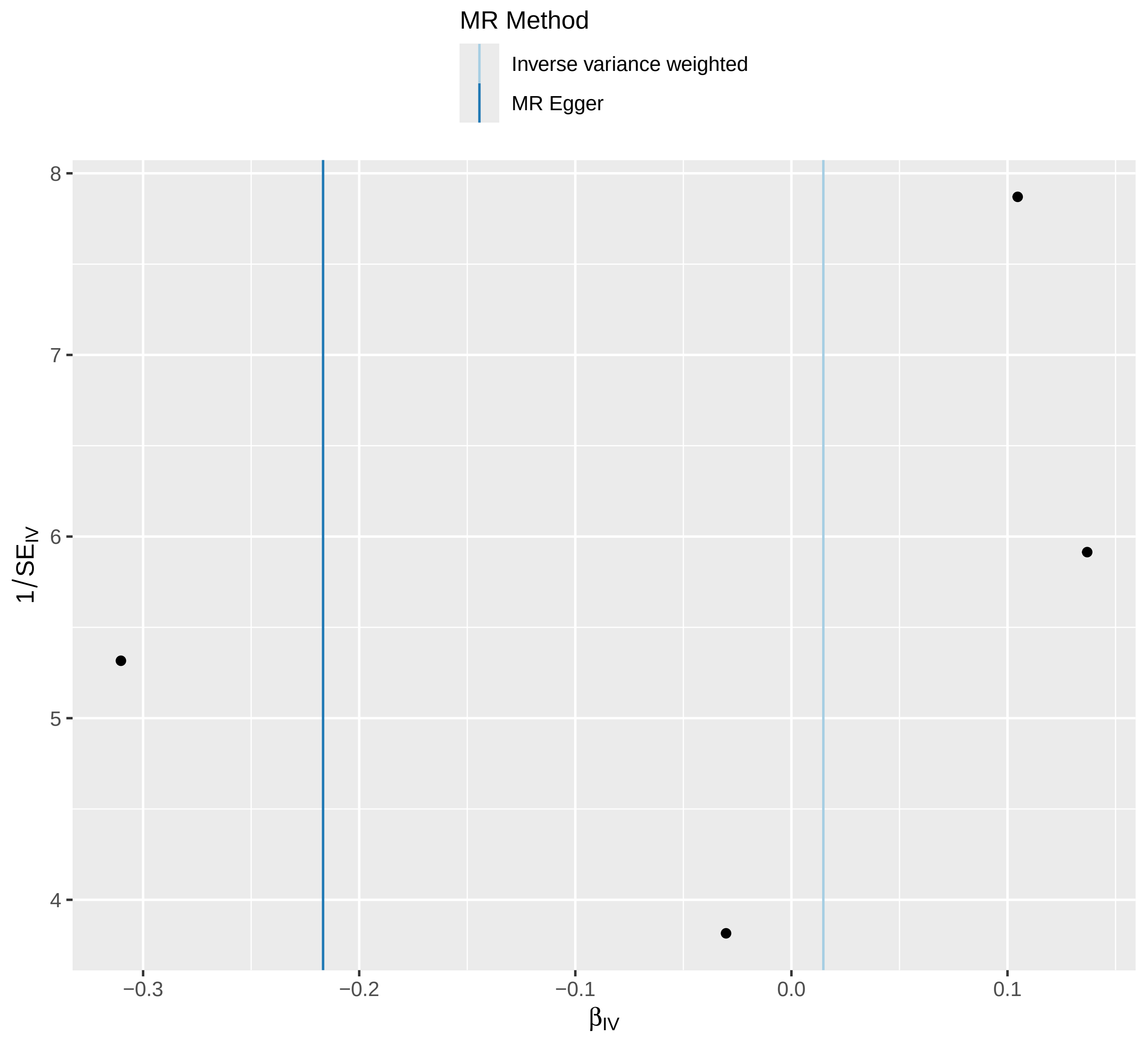
MR Method
Inverse variance weighted
MR Egger
1/SE_IV
β_IV
8
7
6
5
4
−0.3
−0.2
−0.1
0.0
0.1

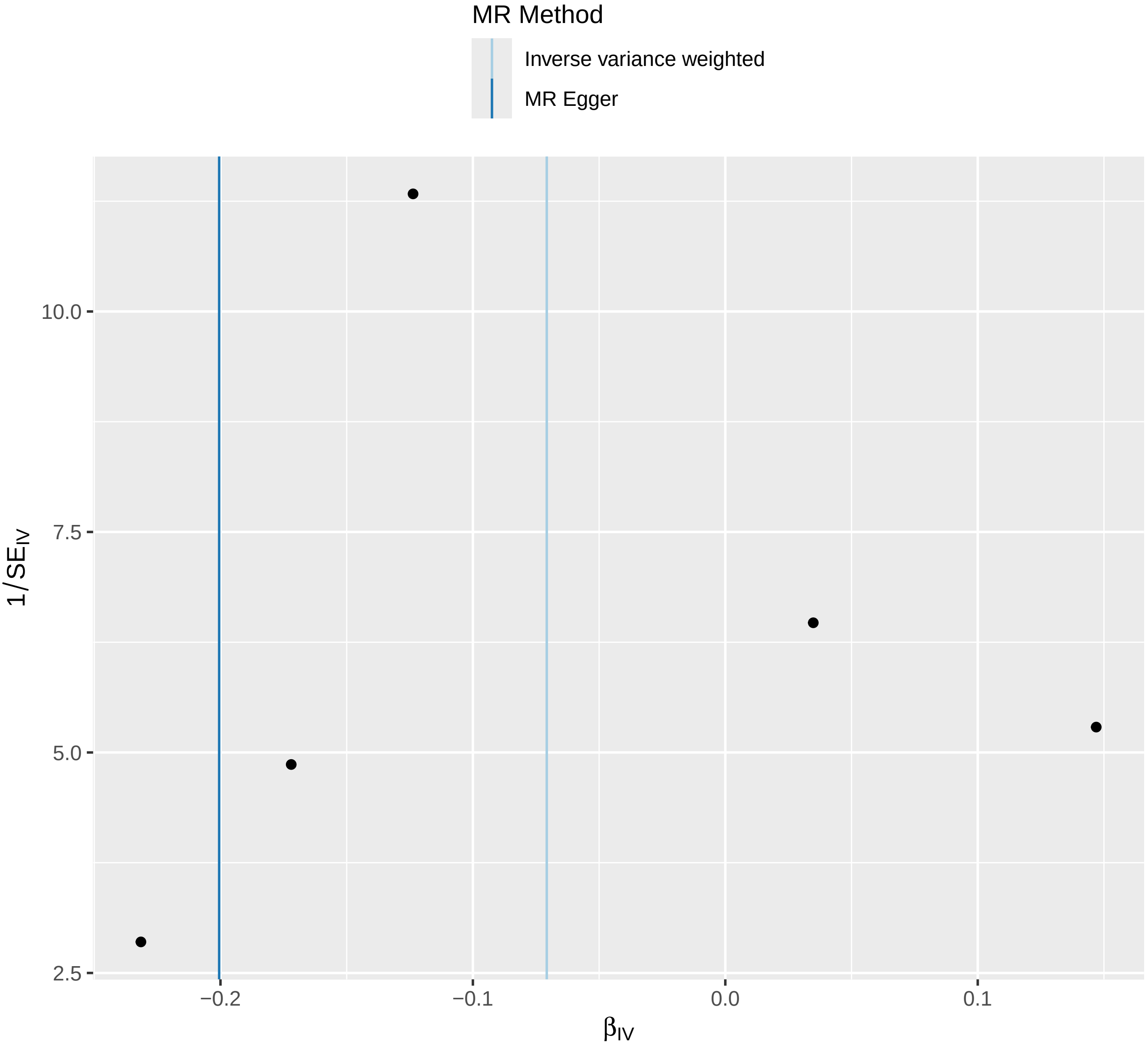
MR Method
Inverse variance weighted
MR Egger
1/SE$_{IV}$
10.0
7.5
5.0
2.5
−0.2
−0.1
0.0
0.1
$\beta_{IV}$

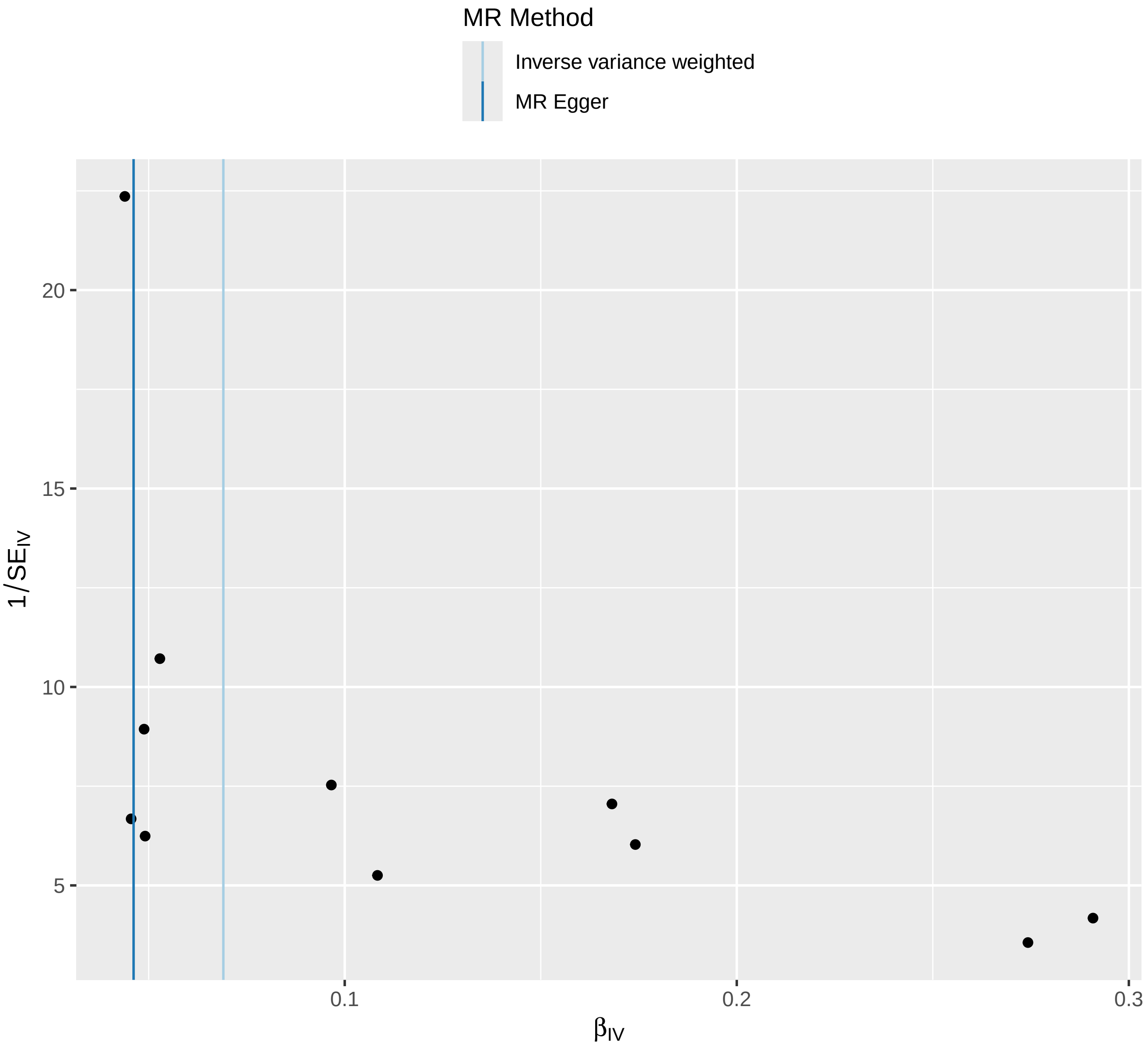
MR Method
Inverse variance weighted
MR Egger
20
15
10
5
0.1
0.2
0.3
$1/SE_{IV}$
$\beta_{IV}$

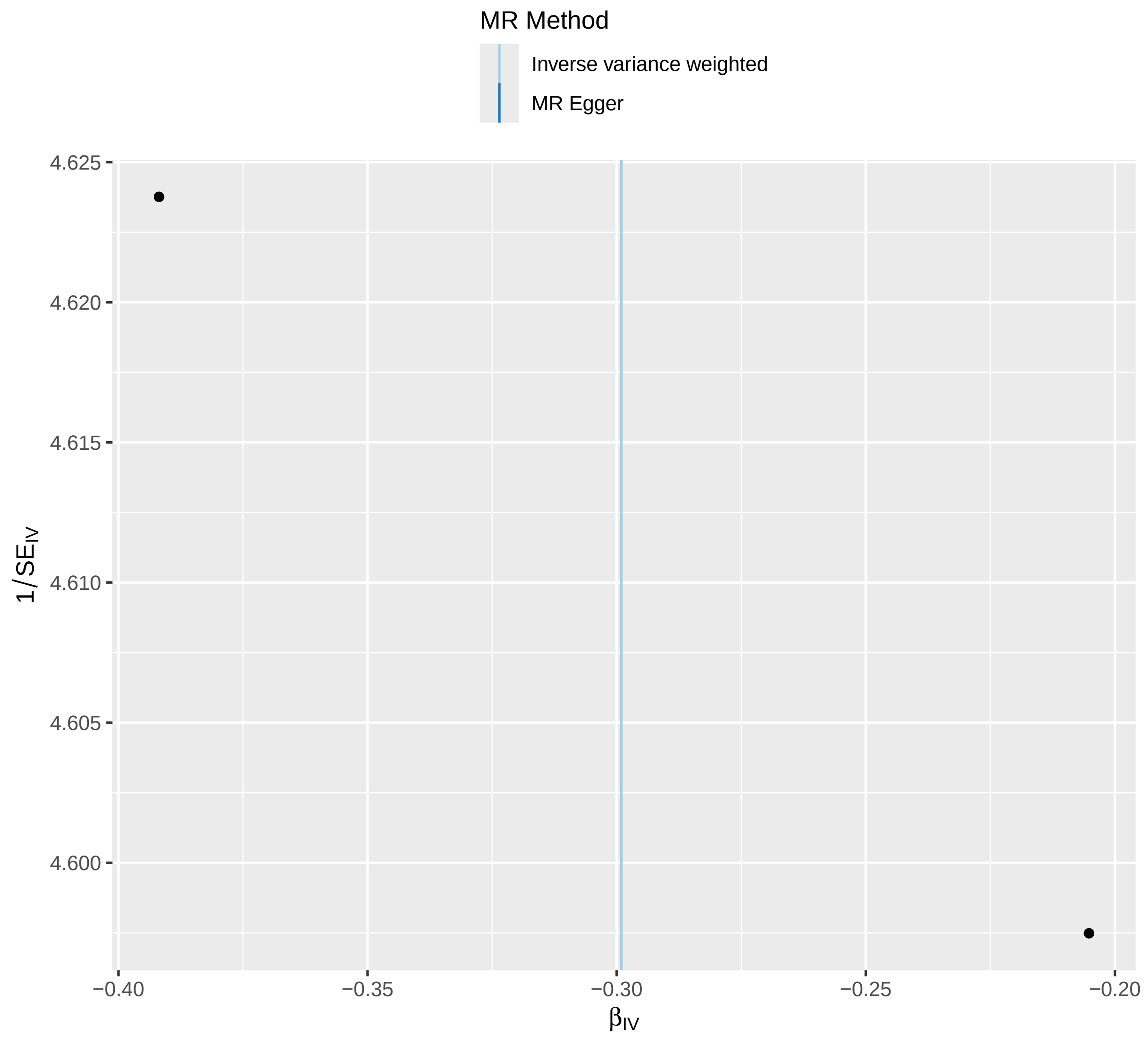
MR Method
Inverse variance weighted
MR Egger
4.625
4.620
4.615
4.610
4.605
4.600
1/SE$_{IV}$
−0.40
−0.35
−0.30
−0.25
−0.20
β$_{IV}$

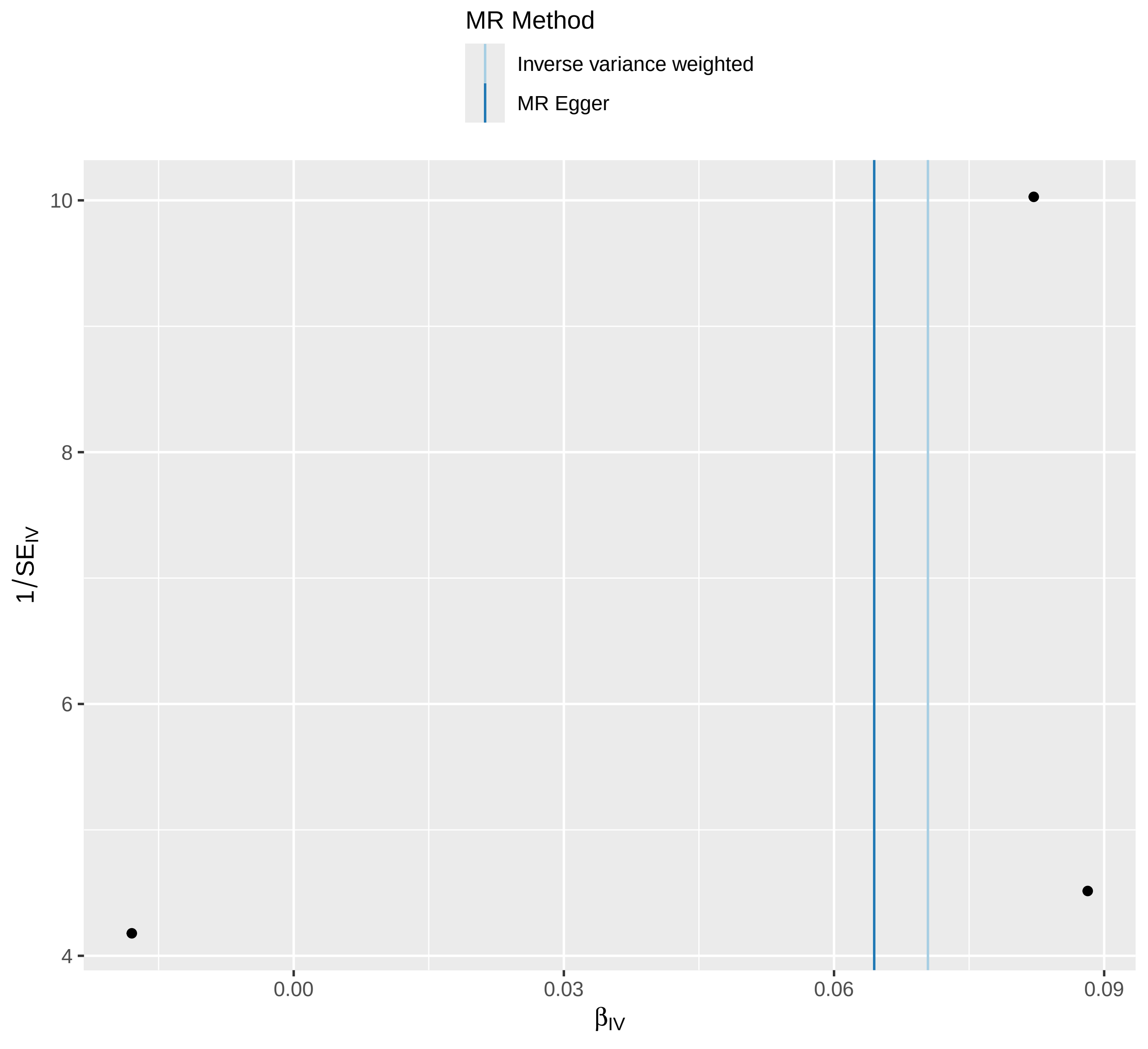
MR Method
Inverse variance weighted
MR Egger
10
8
6
4
1/SE_IV
0.00
0.03
0.06
0.09
β_IV

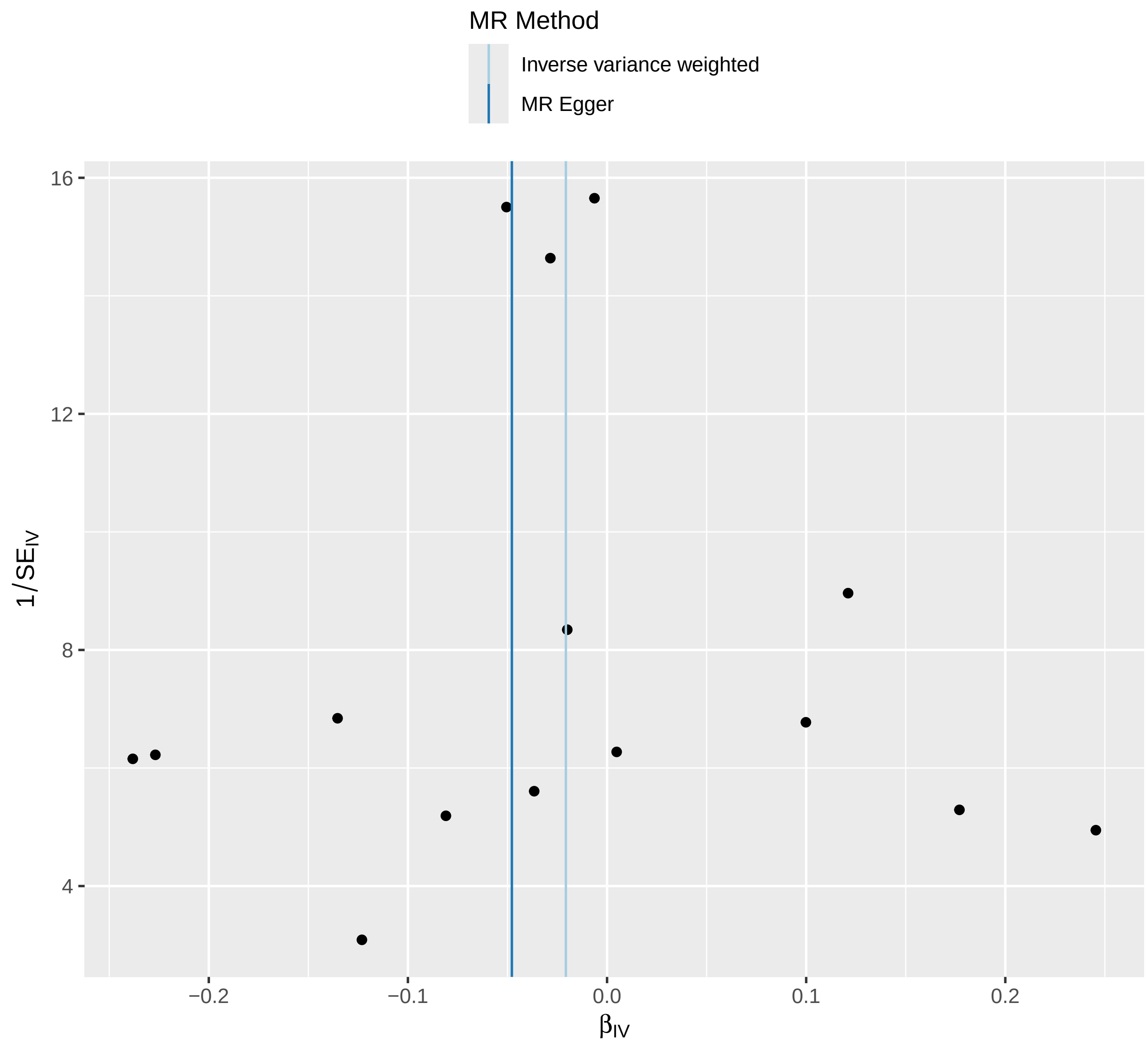
MR Method
Inverse variance weighted
MR Egger
16
12
8
4
$1/SE_{IV}$
−0.2
−0.1
0.0
0.1
0.2
$\beta_{IV}$

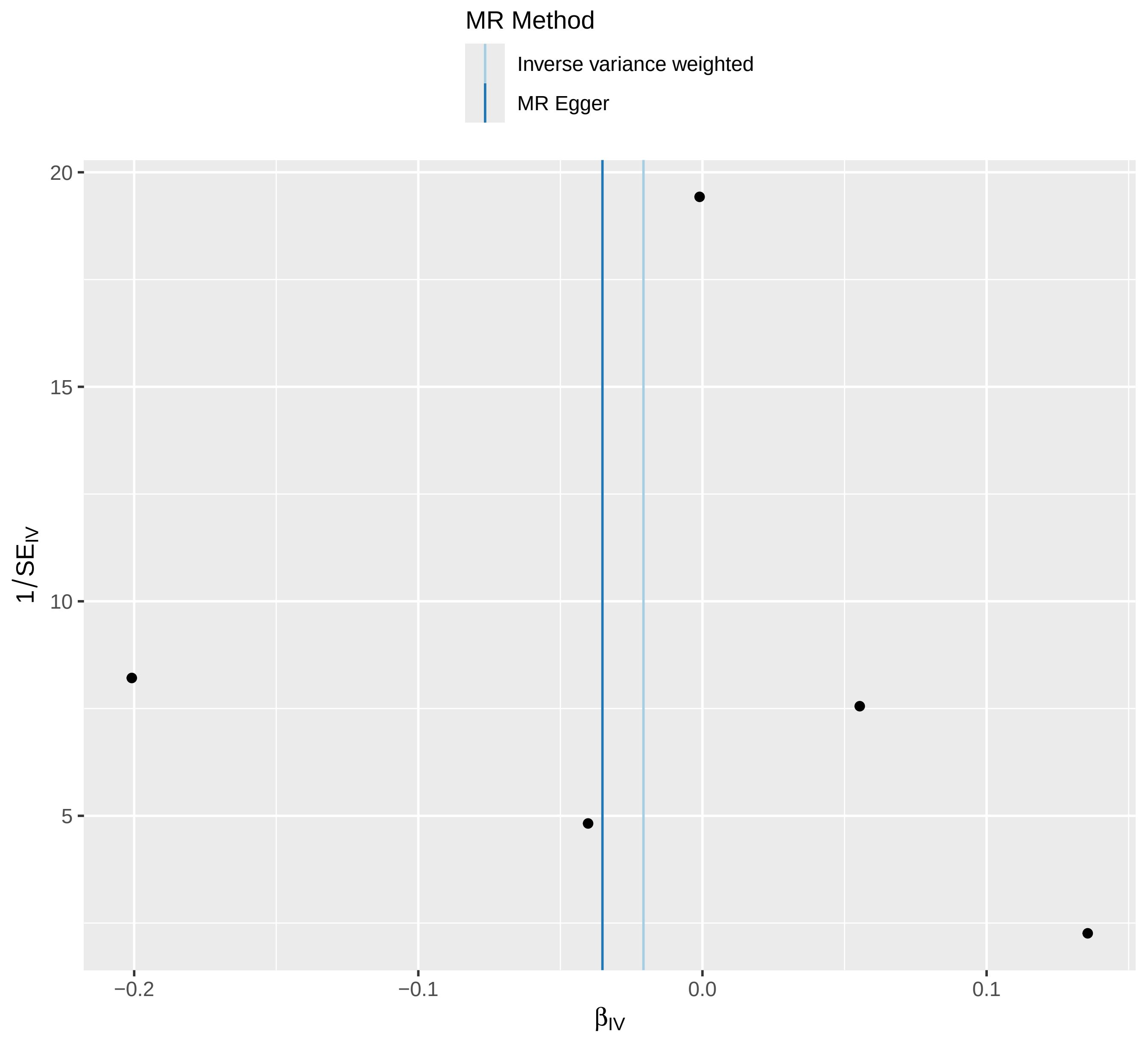

MR Method
Inverse variance weighted
MR Egger
20
15
10
5
$1/SE_{IV}$
−0.2
−0.1
0.0
0.1
$\beta_{IV}$

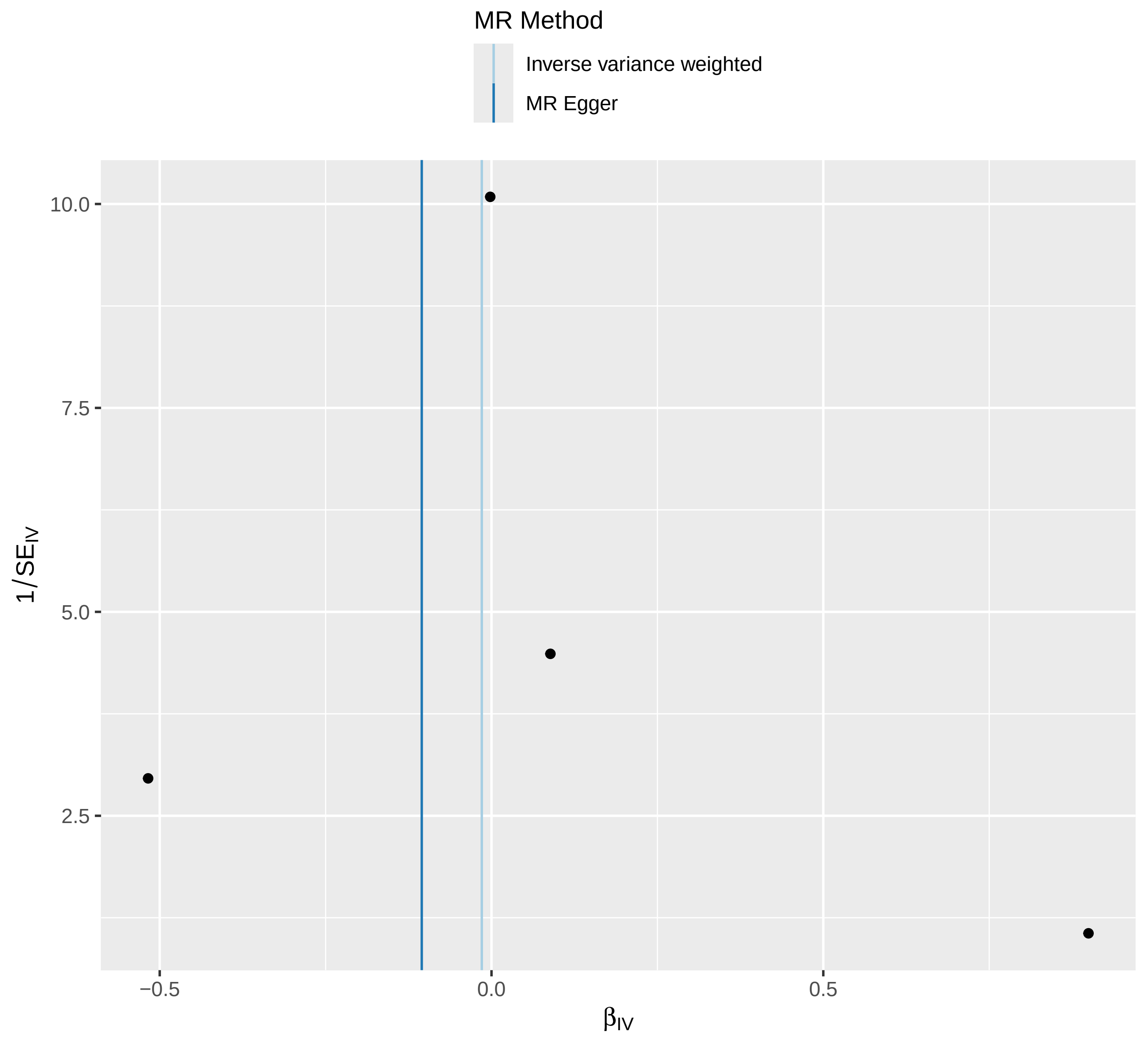
MR Method
Inverse variance weighted
MR Egger
10.0
7.5
5.0
2.5
1/SE_IV
−0.5
0.0
0.5
β_IV

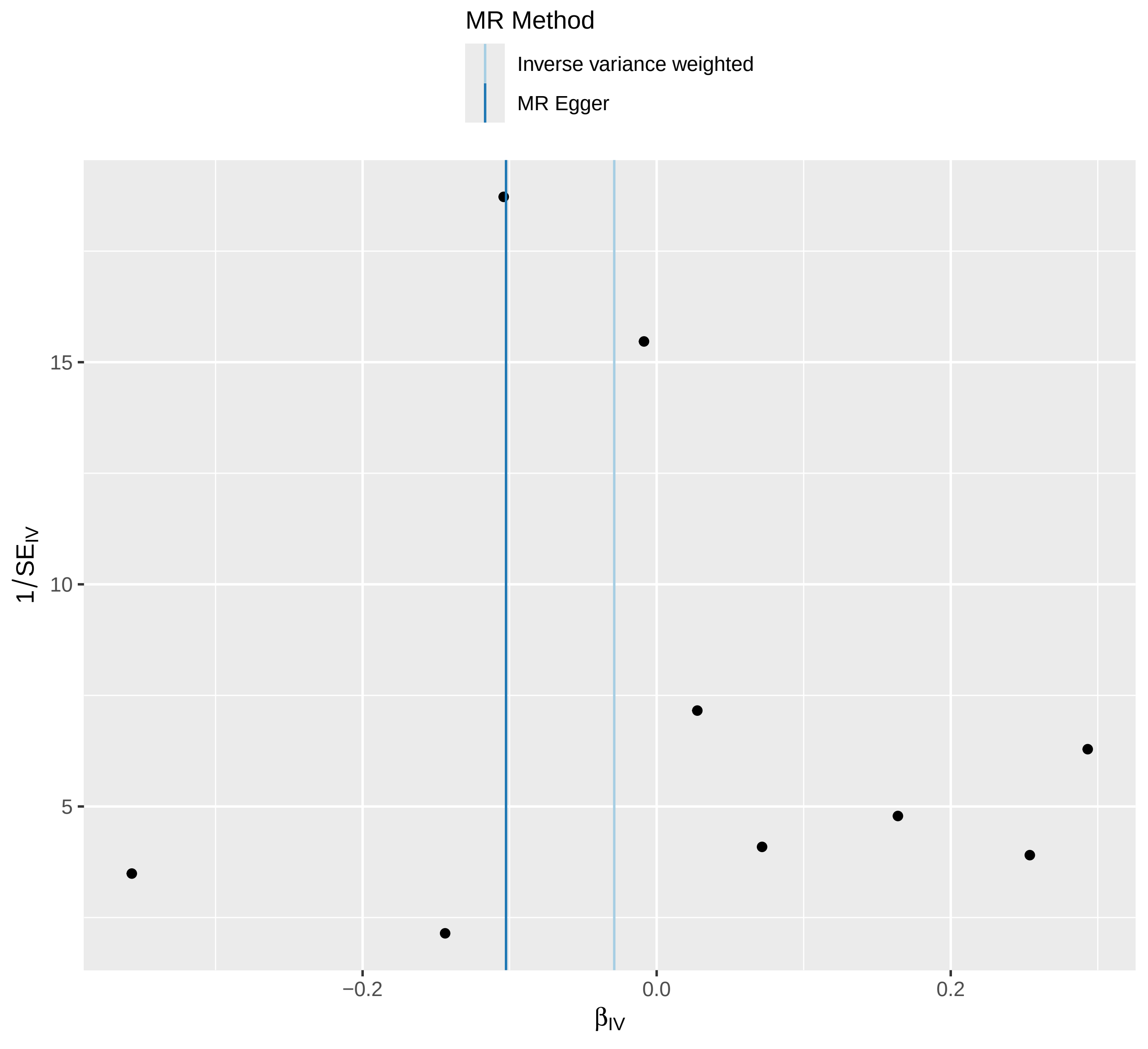
MR Method
Inverse variance weighted
MR Egger
15
10
5
1/SE$_{IV}$
−0.2
0.0
0.2
β$_{IV}$

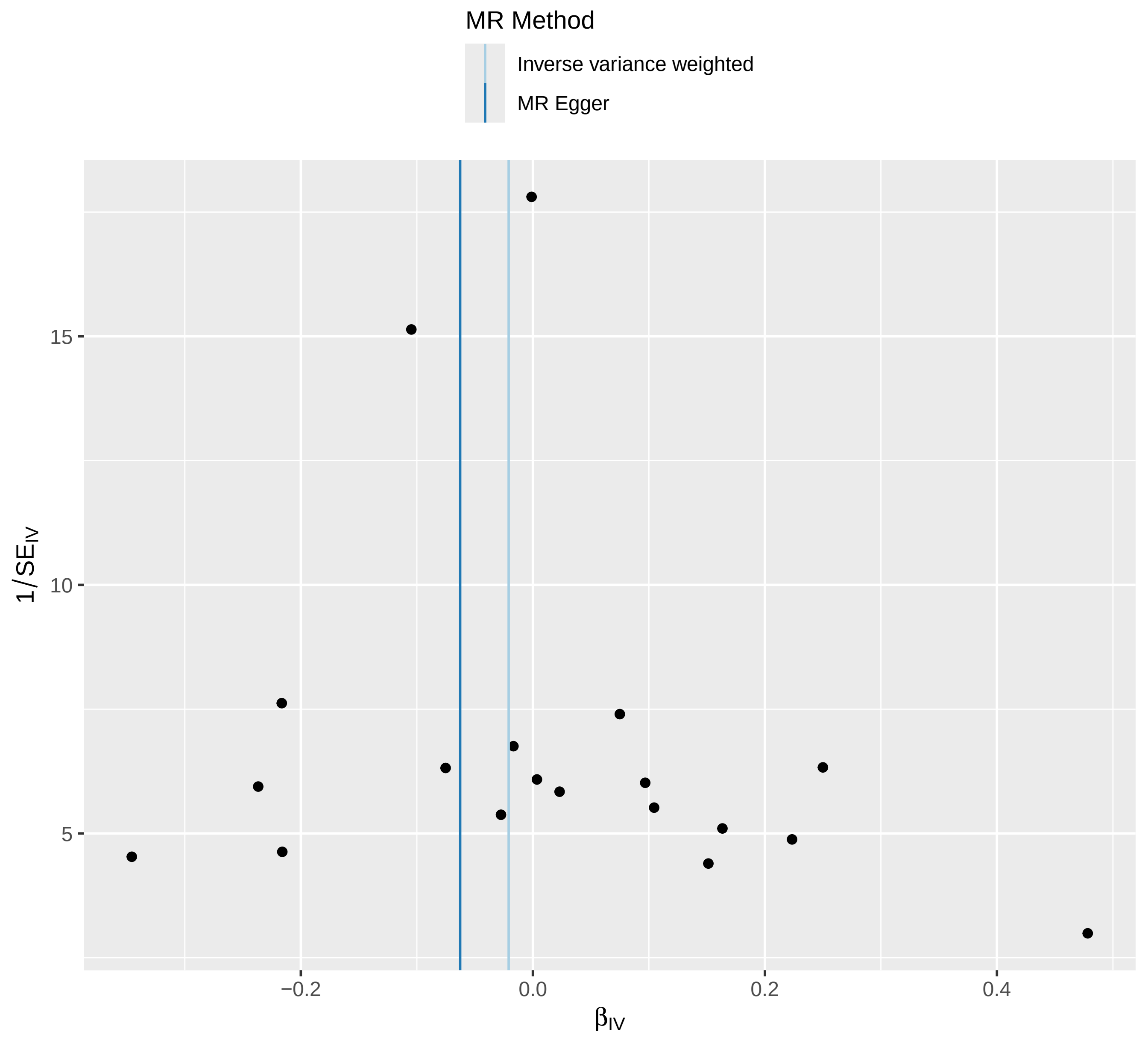

MR Method
Inverse variance weighted
MR Egger
15
10
5
1/SE$_{IV}$
−0.2
0.0
0.2
0.4
β$_{IV}$

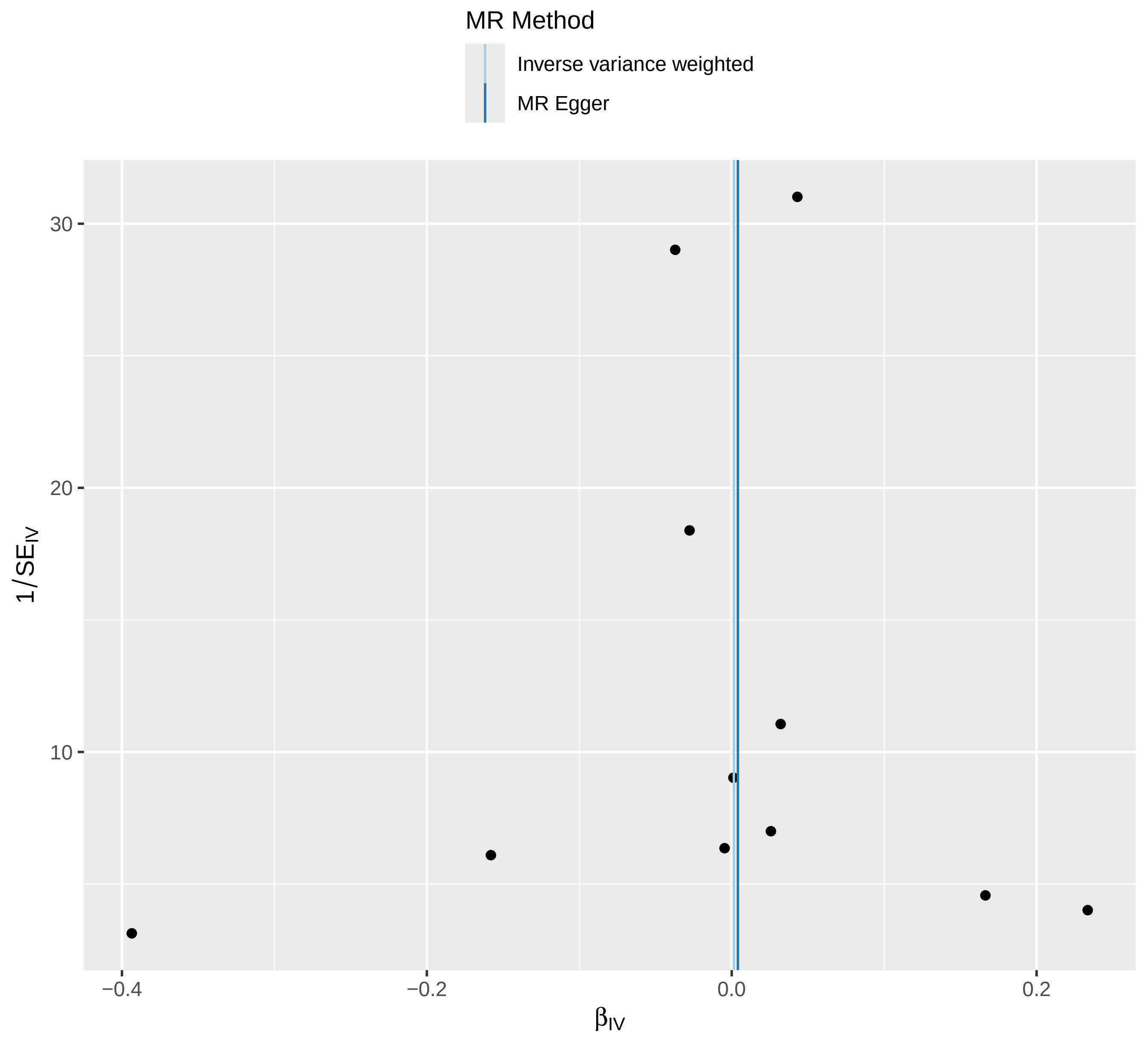
MR Method
Inverse variance weighted
MR Egger
30
20
10
1/SE$_{IV}$
−0.4
−0.2
0.0
0.2
β$_{IV}$

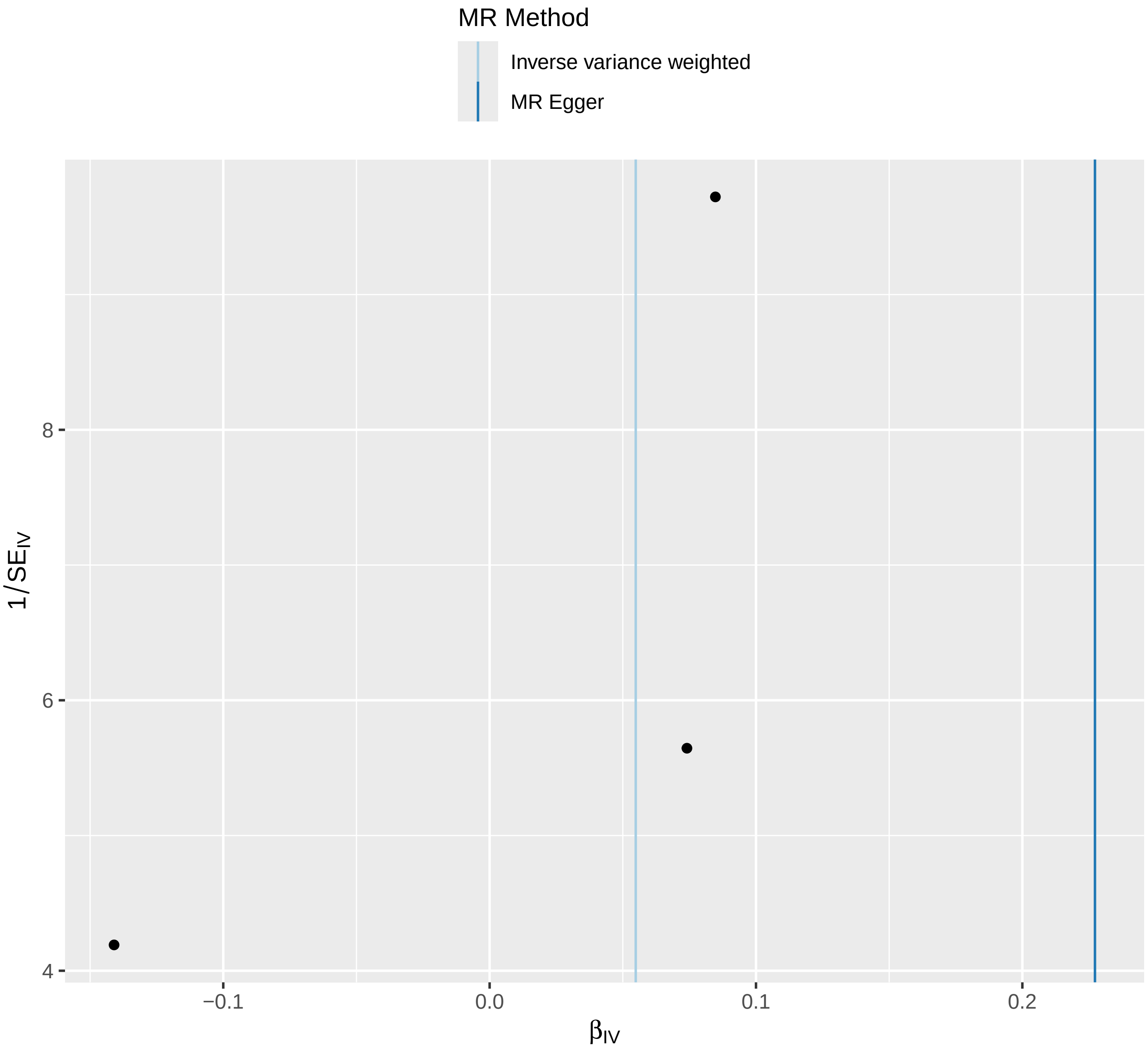
MR Method
Inverse variance weighted
MR Egger
8
6
4
−0.1
0.0
0.1
0.2
$1/SE_{IV}$
$\beta_{IV}$

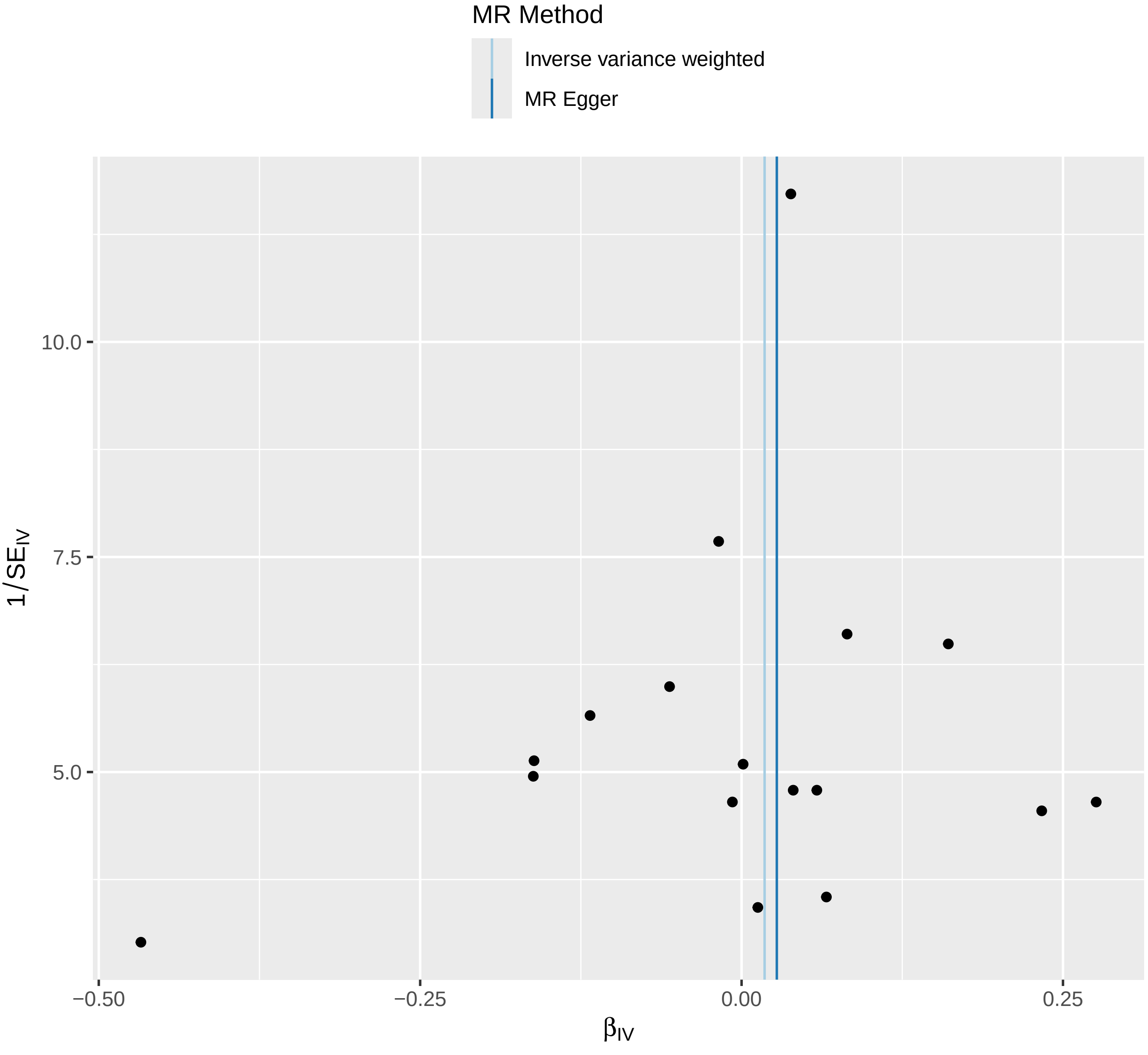

MR Method
Inverse variance weighted
MR Egger
10.0
7.5
5.0
$1/SE_{IV}$
−0.50
−0.25
0.00
0.25
$\beta_{IV}$

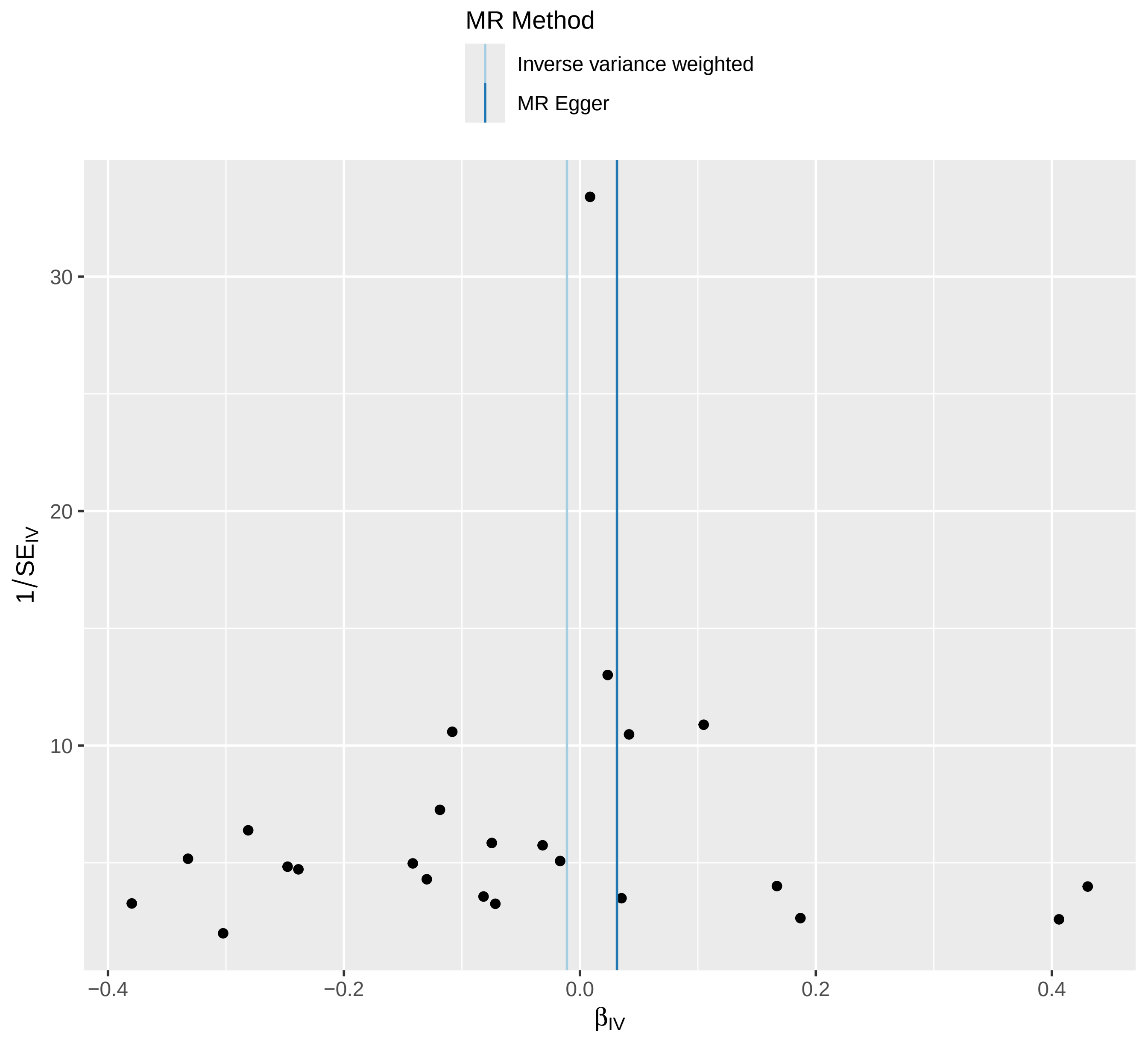
MR Method
Inverse variance weighted
MR Egger
30
20
10
1/SE$_{IV}$
−0.4
−0.2
0.0
0.2
0.4
β$_{IV}$

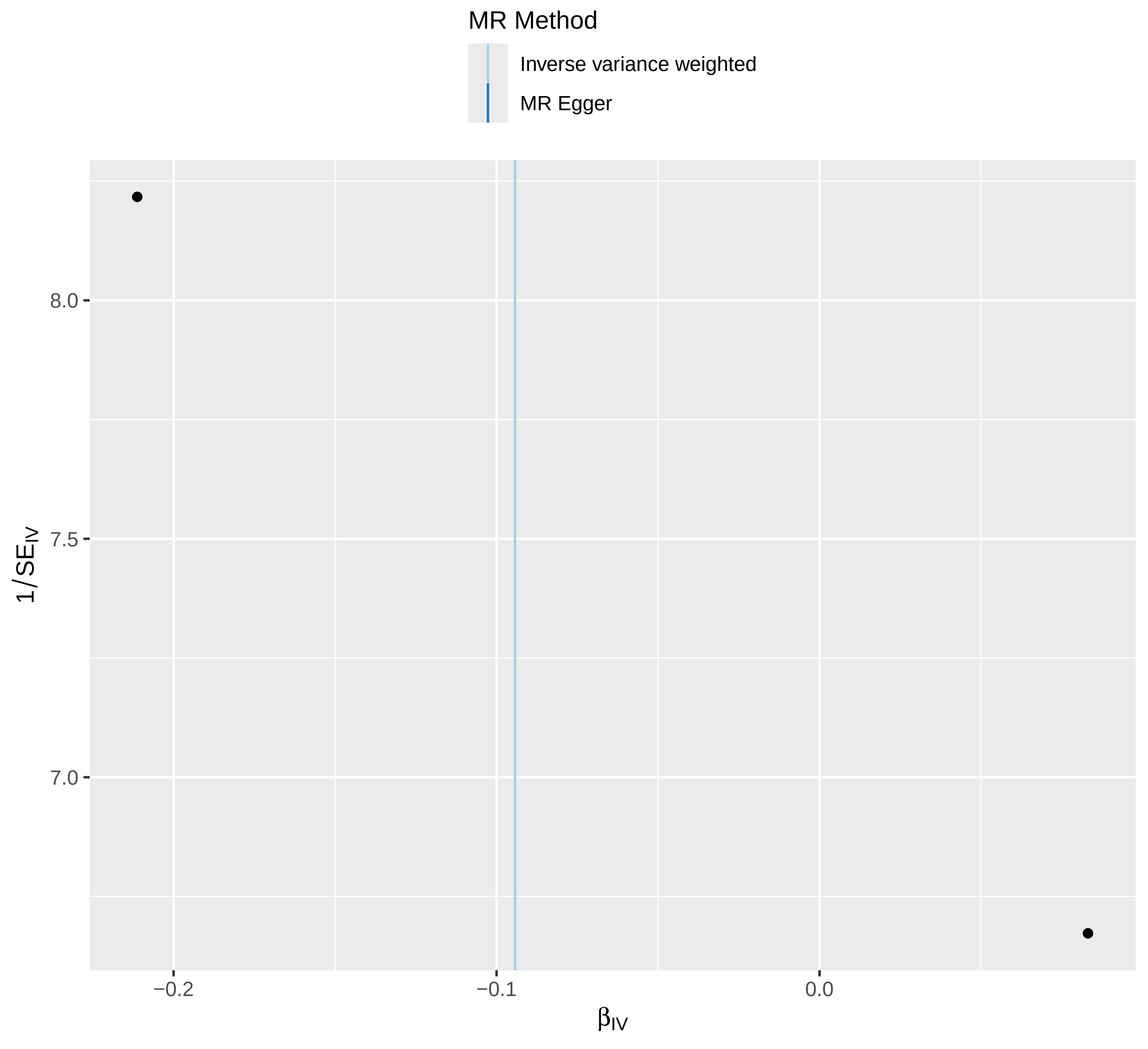
MR Method
Inverse variance weighted
MR Egger
8.0
7.5
7.0
1/$SE_{IV}$
−0.2
−0.1
0.0
$\beta_{IV}$

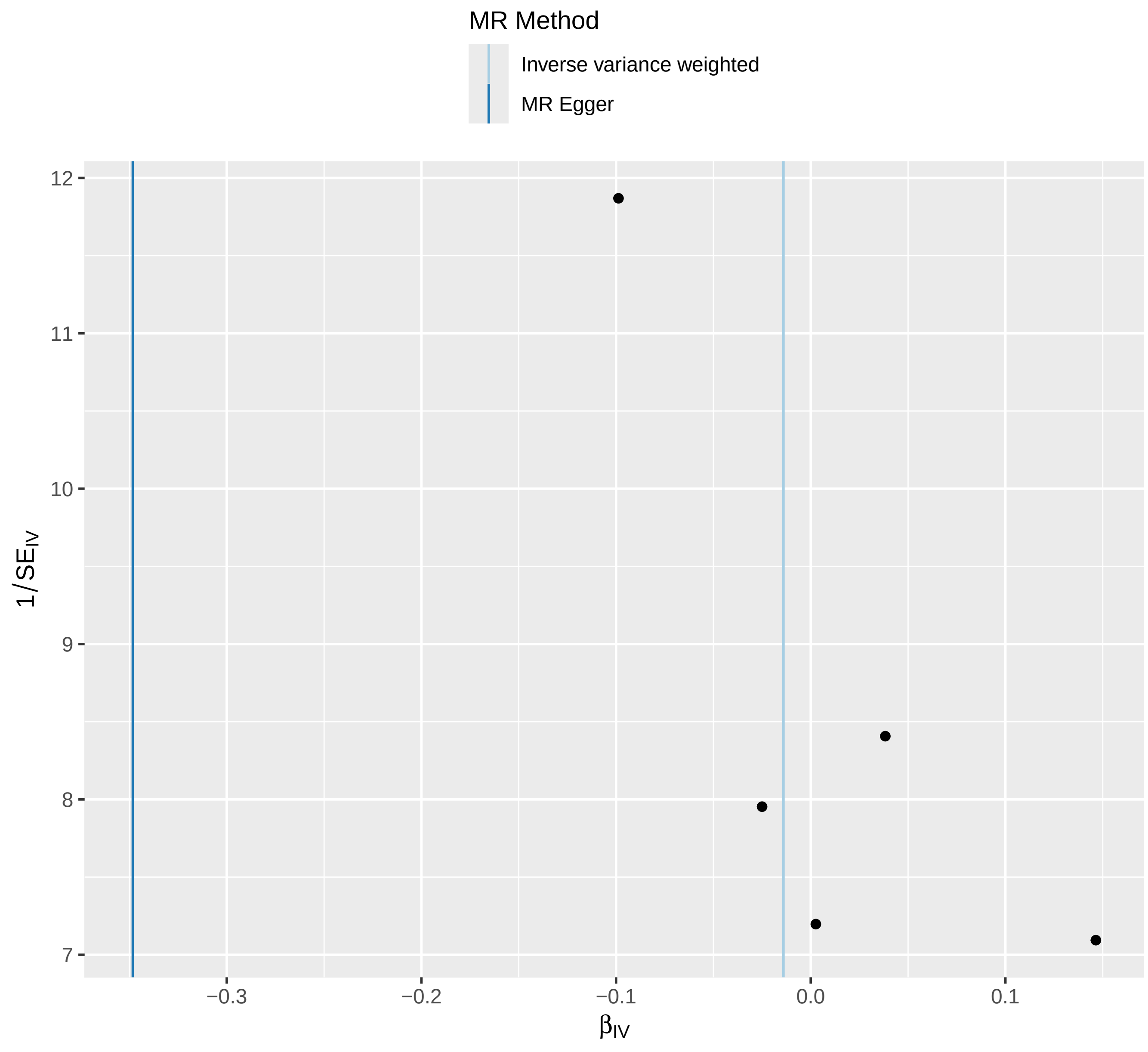
MR Method
Inverse variance weighted
MR Egger
12
11
10
9
8
7
1/SE$_{IV}$
−0.3
−0.2
−0.1
0.0
0.1
β$_{IV}$

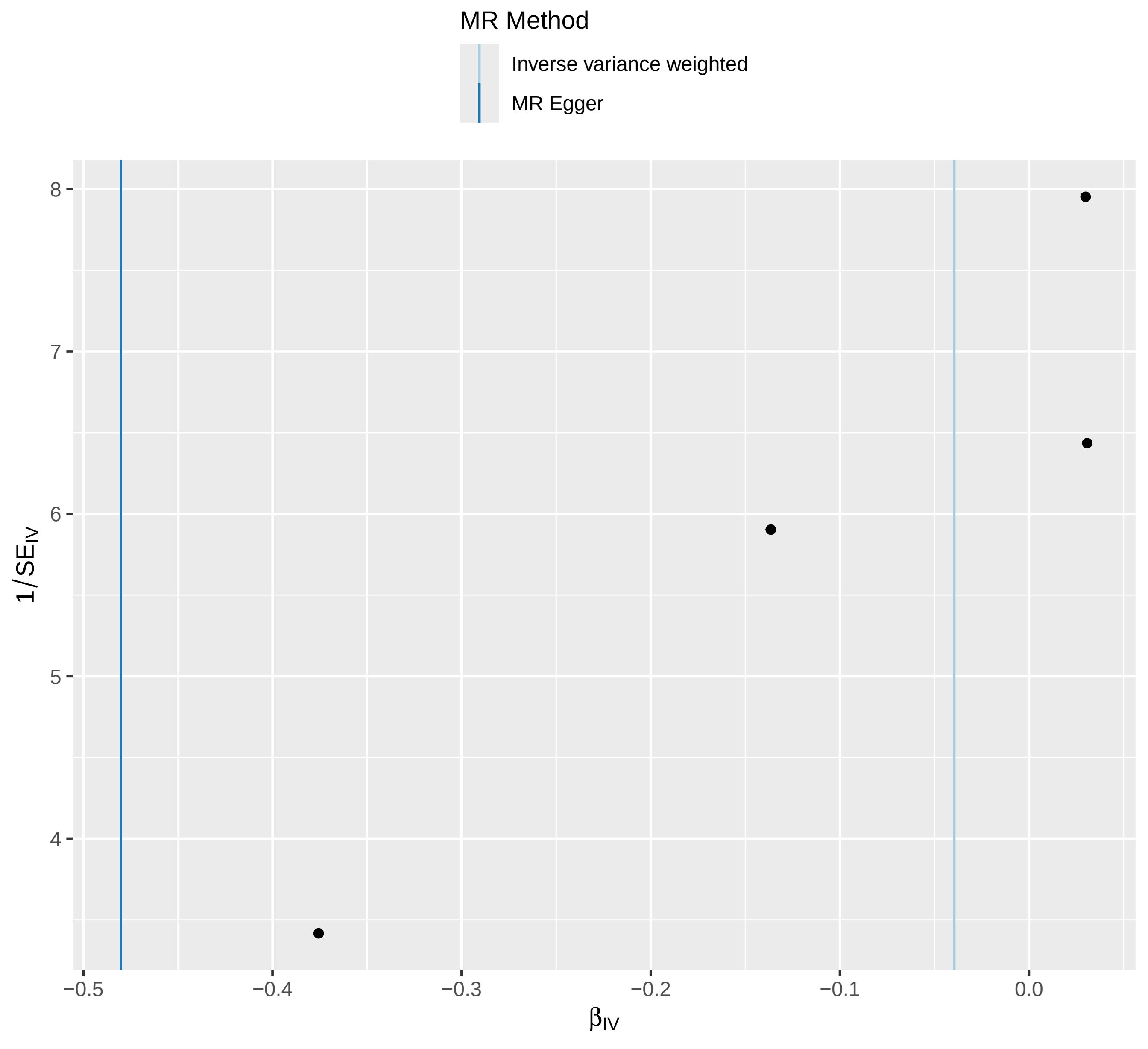
MR Method
Inverse variance weighted
MR Egger
8
7
6
5
4
1/SE$_{IV}$
−0.5
−0.4
−0.3
−0.2
−0.1
0.0
$\beta_{IV}$

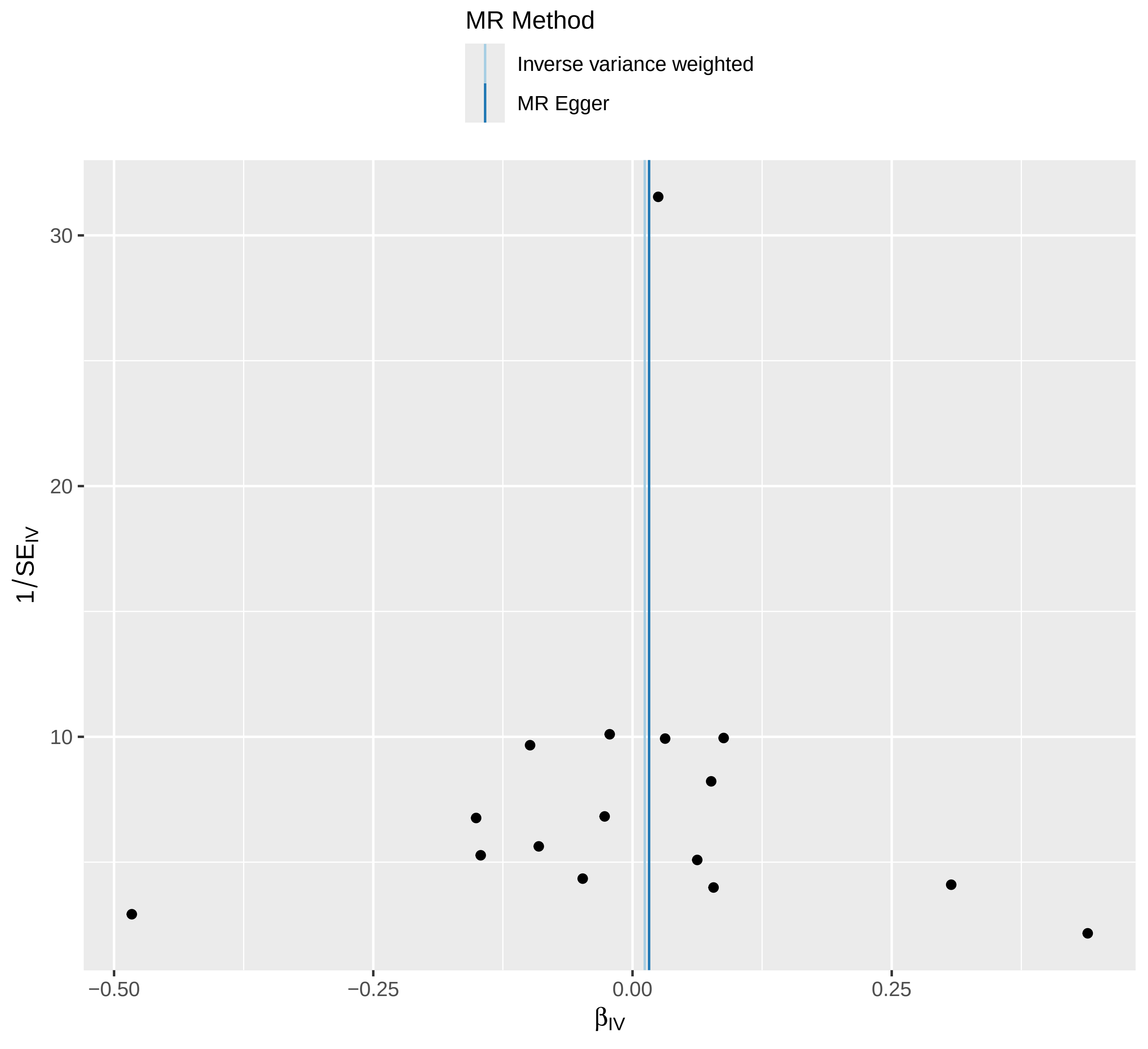
MR Method
Inverse variance weighted
MR Egger
30
20
10
1/SE$_{IV}$
−0.50
−0.25
0.00
0.25
β$_{IV}$

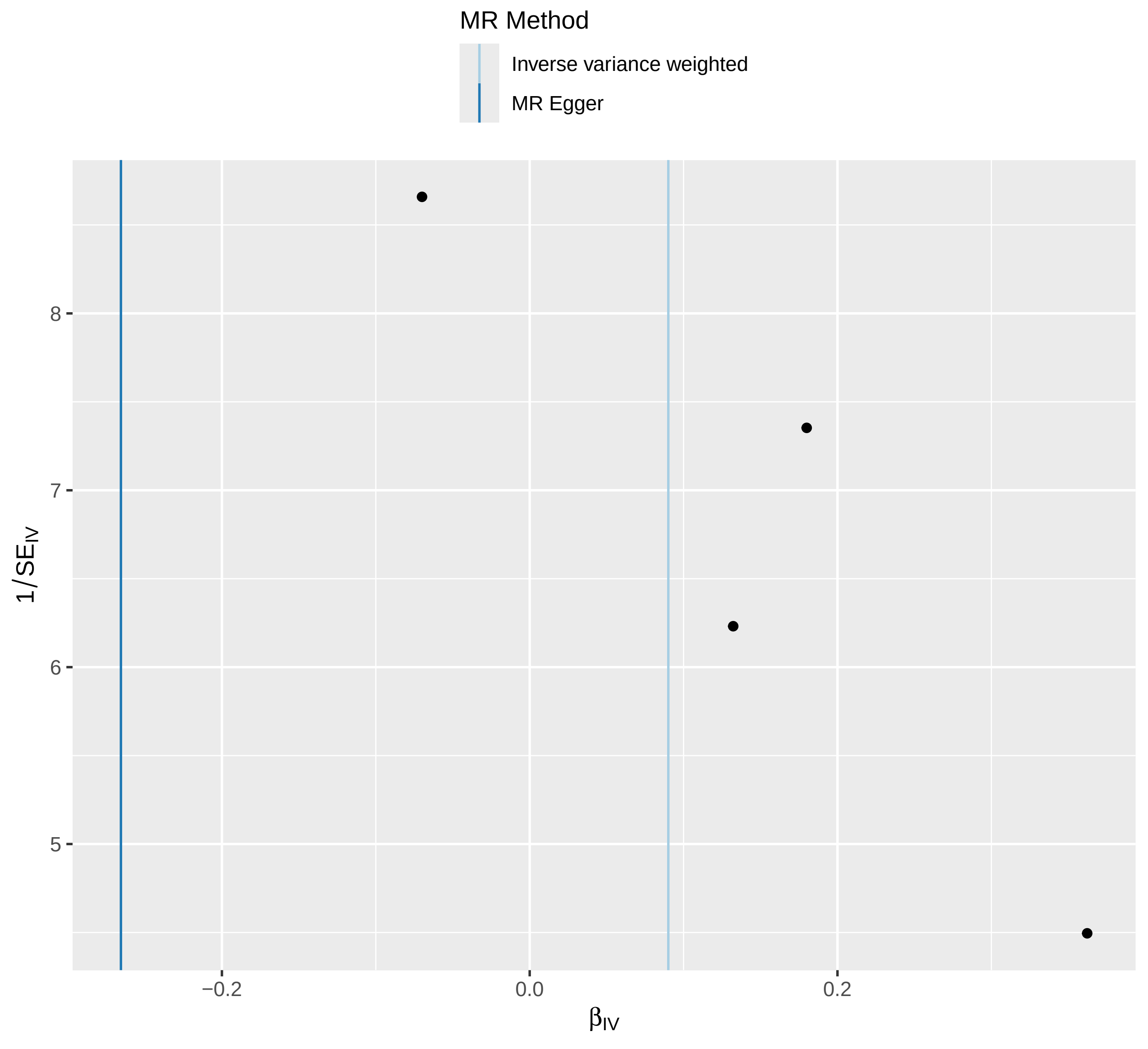

MR Method
Inverse variance weighted
MR Egger
8
7
6
5
1/SE$_{IV}$
−0.2
0.0
0.2
β$_{IV}$

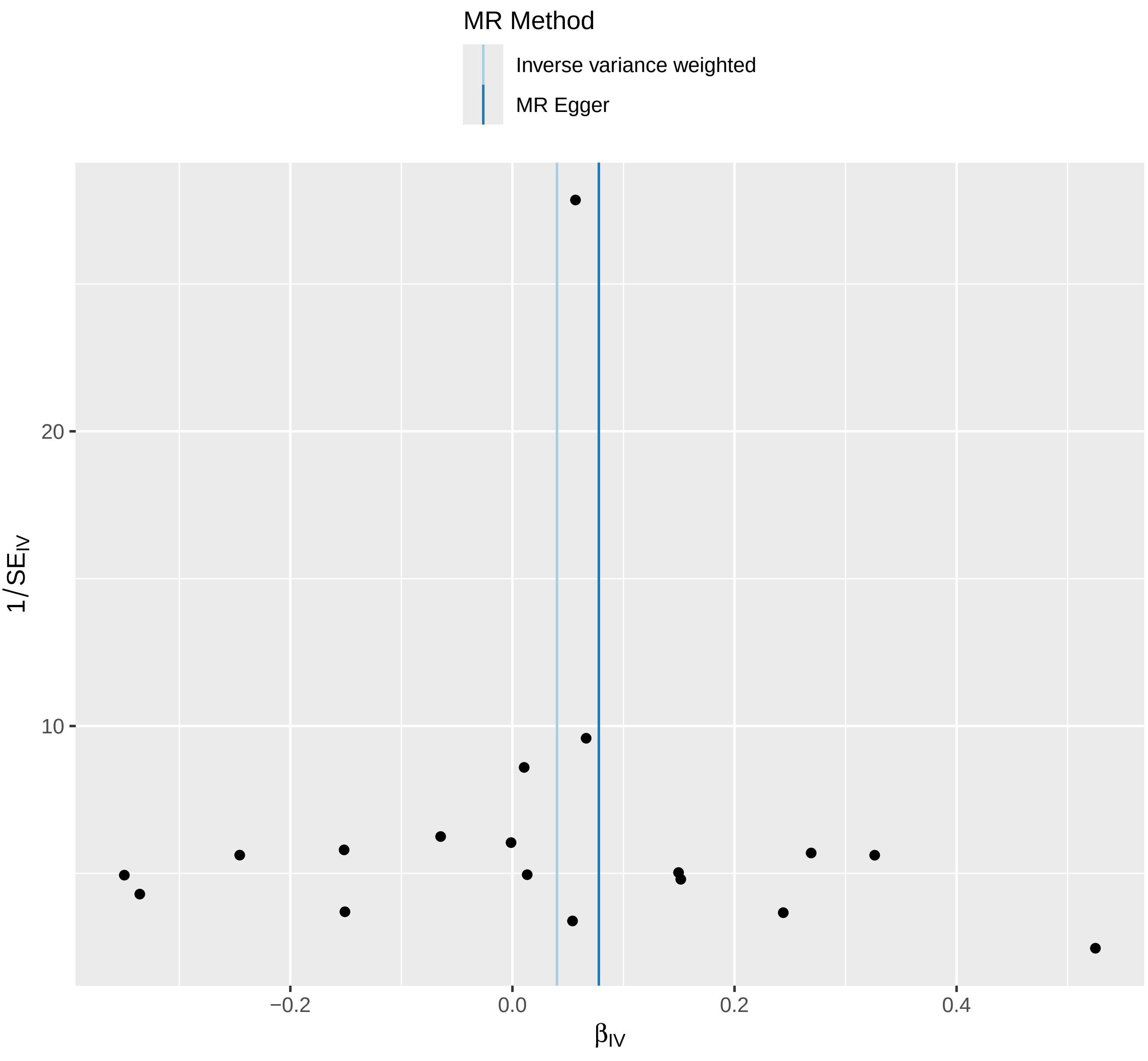
MR Method
Inverse variance weighted
MR Egger
20
10
1/SE$_{IV}$
−0.2
0.0
0.2
0.4
β$_{IV}$

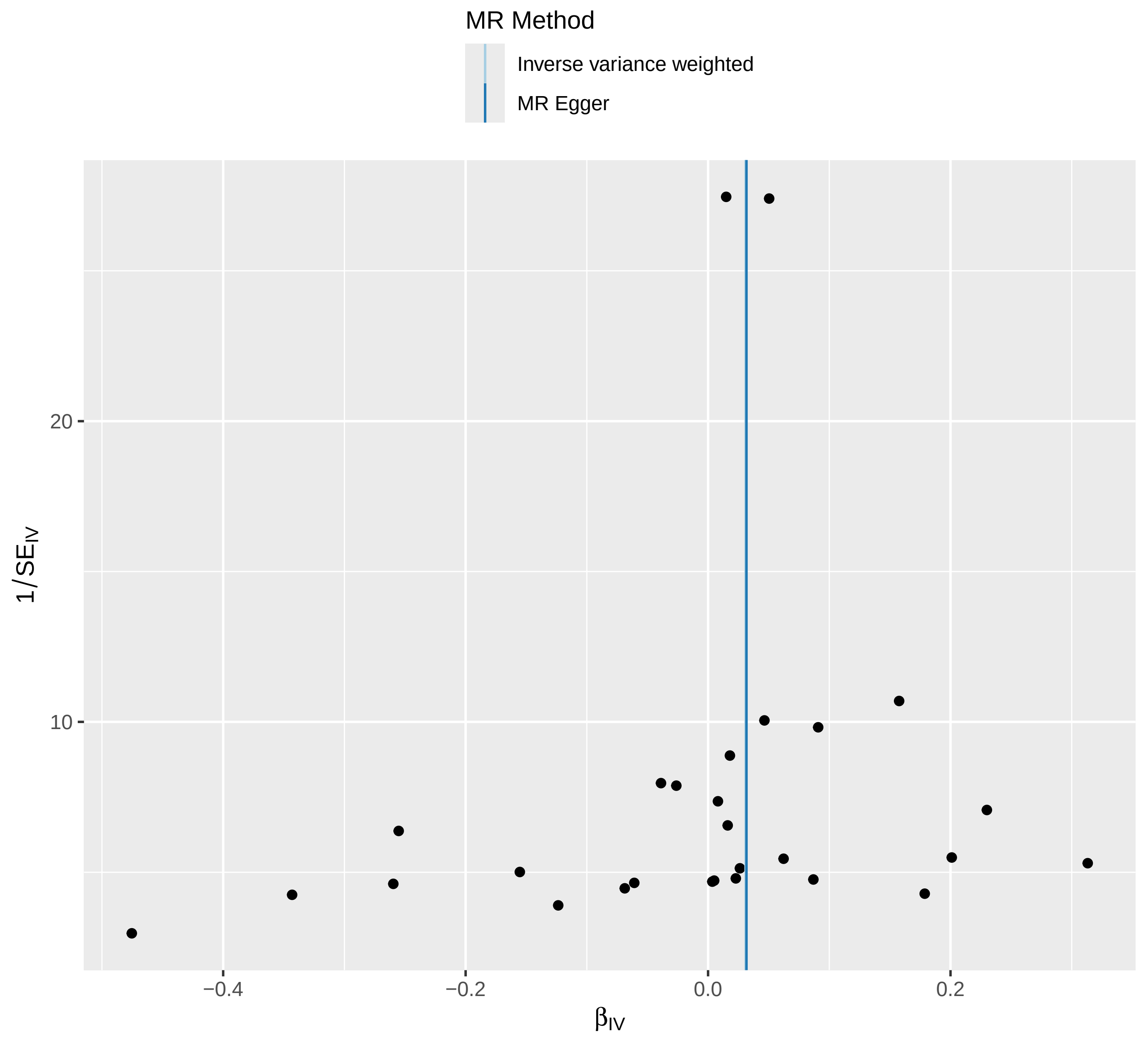

MR Method
Inverse variance weighted
MR Egger
20
10
1/SE_IV
−0.4
−0.2
0.0
0.2
β_IV

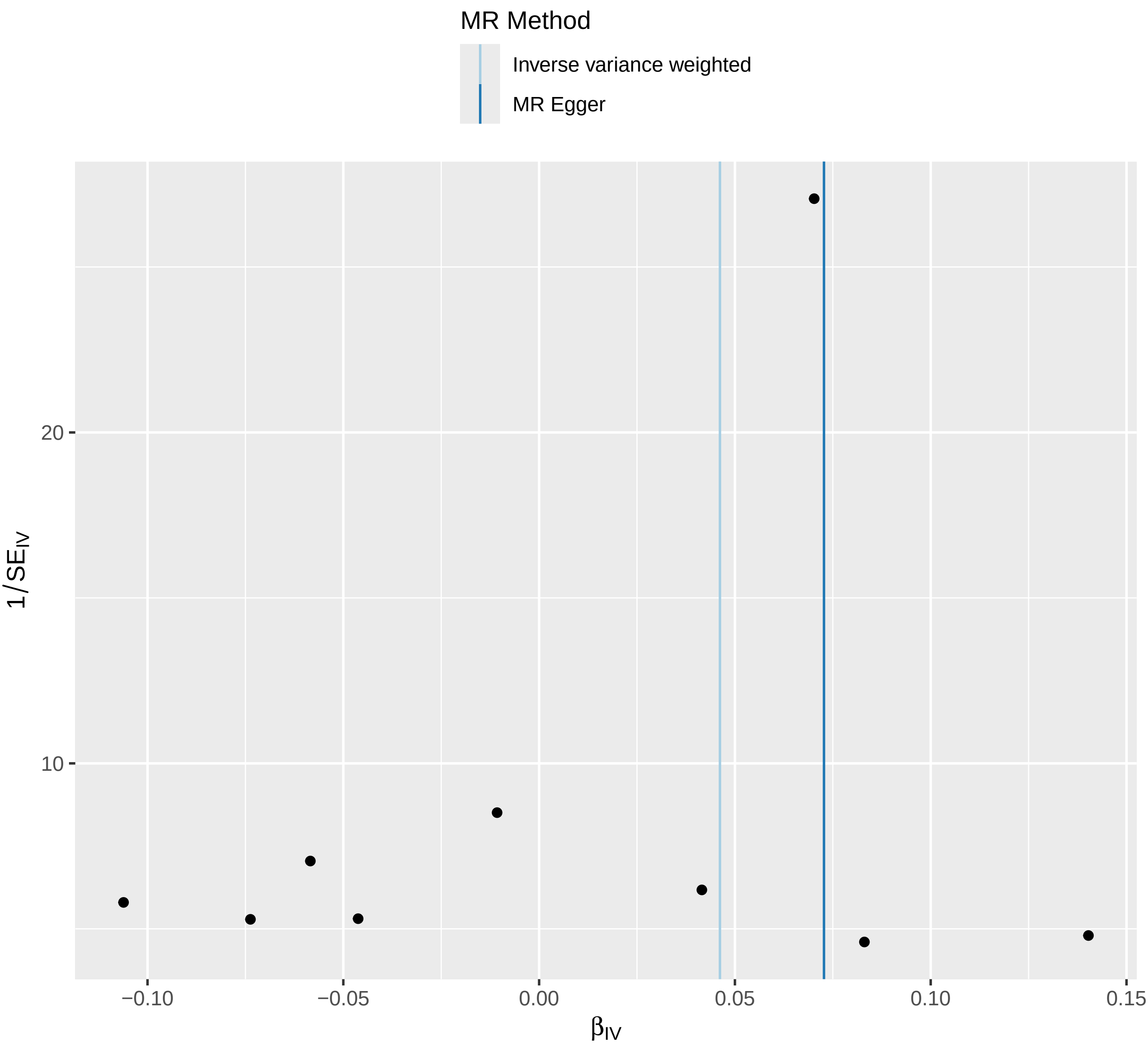
MR Method
Inverse variance weighted
MR Egger
20
10
$1/SE_{IV}$
−0.10
−0.05
0.00
0.05
0.10
0.15
$\beta_{IV}$

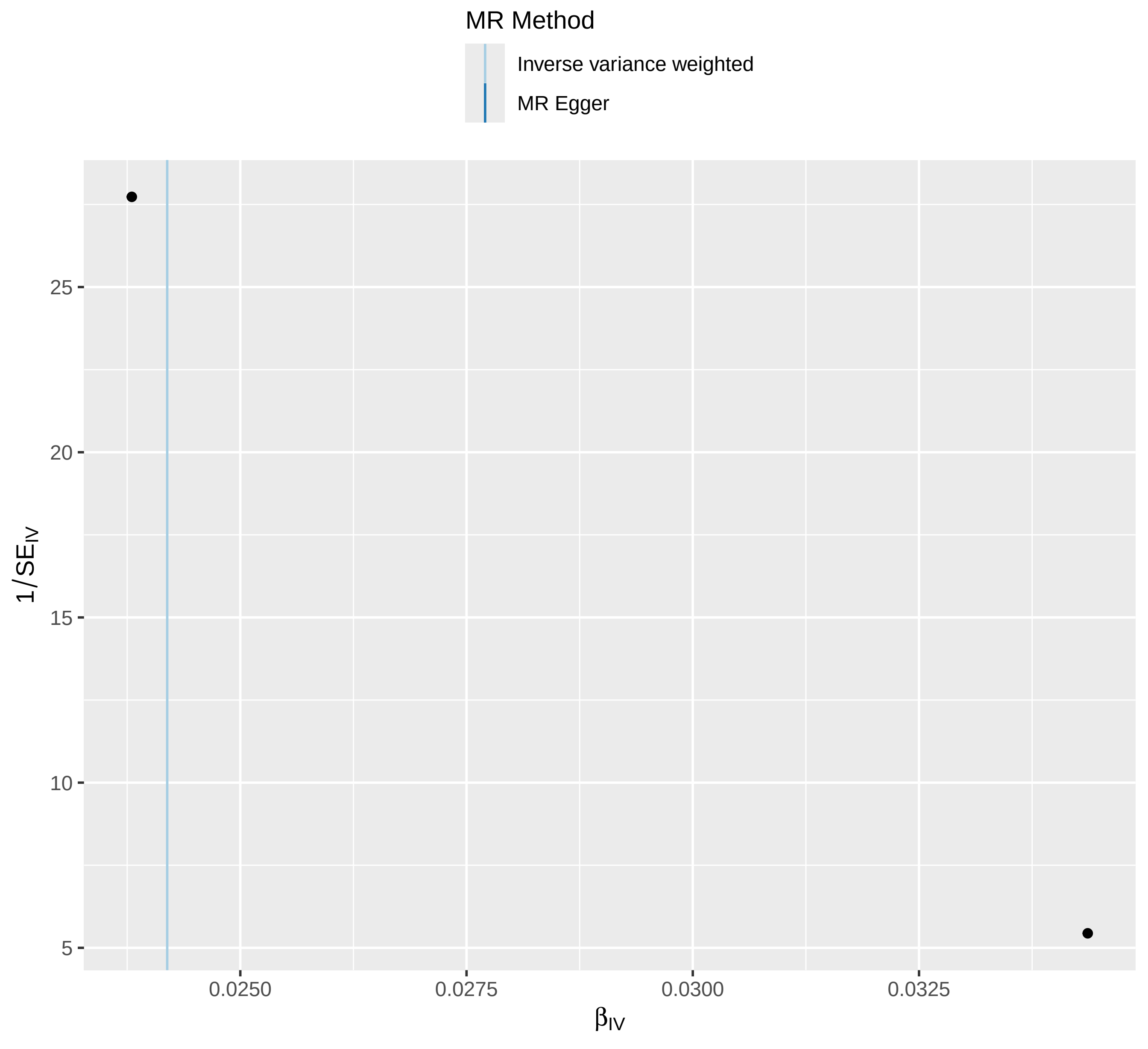
MR Method
Inverse variance weighted
MR Egger
1/SE_IV
25
20
15
10
5
0.0250
0.0275
0.0300
0.0325
β_IV

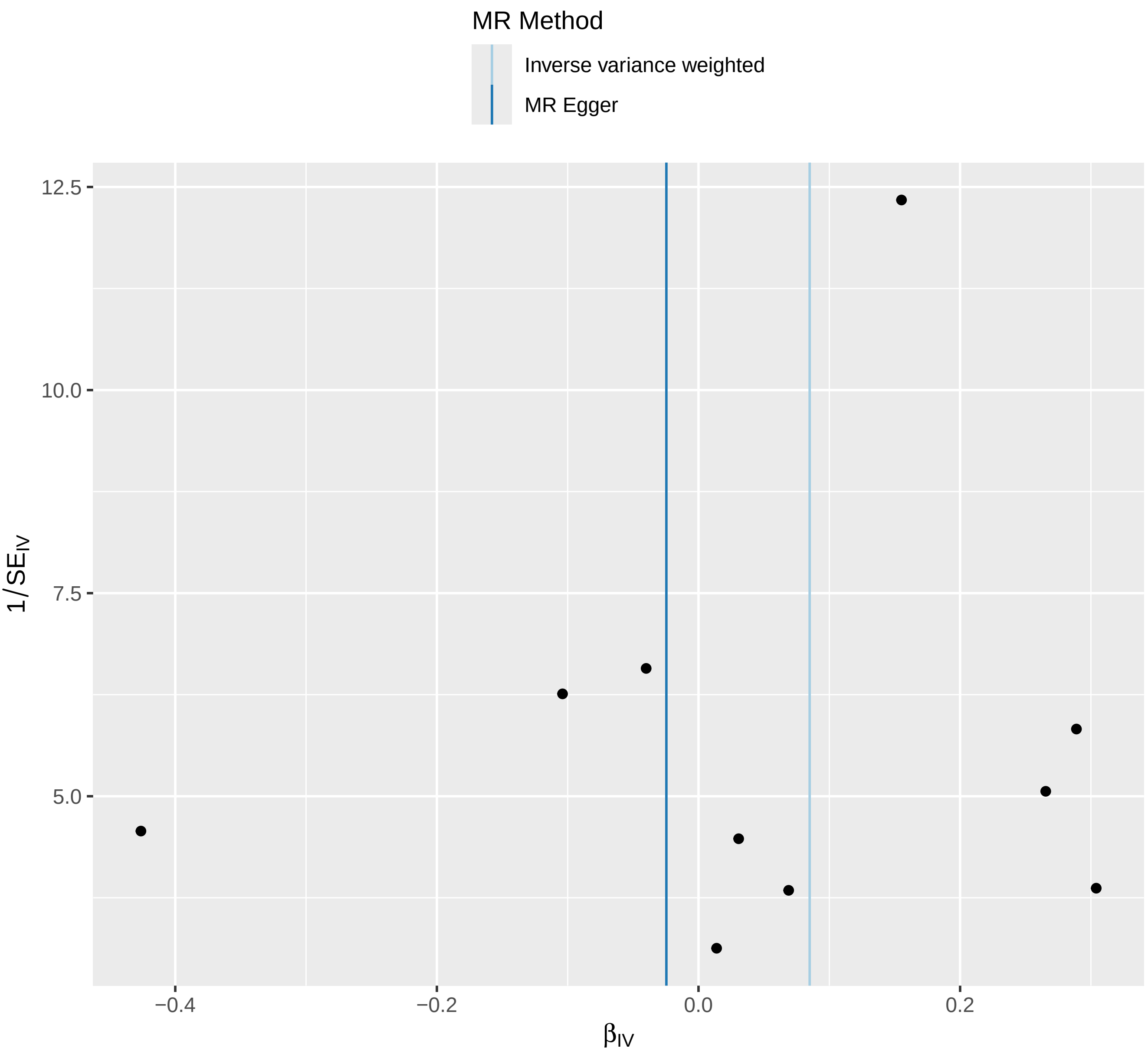

MR Method
Inverse variance weighted
MR Egger
12.5
10.0
7.5
5.0
1/SE$_{IV}$
−0.4
−0.2
0.0
0.2
β$_{IV}$

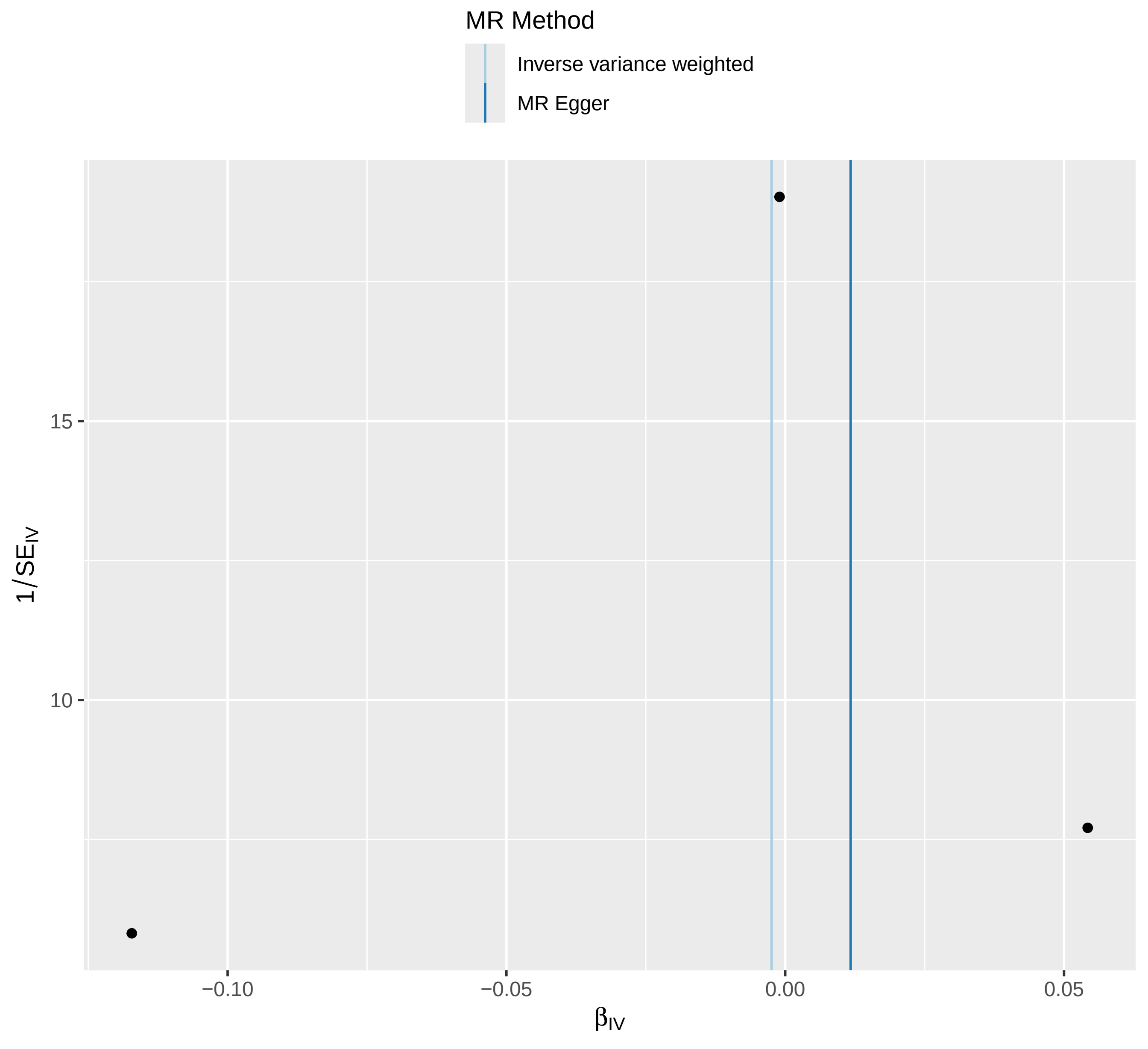

MR Method
Inverse variance weighted
MR Egger
15
10
1/SE_IV
−0.10
−0.05
0.00
0.05
β_IV

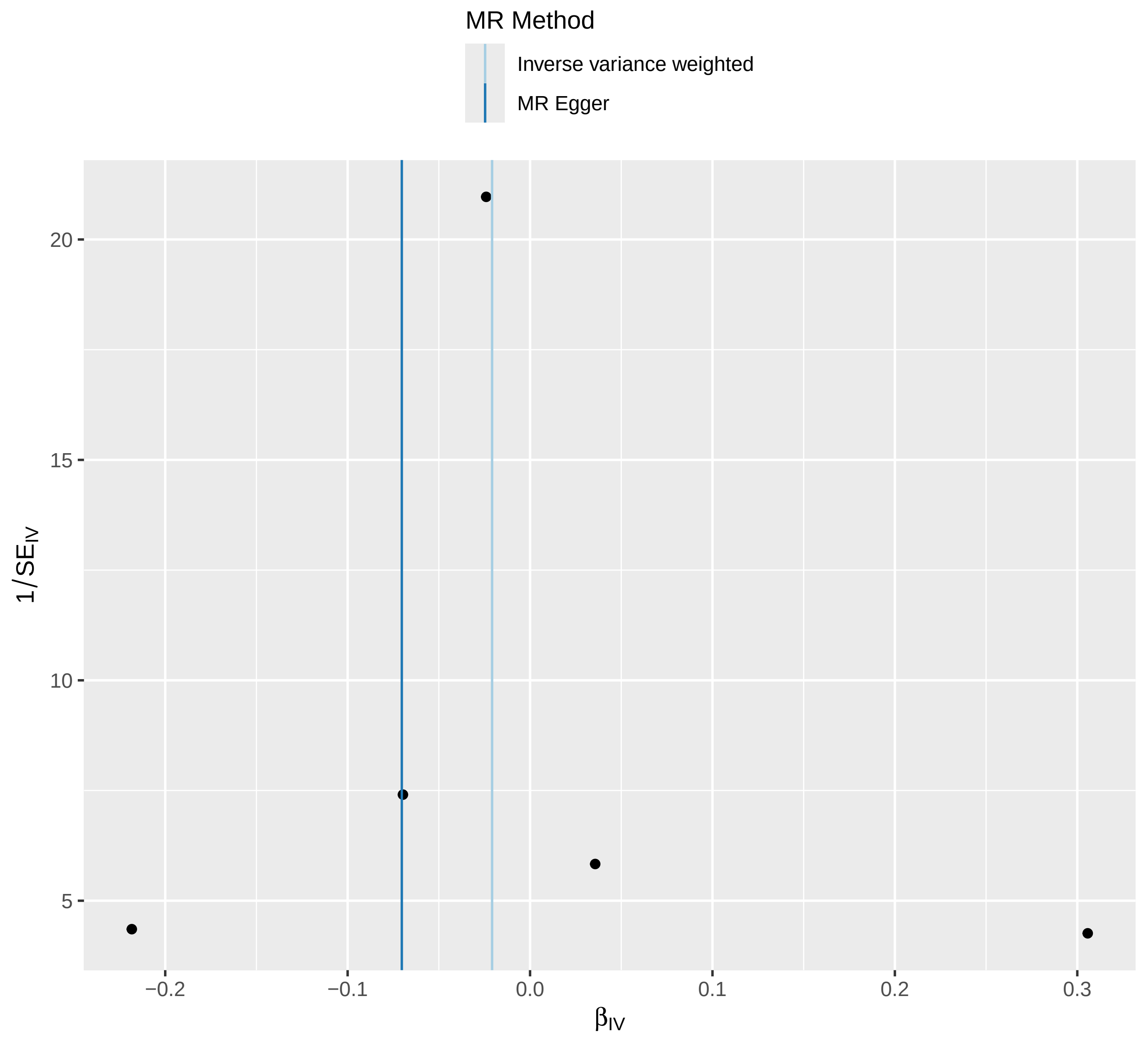
MR Method
Inverse variance weighted
MR Egger
20
15
10
5
1/SE_IV
−0.2
−0.1
0.0
0.1
0.2
0.3
β_IV

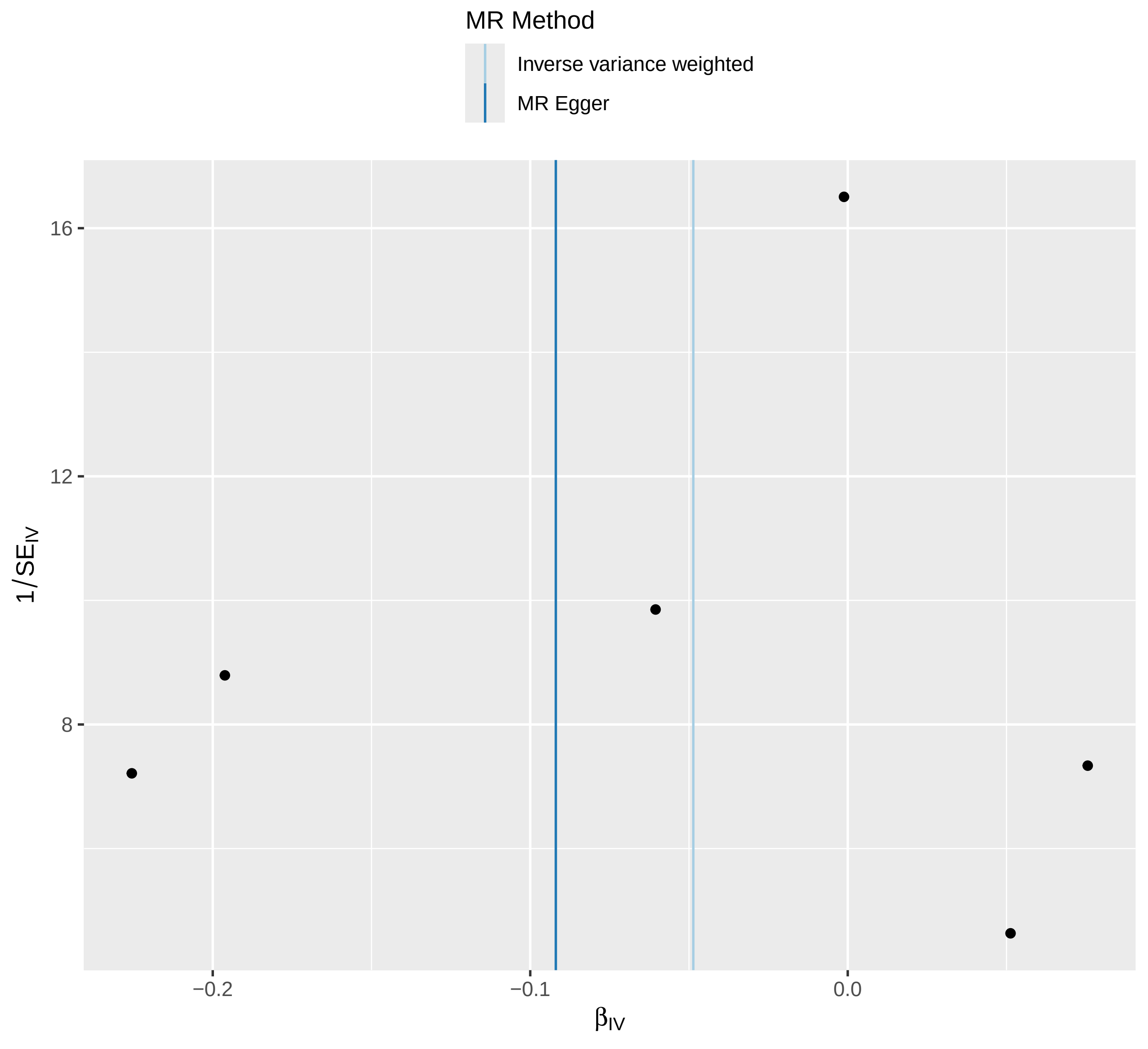
MR Method
Inverse variance weighted
MR Egger
16
12
8
1/SE$_{IV}$
−0.2
−0.1
0.0
β$_{IV}$

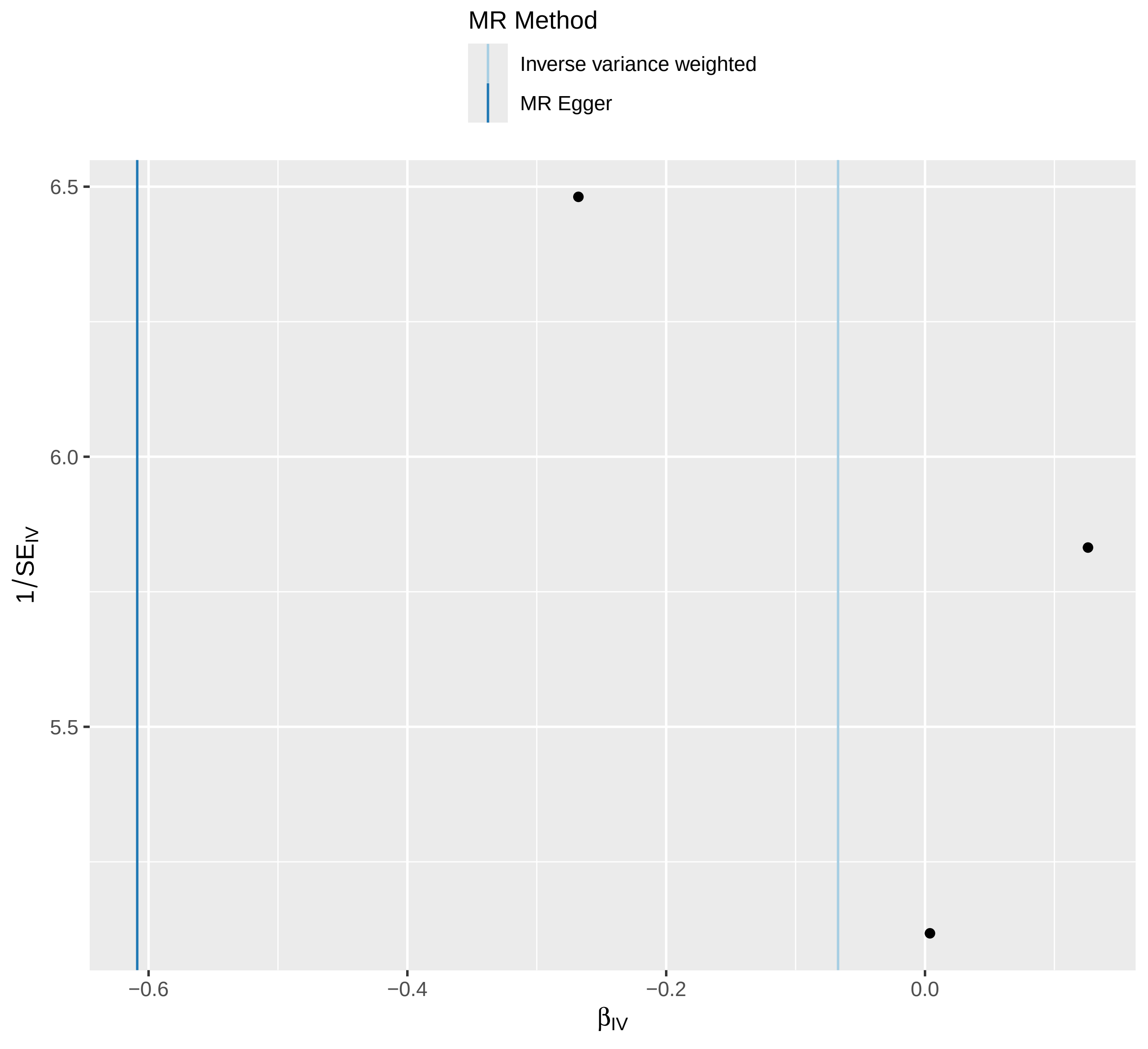
MR Method
Inverse variance weighted
MR Egger
6.5
6.0
5.5
$1/SE_{IV}$
−0.6
−0.4
−0.2
0.0
$\beta_{IV}$

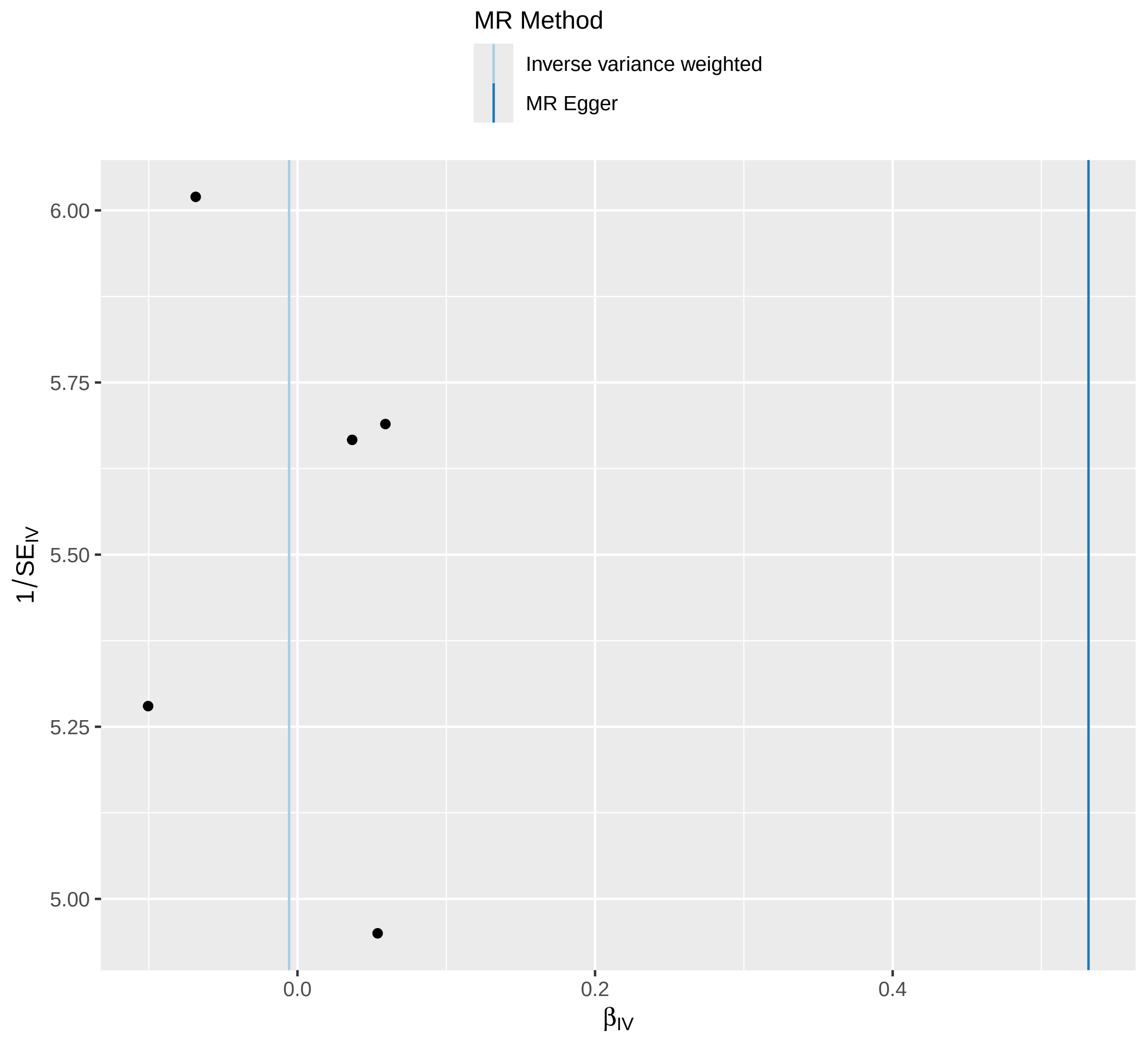

MR Method
Inverse variance weighted
MR Egger
6.00
5.75
5.50
5.25
5.00
1/SE_IV
0.0
0.2
0.4
β_IV

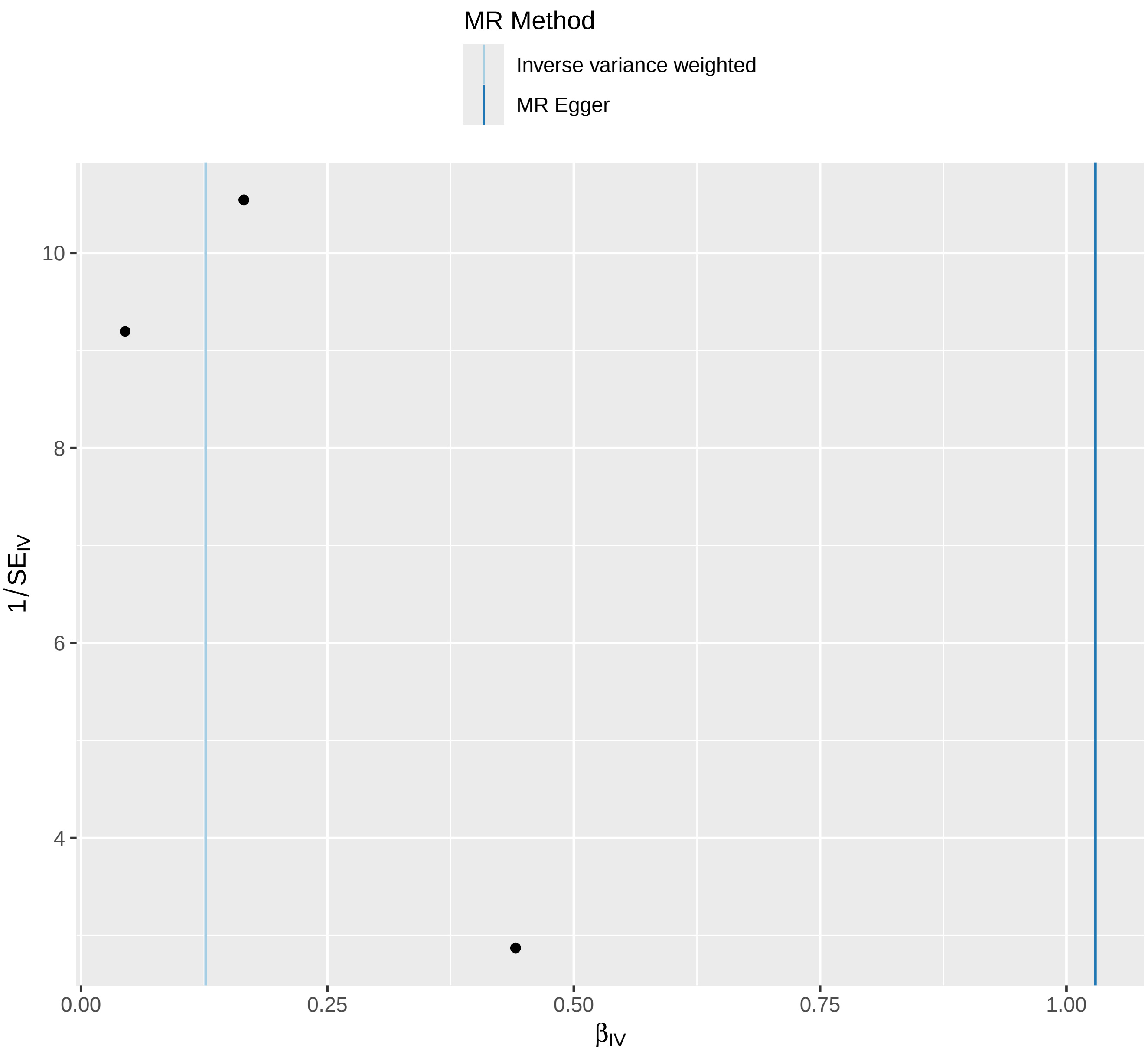
MR Method
Inverse variance weighted
MR Egger
10
8
6
4
0.00
0.25
0.50
0.75
1.00
$1/SE_{IV}$
$\beta_{IV}$

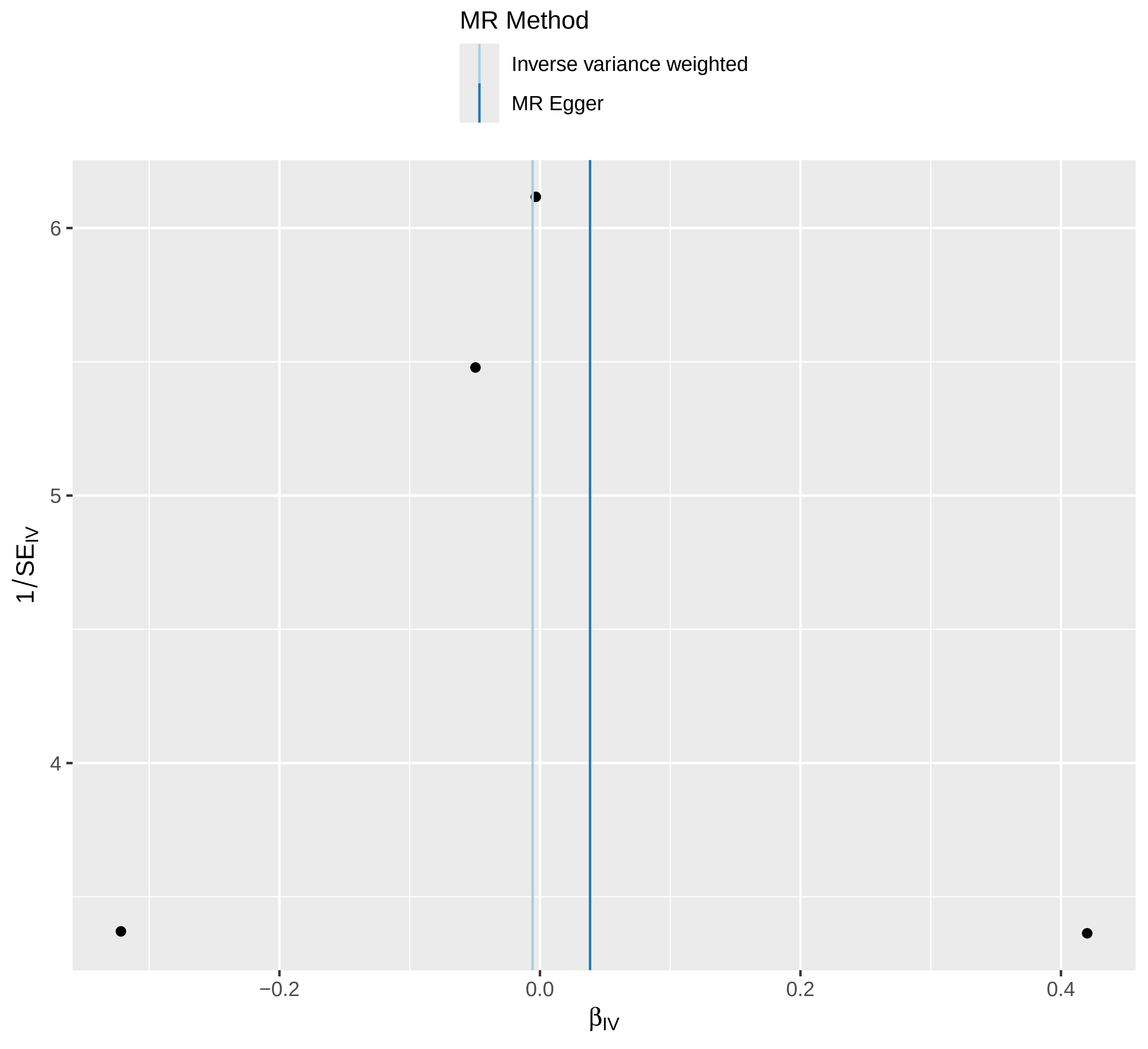
MR Method
Inverse variance weighted
MR Egger
6
5
4
1/SE_IV
−0.2
0.0
0.2
0.4
β_IV

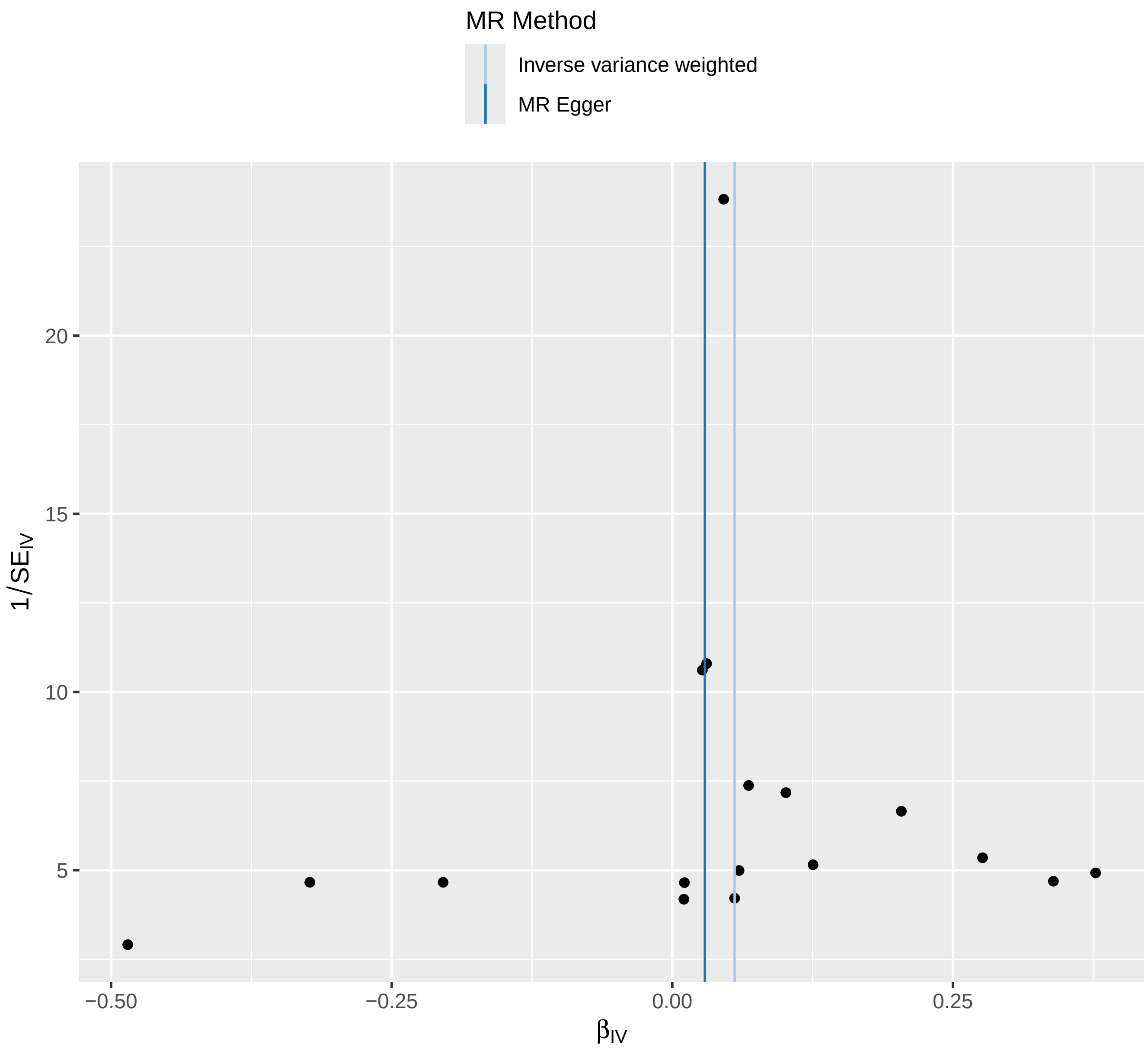
MR Method
Inverse variance weighted
MR Egger
1/SE$_{IV}$
20
15
10
5
−0.50
−0.25
0.00
0.25
β$_{IV}$

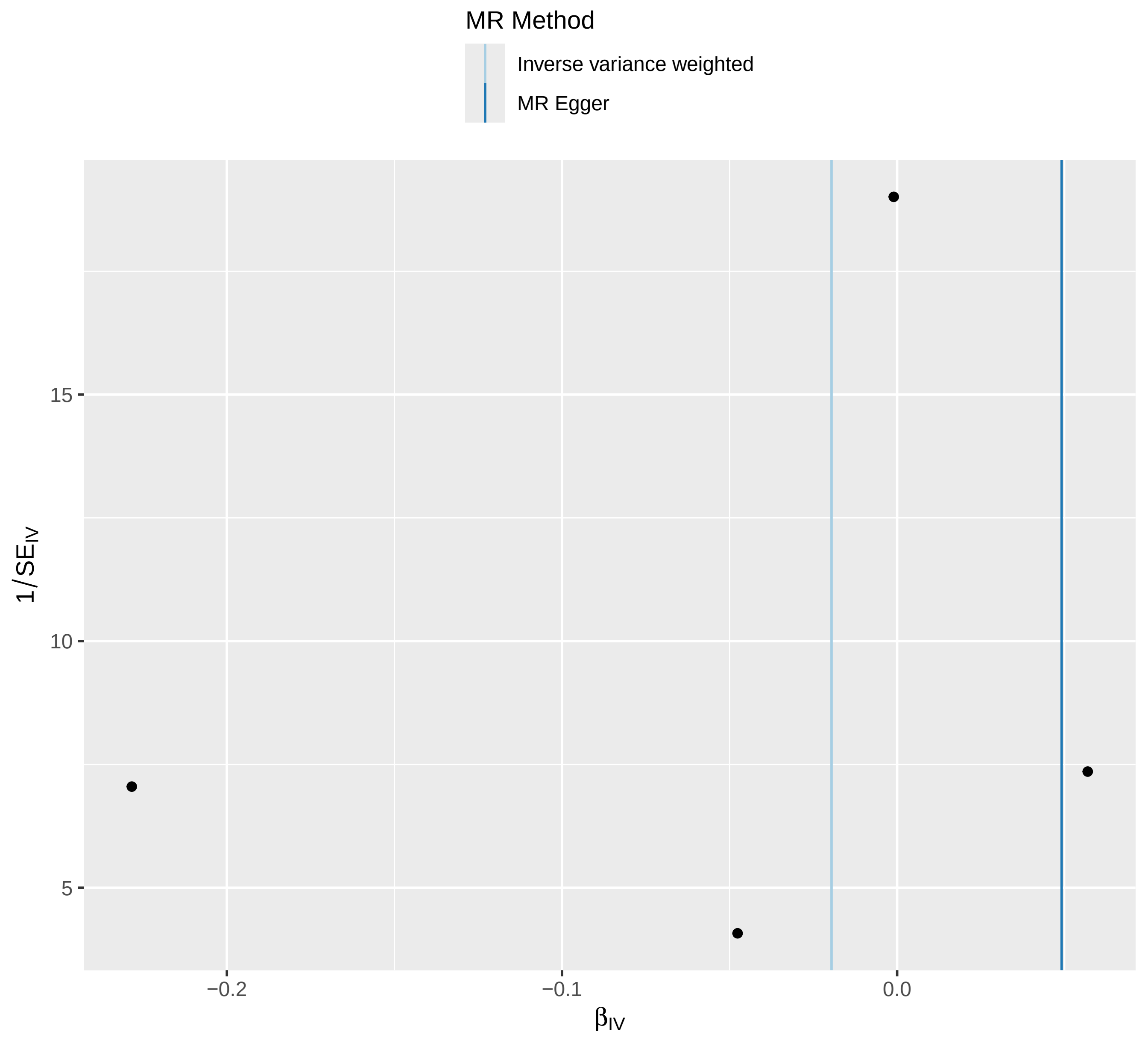
MR Method
Inverse variance weighted
MR Egger
15
10
5
$1/SE_{IV}$
−0.2
−0.1
0.0
$\beta_{IV}$

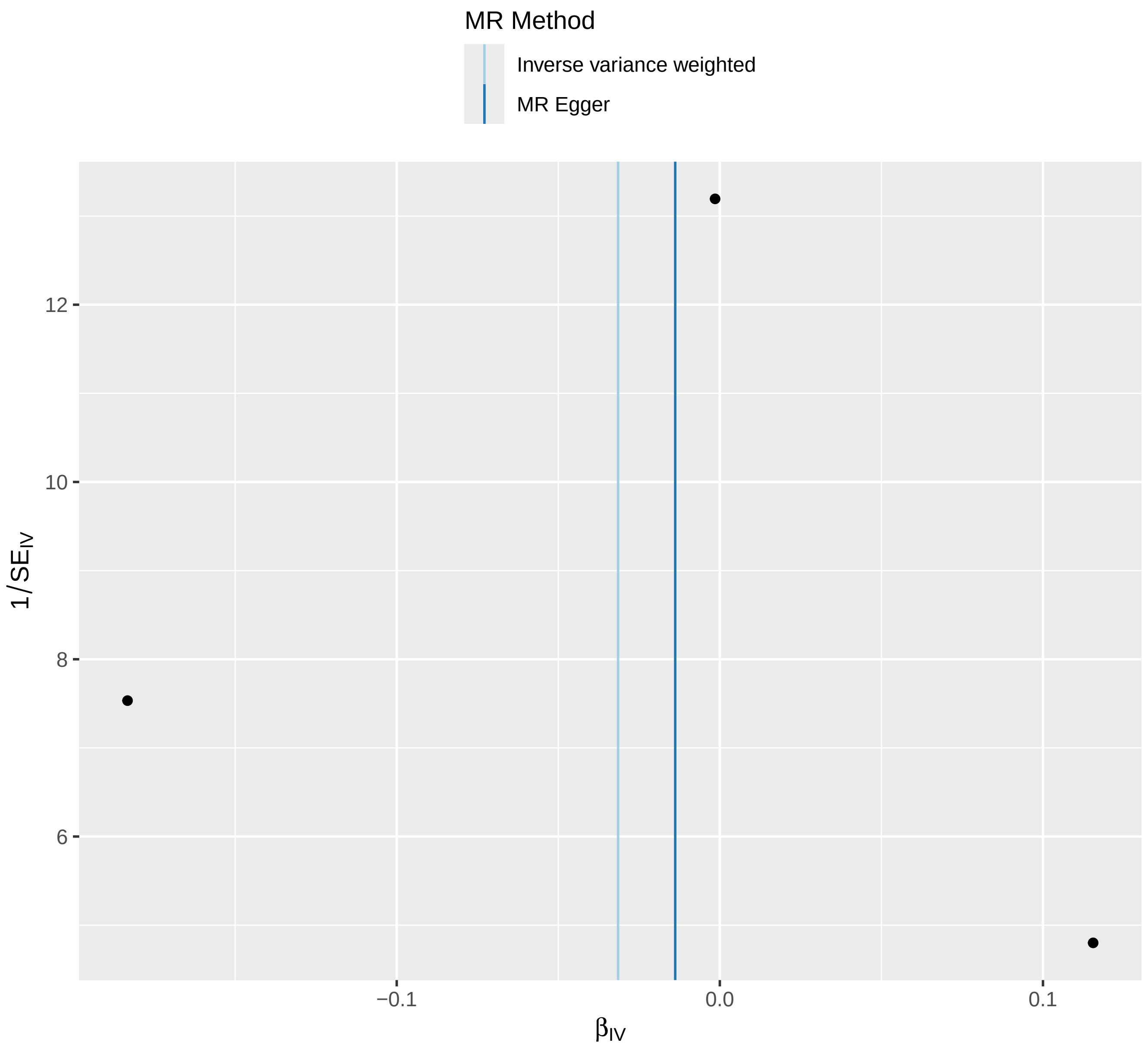

MR Method
Inverse variance weighted
MR Egger
12
10
8
6
1/SE_IV
−0.1
0.0
0.1
β_IV

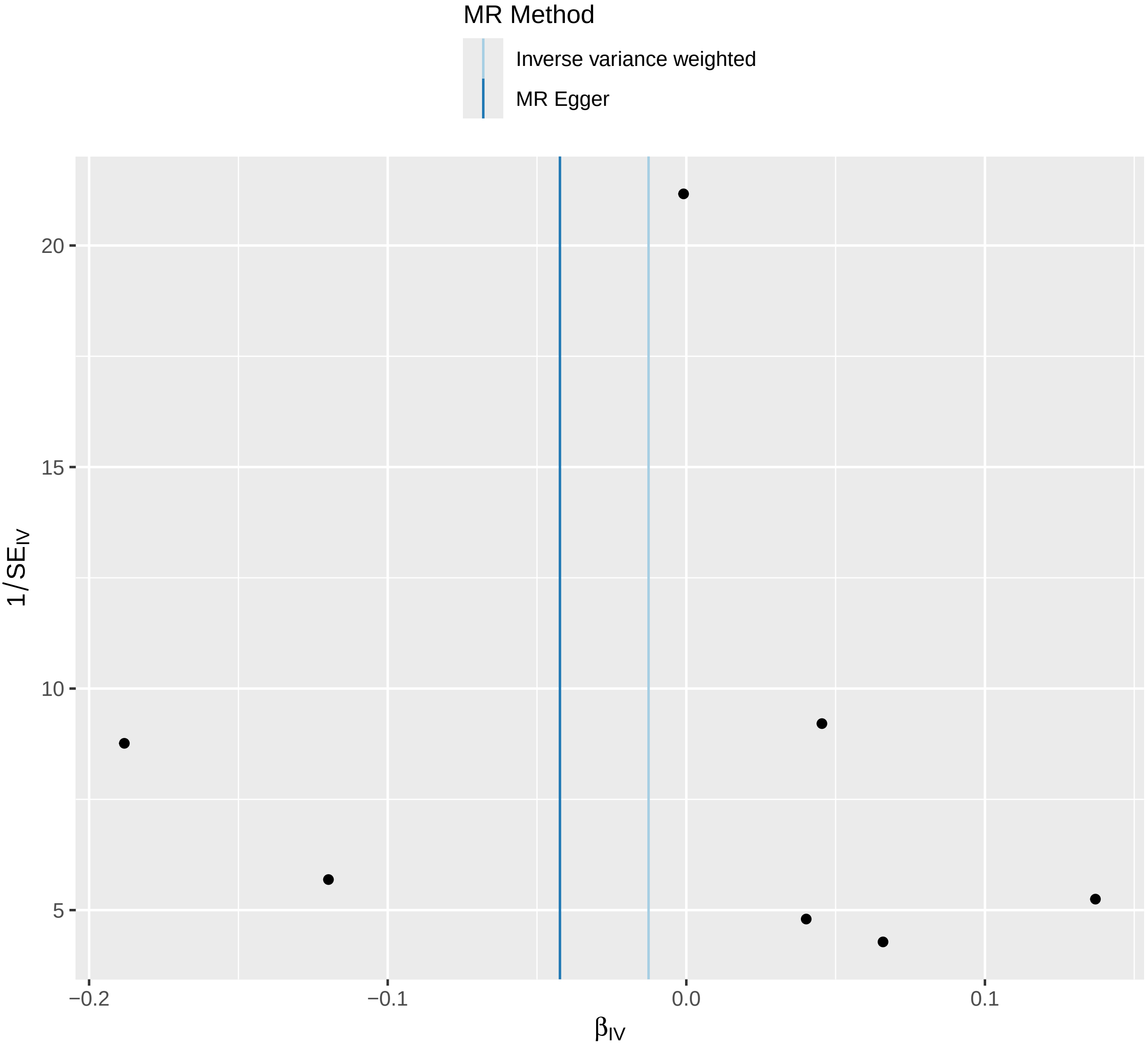

MR Method
Inverse variance weighted
MR Egger
1/SE$_{IV}$
20
15
10
5
β$_{IV}$
−0.2
−0.1
0.0
0.1

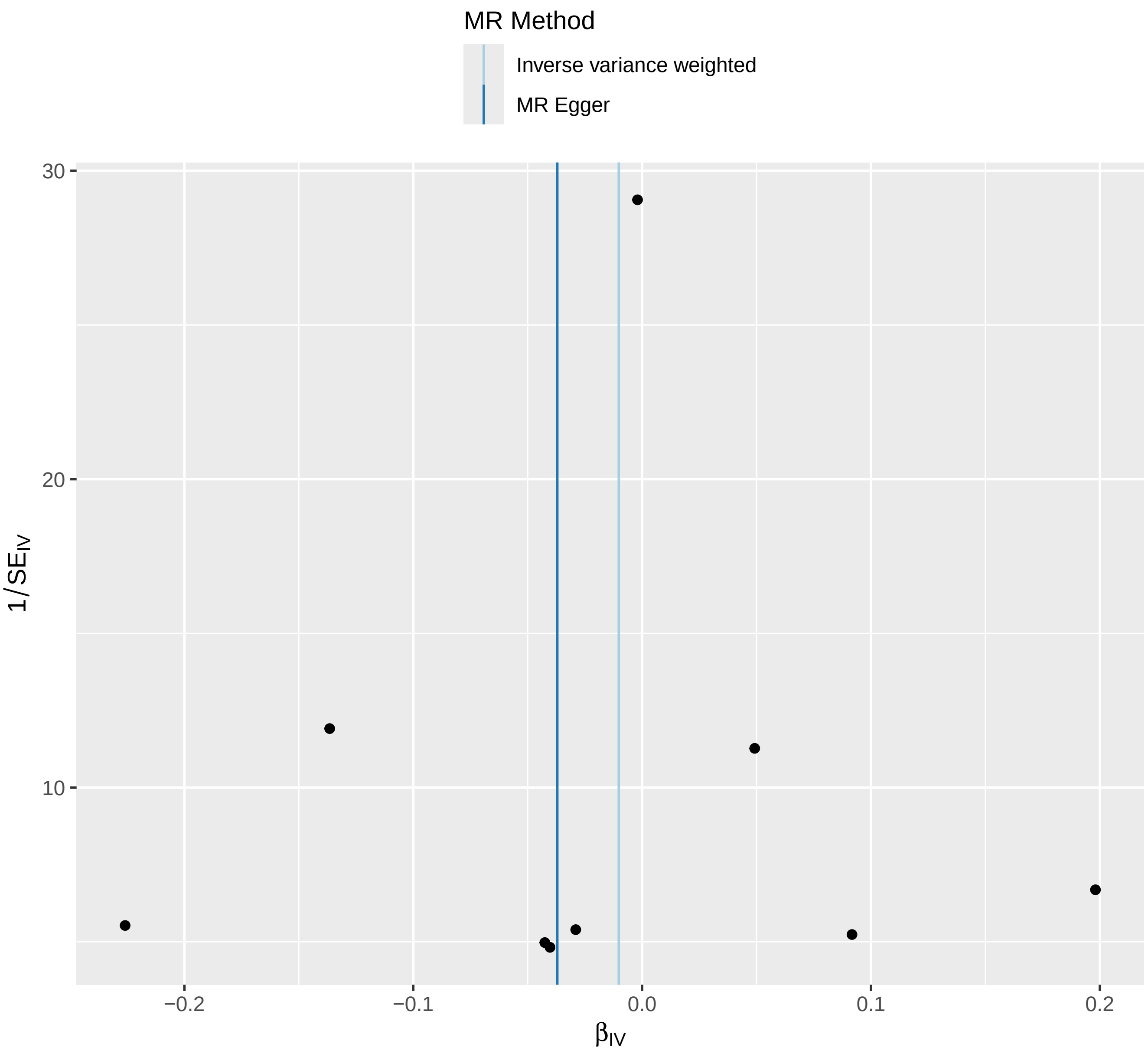
MR Method
Inverse variance weighted
MR Egger
30
20
10
$1/SE_{IV}$
−0.2
−0.1
0.0
0.1
0.2
$\beta_{IV}$

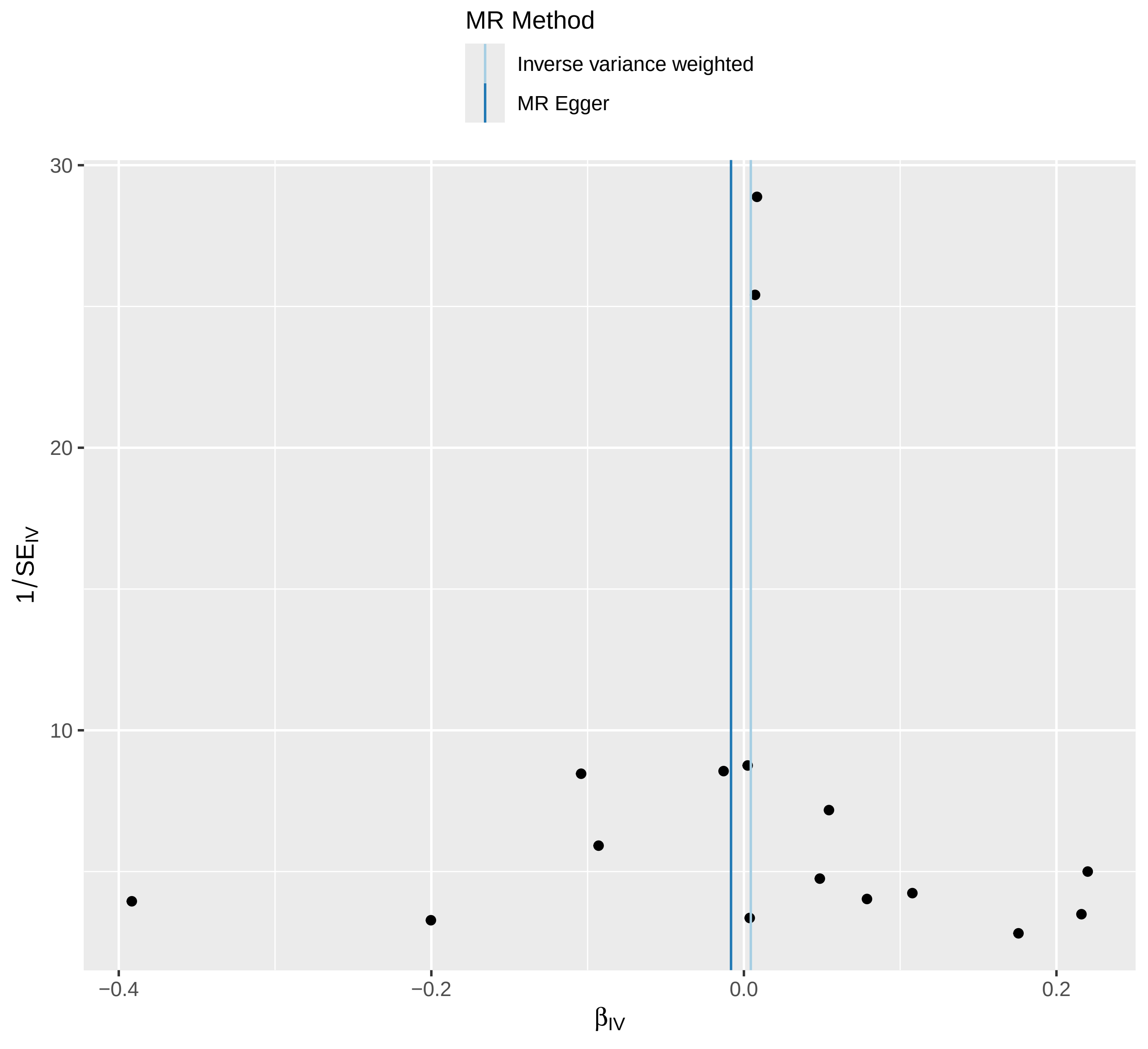
MR Method
Inverse variance weighted
MR Egger
30
20
10
$1/SE_{IV}$
−0.4
−0.2
0.0
0.2
$\beta_{IV}$

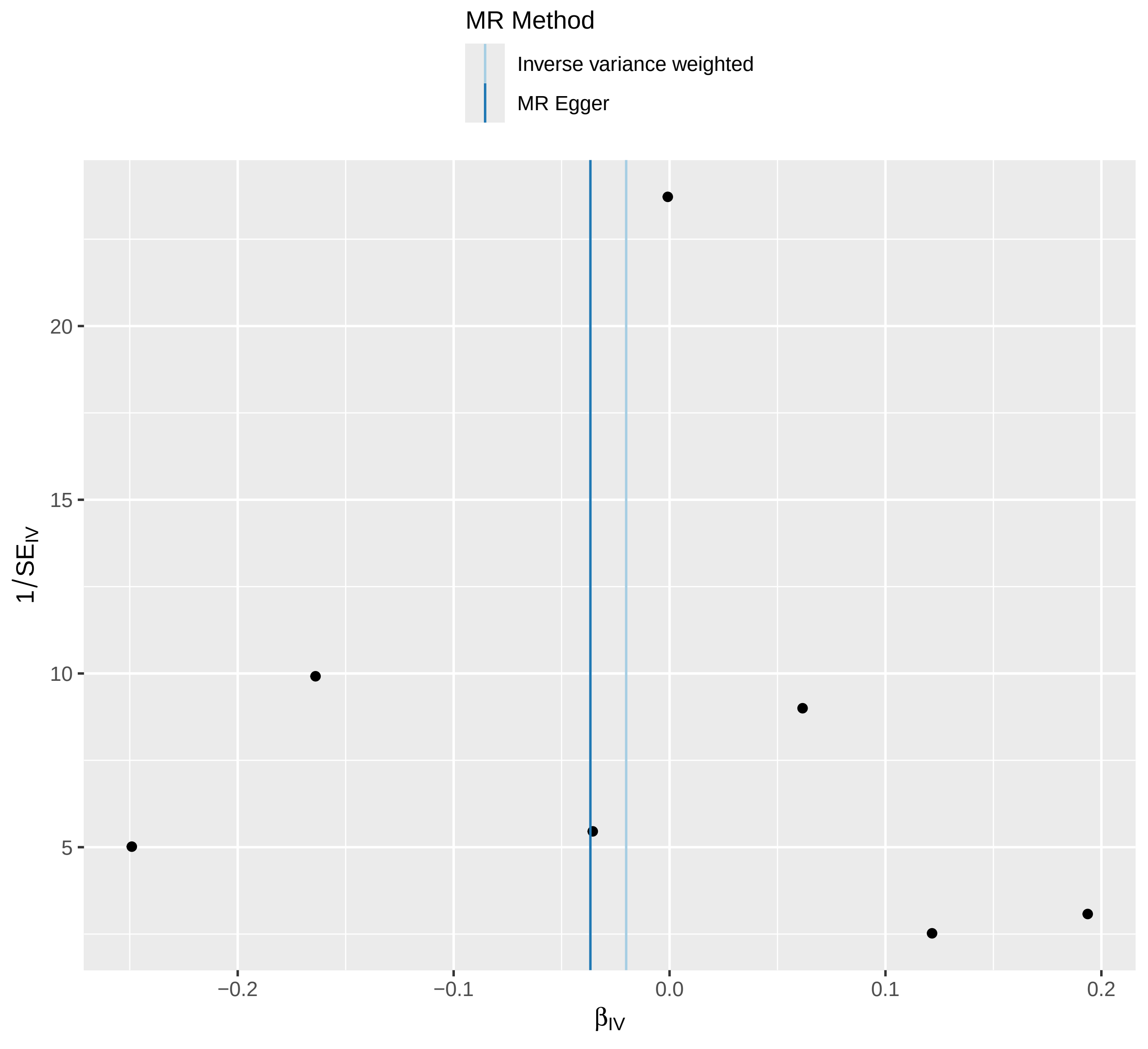
MR Method
Inverse variance weighted
MR Egger
1/SE_IV
20
15
10
5
−0.2
−0.1
0.0
0.1
0.2
β_IV

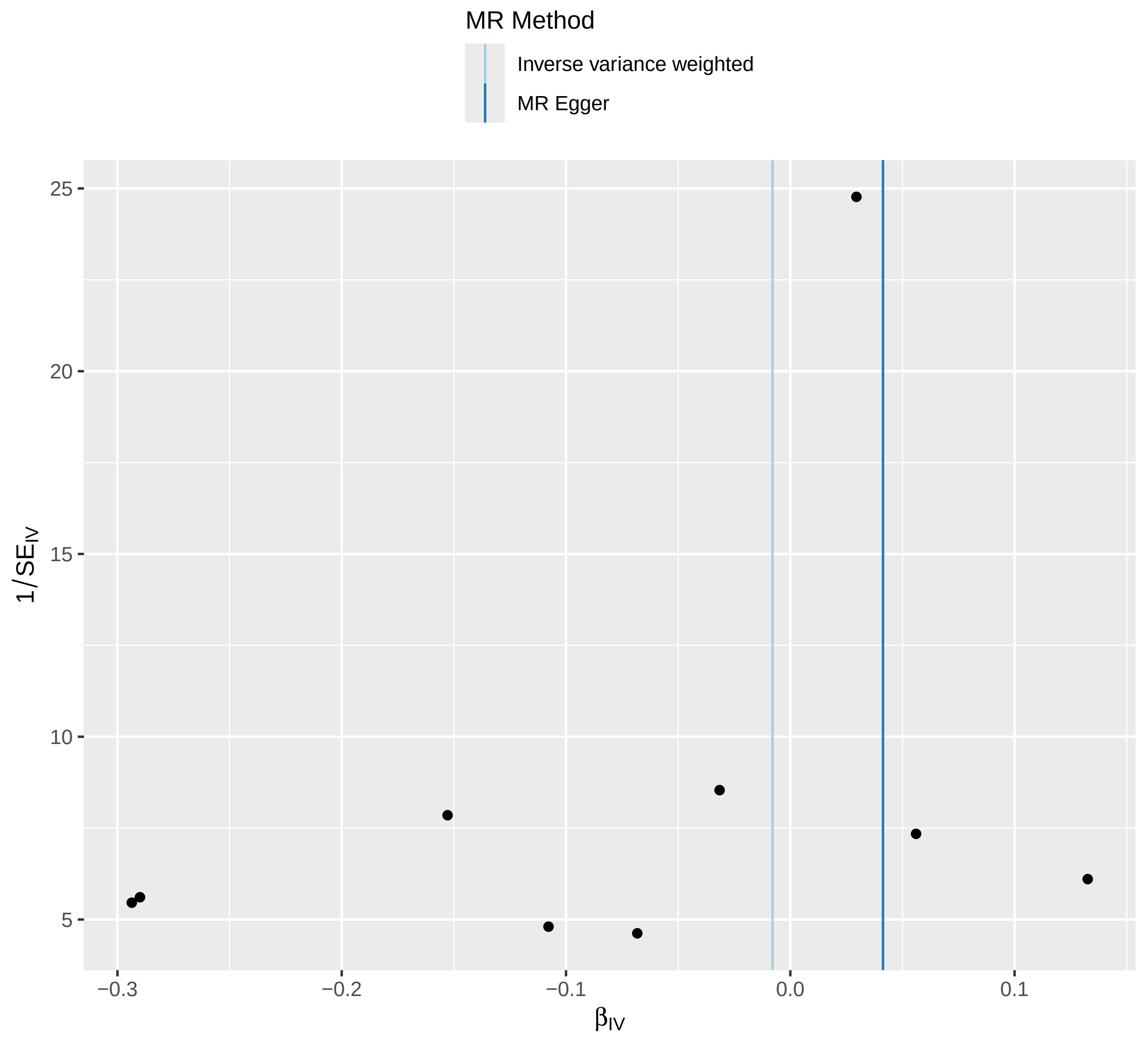
MR Method
Inverse variance weighted
MR Egger
25
20
15
10
5
1/SE$_{IV}$
−0.3
−0.2
−0.1
0.0
0.1
β$_{IV}$

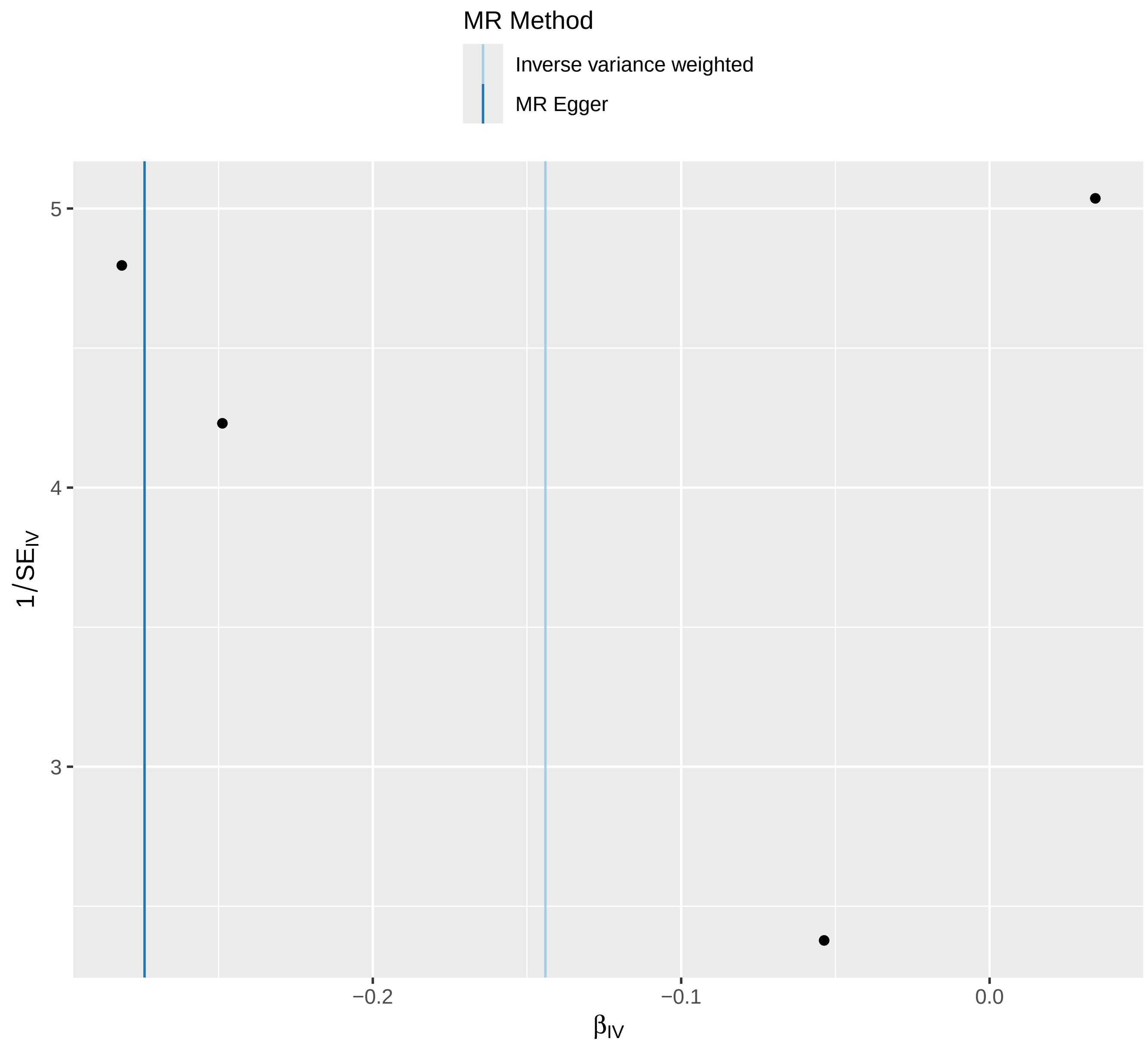

MR Method
Inverse variance weighted
MR Egger
5
4
3
1/SE$_{IV}$
−0.2
−0.1
0.0
β$_{IV}$

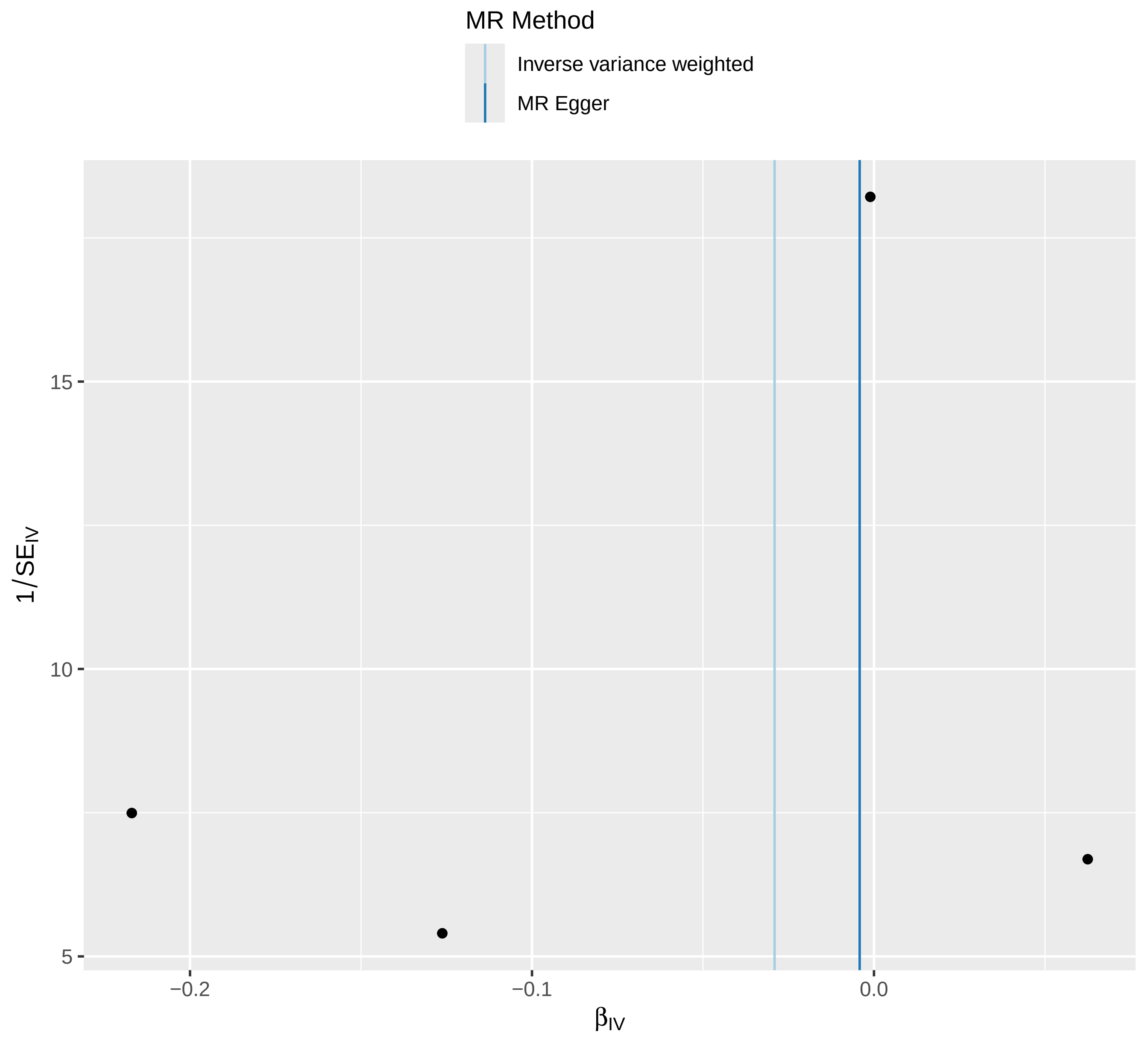
MR Method
Inverse variance weighted
MR Egger
15
10
5
−0.2
−0.1
0.0
$1/SE_{IV}$
$\beta_{IV}$

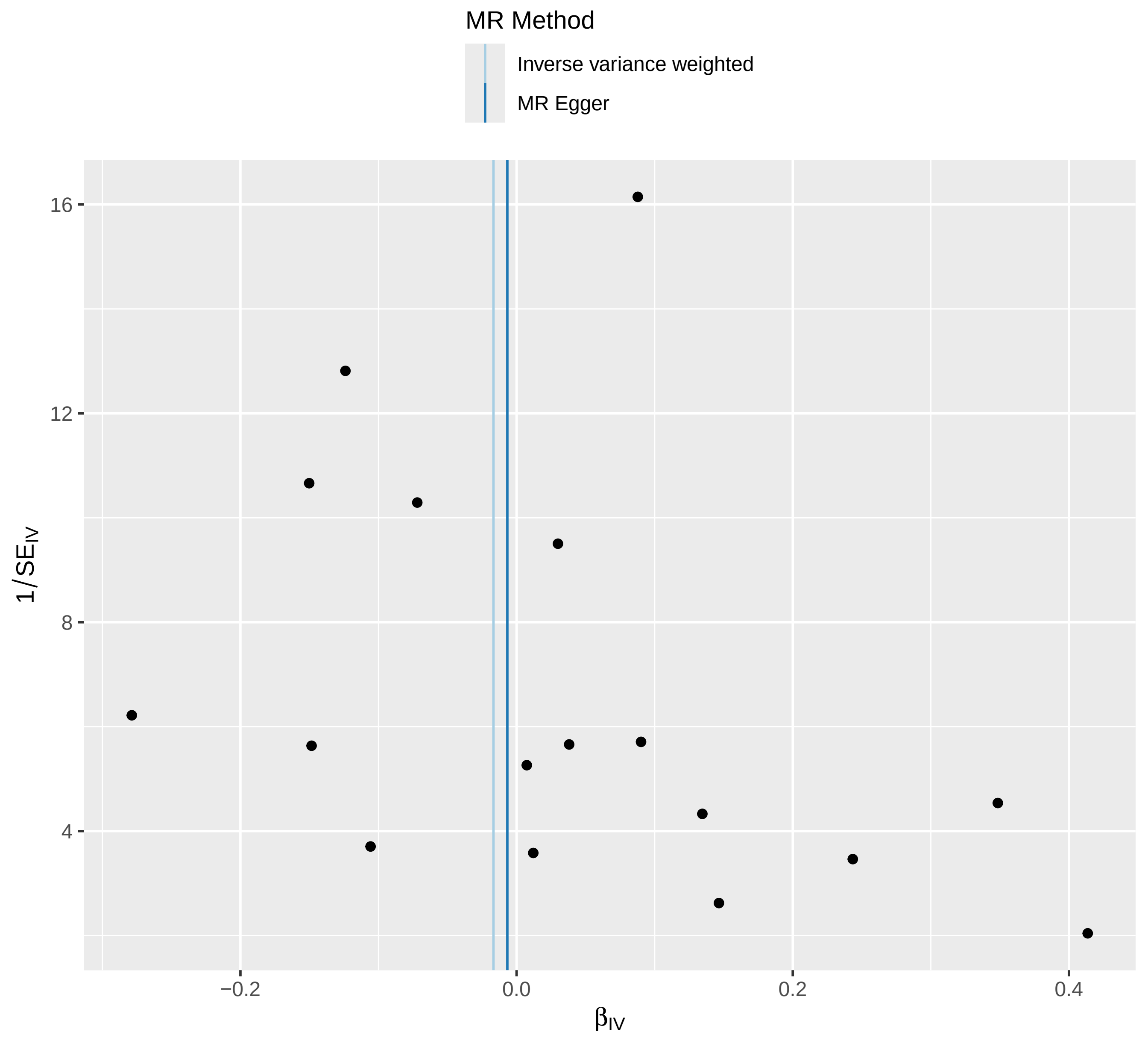

MR Method
Inverse variance weighted
MR Egger
16
12
8
4
1/SE$_{IV}$
−0.2
0.0
0.2
0.4
β$_{IV}$

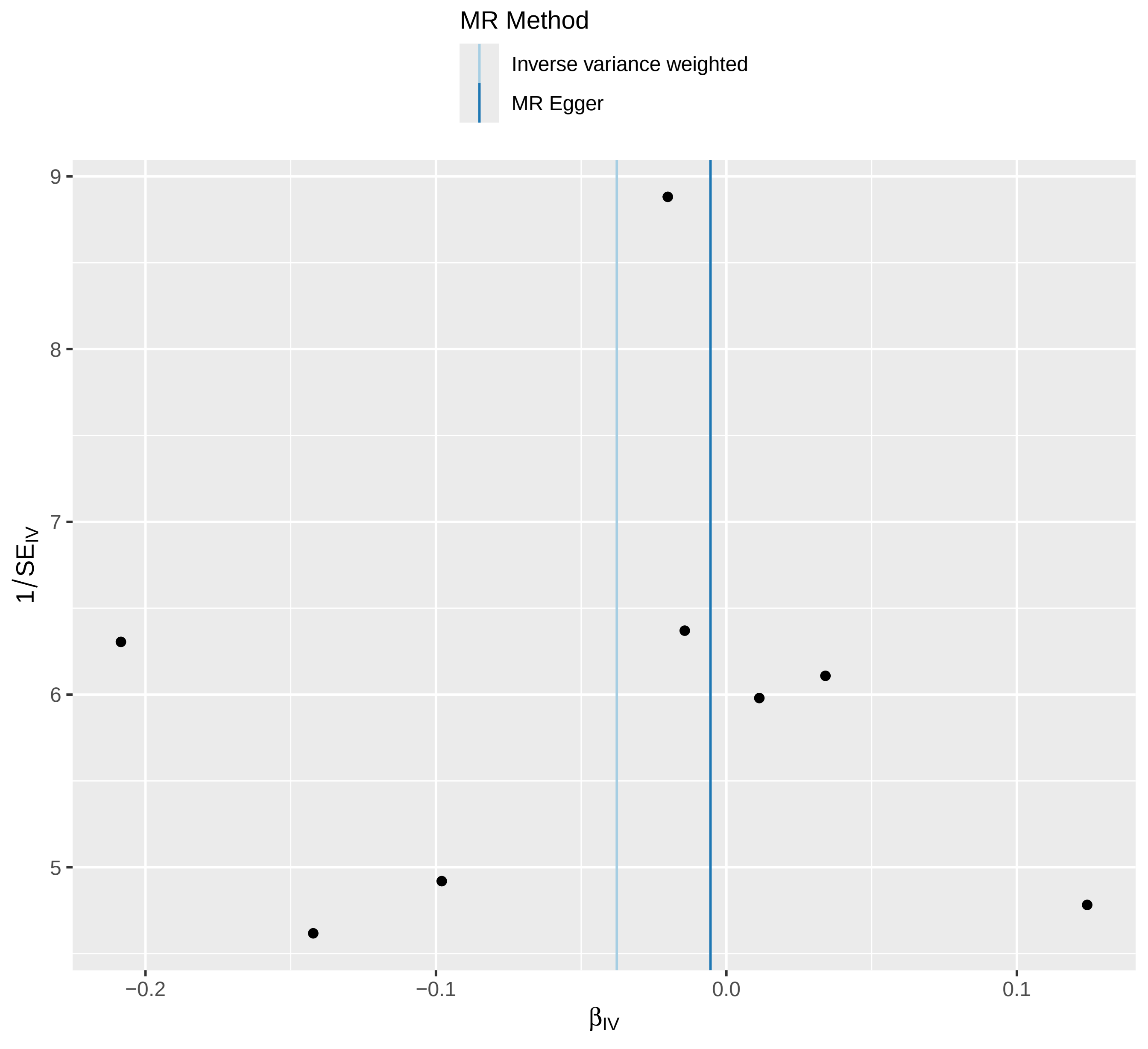
MR Method
Inverse variance weighted
MR Egger
9
8
7
6
5
1/SE_IV
−0.2
−0.1
0.0
0.1
β_IV

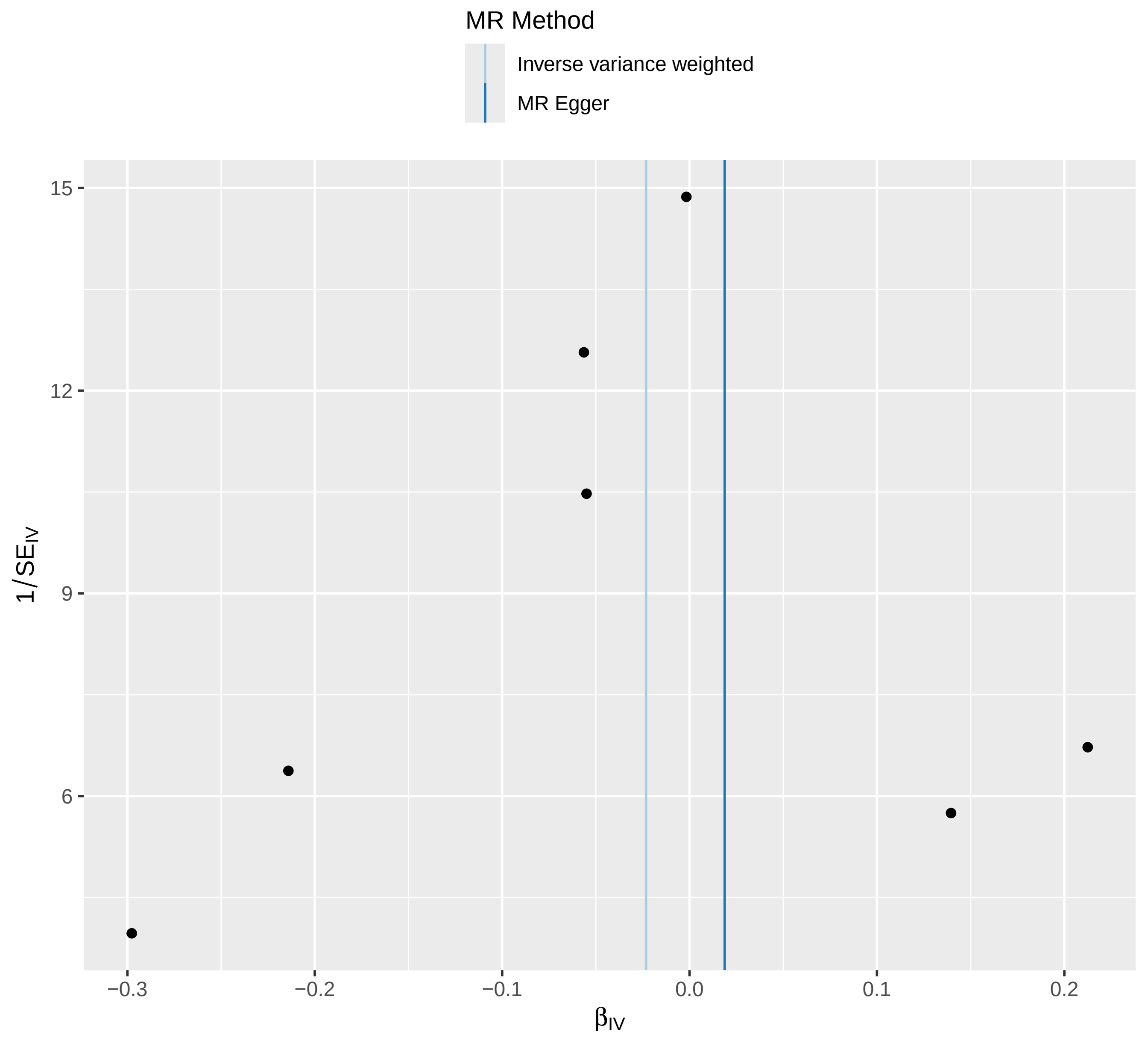
MR Method
Inverse variance weighted
MR Egger
15
12
9
6
1/SE$_{IV}$
−0.3
−0.2
−0.1
0.0
0.1
0.2
β$_{IV}$

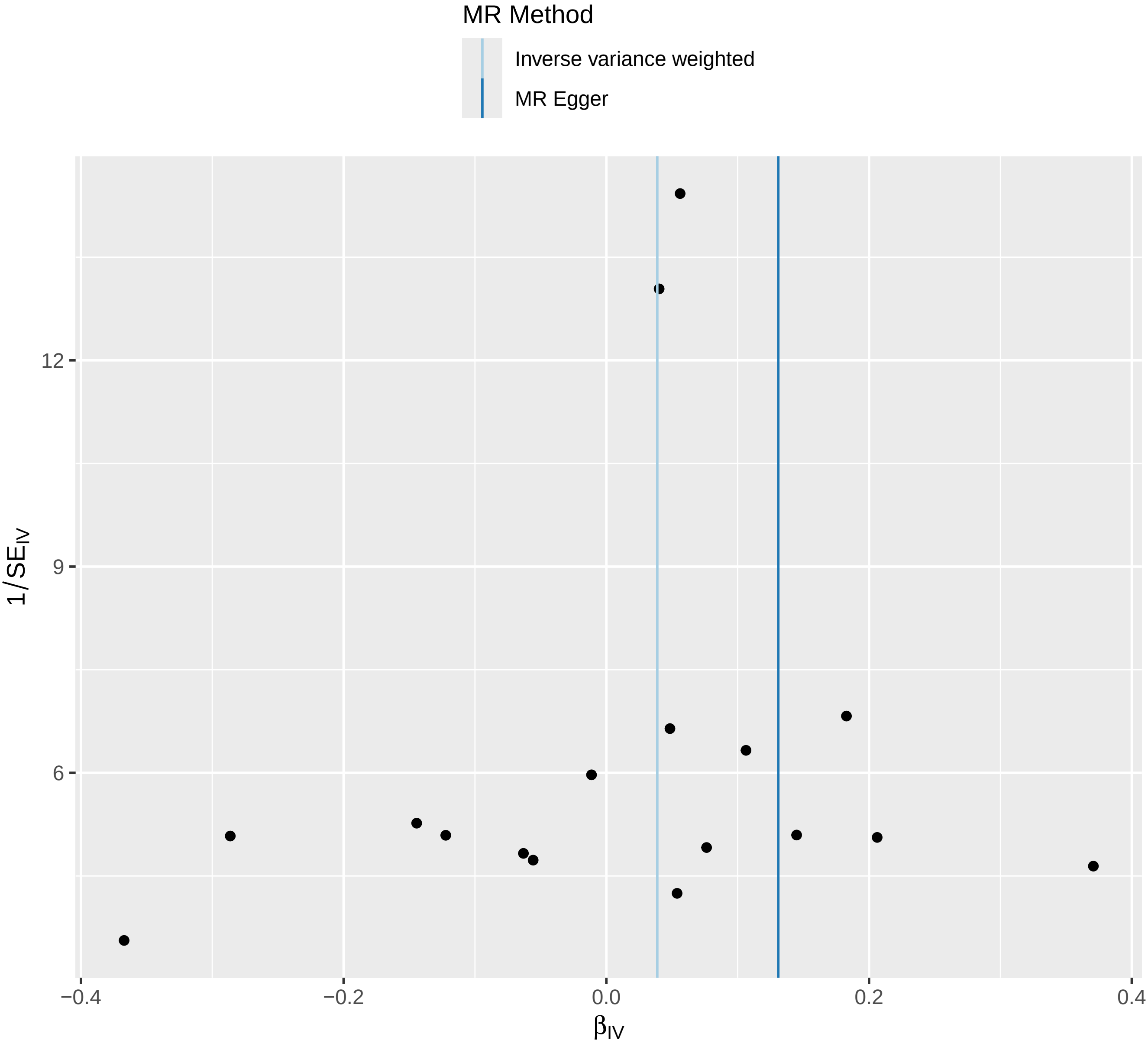

MR Method
Inverse variance weighted
MR Egger
12
9
6
1/SE$_{IV}$
−0.4
−0.2
0.0
0.2
0.4
β$_{IV}$

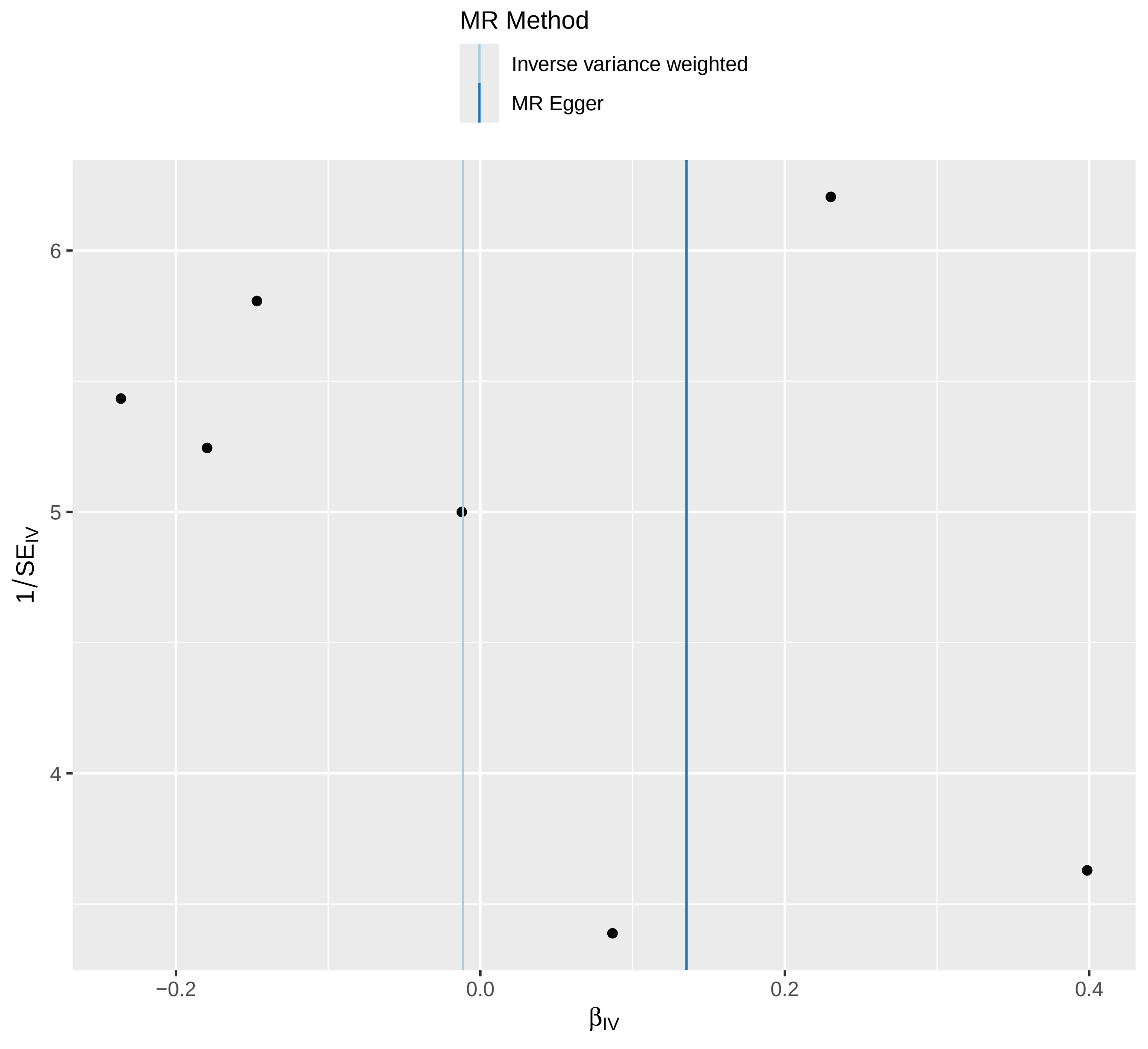
MR Method
Inverse variance weighted
MR Egger
6
5
4
1/SE$_{IV}$
−0.2
0.0
0.2
0.4
β$_{IV}$

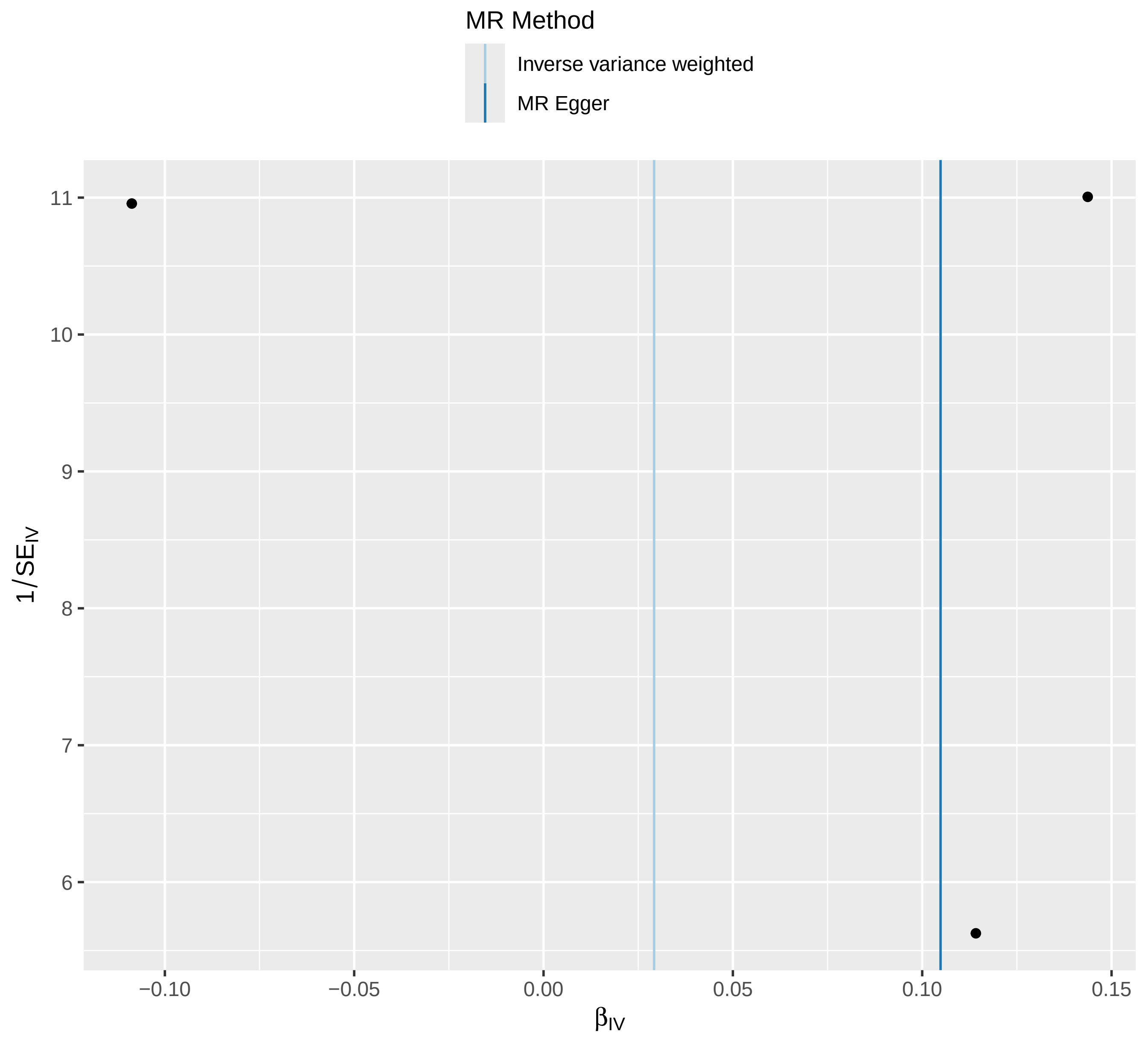
MR Method
Inverse variance weighted
MR Egger
11
10
9
8
7
6
1/SE_IV
−0.10
−0.05
0.00
0.05
0.10
0.15
β_IV

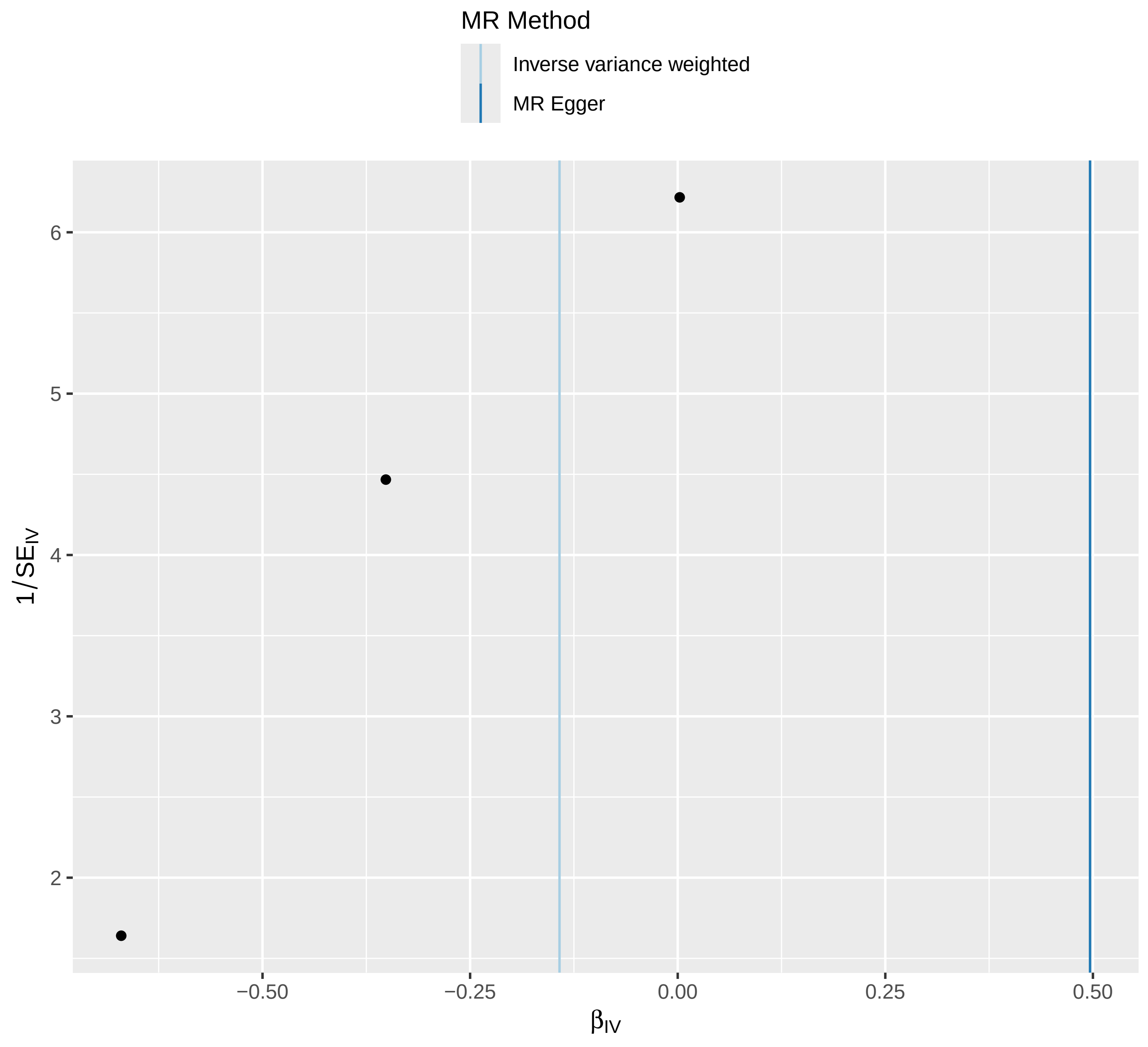
MR Method
Inverse variance weighted
MR Egger
6
5
4
3
2
1/SE$_{IV}$
−0.50
−0.25
0.00
0.25
0.50
β$_{IV}$

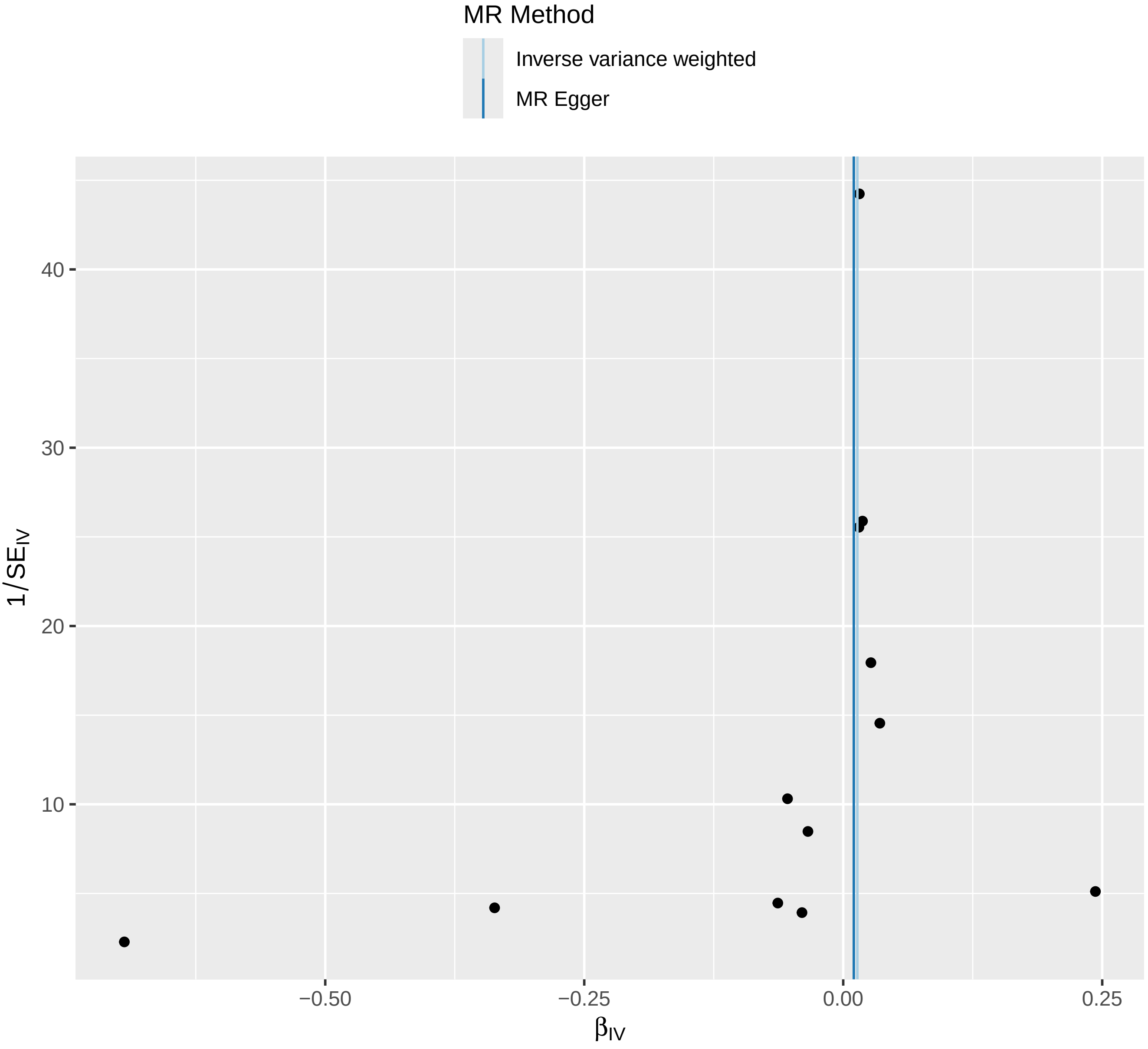
MR Method
Inverse variance weighted
MR Egger
40
30
20
10
1/SE_IV
−0.50
−0.25
0.00
0.25
β_IV

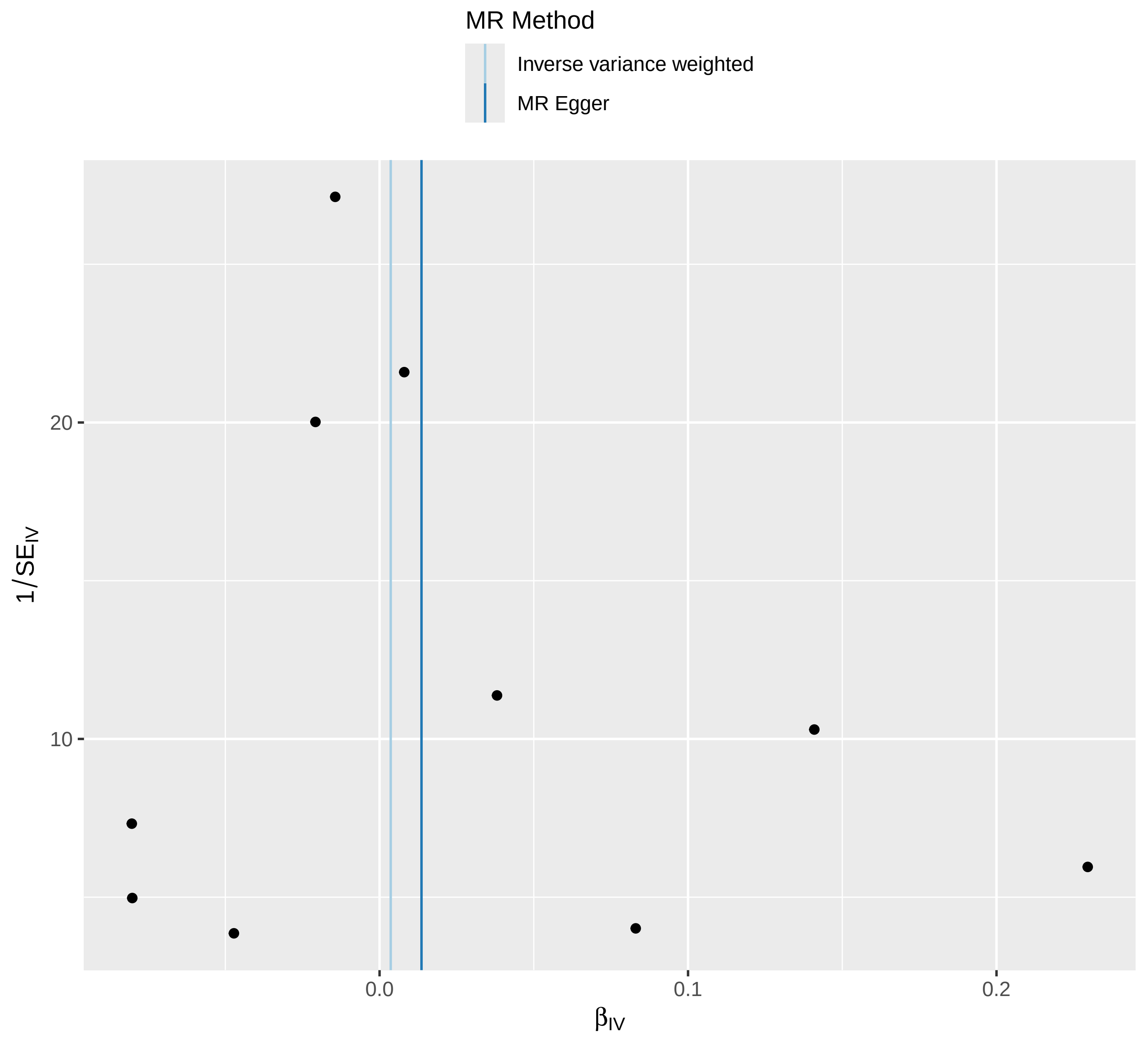
MR Method
Inverse variance weighted
MR Egger
20
10
$1/SE_{IV}$
0.0
0.1
0.2
$\beta_{IV}$

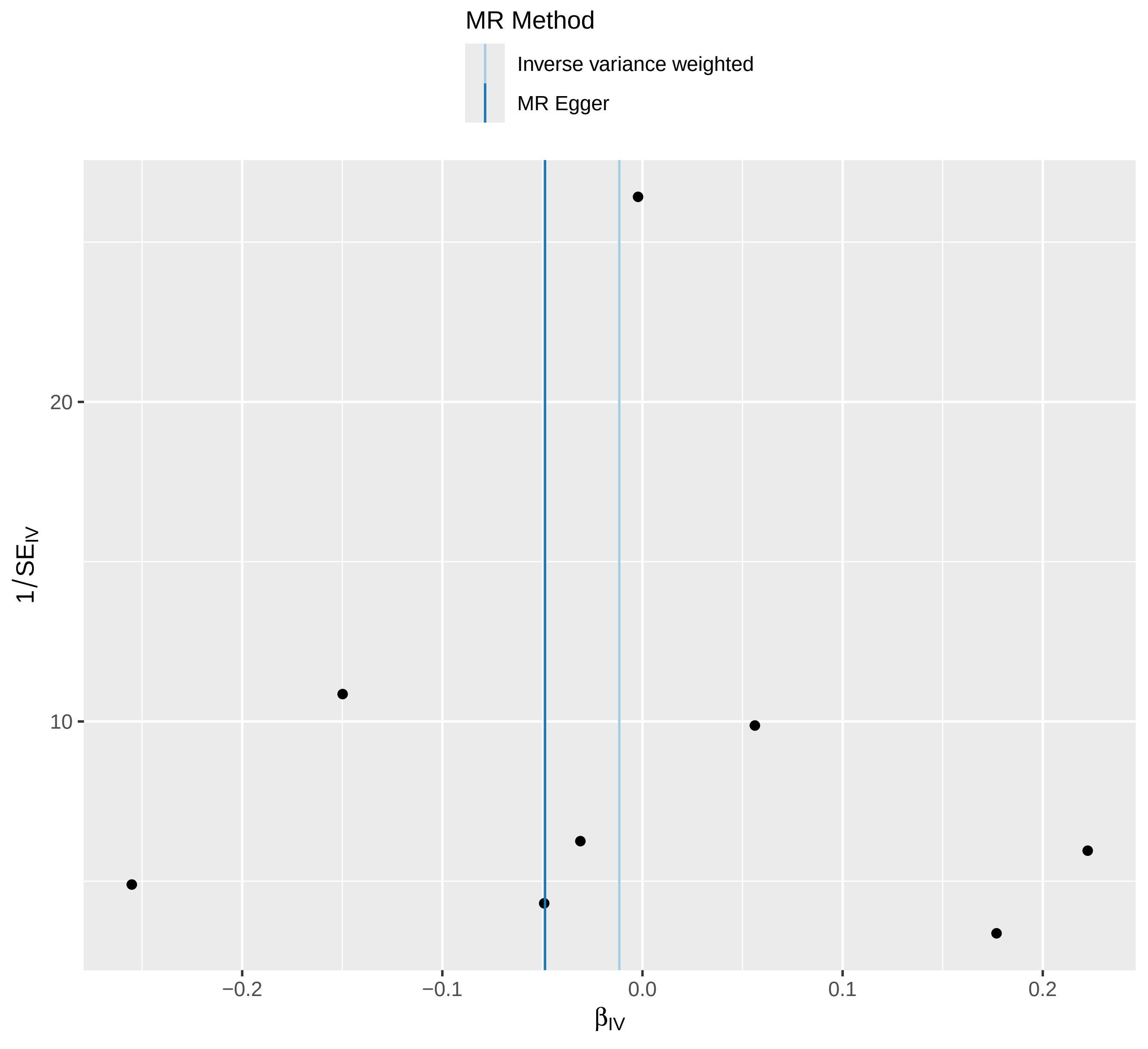
MR Method
Inverse variance weighted
MR Egger
20
10
1/SE_IV
−0.2
−0.1
0.0
0.1
0.2
β_IV

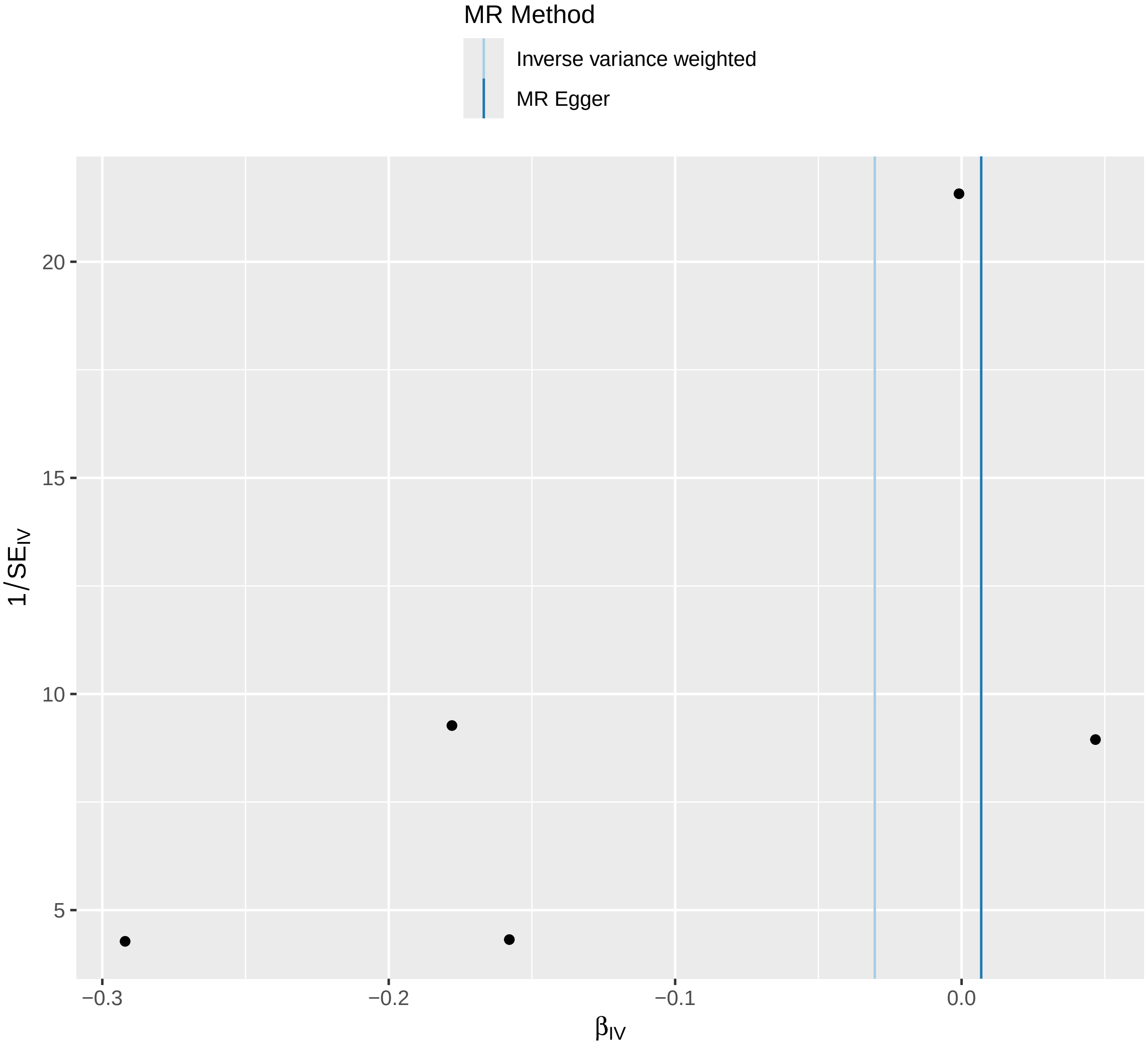
MR Method
Inverse variance weighted
MR Egger
20
15
10
5
1/SE_IV
−0.3
−0.2
−0.1
0.0
β_IV

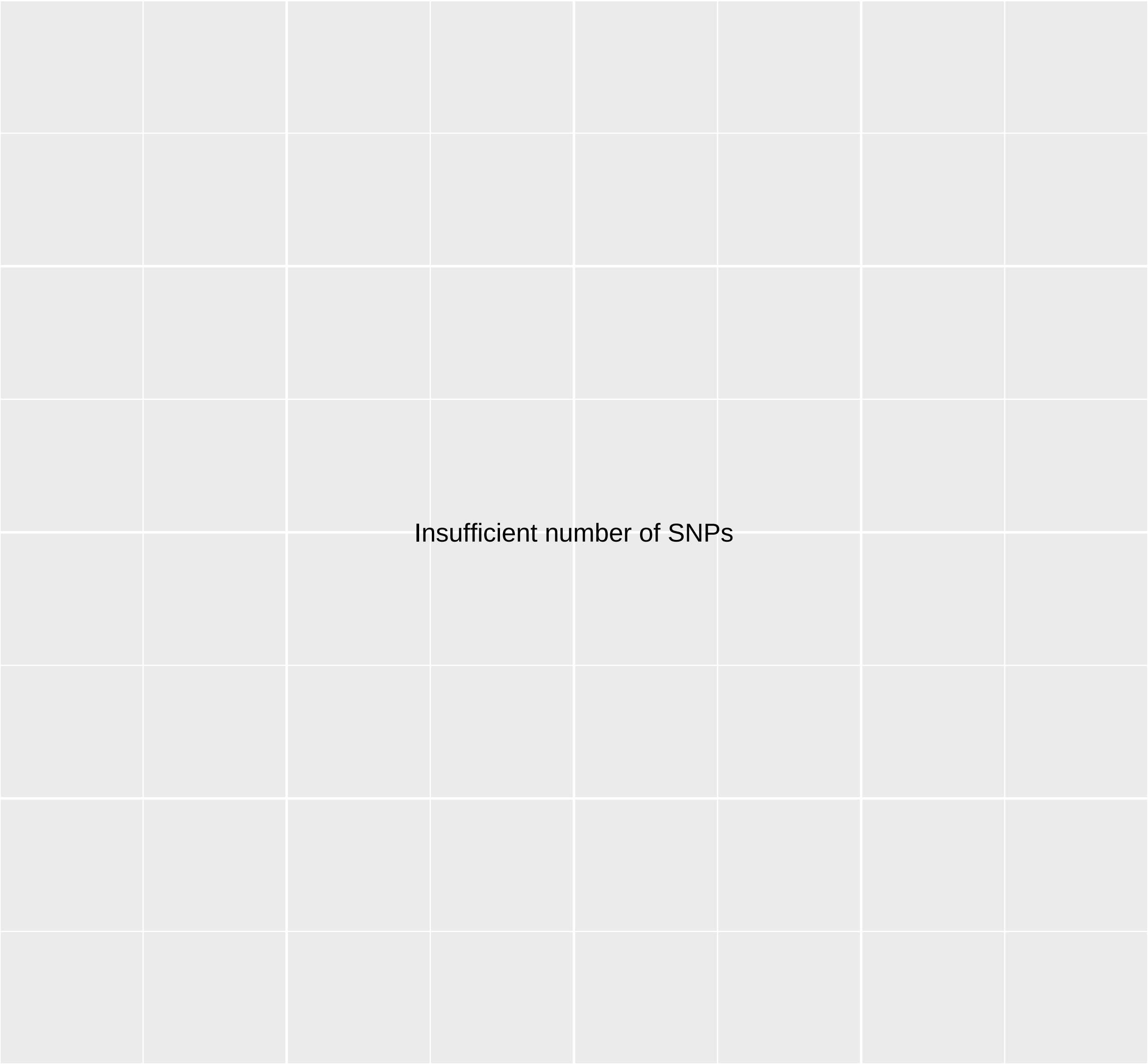
Insufficient number of SNPs

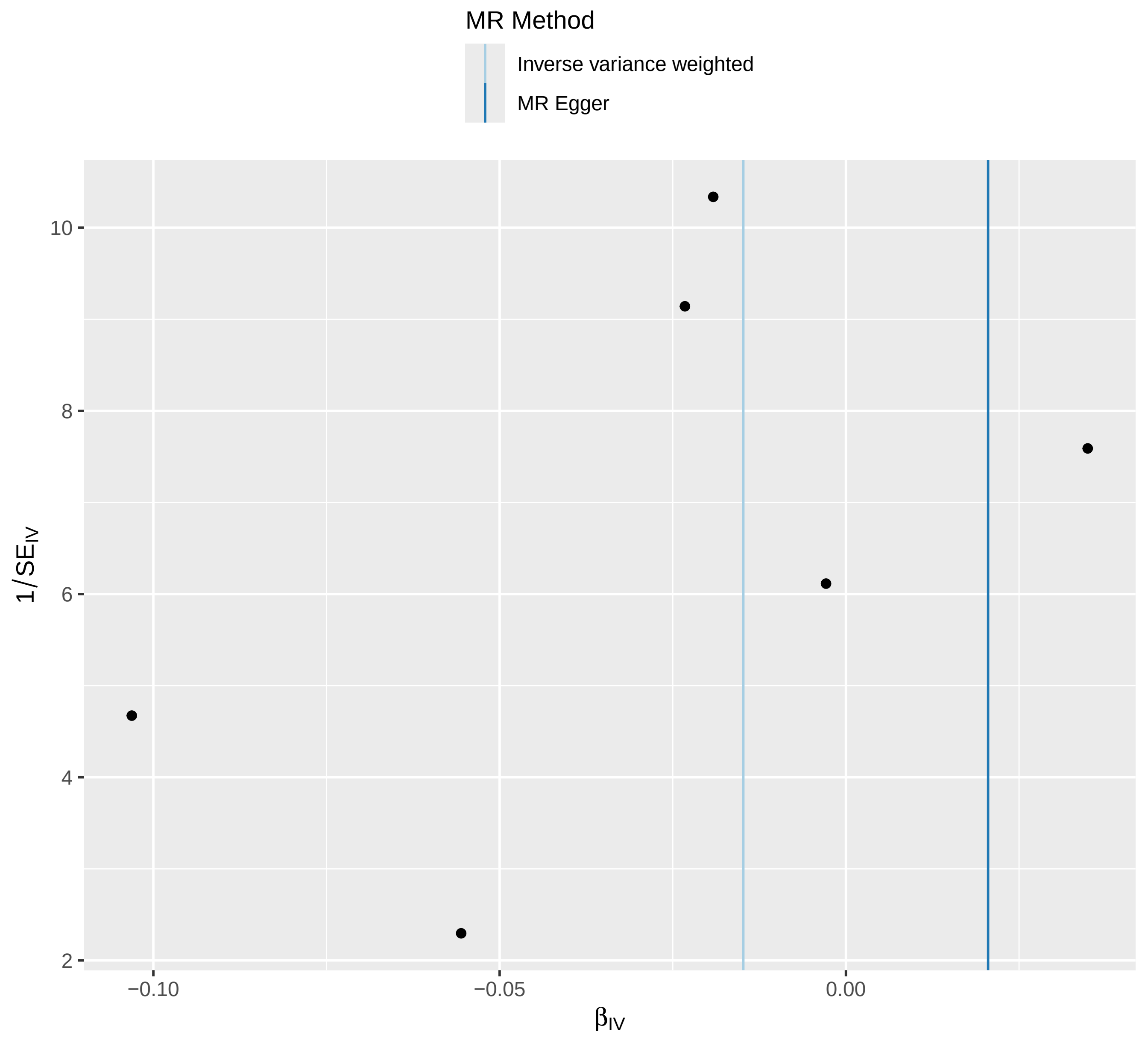
MR Method
Inverse variance weighted
MR Egger
10
8
6
4
2
1/SE_IV
−0.10
−0.05
0.00
β_IV

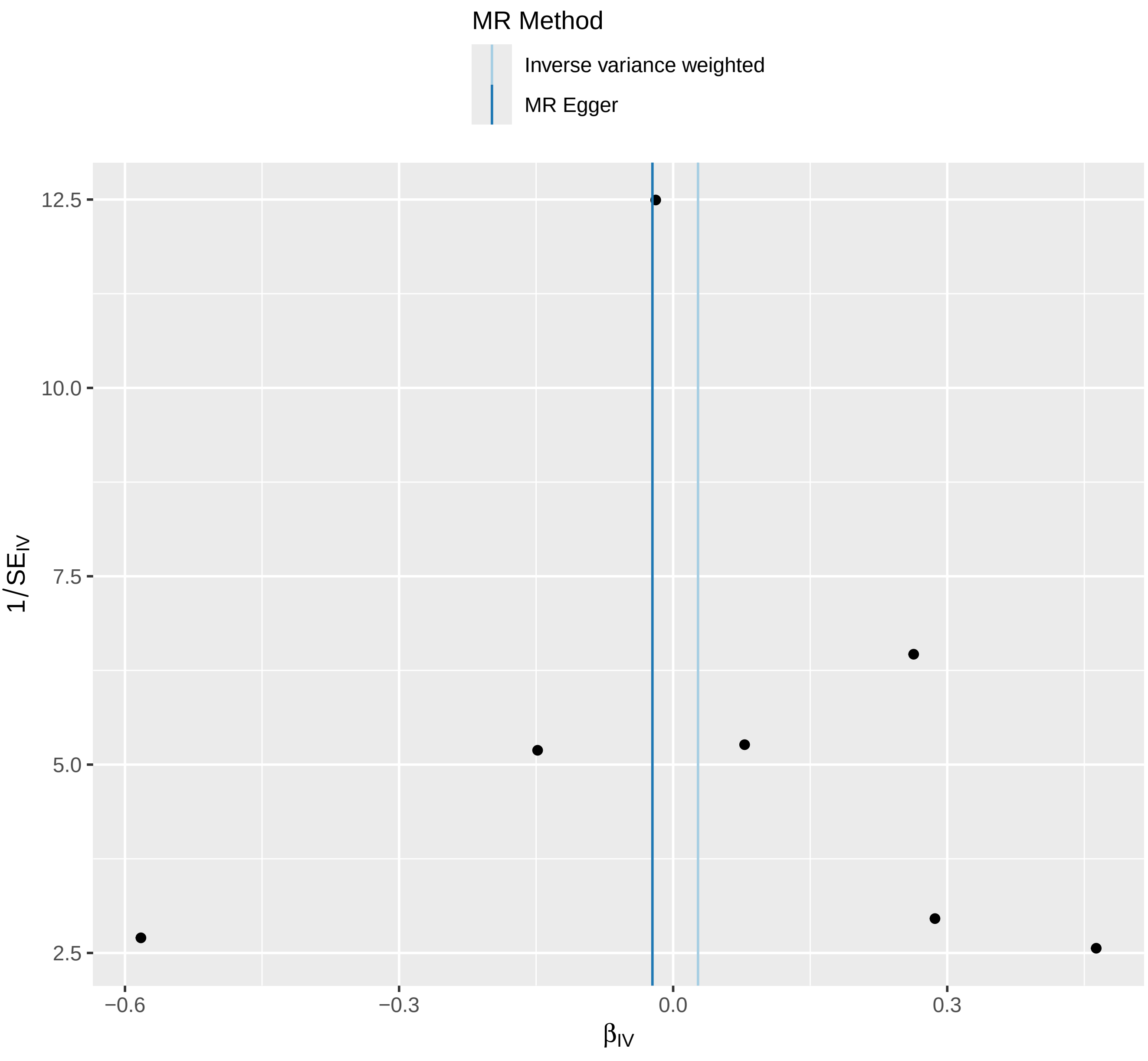
MR Method
Inverse variance weighted
MR Egger
12.5
10.0
7.5
5.0
2.5
1/SE_IV
−0.6
−0.3
0.0
0.3
β_IV

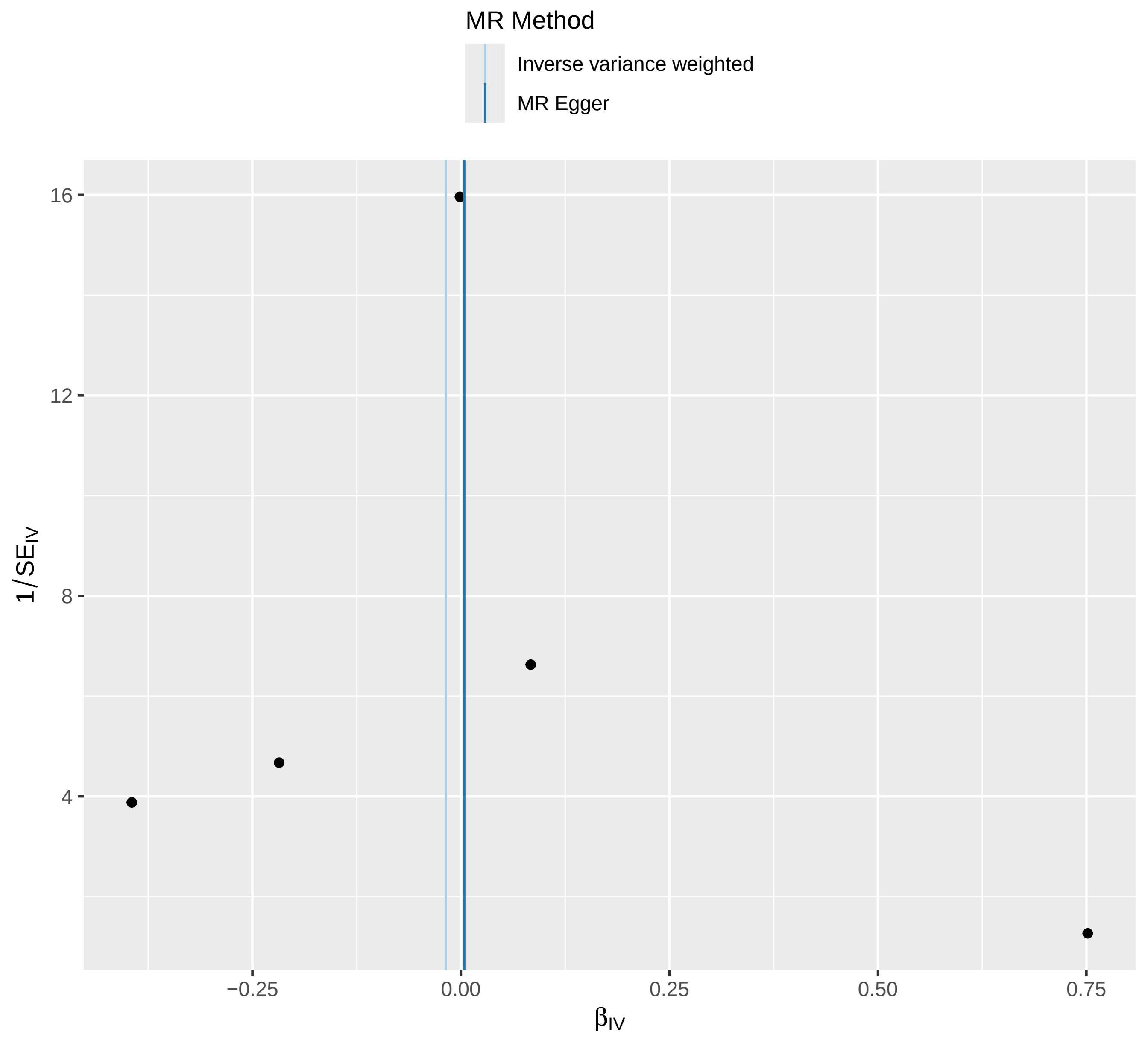
MR Method
Inverse variance weighted
MR Egger
16
12
8
4
1/SE_IV
−0.25
0.00
0.25
0.50
0.75
β_IV

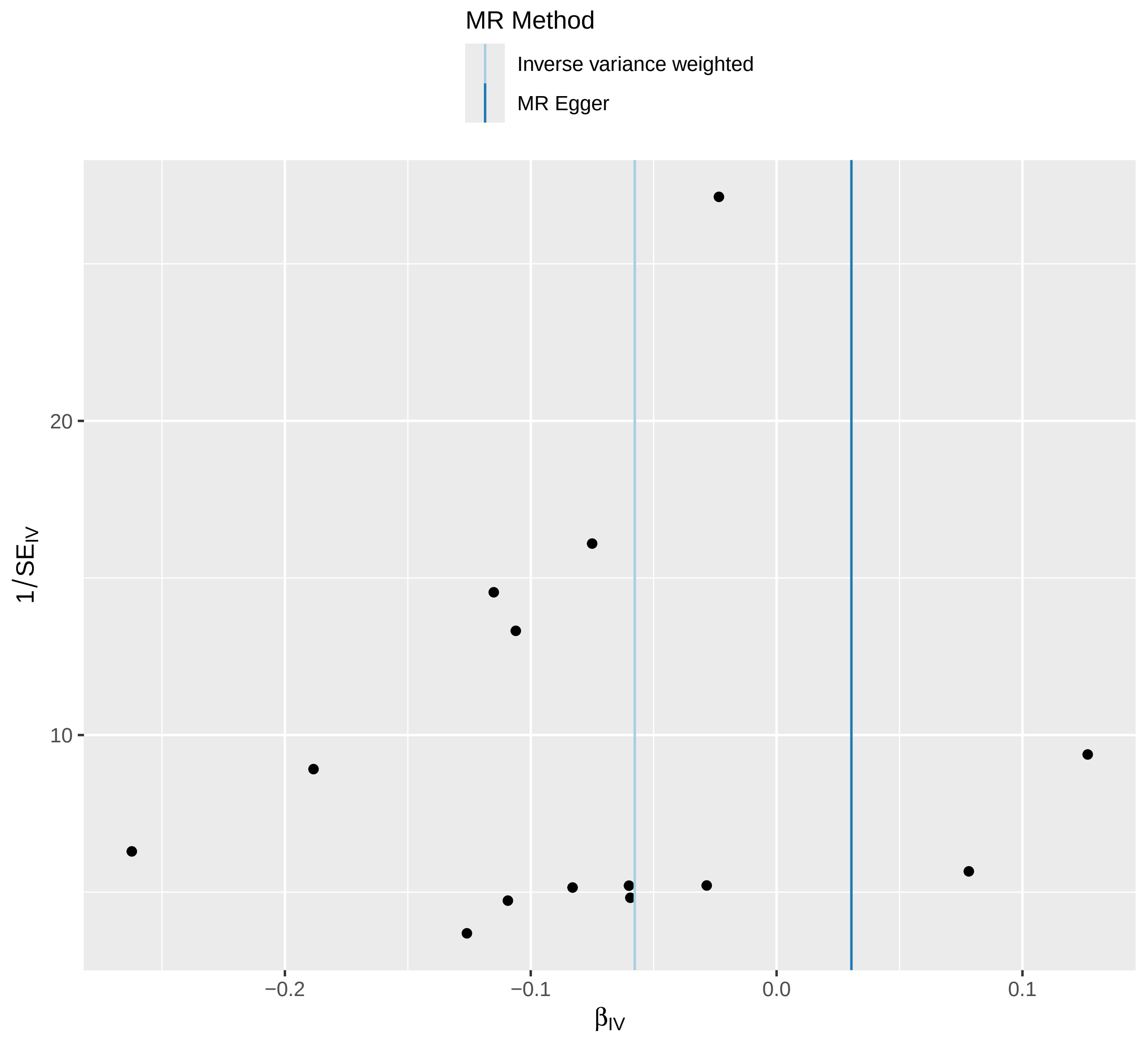
MR Method
Inverse variance weighted
MR Egger
20
10
$1/SE_{IV}$
−0.2
−0.1
0.0
0.1
$\beta_{IV}$

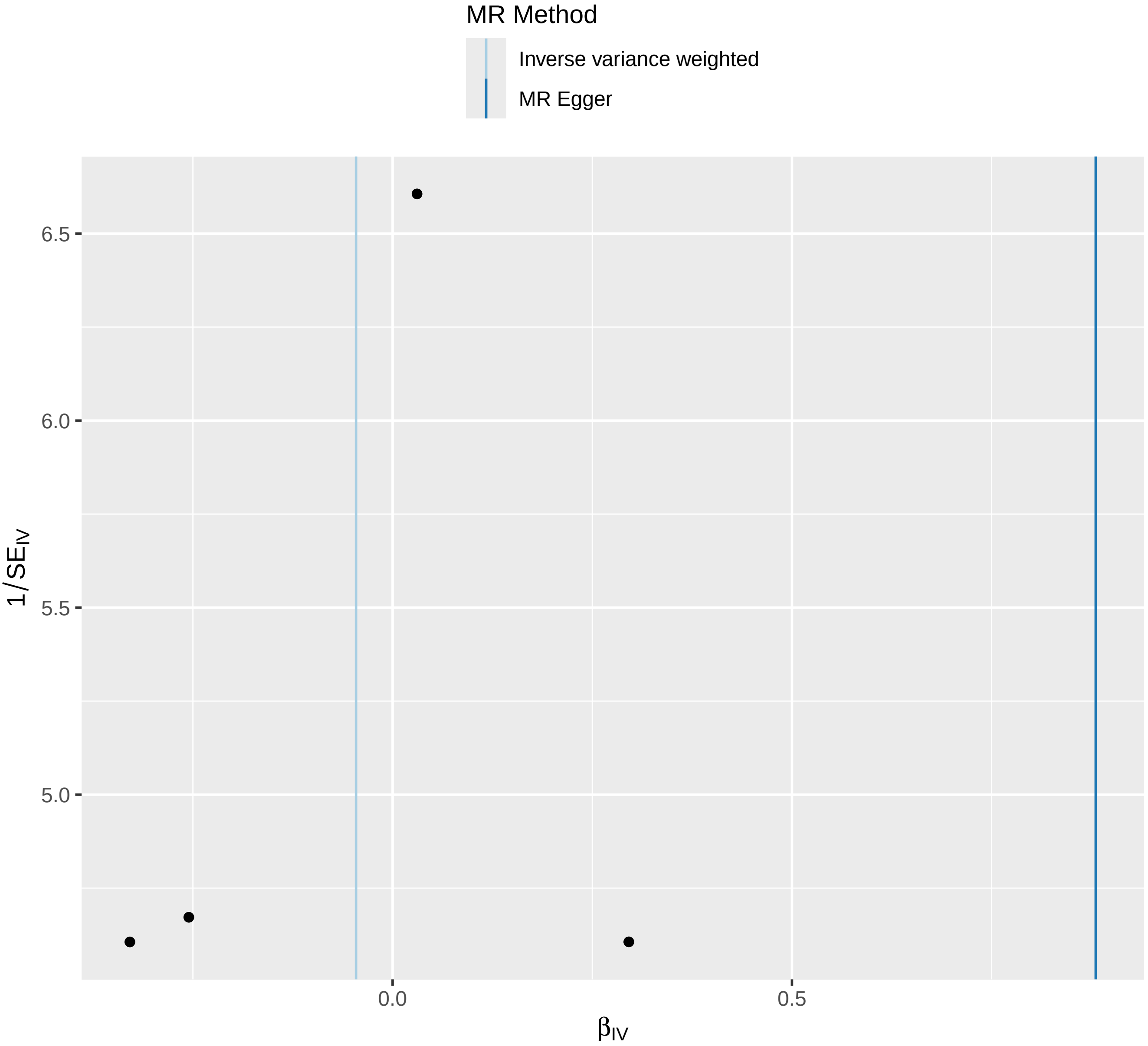

MR Method
Inverse variance weighted
MR Egger
6.5
6.0
5.5
5.0
1/SE_IV
0.0
0.5
β_IV

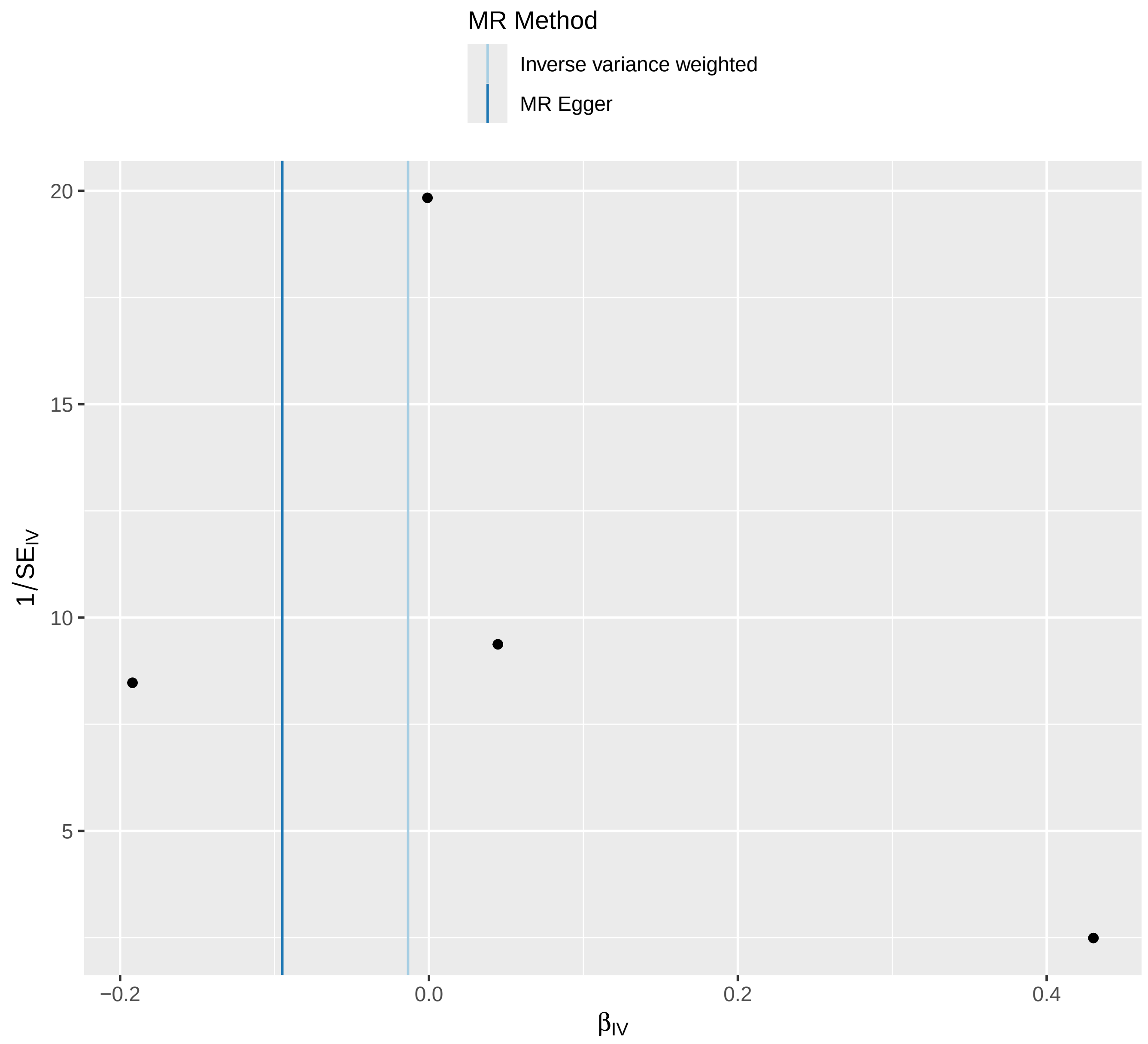
MR Method
Inverse variance weighted
MR Egger
20
15
10
5
−0.2
0.0
0.2
0.4
$1/SE_{IV}$
$\beta_{IV}$

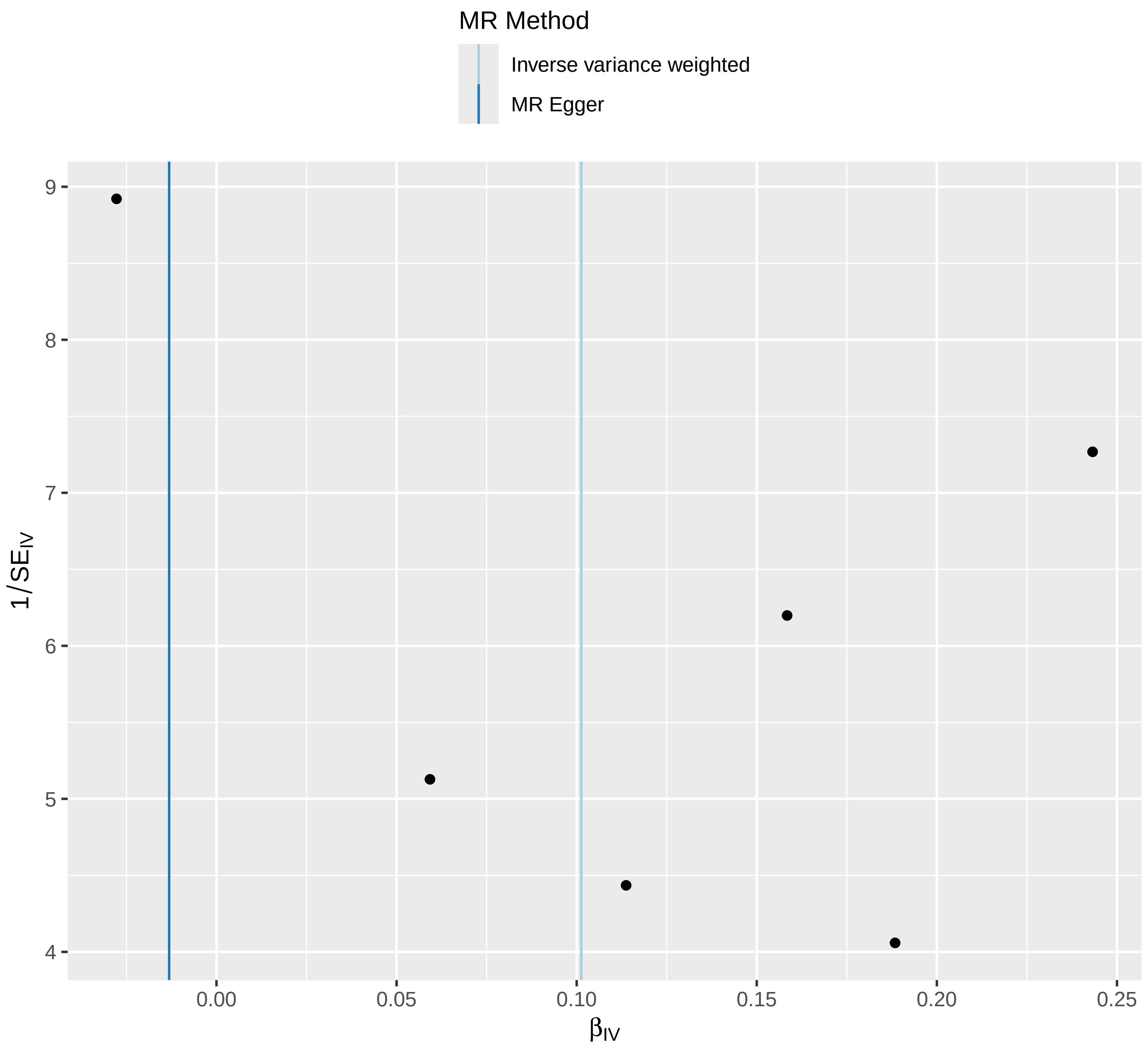

MR Method
Inverse variance weighted
MR Egger
9
8
7
6
5
4
1/SE_IV
0.00
0.05
0.10
0.15
0.20
0.25
β_IV

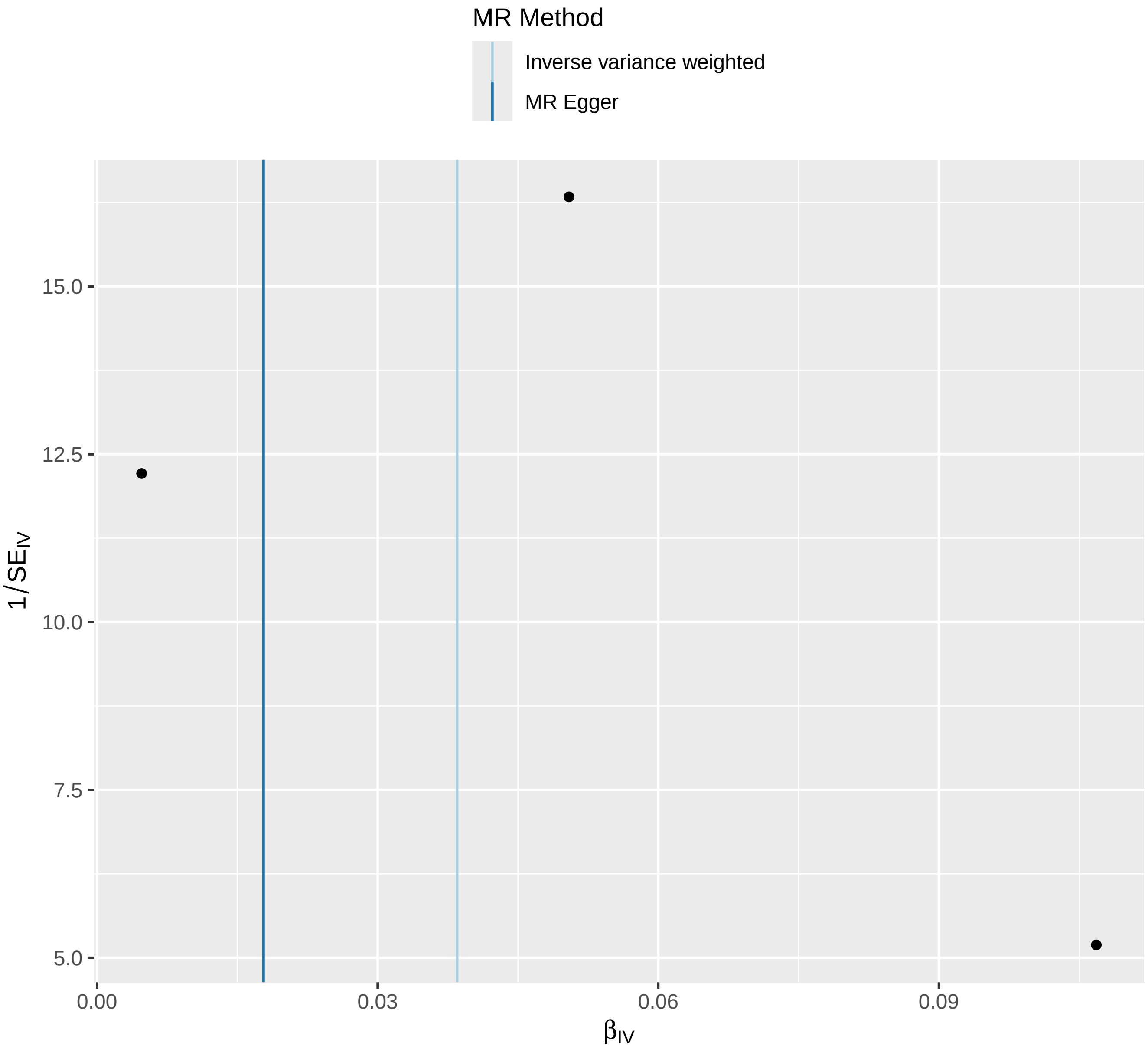

MR Method
Inverse variance weighted
MR Egger
15.0
12.5
10.0
7.5
5.0
1/SE$_{IV}$
0.00
0.03
0.06
0.09
β$_{IV}$

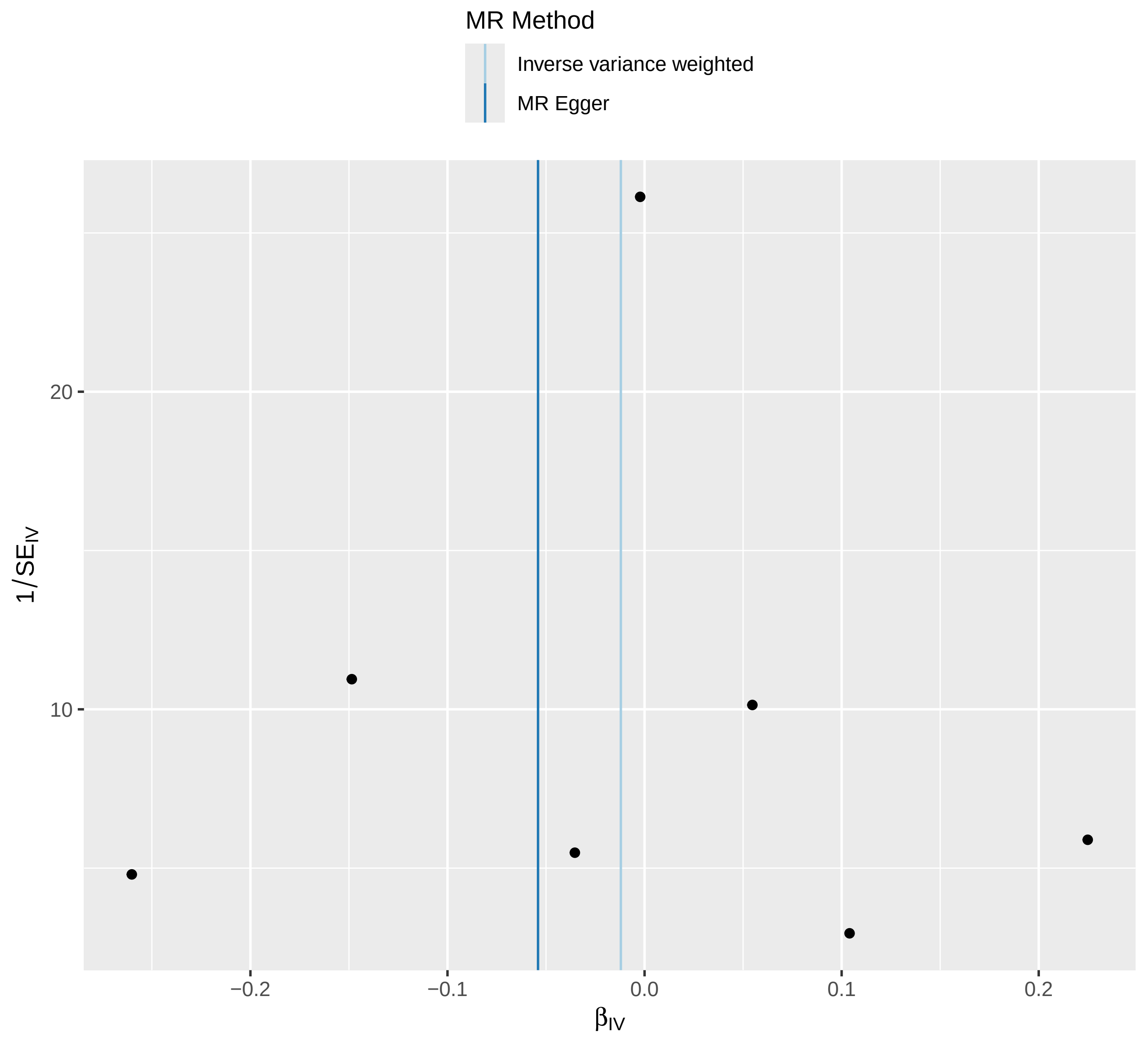

MR Method
Inverse variance weighted
MR Egger
20
10
1/SE$_{IV}$
−0.2
−0.1
0.0
0.1
0.2
β$_{IV}$

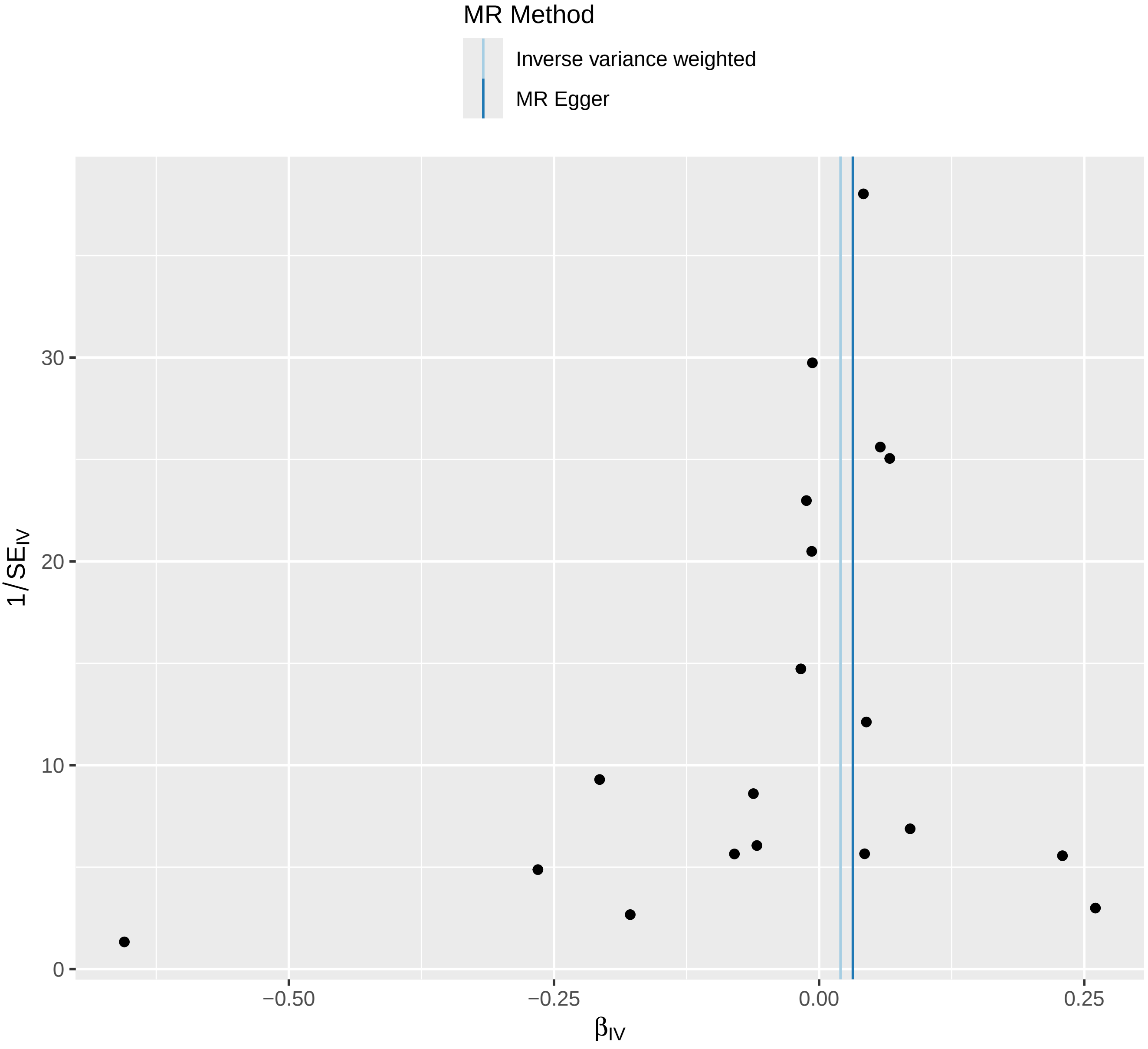

MR Method
Inverse variance weighted
MR Egger
30
20
10
0
−0.50
−0.25
0.00
0.25
1/SE$_{IV}$
β$_{IV}$

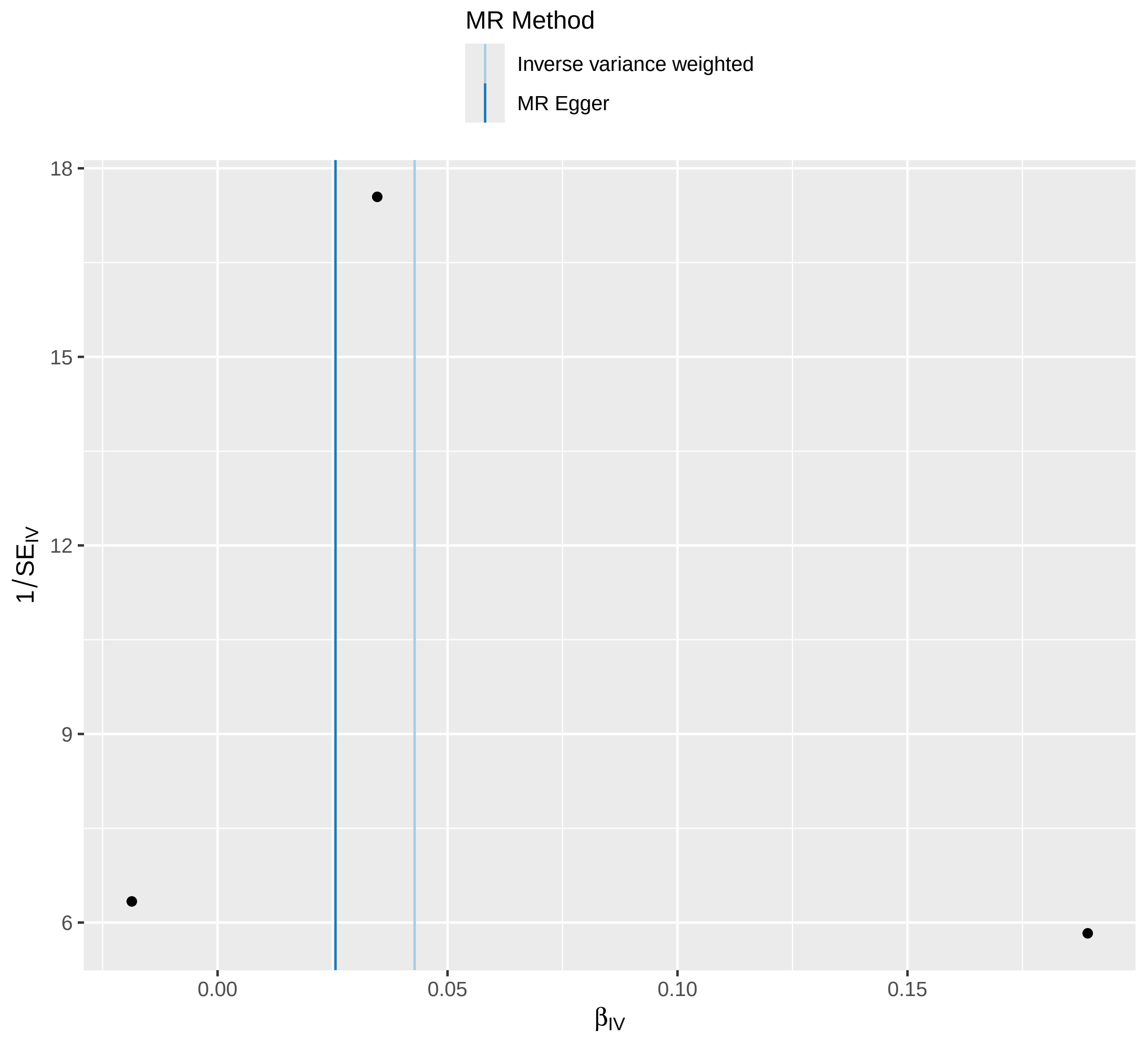
MR Method
Inverse variance weighted
MR Egger
18
15
12
9
6
$1/SE_{IV}$
0.00
0.05
0.10
0.15
$\beta_{IV}$

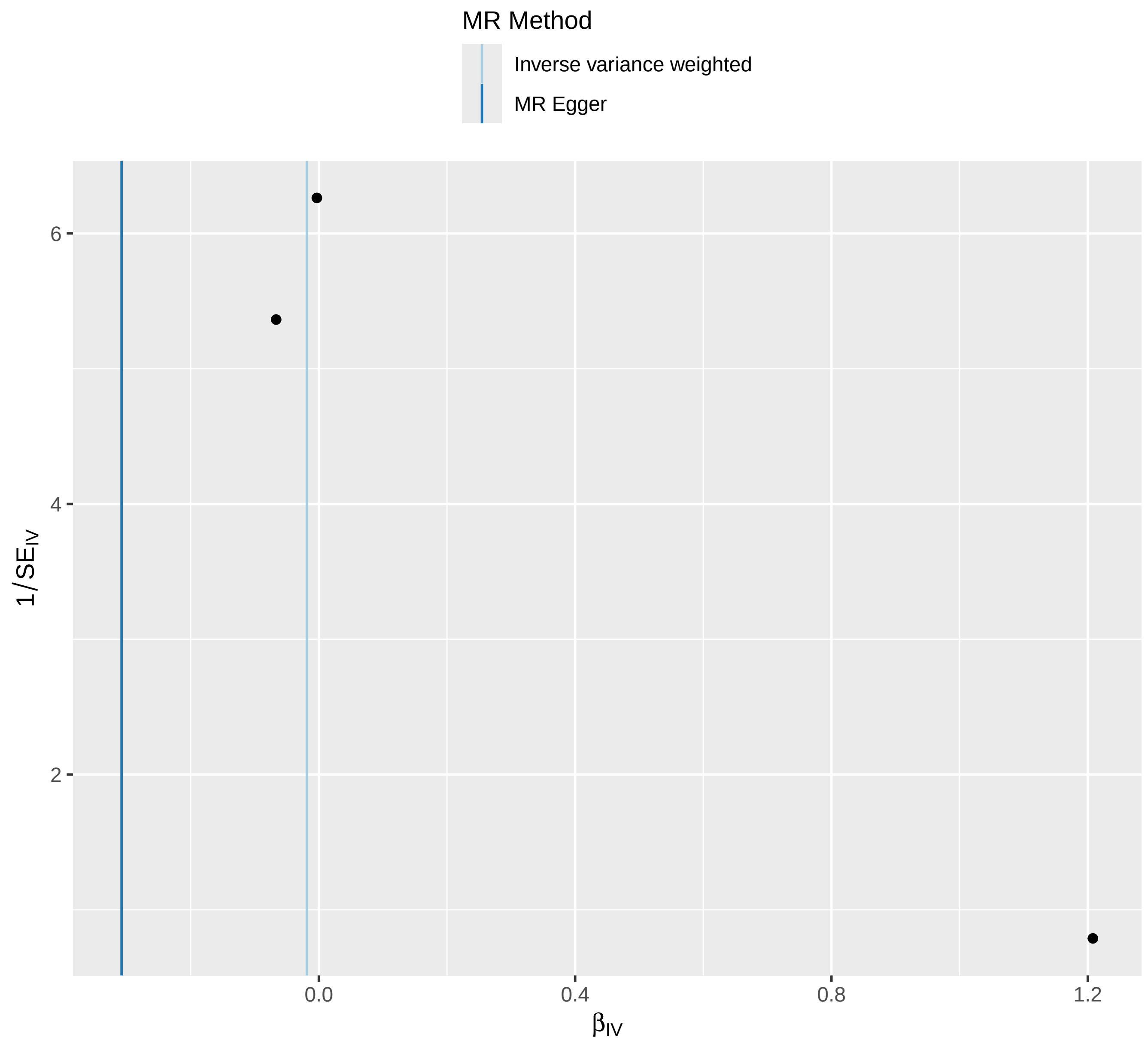

MR Method
Inverse variance weighted
MR Egger
6
4
2
1/SE_IV
0.0
0.4
0.8
1.2
β_IV

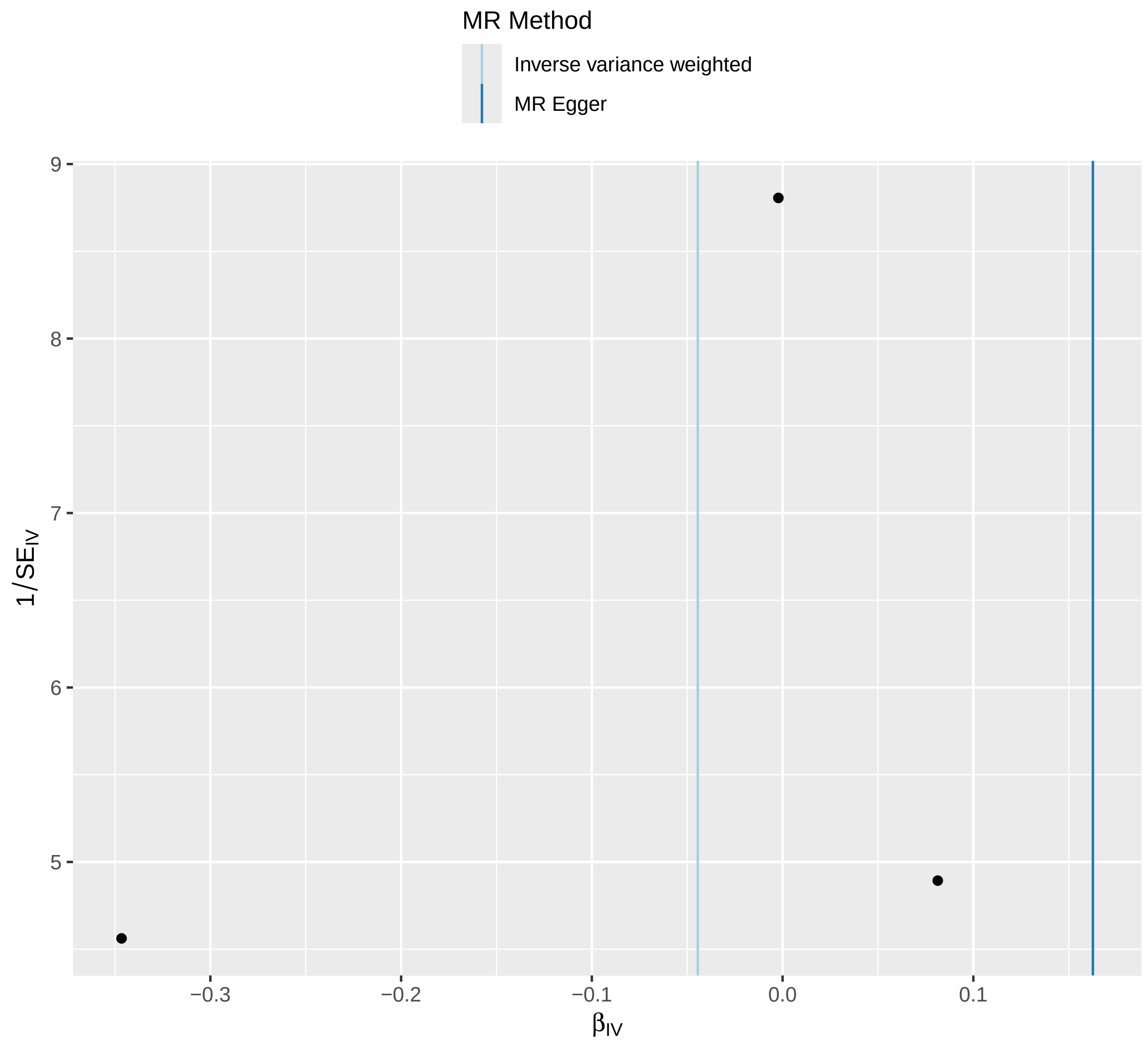

MR Method
Inverse variance weighted
MR Egger
9
8
7
6
5
1/SE$_{IV}$
−0.3
−0.2
−0.1
0.0
0.1
β$_{IV}$

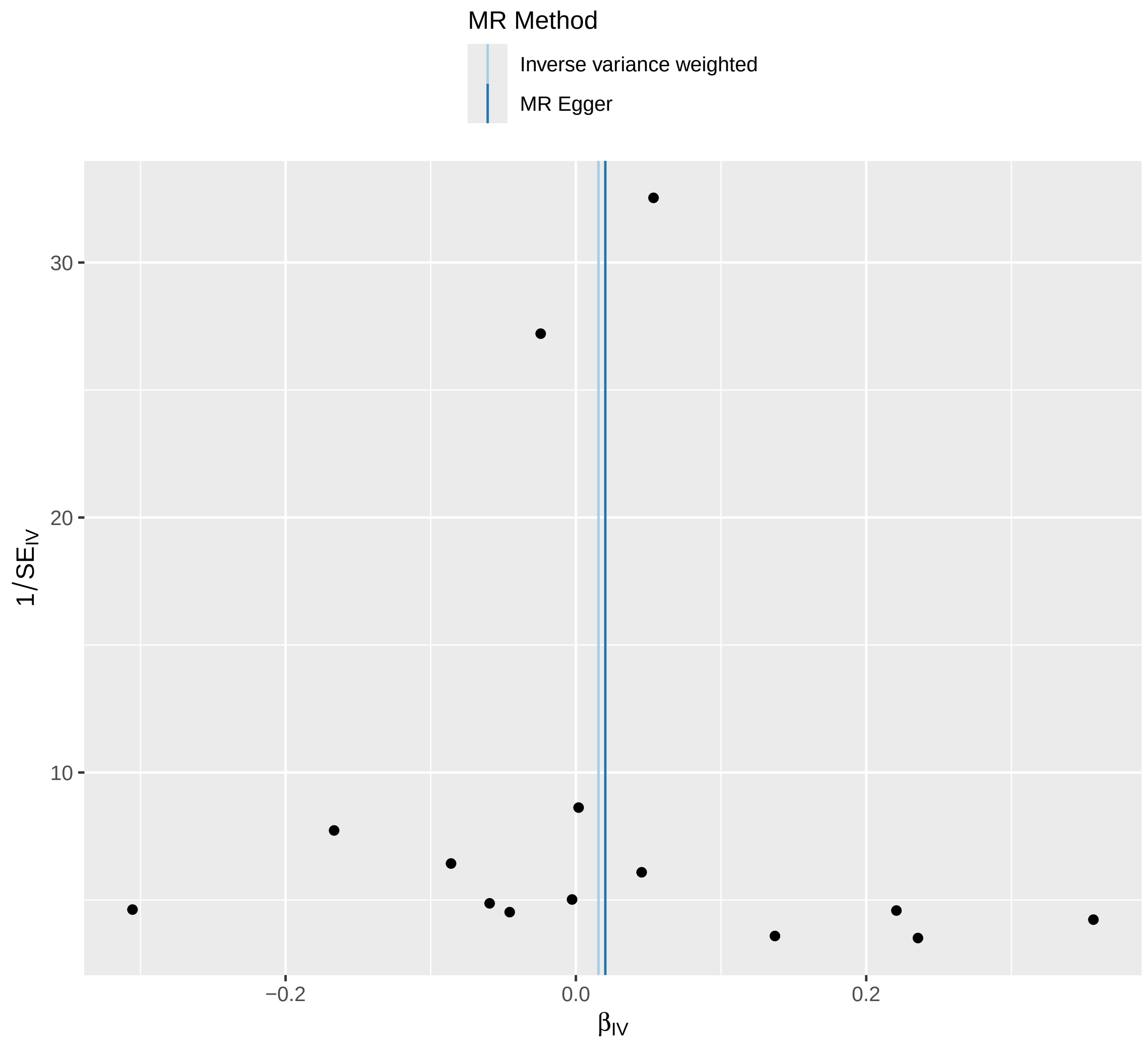
MR Method
Inverse variance weighted
MR Egger
30
20
10
1/SE$_{IV}$
−0.2
0.0
0.2
β$_{IV}$

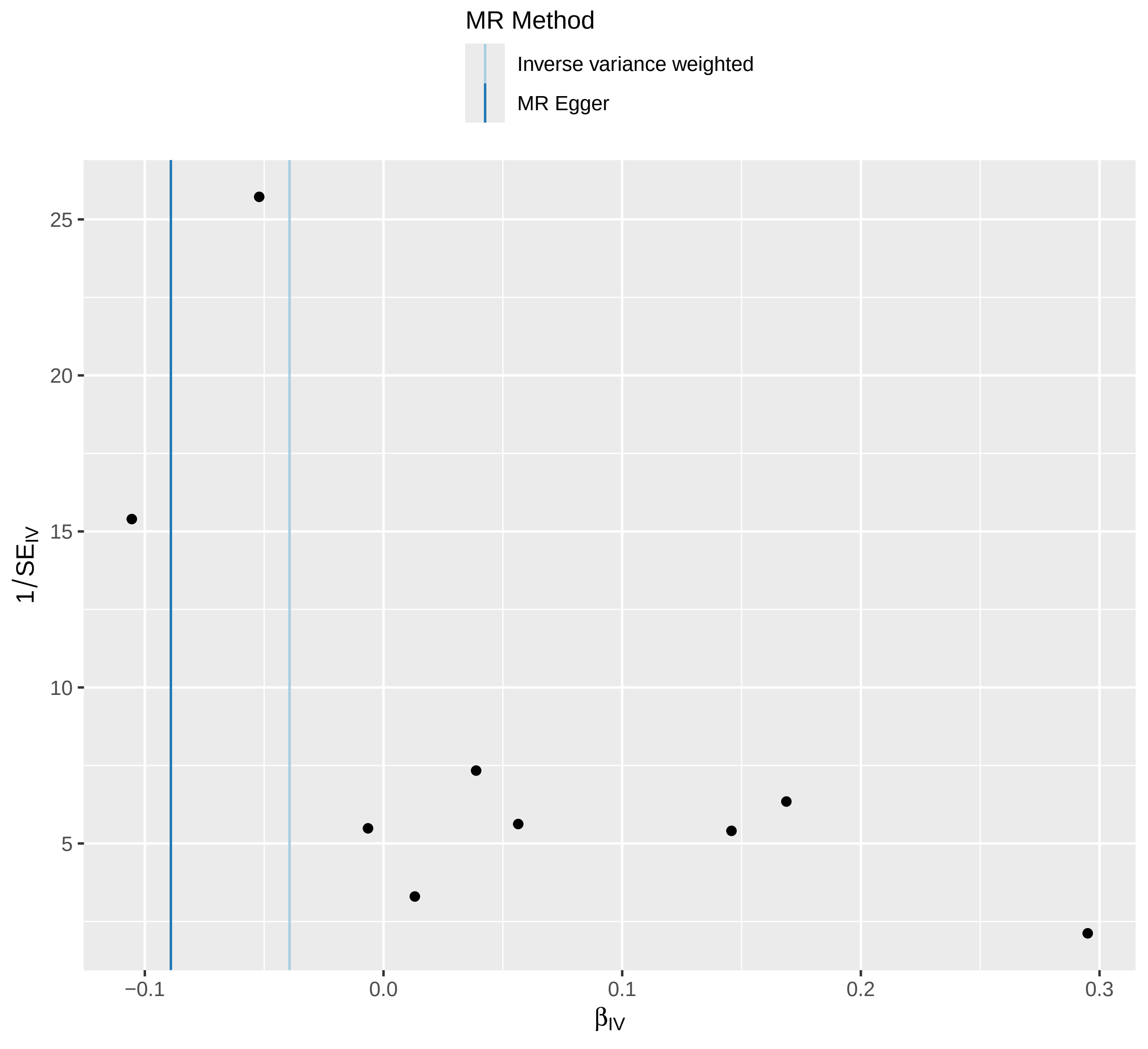
MR Method
Inverse variance weighted
MR Egger
25
20
15
10
5
$1/SE_{IV}$
−0.1
0.0
0.1
0.2
0.3
$\beta_{IV}$

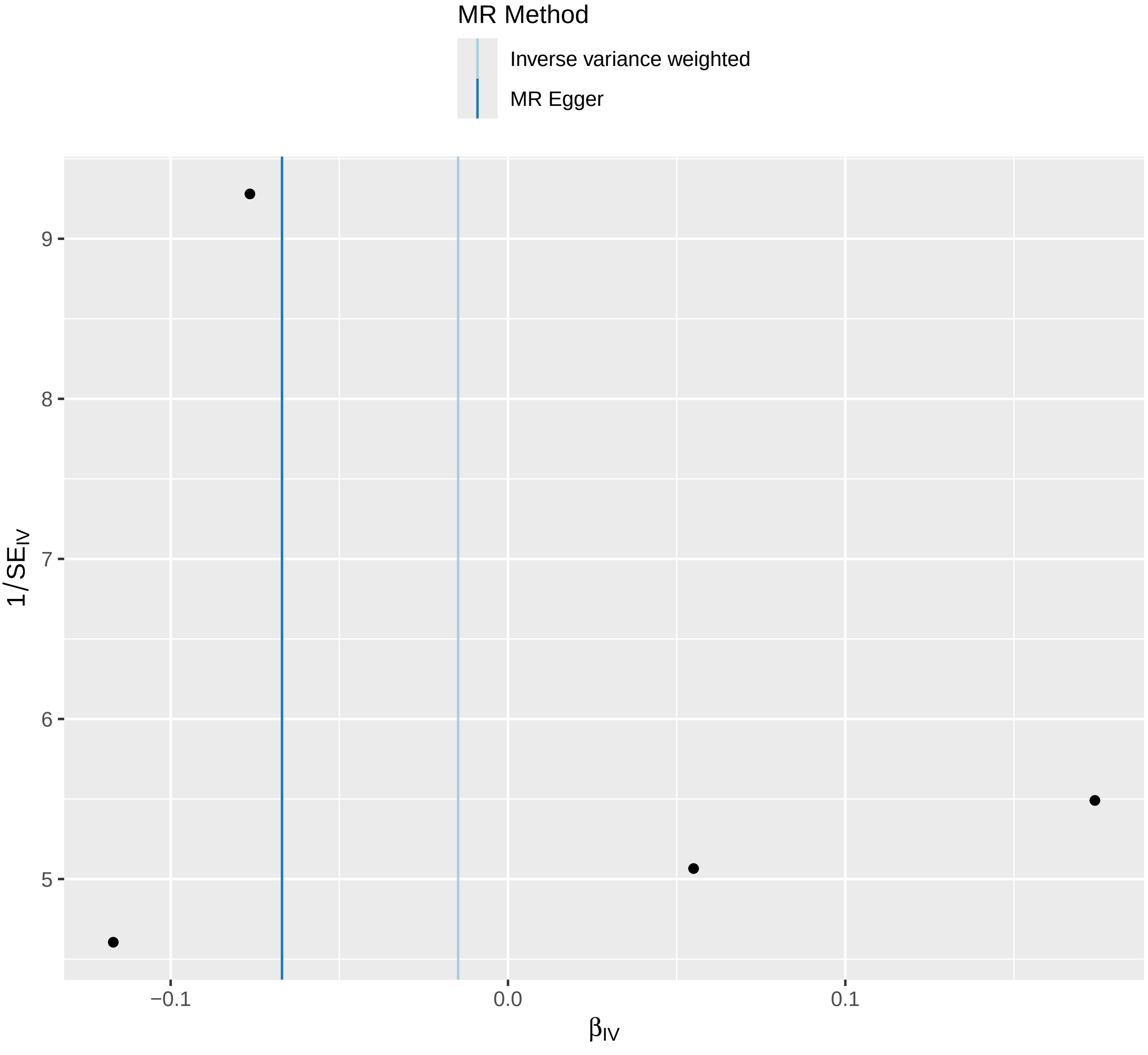

MR Method
Inverse variance weighted
MR Egger
9
8
7
6
5
1/SE_IV
−0.1
0.0
0.1
β_IV

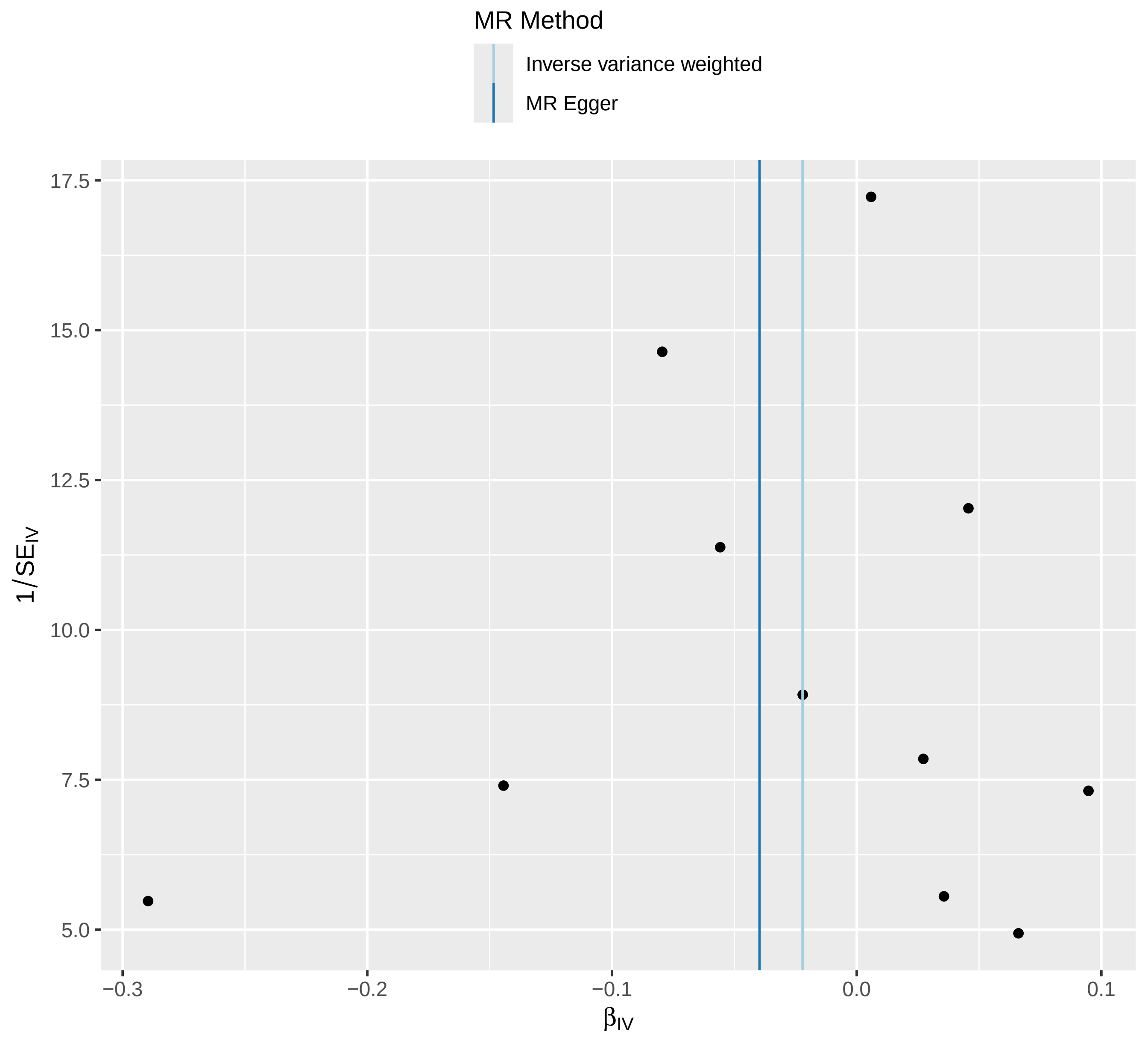

MR Method
Inverse variance weighted
MR Egger
17.5
15.0
12.5
10.0
7.5
5.0
1/SE_IV
−0.3
−0.2
−0.1
0.0
0.1
β_IV

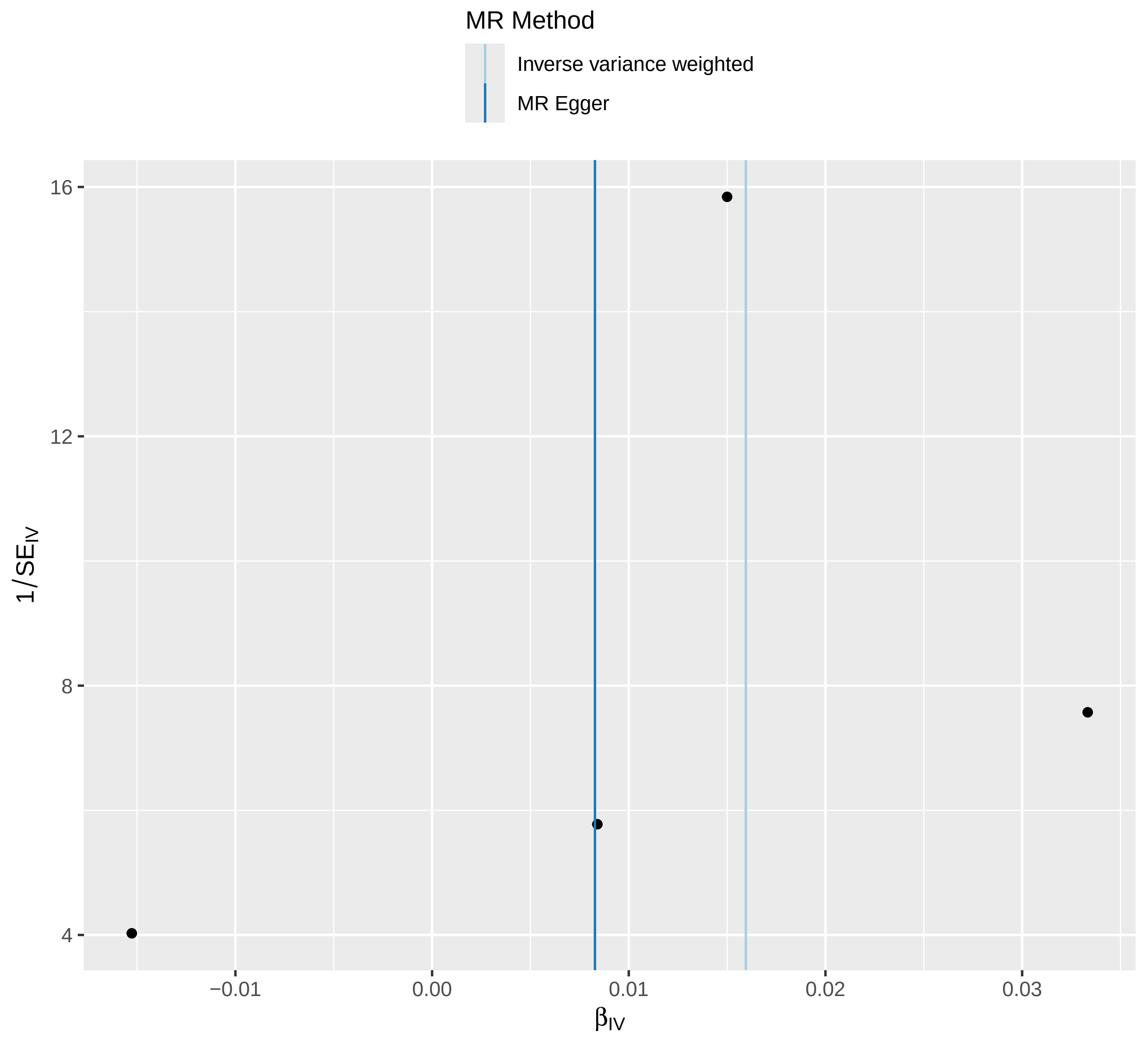
MR Method
Inverse variance weighted
MR Egger
16
12
8
4
1/SE$_{IV}$
−0.01
0.00
0.01
0.02
0.03
β$_{IV}$

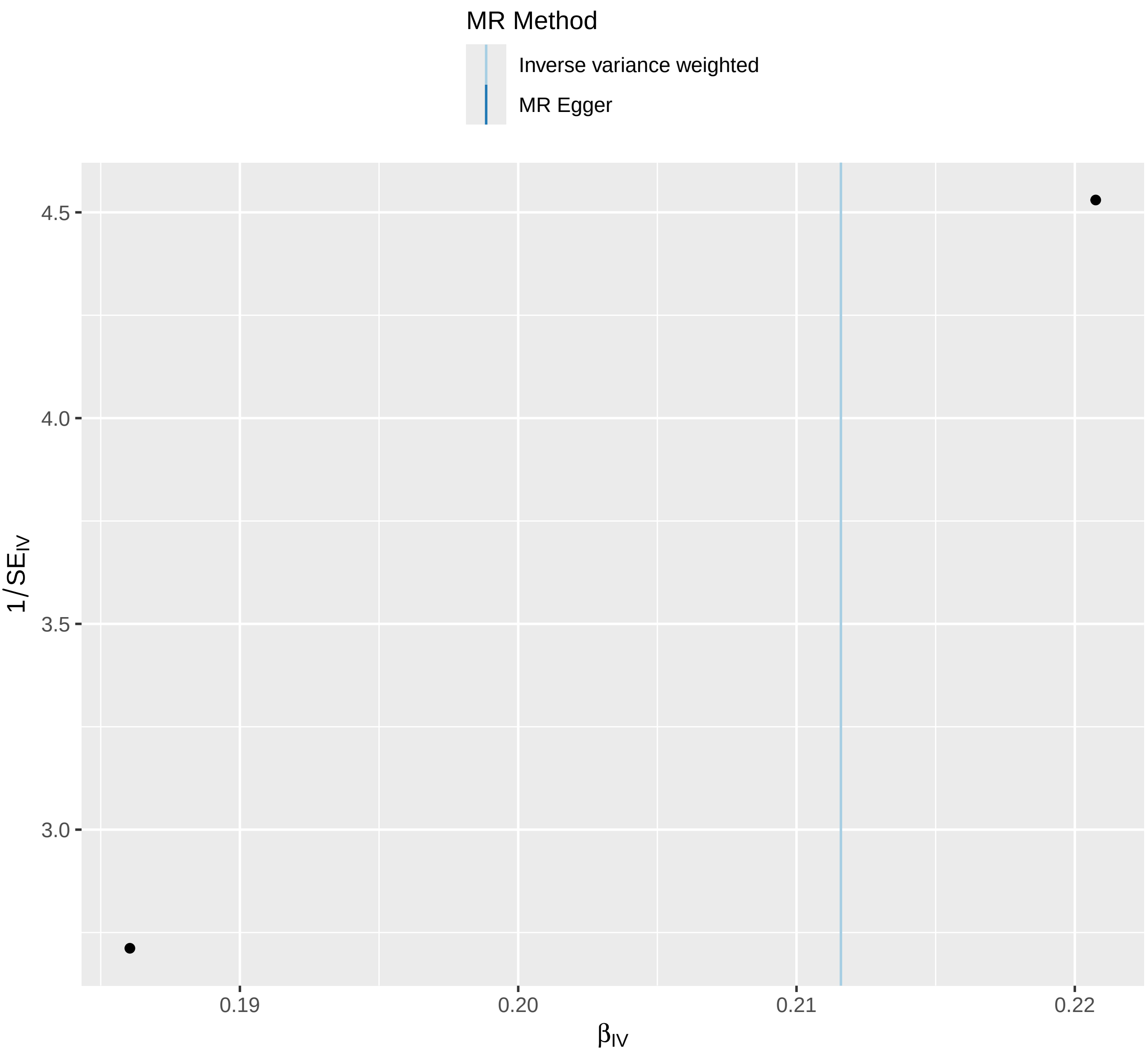
MR Method
Inverse variance weighted
MR Egger
4.5
4.0
3.5
3.0
1/SE$_{IV}$
0.19
0.20
0.21
0.22
β$_{IV}$

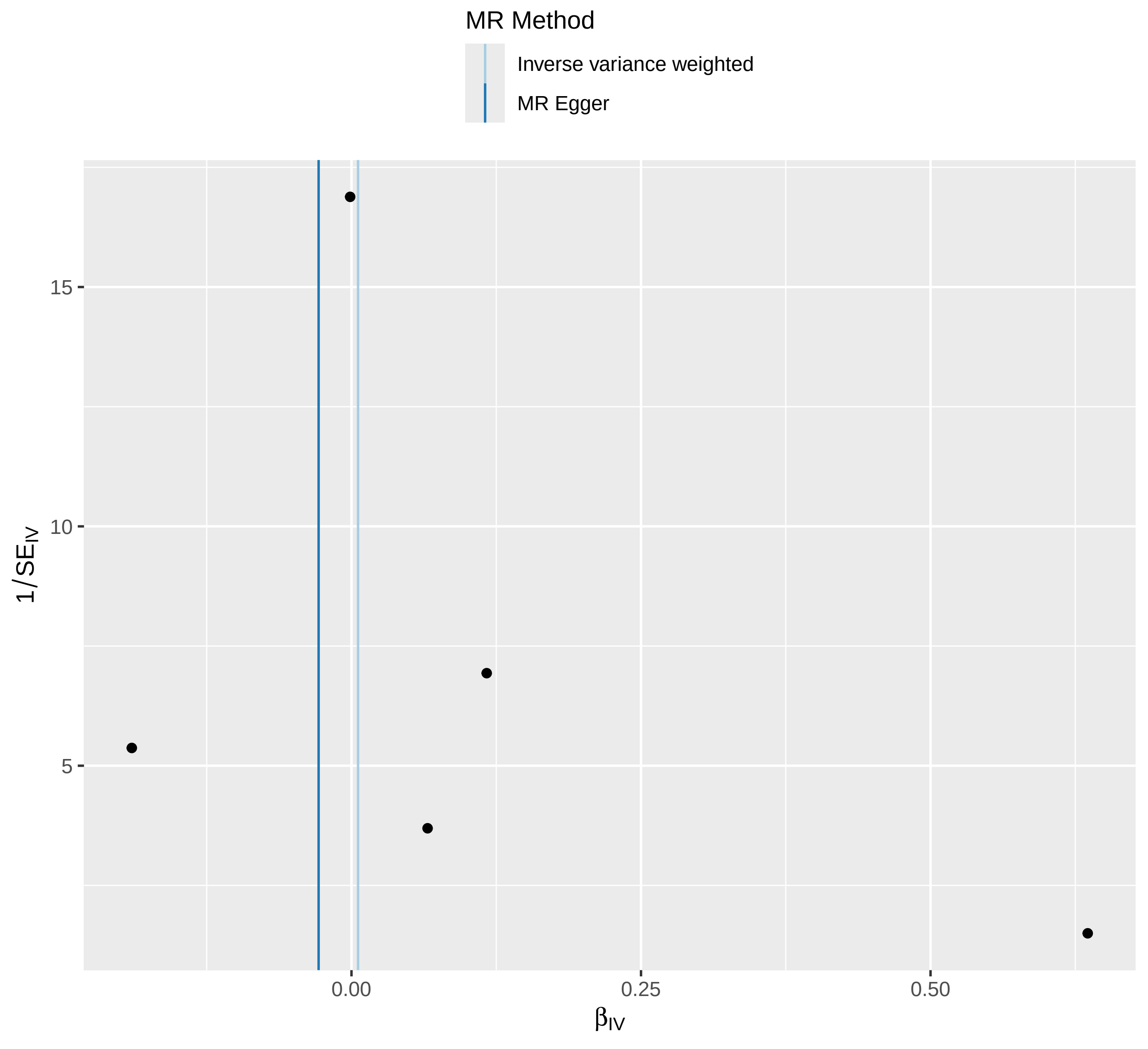

MR Method
Inverse variance weighted
MR Egger
15
10
5
1/SE$_{IV}$
0.00
0.25
0.50
β$_{IV}$

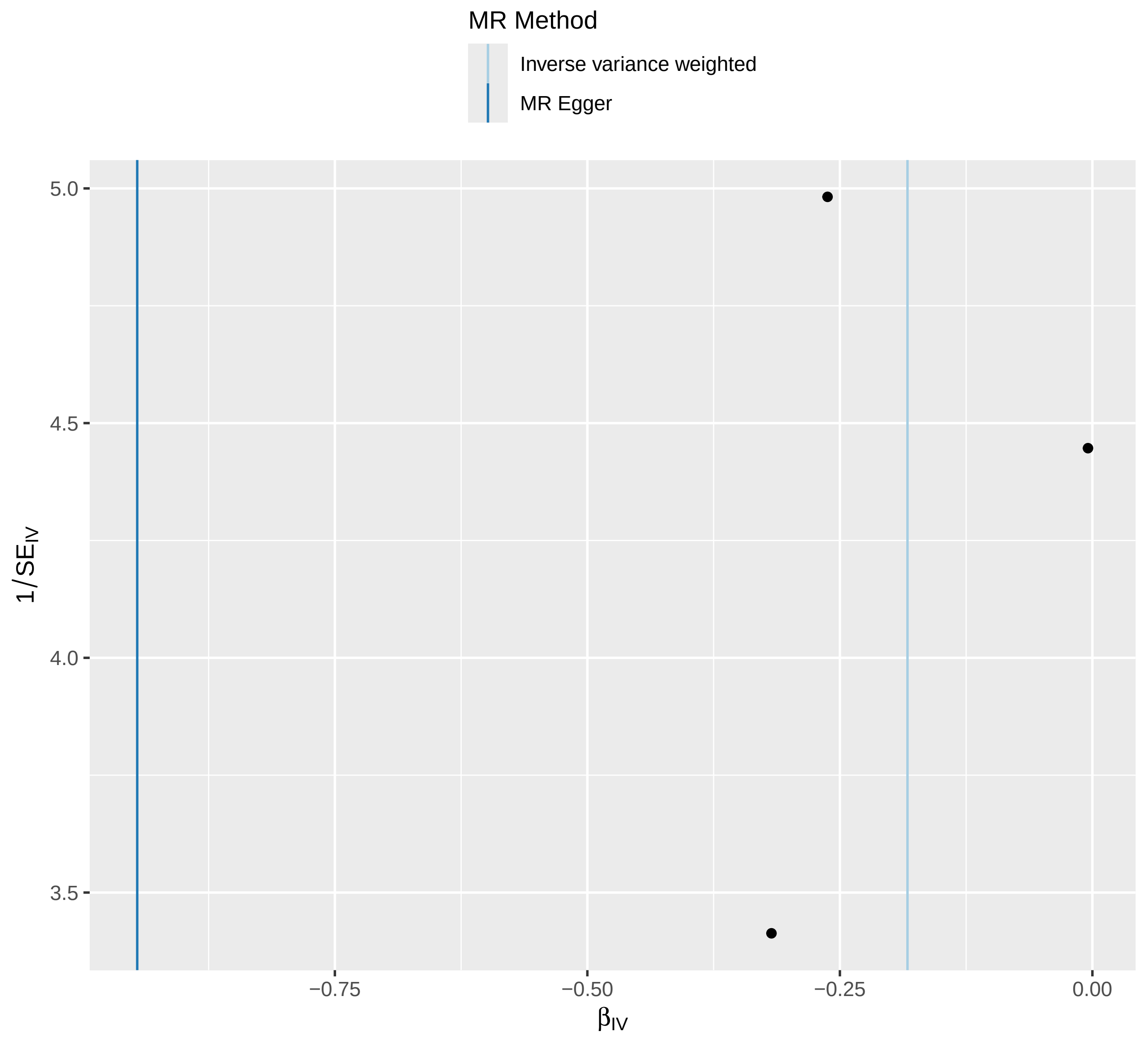
MR Method
Inverse variance weighted
MR Egger
5.0
4.5
4.0
3.5
$1/SE_{IV}$
−0.75
−0.50
−0.25
0.00
$\beta_{IV}$

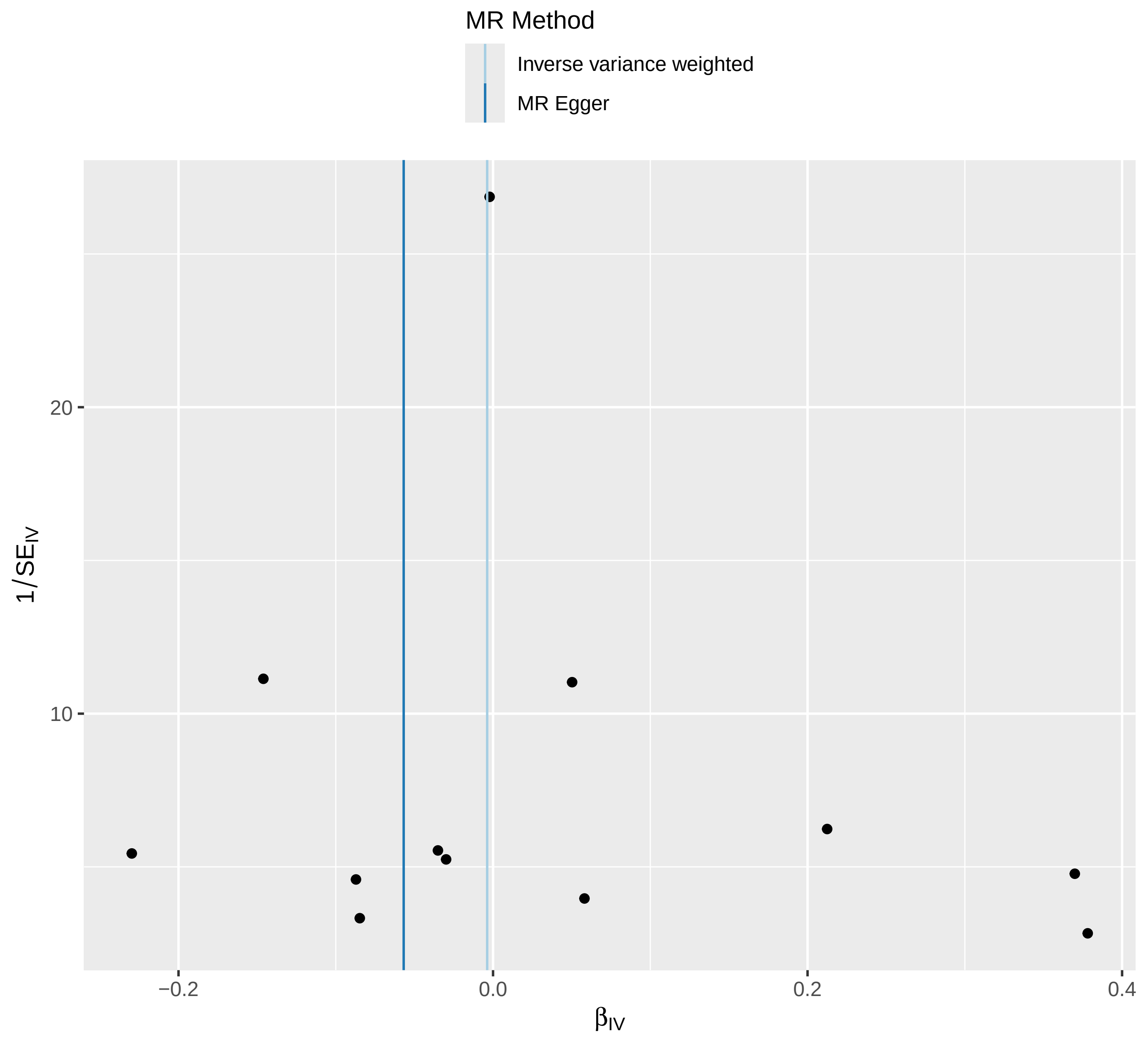

MR Method
Inverse variance weighted
MR Egger
20
10
−0.2
0.0
0.2
0.4
1/SE_IV
β_IV

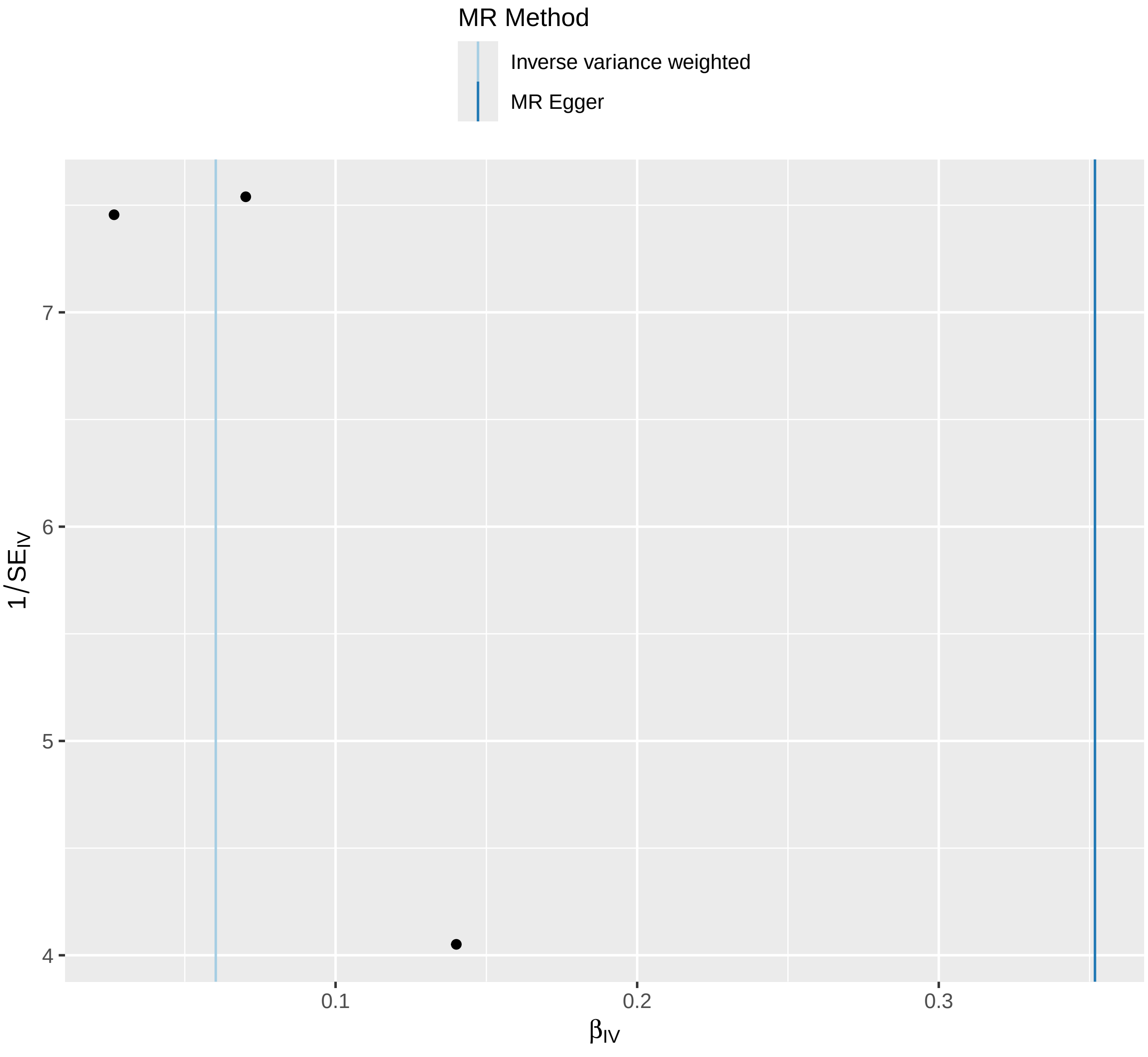
MR Method
Inverse variance weighted
MR Egger
7
6
5
4
1/SE_IV
0.1
0.2
0.3
β_IV

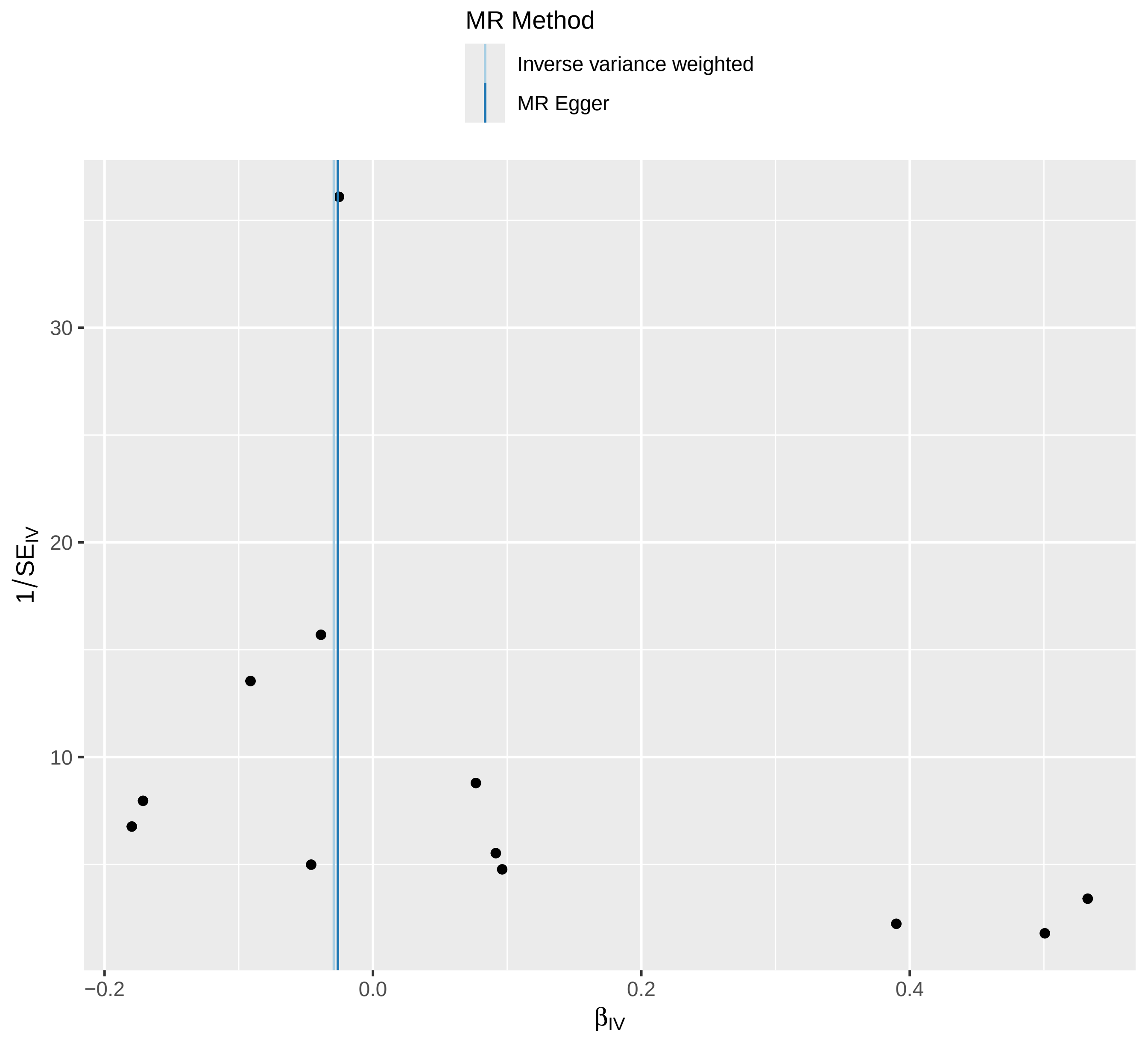

MR Method
Inverse variance weighted
MR Egger
30
20
10
1/SE_IV
−0.2
0.0
0.2
0.4
β_IV

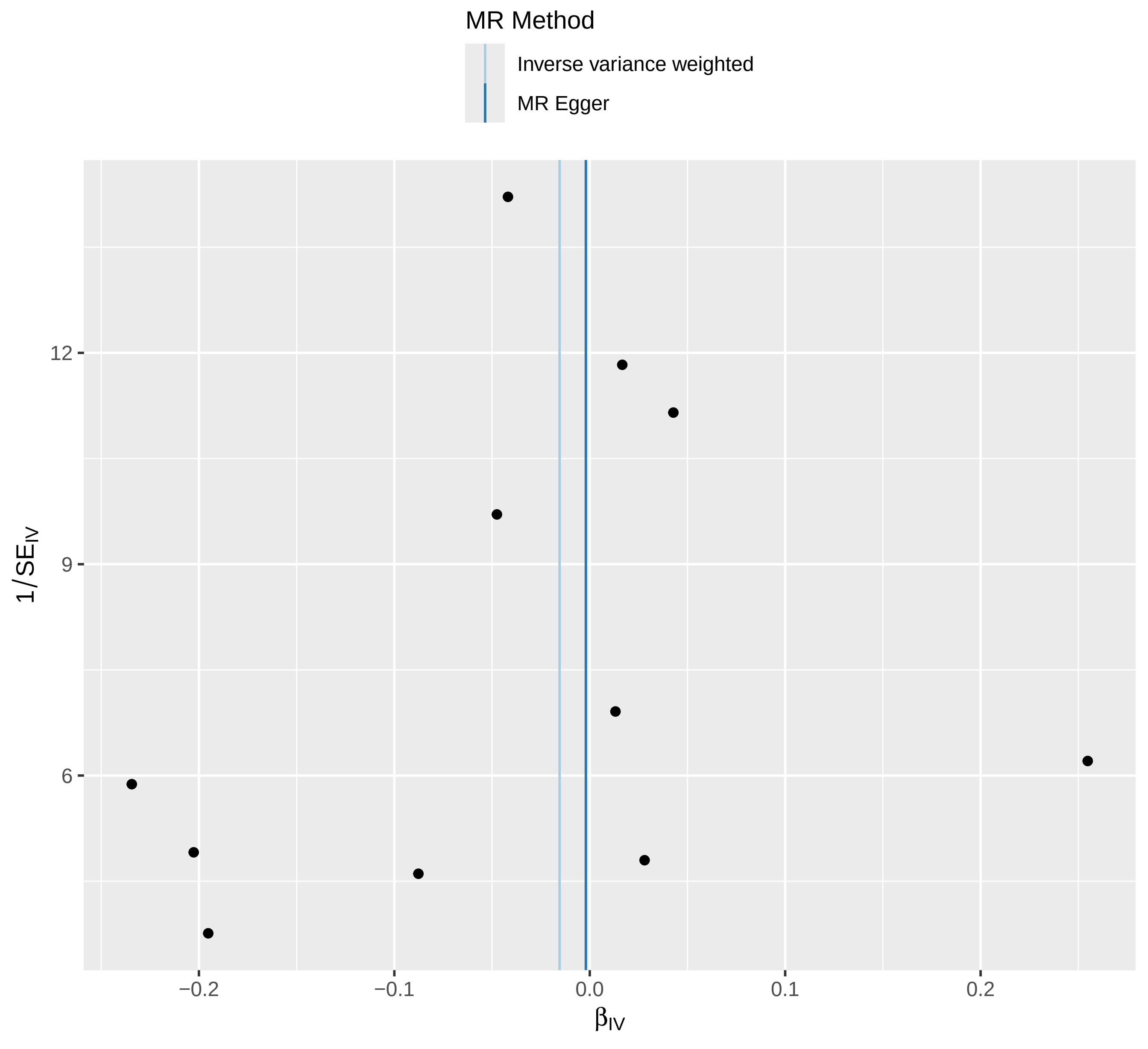
MR Method
Inverse variance weighted
MR Egger
1/SE_IV
12
9
6
−0.2
−0.1
0.0
0.1
0.2
β_IV

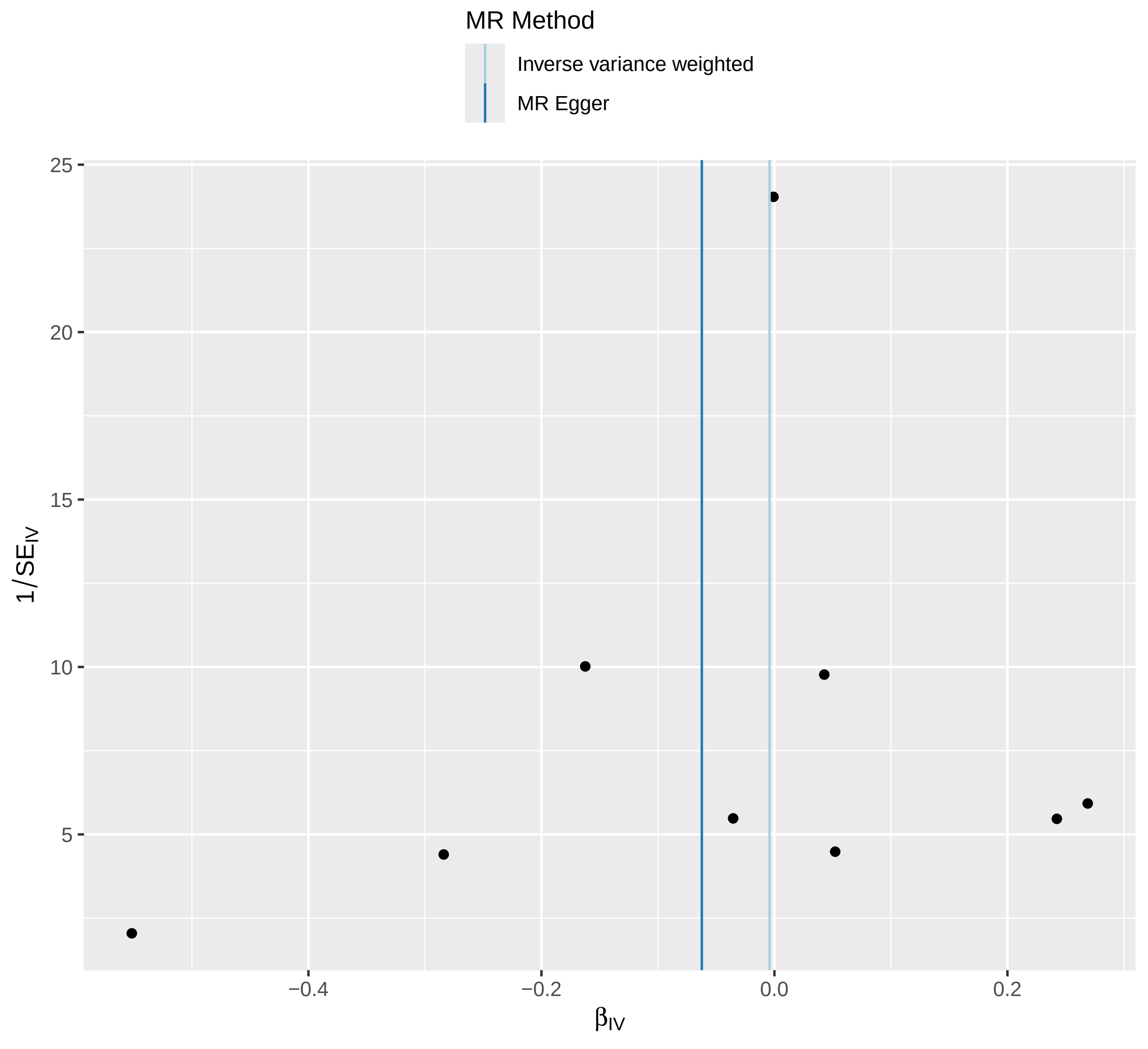

MR Method
Inverse variance weighted
MR Egger
25
20
15
10
5
1/SE$_{IV}$
−0.4
−0.2
0.0
0.2
β$_{IV}$

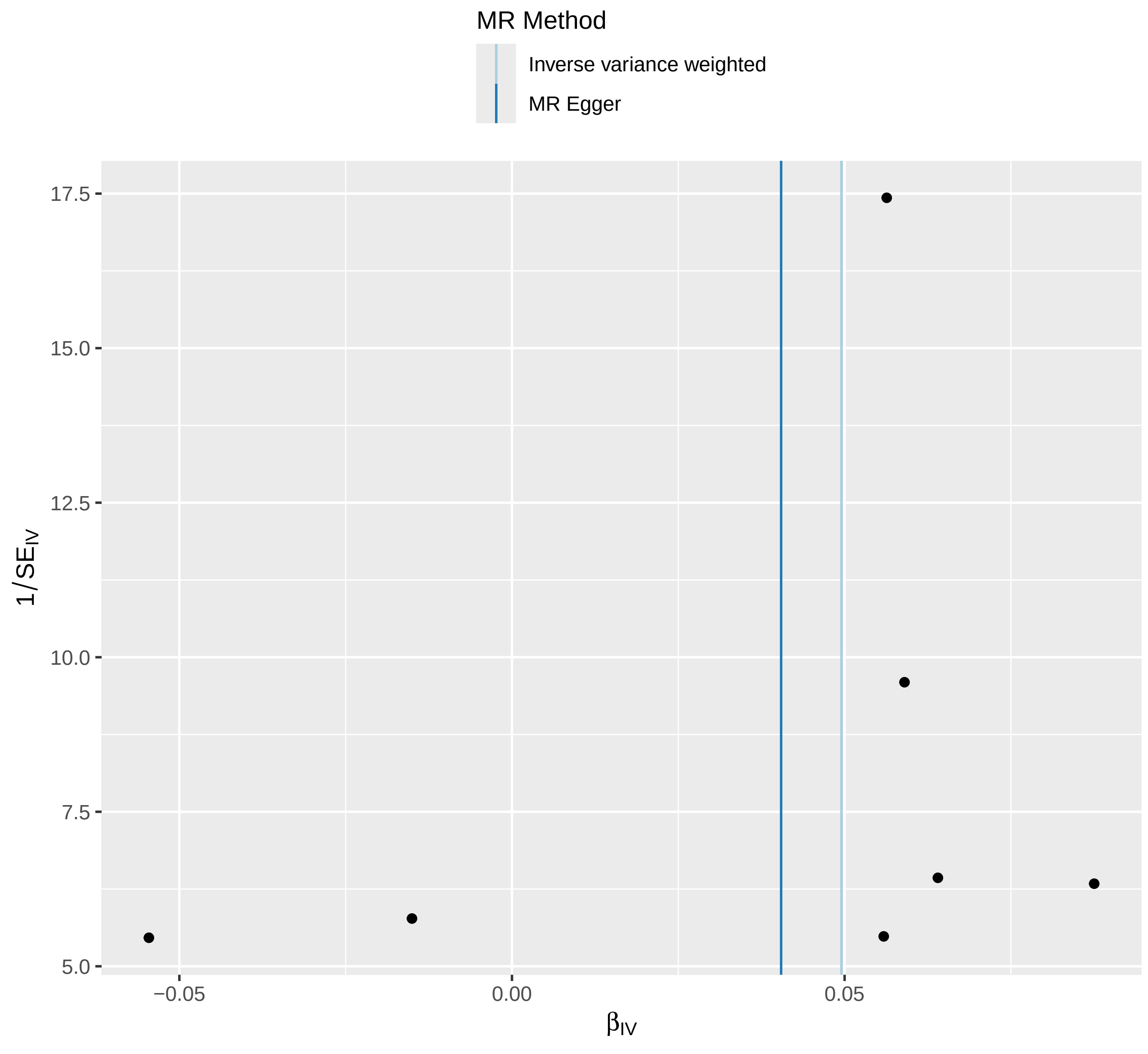
MR Method
Inverse variance weighted
MR Egger
17.5
15.0
12.5
10.0
7.5
5.0
1/SE$_{IV}$
−0.05
0.00
0.05
β$_{IV}$

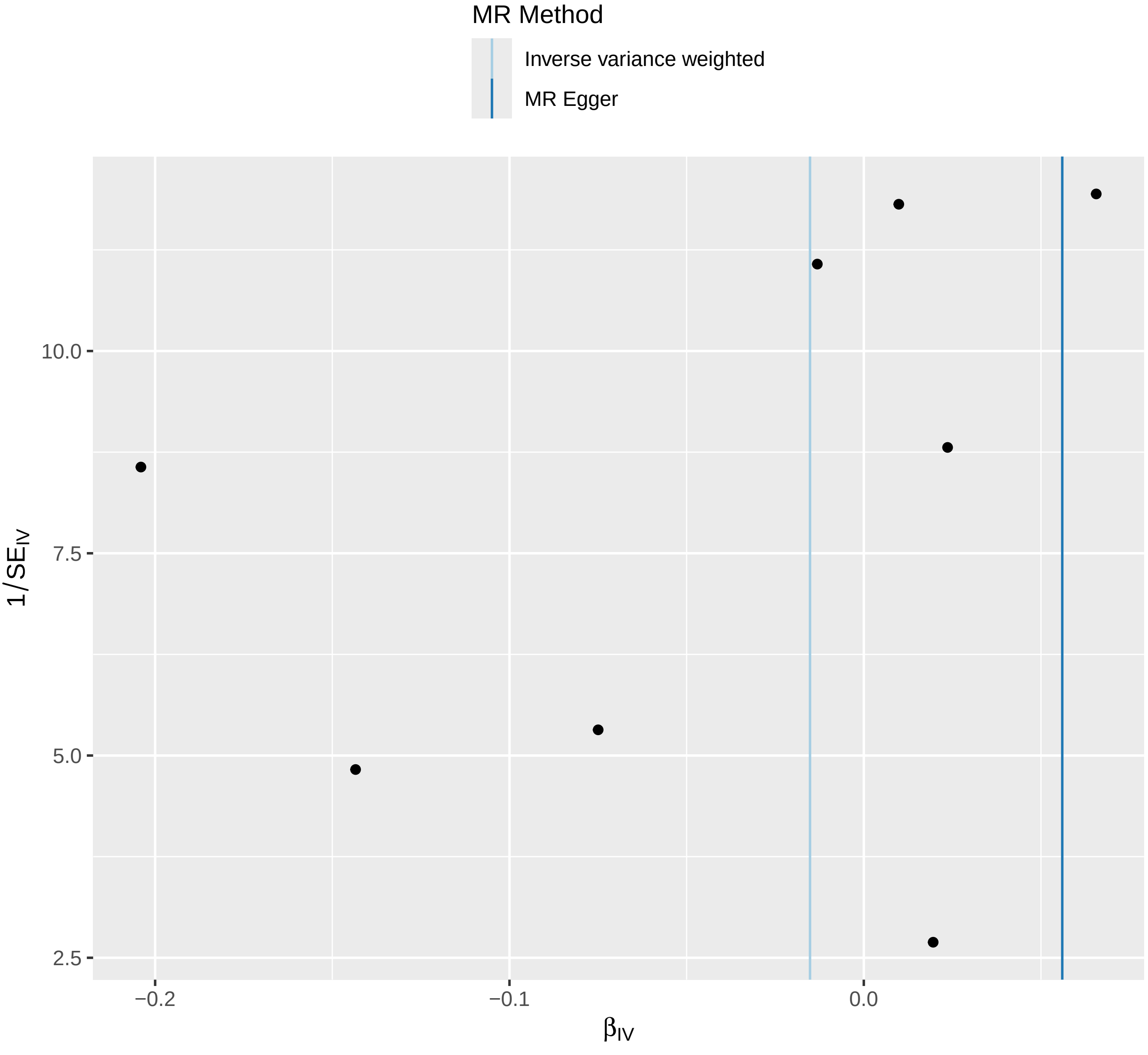

MR Method
Inverse variance weighted
MR Egger
10.0
7.5
5.0
2.5
$1/SE_{IV}$
−0.2
−0.1
0.0
$\beta_{IV}$

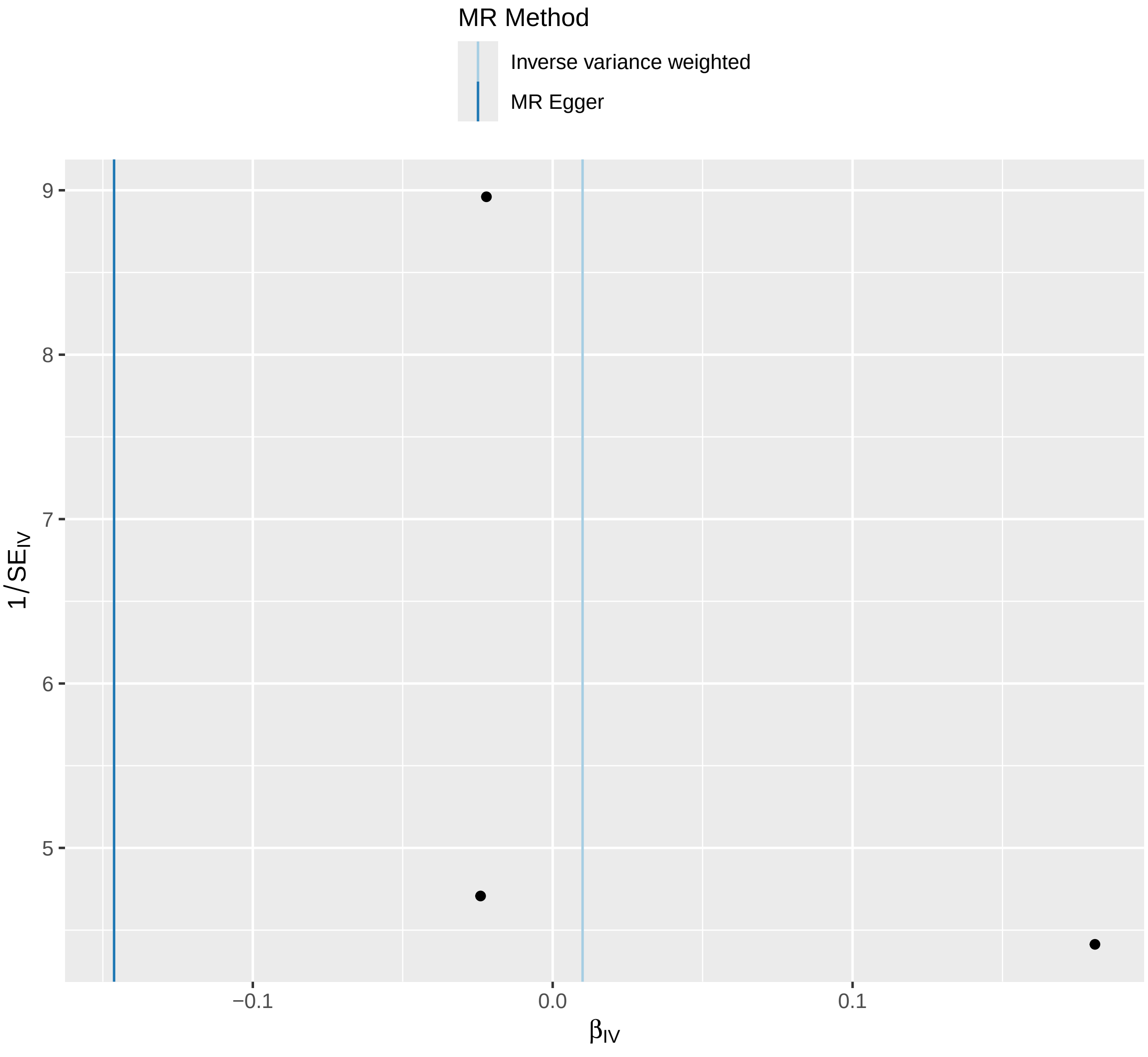
MR Method
Inverse variance weighted
MR Egger
9
8
7
6
5
$1/SE_{IV}$
−0.1
0.0
0.1
$\beta_{IV}$

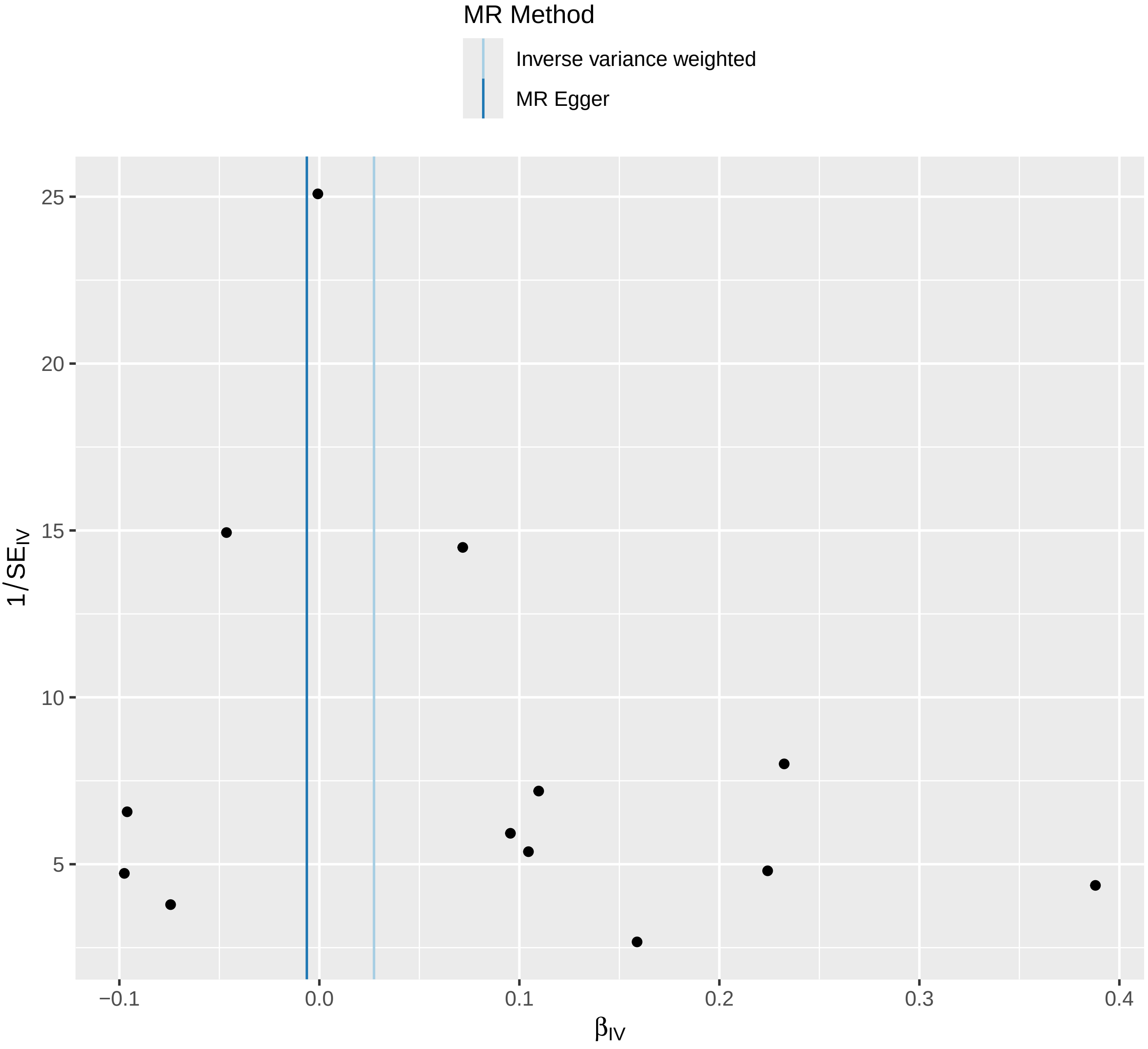

MR Method
Inverse variance weighted
MR Egger
25
20
15
10
5
1/SE_IV
−0.1
0.0
0.1
0.2
0.3
0.4
β_IV

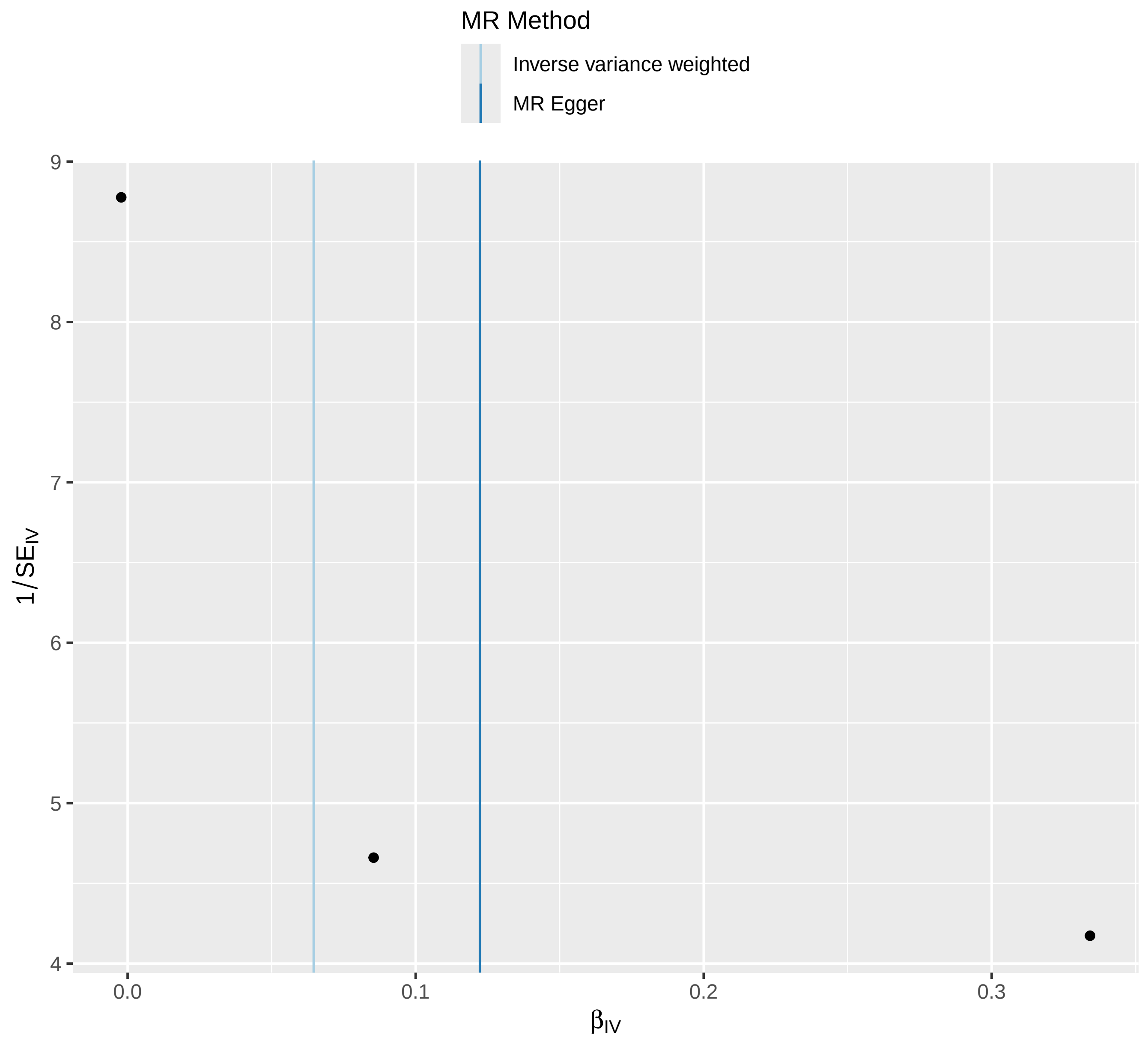
MR Method
Inverse variance weighted
MR Egger
9
8
7
6
5
4
1/SE_IV
0.0
0.1
0.2
0.3
β_IV

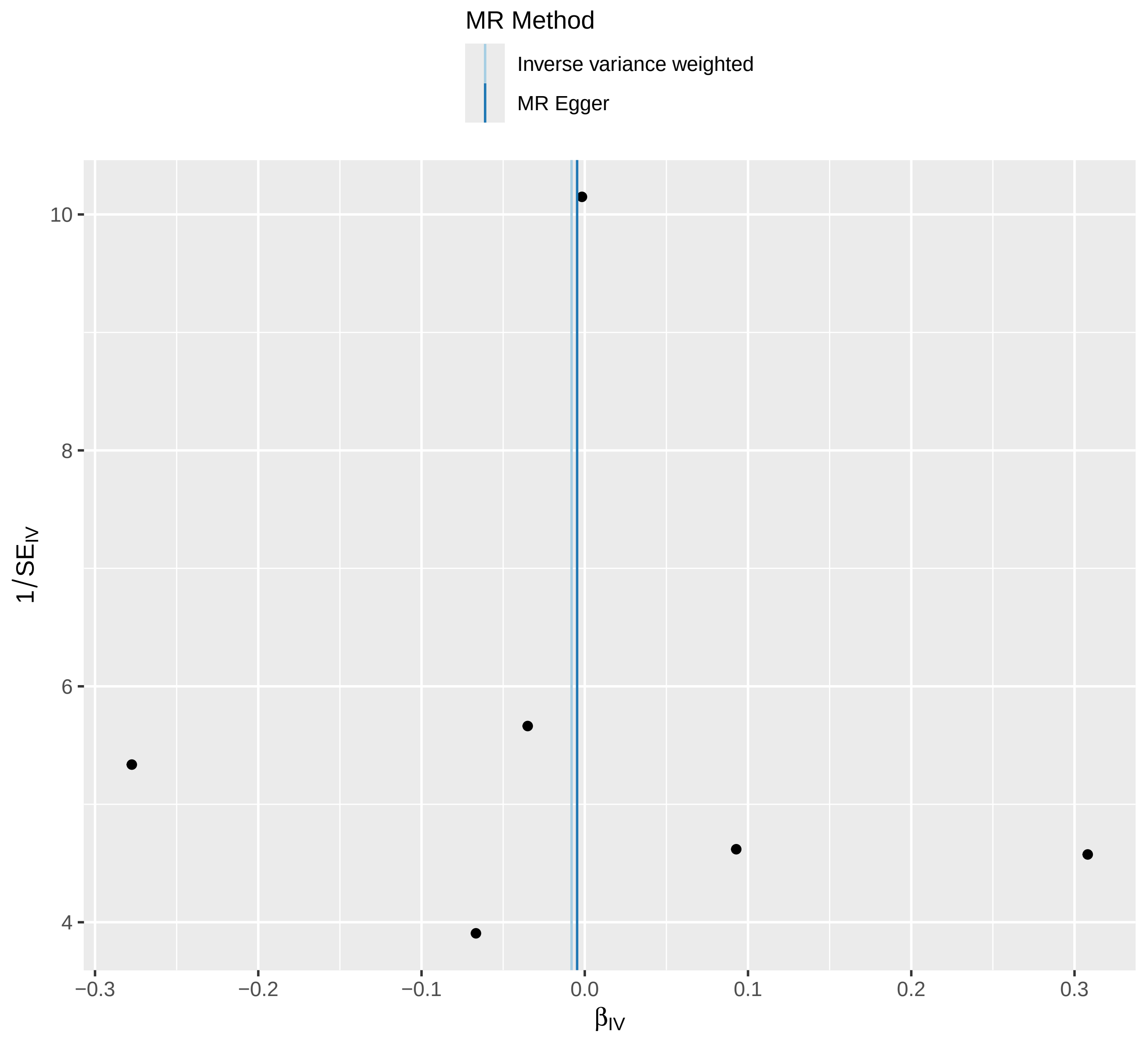
MR Method
Inverse variance weighted
MR Egger
1/SE_IV
10
8
6
4
−0.3
−0.2
−0.1
0.0
0.1
0.2
0.3
β_IV

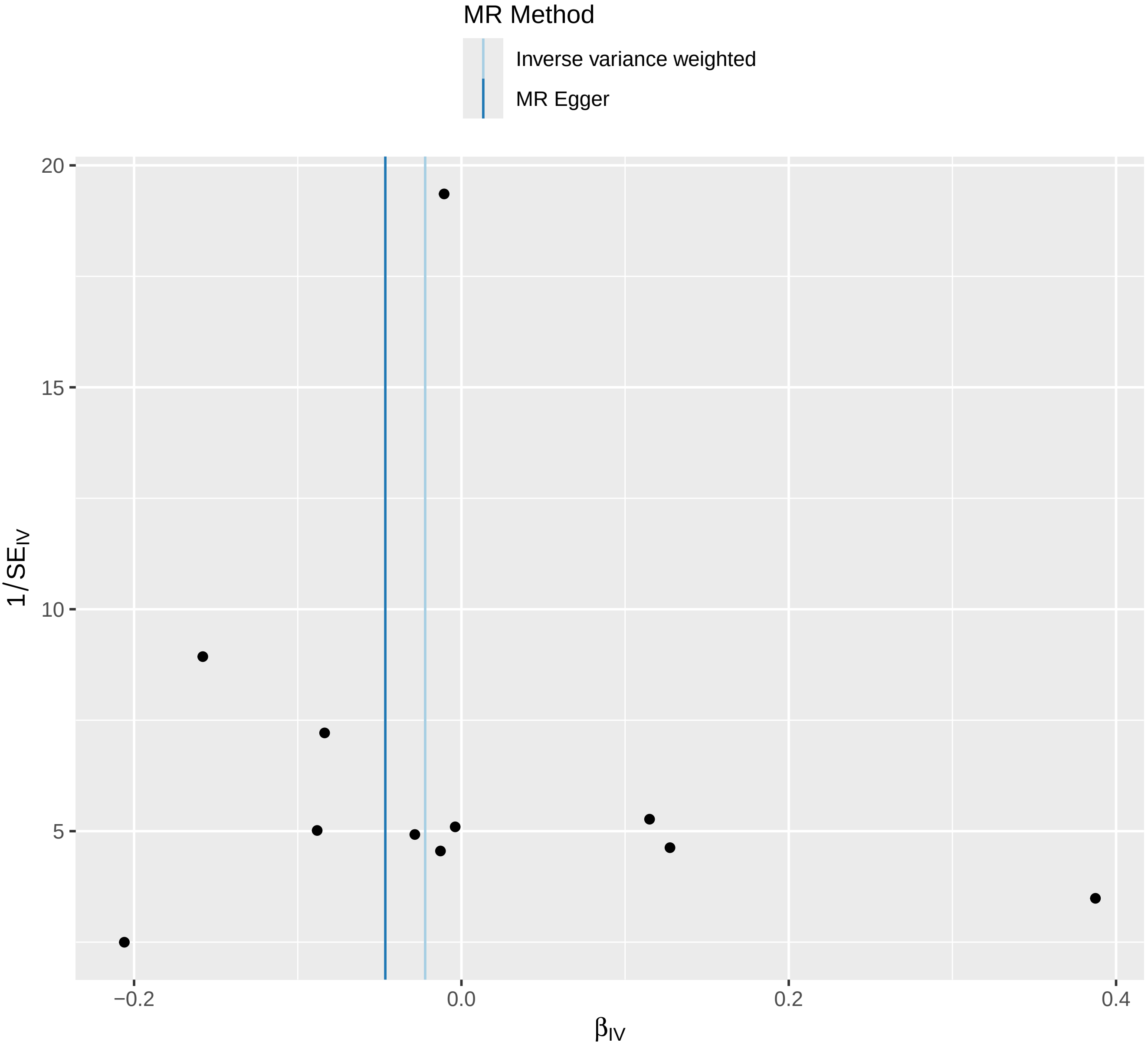
MR Method
Inverse variance weighted
MR Egger
20
15
10
5
1/$SE_{IV}$
−0.2
0.0
0.2
0.4
$\beta_{IV}$

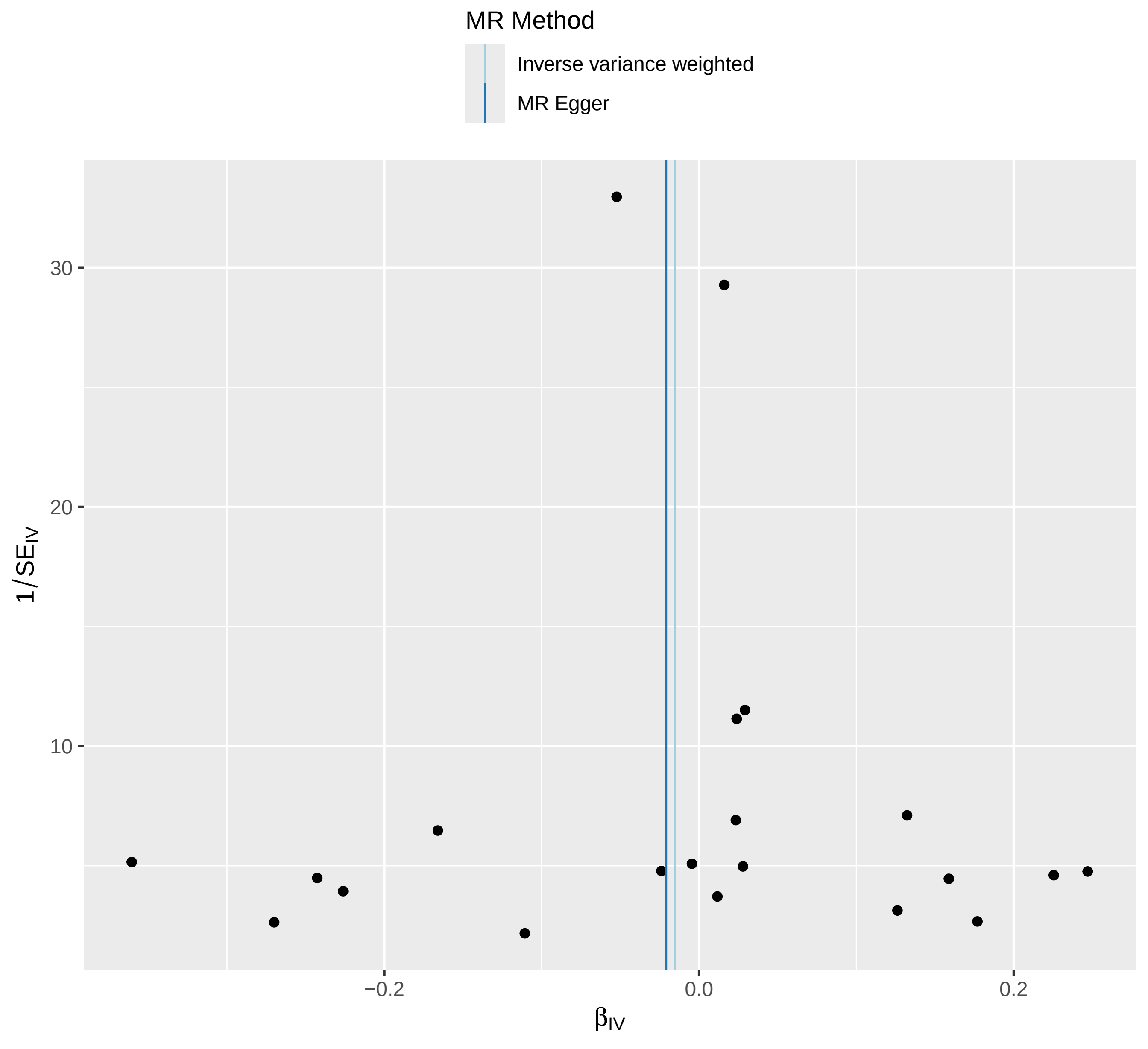
MR Method
Inverse variance weighted
MR Egger
30
20
10
1/SE_IV
−0.2
0.0
0.2
β_IV

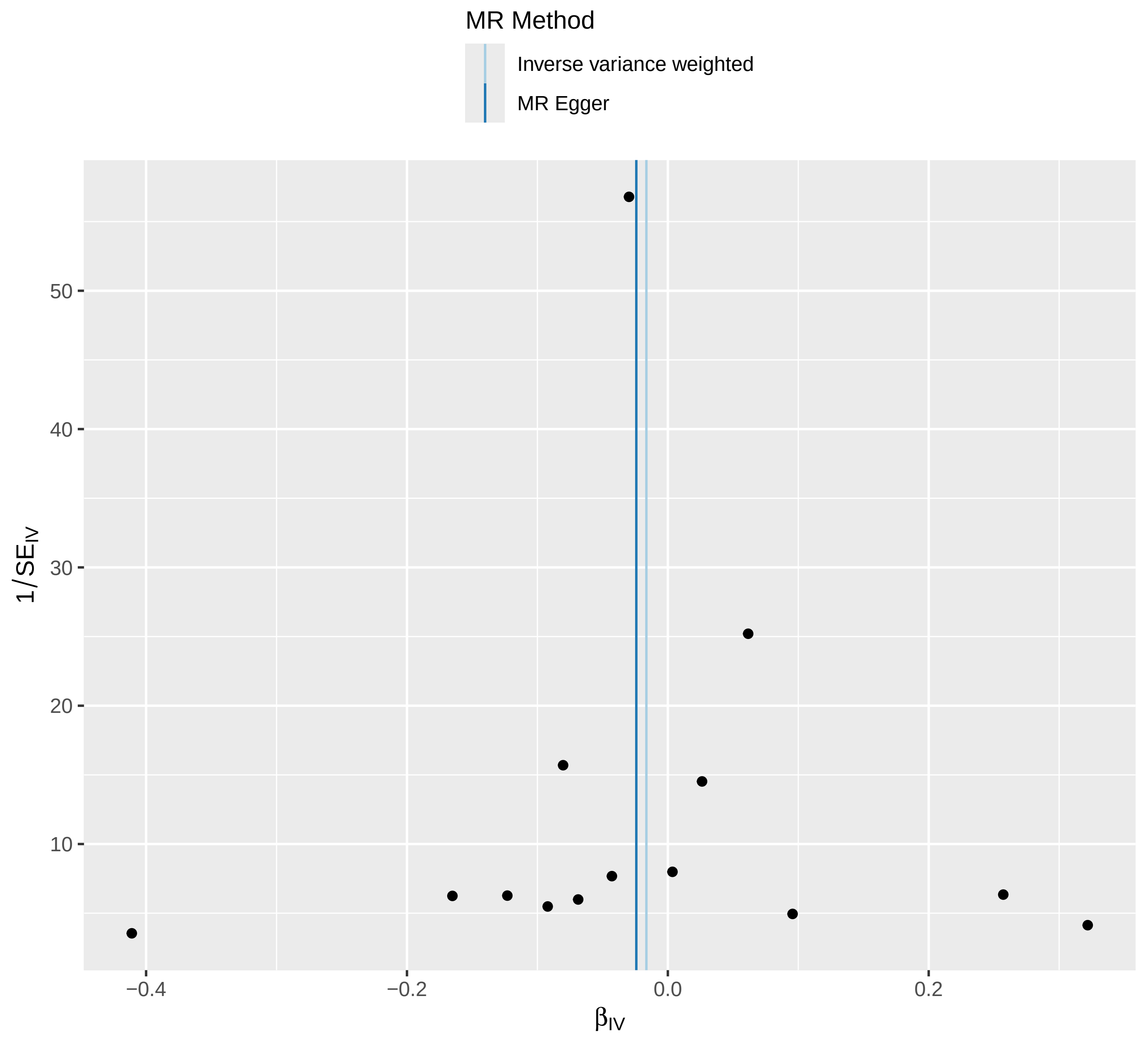
MR Method
Inverse variance weighted
MR Egger
50
40
30
20
10
1/SE$_{IV}$
−0.4
−0.2
0.0
0.2
β$_{IV}$

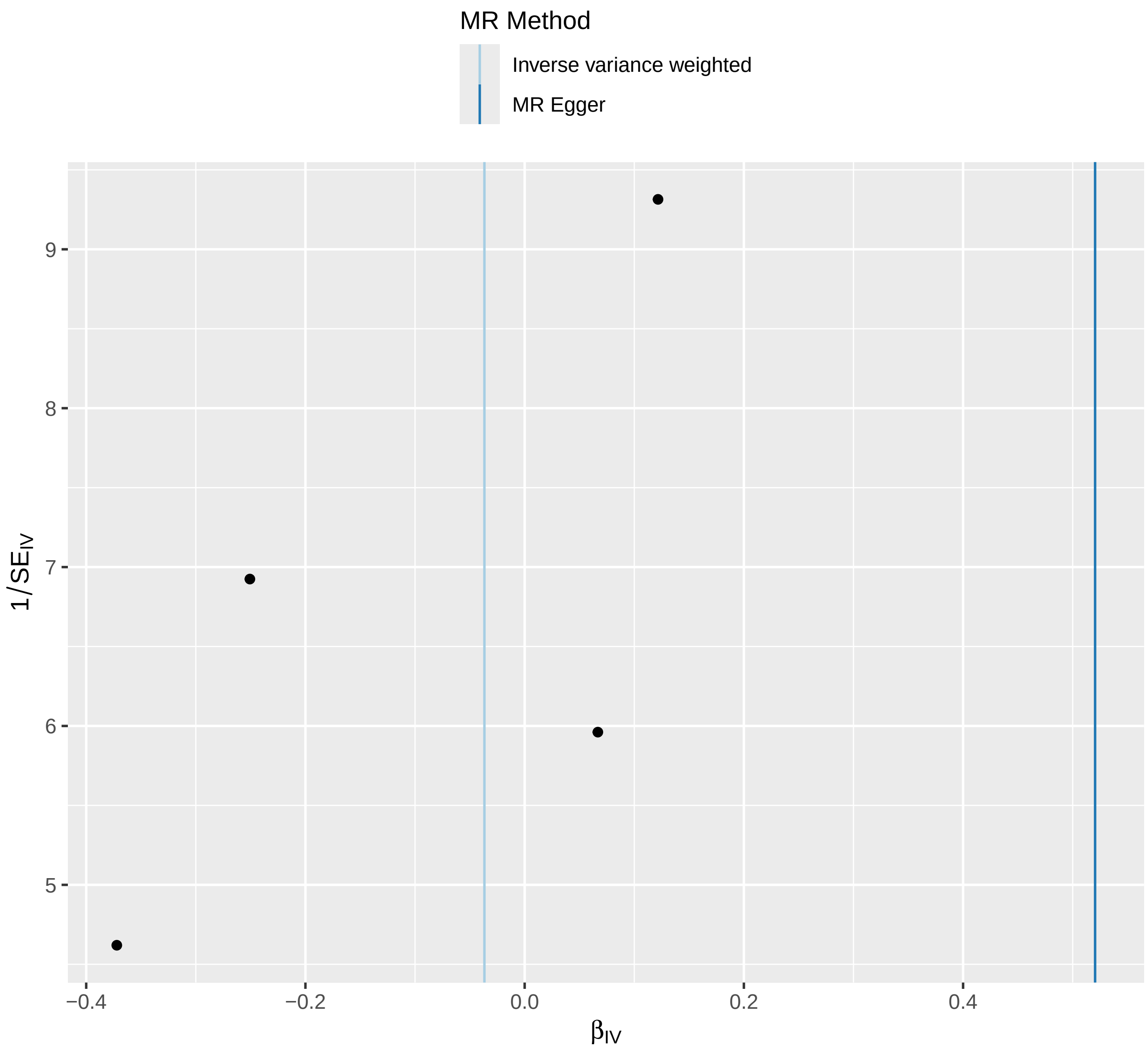
MR Method
Inverse variance weighted
MR Egger
9
8
7
6
5
1/SE_IV
−0.4
−0.2
0.0
0.2
0.4
β_IV

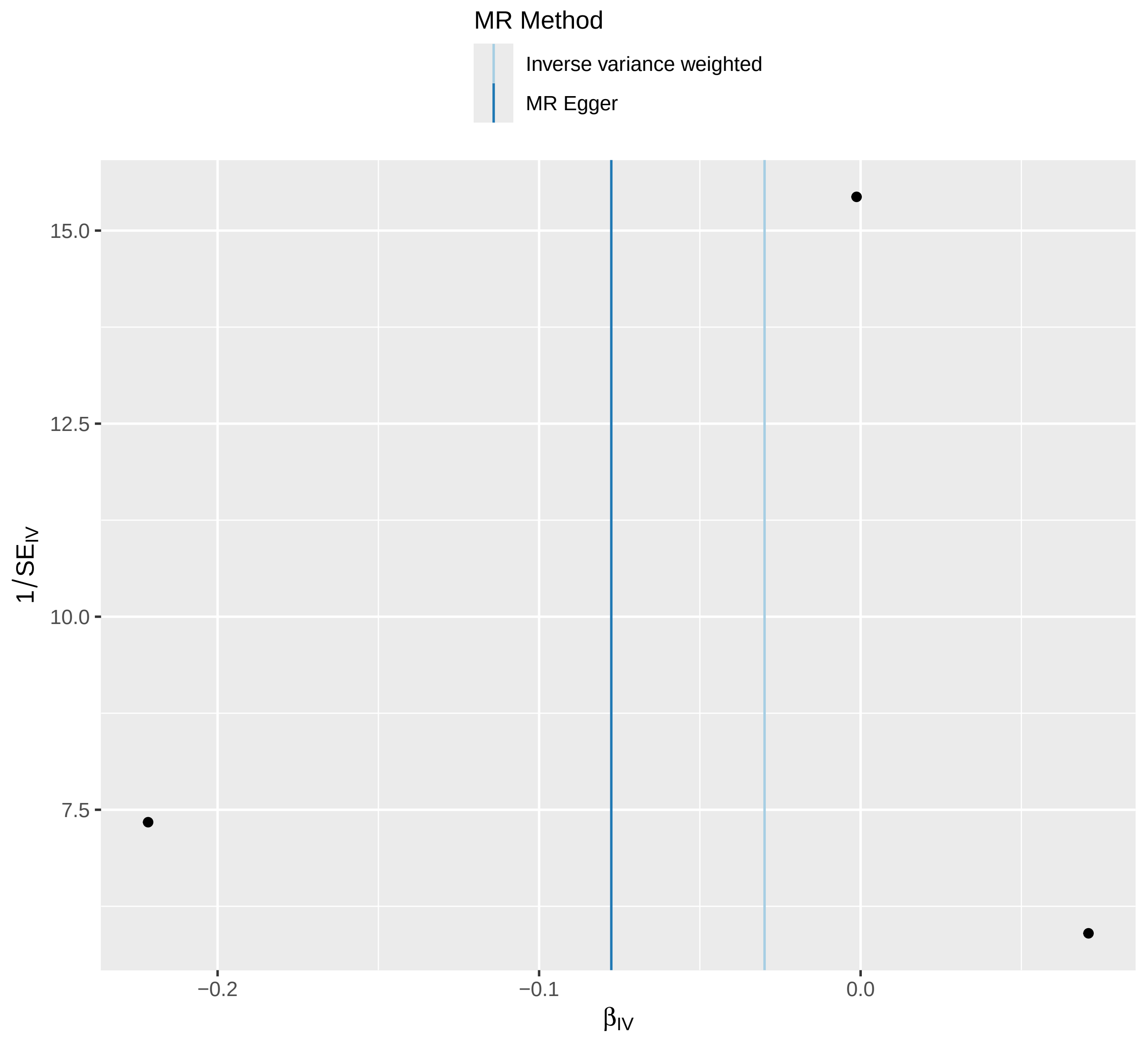
MR Method
Inverse variance weighted
MR Egger
15.0
12.5
10.0
7.5
$1/SE_{IV}$
−0.2
−0.1
0.0
$\beta_{IV}$

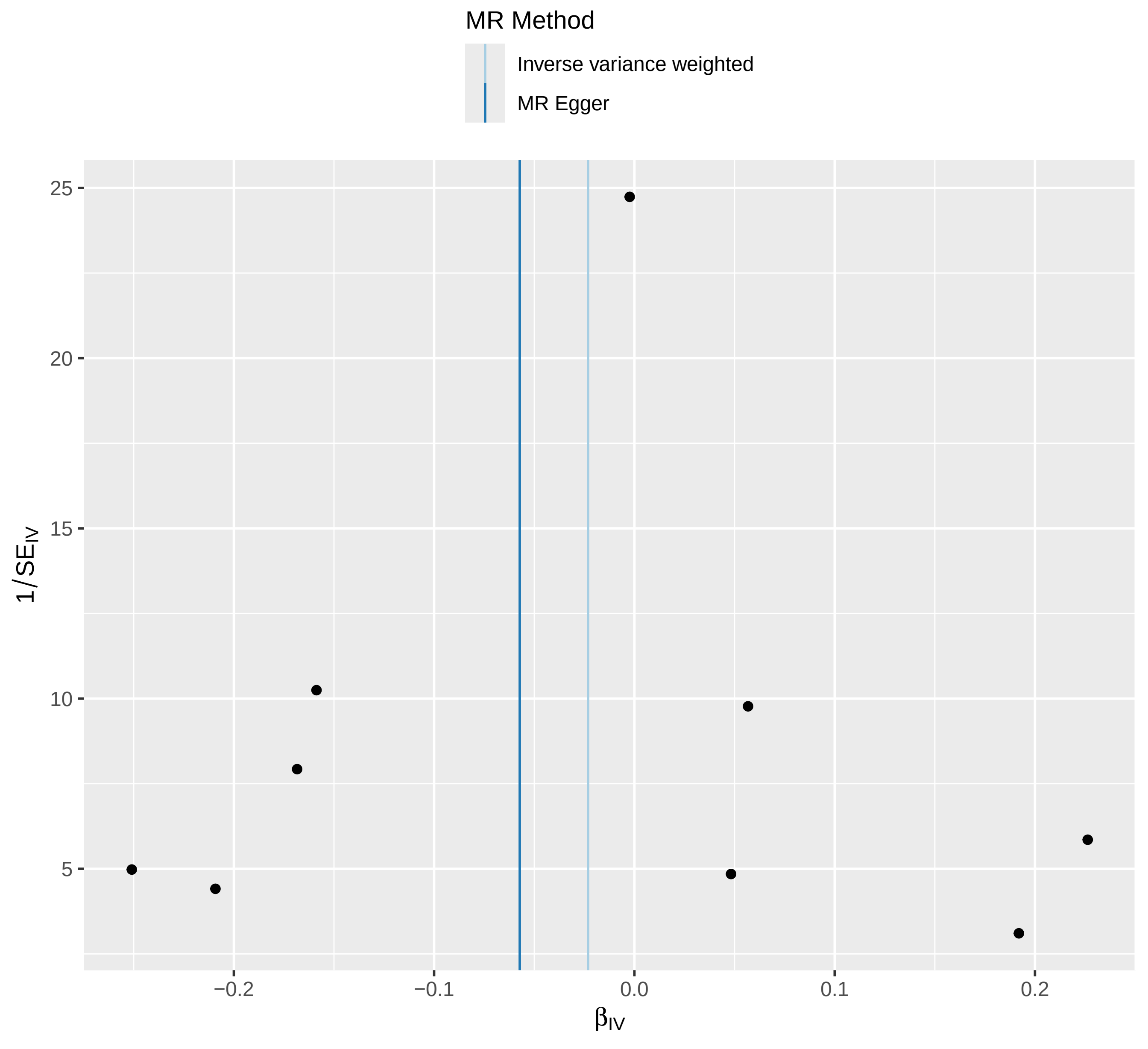
MR Method
Inverse variance weighted
MR Egger
25
20
15
10
5
1/SE$_{IV}$
−0.2
−0.1
0.0
0.1
0.2
β$_{IV}$

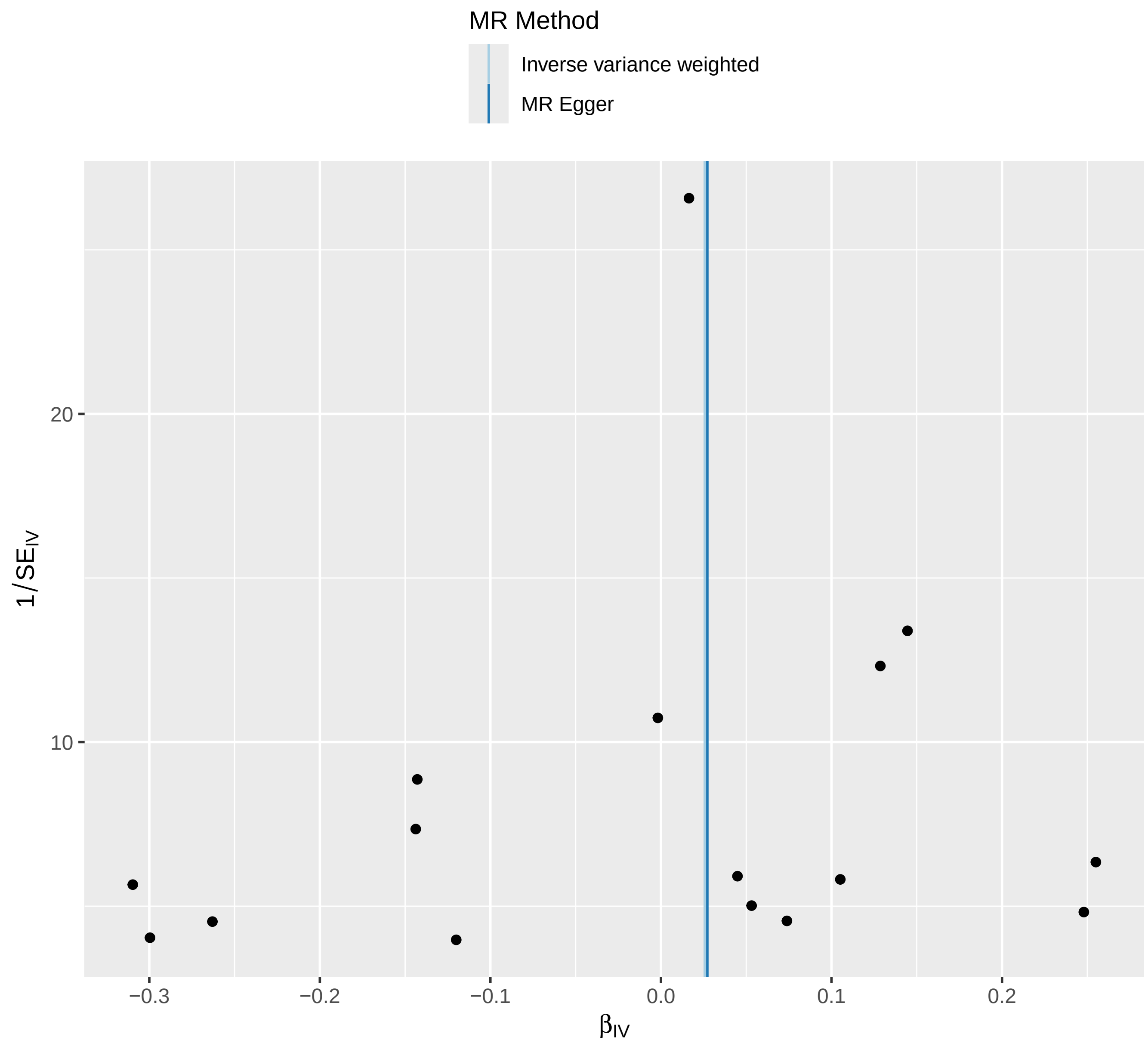
MR Method
Inverse variance weighted
MR Egger
20
10
1/SE$_{IV}$
−0.3
−0.2
−0.1
0.0
0.1
0.2
β$_{IV}$

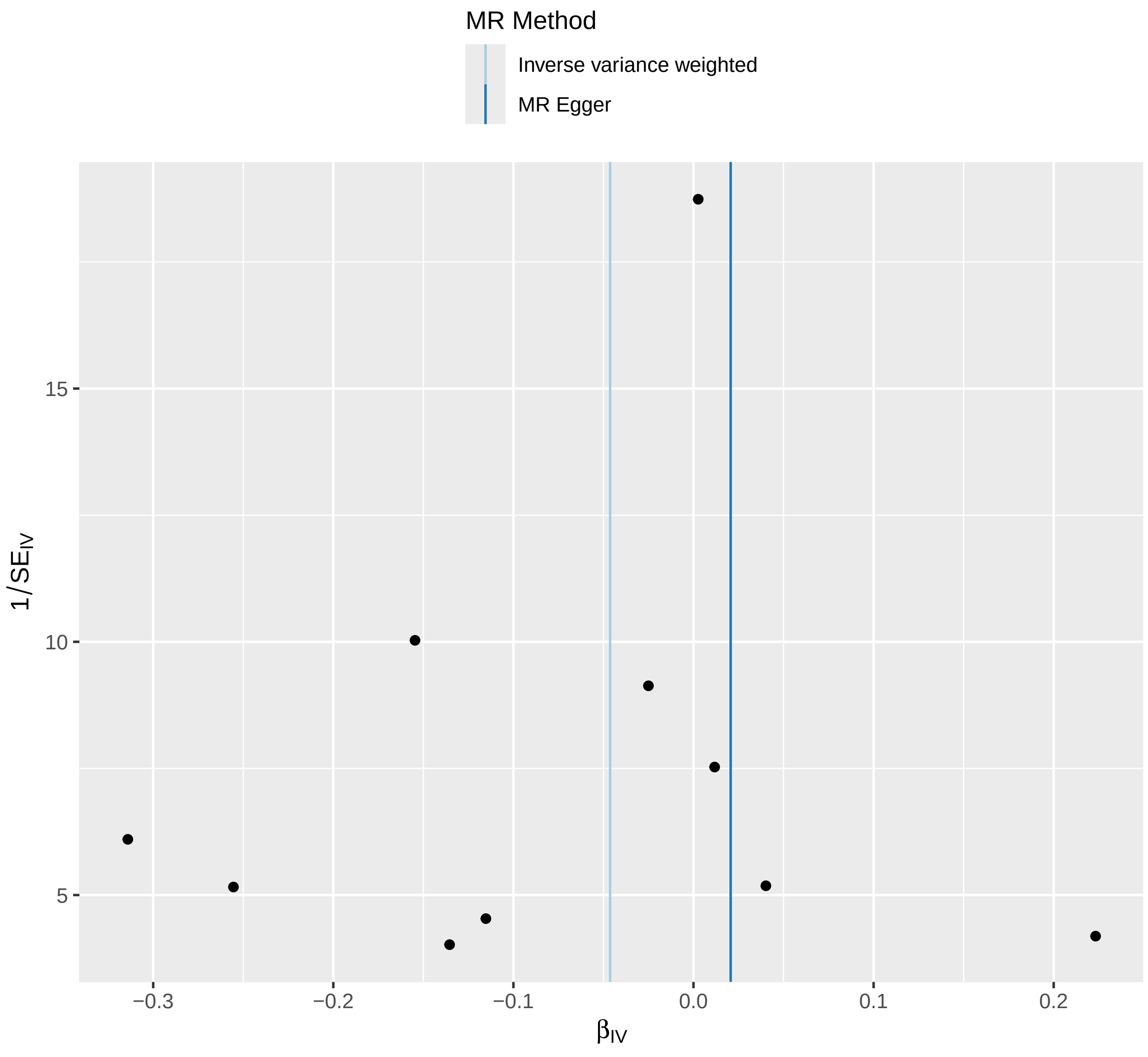

MR Method
Inverse variance weighted
MR Egger
15
10
5
1/SE_IV
−0.3
−0.2
−0.1
0.0
0.1
0.2
β_IV

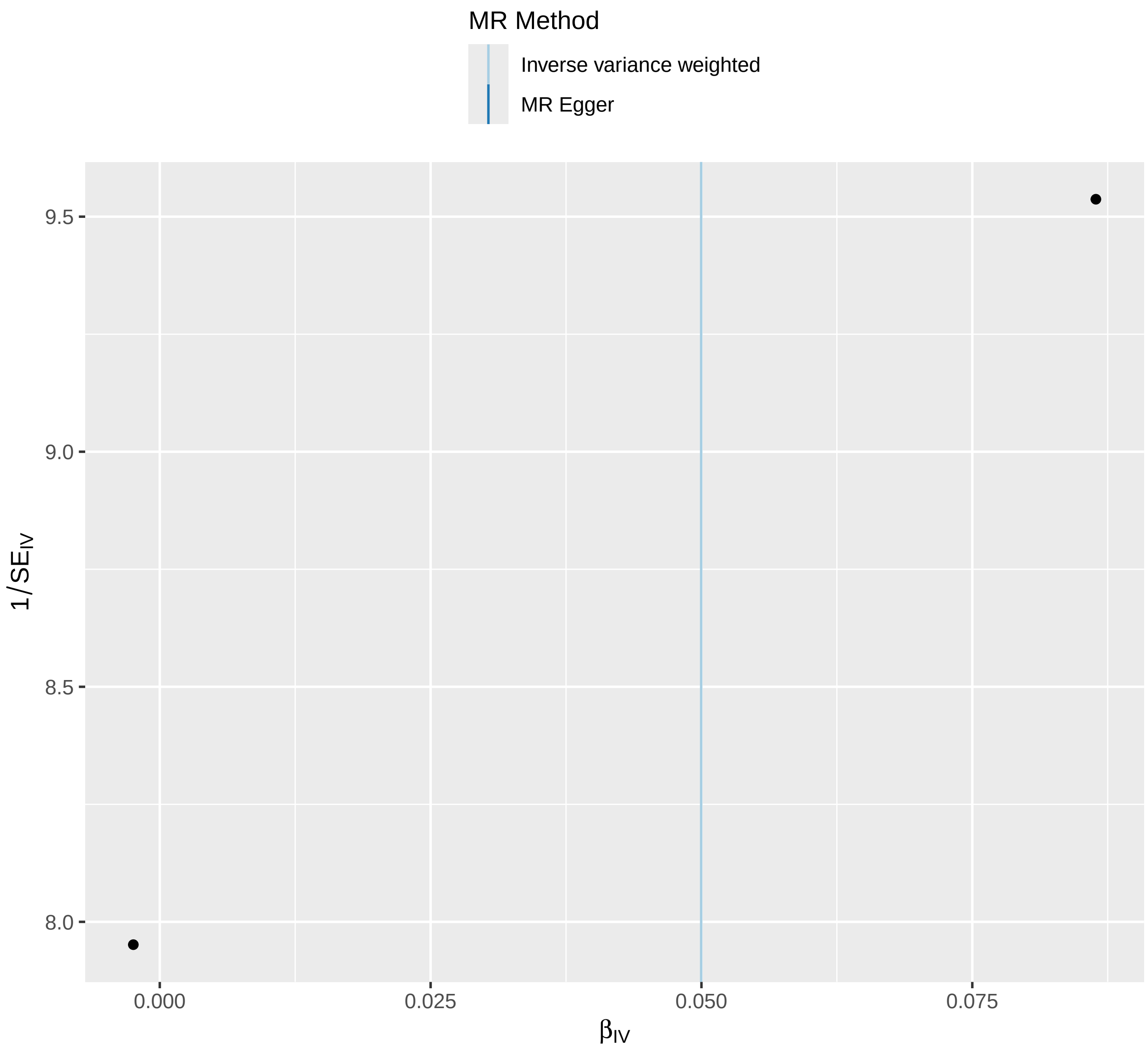
MR Method
Inverse variance weighted
MR Egger
9.5
9.0
8.5
8.0
1/SE_IV
0.000
0.025
0.050
0.075
β_IV

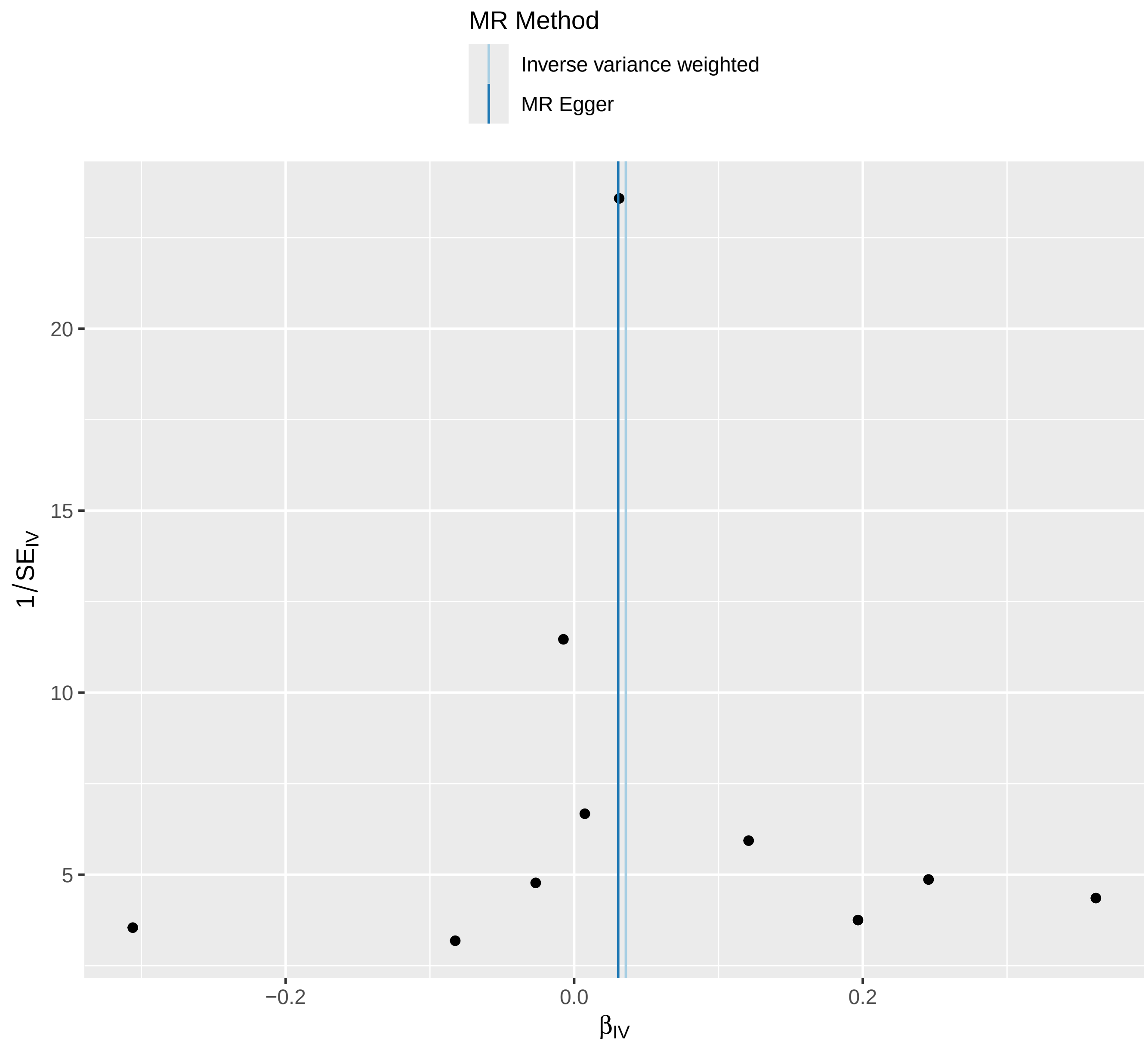

MR Method
Inverse variance weighted
MR Egger
20
15
10
5
1/SE_IV
−0.2
0.0
0.2
β_IV

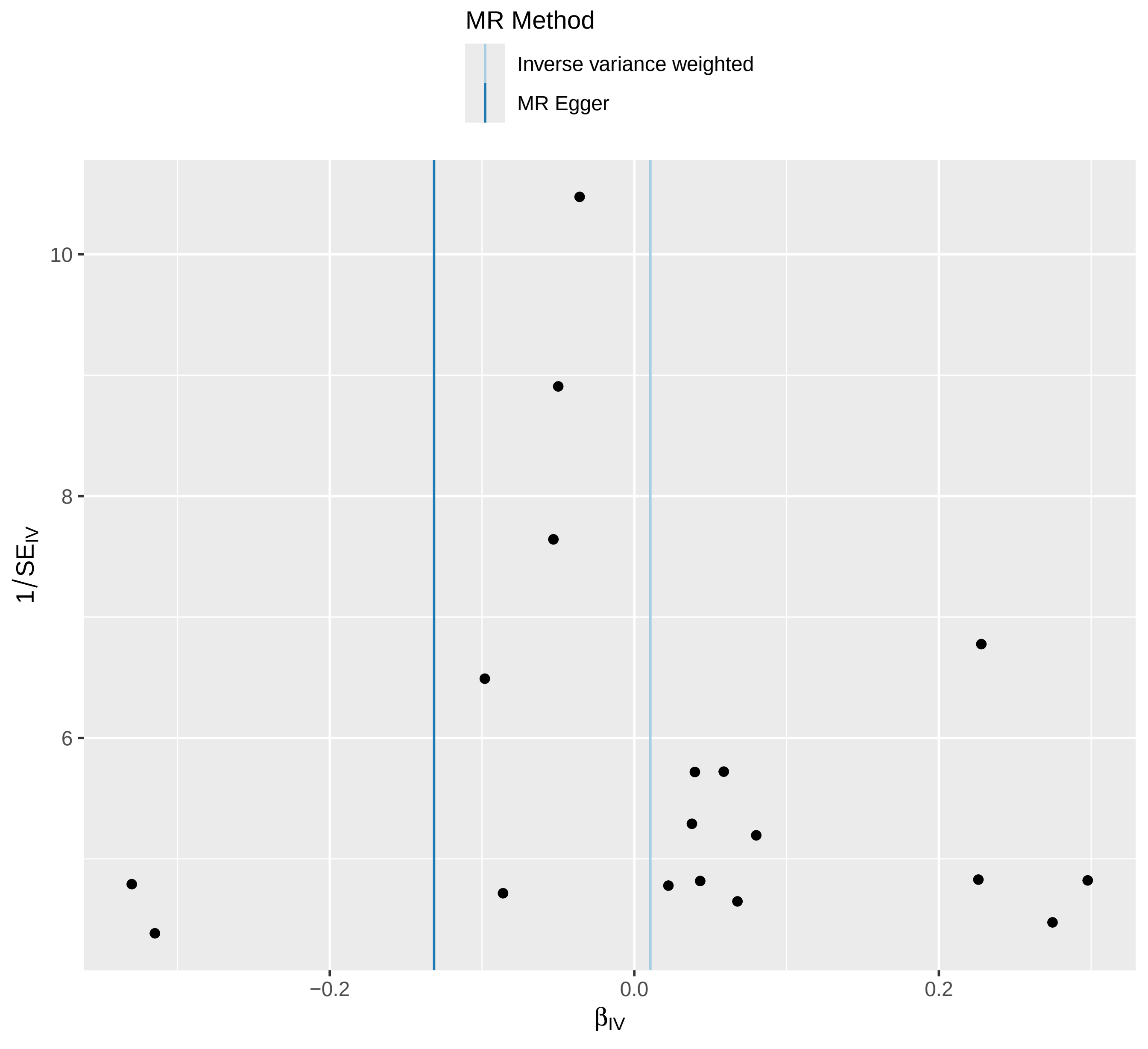
MR Method
Inverse variance weighted
MR Egger
10
8
6
1/SE_IV
−0.2
0.0
0.2
β_IV

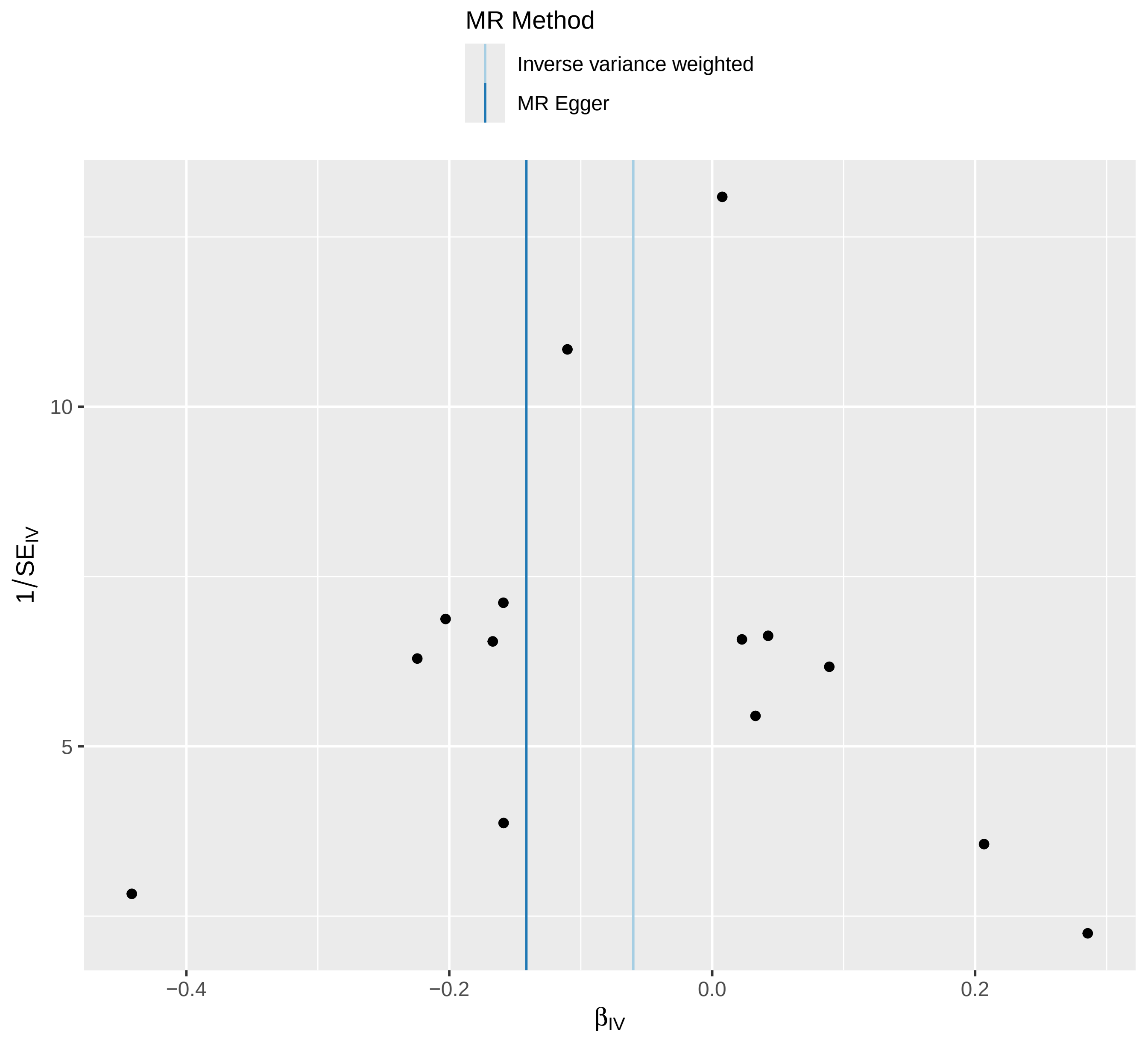
MR Method
Inverse variance weighted
MR Egger
10
5
1/SE$_{IV}$
−0.4
−0.2
0.0
0.2
β$_{IV}$

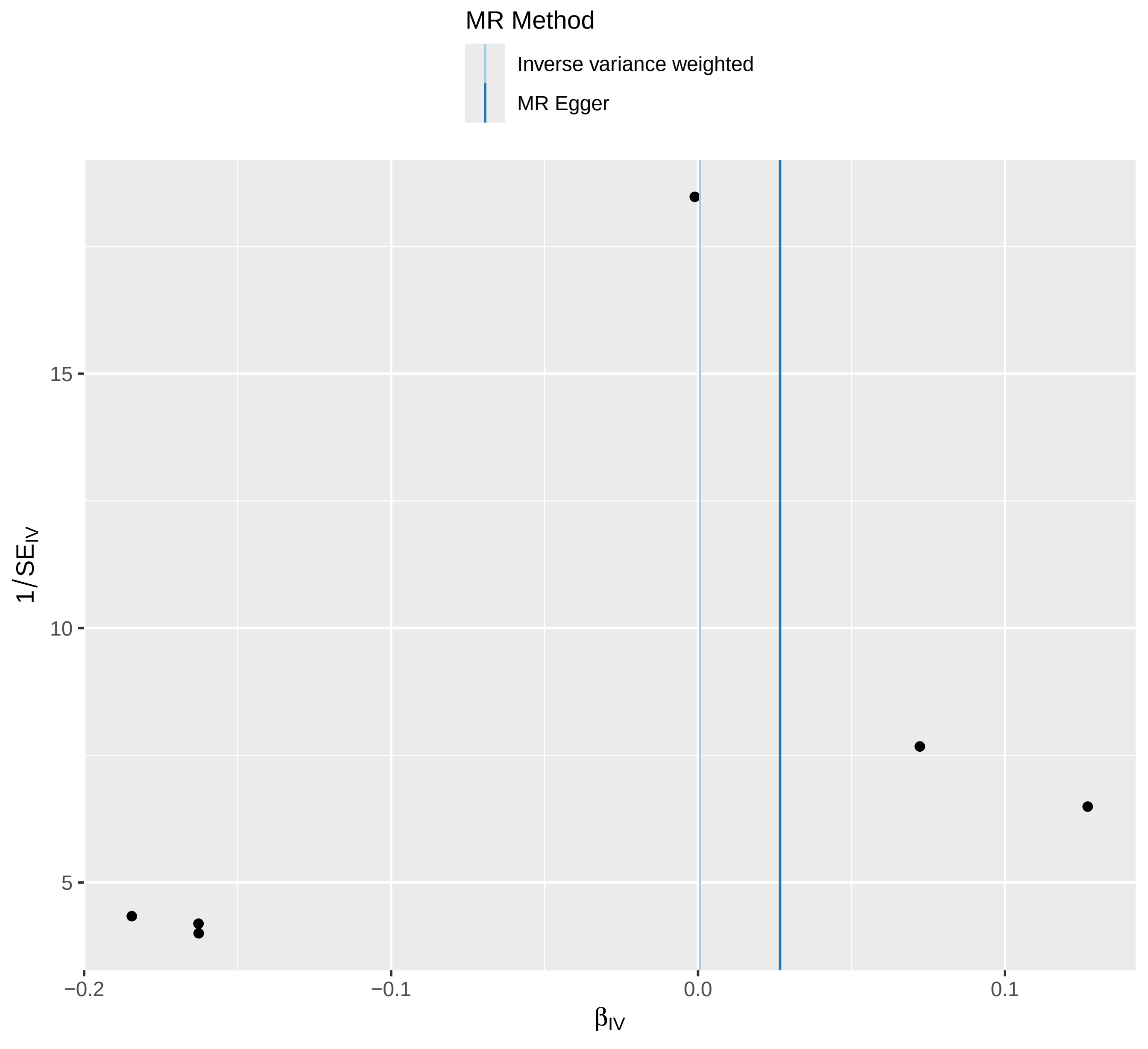
MR Method
Inverse variance weighted
MR Egger
1/SE_IV
15
10
5
−0.2
−0.1
0.0
0.1
β_IV

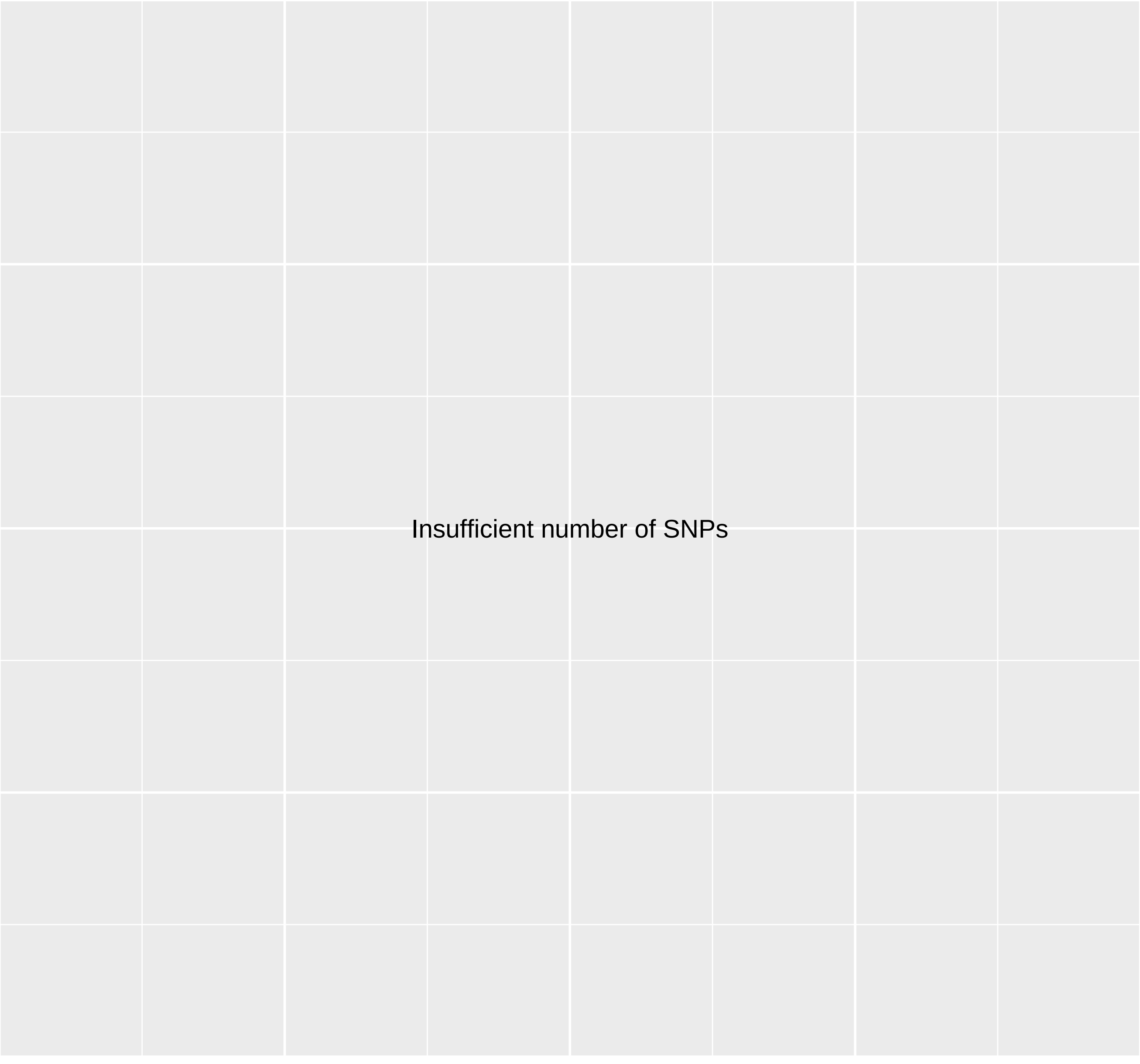
Insufficient number of SNPs

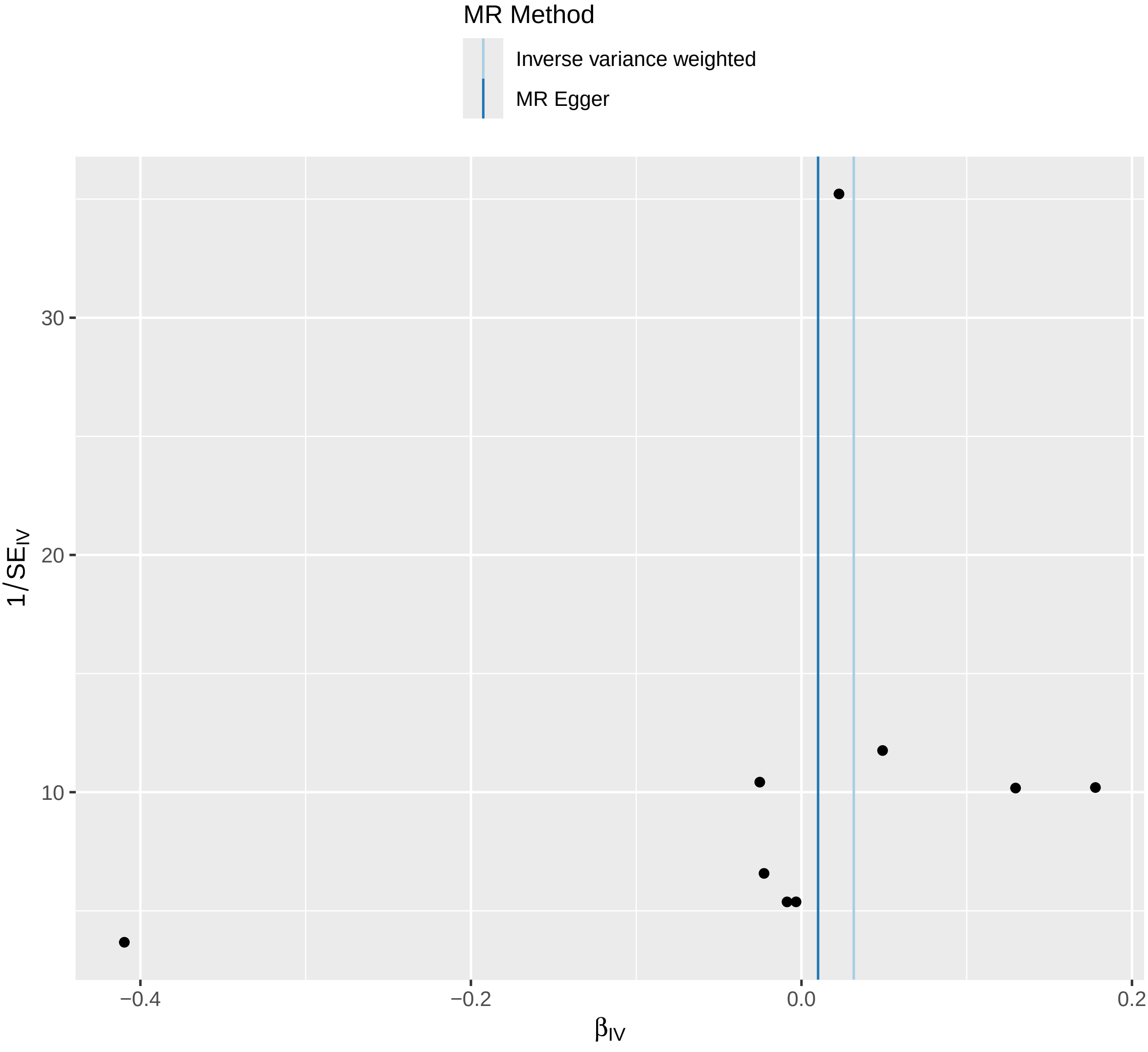
MR Method
Inverse variance weighted
MR Egger
30
20
10
1/SE_IV
−0.4
−0.2
0.0
0.2
β_IV

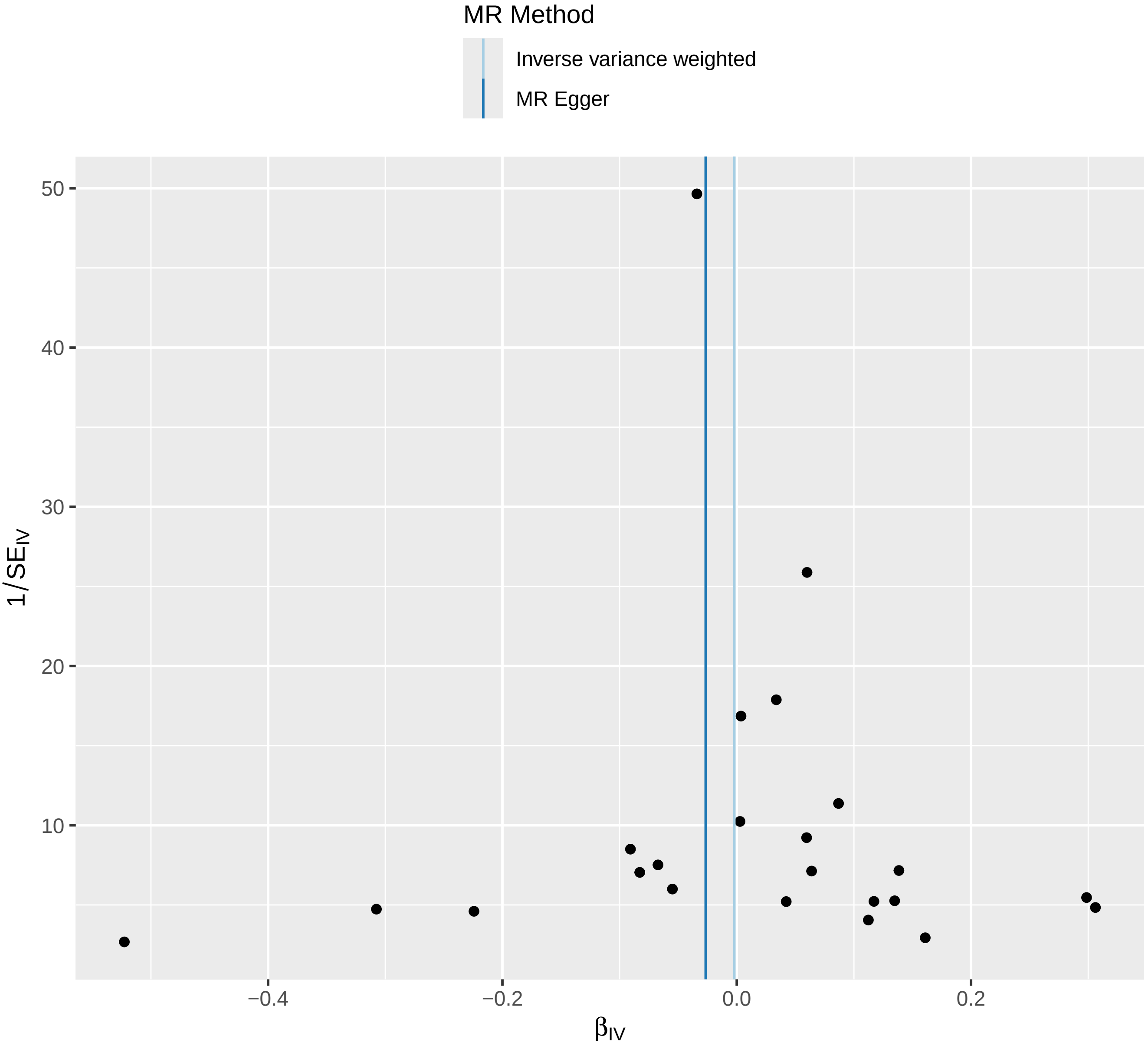

MR Method
Inverse variance weighted
MR Egger
50
40
30
20
10
1/SE$_{IV}$
−0.4
−0.2
0.0
0.2
β$_{IV}$

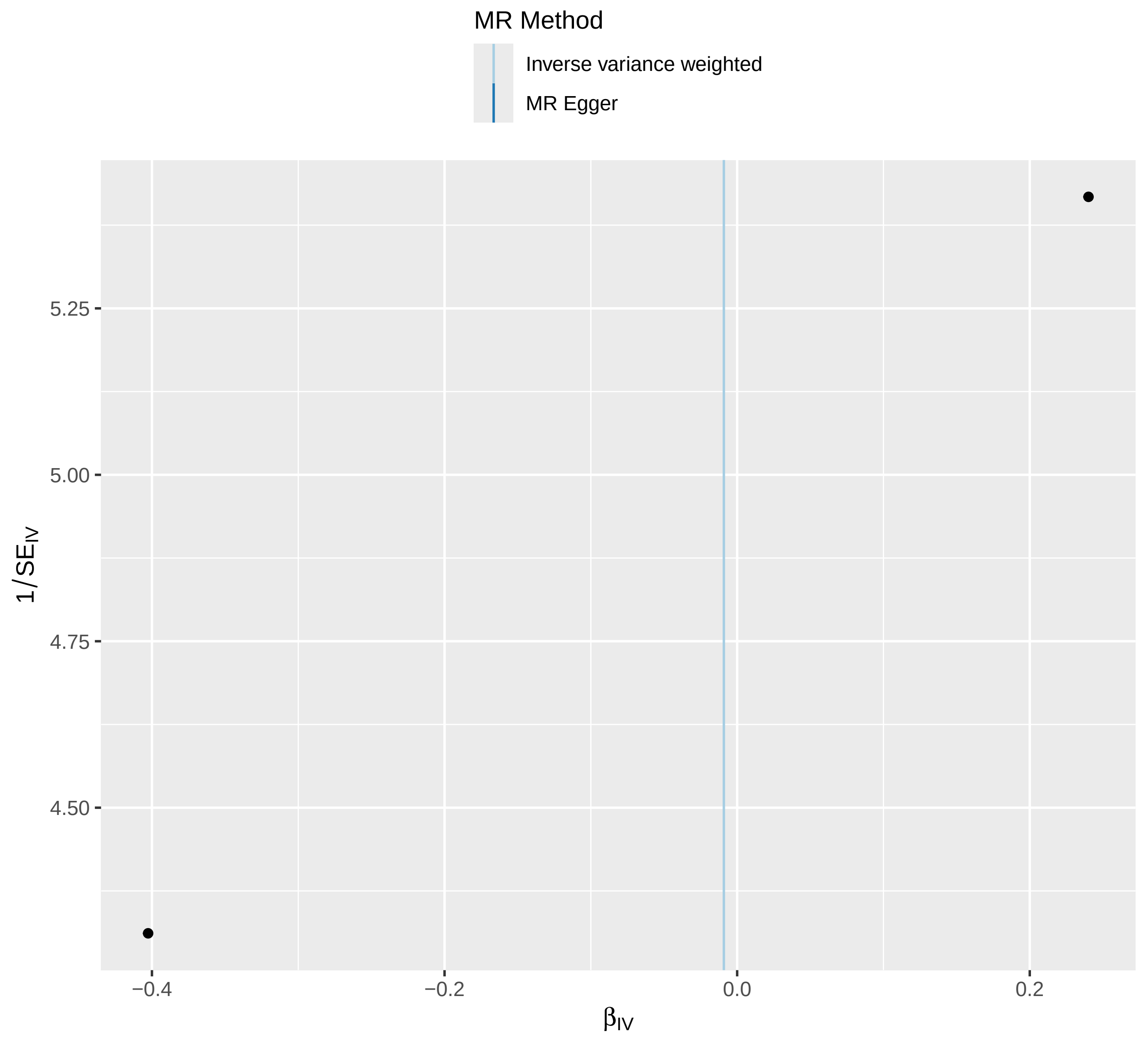
MR Method
Inverse variance weighted
MR Egger
5.25
5.00
4.75
4.50
1/SE_IV
−0.4
−0.2
0.0
0.2
β_IV

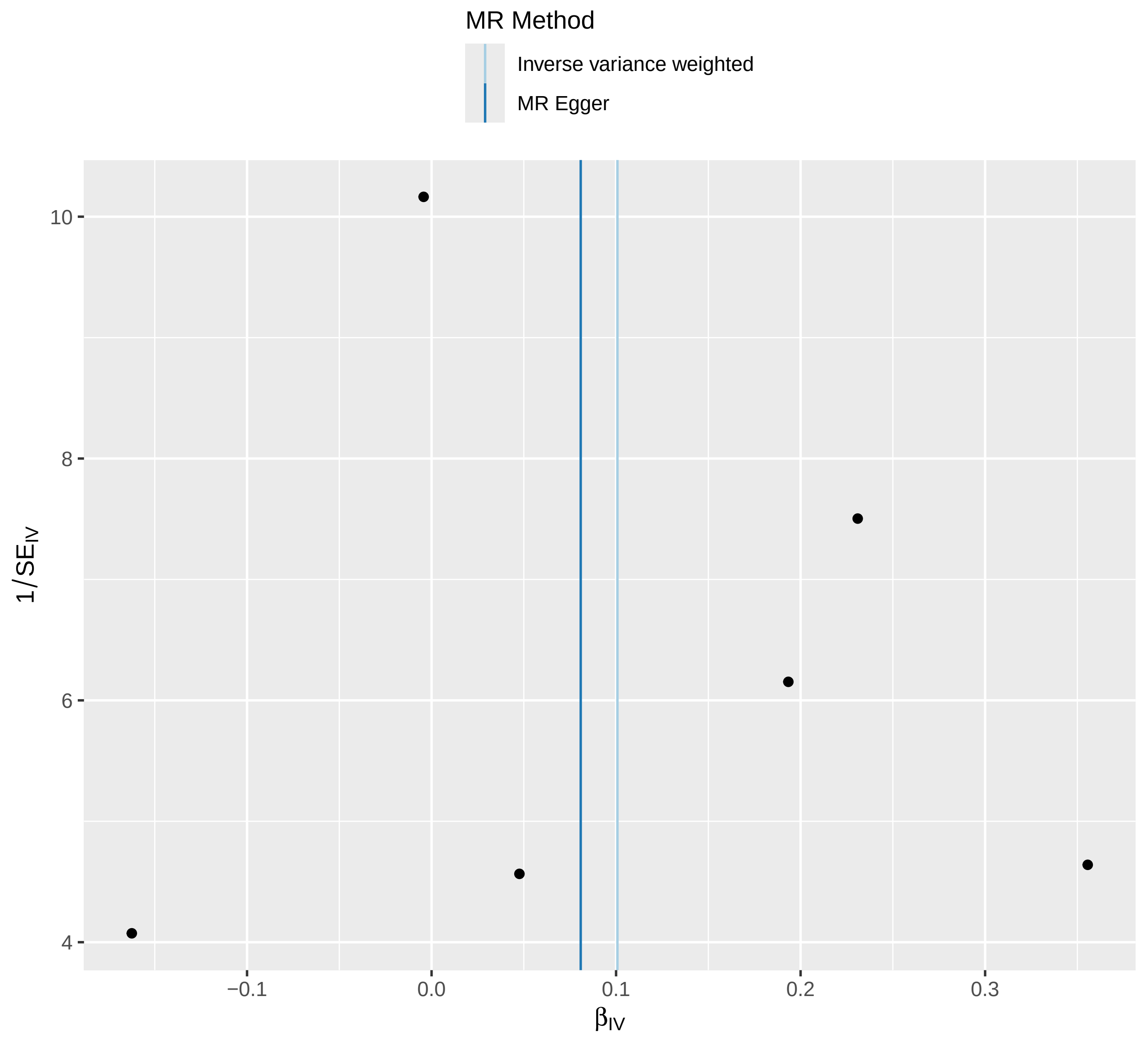
MR Method
Inverse variance weighted
MR Egger
10
8
6
4
1/$SE_{IV}$
−0.1
0.0
0.1
0.2
0.3
$\beta_{IV}$

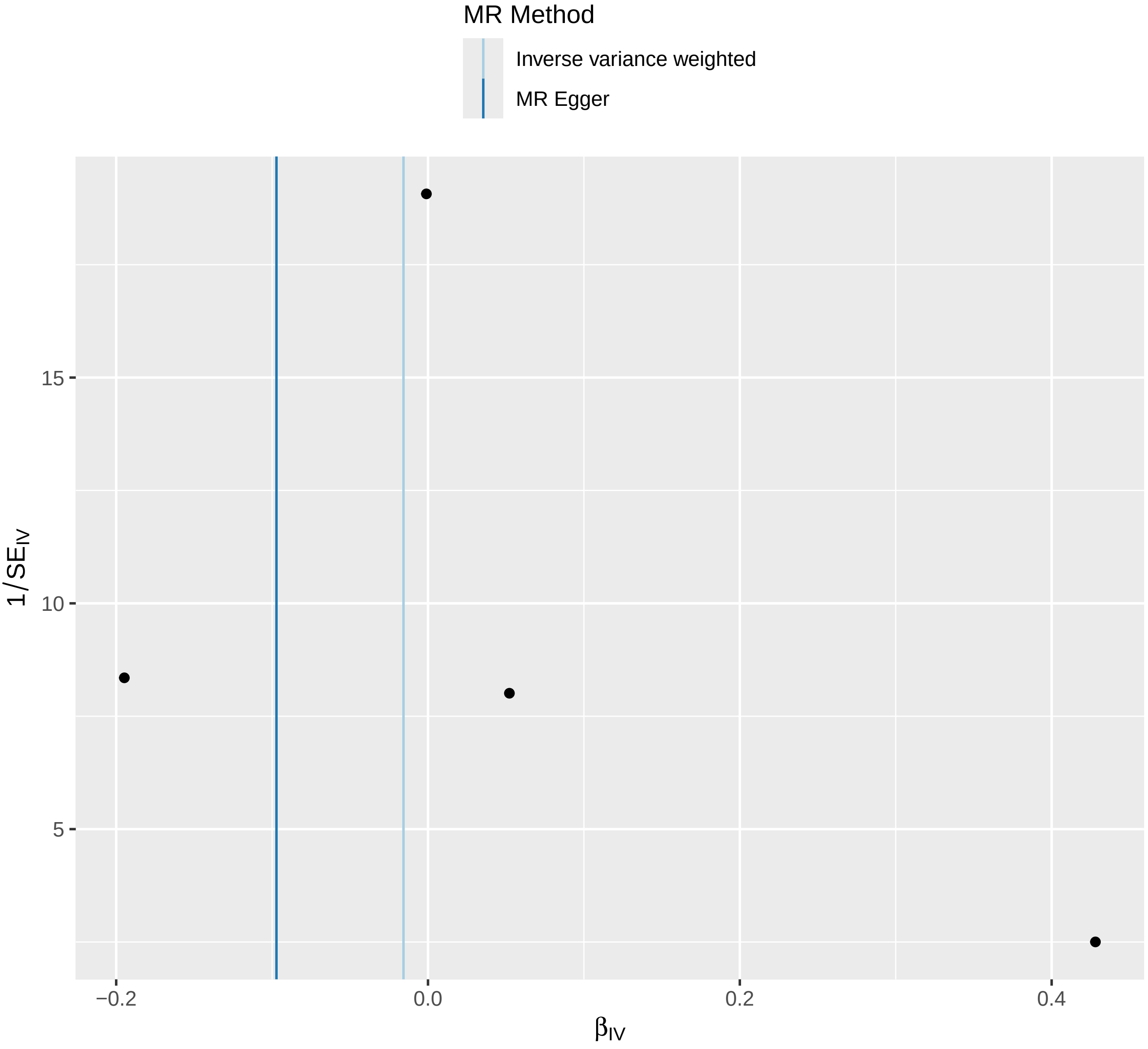
MR Method
Inverse variance weighted
MR Egger
15
10
5
1/SE_IV
−0.2
0.0
0.2
0.4
β_IV

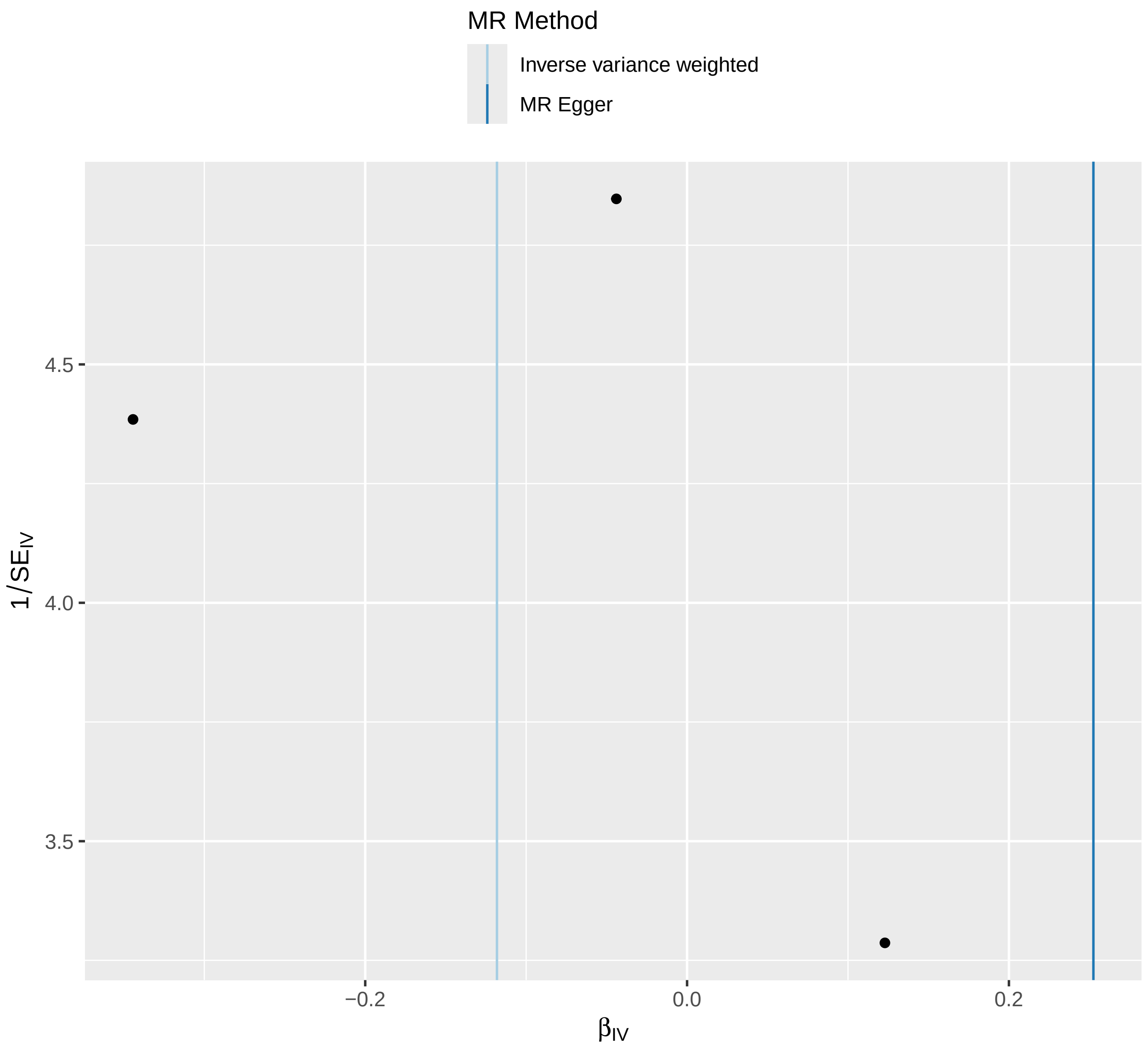
MR Method
Inverse variance weighted
MR Egger
4.5
4.0
3.5
1/SE_IV
−0.2
0.0
0.2
β_IV

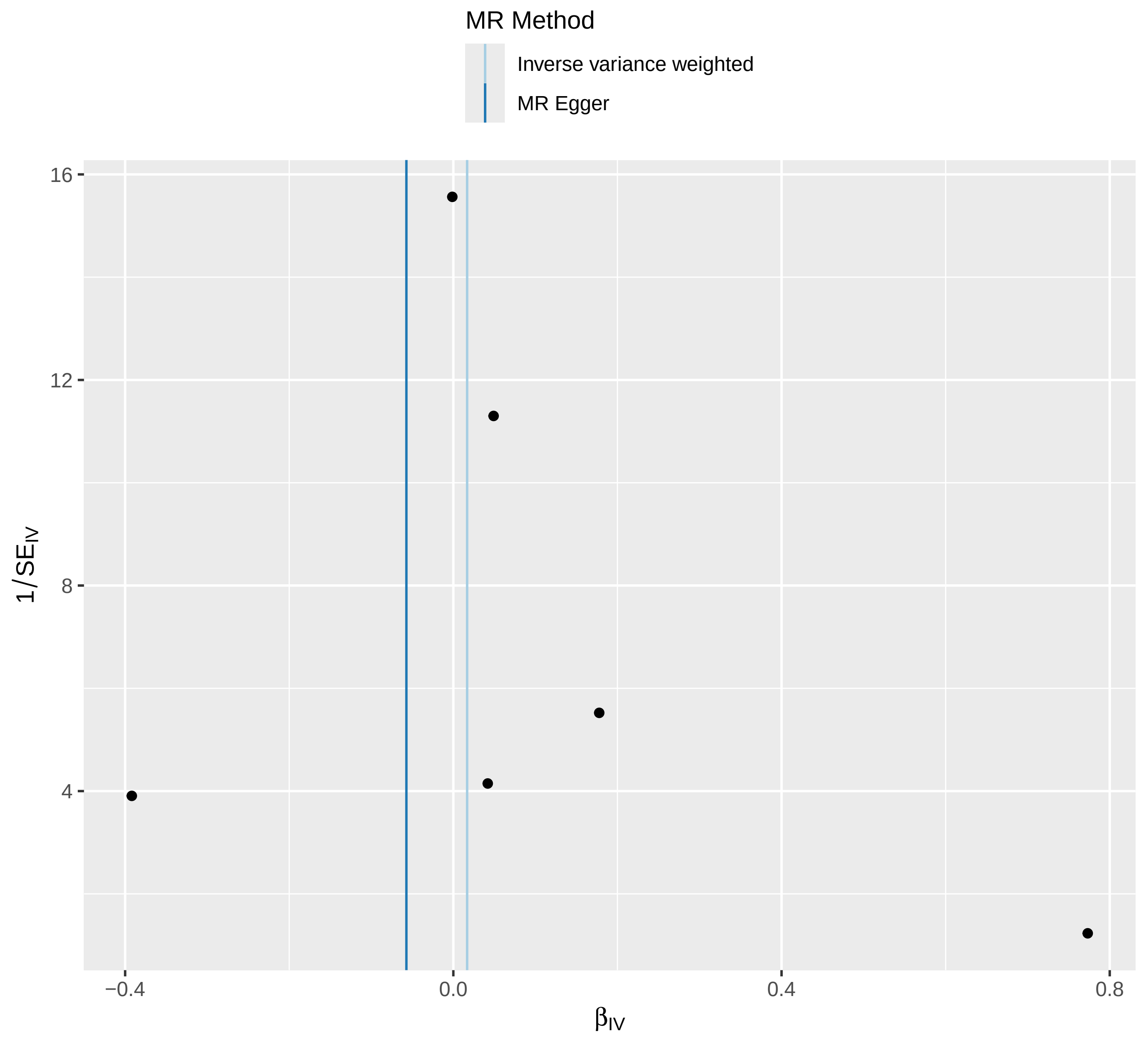

MR Method
Inverse variance weighted
MR Egger
16
12
8
4
1/SE$_{IV}$
−0.4
0.0
0.4
0.8
β$_{IV}$

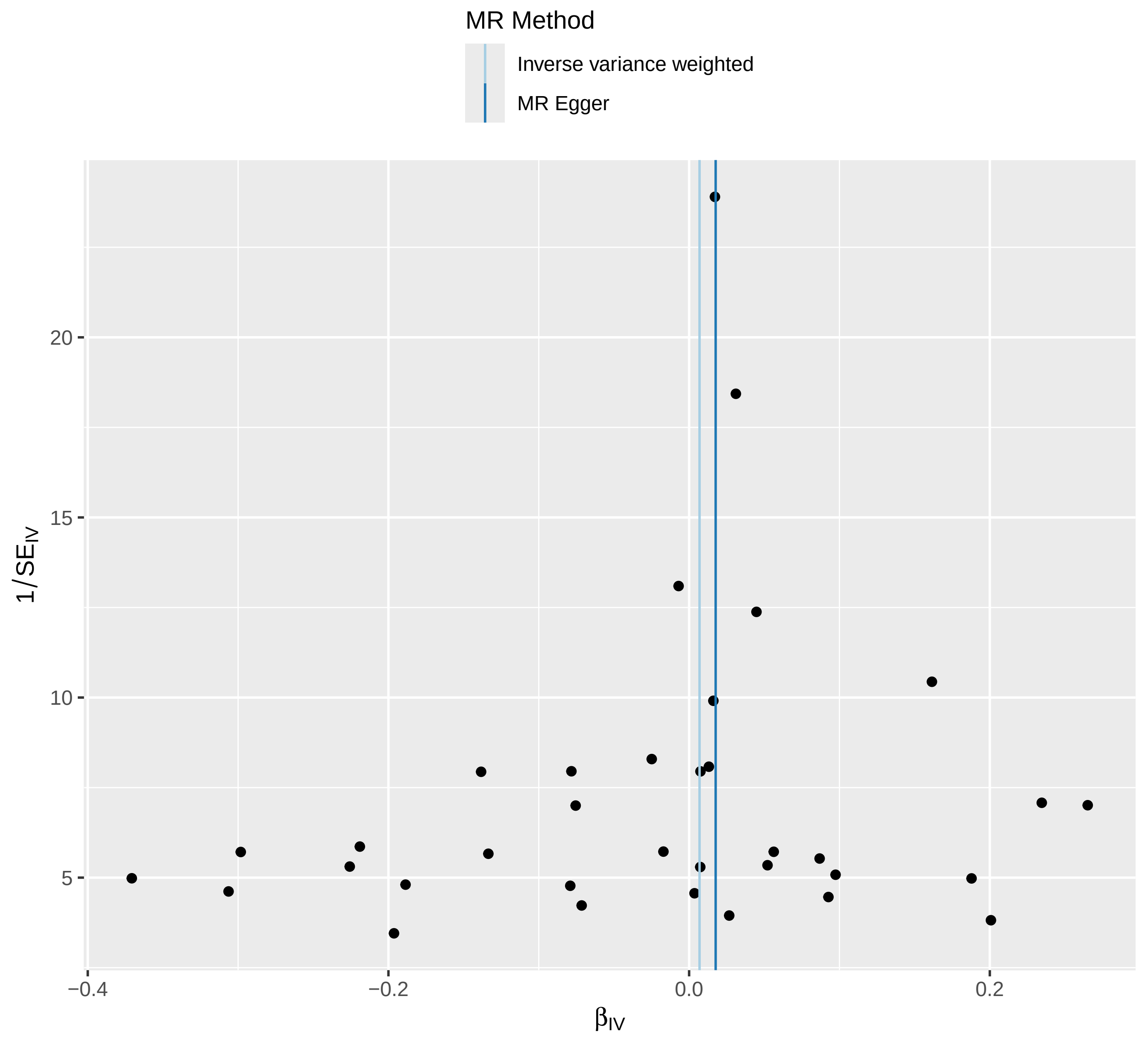
MR Method
Inverse variance weighted
MR Egger
1/SE_IV
20
15
10
5
−0.4
−0.2
0.0
0.2
β_IV

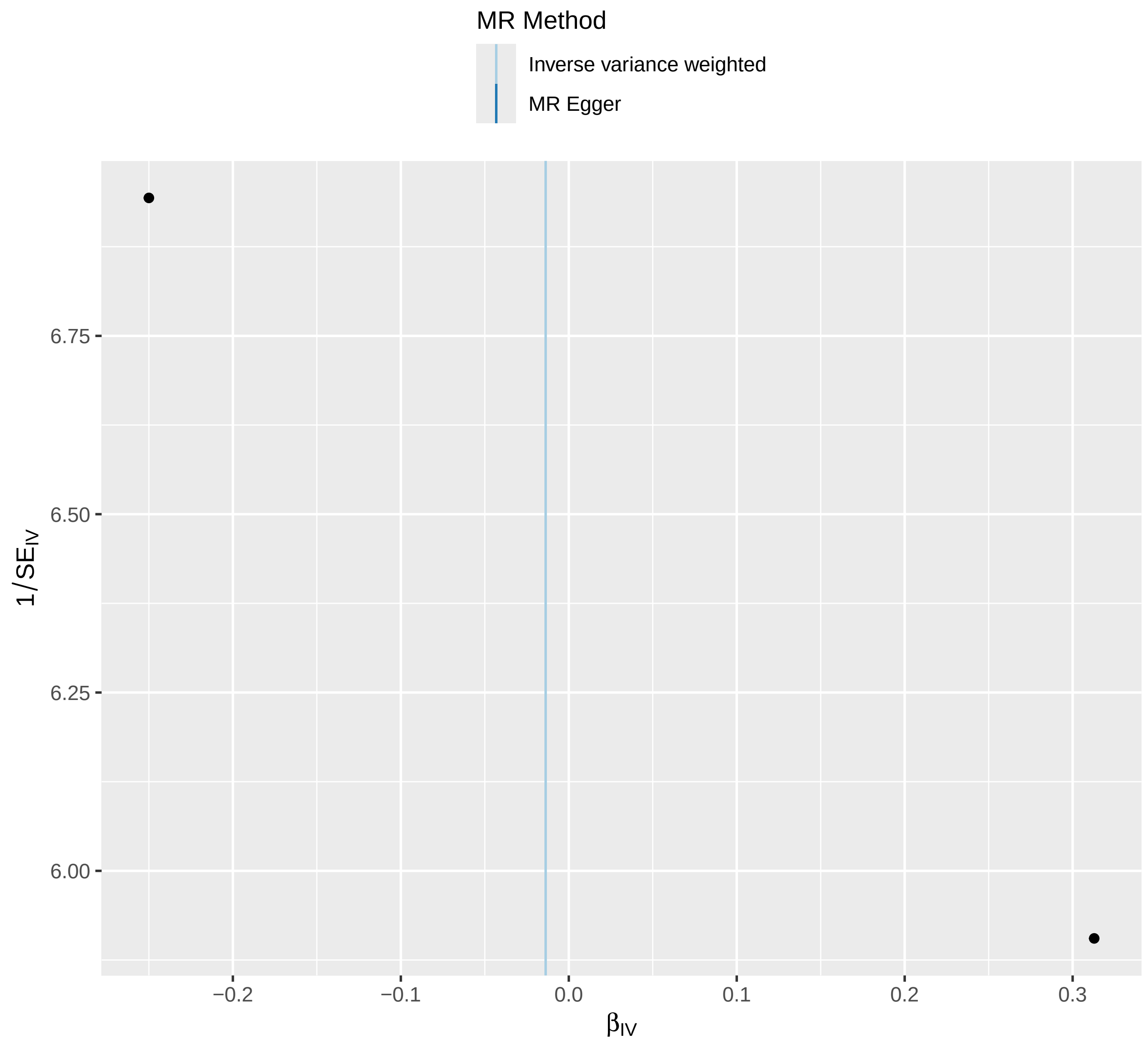

MR Method
Inverse variance weighted
MR Egger
6.75
6.50
6.25
6.00
1/SE$_{IV}$
−0.2
−0.1
0.0
0.1
0.2
0.3
β$_{IV}$

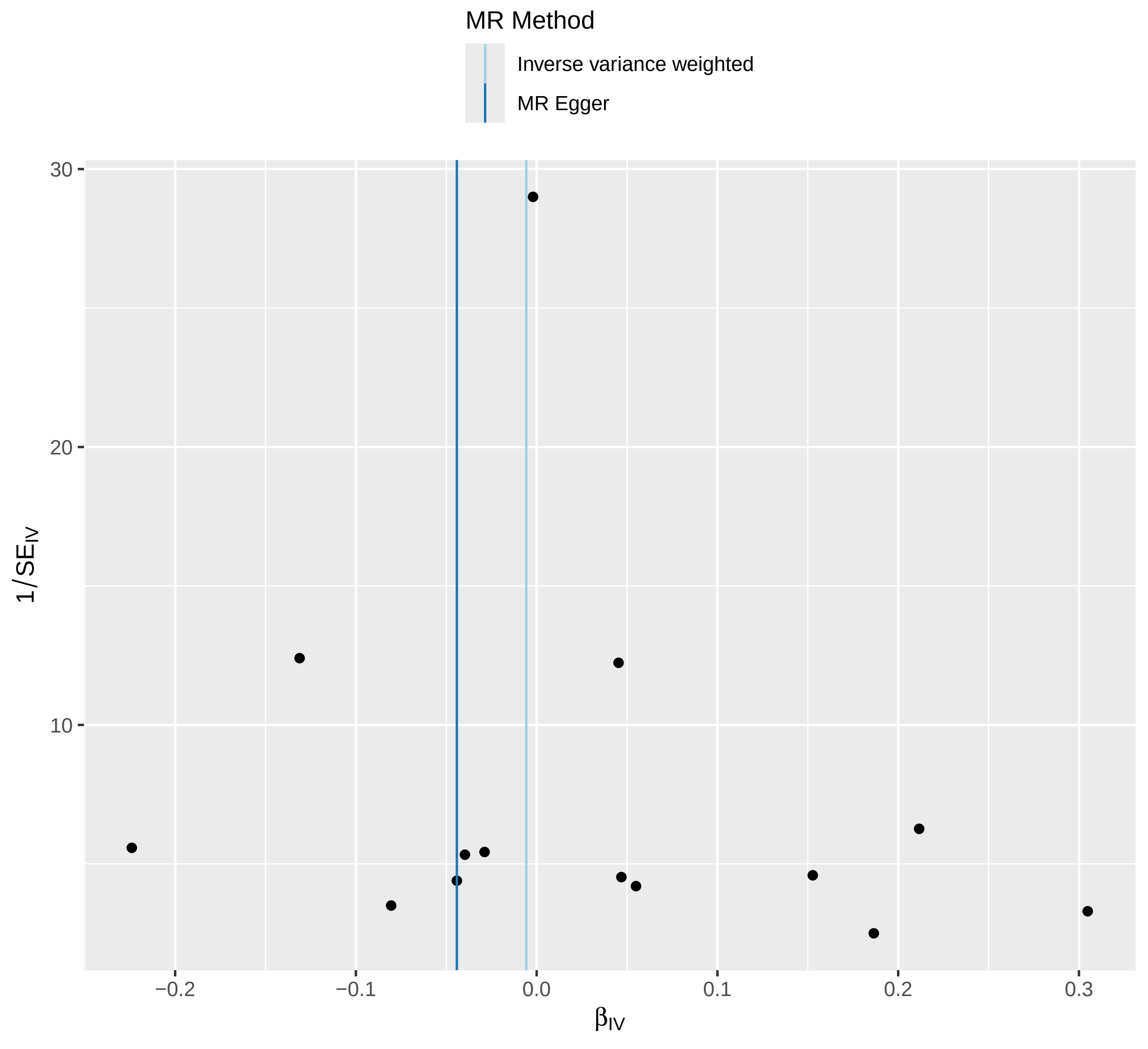

MR Method
Inverse variance weighted
MR Egger
30
20
10
1/SE$_{IV}$
−0.2
−0.1
0.0
0.1
0.2
0.3
$\beta_{IV}$

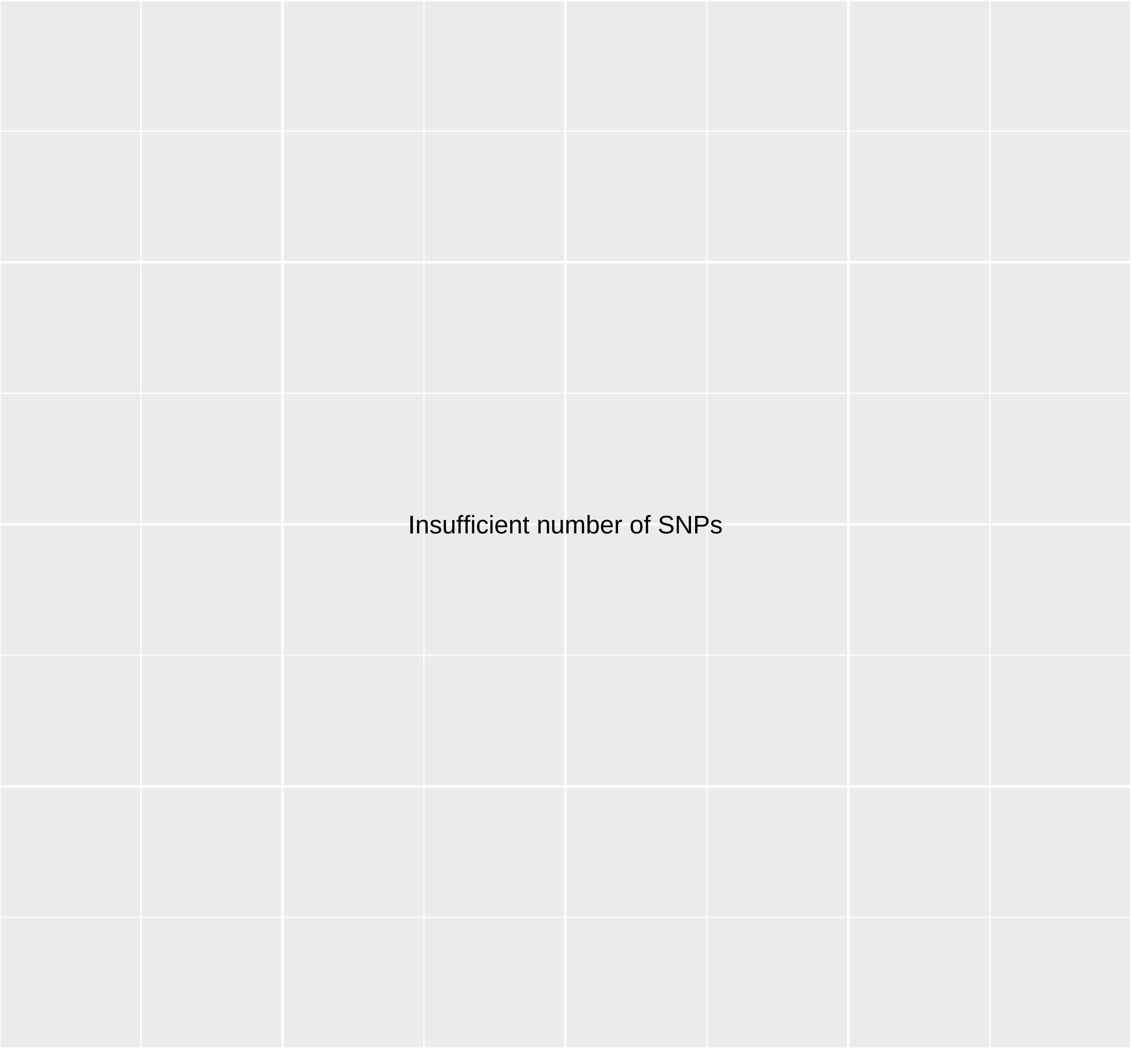
Insufficient number of SNPs

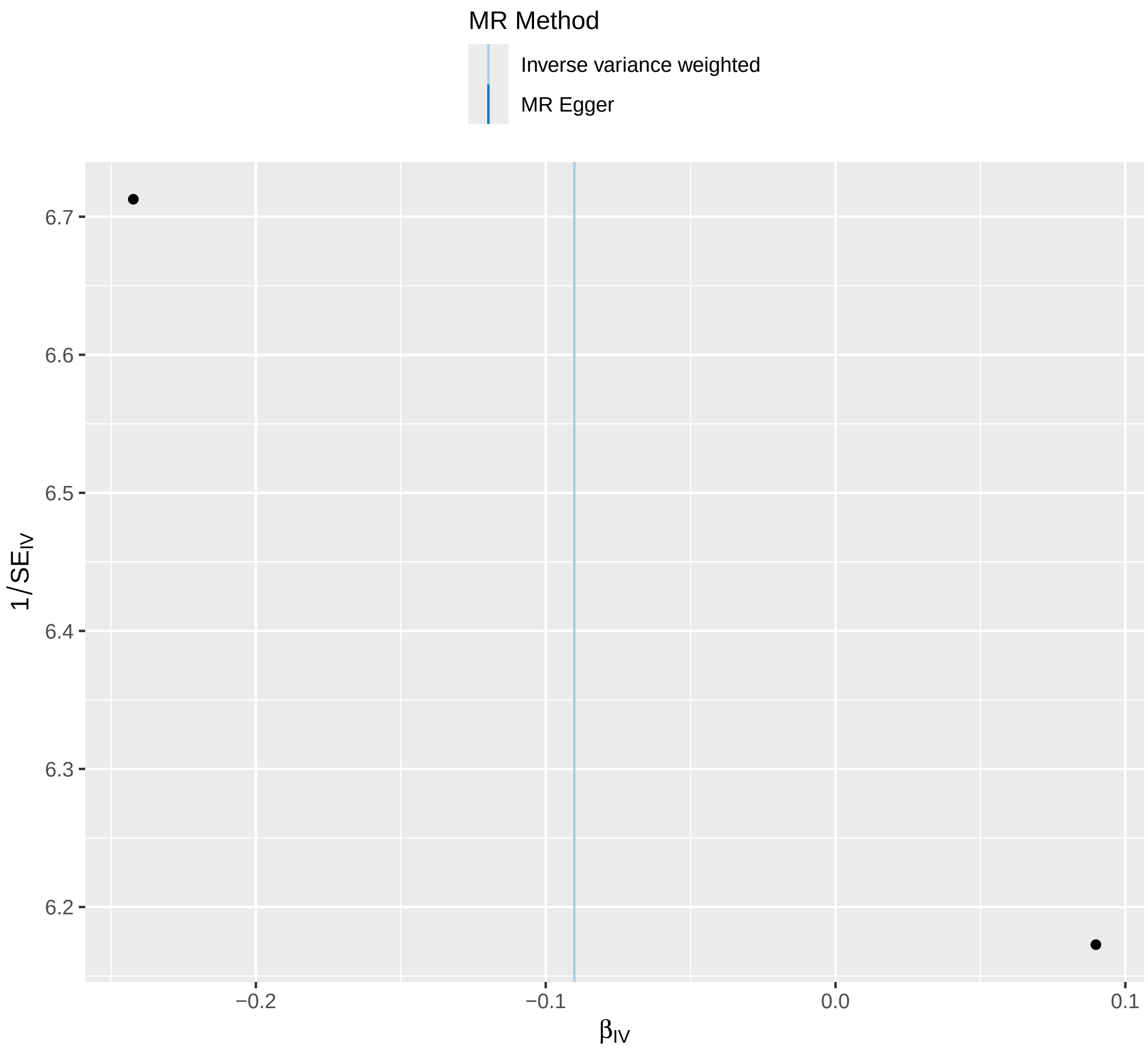

MR Method
Inverse variance weighted
MR Egger
6.7
6.6
6.5
6.4
6.3
6.2
1/SE_IV
−0.2
−0.1
0.0
0.1
β_IV

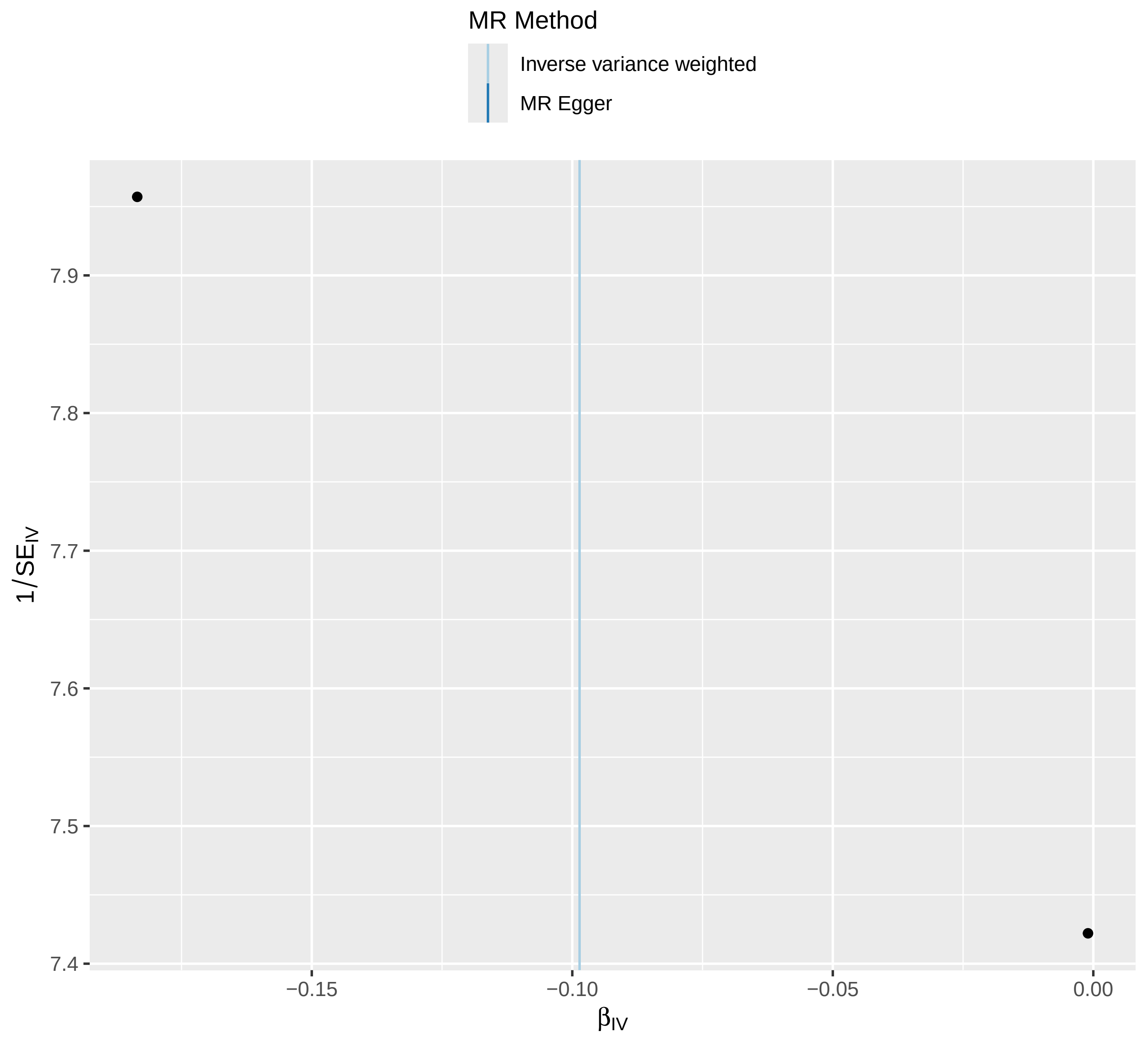
MR Method
Inverse variance weighted
MR Egger
7.9
7.8
7.7
7.6
7.5
7.4
$1/SE_{IV}$
−0.15
−0.10
−0.05
0.00
$\beta_{IV}$

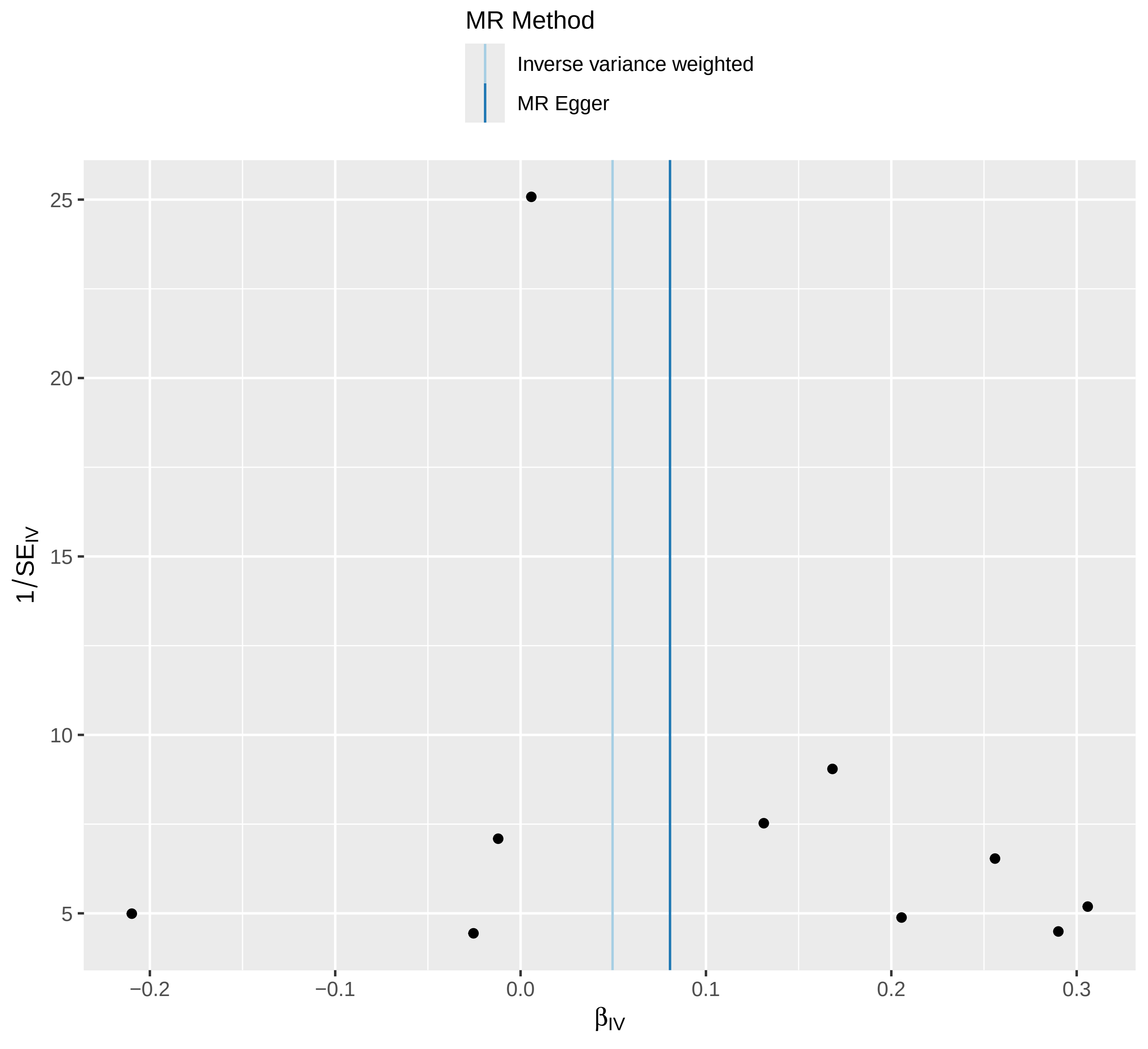
MR Method
Inverse variance weighted
MR Egger
25
20
15
10
5
1/SE_IV
−0.2
−0.1
0.0
0.1
0.2
0.3
β_IV

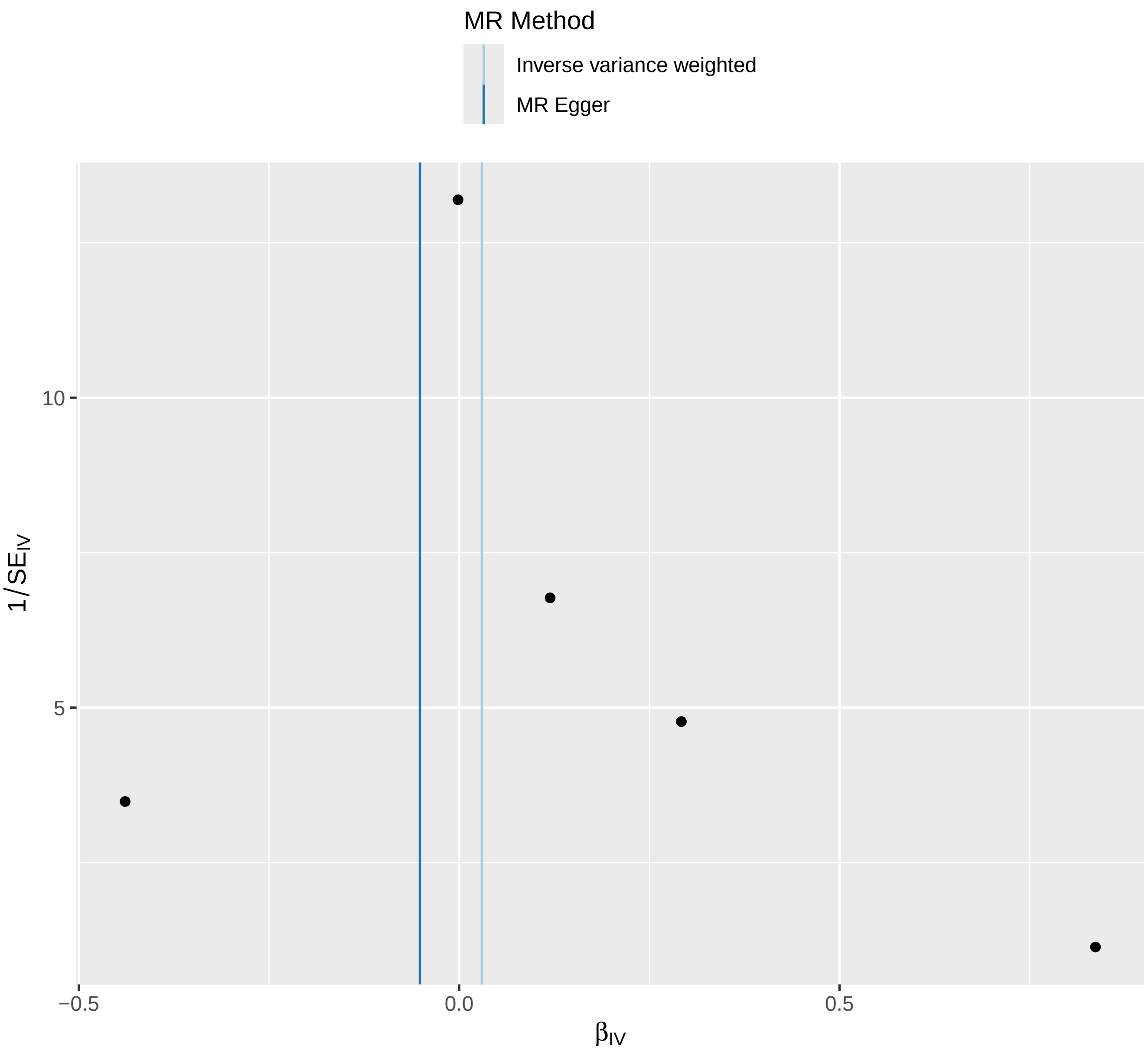
MR Method
Inverse variance weighted
MR Egger
10
5
1/SE_IV
−0.5
0.0
0.5
β_IV

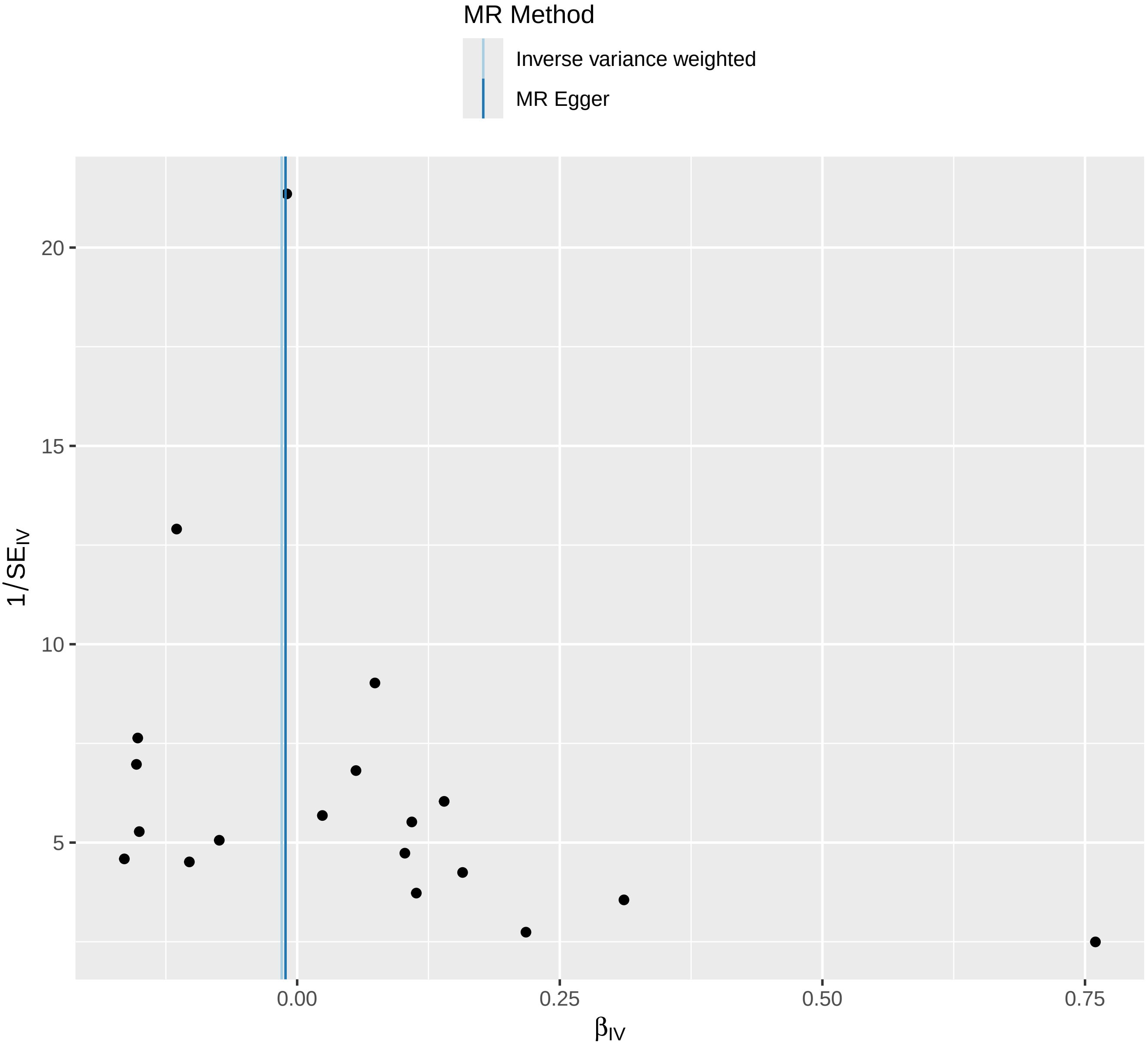

MR Method
Inverse variance weighted
MR Egger
20
15
10
5
1/SE_IV
0.00
0.25
0.50
0.75
β_IV

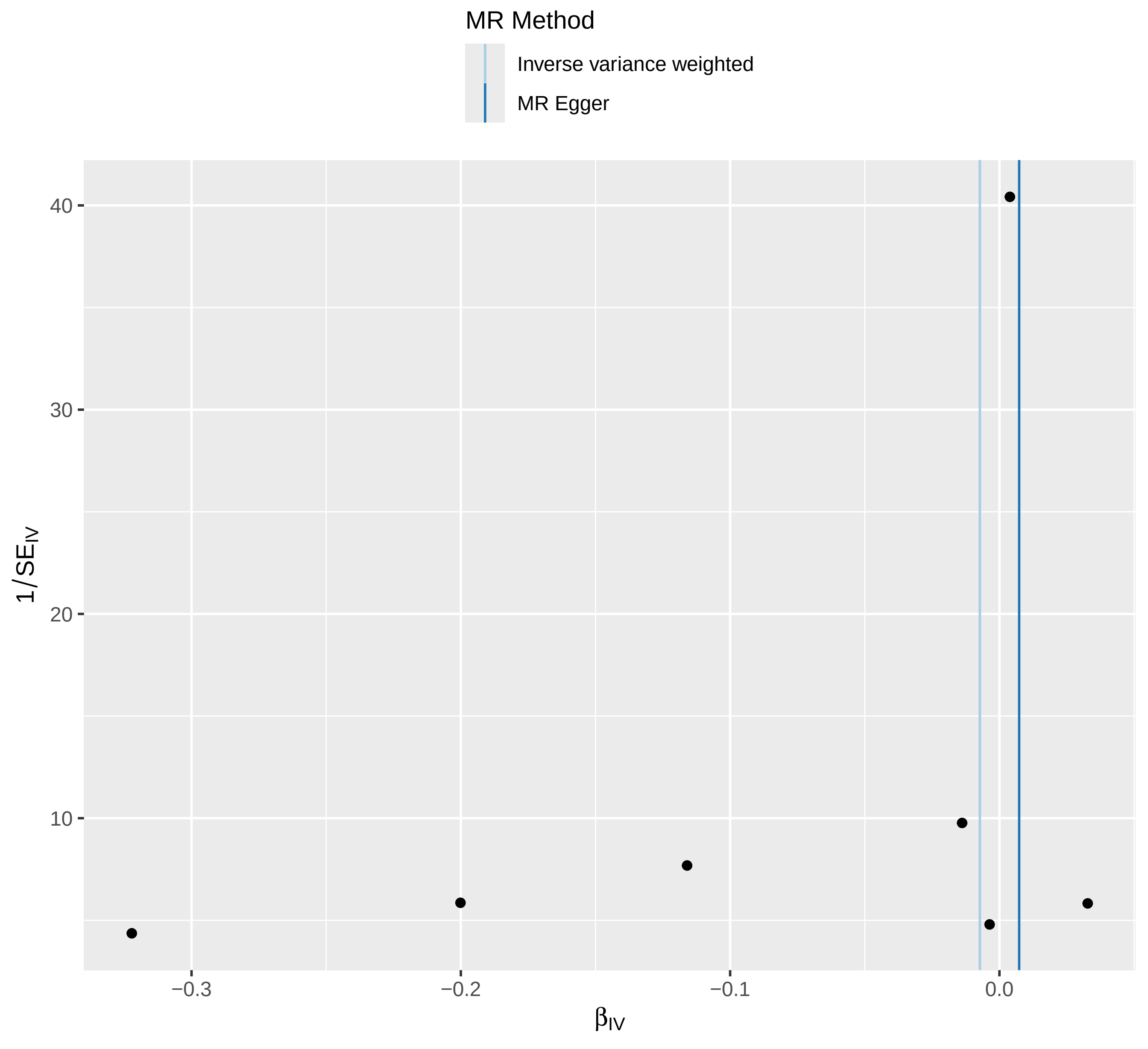

MR Method
Inverse variance weighted
MR Egger
40
30
20
10
1/SE_IV
−0.3
−0.2
−0.1
0.0
β_IV

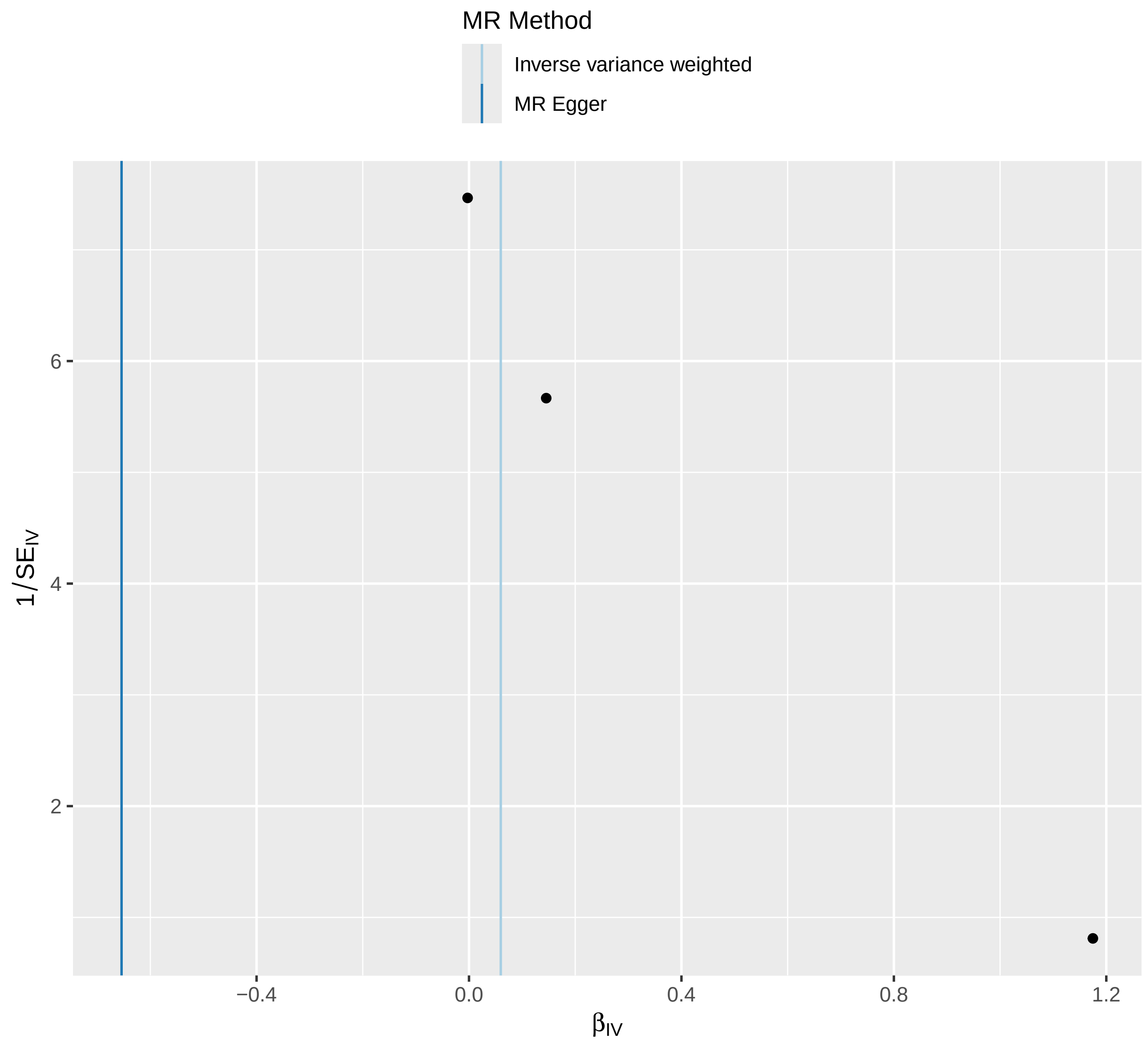
MR Method
Inverse variance weighted
MR Egger
6
4
2
1/SE$_{IV}$
−0.4
0.0
0.4
0.8
1.2
β$_{IV}$

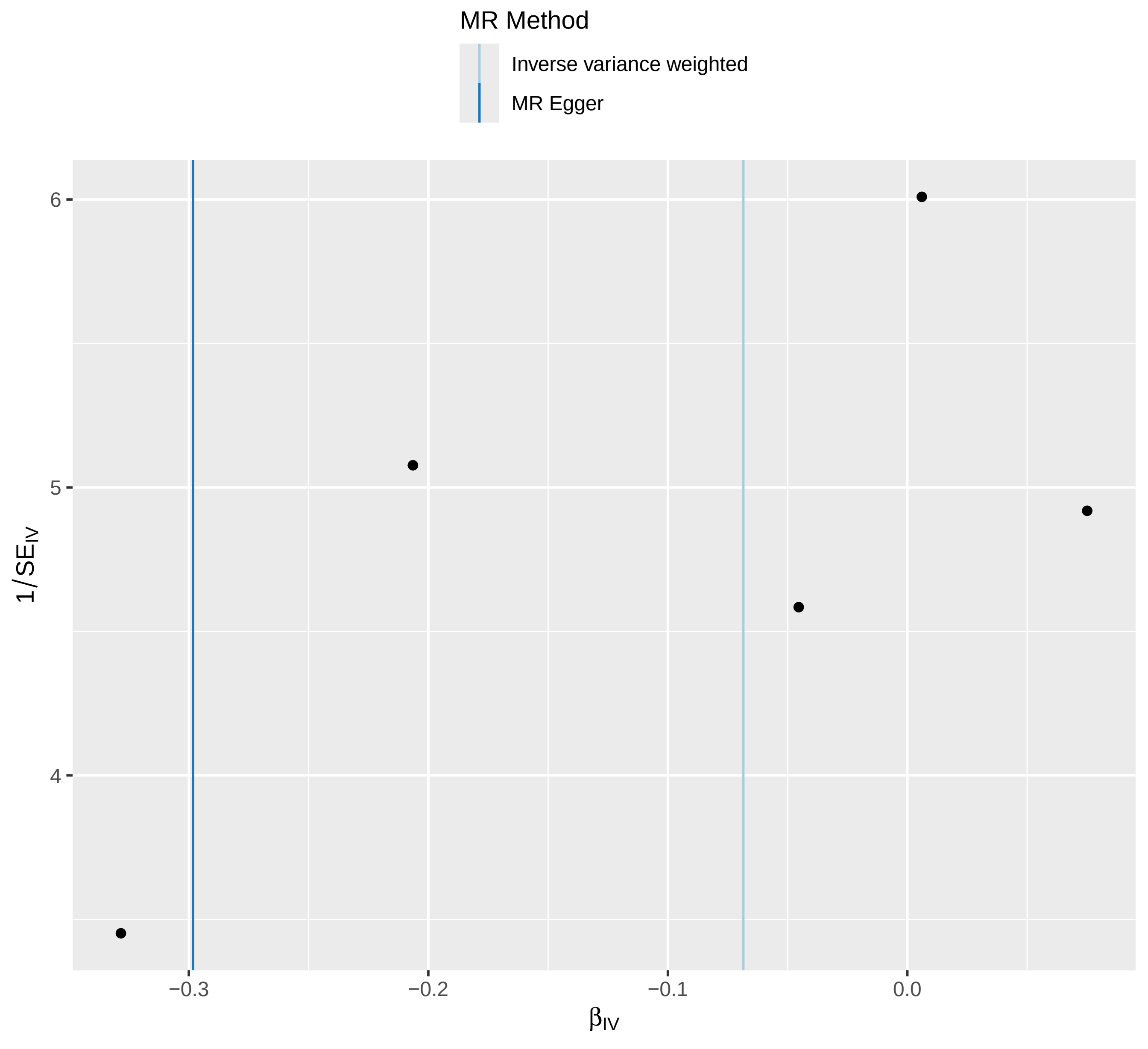

MR Method
Inverse variance weighted
MR Egger
6
5
4
1/SE_IV
−0.3
−0.2
−0.1
0.0
β_IV

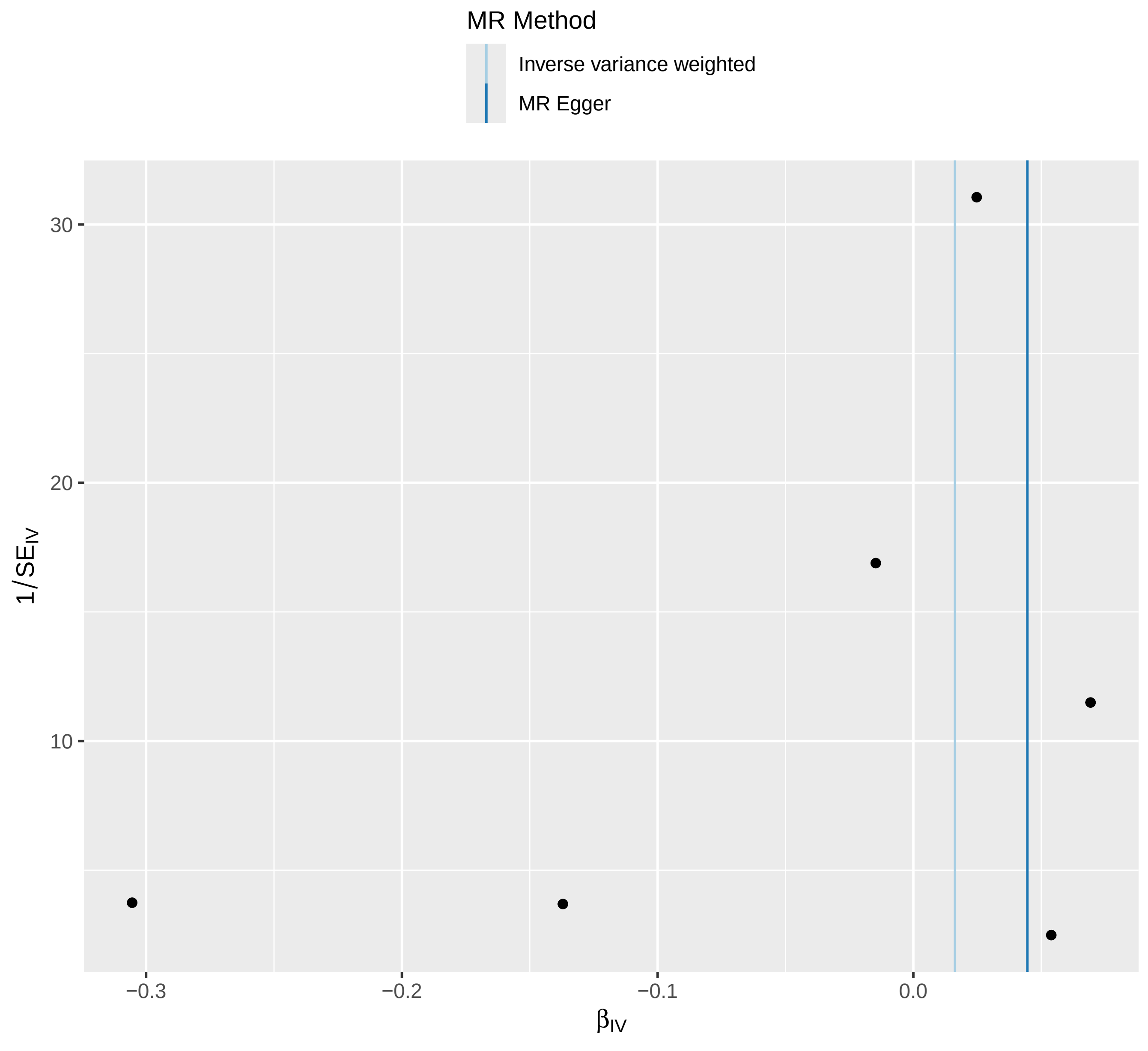

MR Method
Inverse variance weighted
MR Egger
30
20
10
$1/SE_{IV}$
−0.3
−0.2
−0.1
0.0
$\beta_{IV}$

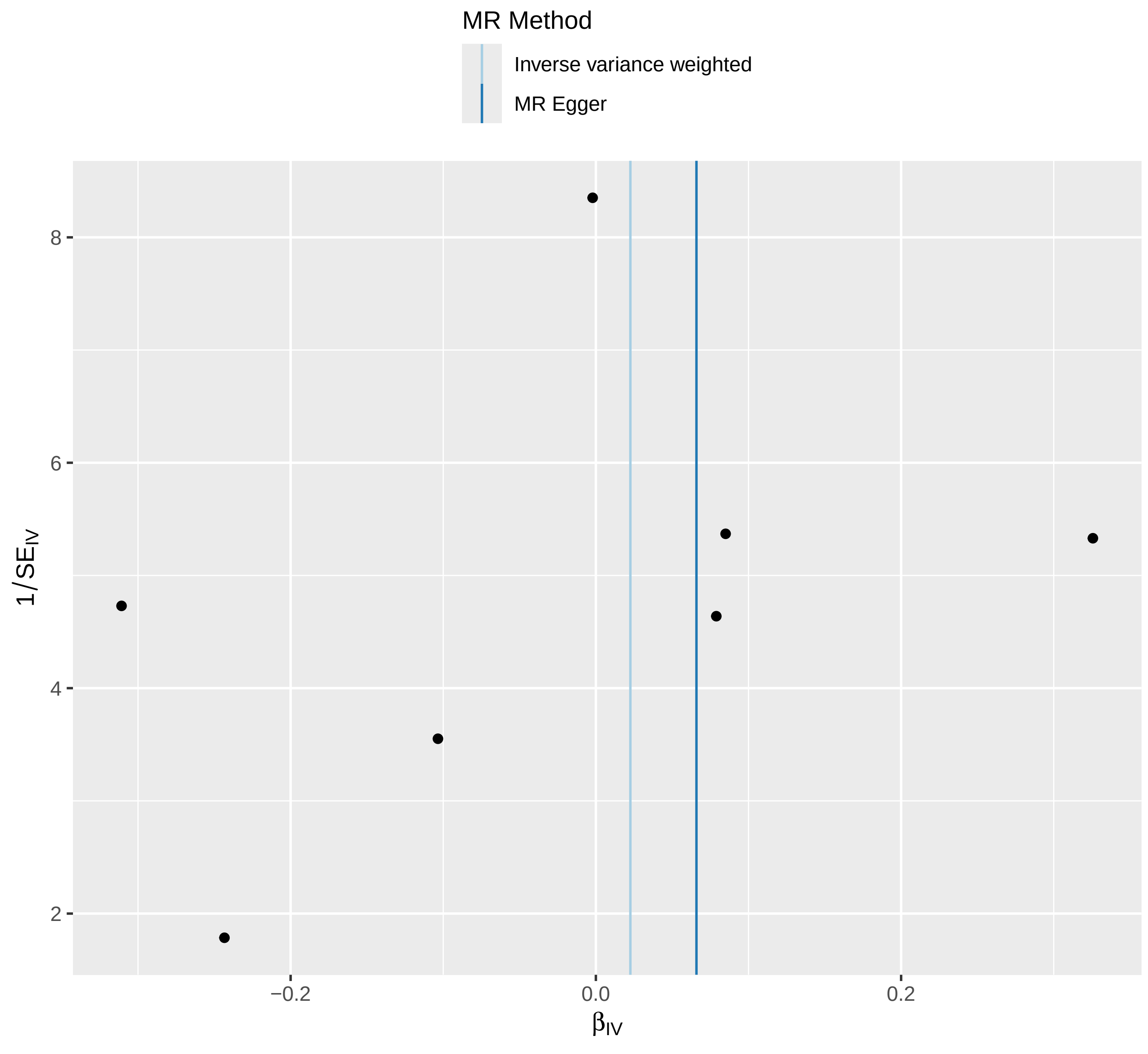

MR Method
Inverse variance weighted
MR Egger
8
6
4
2
−0.2
0.0
0.2
$1/SE_{IV}$
$\beta_{IV}$

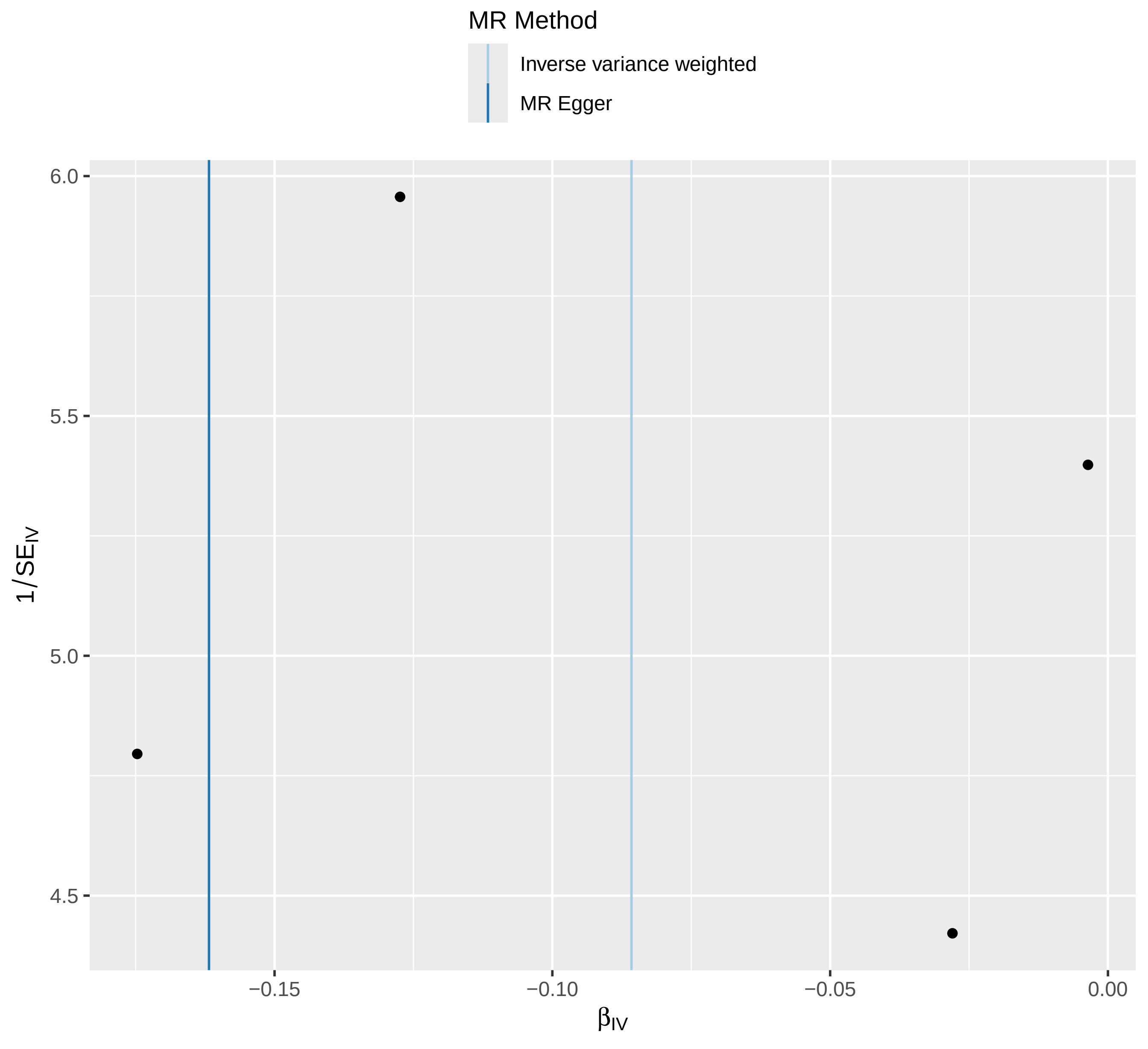
MR Method
Inverse variance weighted
MR Egger
6.0
5.5
5.0
4.5
1/SE$_{IV}$
−0.15
−0.10
−0.05
0.00
β$_{IV}$

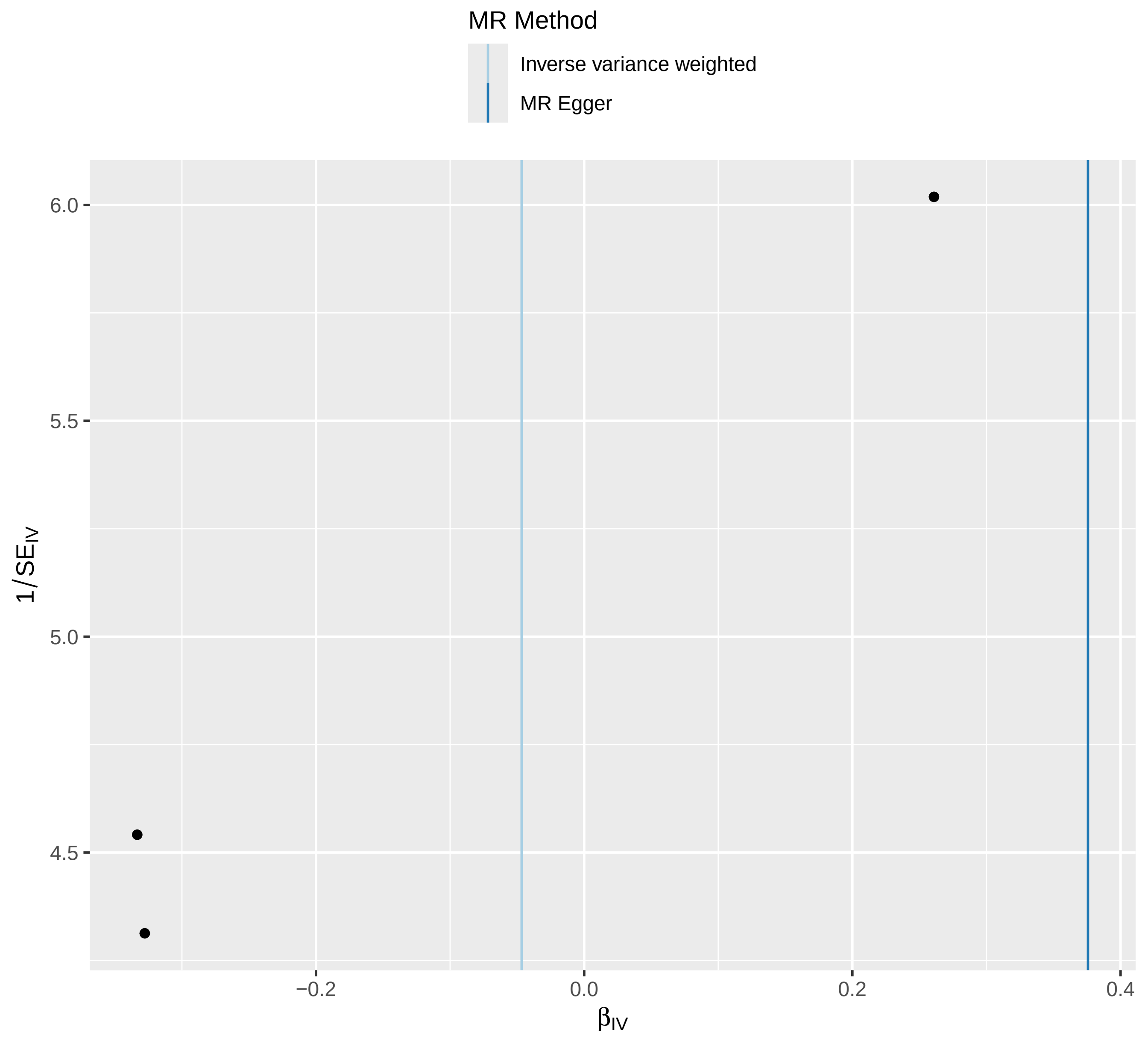

MR Method
Inverse variance weighted
MR Egger
6.0
5.5
5.0
4.5
1/SE_IV
−0.2
0.0
0.2
0.4
β_IV

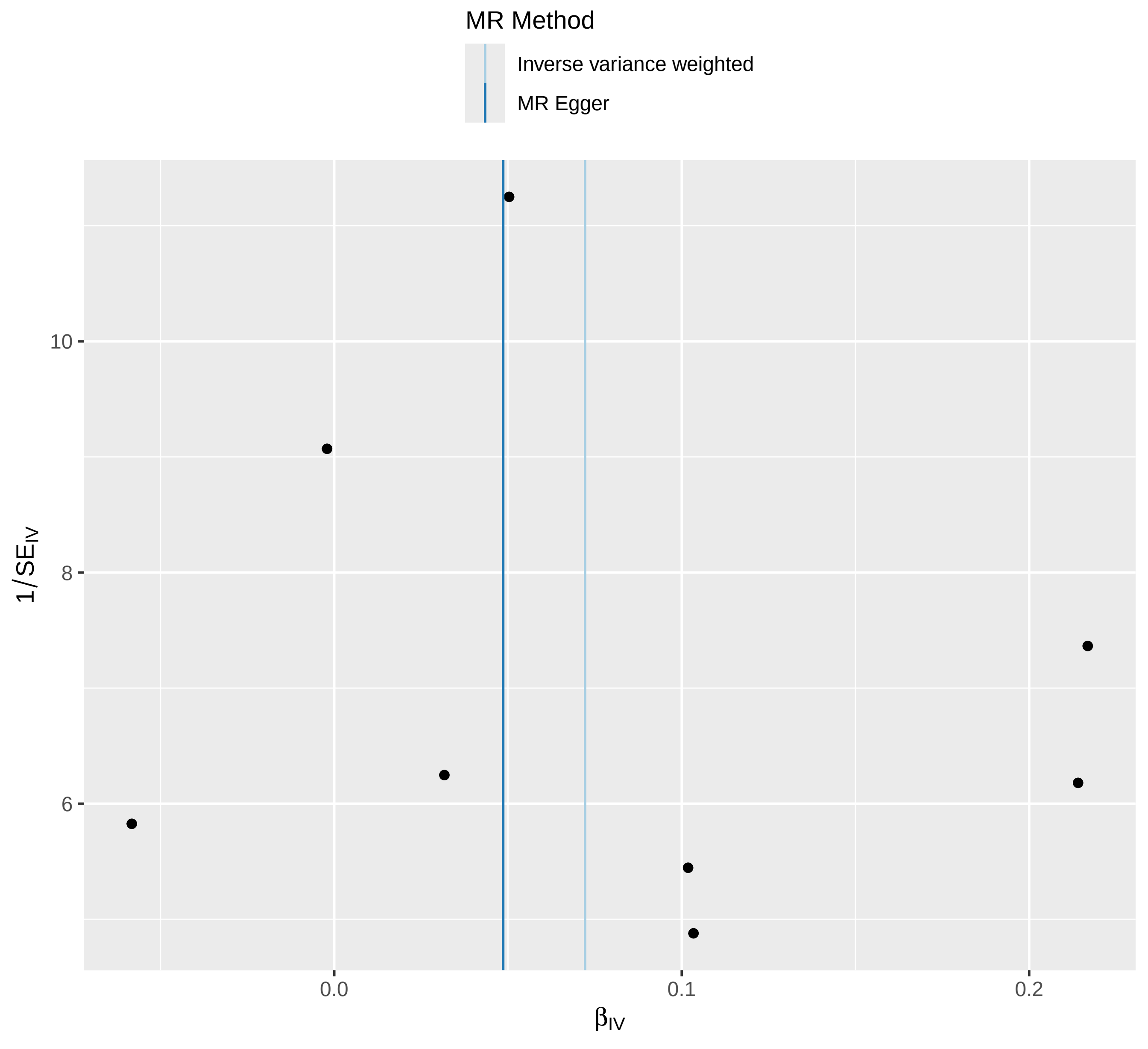
MR Method
Inverse variance weighted
MR Egger
10
8
6
0.0
0.1
0.2
$\beta_{IV}$
$1/SE_{IV}$

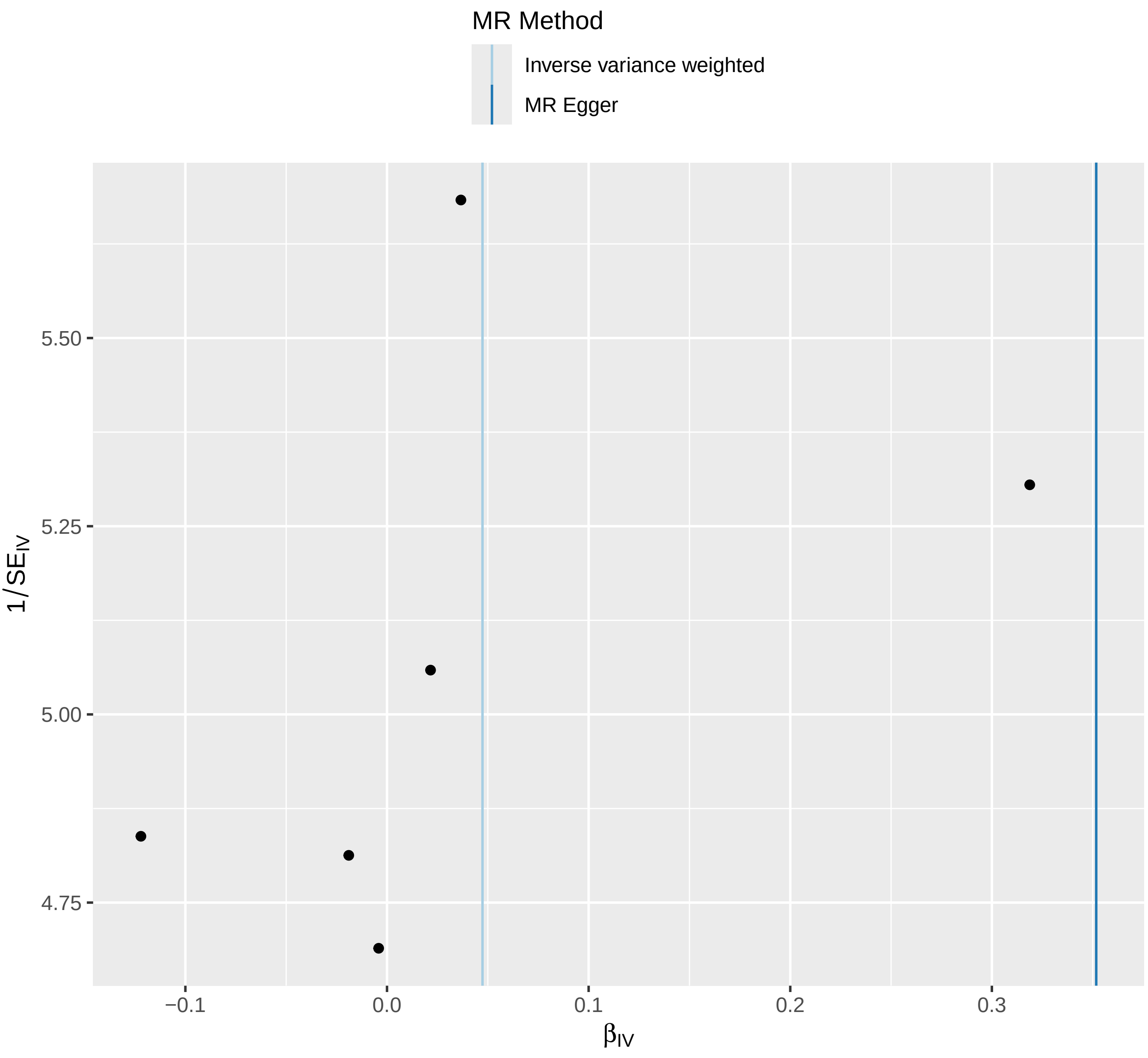
MR Method
Inverse variance weighted
MR Egger
5.50
5.25
5.00
4.75
1/SE_IV
−0.1
0.0
0.1
0.2
0.3
β_IV

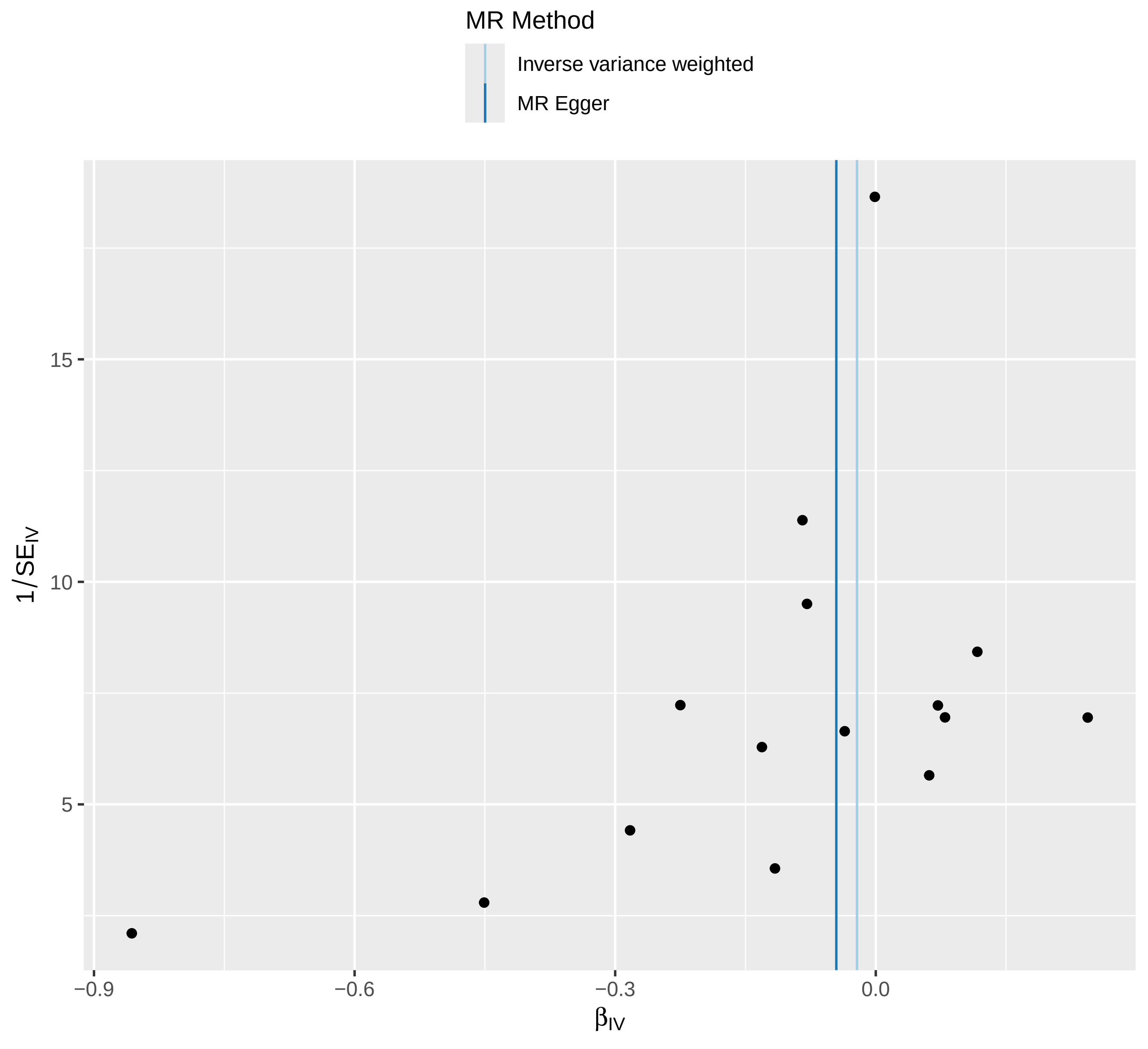

MR Method
Inverse variance weighted
MR Egger
1/SE_IV
15
10
5
−0.9
−0.6
−0.3
0.0
β_IV

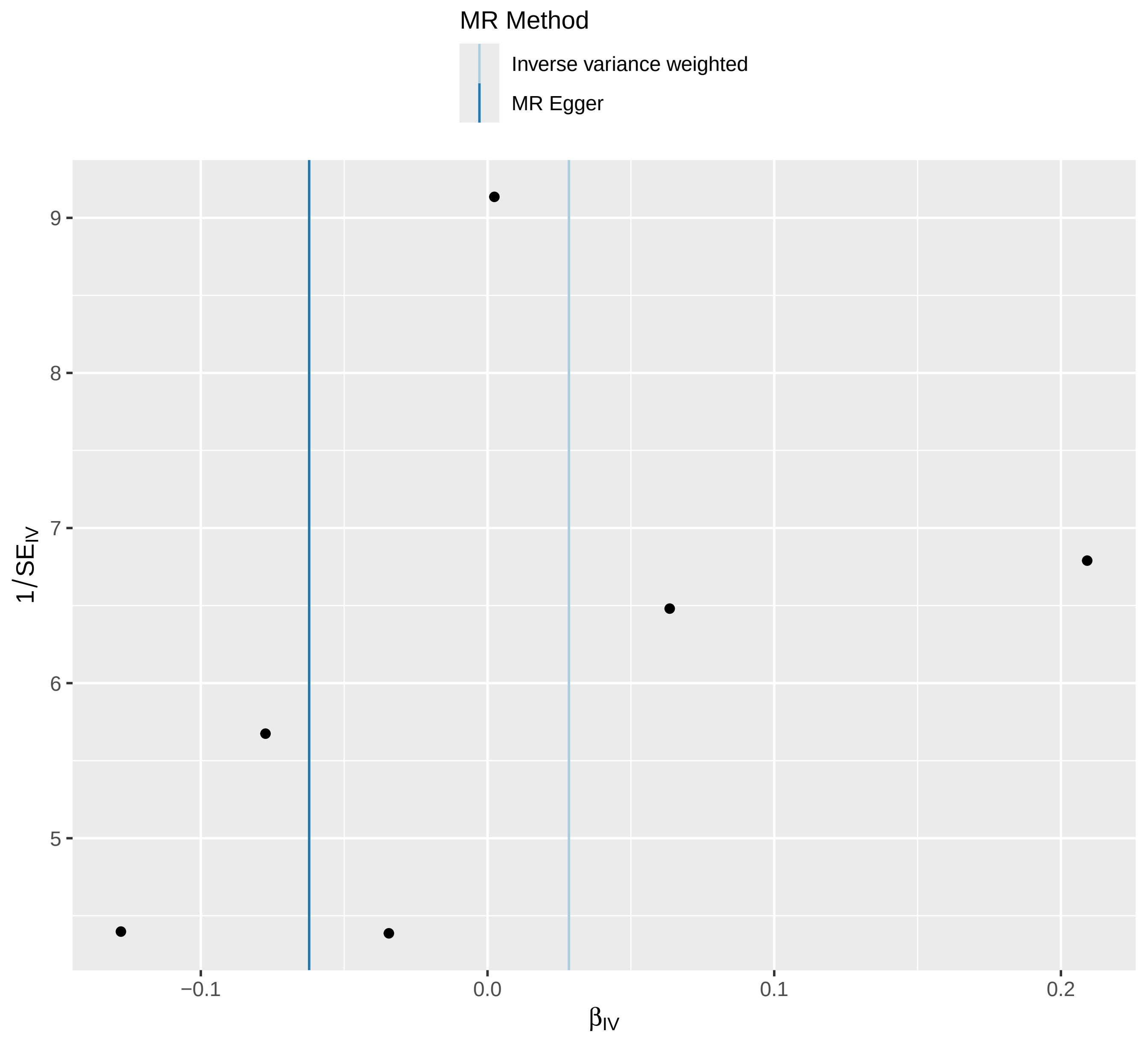
MR Method
Inverse variance weighted
MR Egger
9
8
7
6
5
1/SE_IV
−0.1
0.0
0.1
0.2
β_IV

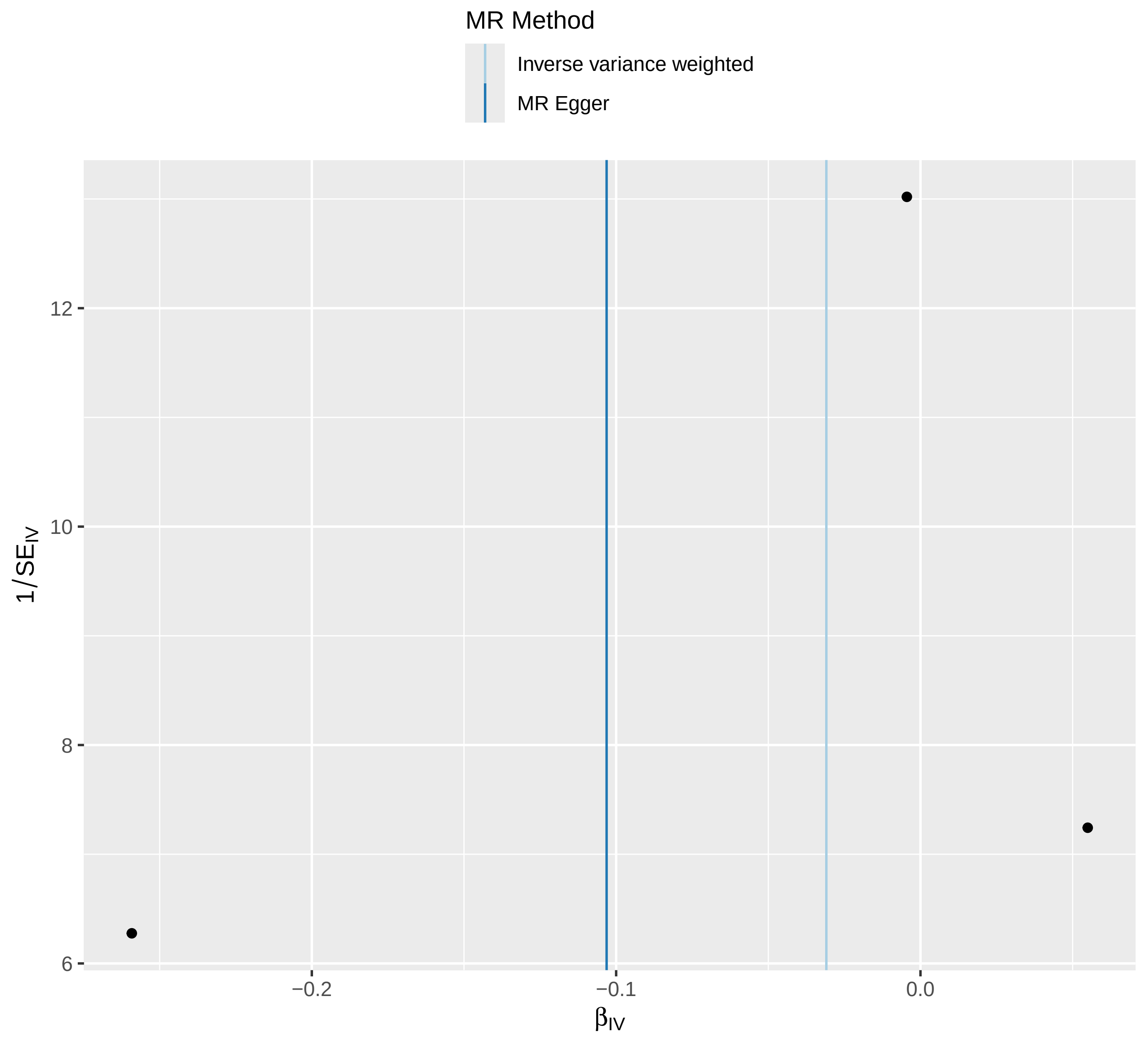
MR Method
Inverse variance weighted
MR Egger
12
10
8
6
1/SE_IV
−0.2
−0.1
0.0
β_IV

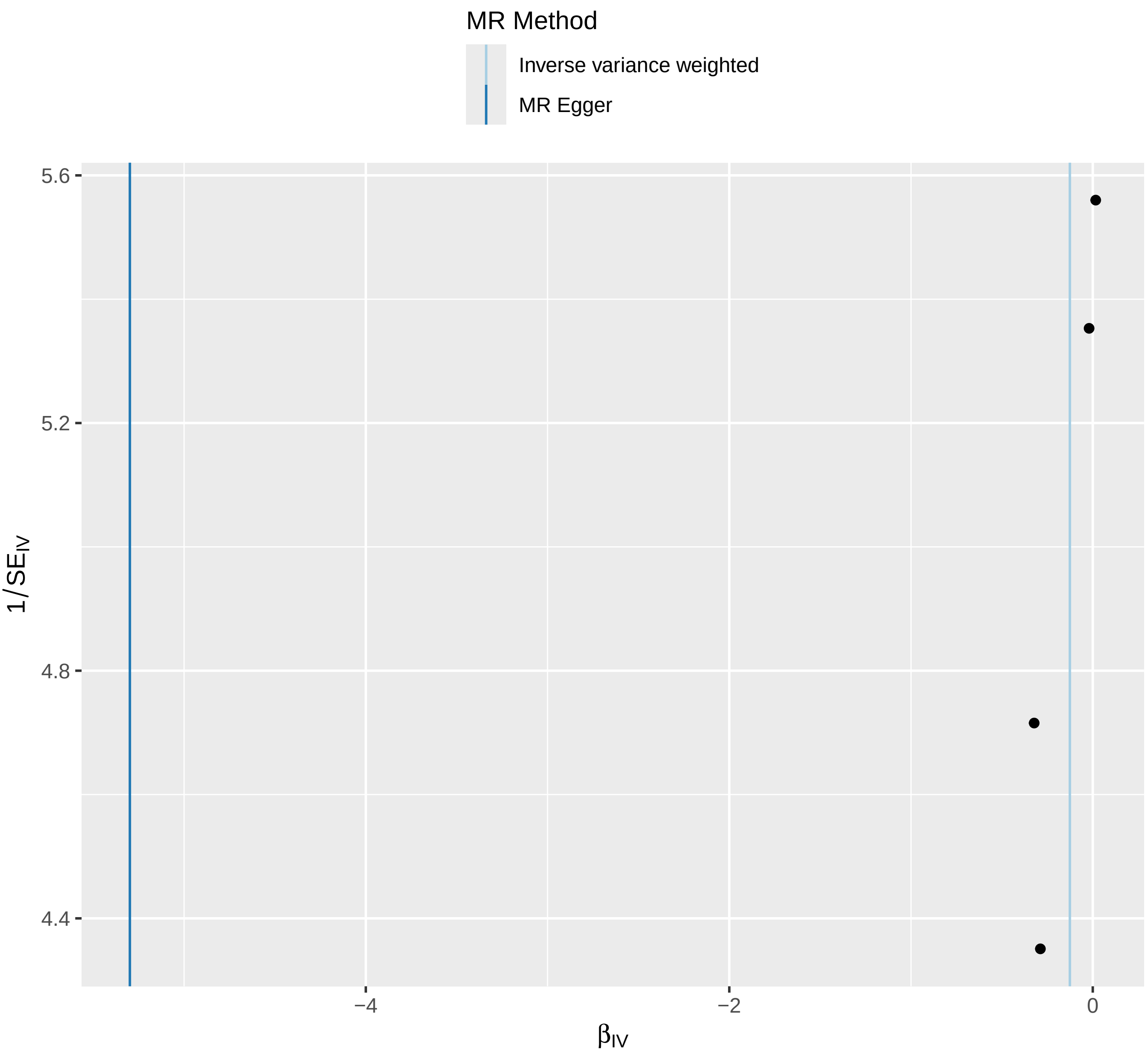

MR Method
Inverse variance weighted
MR Egger
5.6
5.2
4.8
4.4
1/SE$_{IV}$
−4
−2
0
β$_{IV}$

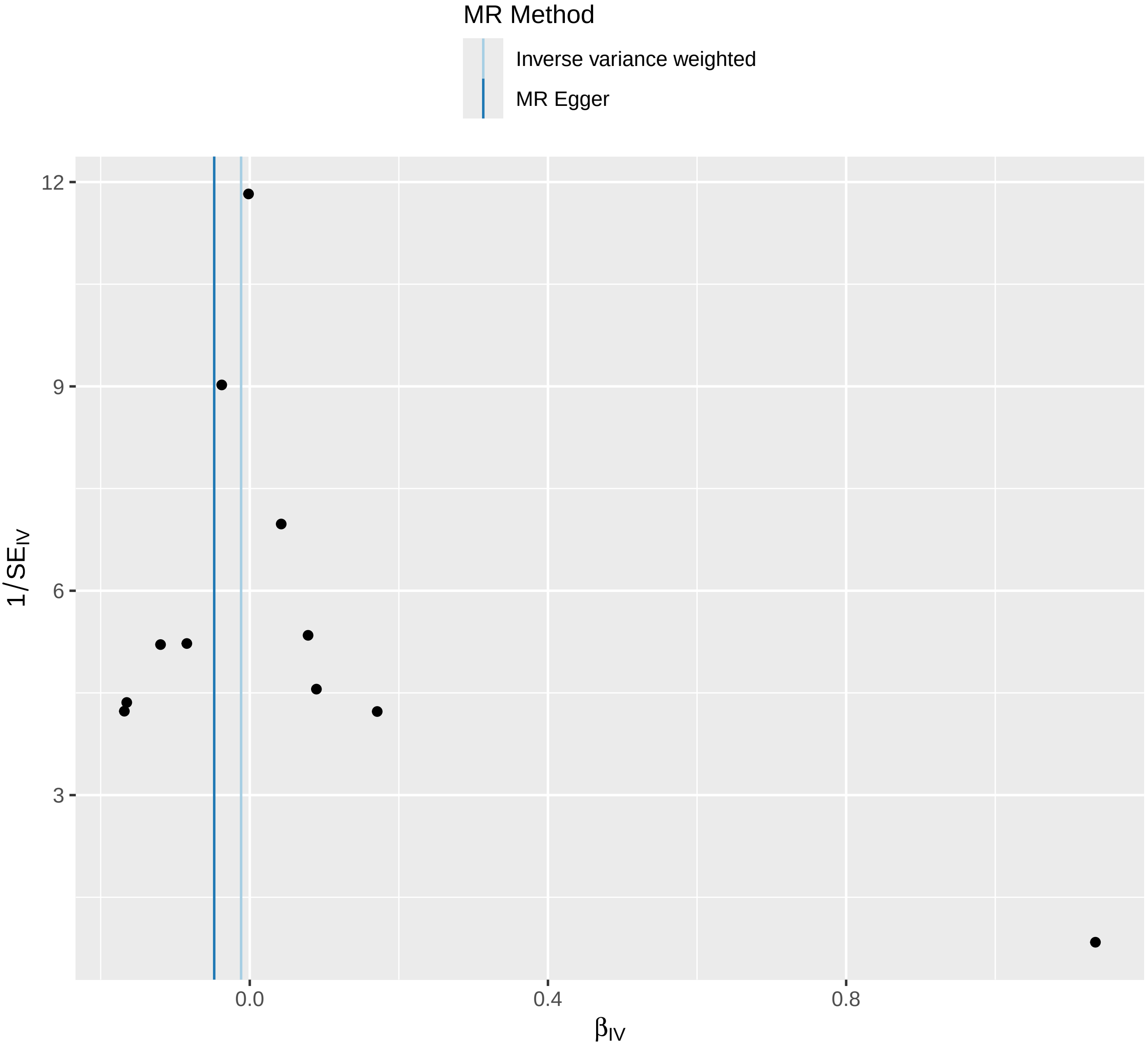
MR Method
Inverse variance weighted
MR Egger
12
9
6
3
$1/SE_{IV}$
0.0
0.4
0.8
$\beta_{IV}$

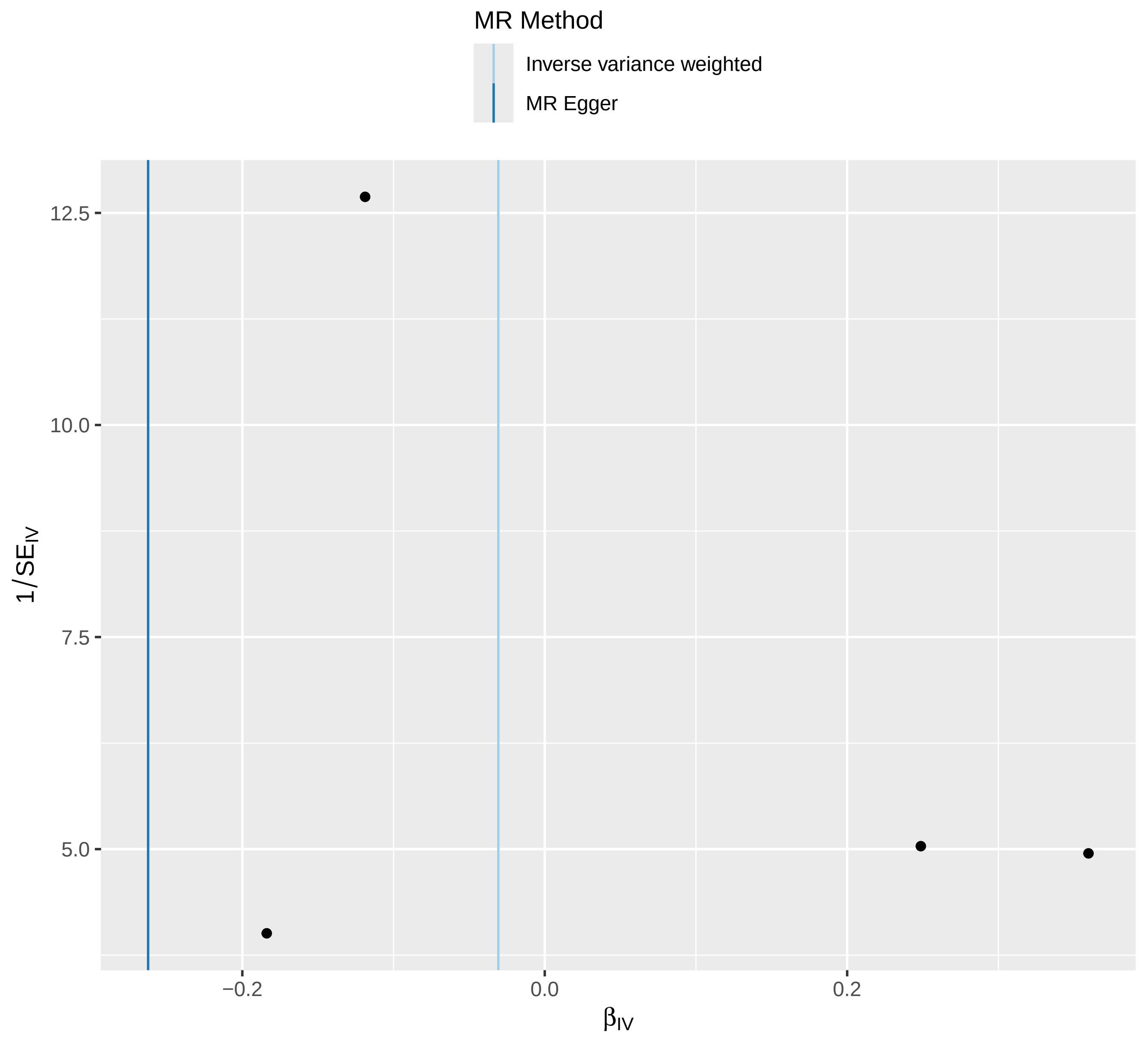

MR Method
Inverse variance weighted
MR Egger
12.5
10.0
7.5
5.0
1/SE_IV
−0.2
0.0
0.2
β_IV

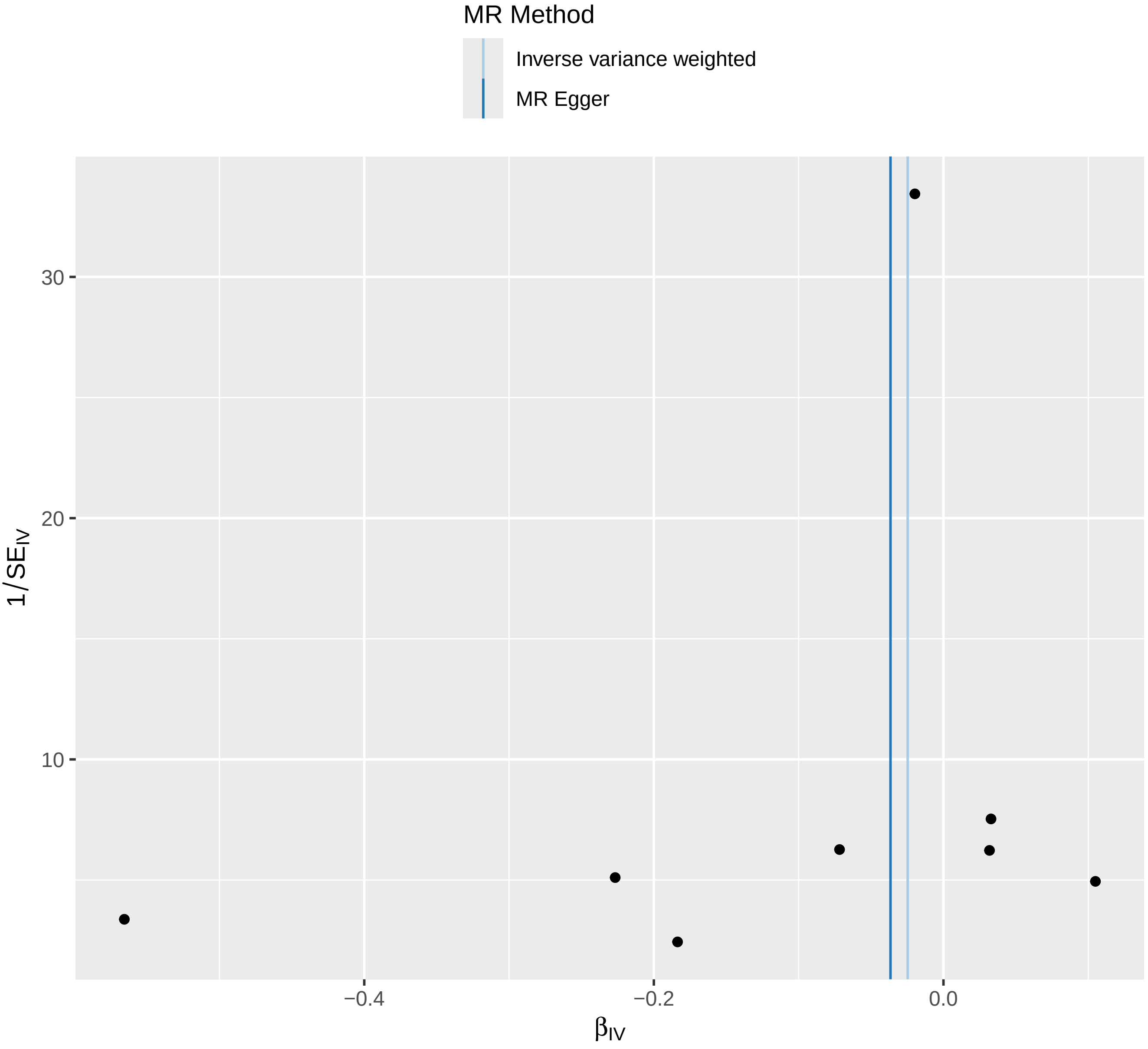

MR Method
Inverse variance weighted
MR Egger
30
20
10
1/SE$_{IV}$
−0.4
−0.2
0.0
β$_{IV}$

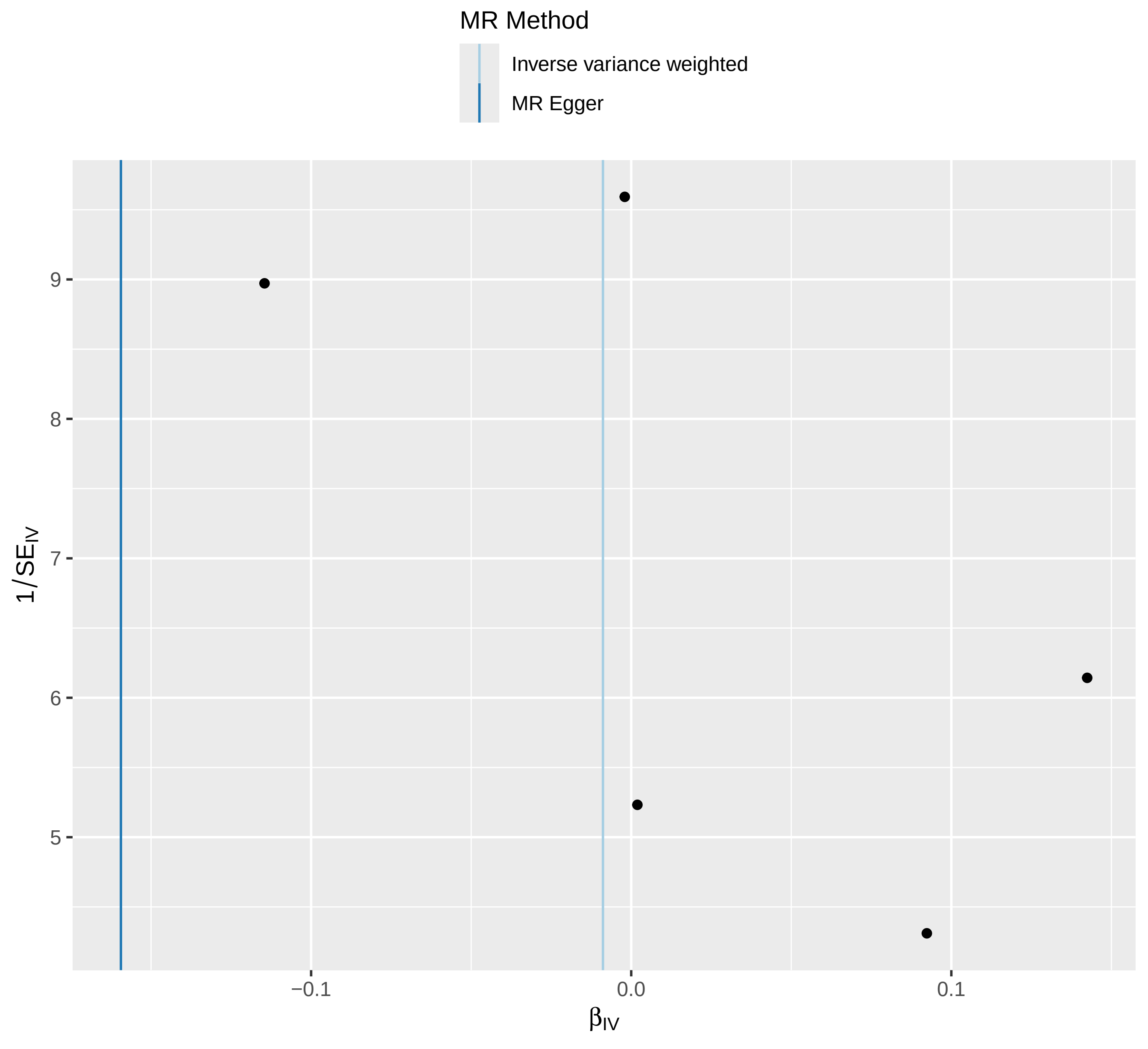
MR Method
Inverse variance weighted
MR Egger
1/SE_IV
9
8
7
6
5
−0.1
0.0
0.1
β_IV

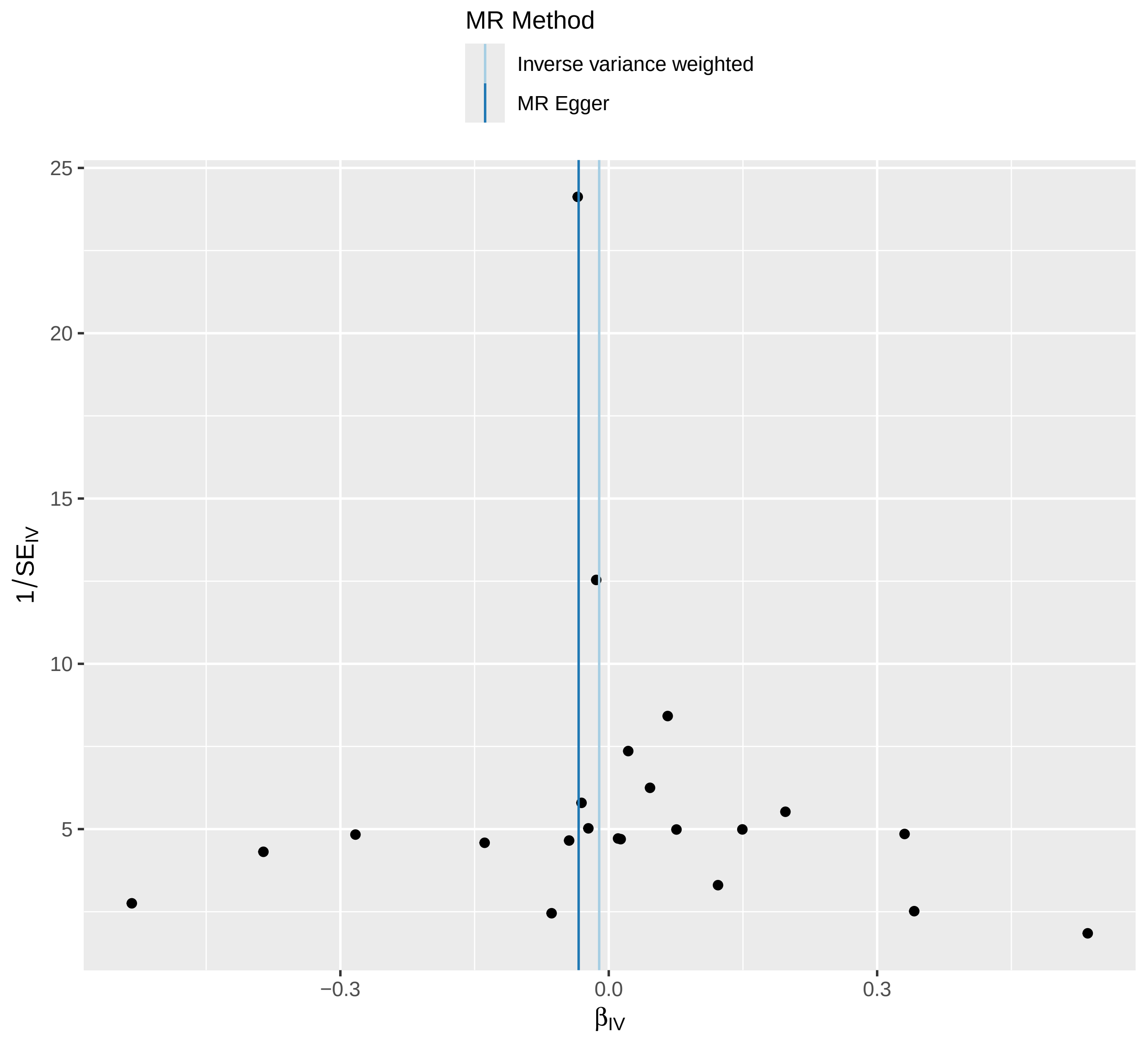
MR Method
Inverse variance weighted
MR Egger
25
20
15
10
5
1/SE$_{IV}$
−0.3
0.0
0.3
β$_{IV}$

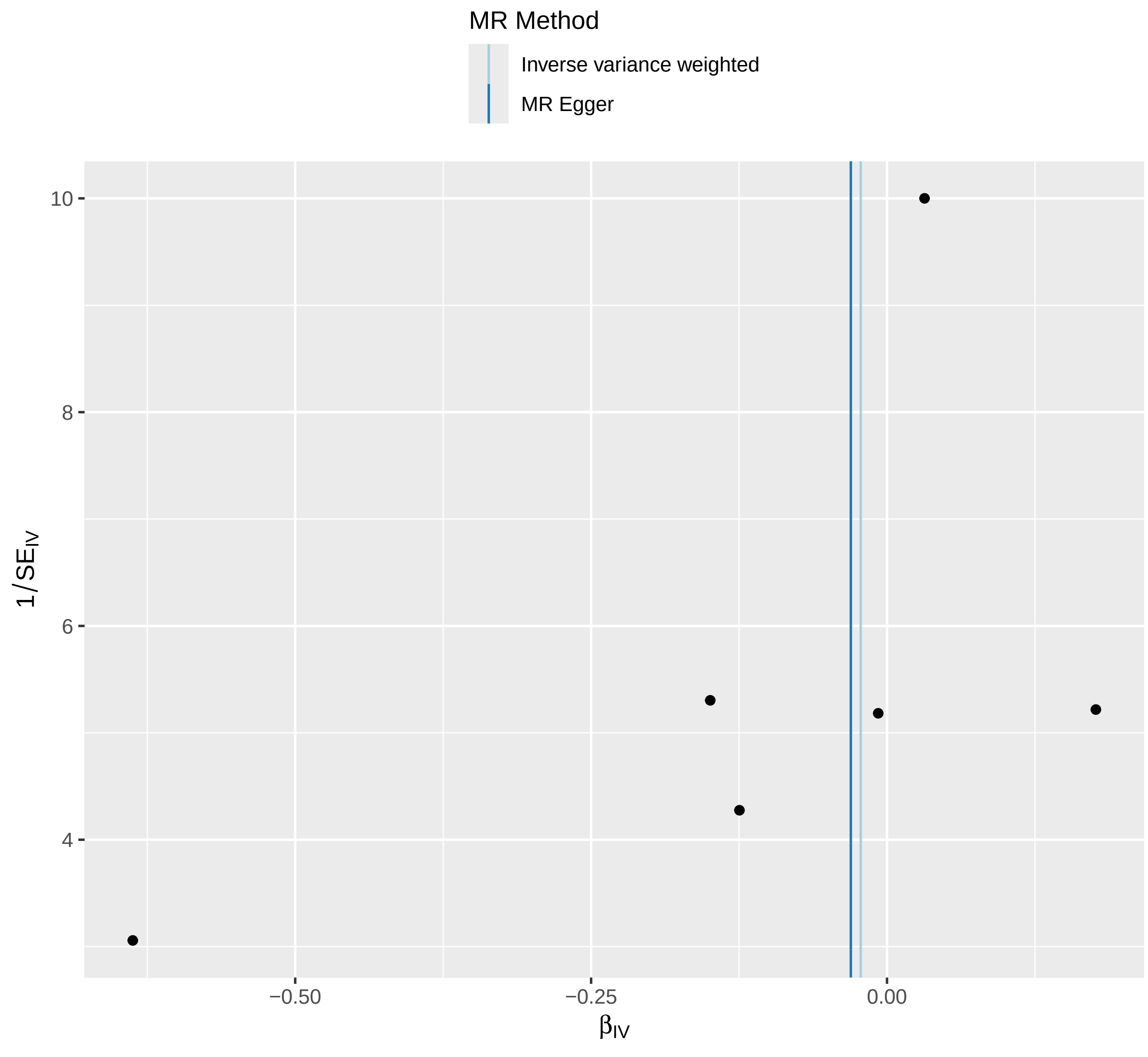

MR Method
Inverse variance weighted
MR Egger
10
8
6
4
1/SE_IV
−0.50
−0.25
0.00
β_IV

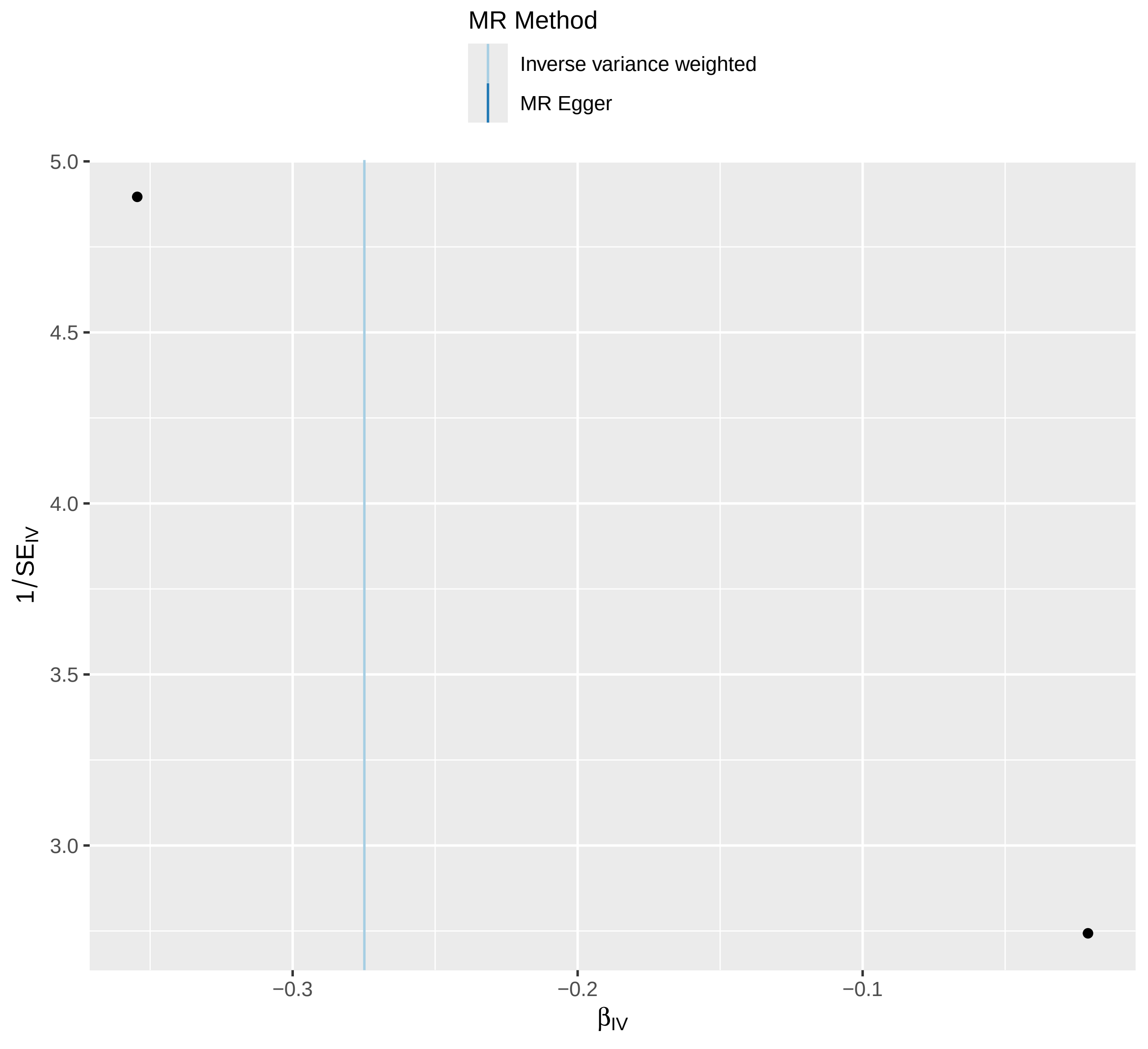

MR Method
Inverse variance weighted
MR Egger
5.0
4.5
4.0
3.5
3.0
1/SE$_{IV}$
−0.3
−0.2
−0.1
β$_{IV}$

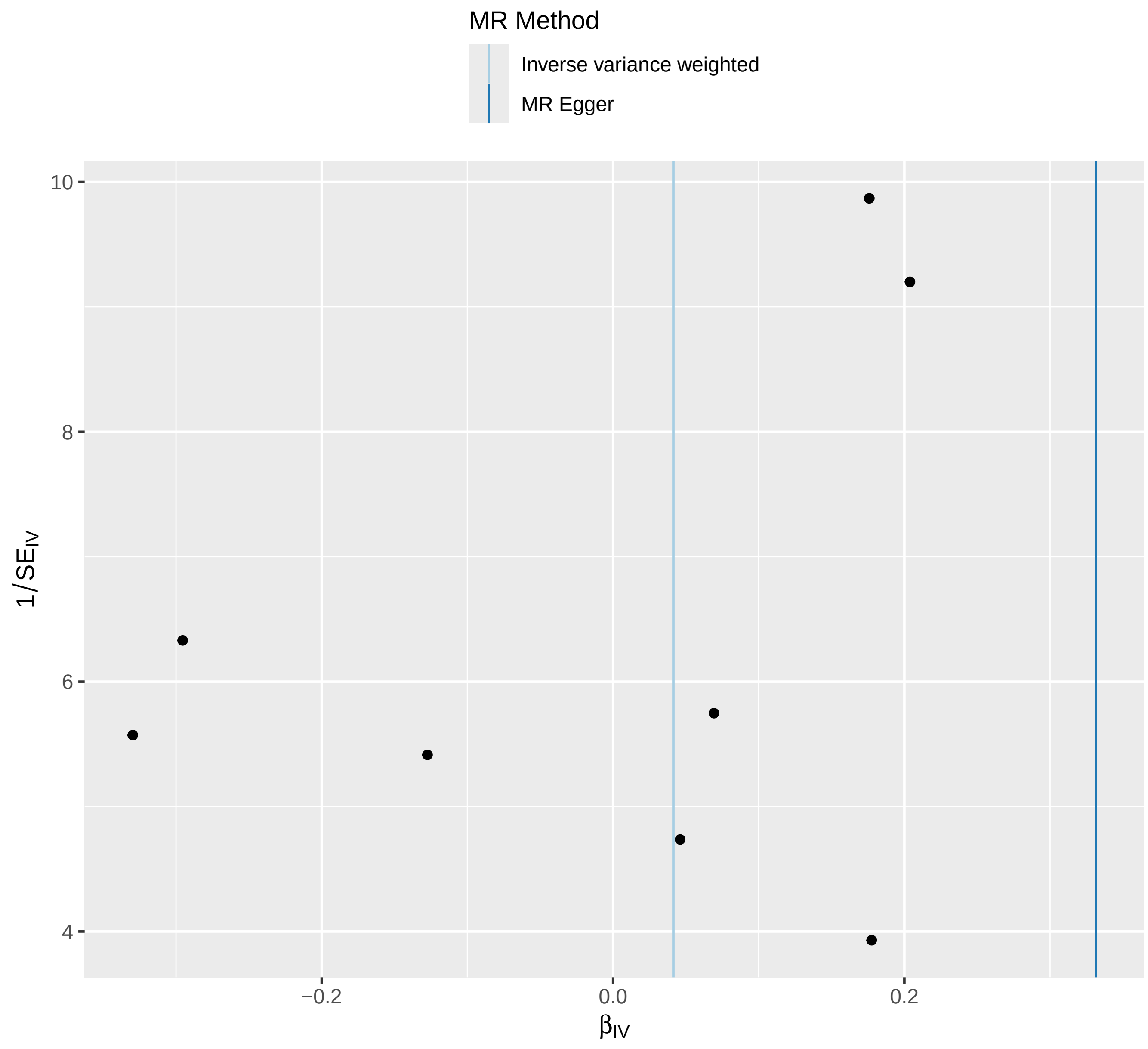

MR Method
Inverse variance weighted
MR Egger
10
8
6
4
1/SE$_{IV}$
−0.2
0.0
0.2
β$_{IV}$

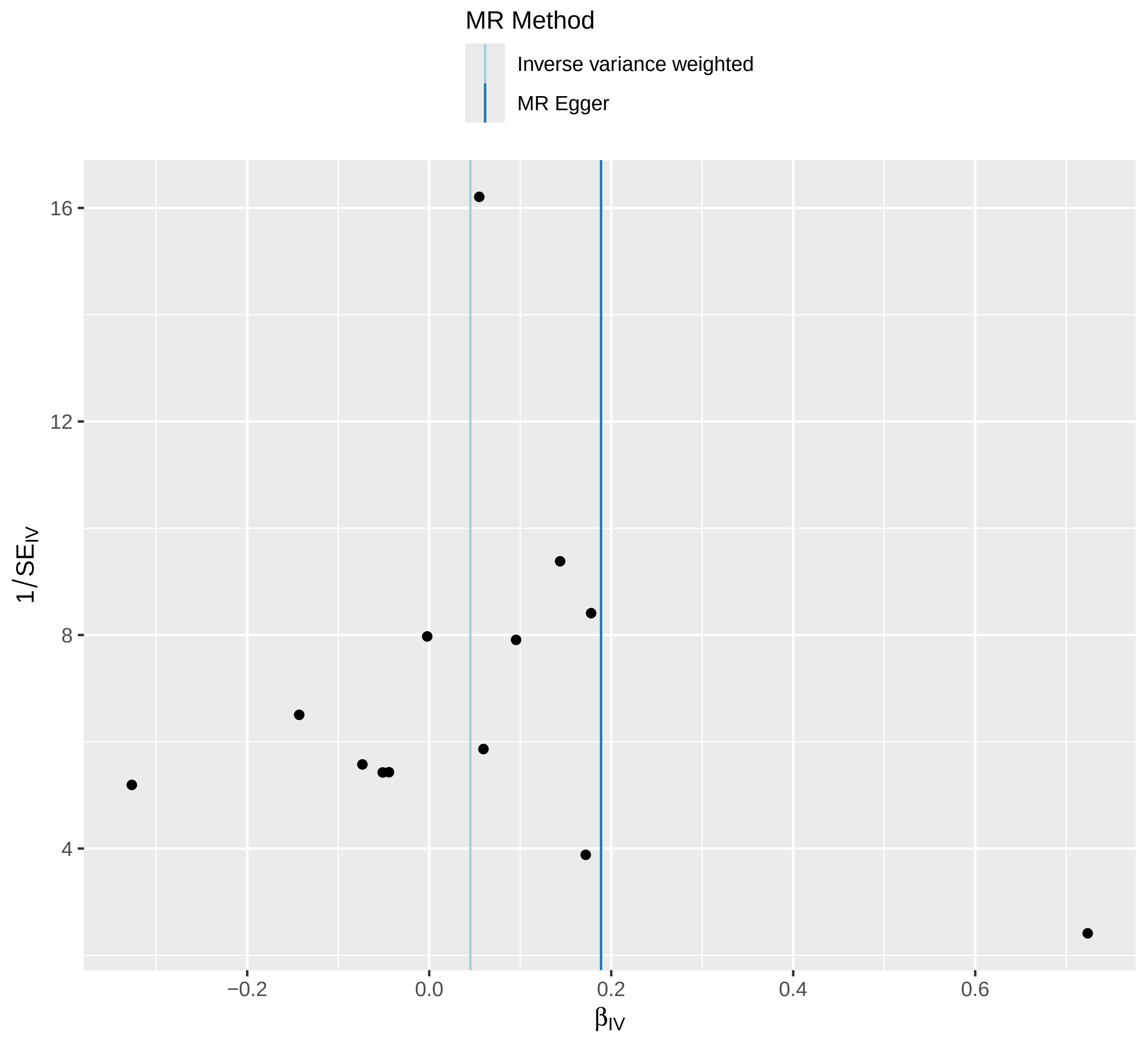

MR Method
Inverse variance weighted
MR Egger
16
12
8
4
1/SE_IV
−0.2
0.0
0.2
0.4
0.6
β_IV

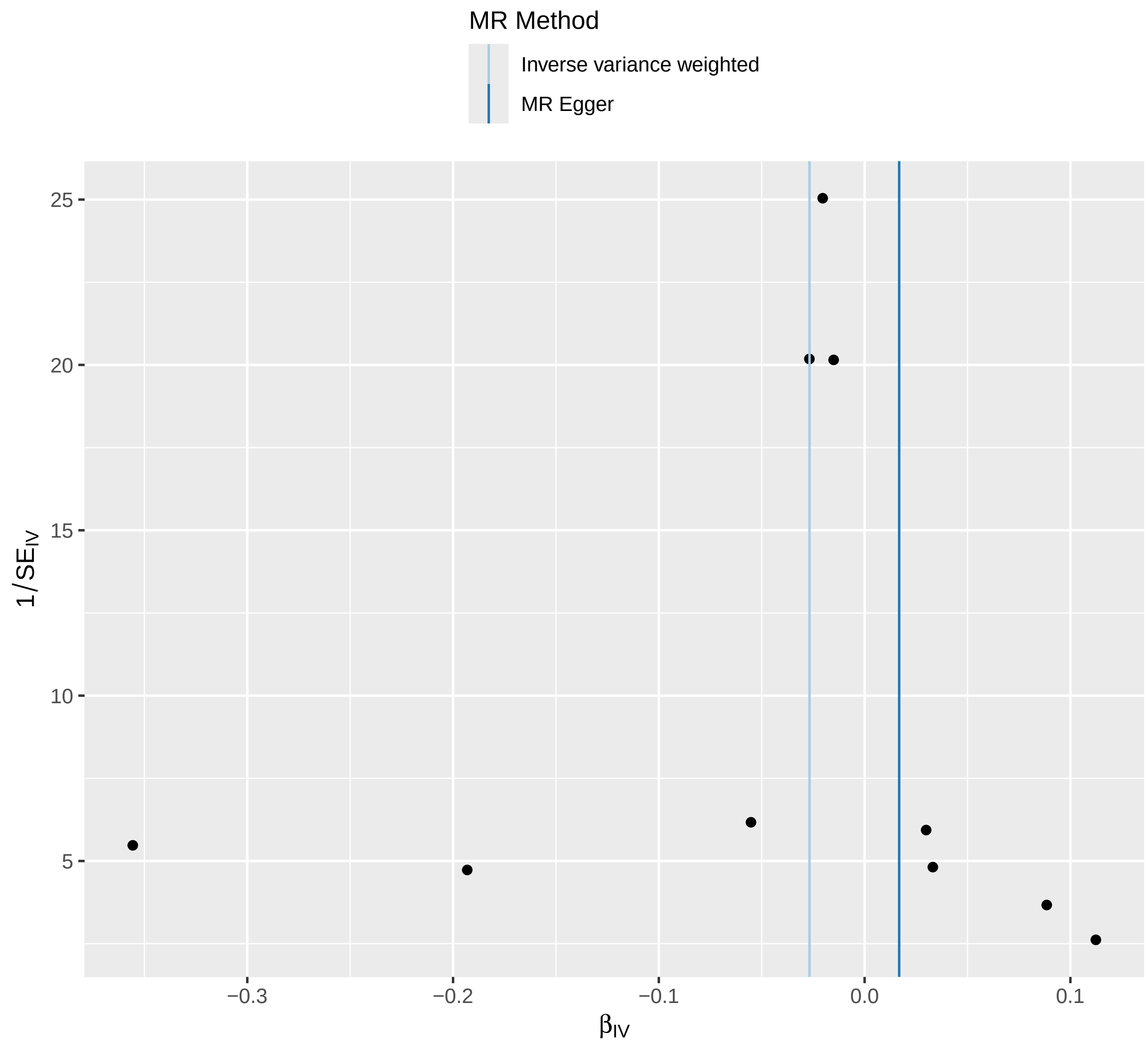

MR Method
Inverse variance weighted
MR Egger
25
20
15
10
5
1/SE$_{IV}$
−0.3
−0.2
−0.1
0.0
0.1
β$_{IV}$

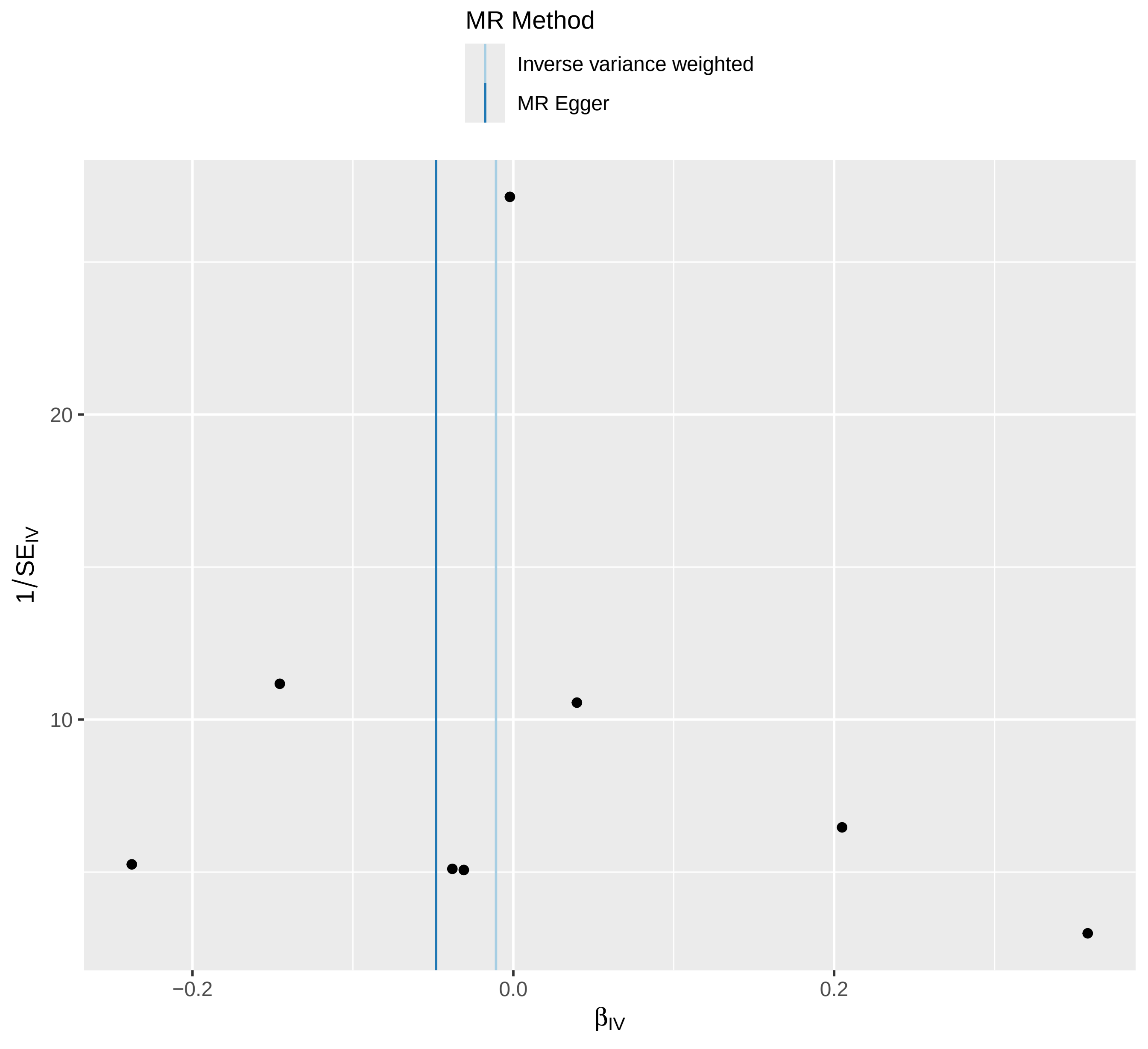

MR Method
Inverse variance weighted
MR Egger
20
10
1/SE$_{IV}$
−0.2
0.0
0.2
β$_{IV}$

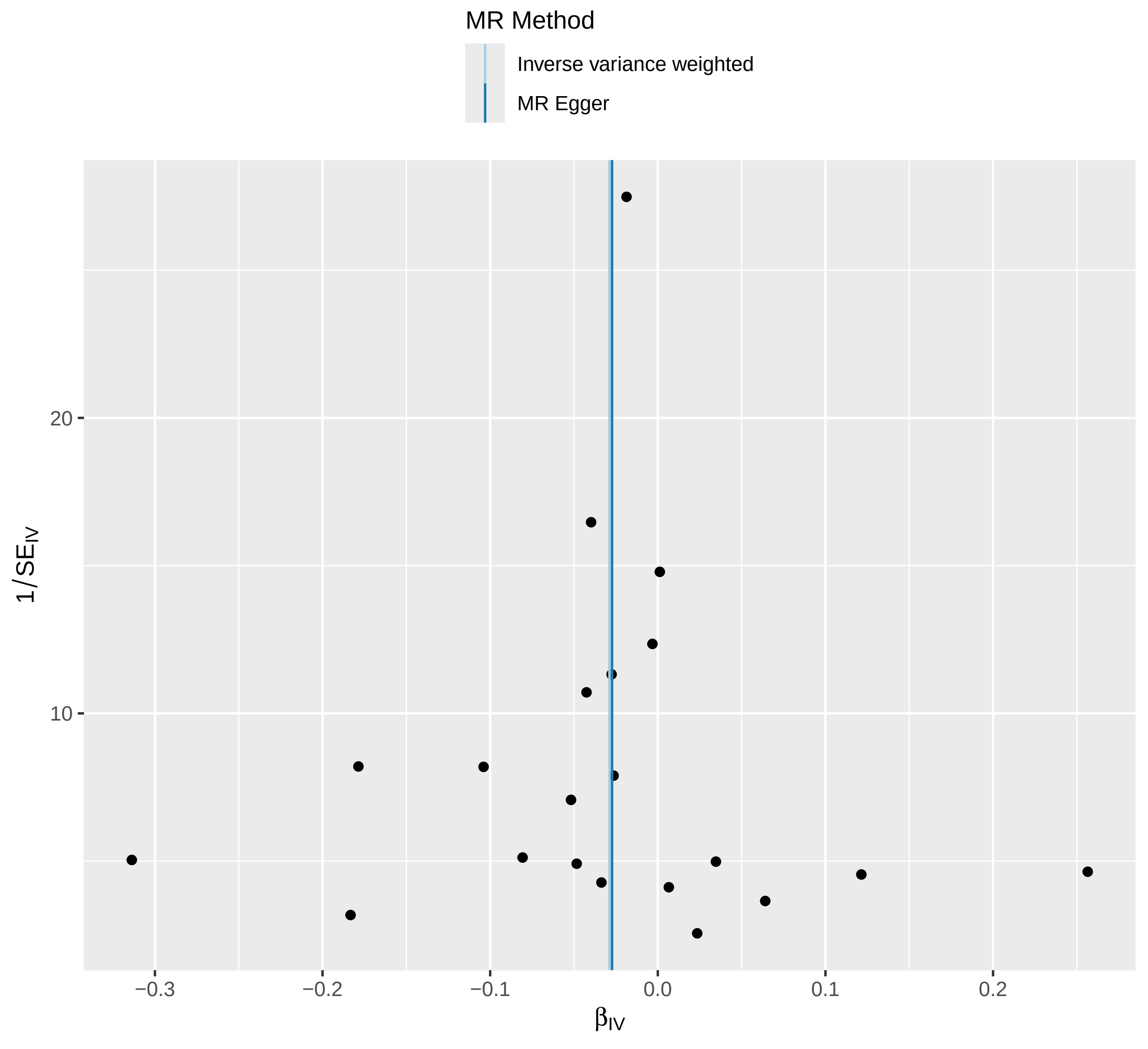

MR Method
Inverse variance weighted
MR Egger
20
10
−0.3
−0.2
−0.1
0.0
0.1
0.2
1/SE$_{IV}$
β$_{IV}$

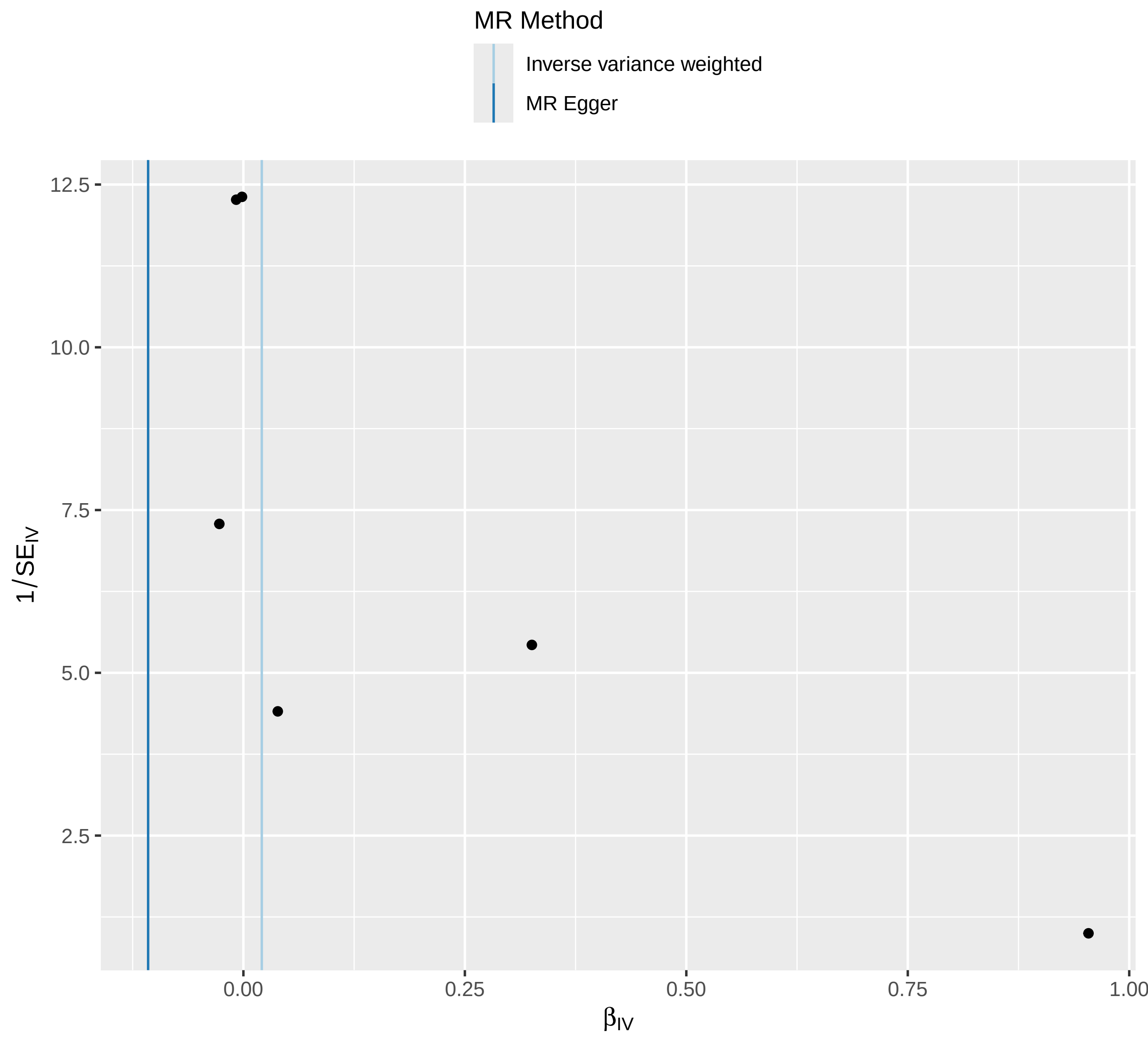
MR Method
Inverse variance weighted
MR Egger
12.5
10.0
7.5
5.0
2.5
1/SE_IV
0.00
0.25
0.50
0.75
1.00
β_IV

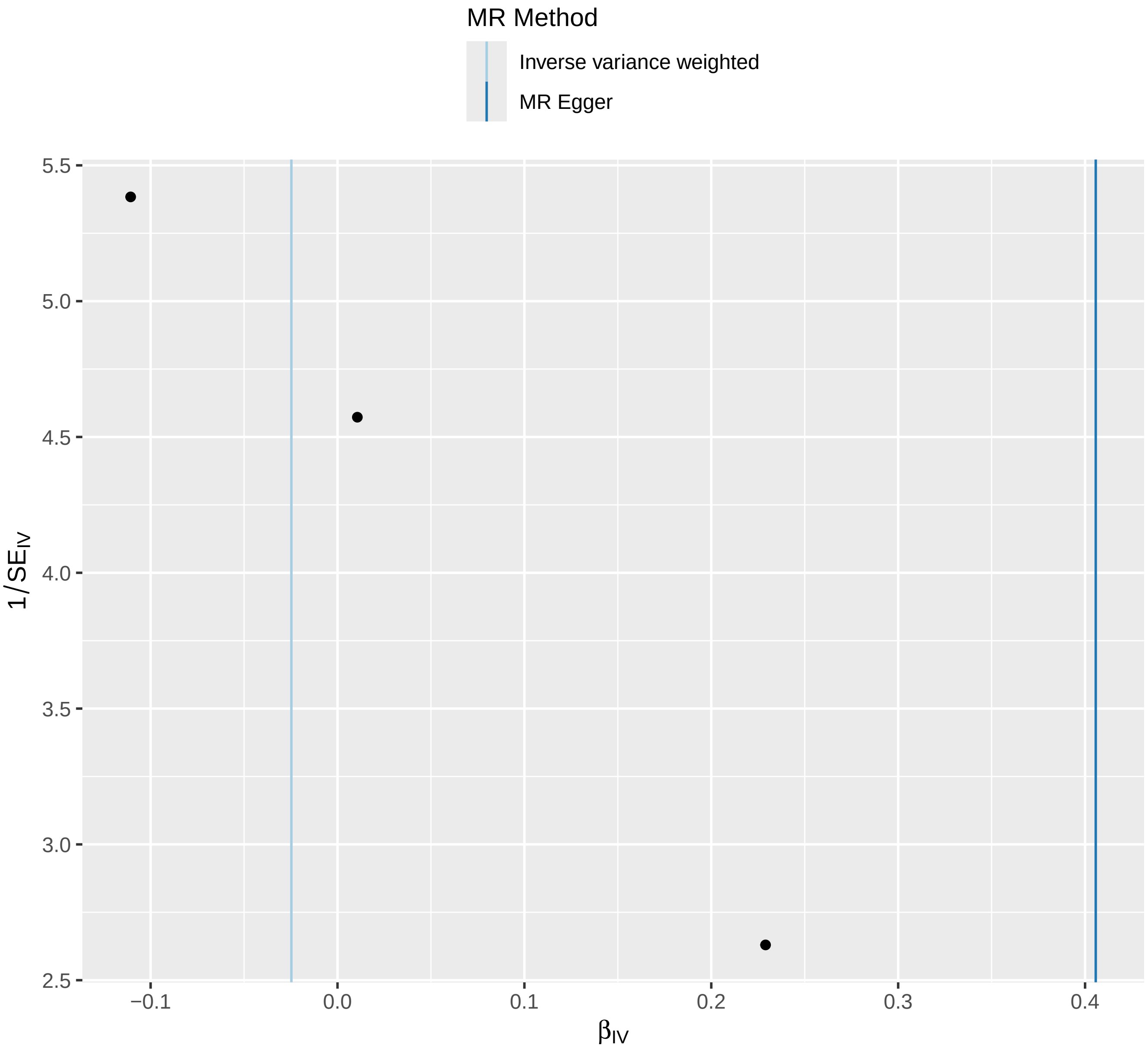

MR Method
Inverse variance weighted
MR Egger
5.5
5.0
4.5
4.0
3.5
3.0
2.5
$1/SE_{IV}$
−0.1
0.0
0.1
0.2
0.3
0.4
$\beta_{IV}$

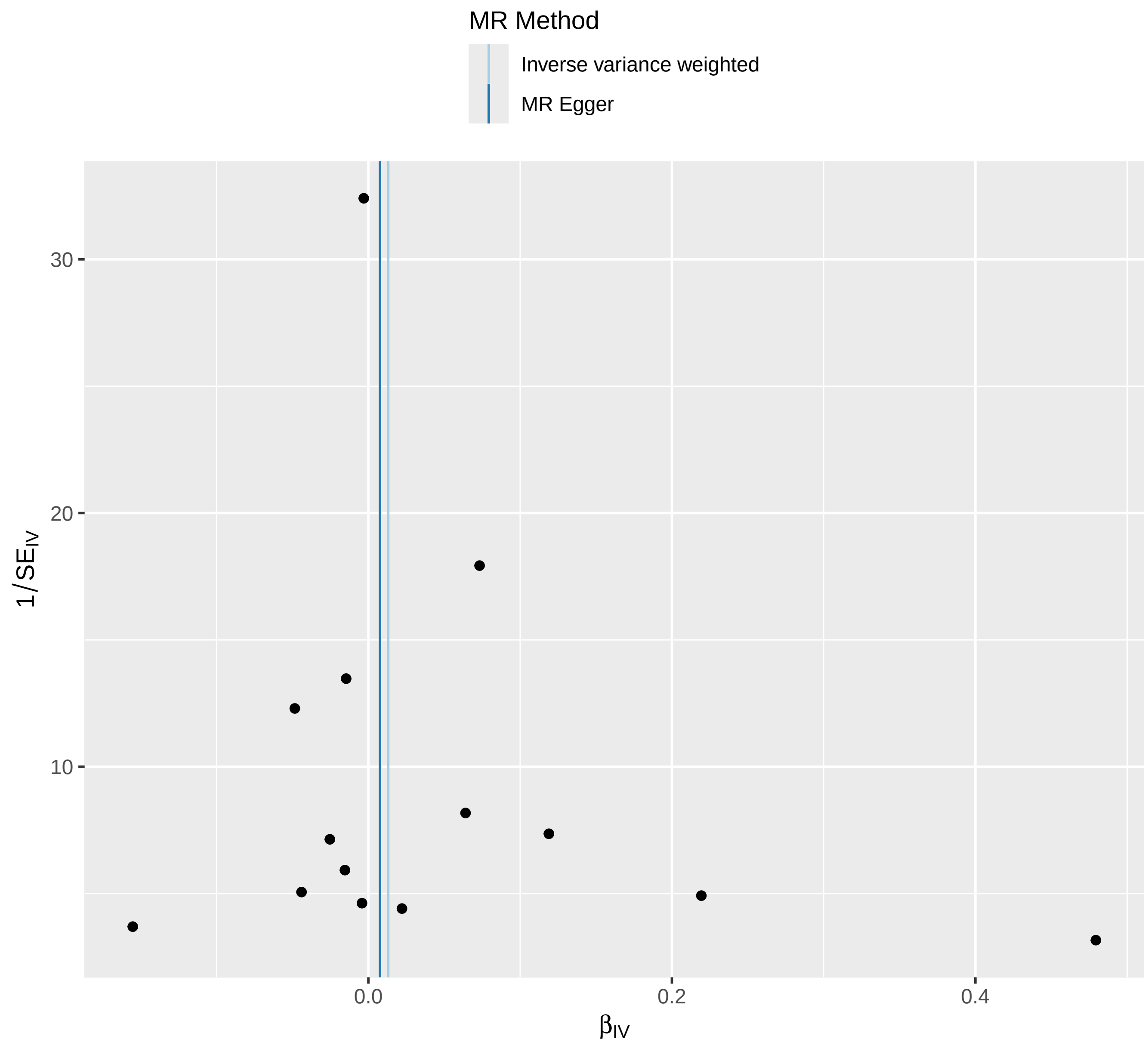
MR Method
Inverse variance weighted
MR Egger
30
20
10
1/SE$_{IV}$
0.0
0.2
0.4
β$_{IV}$

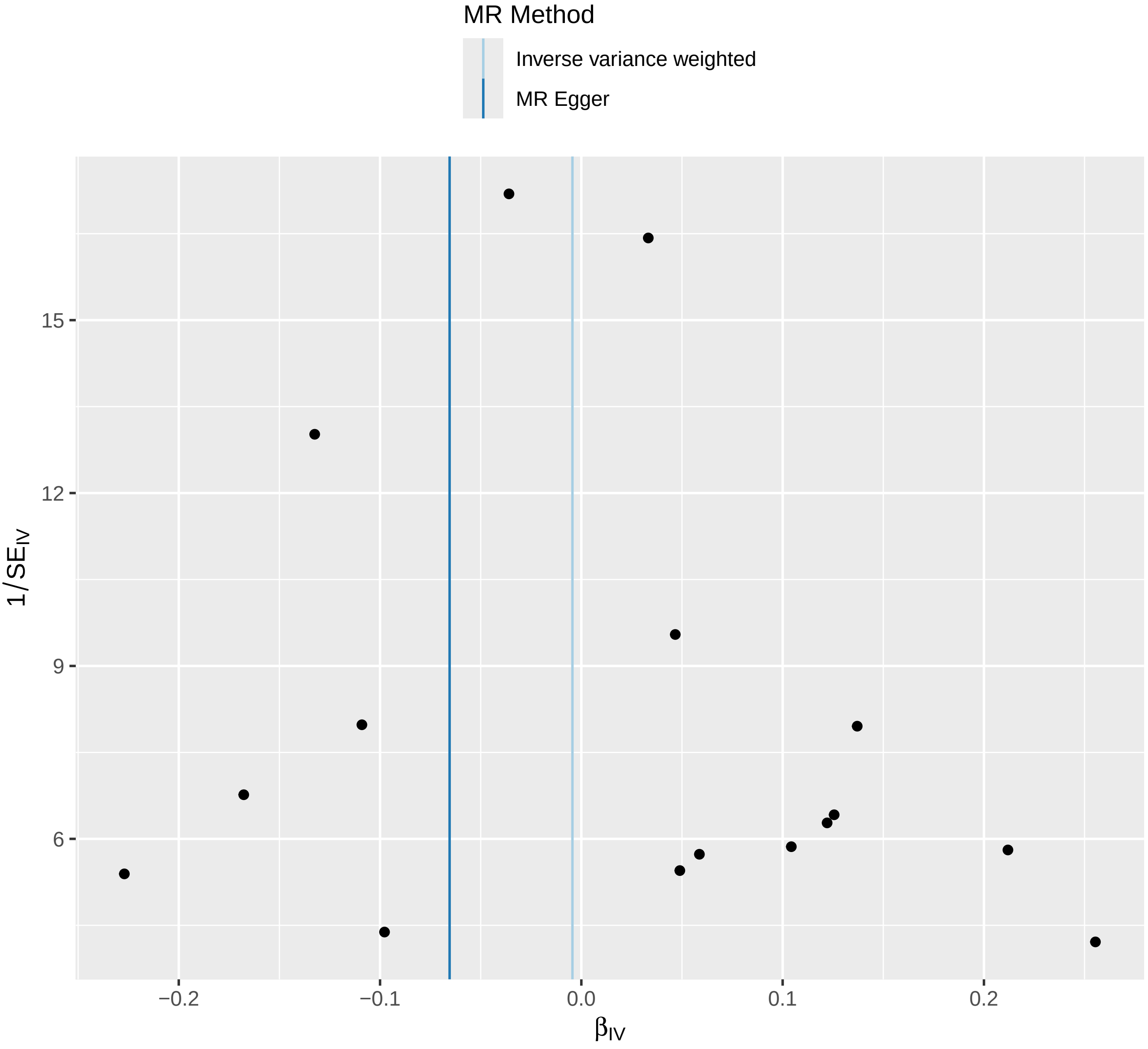

MR Method
Inverse variance weighted
MR Egger
15
12
9
6
1/SE$_{IV}$
−0.2
−0.1
0.0
0.1
0.2
β$_{IV}$

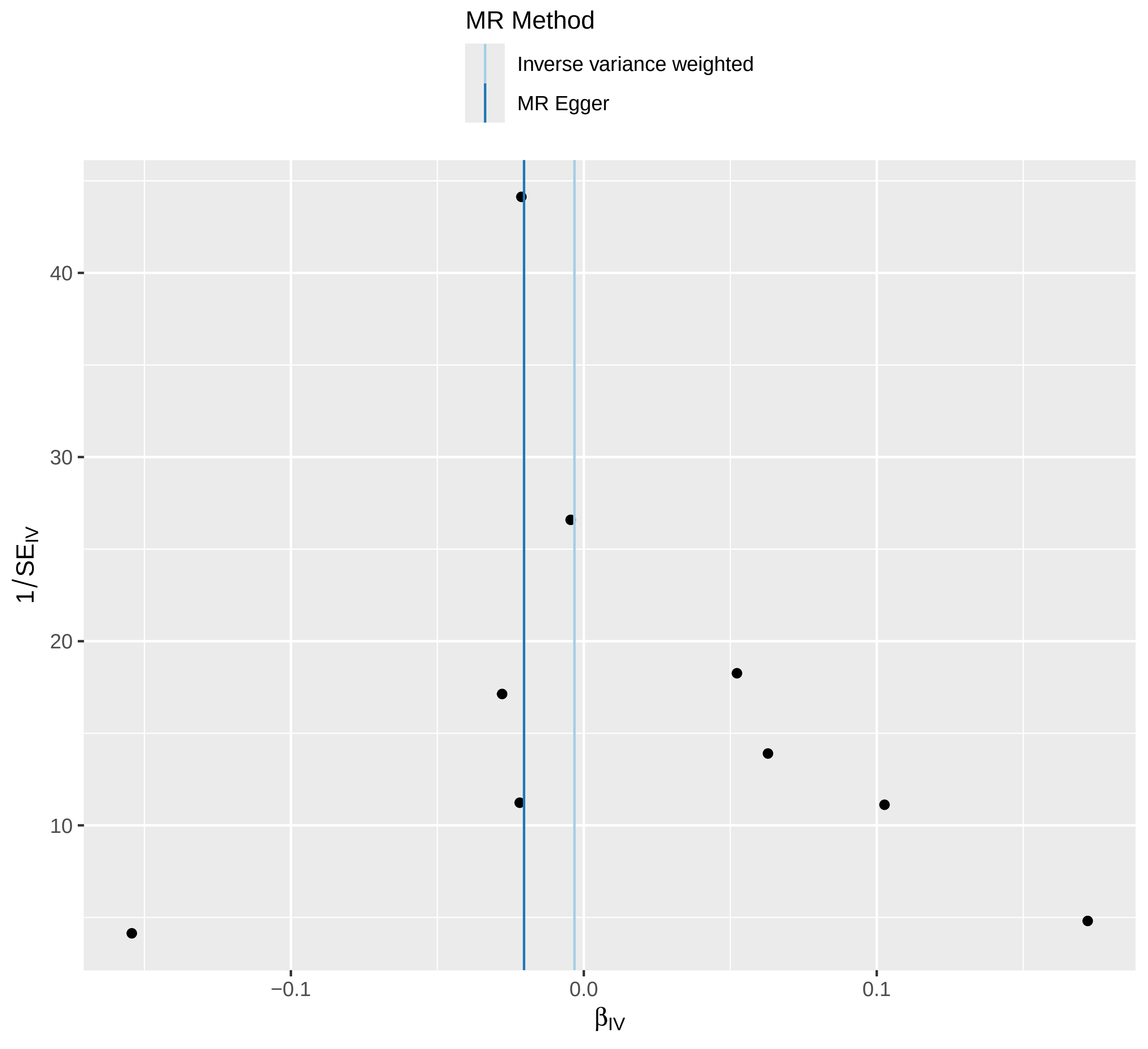
MR Method
Inverse variance weighted
MR Egger
40
30
20
10
1/SE_IV
−0.1
0.0
0.1
β_IV

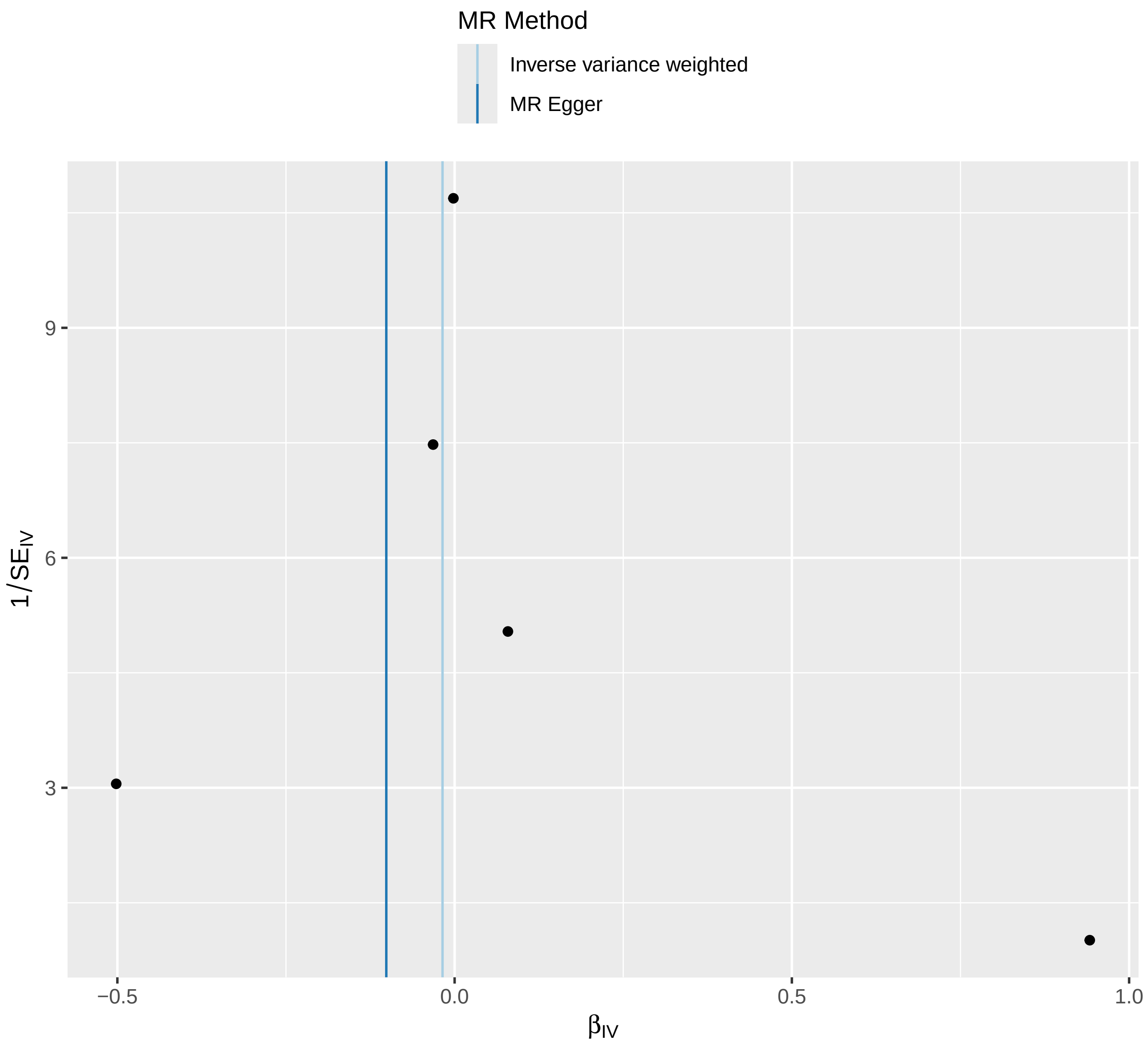
MR Method
Inverse variance weighted
MR Egger
9
6
3
1/SE$_{IV}$
−0.5
0.0
0.5
1.0
β$_{IV}$

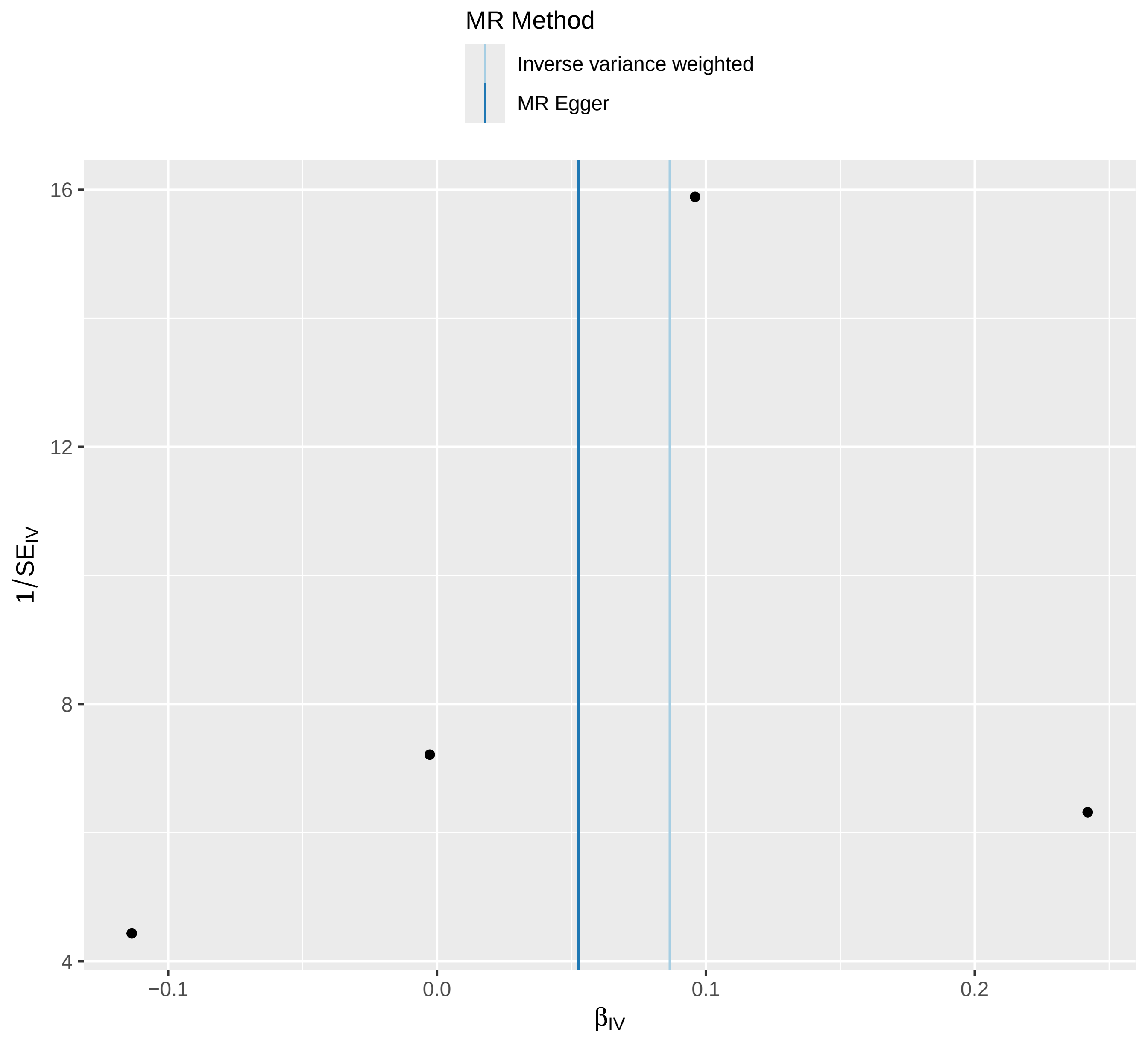
MR Method
Inverse variance weighted
MR Egger
16
12
8
4
1/SE_IV
−0.1
0.0
0.1
0.2
β_IV

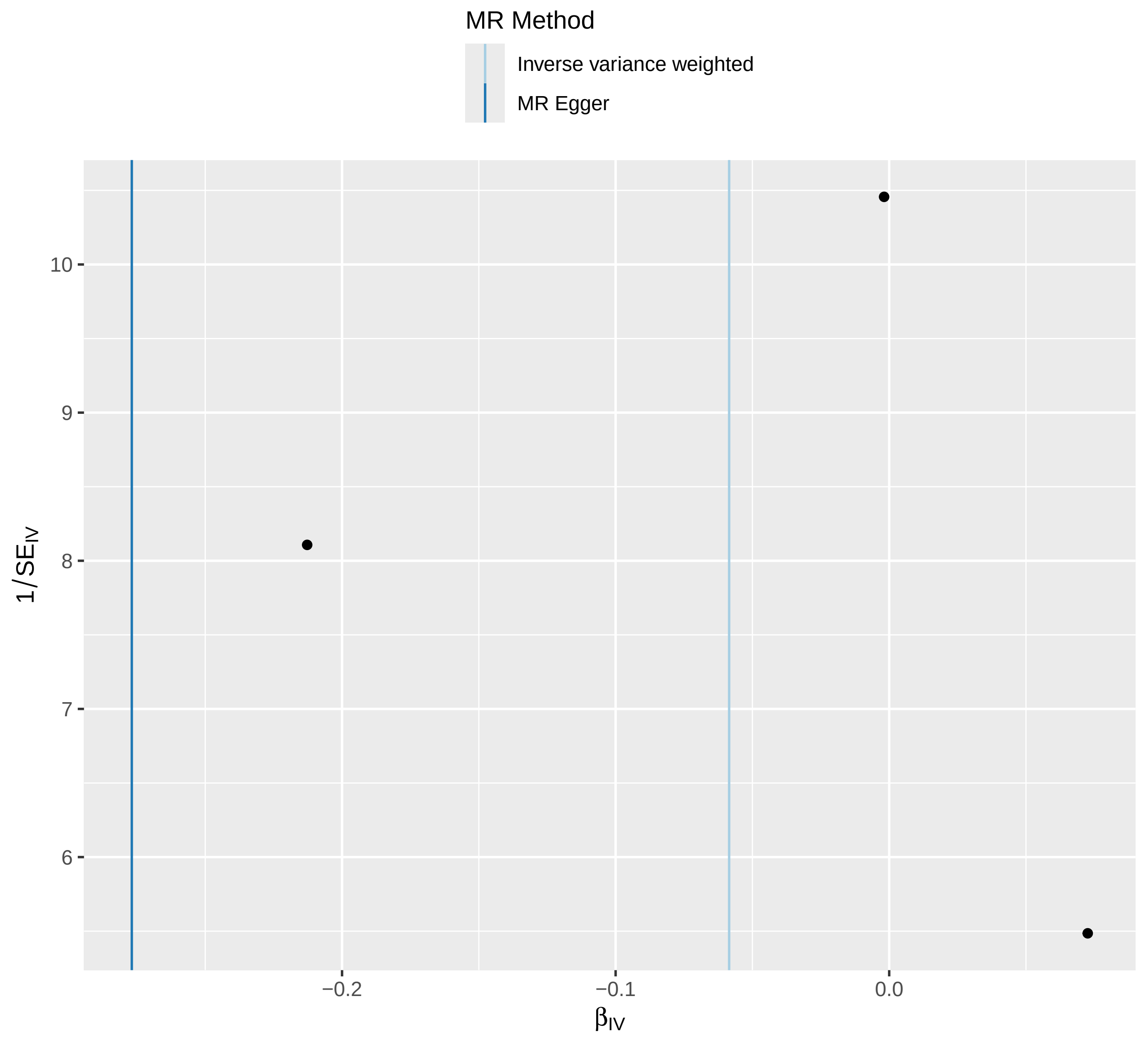

MR Method
Inverse variance weighted
MR Egger
10
9
8
7
6
$1/SE_{IV}$
−0.2
−0.1
0.0
$\beta_{IV}$

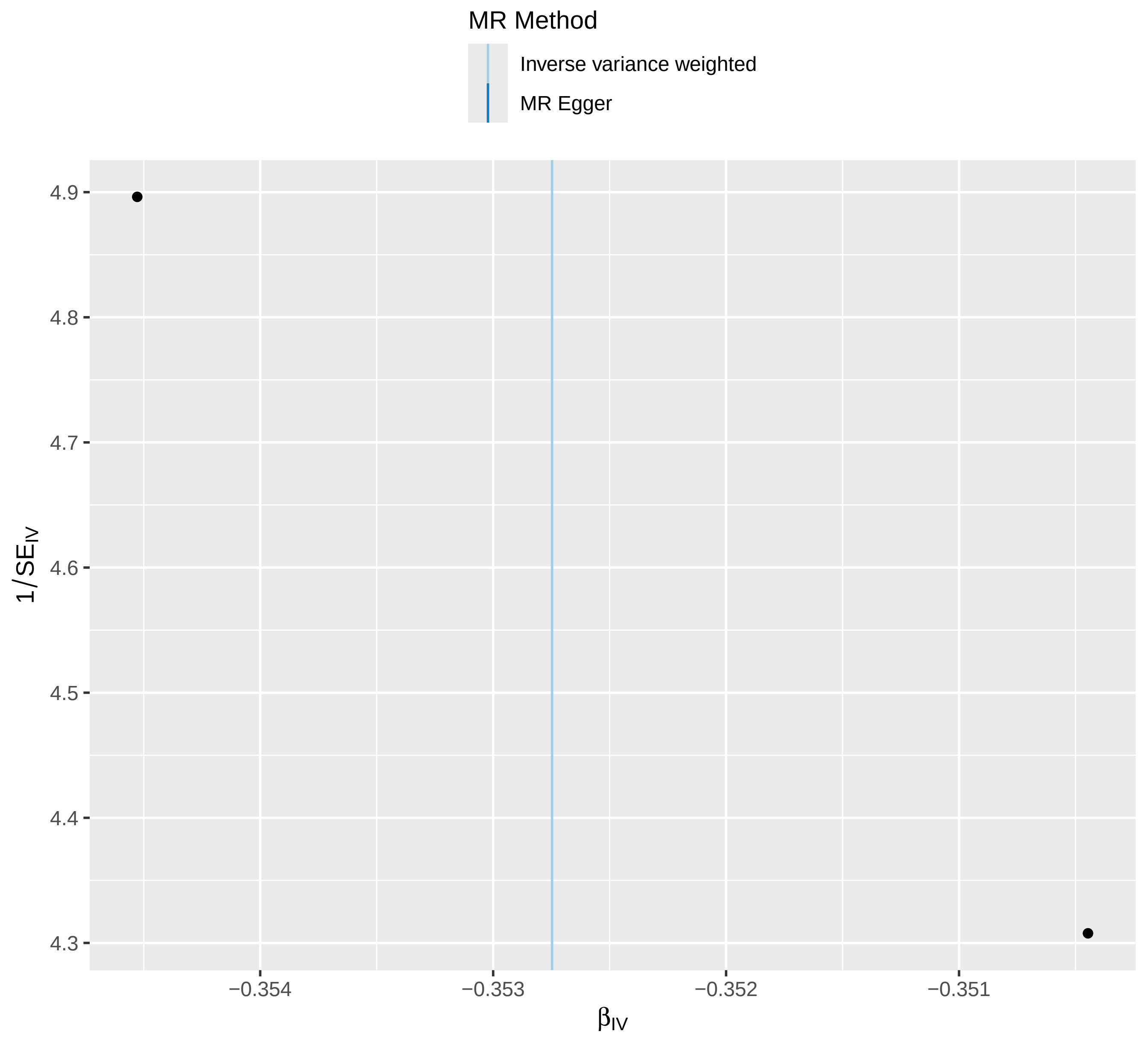
MR Method
Inverse variance weighted
MR Egger
4.9
4.8
4.7
4.6
4.5
4.4
4.3
1/SE_IV
−0.354
−0.353
−0.352
−0.351
β_IV

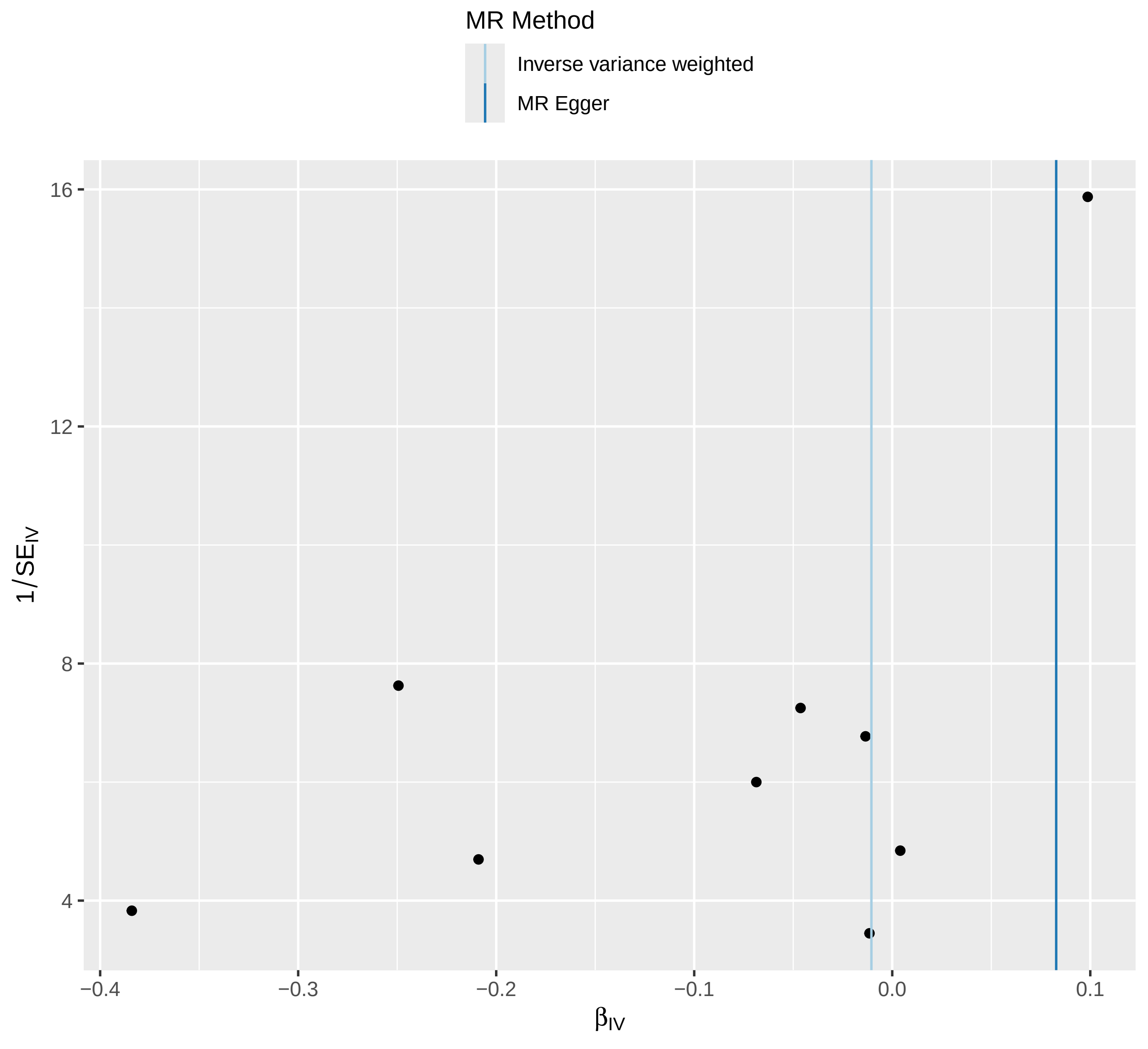

MR Method
Inverse variance weighted
MR Egger
16
12
8
4
1/SE_IV
−0.4
−0.3
−0.2
−0.1
0.0
0.1
β_IV

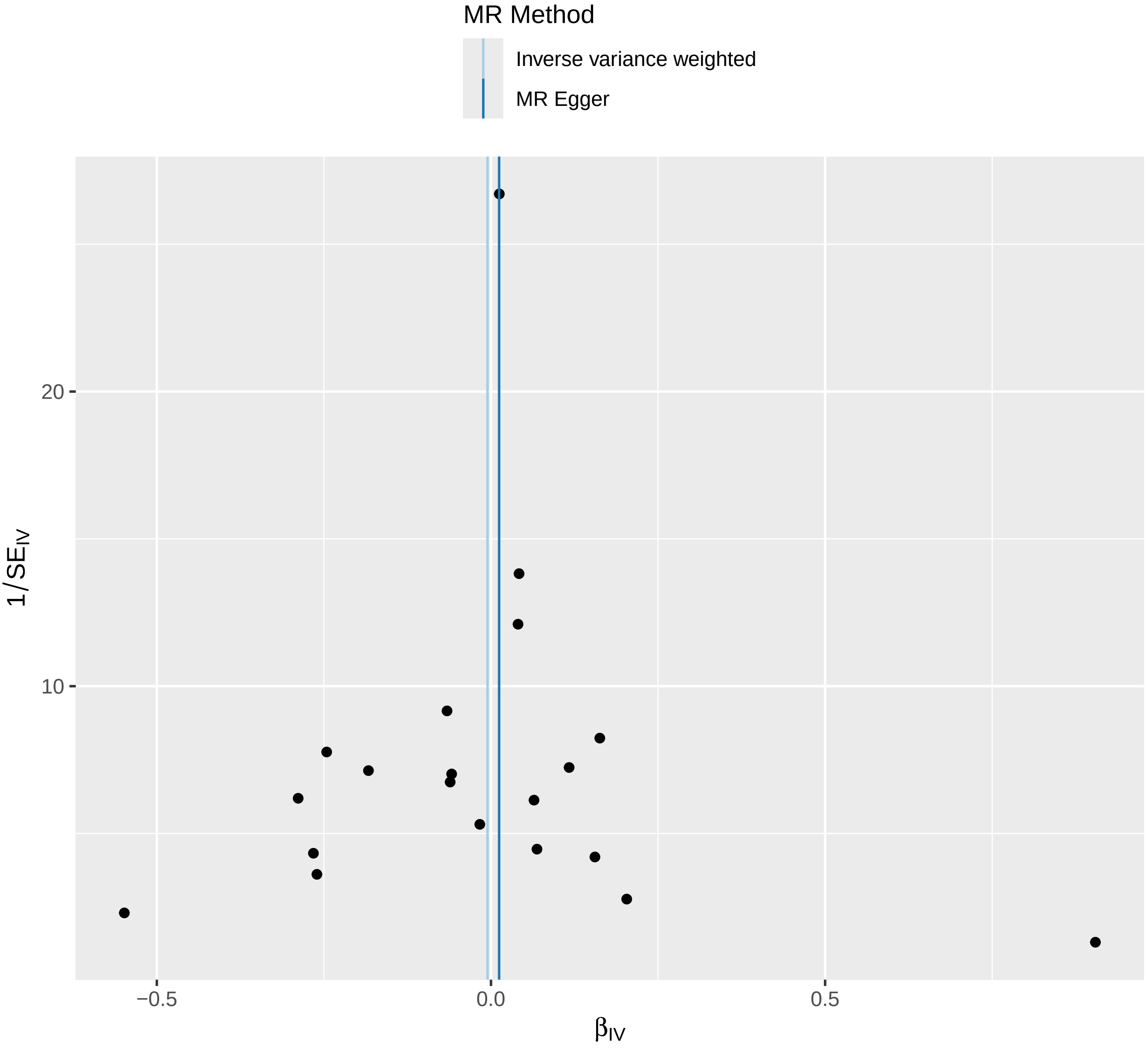
MR Method
Inverse variance weighted
MR Egger
20
10
1/SE_IV
−0.5
0.0
0.5
β_IV

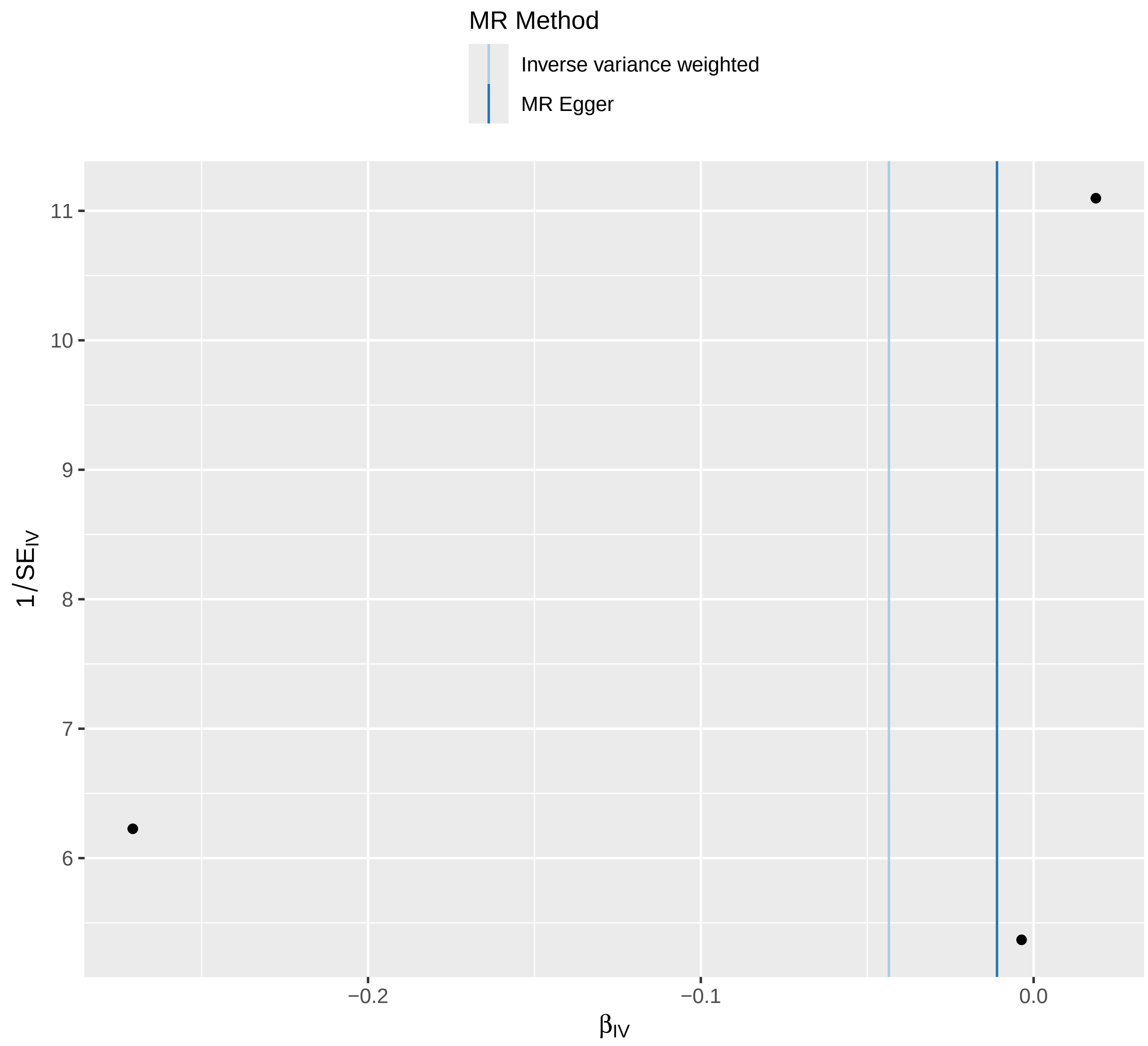

MR Method
Inverse variance weighted
MR Egger
11
10
9
8
7
6
1/SE$_{IV}$
−0.2
−0.1
0.0
β$_{IV}$

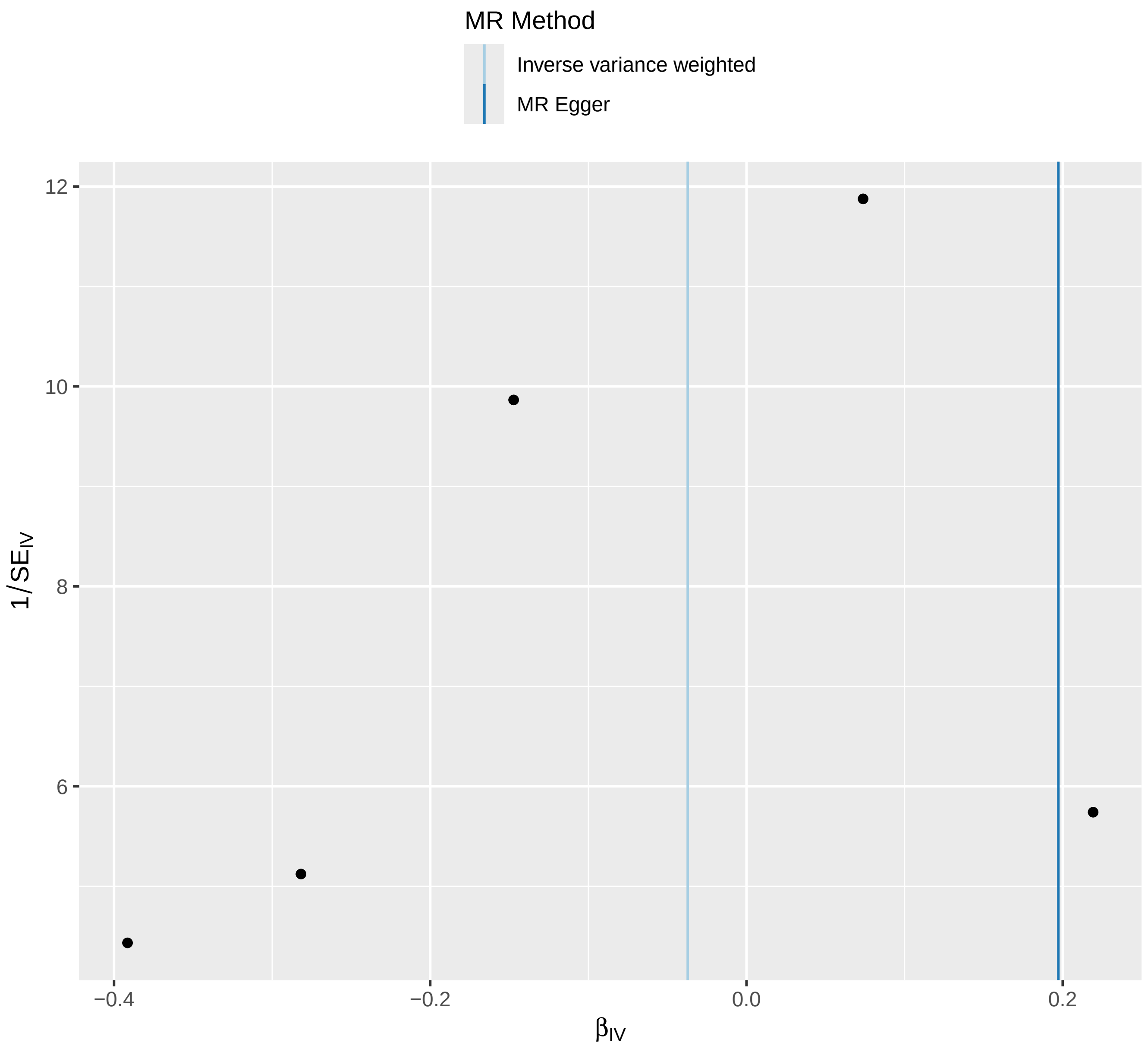
MR Method
Inverse variance weighted
MR Egger
12
10
8
6
1/SE$_{IV}$
−0.4
−0.2
0.0
0.2
β$_{IV}$

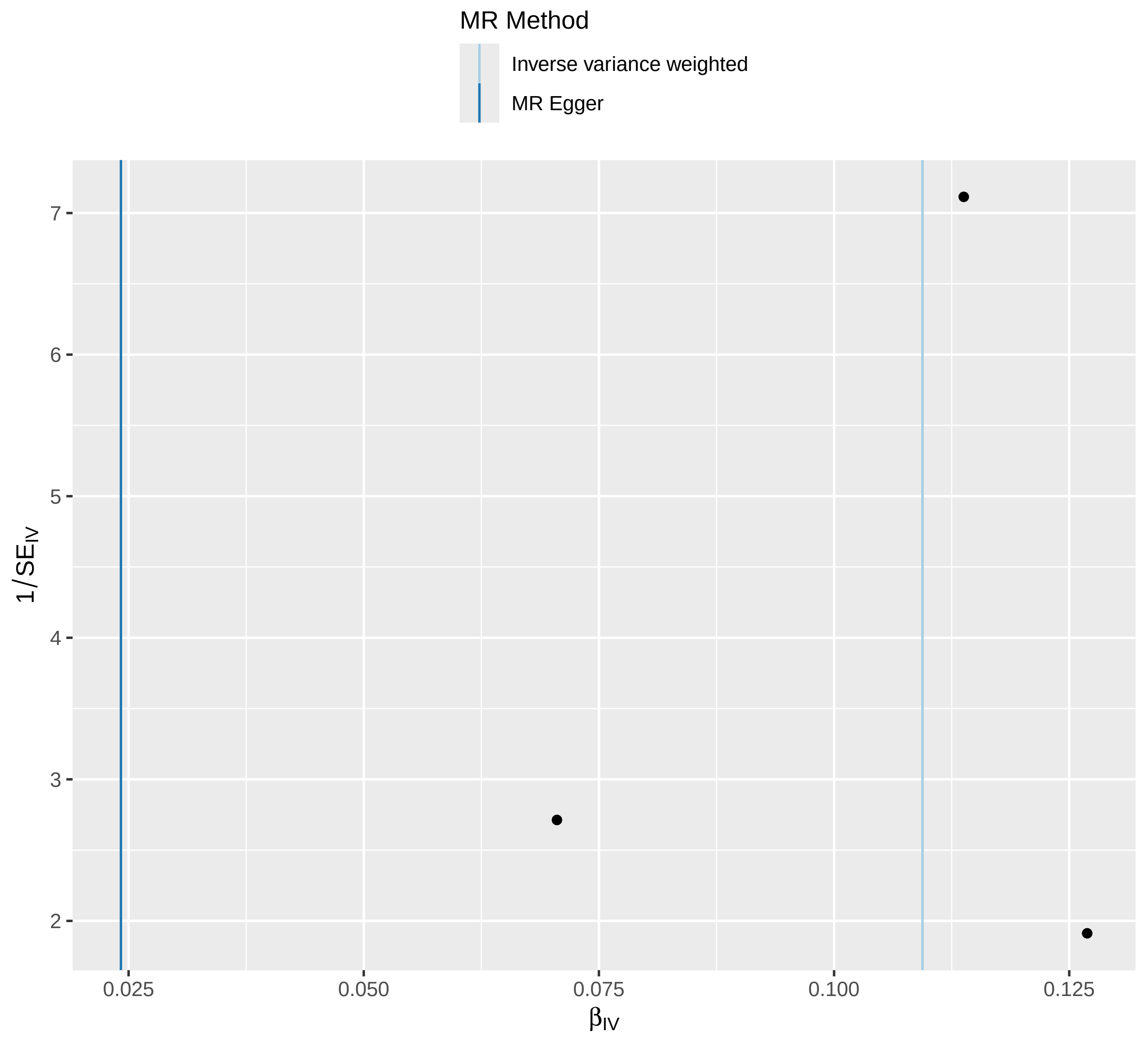
MR Method
Inverse variance weighted
MR Egger
1/SE_IV
β_IV
0.025
0.050
0.075
0.100
0.125
2
3
4
5
6
7

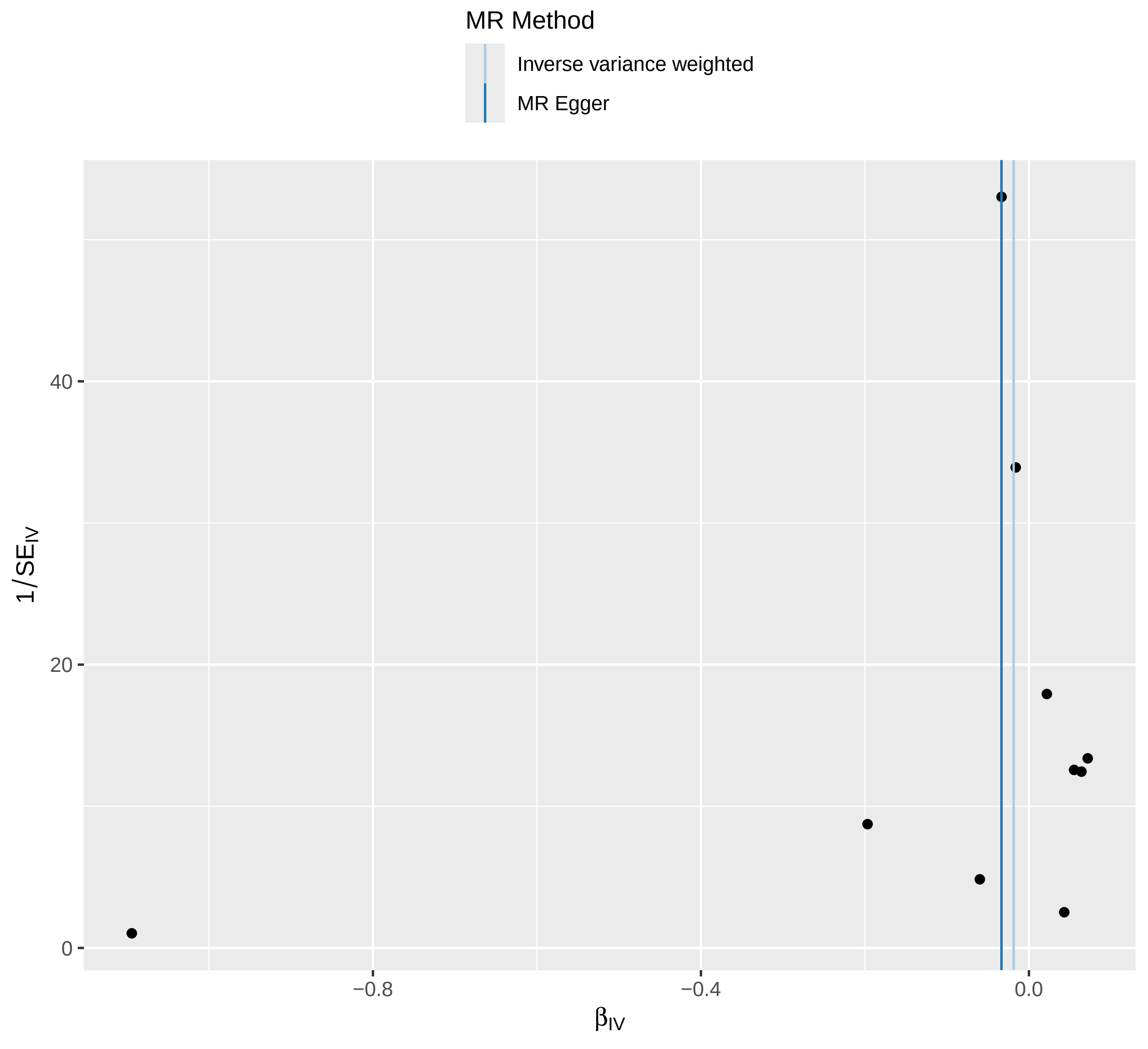

MR Method
Inverse variance weighted
MR Egger
40
20
0
1/SE_IV
−0.8
−0.4
0.0
β_IV

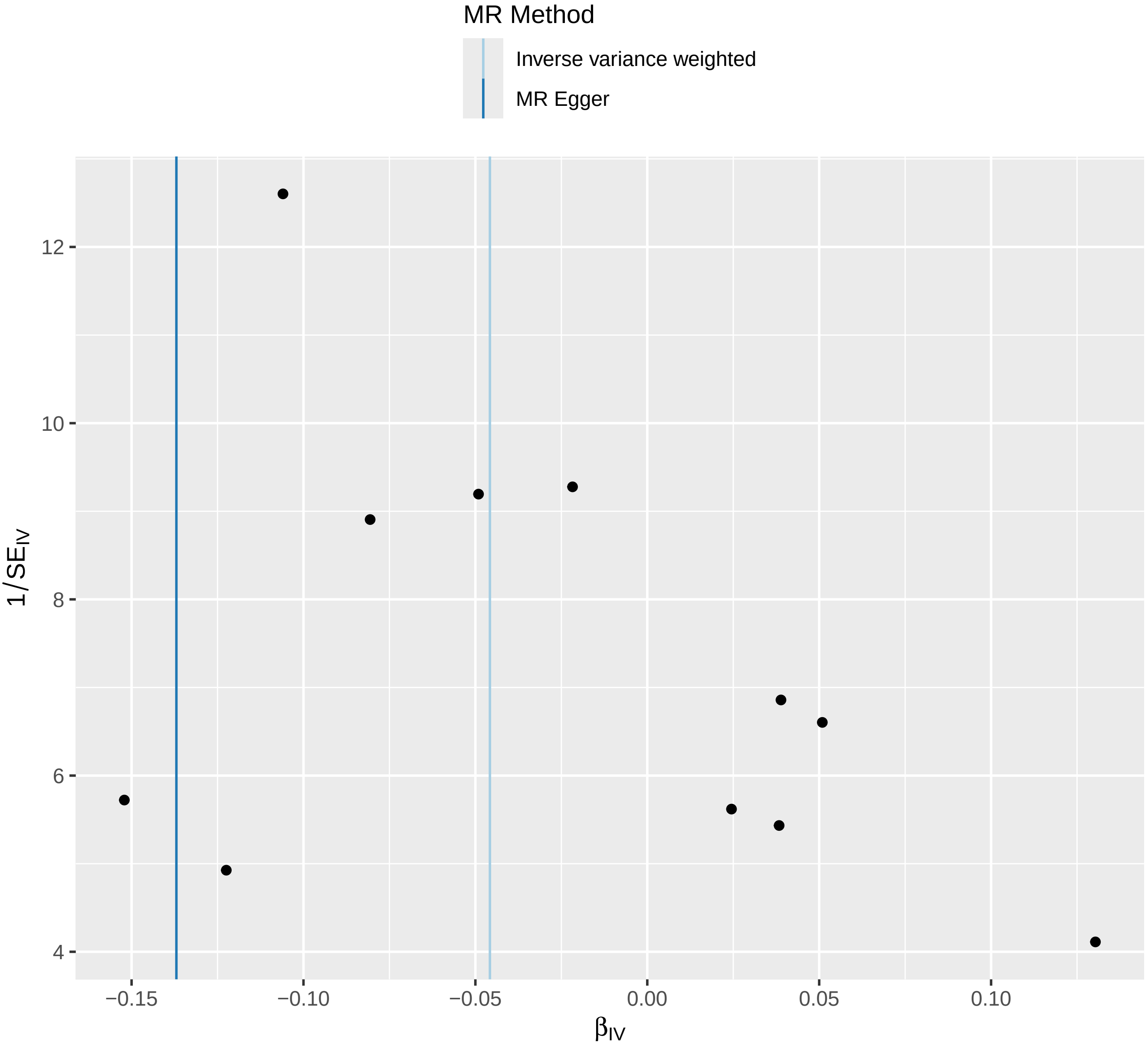
MR Method
Inverse variance weighted
MR Egger
12
10
8
6
4
1/SE_IV
−0.15
−0.10
−0.05
0.00
0.05
0.10
β_IV

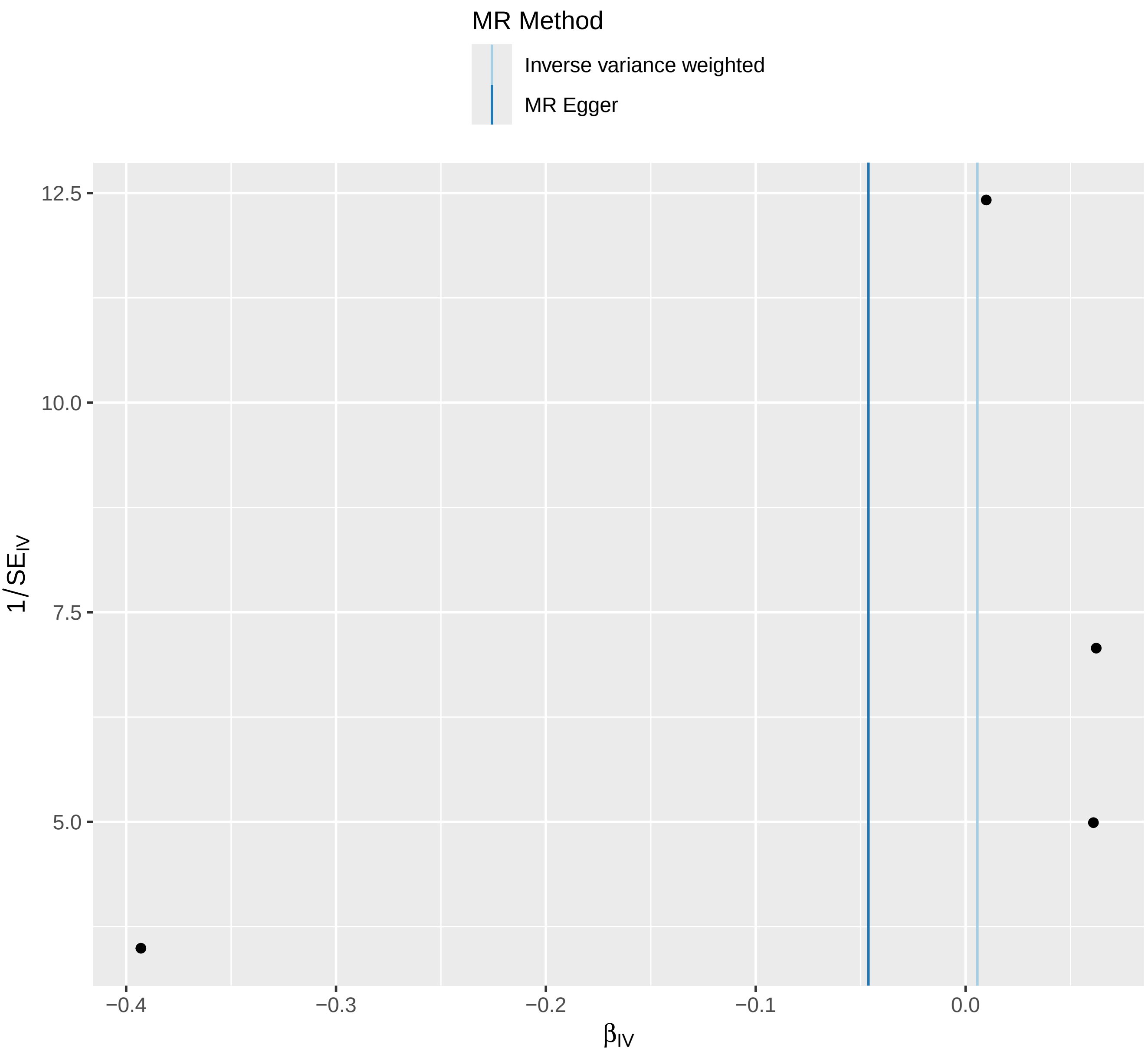
MR Method
Inverse variance weighted
MR Egger
12.5
10.0
7.5
5.0
1/SE_IV
−0.4
−0.3
−0.2
−0.1
0.0
β_IV

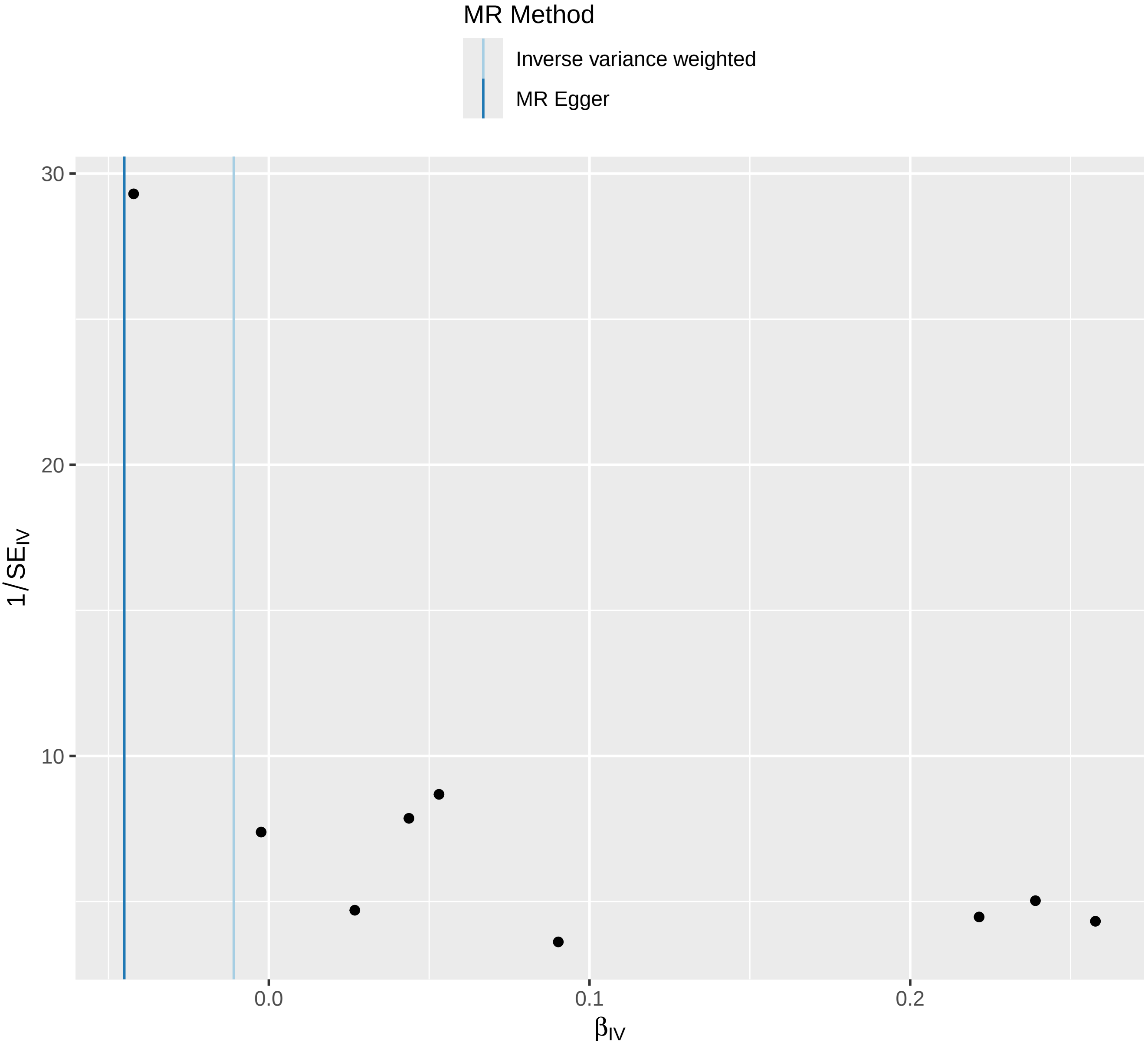
MR Method
Inverse variance weighted
MR Egger
30
20
10
0.0
0.1
0.2
$1/SE_{IV}$
$\beta_{IV}$

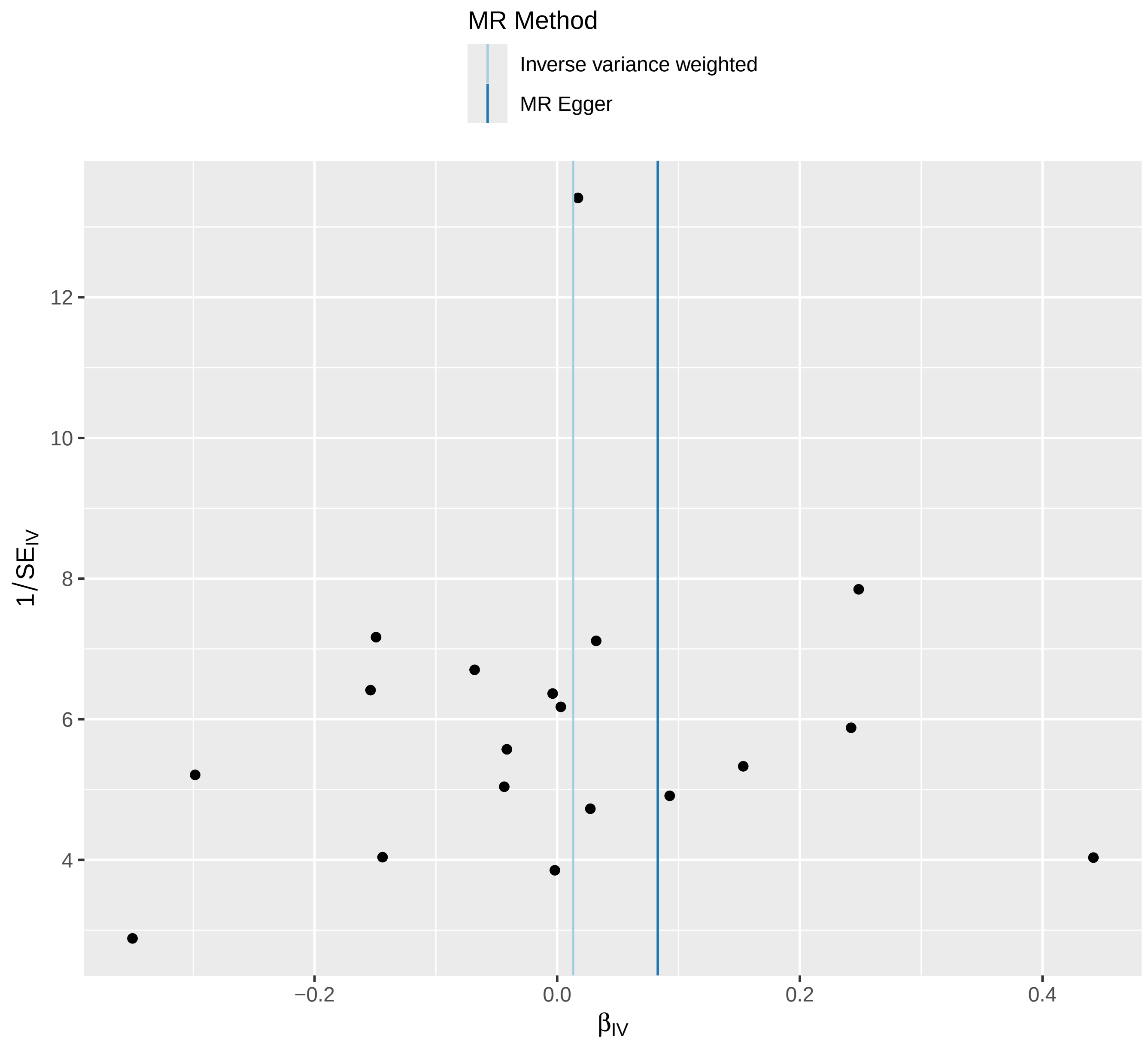
MR Method
Inverse variance weighted
MR Egger
1/SE_IV
12
10
8
6
4
−0.2
0.0
0.2
0.4
β_IV

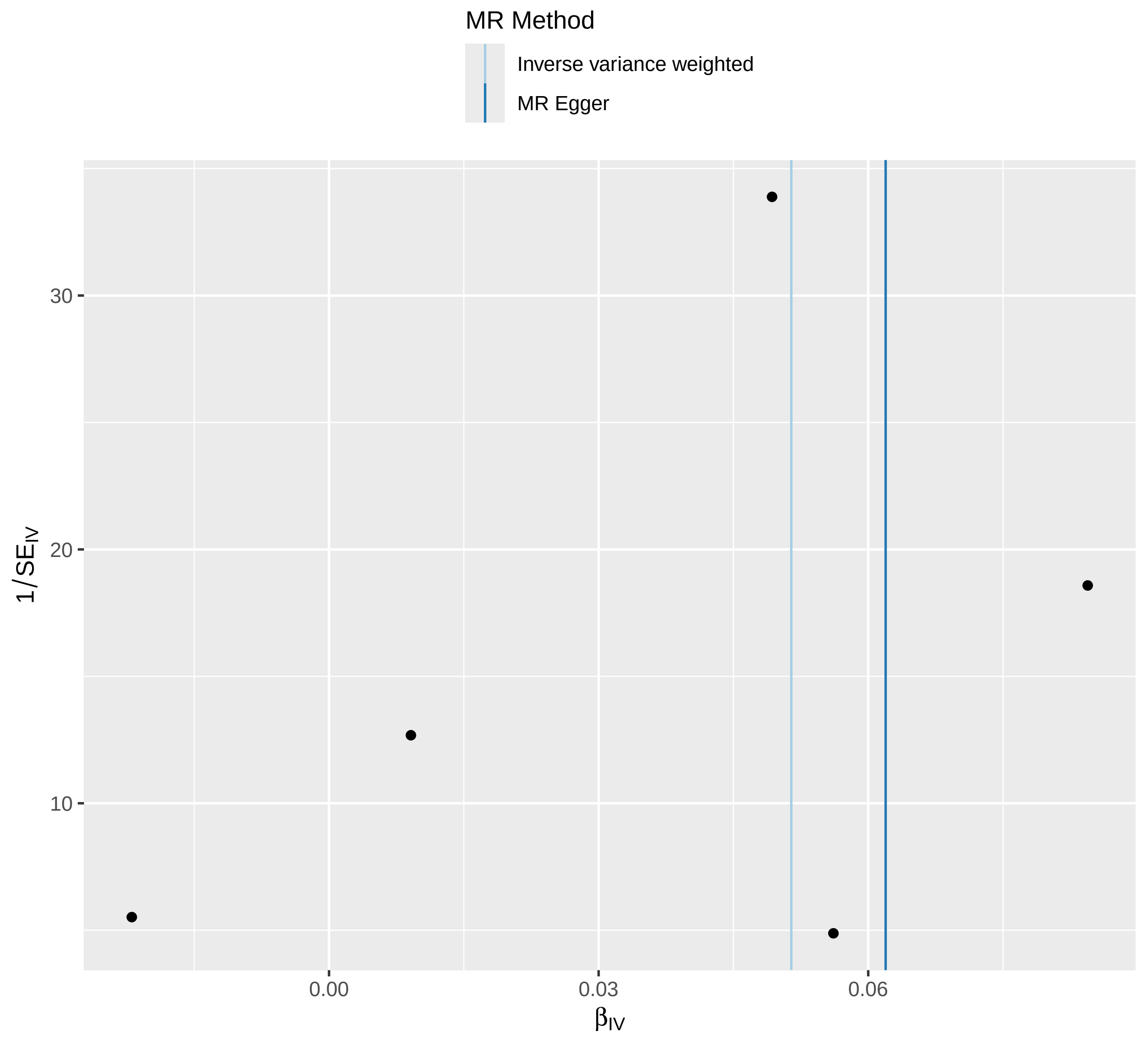
MR Method
Inverse variance weighted
MR Egger
30
20
10
0.00
0.03
0.06
$\beta_{IV}$
$1/SE_{IV}$

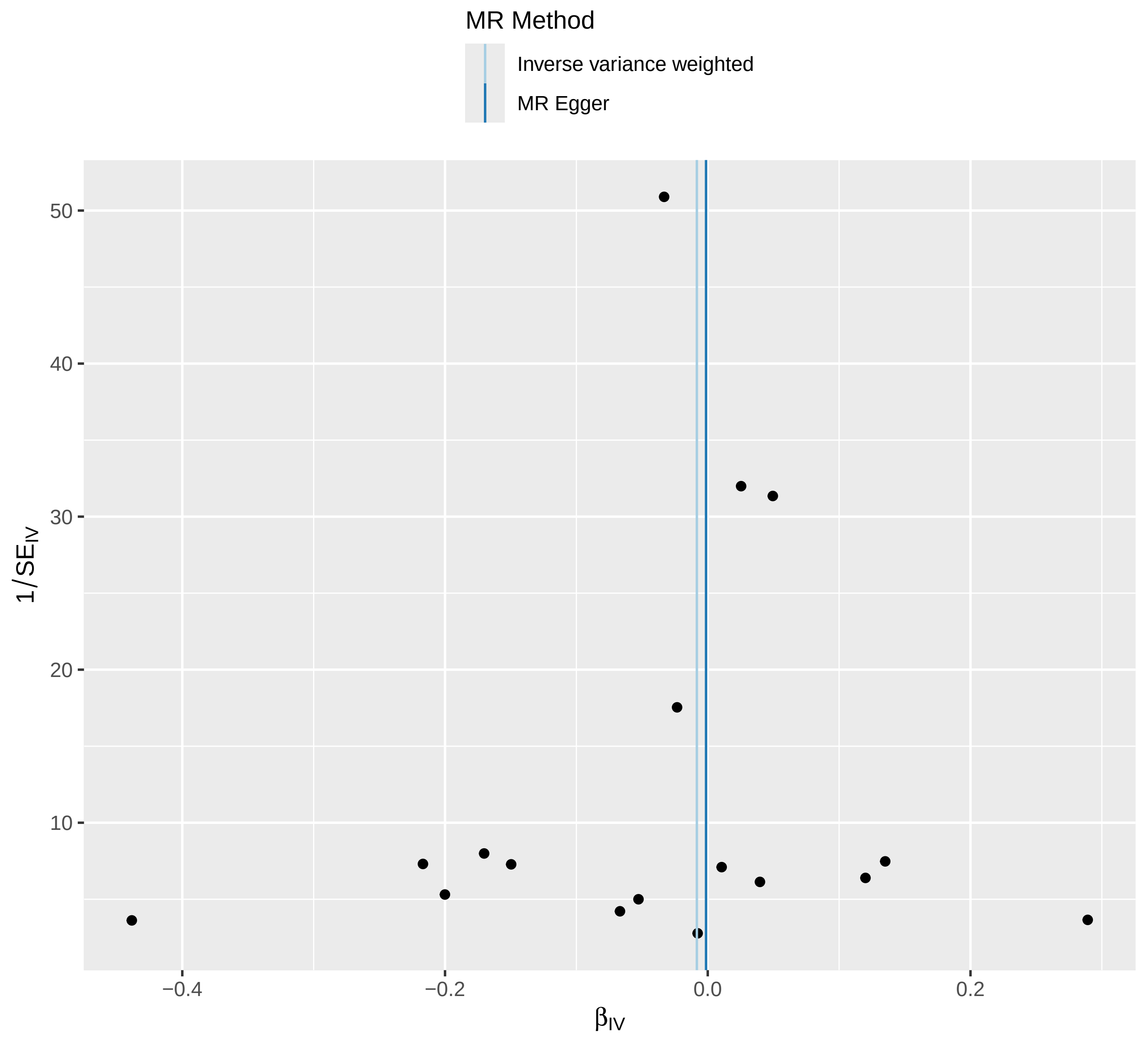
MR Method
Inverse variance weighted
MR Egger
50
40
30
20
10
$1/SE_{IV}$
−0.4
−0.2
0.0
0.2
$\beta_{IV}$

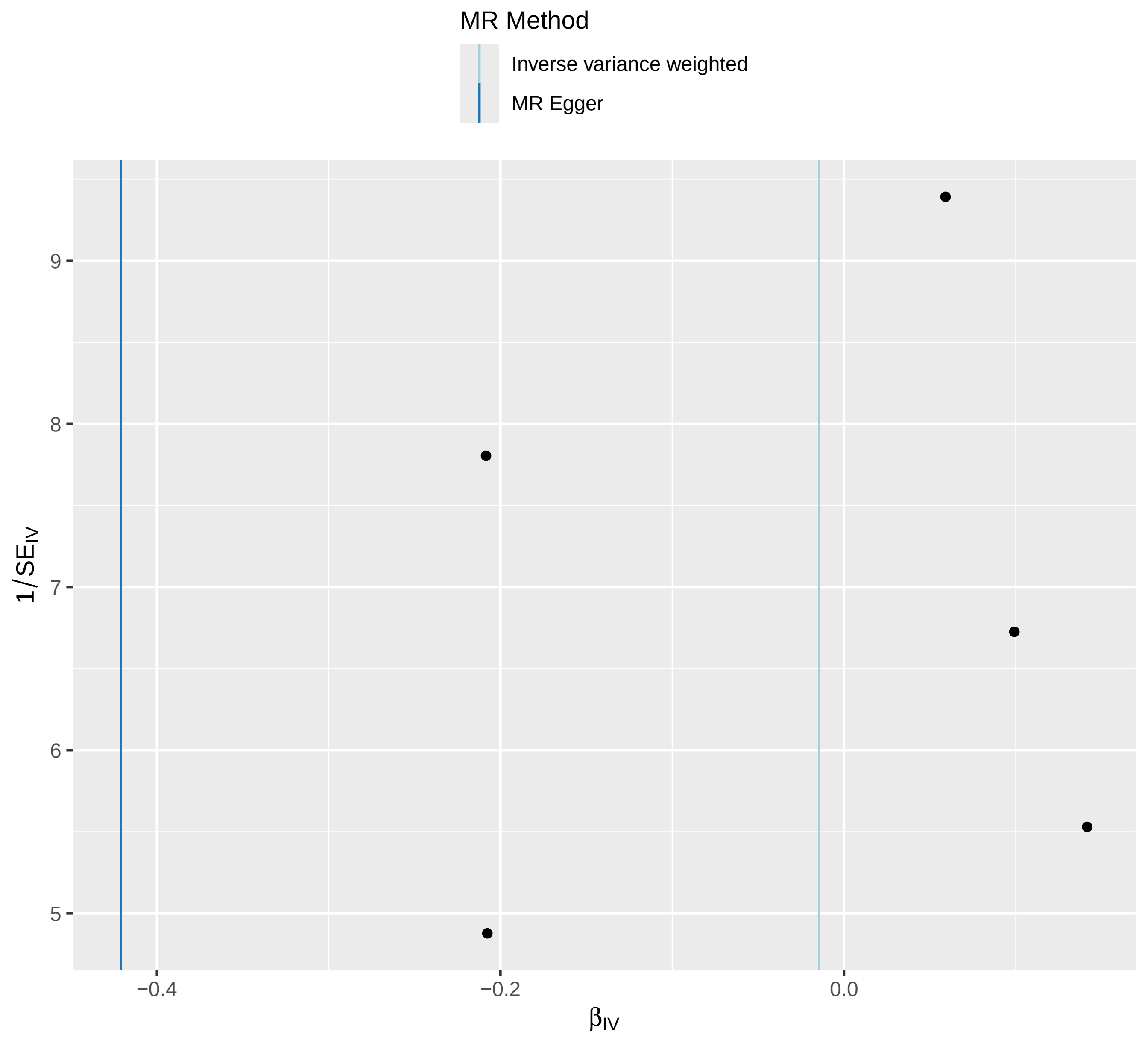

MR Method
Inverse variance weighted
MR Egger
9
8
7
6
5
1/SE_IV
−0.4
−0.2
0.0
β_IV

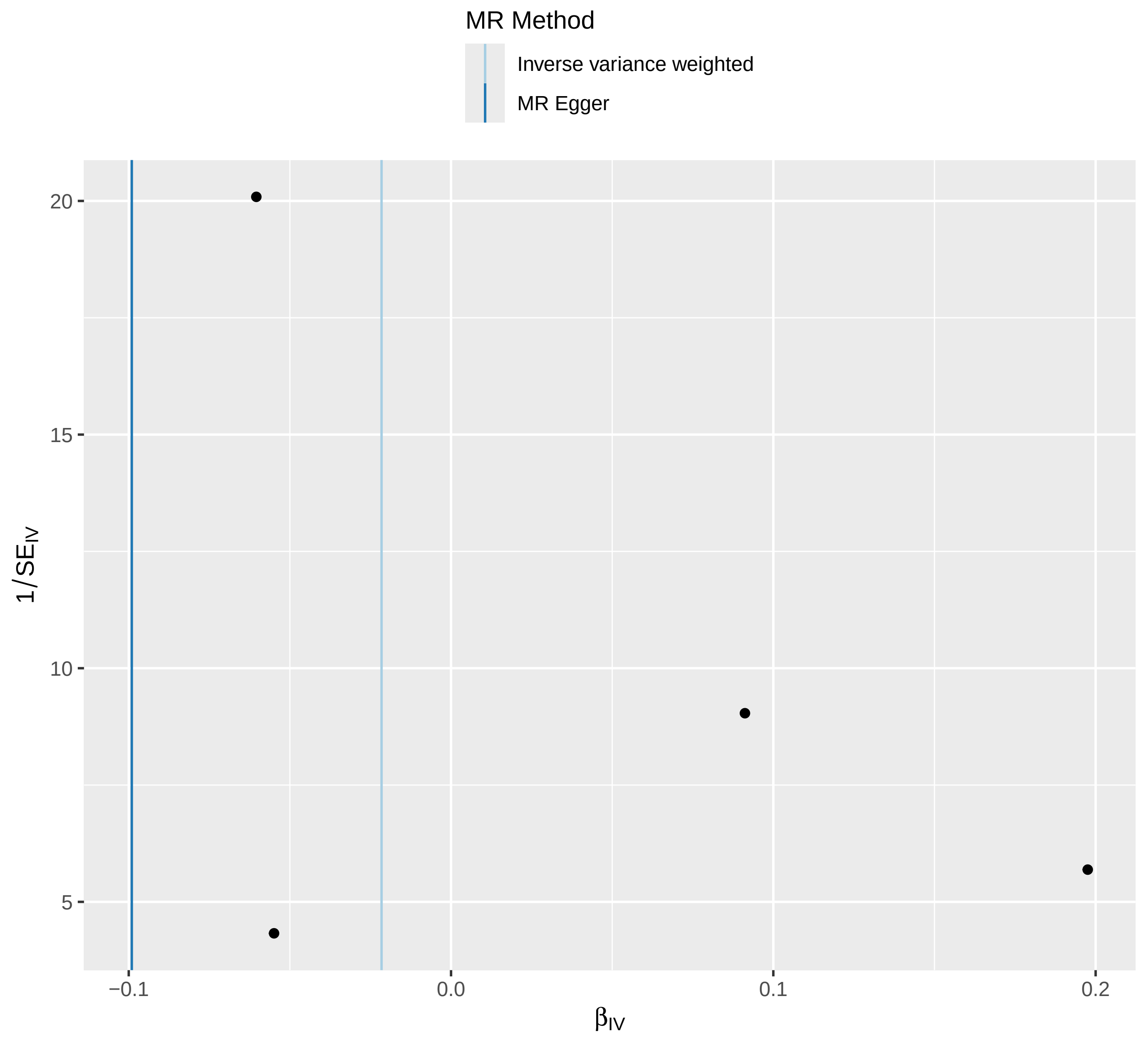
MR Method
Inverse variance weighted
MR Egger
20
15
10
5
1/SE$_{IV}$
−0.1
0.0
0.1
0.2
β$_{IV}$

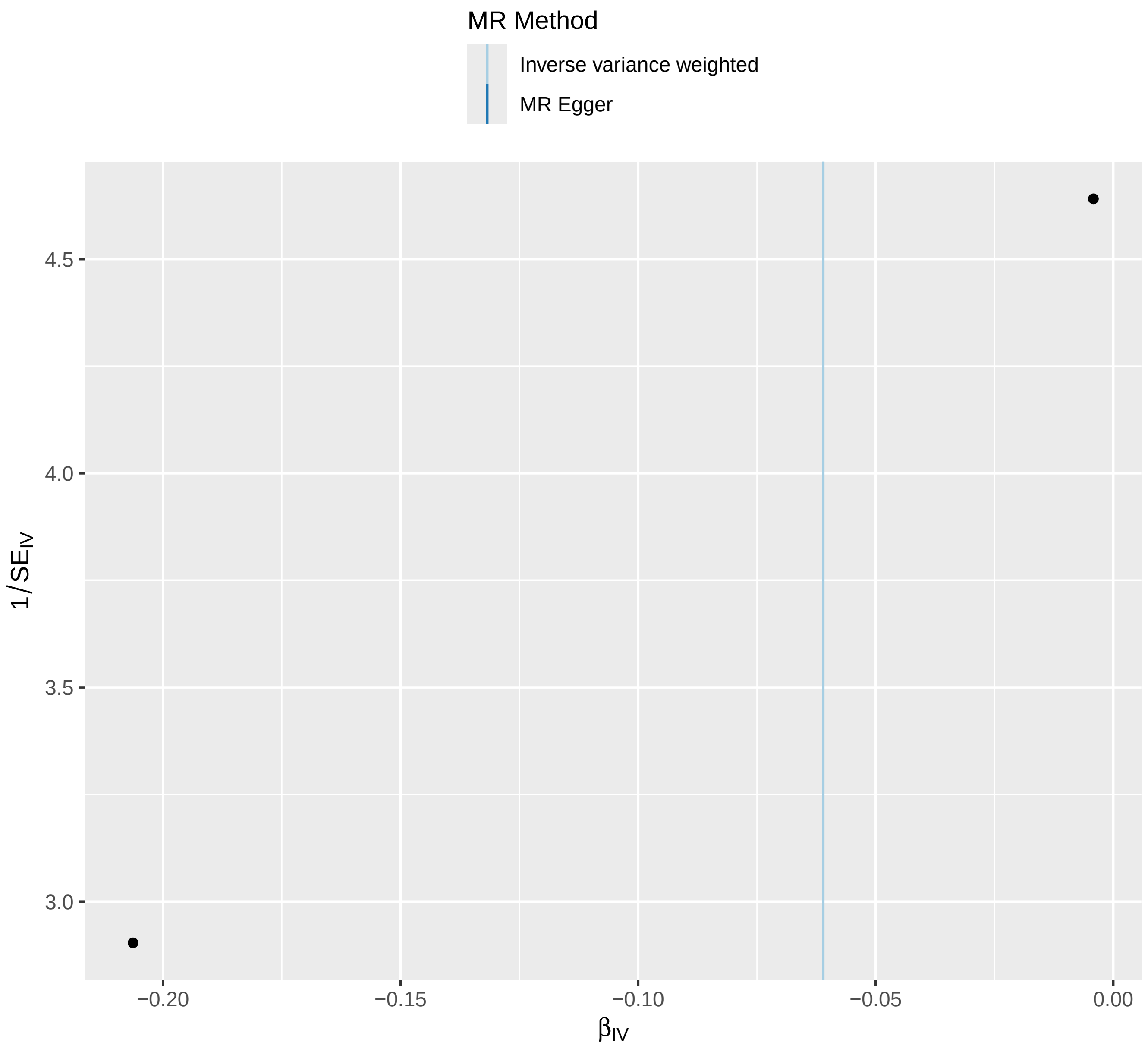
MR Method
Inverse variance weighted
MR Egger
4.5
4.0
3.5
3.0
1/SE_IV
−0.20
−0.15
−0.10
−0.05
0.00
β_IV

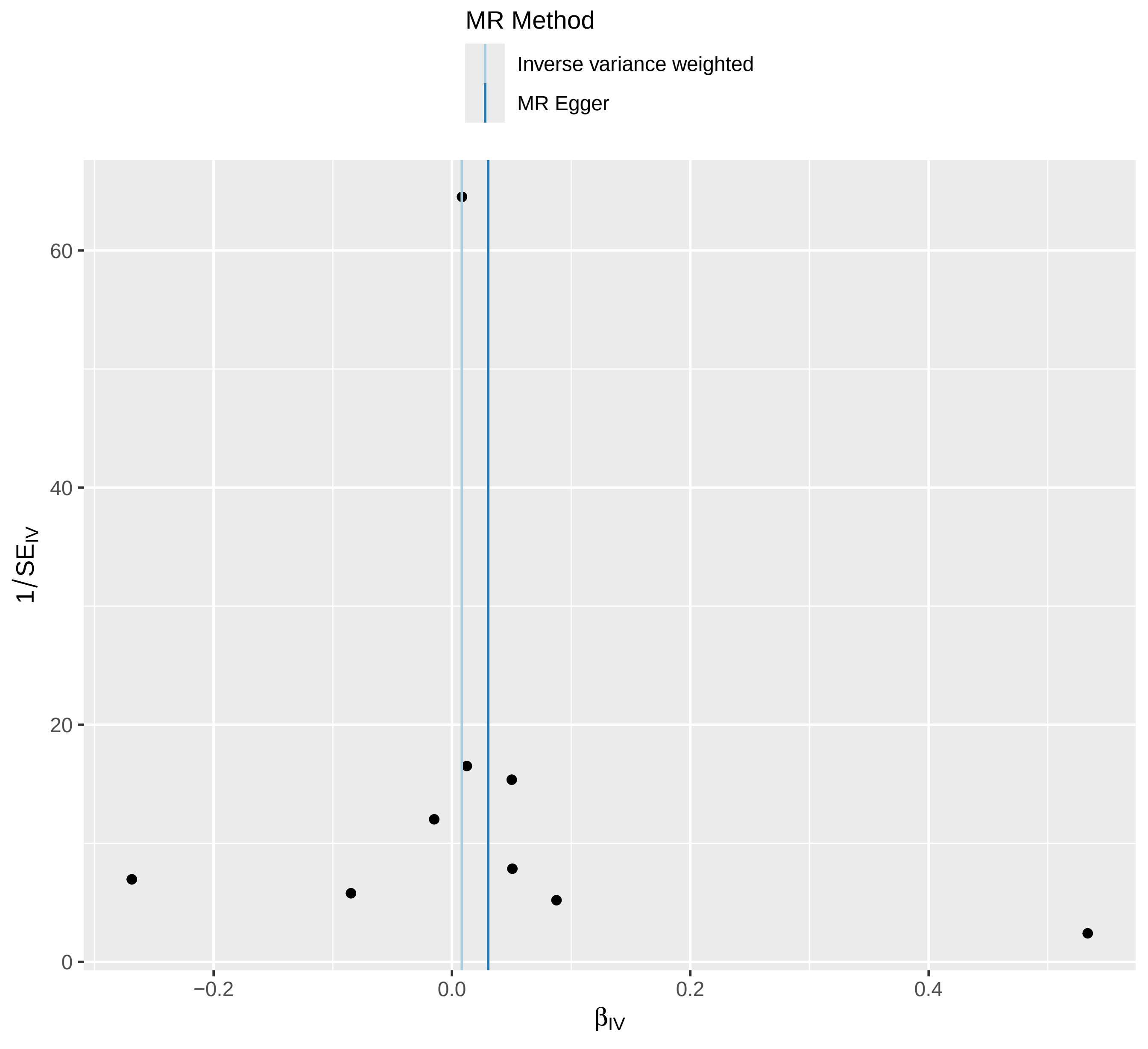
MR Method
Inverse variance weighted
MR Egger
60
40
20
0
1/SE$_{IV}$
−0.2
0.0
0.2
0.4
β$_{IV}$

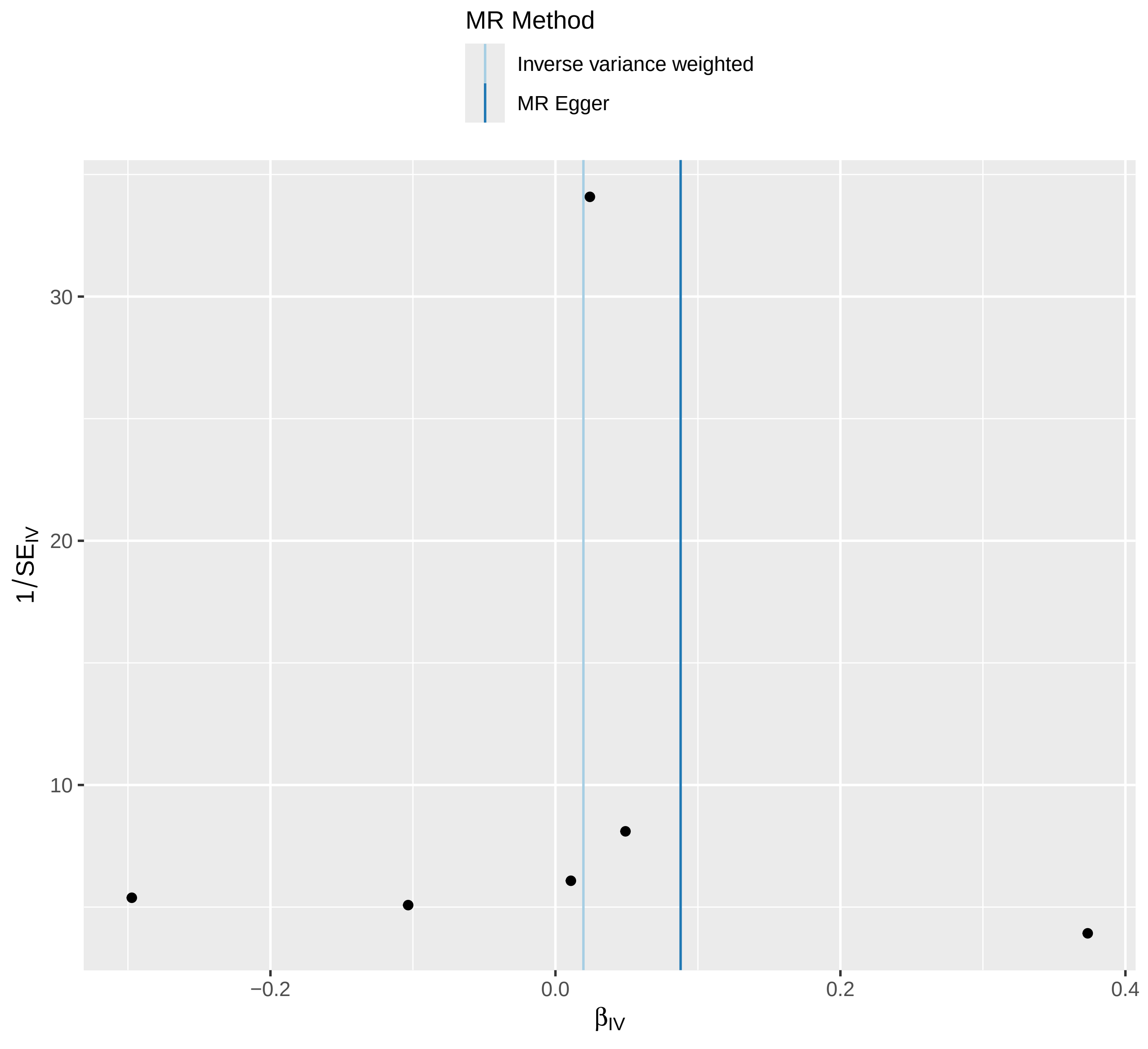

MR Method
Inverse variance weighted
MR Egger
30
20
10
1/SE$_{IV}$
−0.2
0.0
0.2
0.4
β$_{IV}$

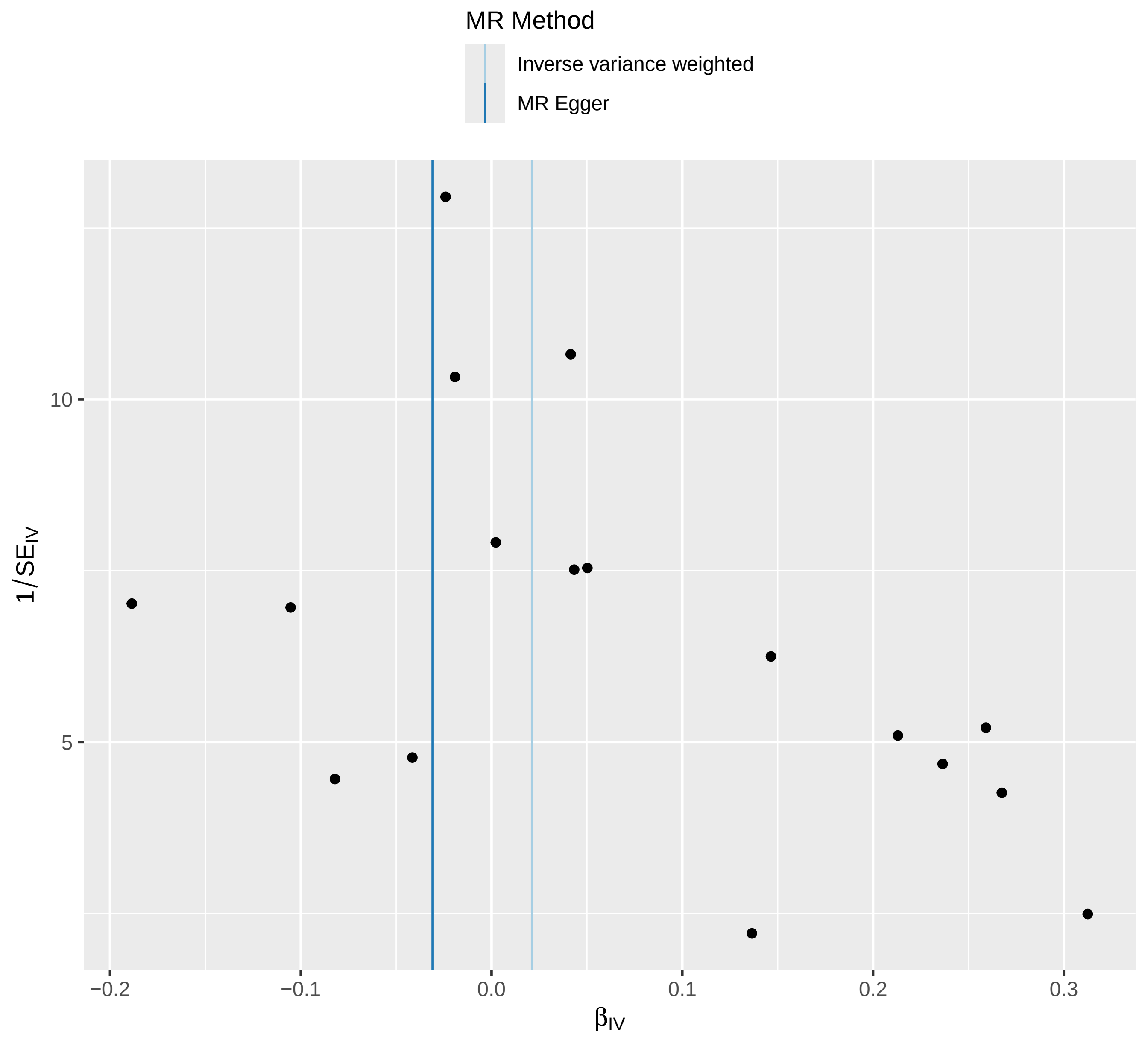
MR Method
Inverse variance weighted
MR Egger
10
5
1/SE_IV
−0.2
−0.1
0.0
0.1
0.2
0.3
β_IV

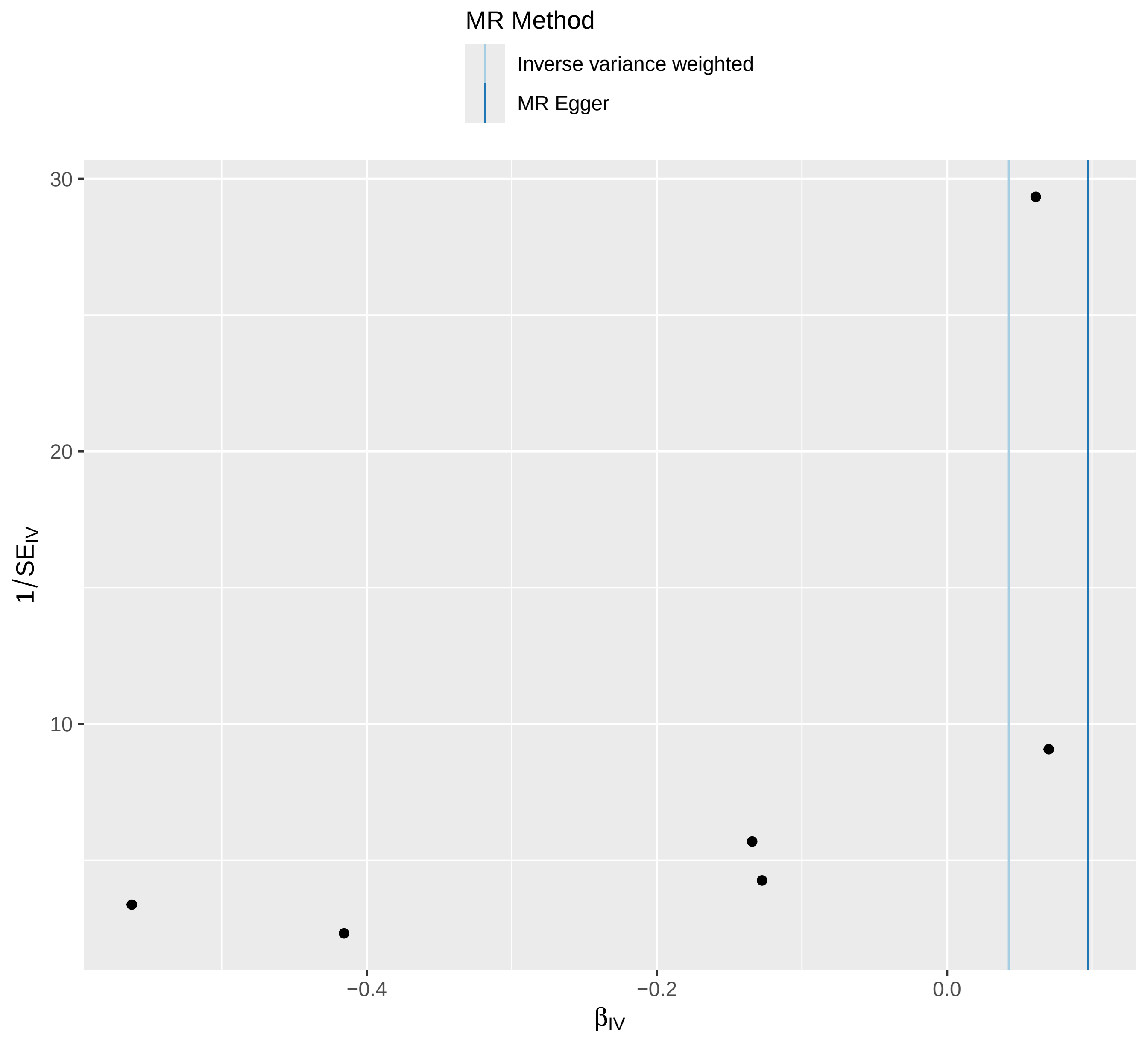
MR Method
Inverse variance weighted
MR Egger
30
20
10
1/SE$_{IV}$
−0.4
−0.2
0.0
β$_{IV}$

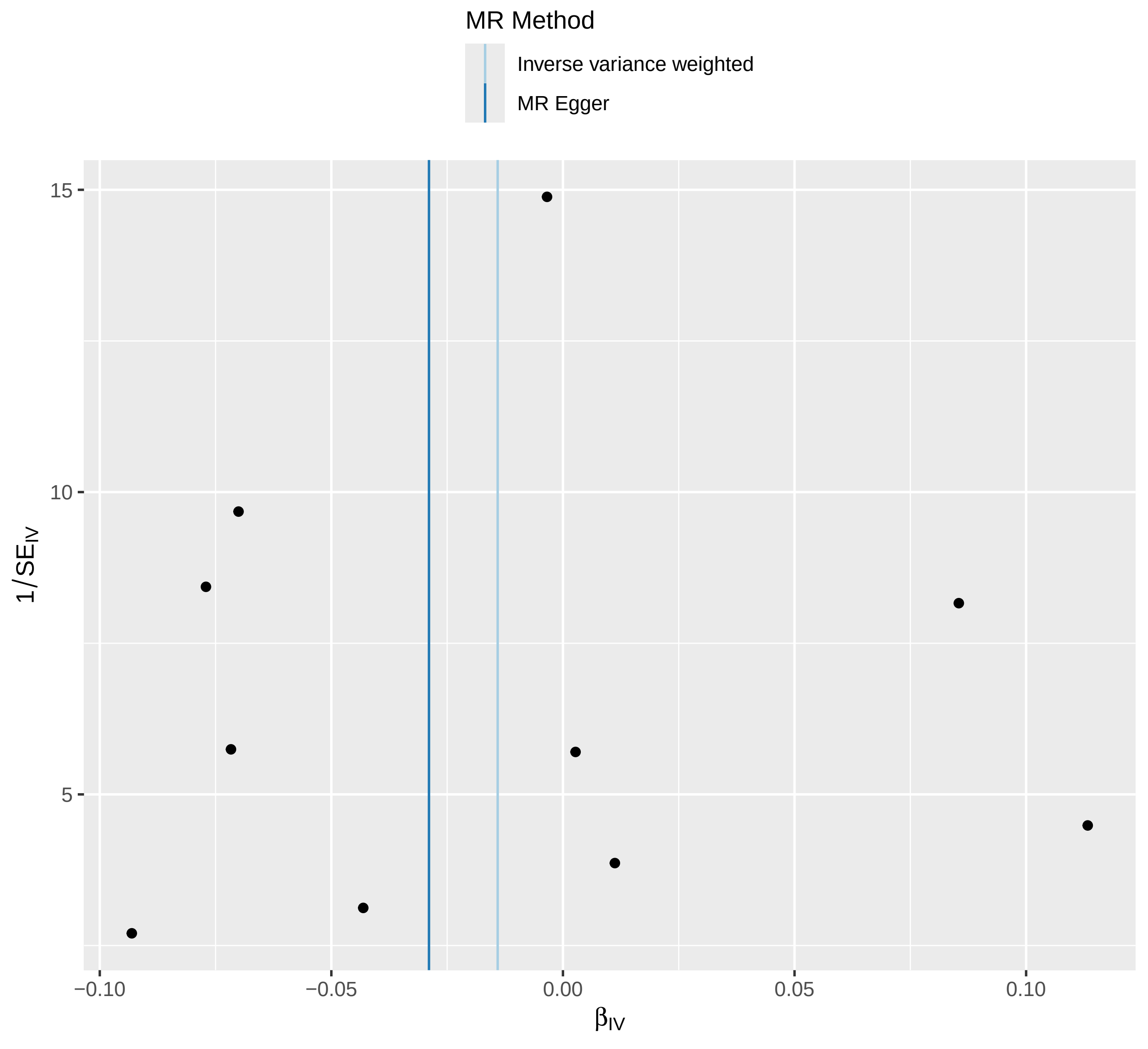
MR Method
Inverse variance weighted
MR Egger
15
10
5
1/SE_IV
−0.10
−0.05
0.00
0.05
0.10
β_IV

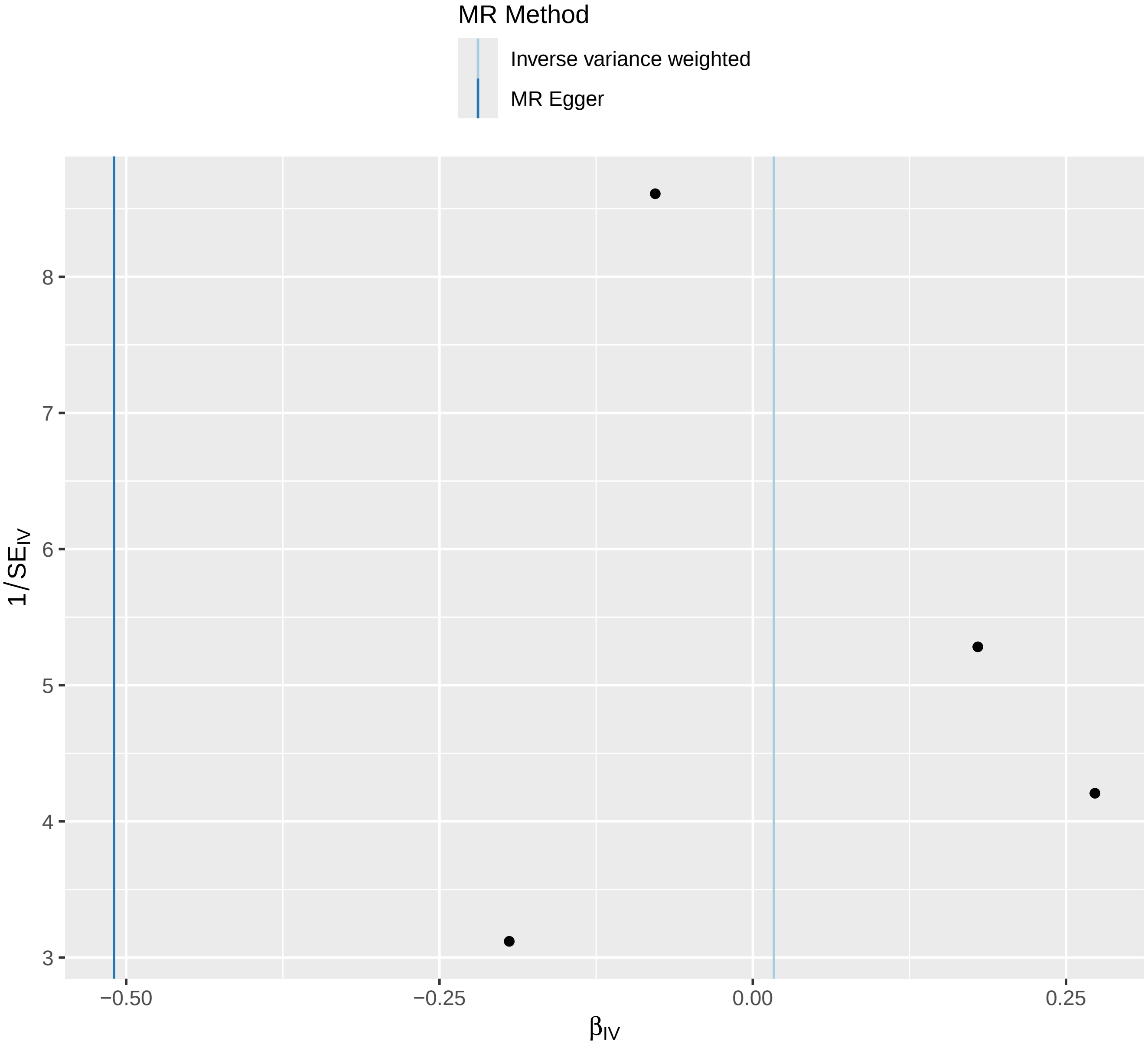

MR Method
Inverse variance weighted
MR Egger
8
7
6
5
4
3
$1/SE_{IV}$
−0.50
−0.25
0.00
0.25
$\beta_{IV}$

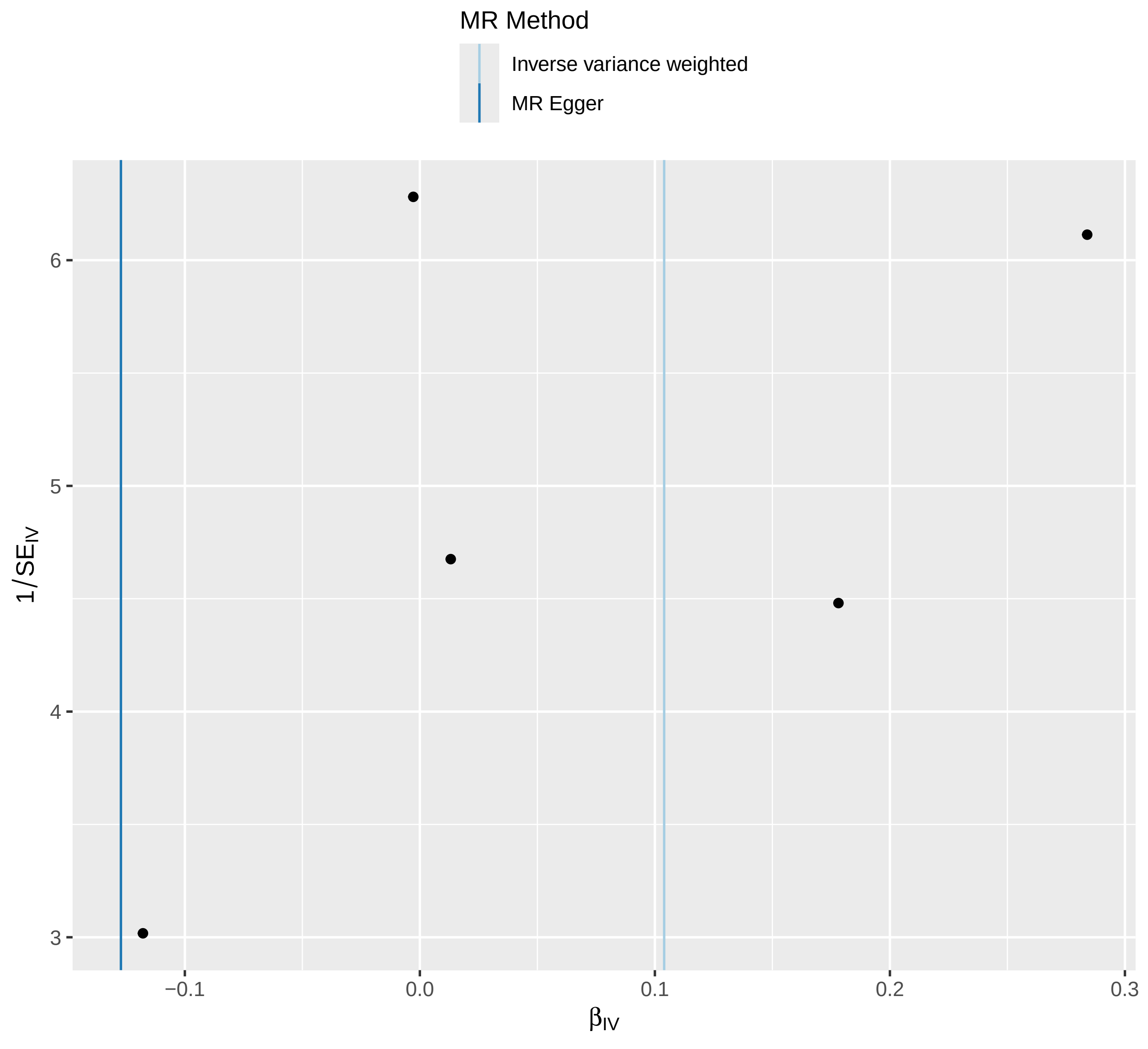
MR Method
Inverse variance weighted
MR Egger
1/SE_IV
6
5
4
3
−0.1
0.0
0.1
0.2
0.3
β_IV

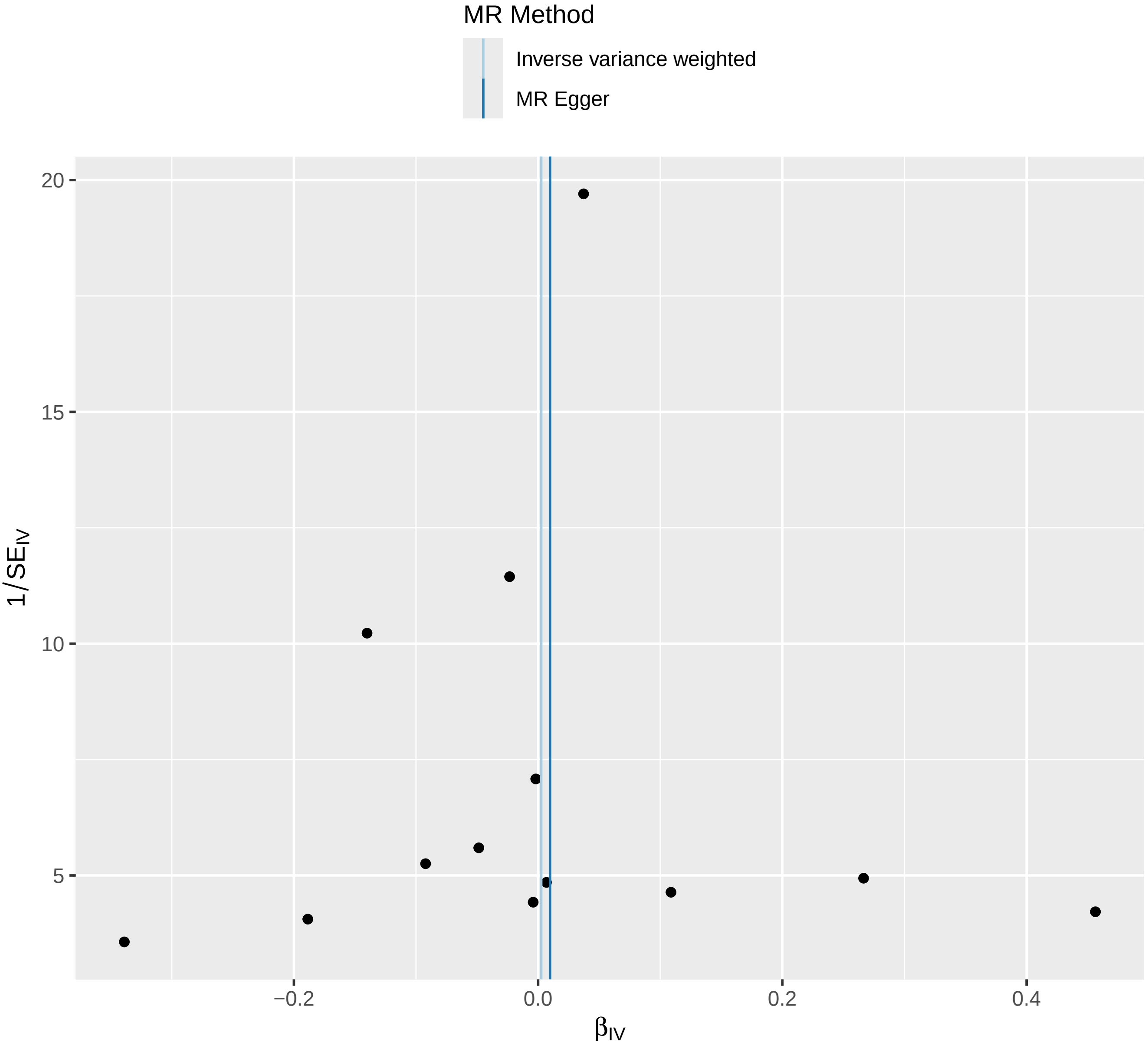
MR Method
Inverse variance weighted
MR Egger
$1/SE_{IV}$
20
15
10
5
−0.2
0.0
0.2
0.4
$\beta_{IV}$

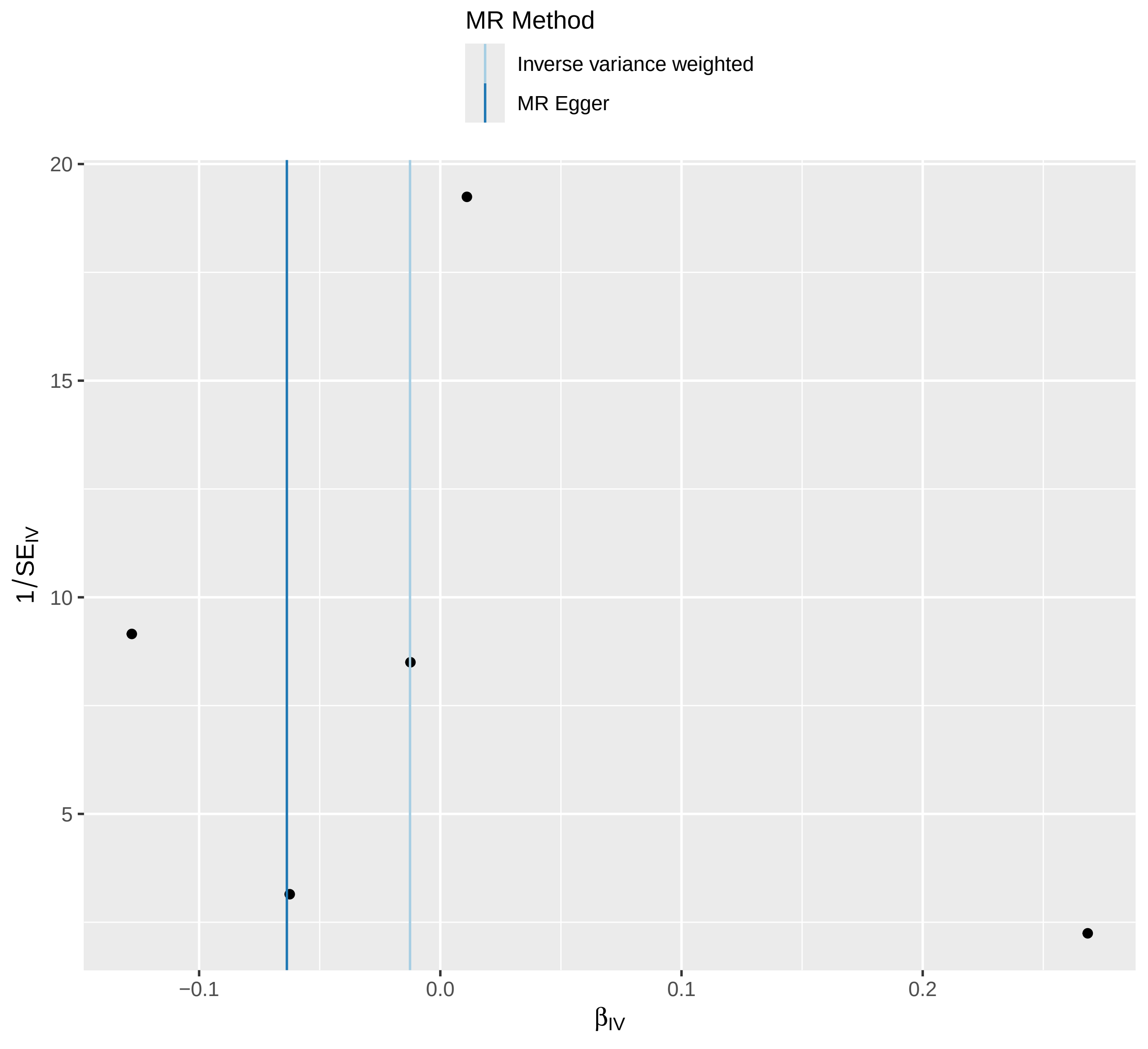

MR Method
Inverse variance weighted
MR Egger
20
15
10
5
1/SE$_{IV}$
−0.1
0.0
0.1
0.2
β$_{IV}$

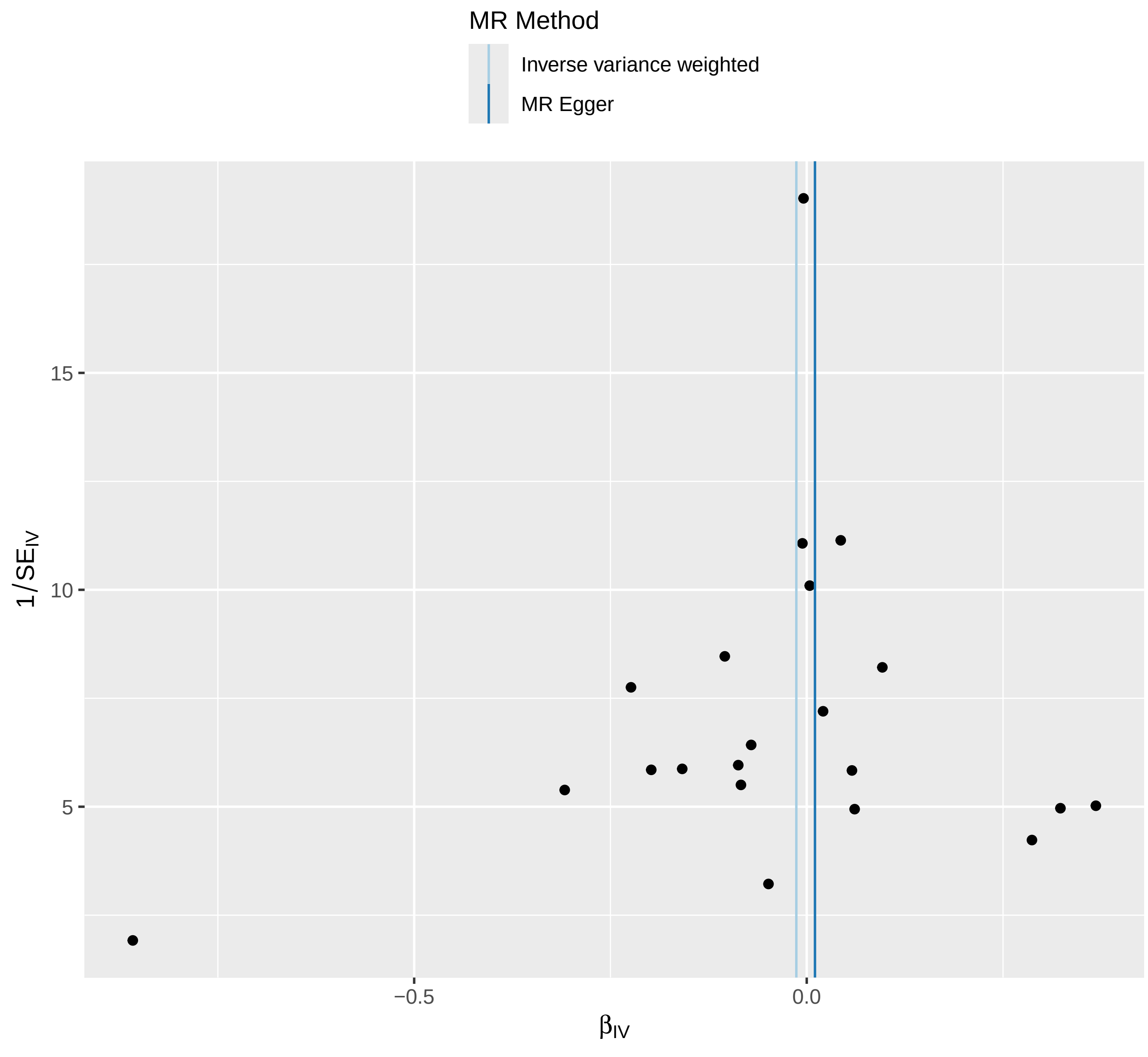
MR Method
Inverse variance weighted
MR Egger
15
10
5
−0.5
0.0
$1/SE_{IV}$
$\beta_{IV}$

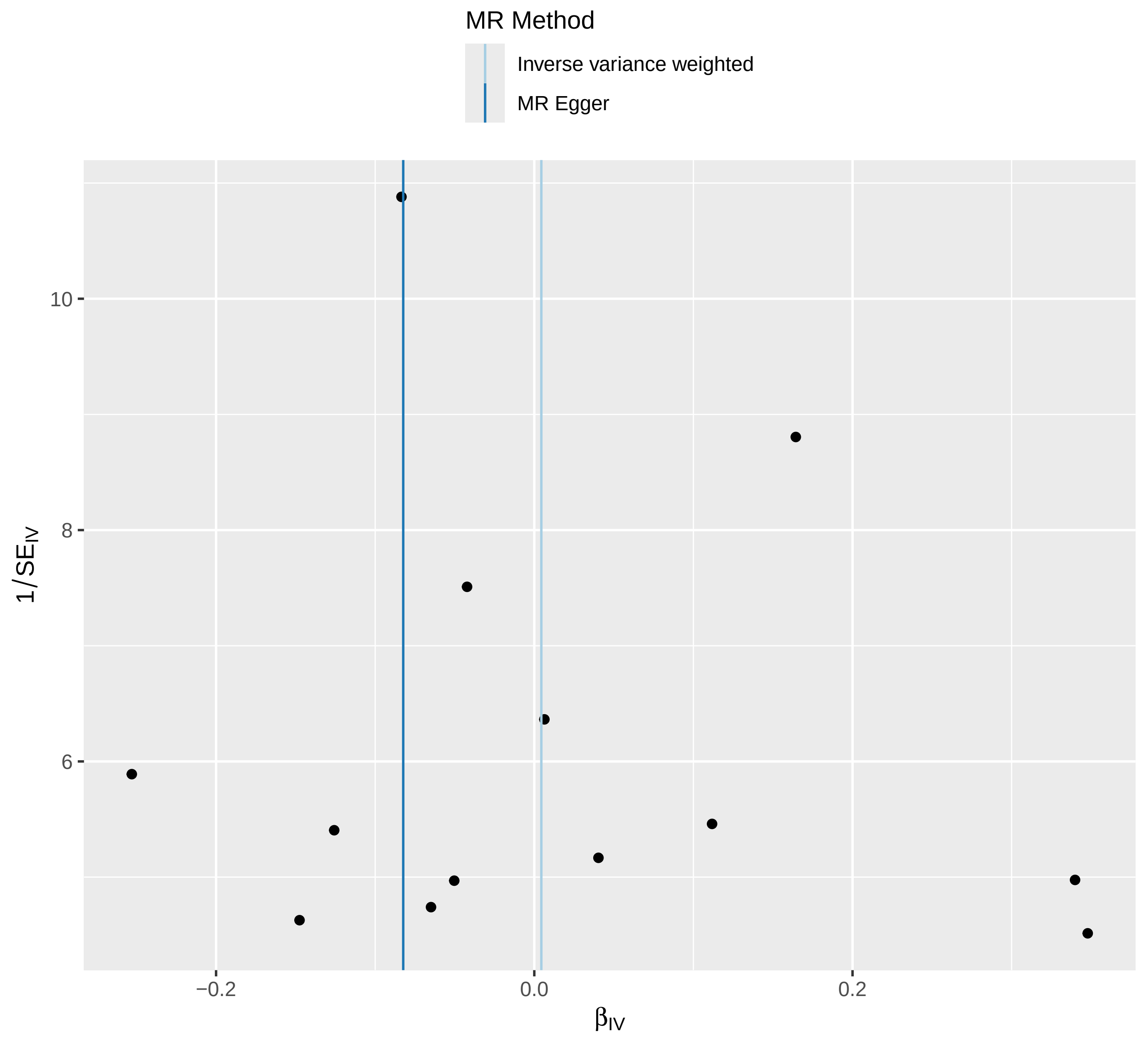
MR Method
Inverse variance weighted
MR Egger
1/SE_IV
10
8
6
β_IV
−0.2
0.0
0.2

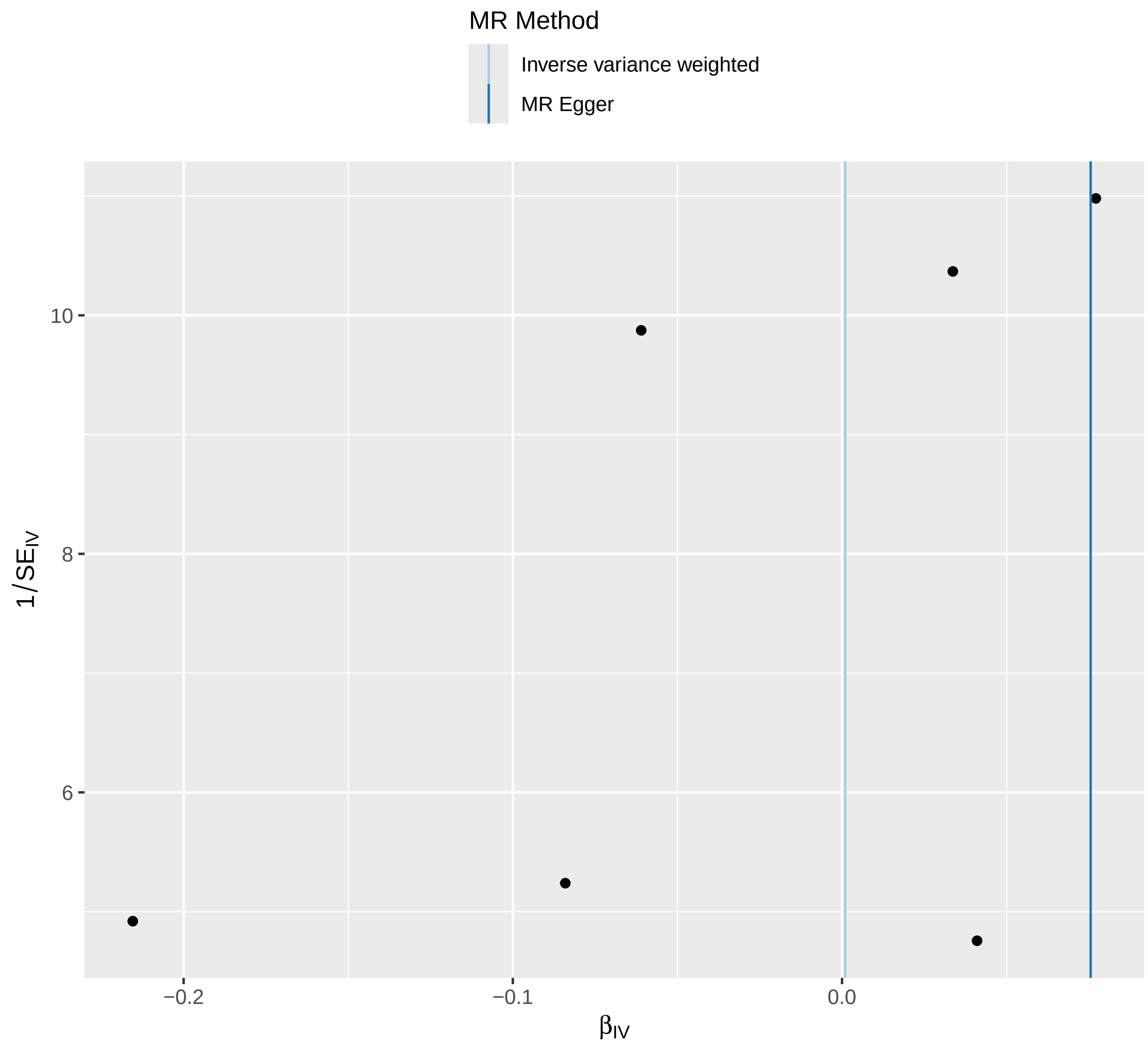
MR Method
Inverse variance weighted
MR Egger
10
8
6
1/SE_IV
−0.2
−0.1
0.0
β_IV

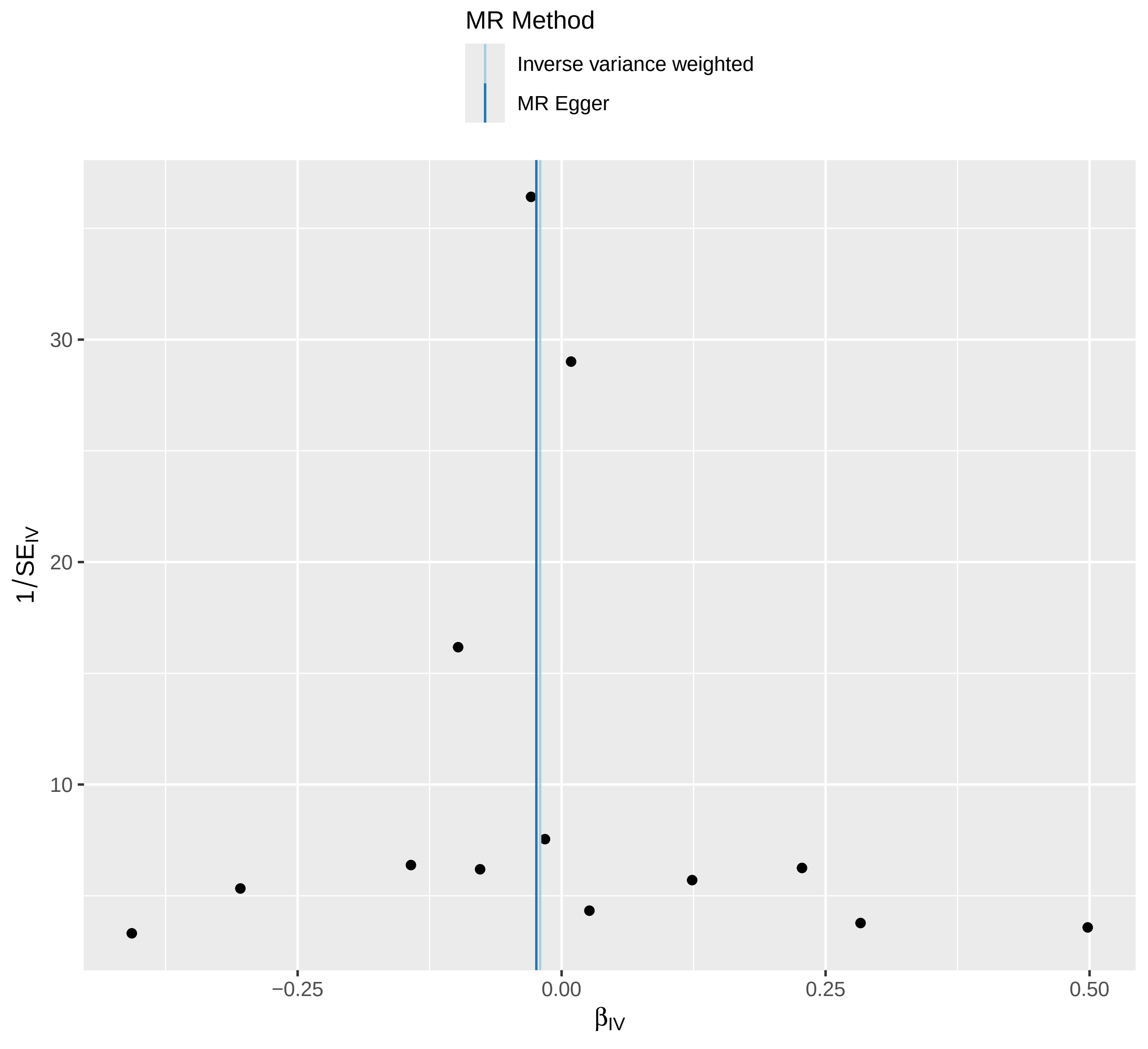

MR Method
Inverse variance weighted
MR Egger
30
20
10
1/SE$_{IV}$
−0.25
0.00
0.25
0.50
β$_{IV}$

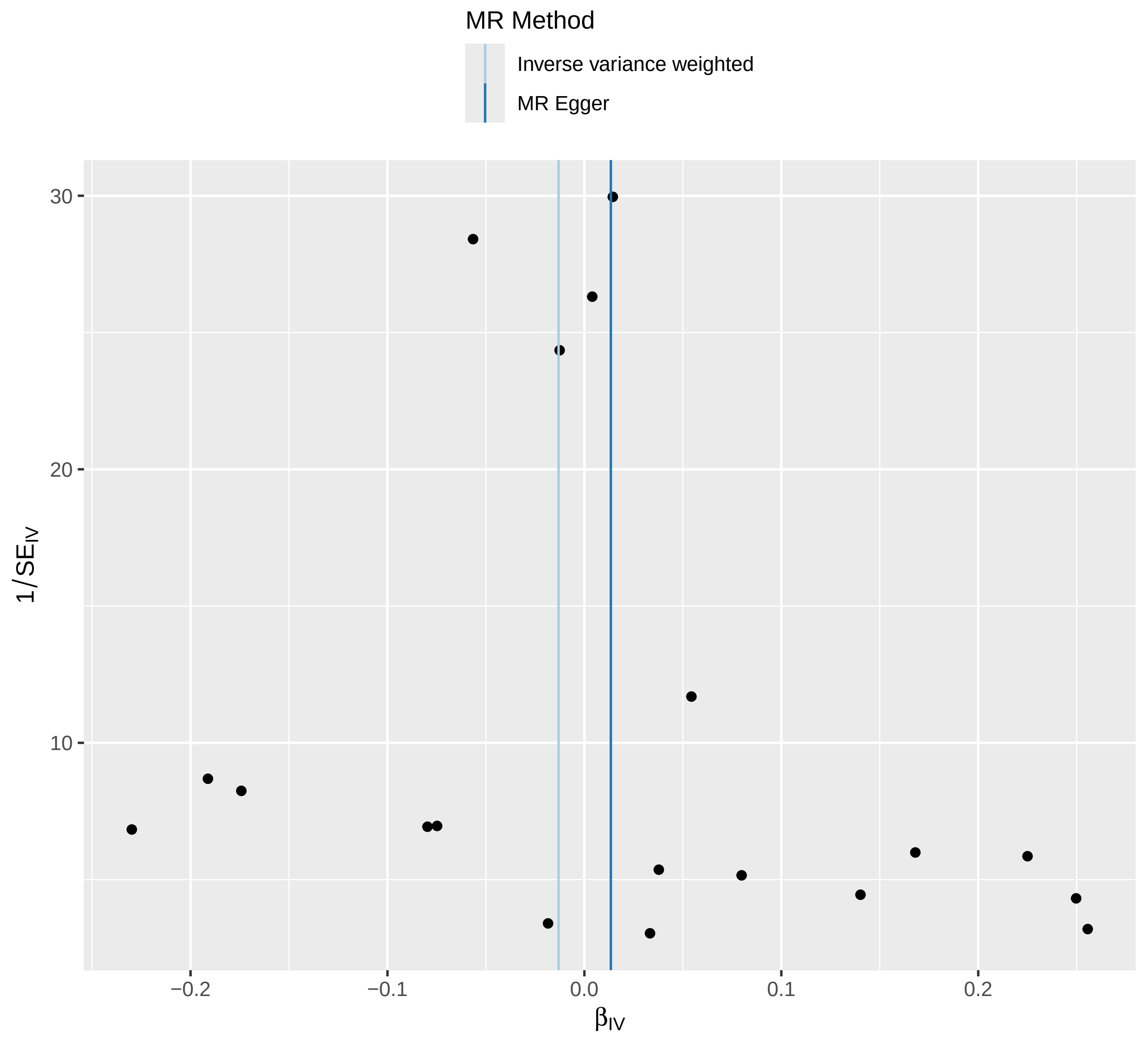
MR Method
Inverse variance weighted
MR Egger
30
20
10
1/SE_IV
−0.2
−0.1
0.0
0.1
0.2
β_IV

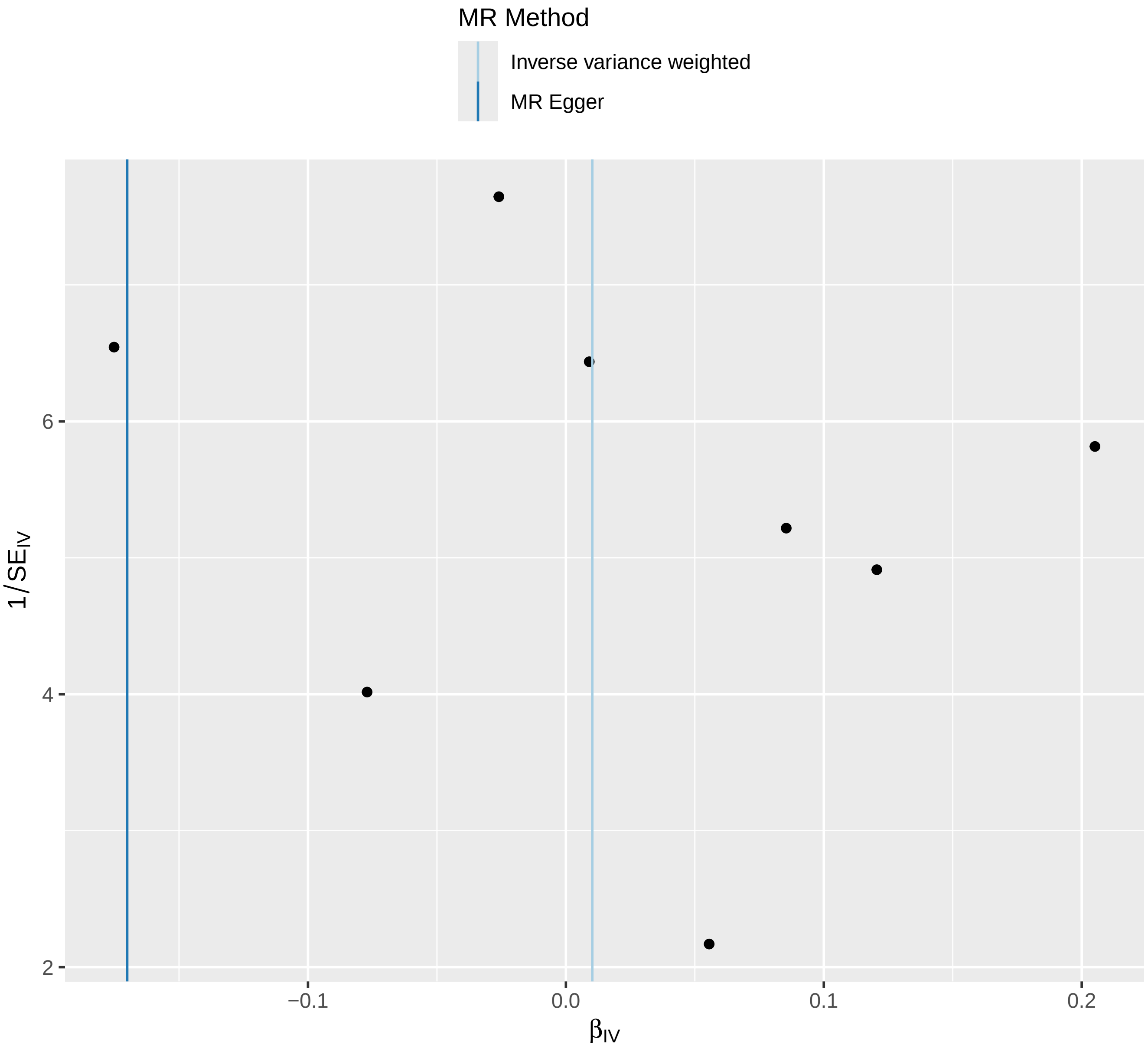
MR Method
Inverse variance weighted
MR Egger
6
4
2
1/SE$_{IV}$
−0.1
0.0
0.1
0.2
β$_{IV}$

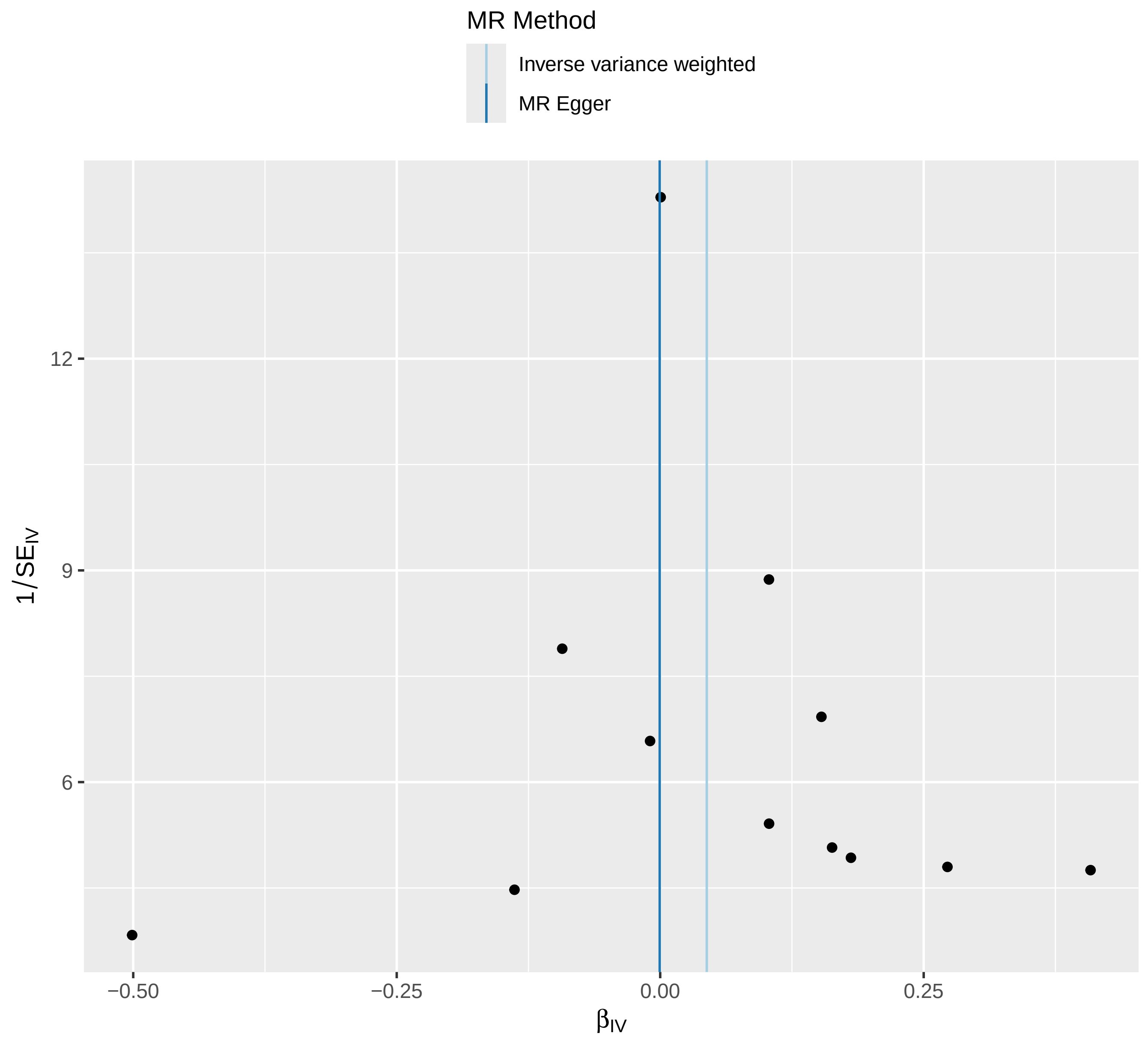
MR Method
Inverse variance weighted
MR Egger
12
9
6
$1/SE_{IV}$
−0.50
−0.25
0.00
0.25
$\beta_{IV}$

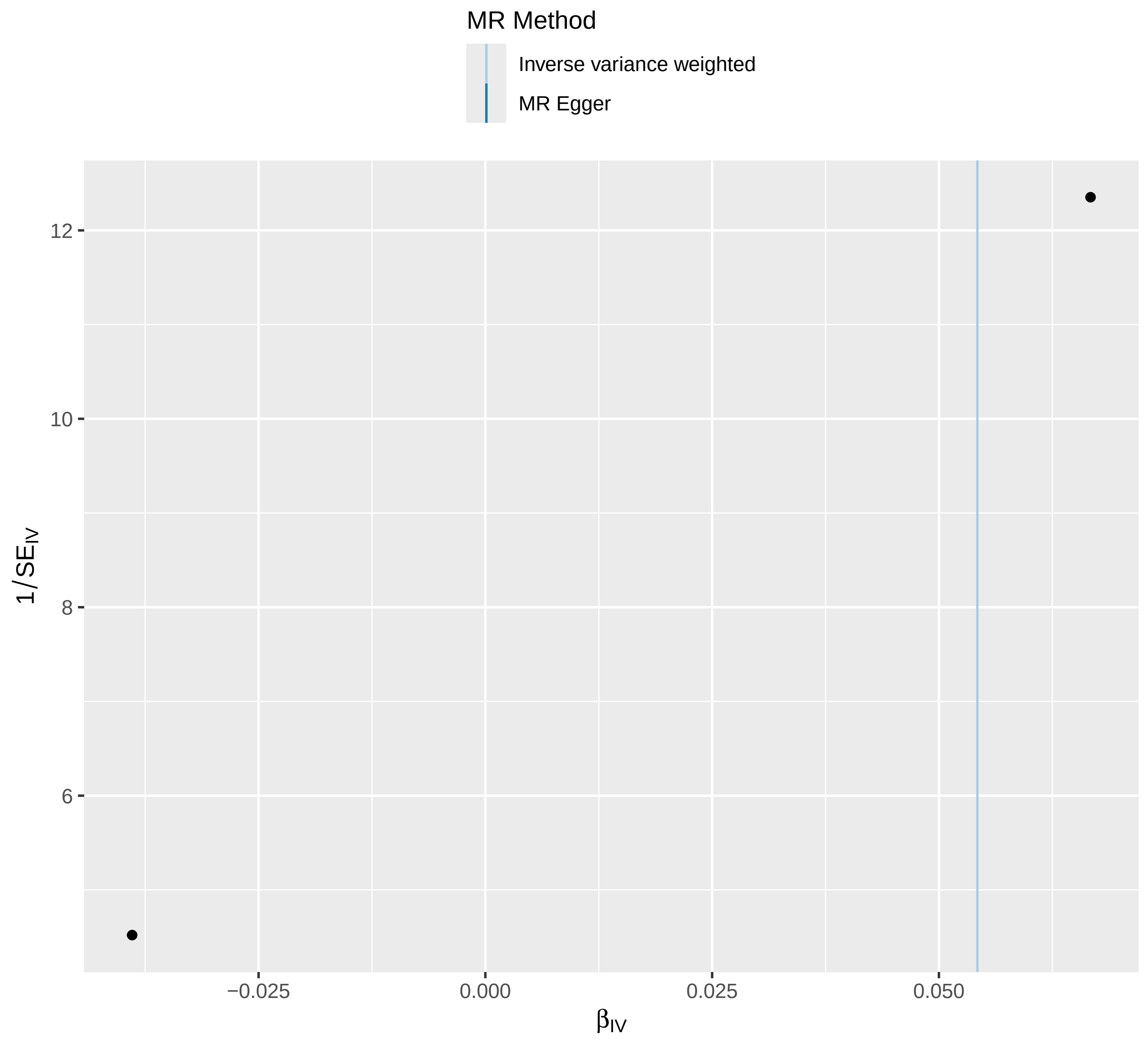
MR Method
Inverse variance weighted
MR Egger
12
10
8
6
−0.025
0.000
0.025
0.050
1/SE$_{IV}$
β$_{IV}$

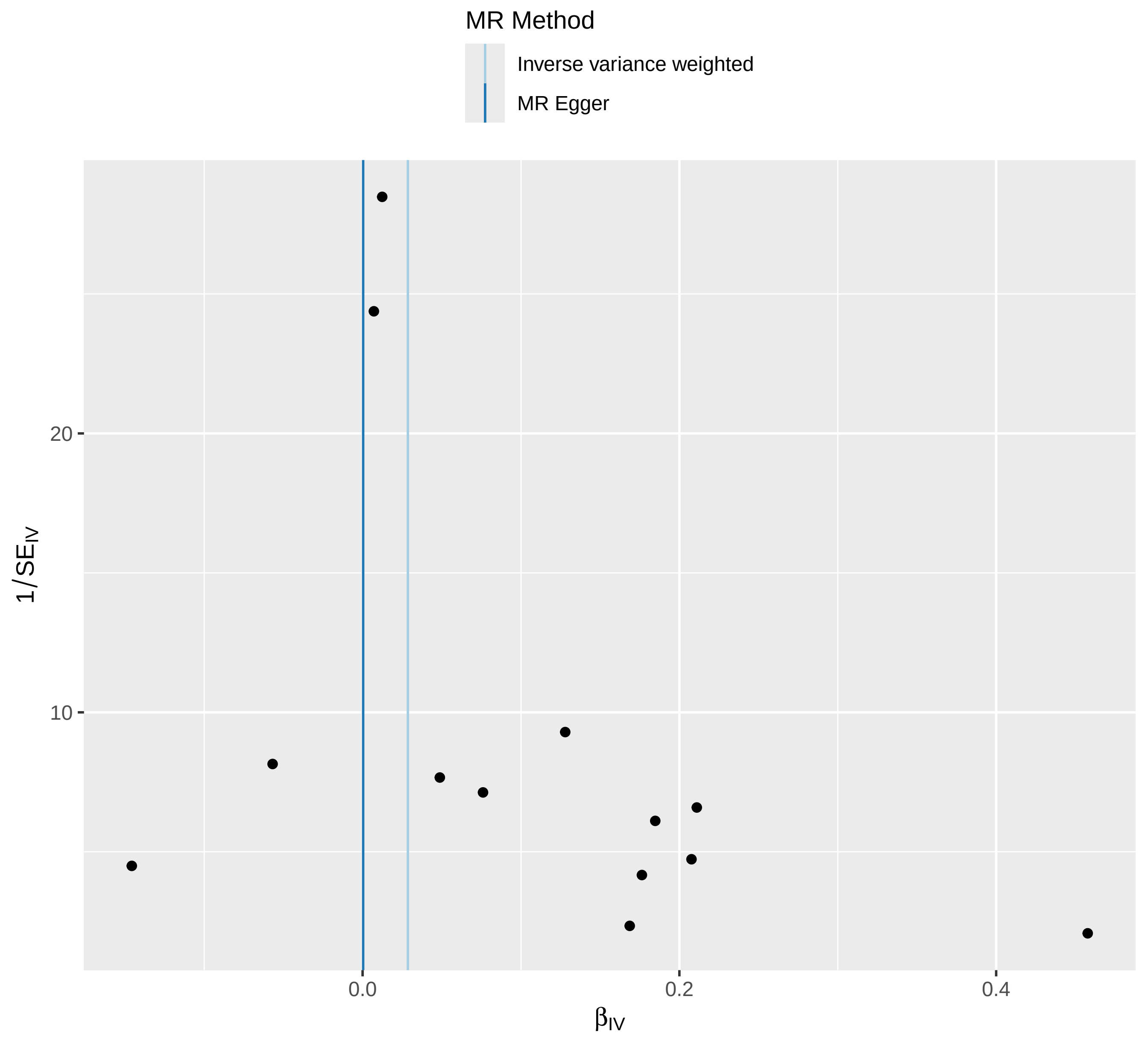
MR Method
Inverse variance weighted
MR Egger
20
10
0.0
0.2
0.4
$1/SE_{IV}$
$\beta_{IV}$

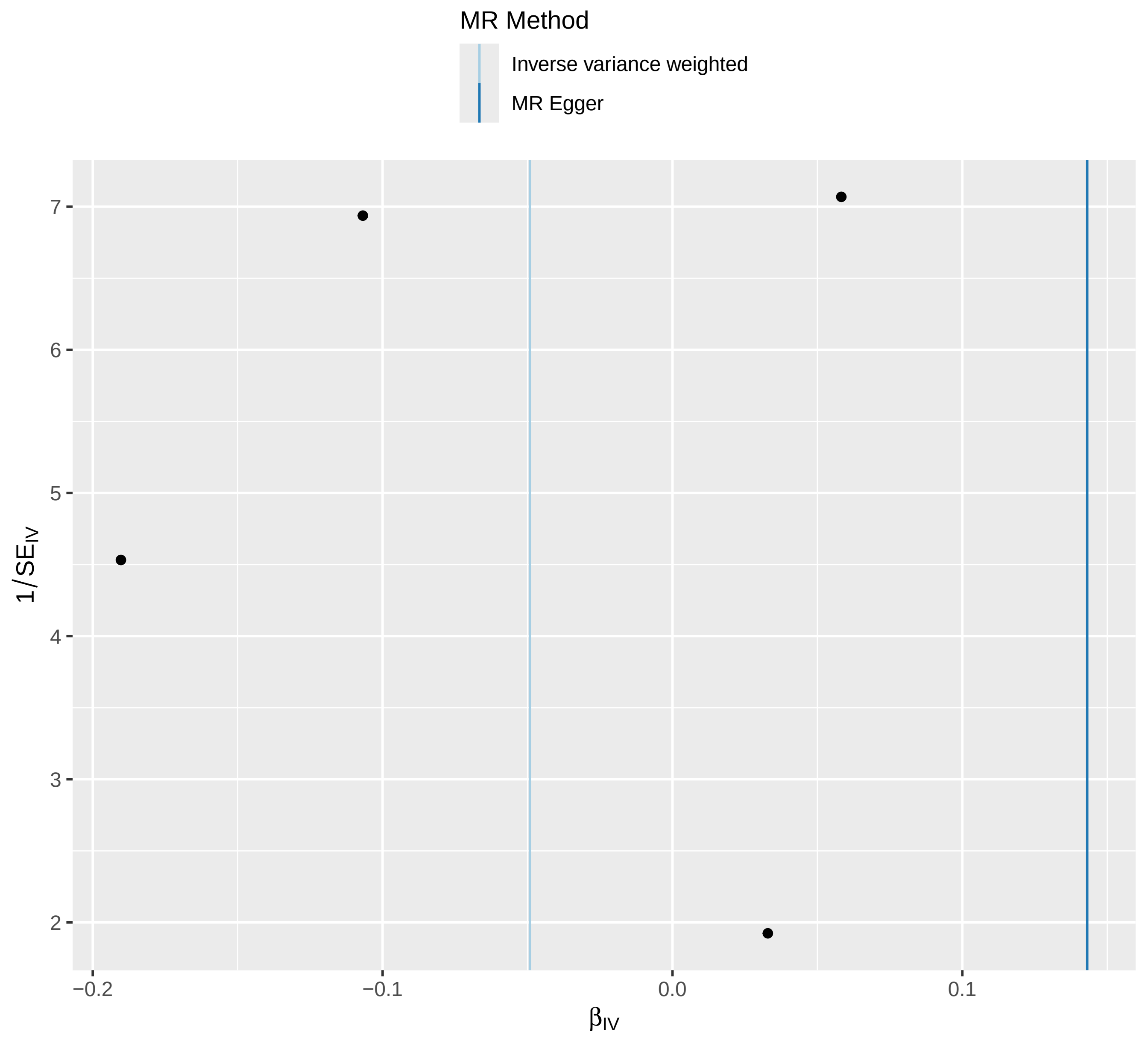

MR Method
Inverse variance weighted
MR Egger
1/SE$_{IV}$
7
6
5
4
3
2
−0.2
−0.1
0.0
0.1
β$_{IV}$

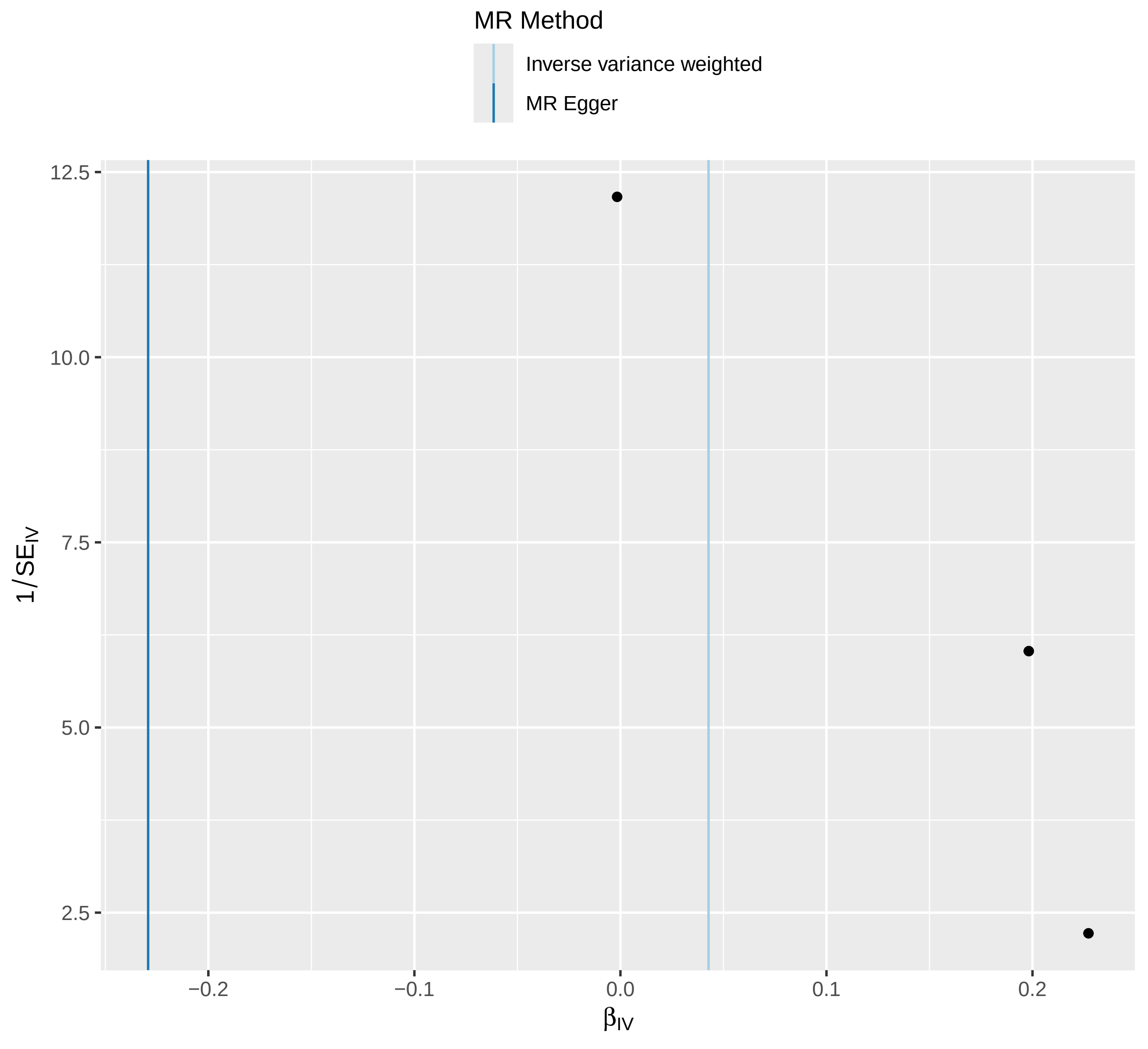

MR Method
Inverse variance weighted
MR Egger
12.5
10.0
7.5
5.0
2.5
1/SE_IV
−0.2
−0.1
0.0
0.1
0.2
β_IV

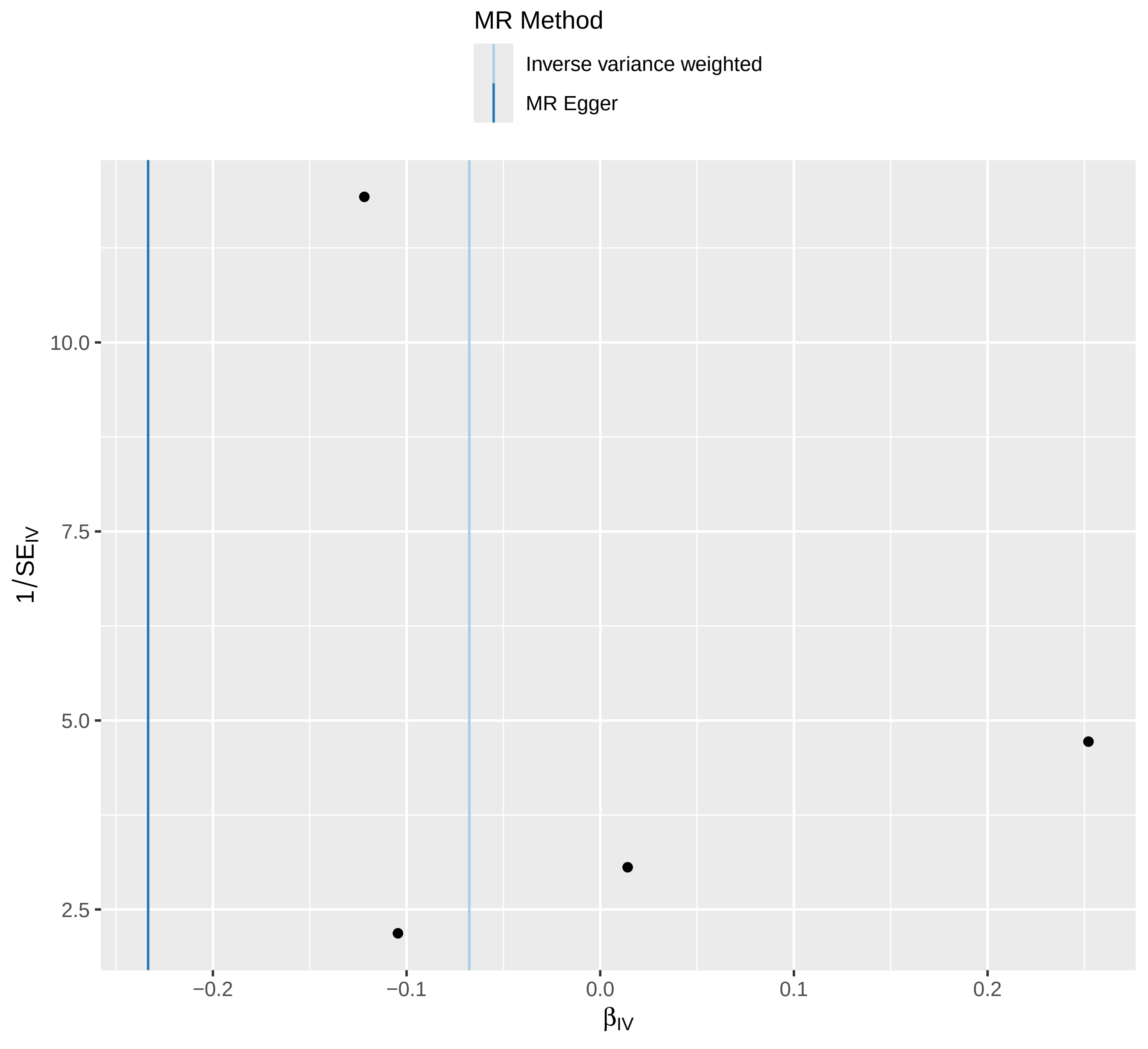

MR Method
Inverse variance weighted
MR Egger
10.0
7.5
5.0
2.5
1/SE_IV
−0.2
−0.1
0.0
0.1
0.2
β_IV

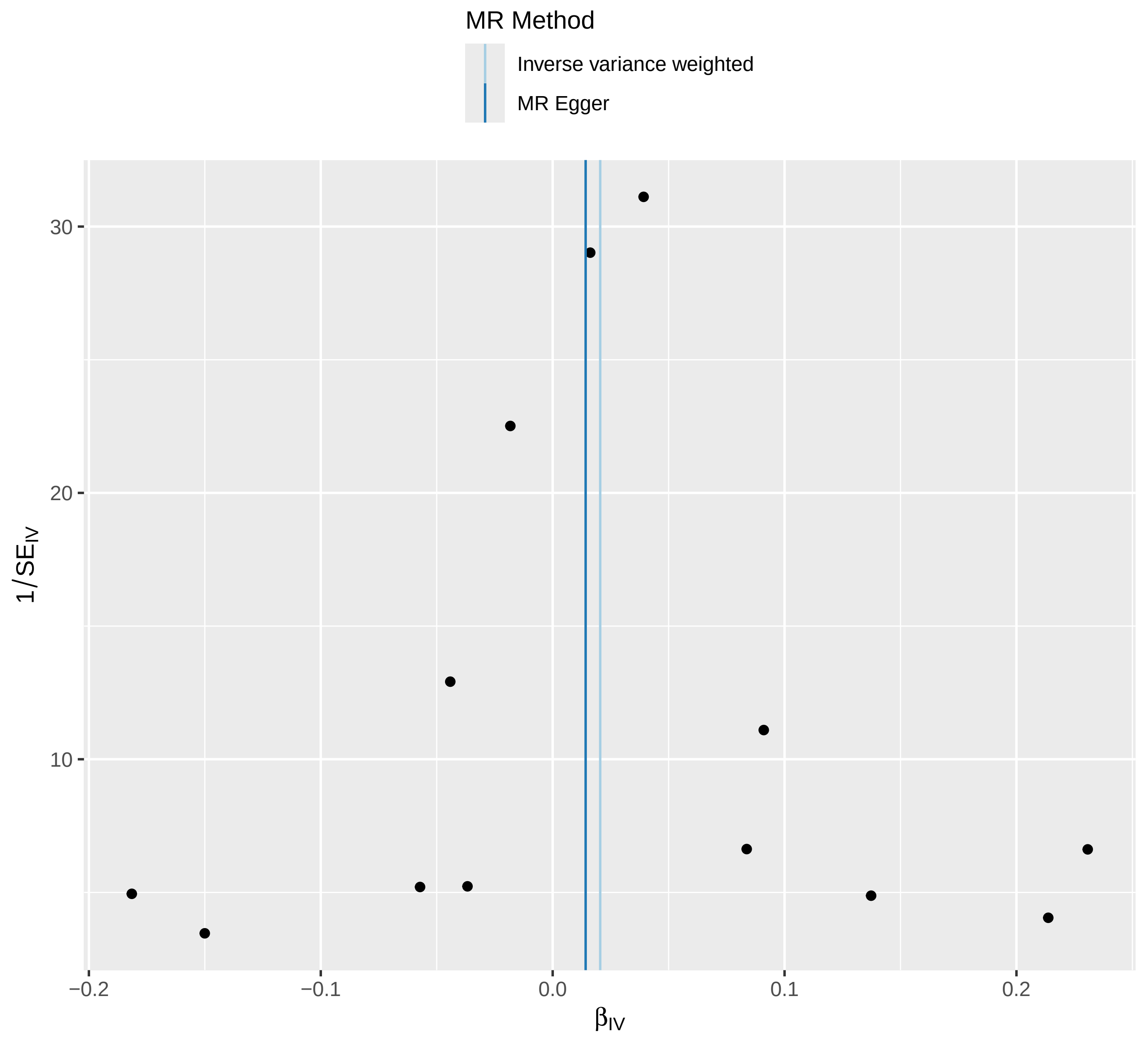
MR Method
Inverse variance weighted
MR Egger
30
20
10
1/SE_IV
−0.2
−0.1
0.0
0.1
0.2
β_IV

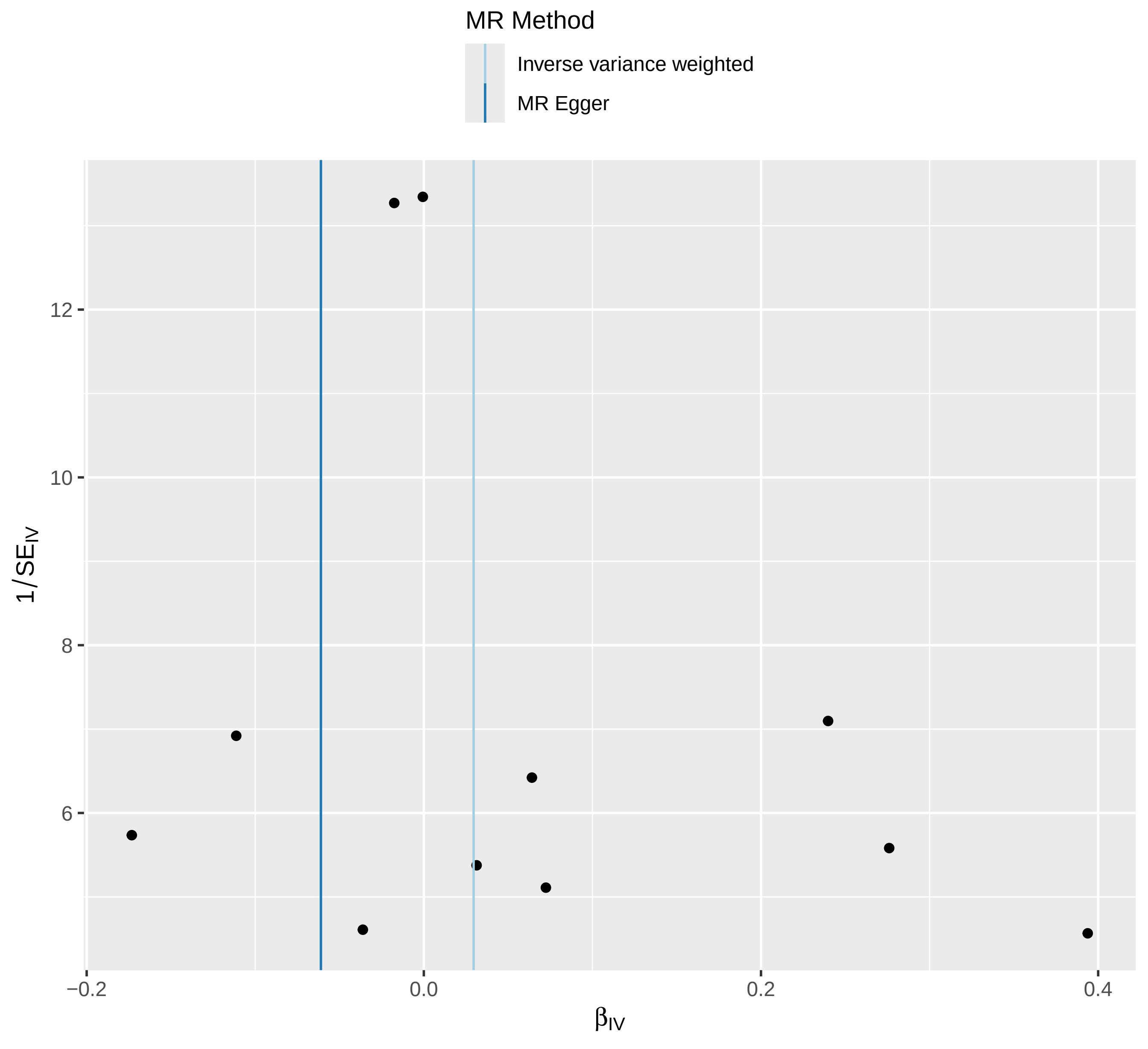
MR Method
Inverse variance weighted
MR Egger
12
10
8
6
$1/SE_{IV}$
−0.2
0.0
0.2
0.4
$\beta_{IV}$

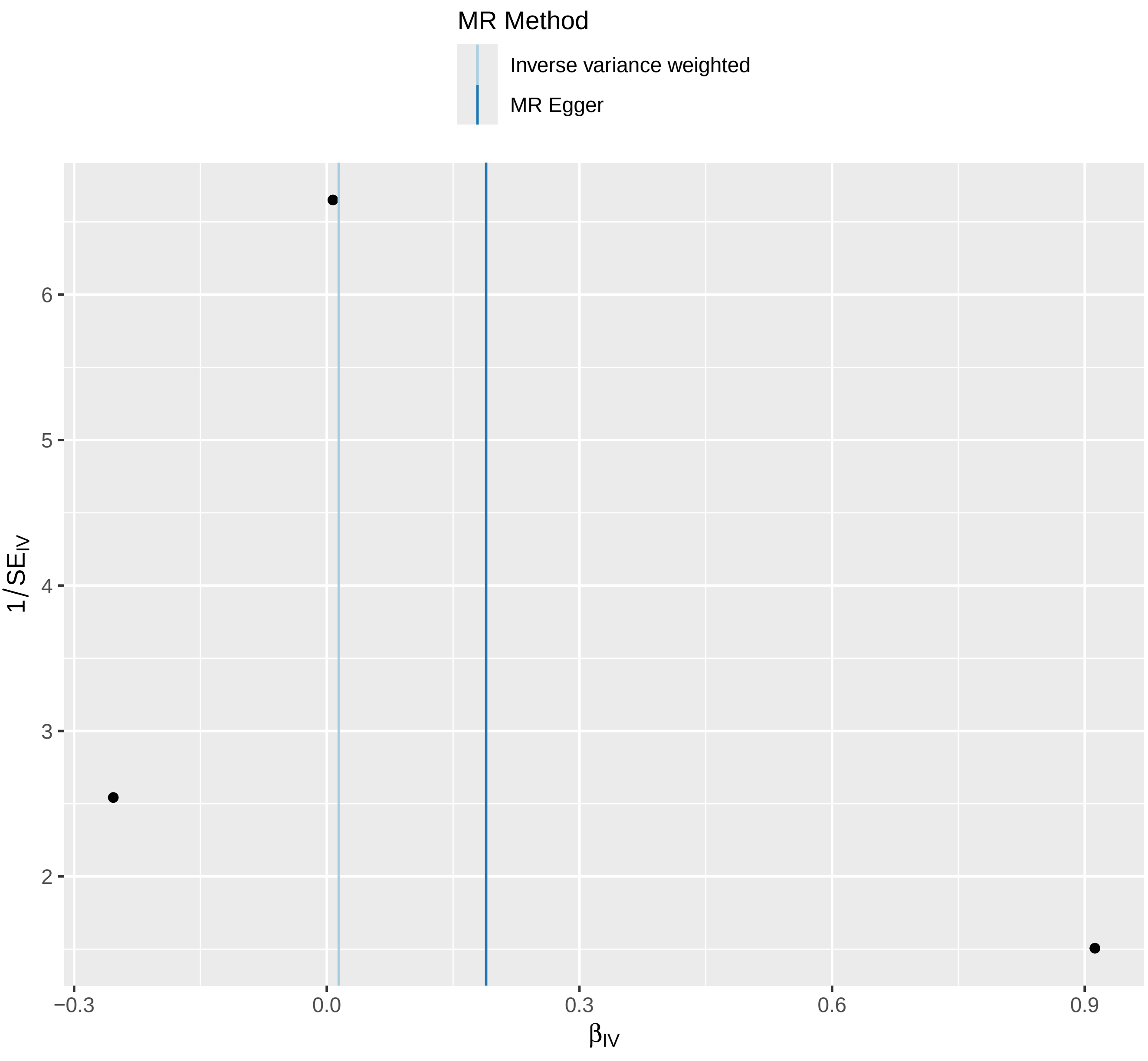
MR Method
Inverse variance weighted
MR Egger
1/SE_IV
β_IV
6
5
4
3
2
−0.3
0.0
0.3
0.6
0.9

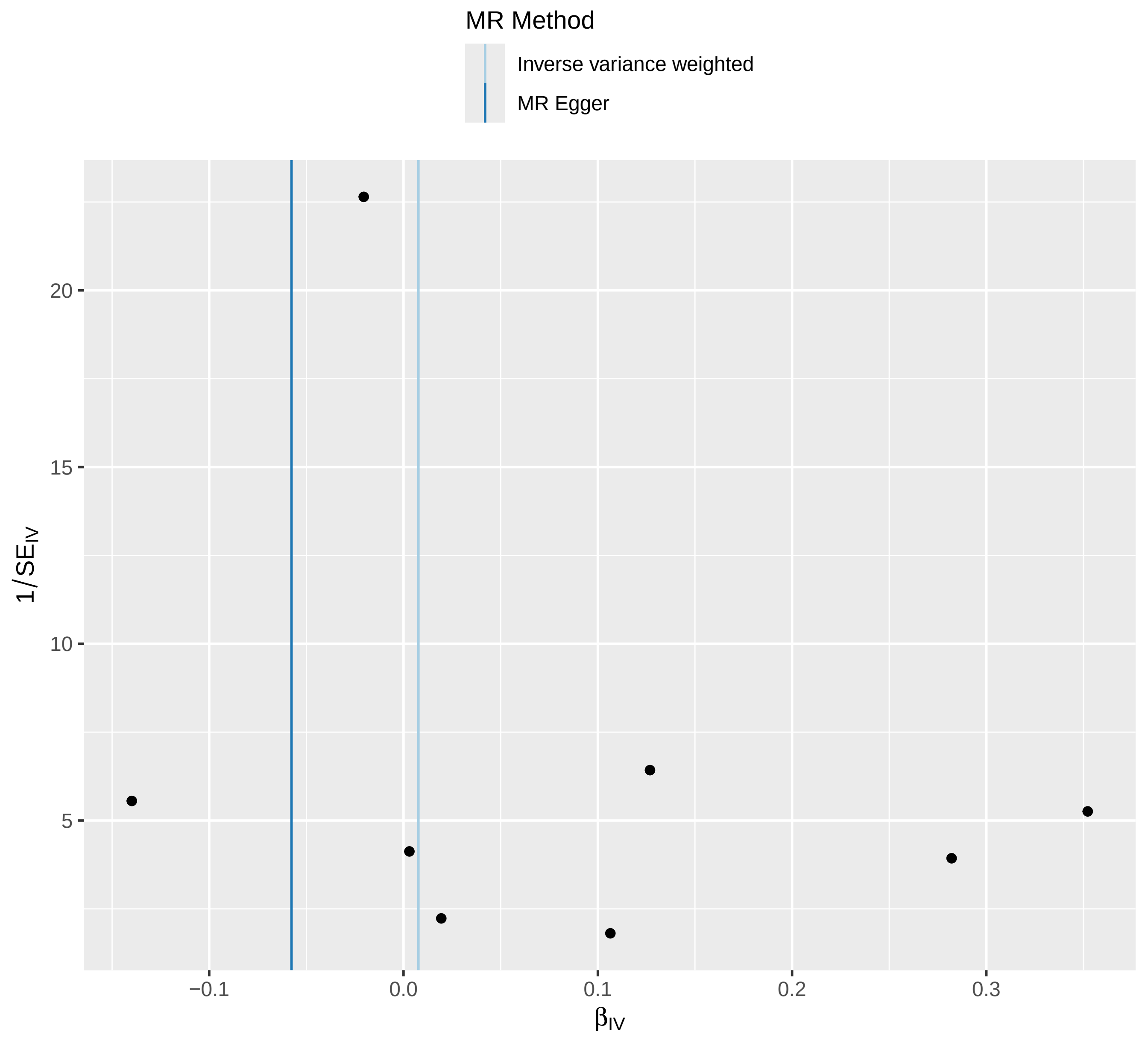

MR Method
Inverse variance weighted
MR Egger
20
15
10
5
1/SE$_{IV}$
−0.1
0.0
0.1
0.2
0.3
β$_{IV}$

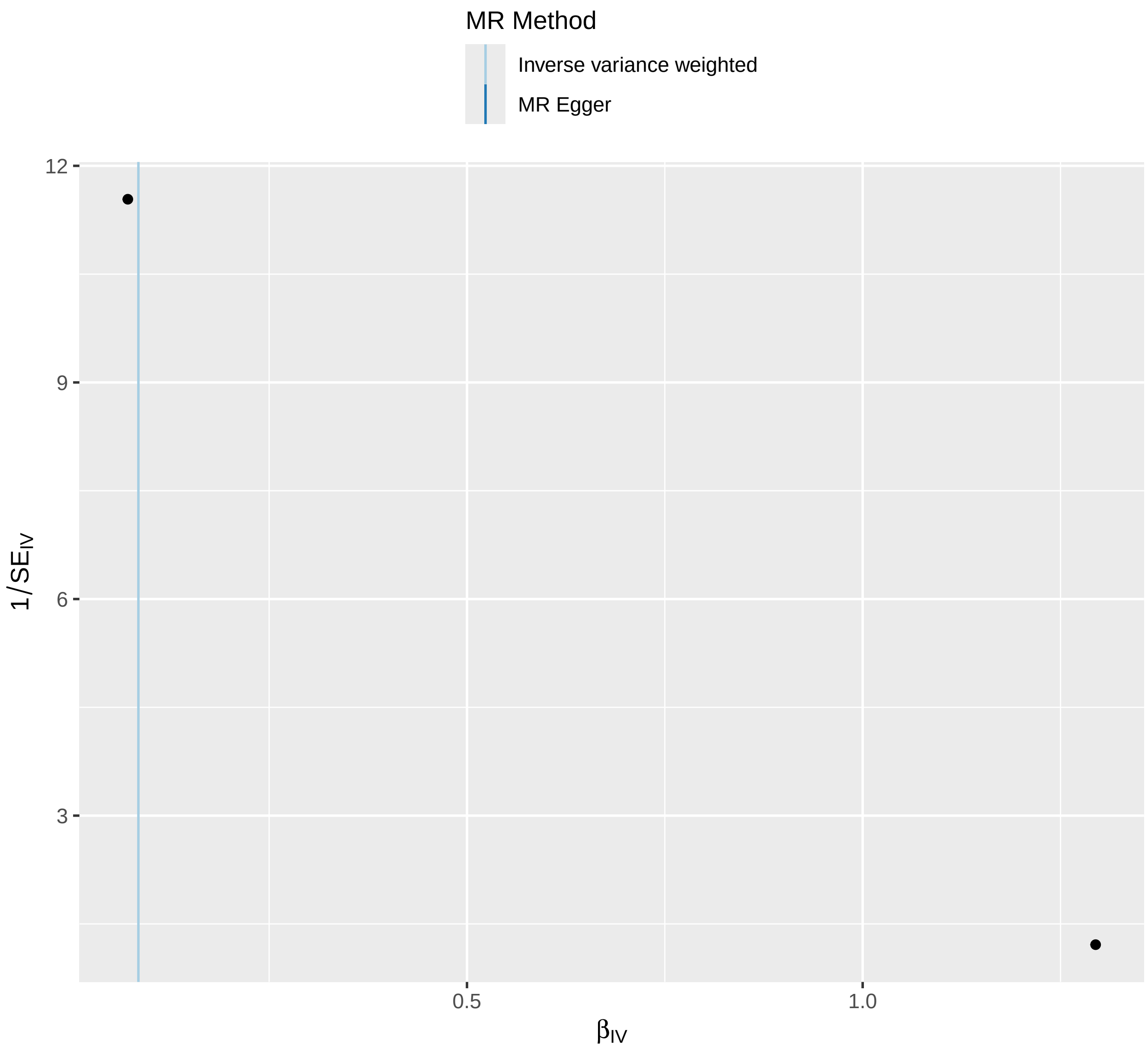
MR Method
Inverse variance weighted
MR Egger
12
9
6
3
0.5
1.0
$\beta_{IV}$
$1/SE_{IV}$

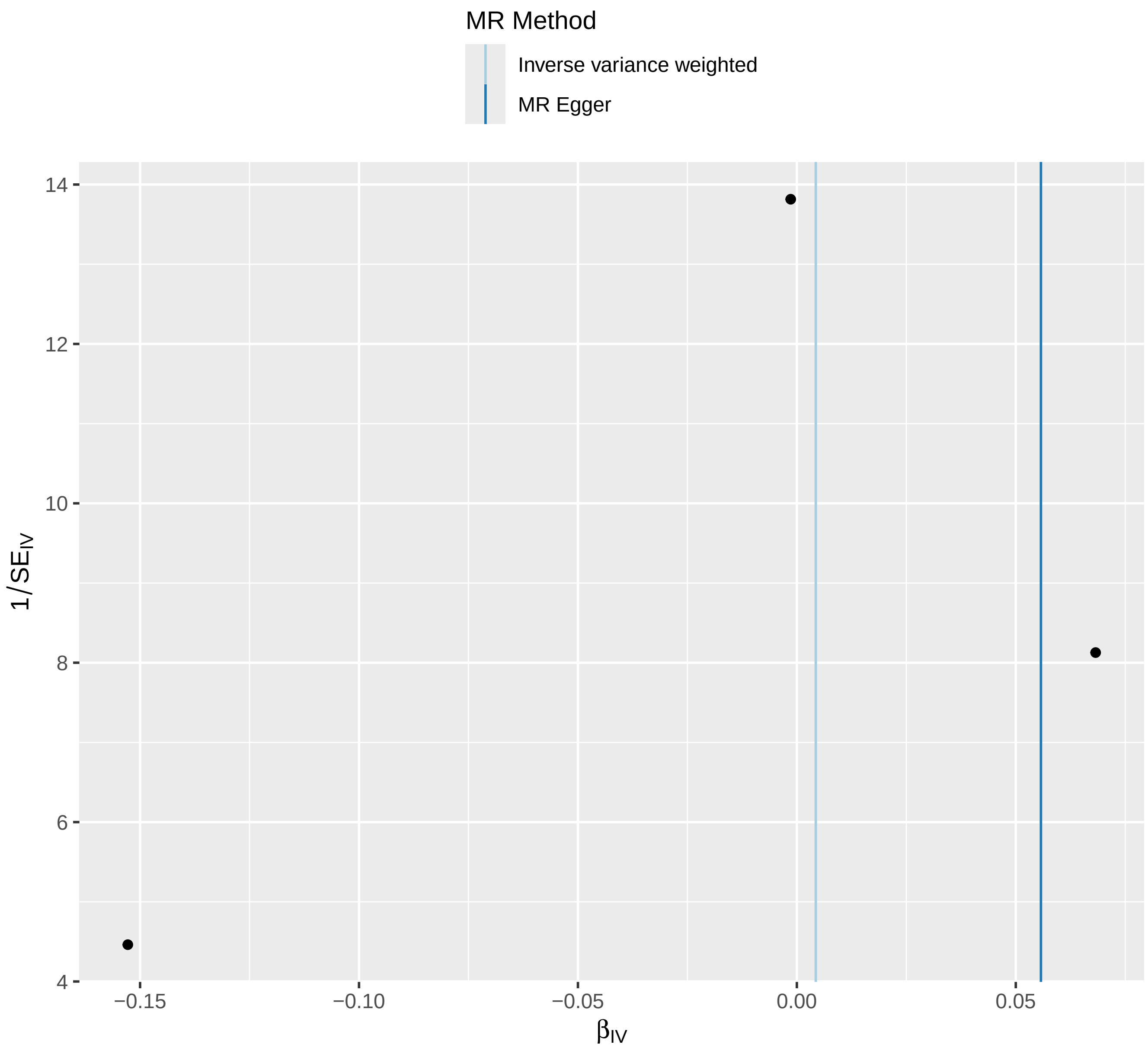
MR Method
Inverse variance weighted
MR Egger
14
12
10
8
6
4
1/SE_IV
−0.15
−0.10
−0.05
0.00
0.05
β_IV

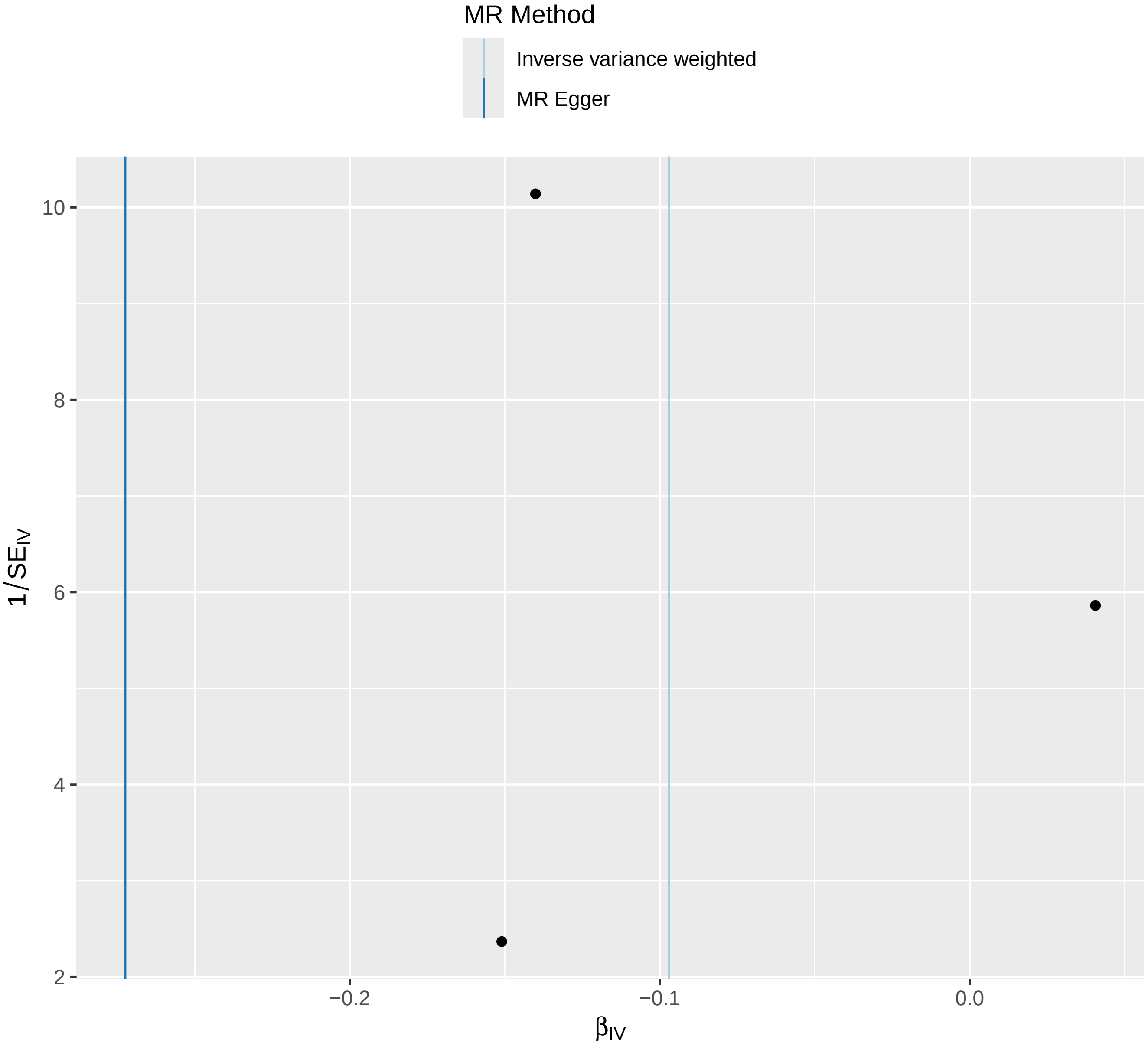
MR Method
Inverse variance weighted
MR Egger
1/SE_IV
β_IV
10
8
6
4
2
−0.2
−0.1
0.0

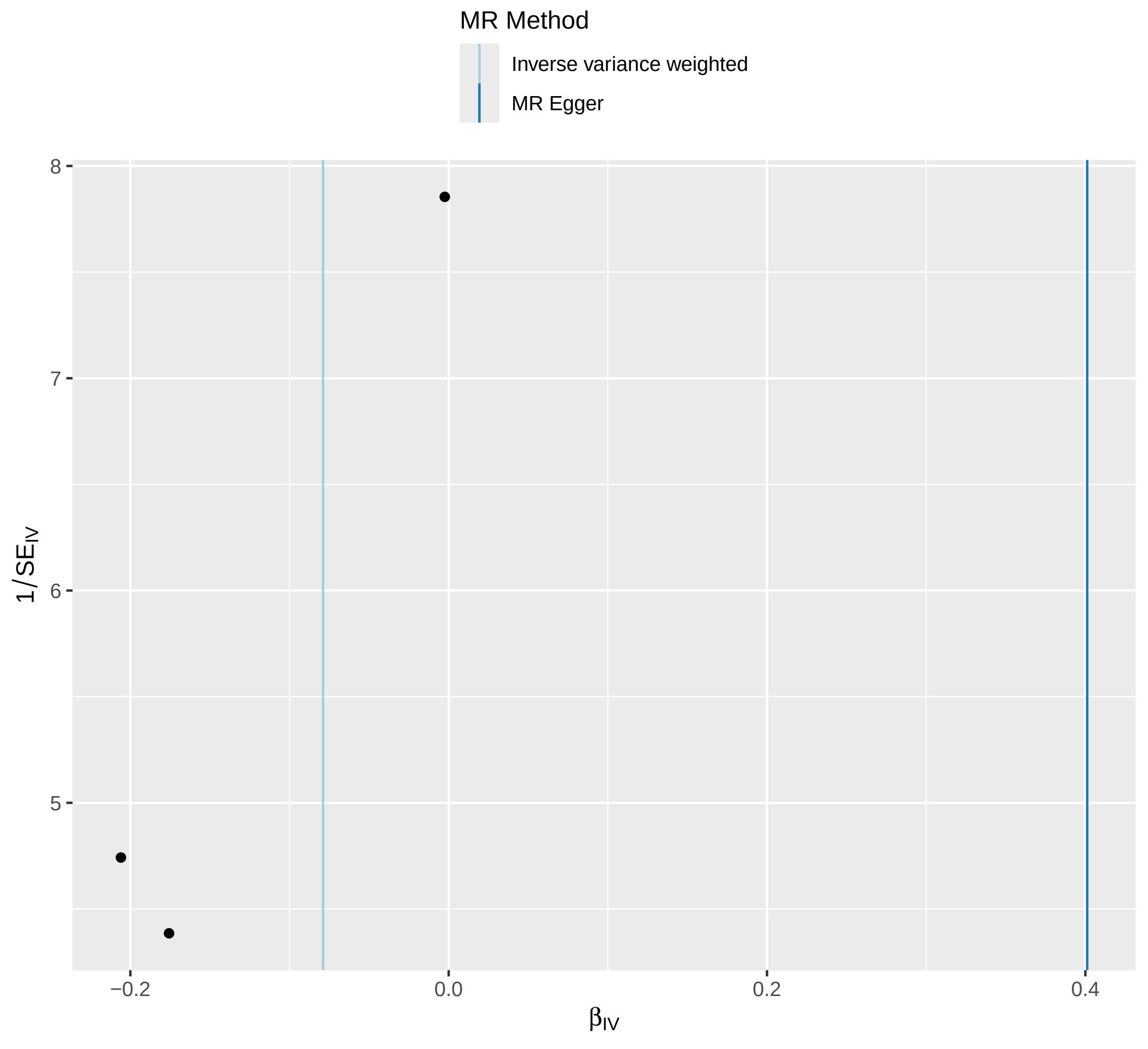

MR Method
Inverse variance weighted
MR Egger
8
7
6
5
1/SE_IV
−0.2
0.0
0.2
0.4
β_IV

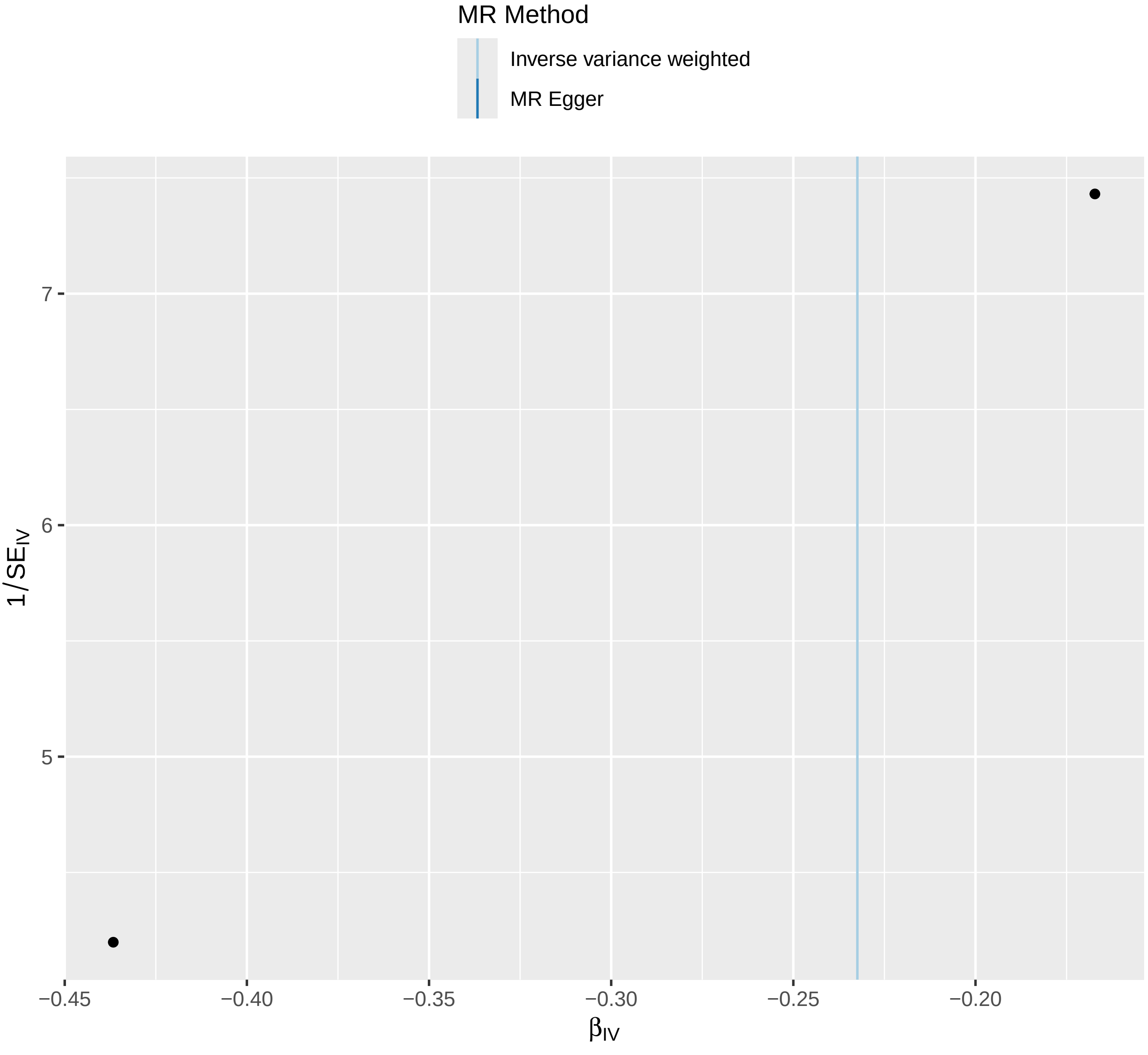
MR Method
Inverse variance weighted
MR Egger
7
6
5
$1/SE_{IV}$
−0.45
−0.40
−0.35
−0.30
−0.25
−0.20
$\beta_{IV}$

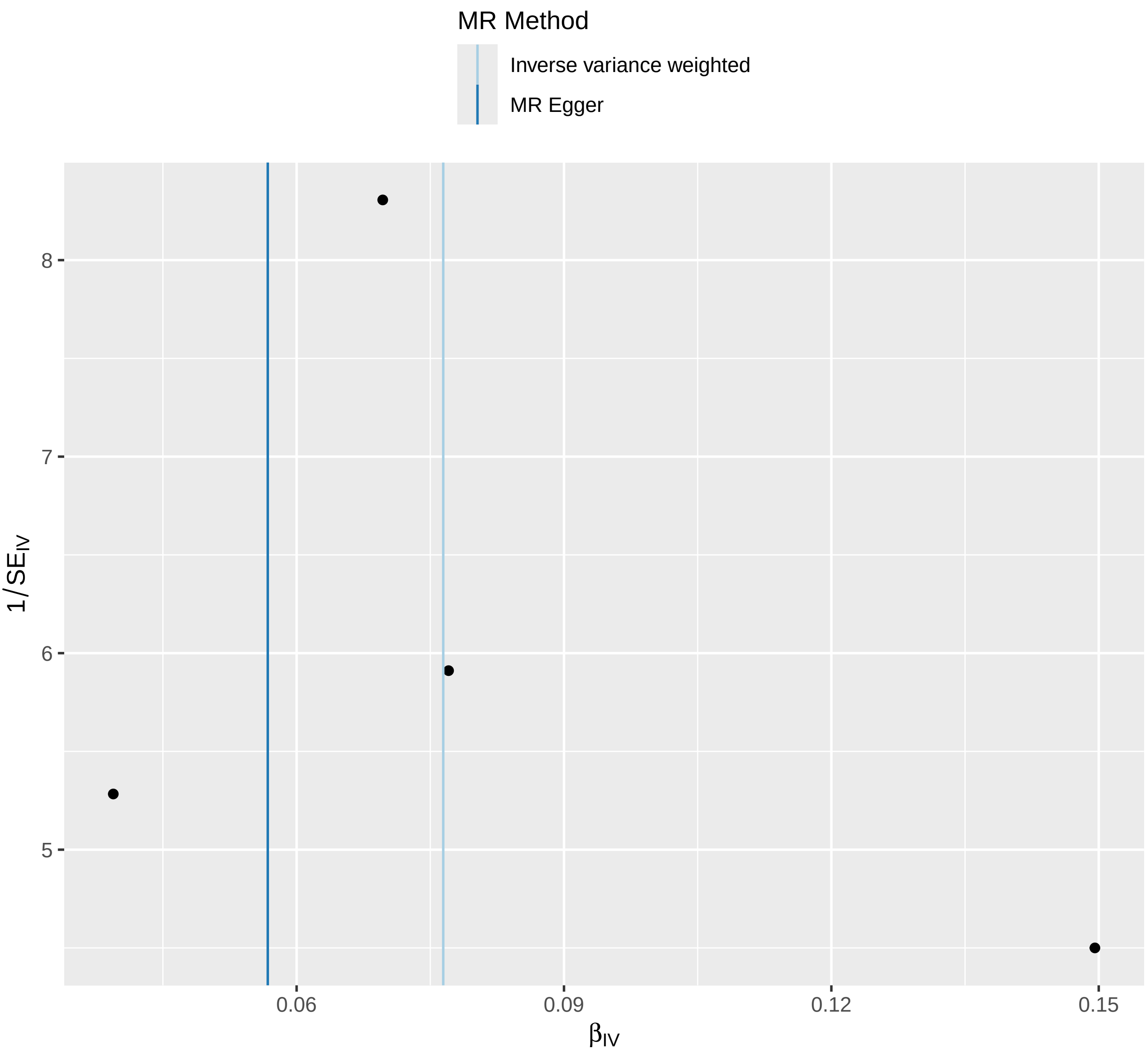

MR Method
Inverse variance weighted
MR Egger
8
7
6
5
$1/SE_{IV}$
0.06
0.09
0.12
0.15
$\beta_{IV}$

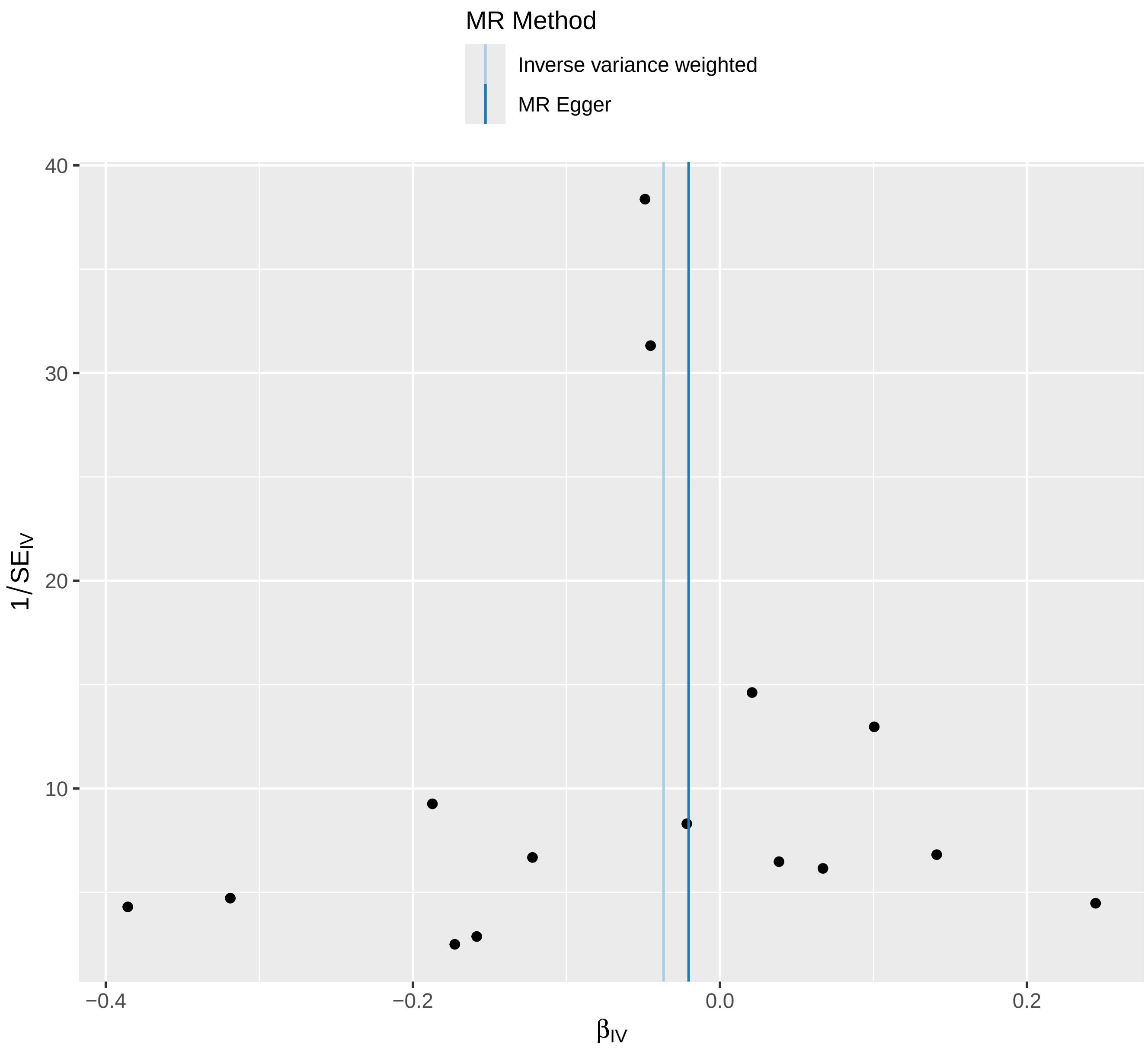

MR Method
Inverse variance weighted
MR Egger
40
30
20
10
1/SE$_{IV}$
−0.4
−0.2
0.0
0.2
β$_{IV}$

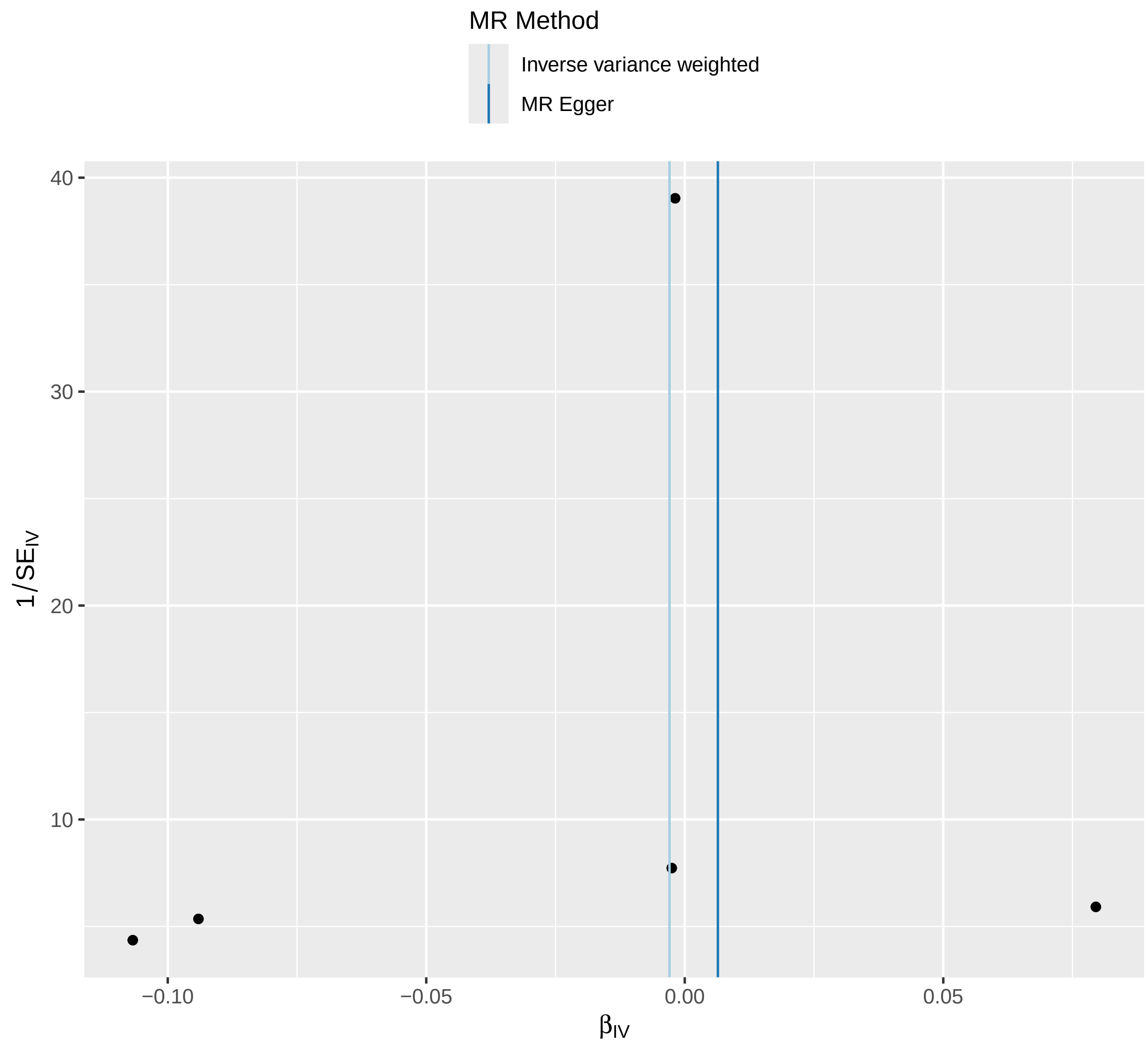
MR Method
Inverse variance weighted
MR Egger
40
30
20
10
−0.10
−0.05
0.00
0.05
$1/SE_{IV}$
$\beta_{IV}$

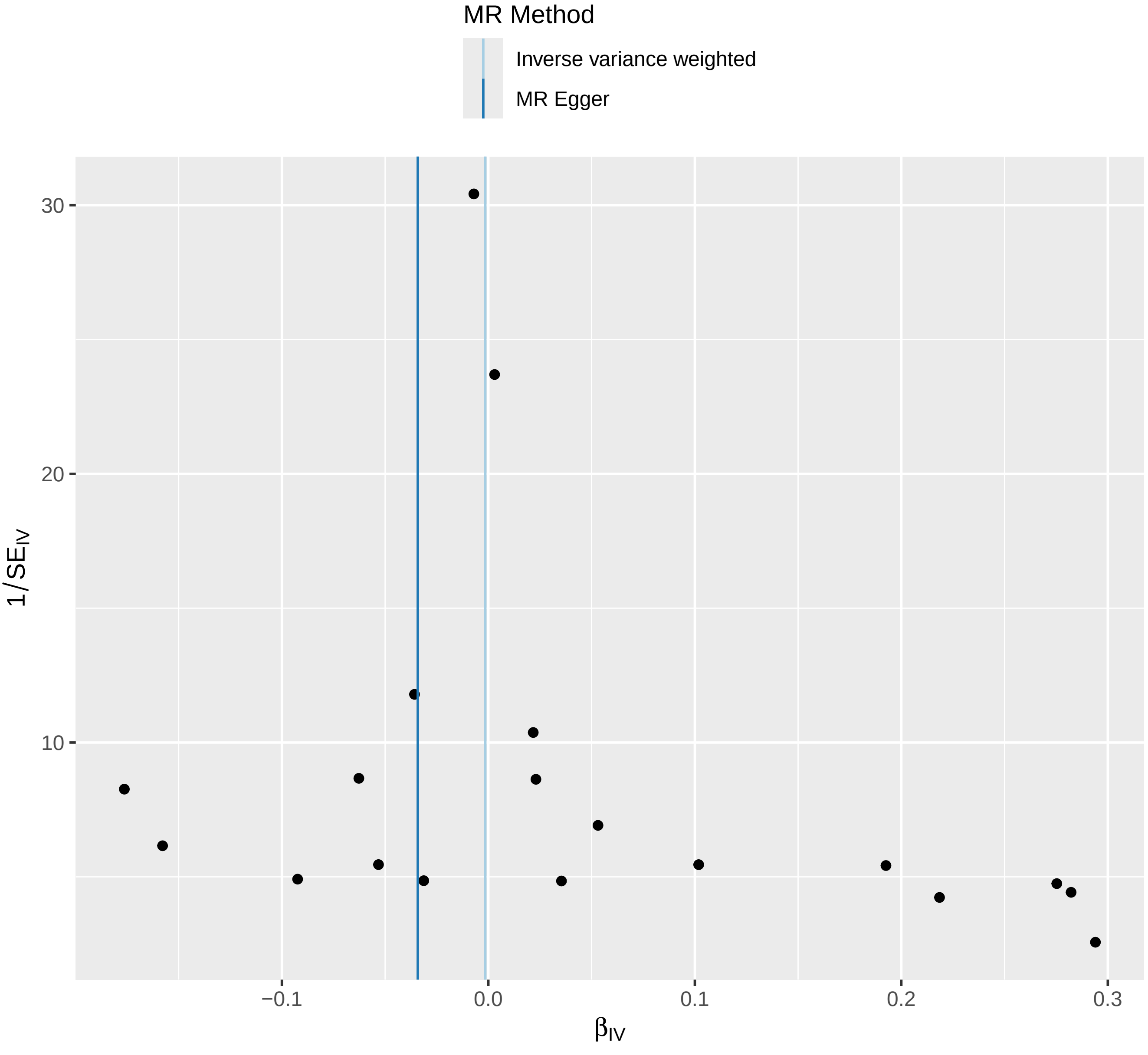

MR Method
Inverse variance weighted
MR Egger
30
20
10
1/SE_IV
−0.1
0.0
0.1
0.2
0.3
β_IV

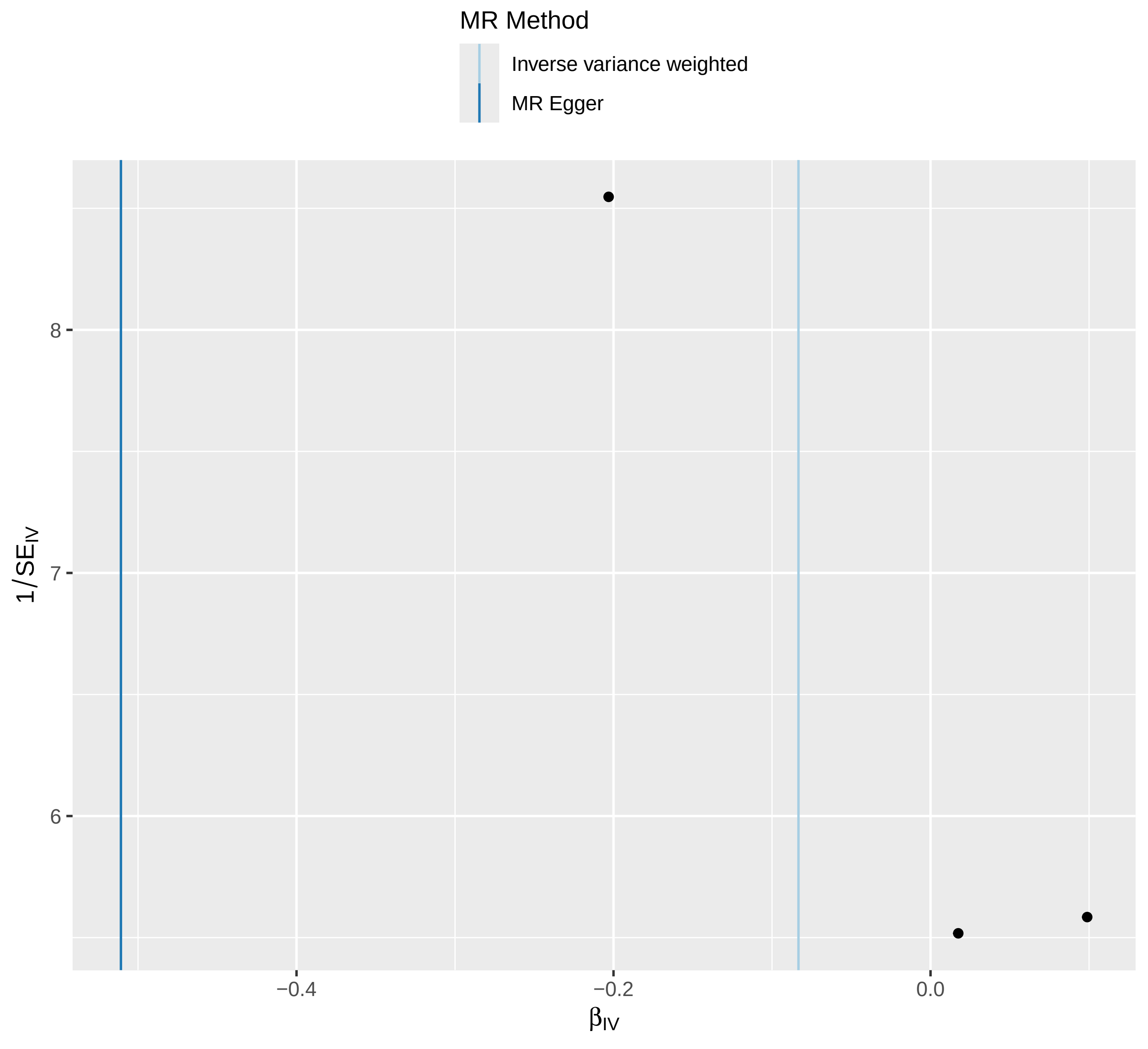

MR Method
Inverse variance weighted
MR Egger
1/SE$_{IV}$
8
7
6
−0.4
−0.2
0.0
β$_{IV}$

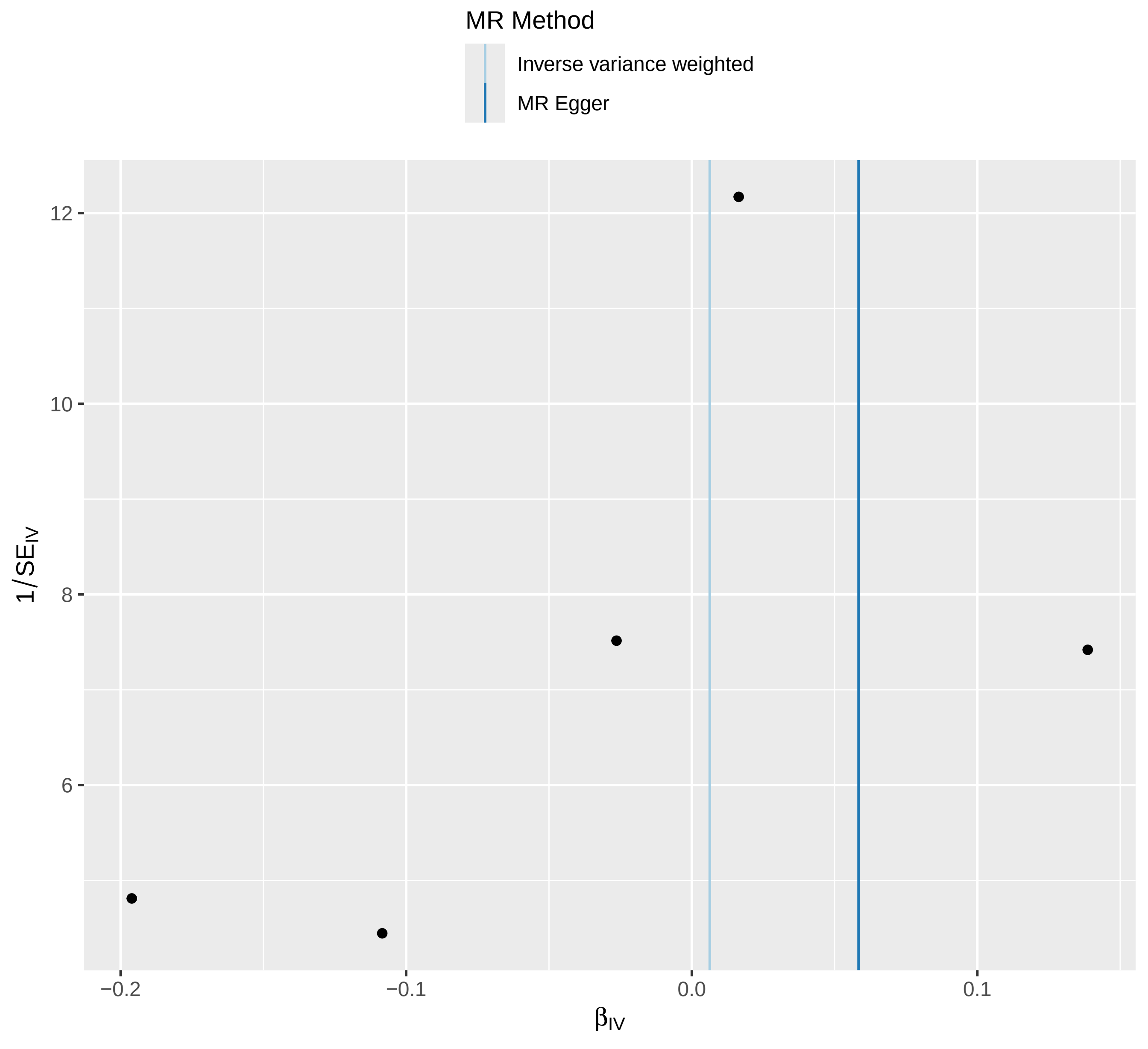
MR Method
Inverse variance weighted
MR Egger
12
10
8
6
1/SE_IV
−0.2
−0.1
0.0
0.1
β_IV

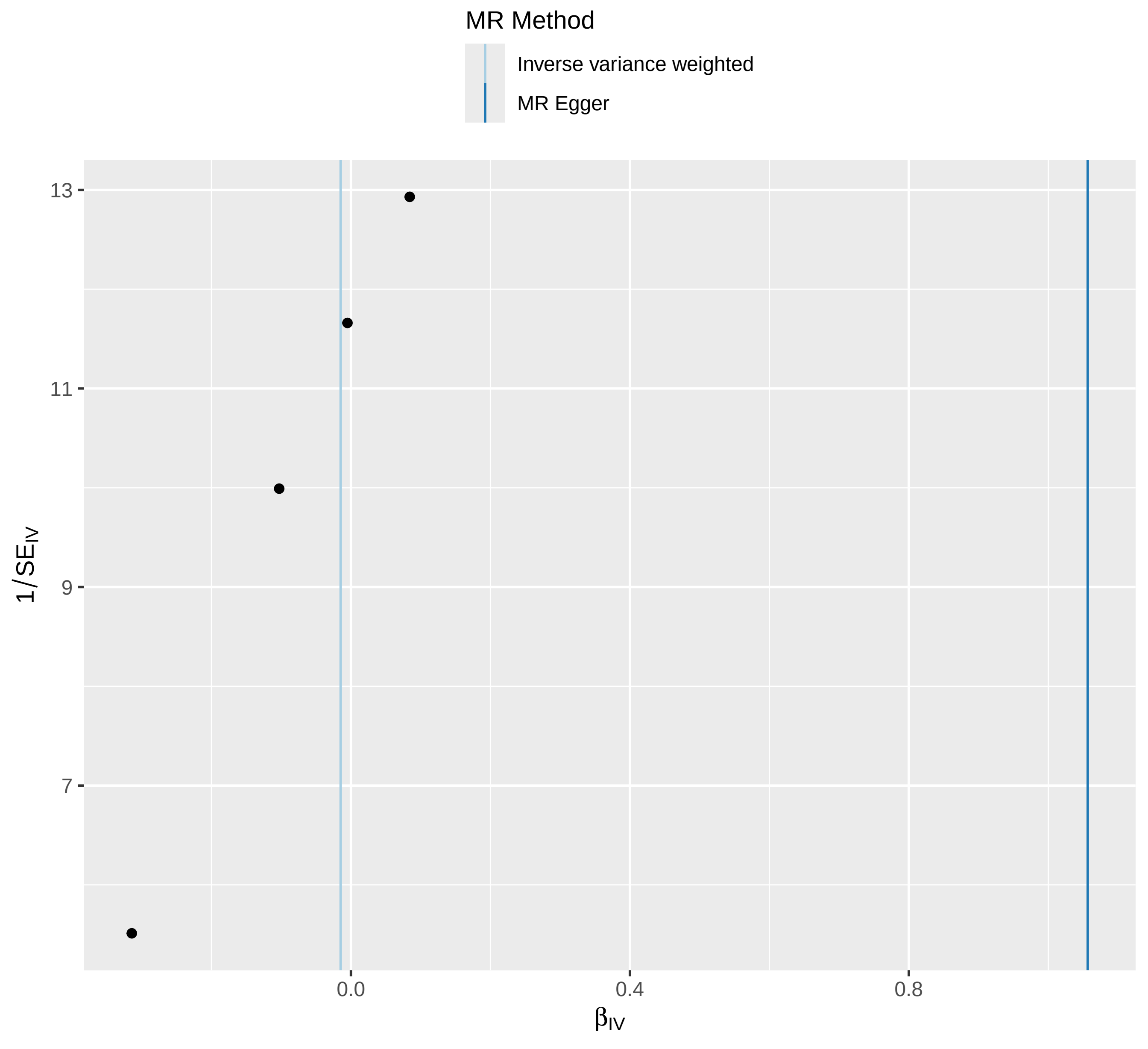
MR Method
Inverse variance weighted
MR Egger
13
11
9
7
1/SE_IV
0.0
0.4
0.8
β_IV

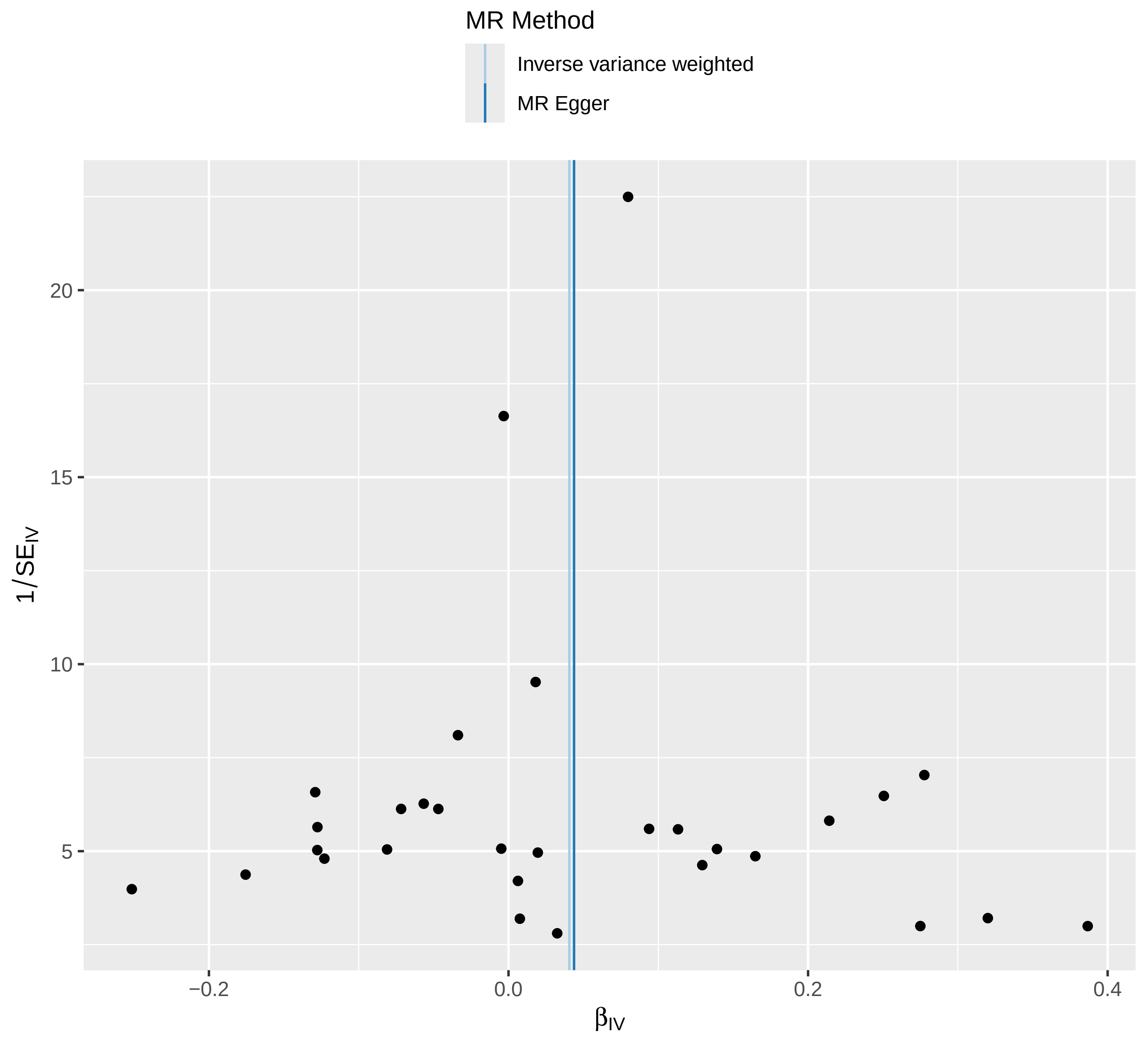
MR Method
Inverse variance weighted
MR Egger
20
15
10
5
1/SE_IV
−0.2
0.0
0.2
0.4
β_IV

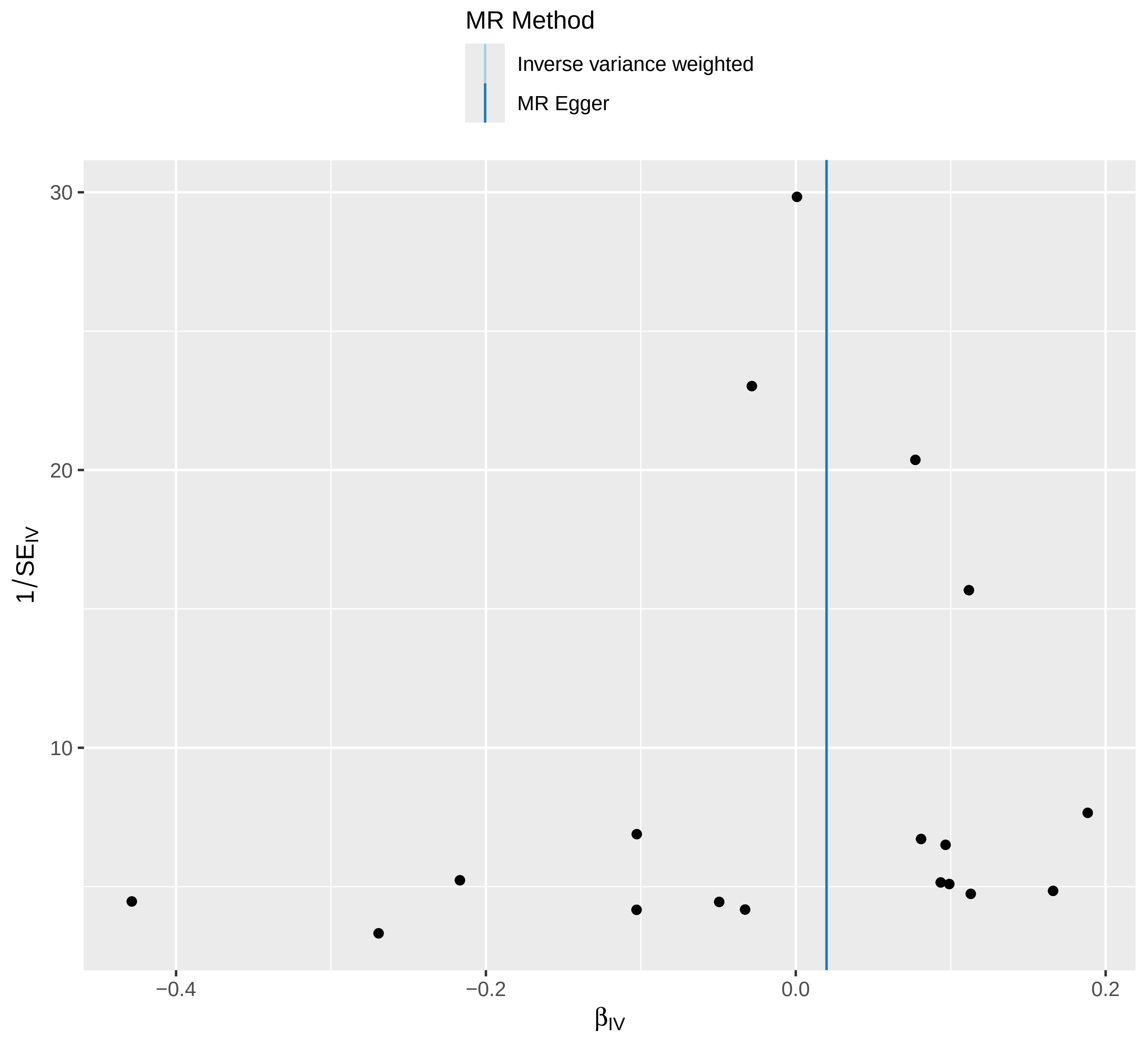
MR Method
Inverse variance weighted
MR Egger
30
20
10
1/SE$_{IV}$
−0.4
−0.2
0.0
0.2
β$_{IV}$

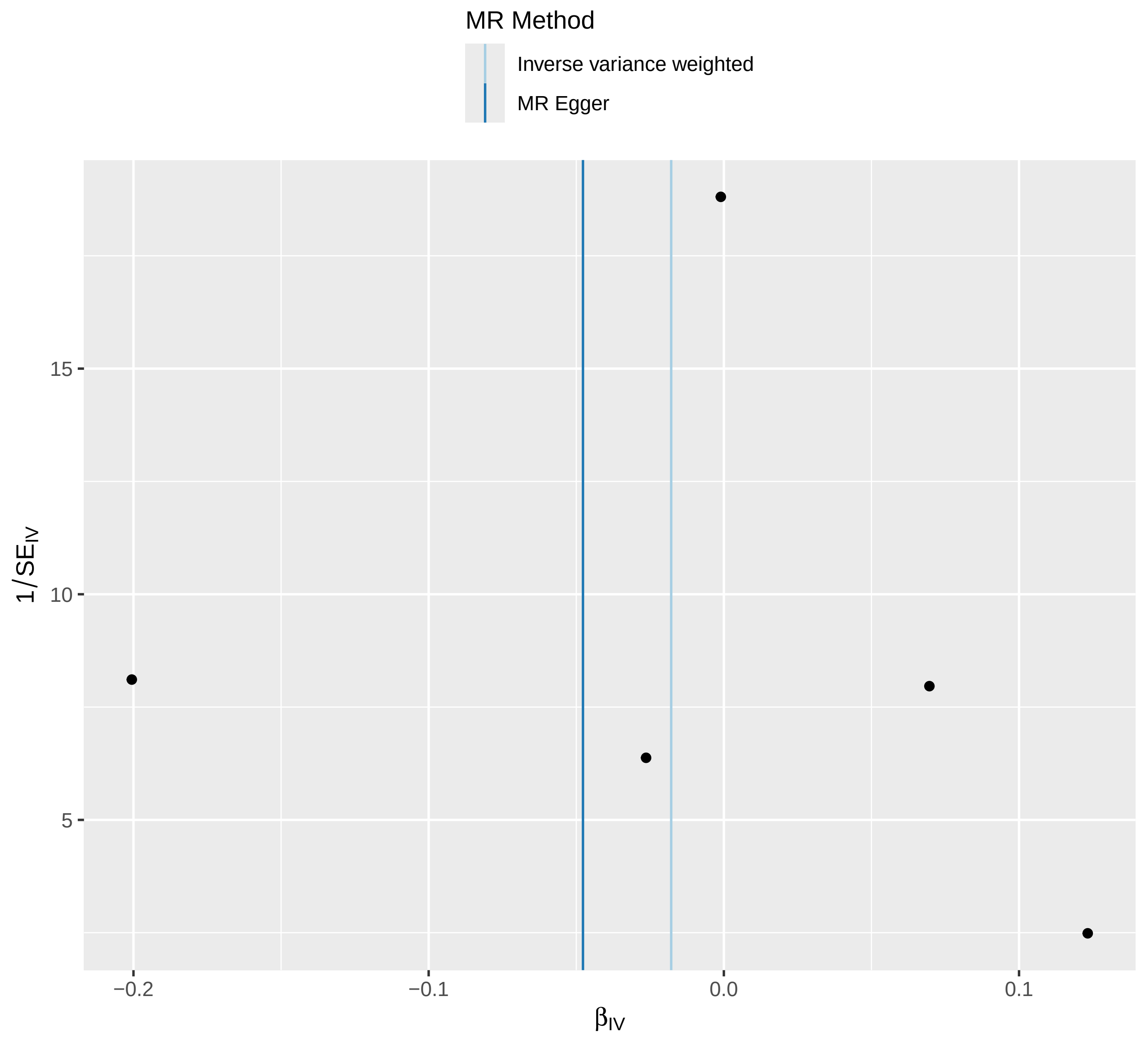

MR Method
Inverse variance weighted
MR Egger
1/SE$_{IV}$
15
10
5
−0.2
−0.1
0.0
0.1
β$_{IV}$

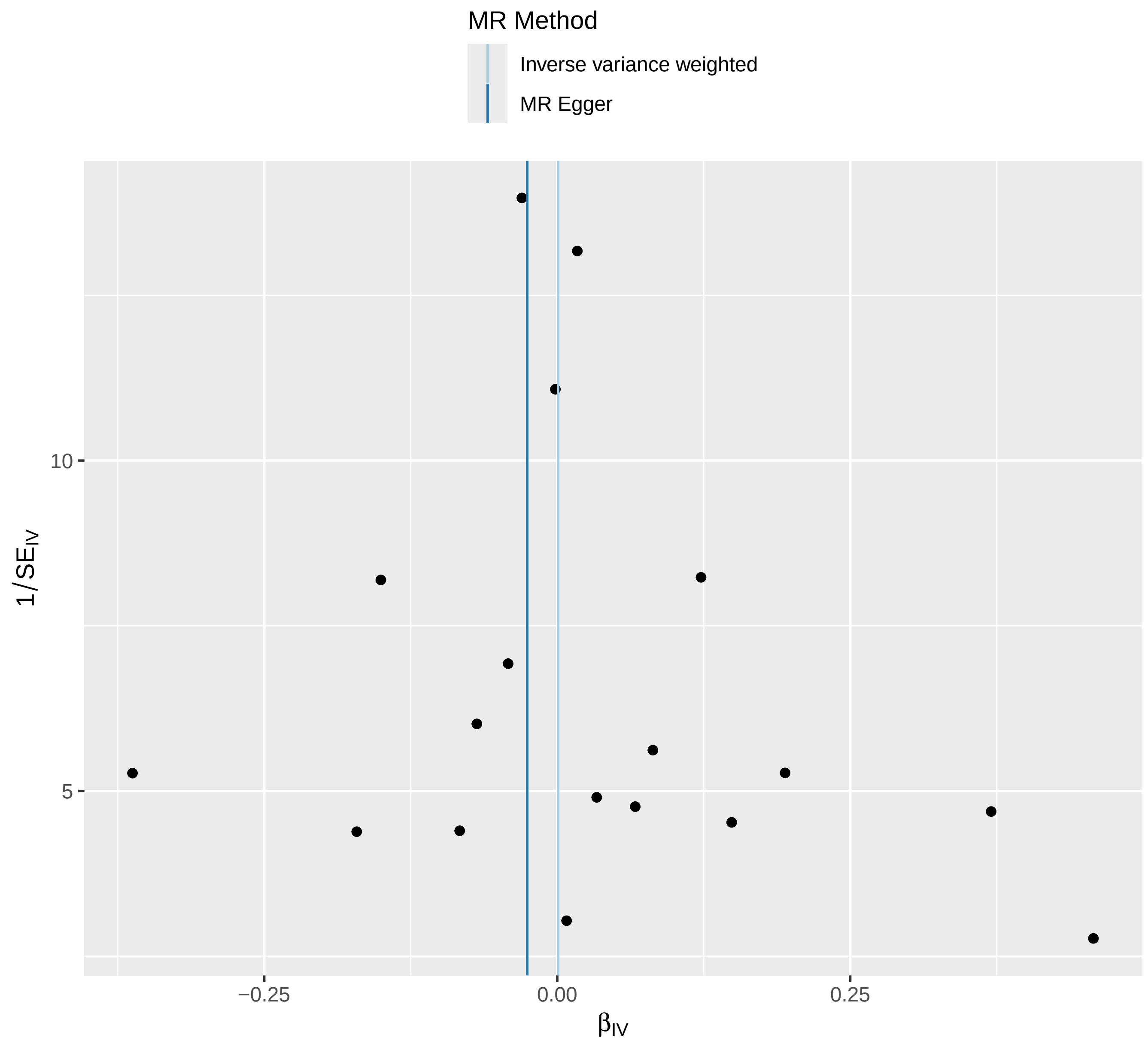

MR Method
Inverse variance weighted
MR Egger
10
5
$1/SE_{IV}$
−0.25
0.00
0.25
$\beta_{IV}$

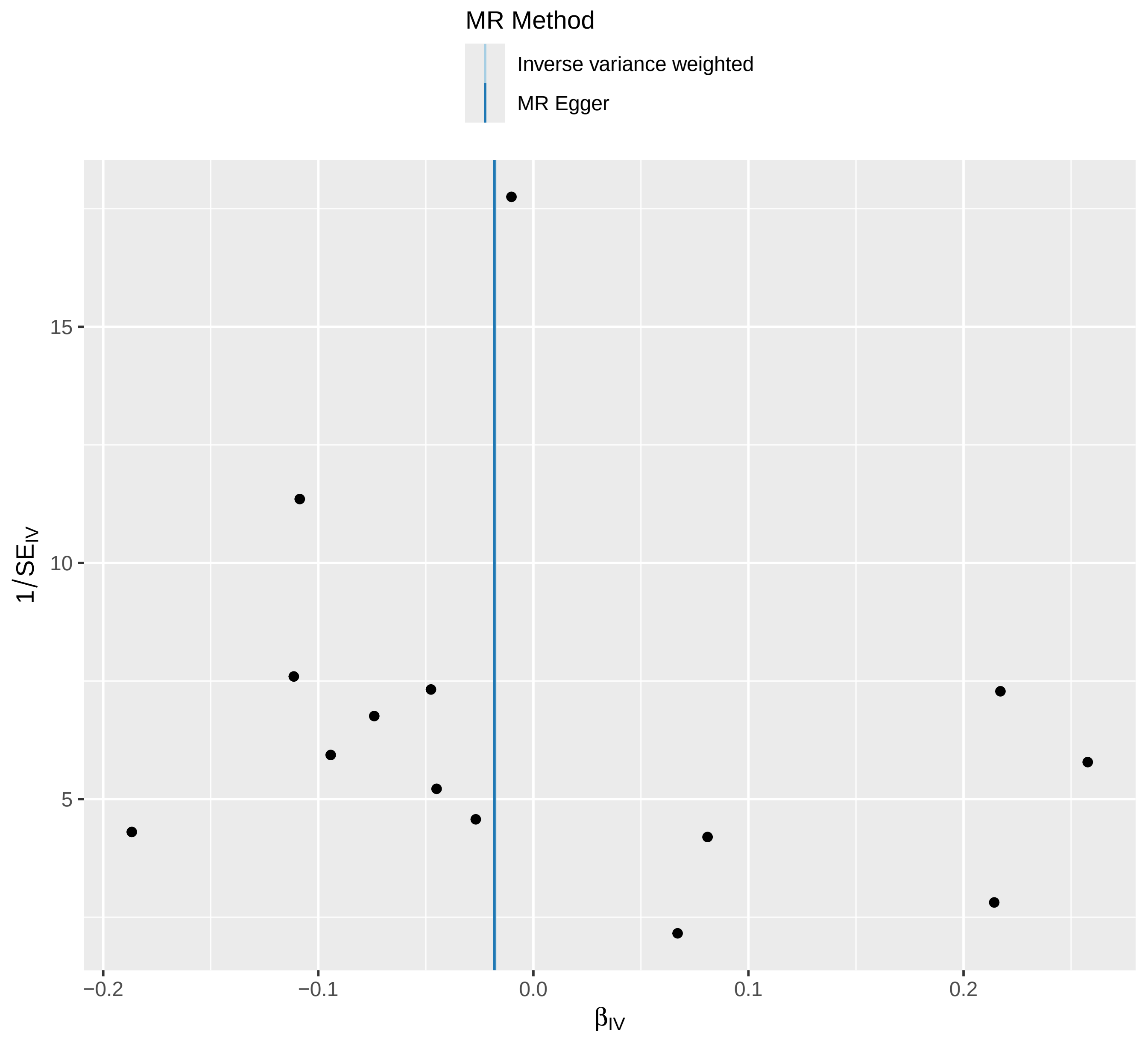
MR Method
Inverse variance weighted
MR Egger
1/SE_IV
15
10
5
−0.2
−0.1
0.0
0.1
0.2
β_IV

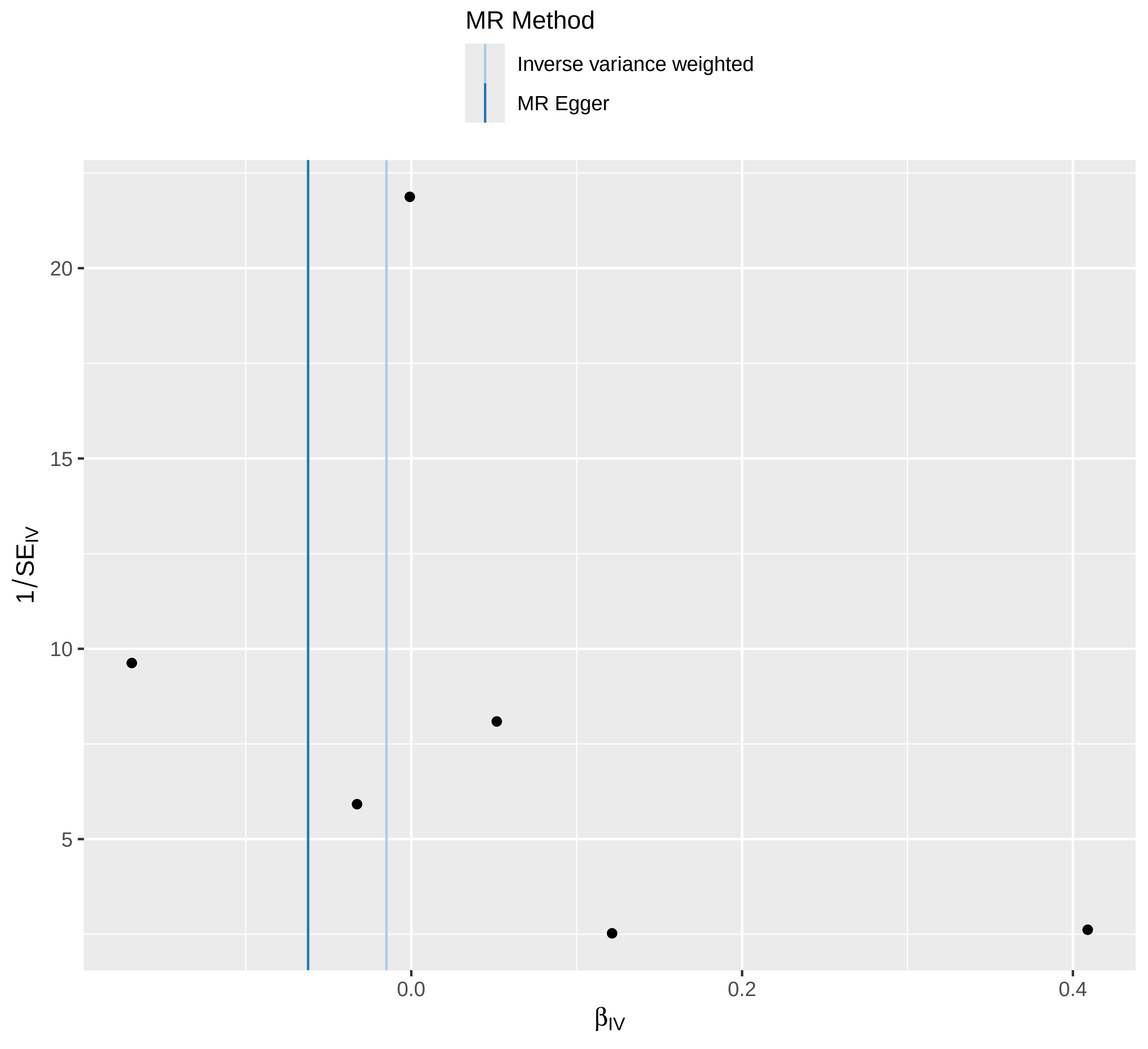

MR Method
Inverse variance weighted
MR Egger
20
15
10
5
1/SE_IV
0.0
0.2
0.4
β_IV

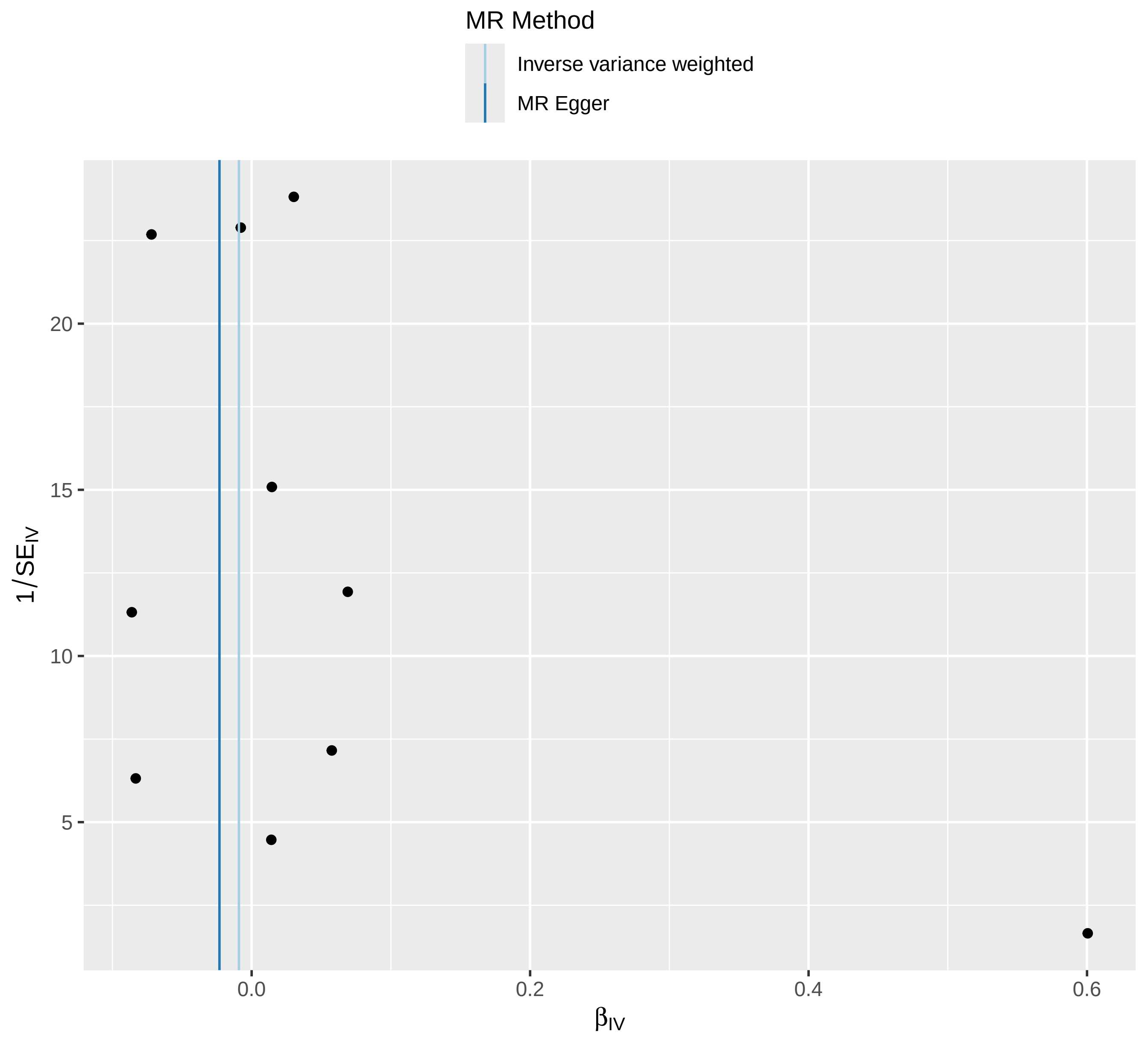
MR Method
Inverse variance weighted
MR Egger
20
15
10
5
$1/SE_{IV}$
0.0
0.2
0.4
0.6
$\beta_{IV}$

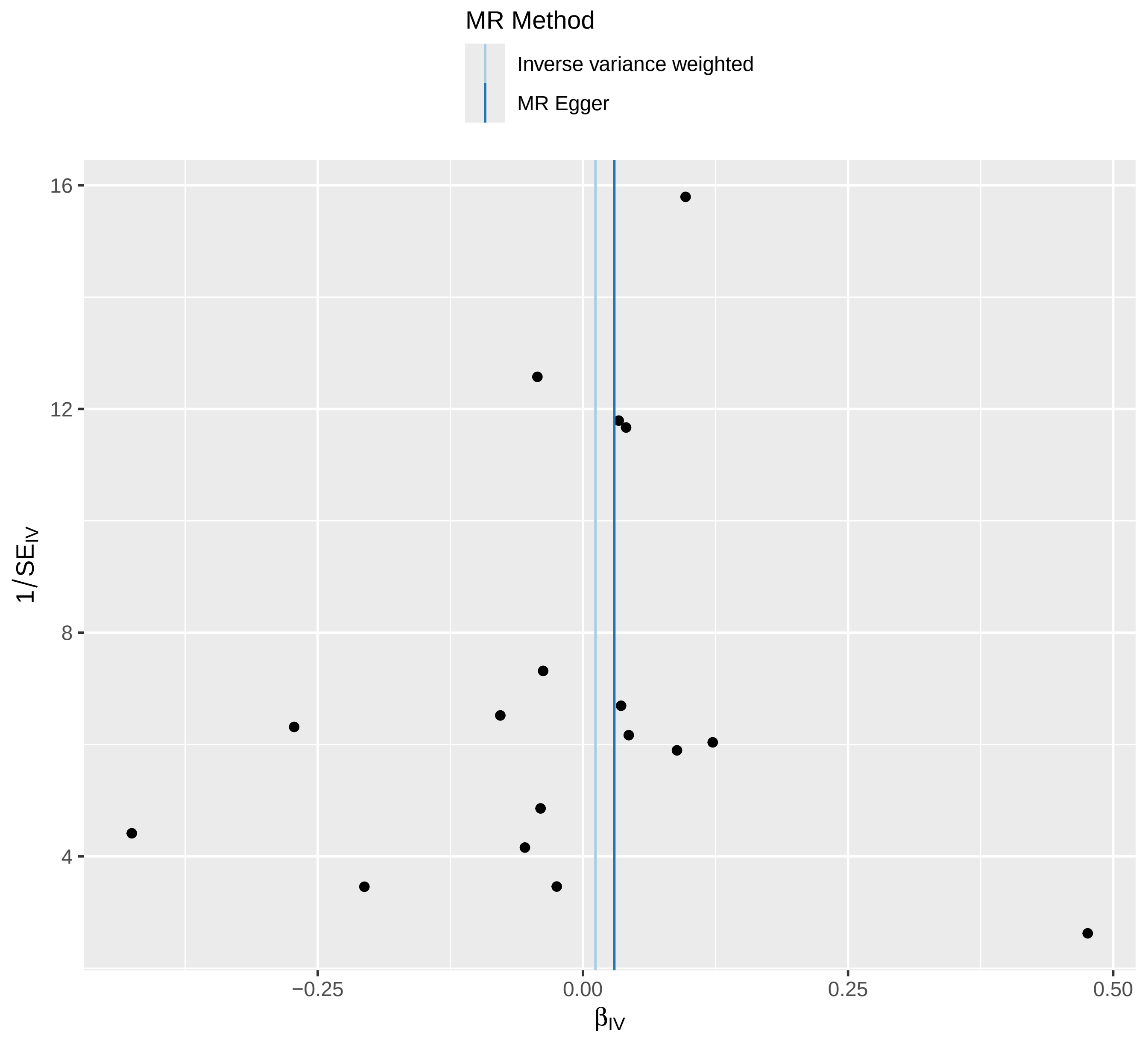

MR Method
Inverse variance weighted
MR Egger
16
12
8
4
1/SE_IV
−0.25
0.00
0.25
0.50
β_IV

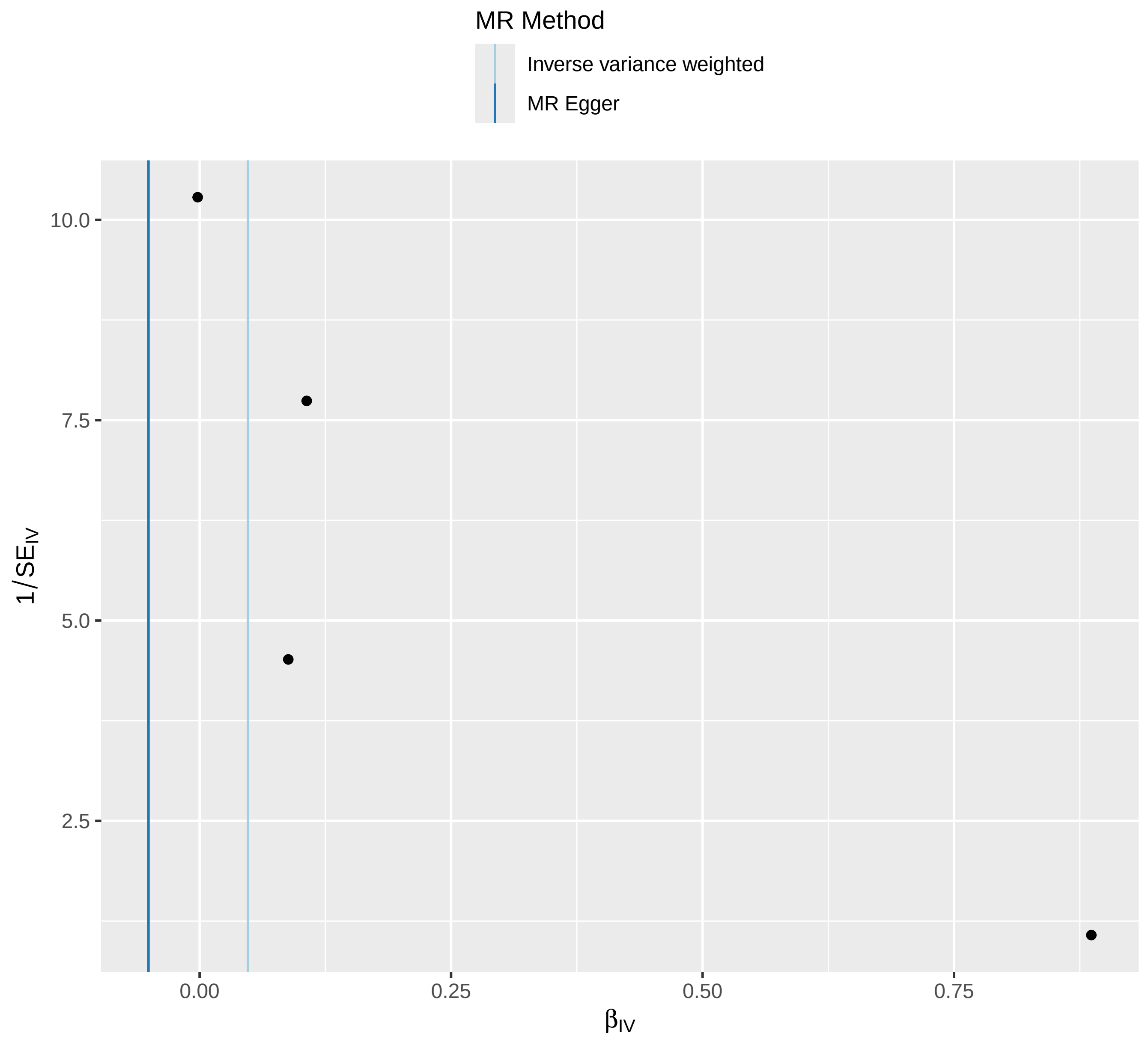
MR Method
Inverse variance weighted
MR Egger
10.0
7.5
5.0
2.5
$1/SE_{IV}$
0.00
0.25
0.50
0.75
$\beta_{IV}$

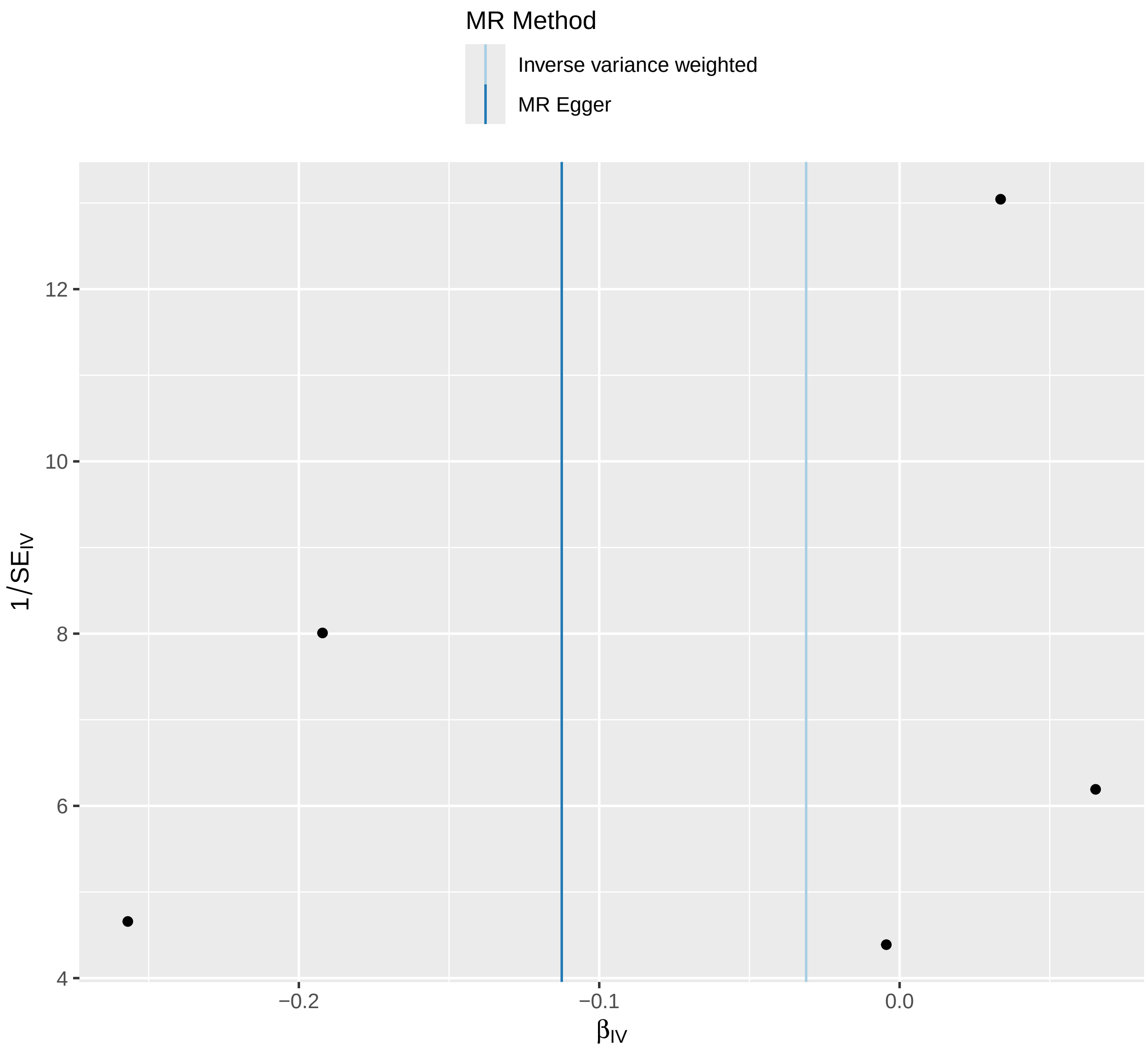
MR Method
Inverse variance weighted
MR Egger
1/SE_IV
12
10
8
6
4
−0.2
−0.1
0.0
β_IV

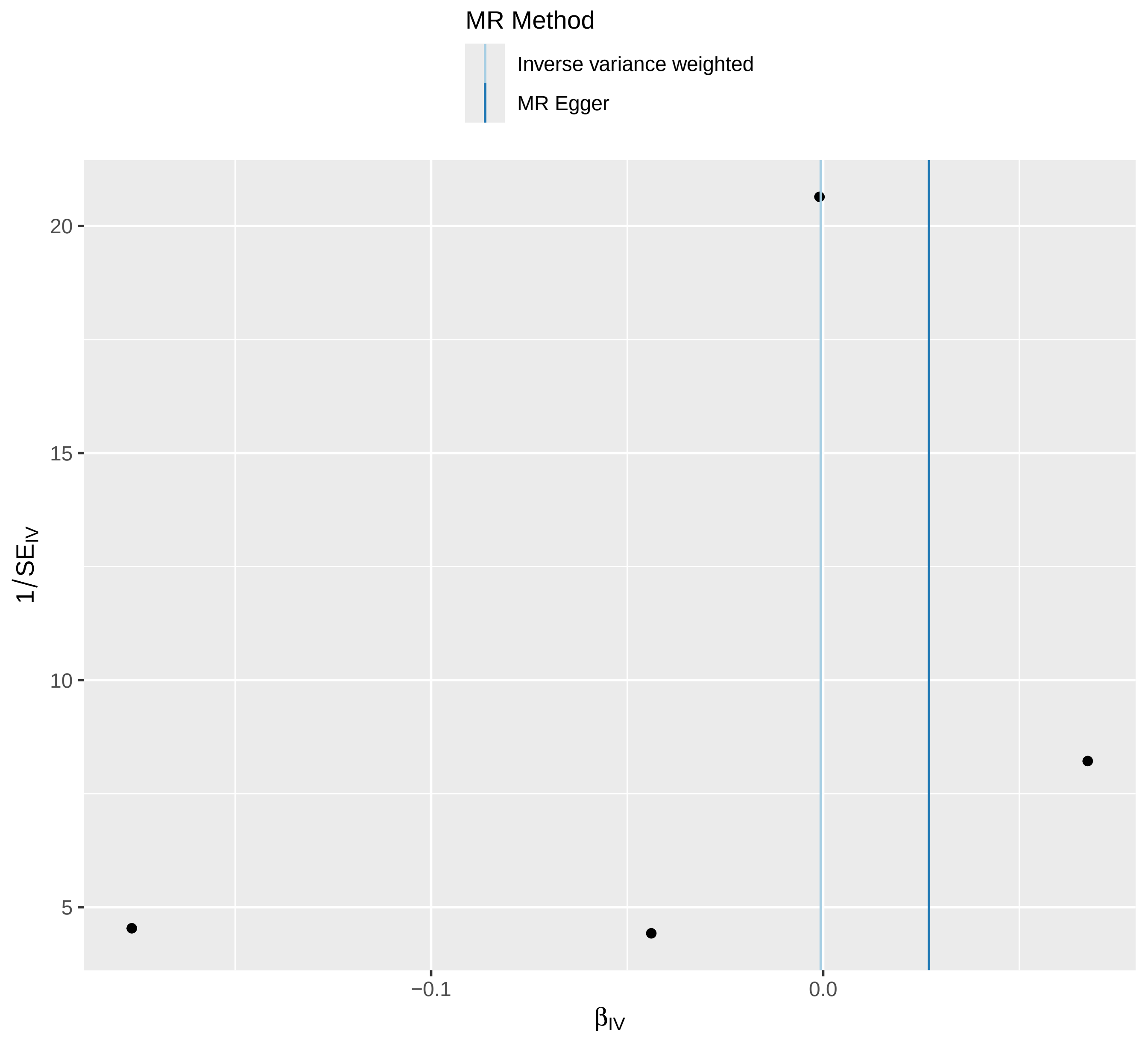

MR Method
Inverse variance weighted
MR Egger
20
15
10
5
1/SE_IV
−0.1
0.0
β_IV

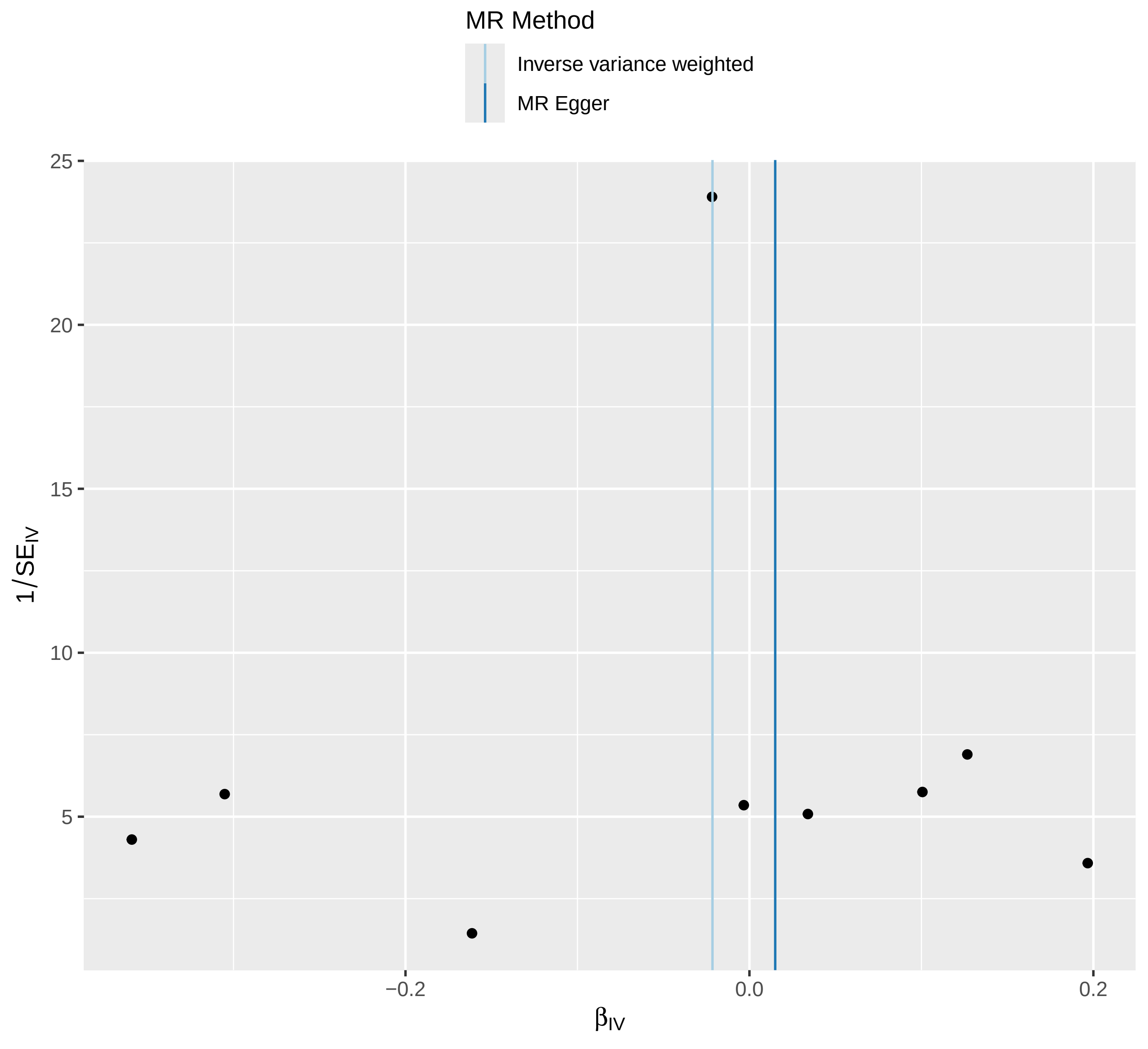
MR Method
Inverse variance weighted
MR Egger
25
20
15
10
5
1/SE$_{IV}$
−0.2
0.0
0.2
β$_{IV}$

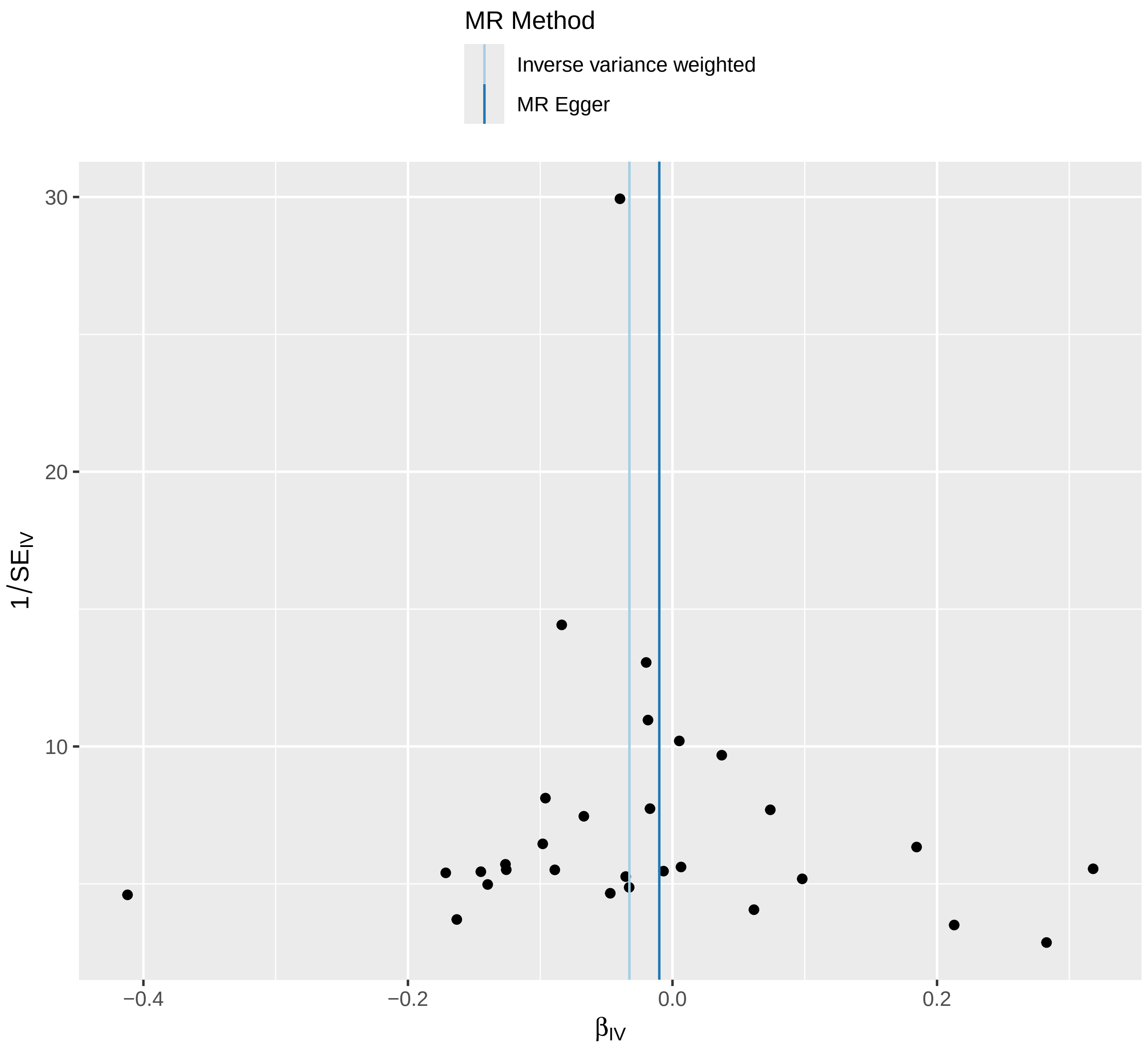
MR Method
Inverse variance weighted
MR Egger
30
20
10
1/SE$_{IV}$
−0.4
−0.2
0.0
0.2
β$_{IV}$

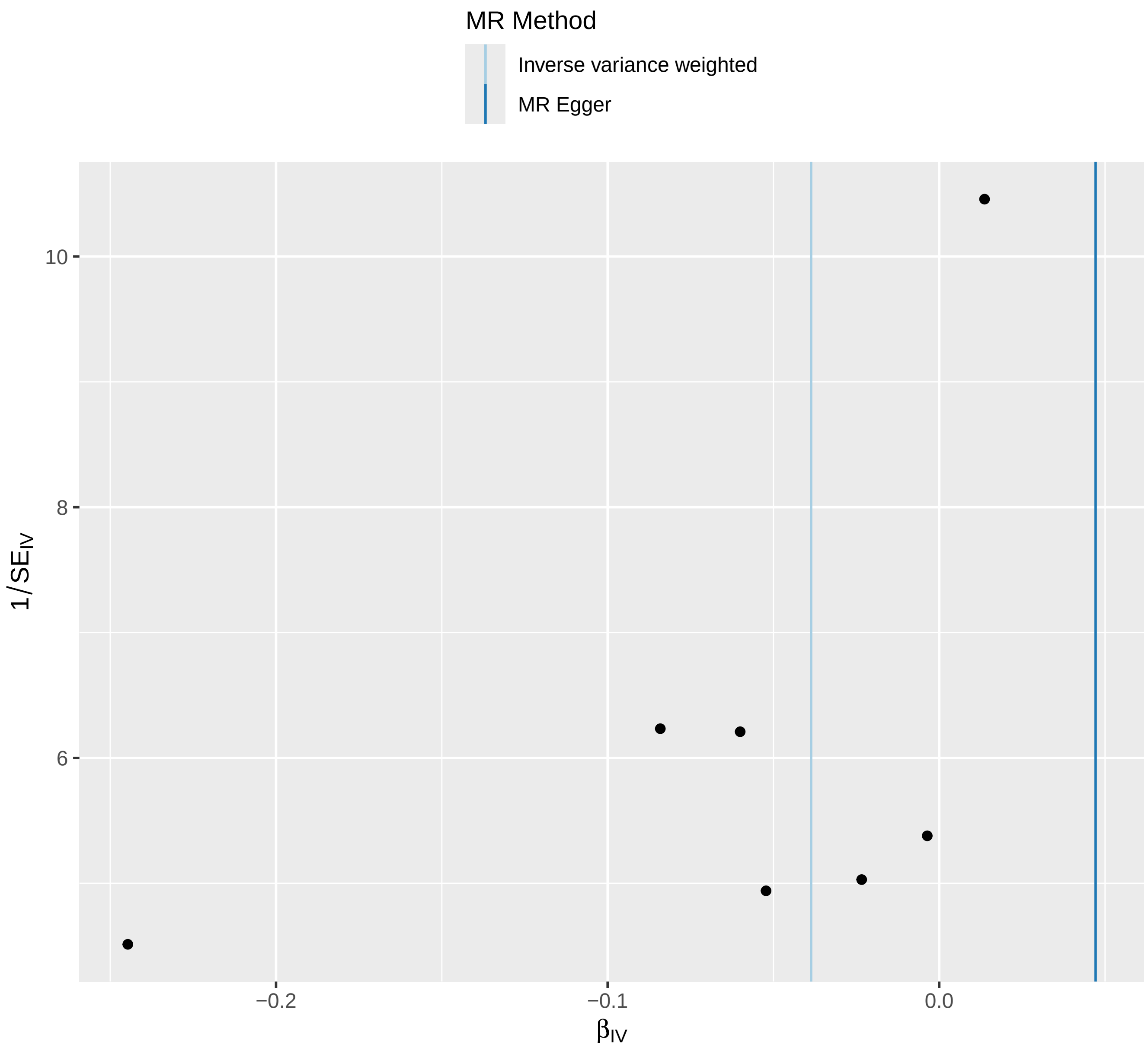

MR Method
Inverse variance weighted
MR Egger
10
8
6
1/SE$_{IV}$
−0.2
−0.1
0.0
β$_{IV}$

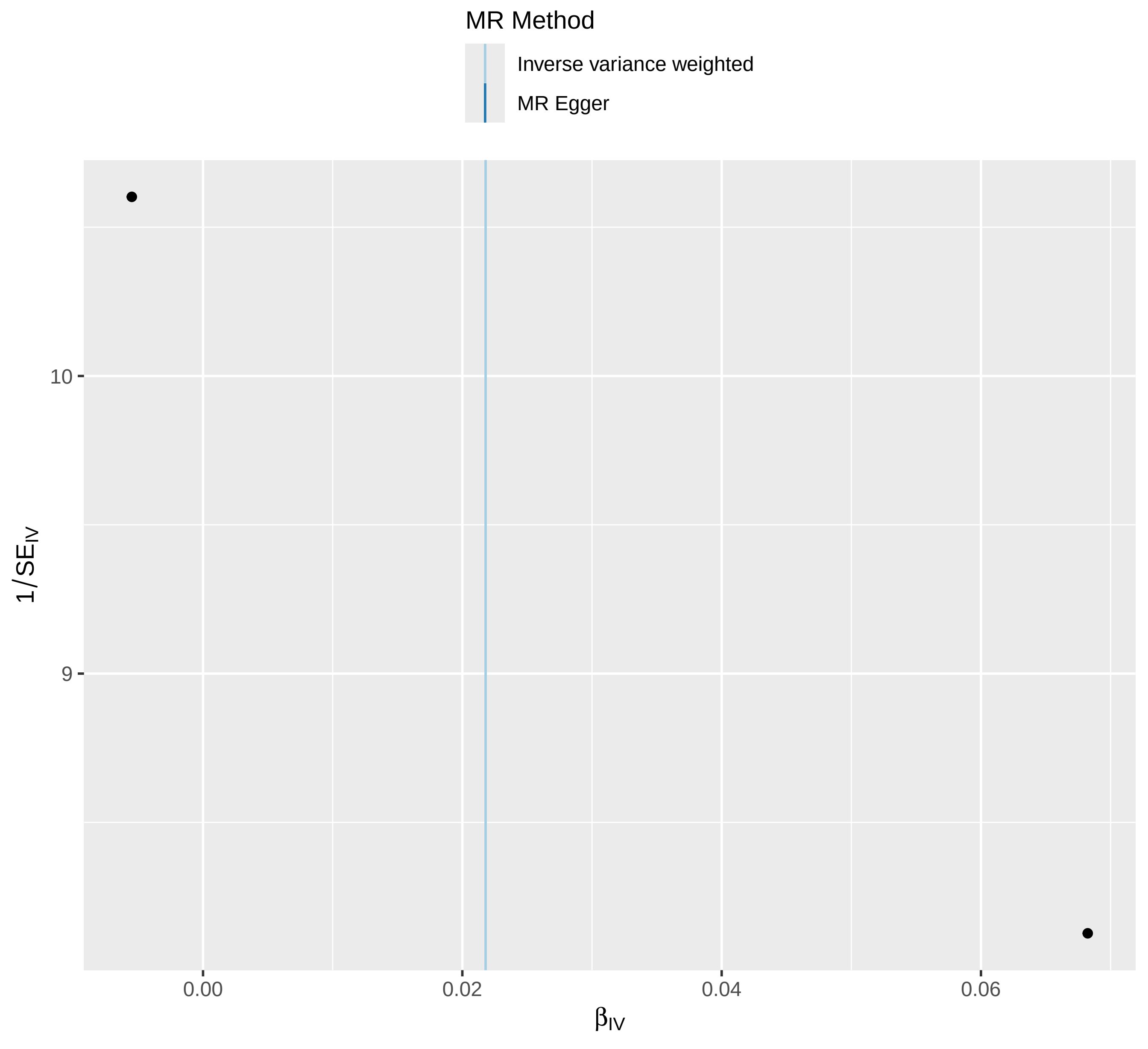
MR Method
Inverse variance weighted
MR Egger
10
9
1/SE_IV
0.00
0.02
0.04
0.06
β_IV

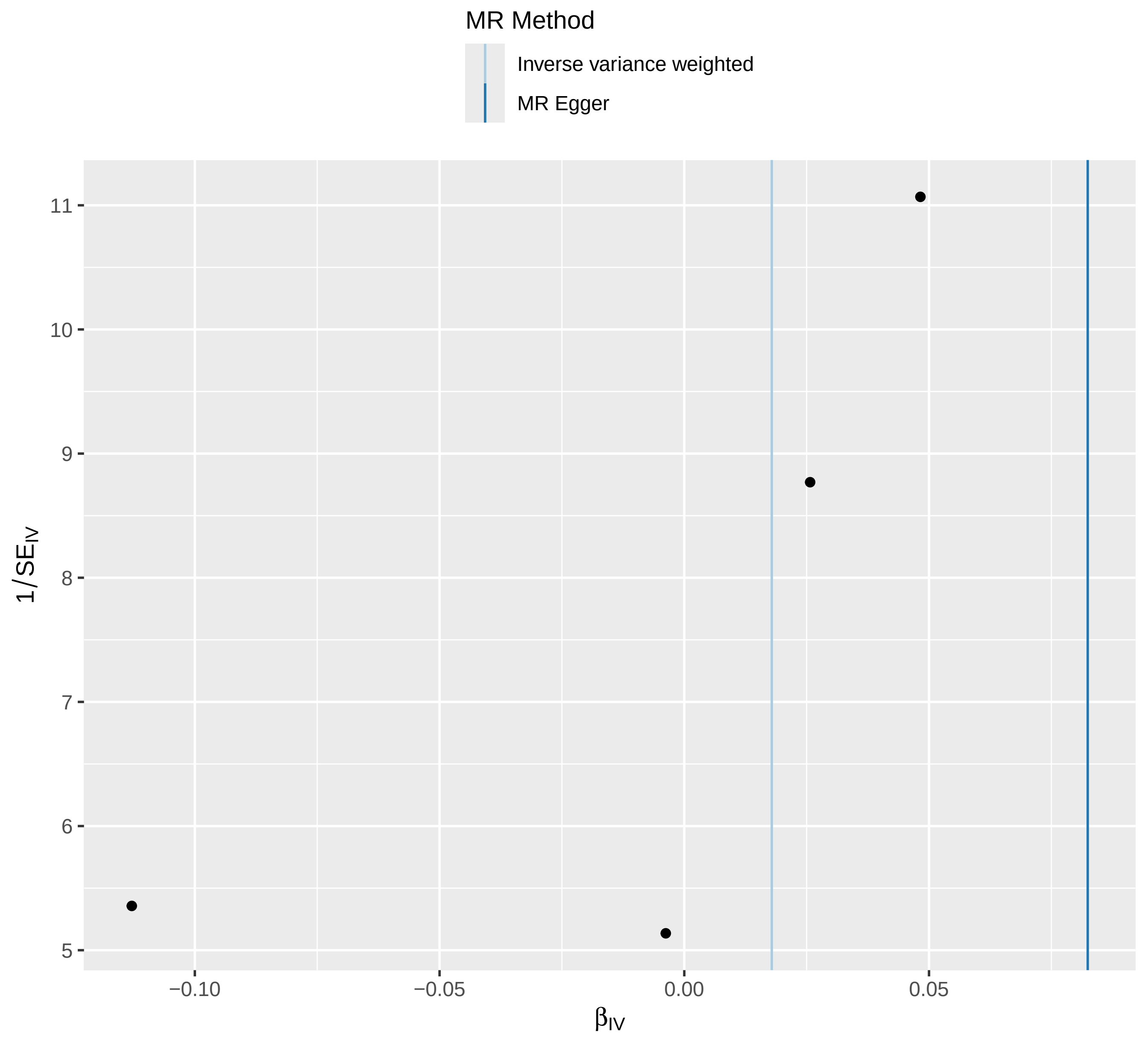
MR Method
Inverse variance weighted
MR Egger
11
10
9
8
7
6
5
$1/SE_{IV}$
−0.10
−0.05
0.00
0.05
$\beta_{IV}$

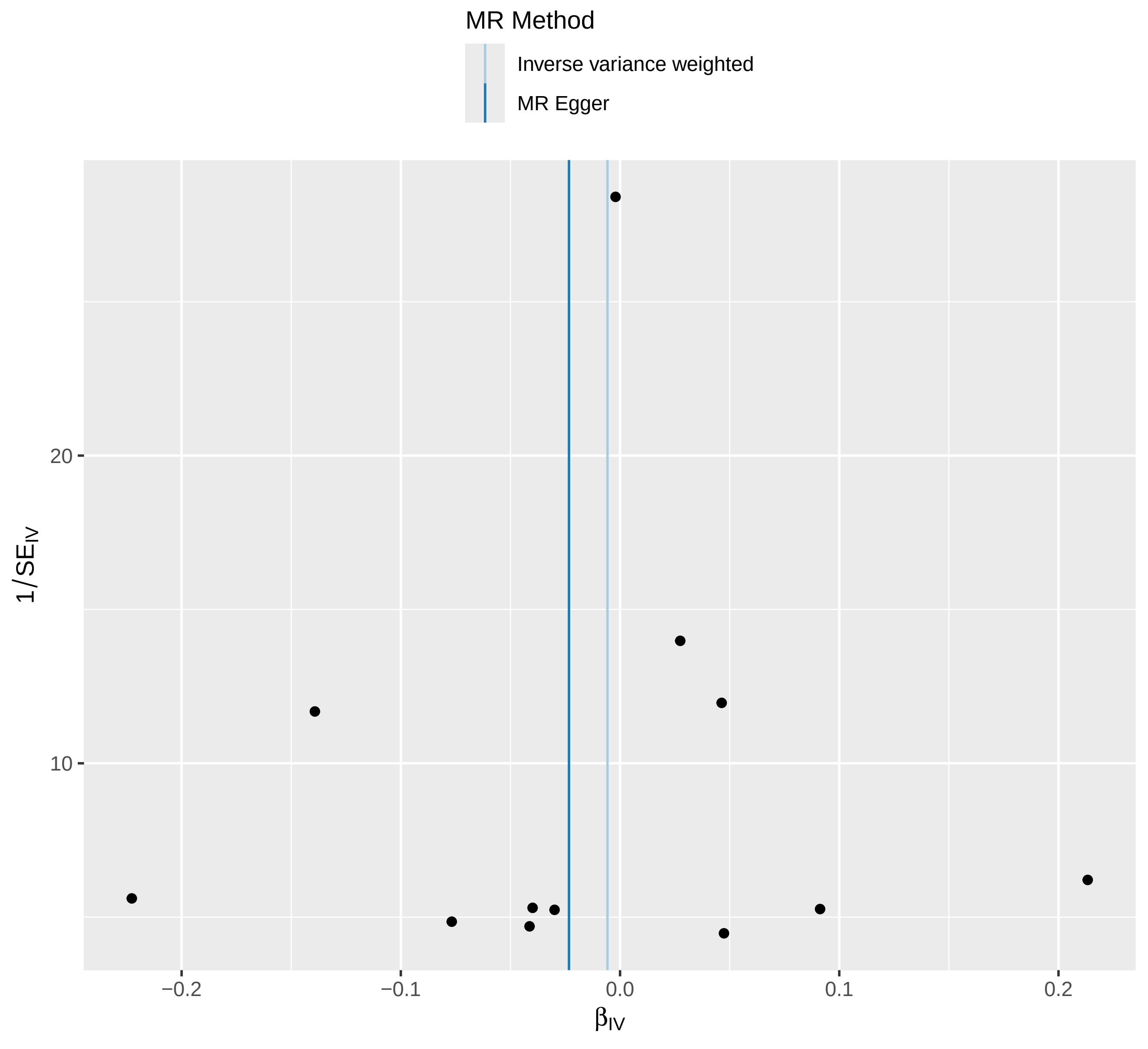
MR Method
Inverse variance weighted
MR Egger
20
10
1/SE_IV
−0.2
−0.1
0.0
0.1
0.2
β_IV

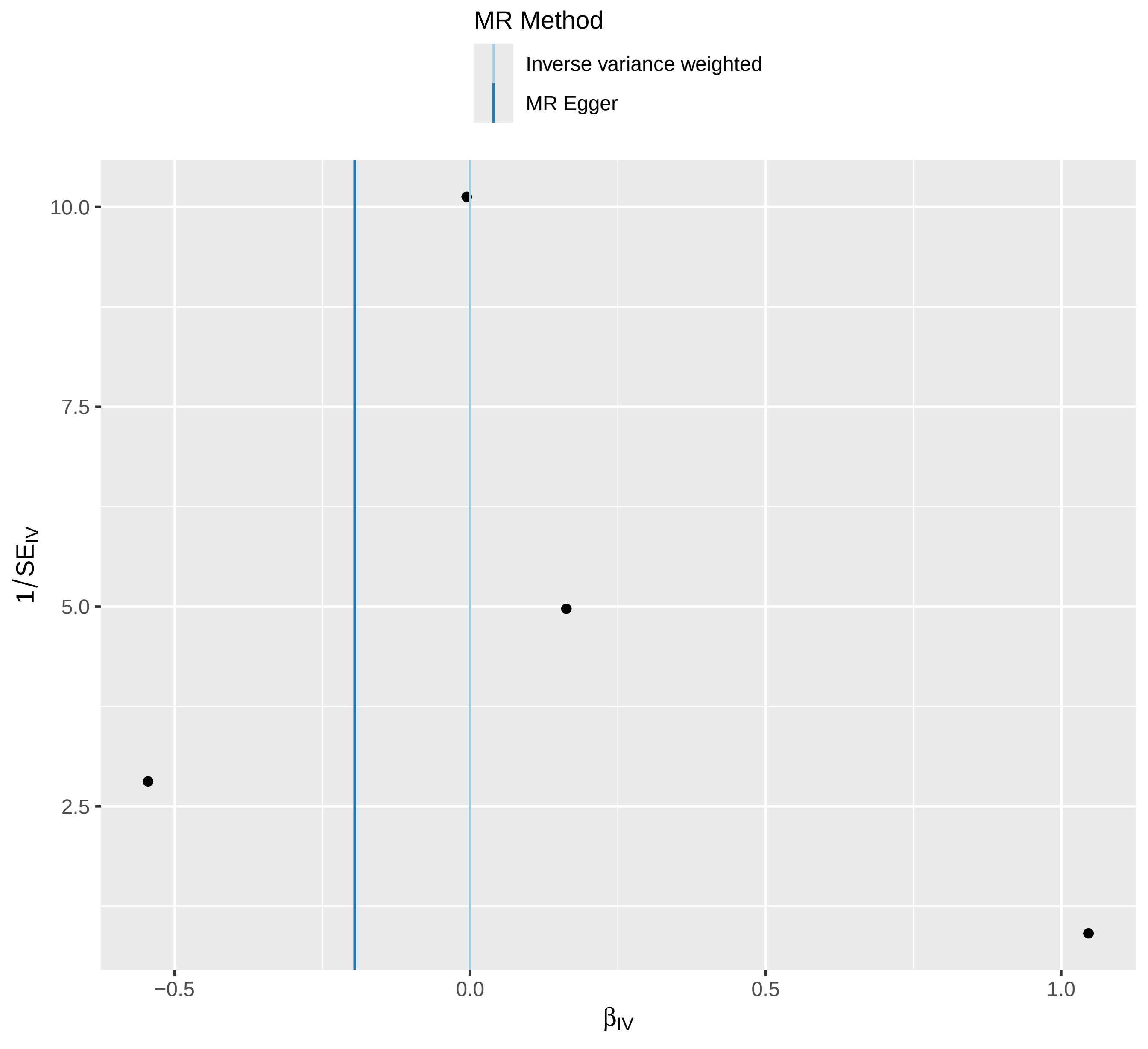

MR Method
Inverse variance weighted
MR Egger
10.0
7.5
5.0
2.5
1/SE$_{IV}$
−0.5
0.0
0.5
1.0
β$_{IV}$

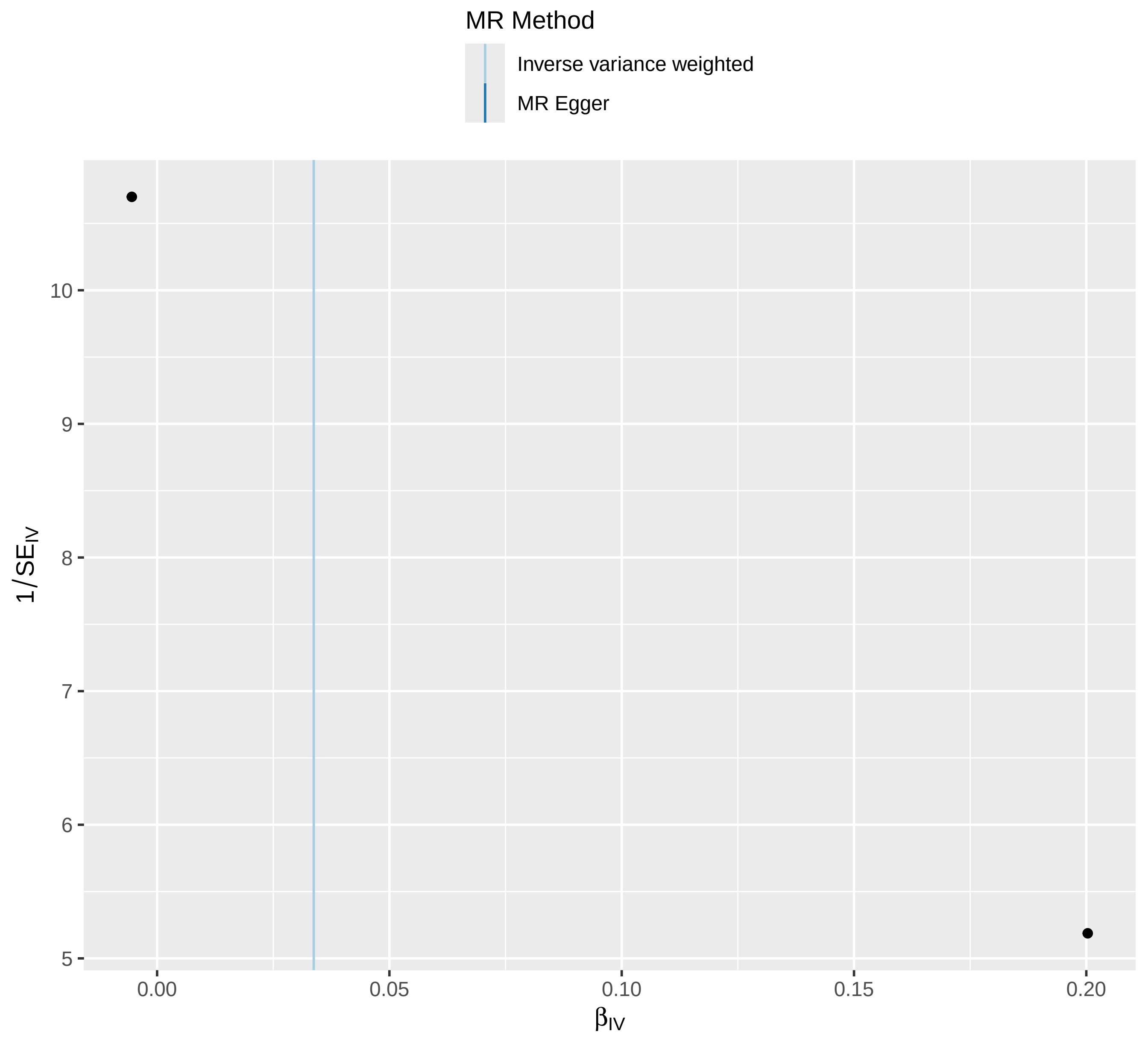

MR Method
Inverse variance weighted
MR Egger
10
9
8
7
6
5
1/SE$_{IV}$
0.00
0.05
0.10
0.15
0.20
β$_{IV}$

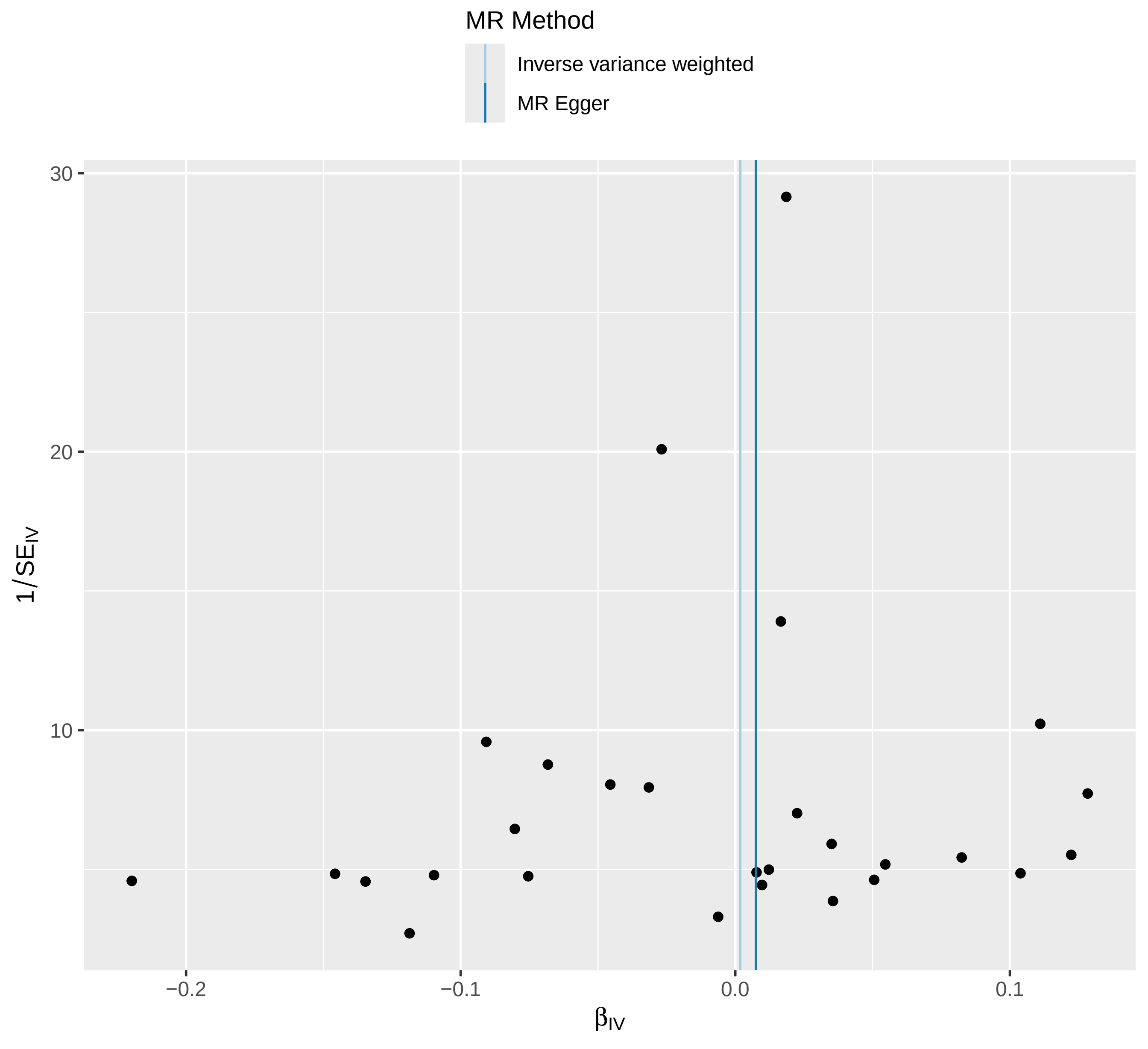
MR Method
Inverse variance weighted
MR Egger
30
20
10
1/SE$_{IV}$
−0.2
−0.1
0.0
0.1
β$_{IV}$

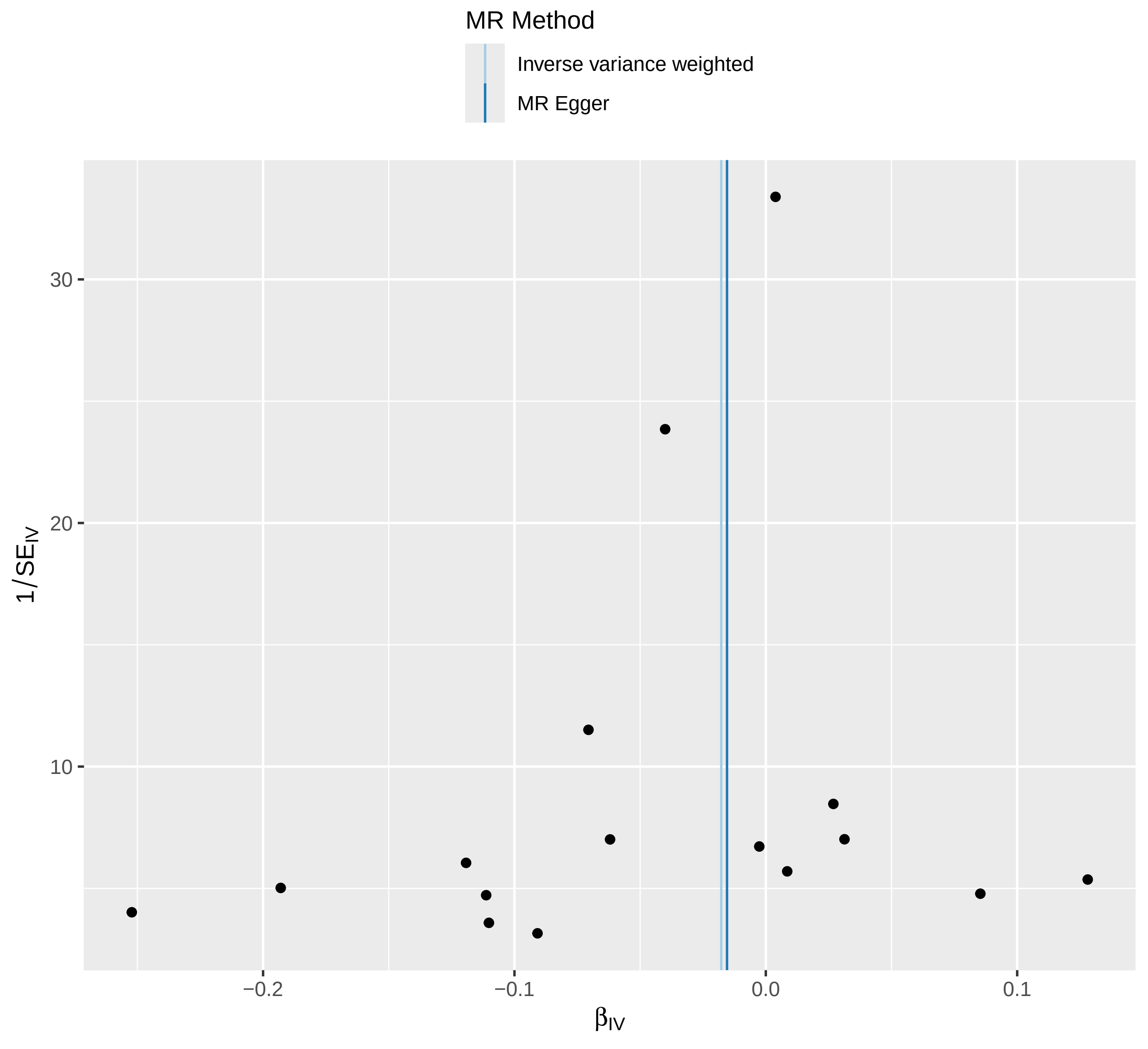

MR Method
Inverse variance weighted
MR Egger
30
20
10
1/SE$_{IV}$
−0.2
−0.1
0.0
0.1
β$_{IV}$

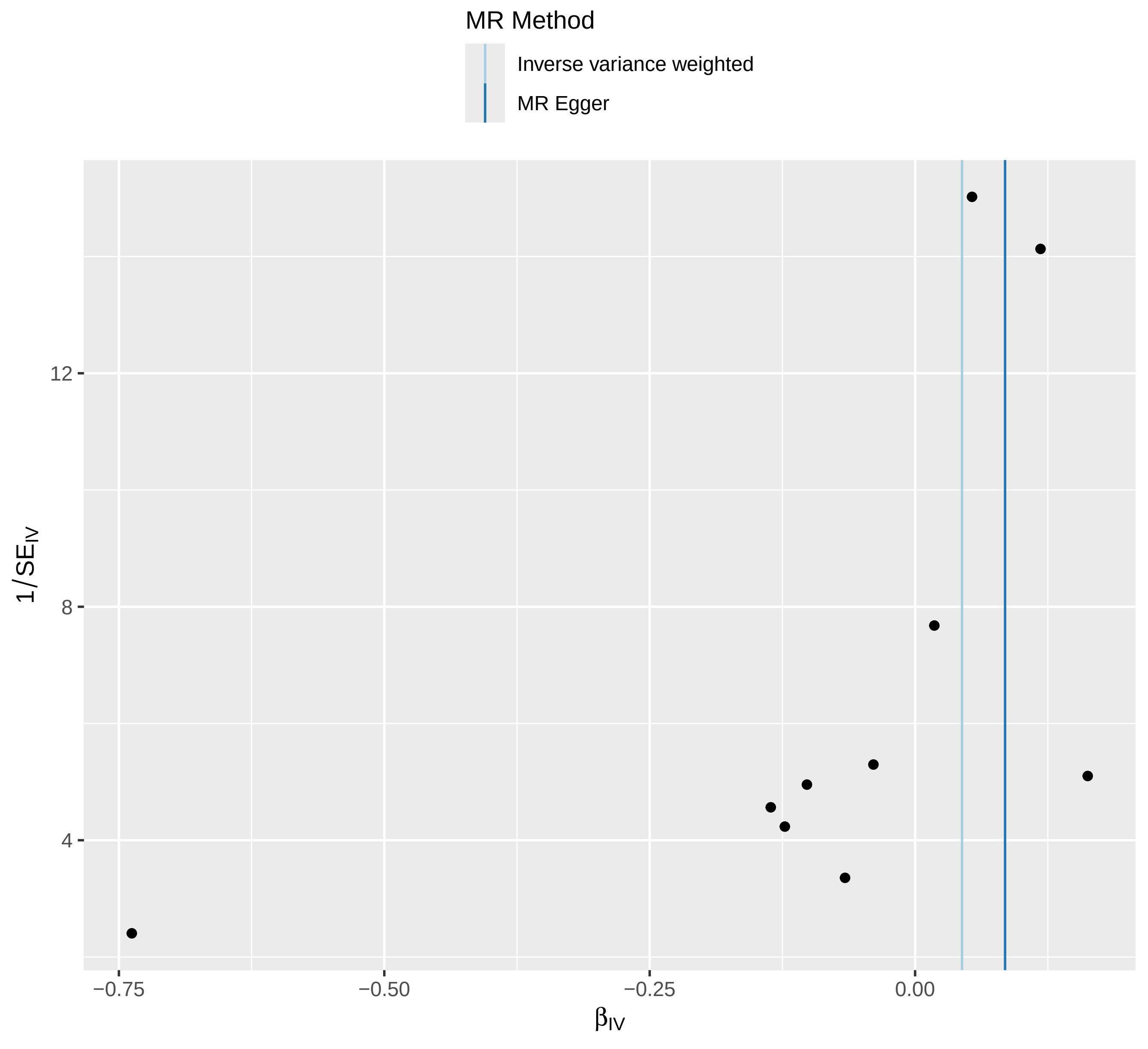
MR Method
Inverse variance weighted
MR Egger
1/SE_IV
12
8
4
−0.75
−0.50
−0.25
0.00
β_IV

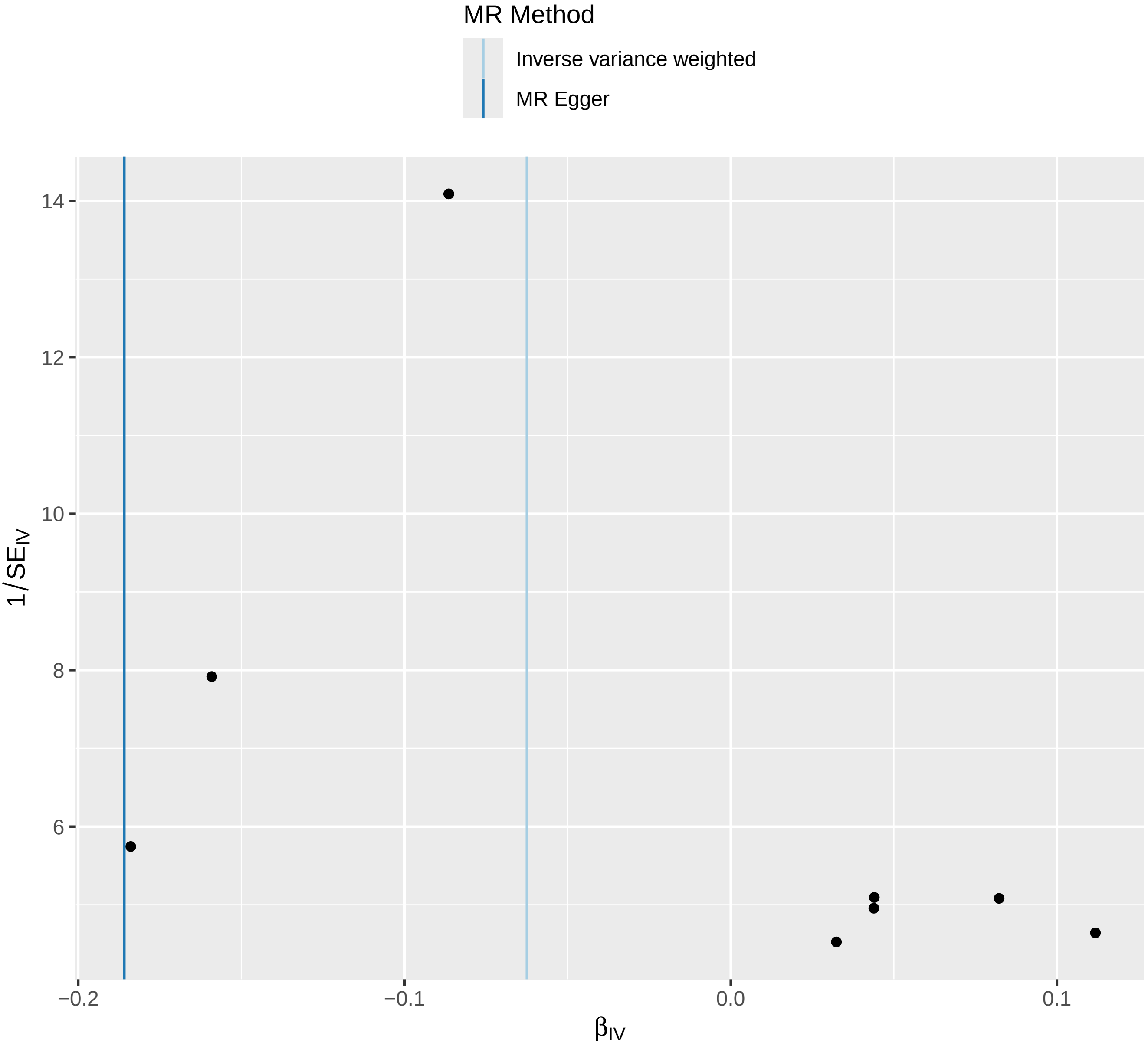
MR Method
Inverse variance weighted
MR Egger
14
12
10
8
6
$1/SE_{IV}$
−0.2
−0.1
0.0
0.1
$\beta_{IV}$

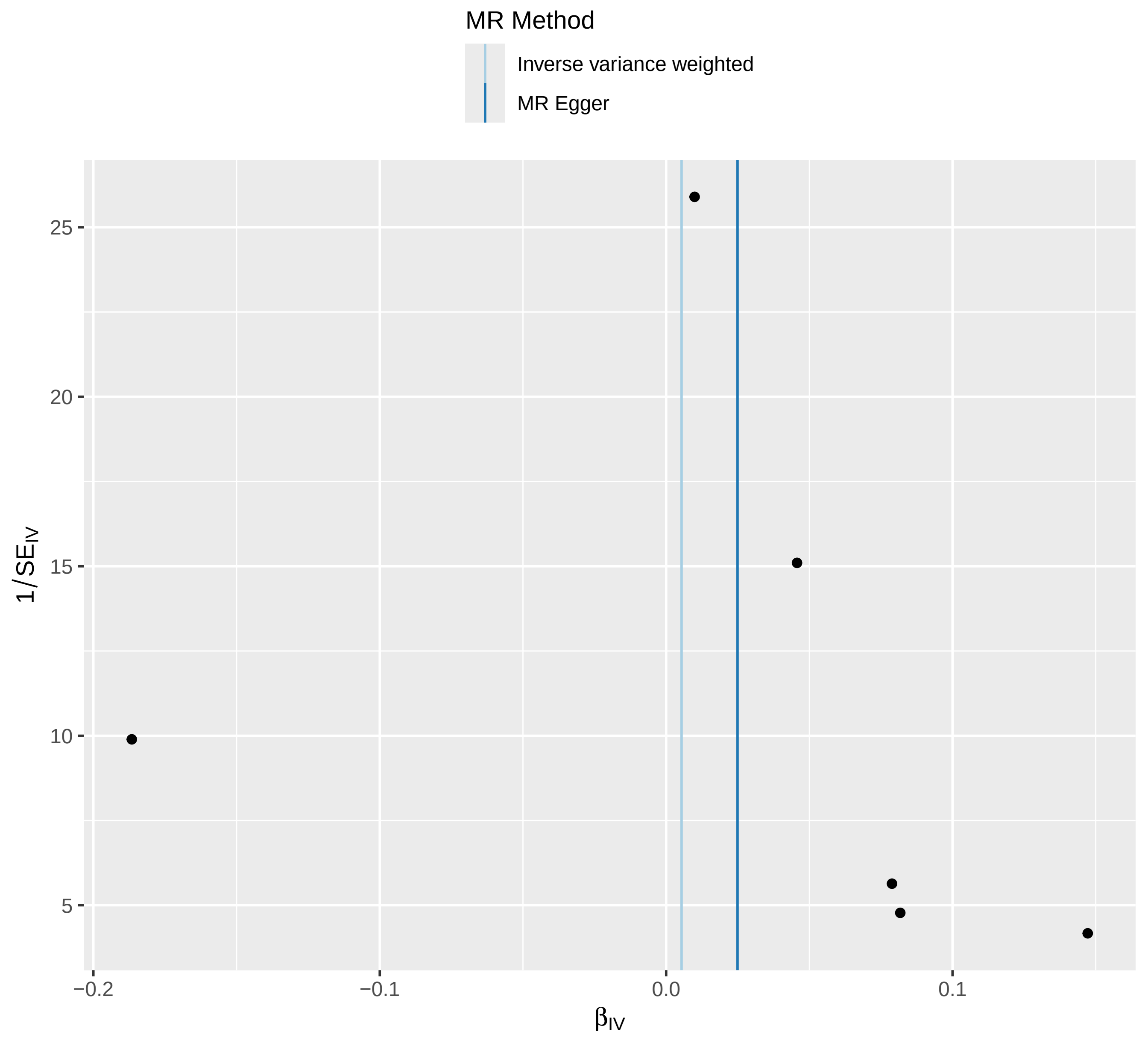
MR Method
Inverse variance weighted
MR Egger
25
20
15
10
5
1/SE$_{IV}$
−0.2
−0.1
0.0
0.1
β$_{IV}$

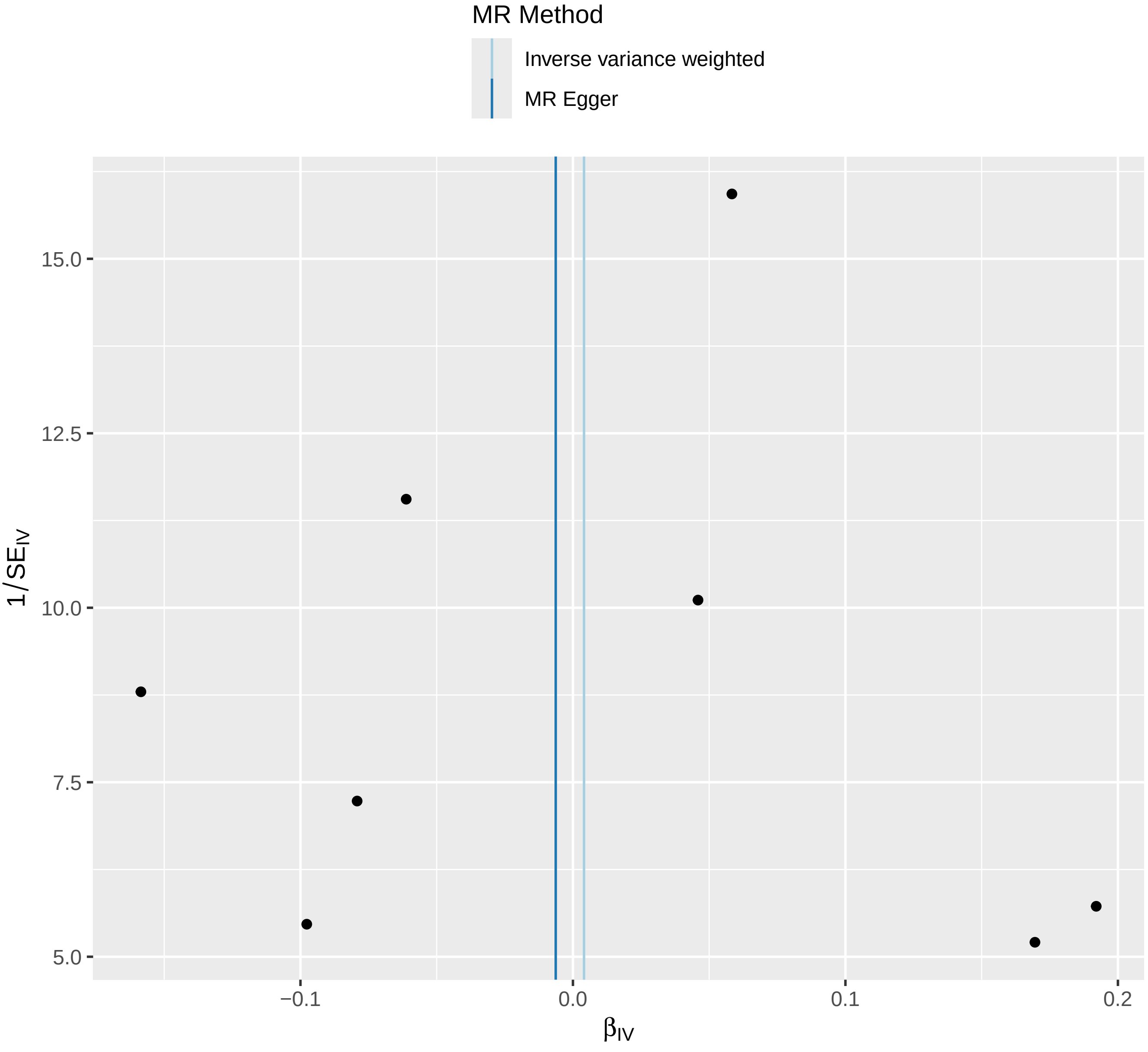
MR Method
Inverse variance weighted
MR Egger
15.0
12.5
10.0
7.5
5.0
$1/SE_{IV}$
−0.1
0.0
0.1
0.2
$\beta_{IV}$

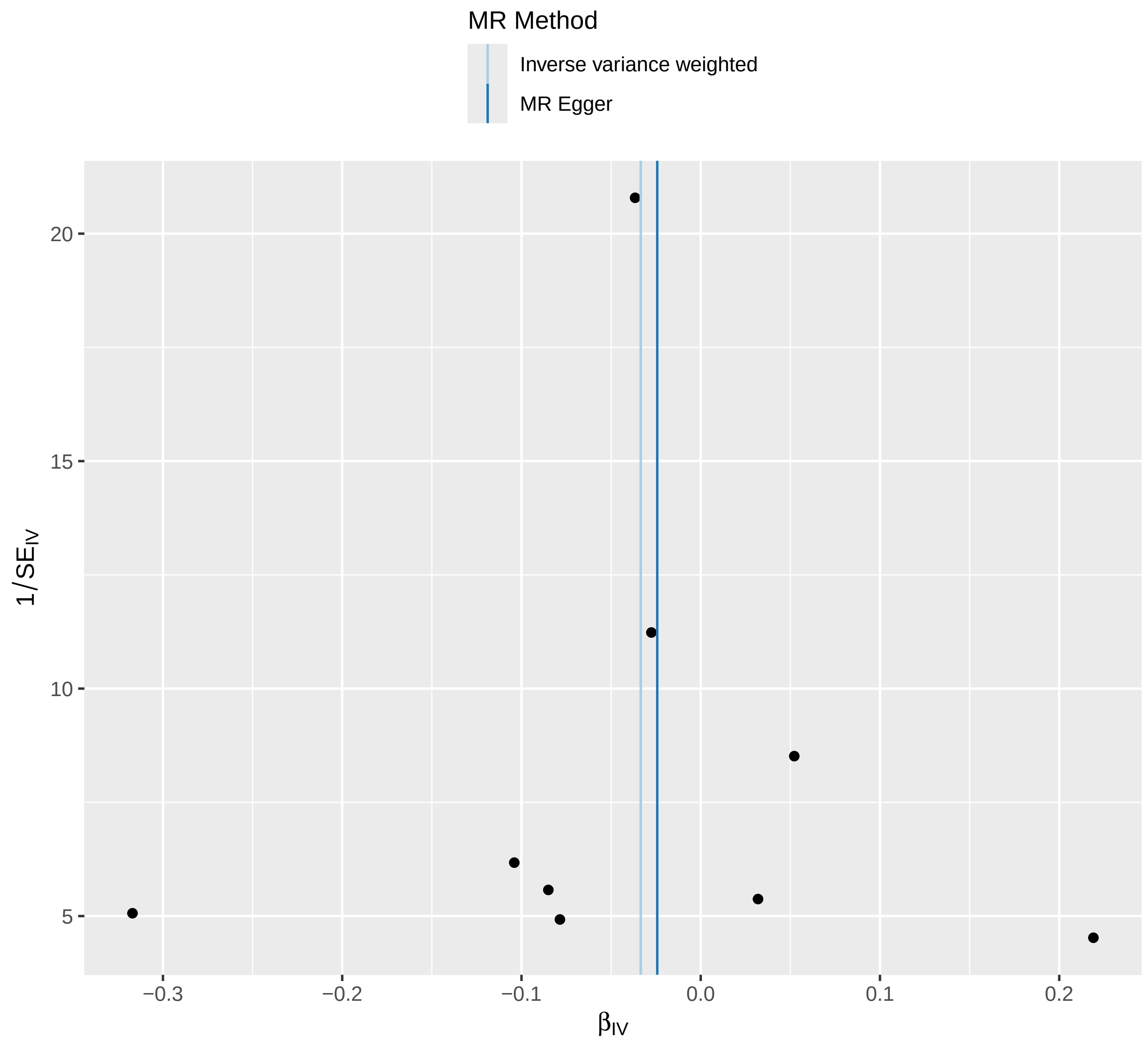
MR Method
Inverse variance weighted
MR Egger
20
15
10
5
1/SE_IV
−0.3
−0.2
−0.1
0.0
0.1
0.2
β_IV

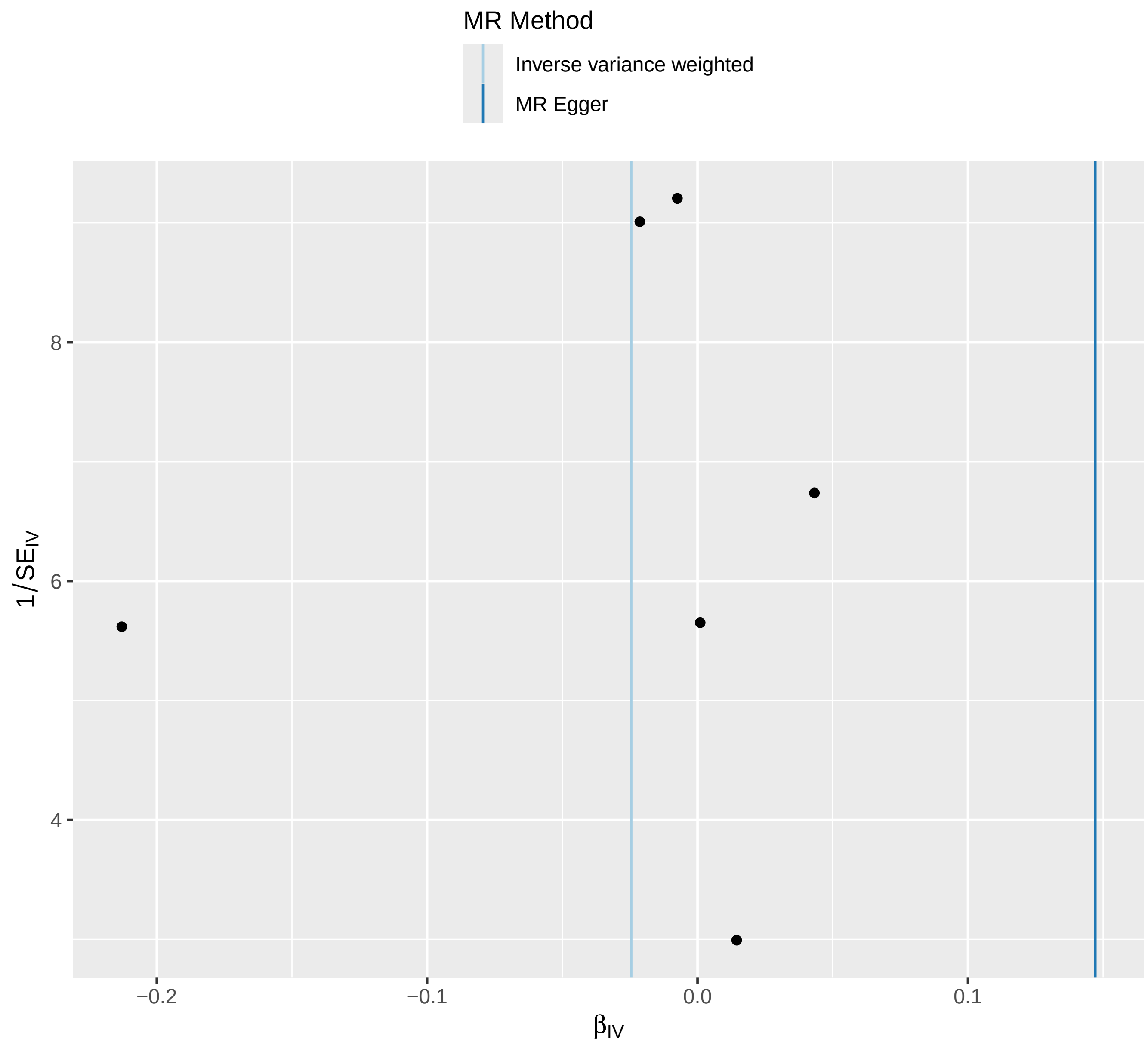

MR Method
Inverse variance weighted
MR Egger
8
6
4
$1/SE_{IV}$
−0.2
−0.1
0.0
0.1
$\beta_{IV}$

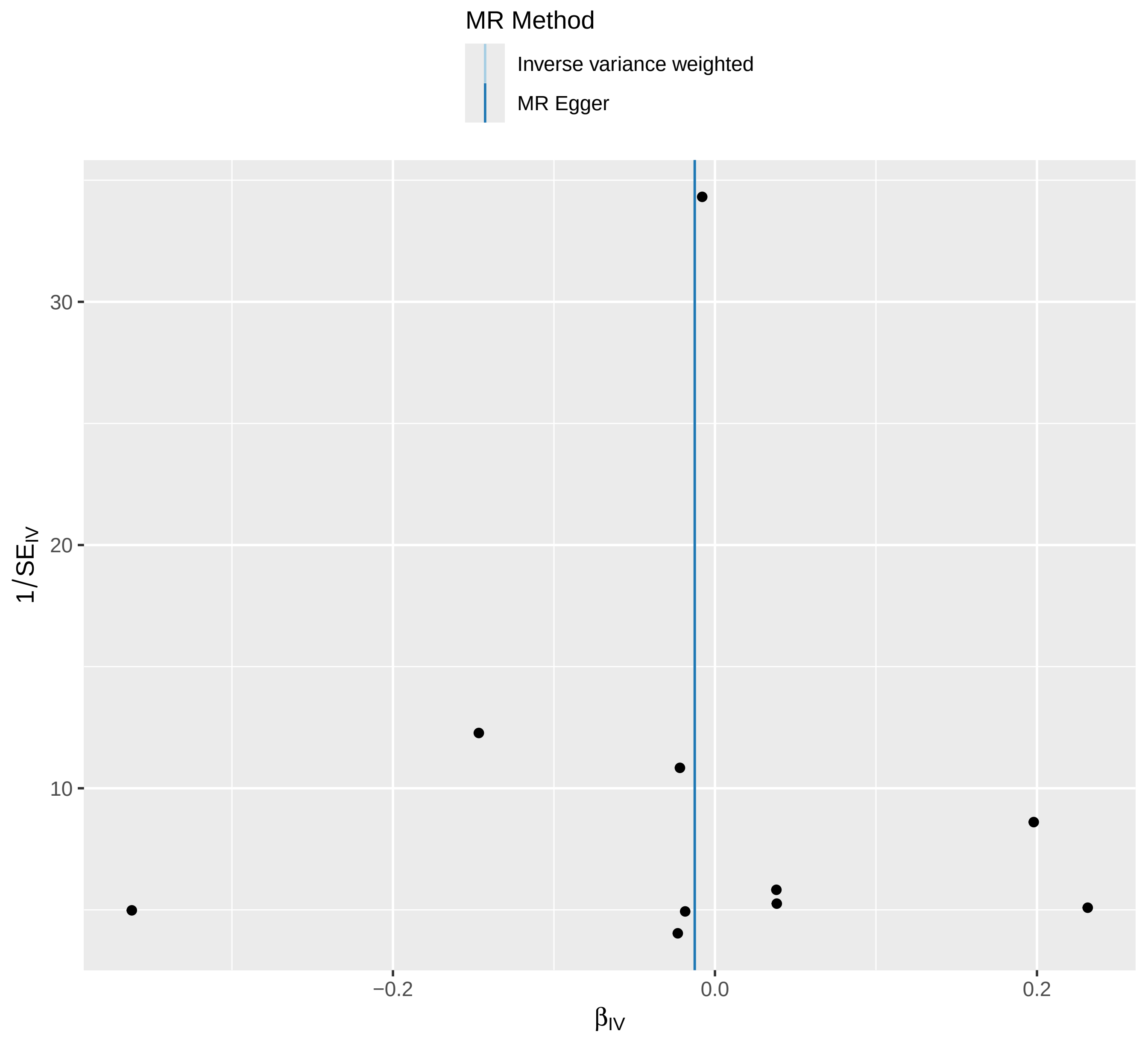
MR Method
Inverse variance weighted
MR Egger
30
20
10
1/SE_IV
−0.2
0.0
0.2
β_IV

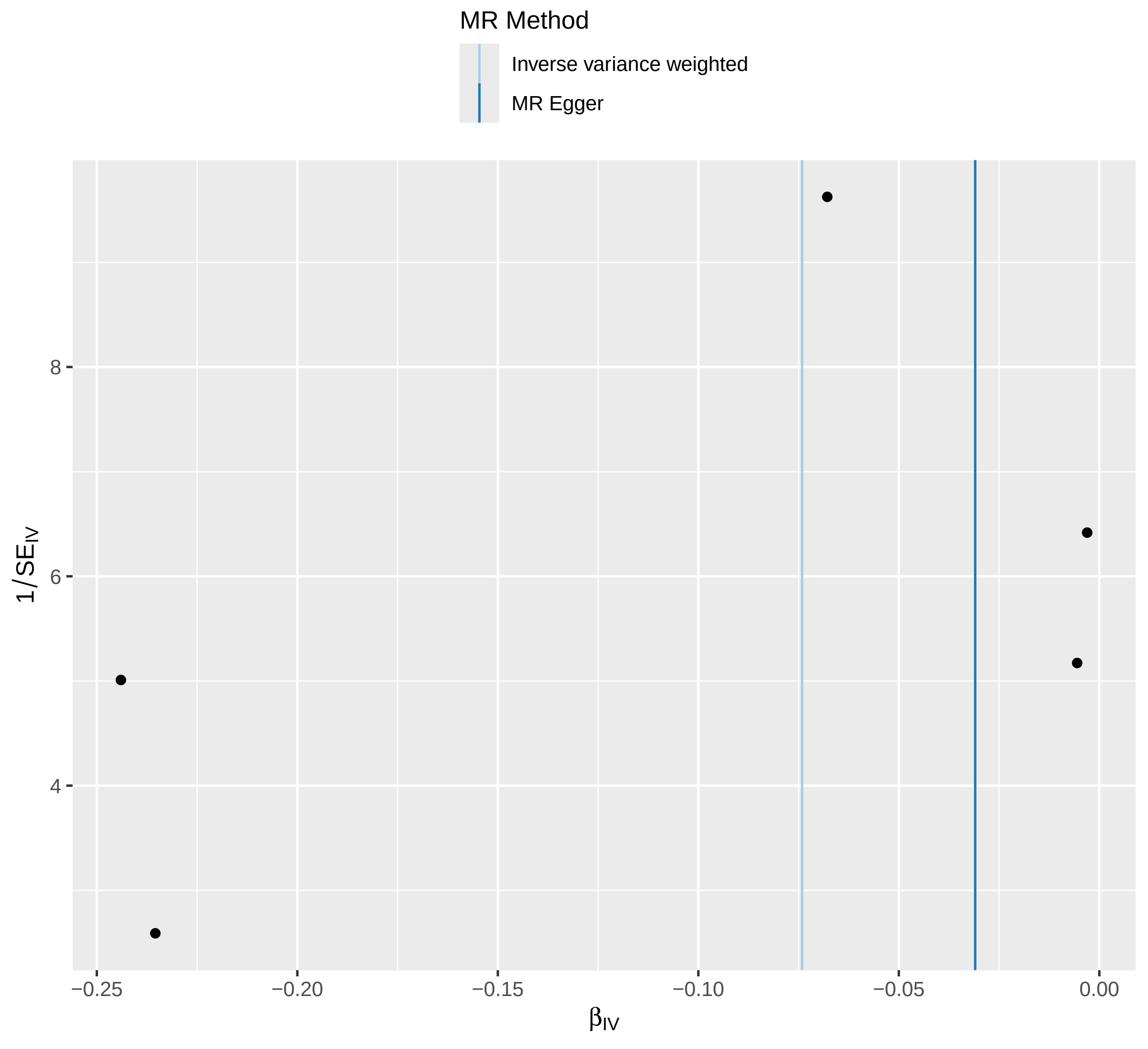
MR Method
Inverse variance weighted
MR Egger
8
6
4
1/SE$_{IV}$
−0.25
−0.20
−0.15
−0.10
−0.05
0.00
β$_{IV}$

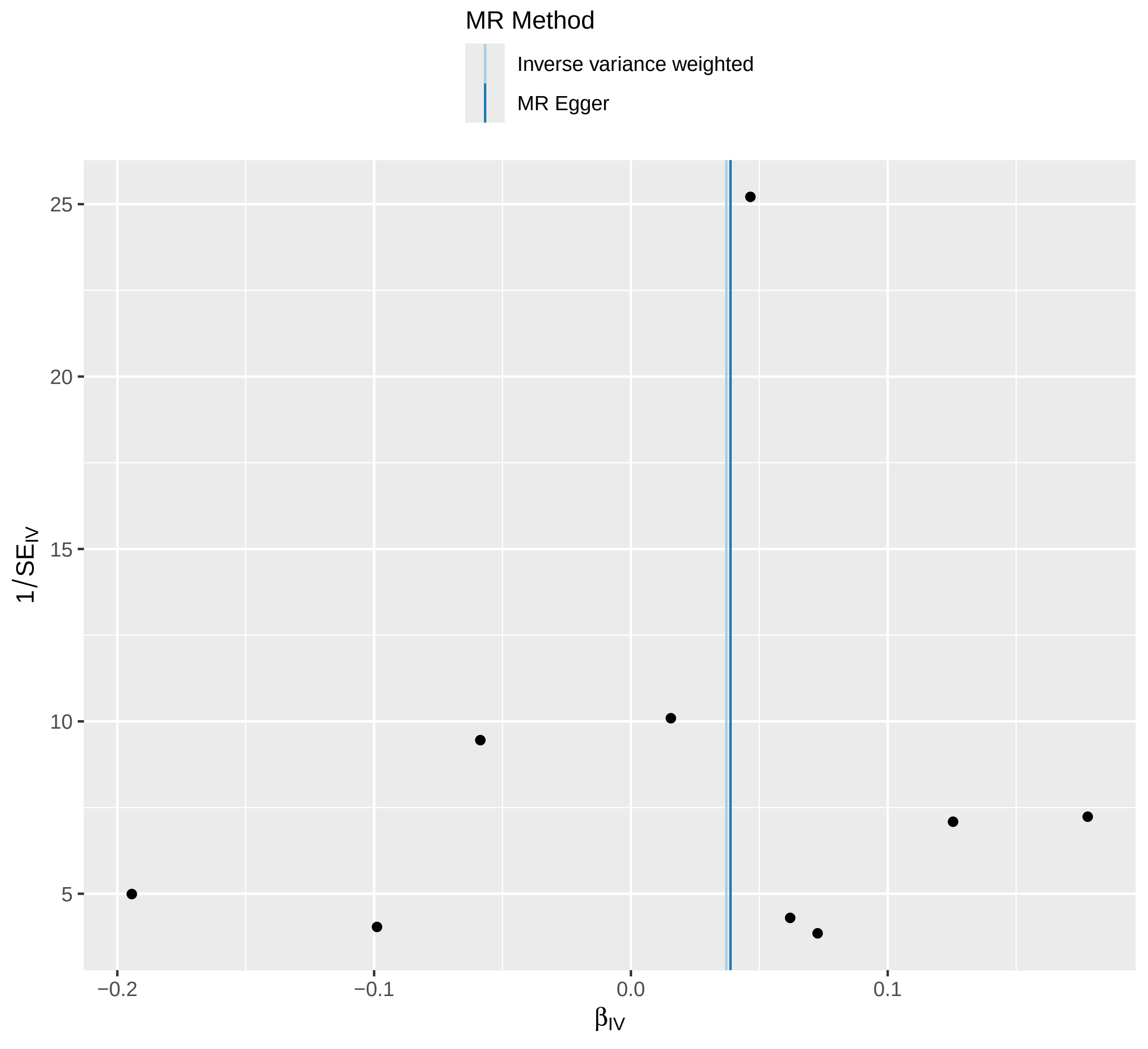

MR Method
Inverse variance weighted
MR Egger
25
20
15
10
5
1/SE_IV
−0.2
−0.1
0.0
0.1
β_IV

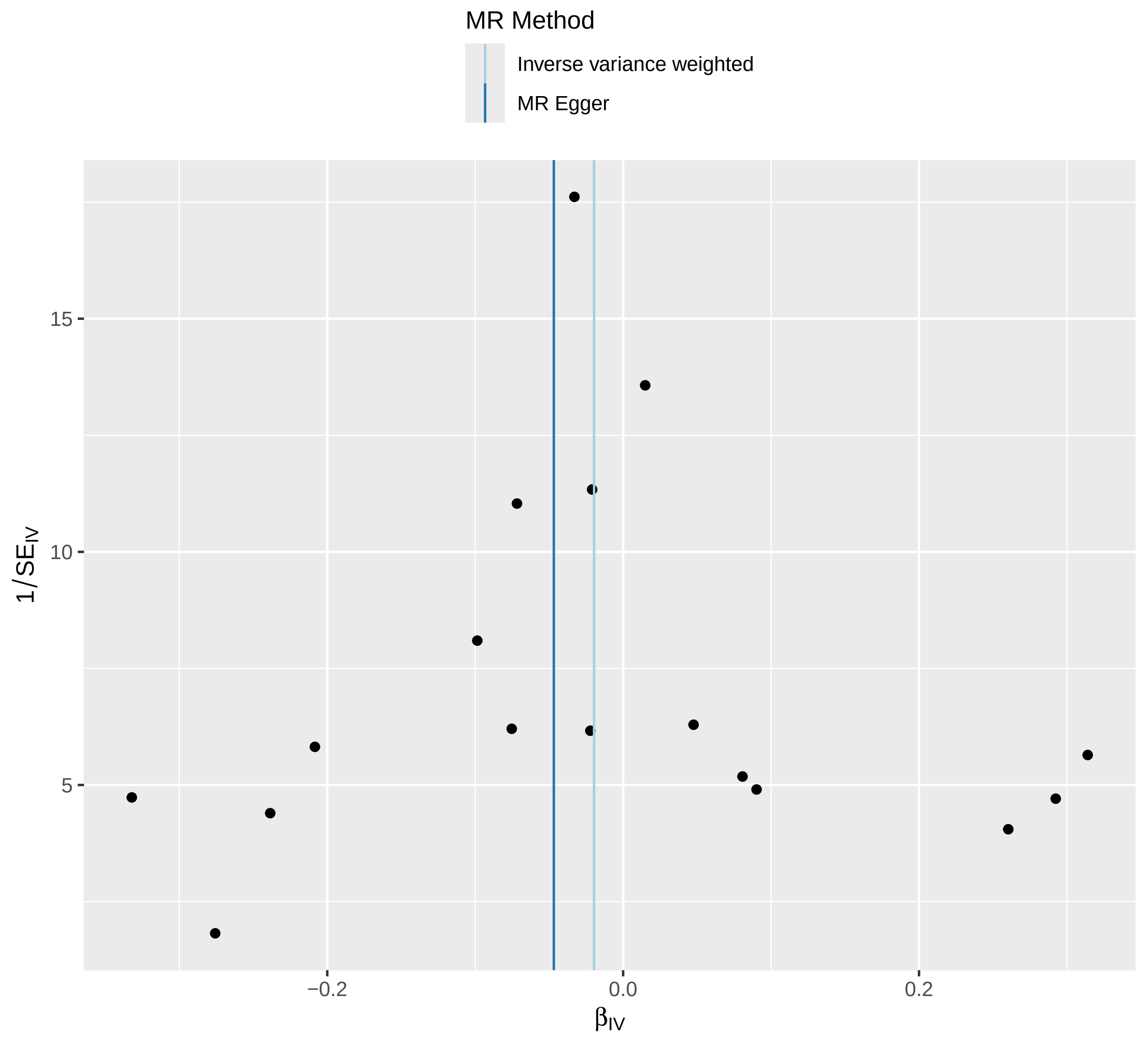
MR Method
Inverse variance weighted
MR Egger
15
10
5
1/SE$_{IV}$
−0.2
0.0
0.2
β$_{IV}$

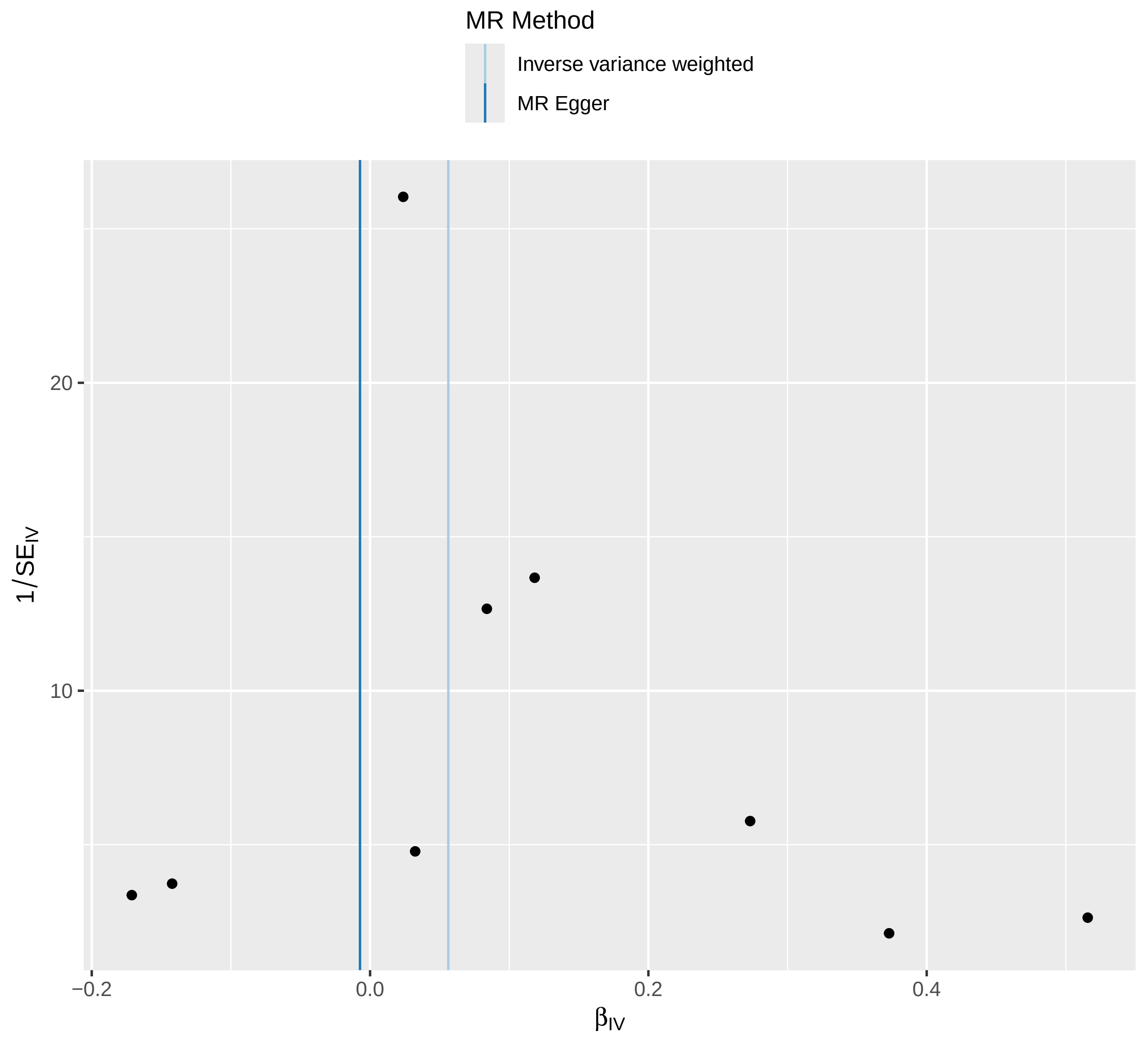
MR Method
Inverse variance weighted
MR Egger
20
10
−0.2
0.0
0.2
0.4
1/SE_IV
β_IV

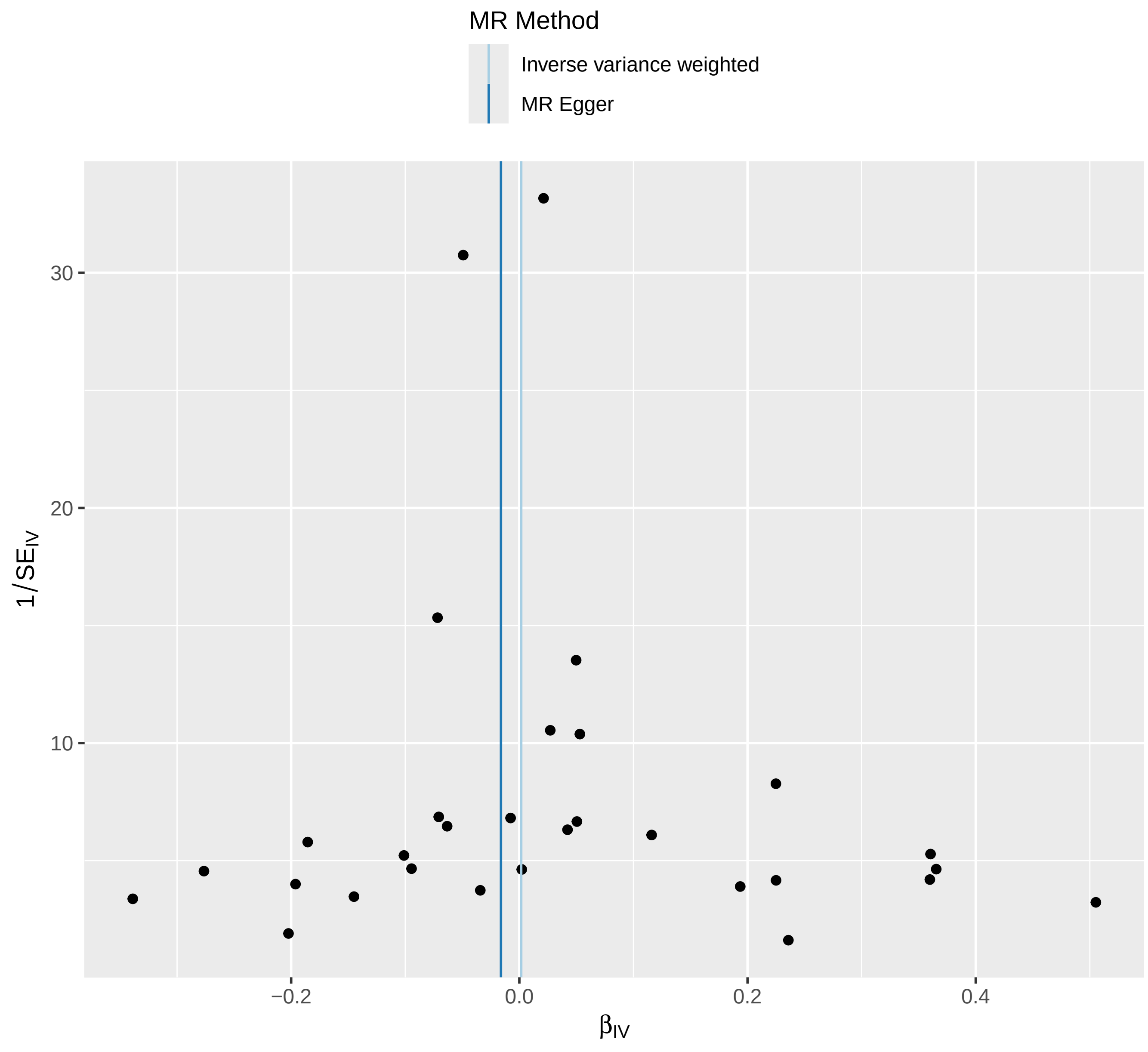
MR Method
Inverse variance weighted
MR Egger
30
20
10
1/SE_IV
−0.2
0.0
0.2
0.4
β_IV

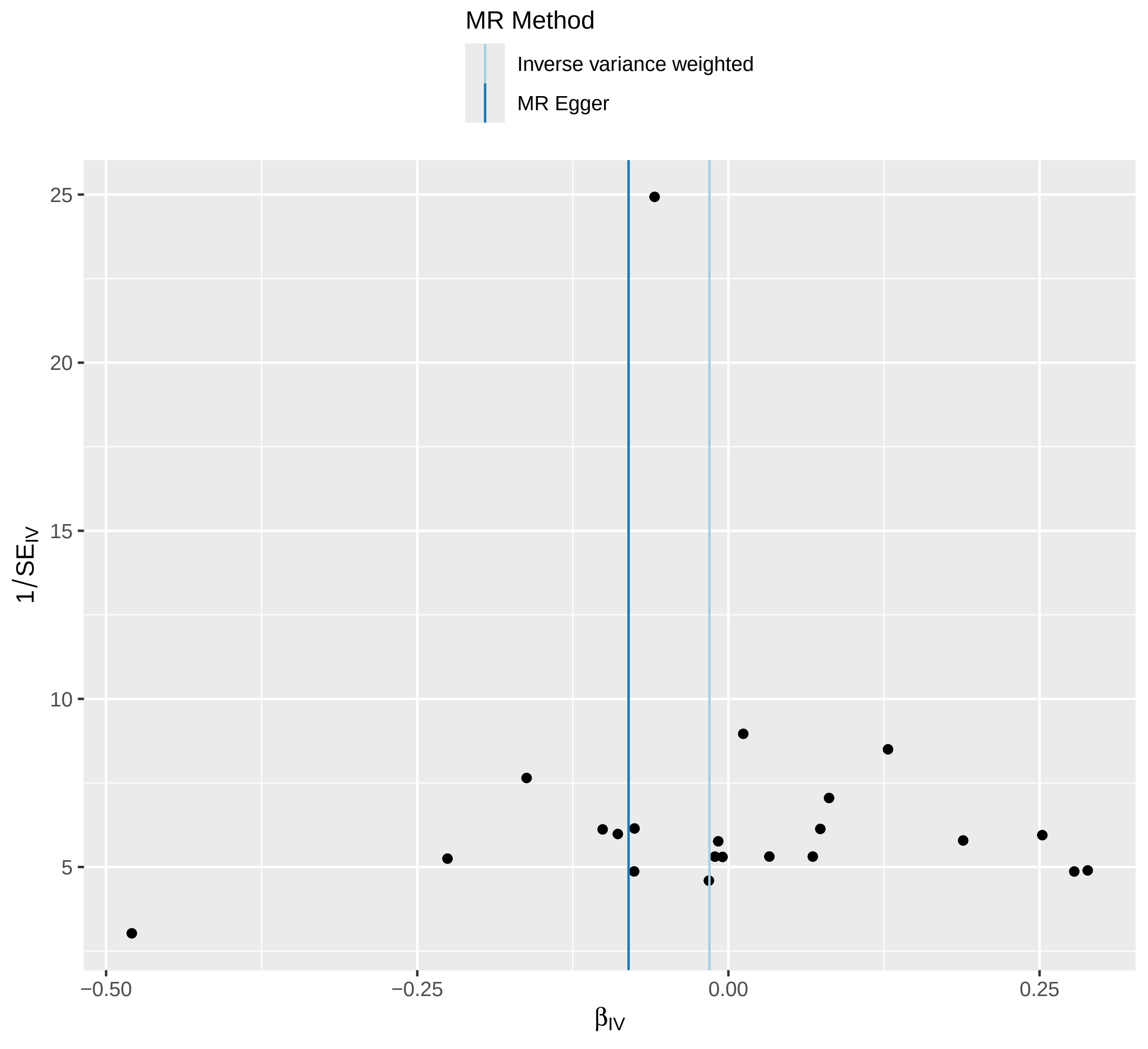
MR Method
Inverse variance weighted
MR Egger
25
20
15
10
5
1/SE$_{IV}$
−0.50
−0.25
0.00
0.25
β$_{IV}$

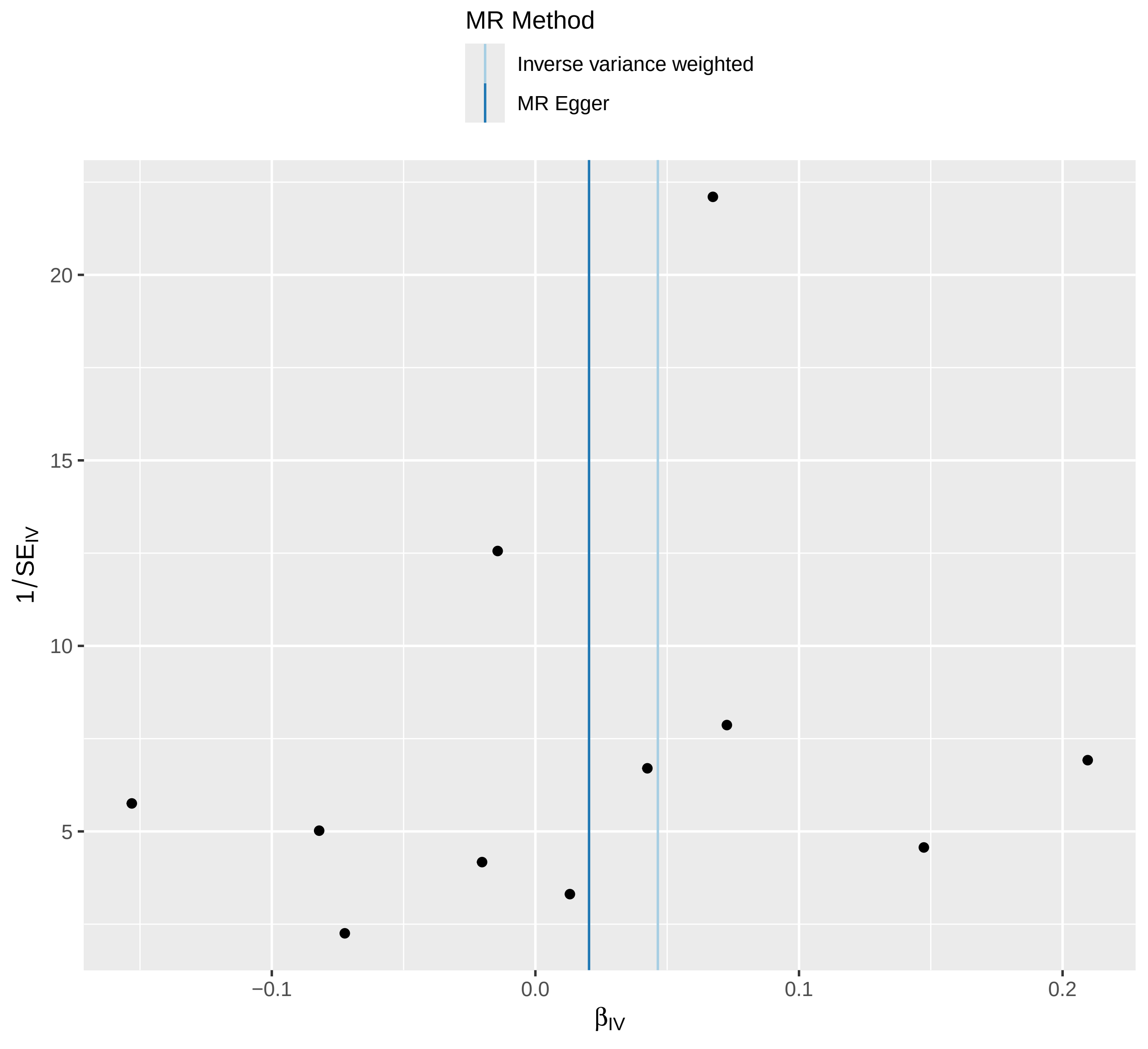

MR Method
Inverse variance weighted
MR Egger
20
15
10
5
1/SE$_{IV}$
−0.1
0.0
0.1
0.2
β$_{IV}$

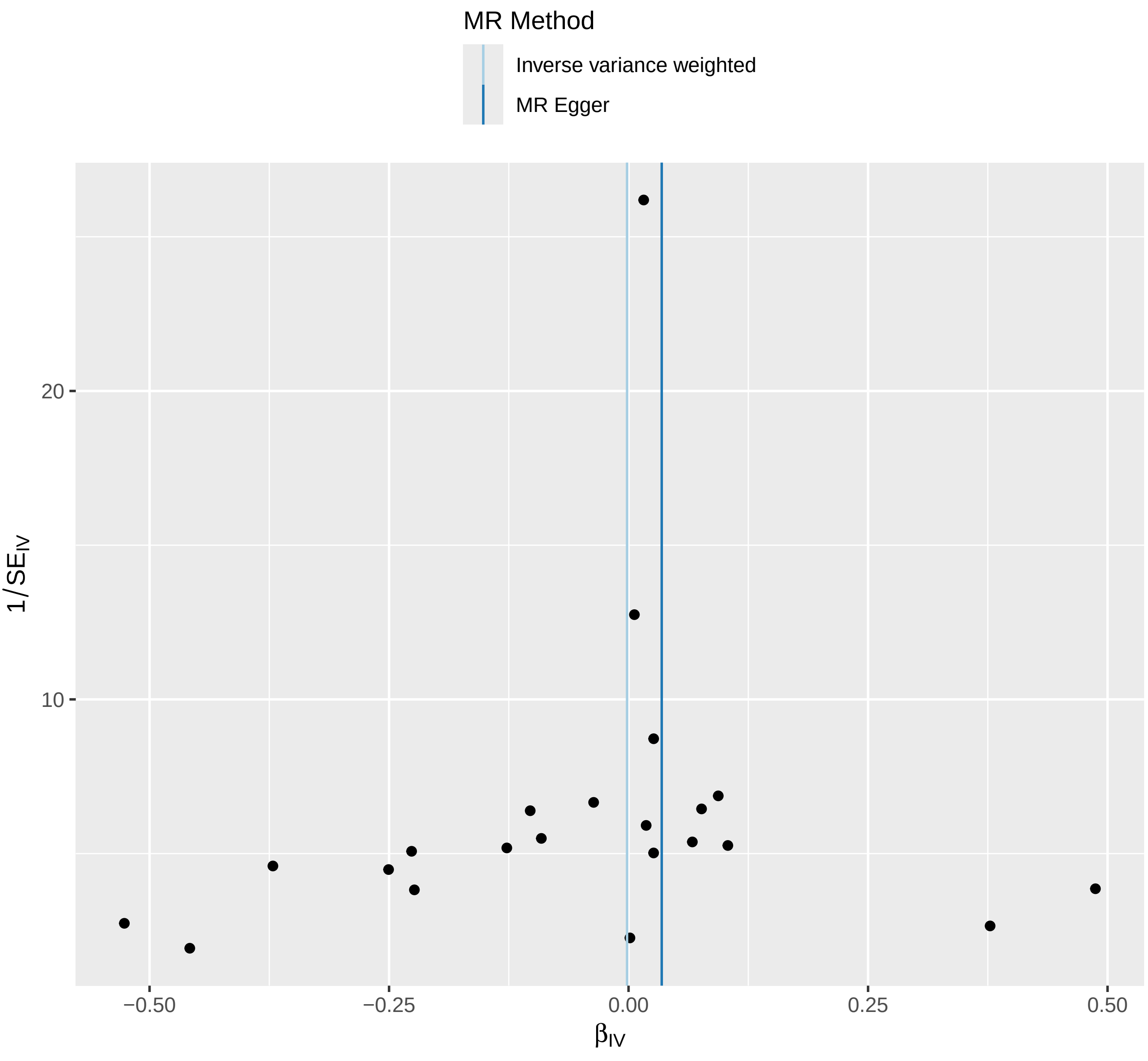
MR Method
Inverse variance weighted
MR Egger
20
10
1/SE$_{IV}$
−0.50
−0.25
0.00
0.25
0.50
β$_{IV}$

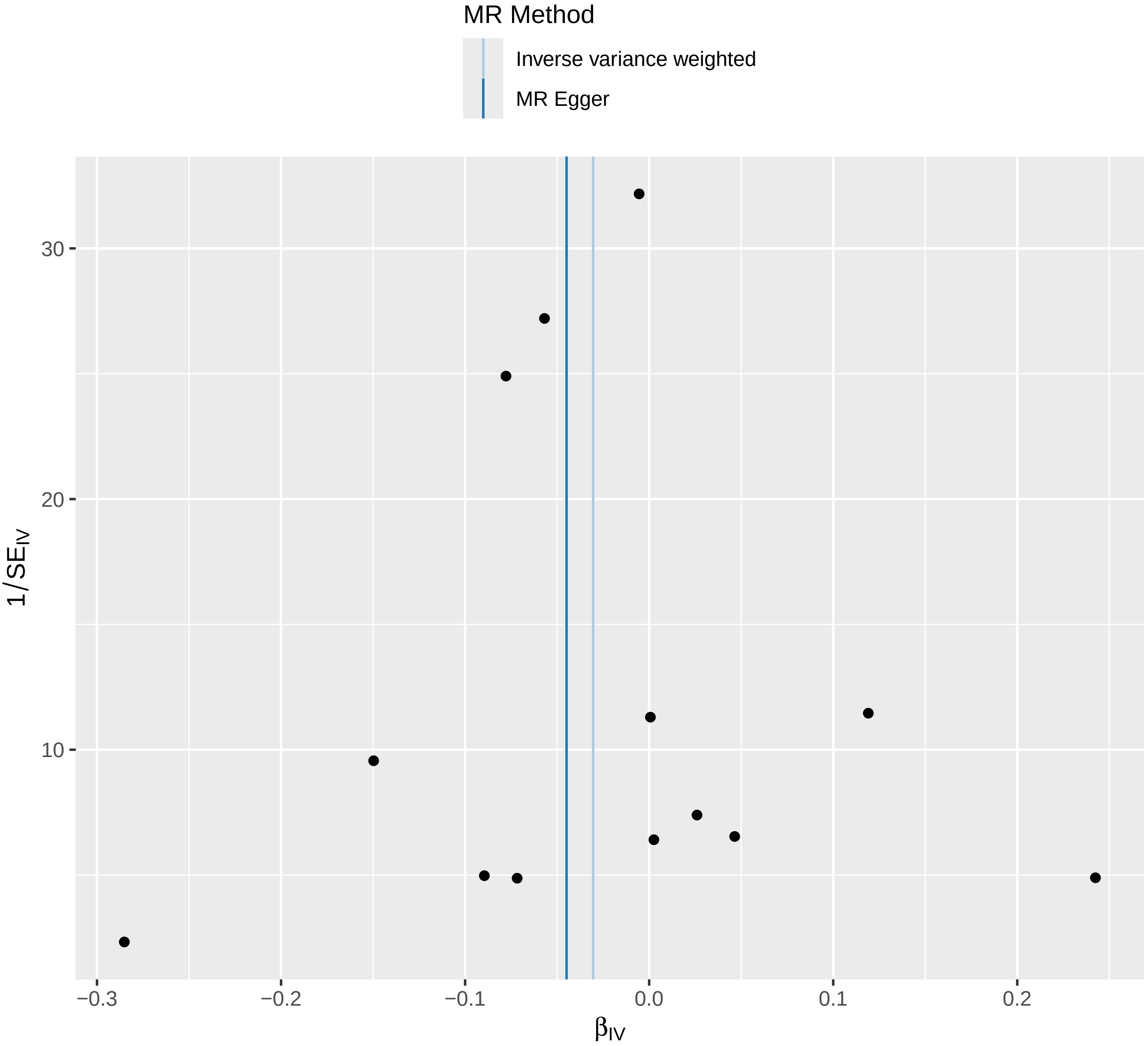

MR Method
Inverse variance weighted
MR Egger
30
20
10
1/SE_IV
−0.3
−0.2
−0.1
0.0
0.1
0.2
β_IV

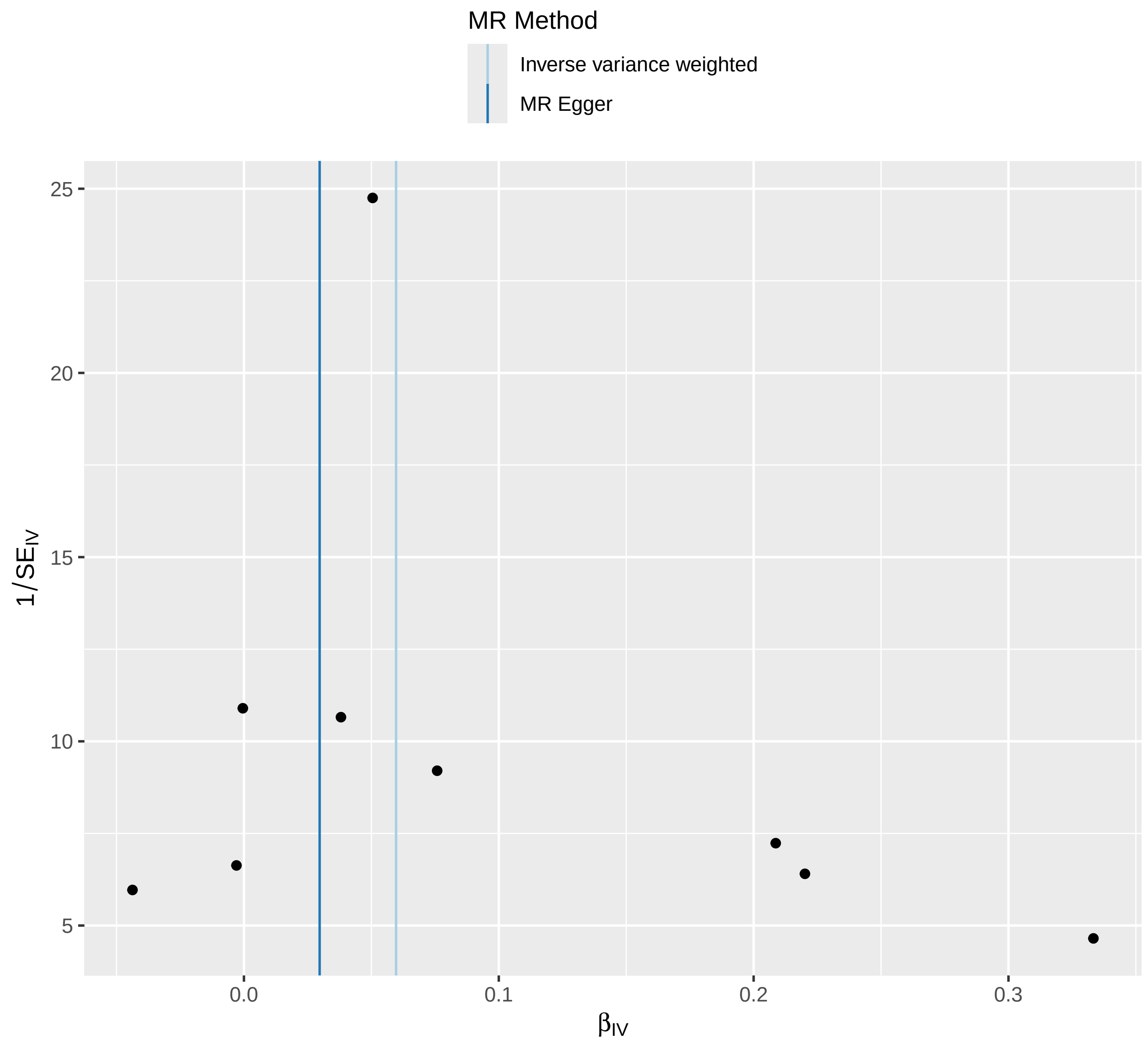
MR Method
Inverse variance weighted
MR Egger
25
20
15
10
5
1/SE_IV
0.0
0.1
0.2
0.3
β_IV

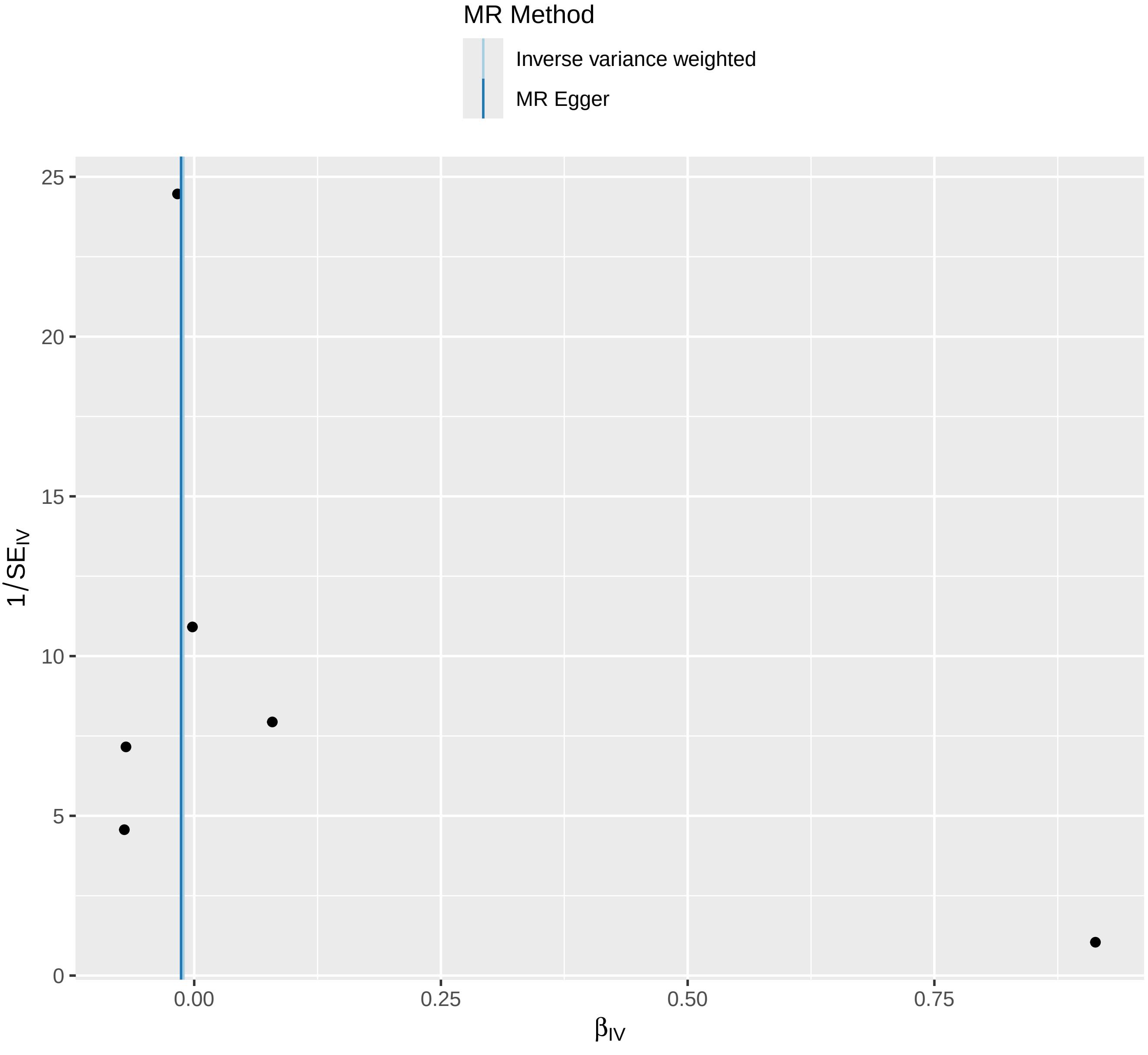
MR Method
Inverse variance weighted
MR Egger
25
20
15
10
5
0
0.00
0.25
0.50
0.75
$1/SE_{IV}$
$\beta_{IV}$

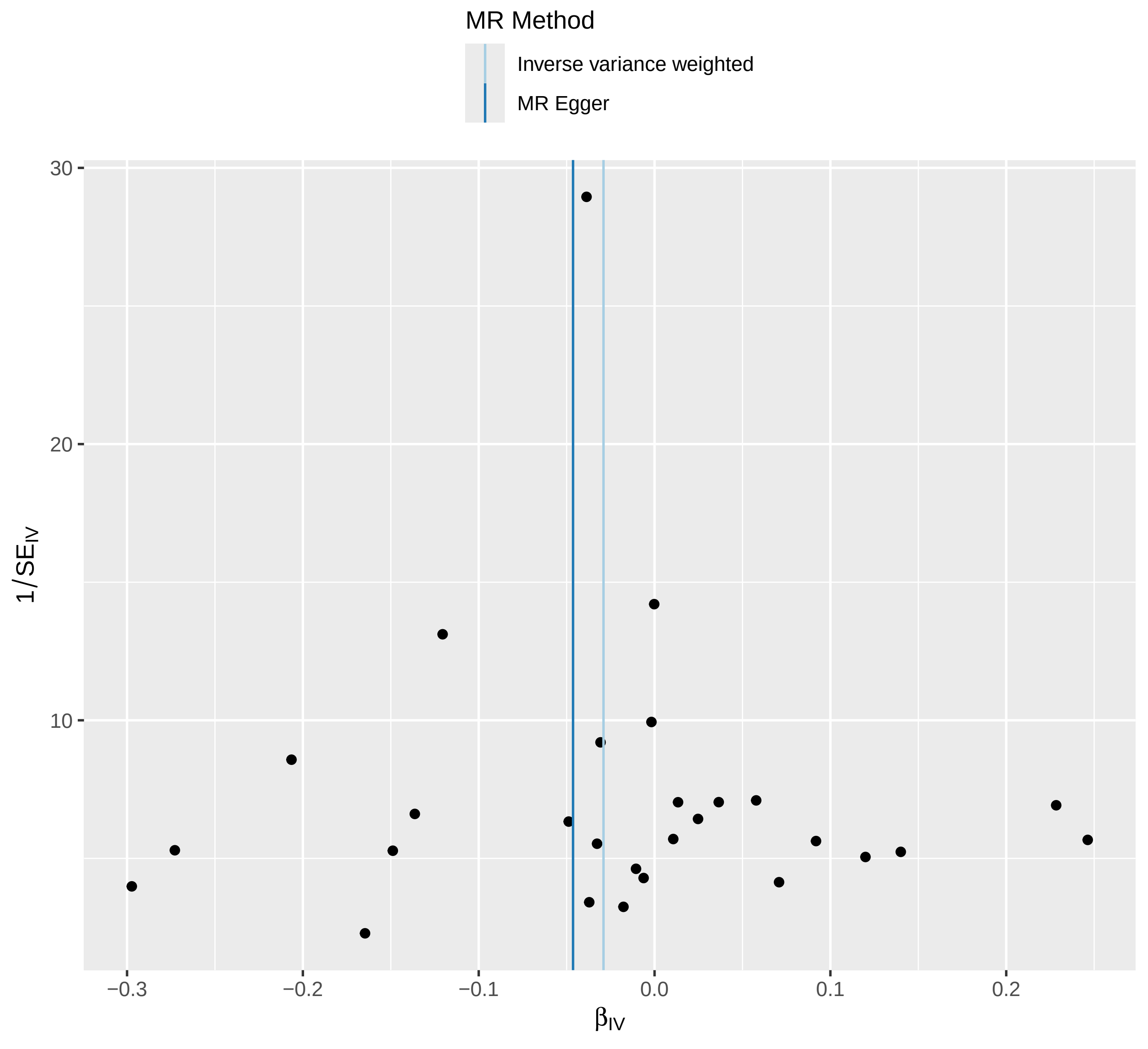
MR Method
Inverse variance weighted
MR Egger
30
20
10
1/SE$_{IV}$
−0.3
−0.2
−0.1
0.0
0.1
0.2
β$_{IV}$

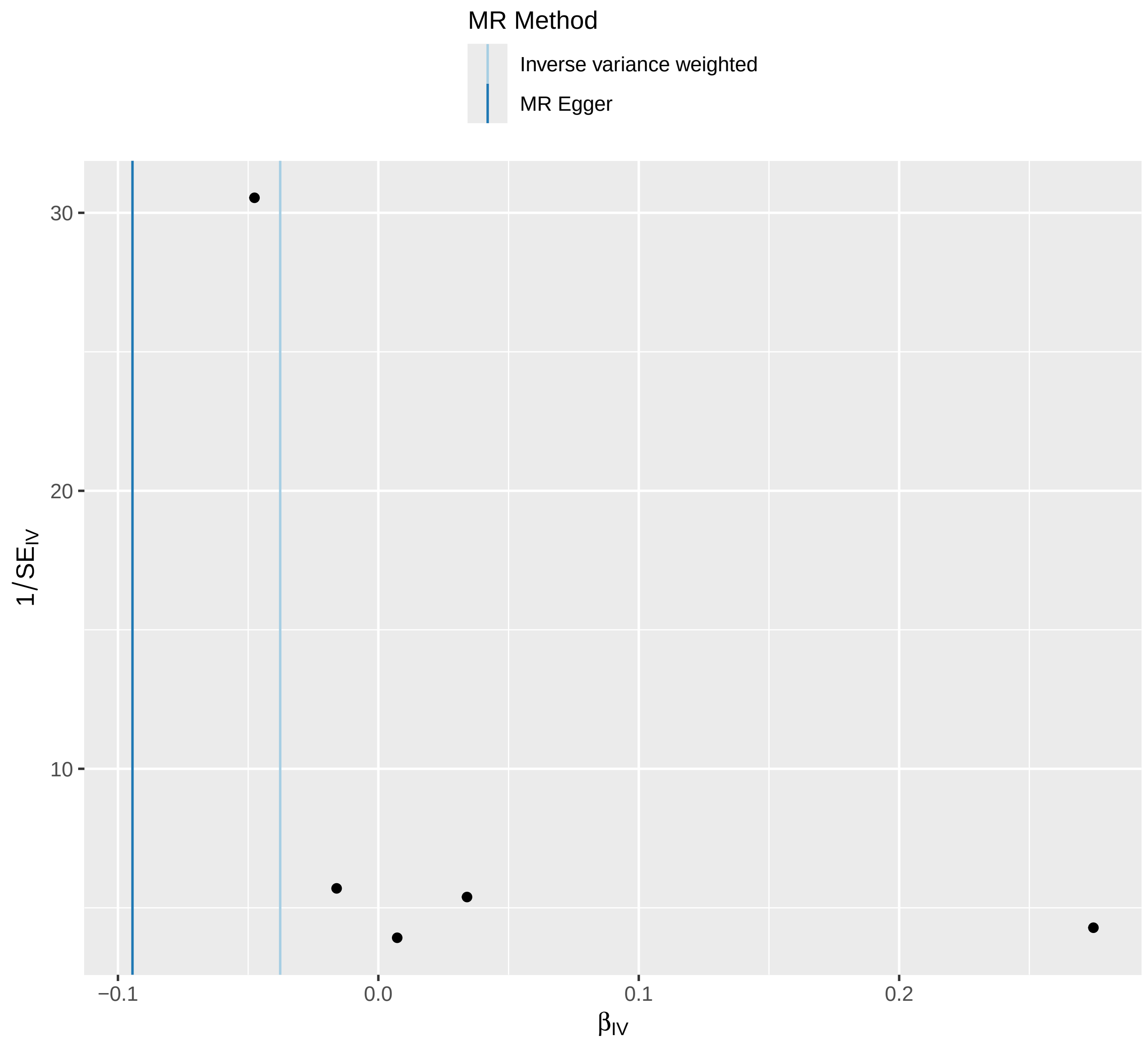
MR Method
Inverse variance weighted
MR Egger
30
20
10
1/SE_IV
−0.1
0.0
0.1
0.2
β_IV

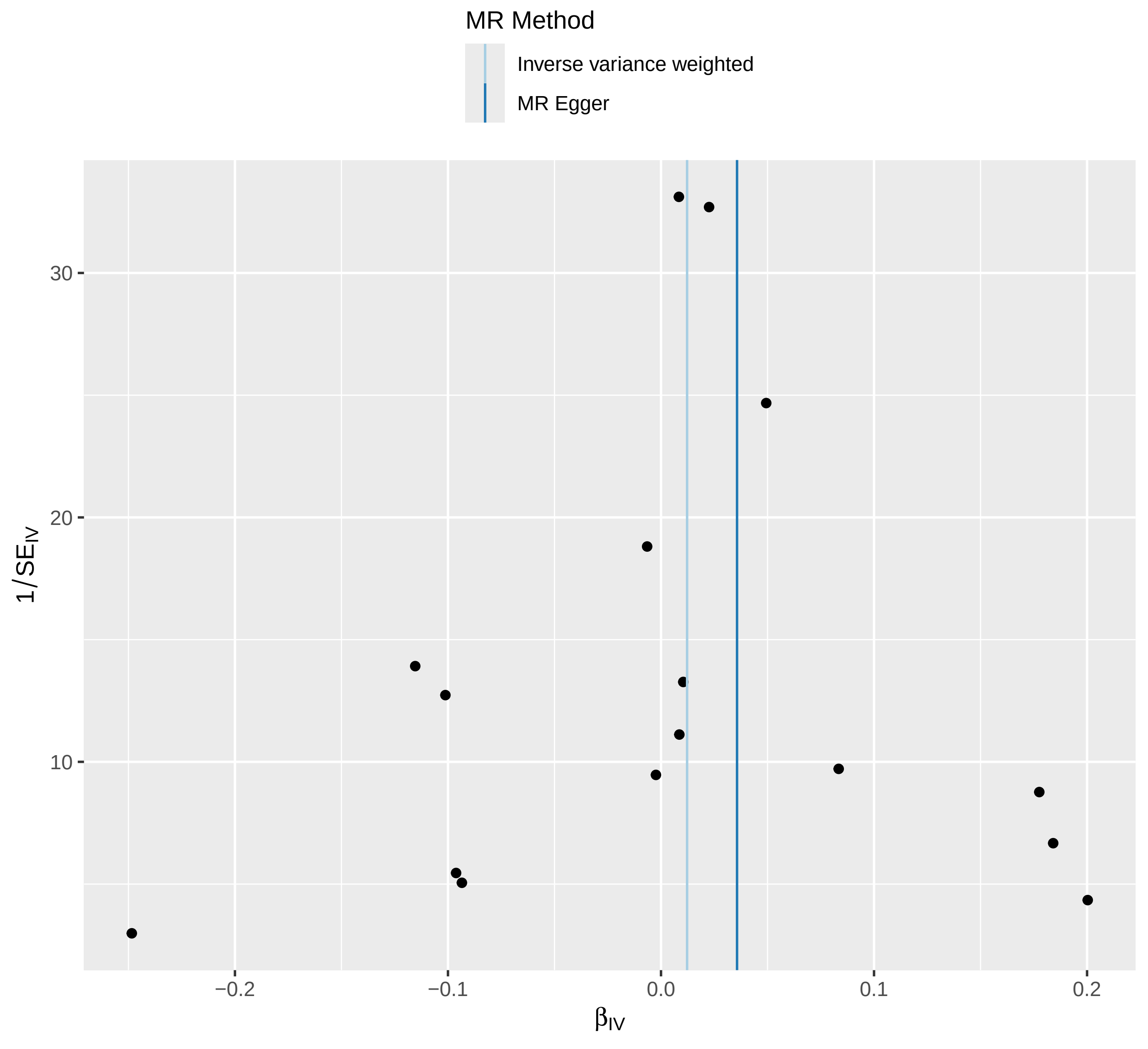
MR Method
Inverse variance weighted
MR Egger
30
20
10
1/SE_IV
−0.2
−0.1
0.0
0.1
0.2
β_IV

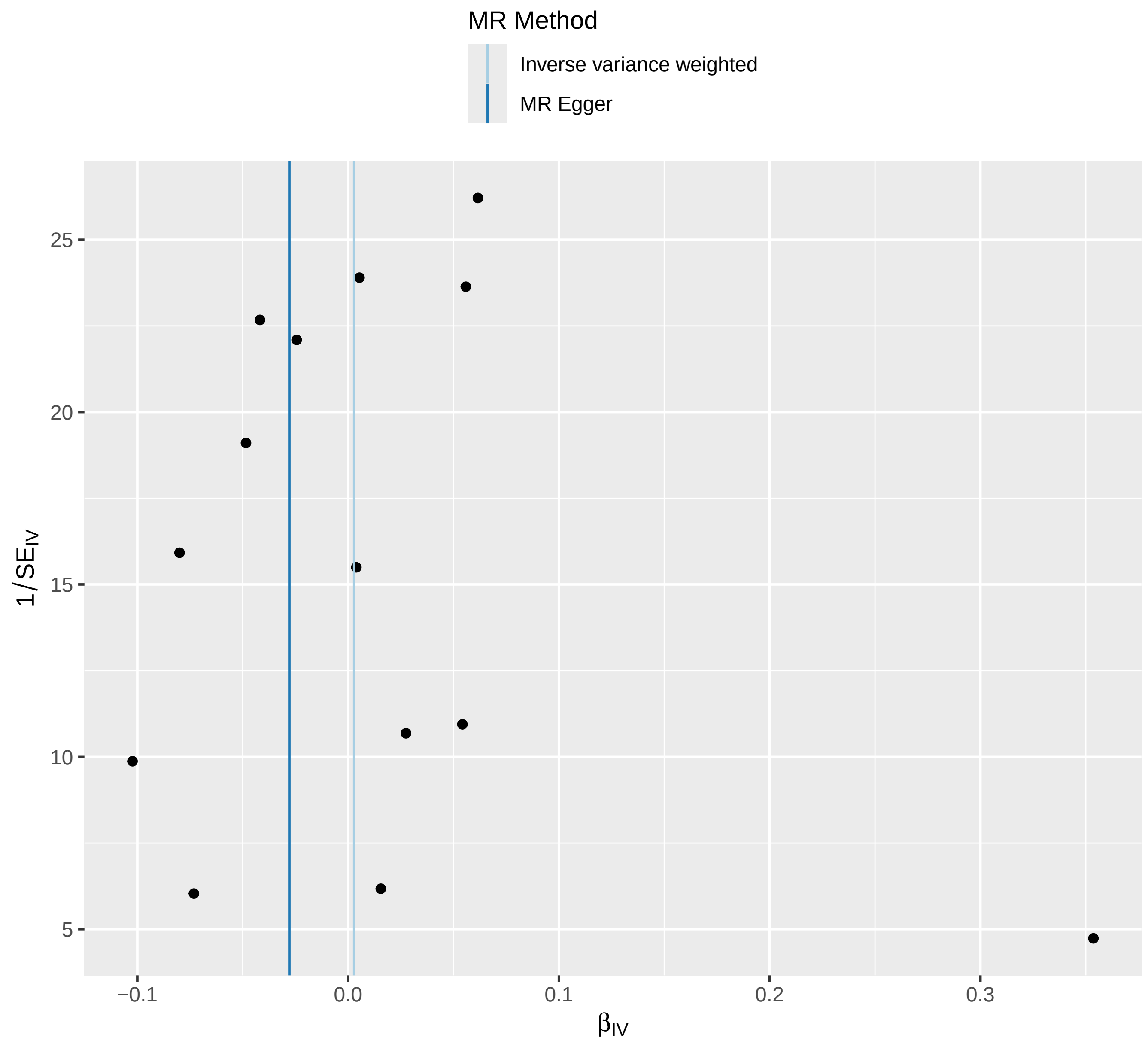

MR Method
Inverse variance weighted
MR Egger
25
20
15
10
5
1/SE_IV
−0.1
0.0
0.1
0.2
0.3
β_IV

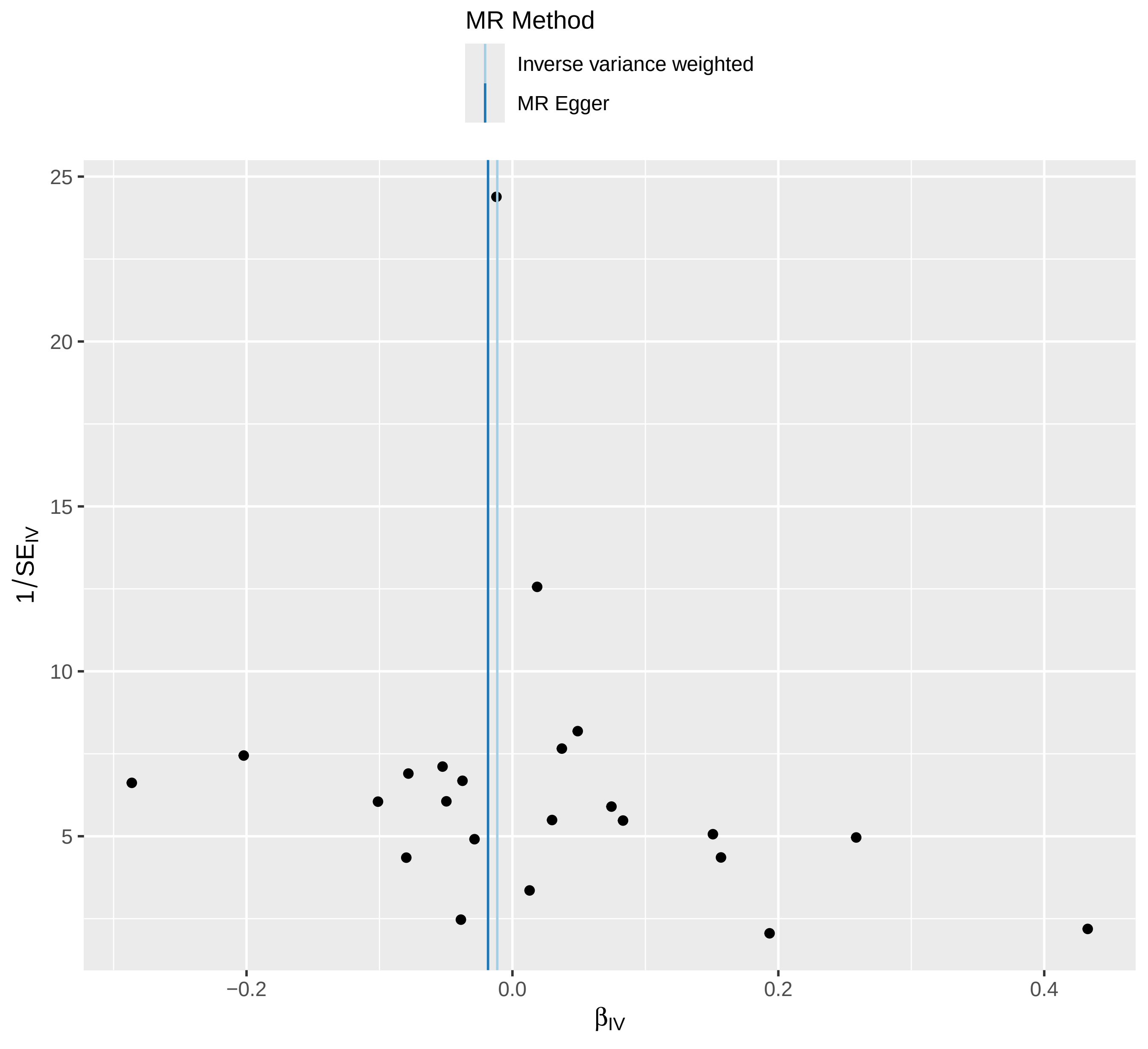
MR Method
Inverse variance weighted
MR Egger
25
20
15
10
5
1/SE$_{IV}$
−0.2
0.0
0.2
0.4
β$_{IV}$

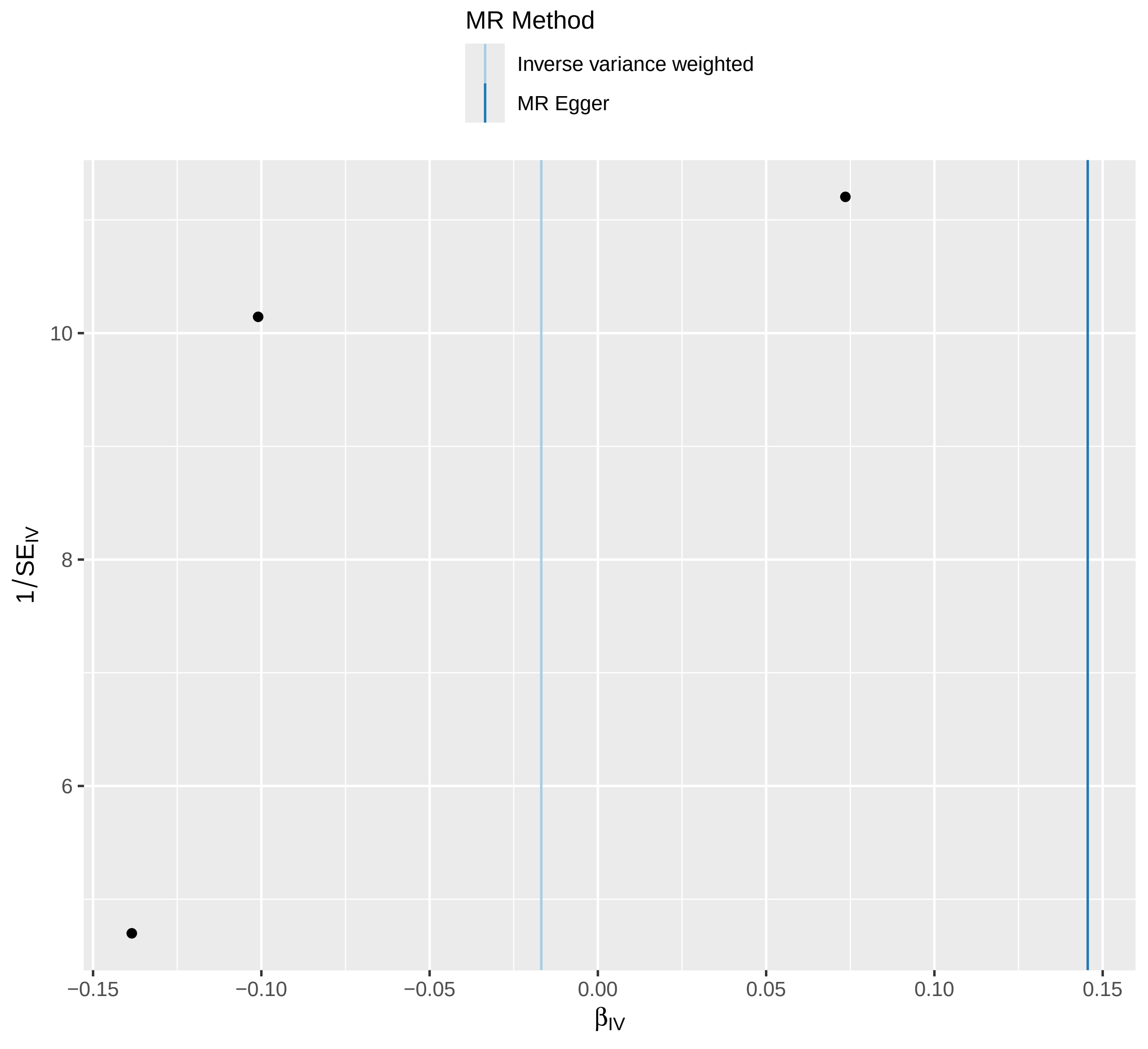

MR Method
Inverse variance weighted
MR Egger
1/SE_IV
10
8
6
−0.15
−0.10
−0.05
0.00
0.05
0.10
0.15
β_IV

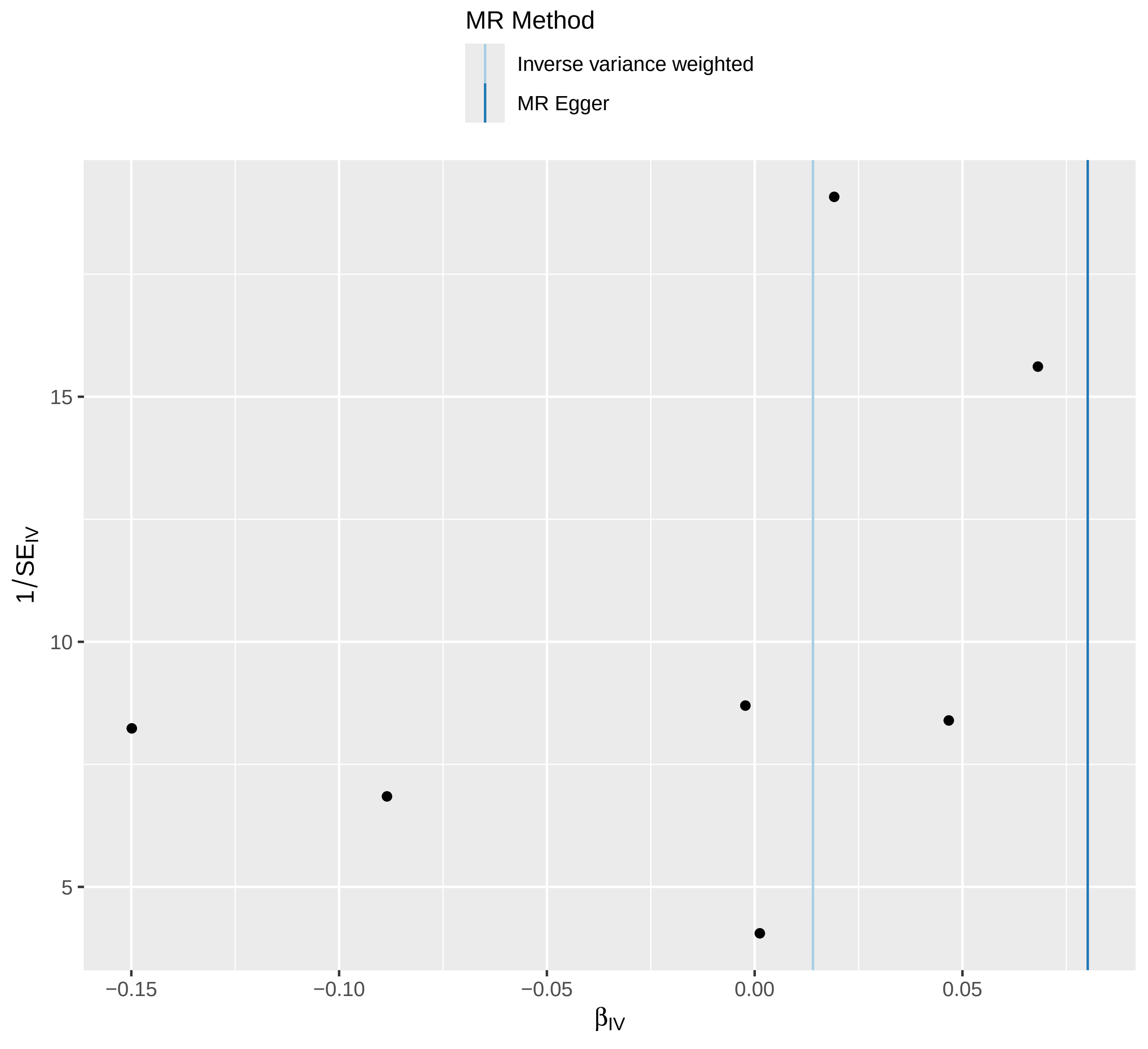
MR Method
Inverse variance weighted
MR Egger
15
10
5
1/$SE_{IV}$
−0.15
−0.10
−0.05
0.00
0.05
$\beta_{IV}$

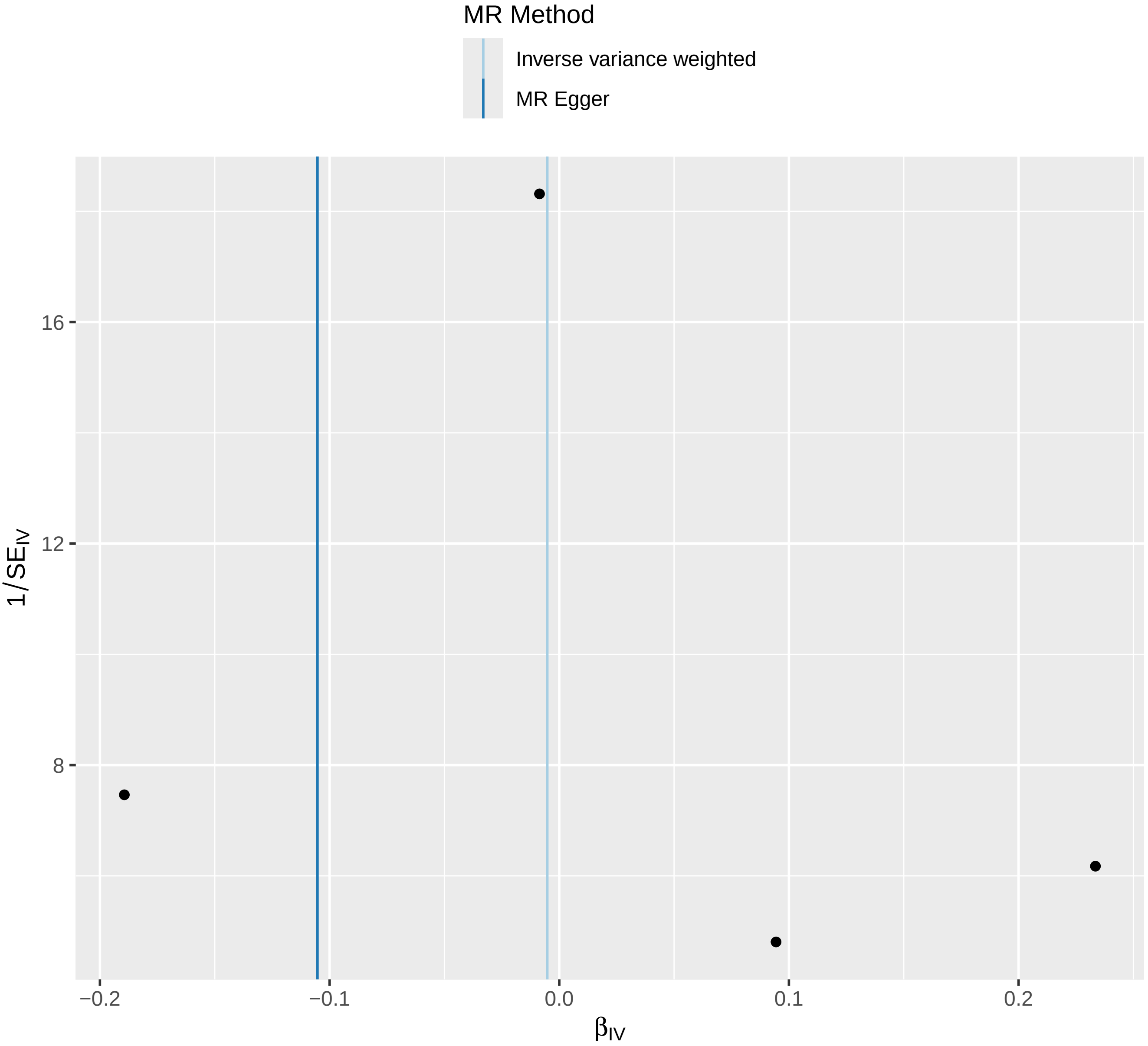
MR Method
Inverse variance weighted
MR Egger
16
12
8
$1/SE_{IV}$
−0.2
−0.1
0.0
0.1
0.2
$\beta_{IV}$

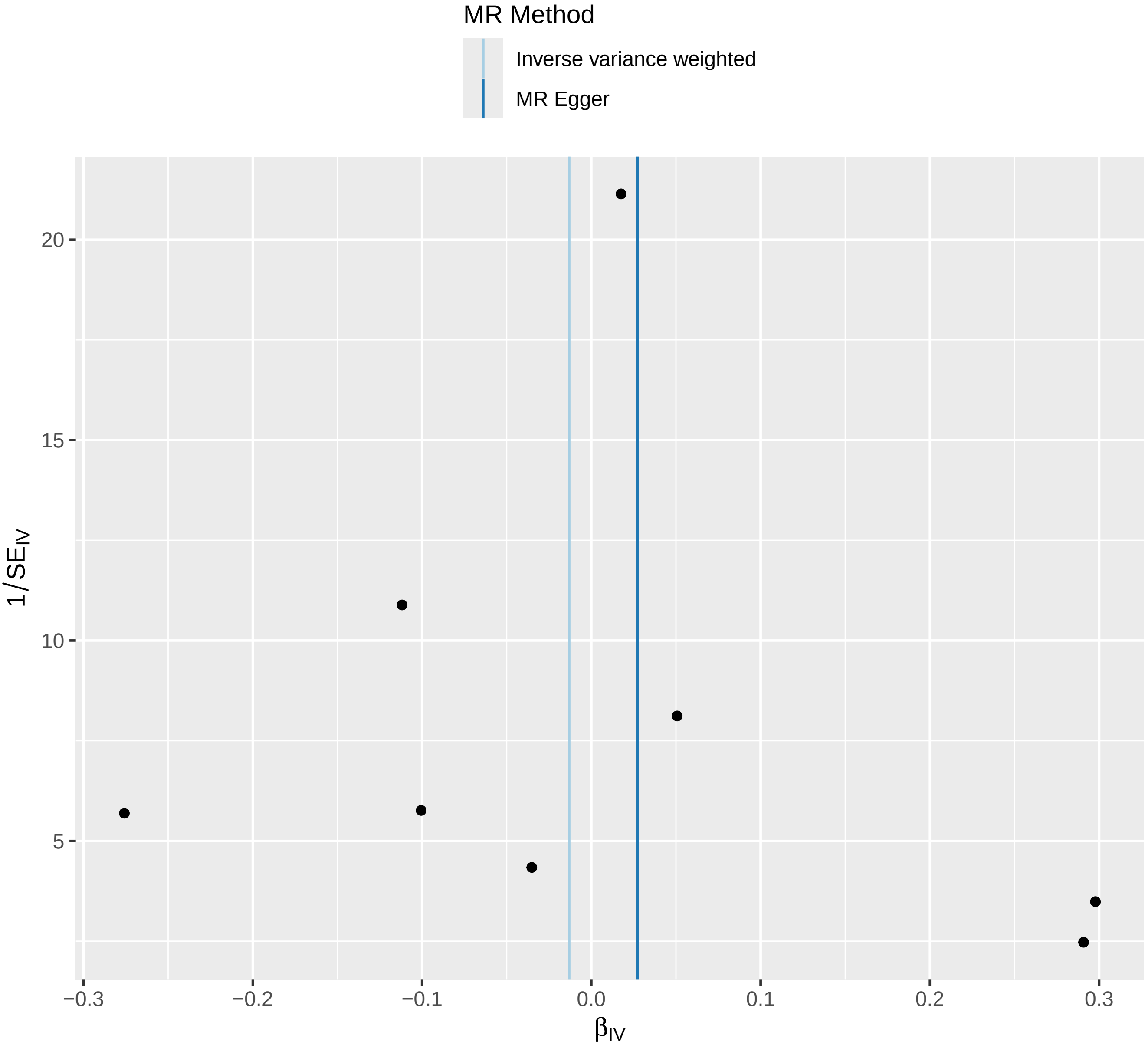

MR Method
Inverse variance weighted
MR Egger
20
15
10
5
−0.3
−0.2
−0.1
0.0
0.1
0.2
0.3
$1/SE_{IV}$
$\beta_{IV}$

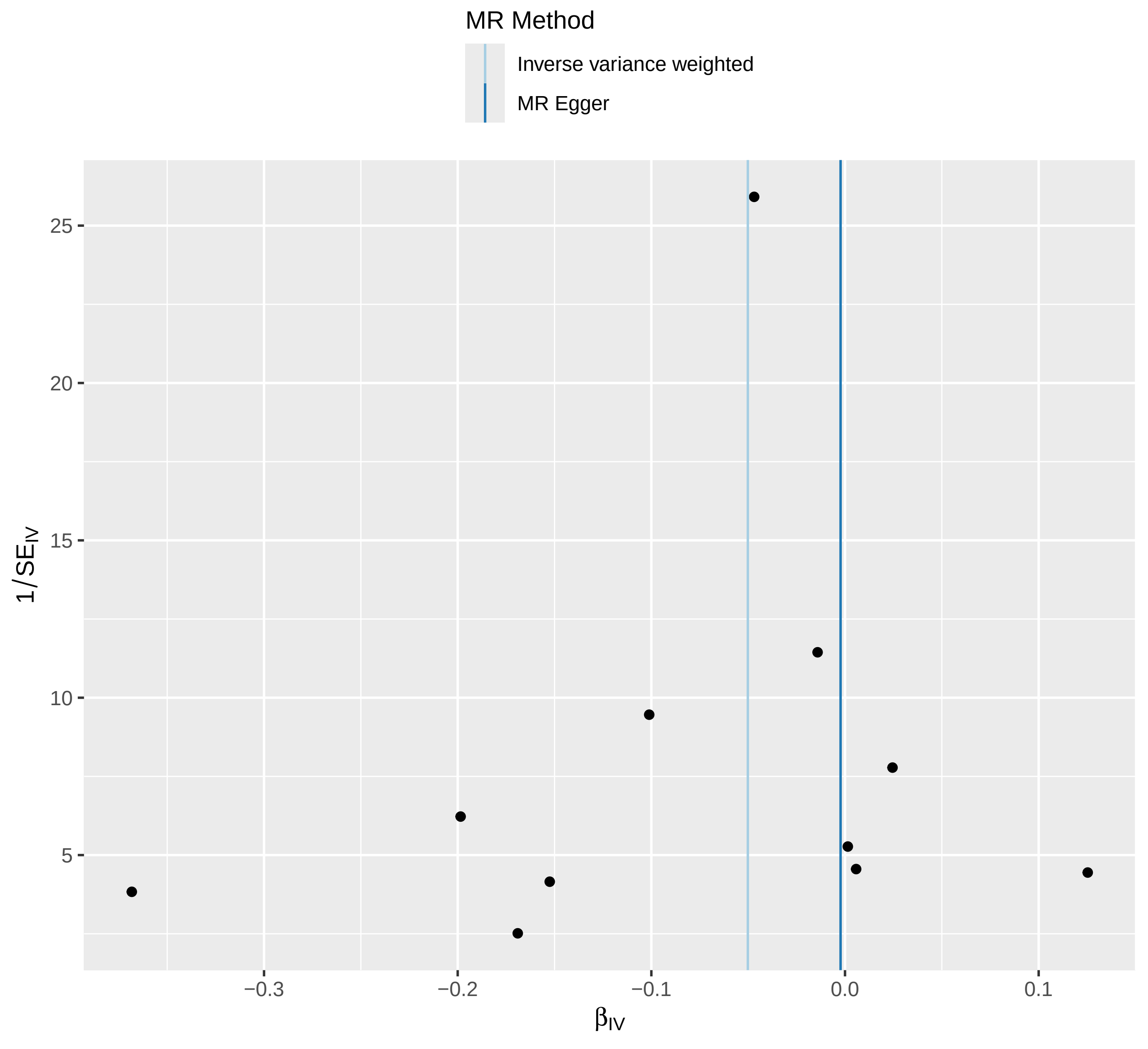

MR Method
Inverse variance weighted
MR Egger
25
20
15
10
5
1/SE$_{IV}$
−0.3
−0.2
−0.1
0.0
0.1
β$_{IV}$

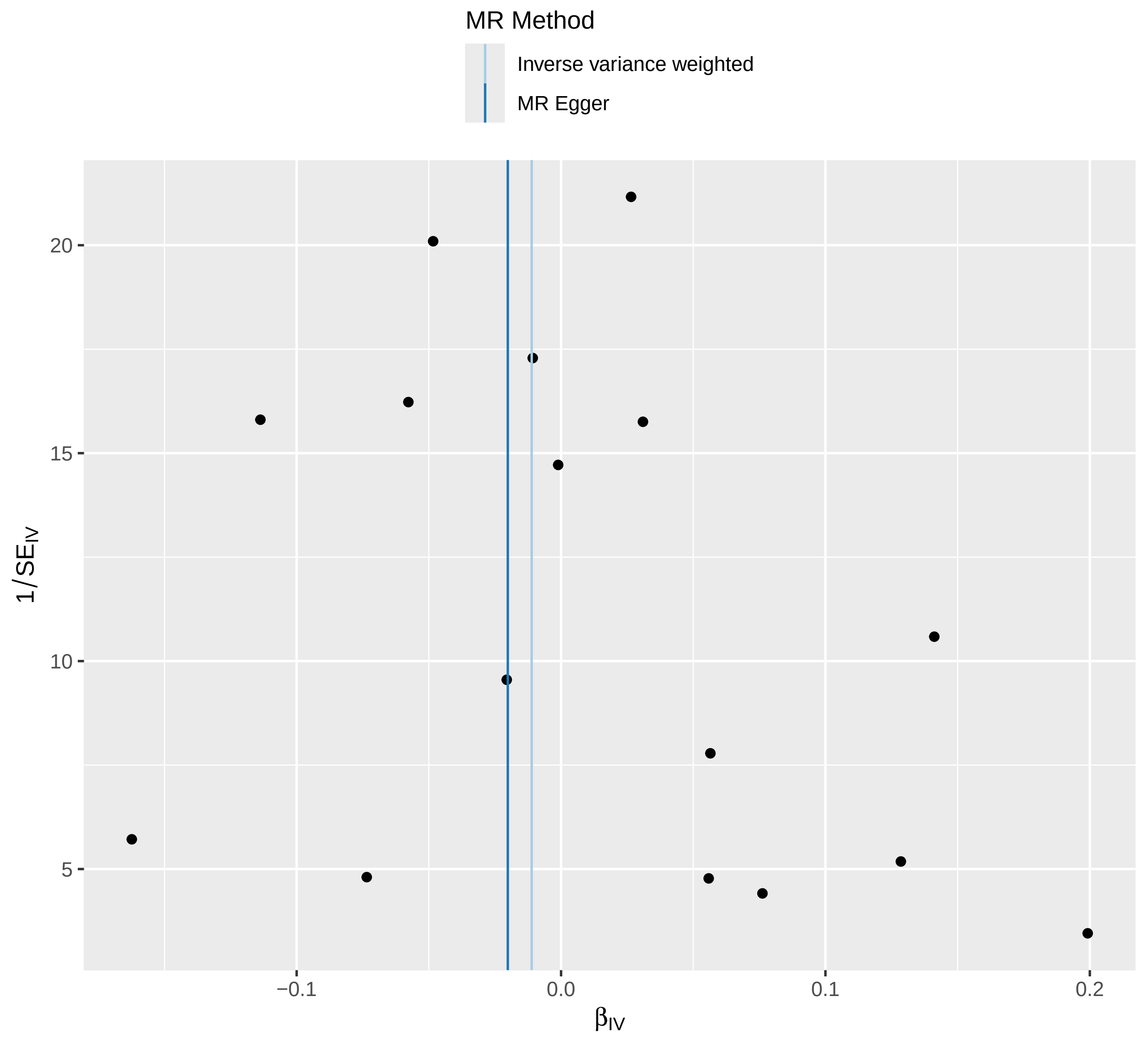
MR Method
Inverse variance weighted
MR Egger
20
15
10
5
1/SE_IV
−0.1
0.0
0.1
0.2
β_IV

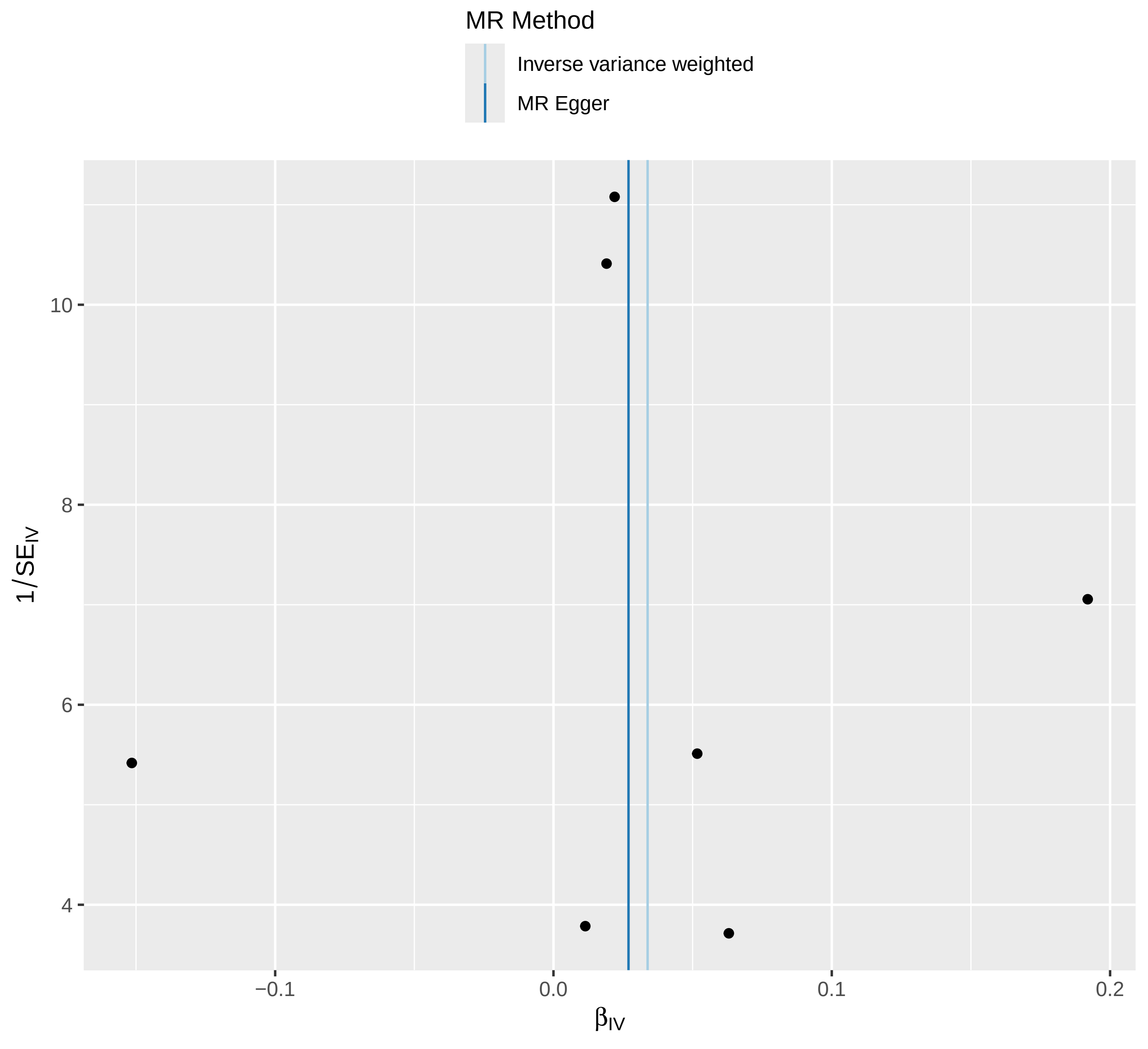

MR Method
Inverse variance weighted
MR Egger
10
8
6
4
1/SE_IV
−0.1
0.0
0.1
0.2
β_IV

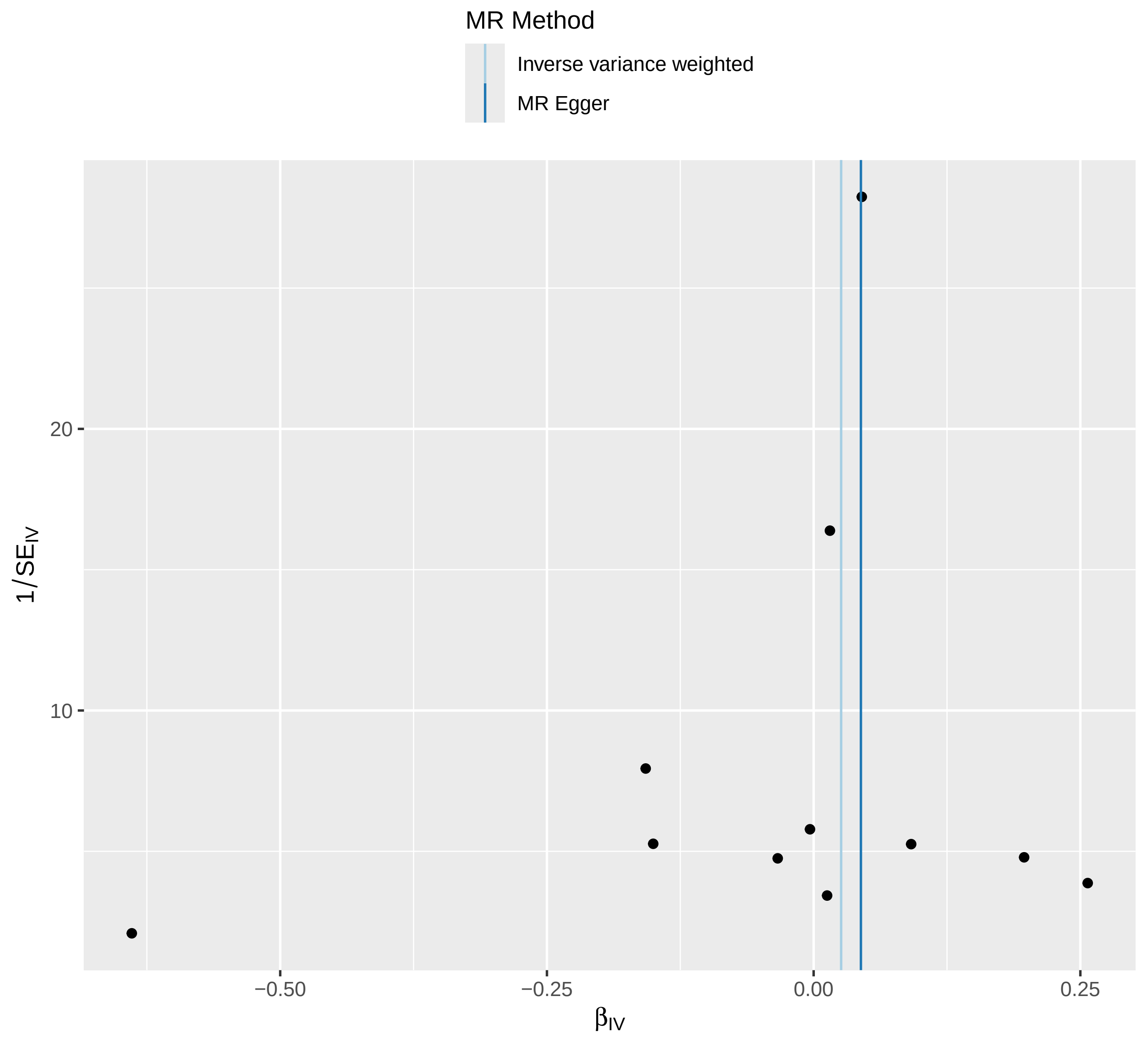
MR Method
Inverse variance weighted
MR Egger
20
10
1/SE_IV
−0.50
−0.25
0.00
0.25
β_IV

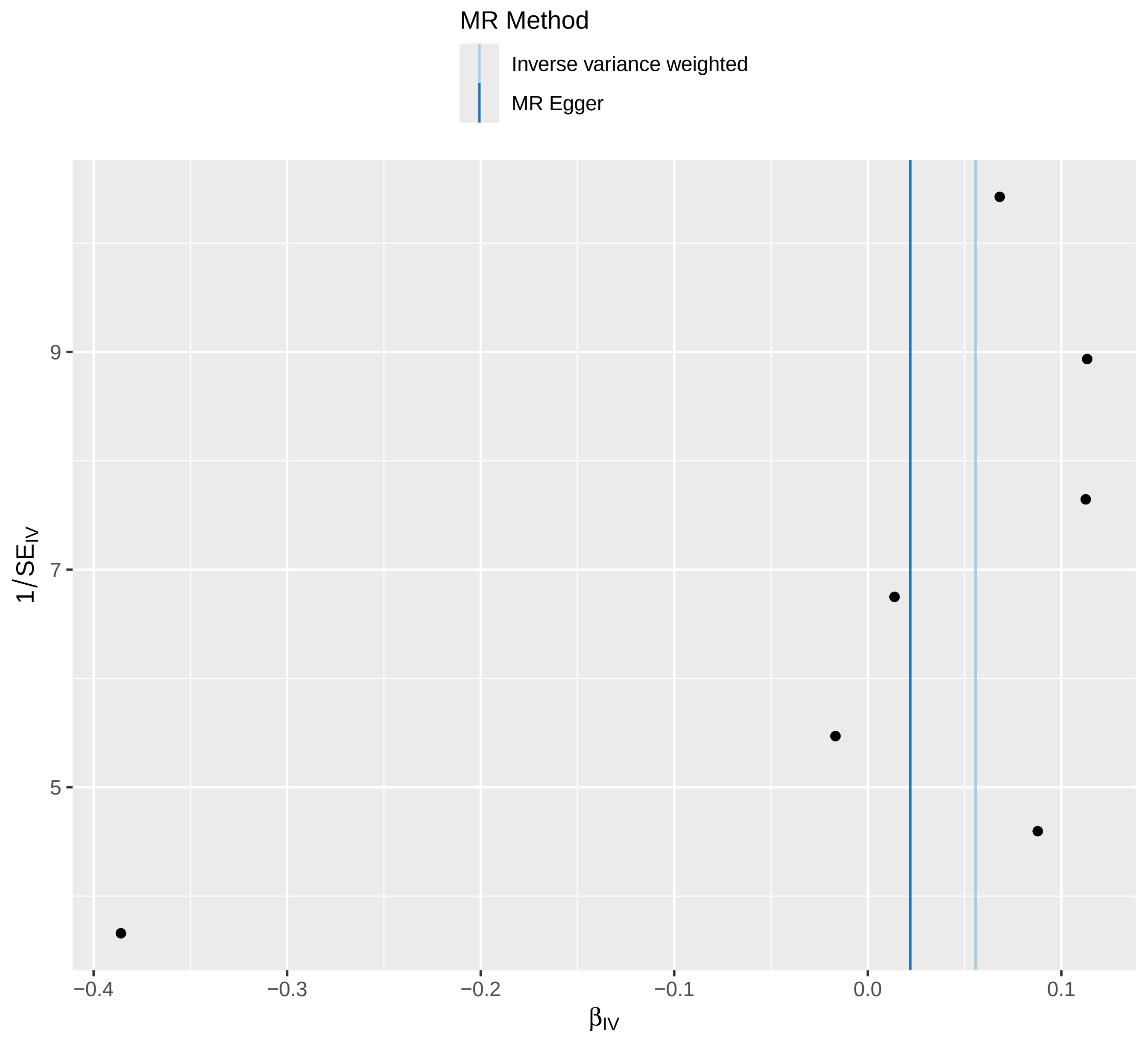

MR Method
Inverse variance weighted
MR Egger
1/SE_IV
9
7
5
−0.4
−0.3
−0.2
−0.1
0.0
0.1
β_IV

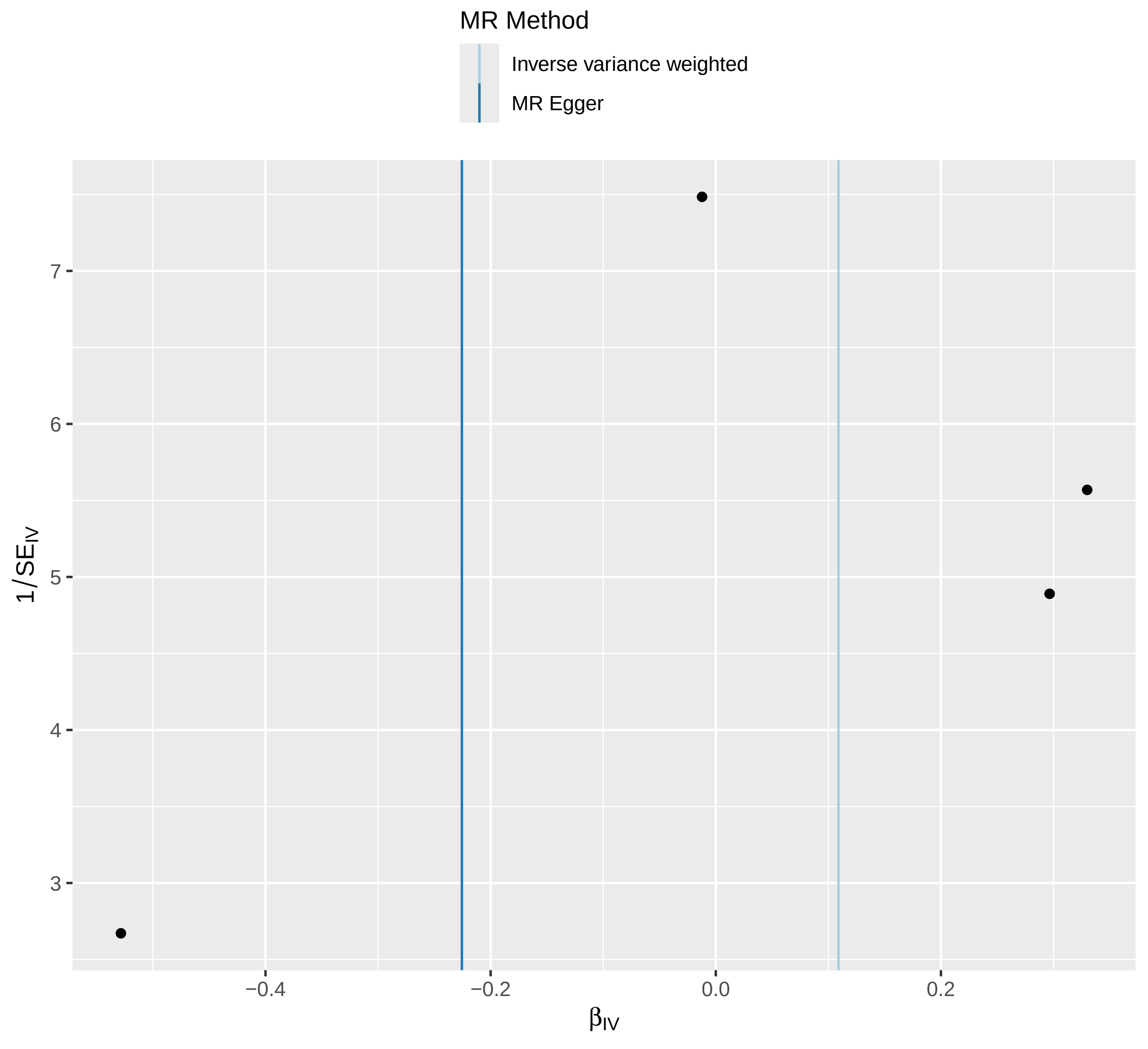
MR Method
Inverse variance weighted
MR Egger
7
6
5
4
3
−0.4
−0.2
0.0
0.2
$1/SE_{IV}$
$\beta_{IV}$

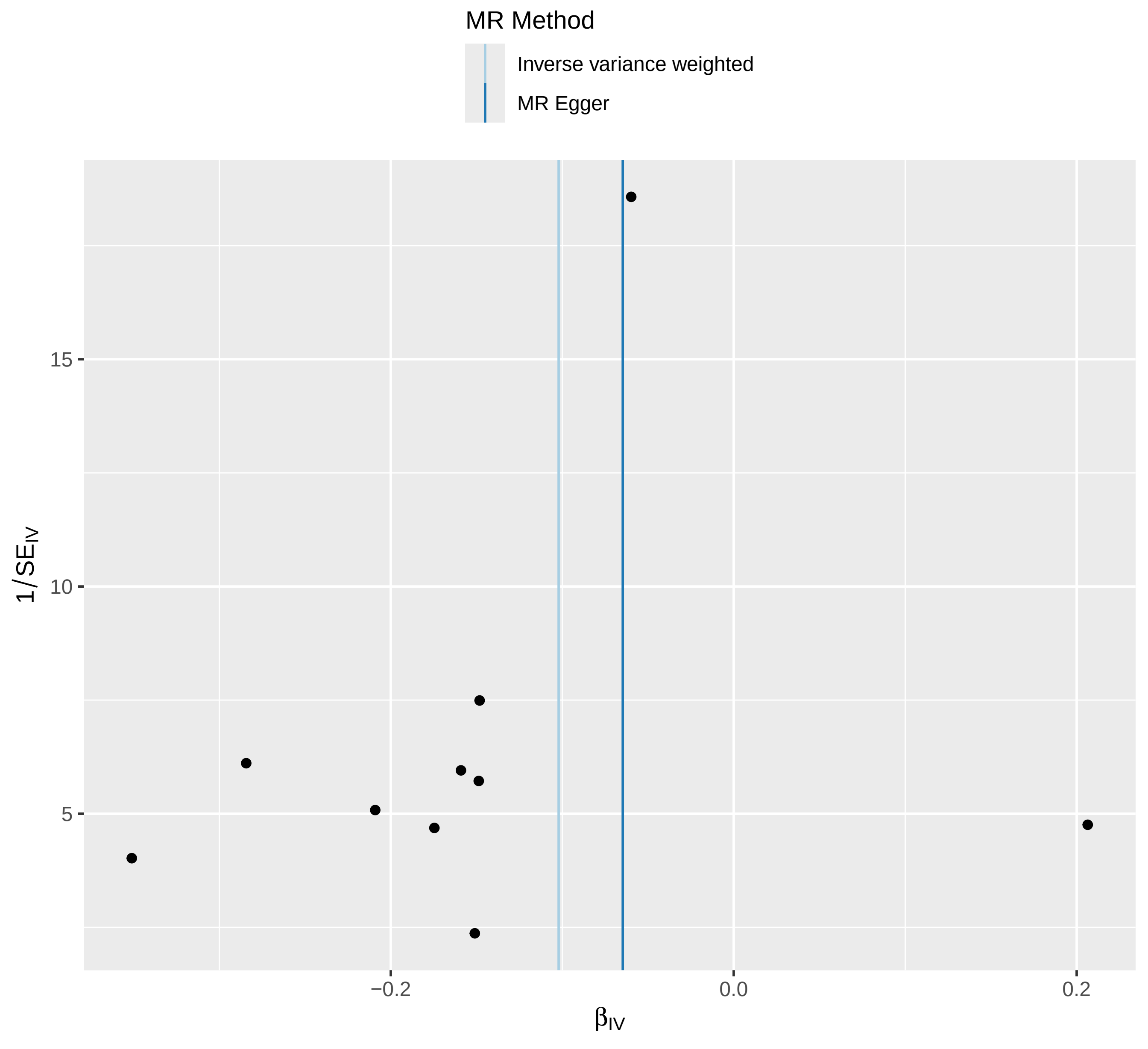

MR Method
Inverse variance weighted
MR Egger
15
10
5
1/SE_IV
−0.2
0.0
0.2
β_IV

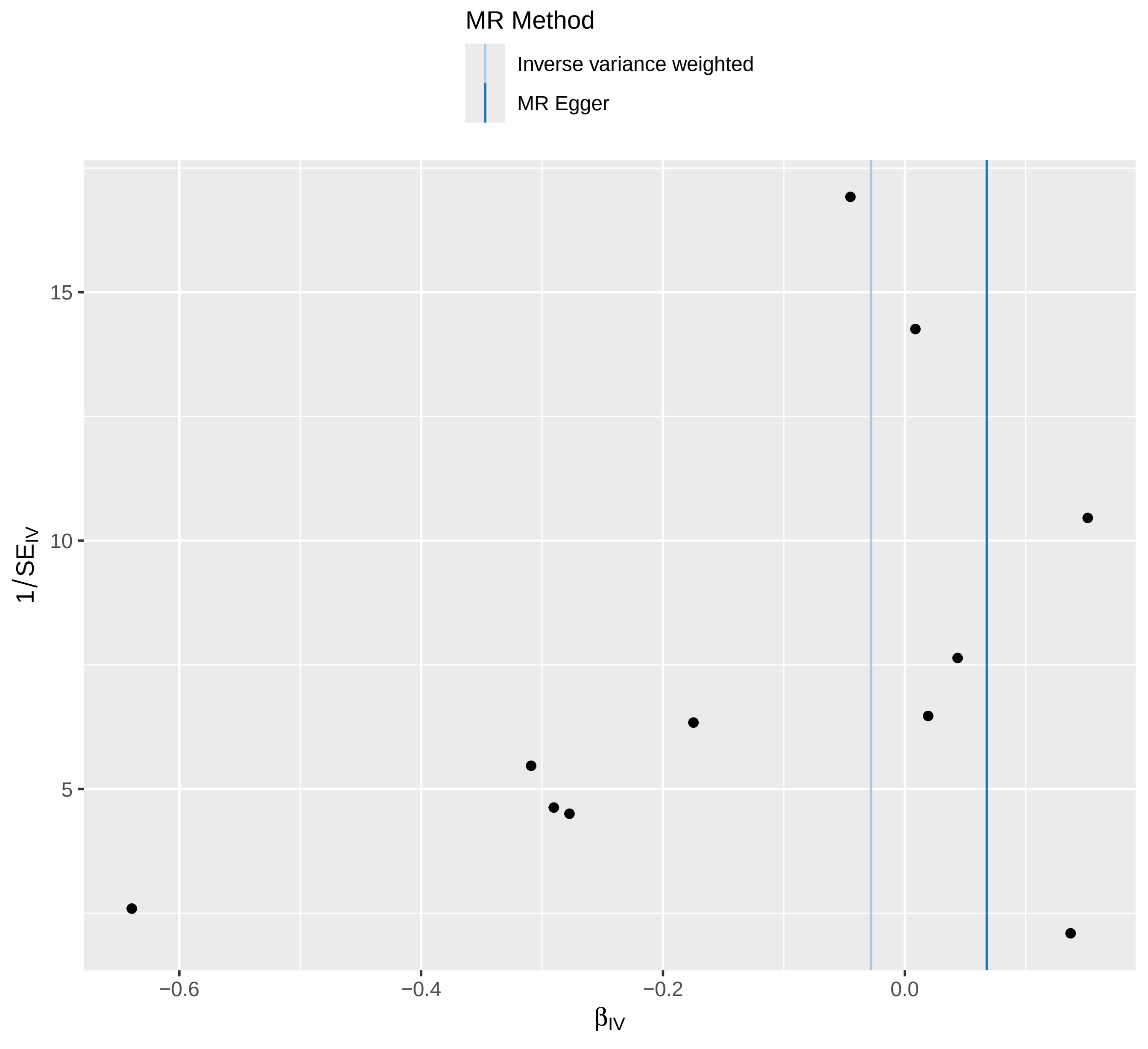

MR Method
Inverse variance weighted
MR Egger
15
10
5
1/SE_IV
−0.6
−0.4
−0.2
0.0
β_IV

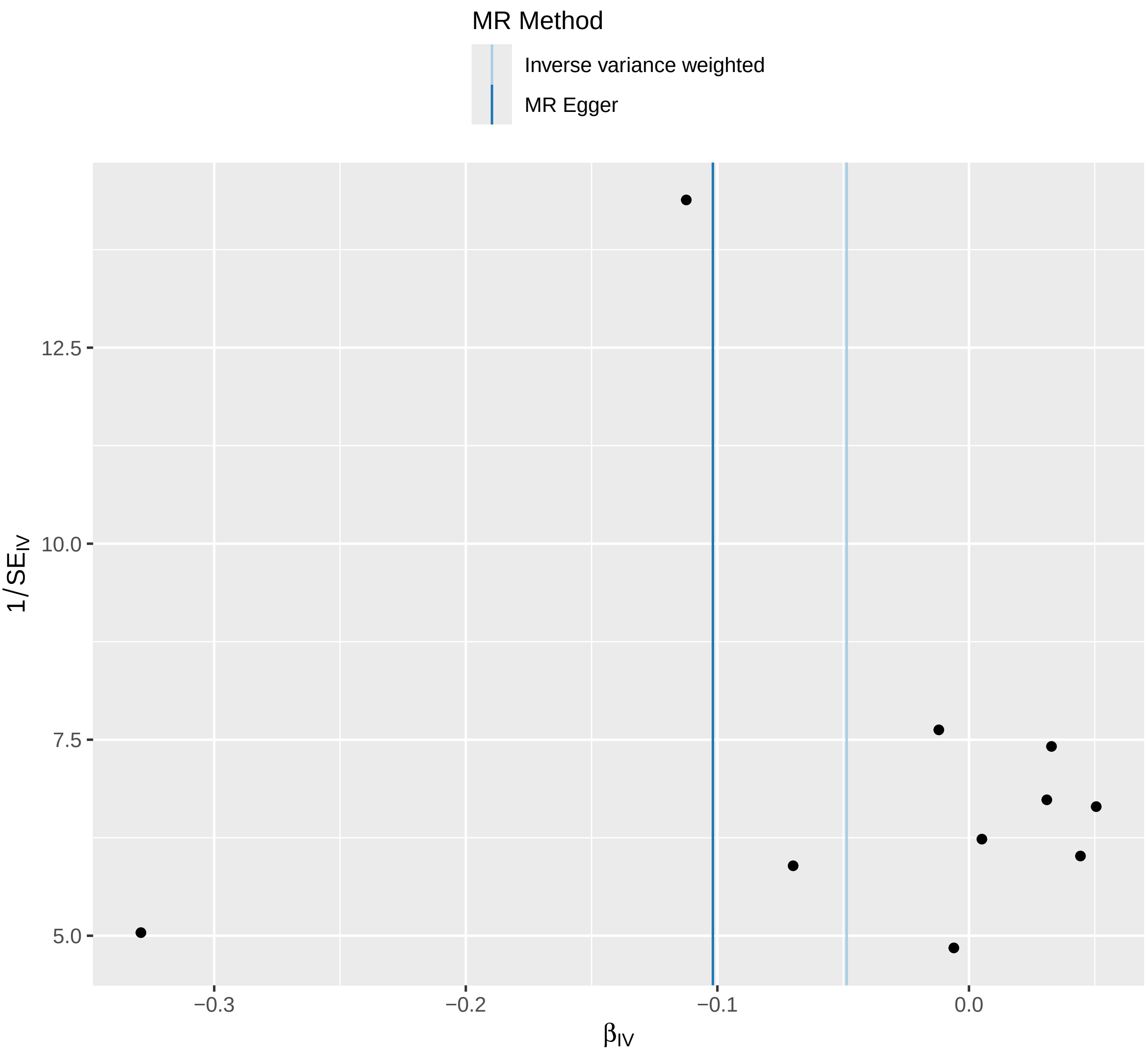

MR Method
Inverse variance weighted
MR Egger
12.5
10.0
7.5
5.0
1/SE_IV
−0.3
−0.2
−0.1
0.0
β_IV

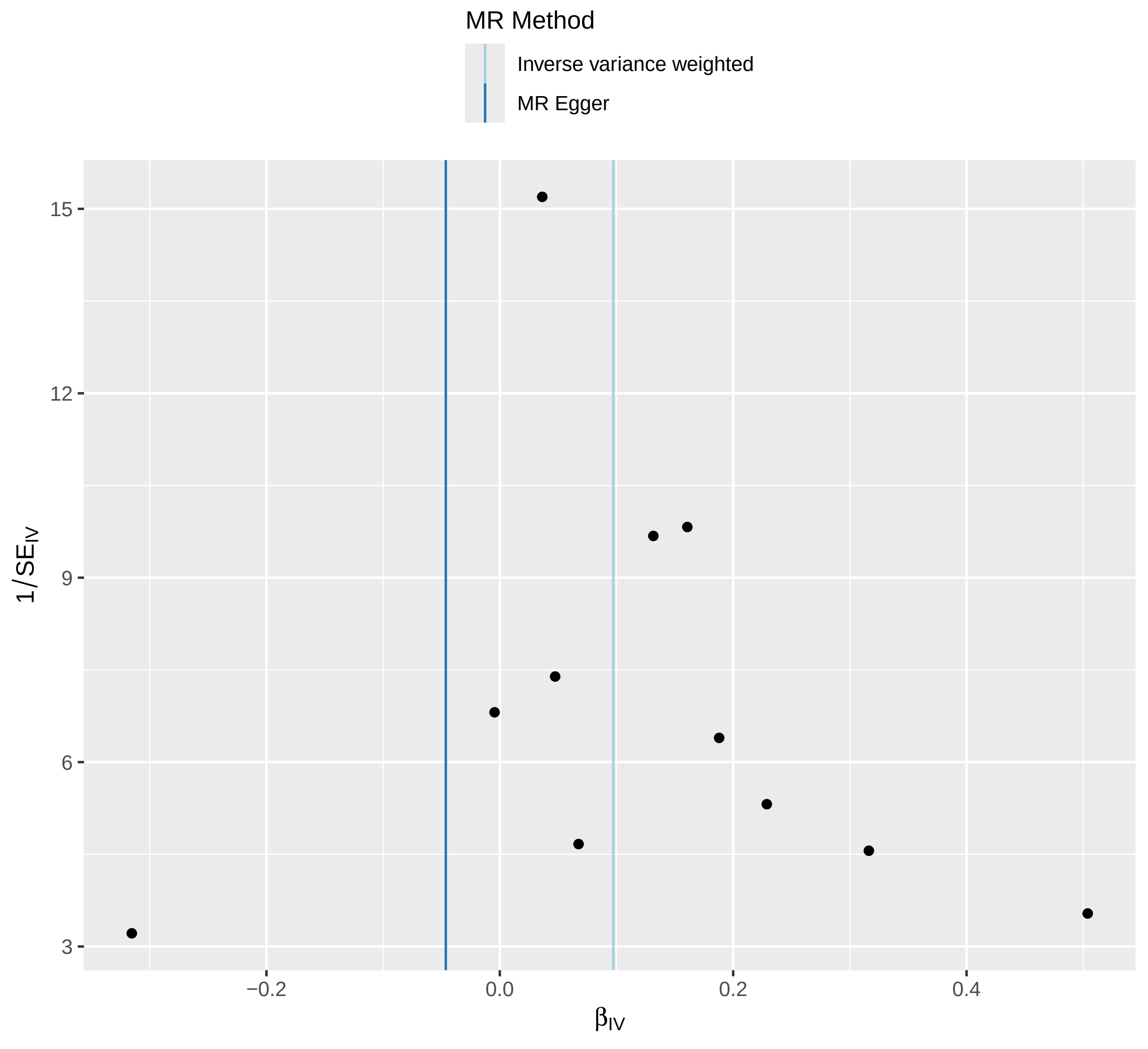
MR Method
Inverse variance weighted
MR Egger
15
12
9
6
3
1/SE_IV
−0.2
0.0
0.2
0.4
β_IV

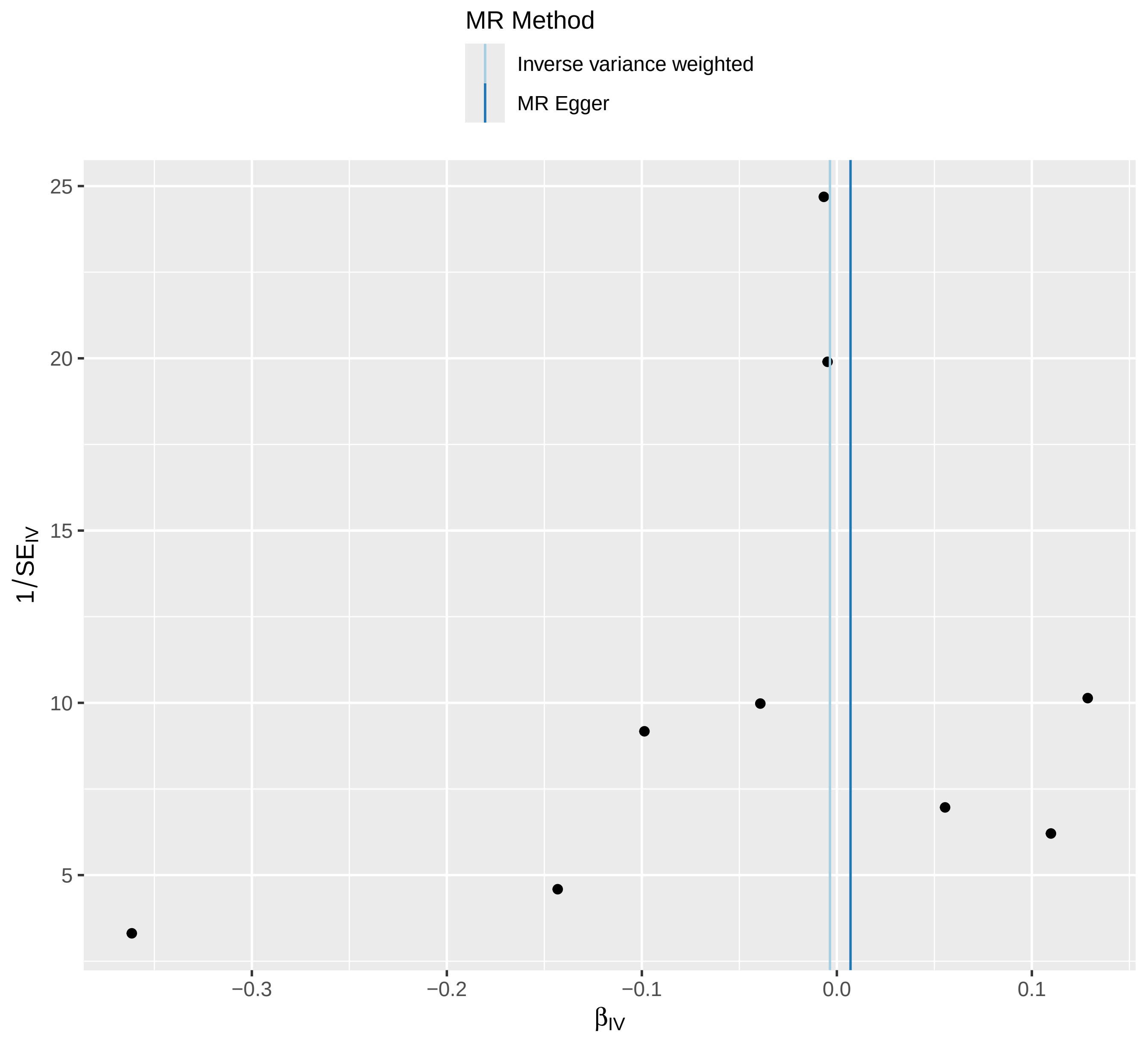
MR Method
Inverse variance weighted
MR Egger
25
20
15
10
5
$1/SE_{IV}$
−0.3
−0.2
−0.1
0.0
0.1
$\beta_{IV}$

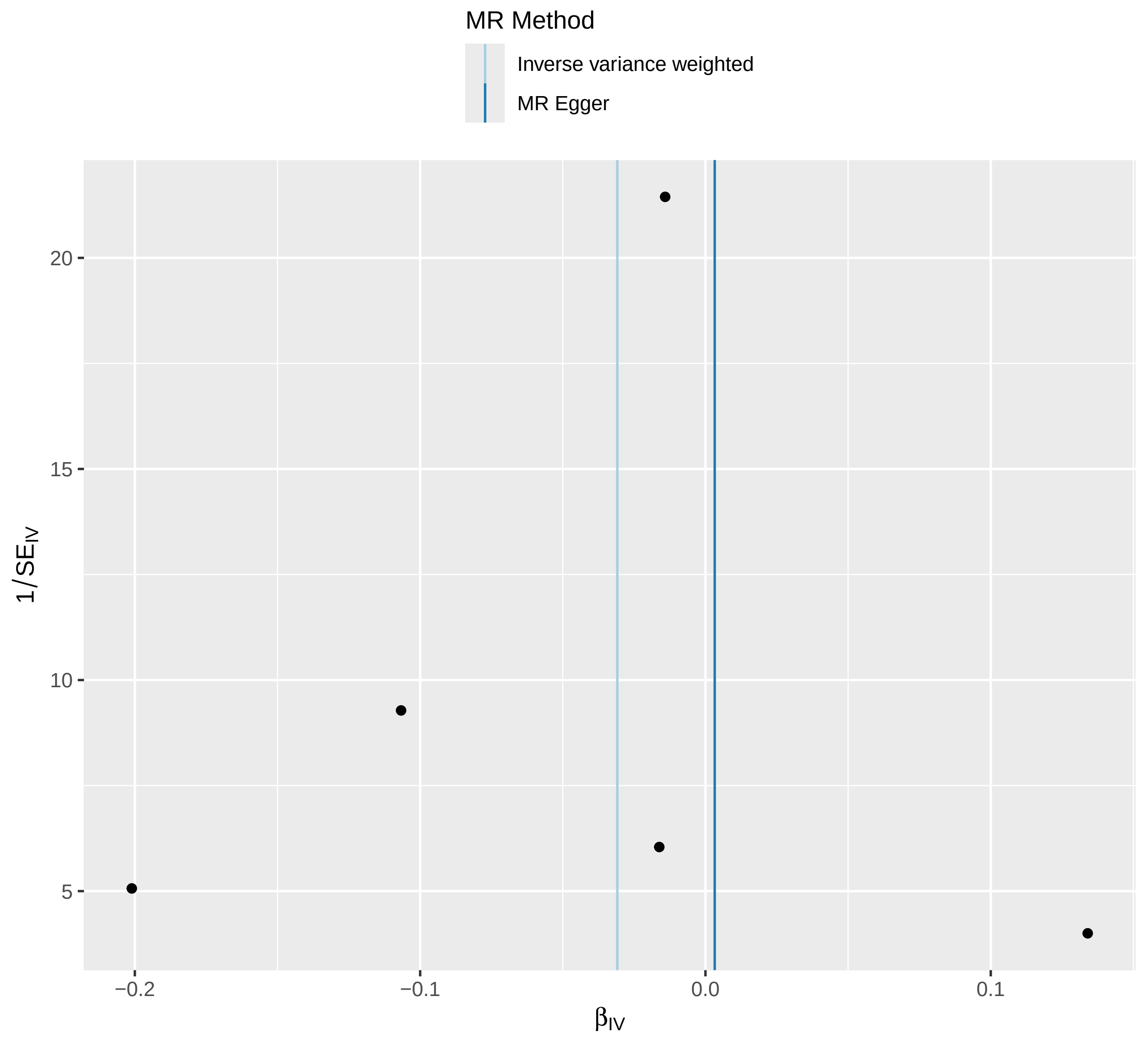

MR Method
Inverse variance weighted
MR Egger
20
15
10
5
$1/SE_{IV}$
−0.2
−0.1
0.0
0.1
$\beta_{IV}$

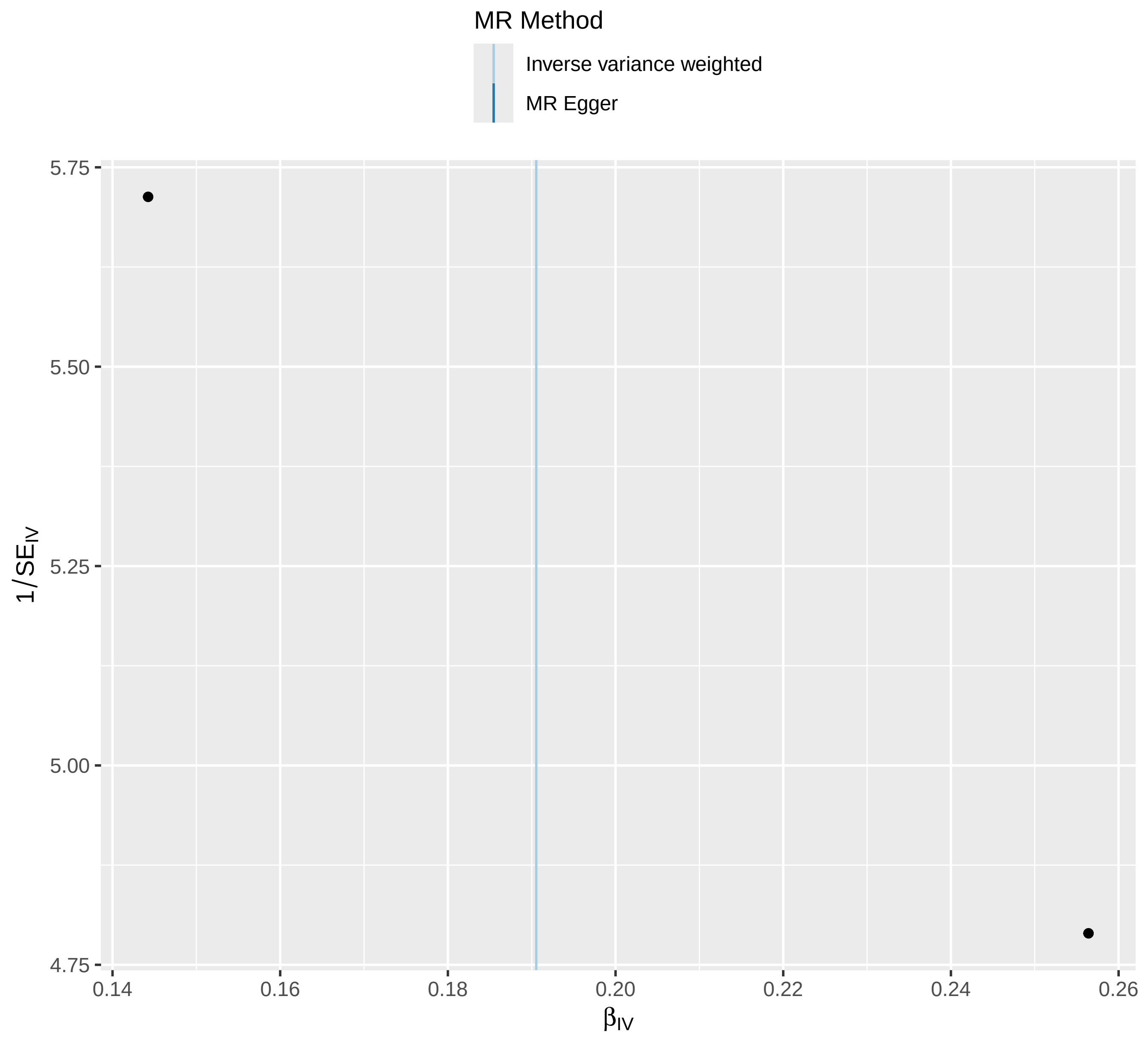
MR Method
Inverse variance weighted
MR Egger
5.75
5.50
5.25
5.00
4.75
1/SE_IV
0.14
0.16
0.18
0.20
0.22
0.24
0.26
β_IV

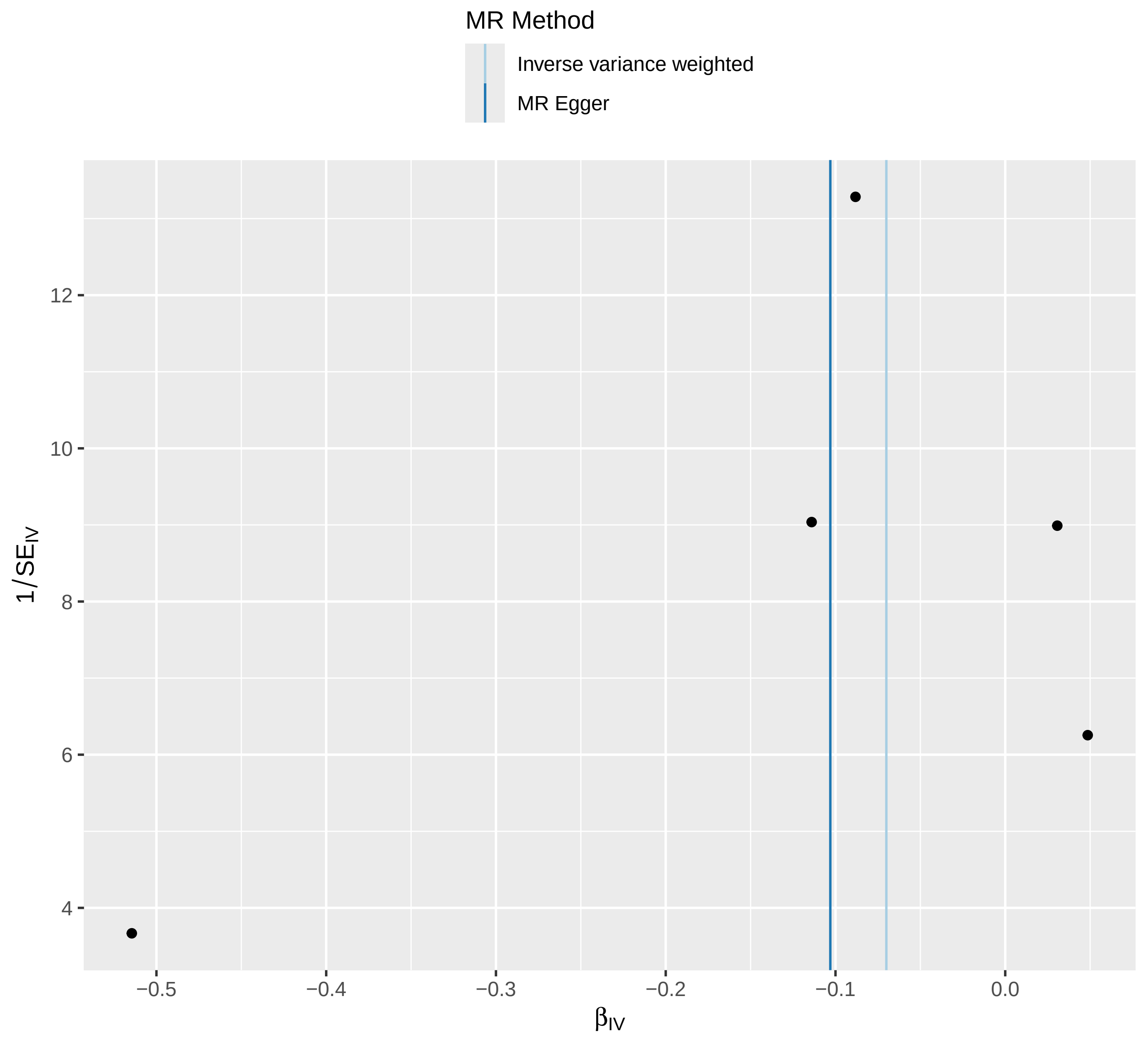

MR Method
Inverse variance weighted
MR Egger
1/SE$_{IV}$
12
10
8
6
4
−0.5
−0.4
−0.3
−0.2
−0.1
0.0
β$_{IV}$

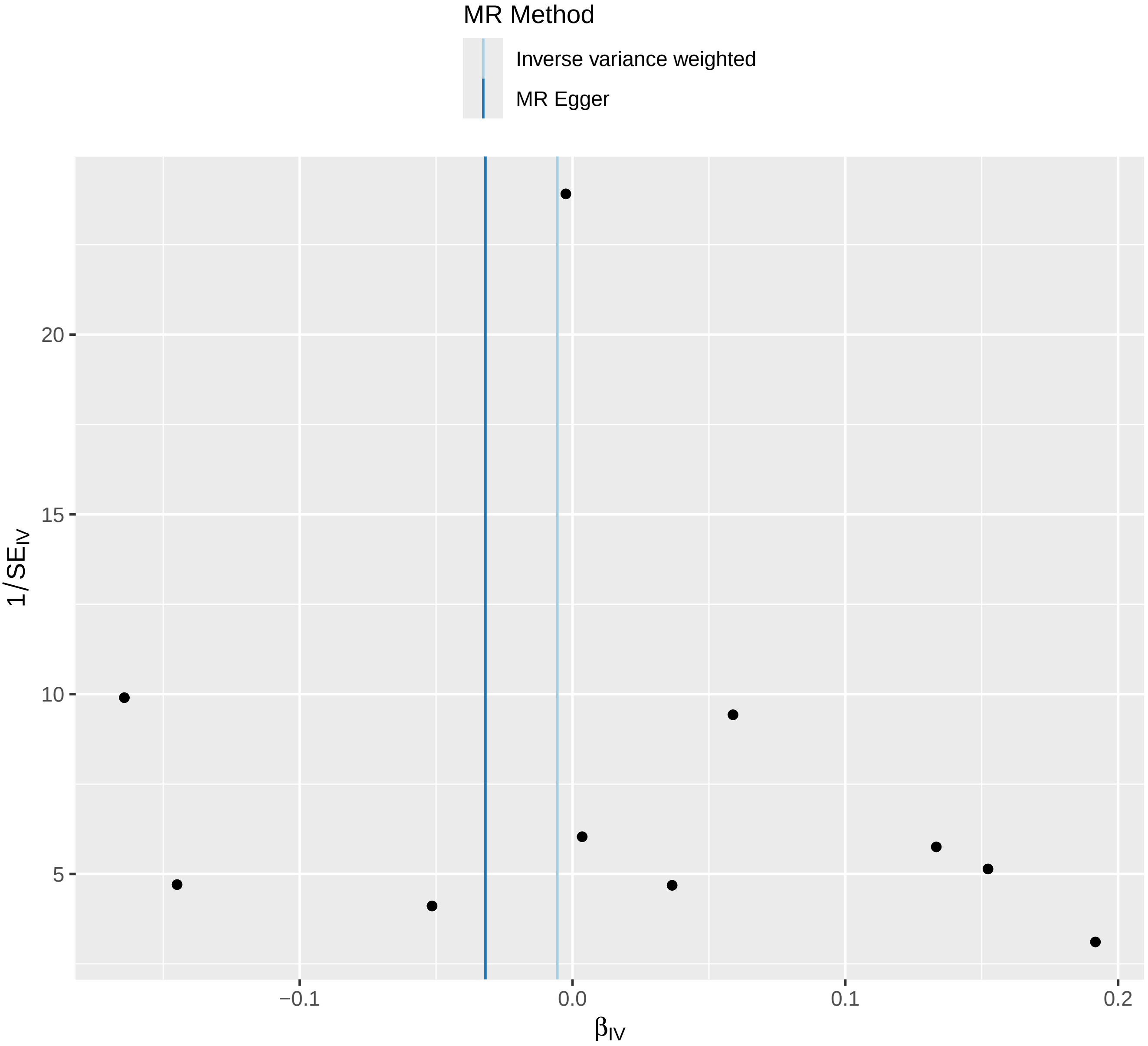

MR Method
Inverse variance weighted
MR Egger
20
15
10
5
$1/SE_{IV}$
−0.1
0.0
0.1
0.2
$\beta_{IV}$

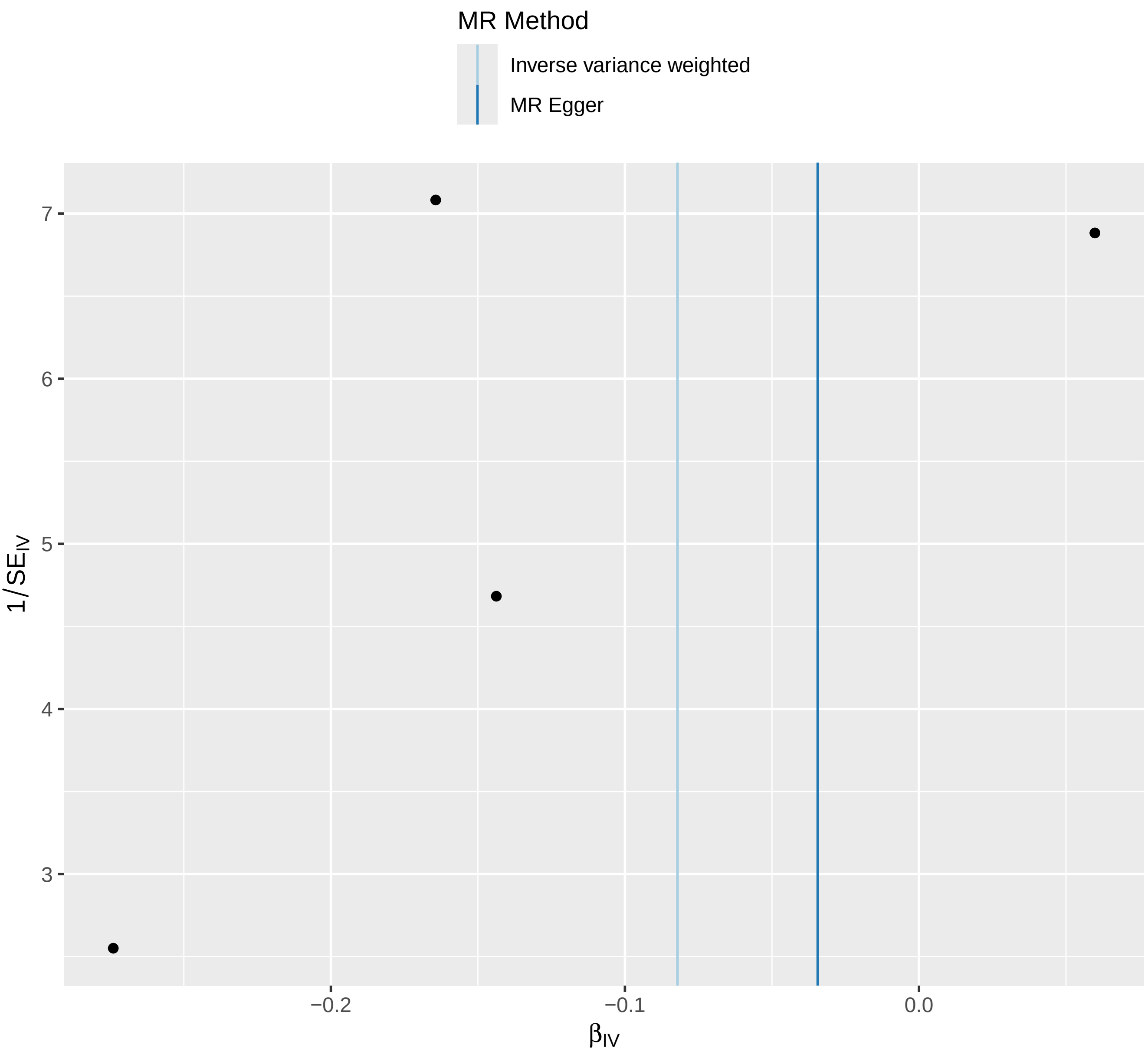
MR Method
Inverse variance weighted
MR Egger
7
6
5
4
3
1/SE_IV
−0.2
−0.1
0.0
β_IV

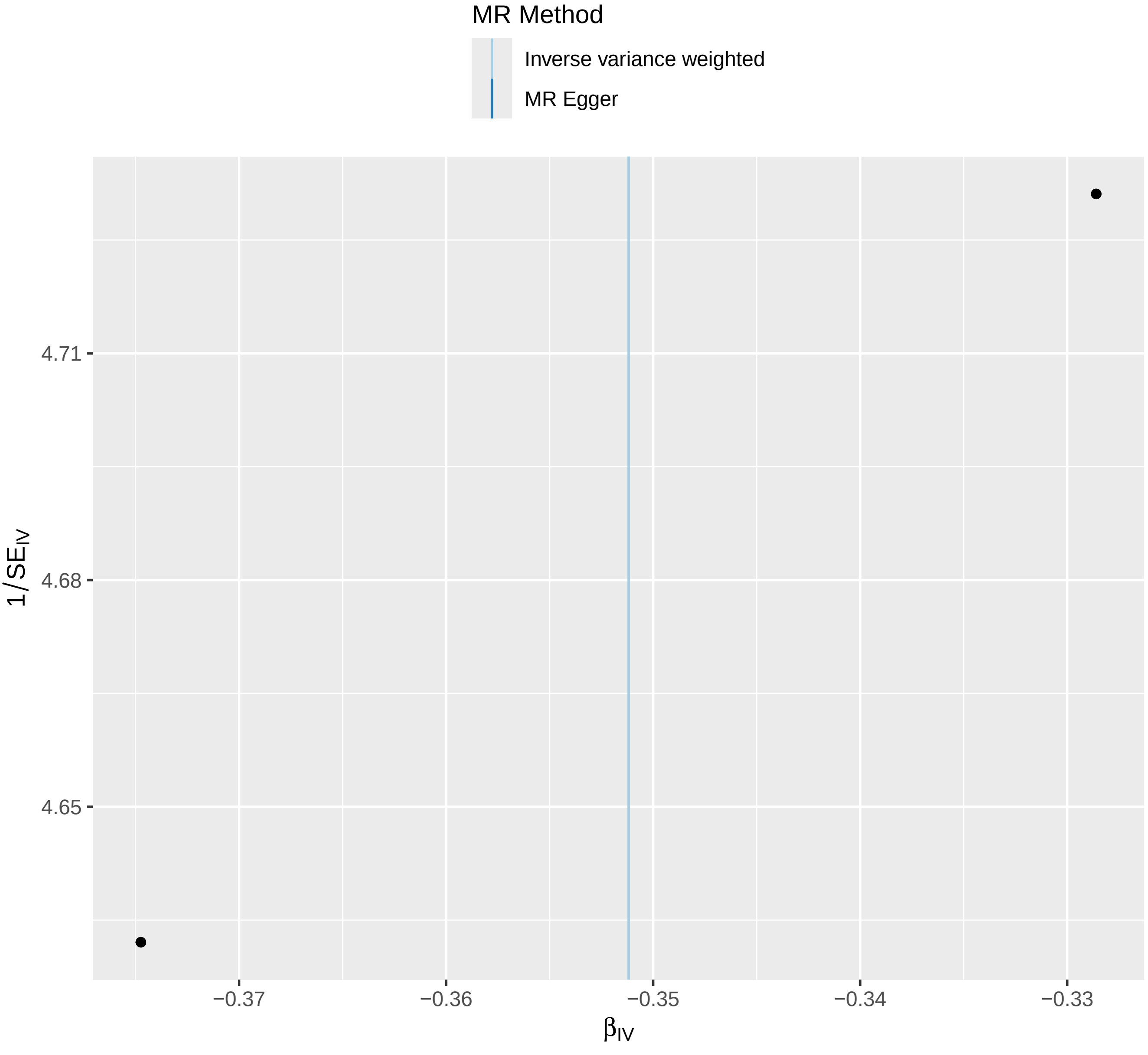
MR Method
Inverse variance weighted
MR Egger
4.71
4.68
4.65
1/SE$_{IV}$
−0.37
−0.36
−0.35
−0.34
−0.33
β$_{IV}$

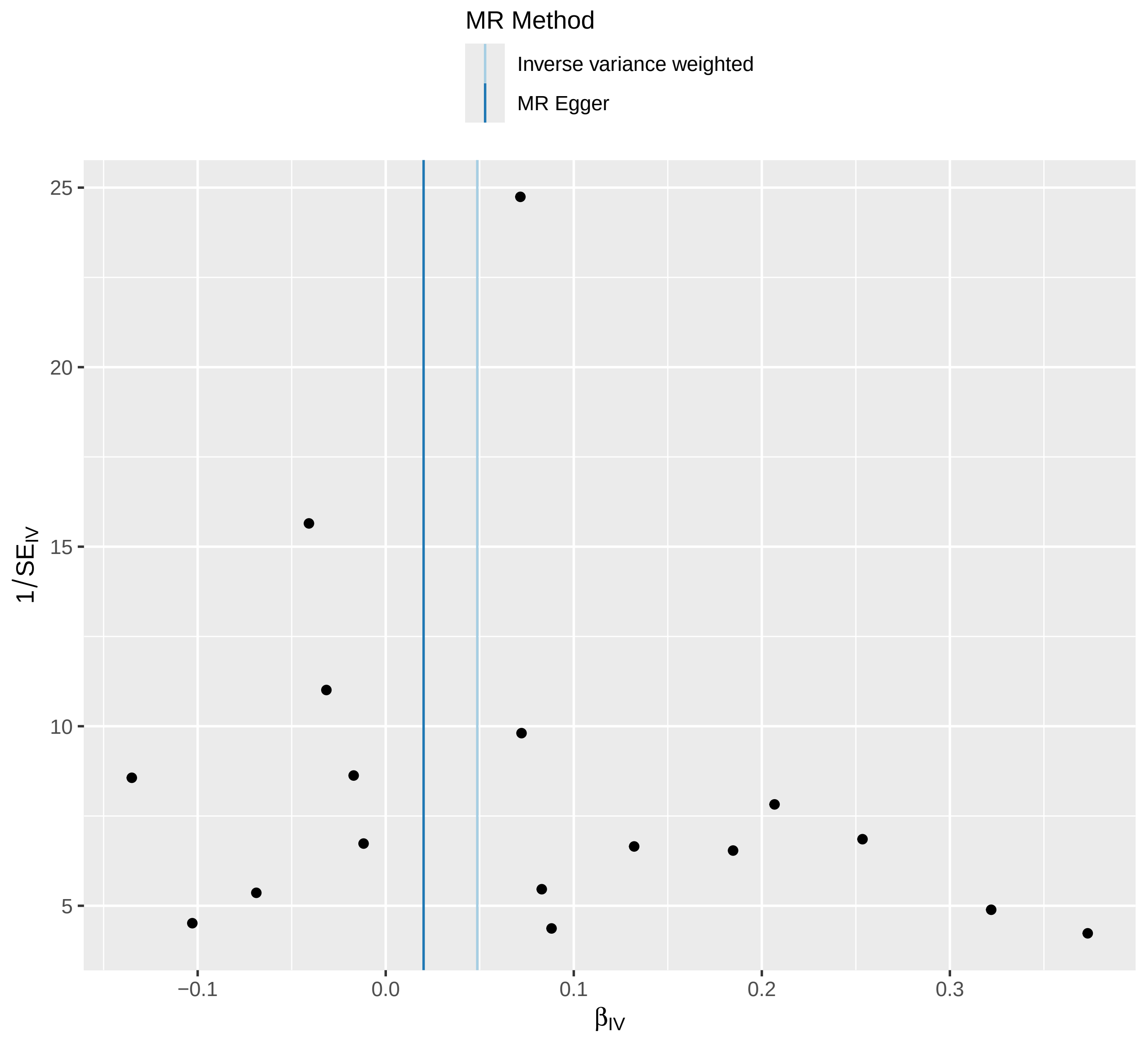

MR Method
Inverse variance weighted
MR Egger
25
20
15
10
5
1/SE_IV
−0.1
0.0
0.1
0.2
0.3
β_IV

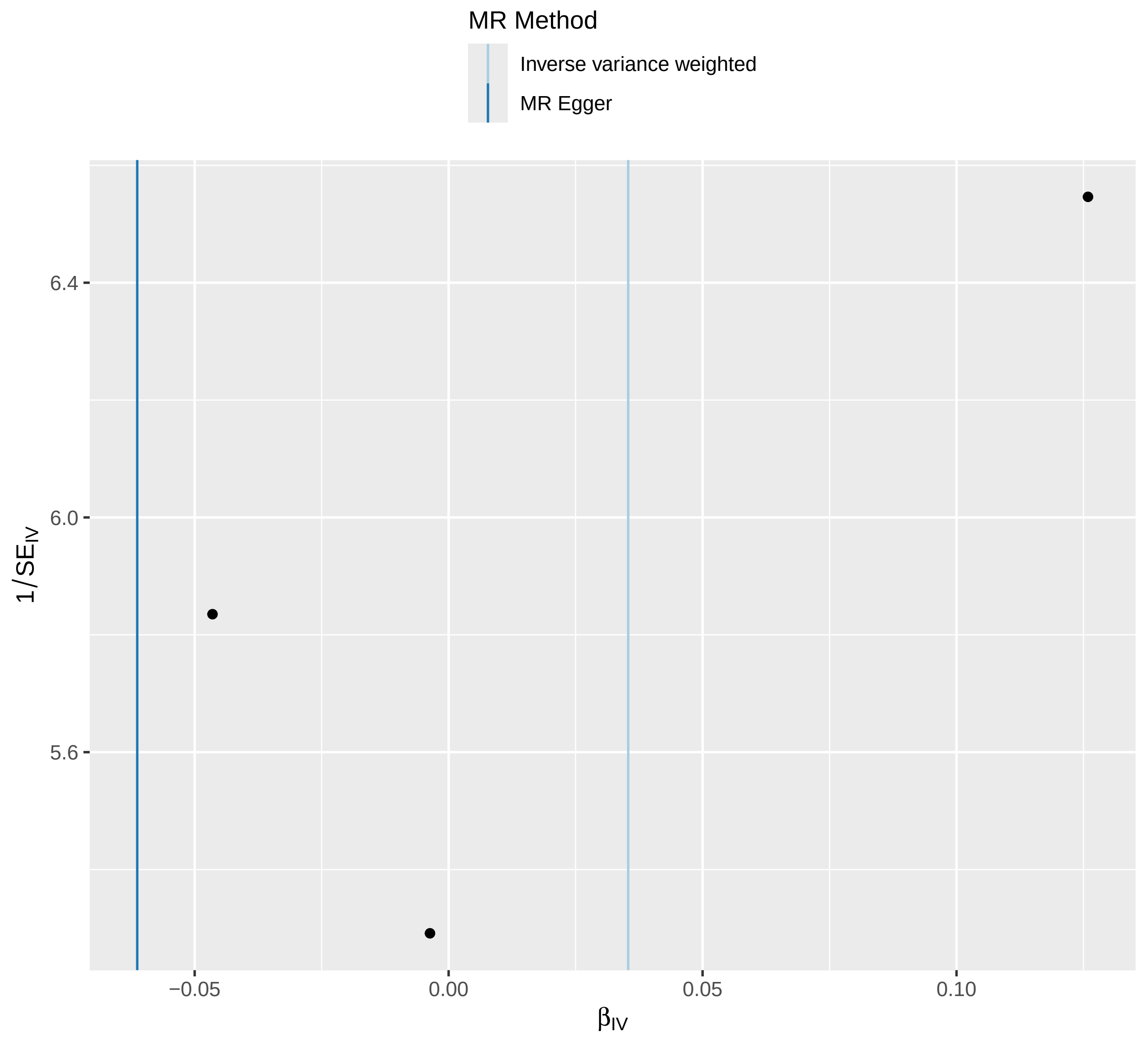

MR Method
Inverse variance weighted
MR Egger
6.4
6.0
5.6
1/SE_IV
−0.05
0.00
0.05
0.10
β_IV

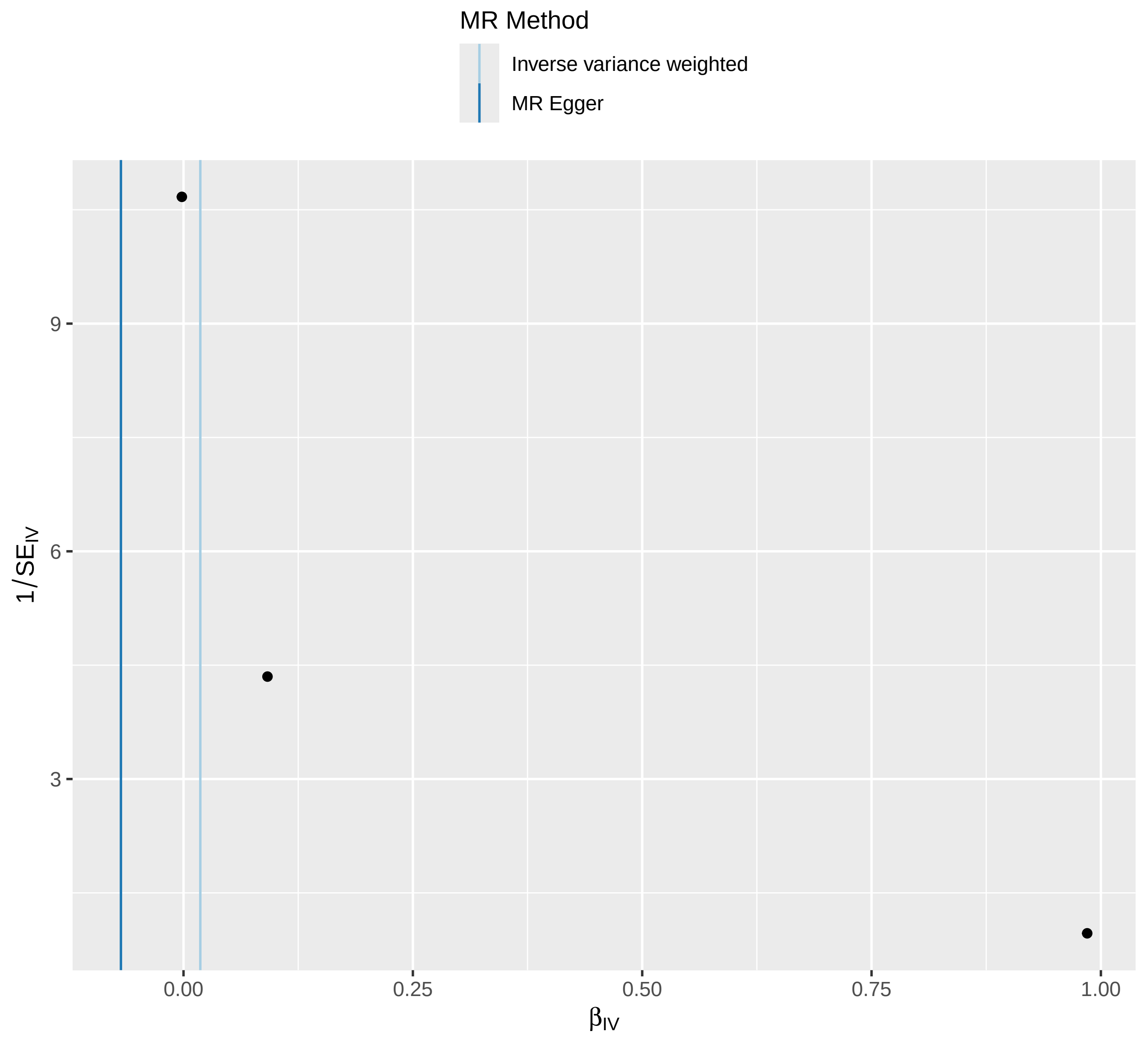
MR Method
Inverse variance weighted
MR Egger
9
6
3
1/SE_IV
0.00
0.25
0.50
0.75
1.00
β_IV

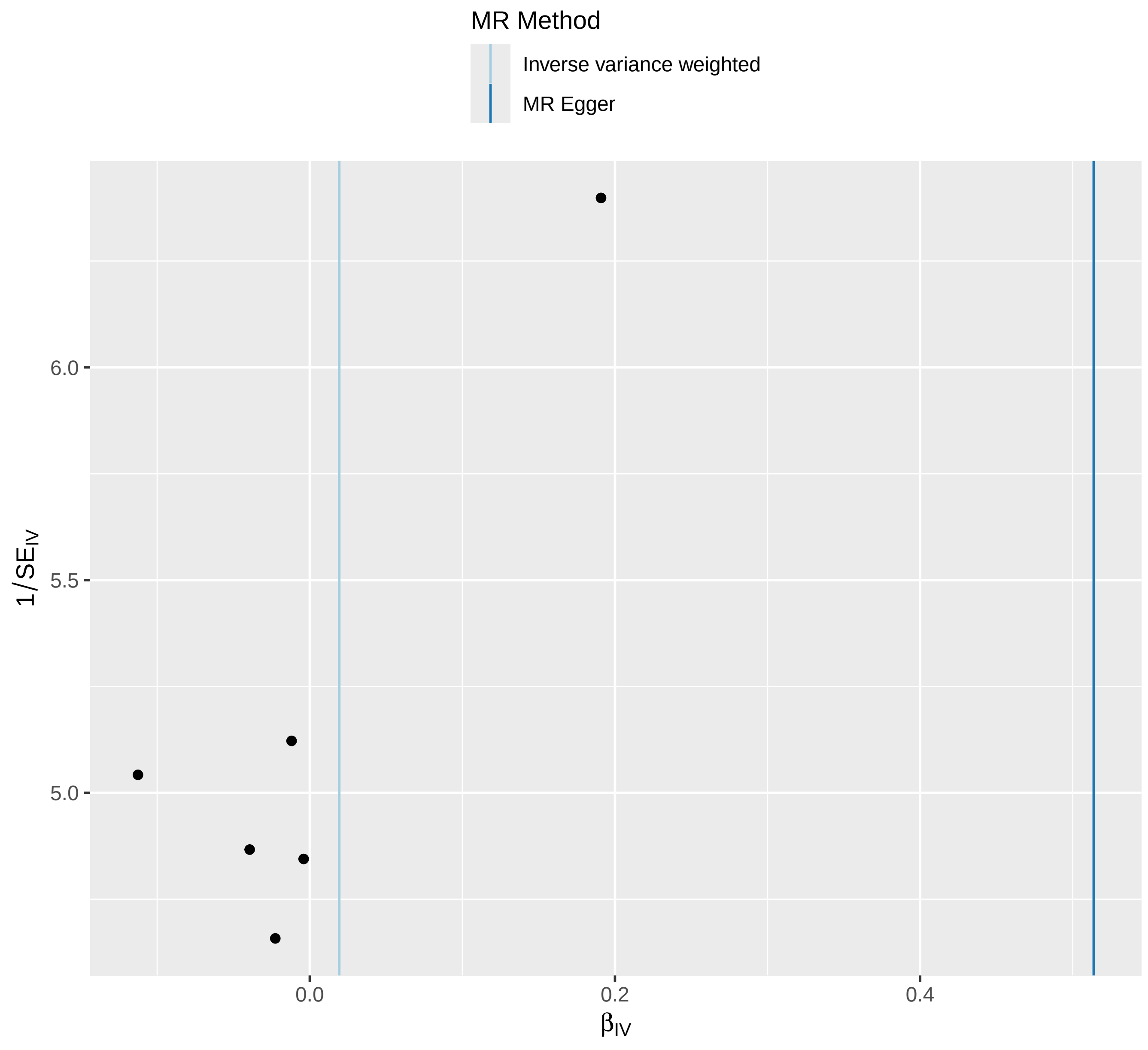
MR Method
Inverse variance weighted
MR Egger
6.0
5.5
5.0
$1/SE_{IV}$
0.0
0.2
0.4
$\beta_{IV}$

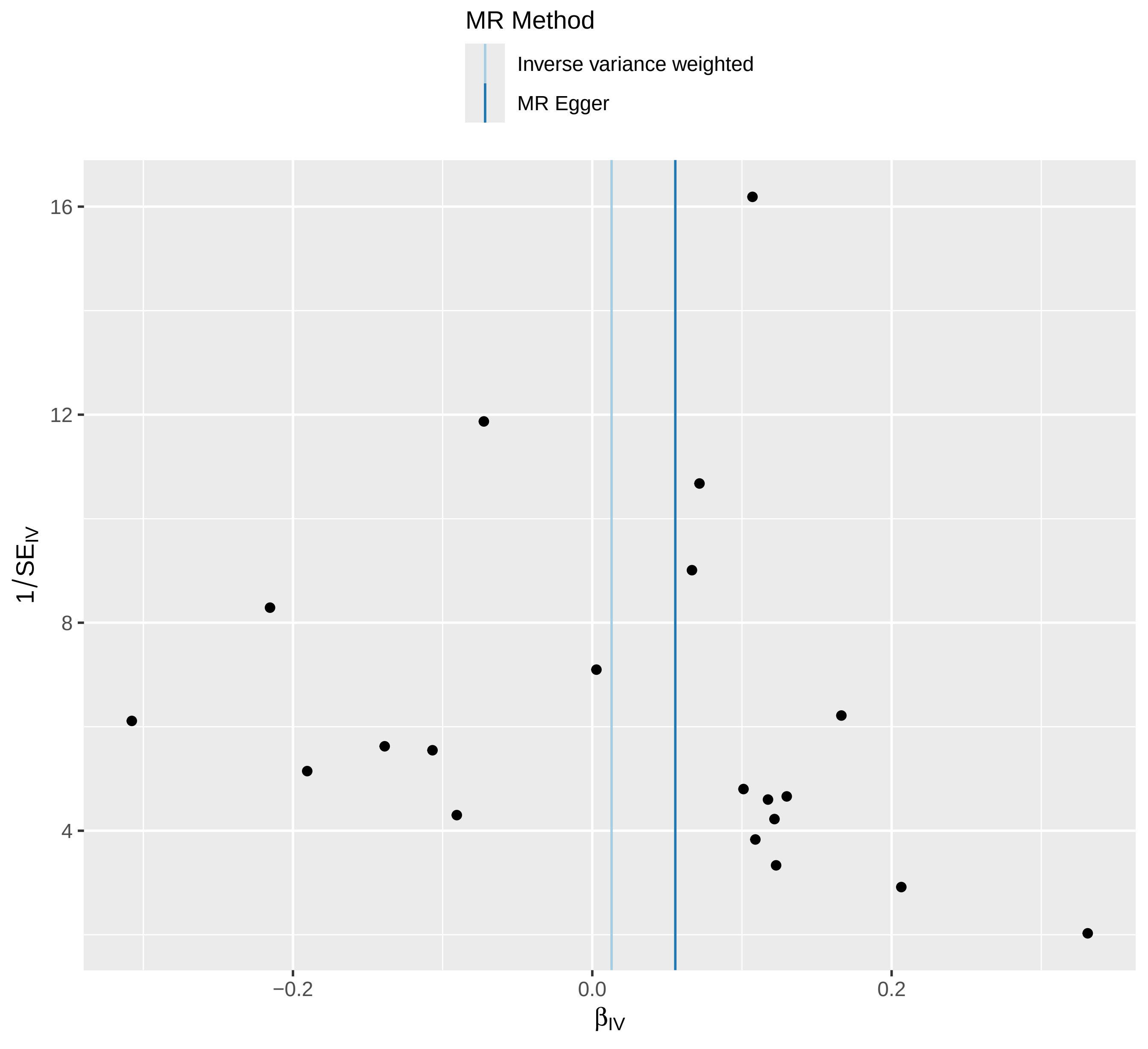
MR Method
Inverse variance weighted
MR Egger
16
12
8
4
1/SE$_{IV}$
−0.2
0.0
0.2
β$_{IV}$

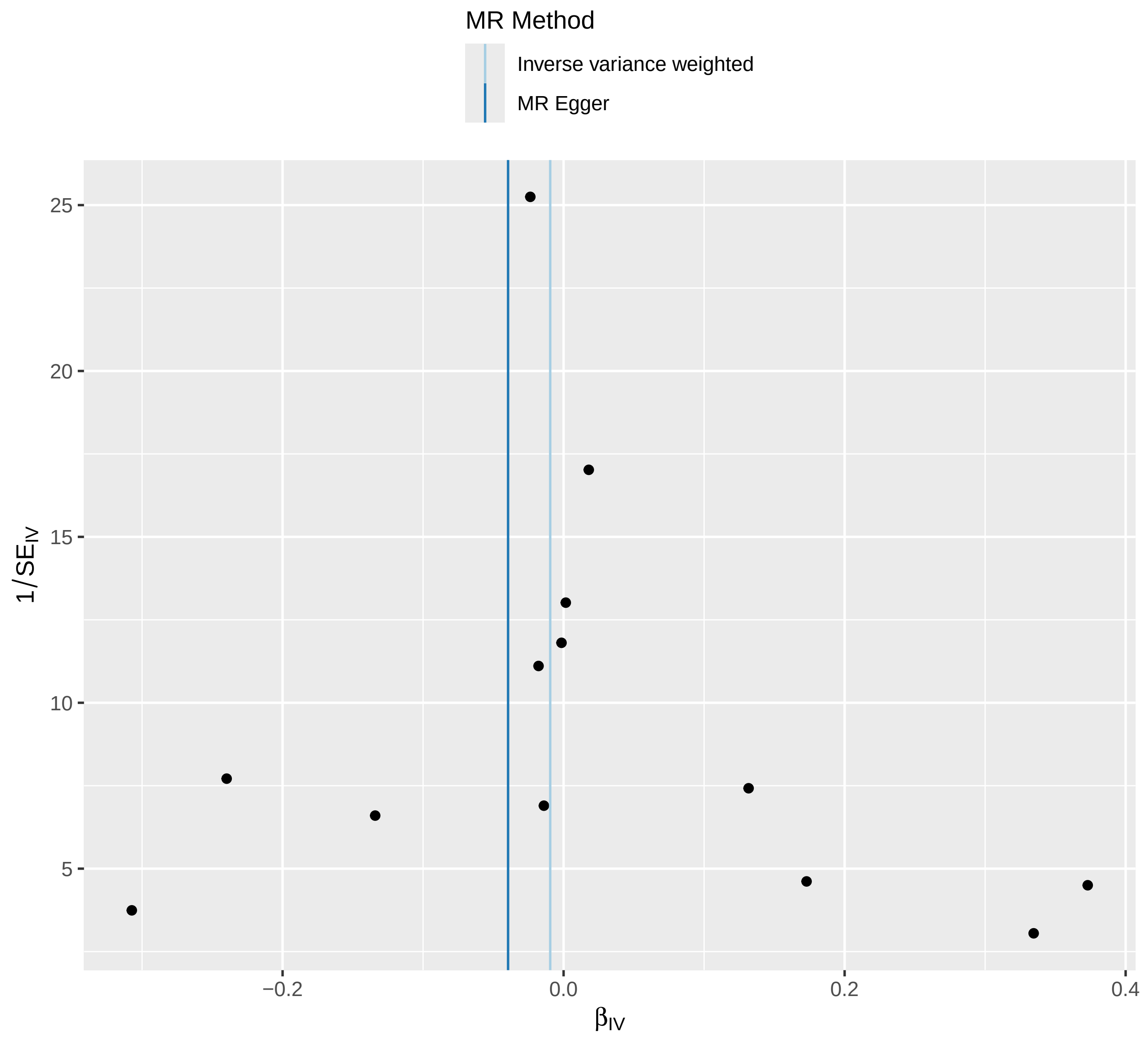

MR Method
Inverse variance weighted
MR Egger
25
20
15
10
5
1/SE_IV
−0.2
0.0
0.2
0.4
β_IV

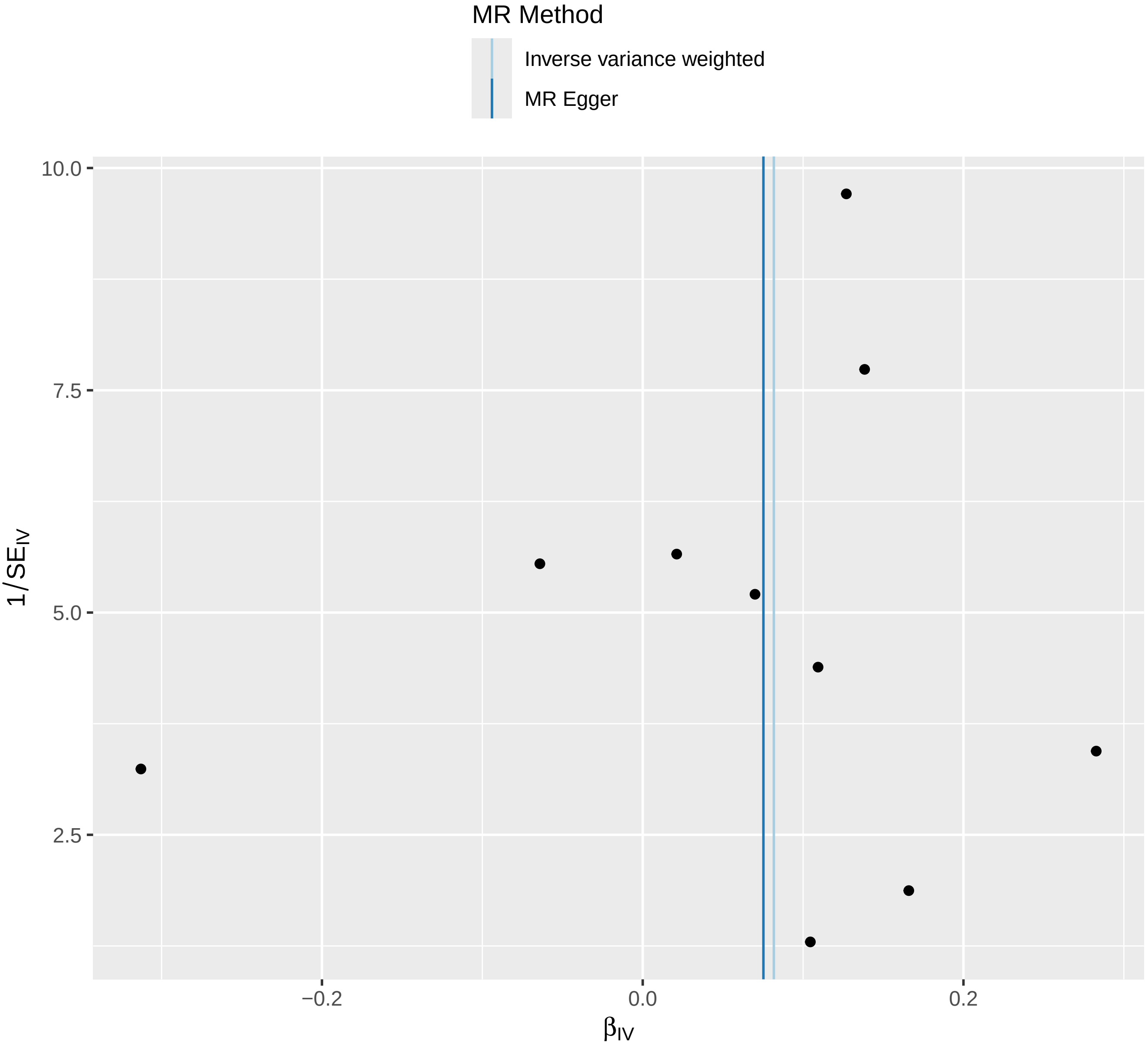

MR Method
Inverse variance weighted
MR Egger
10.0
7.5
5.0
2.5
1/SE$_{IV}$
−0.2
0.0
0.2
β$_{IV}$

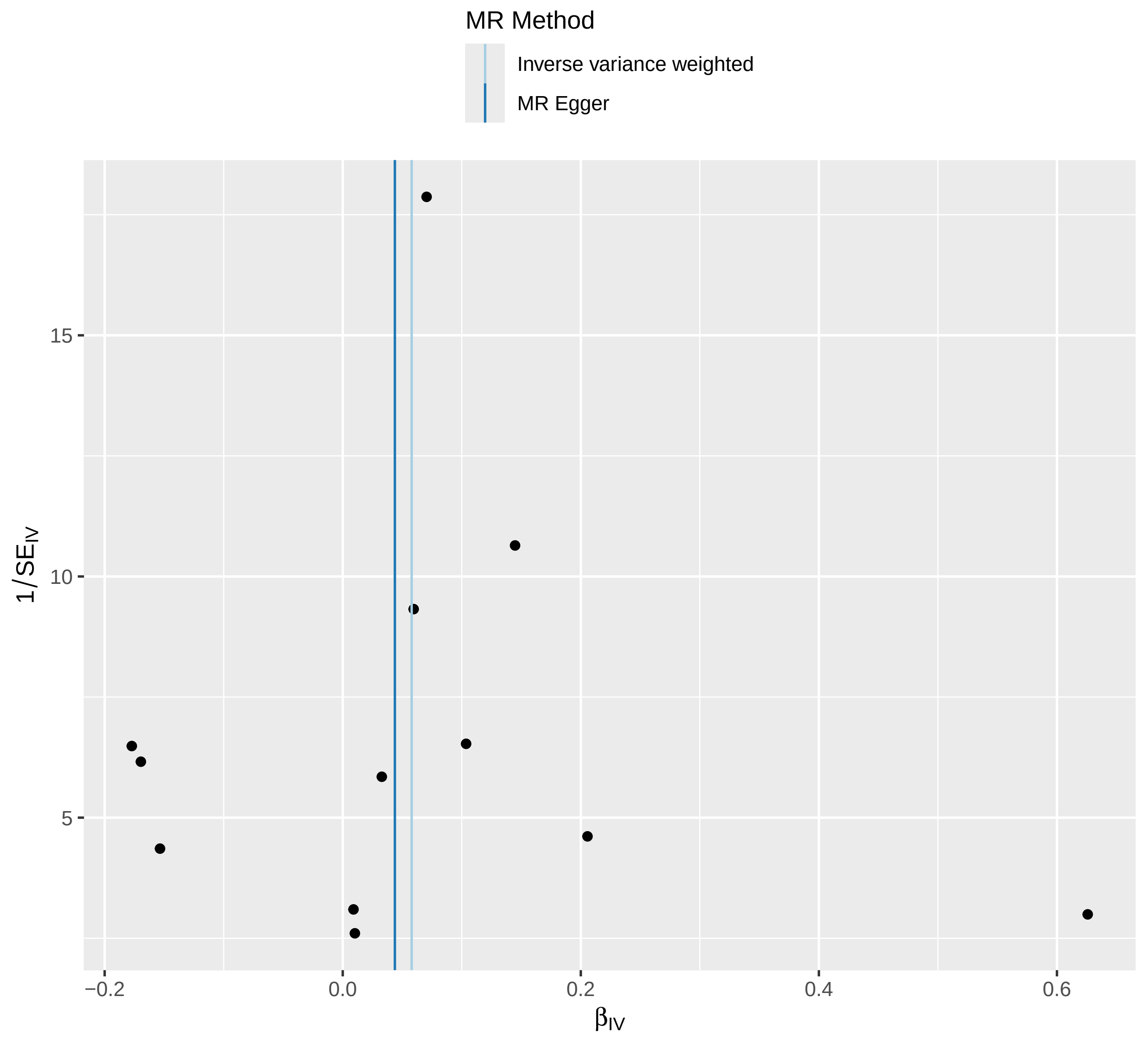
MR Method
Inverse variance weighted
MR Egger
1/SE_IV
β_IV
15
10
5
−0.2
0.0
0.2
0.4
0.6

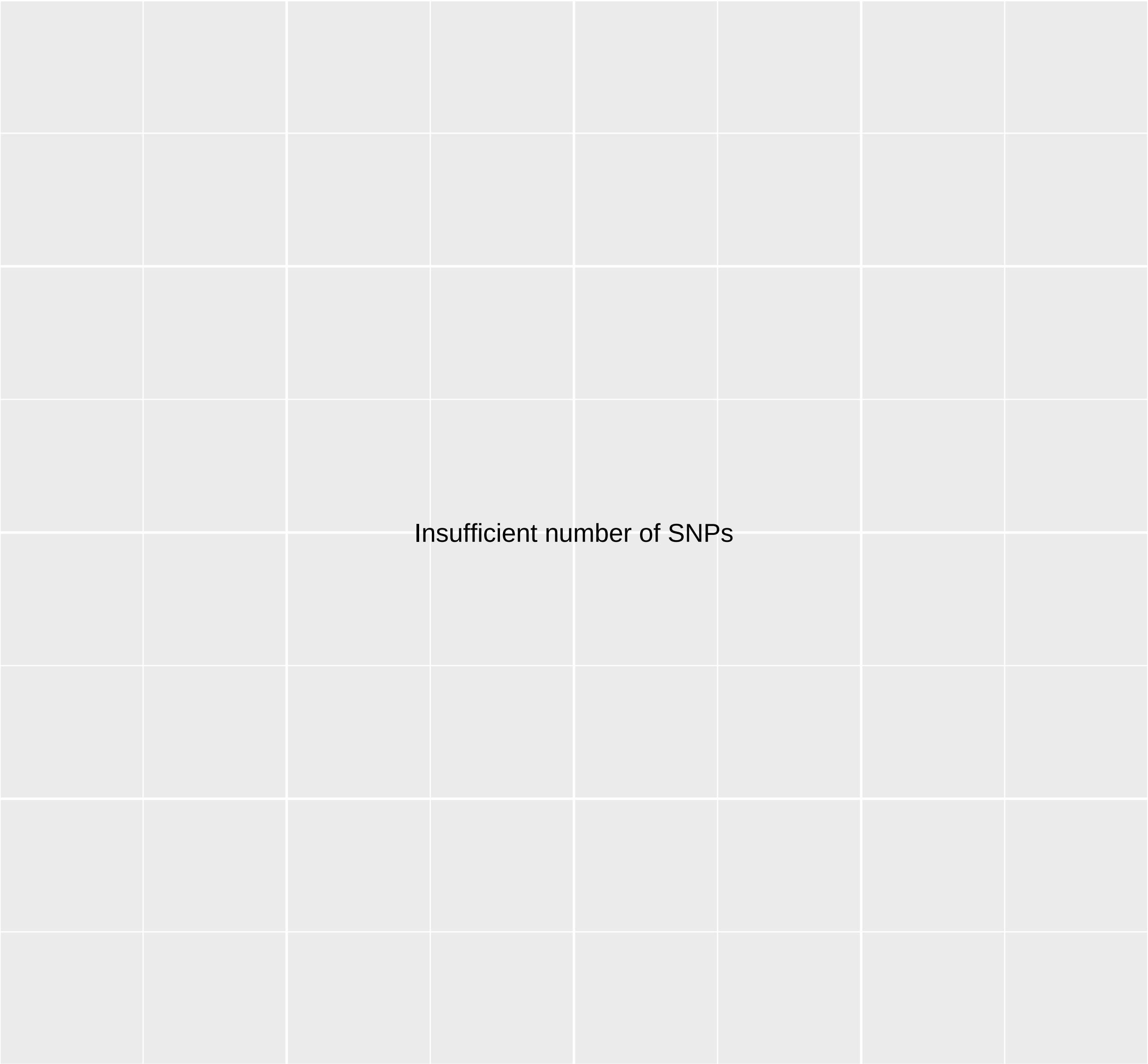
Insufficient number of SNPs

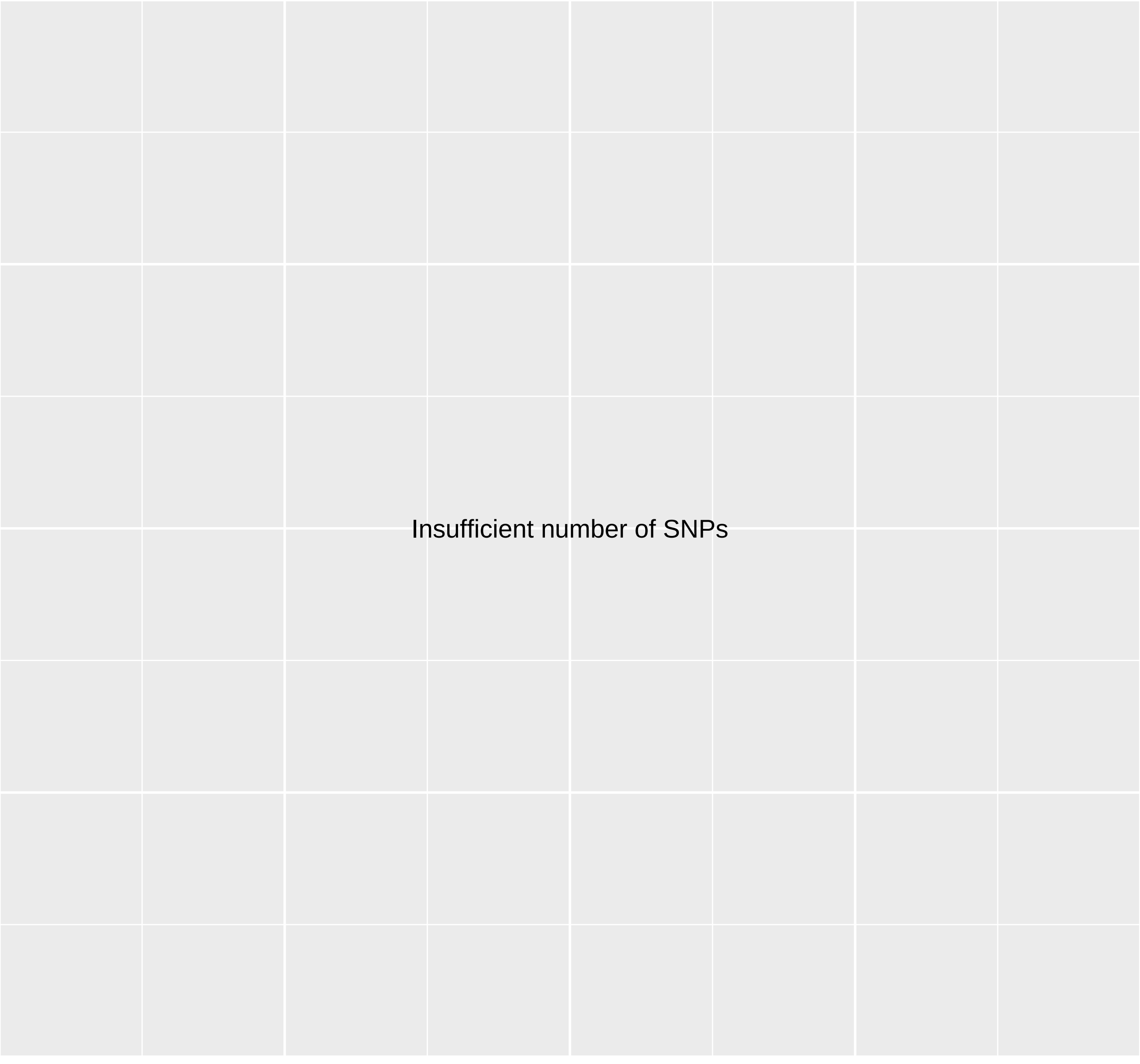
Insufficient number of SNPs

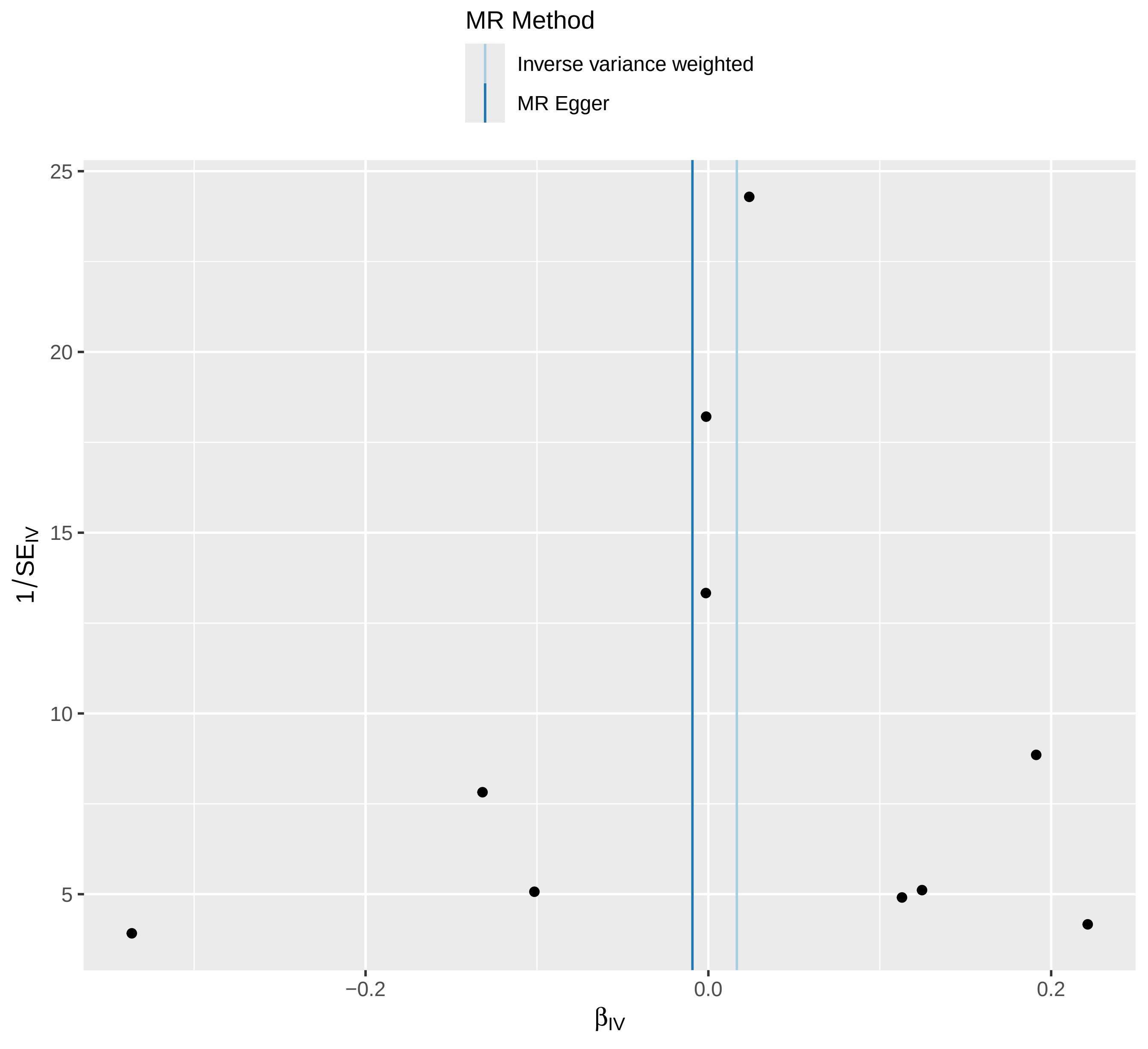
MR Method
Inverse variance weighted
MR Egger
25
20
15
10
5
1/SE_IV
−0.2
0.0
0.2
β_IV

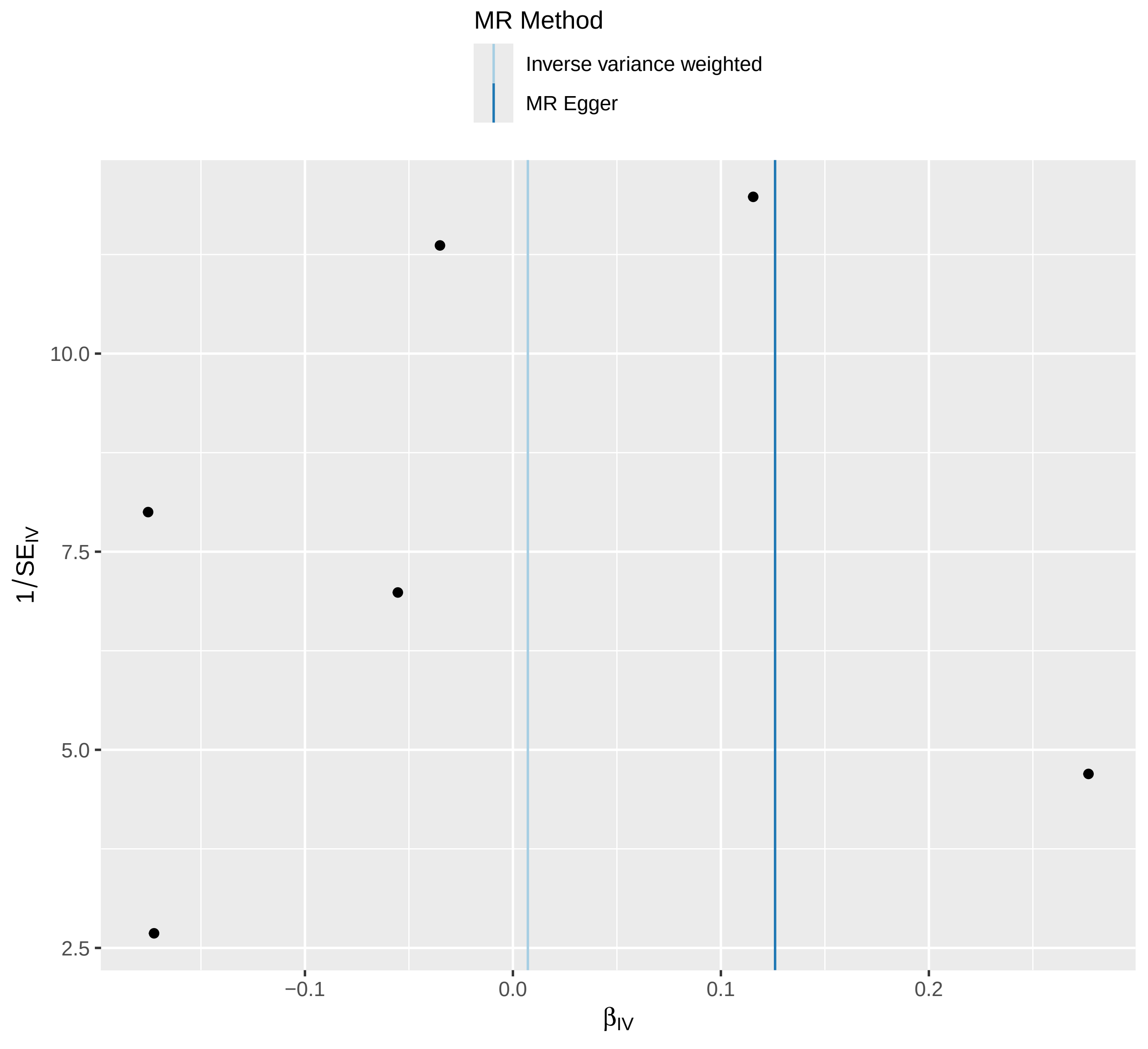
MR Method
Inverse variance weighted
MR Egger
10.0
7.5
5.0
2.5
1/SE$_{IV}$
−0.1
0.0
0.1
0.2
β$_{IV}$

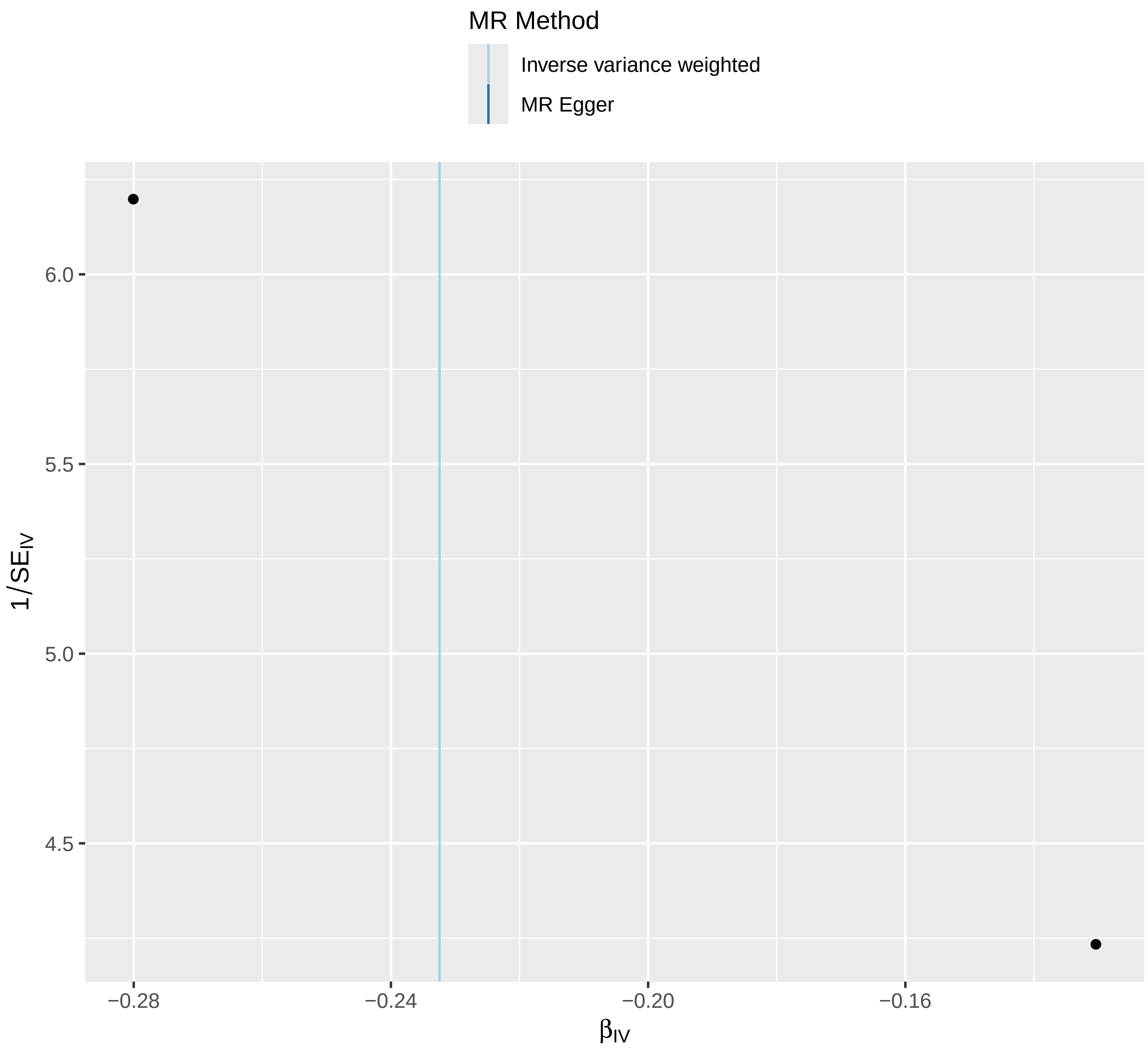
MR Method
Inverse variance weighted
MR Egger
6.0
5.5
5.0
4.5
1/SE_IV
−0.28
−0.24
−0.20
−0.16
β_IV

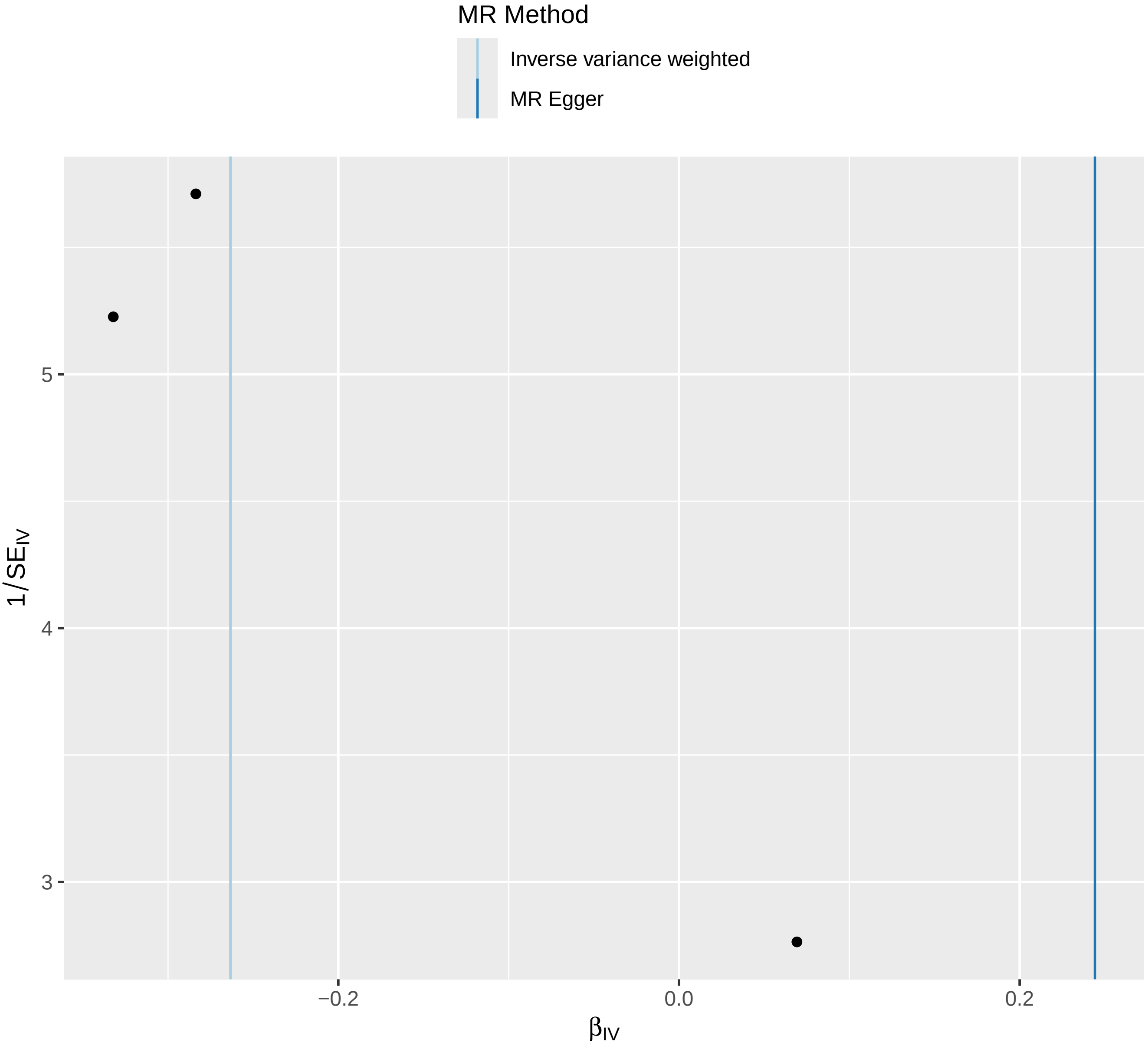
MR Method
Inverse variance weighted
MR Egger
5
4
3
1/SE$_{IV}$
−0.2
0.0
0.2
β$_{IV}$

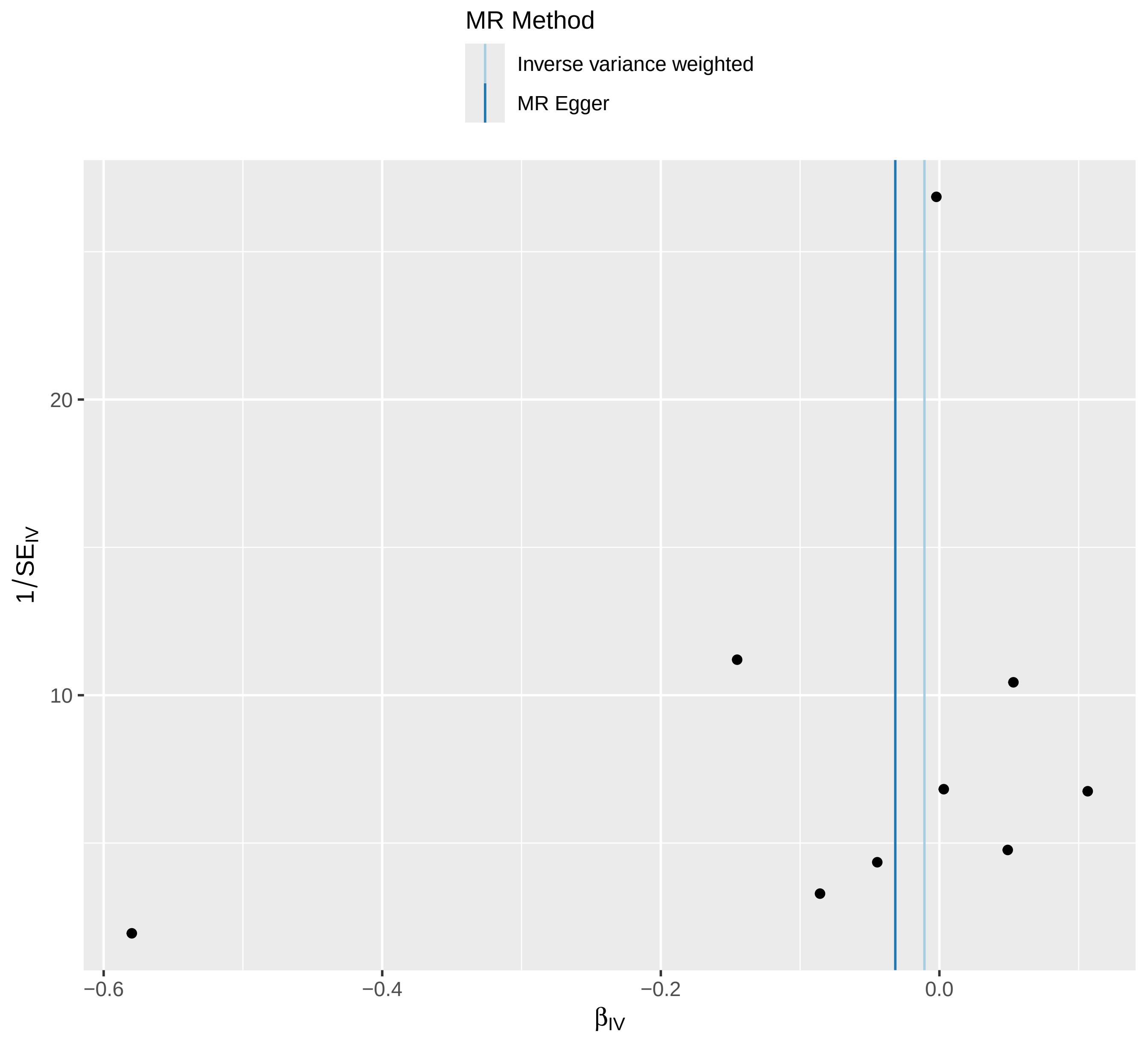

MR Method
Inverse variance weighted
MR Egger
20
10
1/SE$_{IV}$
−0.6
−0.4
−0.2
0.0
β$_{IV}$

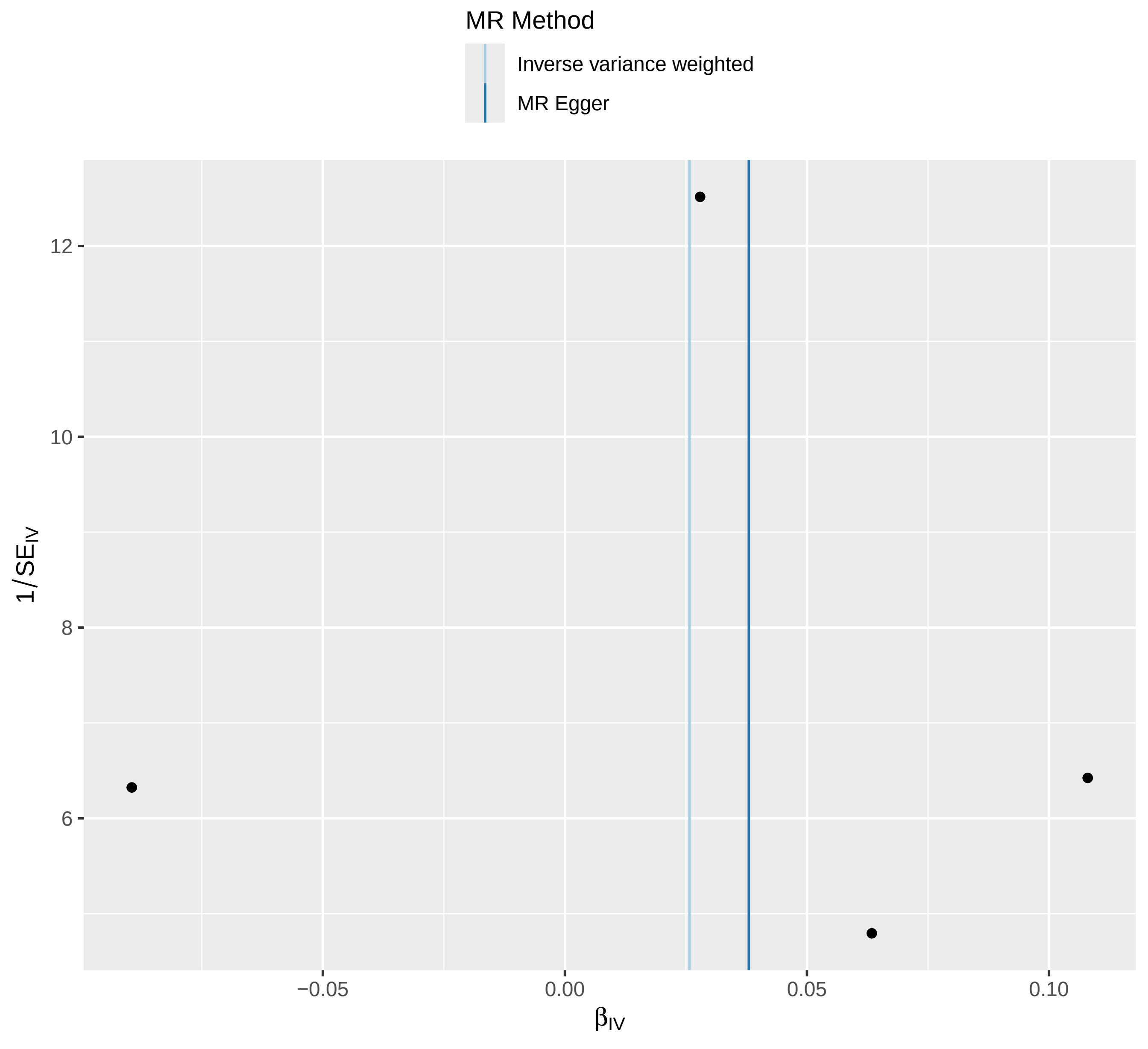
MR Method
Inverse variance weighted
MR Egger
12
10
8
6
1/SE$_{IV}$
−0.05
0.00
0.05
0.10
β$_{IV}$

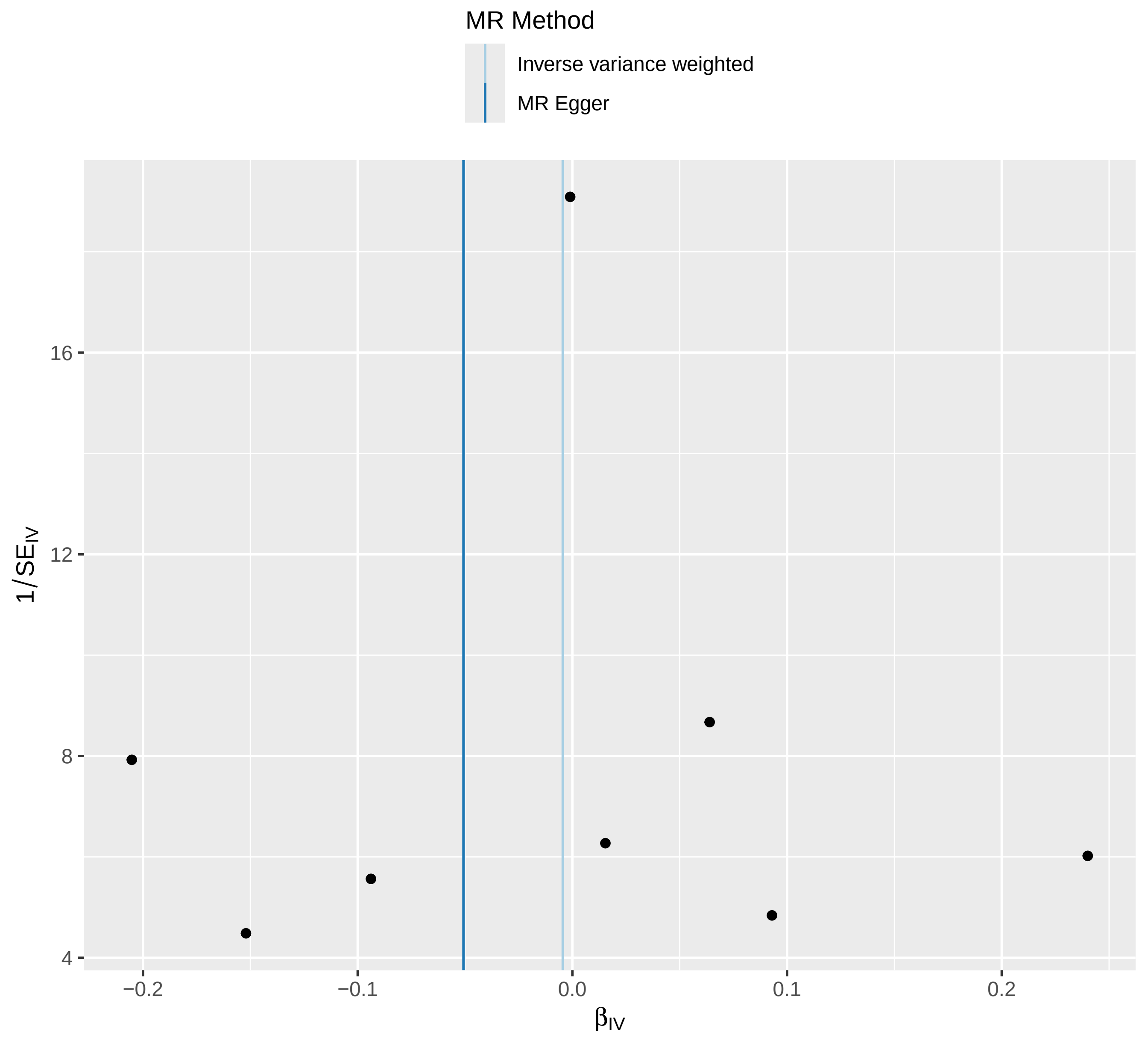
MR Method
Inverse variance weighted
MR Egger
16
12
8
4
1/SE_IV
−0.2
−0.1
0.0
0.1
0.2
β_IV

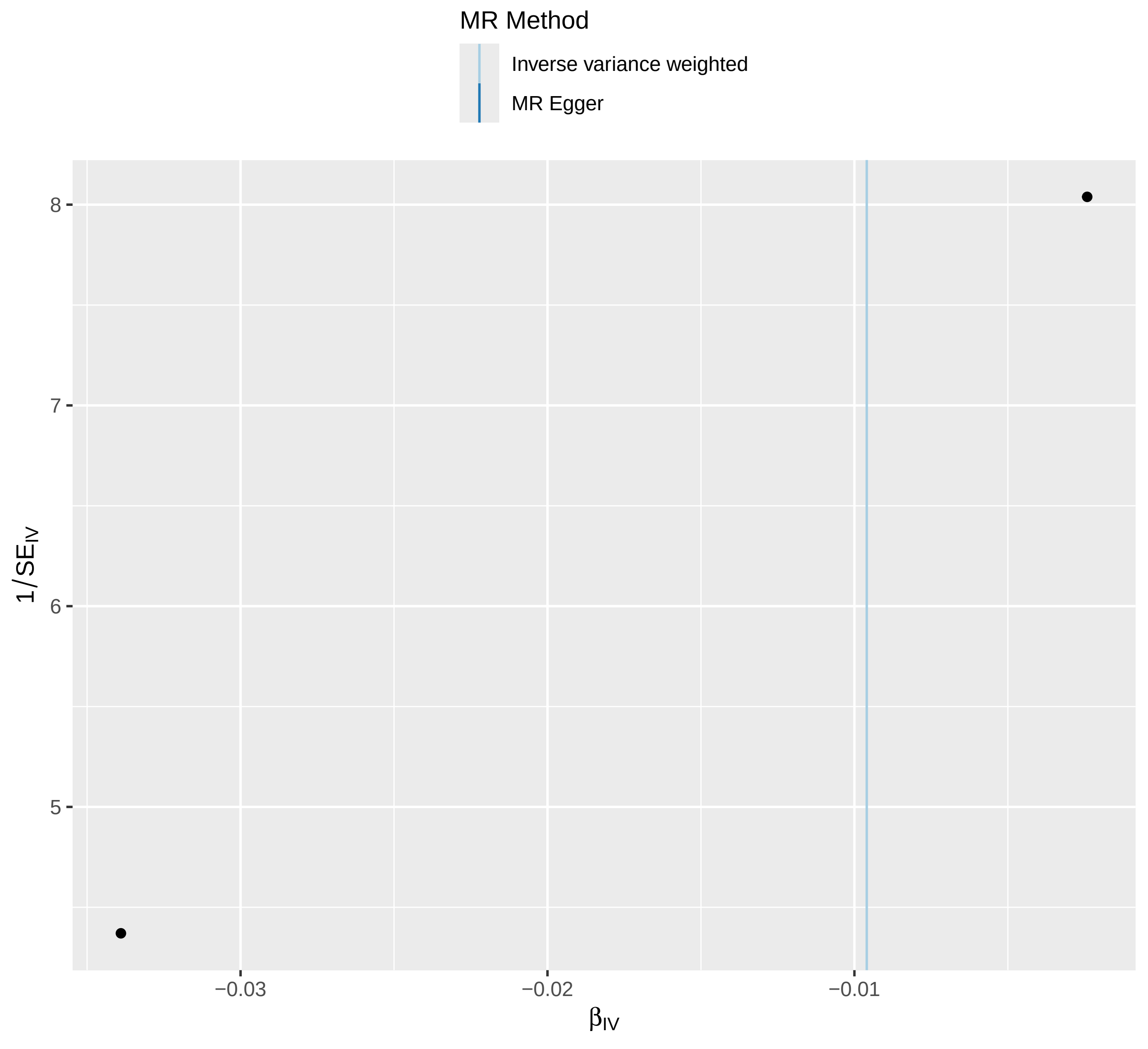
MR Method
Inverse variance weighted
MR Egger
1/SE_IV
8
7
6
5
−0.03
−0.02
−0.01
β_IV

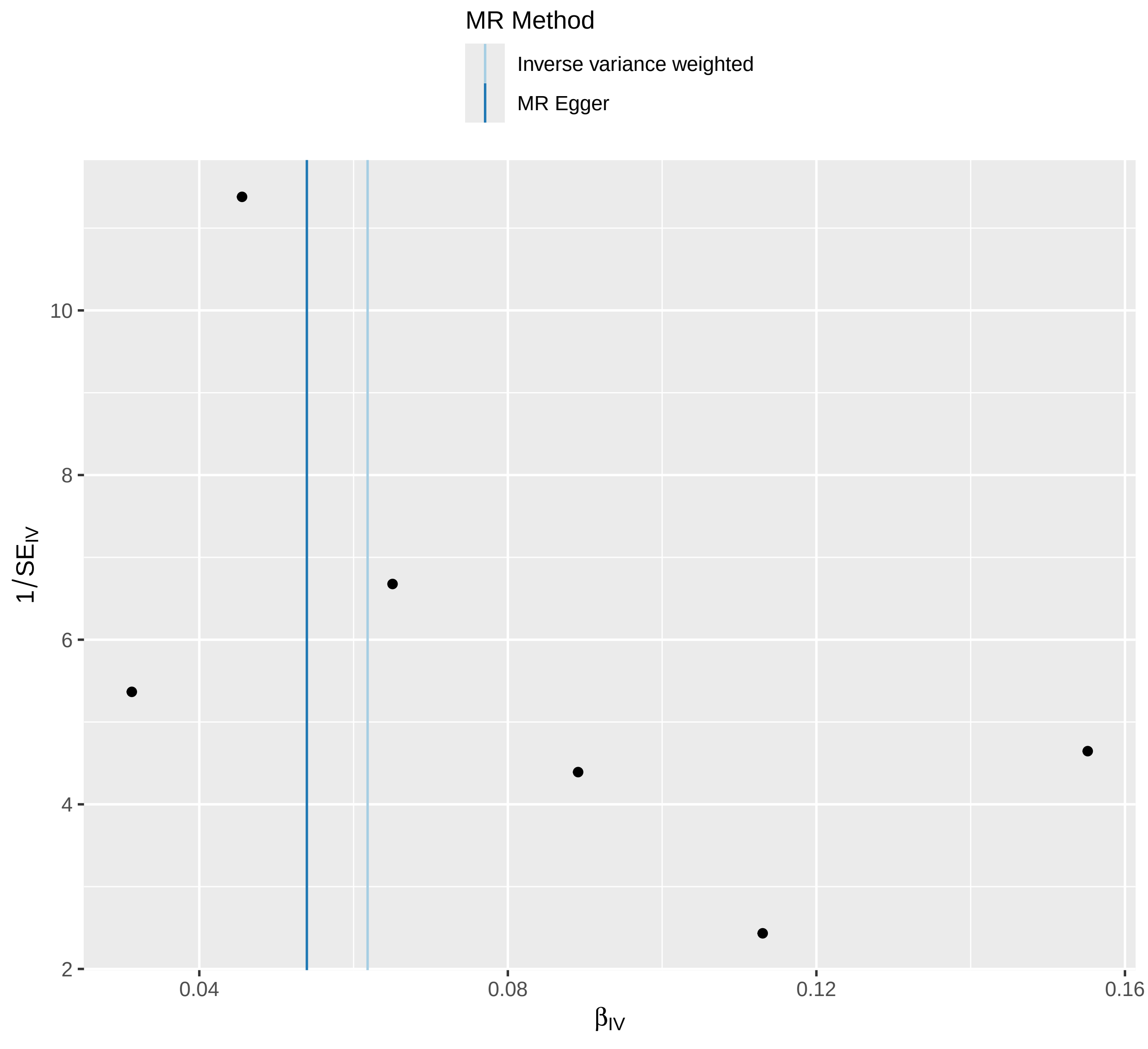

MR Method
Inverse variance weighted
MR Egger
10
8
6
4
2
1/SE_IV
0.04
0.08
0.12
0.16
β_IV

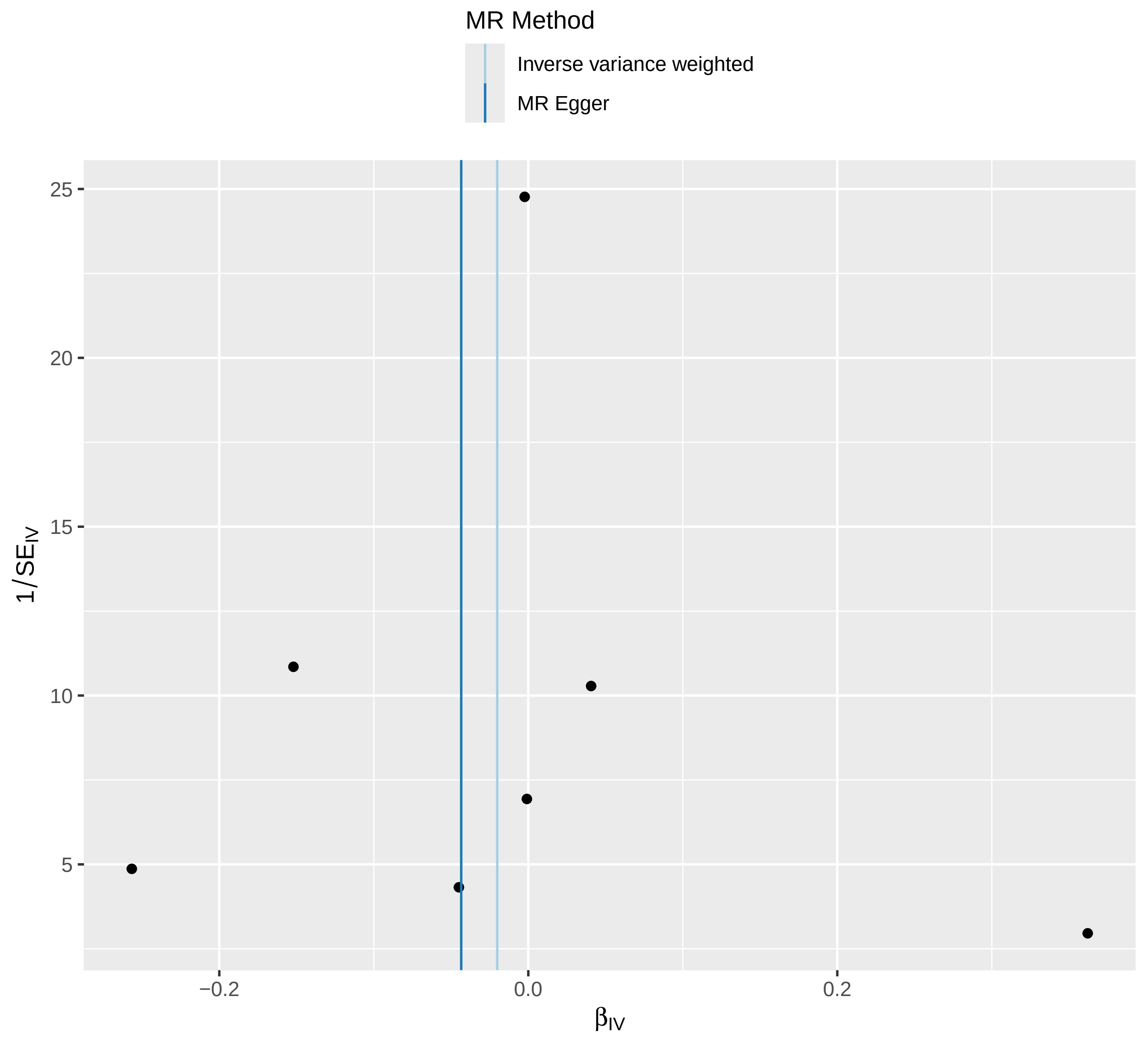
MR Method
Inverse variance weighted
MR Egger
25
20
15
10
5
1/SE_IV
−0.2
0.0
0.2
β_IV

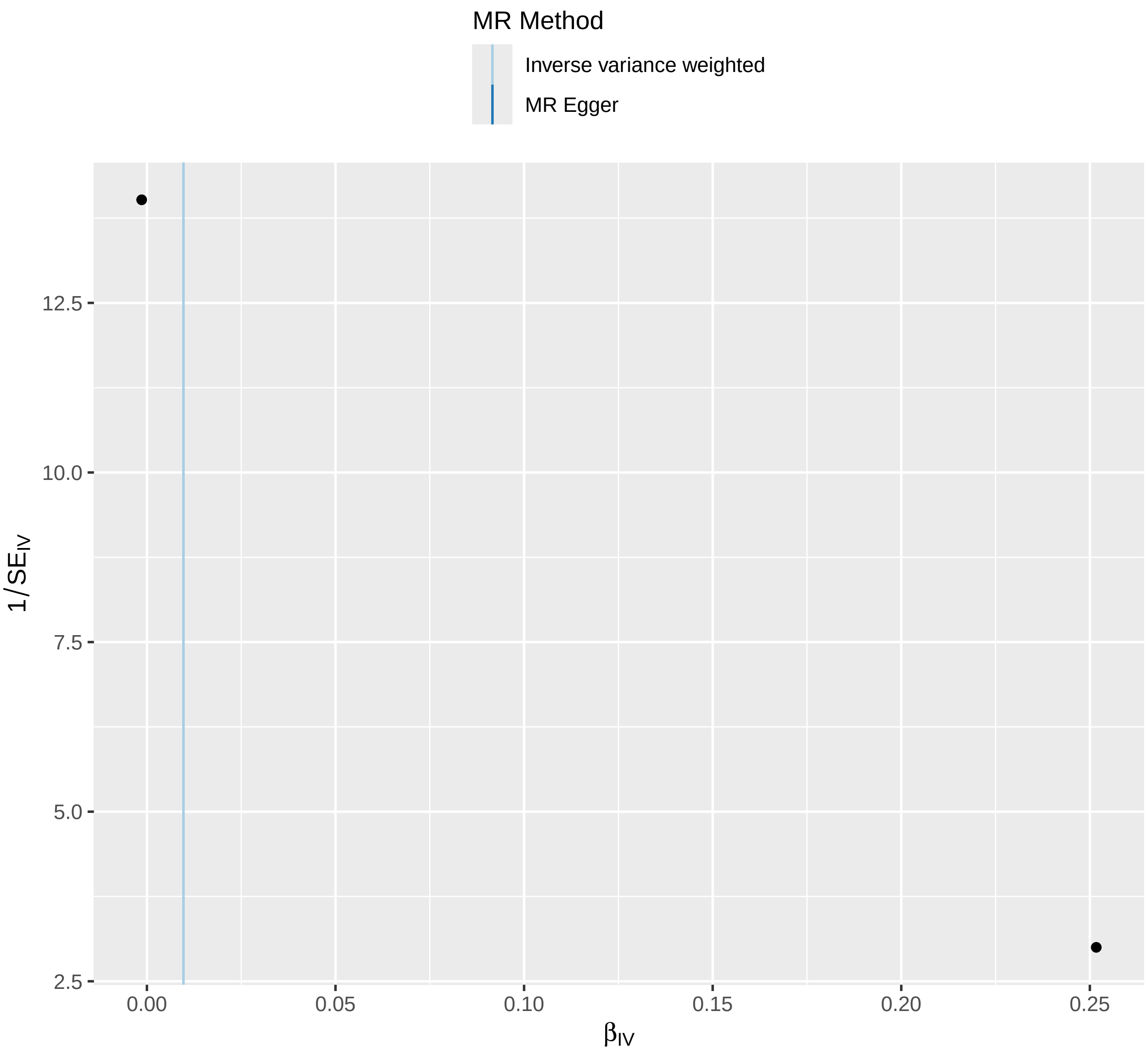
MR Method
Inverse variance weighted
MR Egger
12.5
10.0
7.5
5.0
2.5
$1/SE_{IV}$
0.00
0.05
0.10
0.15
0.20
0.25
$\beta_{IV}$

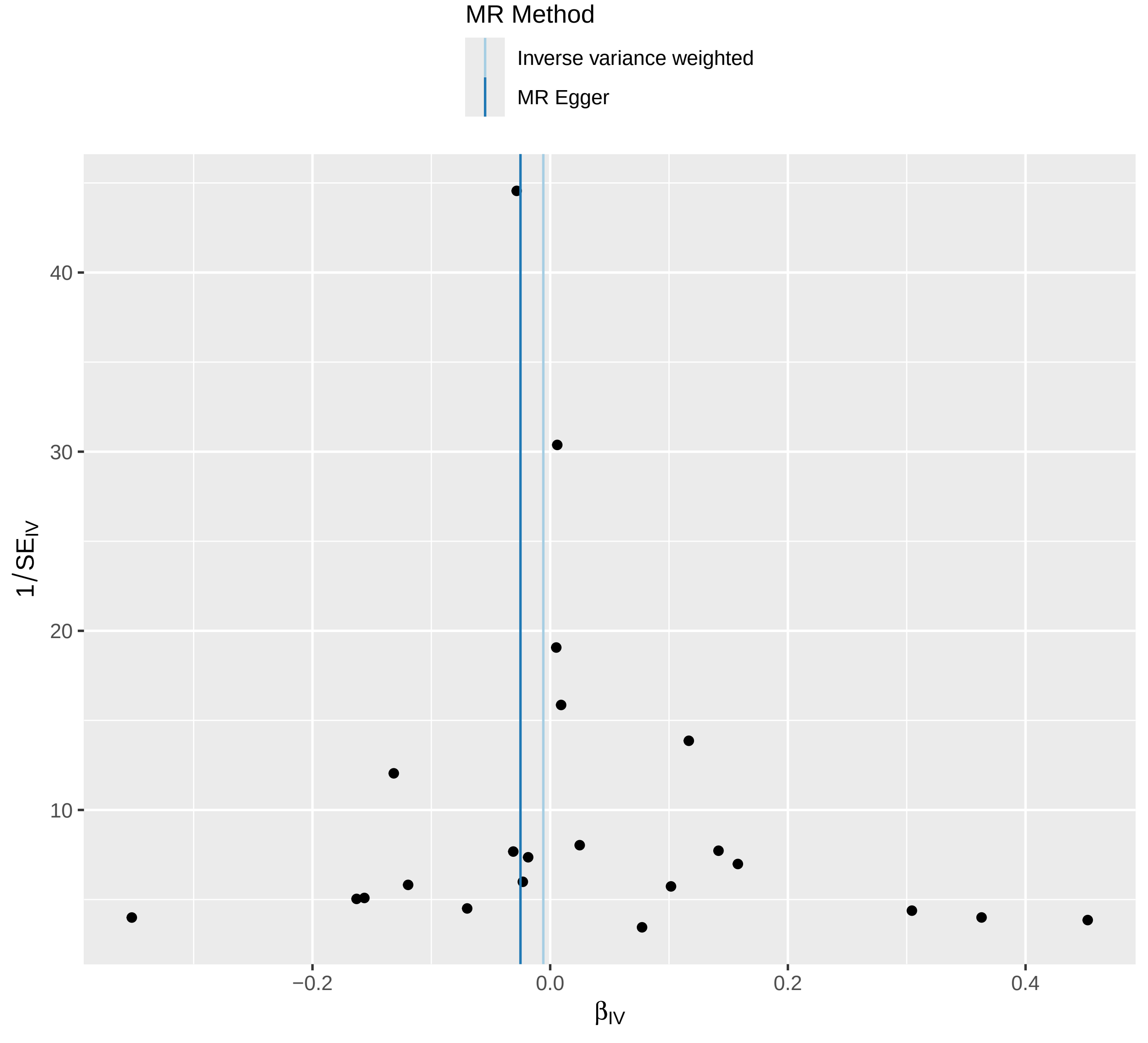
MR Method
Inverse variance weighted
MR Egger
1/SE$_{IV}$
40
30
20
10
−0.2
0.0
0.2
0.4
β$_{IV}$

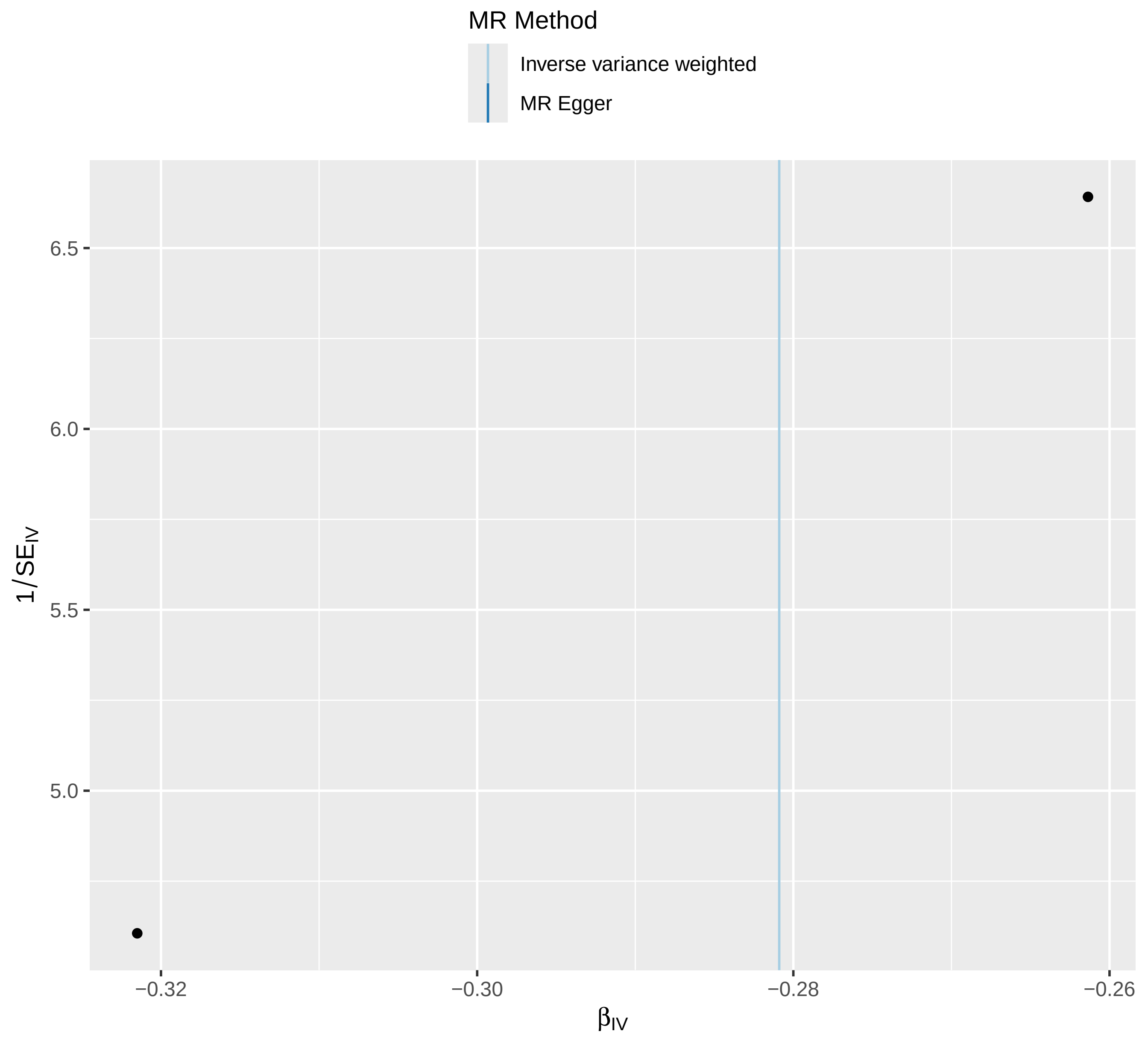
MR Method
Inverse variance weighted
MR Egger
6.5
6.0
5.5
5.0
1/SE_IV
−0.32
−0.30
−0.28
−0.26
β_IV

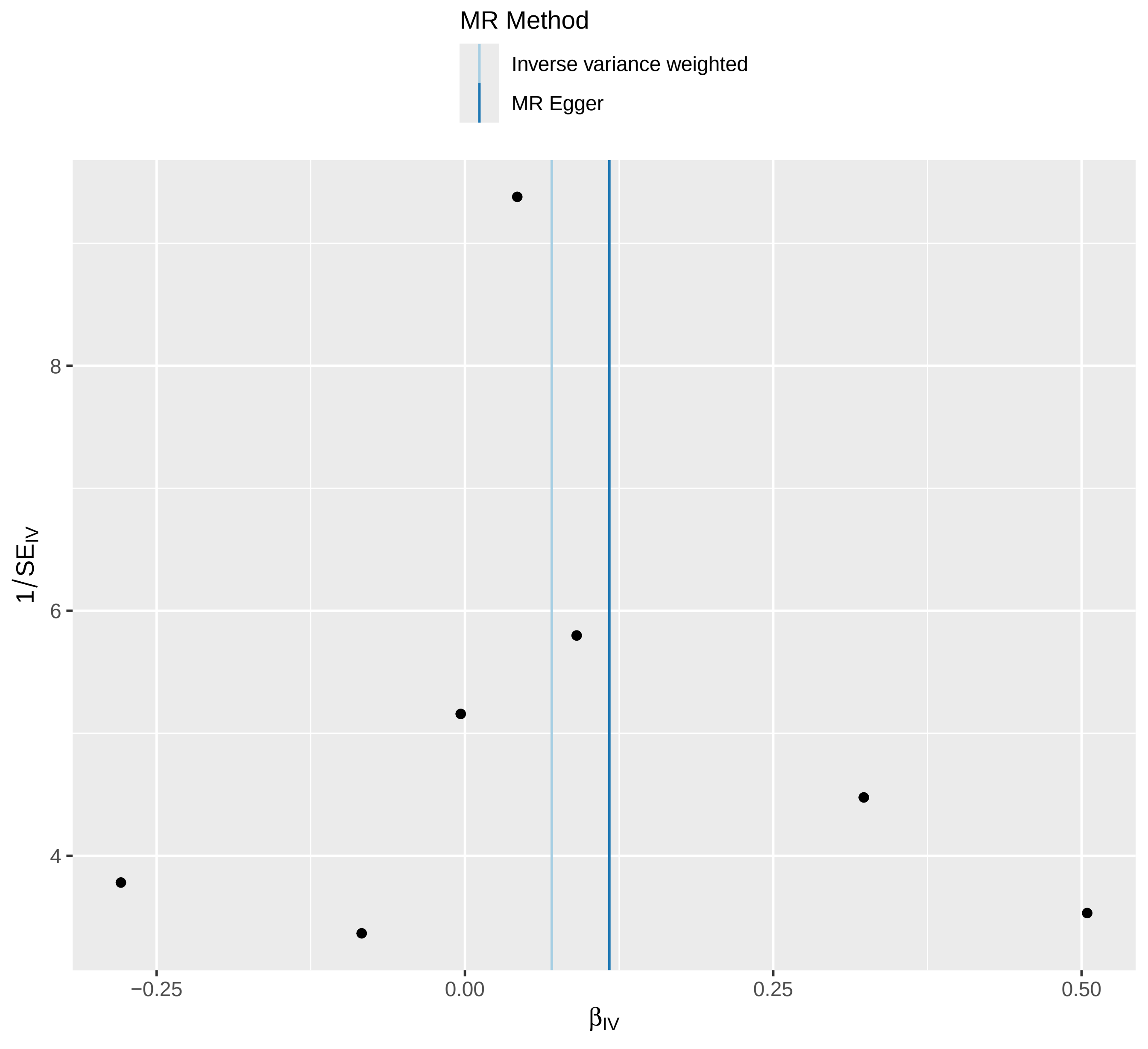
MR Method
Inverse variance weighted
MR Egger
8
6
4
1/SE$_{IV}$
−0.25
0.00
0.25
0.50
β$_{IV}$

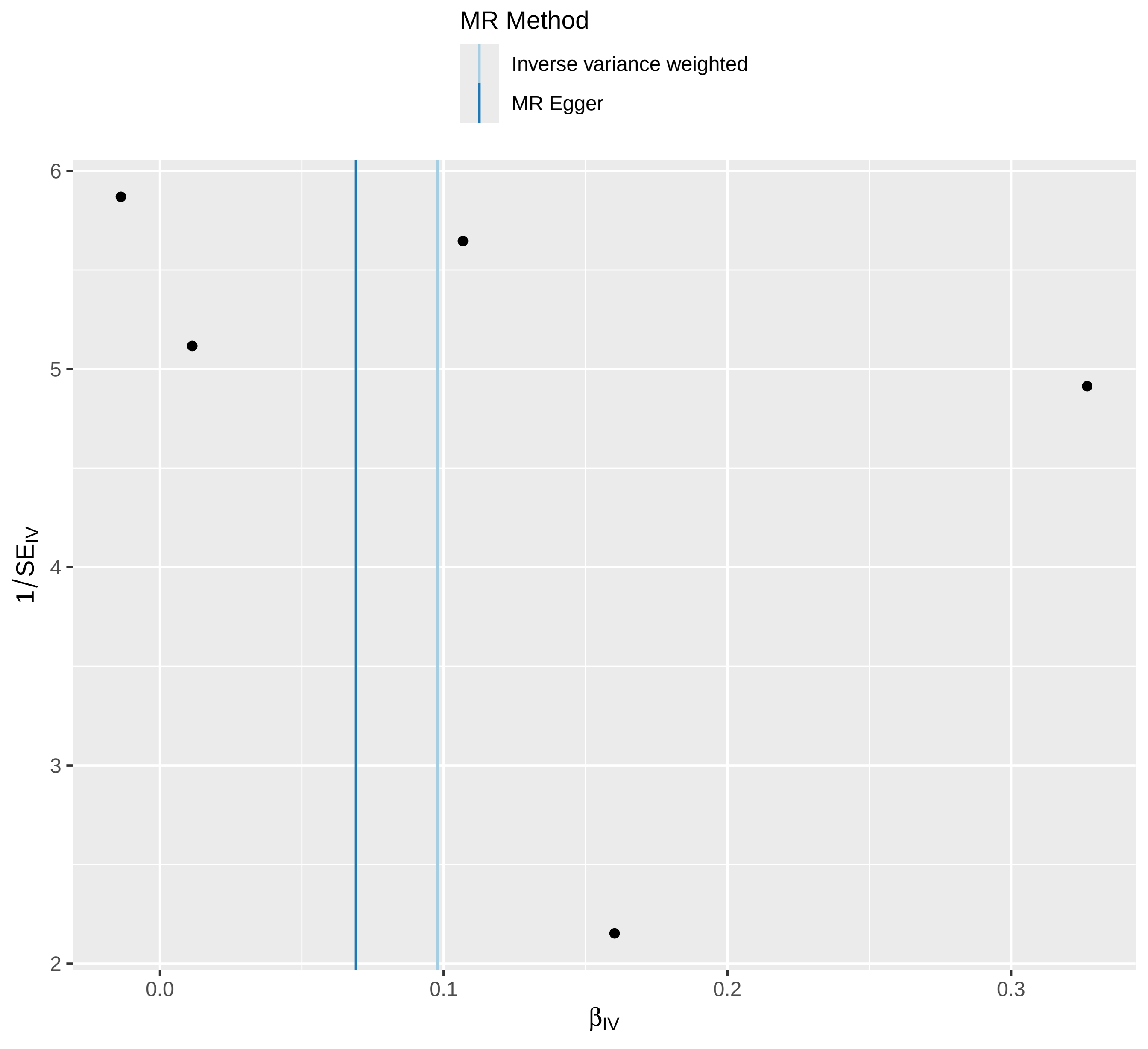

MR Method
Inverse variance weighted
MR Egger
6
5
4
3
2
1/SE_IV
0.0
0.1
0.2
0.3
β_IV

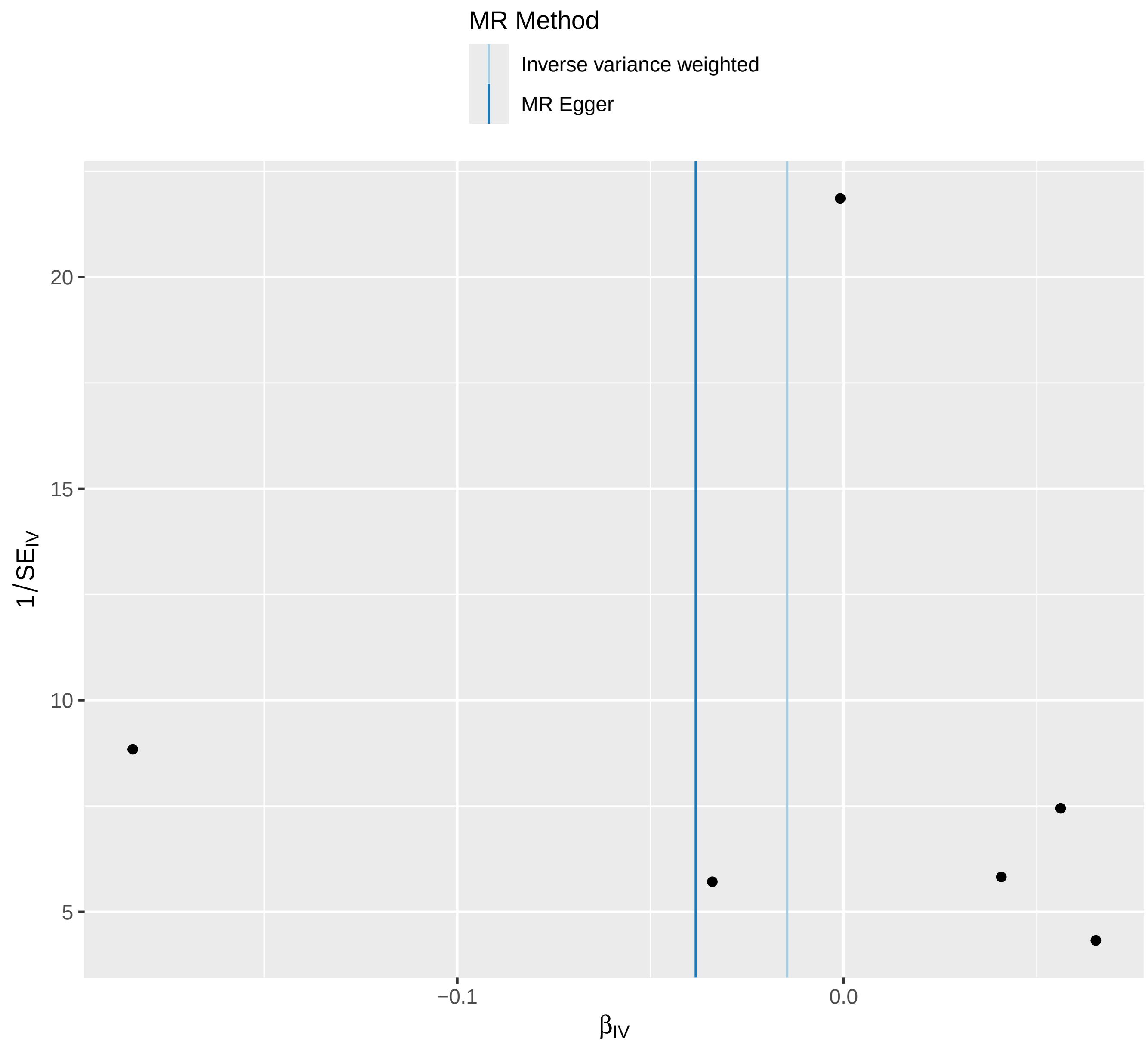
MR Method
Inverse variance weighted
MR Egger
20
15
10
5
−0.1
0.0
1/SE_IV
β_IV

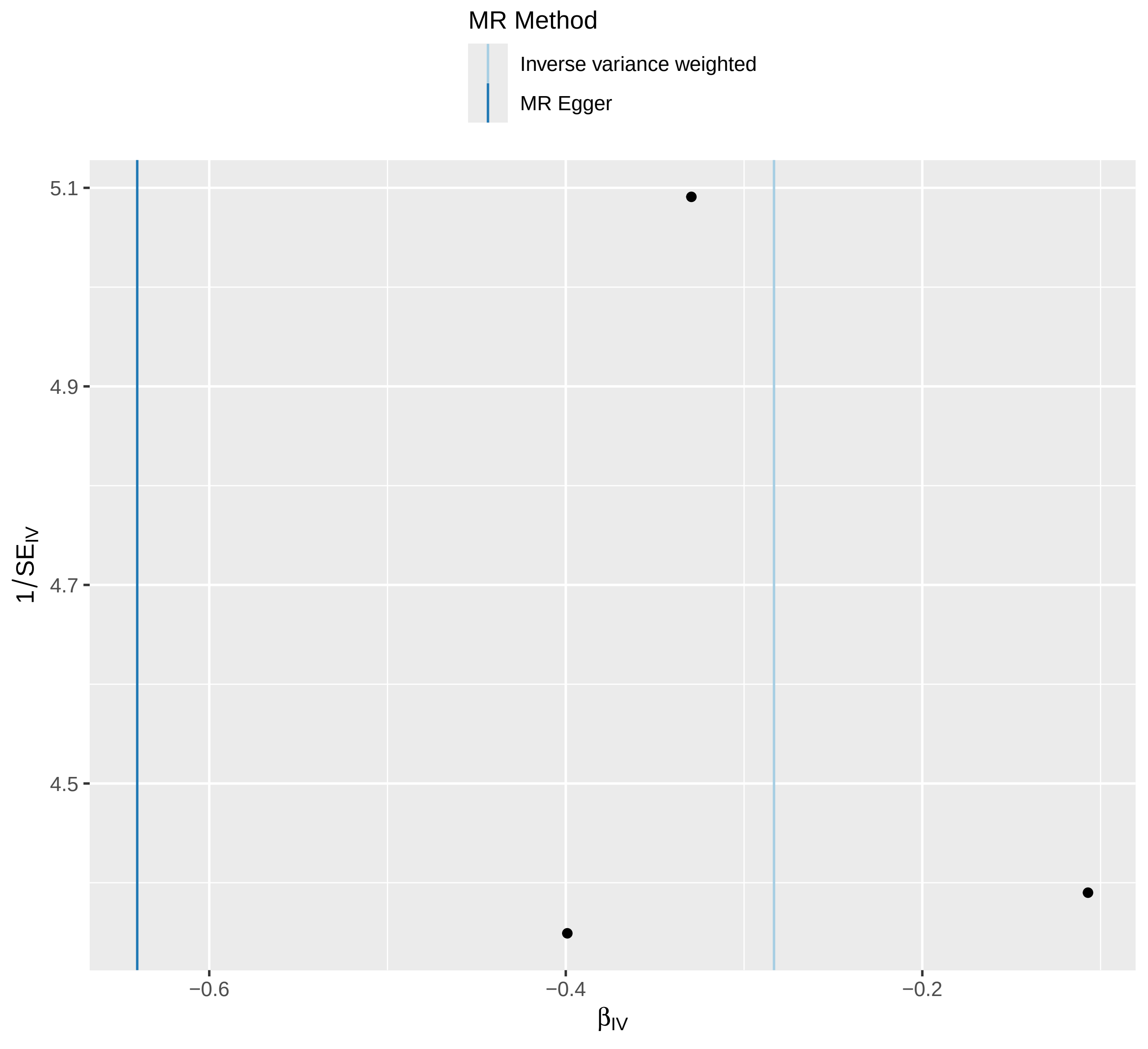

MR Method
Inverse variance weighted
MR Egger
5.1
4.9
4.7
4.5
1/SE$_{IV}$
−0.6
−0.4
−0.2
β$_{IV}$

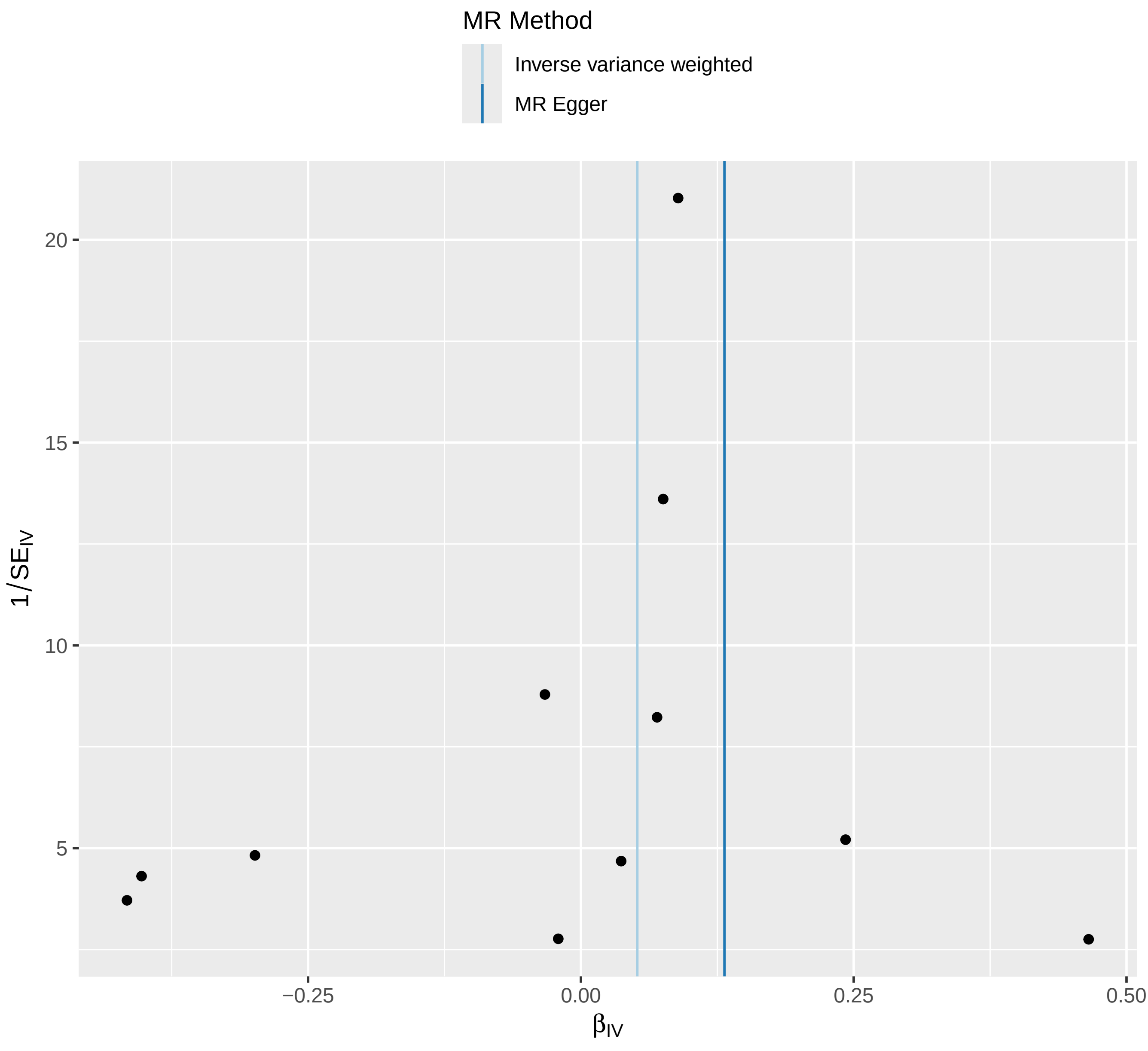
MR Method
Inverse variance weighted
MR Egger
20
15
10
5
$1/SE_{IV}$
−0.25
0.00
0.25
0.50
$\beta_{IV}$

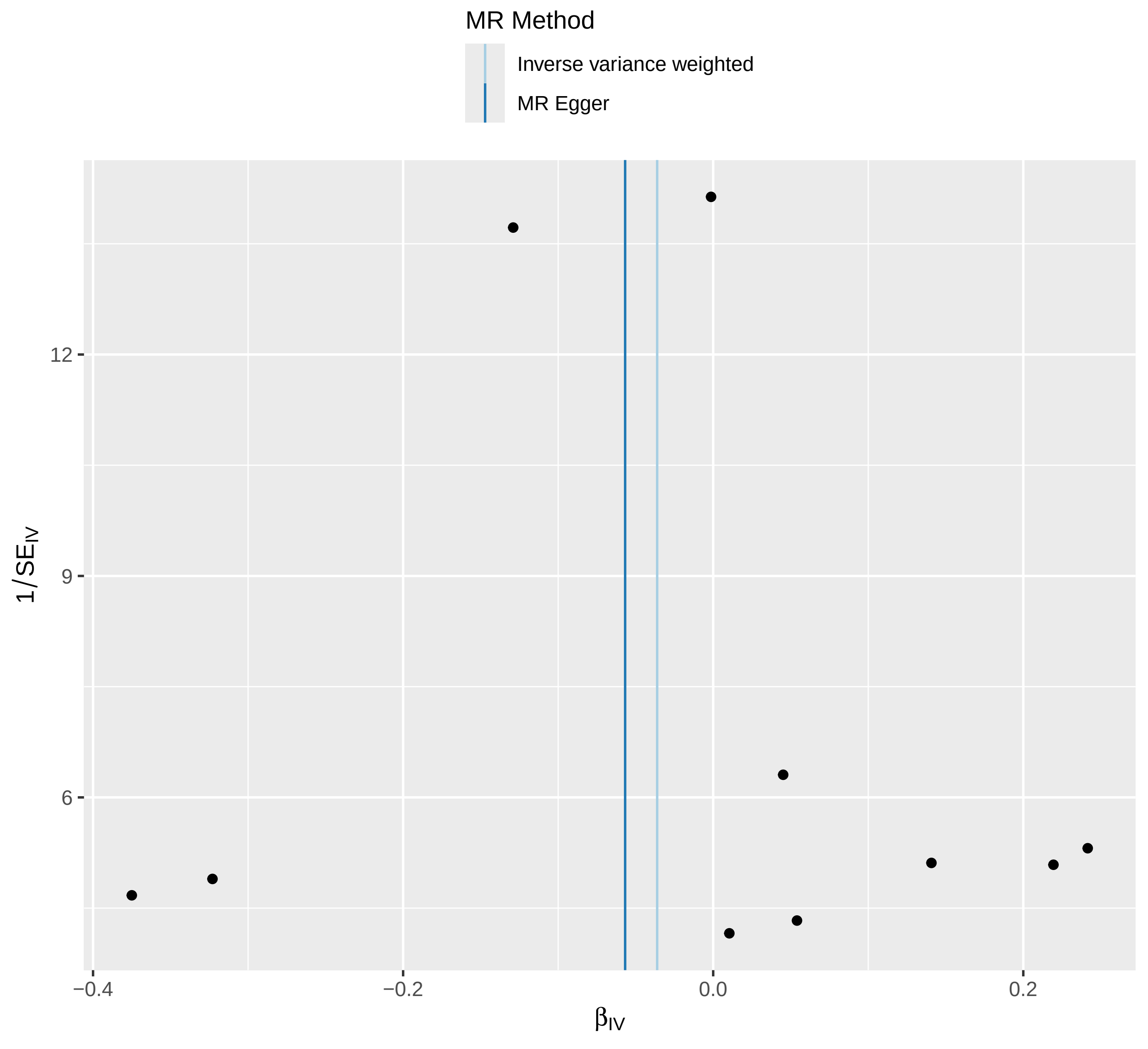

MR Method
Inverse variance weighted
MR Egger
12
9
6
1/SE$_{IV}$
−0.4
−0.2
0.0
0.2
β$_{IV}$

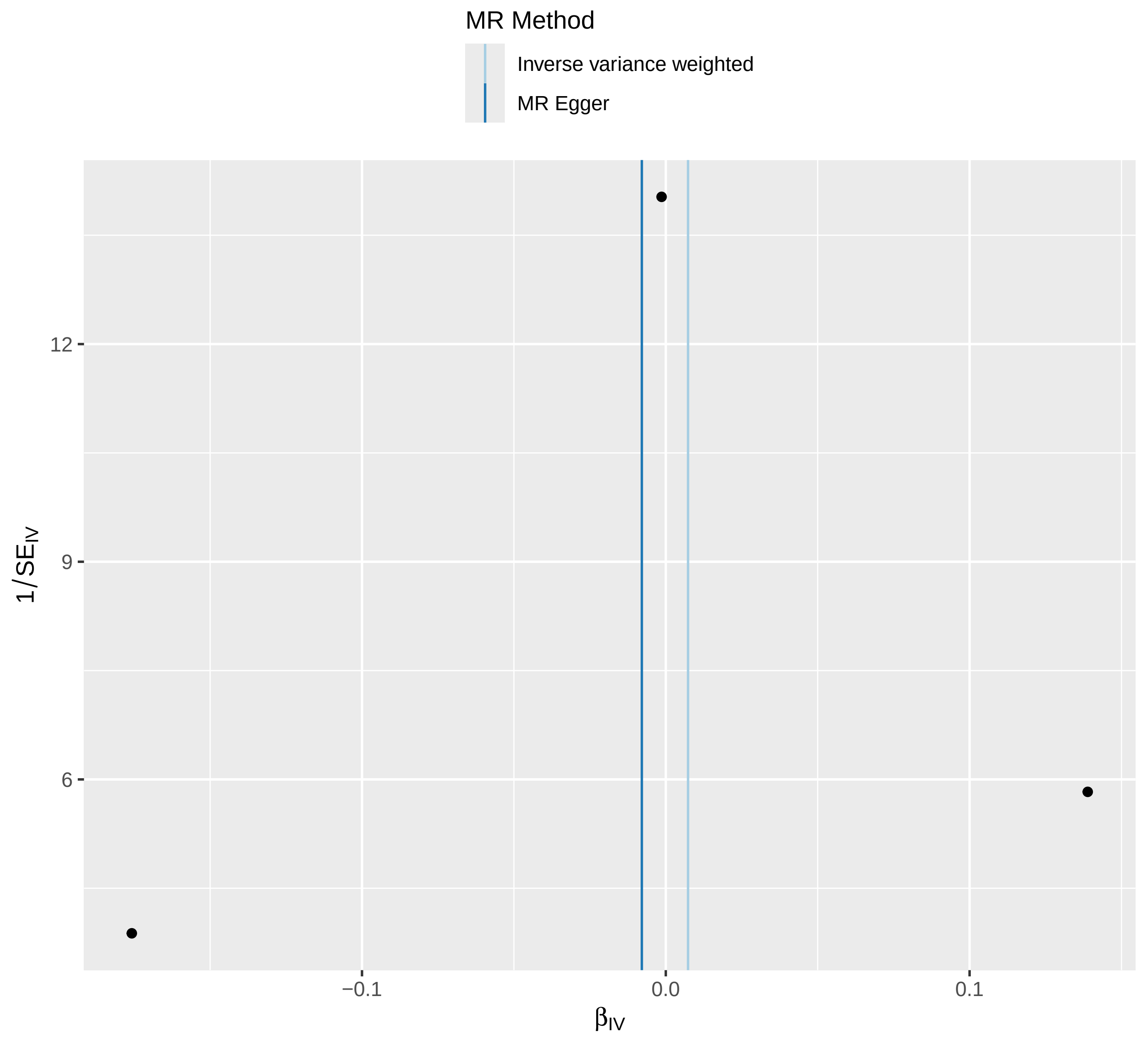

MR Method
Inverse variance weighted
MR Egger
12
9
6
1/SE_IV
−0.1
0.0
0.1
β_IV

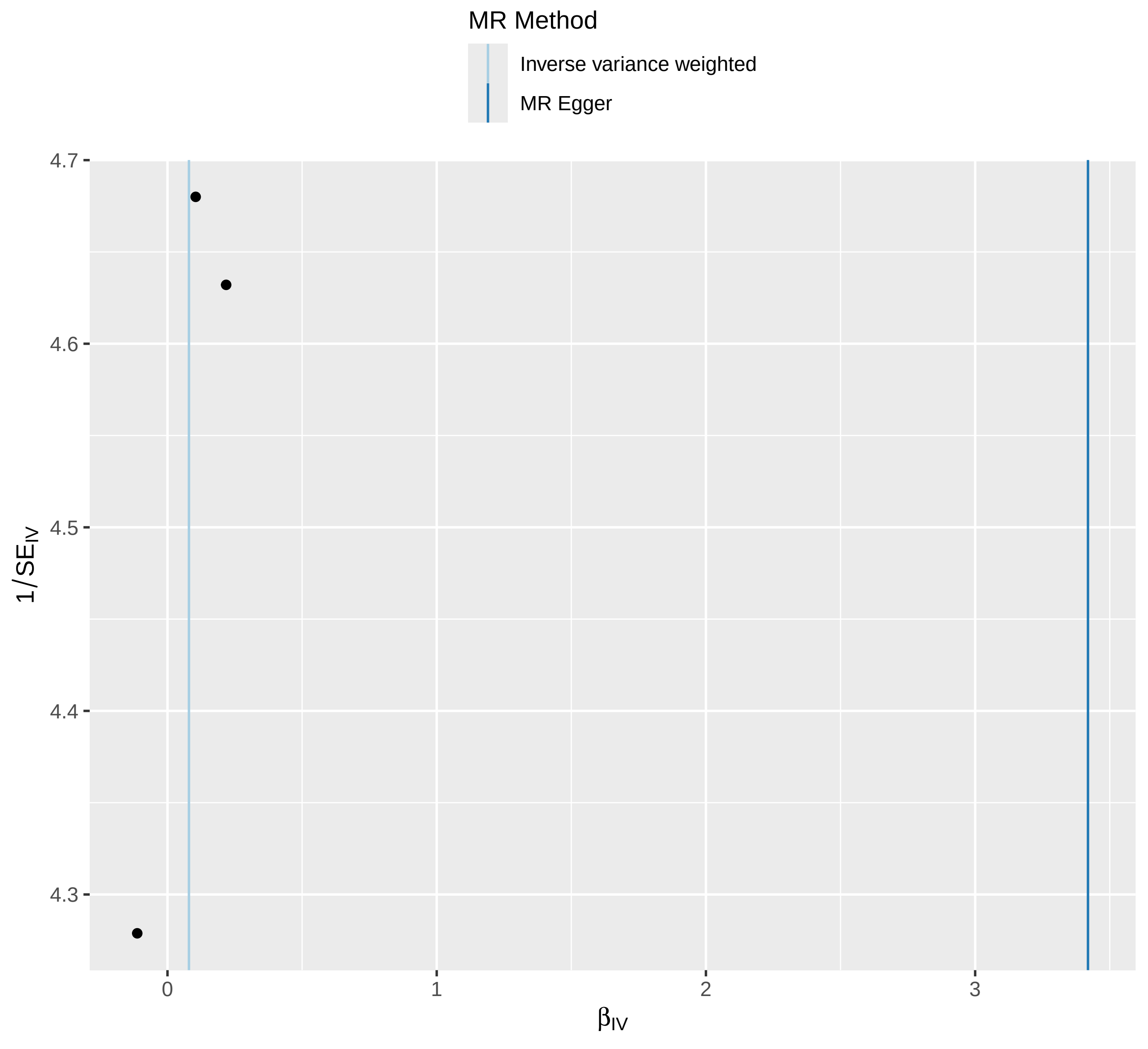

MR Method
Inverse variance weighted
MR Egger
4.7
4.6
4.5
4.4
4.3
$1/SE_{IV}$
0
1
2
3
$\beta_{IV}$

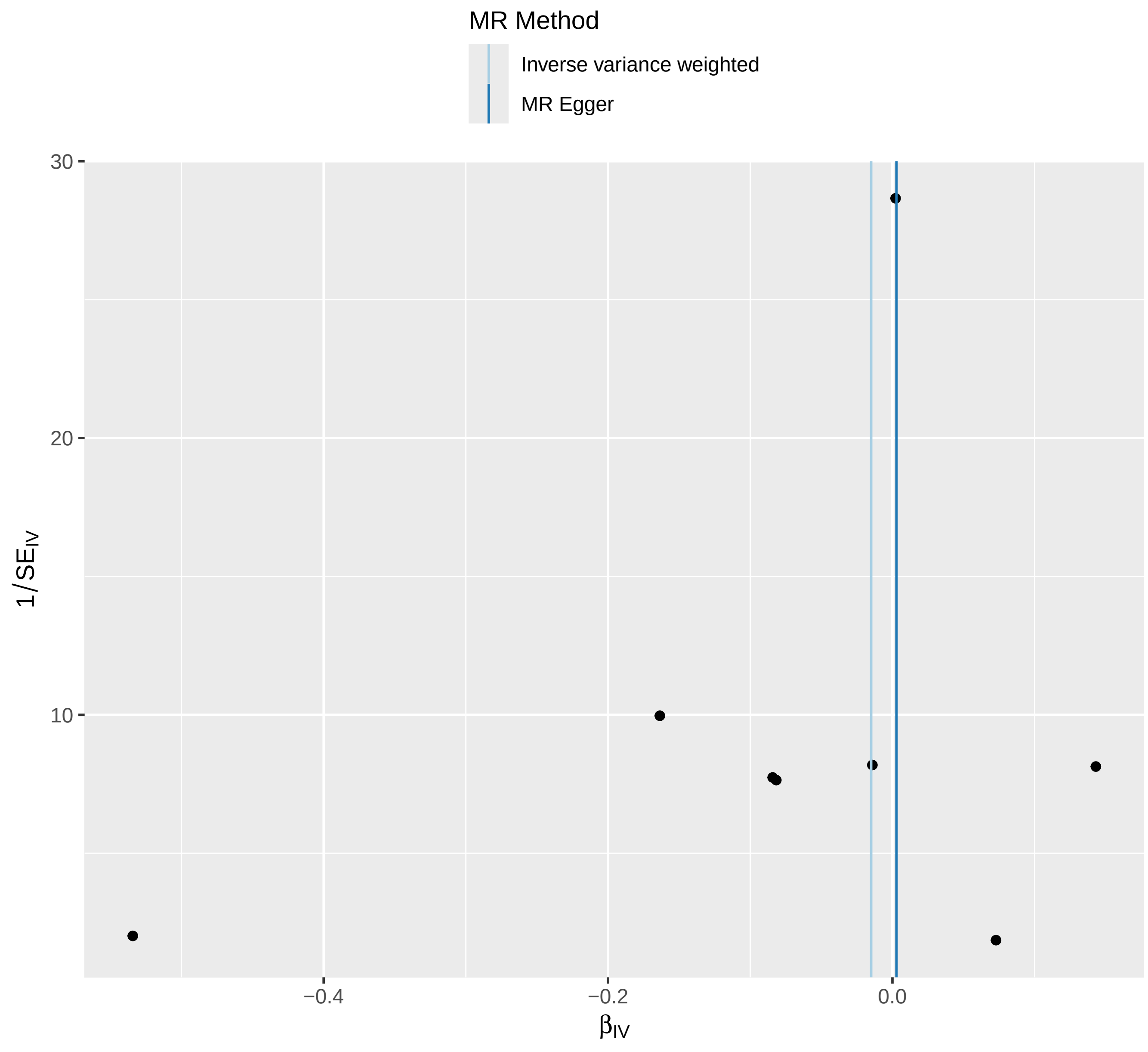

MR Method
Inverse variance weighted
MR Egger
30
20
10
−0.4
−0.2
0.0
1/SE$_{IV}$
β$_{IV}$

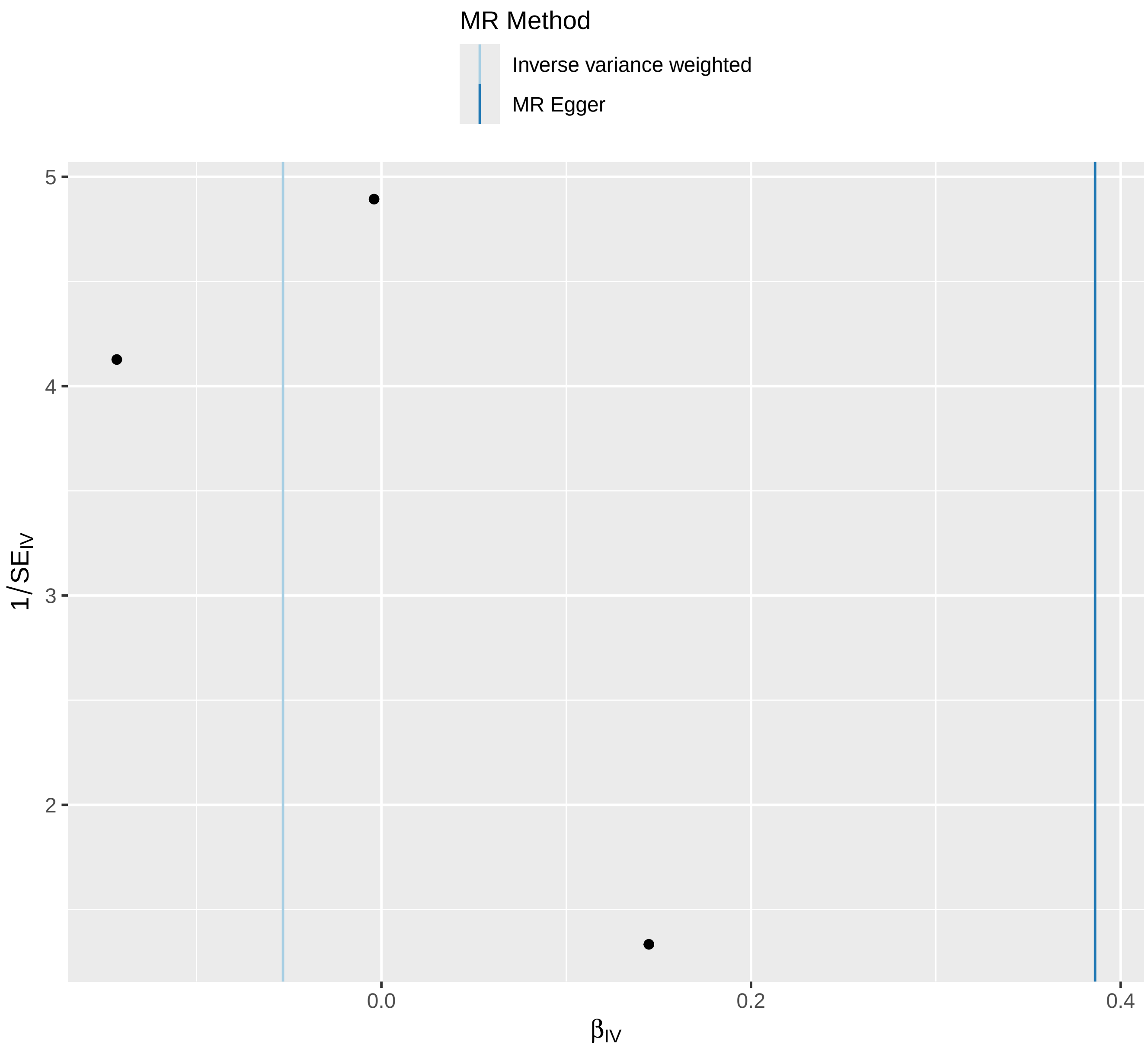
MR Method
Inverse variance weighted
MR Egger
5
4
3
2
1/SE_IV
0.0
0.2
0.4
β_IV

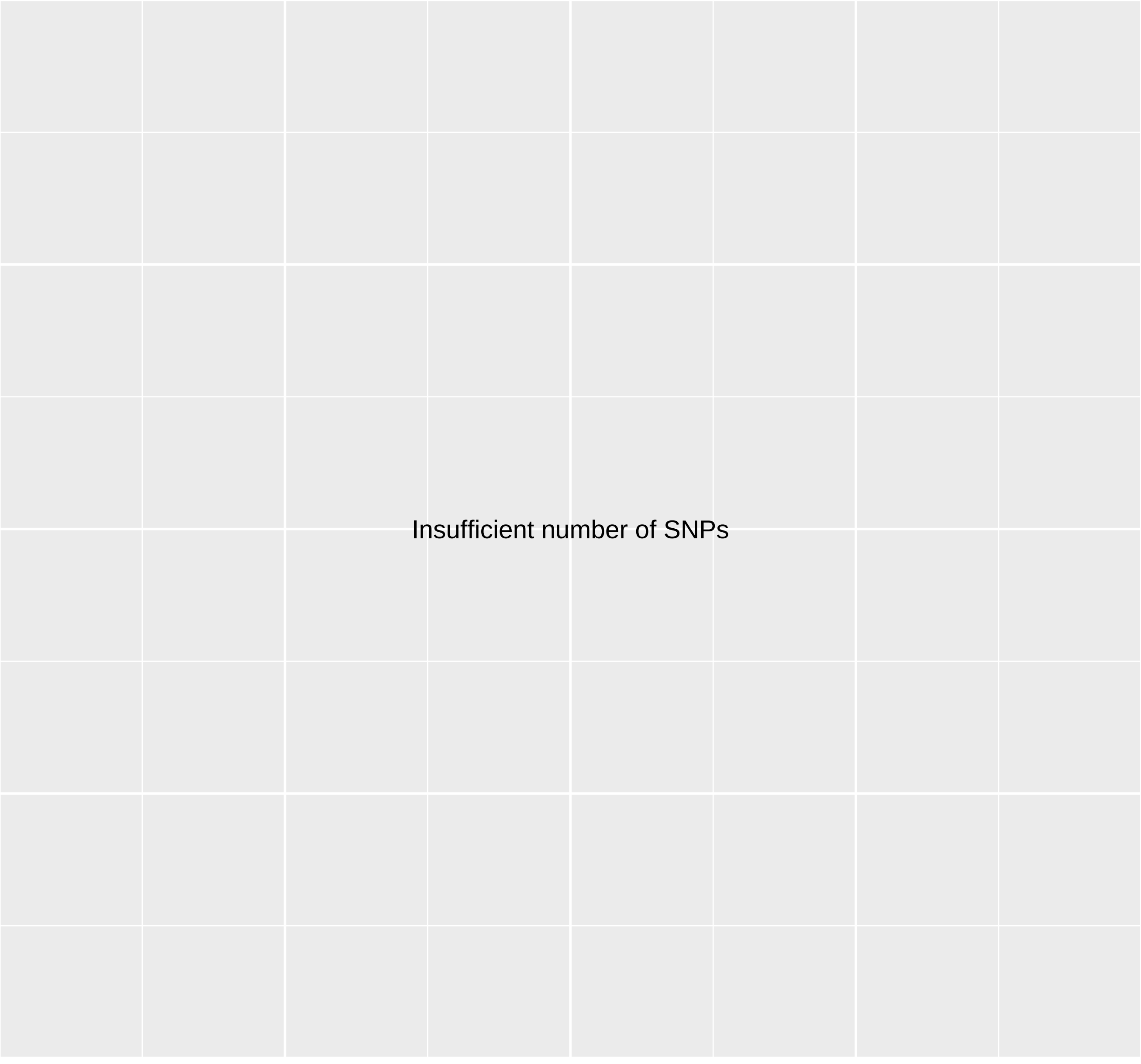
Insufficient number of SNPs

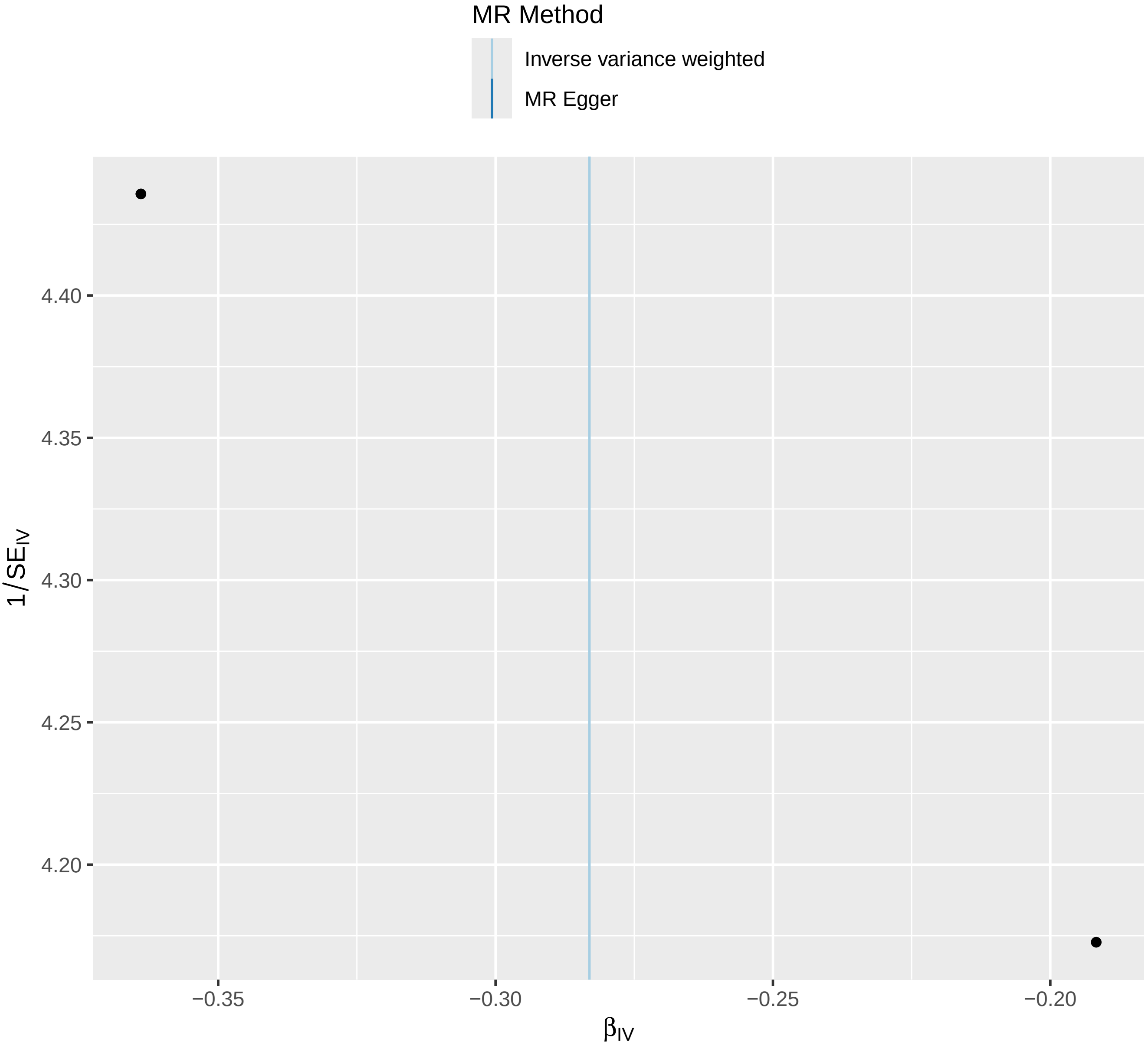
MR Method
Inverse variance weighted
MR Egger
4.40
4.35
4.30
4.25
4.20
−0.35
−0.30
−0.25
−0.20
$\beta_{IV}$
$1/SE_{IV}$

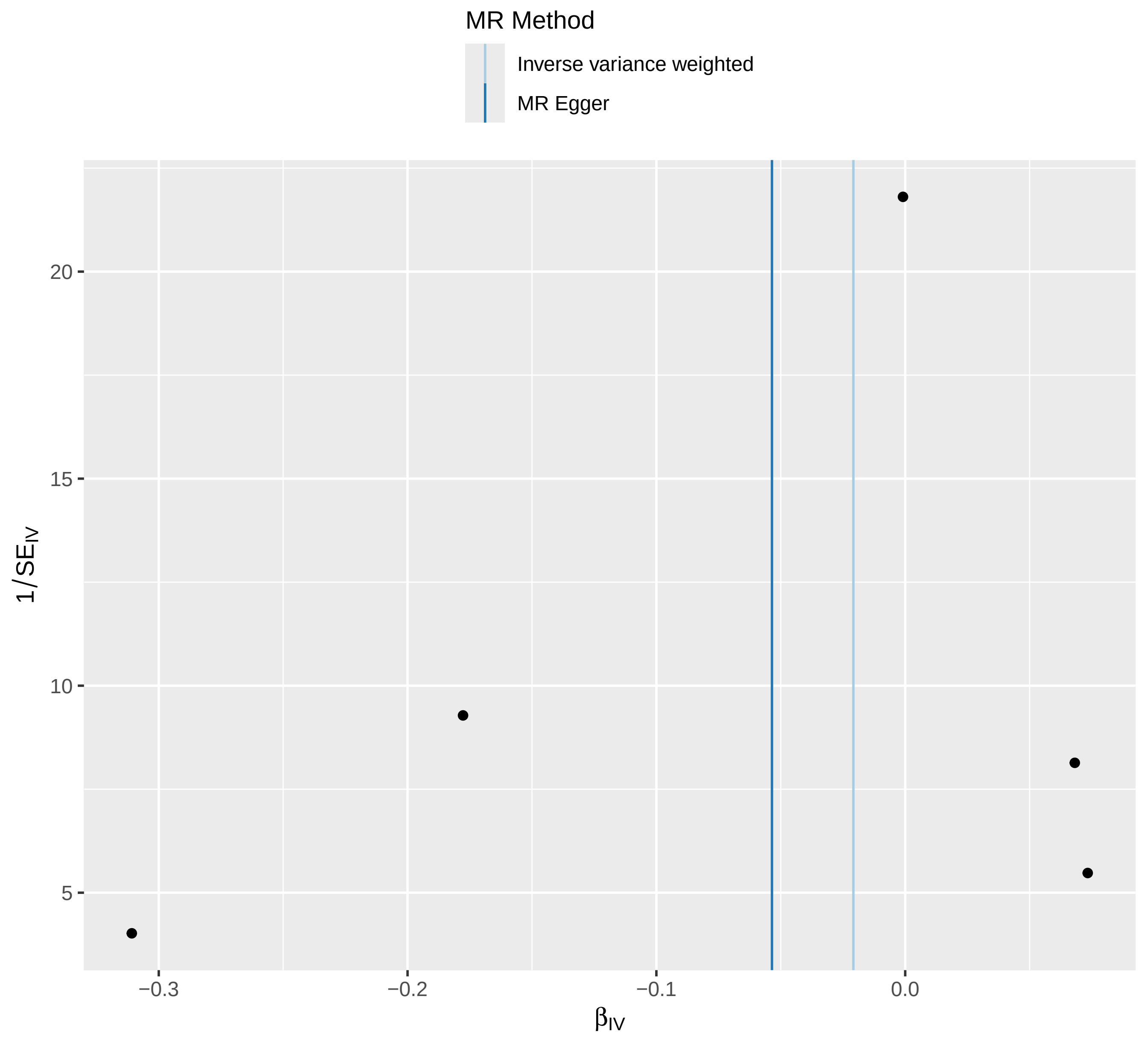
MR Method
Inverse variance weighted
MR Egger
20
15
10
5
1/SE_IV
−0.3
−0.2
−0.1
0.0
β_IV

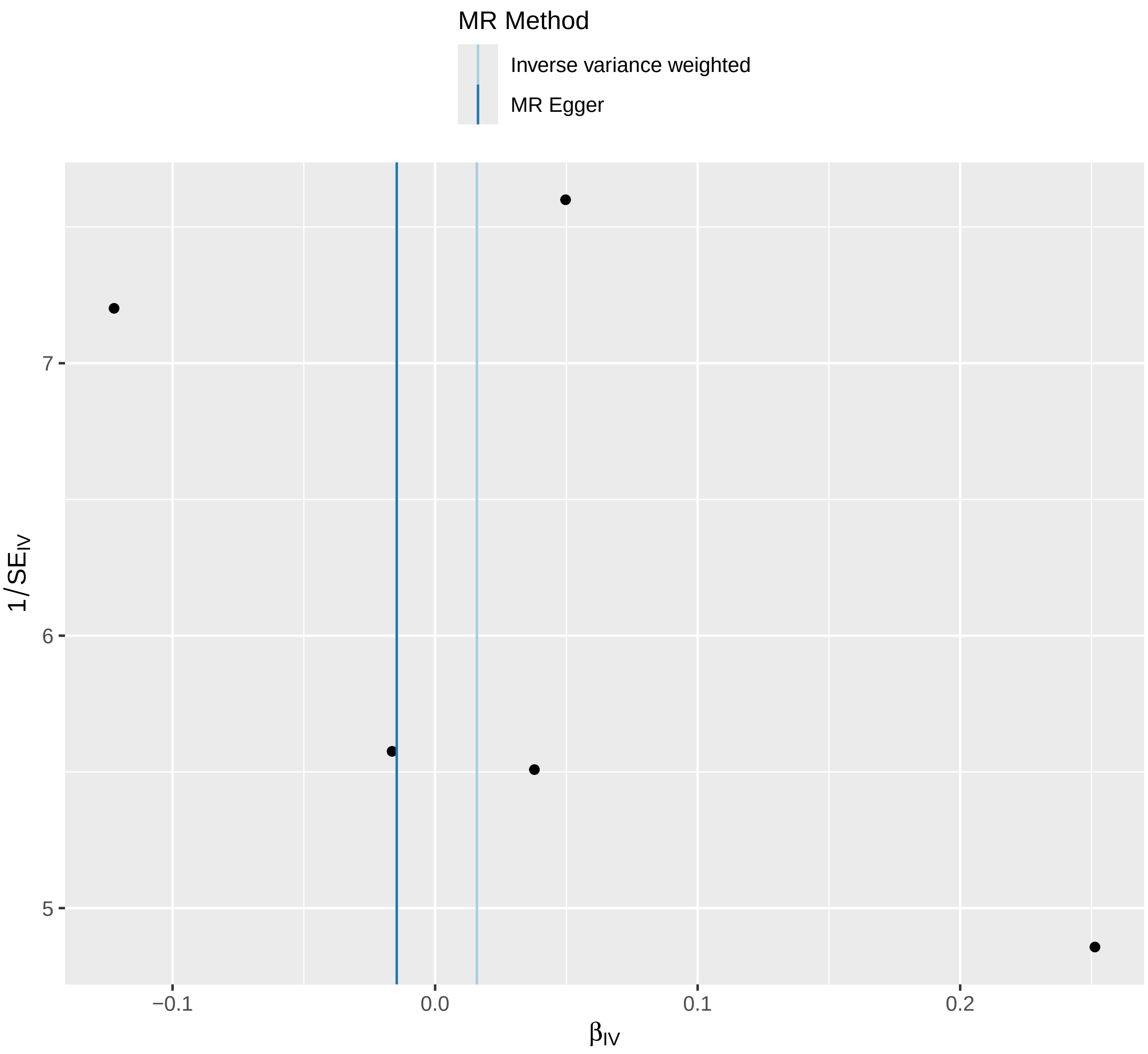

MR Method
Inverse variance weighted
MR Egger
7
6
5
−0.1
0.0
0.1
0.2
1/SE_IV
β_IV

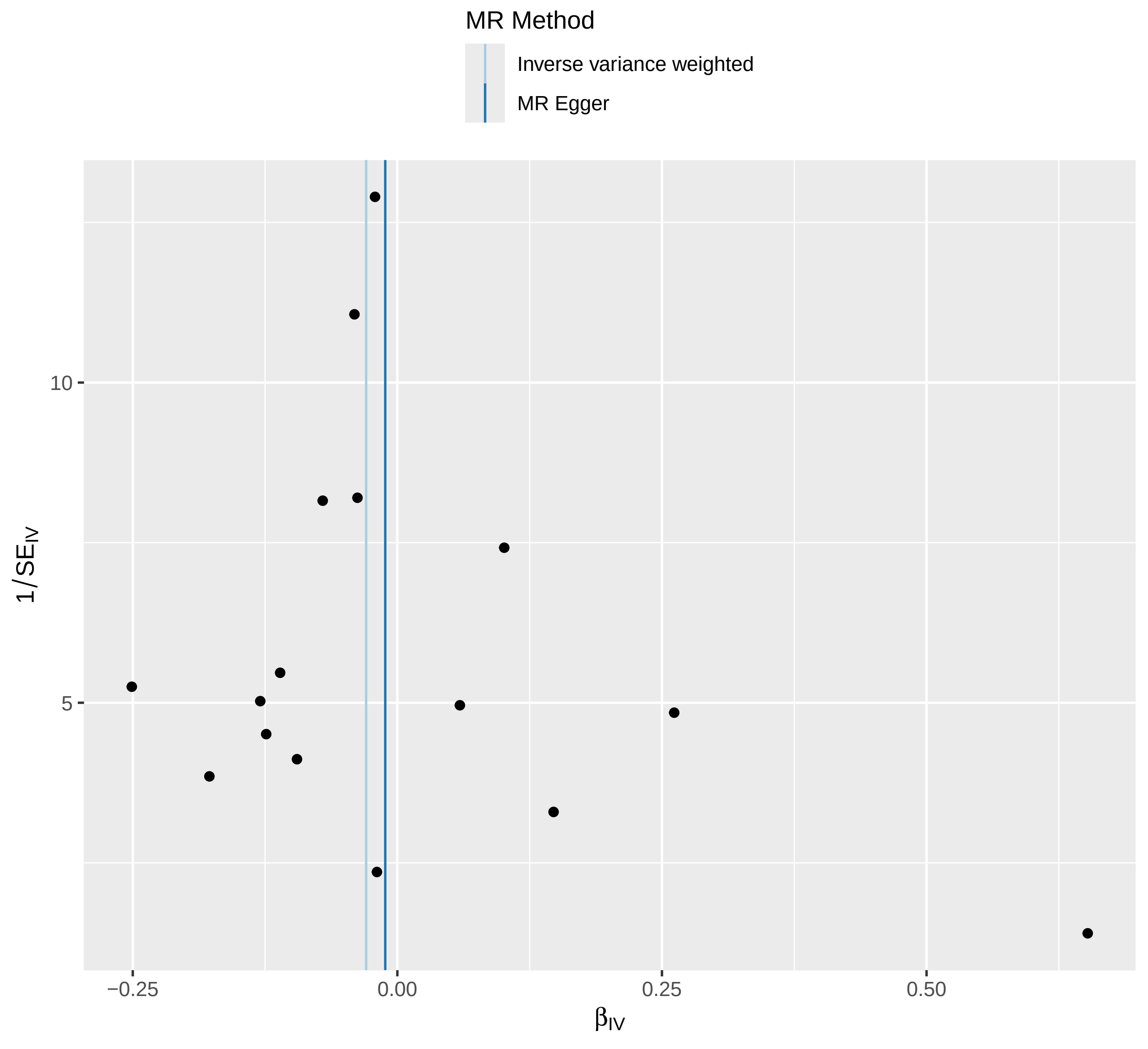
MR Method
Inverse variance weighted
MR Egger
10
5
1/SE$_{IV}$
−0.25
0.00
0.25
0.50
β$_{IV}$

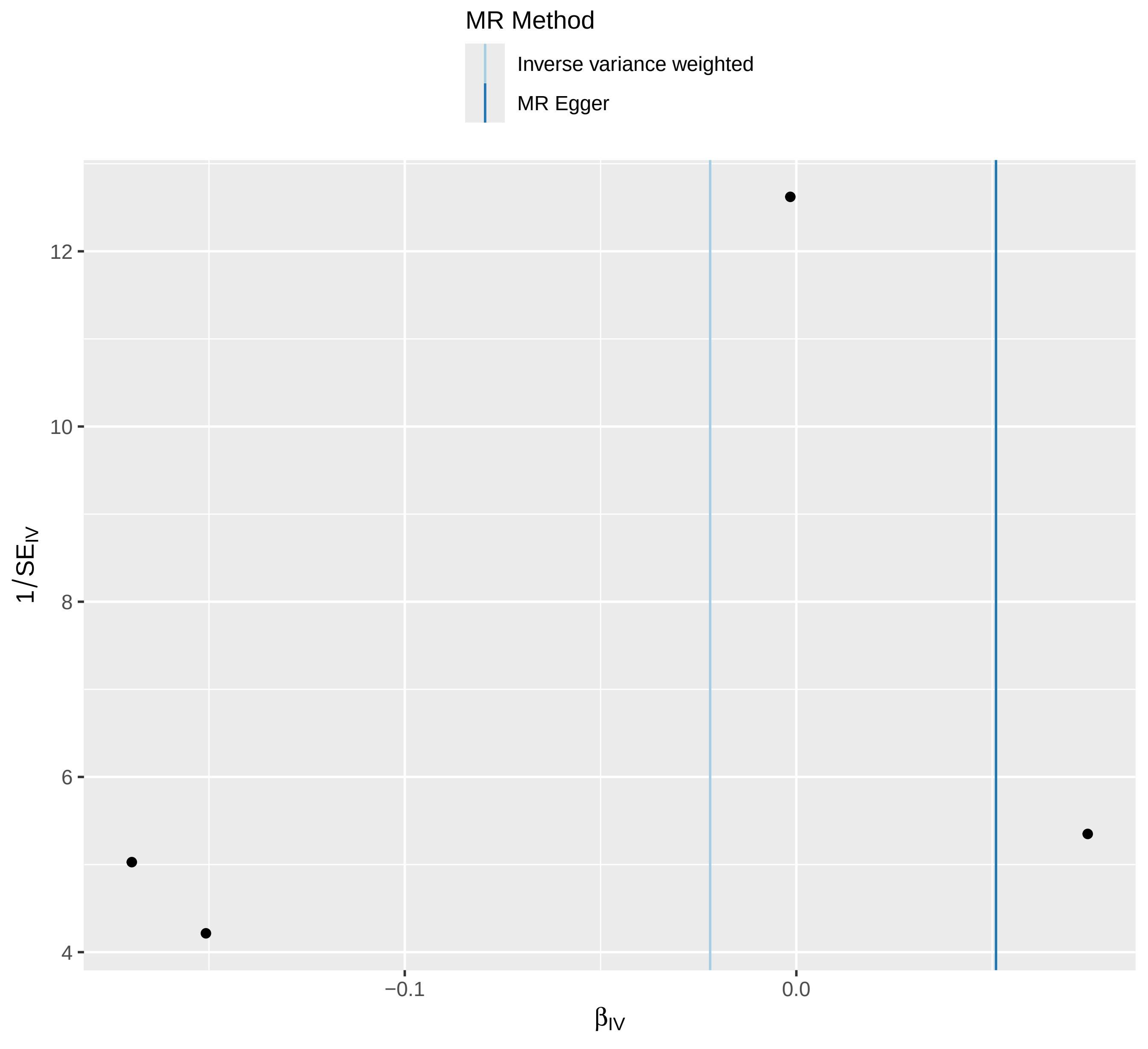
MR Method
Inverse variance weighted
MR Egger
12
10
8
6
4
−0.1
0.0
1/SE_IV
β_IV

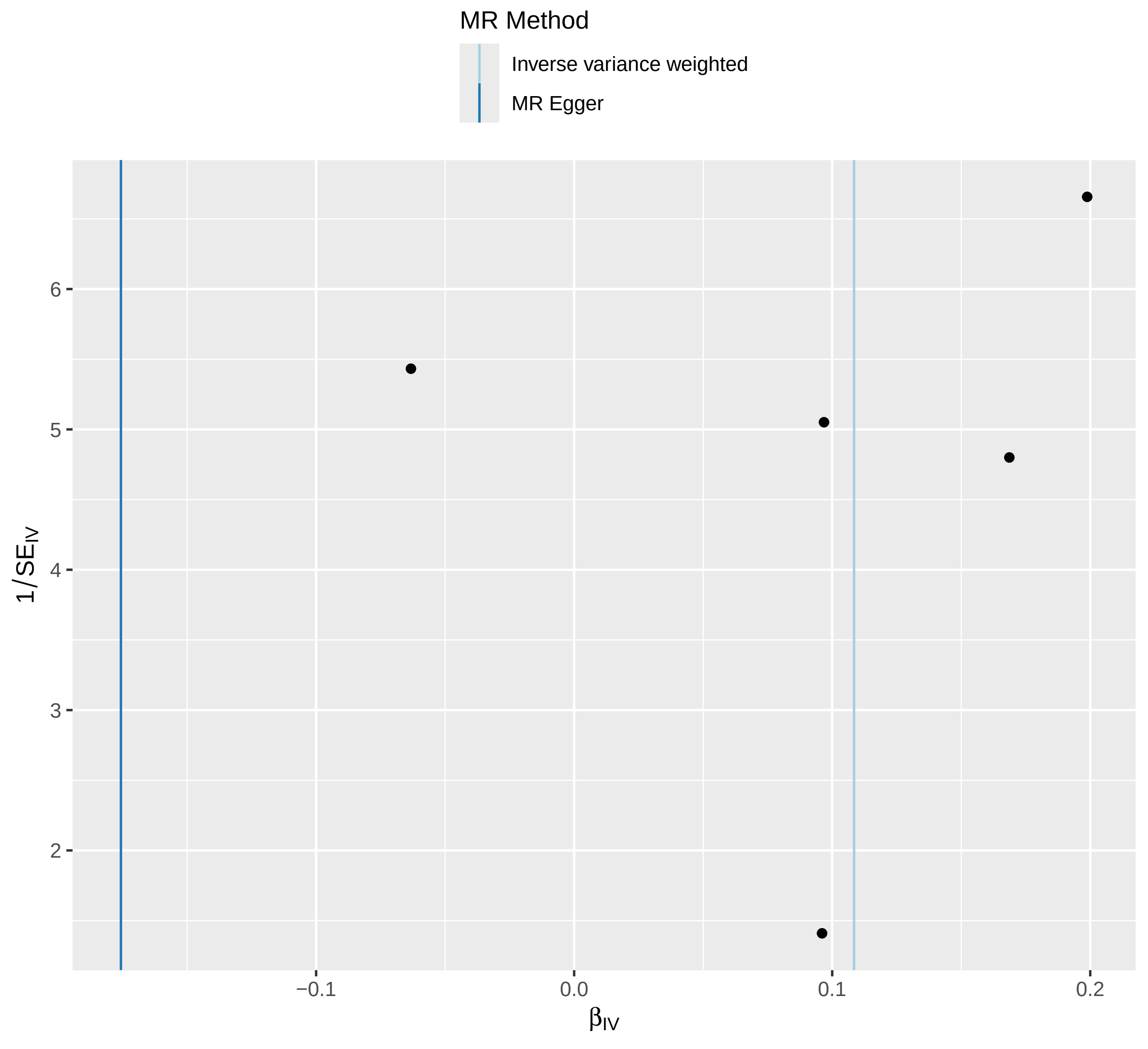
MR Method
Inverse variance weighted
MR Egger
6
5
4
3
2
1/SE_IV
−0.1
0.0
0.1
0.2
β_IV

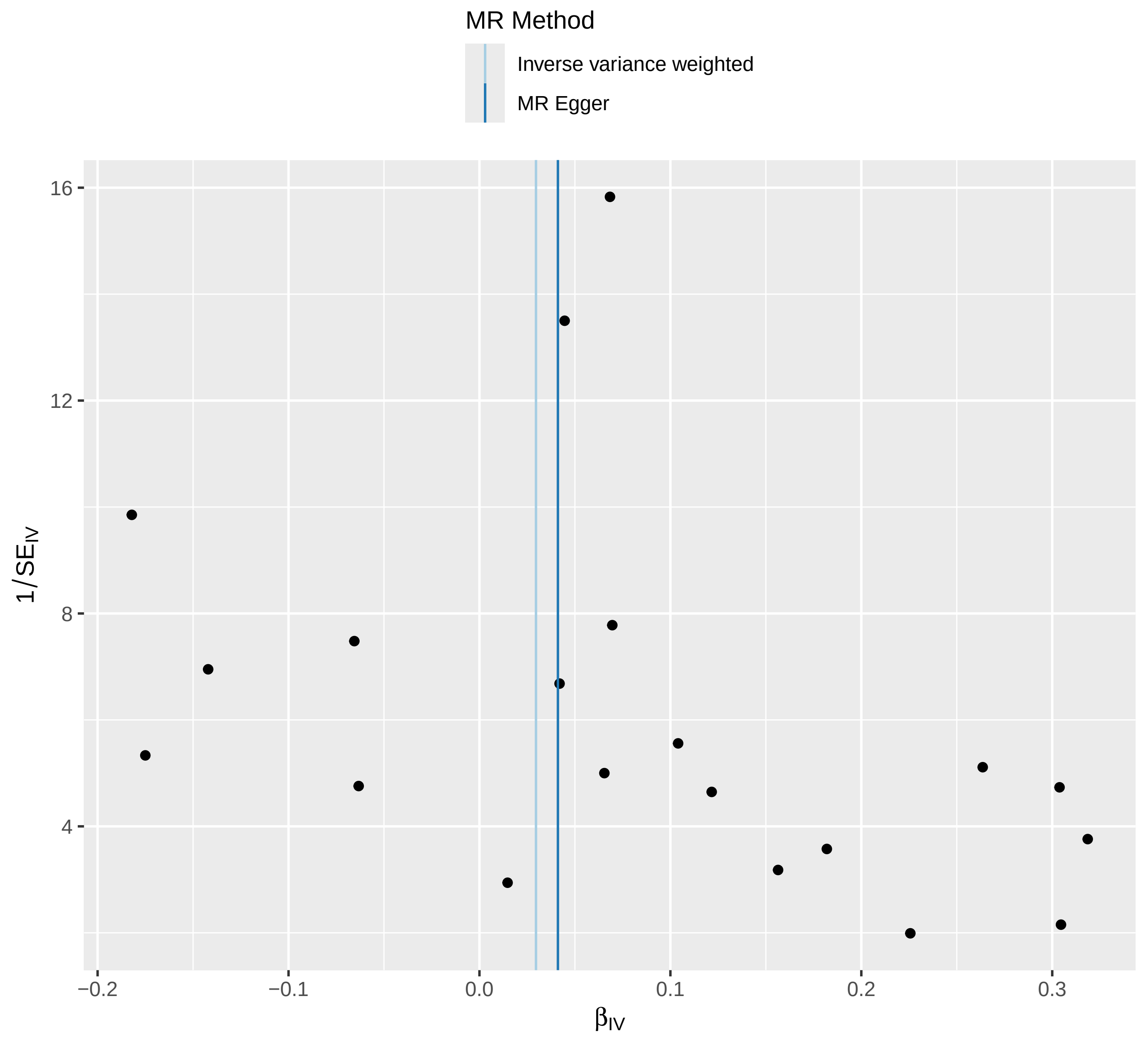
MR Method
Inverse variance weighted
MR Egger
16
12
8
4
1/SE$_{IV}$
−0.2
−0.1
0.0
0.1
0.2
0.3
β$_{IV}$

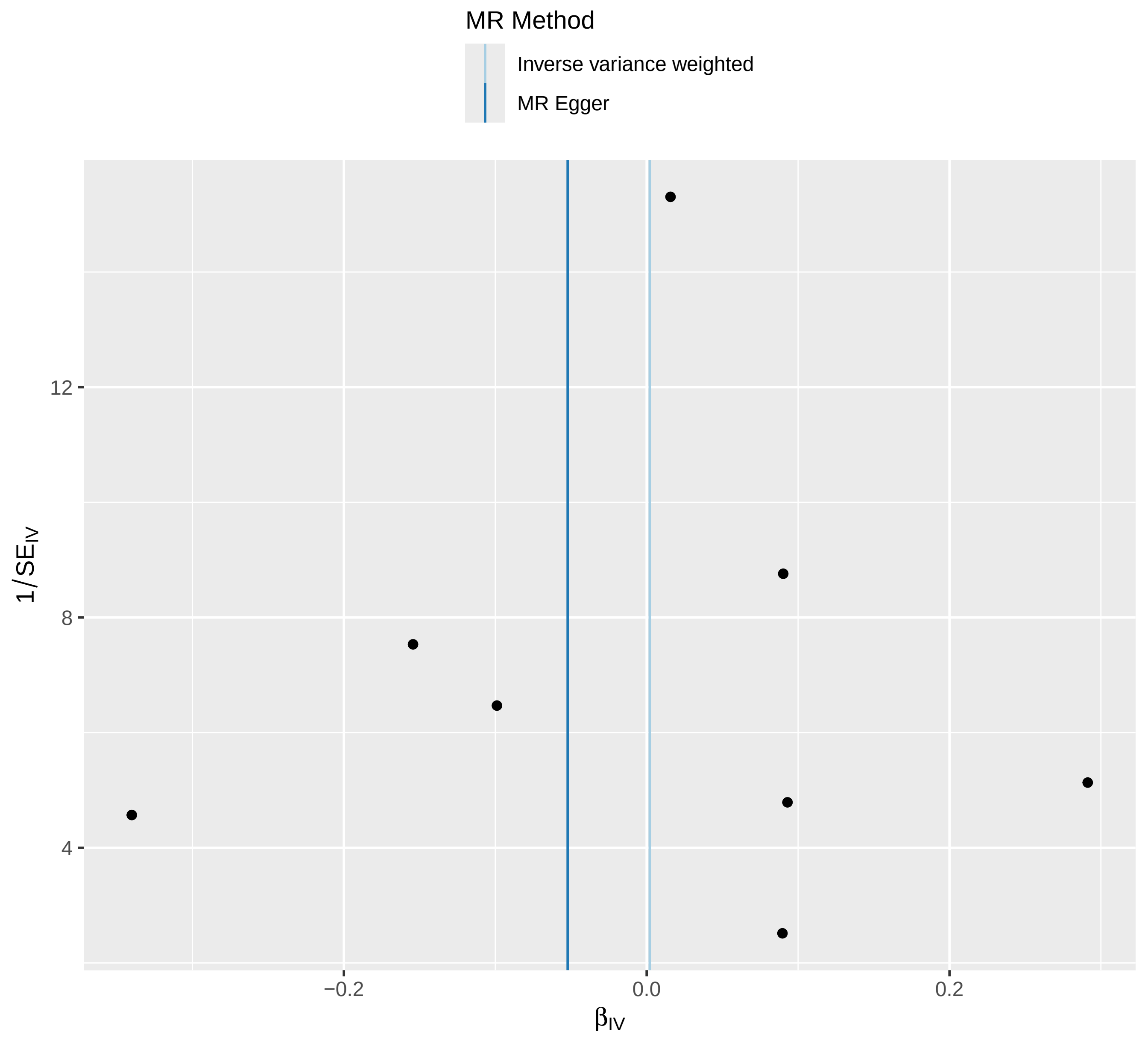

MR Method
Inverse variance weighted
MR Egger
12
8
4
1/SE_IV
−0.2
0.0
0.2
β_IV

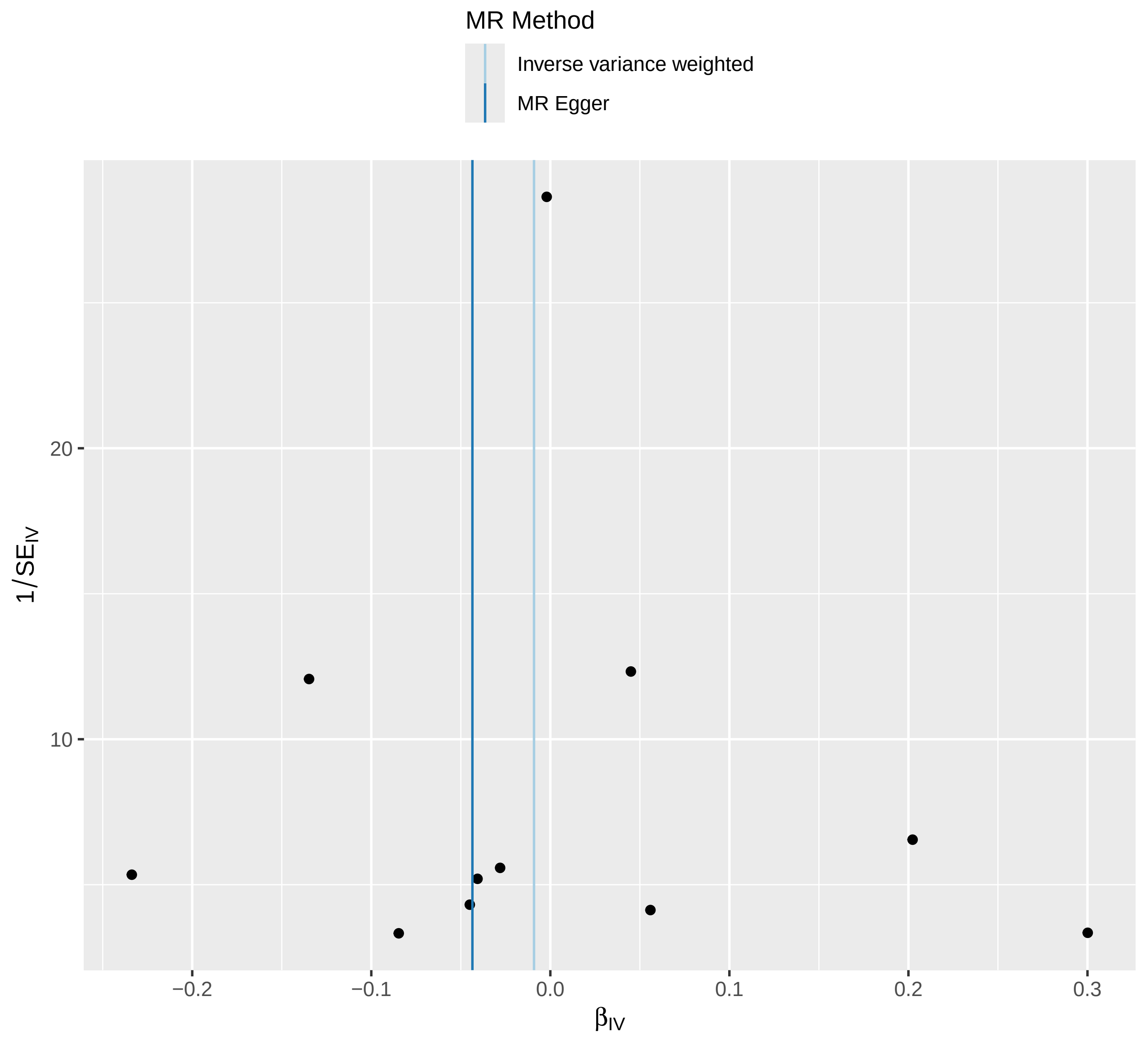

MR Method
Inverse variance weighted
MR Egger
20
10
$1/SE_{IV}$
−0.2
−0.1
0.0
0.1
0.2
0.3
$\beta_{IV}$

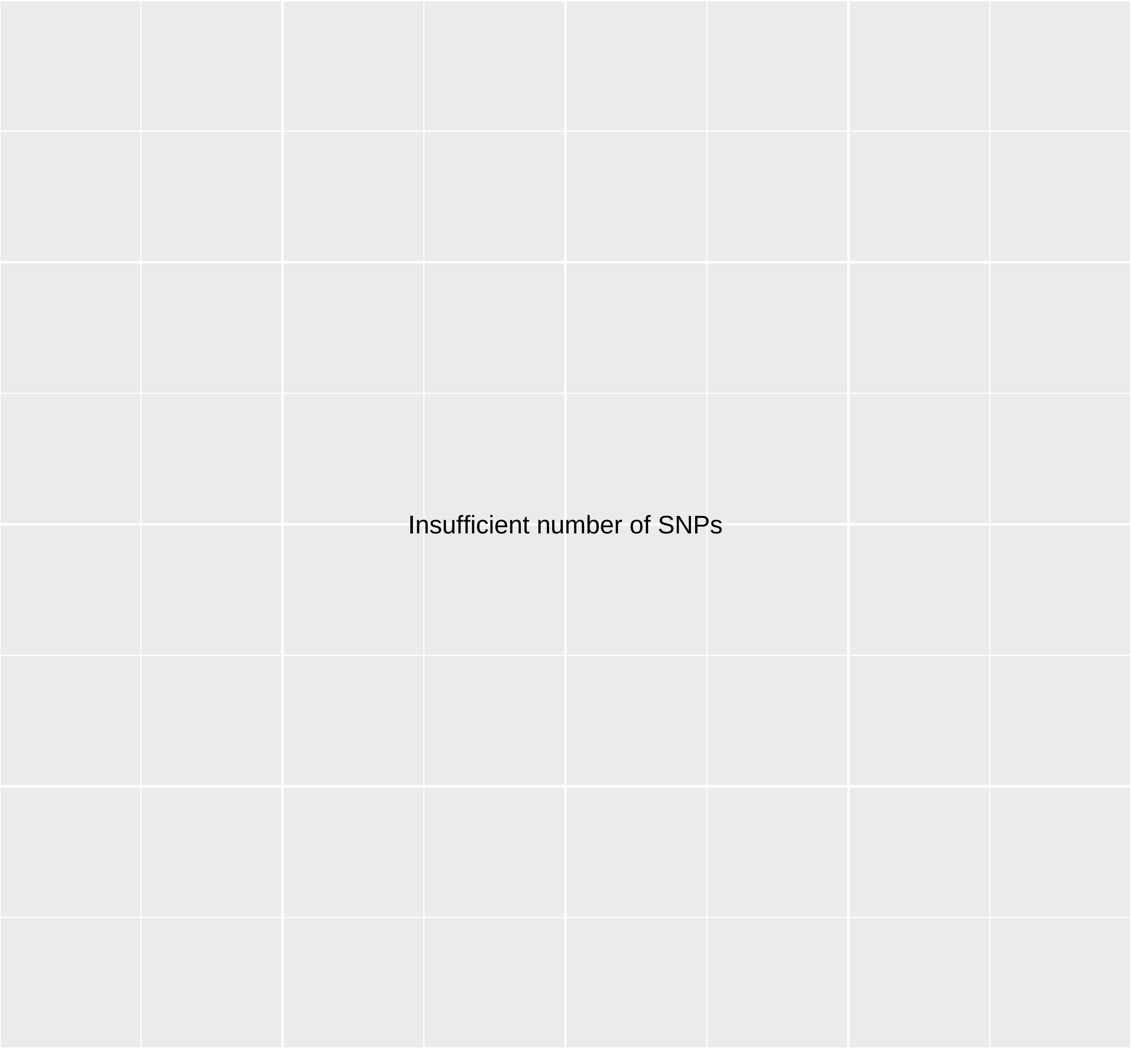
Insufficient number of SNPs

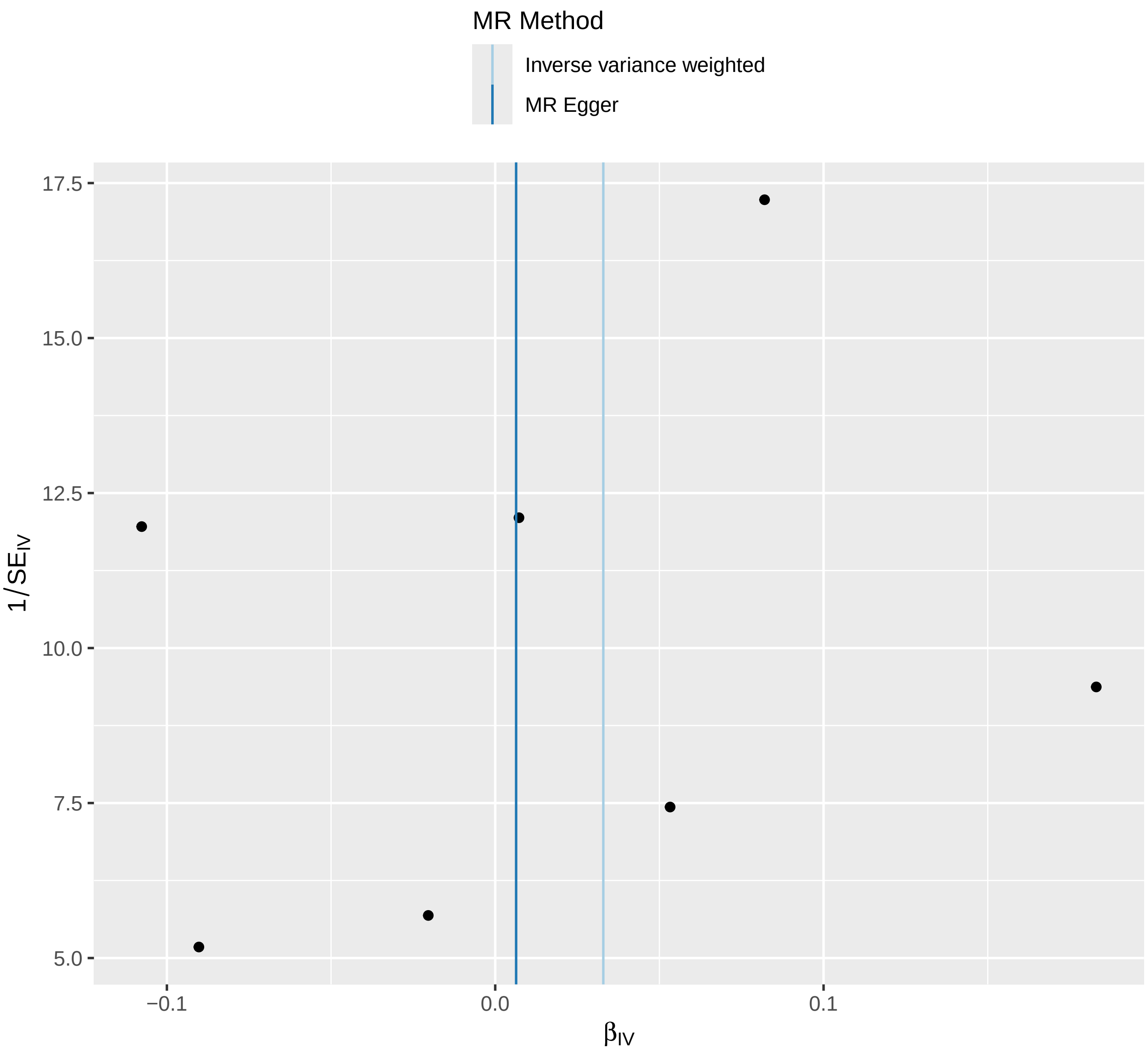

MR Method
Inverse variance weighted
MR Egger
17.5
15.0
12.5
10.0
7.5
5.0
1/SE_IV
−0.1
0.0
0.1
β_IV

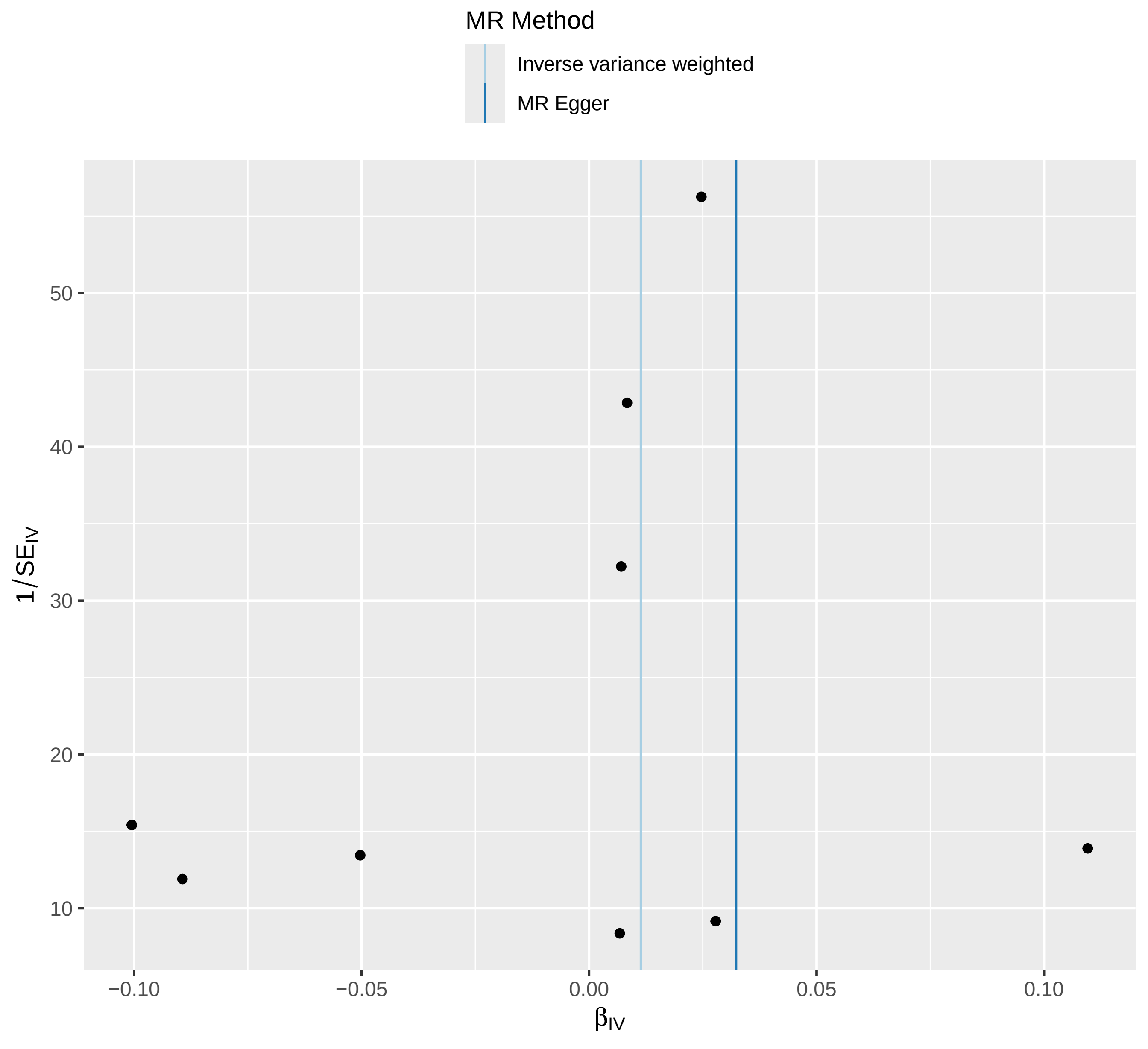
MR Method
Inverse variance weighted
MR Egger
1/SE_IV
50
40
30
20
10
−0.10
−0.05
0.00
0.05
0.10
β_IV

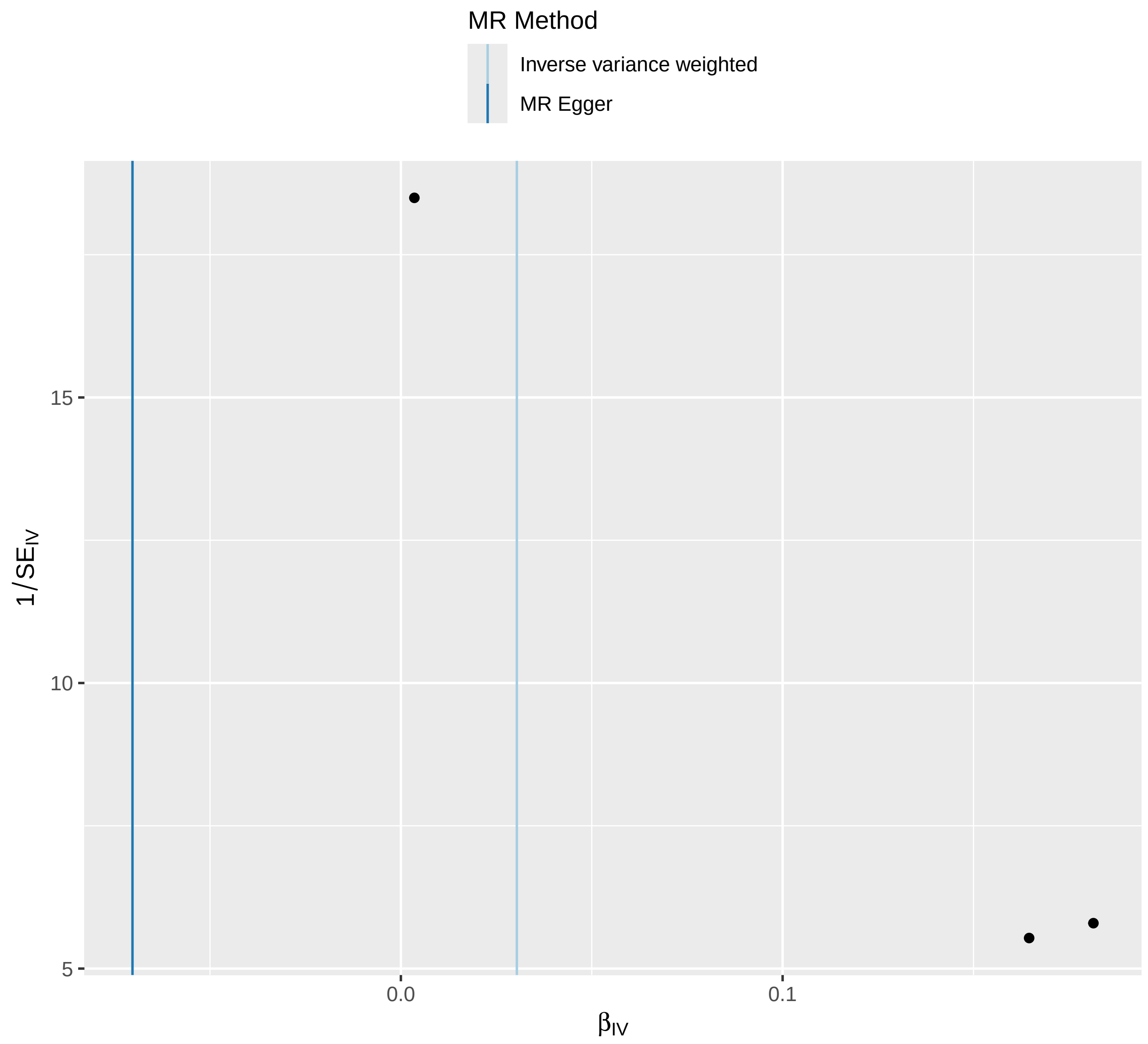
MR Method
Inverse variance weighted
MR Egger
15
10
5
1/SE_IV
0.0
0.1
β_IV

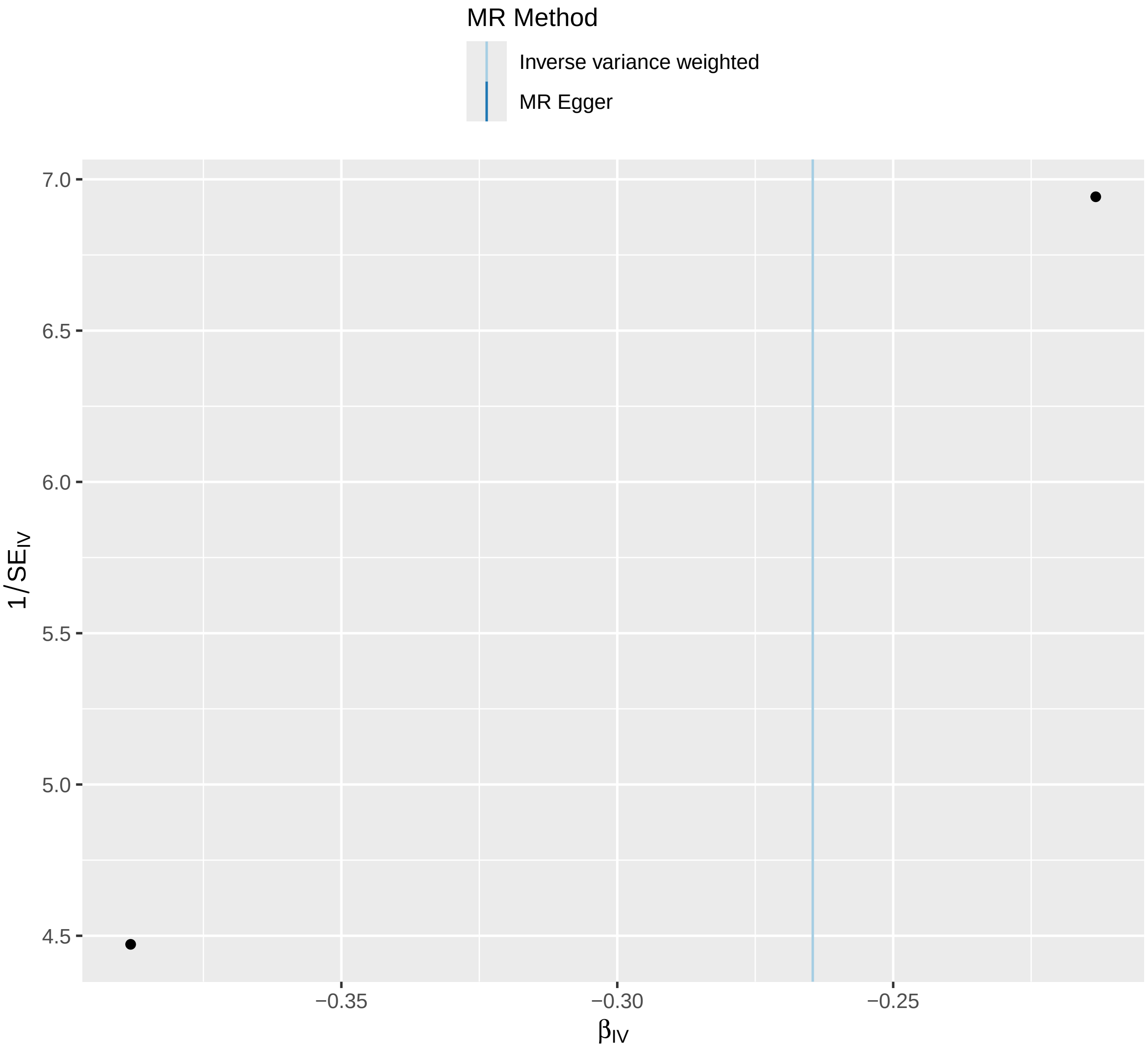
MR Method
Inverse variance weighted
MR Egger
7.0
6.5
6.0
5.5
5.0
4.5
1/SE_IV
−0.35
−0.30
−0.25
β_IV

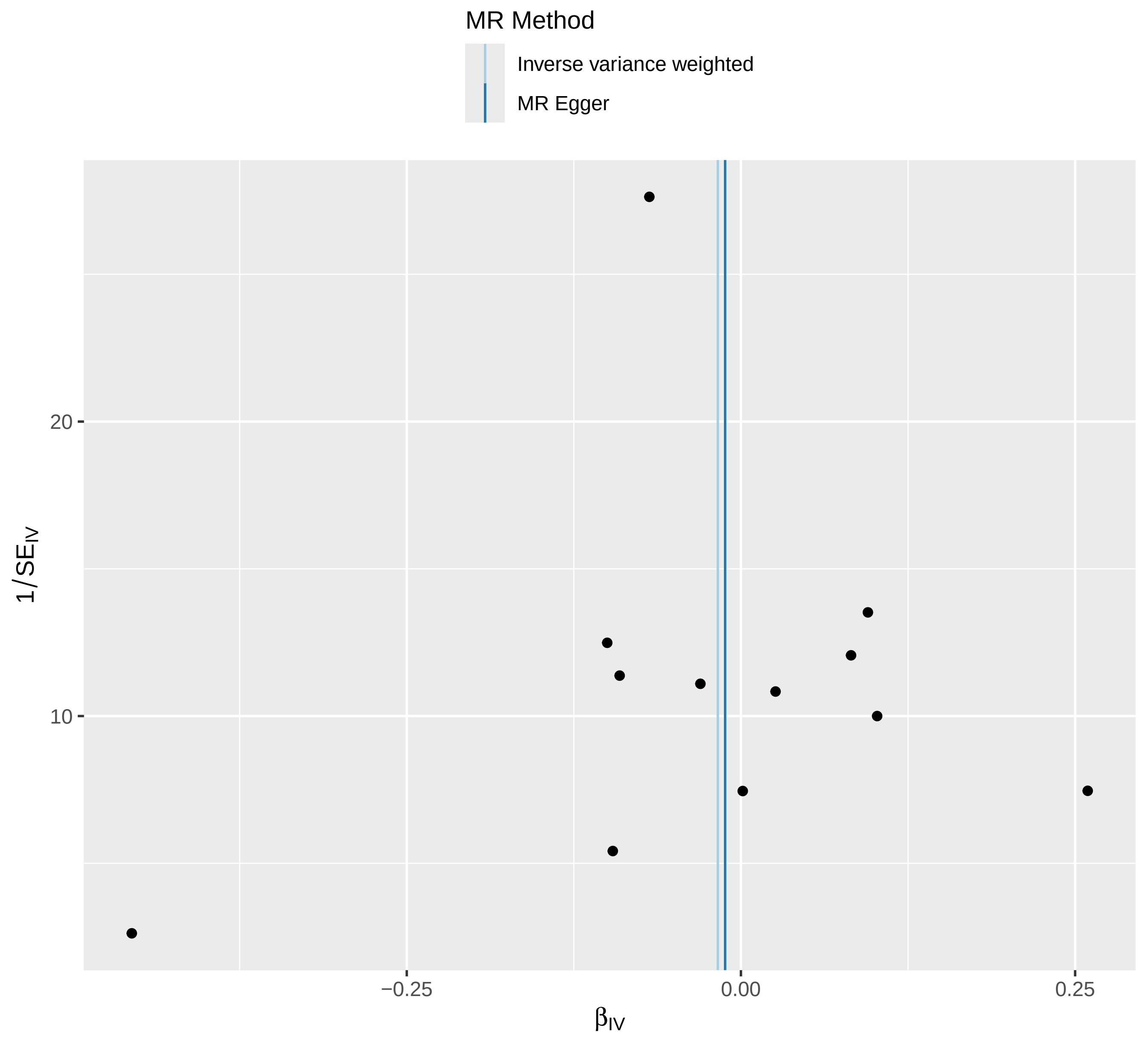
MR Method
Inverse variance weighted
MR Egger
20
10
1/SE$_{IV}$
−0.25
0.00
0.25
β$_{IV}$

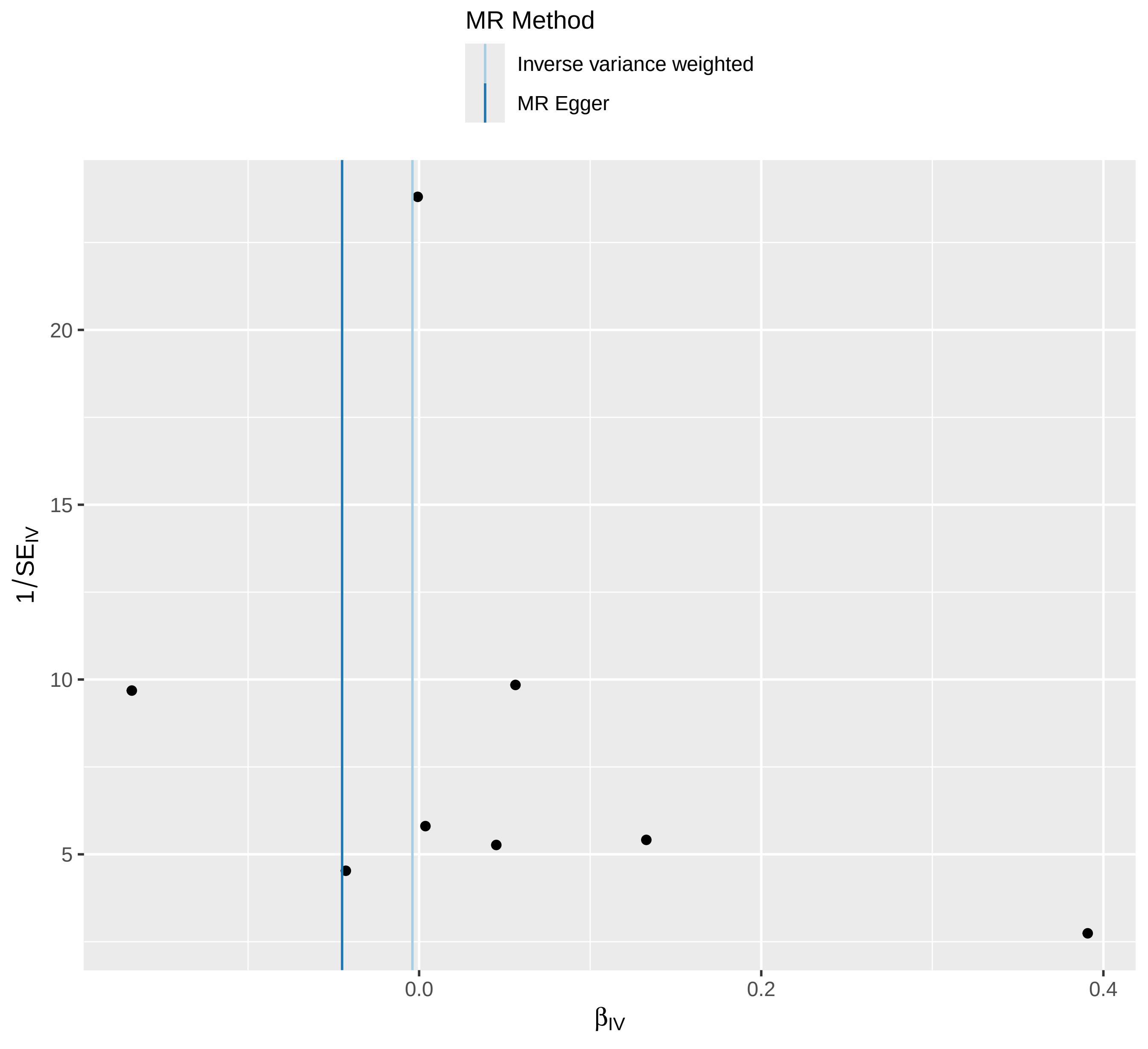

MR Method
Inverse variance weighted
MR Egger
20
15
10
5
1/SE_IV
0.0
0.2
0.4
β_IV

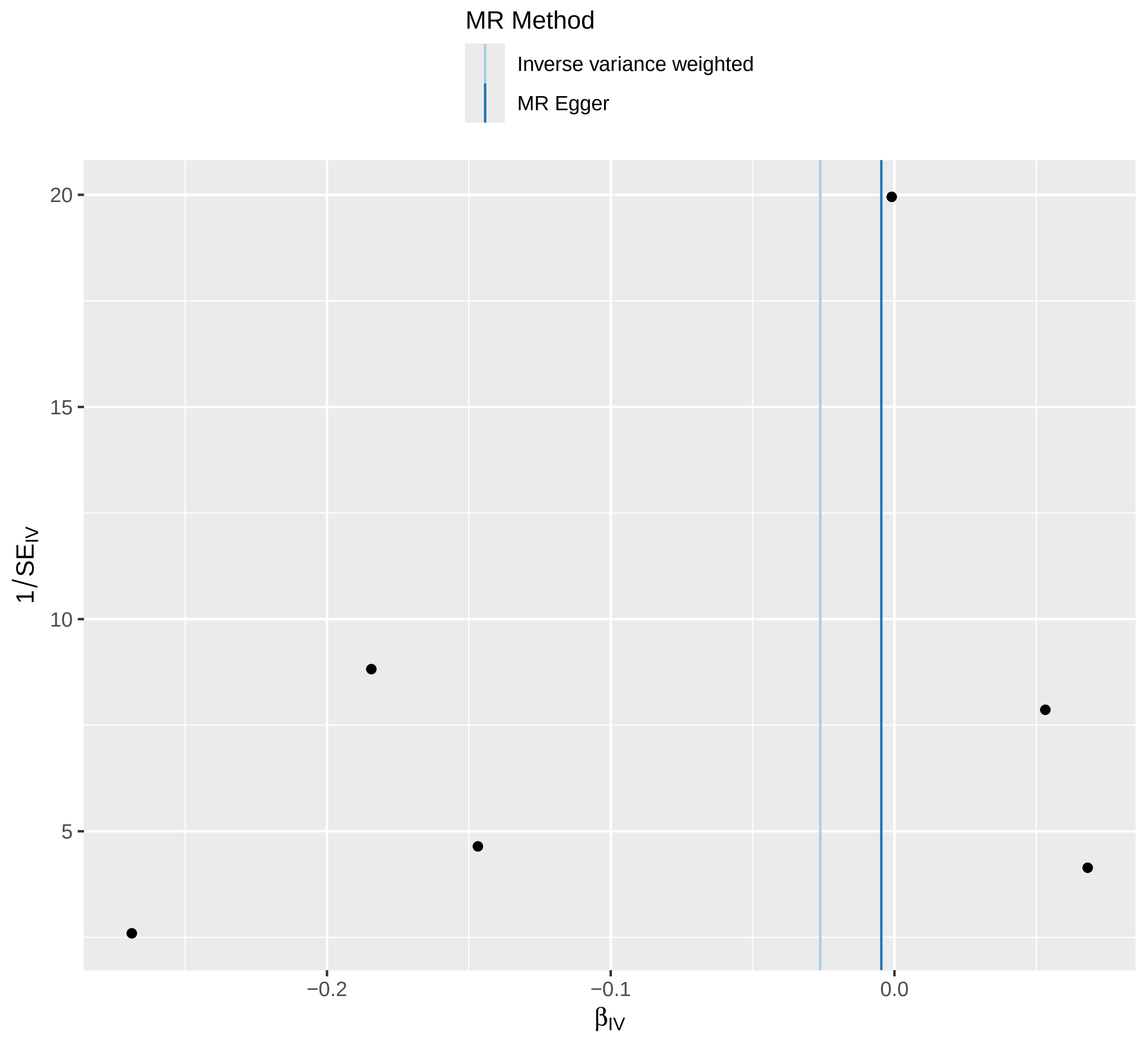
MR Method
Inverse variance weighted
MR Egger
20
15
10
5
1/SE$_{IV}$
−0.2
−0.1
0.0
β$_{IV}$

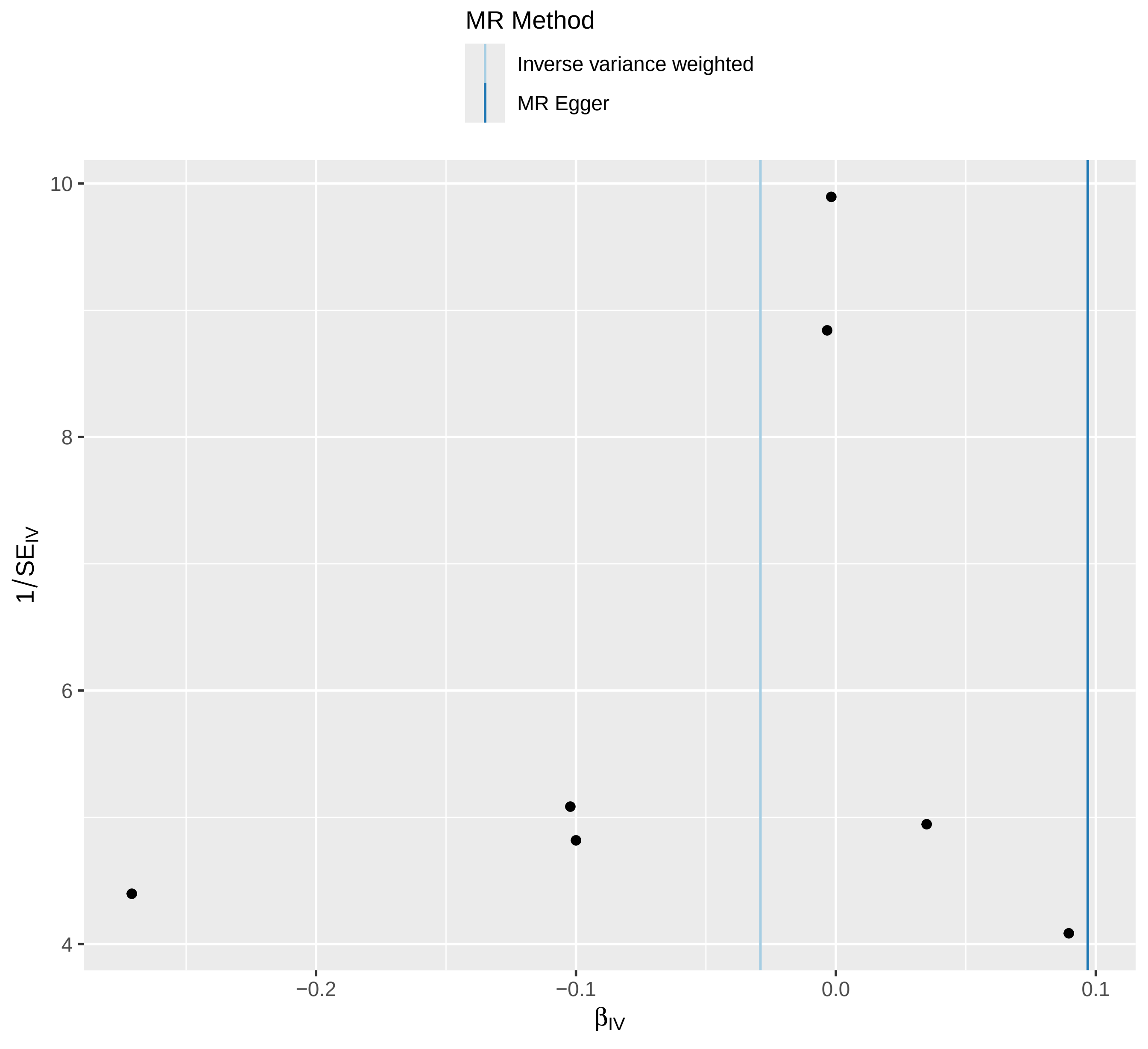
MR Method
Inverse variance weighted
MR Egger
10
8
6
4
1/SE$_{IV}$
−0.2
−0.1
0.0
0.1
β$_{IV}$

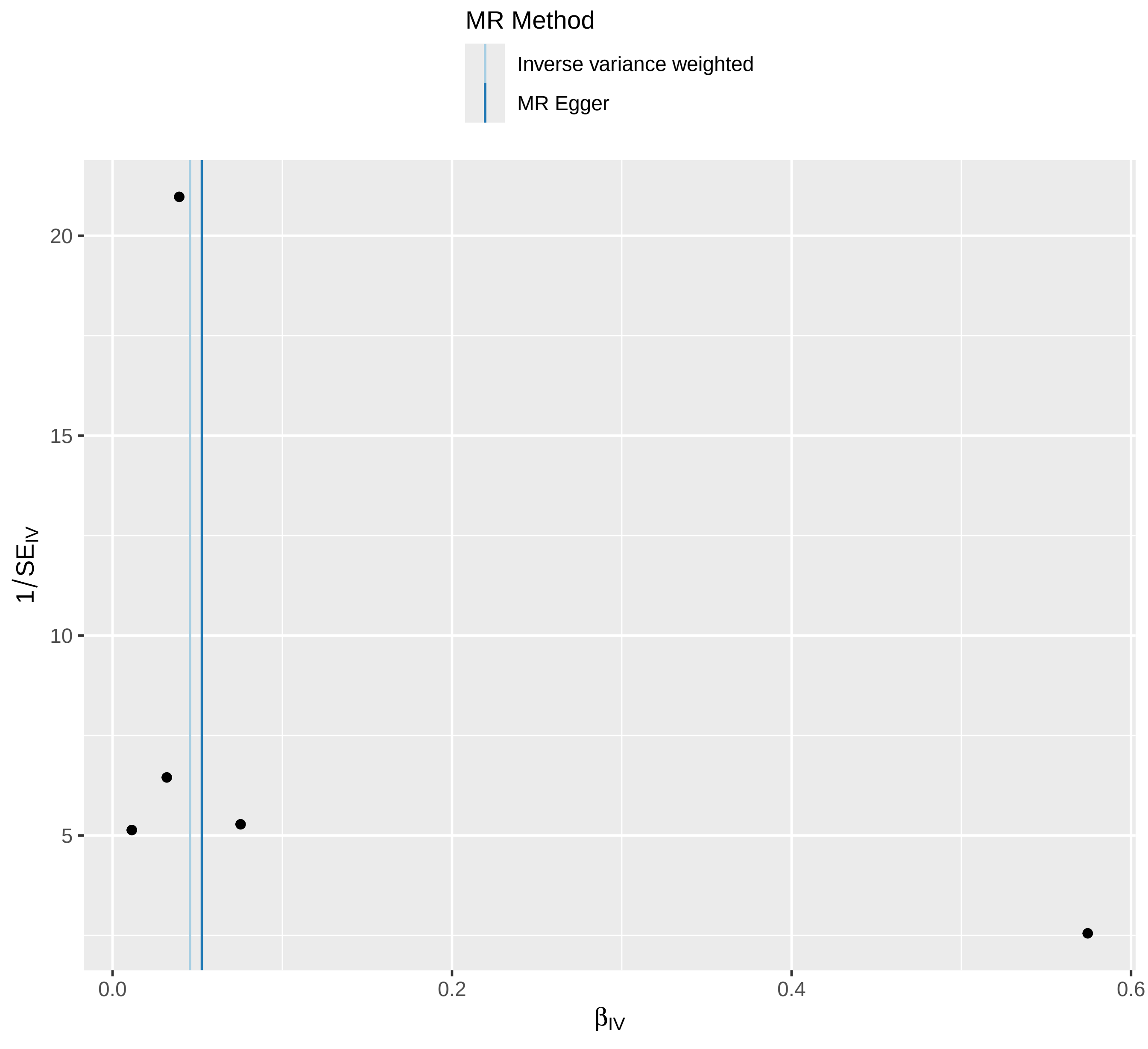
MR Method
Inverse variance weighted
MR Egger
20
15
10
5
$1/SE_{IV}$
0.0
0.2
0.4
0.6
$\beta_{IV}$

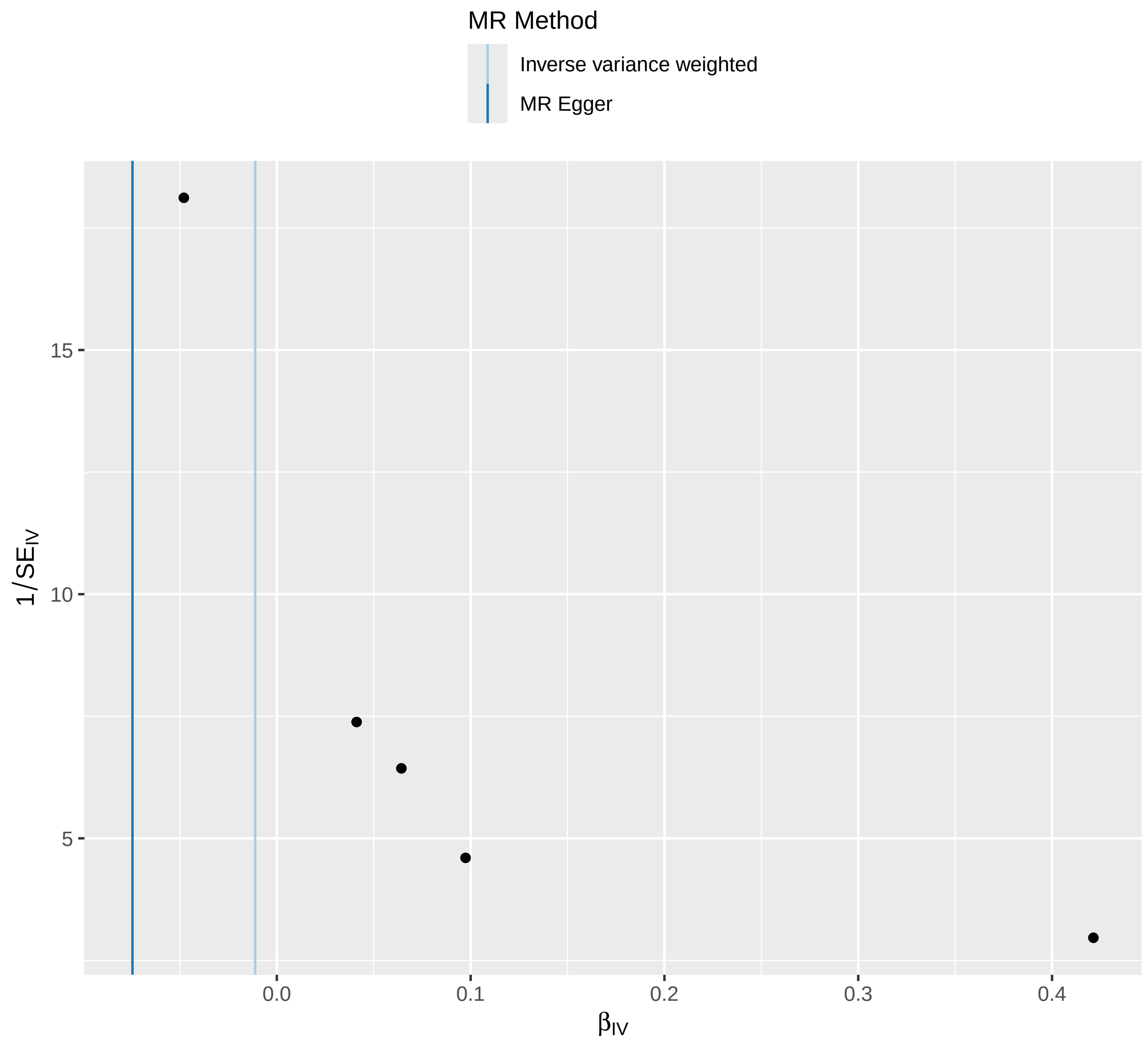

MR Method
Inverse variance weighted
MR Egger
15
10
5
0.0
0.1
0.2
0.3
0.4
$1/SE_{IV}$
$\beta_{IV}$

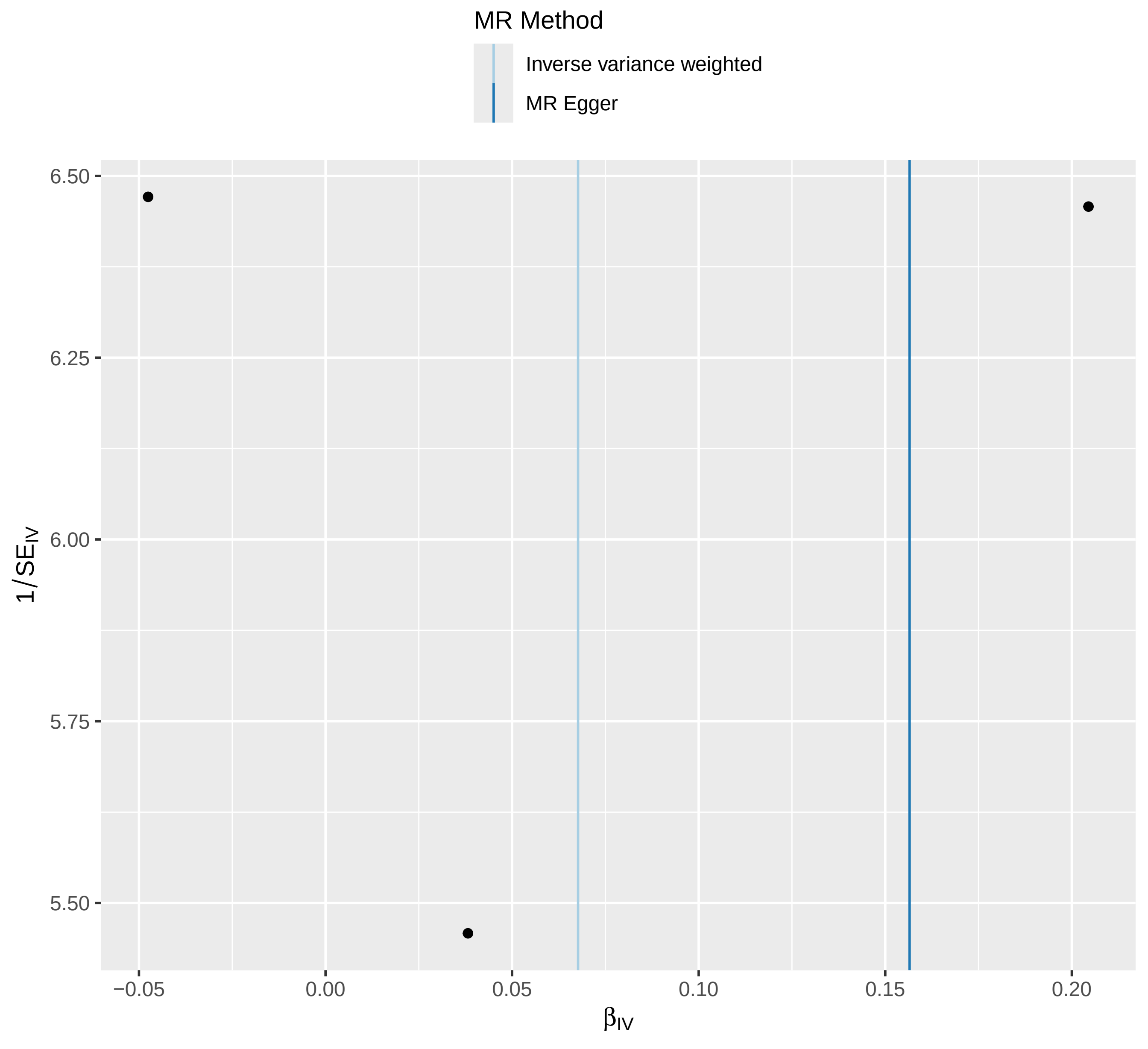

MR Method
Inverse variance weighted
MR Egger
6.50
6.25
6.00
5.75
5.50
1/SE$_{IV}$
−0.05
0.00
0.05
0.10
0.15
0.20
β$_{IV}$

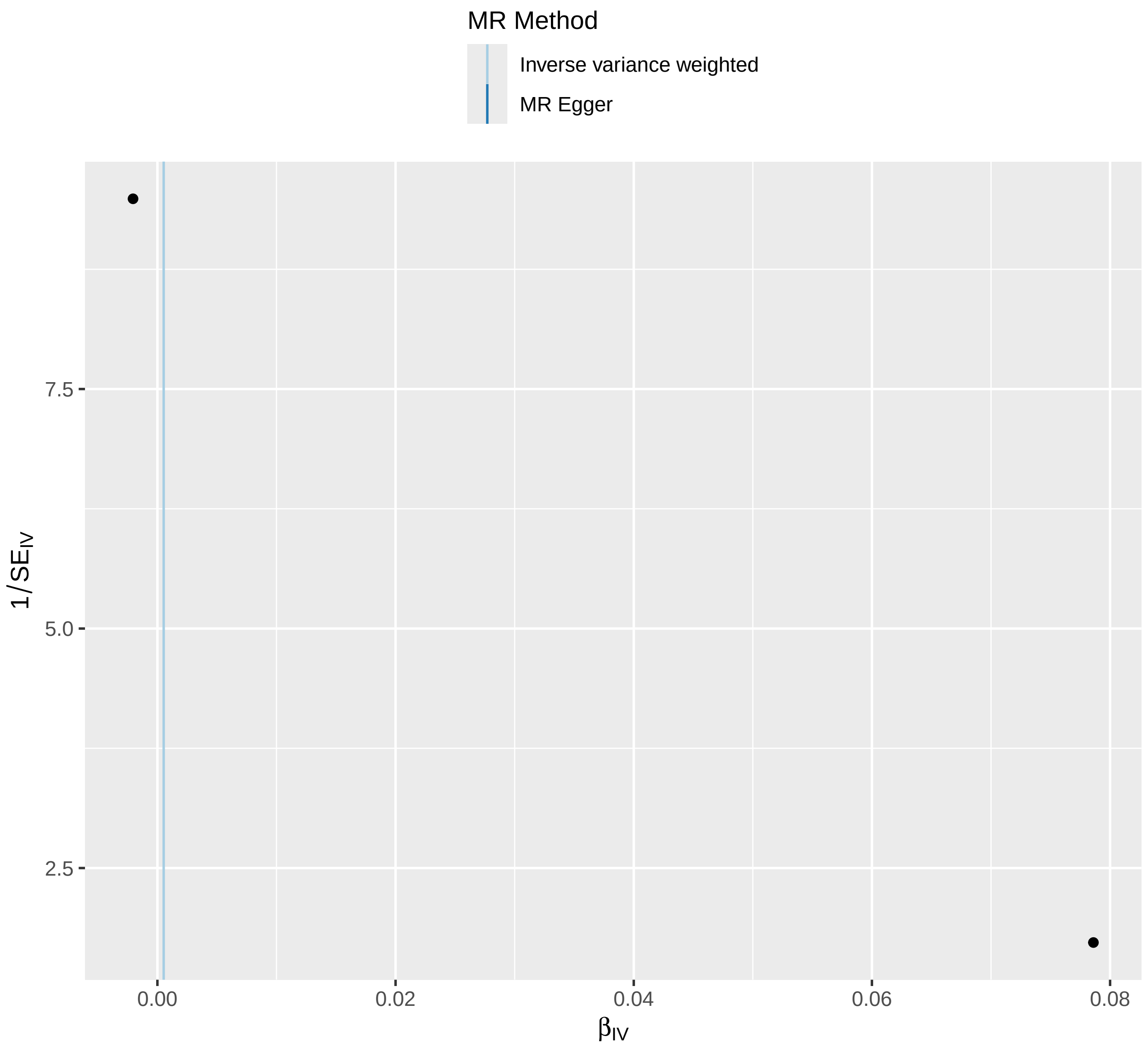

MR Method
Inverse variance weighted
MR Egger
7.5
5.0
2.5
$1/SE_{IV}$
0.00
0.02
0.04
0.06
0.08
$\beta_{IV}$

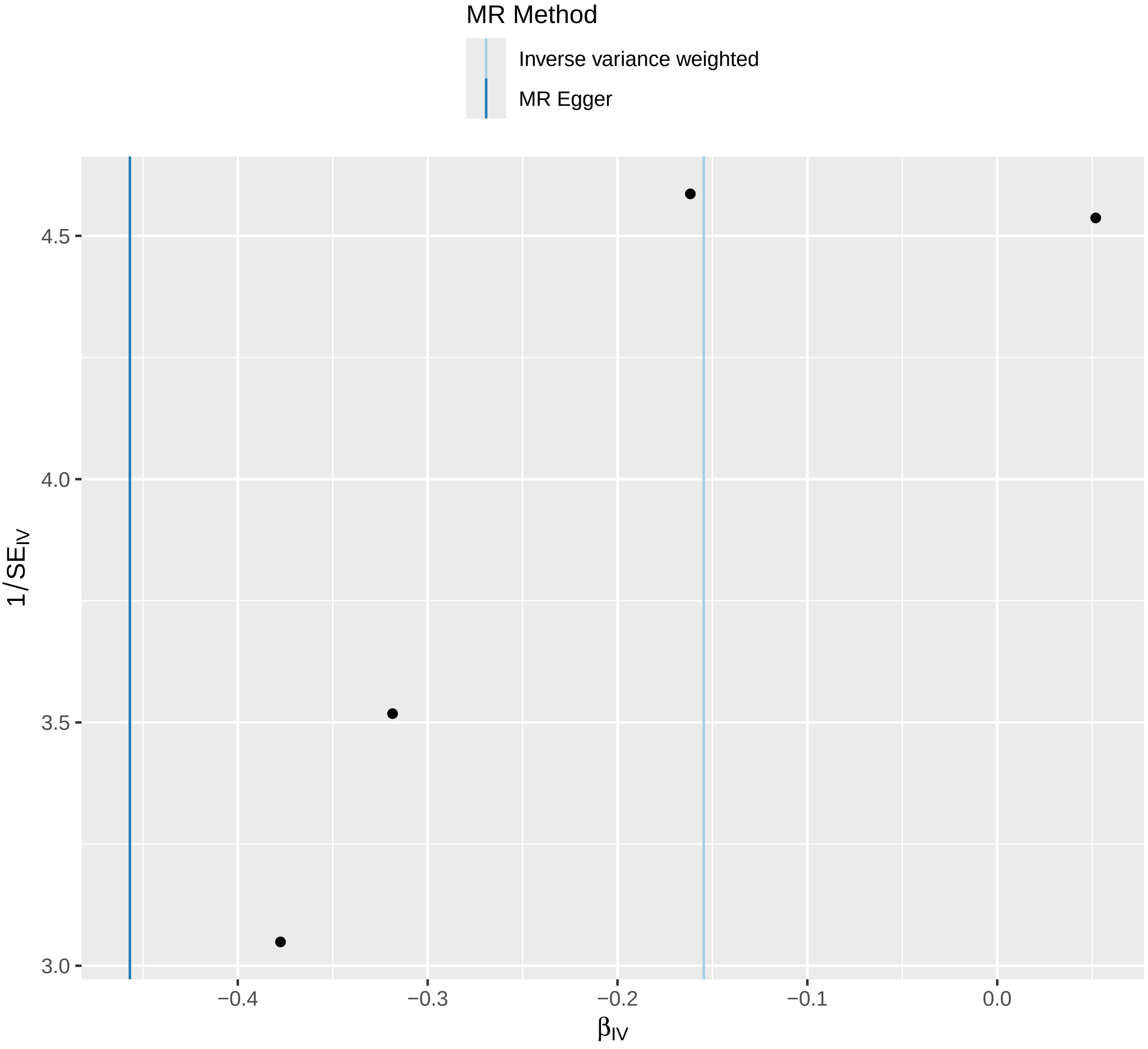
MR Method
Inverse variance weighted
MR Egger
4.5
4.0
3.5
3.0
1/SE_IV
−0.4
−0.3
−0.2
−0.1
0.0
β_IV

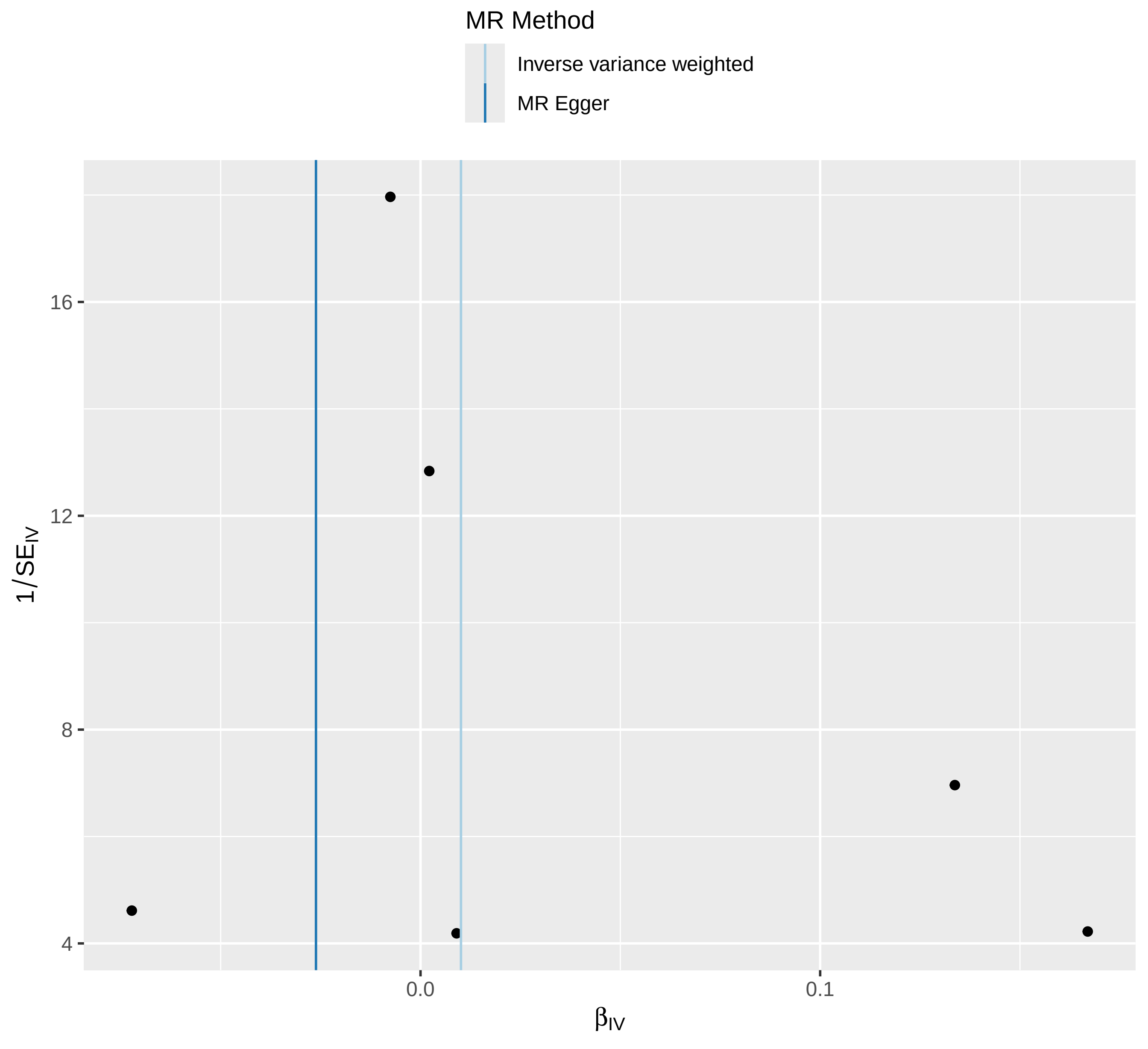

MR Method
Inverse variance weighted
MR Egger
16
12
8
4
0.0
0.1
$1/SE_{IV}$
$\beta_{IV}$

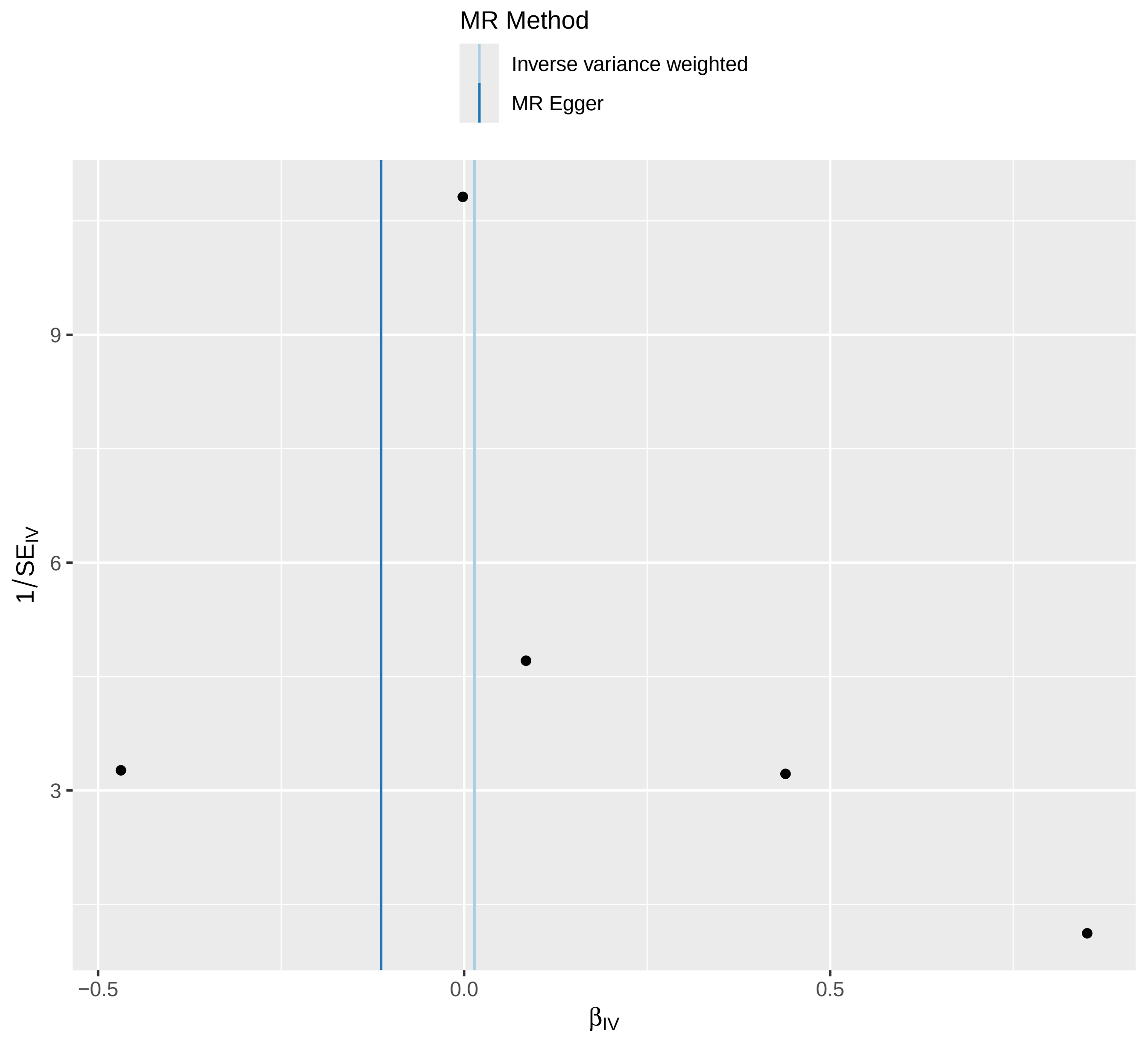

MR Method
Inverse variance weighted
MR Egger
9
6
3
1/SE_IV
−0.5
0.0
0.5
β_IV

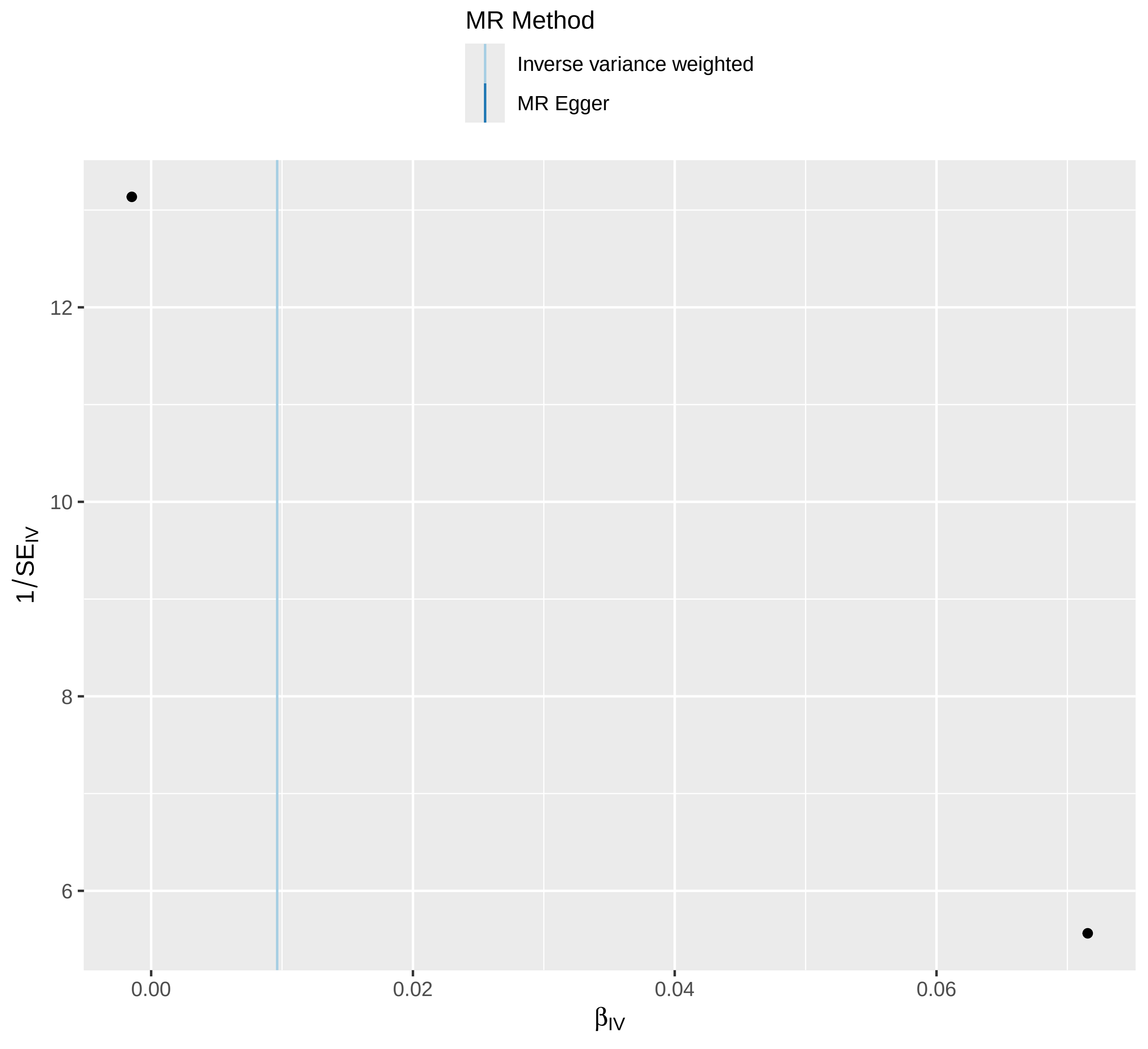

MR Method
Inverse variance weighted
MR Egger
1/SE_IV
12
10
8
6
0.00
0.02
0.04
0.06
β_IV

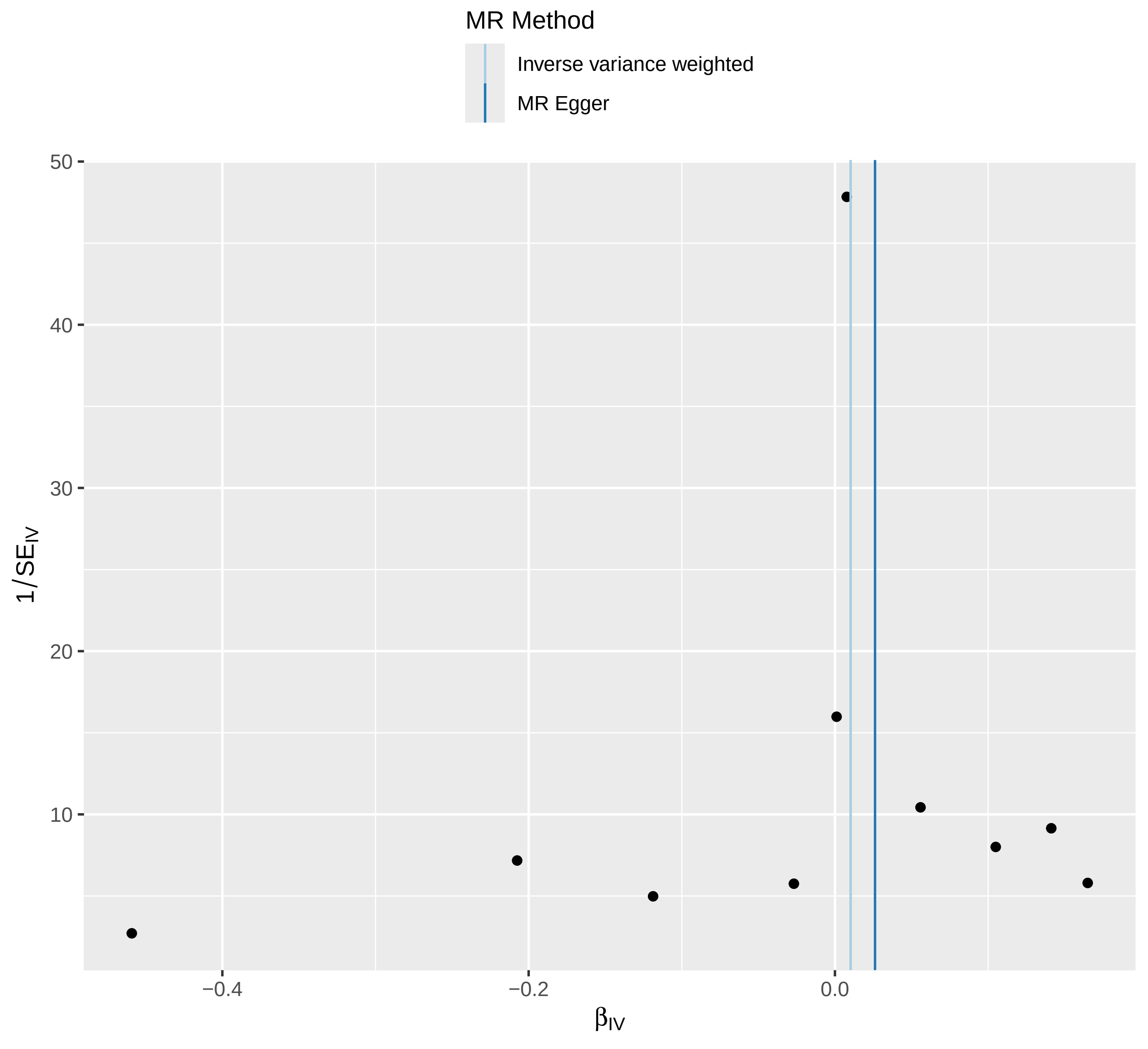
MR Method
Inverse variance weighted
MR Egger
50
40
30
20
10
1/SE_IV
−0.4
−0.2
0.0
β_IV

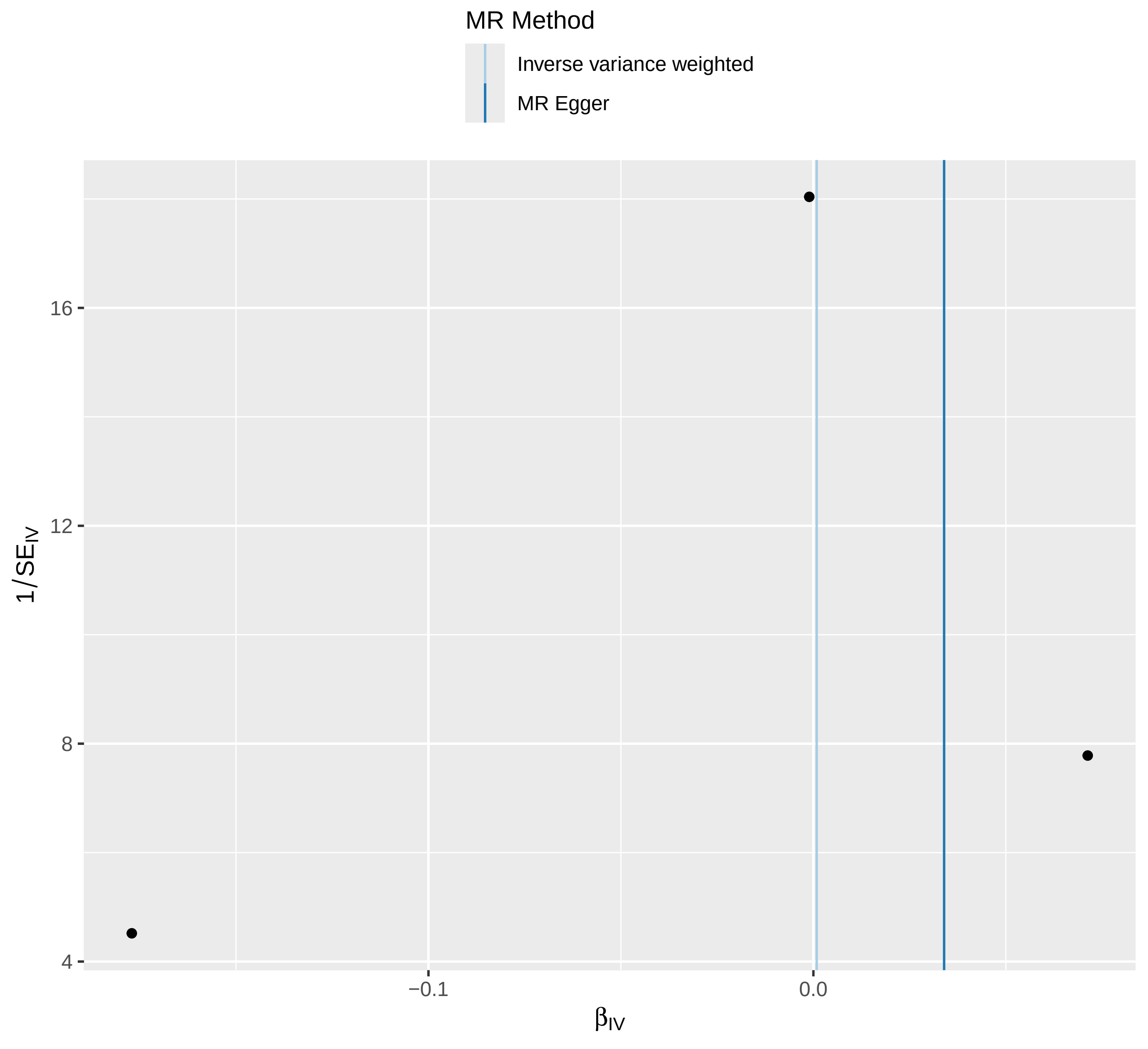
MR Method
Inverse variance weighted
MR Egger
16
12
8
4
1/SE_IV
−0.1
0.0
β_IV

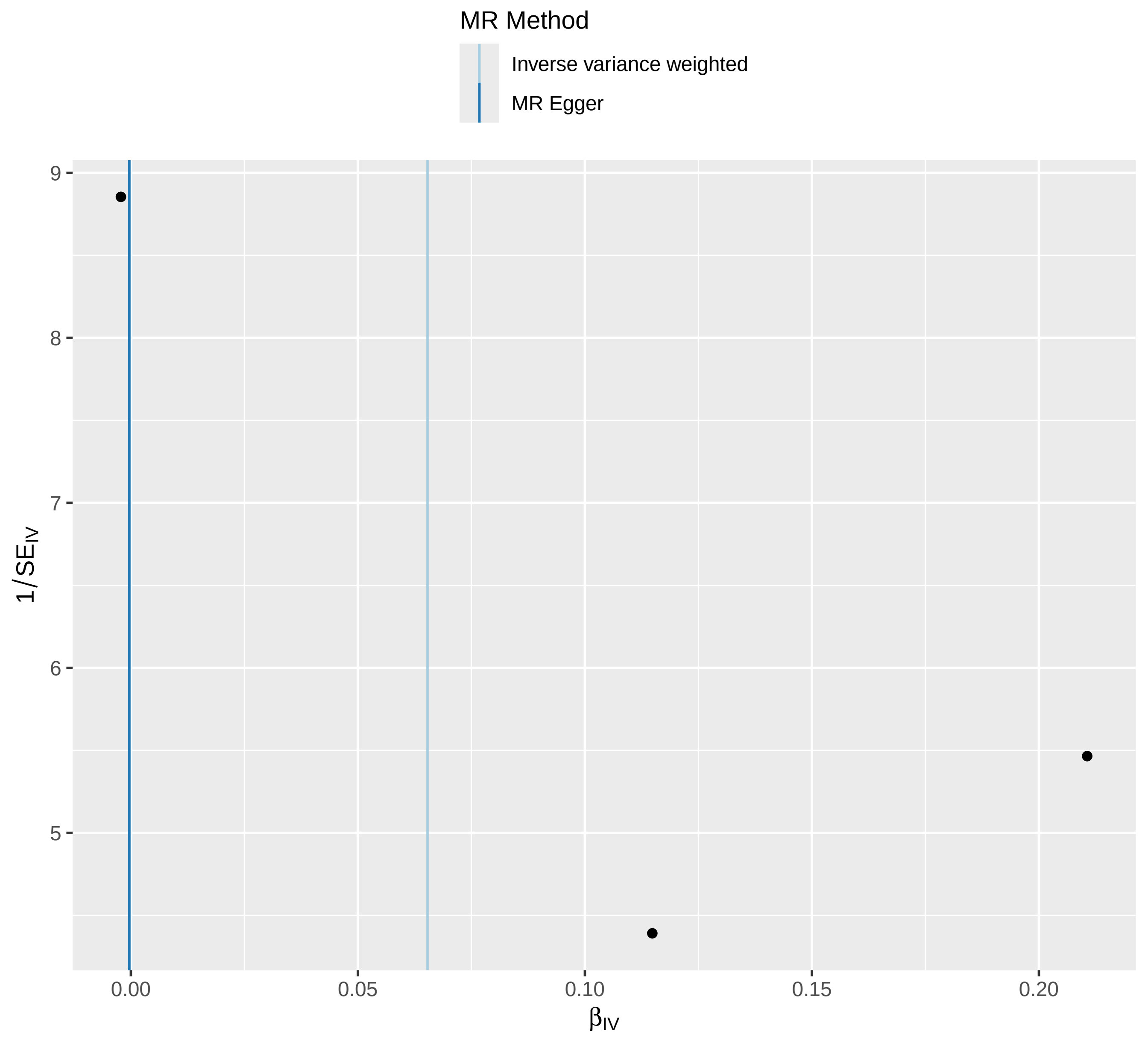
MR Method
Inverse variance weighted
MR Egger
9
8
7
6
5
1/SE_IV
0.00
0.05
0.10
0.15
0.20
β_IV

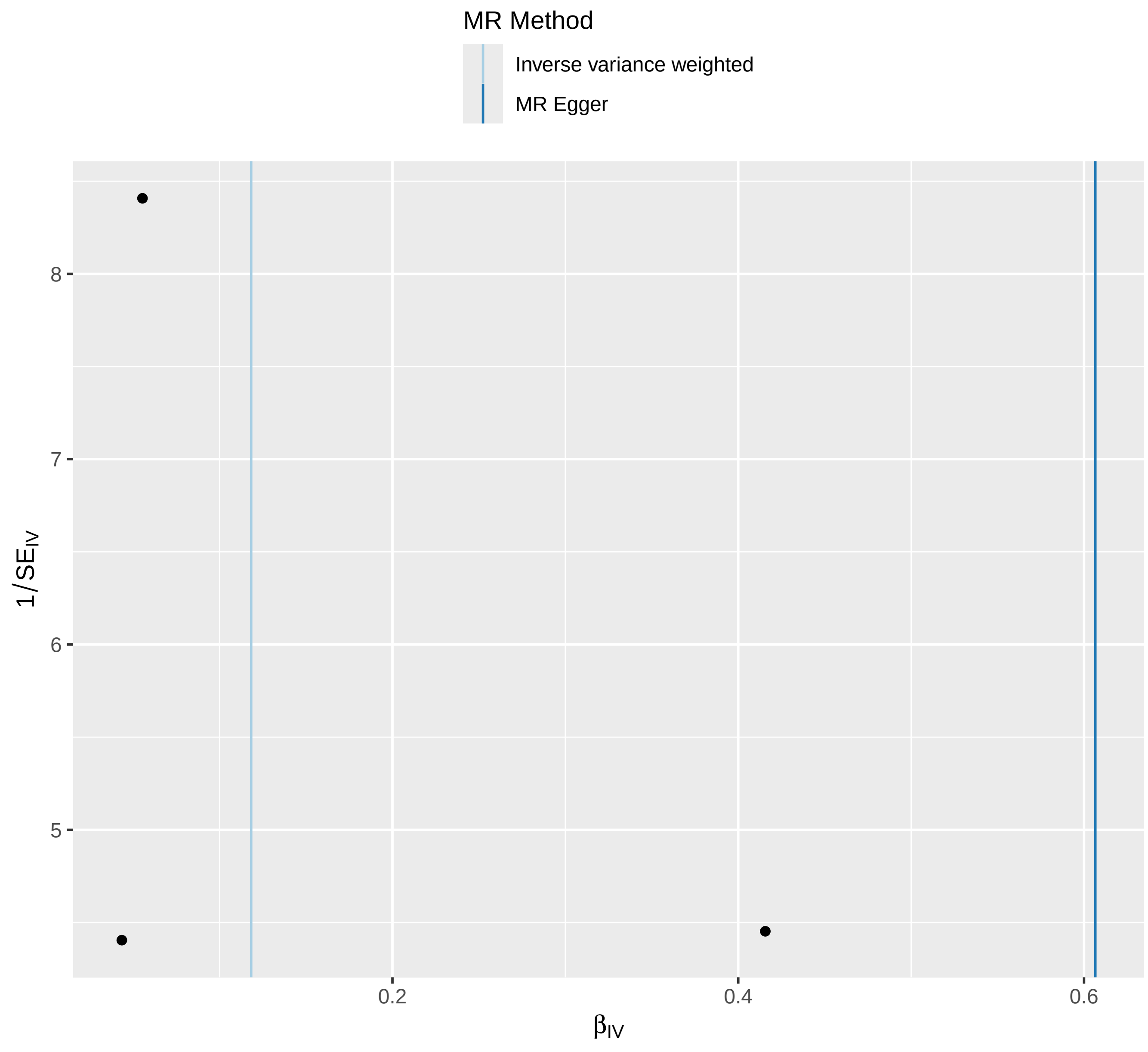
MR Method
Inverse variance weighted
MR Egger
8
7
6
5
1/SE_IV
0.2
0.4
0.6
β_IV

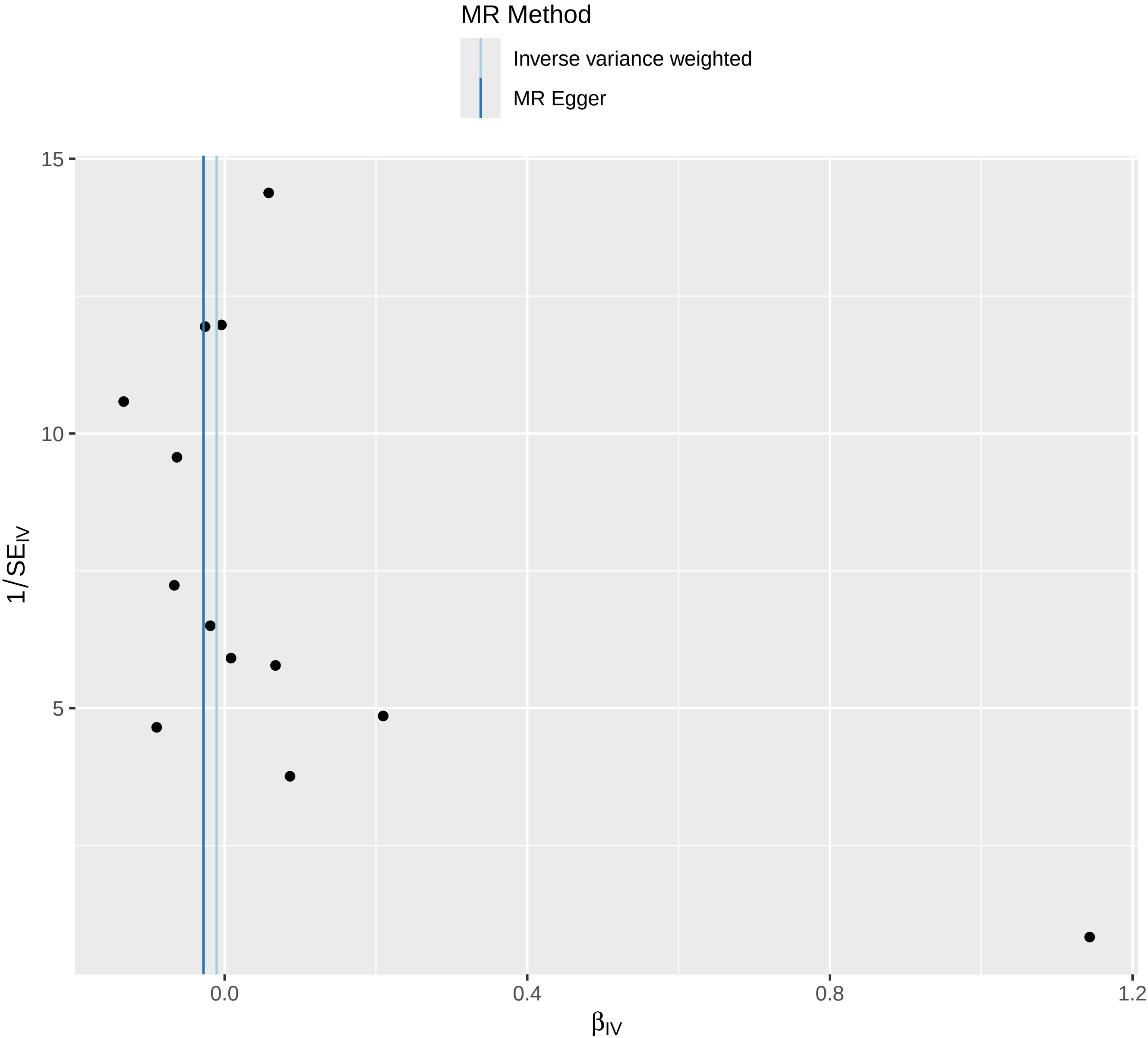
MR Method
Inverse variance weighted
MR Egger
15
10
5
0.0
0.4
0.8
1.2
$1/SE_{IV}$
$\beta_{IV}$

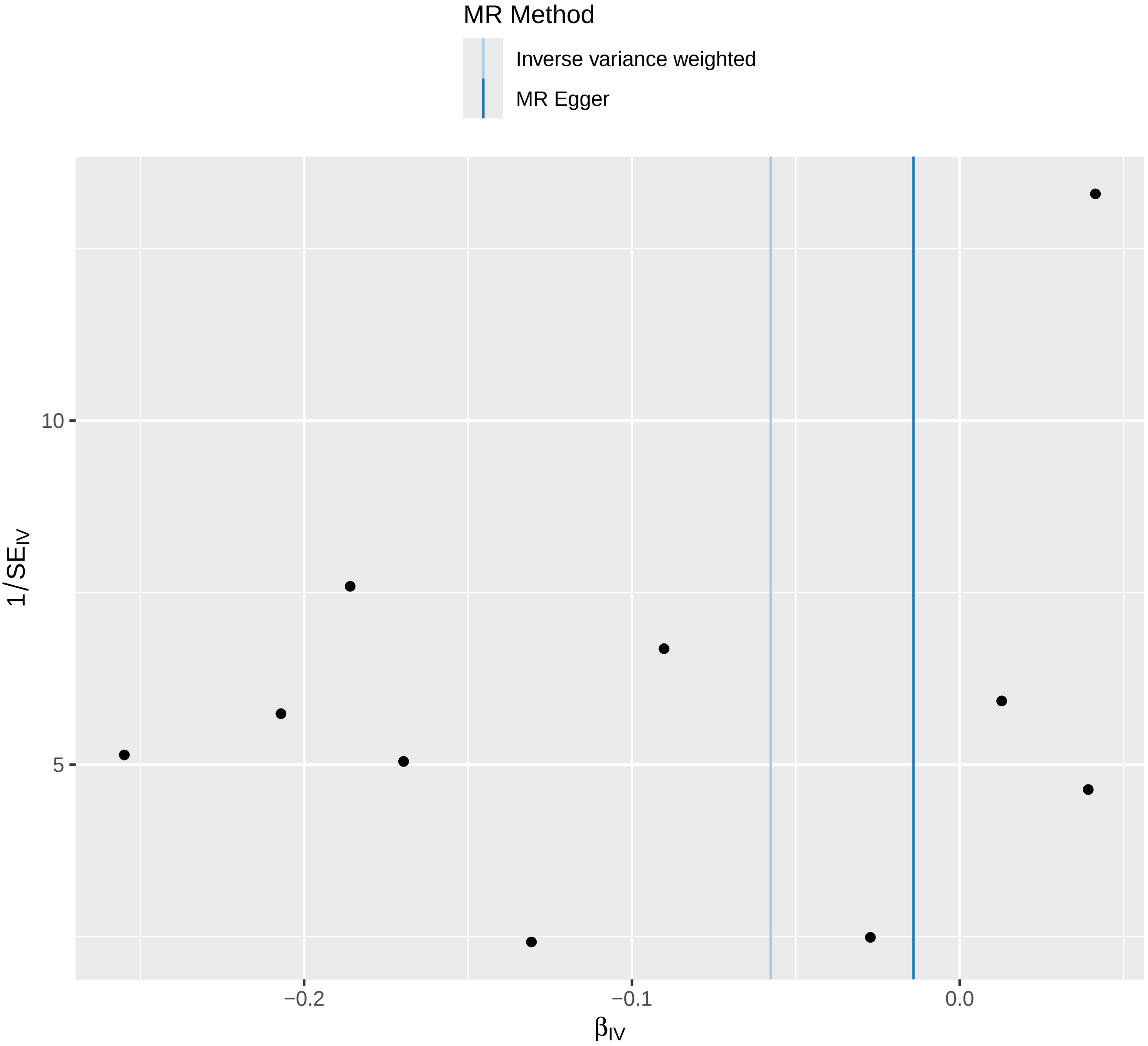
MR Method
Inverse variance weighted
MR Egger
10
5
1/SE$_{IV}$
−0.2
−0.1
0.0
β$_{IV}$

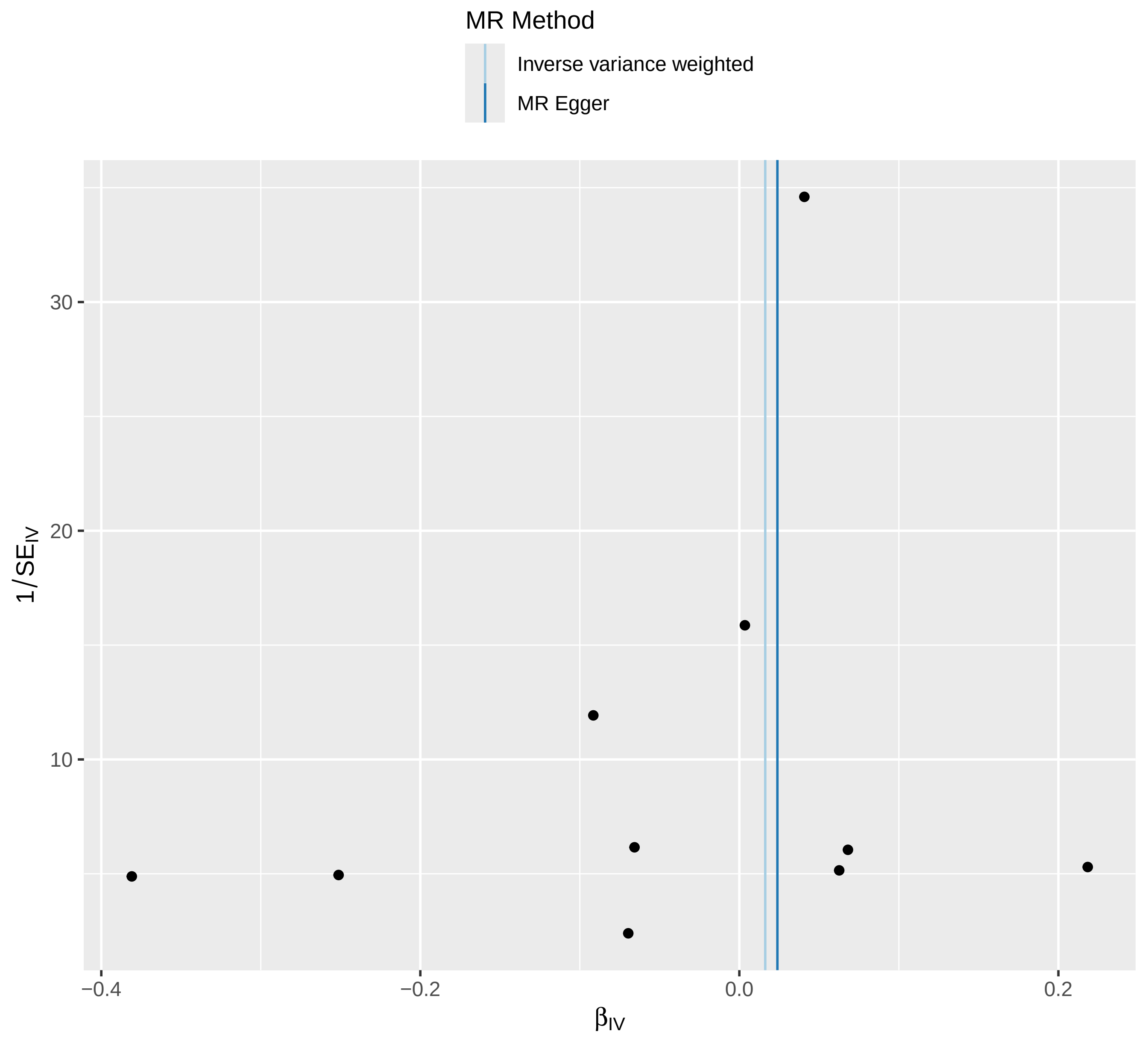
MR Method
Inverse variance weighted
MR Egger
30
20
10
1/SE$_{IV}$
−0.4
−0.2
0.0
0.2
β$_{IV}$

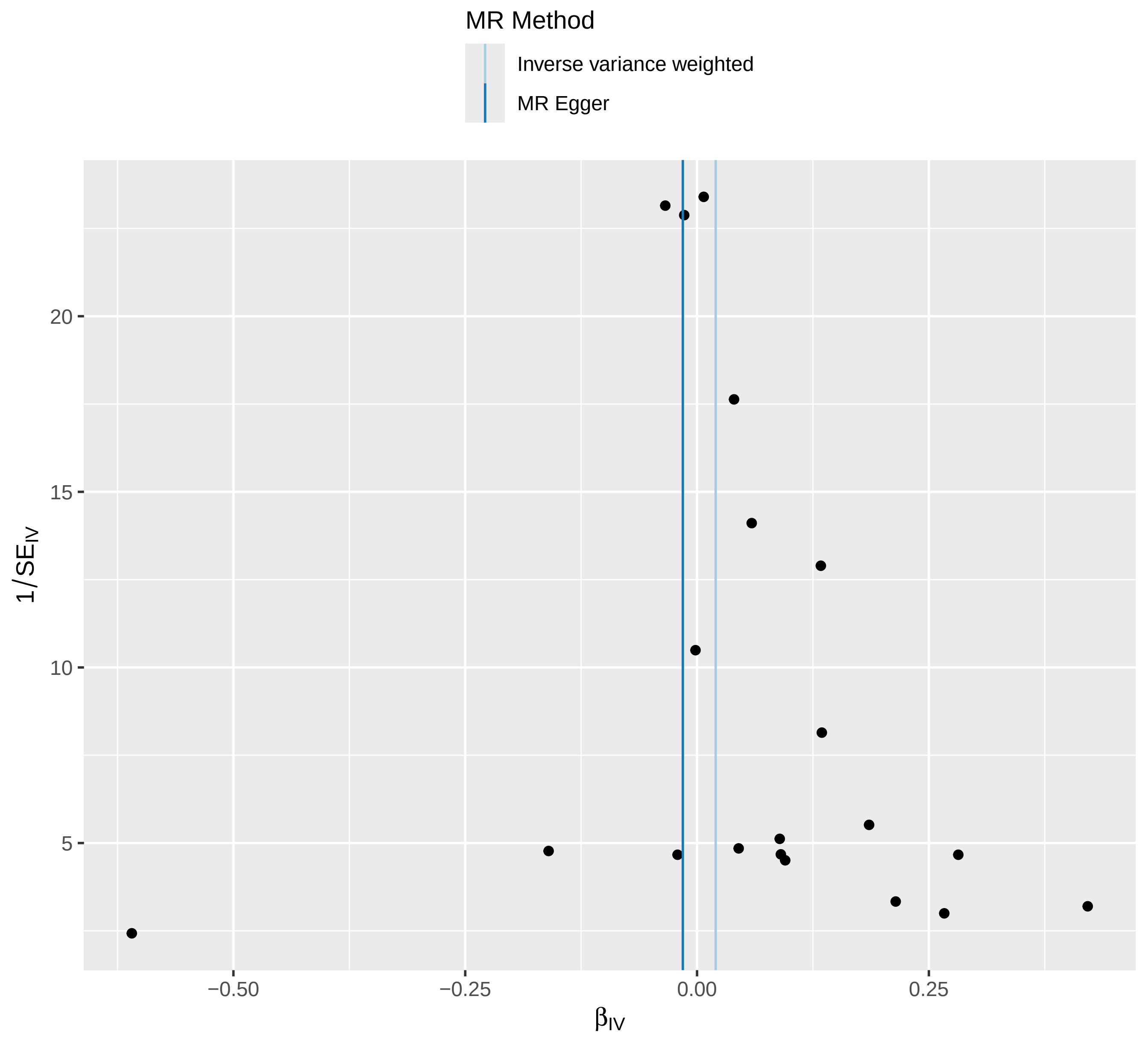

MR Method
Inverse variance weighted
MR Egger
20
15
10
5
1/SE_IV
−0.50
−0.25
0.00
0.25
β_IV

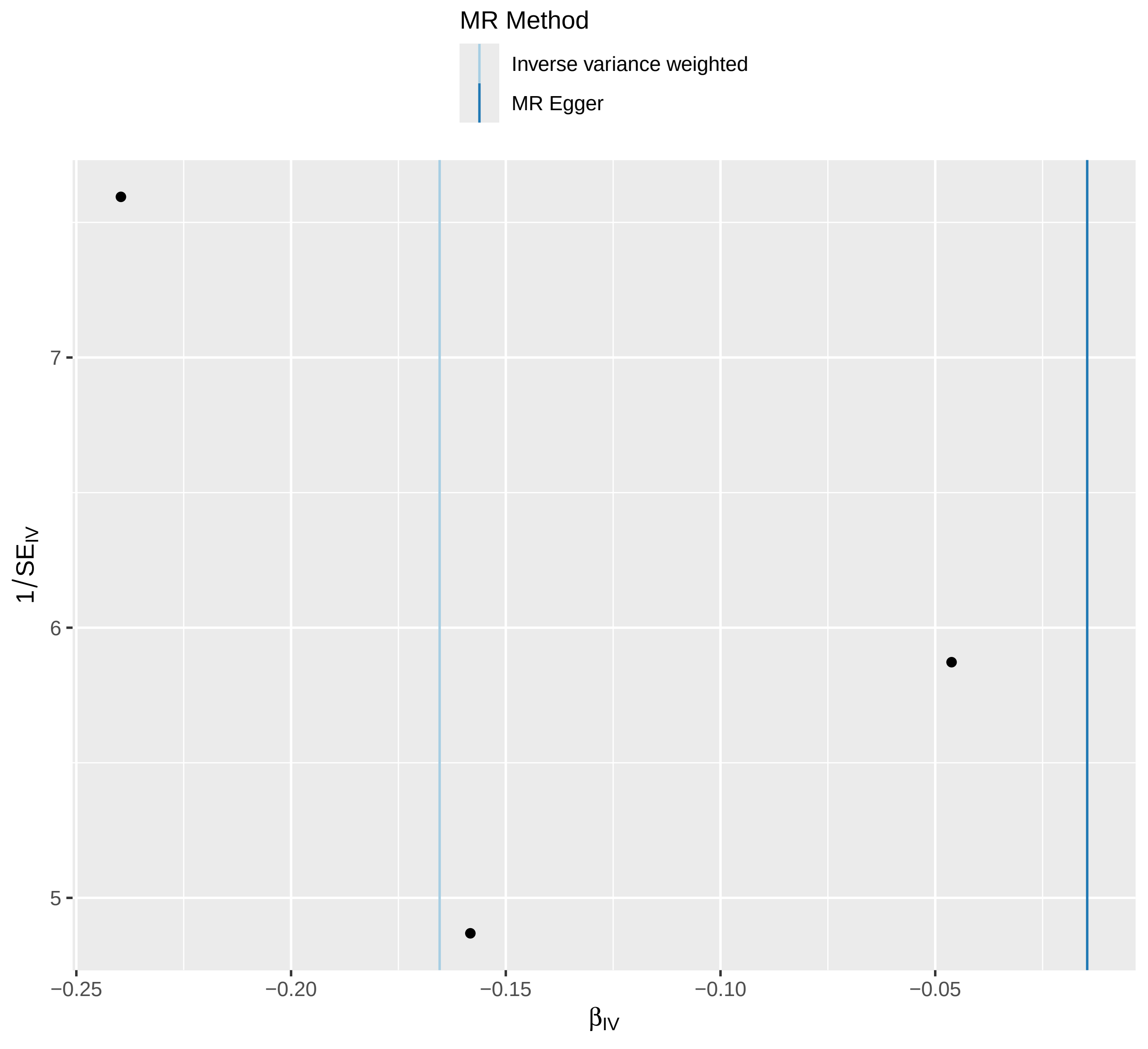
MR Method
Inverse variance weighted
MR Egger
1/SE_IV
7
6
5
−0.25
−0.20
−0.15
−0.10
−0.05
β_IV

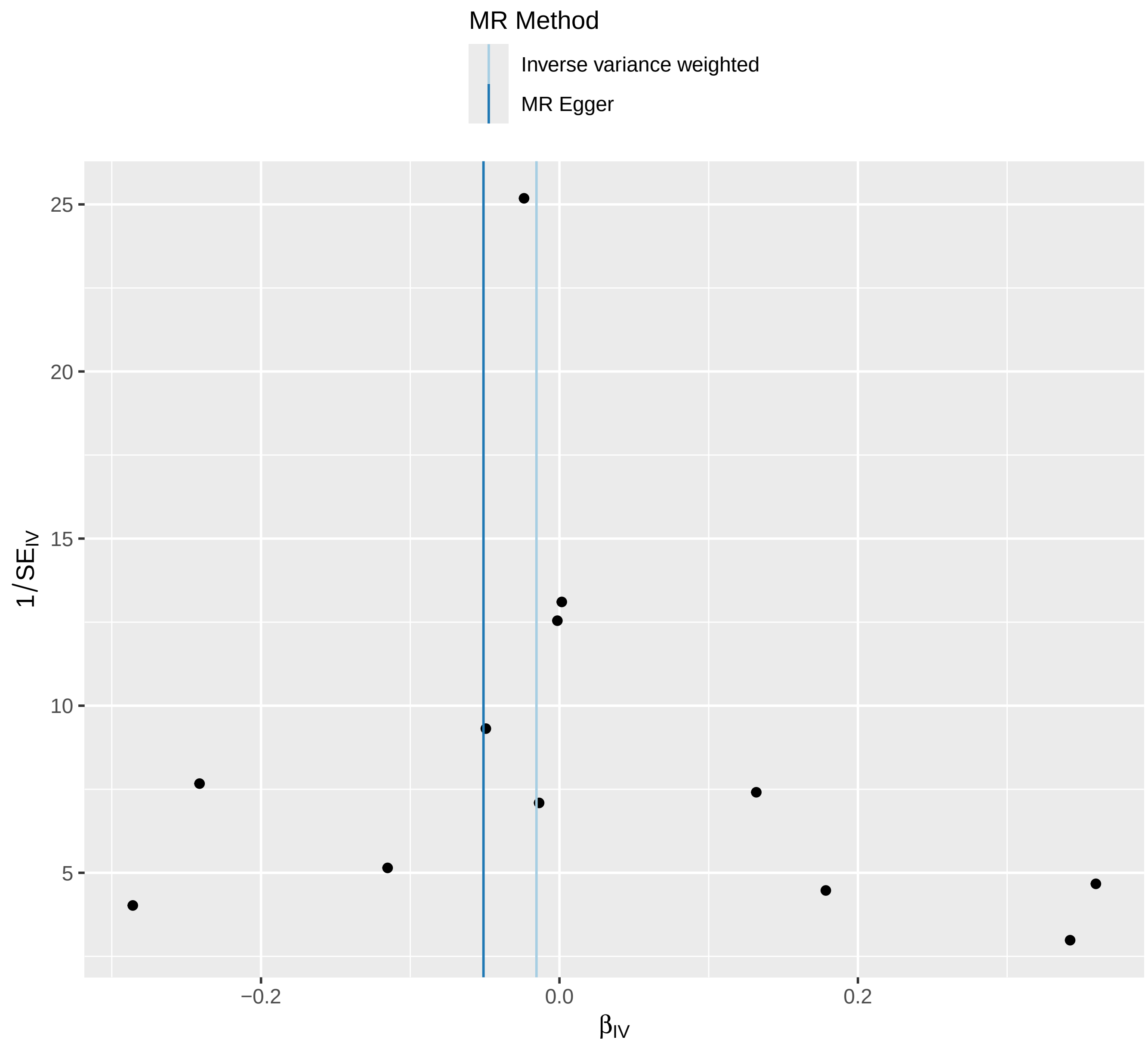
MR Method
Inverse variance weighted
MR Egger
25
20
15
10
5
1/SE$_{IV}$
−0.2
0.0
0.2
β$_{IV}$

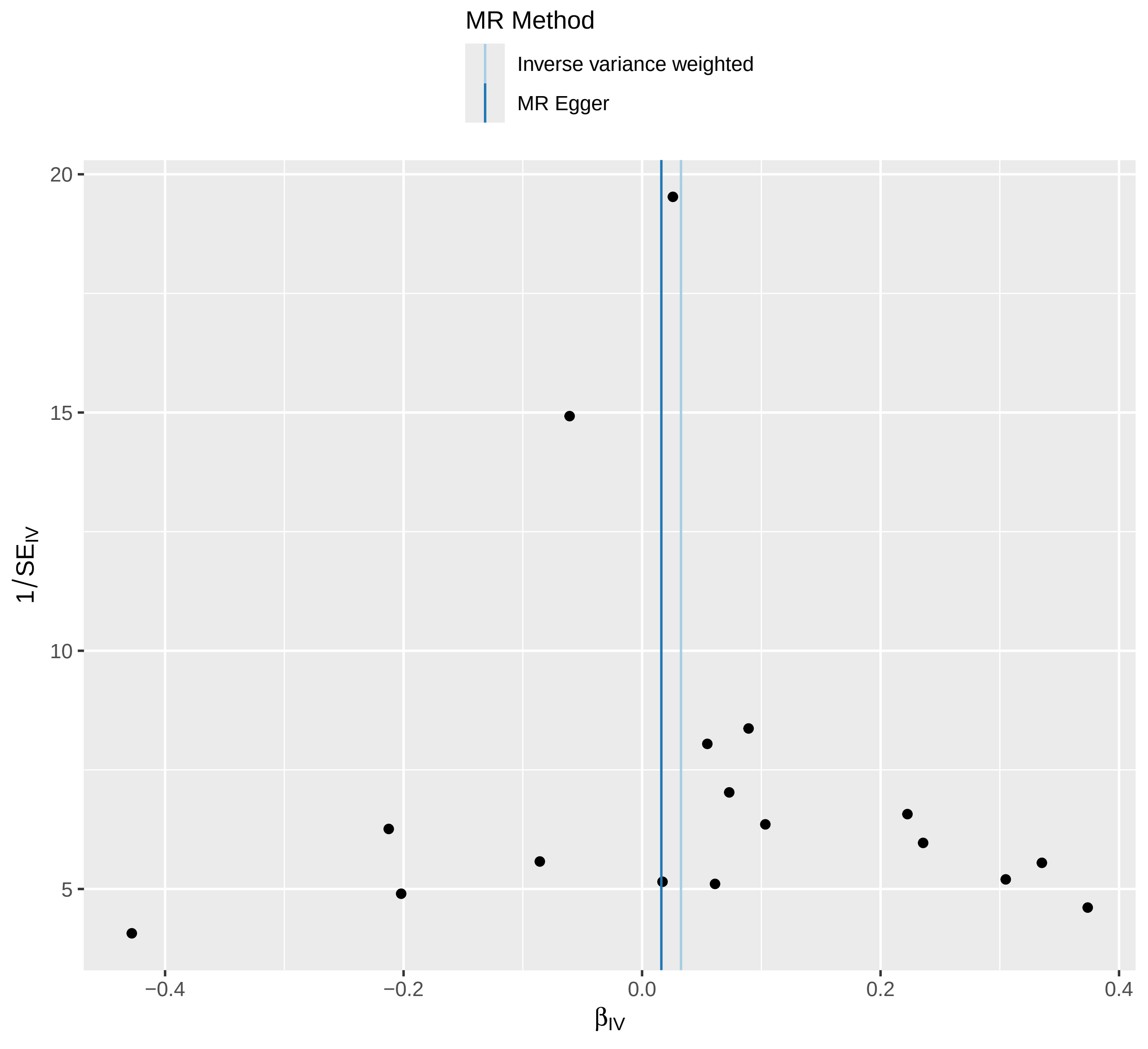

MR Method
Inverse variance weighted
MR Egger
20
15
10
5
1/SE_IV
−0.4
−0.2
0.0
0.2
0.4
β_IV

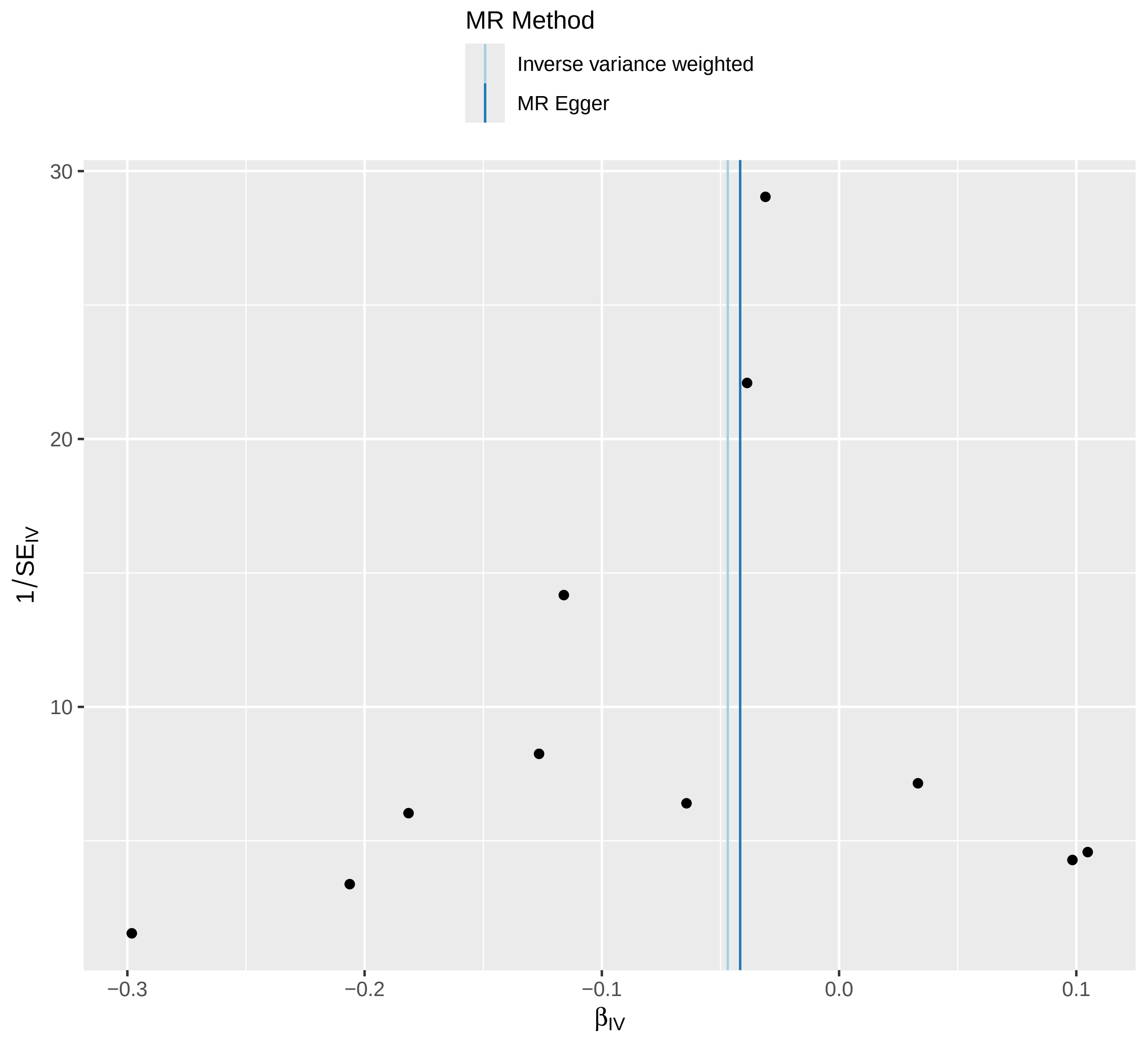
MR Method
Inverse variance weighted
MR Egger
30
20
10
1/SE$_{IV}$
−0.3
−0.2
−0.1
0.0
0.1
β$_{IV}$

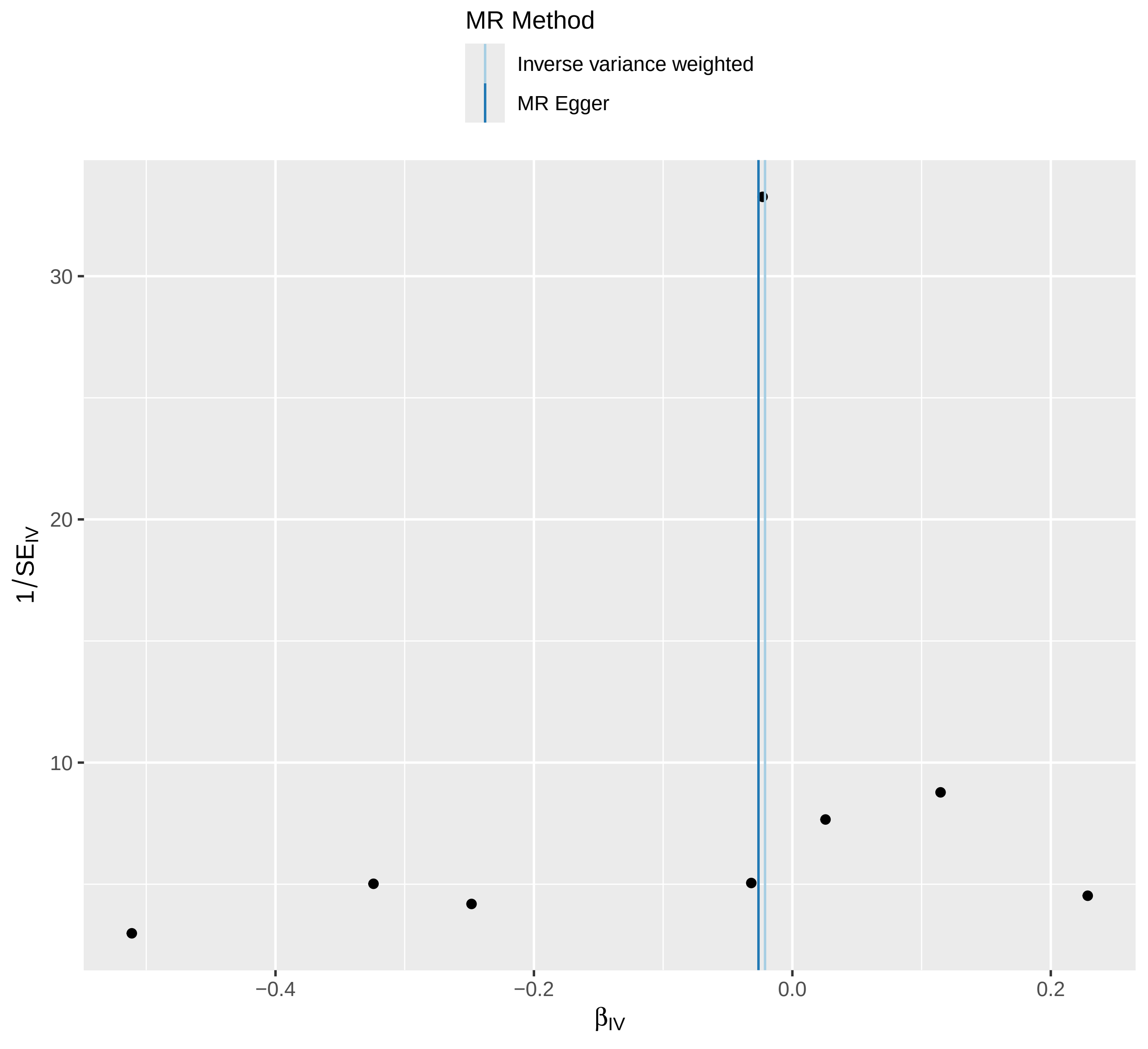

MR Method
Inverse variance weighted
MR Egger
30
20
10
1/SE_IV
−0.4
−0.2
0.0
0.2
β_IV

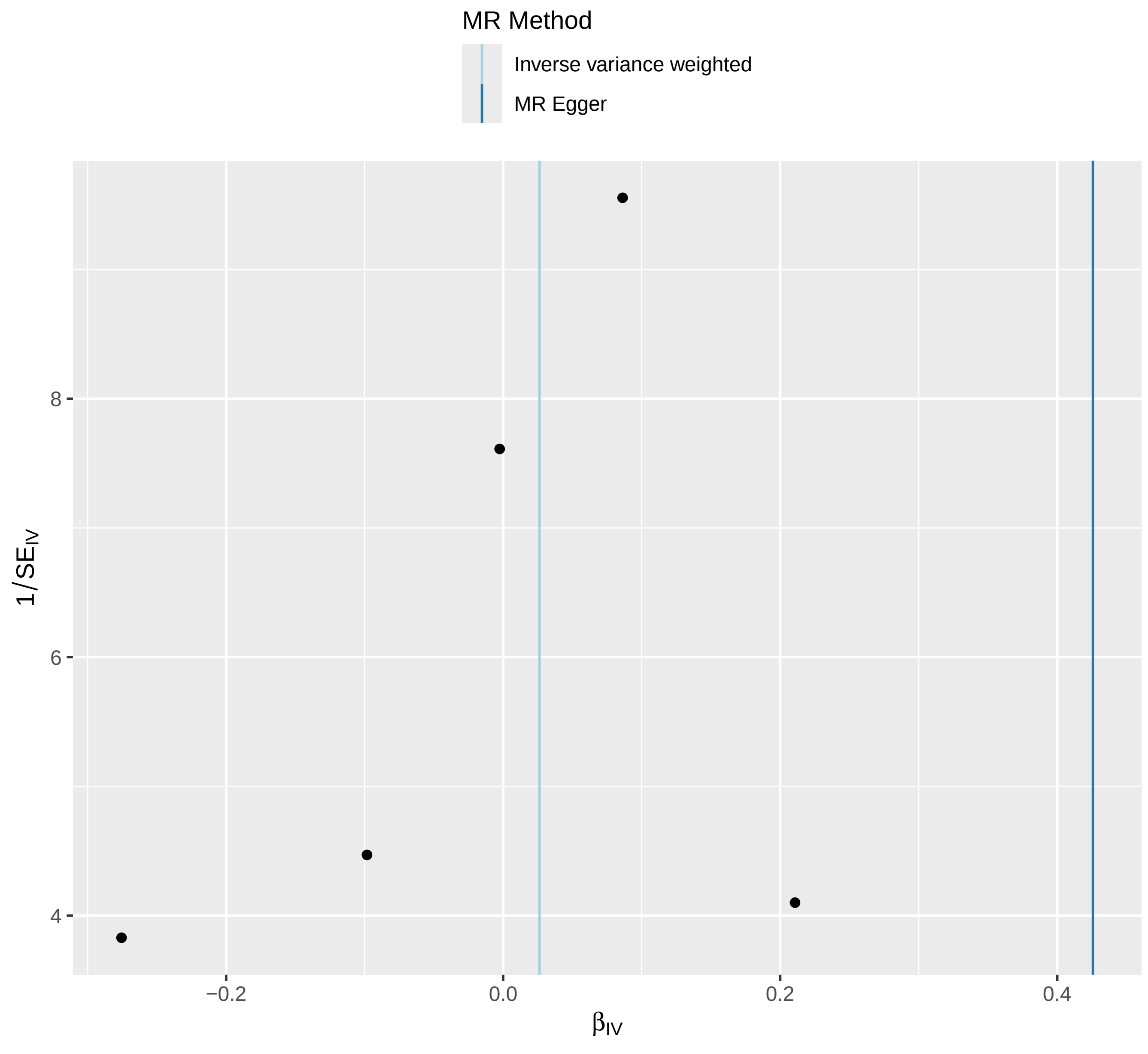
MR Method
Inverse variance weighted
MR Egger
1/SE_IV
8
6
4
−0.2
0.0
0.2
0.4
β_IV

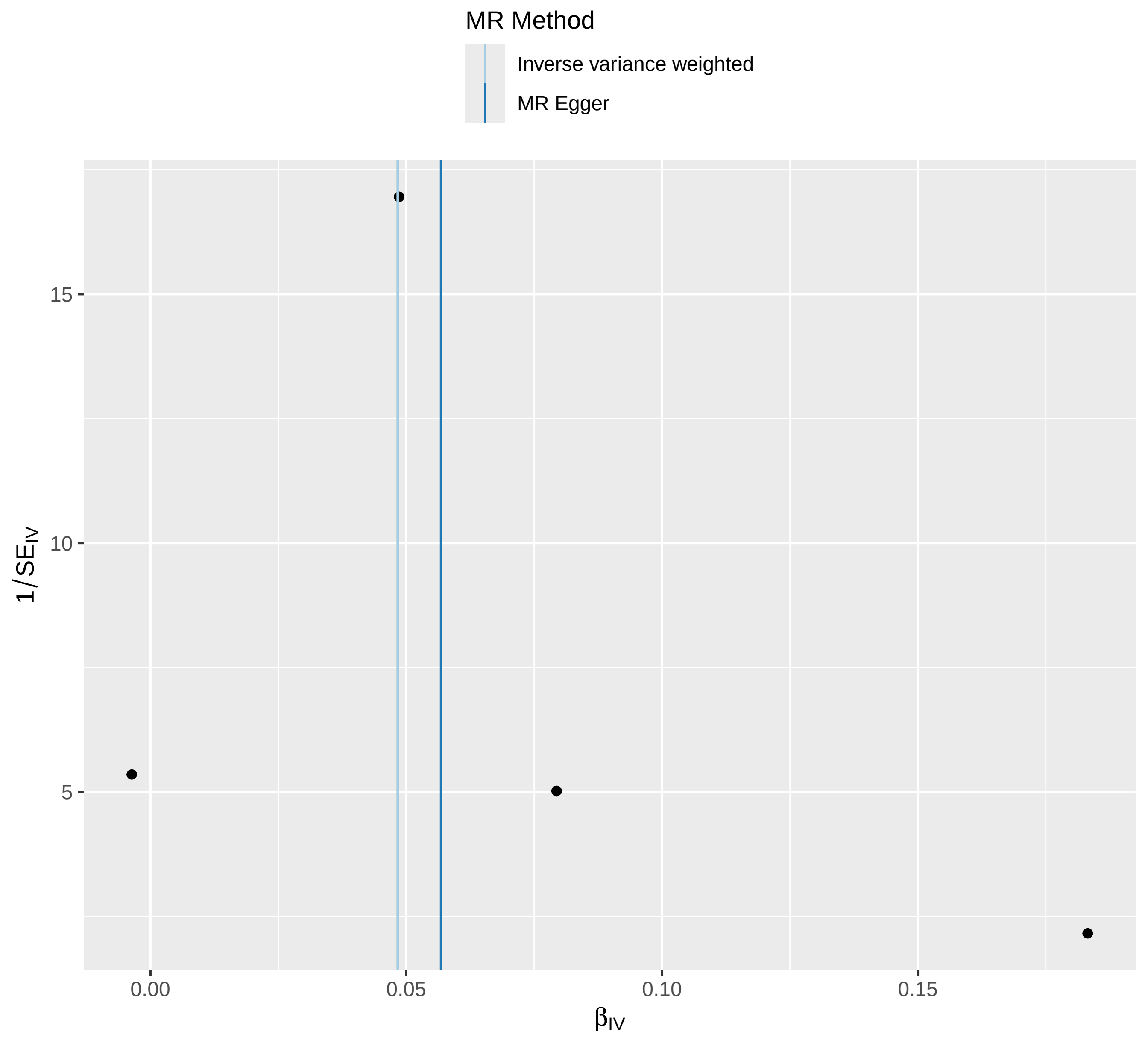
MR Method
Inverse variance weighted
MR Egger
15
10
5
1/SE_IV
0.00
0.05
0.10
0.15
β_IV

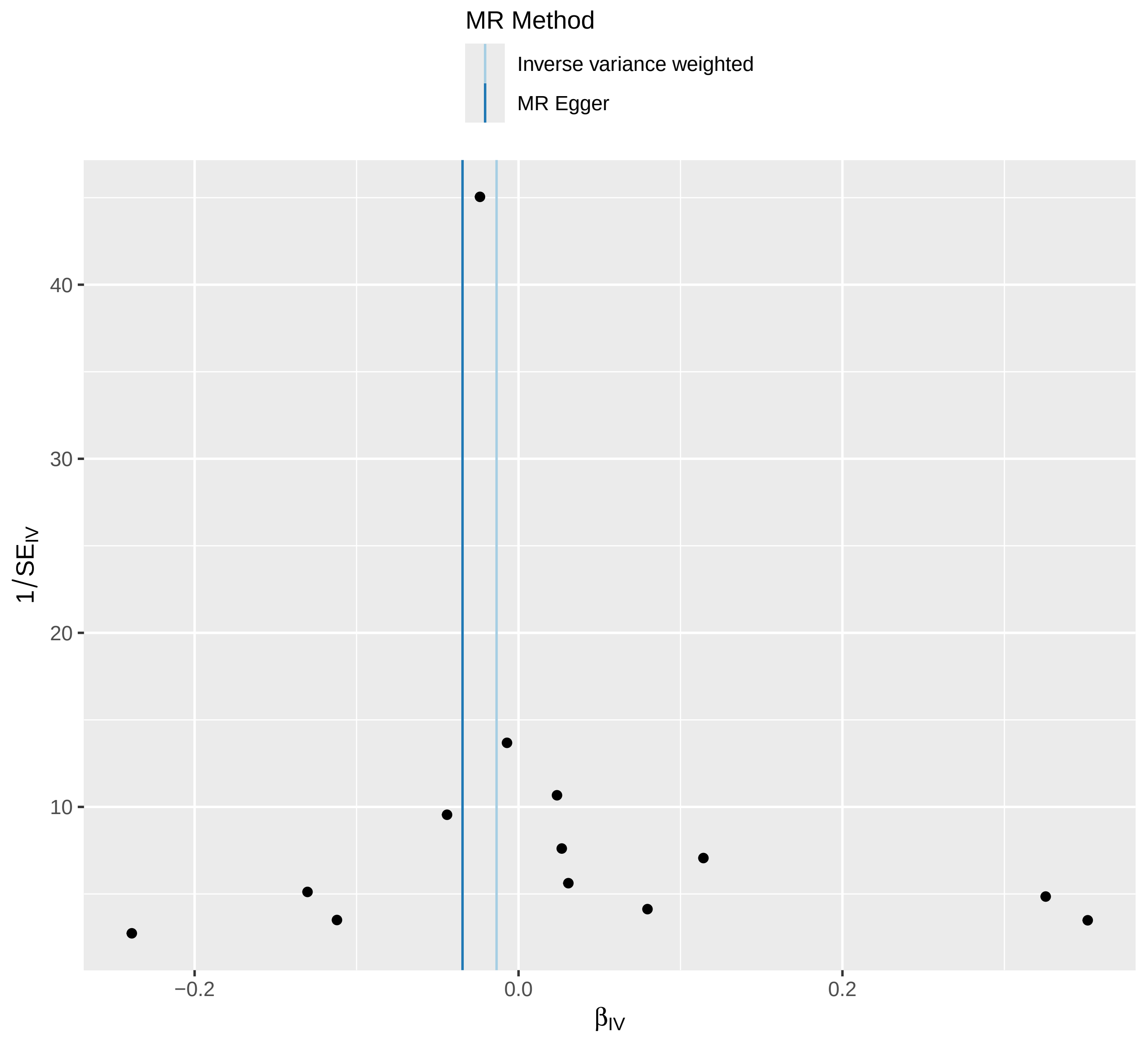

MR Method
Inverse variance weighted
MR Egger
40
30
20
10
1/SE_IV
−0.2
0.0
0.2
β_IV

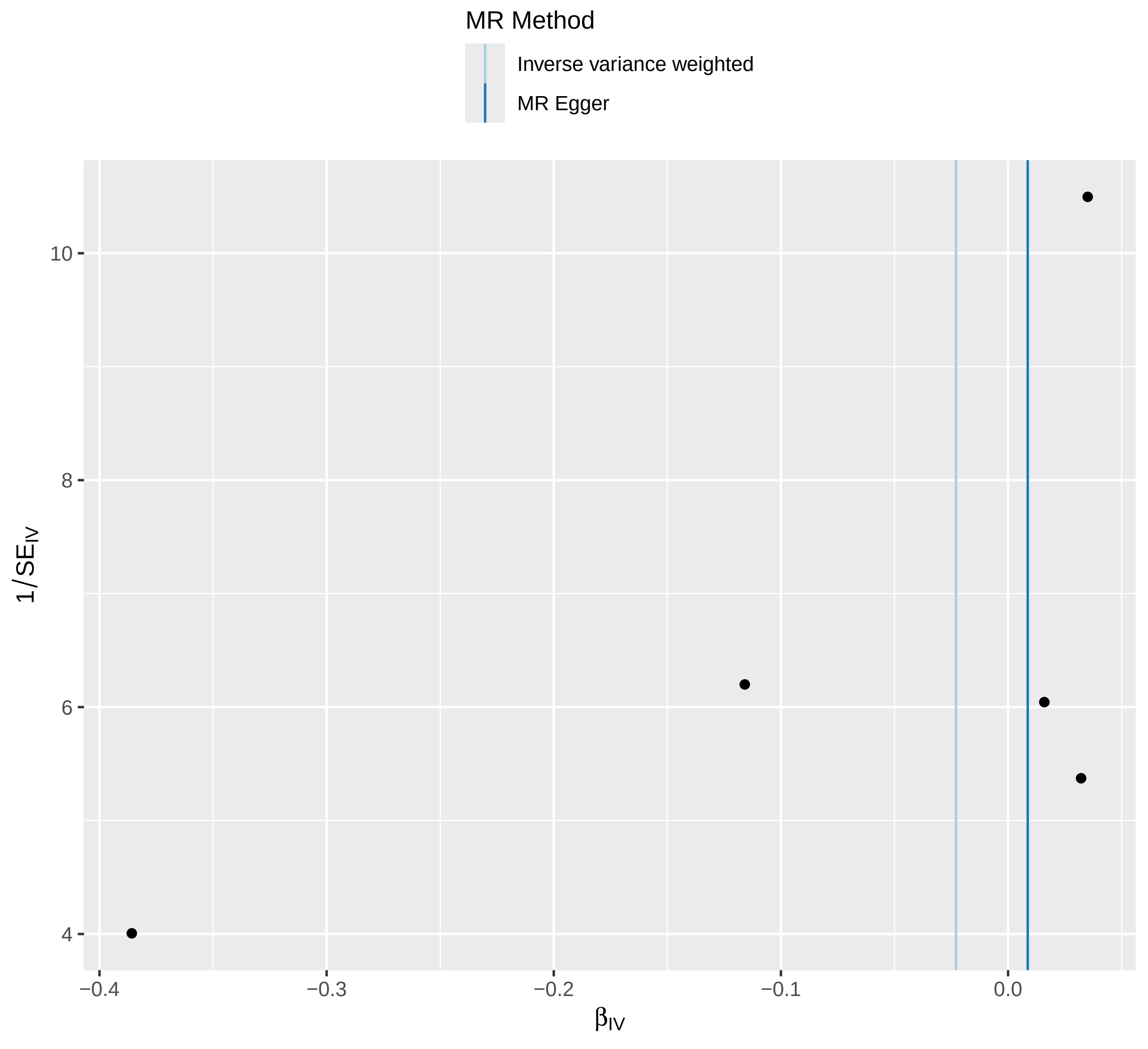

MR Method
Inverse variance weighted
MR Egger
10
8
6
4
1/SE_IV
−0.4
−0.3
−0.2
−0.1
0.0
β_IV

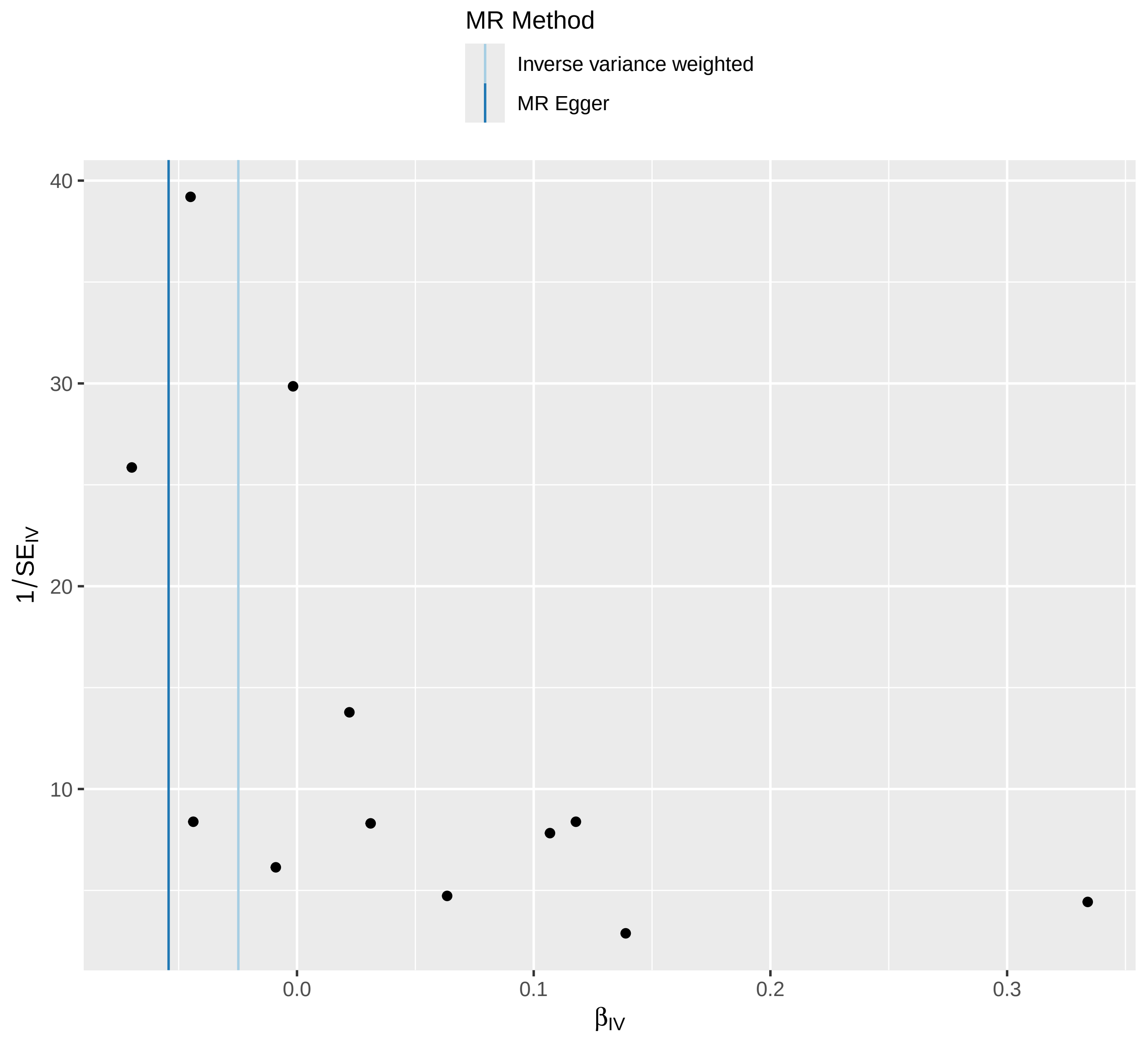
MR Method
Inverse variance weighted
MR Egger
40
30
20
10
0.0
0.1
0.2
0.3
1/SE_IV
β_IV

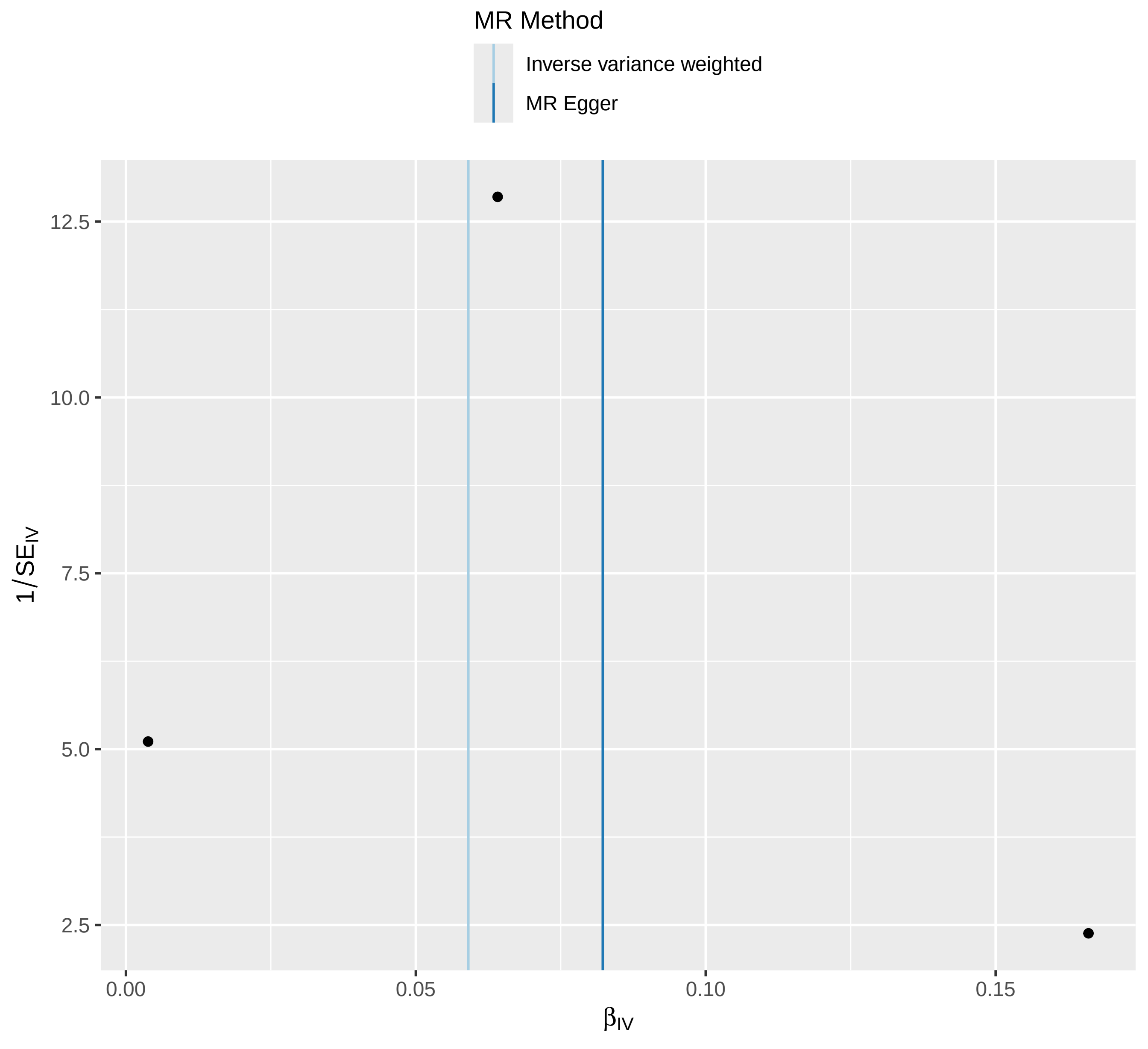
MR Method
Inverse variance weighted
MR Egger
12.5
10.0
7.5
5.0
2.5
1/SE_IV
0.00
0.05
0.10
0.15
β_IV

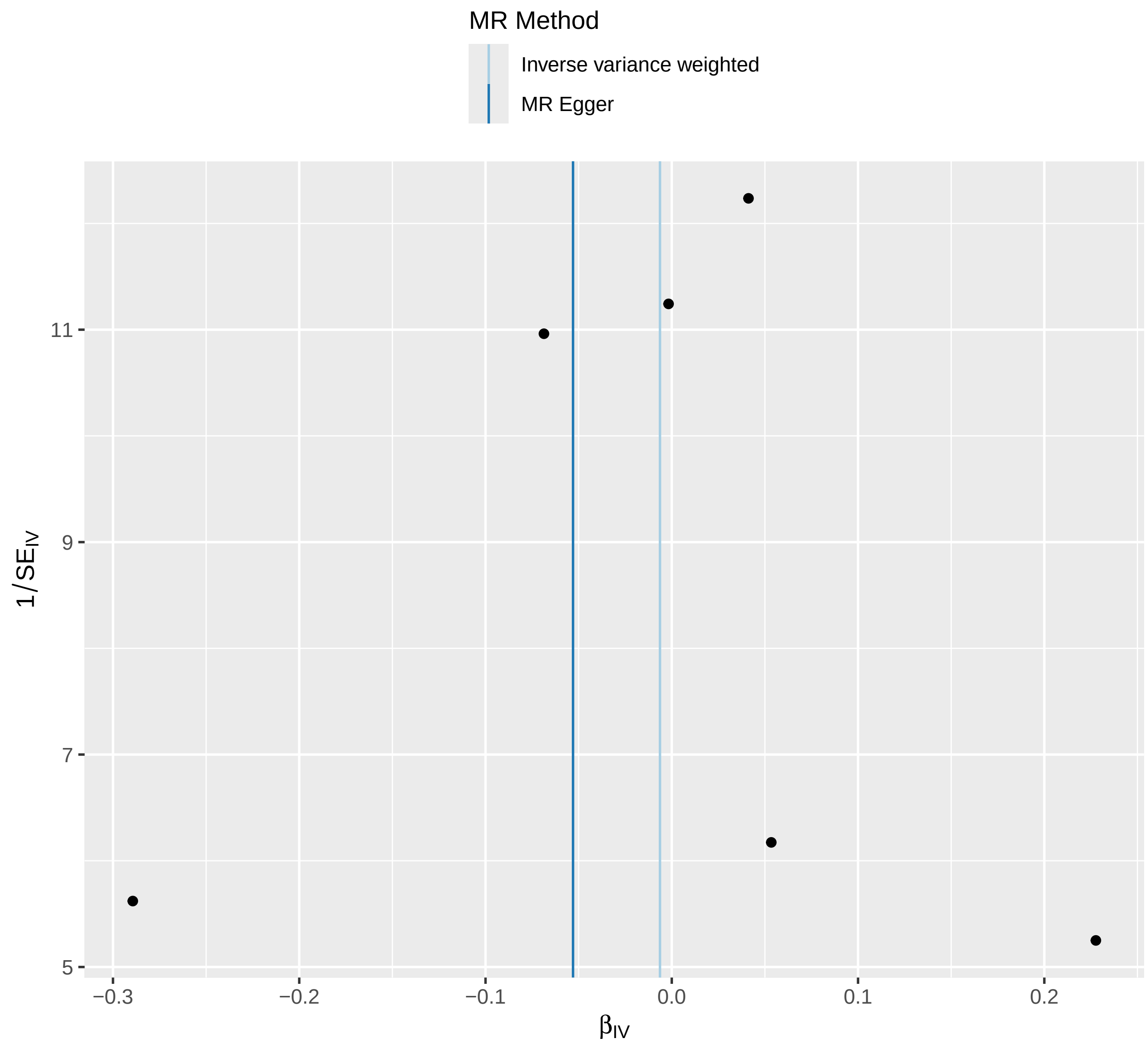
MR Method
Inverse variance weighted
MR Egger
11
9
7
5
1/SE_IV
−0.3
−0.2
−0.1
0.0
0.1
0.2
β_IV

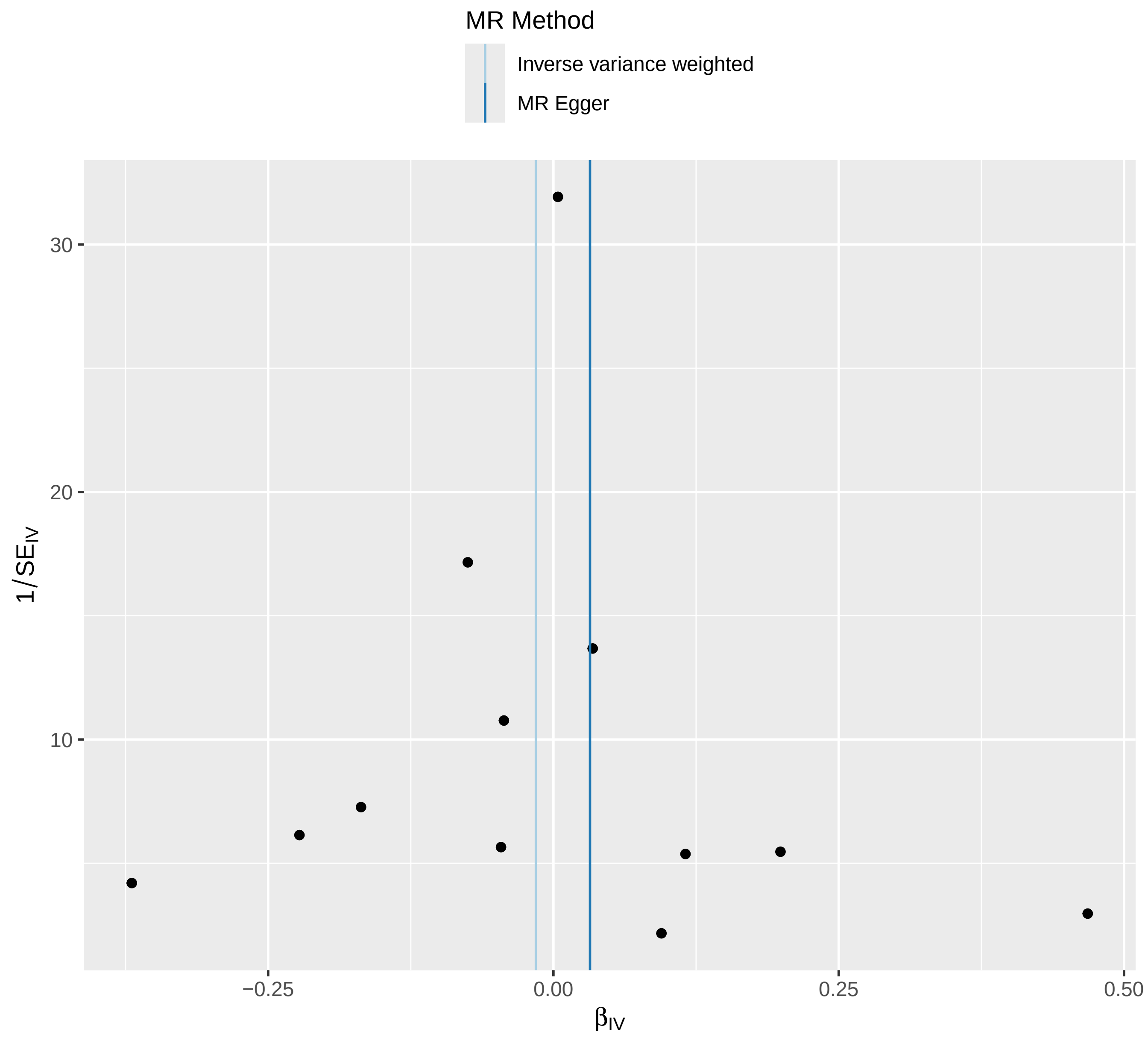

MR Method
Inverse variance weighted
MR Egger
30
20
10
1/SE$_{IV}$
−0.25
0.00
0.25
0.50
β$_{IV}$

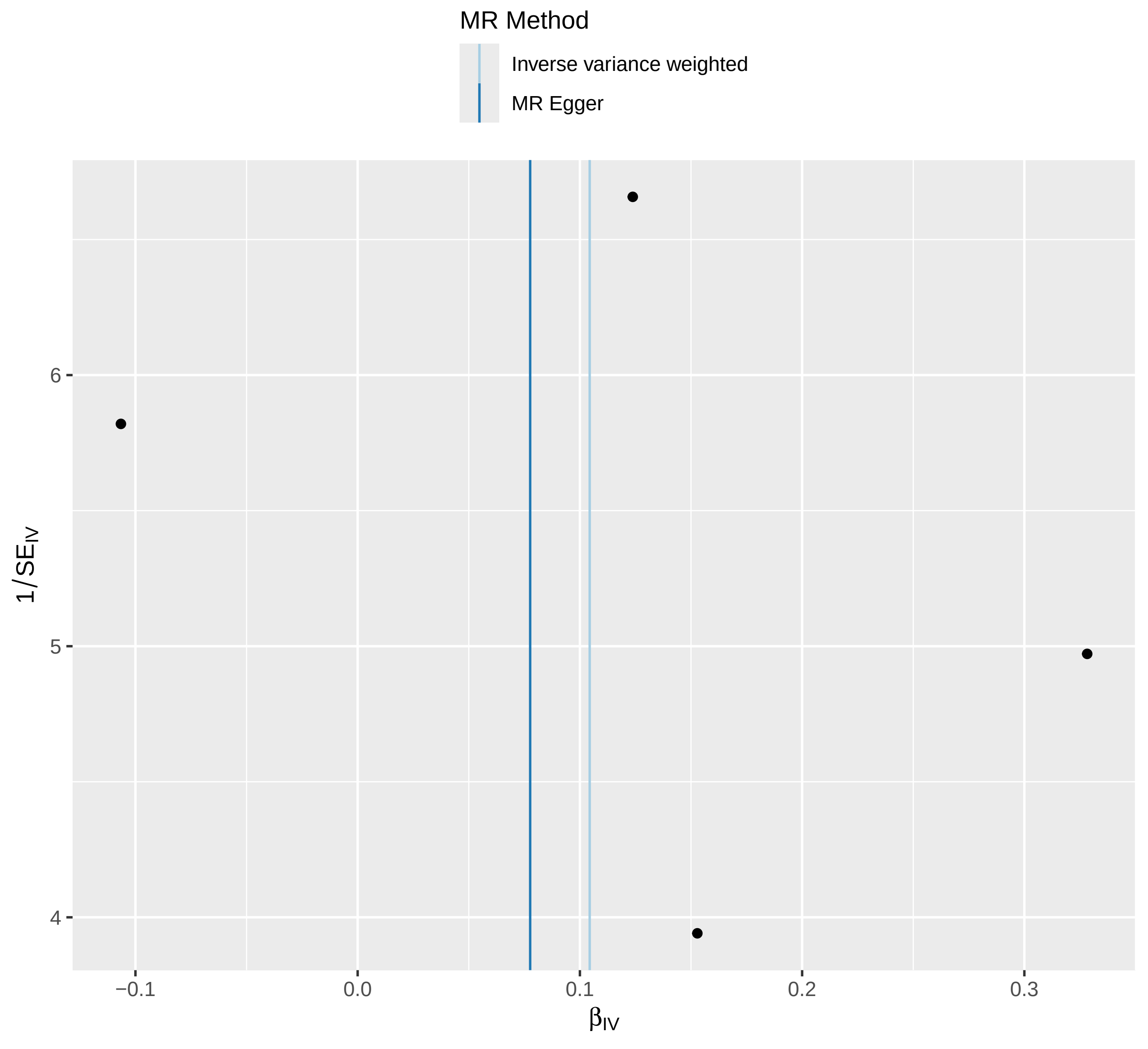

MR Method
Inverse variance weighted
MR Egger
6
5
4
1/SE_IV
−0.1
0.0
0.1
0.2
0.3
β_IV

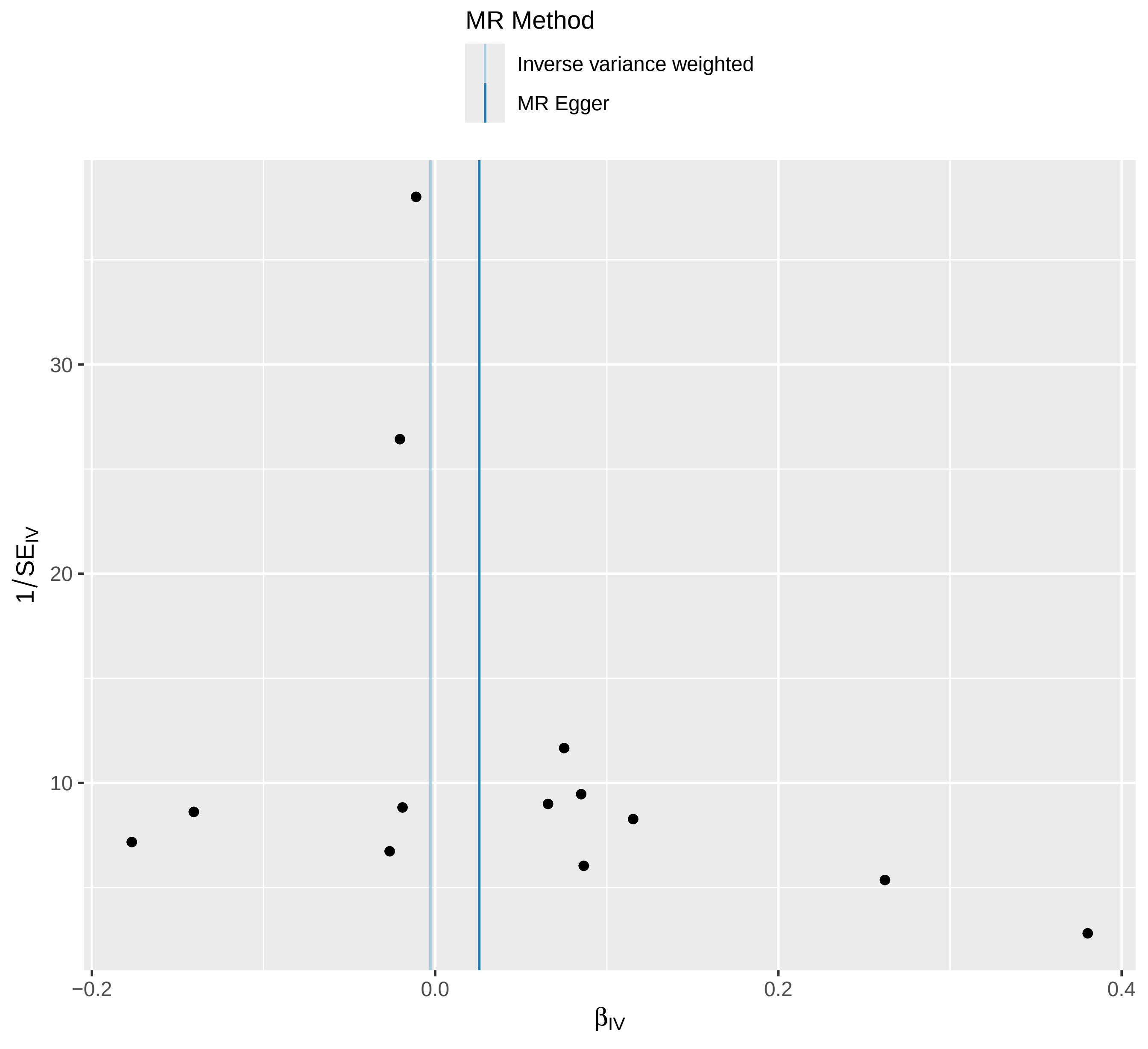

MR Method
Inverse variance weighted
MR Egger
30
20
10
1/SE_IV
−0.2
0.0
0.2
0.4
β_IV

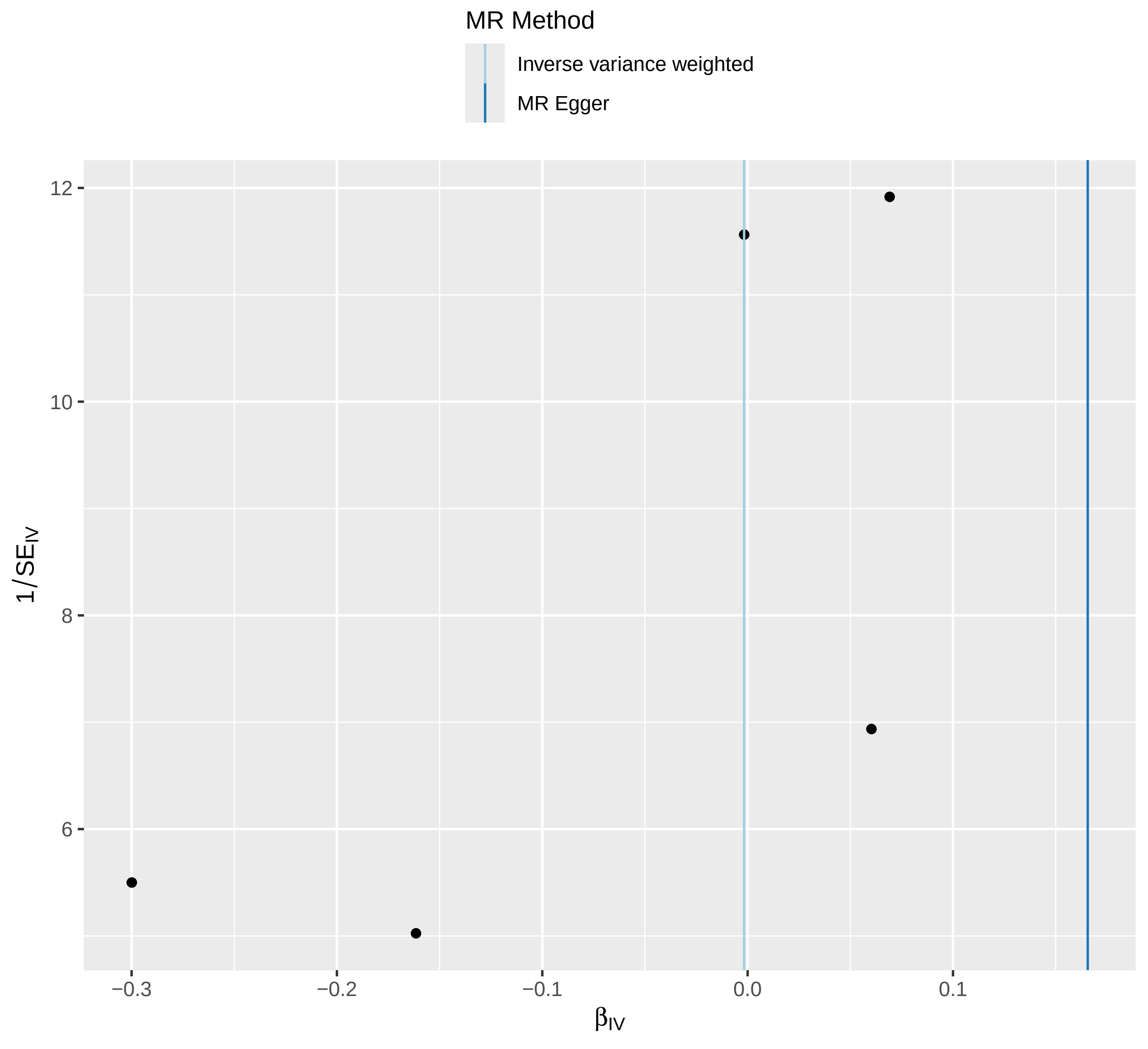

MR Method
Inverse variance weighted
MR Egger
12
10
8
6
1/SE_IV
−0.3
−0.2
−0.1
0.0
0.1
β_IV

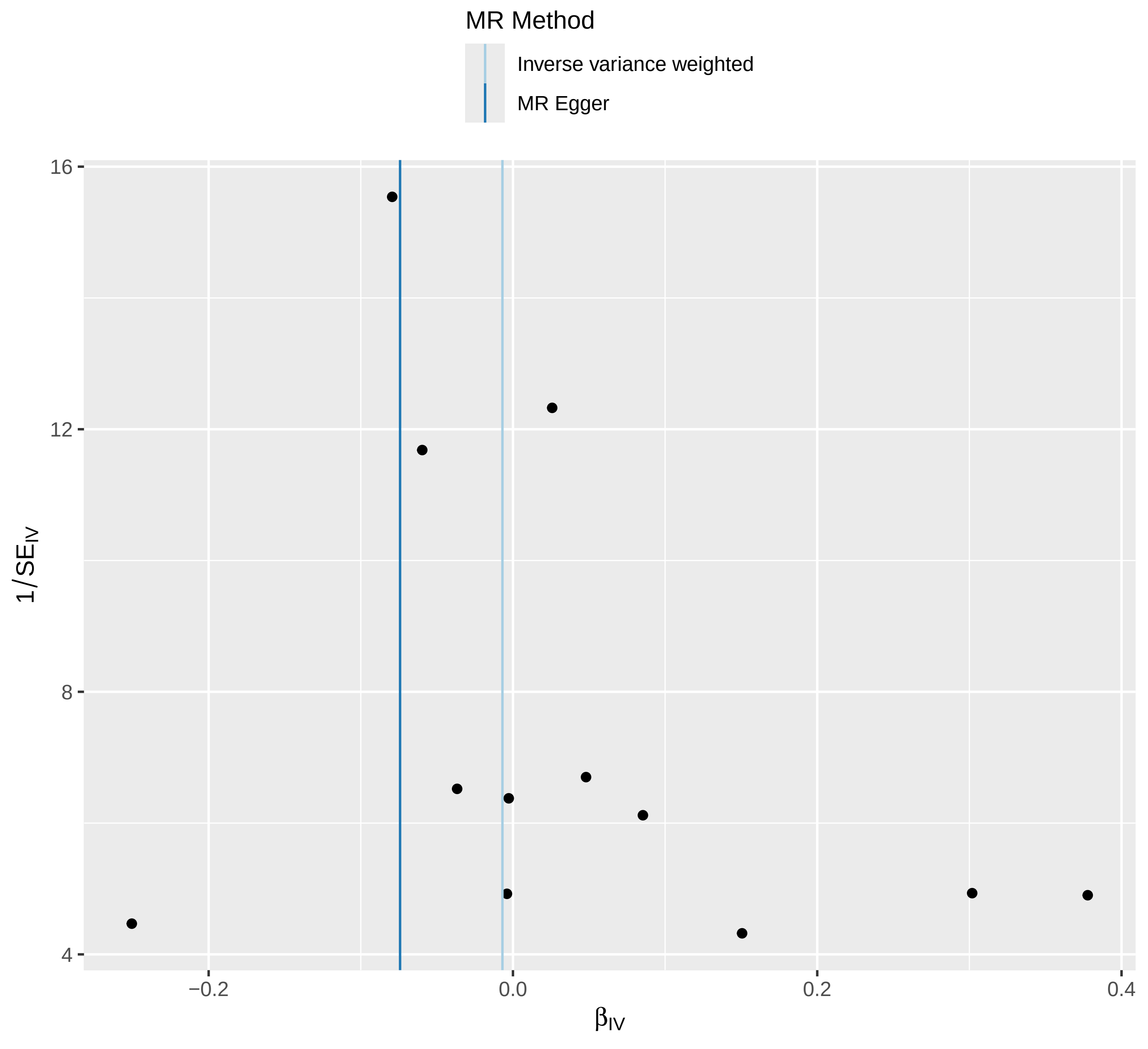
MR Method
Inverse variance weighted
MR Egger
16
12
8
4
1/SE$_{IV}$
−0.2
0.0
0.2
0.4
β$_{IV}$

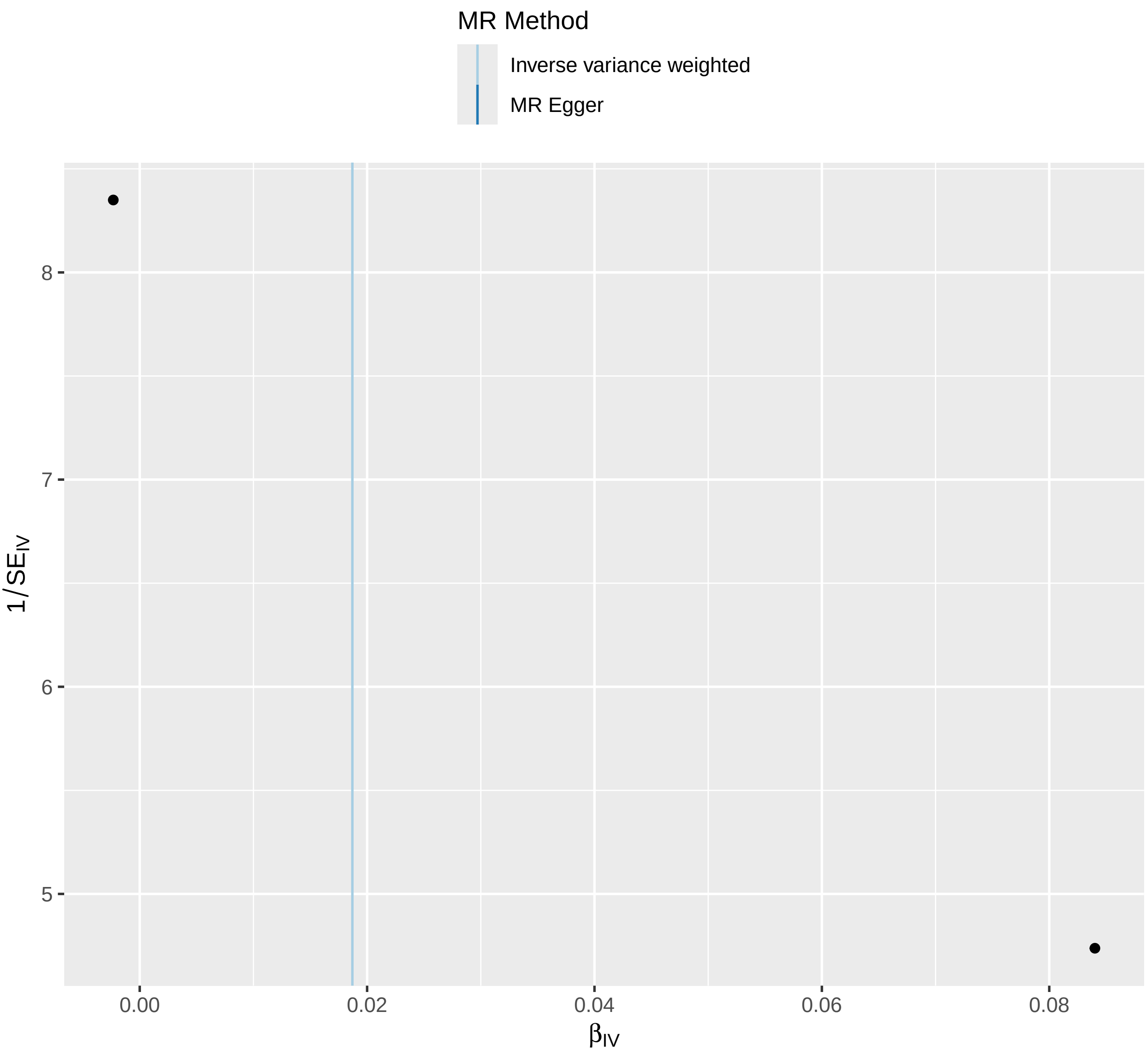
MR Method
Inverse variance weighted
MR Egger
8
7
6
5
1/SE_IV
0.00
0.02
0.04
0.06
0.08
β_IV

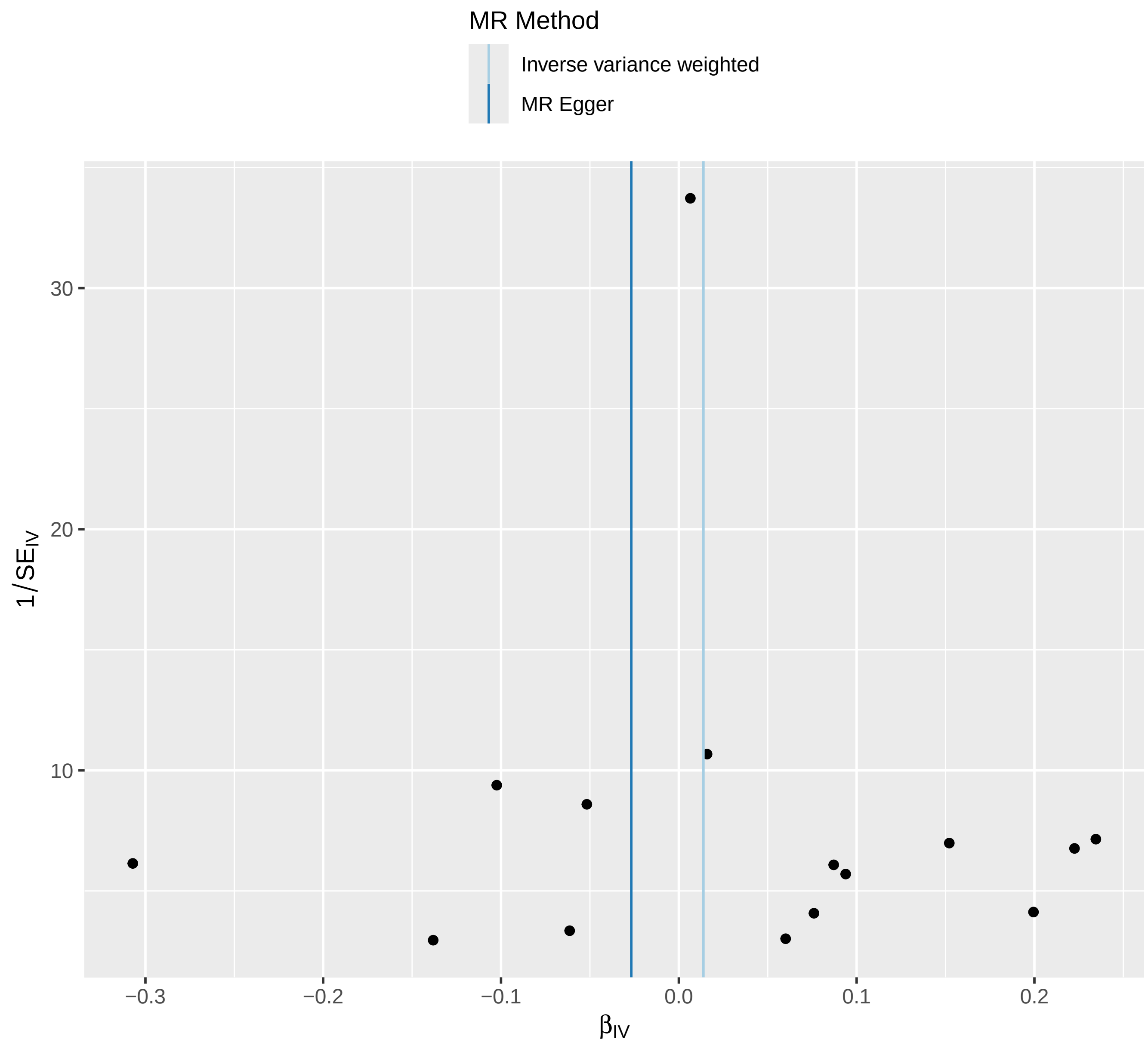

MR Method
Inverse variance weighted
MR Egger
30
20
10
1/SE$_{IV}$
−0.3
−0.2
−0.1
0.0
0.1
0.2
β$_{IV}$

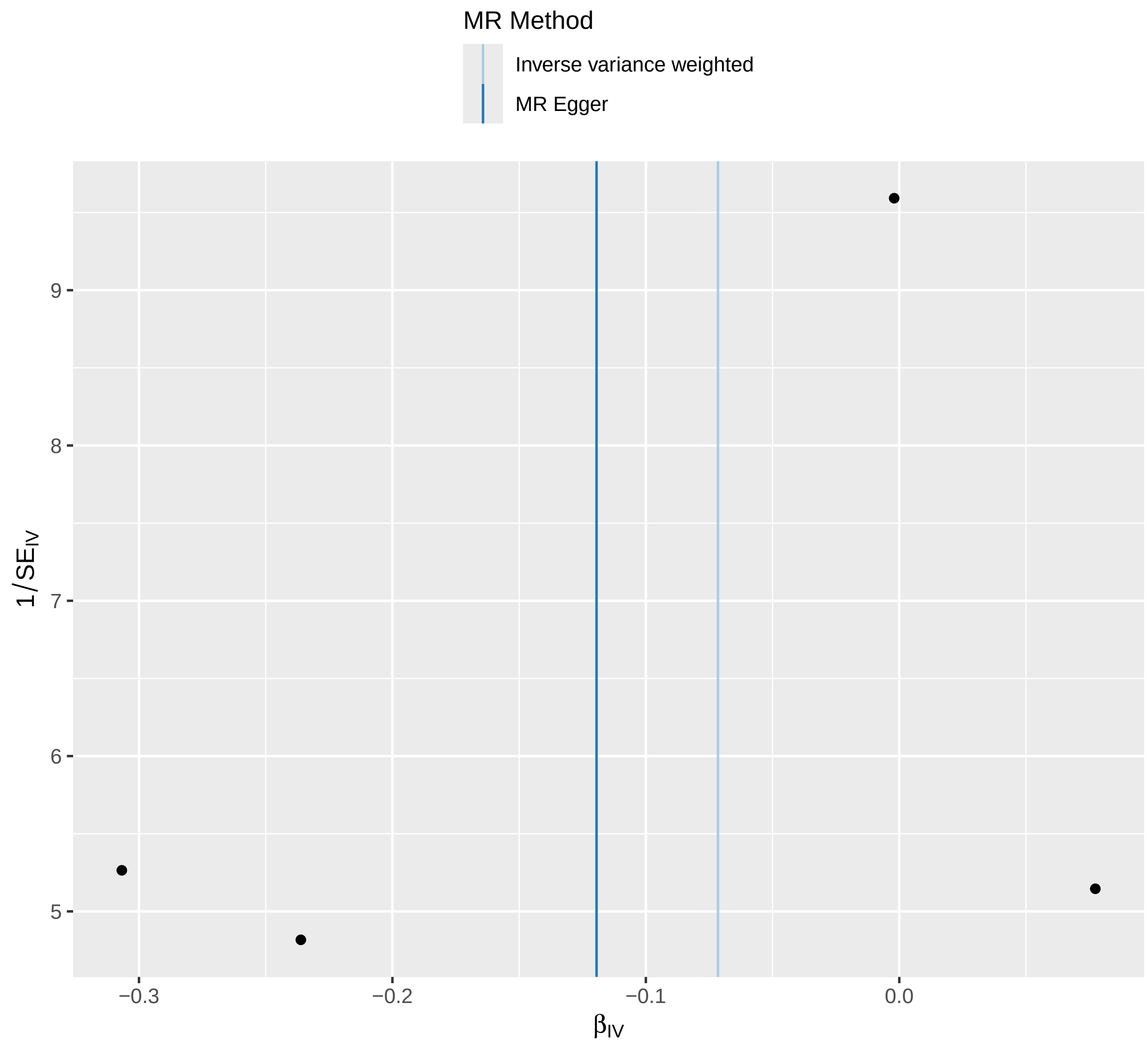
MR Method
Inverse variance weighted
MR Egger
1/SE_IV
β_IV
9
8
7
6
5
−0.3
−0.2
−0.1
0.0

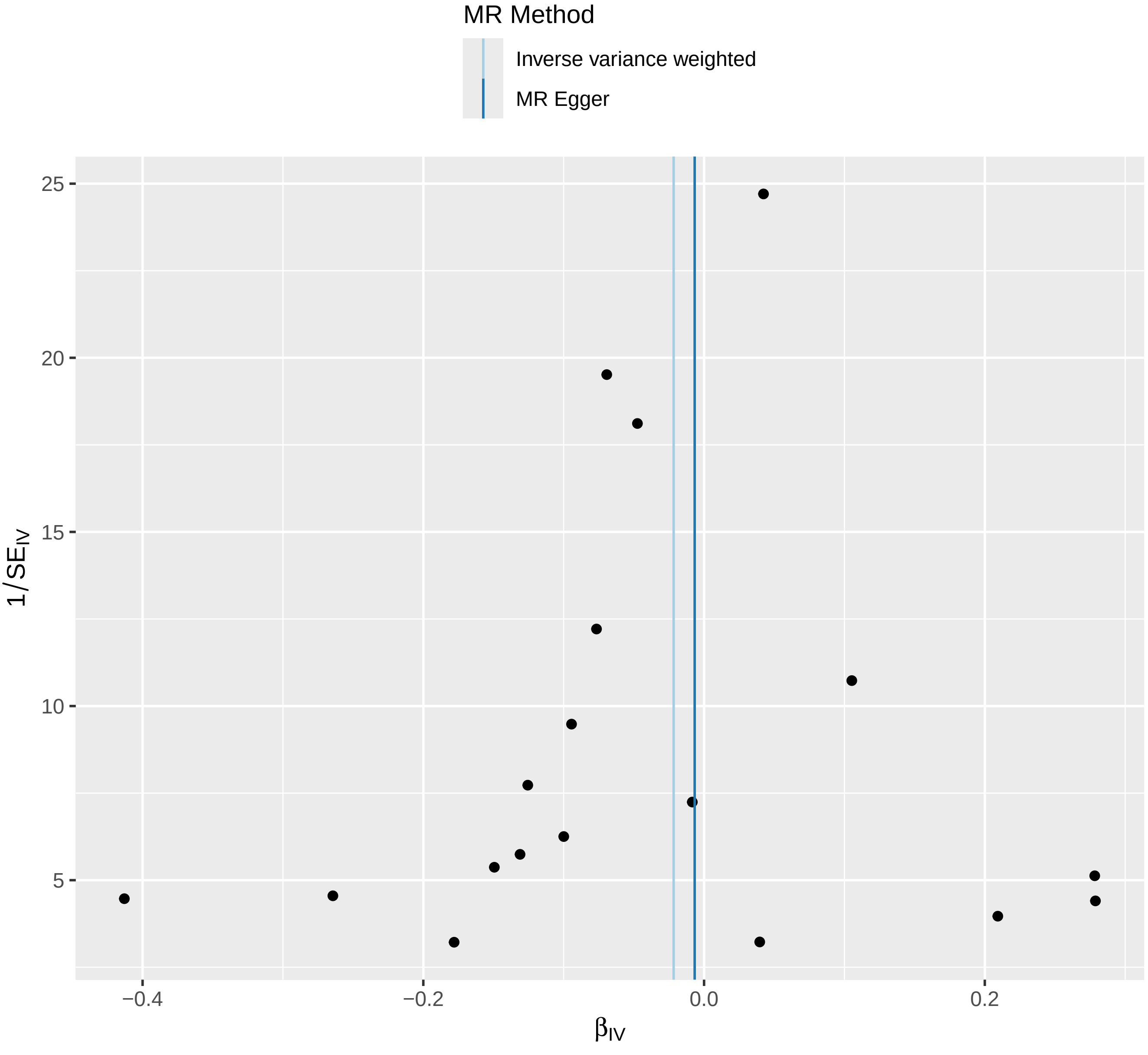

MR Method
Inverse variance weighted
MR Egger
25
20
15
10
5
$1/SE_{IV}$
−0.4
−0.2
0.0
0.2
$\beta_{IV}$

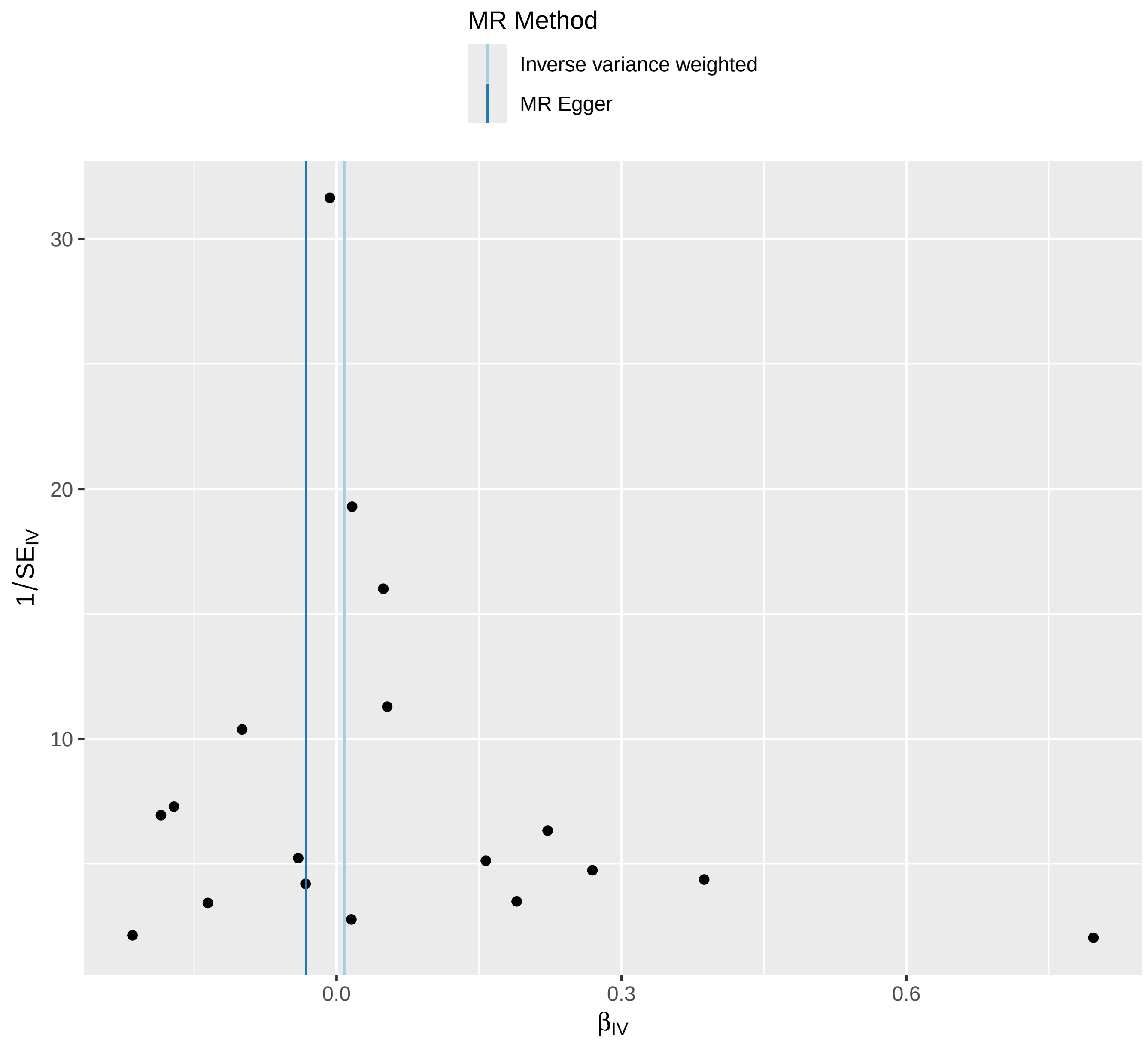

MR Method
Inverse variance weighted
MR Egger
30
20
10
1/SE$_{IV}$
0.0
0.3
0.6
β$_{IV}$

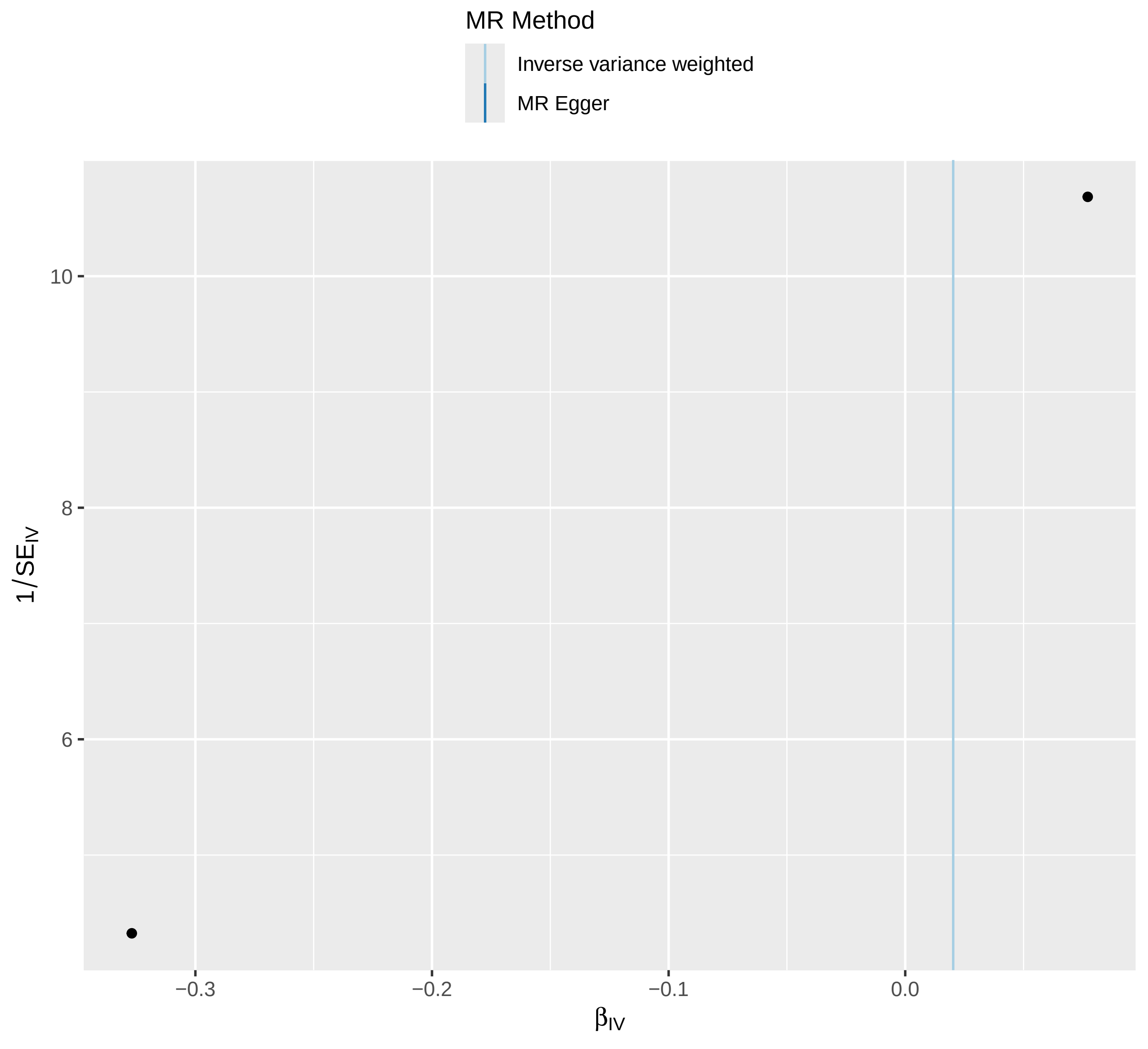
MR Method
Inverse variance weighted
MR Egger
10
8
6
1/SE$_{IV}$
−0.3
−0.2
−0.1
0.0
β$_{IV}$

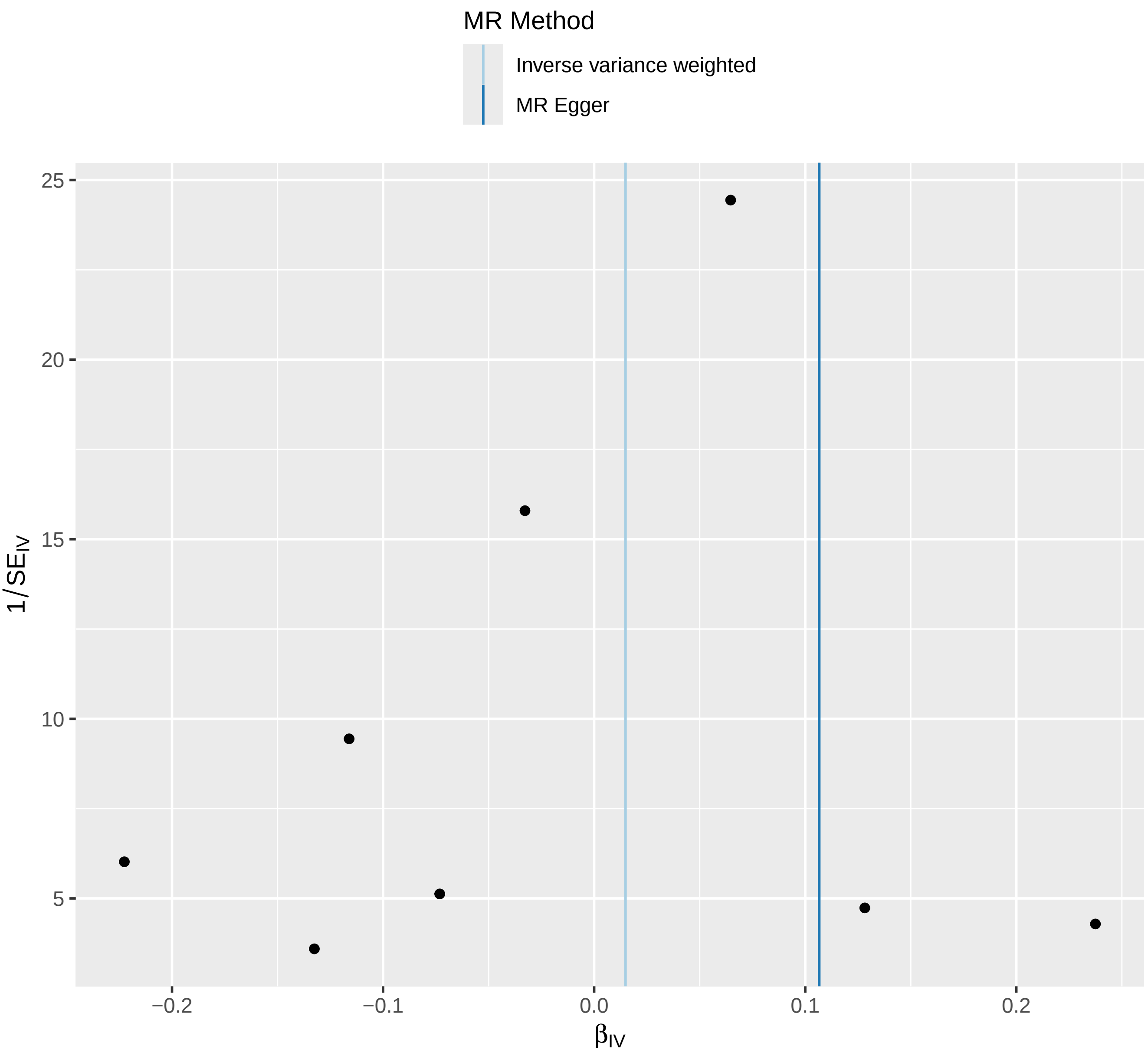
MR Method
Inverse variance weighted
MR Egger
25
20
15
10
5
1/SE_IV
−0.2
−0.1
0.0
0.1
0.2
β_IV

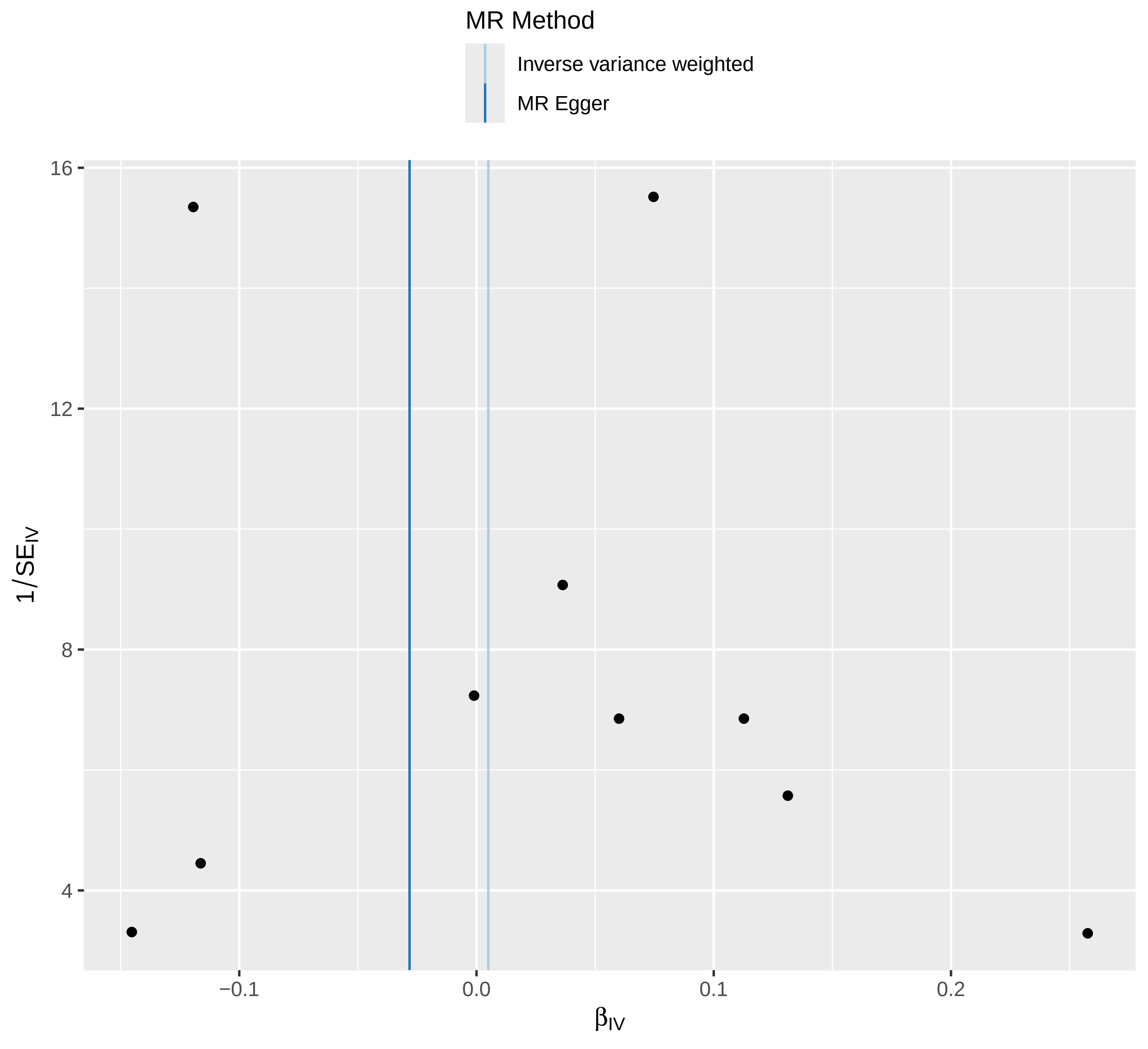
MR Method
Inverse variance weighted
MR Egger
16
12
8
4
$1/SE_{IV}$
−0.1
0.0
0.1
0.2
$\beta_{IV}$

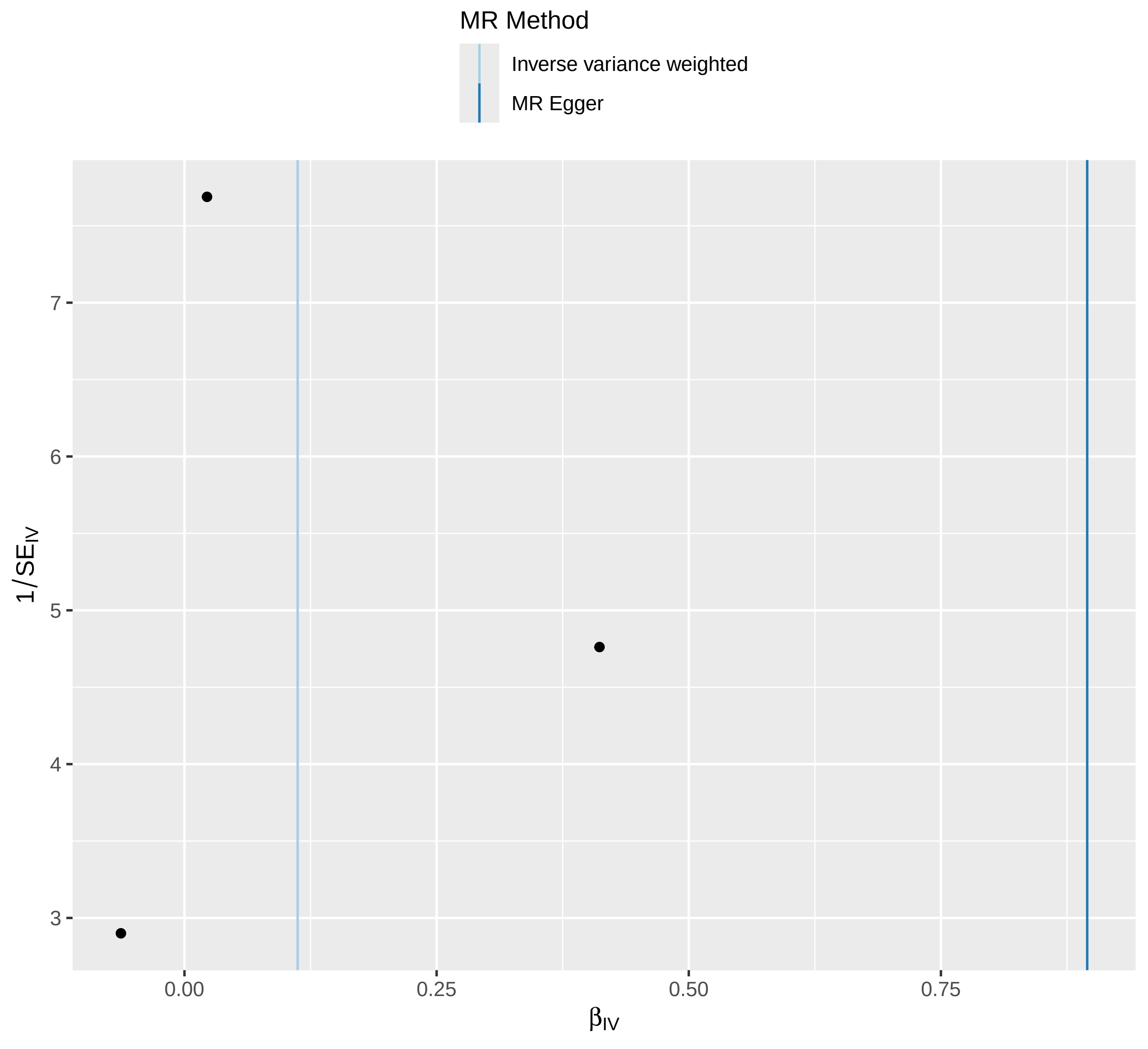

MR Method
Inverse variance weighted
MR Egger
7
6
5
4
3
1/SE$_{IV}$
0.00
0.25
0.50
0.75
β$_{IV}$

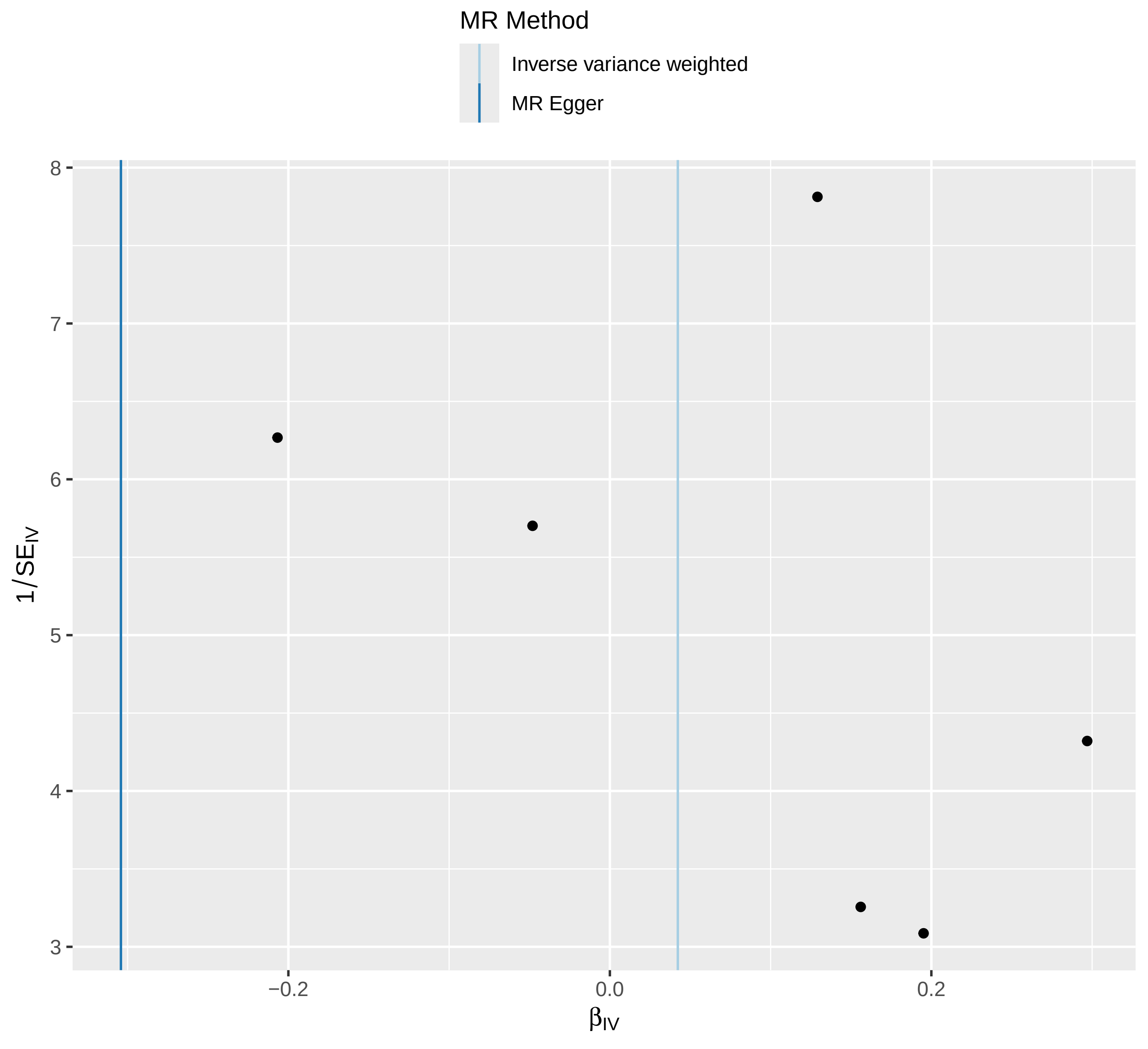
MR Method
Inverse variance weighted
MR Egger
8
7
6
5
4
3
1/SE$_{IV}$
−0.2
0.0
0.2
β$_{IV}$

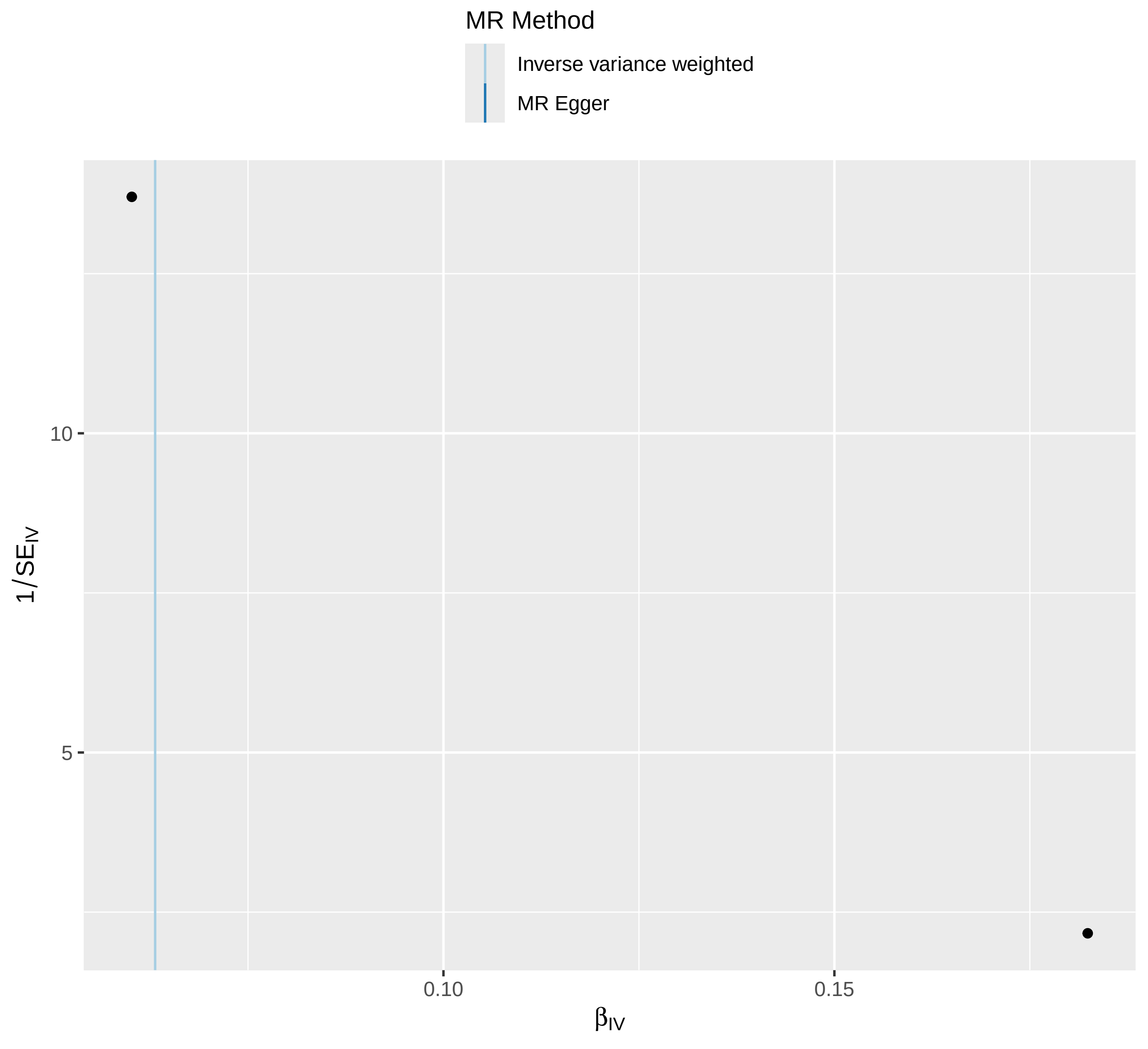
MR Method
Inverse variance weighted
MR Egger
10
5
$1/SE_{IV}$
0.10
0.15
$\beta_{IV}$

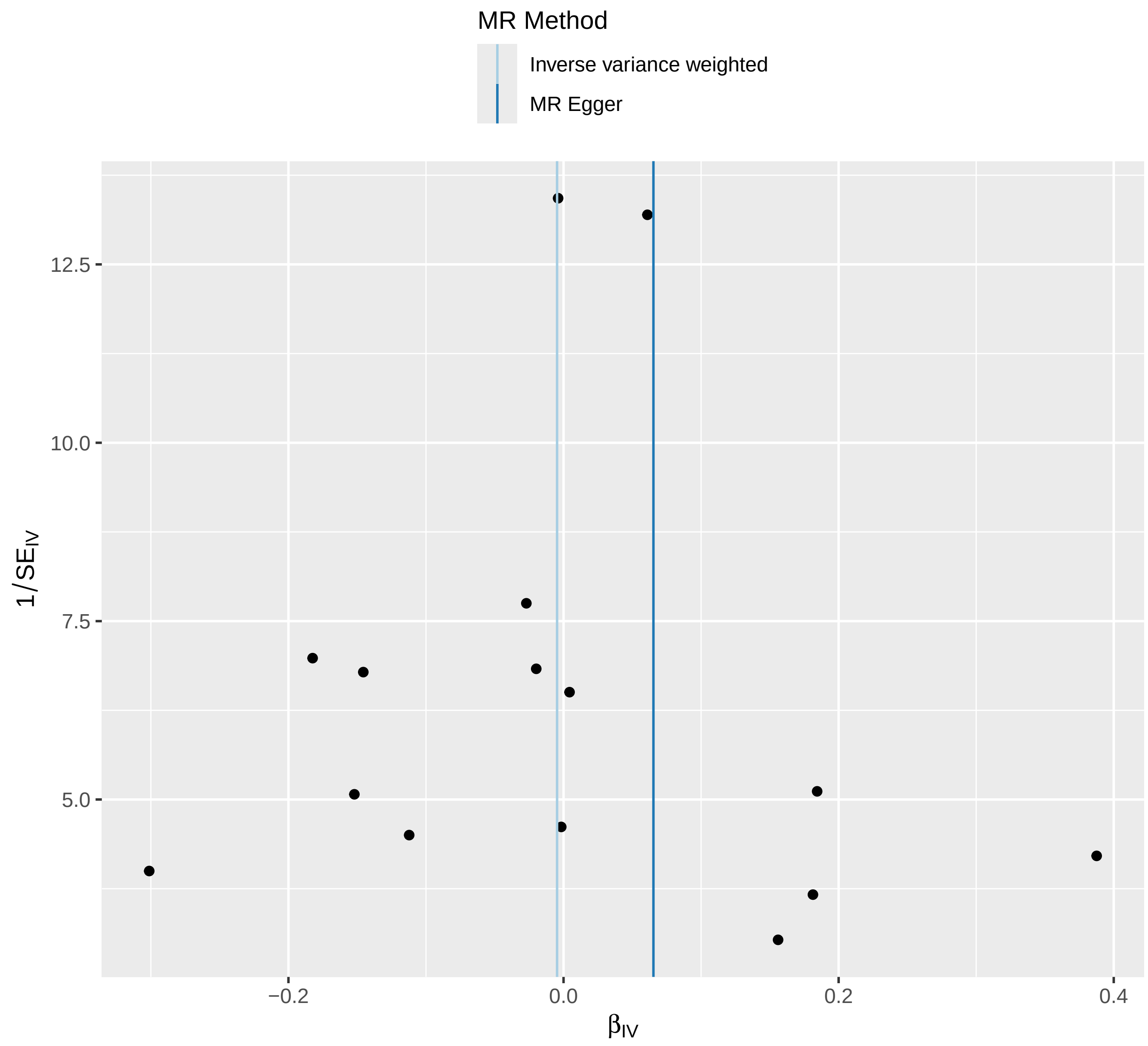

MR Method
Inverse variance weighted
MR Egger
12.5
10.0
7.5
5.0
1/SE$_{IV}$
−0.2
0.0
0.2
0.4
β$_{IV}$

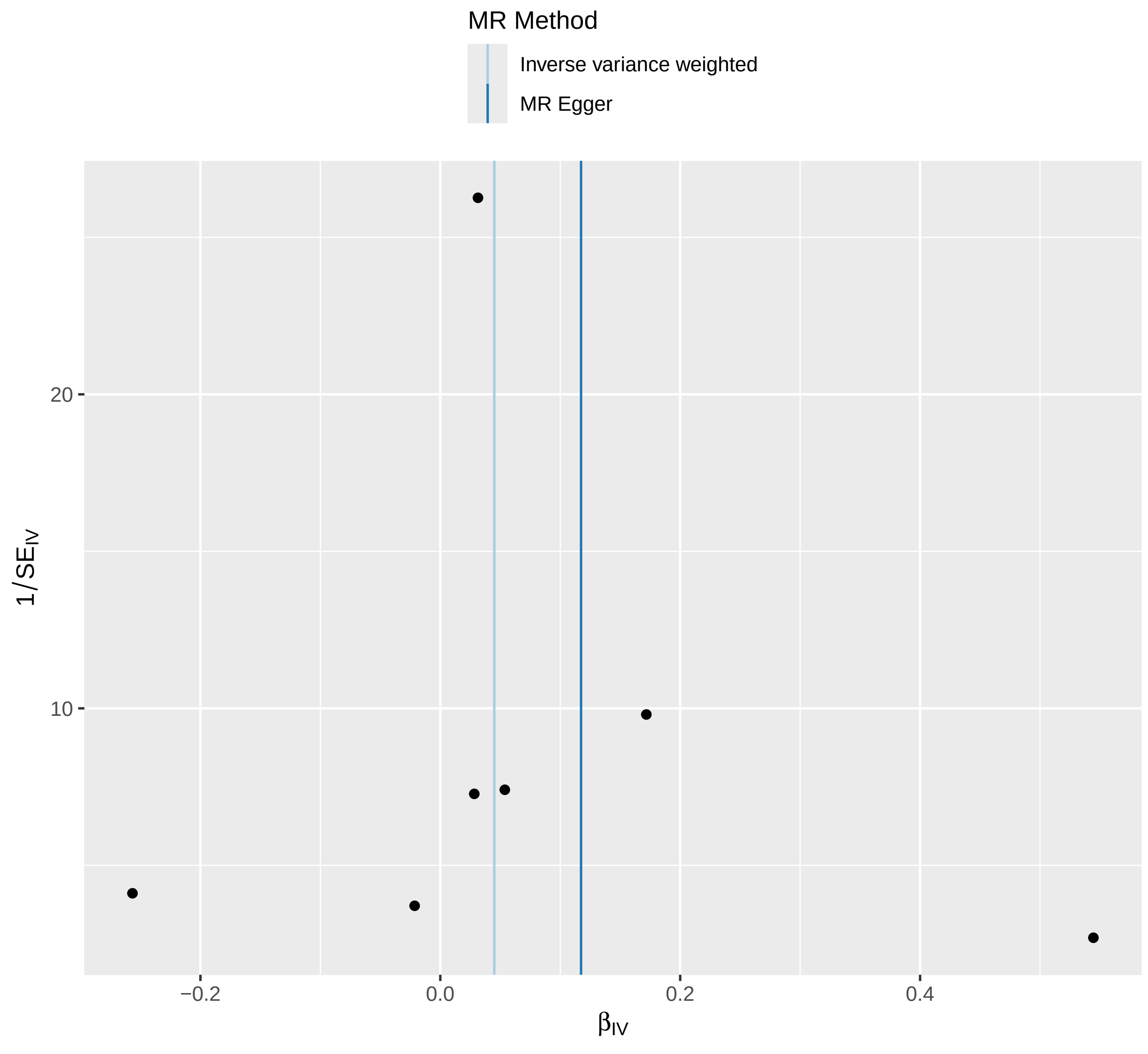
MR Method
Inverse variance weighted
MR Egger
20
10
1/SE$_{IV}$
−0.2
0.0
0.2
0.4
β$_{IV}$

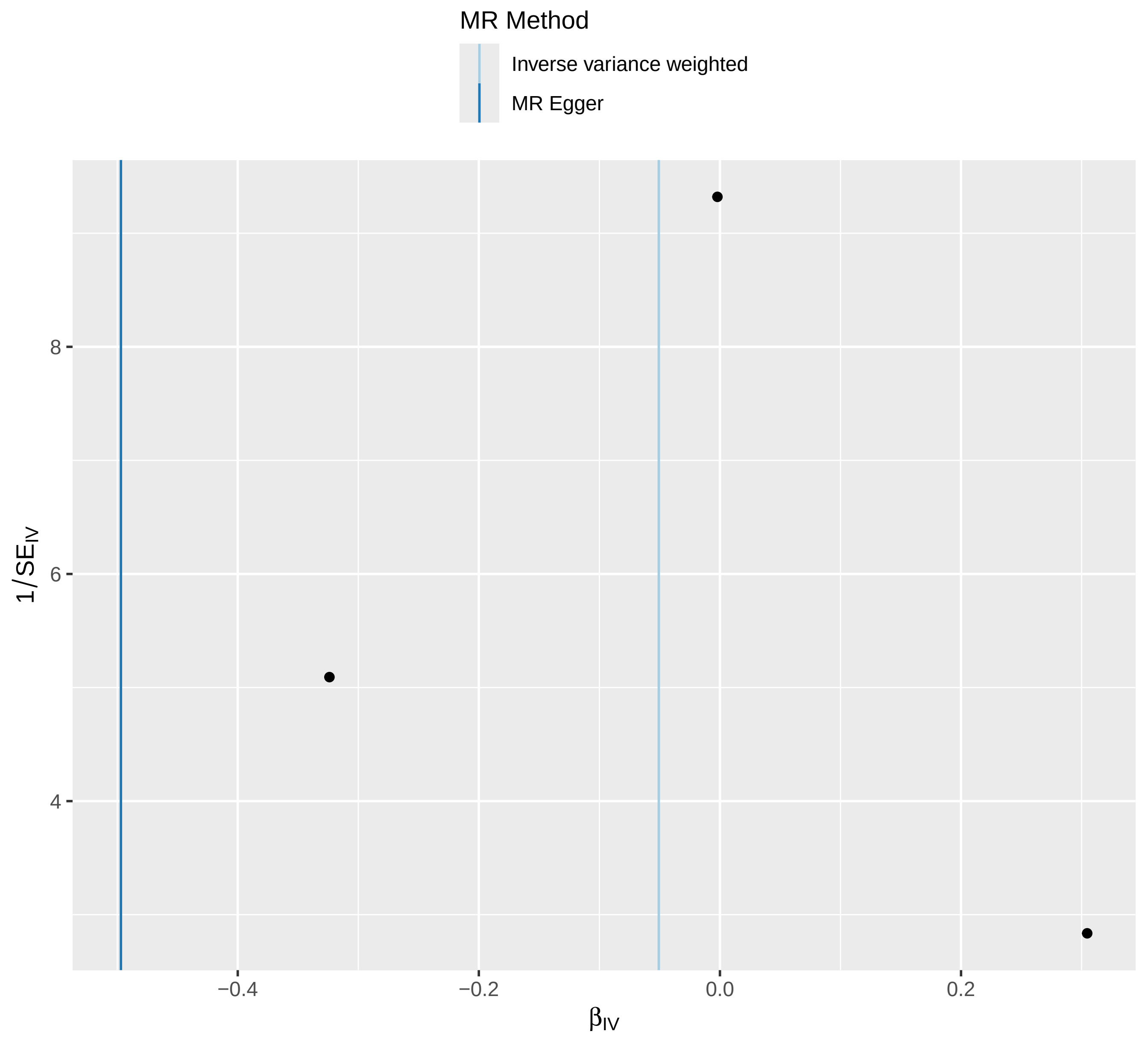
MR Method
Inverse variance weighted
MR Egger
8
6
4
1/SE$_{IV}$
−0.4
−0.2
0.0
0.2
β$_{IV}$

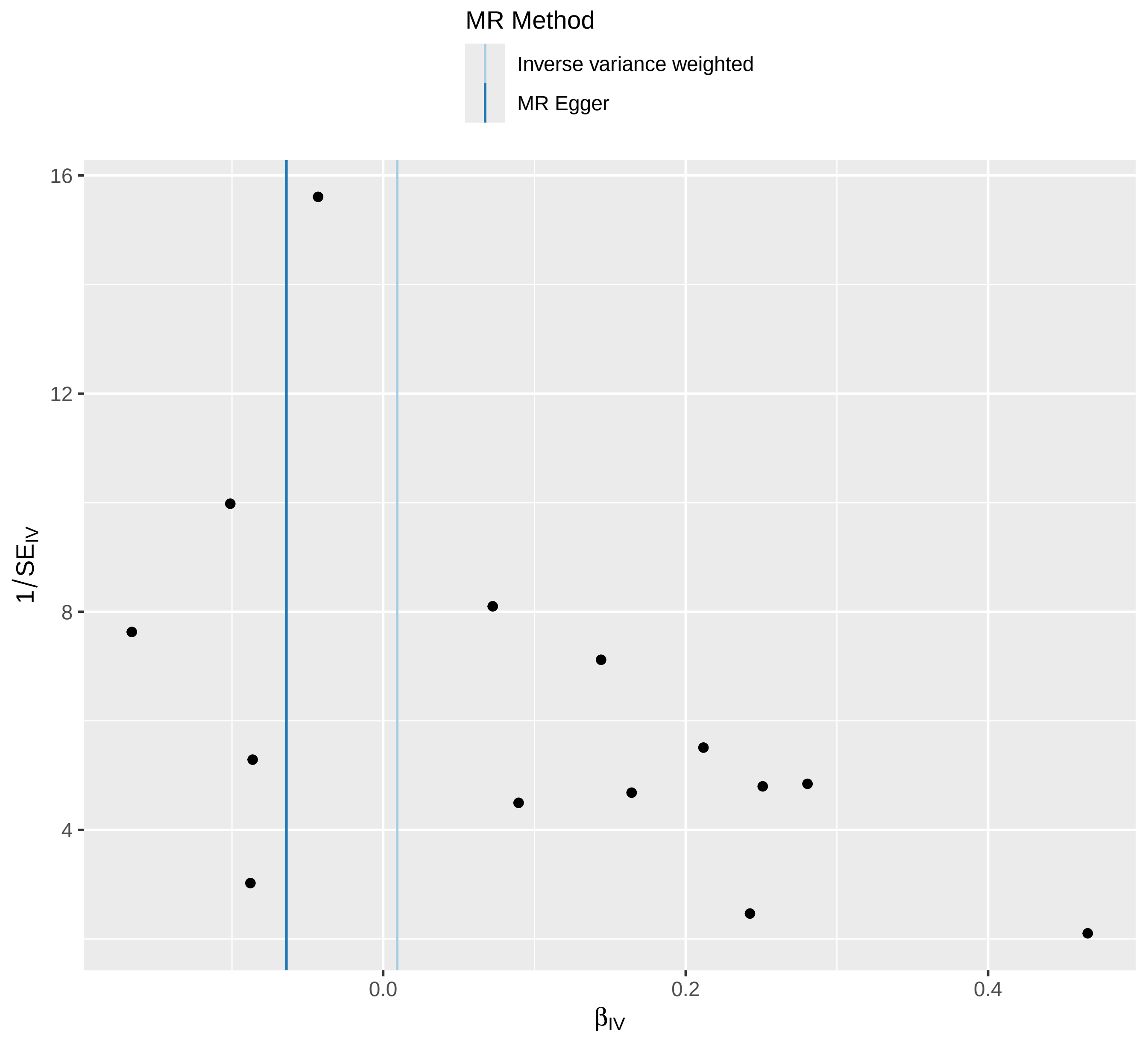

MR Method
Inverse variance weighted
MR Egger
16
12
8
4
1/SE_IV
0.0
0.2
0.4
β_IV

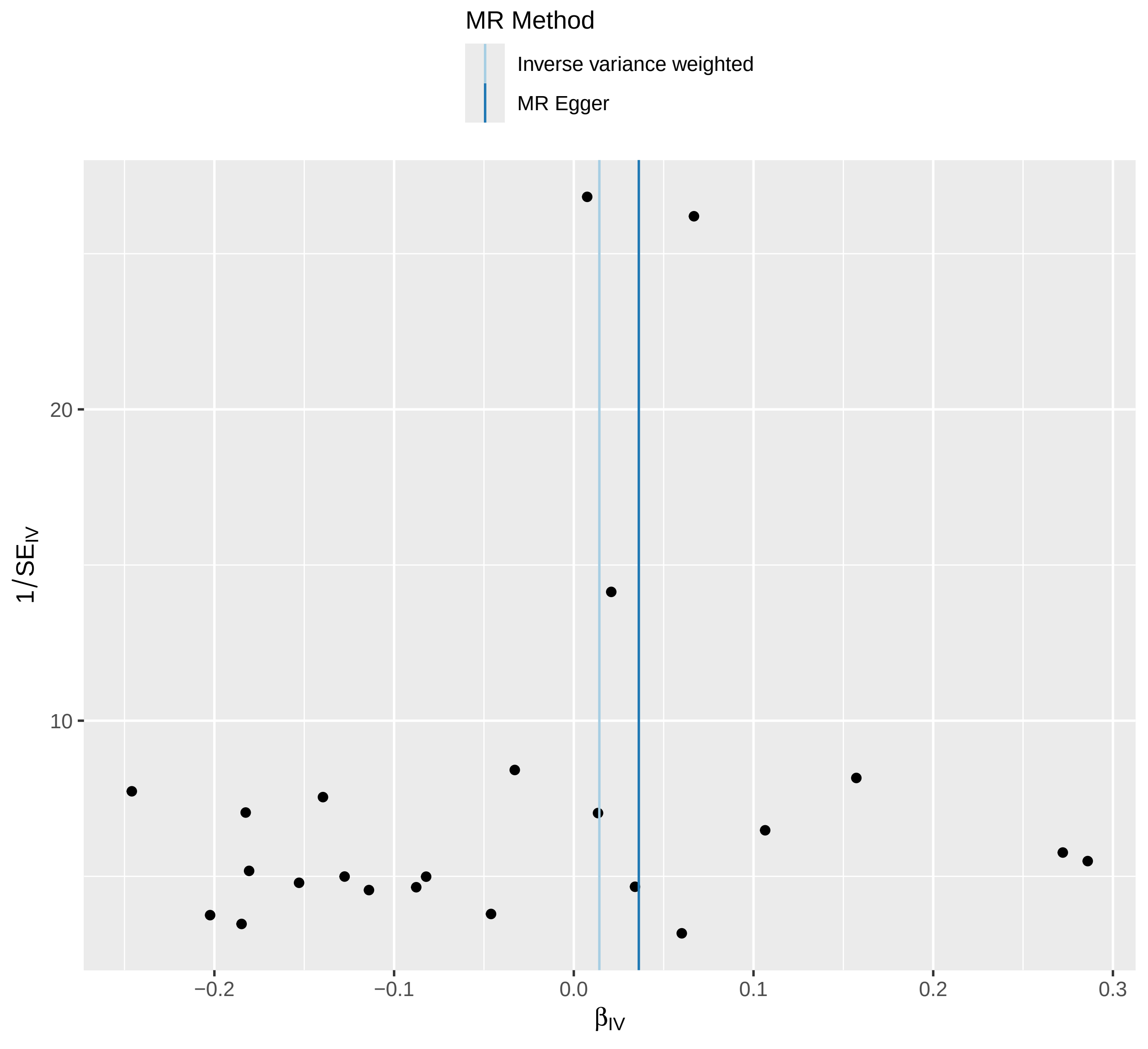

MR Method
Inverse variance weighted
MR Egger
20
10
1/SE_IV
−0.2
−0.1
0.0
0.1
0.2
0.3
β_IV

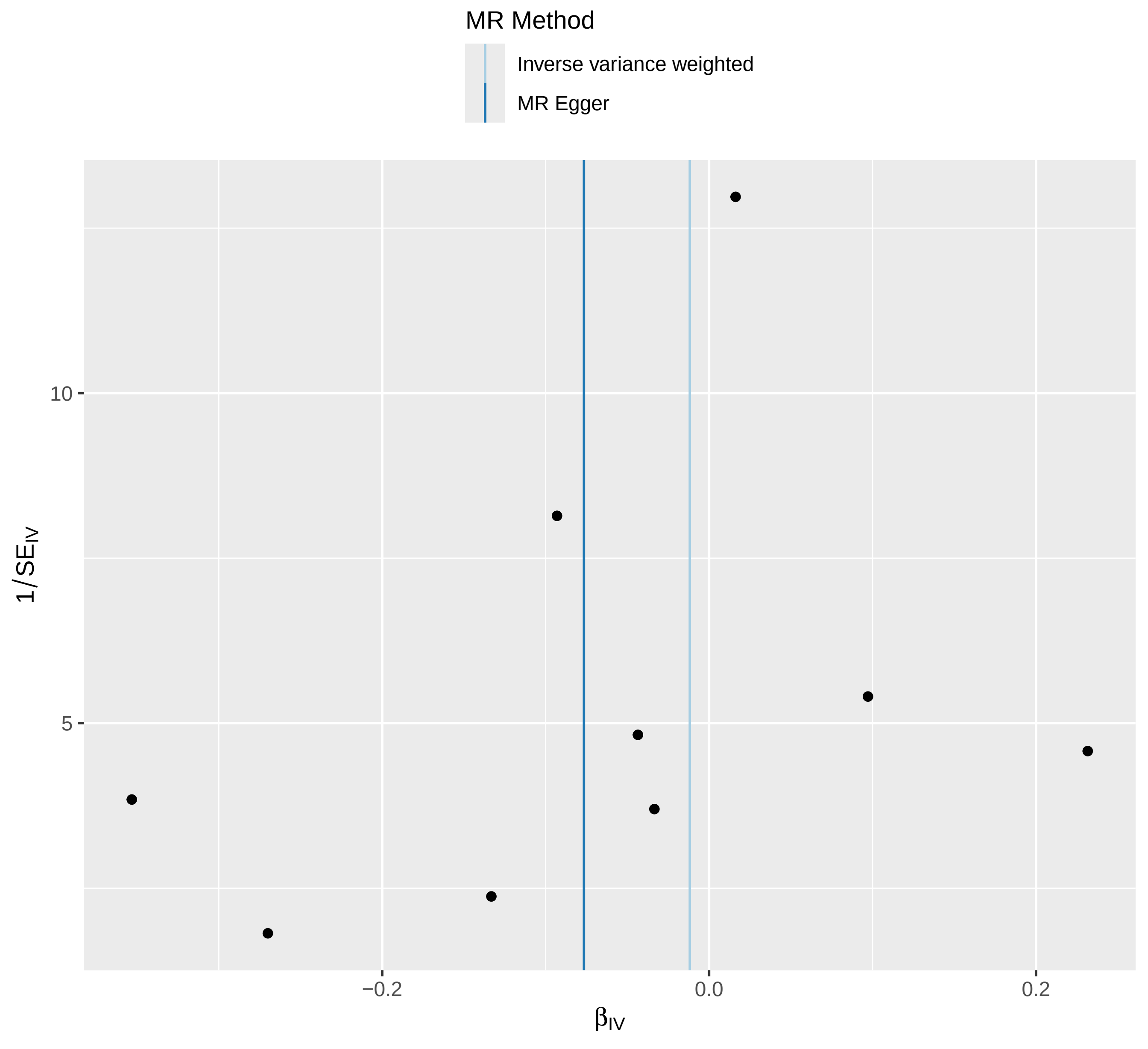

MR Method
Inverse variance weighted
MR Egger
10
5
1/SE$_{IV}$
−0.2
0.0
0.2
β$_{IV}$

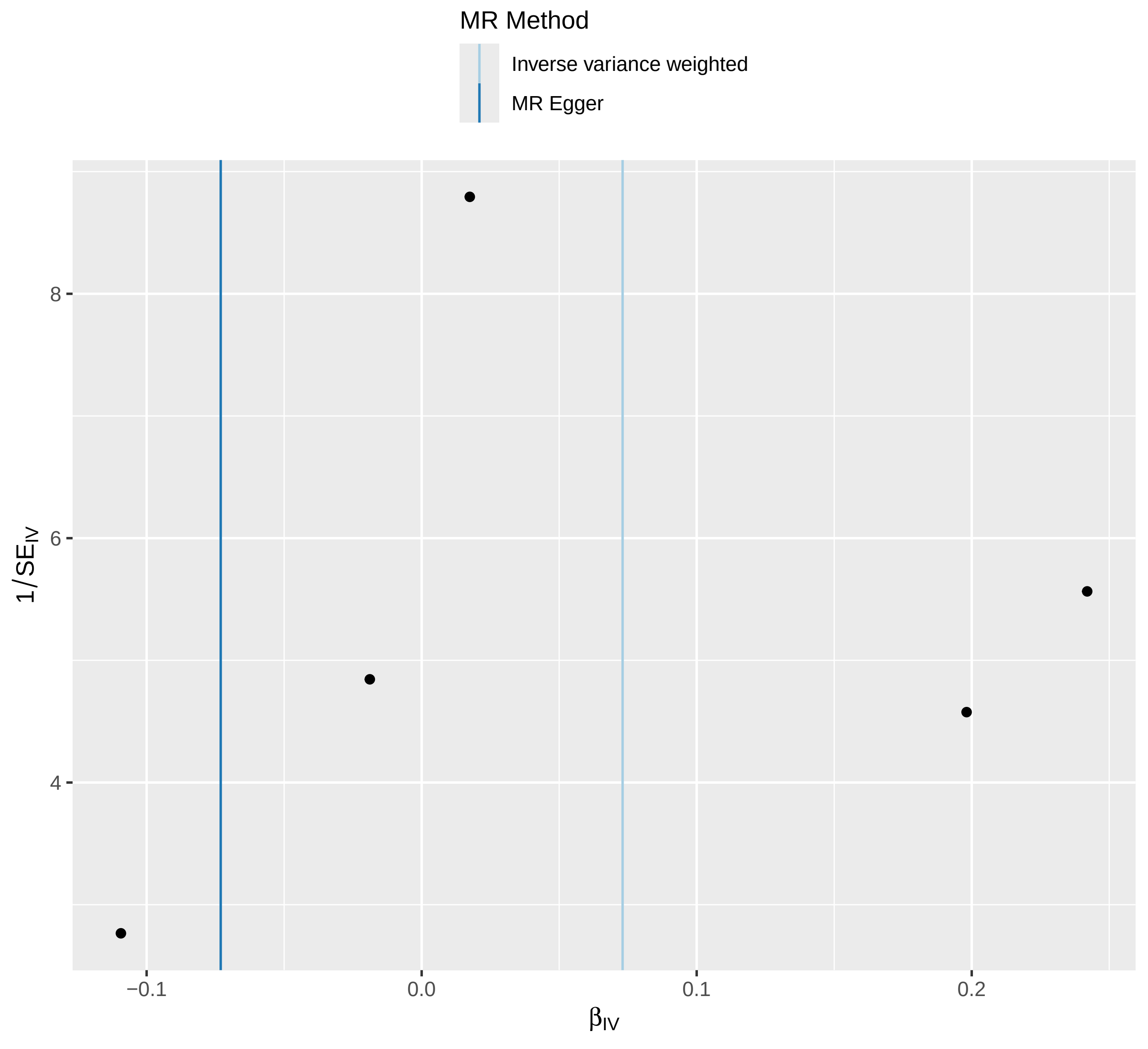

MR Method
Inverse variance weighted
MR Egger
8
6
4
1/SE$_{IV}$
−0.1
0.0
0.1
0.2
β$_{IV}$

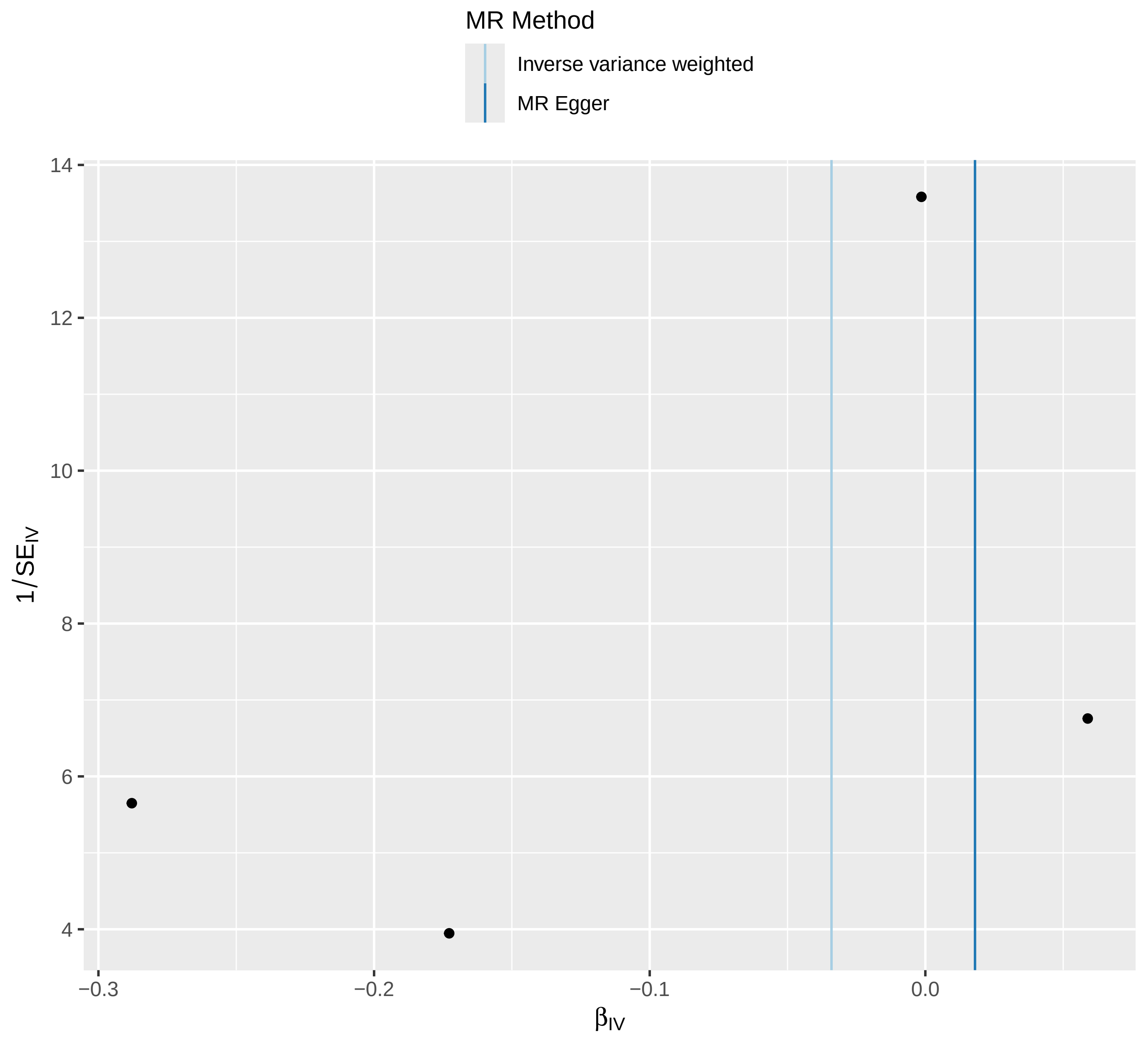

MR Method
Inverse variance weighted
MR Egger
14
12
10
8
6
4
1/SE_IV
−0.3
−0.2
−0.1
0.0
β_IV

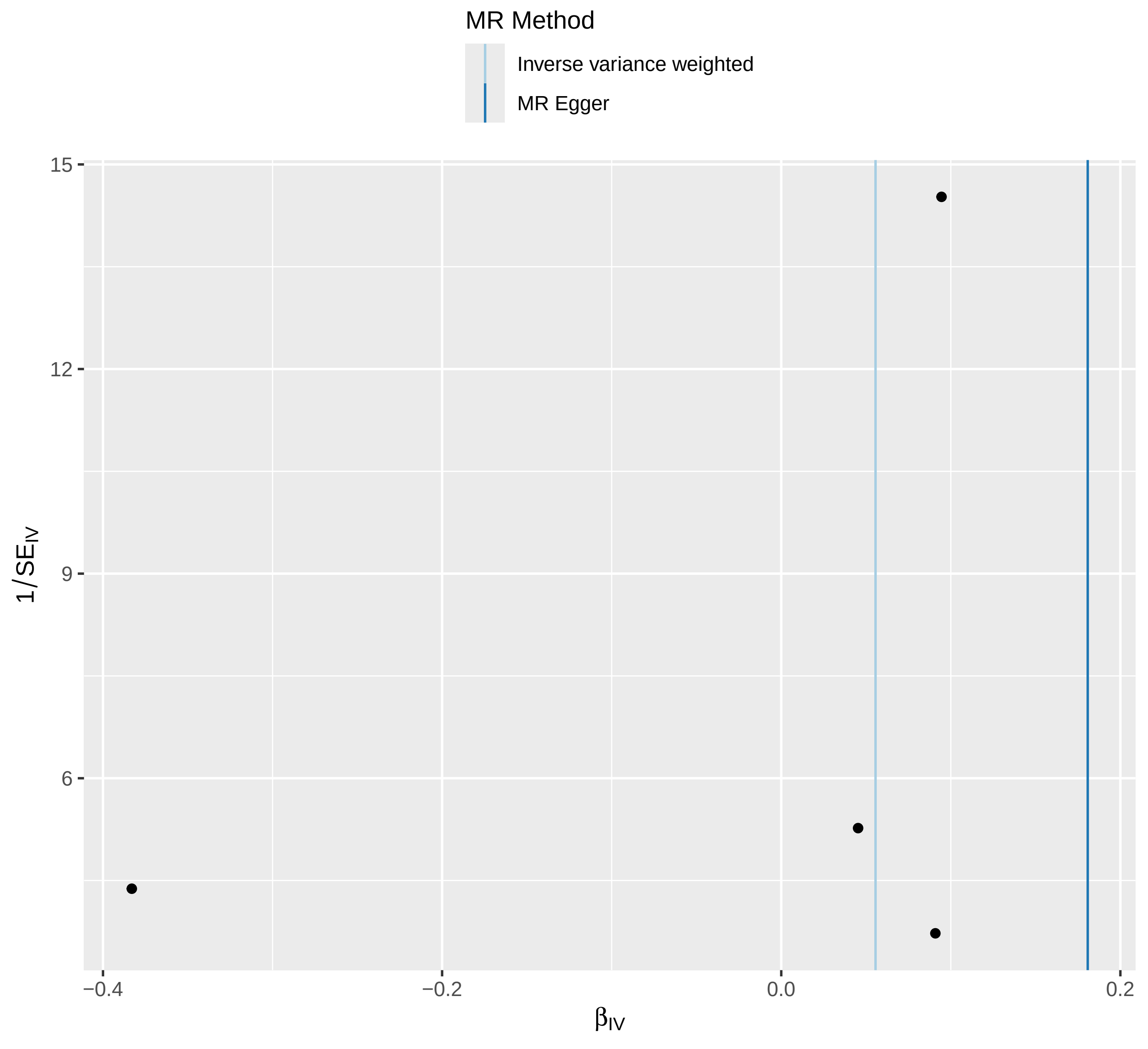
MR Method
Inverse variance weighted
MR Egger
15
12
9
6
1/SE$_{IV}$
−0.4
−0.2
0.0
0.2
β$_{IV}$

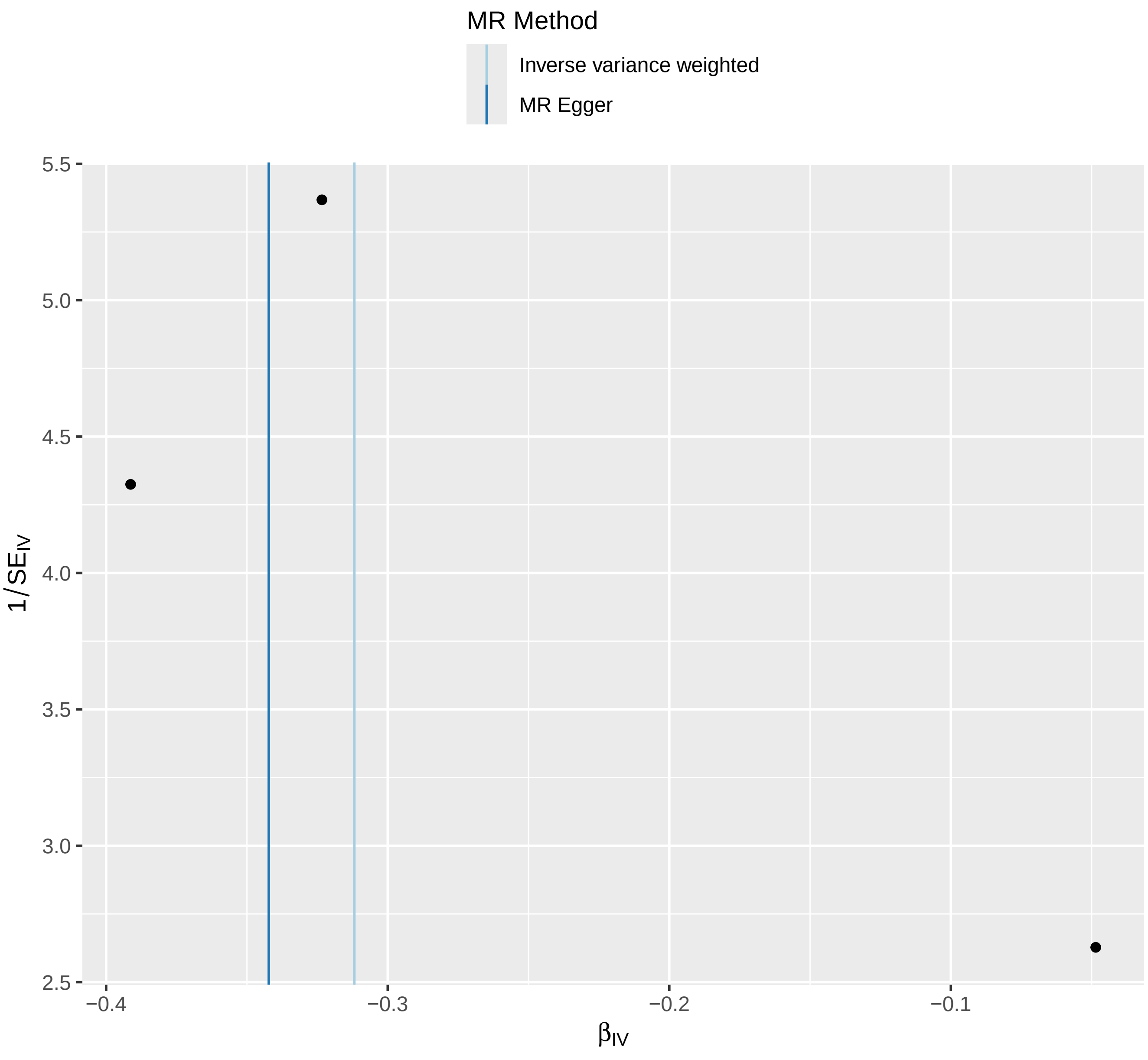
MR Method
Inverse variance weighted
MR Egger
5.5
5.0
4.5
4.0
3.5
3.0
2.5
1/SE_IV
−0.4
−0.3
−0.2
−0.1
β_IV

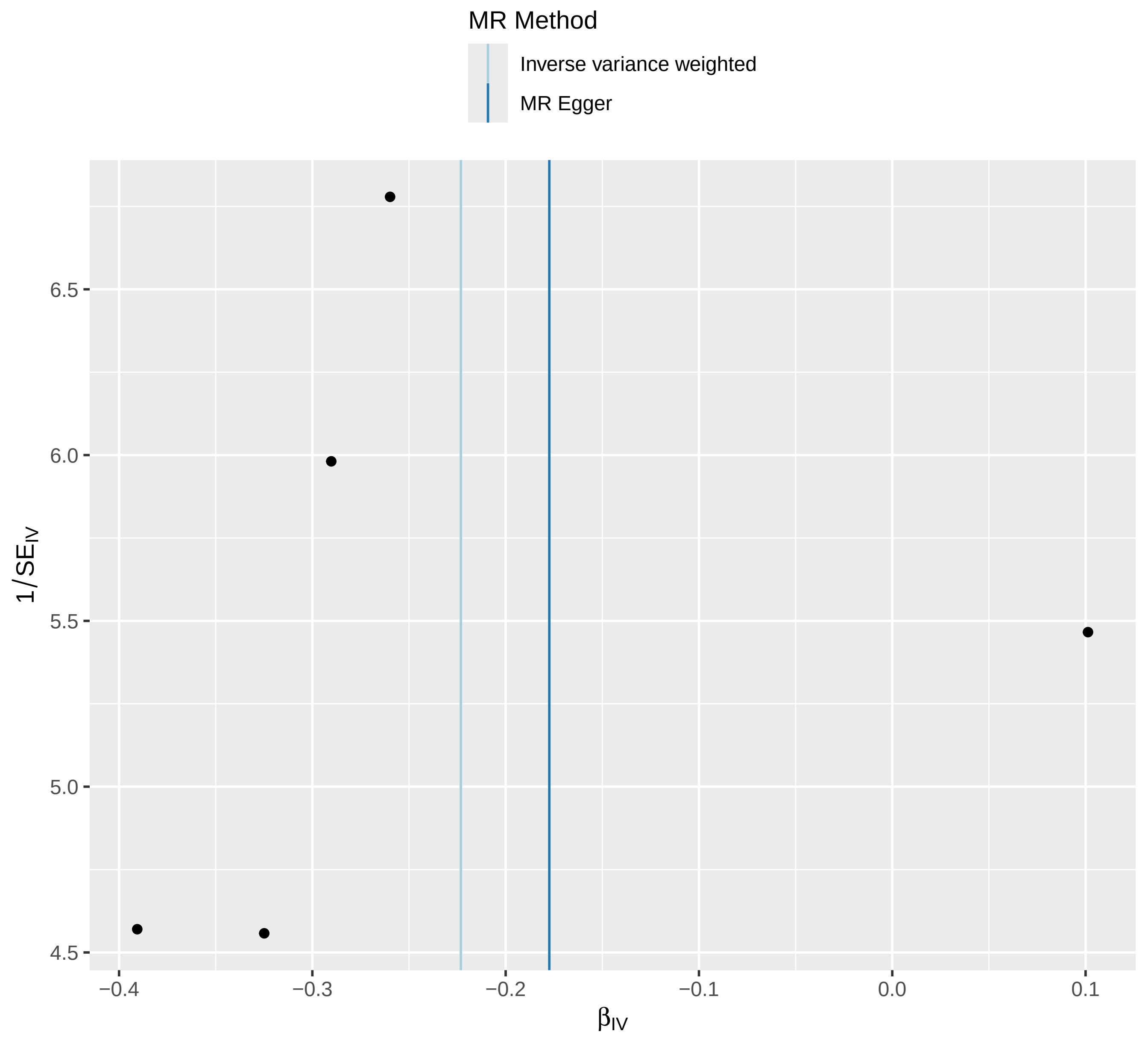

MR Method
Inverse variance weighted
MR Egger
6.5
6.0
5.5
5.0
4.5
1/SE_IV
−0.4
−0.3
−0.2
−0.1
0.0
0.1
β_IV

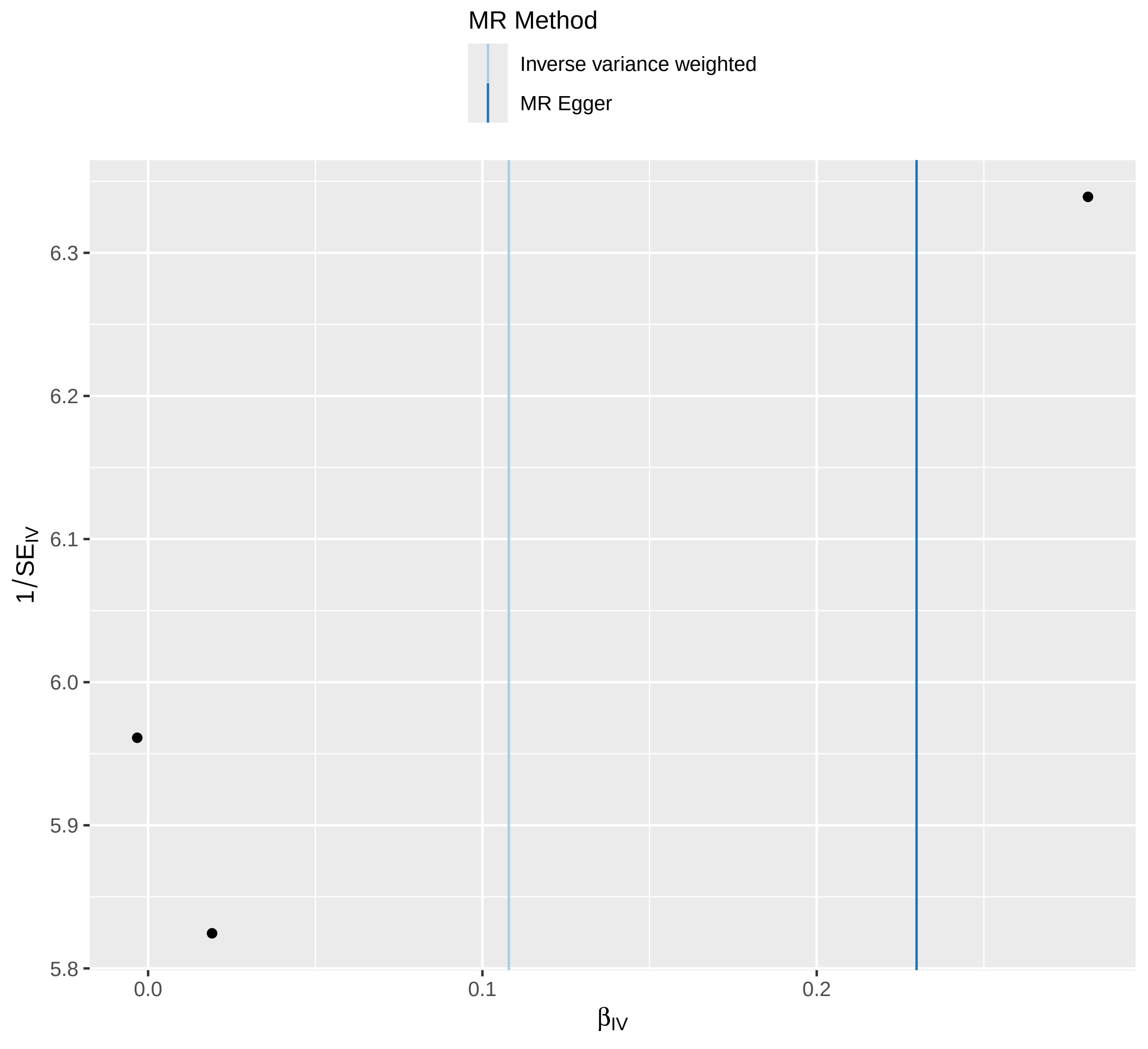

MR Method
Inverse variance weighted
MR Egger
6.3
6.2
6.1
6.0
5.9
5.8
1/SE$_{IV}$
0.0
0.1
0.2
β$_{IV}$

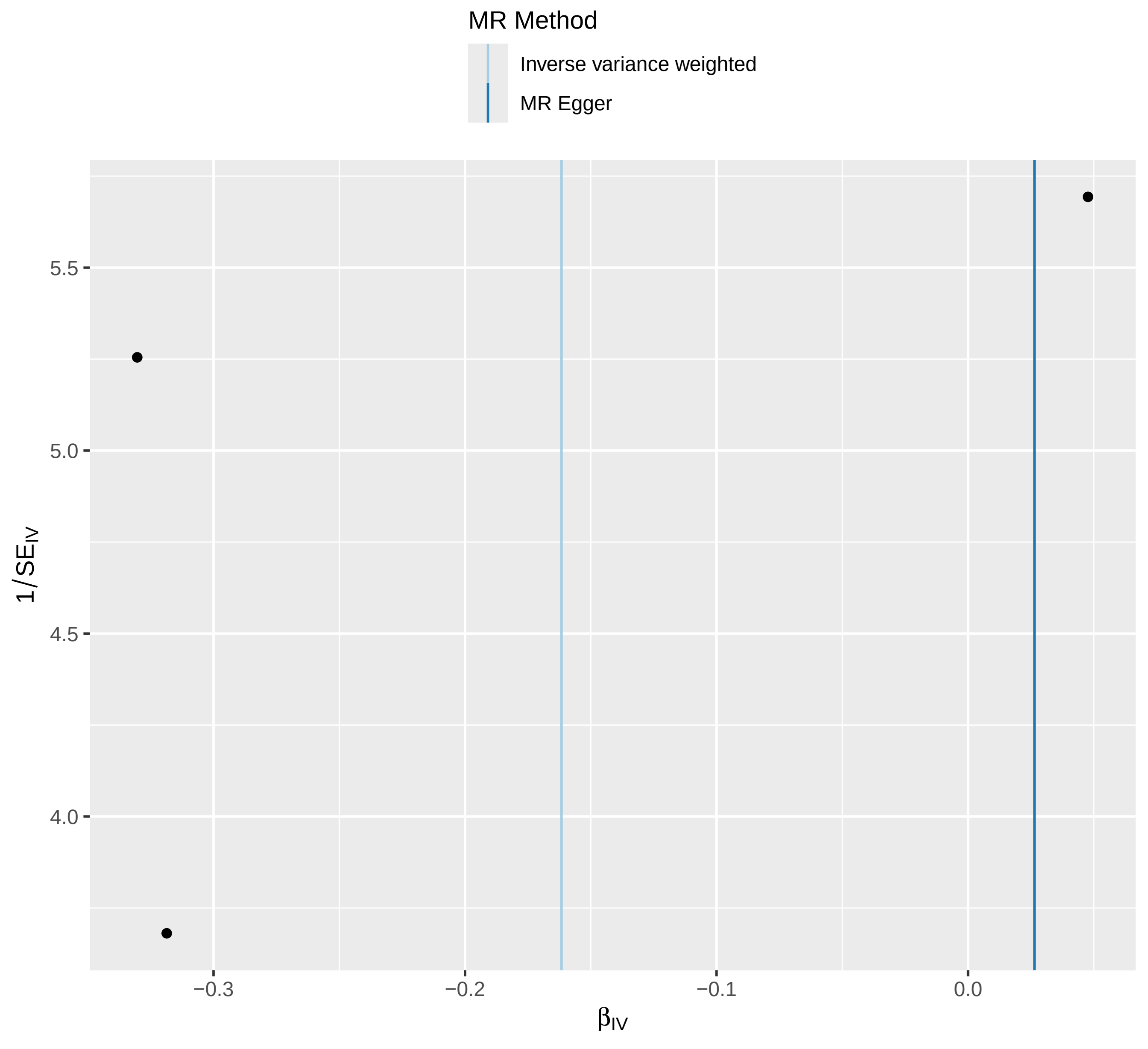

MR Method
Inverse variance weighted
MR Egger
5.5
5.0
4.5
4.0
1/SE_IV
−0.3
−0.2
−0.1
0.0
β_IV

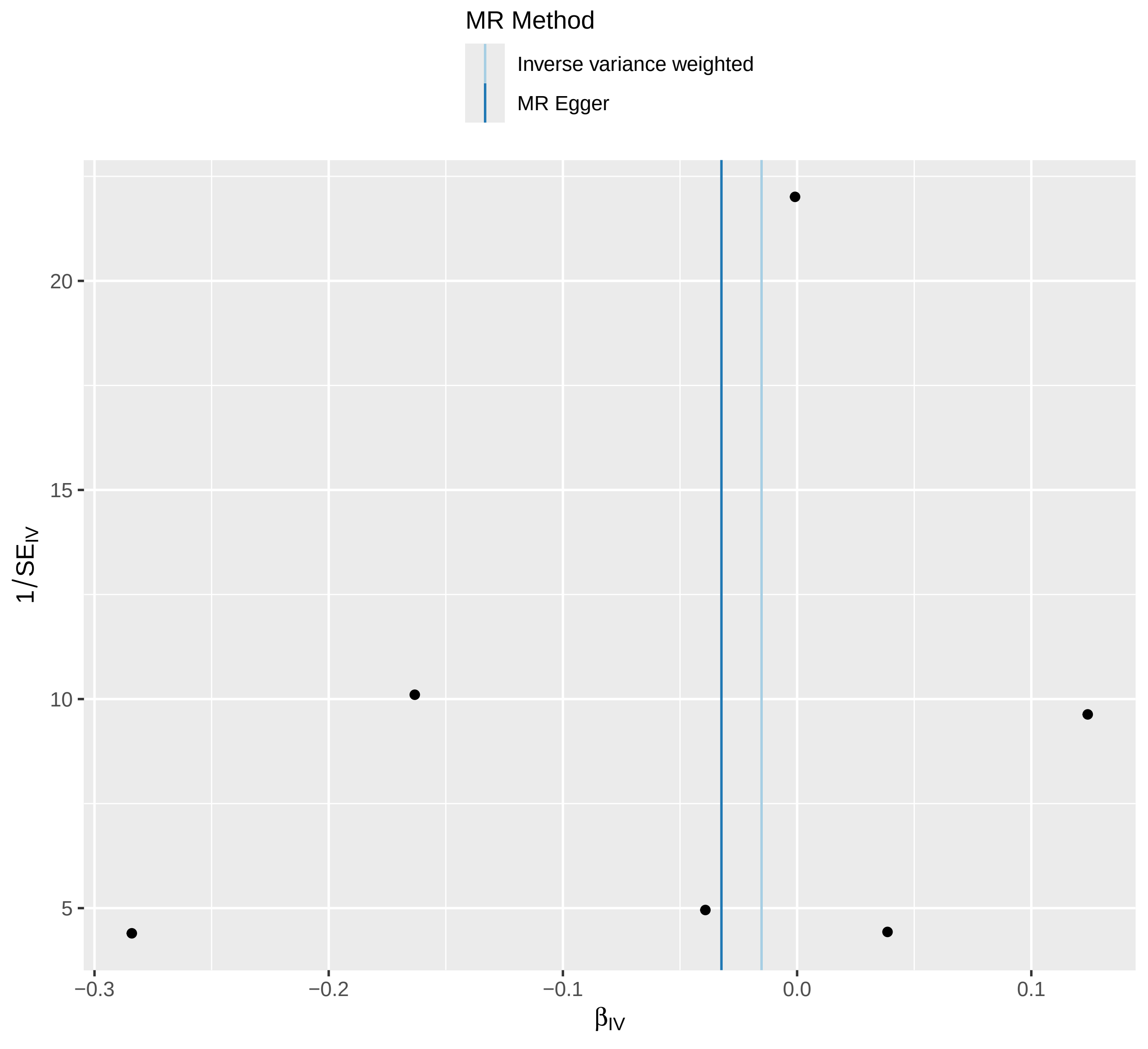

MR Method
Inverse variance weighted
MR Egger
20
15
10
5
1/SE_IV
−0.3
−0.2
−0.1
0.0
0.1
β_IV

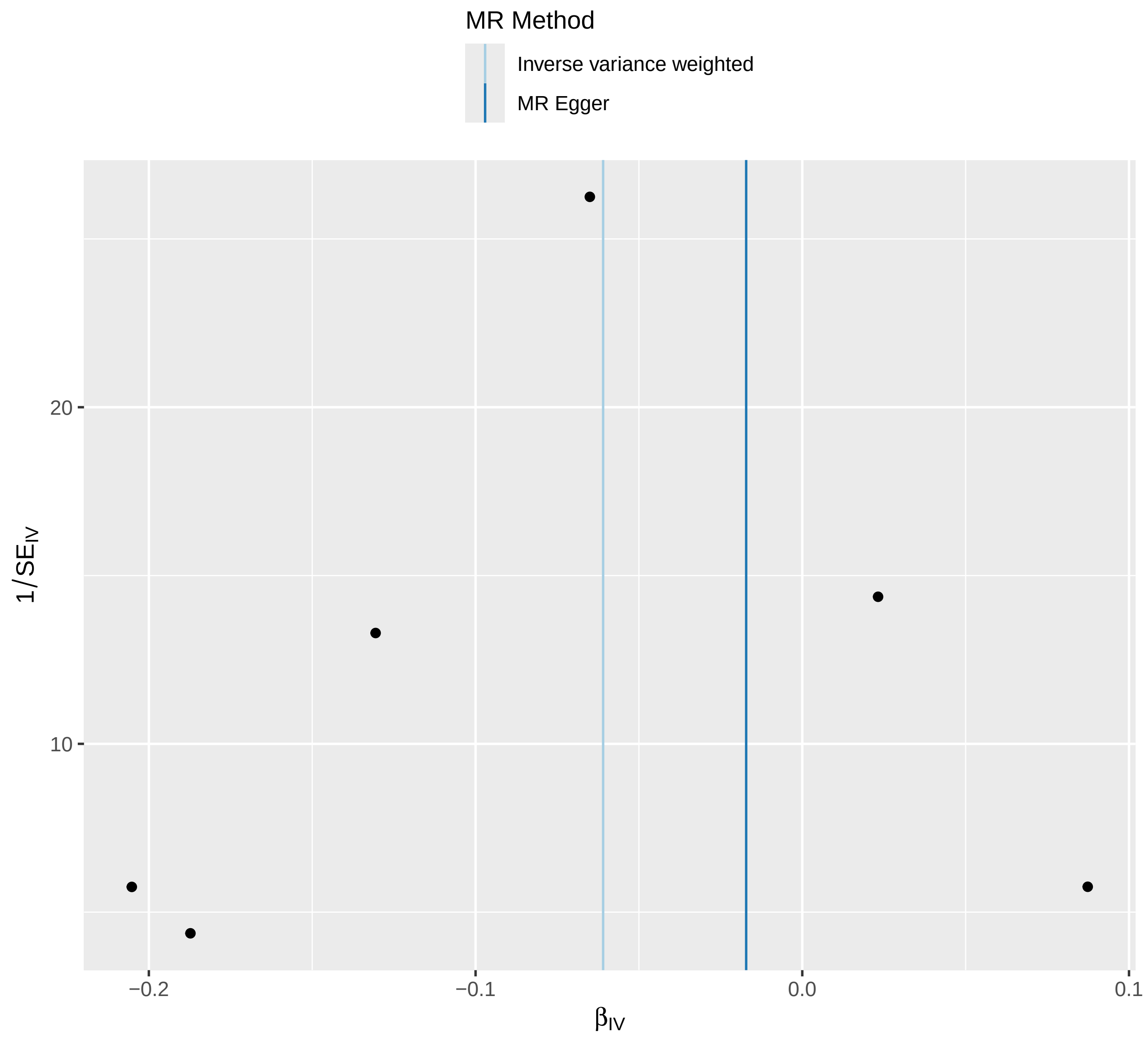
MR Method
Inverse variance weighted
MR Egger
20
10
1/SE_IV
−0.2
−0.1
0.0
0.1
β_IV

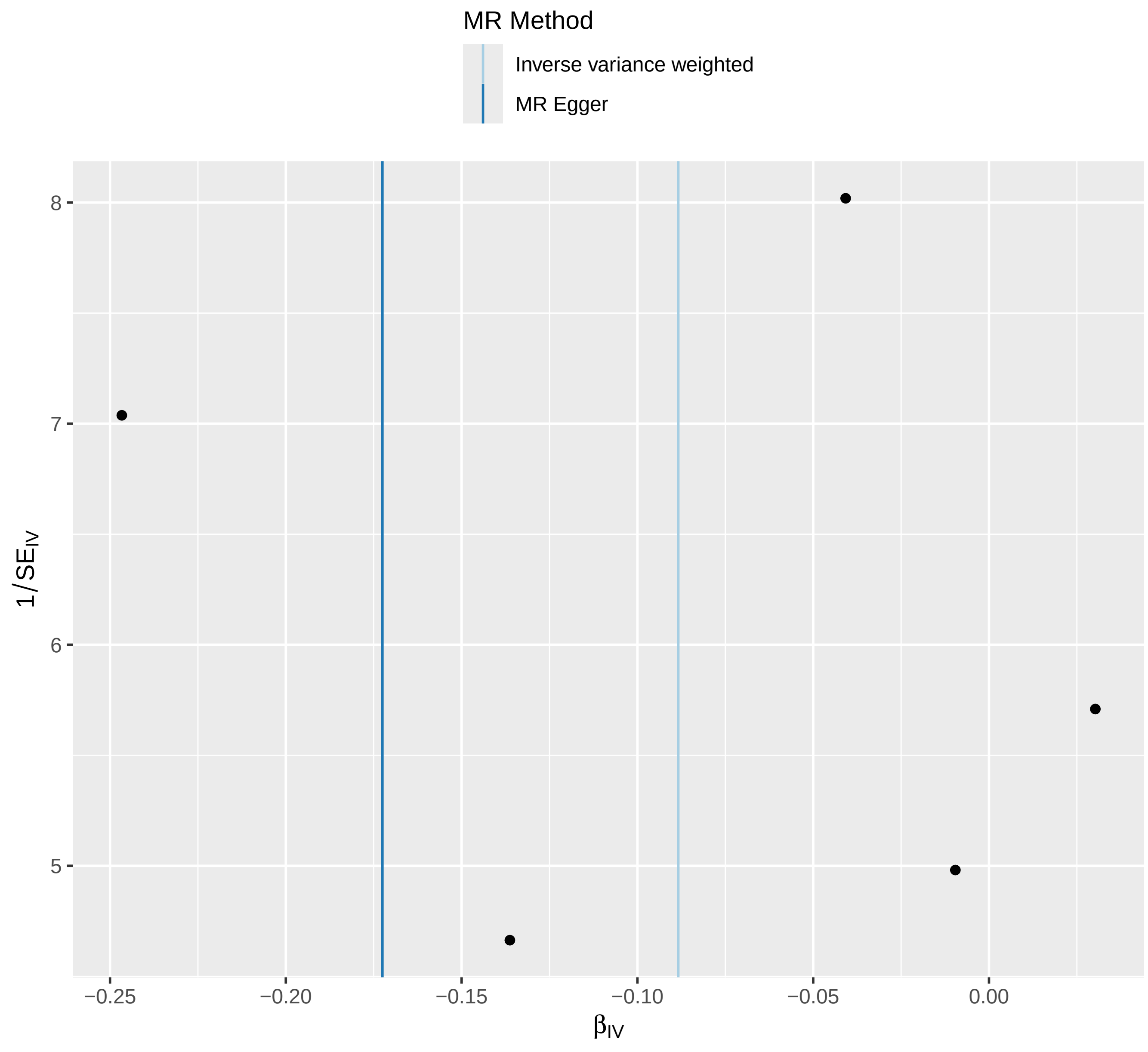

MR Method
Inverse variance weighted
MR Egger
8
7
6
5
1/SE_IV
−0.25
−0.20
−0.15
−0.10
−0.05
0.00
β_IV

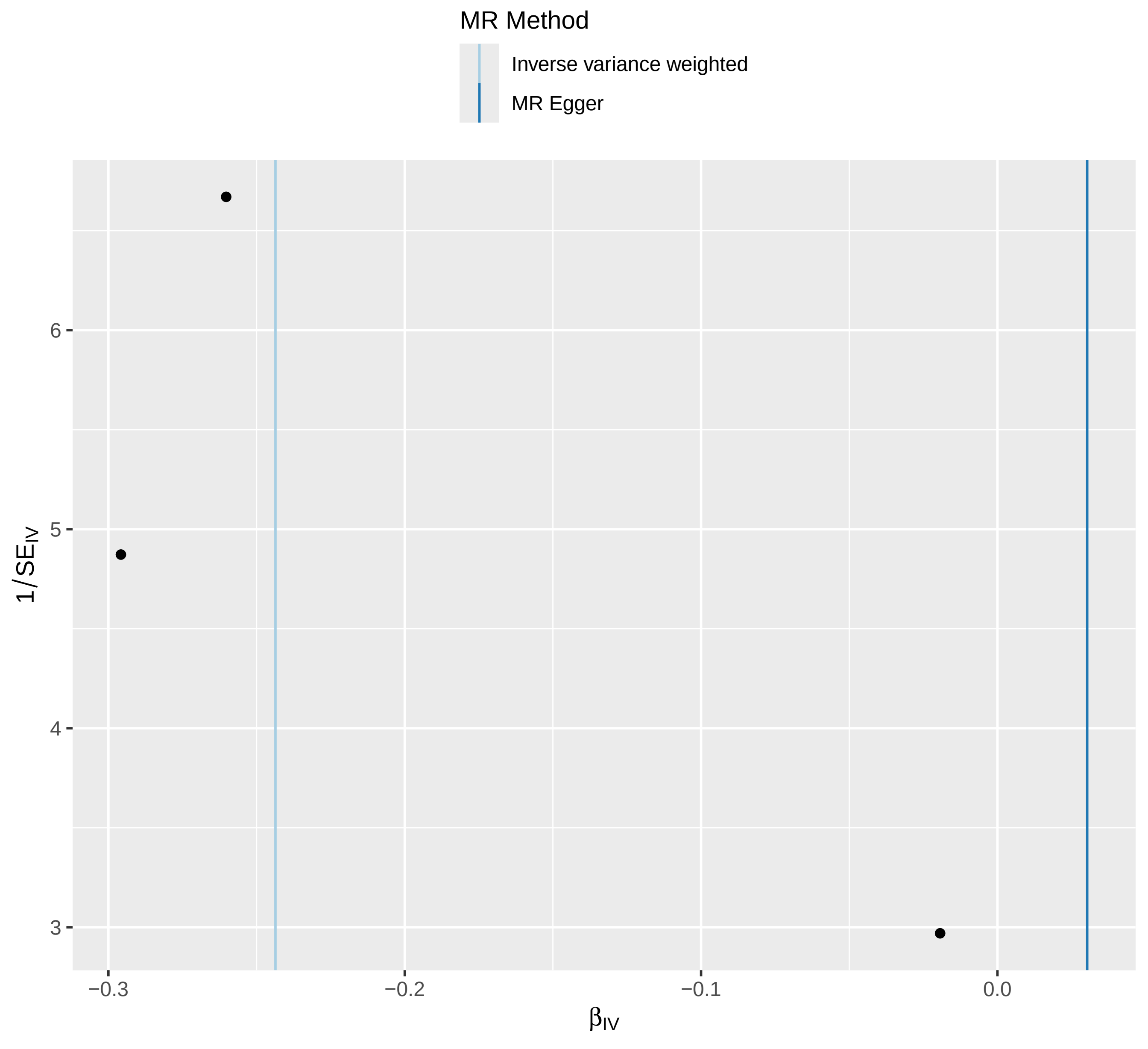
MR Method
Inverse variance weighted
MR Egger
1/SE_IV
6
5
4
3
−0.3
−0.2
−0.1
0.0
β_IV

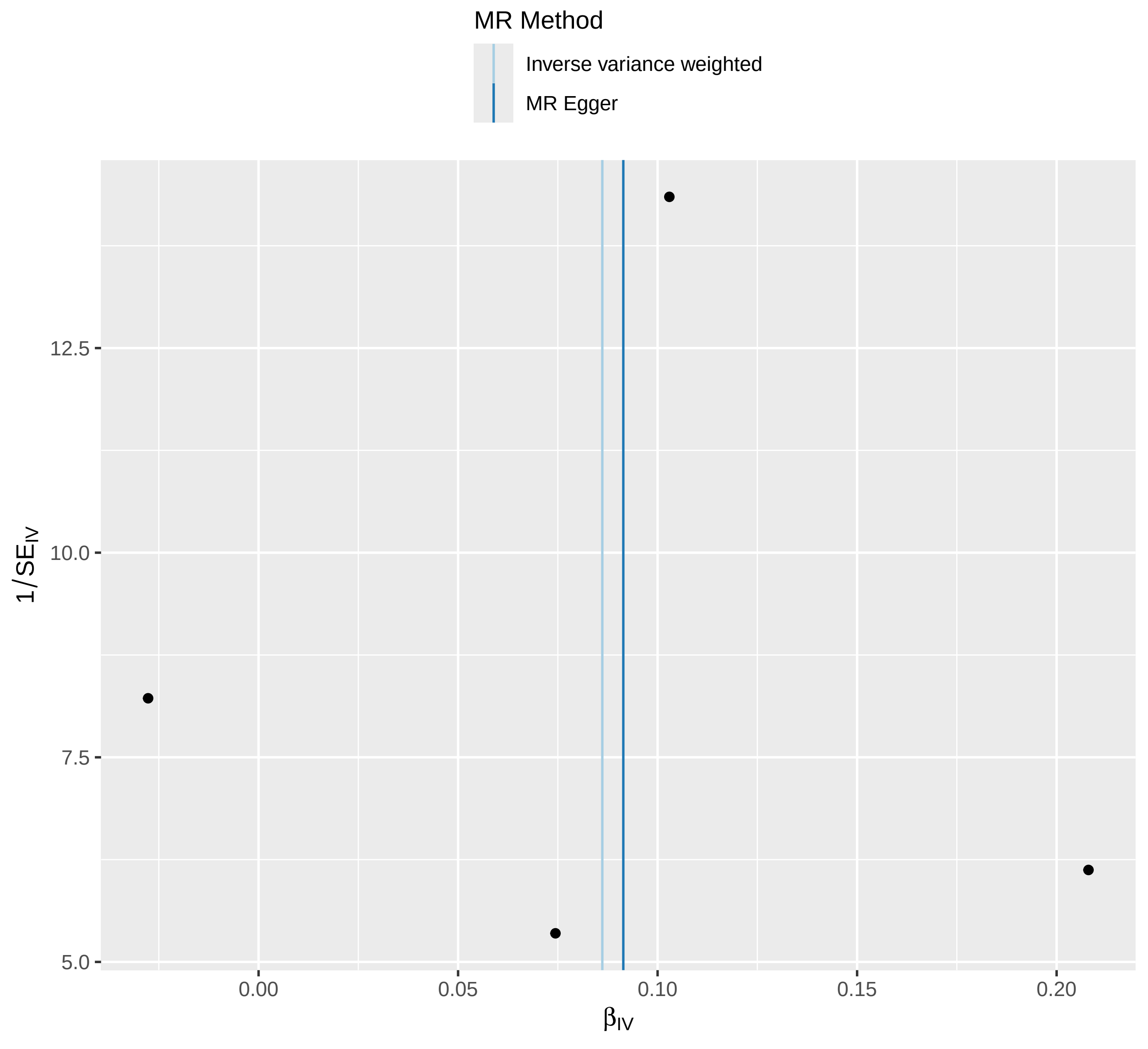

MR Method
Inverse variance weighted
MR Egger
12.5
10.0
7.5
5.0
1/SE_IV
0.00
0.05
0.10
0.15
0.20
β_IV

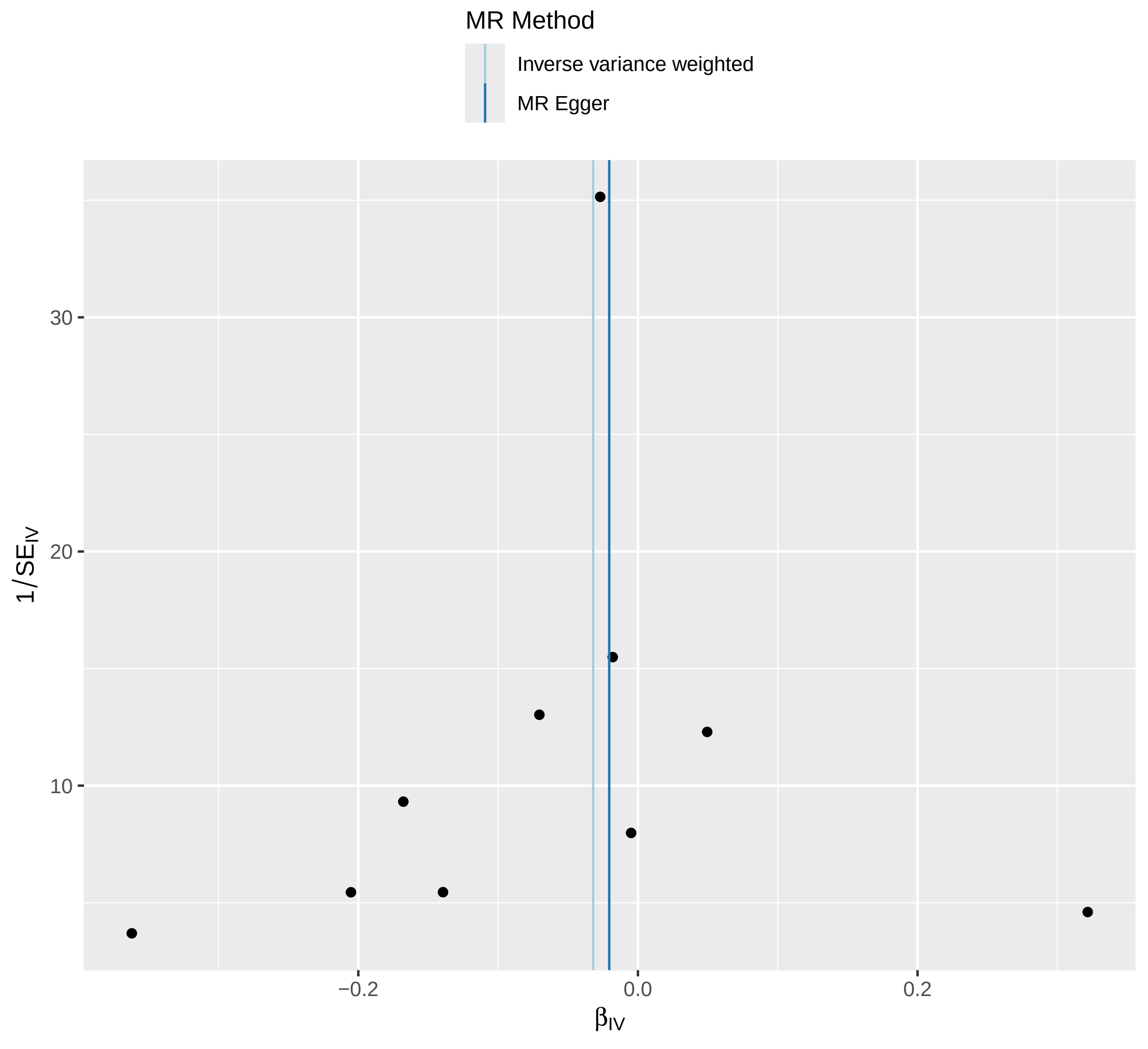
MR Method
Inverse variance weighted
MR Egger
30
20
10
1/SE$_{IV}$
−0.2
0.0
0.2
β$_{IV}$

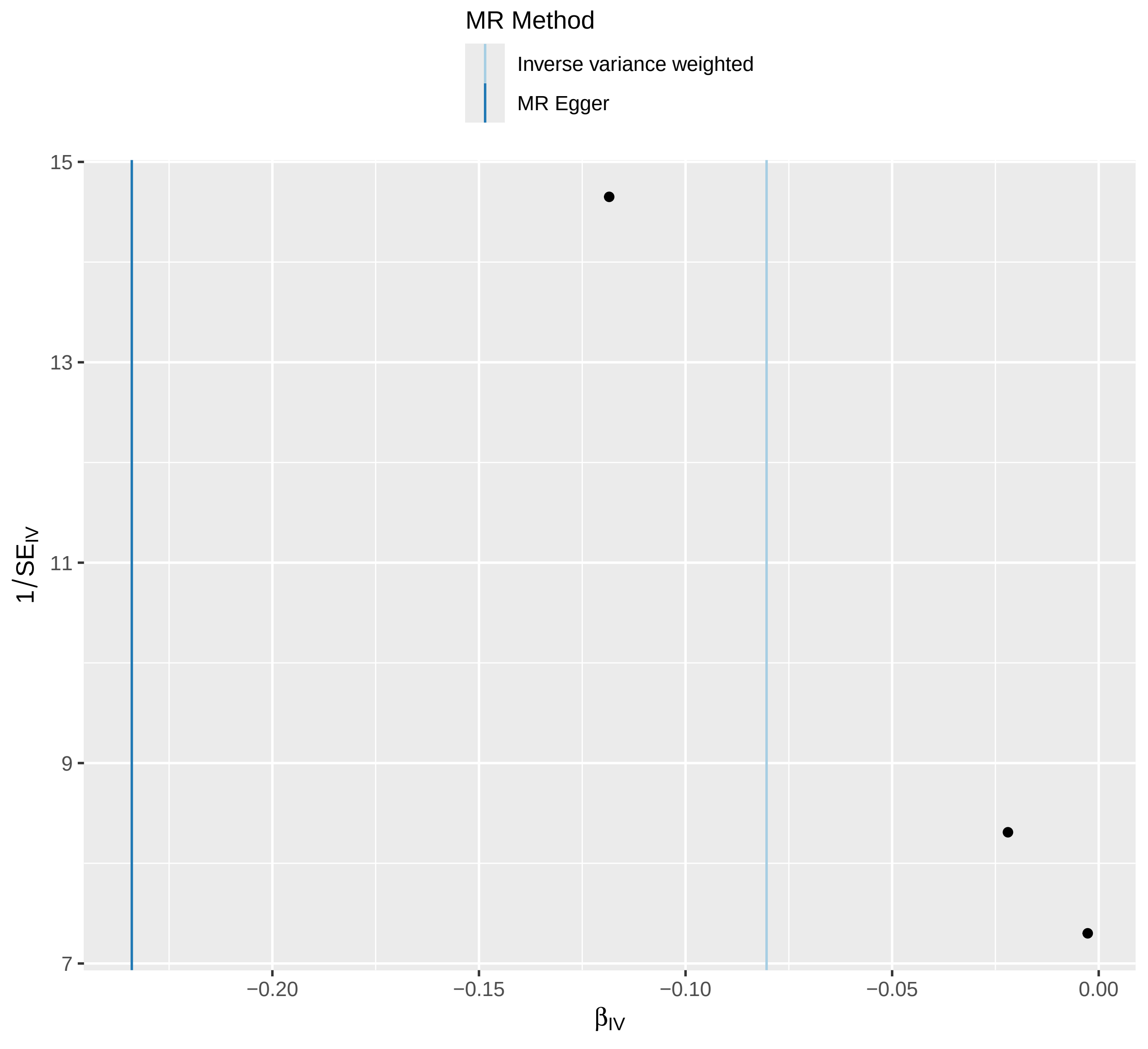
MR Method
Inverse variance weighted
MR Egger
15
13
11
9
7
1/SE$_{IV}$
−0.20
−0.15
−0.10
−0.05
0.00
β$_{IV}$

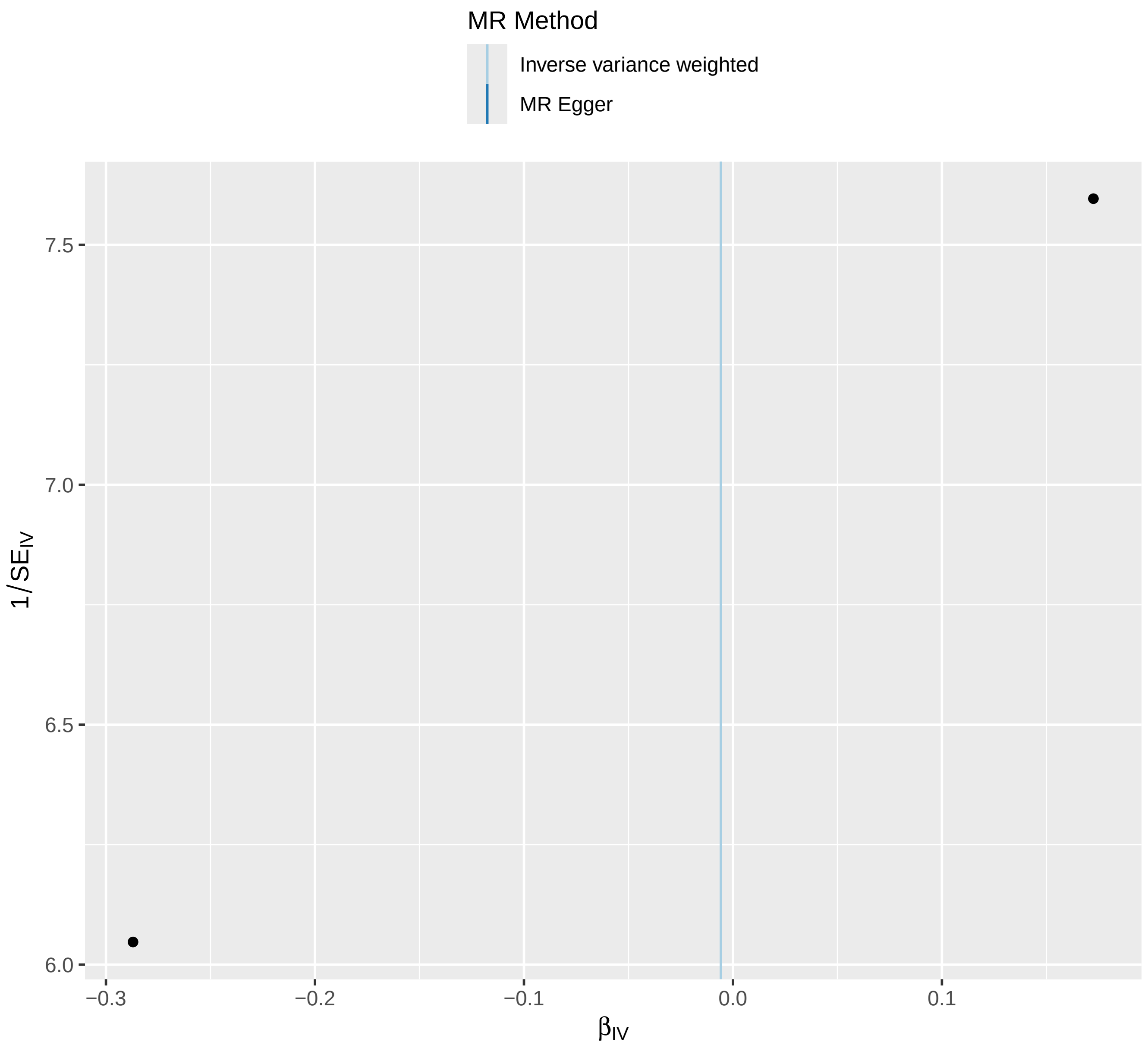

MR Method
Inverse variance weighted
MR Egger
7.5
7.0
6.5
6.0
1/SE$_{IV}$
−0.3
−0.2
−0.1
0.0
0.1
β$_{IV}$

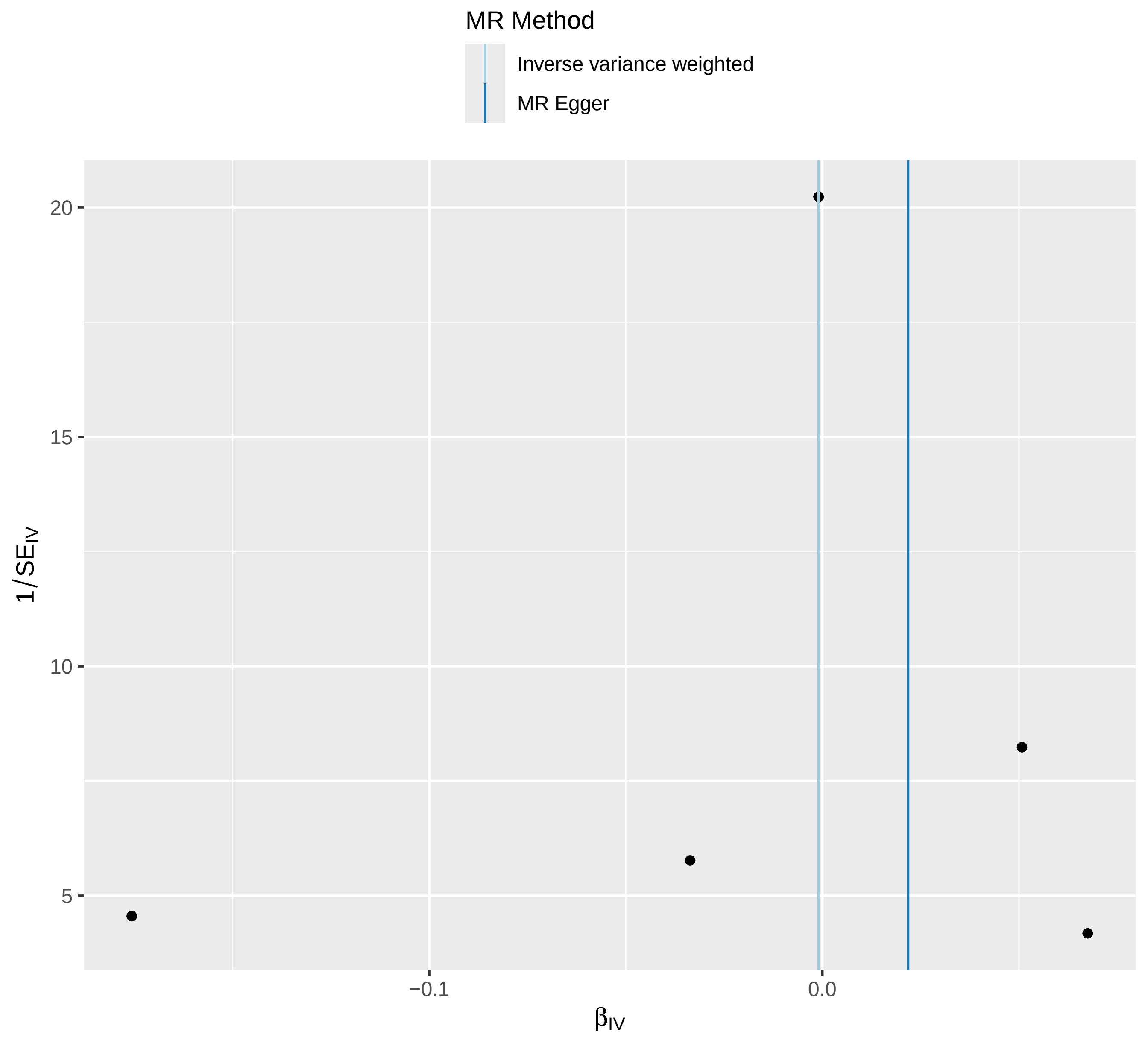
MR Method
Inverse variance weighted
MR Egger
20
15
10
5
1/SE_IV
−0.1
0.0
β_IV

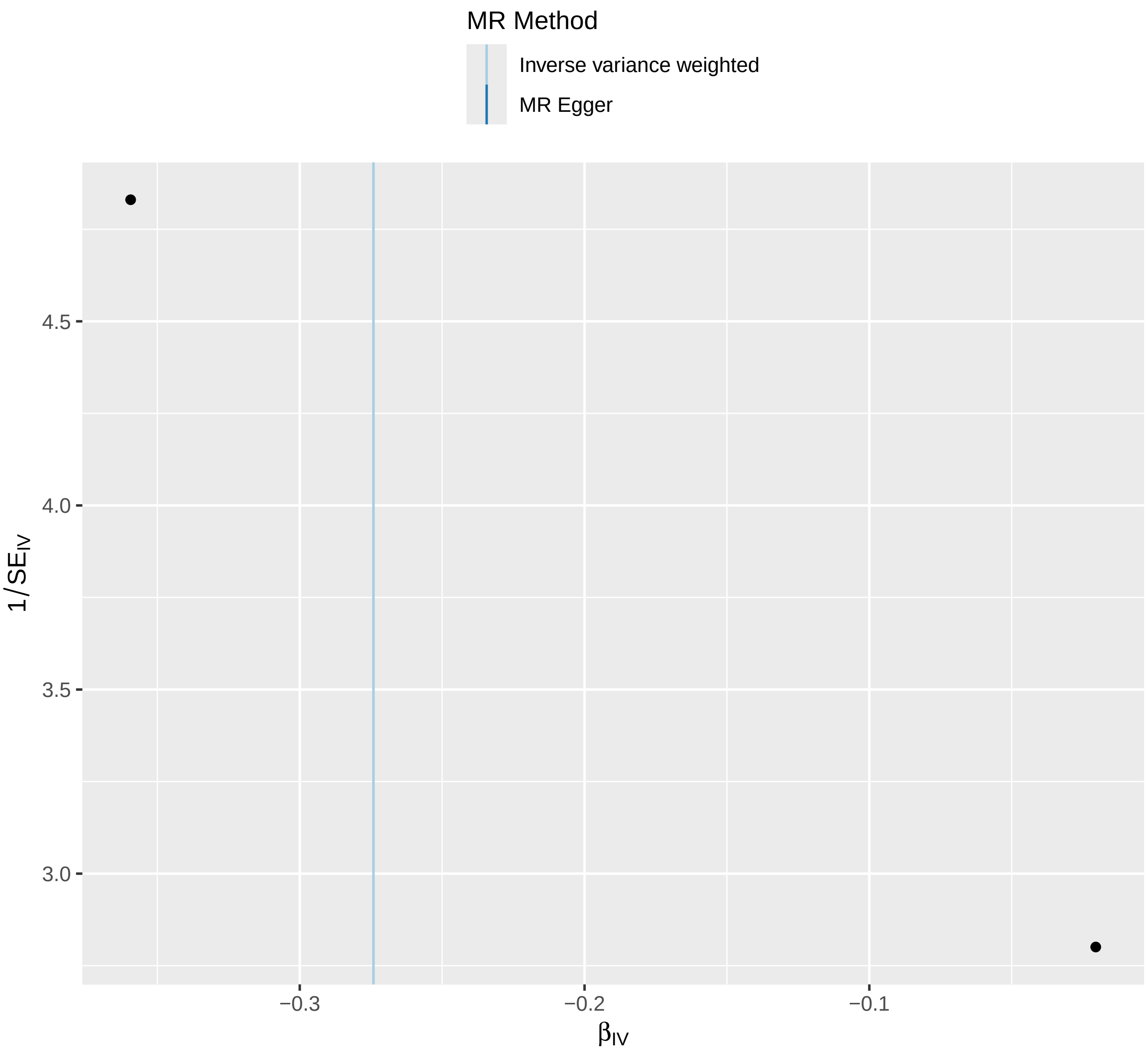

MR Method
Inverse variance weighted
MR Egger
4.5
4.0
3.5
3.0
1/SE_IV
−0.3
−0.2
−0.1
β_IV

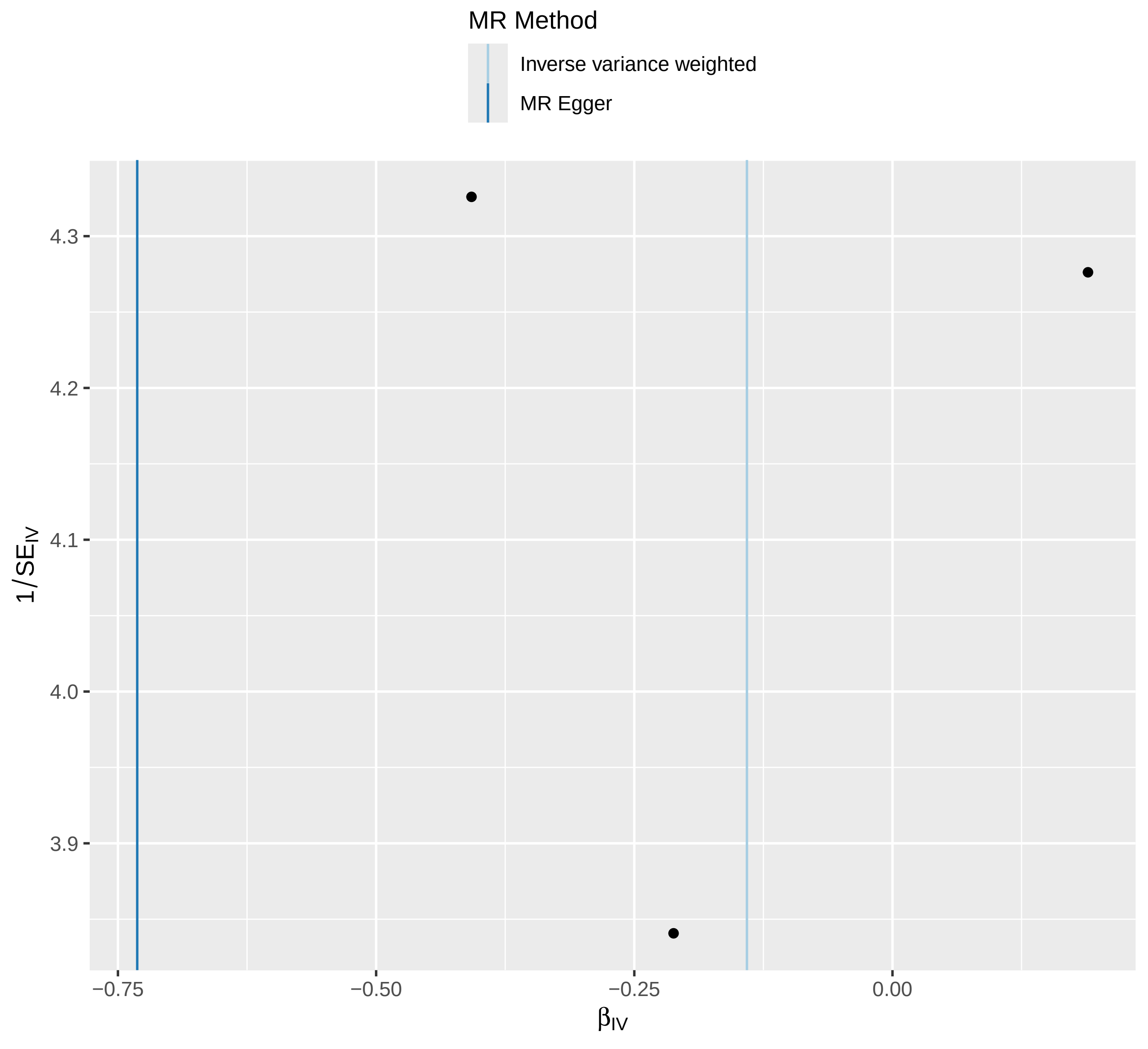
MR Method
Inverse variance weighted
MR Egger
4.3
4.2
4.1
4.0
3.9
1/SE_IV
−0.75
−0.50
−0.25
0.00
β_IV

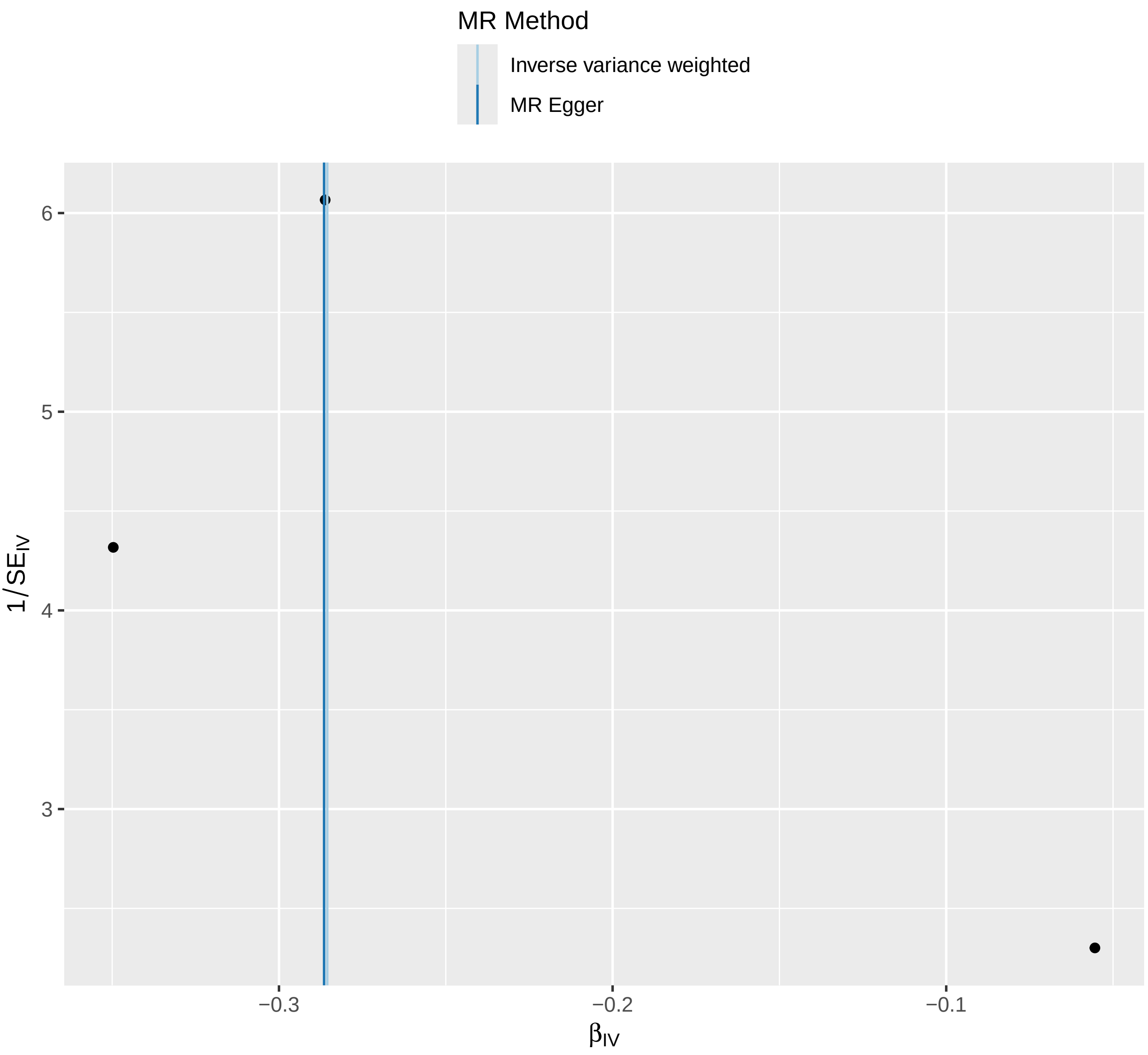
MR Method
Inverse variance weighted
MR Egger
1/SE$_{IV}$
6
5
4
3
−0.3
−0.2
−0.1
β$_{IV}$

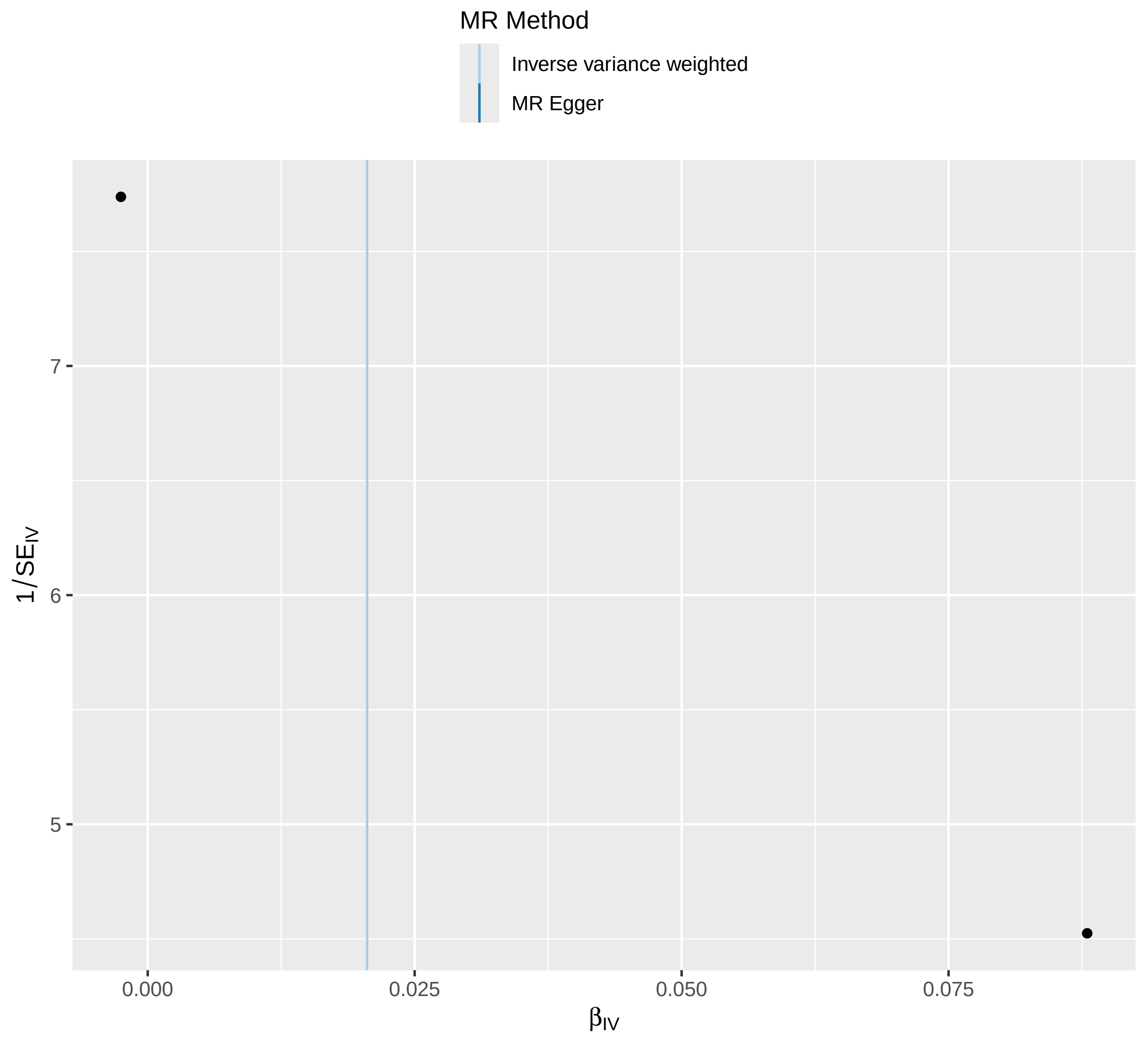
MR Method
Inverse variance weighted
MR Egger
1/SE_IV
7
6
5
0.000
0.025
0.050
0.075
β_IV

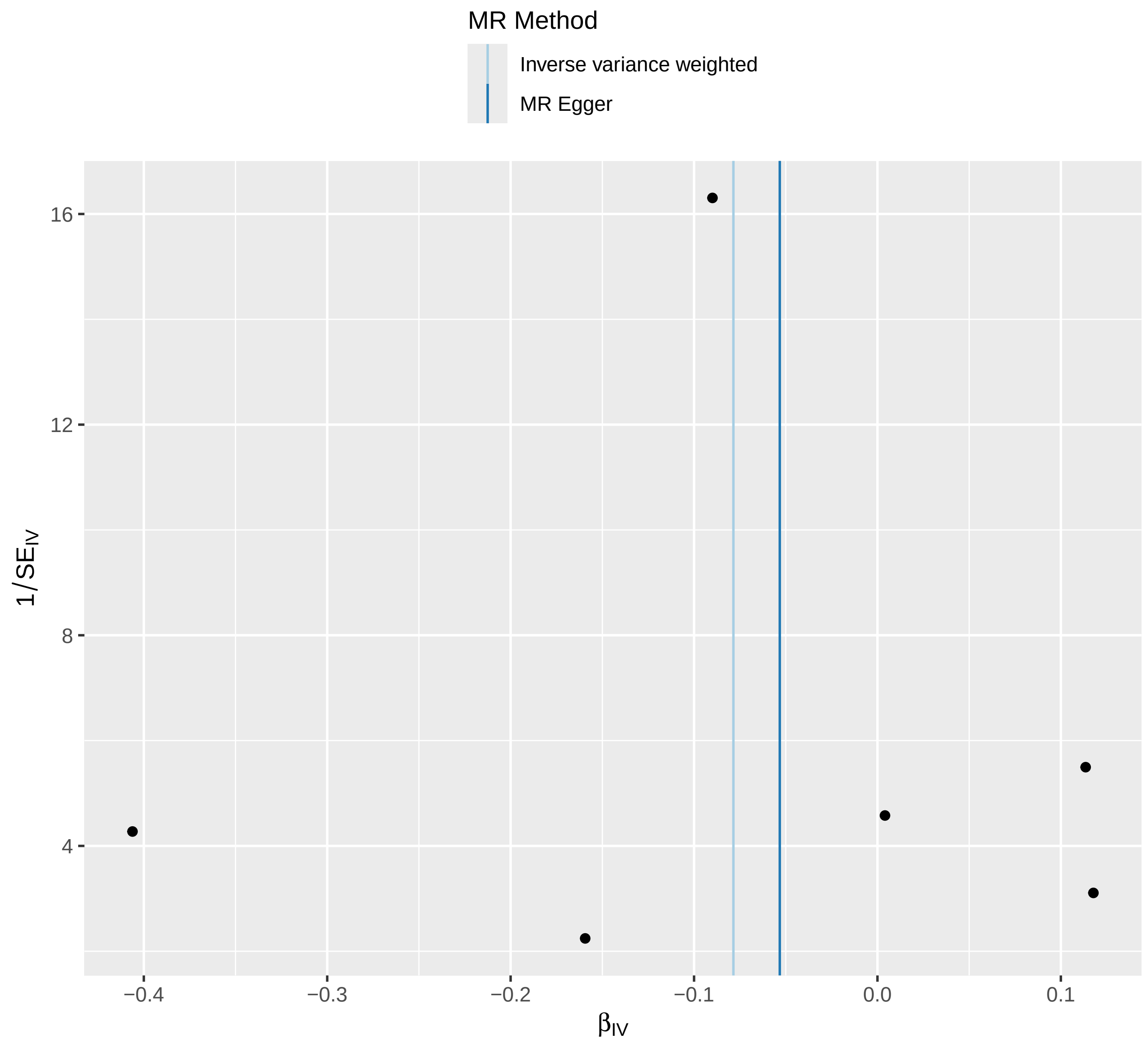

MR Method
Inverse variance weighted
MR Egger
16
12
8
4
1/SE$_{IV}$
−0.4
−0.3
−0.2
−0.1
0.0
0.1
β$_{IV}$

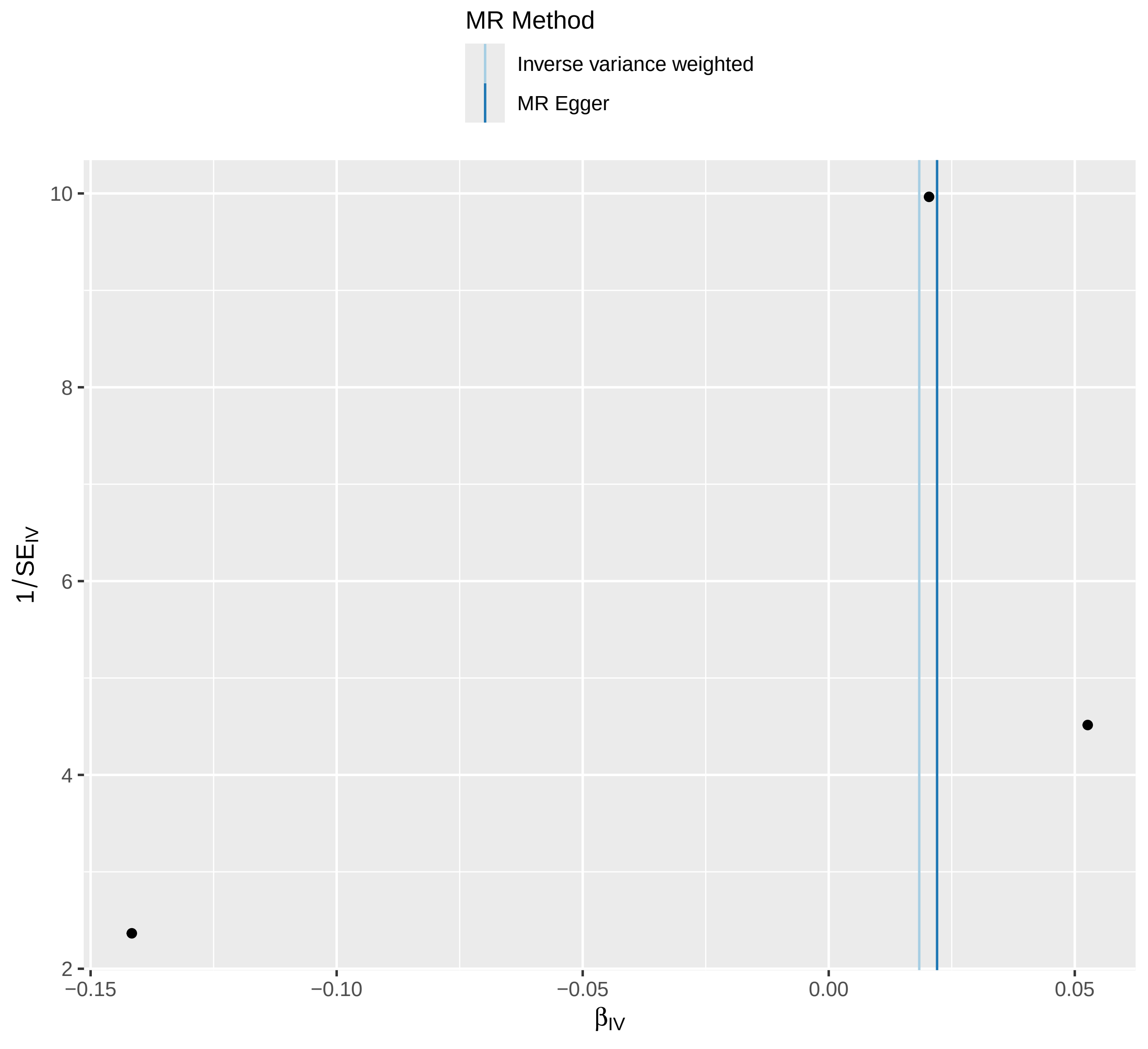

MR Method
Inverse variance weighted
MR Egger
10
8
6
4
2
1/SE_IV
−0.15
−0.10
−0.05
0.00
0.05
β_IV

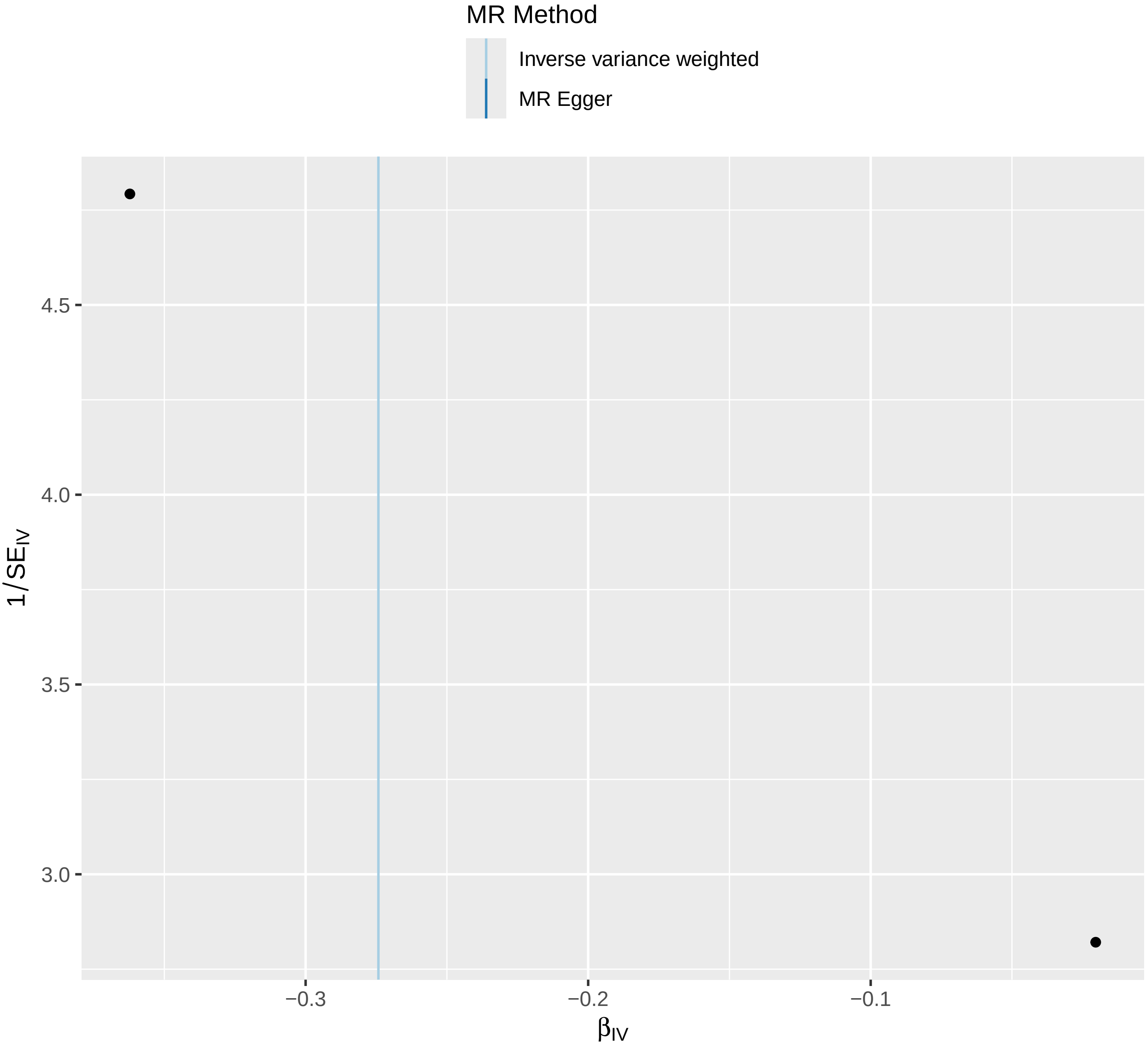

MR Method
Inverse variance weighted
MR Egger
4.5
4.0
3.5
3.0
1/SE$_{IV}$
−0.3
−0.2
−0.1
$\beta_{IV}$

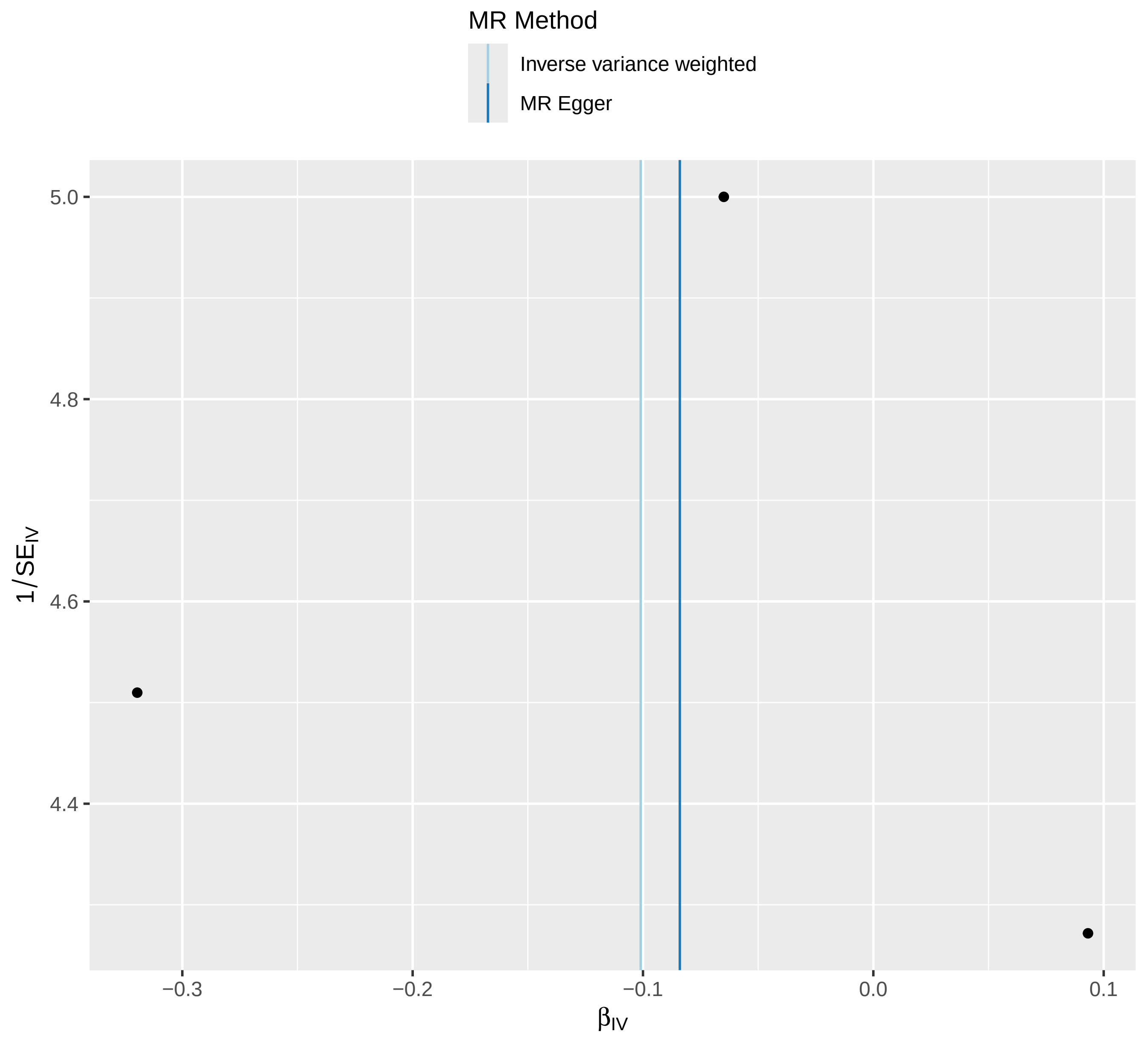

MR Method
Inverse variance weighted
MR Egger
5.0
4.8
4.6
4.4
1/SE_IV
−0.3
−0.2
−0.1
0.0
0.1
β_IV

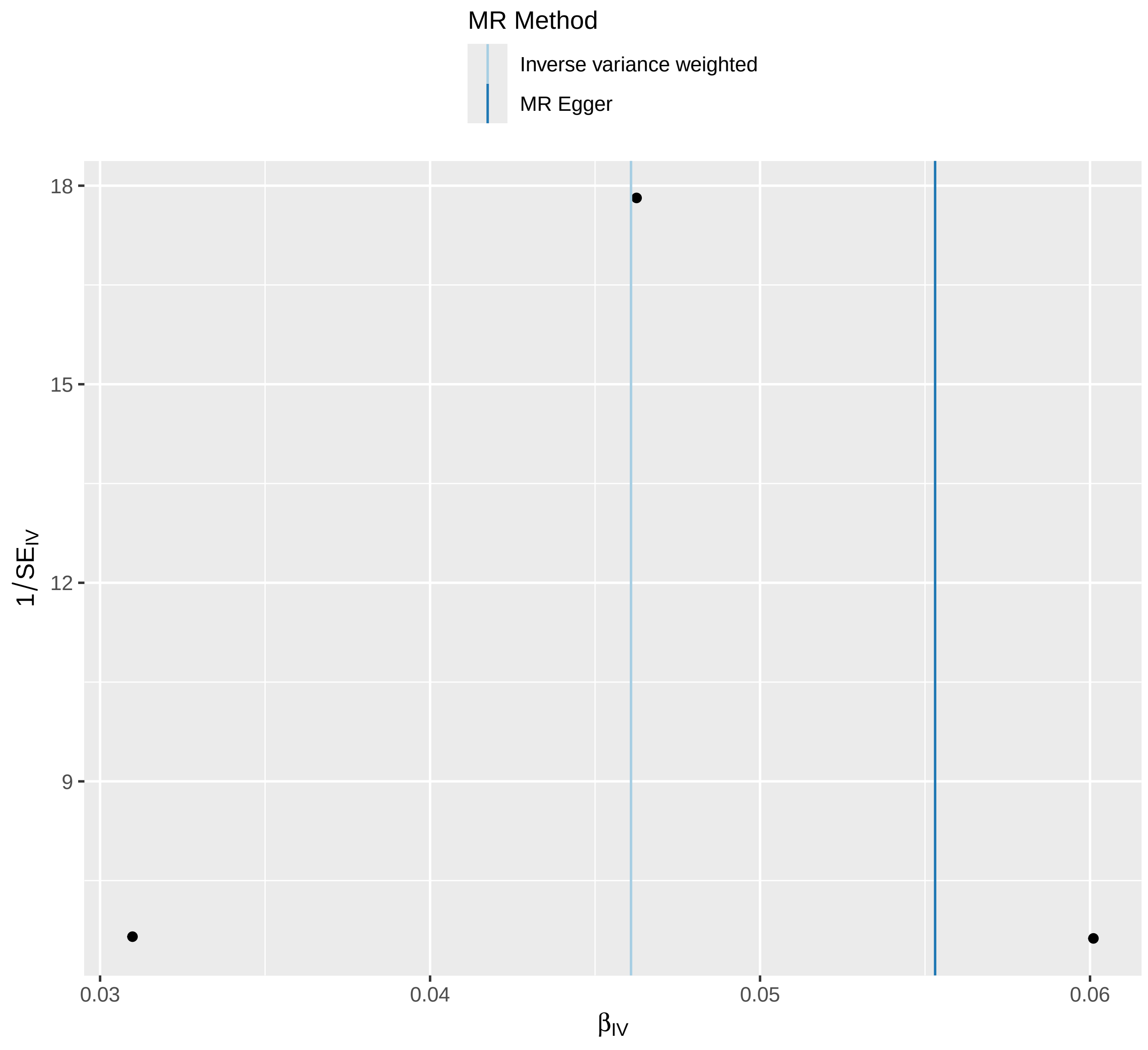
MR Method
Inverse variance weighted
MR Egger
18
15
12
9
1/SE_IV
0.03
0.04
0.05
0.06
β_IV

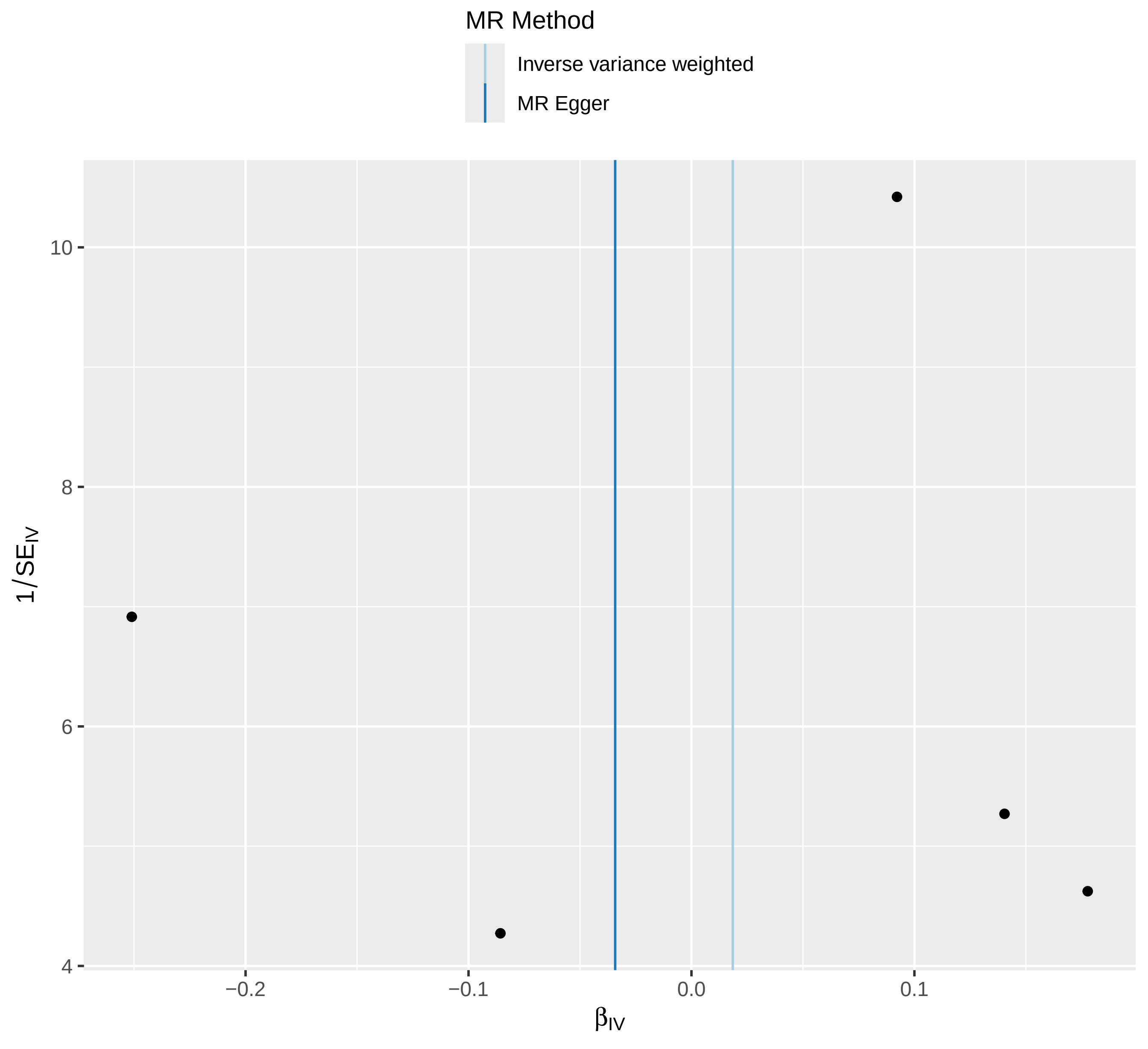
MR Method
Inverse variance weighted
MR Egger
10
8
6
4
1/SE$_{IV}$
−0.2
−0.1
0.0
0.1
β$_{IV}$

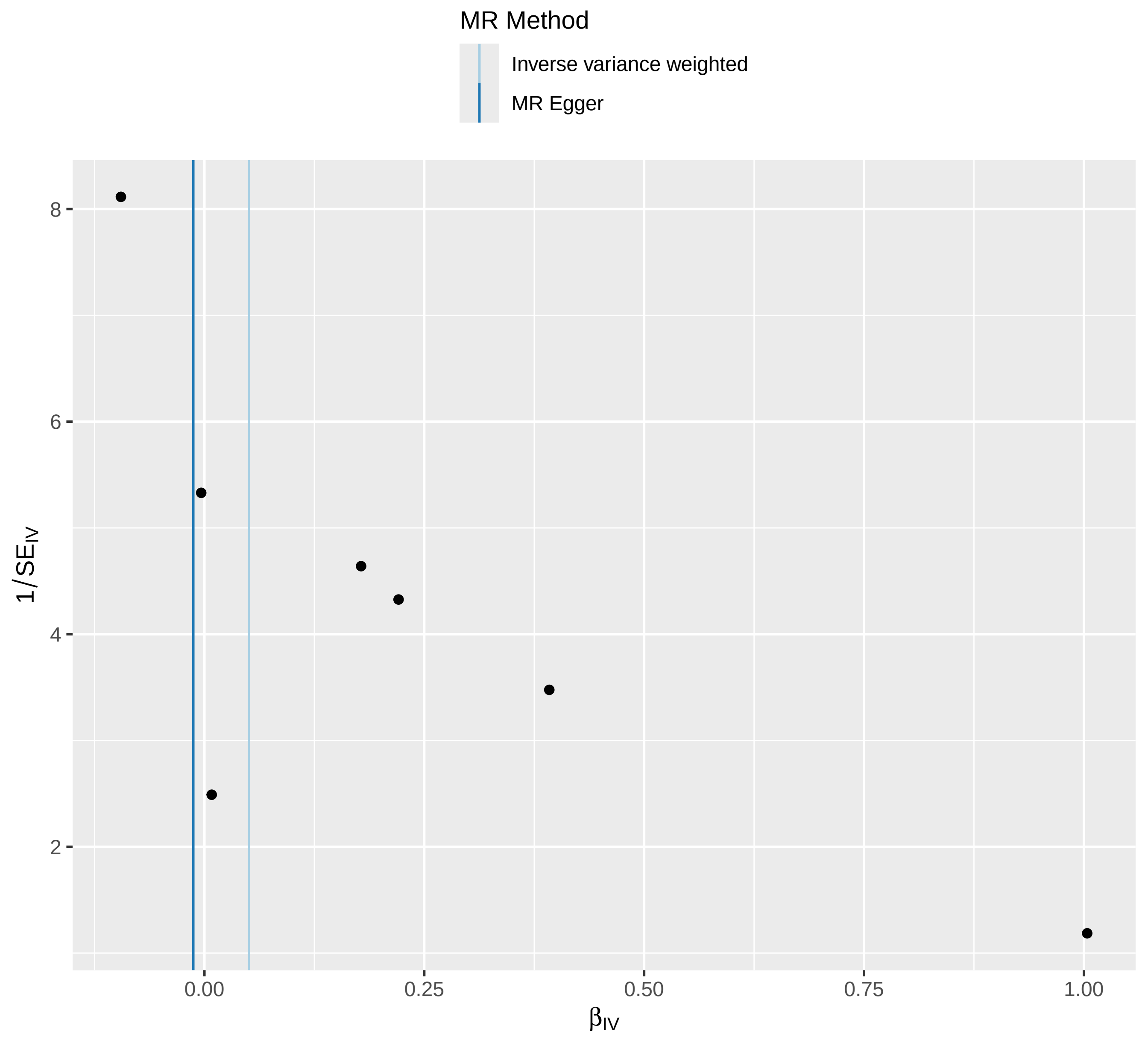
MR Method
Inverse variance weighted
MR Egger
8
6
4
2
1/SE_IV
0.00
0.25
0.50
0.75
1.00
β_IV

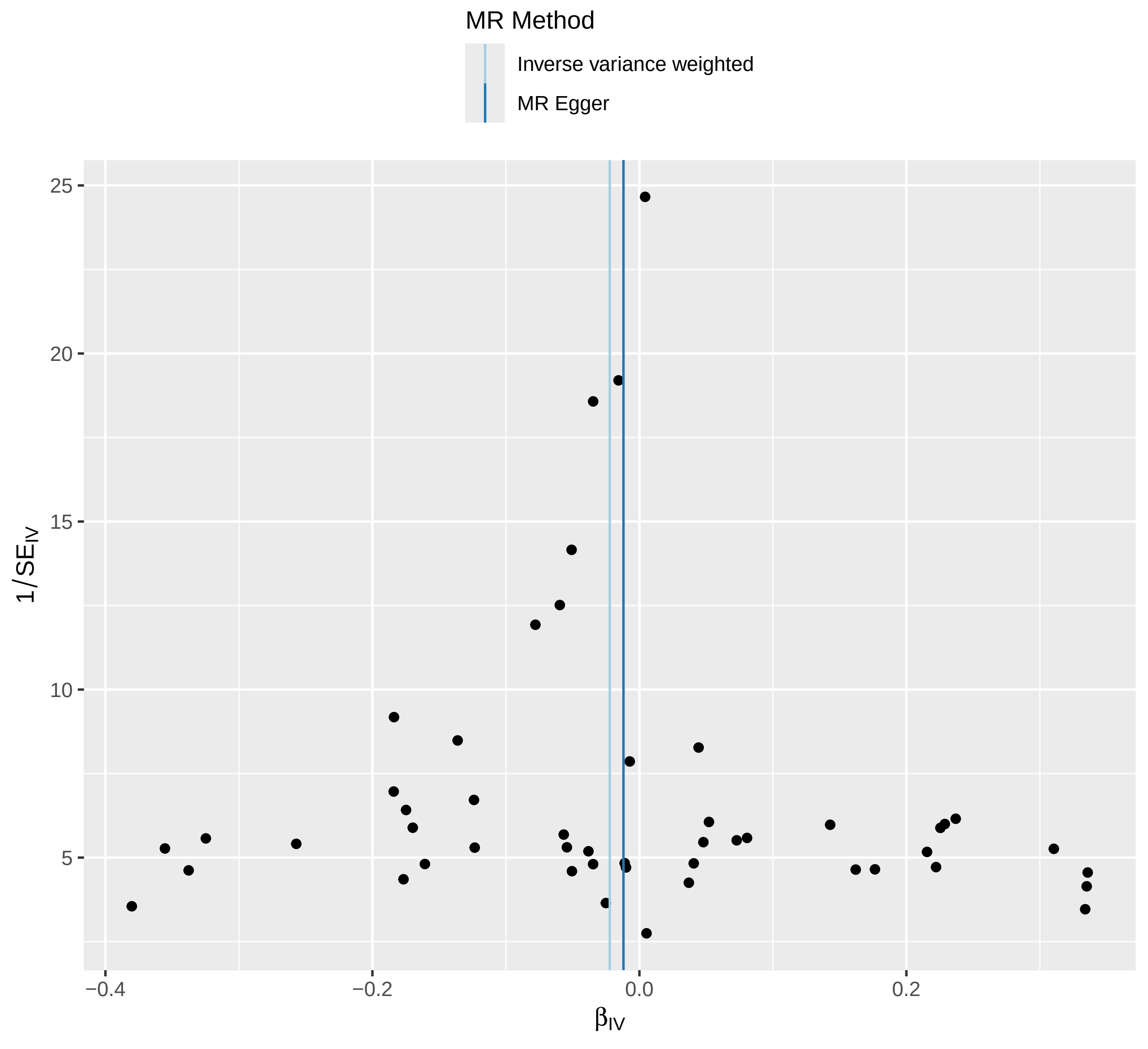
MR Method
Inverse variance weighted
MR Egger
25
20
15
10
5
$1/SE_{IV}$
−0.4
−0.2
0.0
0.2
$\beta_{IV}$

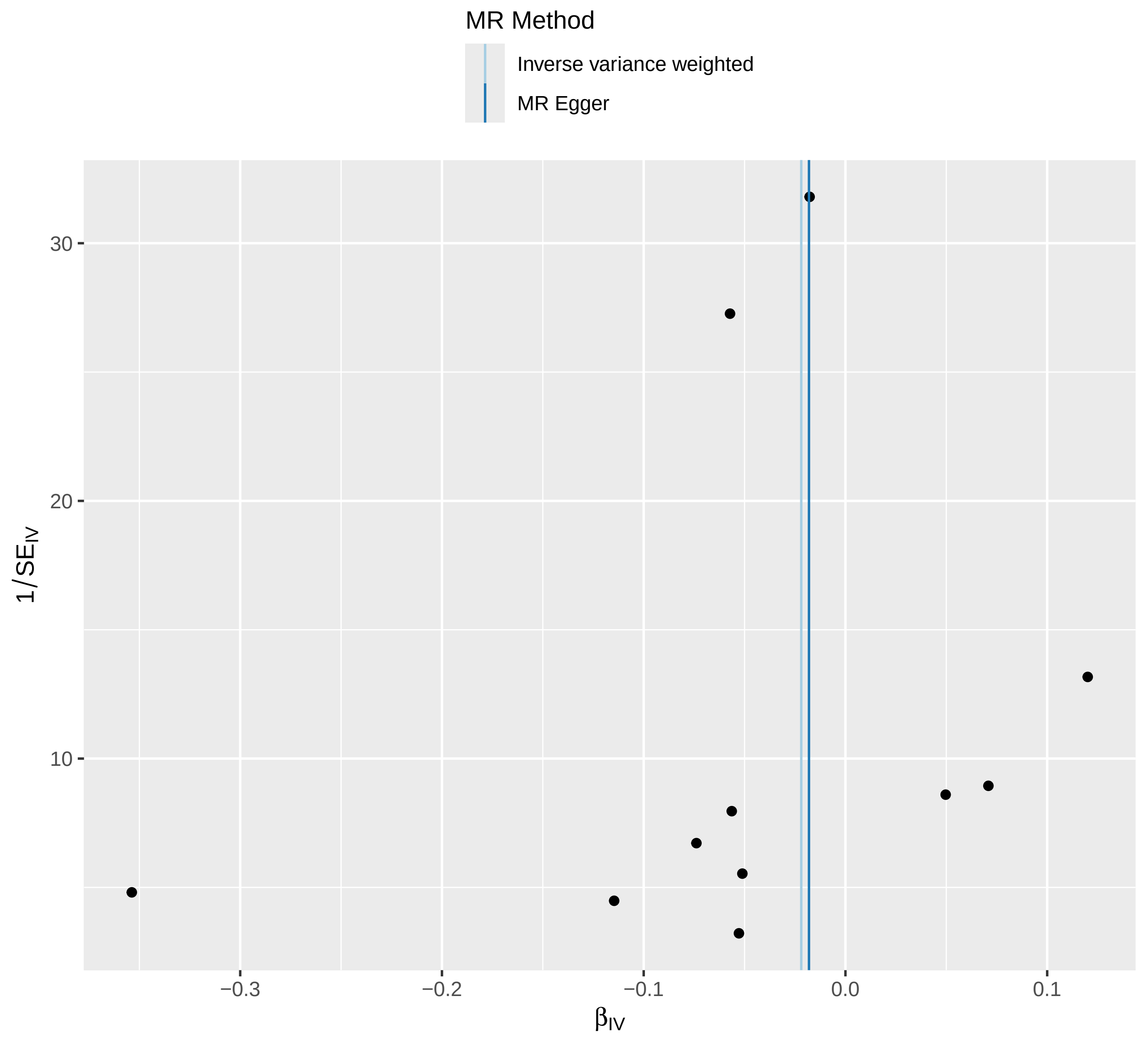
MR Method
Inverse variance weighted
MR Egger
30
20
10
1/$SE_{IV}$
−0.3
−0.2
−0.1
0.0
0.1
$β_{IV}$

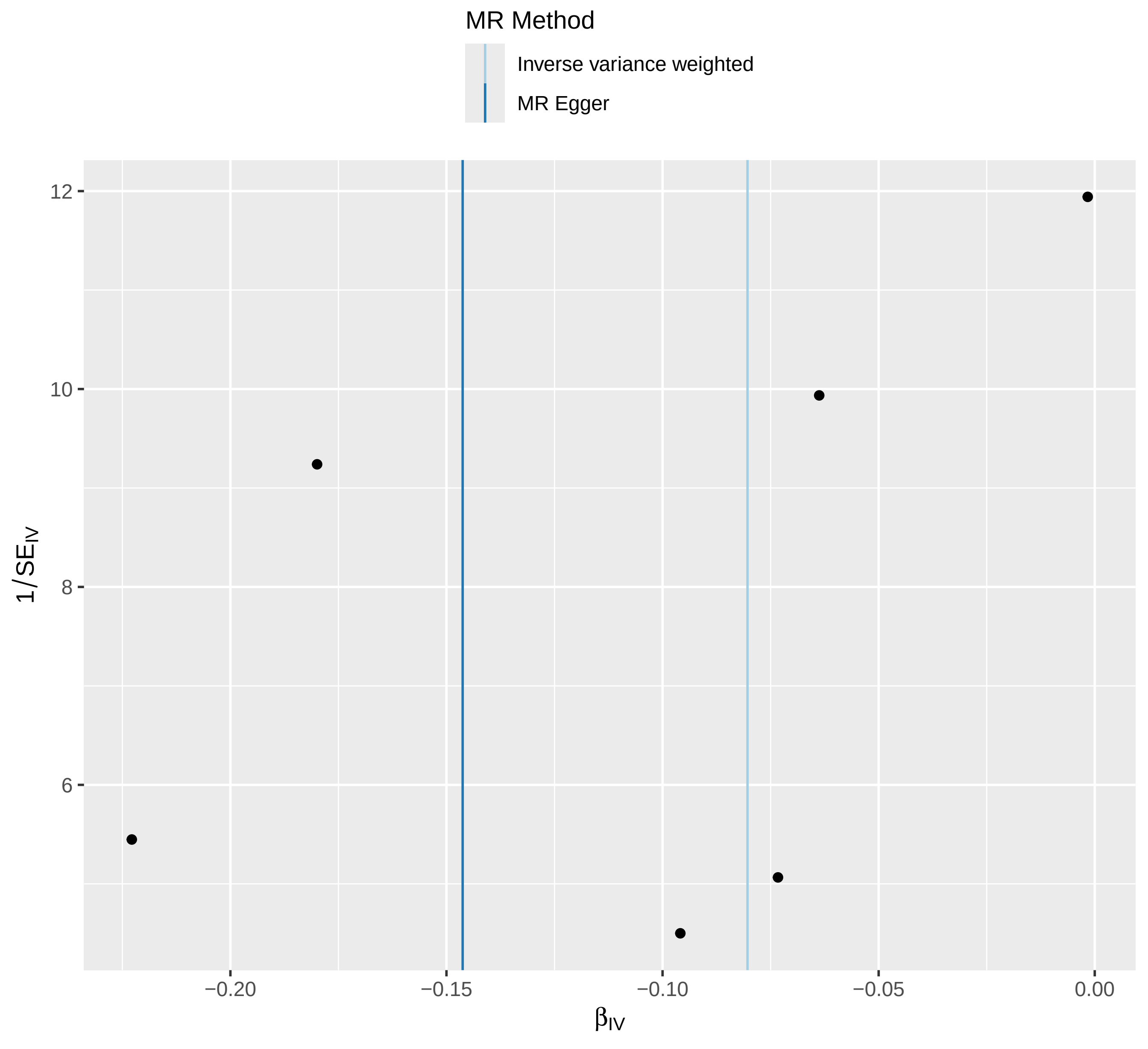

MR Method
Inverse variance weighted
MR Egger
12
10
8
6
$1/SE_{IV}$
−0.20
−0.15
−0.10
−0.05
0.00
$\beta_{IV}$

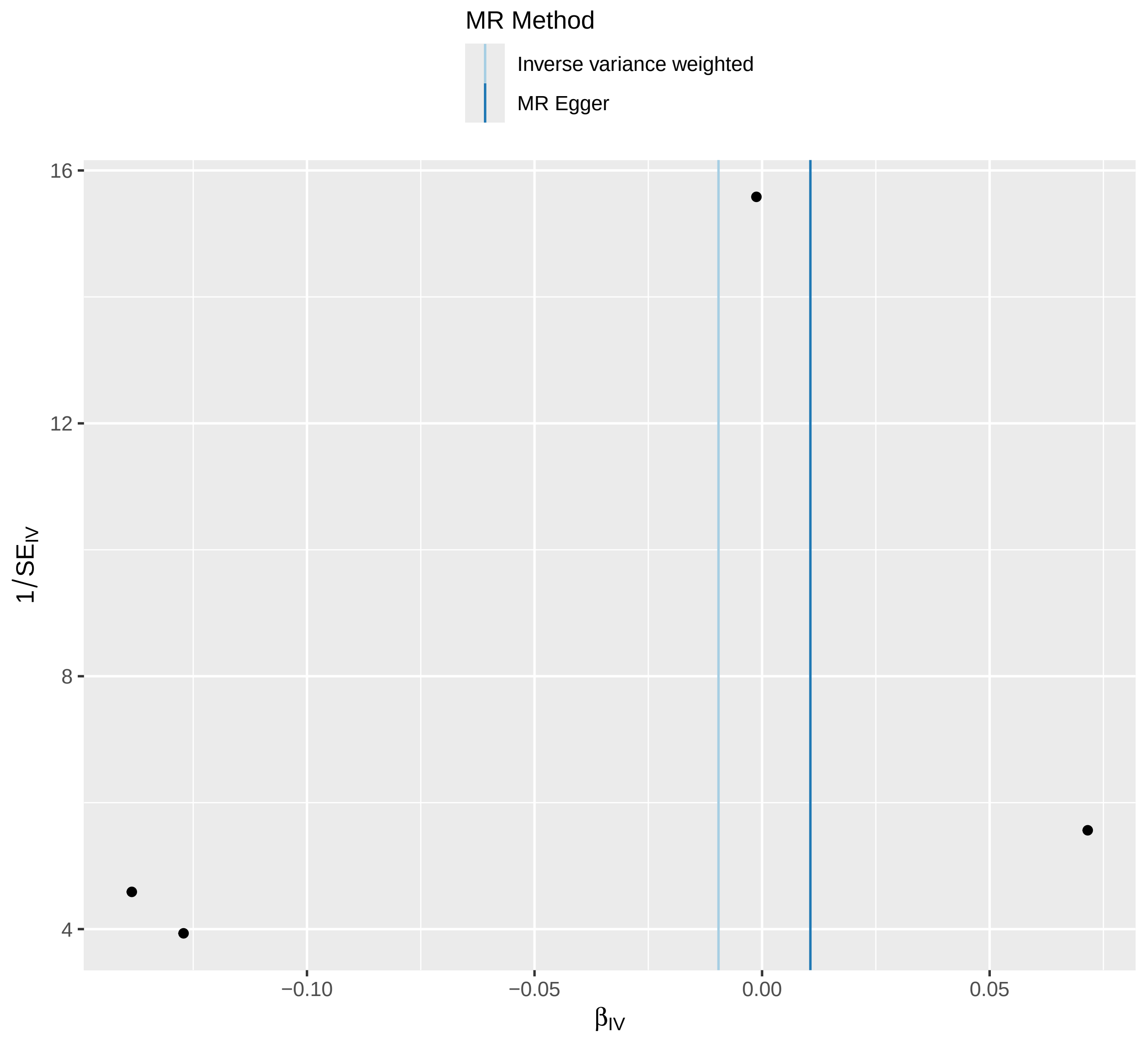
MR Method
Inverse variance weighted
MR Egger
16
12
8
4
−0.10
−0.05
0.00
0.05
$1/SE_{IV}$
$\beta_{IV}$

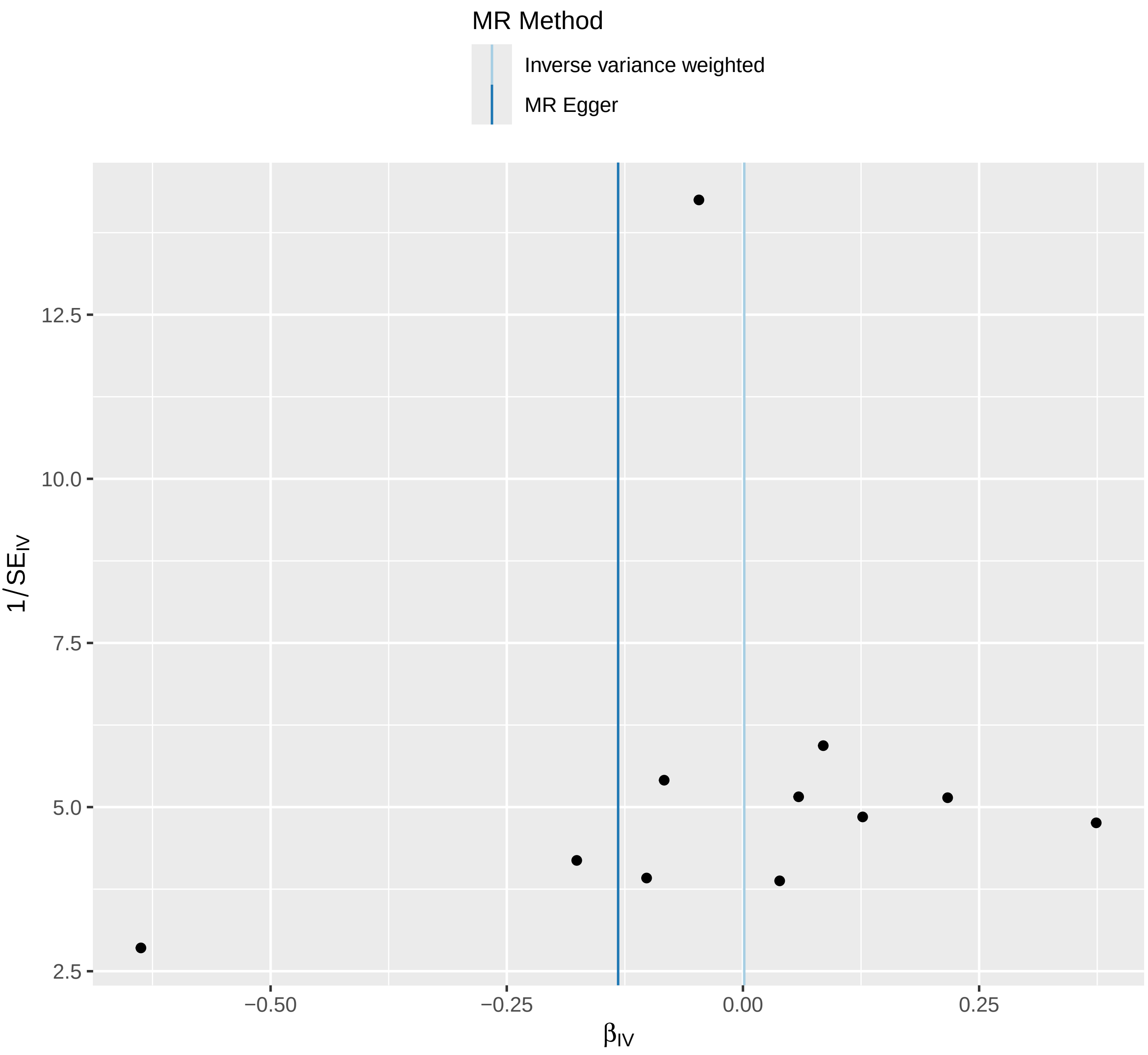
MR Method
Inverse variance weighted
MR Egger
12.5
10.0
7.5
5.0
2.5
1/SE$_{IV}$
−0.50
−0.25
0.00
0.25
β$_{IV}$

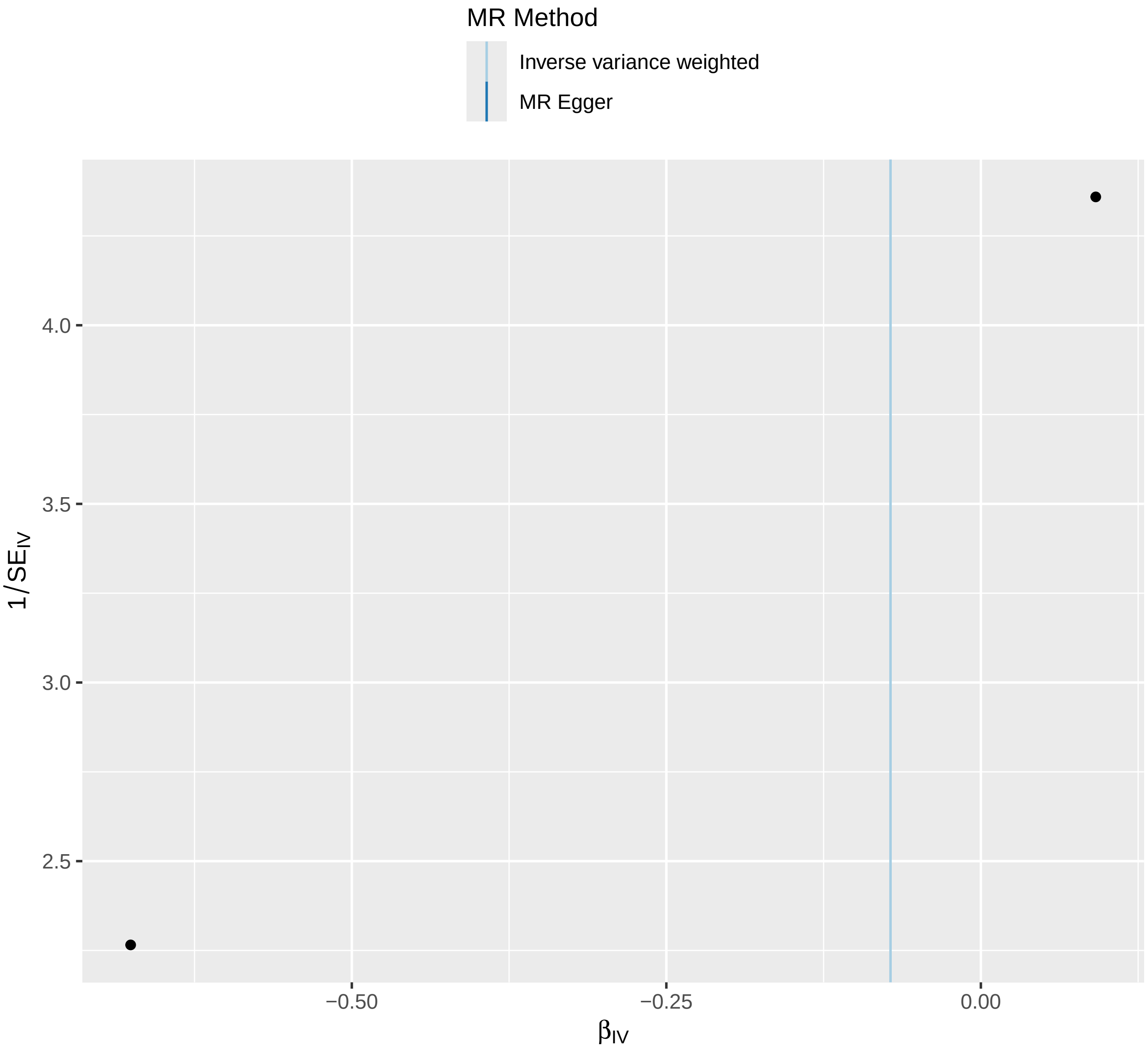
MR Method
Inverse variance weighted
MR Egger
1/SE_IV
4.0
3.5
3.0
2.5
−0.50
−0.25
0.00
β_IV

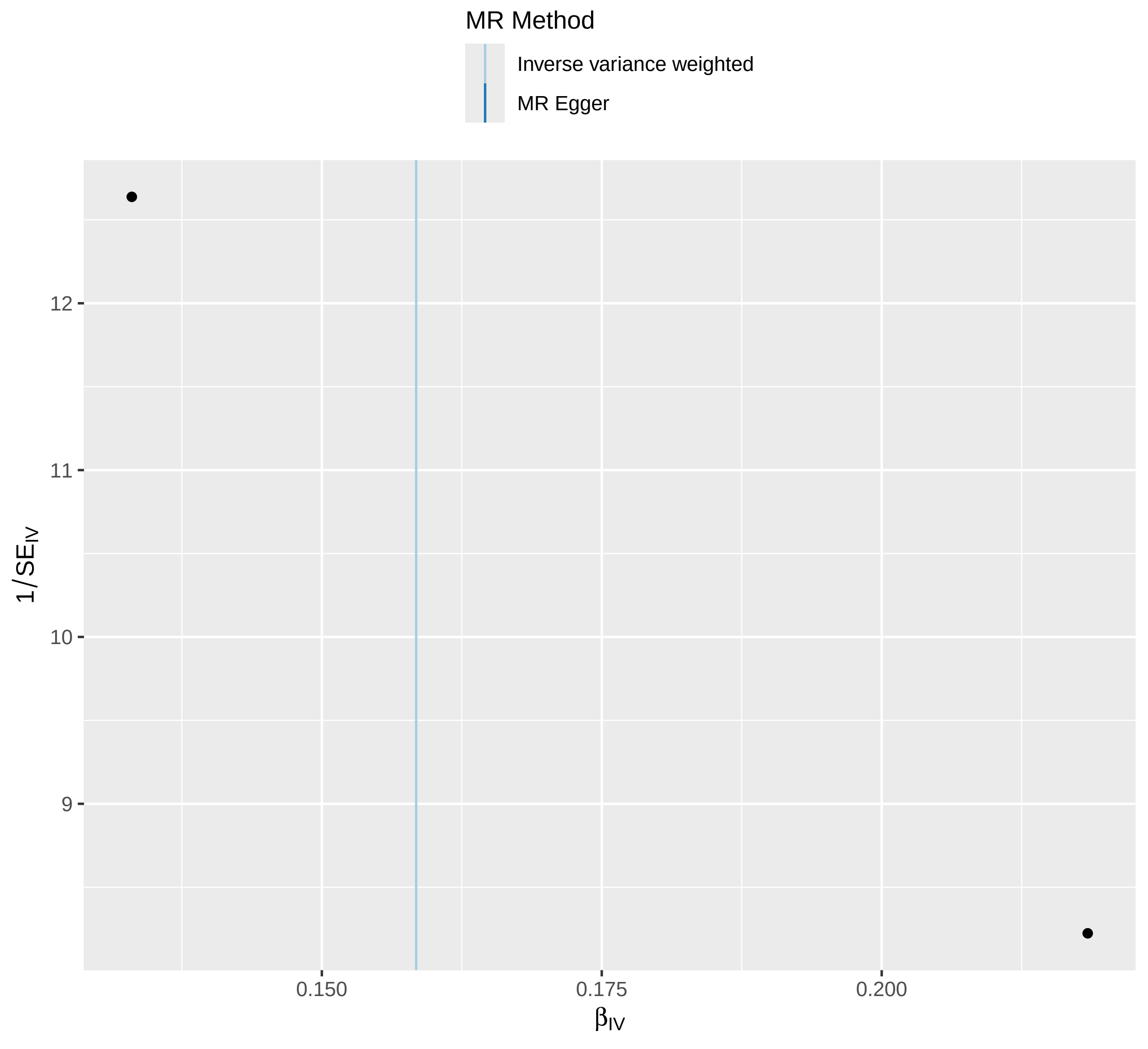

MR Method
Inverse variance weighted
MR Egger
12
11
10
9
1/SE$_{IV}$
0.150
0.175
0.200
β$_{IV}$

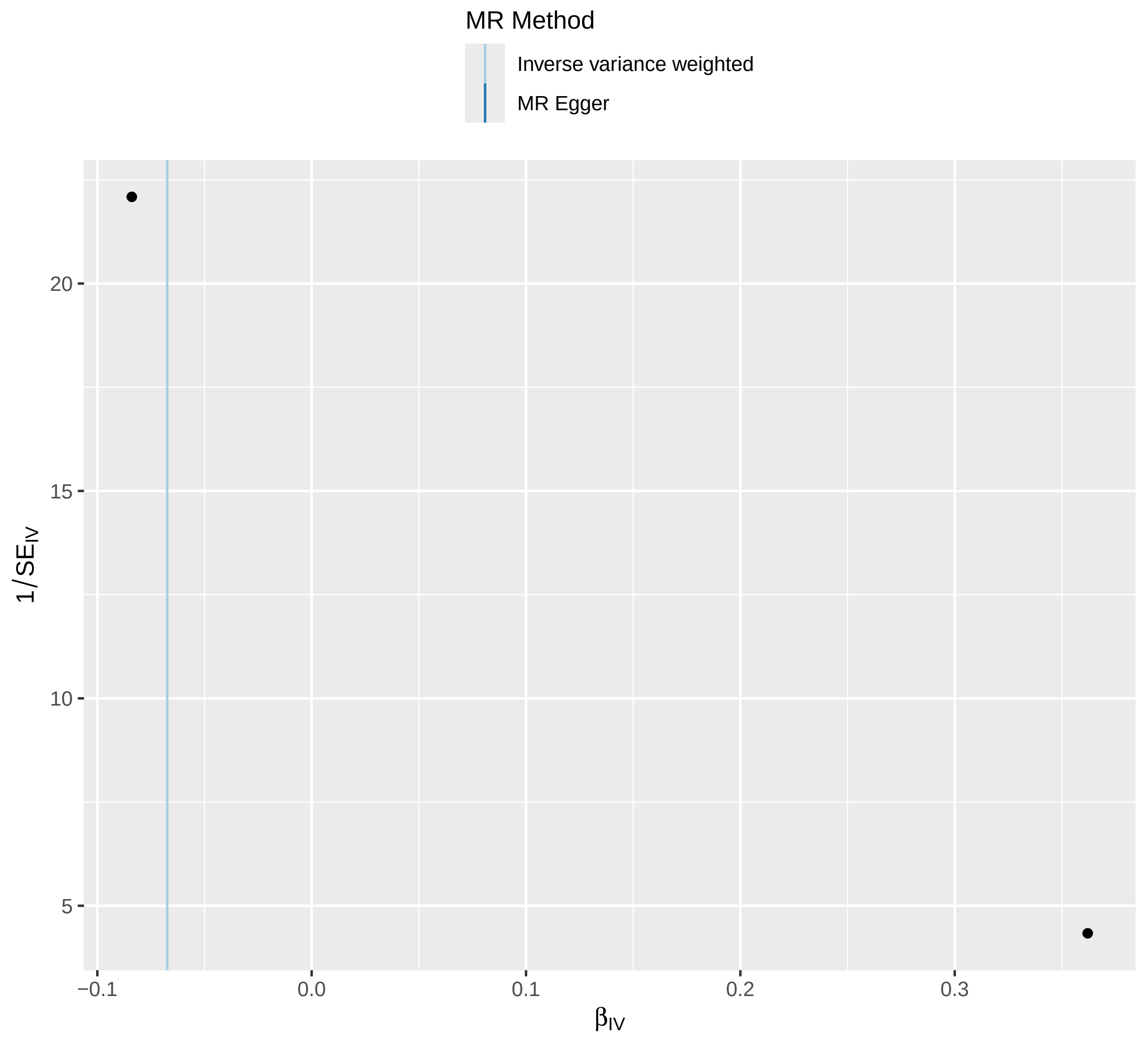

MR Method
Inverse variance weighted
MR Egger
20
15
10
5
−0.1
0.0
0.1
0.2
0.3
1/SE_IV
β_IV

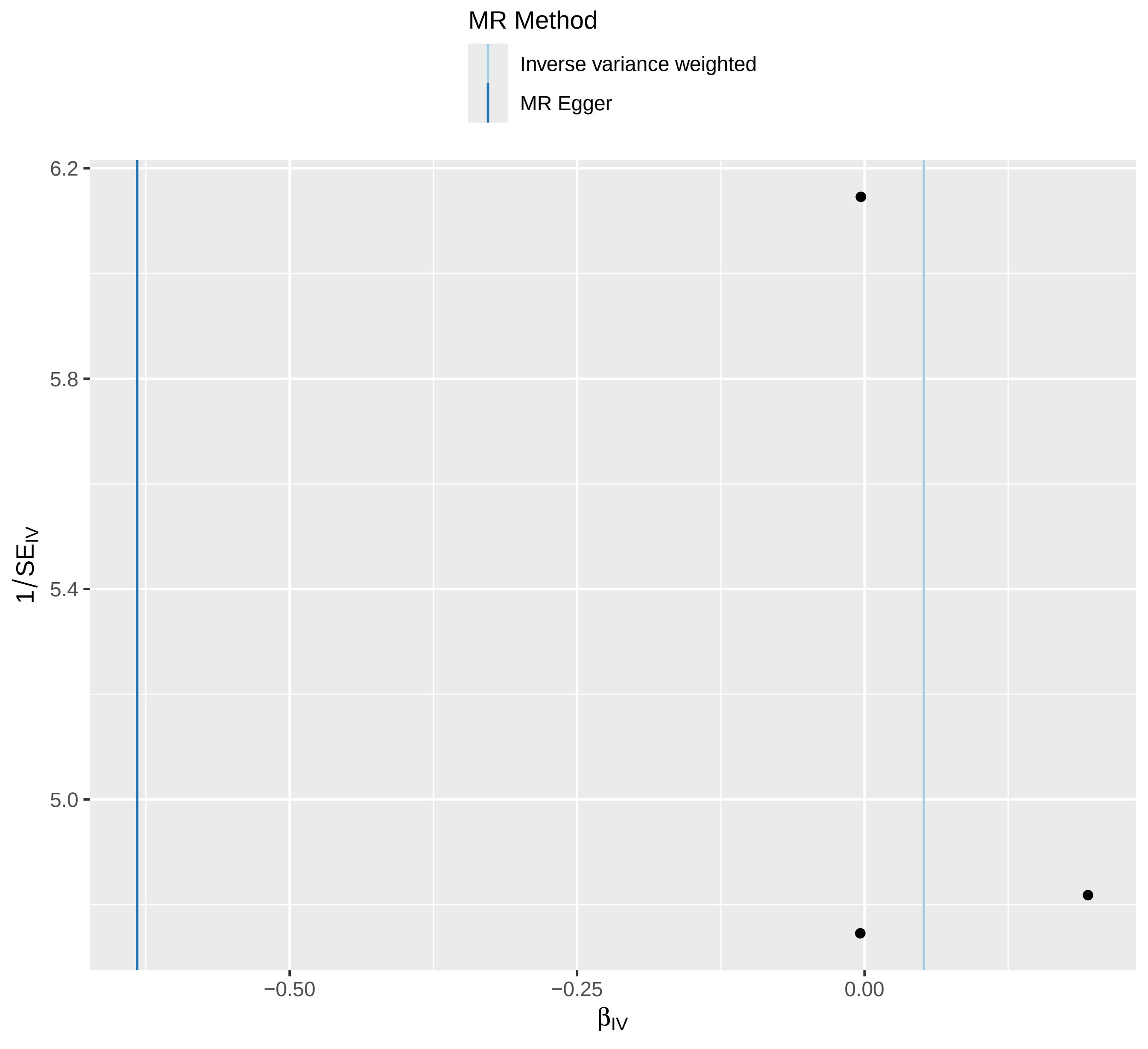
MR Method
Inverse variance weighted
MR Egger
6.2
5.8
5.4
5.0
1/SE_IV
−0.50
−0.25
0.00
β_IV

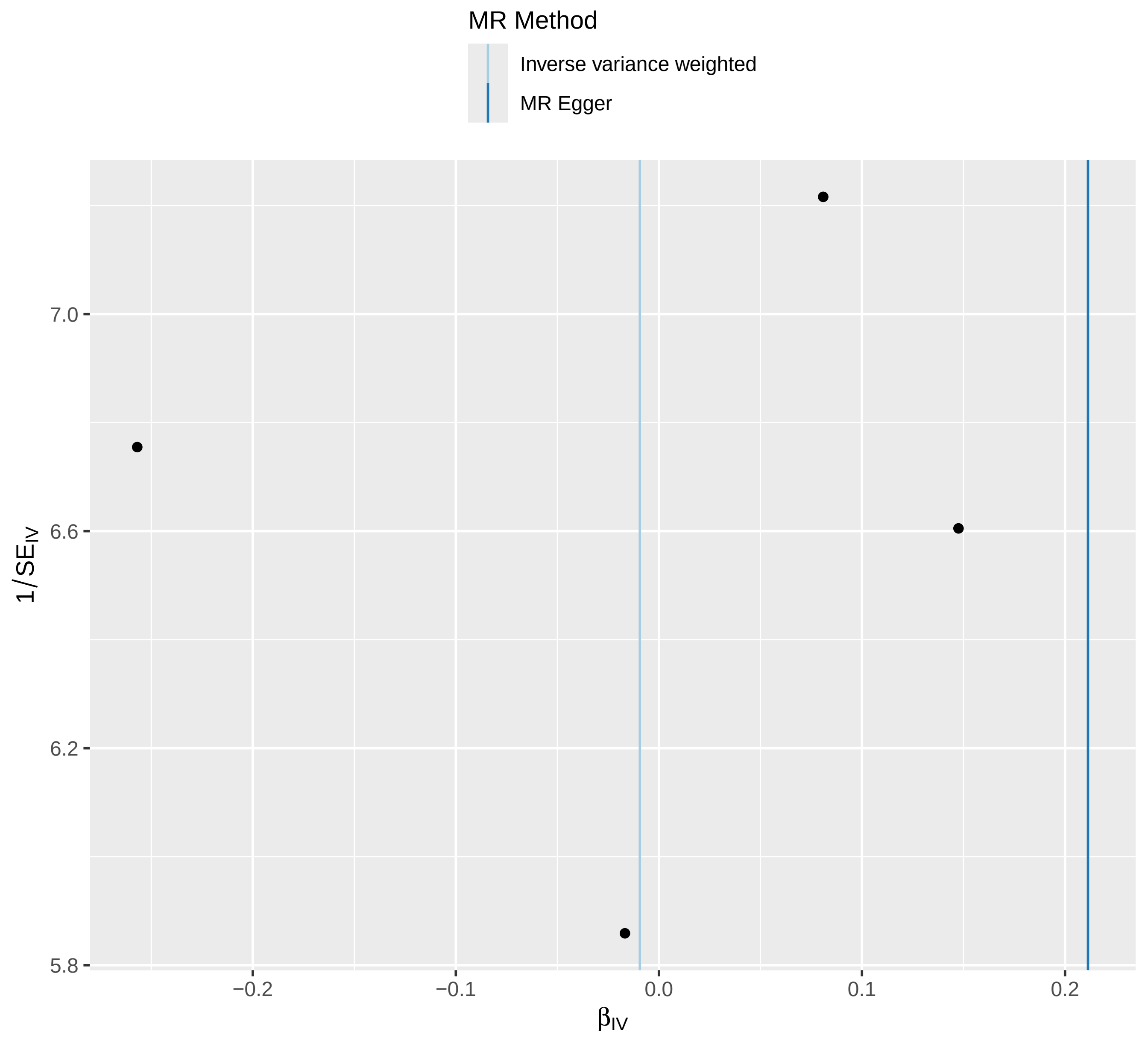
MR Method
Inverse variance weighted
MR Egger
7.0
6.6
6.2
5.8
1/SE_IV
−0.2
−0.1
0.0
0.1
0.2
β_IV

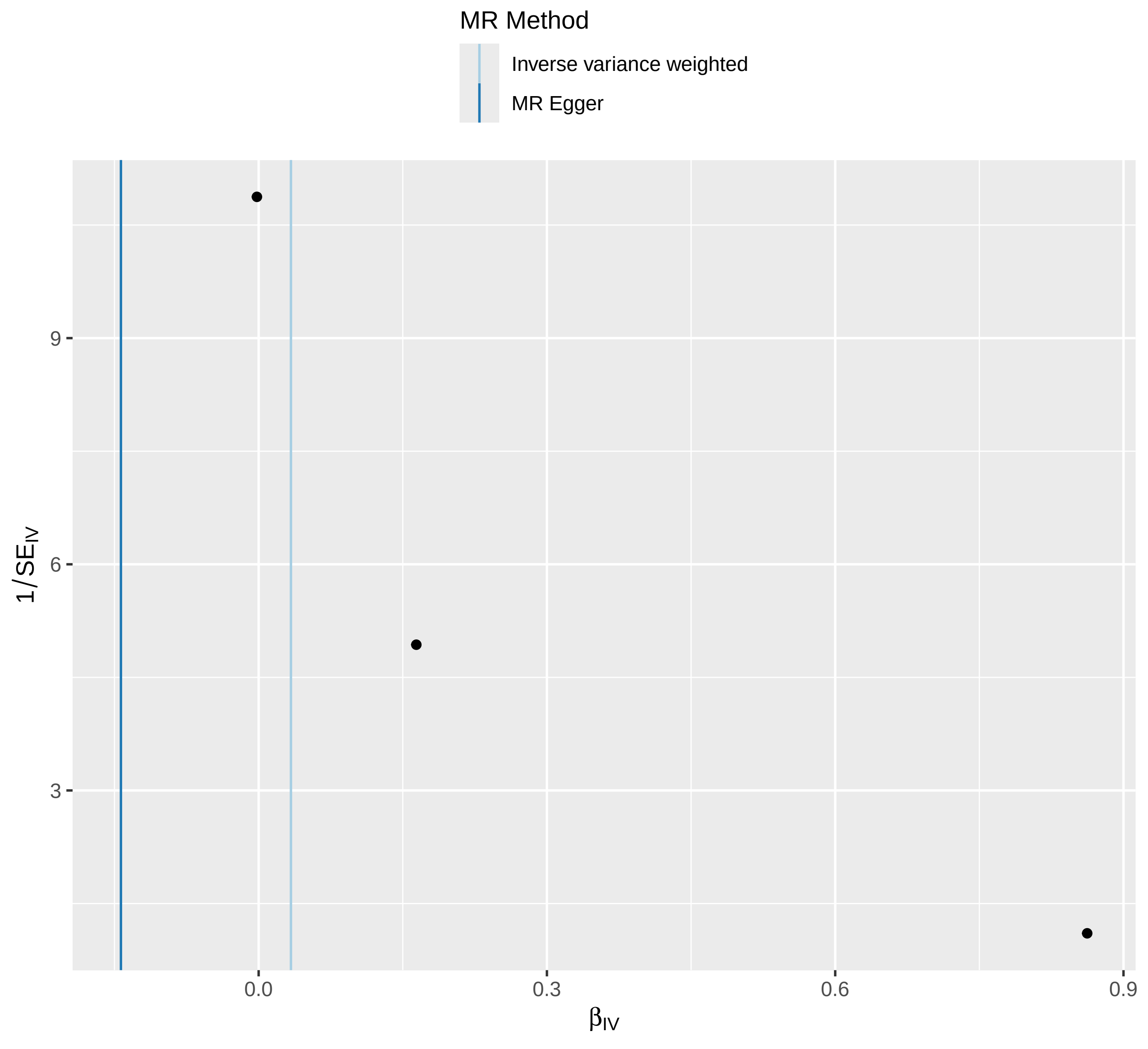
MR Method
Inverse variance weighted
MR Egger
9
6
3
1/SE$_{IV}$
0.0
0.3
0.6
0.9
β$_{IV}$

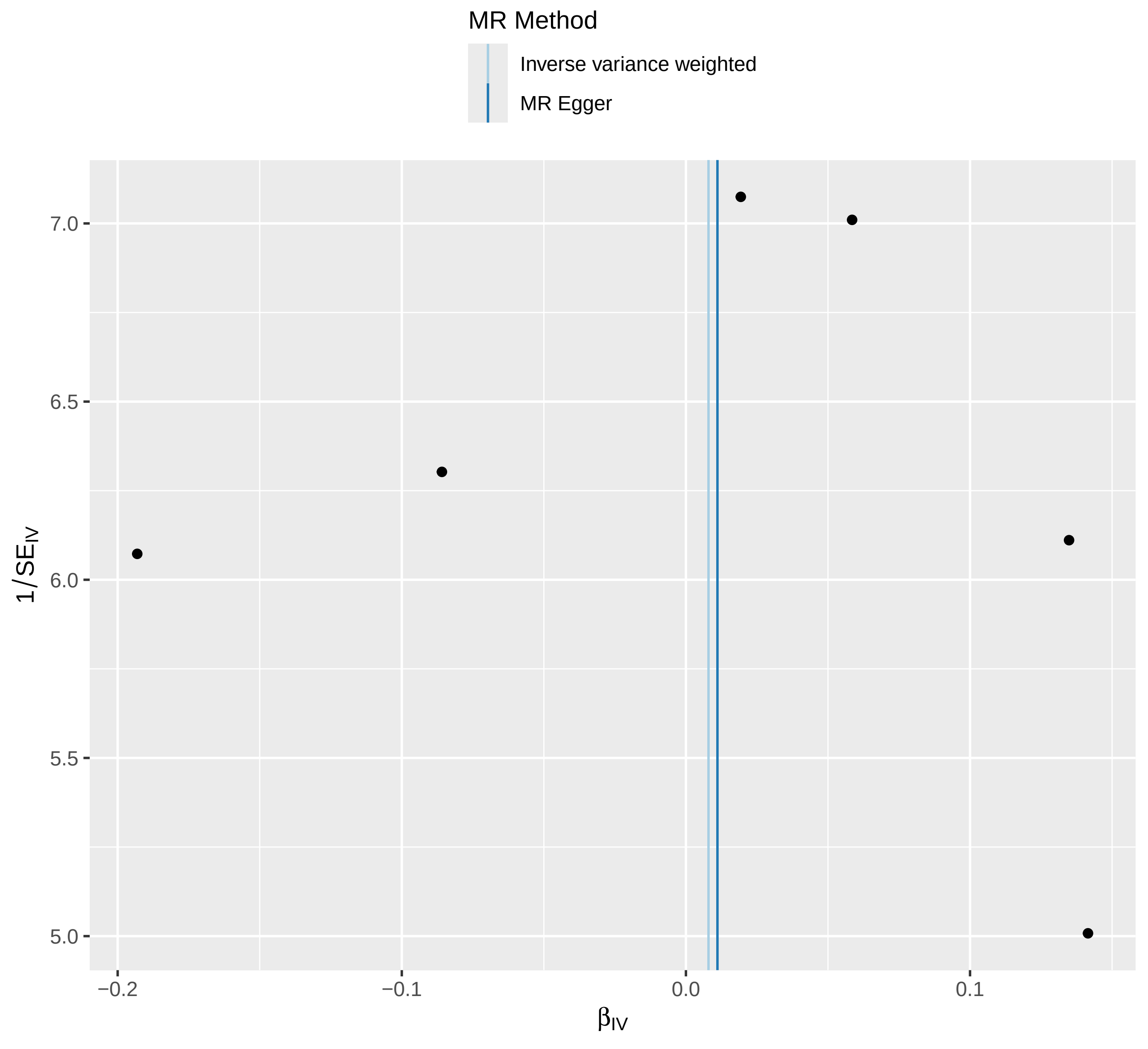

MR Method
Inverse variance weighted
MR Egger
7.0
6.5
6.0
5.5
5.0
1/SE_IV
−0.2
−0.1
0.0
0.1
β_IV

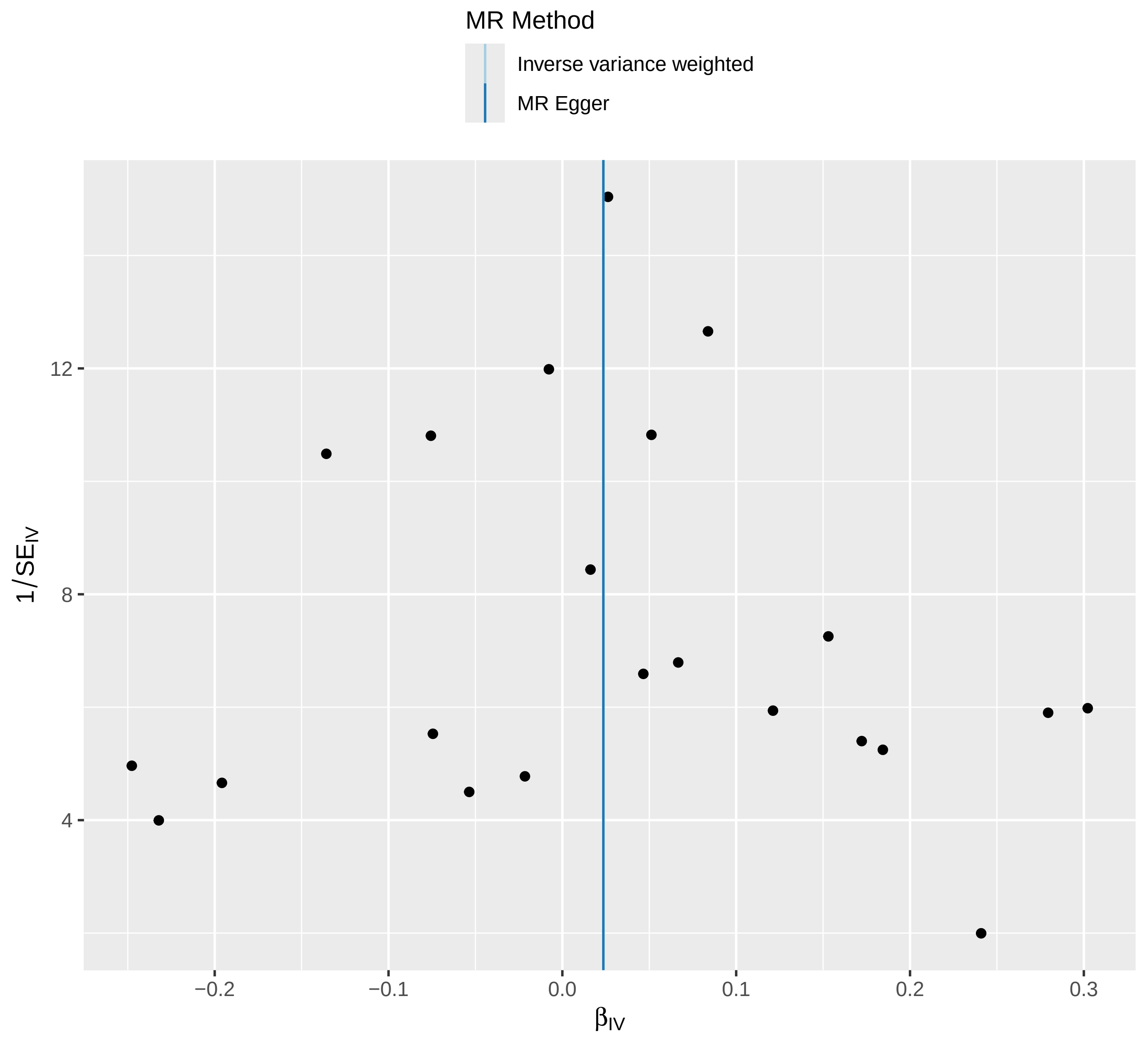

MR Method
Inverse variance weighted
MR Egger
1/SE_IV
12
8
4
−0.2
−0.1
0.0
0.1
0.2
0.3
β_IV

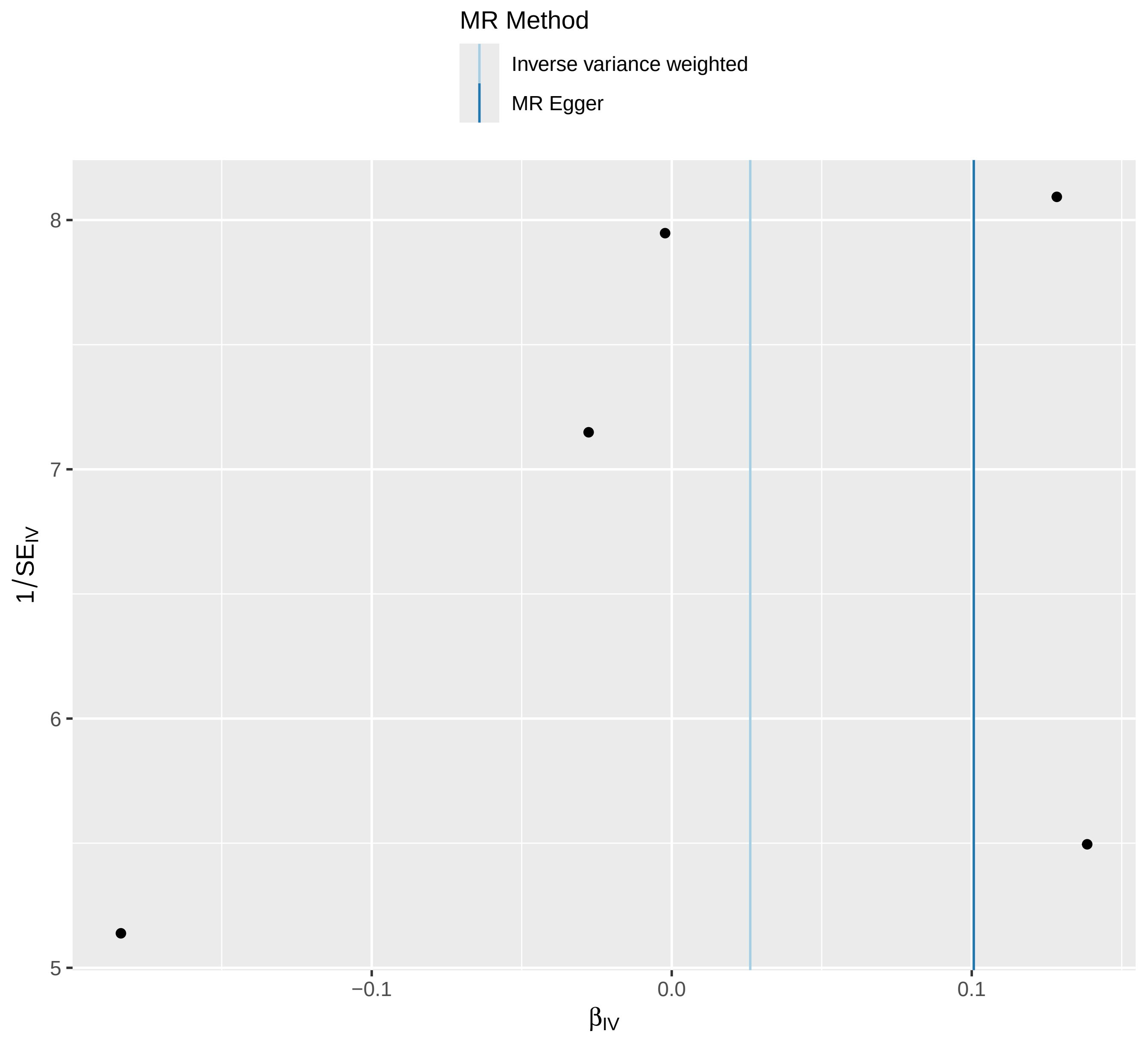
MR Method
Inverse variance weighted
MR Egger
8
7
6
5
1/SE$_{IV}$
−0.1
0.0
0.1
β$_{IV}$

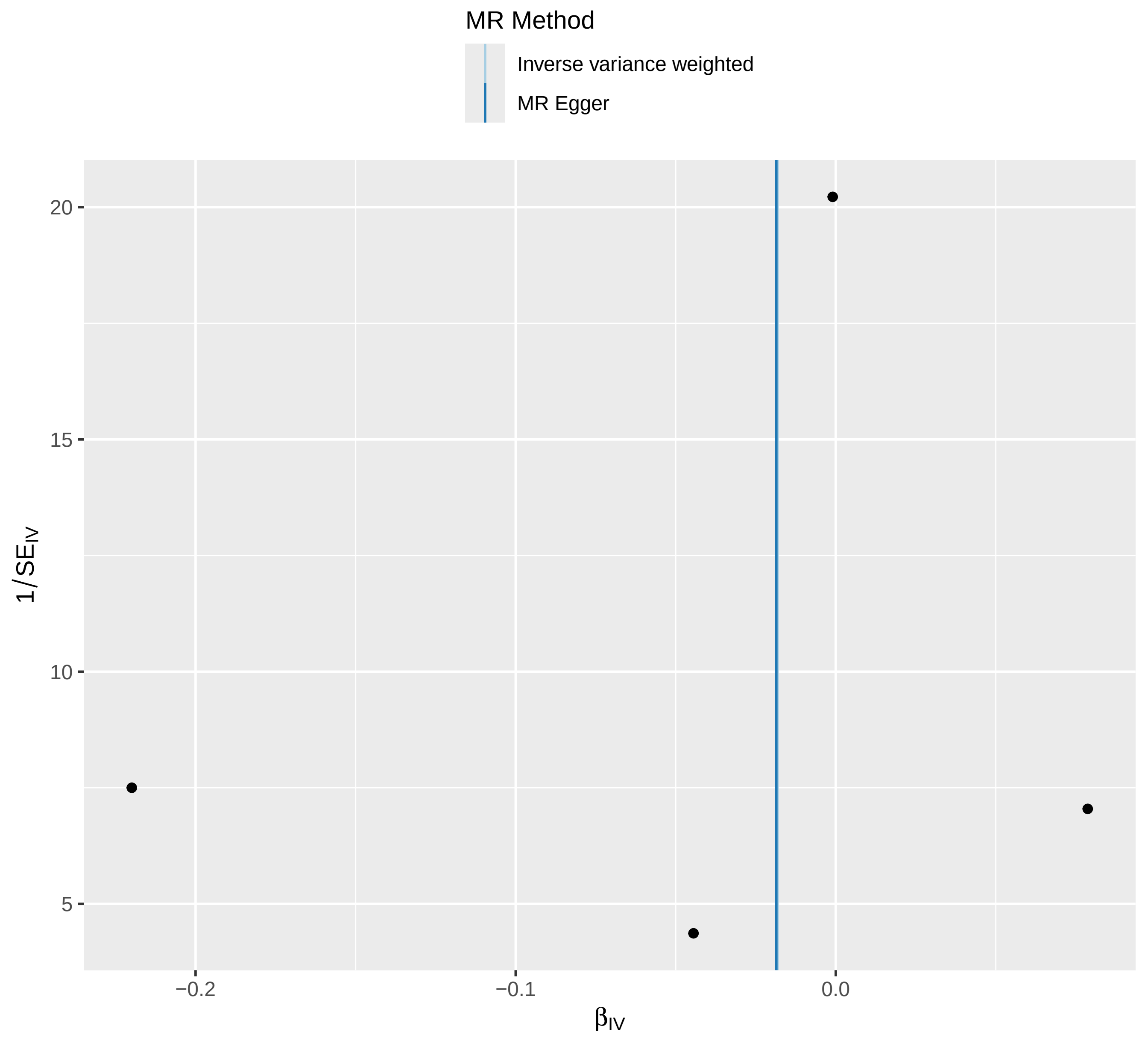

MR Method
Inverse variance weighted
MR Egger
20
15
10
5
1/SE$_{IV}$
−0.2
−0.1
0.0
$\beta_{IV}$

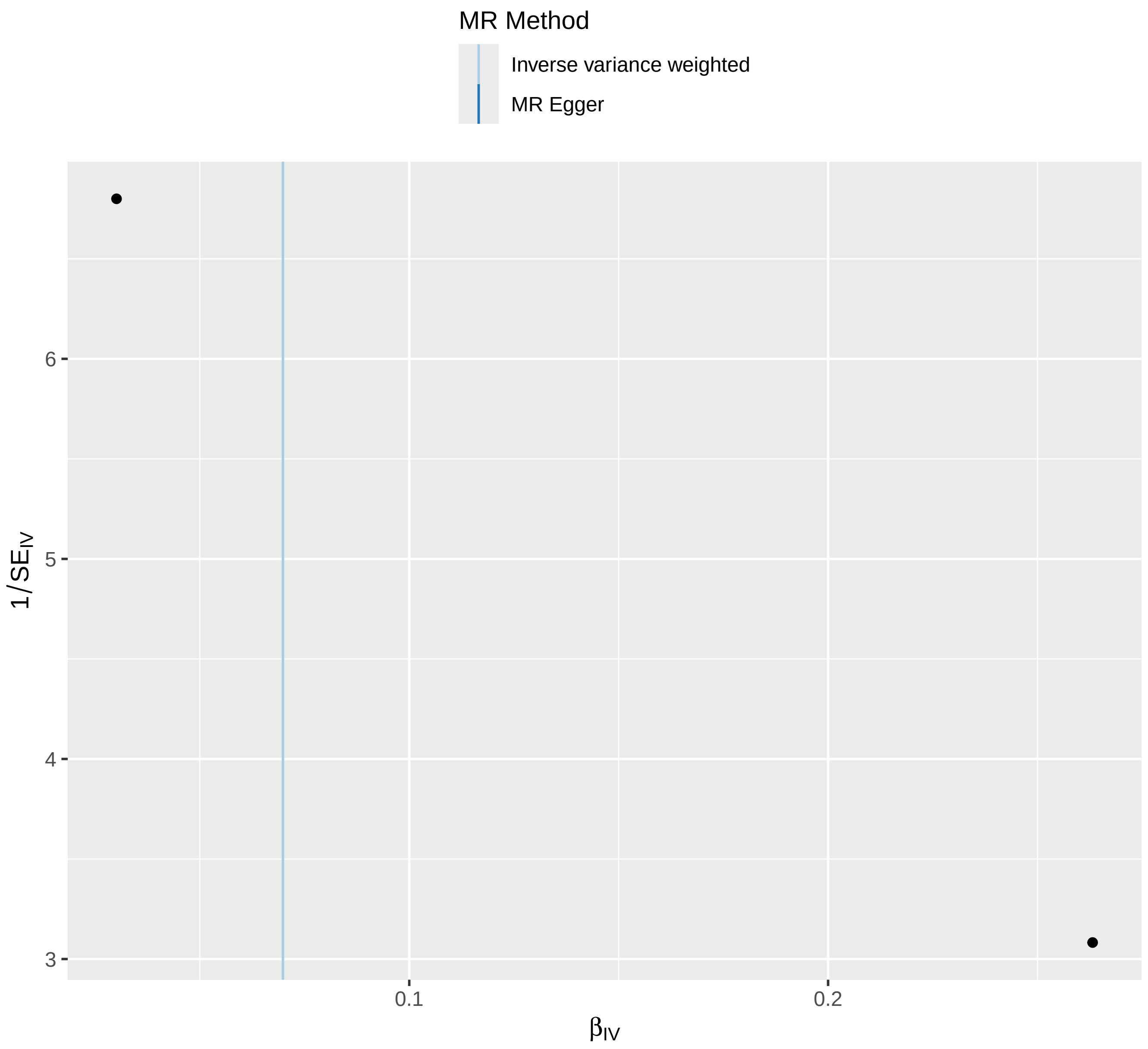

MR Method
Inverse variance weighted
MR Egger
6
5
4
3
1/SE_IV
0.1
0.2
β_IV

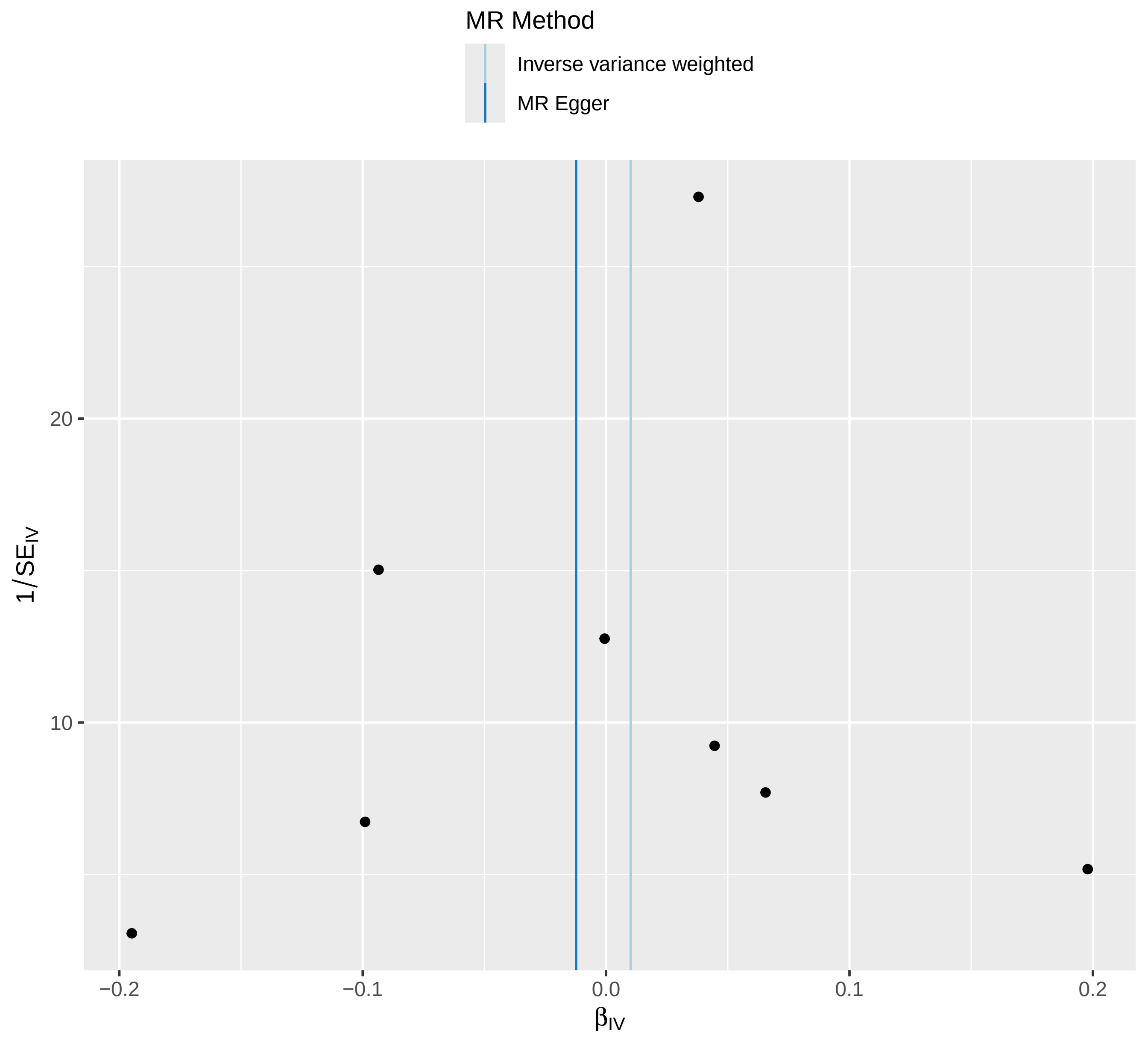

MR Method
Inverse variance weighted
MR Egger
20
10
1/SE_IV
−0.2
−0.1
0.0
0.1
0.2
β_IV

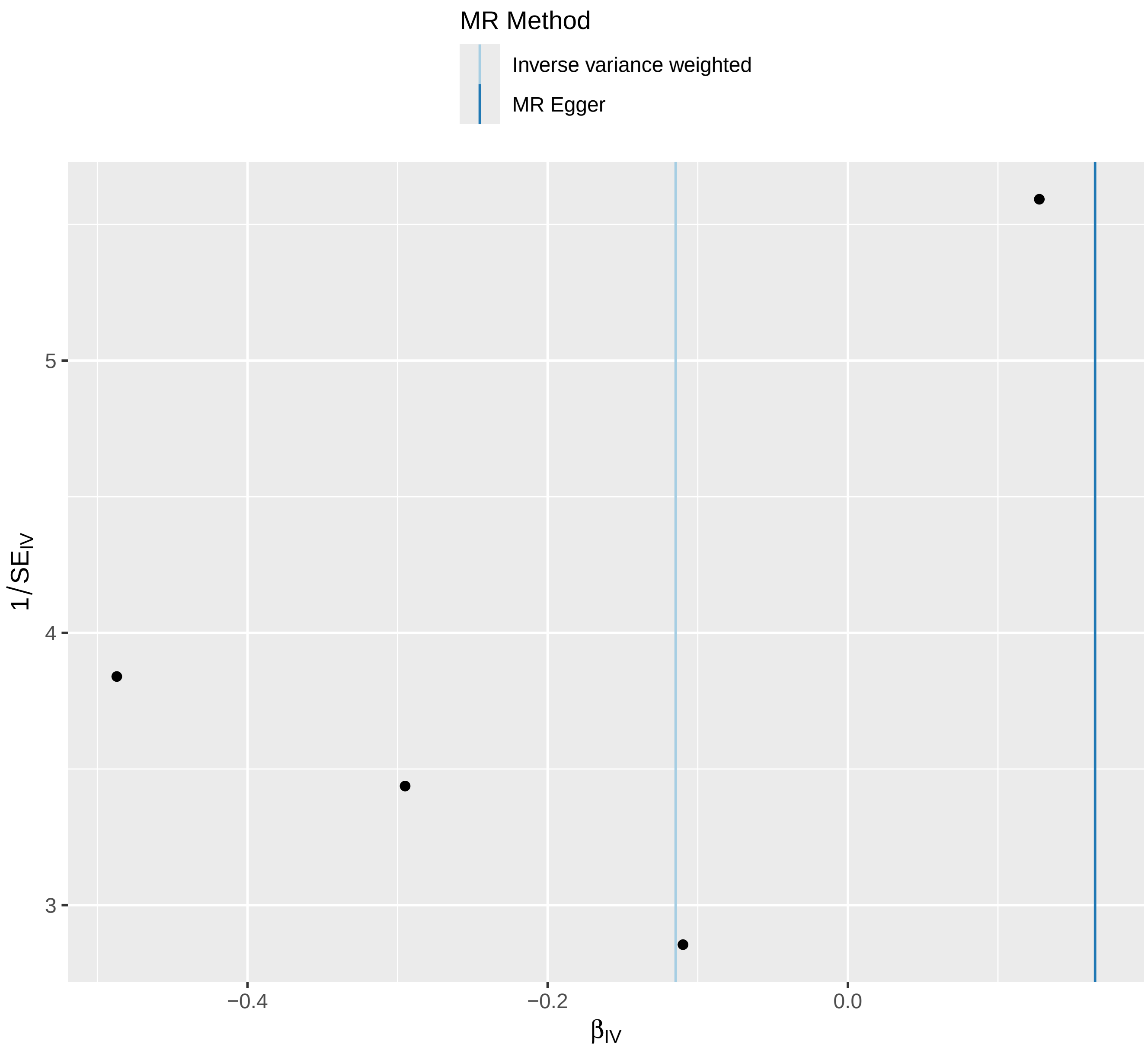
MR Method
Inverse variance weighted
MR Egger
5
4
3
1/SE_IV
−0.4
−0.2
0.0
β_IV

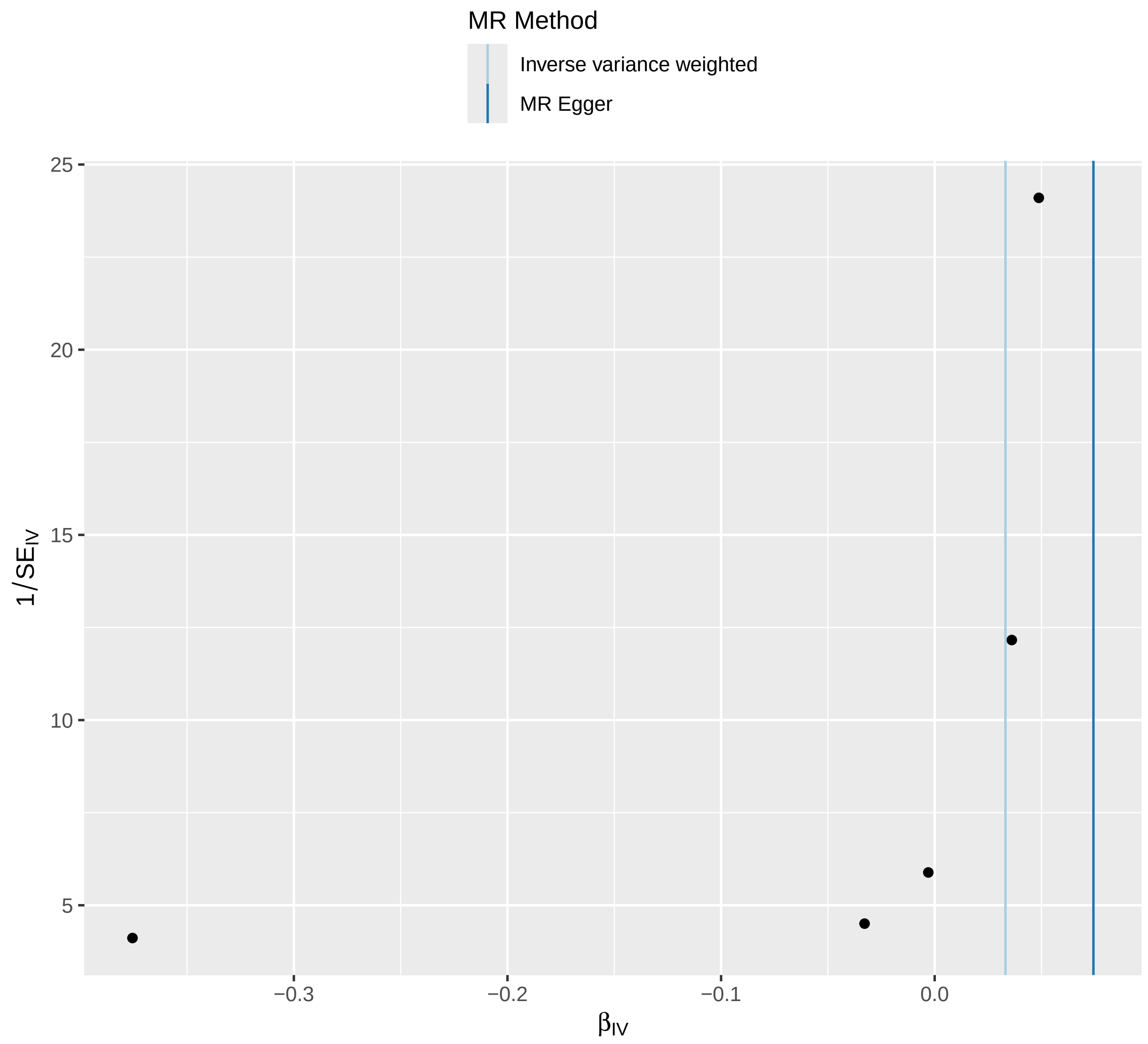
MR Method
Inverse variance weighted
MR Egger
25
20
15
10
5
1/SE_IV
−0.3
−0.2
−0.1
0.0
β_IV

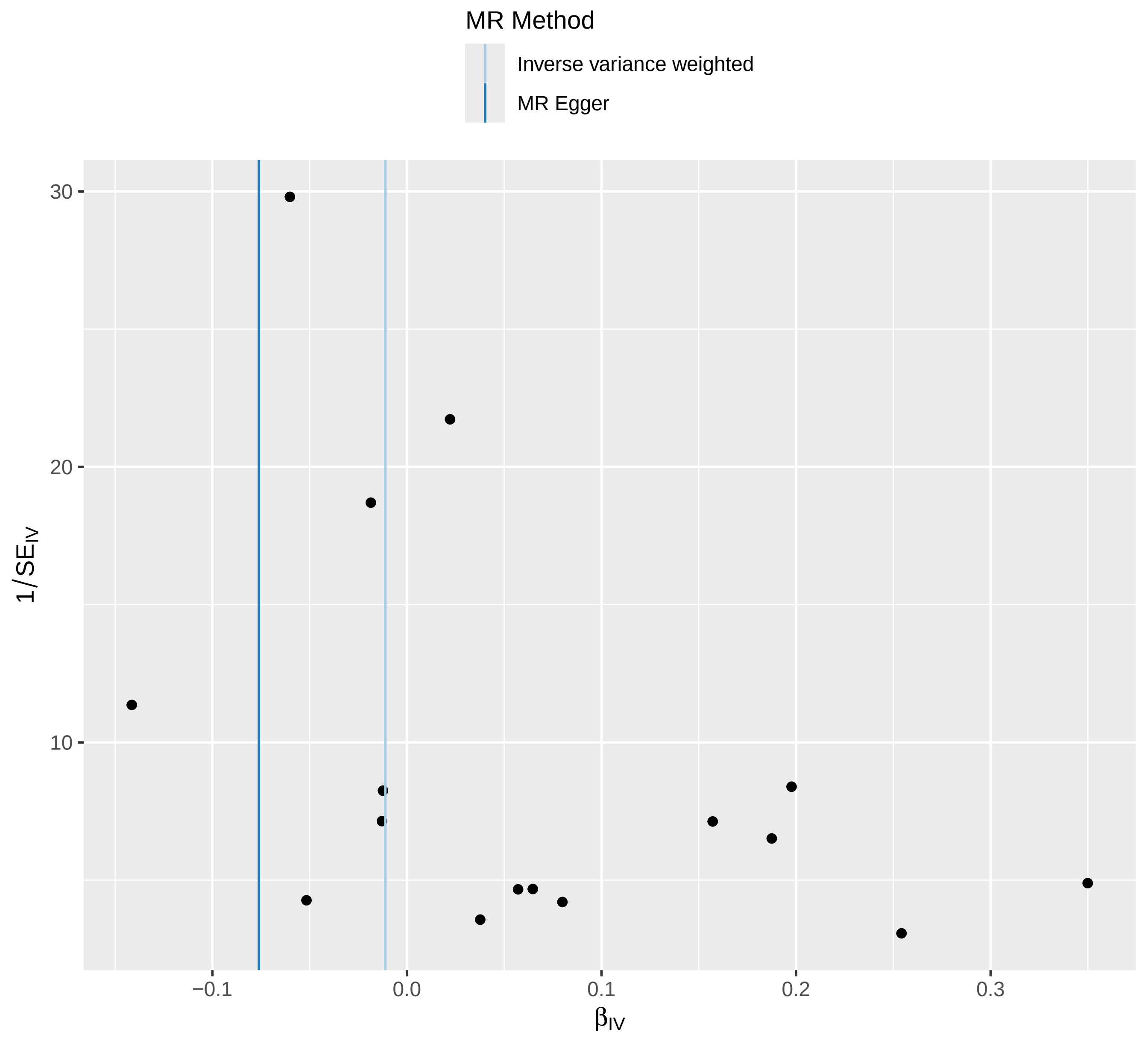

MR Method
Inverse variance weighted
MR Egger
30
20
10
1/SE_IV
−0.1
0.0
0.1
0.2
0.3
β_IV

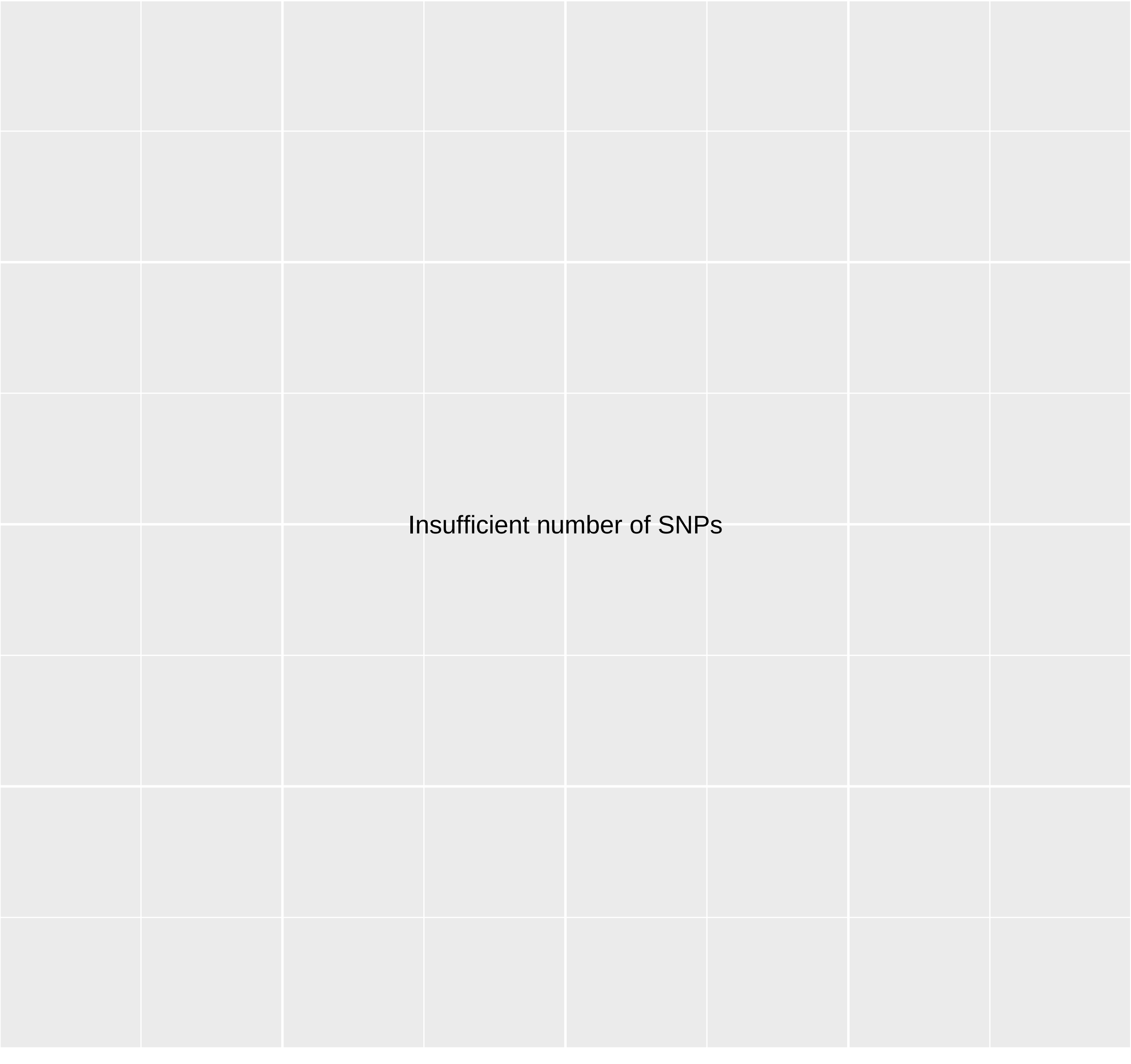
Insufficient number of SNPs

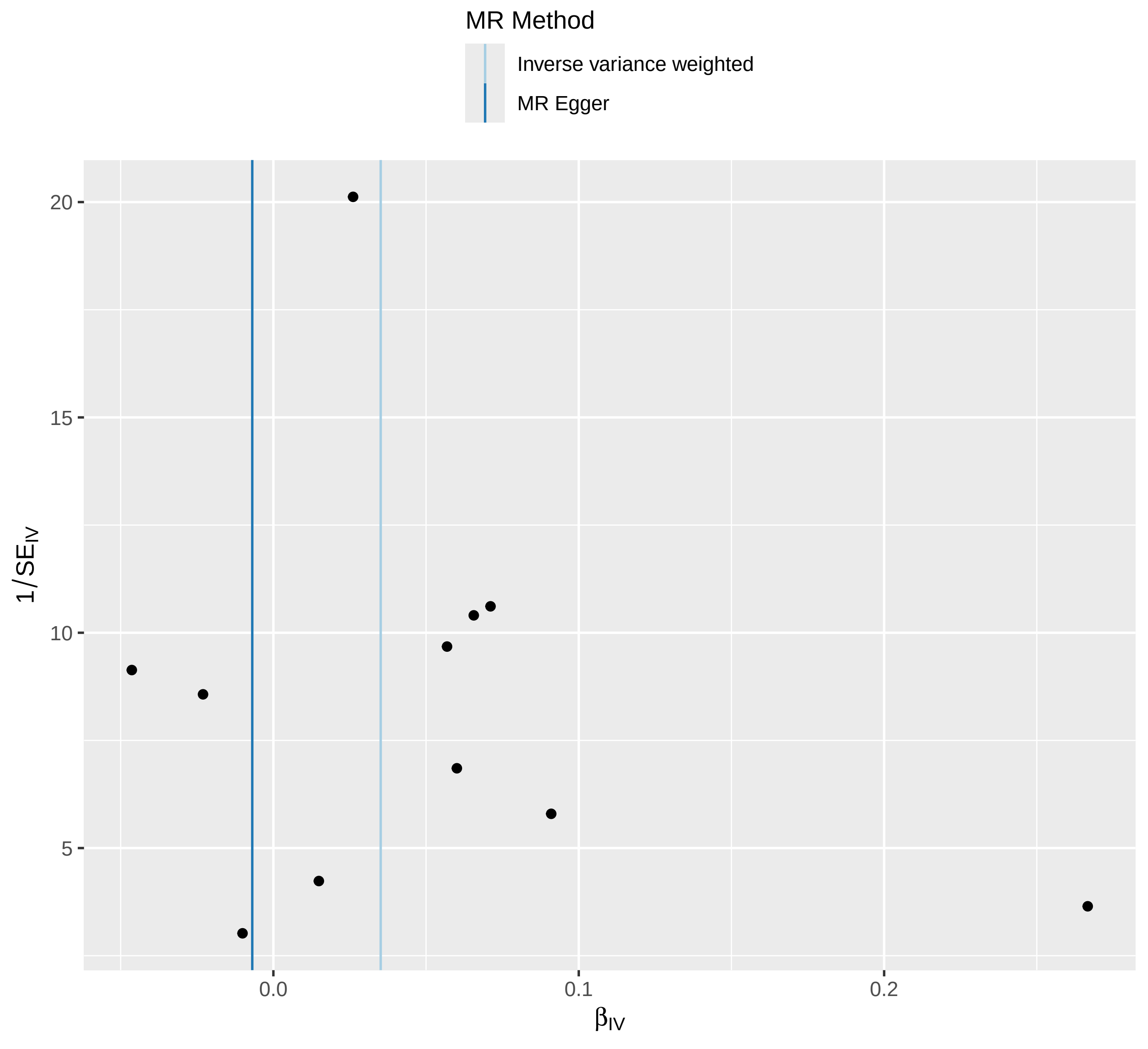

MR Method
Inverse variance weighted
MR Egger
20
15
10
5
0.0
0.1
0.2
1/SE_IV
β_IV

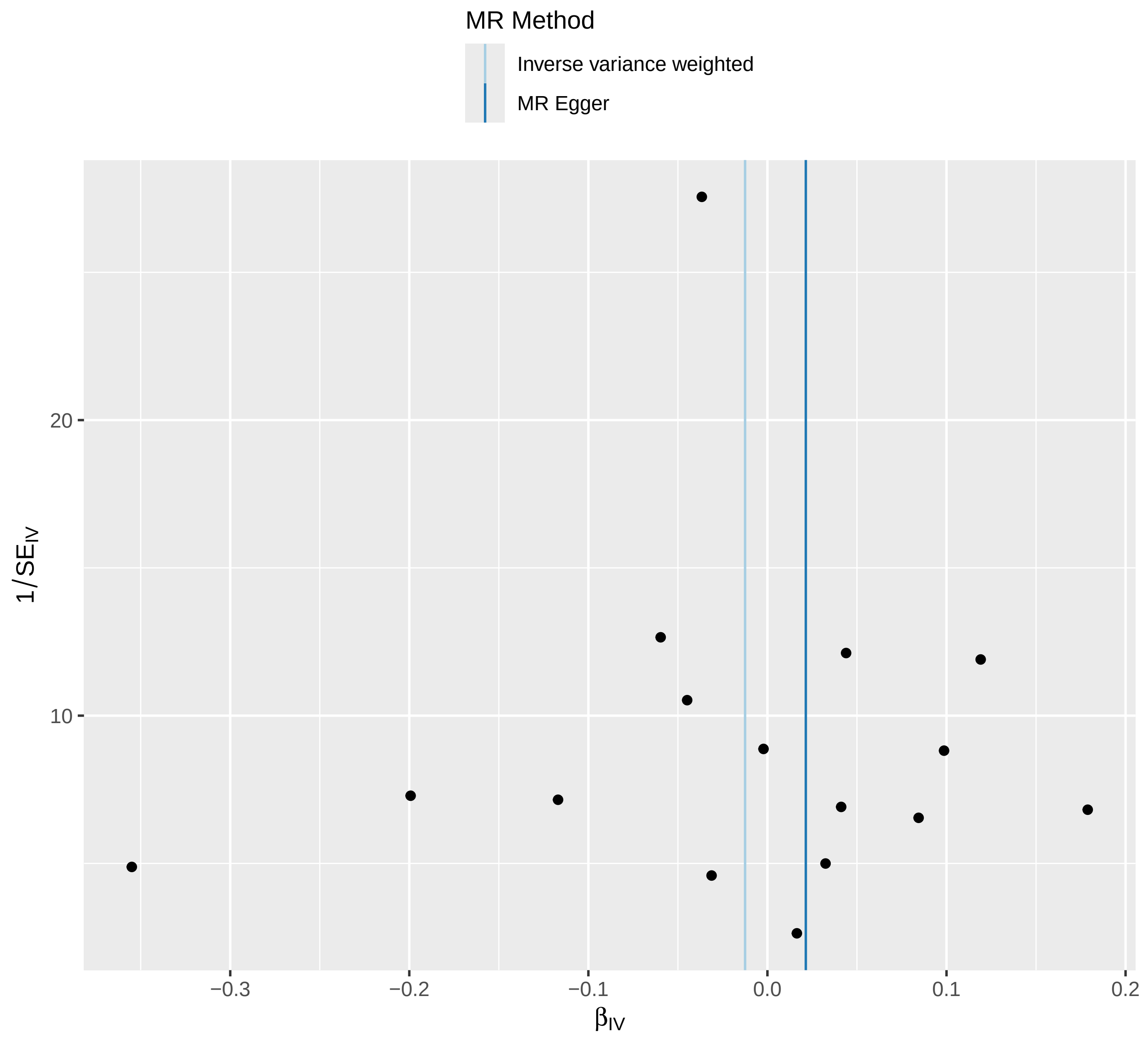

MR Method
Inverse variance weighted
MR Egger
20
10
1/SE_IV
−0.3
−0.2
−0.1
0.0
0.1
0.2
β_IV

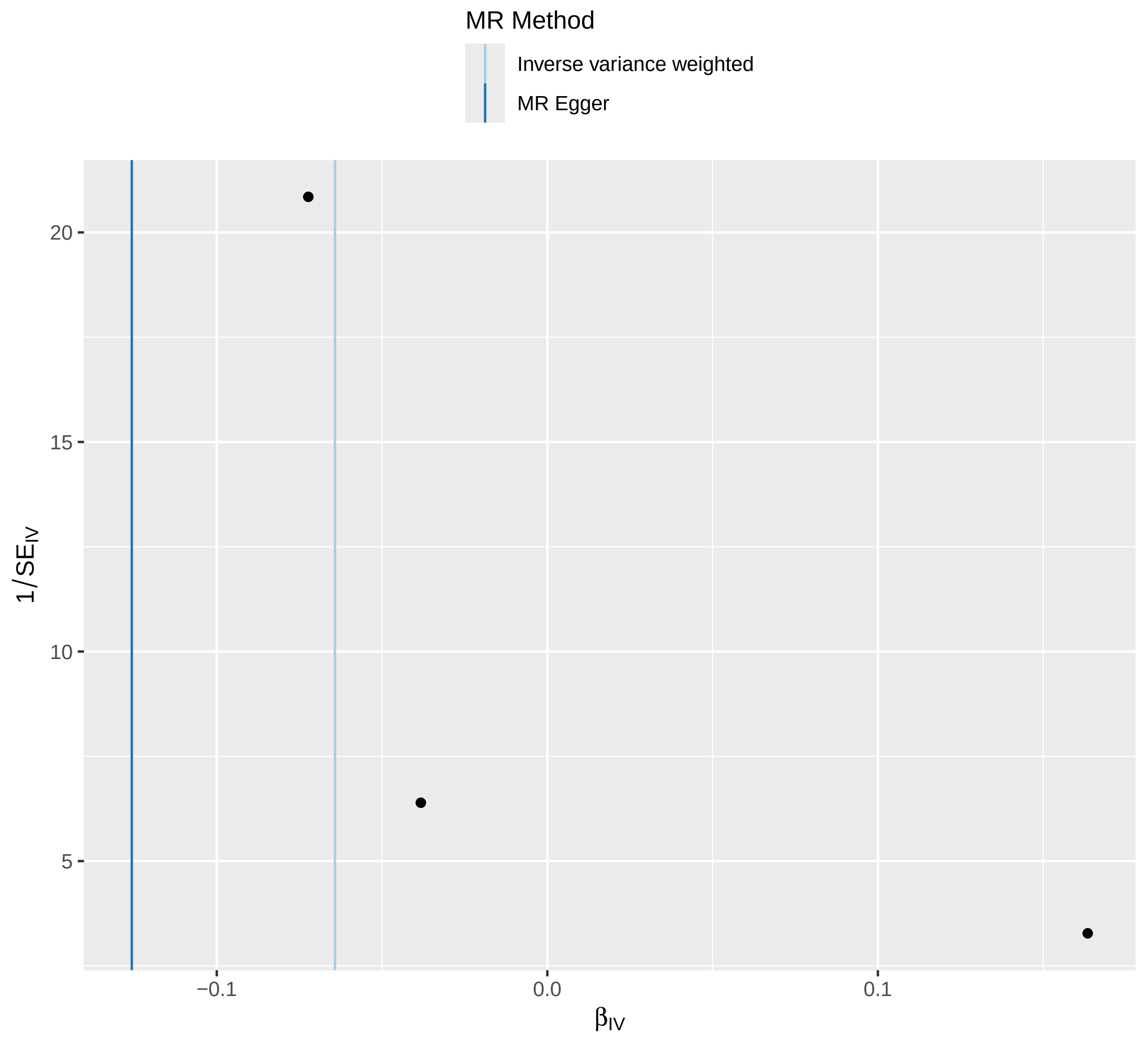
MR Method
Inverse variance weighted
MR Egger
20
15
10
5
1/SE_IV
−0.1
0.0
0.1
β_IV

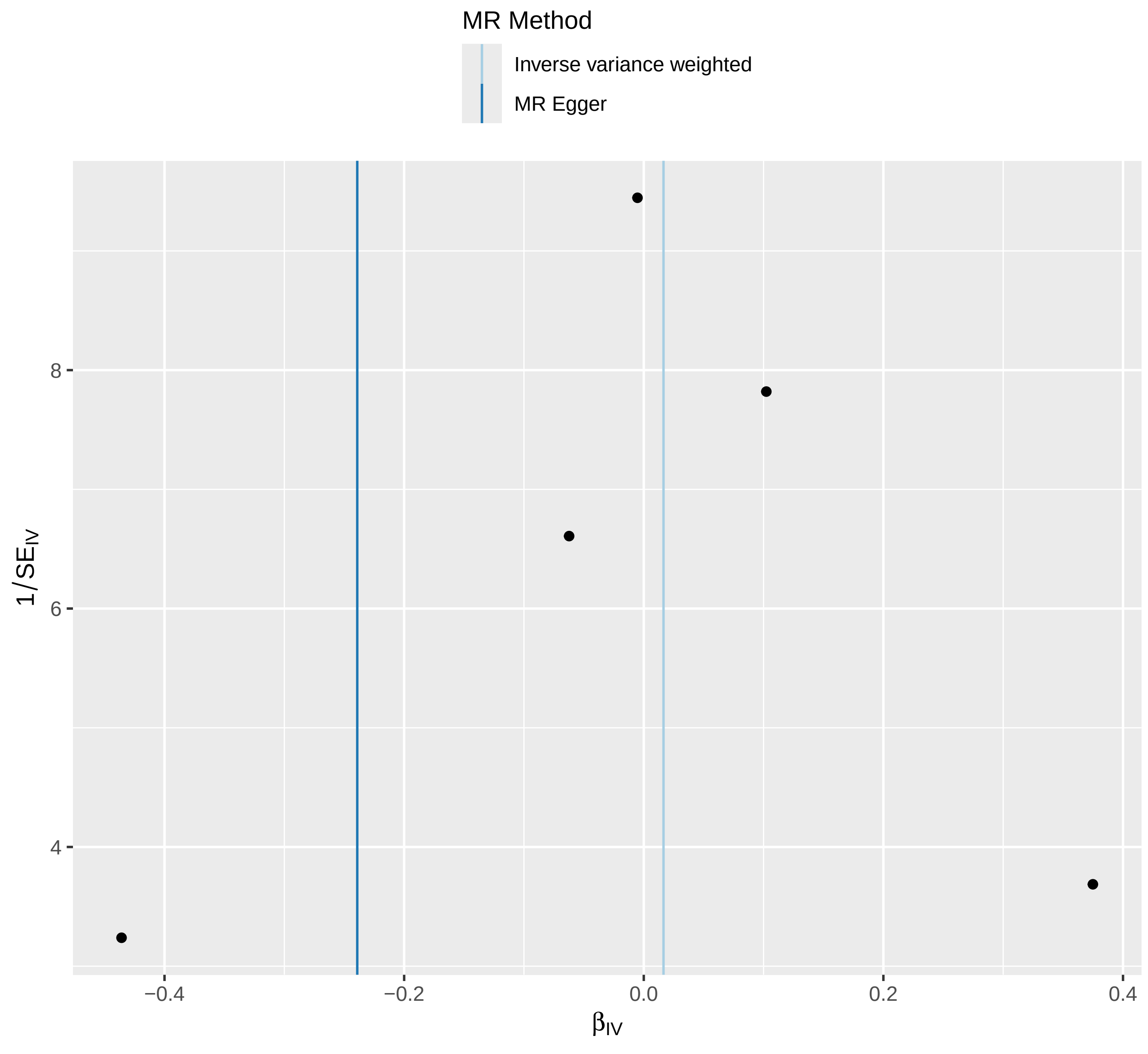

MR Method
Inverse variance weighted
MR Egger
8
6
4
1/SE$_{IV}$
−0.4
−0.2
0.0
0.2
0.4
β$_{IV}$

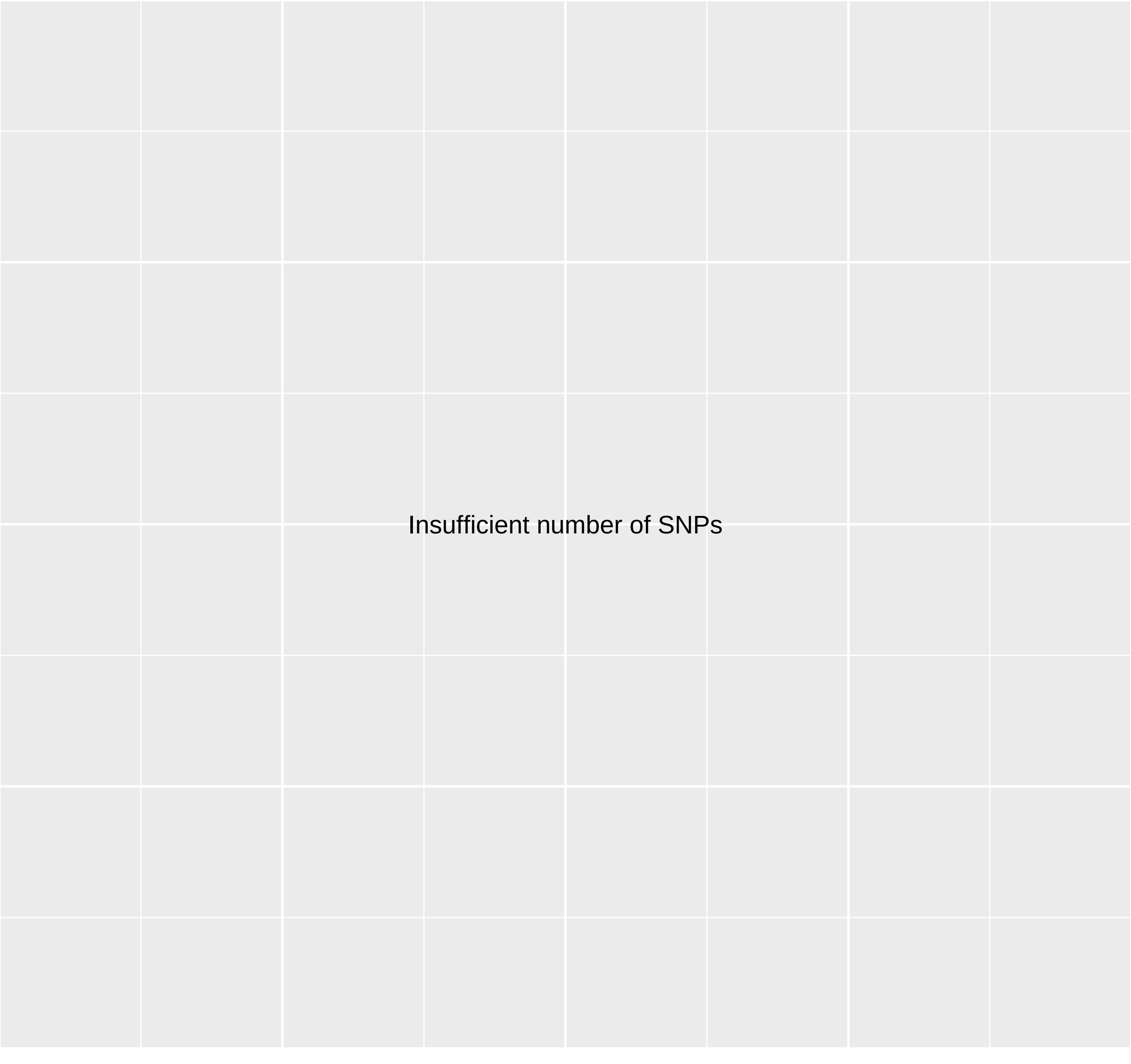
Insufficient number of SNPs

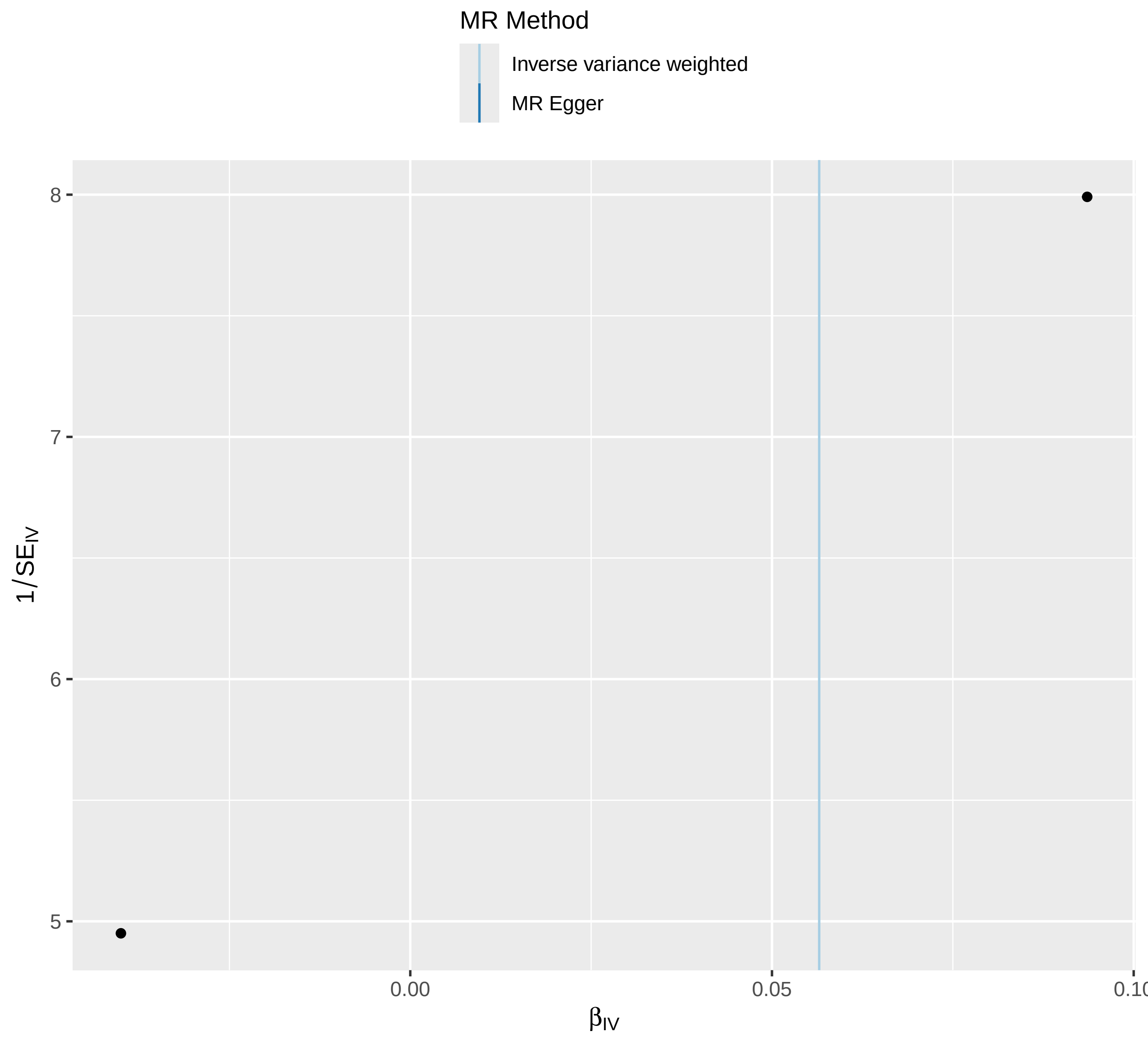

MR Method
Inverse variance weighted
MR Egger
8
7
6
5
0.00
0.05
0.10
1/SE$_{IV}$
β$_{IV}$

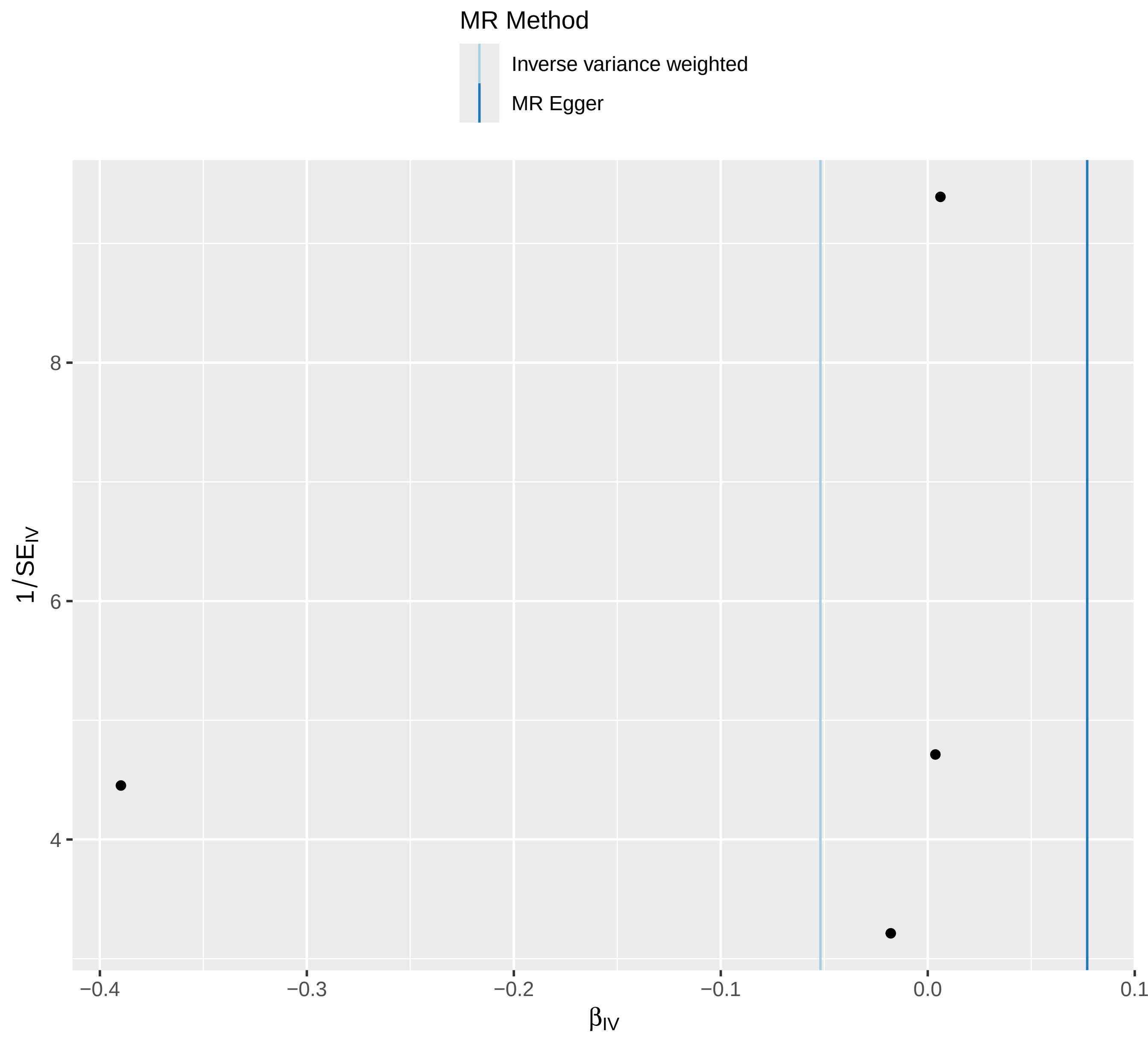

MR Method
Inverse variance weighted
MR Egger
8
6
4
1/SE$_{IV}$
−0.4
−0.3
−0.2
−0.1
0.0
0.1
β$_{IV}$

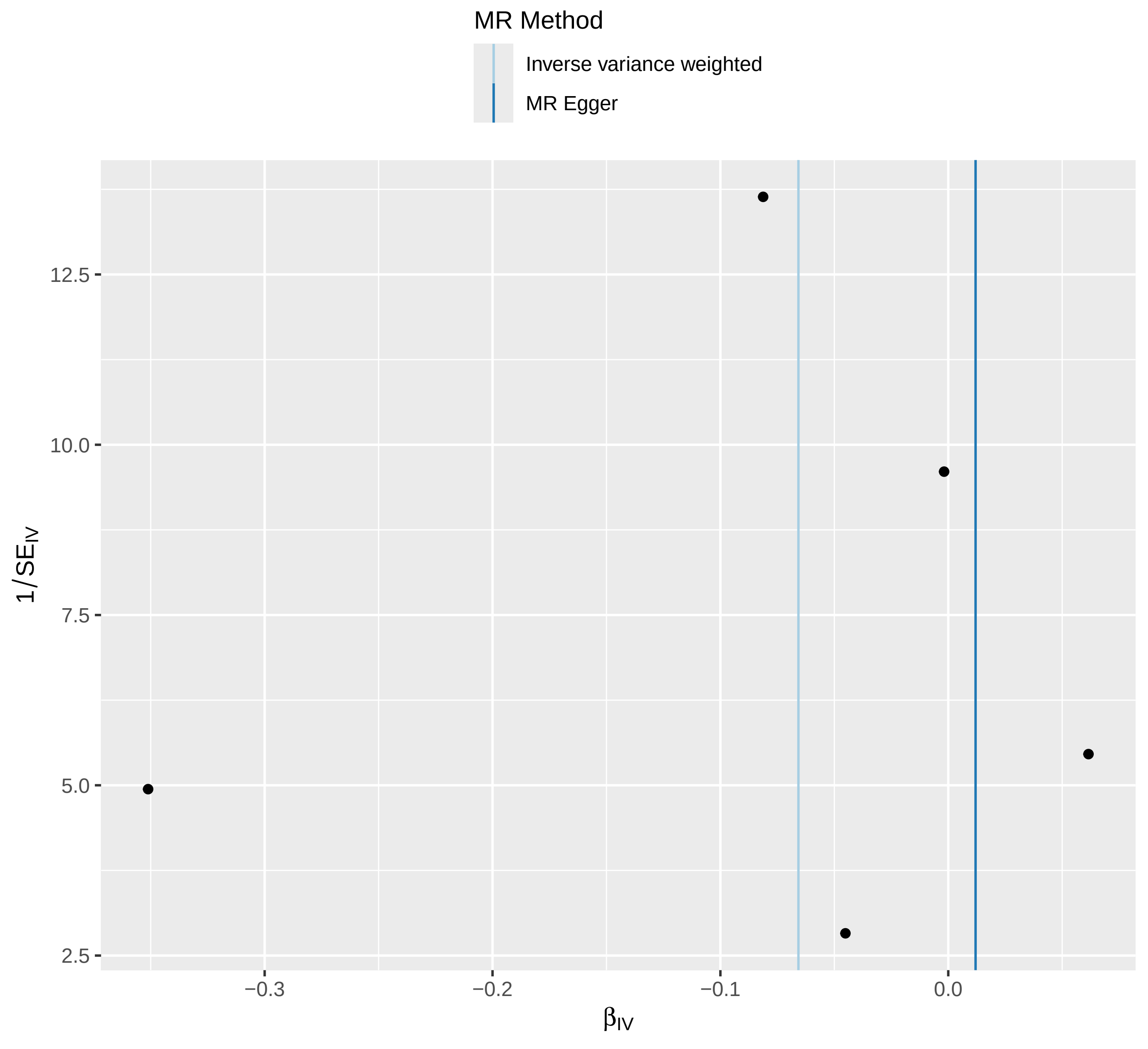

MR Method
Inverse variance weighted
MR Egger
12.5
10.0
7.5
5.0
2.5
1/SE$_{IV}$
−0.3
−0.2
−0.1
0.0
β$_{IV}$

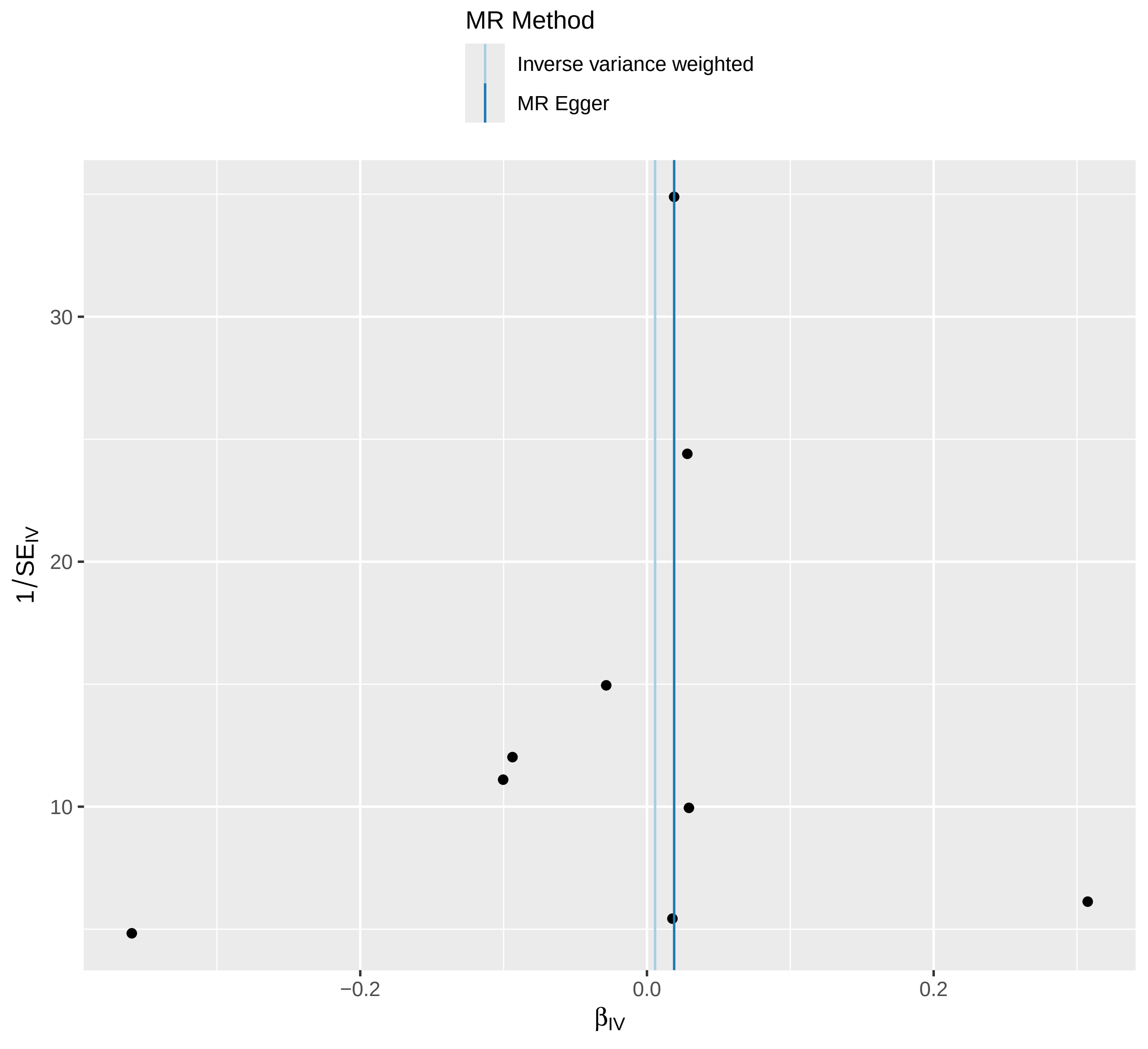

MR Method
Inverse variance weighted
MR Egger
30
20
10
1/SE$_{IV}$
−0.2
0.0
0.2
β$_{IV}$

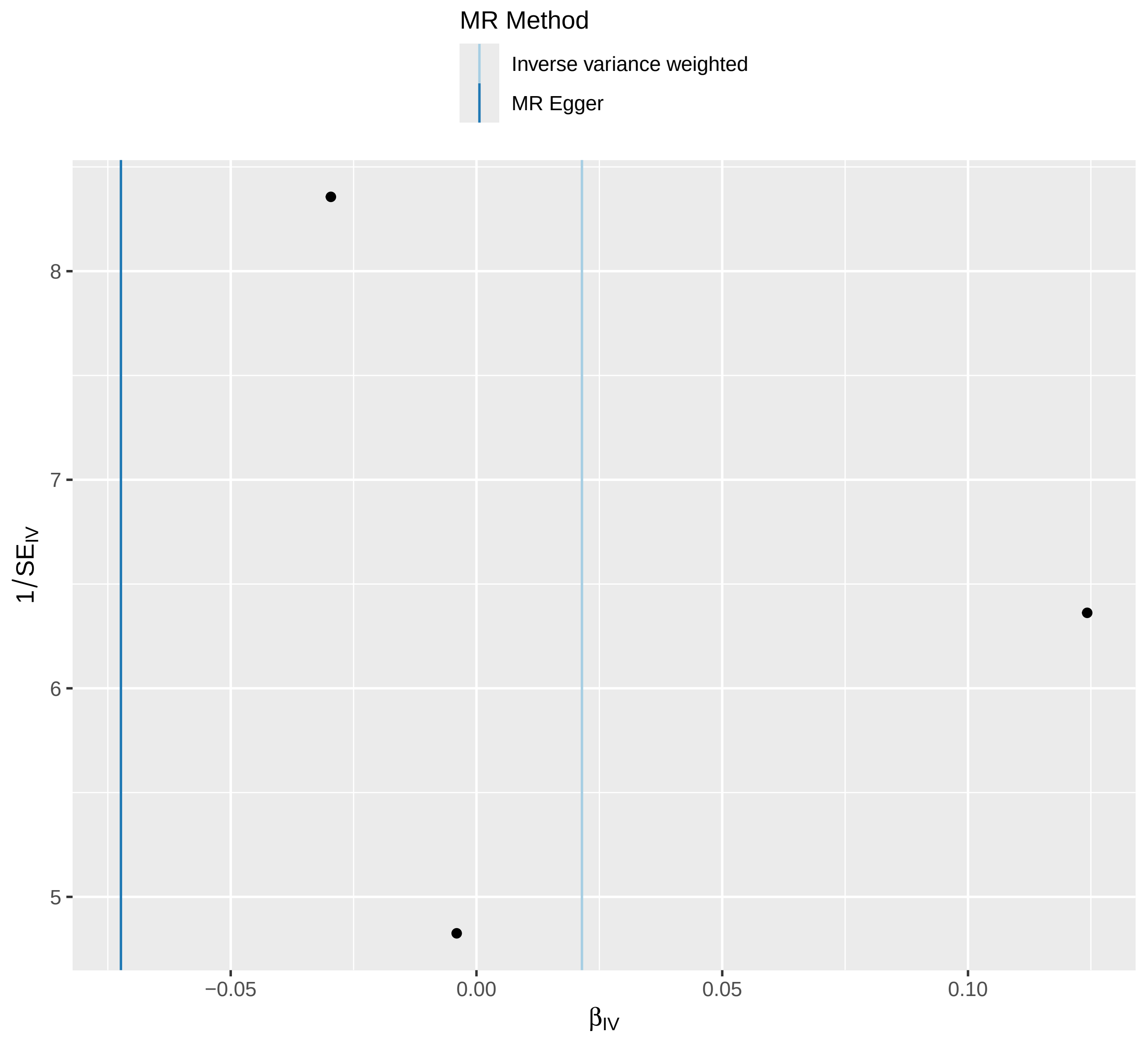
MR Method
Inverse variance weighted
MR Egger
1/SE$_{IV}$
8
7
6
5
−0.05
0.00
0.05
0.10
β$_{IV}$

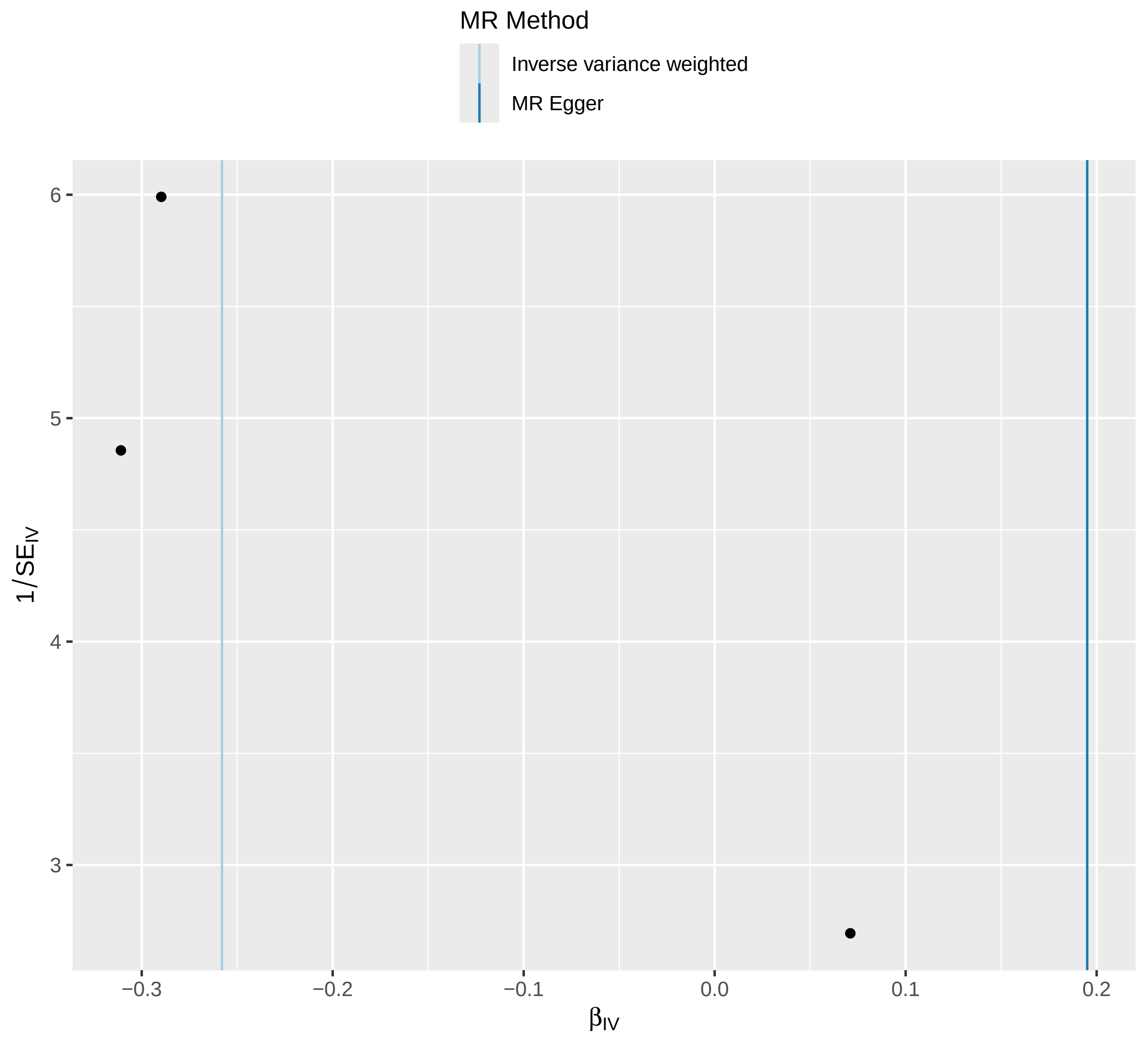

MR Method
Inverse variance weighted
MR Egger
6
5
4
3
1/SE$_{IV}$
−0.3
−0.2
−0.1
0.0
0.1
0.2
β$_{IV}$

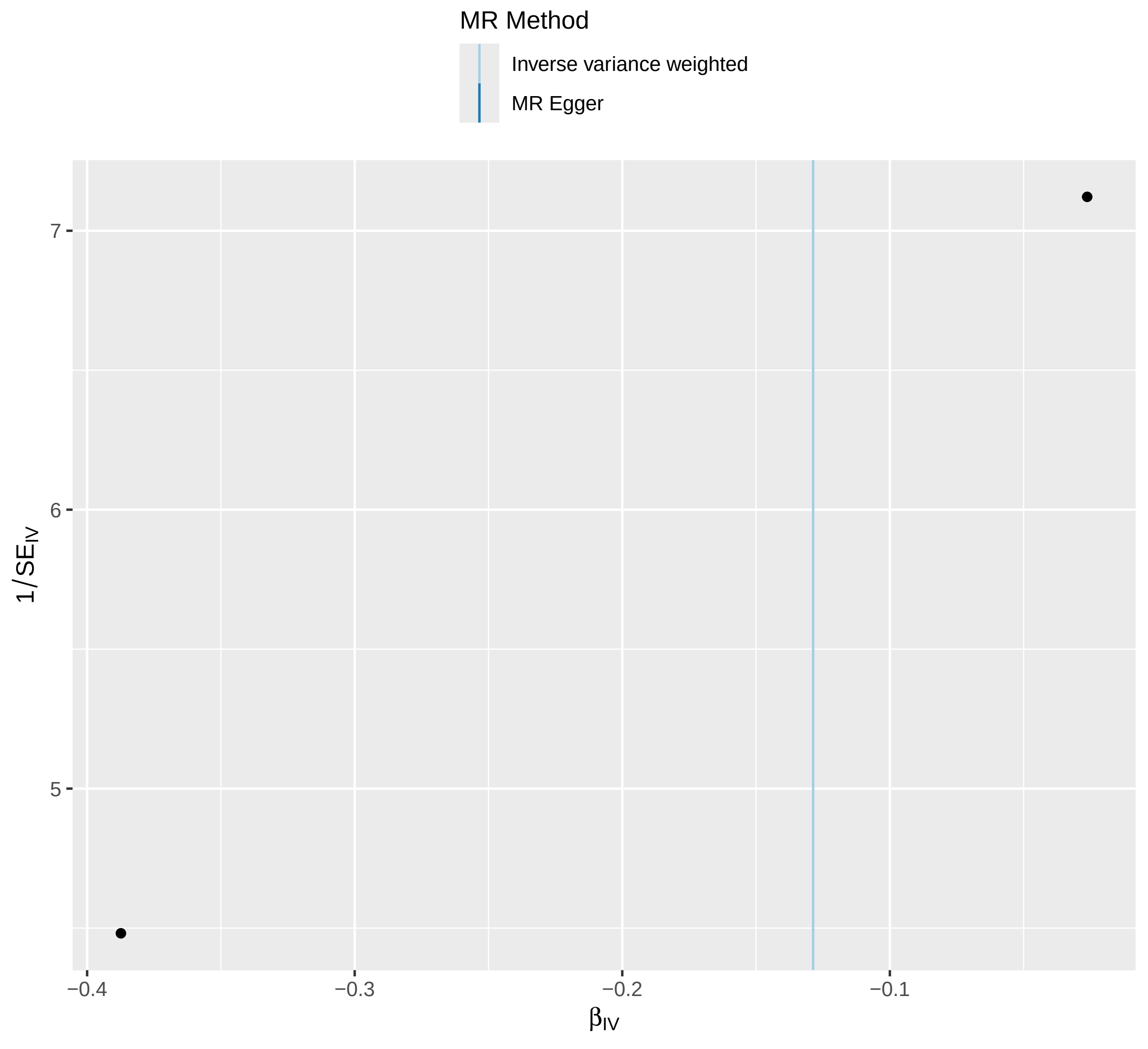
MR Method
Inverse variance weighted
MR Egger
7
6
5
1/SE$_{IV}$
−0.4
−0.3
−0.2
−0.1
β$_{IV}$

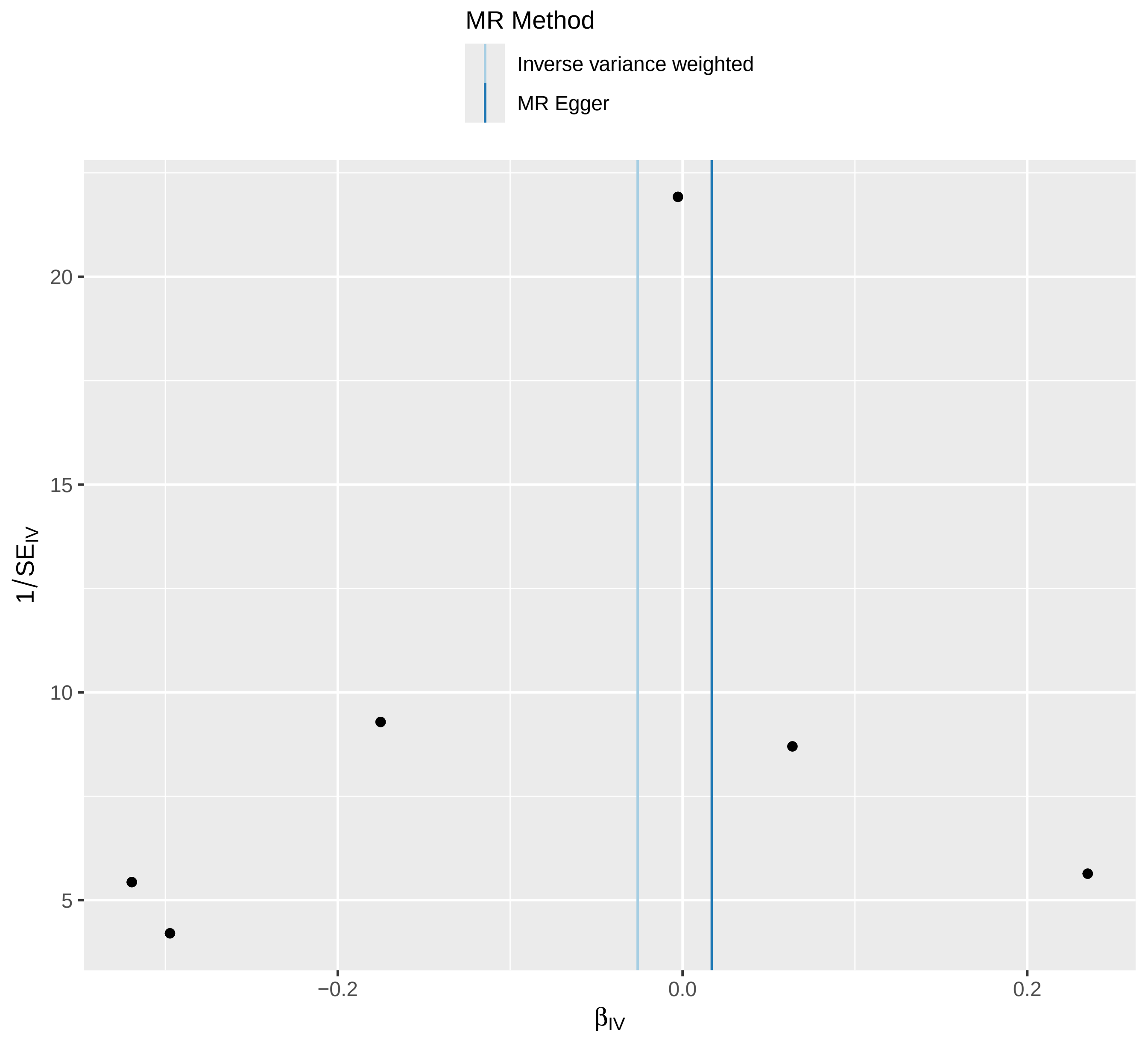

MR Method
Inverse variance weighted
MR Egger
20
15
10
5
1/SE_IV
−0.2
0.0
0.2
β_IV

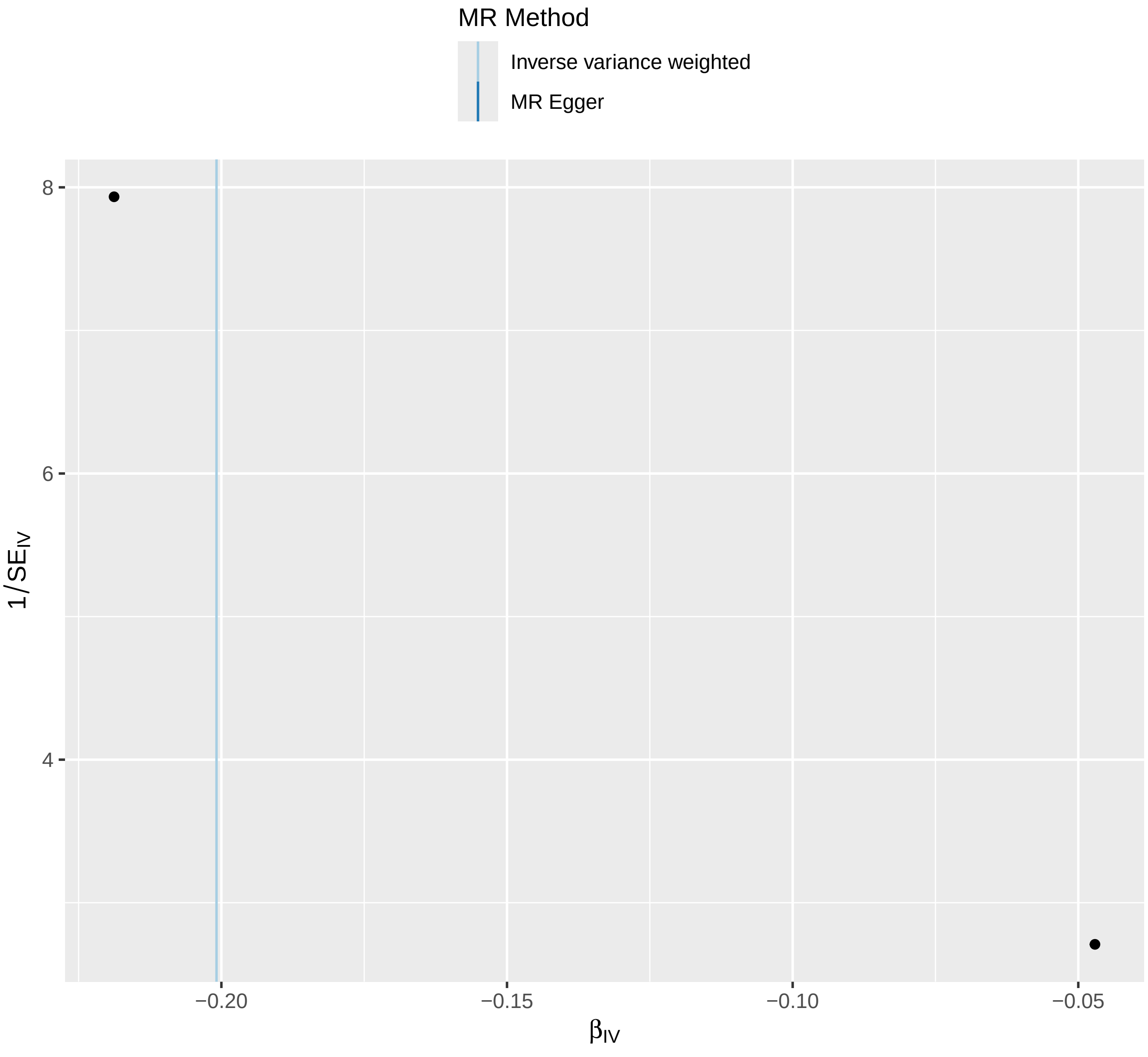
MR Method
Inverse variance weighted
MR Egger
8
6
4
1/SE_IV
−0.20
−0.15
−0.10
−0.05
β_IV

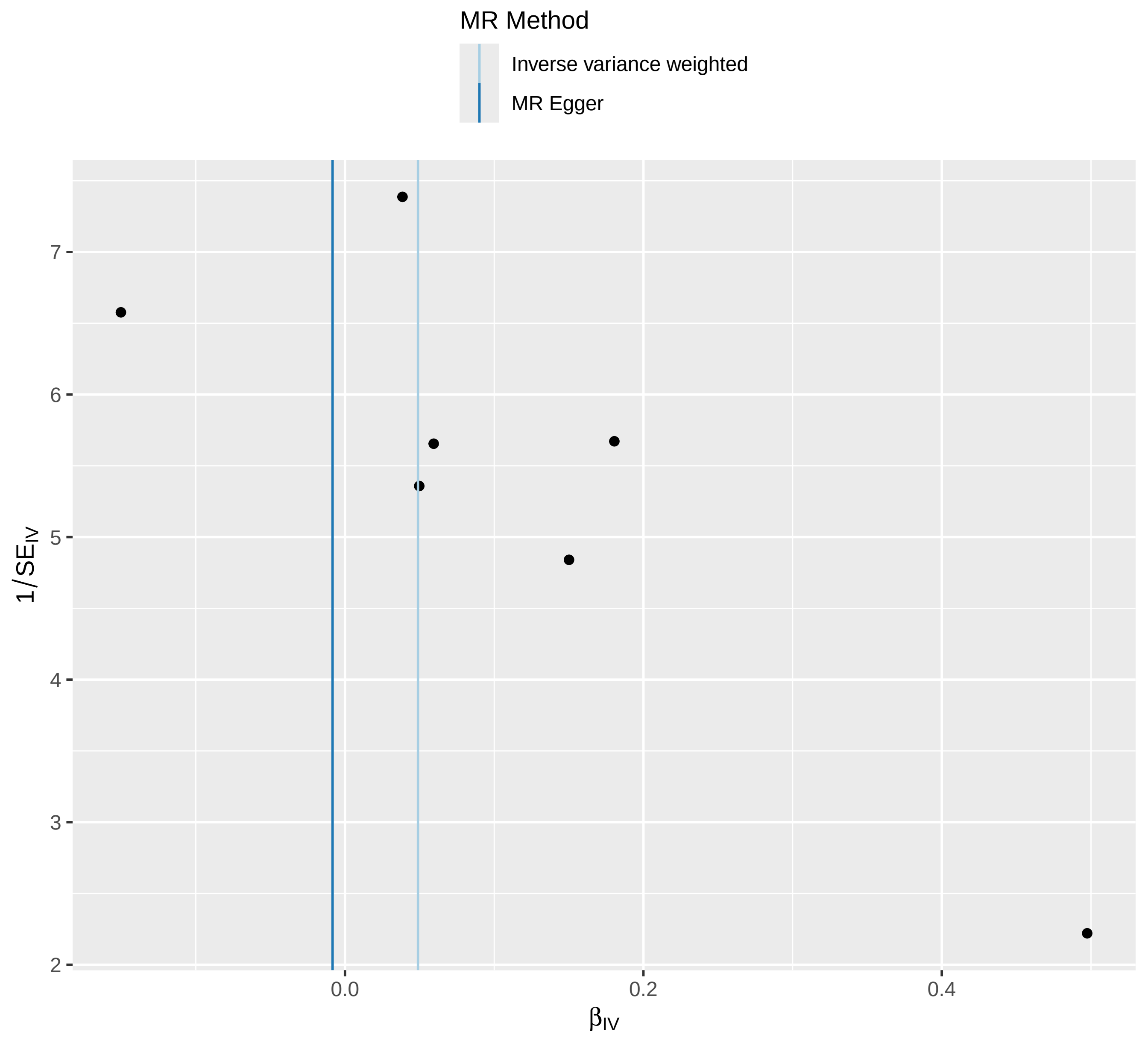
MR Method
Inverse variance weighted
MR Egger
1/SE$_{IV}$
β$_{IV}$
7
6
5
4
3
2
0.0
0.2
0.4

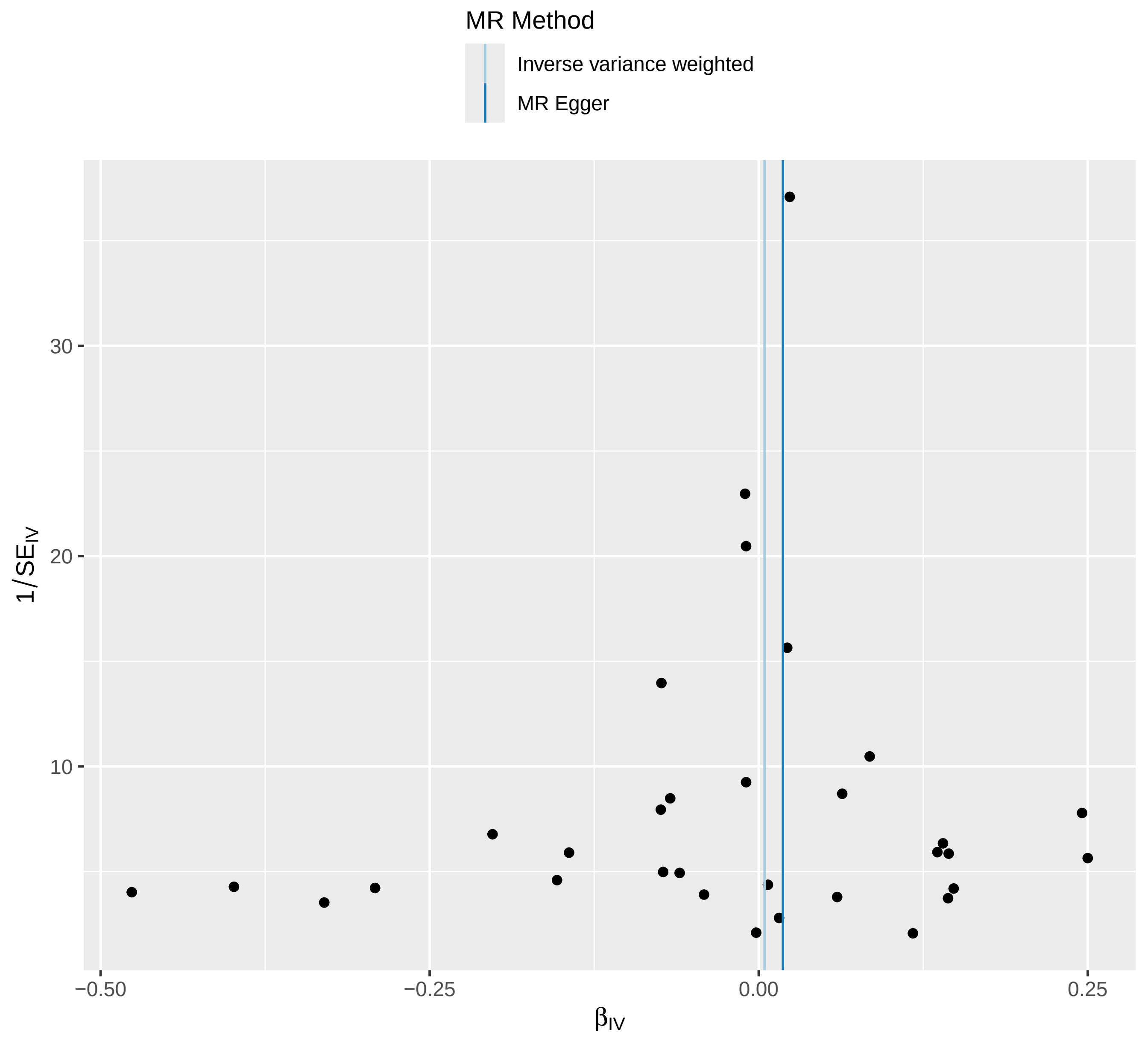
MR Method
Inverse variance weighted
MR Egger
30
20
10
1/SE$_{IV}$
−0.50
−0.25
0.00
0.25
β$_{IV}$

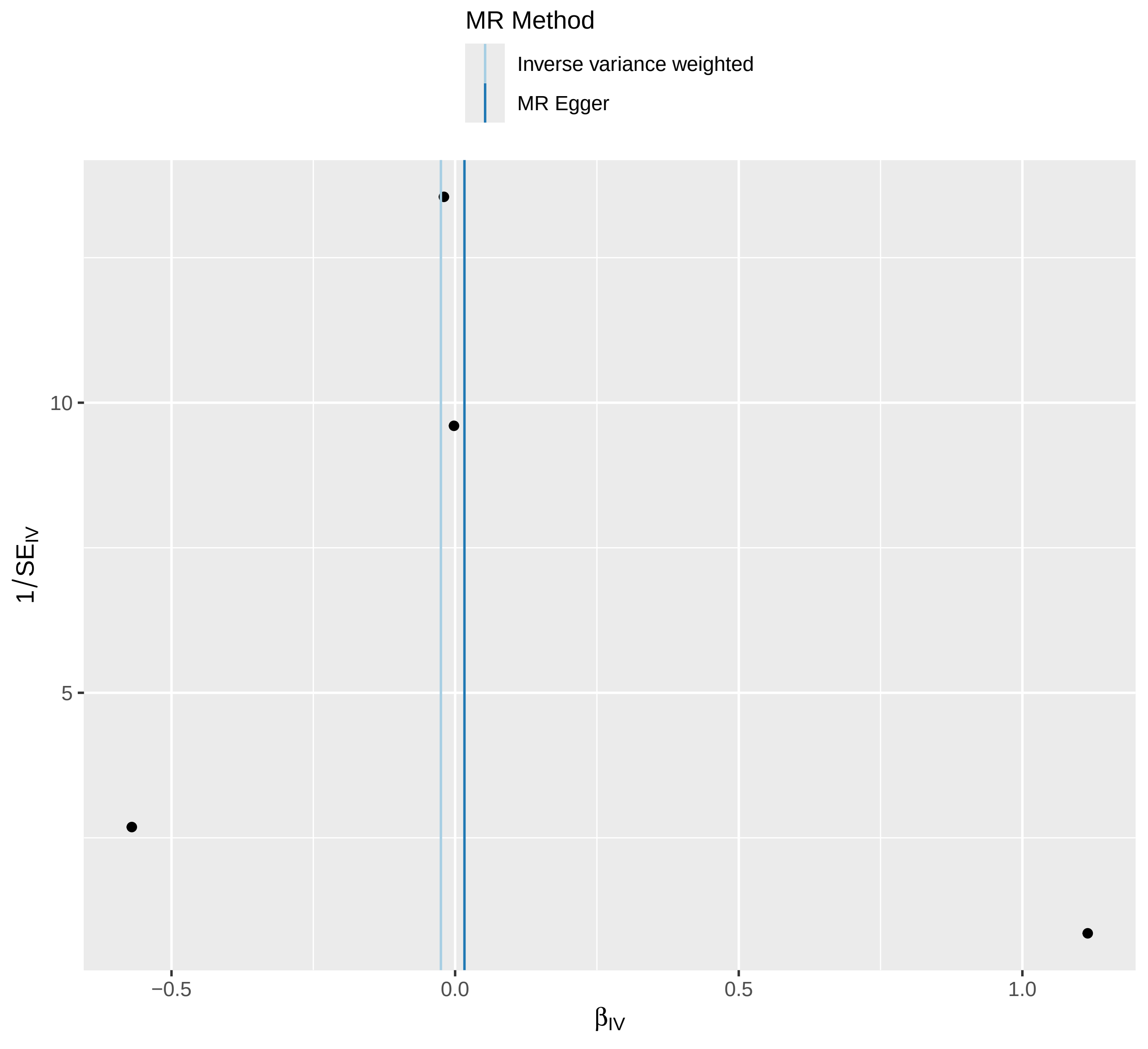
MR Method
Inverse variance weighted
MR Egger
10
5
1/SE_IV
−0.5
0.0
0.5
1.0
β_IV

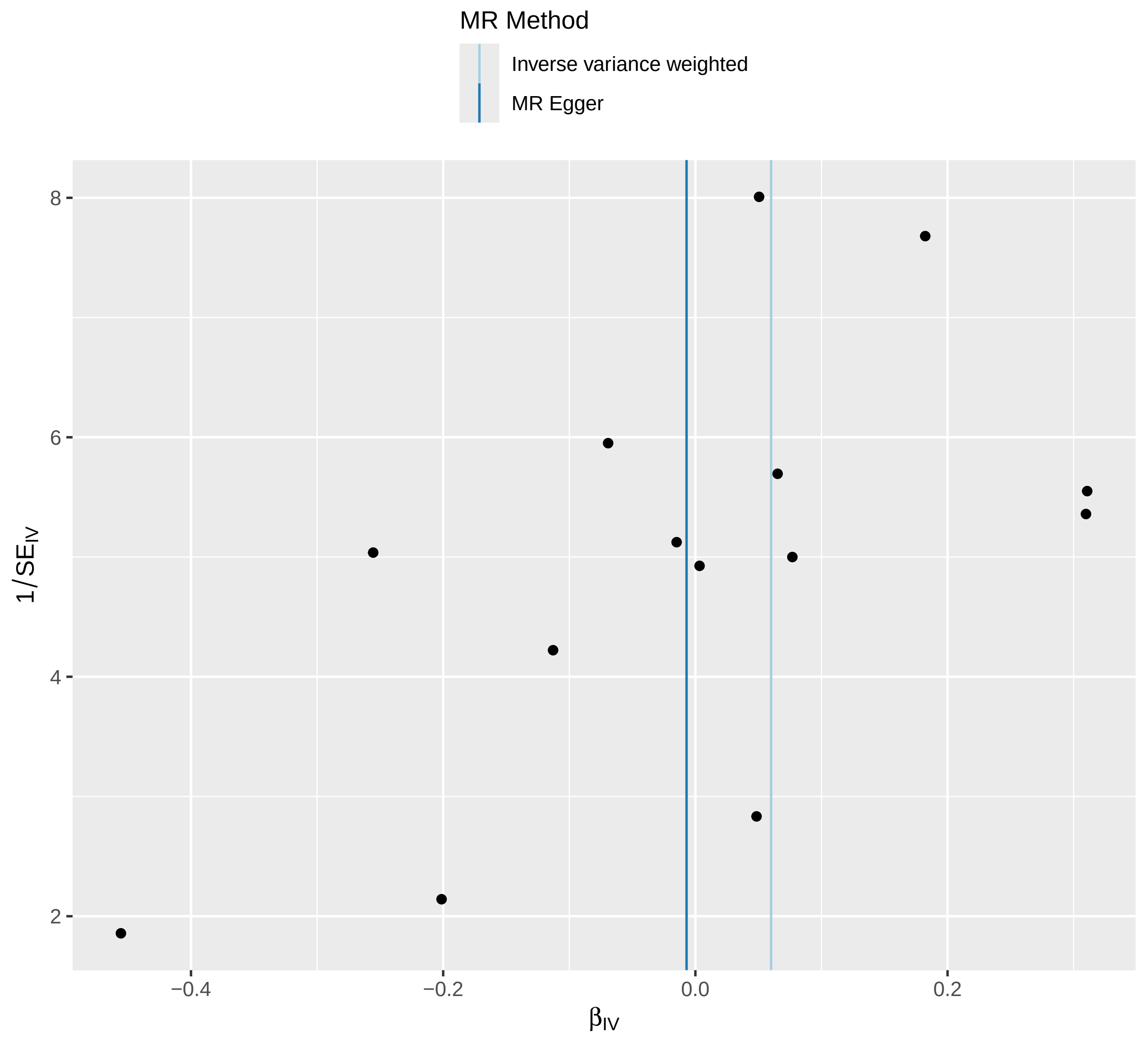
MR Method
Inverse variance weighted
MR Egger
8
6
4
2
1/SE$_{IV}$
−0.4
−0.2
0.0
0.2
β$_{IV}$

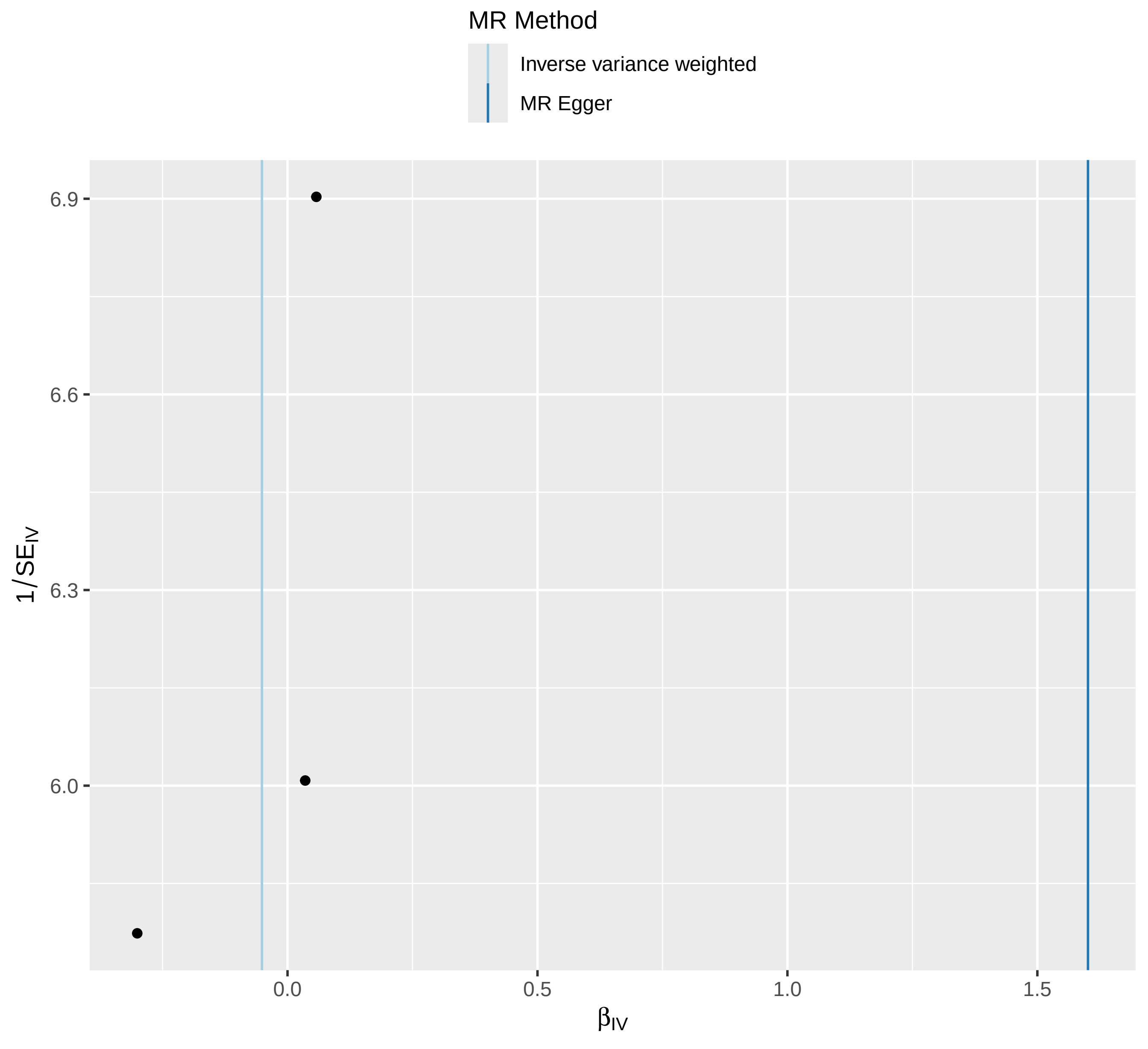

MR Method
Inverse variance weighted
MR Egger
6.9
6.6
6.3
6.0
1/SE_IV
0.0
0.5
1.0
1.5
β_IV

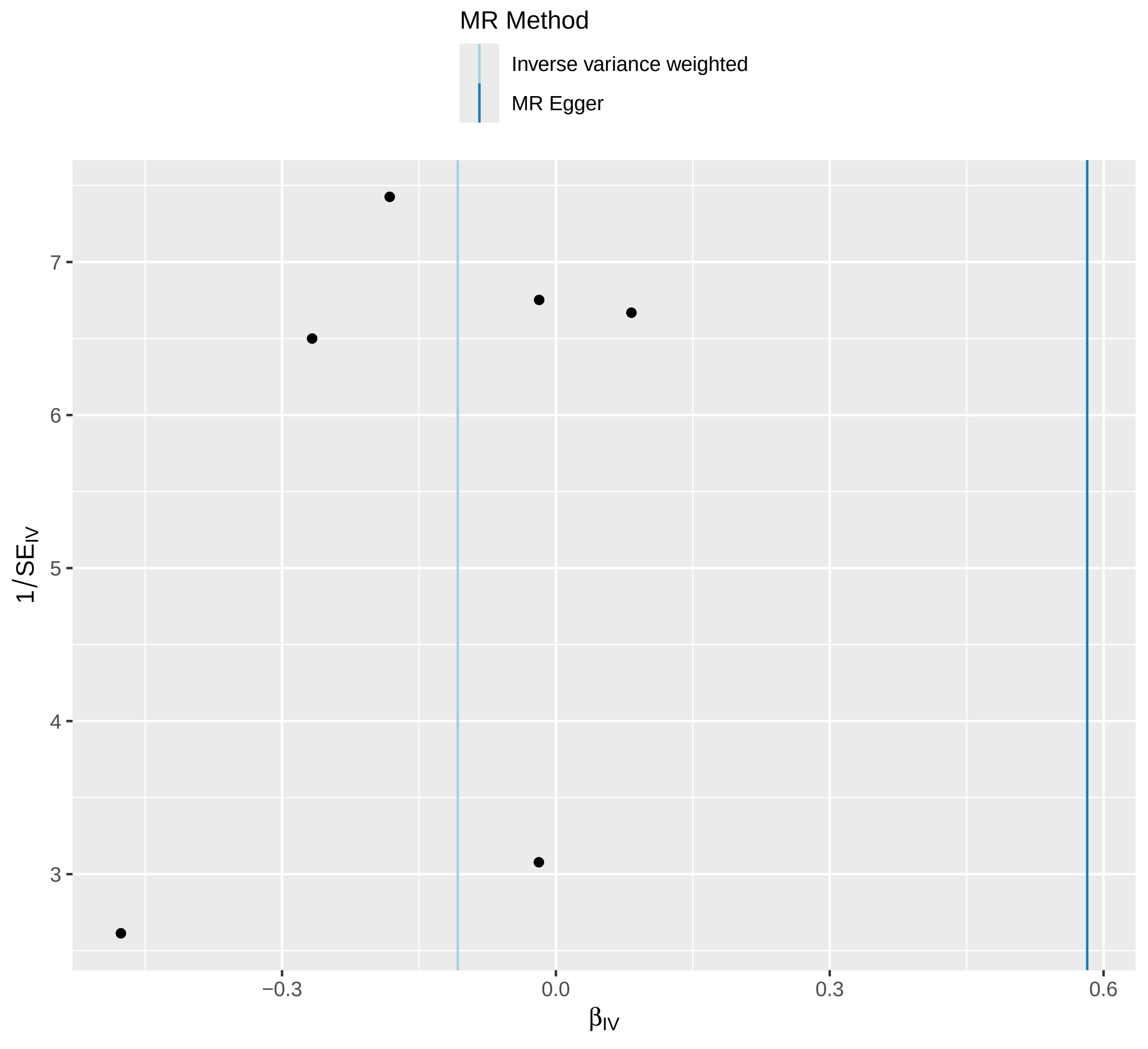
MR Method
Inverse variance weighted
MR Egger
7
6
5
4
3
1/SE$_{IV}$
−0.3
0.0
0.3
0.6
β$_{IV}$

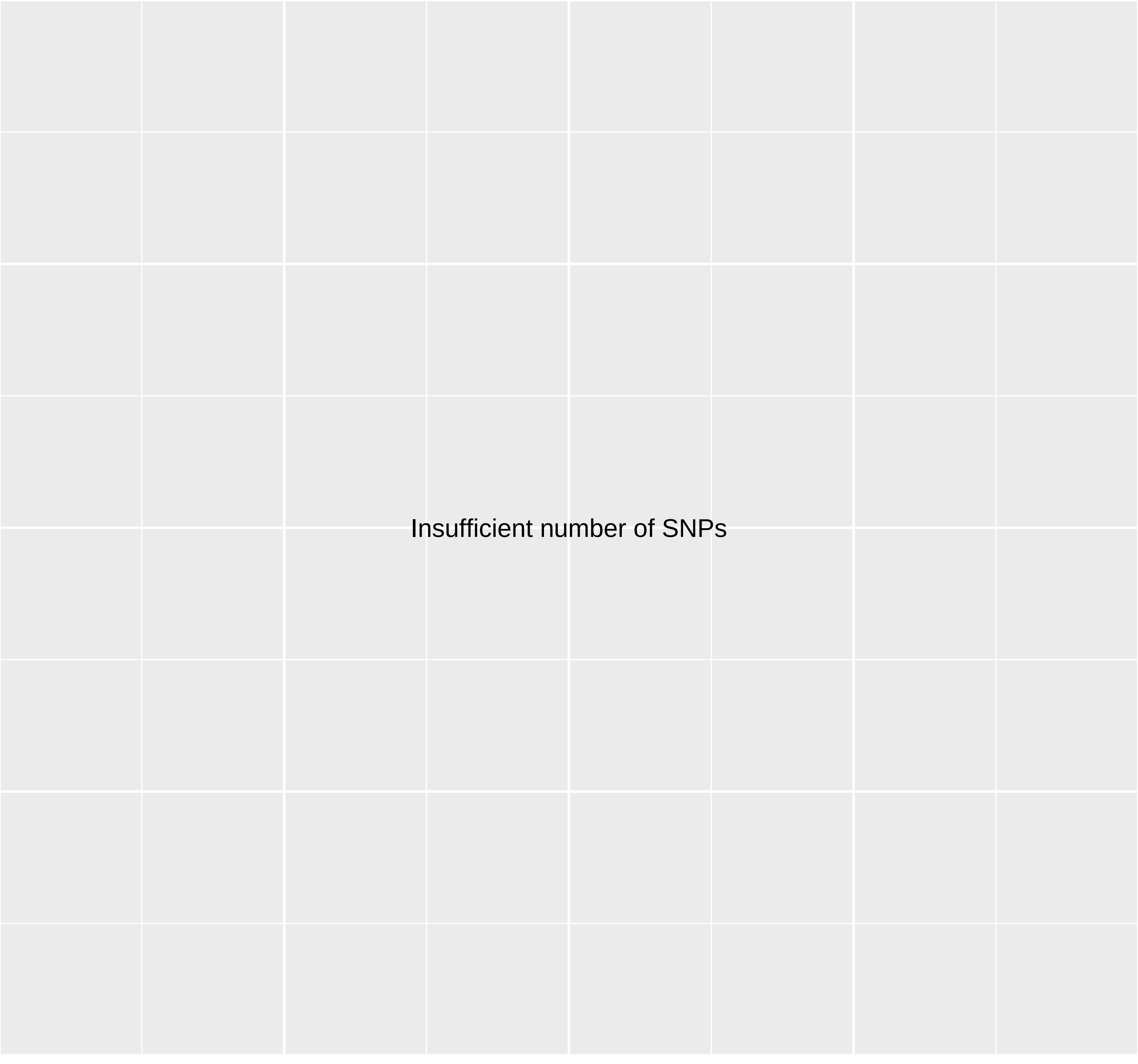
Insufficient number of SNPs

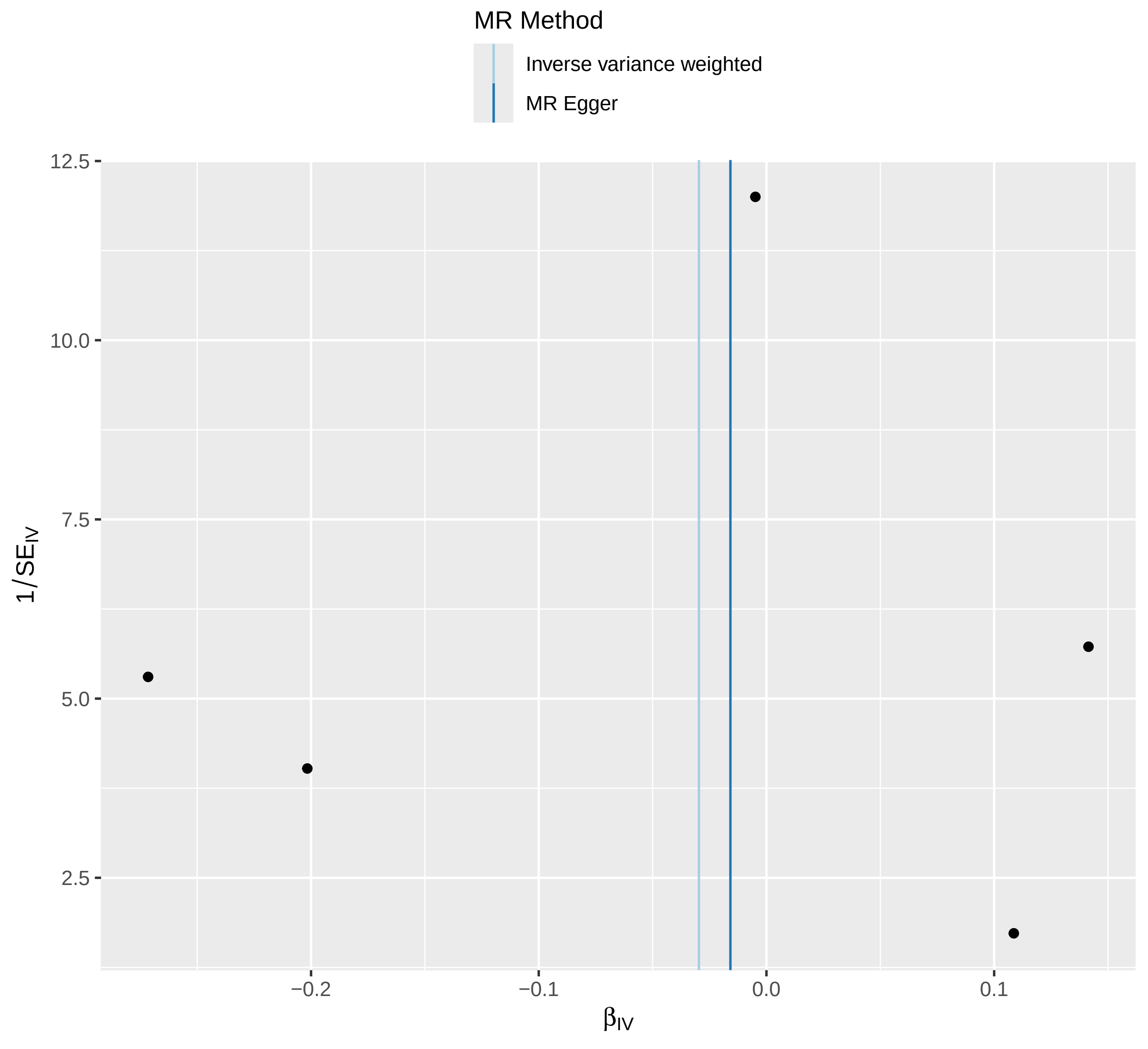

MR Method
Inverse variance weighted
MR Egger
12.5
10.0
7.5
5.0
2.5
1/SE_IV
−0.2
−0.1
0.0
0.1
β_IV

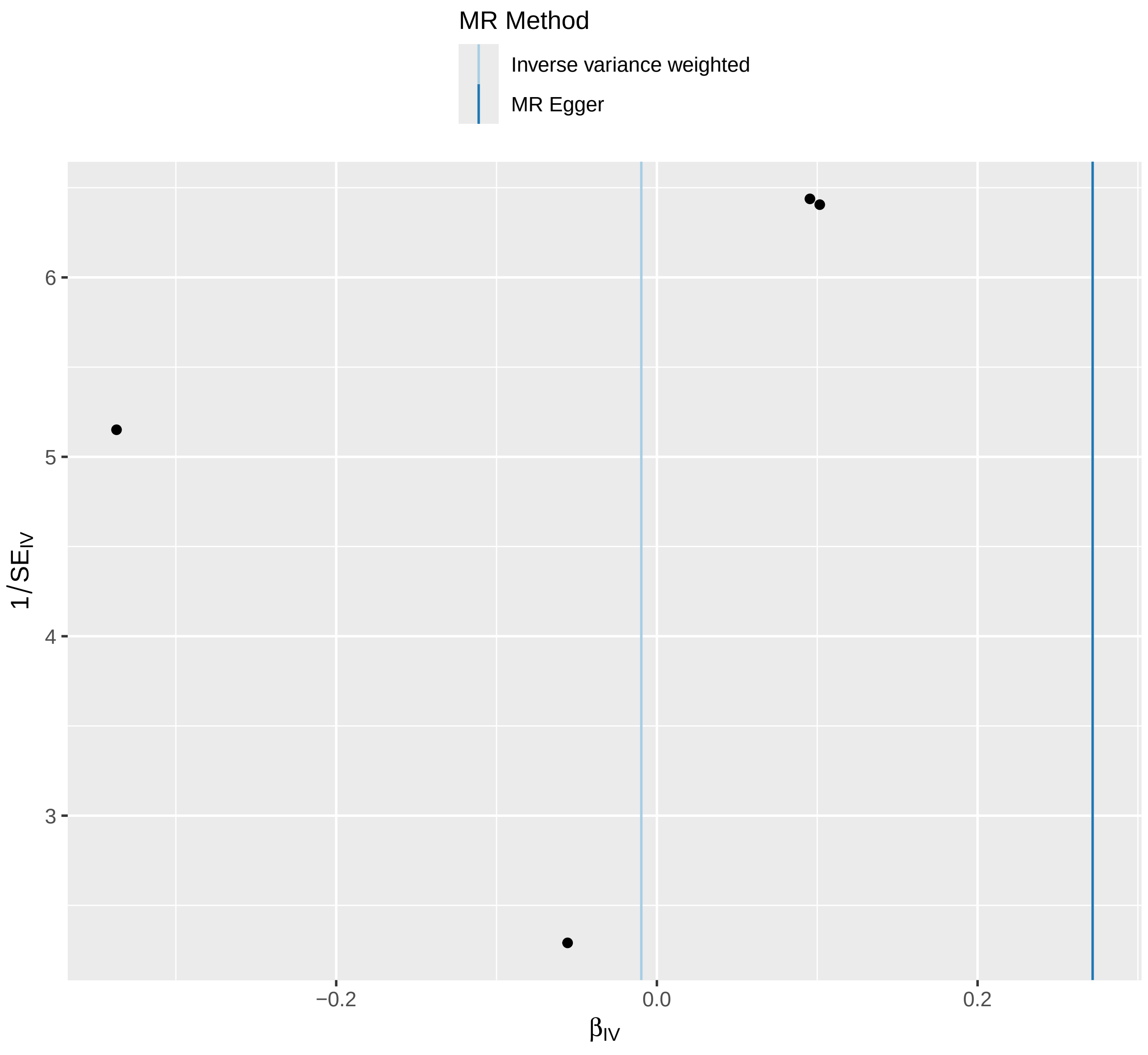
MR Method
Inverse variance weighted
MR Egger
6
5
4
3
1/SE$_{IV}$
−0.2
0.0
0.2
β$_{IV}$

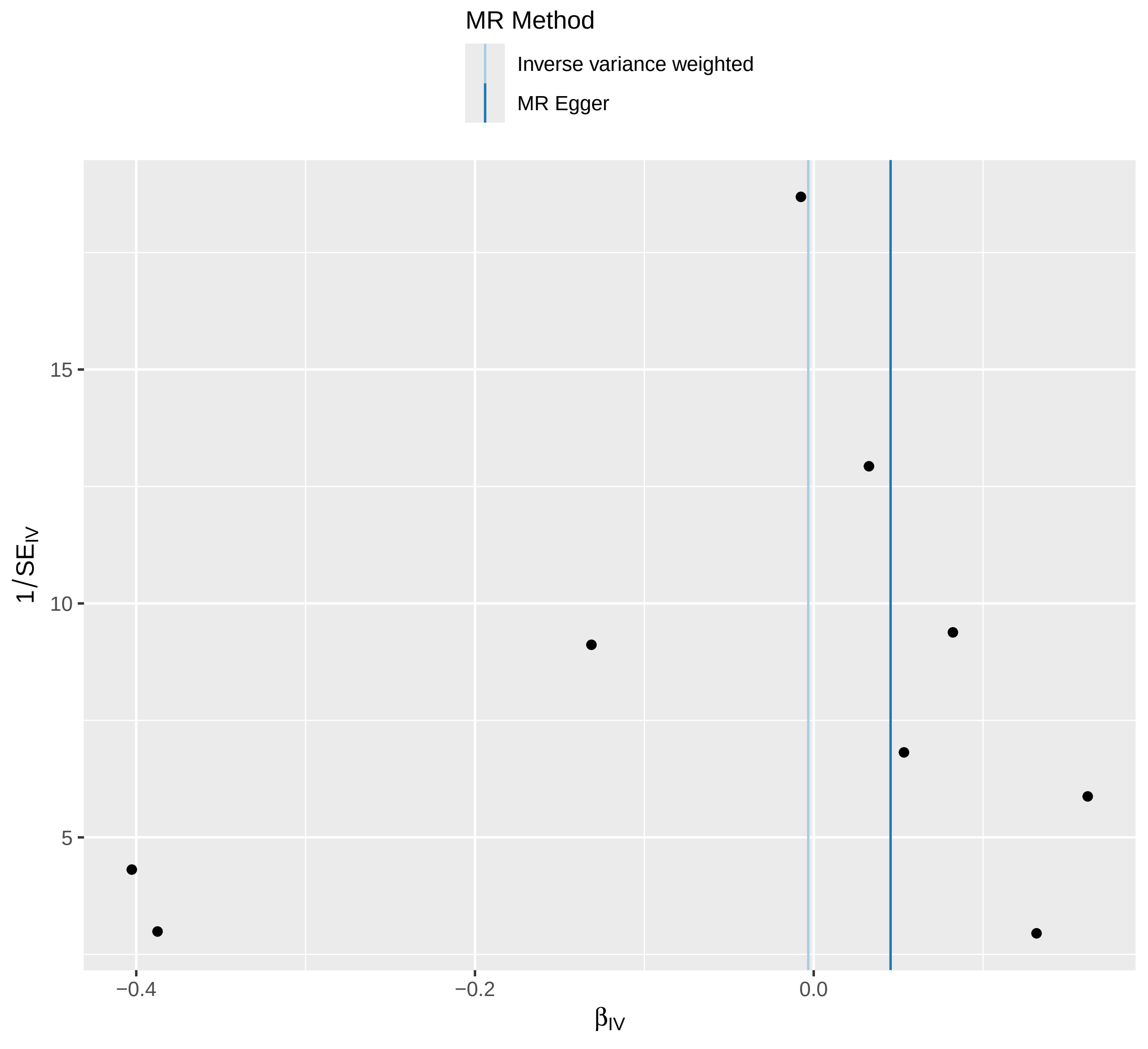
MR Method
Inverse variance weighted
MR Egger
15
10
5
$1/SE_{IV}$
−0.4
−0.2
0.0
$\beta_{IV}$

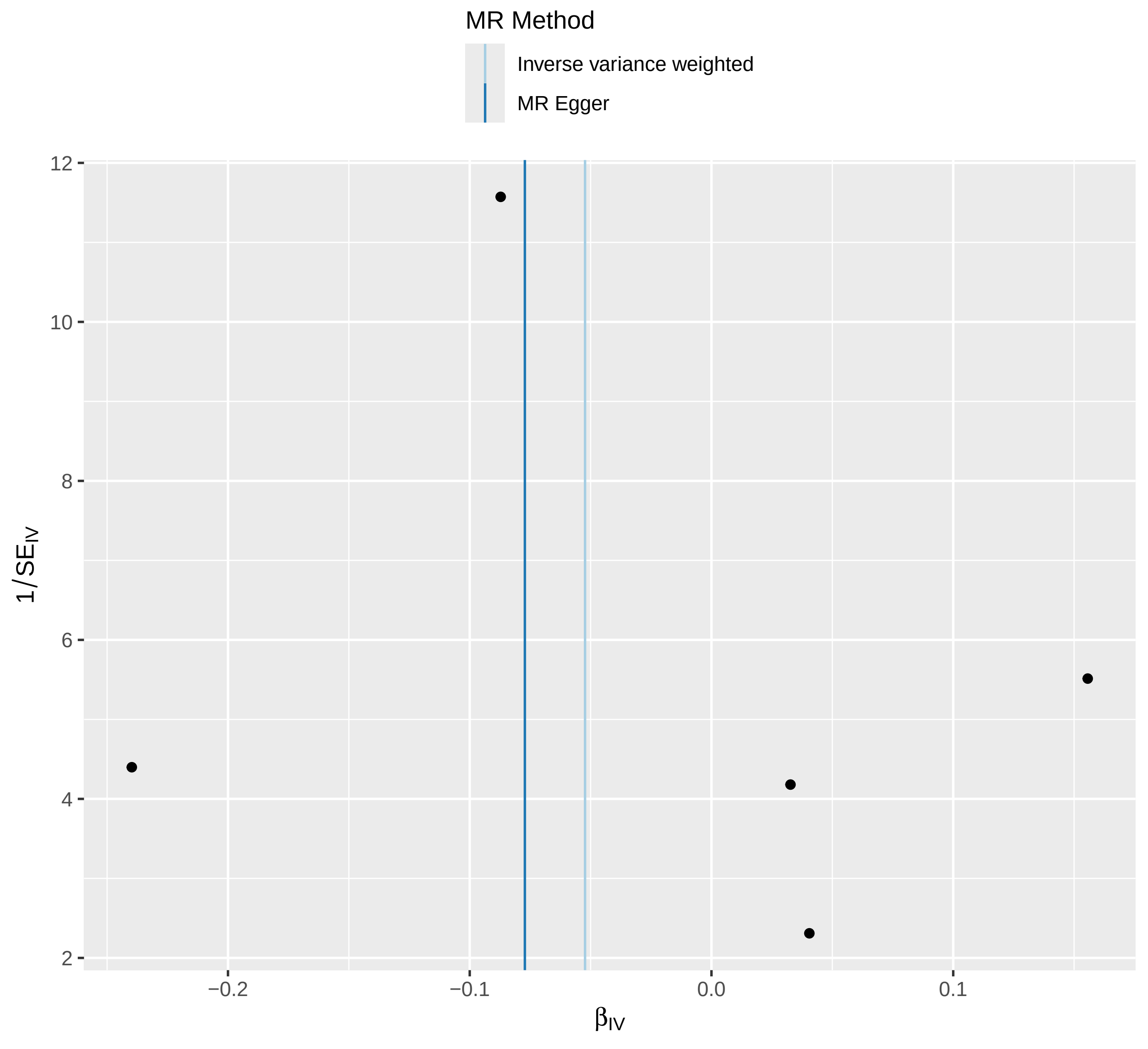

MR Method
Inverse variance weighted
MR Egger
12
10
8
6
4
2
$1/SE_{IV}$
−0.2
−0.1
0.0
0.1
$\beta_{IV}$

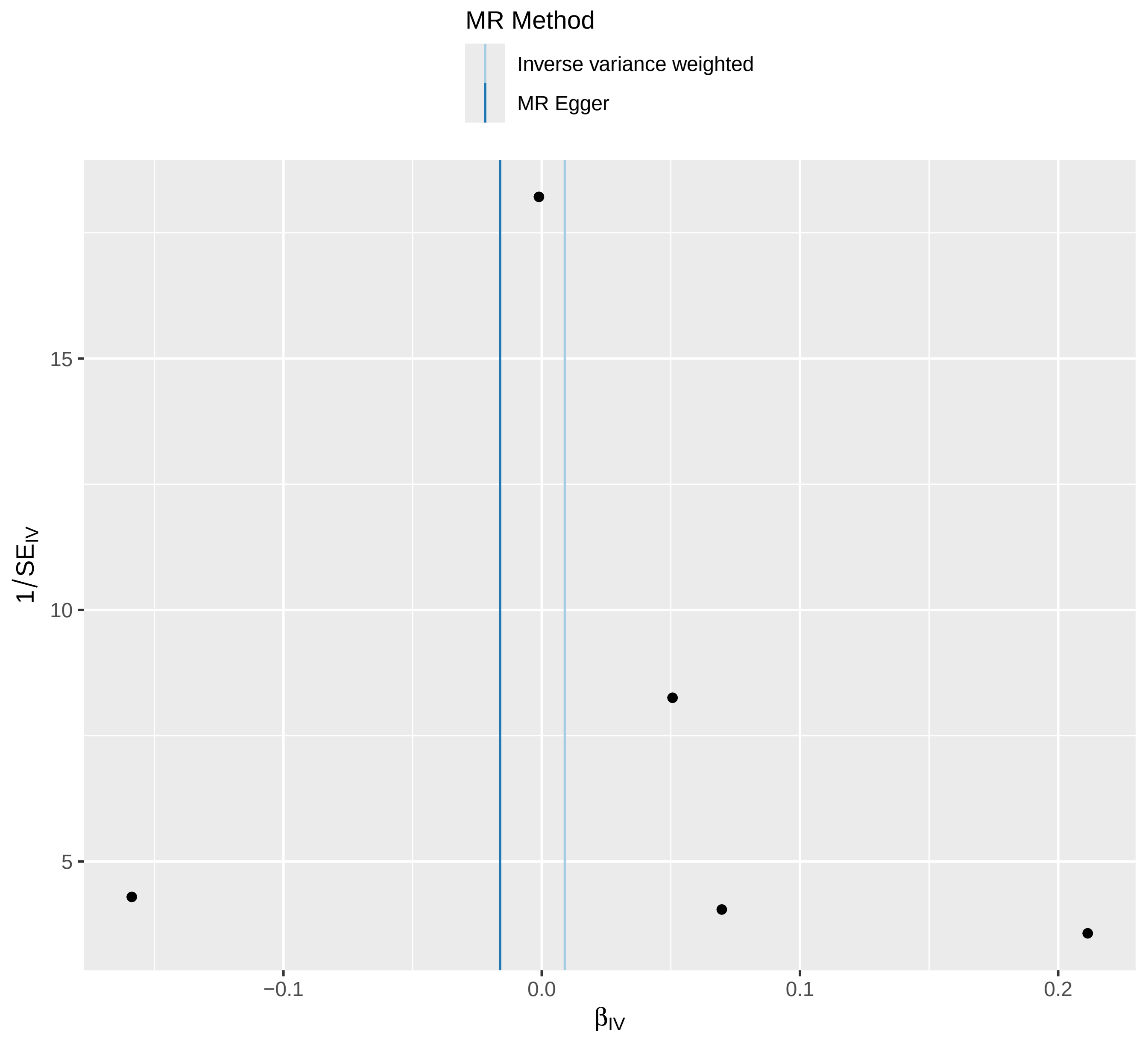
MR Method
Inverse variance weighted
MR Egger
15
10
5
1/SE$_{IV}$
−0.1
0.0
0.1
0.2
β$_{IV}$

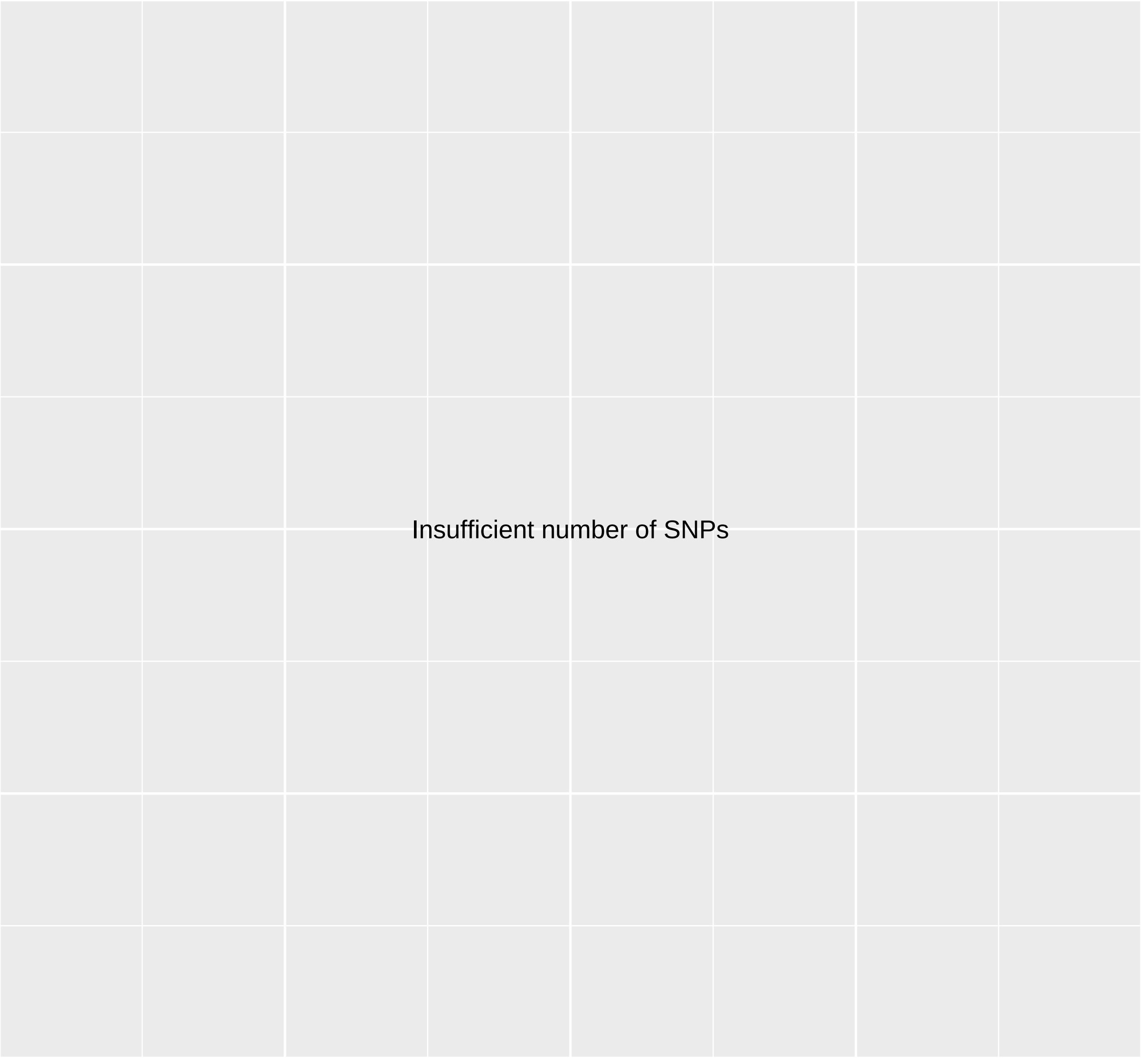
Insufficient number of SNPs

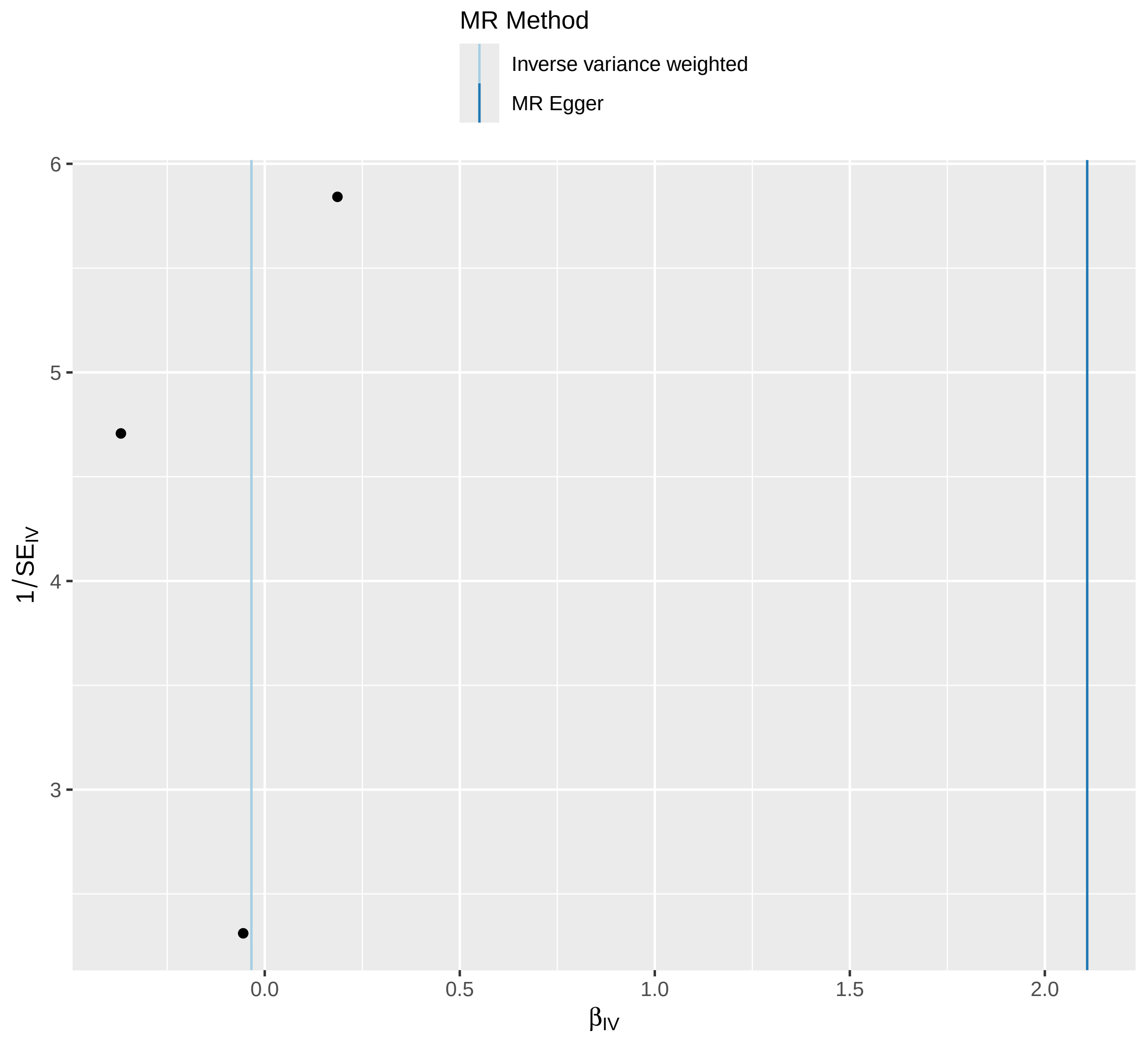
MR Method
Inverse variance weighted
MR Egger
6
5
4
3
$1/SE_{IV}$
0.0
0.5
1.0
1.5
2.0
$\beta_{IV}$

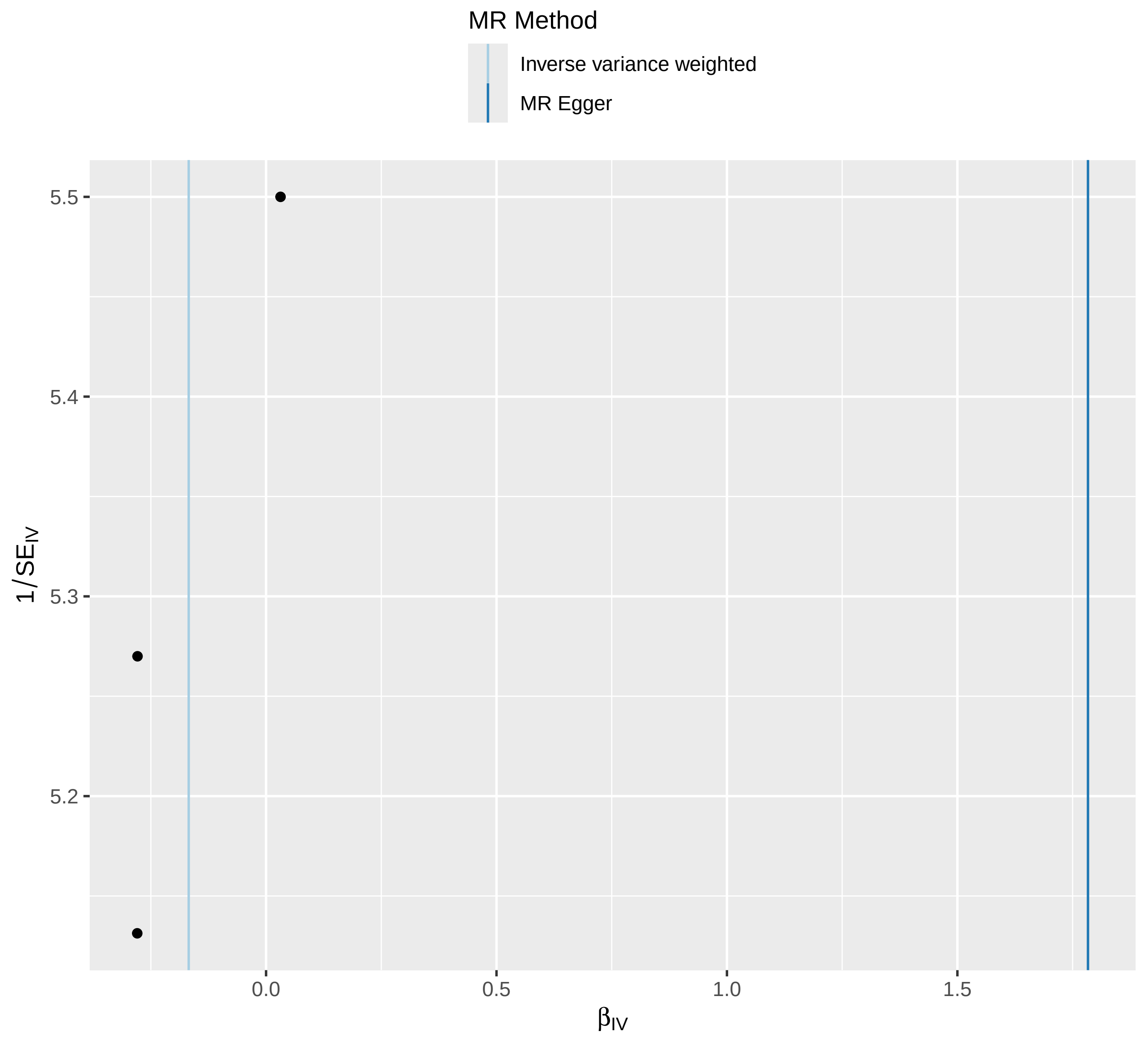
MR Method
Inverse variance weighted
MR Egger
5.5
5.4
5.3
5.2
1/SE_IV
0.0
0.5
1.0
1.5
β_IV

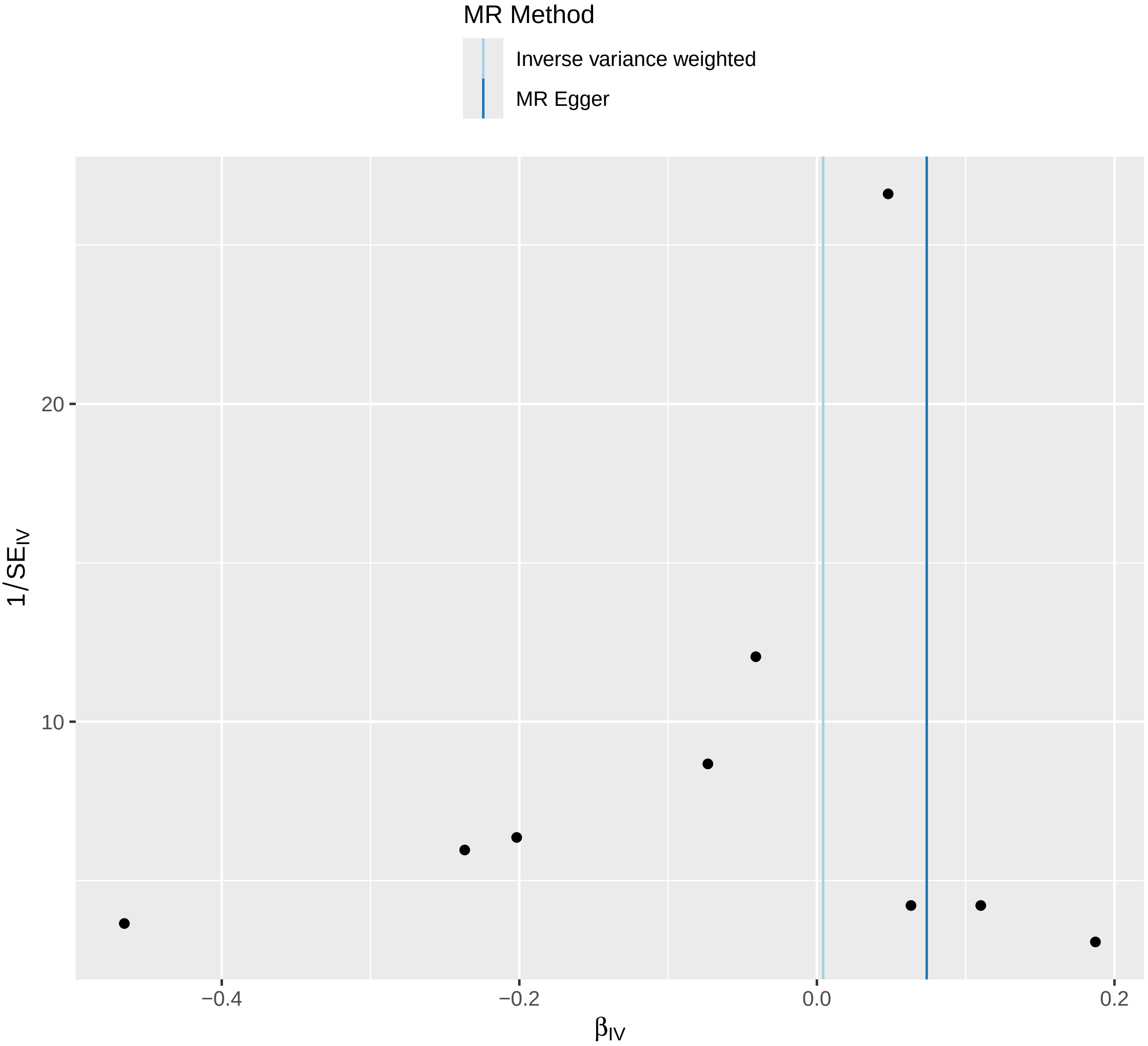

MR Method
Inverse variance weighted
MR Egger
20
10
1/SE_IV
−0.4
−0.2
0.0
0.2
β_IV

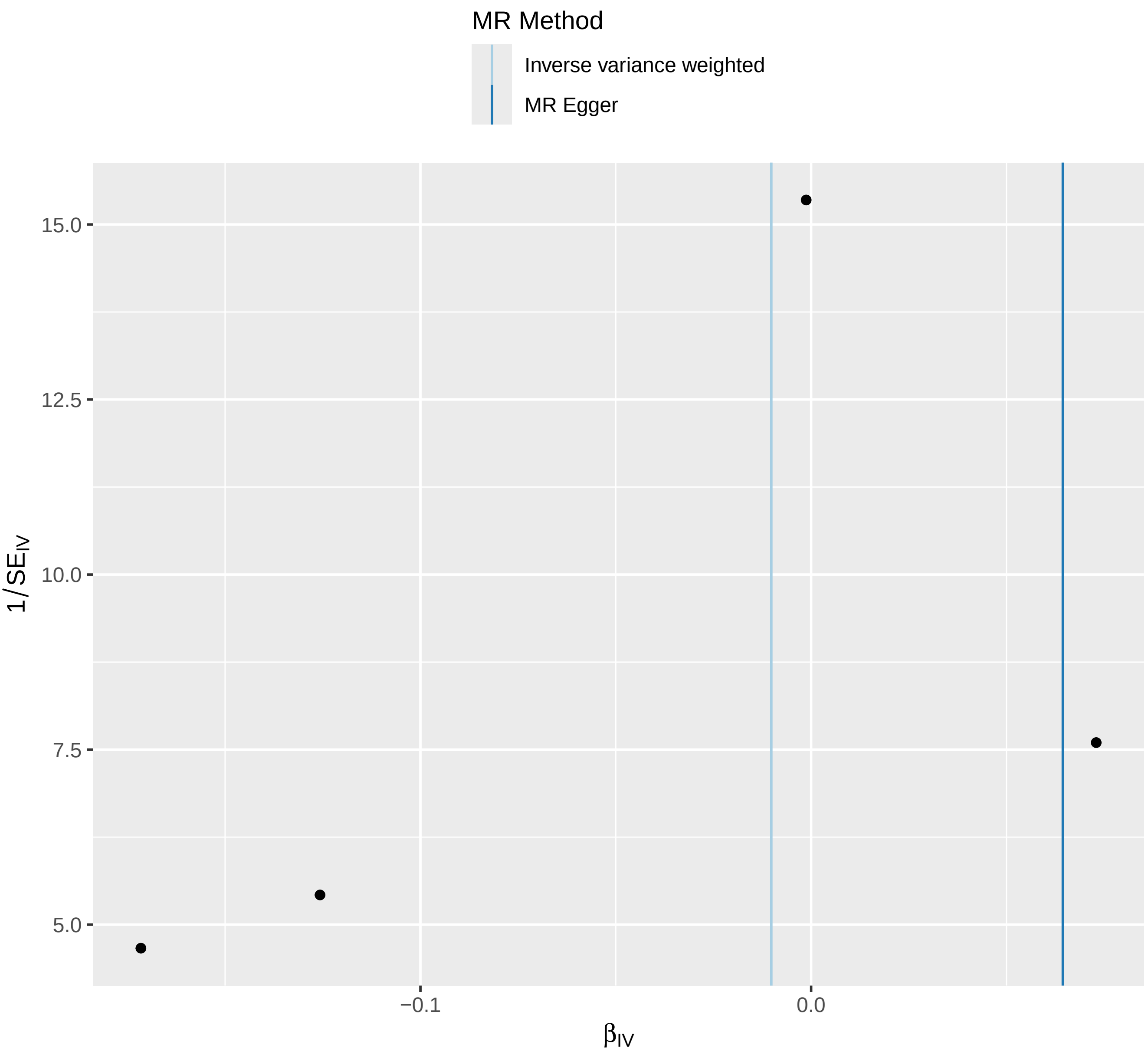
MR Method
Inverse variance weighted
MR Egger
15.0
12.5
10.0
7.5
5.0
−0.1
0.0
βIV
1/SEIV

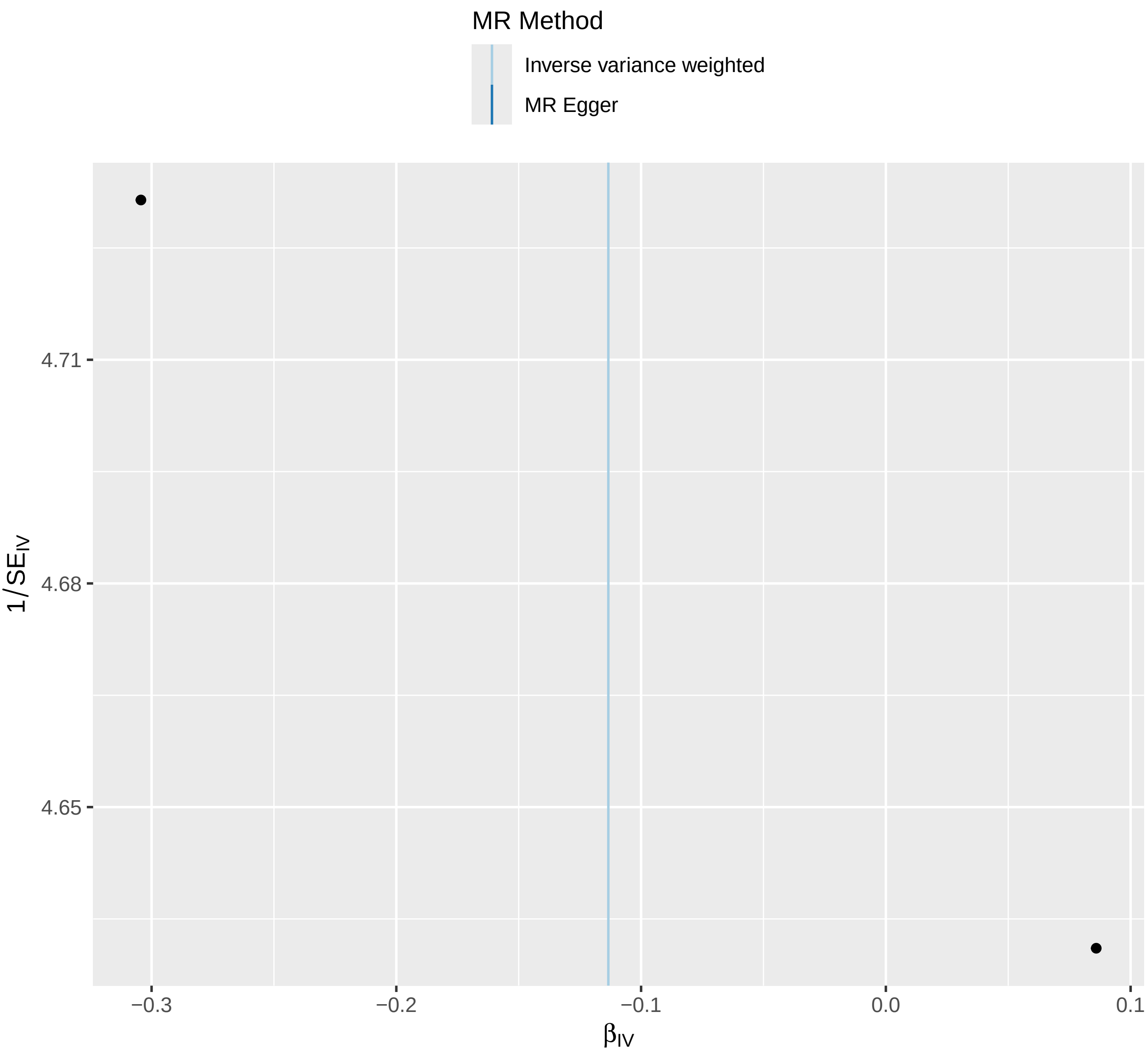

MR Method
Inverse variance weighted
MR Egger
4.71
4.68
4.65
1/SE_IV
−0.3
−0.2
−0.1
0.0
0.1
β_IV

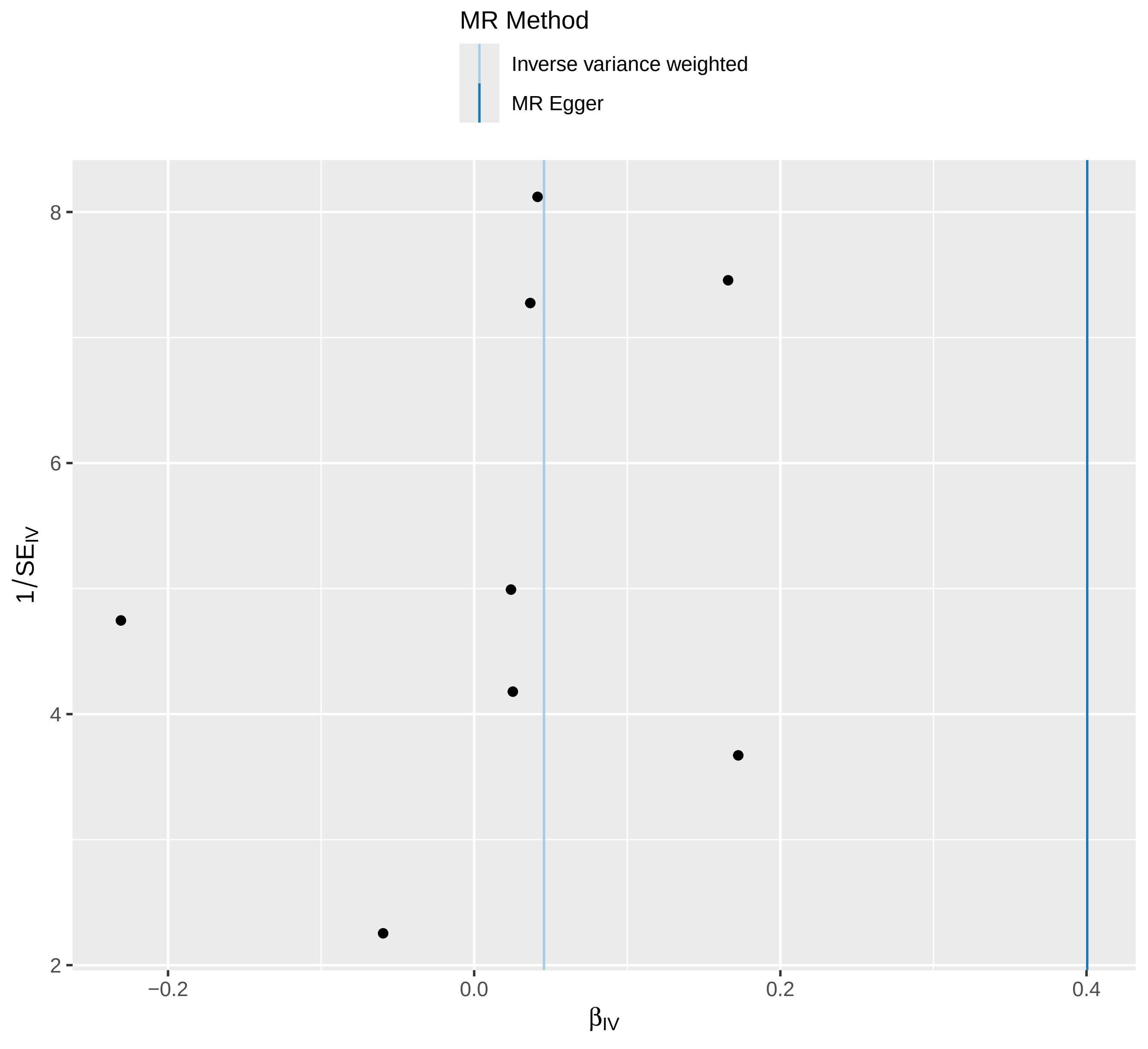

MR Method
Inverse variance weighted
MR Egger
8
6
4
2
1/SE_IV
−0.2
0.0
0.2
0.4
β_IV

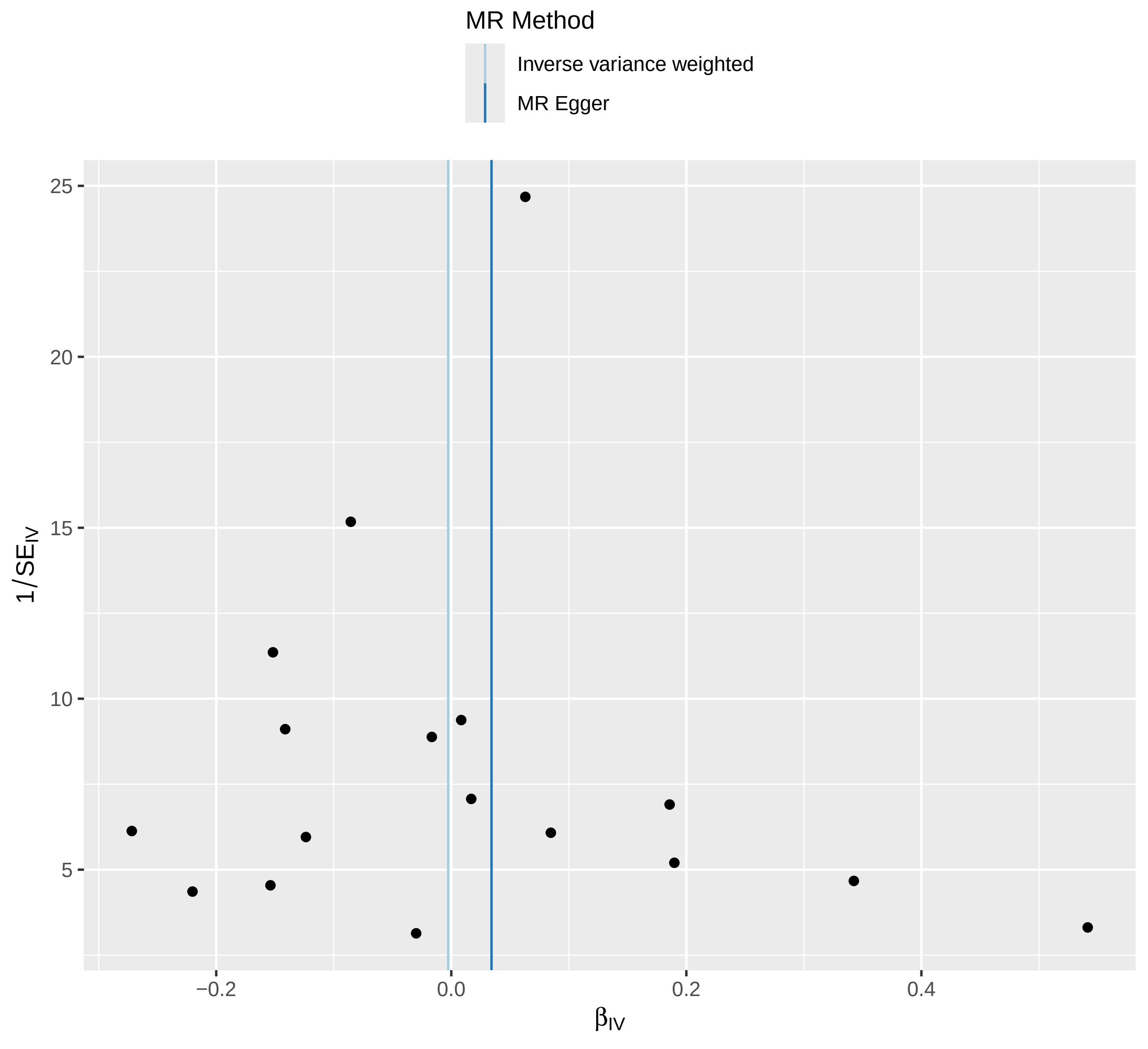

MR Method
Inverse variance weighted
MR Egger
25
20
15
10
5
1/SE$_{IV}$
−0.2
0.0
0.2
0.4
β$_{IV}$

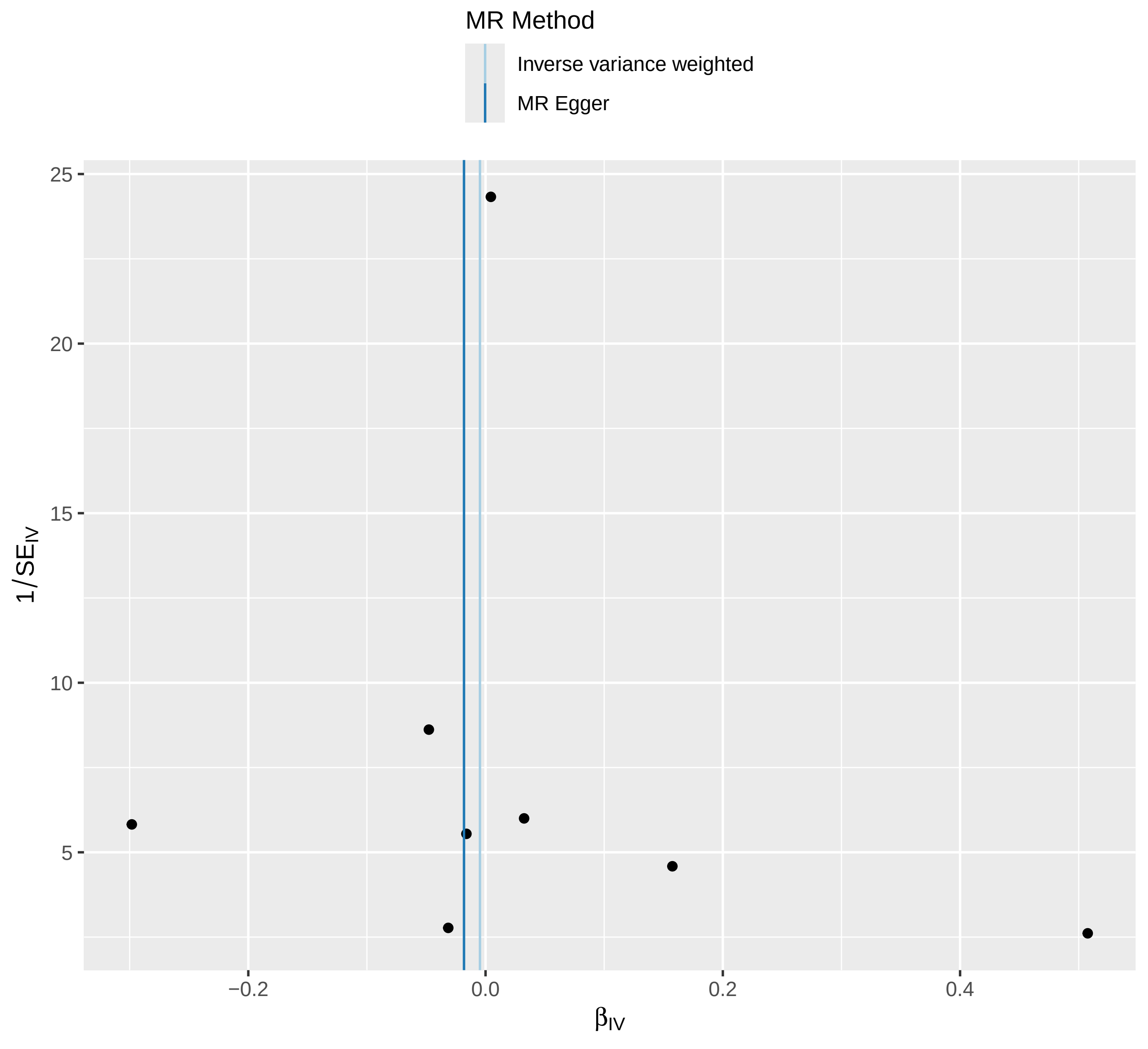

MR Method
Inverse variance weighted
MR Egger
25
20
15
10
5
1/SE_IV
−0.2
0.0
0.2
0.4
β_IV

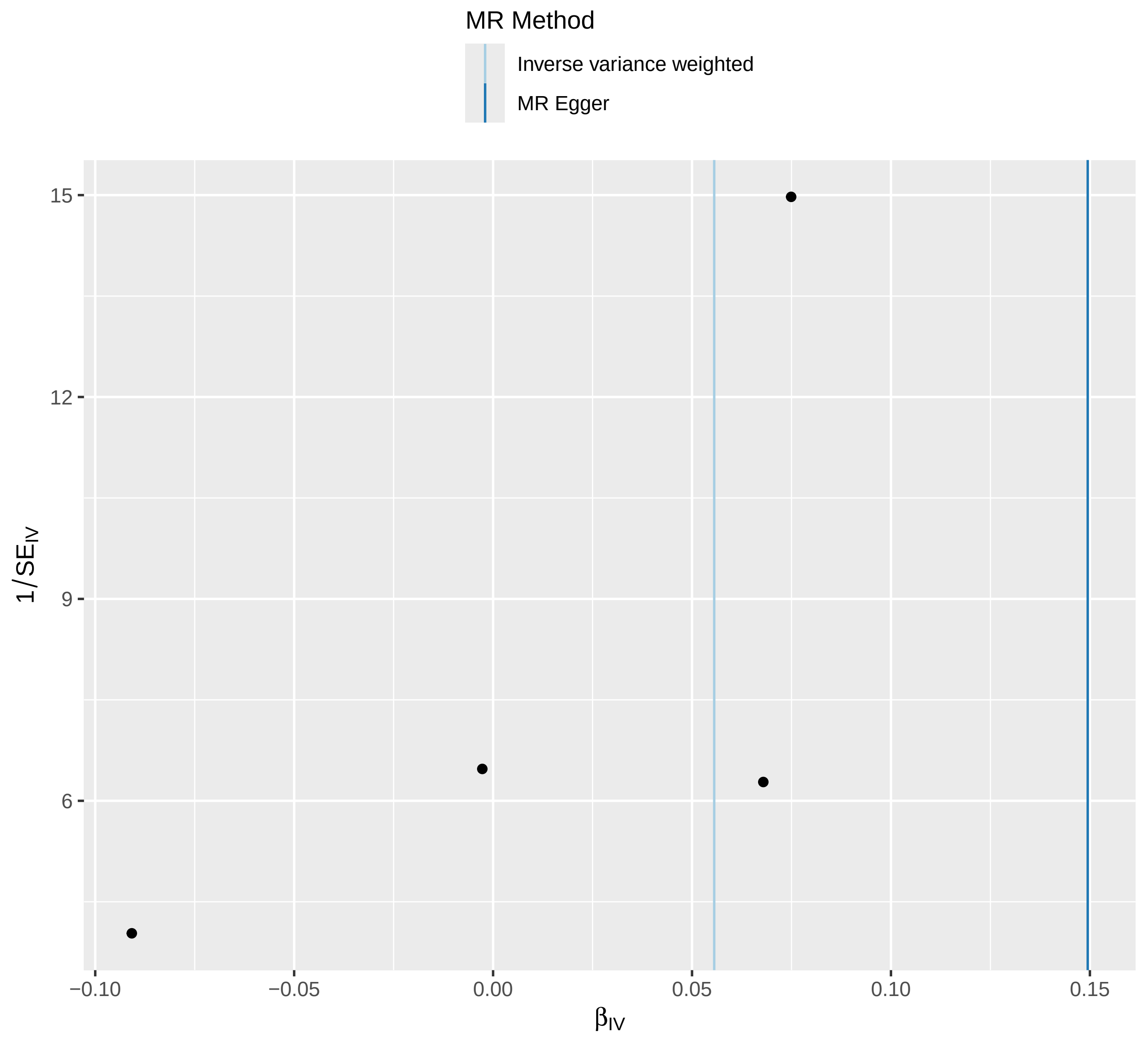

MR Method
Inverse variance weighted
MR Egger
15
12
9
6
1/SE$_{IV}$
−0.10
−0.05
0.00
0.05
0.10
0.15
β$_{IV}$

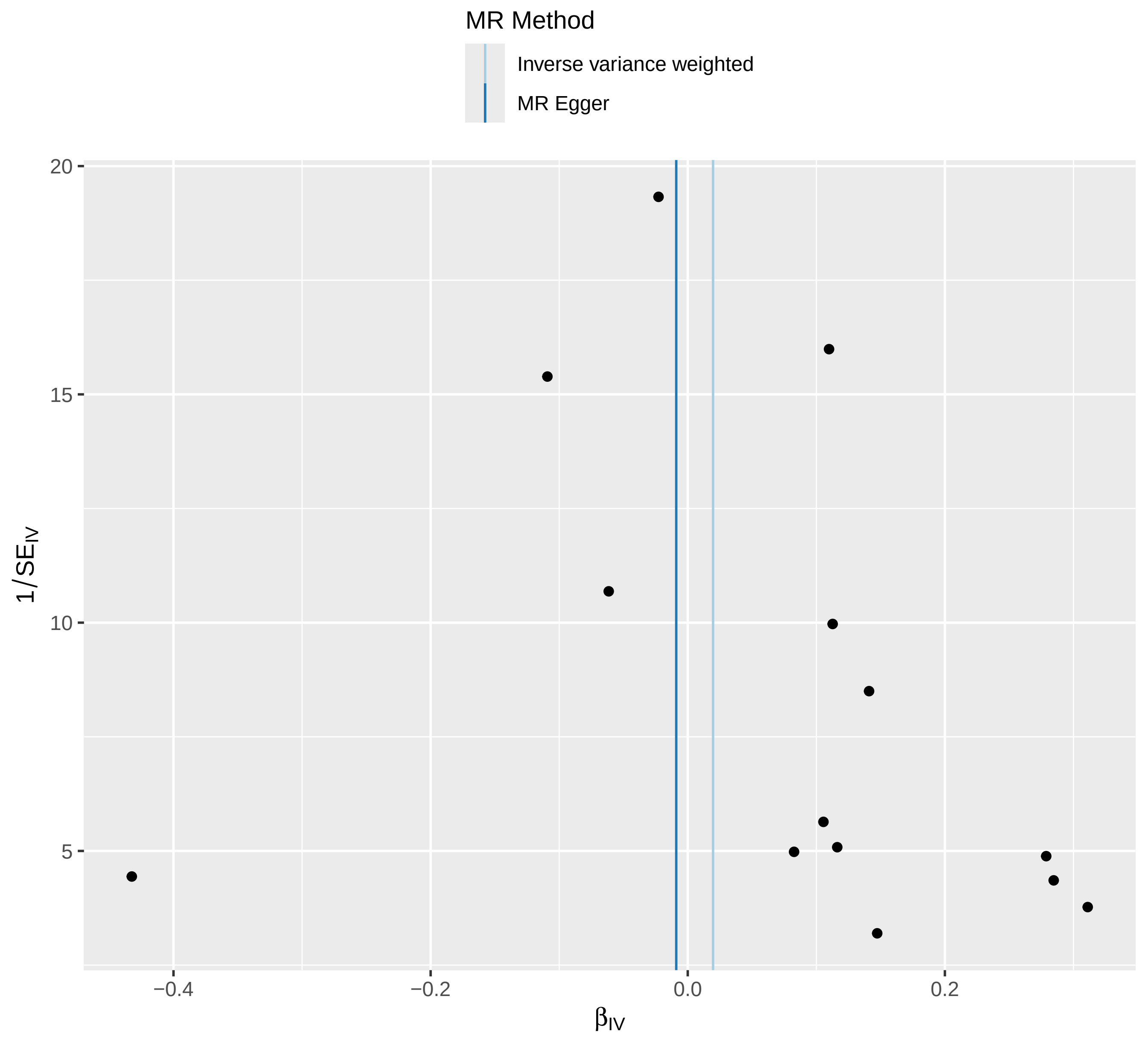

MR Method
Inverse variance weighted
MR Egger
20
15
10
5
1/SE_IV
−0.4
−0.2
0.0
0.2
β_IV

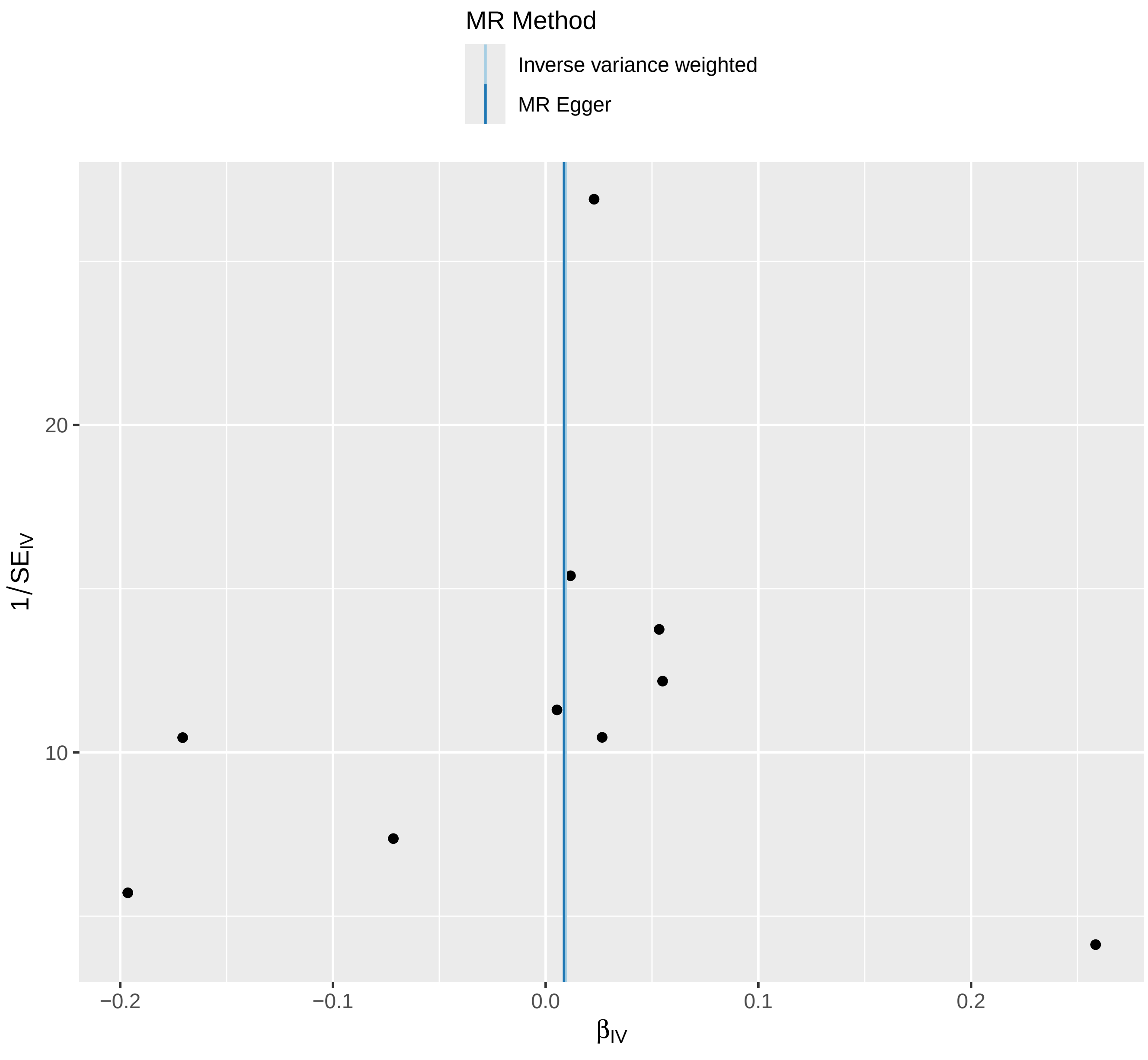
MR Method
Inverse variance weighted
MR Egger
20
10
1/SE_IV
−0.2
−0.1
0.0
0.1
0.2
β_IV

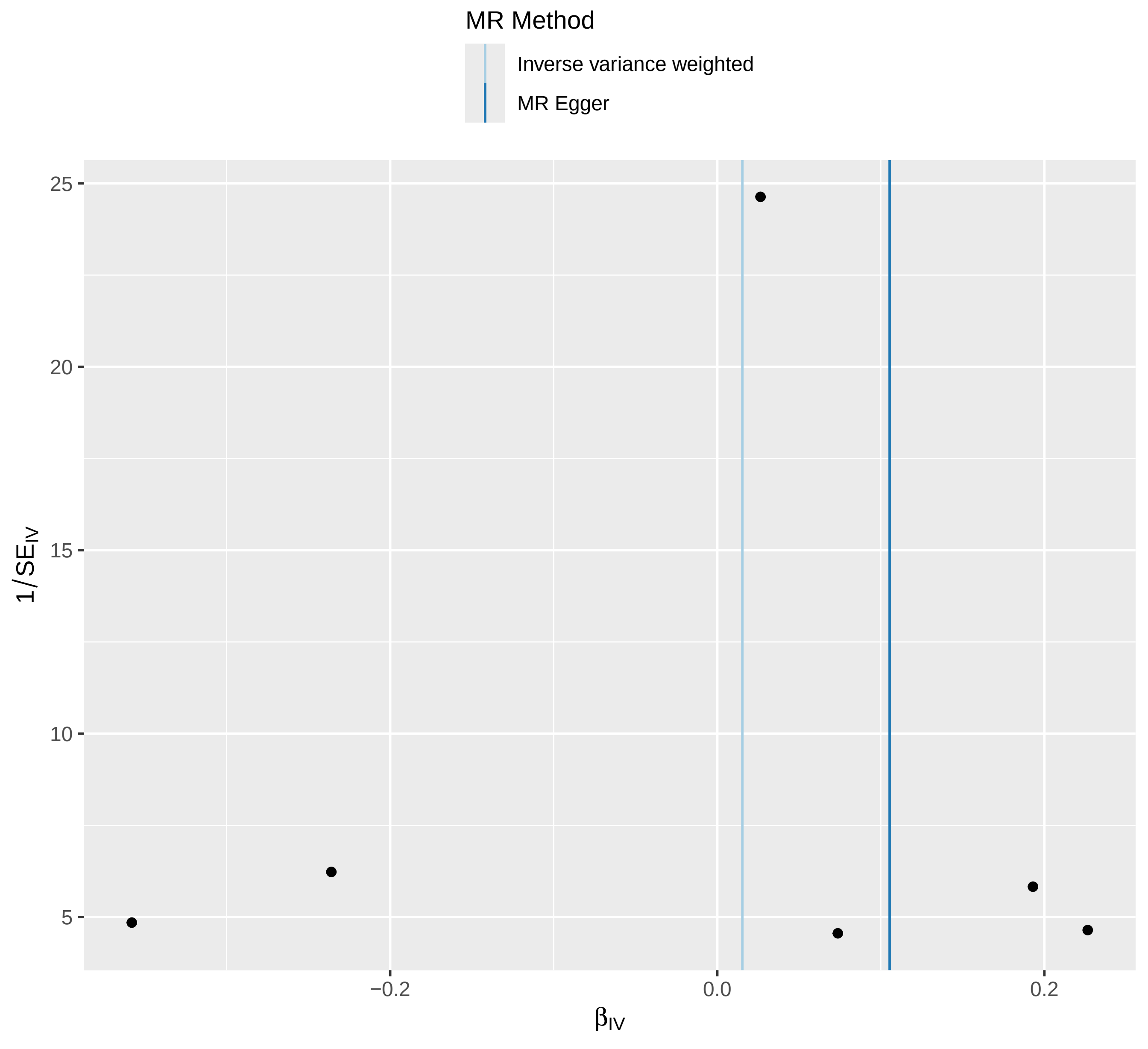

MR Method
Inverse variance weighted
MR Egger
25
20
15
10
5
1/SE_IV
−0.2
0.0
0.2
β_IV

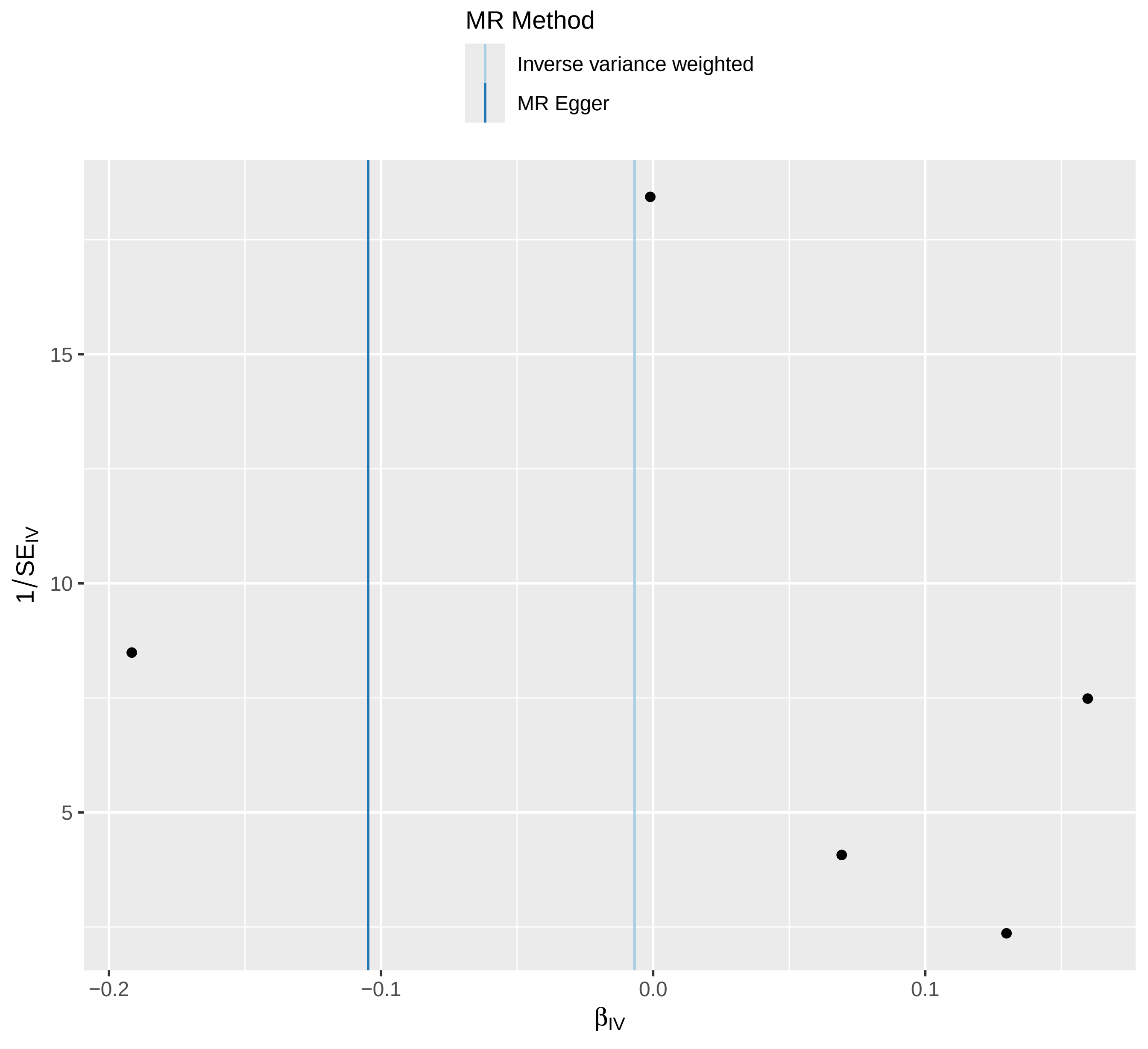

MR Method
Inverse variance weighted
MR Egger
15
10
5
$1/SE_{IV}$
−0.2
−0.1
0.0
0.1
$\beta_{IV}$

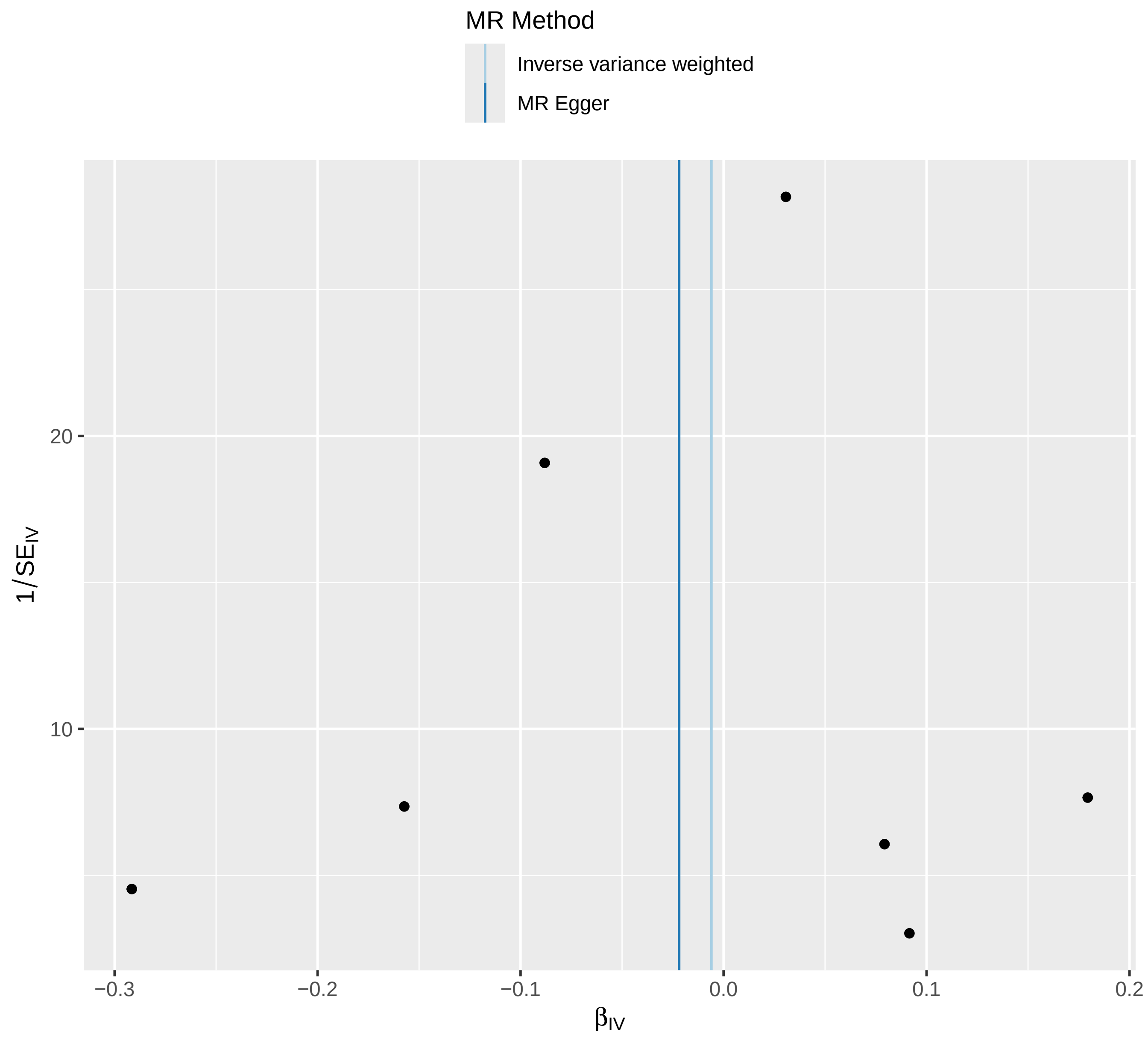
MR Method
Inverse variance weighted
MR Egger
20
10
1/SE_IV
−0.3
−0.2
−0.1
0.0
0.1
0.2
β_IV

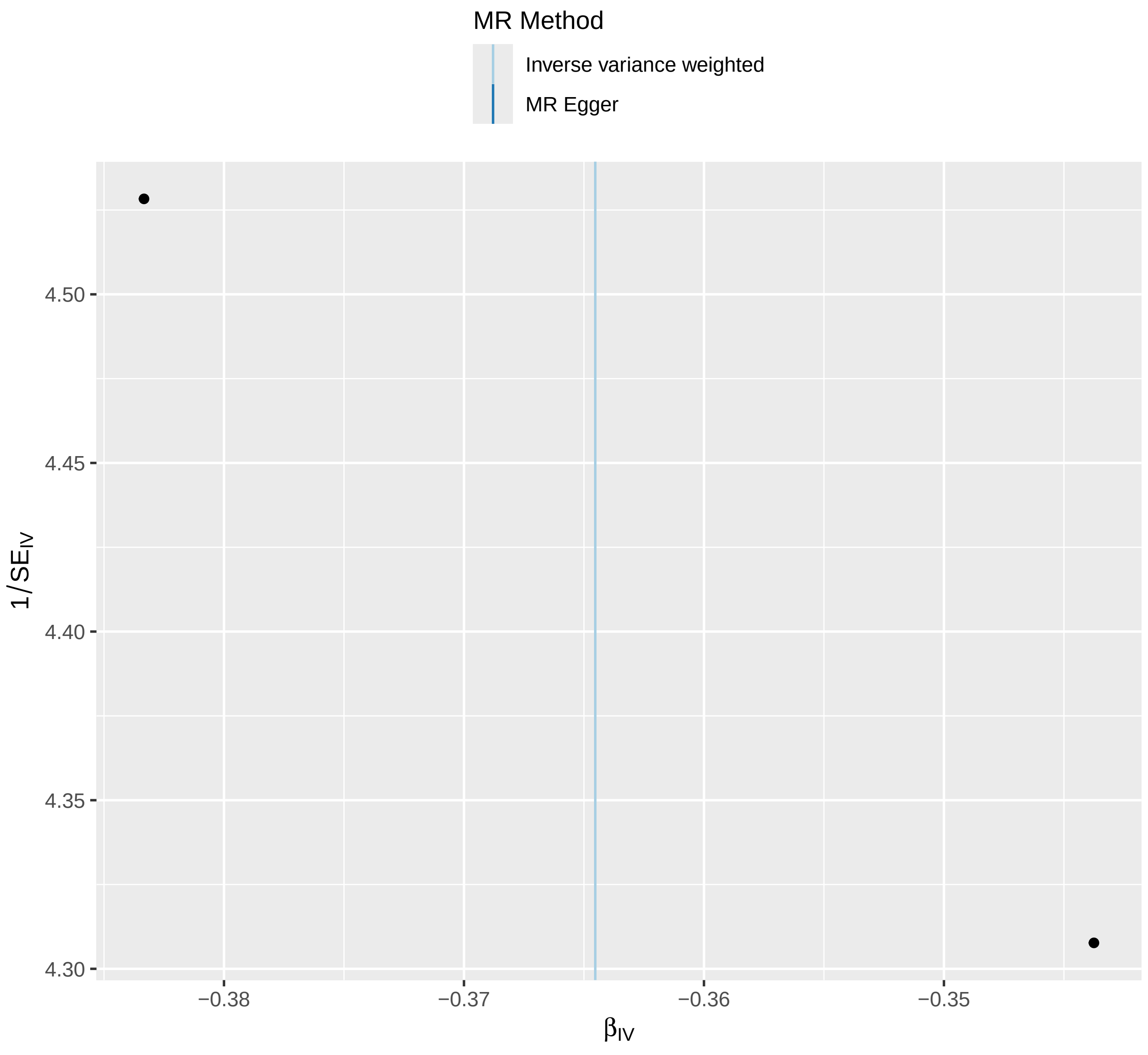
MR Method
Inverse variance weighted
MR Egger
$1/SE_{IV}$
4.50
4.45
4.40
4.35
4.30
−0.38
−0.37
−0.36
−0.35
$\beta_{IV}$

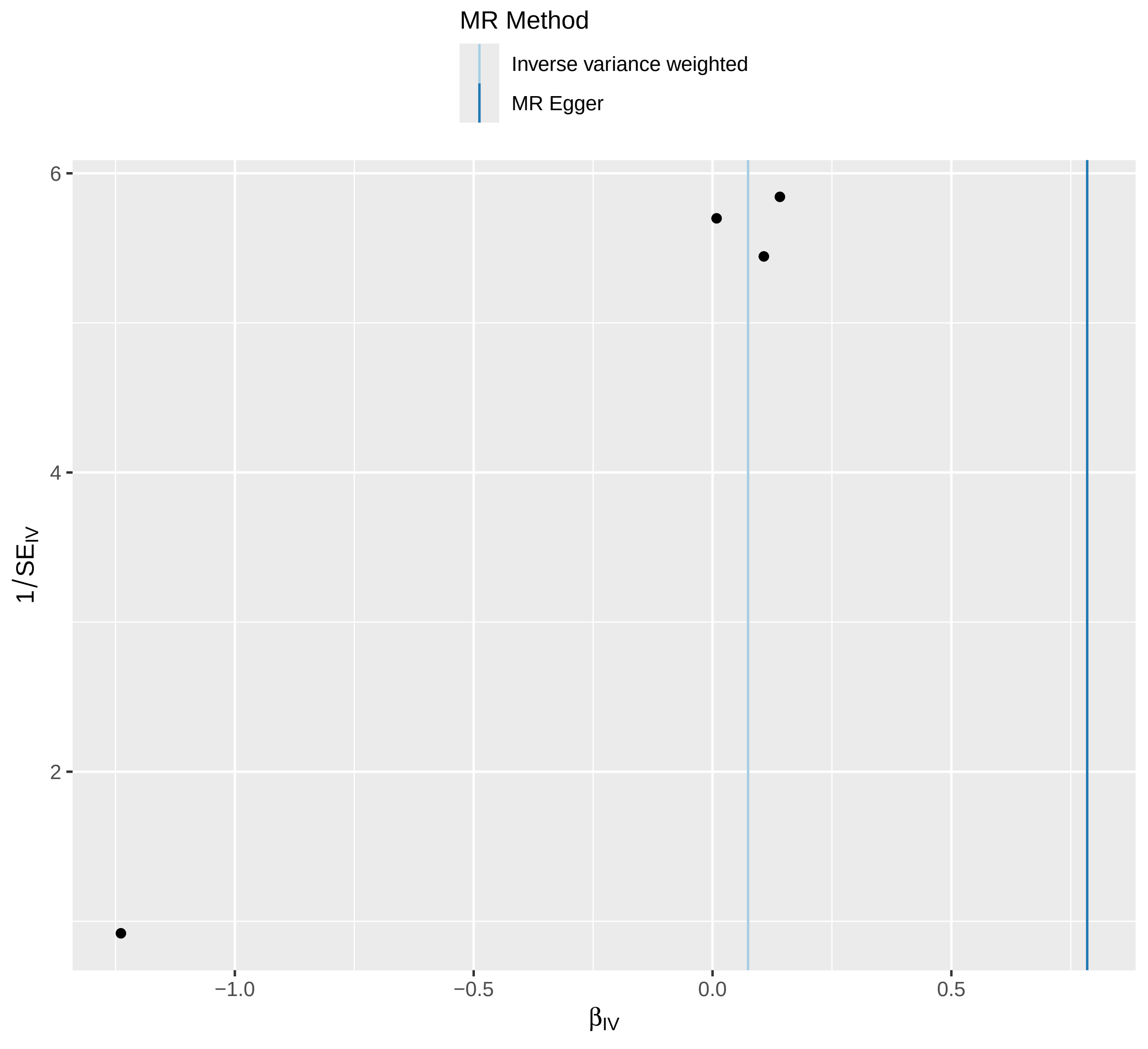

MR Method
Inverse variance weighted
MR Egger
6
4
2
1/SE$_{IV}$
−1.0
−0.5
0.0
0.5
β$_{IV}$

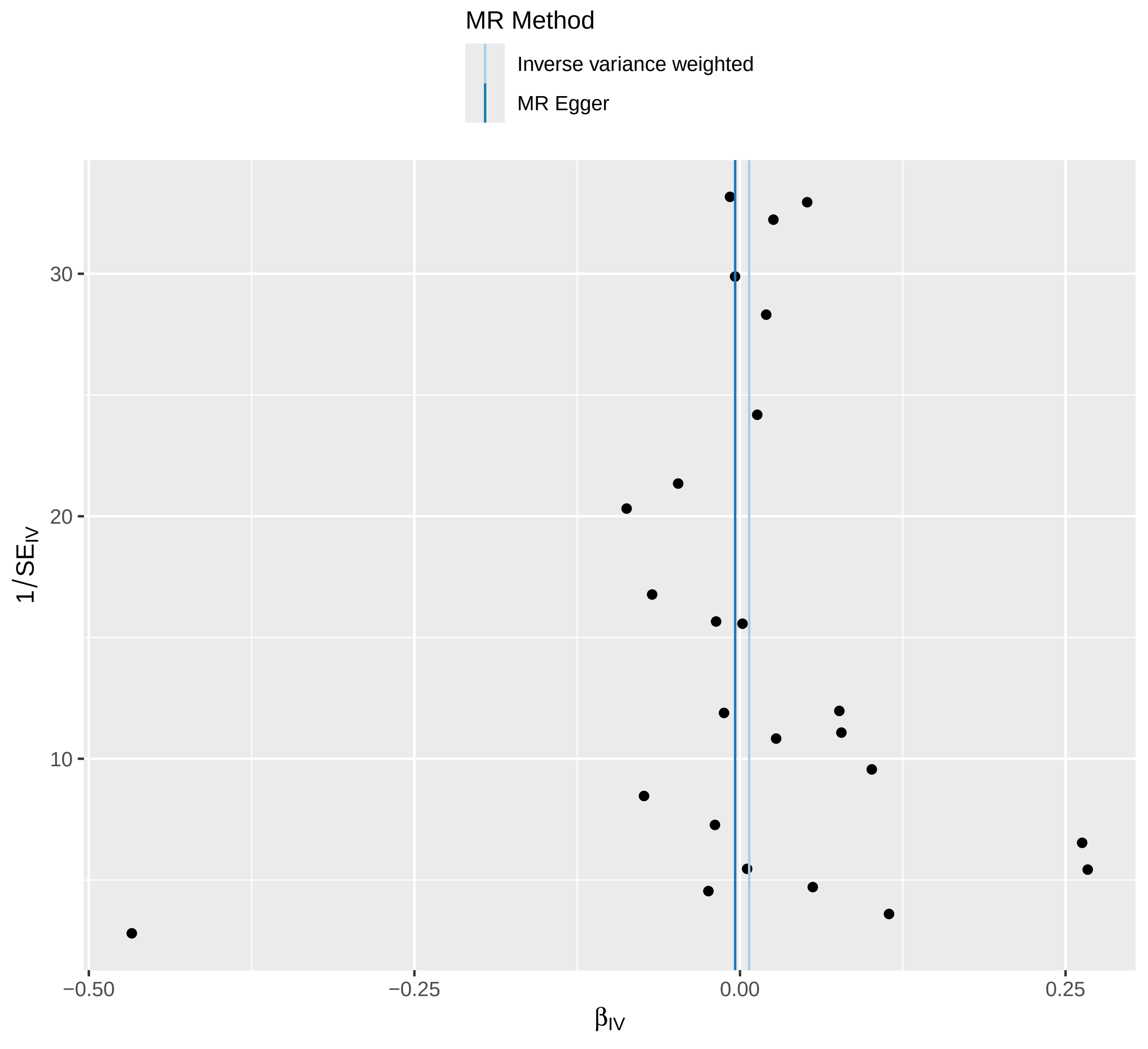
MR Method
Inverse variance weighted
MR Egger
30
20
10
1/SE$_{IV}$
−0.50
−0.25
0.00
0.25
β$_{IV}$

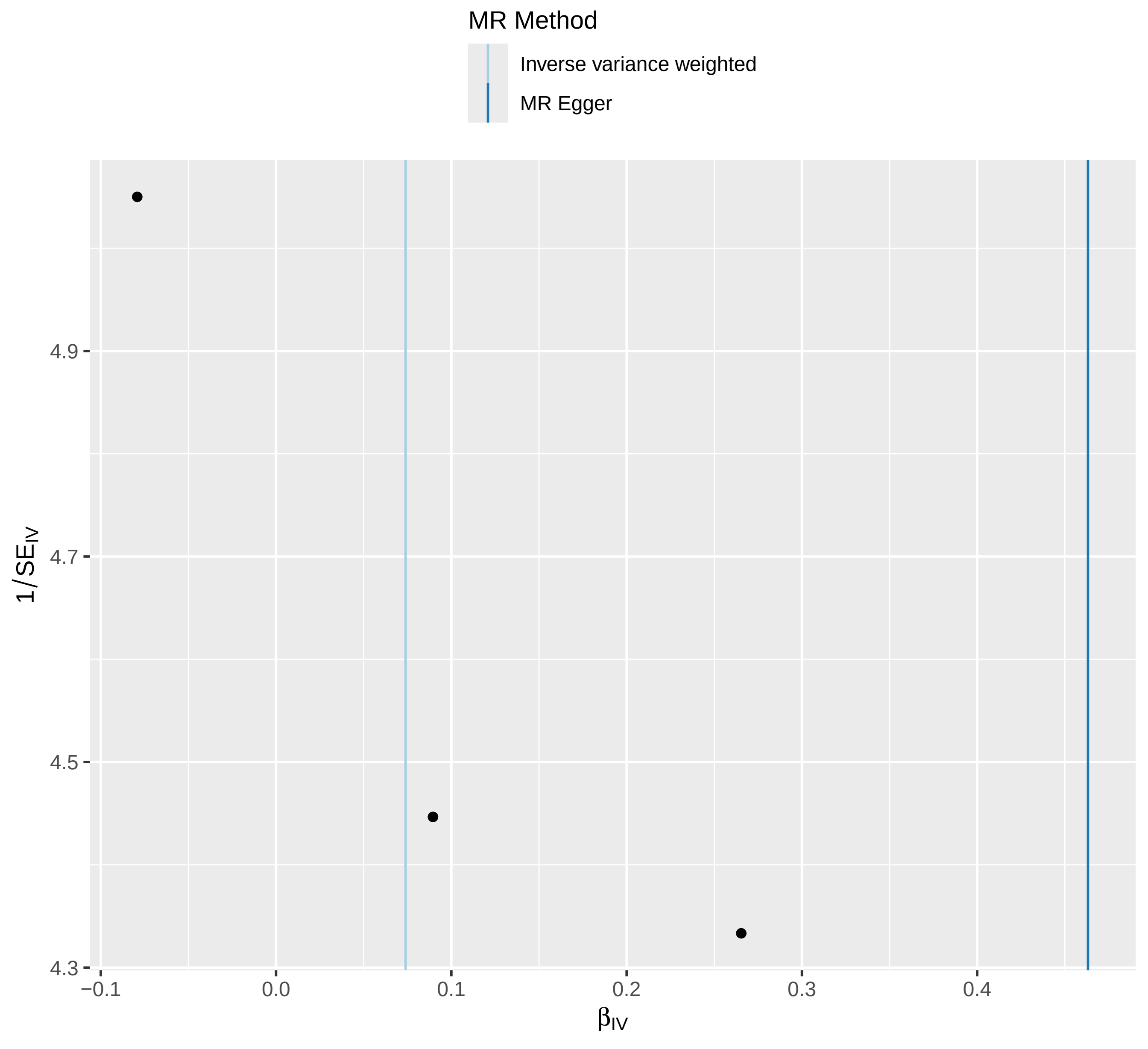
MR Method
Inverse variance weighted
MR Egger
1/SE$_{IV}$
4.9
4.7
4.5
4.3
−0.1
0.0
0.1
0.2
0.3
0.4
β$_{IV}$

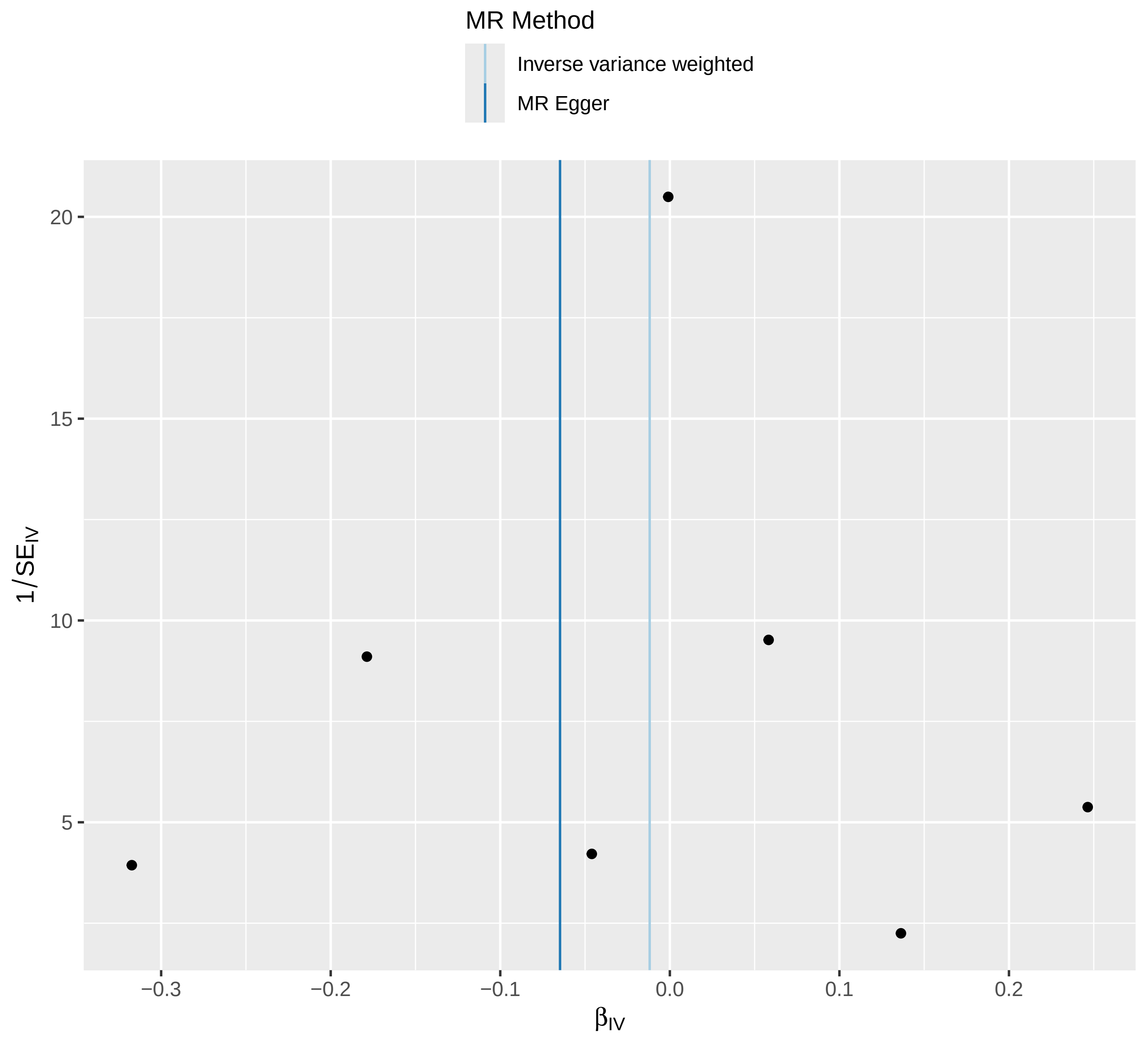

MR Method
Inverse variance weighted
MR Egger
20
15
10
5
1/SE$_{IV}$
−0.3
−0.2
−0.1
0.0
0.1
0.2
β$_{IV}$

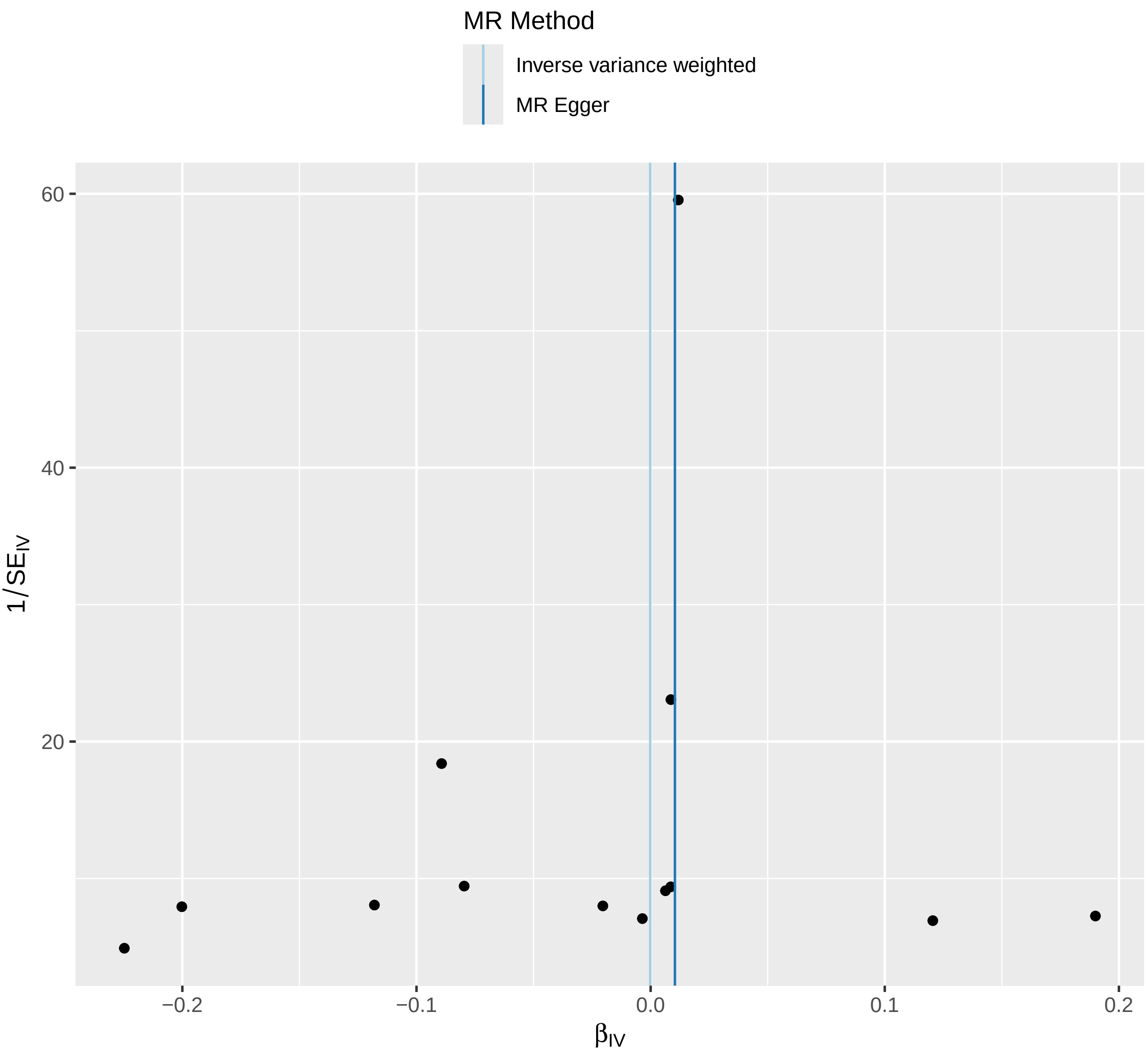
MR Method
Inverse variance weighted
MR Egger
60
40
20
1/SE$_{IV}$
−0.2
−0.1
0.0
0.1
0.2
β$_{IV}$

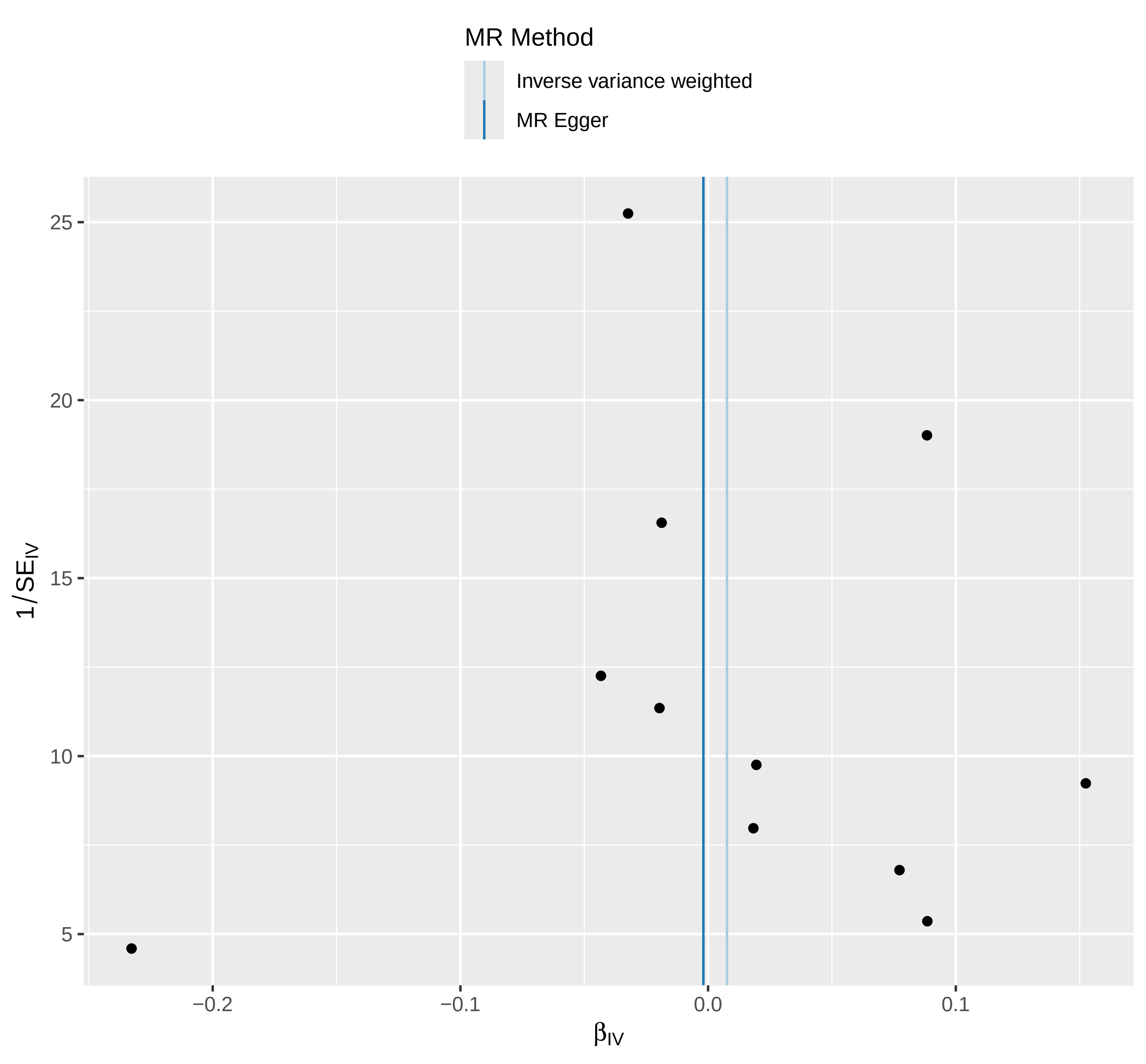

MR Method
Inverse variance weighted
MR Egger
25
20
15
10
5
1/SE_IV
−0.2
−0.1
0.0
0.1
β_IV

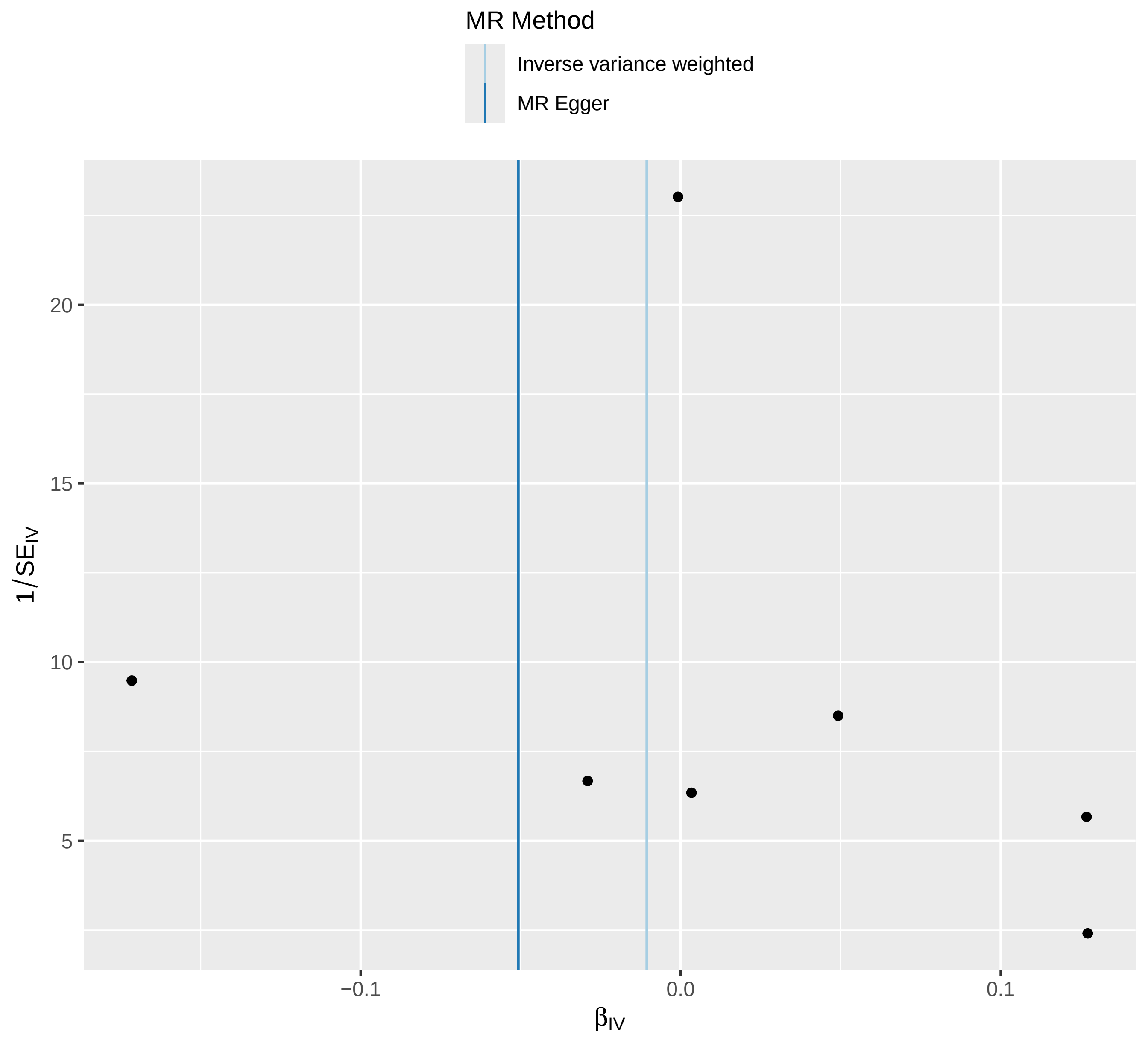
MR Method
Inverse variance weighted
MR Egger
20
15
10
5
1/SE$_{IV}$
−0.1
0.0
0.1
β$_{IV}$

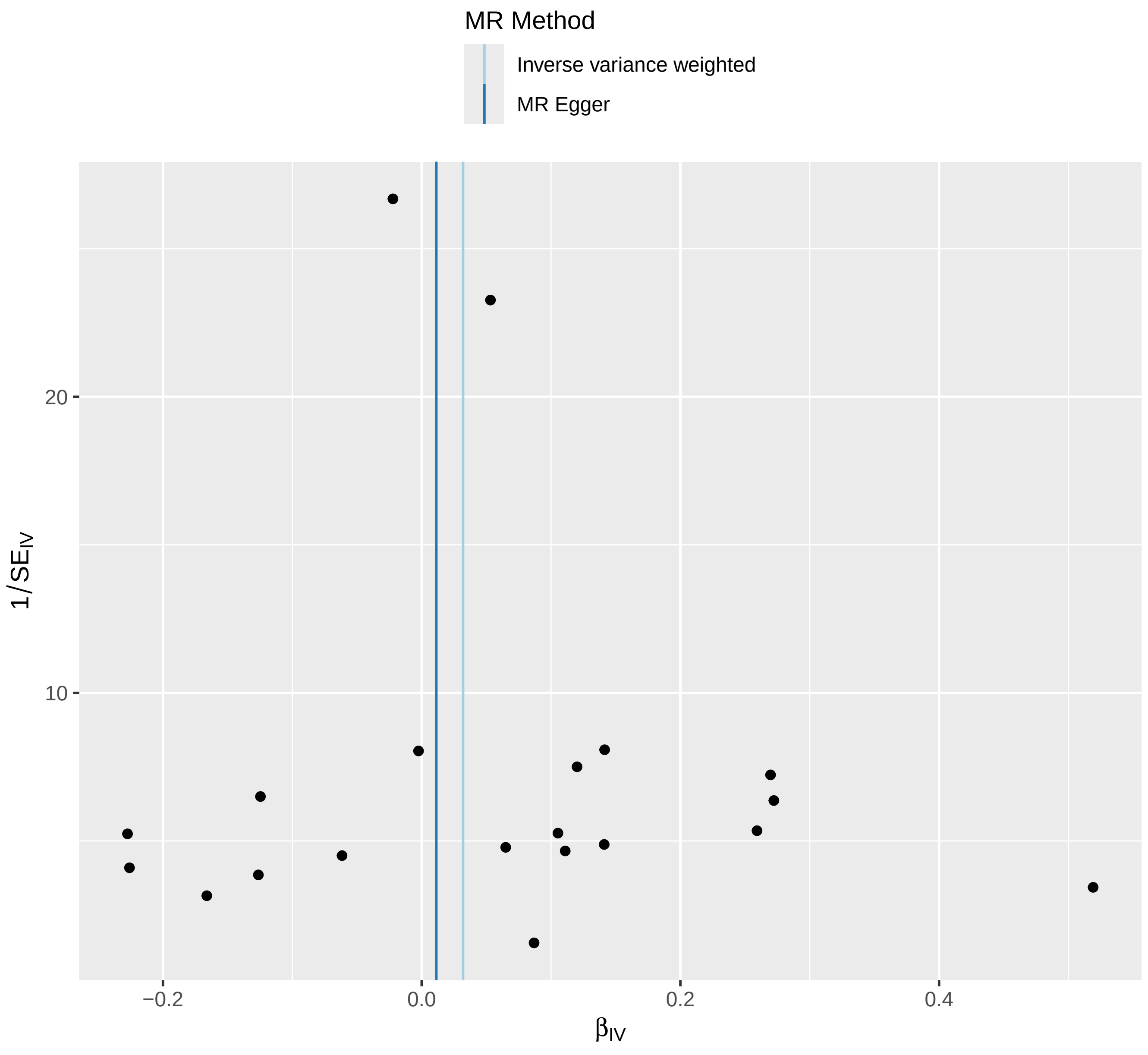

MR Method
Inverse variance weighted
MR Egger
20
10
1/SE_IV
−0.2
0.0
0.2
0.4
β_IV

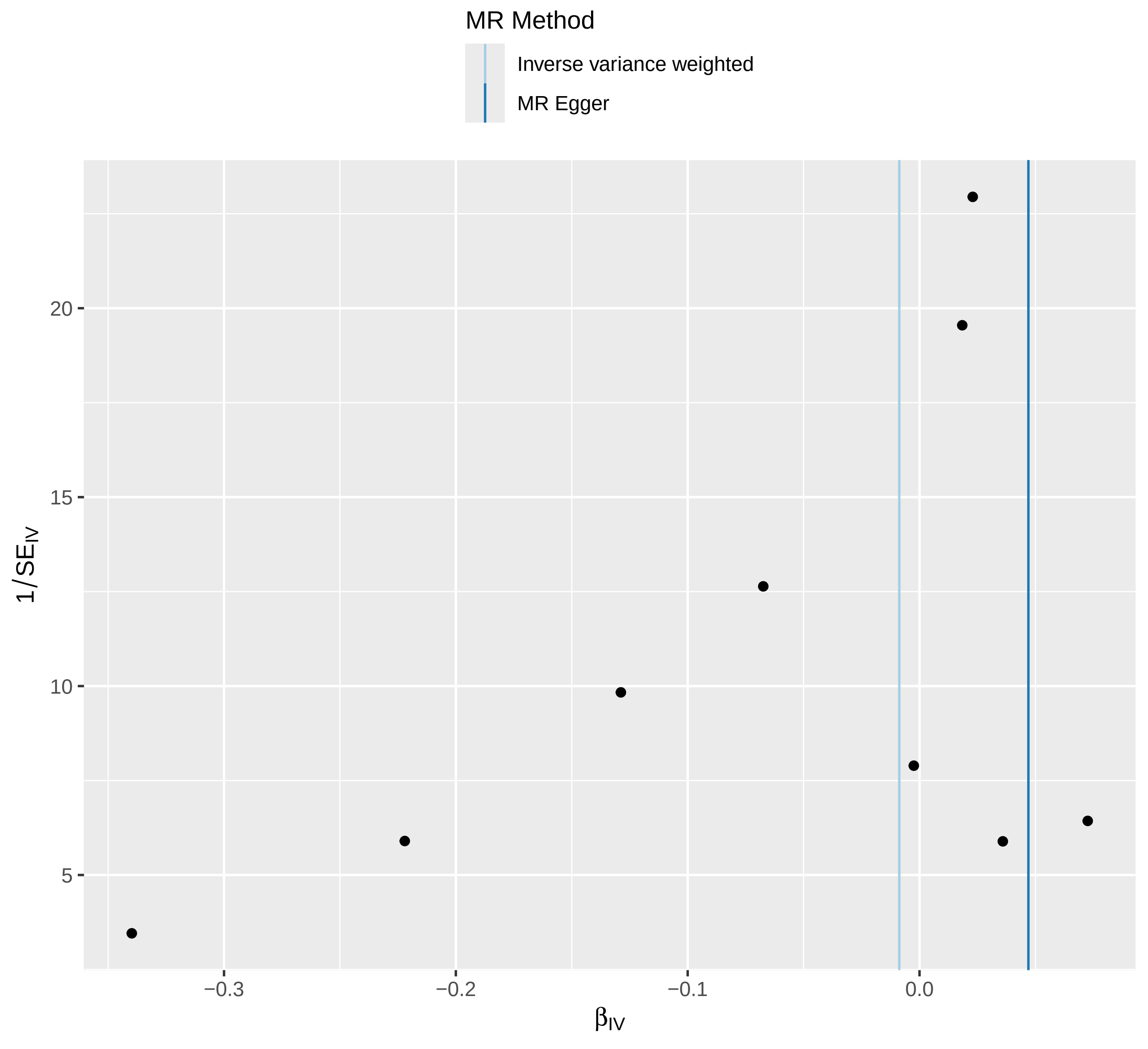

MR Method
Inverse variance weighted
MR Egger
20
15
10
5
1/SE_IV
−0.3
−0.2
−0.1
0.0
β_IV

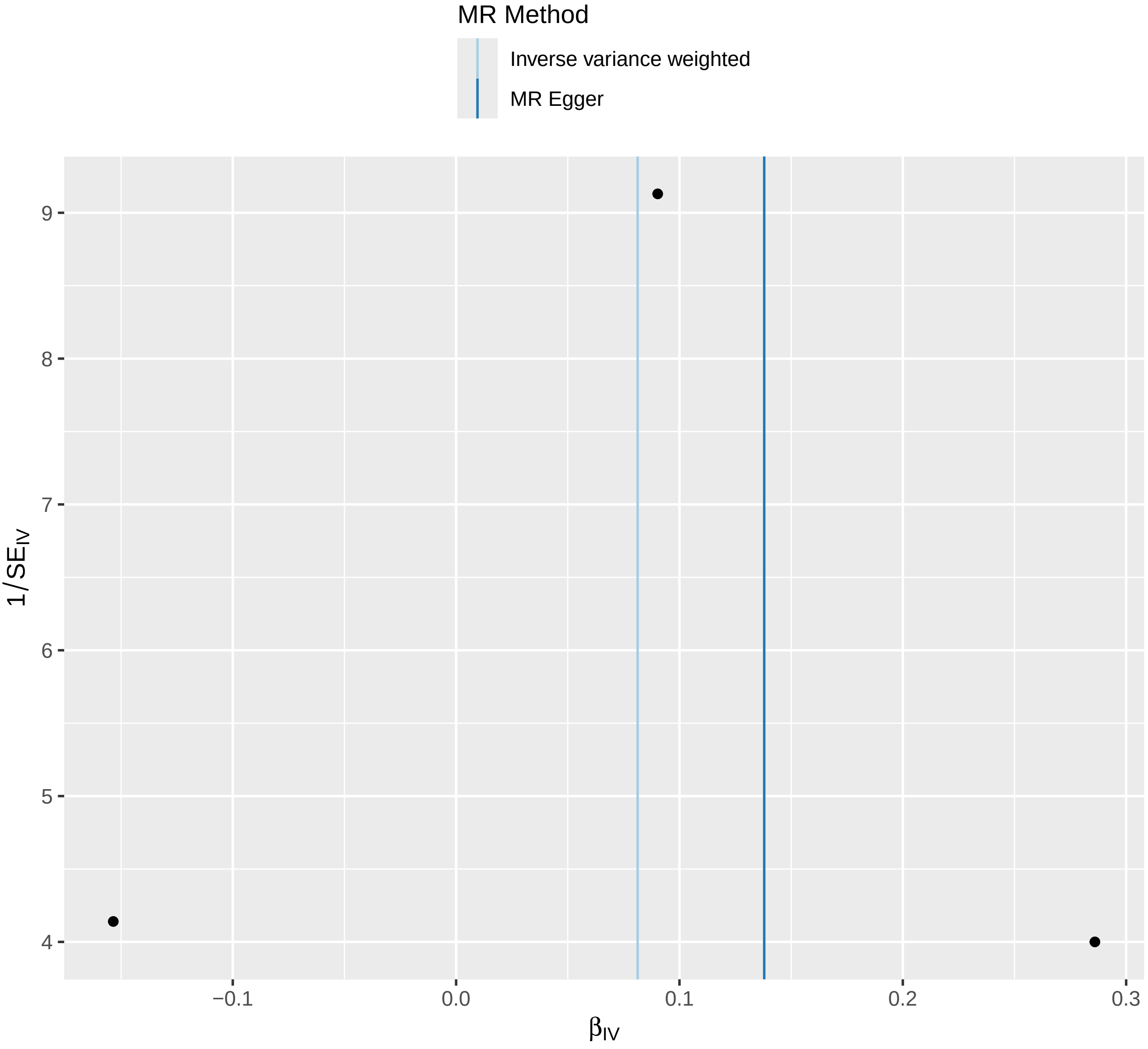
MR Method
Inverse variance weighted
MR Egger
9
8
7
6
5
4
$1/SE_{IV}$
−0.1
0.0
0.1
0.2
0.3
$\beta_{IV}$

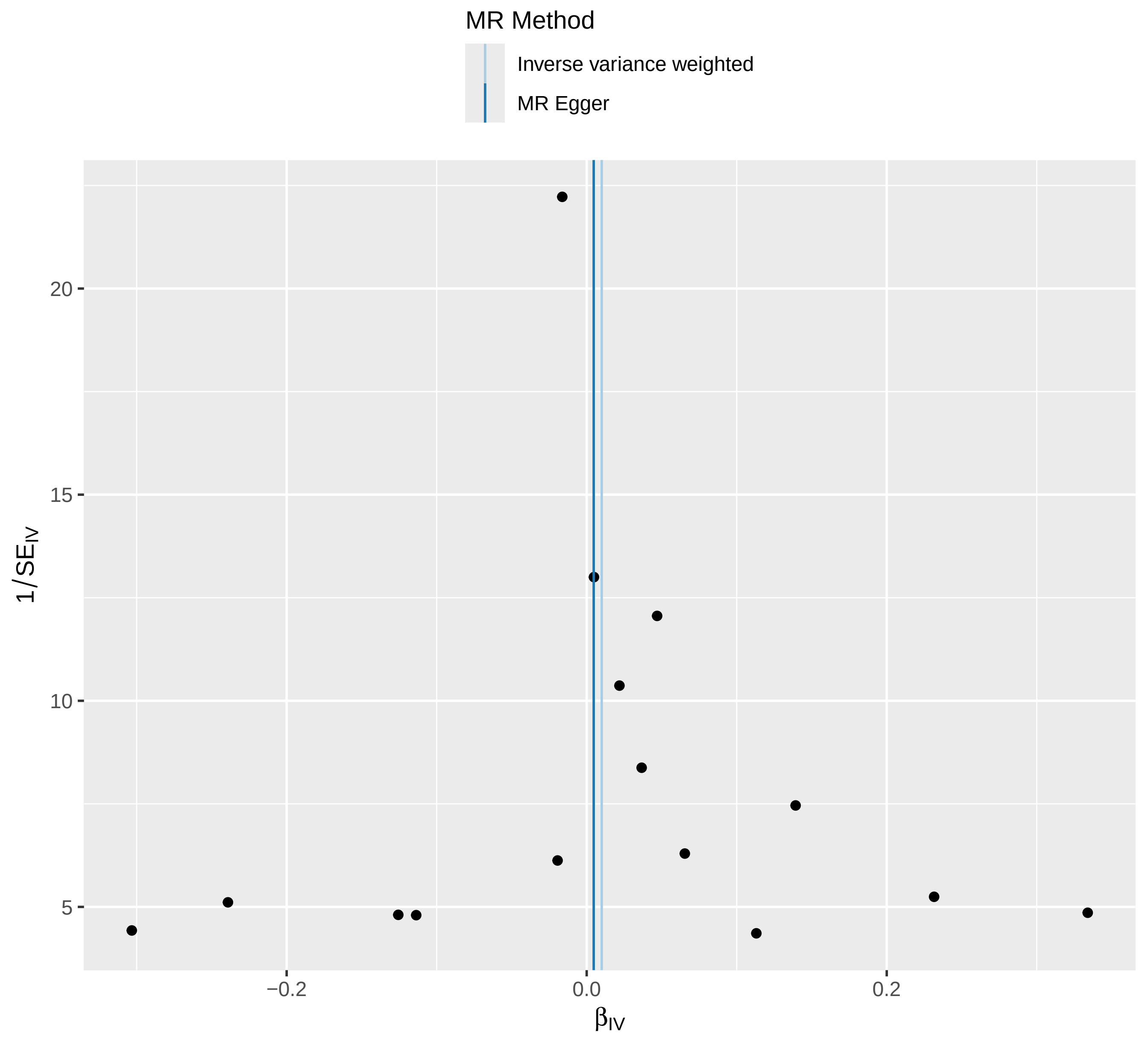

MR Method
Inverse variance weighted
MR Egger
20
15
10
5
$1/SE_{IV}$
−0.2
0.0
0.2
$\beta_{IV}$

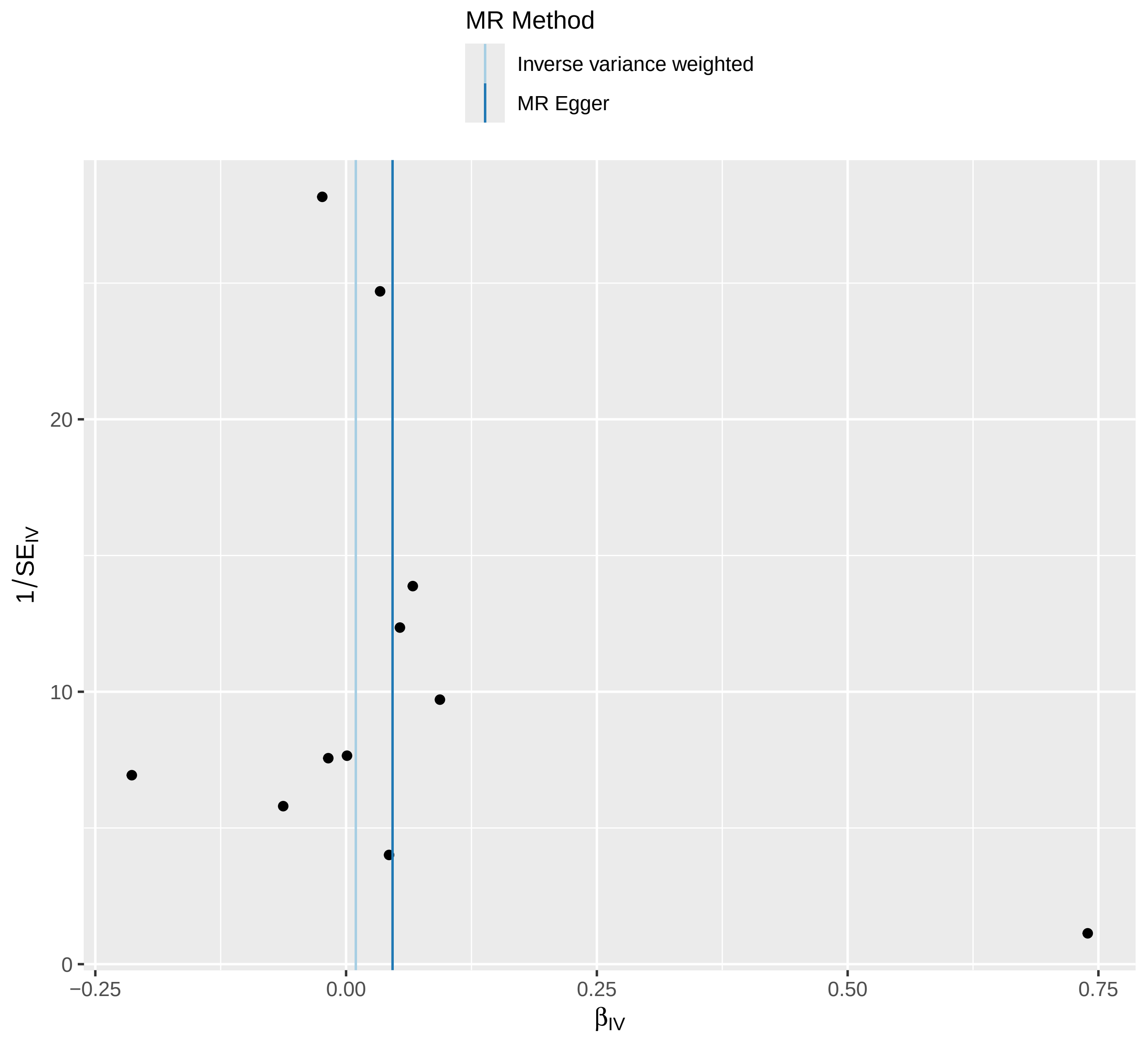

MR Method
Inverse variance weighted
MR Egger
1/SE_IV
20
10
0
−0.25
0.00
0.25
0.50
0.75
β_IV

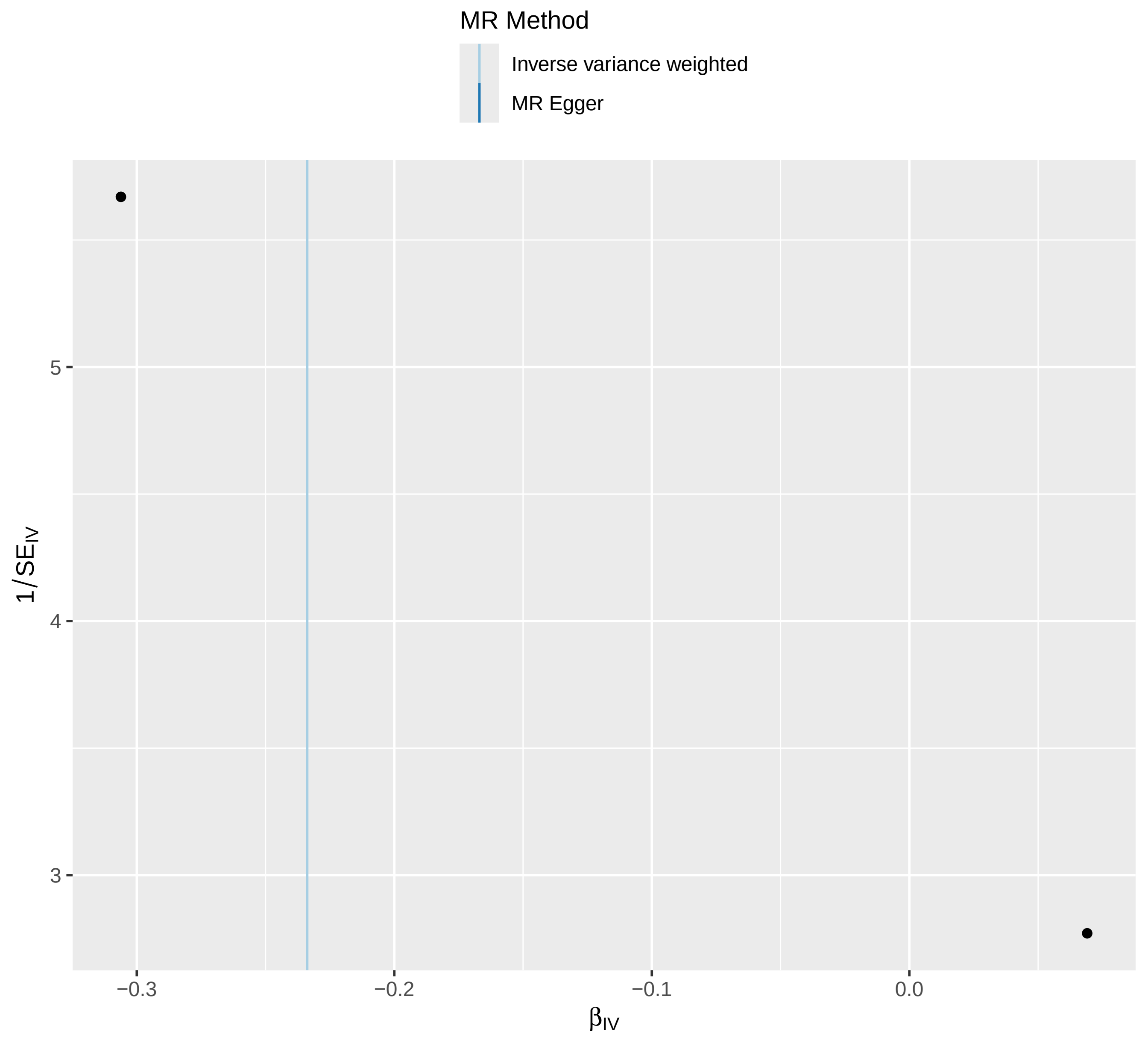

MR Method
Inverse variance weighted
MR Egger
1/SE_IV
5
4
3
−0.3
−0.2
−0.1
0.0
β_IV

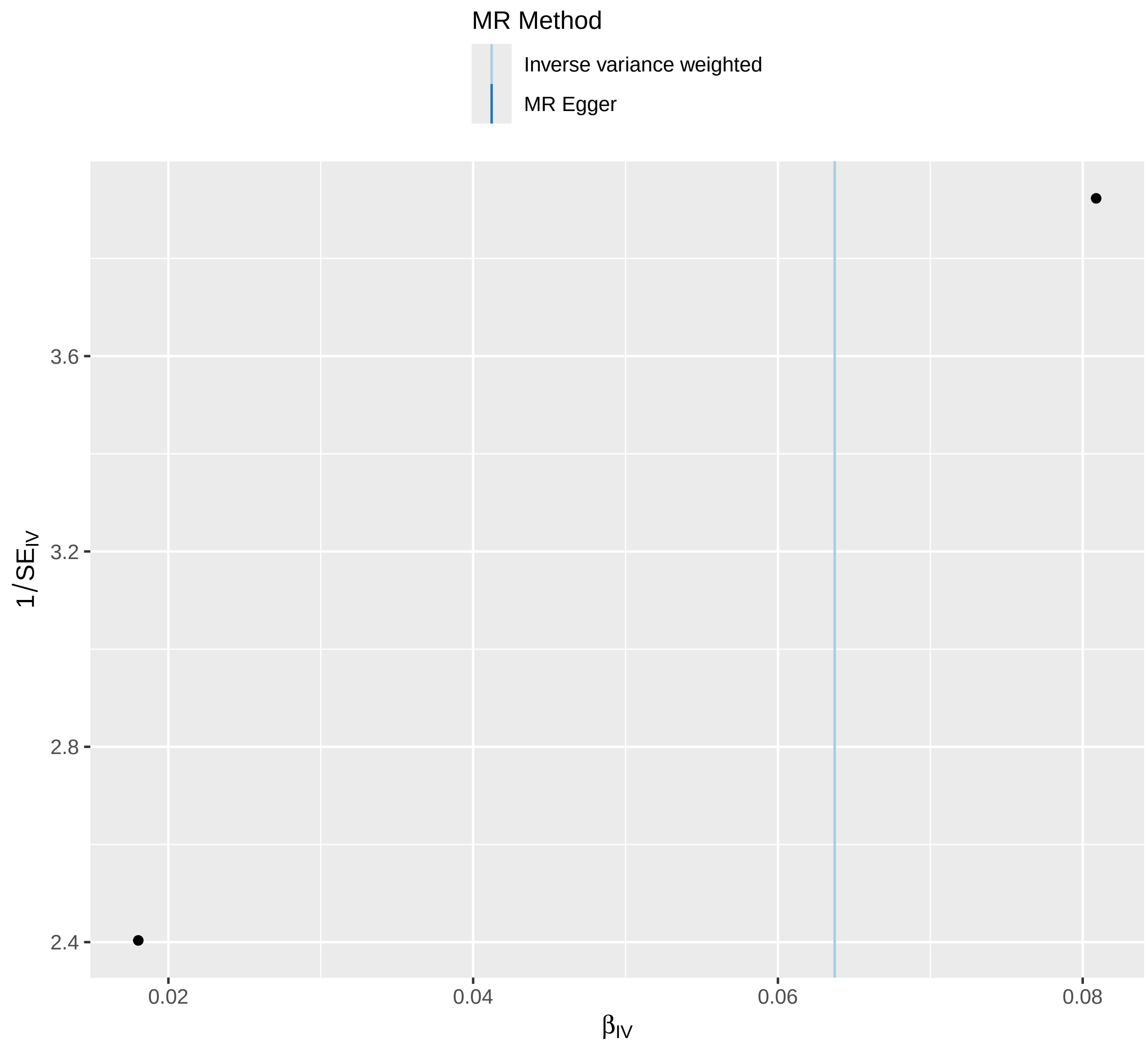

MR Method
Inverse variance weighted
MR Egger
3.6
3.2
2.8
2.4
1/SE$_{IV}$
0.02
0.04
0.06
0.08
β$_{IV}$

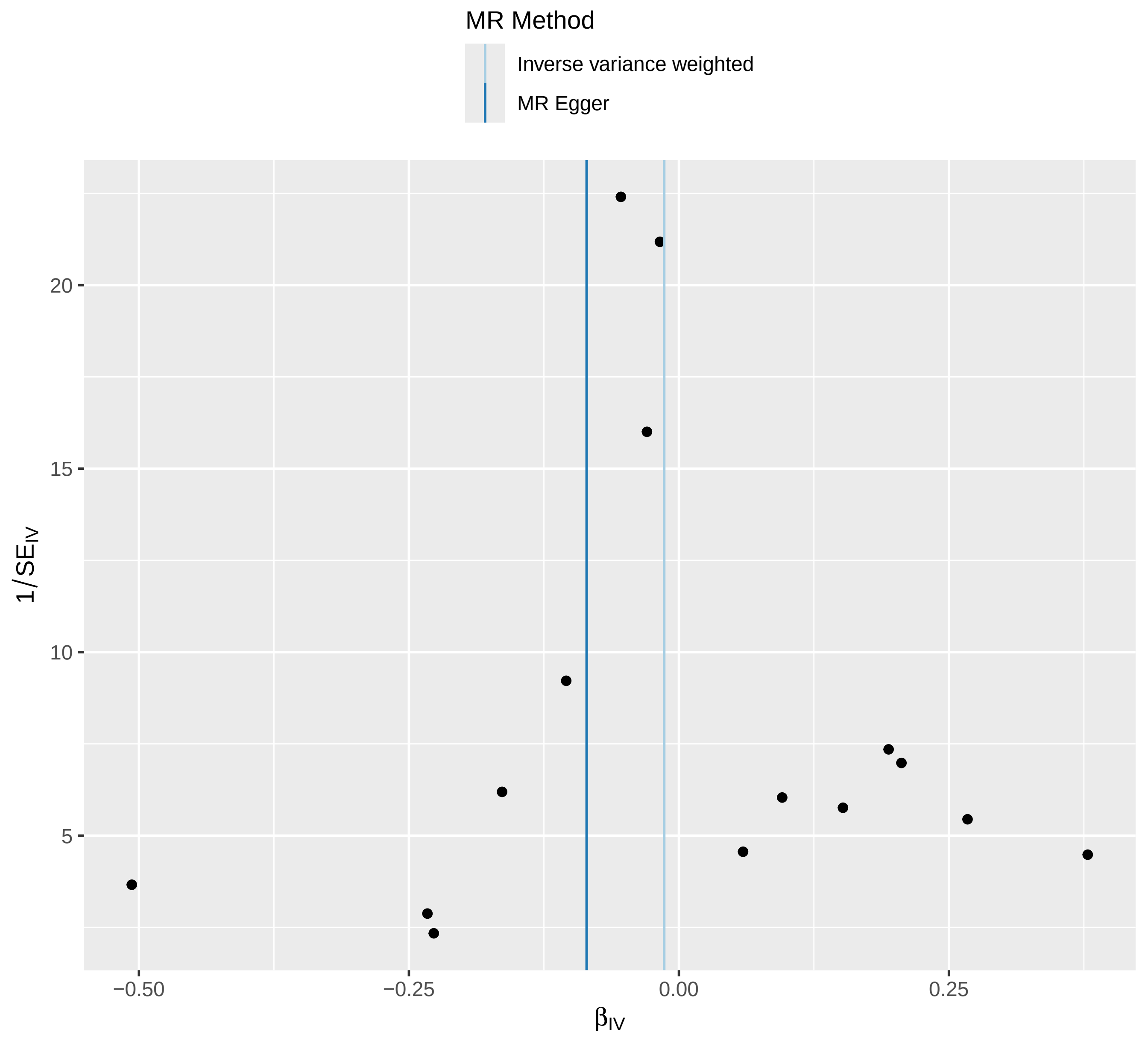
MR Method
Inverse variance weighted
MR Egger
20
15
10
5
1/SE$_{IV}$
−0.50
−0.25
0.00
0.25
β$_{IV}$

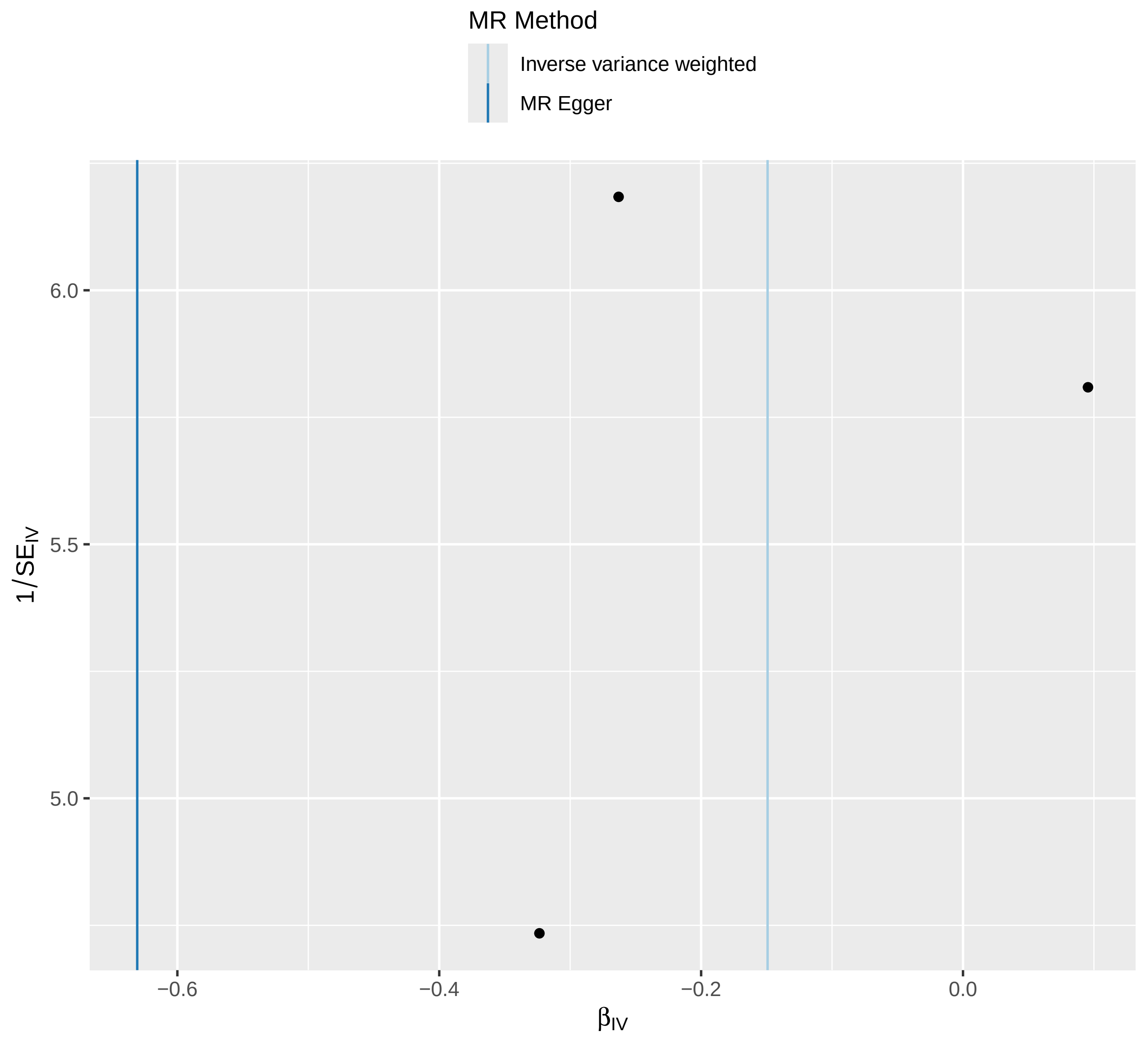

MR Method
Inverse variance weighted
MR Egger
6.0
5.5
5.0
1/SE$_{IV}$
−0.6
−0.4
−0.2
0.0
β$_{IV}$

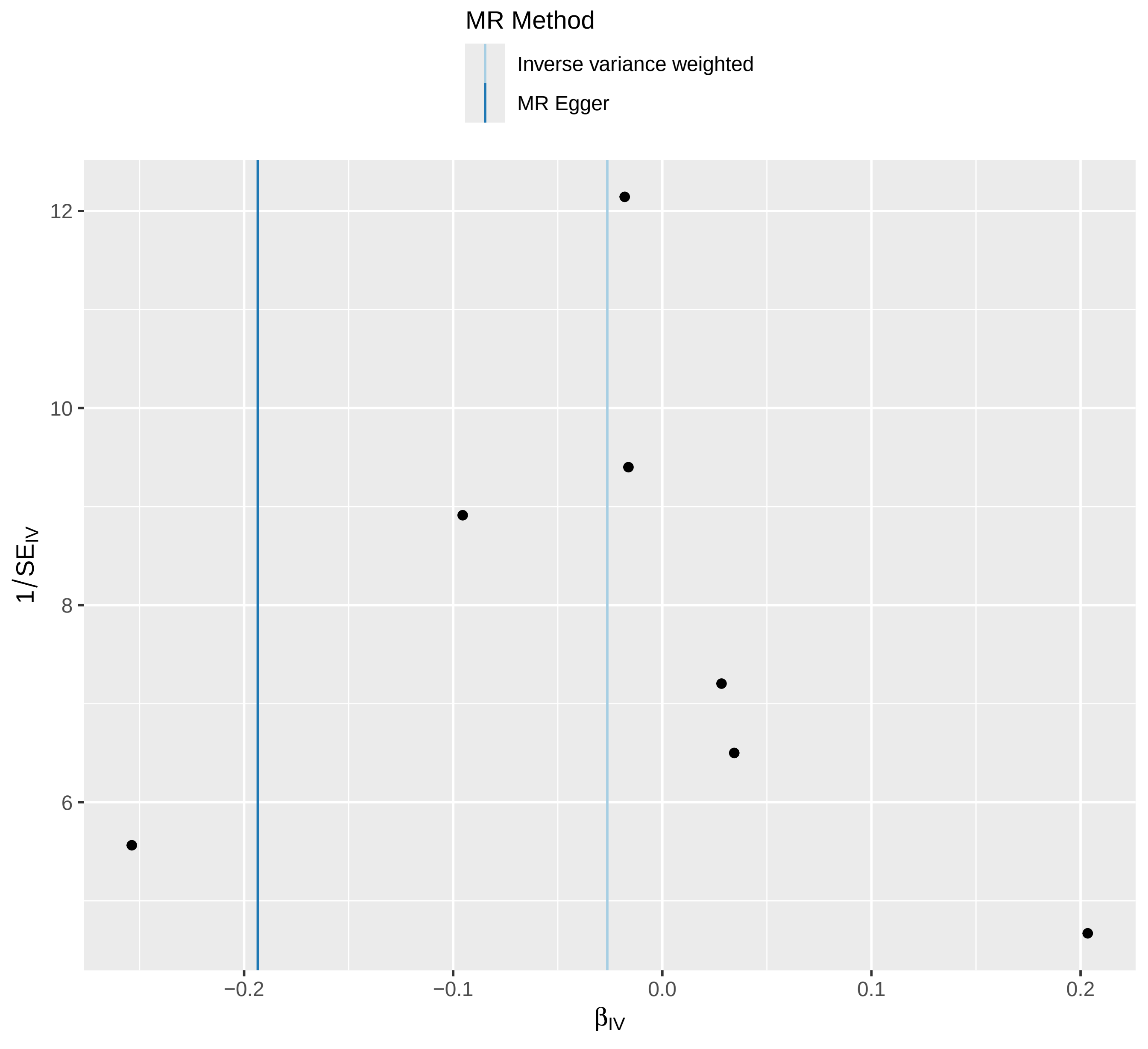
MR Method
Inverse variance weighted
MR Egger
12
10
8
6
1/SE_IV
−0.2
−0.1
0.0
0.1
0.2
β_IV

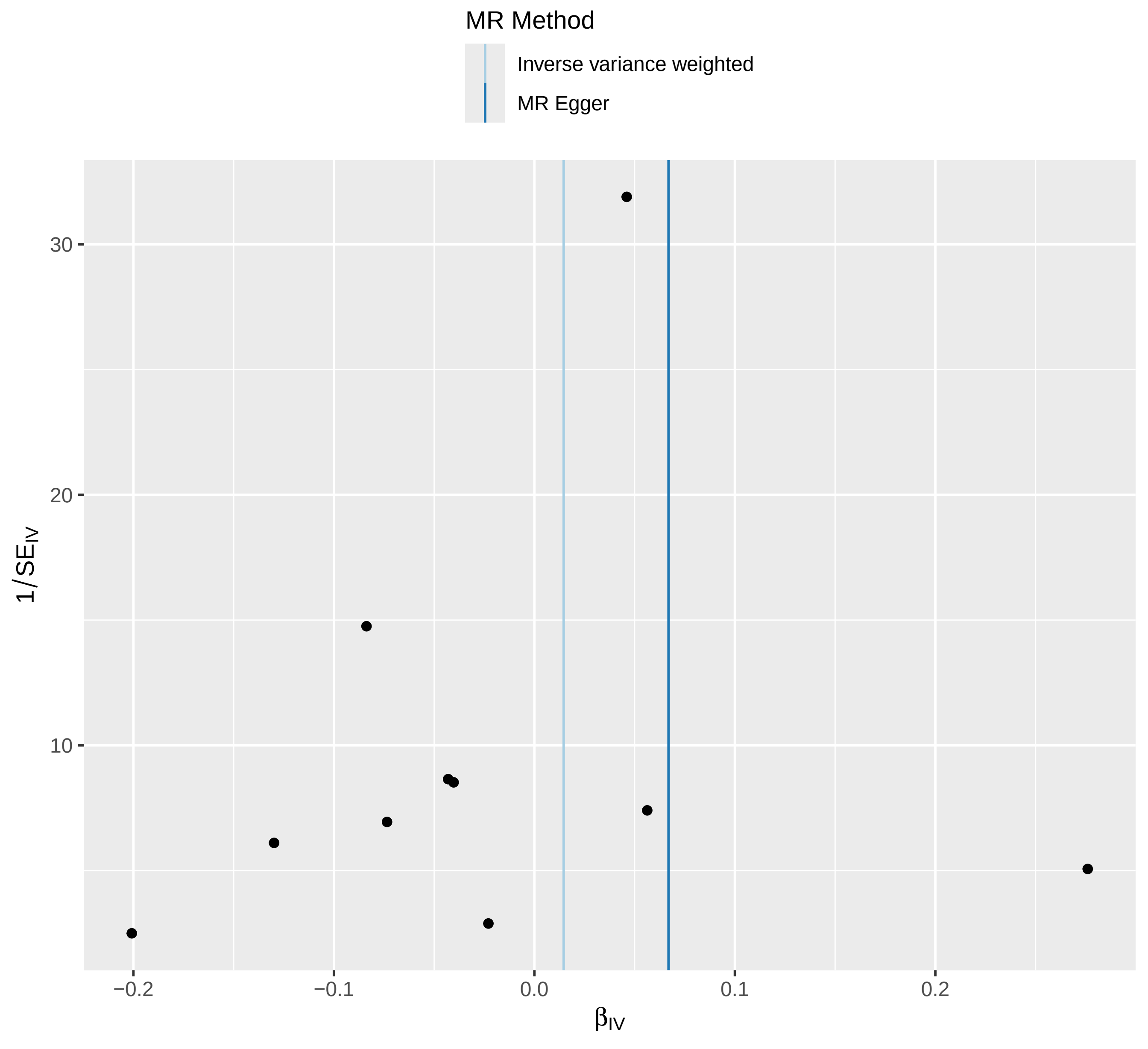
MR Method
Inverse variance weighted
MR Egger
30
20
10
1/SE$_{IV}$
−0.2
−0.1
0.0
0.1
0.2
β$_{IV}$

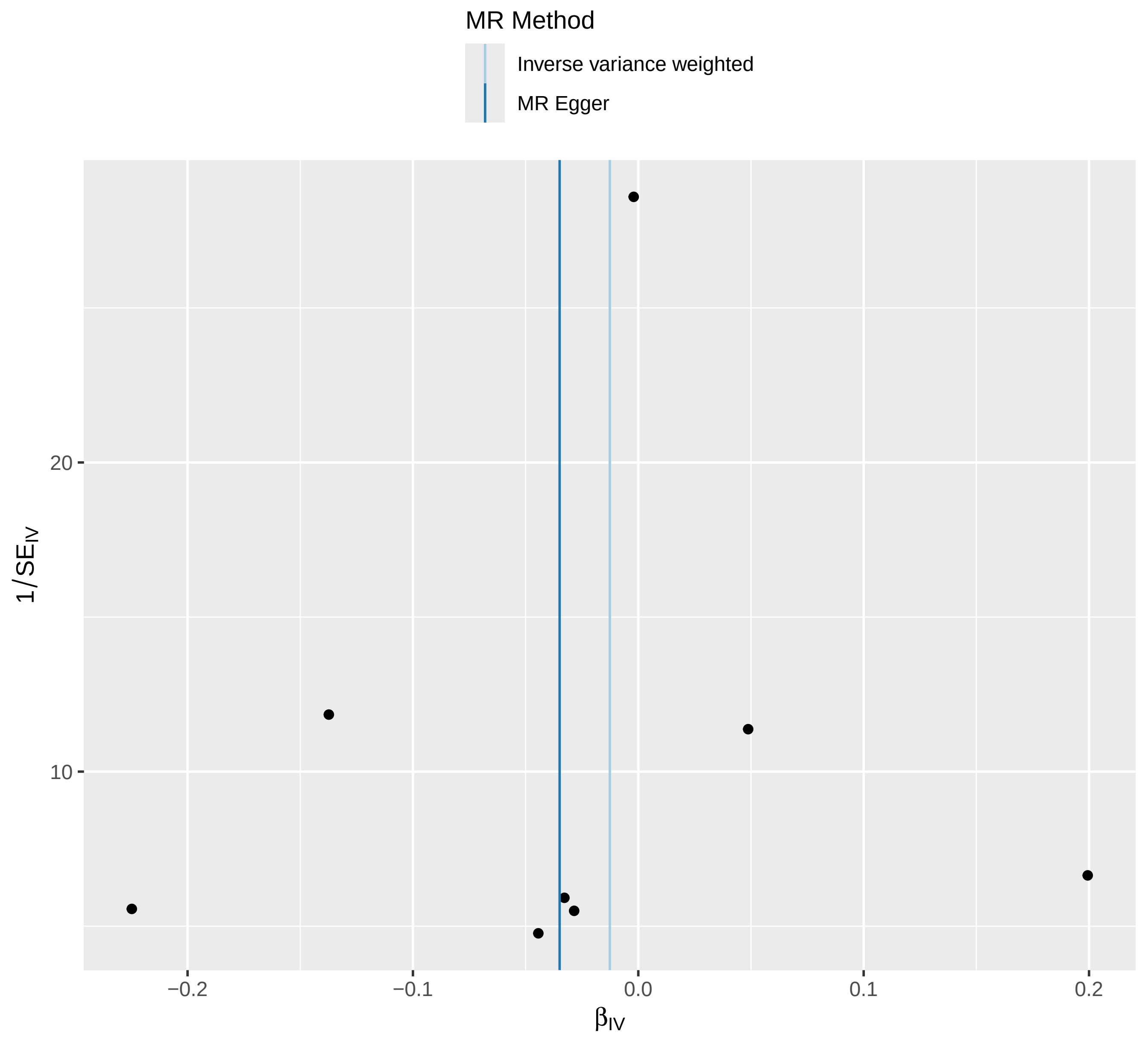

MR Method
Inverse variance weighted
MR Egger
20
10
$1/SE_{IV}$
−0.2
−0.1
0.0
0.1
0.2
$\beta_{IV}$

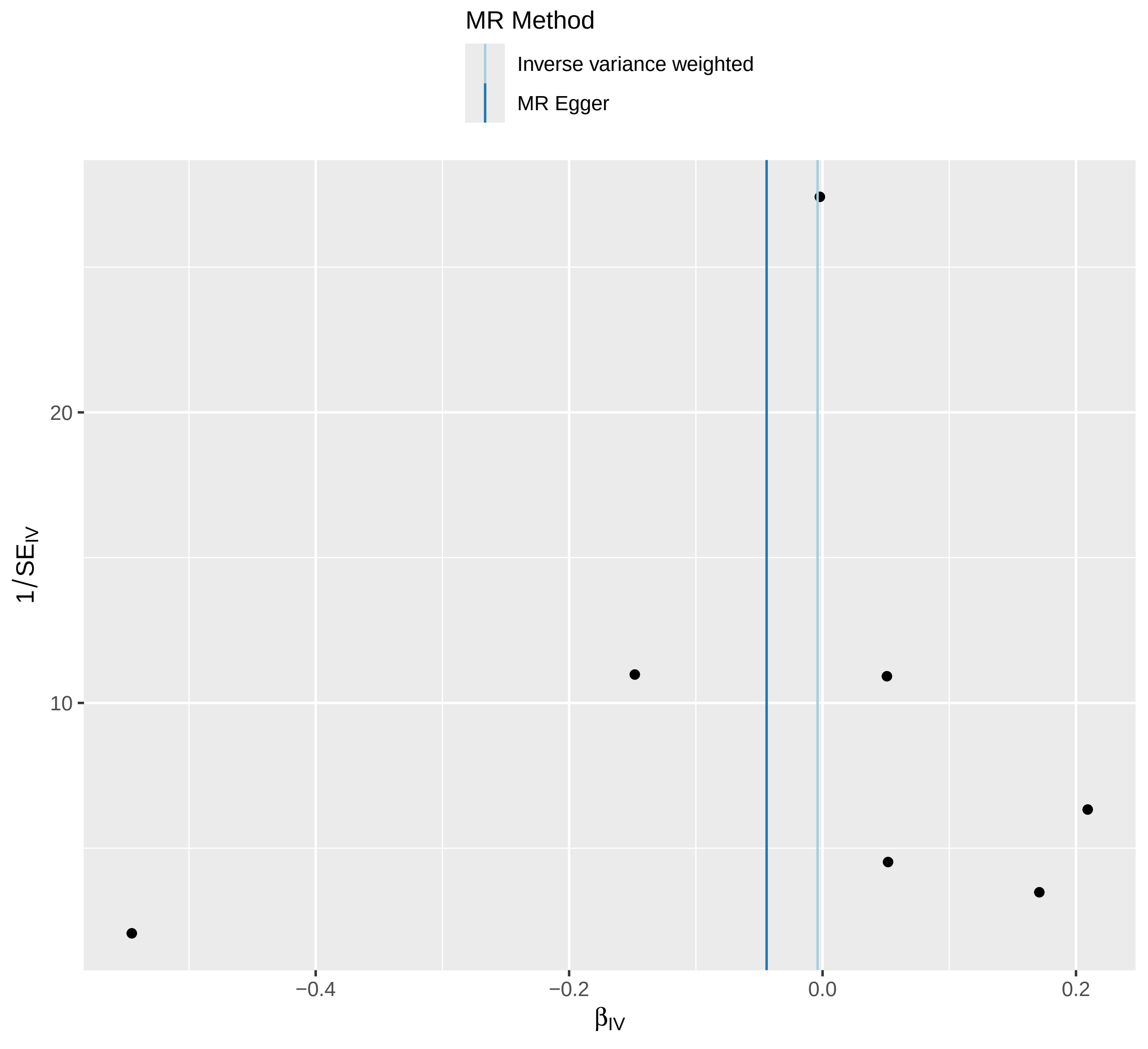

MR Method
Inverse variance weighted
MR Egger
20
10
1/SE$_{IV}$
−0.4
−0.2
0.0
0.2
β$_{IV}$

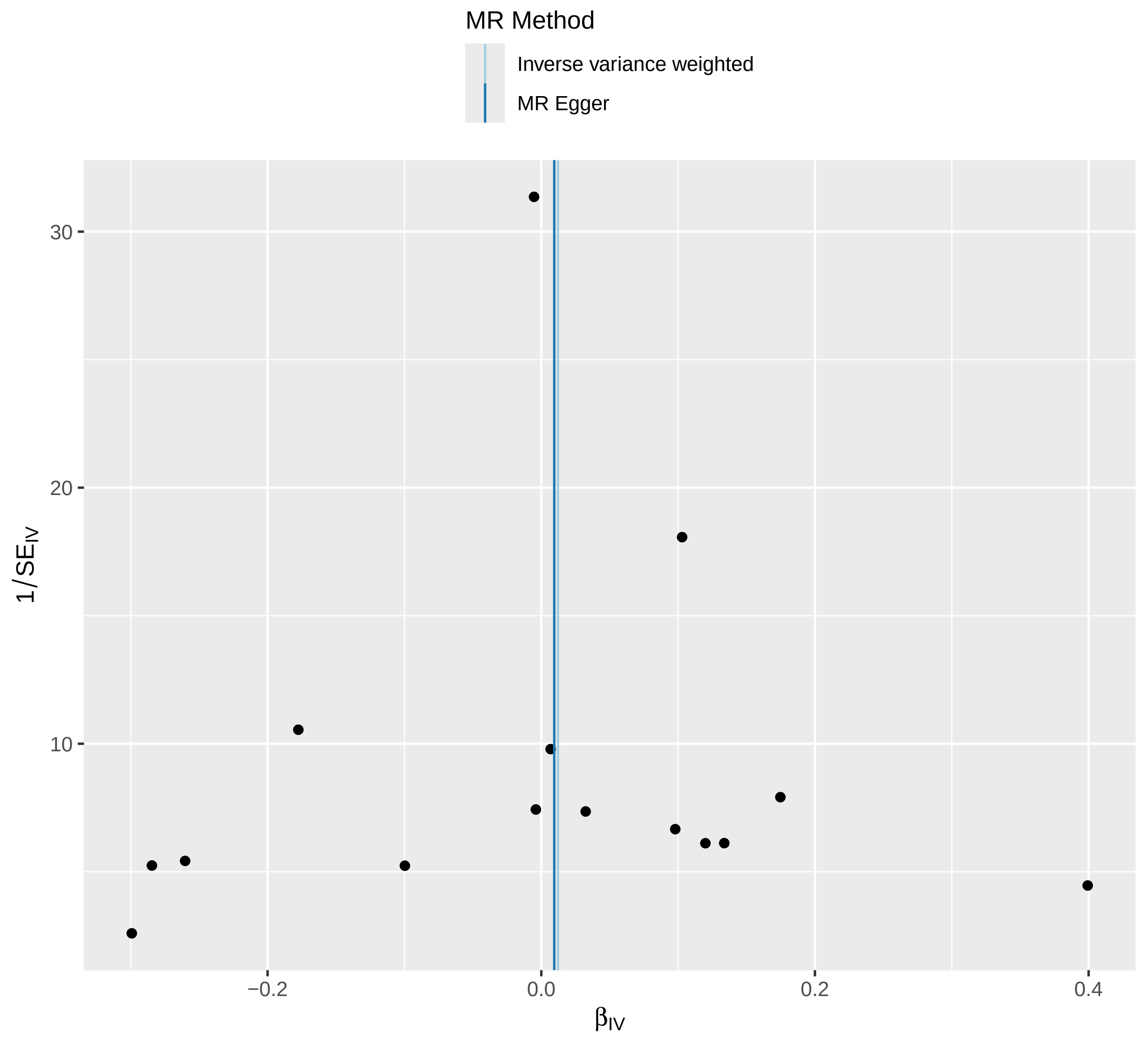

MR Method
Inverse variance weighted
MR Egger
30
20
10
1/SE$_{IV}$
−0.2
0.0
0.2
0.4
β$_{IV}$

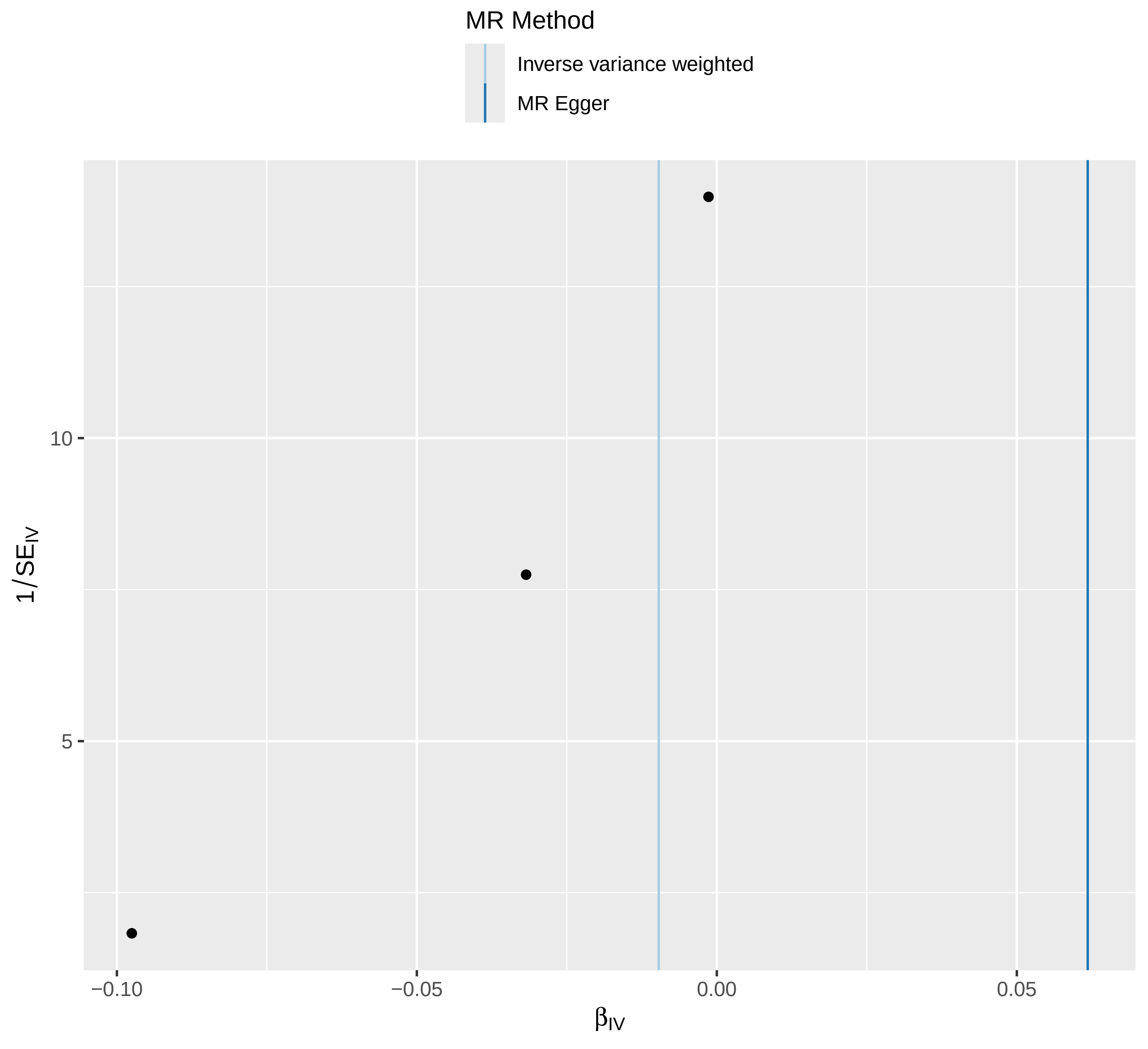
MR Method
Inverse variance weighted
MR Egger
10
5
1/SE_IV
−0.10
−0.05
0.00
0.05
β_IV

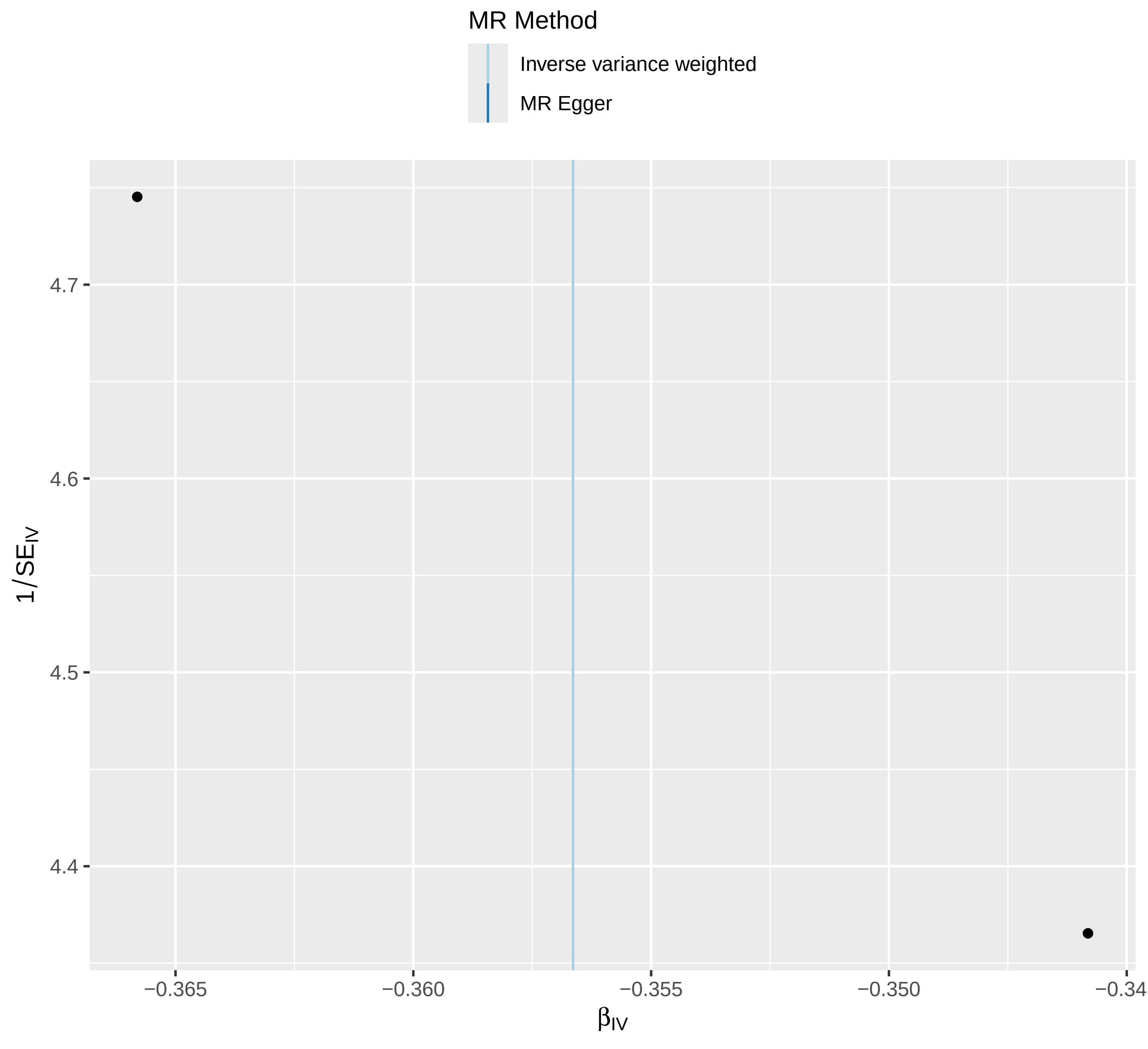

MR Method
Inverse variance weighted
MR Egger
4.7
4.6
4.5
4.4
1/SE$_{IV}$
−0.365
−0.360
−0.355
−0.350
−0.34
β$_{IV}$

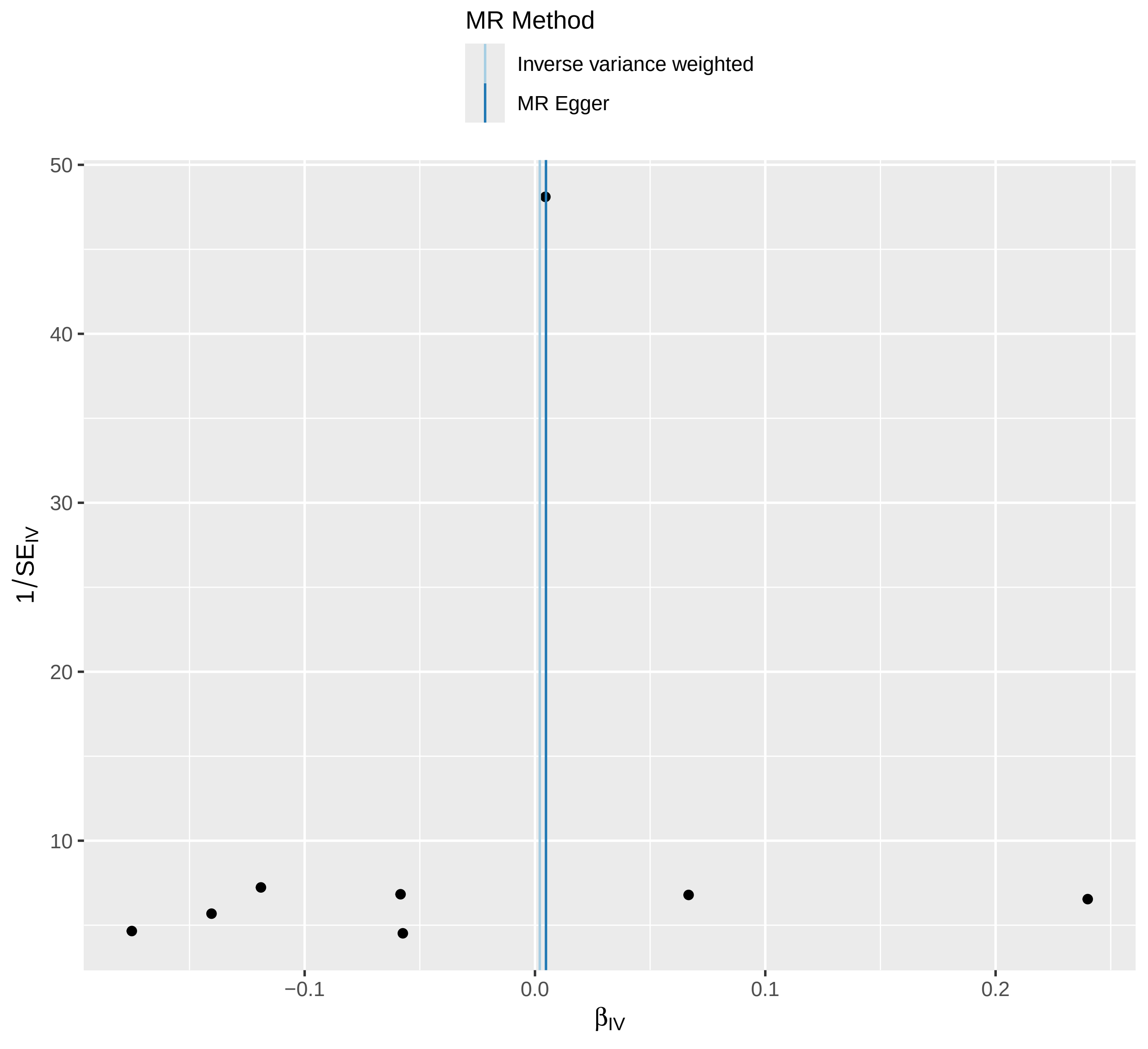
MR Method
Inverse variance weighted
MR Egger
50
40
30
20
10
1/SE$_{IV}$
−0.1
0.0
0.1
0.2
β$_{IV}$

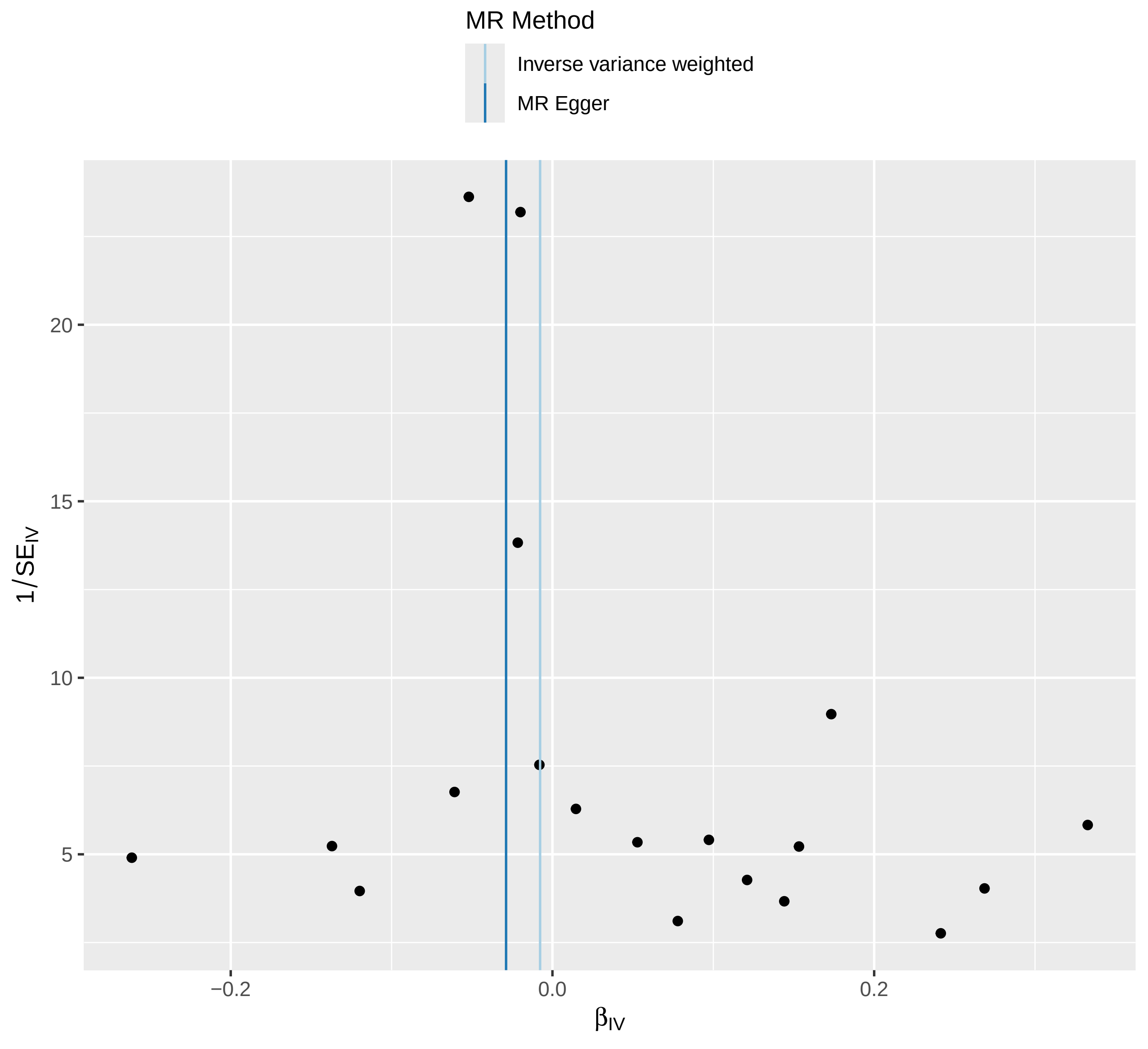

MR Method
Inverse variance weighted
MR Egger
20
15
10
5
1/SE_IV
−0.2
0.0
0.2
β_IV

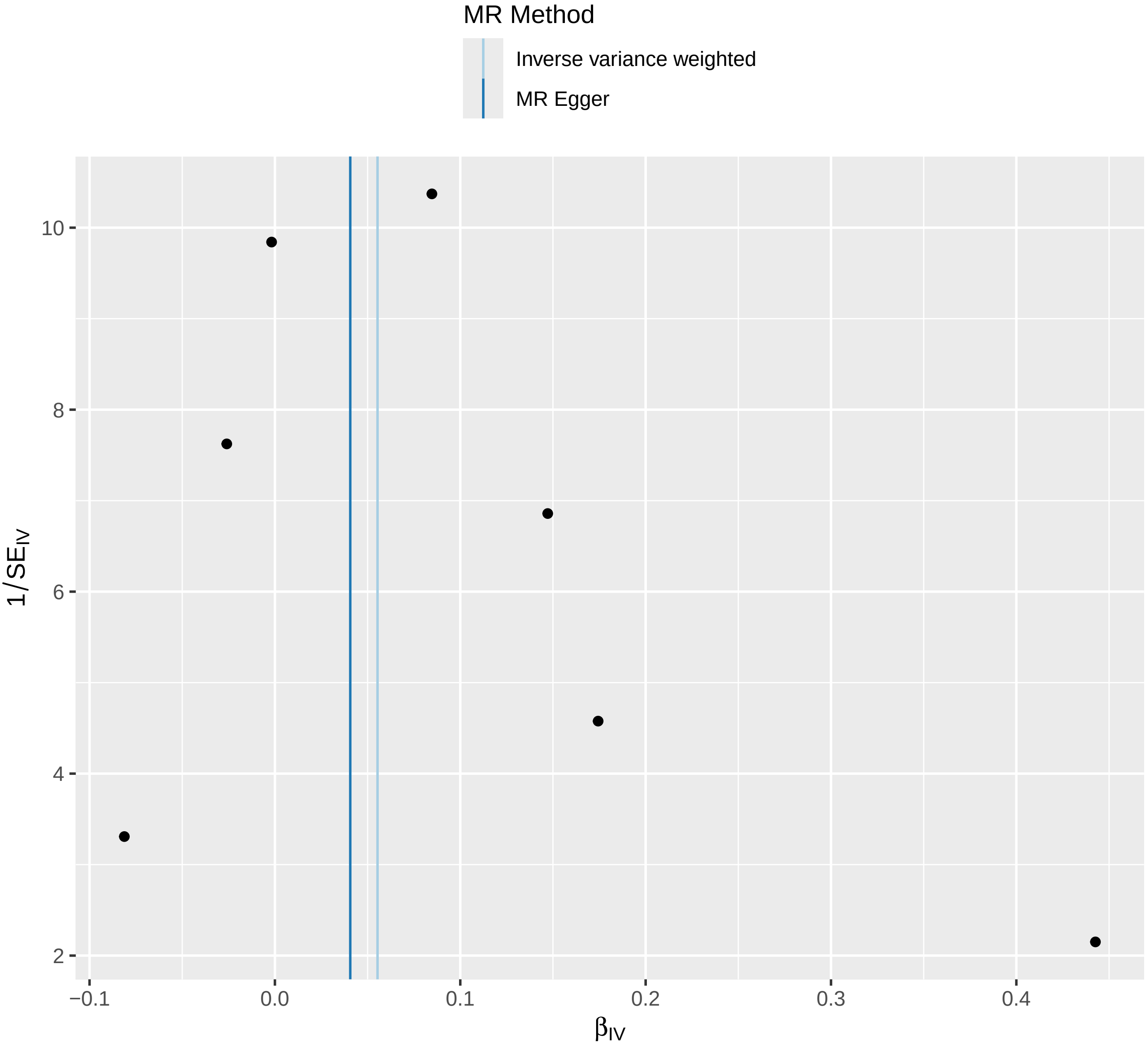
MR Method
Inverse variance weighted
MR Egger
10
8
6
4
2
1/SE$_{IV}$
−0.1
0.0
0.1
0.2
0.3
0.4
β$_{IV}$

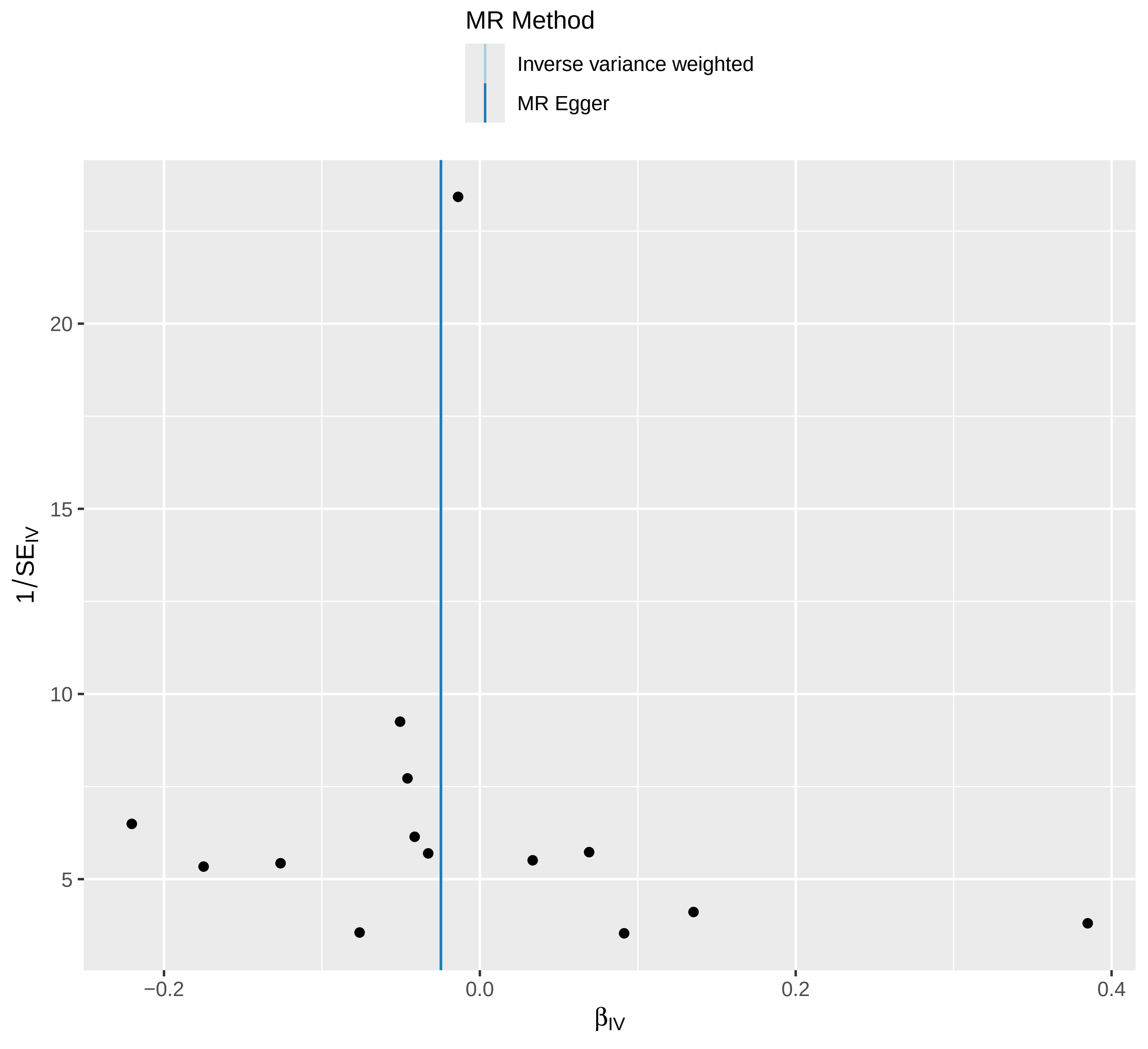
MR Method
Inverse variance weighted
MR Egger
20
15
10
5
1/SE$_{IV}$
−0.2
0.0
0.2
0.4
β$_{IV}$

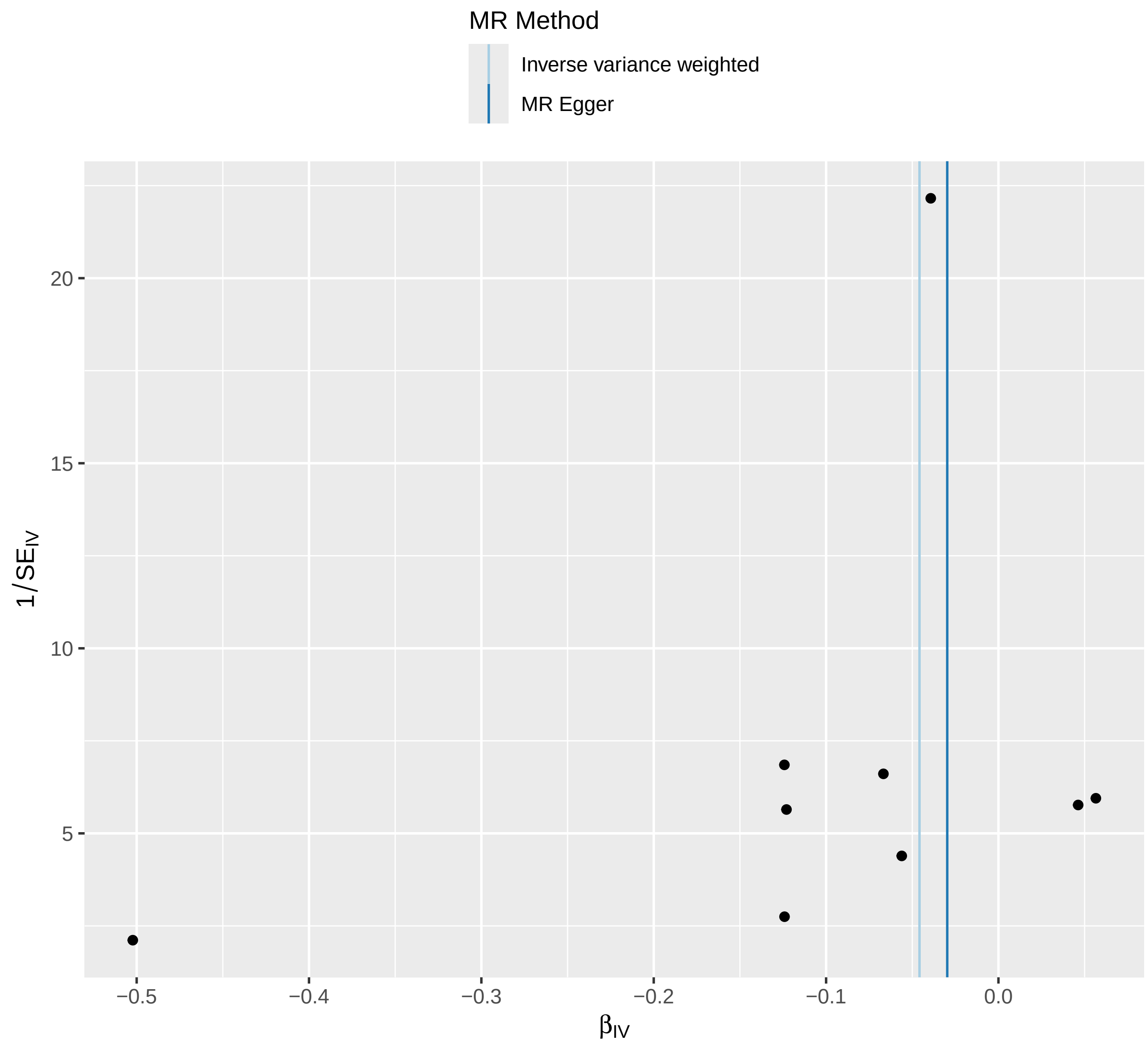

MR Method
Inverse variance weighted
MR Egger
20
15
10
5
1/SE$_{IV}$
−0.5
−0.4
−0.3
−0.2
−0.1
0.0
β$_{IV}$

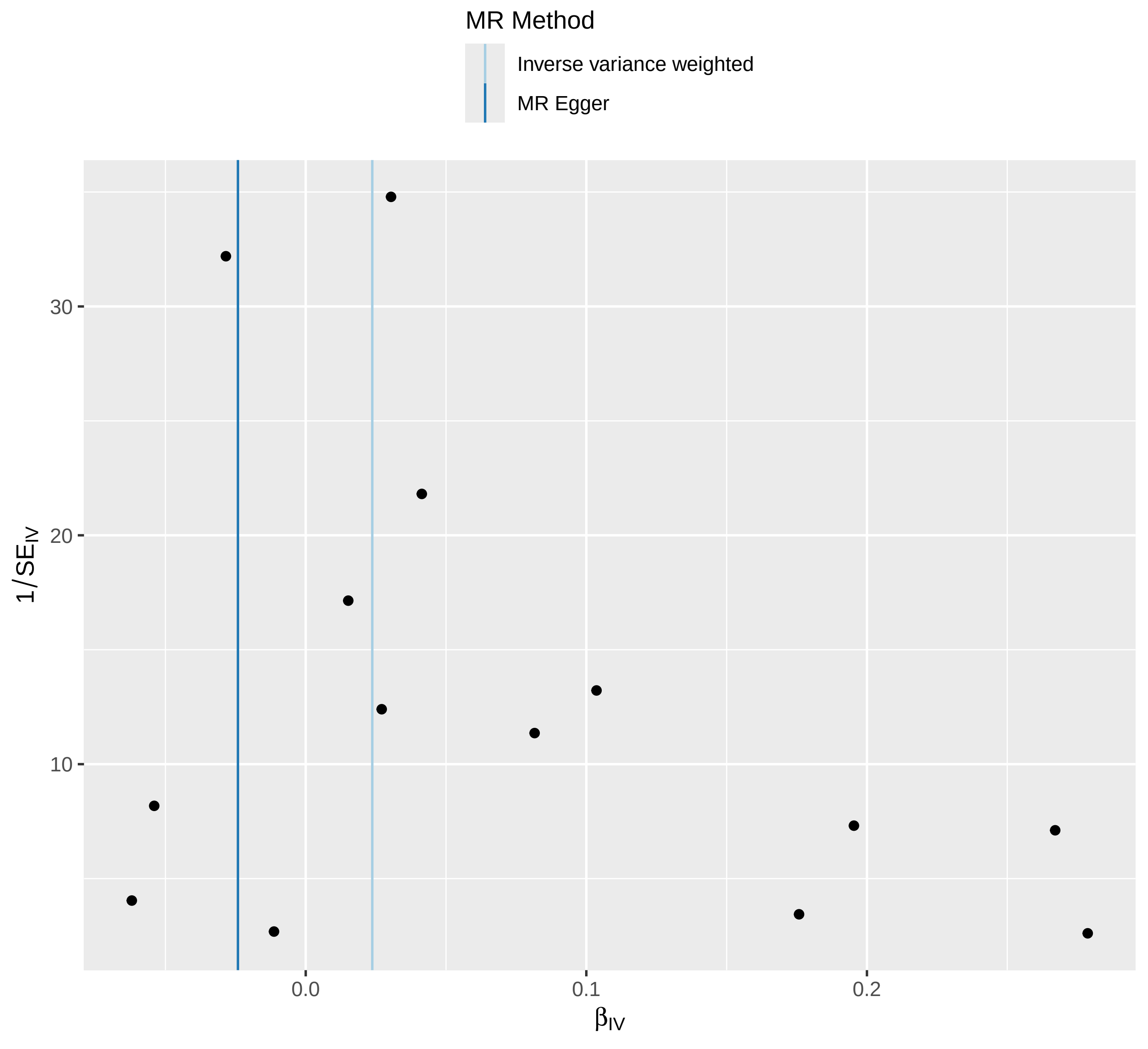
MR Method
Inverse variance weighted
MR Egger
30
20
10
1/SE$_{IV}$
0.0
0.1
0.2
β$_{IV}$

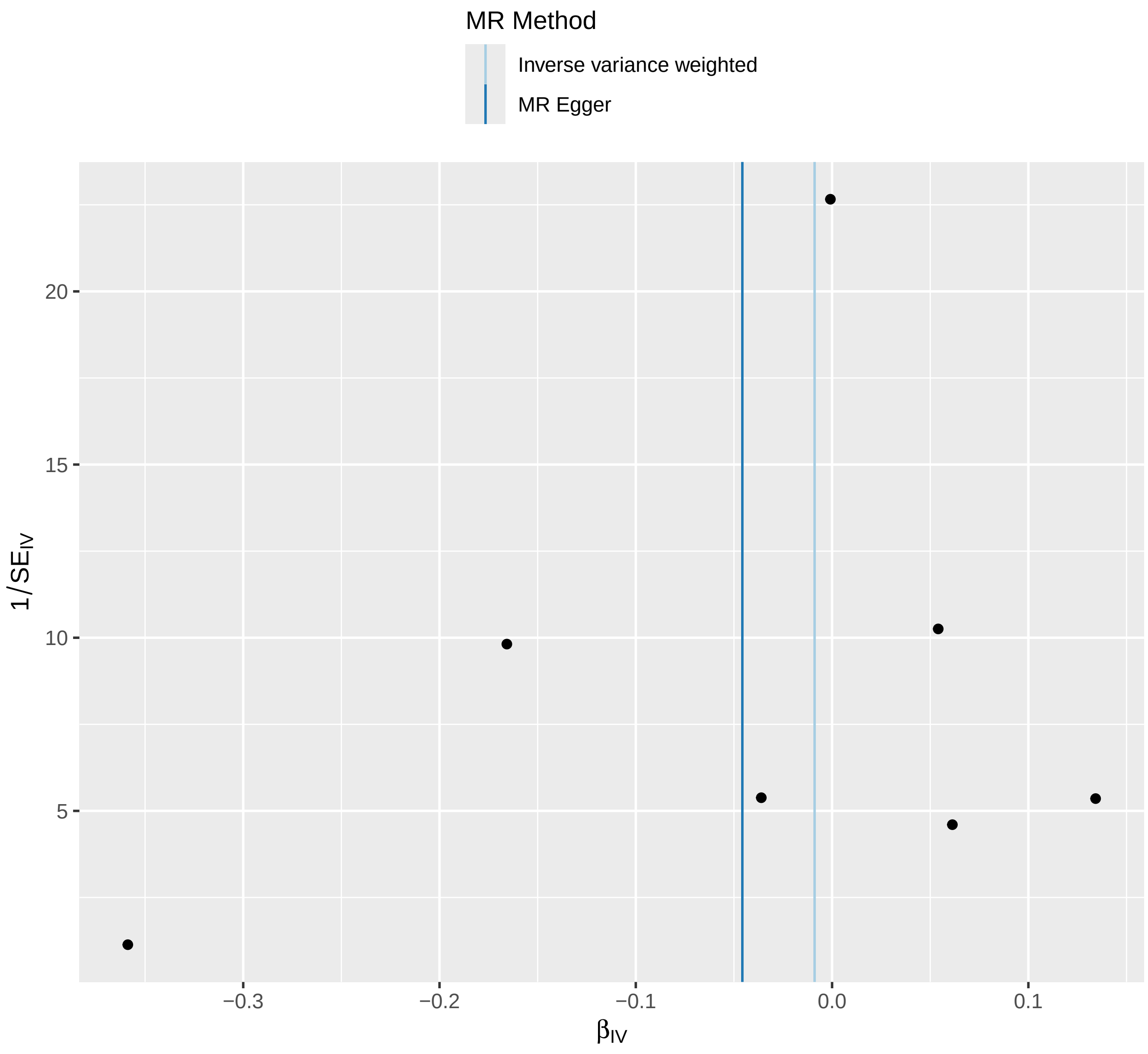
MR Method
Inverse variance weighted
MR Egger
1/SE$_{IV}$
20
15
10
5
−0.3
−0.2
−0.1
0.0
0.1
β$_{IV}$

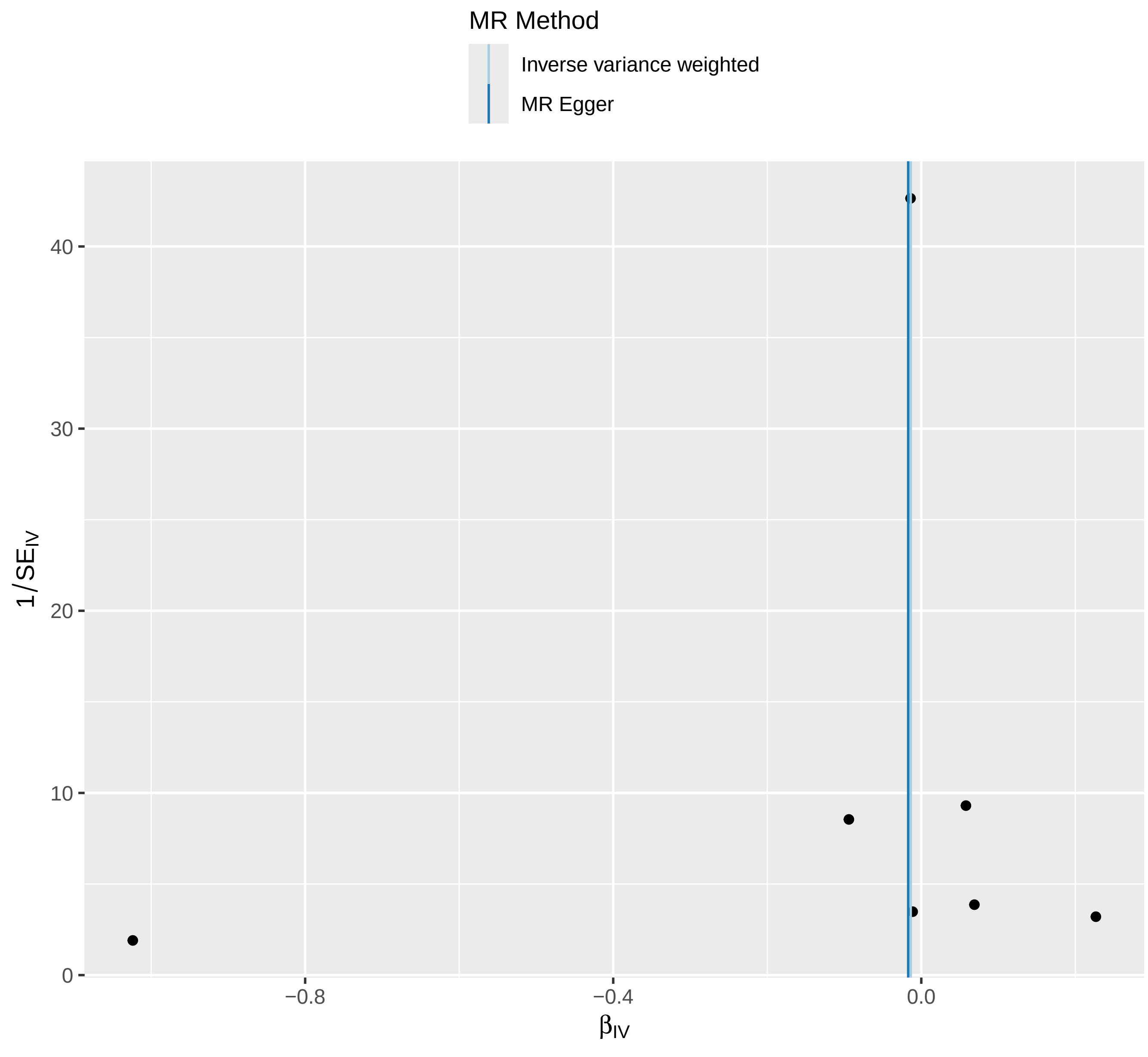

MR Method
Inverse variance weighted
MR Egger
40
30
20
10
0
1/SE$_{IV}$
−0.8
−0.4
0.0
β$_{IV}$

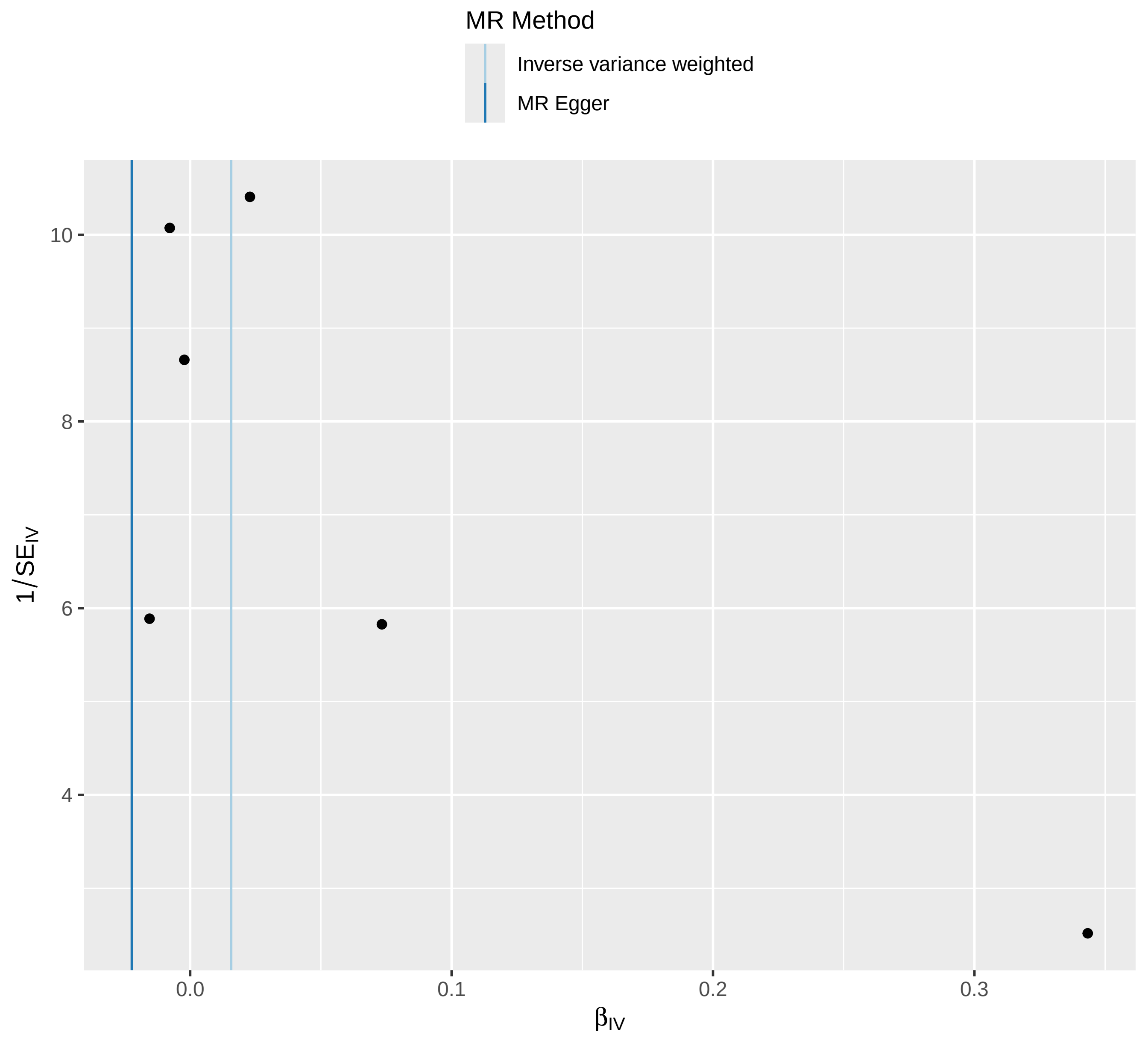

MR Method
Inverse variance weighted
MR Egger
10
8
6
4
1/SE_IV
0.0
0.1
0.2
0.3
β_IV

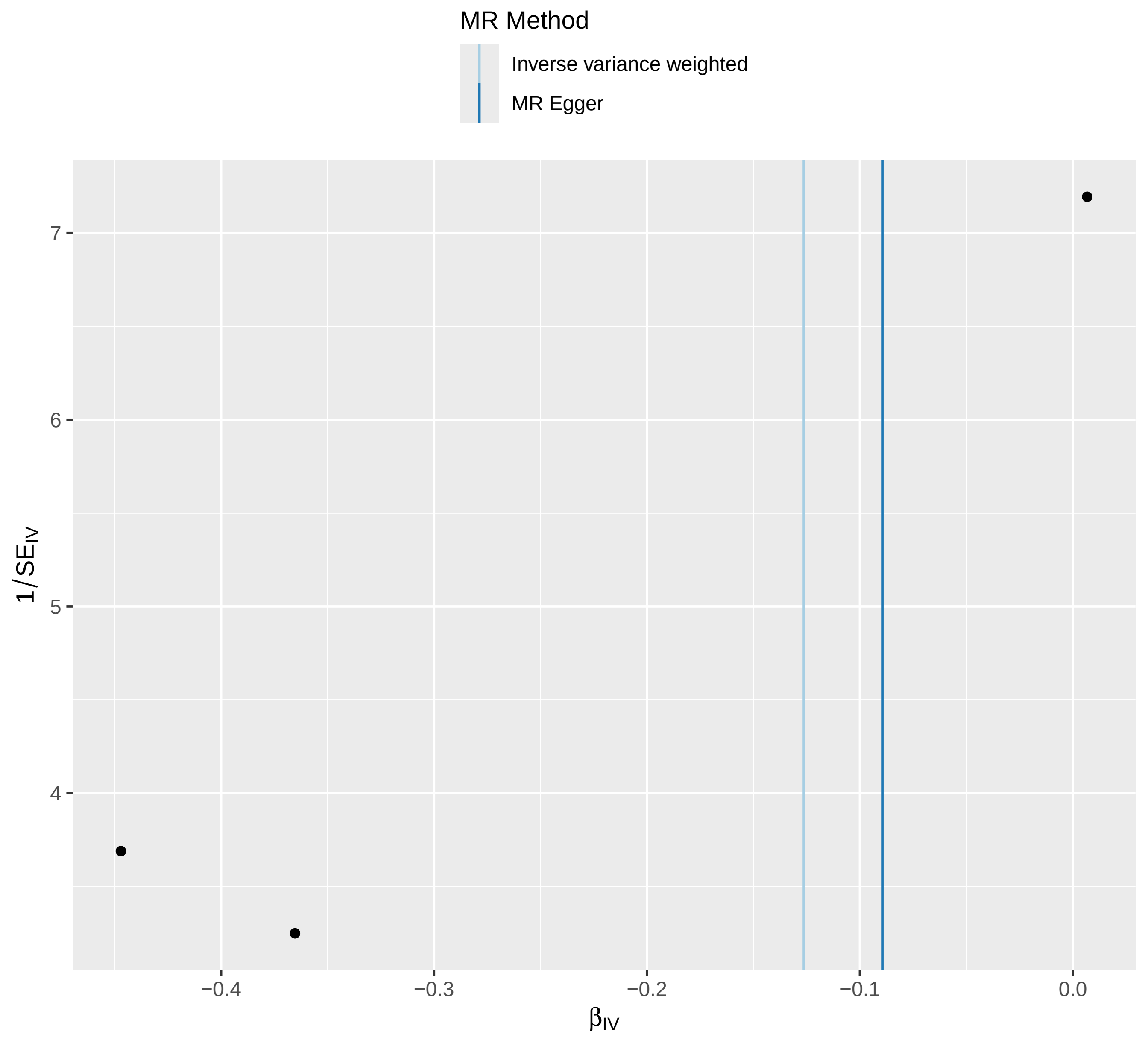

MR Method
Inverse variance weighted
MR Egger
7
6
5
4
1/SE_IV
−0.4
−0.3
−0.2
−0.1
0.0
β_IV

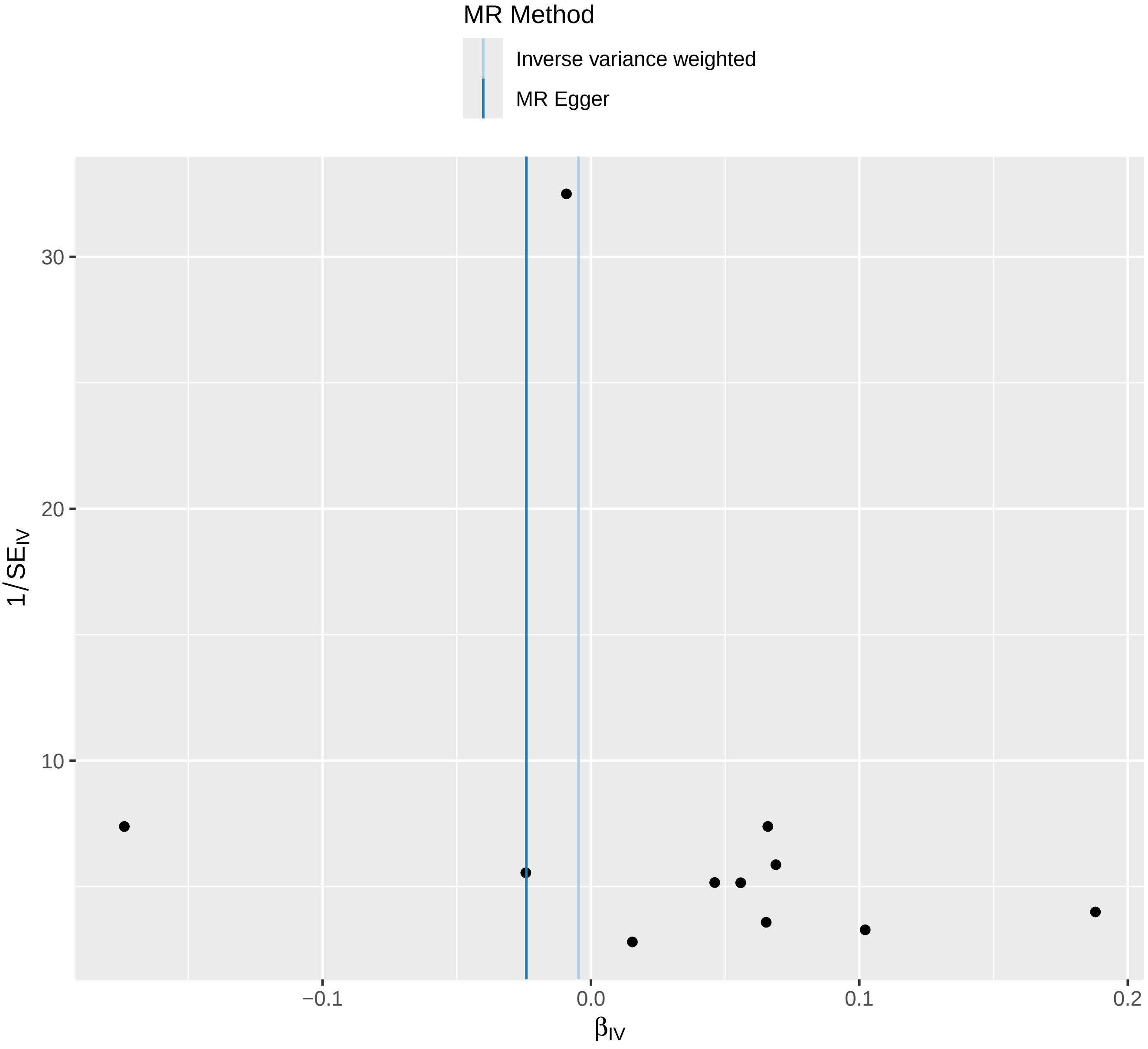
MR Method
Inverse variance weighted
MR Egger
30
20
10
1/SE_IV
−0.1
0.0
0.1
0.2
β_IV

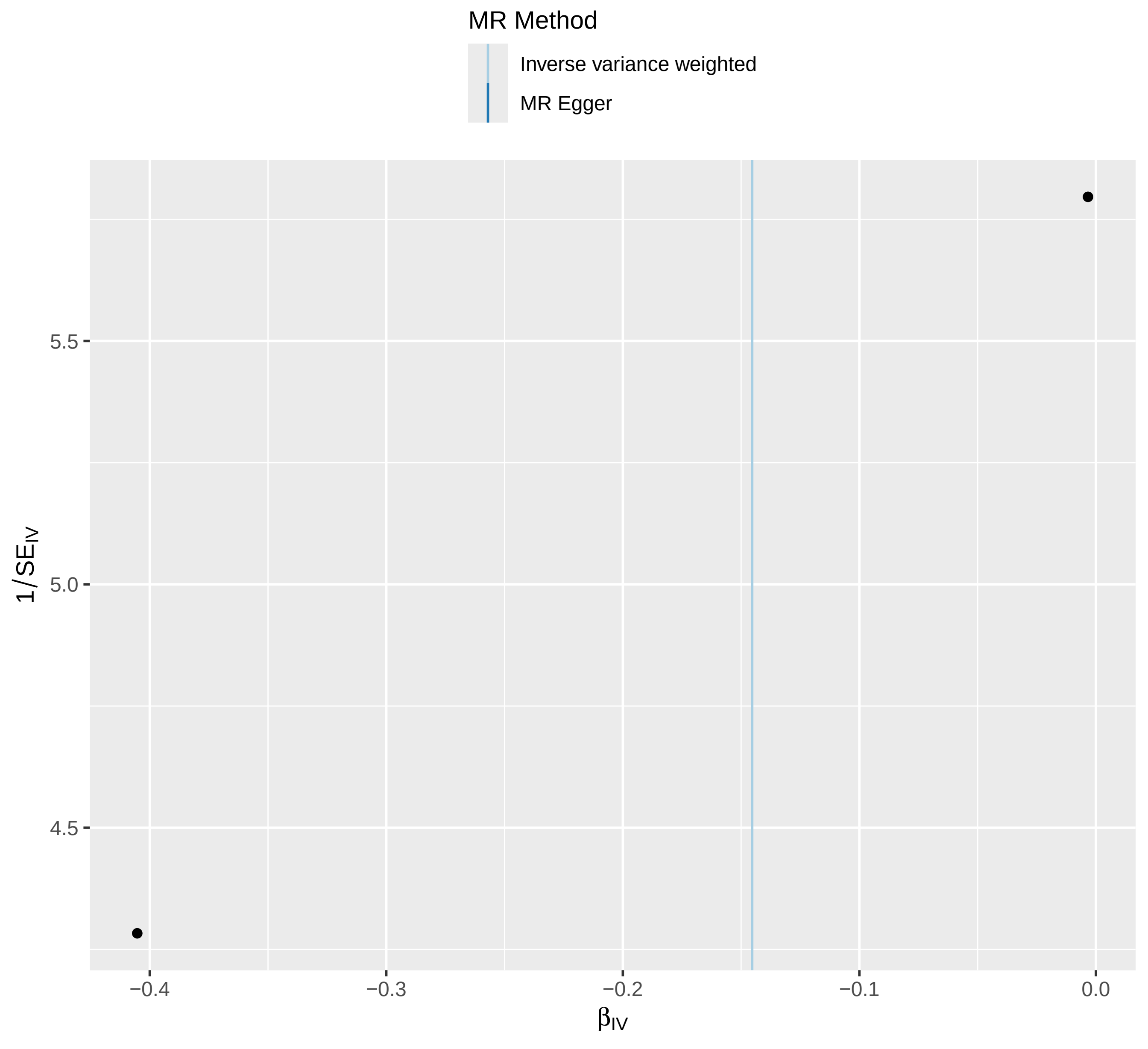
MR Method
Inverse variance weighted
MR Egger
1/SE_IV
5.5
5.0
4.5
−0.4
−0.3
−0.2
−0.1
0.0
β_IV

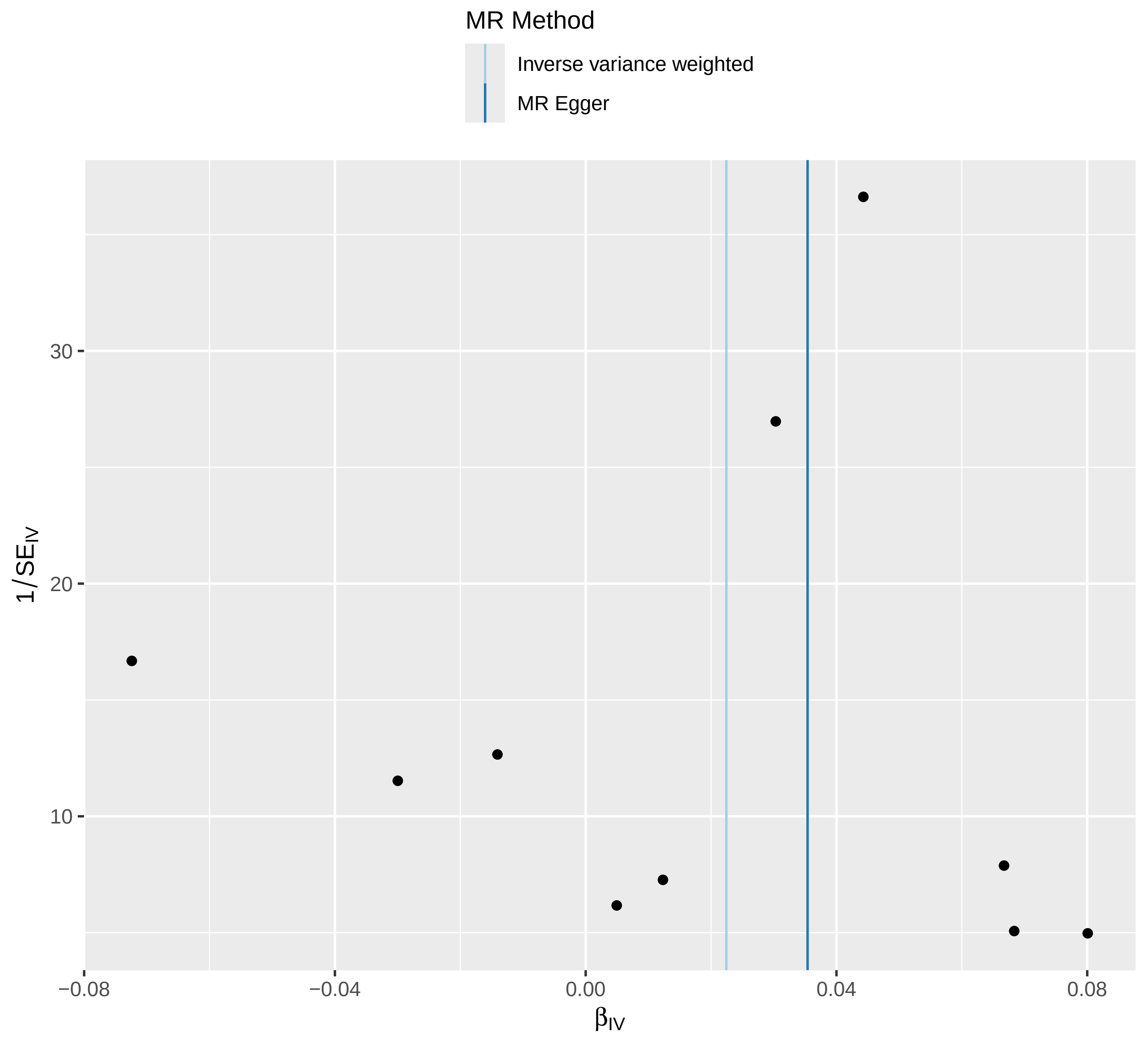

MR Method
Inverse variance weighted
MR Egger
30
20
10
1/SE$_{IV}$
−0.08
−0.04
0.00
0.04
0.08
β$_{IV}$

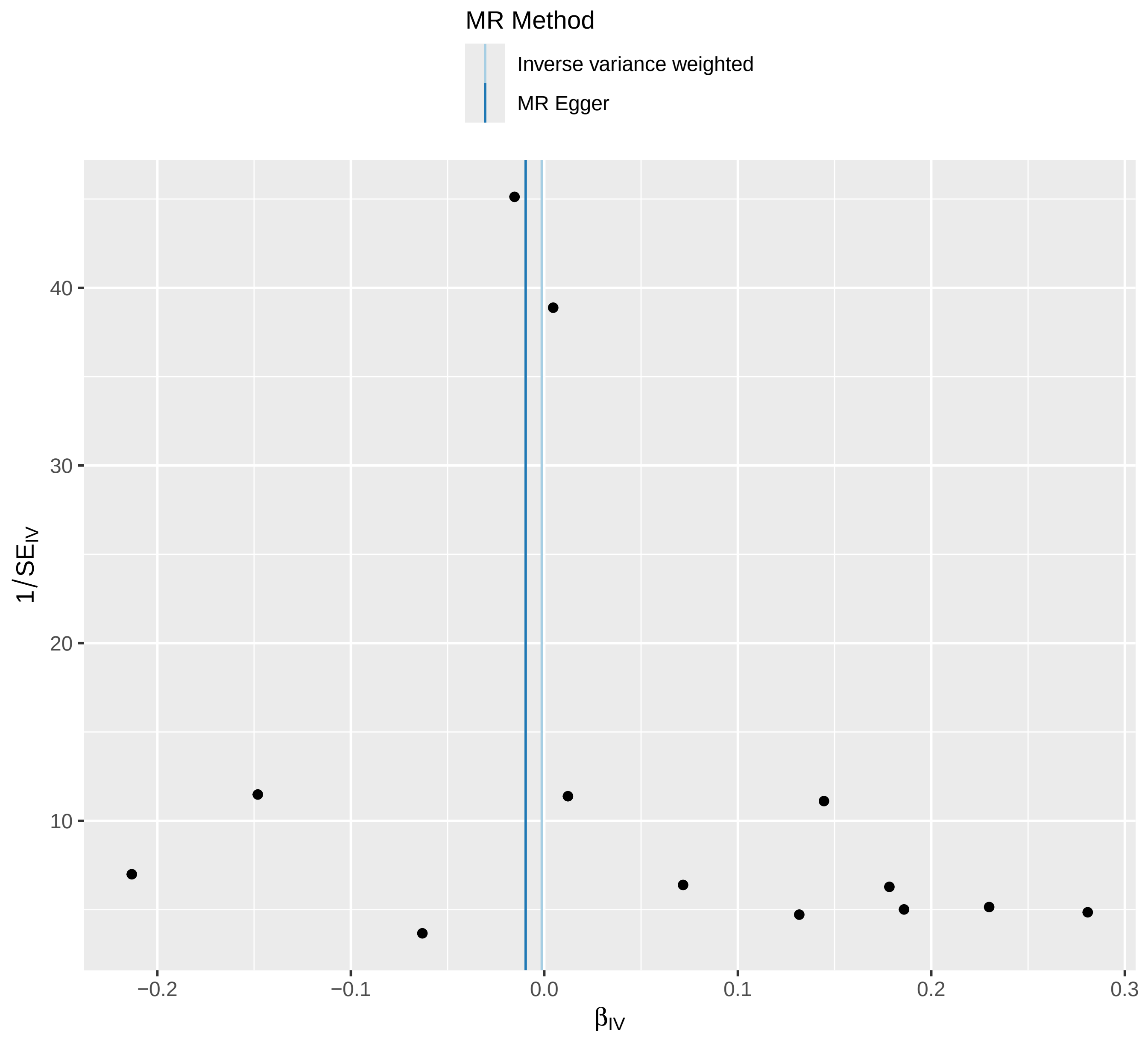
MR Method
Inverse variance weighted
MR Egger
40
30
20
10
−0.2
−0.1
0.0
0.1
0.2
0.3
1/SE$_{IV}$
β$_{IV}$

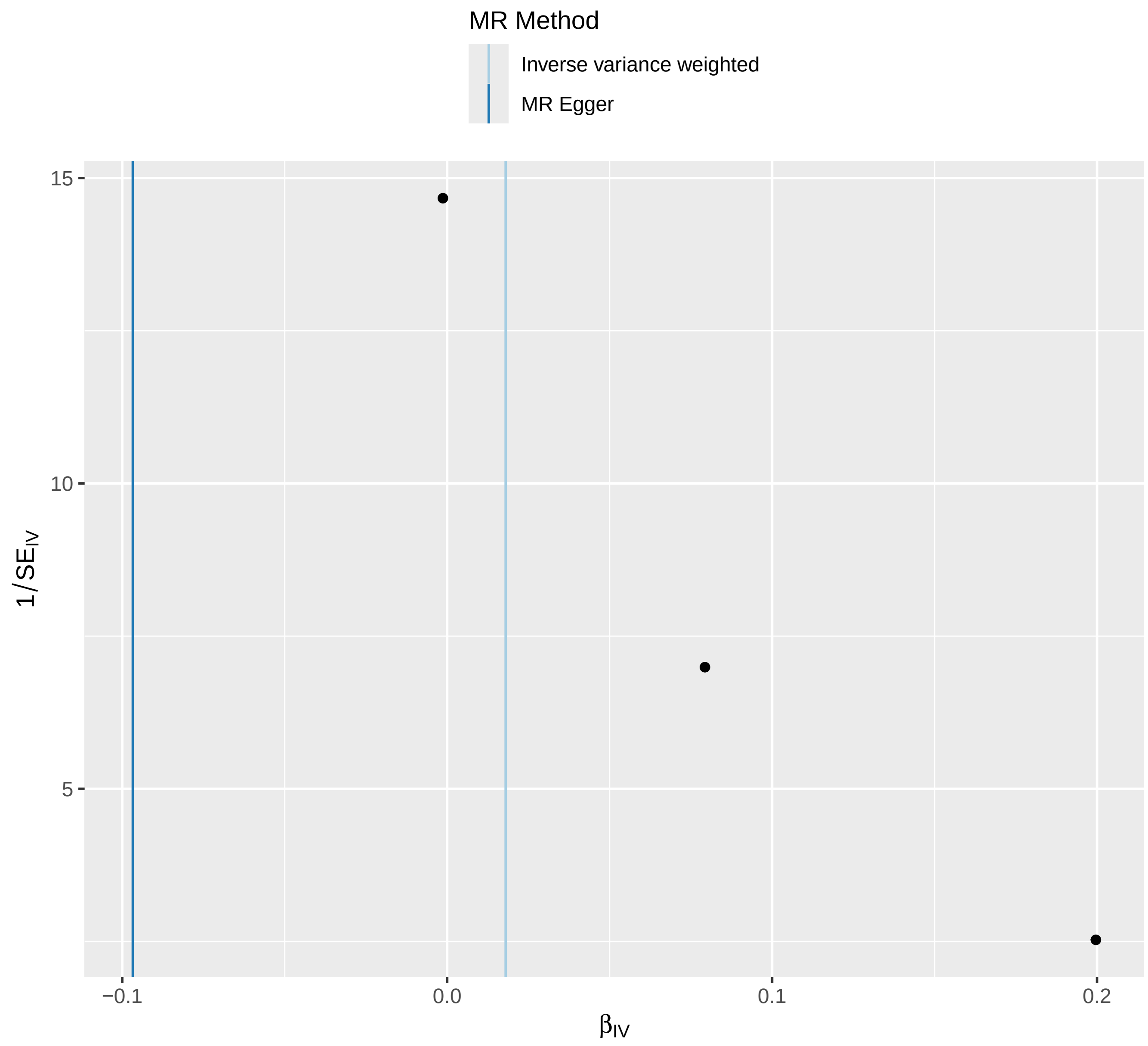

MR Method
Inverse variance weighted
MR Egger
15
10
5
$1/SE_{IV}$
−0.1
0.0
0.1
0.2
$\beta_{IV}$

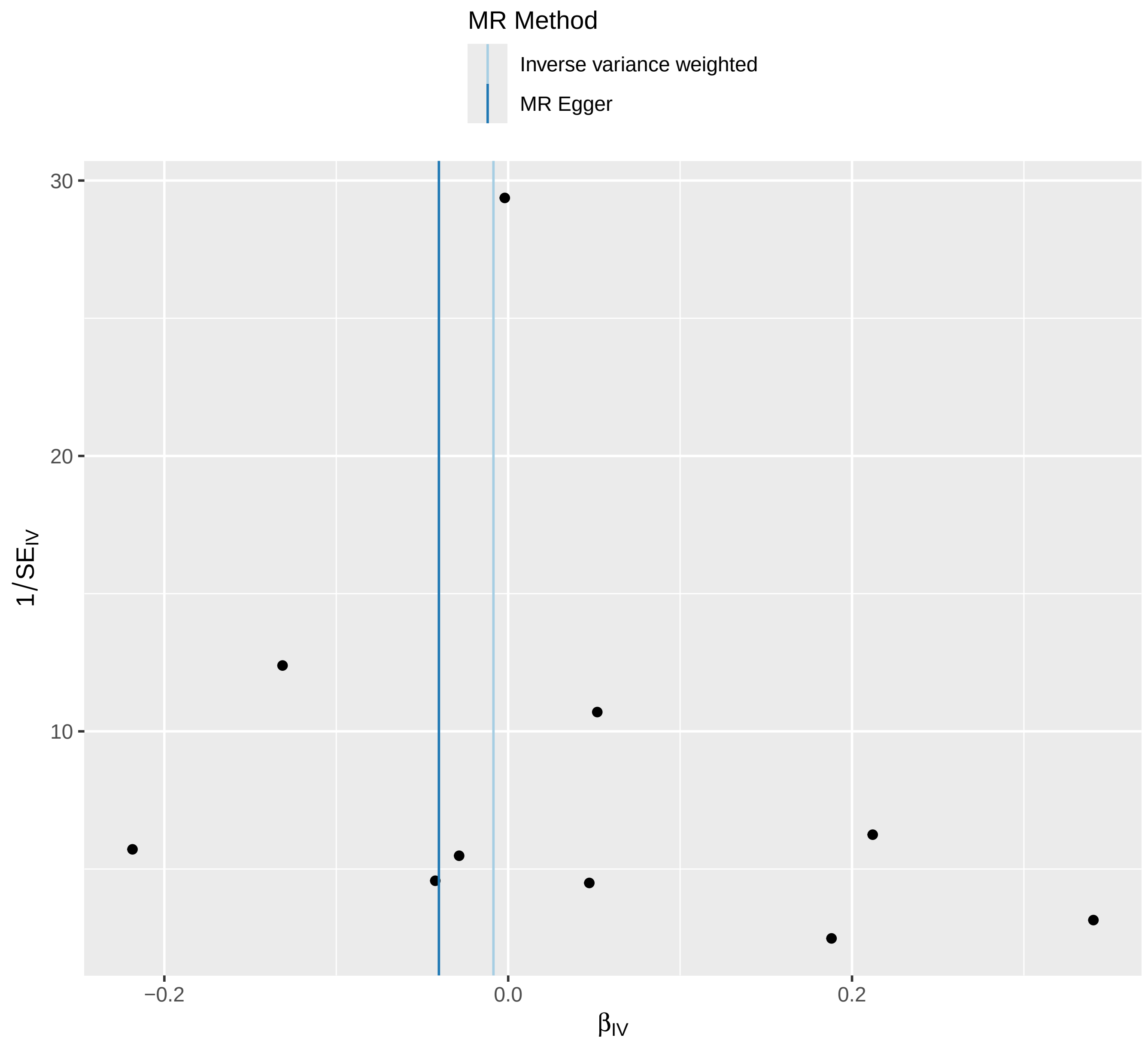

MR Method
Inverse variance weighted
MR Egger
30
20
10
$1/SE_{IV}$
−0.2
0.0
0.2
$\beta_{IV}$

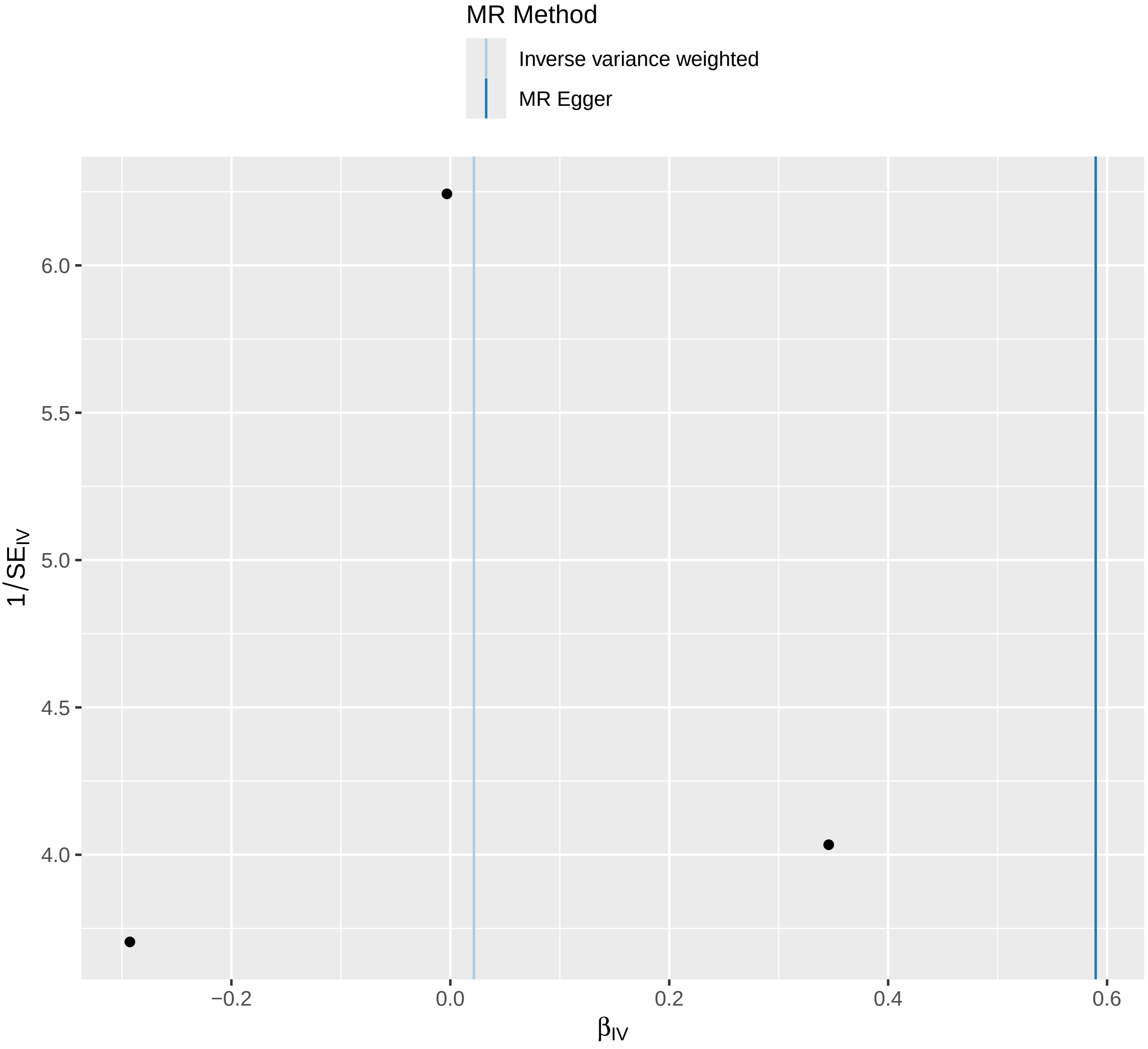

MR Method
Inverse variance weighted
MR Egger
6.0
5.5
5.0
4.5
4.0
1/SE_IV
−0.2
0.0
0.2
0.4
0.6
β_IV

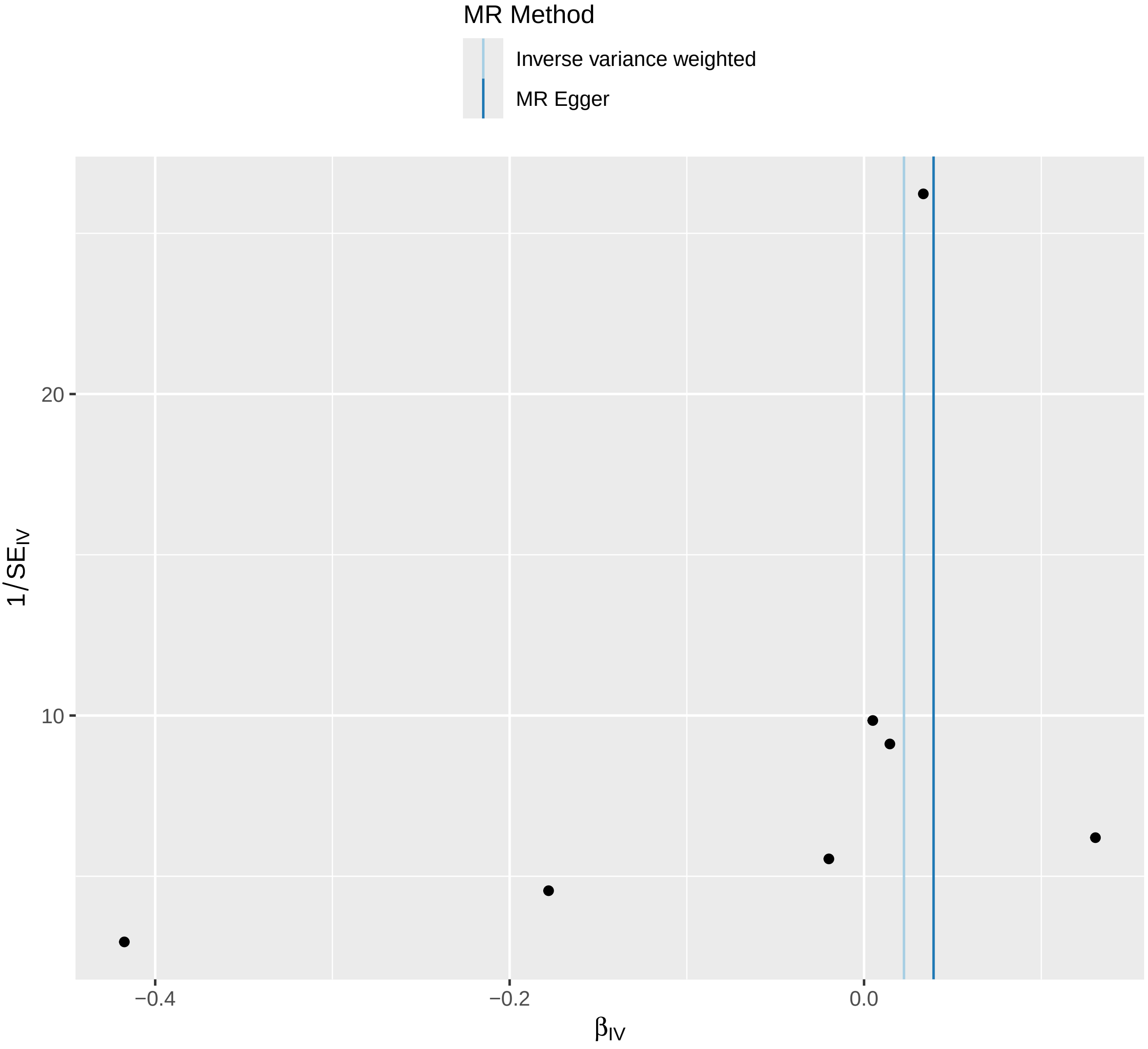

MR Method
Inverse variance weighted
MR Egger
20
10
1/SE_IV
−0.4
−0.2
0.0
β_IV

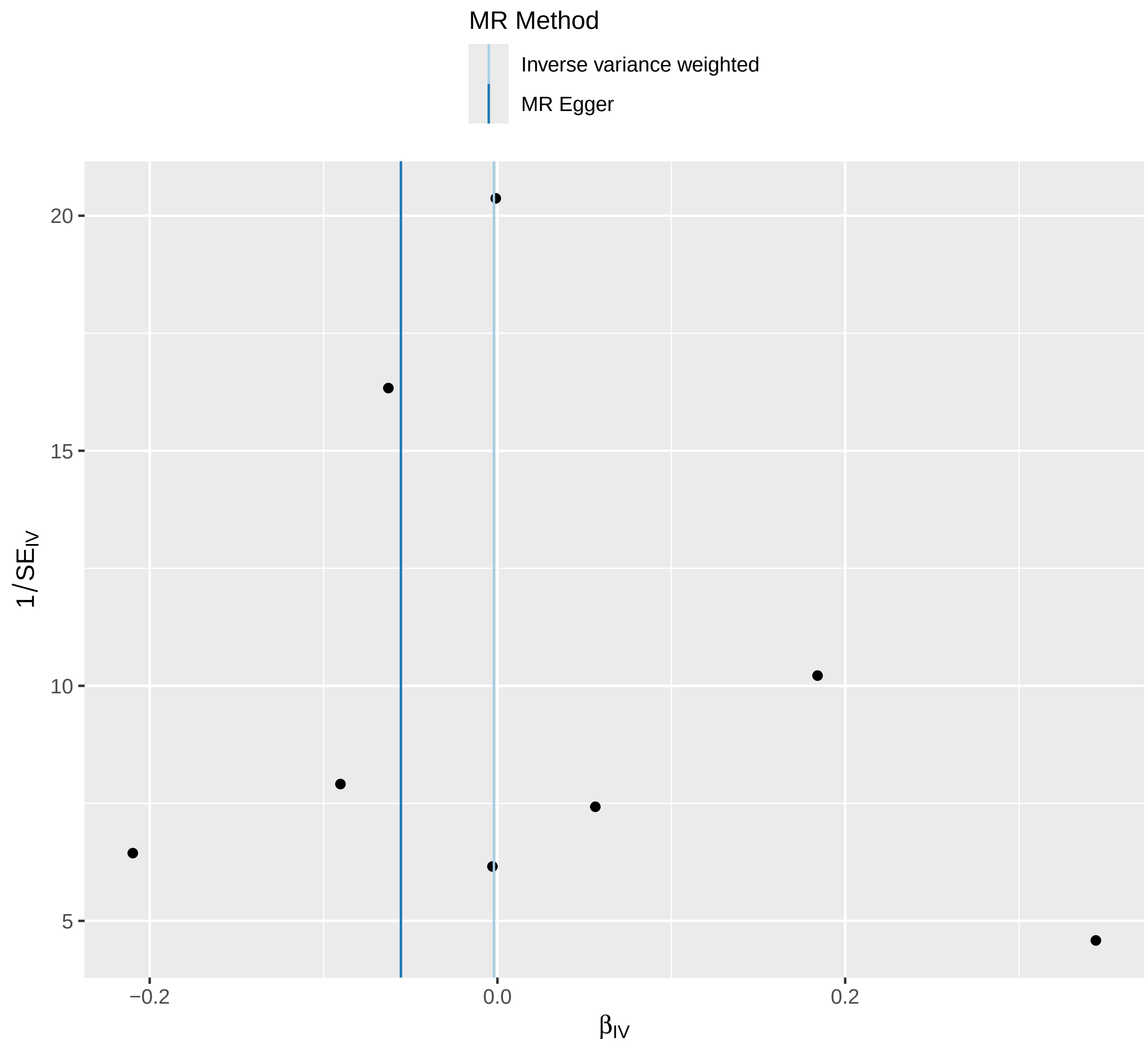
MR Method
Inverse variance weighted
MR Egger
20
15
10
5
1/SE_IV
−0.2
0.0
0.2
β_IV

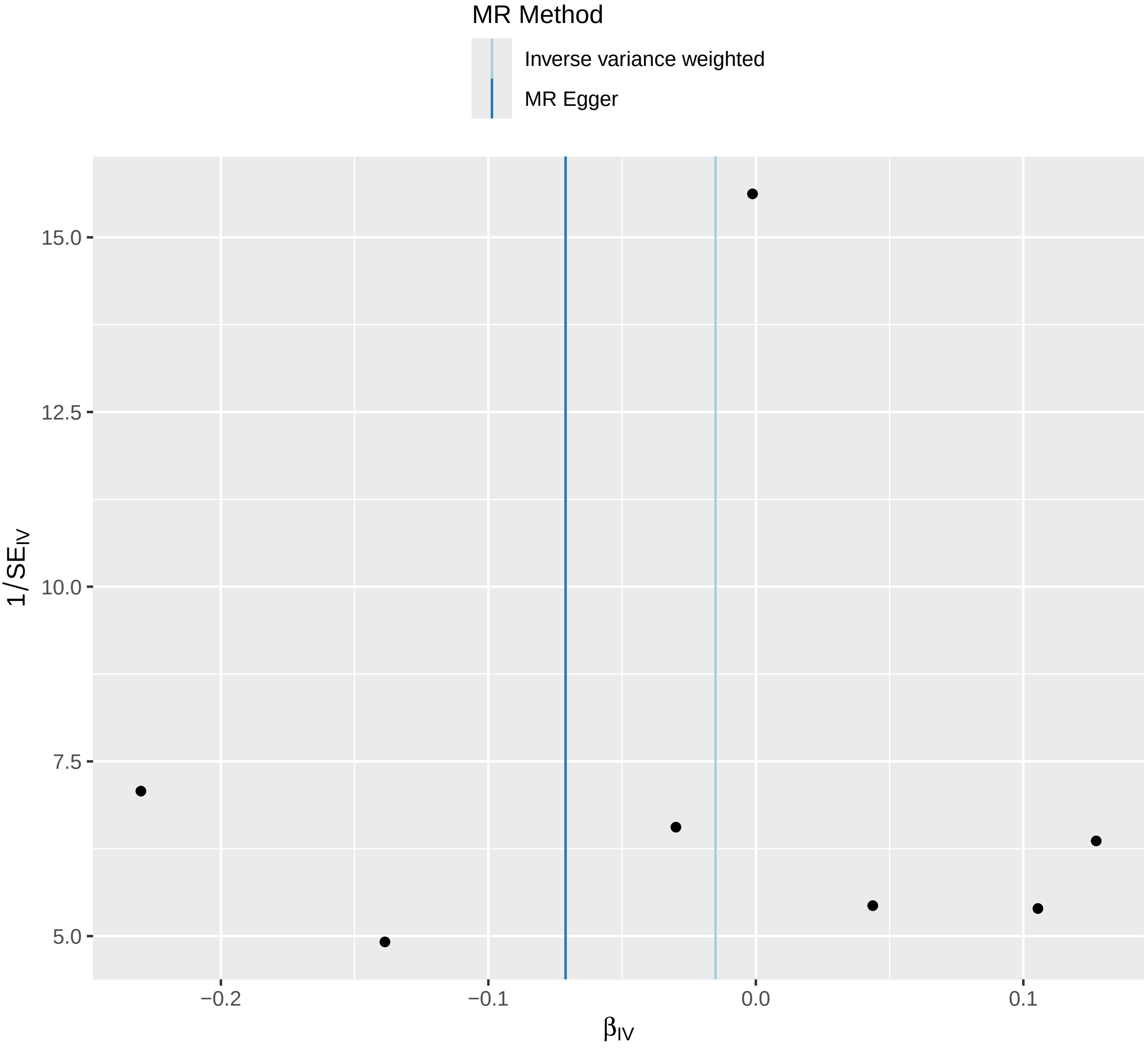

MR Method
Inverse variance weighted
MR Egger
15.0
12.5
10.0
7.5
5.0
1/SE$_{IV}$
−0.2
−0.1
0.0
0.1
β$_{IV}$

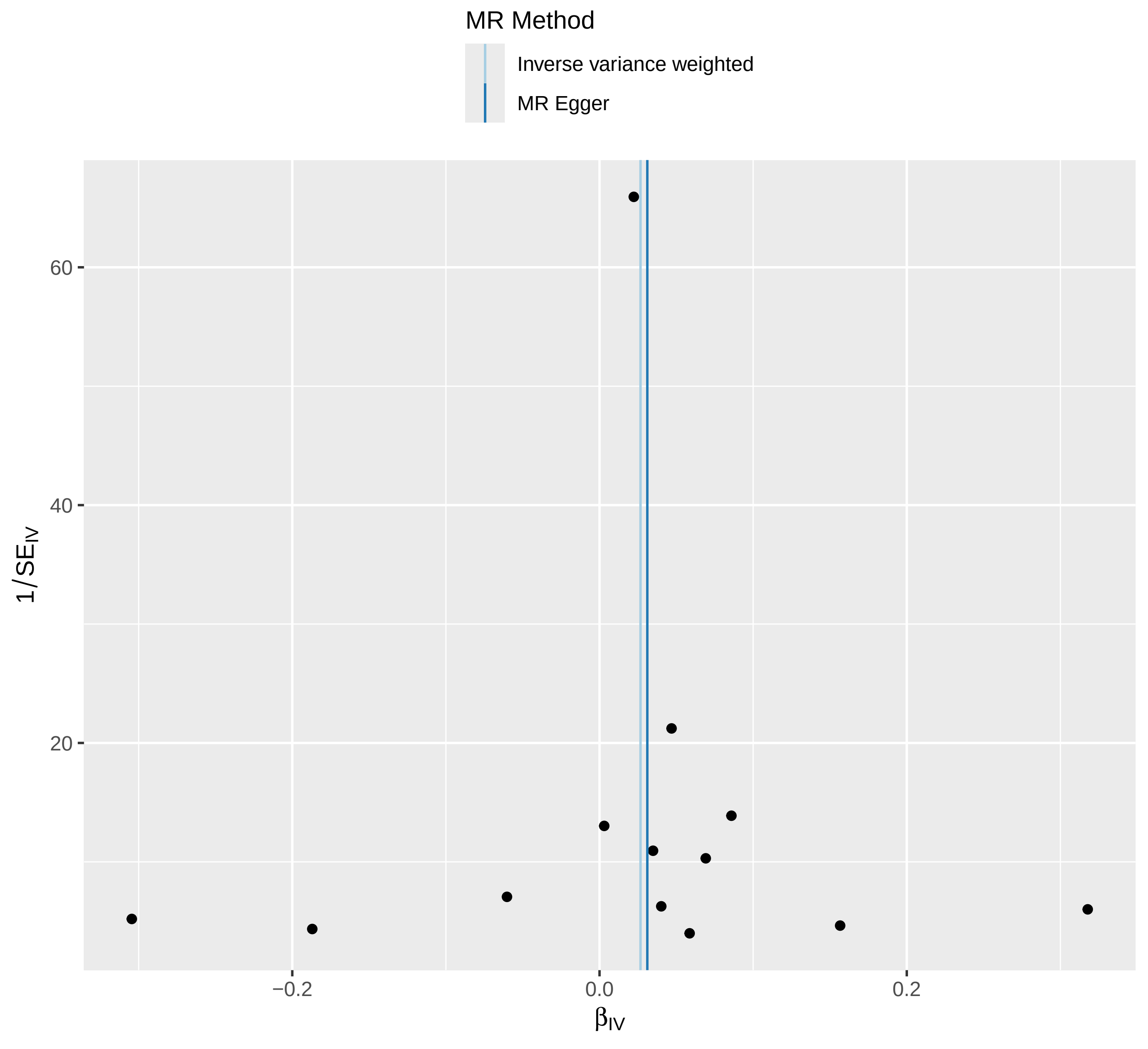

MR Method
Inverse variance weighted
MR Egger
60
40
20
−0.2
0.0
0.2
$1/SE_{IV}$
$\beta_{IV}$

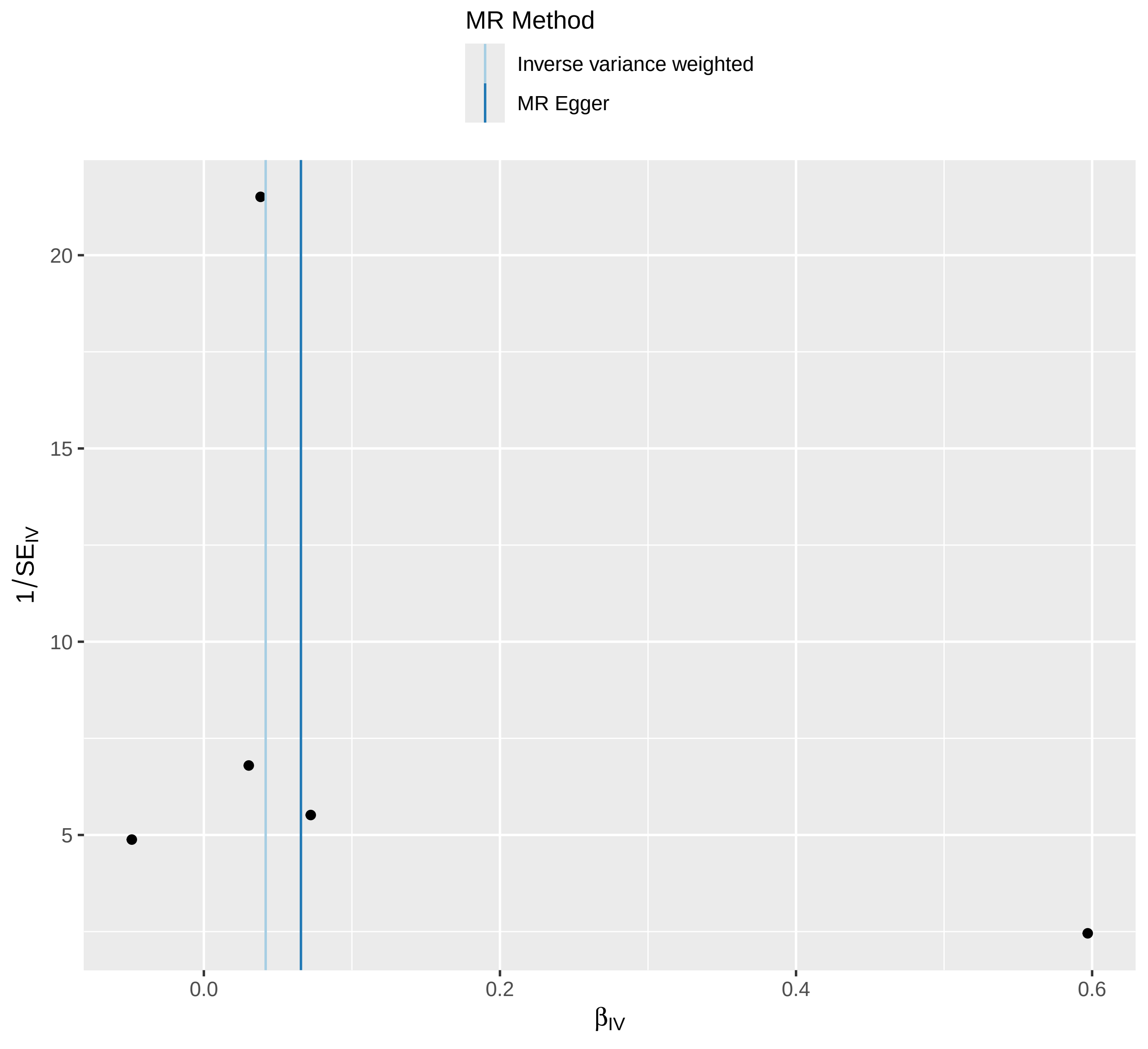

MR Method
Inverse variance weighted
MR Egger
20
15
10
5
$1/SE_{IV}$
0.0
0.2
0.4
0.6
$\beta_{IV}$

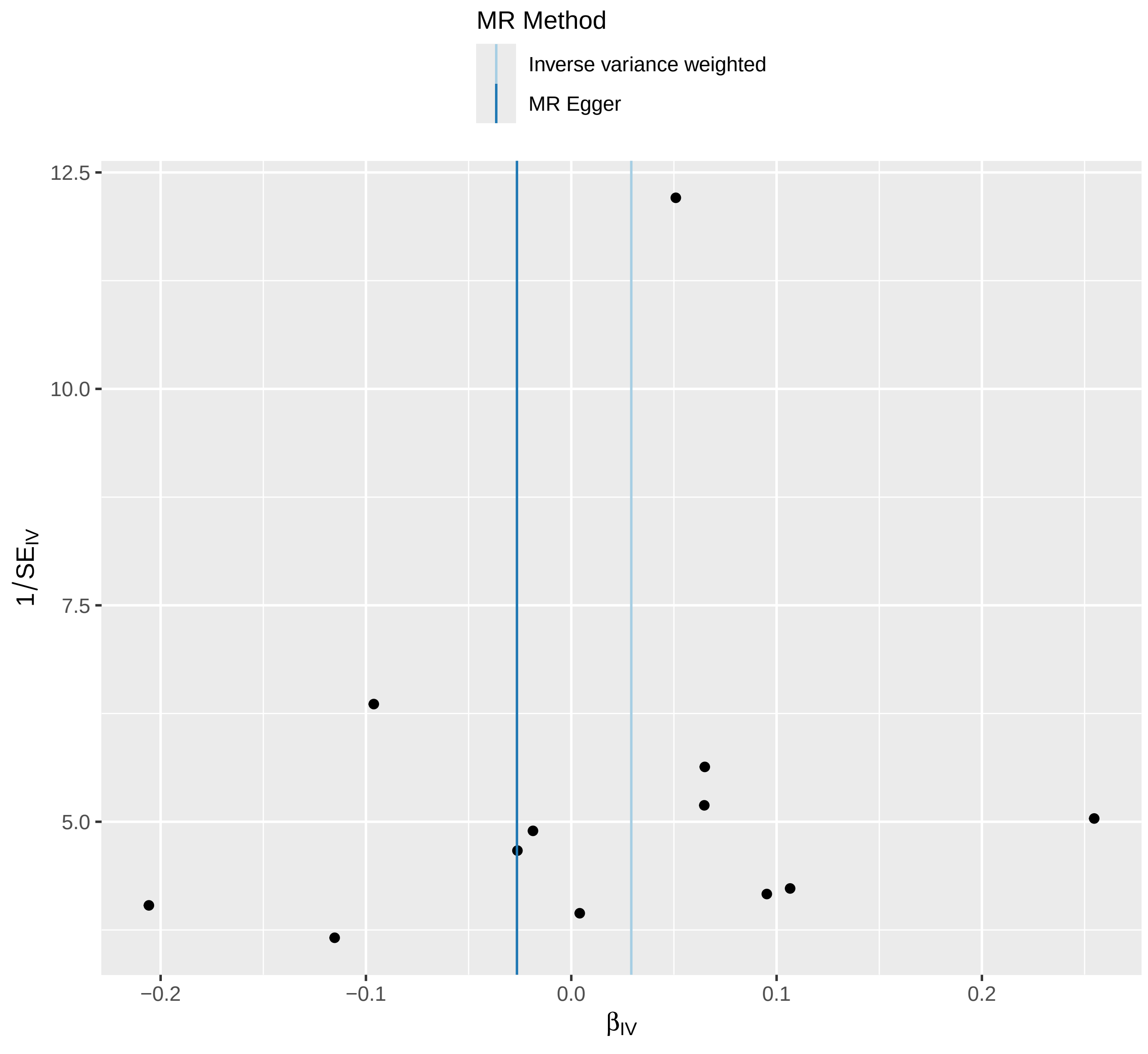
MR Method
Inverse variance weighted
MR Egger
12.5
10.0
7.5
5.0
1/SE_IV
−0.2
−0.1
0.0
0.1
0.2
β_IV

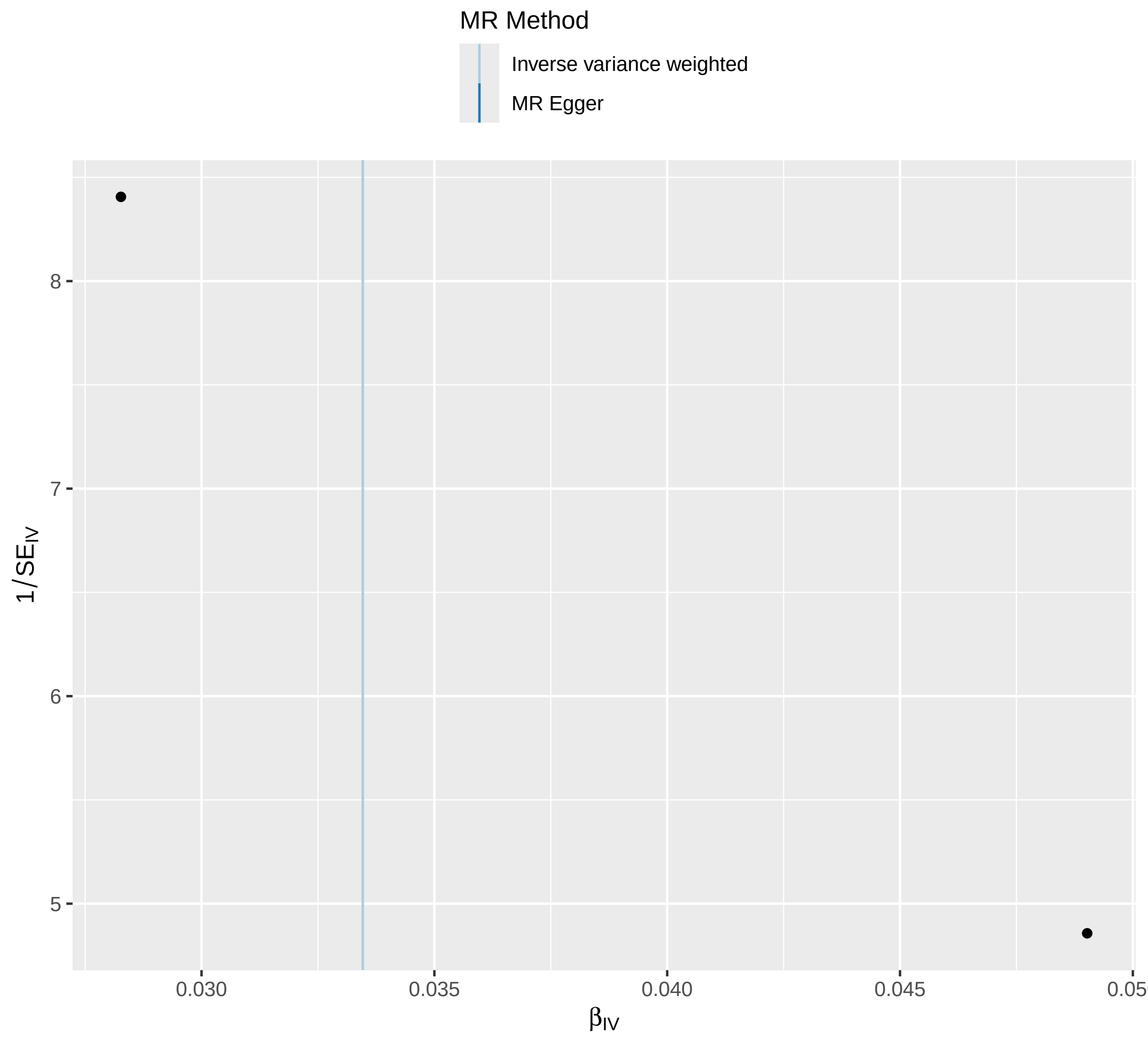

MR Method
Inverse variance weighted
MR Egger
1/SE_IV
8
7
6
5
0.030
0.035
0.040
0.045
0.050
β_IV

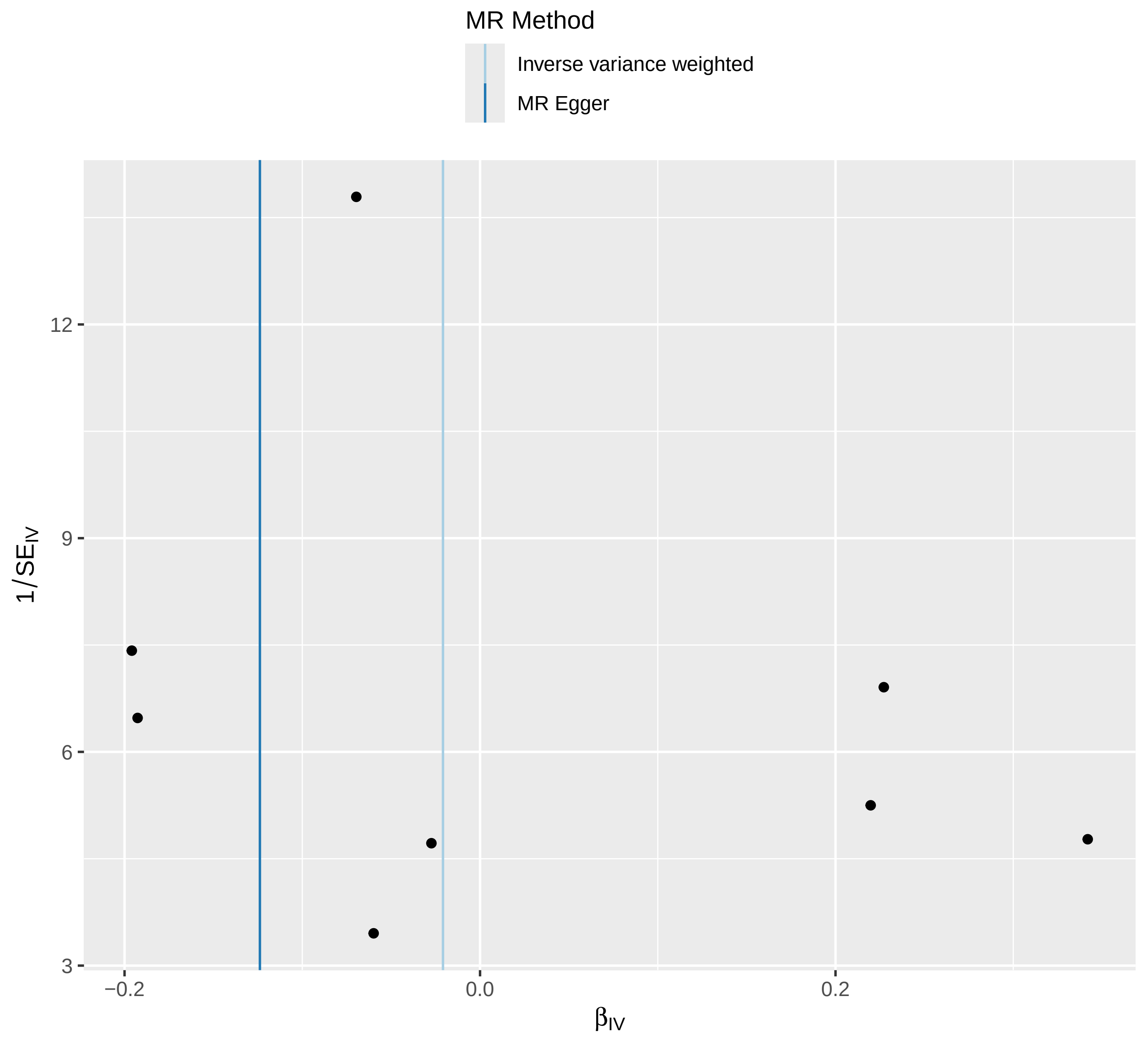
MR Method
Inverse variance weighted
MR Egger
12
9
6
3
1/SE_IV
−0.2
0.0
0.2
β_IV

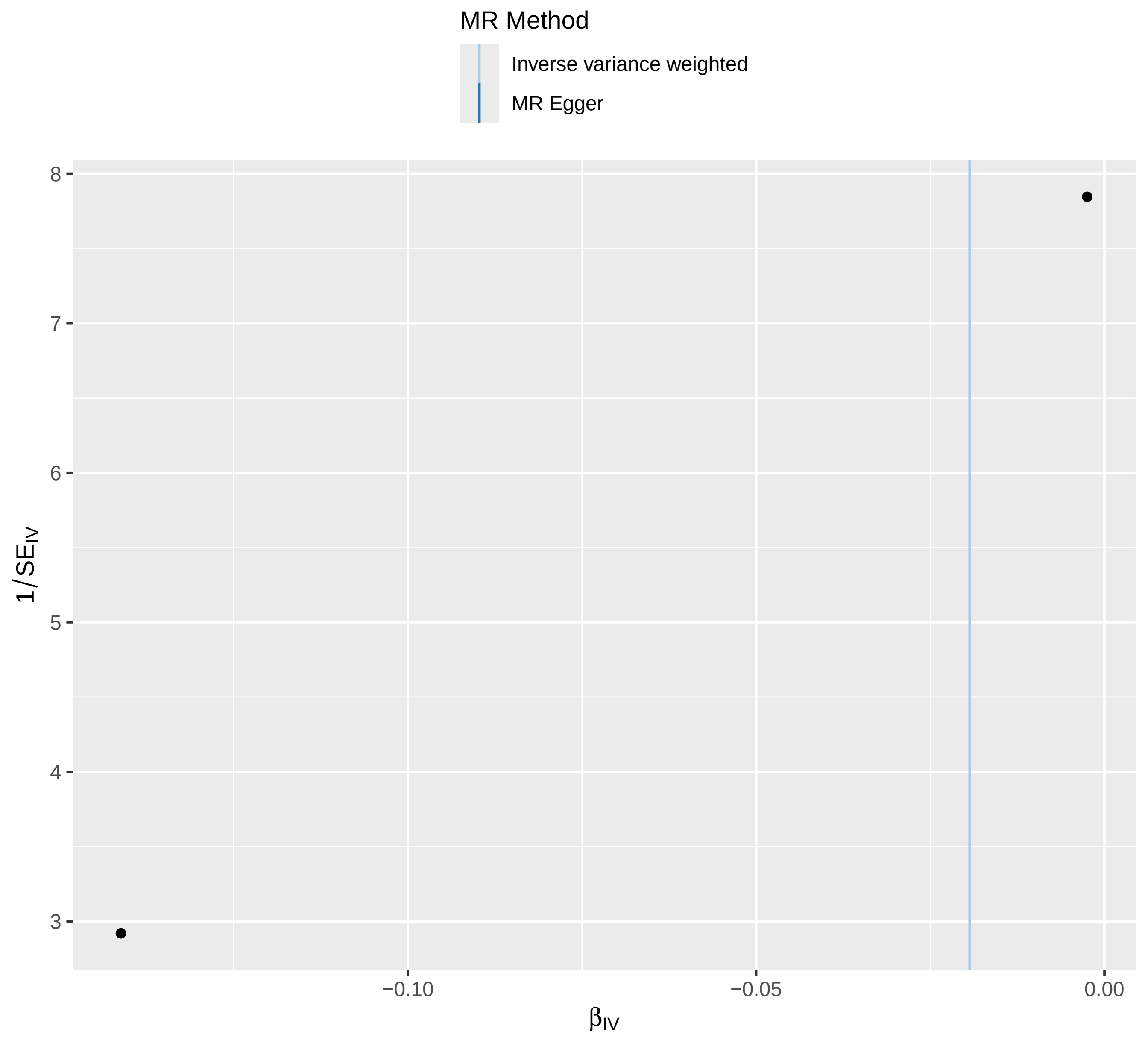

MR Method
Inverse variance weighted
MR Egger
8
7
6
5
4
3
1/SE_IV
−0.10
−0.05
0.00
β_IV

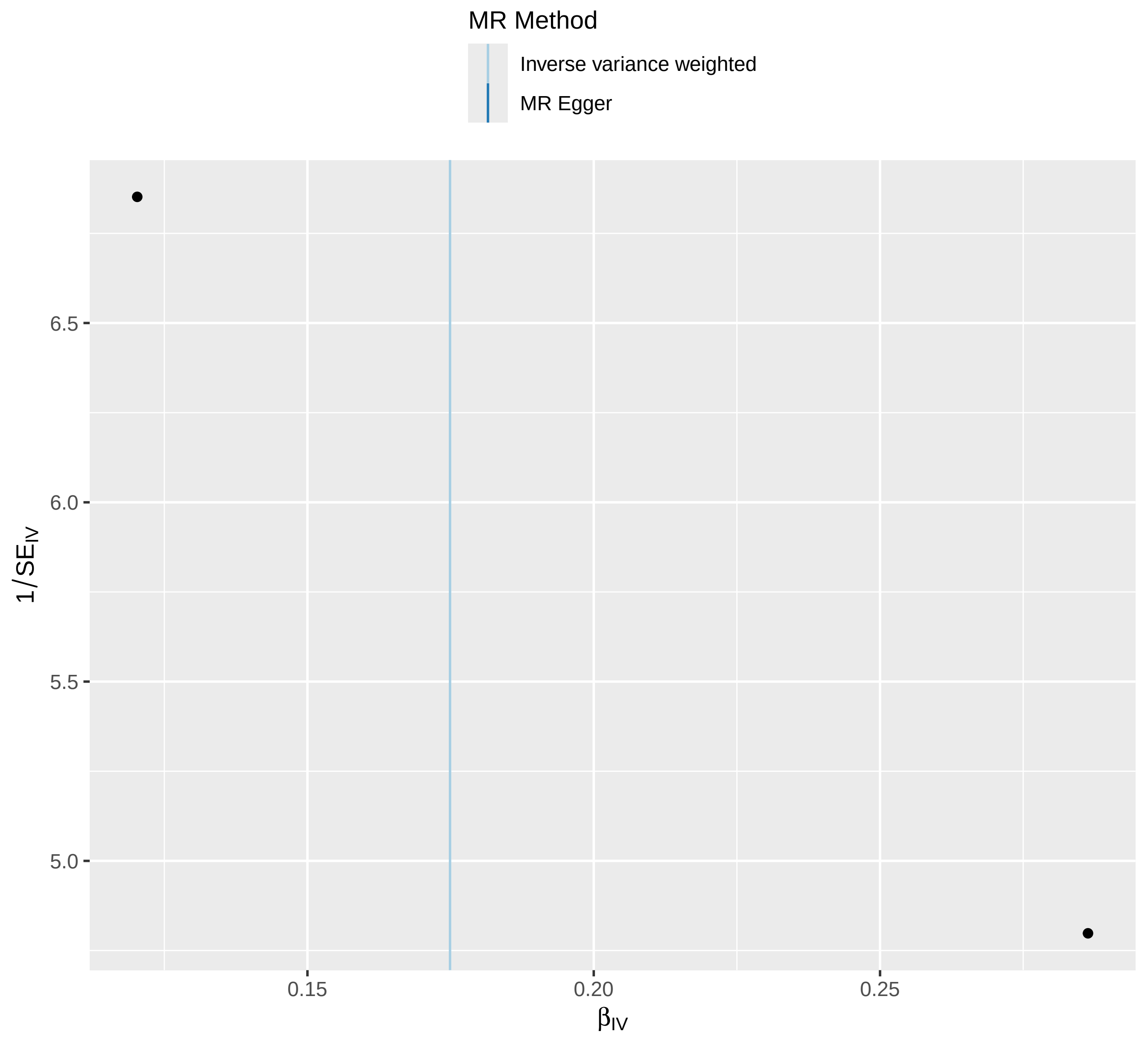

MR Method
Inverse variance weighted
MR Egger
6.5
6.0
5.5
5.0
1/$SE_{IV}$
0.15
0.20
0.25
$\beta_{IV}$

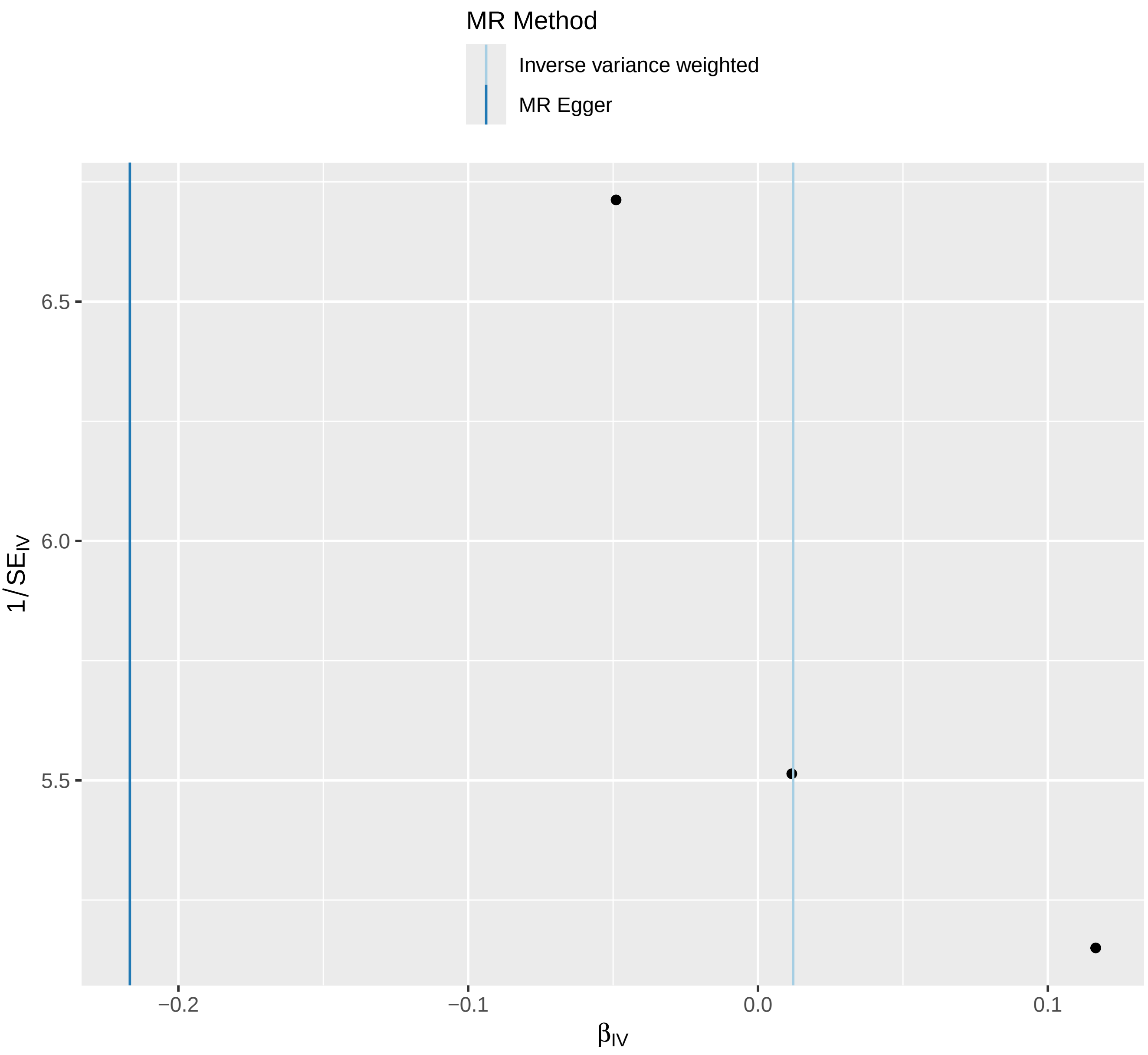

MR Method
Inverse variance weighted
MR Egger
6.5
6.0
5.5
1/SE$_{IV}$
−0.2
−0.1
0.0
0.1
β$_{IV}$

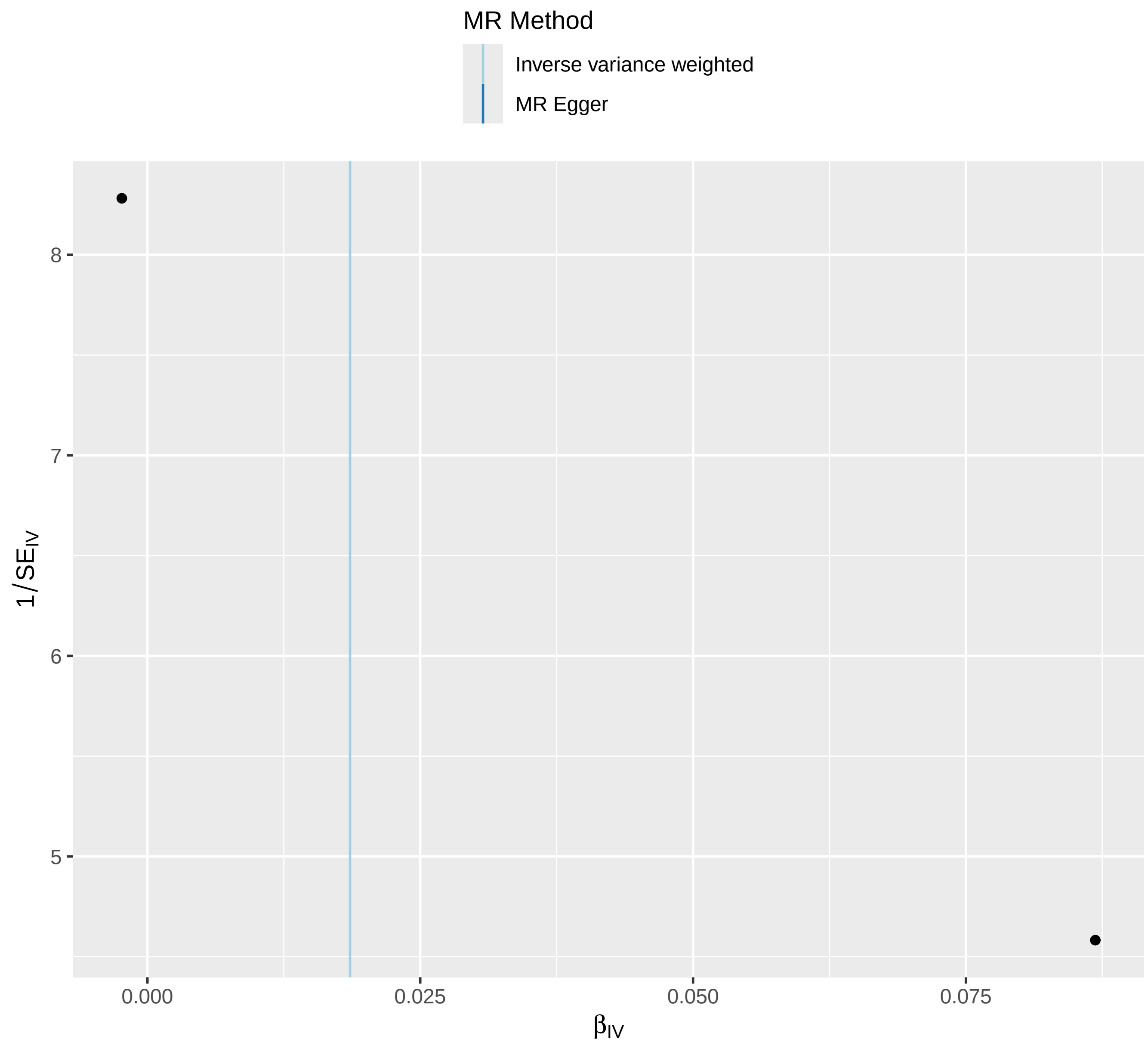
MR Method
Inverse variance weighted
MR Egger
8
7
6
5
1/SE$_{IV}$
0.000
0.025
0.050
0.075
β$_{IV}$

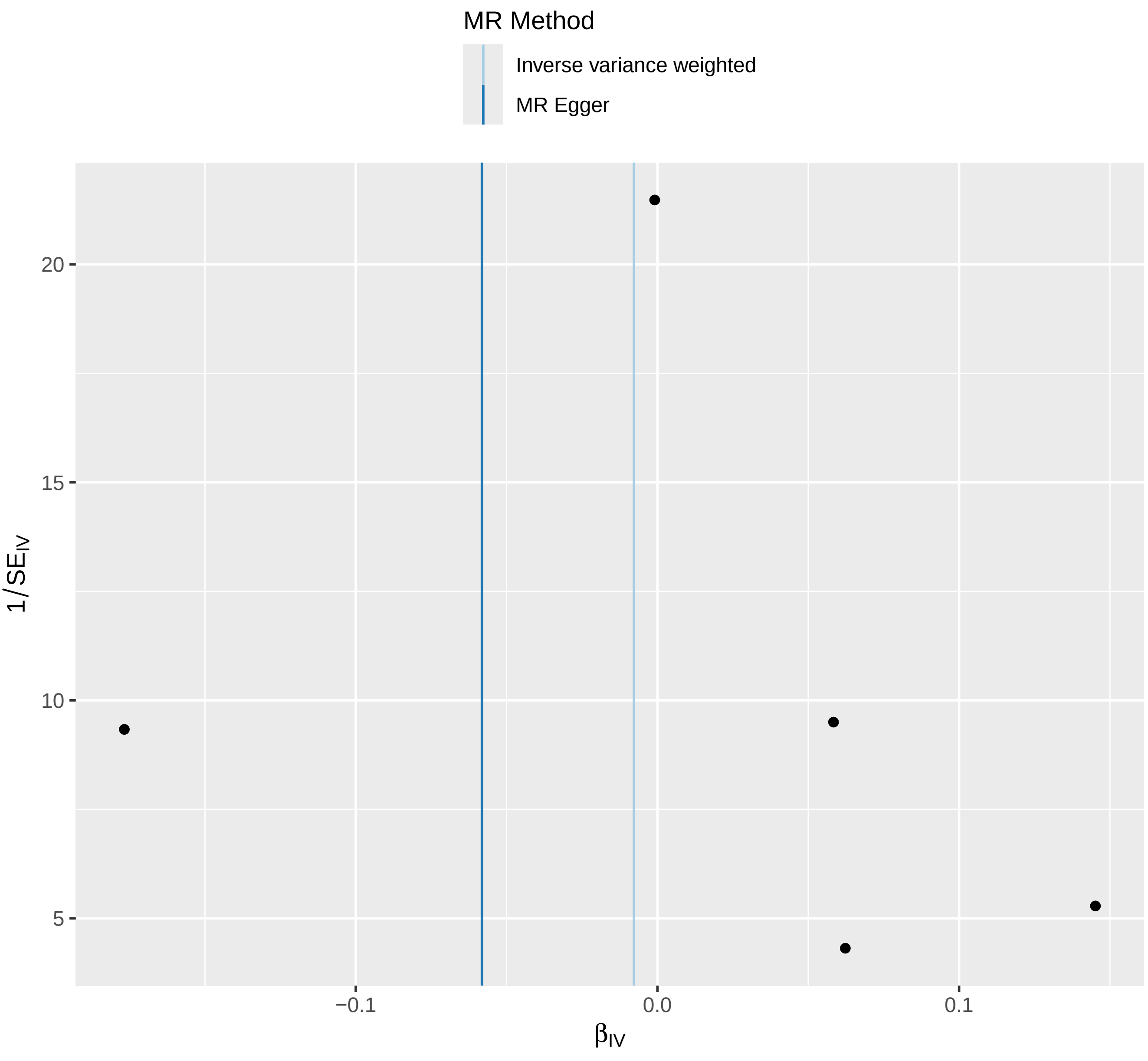

MR Method
Inverse variance weighted
MR Egger
20
15
10
5
1/SE$_{IV}$
−0.1
0.0
0.1
β$_{IV}$

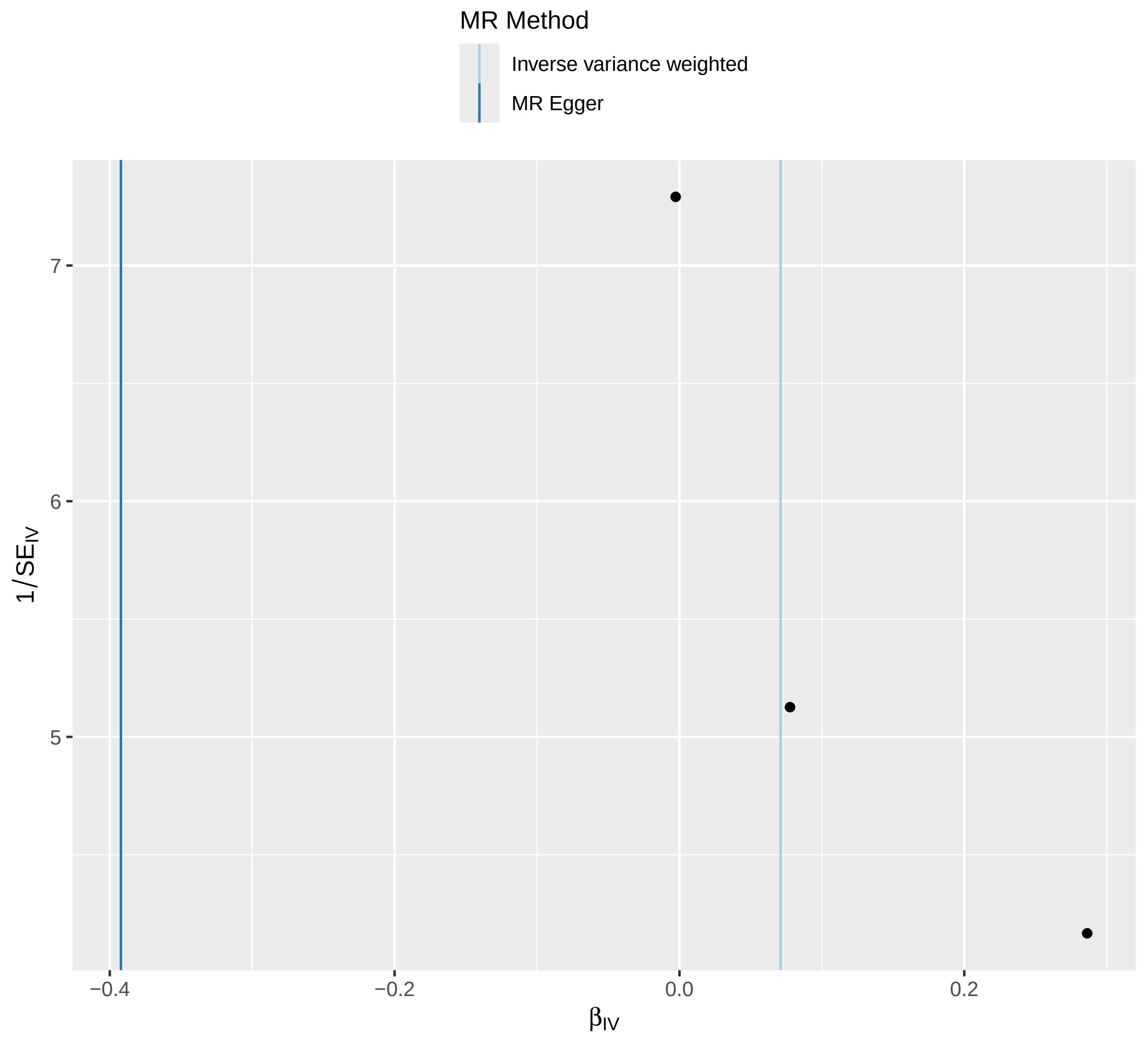
MR Method
Inverse variance weighted
MR Egger
7
6
5
1/SE_IV
−0.4
−0.2
0.0
0.2
β_IV

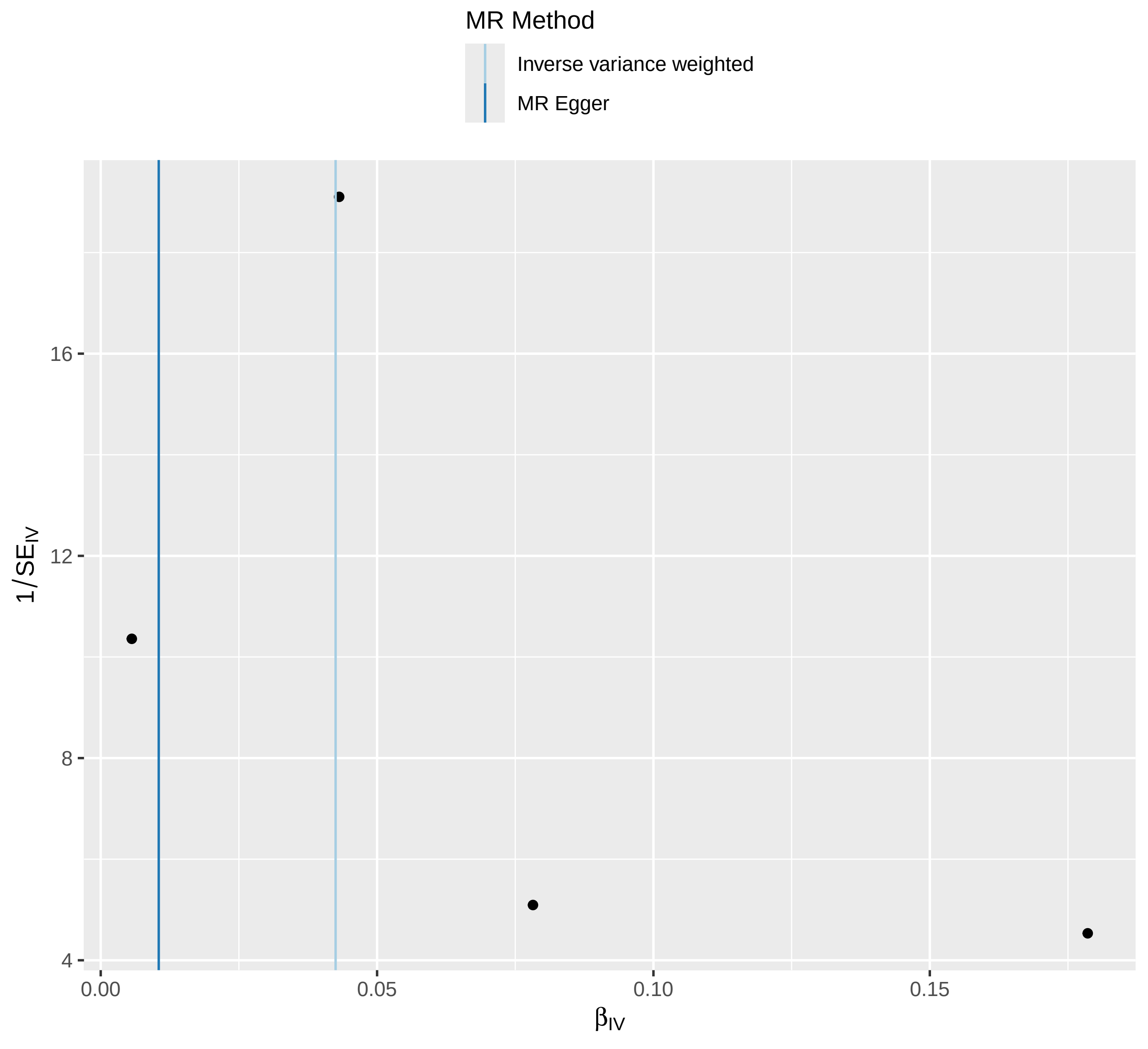

MR Method
Inverse variance weighted
MR Egger
16
12
8
4
0.00
0.05
0.10
0.15
1/SE_IV
β_IV

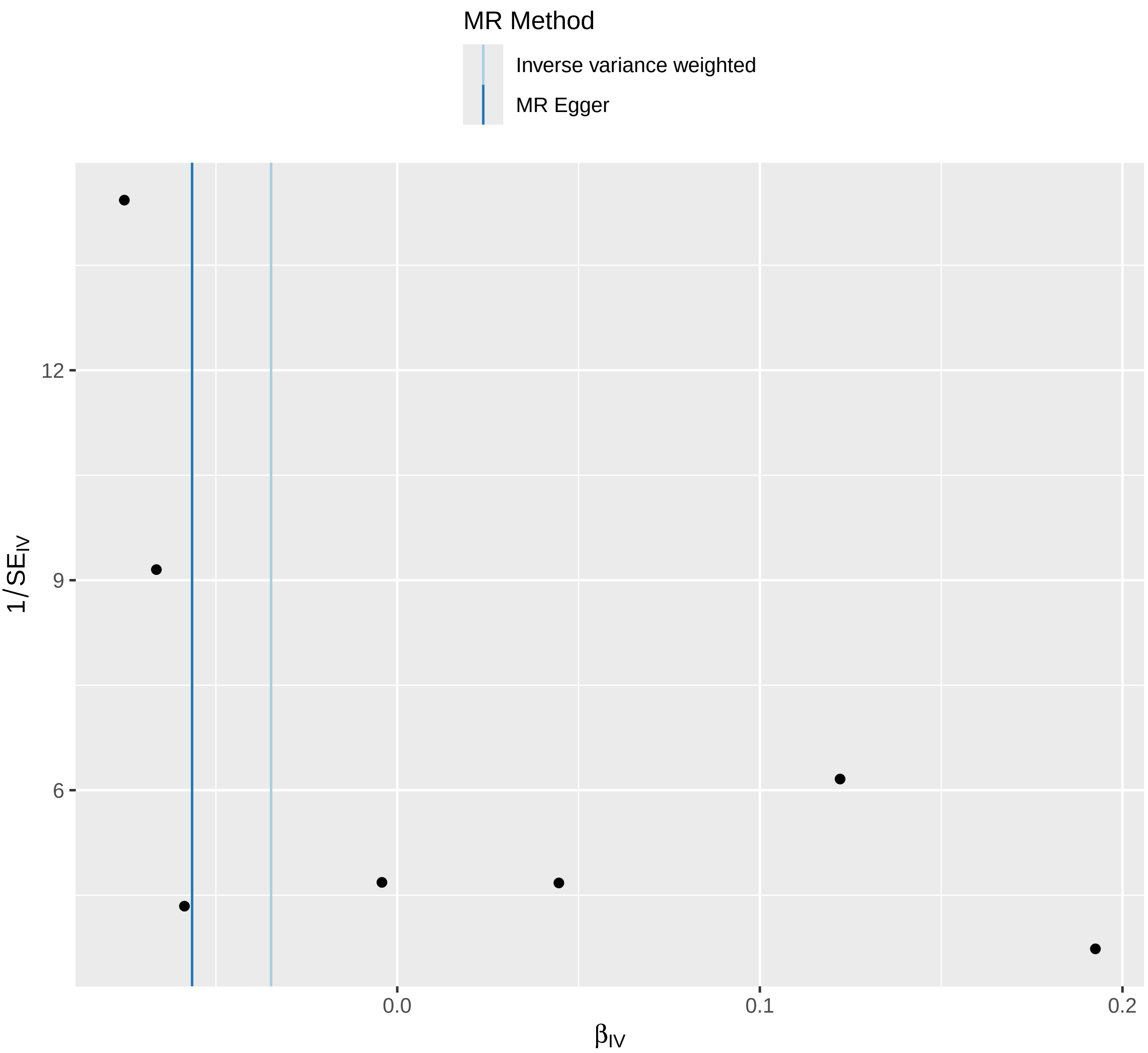
MR Method
Inverse variance weighted
MR Egger
12
9
6
0.0
0.1
0.2
1/SE_IV
β_IV

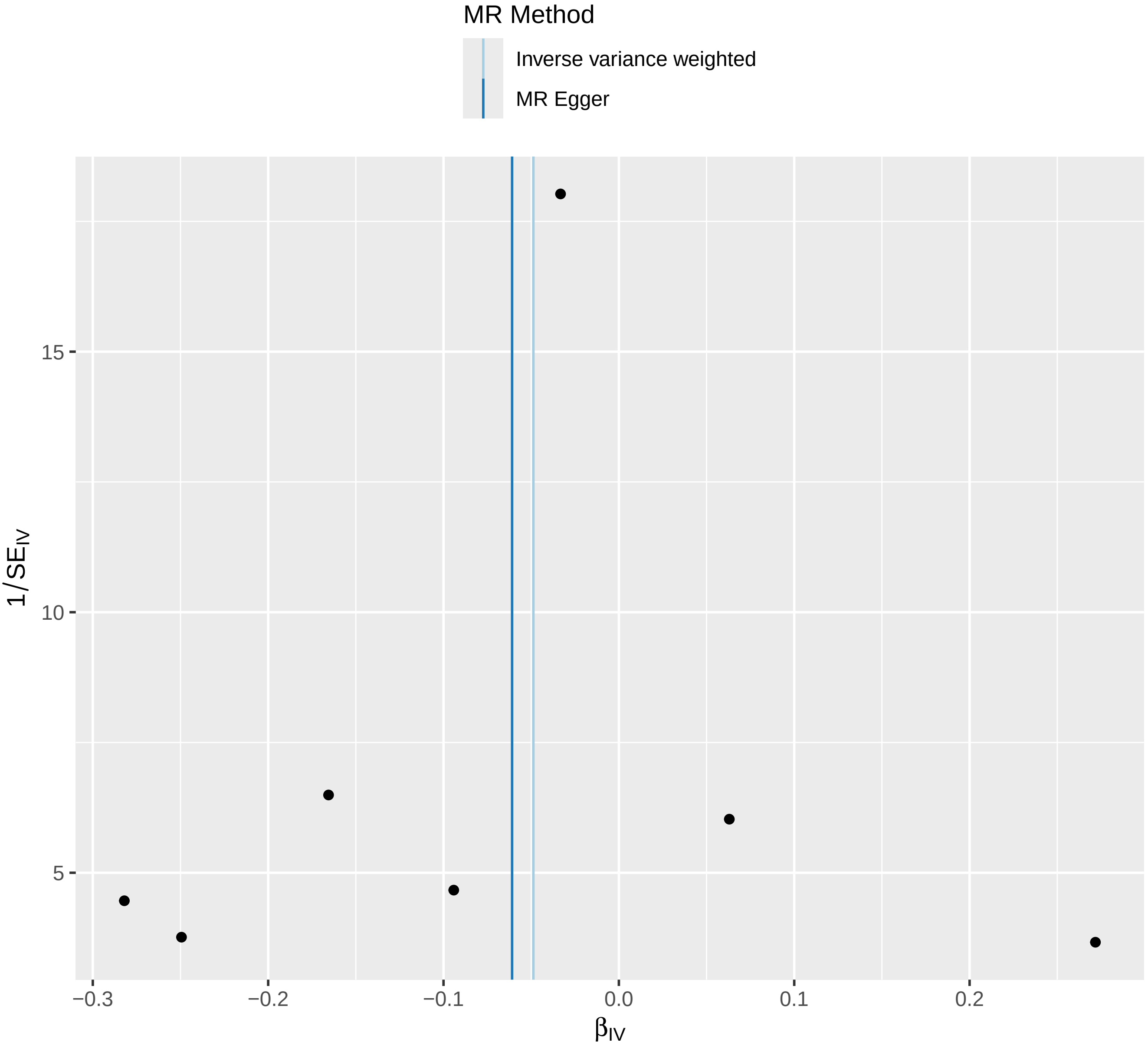

MR Method
Inverse variance weighted
MR Egger
15
10
5
$1/SE_{IV}$
−0.3
−0.2
−0.1
0.0
0.1
0.2
$\beta_{IV}$

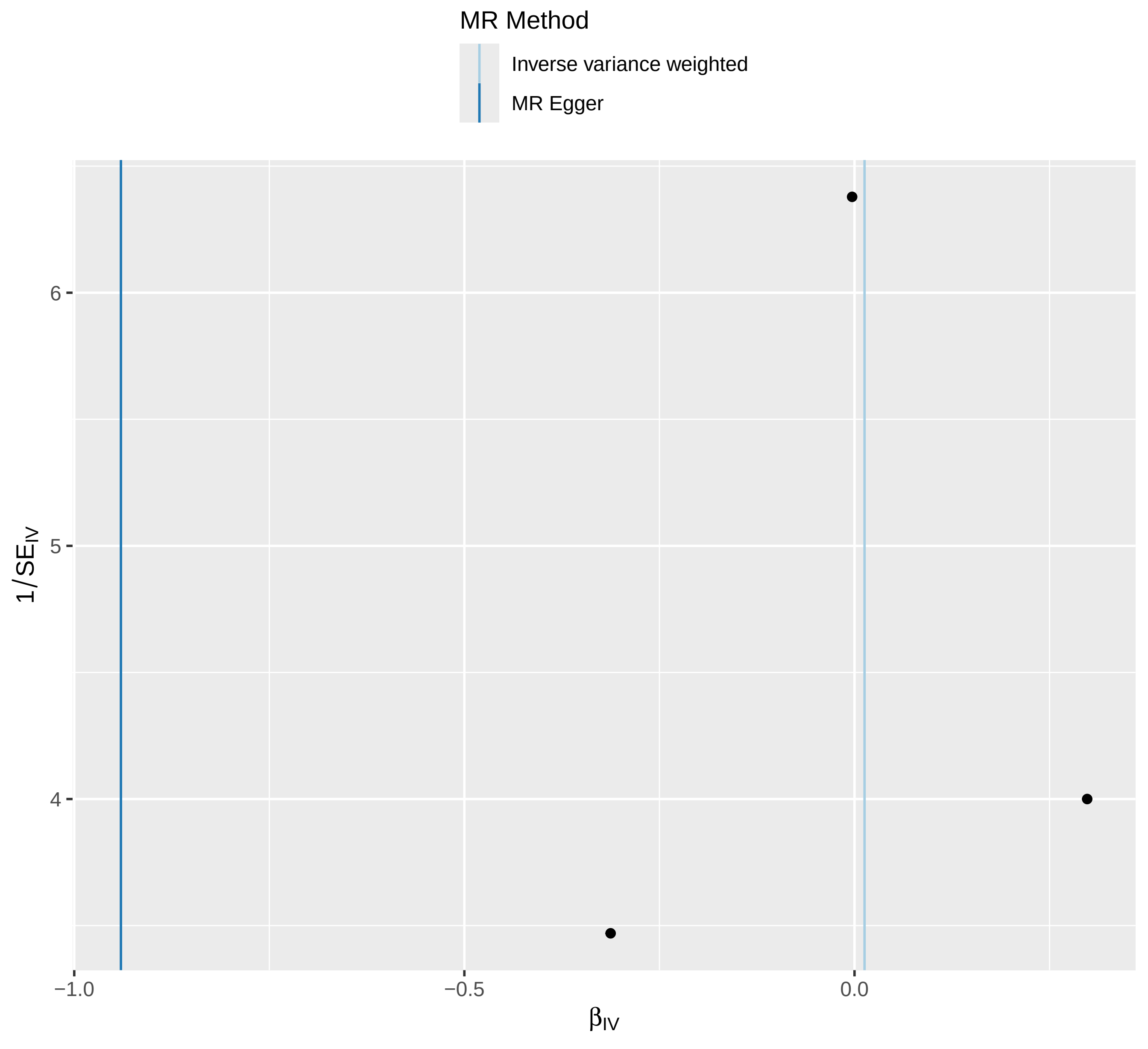
MR Method
Inverse variance weighted
MR Egger
1/SE$_{IV}$
6
5
4
−1.0
−0.5
0.0
β$_{IV}$

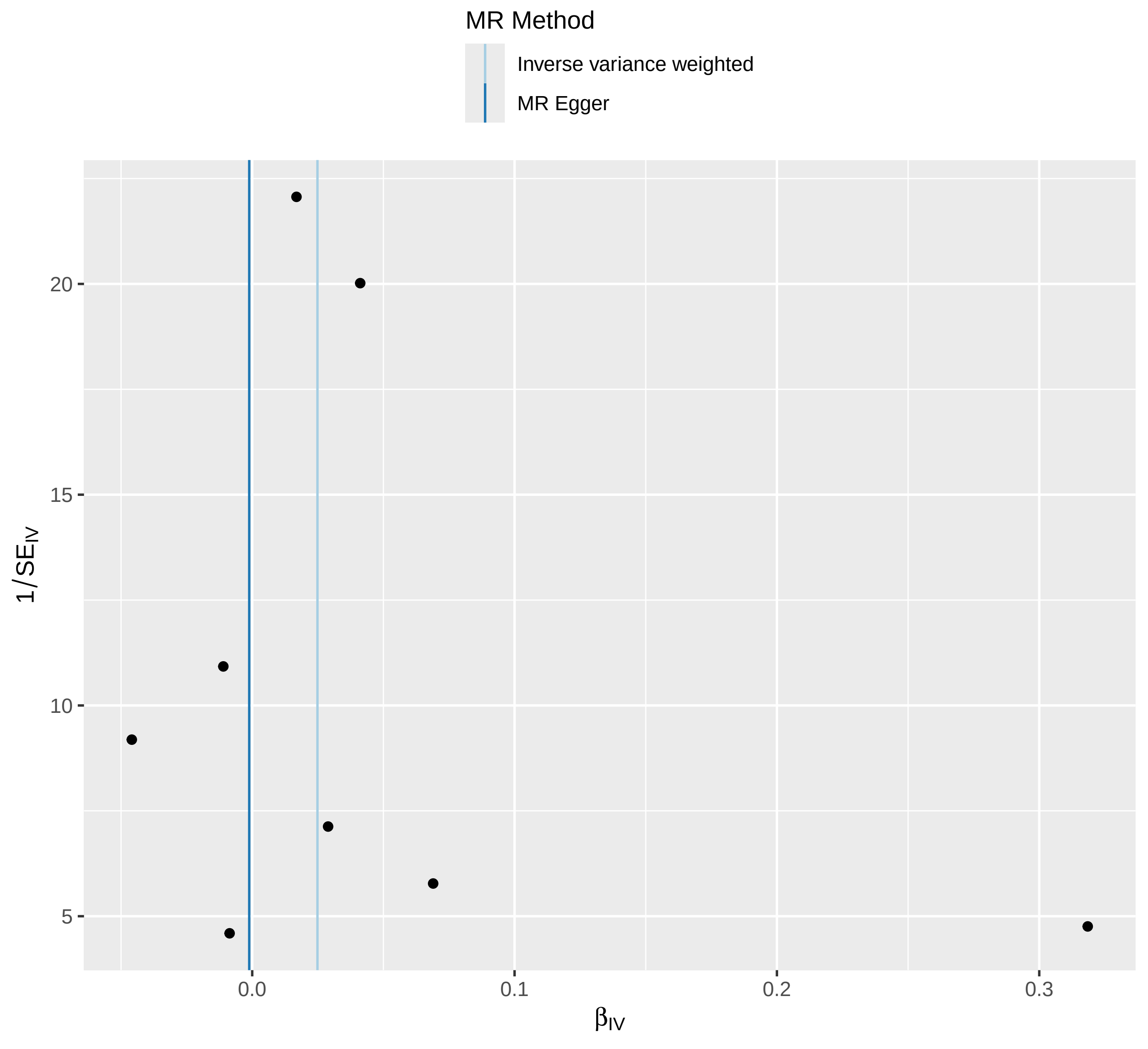

MR Method
Inverse variance weighted
MR Egger
20
15
10
5
1/SE_IV
0.0
0.1
0.2
0.3
β_IV

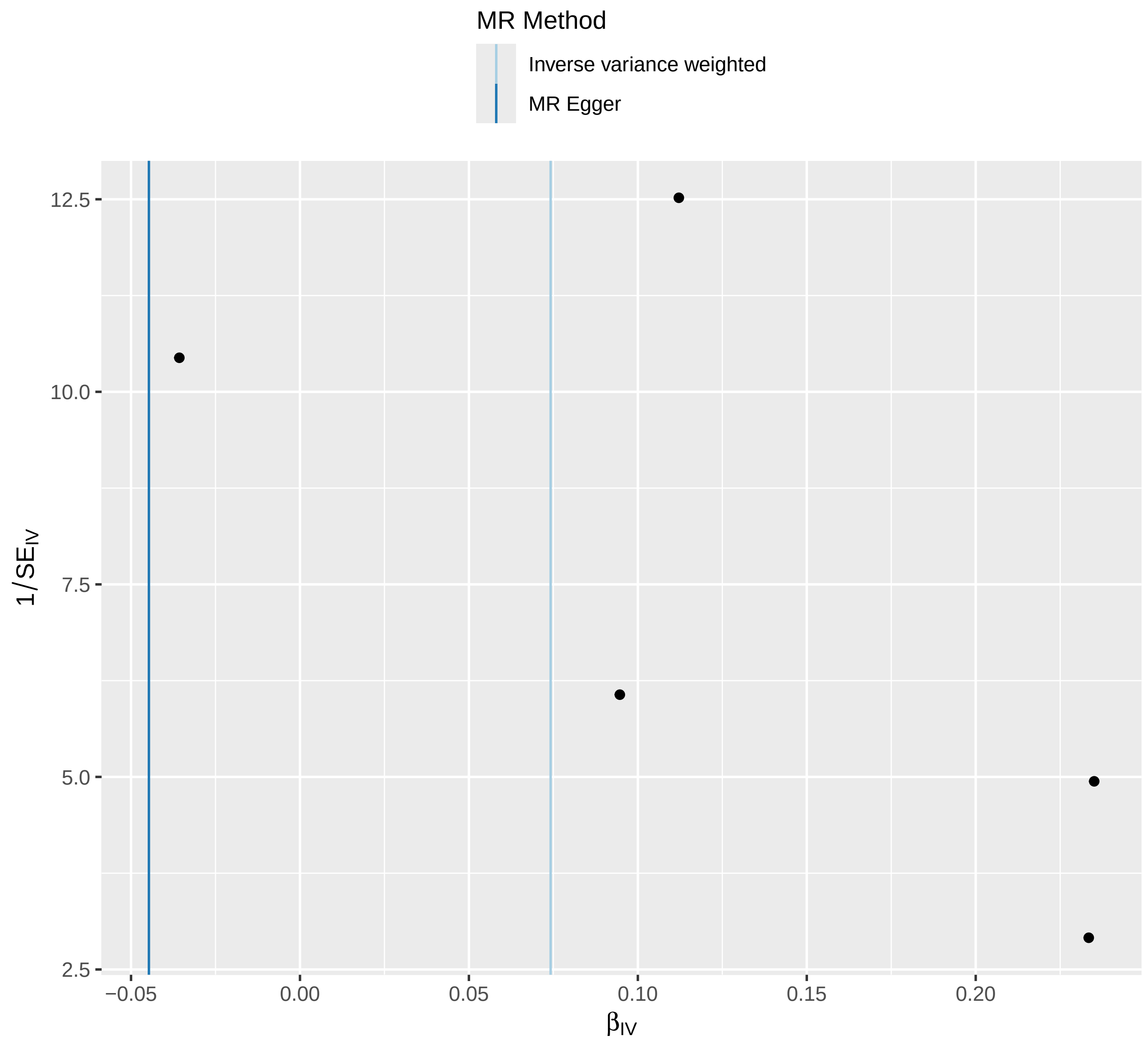

MR Method
Inverse variance weighted
MR Egger
12.5
10.0
7.5
5.0
2.5
1/SE_IV
−0.05
0.00
0.05
0.10
0.15
0.20
β_IV

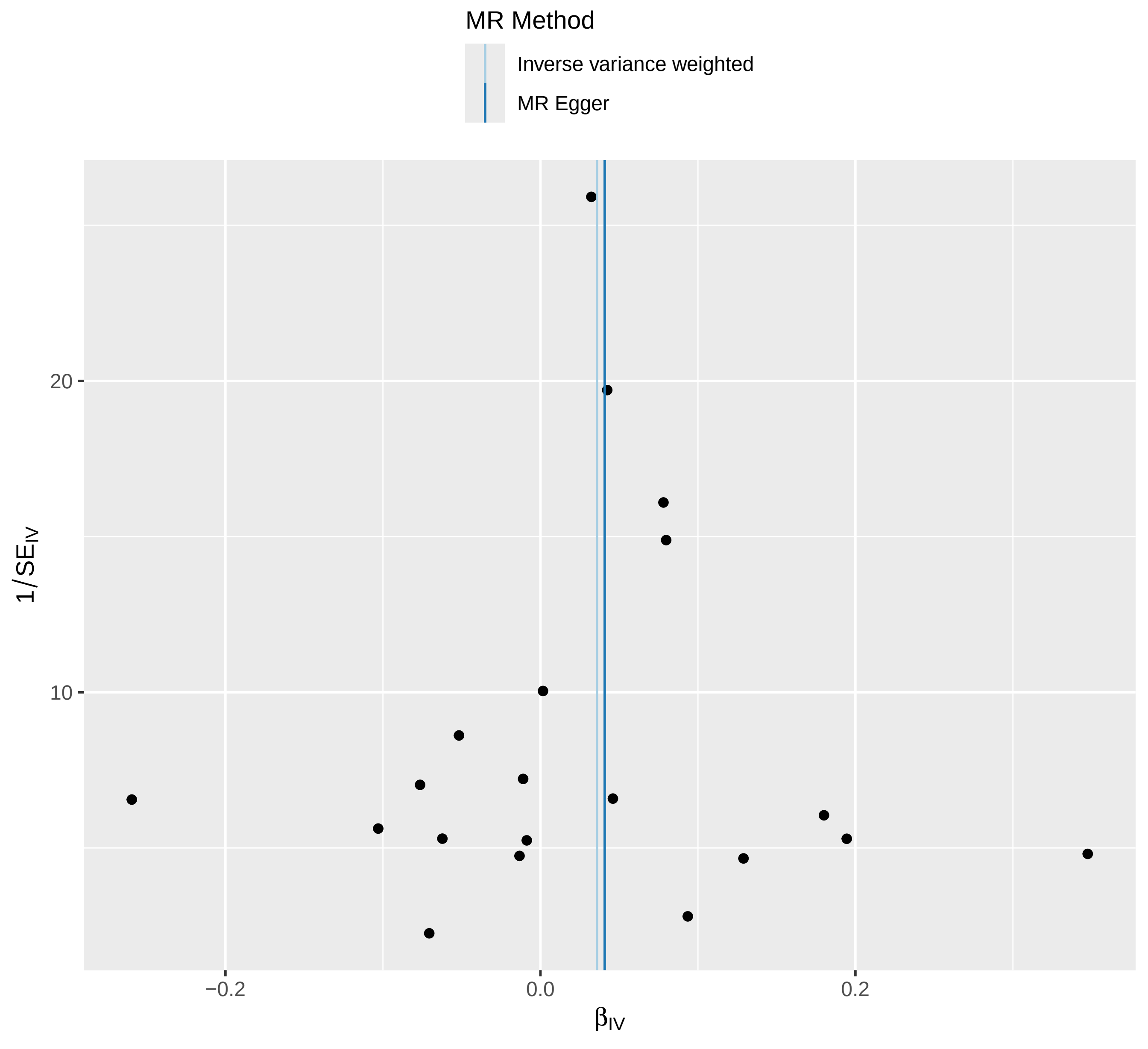
MR Method
Inverse variance weighted
MR Egger
20
10
−0.2
0.0
0.2
1/SE_IV
β_IV

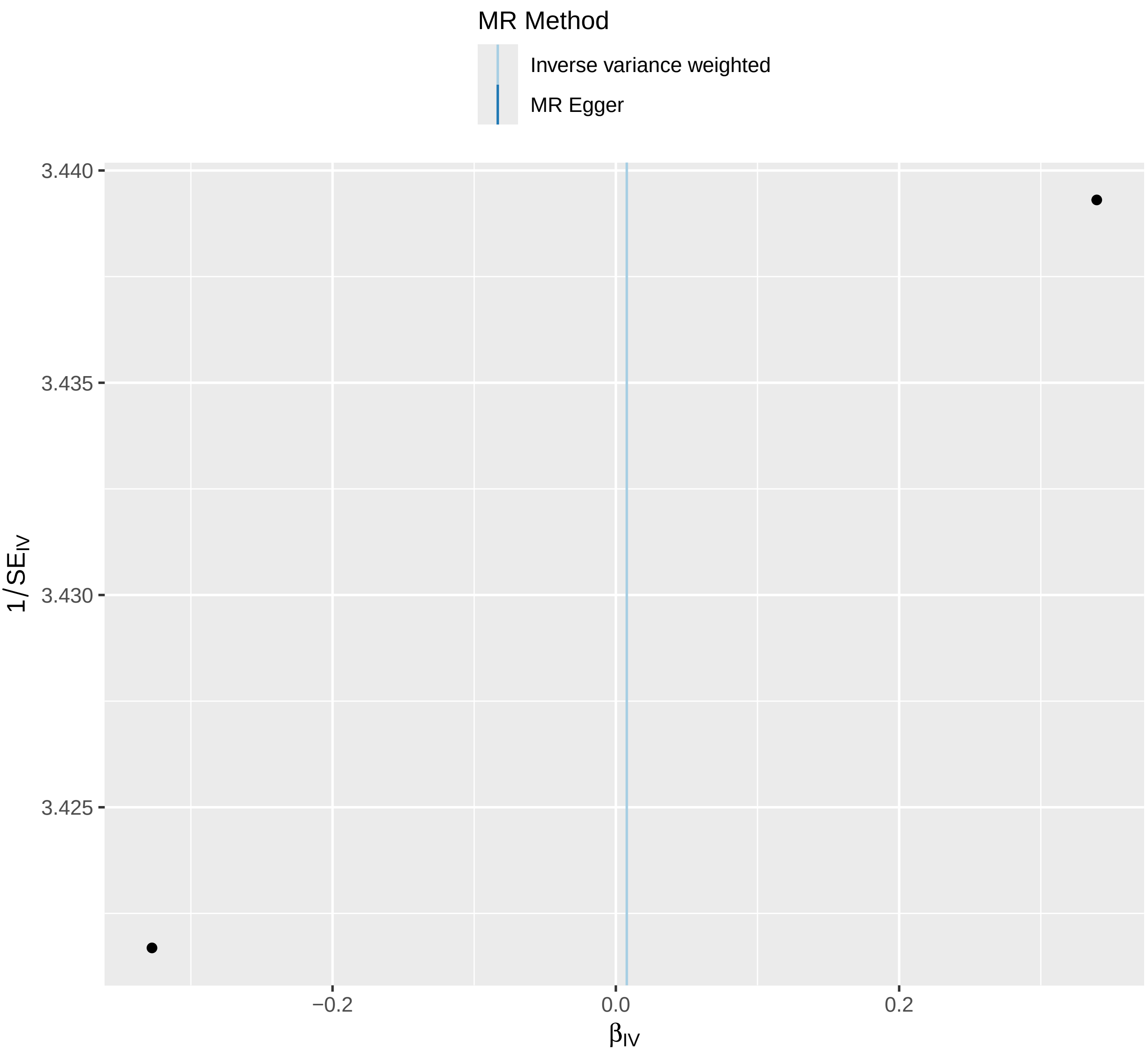
MR Method
Inverse variance weighted
MR Egger
3.440
3.435
3.430
3.425
1/SE_IV
−0.2
0.0
0.2
β_IV

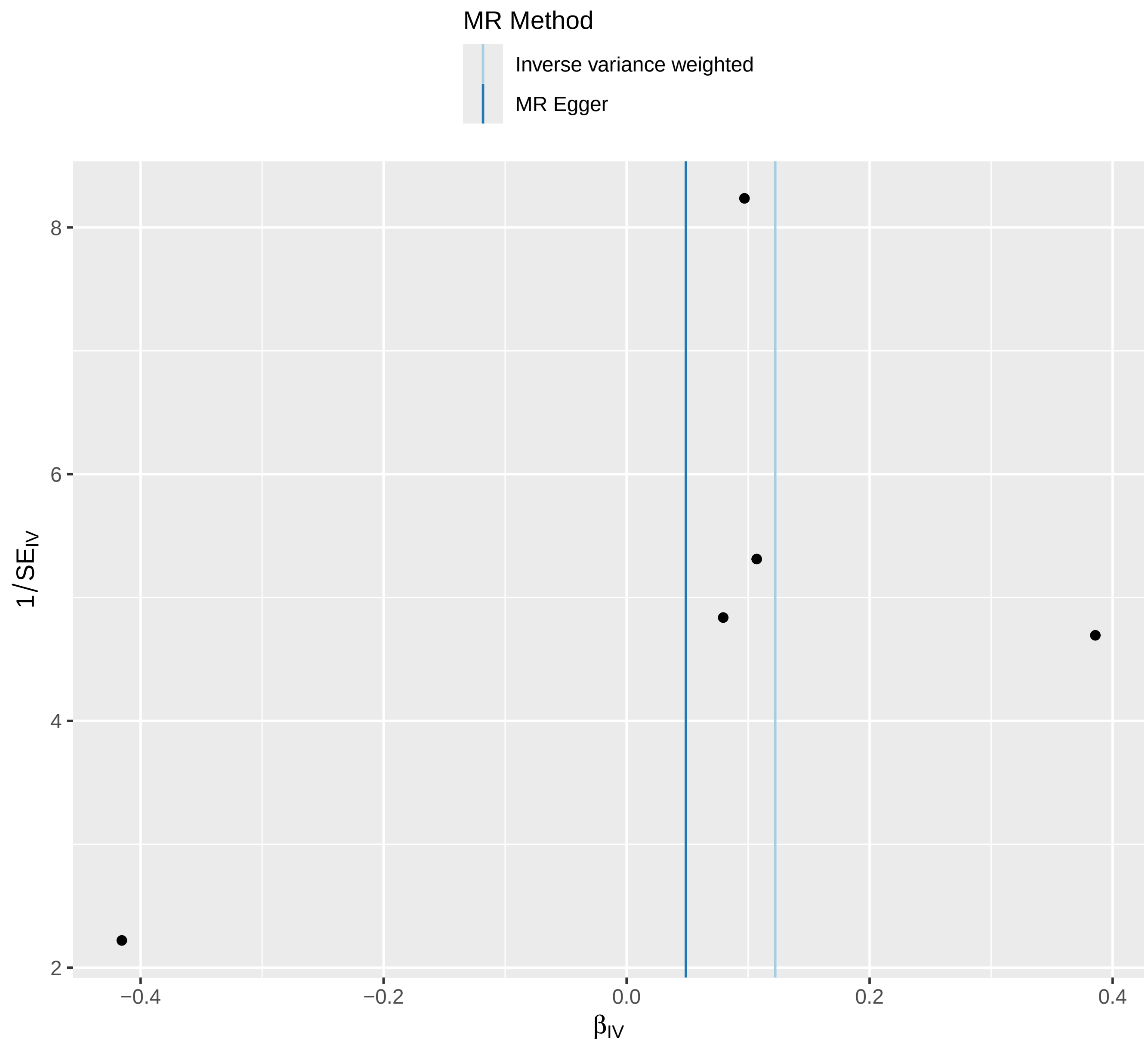
MR Method
Inverse variance weighted
MR Egger
8
6
4
2
1/SE$_{IV}$
−0.4
−0.2
0.0
0.2
0.4
β$_{IV}$

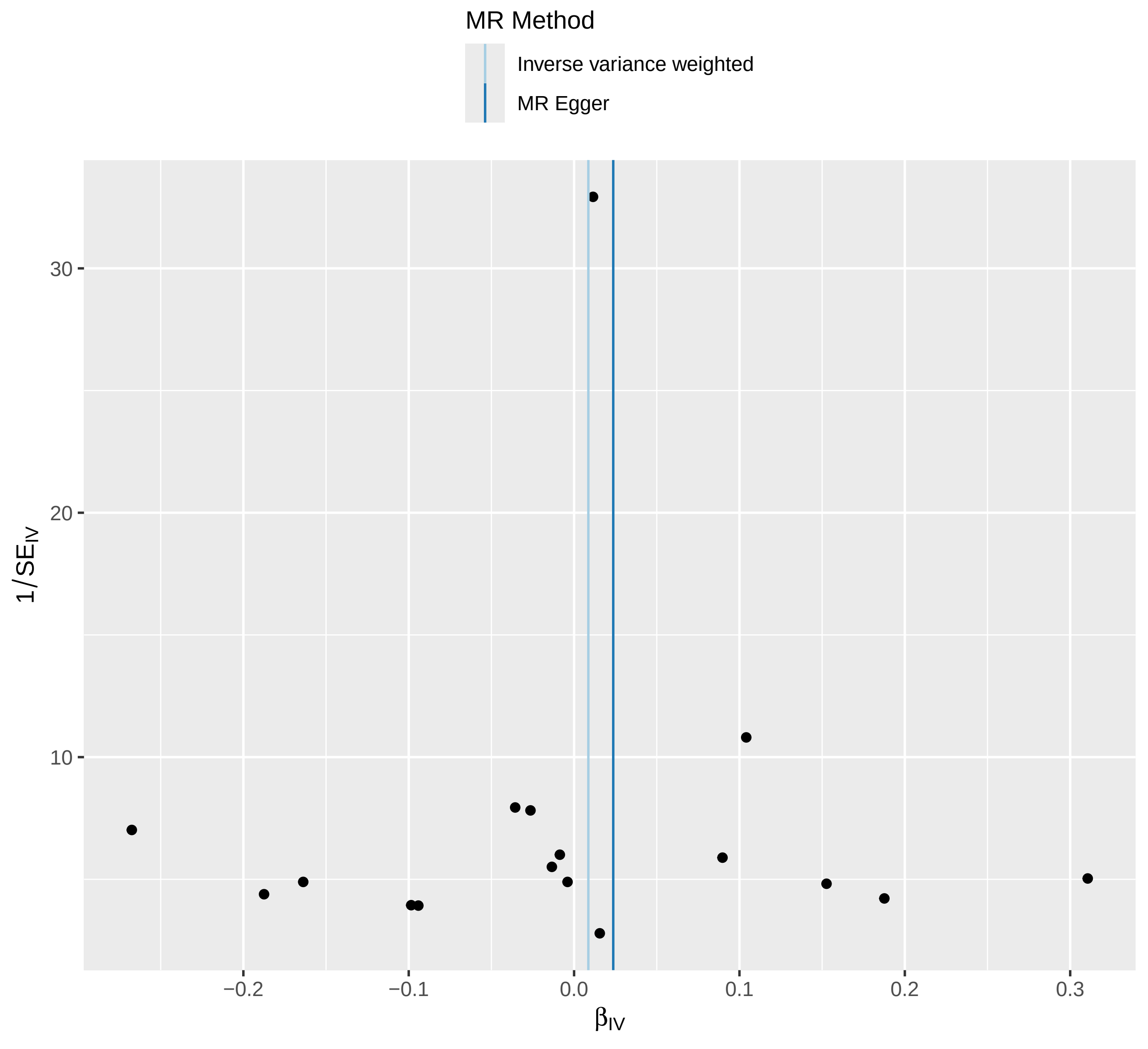
MR Method
Inverse variance weighted
MR Egger
30
20
10
1/SE$_{IV}$
−0.2
−0.1
0.0
0.1
0.2
0.3
β$_{IV}$

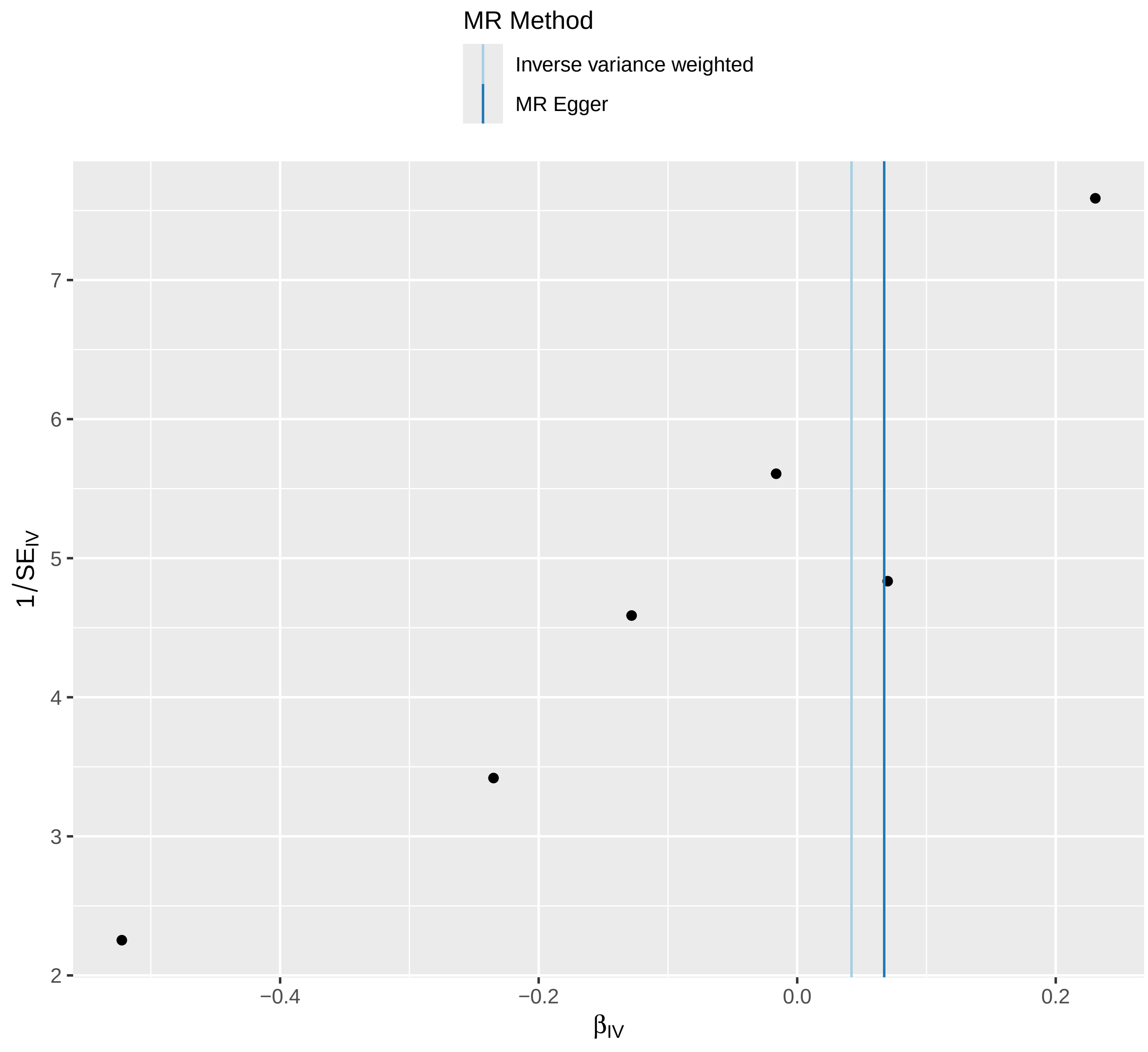
MR Method
Inverse variance weighted
MR Egger
1/$SE_{IV}$
$\beta_{IV}$
7
6
5
4
3
2
−0.4
−0.2
0.0
0.2

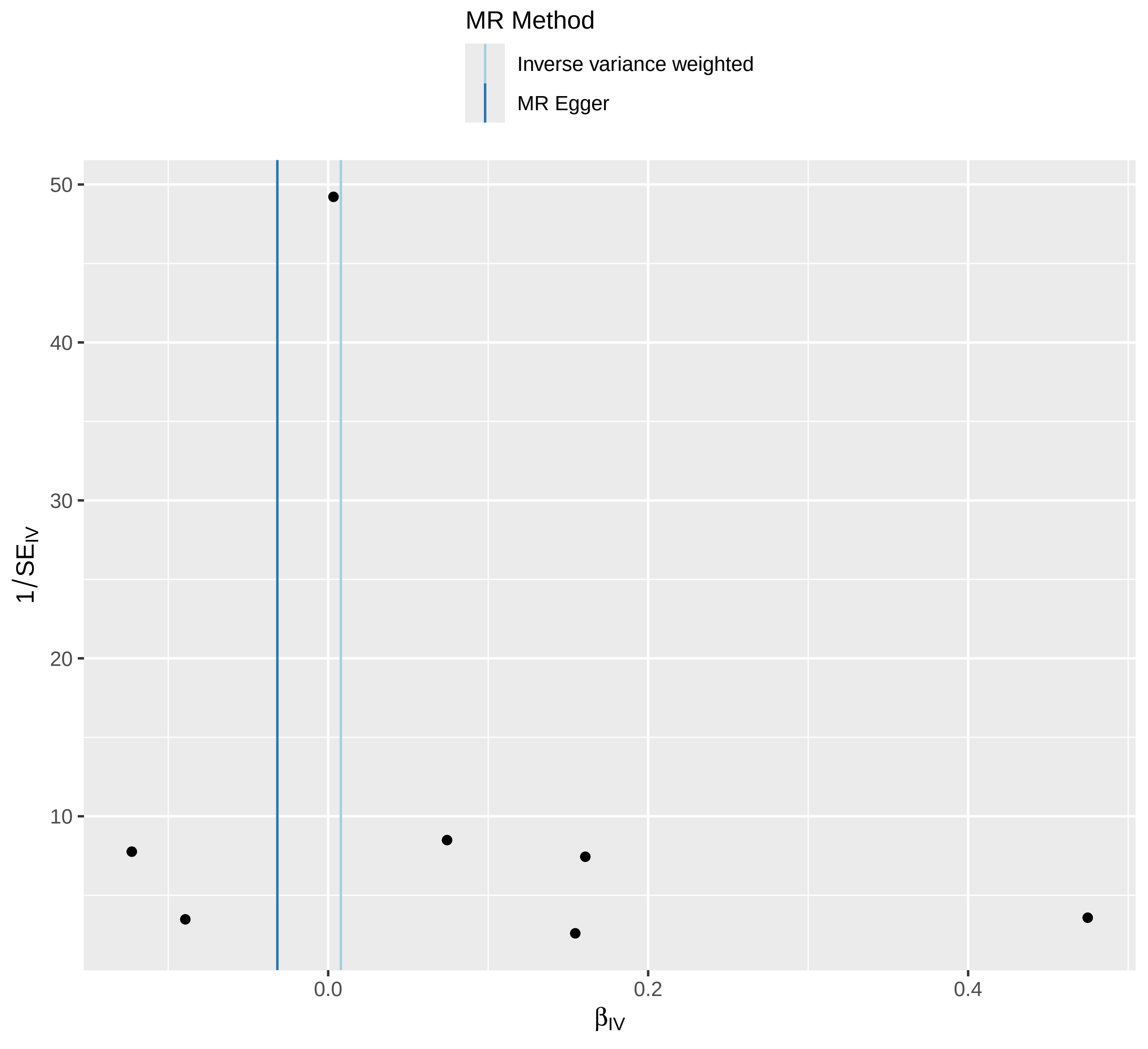
MR Method
Inverse variance weighted
MR Egger
1/$SE_{IV}$
50
40
30
20
10
0.0
0.2
0.4
$\beta_{IV}$

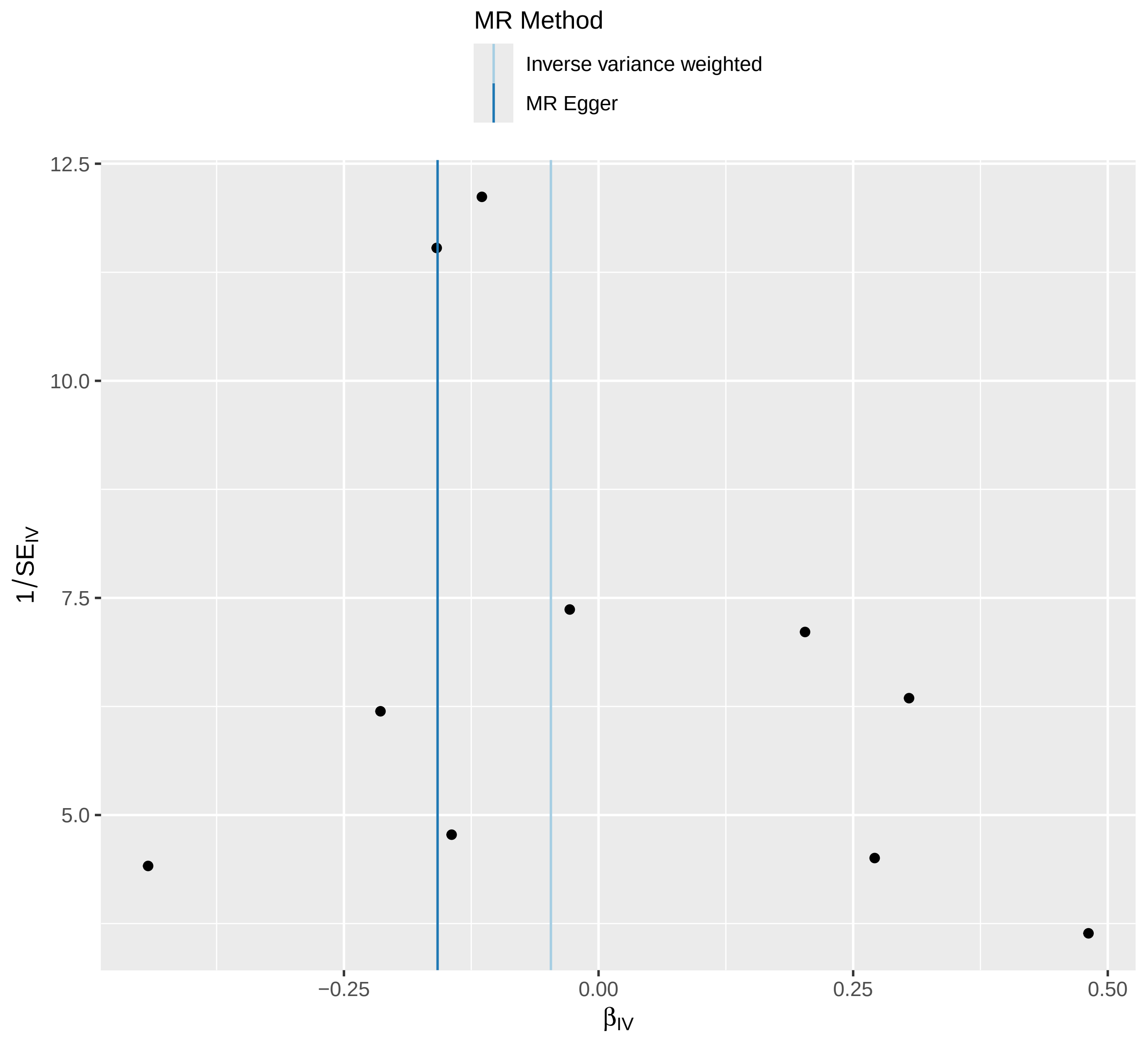

MR Method
Inverse variance weighted
MR Egger
12.5
10.0
7.5
5.0
1/SE_IV
−0.25
0.00
0.25
0.50
β_IV

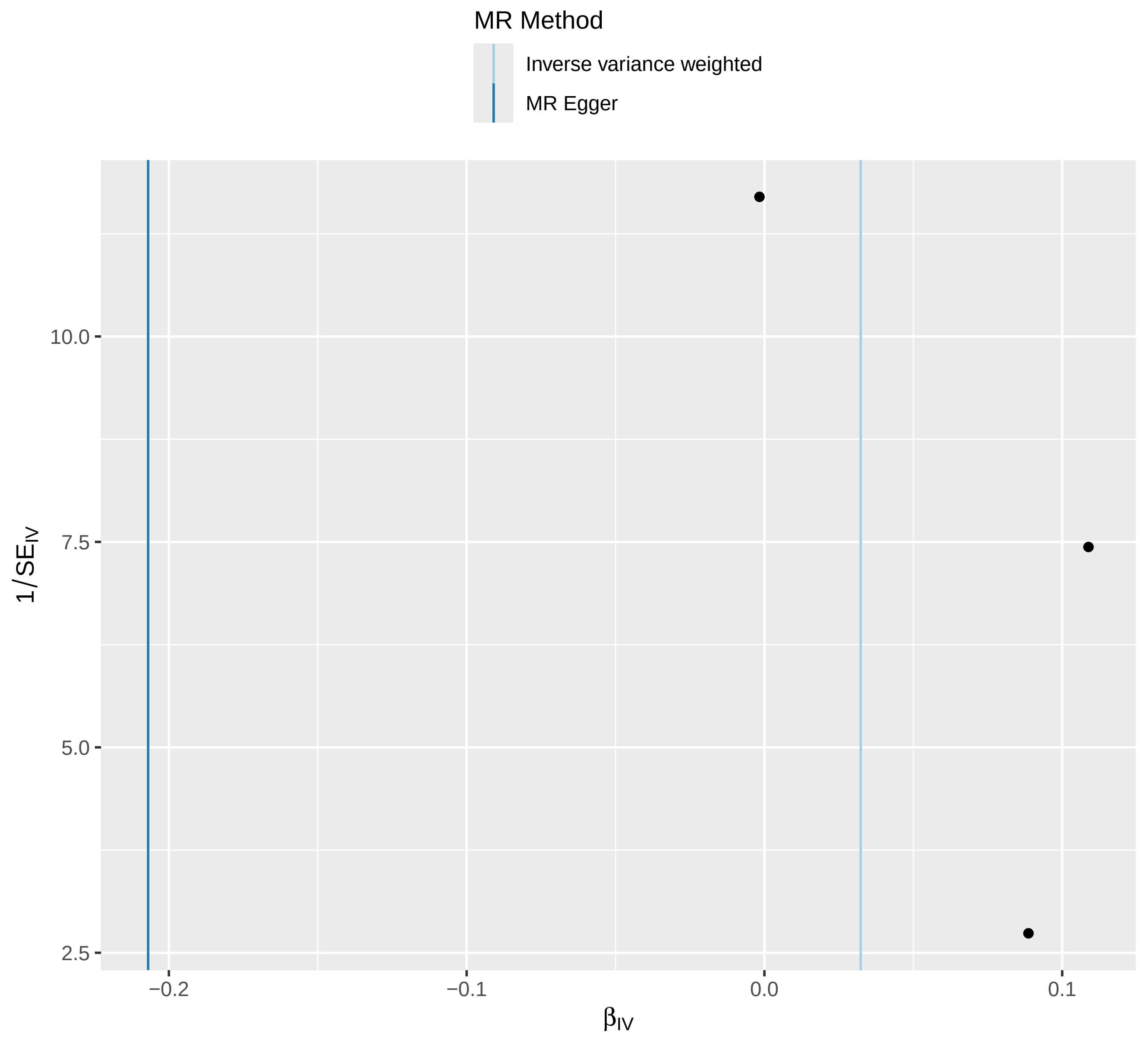

MR Method
Inverse variance weighted
MR Egger
10.0
7.5
5.0
2.5
1/SE_IV
−0.2
−0.1
0.0
0.1
β_IV

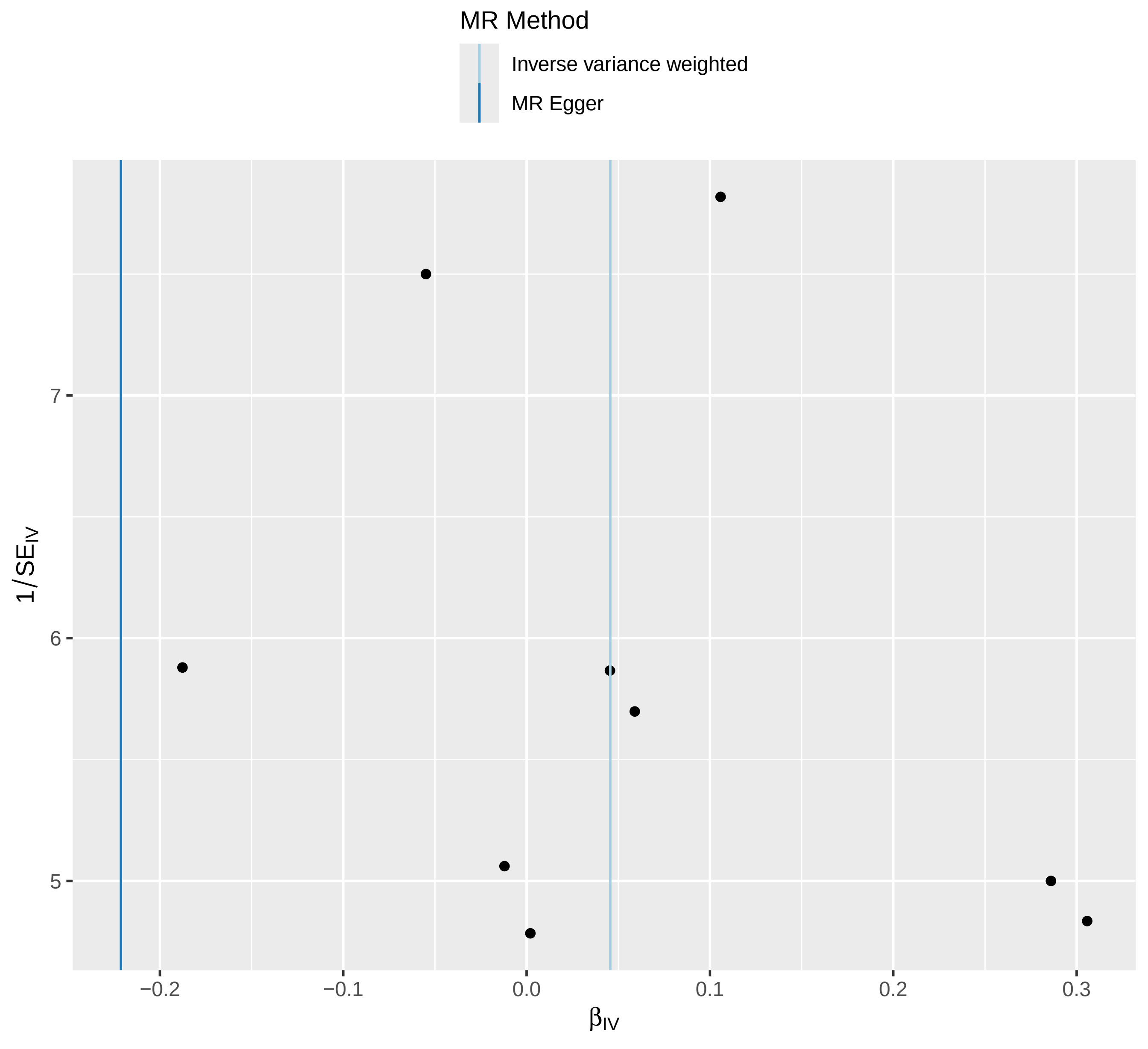
MR Method
Inverse variance weighted
MR Egger
7
6
5
1/SE_IV
−0.2
−0.1
0.0
0.1
0.2
0.3
β_IV

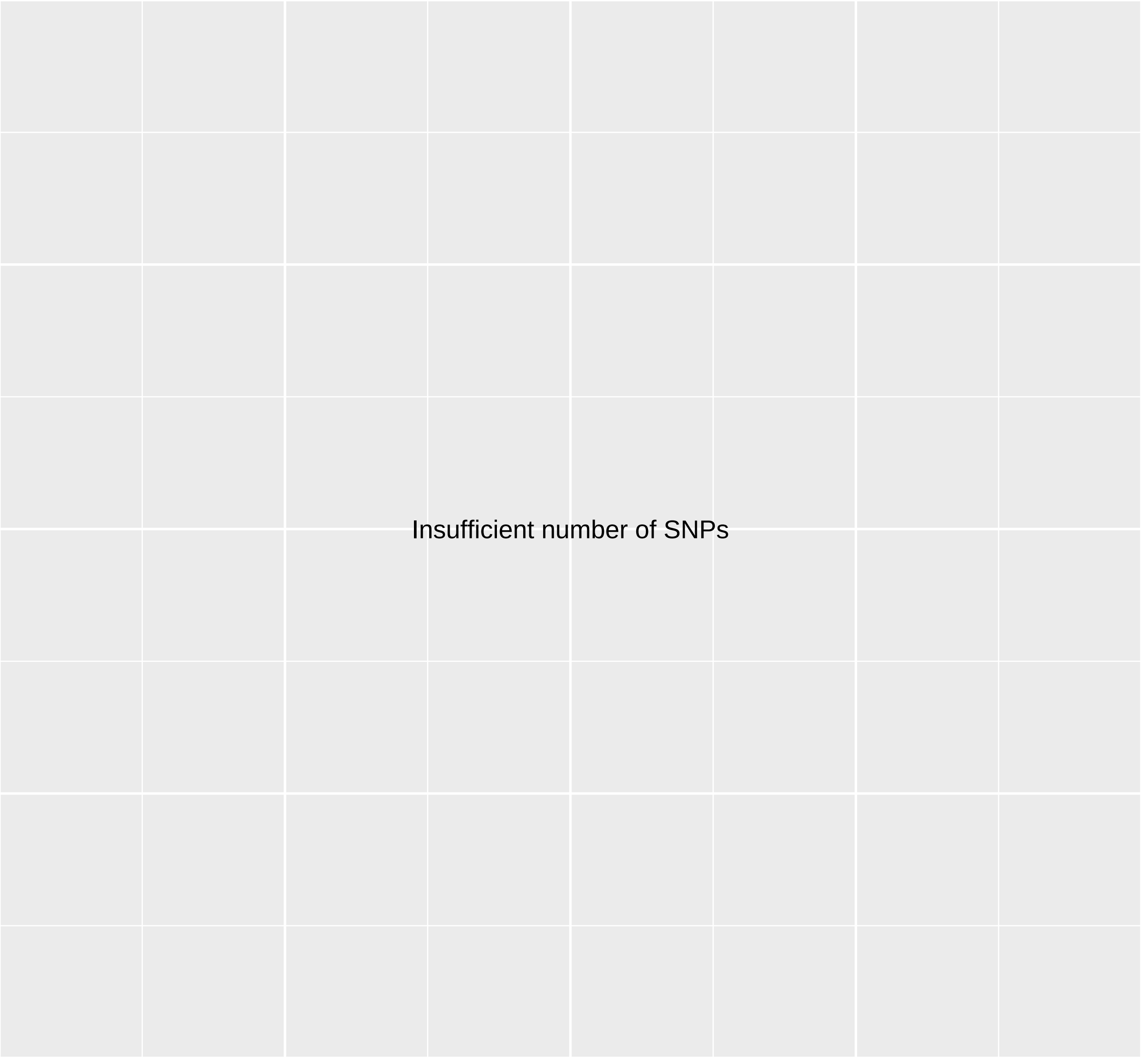
Insufficient number of SNPs

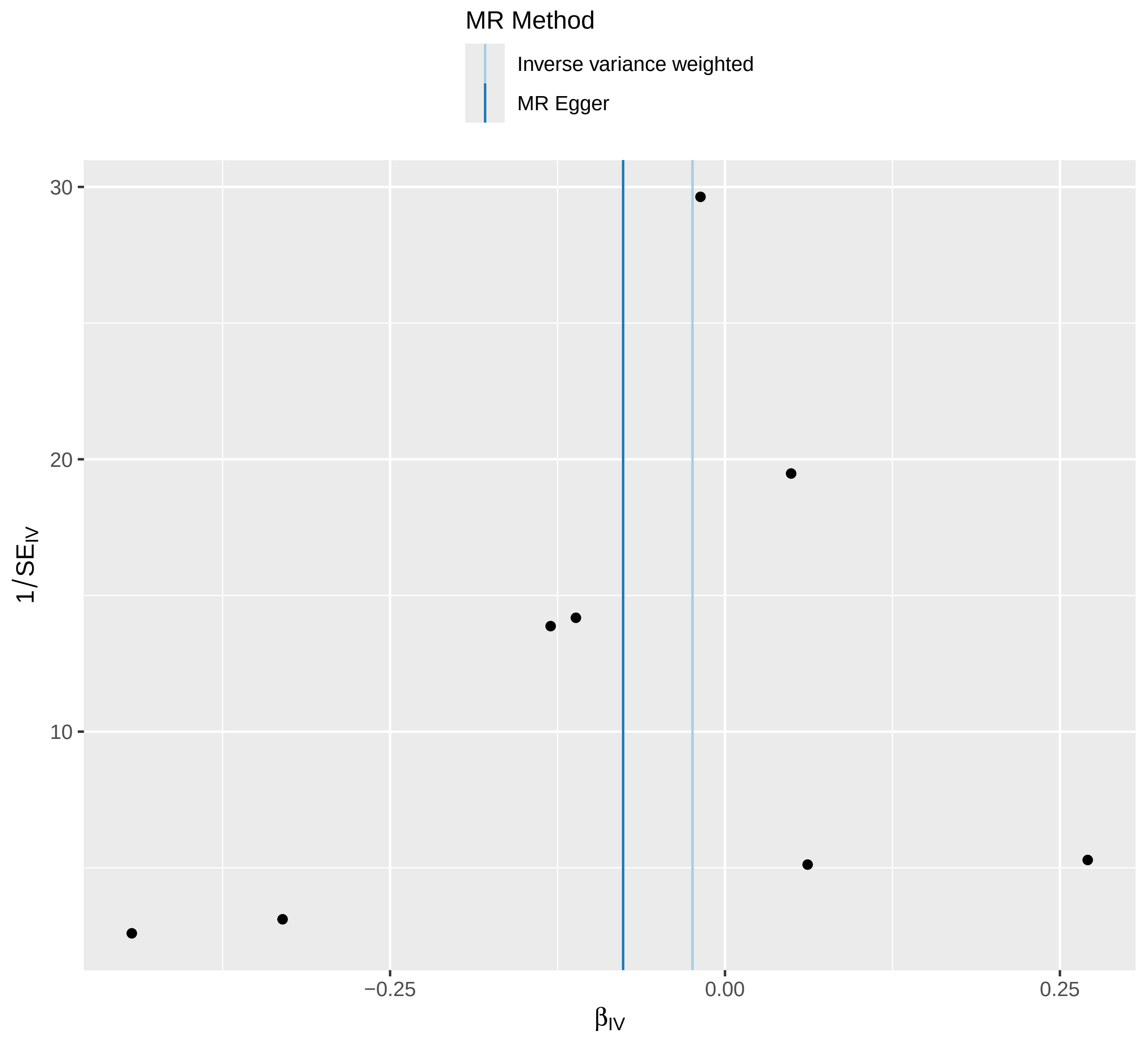
MR Method
Inverse variance weighted
MR Egger
30
20
10
1/SE$_{IV}$
−0.25
0.00
0.25
β$_{IV}$

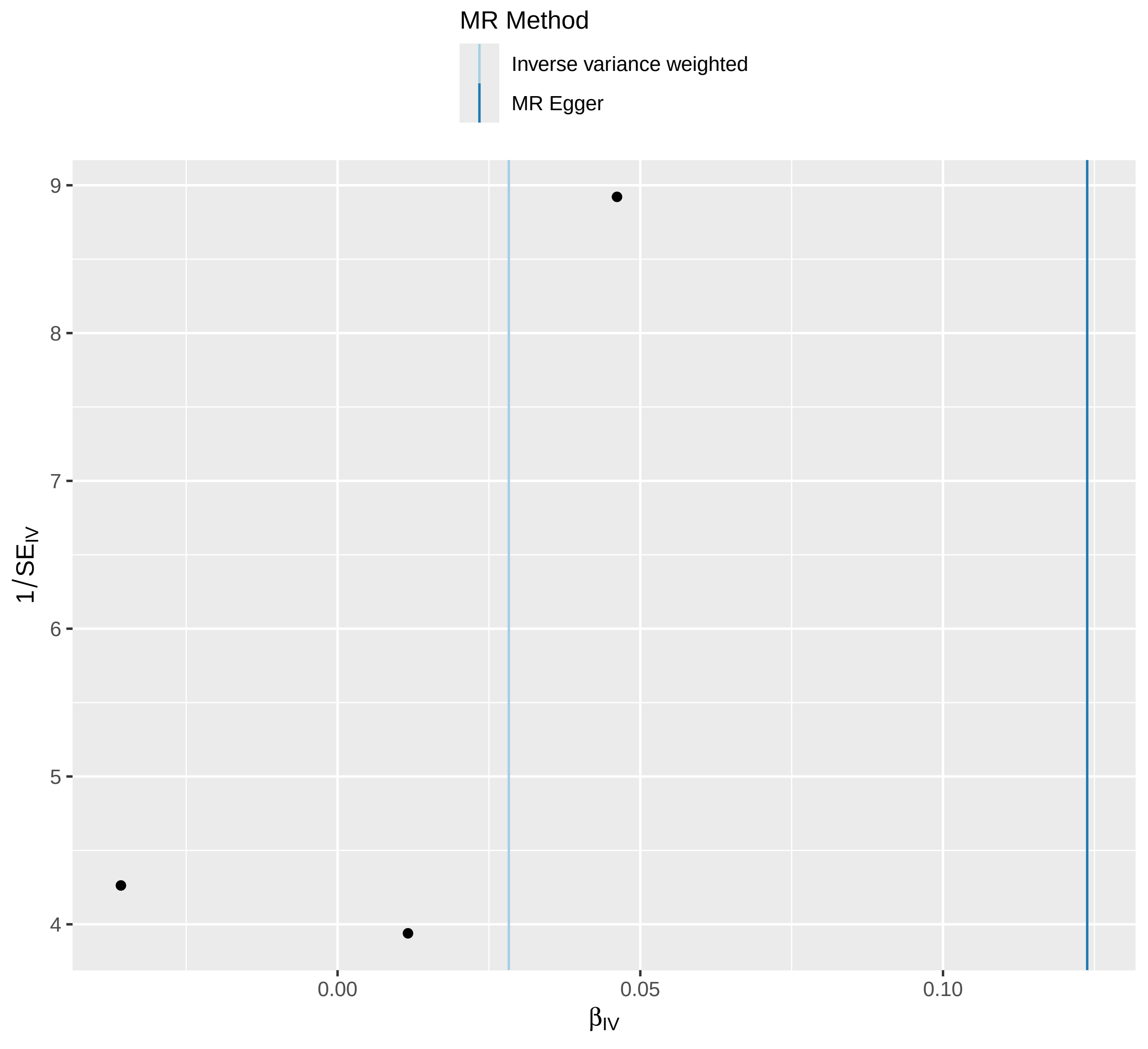
MR Method
Inverse variance weighted
MR Egger
9
8
7
6
5
4
1/SE$_{IV}$
0.00
0.05
0.10
β$_{IV}$

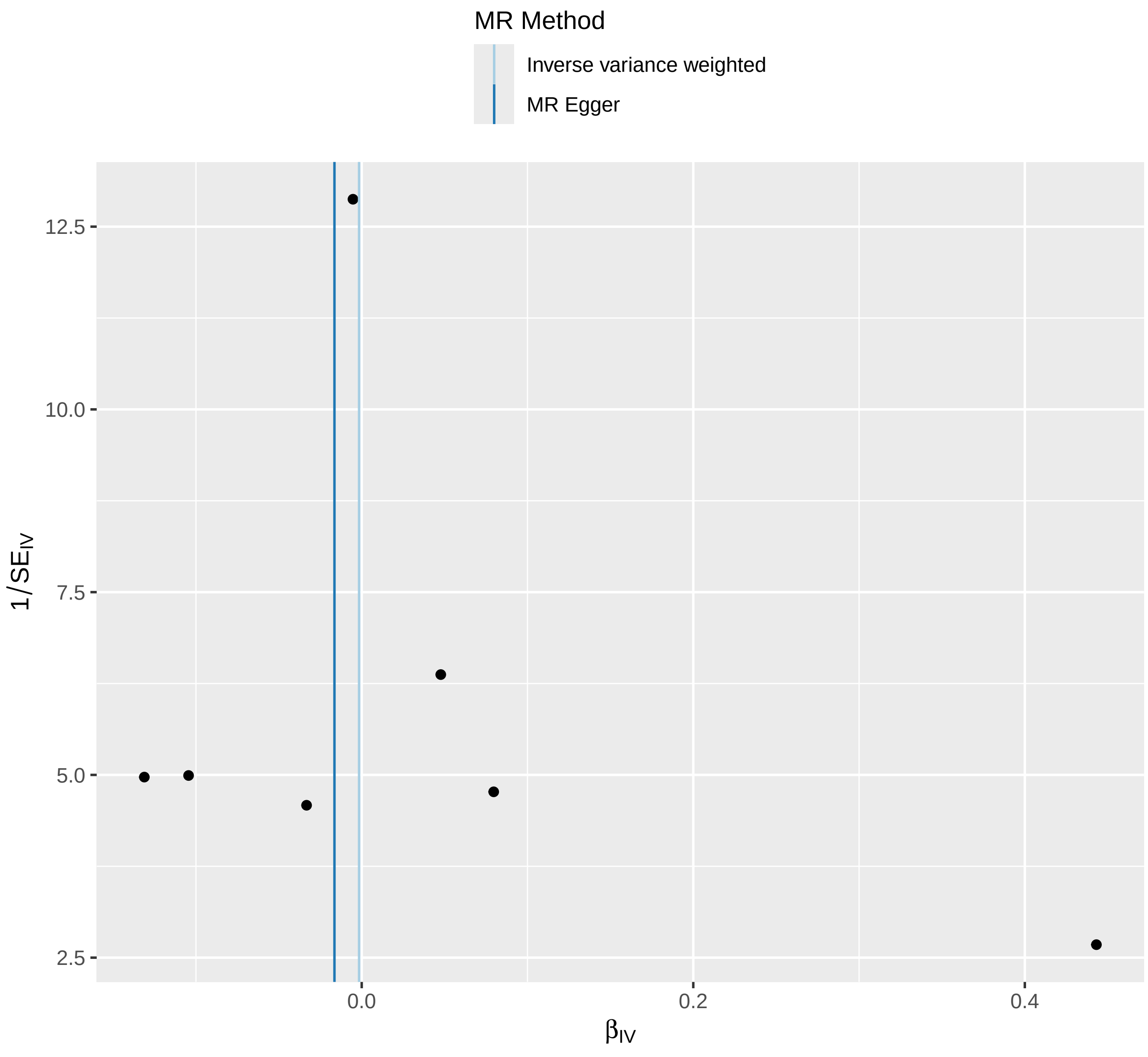
MR Method
Inverse variance weighted
MR Egger
12.5
10.0
7.5
5.0
2.5
1/SE$_{IV}$
0.0
0.2
0.4
β$_{IV}$

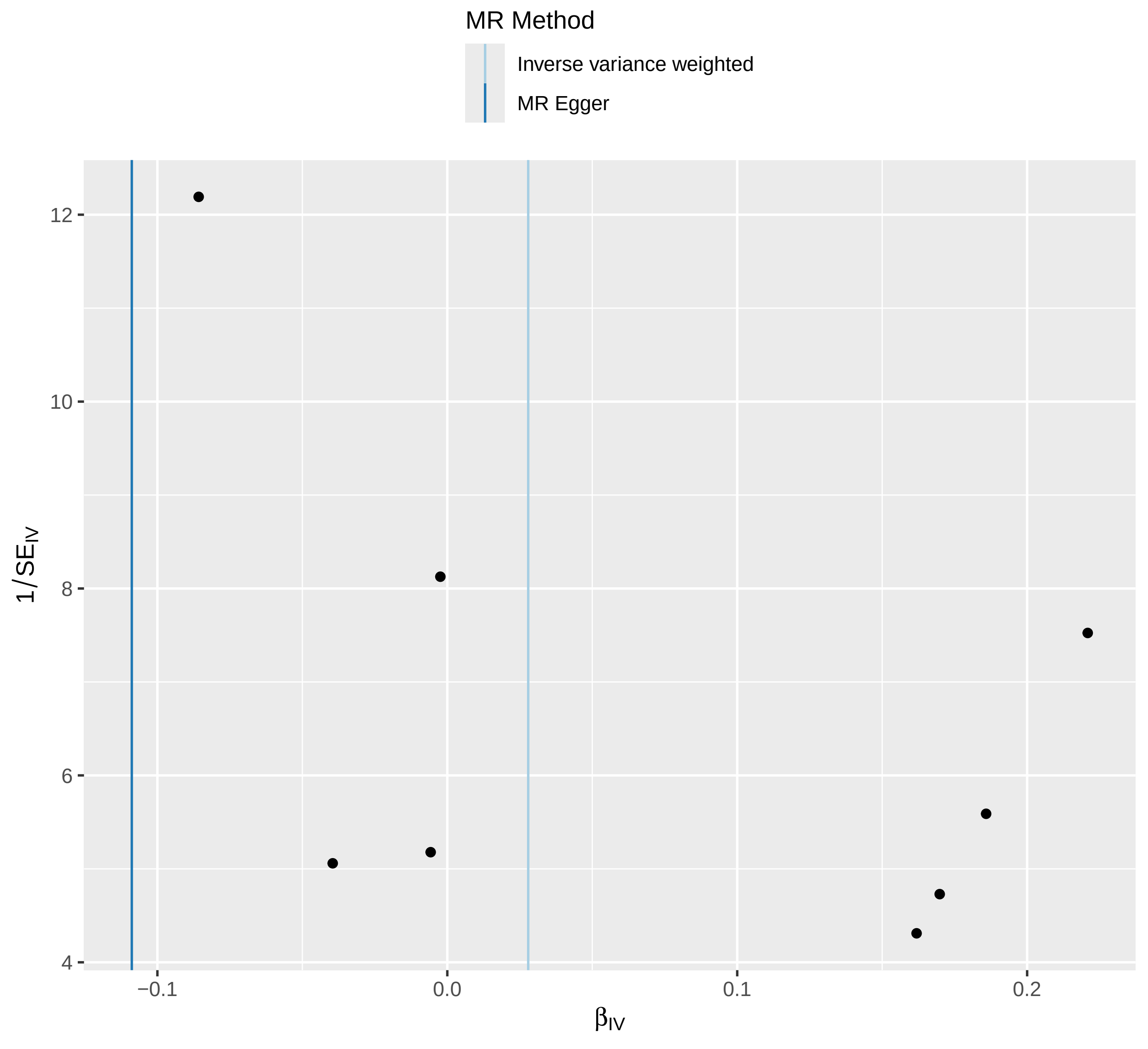

MR Method
Inverse variance weighted
MR Egger
12
10
8
6
4
1/SE$_{IV}$
−0.1
0.0
0.1
0.2
β$_{IV}$

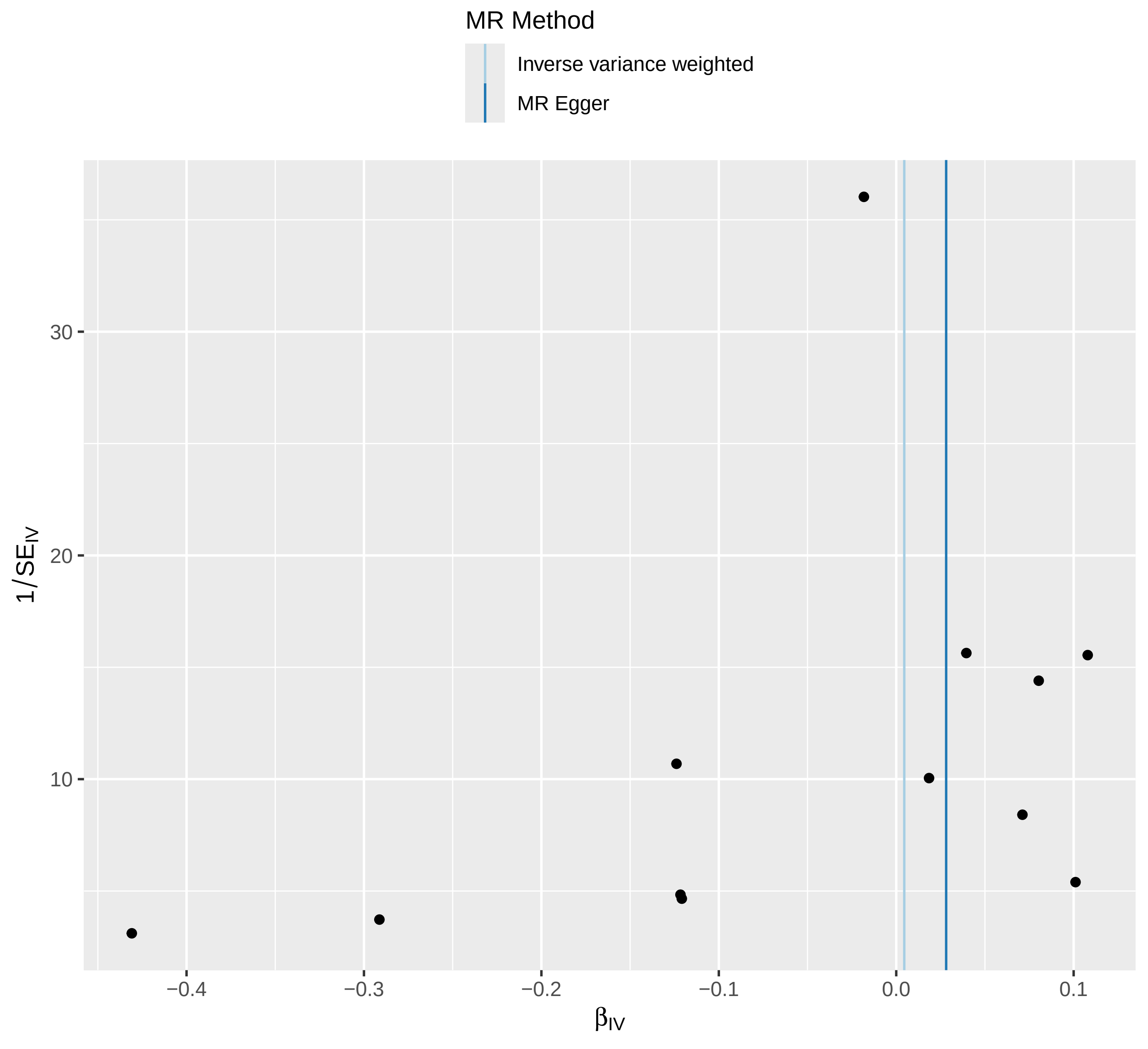
MR Method
Inverse variance weighted
MR Egger
30
20
10
1/SE$_{IV}$
−0.4
−0.3
−0.2
−0.1
0.0
0.1
β$_{IV}$

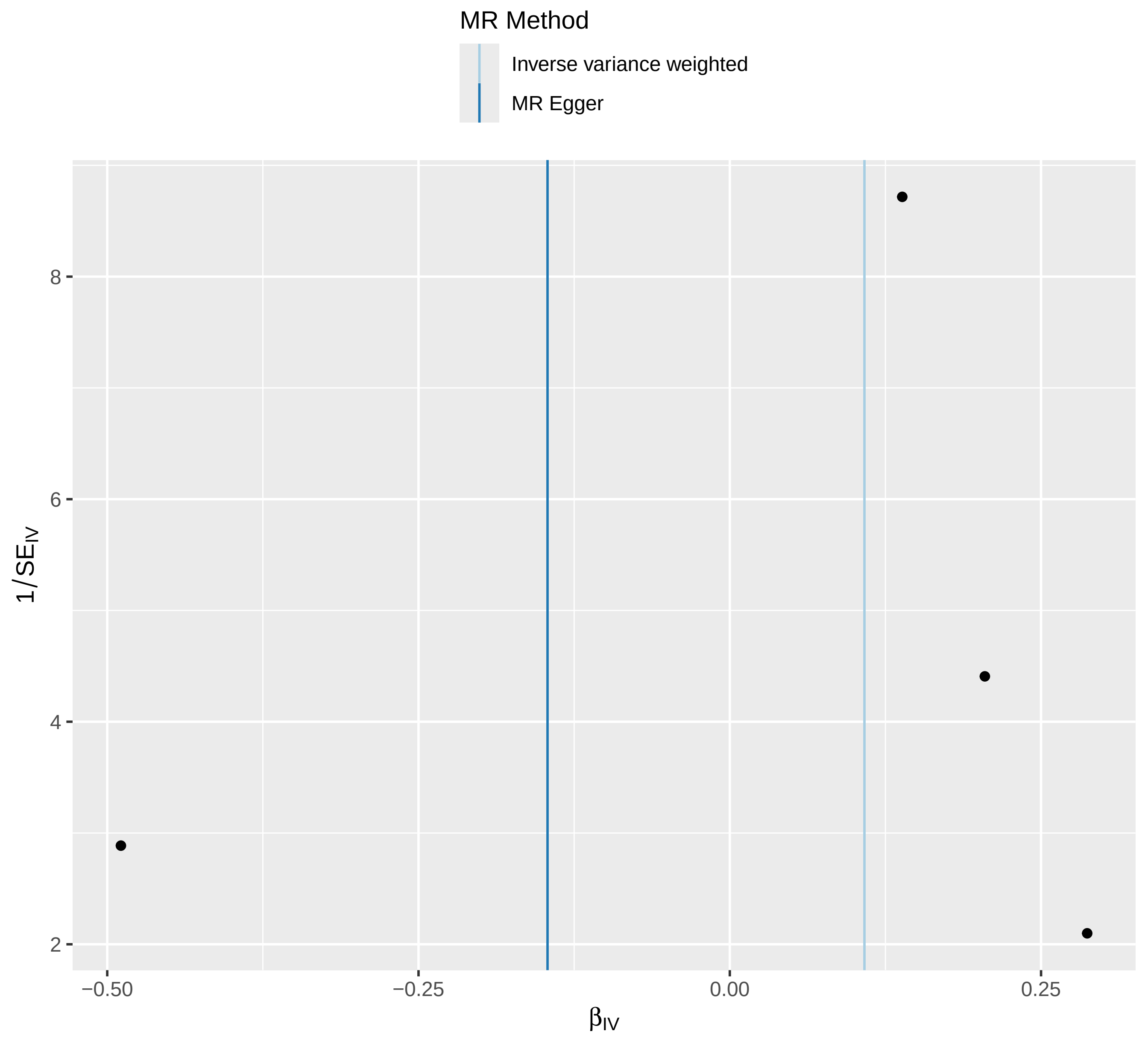
MR Method
Inverse variance weighted
MR Egger
8
6
4
2
1/SE_IV
−0.50
−0.25
0.00
0.25
β_IV

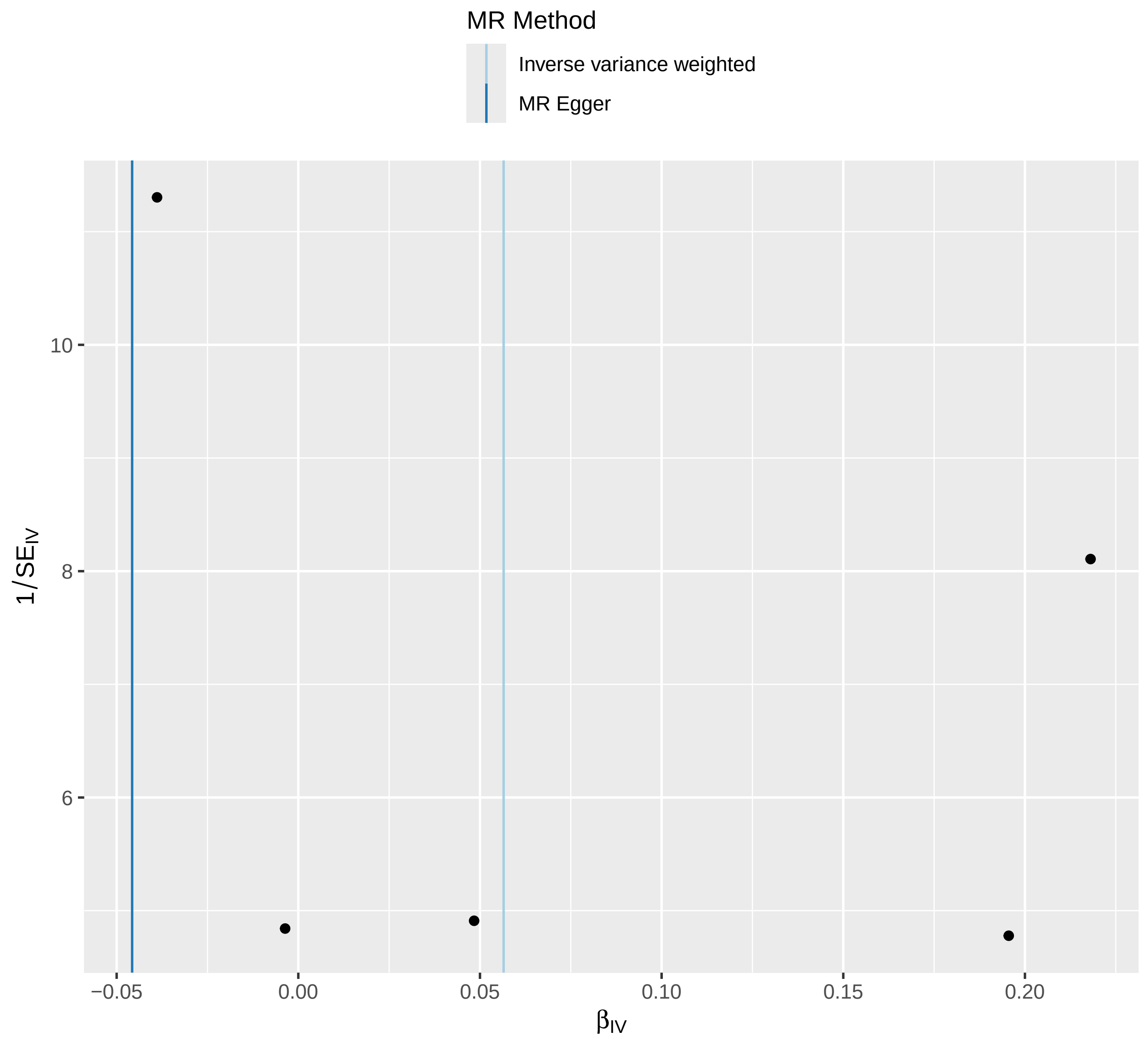
MR Method
Inverse variance weighted
MR Egger
10
8
6
$1/SE_{IV}$
−0.05
0.00
0.05
0.10
0.15
0.20
$\beta_{IV}$

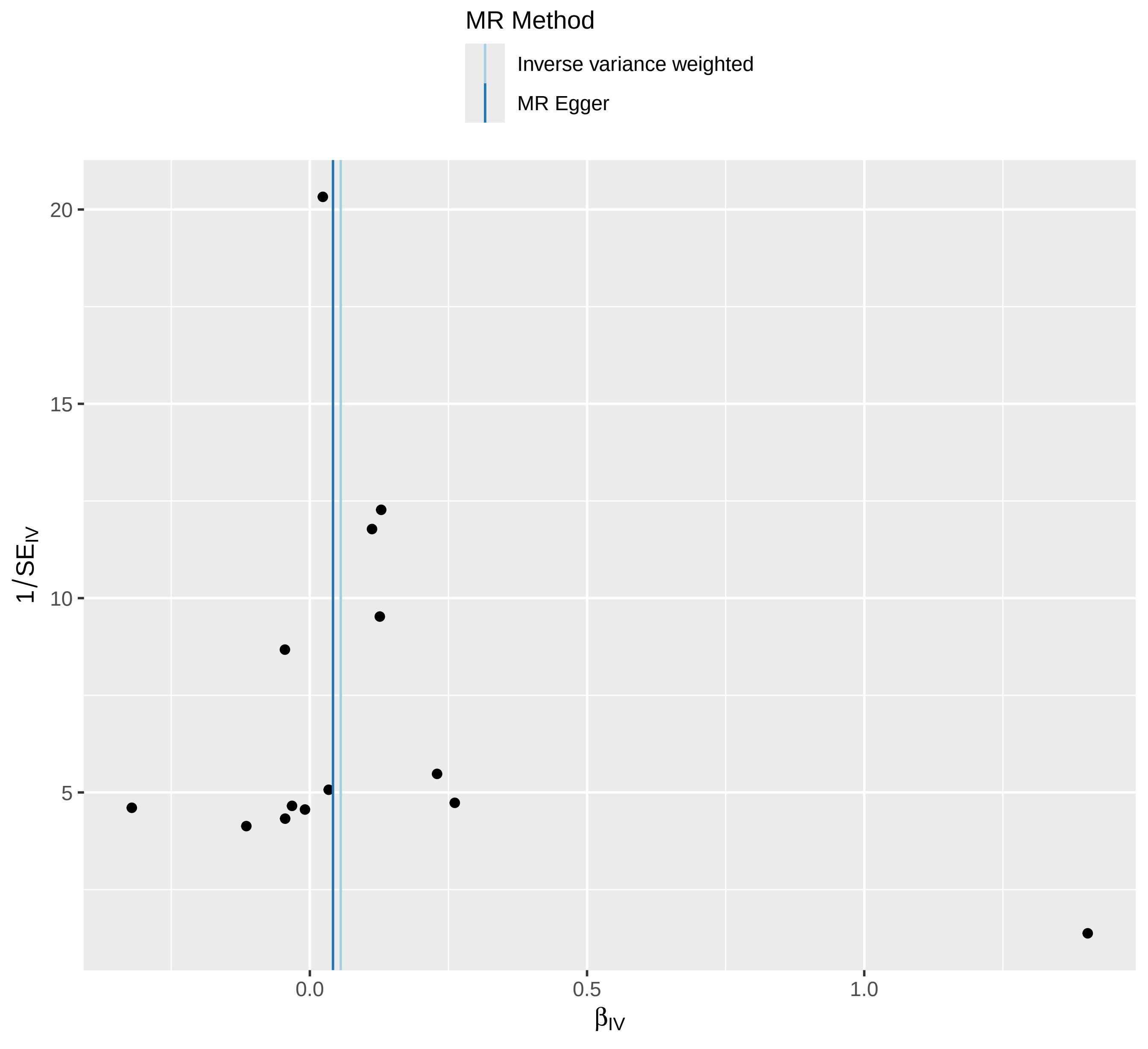

MR Method
Inverse variance weighted
MR Egger
20
15
10
5
1/SE_IV
0.0
0.5
1.0
β_IV

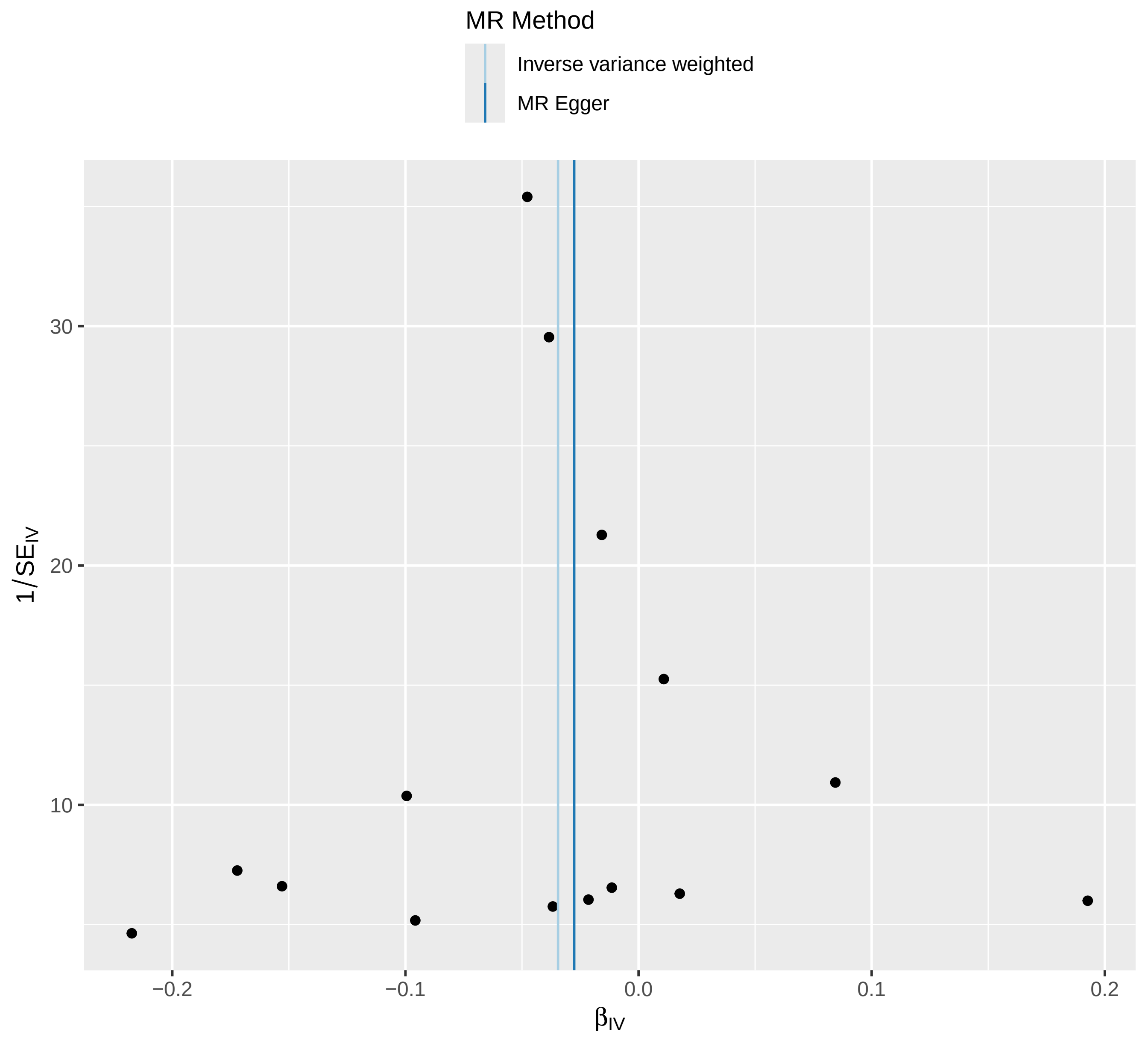
MR Method
Inverse variance weighted
MR Egger
1/SE_IV
30
20
10
−0.2
−0.1
0.0
0.1
0.2
β_IV

Insufficient number of SNPs

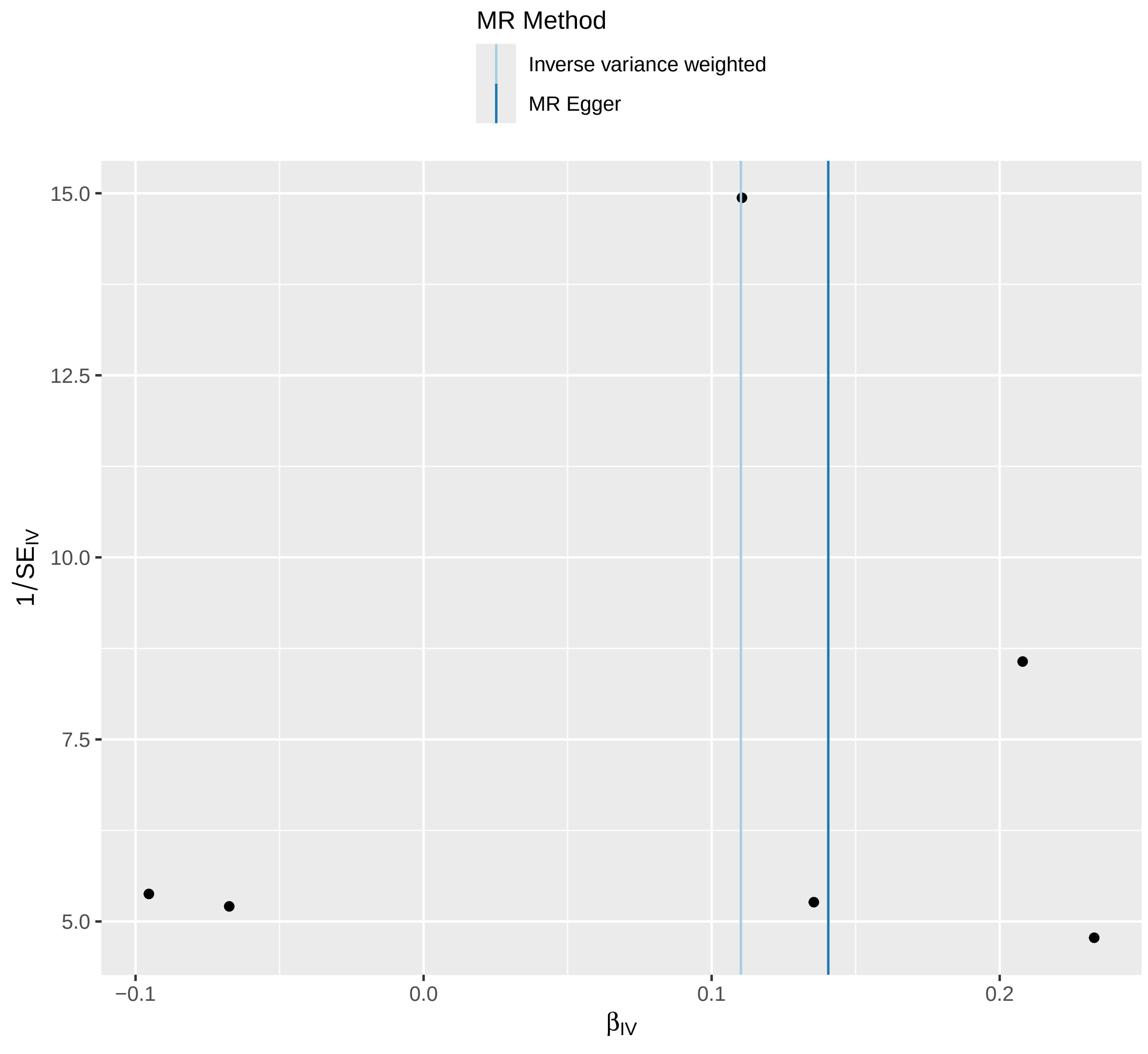
MR Method
Inverse variance weighted
MR Egger
15.0
12.5
10.0
7.5
5.0
$1/SE_{IV}$
−0.1
0.0
0.1
0.2
$\beta_{IV}$

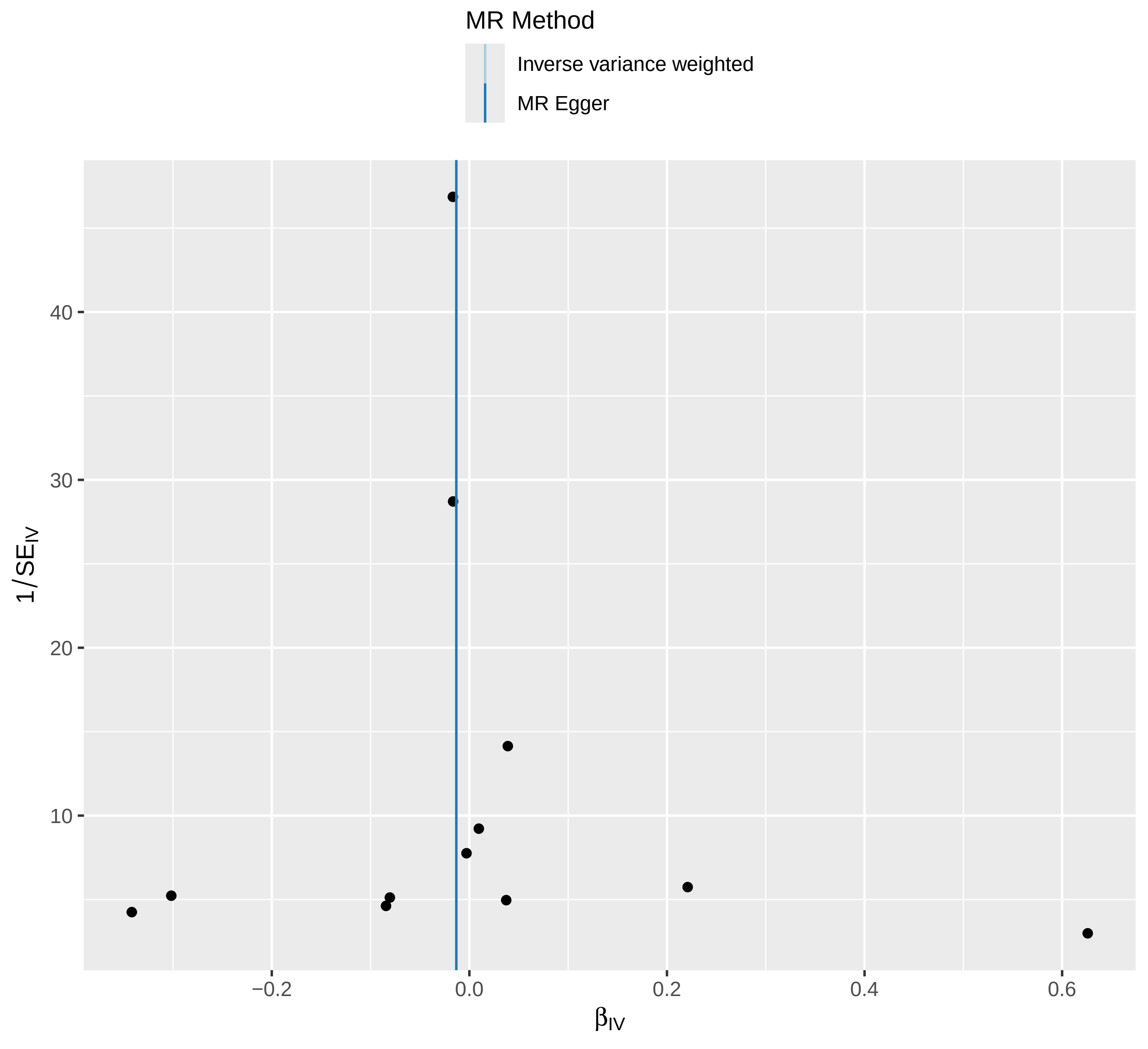
MR Method
Inverse variance weighted
MR Egger
40
30
20
10
$1/SE_{IV}$
−0.2
0.0
0.2
0.4
0.6
$\beta_{IV}$

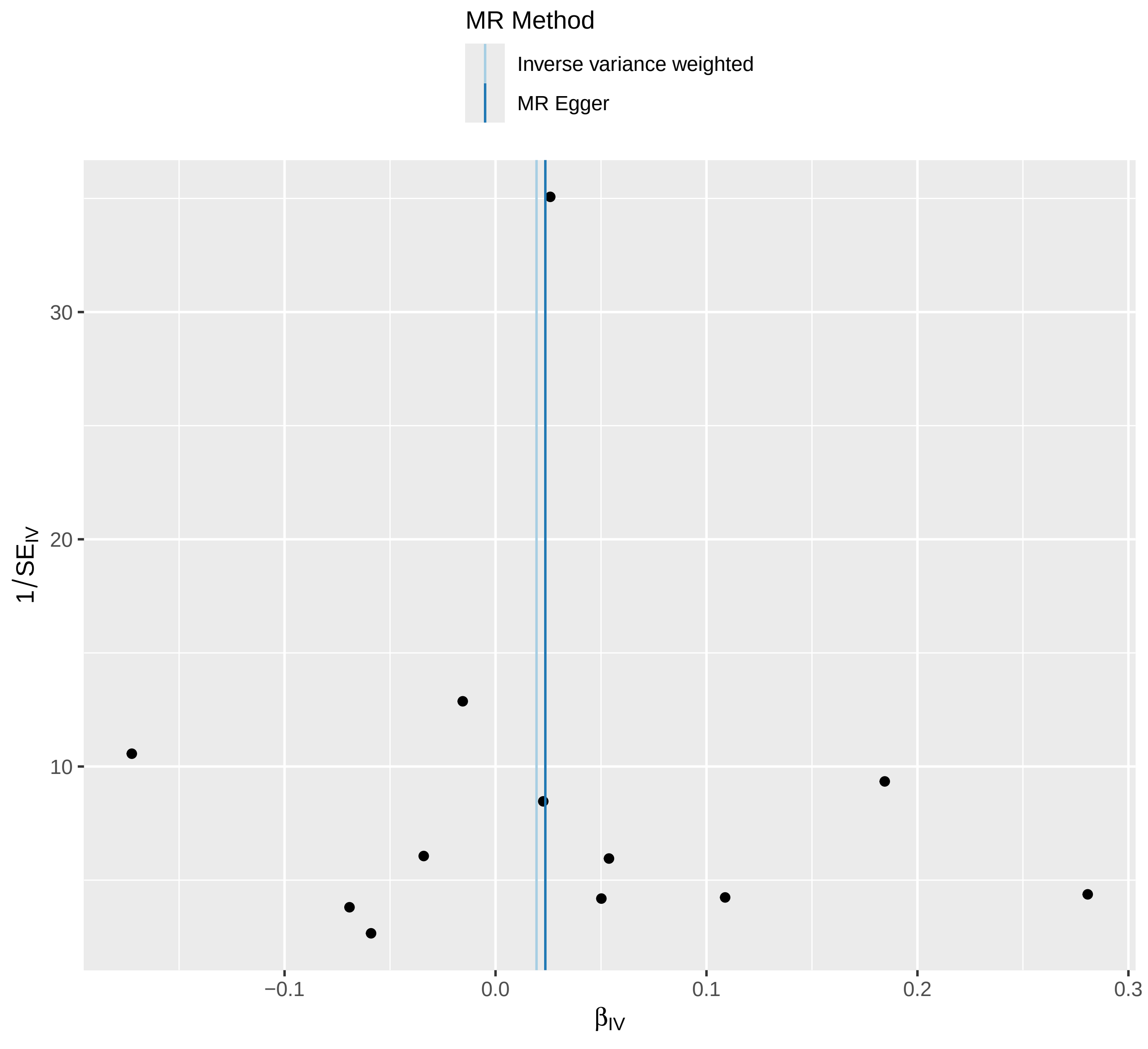

MR Method
Inverse variance weighted
MR Egger
30
20
10
1/SE$_{IV}$
−0.1
0.0
0.1
0.2
0.3
β$_{IV}$

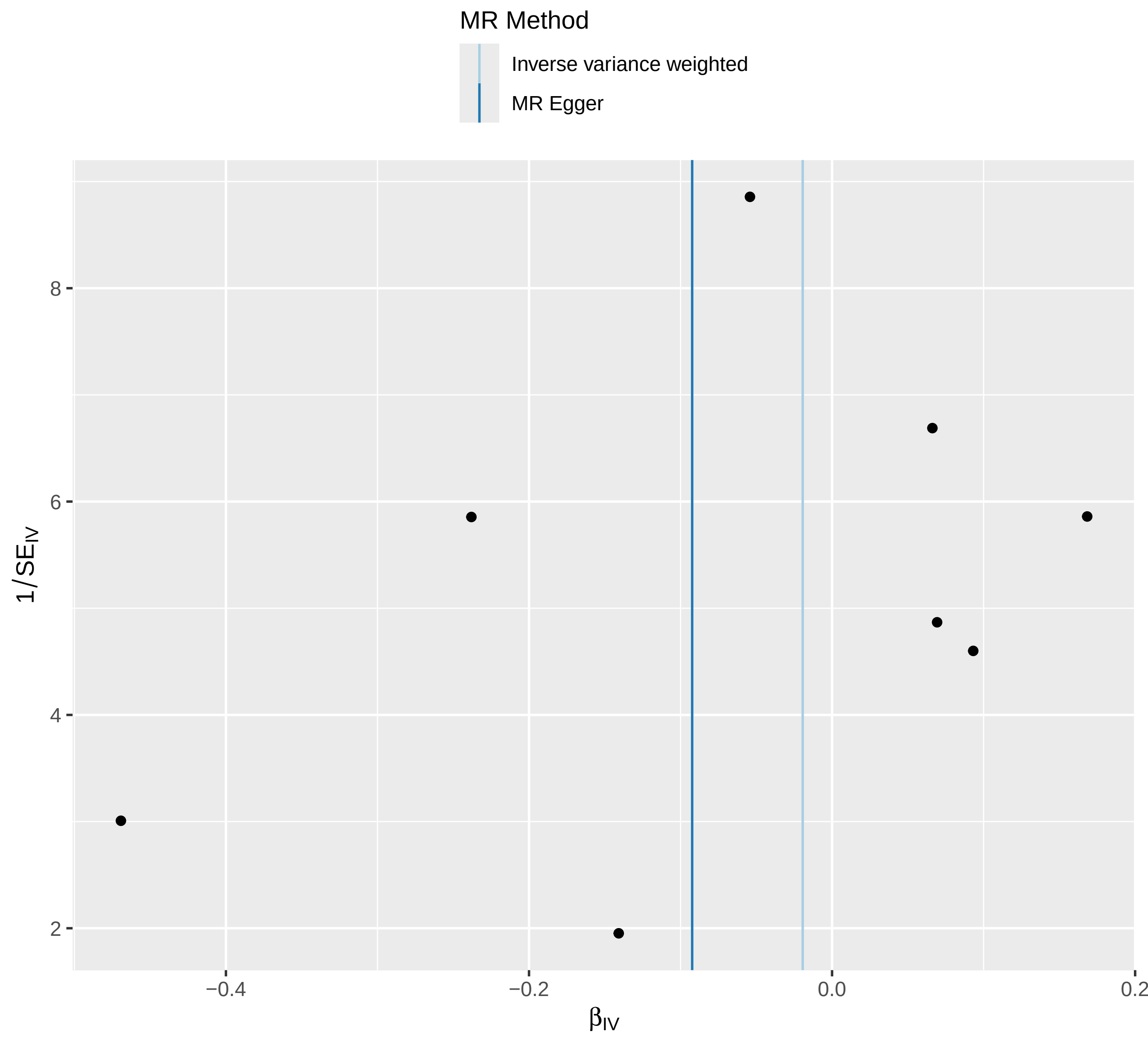

MR Method
Inverse variance weighted
MR Egger
8
6
4
2
$1/SE_{IV}$
−0.4
−0.2
0.0
0.2
$\beta_{IV}$

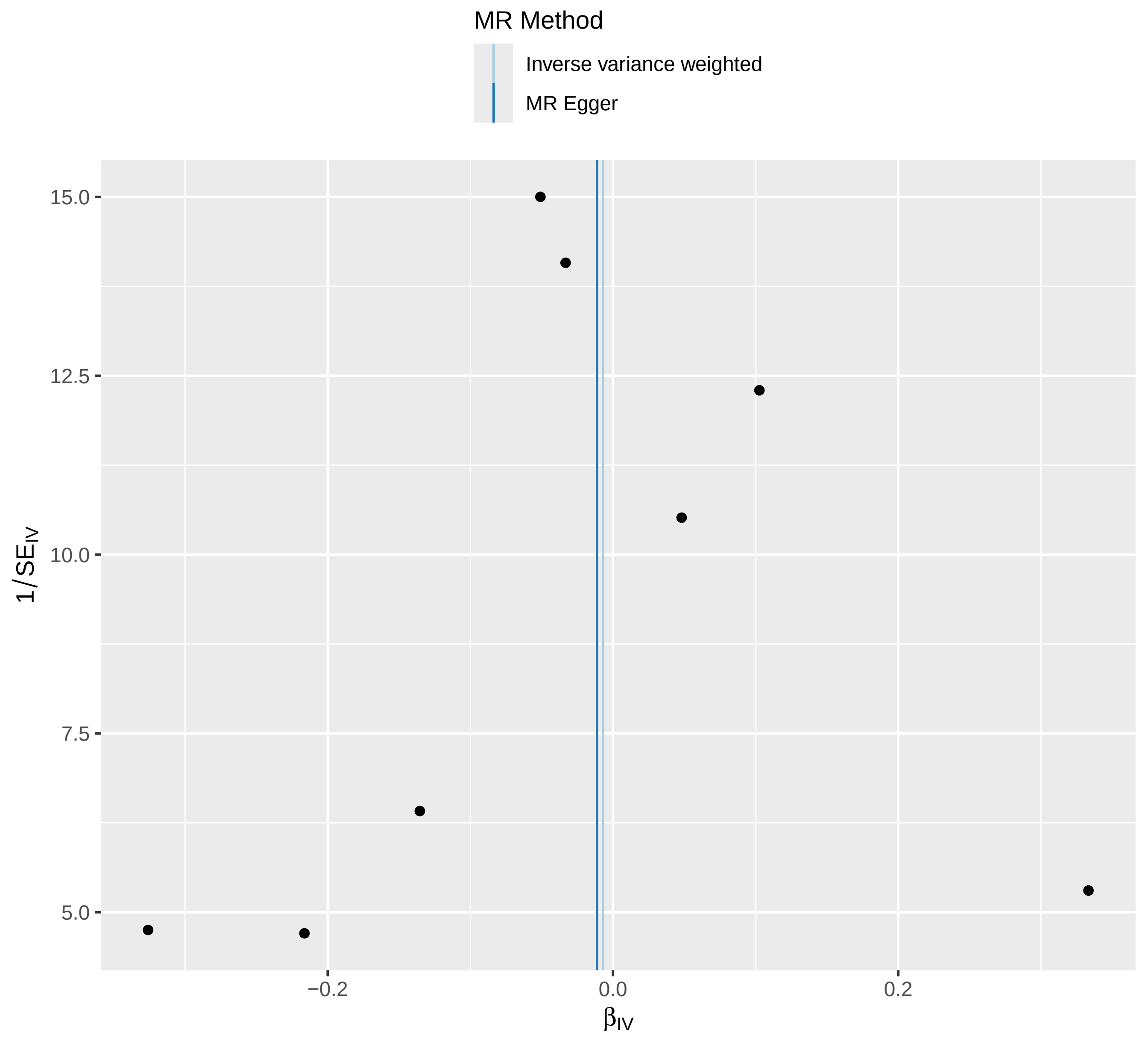
MR Method
Inverse variance weighted
MR Egger
15.0
12.5
10.0
7.5
5.0
1/SE_IV
−0.2
0.0
0.2
β_IV

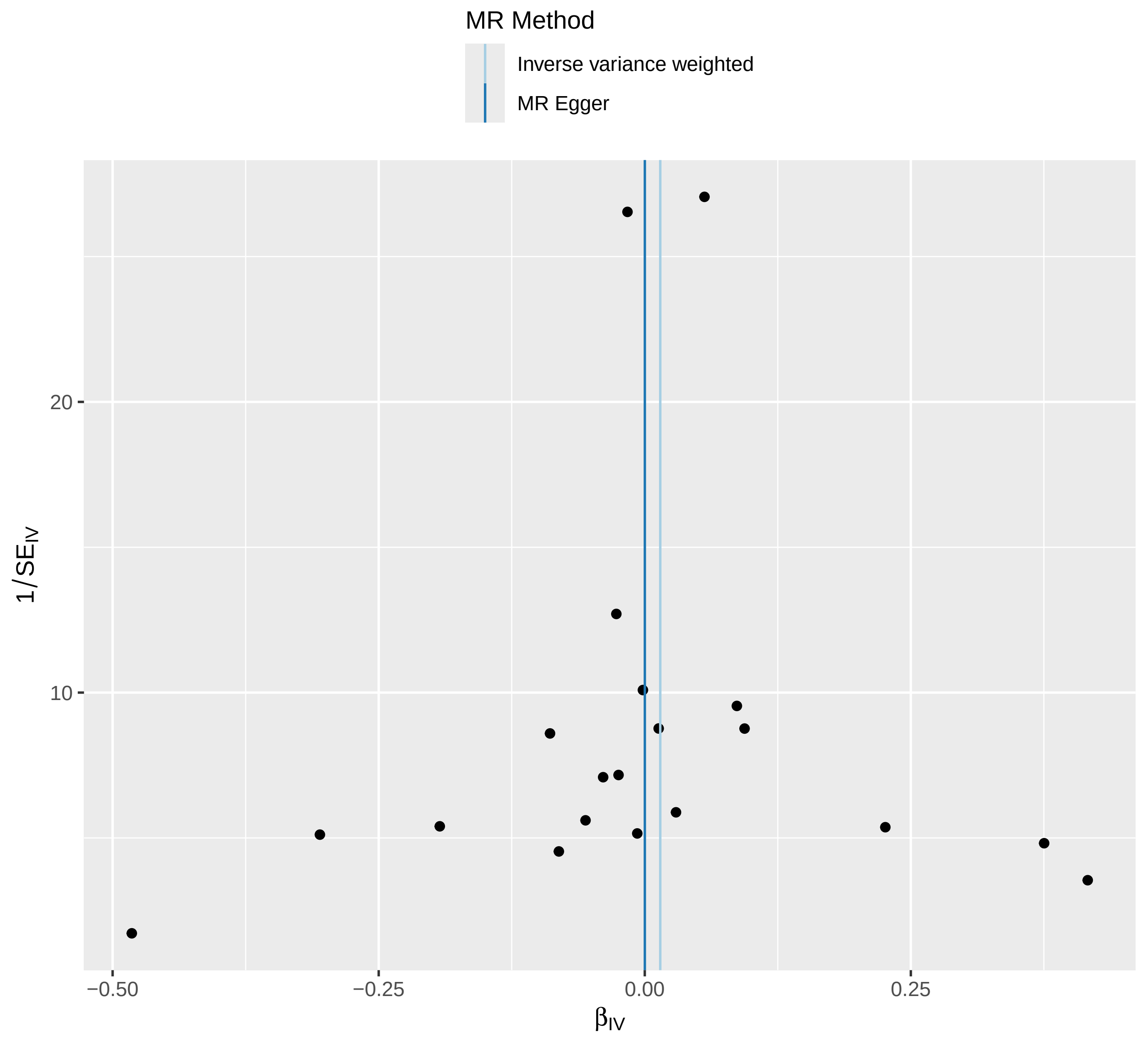

MR Method
Inverse variance weighted
MR Egger
20
10
1/SE_IV
−0.50
−0.25
0.00
0.25
β_IV

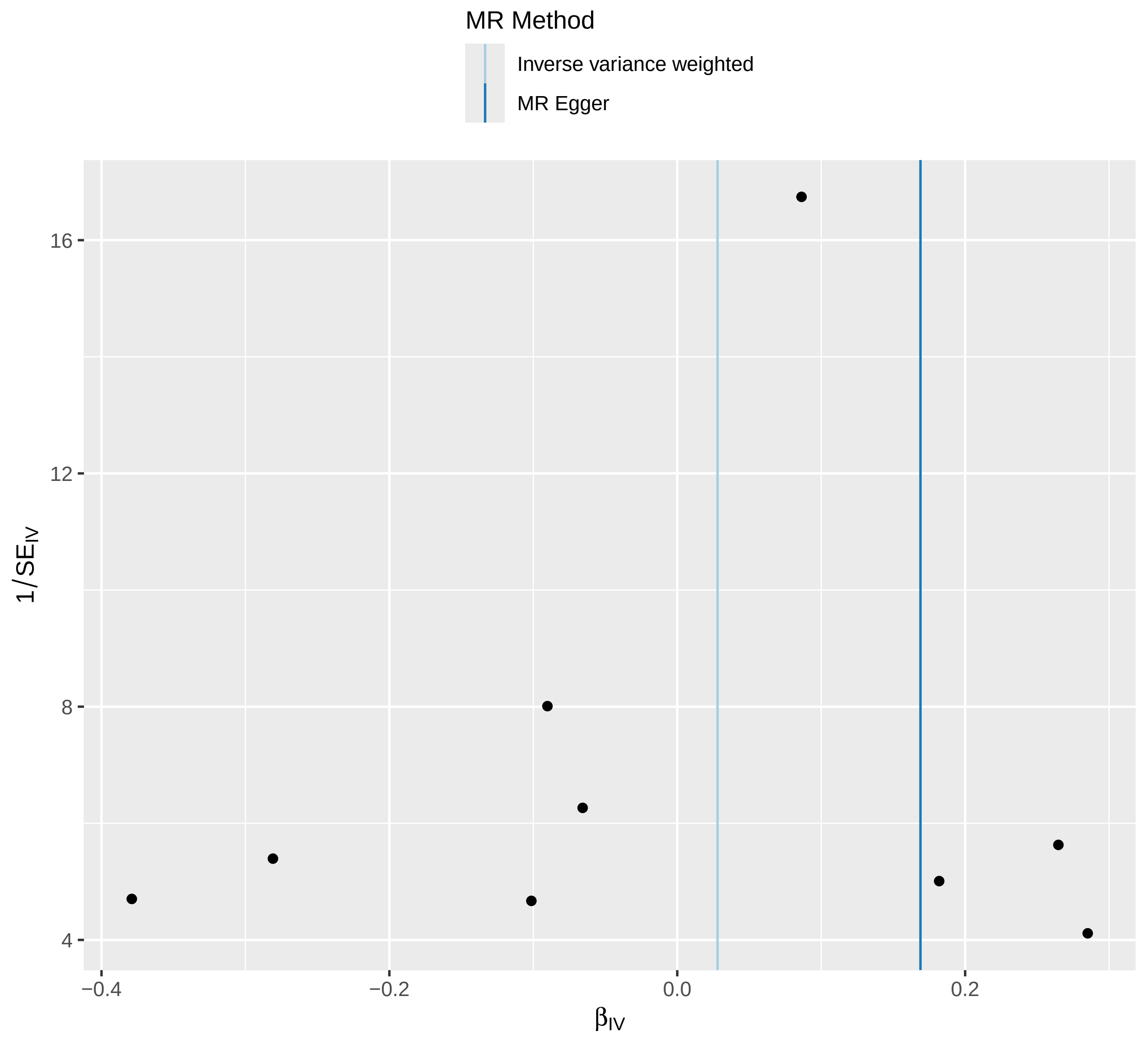

MR Method
Inverse variance weighted
MR Egger
16
12
8
4
1/SE$_{IV}$
−0.4
−0.2
0.0
0.2
β$_{IV}$

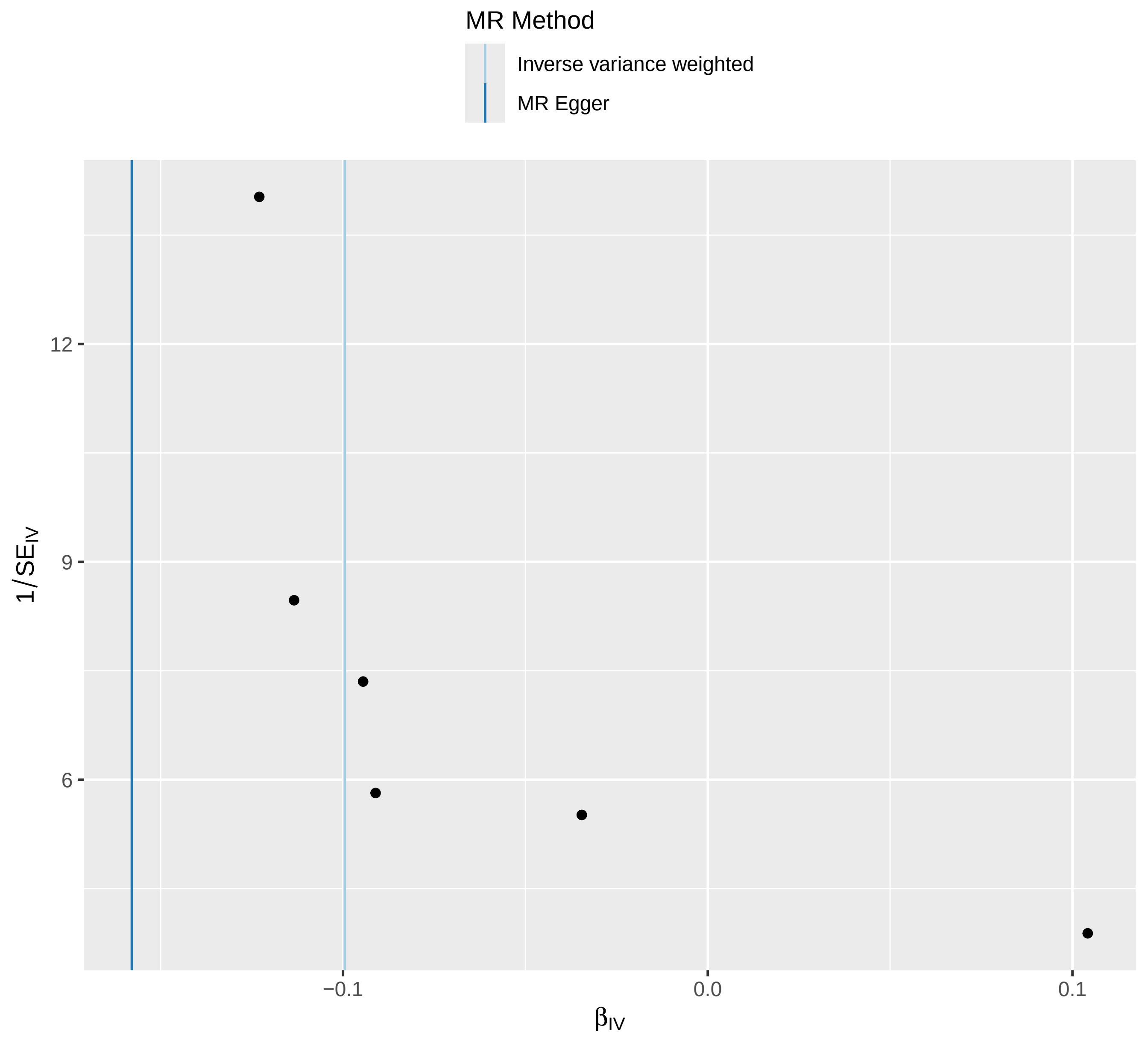

MR Method
Inverse variance weighted
MR Egger
12
9
6
1/SE$_{IV}$
−0.1
0.0
0.1
β$_{IV}$

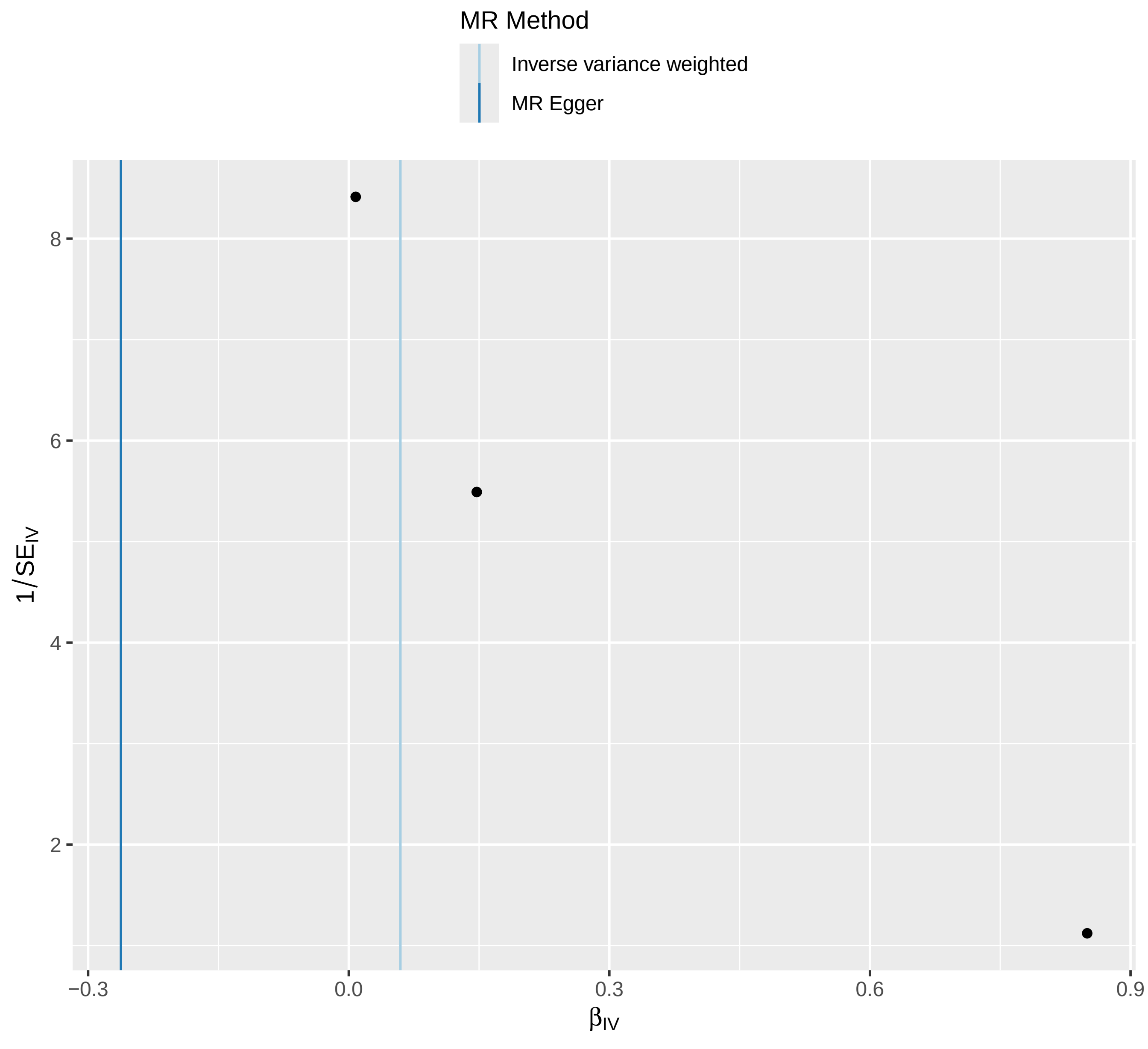
MR Method
Inverse variance weighted
MR Egger
8
6
4
2
1/SE_IV
−0.3
0.0
0.3
0.6
0.9
β_IV

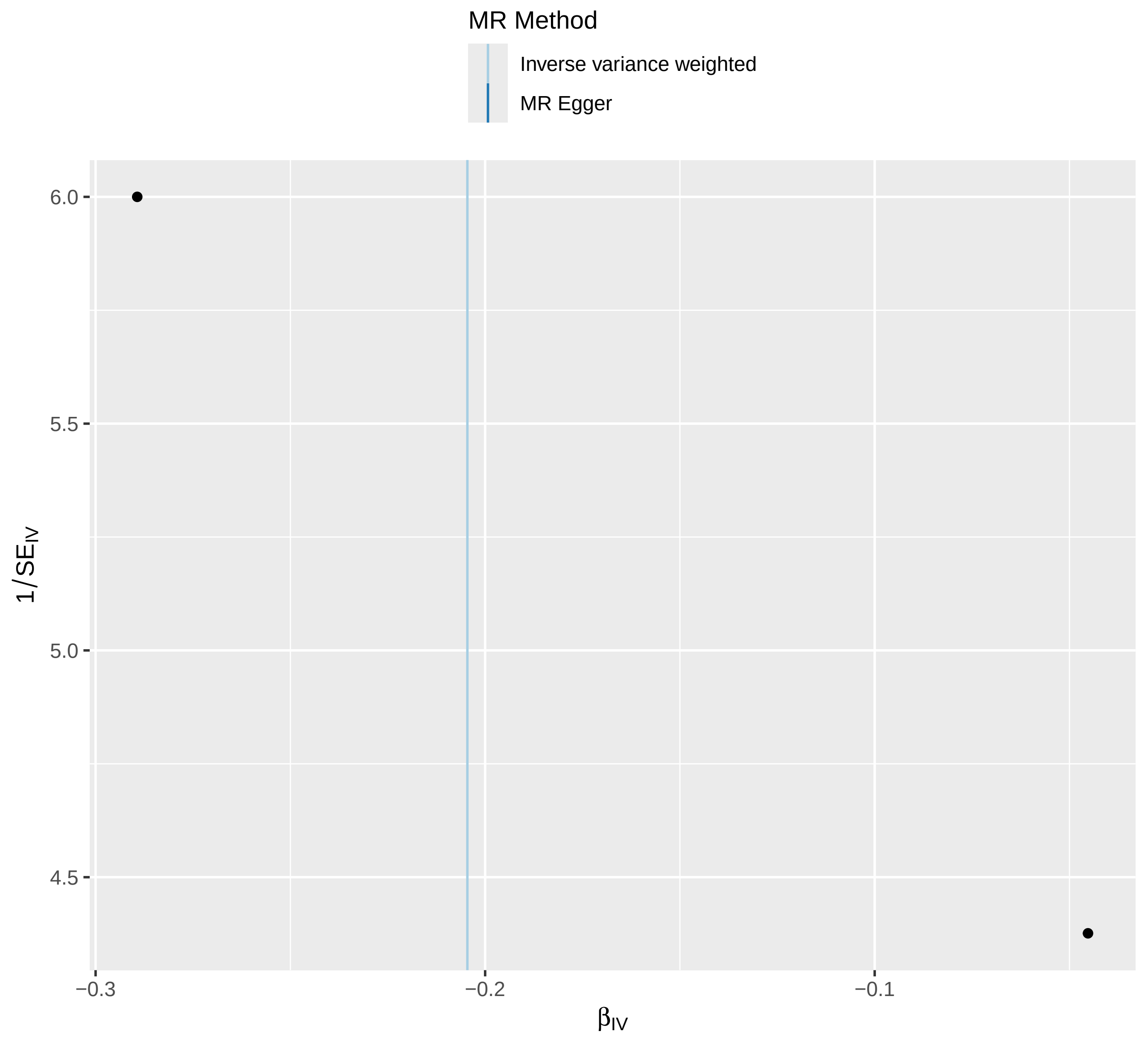

MR Method
Inverse variance weighted
MR Egger
6.0
5.5
5.0
4.5
1/SE_IV
−0.3
−0.2
−0.1
β_IV

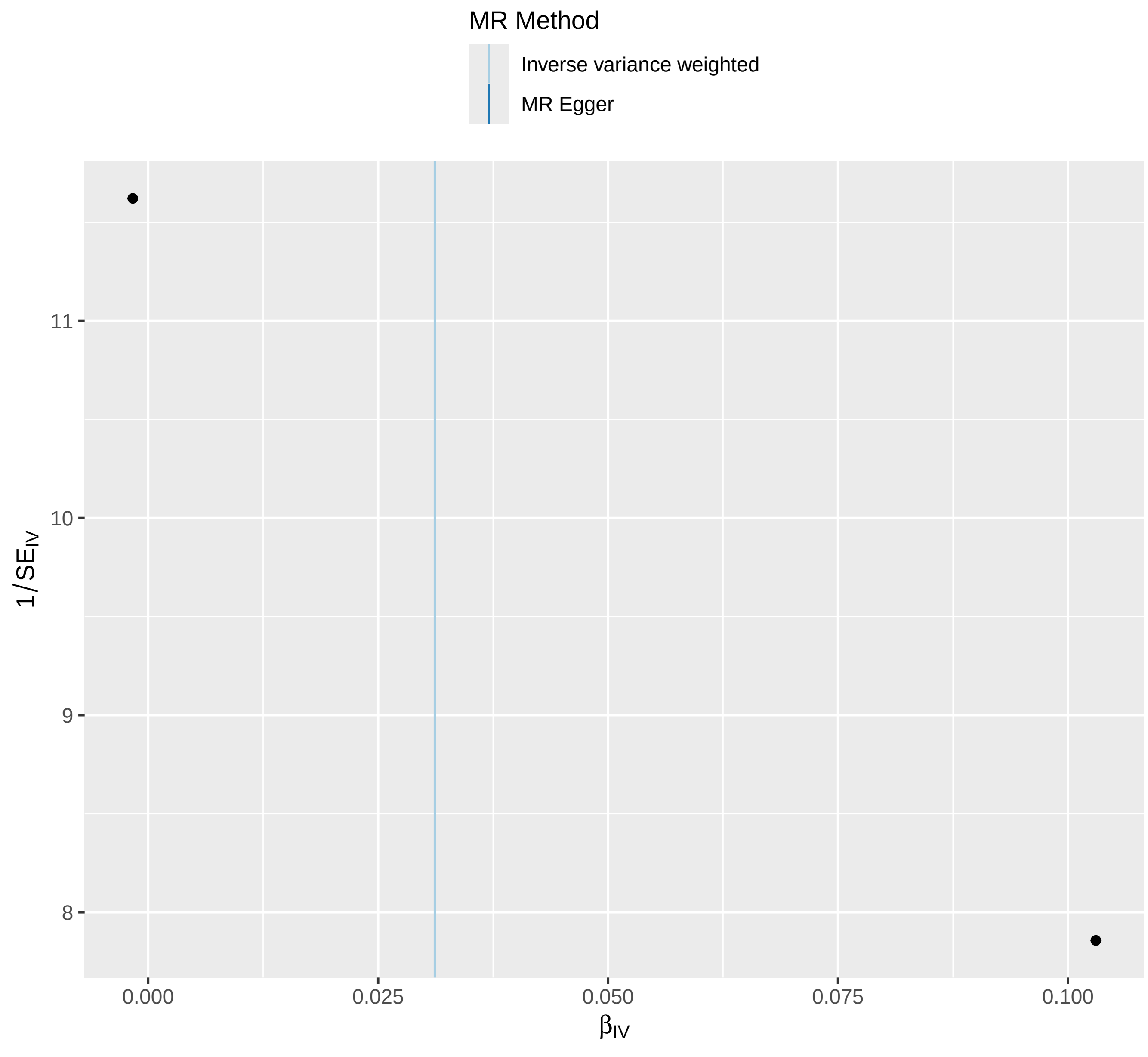
MR Method
Inverse variance weighted
MR Egger
11
10
9
8
1/SE_IV
0.000
0.025
0.050
0.075
0.100
β_IV

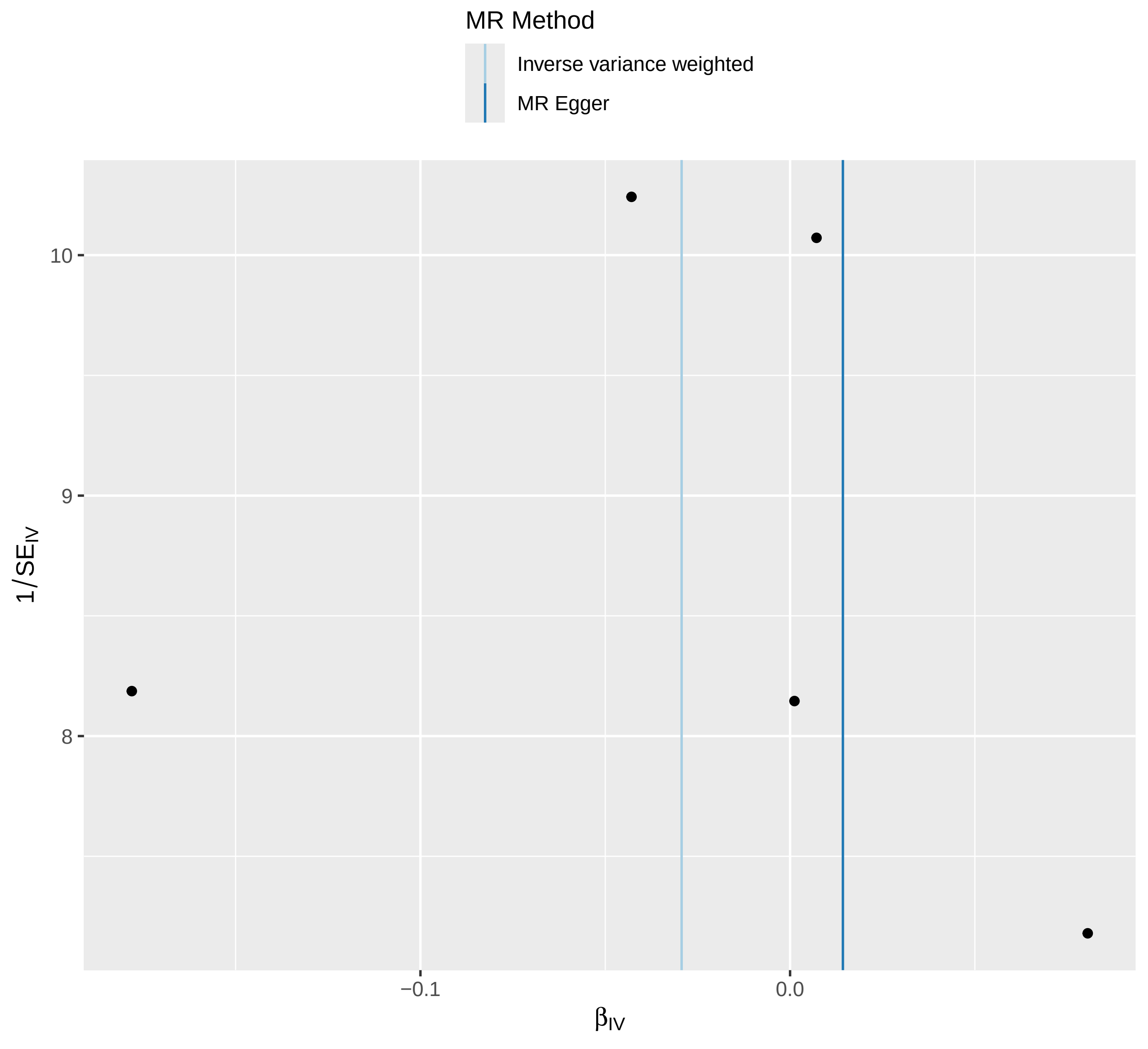

MR Method
Inverse variance weighted
MR Egger
10
9
8
1/SE_IV
−0.1
0.0
β_IV

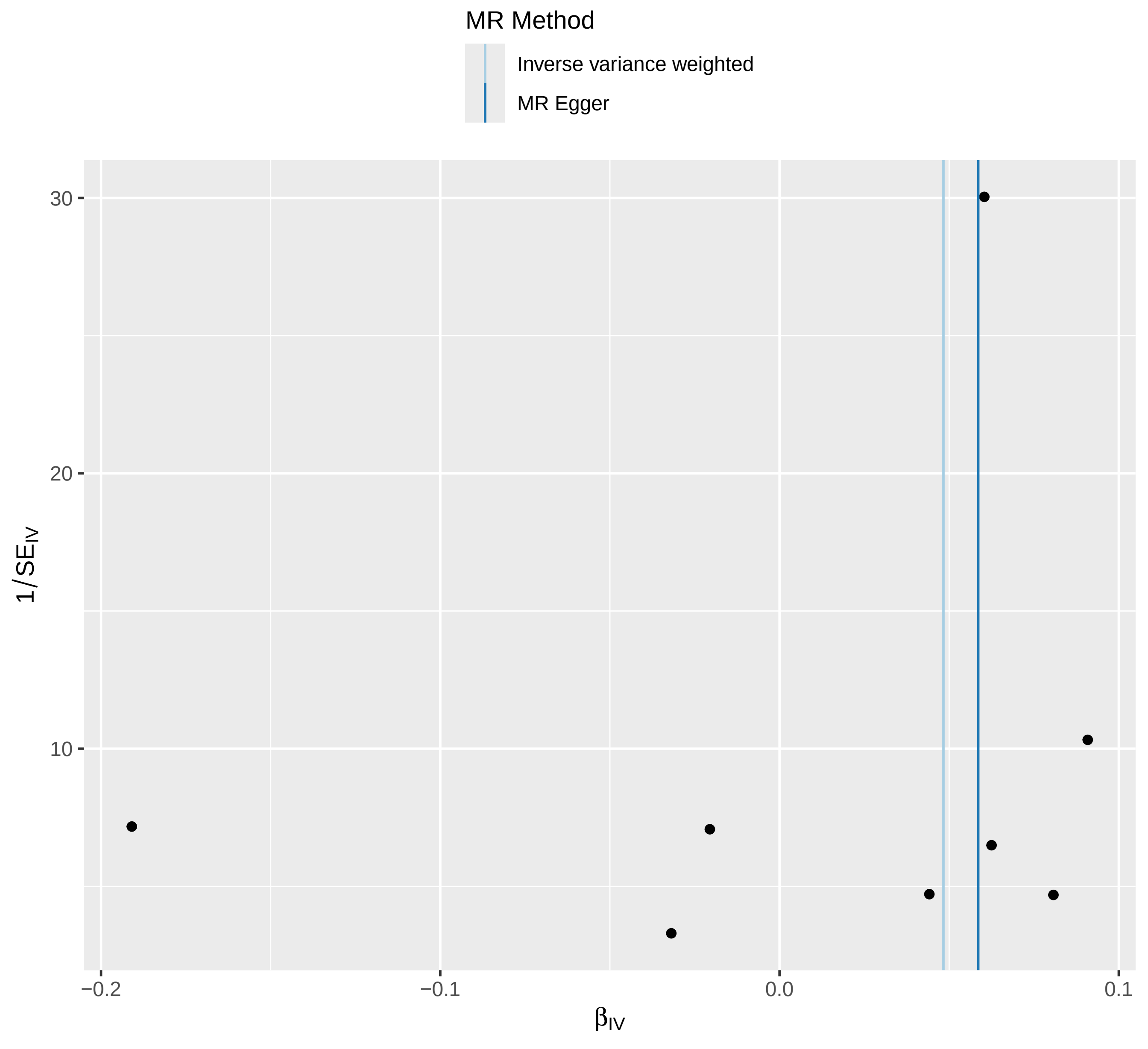
MR Method
Inverse variance weighted
MR Egger
30
20
10
1/SE_IV
−0.2
−0.1
0.0
0.1
β_IV

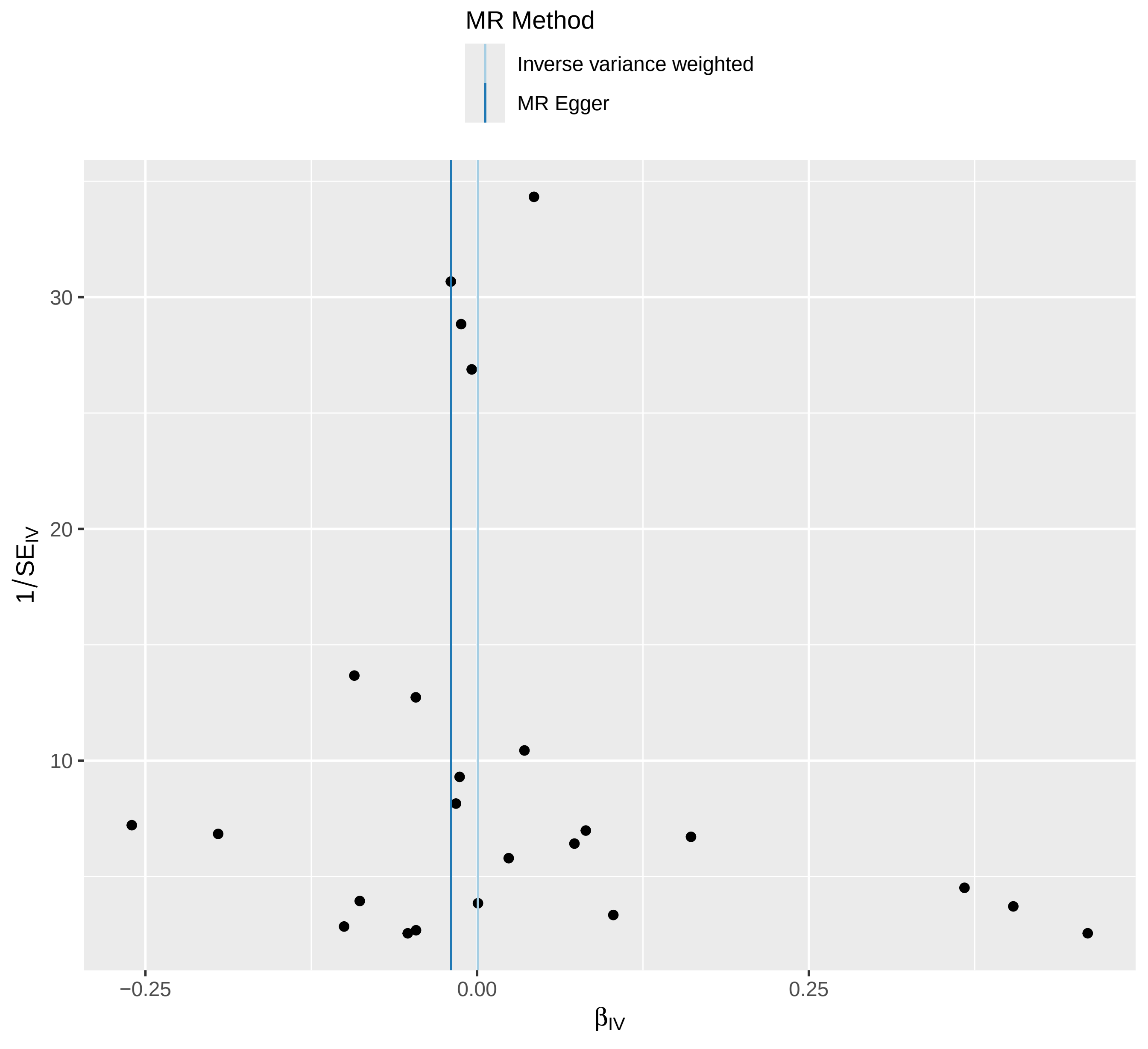

MR Method
Inverse variance weighted
MR Egger
30
20
10
$1/SE_{IV}$
−0.25
0.00
0.25
$\beta_{IV}$

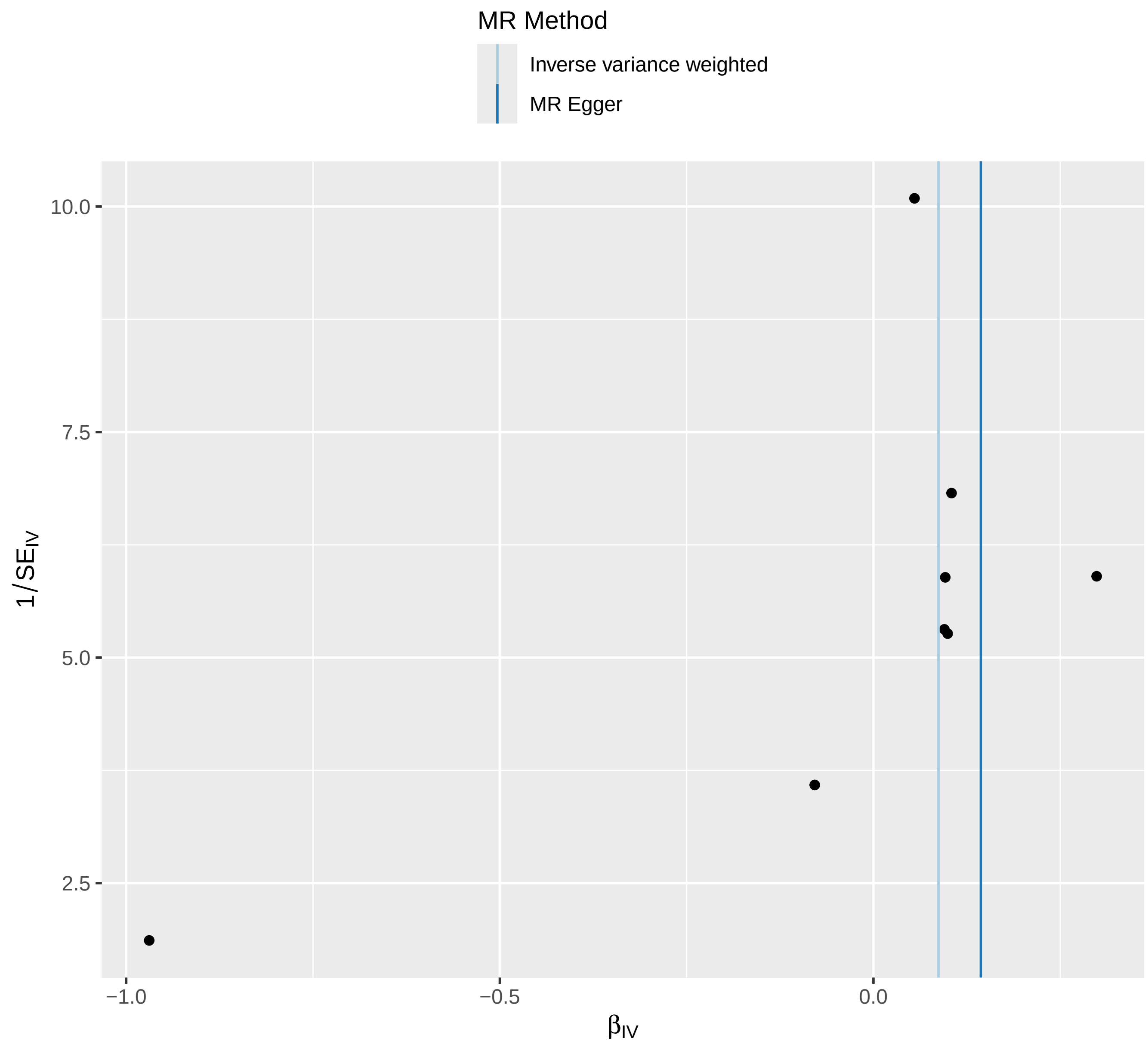
MR Method
Inverse variance weighted
MR Egger
10.0
7.5
5.0
2.5
1/$SE_{IV}$
−1.0
−0.5
0.0
$β_{IV}$

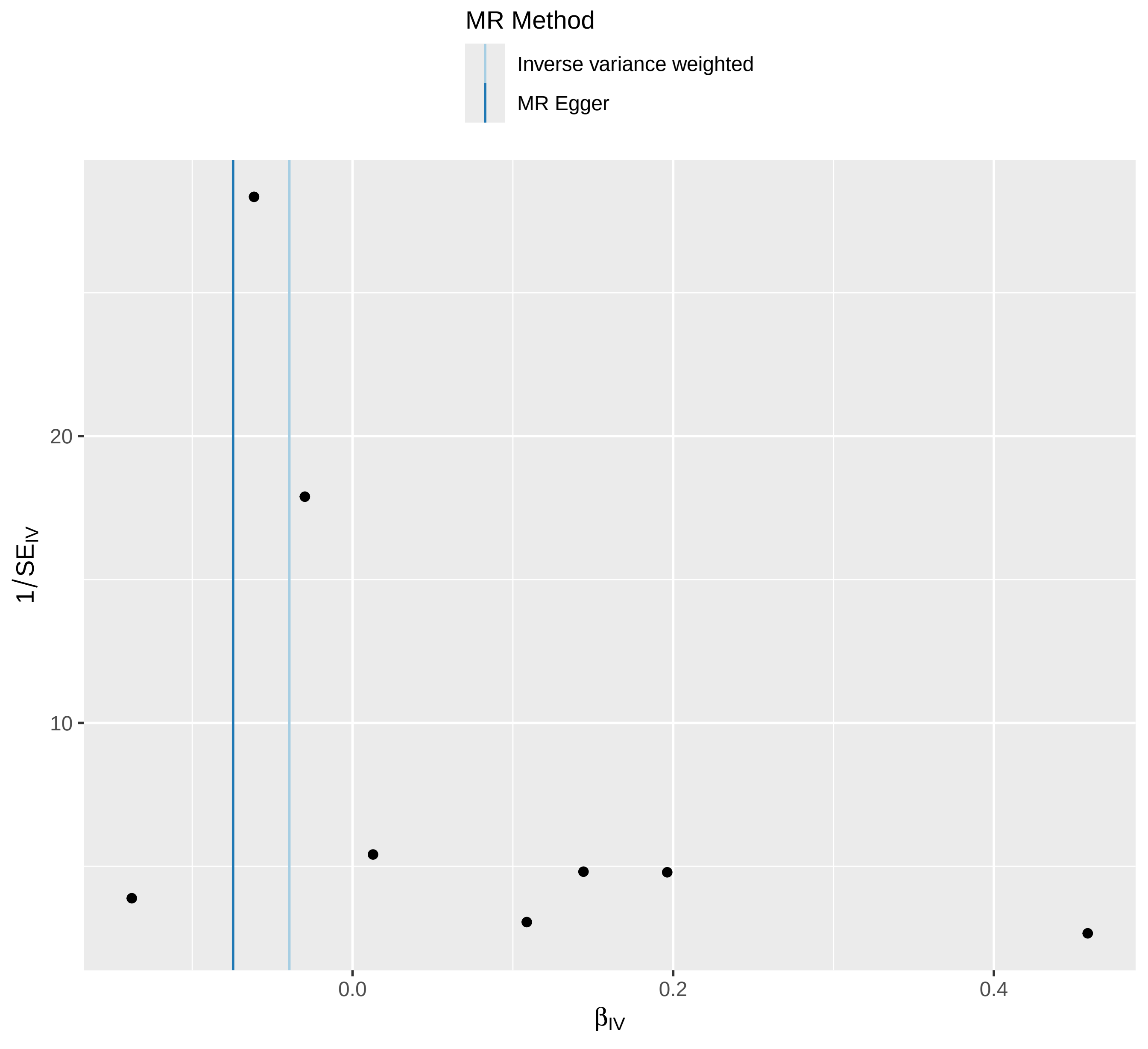
MR Method
Inverse variance weighted
MR Egger
20
10
$1/SE_{IV}$
0.0
0.2
0.4
$\beta_{IV}$

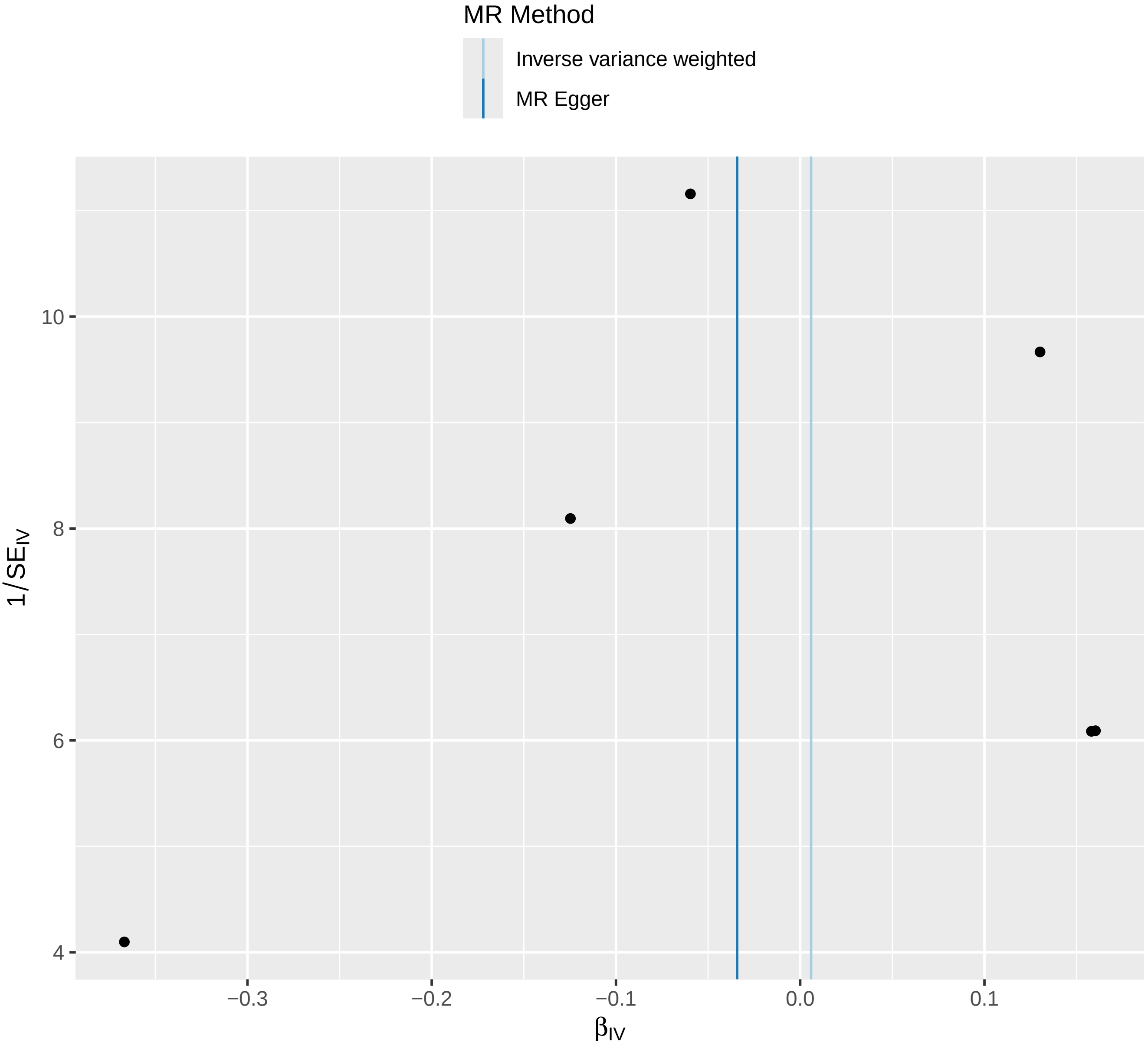

MR Method
Inverse variance weighted
MR Egger
10
8
6
4
1/SE_IV
−0.3
−0.2
−0.1
0.0
0.1
β_IV

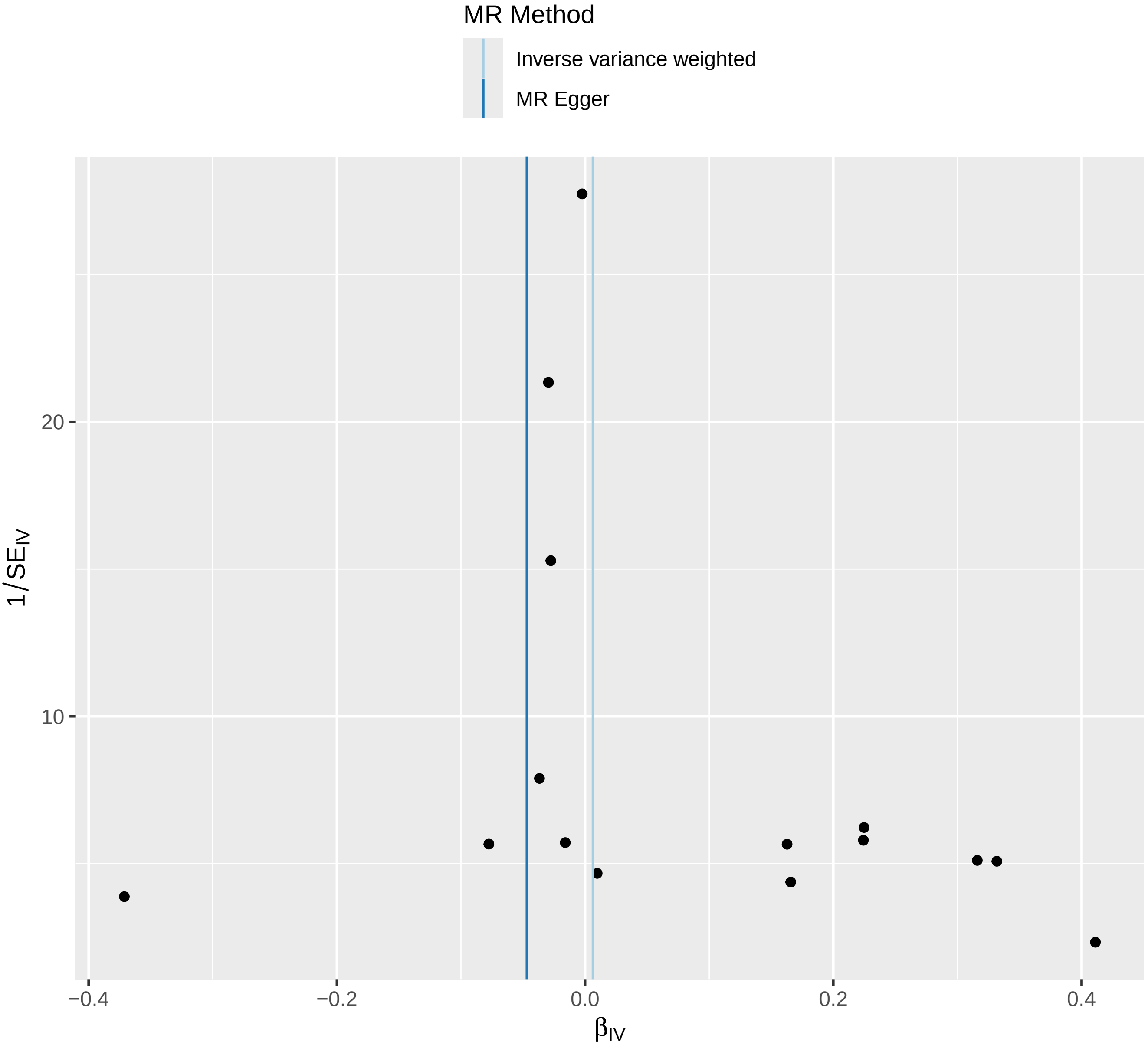
MR Method
Inverse variance weighted
MR Egger
20
10
1/SE$_{IV}$
−0.4
−0.2
0.0
0.2
0.4
β$_{IV}$

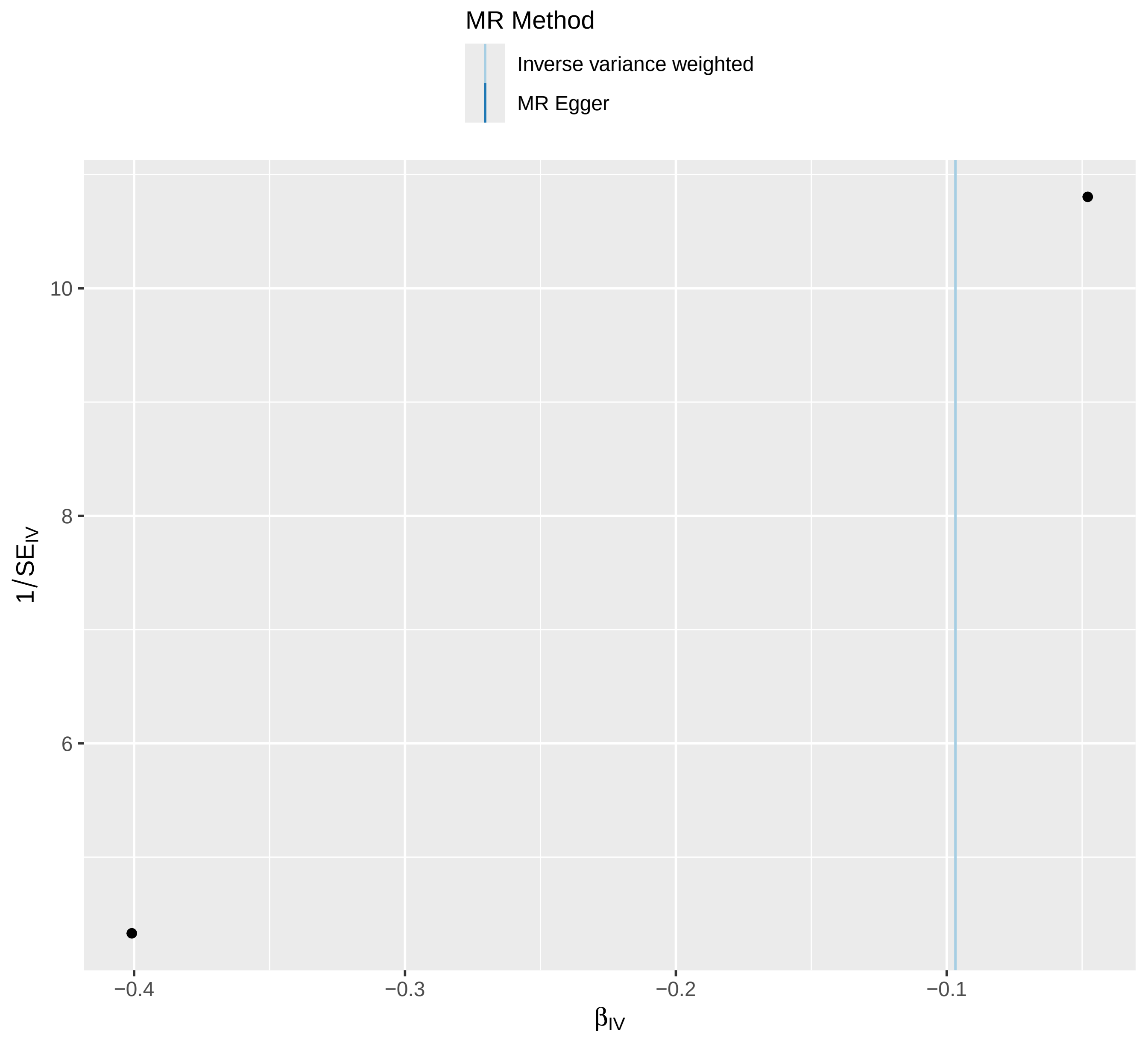

MR Method
Inverse variance weighted
MR Egger
10
8
6
1/SE$_{IV}$
−0.4
−0.3
−0.2
−0.1
β$_{IV}$

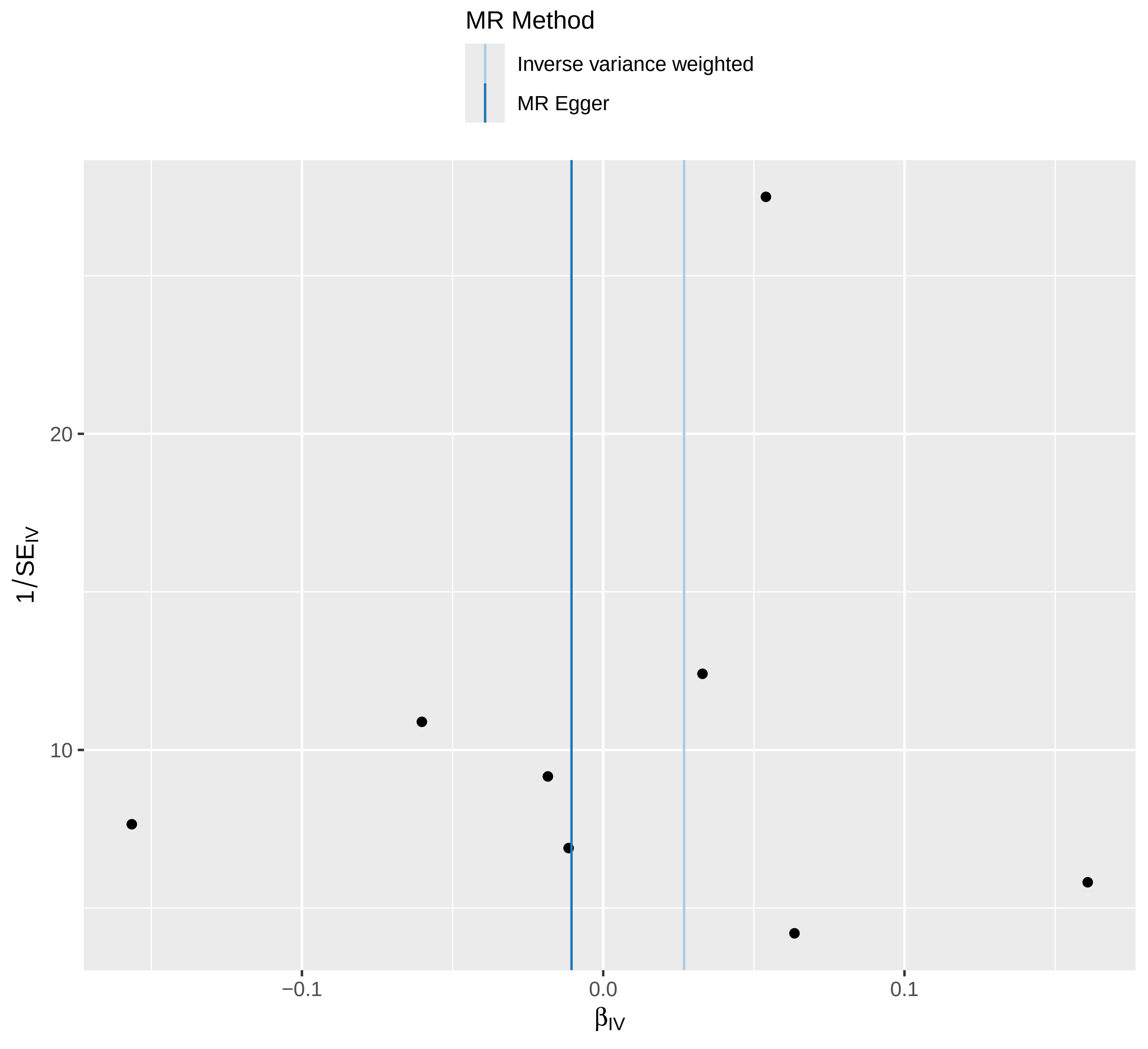

MR Method
Inverse variance weighted
MR Egger
20
10
1/SE_IV
−0.1
0.0
0.1
β_IV

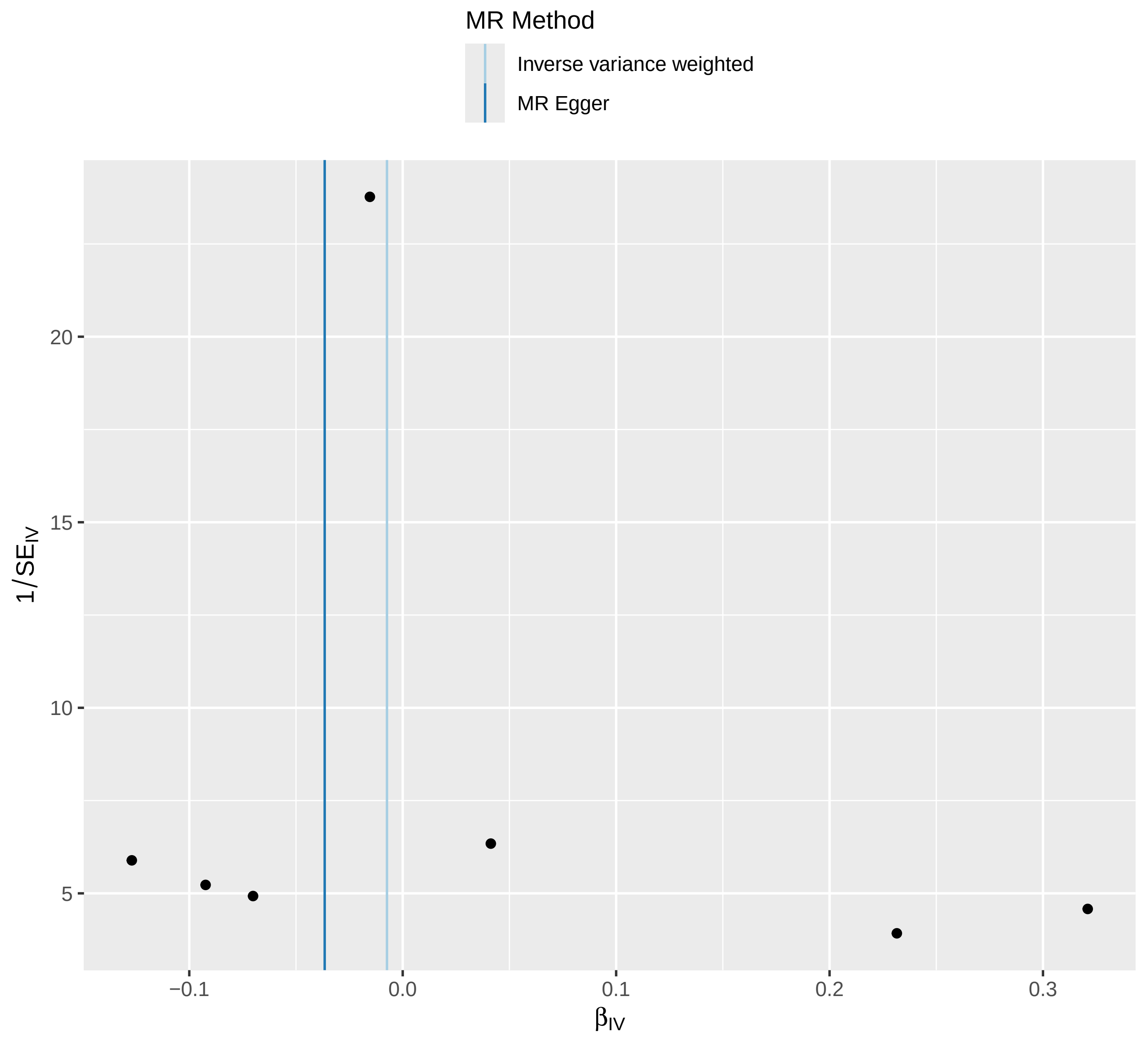

MR Method
Inverse variance weighted
MR Egger
1/SE$_{IV}$
20
15
10
5
−0.1
0.0
0.1
0.2
0.3
β$_{IV}$

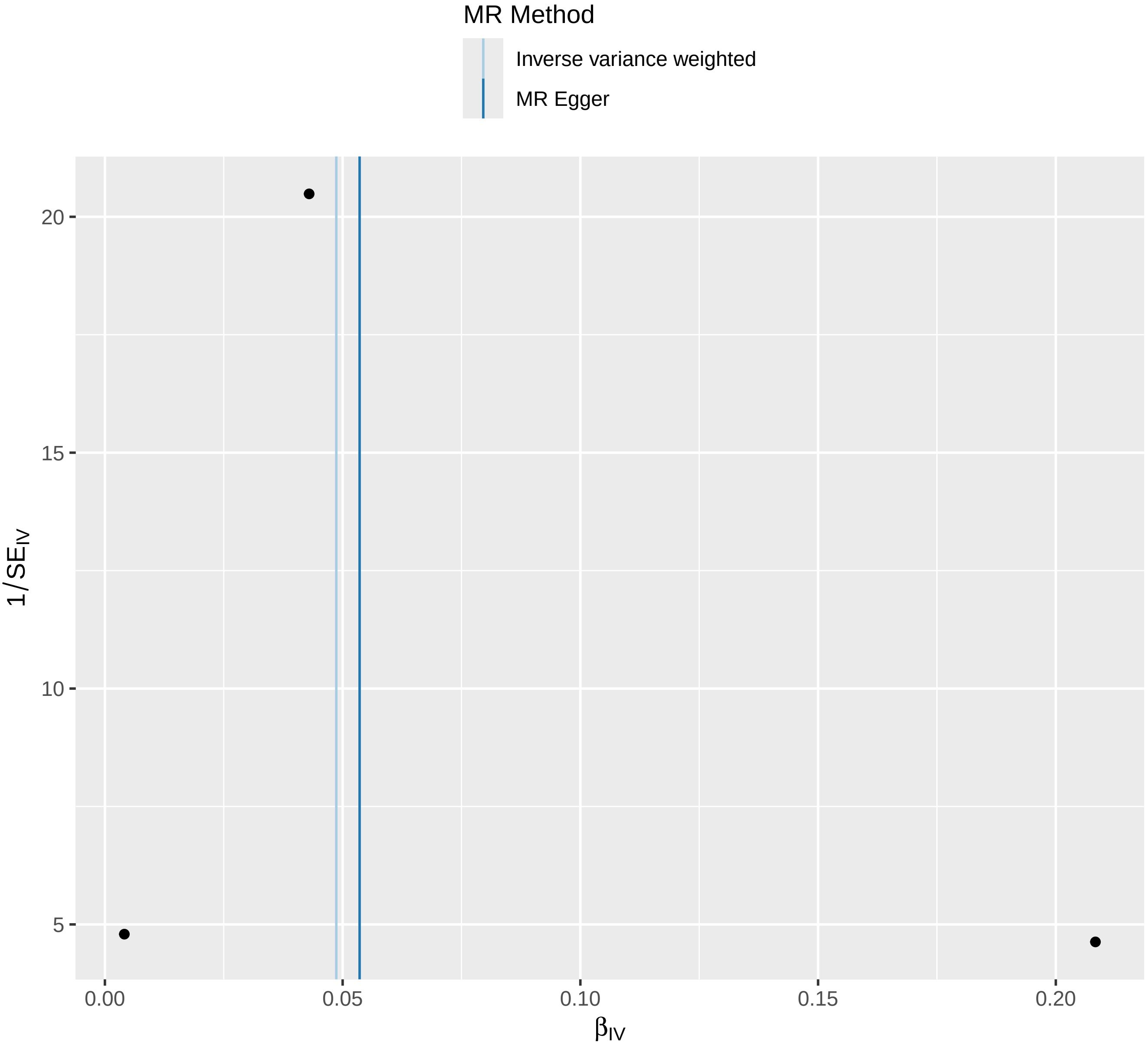

MR Method
Inverse variance weighted
MR Egger
20
15
10
5
$1/SE_{IV}$
0.00
0.05
0.10
0.15
0.20
$\beta_{IV}$

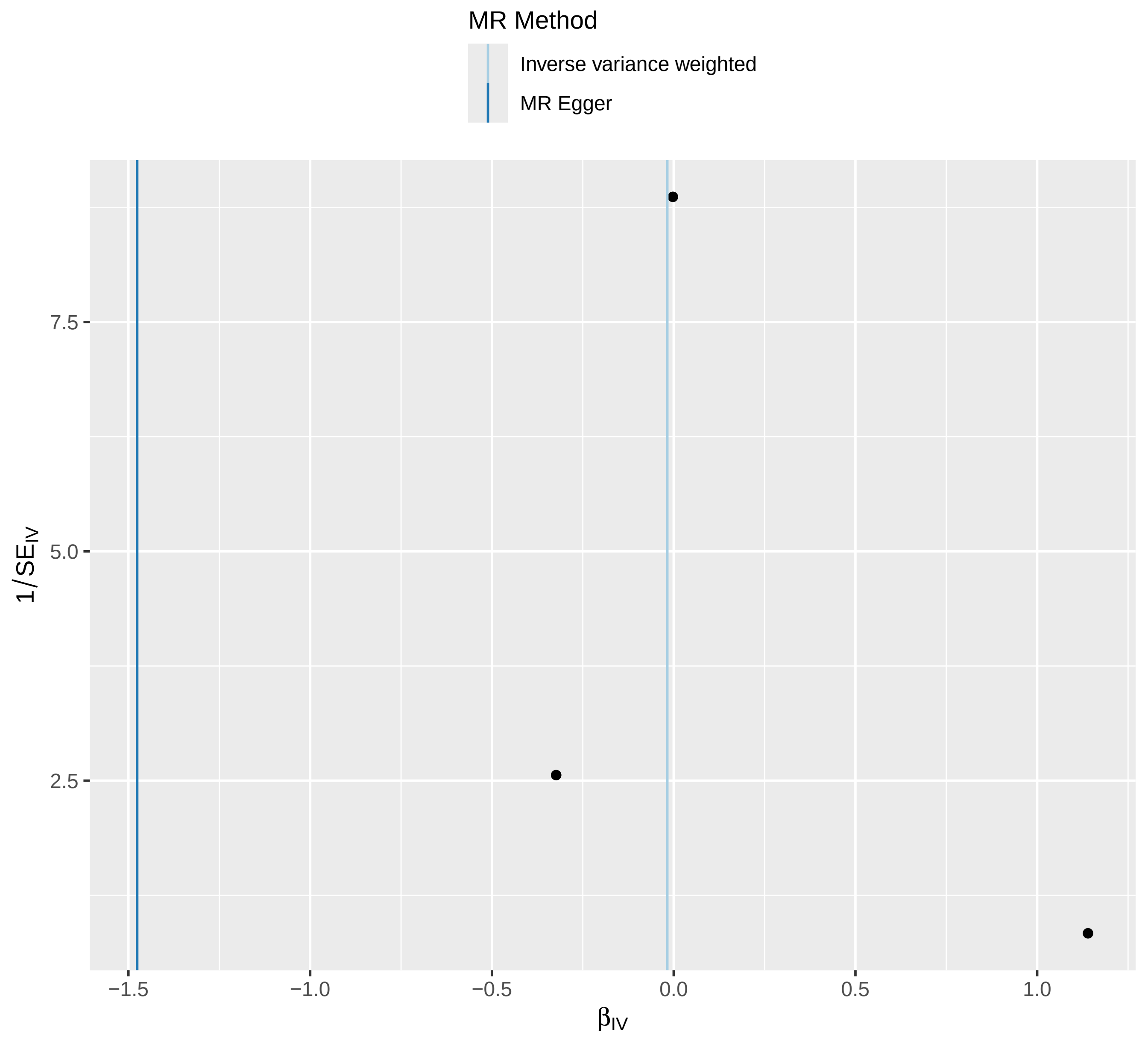

MR Method
Inverse variance weighted
MR Egger
7.5
5.0
2.5
1/SE$_{IV}$
−1.5
−1.0
−0.5
0.0
0.5
1.0
$\beta_{IV}$

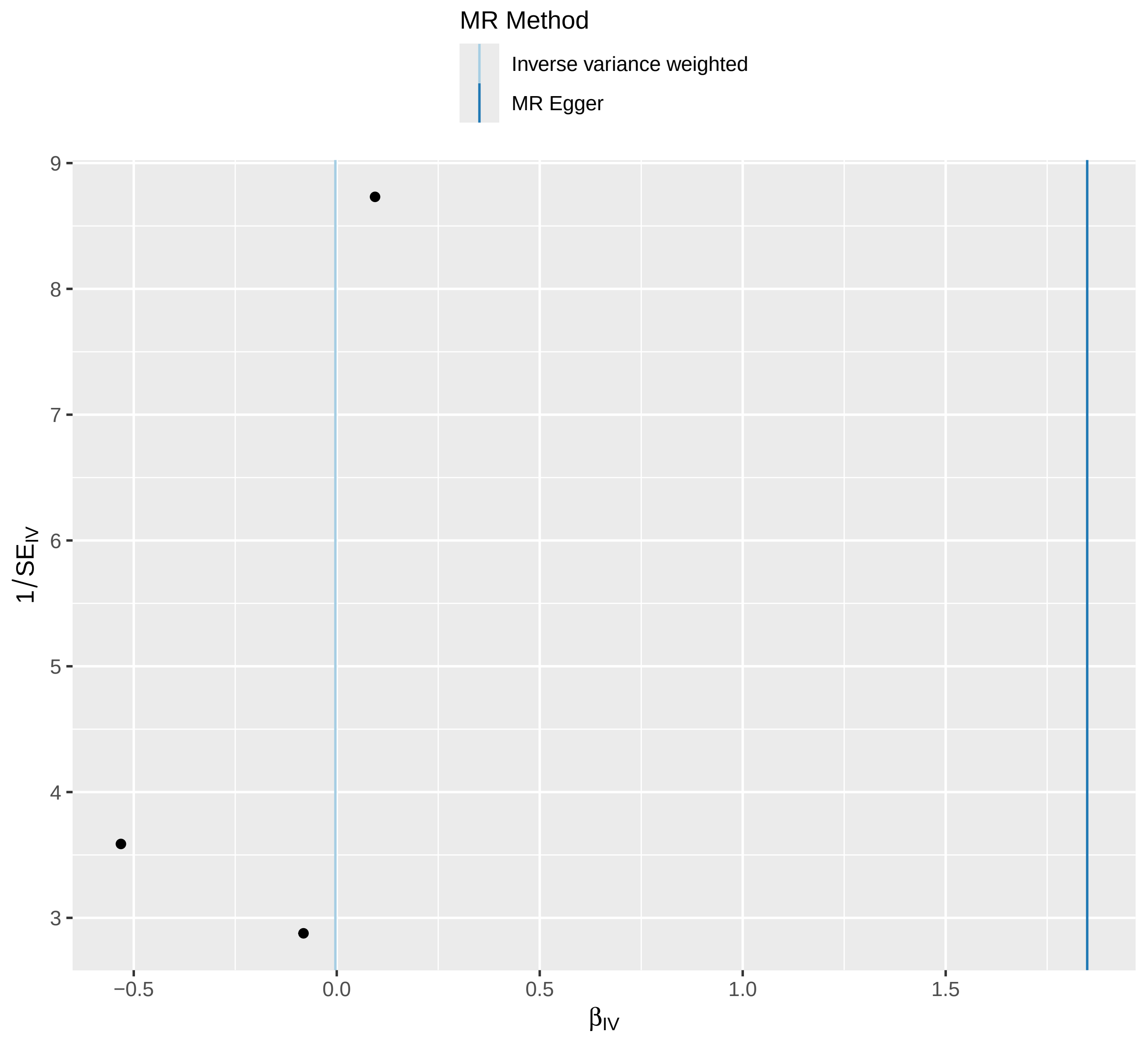

MR Method
Inverse variance weighted
MR Egger
9
8
7
6
5
4
3
1/SE_IV
−0.5
0.0
0.5
1.0
1.5
β_IV

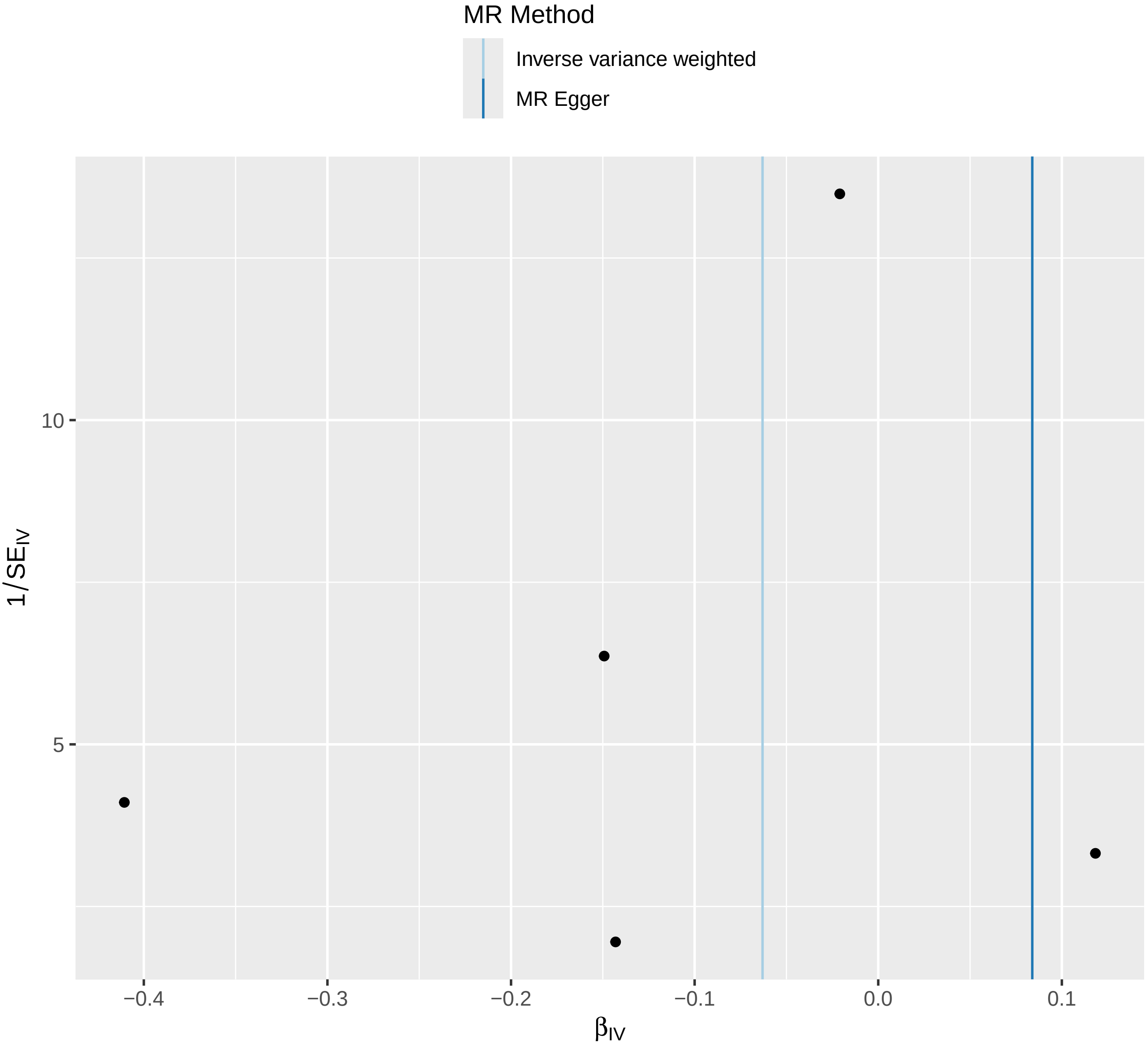
MR Method
Inverse variance weighted
MR Egger
10
5
$1/SE_{IV}$
−0.4
−0.3
−0.2
−0.1
0.0
0.1
$\beta_{IV}$

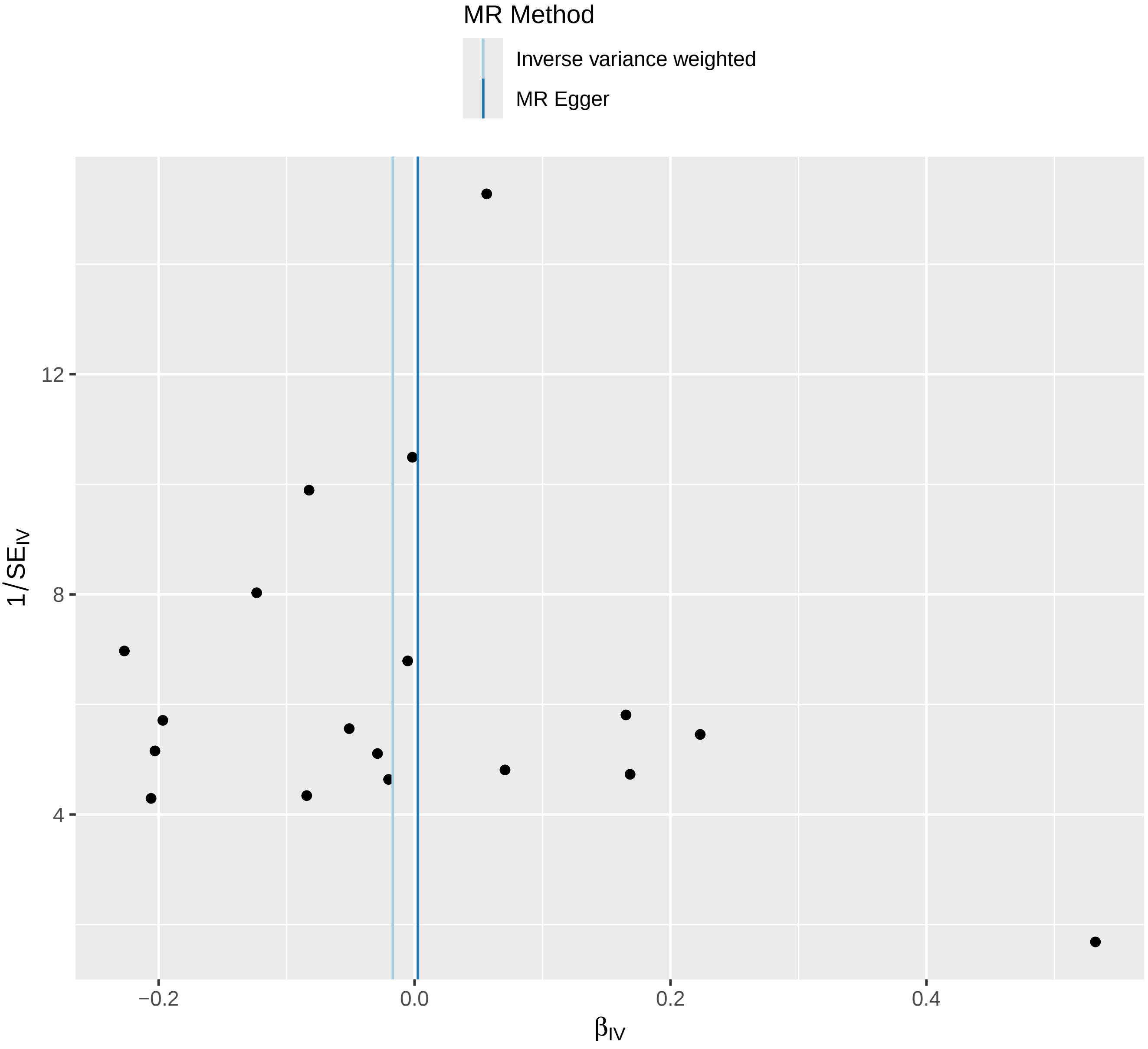
MR Method
Inverse variance weighted
MR Egger
12
8
4
1/SE_IV
−0.2
0.0
0.2
0.4
β_IV

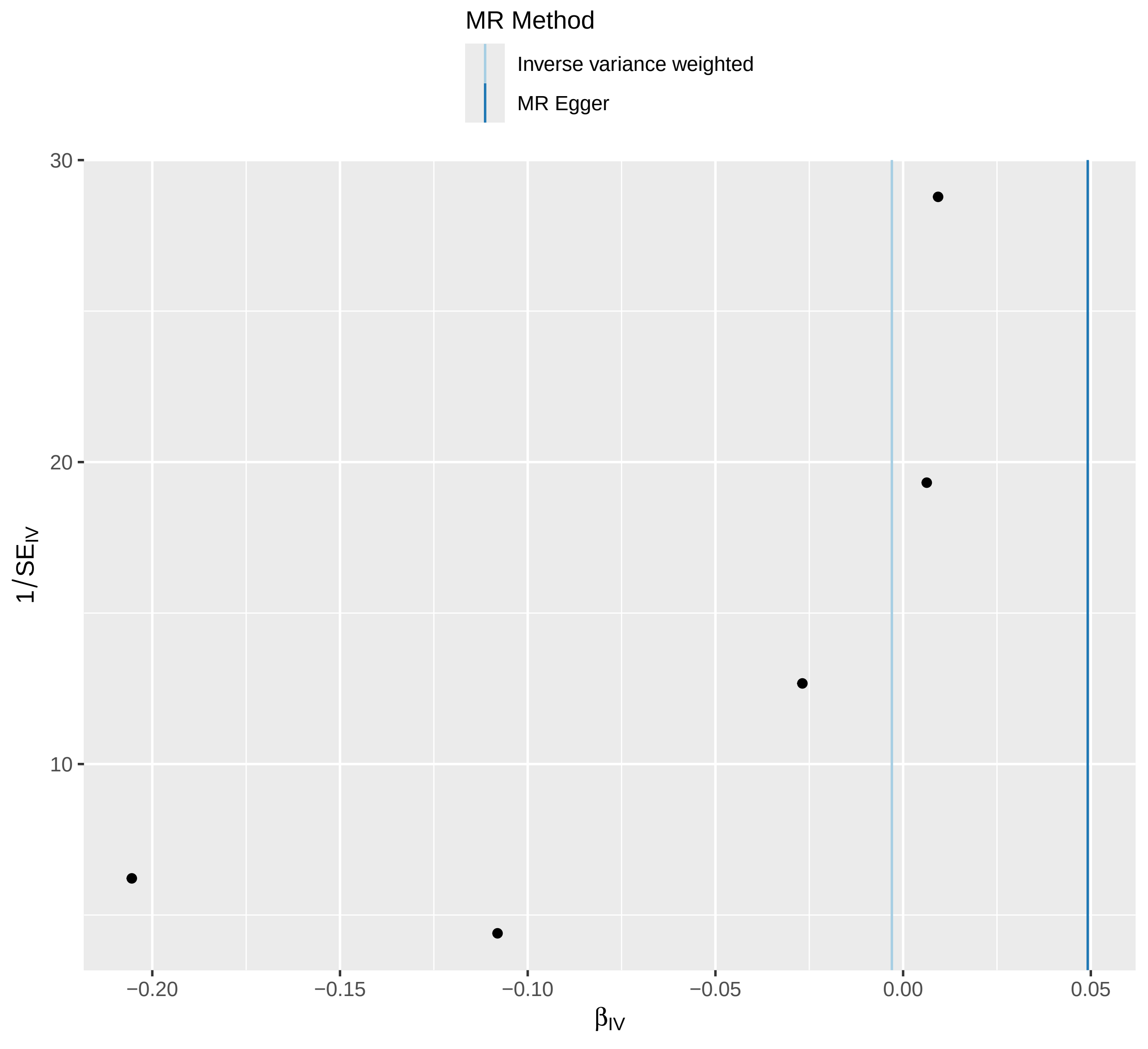
MR Method
Inverse variance weighted
MR Egger
30
20
10
1/SE_IV
−0.20
−0.15
−0.10
−0.05
0.00
0.05
β_IV

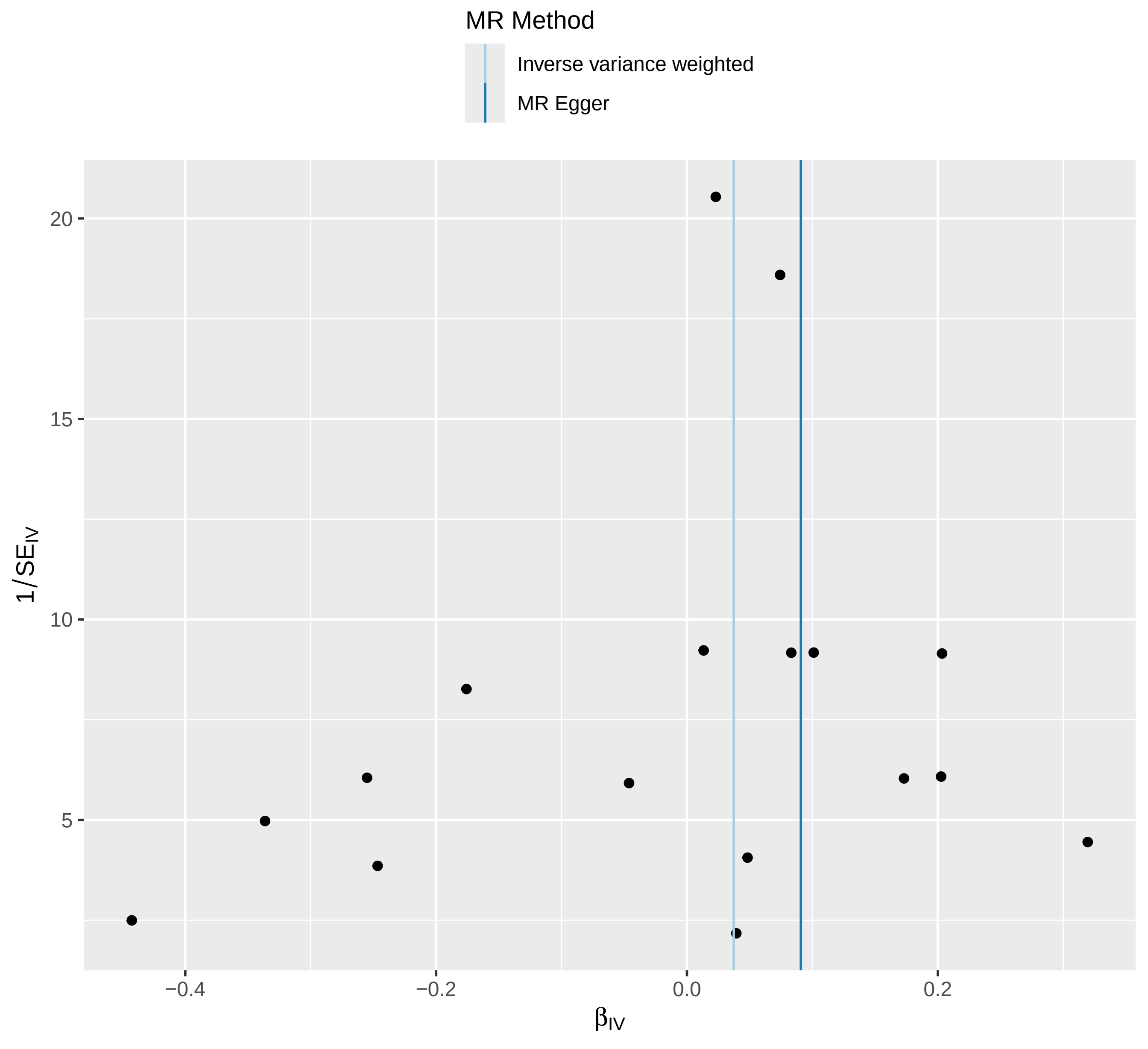

MR Method
Inverse variance weighted
MR Egger
20
15
10
5
1/SE_IV
−0.4
−0.2
0.0
0.2
β_IV

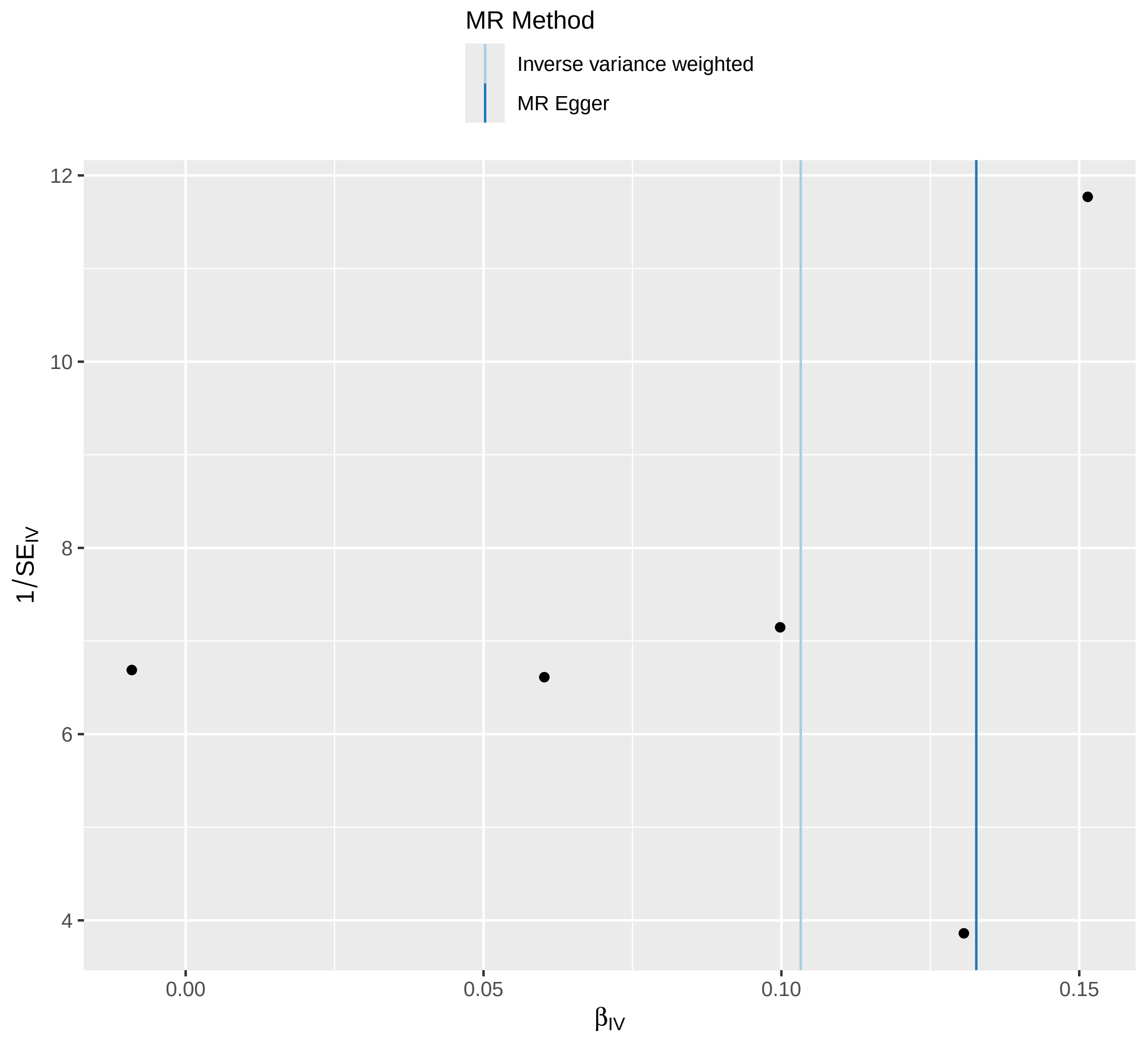
MR Method
Inverse variance weighted
MR Egger
12
10
8
6
4
1/SE_IV
0.00
0.05
0.10
0.15
β_IV

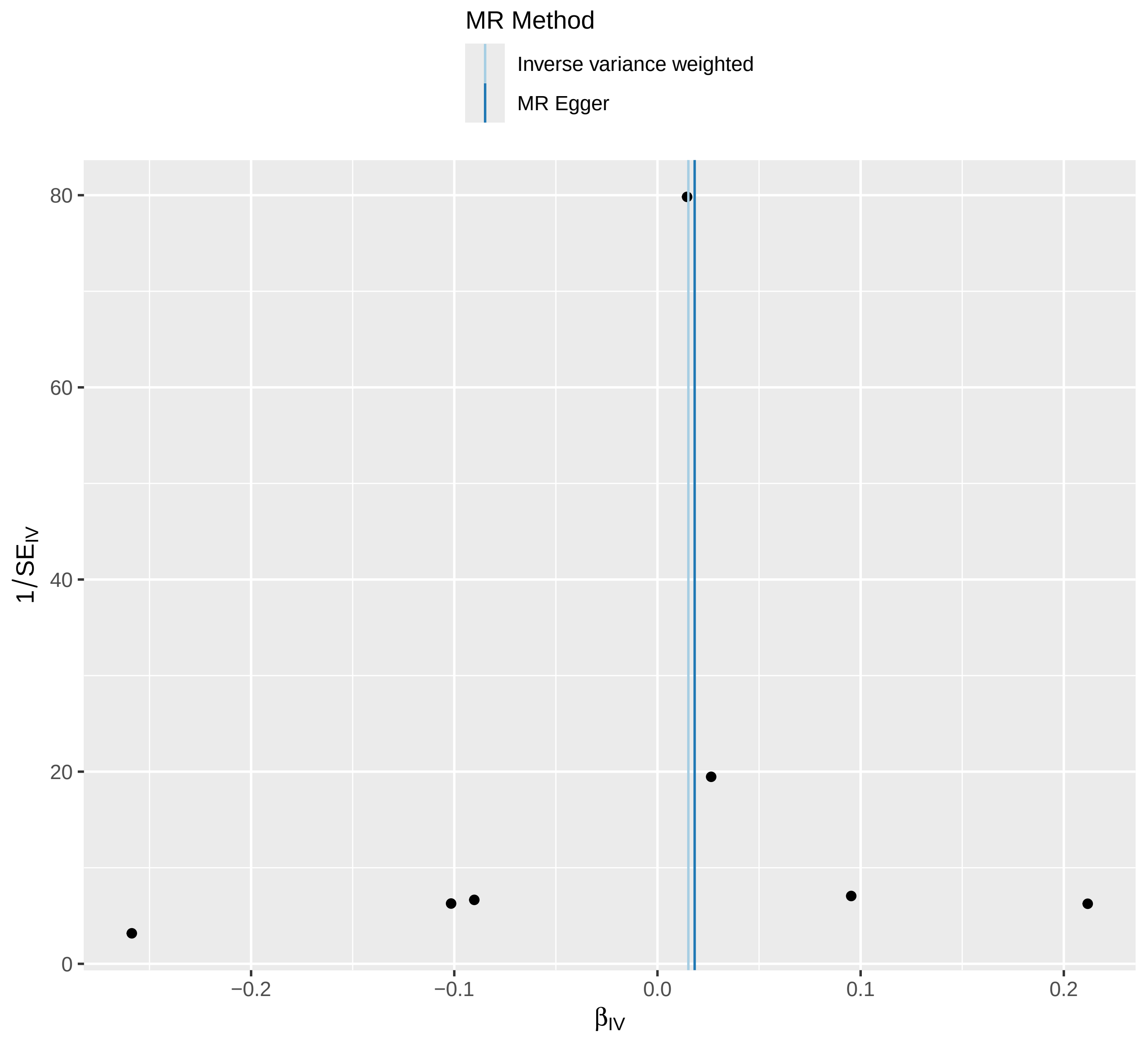
MR Method
Inverse variance weighted
MR Egger
80
60
40
20
0
1/SE_IV
−0.2
−0.1
0.0
0.1
0.2
β_IV

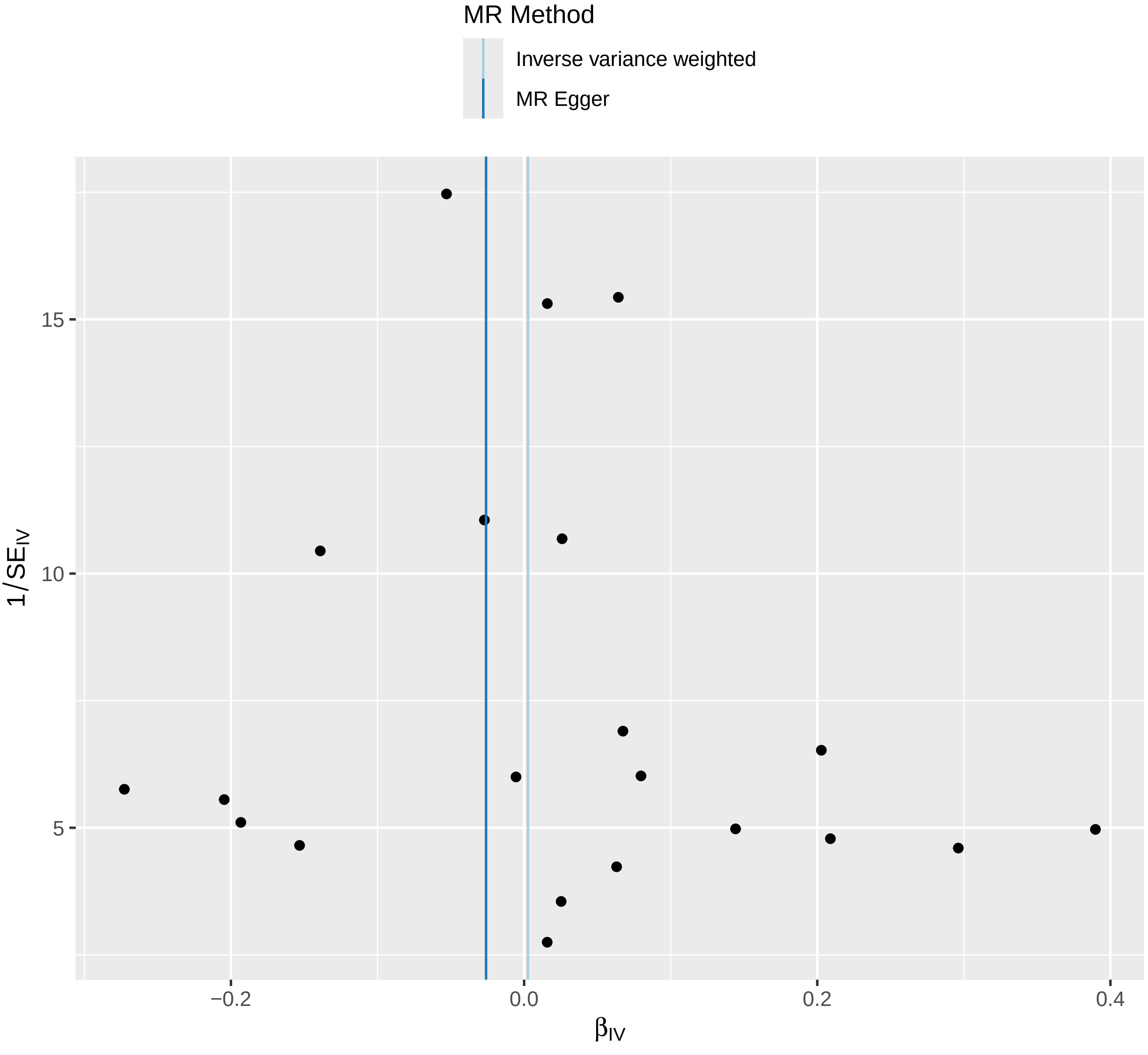
MR Method
Inverse variance weighted
MR Egger
15
10
5
1/SE_IV
−0.2
0.0
0.2
0.4
β_IV

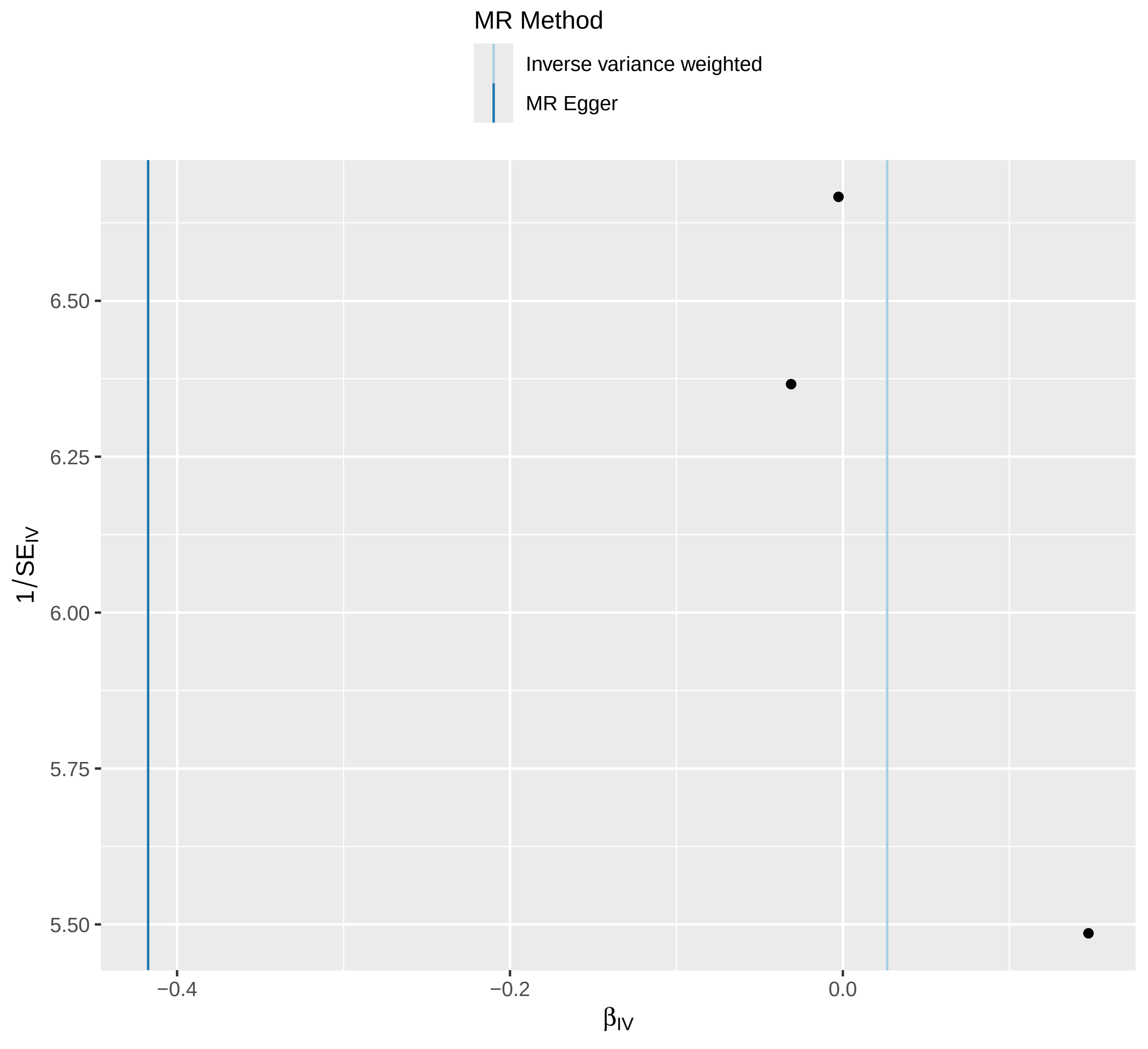
MR Method
Inverse variance weighted
MR Egger
6.50
6.25
6.00
5.75
5.50
1/SE$_{IV}$
−0.4
−0.2
0.0
β$_{IV}$

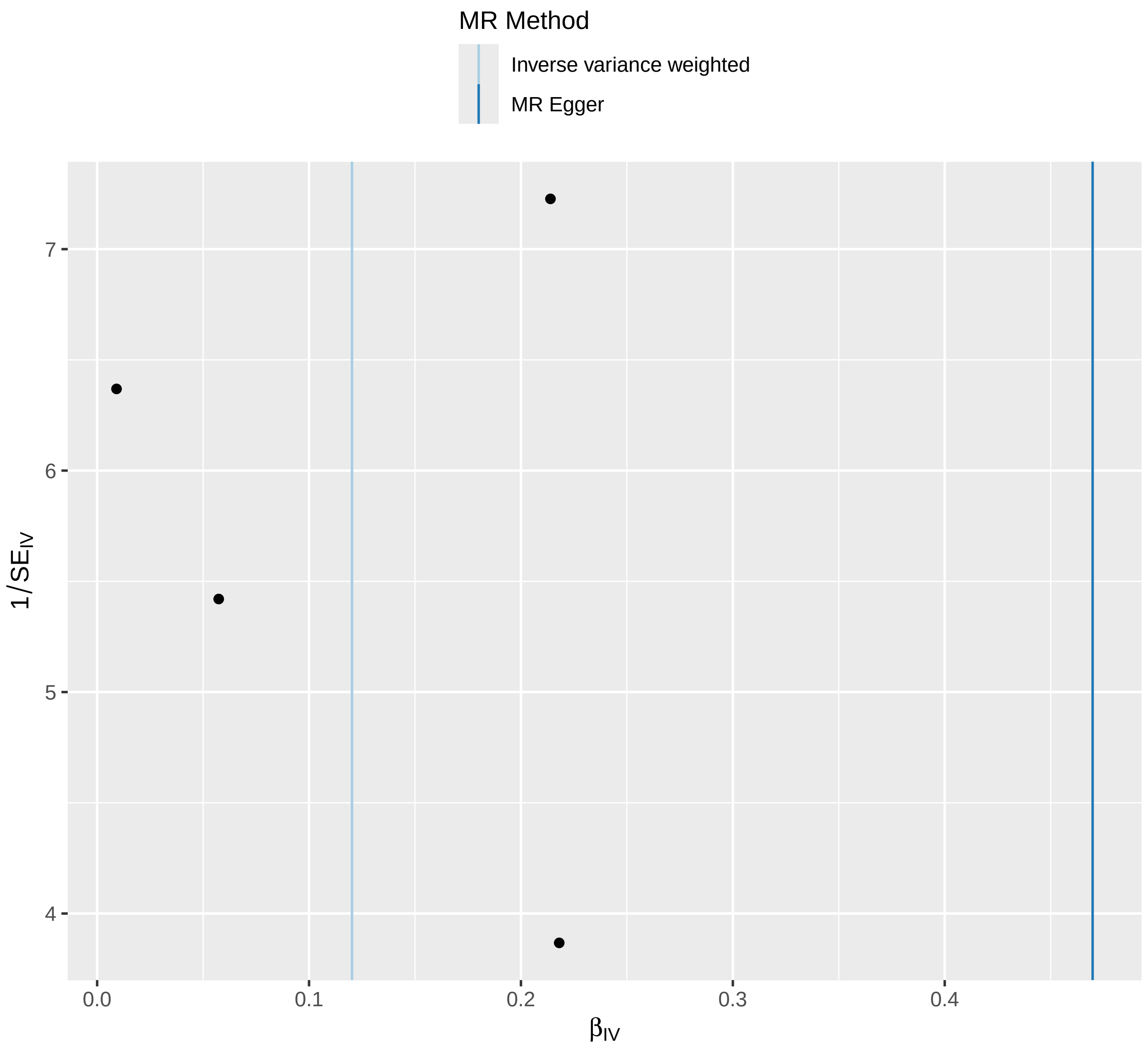

MR Method
Inverse variance weighted
MR Egger
7
6
5
4
1/SE$_{IV}$
0.0
0.1
0.2
0.3
0.4
β$_{IV}$

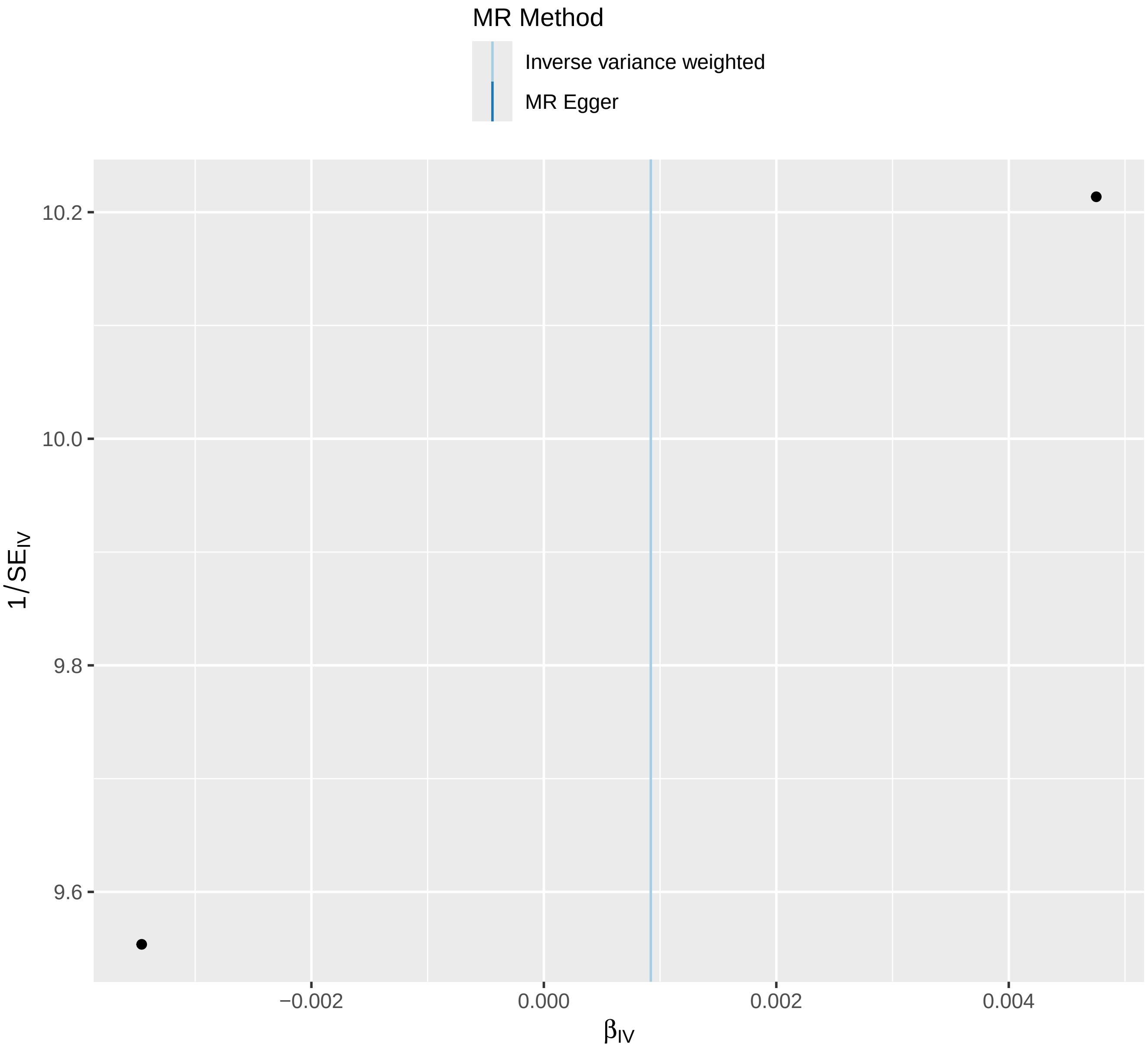
MR Method
Inverse variance weighted
MR Egger
10.2
10.0
9.8
9.6
1/SE$_{IV}$
−0.002
0.000
0.002
0.004
β$_{IV}$

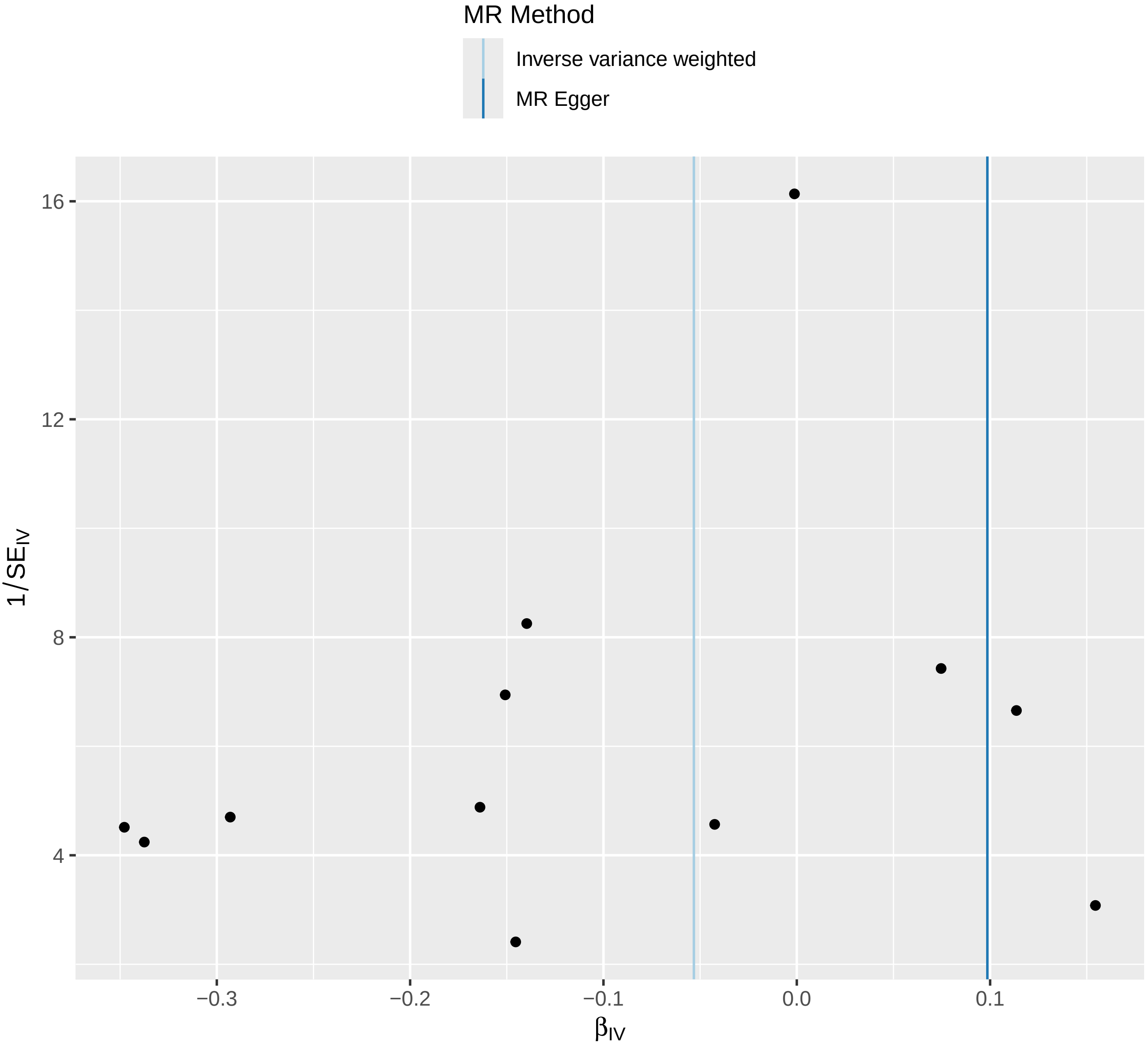

MR Method
Inverse variance weighted
MR Egger
16
12
8
4
1/SE_IV
−0.3
−0.2
−0.1
0.0
0.1
β_IV

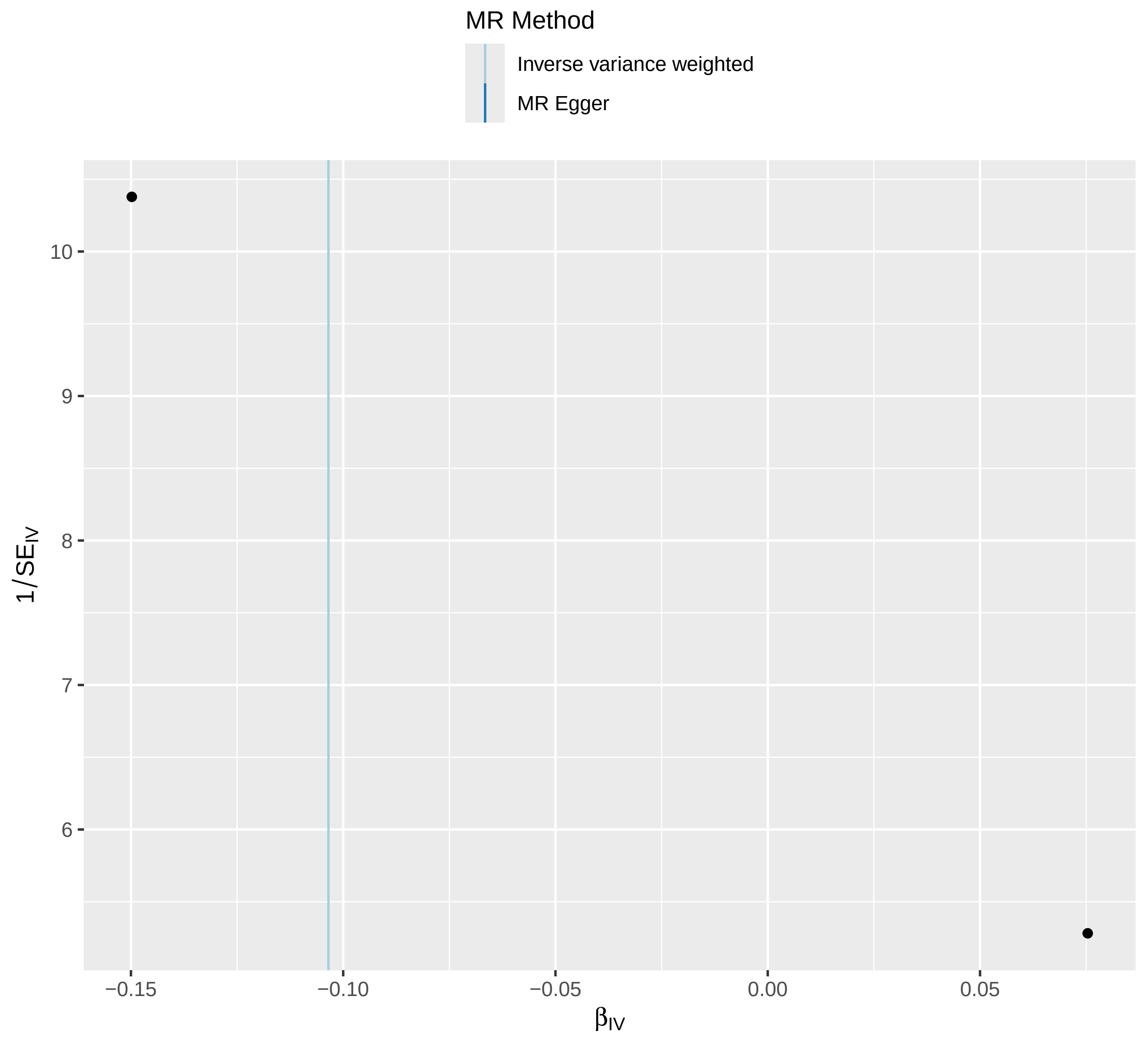
MR Method
Inverse variance weighted
MR Egger
10
9
8
7
6
1/SE_IV
−0.15
−0.10
−0.05
0.00
0.05
β_IV

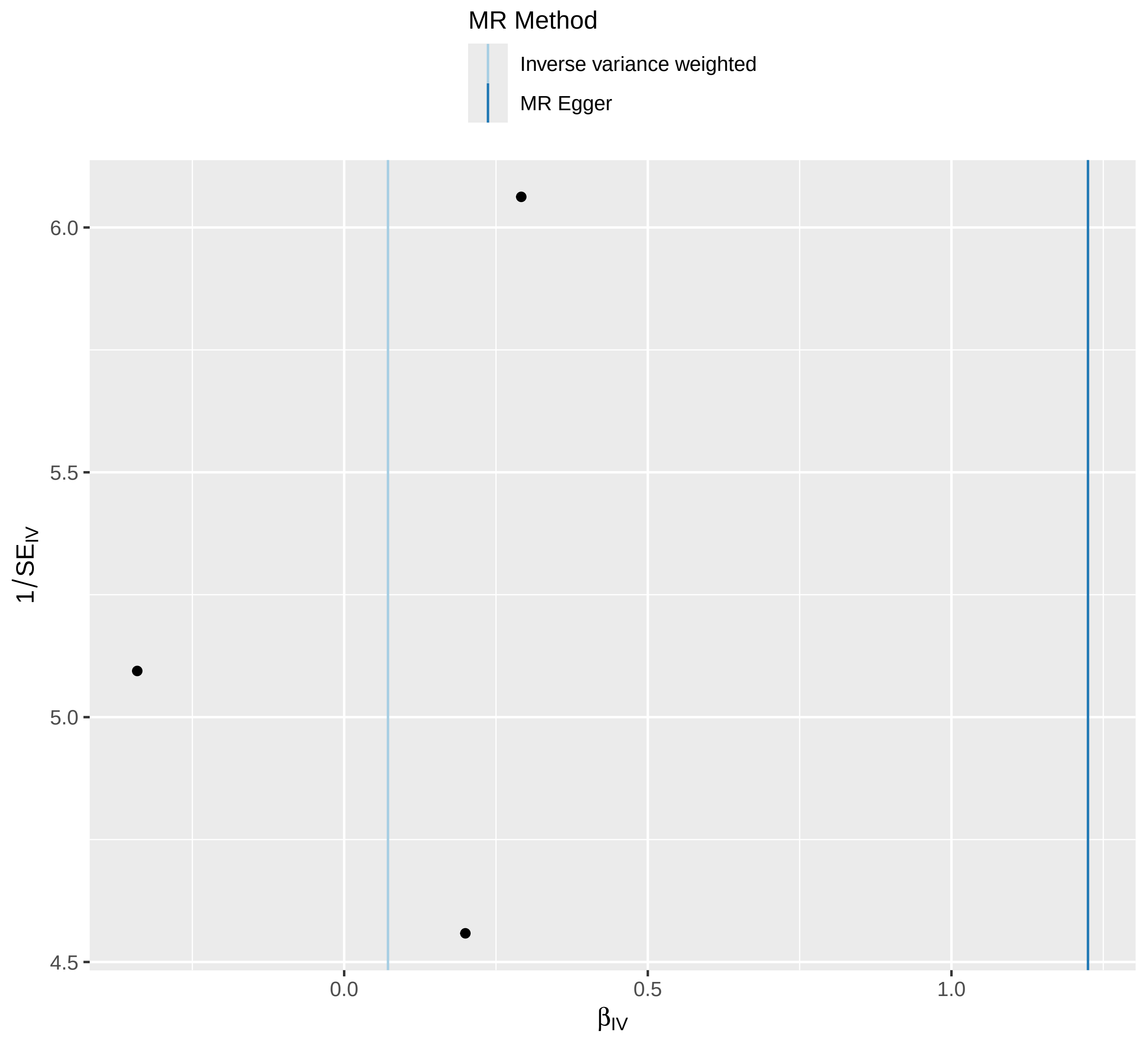

MR Method
Inverse variance weighted
MR Egger
6.0
5.5
5.0
4.5
1/SE$_{IV}$
0.0
0.5
1.0
β$_{IV}$

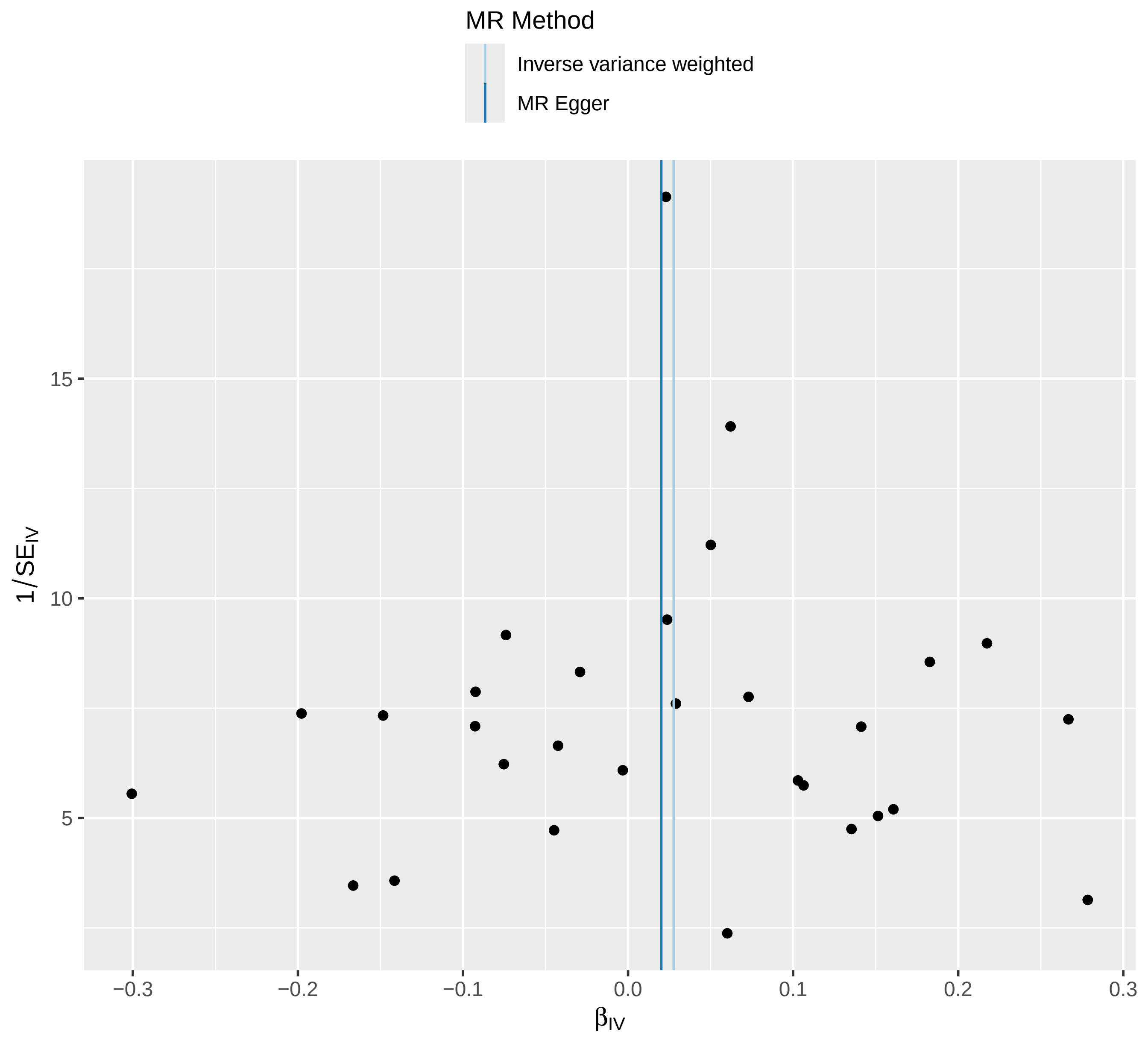
MR Method
Inverse variance weighted
MR Egger
15
10
5
−0.3
−0.2
−0.1
0.0
0.1
0.2
0.3
1/SE_IV
β_IV

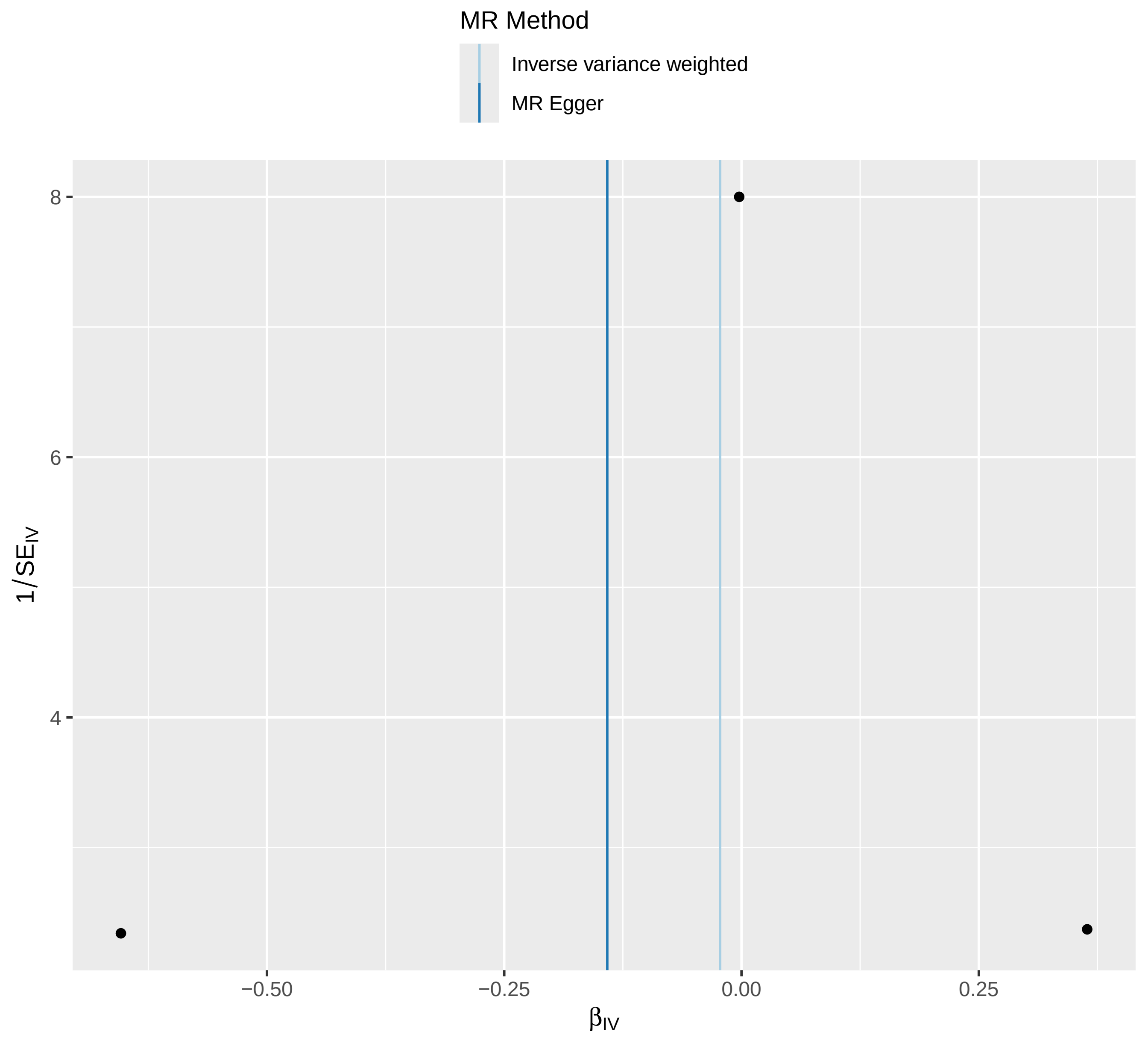
MR Method
Inverse variance weighted
MR Egger
8
6
4
1/SE_IV
−0.50
−0.25
0.00
0.25
β_IV

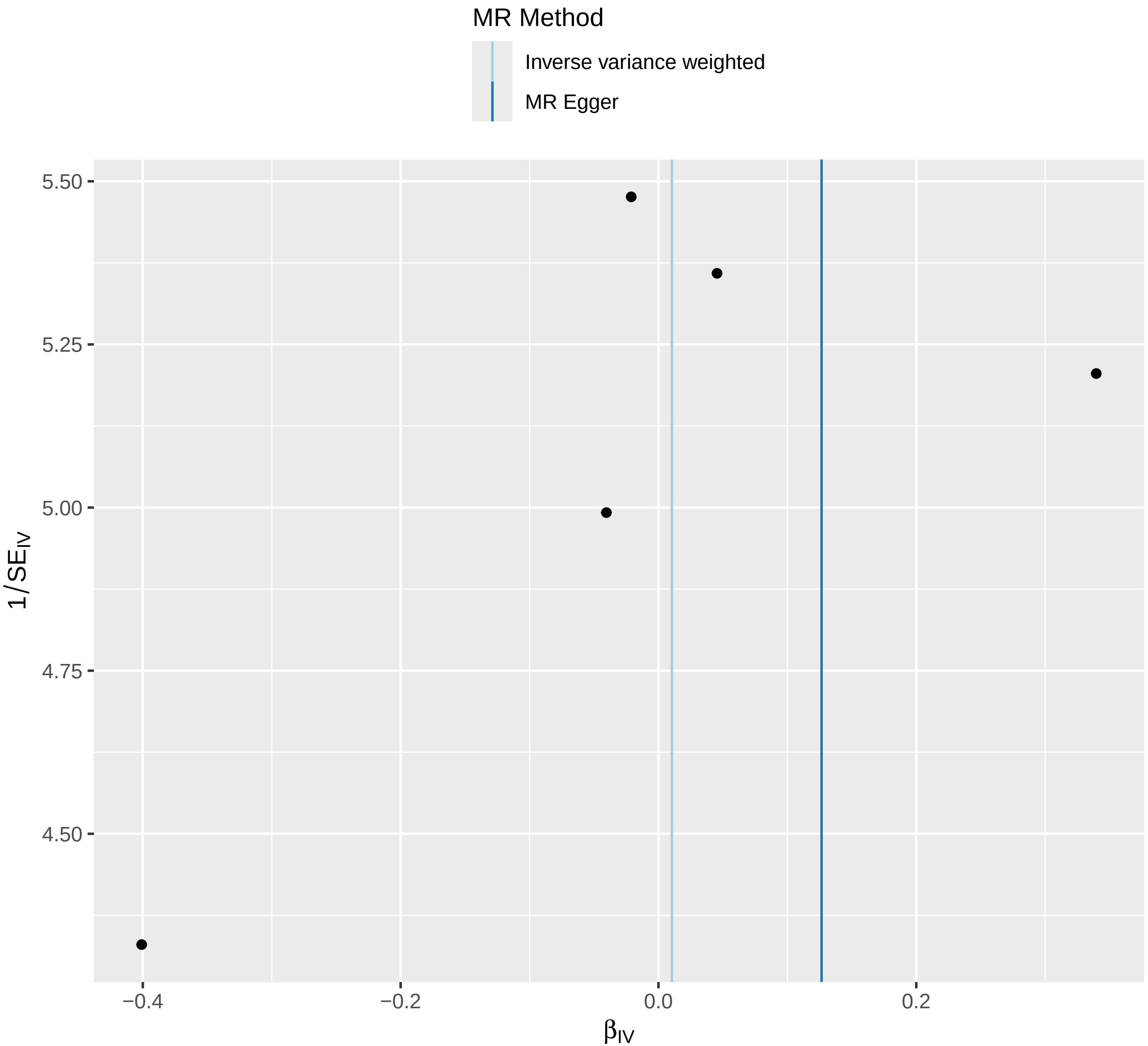

MR Method
Inverse variance weighted
MR Egger
5.50
5.25
5.00
4.75
4.50
$1/SE_{IV}$
−0.4
−0.2
0.0
0.2
$\beta_{IV}$

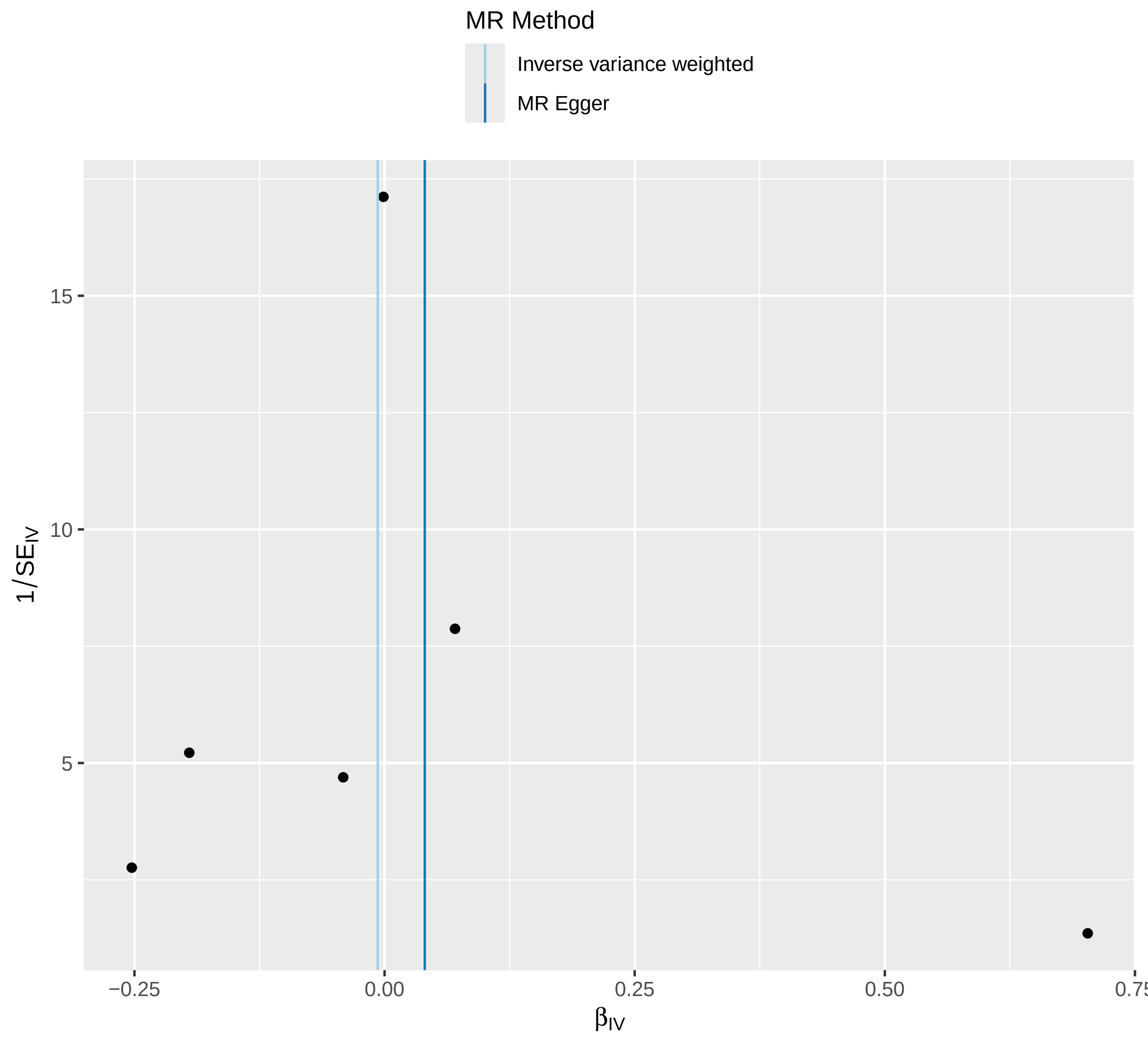

MR Method
Inverse variance weighted
MR Egger
15
10
5
1/SE$_{IV}$
−0.25
0.00
0.25
0.50
0.75
β$_{IV}$

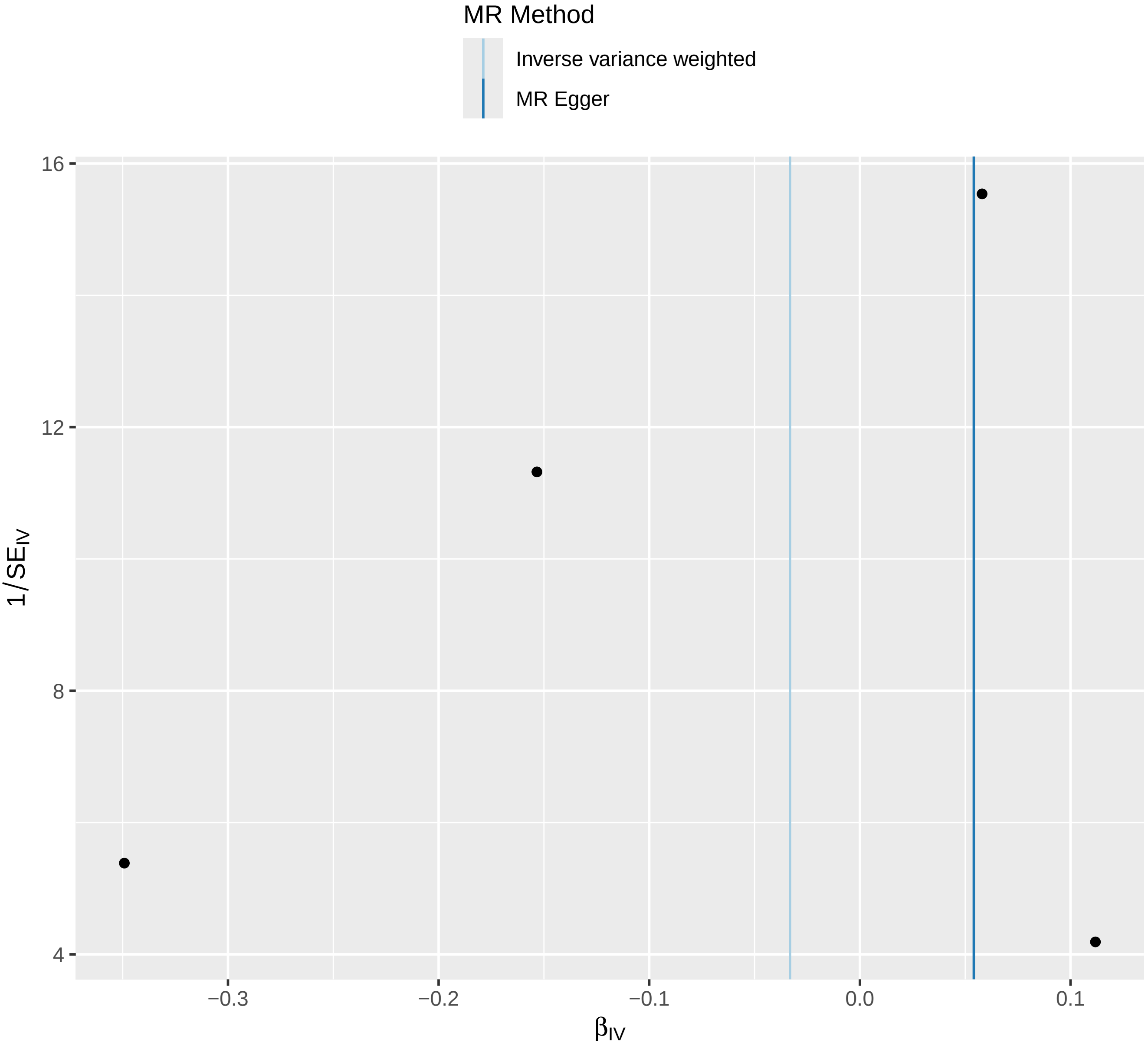

MR Method
Inverse variance weighted
MR Egger
16
12
8
4
1/SE$_{IV}$
−0.3
−0.2
−0.1
0.0
0.1
β$_{IV}$

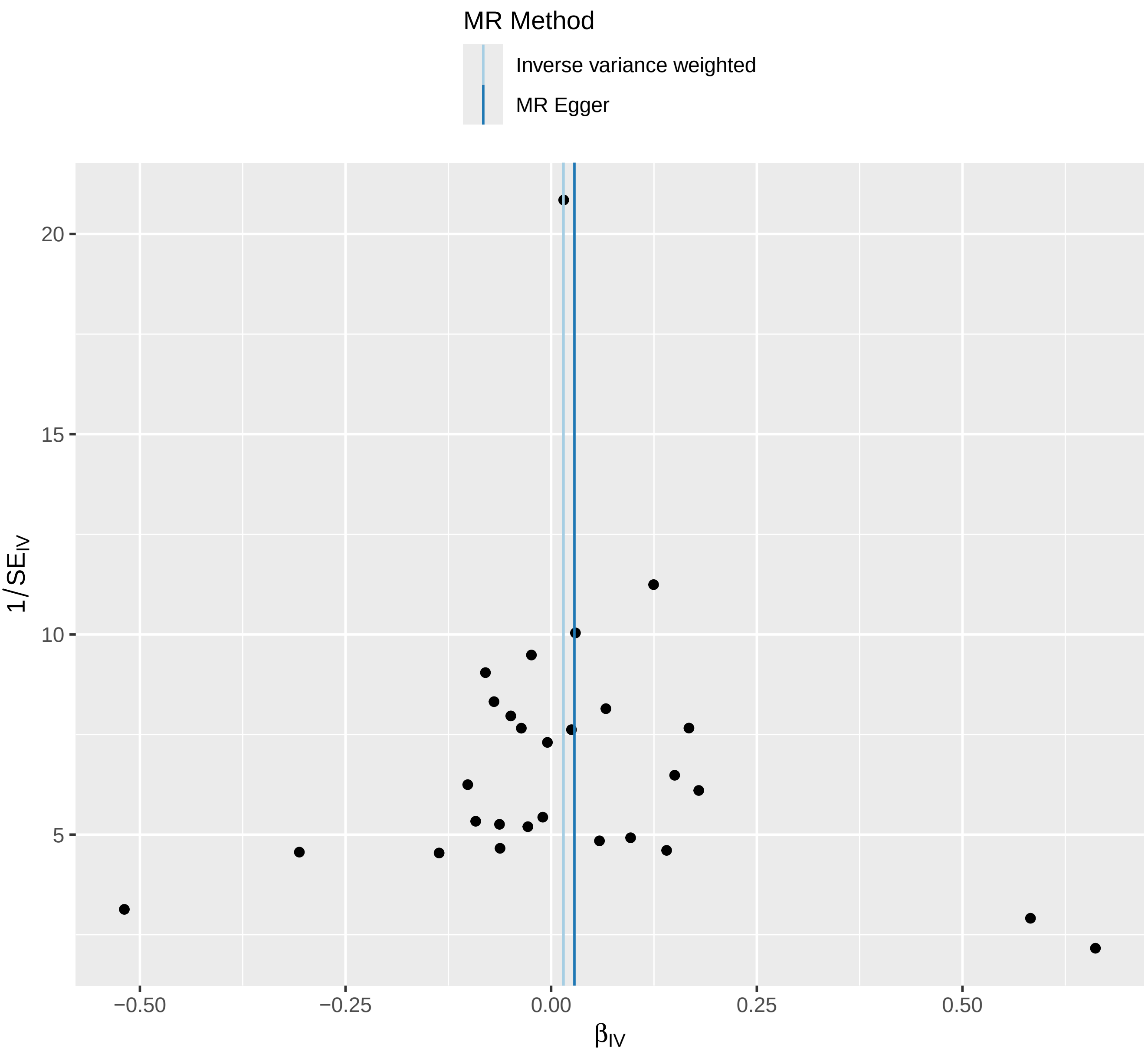
MR Method
Inverse variance weighted
MR Egger
1/SE$_{IV}$
20
15
10
5
−0.50
−0.25
0.00
0.25
0.50
β$_{IV}$

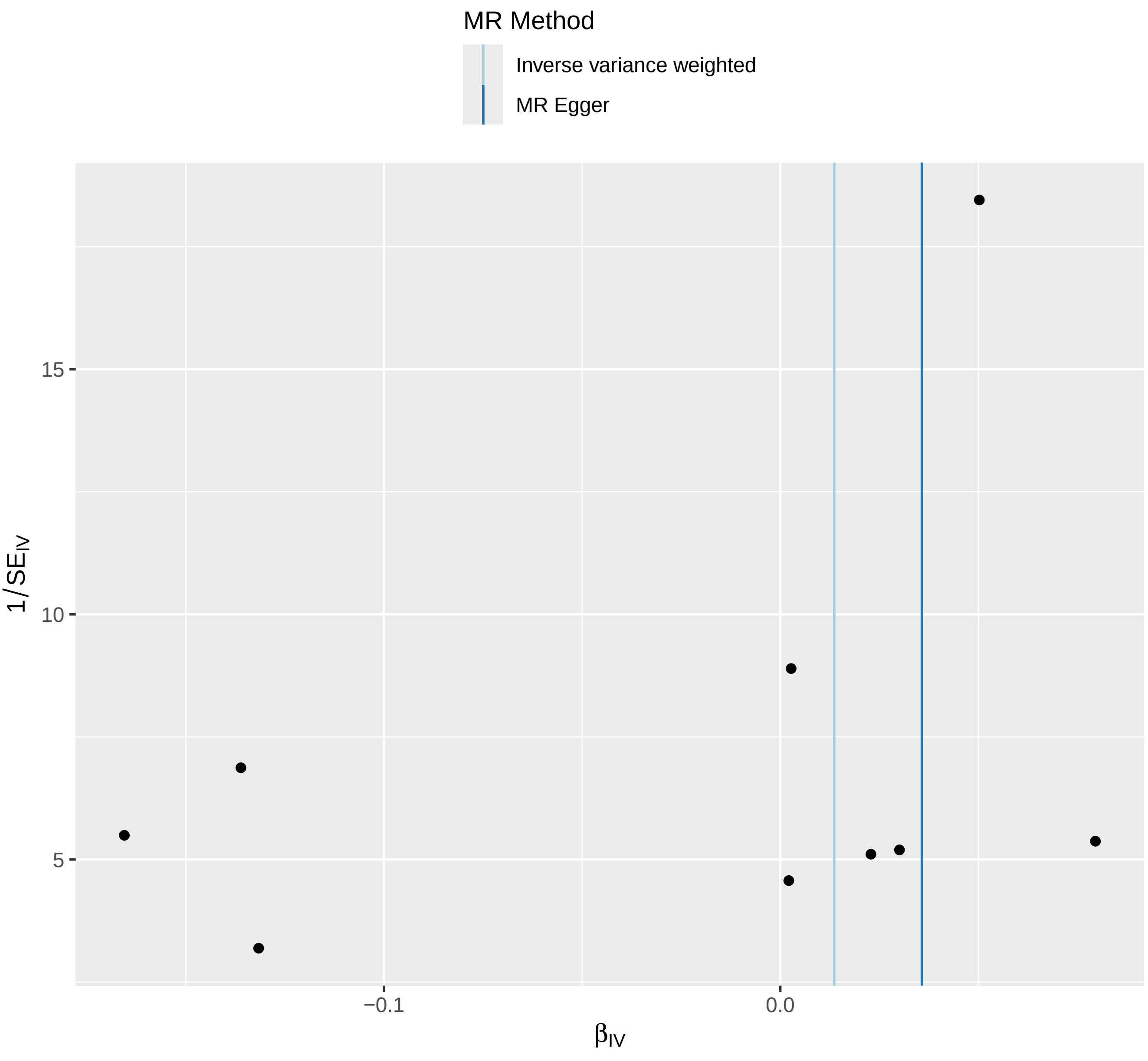
MR Method
Inverse variance weighted
MR Egger
15
10
5
1/SE_IV
−0.1
0.0
β_IV

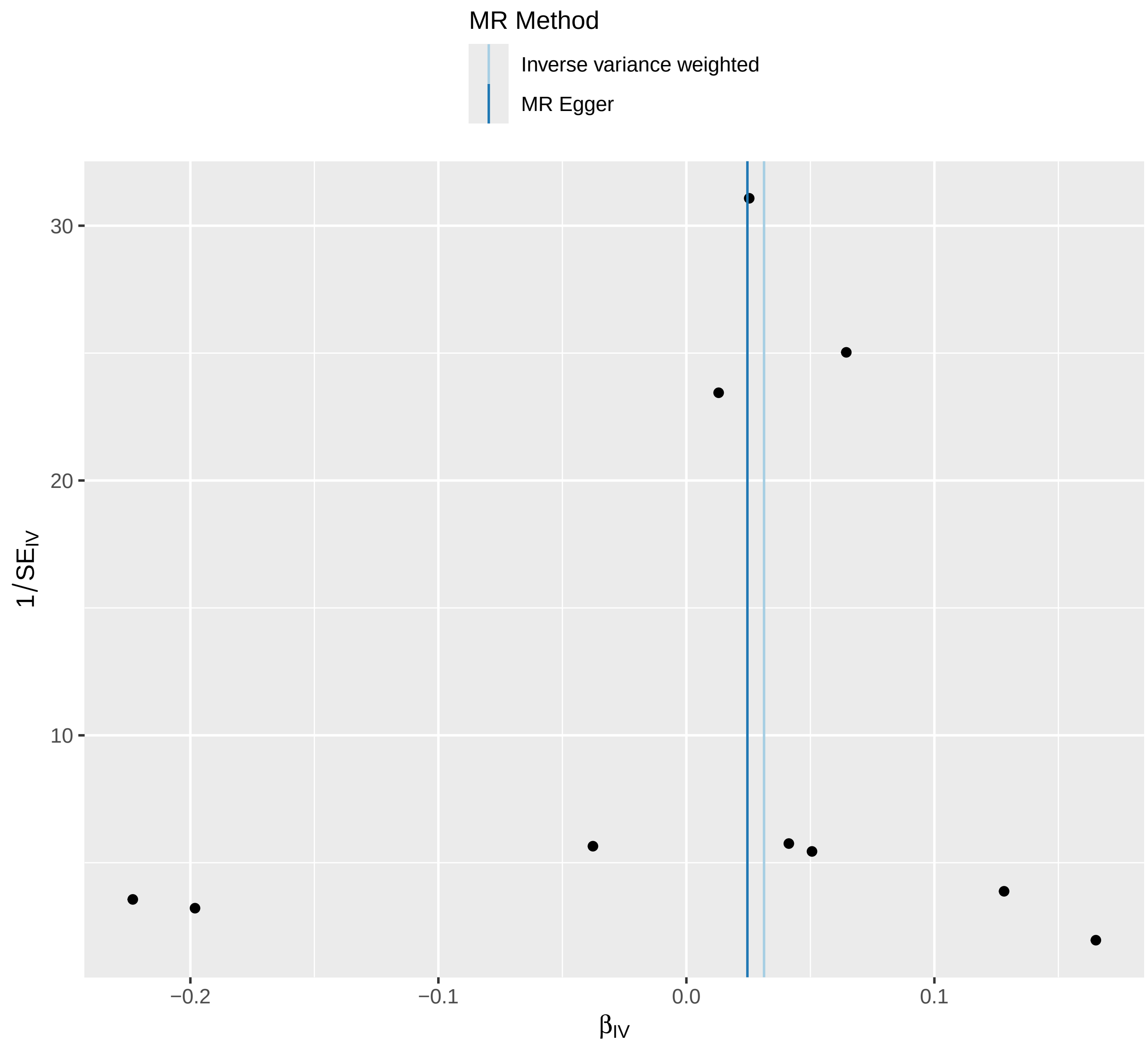

MR Method
Inverse variance weighted
MR Egger
30
20
10
1/SE$_{IV}$
−0.2
−0.1
0.0
0.1
β$_{IV}$

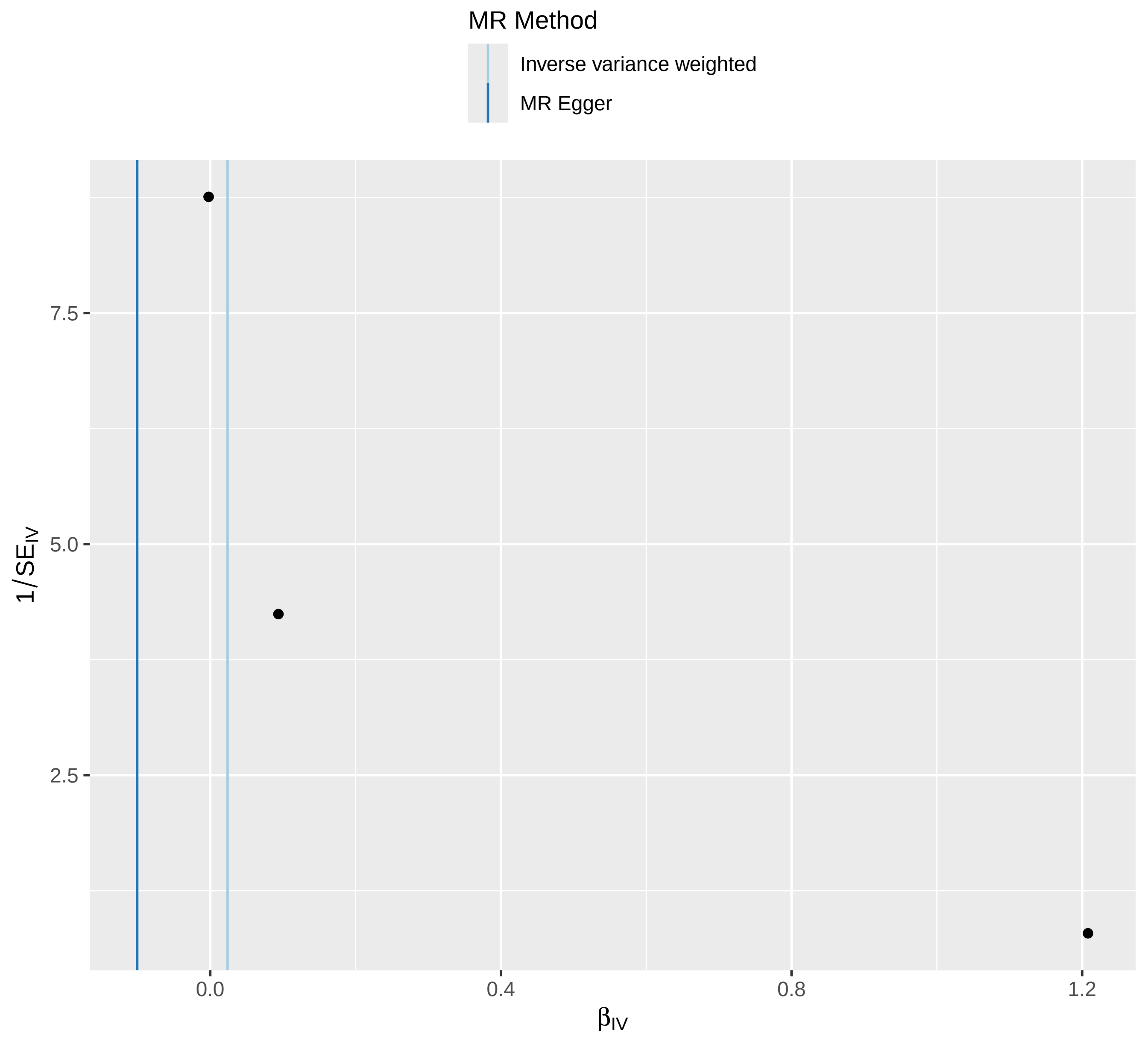

MR Method
Inverse variance weighted
MR Egger
7.5
5.0
2.5
$1/SE_{IV}$
0.0
0.4
0.8
1.2
$\beta_{IV}$

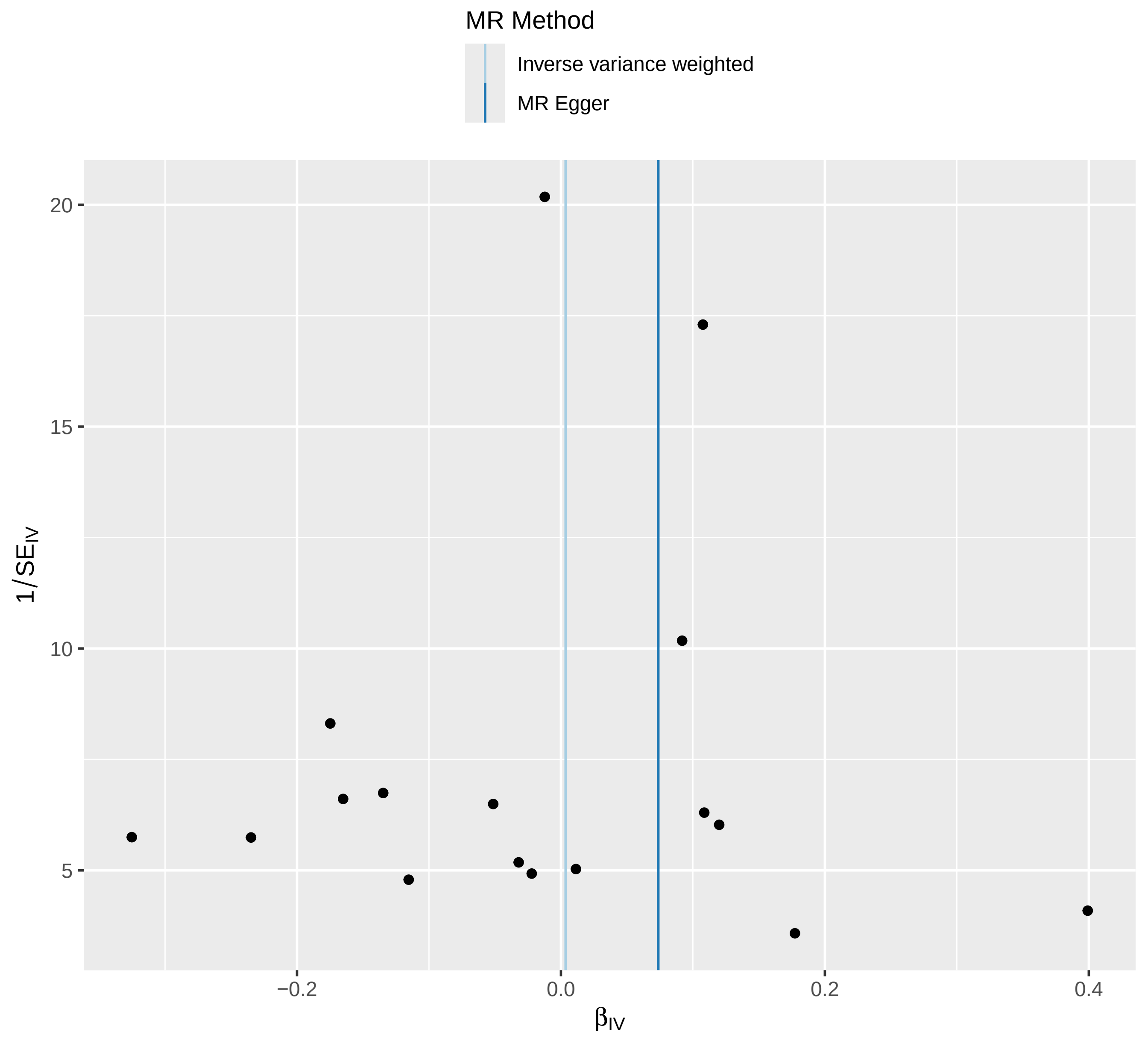
MR Method
Inverse variance weighted
MR Egger
20
15
10
5
1/SE_IV
−0.2
0.0
0.2
0.4
β_IV

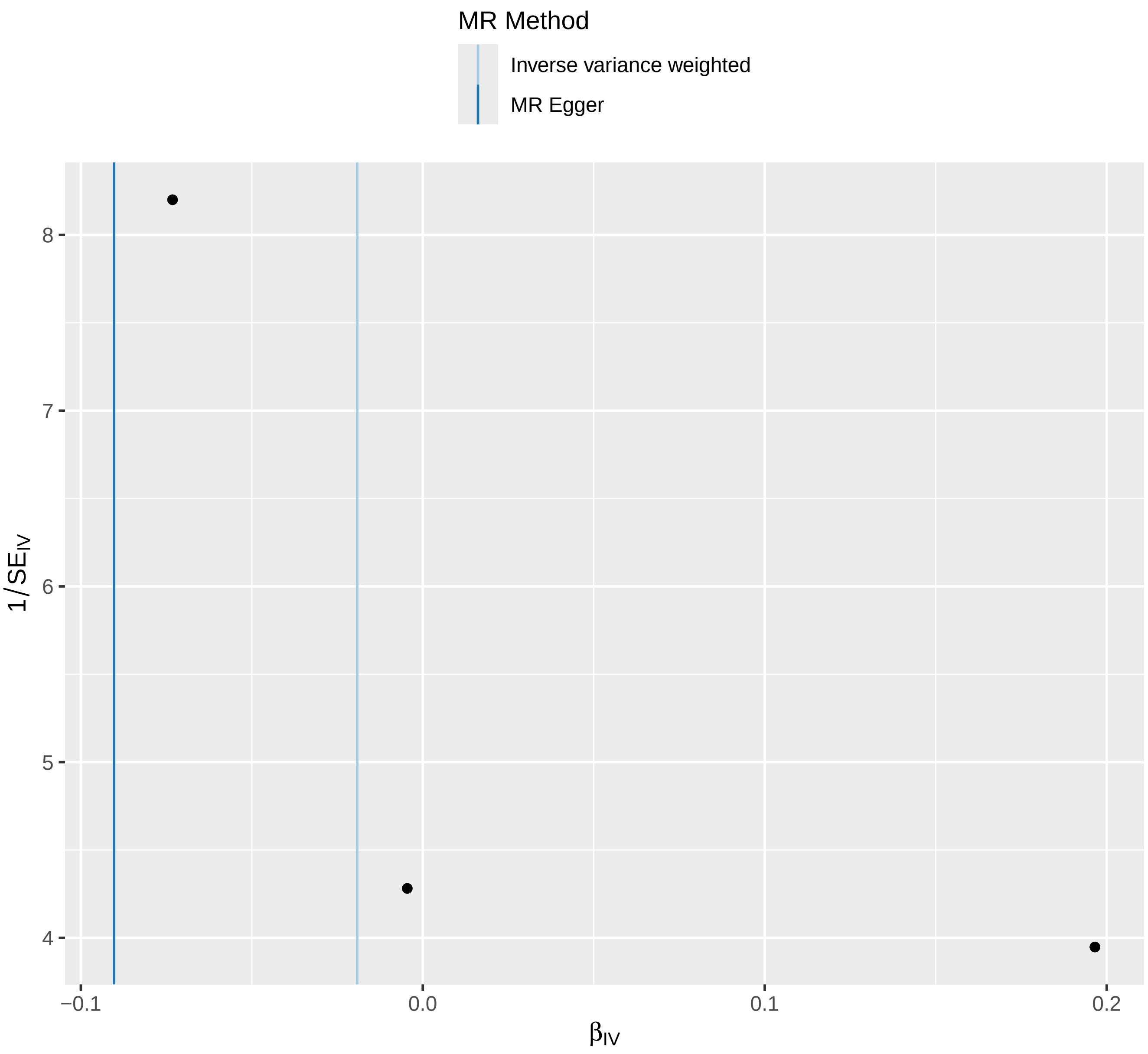

MR Method
Inverse variance weighted
MR Egger
8
7
6
5
4
1/SE_IV
−0.1
0.0
0.1
0.2
β_IV

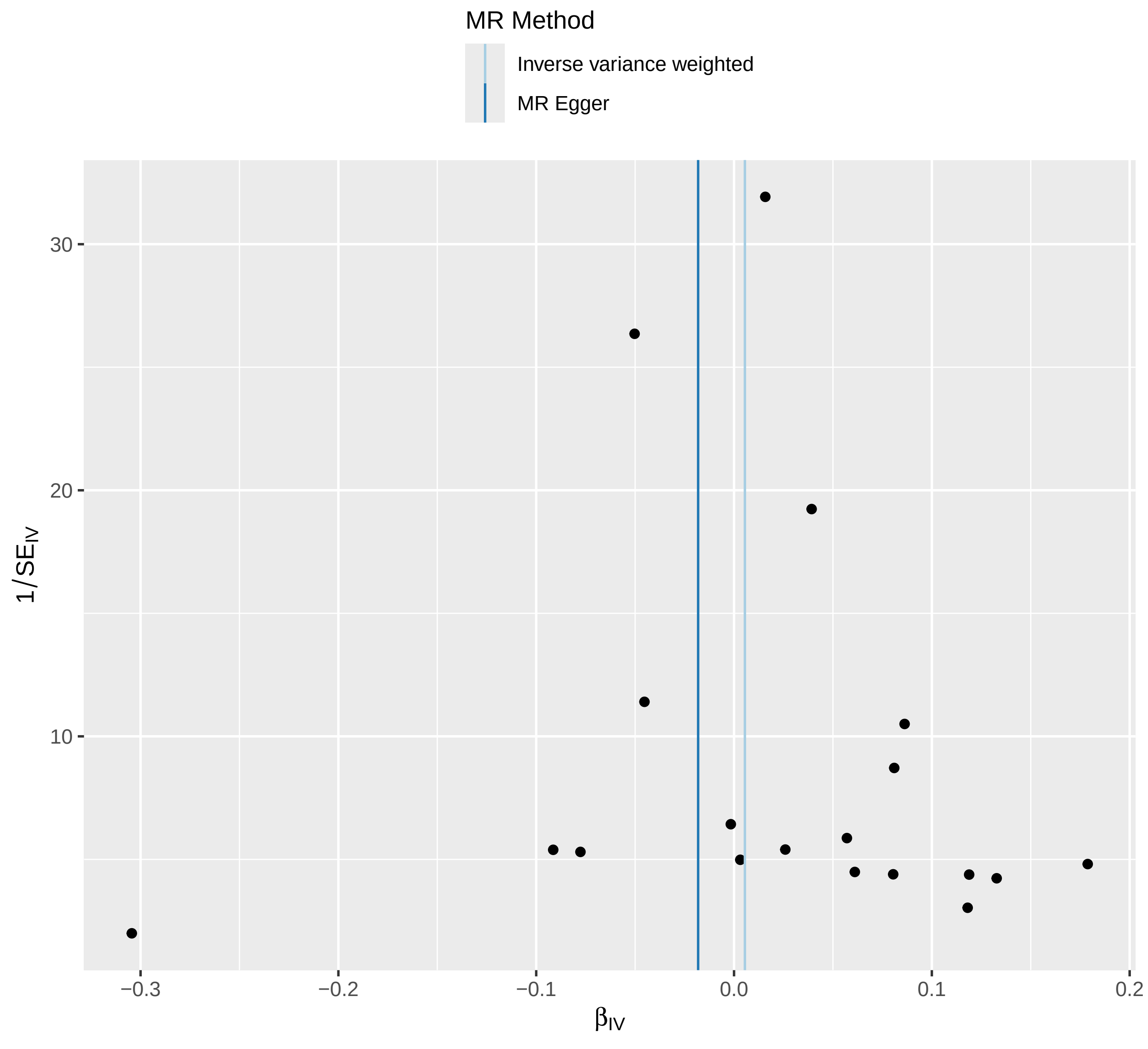
MR Method
Inverse variance weighted
MR Egger
1/SE_IV
30
20
10
β_IV
−0.3
−0.2
−0.1
0.0
0.1
0.2

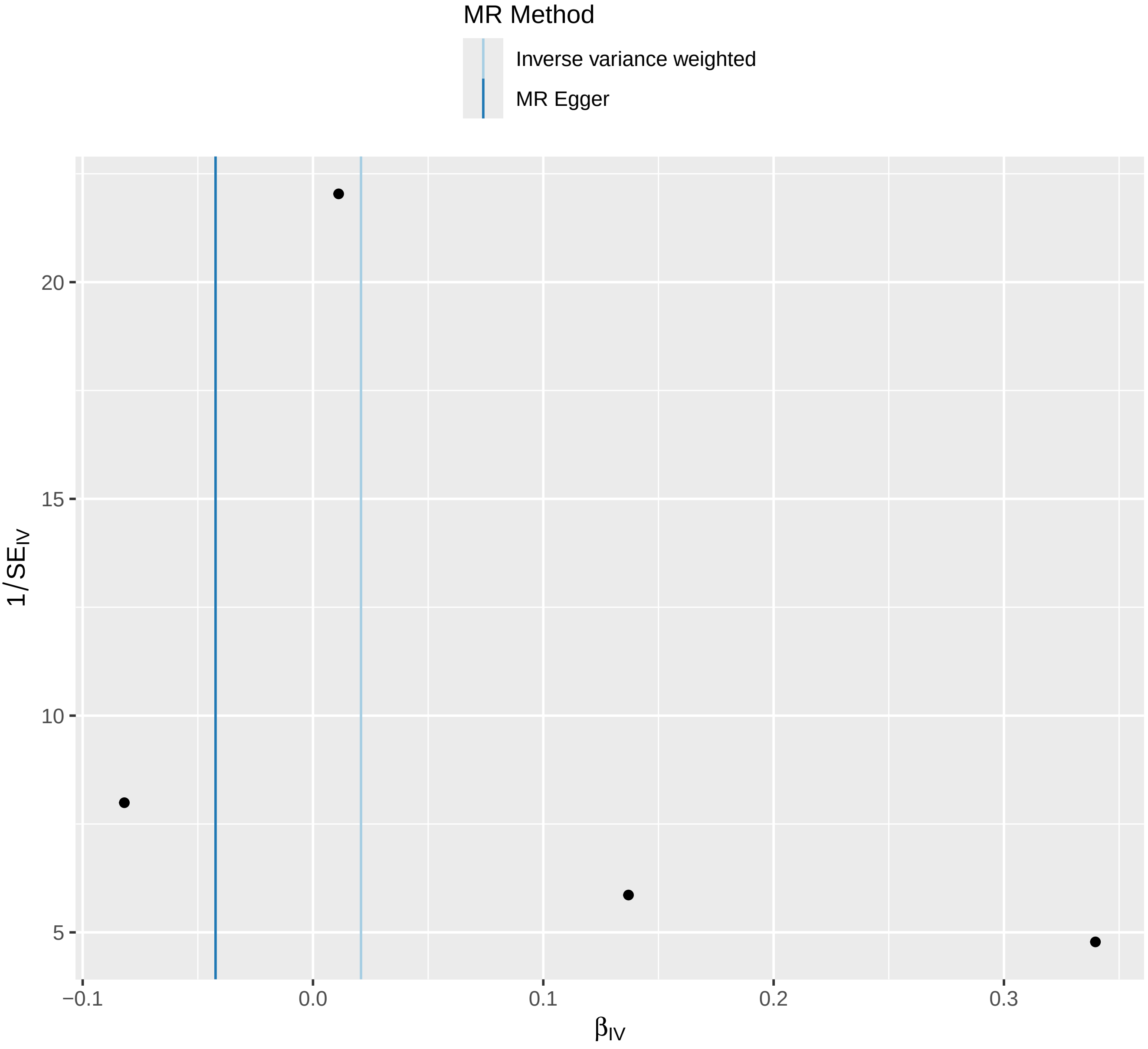
MR Method
Inverse variance weighted
MR Egger
20
15
10
5
1/SE_IV
−0.1
0.0
0.1
0.2
0.3
β_IV

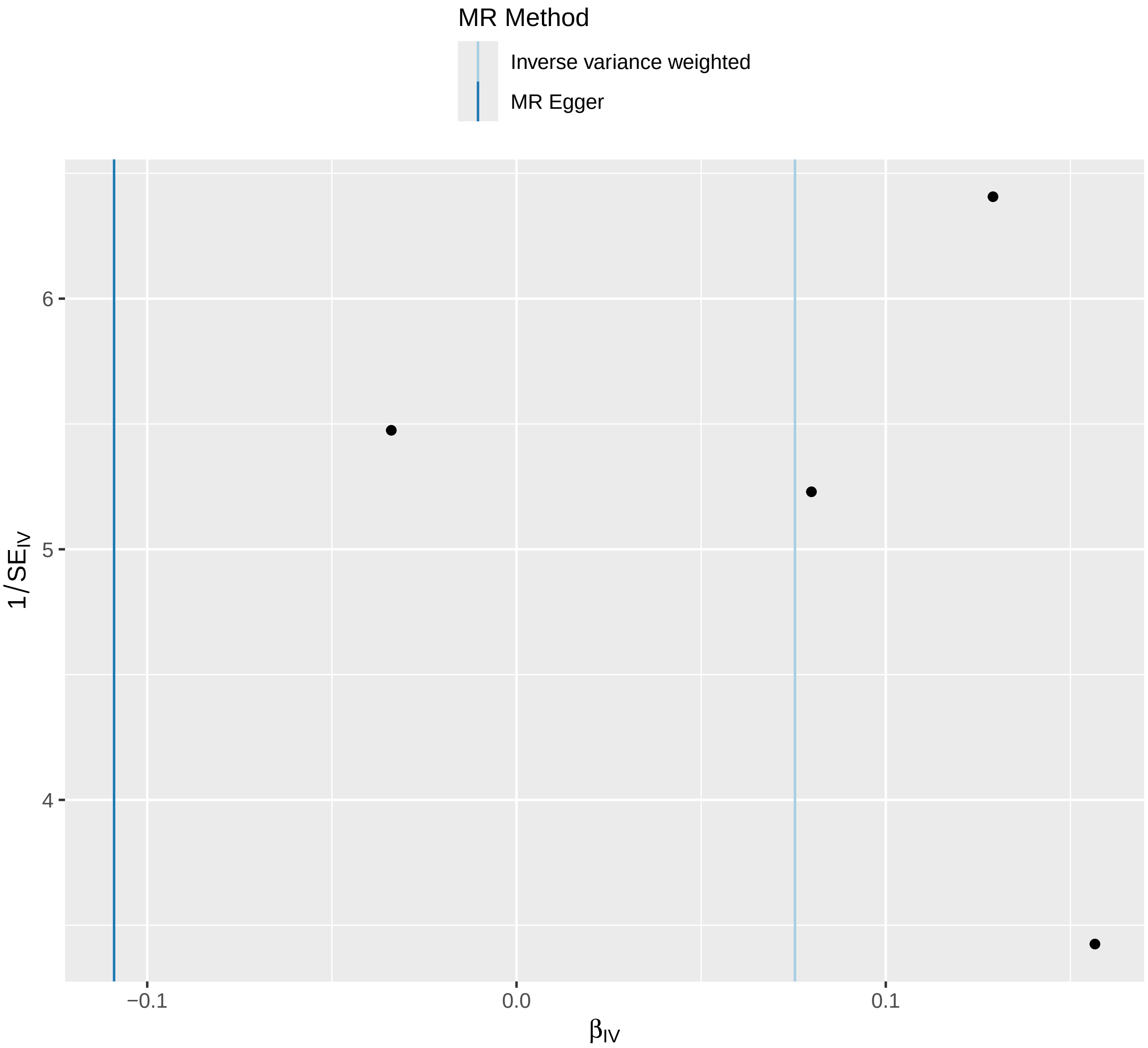

MR Method
Inverse variance weighted
MR Egger
6
5
4
1/SE_IV
−0.1
0.0
0.1
β_IV

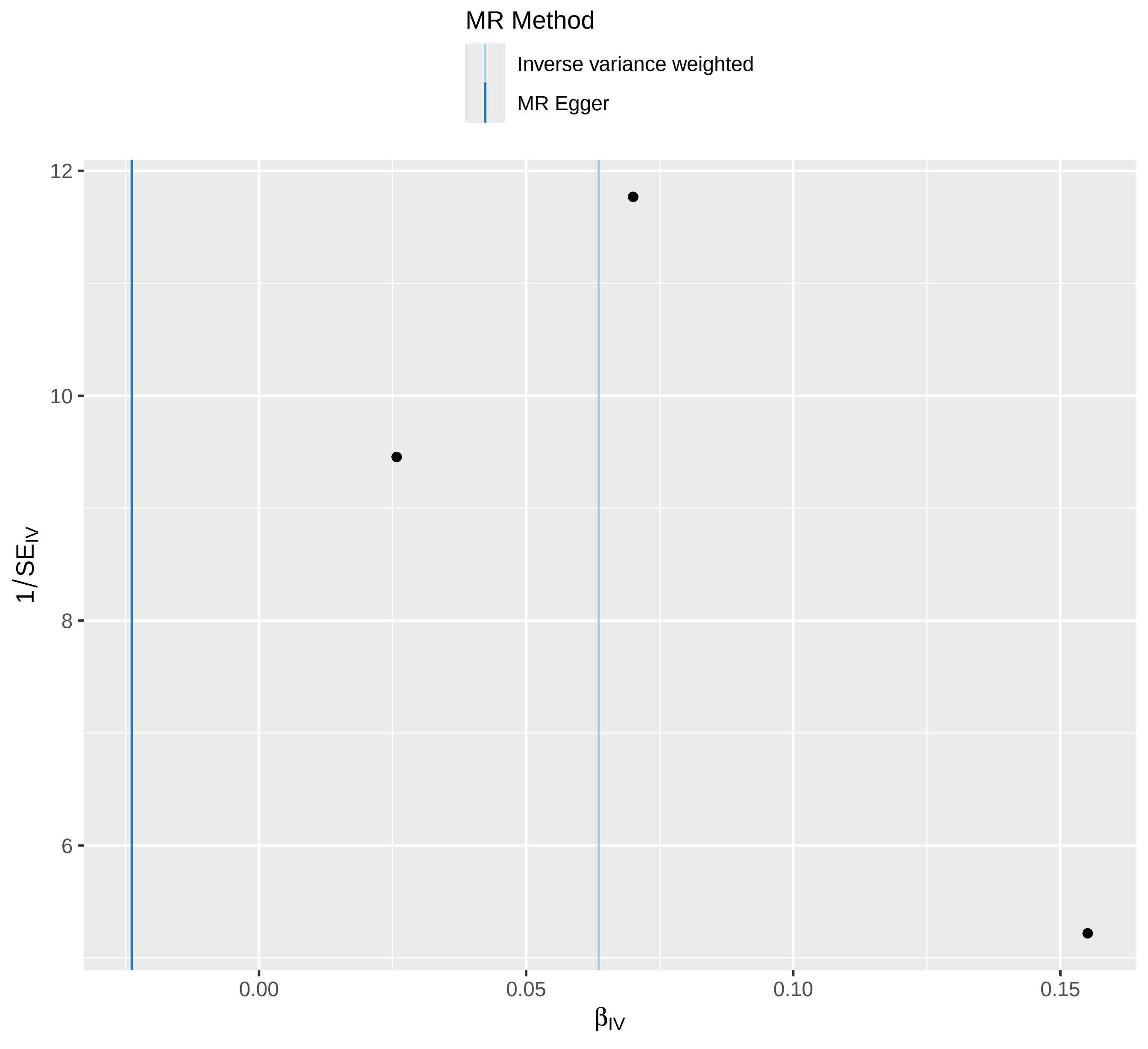
MR Method
Inverse variance weighted
MR Egger
12
10
8
6
1/SE$_{IV}$
0.00
0.05
0.10
0.15
β$_{IV}$

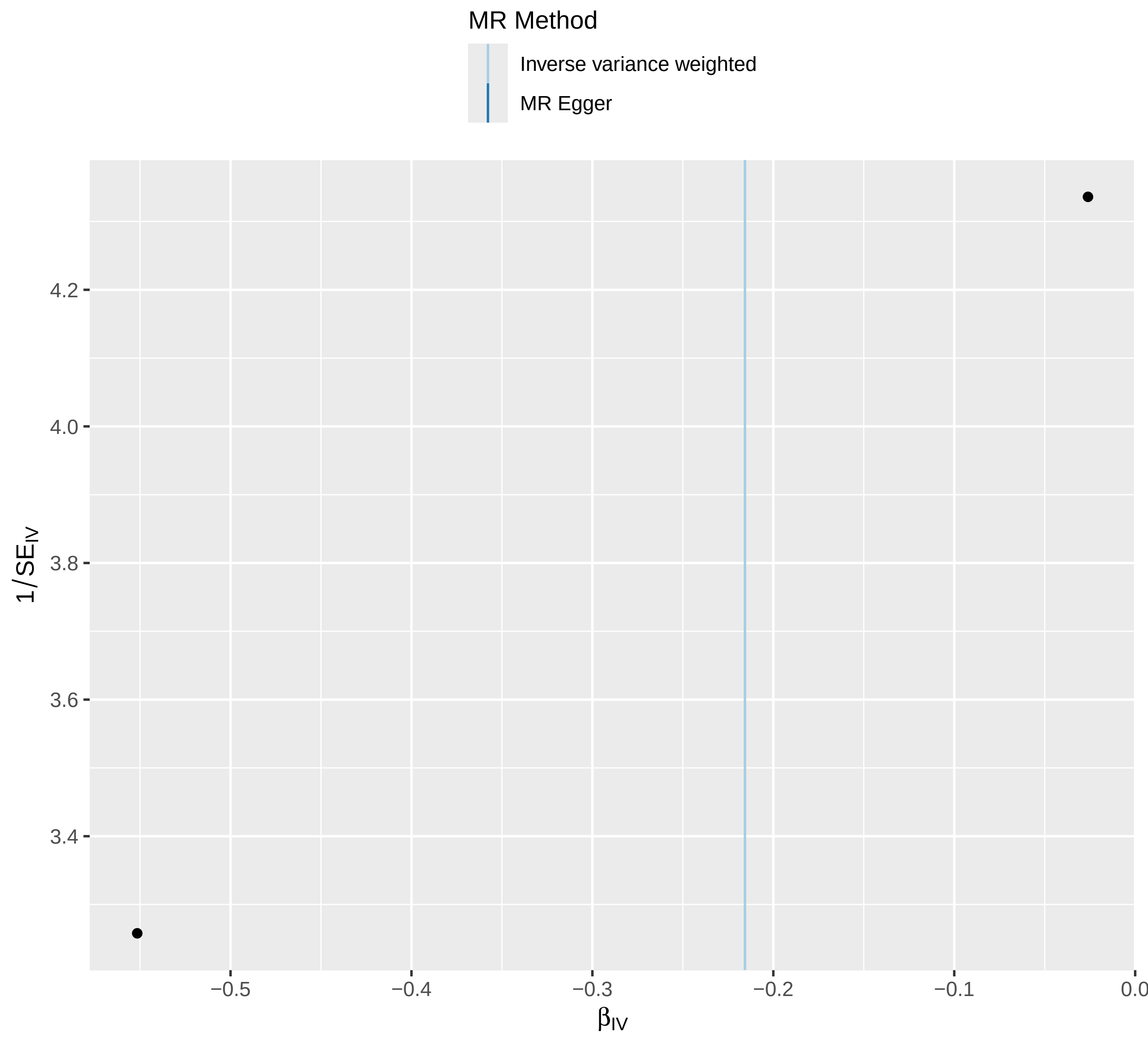

MR Method
Inverse variance weighted
MR Egger
4.2
4.0
3.8
3.6
3.4
1/SE$_{IV}$
−0.5
−0.4
−0.3
−0.2
−0.1
0.0
β$_{IV}$

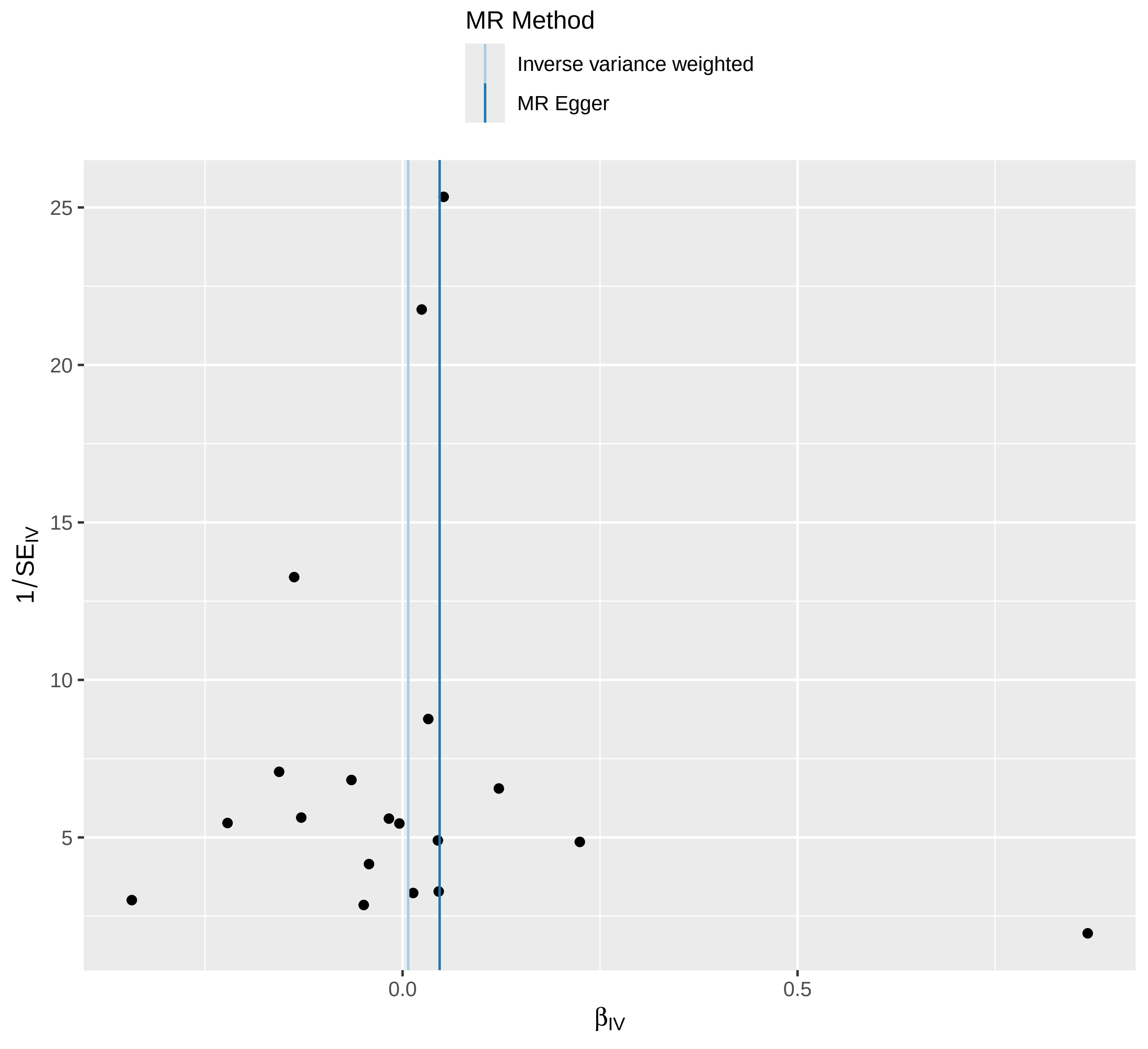
MR Method
Inverse variance weighted
MR Egger
25
20
15
10
5
1/SE_IV
0.0
0.5
β_IV

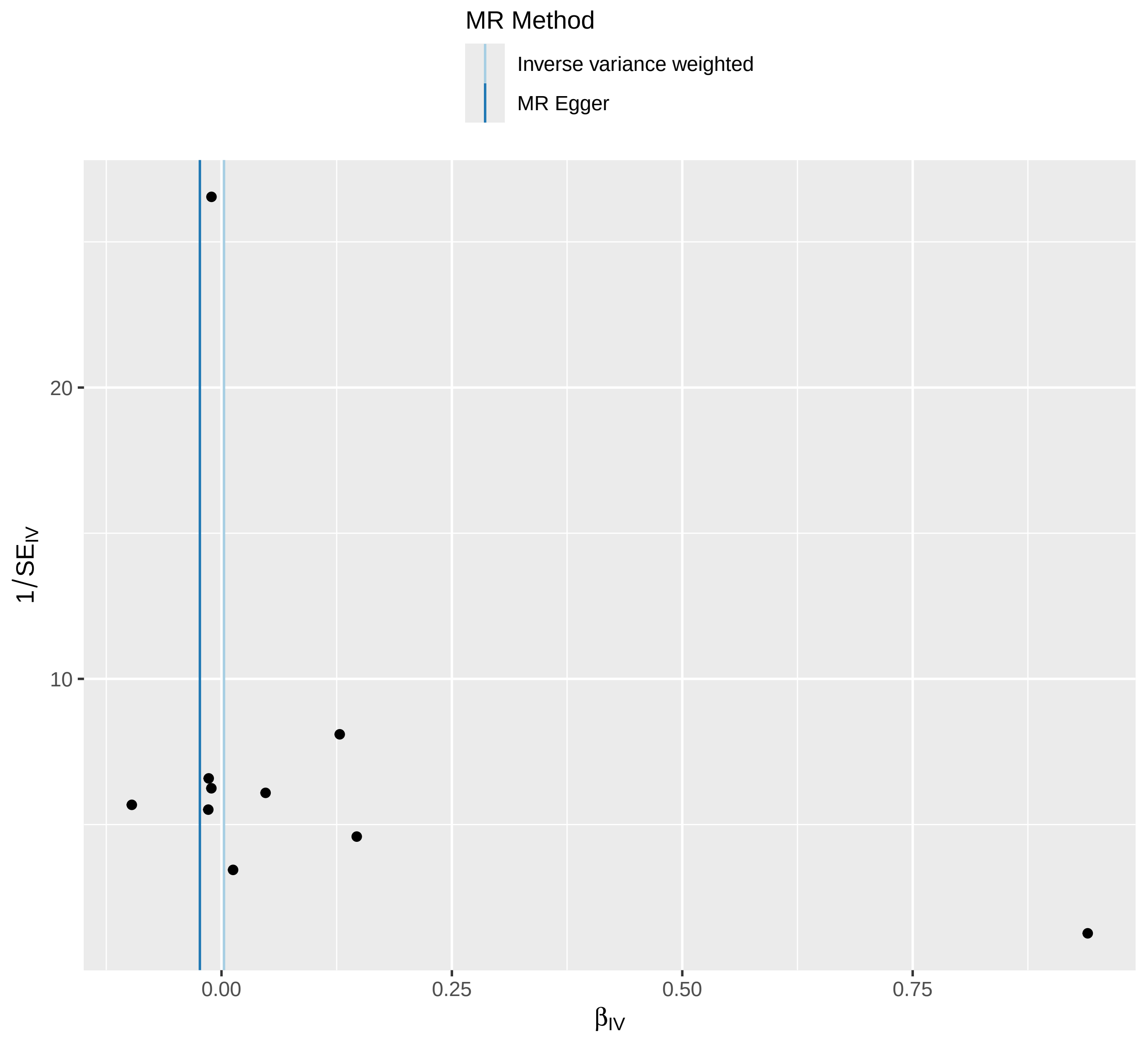

MR Method
Inverse variance weighted
MR Egger
20
10
$1/SE_{IV}$
0.00
0.25
0.50
0.75
$\beta_{IV}$

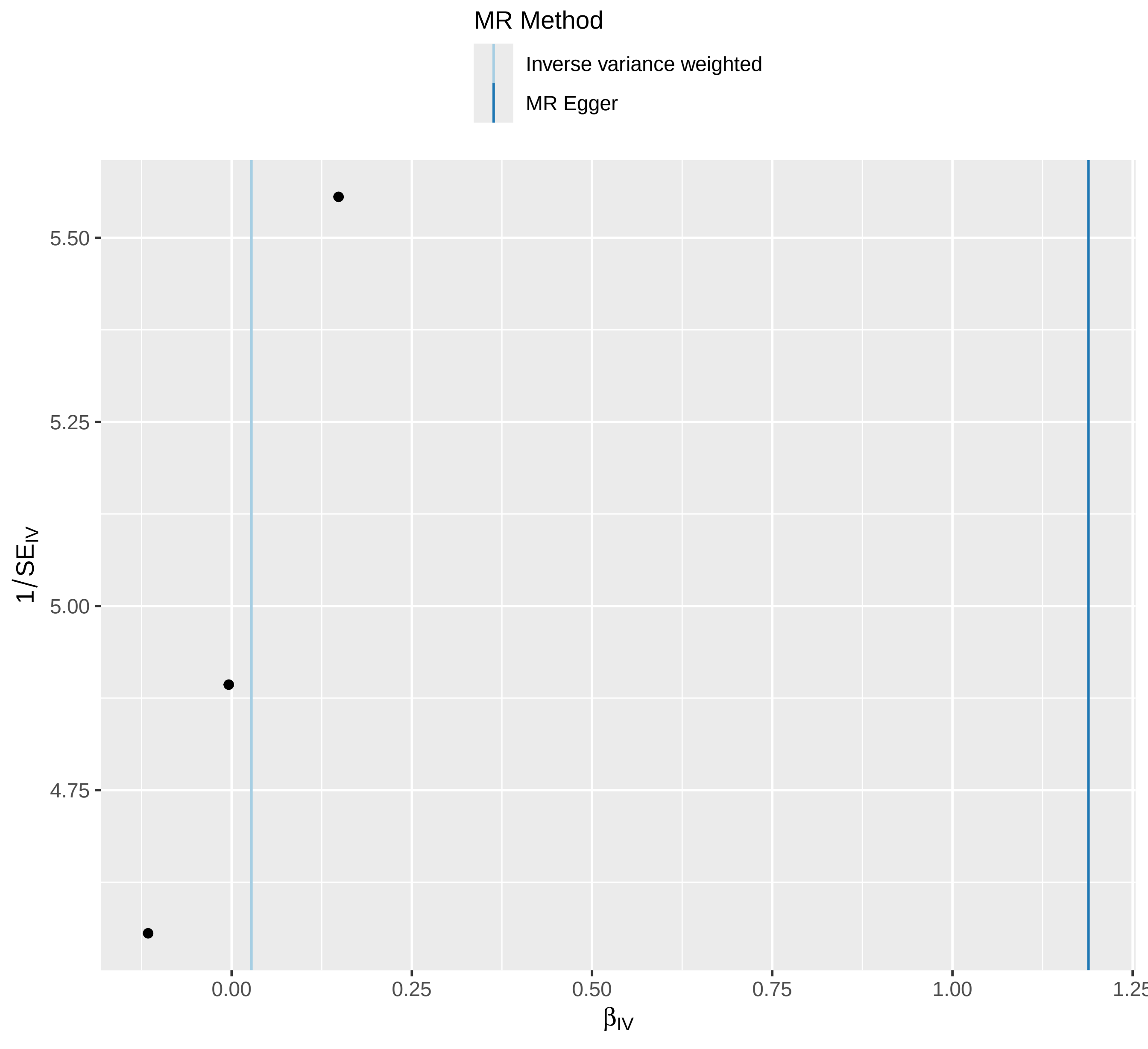

MR Method
Inverse variance weighted
MR Egger
5.50
5.25
5.00
4.75
1/SE$_{IV}$
0.00
0.25
0.50
0.75
1.00
1.25
β$_{IV}$

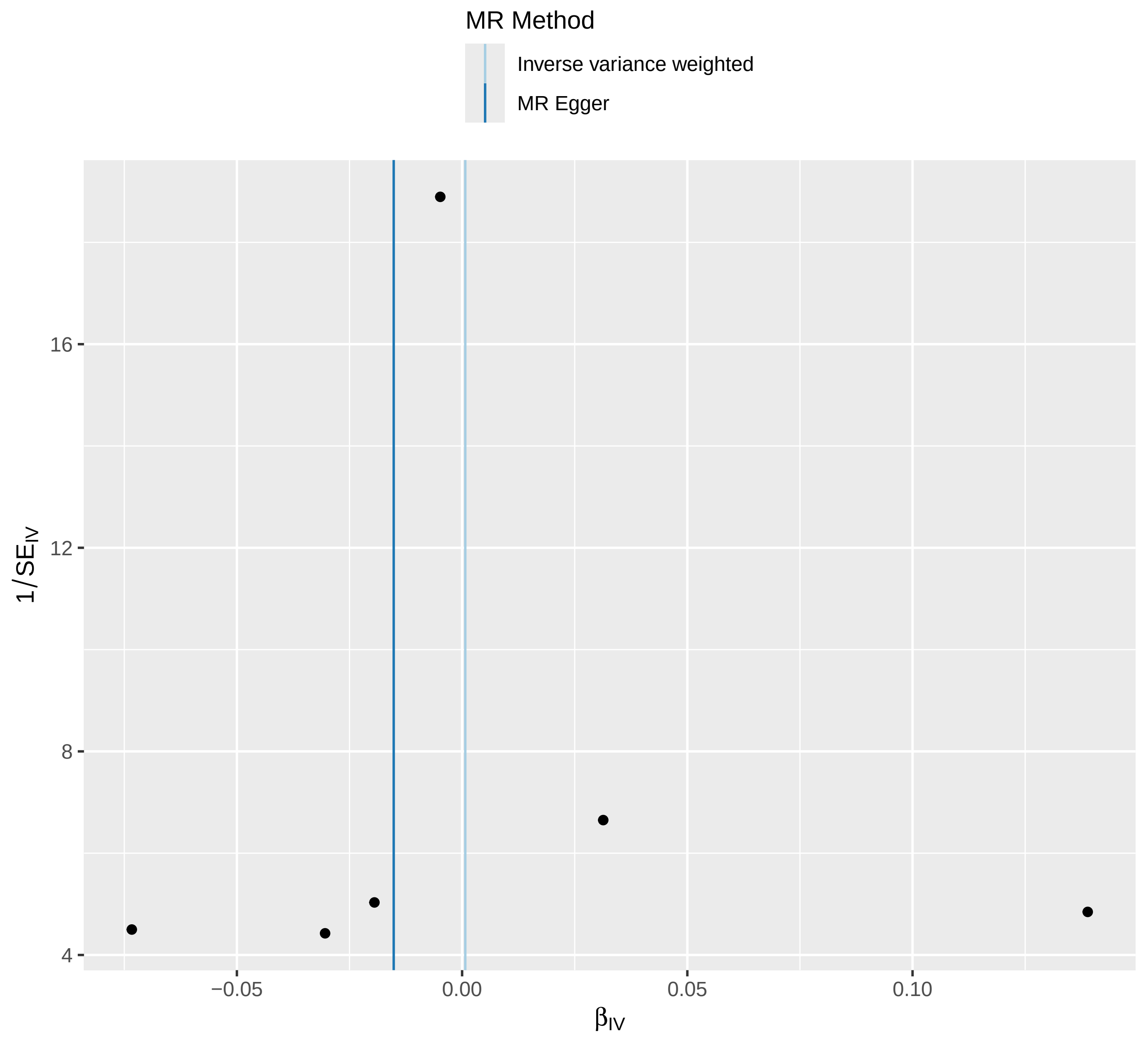
MR Method
Inverse variance weighted
MR Egger
1/SE_IV
16
12
8
4
−0.05
0.00
0.05
0.10
β_IV

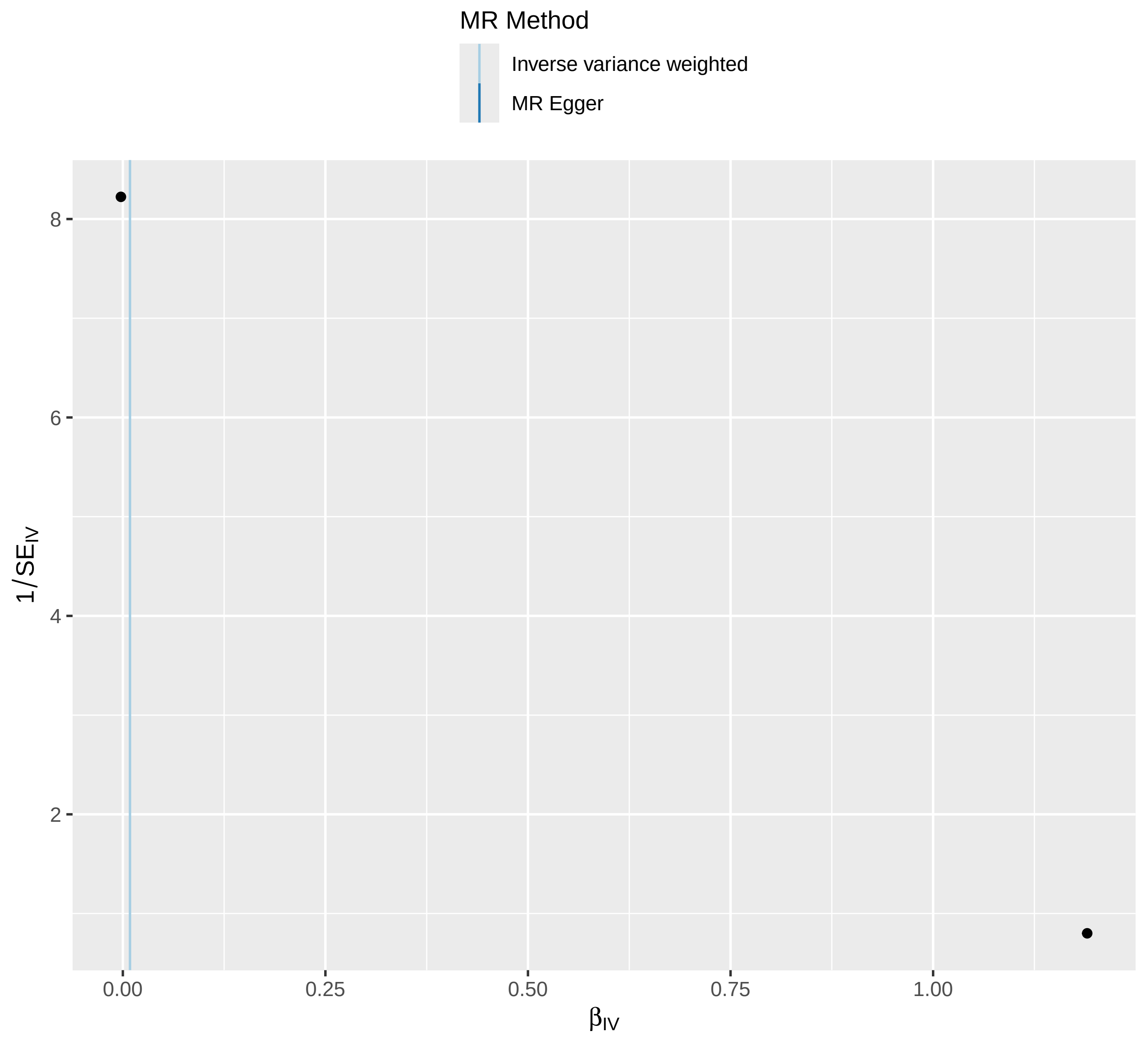

MR Method
Inverse variance weighted
MR Egger
8
6
4
2
$1/SE_{IV}$
0.00
0.25
0.50
0.75
1.00
$\beta_{IV}$

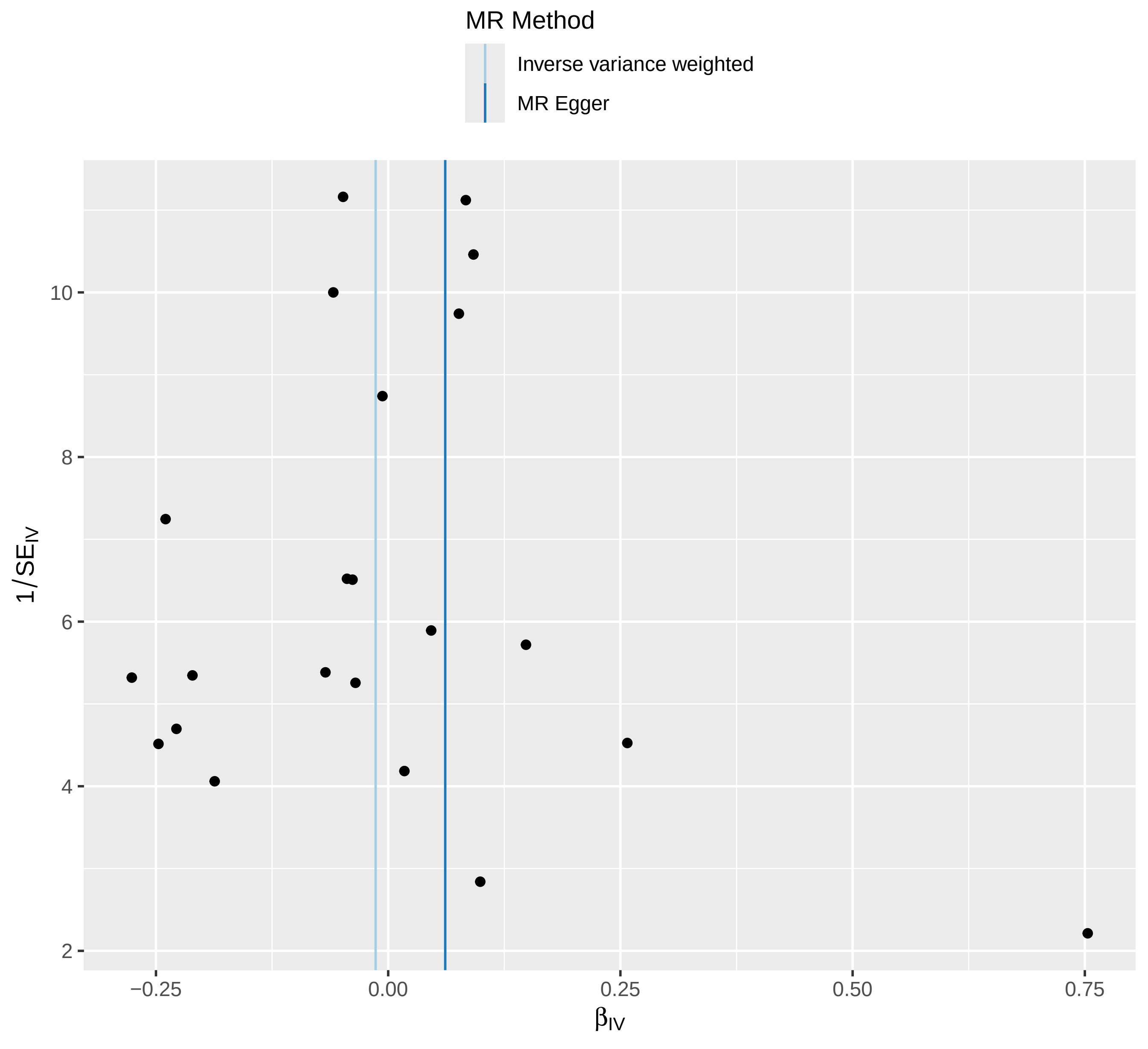

MR Method
Inverse variance weighted
MR Egger
10
8
6
4
2
1/SE$_{IV}$
−0.25
0.00
0.25
0.50
0.75
β$_{IV}$

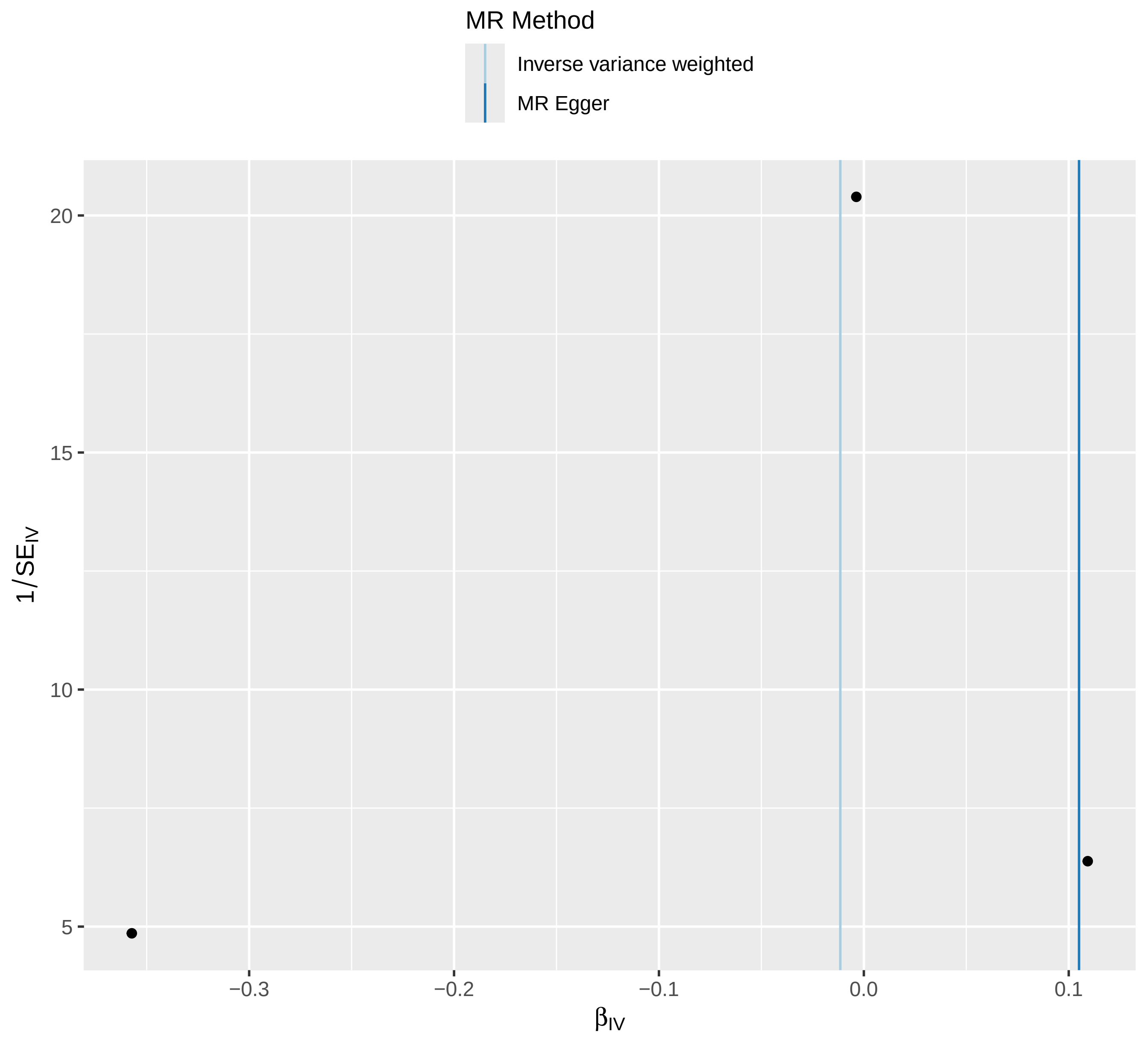
MR Method
Inverse variance weighted
MR Egger
20
15
10
5
−0.3
−0.2
−0.1
0.0
0.1
1/SE_IV
β_IV

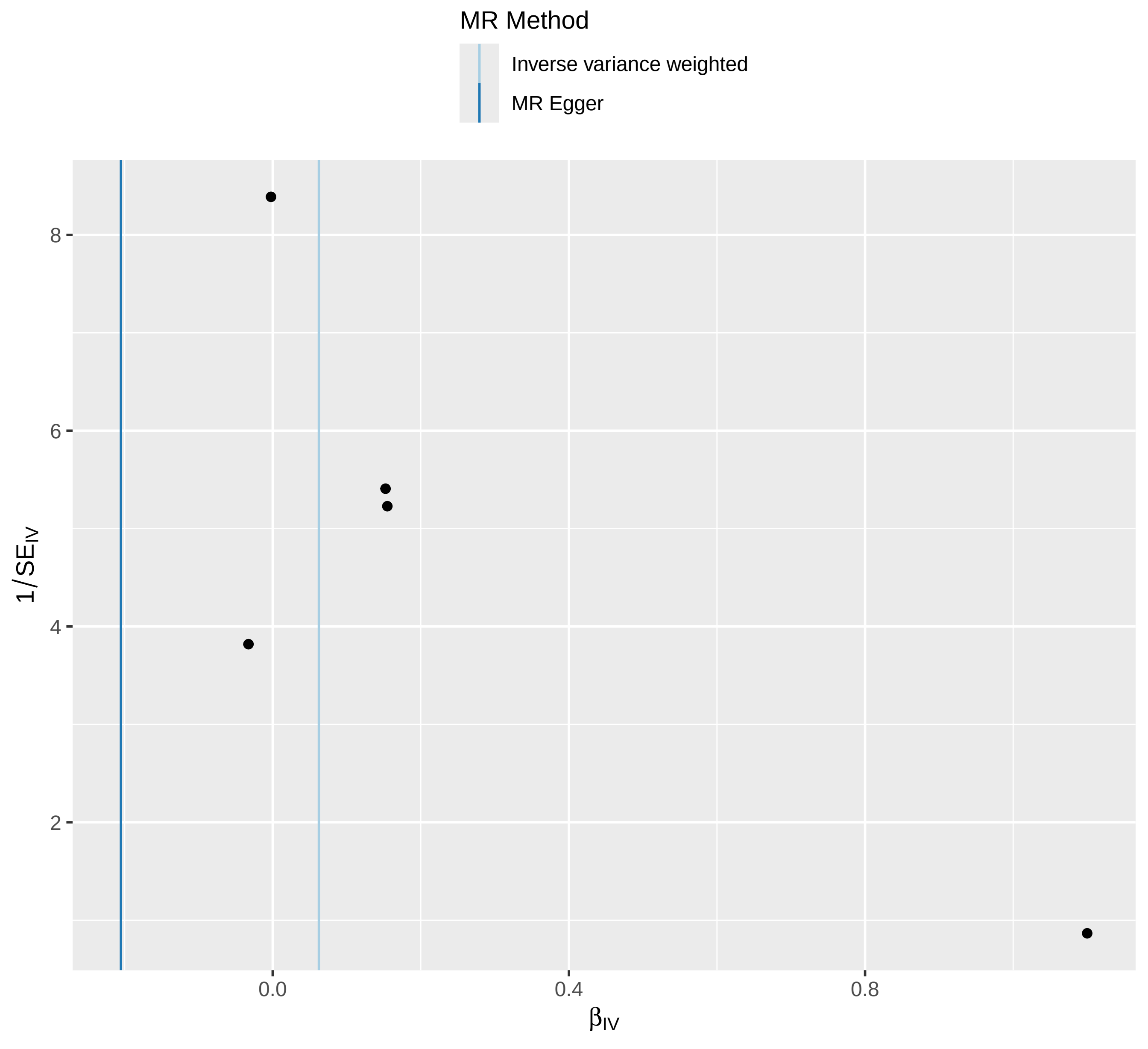

MR Method
Inverse variance weighted
MR Egger
8
6
4
2
1/SE$_{IV}$
0.0
0.4
0.8
β$_{IV}$

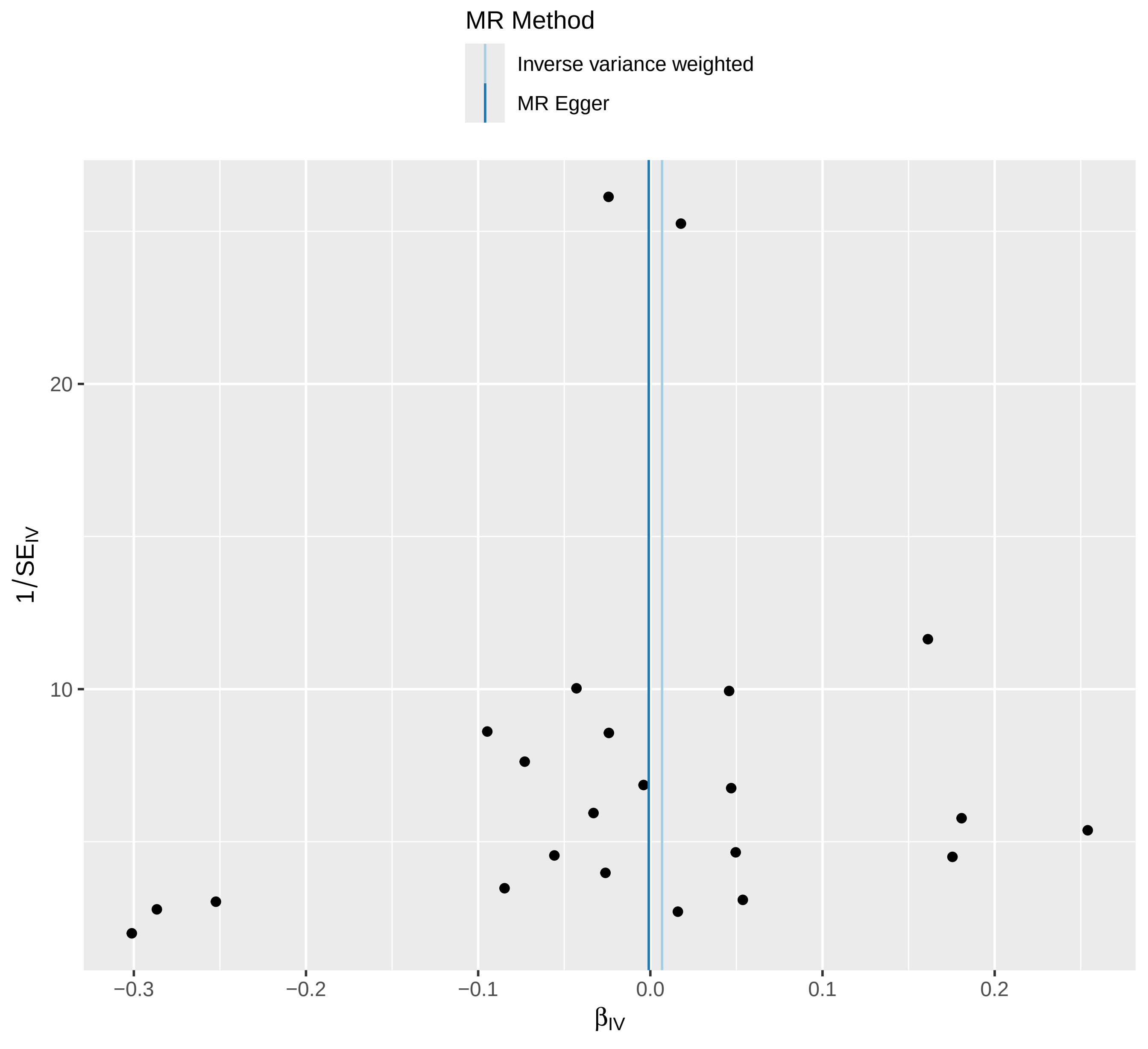
MR Method
Inverse variance weighted
MR Egger
20
10
1/SE_IV
−0.3
−0.2
−0.1
0.0
0.1
0.2
β_IV

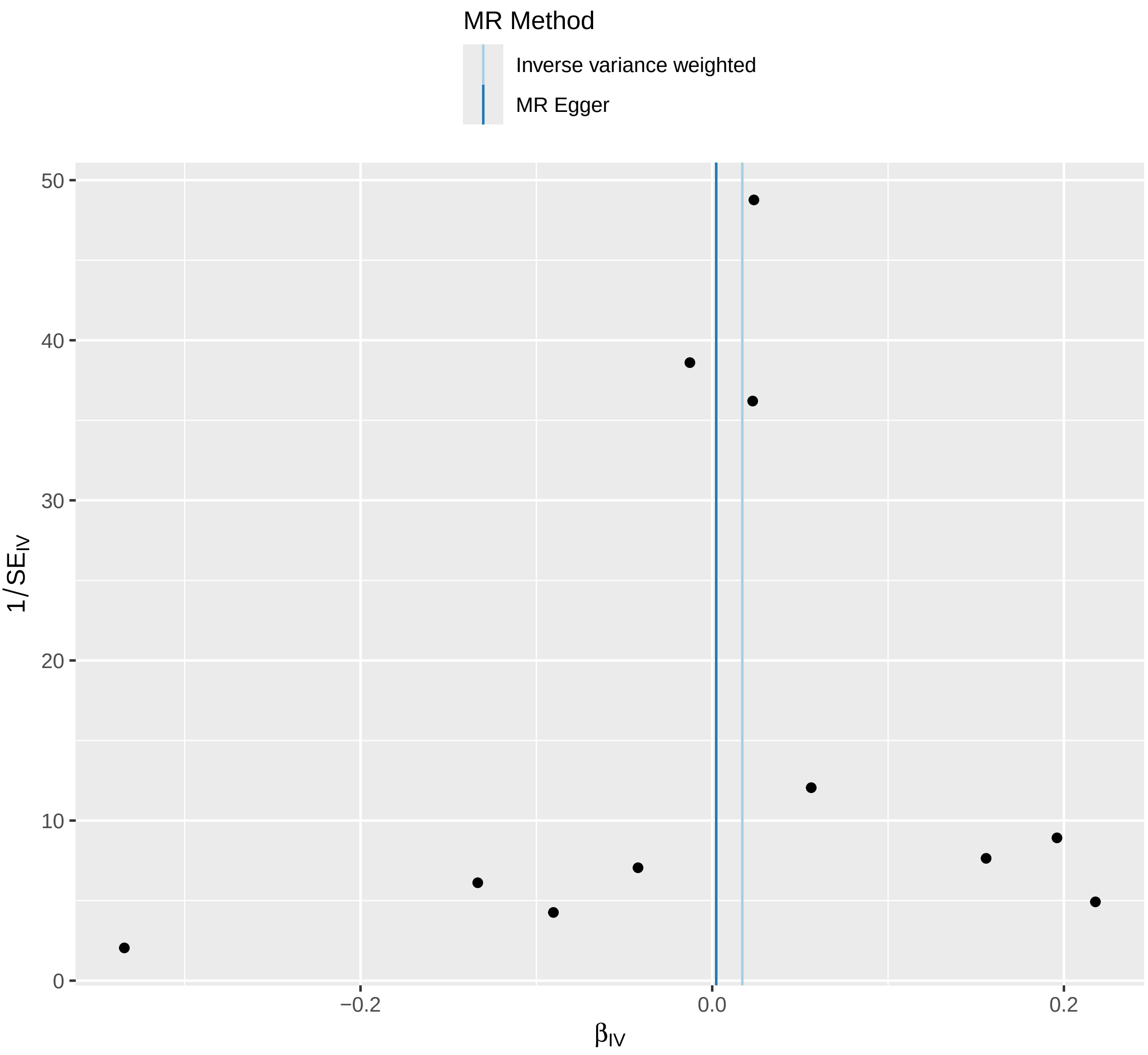
MR Method
Inverse variance weighted
MR Egger
1/SE_IV
β_IV
50
40
30
20
10
0
−0.2
0.0
0.2

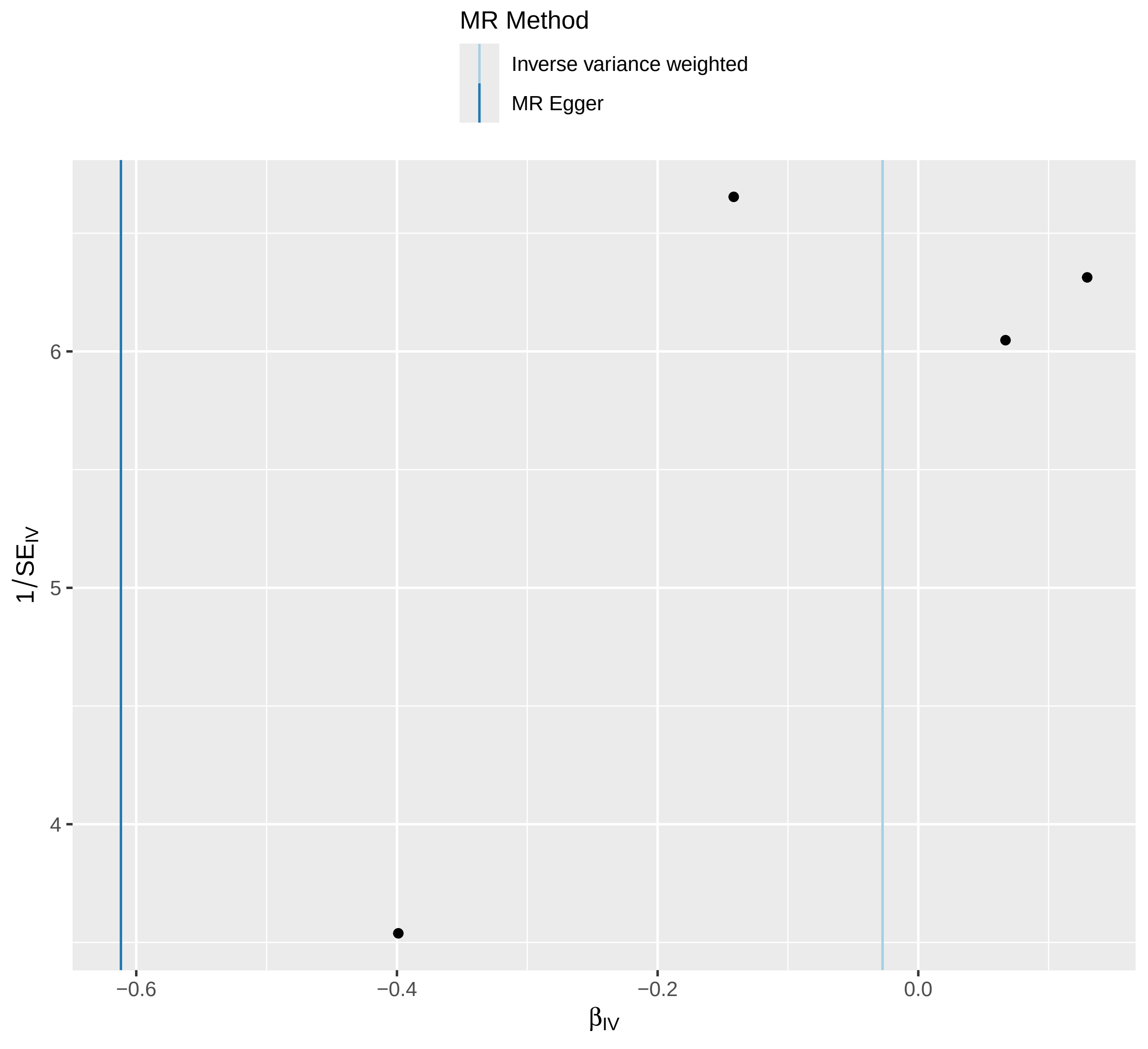
MR Method
Inverse variance weighted
MR Egger
6
5
4
1/SE_IV
−0.6
−0.4
−0.2
0.0
β_IV

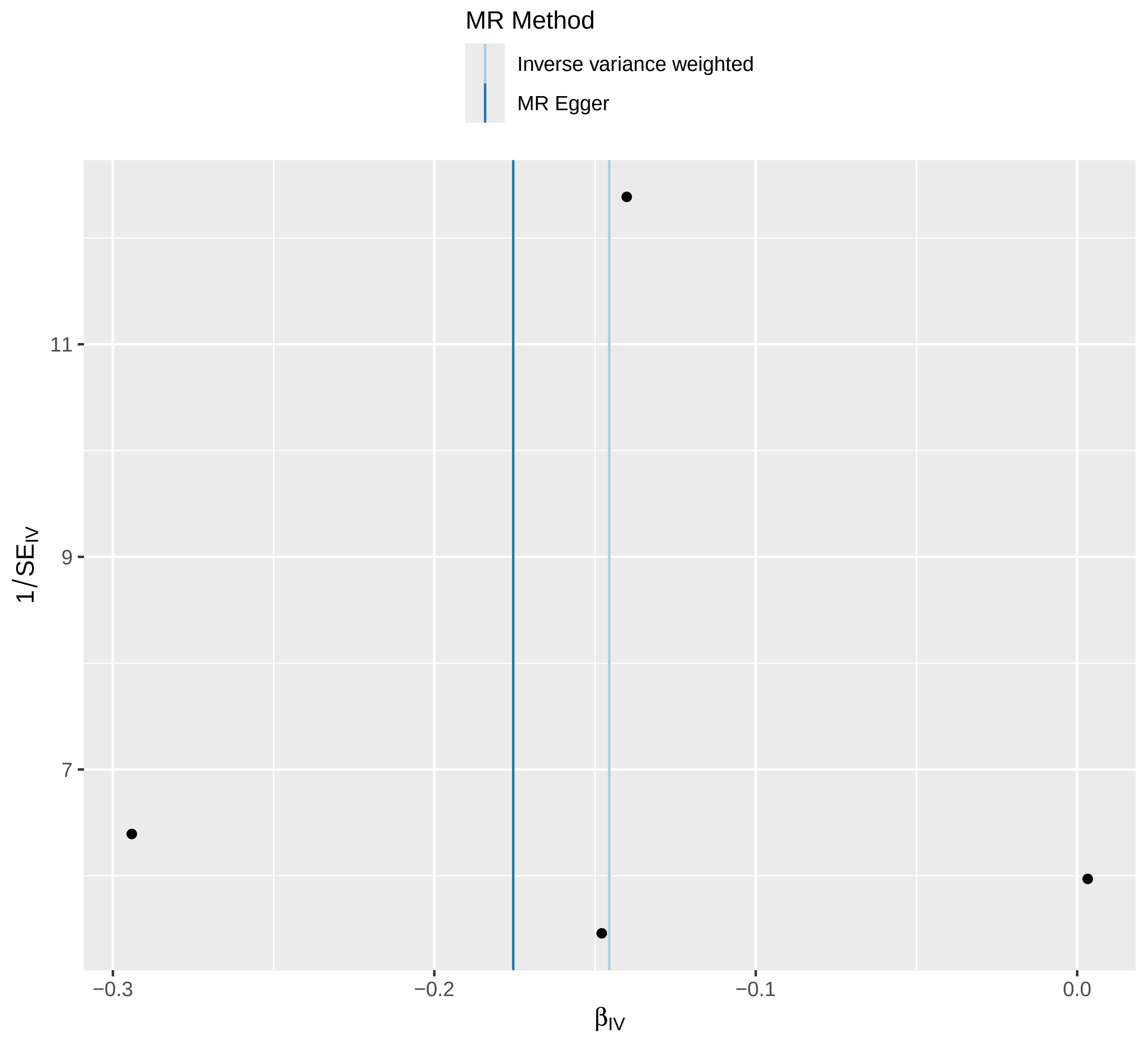

MR Method
Inverse variance weighted
MR Egger
11
9
7
1/SE$_{IV}$
−0.3
−0.2
−0.1
0.0
β$_{IV}$

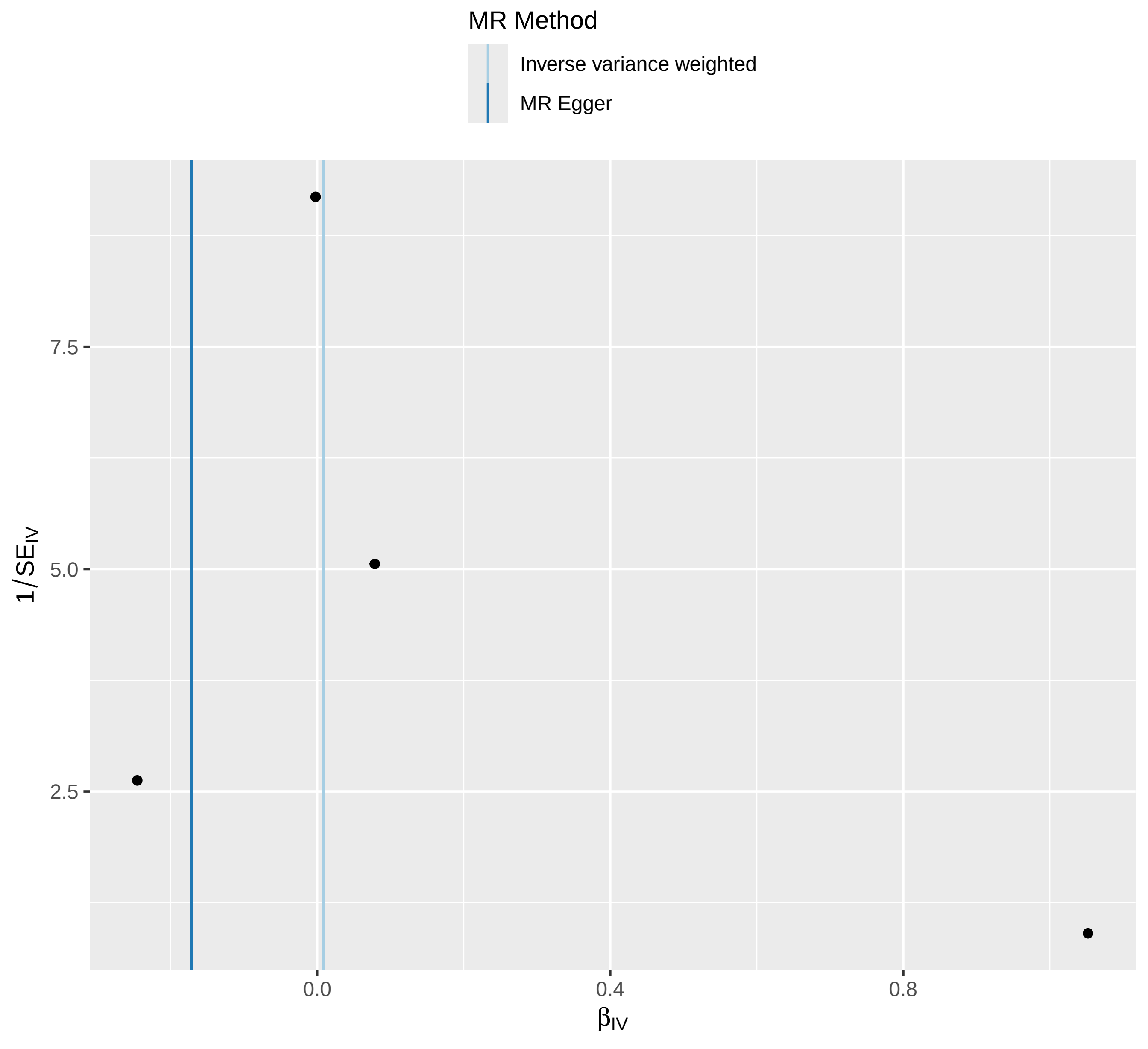
MR Method
Inverse variance weighted
MR Egger
7.5
5.0
2.5
0.0
0.4
0.8
1/SE_IV
β_IV

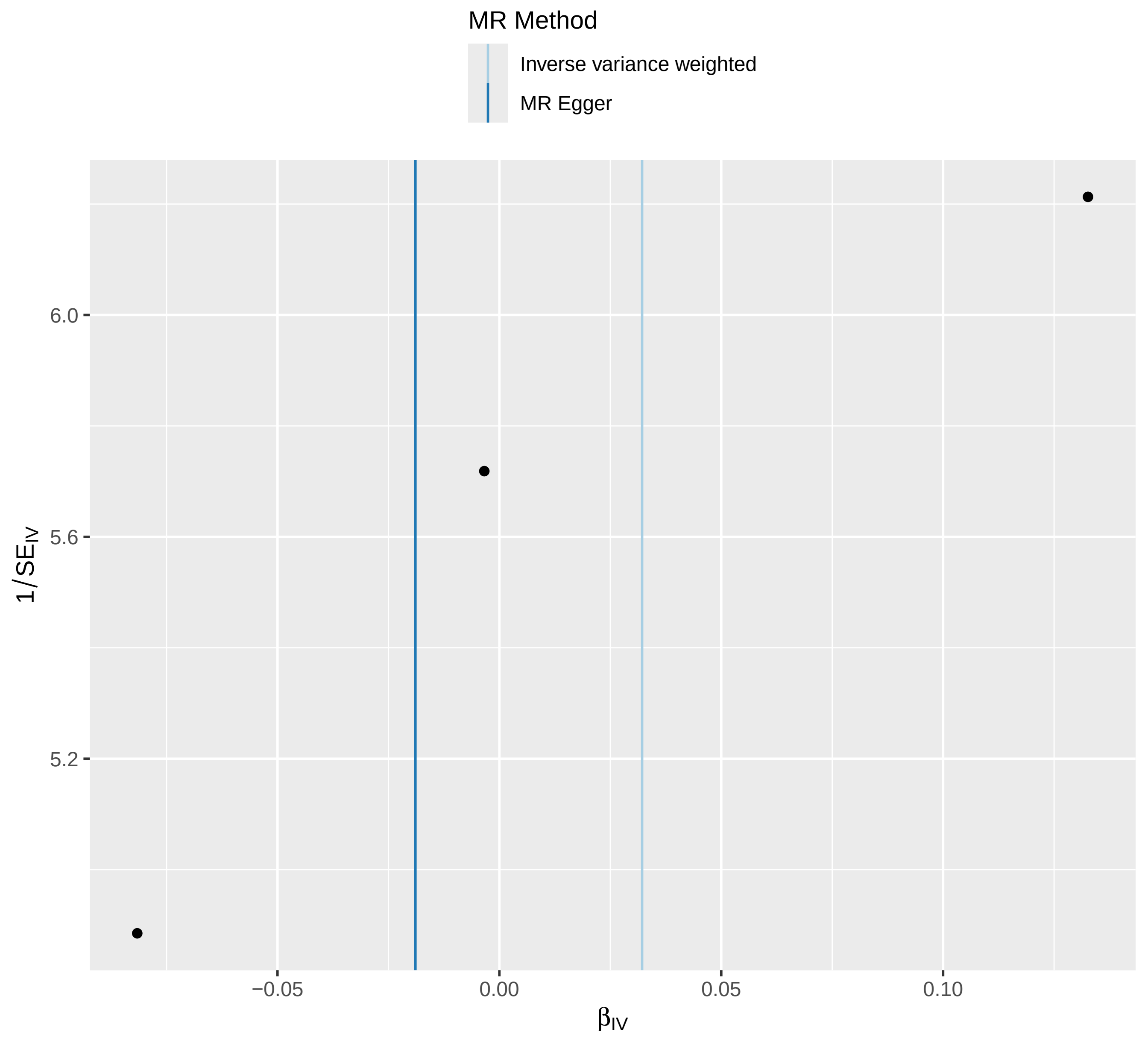

MR Method
Inverse variance weighted
MR Egger
6.0
5.6
5.2
1/SE_IV
−0.05
0.00
0.05
0.10
β_IV

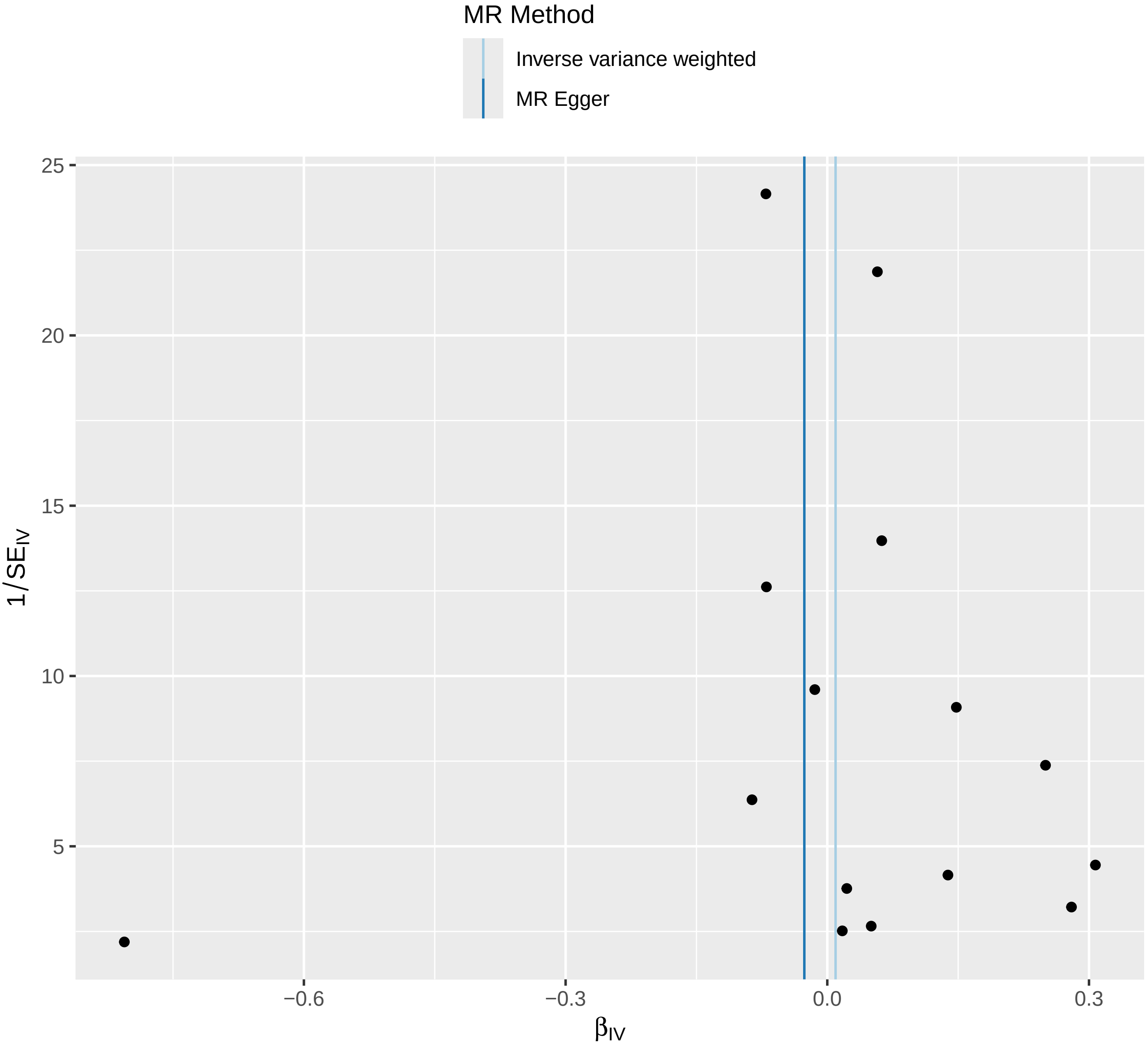

MR Method
Inverse variance weighted
MR Egger
25
20
15
10
5
1/SE$_{IV}$
−0.6
−0.3
0.0
0.3
β$_{IV}$

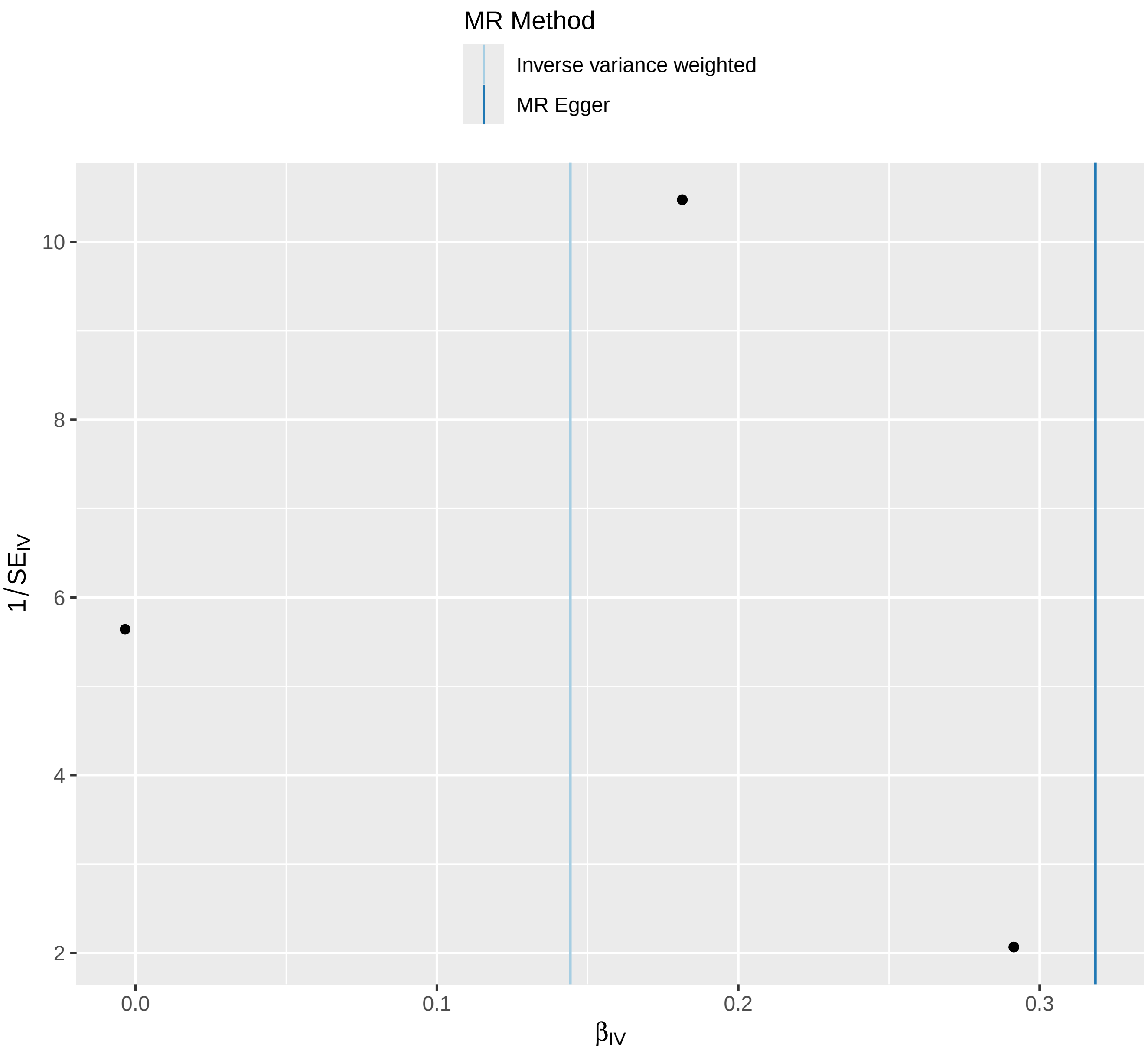
MR Method
Inverse variance weighted
MR Egger
10
8
6
4
2
1/SE_IV
0.0
0.1
0.2
0.3
β_IV

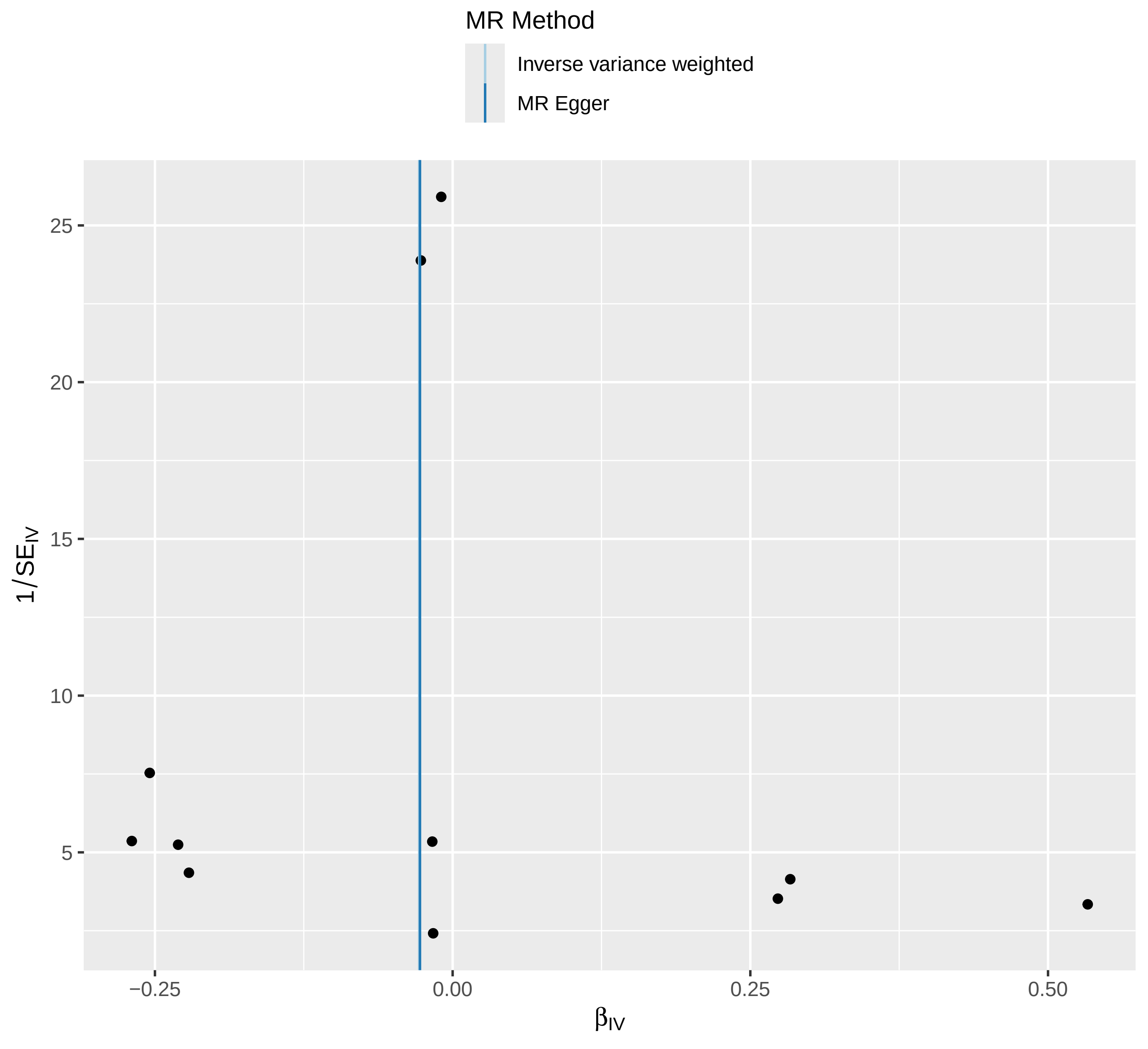

MR Method
Inverse variance weighted
MR Egger
25
20
15
10
5
1/SE_IV
−0.25
0.00
0.25
0.50
β_IV

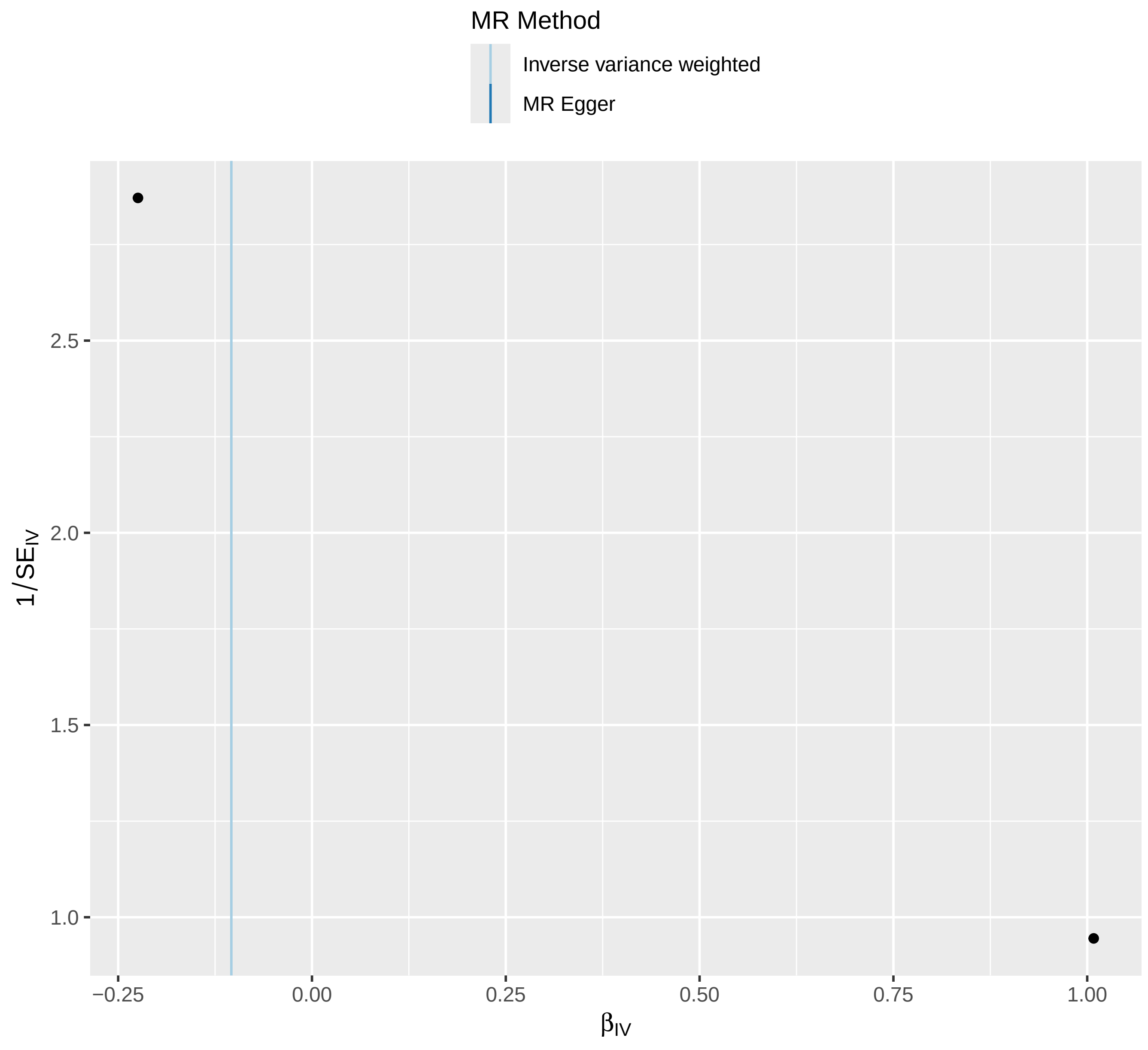

MR Method
Inverse variance weighted
MR Egger
2.5
2.0
1.5
1.0
1/SE_IV
−0.25
0.00
0.25
0.50
0.75
1.00
β_IV

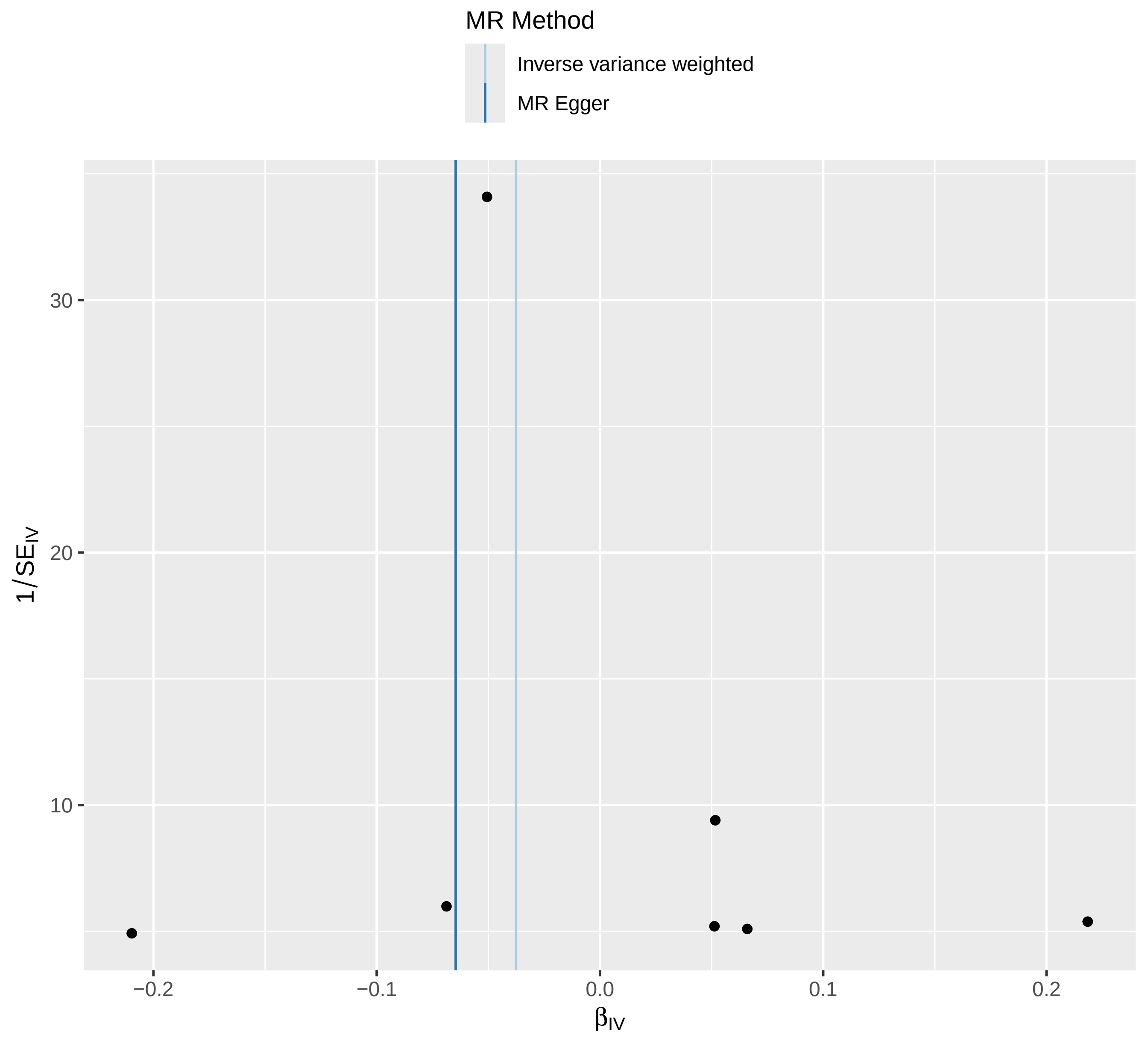
MR Method
Inverse variance weighted
MR Egger
30
20
10
1/SE$_{IV}$
−0.2
−0.1
0.0
0.1
0.2
β$_{IV}$

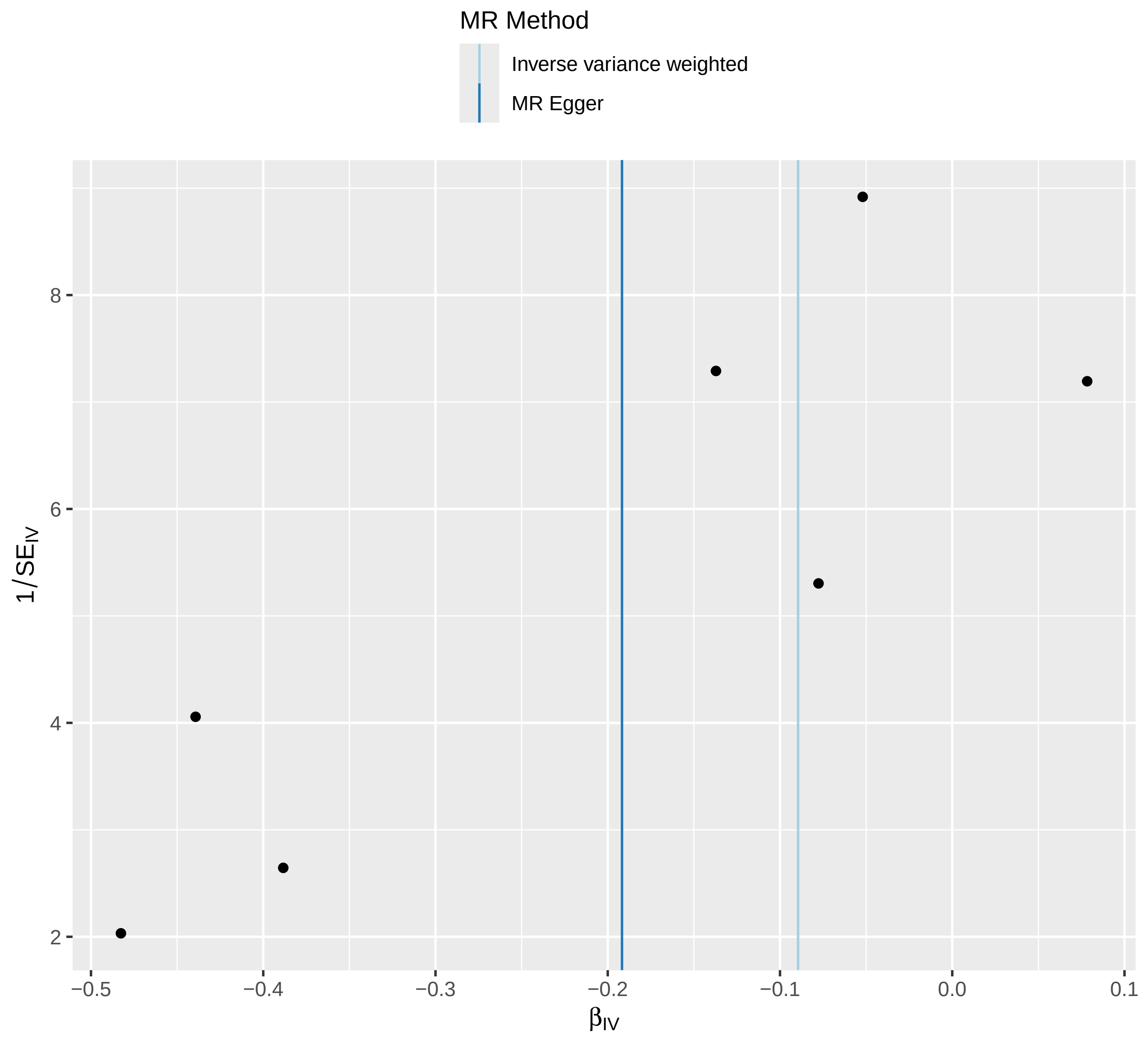

MR Method
Inverse variance weighted
MR Egger
1/SE_IV
β_IV
8
6
4
2
−0.5
−0.4
−0.3
−0.2
−0.1
0.0
0.1

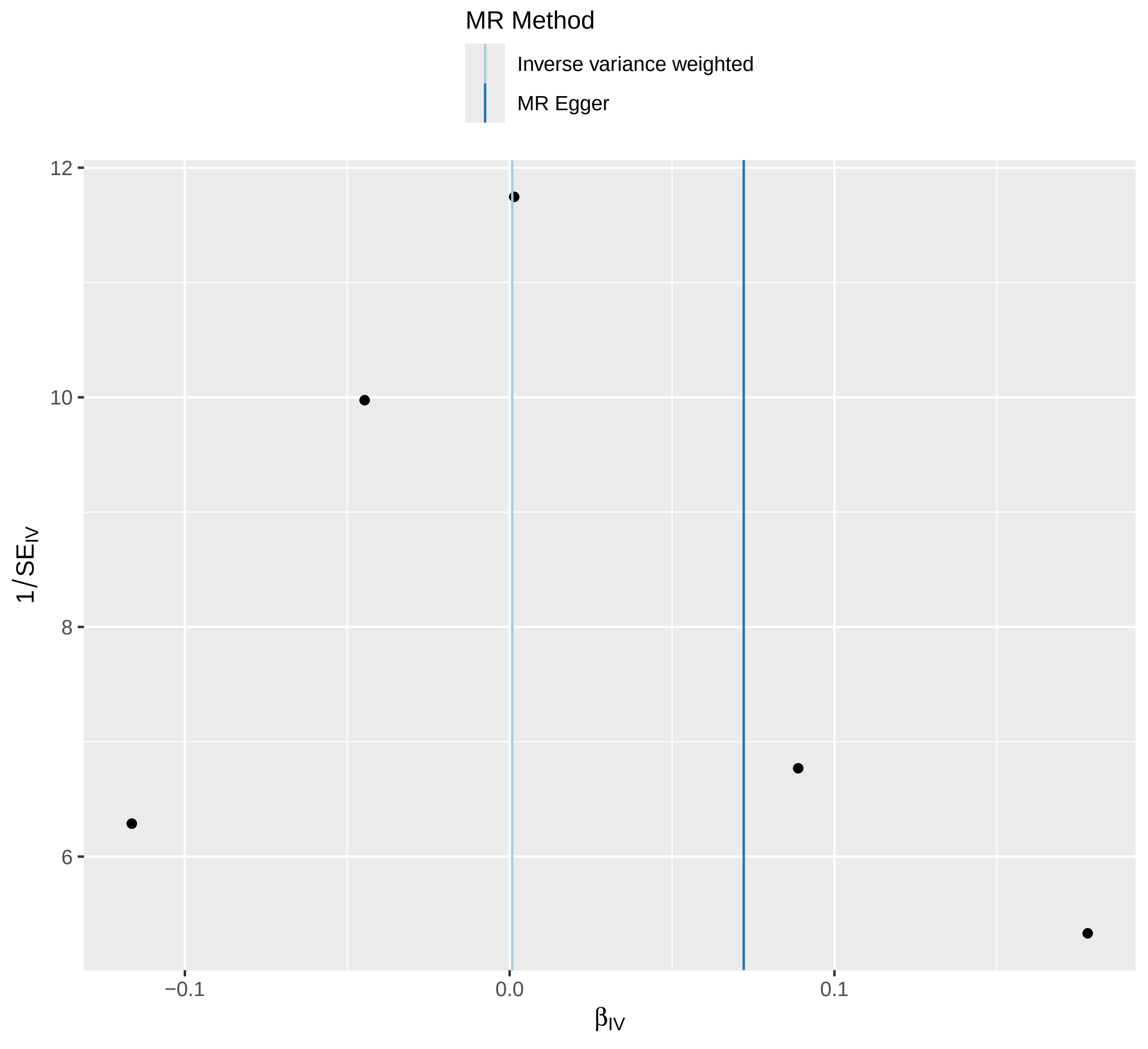
MR Method
Inverse variance weighted
MR Egger
12
10
8
6
1/SE$_{IV}$
−0.1
0.0
0.1
β$_{IV}$

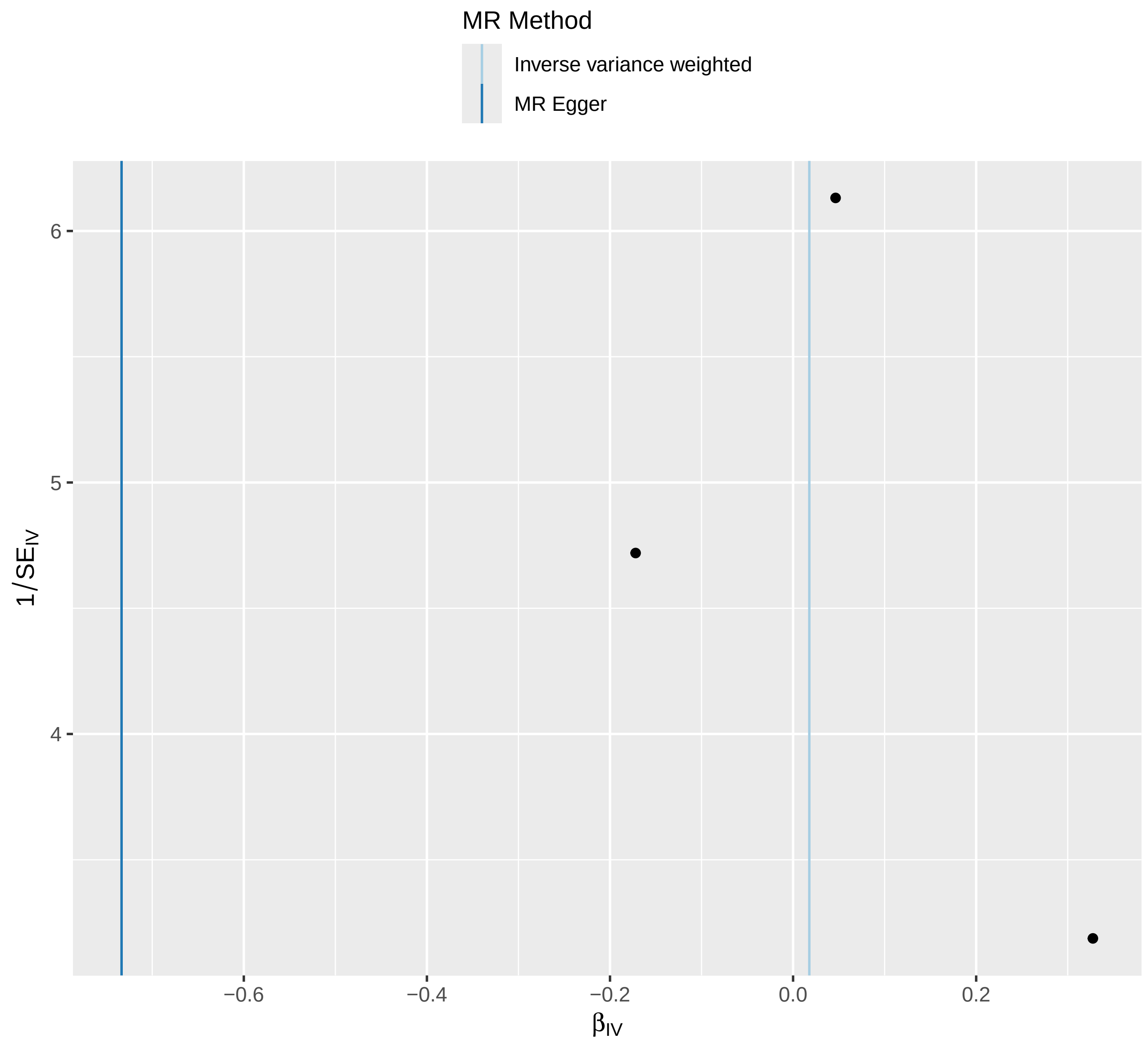

MR Method
Inverse variance weighted
MR Egger
6
5
4
1/SE_IV
−0.6
−0.4
−0.2
0.0
0.2
β_IV

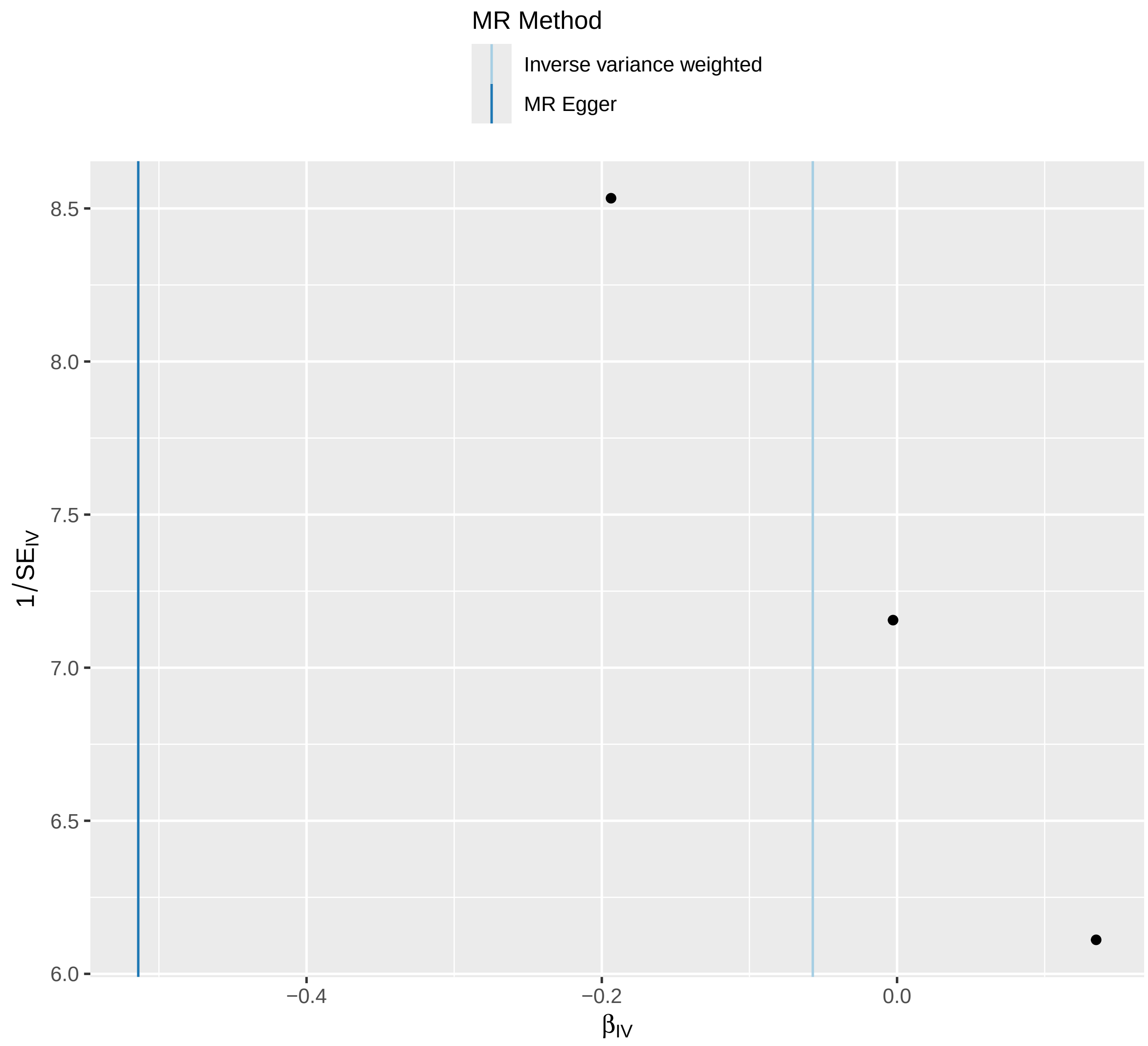
MR Method
Inverse variance weighted
MR Egger
8.5
8.0
7.5
7.0
6.5
6.0
1/SE$_{IV}$
−0.4
−0.2
0.0
β$_{IV}$

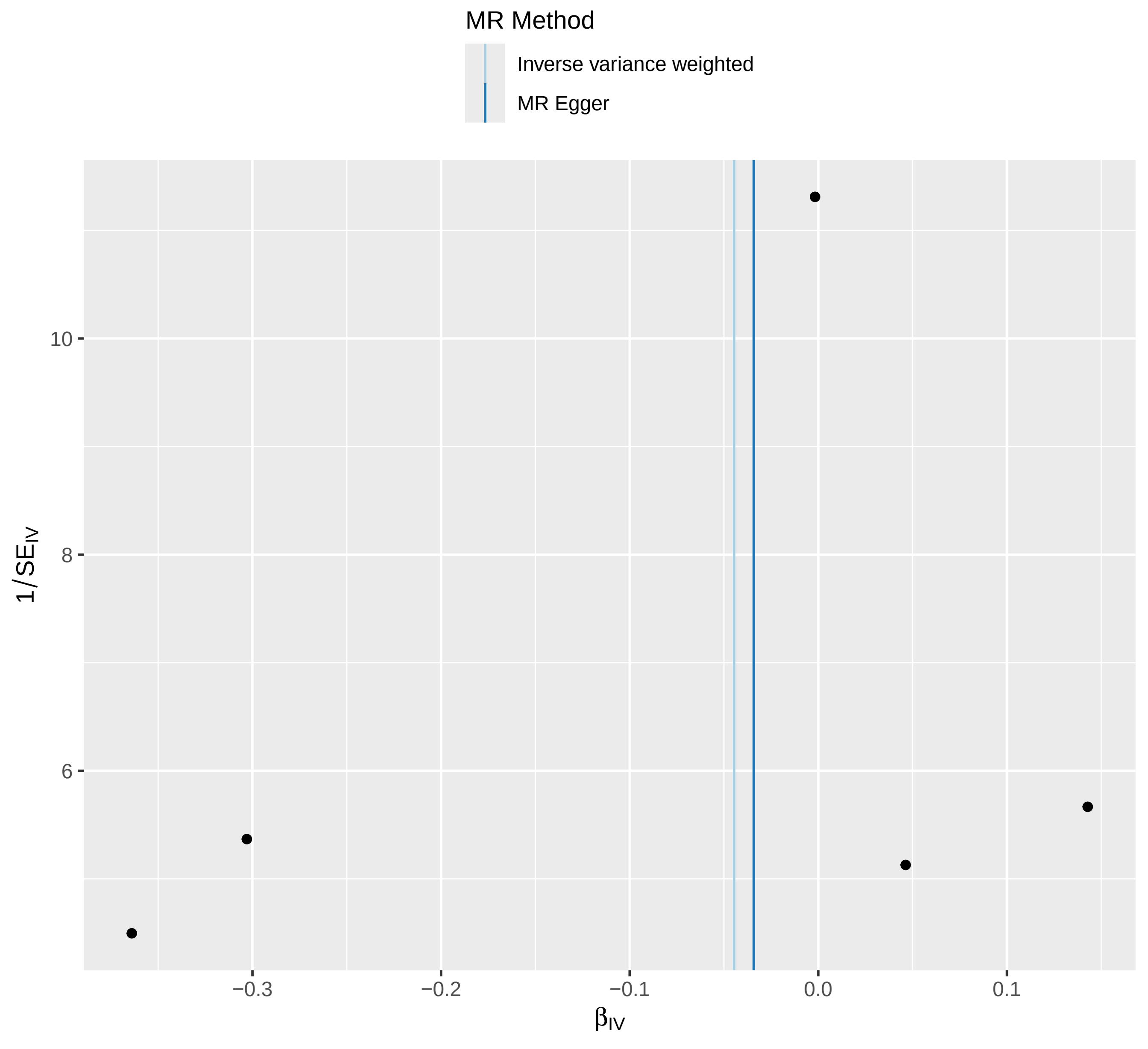
MR Method
Inverse variance weighted
MR Egger
1/$SE_{IV}$
10
8
6
−0.3
−0.2
−0.1
0.0
0.1
$\beta_{IV}$

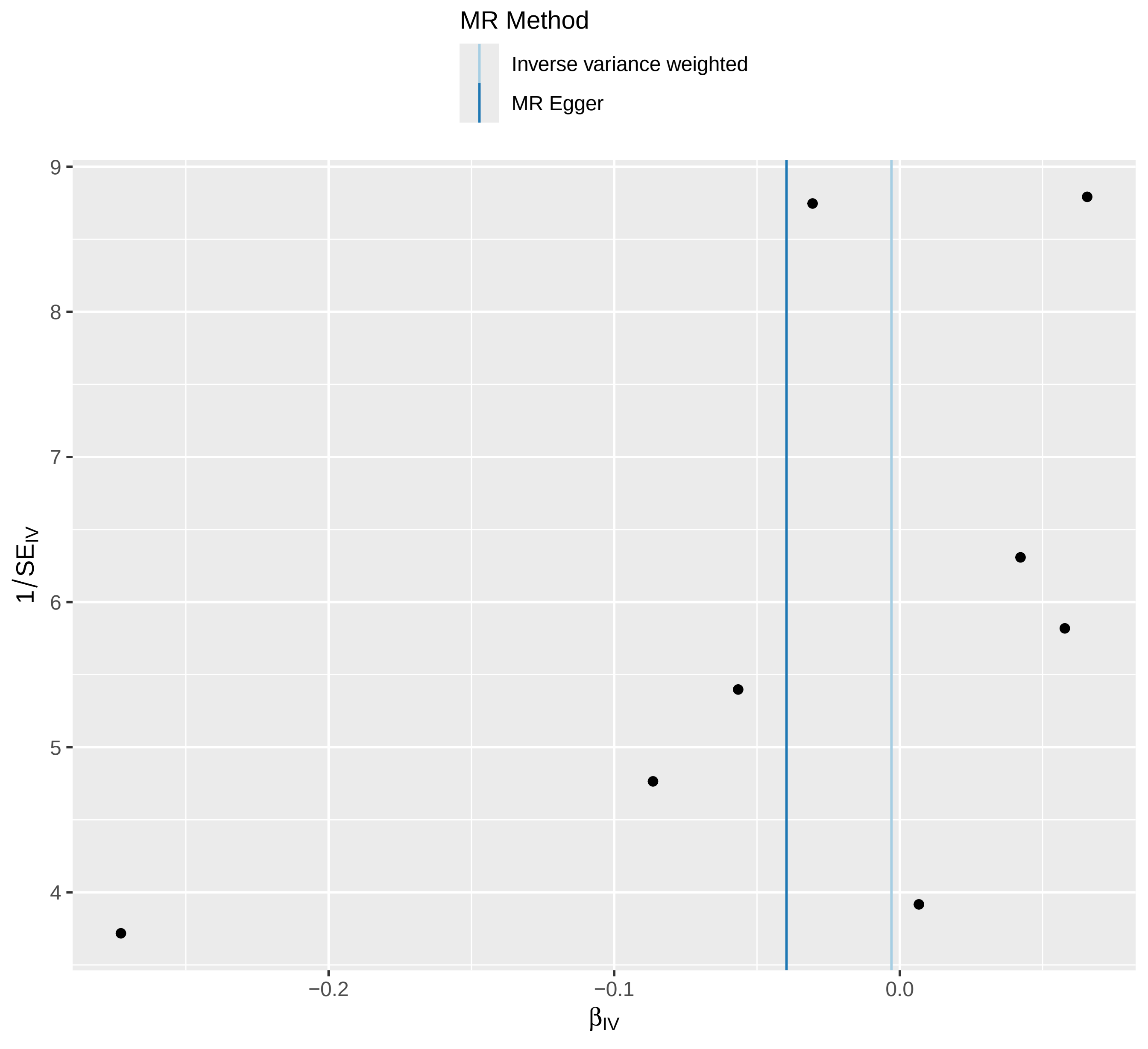

MR Method
Inverse variance weighted
MR Egger
9
8
7
6
5
4
1/SE_IV
−0.2
−0.1
0.0
β_IV

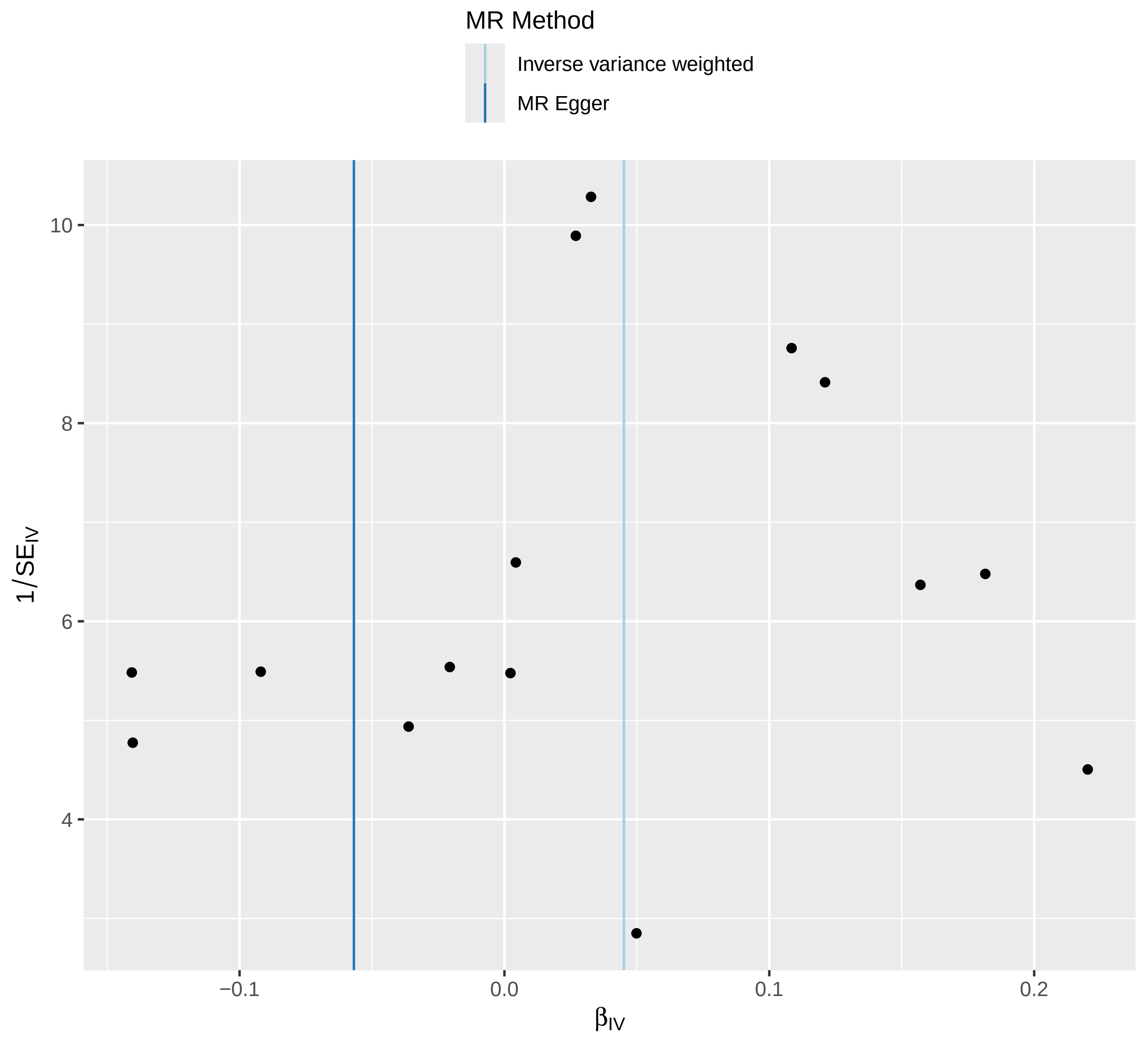
MR Method
Inverse variance weighted
MR Egger
10
8
6
4
1/SE_IV
−0.1
0.0
0.1
0.2
β_IV

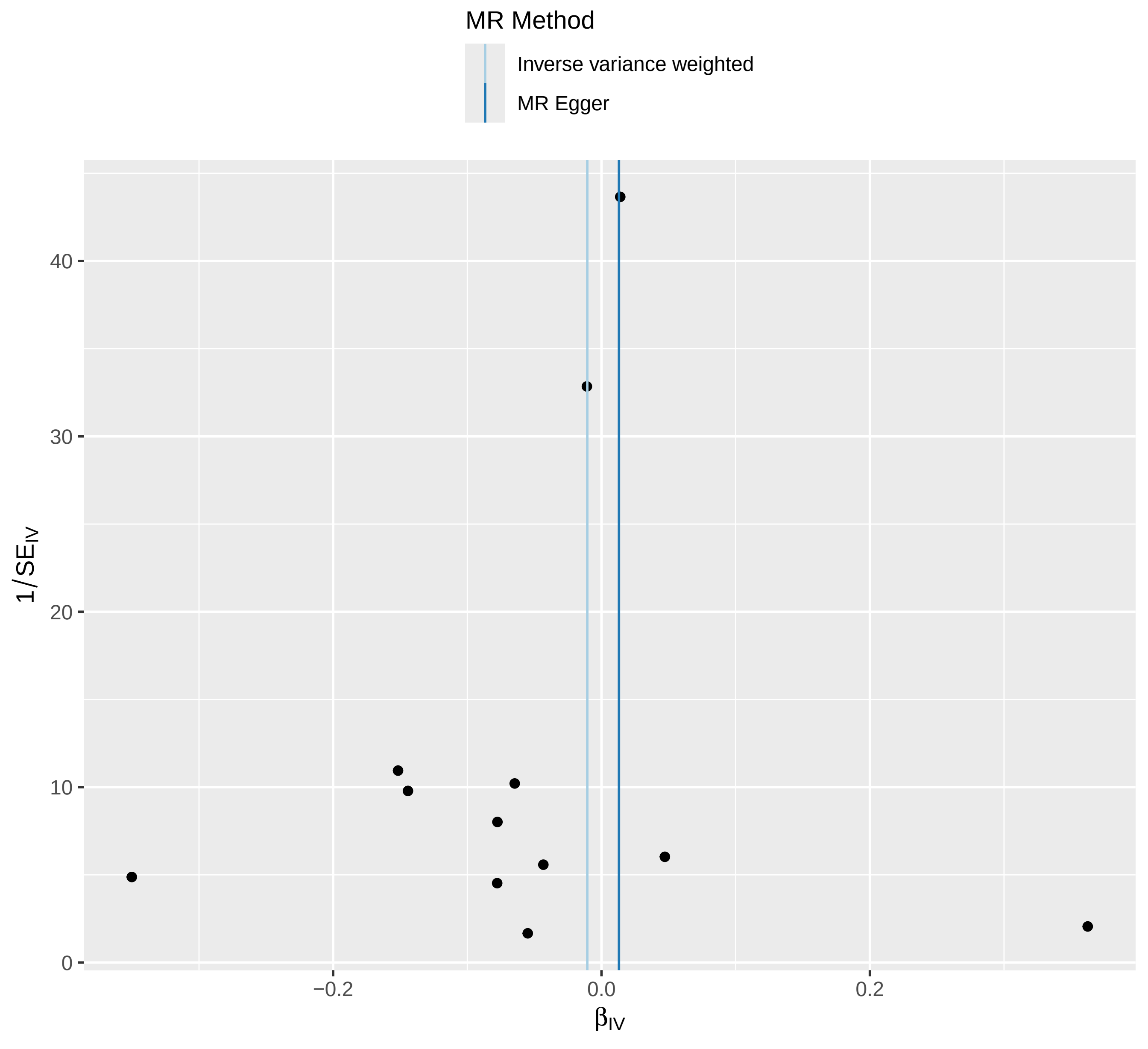
MR Method
Inverse variance weighted
MR Egger
40
30
20
10
0
−0.2
0.0
0.2
$1/SE_{IV}$
$\beta_{IV}$

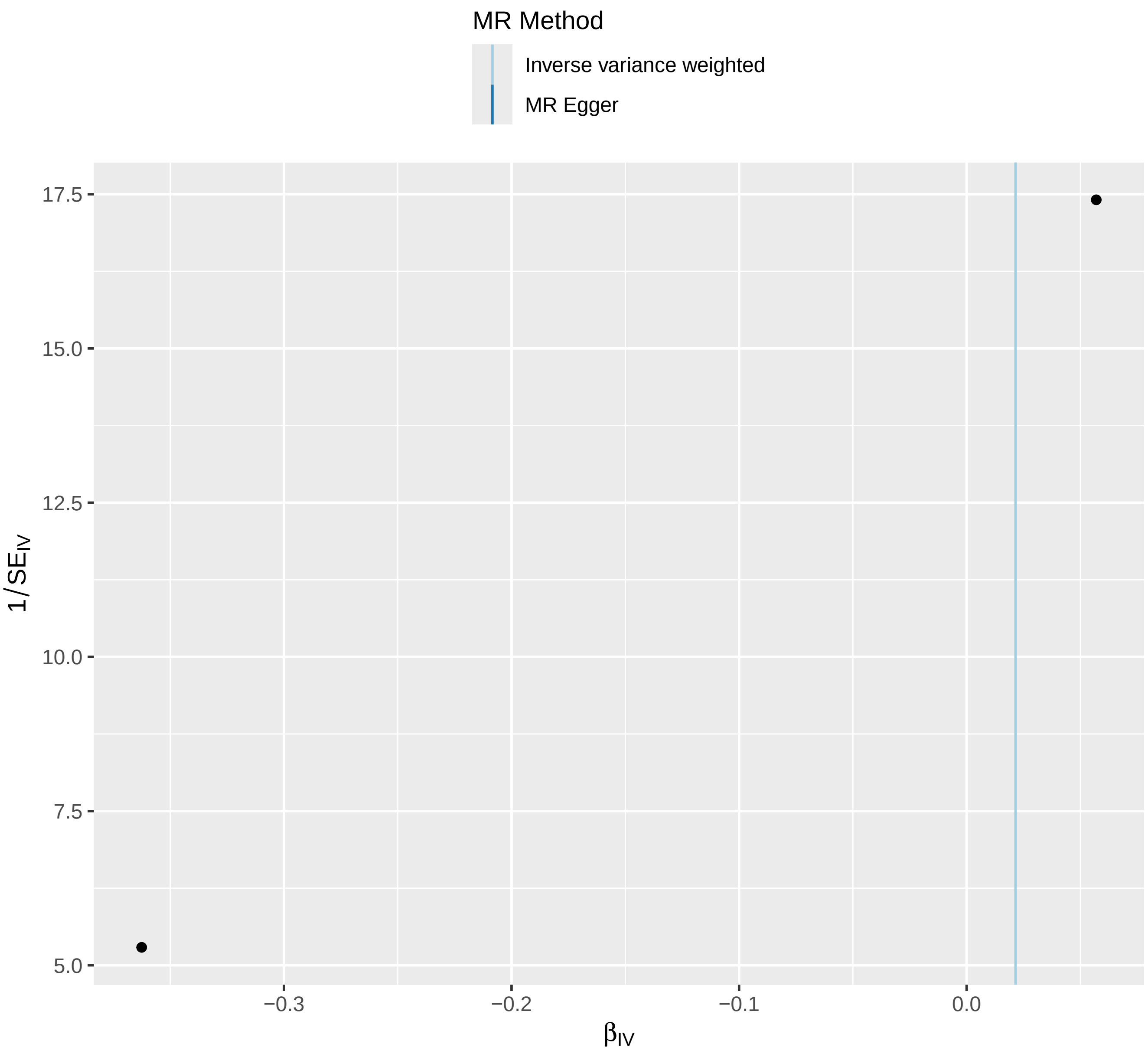
MR Method
Inverse variance weighted
MR Egger
17.5
15.0
12.5
10.0
7.5
5.0
1/SE_IV
−0.3
−0.2
−0.1
0.0
β_IV

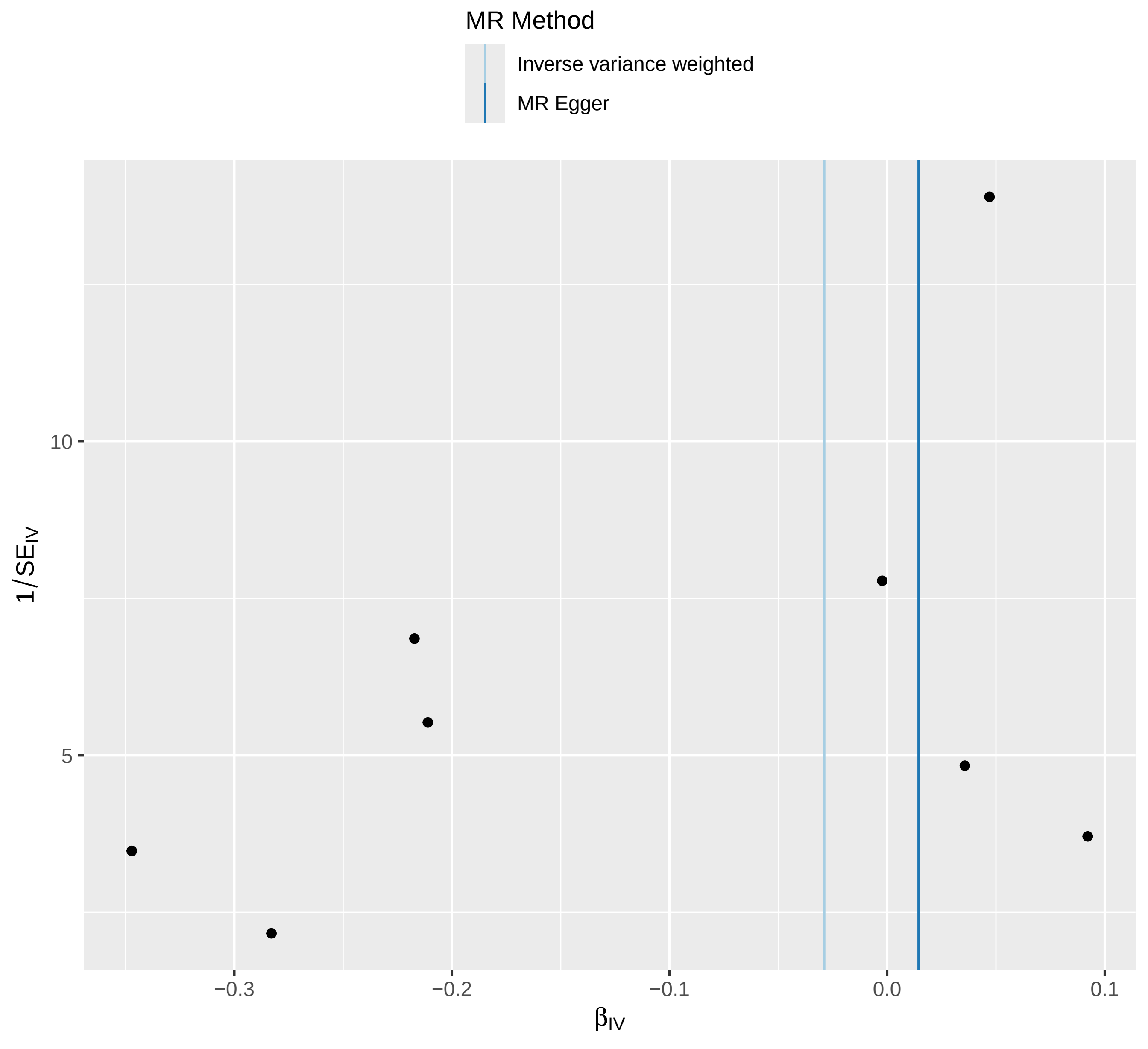
MR Method
Inverse variance weighted
MR Egger
10
5
1/SE_IV
−0.3
−0.2
−0.1
0.0
0.1
β_IV

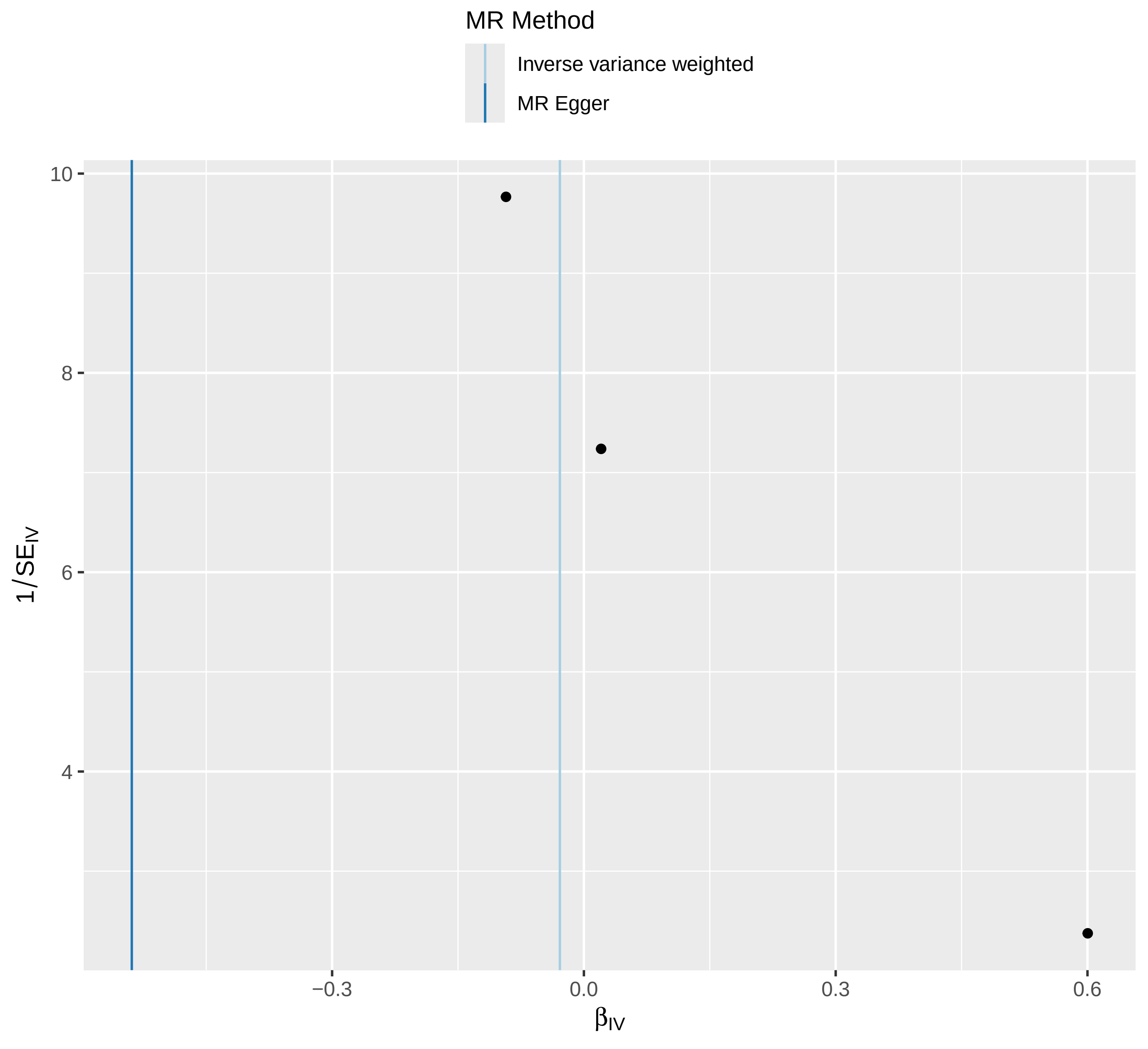
MR Method
Inverse variance weighted
MR Egger
1/SE_IV
β_IV
10
8
6
4
−0.3
0.0
0.3
0.6

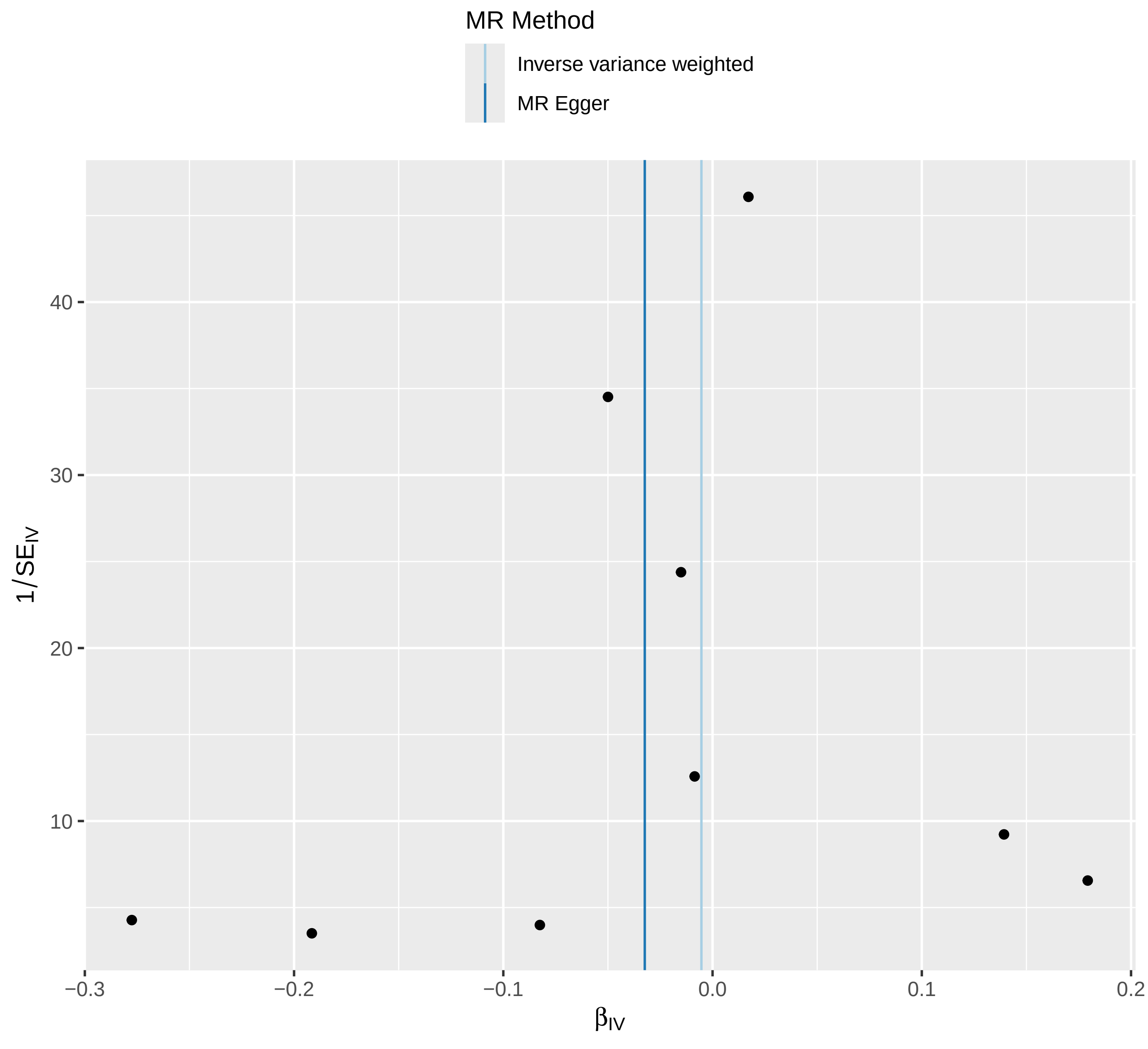
MR Method
Inverse variance weighted
MR Egger
40
30
20
10
1/SE_IV
−0.3
−0.2
−0.1
0.0
0.1
0.2
β_IV

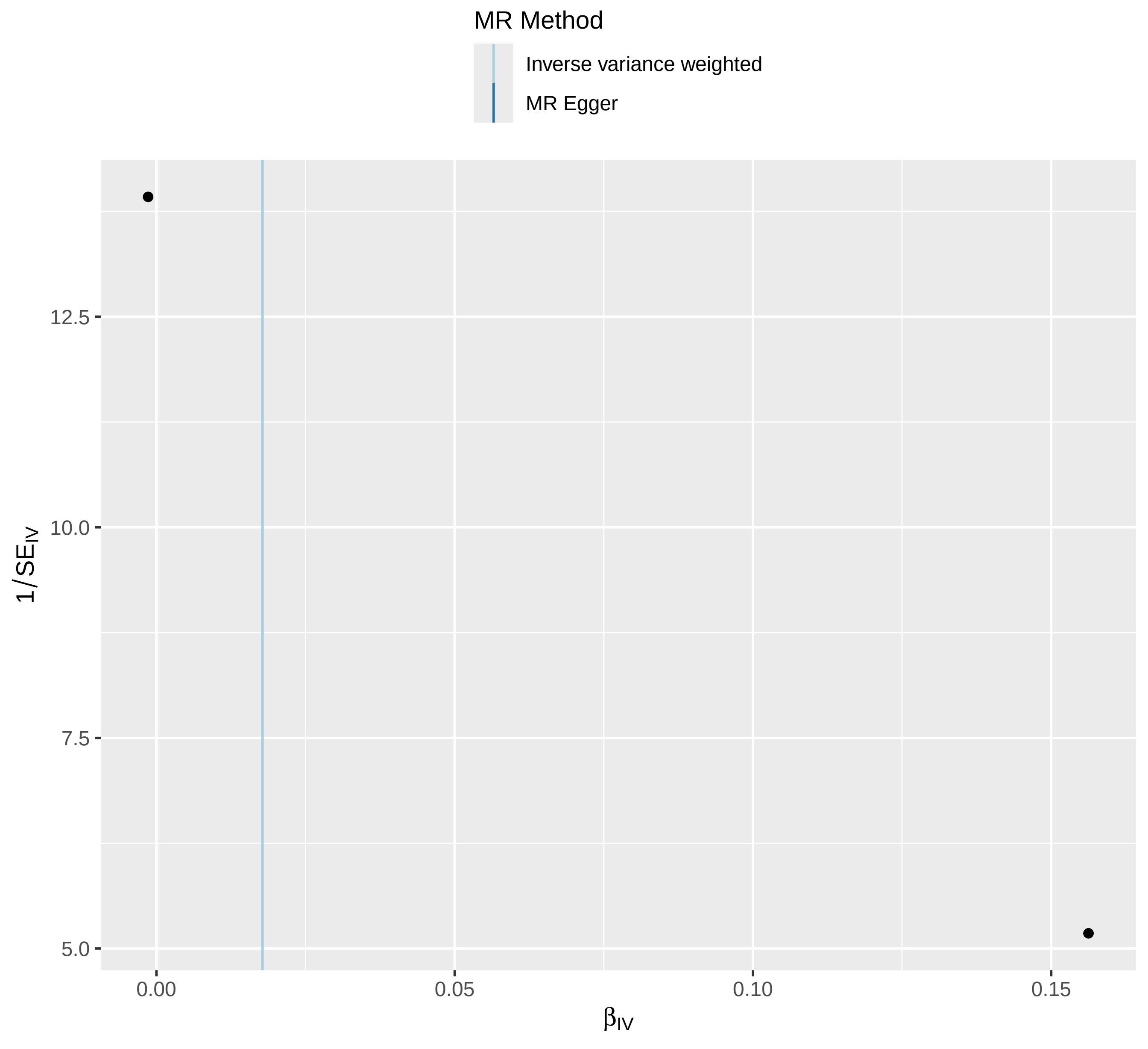
MR Method
Inverse variance weighted
MR Egger
12.5
10.0
7.5
5.0
1/SE_IV
0.00
0.05
0.10
0.15
β_IV

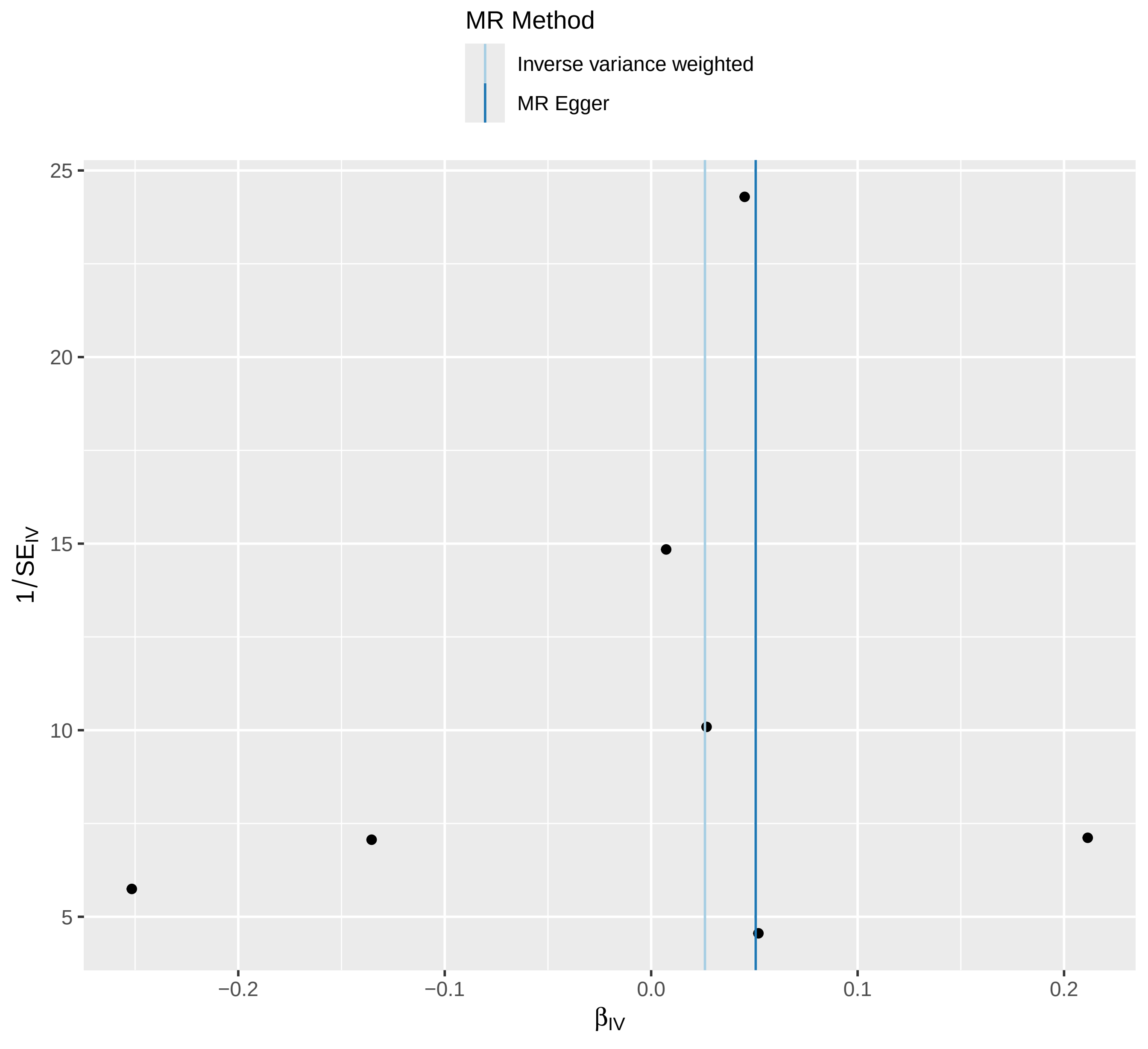
MR Method
Inverse variance weighted
MR Egger
25
20
15
10
5
1/SE$_{IV}$
−0.2
−0.1
0.0
0.1
0.2
β$_{IV}$

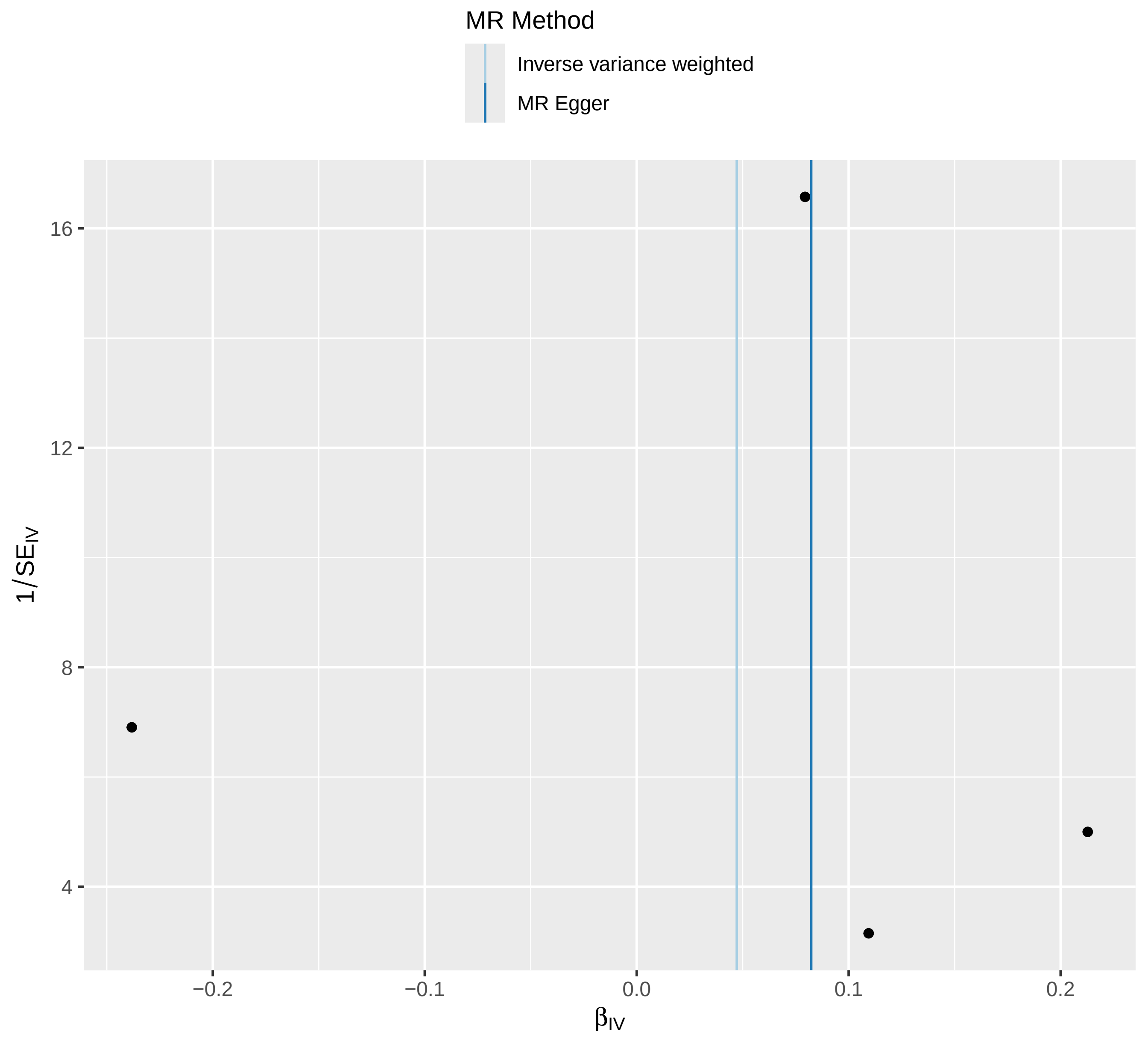
MR Method
Inverse variance weighted
MR Egger
16
12
8
4
1/SE$_{IV}$
−0.2
−0.1
0.0
0.1
0.2
$\beta_{IV}$

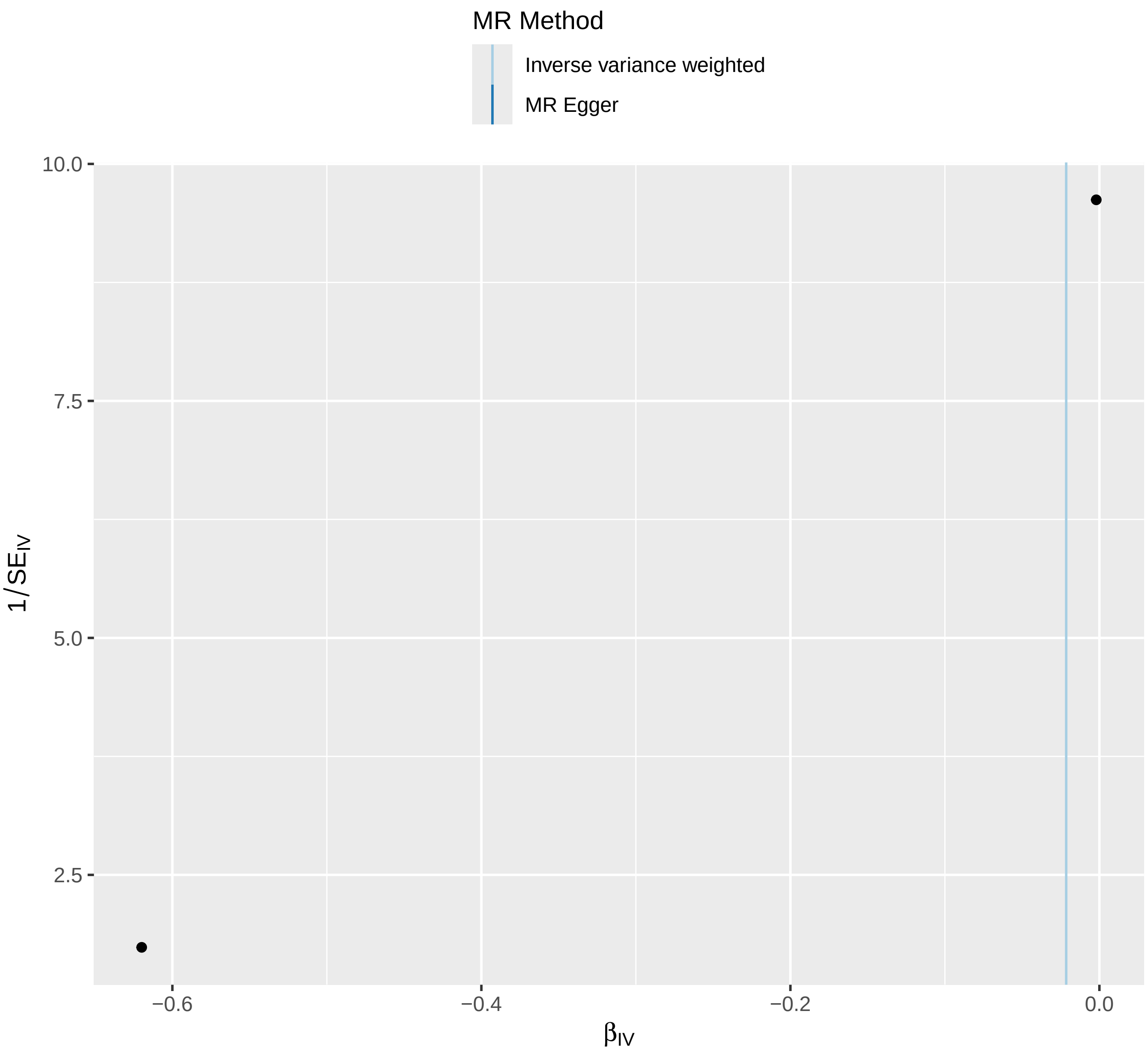

MR Method
Inverse variance weighted
MR Egger
10.0
7.5
5.0
2.5
1/SE_IV
−0.6
−0.4
−0.2
0.0
β_IV

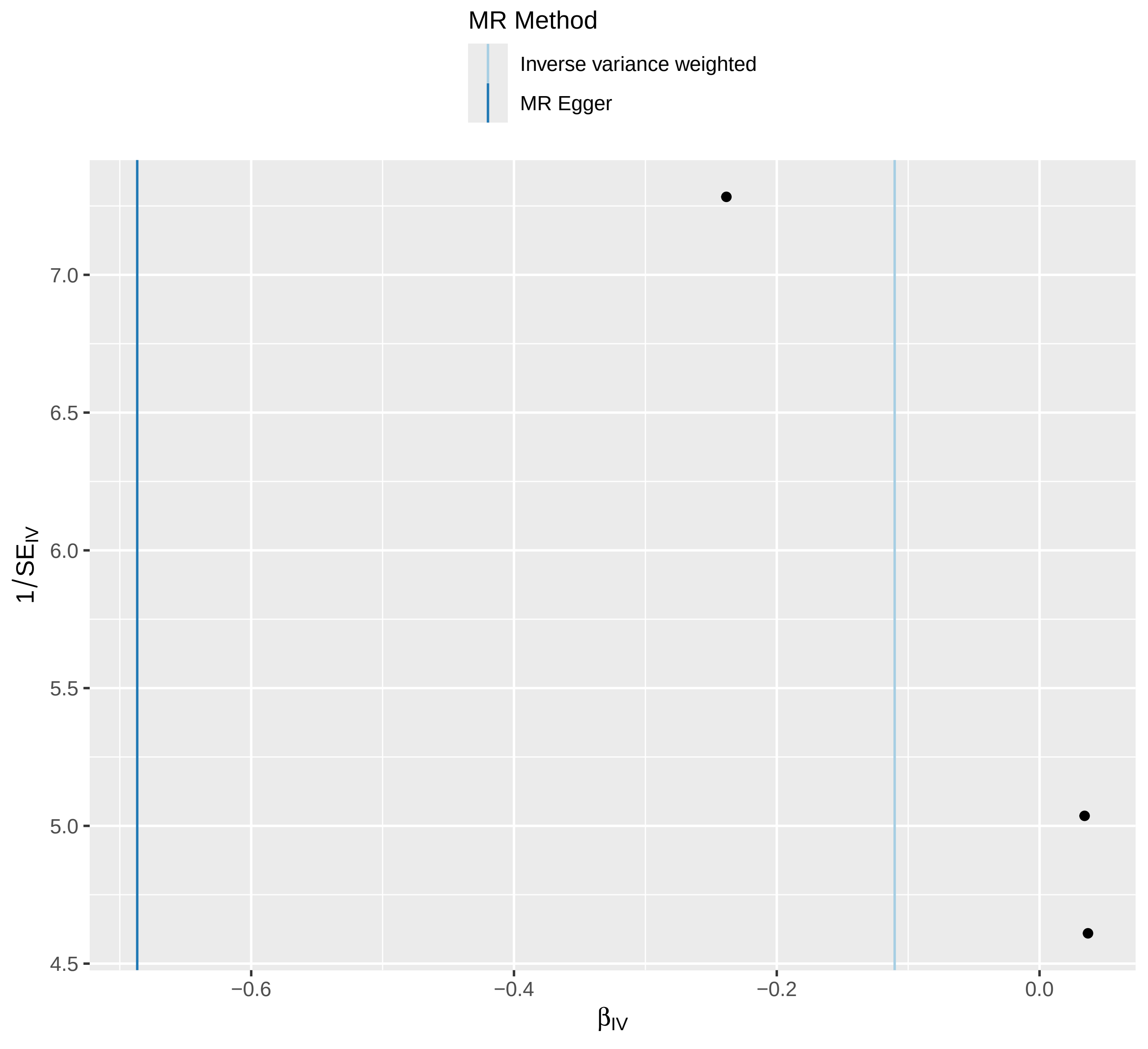

MR Method
Inverse variance weighted
MR Egger
7.0
6.5
6.0
5.5
5.0
4.5
1/SE_IV
−0.6
−0.4
−0.2
0.0
β_IV

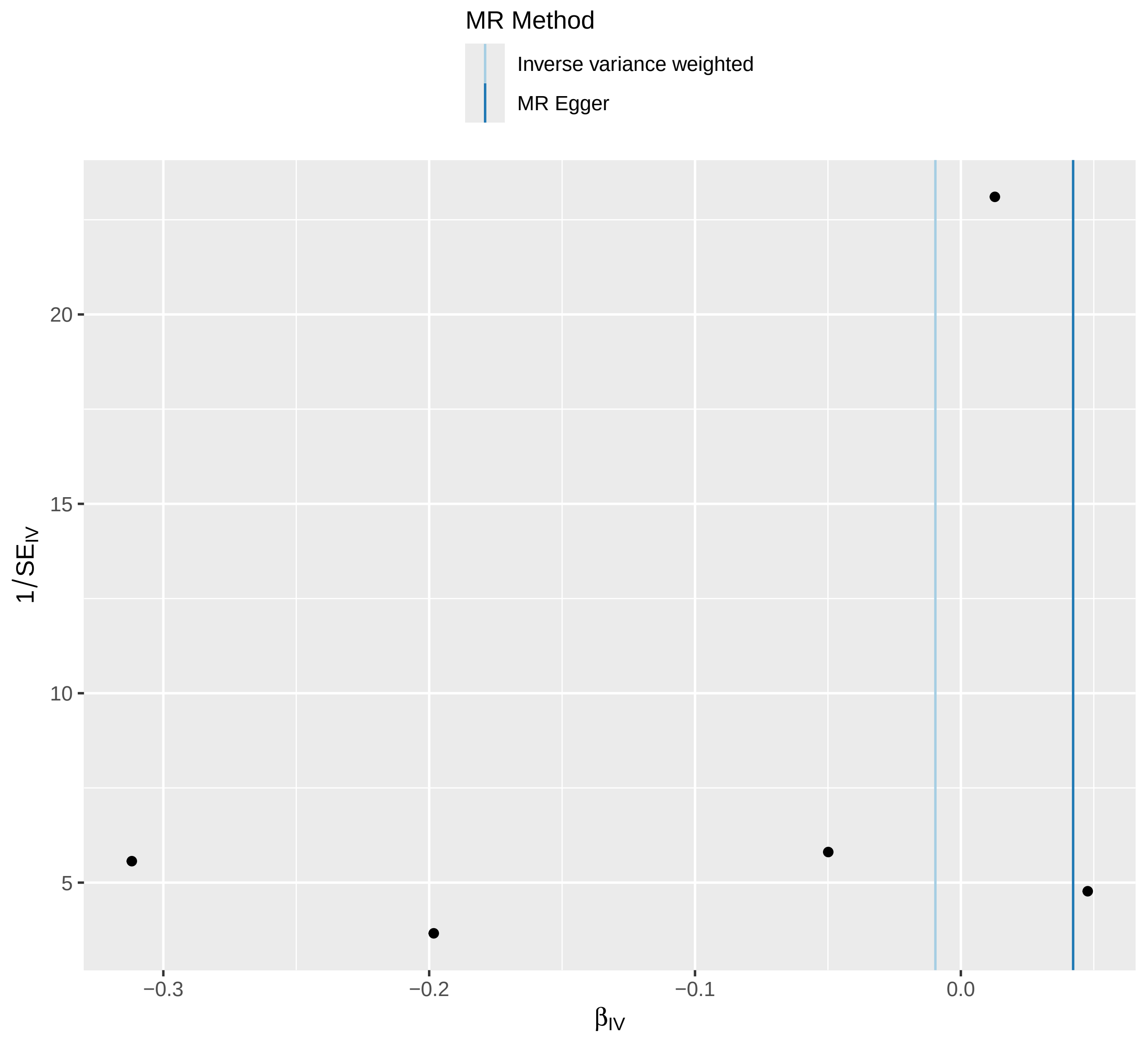

MR Method
Inverse variance weighted
MR Egger
20
15
10
5
−0.3
−0.2
−0.1
0.0
$1/SE_{IV}$
$\beta_{IV}$

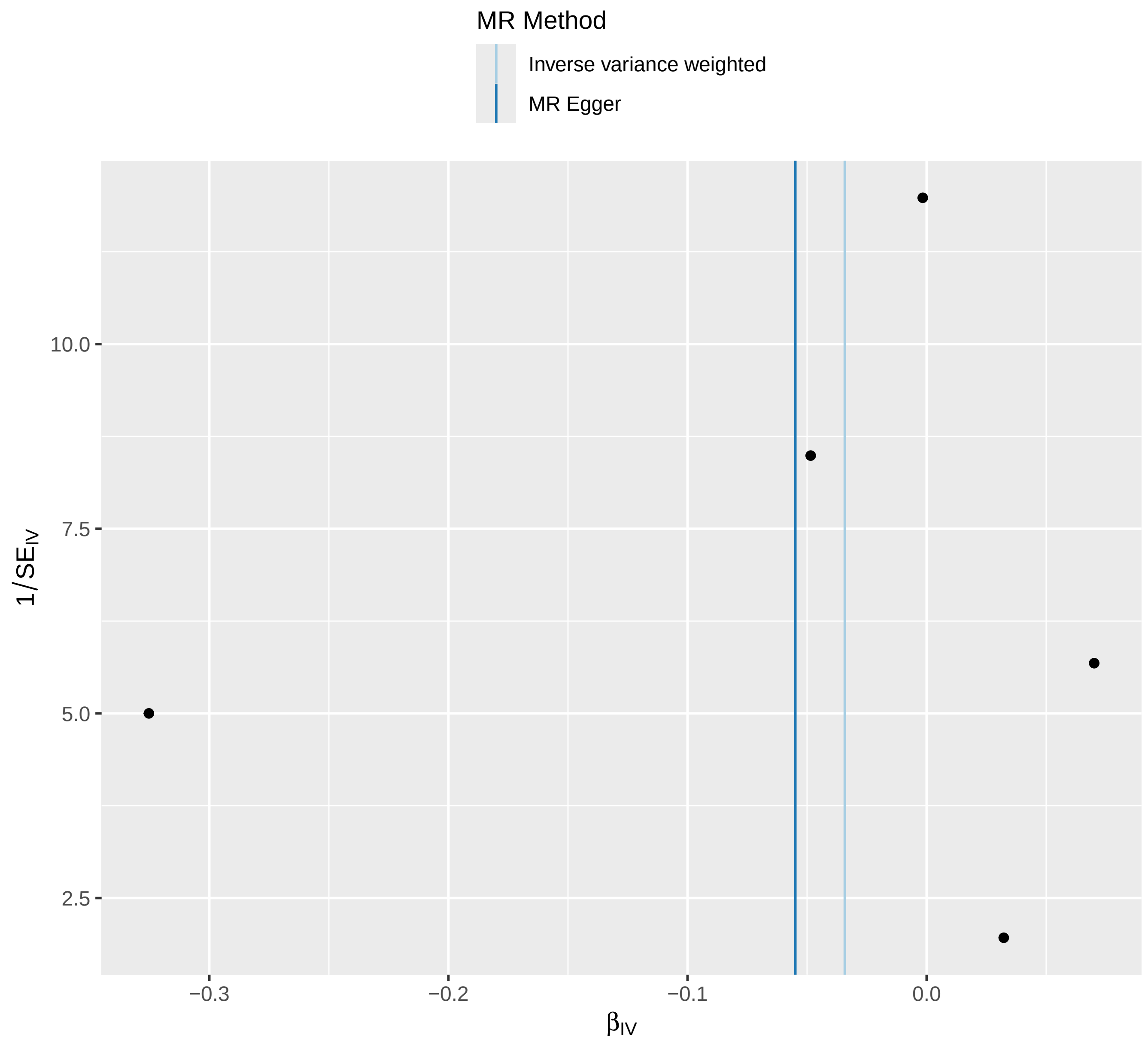
MR Method
Inverse variance weighted
MR Egger
10.0
7.5
5.0
2.5
1/SE_IV
−0.3
−0.2
−0.1
0.0
β_IV

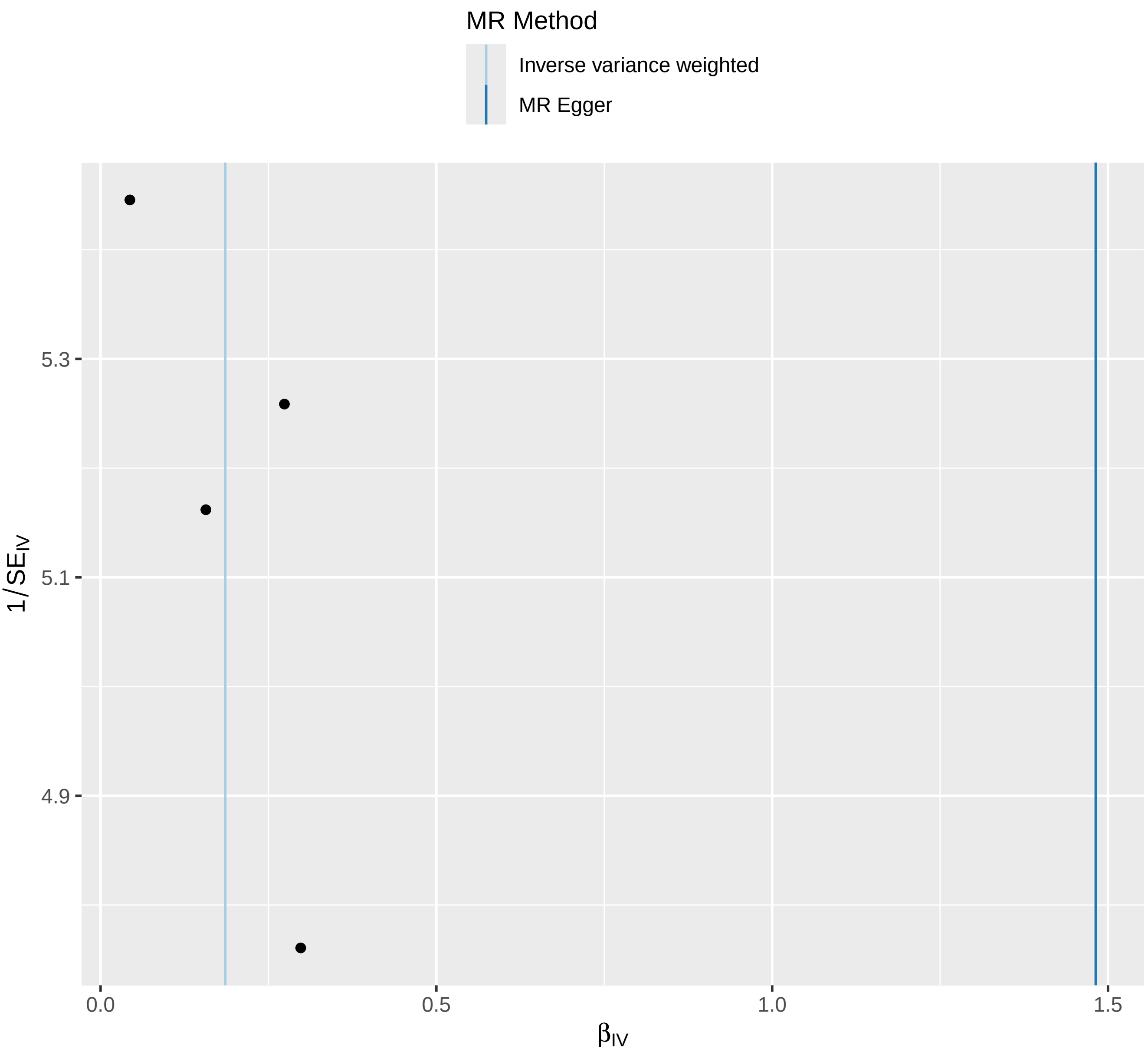
MR Method
Inverse variance weighted
MR Egger
1/SE_IV
5.3
5.1
4.9
0.0
0.5
1.0
1.5
β_IV

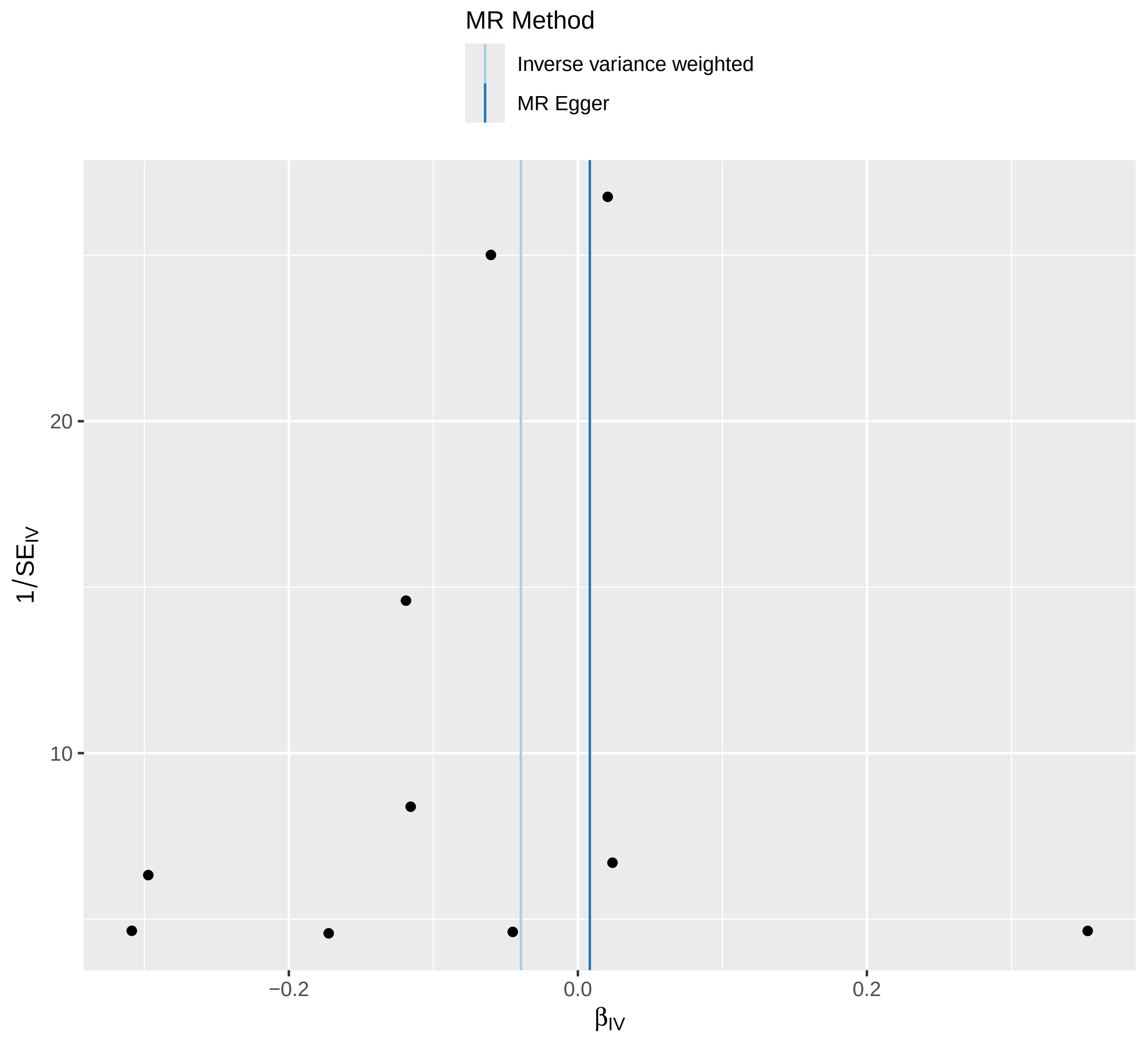

MR Method
Inverse variance weighted
MR Egger
20
10
1/SE_IV
−0.2
0.0
0.2
β_IV

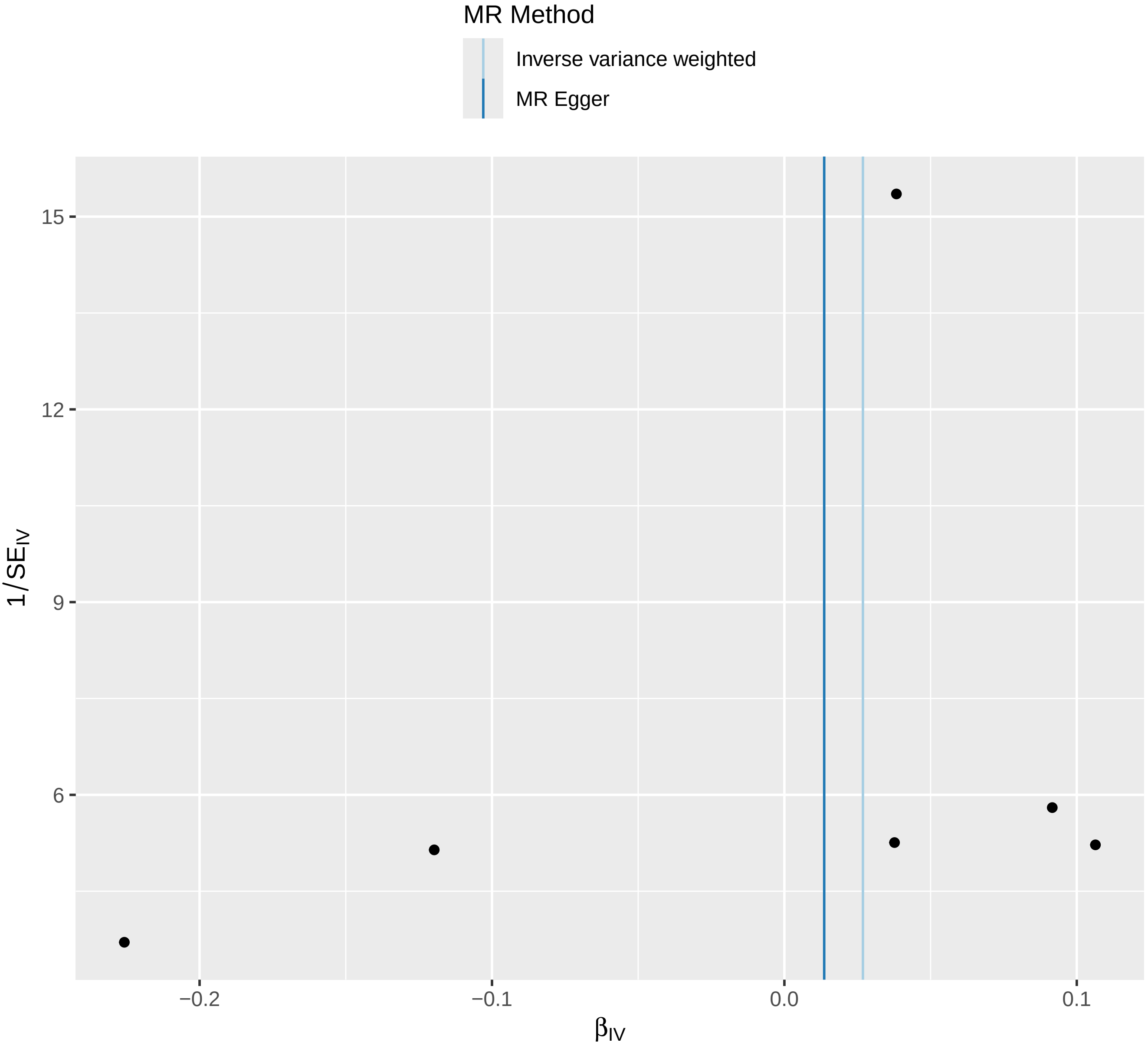
MR Method
Inverse variance weighted
MR Egger
15
12
9
6
$1/SE_{IV}$
−0.2
−0.1
0.0
0.1
$\beta_{IV}$

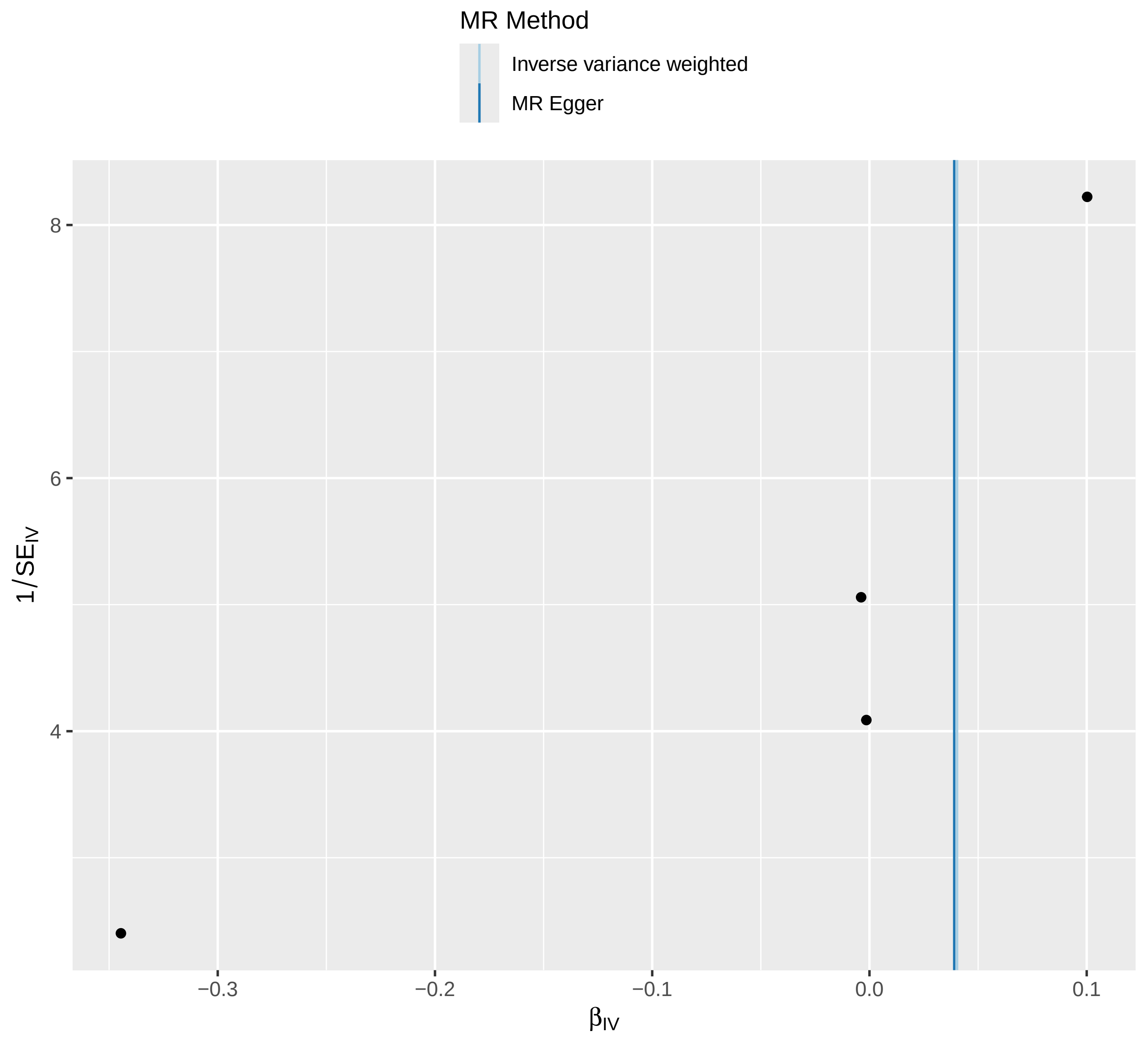
MR Method
Inverse variance weighted
MR Egger
8
6
4
1/SE_IV
−0.3
−0.2
−0.1
0.0
0.1
β_IV

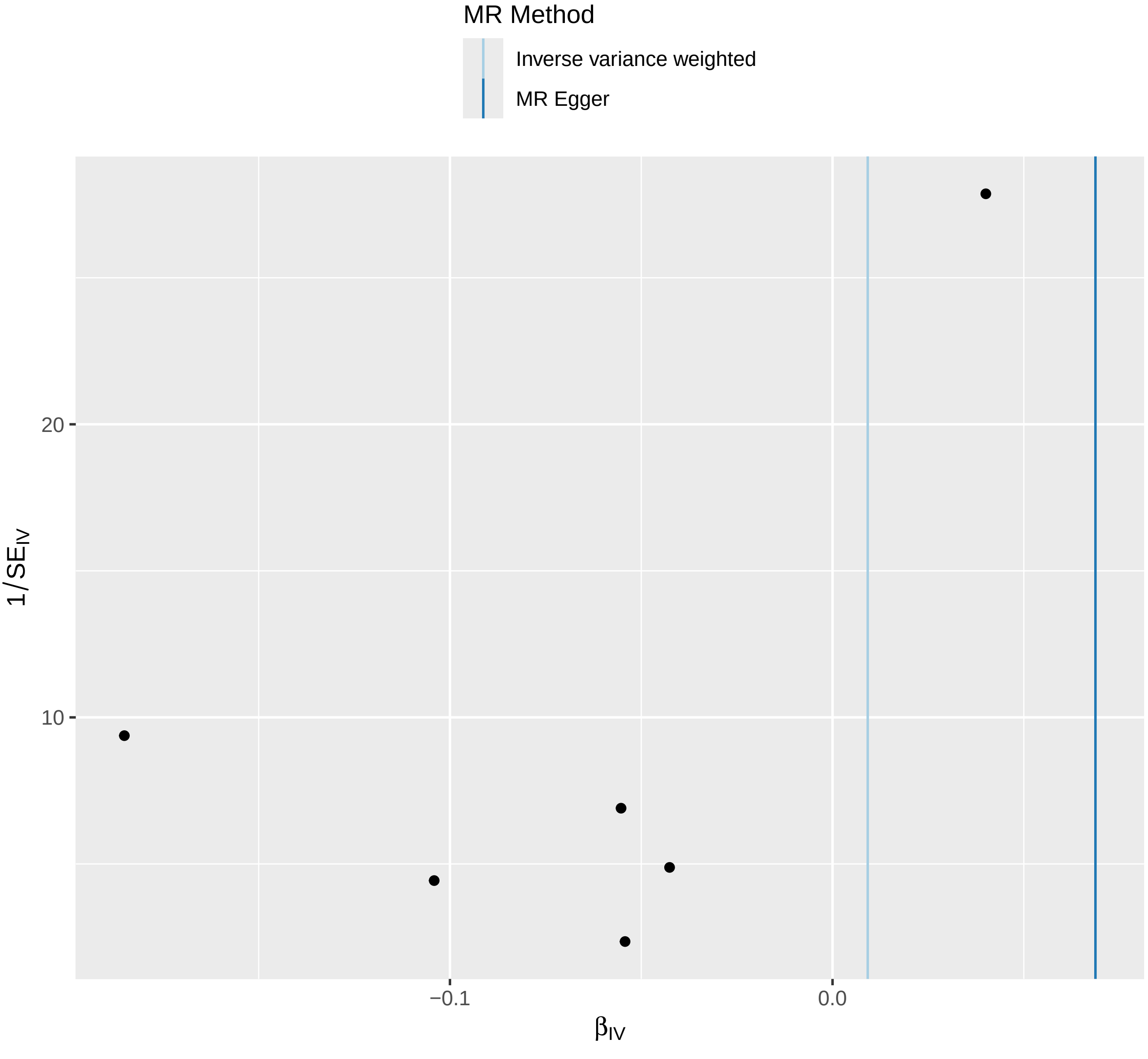

MR Method
Inverse variance weighted
MR Egger
20
10
1/SE$_{IV}$
−0.1
0.0
β$_{IV}$

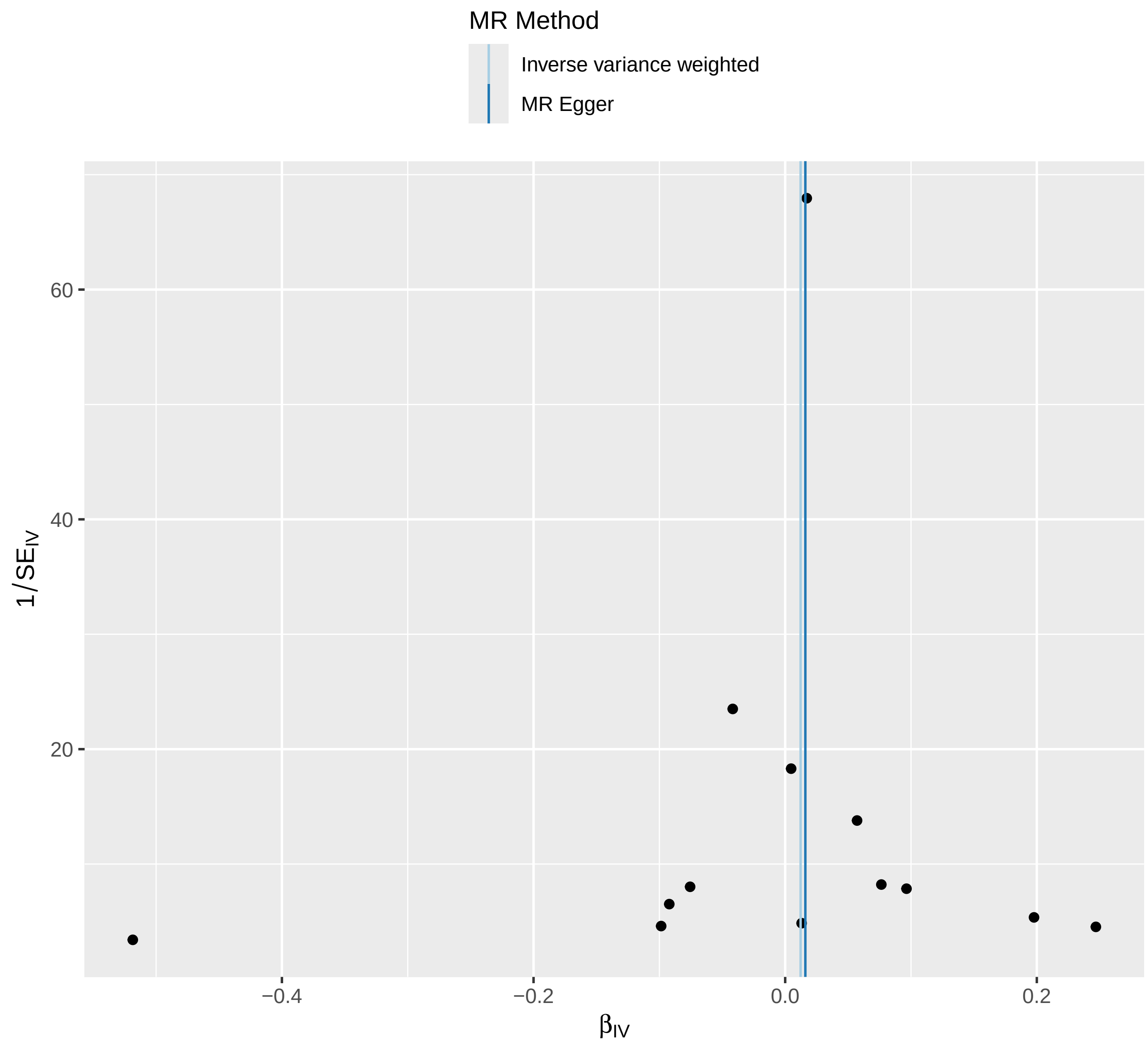

MR Method
Inverse variance weighted
MR Egger
60
40
20
1/SE$_{IV}$
−0.4
−0.2
0.0
0.2
β$_{IV}$

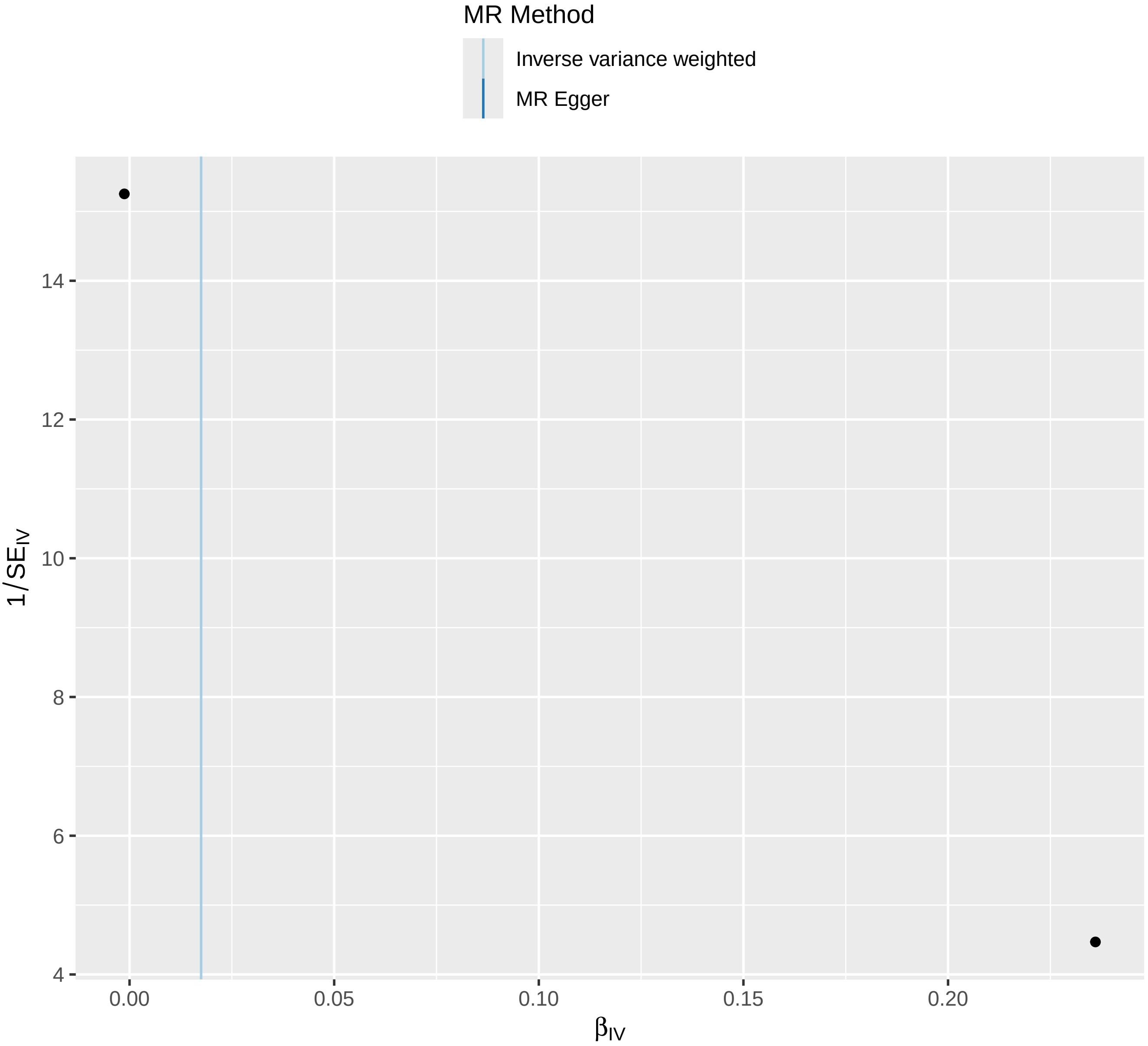
MR Method
Inverse variance weighted
MR Egger
14
12
10
8
6
4
1/SE$_{IV}$
0.00
0.05
0.10
0.15
0.20
β$_{IV}$

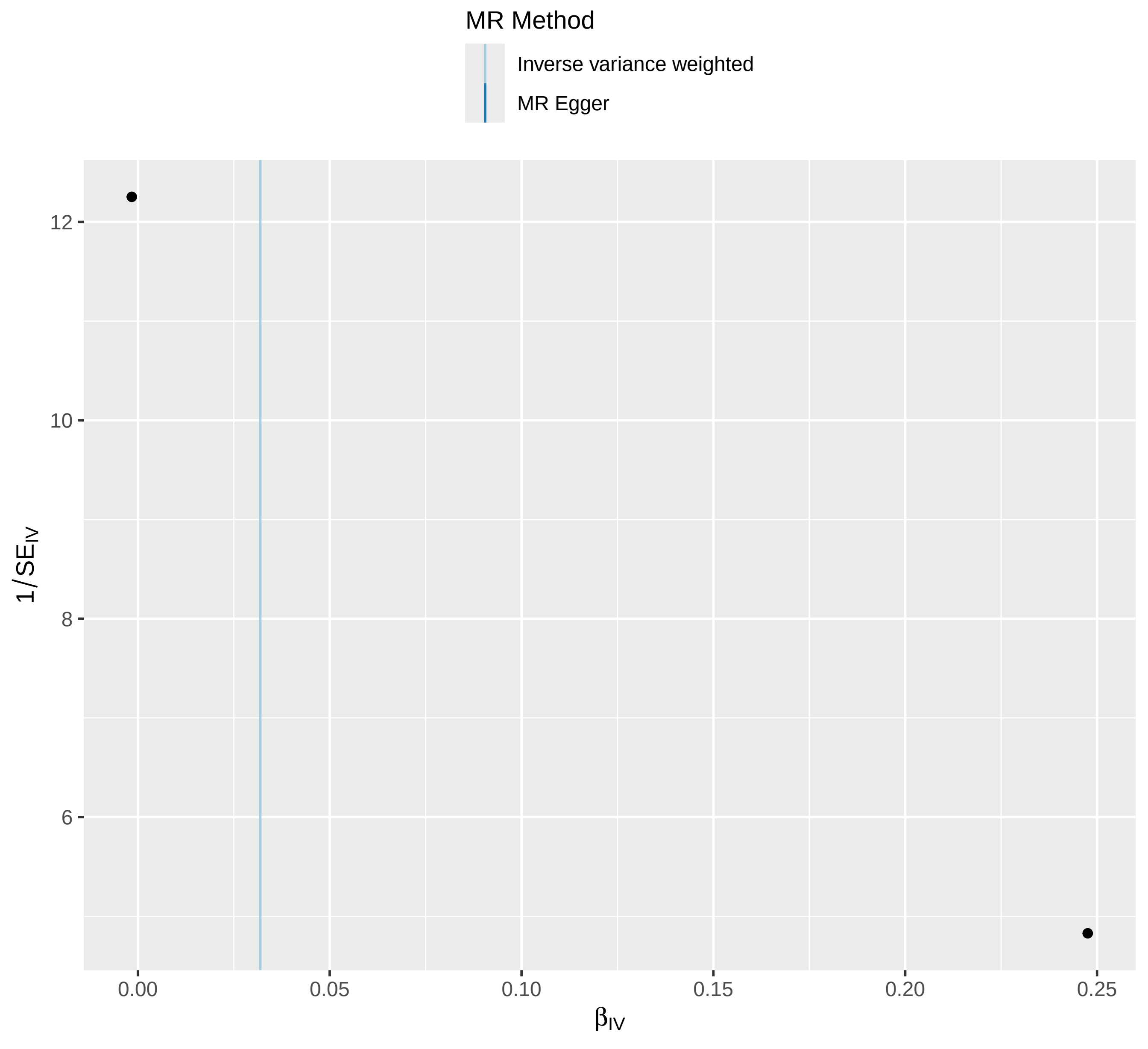
MR Method
Inverse variance weighted
MR Egger
12
10
8
6
1/SE_IV
0.00
0.05
0.10
0.15
0.20
0.25
β_IV

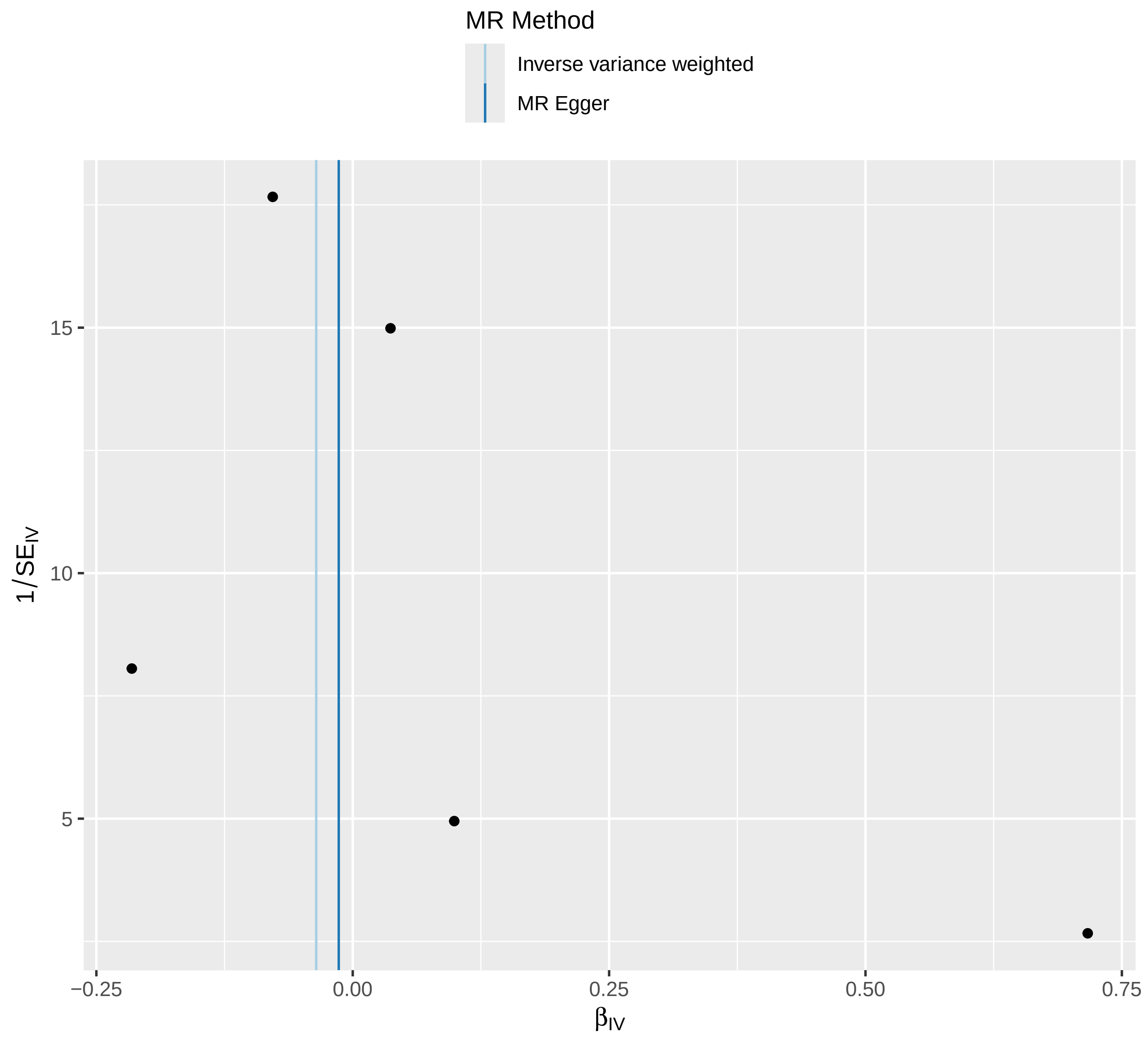

MR Method
Inverse variance weighted
MR Egger
15
10
5
1/SE$_{IV}$
−0.25
0.00
0.25
0.50
0.75
β$_{IV}$

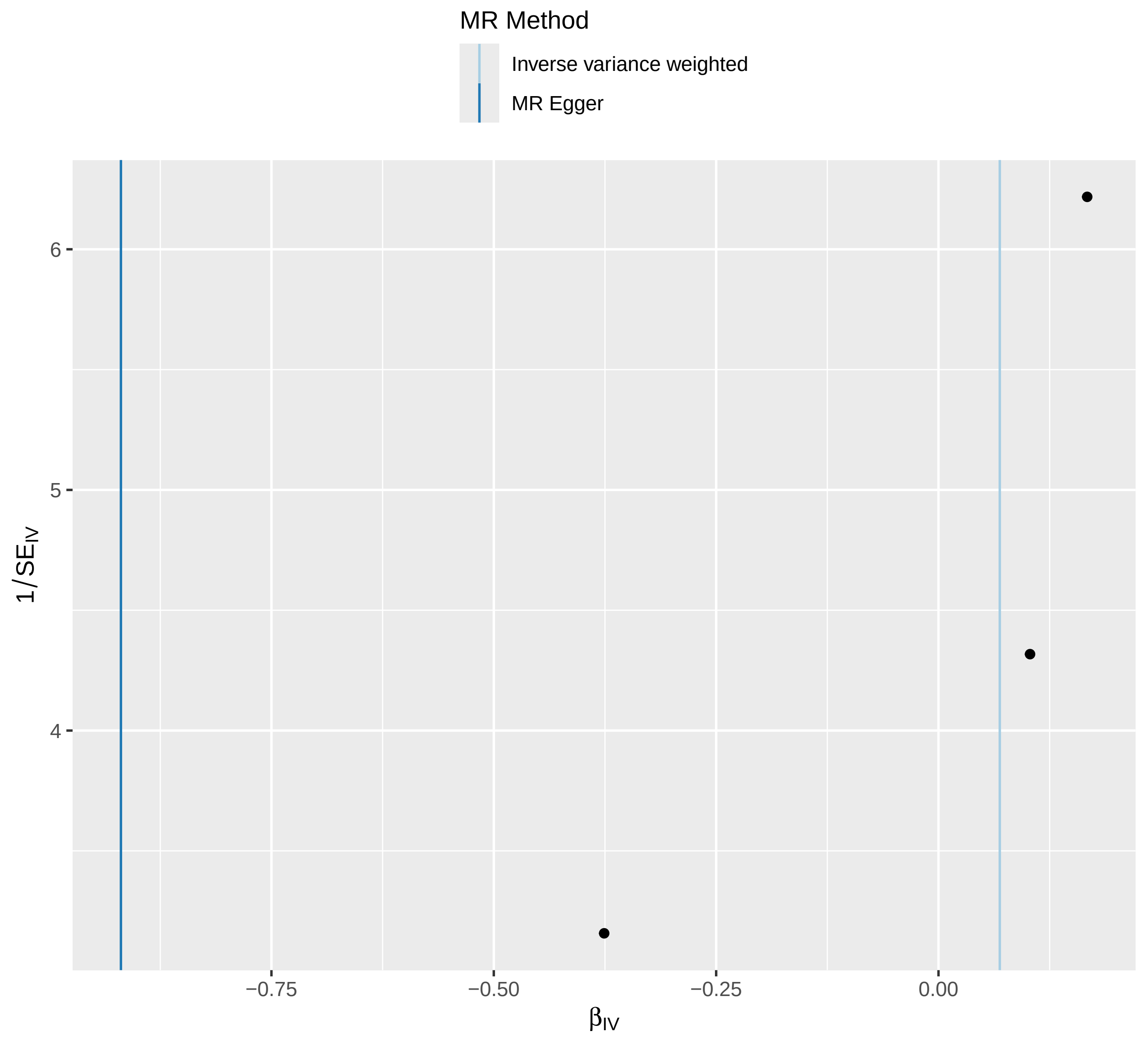
MR Method
Inverse variance weighted
MR Egger
6
5
4
$1/SE_{IV}$
−0.75
−0.50
−0.25
0.00
$\beta_{IV}$

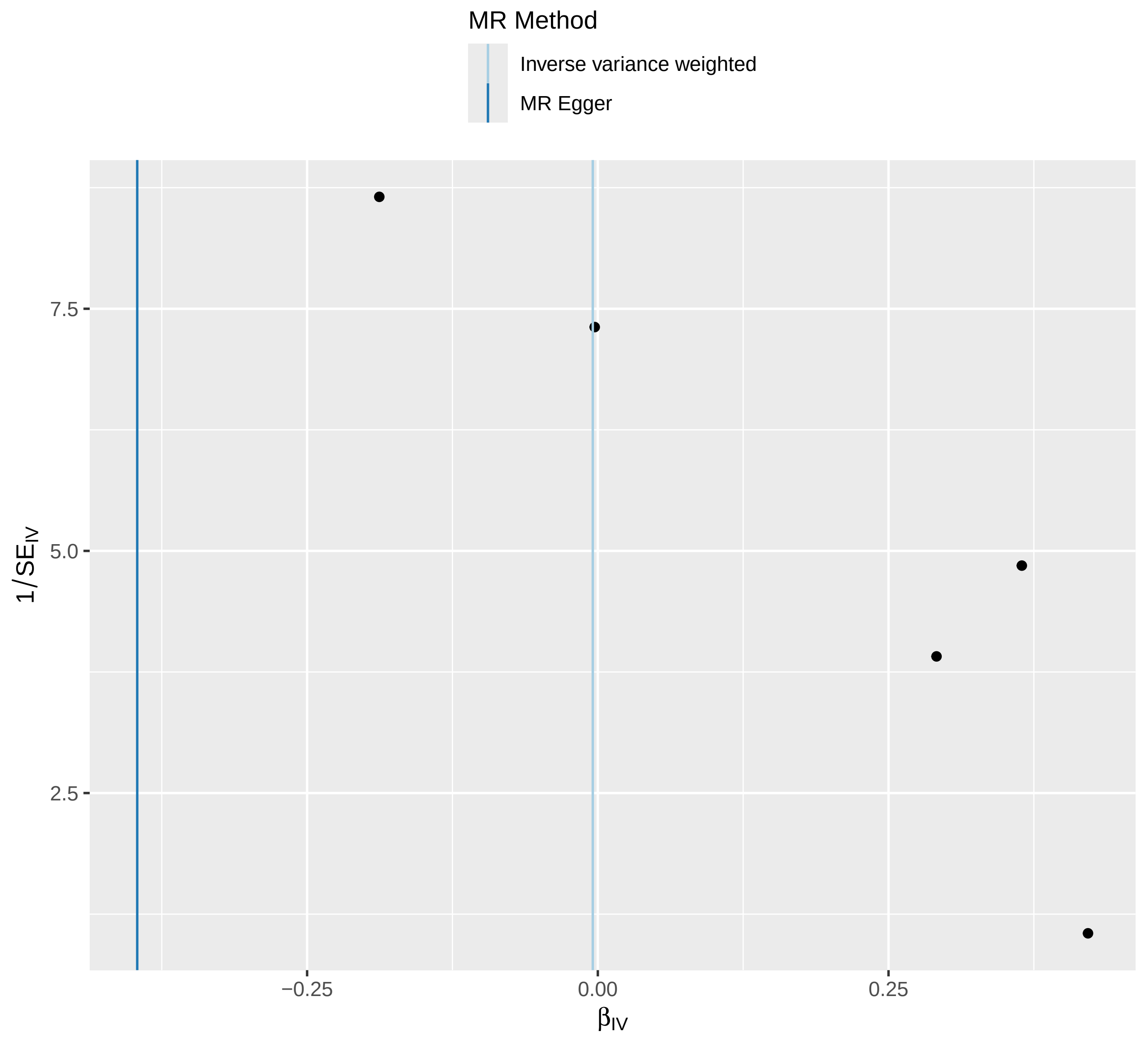
MR Method
Inverse variance weighted
MR Egger
7.5
5.0
2.5
1/SE$_{IV}$
−0.25
0.00
0.25
β$_{IV}$

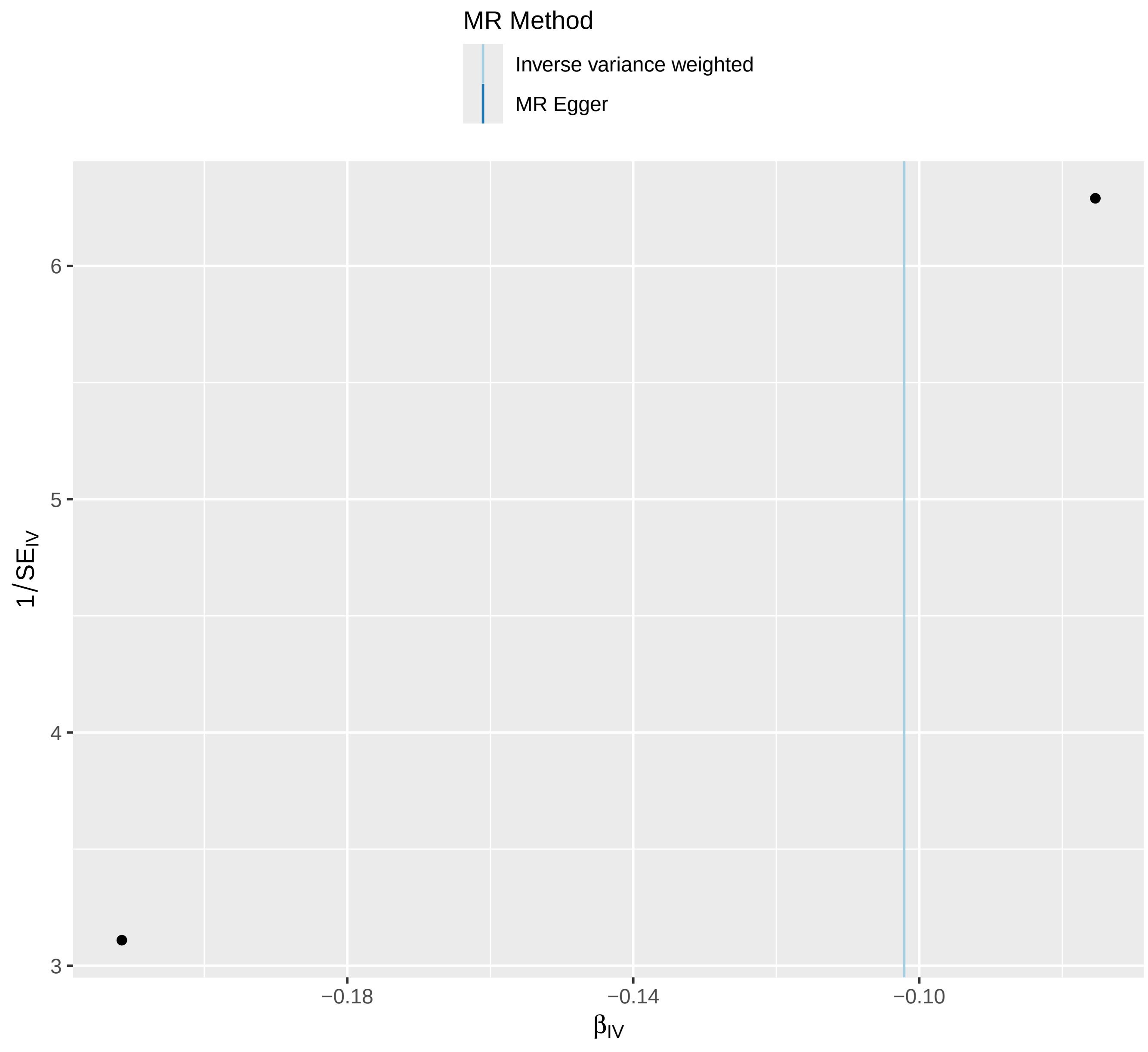
MR Method
Inverse variance weighted
MR Egger
6
5
4
3
1/SE_IV
−0.18
−0.14
−0.10
β_IV

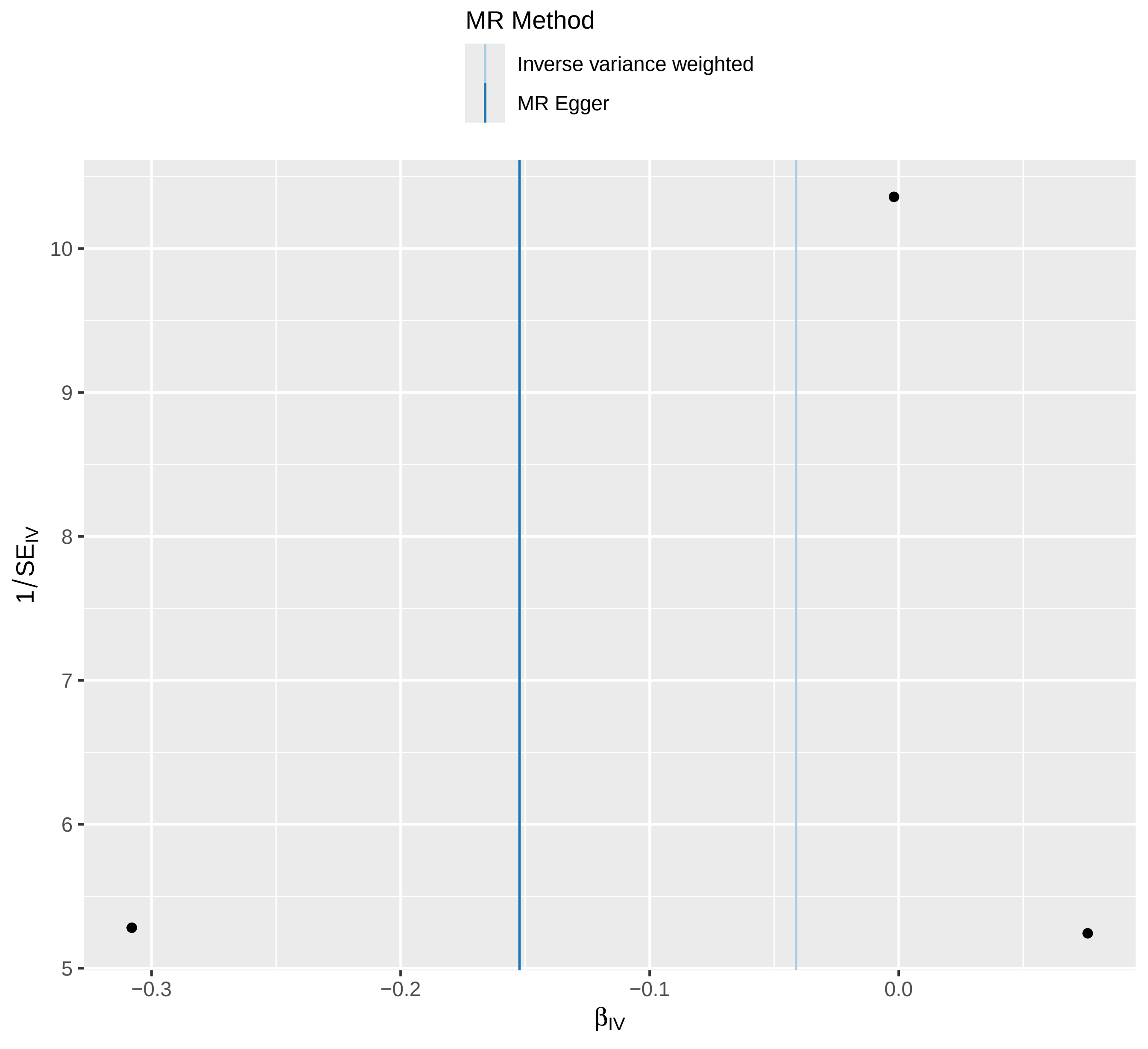

MR Method
Inverse variance weighted
MR Egger
10
9
8
7
6
5
1/SE_IV
−0.3
−0.2
−0.1
0.0
β_IV

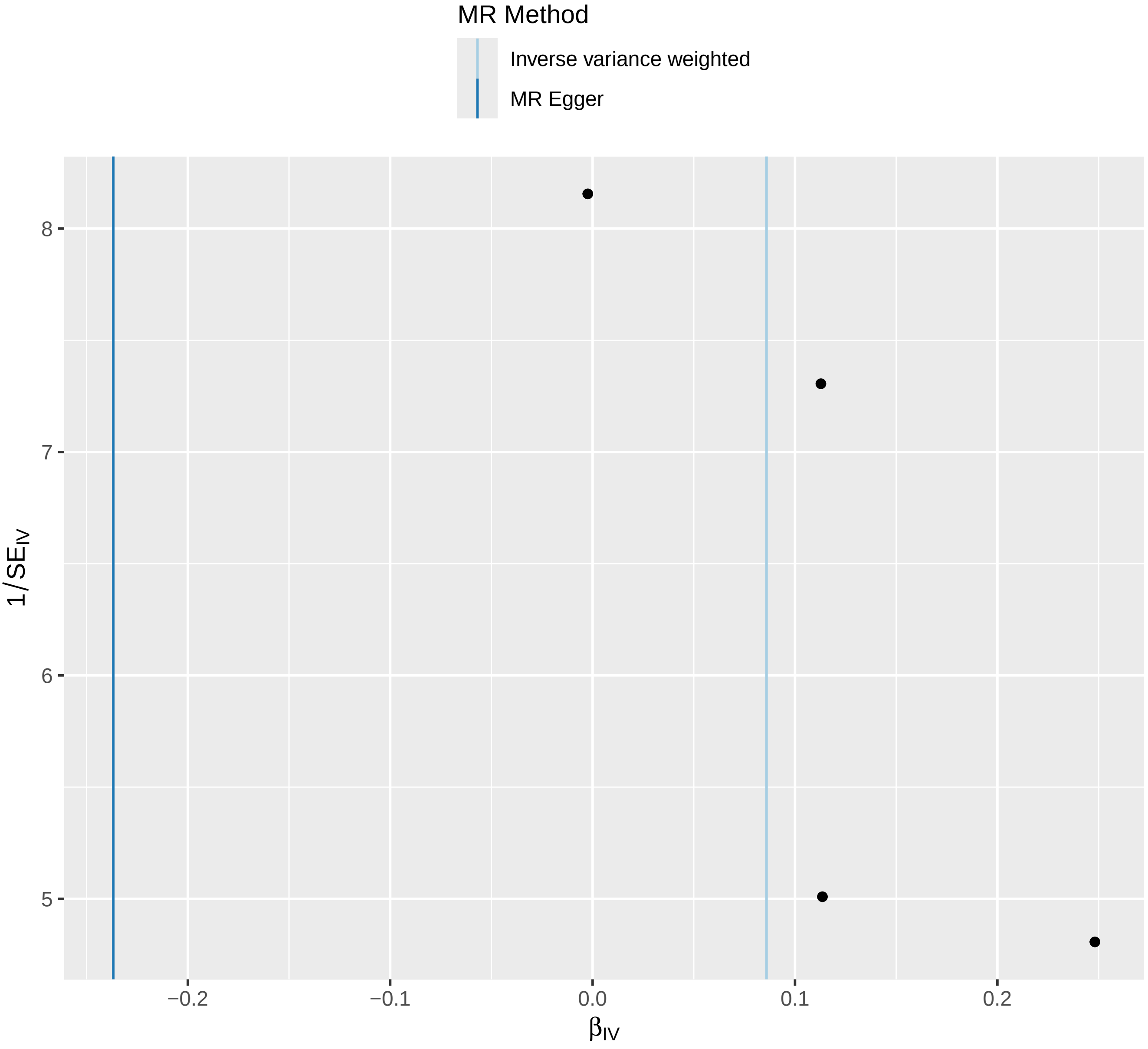
MR Method
Inverse variance weighted
MR Egger
8
7
6
5
1/SE$_{IV}$
−0.2
−0.1
0.0
0.1
0.2
β$_{IV}$

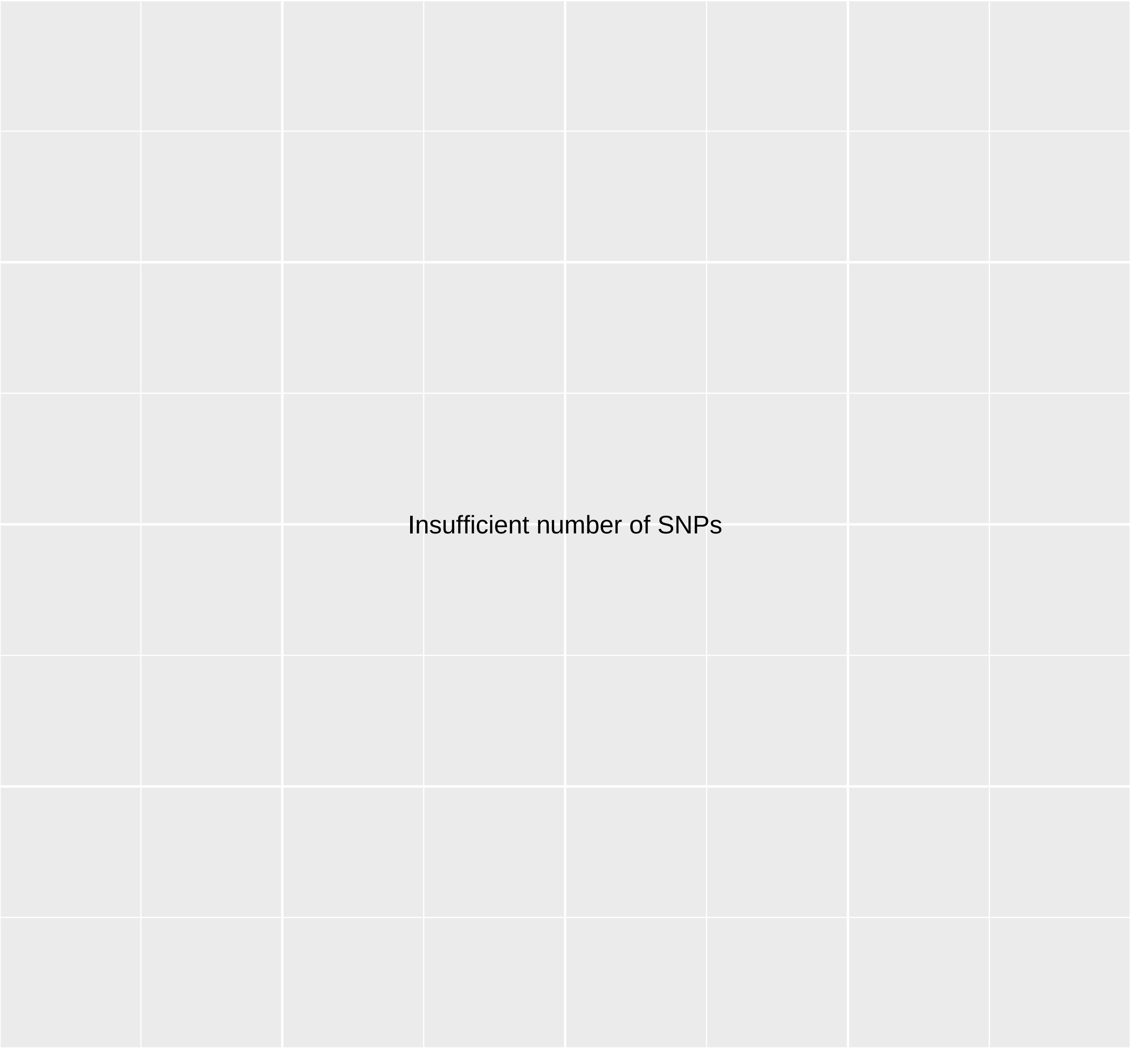
Insufficient number of SNPs

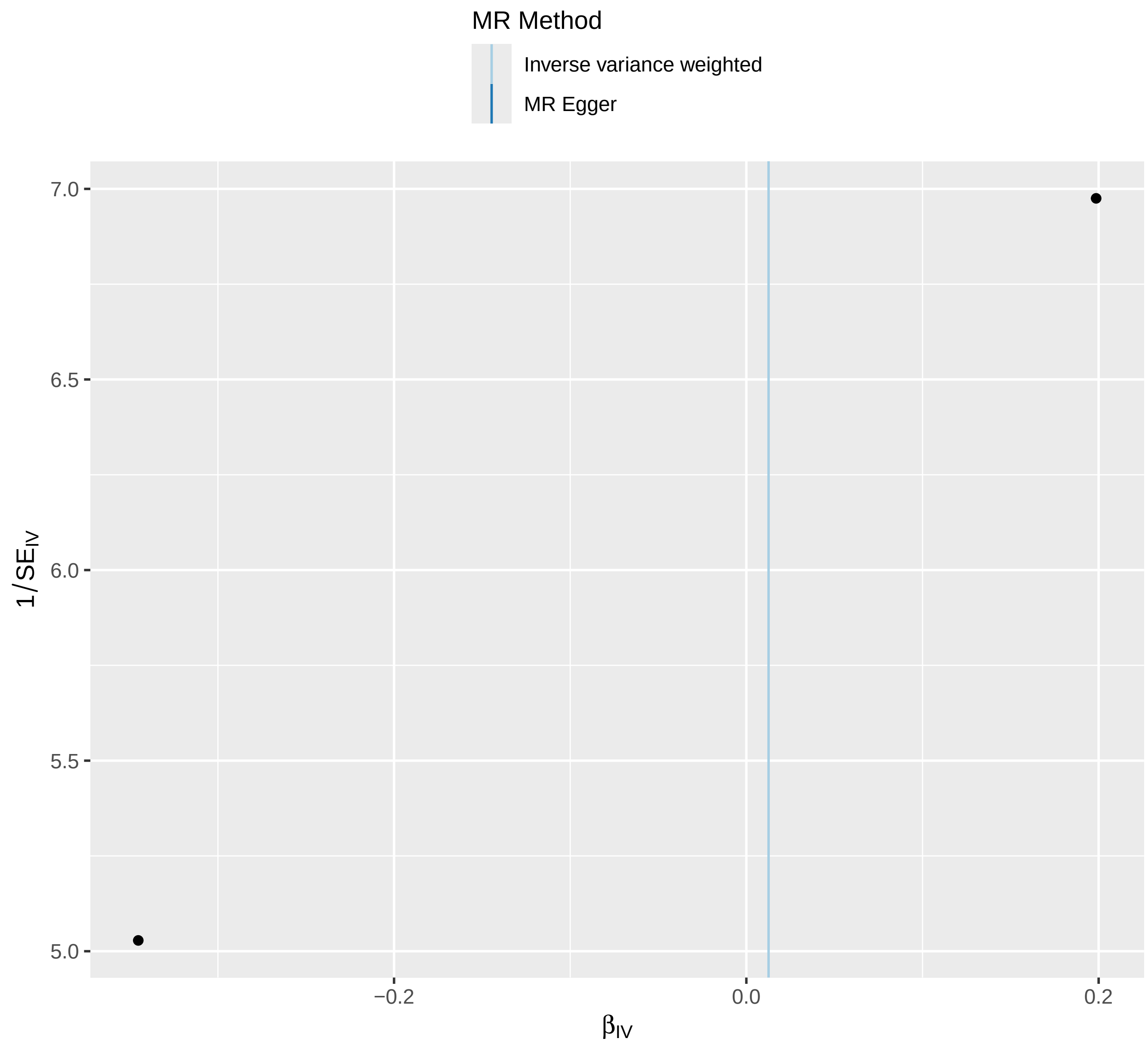
MR Method
Inverse variance weighted
MR Egger
1/SE_IV
7.0
6.5
6.0
5.5
5.0
−0.2
0.0
0.2
β_IV

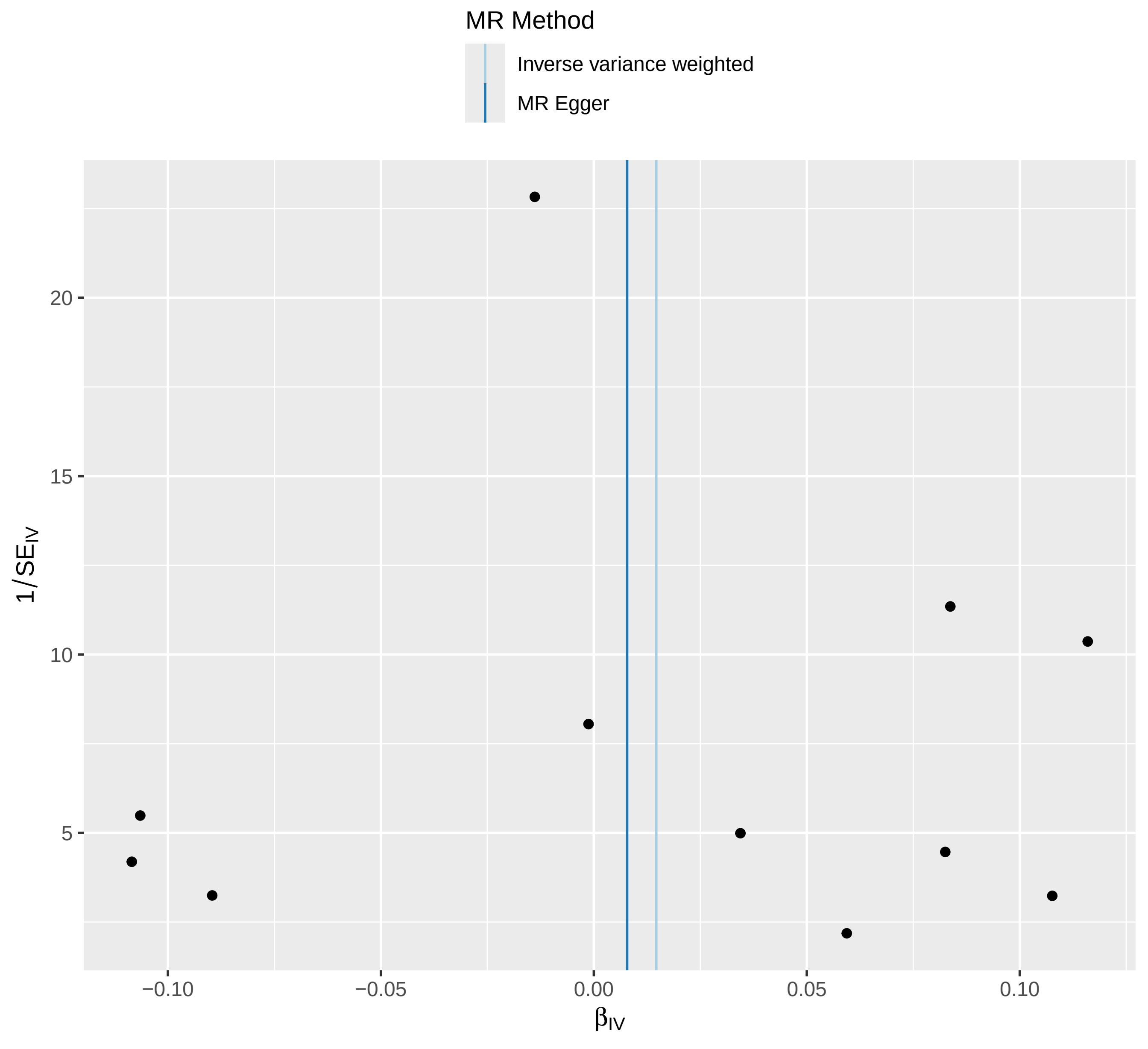
MR Method
Inverse variance weighted
MR Egger
20
15
10
5
1/SE$_{IV}$
−0.10
−0.05
0.00
0.05
0.10
β$_{IV}$

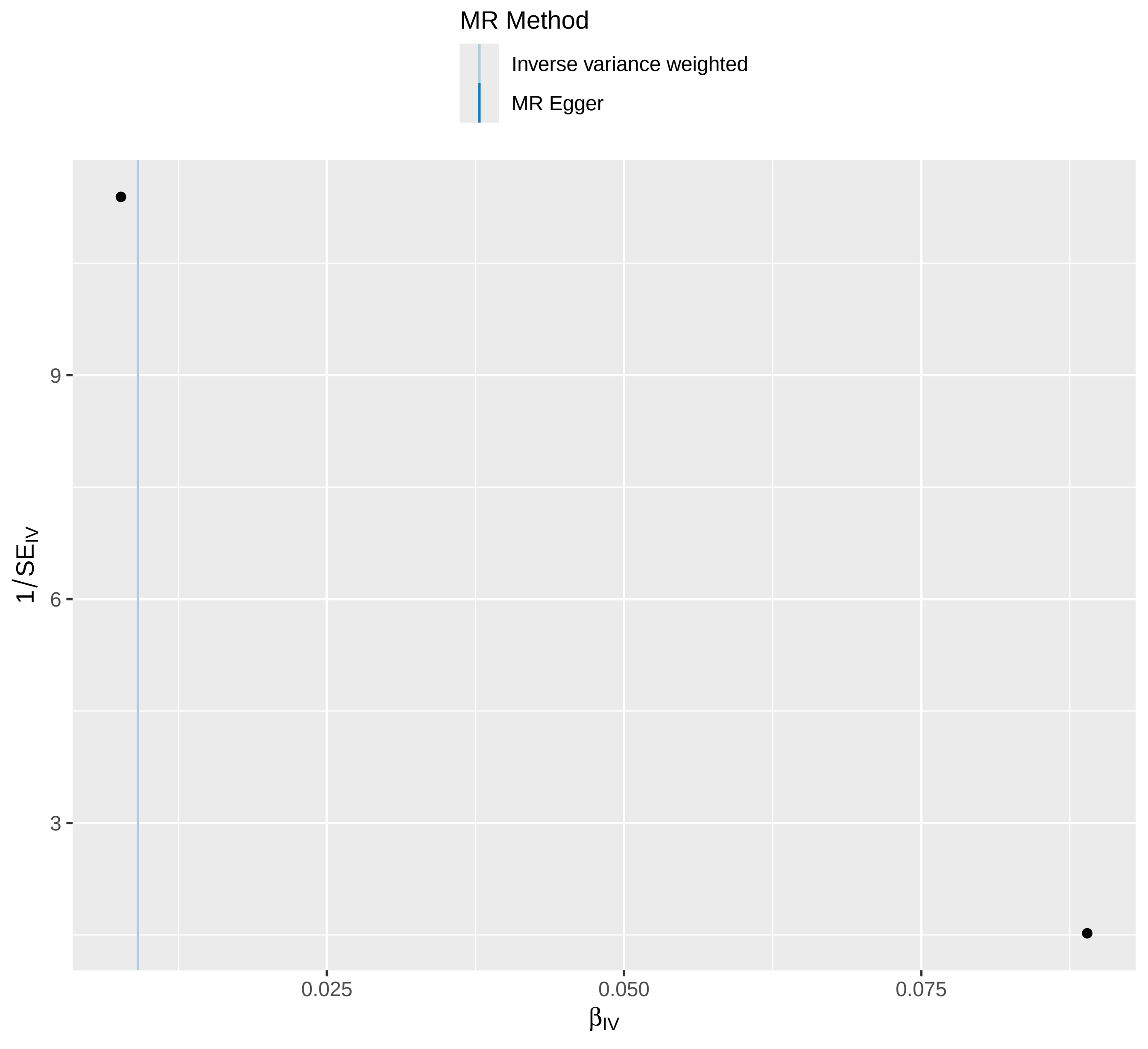
MR Method
Inverse variance weighted
MR Egger
9
6
3
1/SE_IV
0.025
0.050
0.075
β_IV

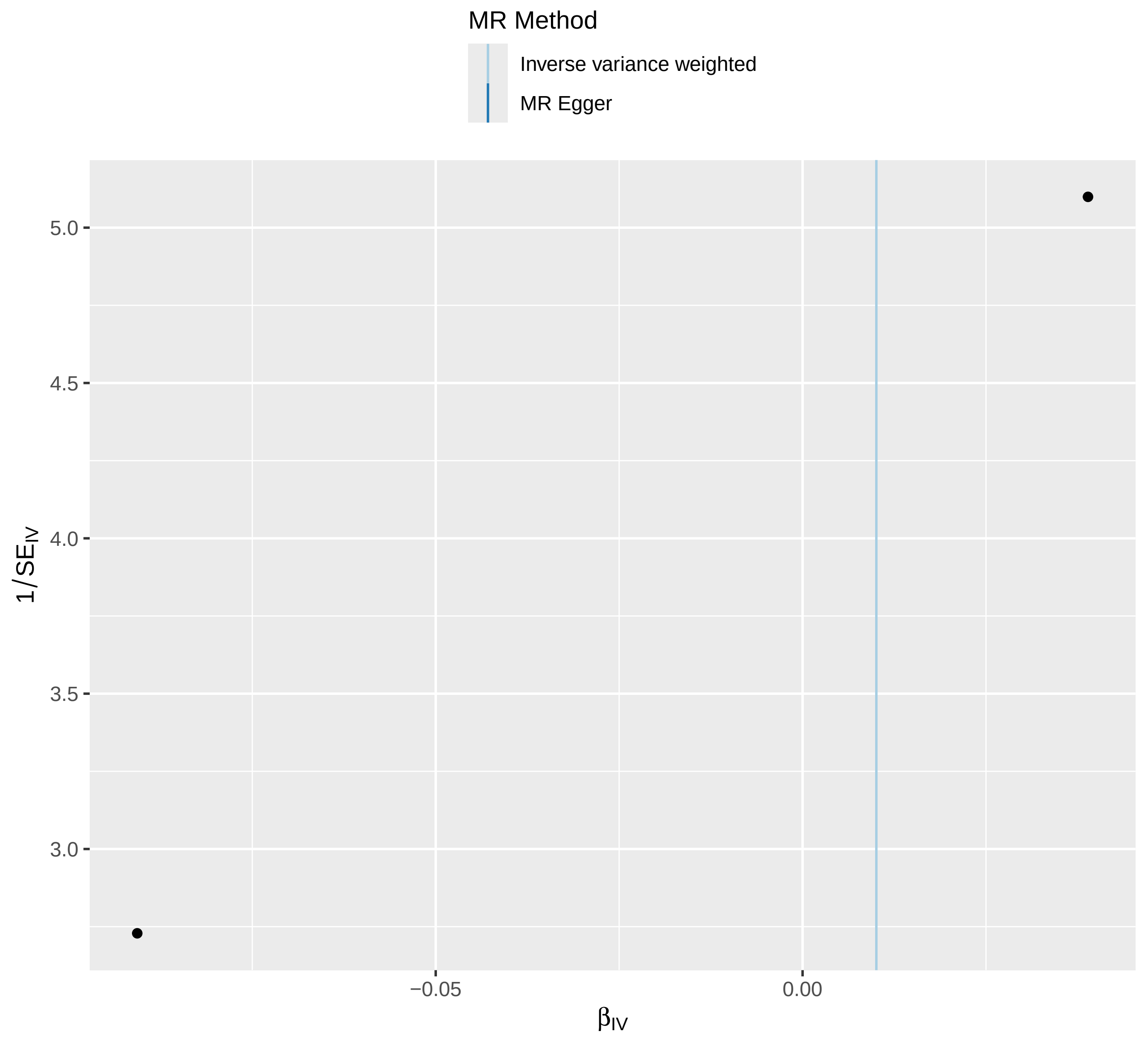
MR Method
Inverse variance weighted
MR Egger
5.0
4.5
4.0
3.5
3.0
1/SE$_{IV}$
−0.05
0.00
β$_{IV}$

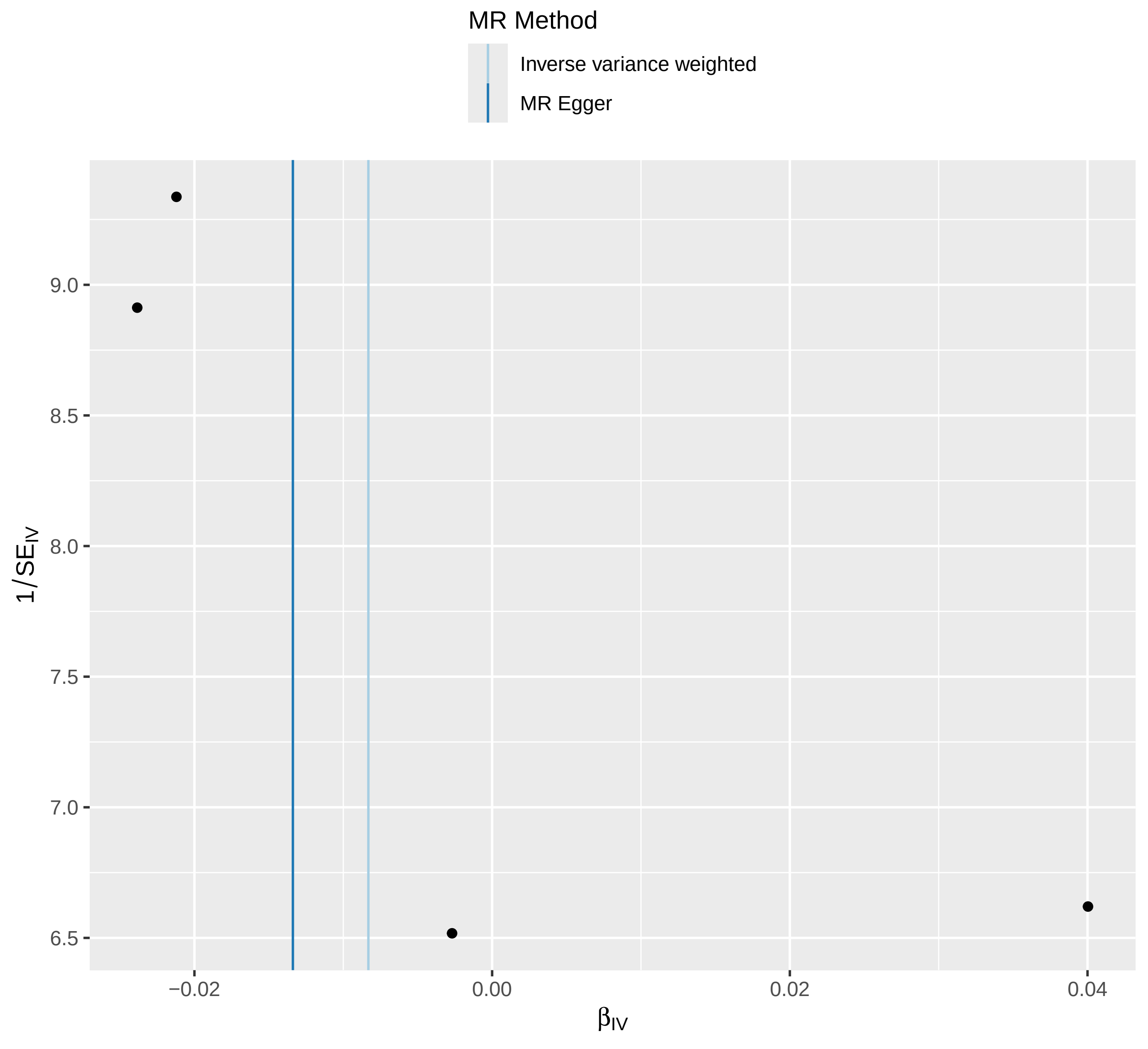

MR Method
Inverse variance weighted
MR Egger
9.0
8.5
8.0
7.5
7.0
6.5
$1/SE_{IV}$
−0.02
0.00
0.02
0.04
$\beta_{IV}$

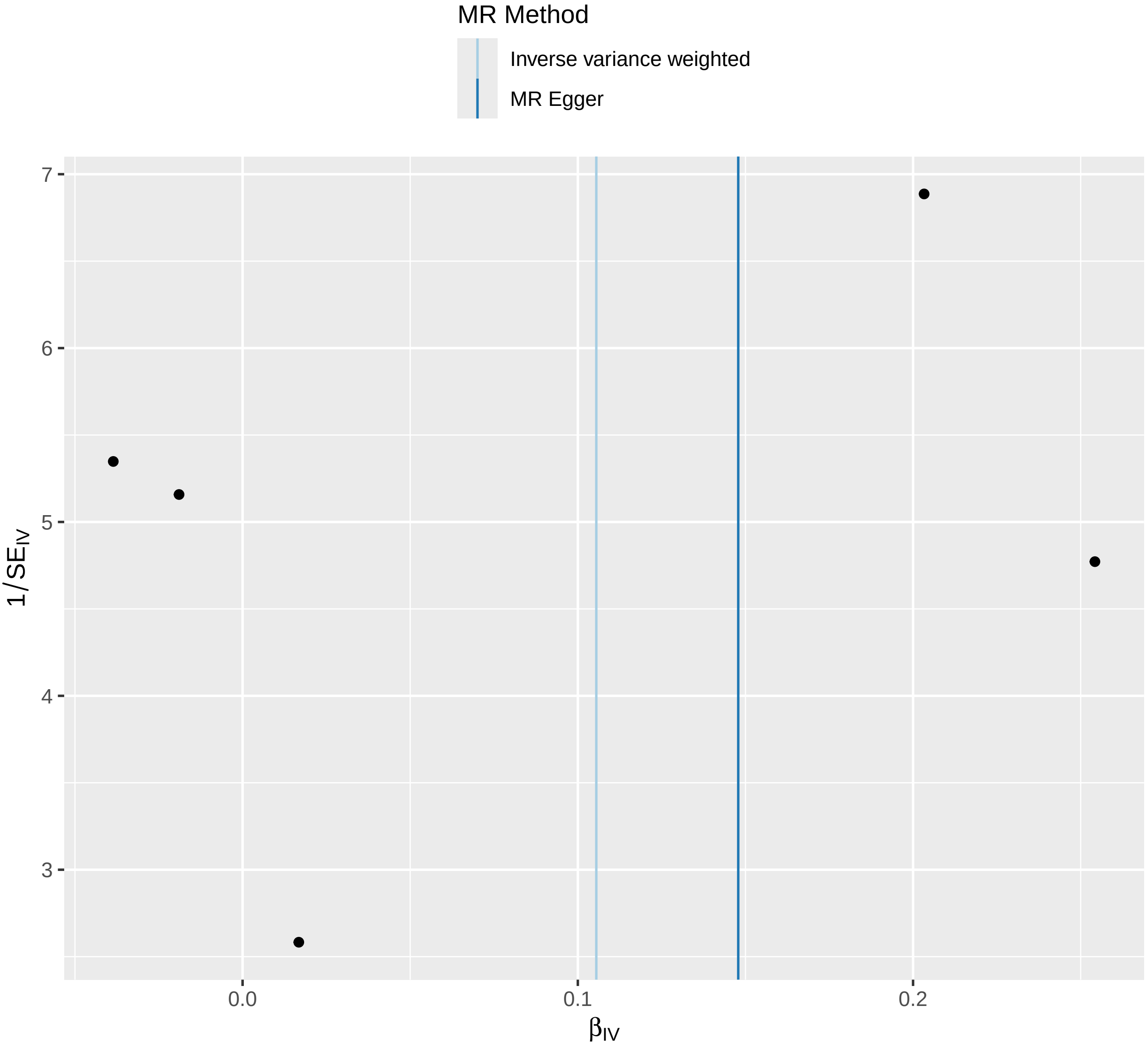
MR Method
Inverse variance weighted
MR Egger
7
6
5
4
3
1/SE$_{IV}$
0.0
0.1
0.2
β$_{IV}$

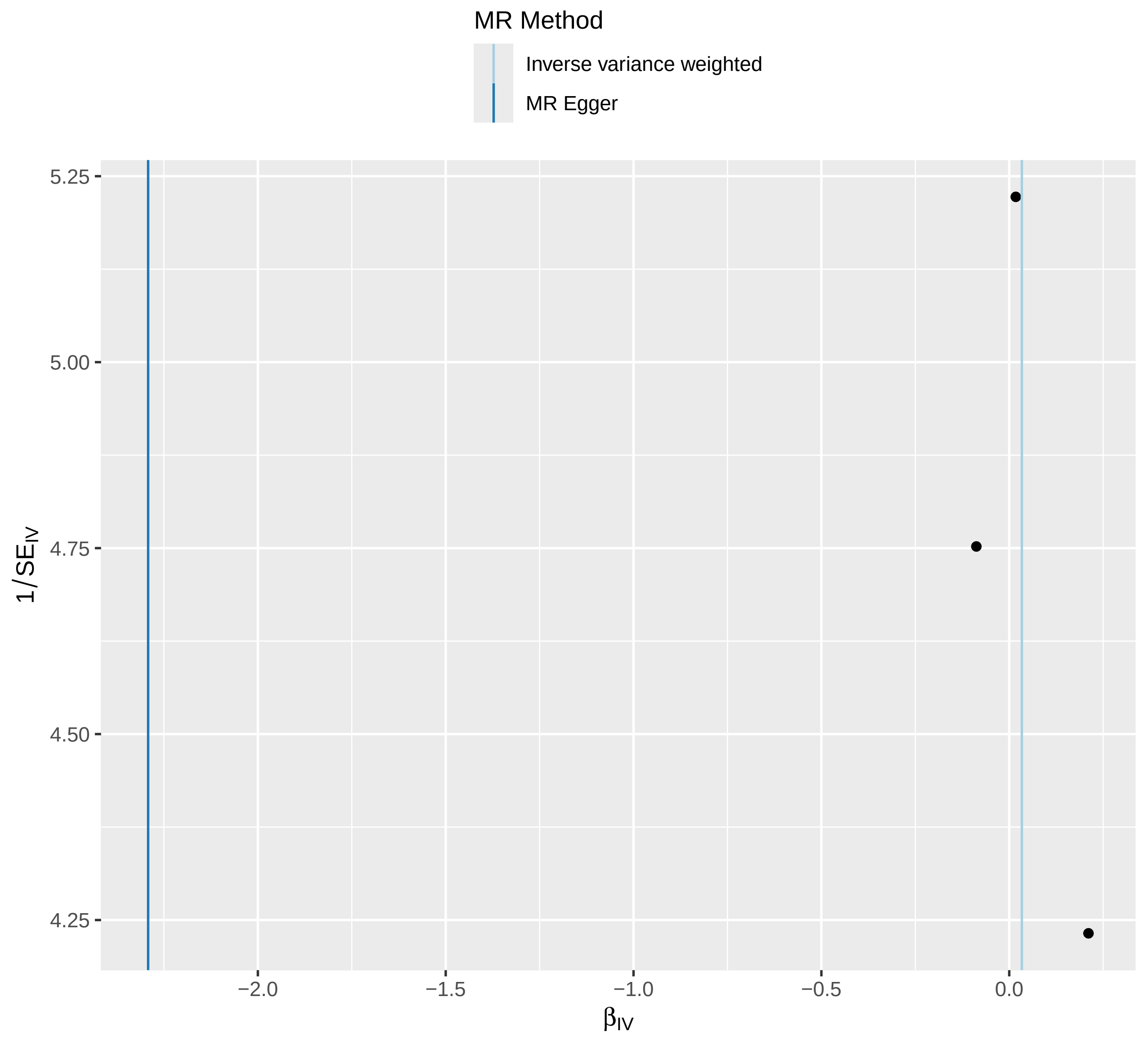
MR Method
Inverse variance weighted
MR Egger
5.25
5.00
4.75
4.50
4.25
1/SE$_{IV}$
−2.0
−1.5
−1.0
−0.5
0.0
β$_{IV}$

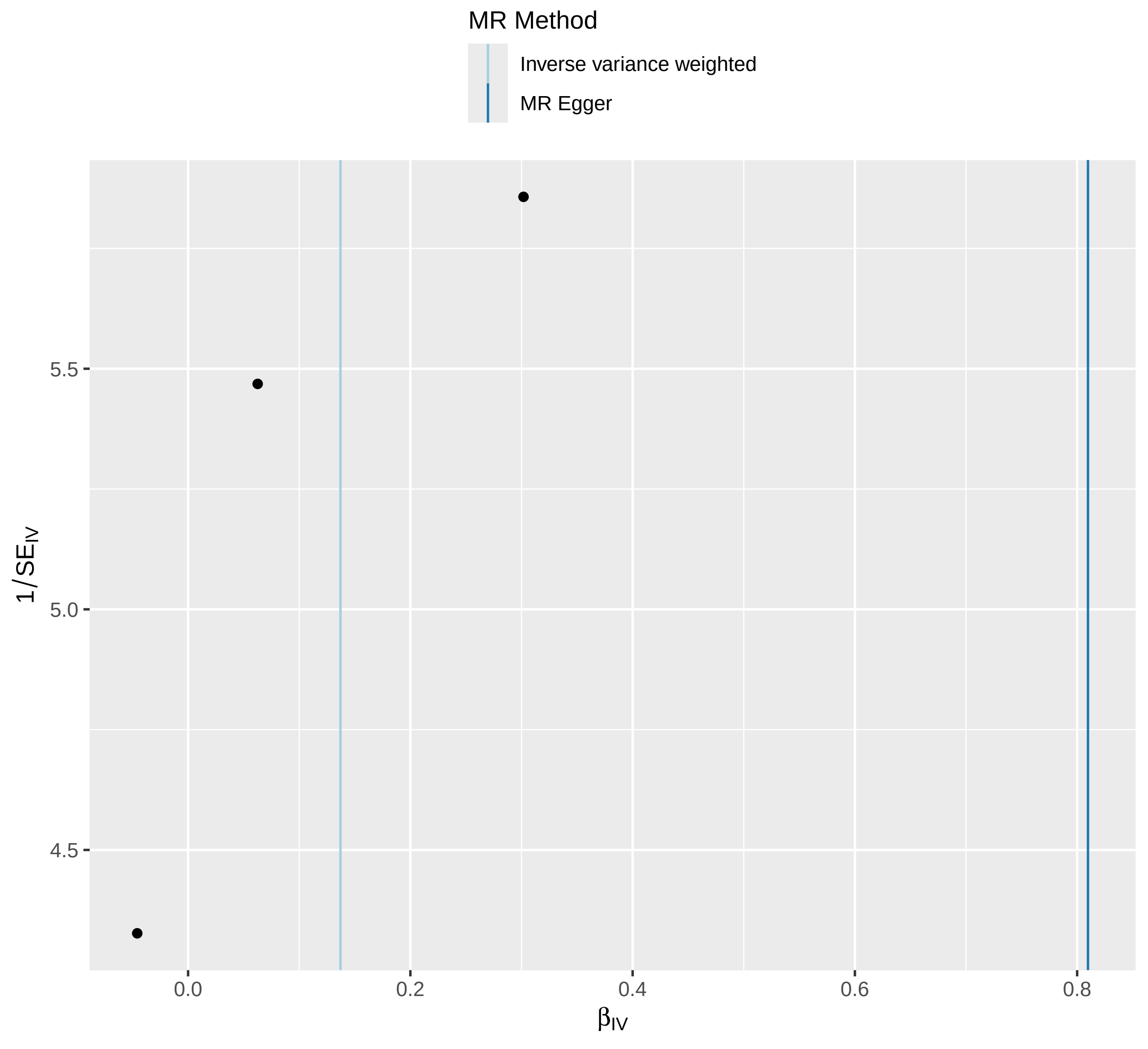
MR Method
Inverse variance weighted
MR Egger
5.5
5.0
4.5
1/SE_IV
0.0
0.2
0.4
0.6
0.8
β_IV

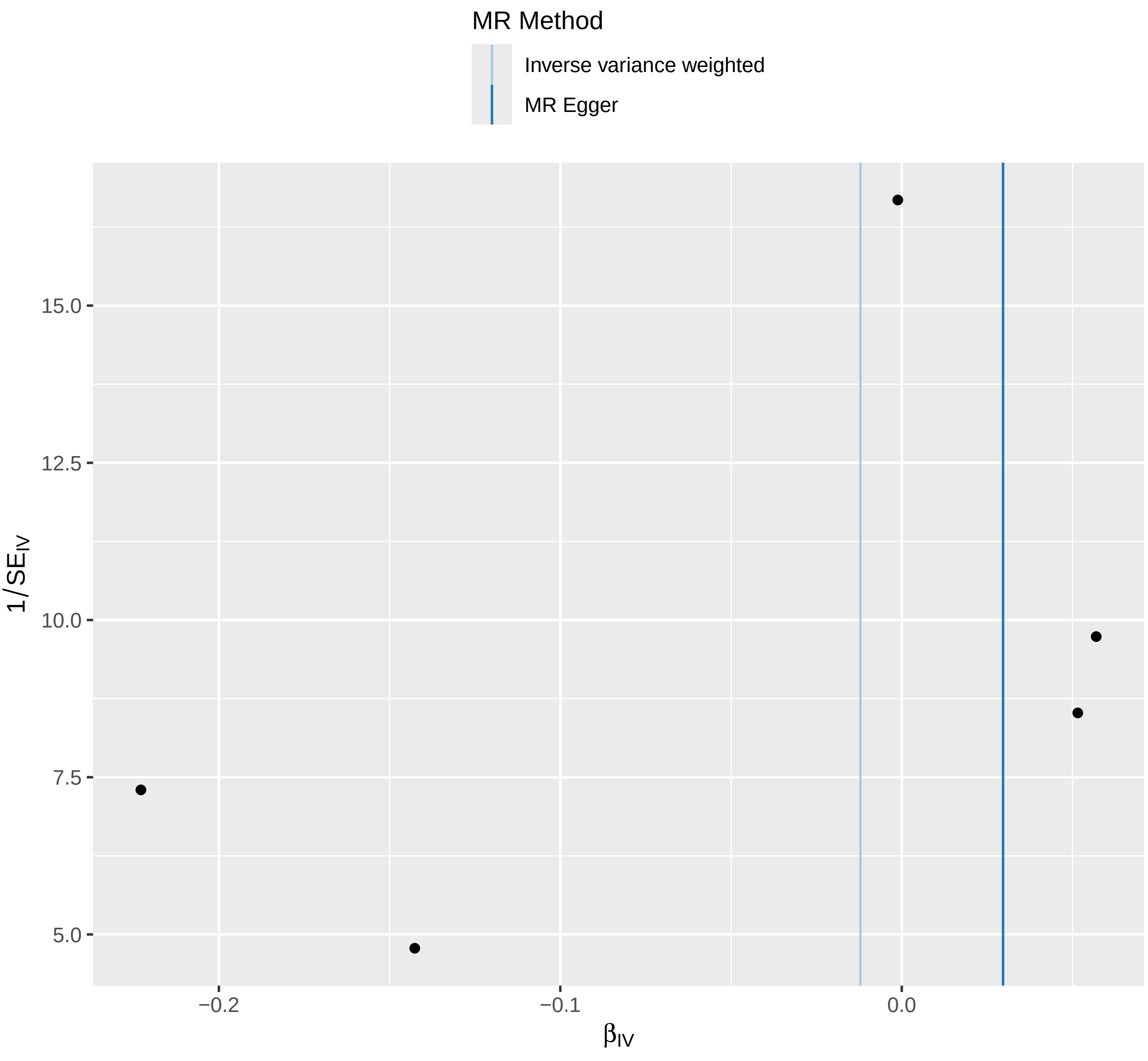

MR Method
Inverse variance weighted
MR Egger
15.0
12.5
10.0
7.5
5.0
$1/SE_{IV}$
−0.2
−0.1
0.0
$\beta_{IV}$

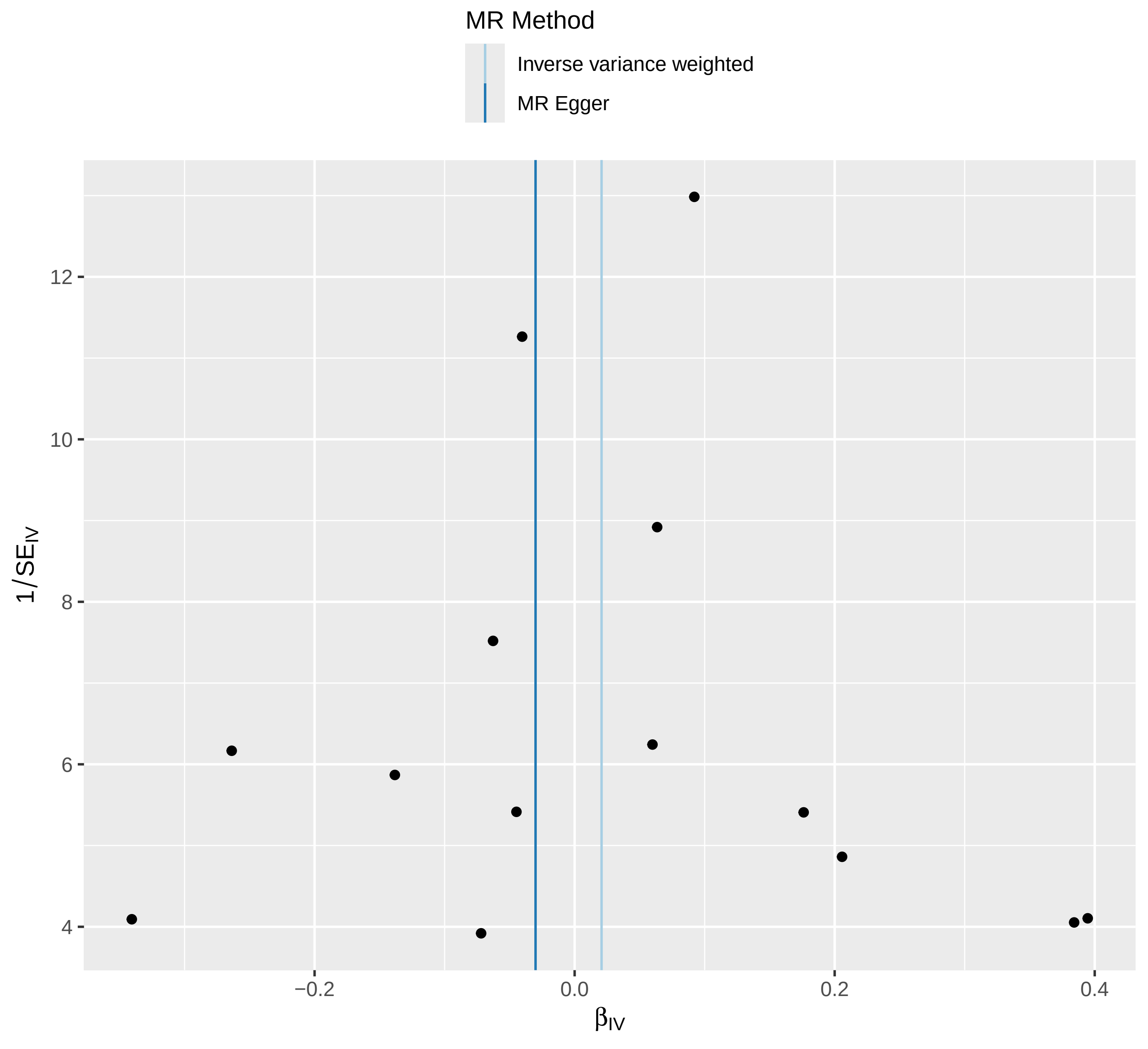

MR Method
Inverse variance weighted
MR Egger
1/SE_IV
12
10
8
6
4
−0.2
0.0
0.2
0.4
β_IV

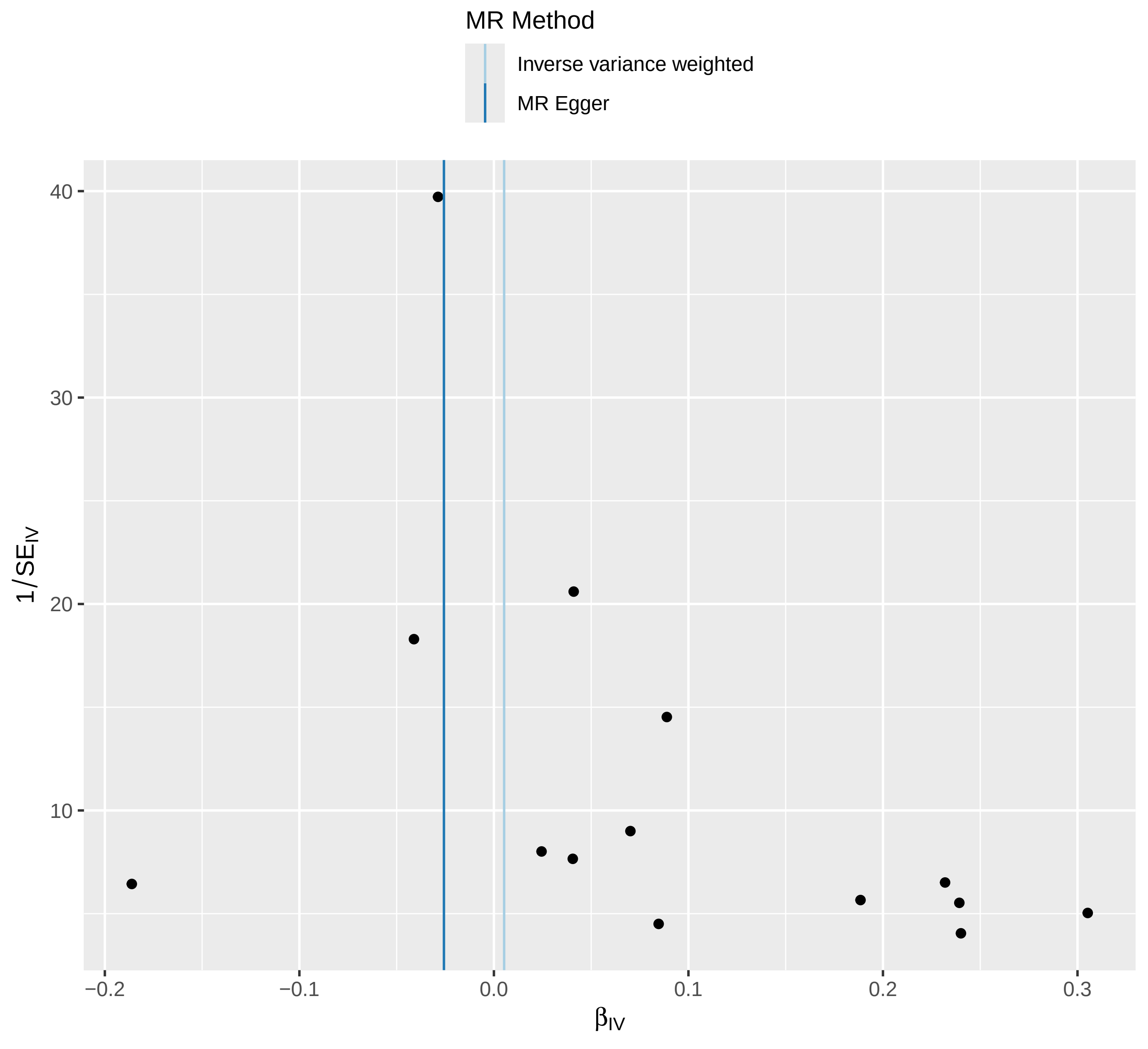

MR Method
Inverse variance weighted
MR Egger
40
30
20
10
1/SE$_{IV}$
−0.2
−0.1
0.0
0.1
0.2
0.3
β$_{IV}$

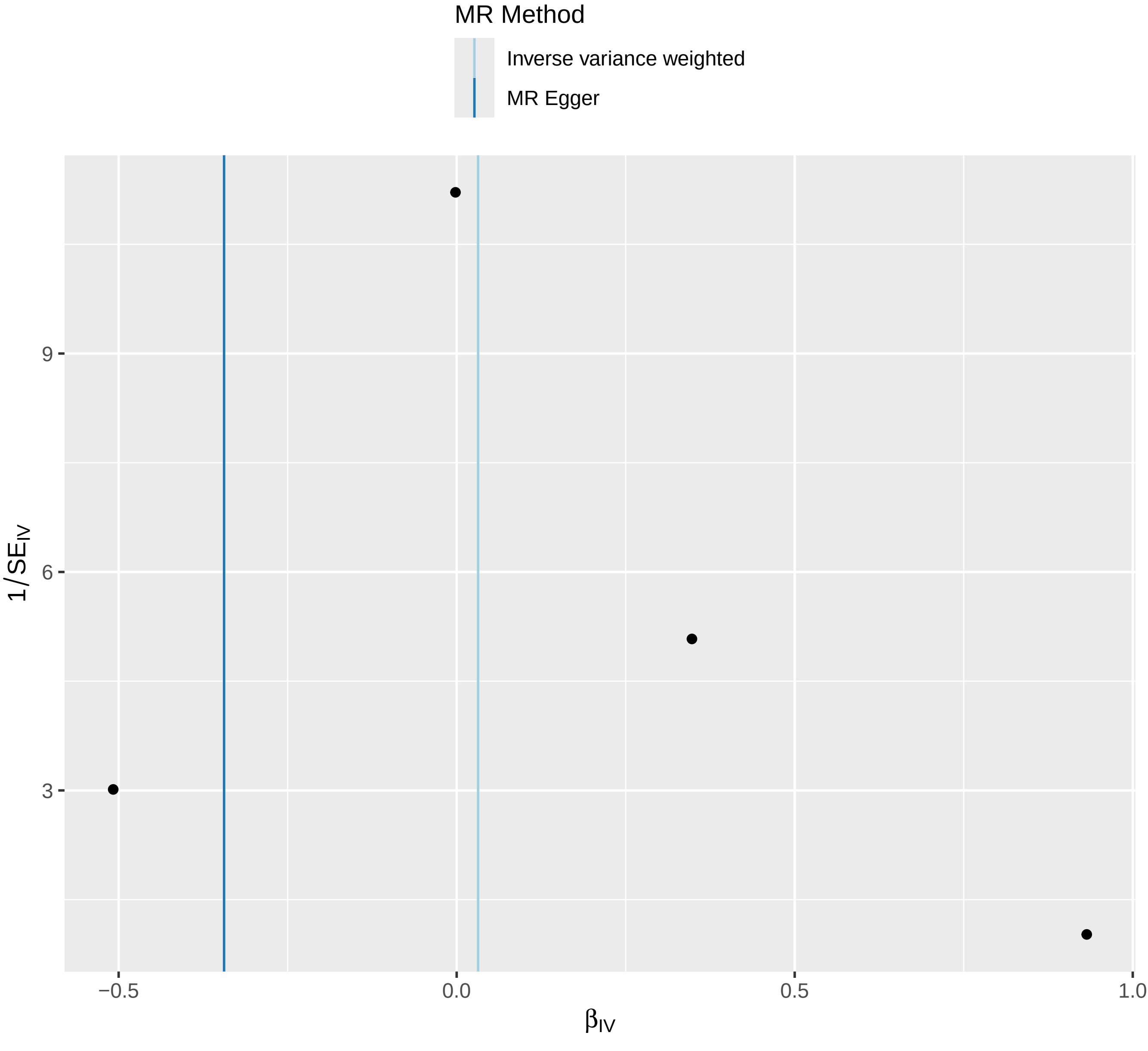
MR Method
Inverse variance weighted
MR Egger
9
6
3
−0.5
0.0
0.5
1.0
1/SE_IV
β_IV

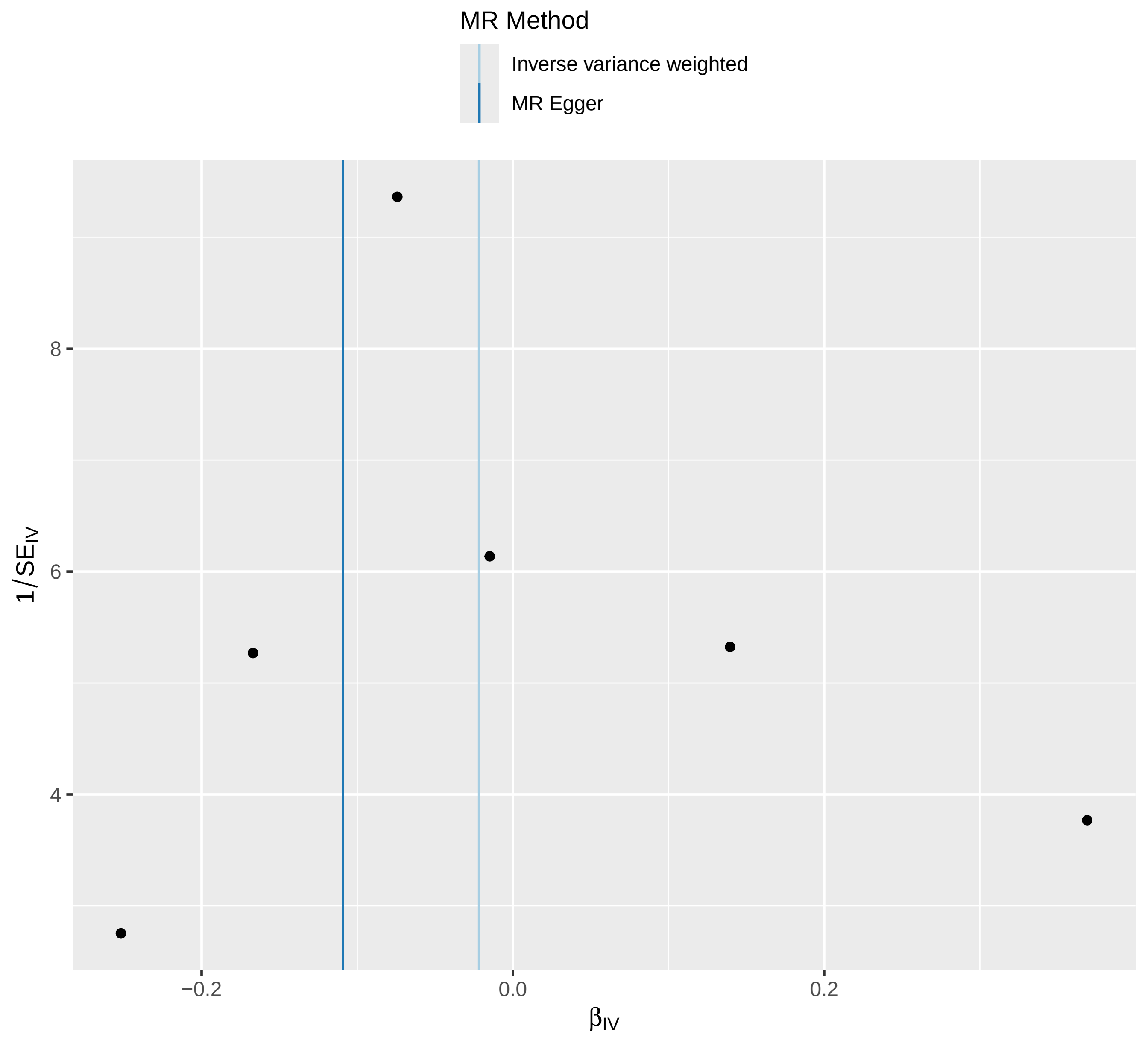
MR Method
Inverse variance weighted
MR Egger
1/SE_IV
8
6
4
−0.2
0.0
0.2
β_IV

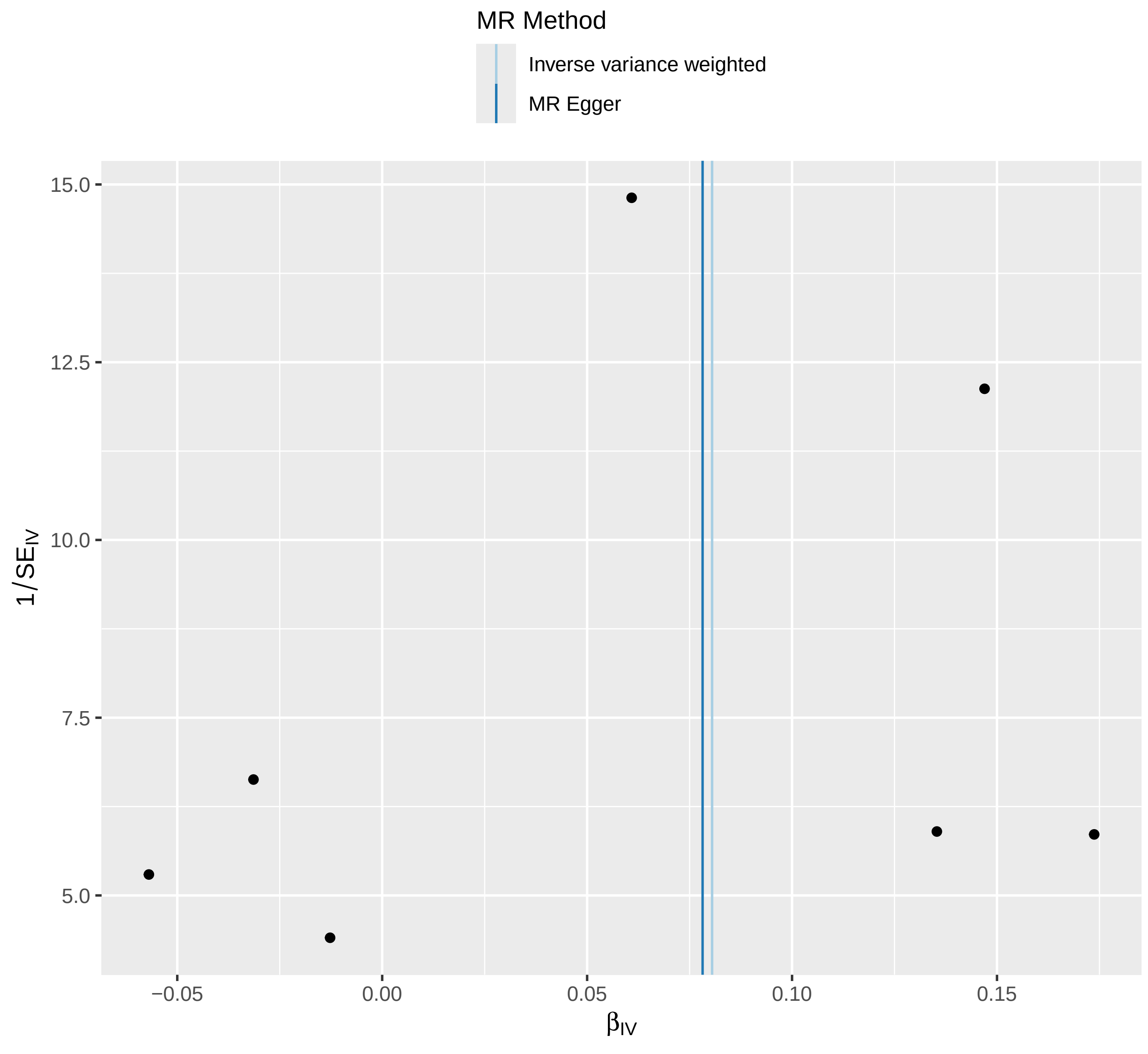

MR Method
Inverse variance weighted
MR Egger
15.0
12.5
10.0
7.5
5.0
1/SE$_{IV}$
−0.05
0.00
0.05
0.10
0.15
β$_{IV}$

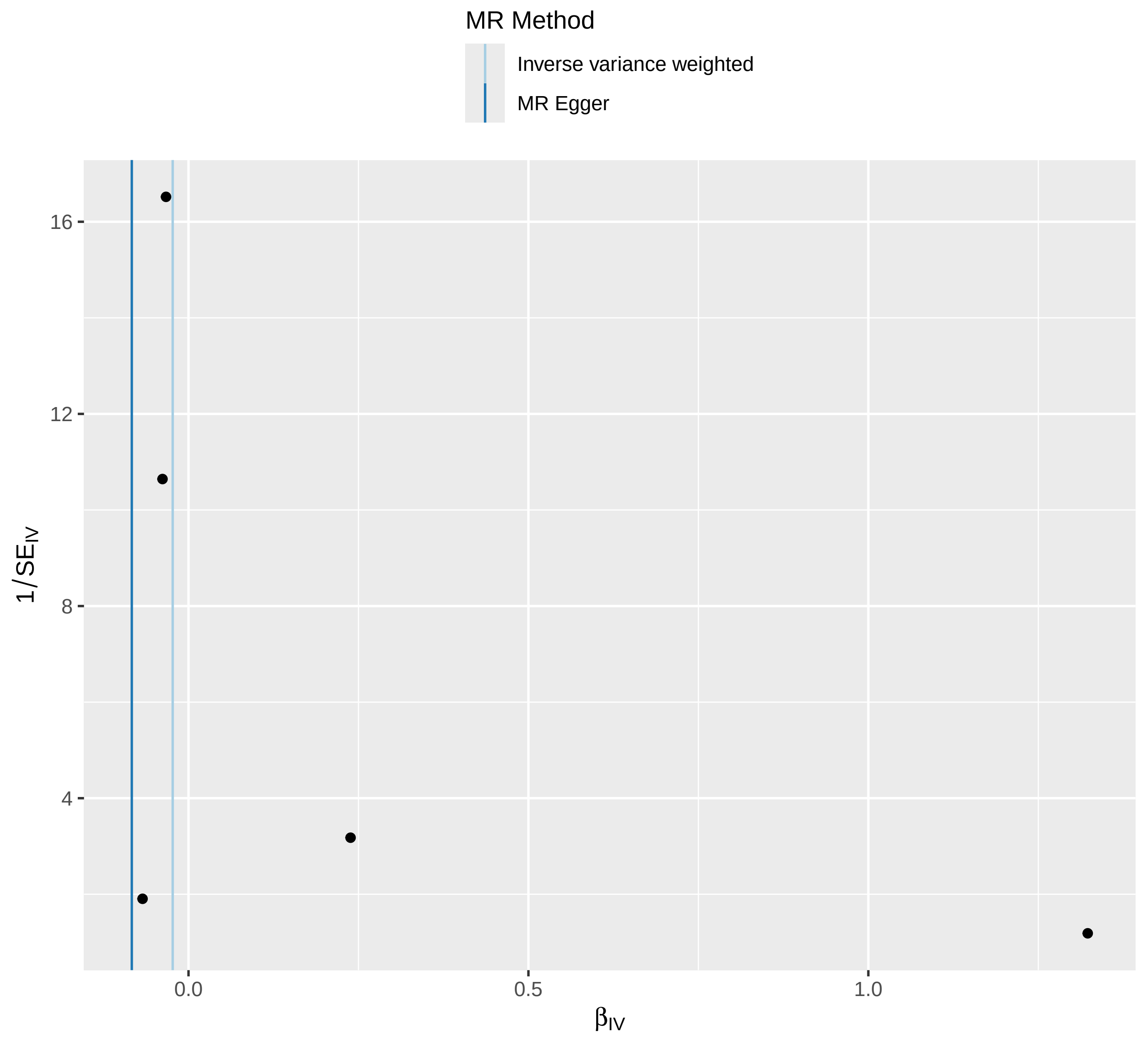

MR Method
Inverse variance weighted
MR Egger
16
12
8
4
1/SE_IV
0.0
0.5
1.0
β_IV

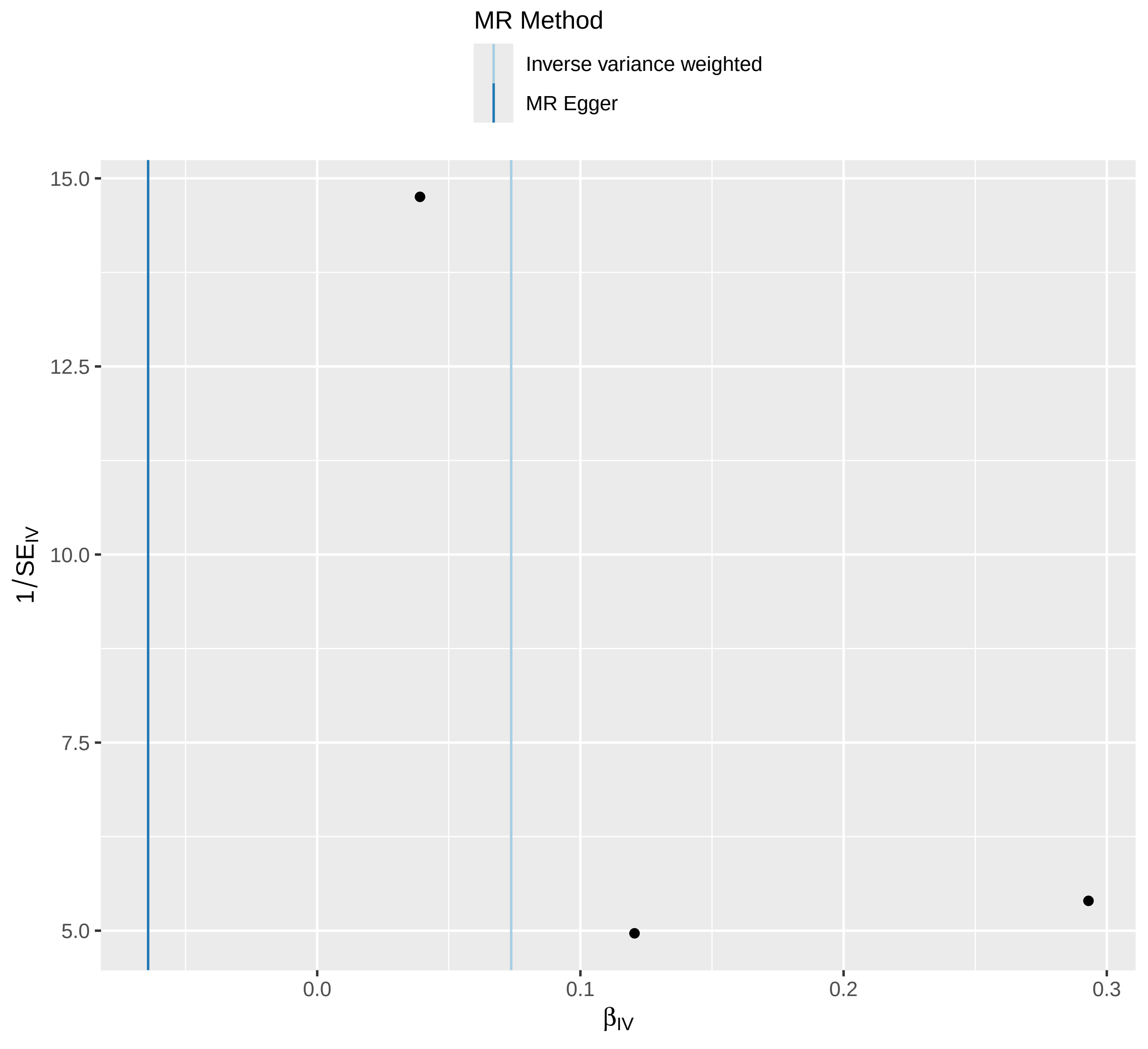
MR Method
Inverse variance weighted
MR Egger
15.0
12.5
10.0
7.5
5.0
1/SE_IV
0.0
0.1
0.2
0.3
β_IV

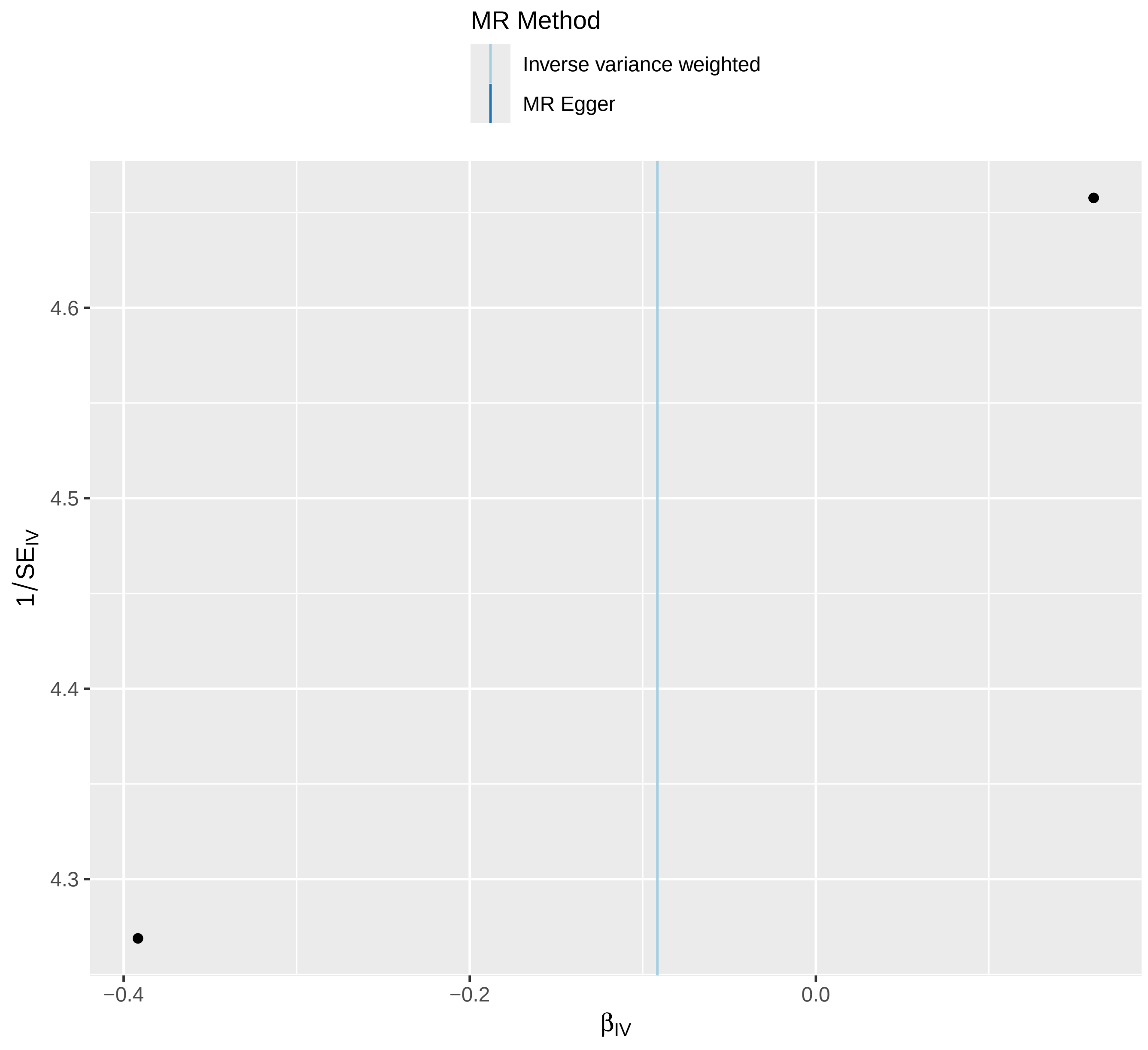
MR Method
Inverse variance weighted
MR Egger
4.6
4.5
4.4
4.3
1/SE_IV
−0.4
−0.2
0.0
β_IV

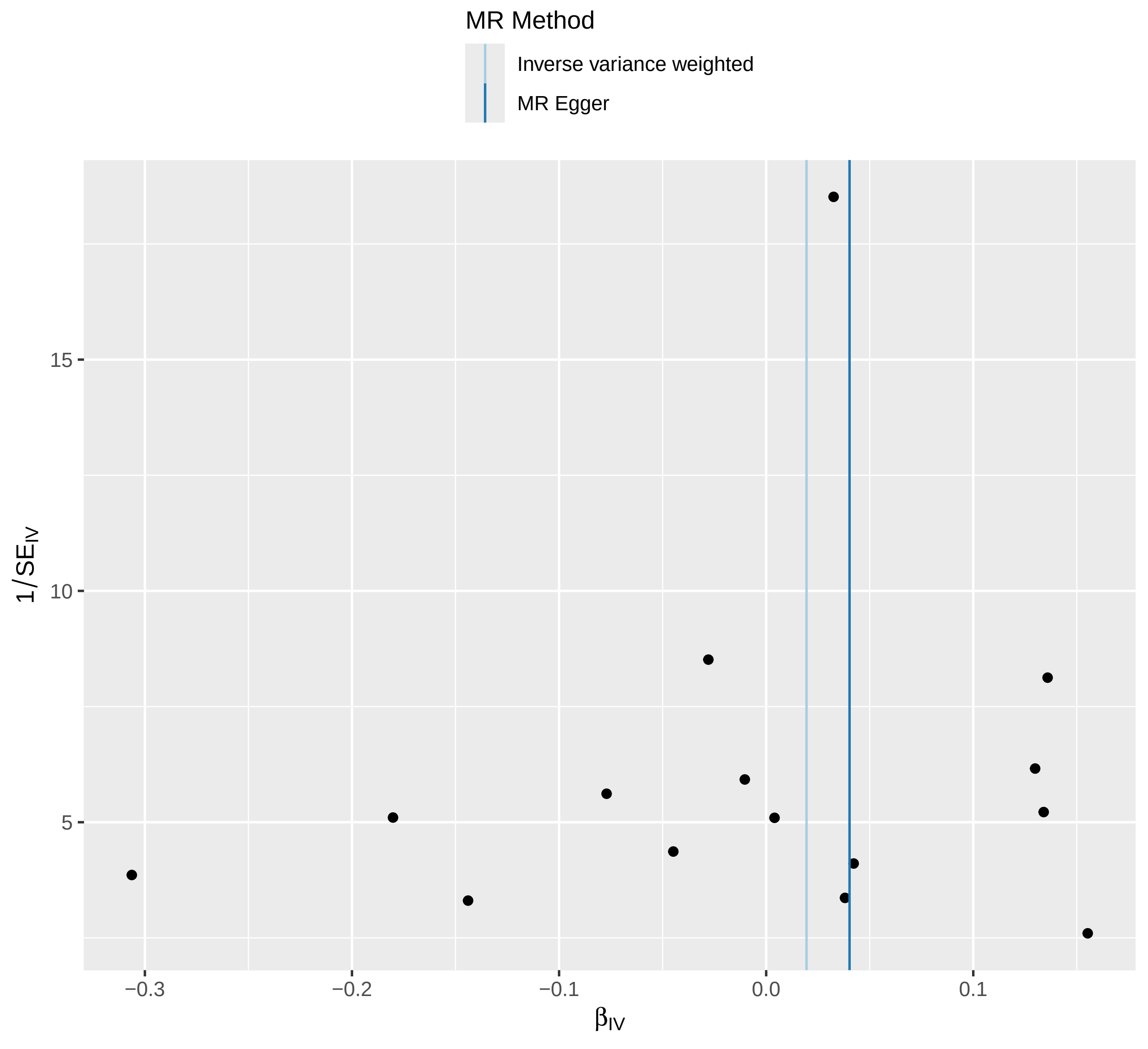

MR Method
Inverse variance weighted
MR Egger
15
10
5
1/SE_IV
−0.3
−0.2
−0.1
0.0
0.1
β_IV

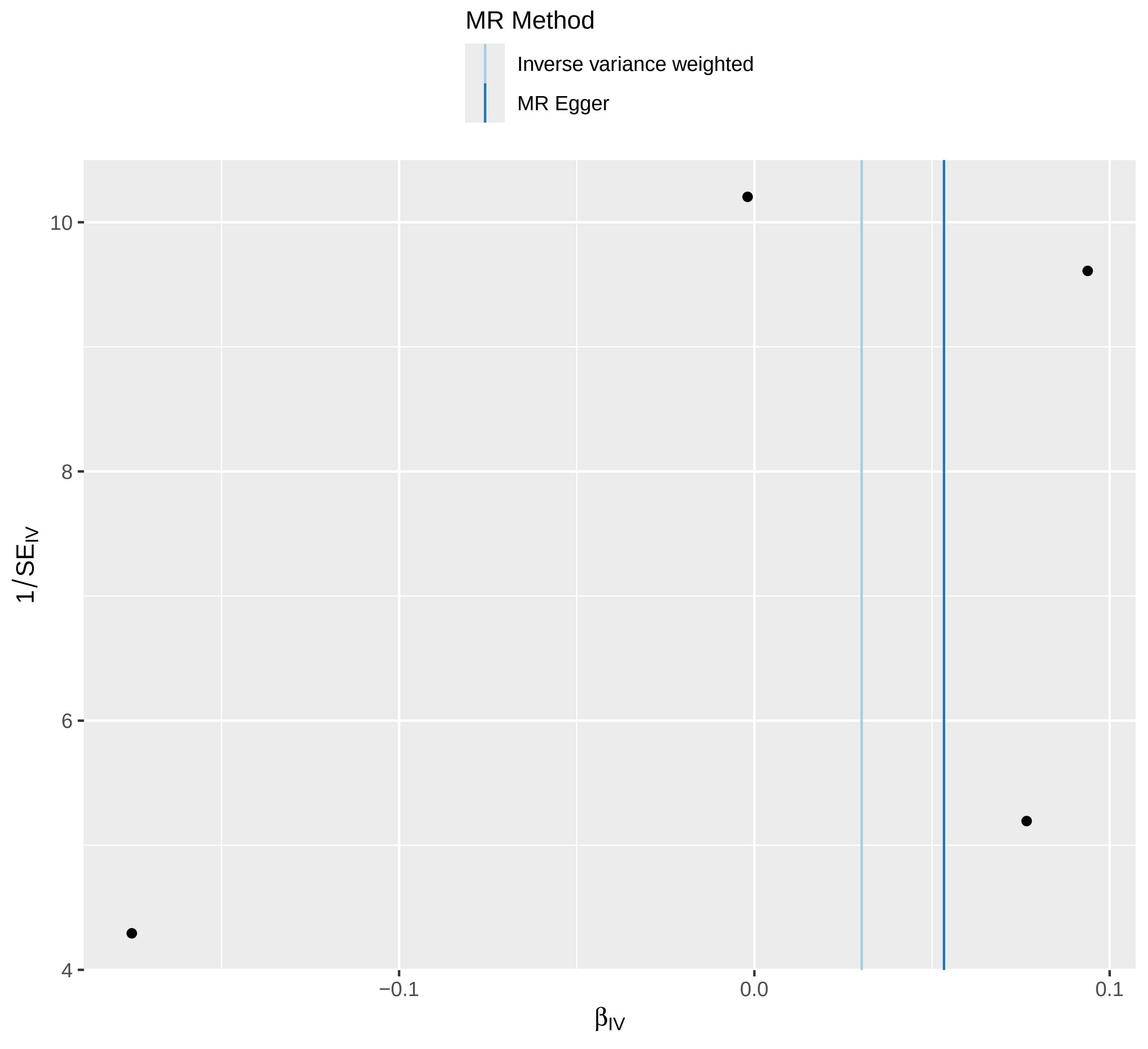
MR Method
Inverse variance weighted
MR Egger
10
8
6
4
1/SE$_{IV}$
−0.1
0.0
0.1
β$_{IV}$

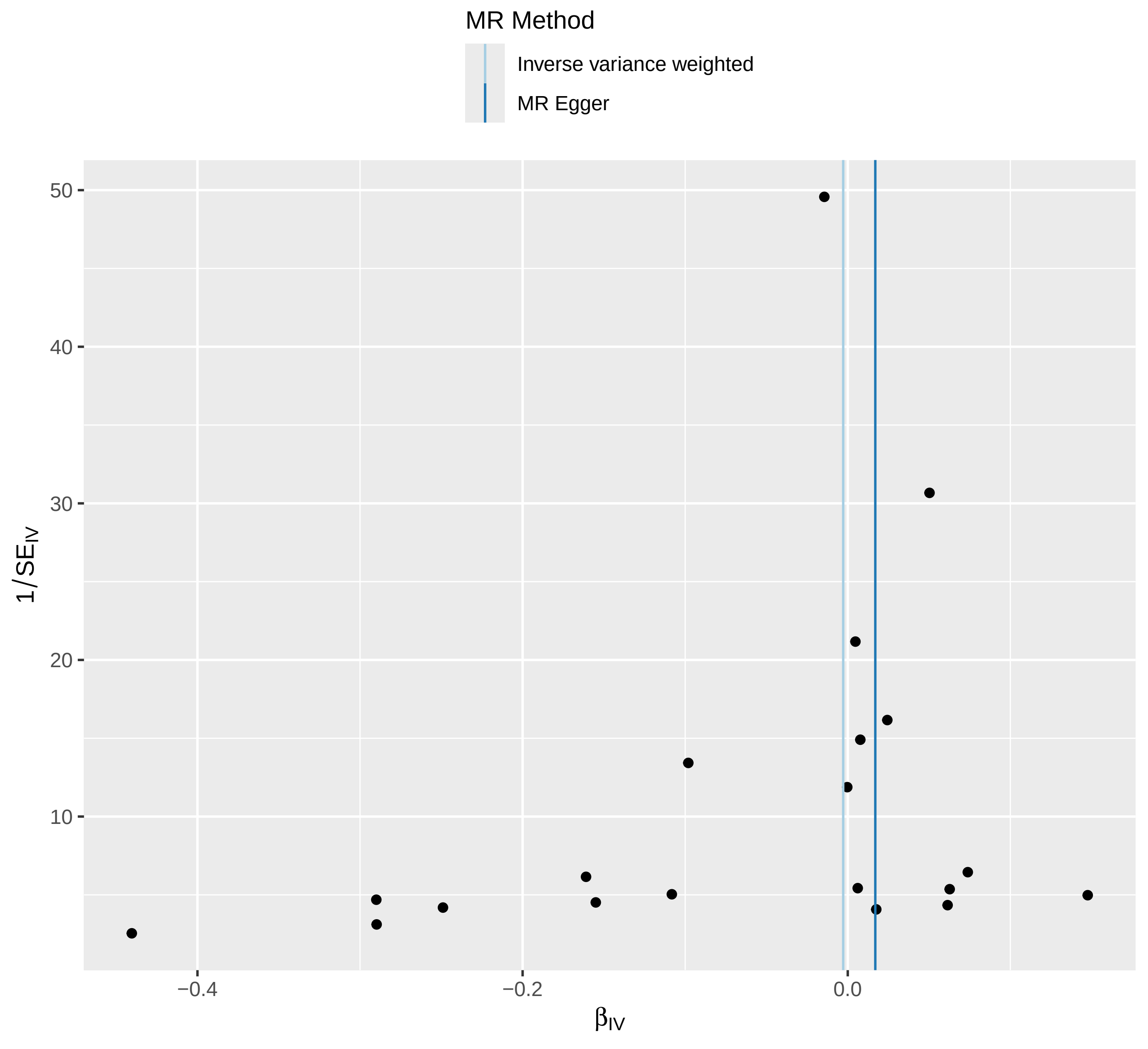
MR Method
Inverse variance weighted
MR Egger
50
40
30
20
10
1/SE_IV
−0.4
−0.2
0.0
β_IV

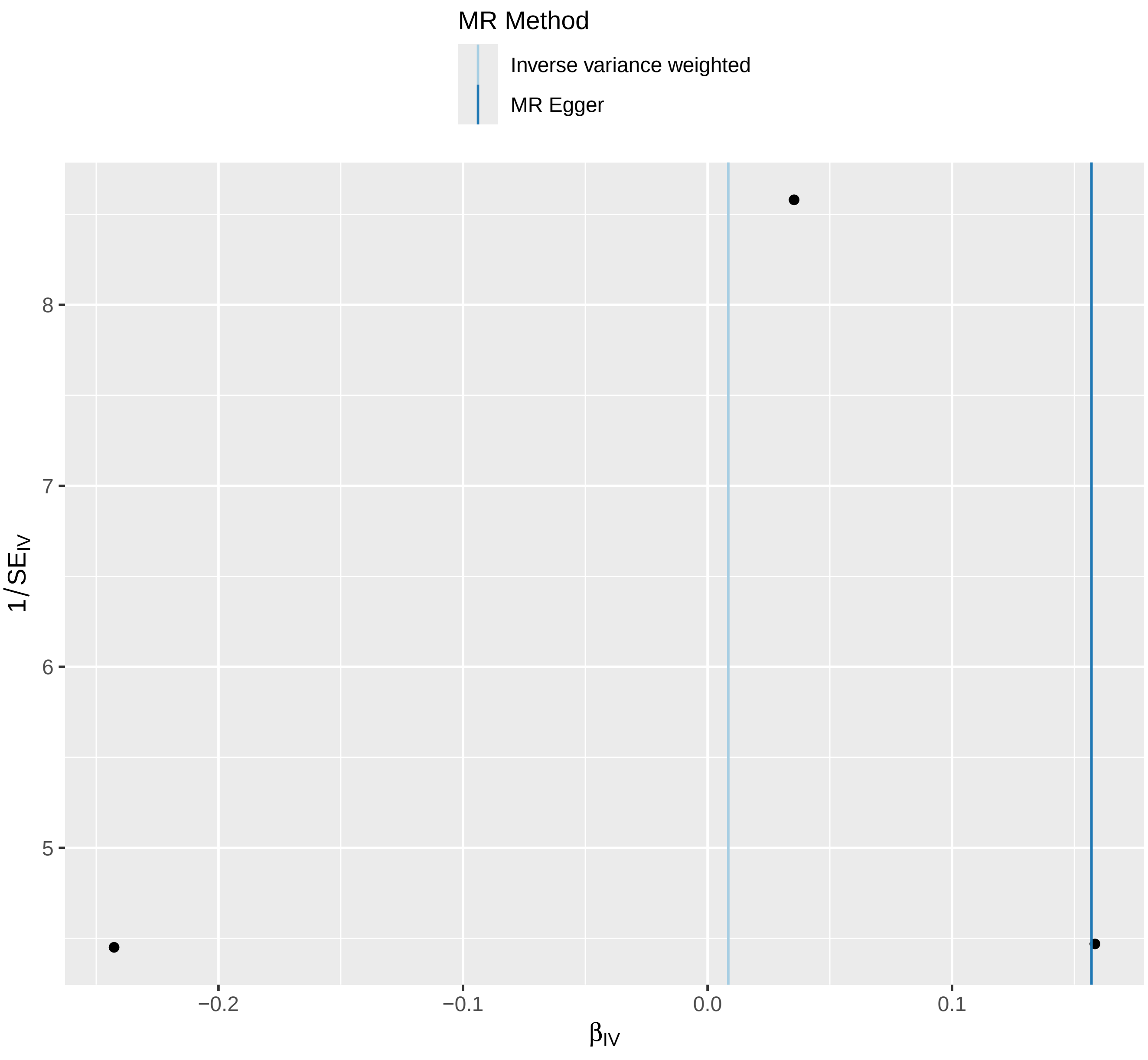

MR Method
Inverse variance weighted
MR Egger
8
7
6
5
1/SE$_{IV}$
−0.2
−0.1
0.0
0.1
β$_{IV}$

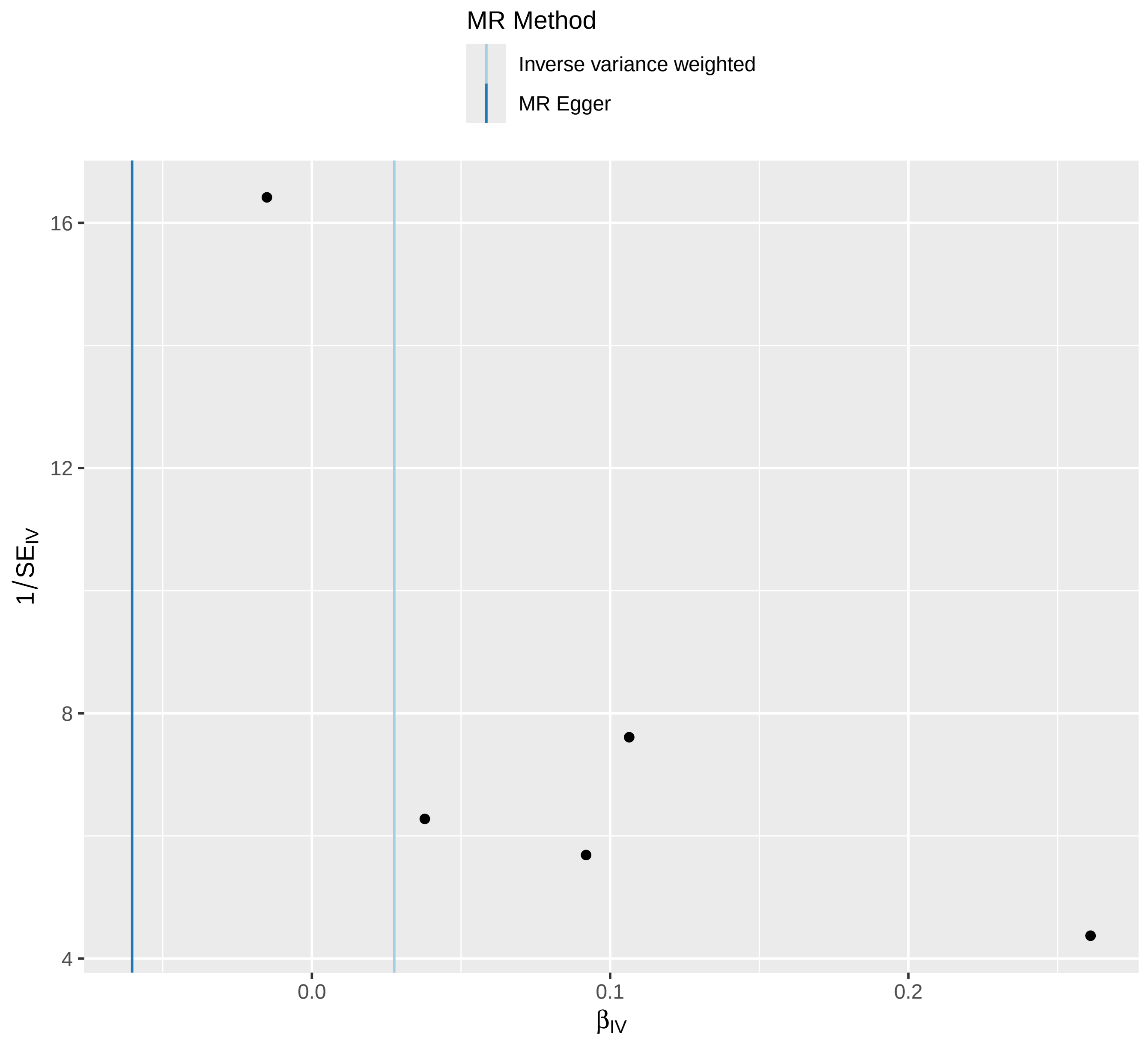

MR Method
Inverse variance weighted
MR Egger
16
12
8
4
$1/SE_{IV}$
0.0
0.1
0.2
$\beta_{IV}$

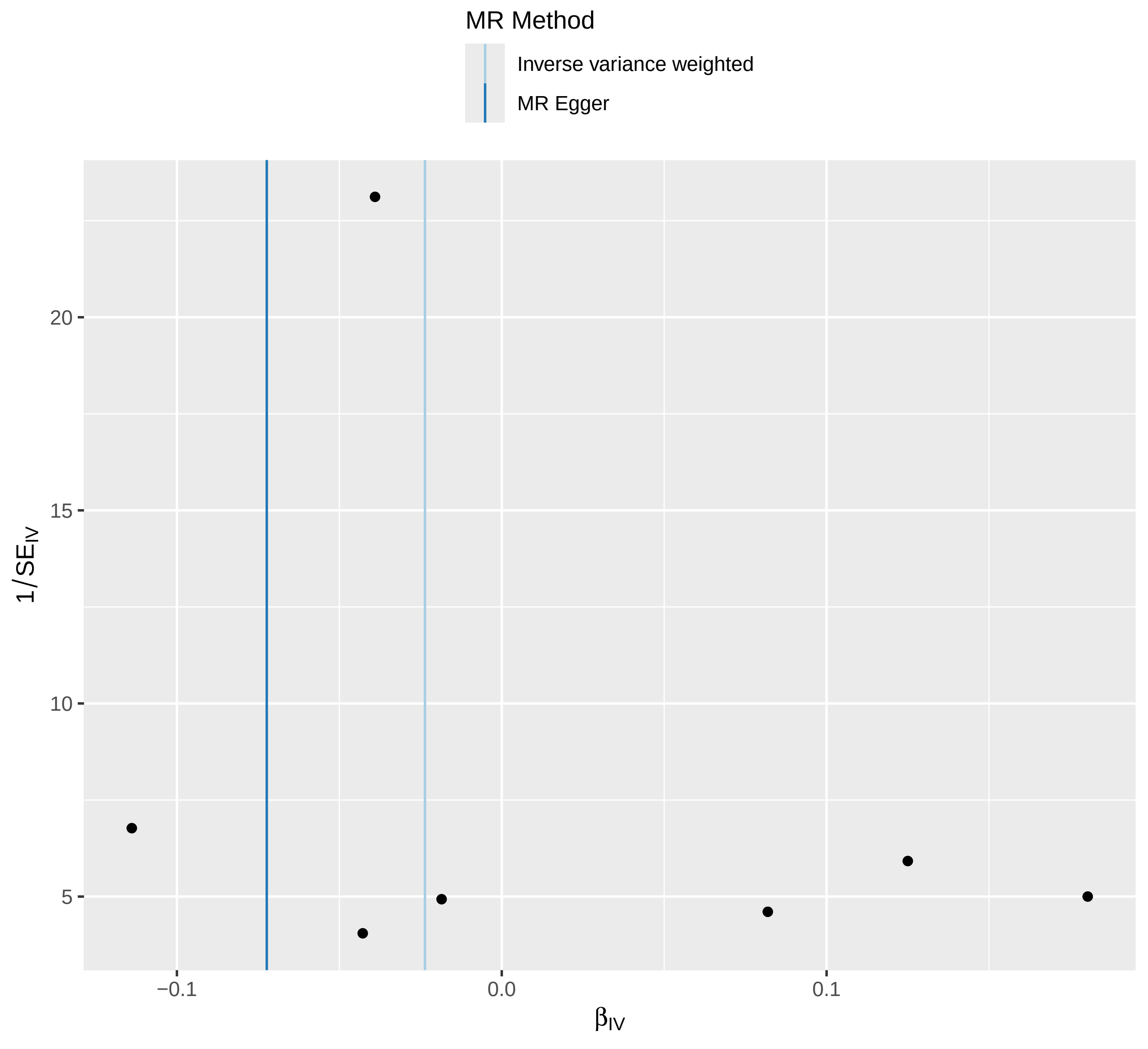

MR Method
Inverse variance weighted
MR Egger
20
15
10
5
$1/SE_{IV}$
−0.1
0.0
0.1
$\beta_{IV}$

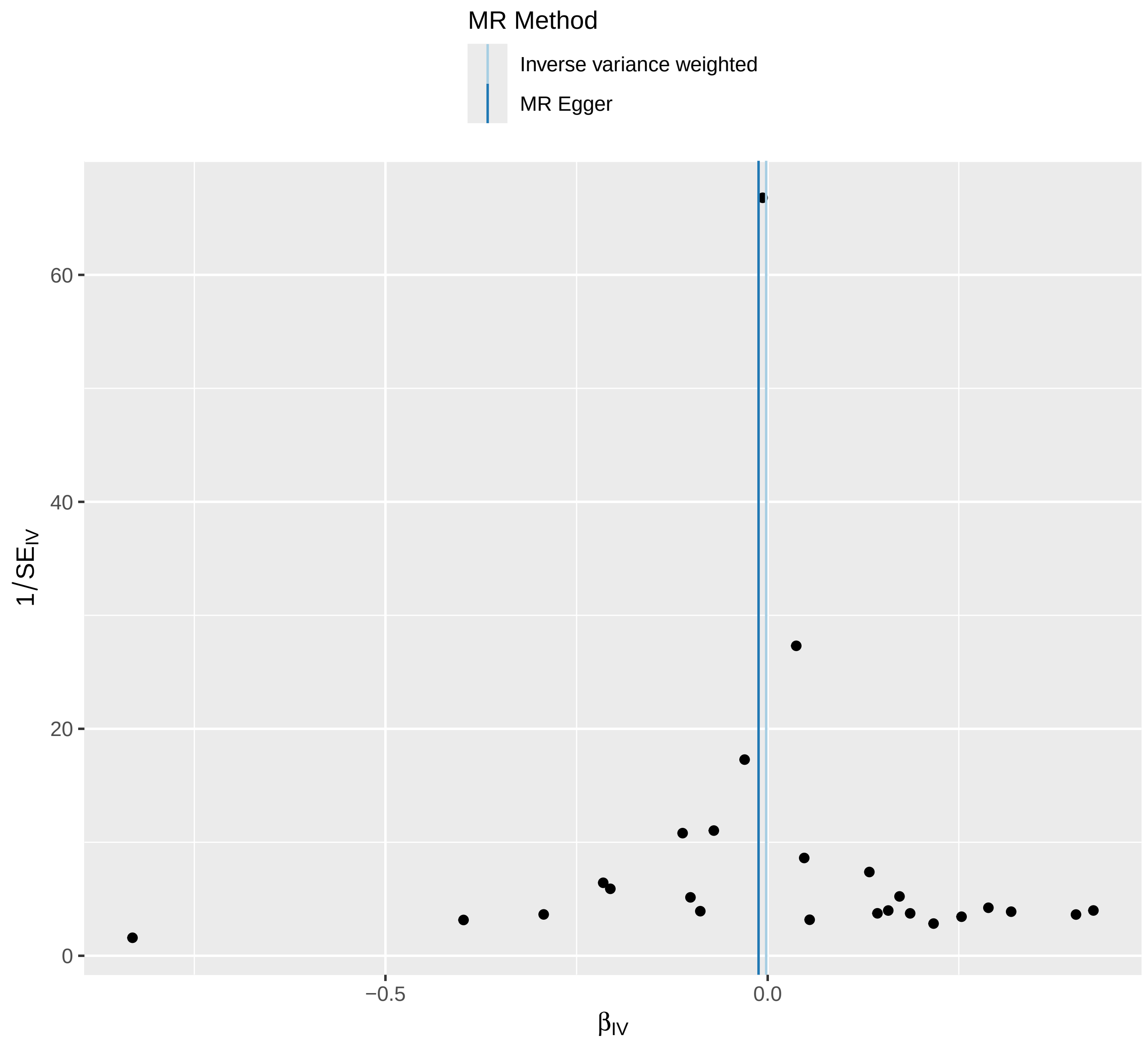

MR Method
Inverse variance weighted
MR Egger
60
40
20
0
$1/SE_{IV}$
−0.5
0.0
$\beta_{IV}$

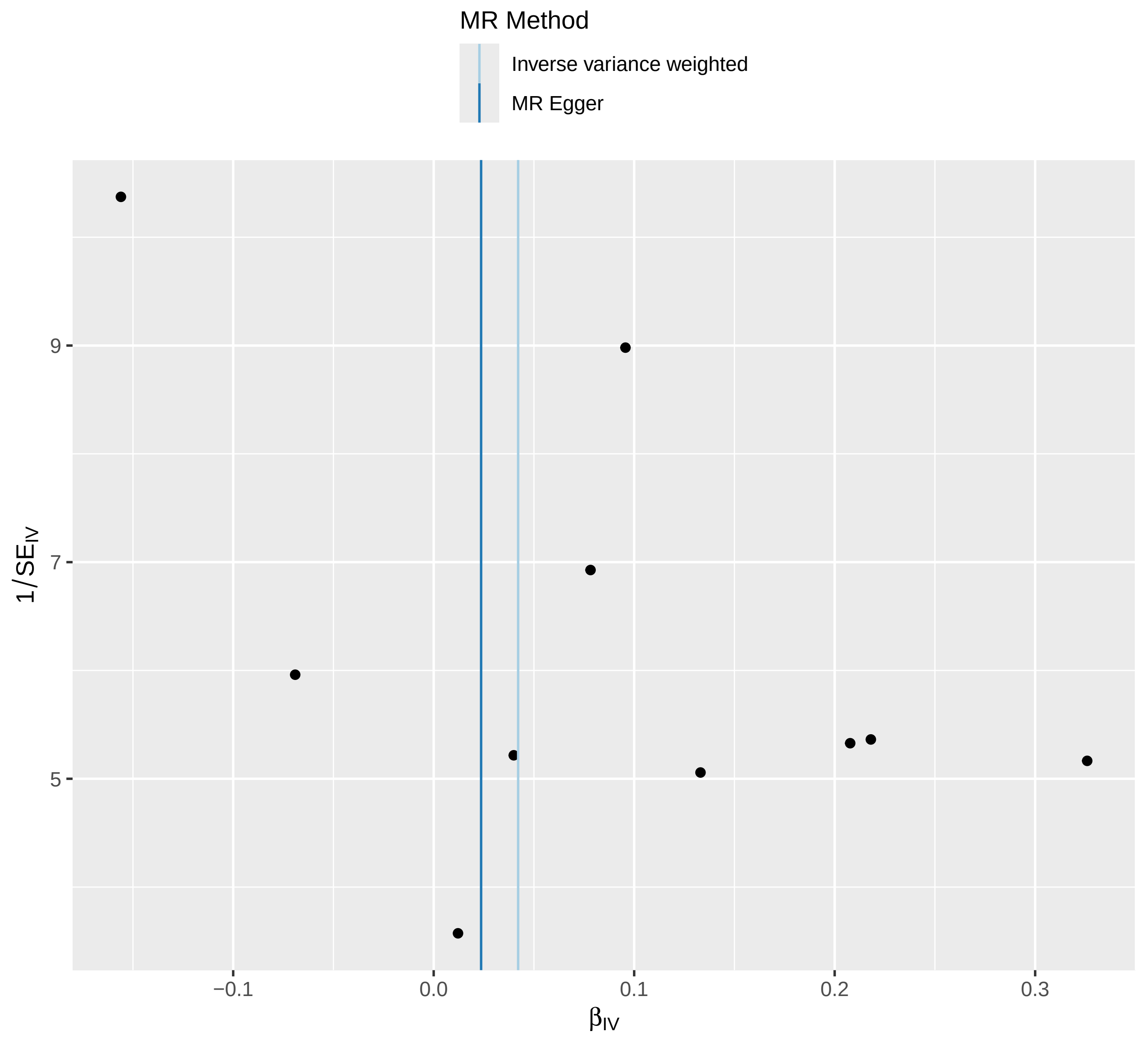

MR Method
Inverse variance weighted
MR Egger
9
7
5
1/SE$_{IV}$
−0.1
0.0
0.1
0.2
0.3
β$_{IV}$

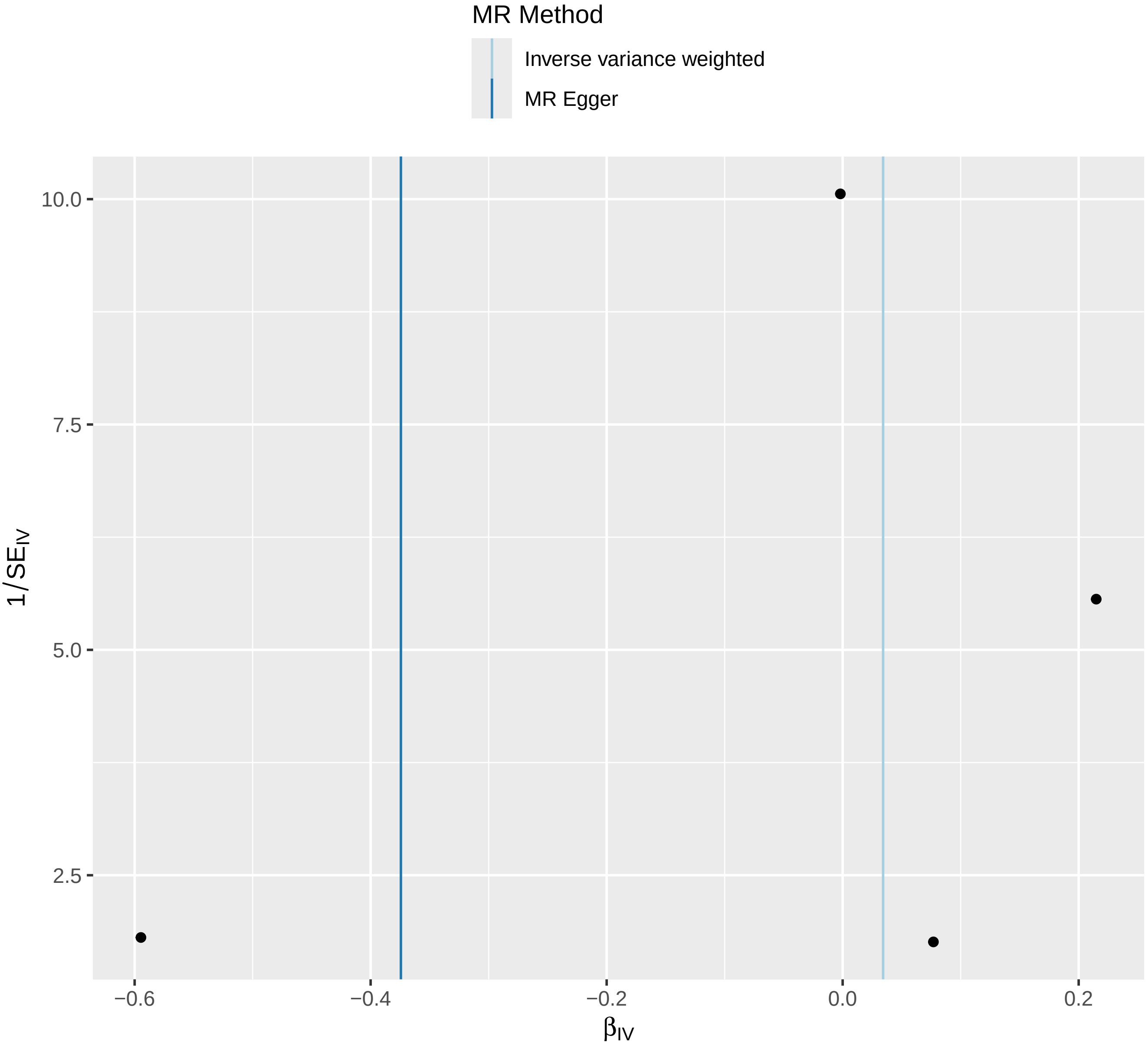

MR Method
Inverse variance weighted
MR Egger
10.0
7.5
5.0
2.5
1/SE$_{IV}$
−0.6
−0.4
−0.2
0.0
0.2
β$_{IV}$

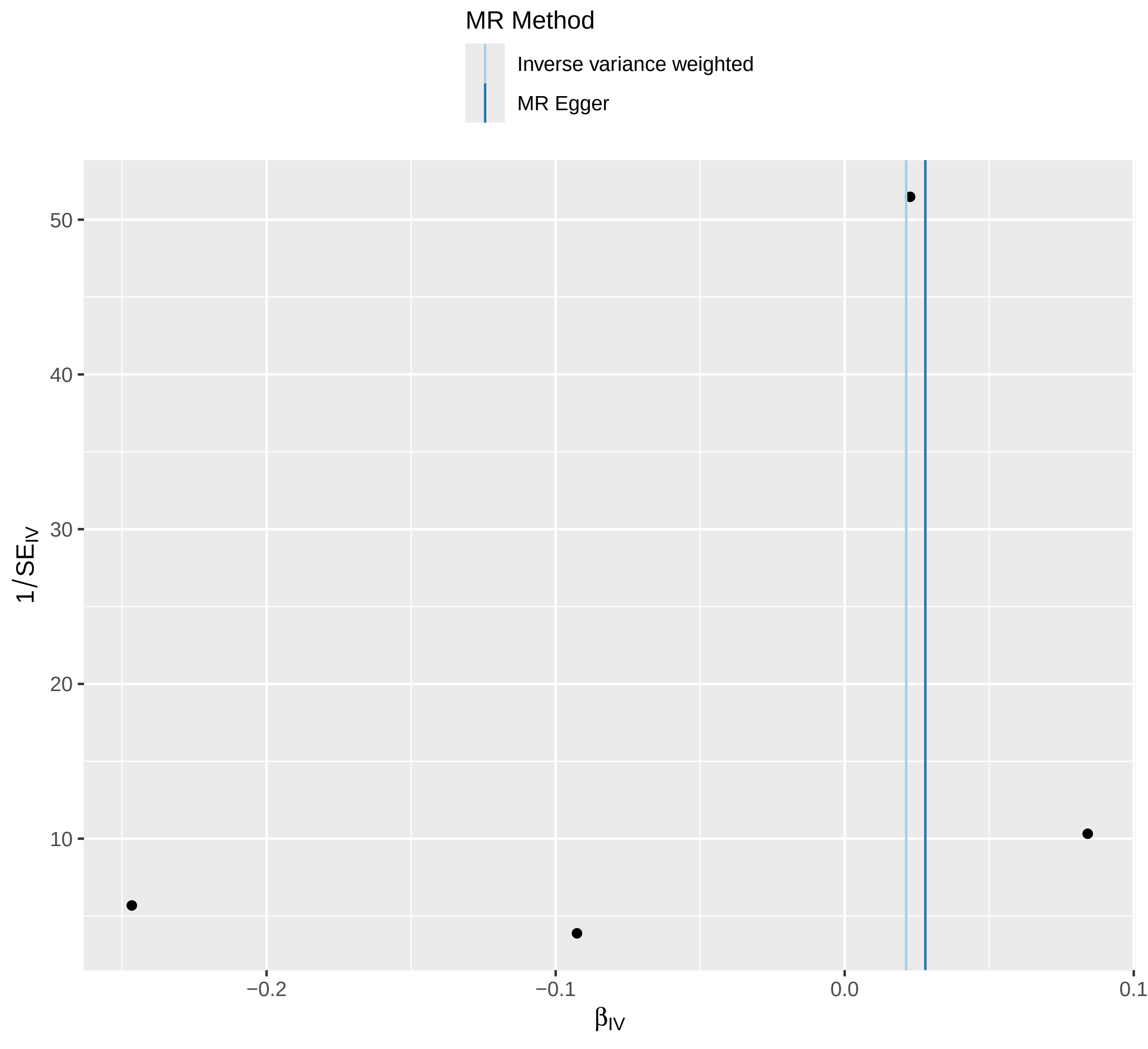

MR Method
Inverse variance weighted
MR Egger
50
40
30
20
10
$1/SE_{IV}$
−0.2
−0.1
0.0
0.1
$\beta_{IV}$

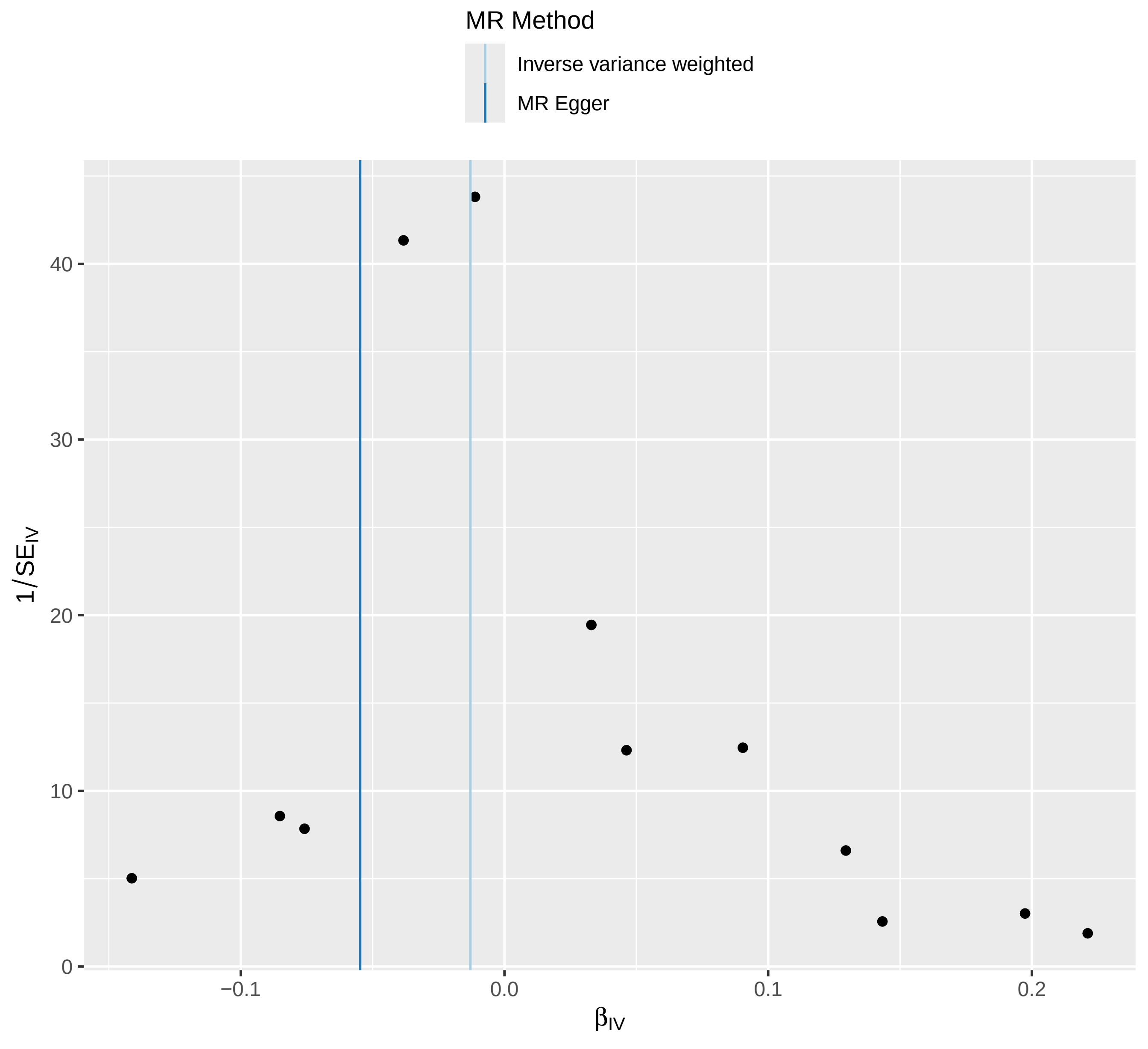

MR Method
Inverse variance weighted
MR Egger
1/SE_IV
β_IV
40
30
20
10
0
−0.1
0.0
0.1
0.2

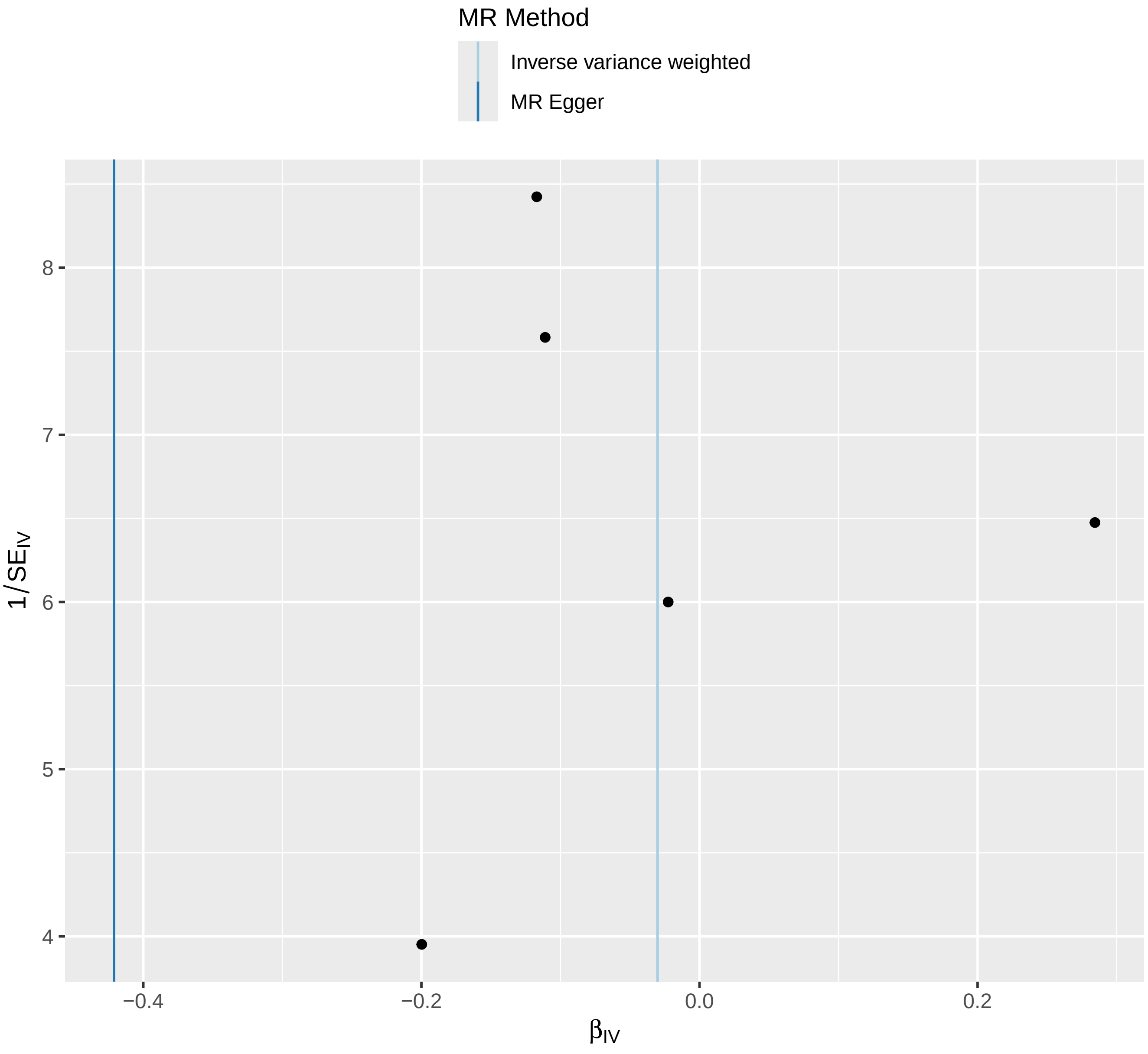

MR Method
Inverse variance weighted
MR Egger
8
7
6
5
4
1/SE_IV
−0.4
−0.2
0.0
0.2
β_IV

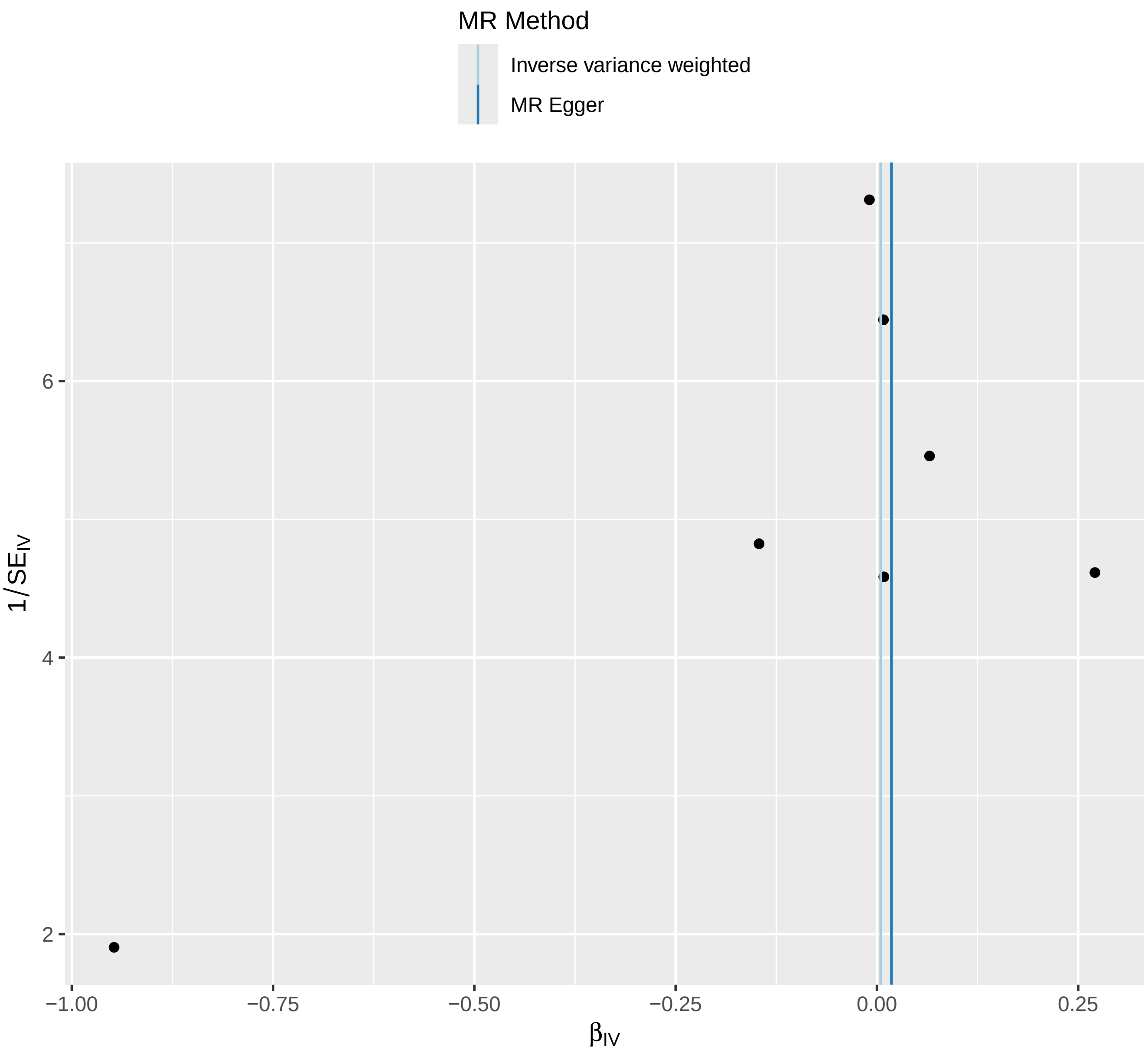
MR Method
Inverse variance weighted
MR Egger
6
4
2
1/SE_IV
−1.00
−0.75
−0.50
−0.25
0.00
0.25
β_IV

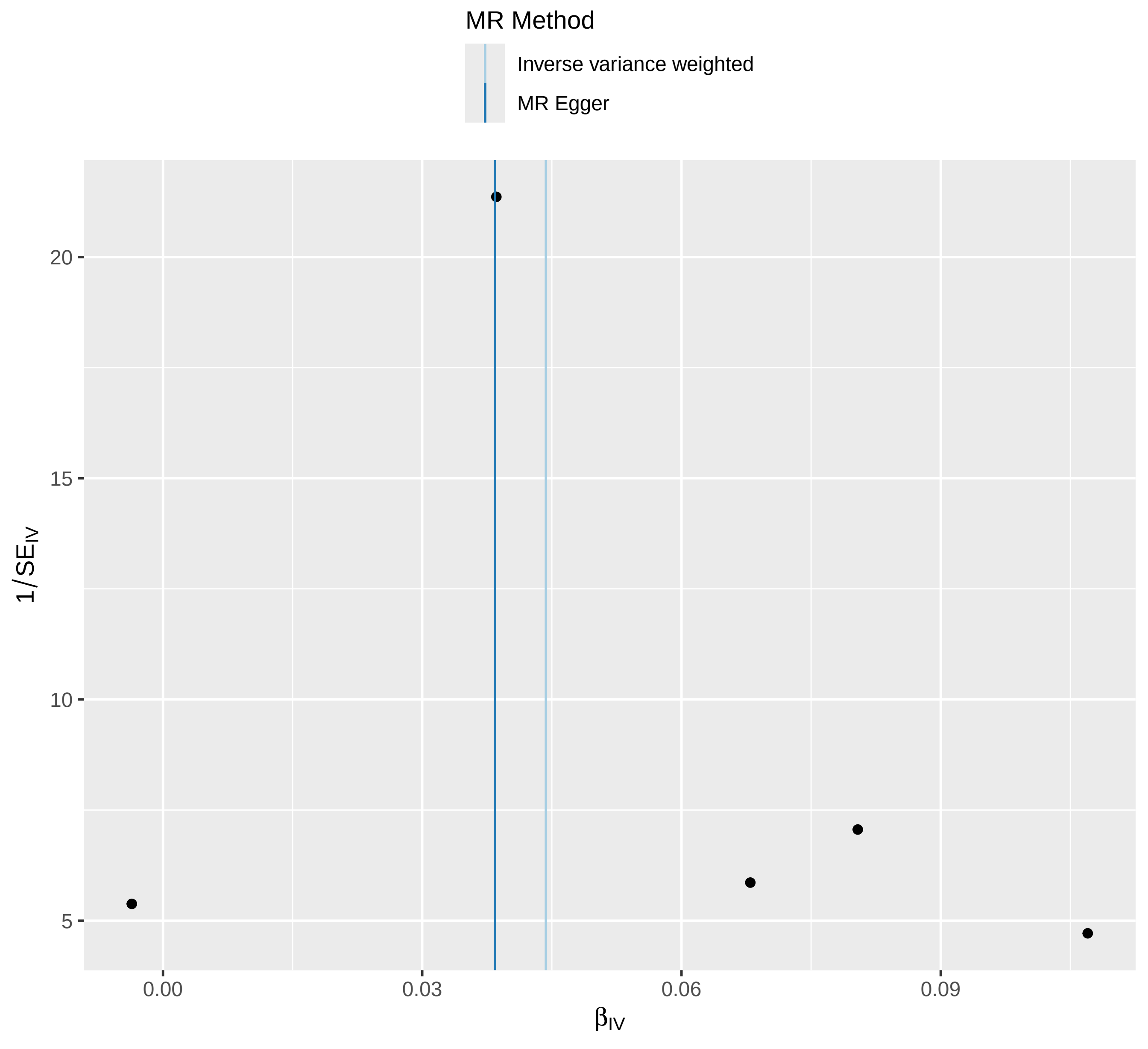
MR Method
Inverse variance weighted
MR Egger
20
15
10
5
1/$SE_{IV}$
0.00
0.03
0.06
0.09
$\beta_{IV}$

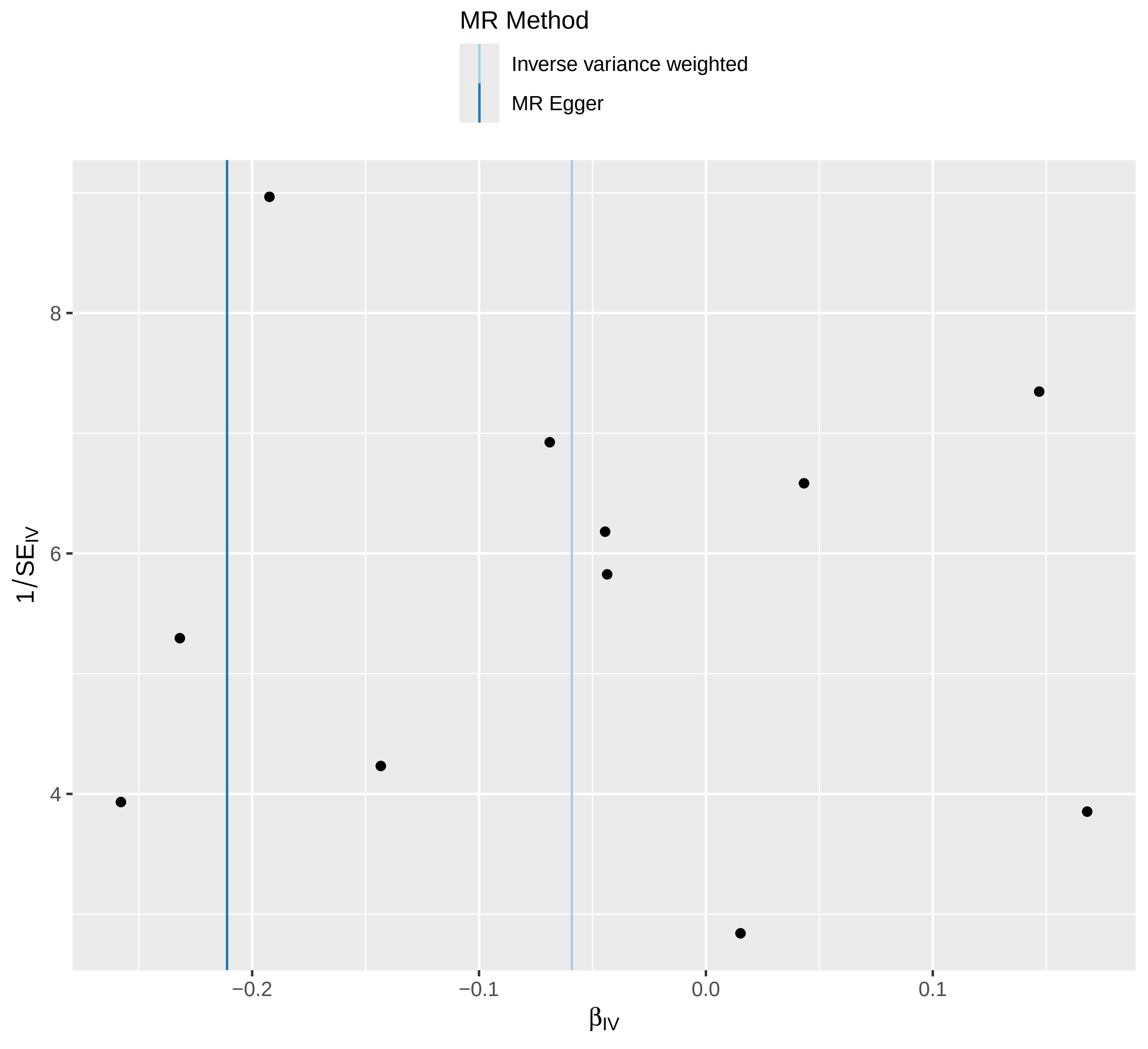
MR Method
Inverse variance weighted
MR Egger
8
6
4
1/SE_IV
−0.2
−0.1
0.0
0.1
β_IV

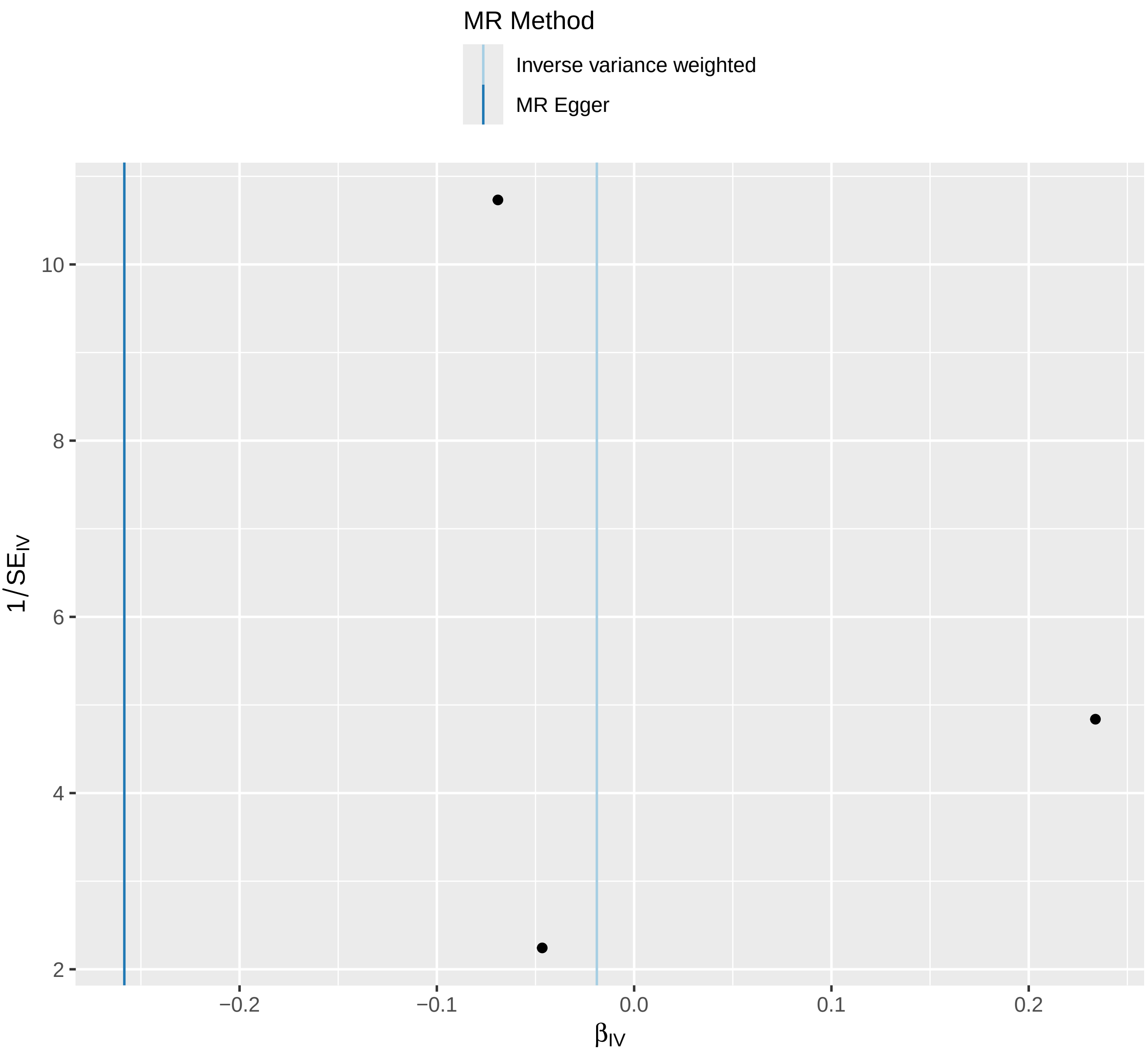
MR Method
Inverse variance weighted
MR Egger
1/SE_IV
10
8
6
4
2
−0.2
−0.1
0.0
0.1
0.2
β_IV

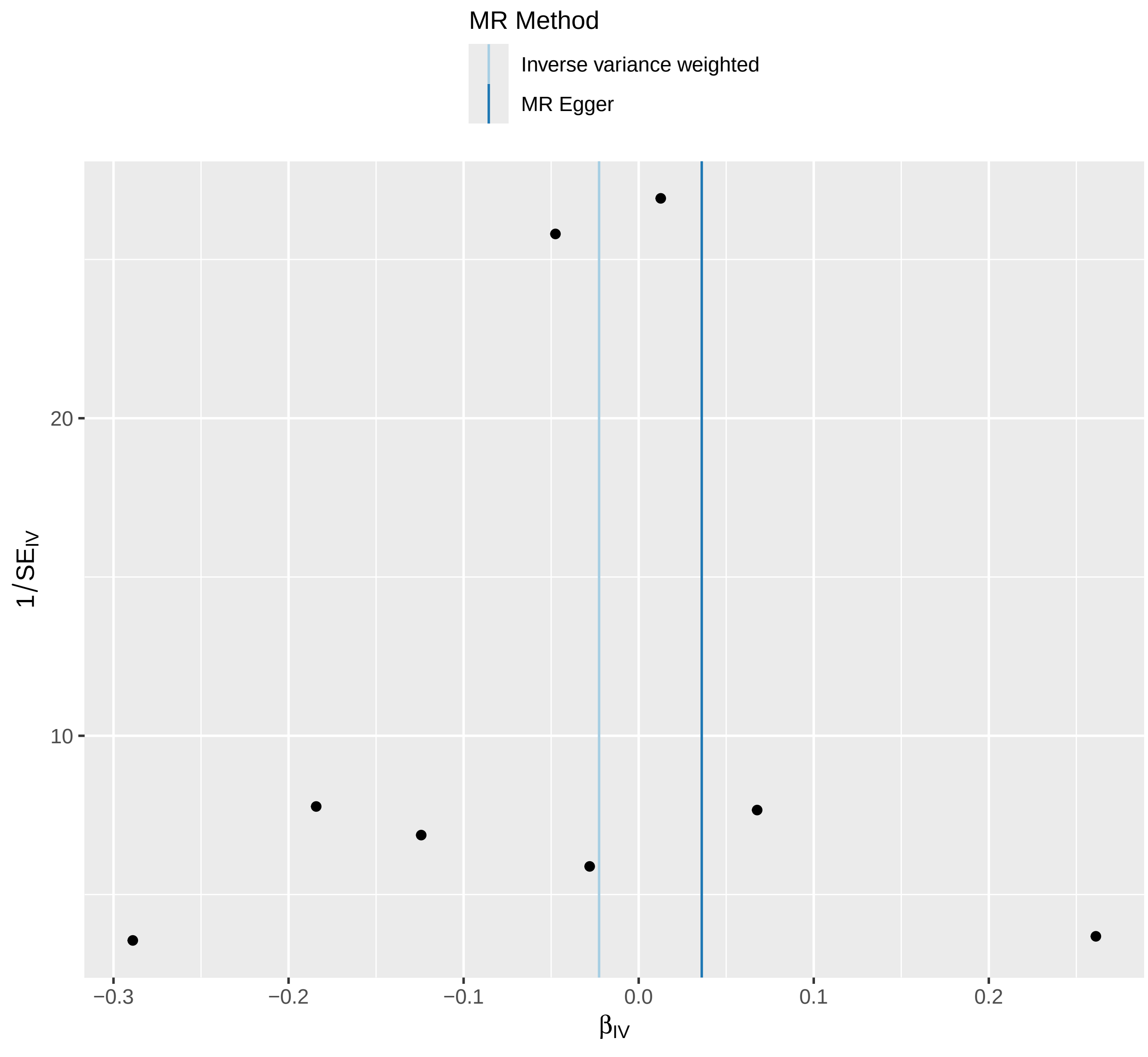

MR Method
Inverse variance weighted
MR Egger
20
10
$1/SE_{IV}$
−0.3
−0.2
−0.1
0.0
0.1
0.2
$\beta_{IV}$

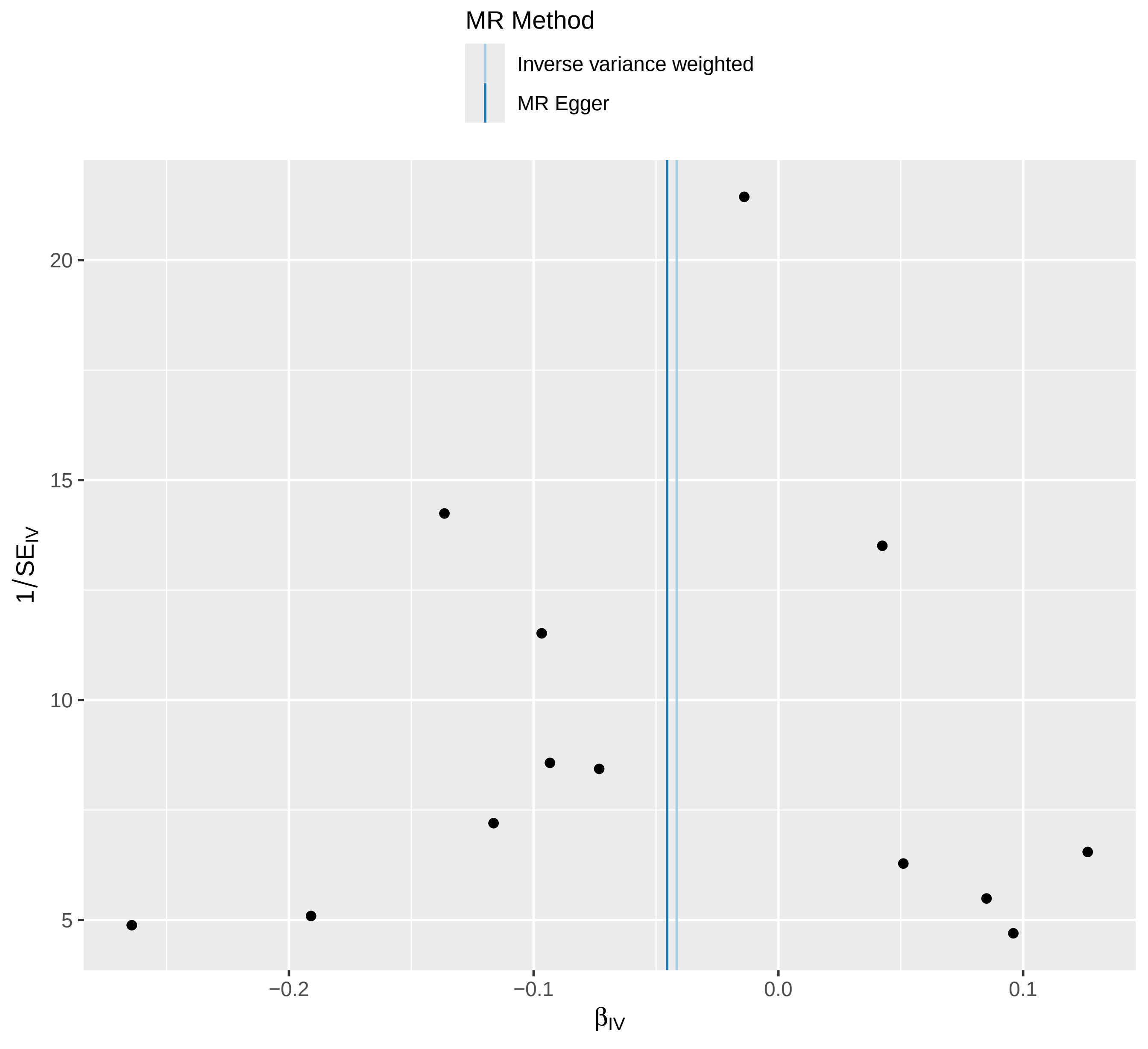

MR Method
Inverse variance weighted
MR Egger
20
15
10
5
1/SE$_{IV}$
−0.2
−0.1
0.0
0.1
β$_{IV}$

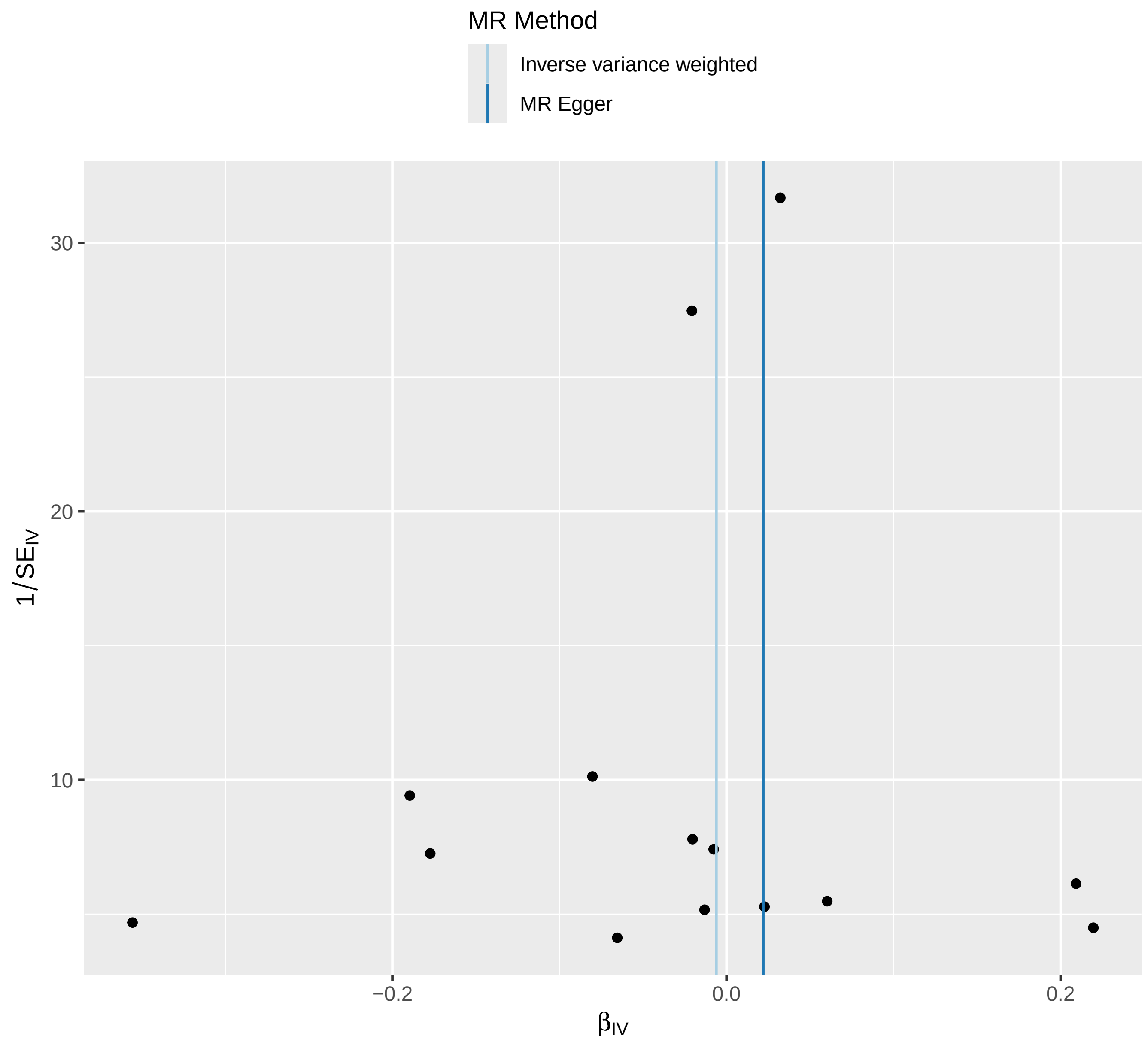

MR Method
Inverse variance weighted
MR Egger
30
20
10
1/SE$_{IV}$
−0.2
0.0
0.2
β$_{IV}$

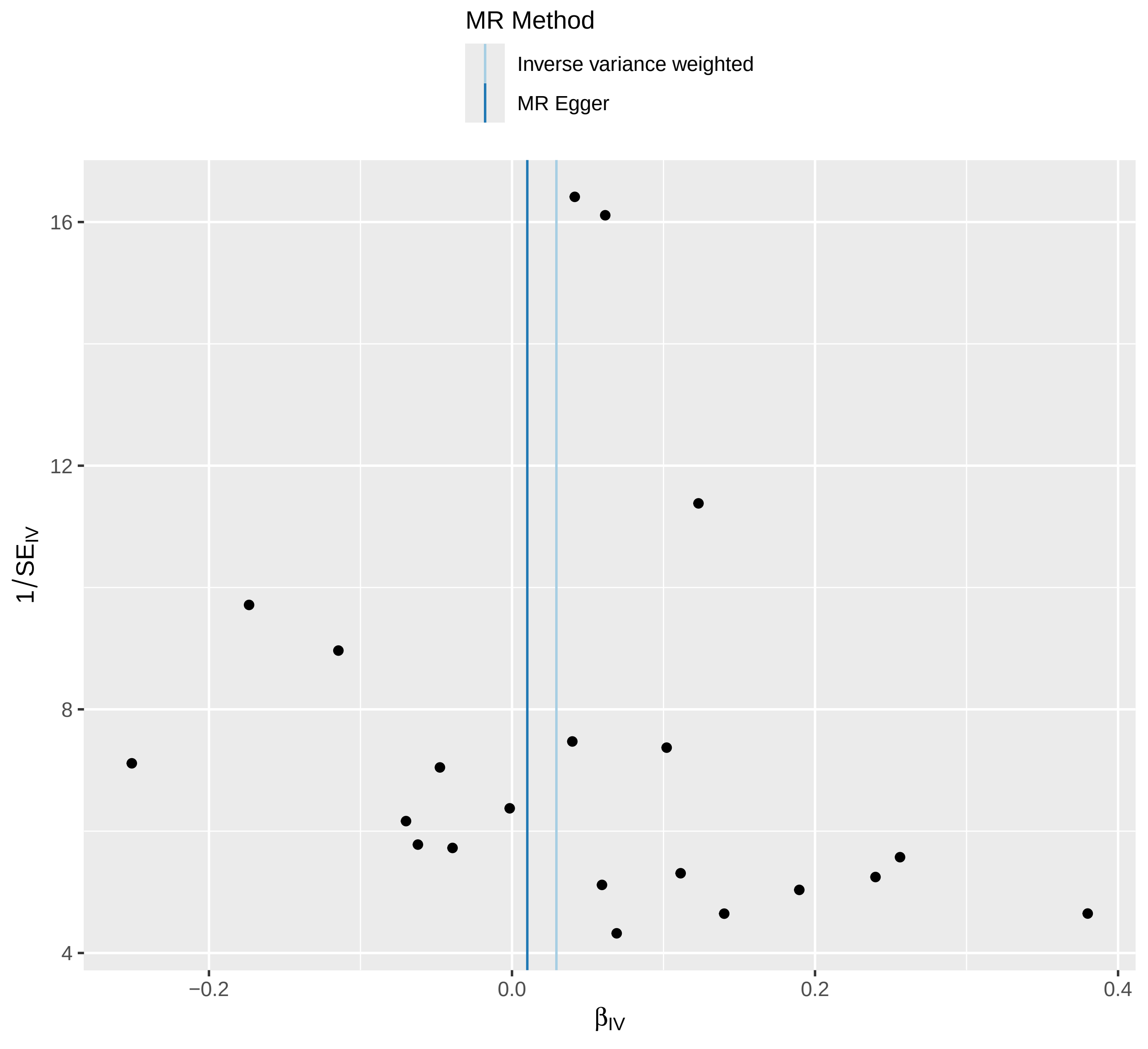

MR Method
Inverse variance weighted
MR Egger
16
12
8
4
1/SE$_{IV}$
−0.2
0.0
0.2
0.4
β$_{IV}$

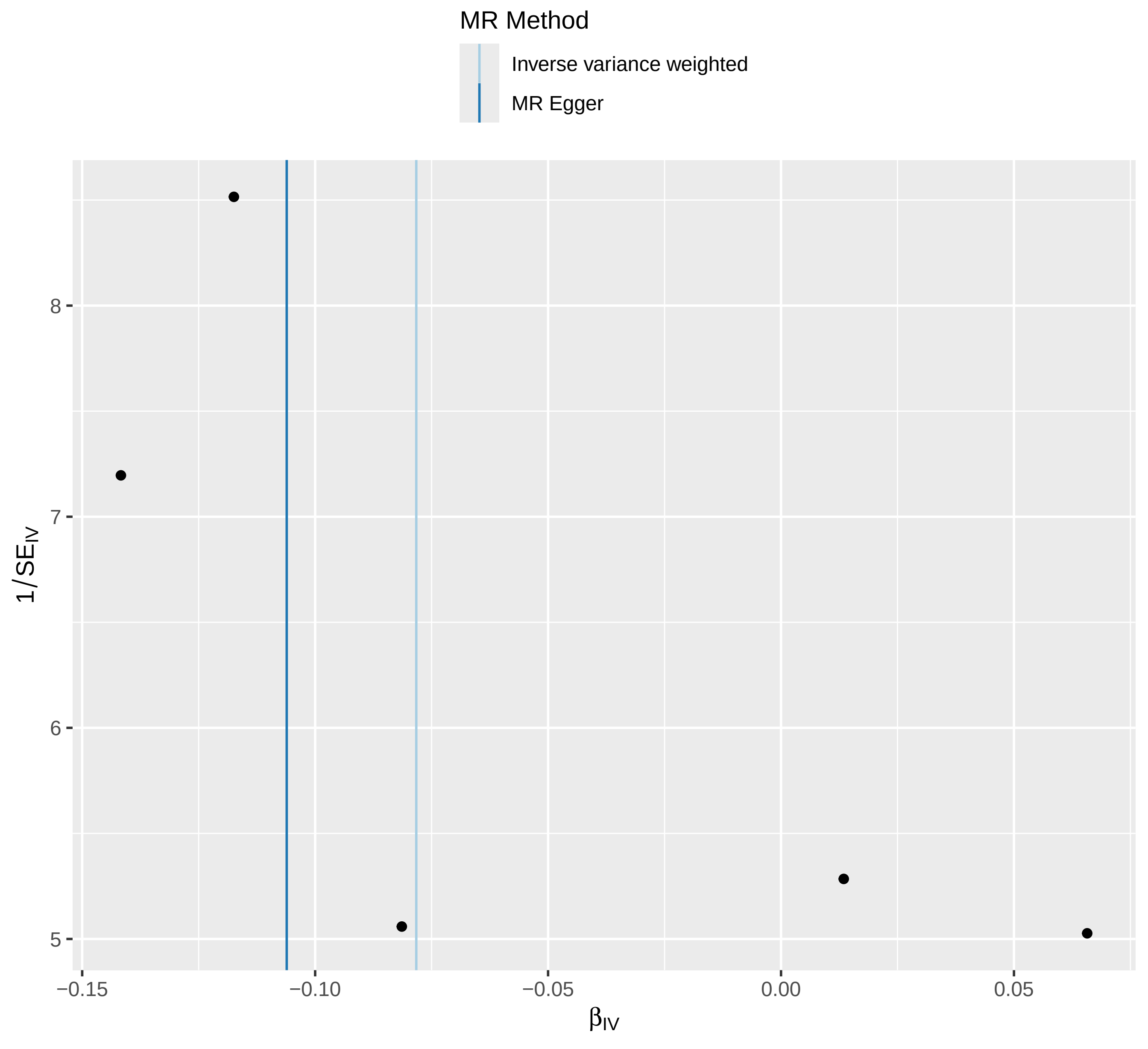

MR Method
Inverse variance weighted
MR Egger
8
7
6
5
1/SE_IV
−0.15
−0.10
−0.05
0.00
0.05
β_IV

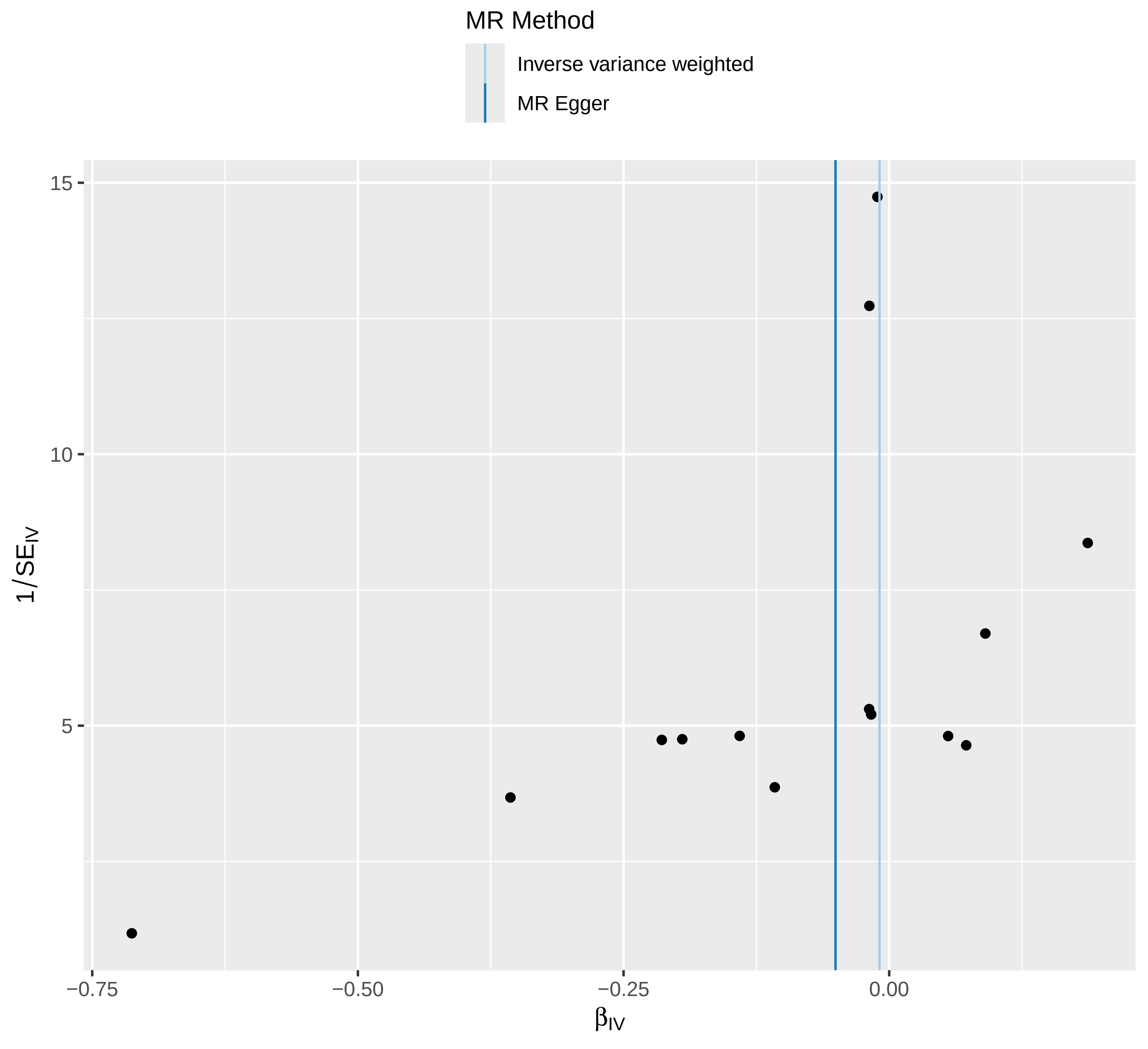
MR Method
Inverse variance weighted
MR Egger
15
10
5
1/SE_IV
−0.75
−0.50
−0.25
0.00
β_IV

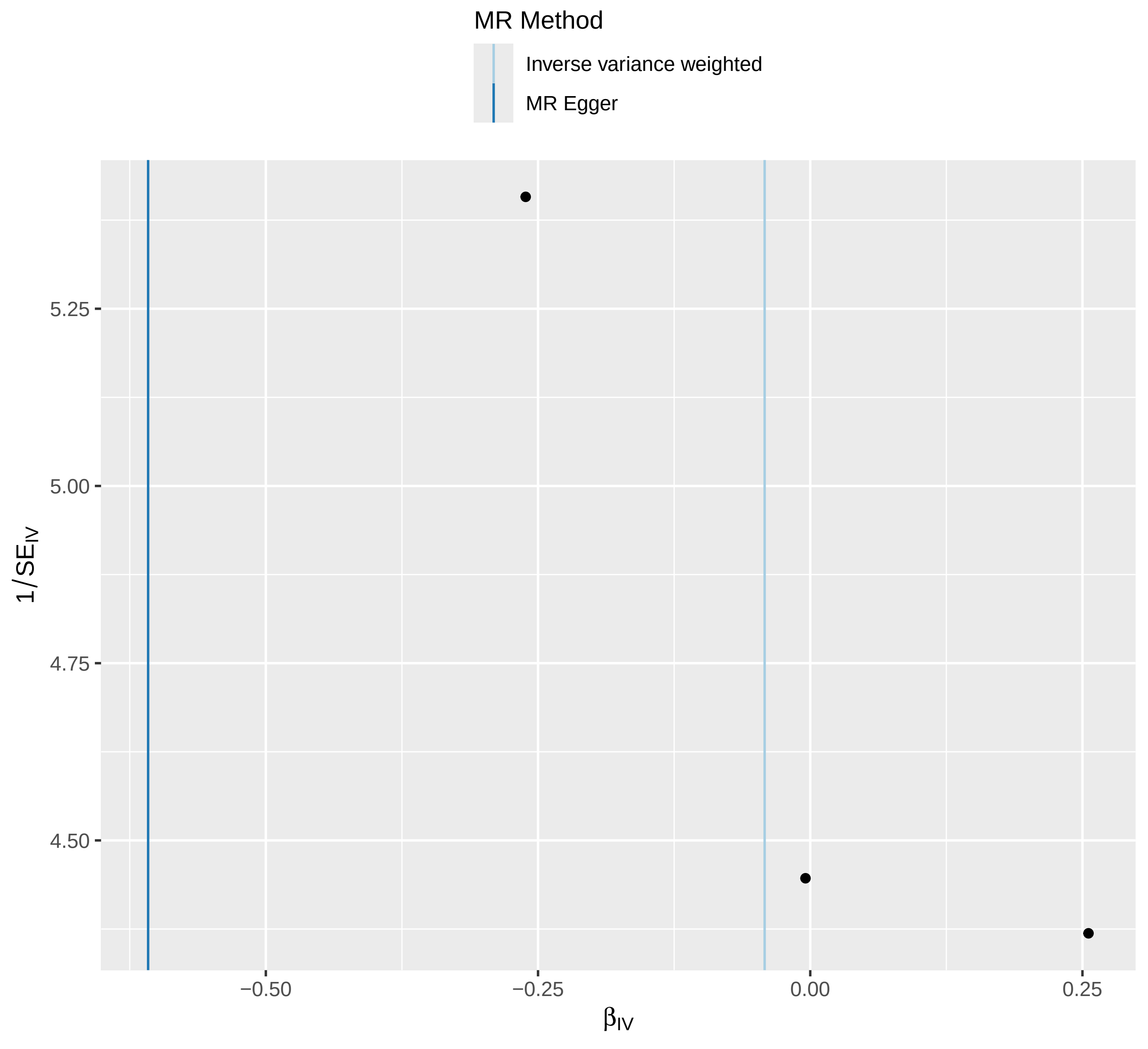
MR Method
Inverse variance weighted
MR Egger
5.25
5.00
4.75
4.50
1/SE$_{IV}$
−0.50
−0.25
0.00
0.25
β$_{IV}$

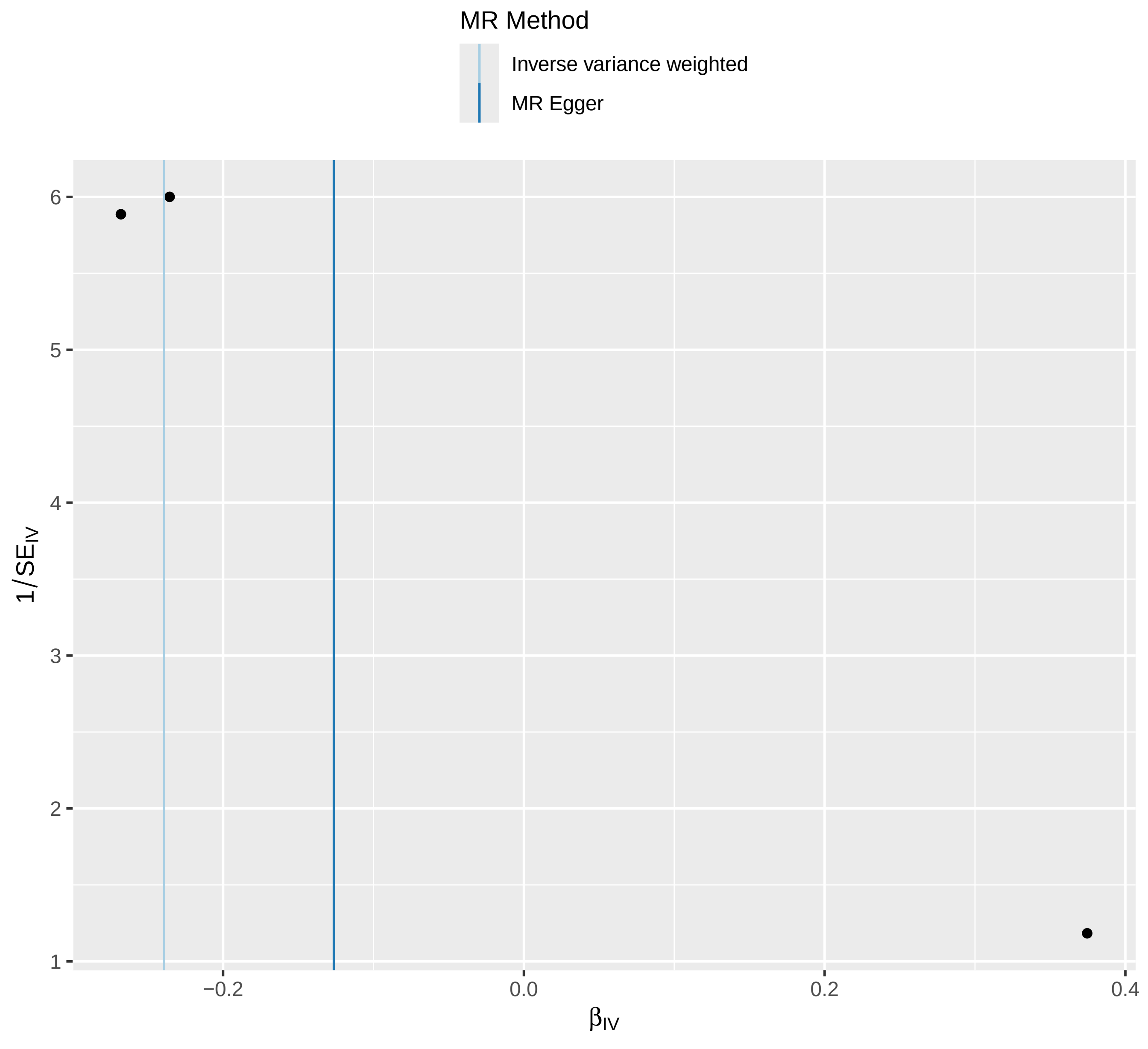
MR Method
Inverse variance weighted
MR Egger
6
5
4
3
2
1
1/SE_IV
−0.2
0.0
0.2
0.4
β_IV

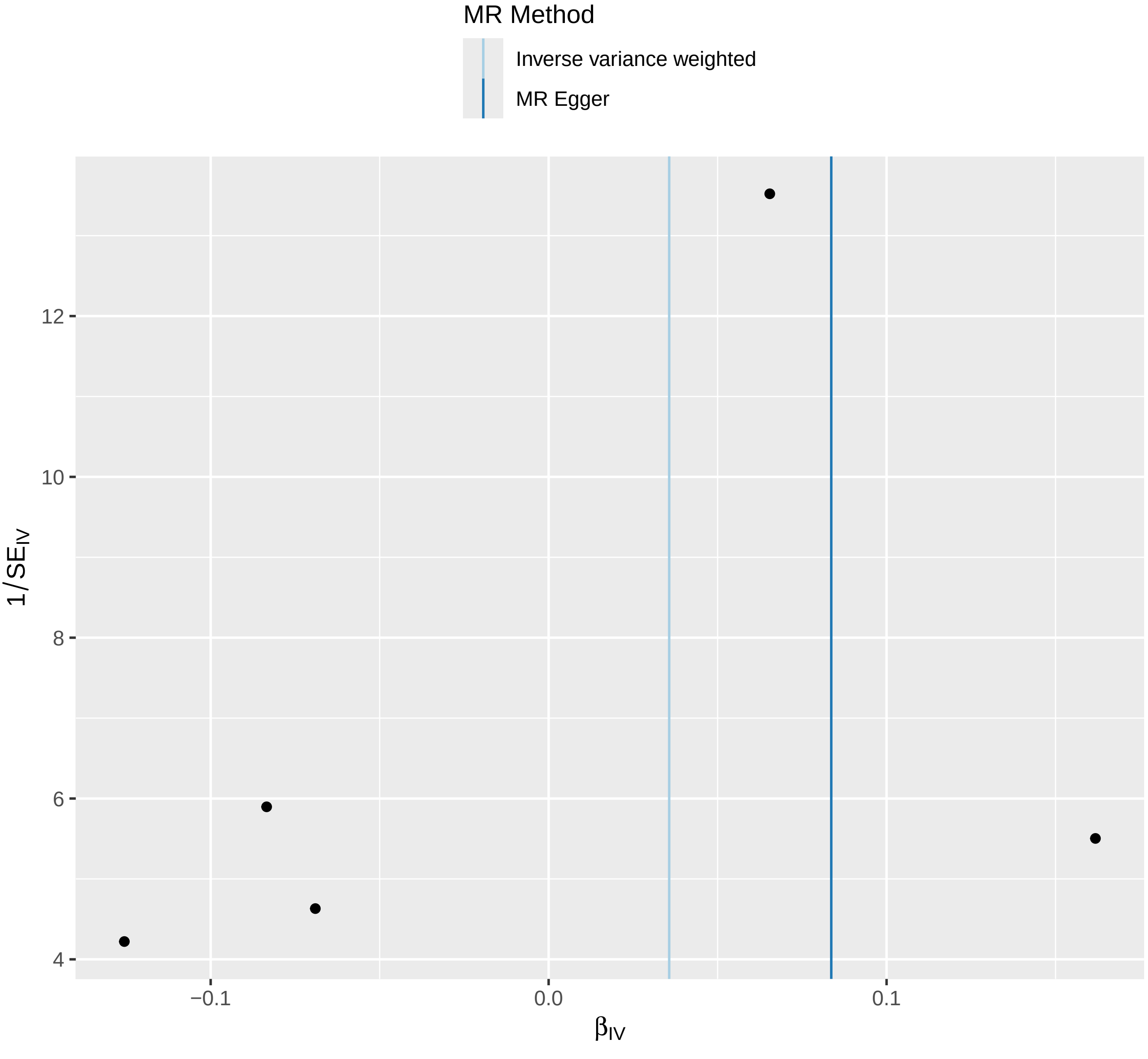
MR Method
Inverse variance weighted
MR Egger
1/SE$_{IV}$
12
10
8
6
4
−0.1
0.0
0.1
β$_{IV}$

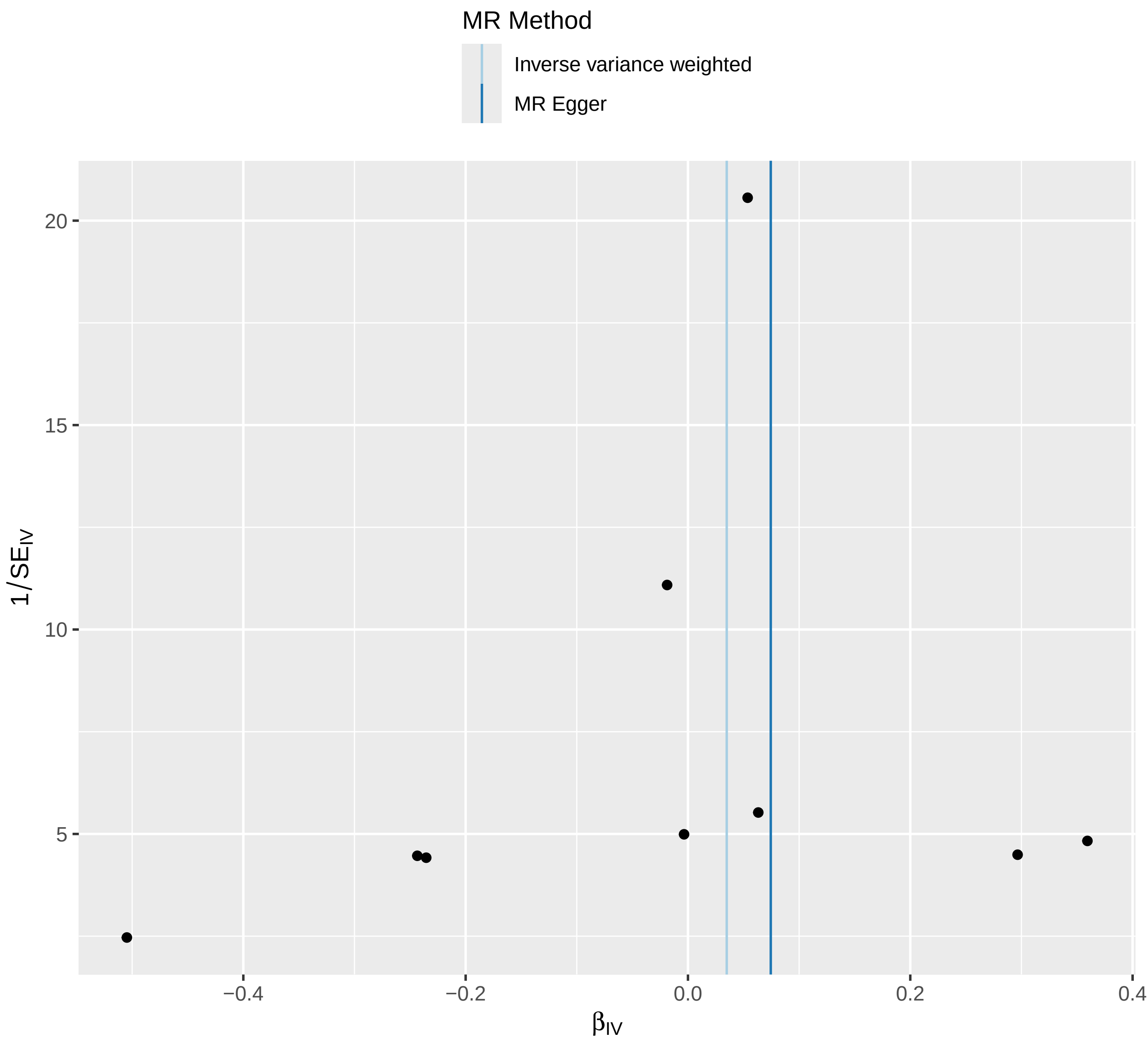

MR Method
Inverse variance weighted
MR Egger
20
15
10
5
$1/SE_{IV}$
−0.4
−0.2
0.0
0.2
0.4
$\beta_{IV}$

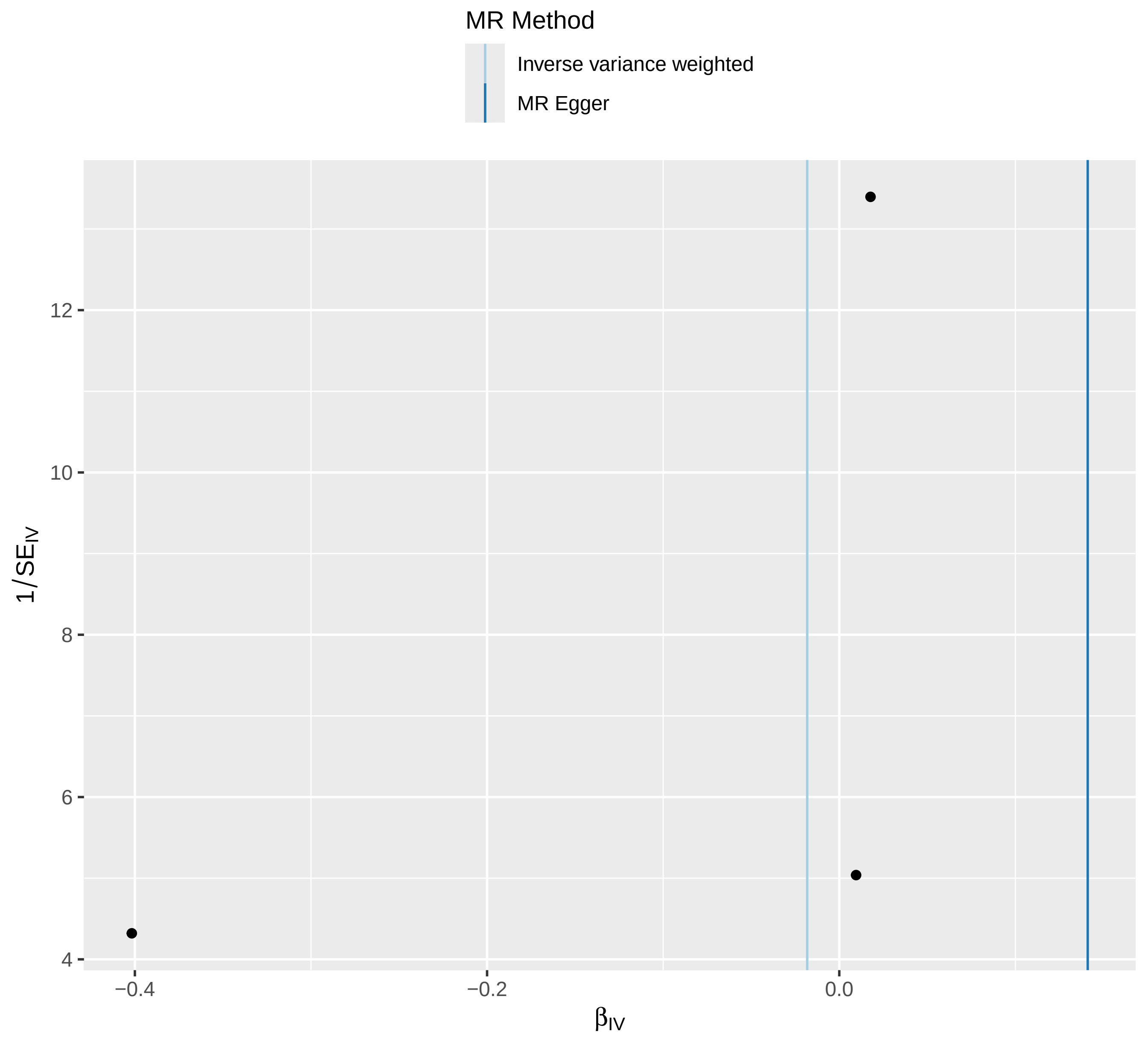

MR Method
Inverse variance weighted
MR Egger
12
10
8
6
4
1/SE_IV
−0.4
−0.2
0.0
β_IV

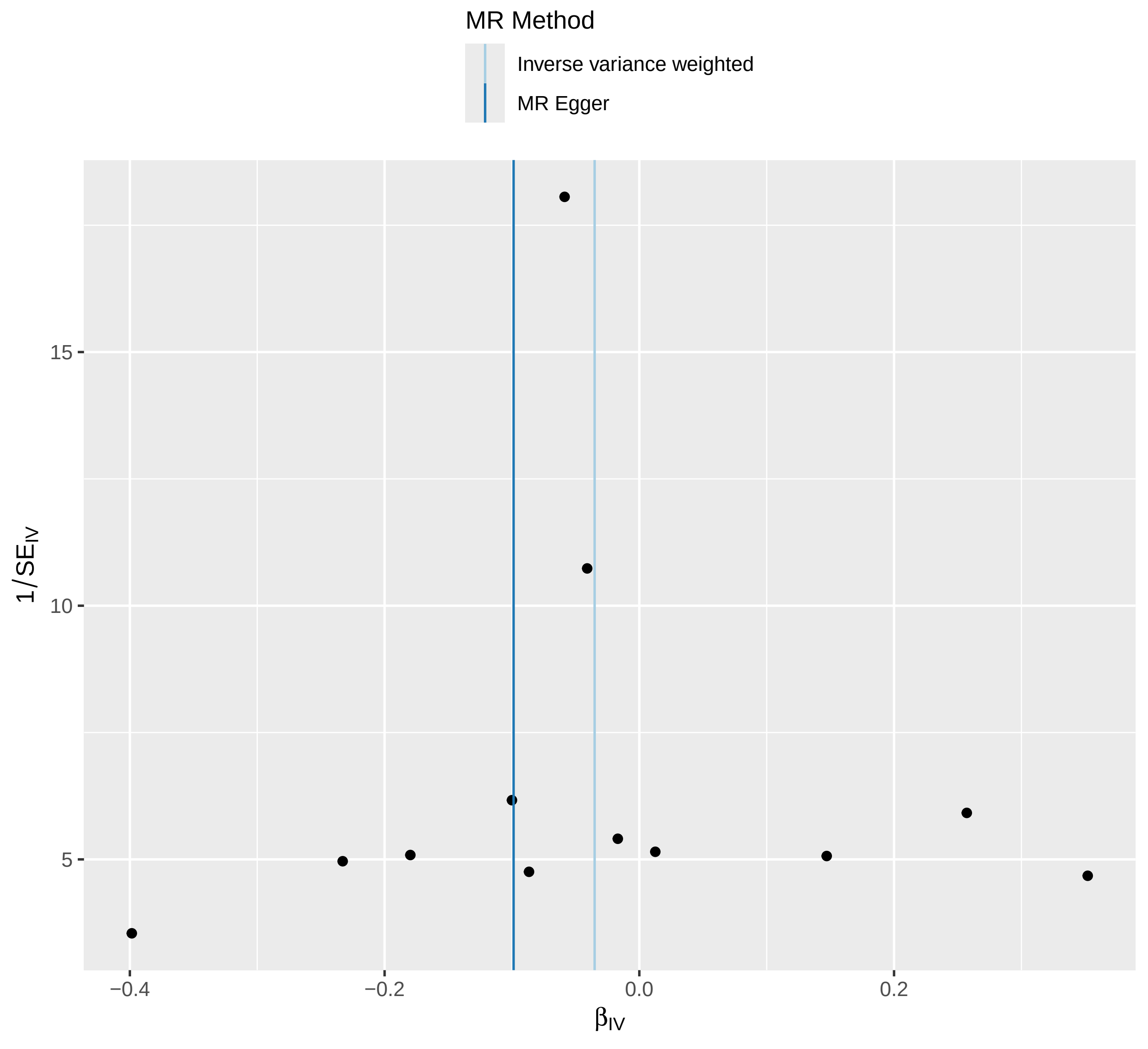

MR Method
Inverse variance weighted
MR Egger
15
10
5
1/SE$_{IV}$
−0.4
−0.2
0.0
0.2
β$_{IV}$

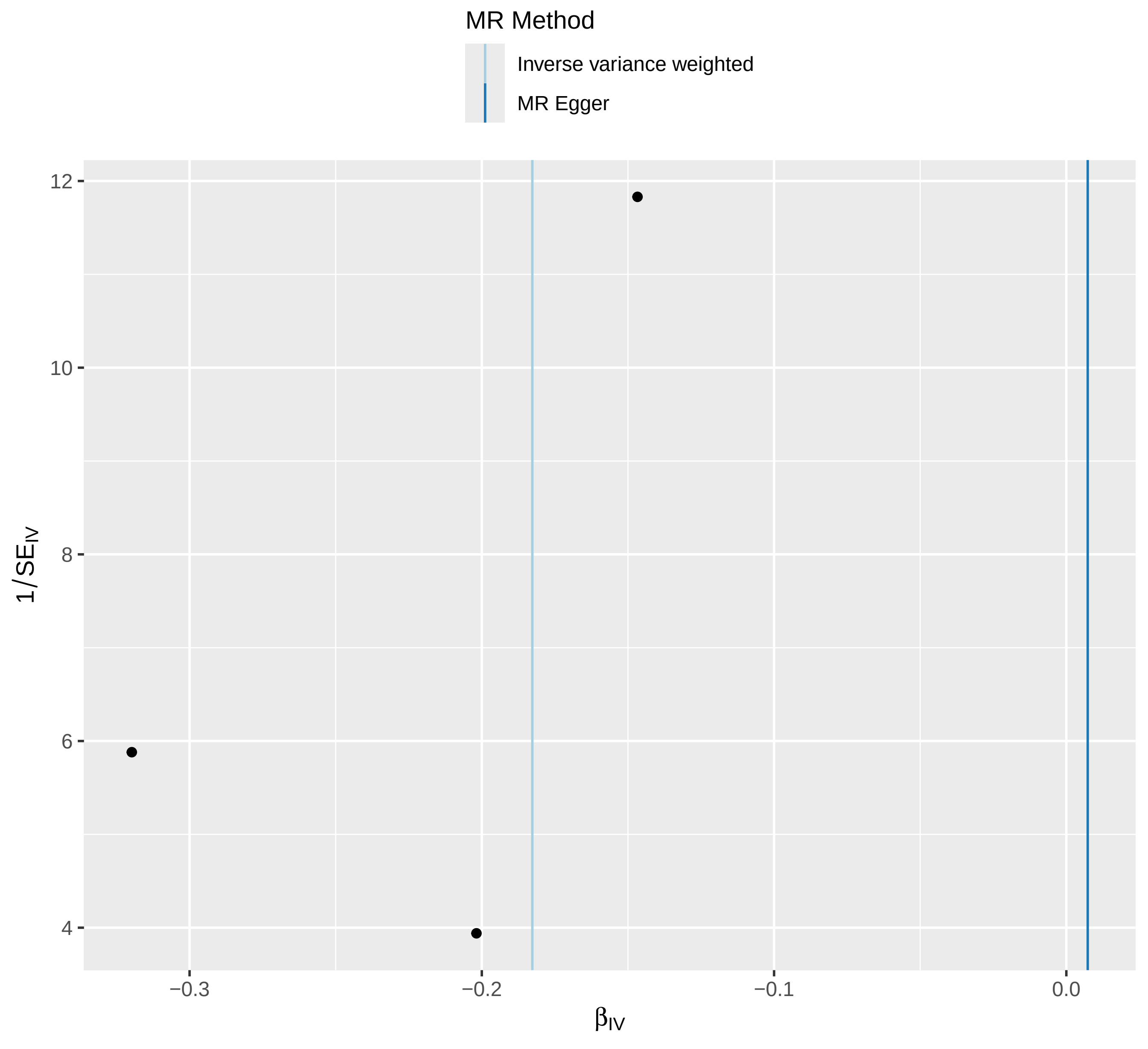
MR Method
Inverse variance weighted
MR Egger
12
10
8
6
4
1/SE_IV
−0.3
−0.2
−0.1
0.0
β_IV

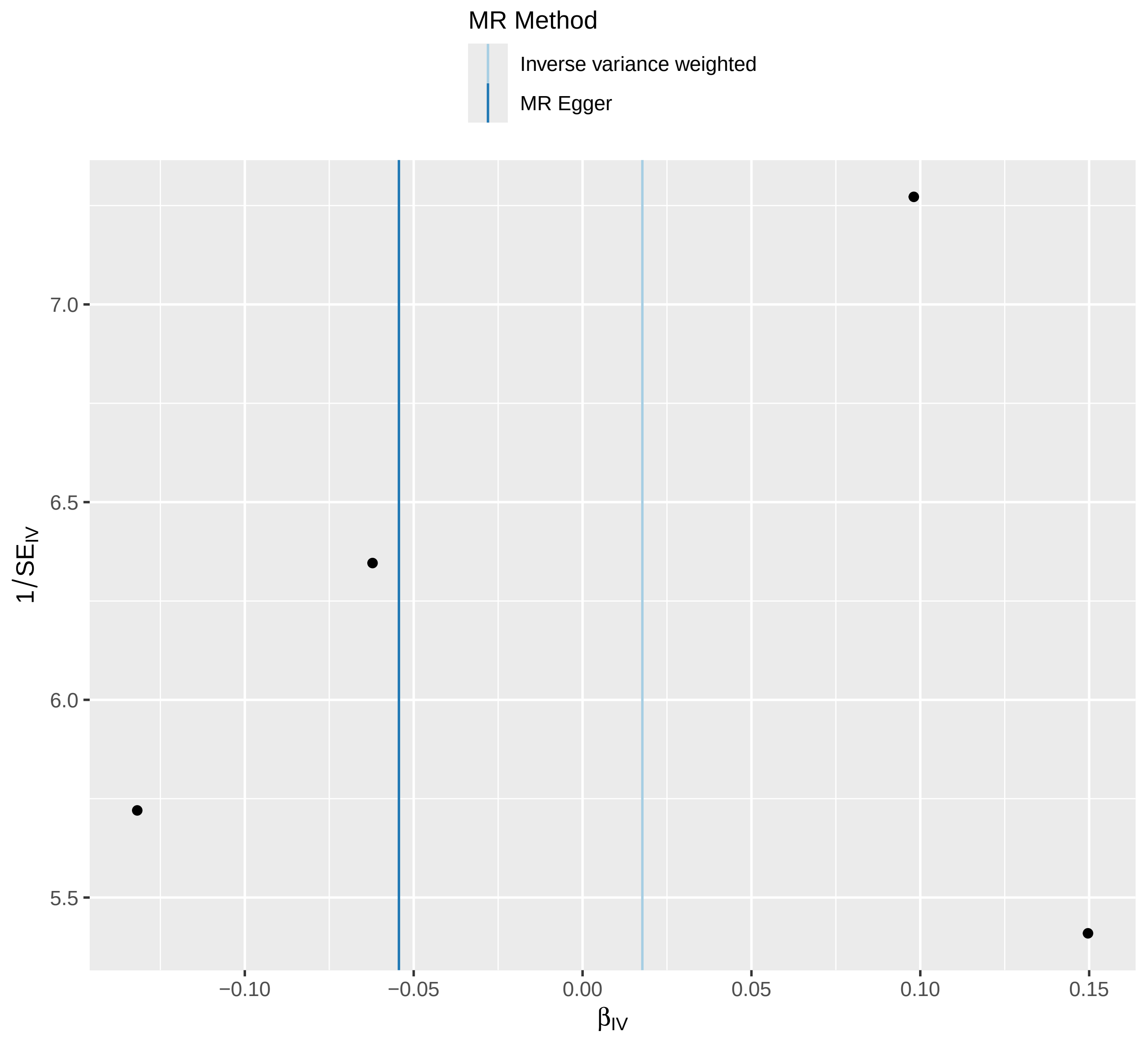
MR Method
Inverse variance weighted
MR Egger
7.0
6.5
6.0
5.5
1/SE_IV
−0.10
−0.05
0.00
0.05
0.10
0.15
β_IV

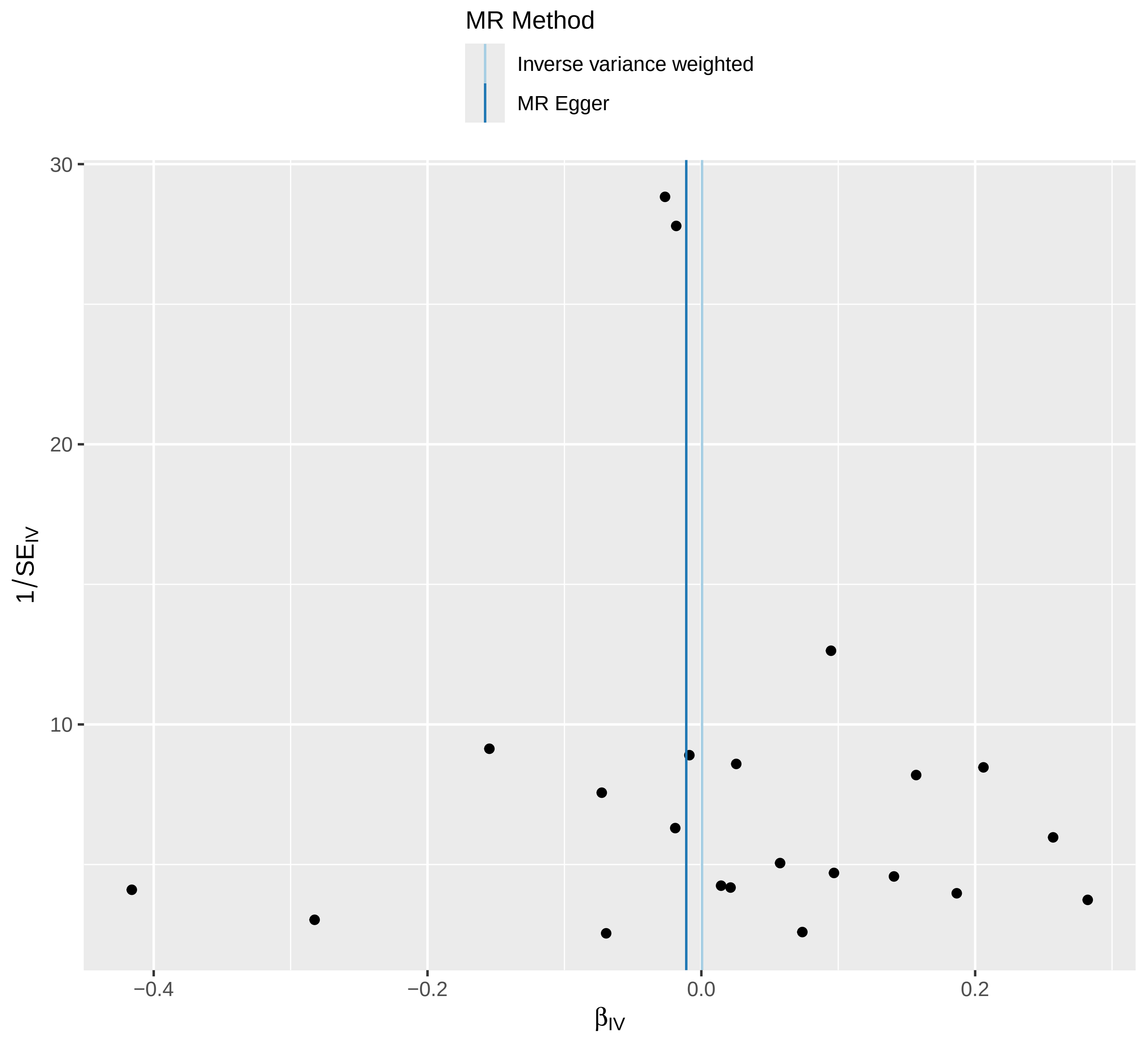

MR Method
Inverse variance weighted
MR Egger
30
20
10
1/SE$_{IV}$
−0.4
−0.2
0.0
0.2
β$_{IV}$

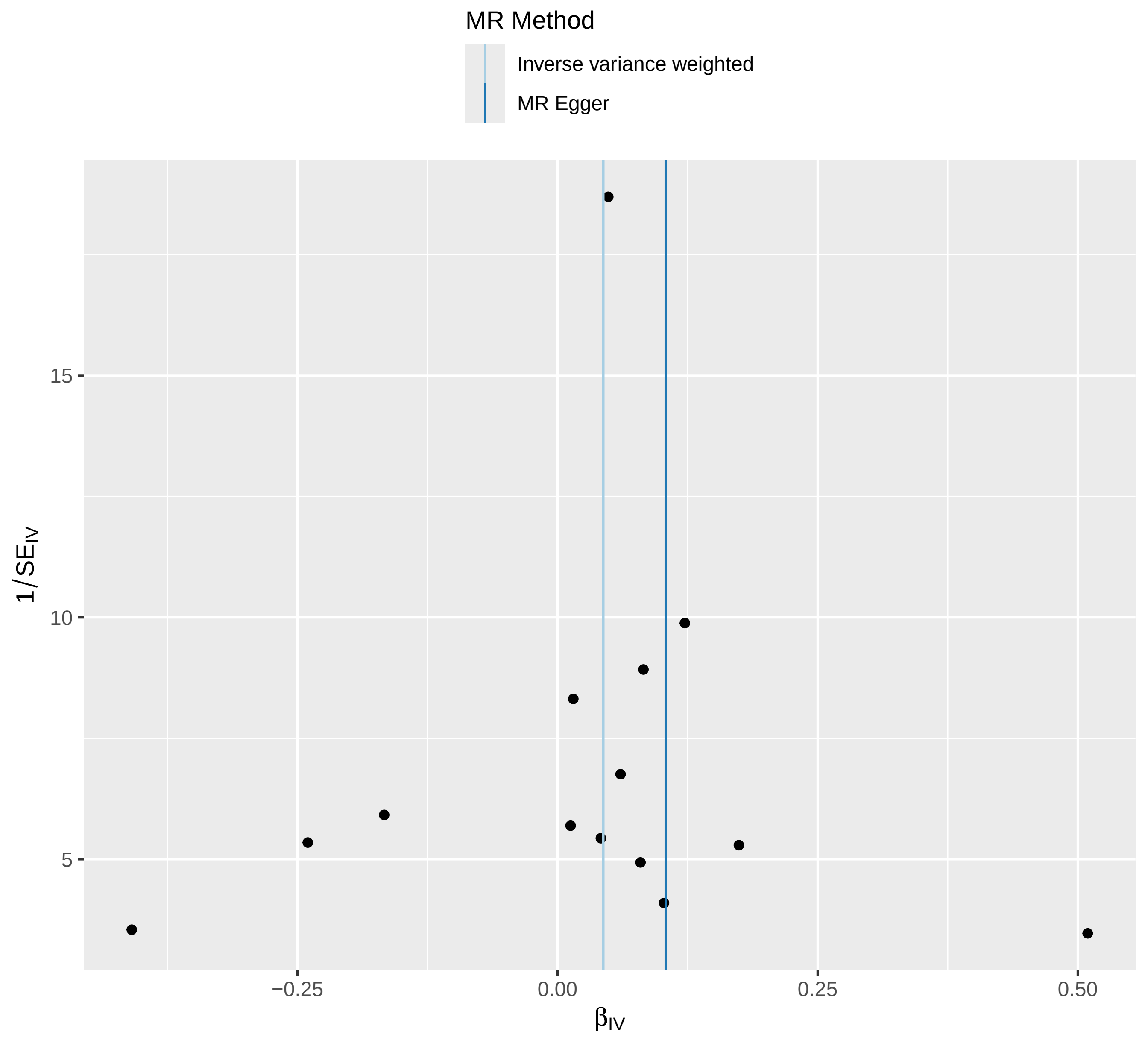

MR Method
Inverse variance weighted
MR Egger
1/SE_IV
15
10
5
−0.25
0.00
0.25
0.50
β_IV

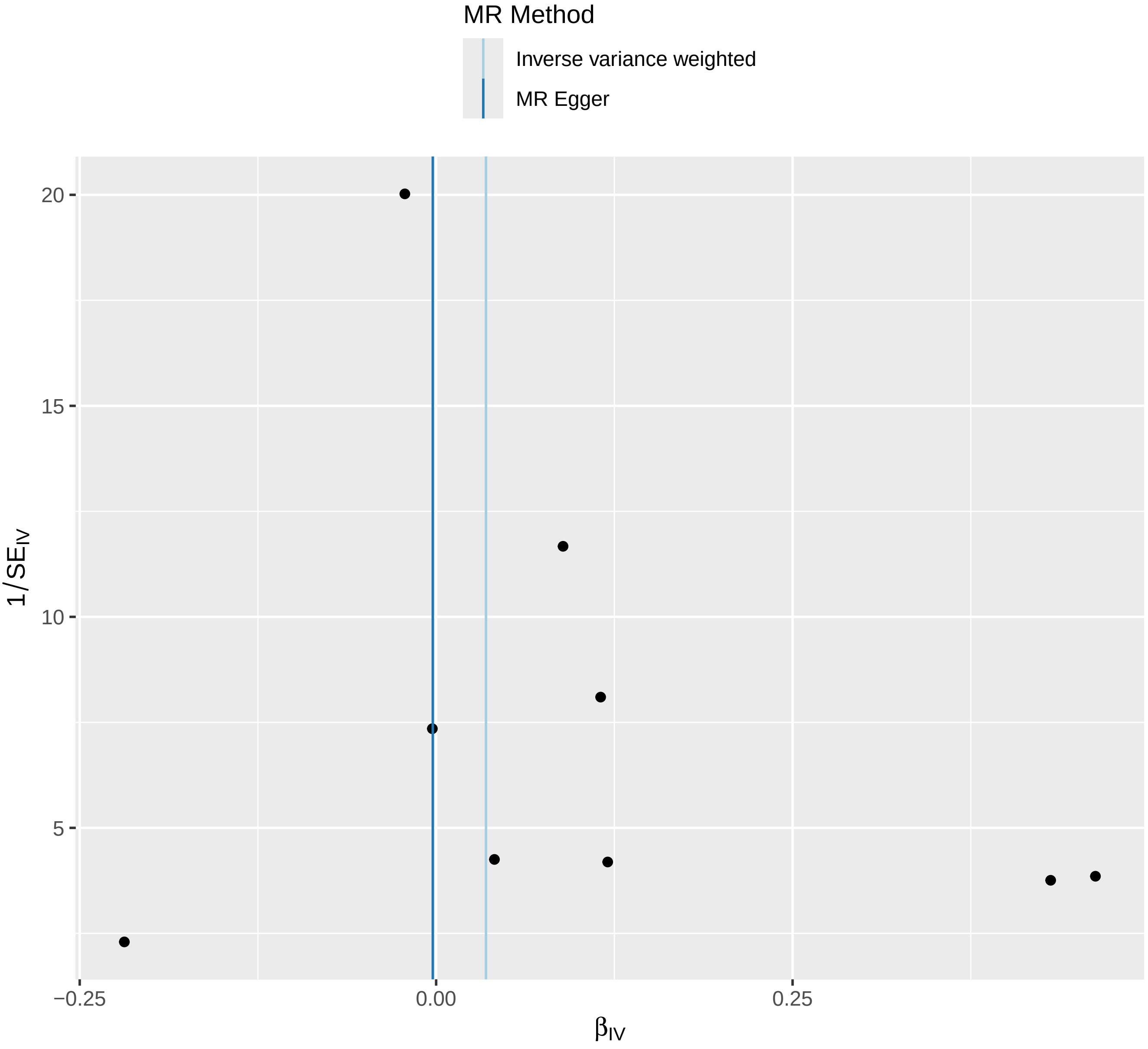

MR Method
Inverse variance weighted
MR Egger
20
15
10
5
−0.25
0.00
0.25
$1/SE_{IV}$
$\beta_{IV}$

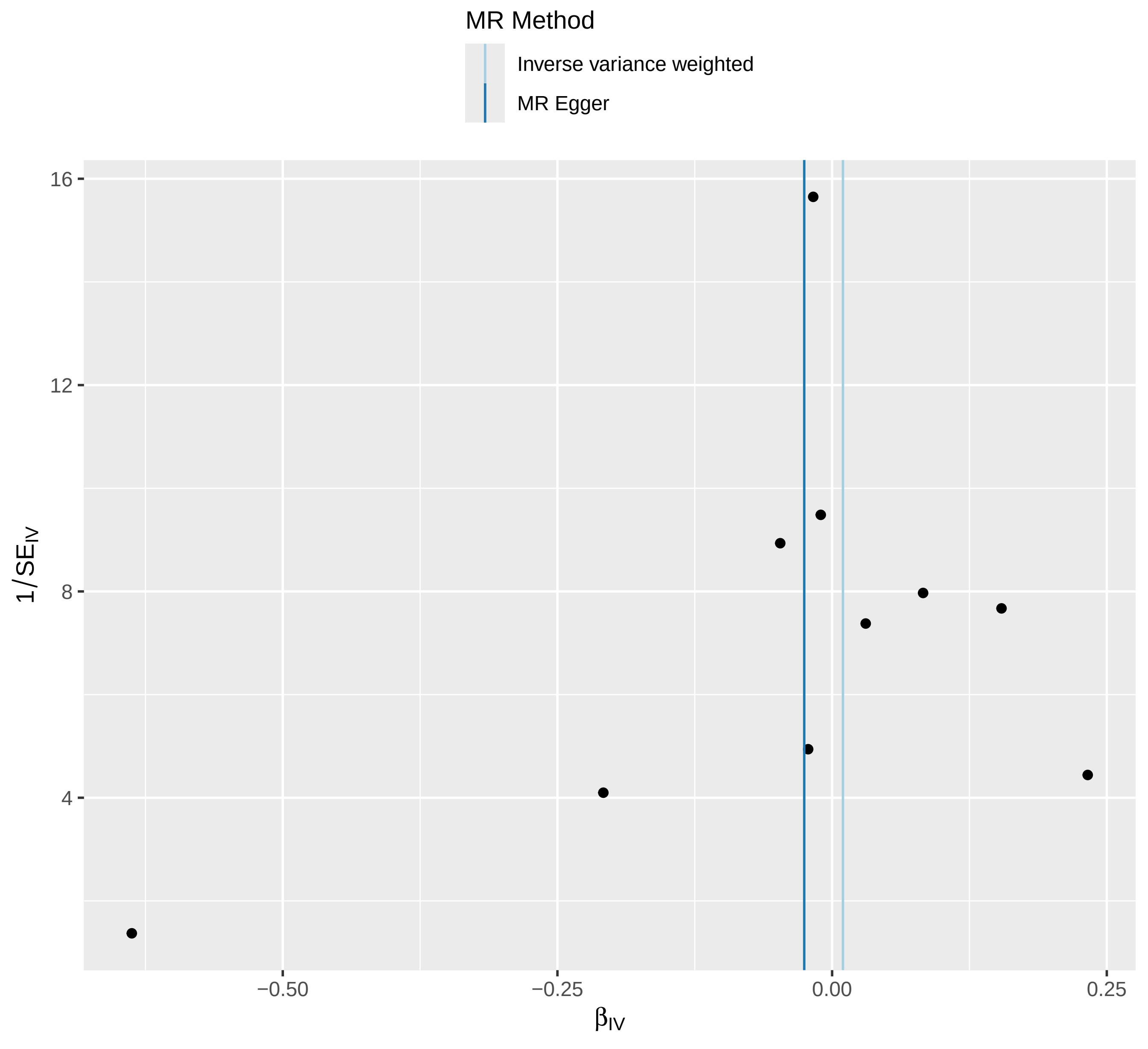
MR Method
Inverse variance weighted
MR Egger
16
12
8
4
1/SE_IV
−0.50
−0.25
0.00
0.25
β_IV

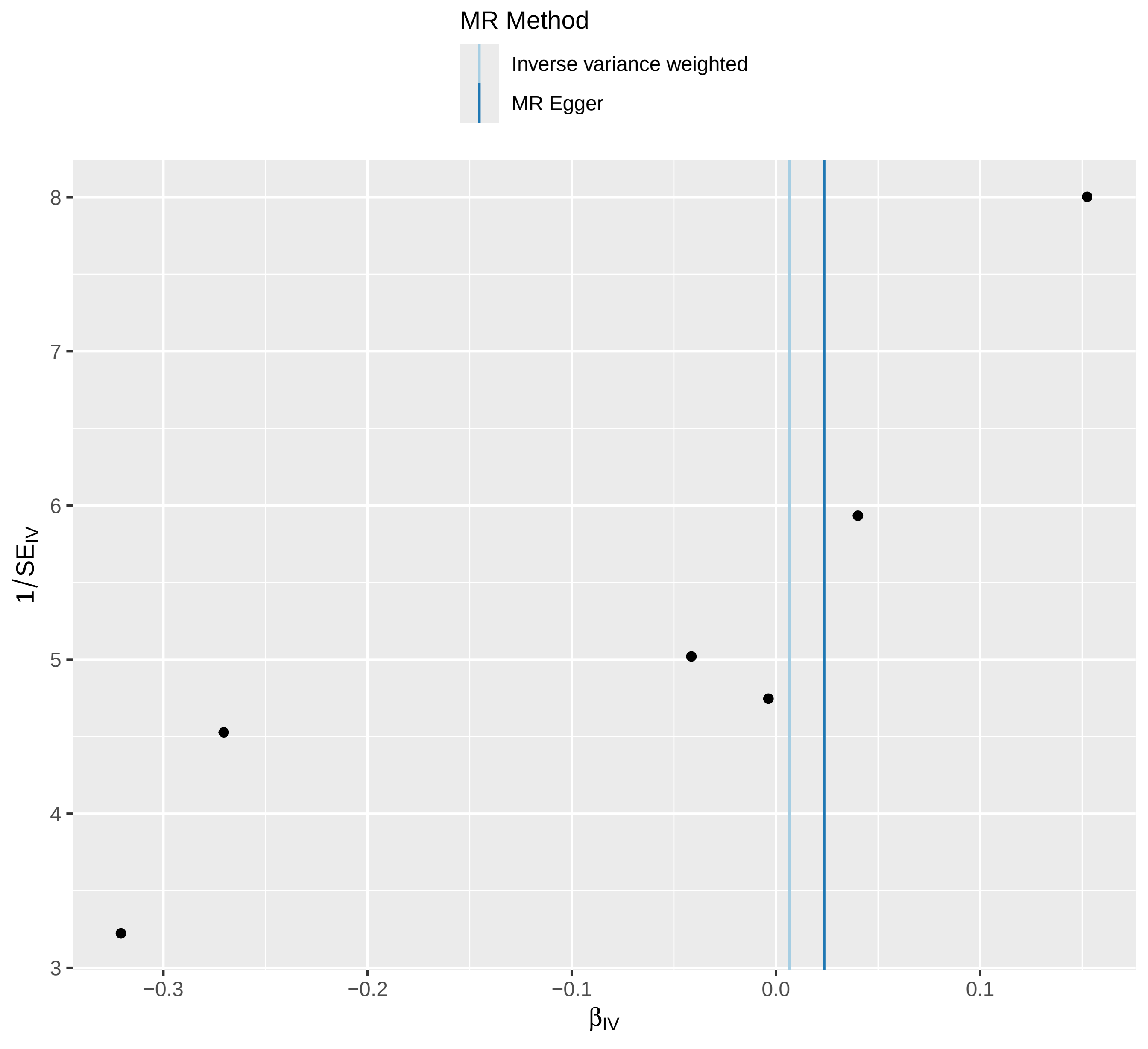
MR Method
Inverse variance weighted
MR Egger
8
7
6
5
4
3
1/SE_IV
−0.3
−0.2
−0.1
0.0
0.1
β_IV

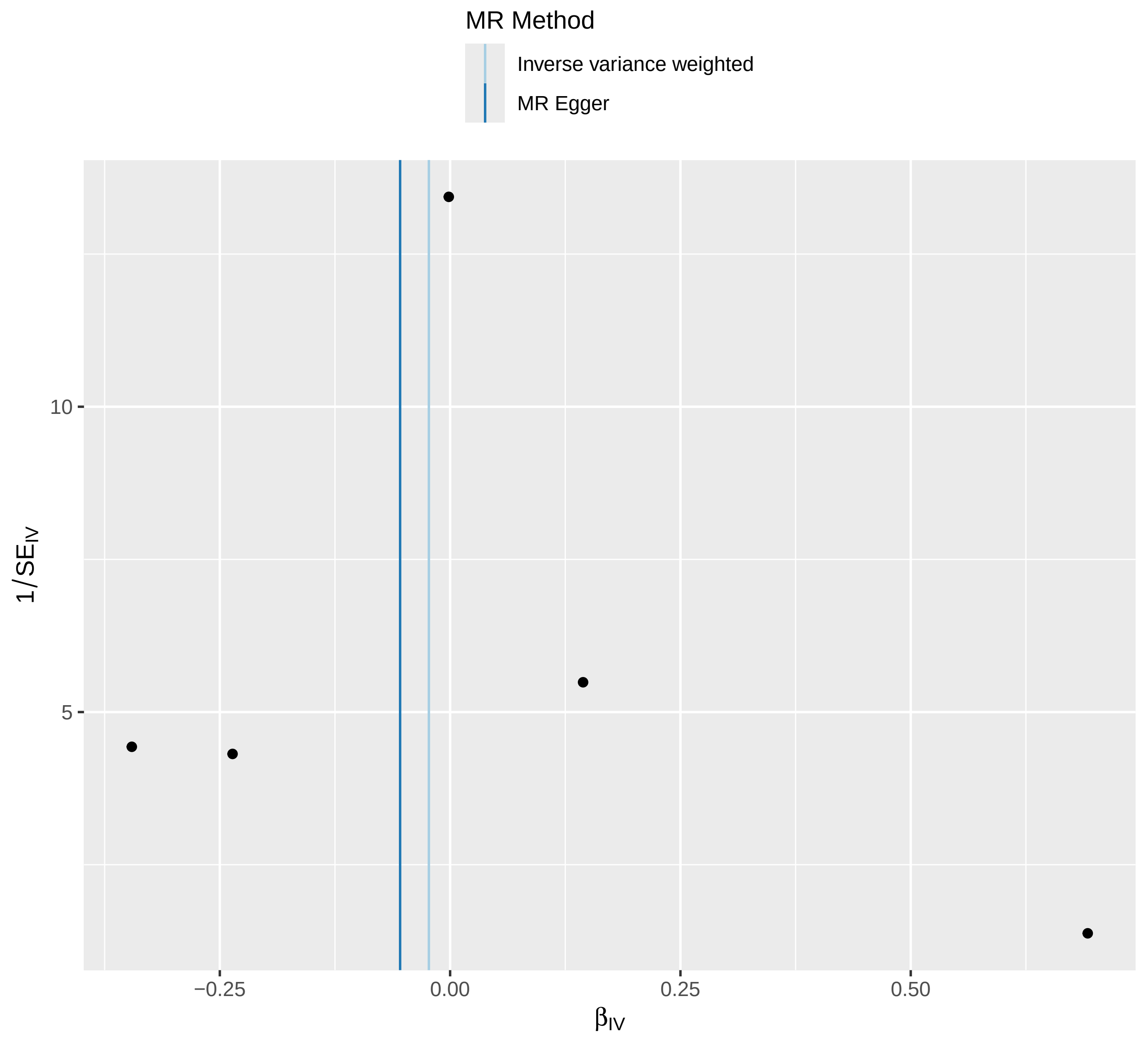
MR Method
Inverse variance weighted
MR Egger
10
5
$1/SE_{IV}$
−0.25
0.00
0.25
0.50
$\beta_{IV}$

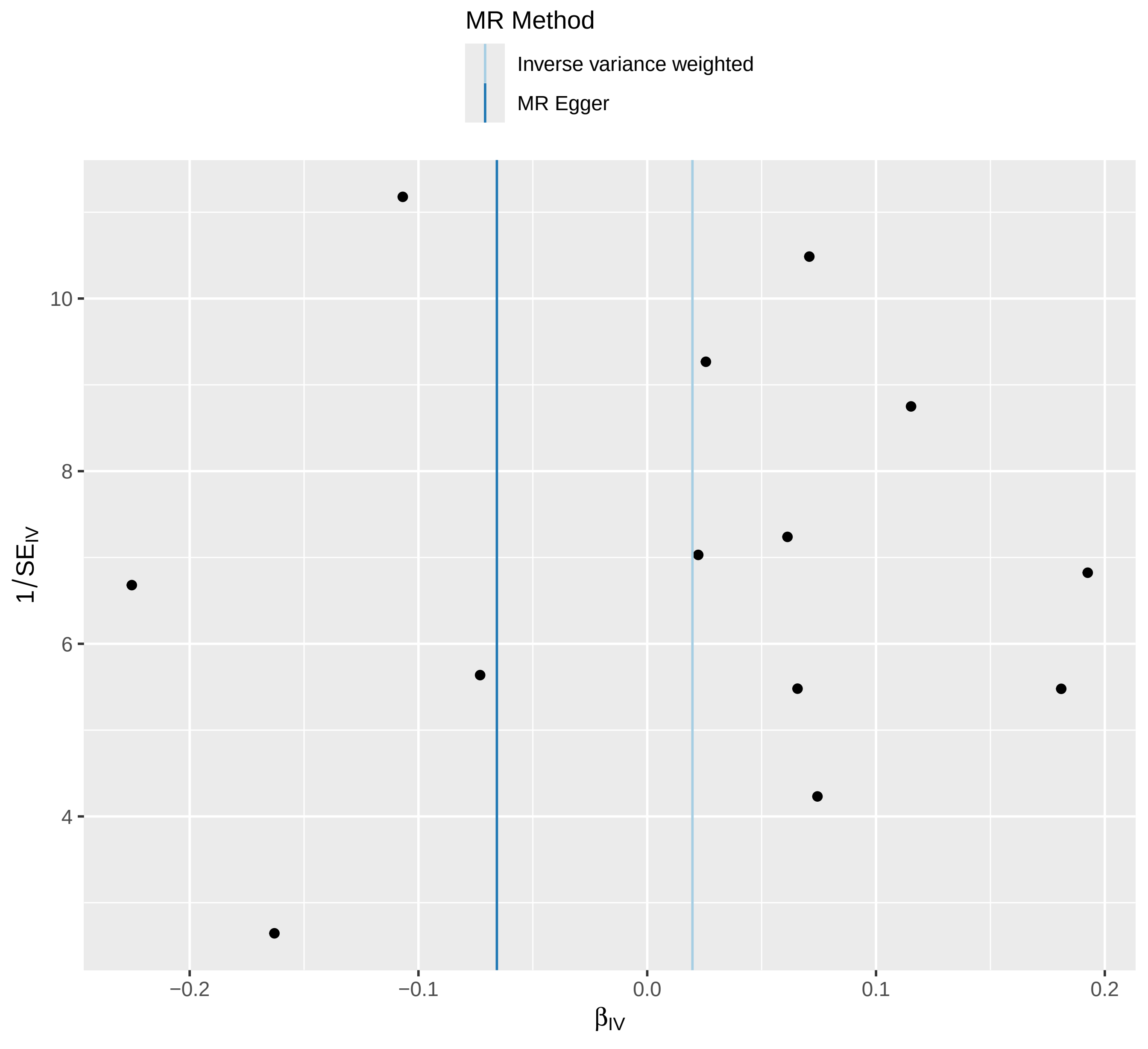

MR Method
Inverse variance weighted
MR Egger
10
8
6
4
1/SE$_{IV}$
−0.2
−0.1
0.0
0.1
0.2
β$_{IV}$

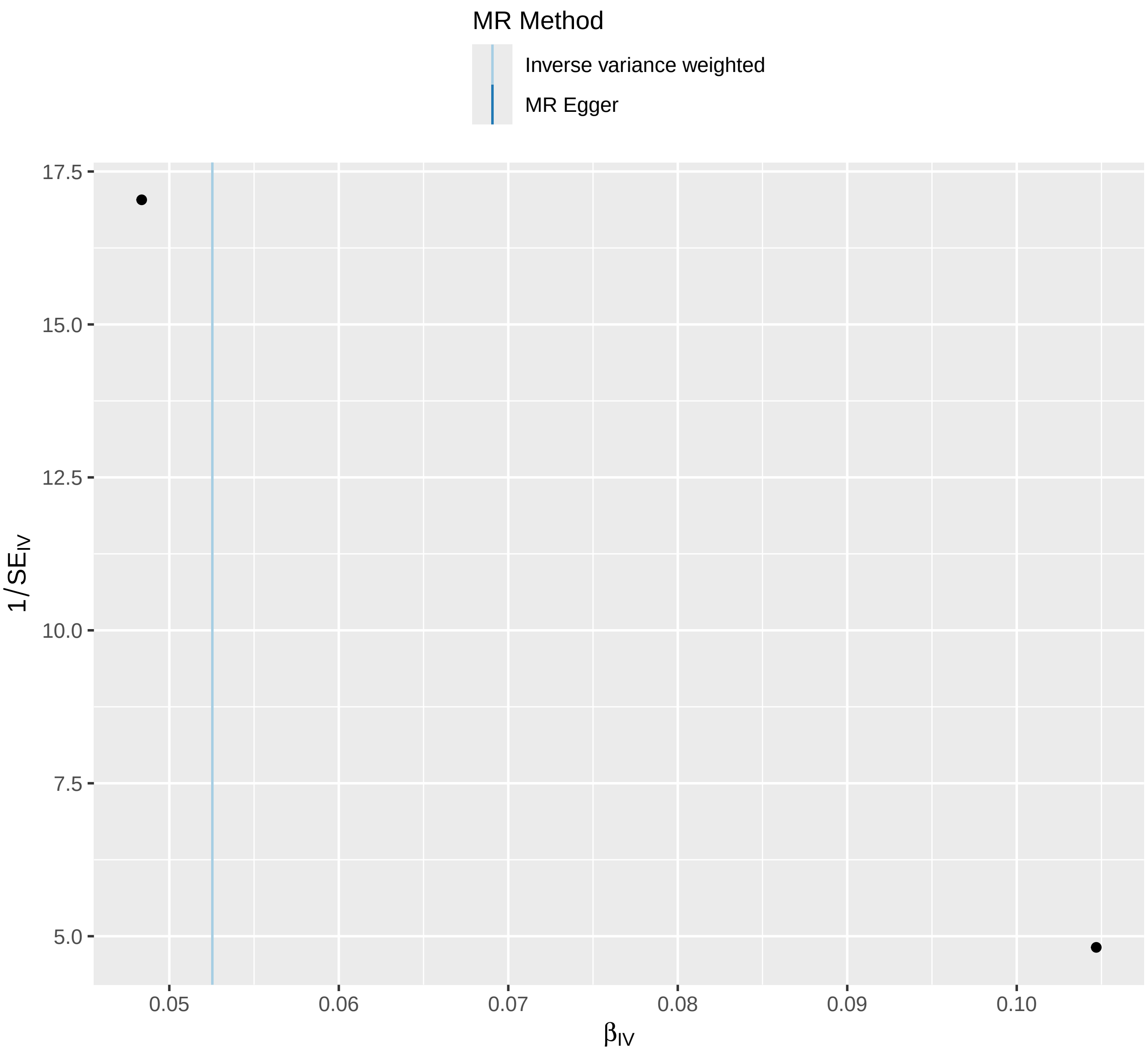

MR Method
Inverse variance weighted
MR Egger
17.5
15.0
12.5
10.0
7.5
5.0
1/SE$_{IV}$
0.05
0.06
0.07
0.08
0.09
0.10
β$_{IV}$

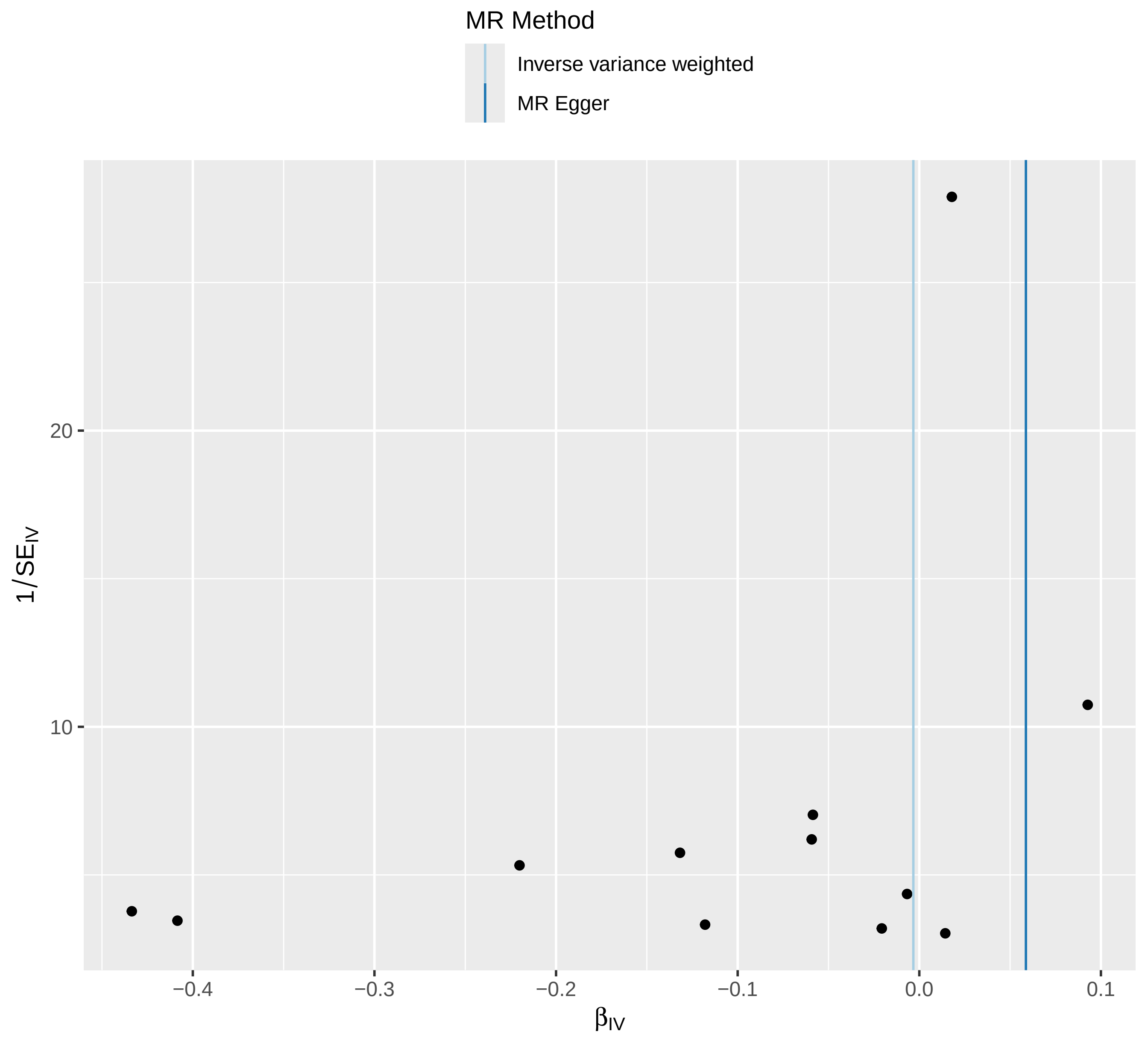

MR Method
Inverse variance weighted
MR Egger
20
10
1/SE_IV
−0.4
−0.3
−0.2
−0.1
0.0
0.1
β_IV

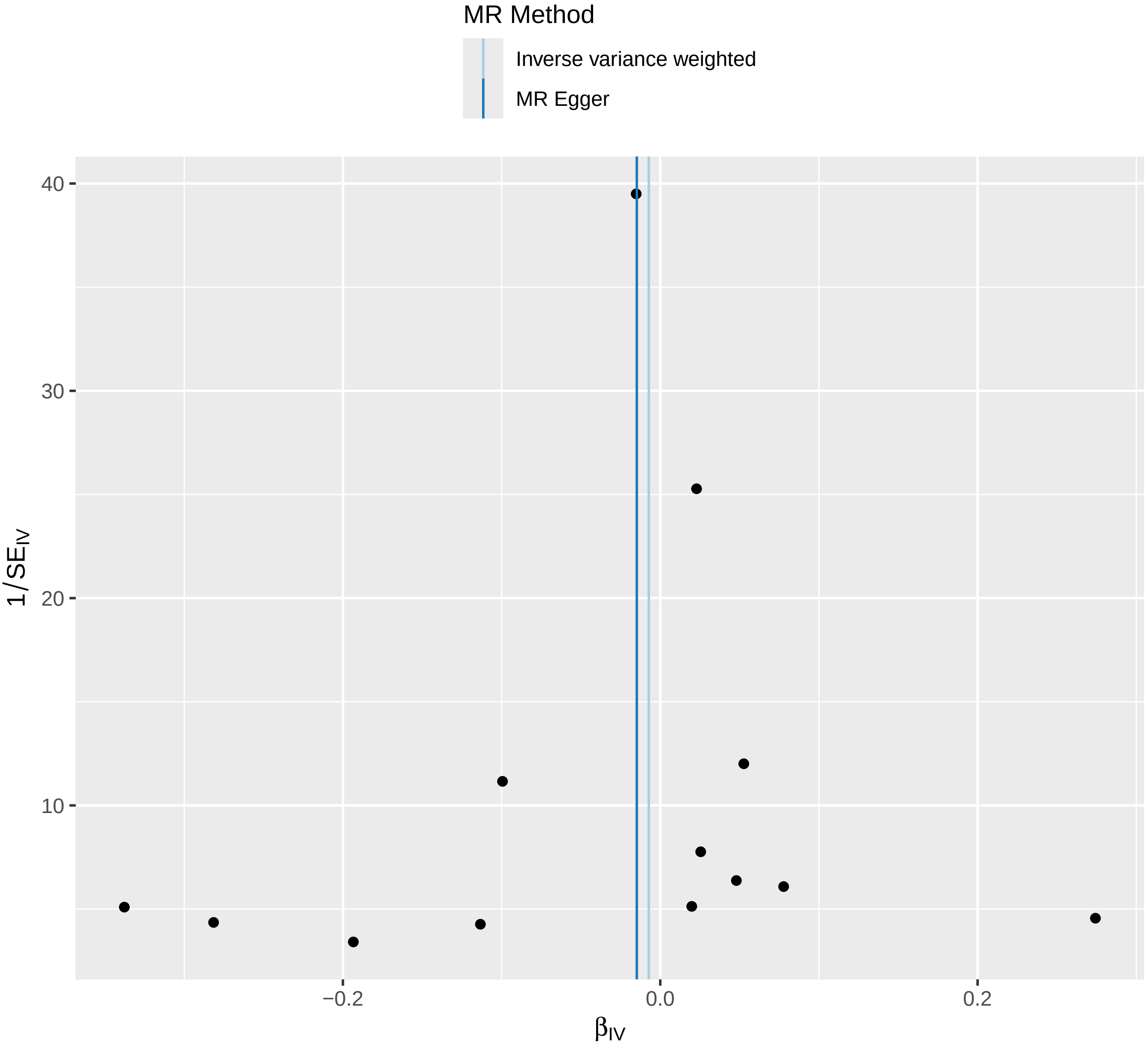

MR Method
Inverse variance weighted
MR Egger
40
30
20
10
1/SE$_{IV}$
−0.2
0.0
0.2
β$_{IV}$

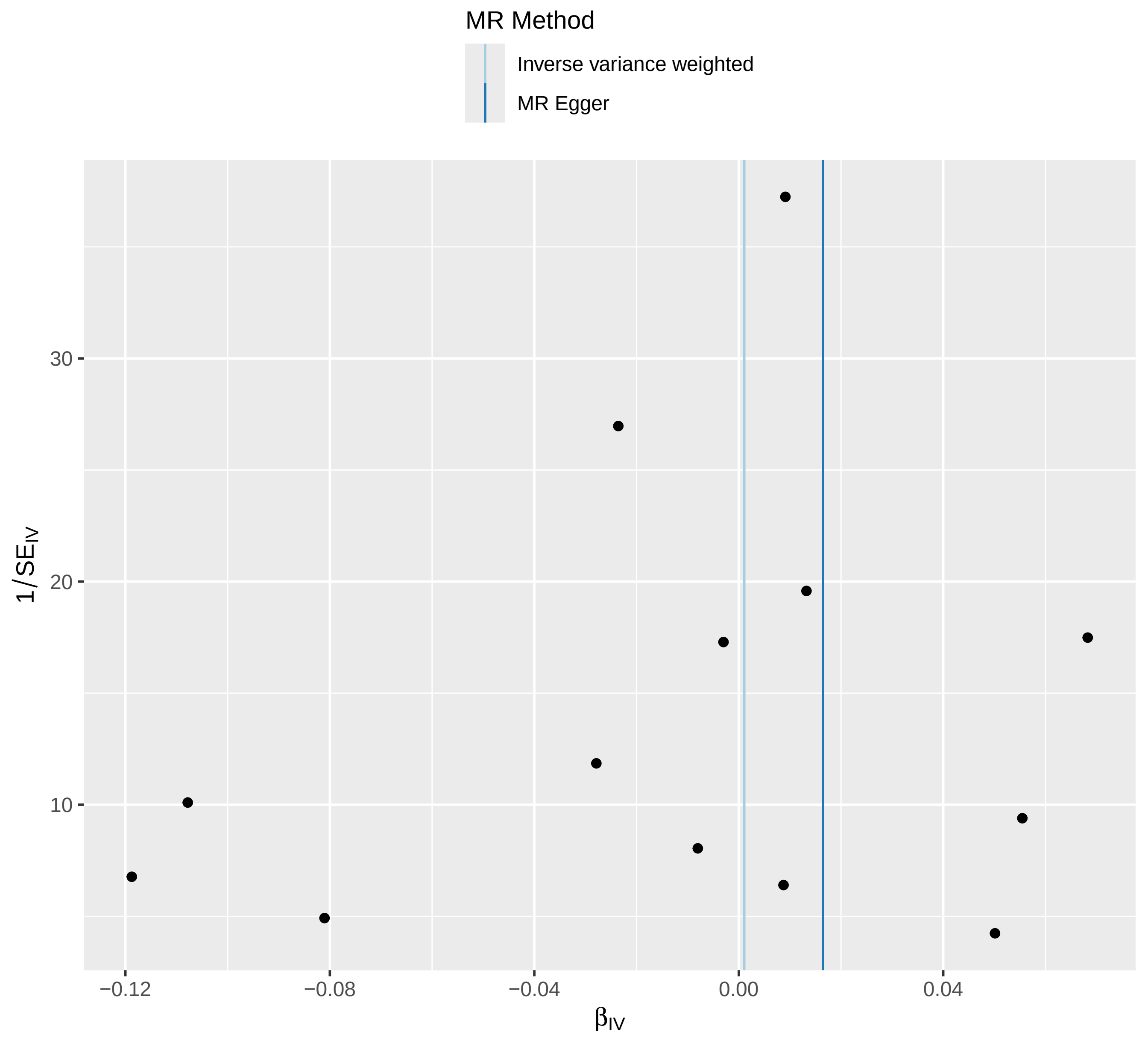
MR Method
Inverse variance weighted
MR Egger
30
20
10
1/SE_IV
−0.12
−0.08
−0.04
0.00
0.04
β_IV

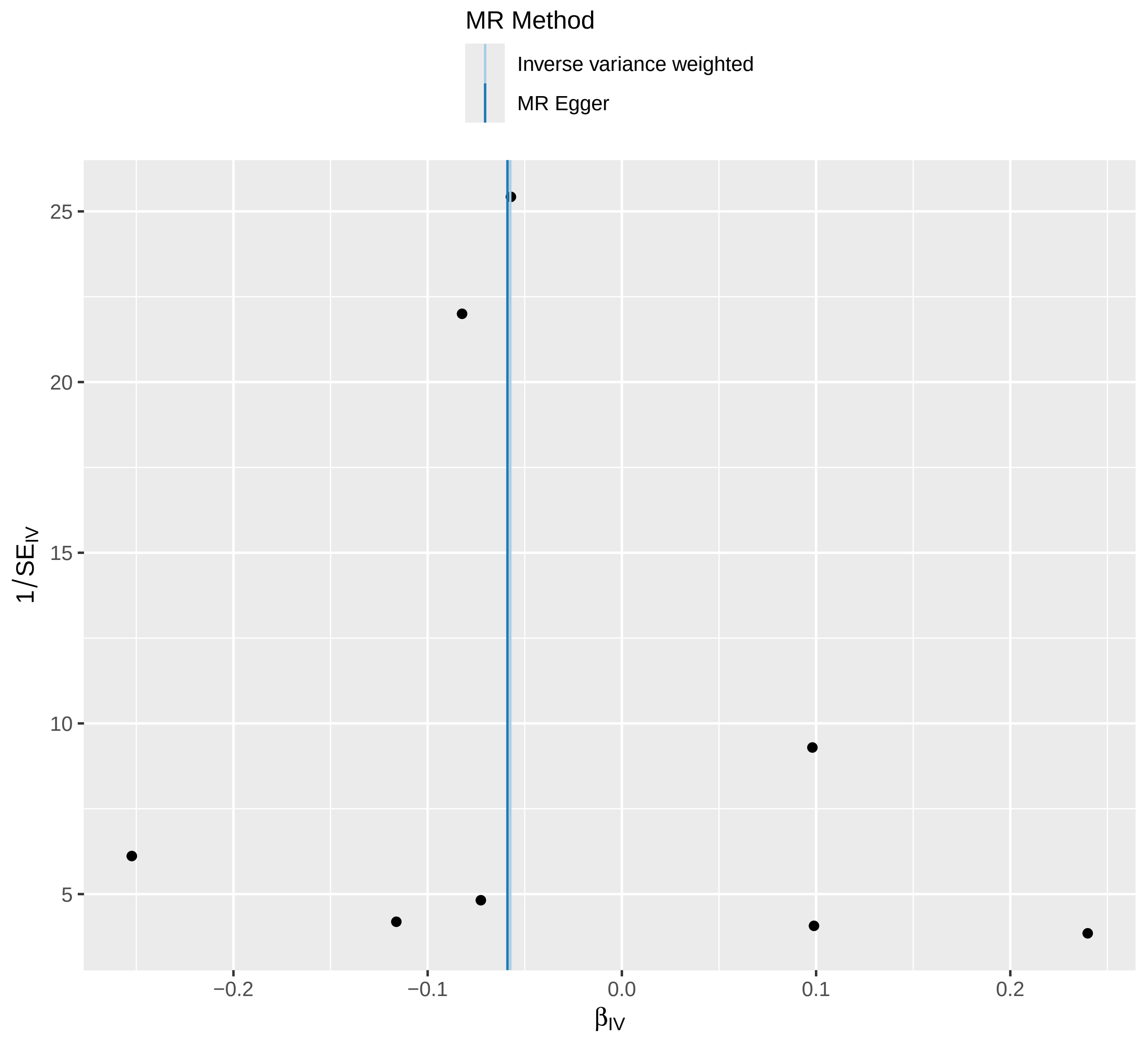

MR Method
Inverse variance weighted
MR Egger
25
20
15
10
5
1/SE_IV
−0.2
−0.1
0.0
0.1
0.2
β_IV

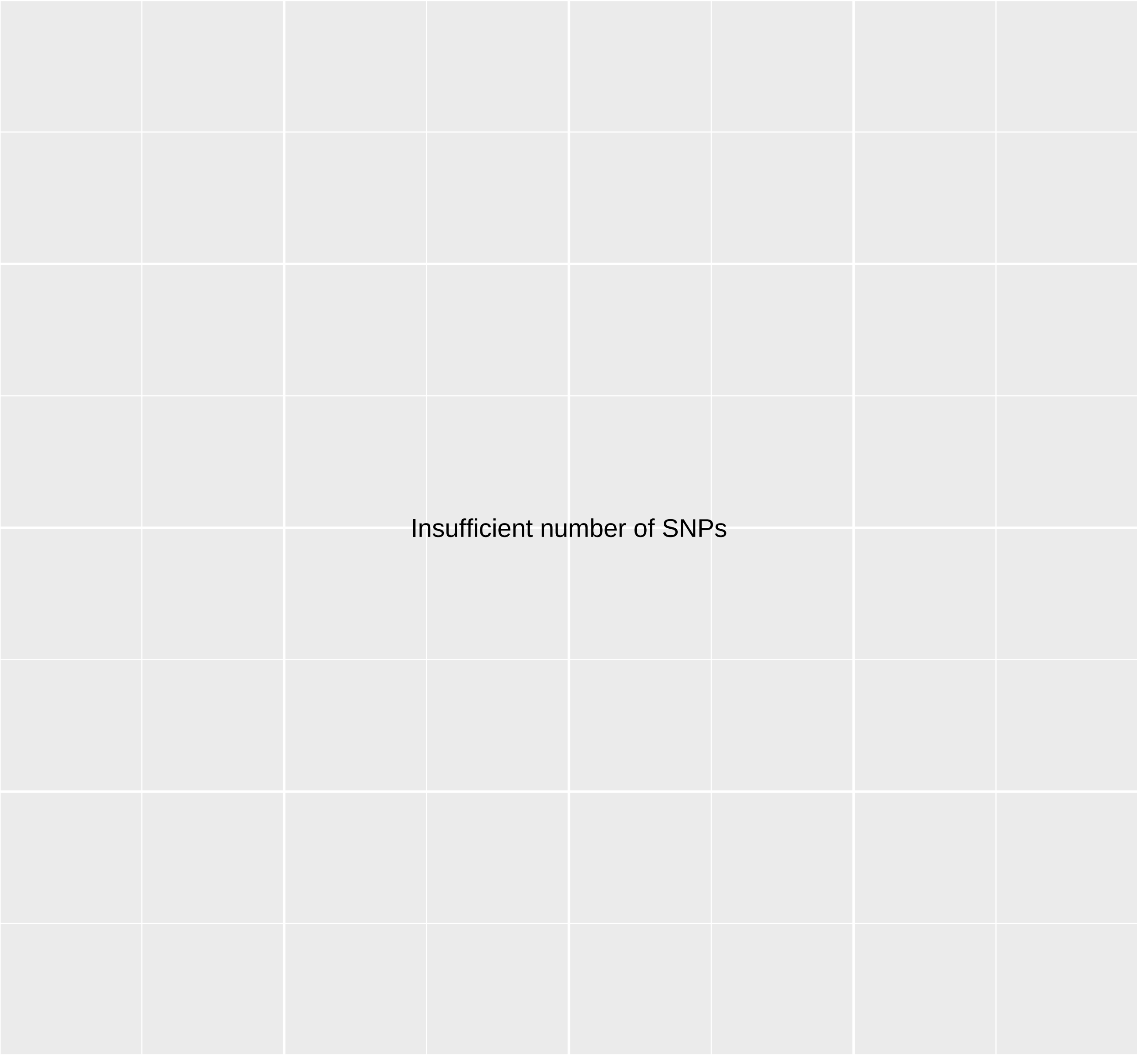
Insufficient number of SNPs

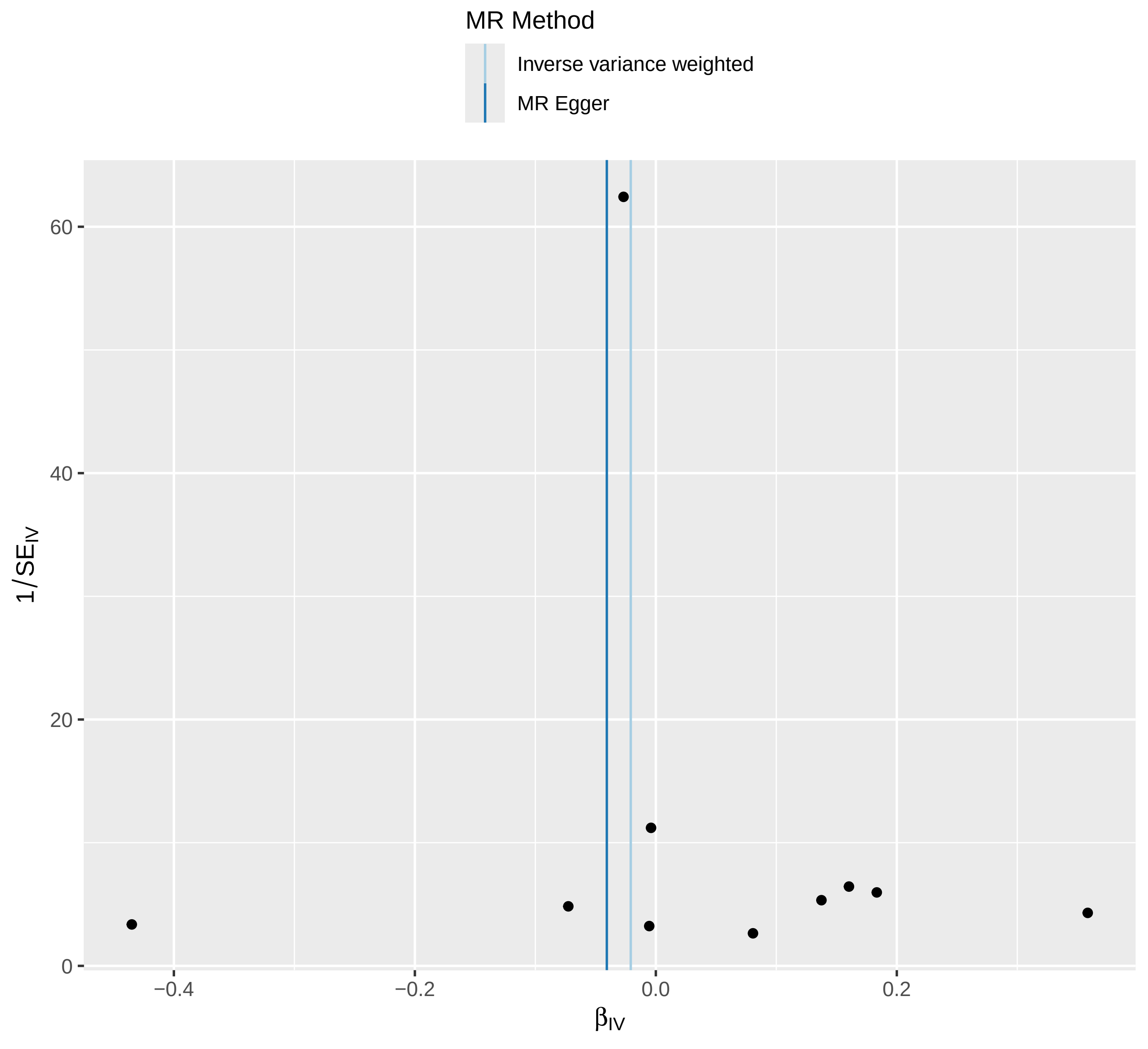

MR Method
Inverse variance weighted
MR Egger
60
40
20
0
−0.4
−0.2
0.0
0.2
1/SE_IV
β_IV

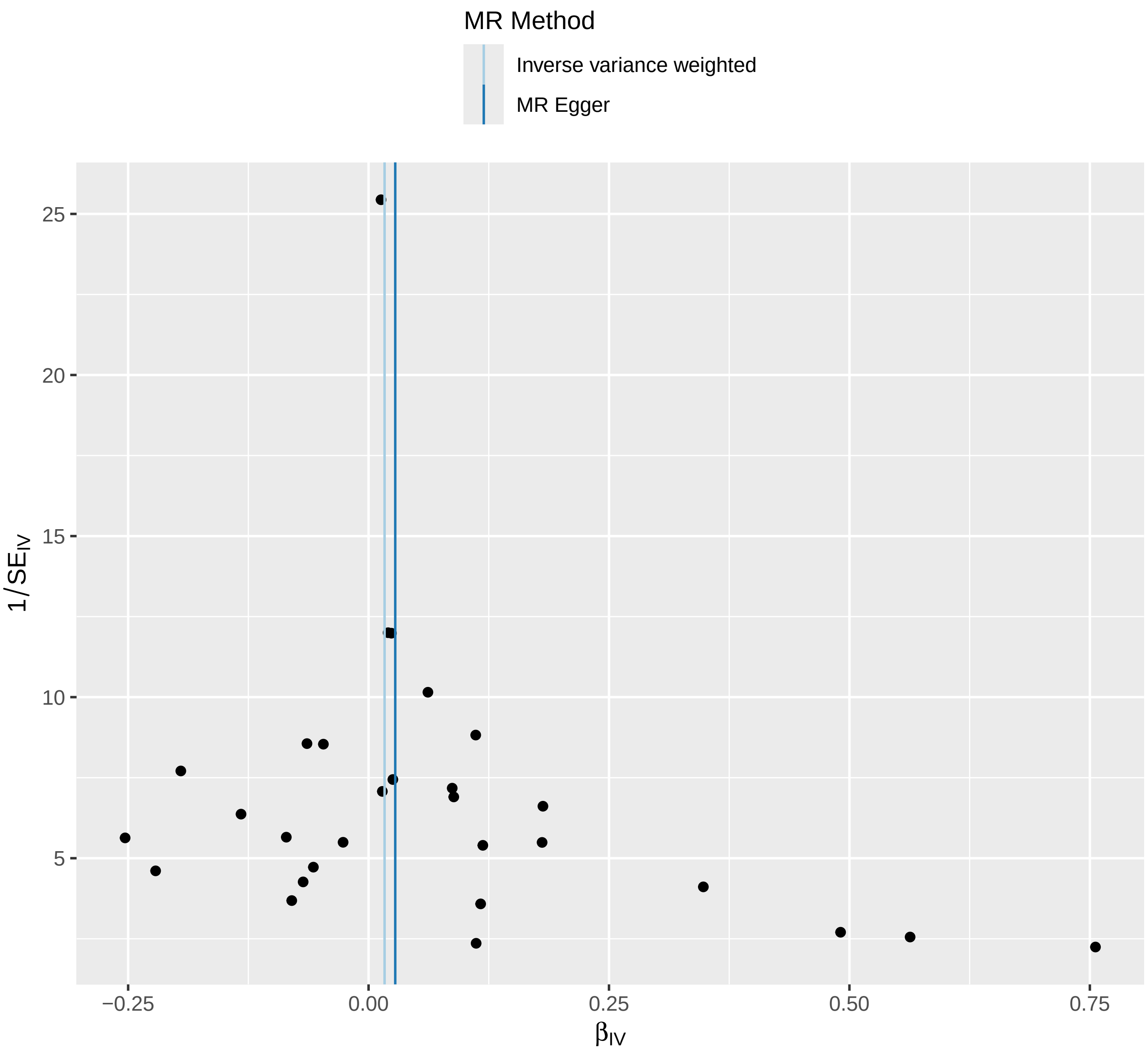
MR Method
Inverse variance weighted
MR Egger
1/SE_IV
25
20
15
10
5
−0.25
0.00
0.25
0.50
0.75
β_IV

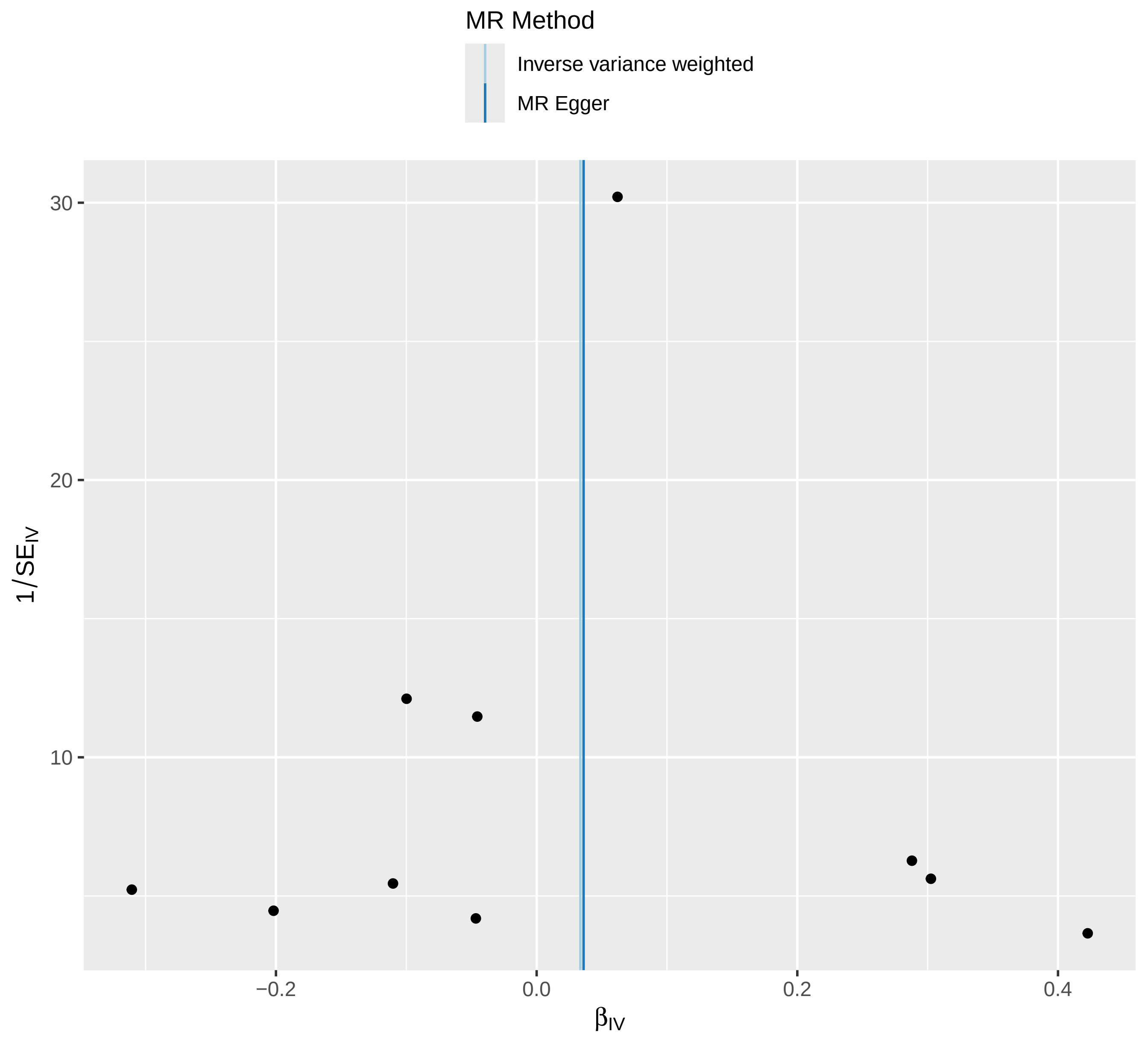

MR Method
Inverse variance weighted
MR Egger
30
20
10
1/SE_IV
−0.2
0.0
0.2
0.4
β_IV

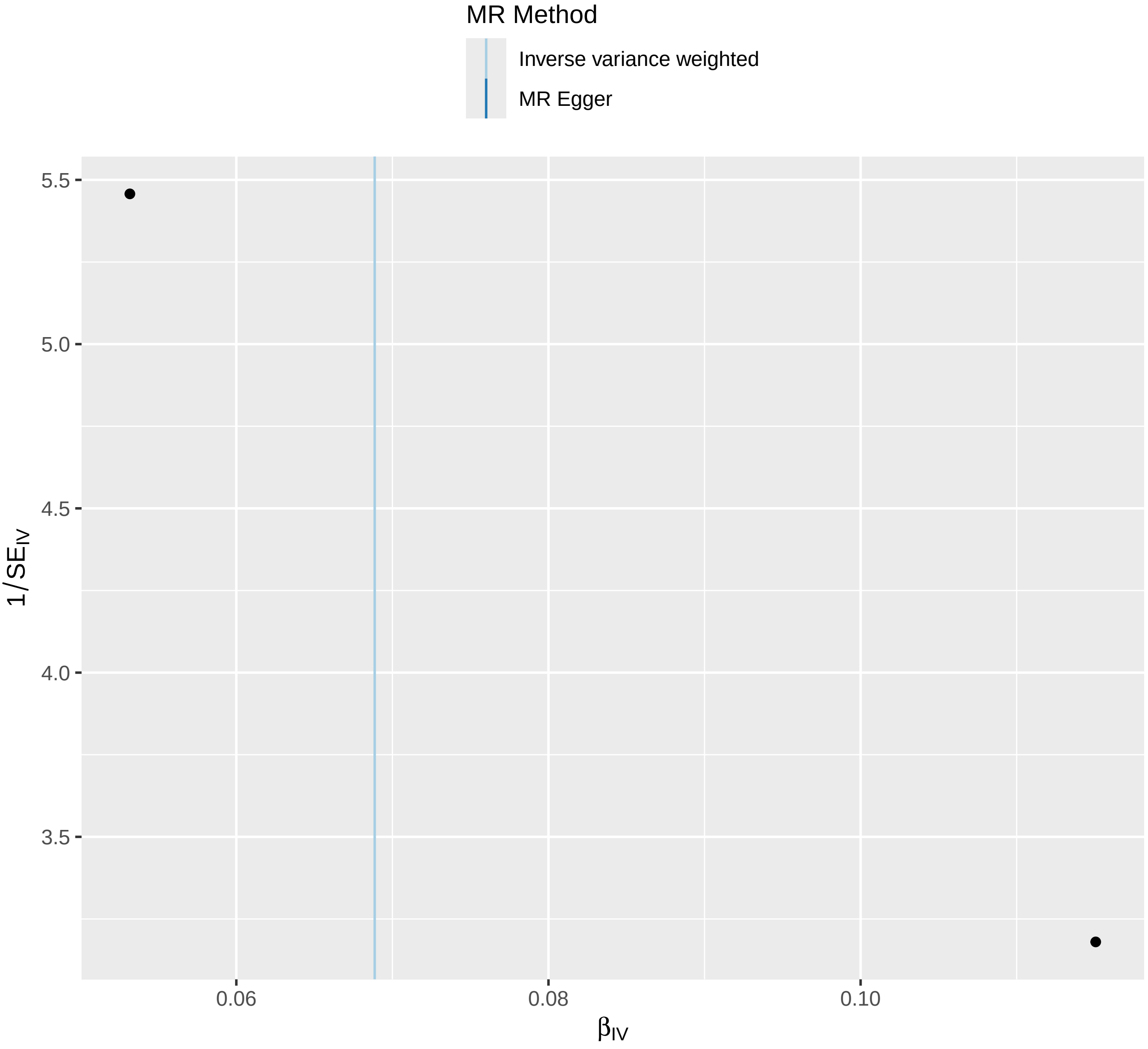
MR Method
Inverse variance weighted
MR Egger
5.5
5.0
4.5
4.0
3.5
$1/SE_{IV}$
0.06
0.08
0.10
$\beta_{IV}$

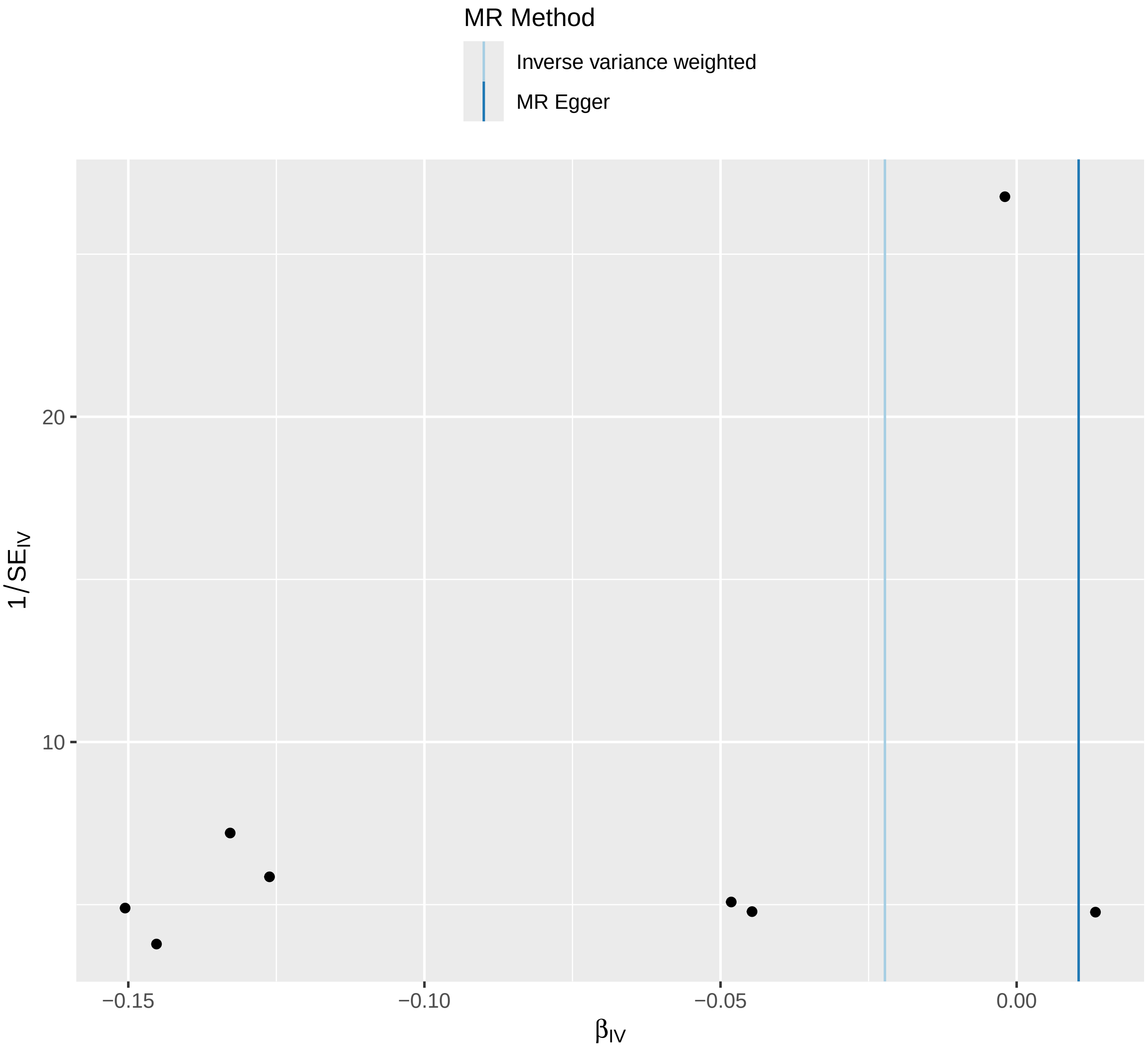
MR Method
Inverse variance weighted
MR Egger
20
10
1/SE$_{IV}$
−0.15
−0.10
−0.05
0.00
β$_{IV}$

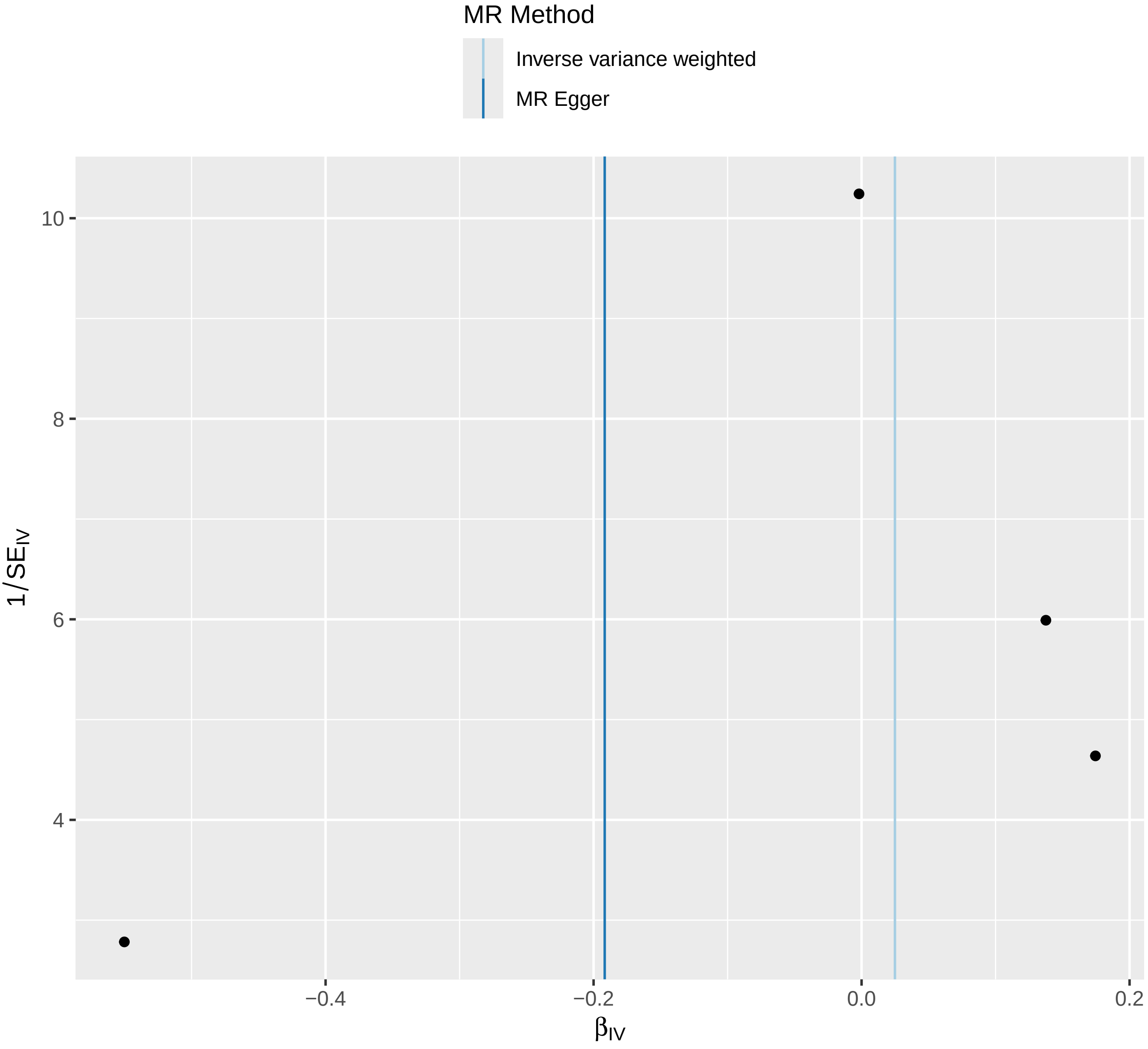

MR Method
Inverse variance weighted
MR Egger
10
8
6
4
1/SE_IV
−0.4
−0.2
0.0
0.2
β_IV

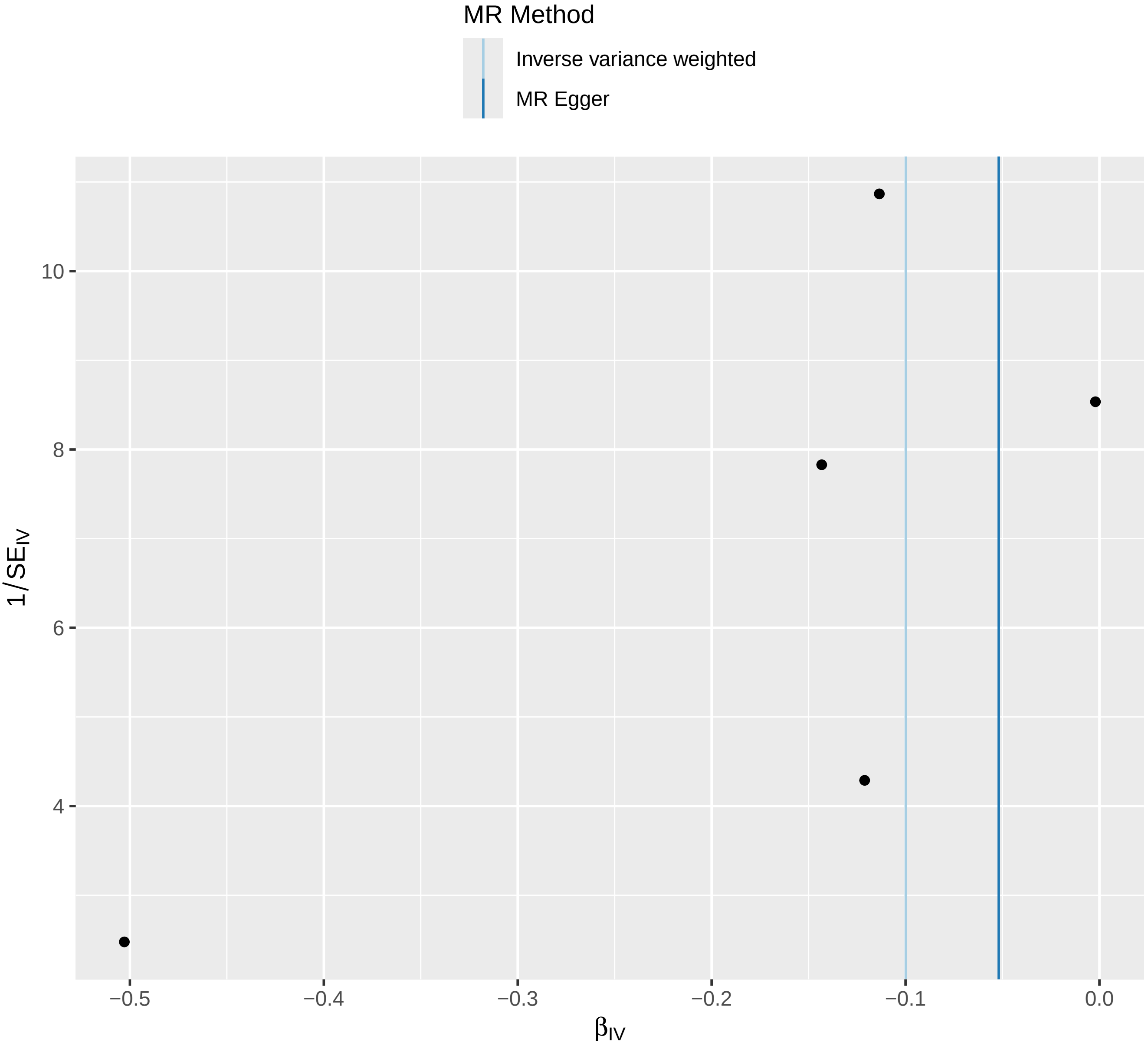

MR Method
Inverse variance weighted
MR Egger
10
8
6
4
1/SE_IV
−0.5
−0.4
−0.3
−0.2
−0.1
0.0
β_IV

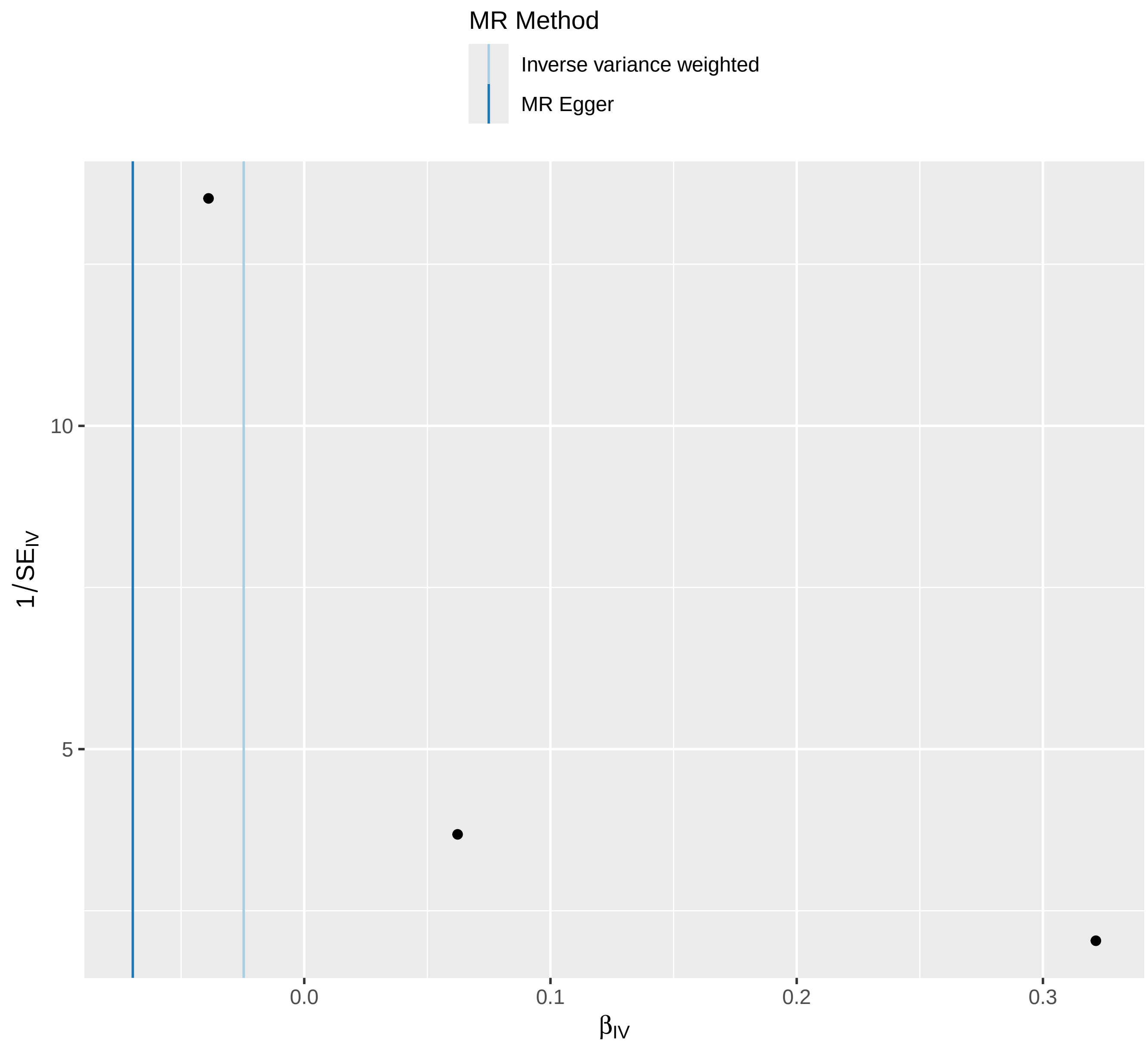
MR Method
Inverse variance weighted
MR Egger
10
5
0.0
0.1
0.2
0.3
$1/SE_{IV}$
$\beta_{IV}$

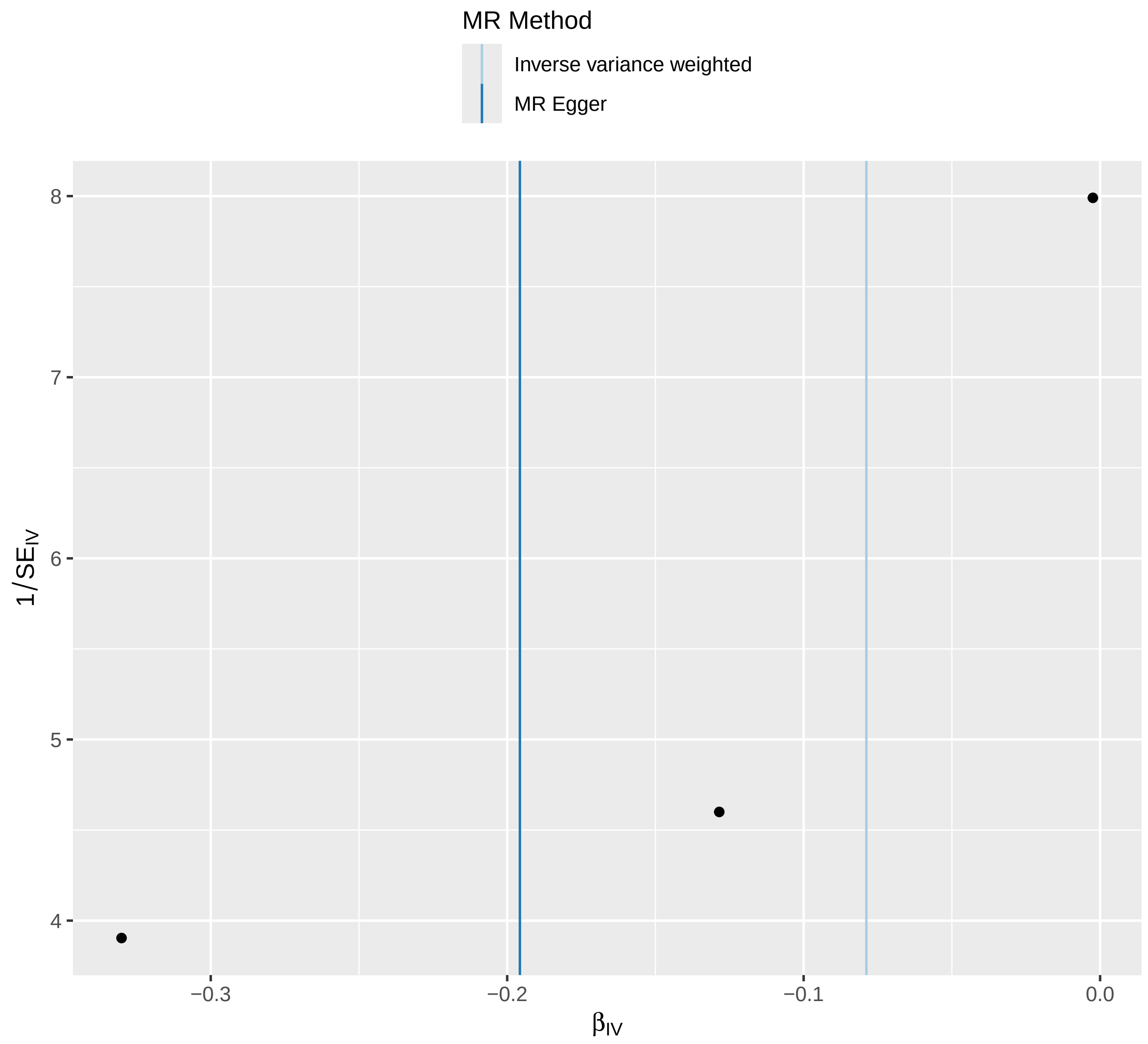
MR Method
Inverse variance weighted
MR Egger
8
7
6
5
4
1/SE_IV
−0.3
−0.2
−0.1
0.0
β_IV

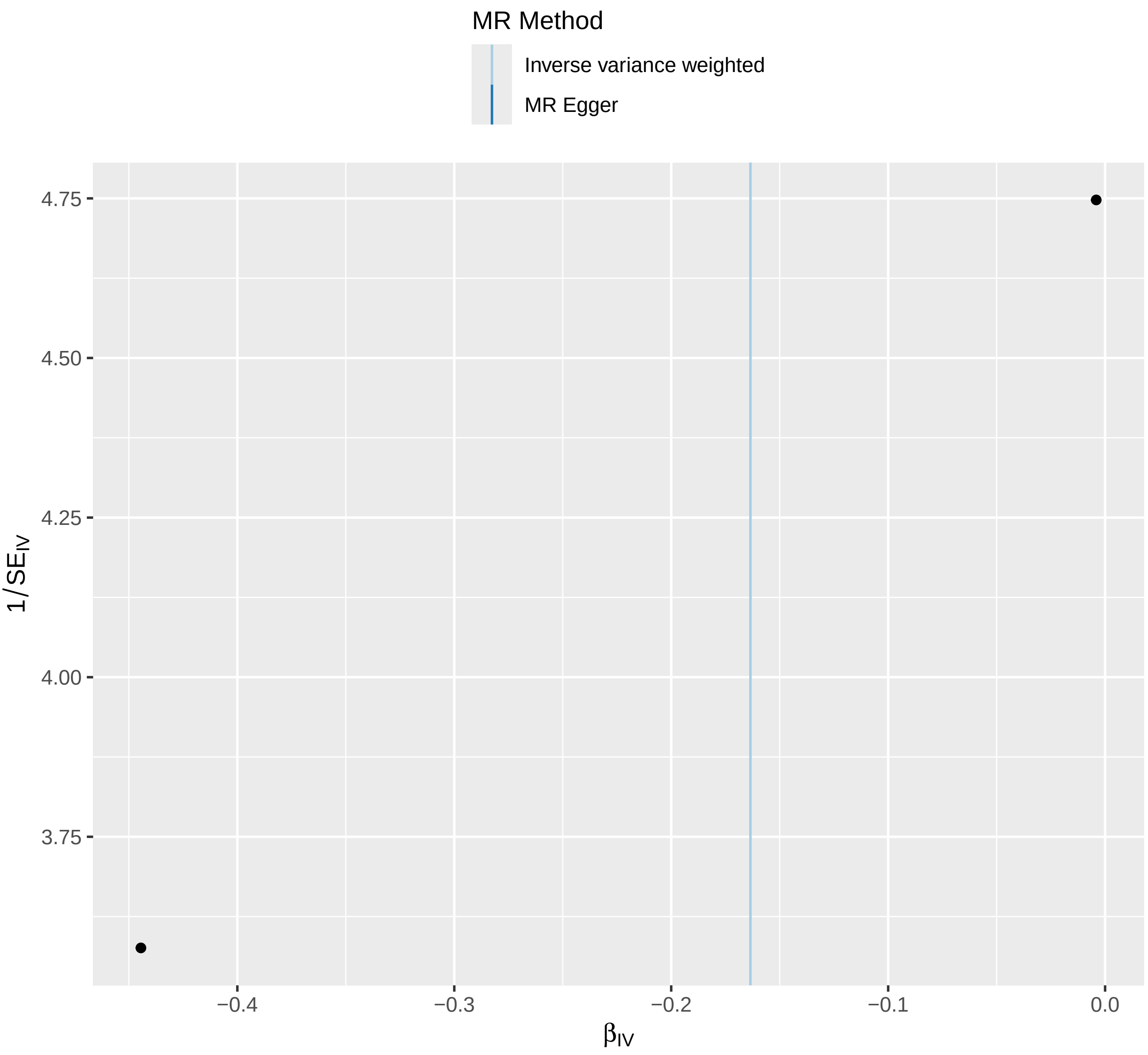
MR Method
Inverse variance weighted
MR Egger
4.75
4.50
4.25
4.00
3.75
1/SE_IV
−0.4
−0.3
−0.2
−0.1
0.0
β_IV

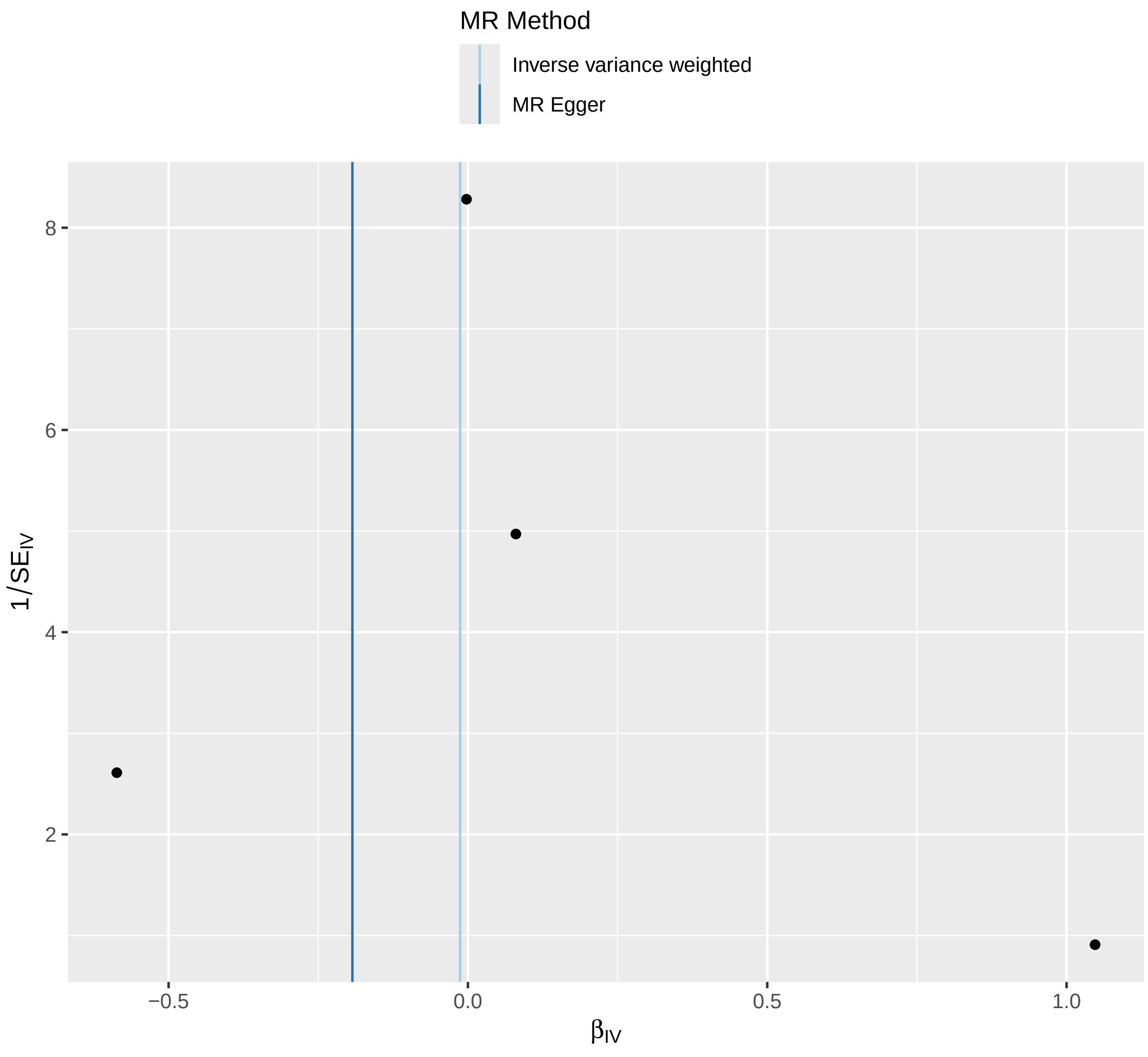

MR Method
Inverse variance weighted
MR Egger
8
6
4
2
1/SE$_{IV}$
−0.5
0.0
0.5
1.0
β$_{IV}$

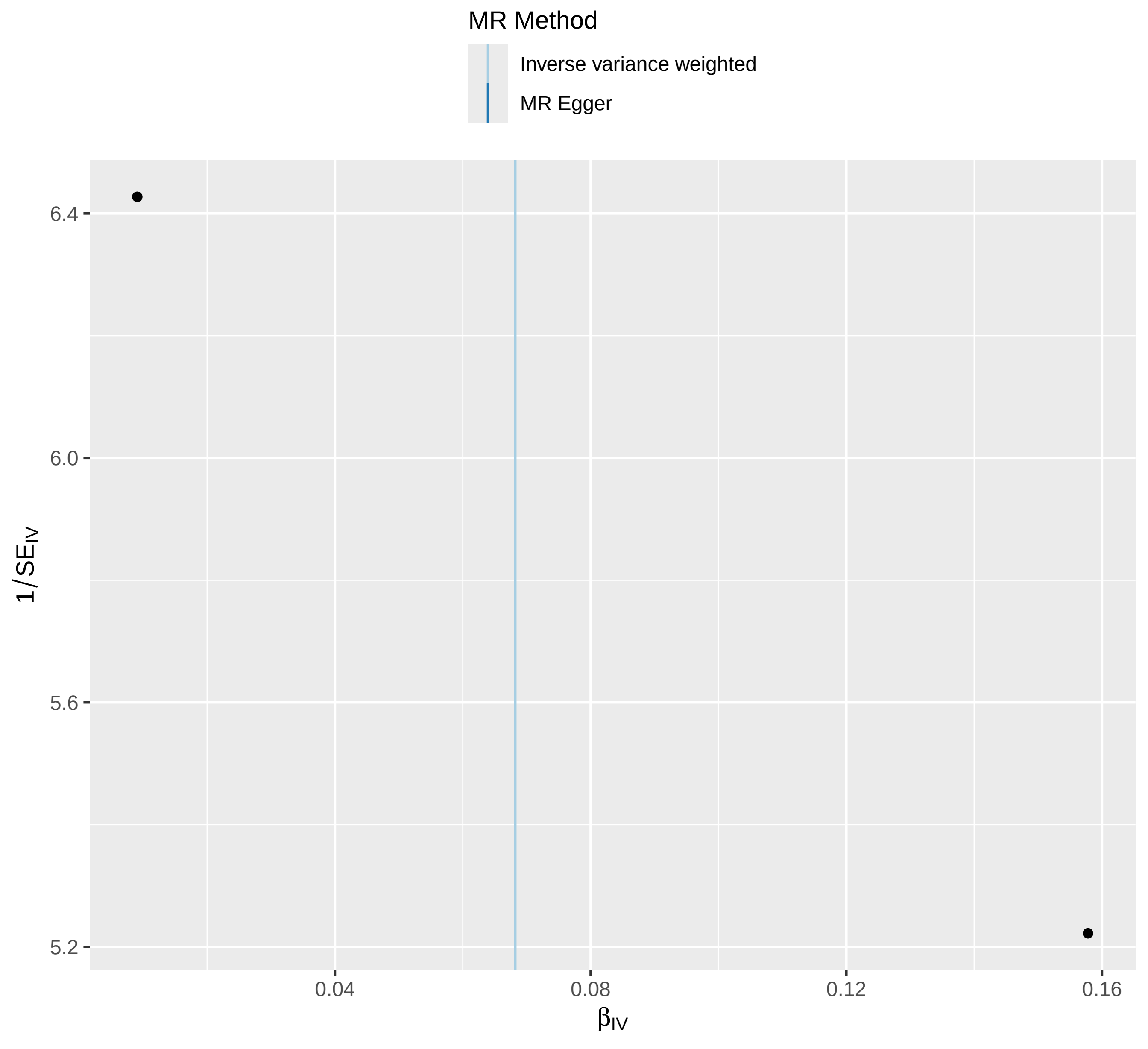
MR Method
Inverse variance weighted
MR Egger
6.4
6.0
5.6
5.2
1/SE_IV
0.04
0.08
0.12
0.16
β_IV

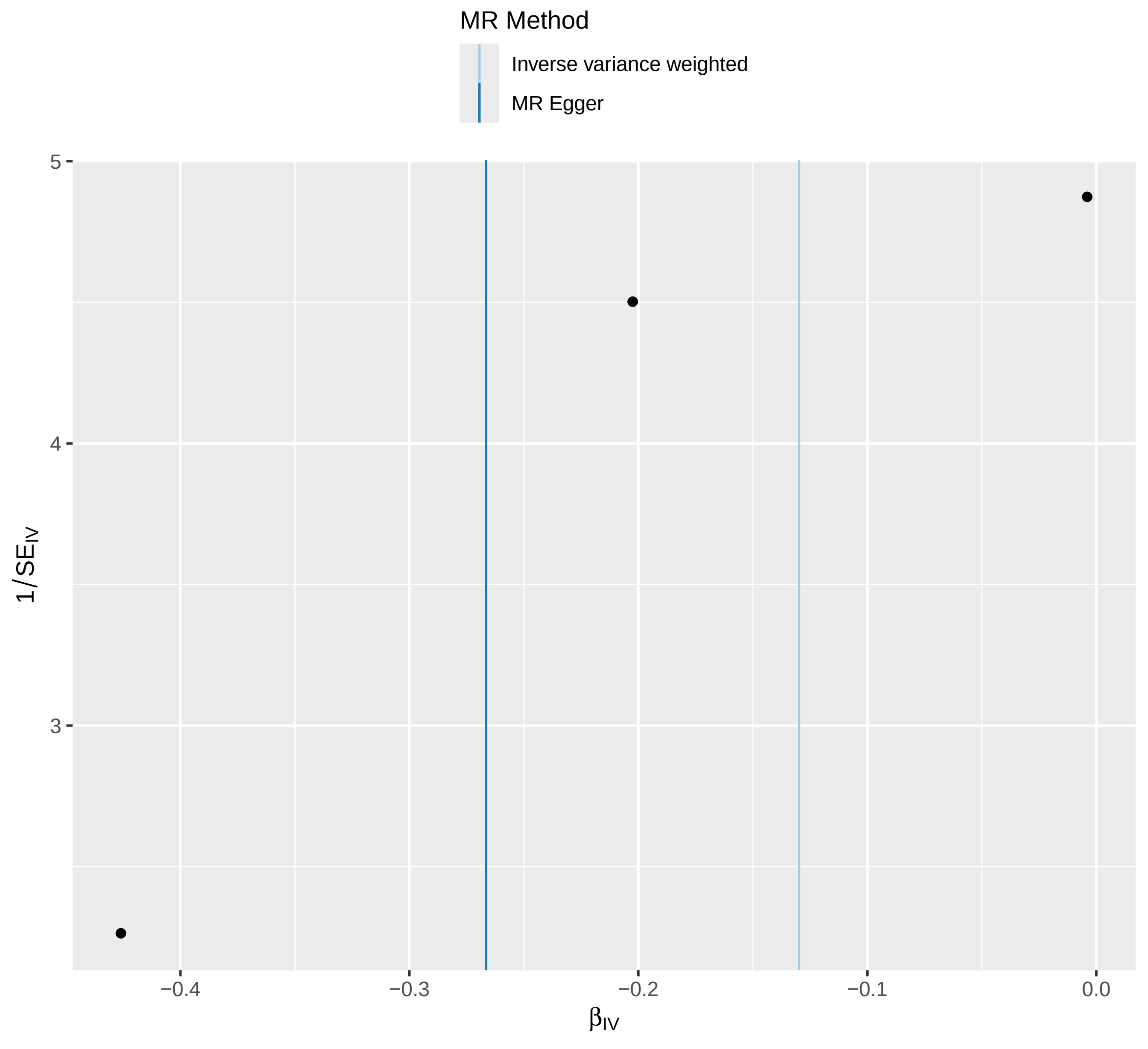
MR Method
Inverse variance weighted
MR Egger
5
4
3
1/SE_IV
−0.4
−0.3
−0.2
−0.1
0.0
β_IV

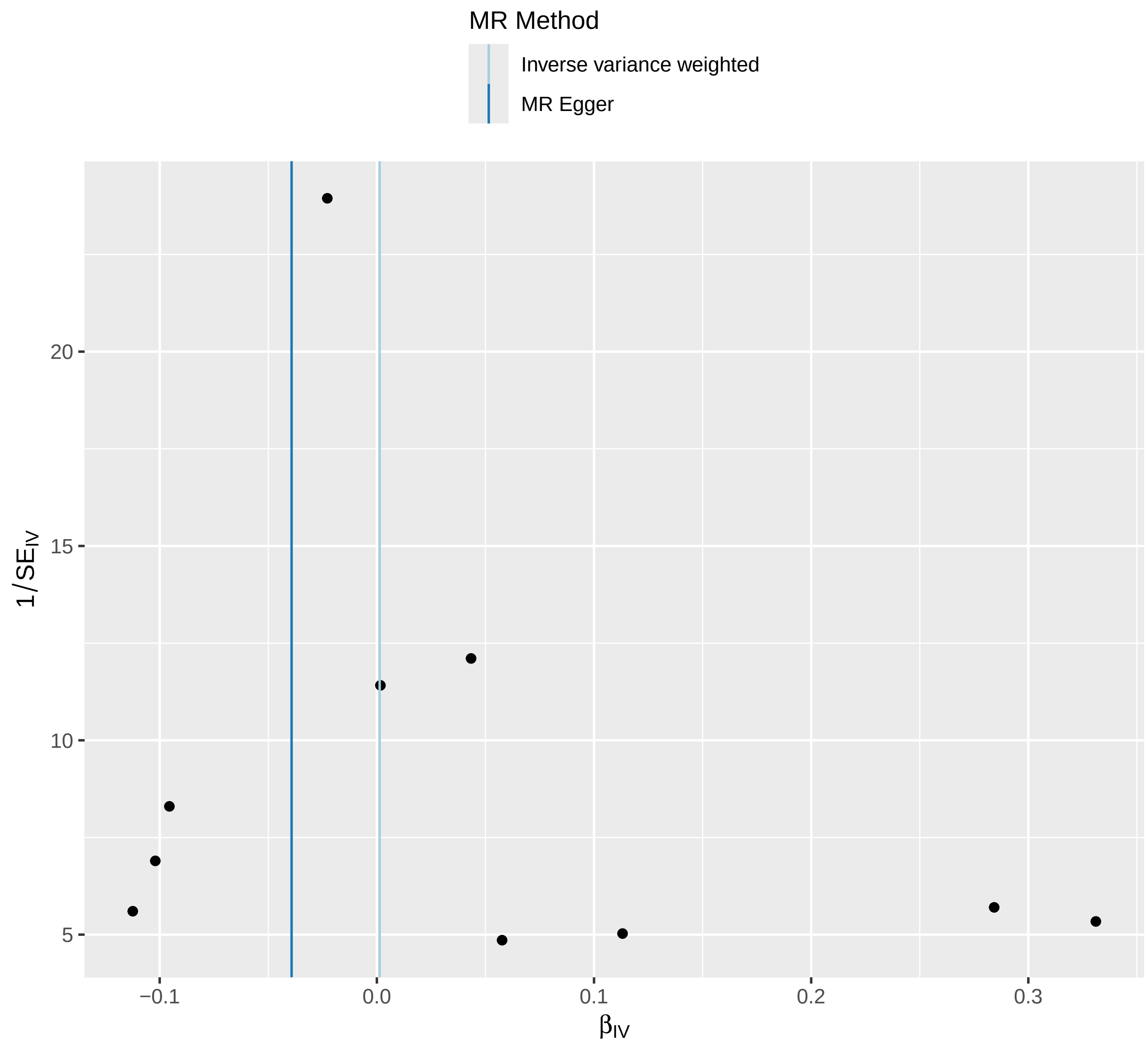

MR Method
Inverse variance weighted
MR Egger
20
15
10
5
$1/SE_{IV}$
−0.1
0.0
0.1
0.2
0.3
$\beta_{IV}$

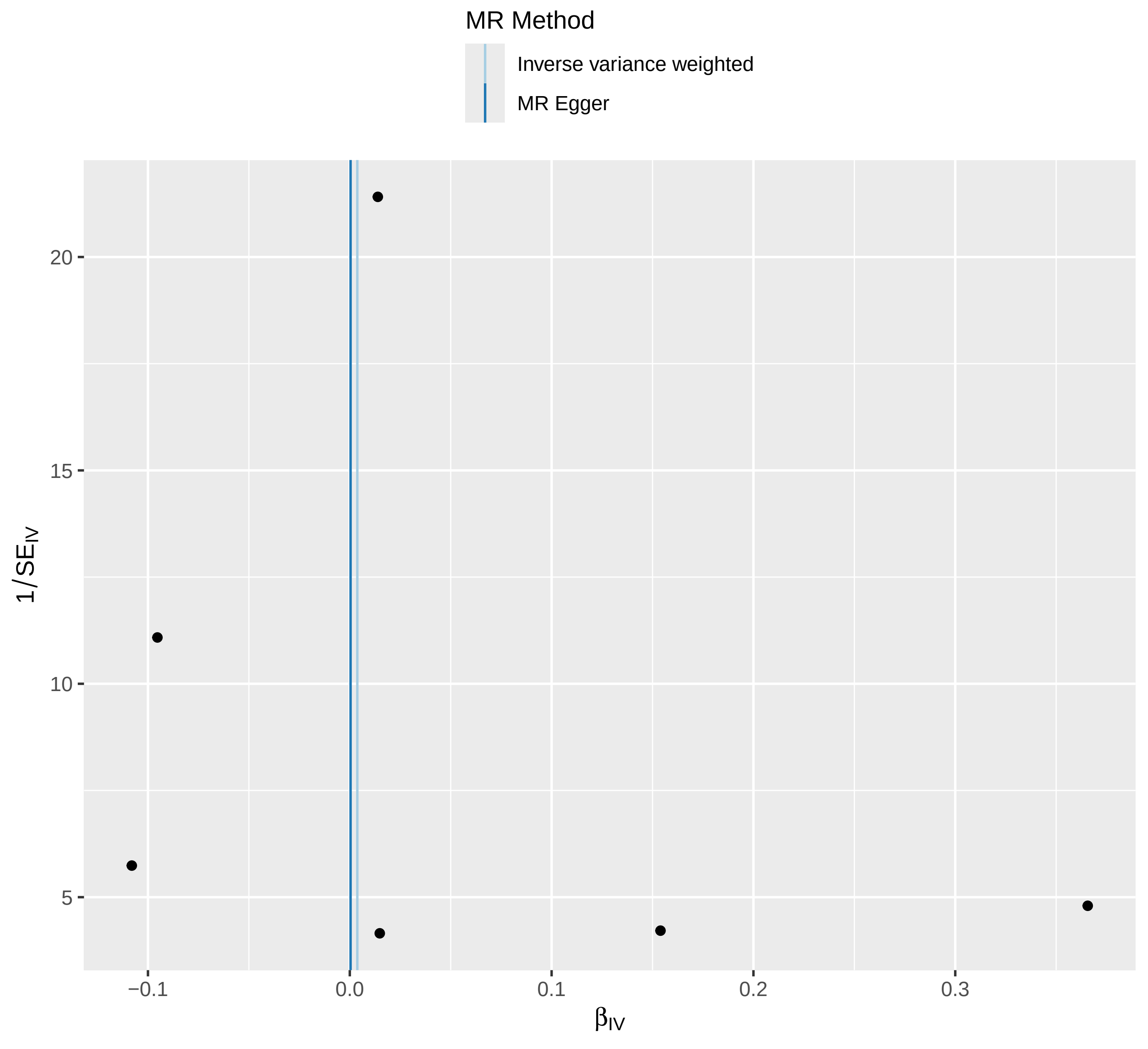

MR Method
Inverse variance weighted
MR Egger
1/SE_IV
20
15
10
5
−0.1
0.0
0.1
0.2
0.3
β_IV

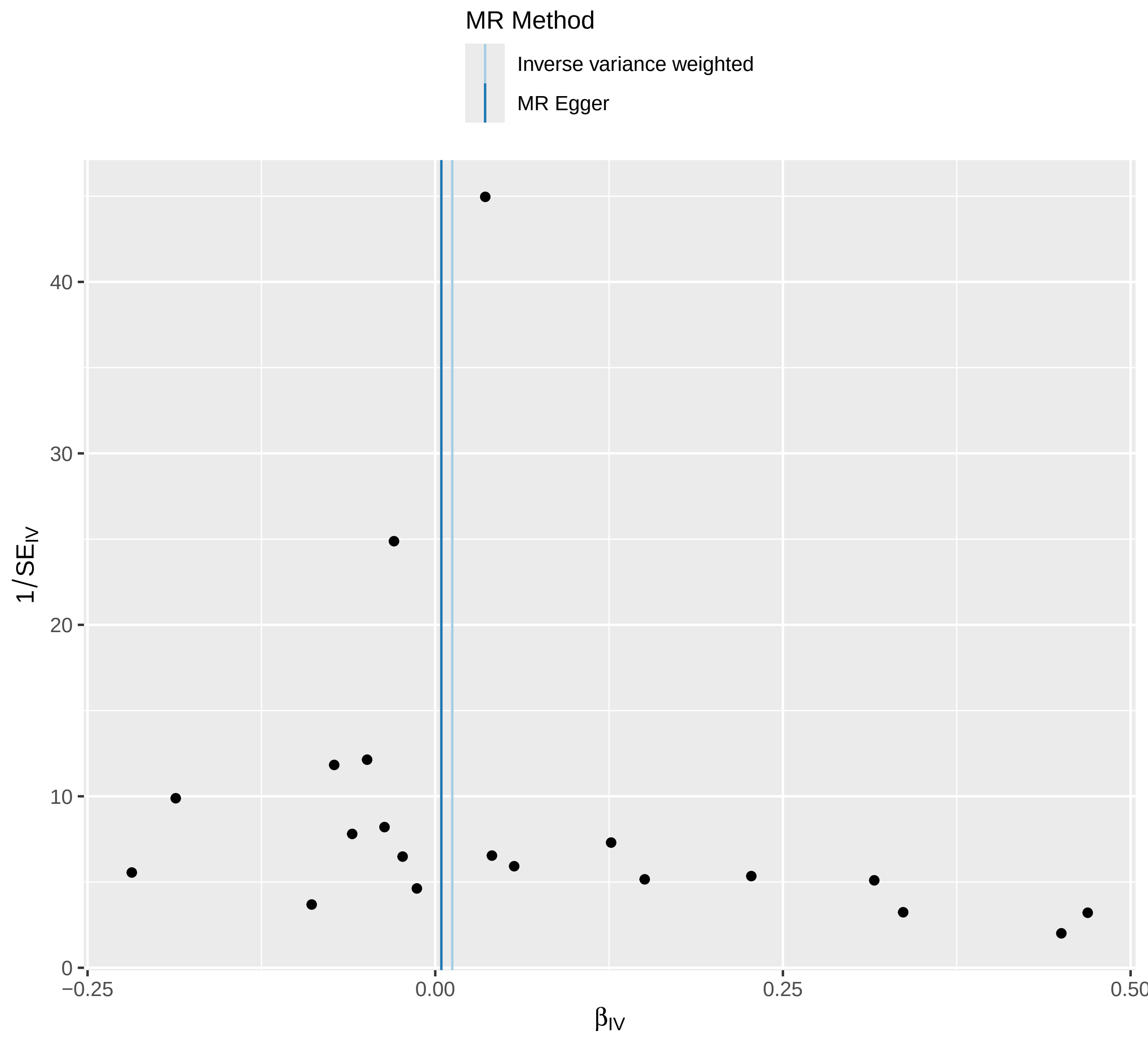
MR Method
Inverse variance weighted
MR Egger
1/$SE_{IV}$
$\beta_{IV}$
0
10
20
30
40
−0.25
0.00
0.25
0.50

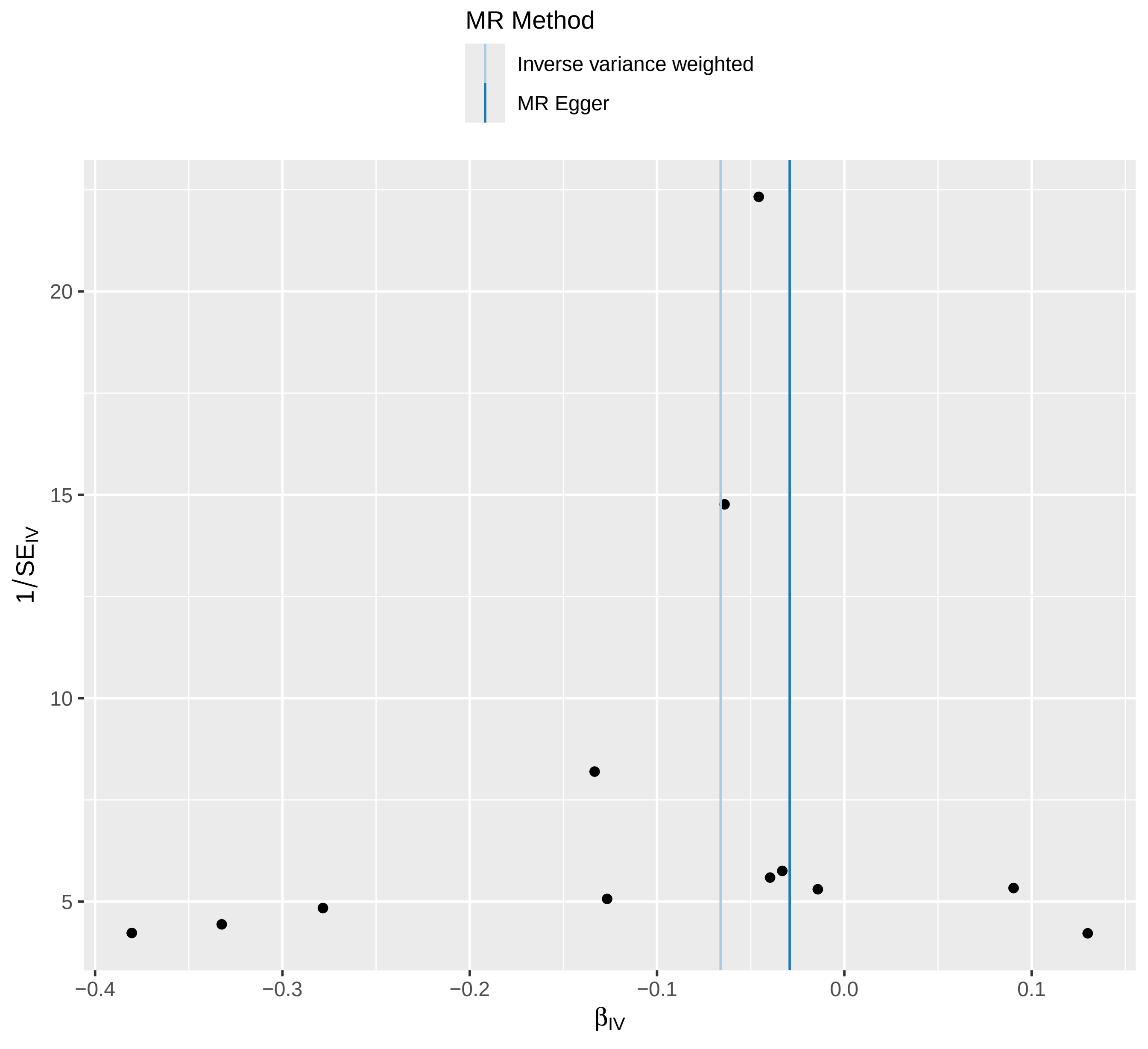
MR Method
Inverse variance weighted
MR Egger
1/SE_IV
20
15
10
5
−0.4
−0.3
−0.2
−0.1
0.0
0.1
β_IV

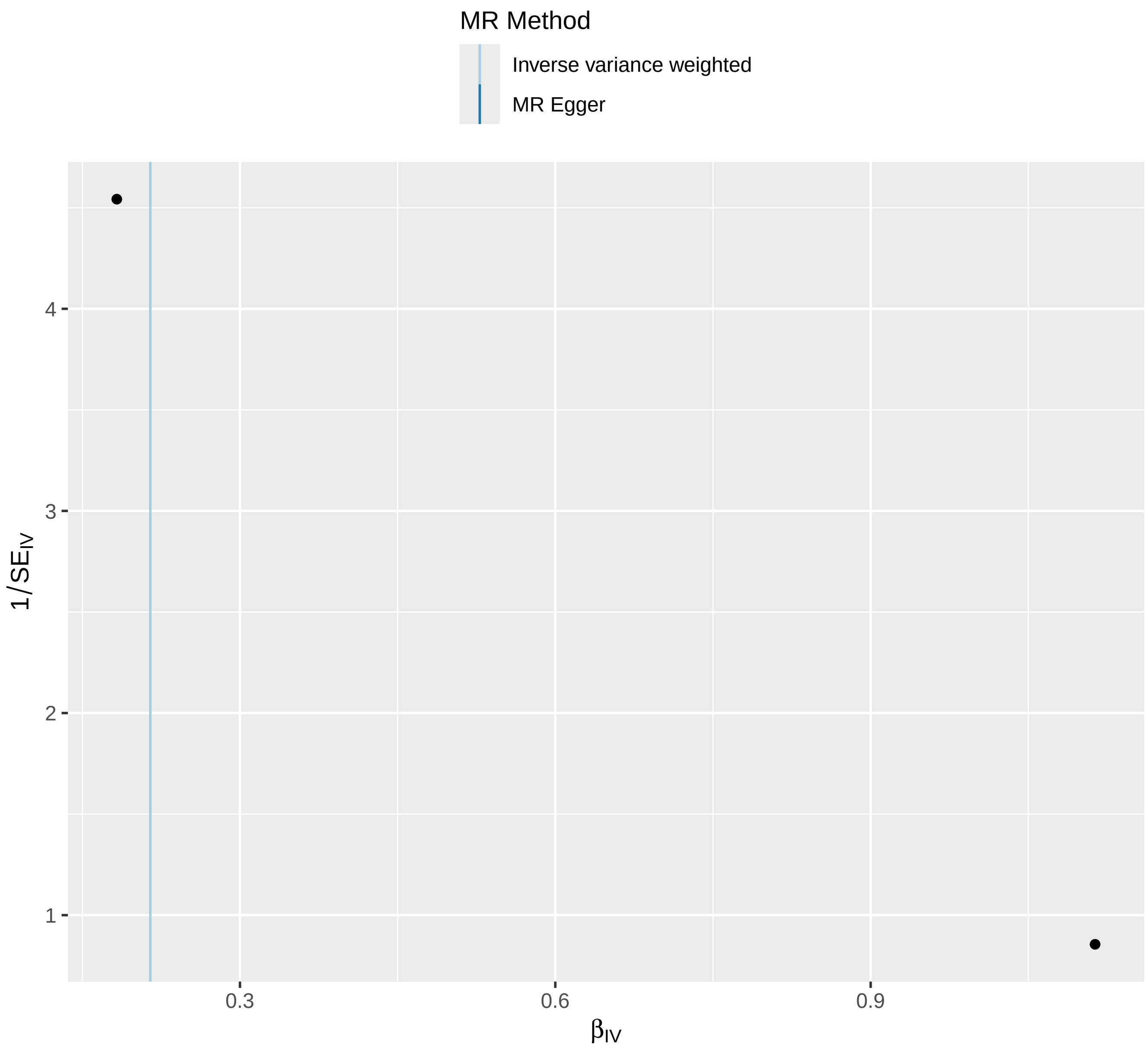

MR Method
Inverse variance weighted
MR Egger
4
3
2
1
$1/SE_{IV}$
0.3
0.6
0.9
$\beta_{IV}$

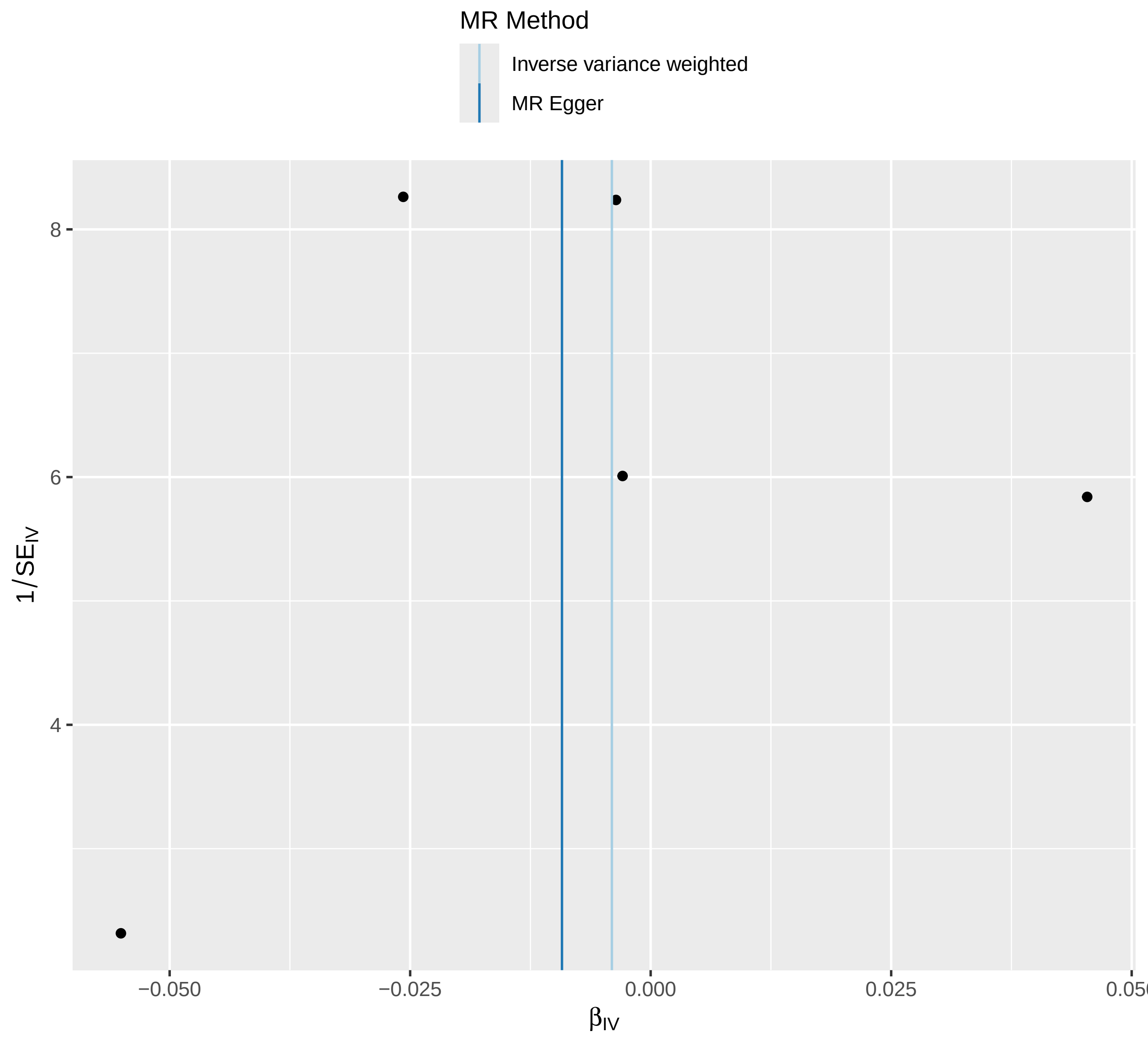

MR Method
Inverse variance weighted
MR Egger
8
6
4
1/SE_IV
−0.050
−0.025
0.000
0.025
0.050
β_IV

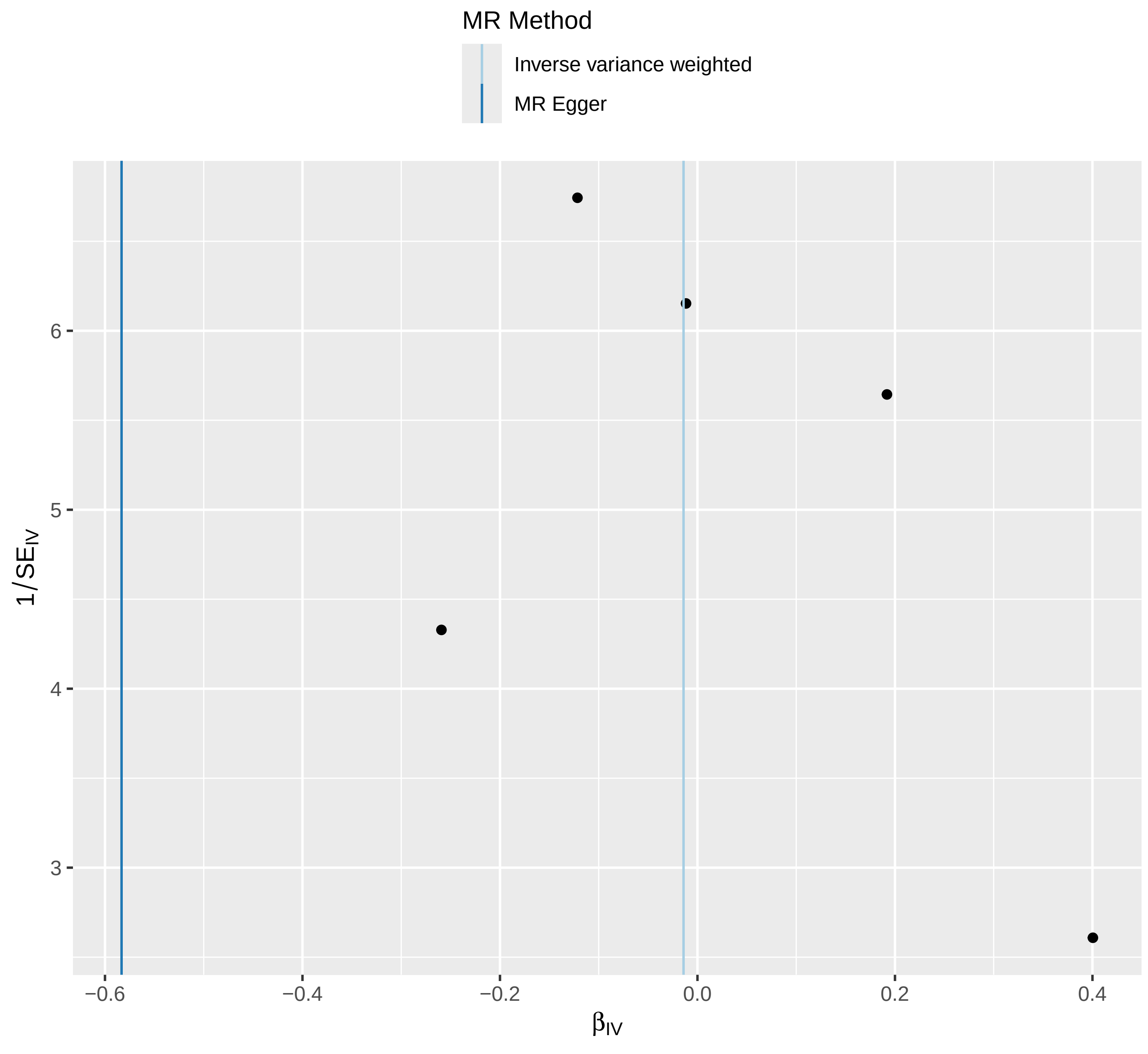

MR Method
Inverse variance weighted
MR Egger
1/SE$_{IV}$
$\beta_{IV}$
−0.6
−0.4
−0.2
0.0
0.2
0.4
3
4
5
6

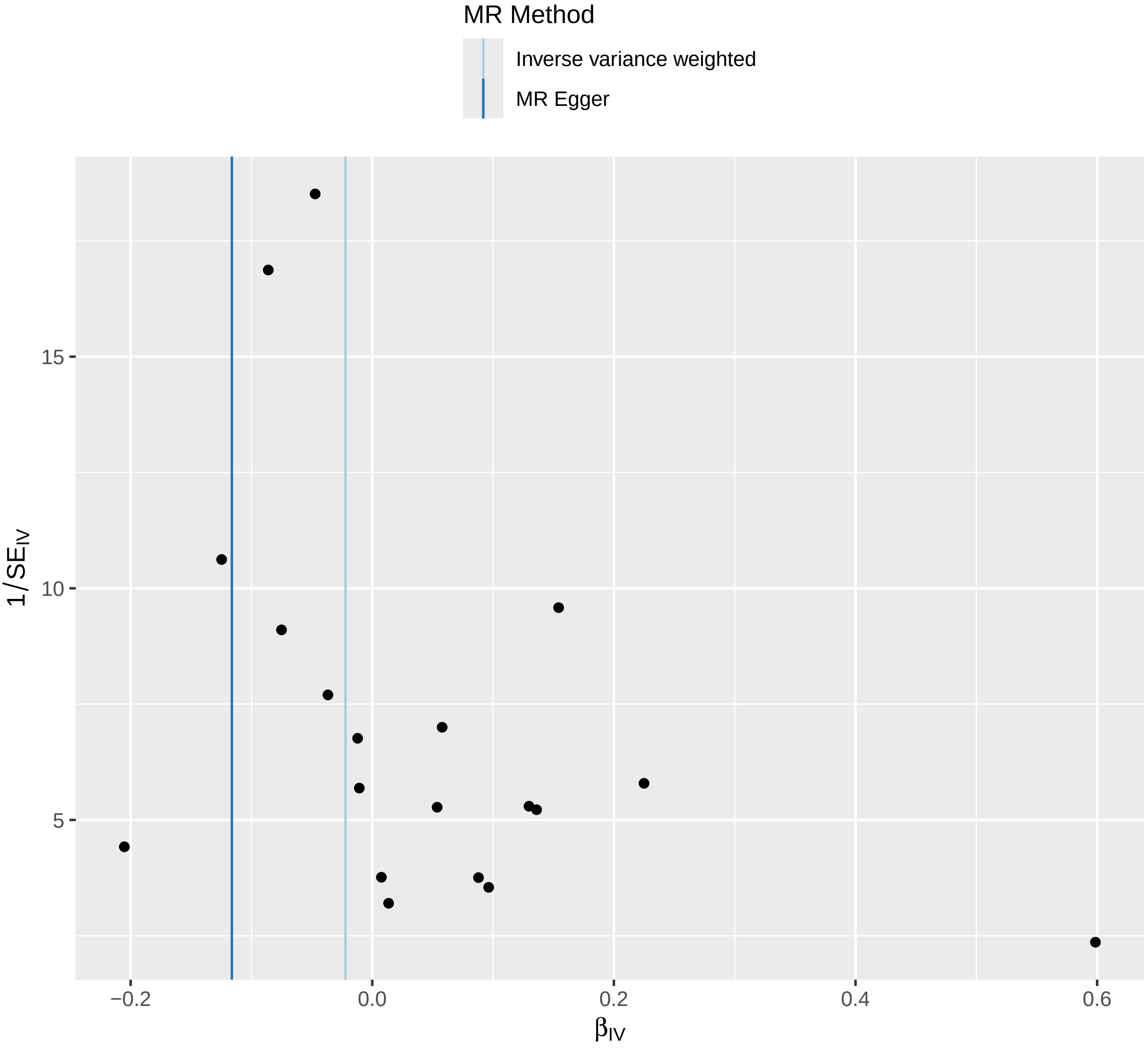
MR Method
Inverse variance weighted
MR Egger
15
10
5
1/SE_IV
−0.2
0.0
0.2
0.4
0.6
β_IV

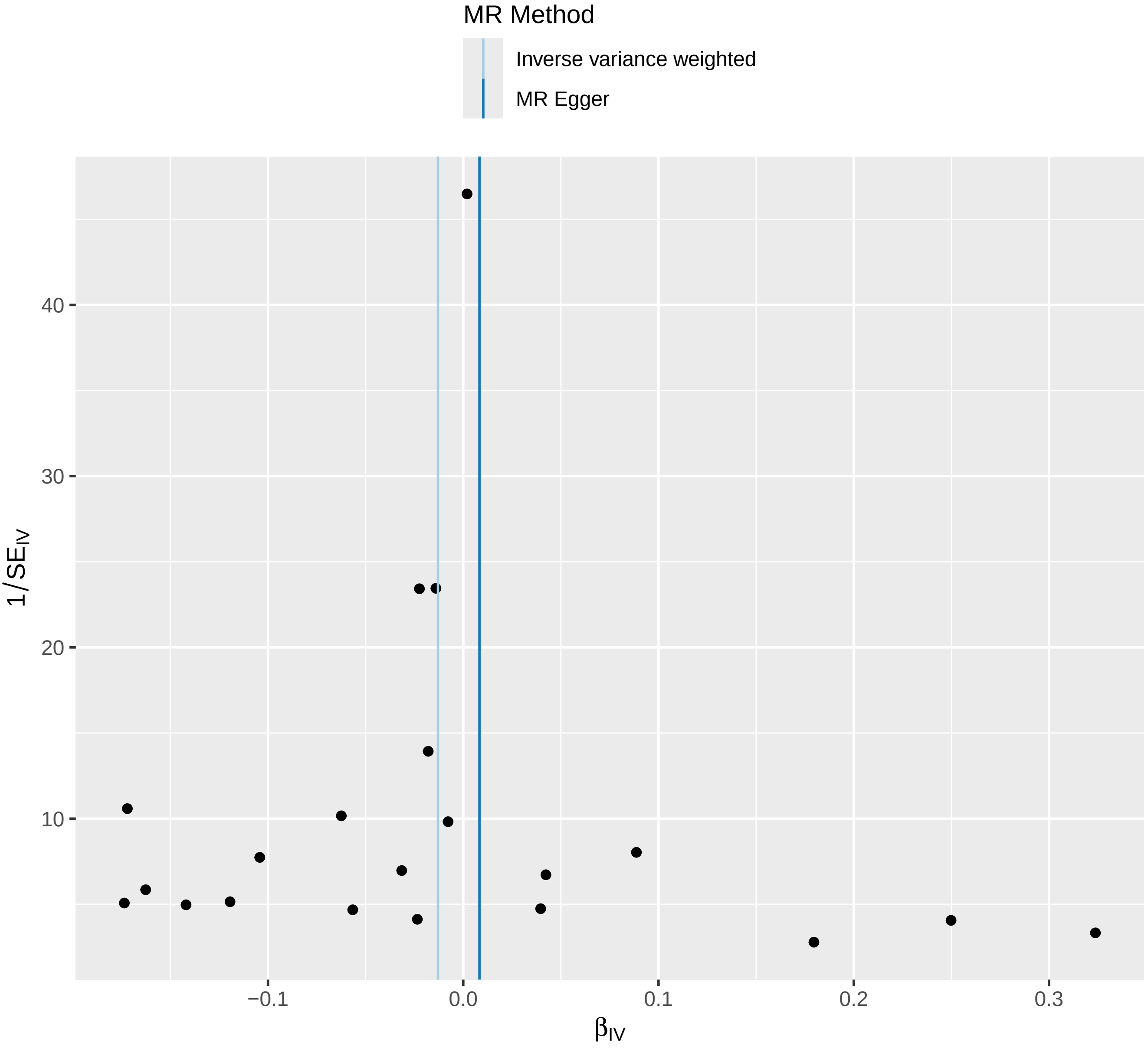

MR Method
Inverse variance weighted
MR Egger
40
30
20
10
1/SE$_{IV}$
−0.1
0.0
0.1
0.2
0.3
β$_{IV}$

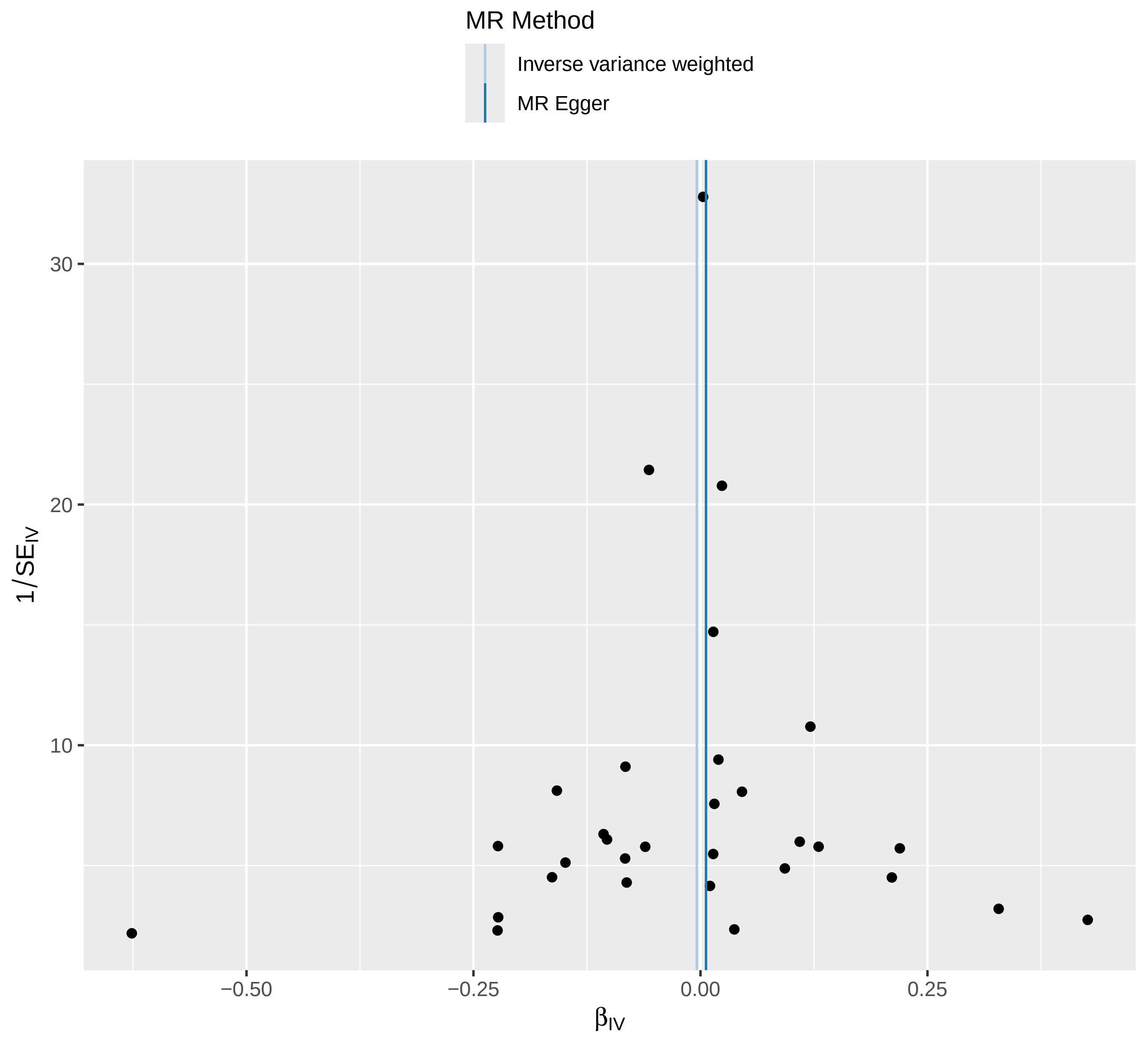
MR Method
Inverse variance weighted
MR Egger
1/SE$_{IV}$
β$_{IV}$
30
20
10
−0.50
−0.25
0.00
0.25

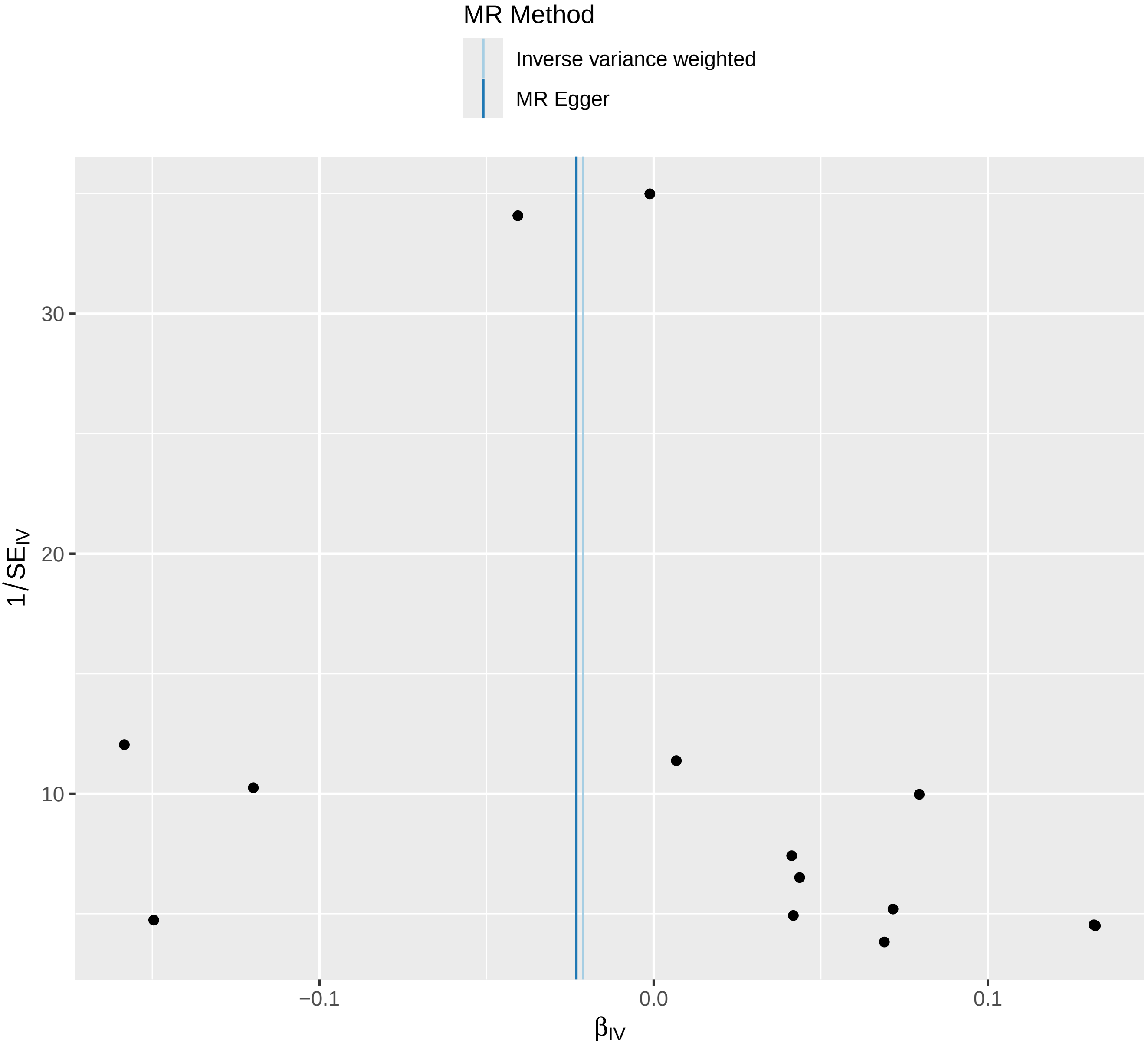
MR Method
Inverse variance weighted
MR Egger
30
20
10
1/SE$_{IV}$
−0.1
0.0
0.1
β$_{IV}$

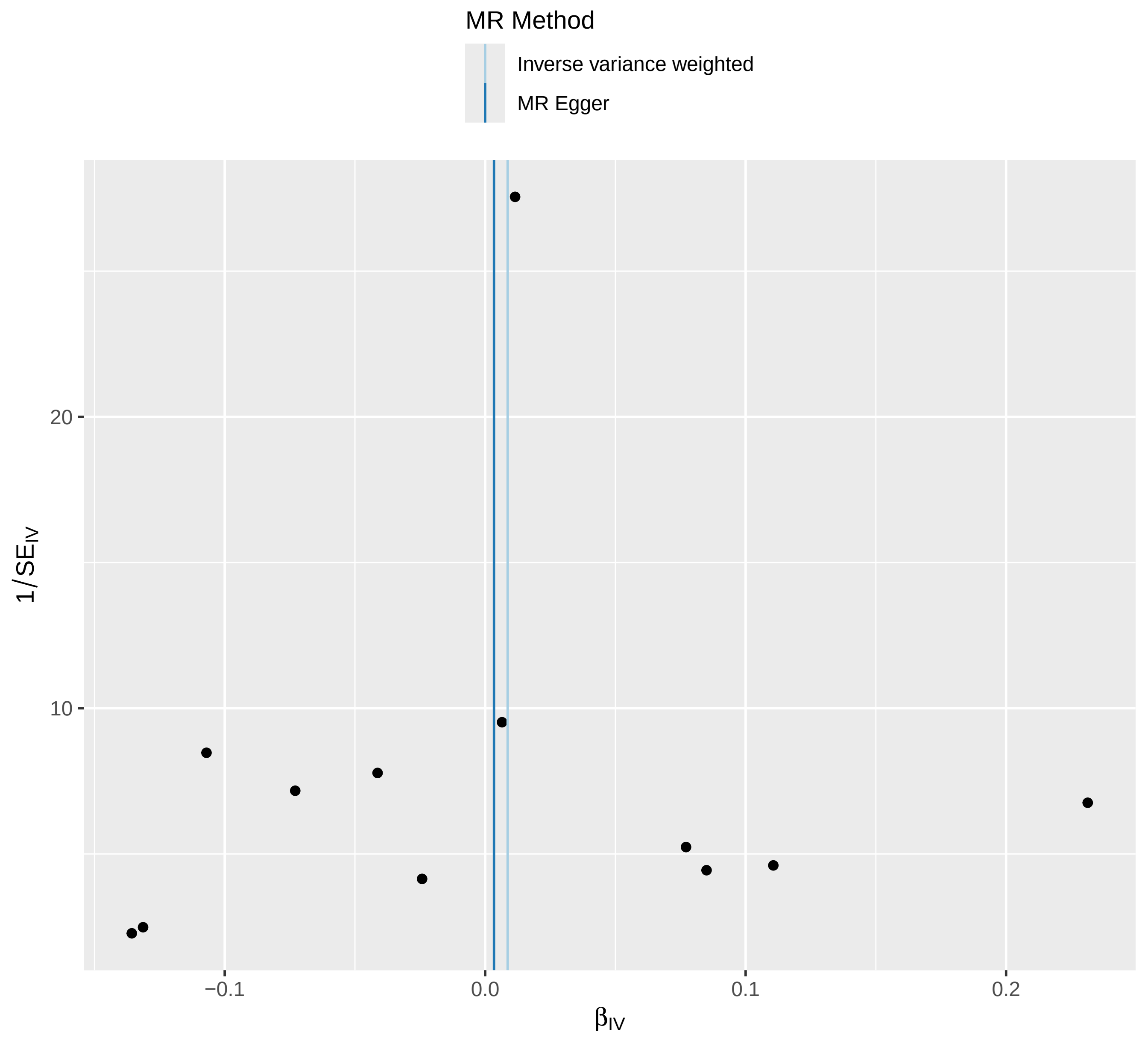
MR Method
Inverse variance weighted
MR Egger
20
10
1/SE$_{IV}$
−0.1
0.0
0.1
0.2
β$_{IV}$

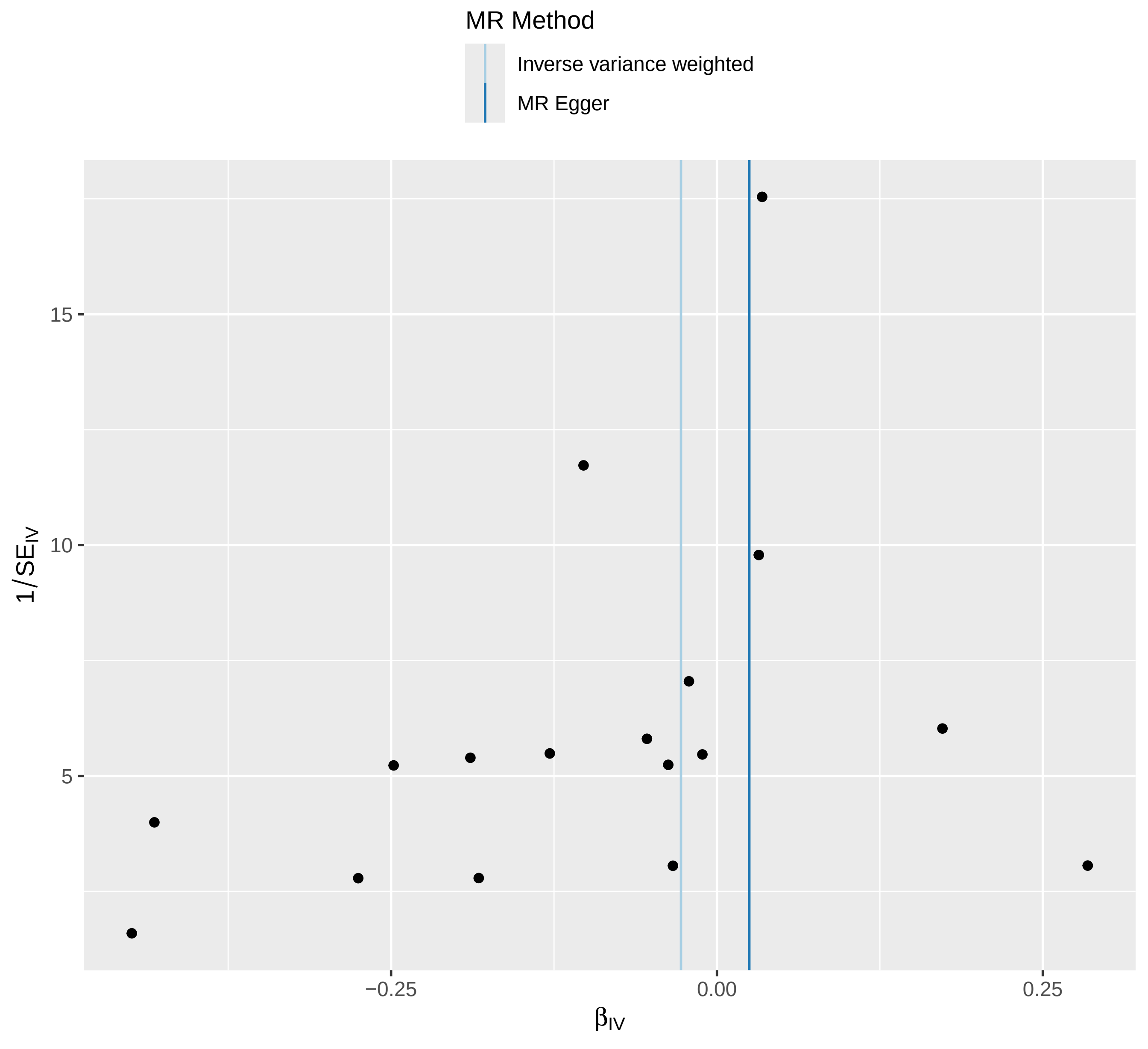

MR Method
Inverse variance weighted
MR Egger
15
10
5
1/SE_IV
−0.25
0.00
0.25
β_IV

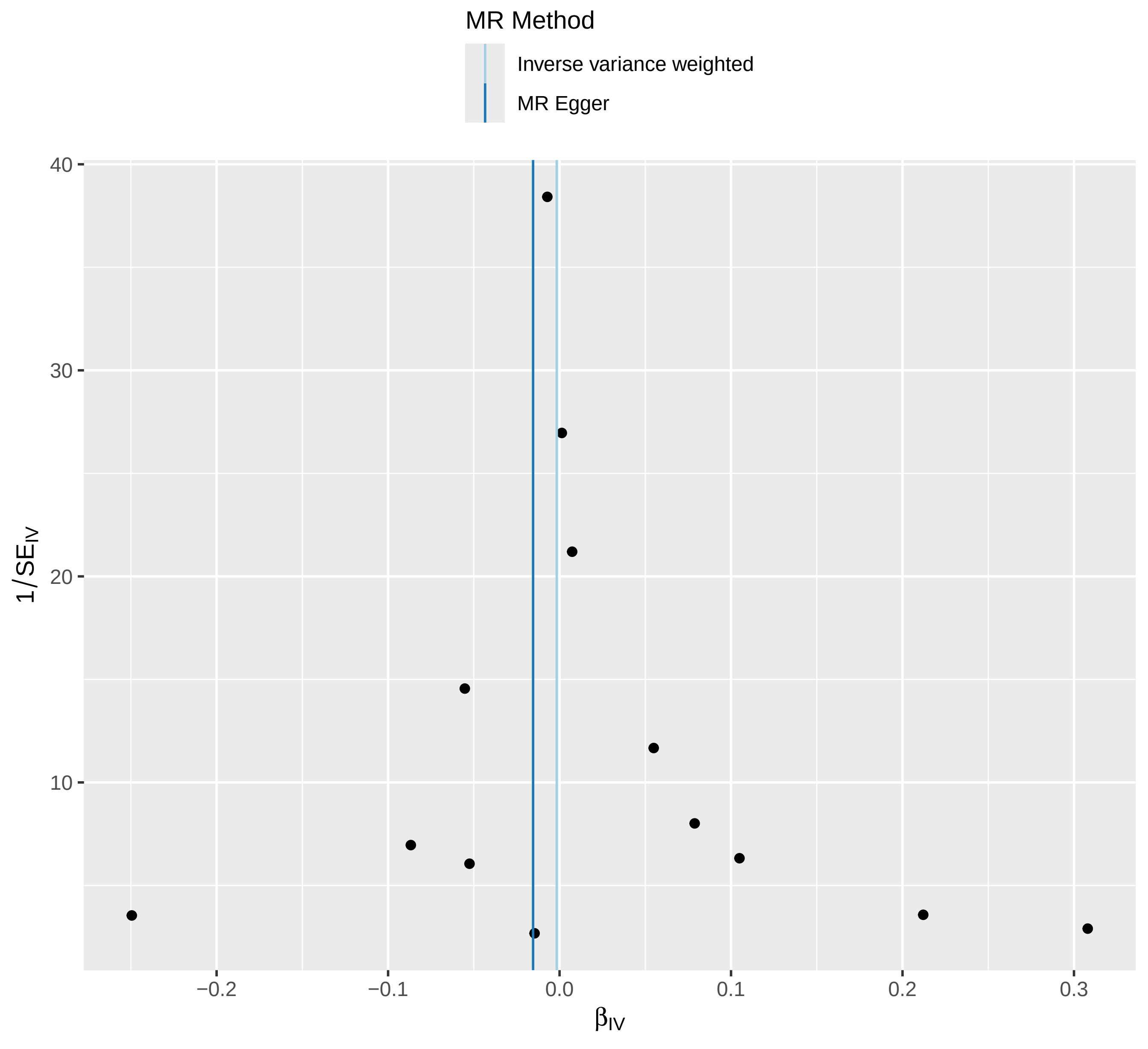
MR Method
Inverse variance weighted
MR Egger
40
30
20
10
1/SE$_{IV}$
−0.2
−0.1
0.0
0.1
0.2
0.3
β$_{IV}$

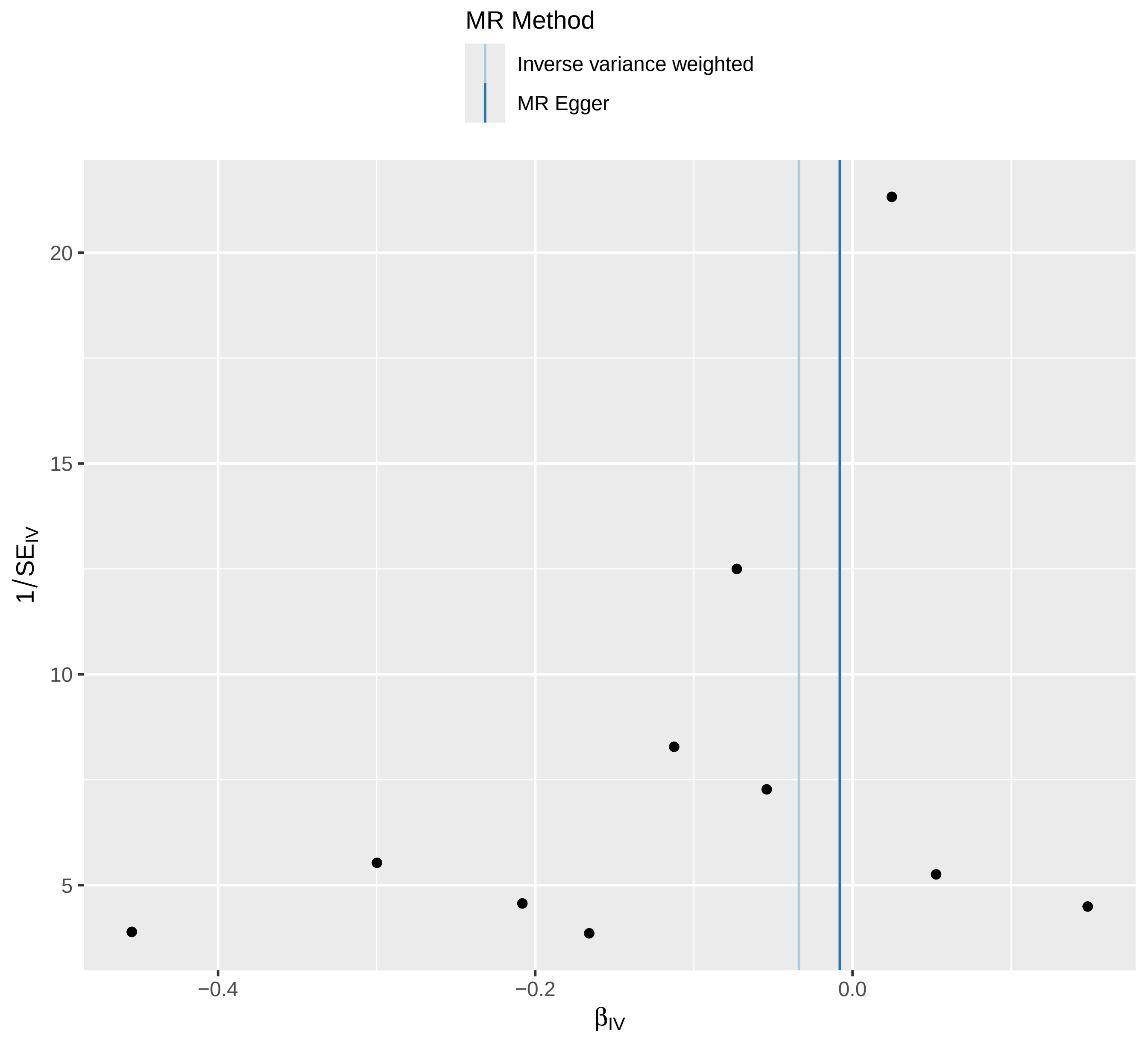

MR Method
Inverse variance weighted
MR Egger
20
15
10
5
1/SE$_{IV}$
−0.4
−0.2
0.0
$\beta_{IV}$

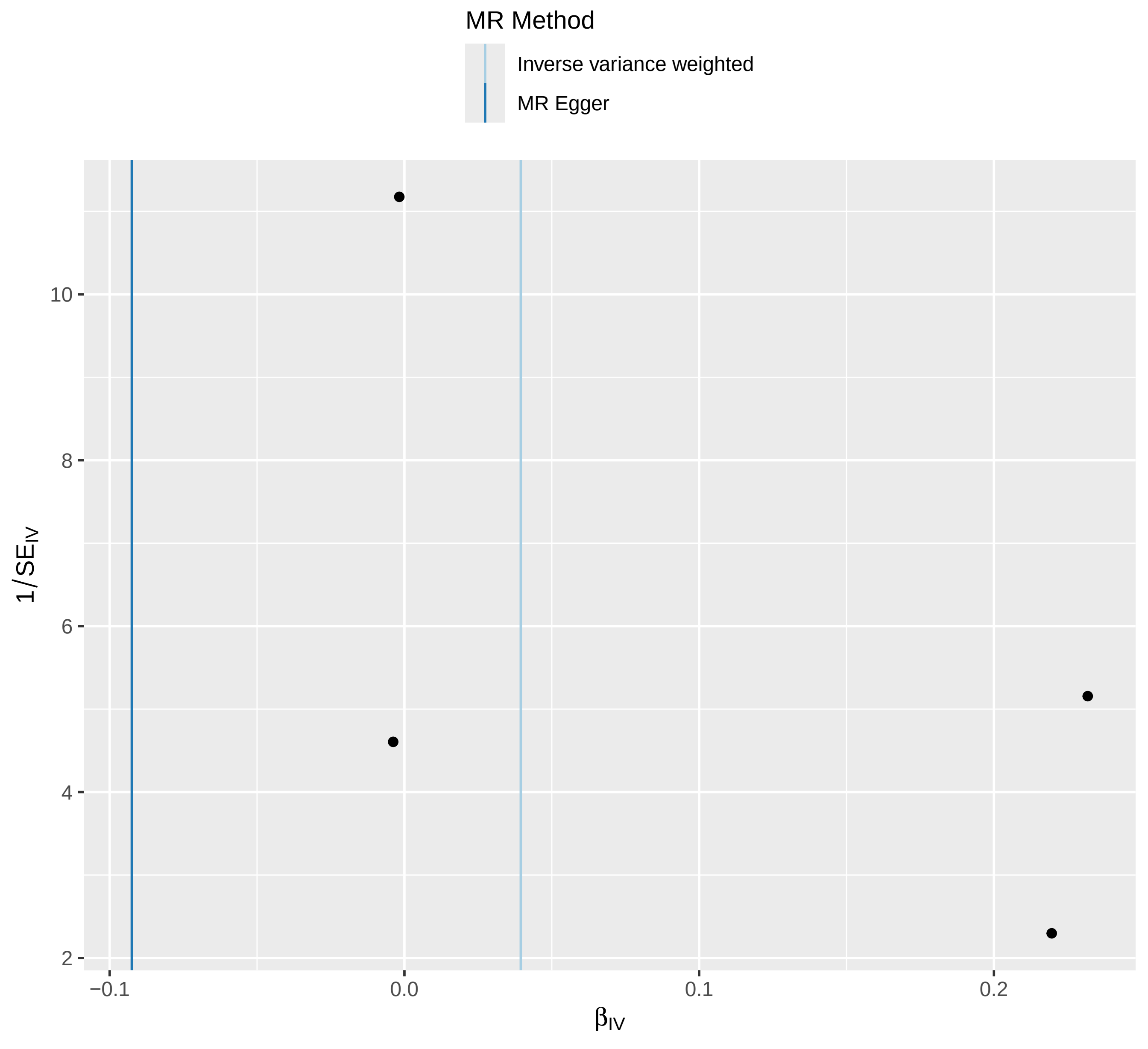

MR Method
Inverse variance weighted
MR Egger
1/SE_IV
β_IV
10
8
6
4
2
−0.1
0.0
0.1
0.2

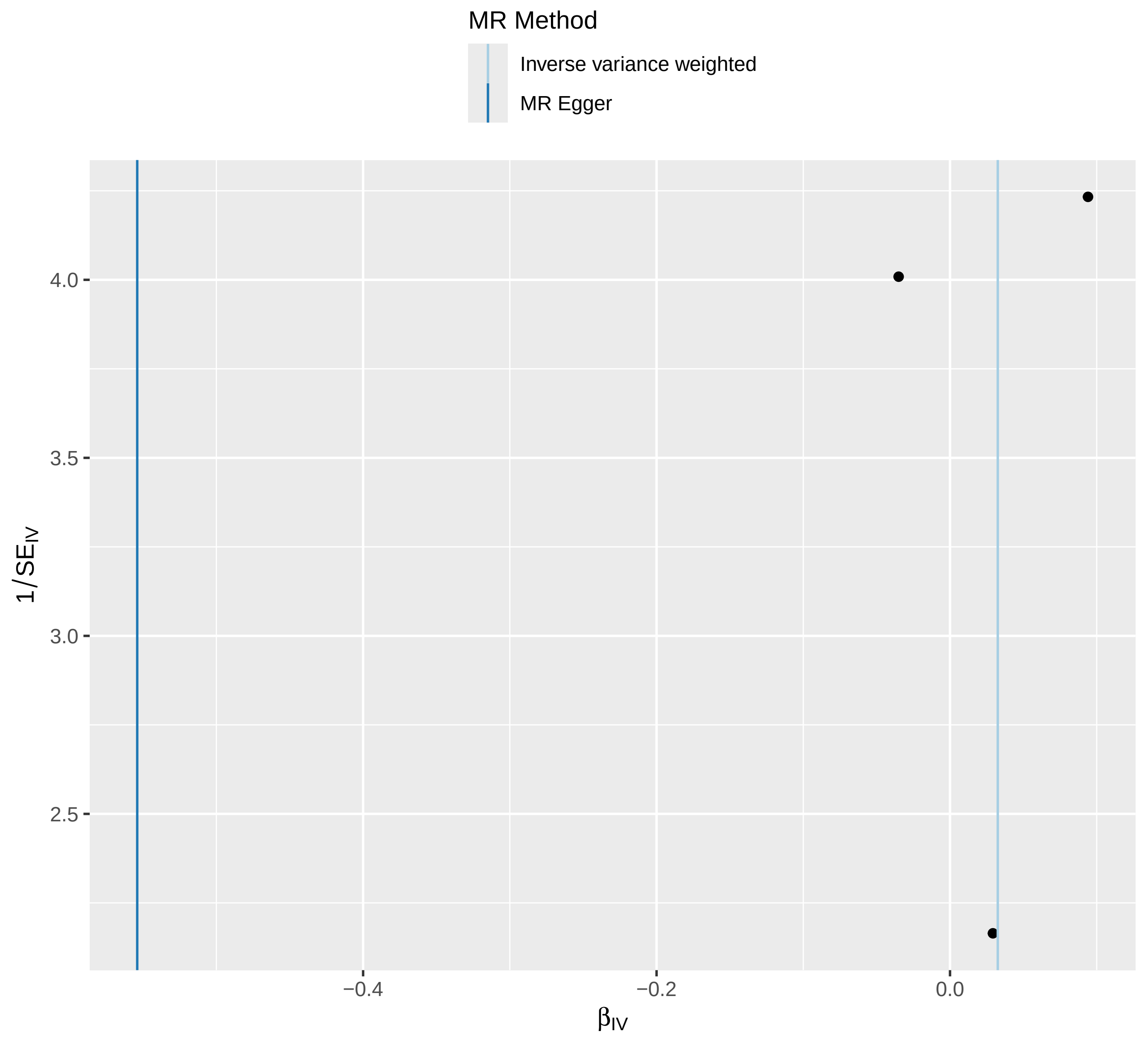

MR Method
Inverse variance weighted
MR Egger
4.0
3.5
3.0
2.5
$1/SE_{IV}$
−0.4
−0.2
0.0
$\beta_{IV}$

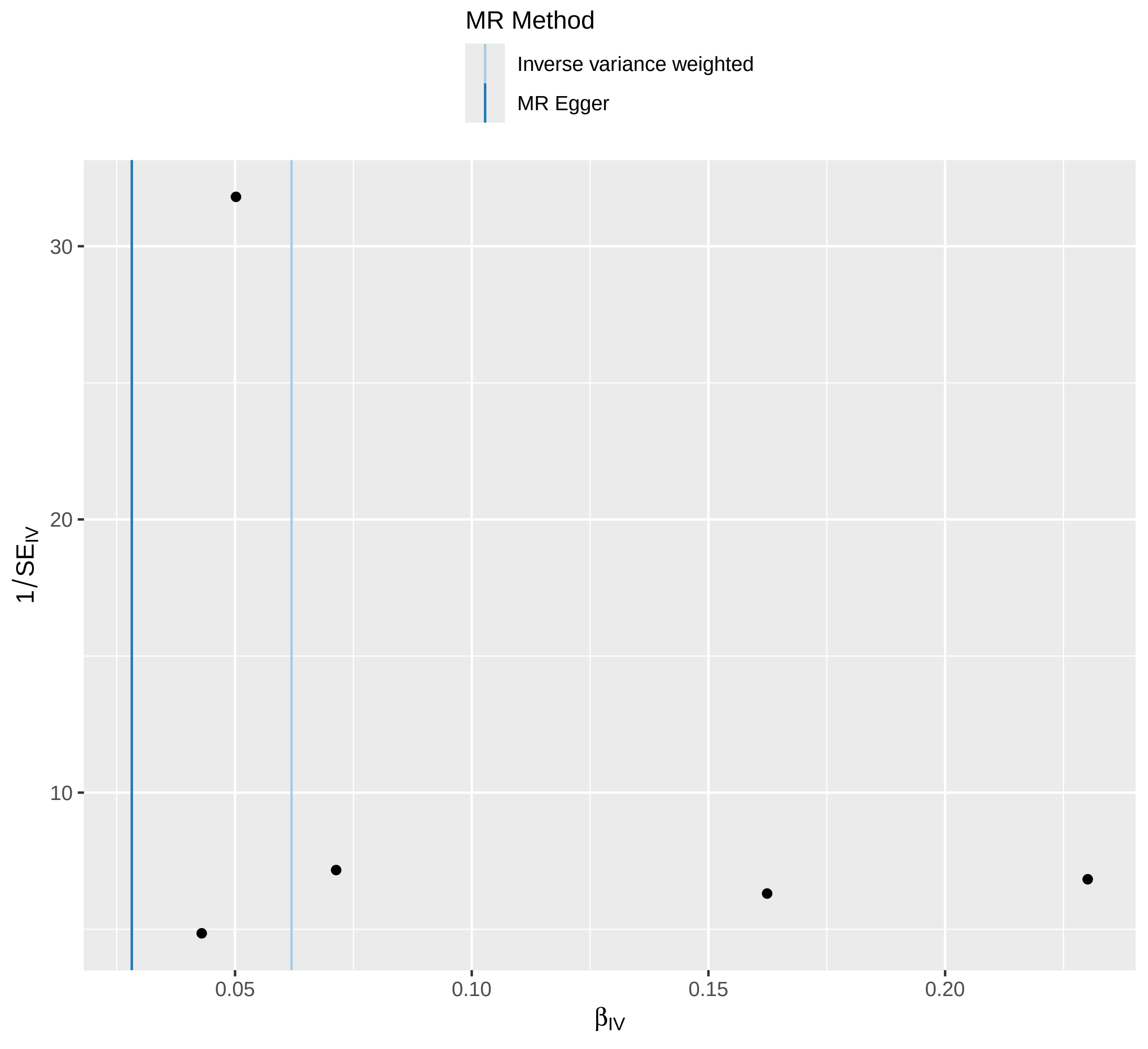

MR Method
Inverse variance weighted
MR Egger
30
20
10
1/SE_IV
0.05
0.10
0.15
0.20
β_IV

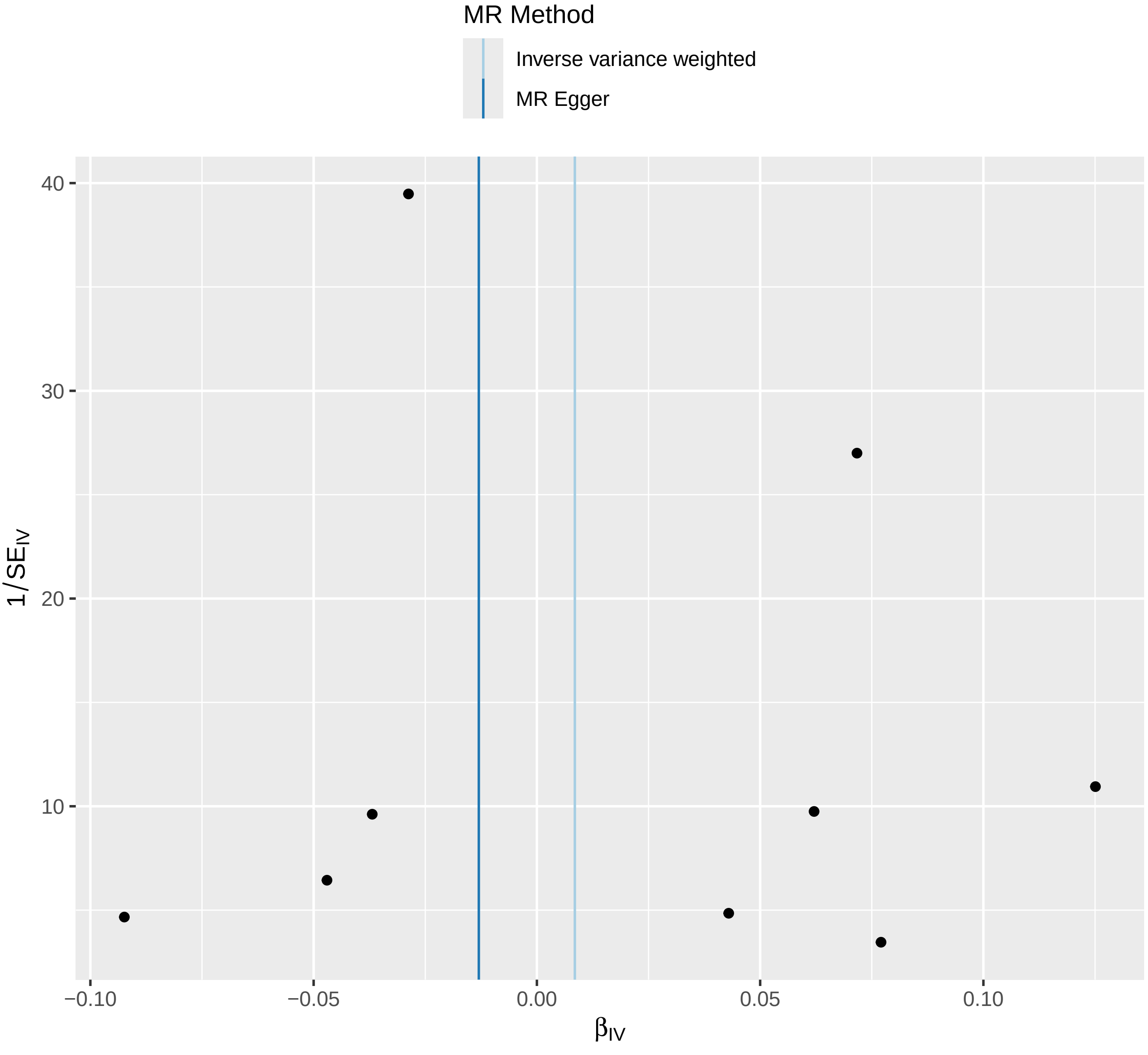
MR Method
Inverse variance weighted
MR Egger
40
30
20
10
$1/SE_{IV}$
−0.10
−0.05
0.00
0.05
0.10
$\beta_{IV}$

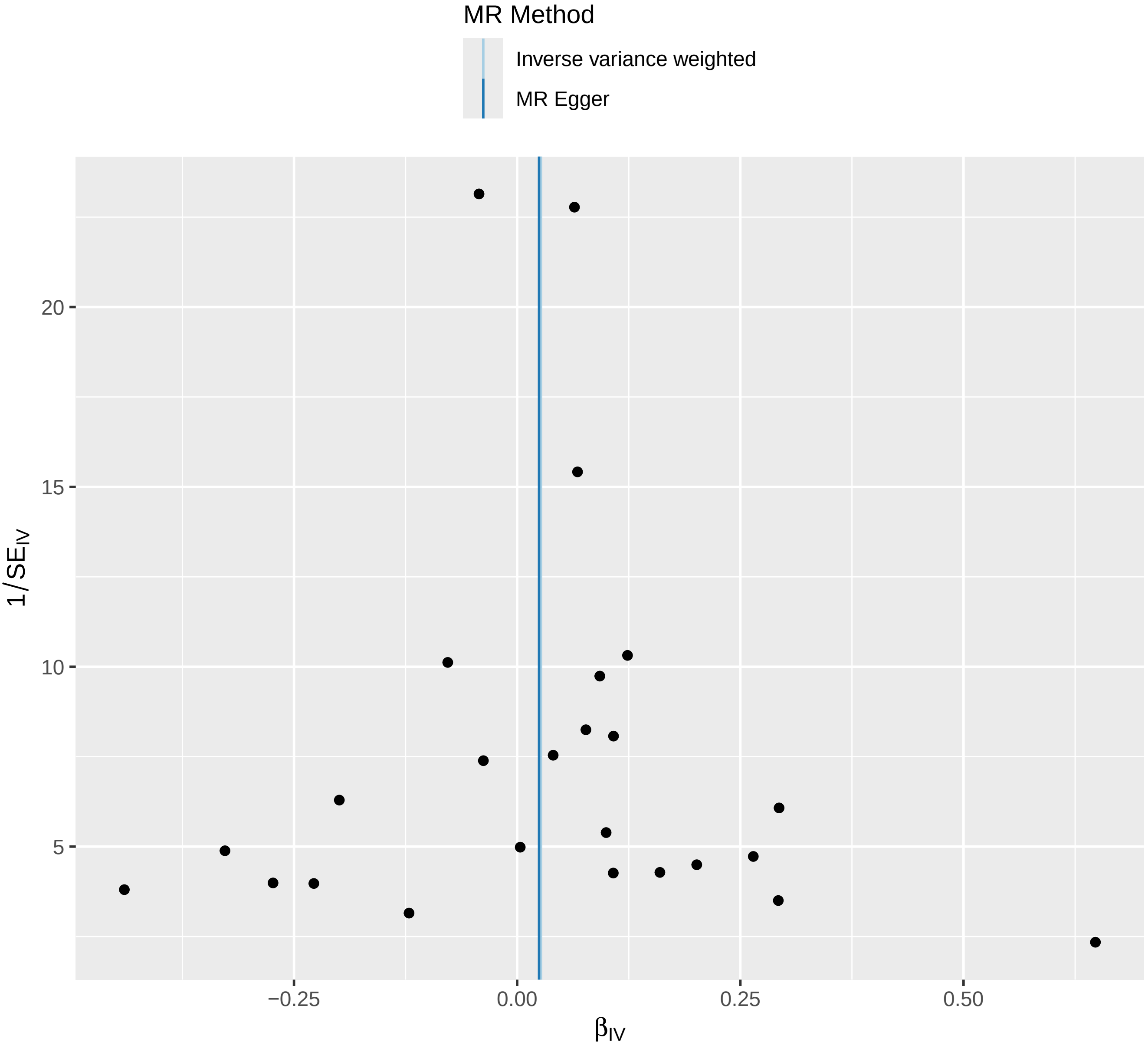

MR Method
Inverse variance weighted
MR Egger
20
15
10
5
1/SE_IV
−0.25
0.00
0.25
0.50
β_IV

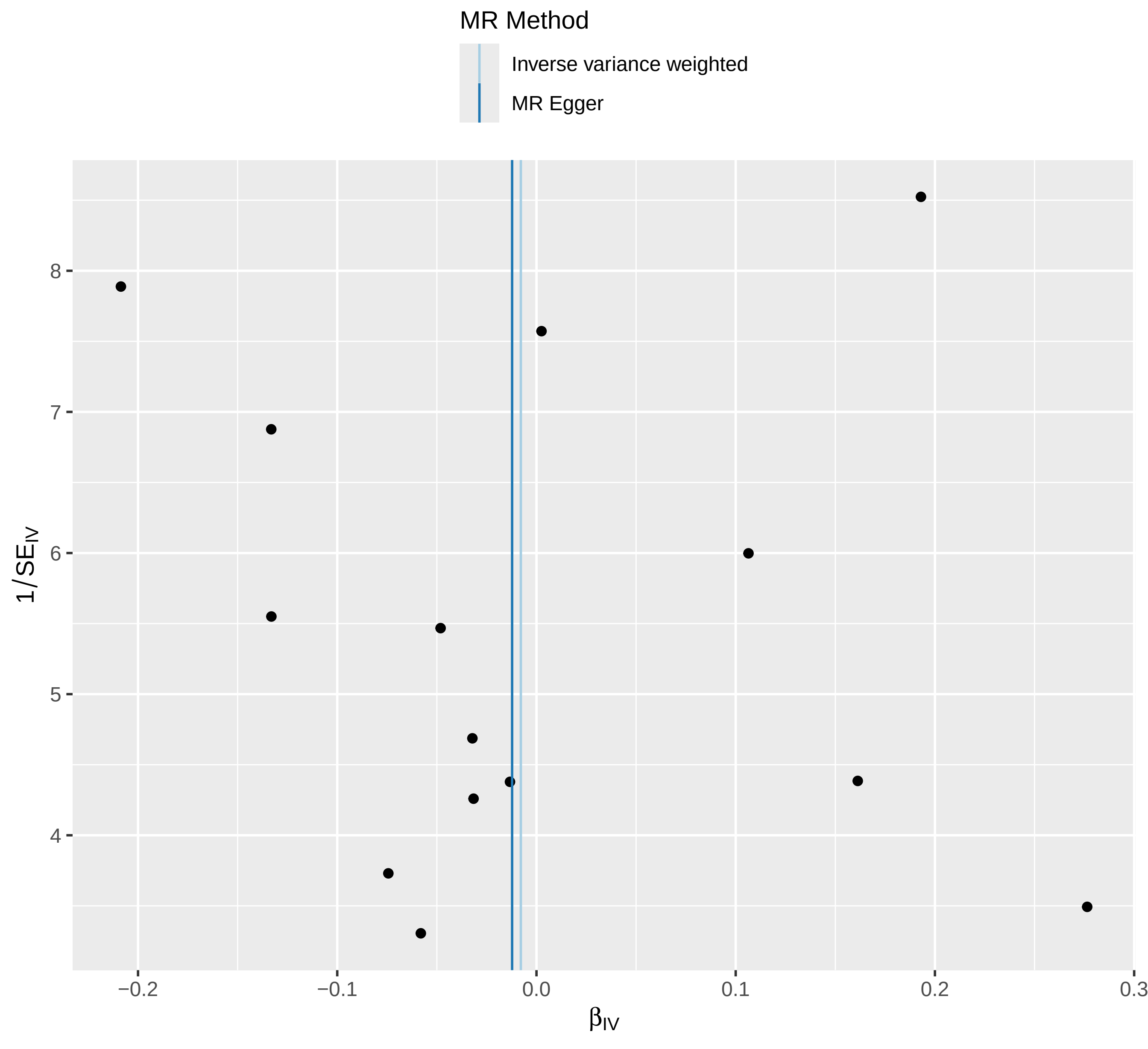

MR Method
Inverse variance weighted
MR Egger
8
7
6
5
4
1/SE$_{IV}$
−0.2
−0.1
0.0
0.1
0.2
0.3
β$_{IV}$

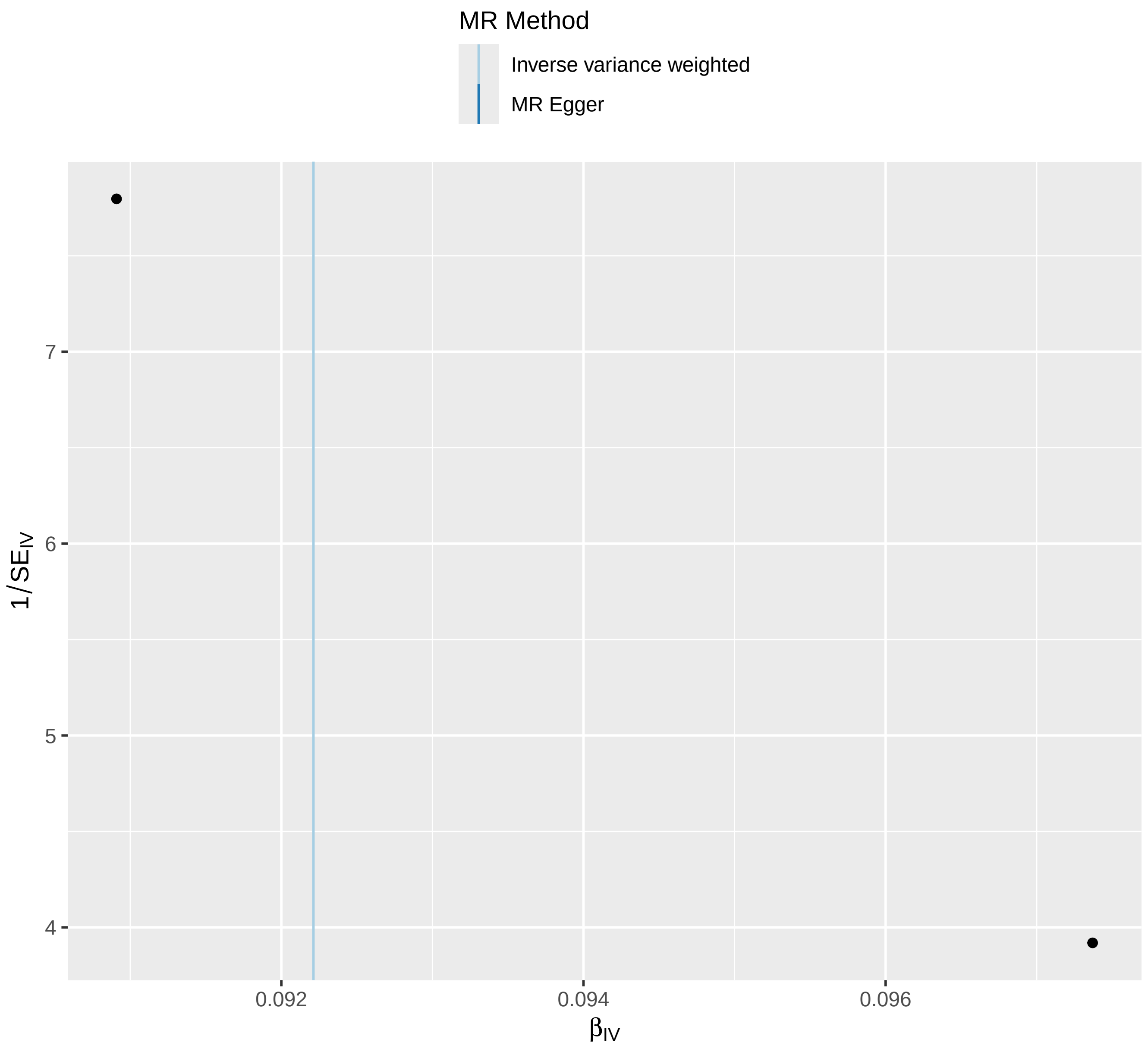
MR Method
Inverse variance weighted
MR Egger
1/SE$_{IV}$
7
6
5
4
0.092
0.094
0.096
$\beta_{IV}$

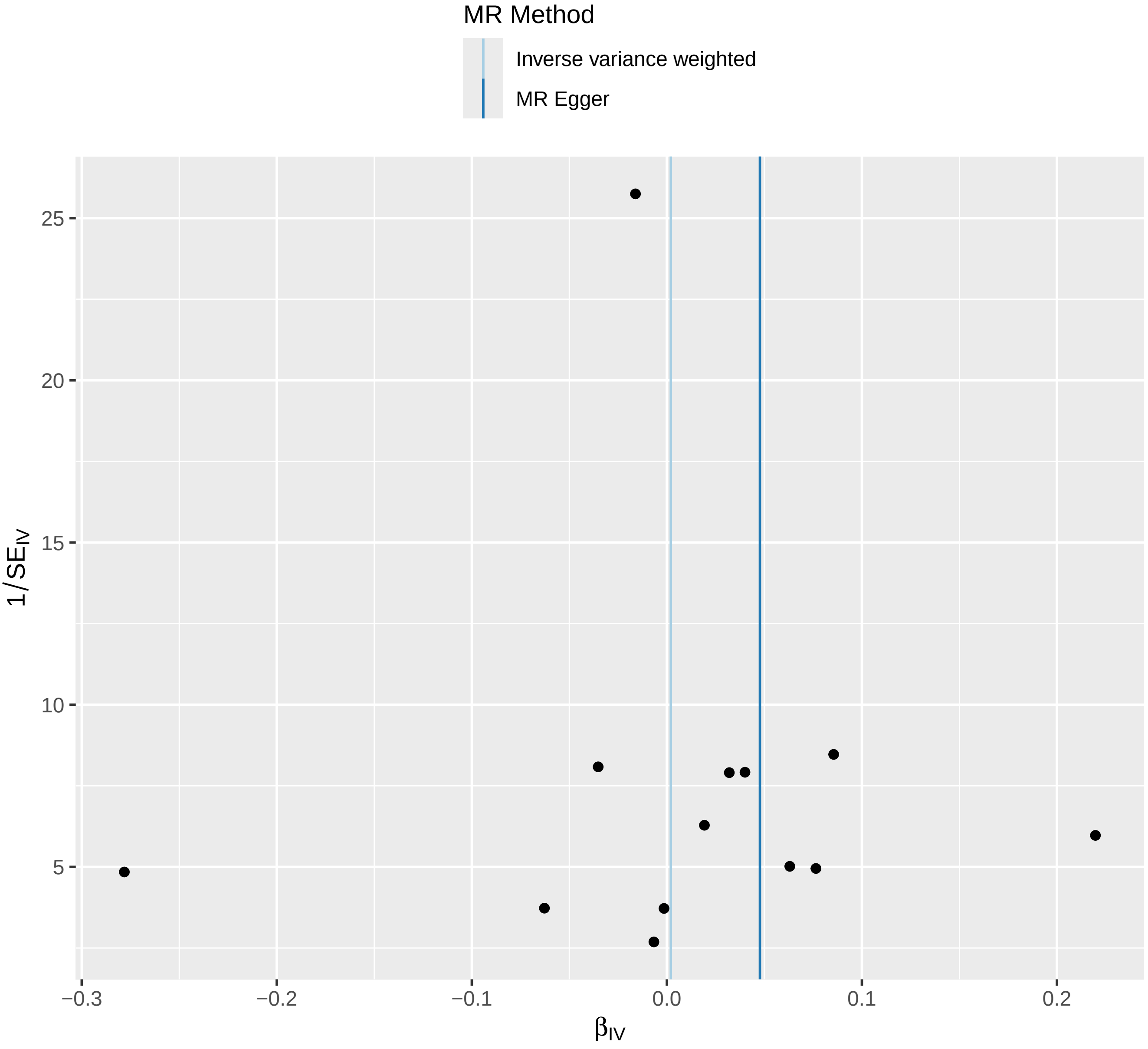

MR Method
Inverse variance weighted
MR Egger
25
20
15
10
5
$1/SE_{IV}$
−0.3
−0.2
−0.1
0.0
0.1
0.2
$\beta_{IV}$

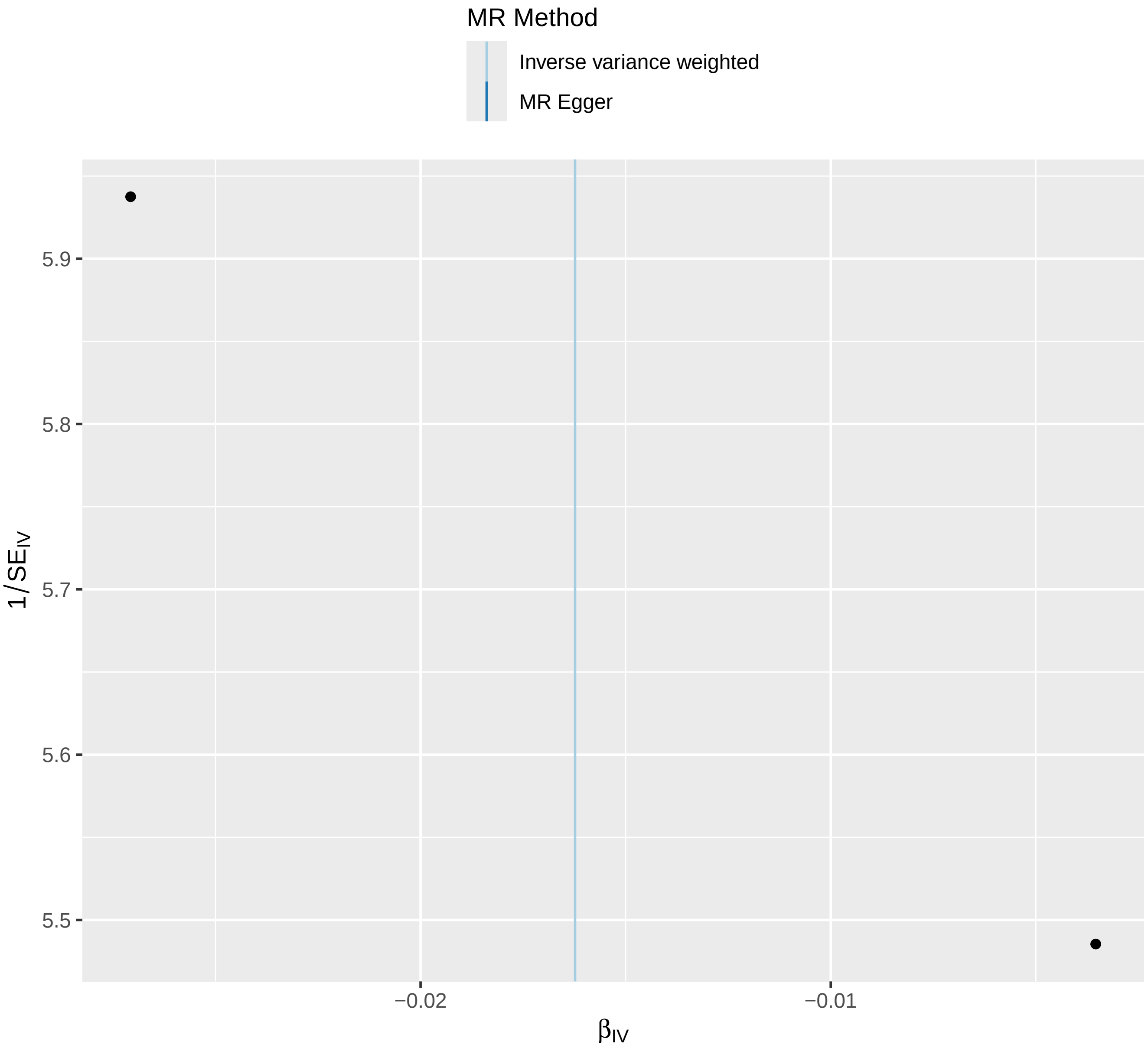

MR Method
Inverse variance weighted
MR Egger
5.9
5.8
5.7
5.6
5.5
1/SE_IV
−0.02
−0.01
β_IV

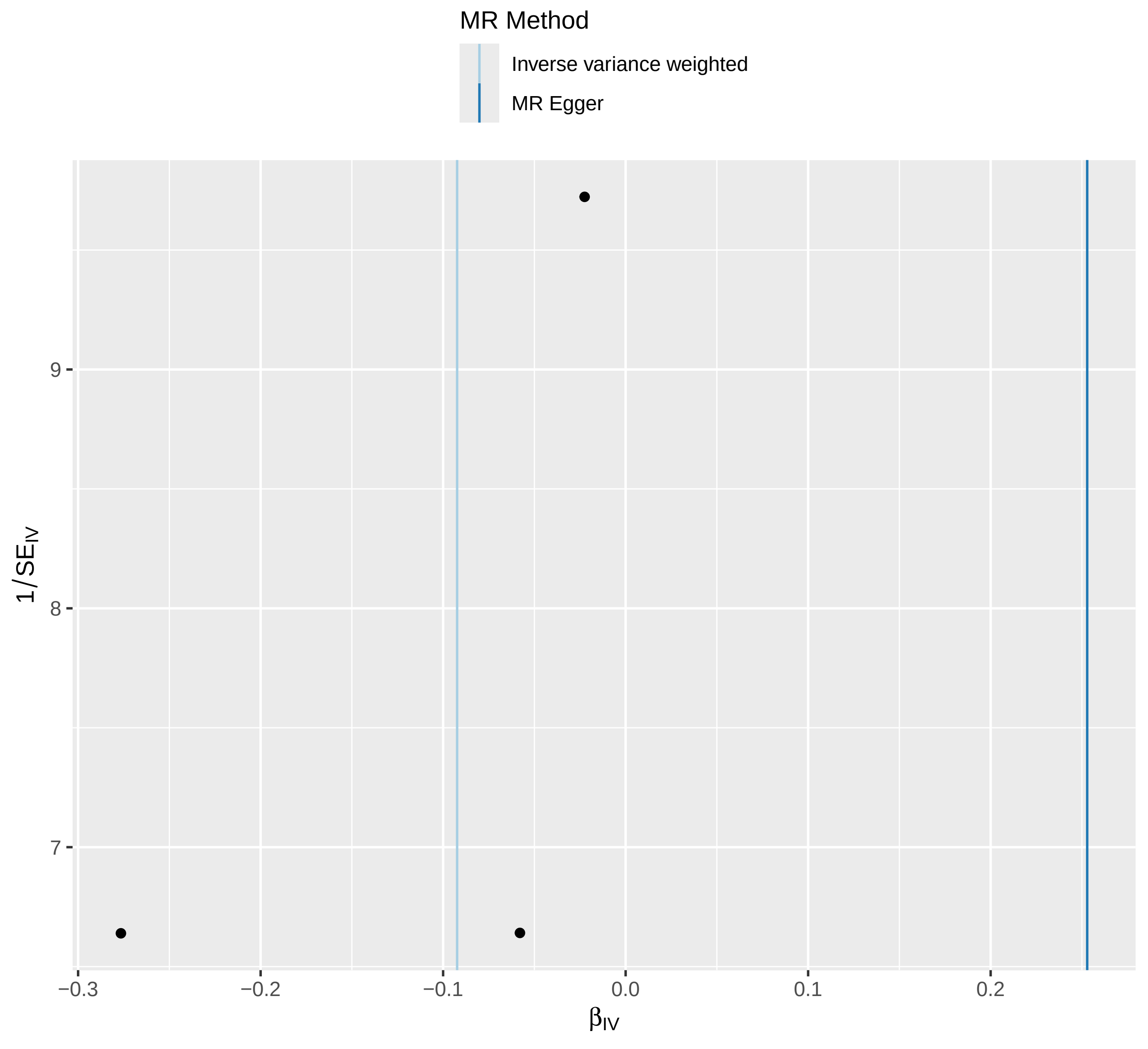
MR Method
Inverse variance weighted
MR Egger
1/SE$_{IV}$
9
8
7
−0.3
−0.2
−0.1
0.0
0.1
0.2
β$_{IV}$

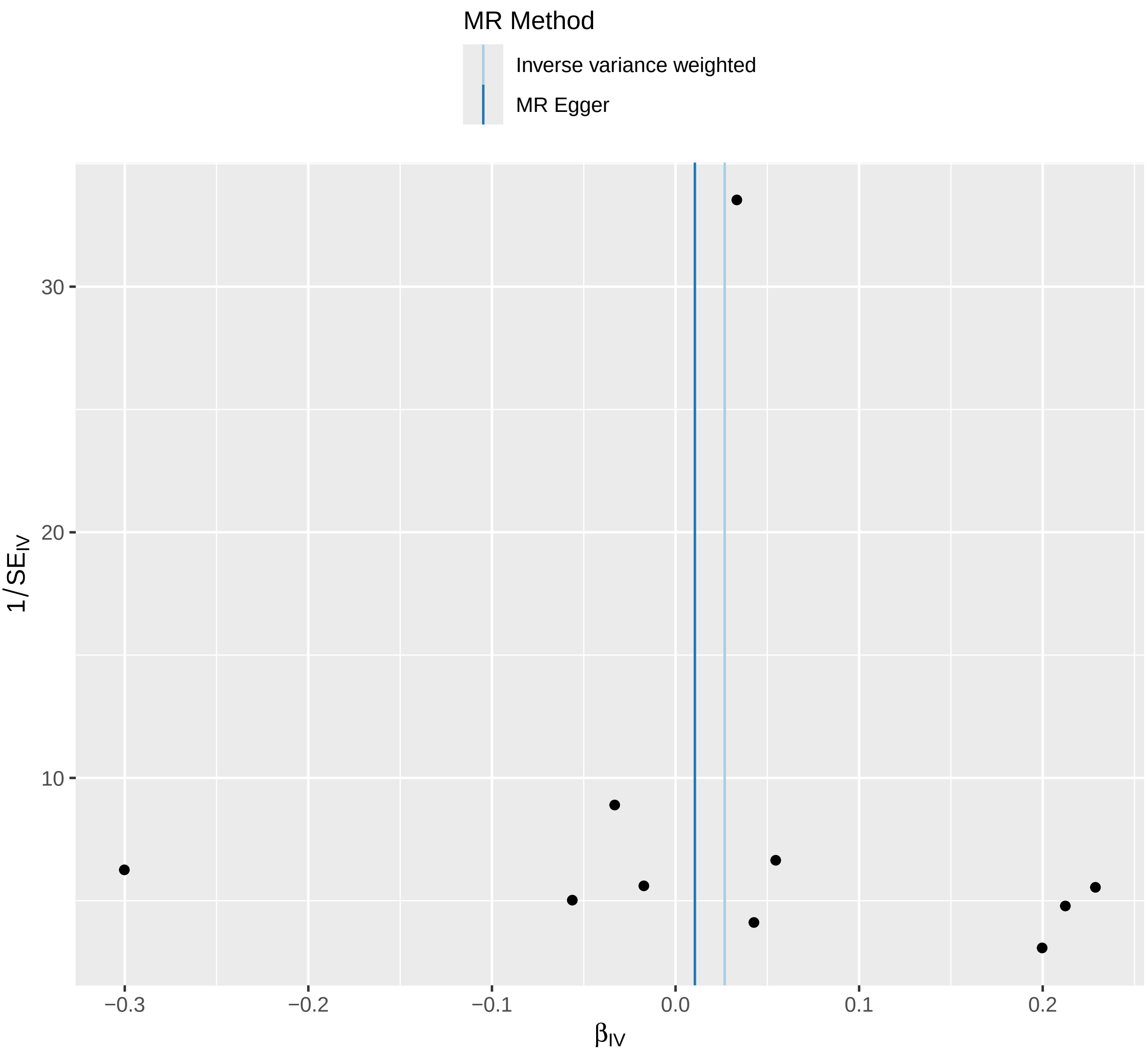
MR Method
Inverse variance weighted
MR Egger
30
20
10
$1/SE_{IV}$
−0.3
−0.2
−0.1
0.0
0.1
0.2
$\beta_{IV}$

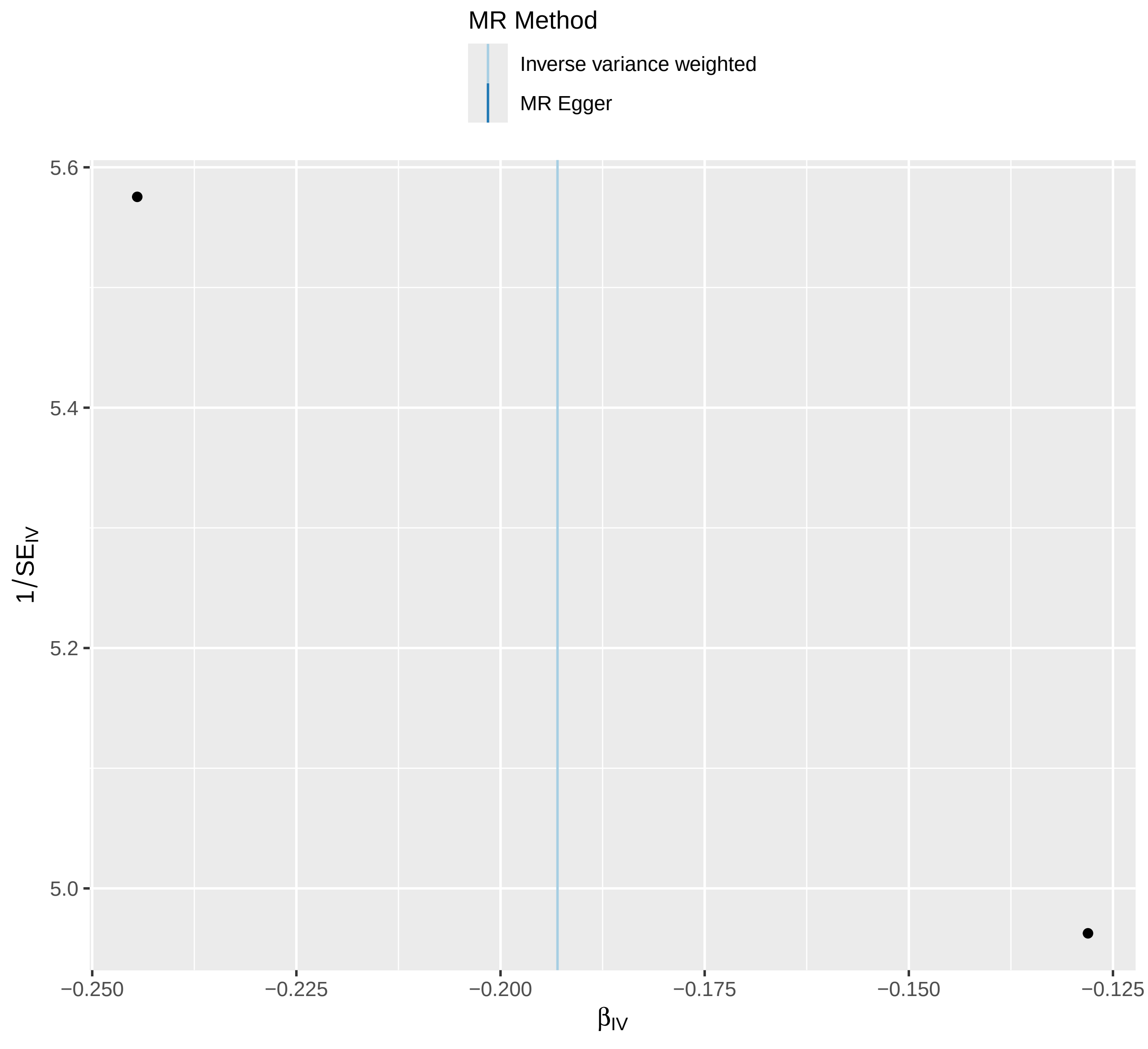

MR Method
Inverse variance weighted
MR Egger
5.6
5.4
5.2
5.0
1/SE$_{IV}$
−0.250
−0.225
−0.200
−0.175
−0.150
−0.125
β$_{IV}$

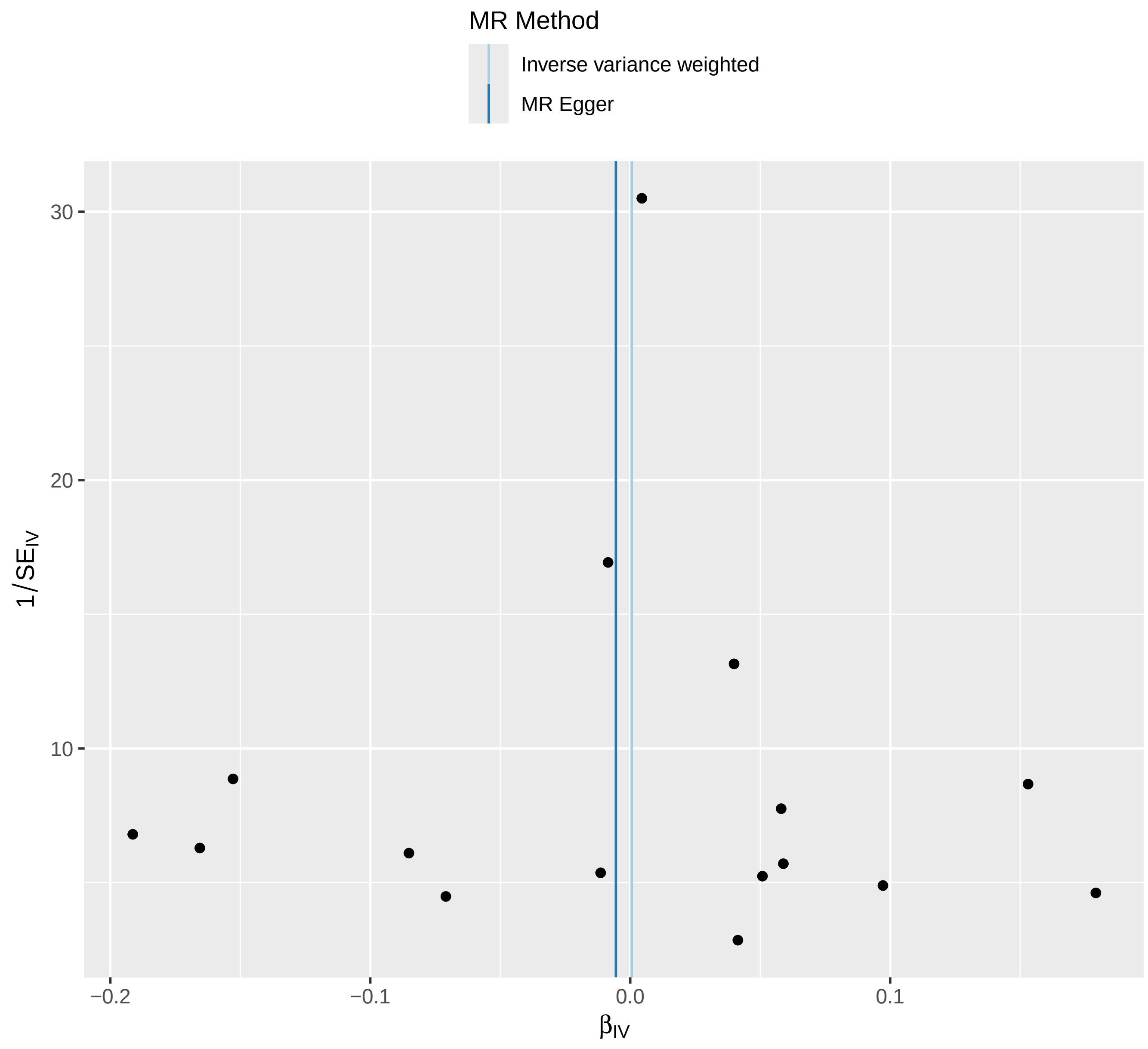
MR Method
Inverse variance weighted
MR Egger
1/SE_IV
30
20
10
−0.2
−0.1
0.0
0.1
β_IV

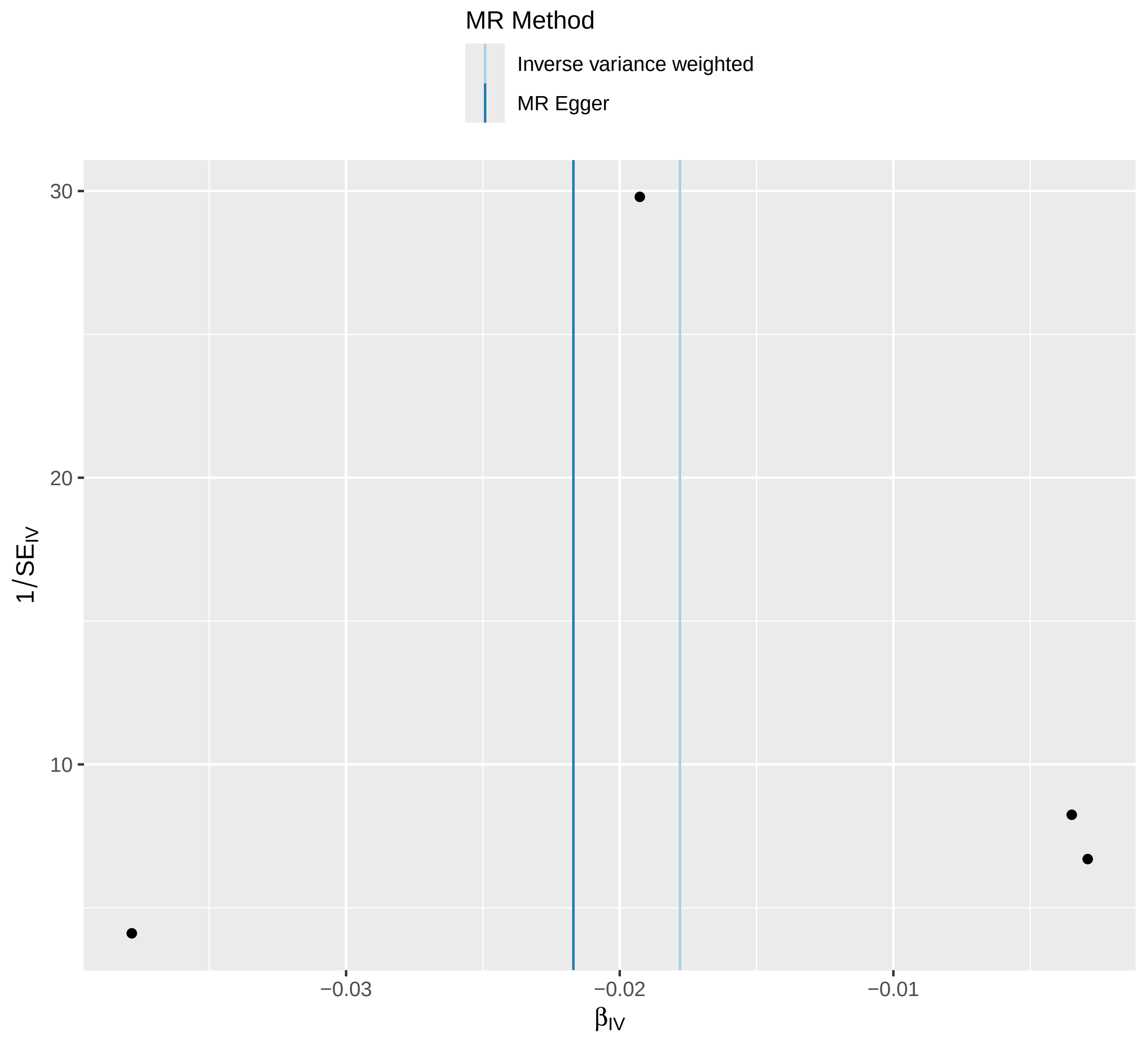
MR Method
Inverse variance weighted
MR Egger
30
20
10
1/$SE_{IV}$
−0.03
−0.02
−0.01
$\beta_{IV}$

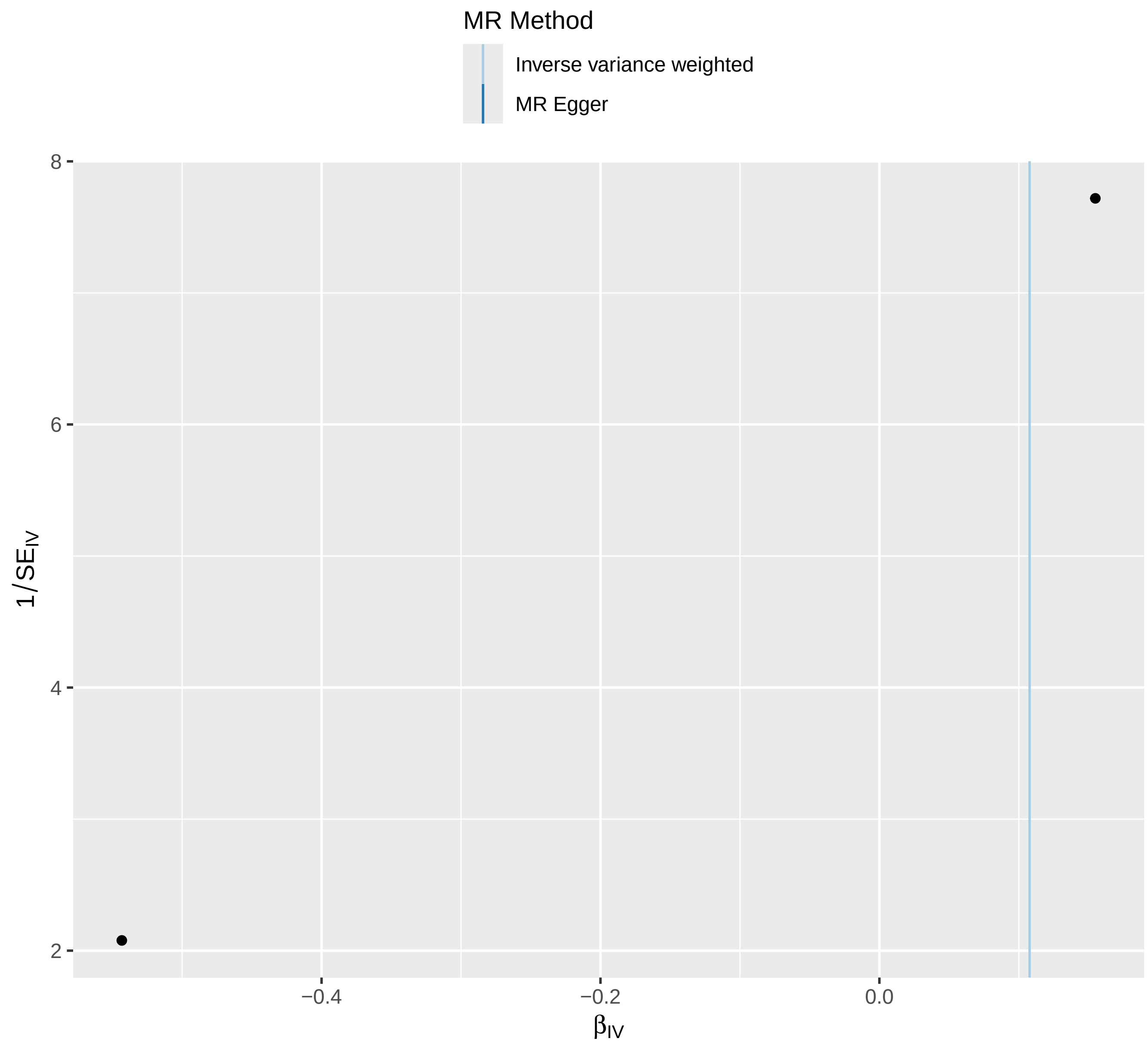
MR Method
Inverse variance weighted
MR Egger
8
6
4
2
1/SE_IV
−0.4
−0.2
0.0
β_IV

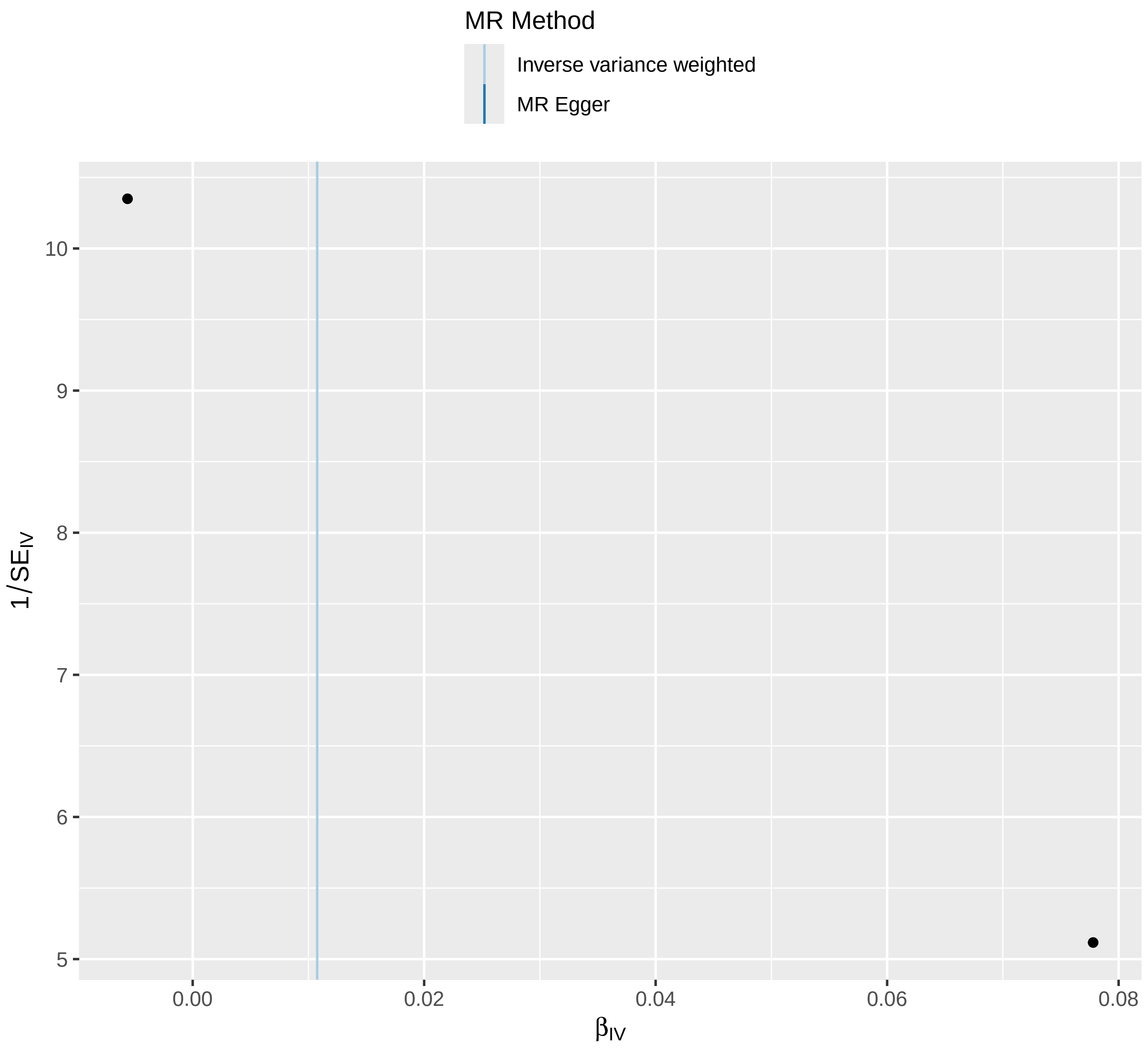

MR Method
Inverse variance weighted
MR Egger
10
9
8
7
6
5
1/SE_IV
0.00
0.02
0.04
0.06
0.08
β_IV

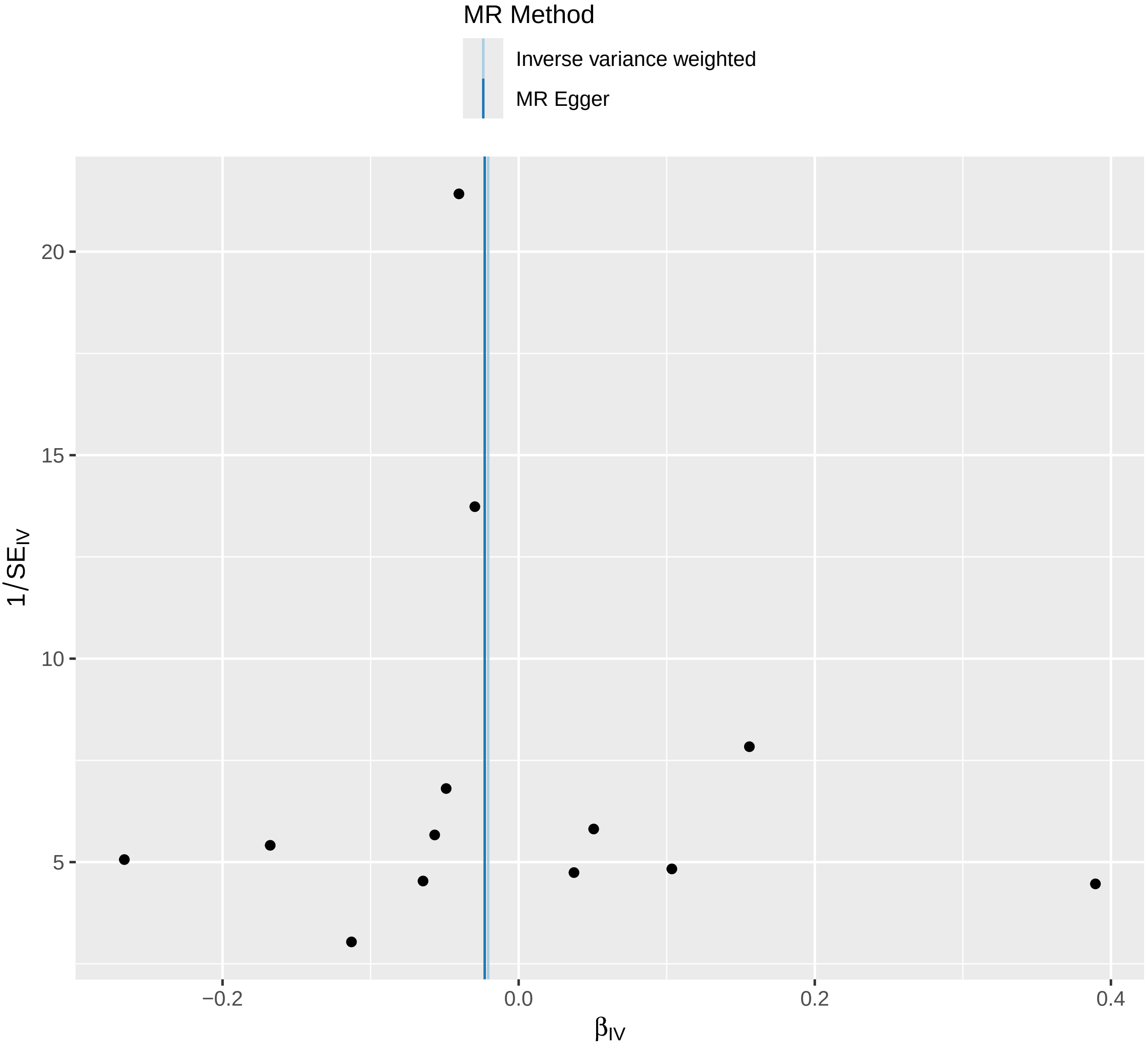

MR Method
Inverse variance weighted
MR Egger
20
15
10
5
1/SE_IV
−0.2
0.0
0.2
0.4
β_IV

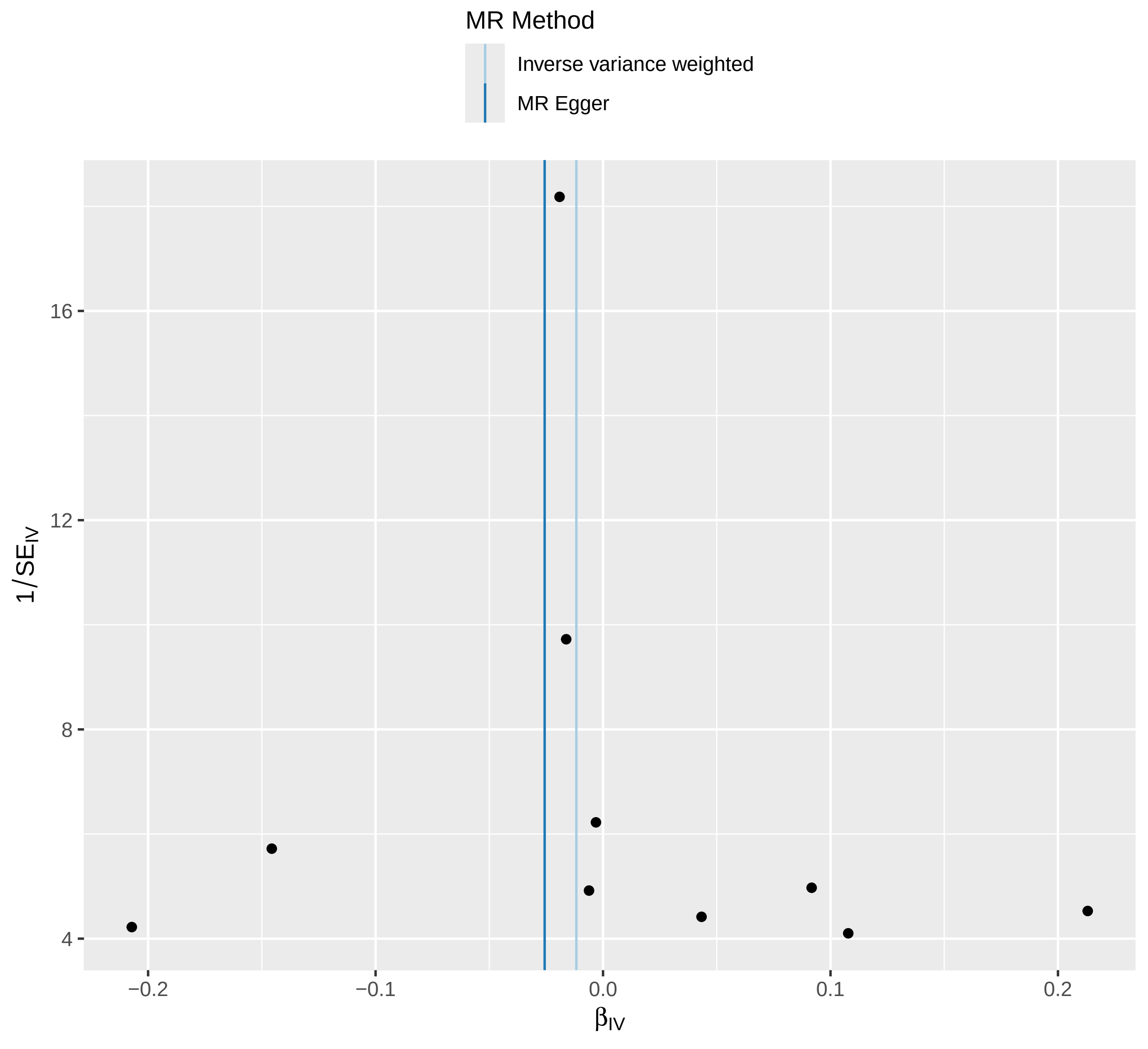
MR Method
Inverse variance weighted
MR Egger
16
12
8
4
1/SE$_{IV}$
−0.2
−0.1
0.0
0.1
0.2
β$_{IV}$

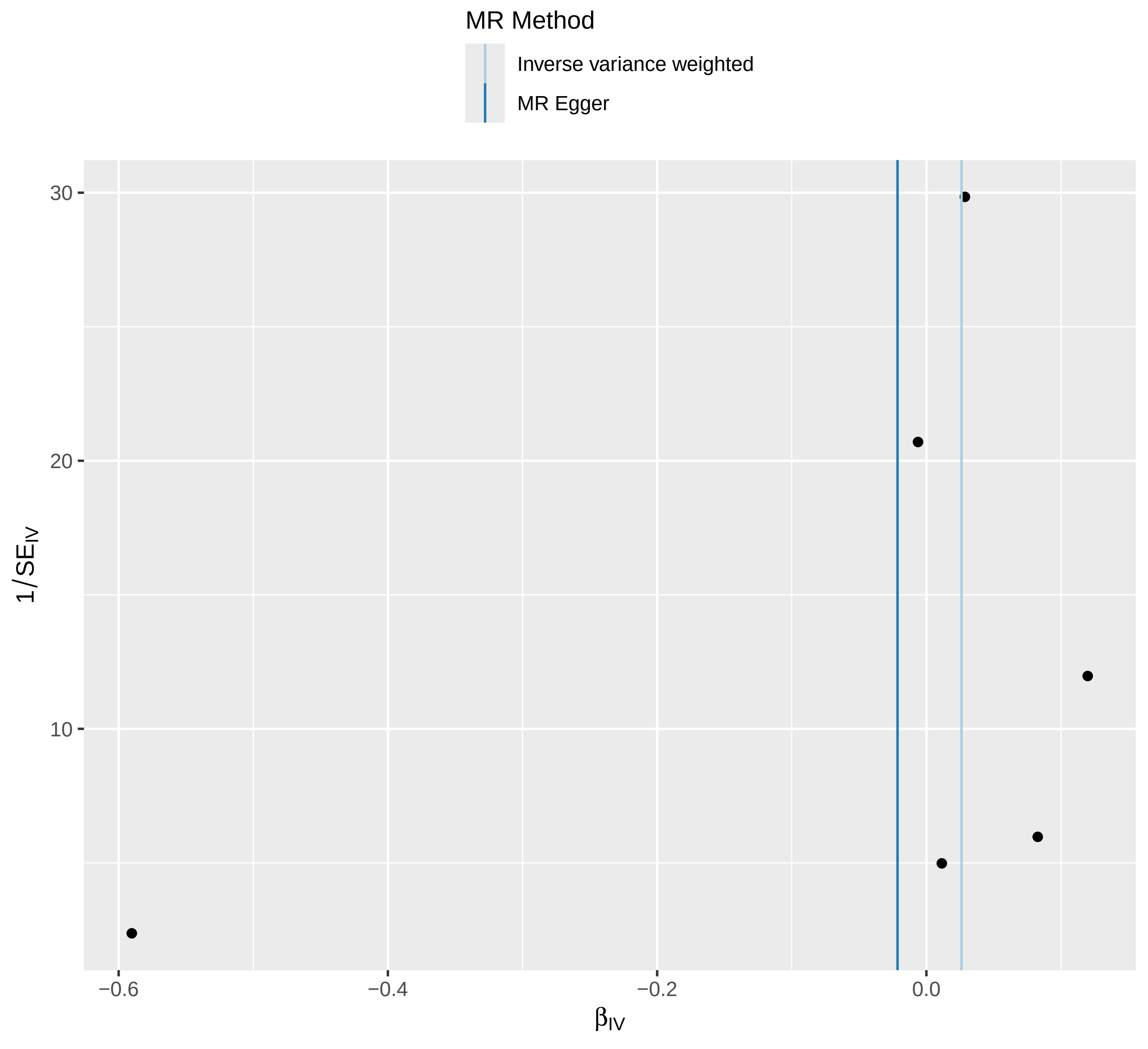
MR Method
Inverse variance weighted
MR Egger
30
20
10
1/SE_IV
−0.6
−0.4
−0.2
0.0
β_IV

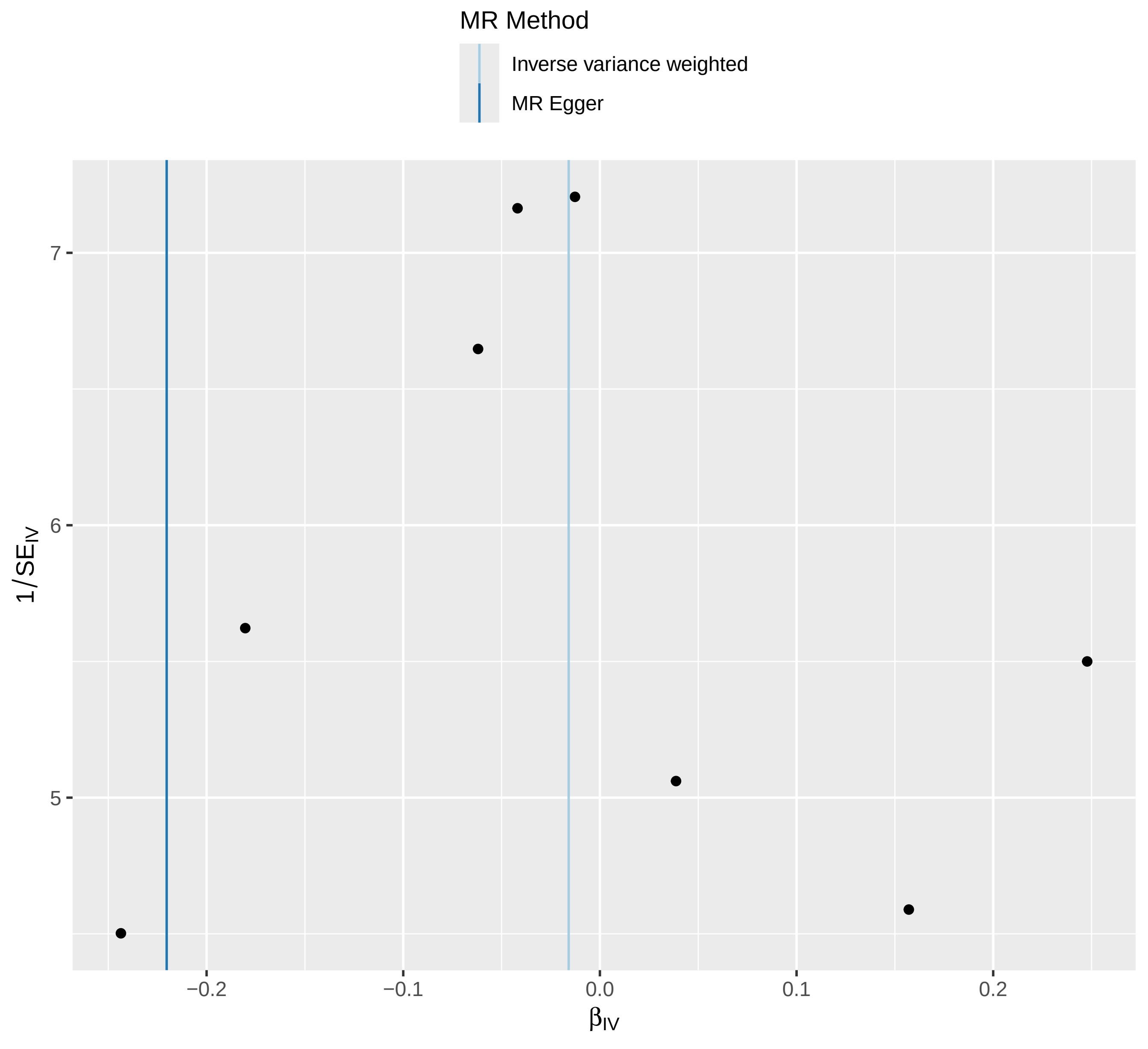

MR Method
Inverse variance weighted
MR Egger
7
6
5
1/SE$_{IV}$
−0.2
−0.1
0.0
0.1
0.2
β$_{IV}$

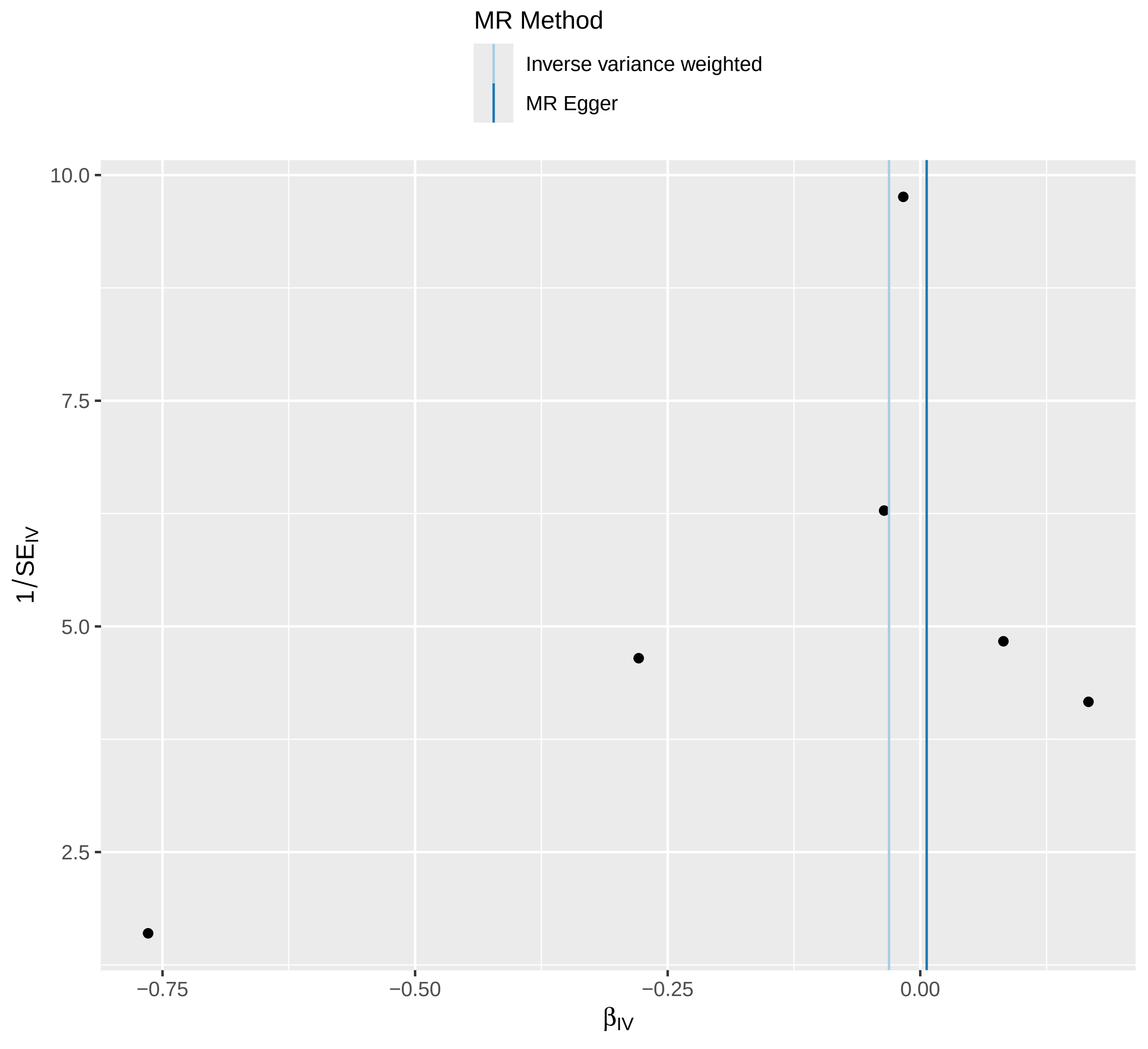
MR Method
Inverse variance weighted
MR Egger
10.0
7.5
5.0
2.5
$1/SE_{IV}$
−0.75
−0.50
−0.25
0.00
$\beta_{IV}$

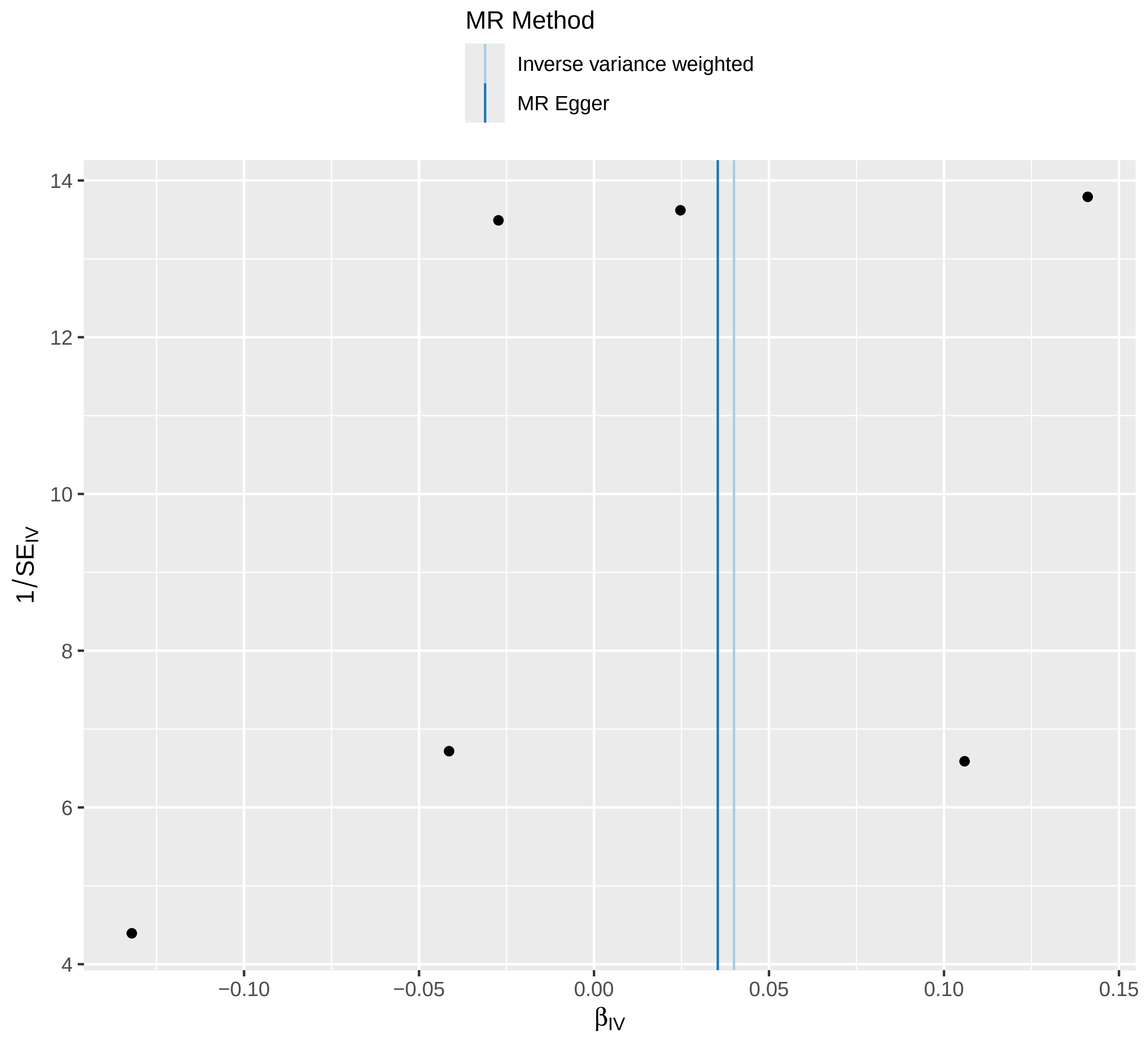
MR Method
Inverse variance weighted
MR Egger
14
12
10
8
6
4
1/SE_IV
−0.10
−0.05
0.00
0.05
0.10
0.15
β_IV

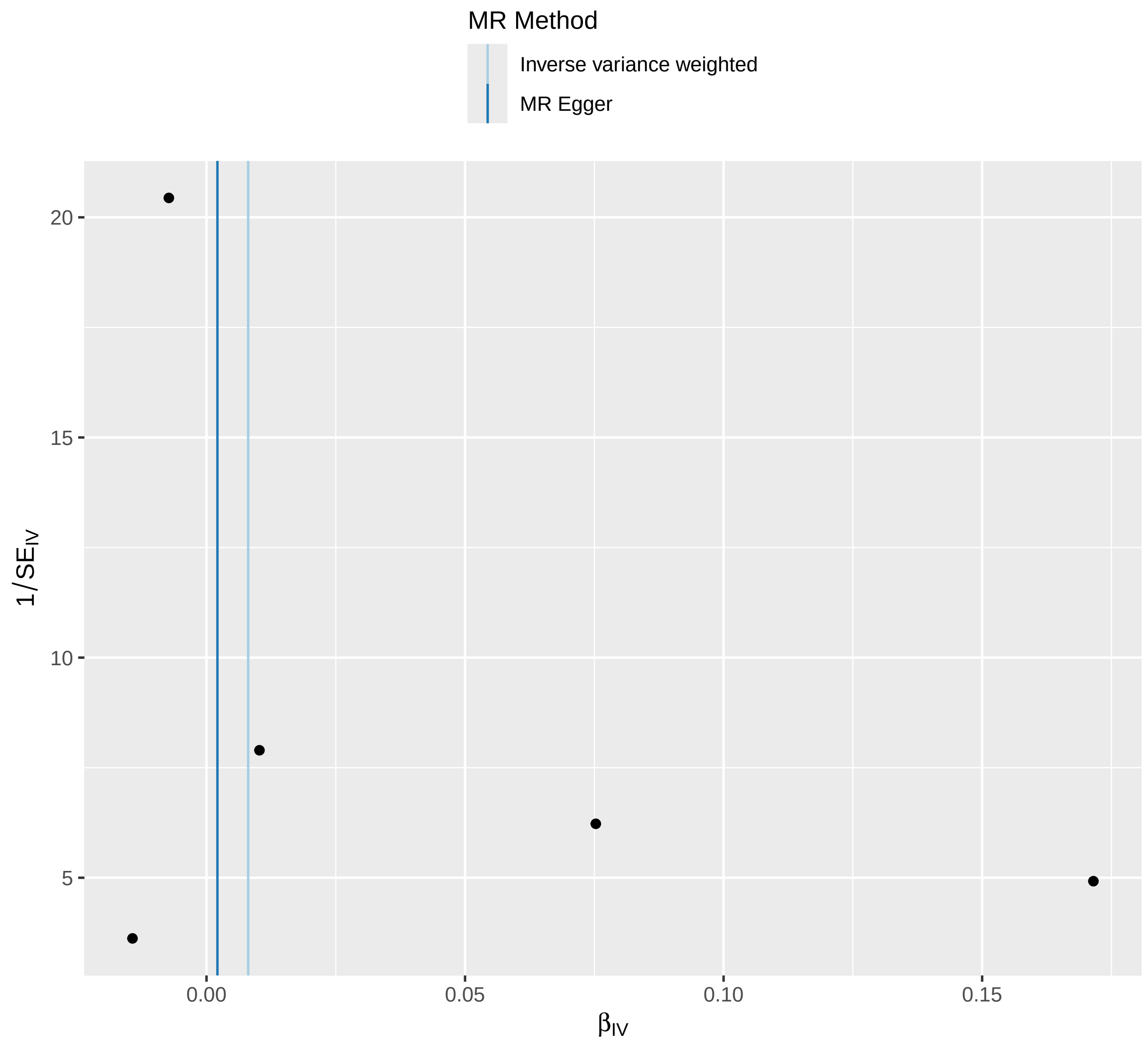

MR Method
Inverse variance weighted
MR Egger
20
15
10
5
1/SE_IV
0.00
0.05
0.10
0.15
β_IV

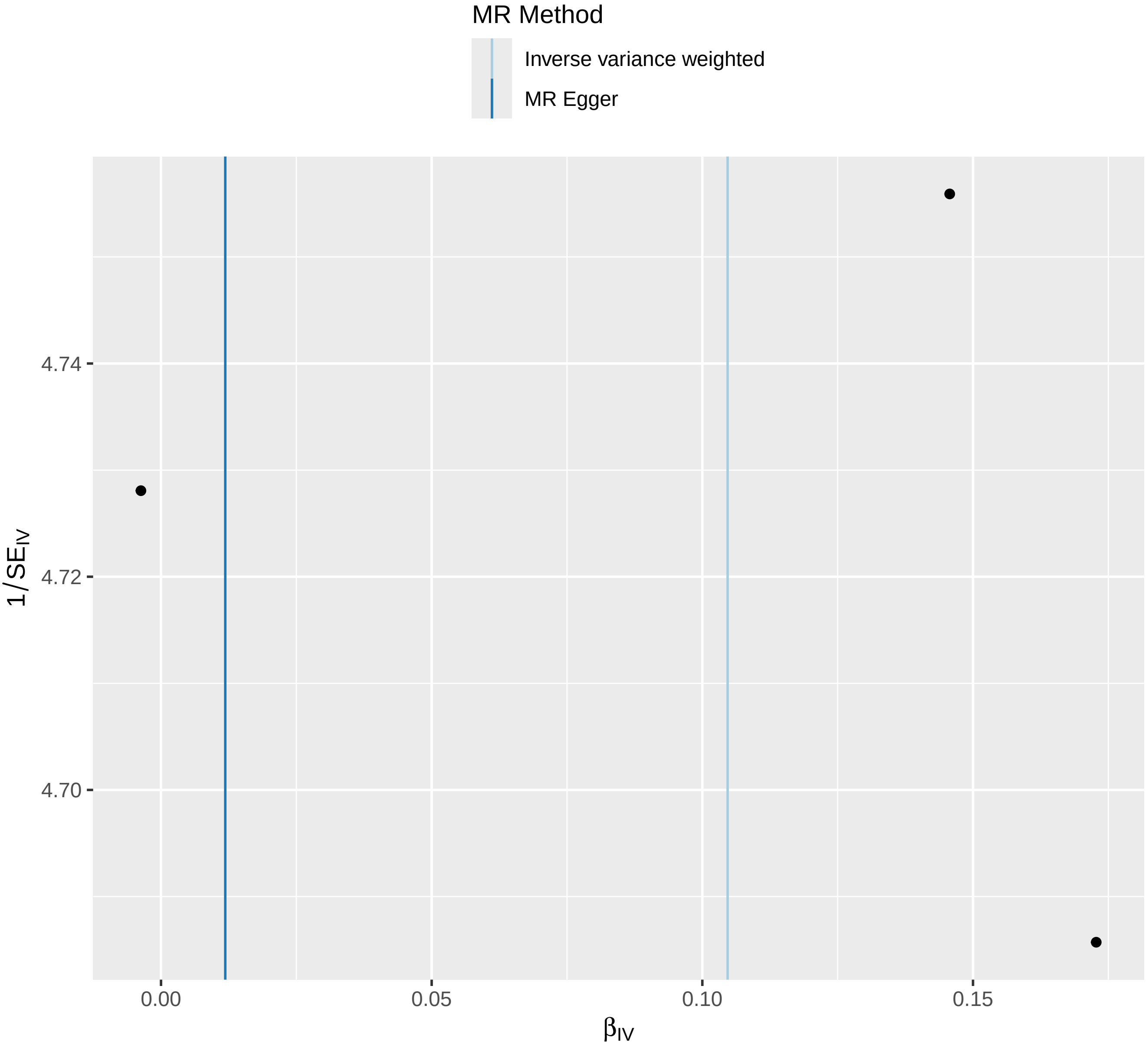

MR Method
Inverse variance weighted
MR Egger
4.74
4.72
4.70
1/SE$_{IV}$
0.00
0.05
0.10
0.15
β$_{IV}$

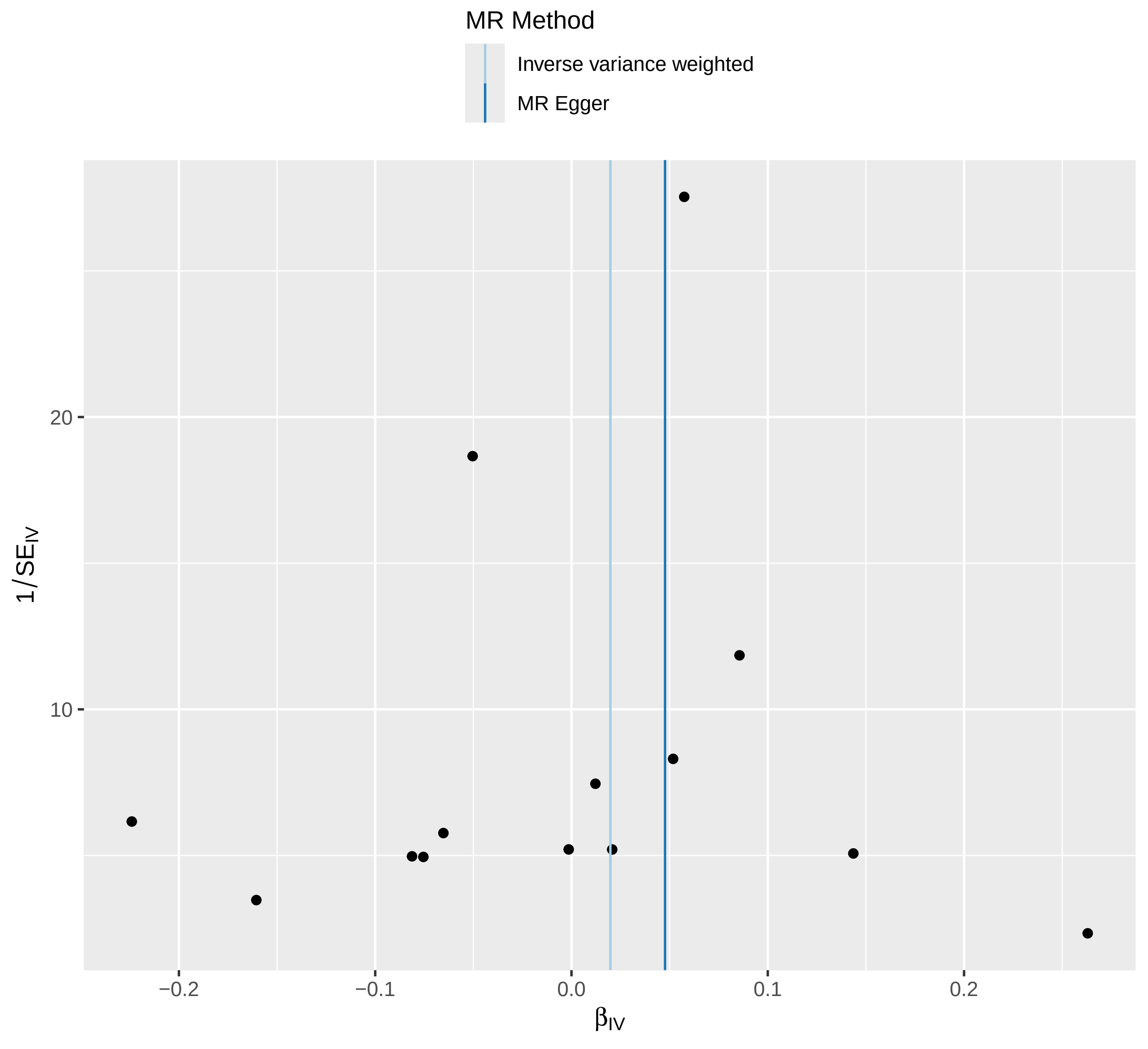

MR Method
Inverse variance weighted
MR Egger
20
10
1/SE_IV
−0.2
−0.1
0.0
0.1
0.2
β_IV

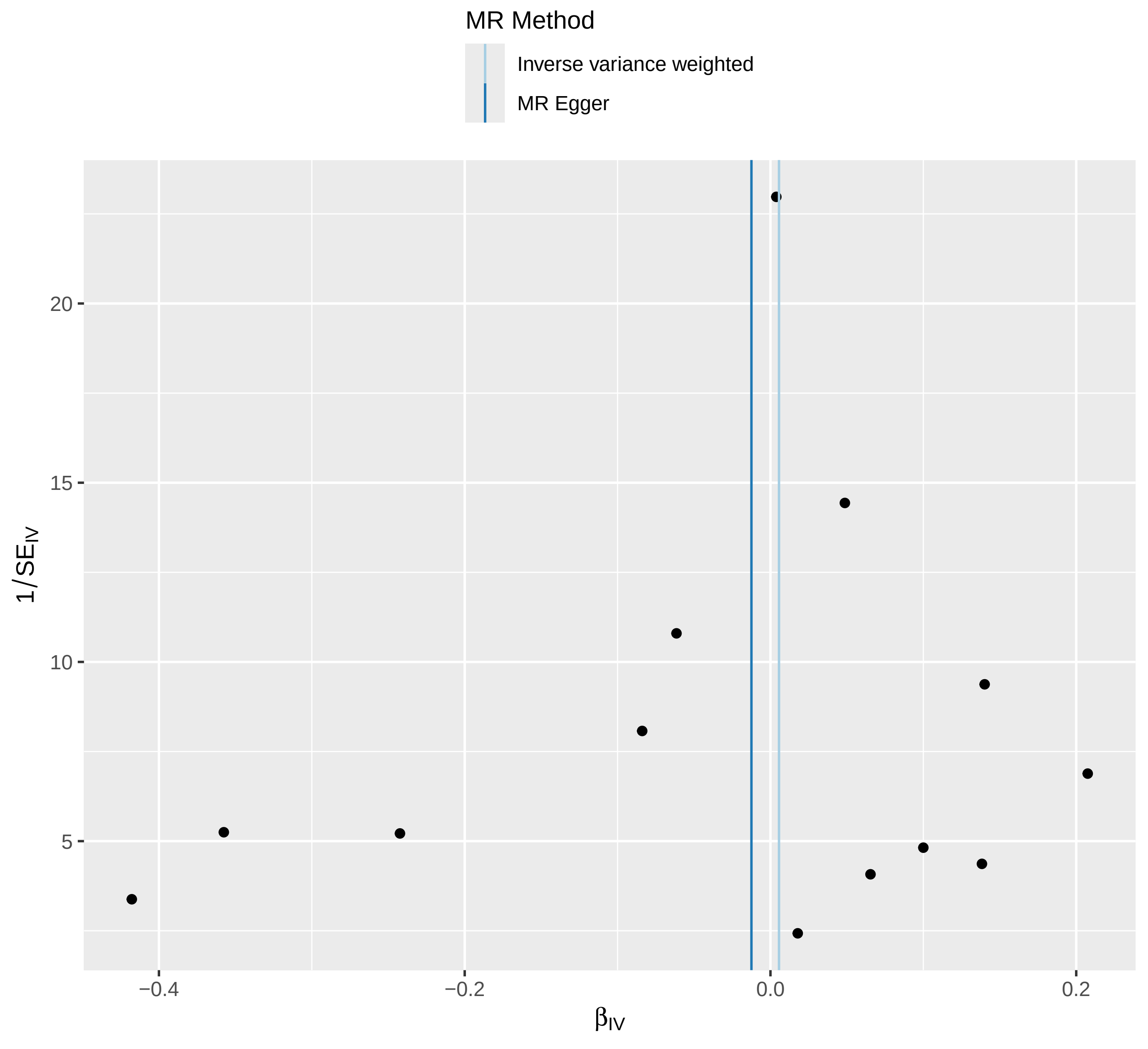
MR Method
Inverse variance weighted
MR Egger
1/SE$_{IV}$
β$_{IV}$
20
15
10
5
−0.4
−0.2
0.0
0.2

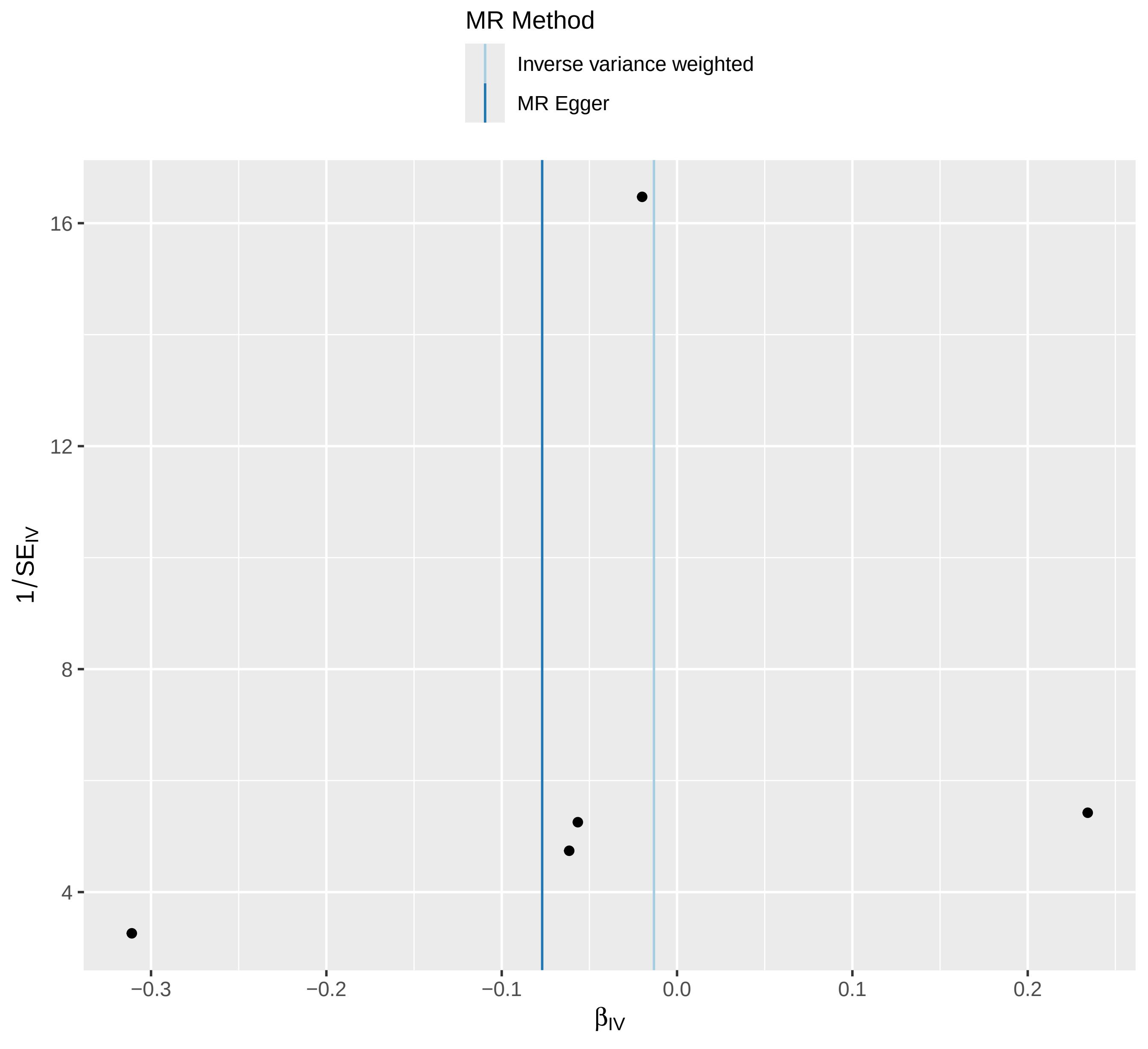
MR Method
Inverse variance weighted
MR Egger
16
12
8
4
1/$SE_{IV}$
−0.3
−0.2
−0.1
0.0
0.1
0.2
$\beta_{IV}$

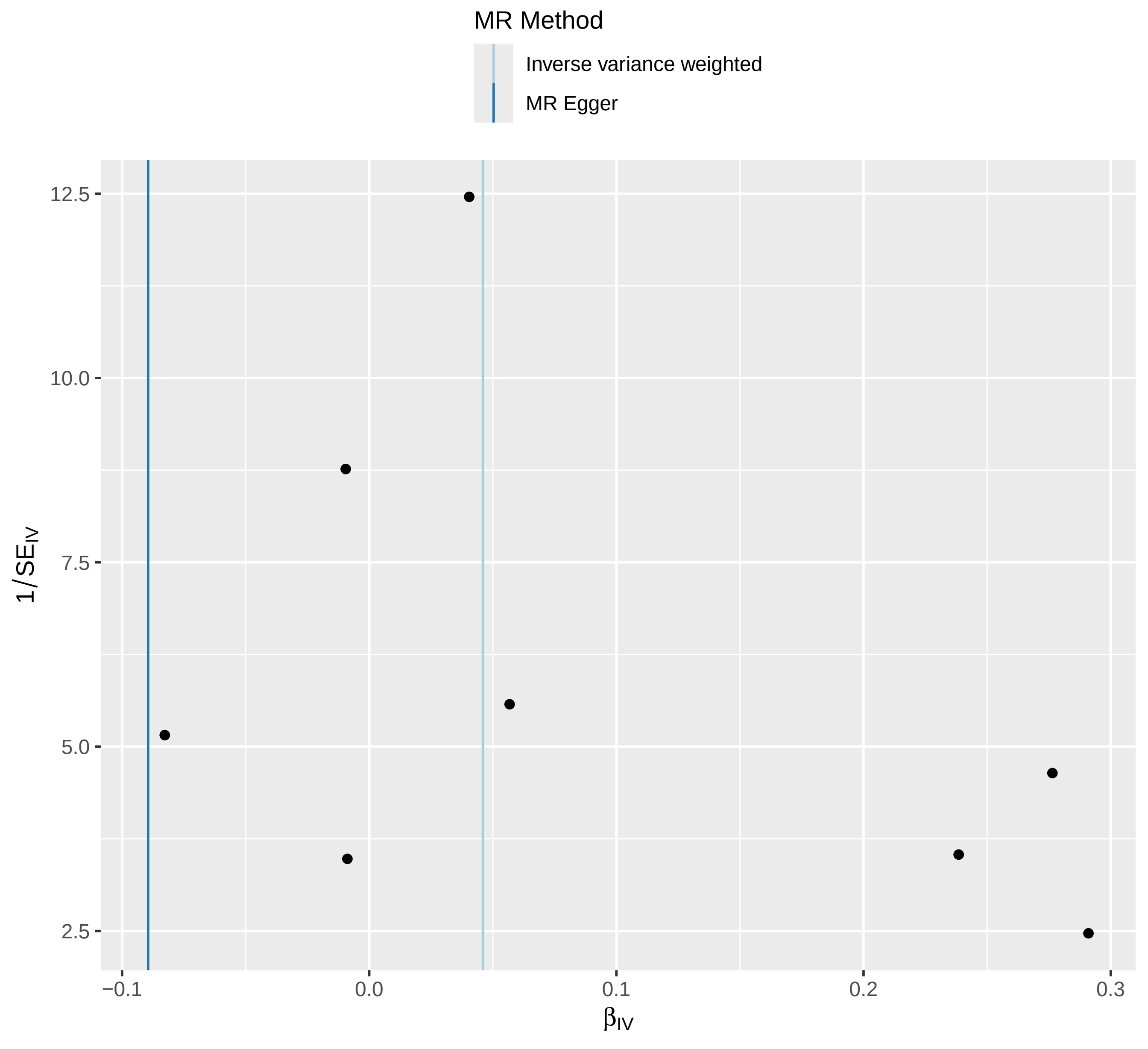
MR Method
Inverse variance weighted
MR Egger
12.5
10.0
7.5
5.0
2.5
1/SE_IV
−0.1
0.0
0.1
0.2
0.3
β_IV

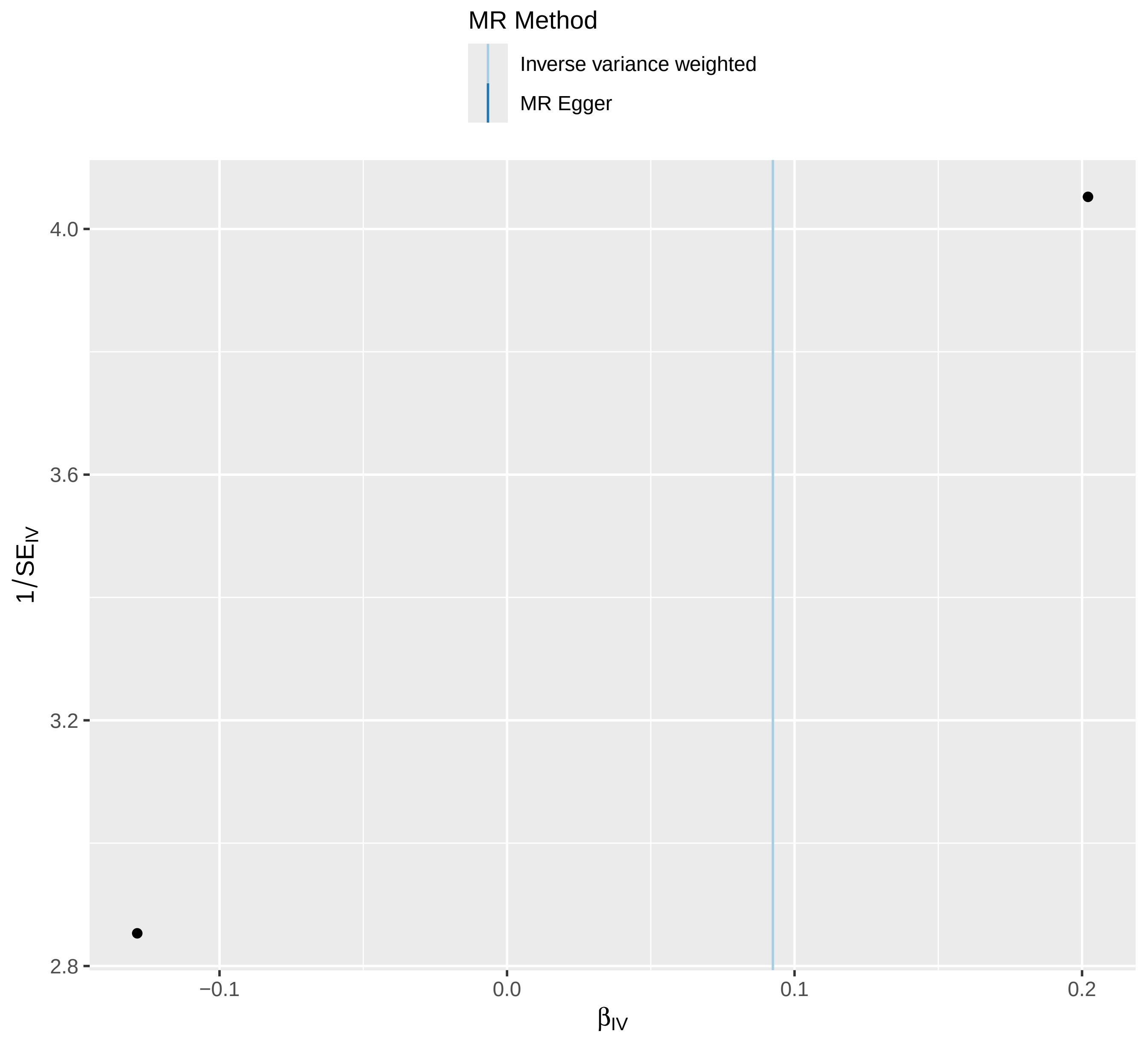
MR Method
Inverse variance weighted
MR Egger
4.0
3.6
3.2
2.8
1/SE_IV
−0.1
0.0
0.1
0.2
β_IV

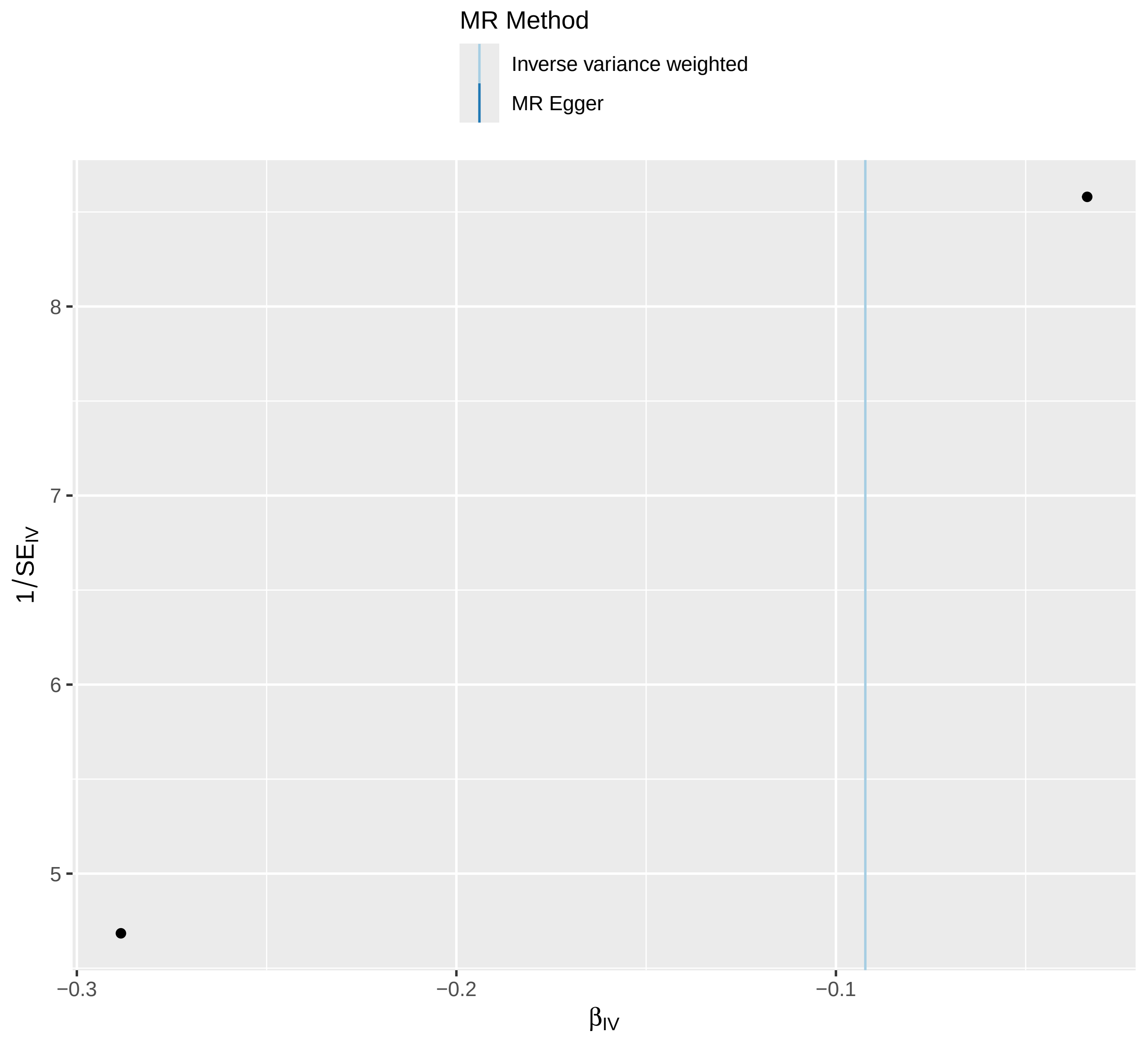
MR Method
Inverse variance weighted
MR Egger
1/SEIV
8
7
6
5
−0.3
−0.2
−0.1
βIV

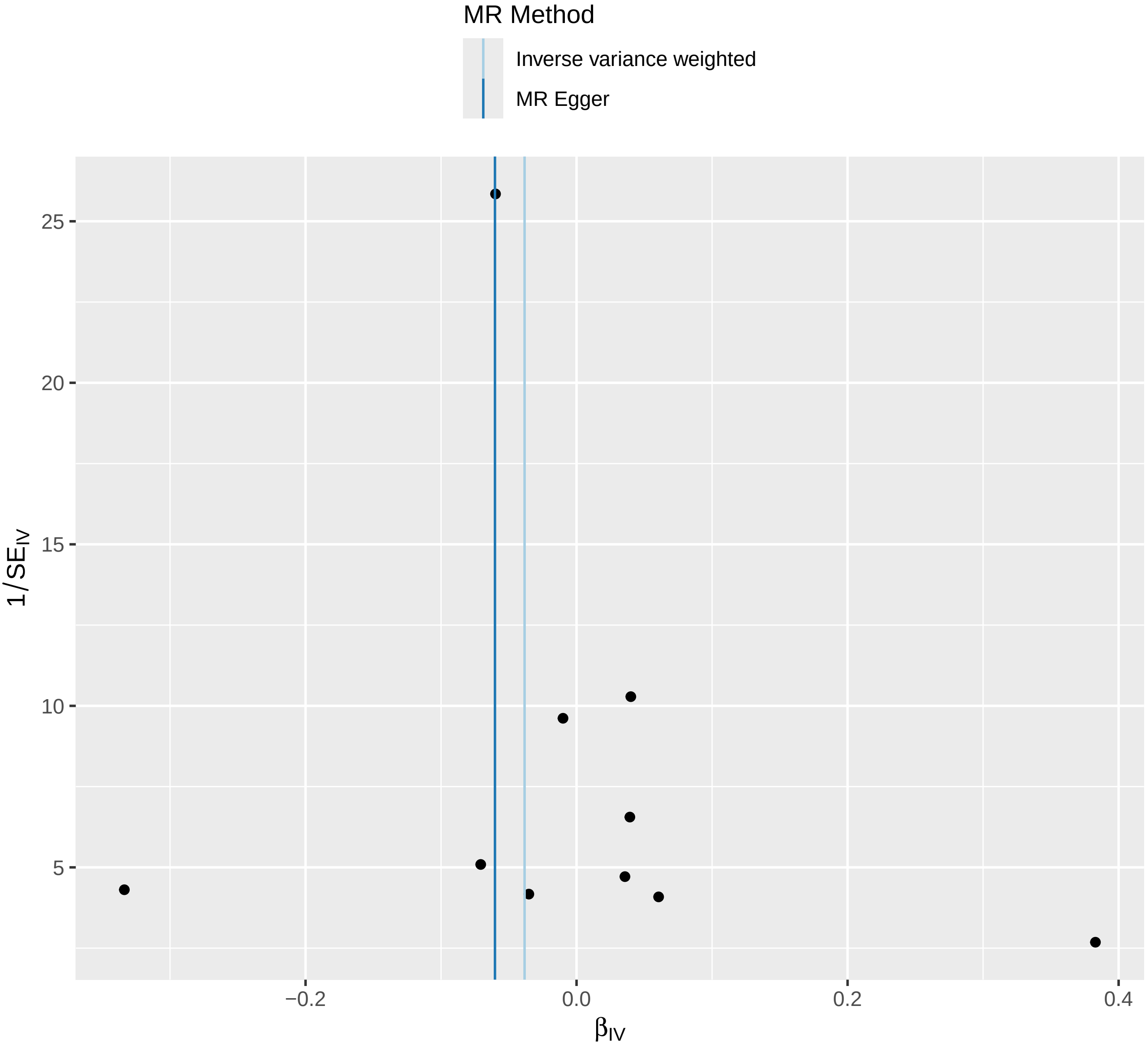
MR Method
Inverse variance weighted
MR Egger
25
20
15
10
5
−0.2
0.0
0.2
0.4
$1/SE_{IV}$
$\beta_{IV}$

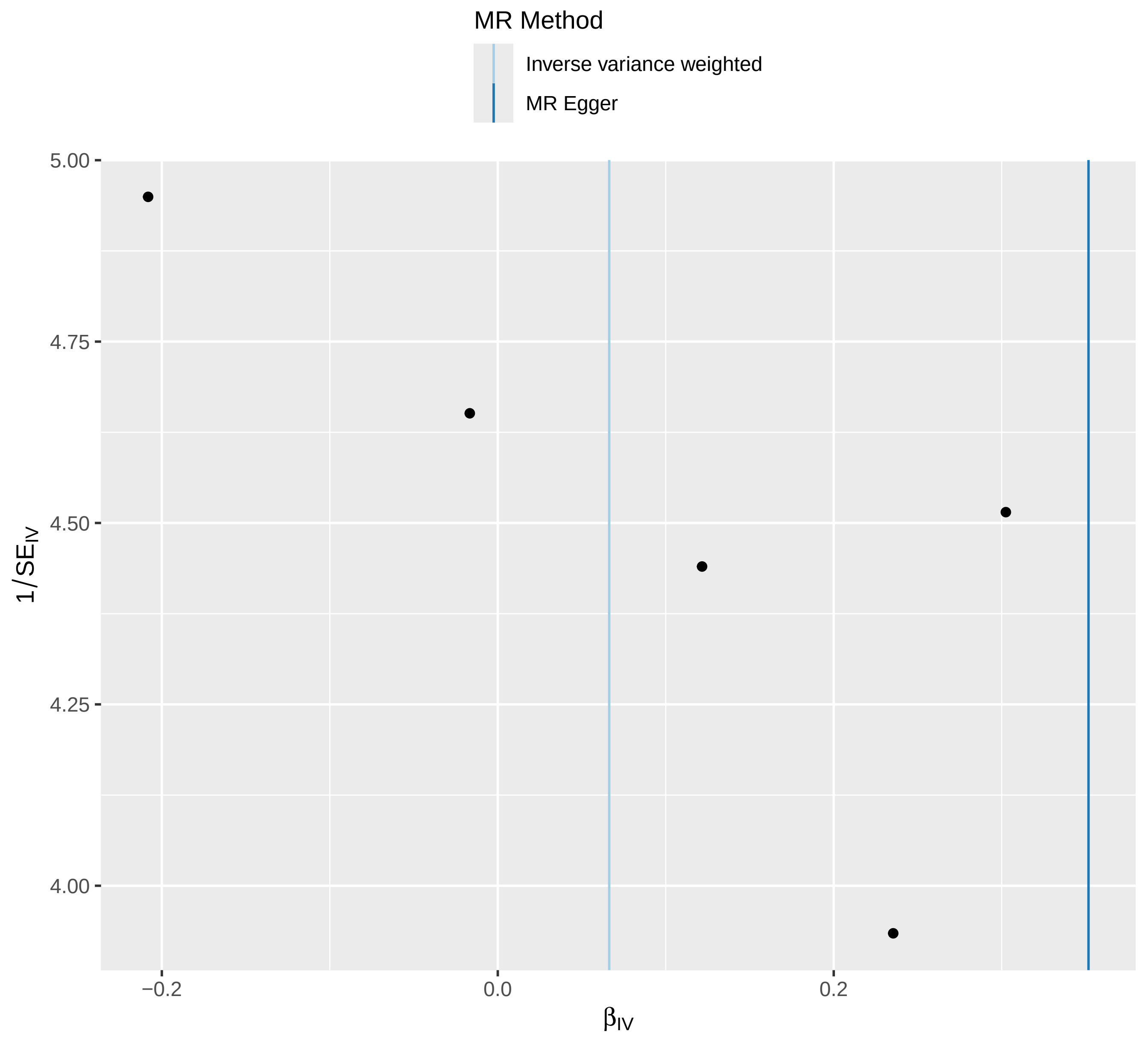

MR Method
Inverse variance weighted
MR Egger
5.00
4.75
4.50
4.25
4.00
1/SE$_{IV}$
−0.2
0.0
0.2
β$_{IV}$

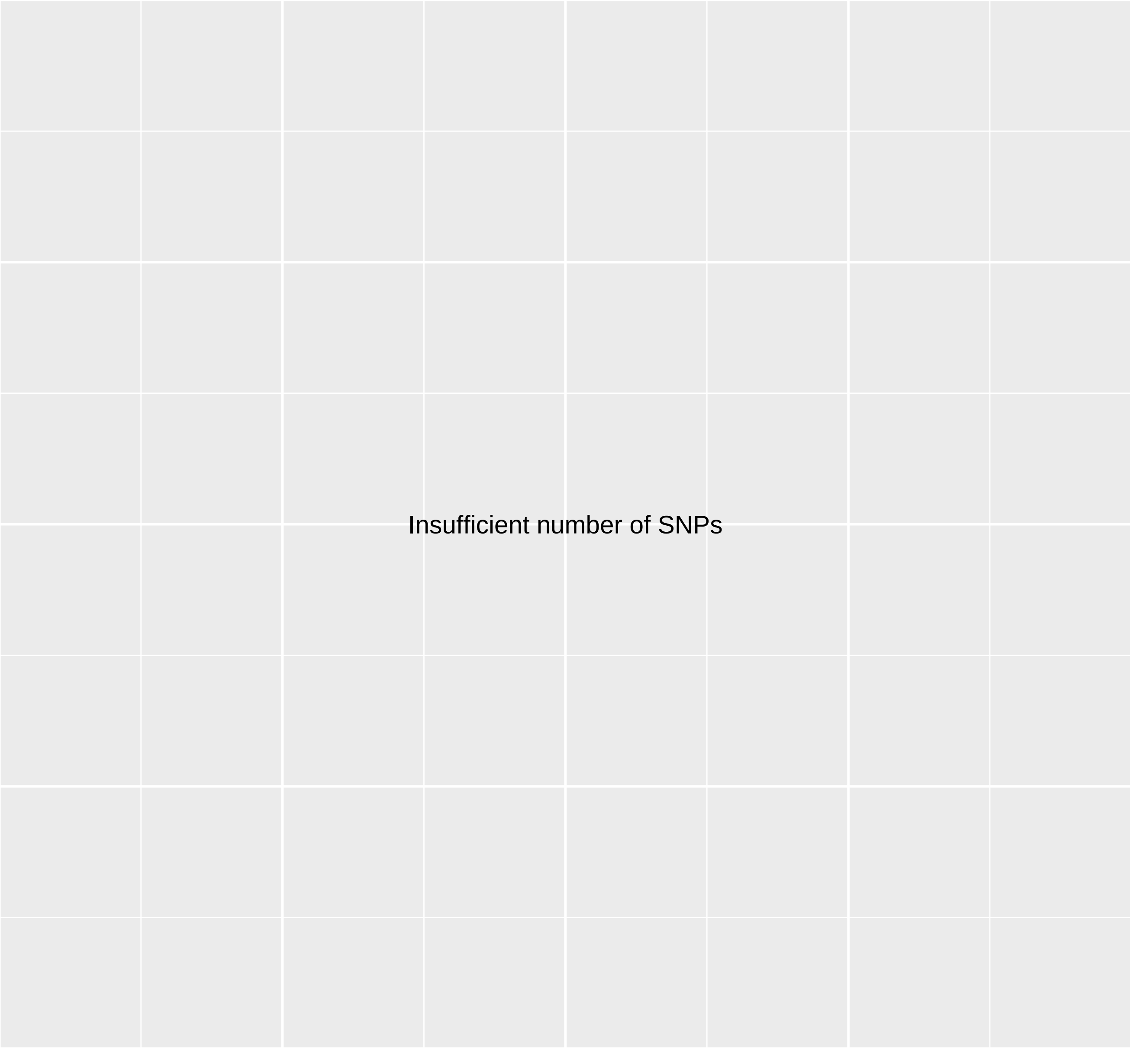
Insufficient number of SNPs

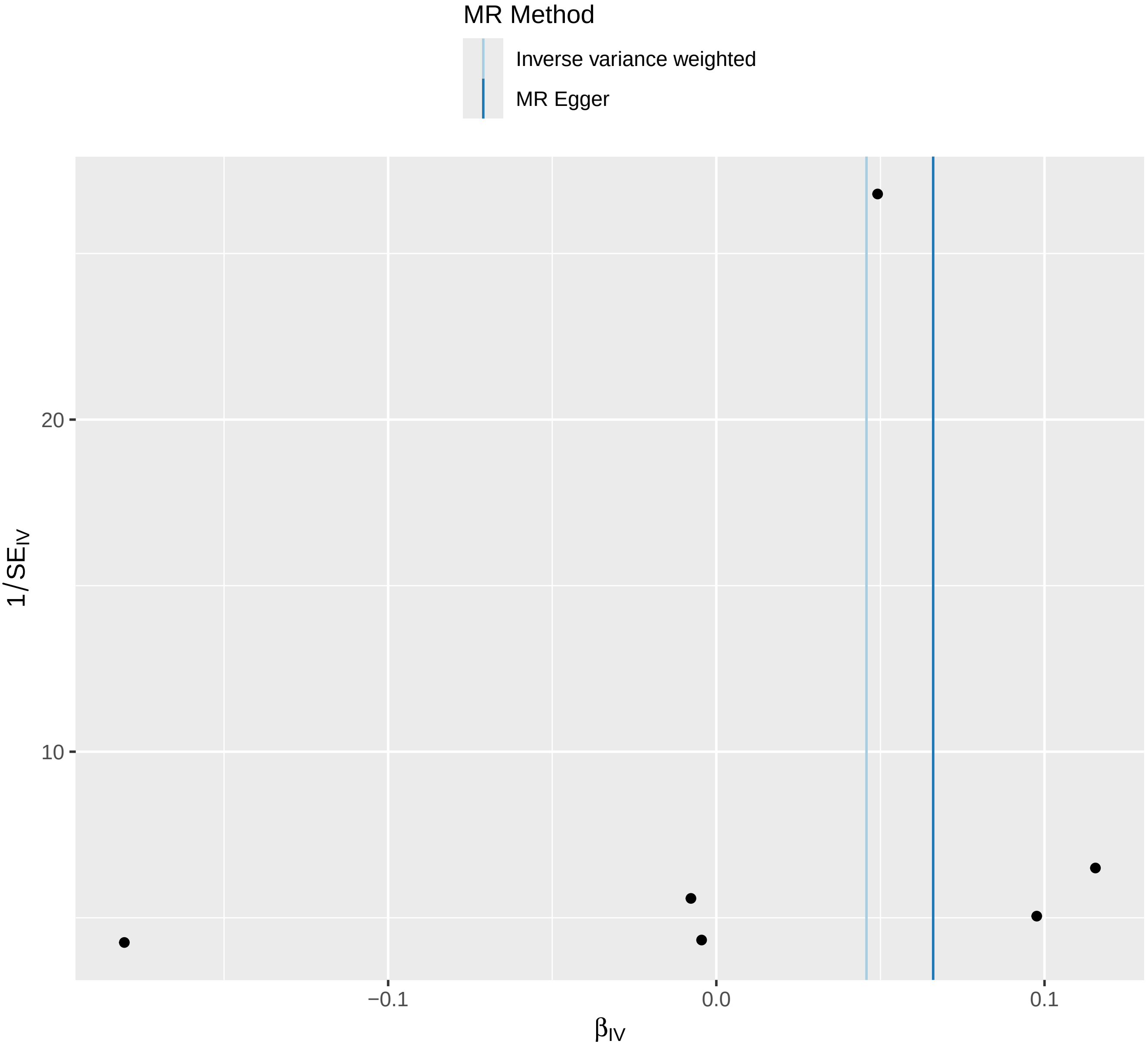
MR Method
Inverse variance weighted
MR Egger
20
10
1/SE$_{IV}$
−0.1
0.0
0.1
β$_{IV}$

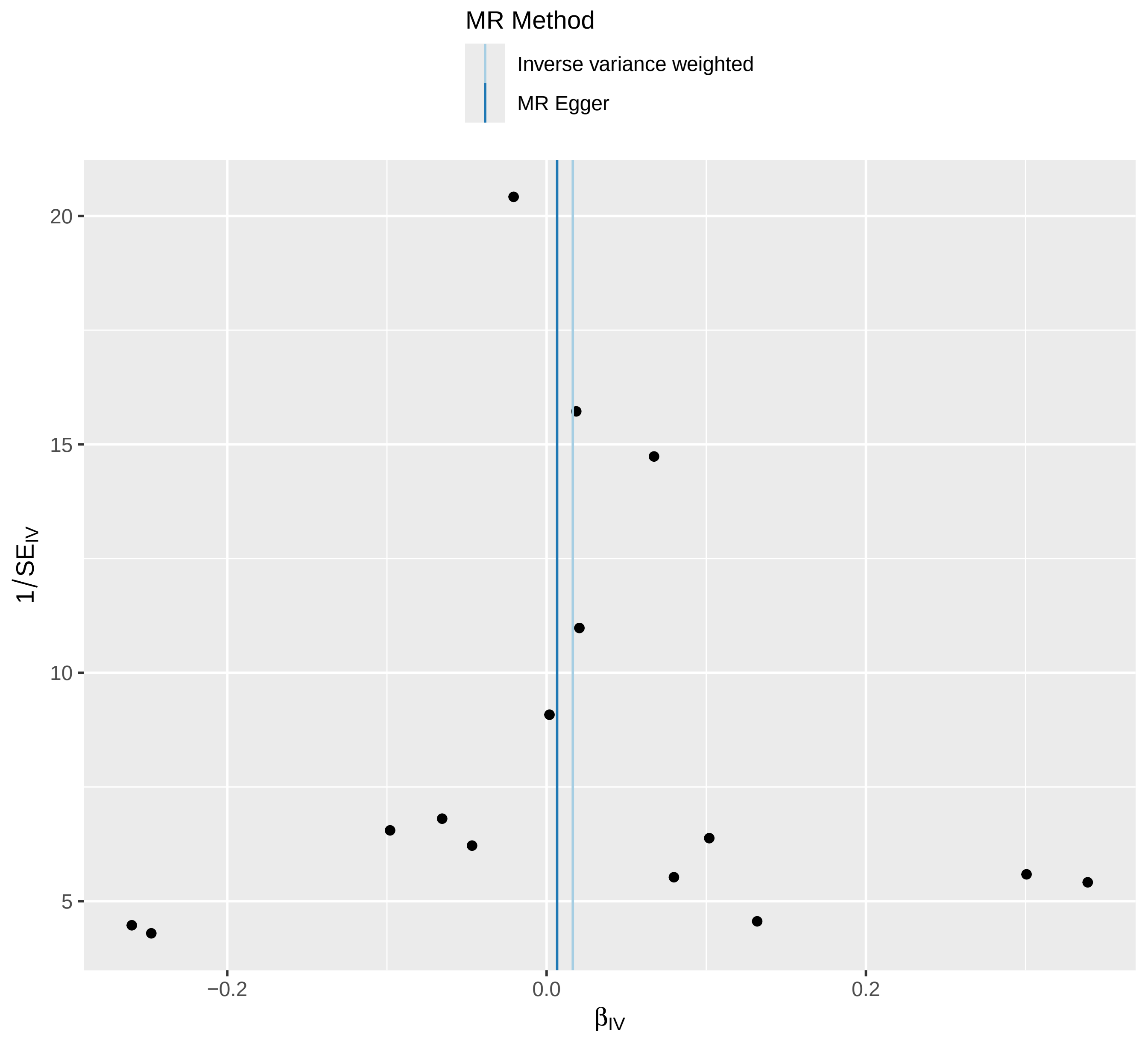

MR Method
Inverse variance weighted
MR Egger
20
15
10
5
$1/SE_{IV}$
−0.2
0.0
0.2
$\beta_{IV}$

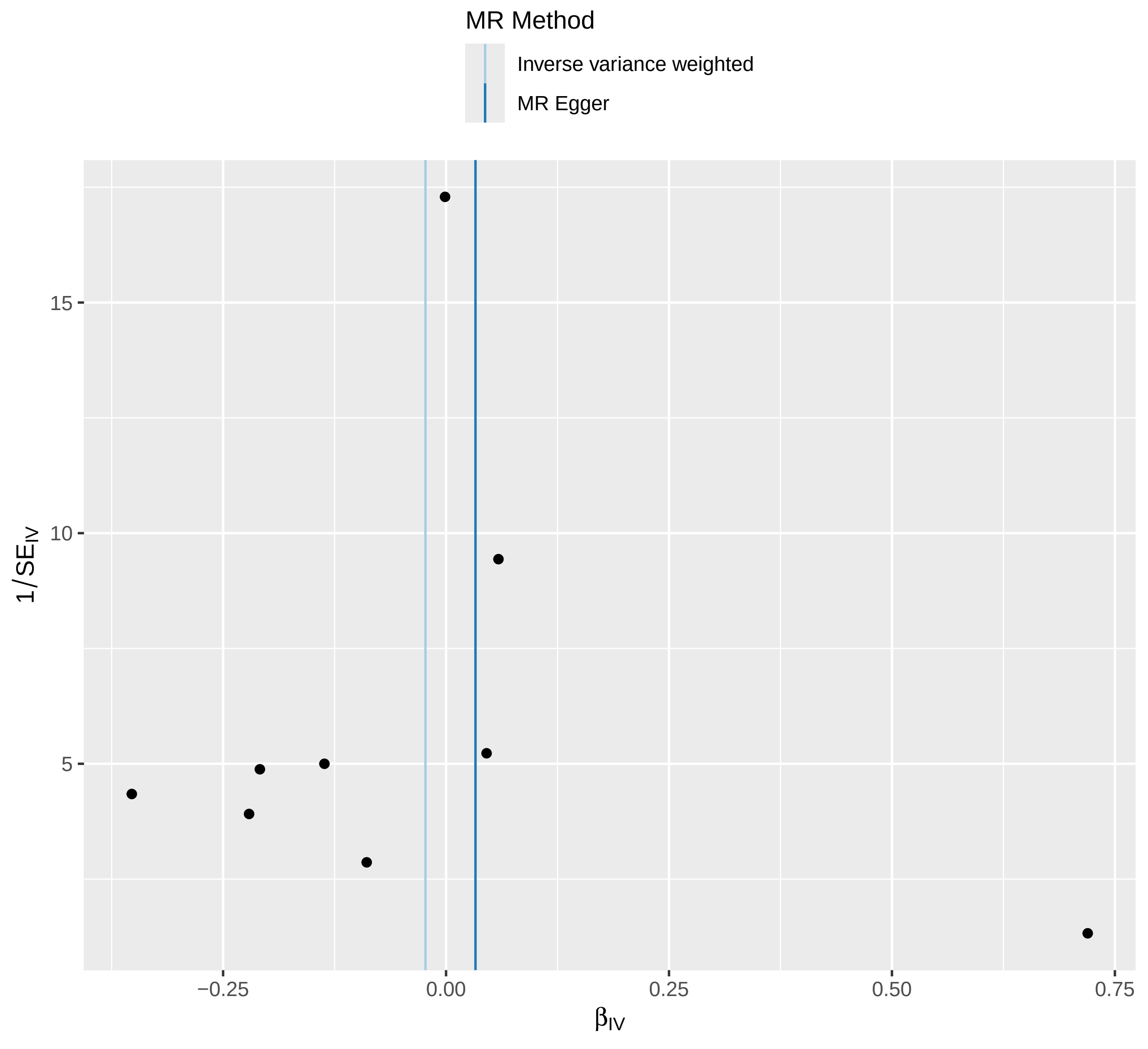
MR Method
Inverse variance weighted
MR Egger
15
10
5
1/SE$_{IV}$
−0.25
0.00
0.25
0.50
0.75
β$_{IV}$

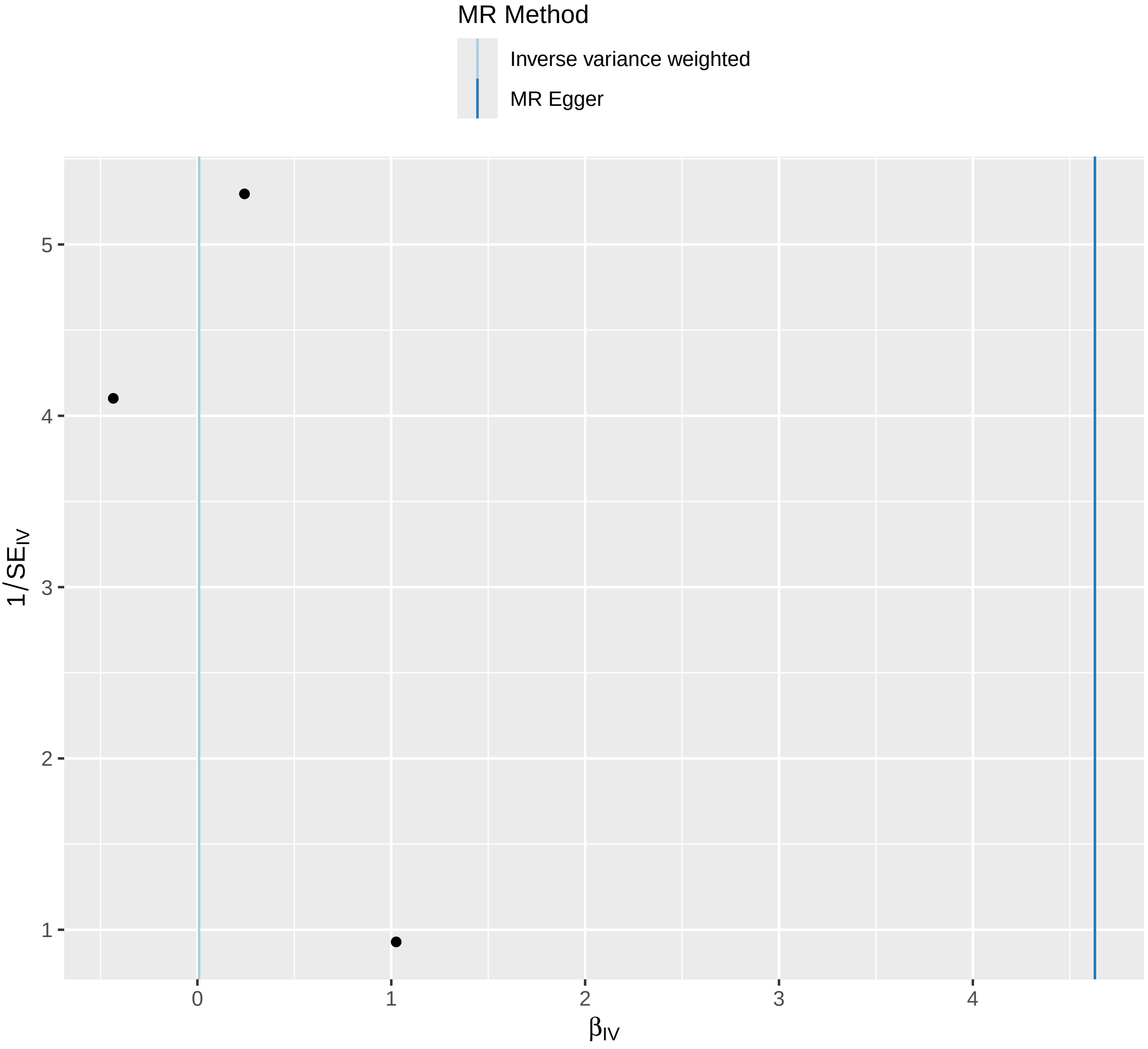
MR Method
Inverse variance weighted
MR Egger
5
4
3
2
1
0
1
2
3
4
$1/SE_{IV}$
$\beta_{IV}$

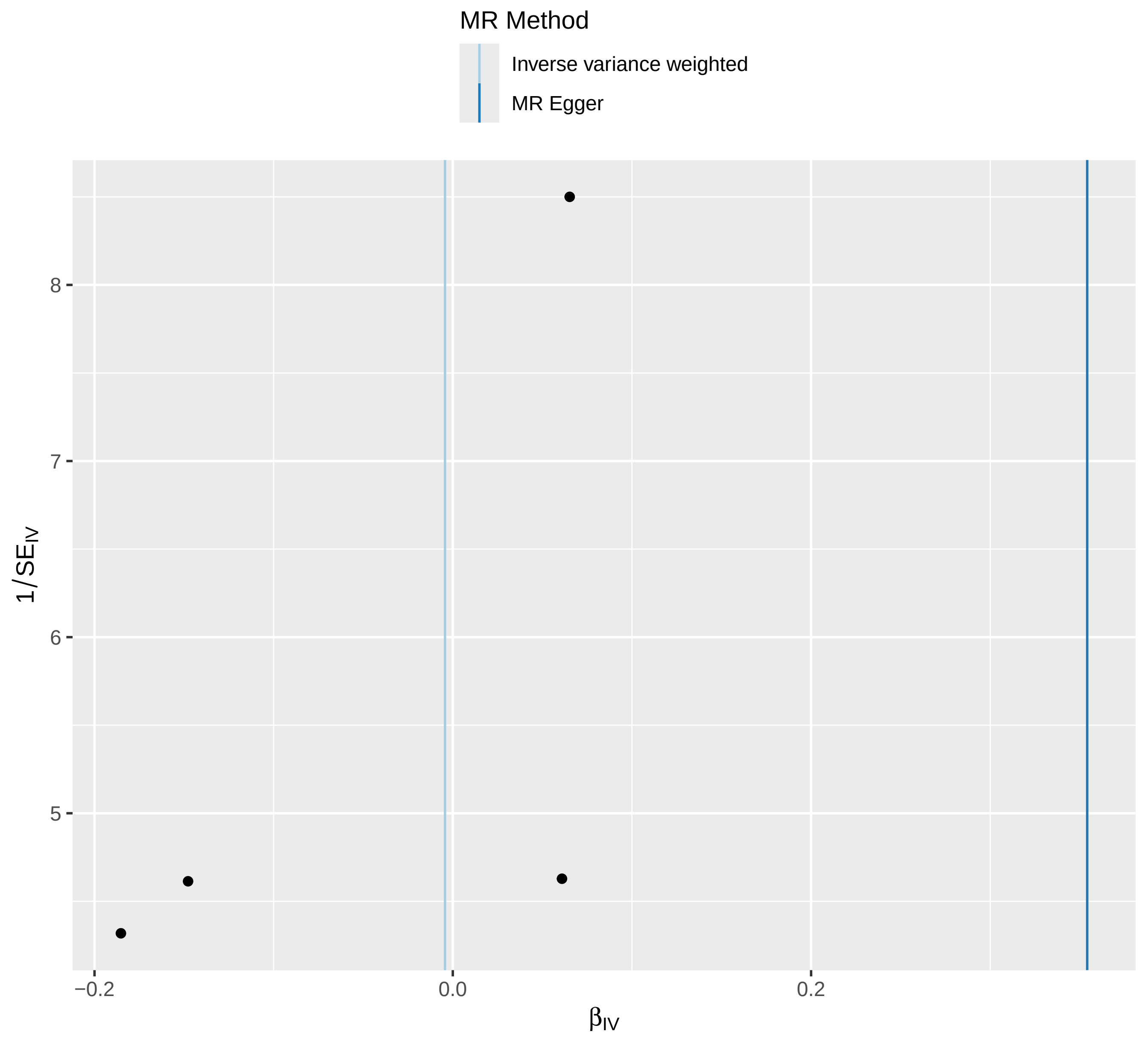
MR Method
Inverse variance weighted
MR Egger
8
7
6
5
1/SE_IV
−0.2
0.0
0.2
β_IV

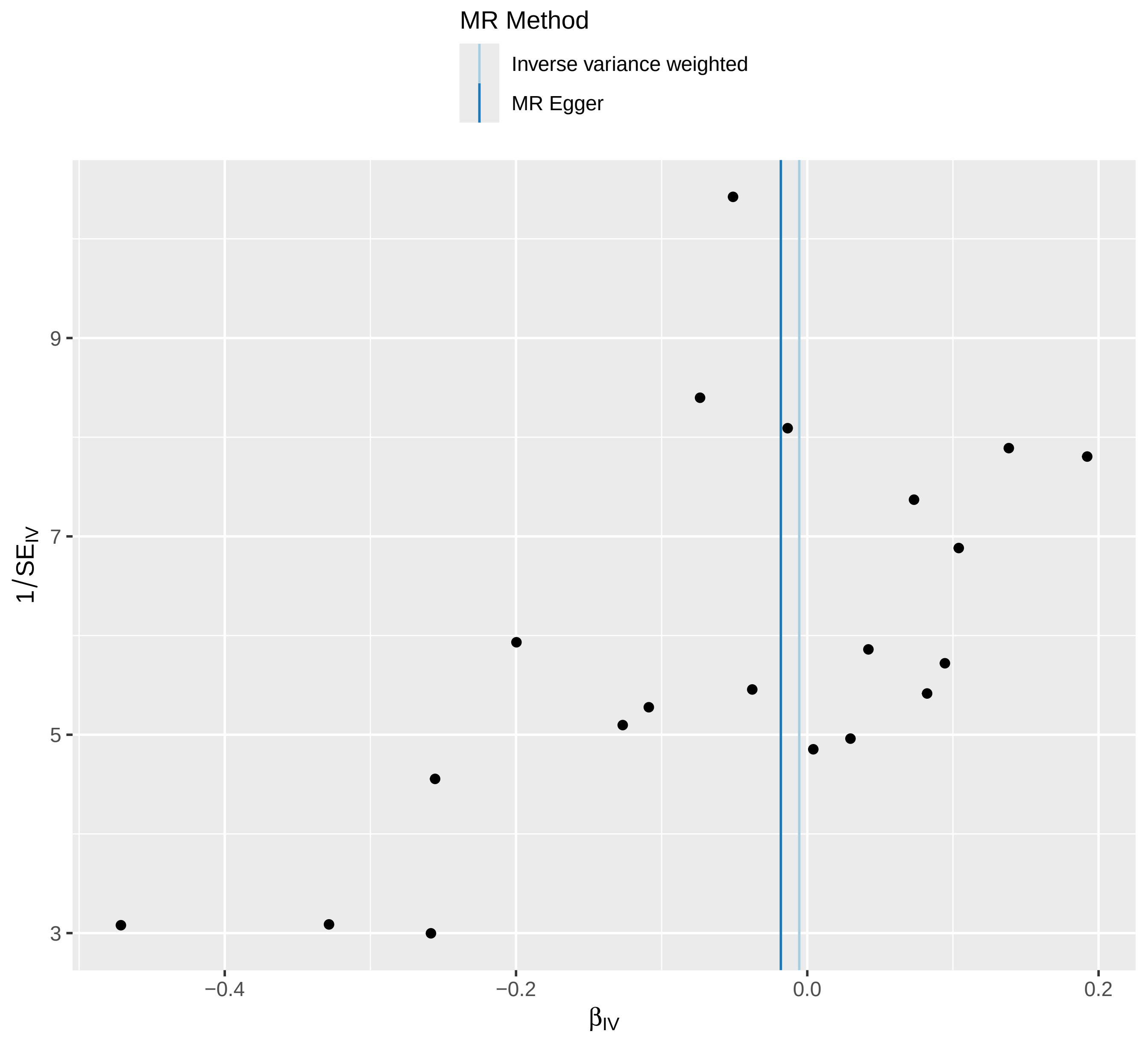
MR Method
Inverse variance weighted
MR Egger
9
7
5
3
1/SE$_{IV}$
−0.4
−0.2
0.0
0.2
β$_{IV}$

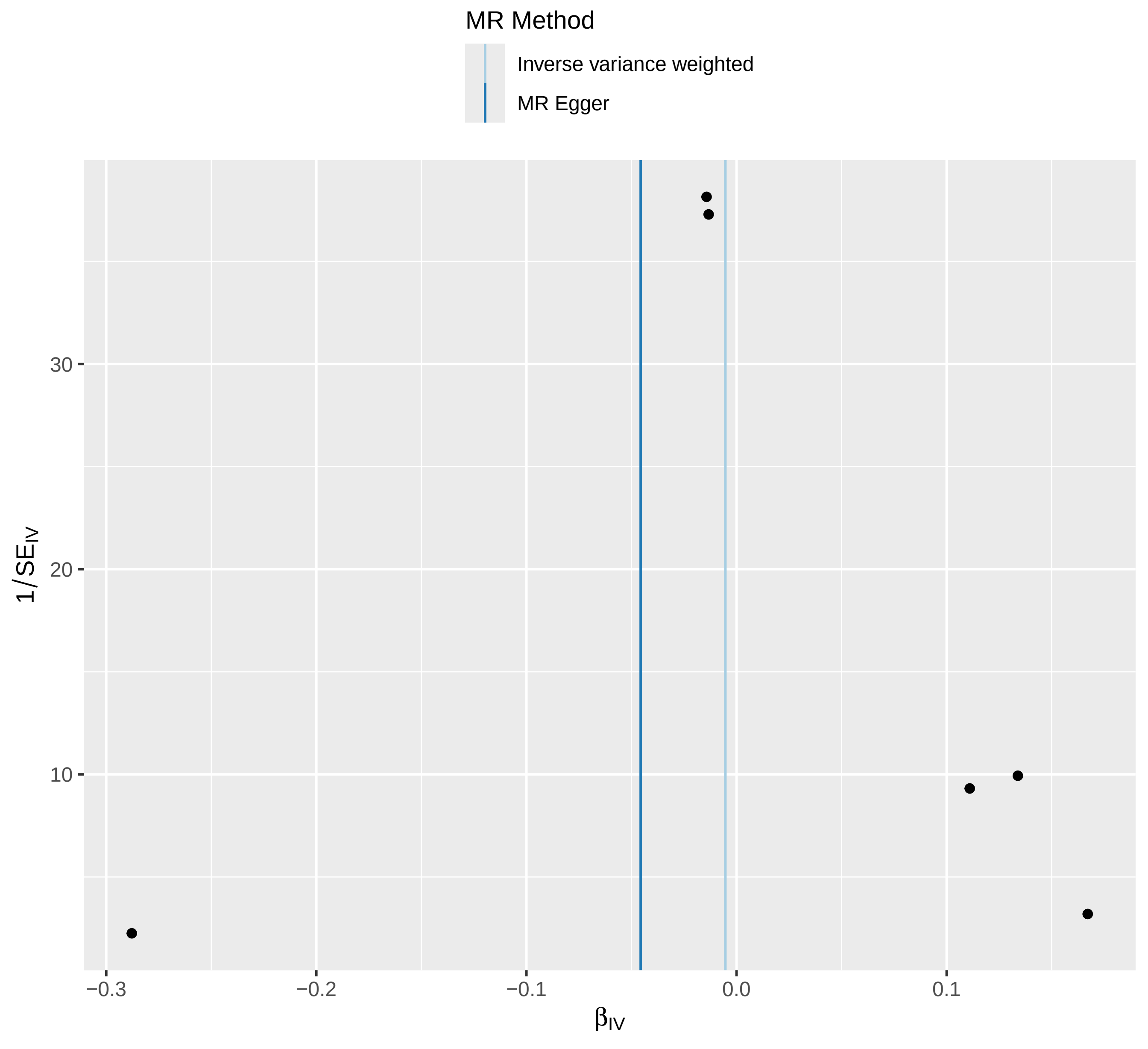

MR Method
Inverse variance weighted
MR Egger
30
20
10
−0.3
−0.2
−0.1
0.0
0.1
$1/SE_{IV}$
$\beta_{IV}$

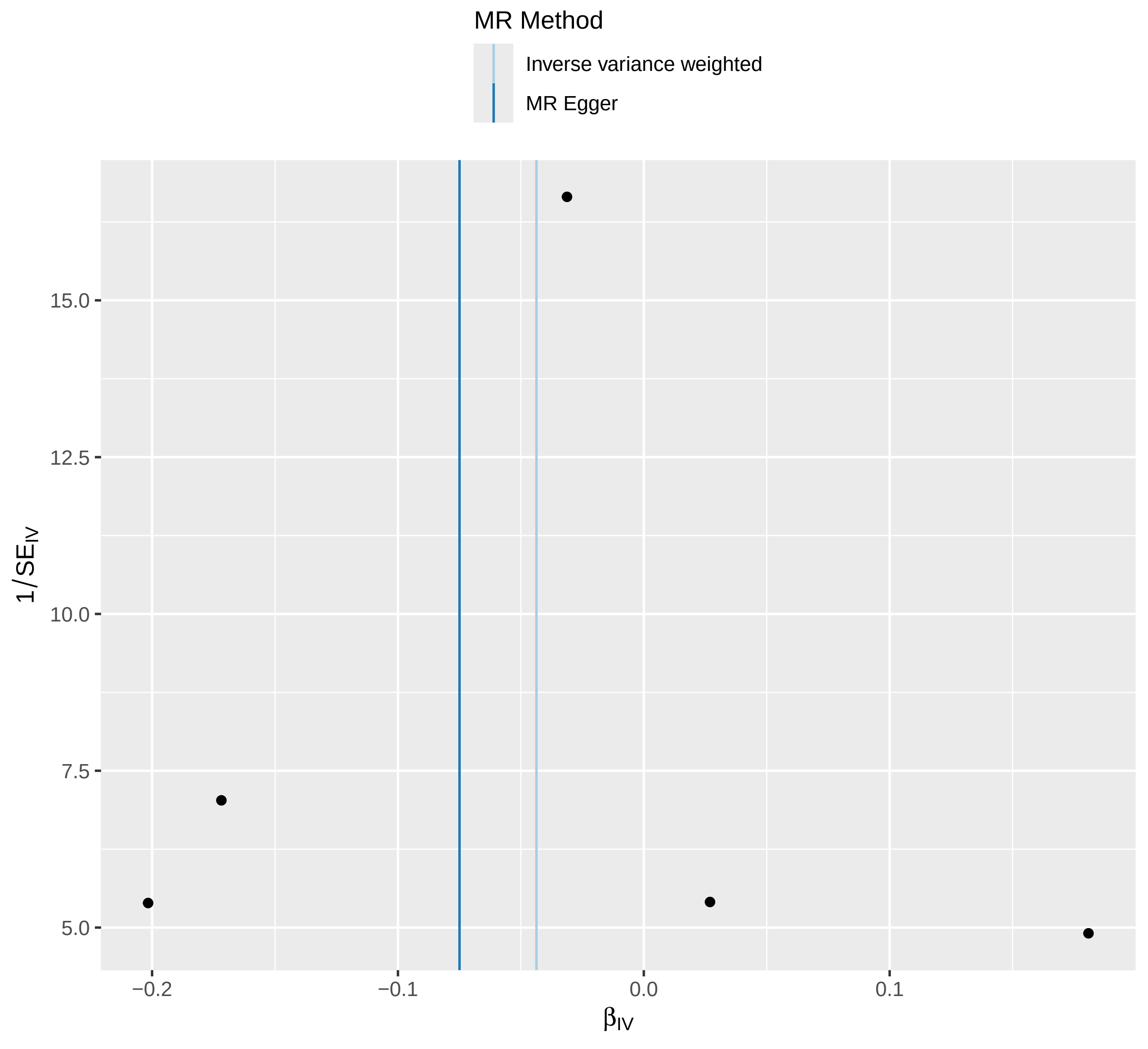

MR Method
Inverse variance weighted
MR Egger
15.0
12.5
10.0
7.5
5.0
1/SE$_{IV}$
−0.2
−0.1
0.0
0.1
β$_{IV}$

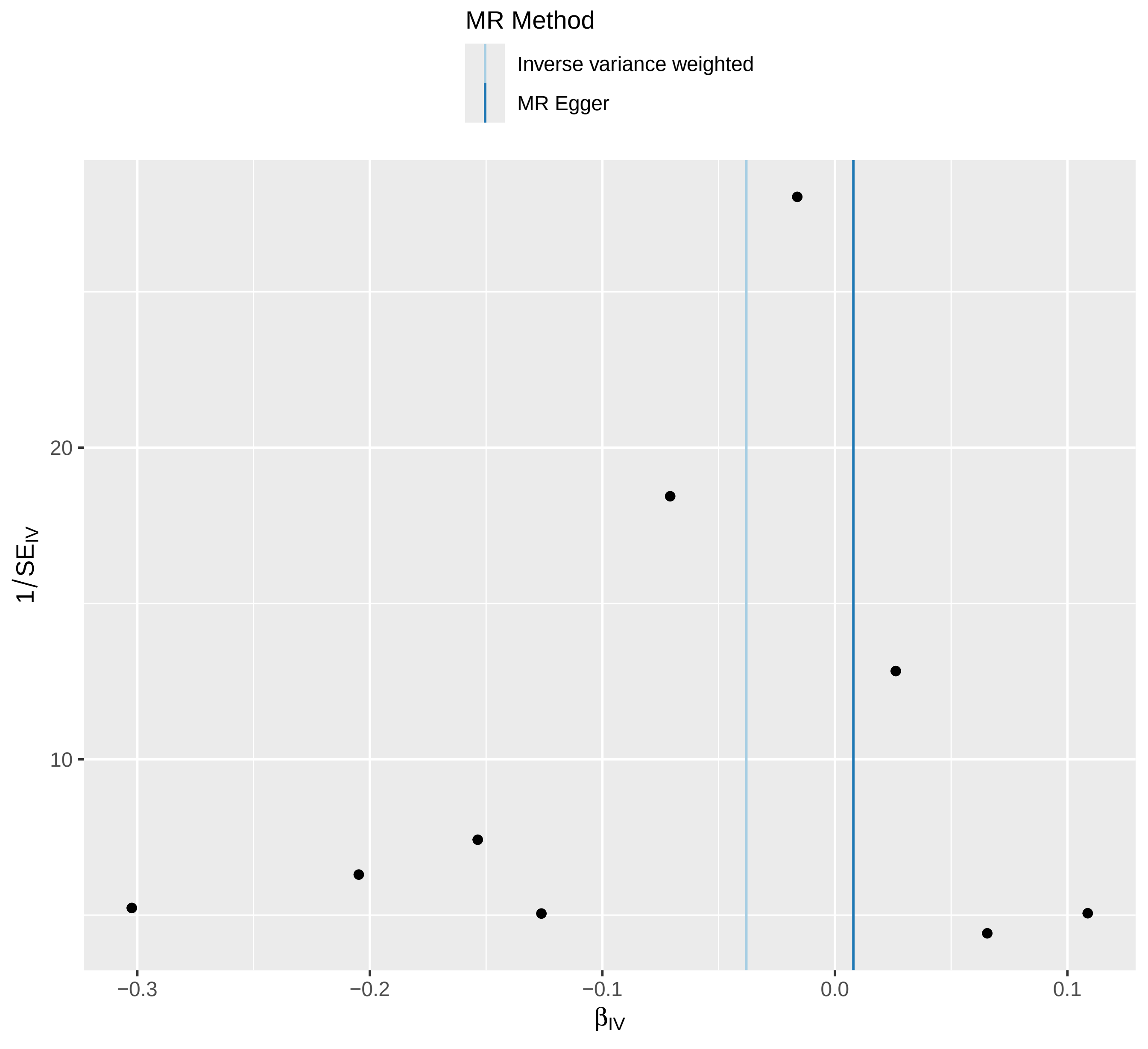

MR Method
Inverse variance weighted
MR Egger
20
10
1/SE_IV
−0.3
−0.2
−0.1
0.0
0.1
β_IV

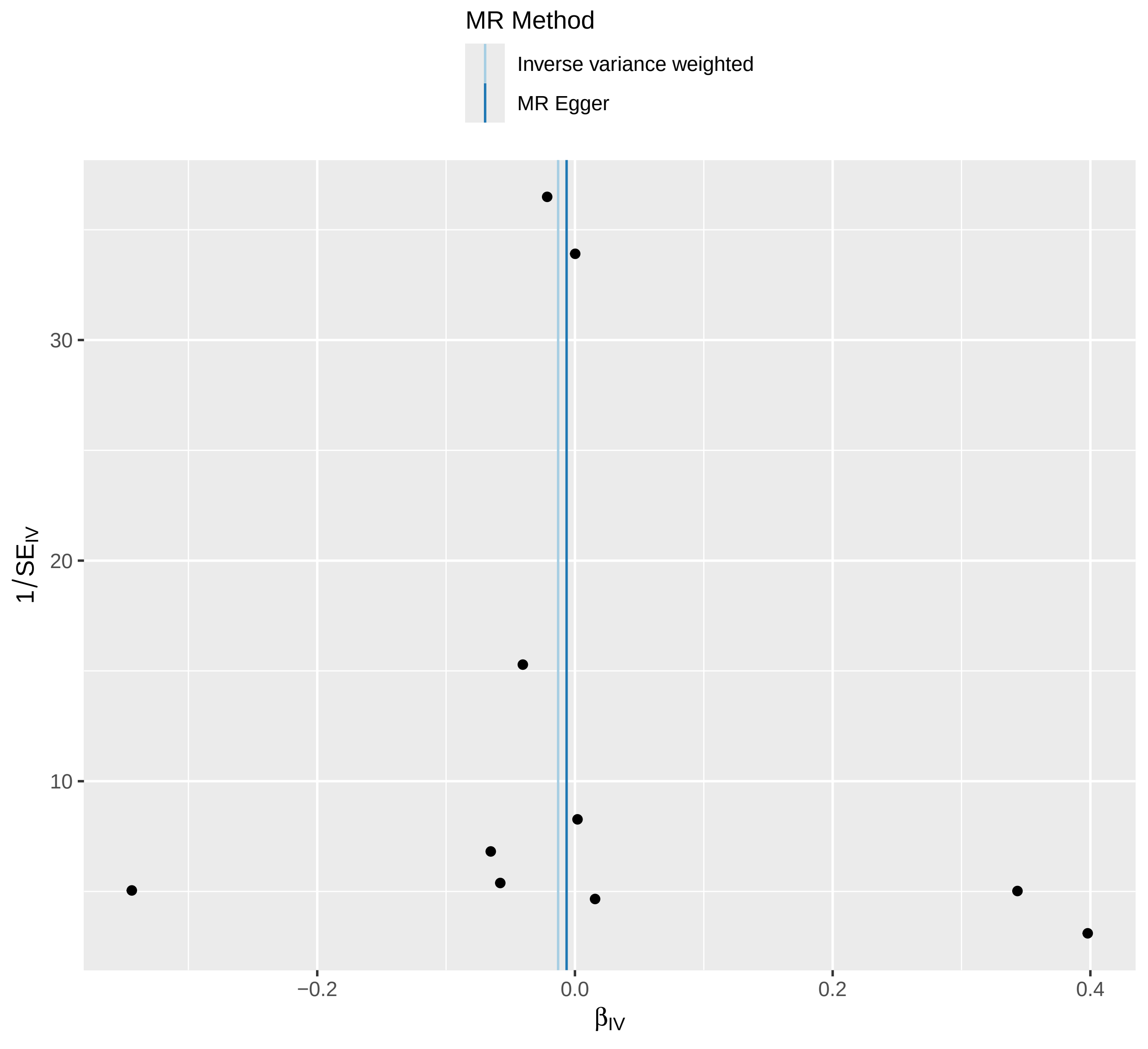

MR Method
Inverse variance weighted
MR Egger
1/SE$_{IV}$
30
20
10
−0.2
0.0
0.2
0.4
β$_{IV}$

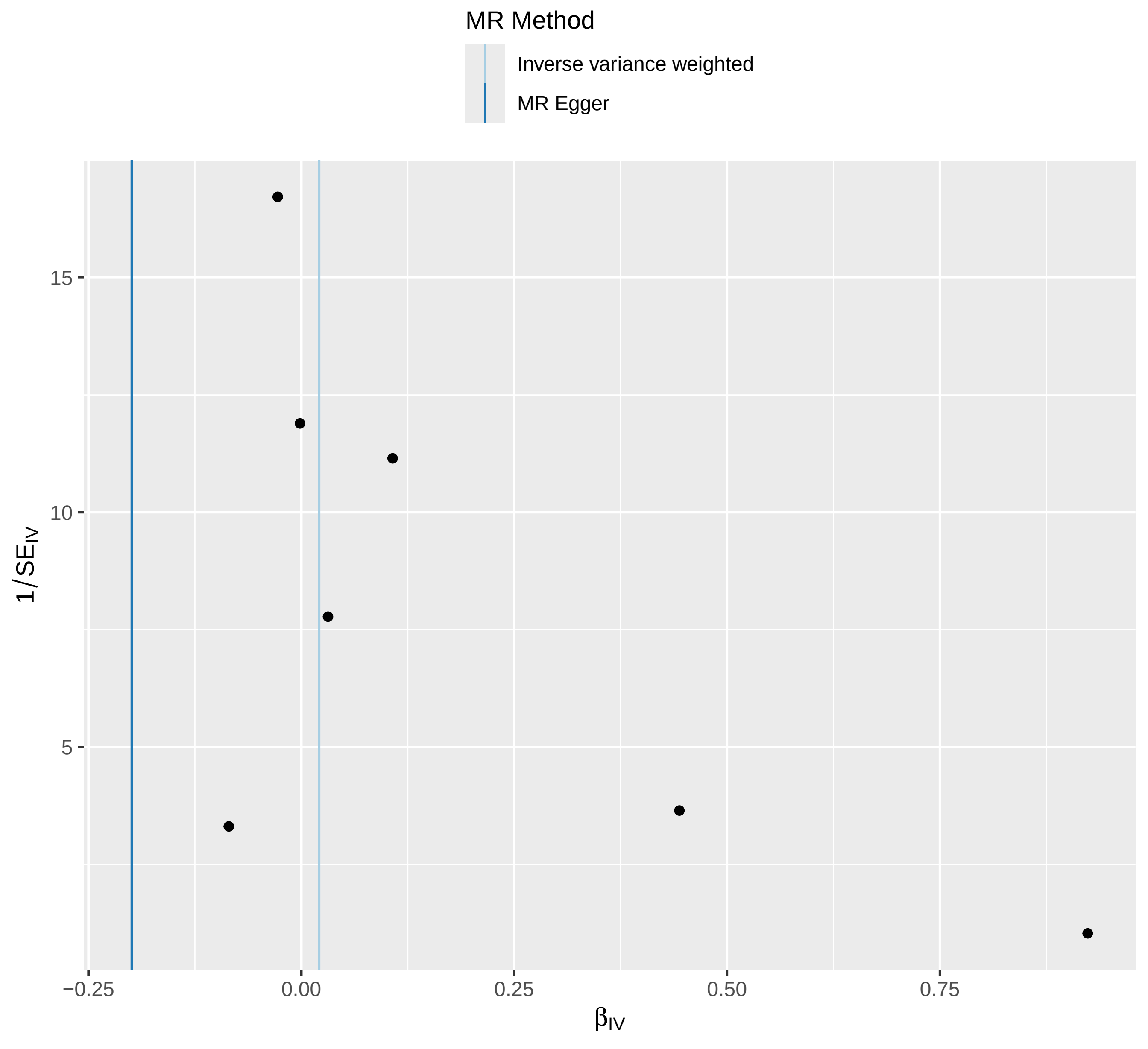
MR Method
Inverse variance weighted
MR Egger
1/SE$_{IV}$
15
10
5
−0.25
0.00
0.25
0.50
0.75
β$_{IV}$

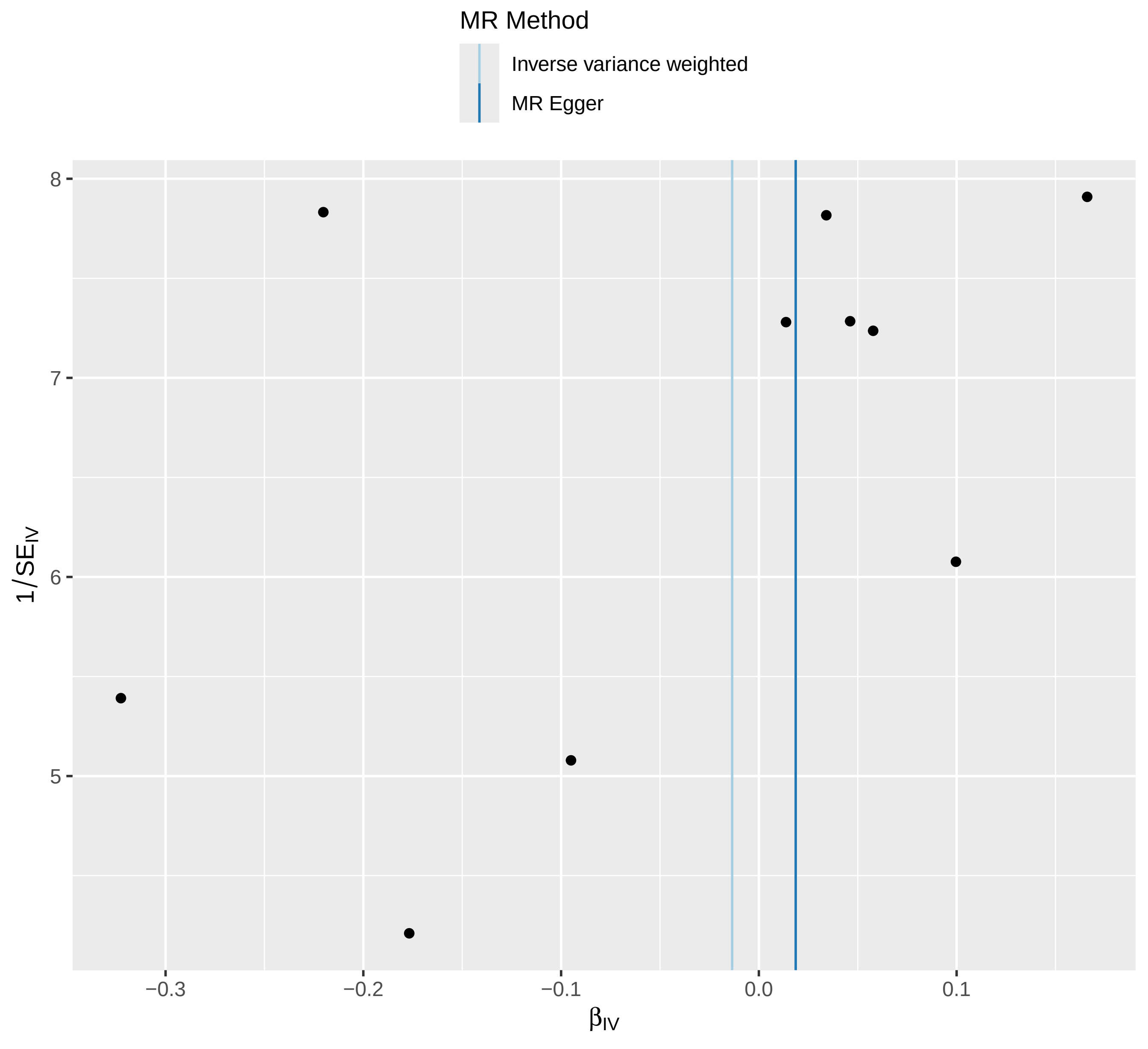

MR Method
Inverse variance weighted
MR Egger
8
7
6
5
1/SE$_{IV}$
−0.3
−0.2
−0.1
0.0
0.1
β$_{IV}$

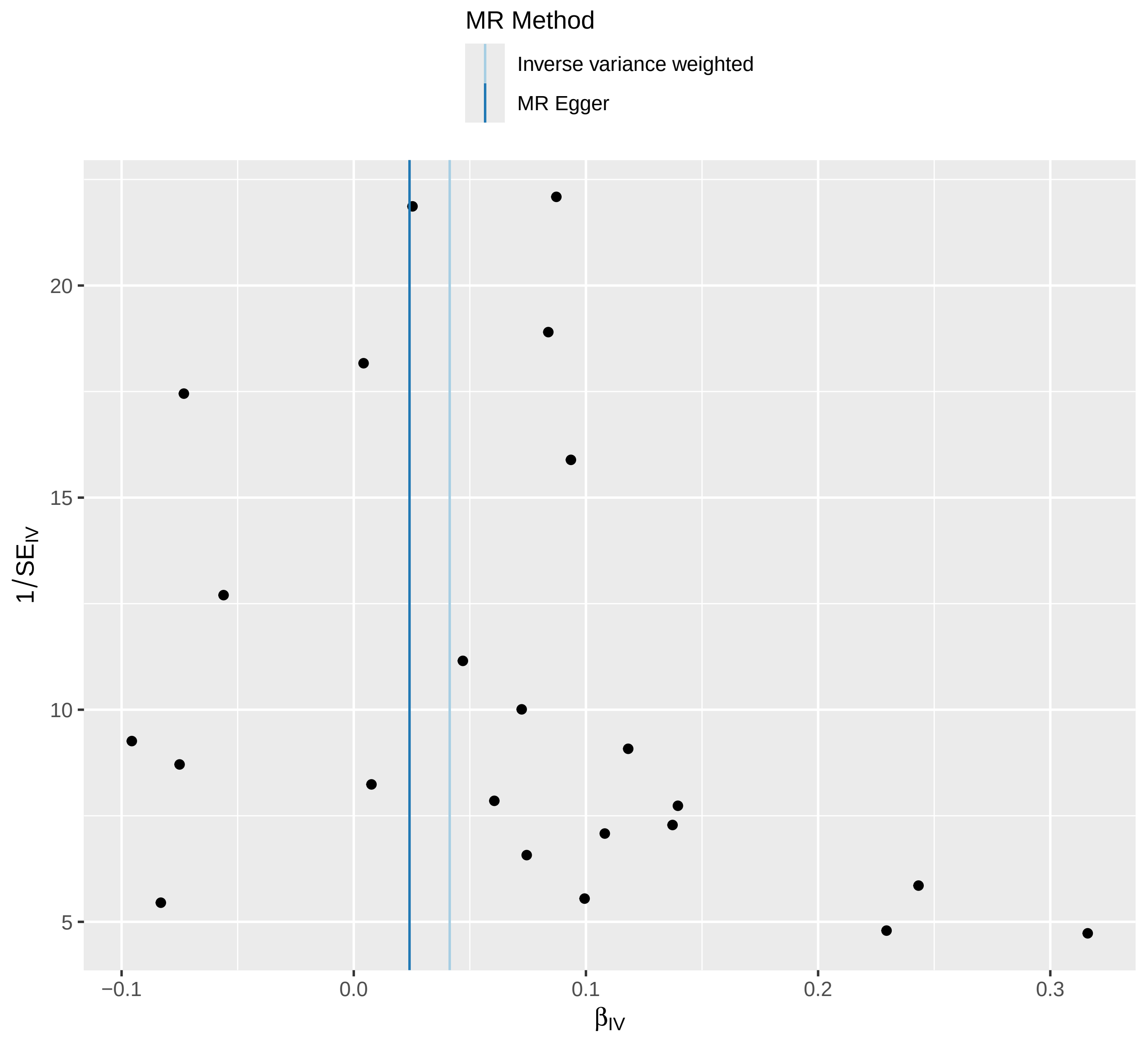
MR Method
Inverse variance weighted
MR Egger
20
15
10
5
1/SE$_{IV}$
−0.1
0.0
0.1
0.2
0.3
β$_{IV}$

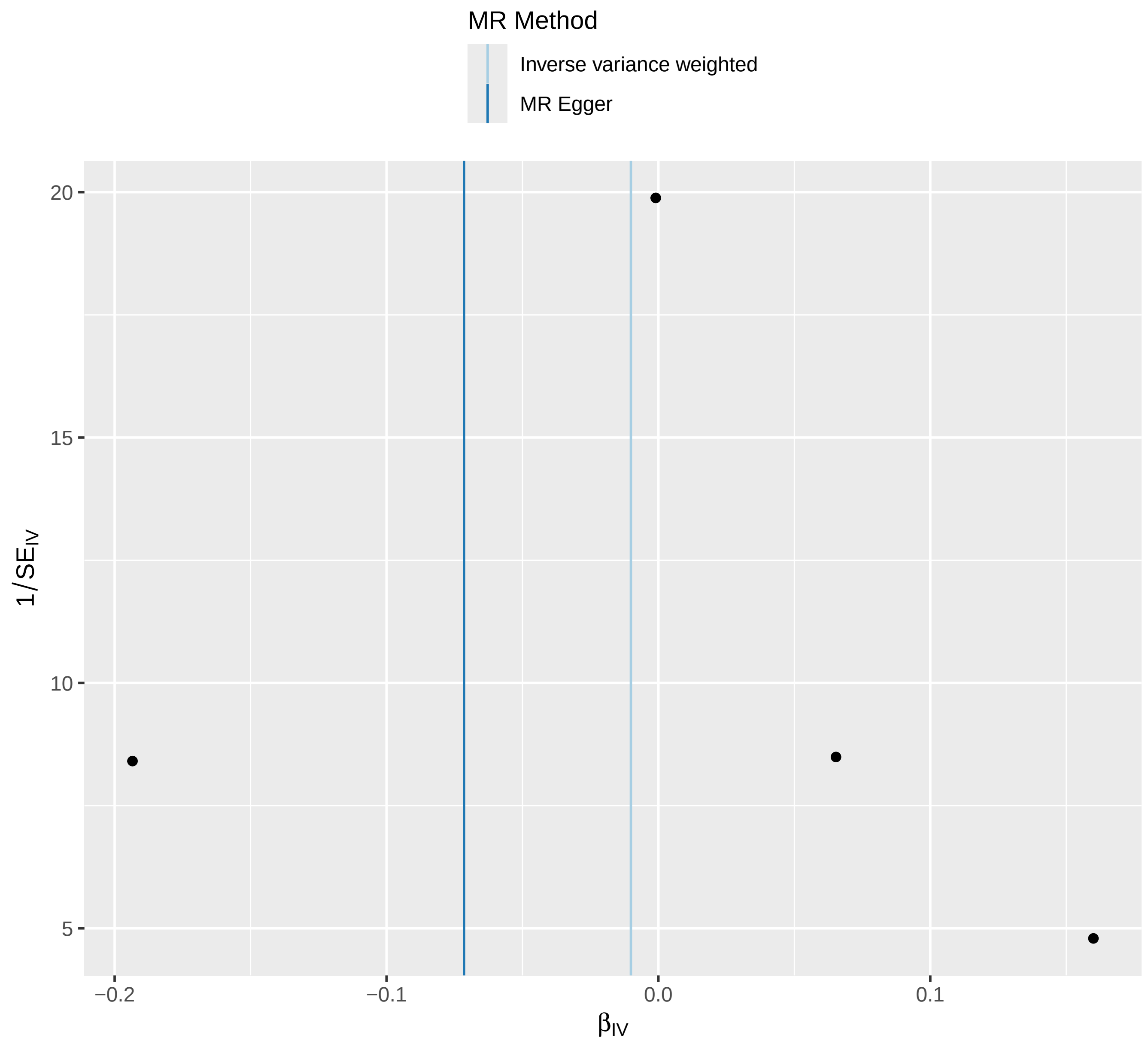

MR Method
Inverse variance weighted
MR Egger
20
15
10
5
1/SE$_{IV}$
−0.2
−0.1
0.0
0.1
β$_{IV}$

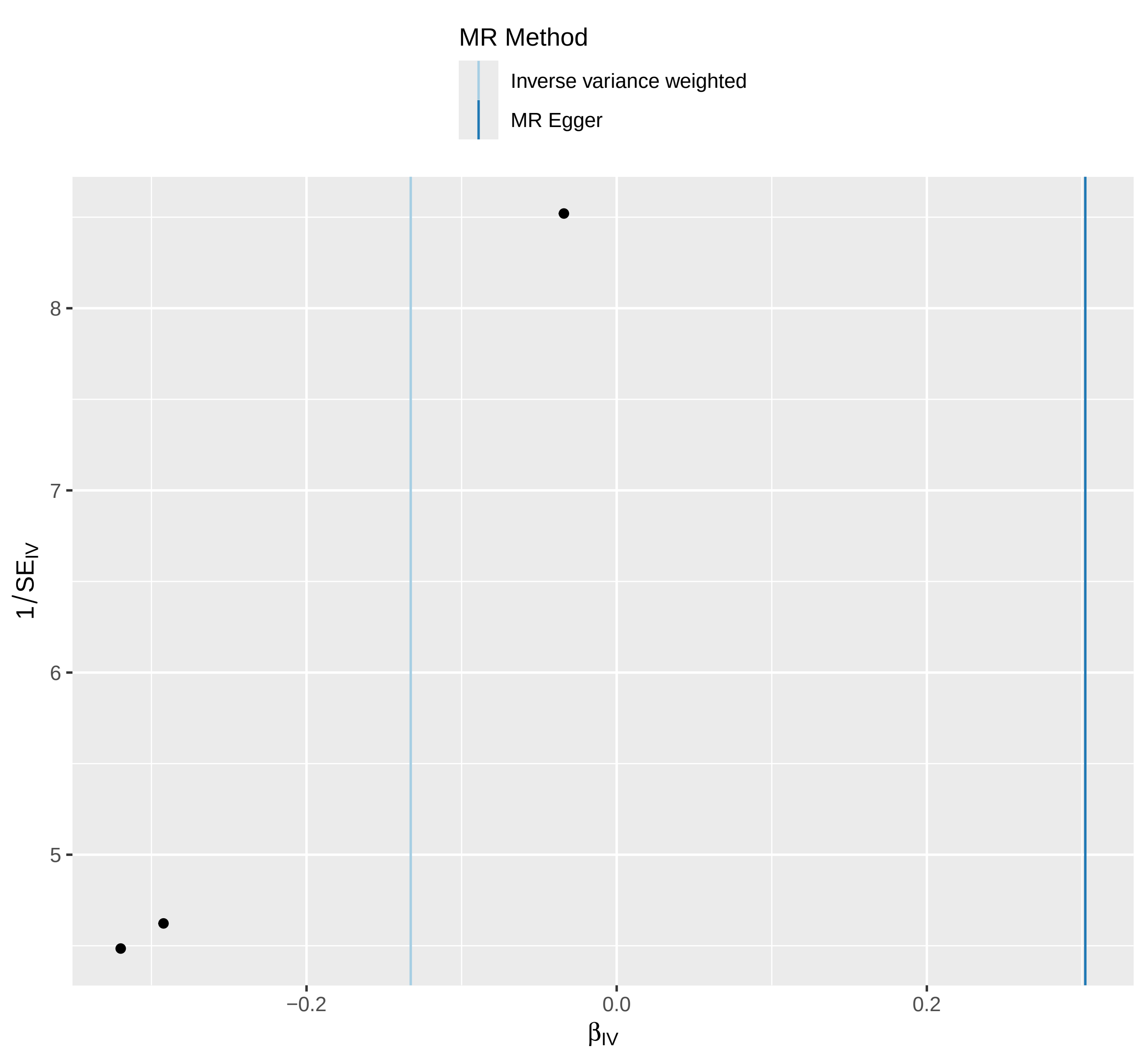
MR Method
Inverse variance weighted
MR Egger
8
7
6
5
1/SE_IV
−0.2
0.0
0.2
β_IV

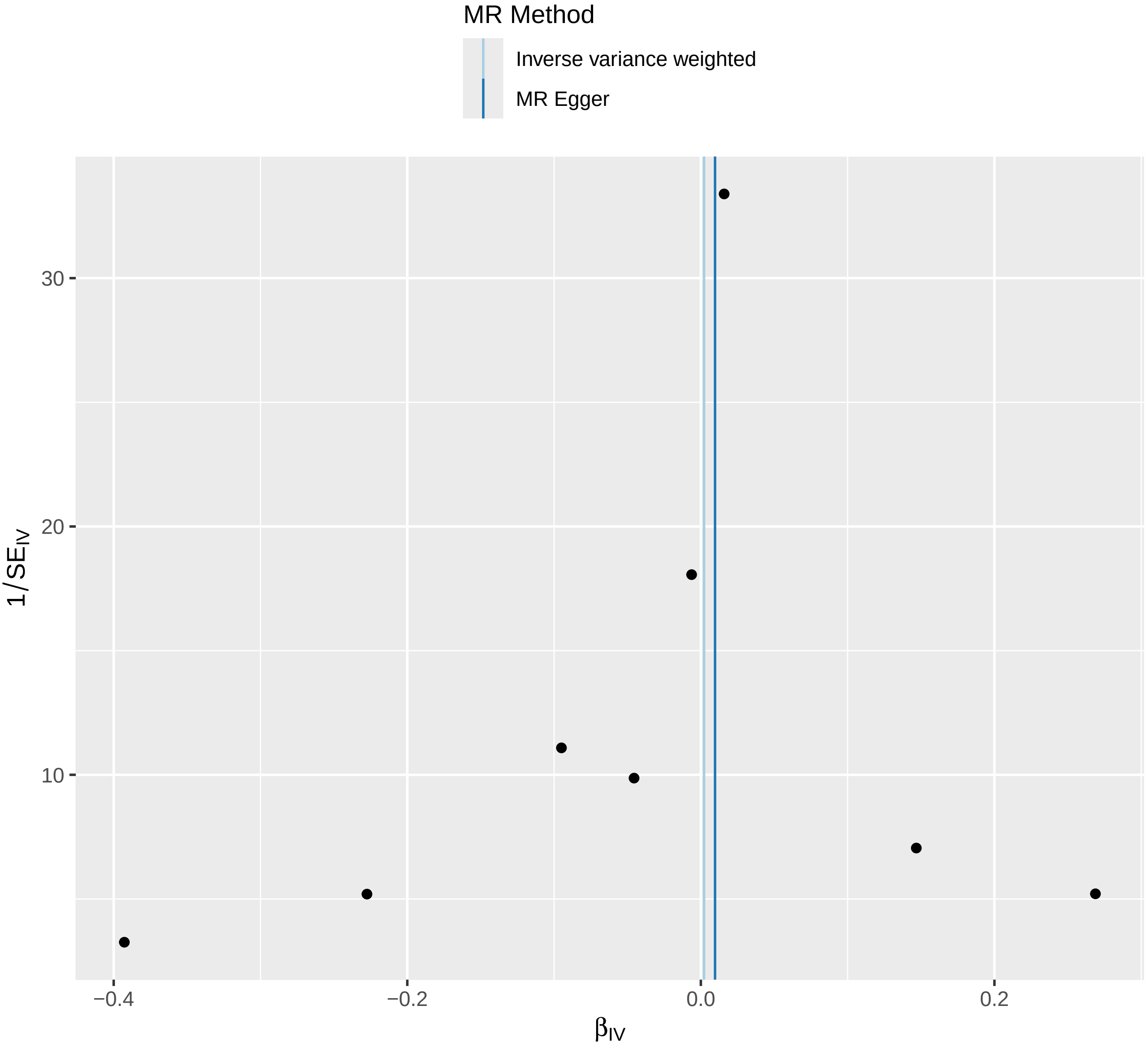

MR Method
Inverse variance weighted
MR Egger
30
20
10
1/SE_IV
−0.4
−0.2
0.0
0.2
β_IV

Insufficient number of SNPs

Insufficient number of SNPs

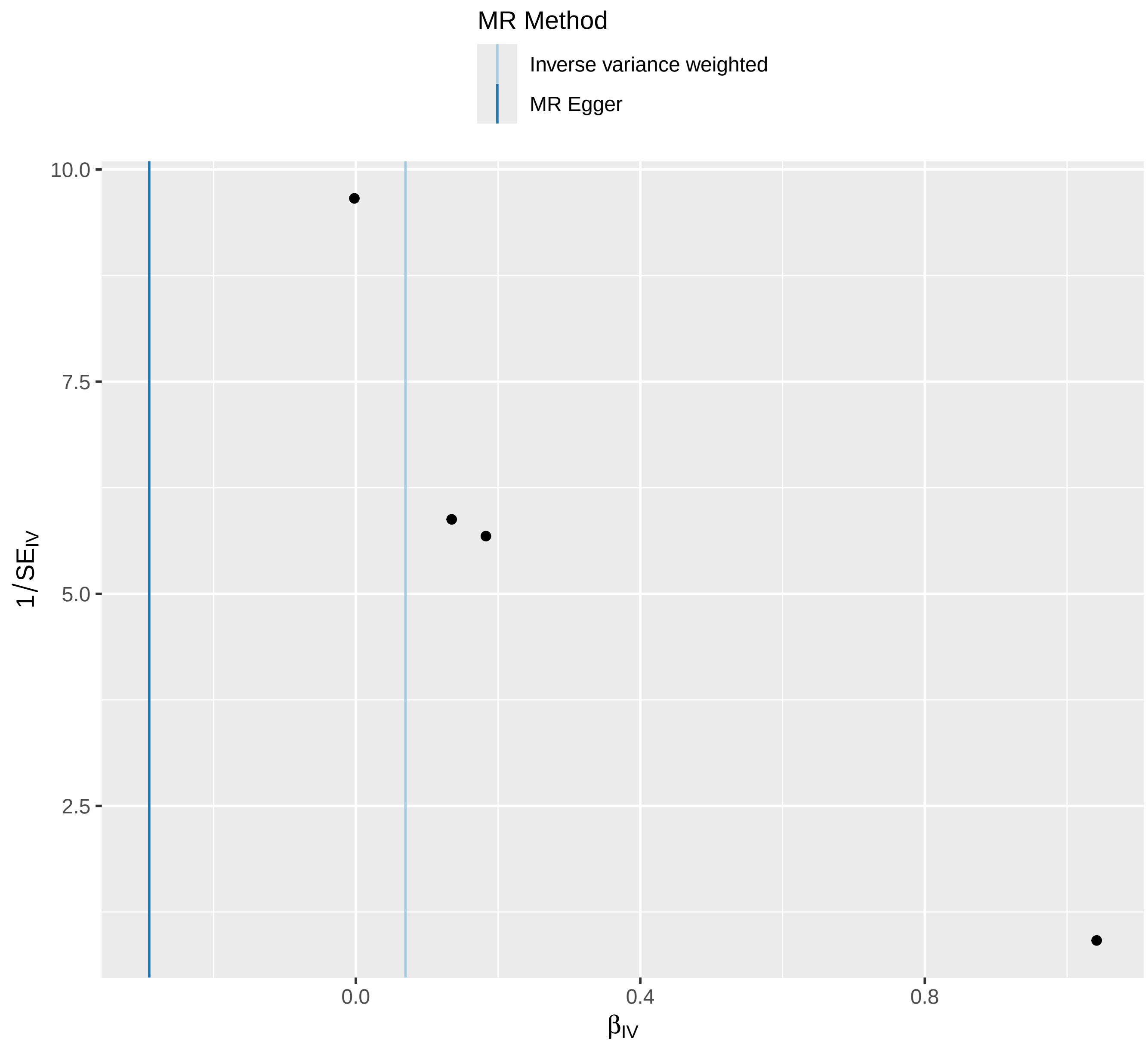
MR Method
Inverse variance weighted
MR Egger
10.0
7.5
5.0
2.5
1/SE_IV
0.0
0.4
0.8
β_IV

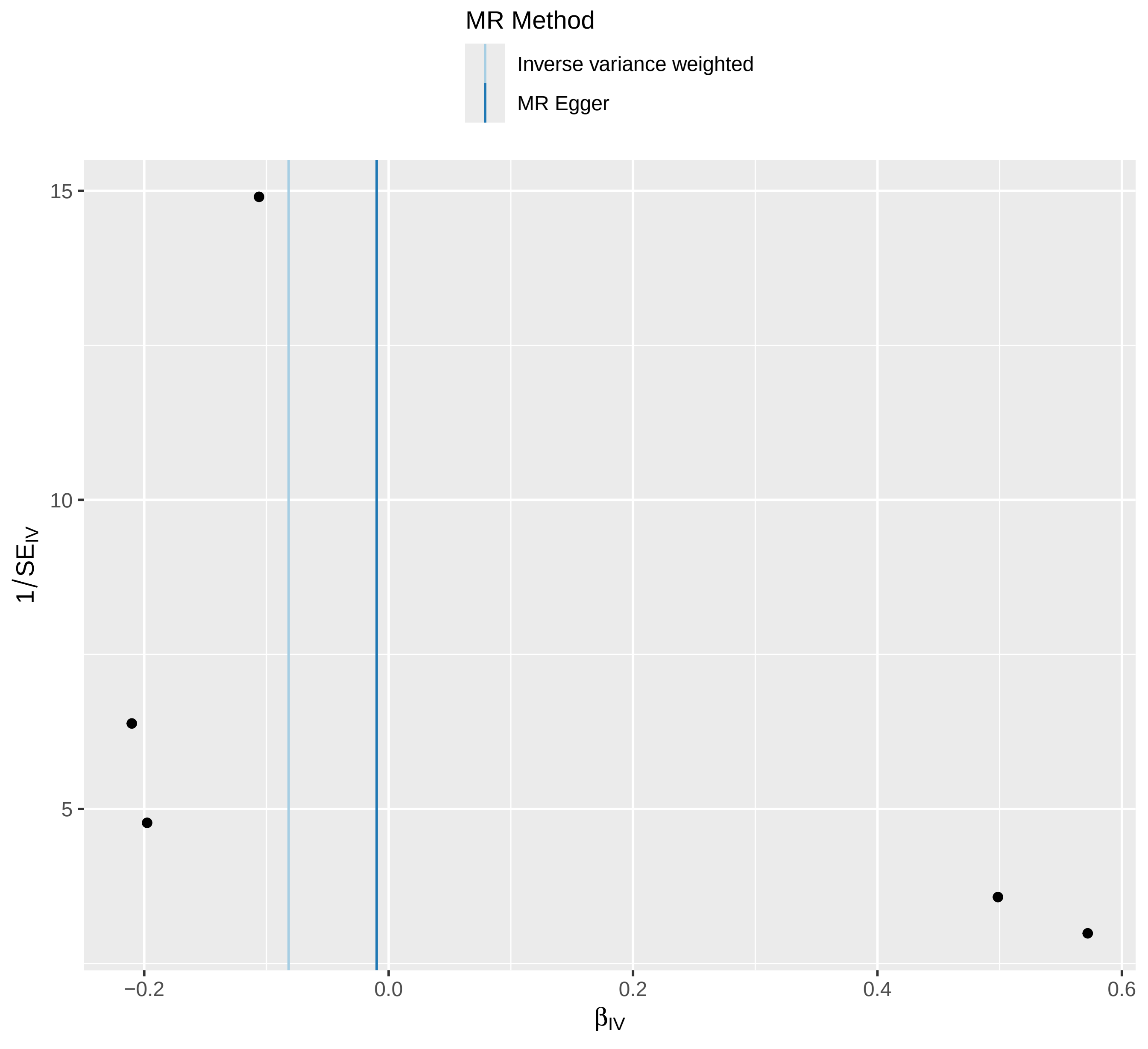

MR Method
Inverse variance weighted
MR Egger
15
10
5
1/SE_IV
−0.2
0.0
0.2
0.4
0.6
β_IV

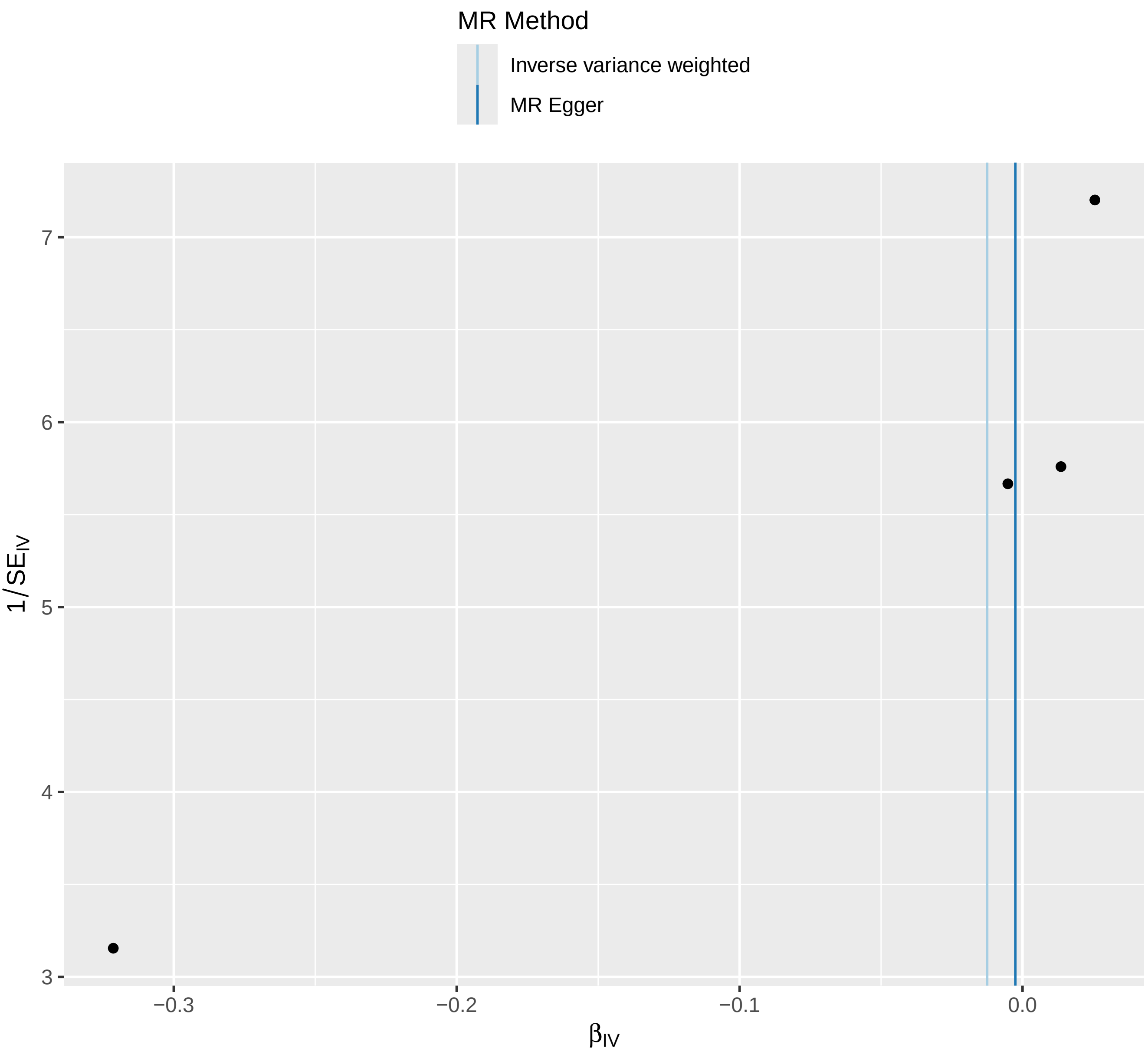
MR Method
Inverse variance weighted
MR Egger
7
6
5
4
3
−0.3
−0.2
−0.1
0.0
$1/SE_{IV}$
$\beta_{IV}$

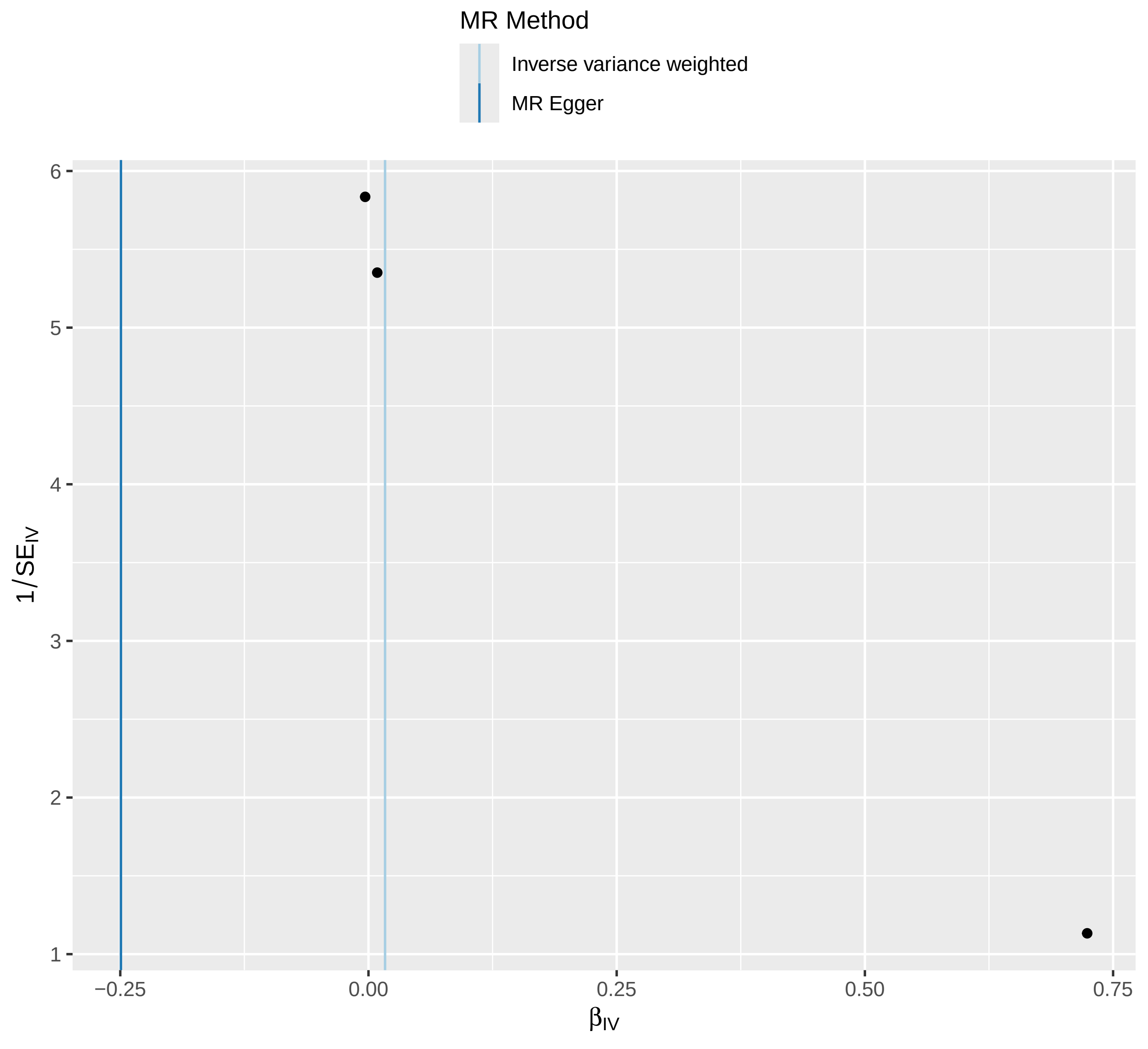
MR Method
Inverse variance weighted
MR Egger
6
5
4
3
2
1
$1/SE_{IV}$
−0.25
0.00
0.25
0.50
0.75
$\beta_{IV}$

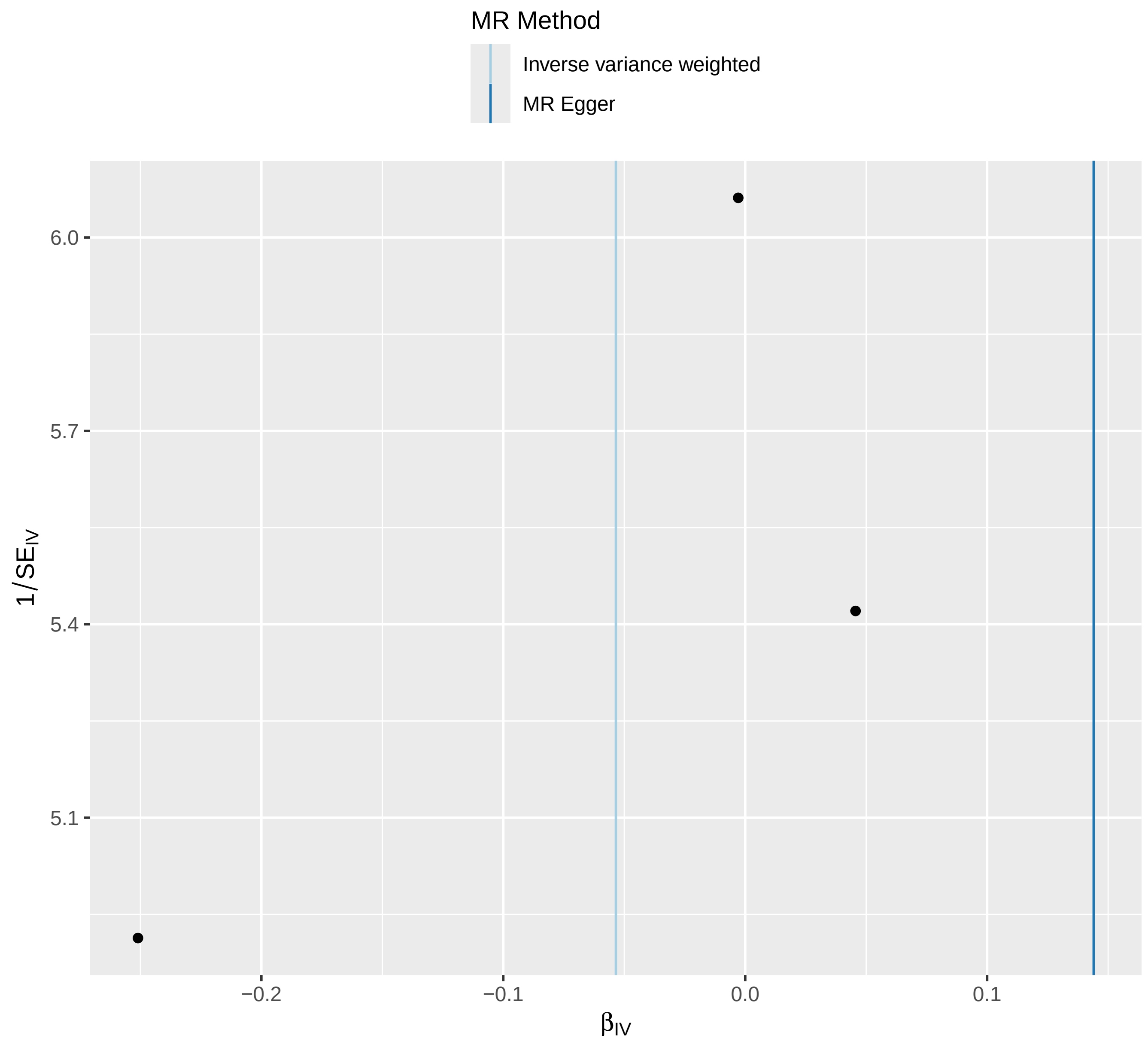

MR Method
Inverse variance weighted
MR Egger
6.0
5.7
5.4
5.1
1/SE$_{IV}$
−0.2
−0.1
0.0
0.1
β$_{IV}$

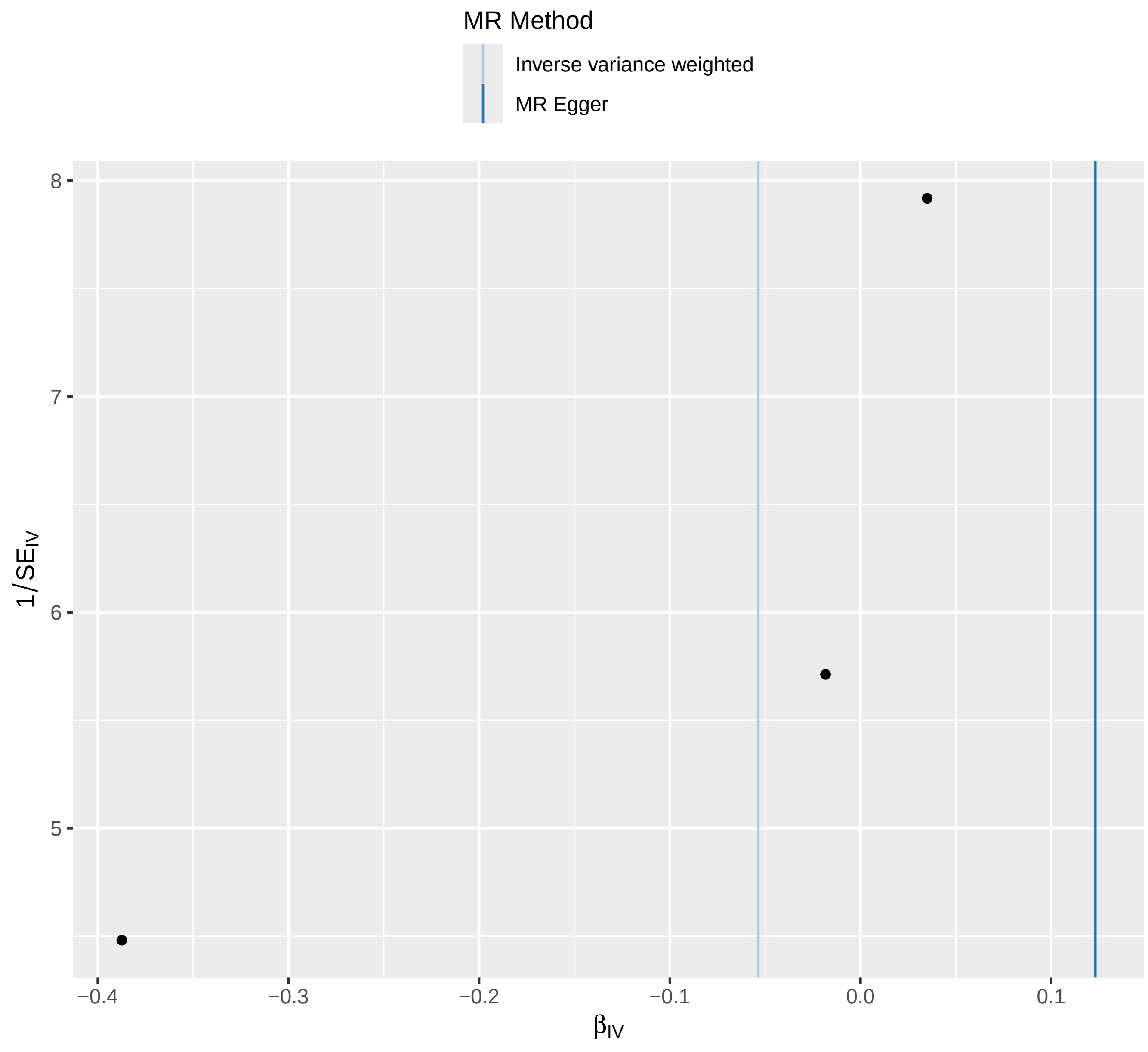
MR Method
Inverse variance weighted
MR Egger
1/SE_IV
β_IV
8
7
6
5
−0.4
−0.3
−0.2
−0.1
0.0
0.1

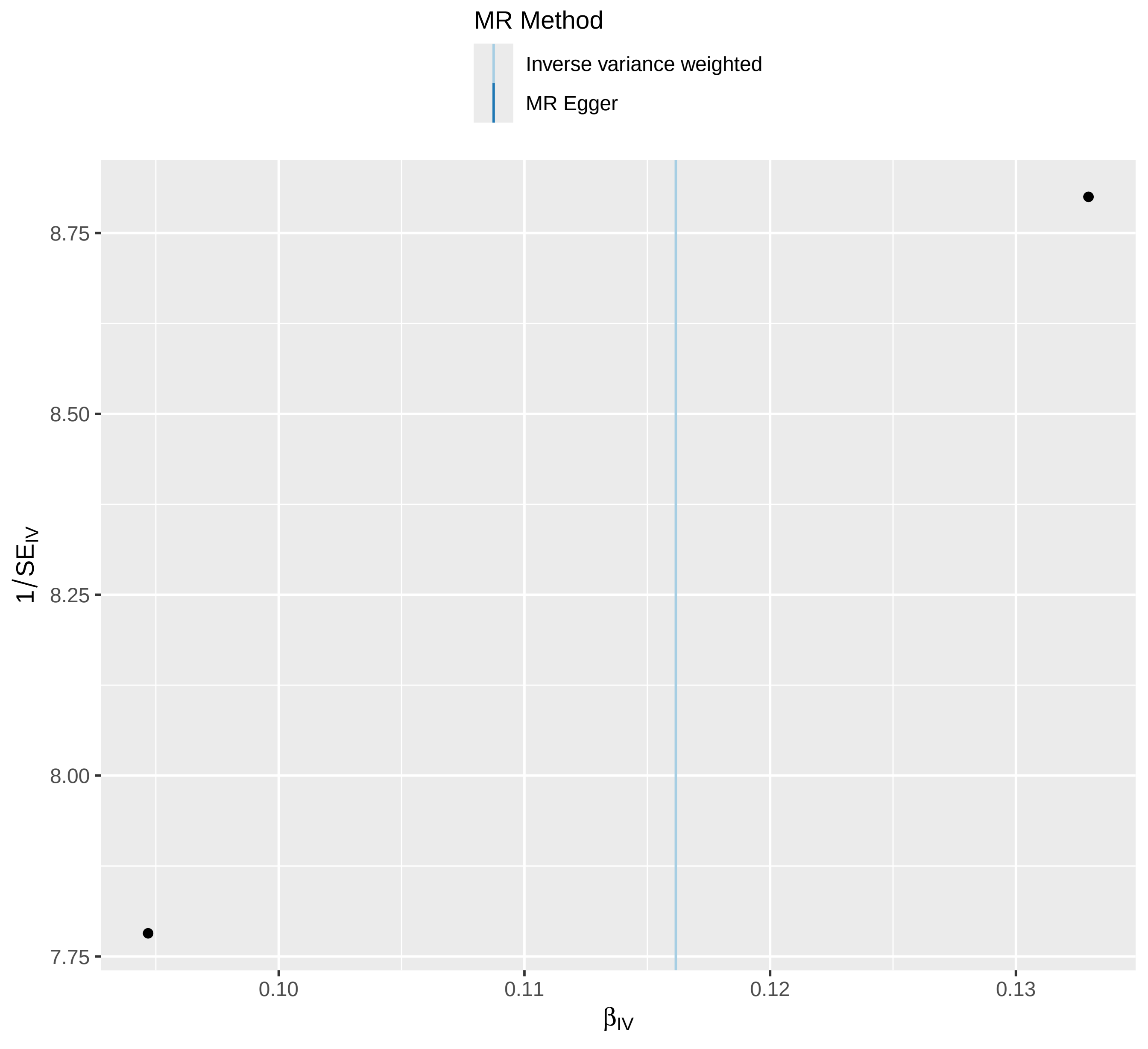
MR Method
Inverse variance weighted
MR Egger
8.75
8.50
8.25
8.00
7.75
$1/SE_{IV}$
0.10
0.11
0.12
0.13
$\beta_{IV}$

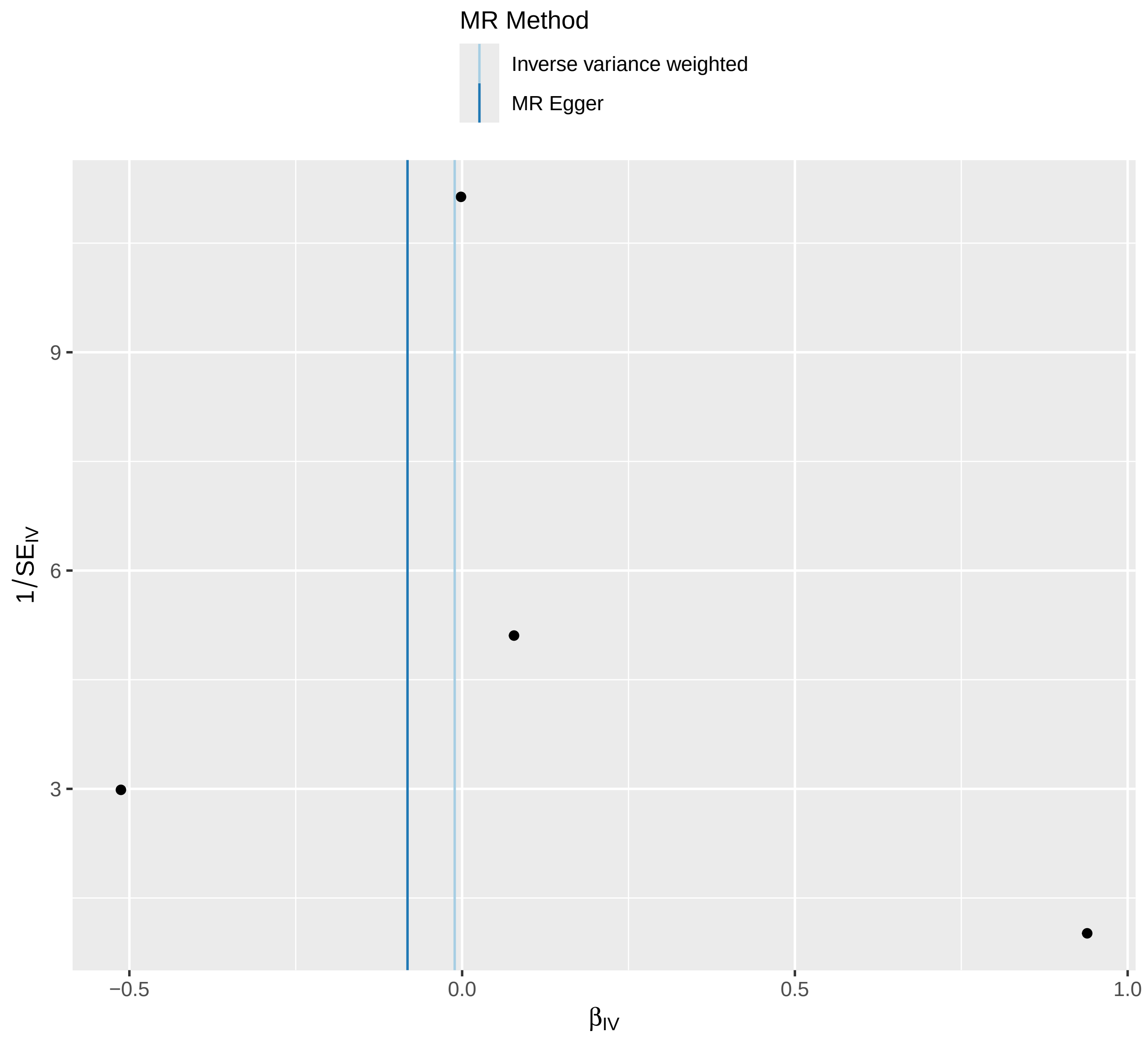
MR Method
Inverse variance weighted
MR Egger
1/SE$_{IV}$
9
6
3
−0.5
0.0
0.5
1.0
β$_{IV}$

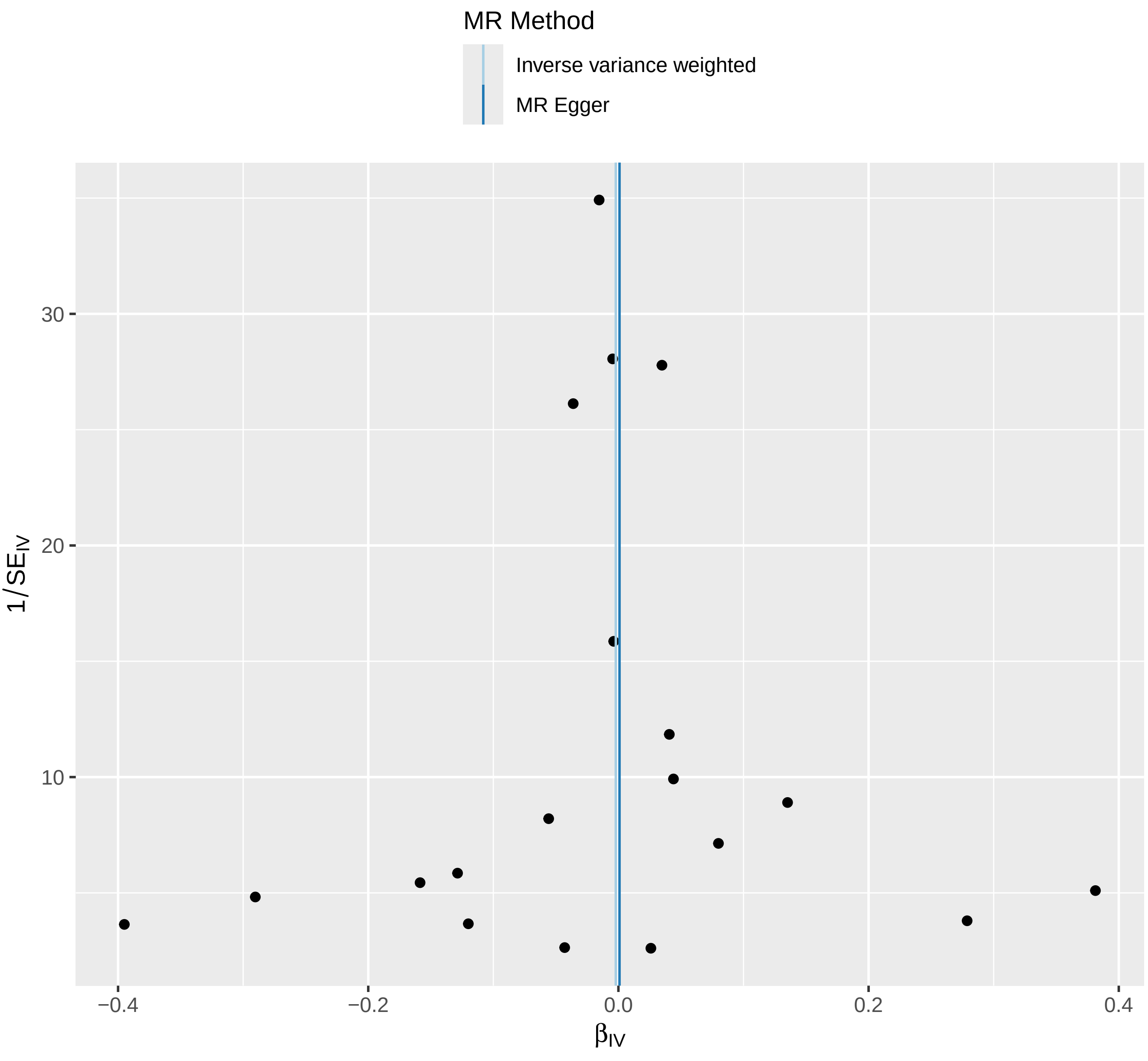
MR Method
Inverse variance weighted
MR Egger
30
20
10
1/SE$_{IV}$
−0.4
−0.2
0.0
0.2
0.4
β$_{IV}$

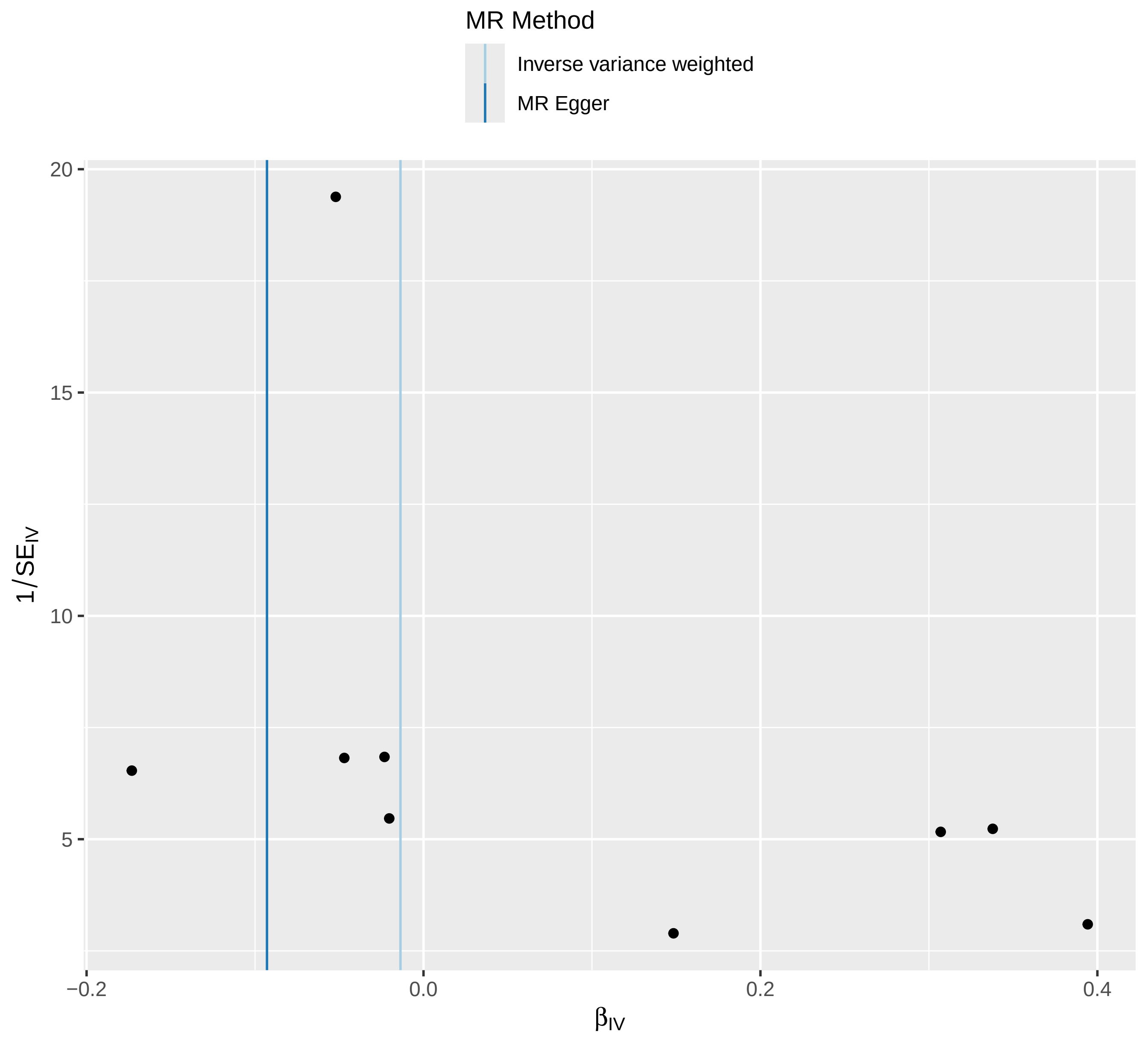
MR Method
Inverse variance weighted
MR Egger
20
15
10
5
1/SE_IV
−0.2
0.0
0.2
0.4
β_IV

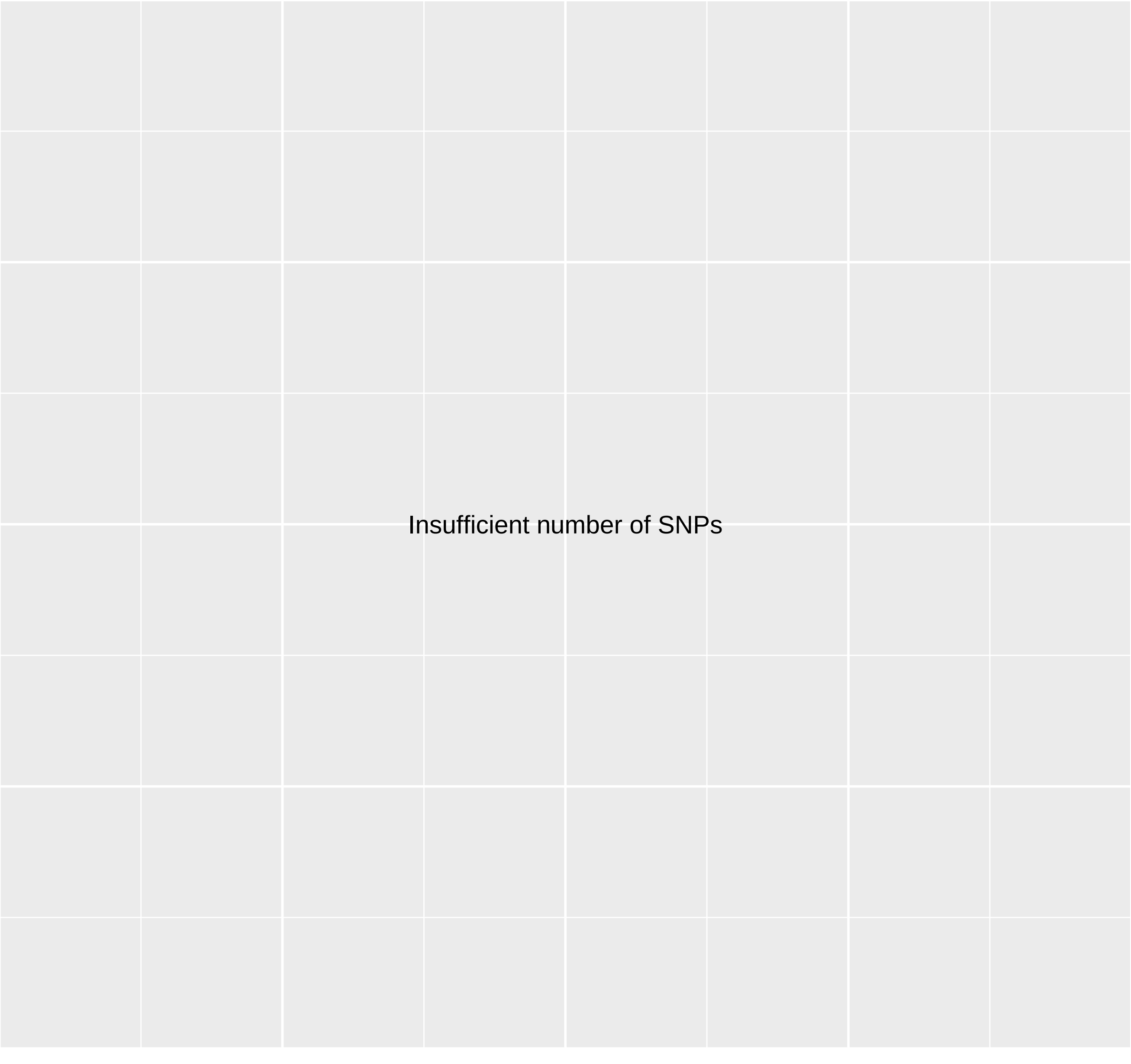
Insufficient number of SNPs

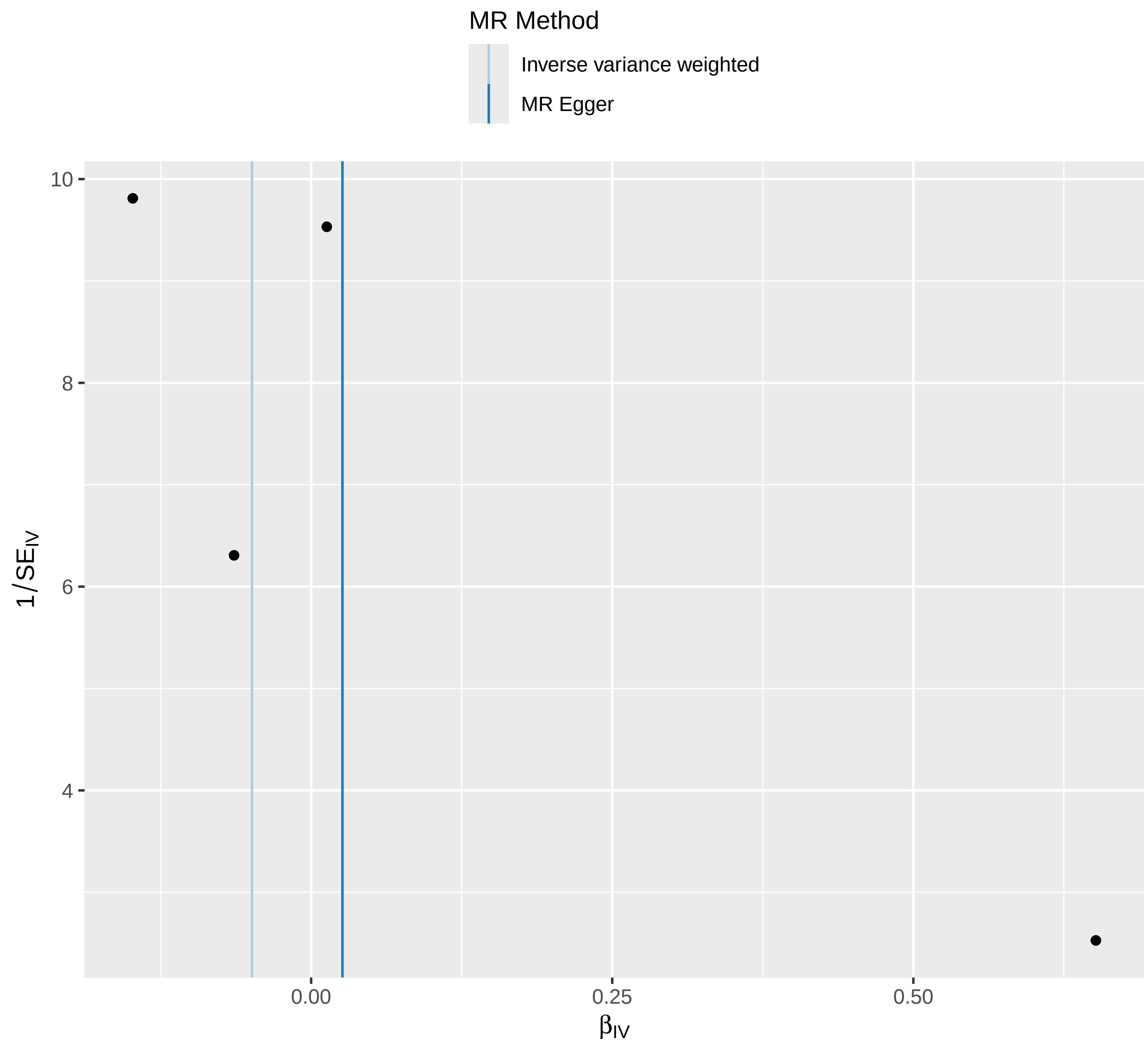
MR Method
Inverse variance weighted
MR Egger
10
8
6
4
1/SE_IV
0.00
0.25
0.50
β_IV

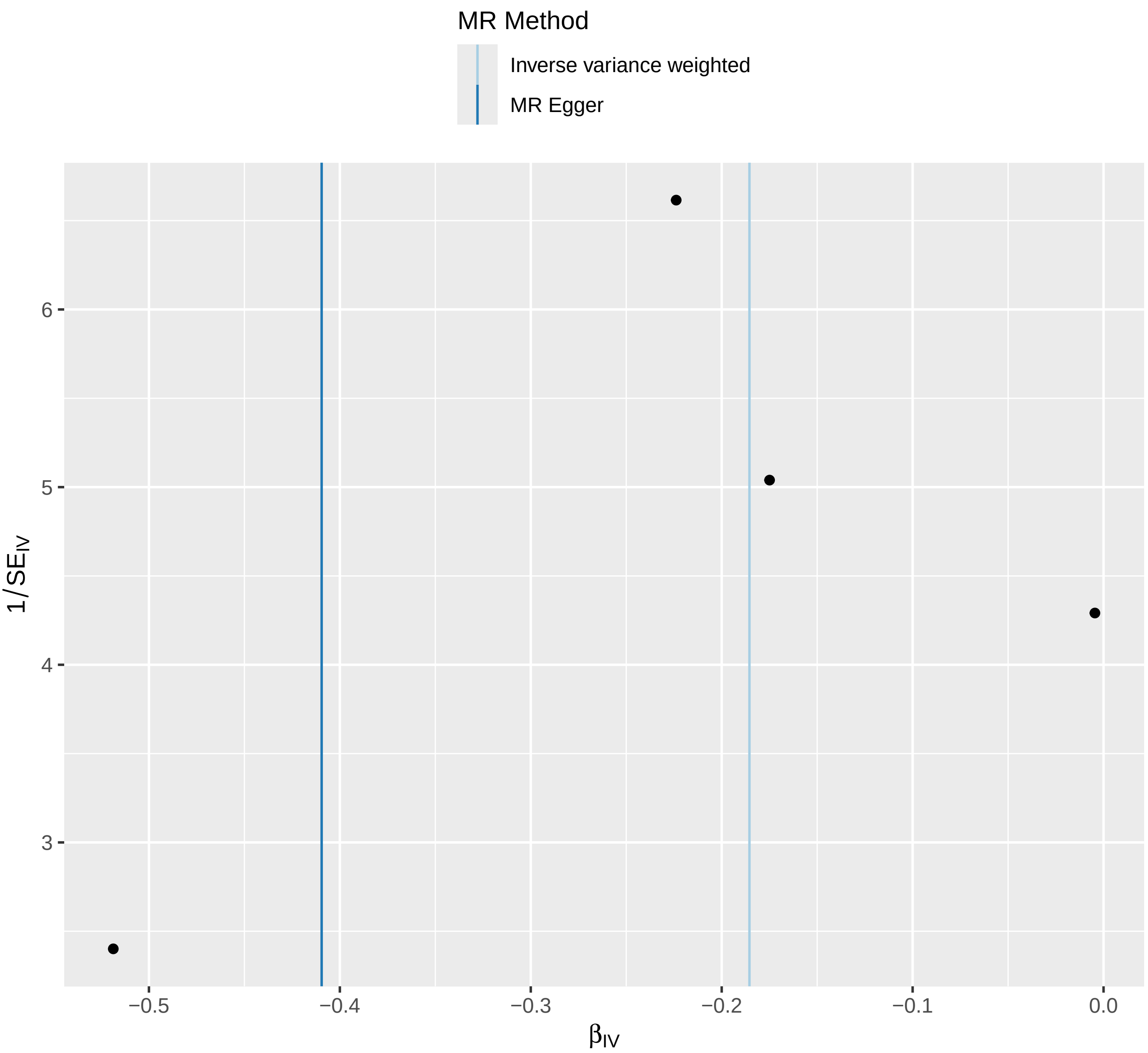
MR Method
Inverse variance weighted
MR Egger
6
5
4
3
1/SE_IV
−0.5
−0.4
−0.3
−0.2
−0.1
0.0
β_IV

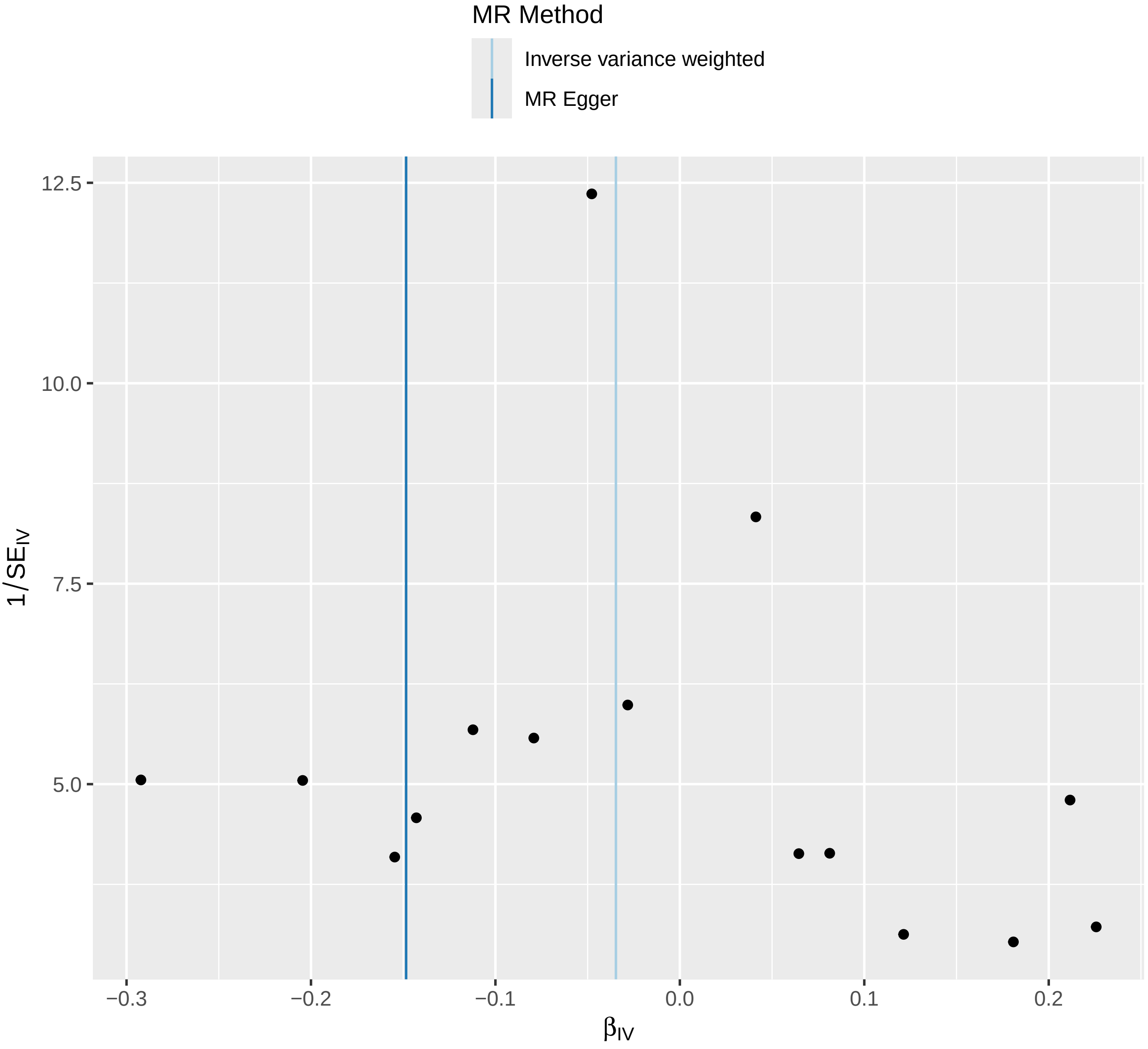
MR Method
Inverse variance weighted
MR Egger
12.5
10.0
7.5
5.0
1/SE$_{IV}$
−0.3
−0.2
−0.1
0.0
0.1
0.2
β$_{IV}$

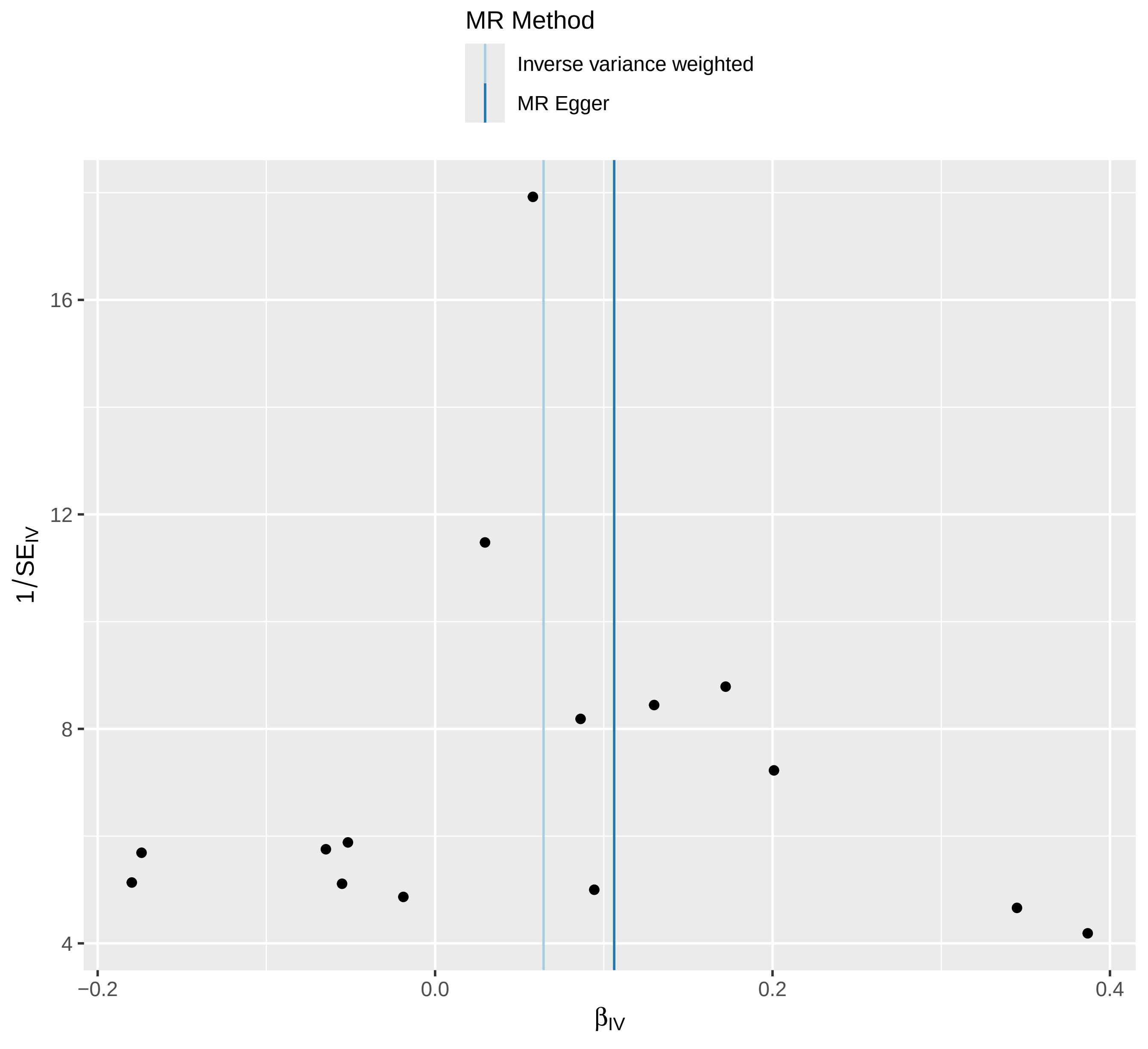
MR Method
Inverse variance weighted
MR Egger
16
12
8
4
−0.2
0.0
0.2
0.4
$1/SE_{IV}$
$\beta_{IV}$

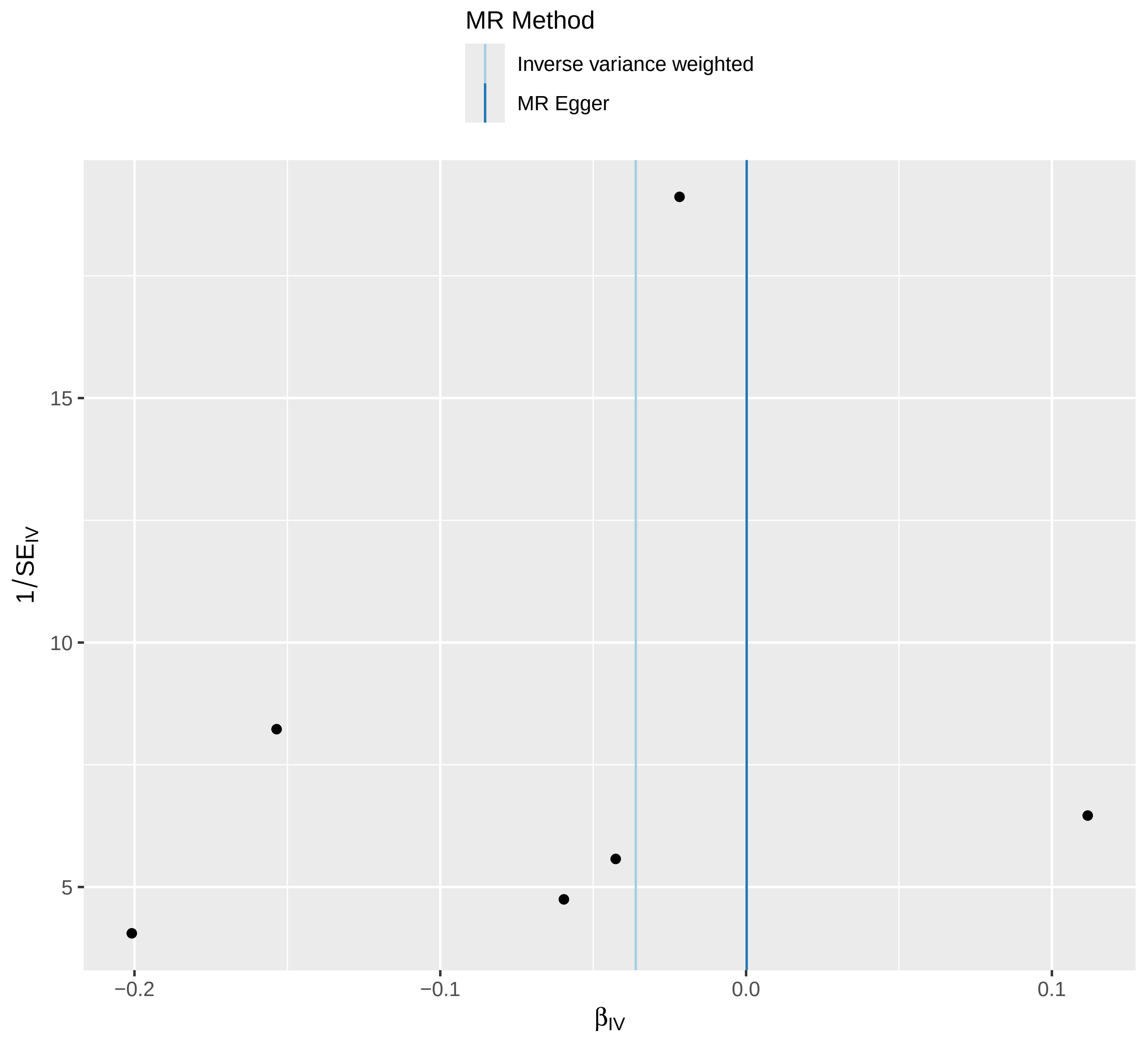

MR Method
Inverse variance weighted
MR Egger
15
10
5
1/SE_IV
−0.2
−0.1
0.0
0.1
β_IV

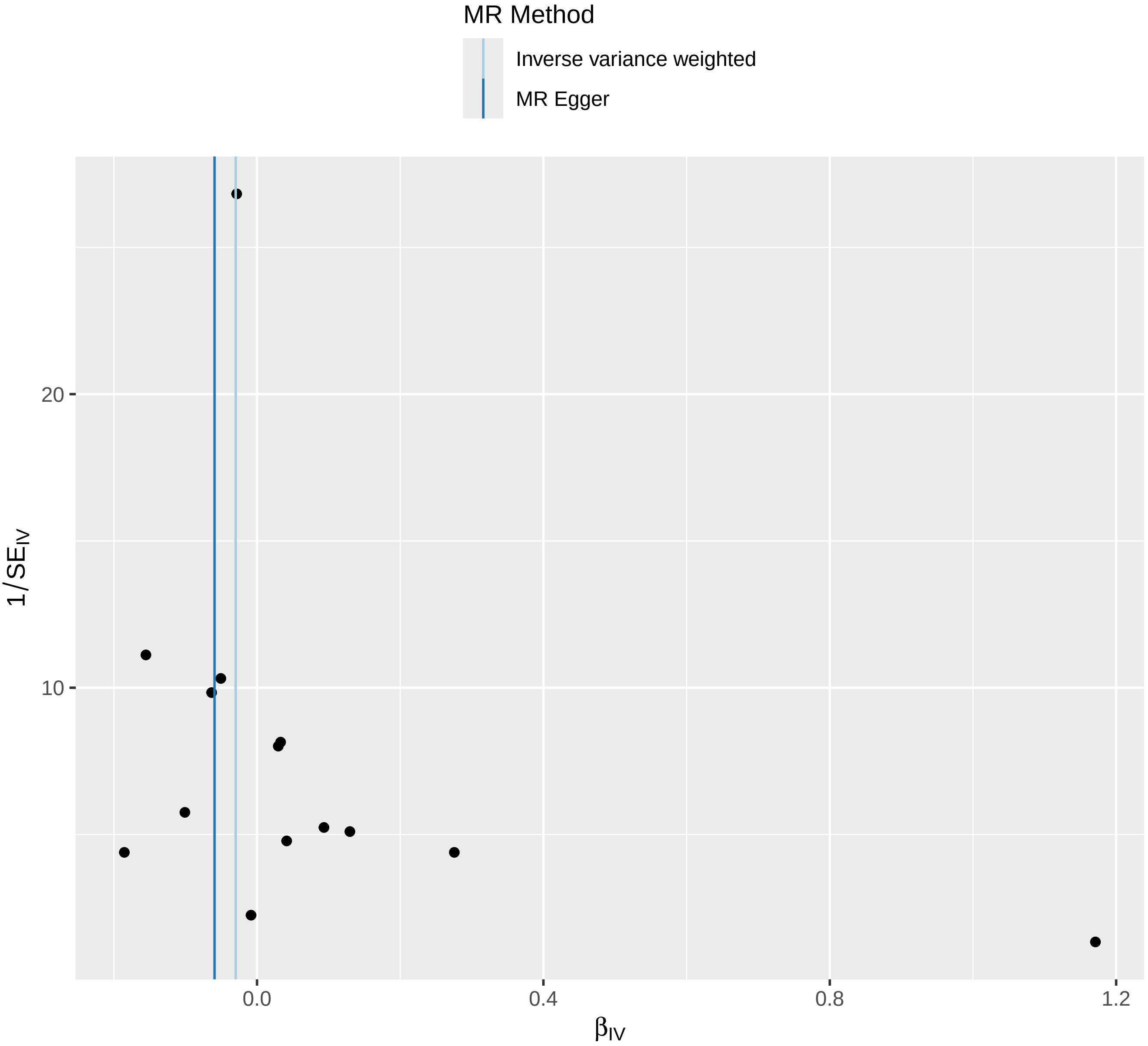
MR Method
Inverse variance weighted
MR Egger
20
10
1/SE$_{IV}$
0.0
0.4
0.8
1.2
β$_{IV}$

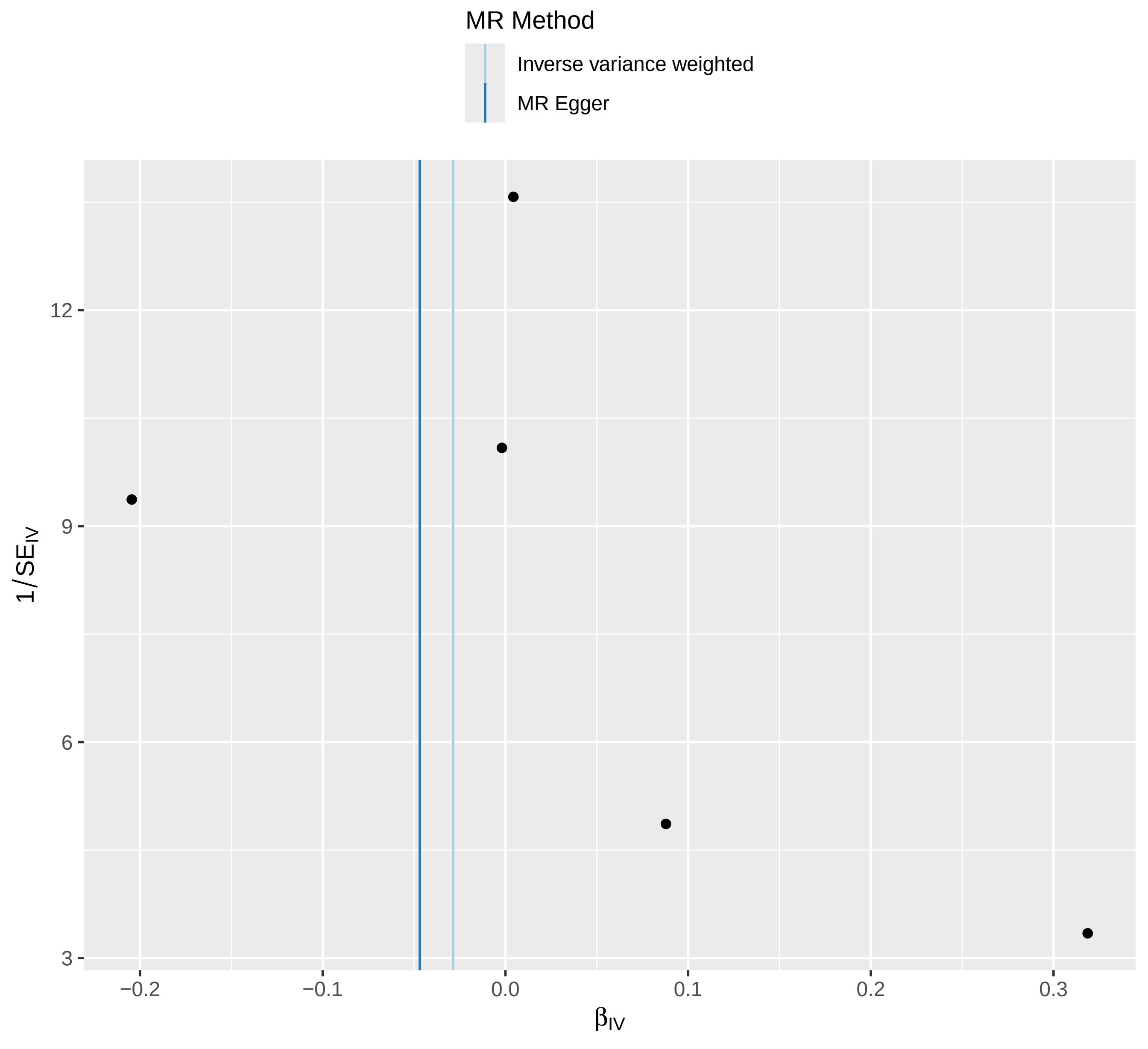

MR Method
Inverse variance weighted
MR Egger
12
9
6
3
1/SE_IV
−0.2
−0.1
0.0
0.1
0.2
0.3
β_IV

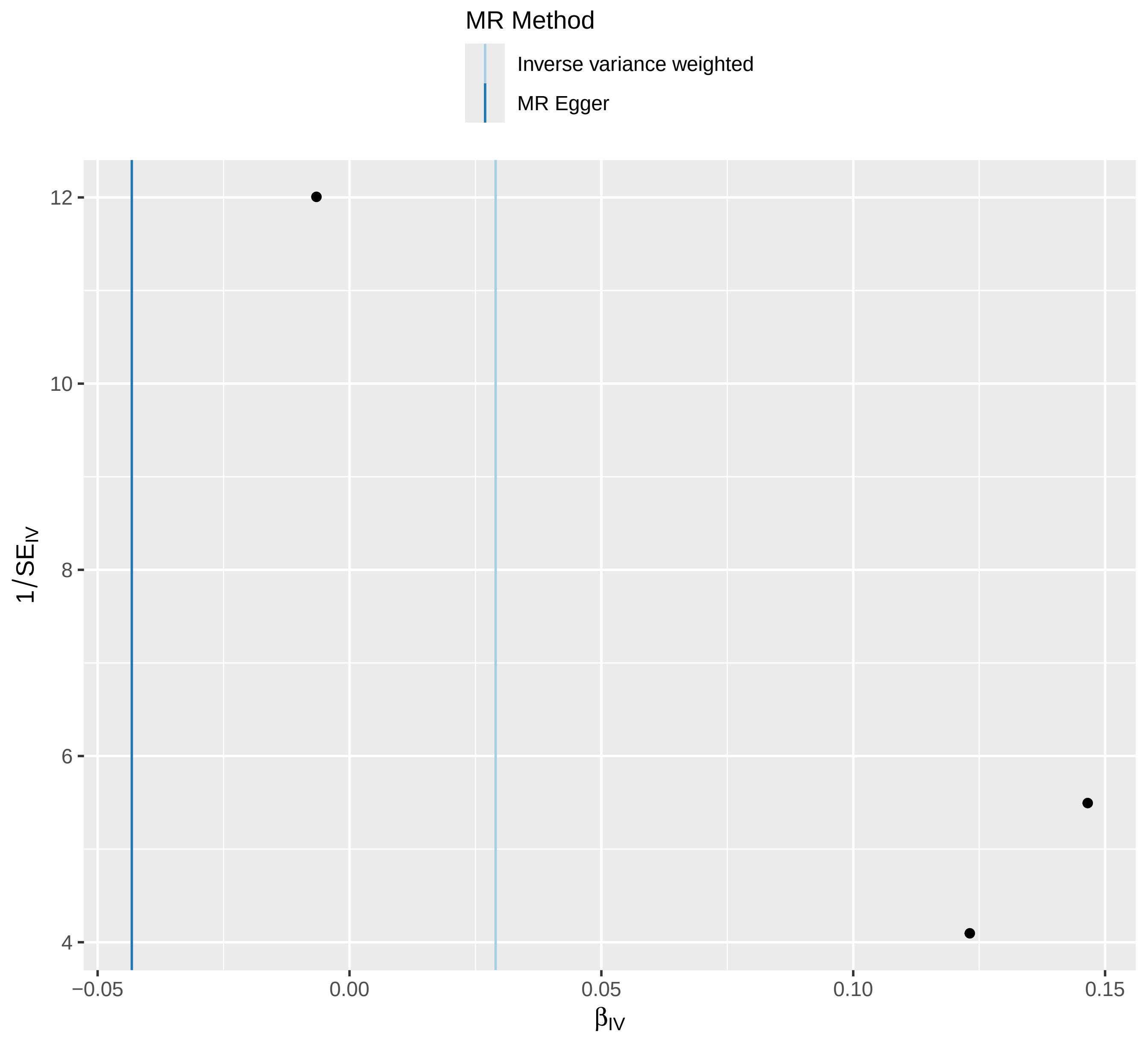

MR Method
Inverse variance weighted
MR Egger
12
10
8
6
4
$1/SE_{IV}$
−0.05
0.00
0.05
0.10
0.15
$\beta_{IV}$

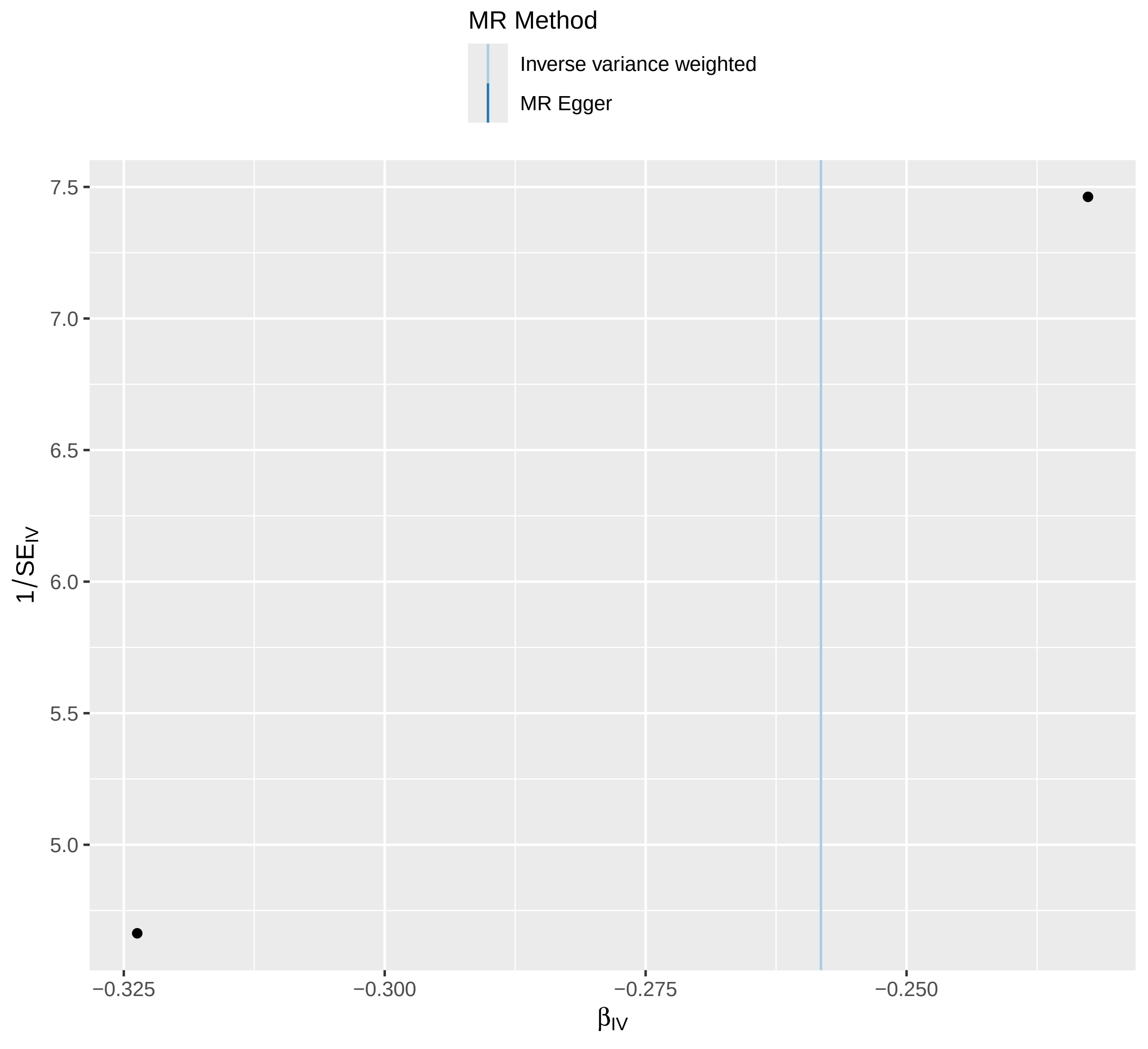
MR Method
Inverse variance weighted
MR Egger
7.5
7.0
6.5
6.0
5.5
5.0
1/SE$_{IV}$
−0.325
−0.300
−0.275
−0.250
β$_{IV}$

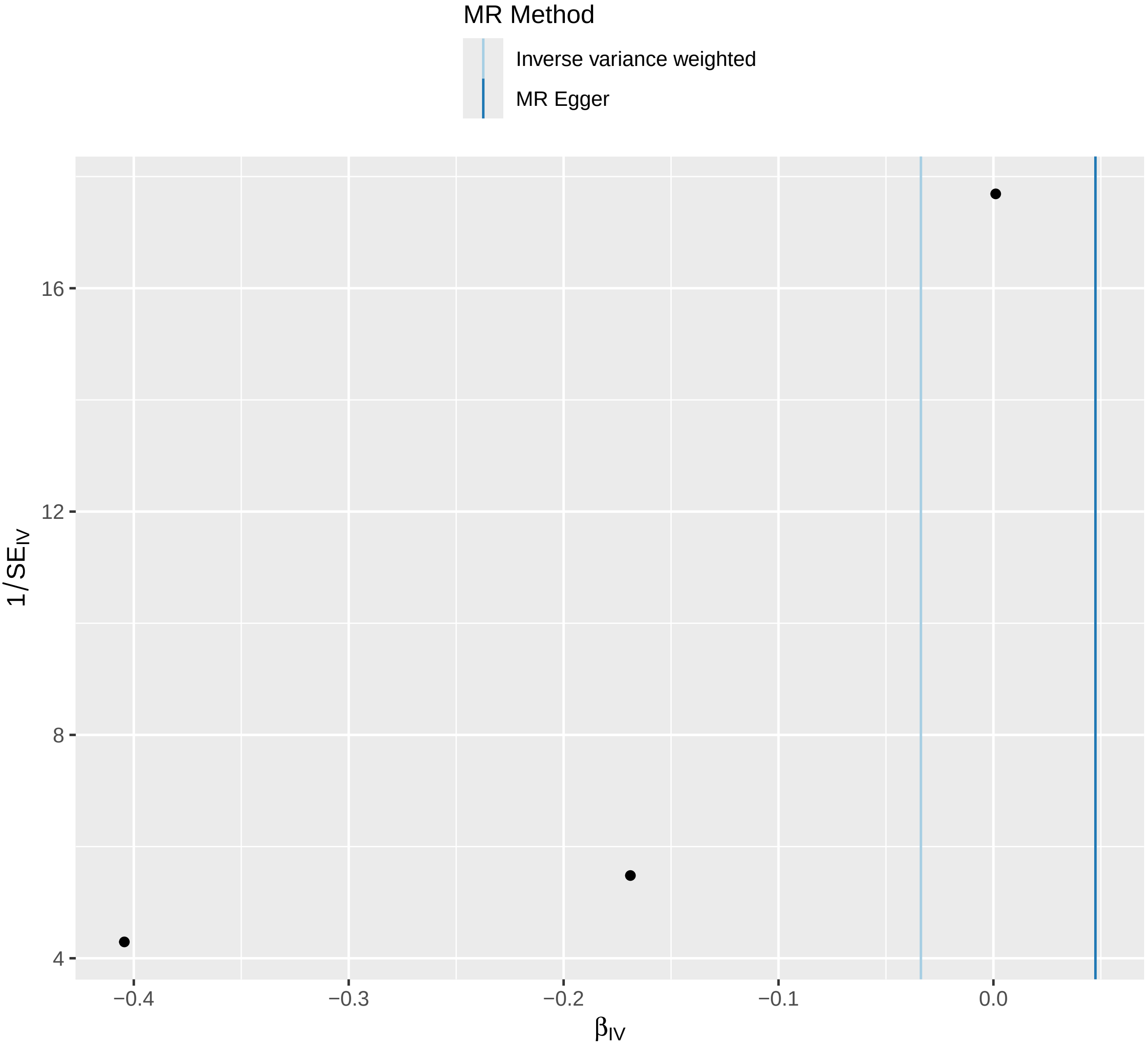
MR Method
Inverse variance weighted
MR Egger
16
12
8
4
1/SE$_{IV}$
−0.4
−0.3
−0.2
−0.1
0.0
β$_{IV}$

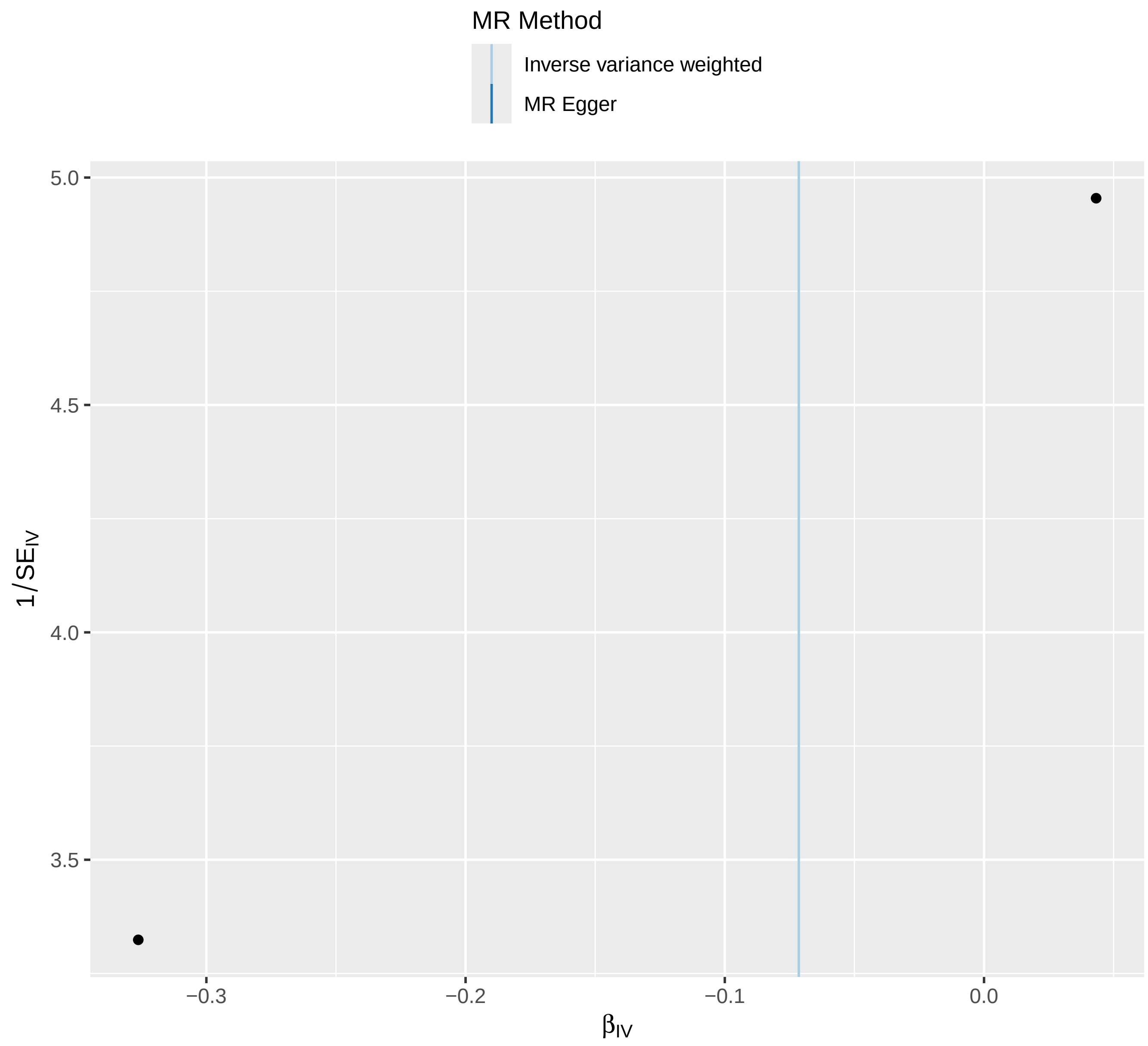
MR Method
Inverse variance weighted
MR Egger
5.0
4.5
4.0
3.5
1/SE_IV
−0.3
−0.2
−0.1
0.0
β_IV

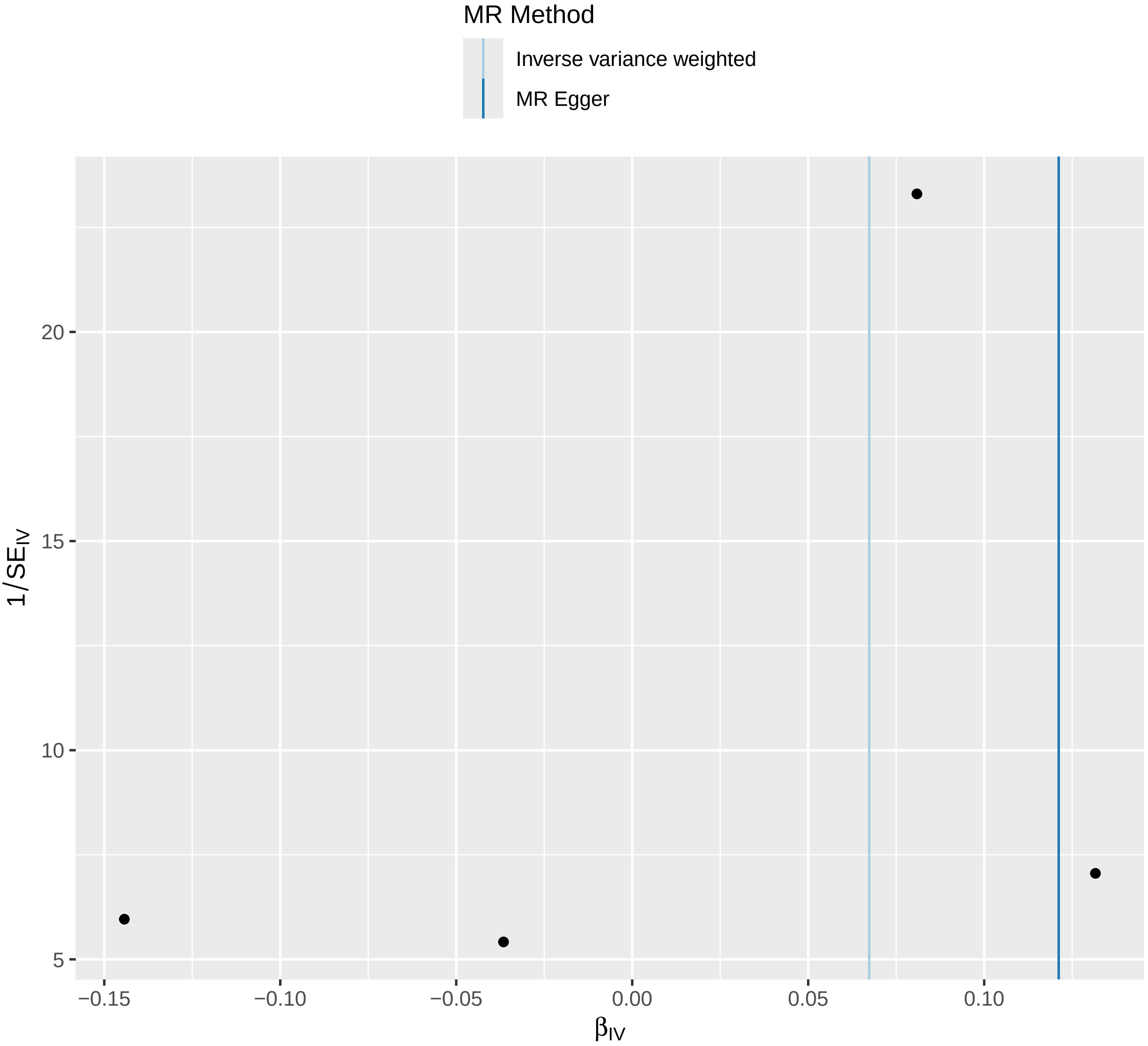
MR Method
Inverse variance weighted
MR Egger
20
15
10
5
$1/SE_{IV}$
−0.15
−0.10
−0.05
0.00
0.05
0.10
$\beta_{IV}$

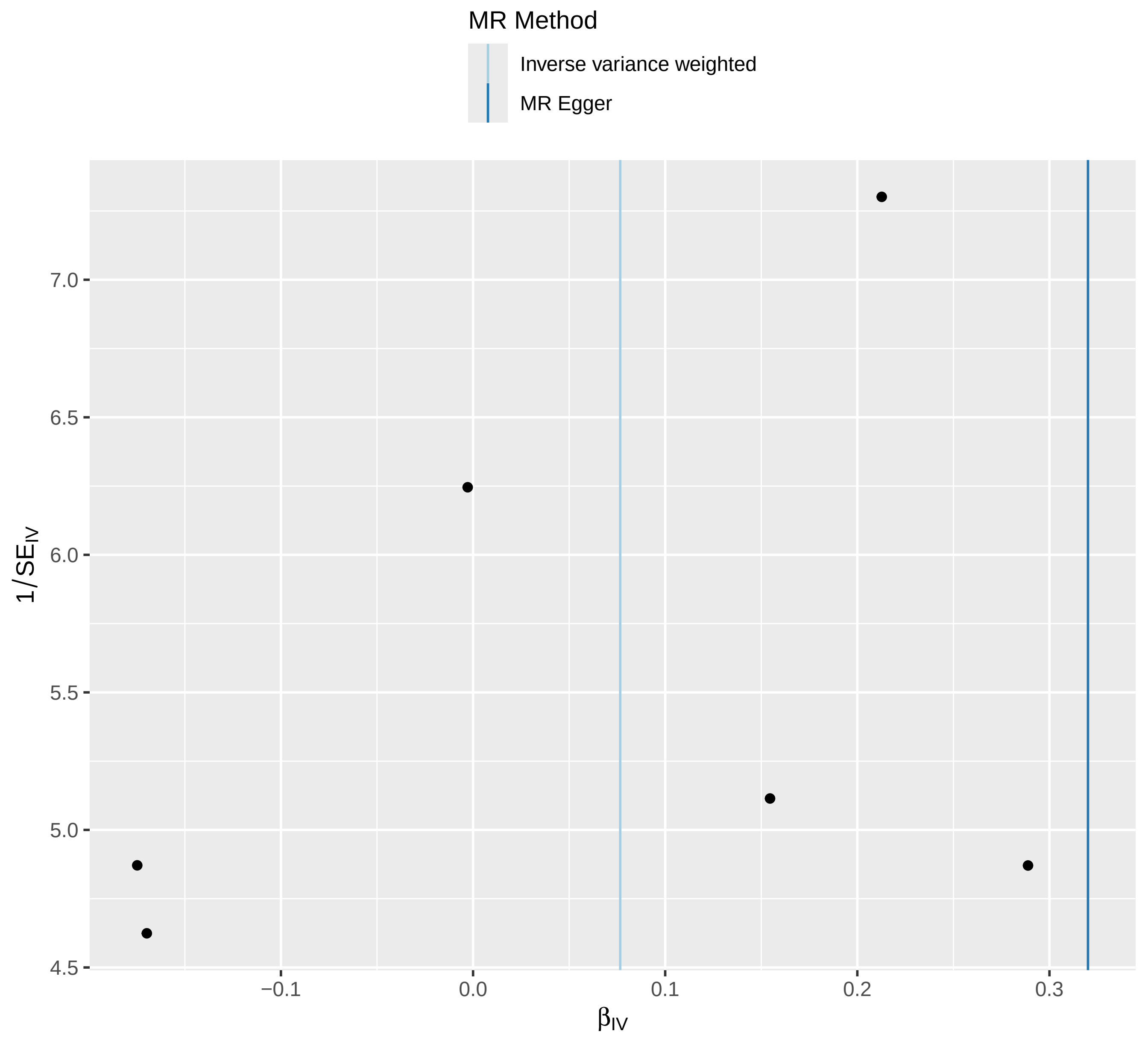
MR Method
Inverse variance weighted
MR Egger
1/SE_IV
β_IV
7.0
6.5
6.0
5.5
5.0
4.5
−0.1
0.0
0.1
0.2
0.3

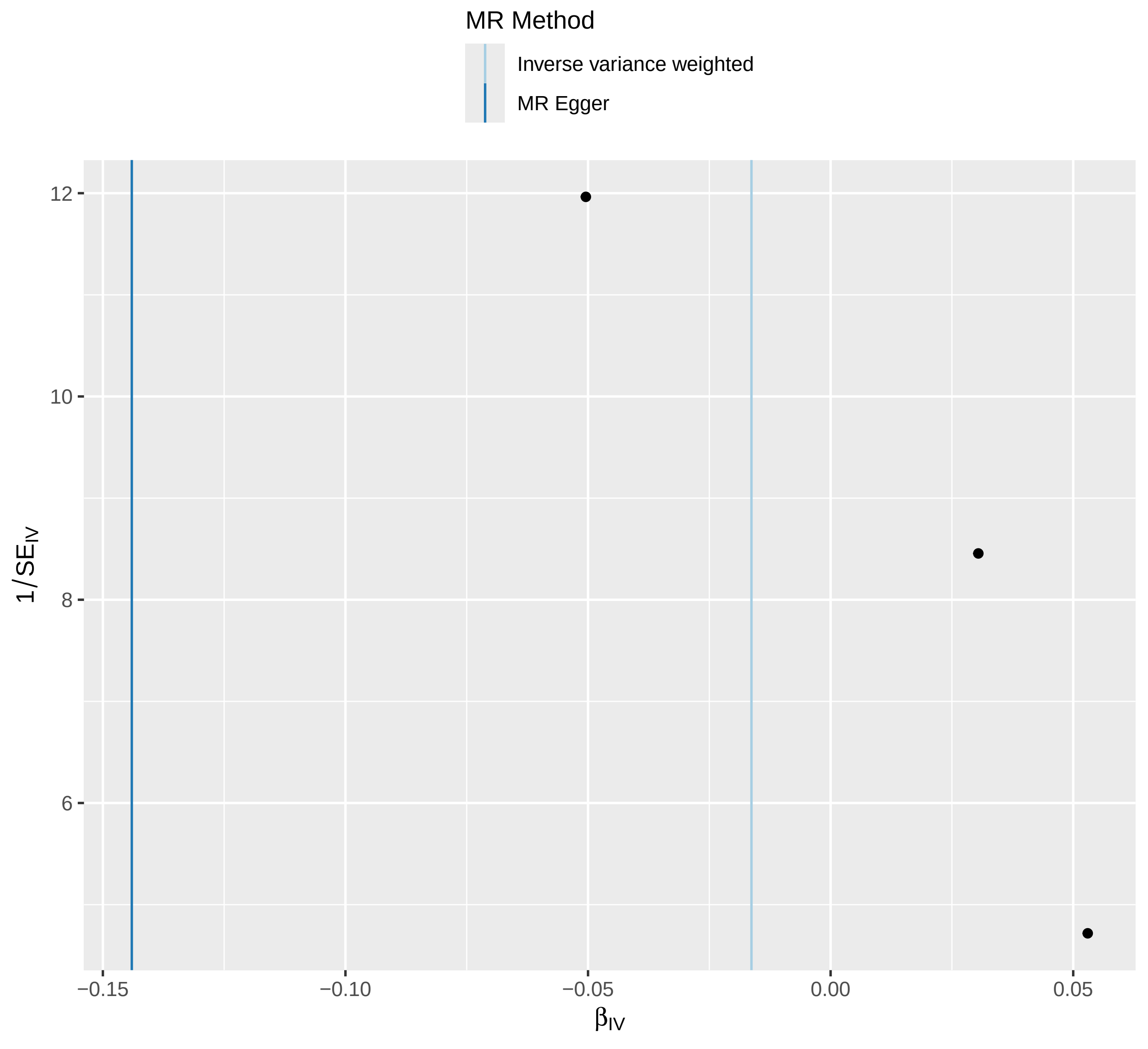
MR Method
Inverse variance weighted
MR Egger
12
10
8
6
1/SE_IV
−0.15
−0.10
−0.05
0.00
0.05
β_IV

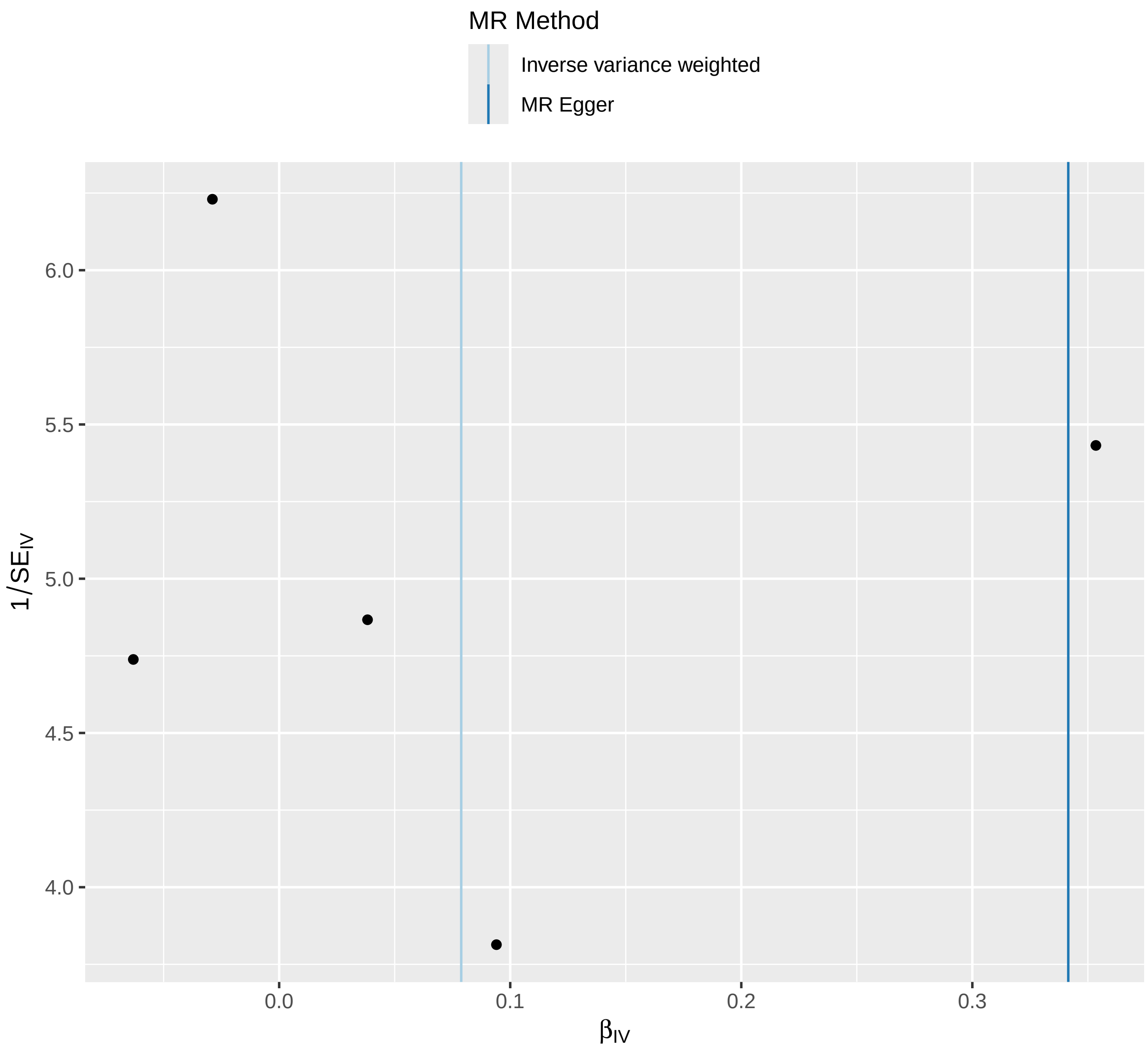
MR Method
Inverse variance weighted
MR Egger
6.0
5.5
5.0
4.5
4.0
$1/SE_{IV}$
0.0
0.1
0.2
0.3
$\beta_{IV}$

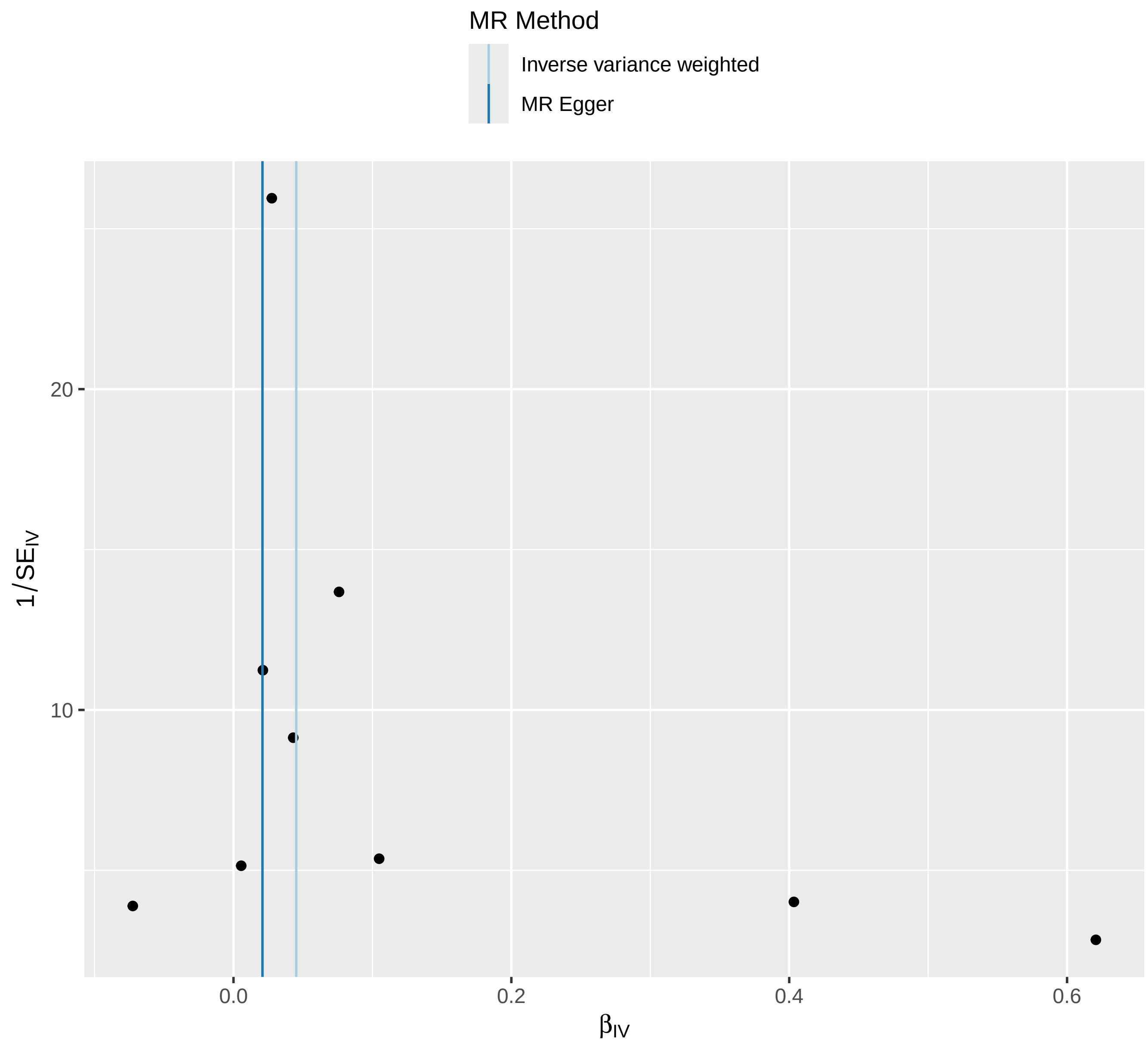
MR Method
Inverse variance weighted
MR Egger
20
10
1/SE$_{IV}$
0.0
0.2
0.4
0.6
β$_{IV}$

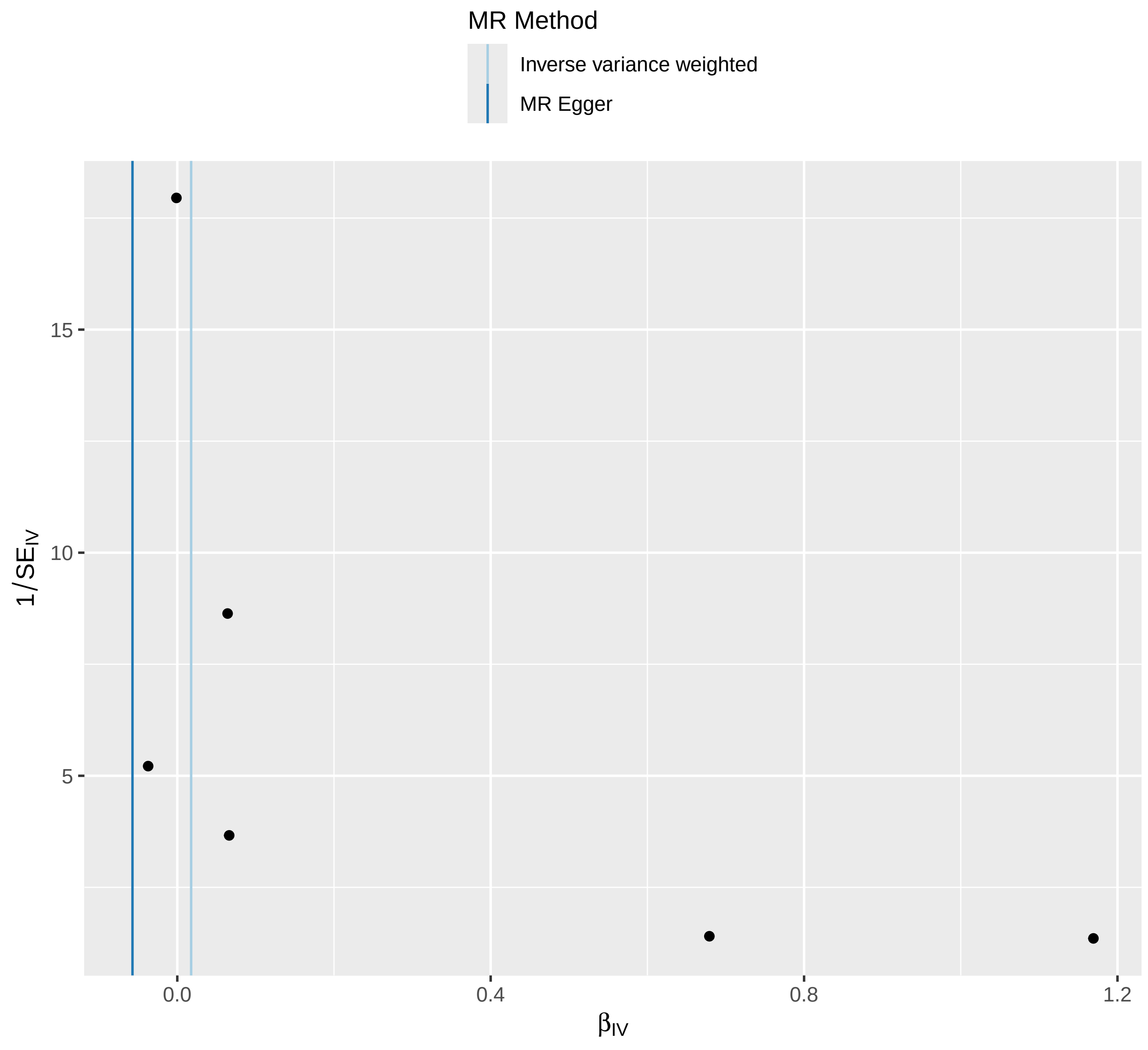
MR Method
Inverse variance weighted
MR Egger
15
10
5
1/SE_IV
0.0
0.4
0.8
1.2
β_IV

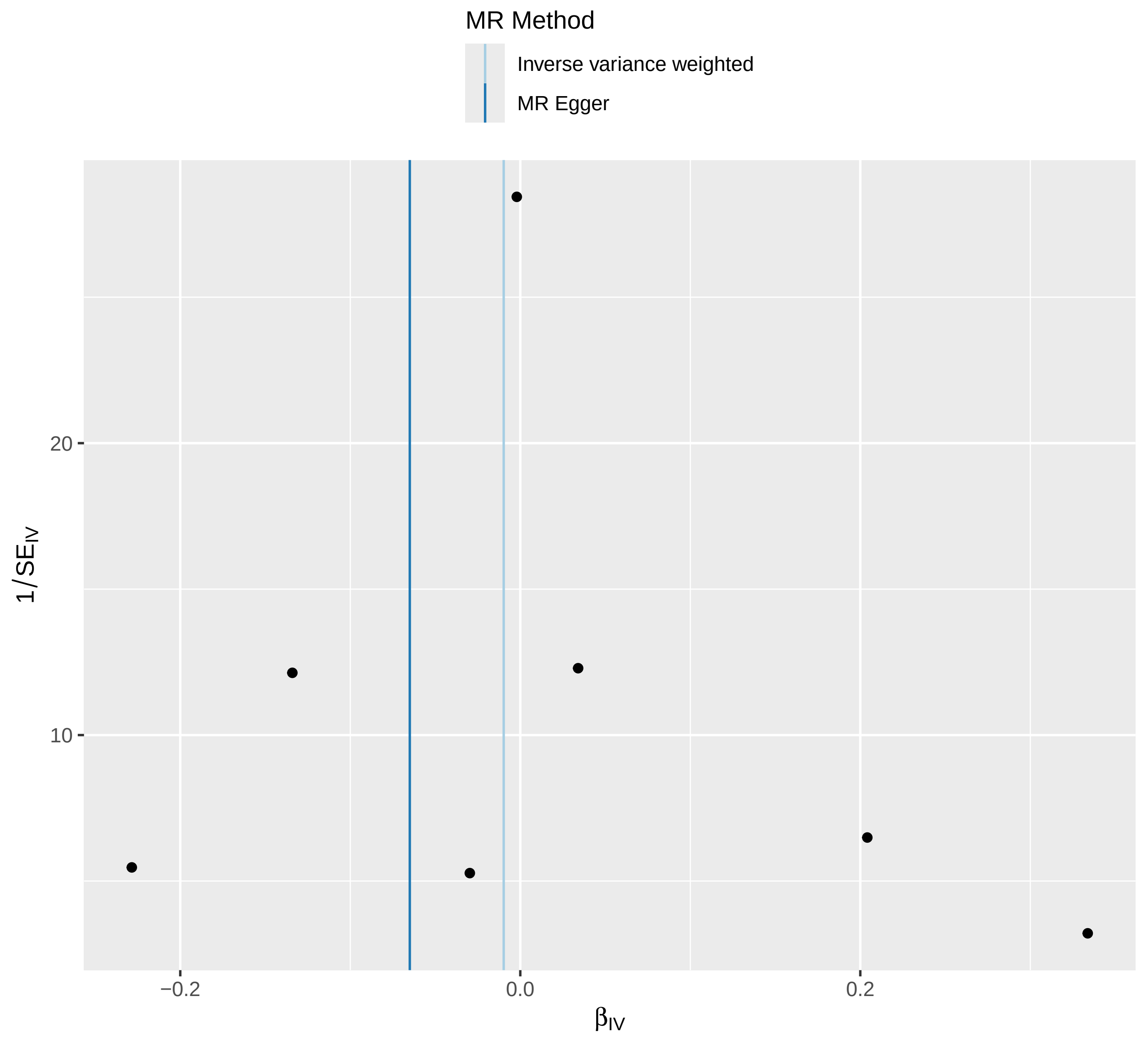
MR Method
Inverse variance weighted
MR Egger
20
10
1/$SE_{IV}$
−0.2
0.0
0.2
$\beta_{IV}$

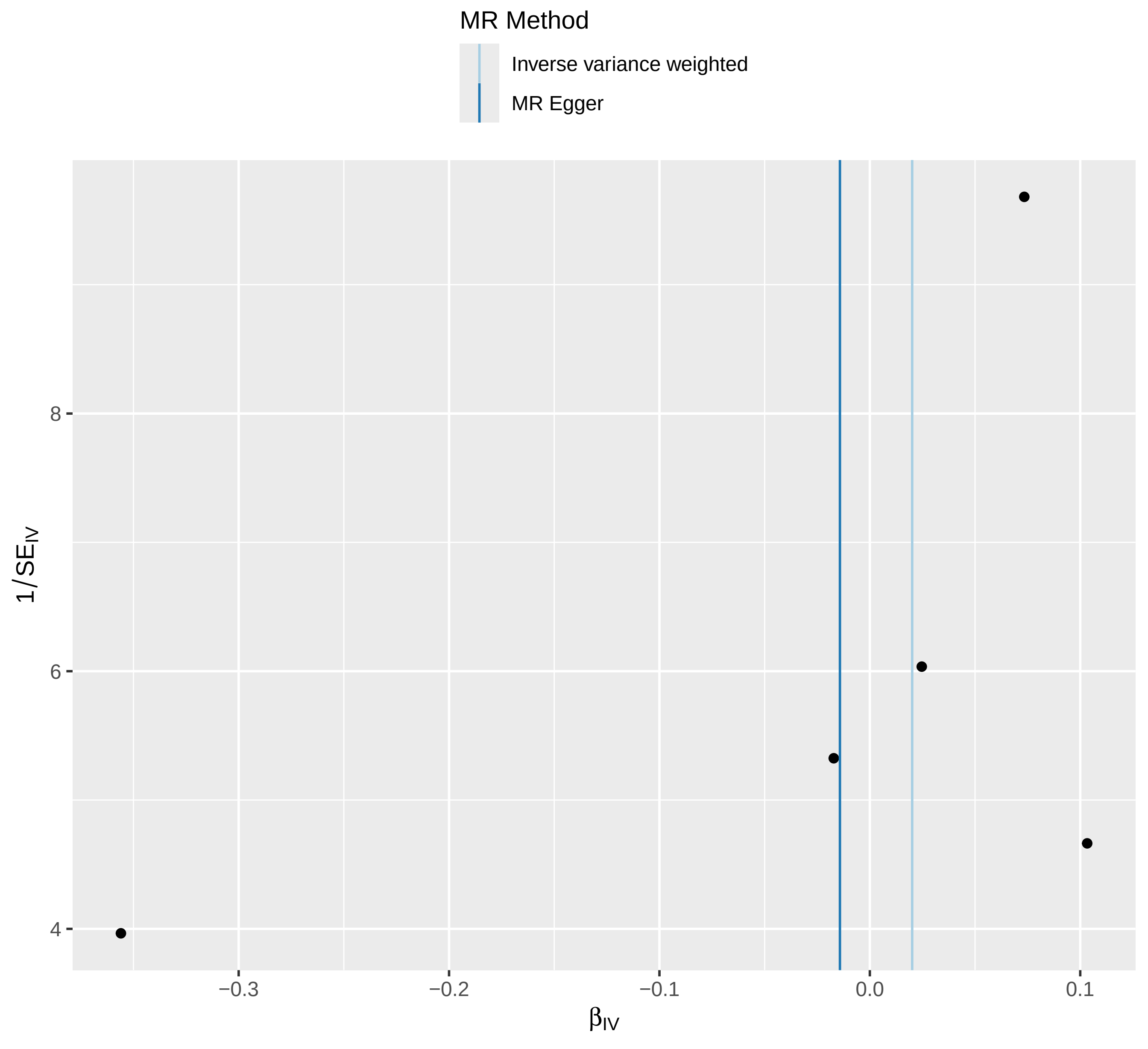
MR Method
Inverse variance weighted
MR Egger
8
6
4
1/SE_IV
−0.3
−0.2
−0.1
0.0
0.1
β_IV

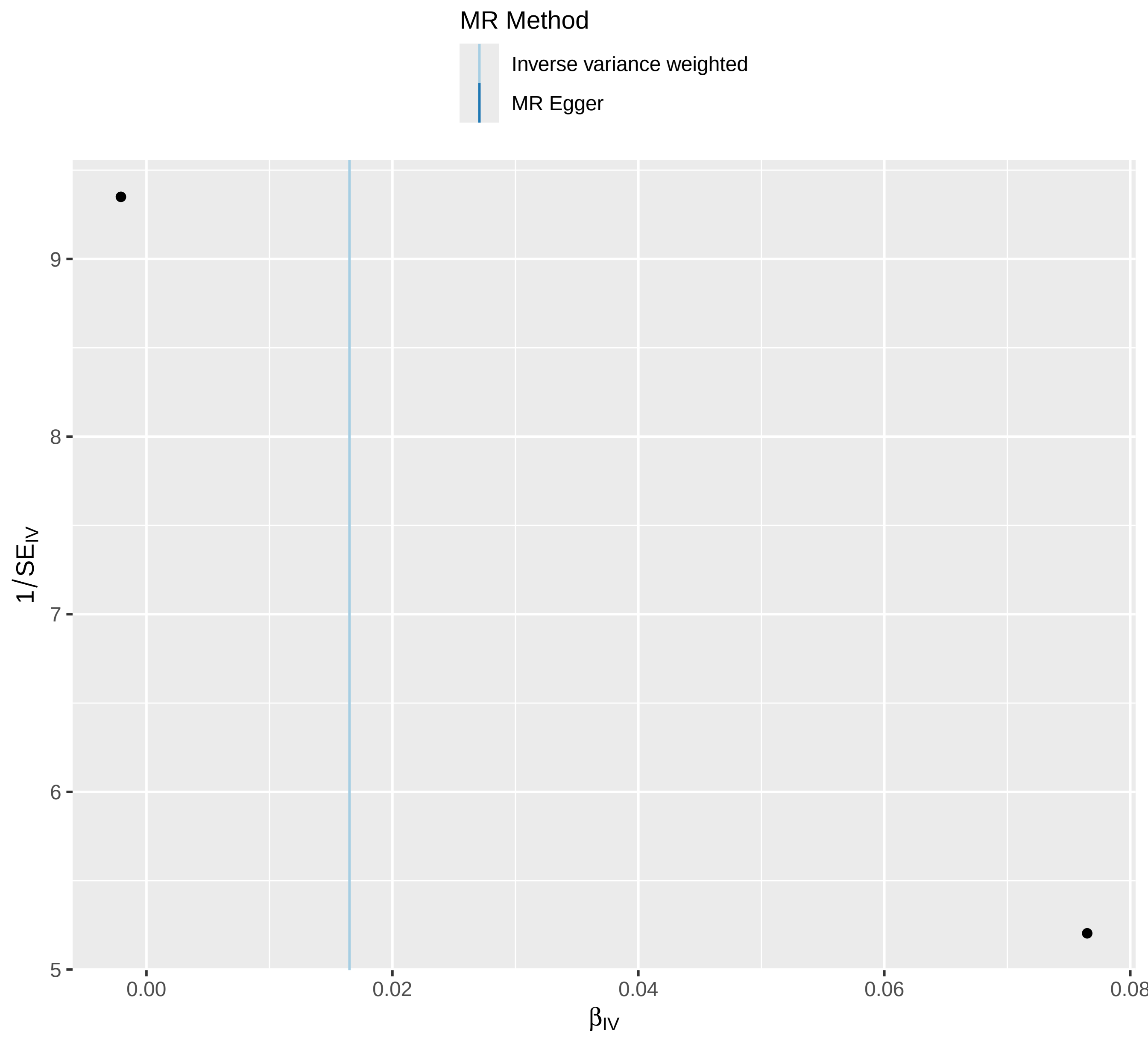

MR Method
Inverse variance weighted
MR Egger
9
8
7
6
5
1/SE$_{IV}$
0.00
0.02
0.04
0.06
0.08
β$_{IV}$

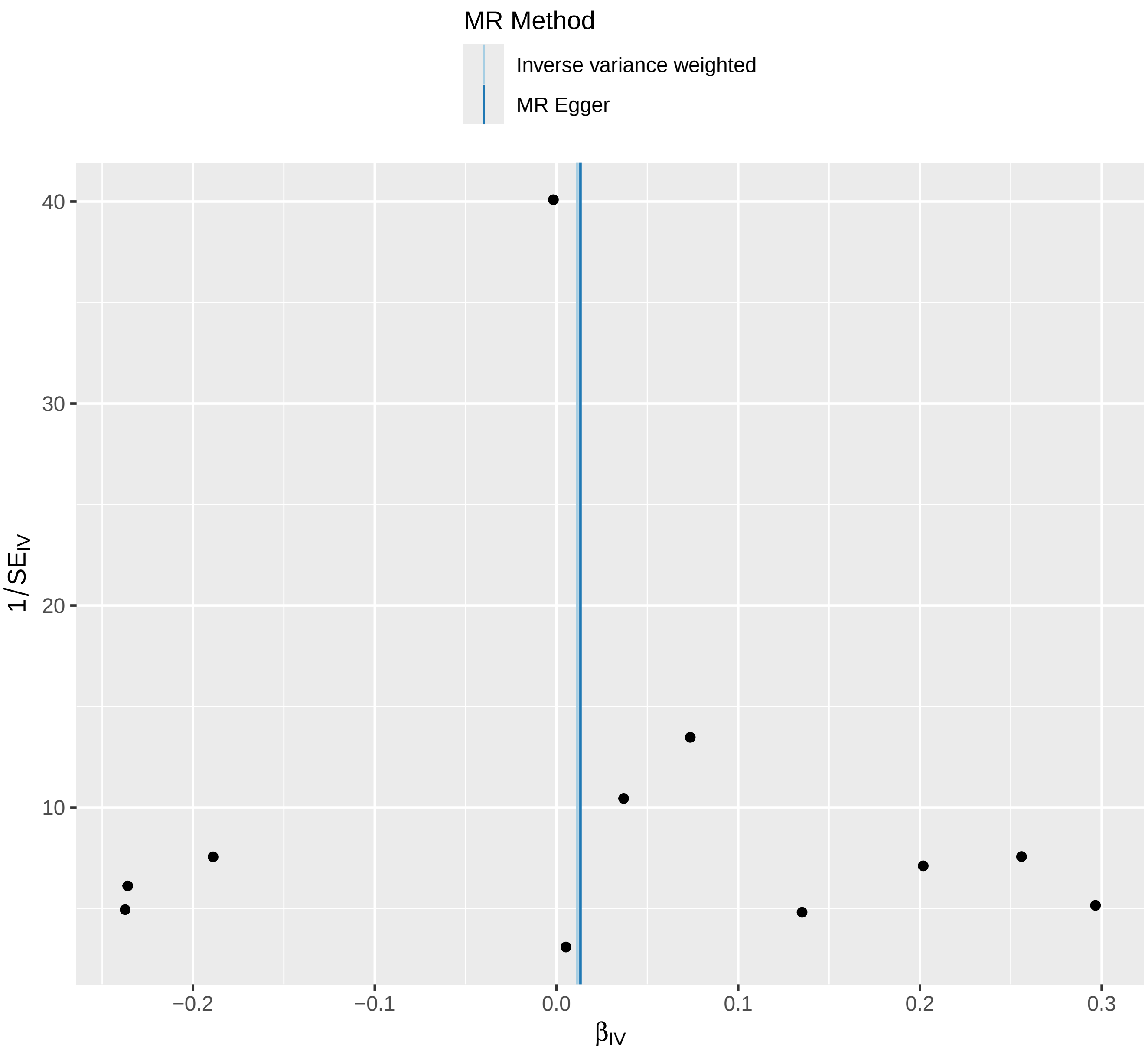
MR Method
Inverse variance weighted
MR Egger
40
30
20
10
−0.2
−0.1
0.0
0.1
0.2
0.3
$\beta_{IV}$
$1/SE_{IV}$

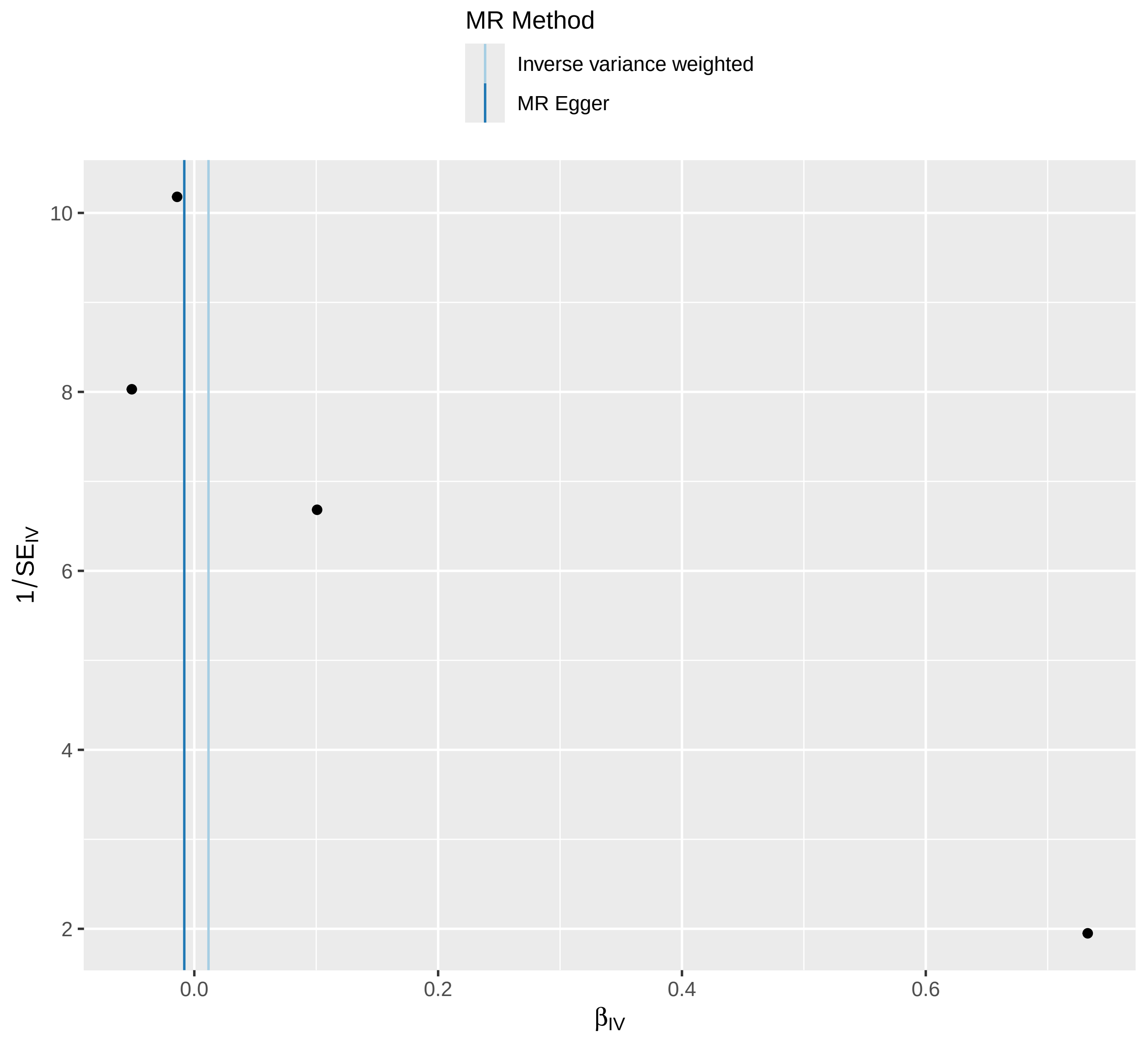
MR Method
Inverse variance weighted
MR Egger
10
8
6
4
2
1/SE_IV
0.0
0.2
0.4
0.6
β_IV

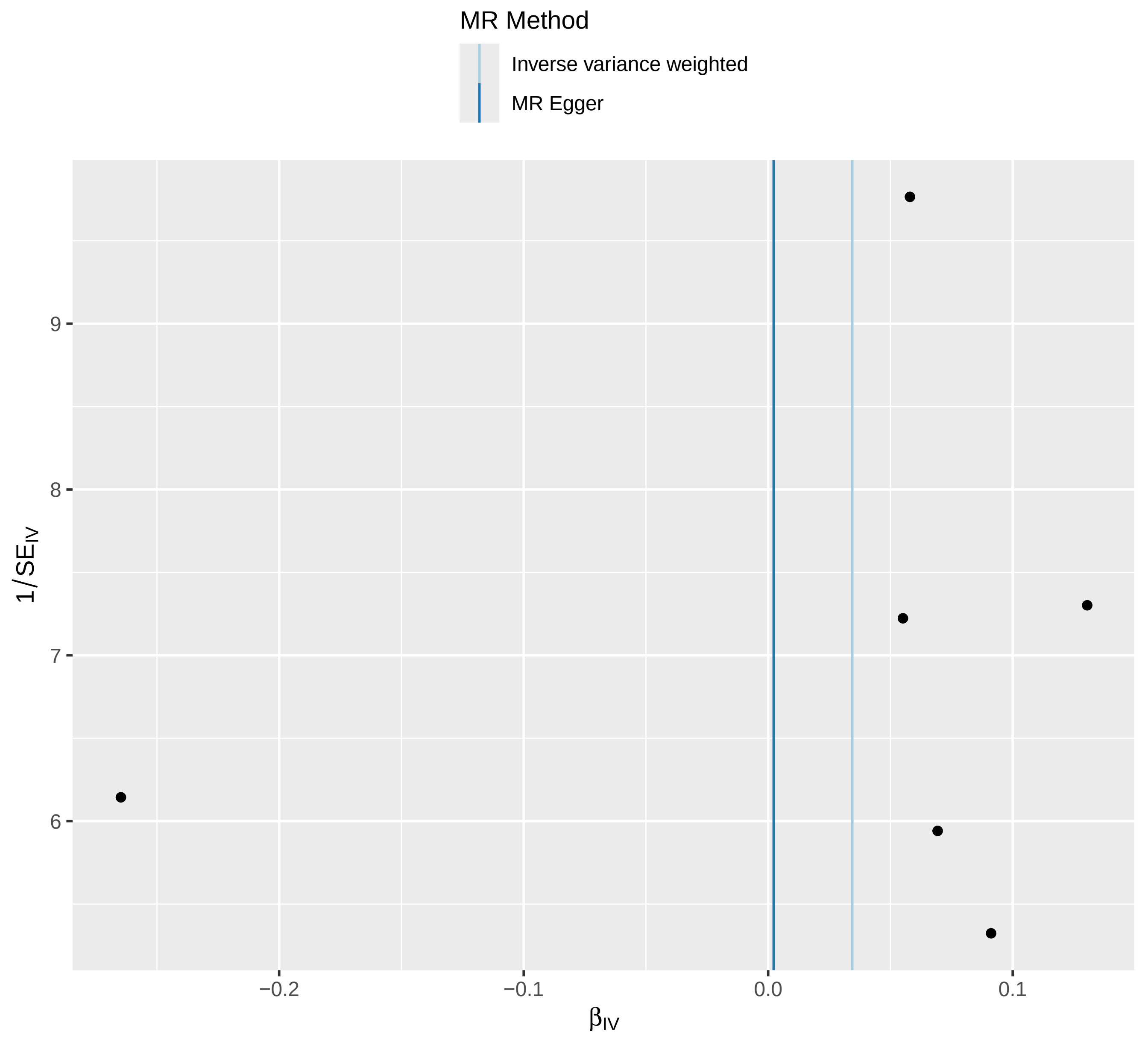
MR Method
Inverse variance weighted
MR Egger
1/SE_IV
9
8
7
6
−0.2
−0.1
0.0
0.1
β_IV

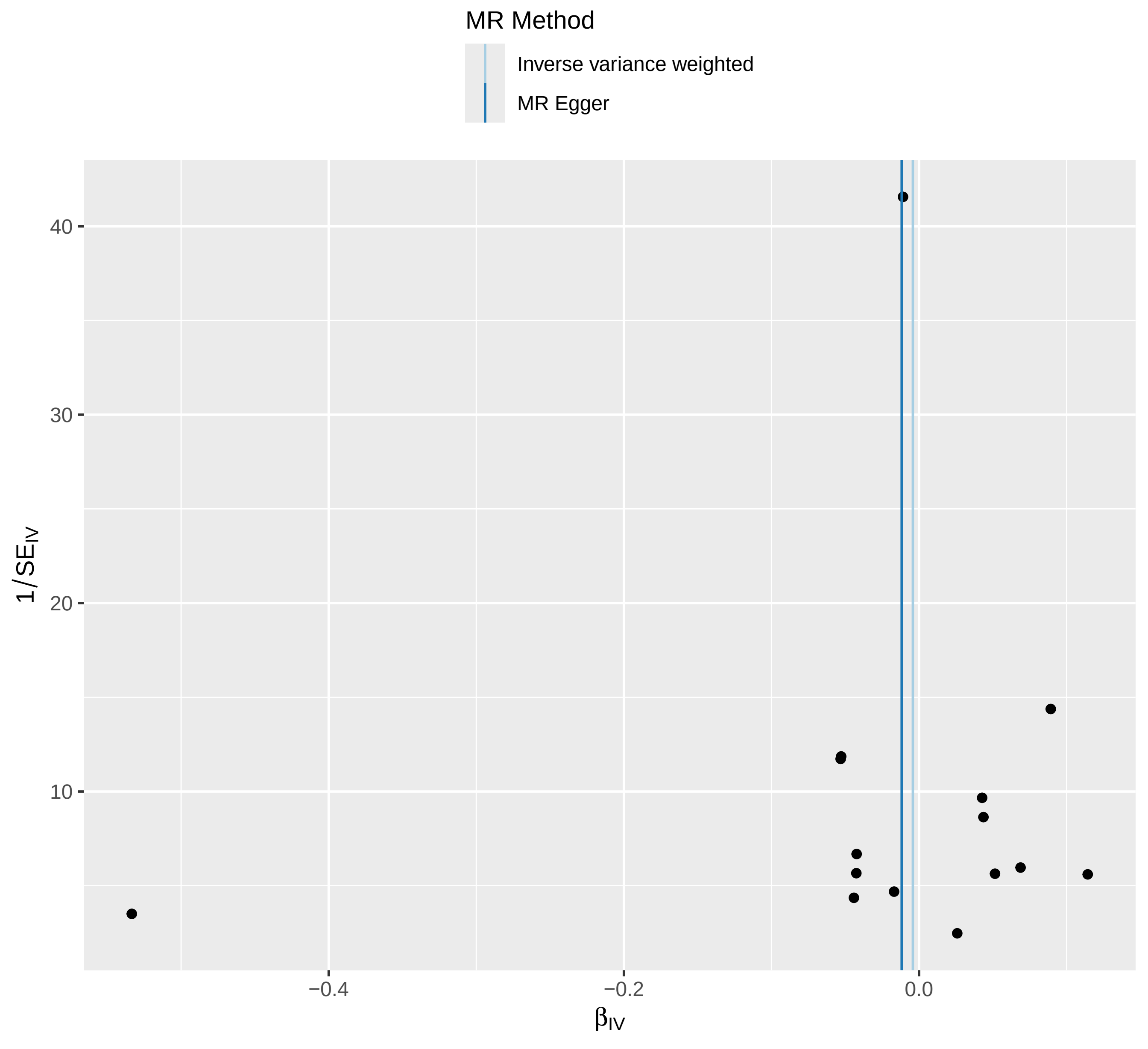
MR Method
Inverse variance weighted
MR Egger
40
30
20
10
−0.4
−0.2
0.0
$1/SE_{IV}$
$\beta_{IV}$

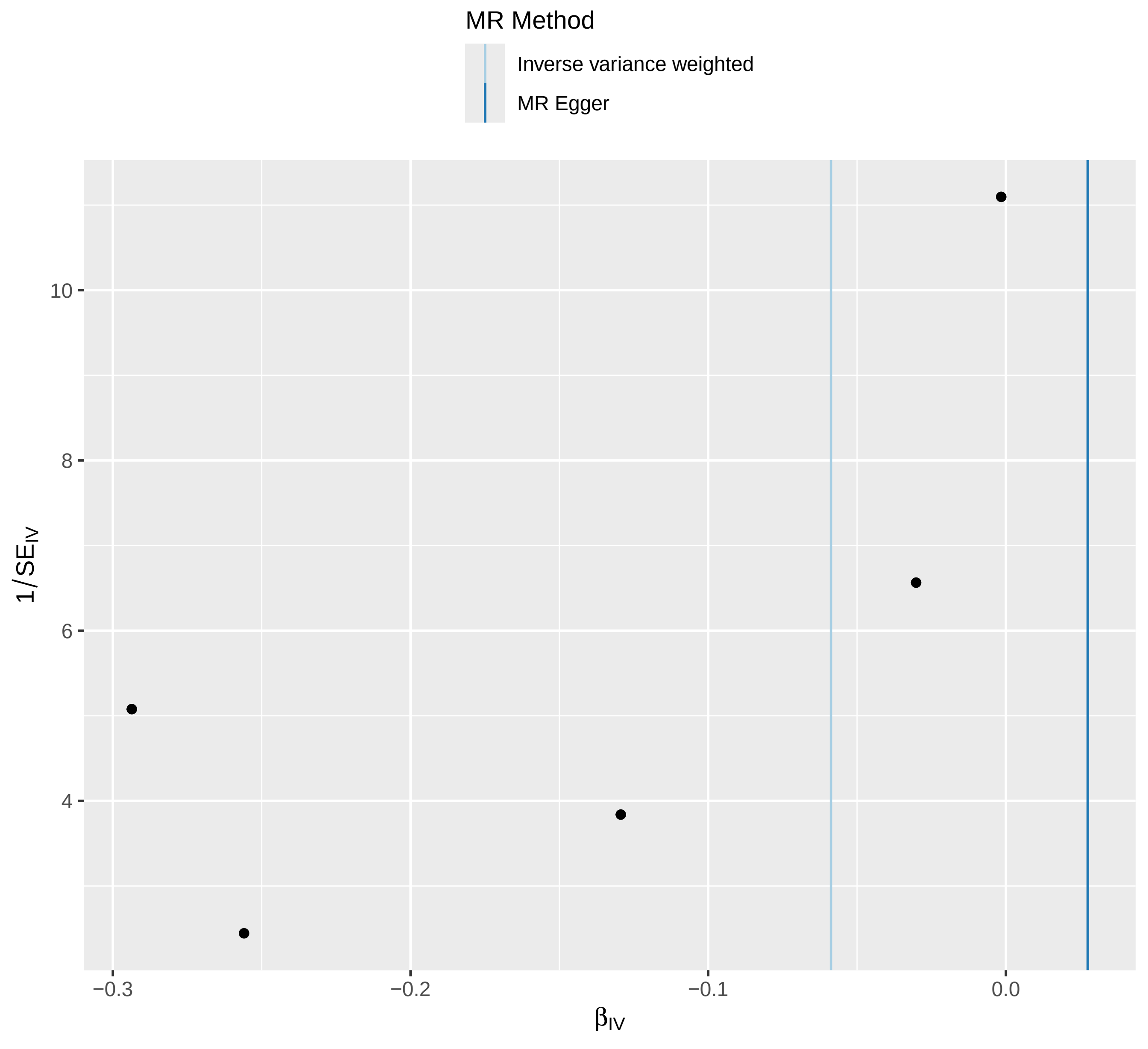
MR Method
Inverse variance weighted
MR Egger
10
8
6
4
1/SE_IV
−0.3
−0.2
−0.1
0.0
β_IV

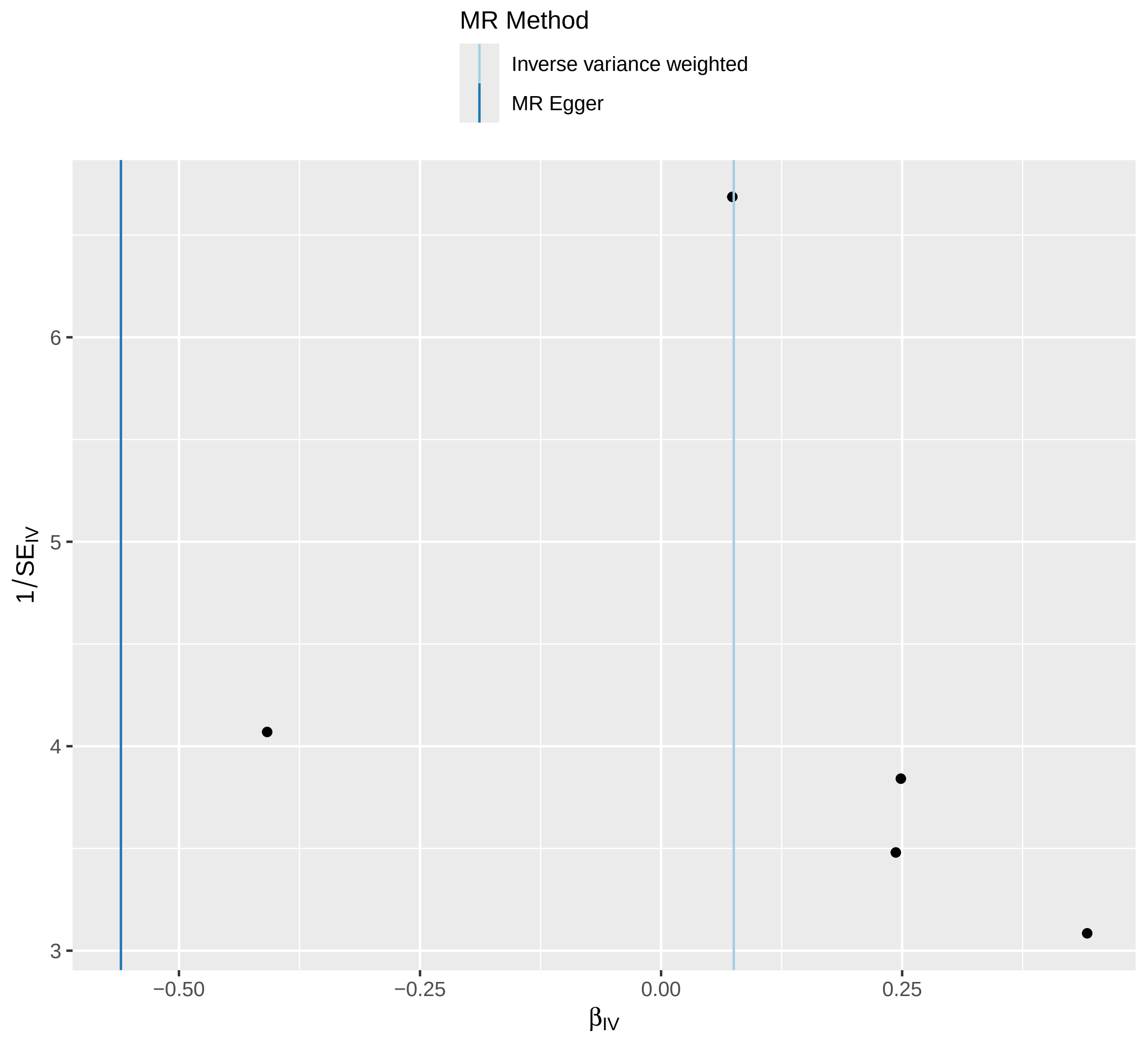

MR Method
Inverse variance weighted
MR Egger
6
5
4
3
1/SE_IV
−0.50
−0.25
0.00
0.25
β_IV

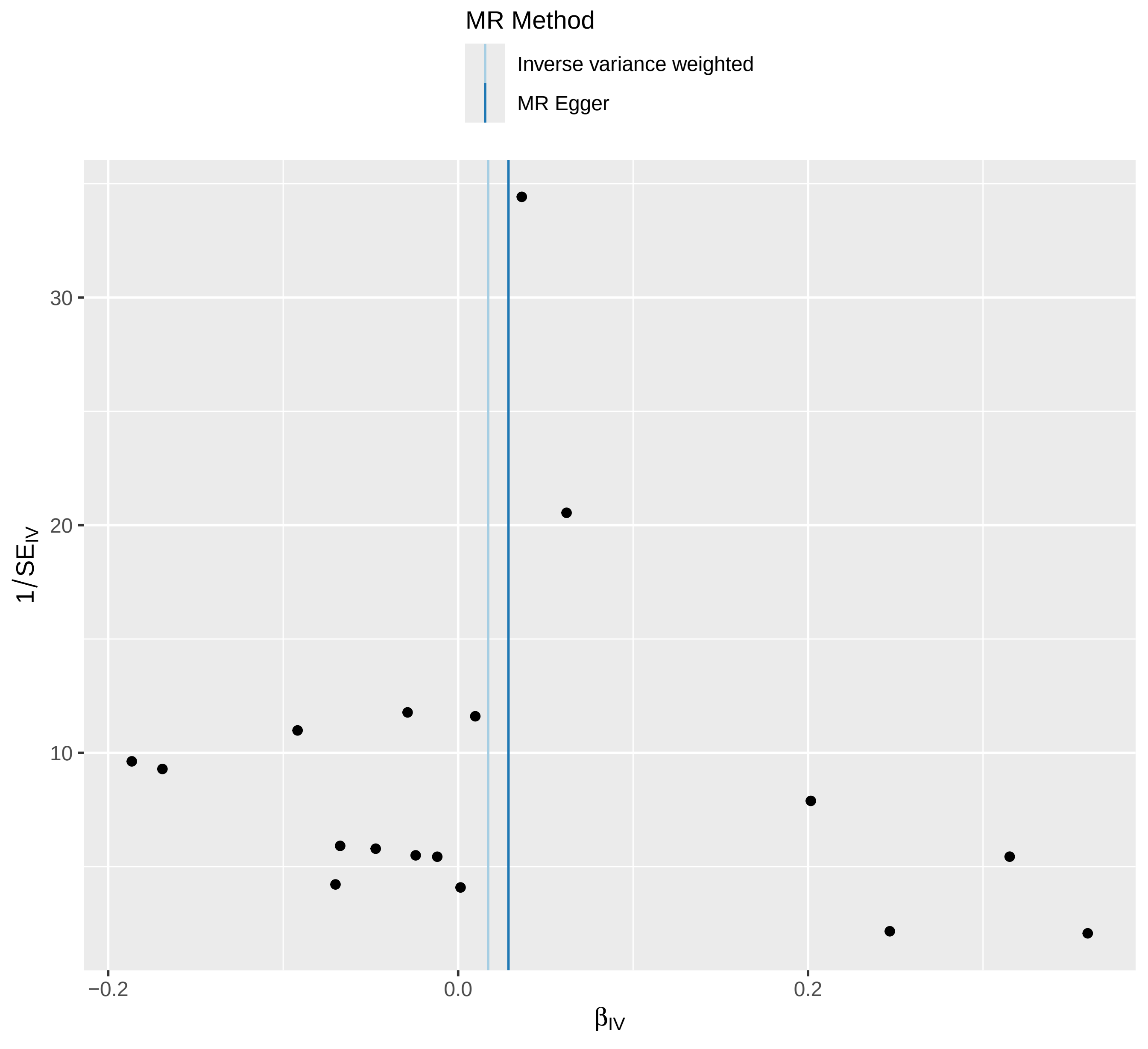
MR Method
Inverse variance weighted
MR Egger
30
20
10
$1/SE_{IV}$
−0.2
0.0
0.2
$\beta_{IV}$

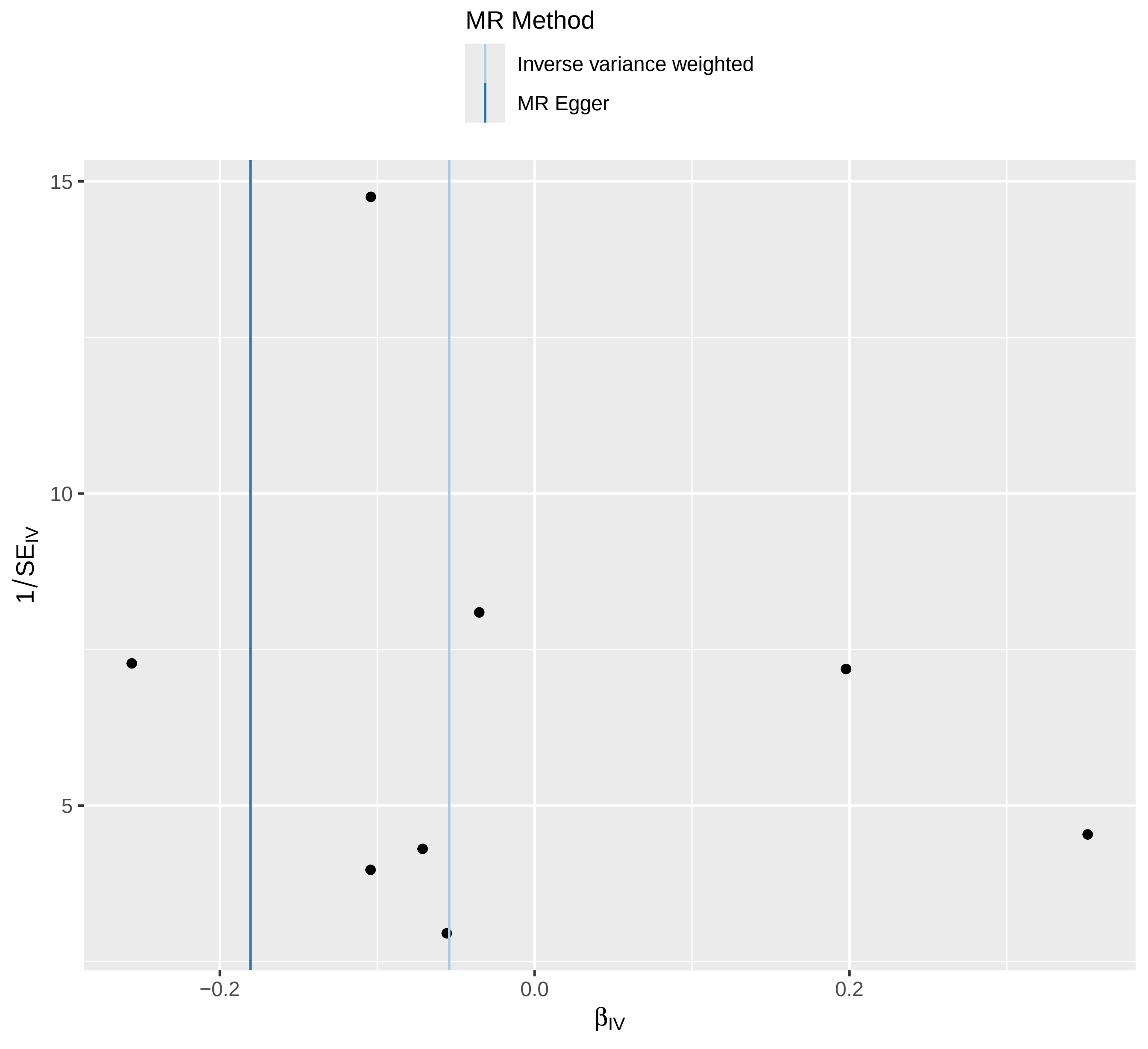

MR Method
Inverse variance weighted
MR Egger
15
10
5
1/SE$_{IV}$
−0.2
0.0
0.2
β$_{IV}$

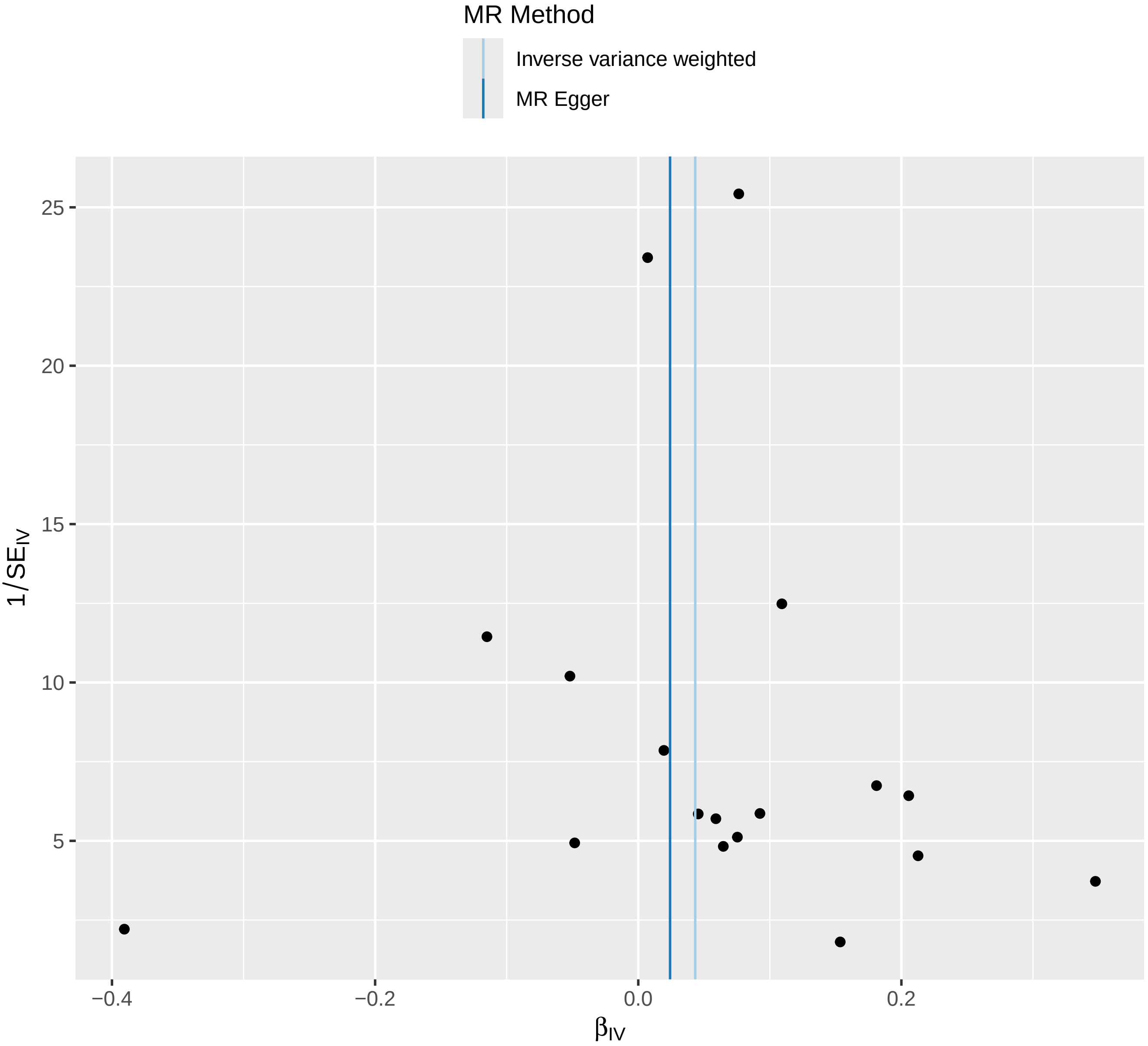
MR Method
Inverse variance weighted
MR Egger
25
20
15
10
5
$1/SE_{IV}$
−0.4
−0.2
0.0
0.2
$\beta_{IV}$

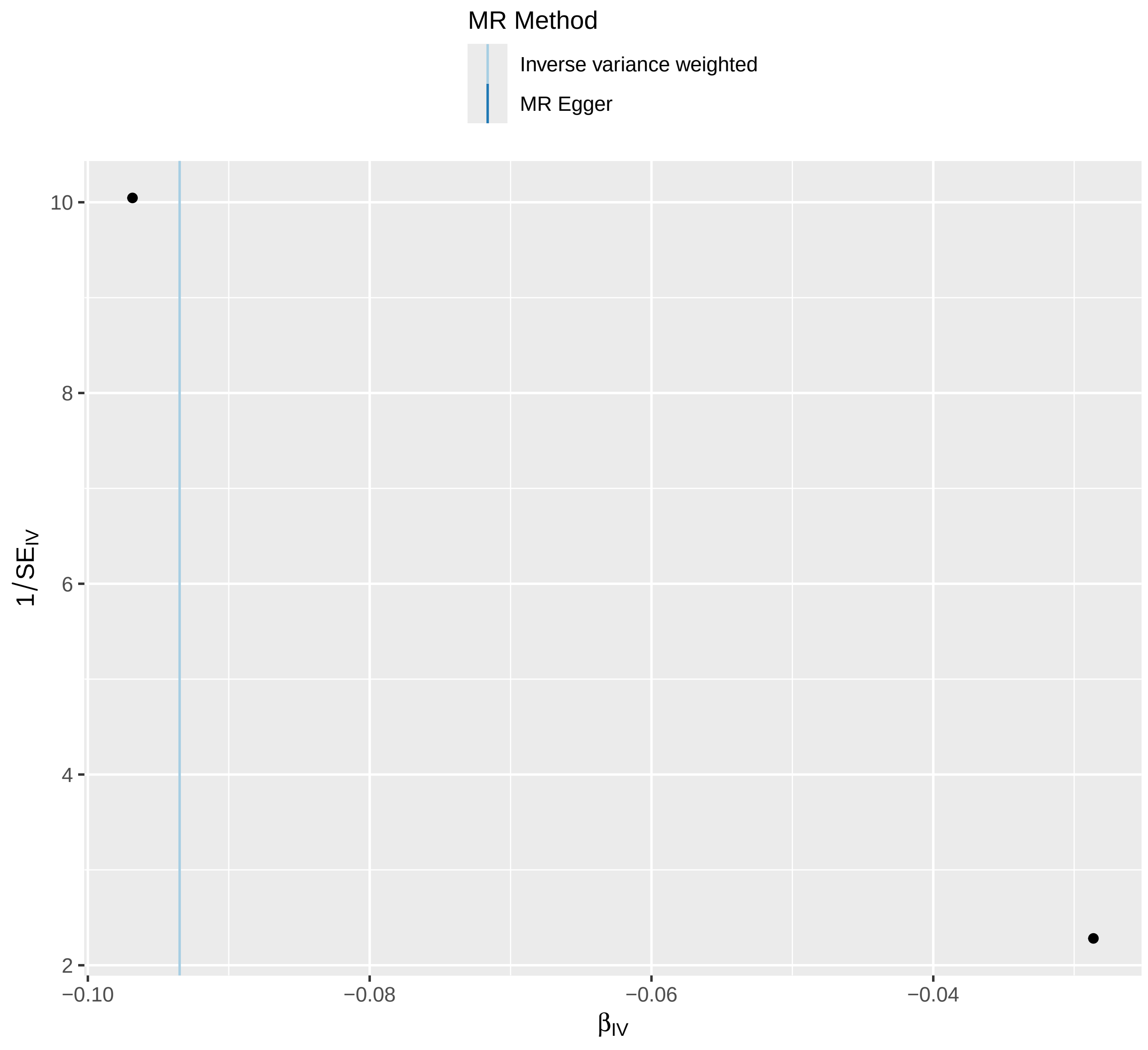
MR Method
Inverse variance weighted
MR Egger
10
8
6
4
2
$1/SE_{IV}$
−0.10
−0.08
−0.06
−0.04
$\beta_{IV}$

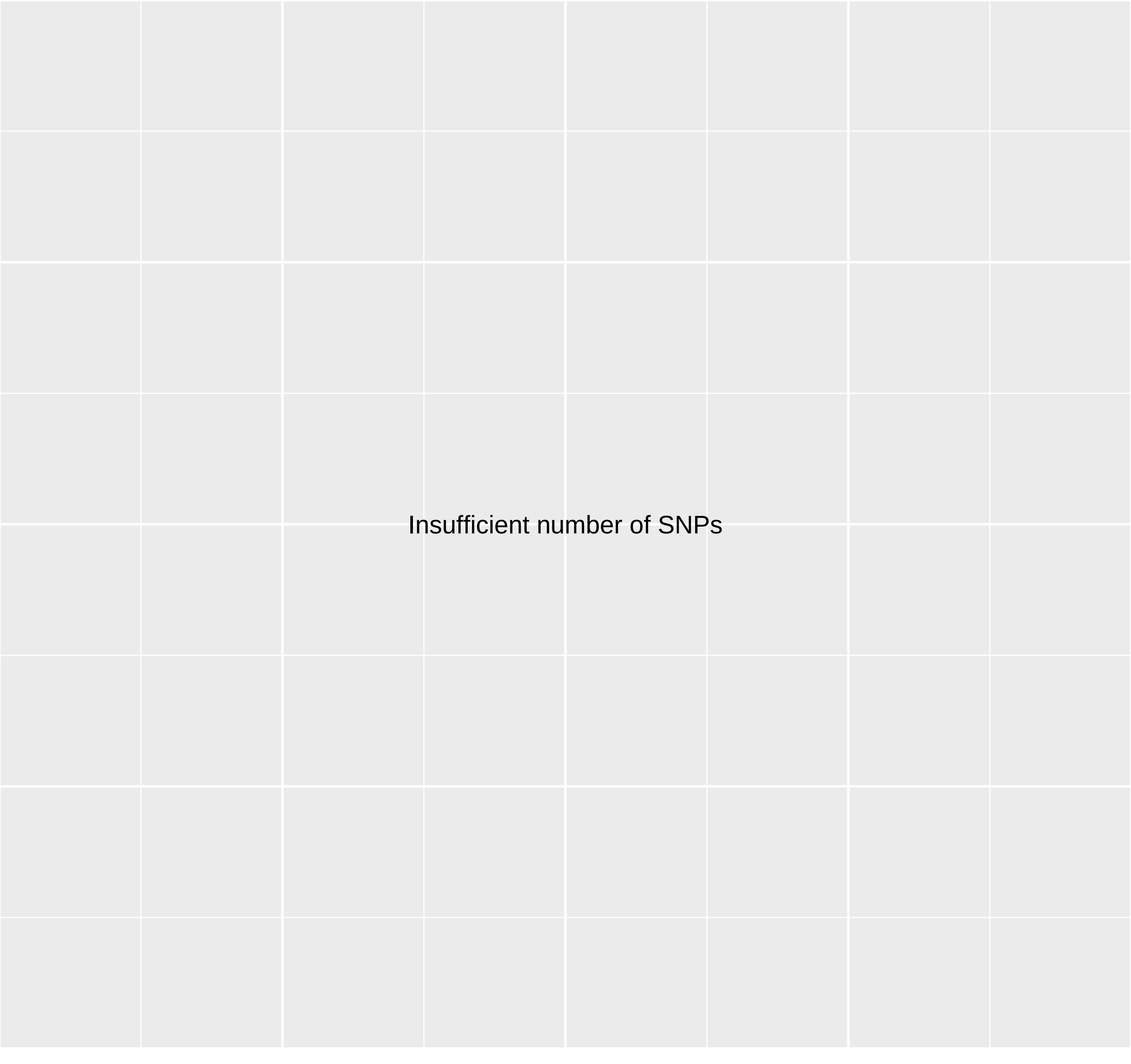
Insufficient number of SNPs

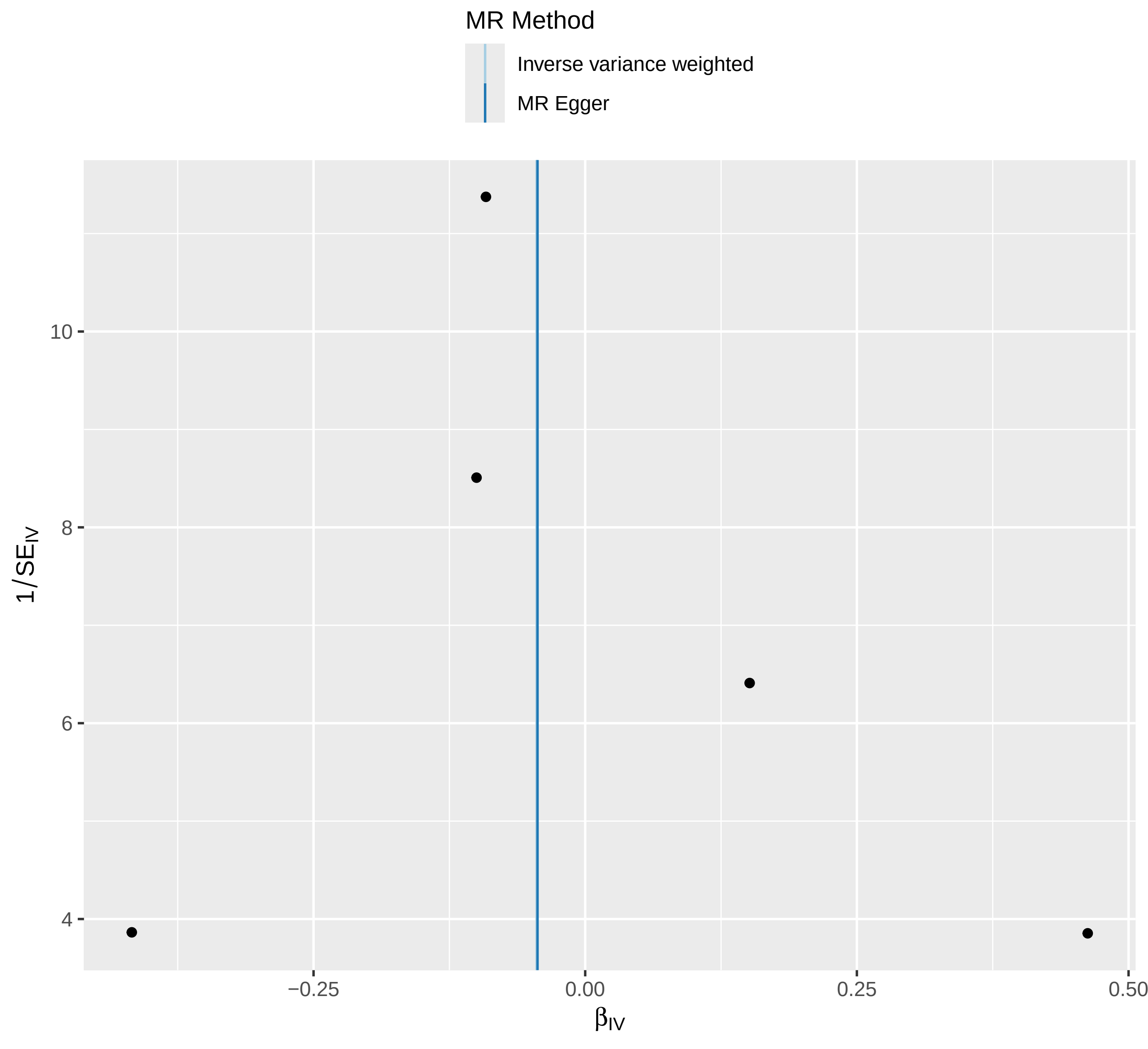

MR Method
Inverse variance weighted
MR Egger
10
8
6
4
1/SE$_{IV}$
−0.25
0.00
0.25
0.50
β$_{IV}$

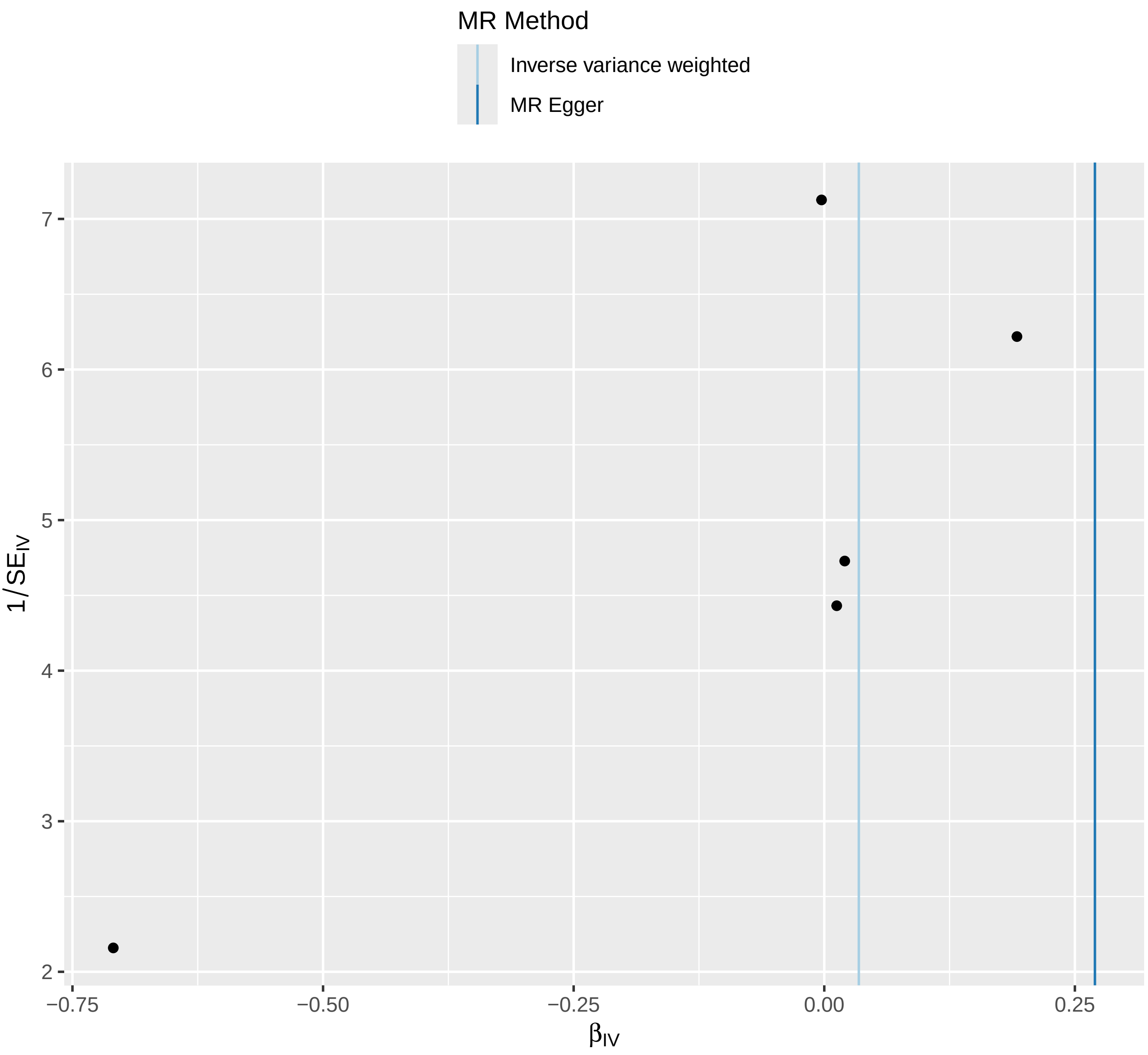

MR Method
Inverse variance weighted
MR Egger
7
6
5
4
3
2
1/SE_IV
−0.75
−0.50
−0.25
0.00
0.25
β_IV

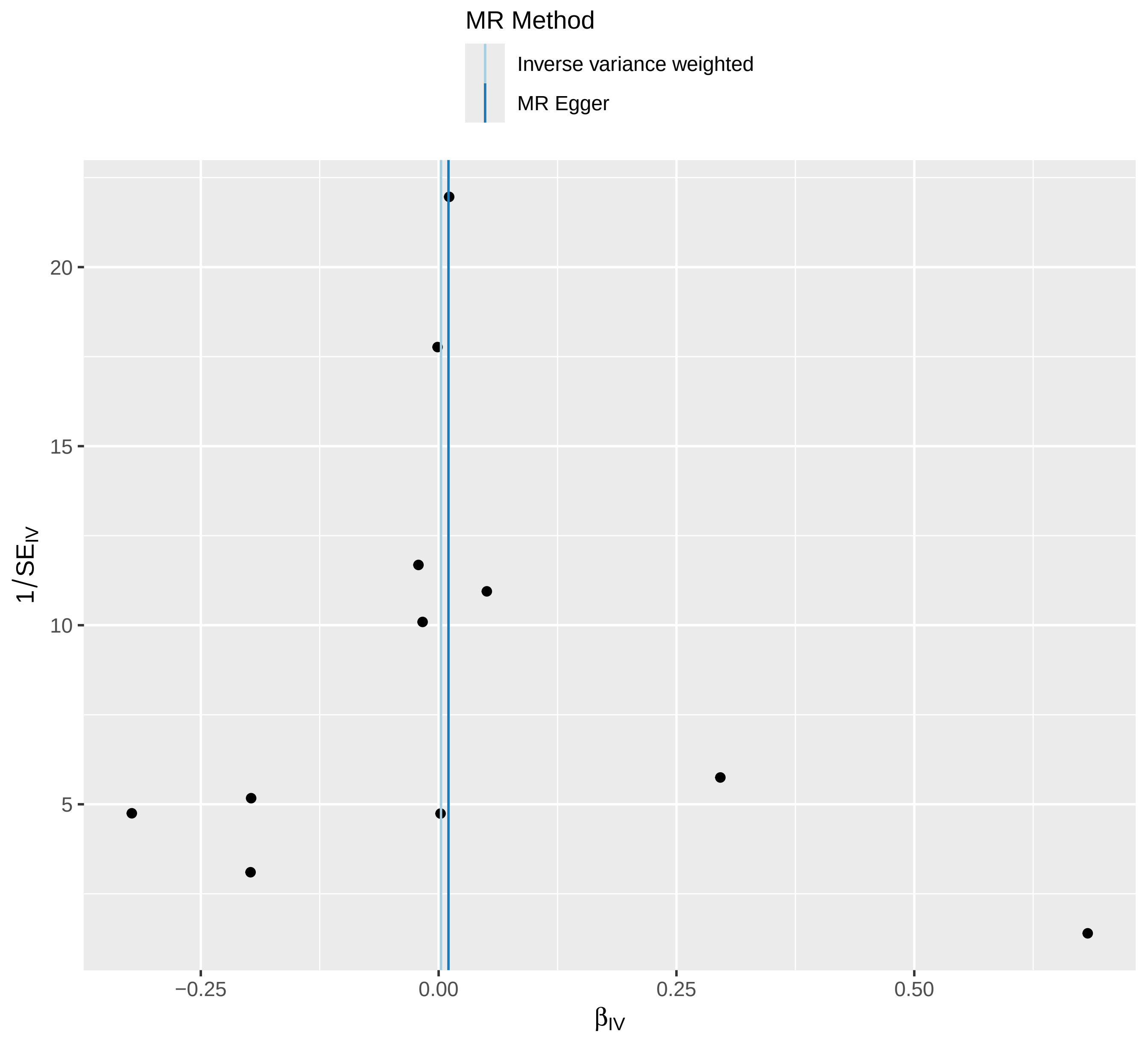

MR Method
Inverse variance weighted
MR Egger
20
15
10
5
1/SE$_{IV}$
−0.25
0.00
0.25
0.50
β$_{IV}$

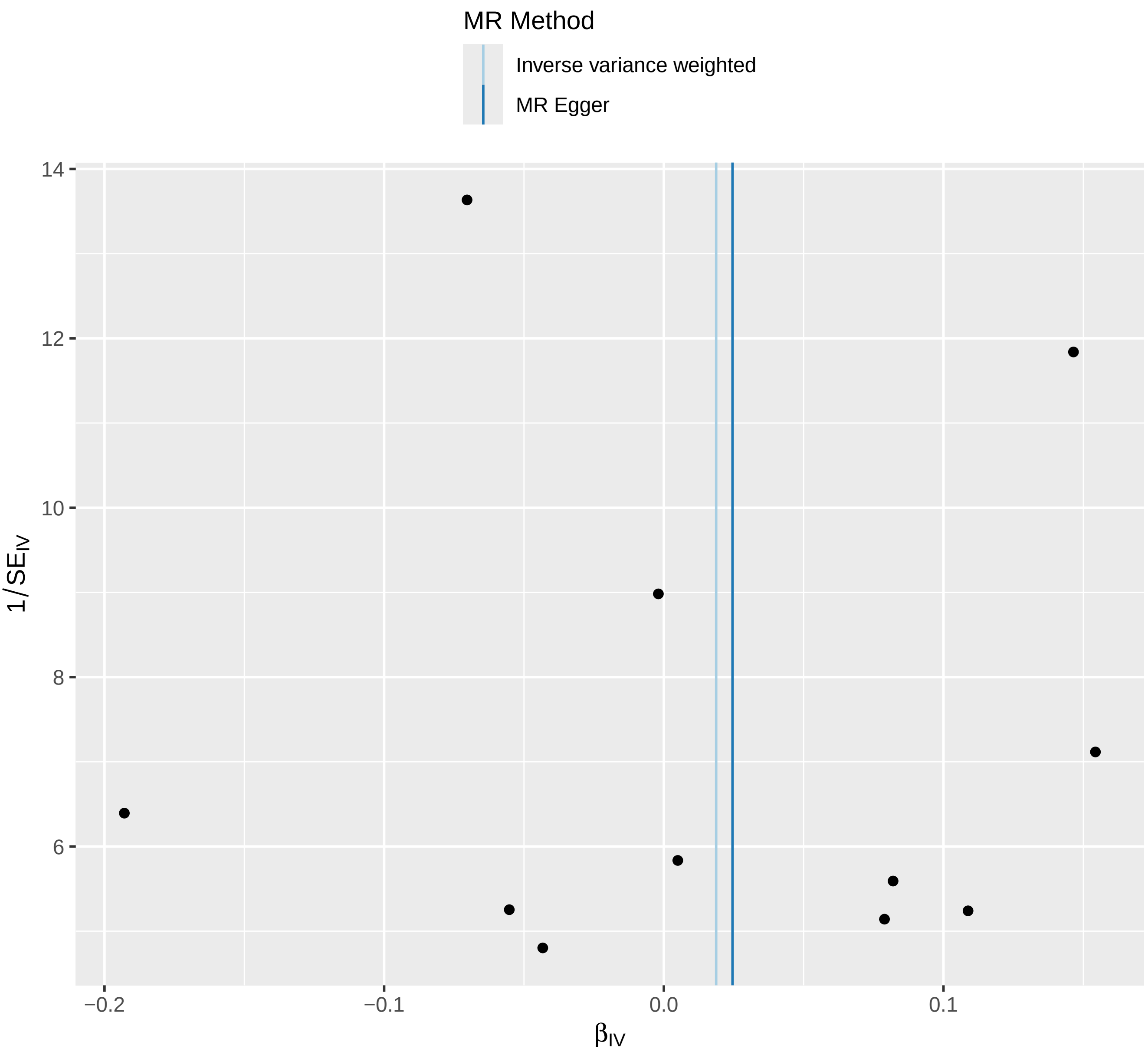
MR Method
Inverse variance weighted
MR Egger
14
12
10
8
6
1/SE$_{IV}$
−0.2
−0.1
0.0
0.1
β$_{IV}$

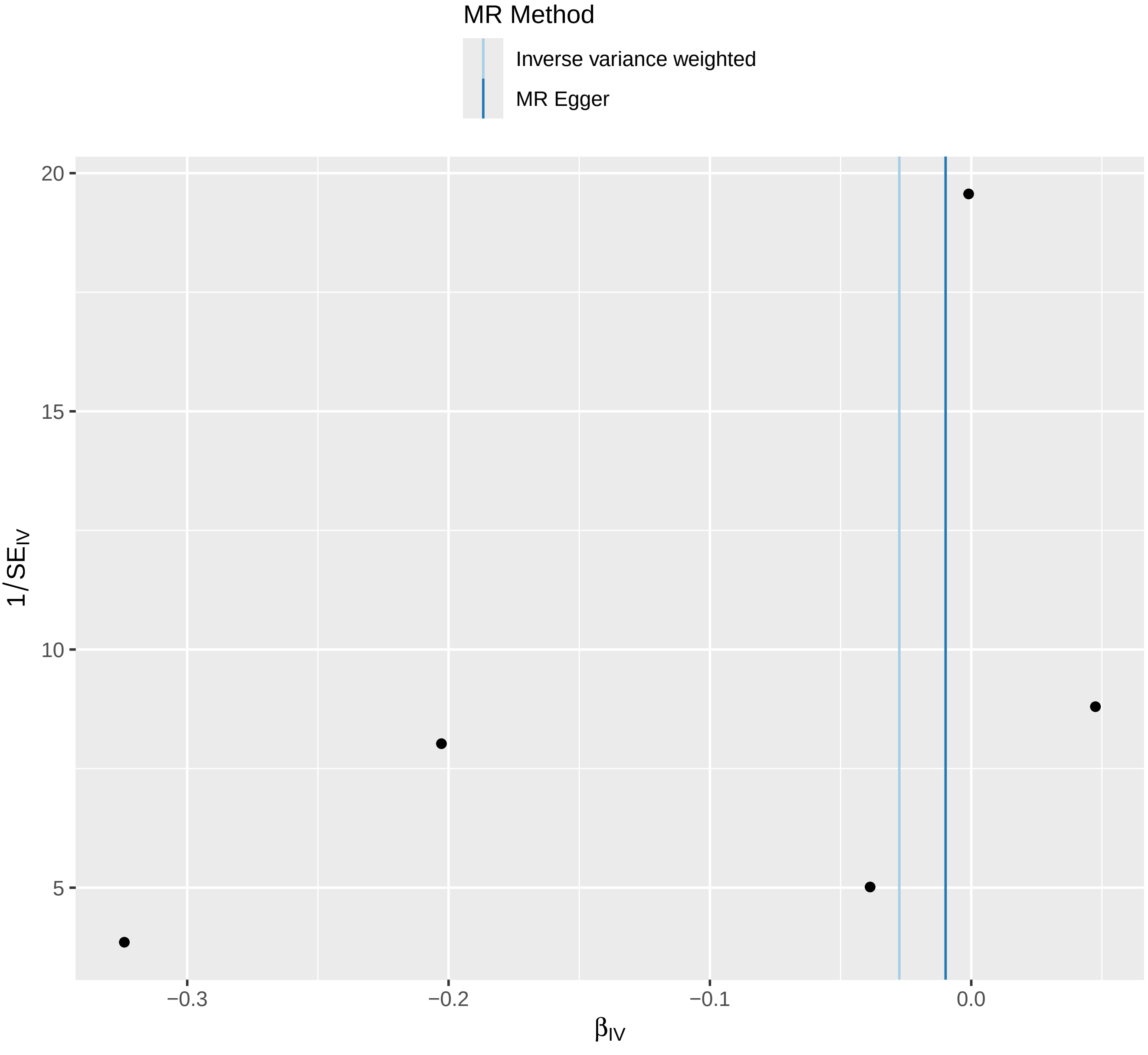
MR Method
Inverse variance weighted
MR Egger
20
15
10
5
1/SE_IV
−0.3
−0.2
−0.1
0.0
β_IV

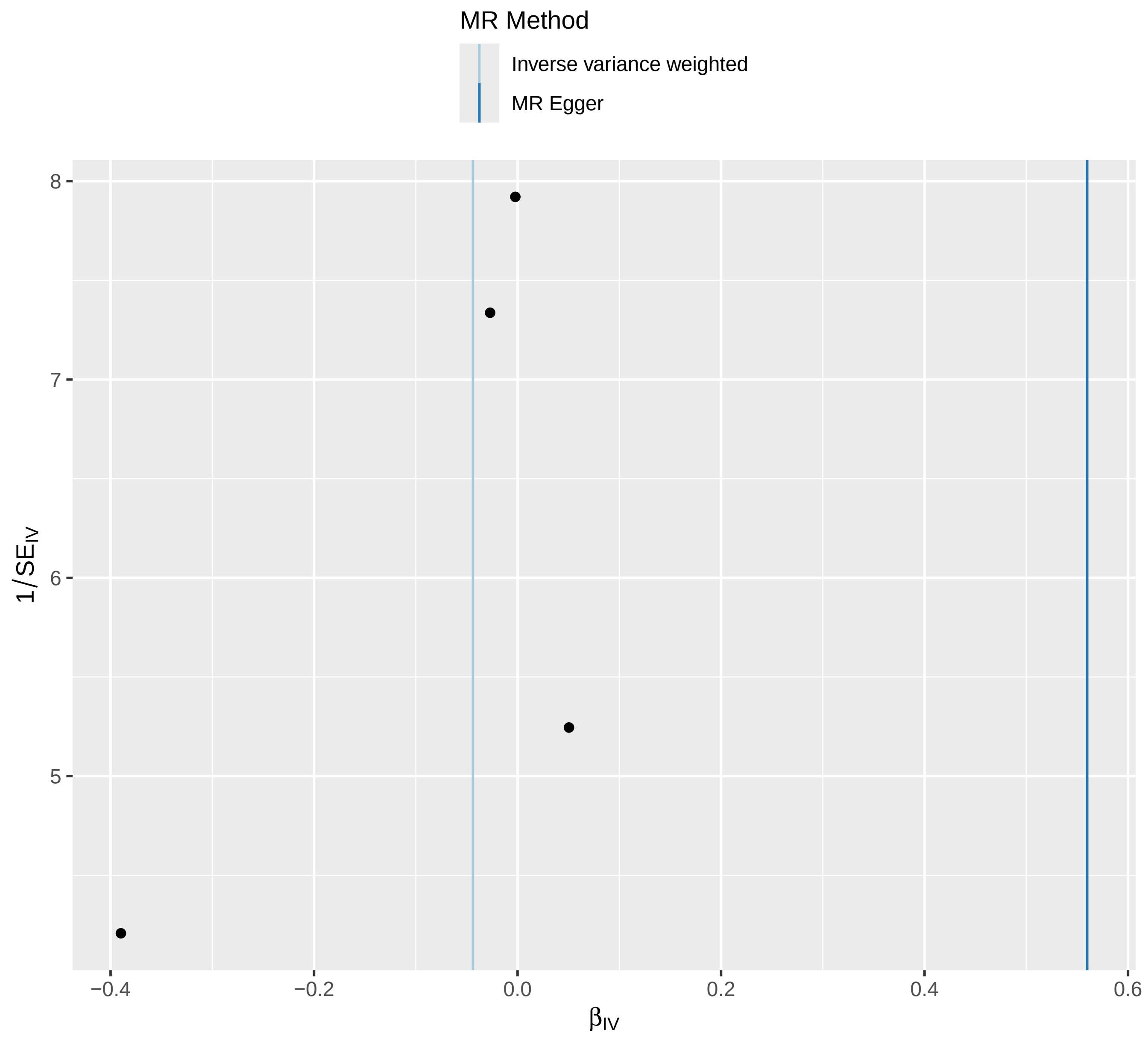
MR Method
Inverse variance weighted
MR Egger
1/SE$_{IV}$
8
7
6
5
−0.4
−0.2
0.0
0.2
0.4
0.6
β$_{IV}$

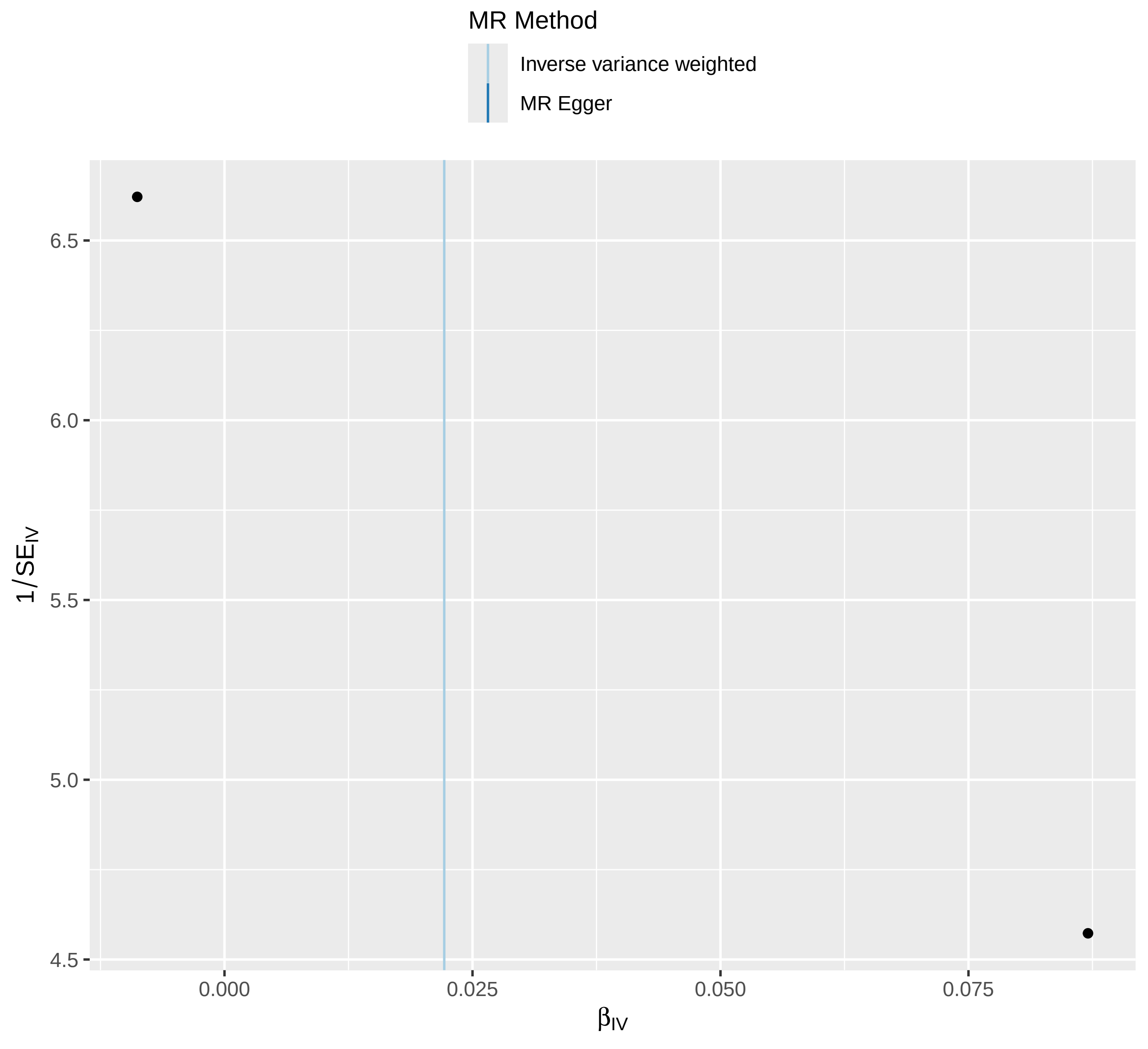
MR Method
Inverse variance weighted
MR Egger
6.5
6.0
5.5
5.0
4.5
1/SE_IV
0.000
0.025
0.050
0.075
β_IV

Insufficient number of SNPs

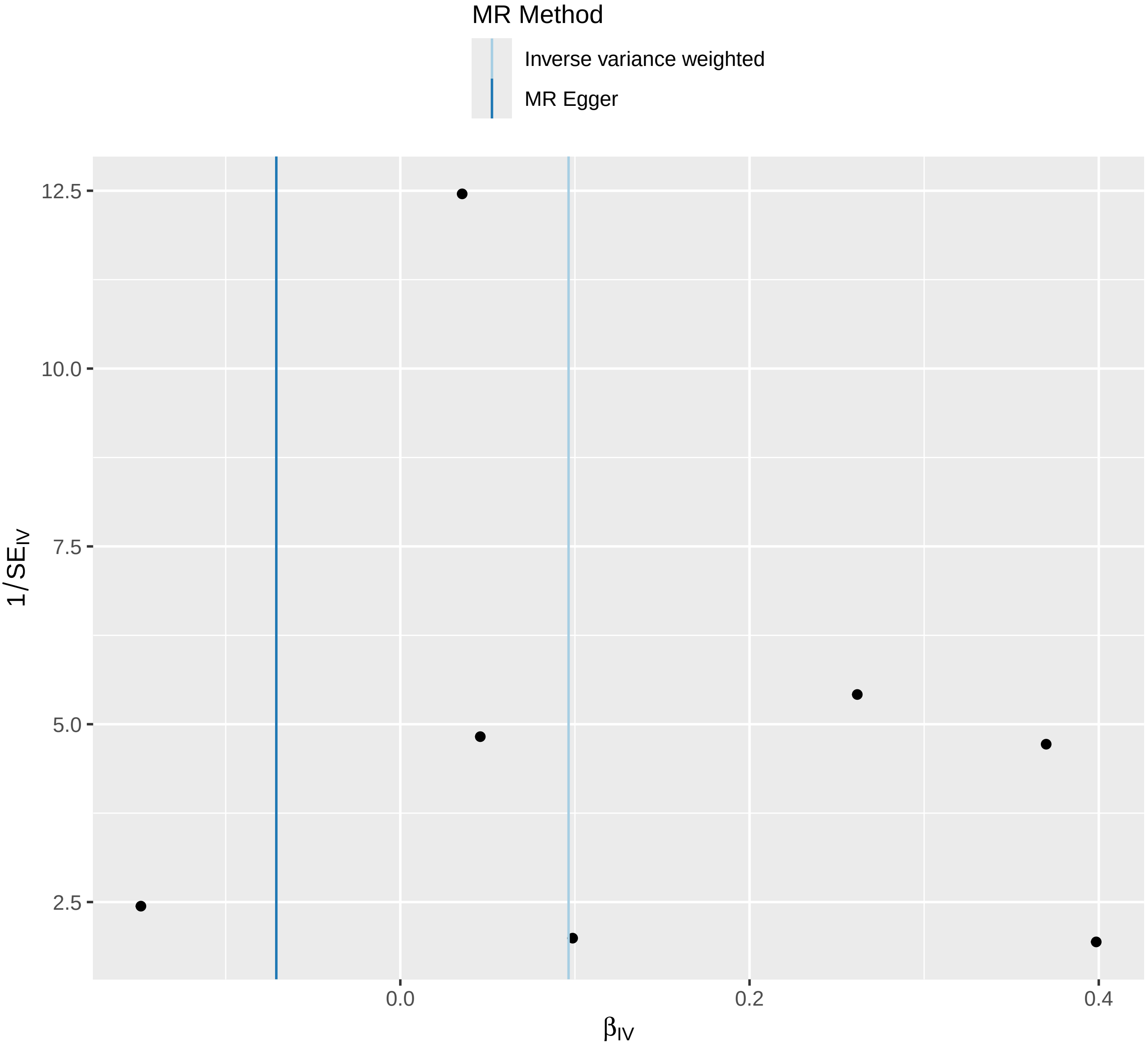

MR Method
Inverse variance weighted
MR Egger
12.5
10.0
7.5
5.0
2.5
$1/SE_{IV}$
0.0
0.2
0.4
$\beta_{IV}$

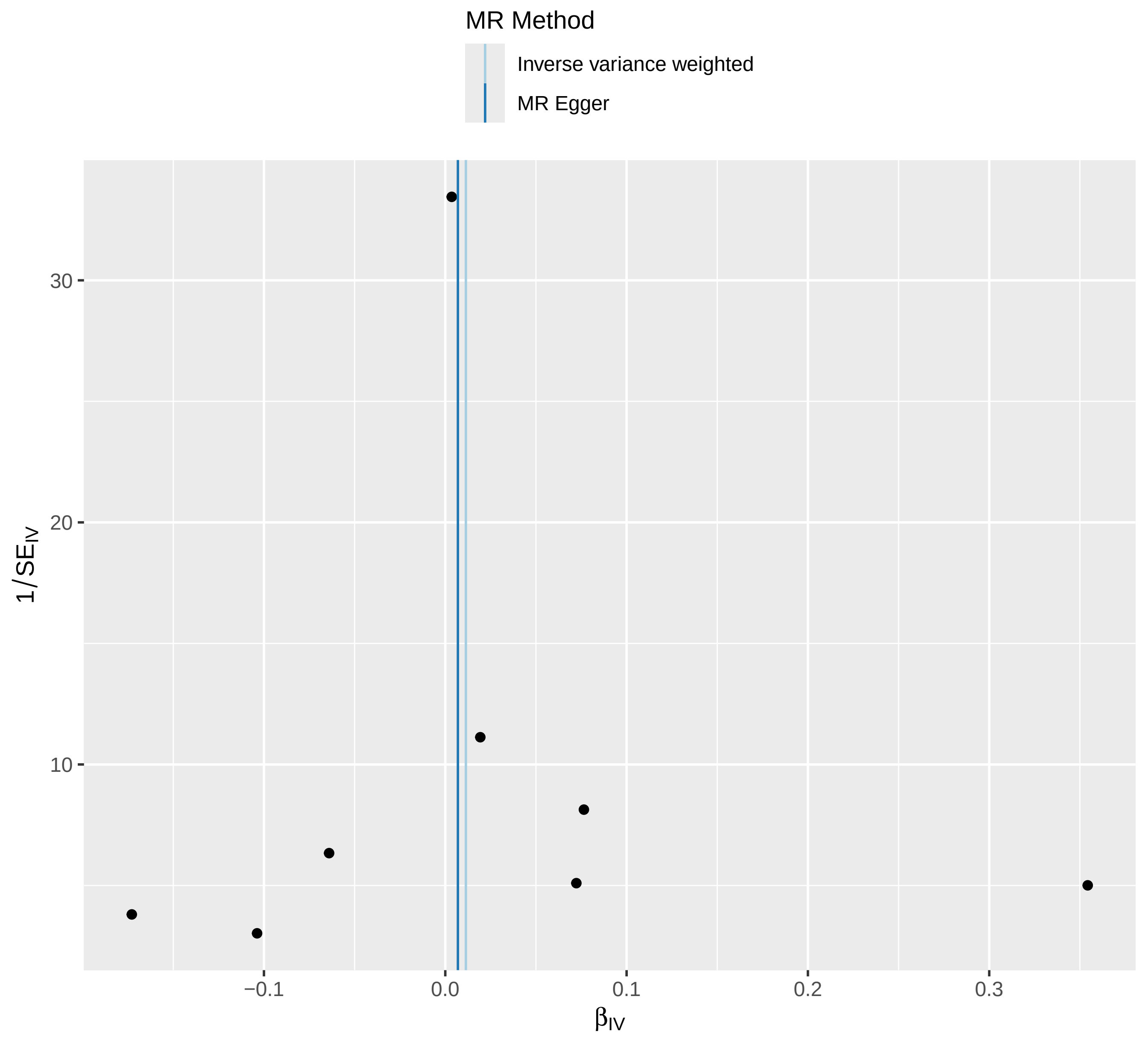

MR Method
Inverse variance weighted
MR Egger
30
20
10
1/SE_IV
−0.1
0.0
0.1
0.2
0.3
β_IV

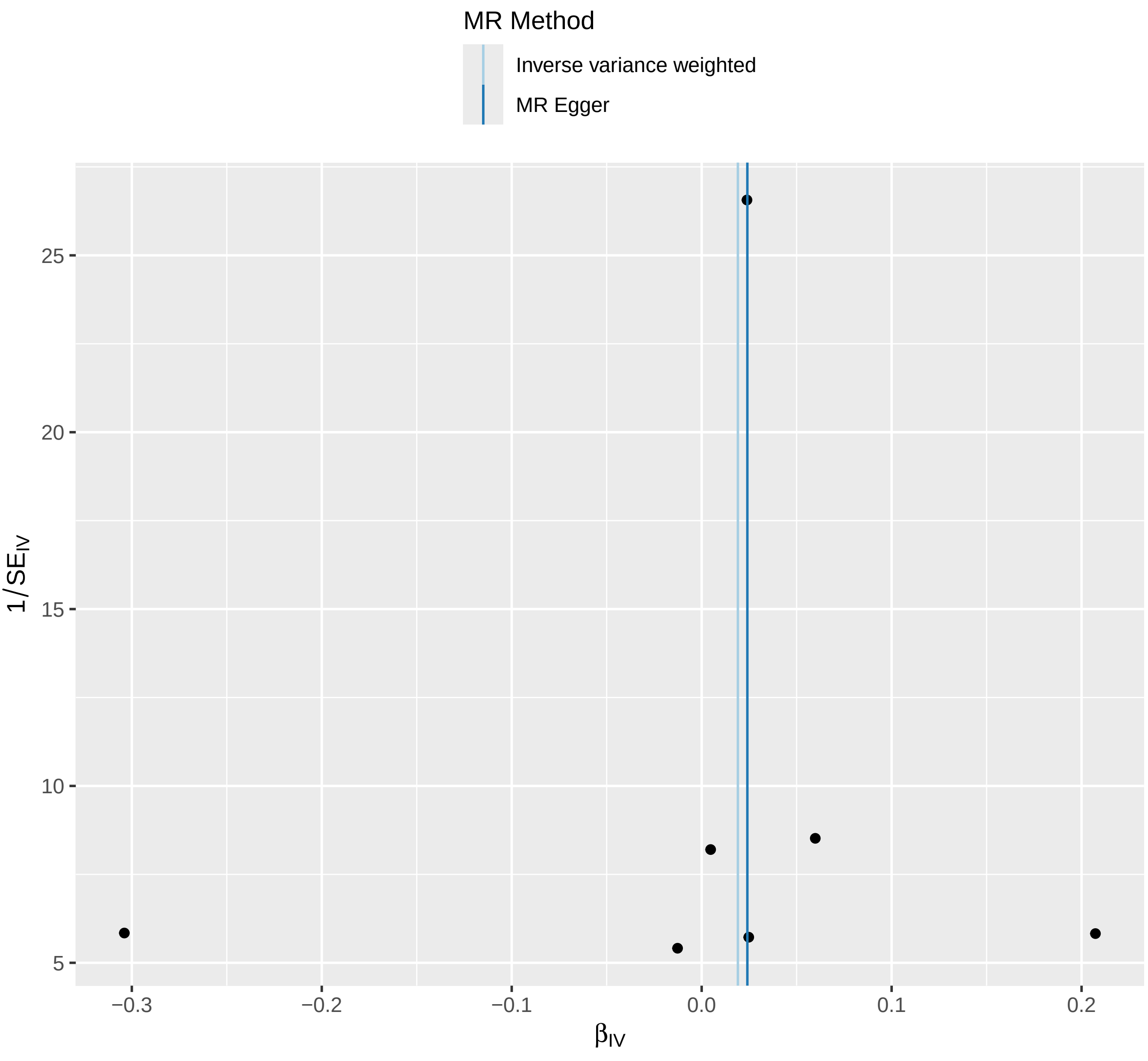

MR Method
Inverse variance weighted
MR Egger
25
20
15
10
5
−0.3
−0.2
−0.1
0.0
0.1
0.2
$1/SE_{IV}$
$β_{IV}$

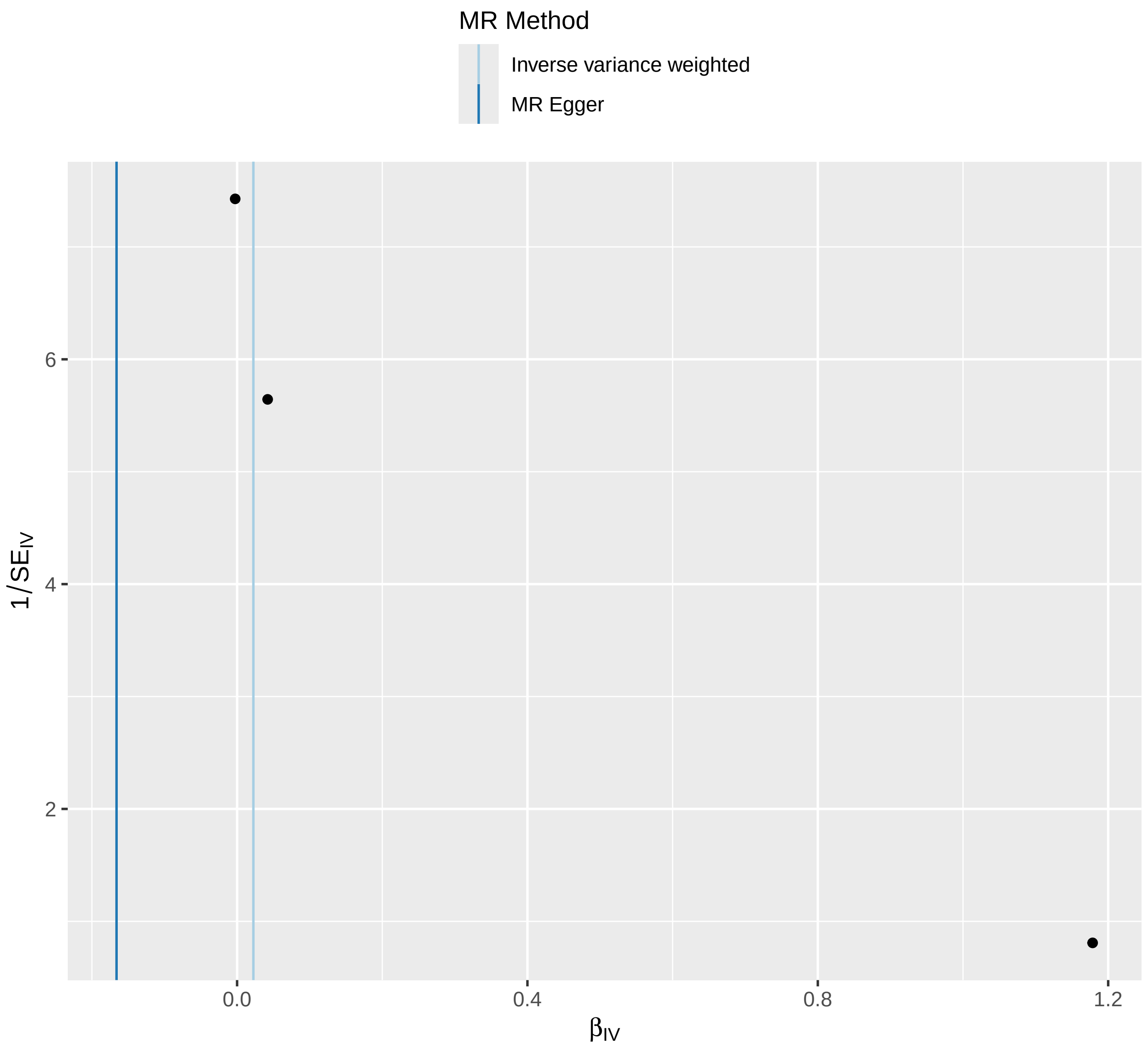

MR Method
Inverse variance weighted
MR Egger
6
4
2
1/SE$_{IV}$
0.0
0.4
0.8
1.2
$\beta_{IV}$

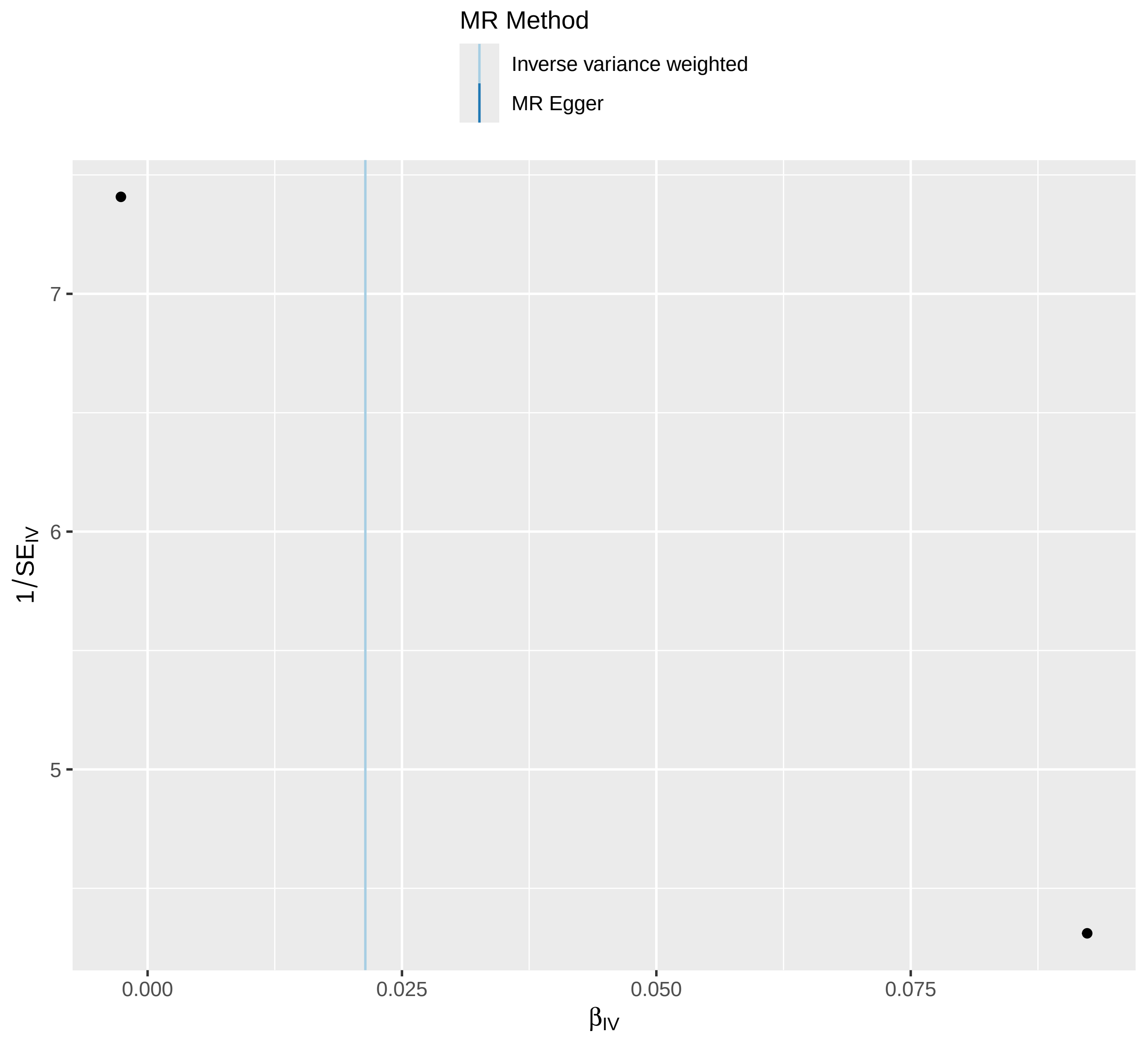
MR Method
Inverse variance weighted
MR Egger
7
6
5
1/SE_IV
0.000
0.025
0.050
0.075
β_IV

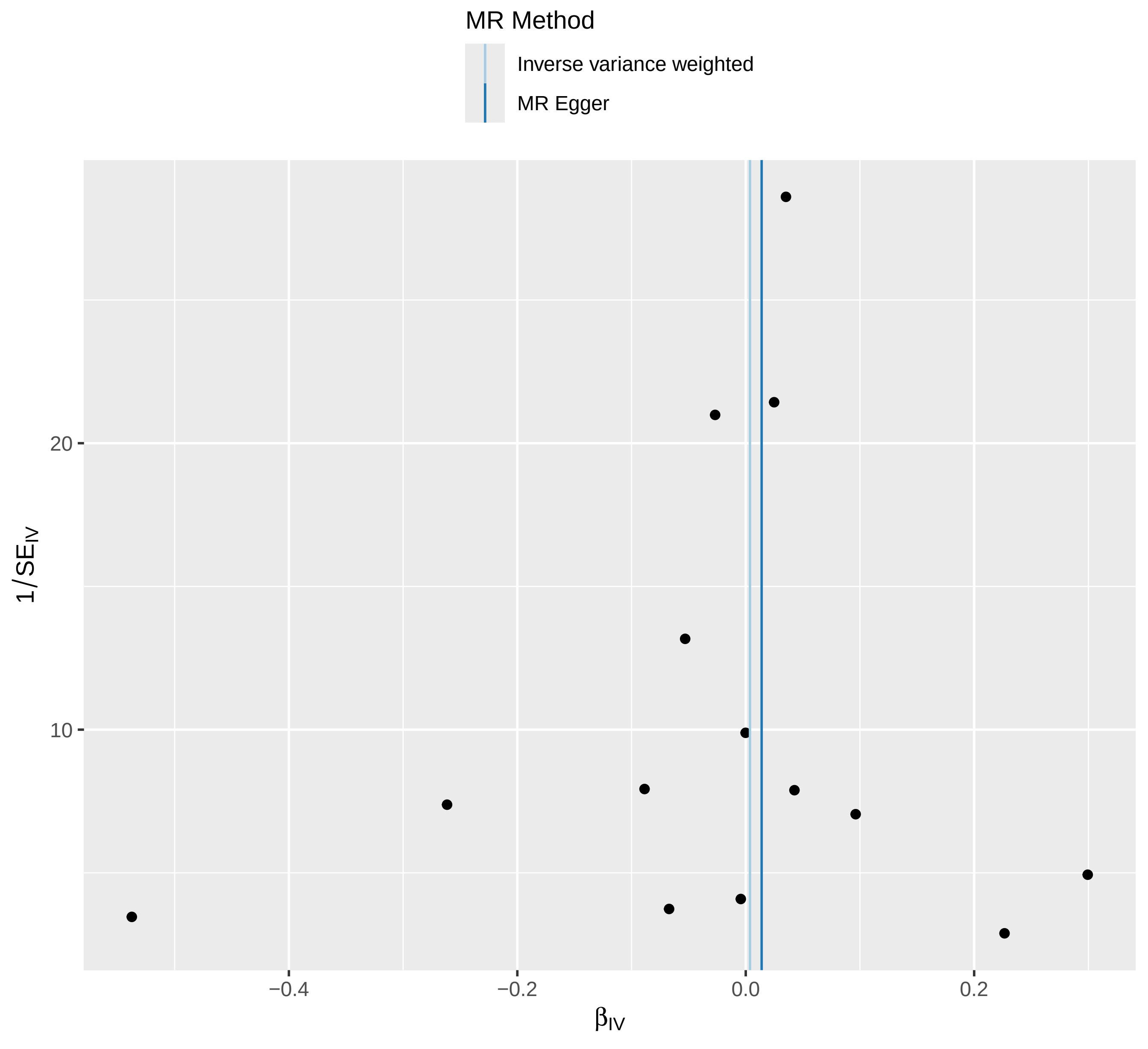

MR Method
Inverse variance weighted
MR Egger
20
10
1/SE$_{IV}$
−0.4
−0.2
0.0
0.2
β$_{IV}$

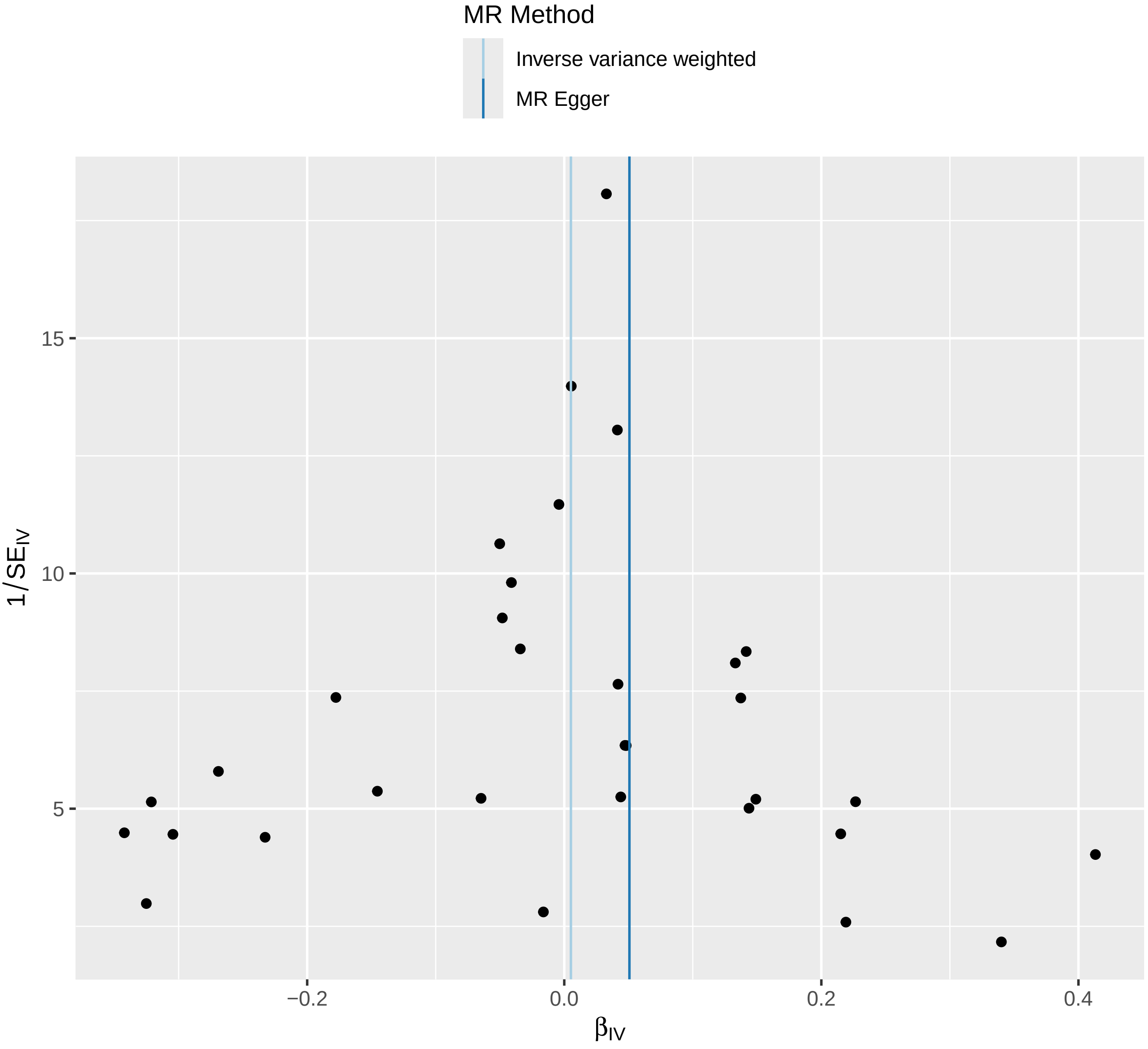

MR Method
Inverse variance weighted
MR Egger
15
10
5
1/SE$_{IV}$
−0.2
0.0
0.2
0.4
β$_{IV}$

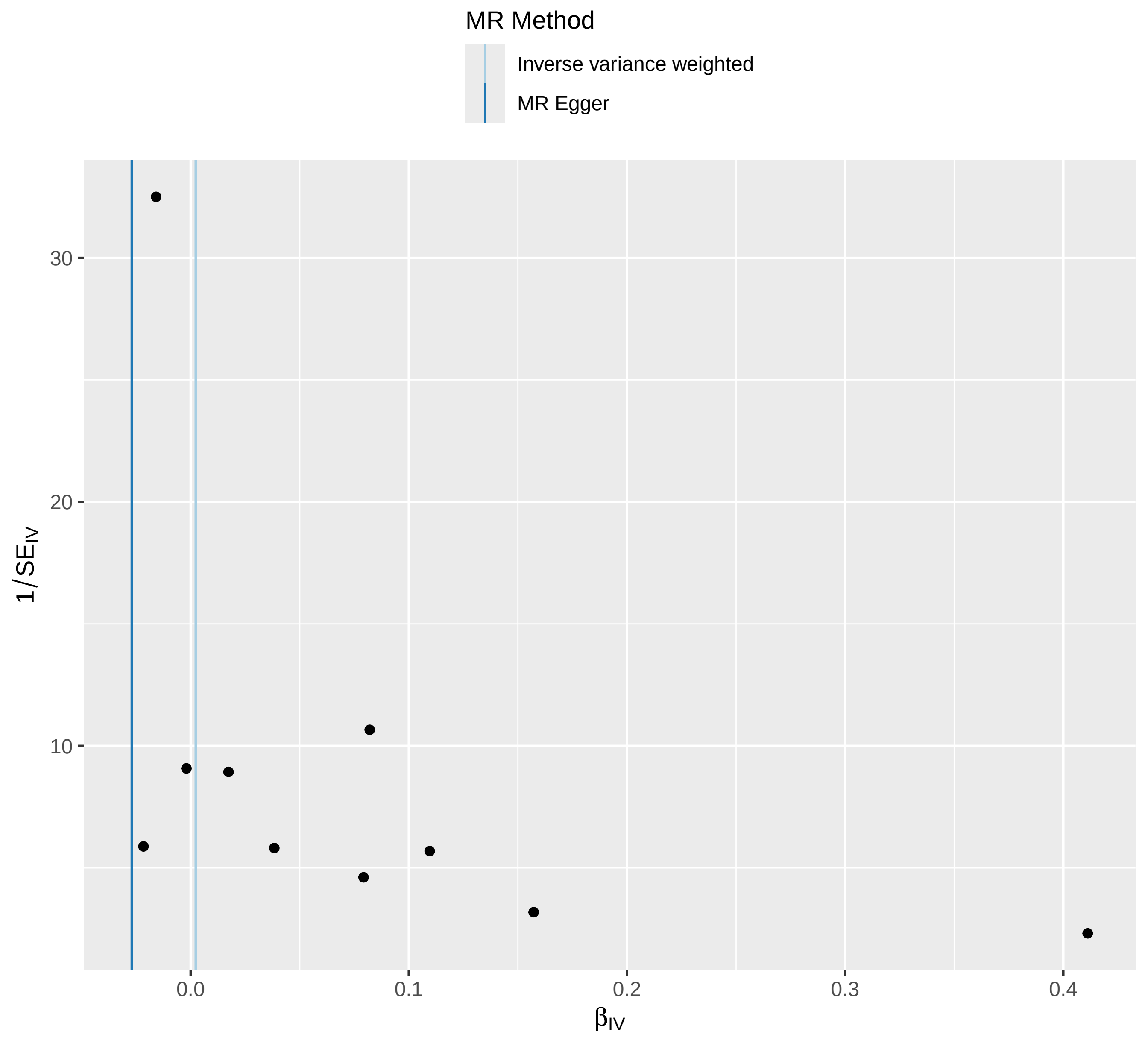
MR Method
Inverse variance weighted
MR Egger
30
20
10
$1/SE_{IV}$
0.0
0.1
0.2
0.3
0.4
$\beta_{IV}$

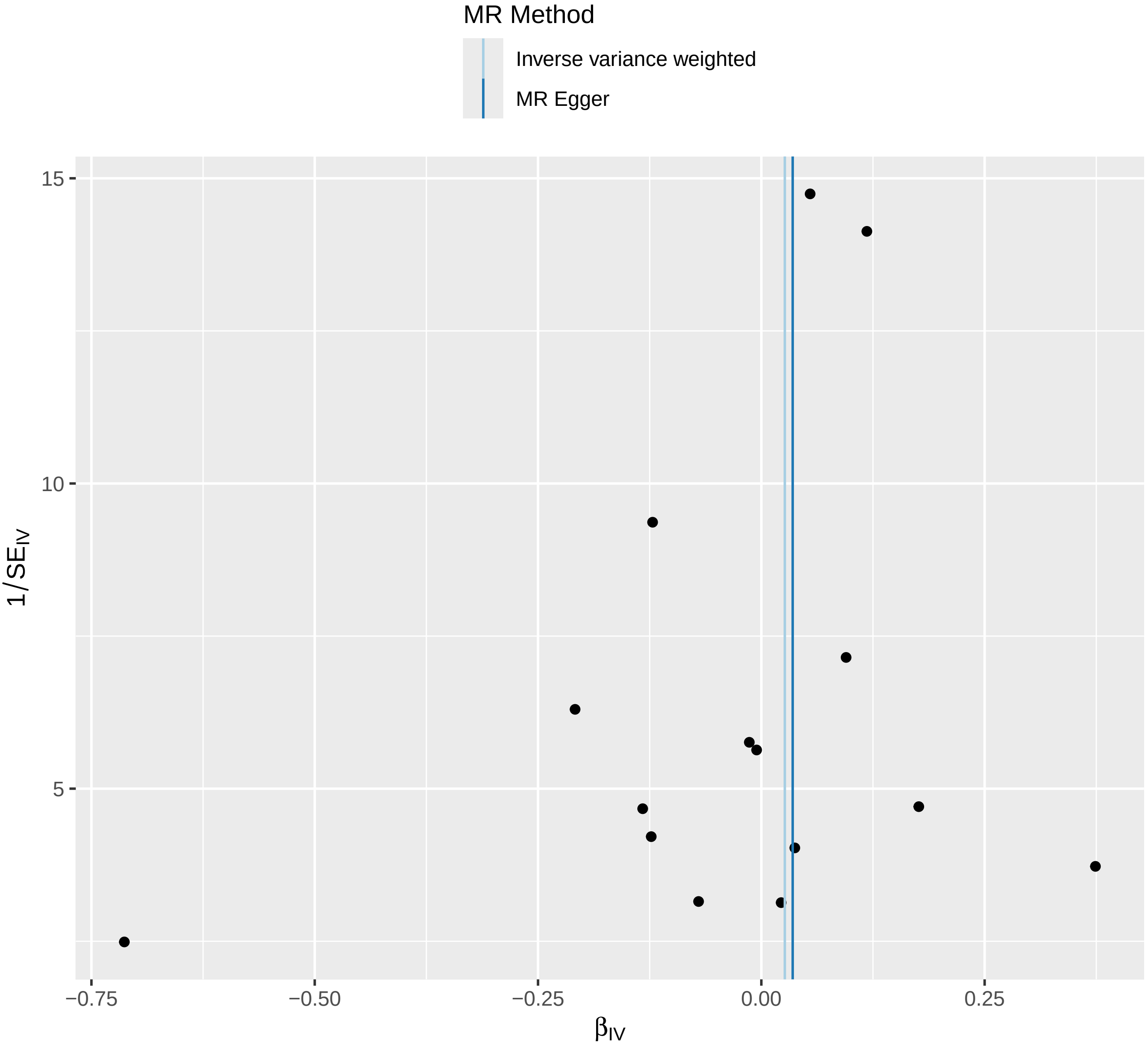

MR Method
Inverse variance weighted
MR Egger
15
10
5
1/SE_IV
−0.75
−0.50
−0.25
0.00
0.25
β_IV

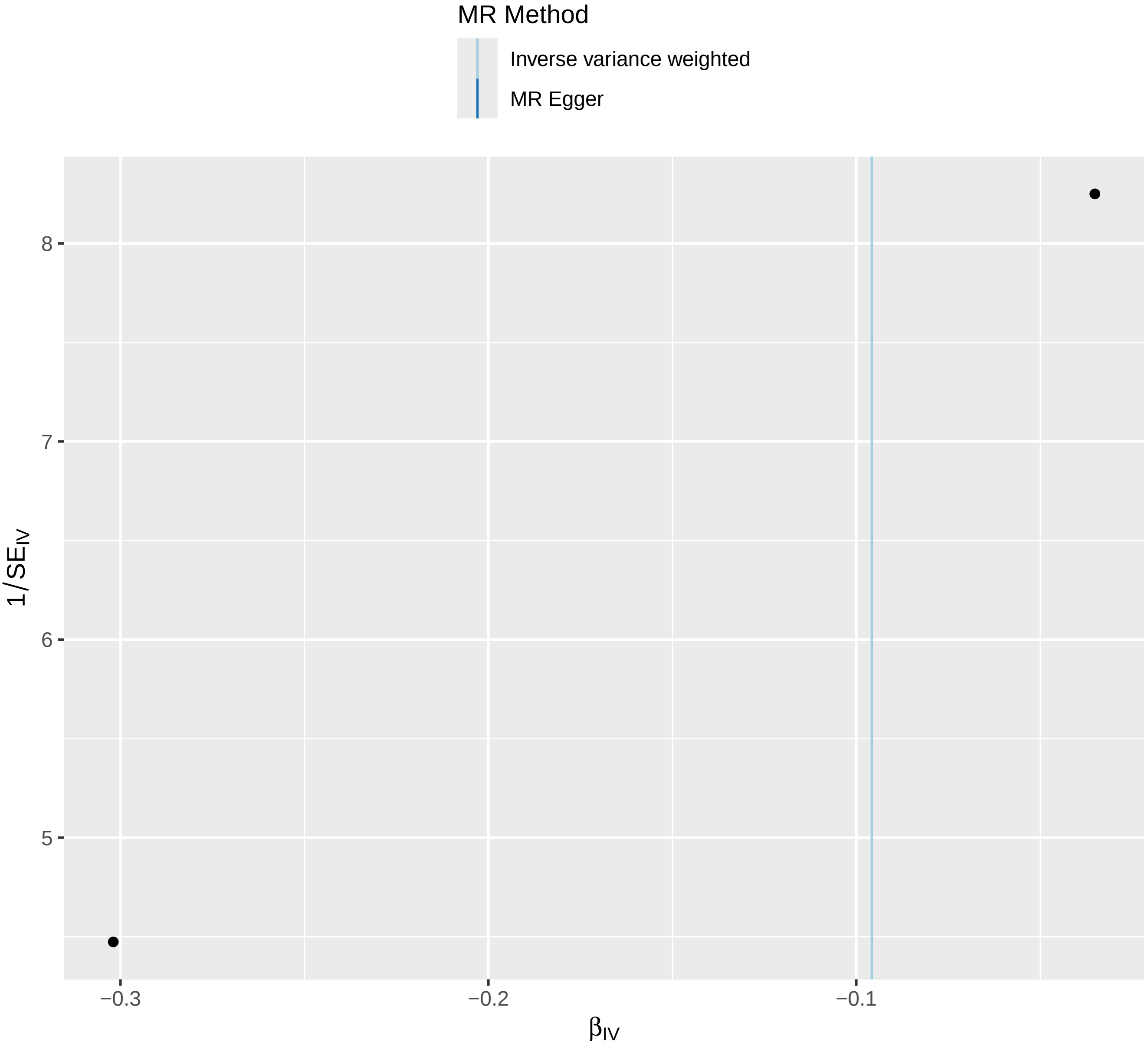
MR Method
Inverse variance weighted
MR Egger
8
7
6
5
1/SE$_{IV}$
−0.3
−0.2
−0.1
β$_{IV}$

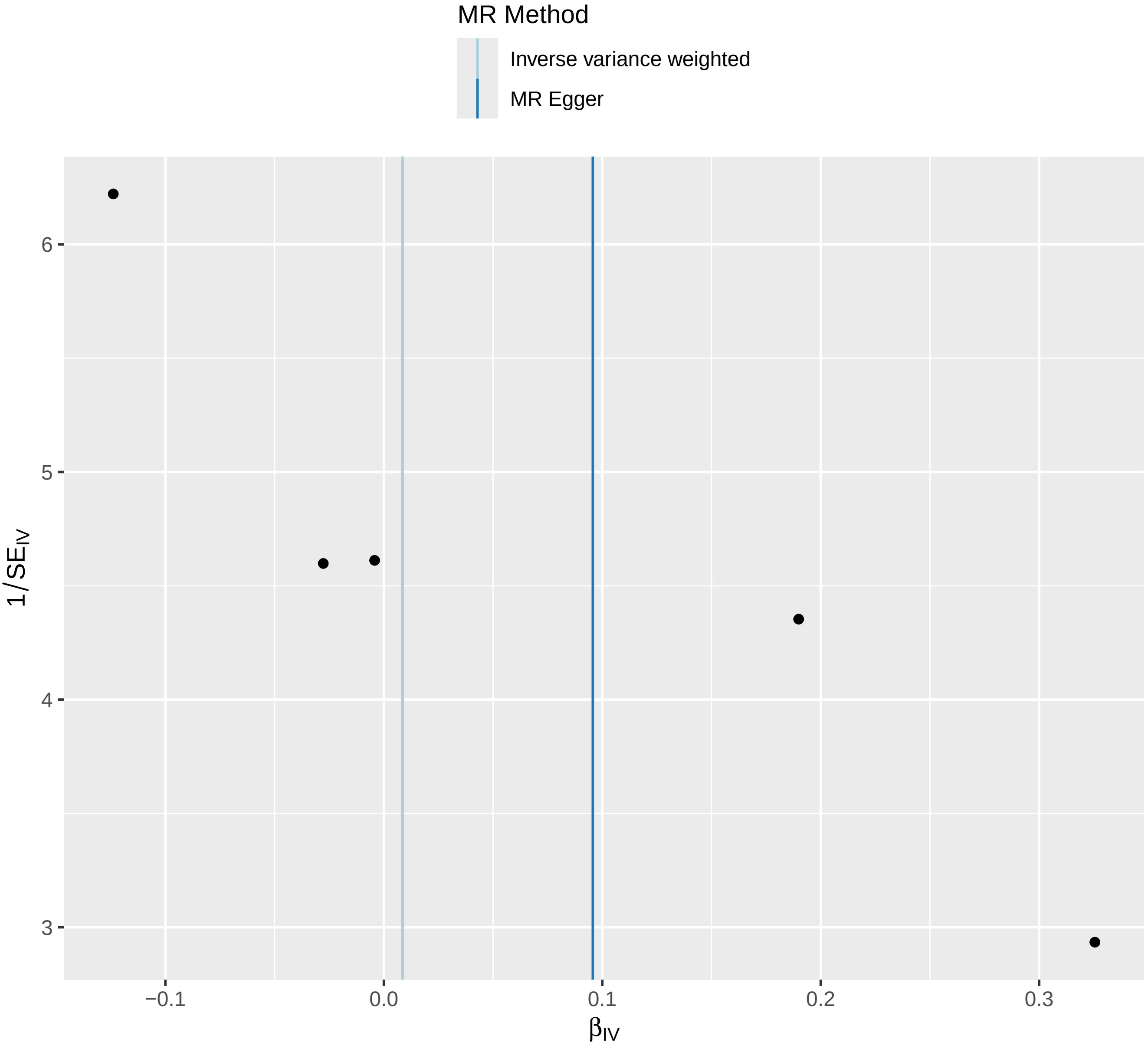
MR Method
Inverse variance weighted
MR Egger
6
5
4
3
1/SE_IV
−0.1
0.0
0.1
0.2
0.3
β_IV

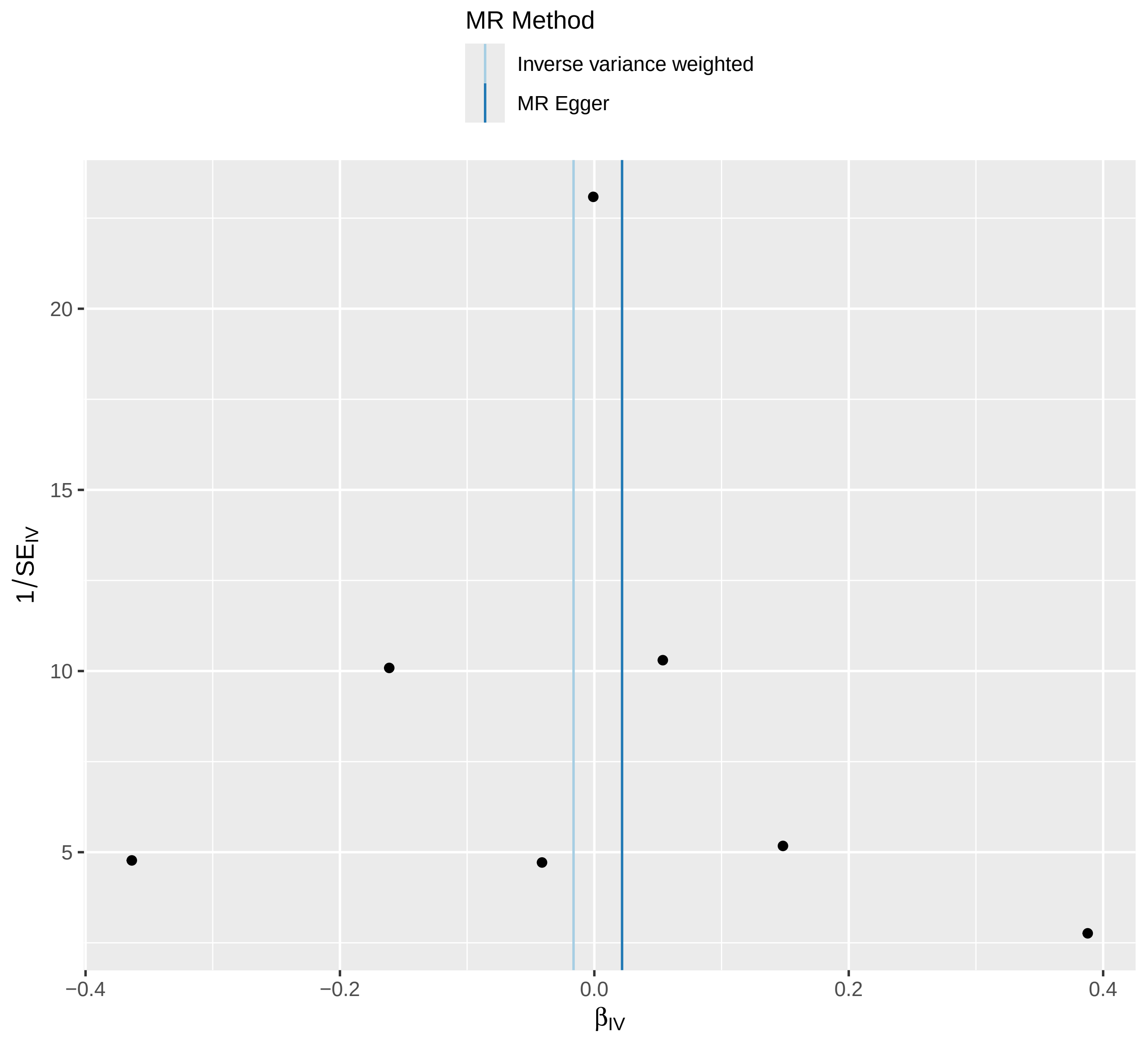
MR Method
Inverse variance weighted
MR Egger
20
15
10
5
1/SE$_{IV}$
−0.4
−0.2
0.0
0.2
0.4
β$_{IV}$

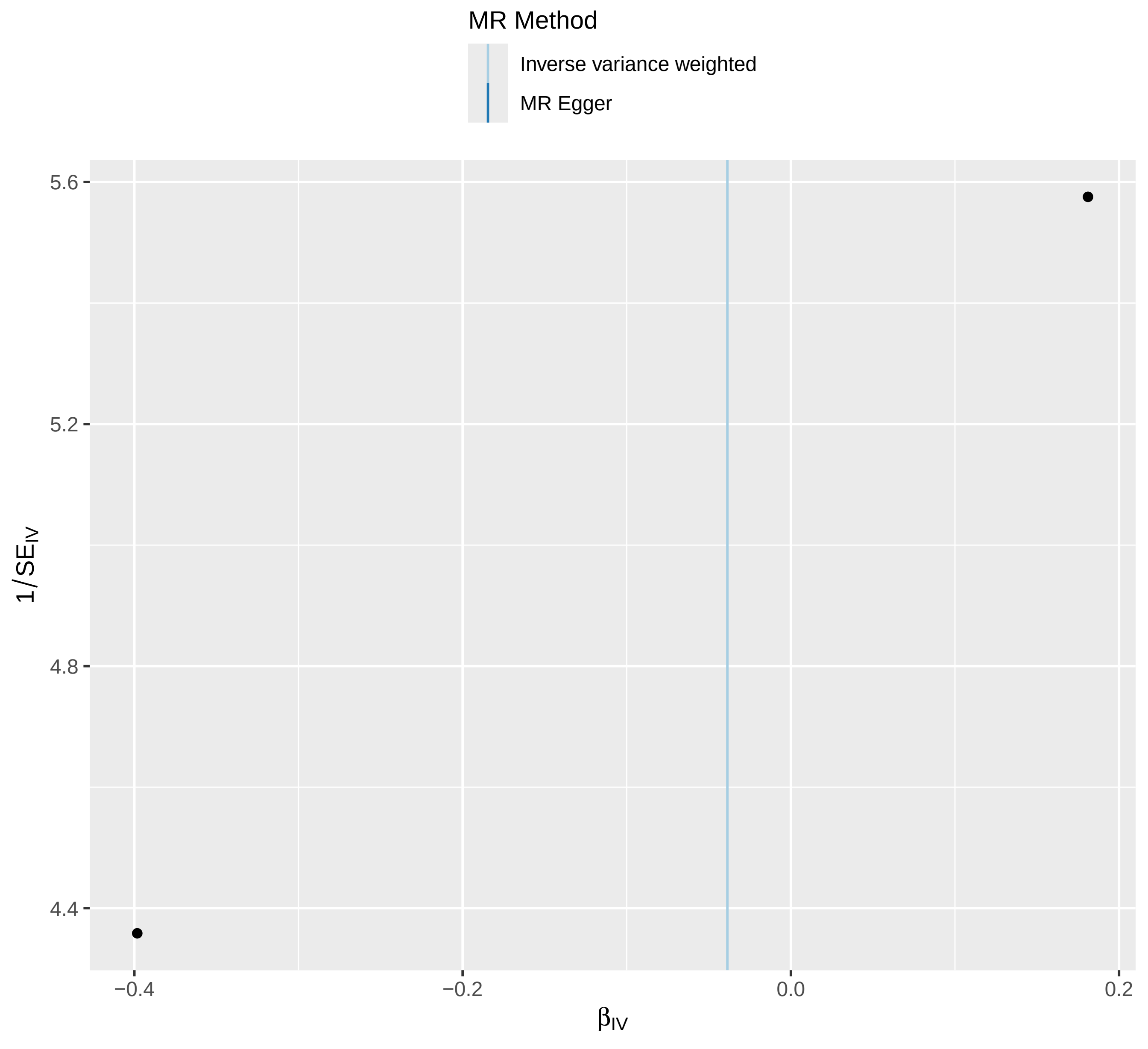
MR Method
Inverse variance weighted
MR Egger
5.6
5.2
4.8
4.4
1/SE$_{IV}$
−0.4
−0.2
0.0
0.2
β$_{IV}$

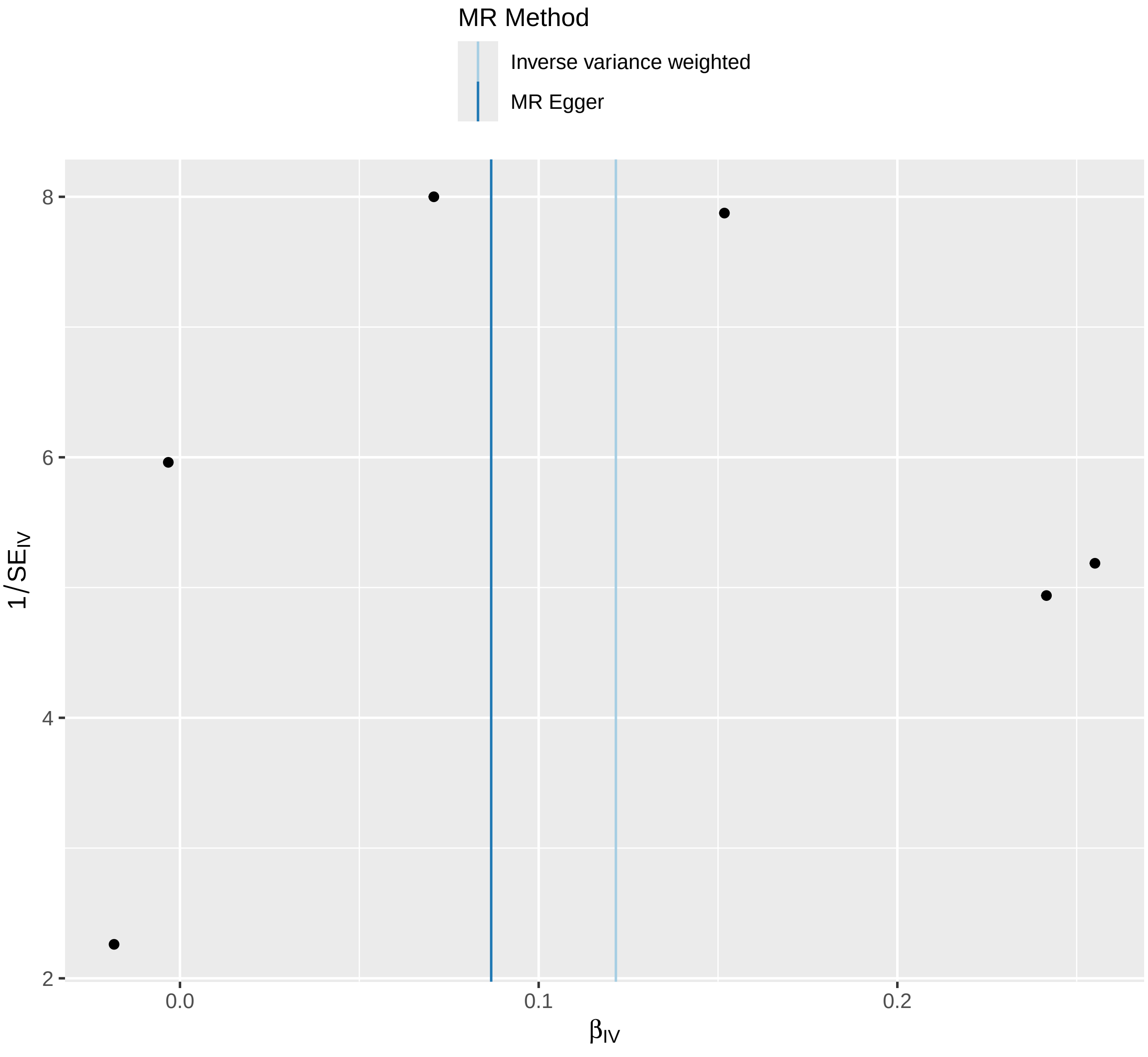

MR Method
Inverse variance weighted
MR Egger
8
6
4
2
1/SE$_{IV}$
0.0
0.1
0.2
β$_{IV}$

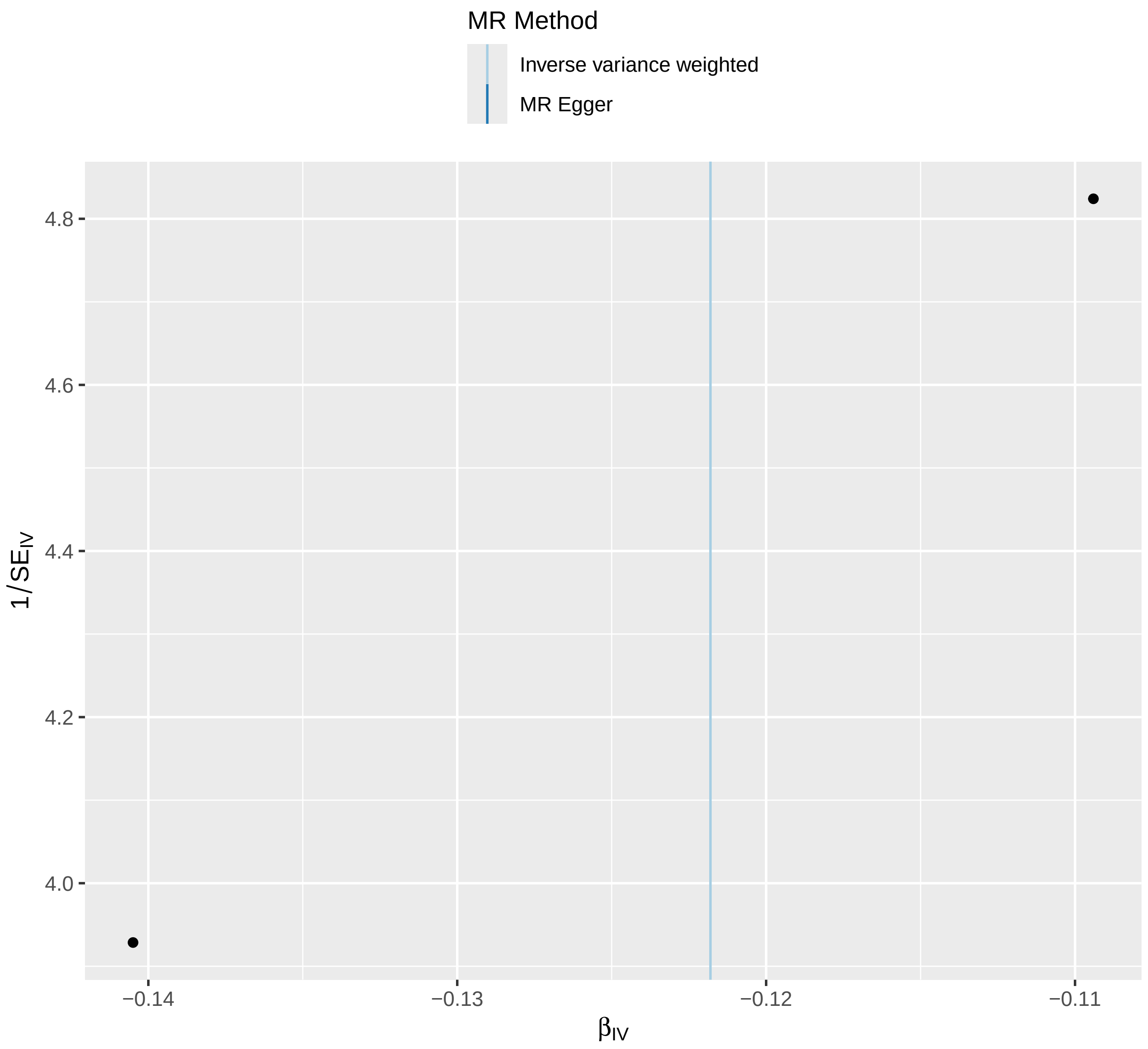
MR Method
Inverse variance weighted
MR Egger
4.8
4.6
4.4
4.2
4.0
1/SE$_{IV}$
−0.14
−0.13
−0.12
−0.11
β$_{IV}$

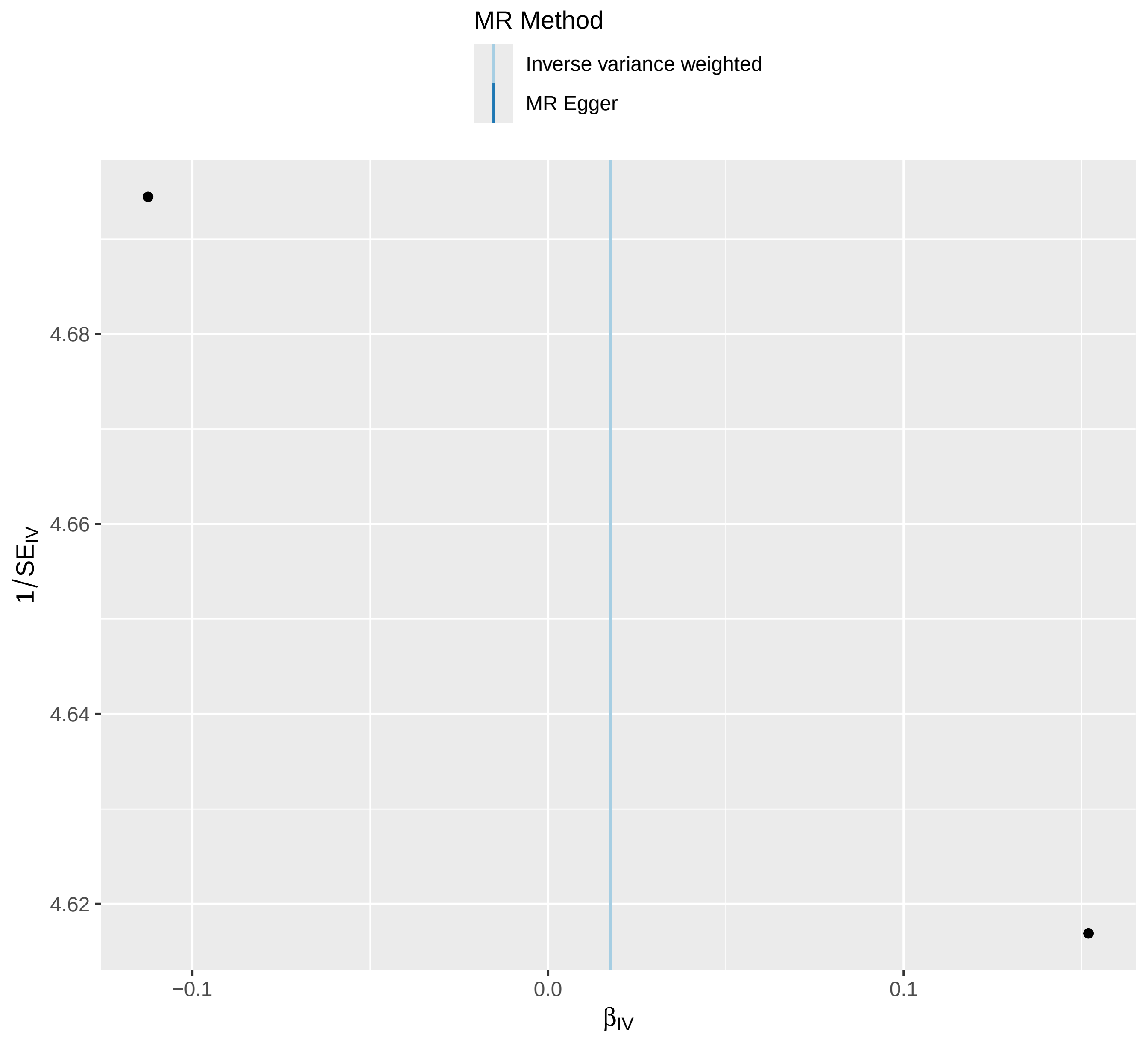

MR Method
Inverse variance weighted
MR Egger
4.68
4.66
4.64
4.62
1/SE$_{IV}$
−0.1
0.0
0.1
β$_{IV}$

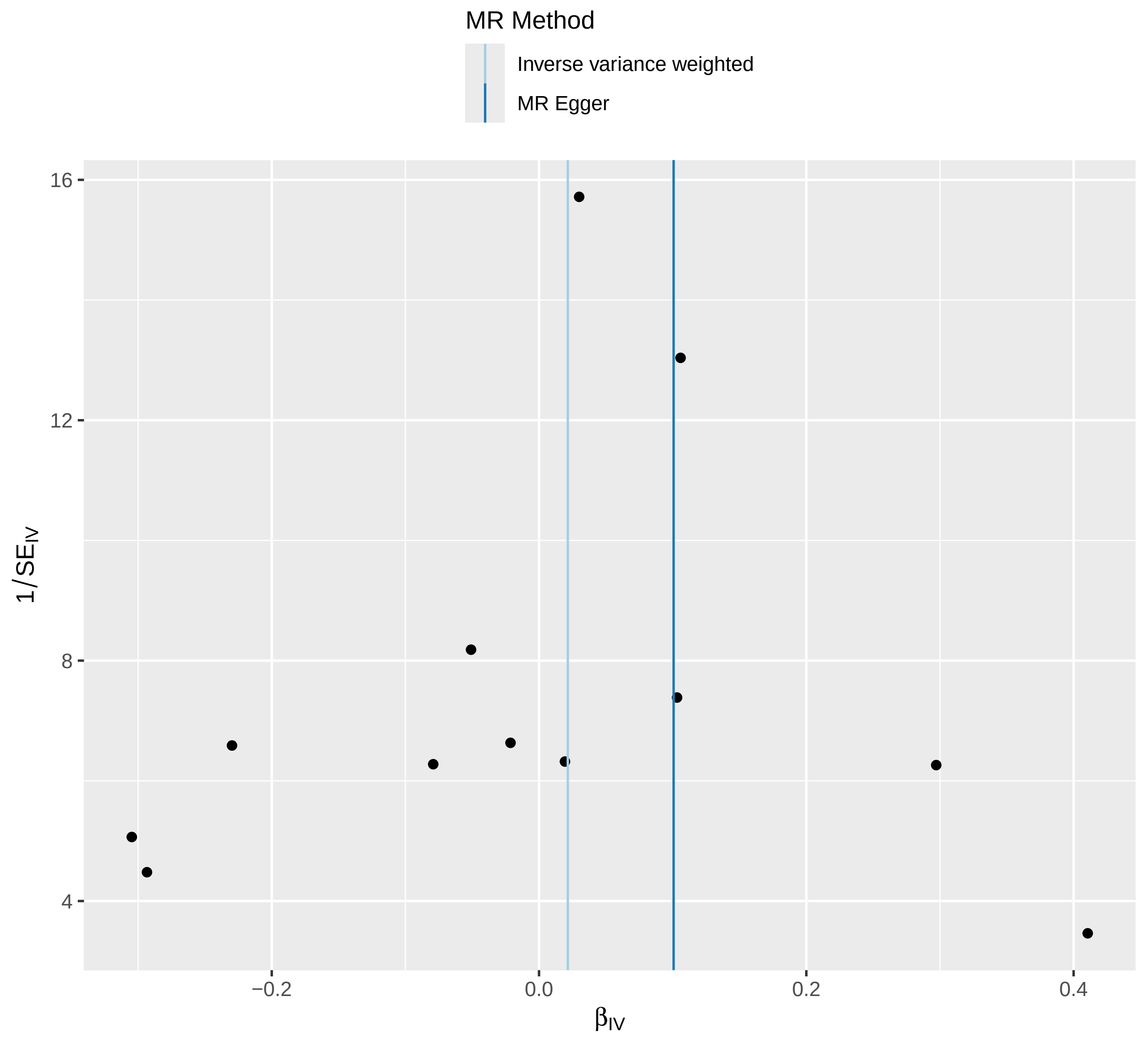
MR Method
Inverse variance weighted
MR Egger
16
12
8
4
1/SE$_{IV}$
−0.2
0.0
0.2
0.4
β$_{IV}$

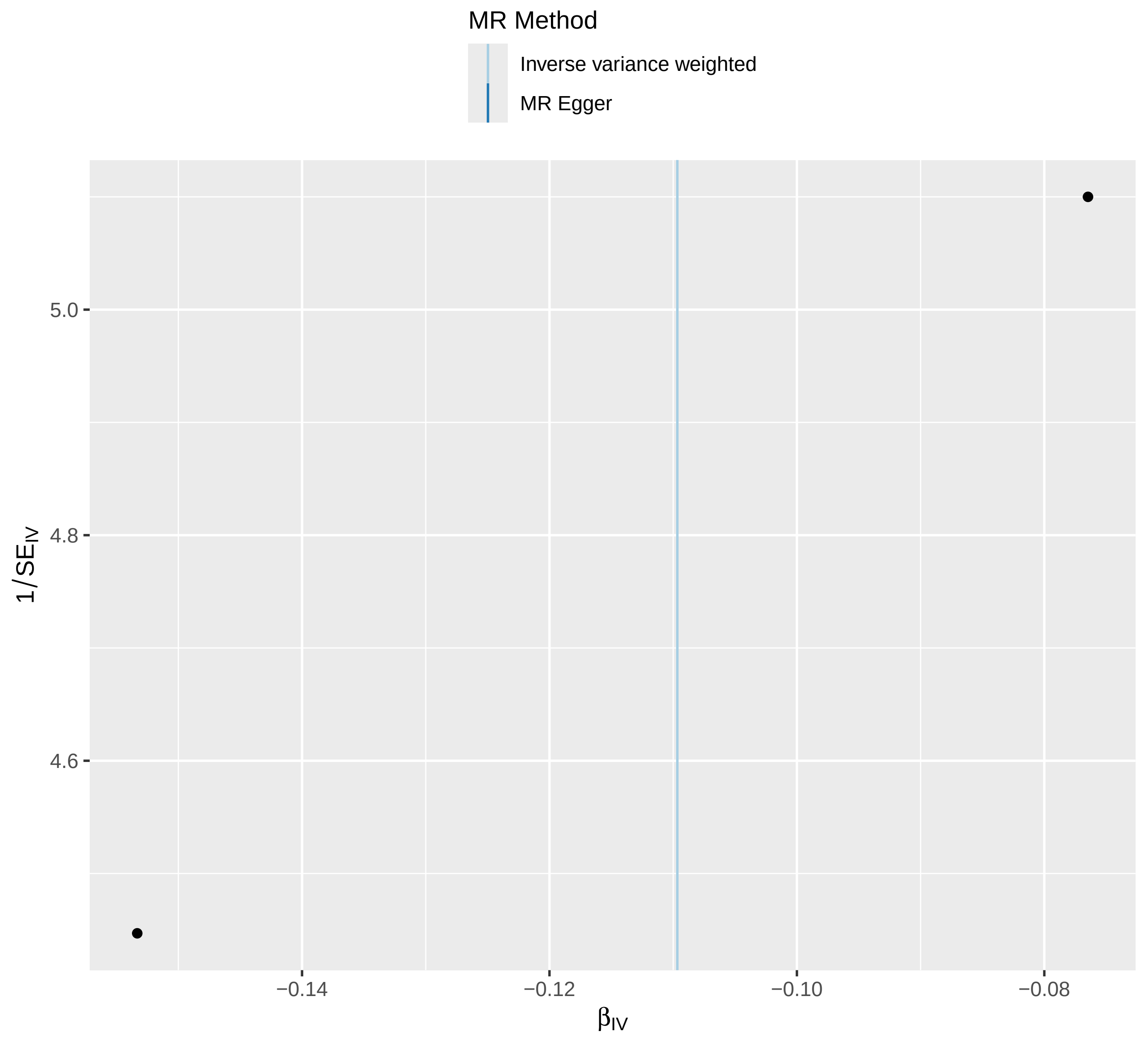
MR Method
Inverse variance weighted
MR Egger
5.0
4.8
4.6
1/SE_IV
−0.14
−0.12
−0.10
−0.08
β_IV

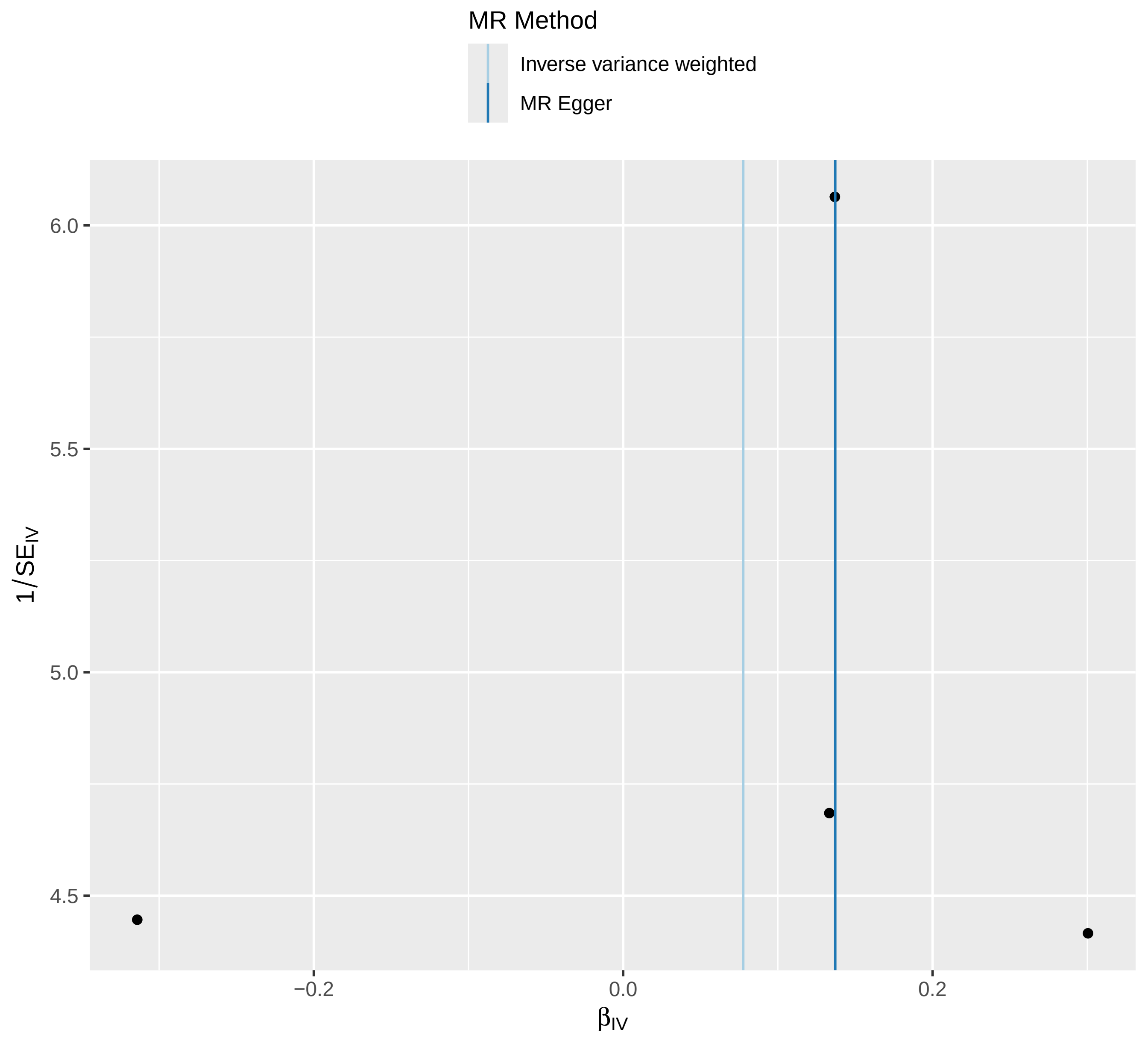
MR Method
Inverse variance weighted
MR Egger
6.0
5.5
5.0
4.5
1/SE$_{IV}$
−0.2
0.0
0.2
β$_{IV}$

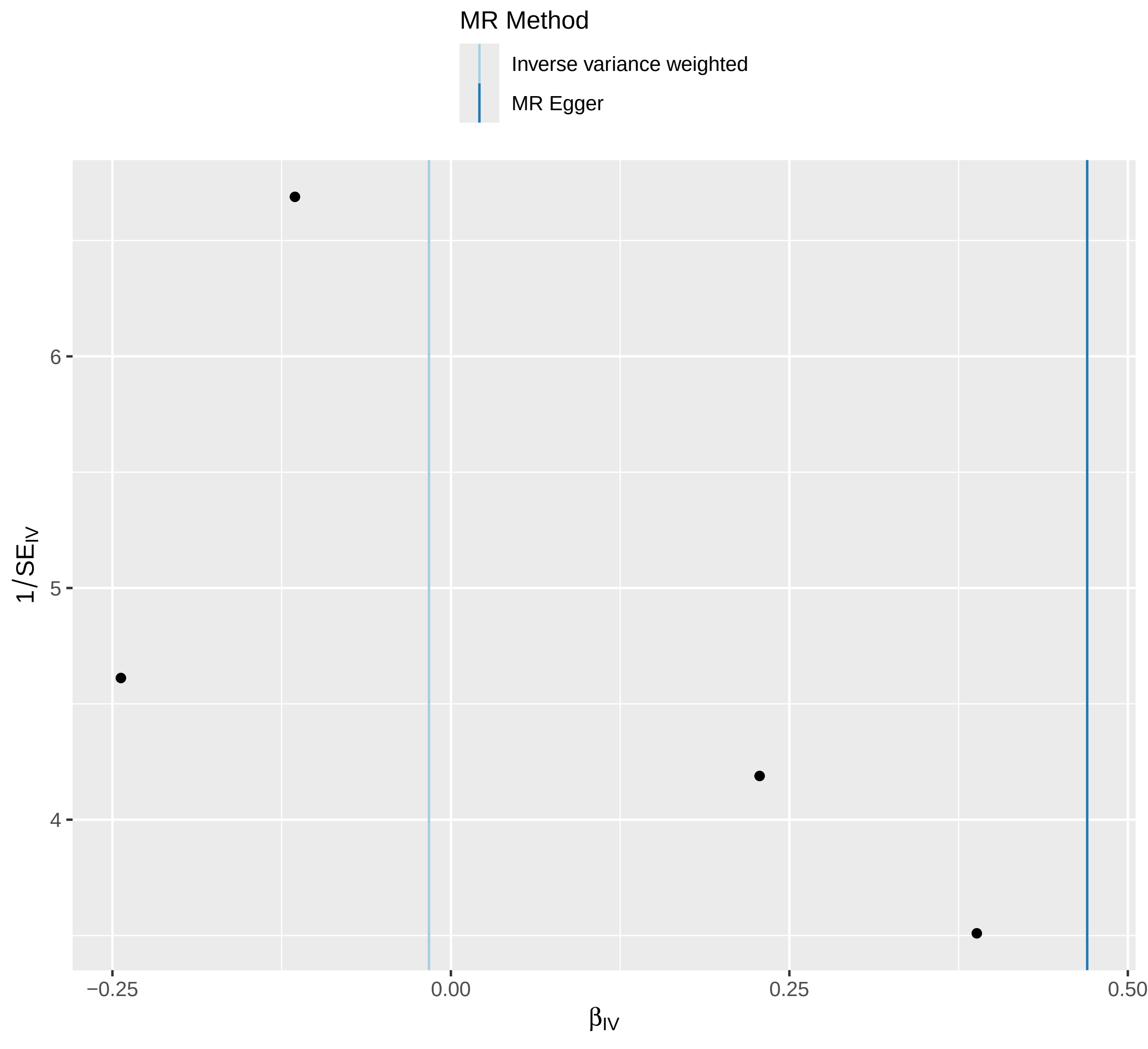

MR Method
Inverse variance weighted
MR Egger
6
5
4
1/SE_IV
−0.25
0.00
0.25
0.50
β_IV

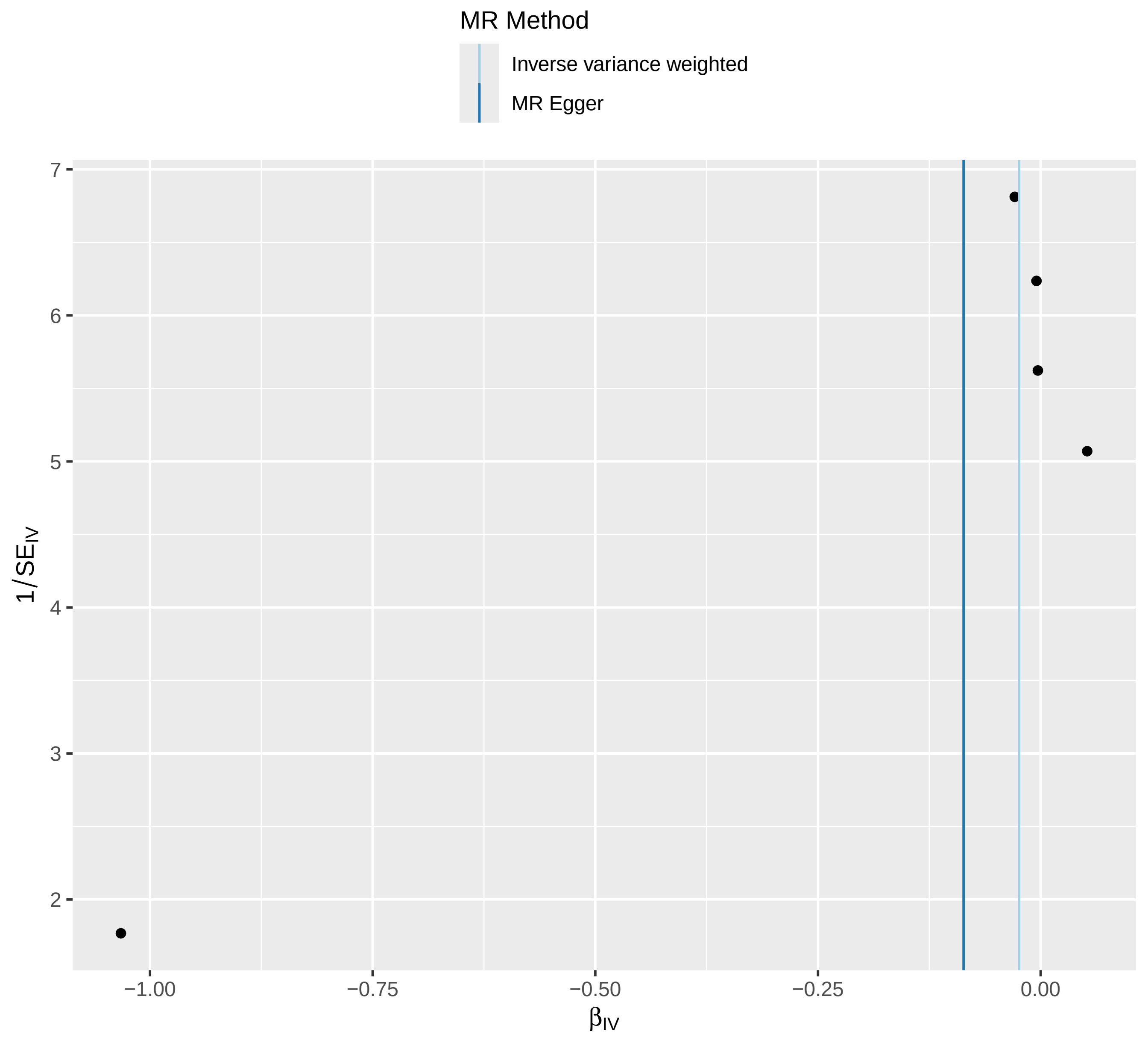
MR Method
Inverse variance weighted
MR Egger
1/SE$_{IV}$
7
6
5
4
3
2
β$_{IV}$
−1.00
−0.75
−0.50
−0.25
0.00

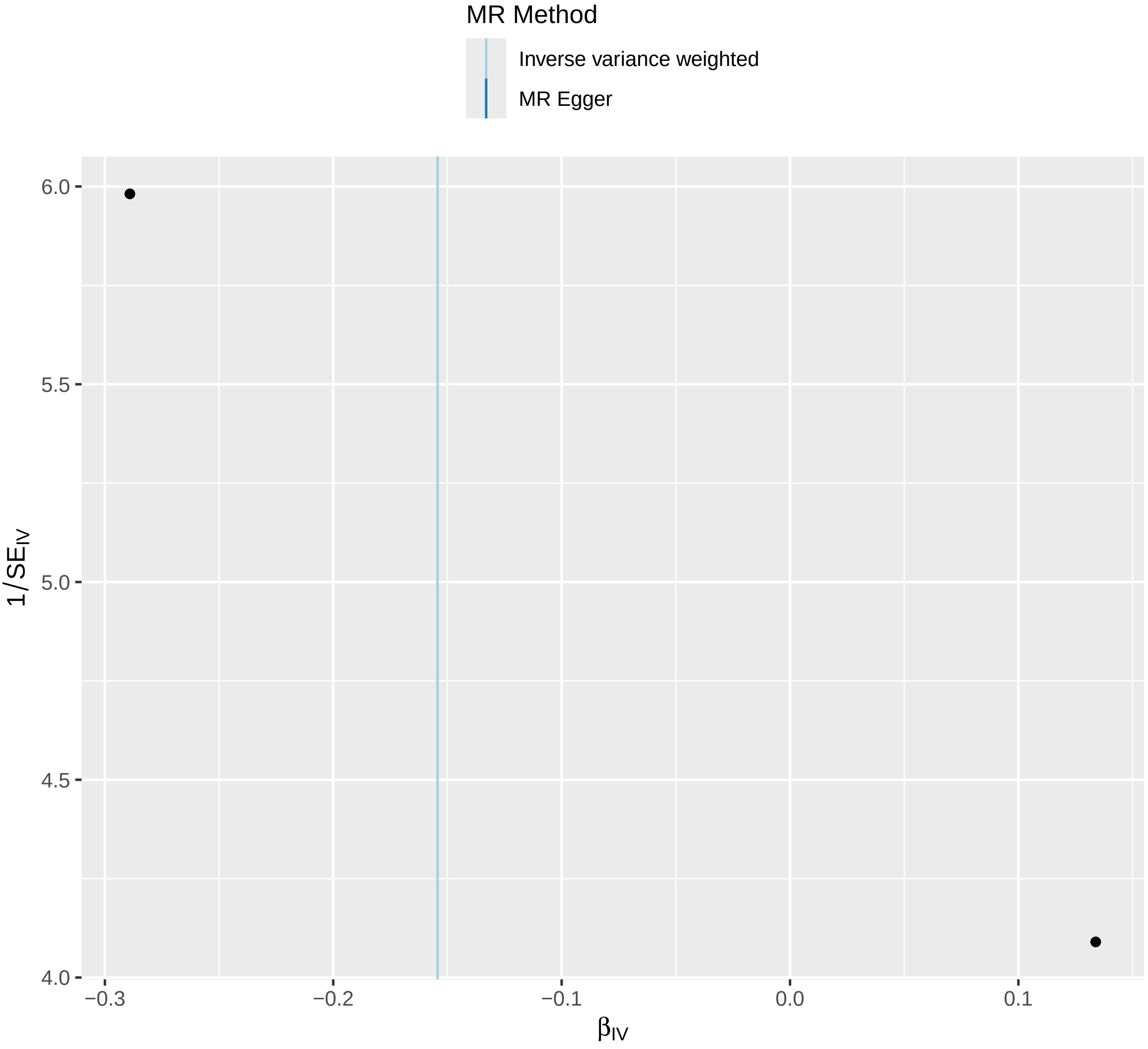
MR Method
Inverse variance weighted
MR Egger
6.0
5.5
5.0
4.5
4.0
1/SE_IV
−0.3
−0.2
−0.1
0.0
0.1
β_IV

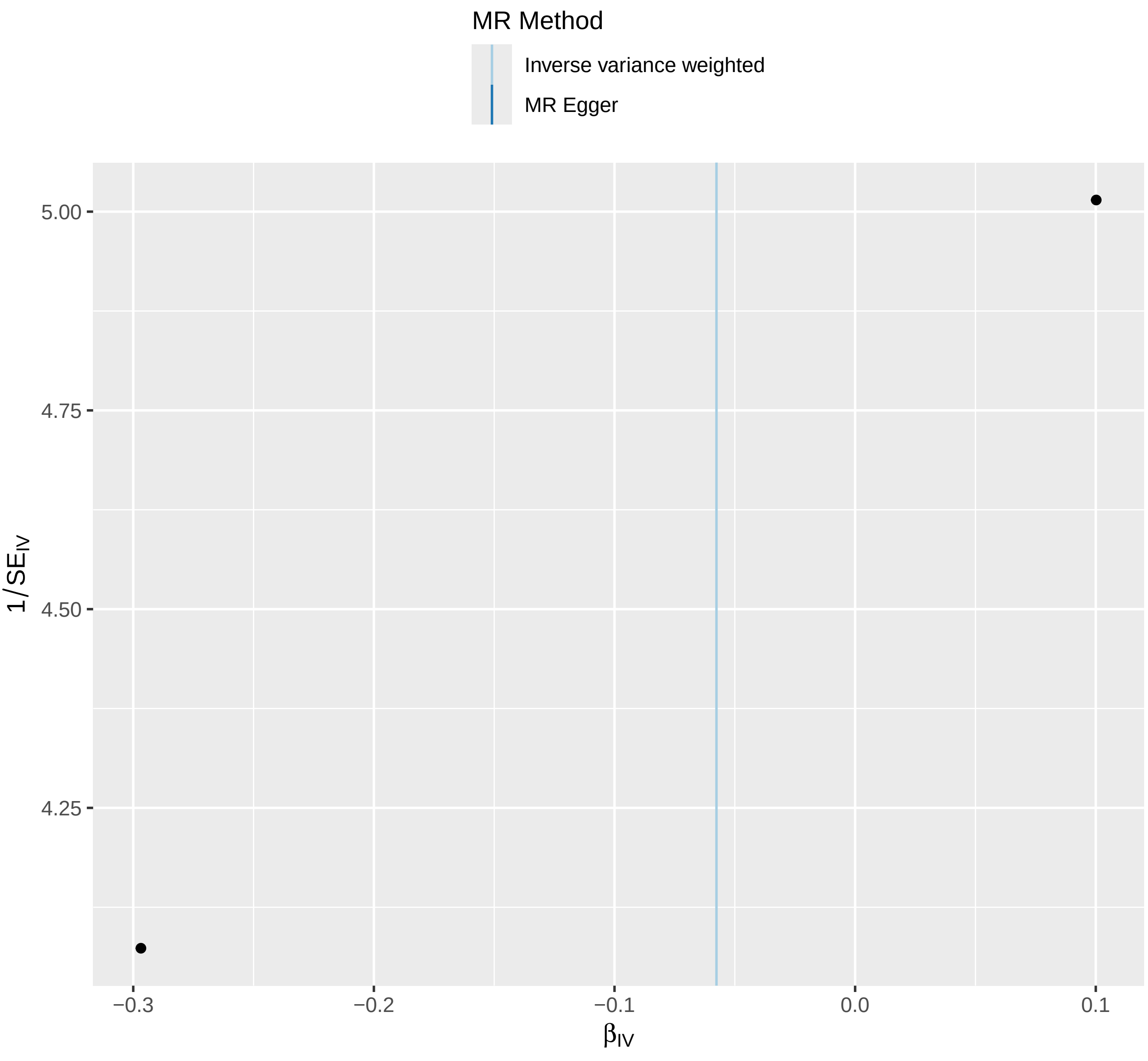
MR Method
Inverse variance weighted
MR Egger
5.00
4.75
4.50
4.25
1/SE_IV
−0.3
−0.2
−0.1
0.0
0.1
β_IV

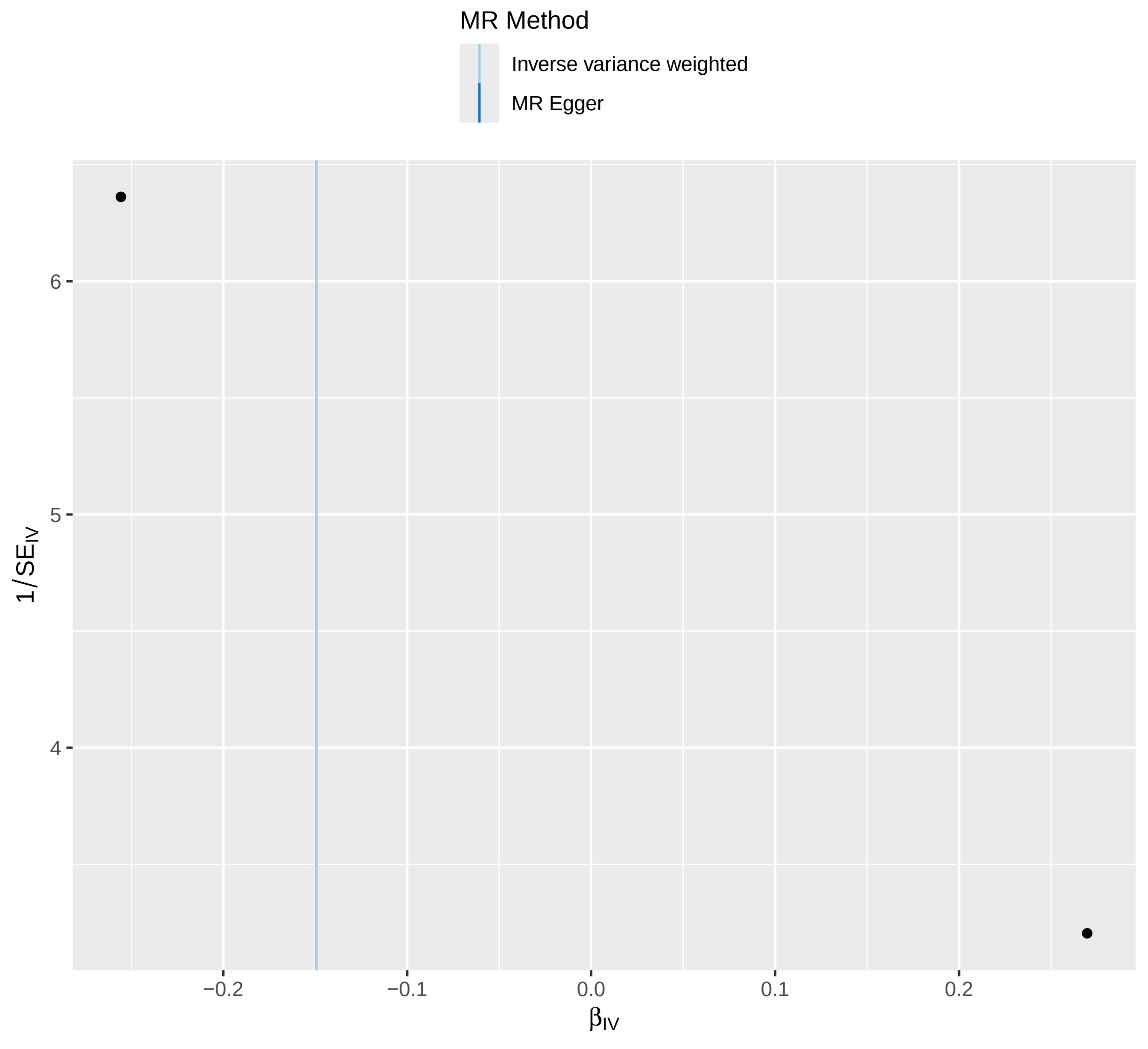
MR Method
Inverse variance weighted
MR Egger
6
5
4
1/SE_IV
−0.2
−0.1
0.0
0.1
0.2
β_IV

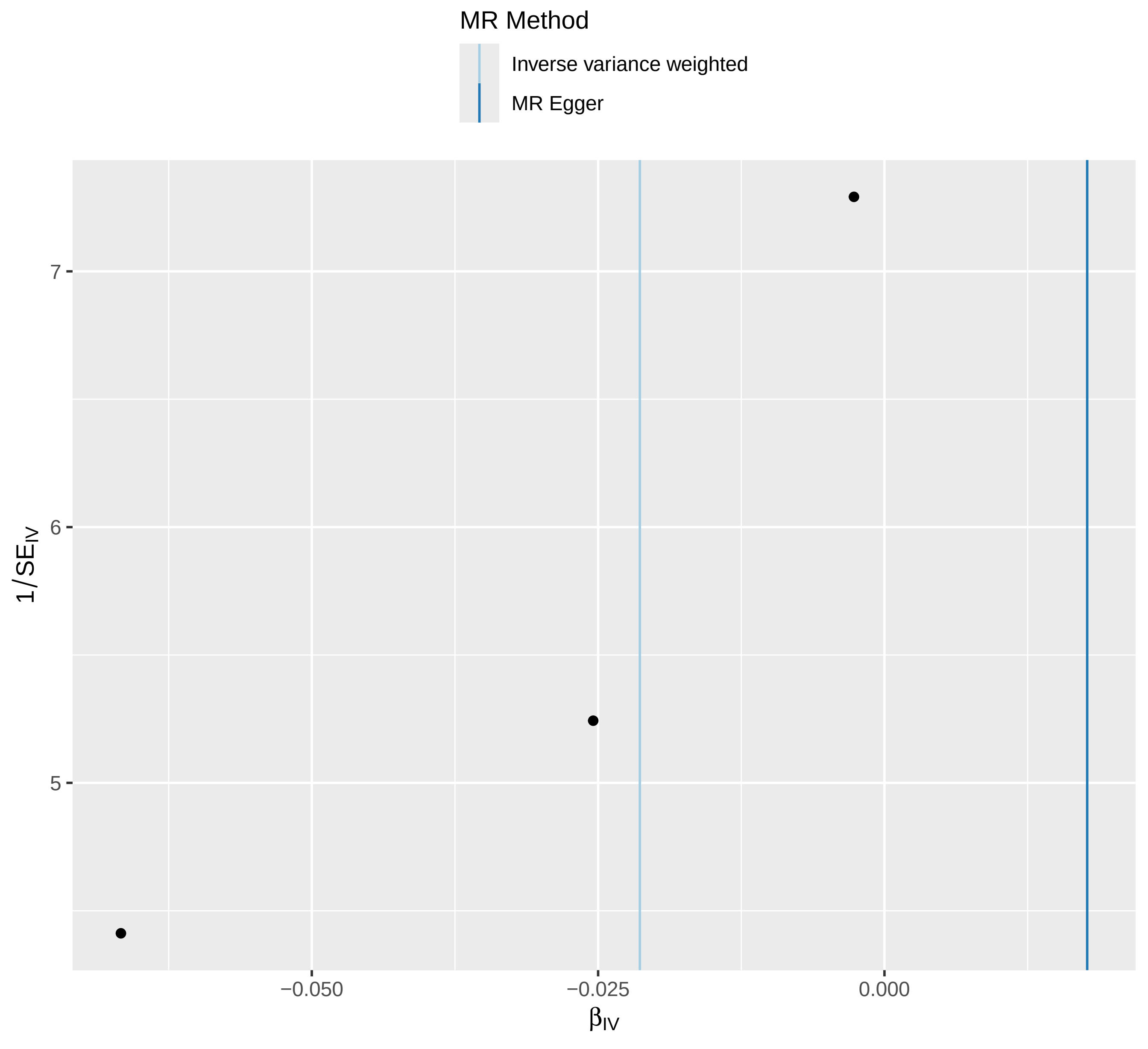
MR Method
Inverse variance weighted
MR Egger
7
6
5
1/SE_IV
−0.050
−0.025
0.000
β_IV

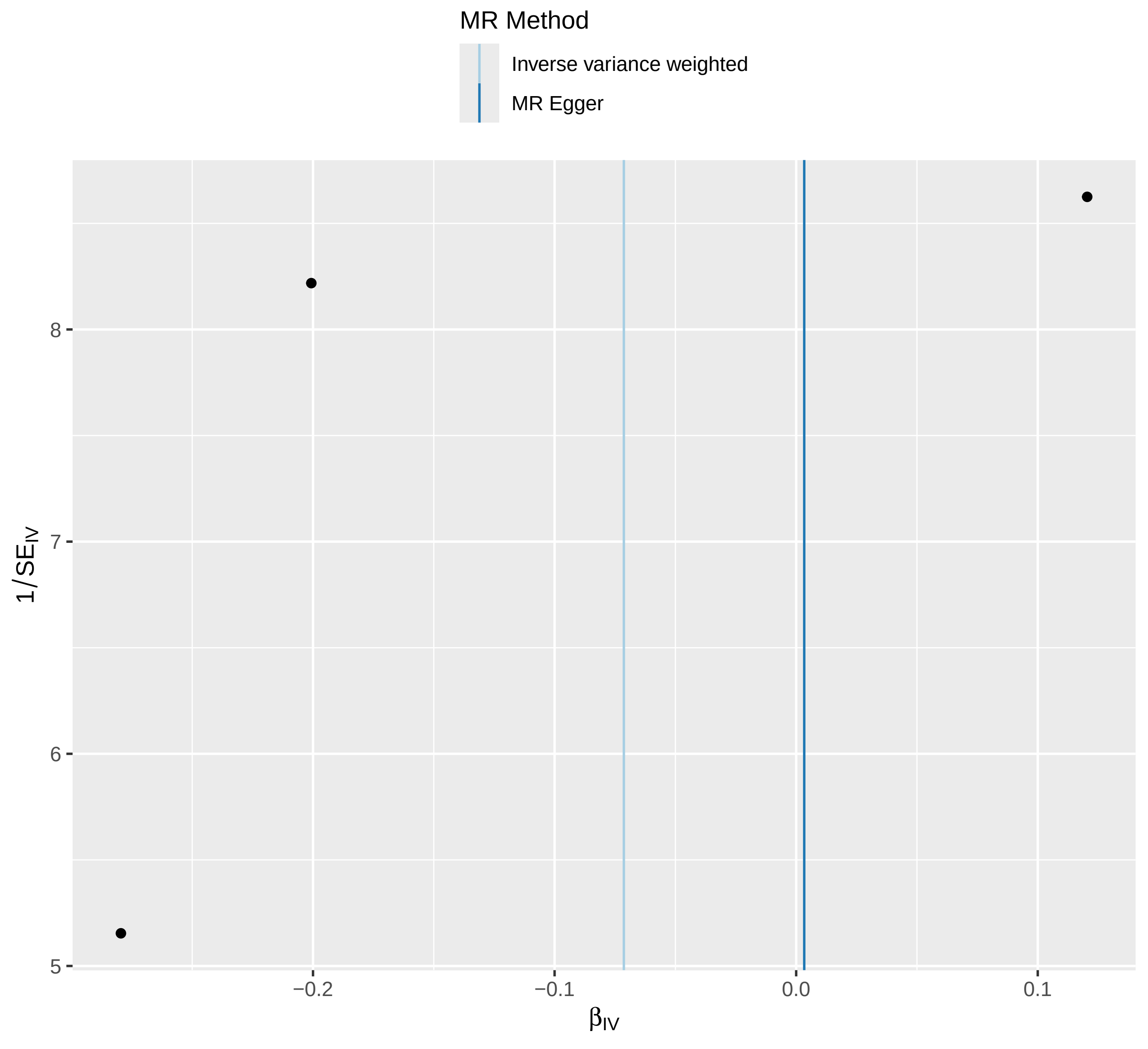
MR Method
Inverse variance weighted
MR Egger
8
7
6
5
1/SE_IV
−0.2
−0.1
0.0
0.1
β_IV

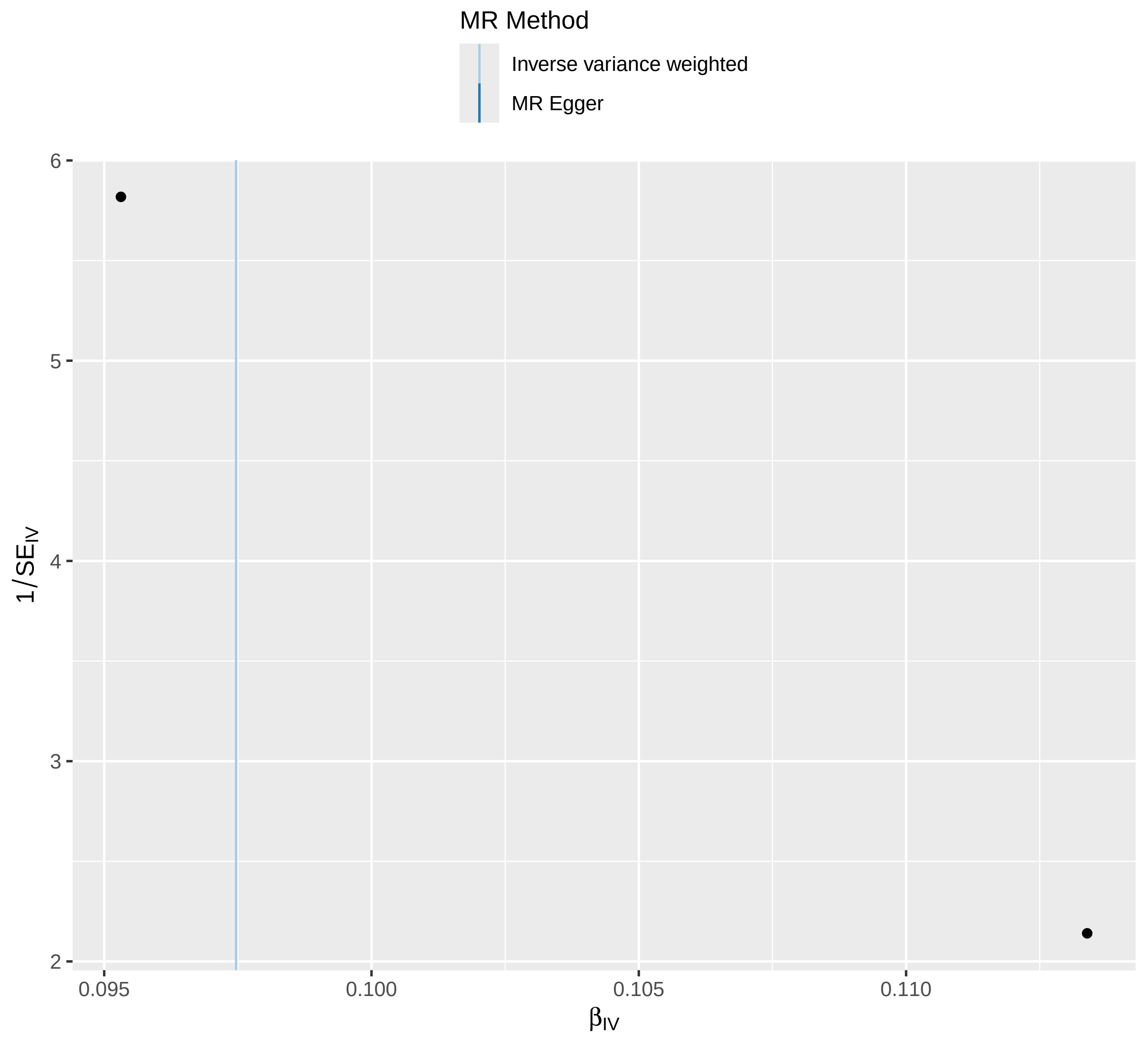
MR Method
Inverse variance weighted
MR Egger
6
5
4
3
2
1/SE_IV
0.095
0.100
0.105
0.110
β_IV

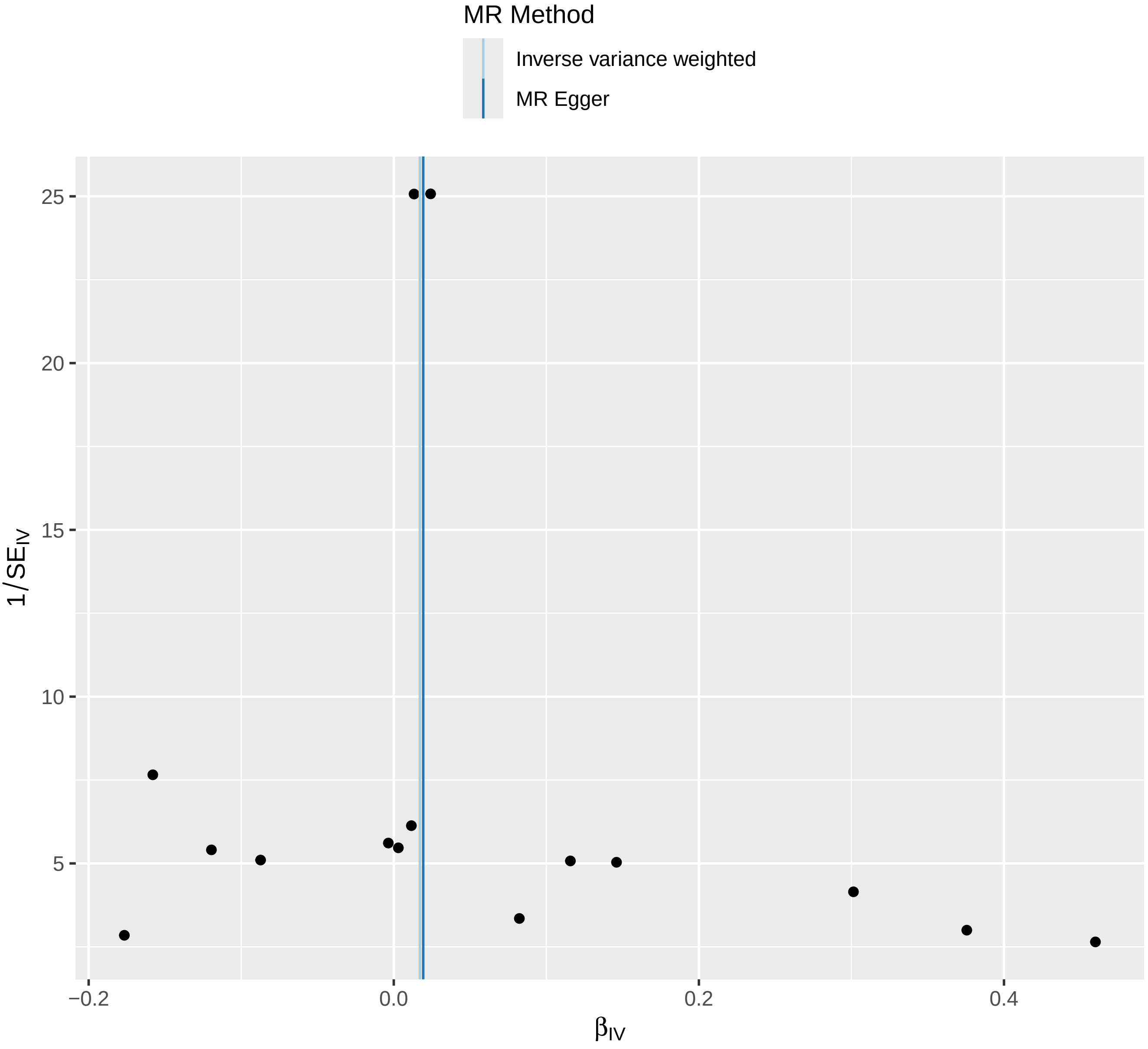
MR Method
Inverse variance weighted
MR Egger
25
20
15
10
5
$1/SE_{IV}$
−0.2
0.0
0.2
0.4
$\beta_{IV}$

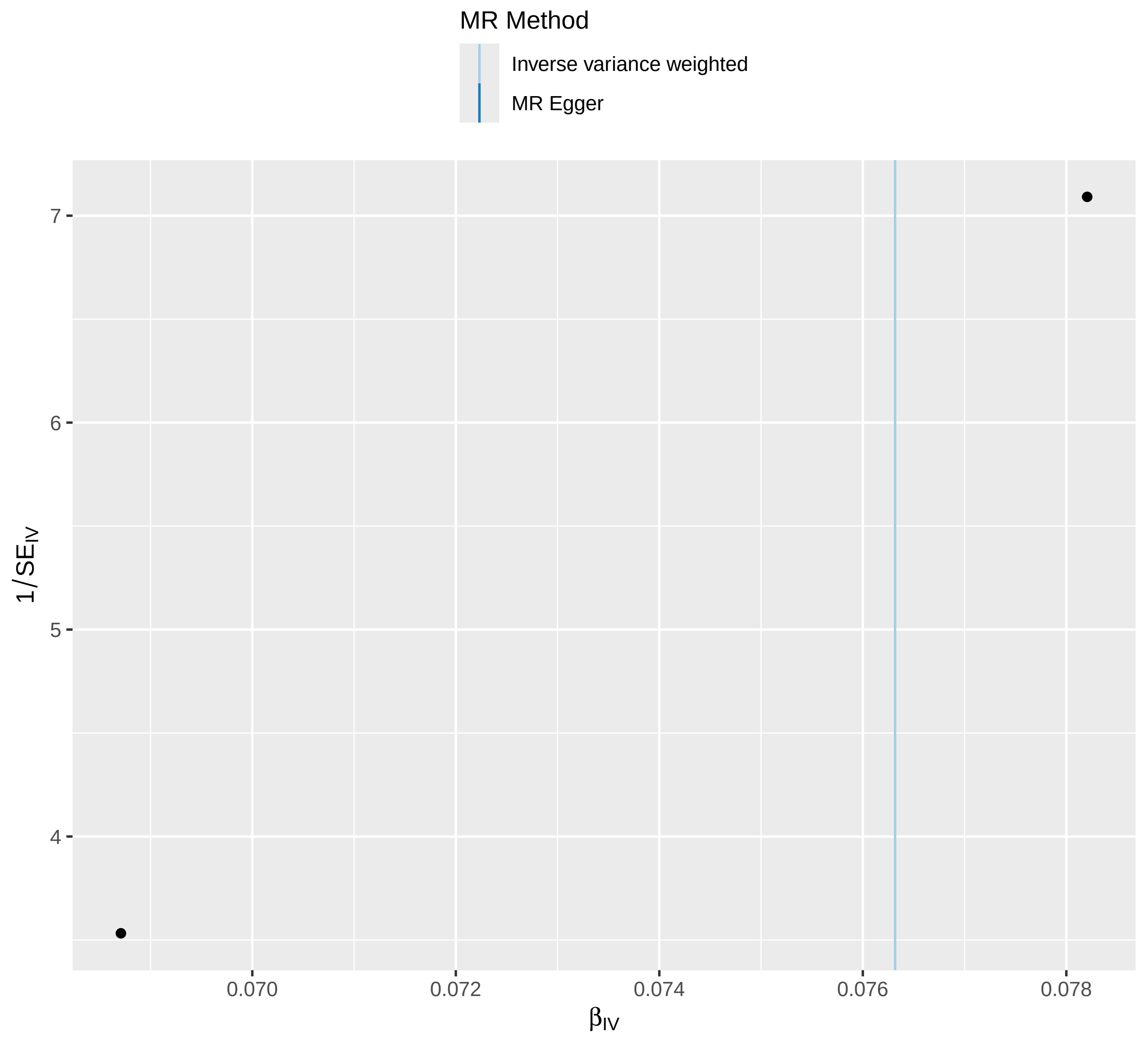
MR Method
Inverse variance weighted
MR Egger
7
6
5
4
1/SE_IV
0.070
0.072
0.074
0.076
0.078
β_IV

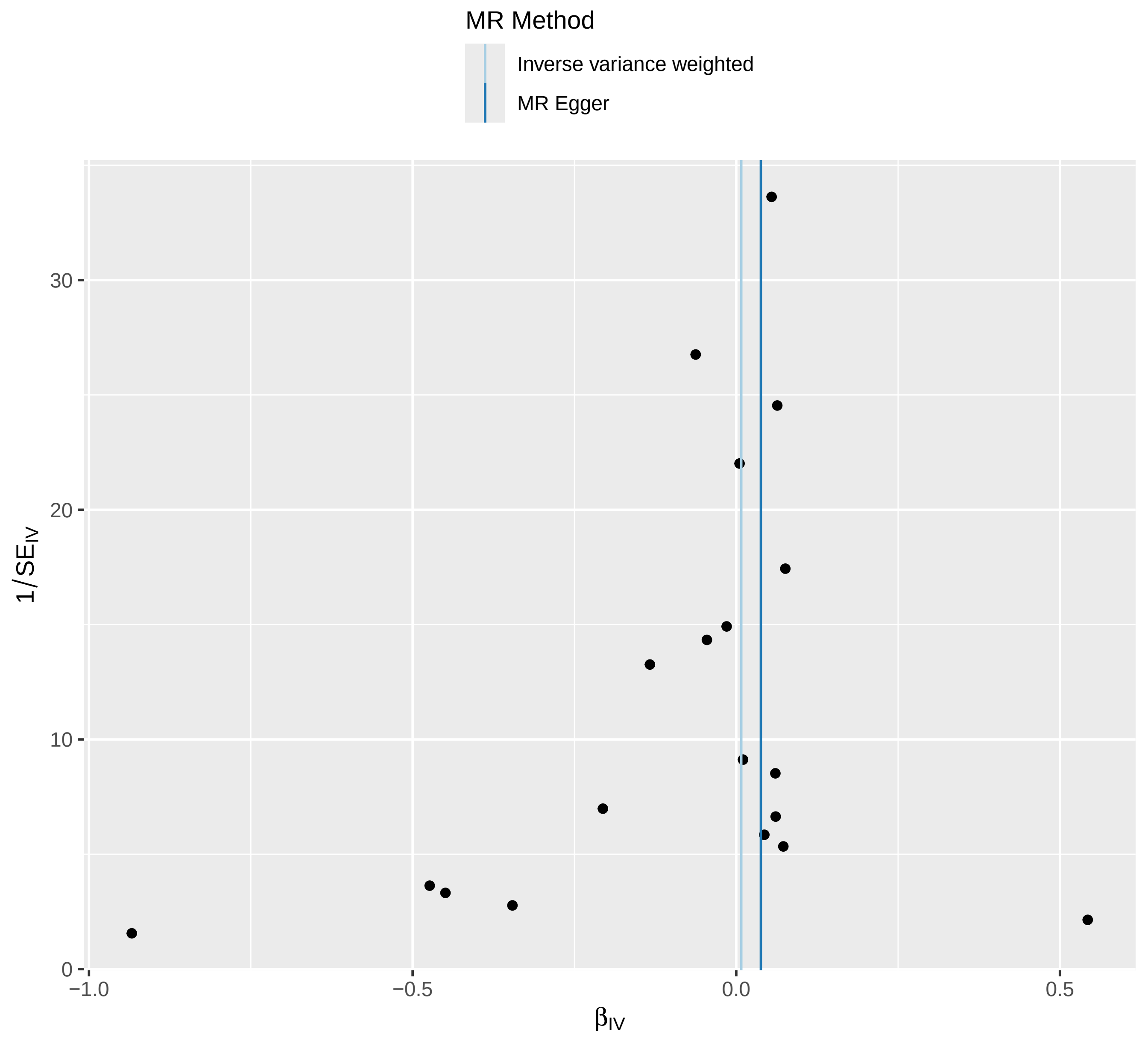
MR Method
Inverse variance weighted
MR Egger
30
20
10
0
1/SE_IV
−1.0
−0.5
0.0
0.5
β_IV

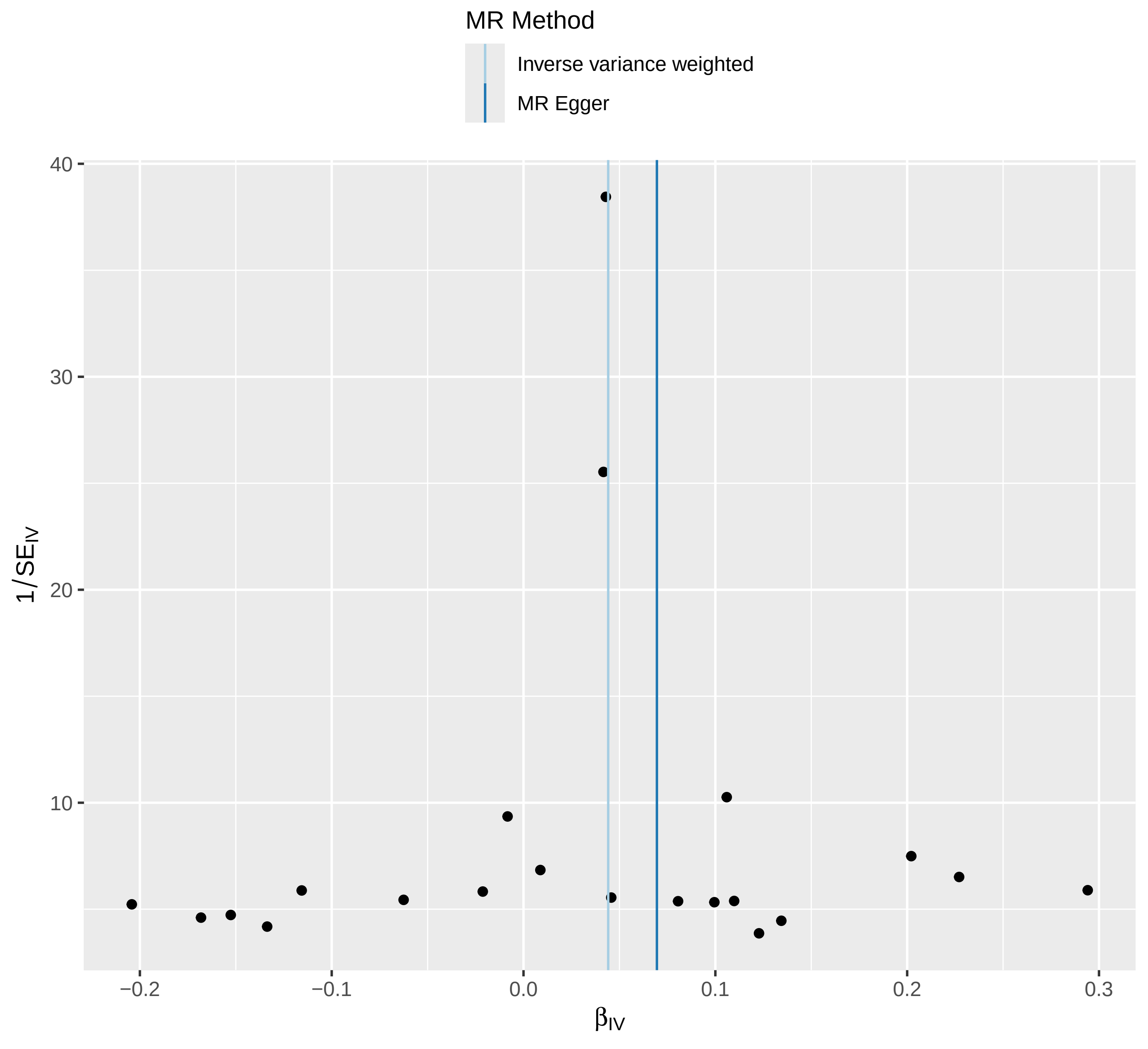
MR Method
Inverse variance weighted
MR Egger
40
30
20
10
1/SE$_{IV}$
−0.2
−0.1
0.0
0.1
0.2
0.3
β$_{IV}$

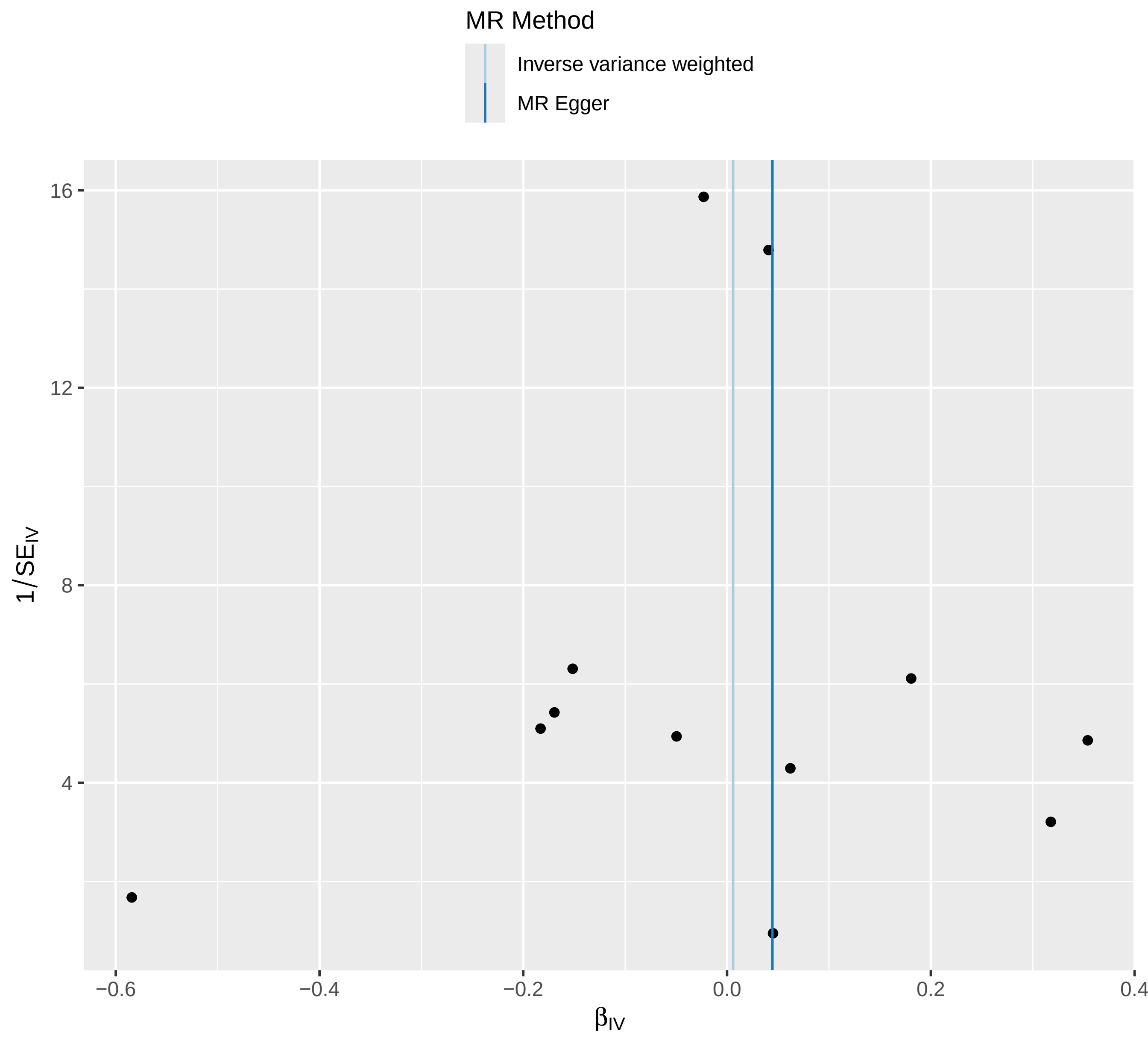

MR Method
Inverse variance weighted
MR Egger
16
12
8
4
1/SE_IV
−0.6
−0.4
−0.2
0.0
0.2
0.4
β_IV

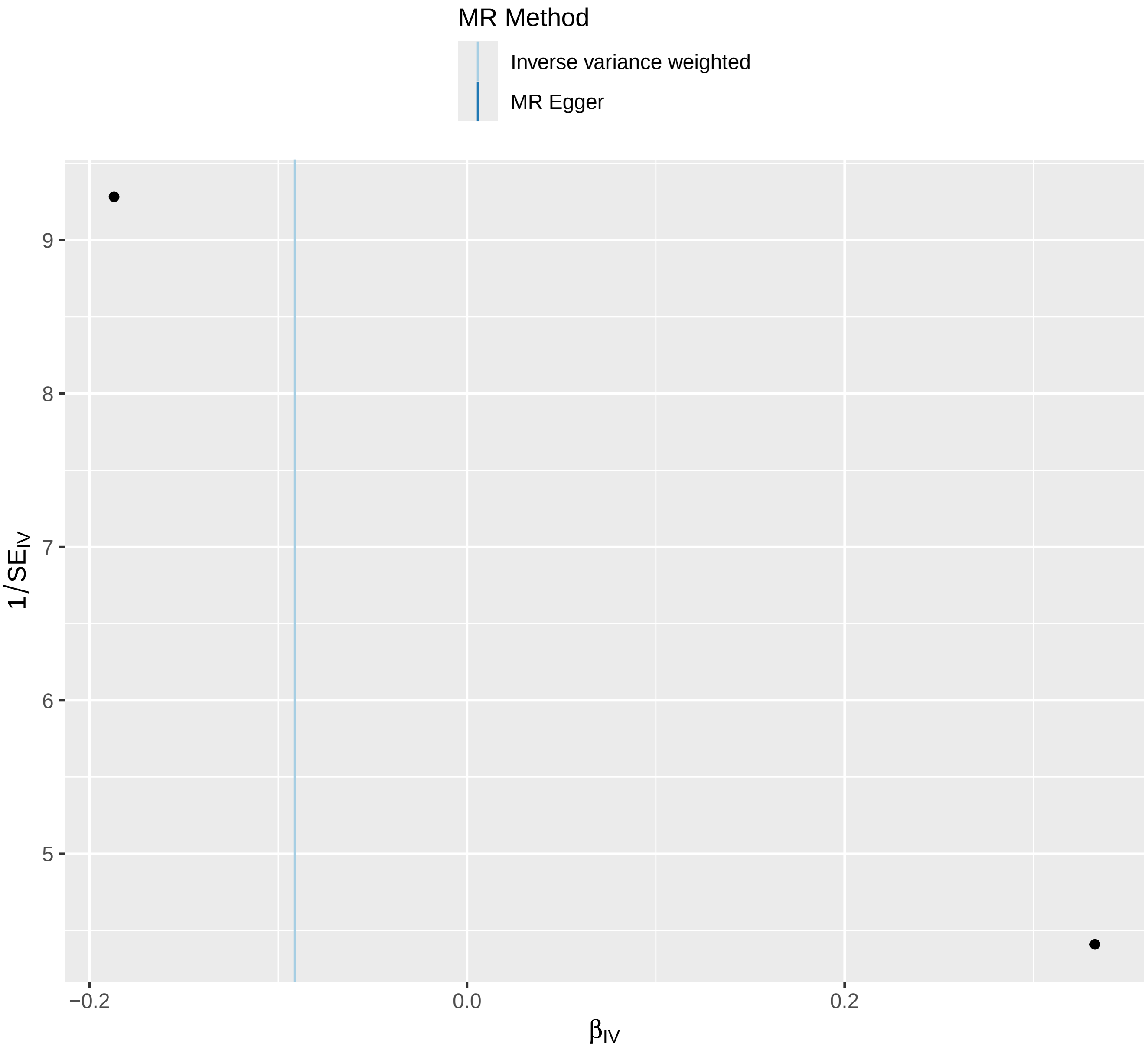

MR Method
Inverse variance weighted
MR Egger
9
8
7
6
5
1/SE_IV
−0.2
0.0
0.2
β_IV

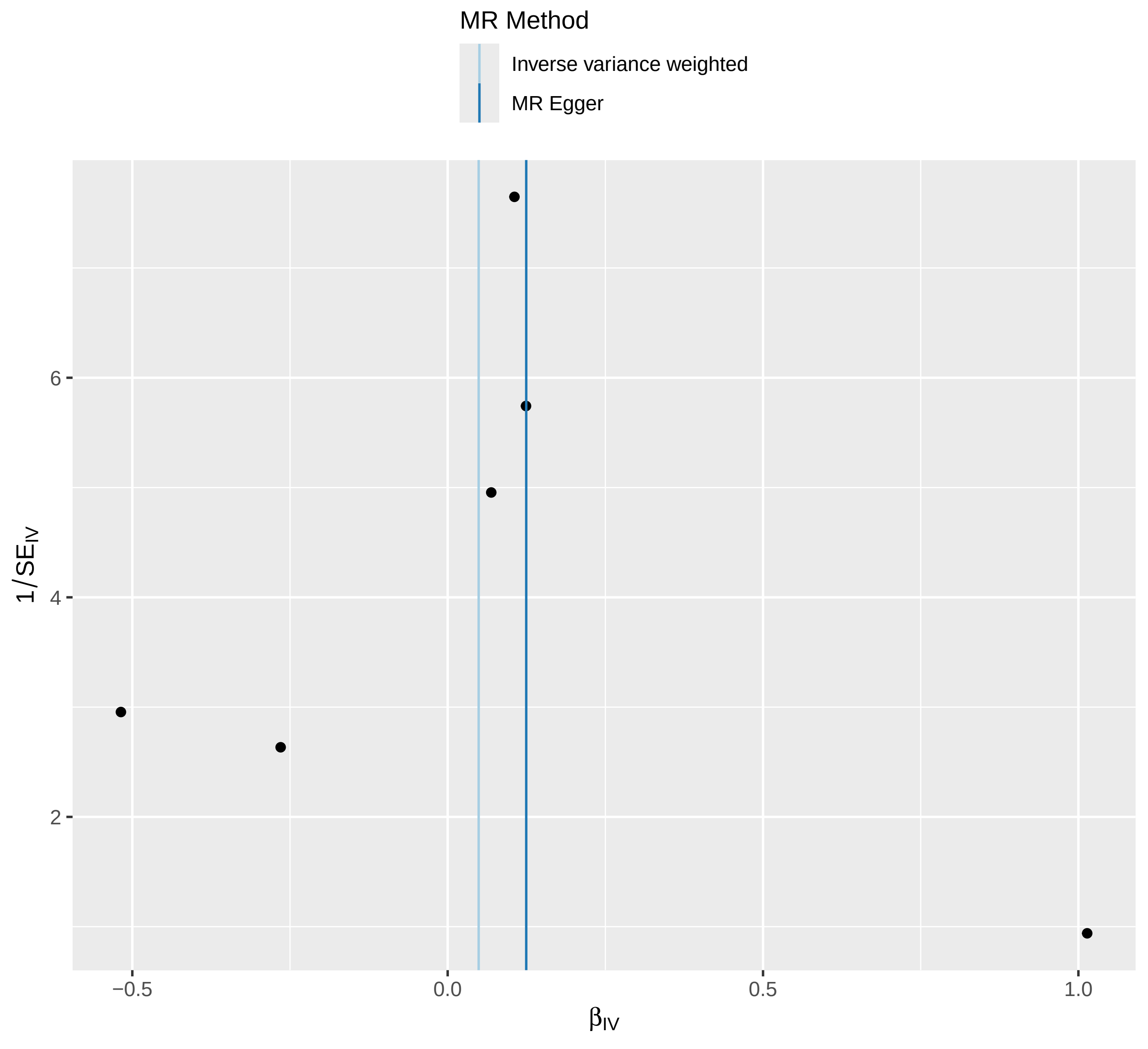

MR Method
Inverse variance weighted
MR Egger
6
4
2
1/SE$_{IV}$
−0.5
0.0
0.5
1.0
β$_{IV}$

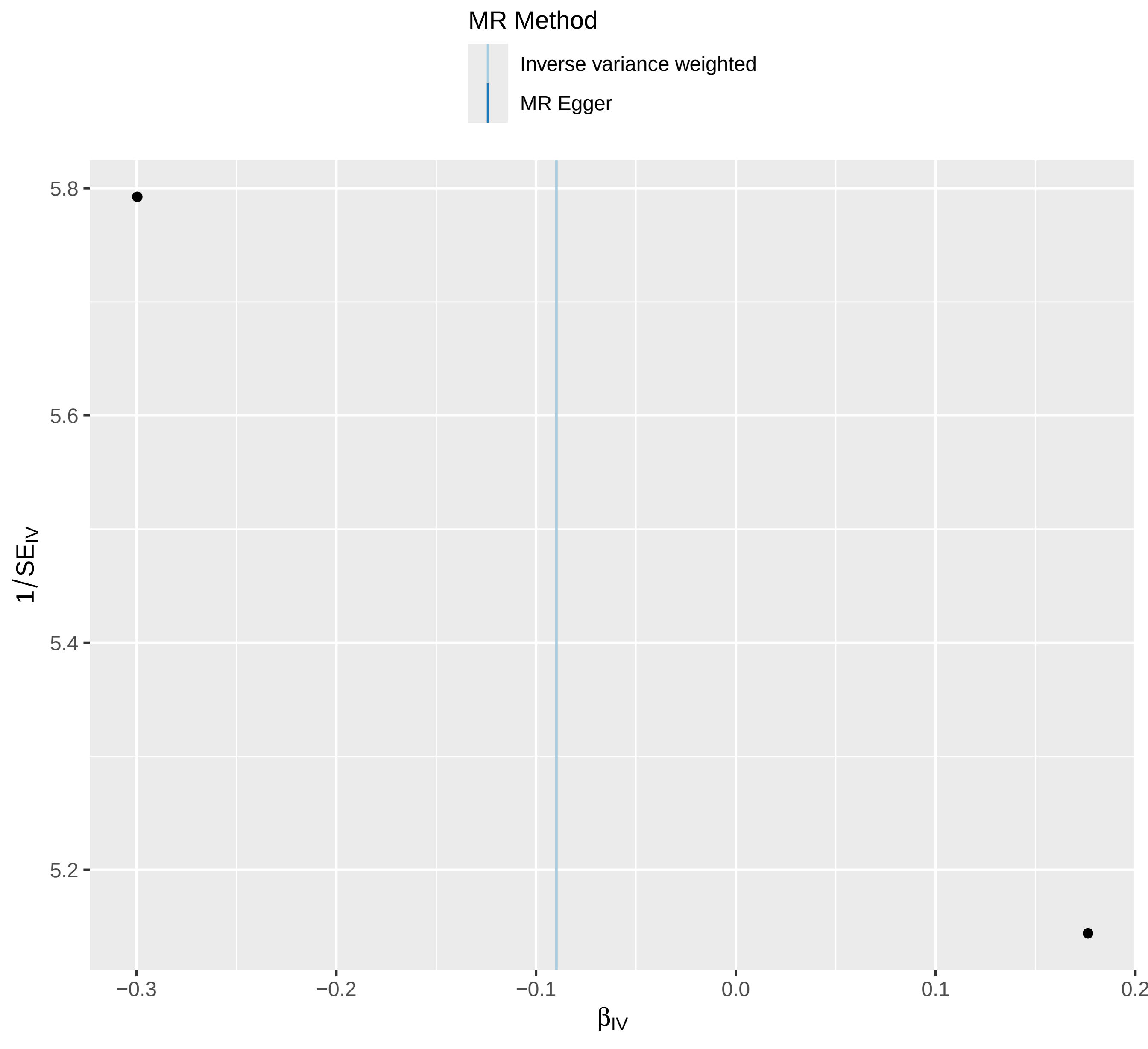

MR Method
Inverse variance weighted
MR Egger
5.8
5.6
5.4
5.2
1/SE_IV
−0.3
−0.2
−0.1
0.0
0.1
0.2
β_IV

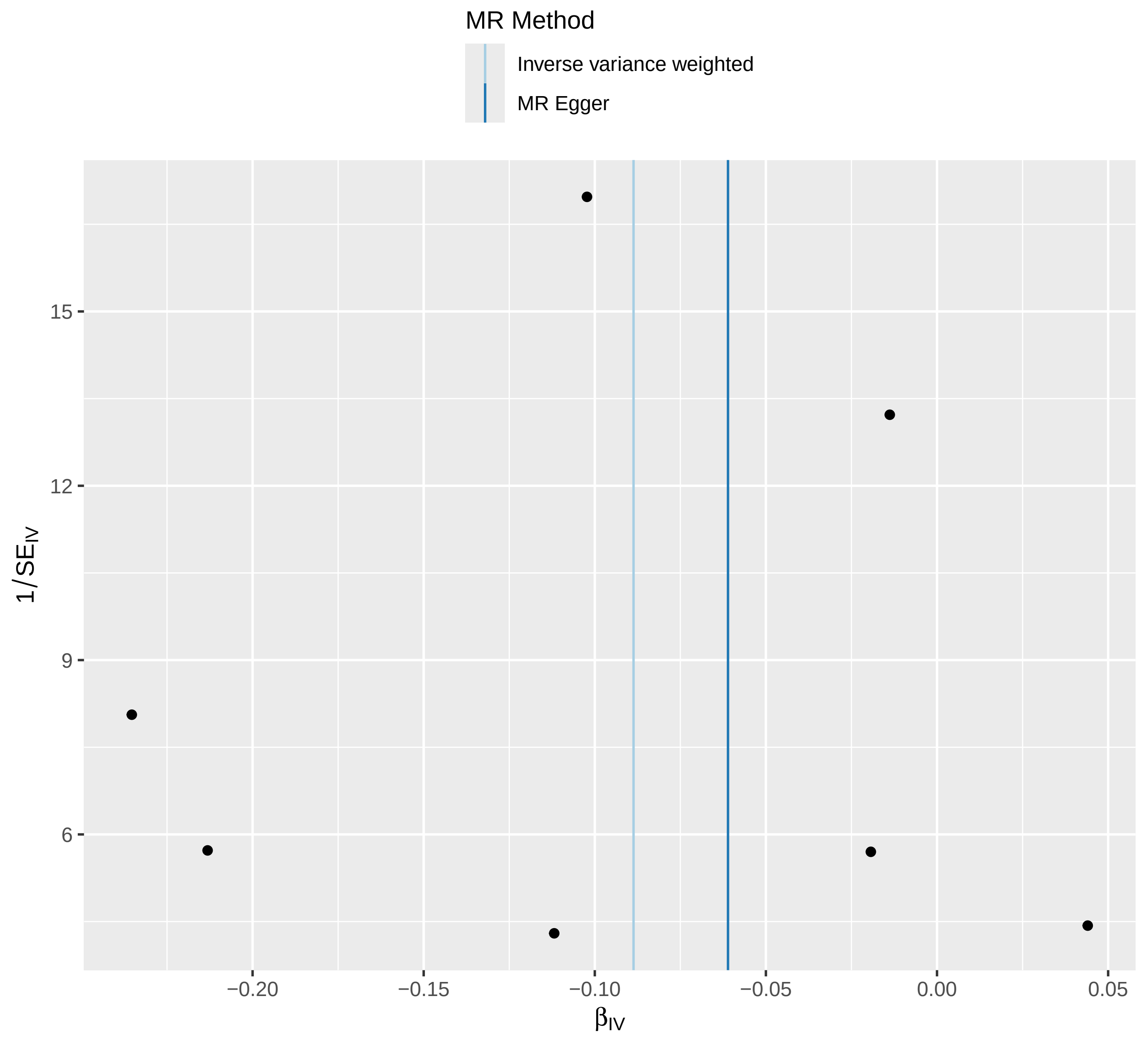
MR Method
Inverse variance weighted
MR Egger
15
12
9
6
1/SE_IV
−0.20
−0.15
−0.10
−0.05
0.00
0.05
β_IV

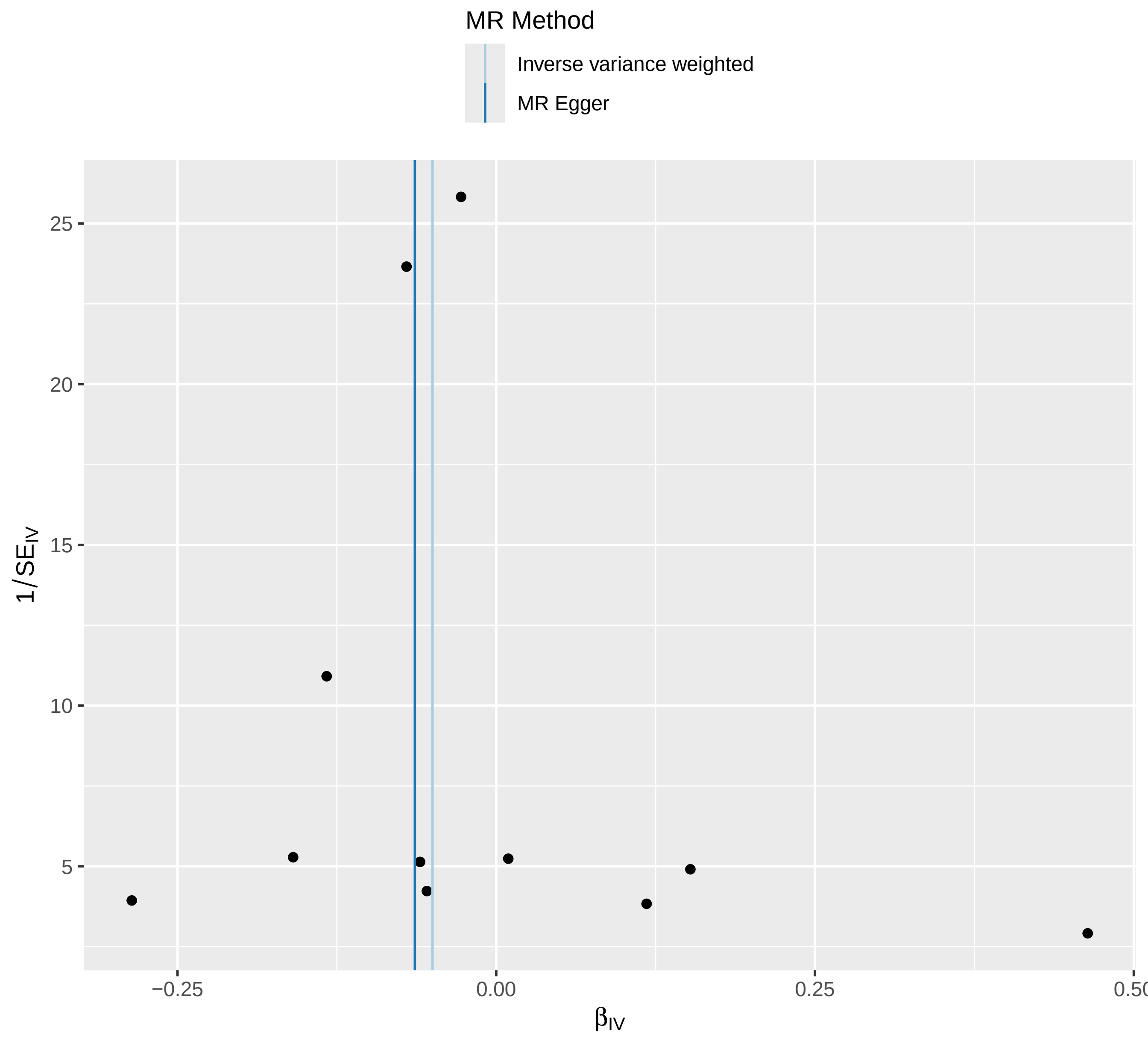
MR Method
Inverse variance weighted
MR Egger
25
20
15
10
5
1/SE$_{IV}$
−0.25
0.00
0.25
0.50
β$_{IV}$

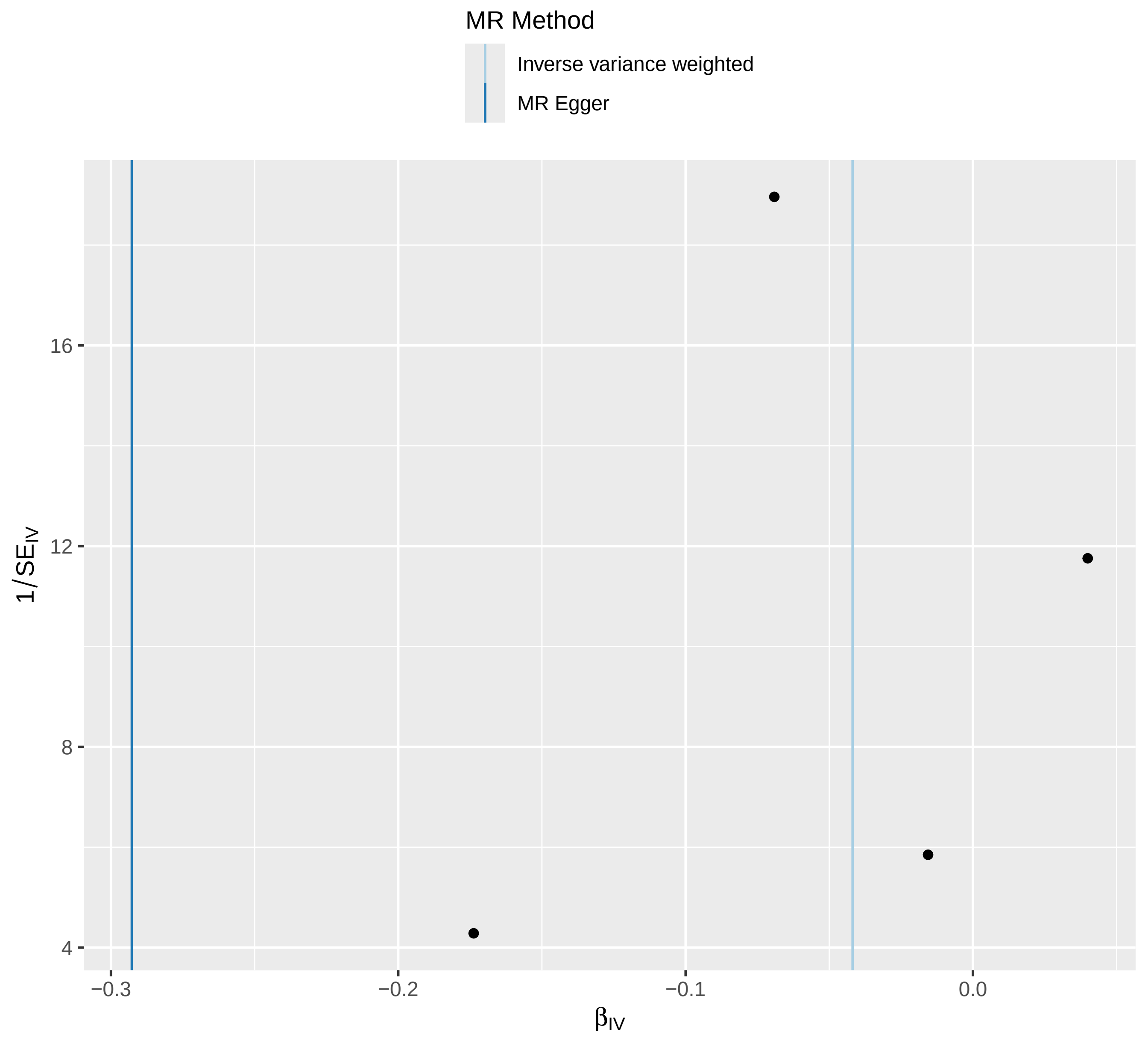

MR Method
Inverse variance weighted
MR Egger
16
12
8
4
1/SE$_{IV}$
−0.3
−0.2
−0.1
0.0
β$_{IV}$

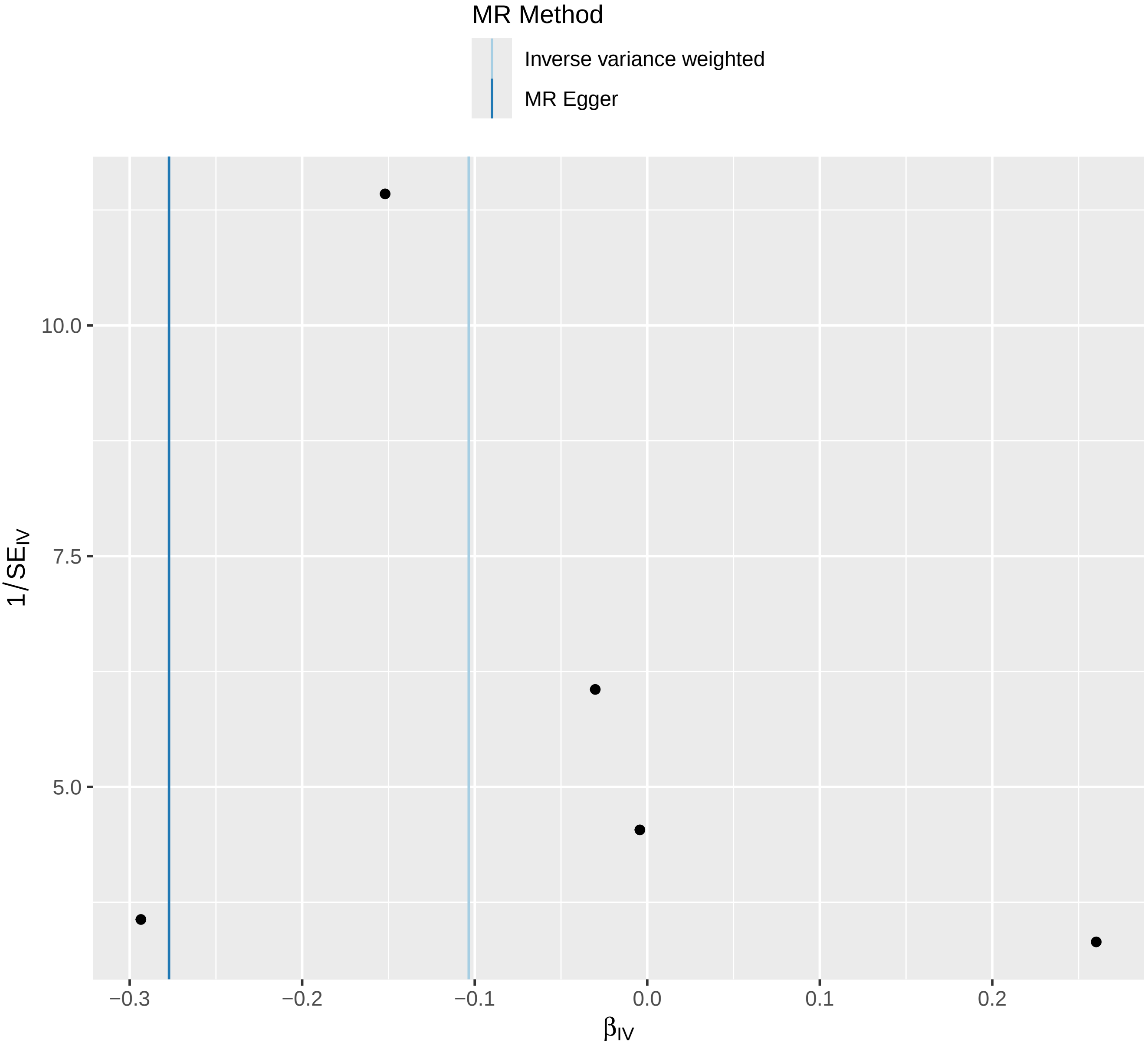

MR Method
Inverse variance weighted
MR Egger
10.0
7.5
5.0
1/SE$_{IV}$
−0.3
−0.2
−0.1
0.0
0.1
0.2
β$_{IV}$

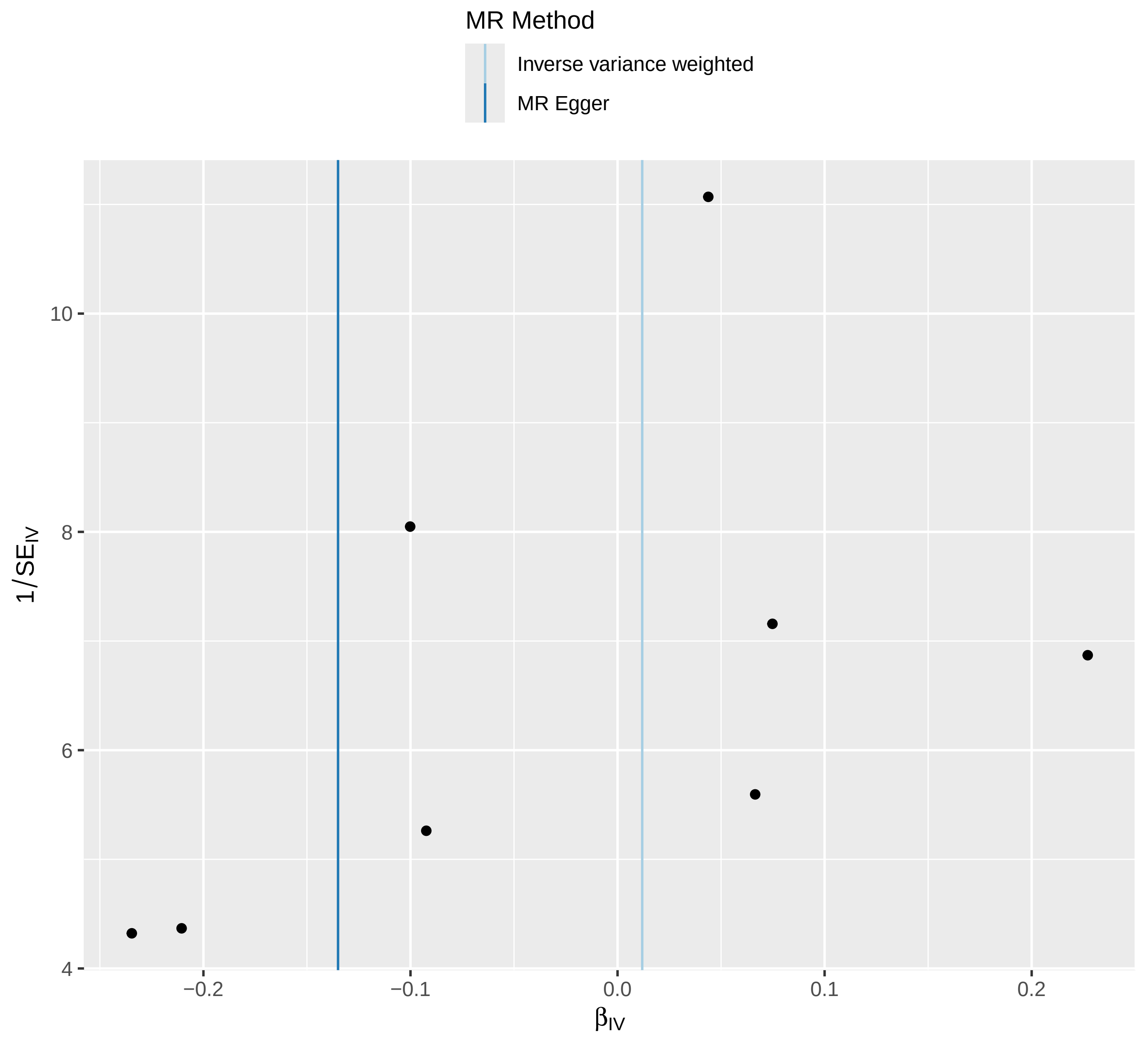

MR Method
Inverse variance weighted
MR Egger
10
8
6
4
1/SE_IV
−0.2
−0.1
0.0
0.1
0.2
β_IV

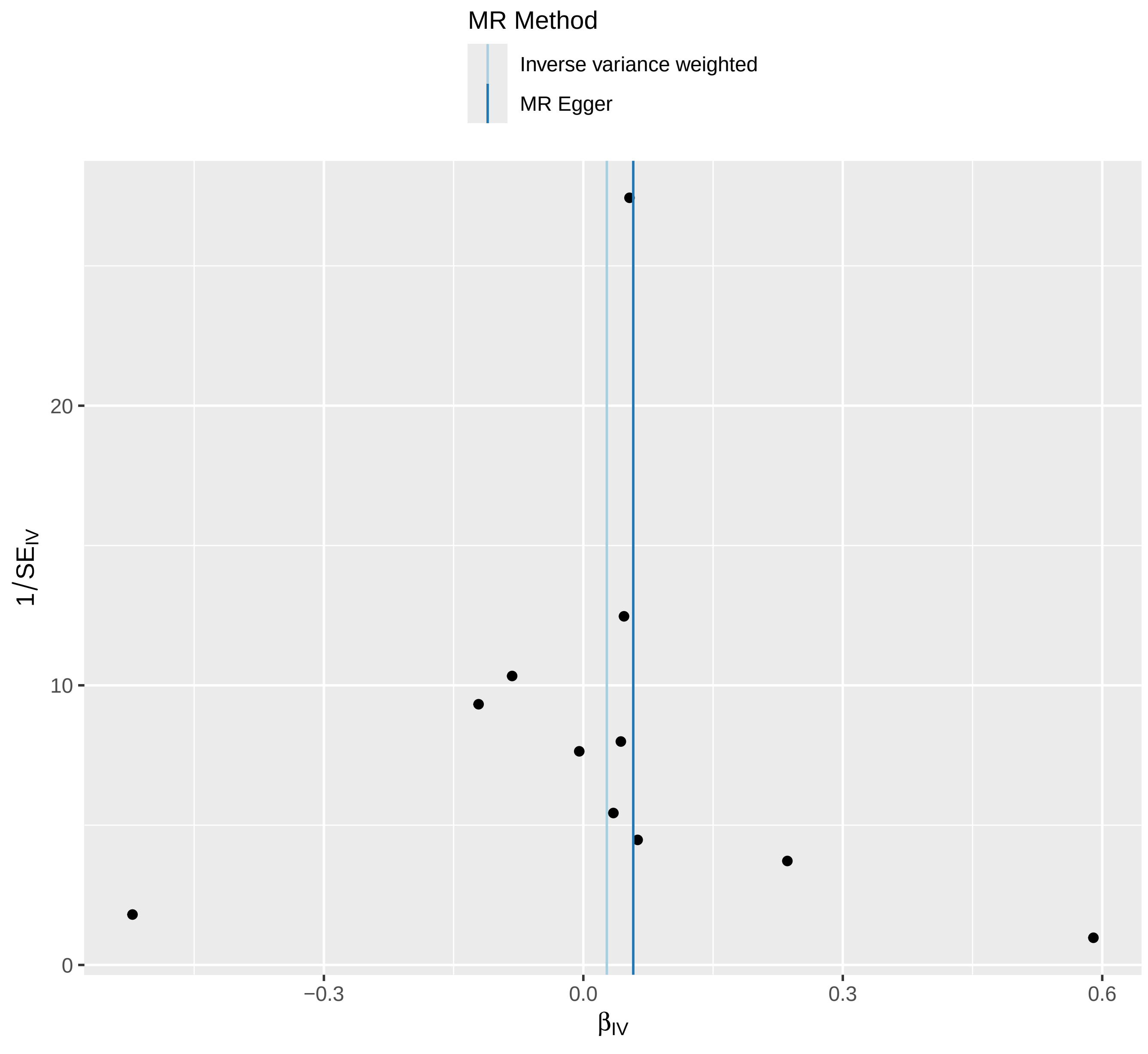
MR Method
Inverse variance weighted
MR Egger
20
10
0
1/$SE_{IV}$
−0.3
0.0
0.3
0.6
$\beta_{IV}$

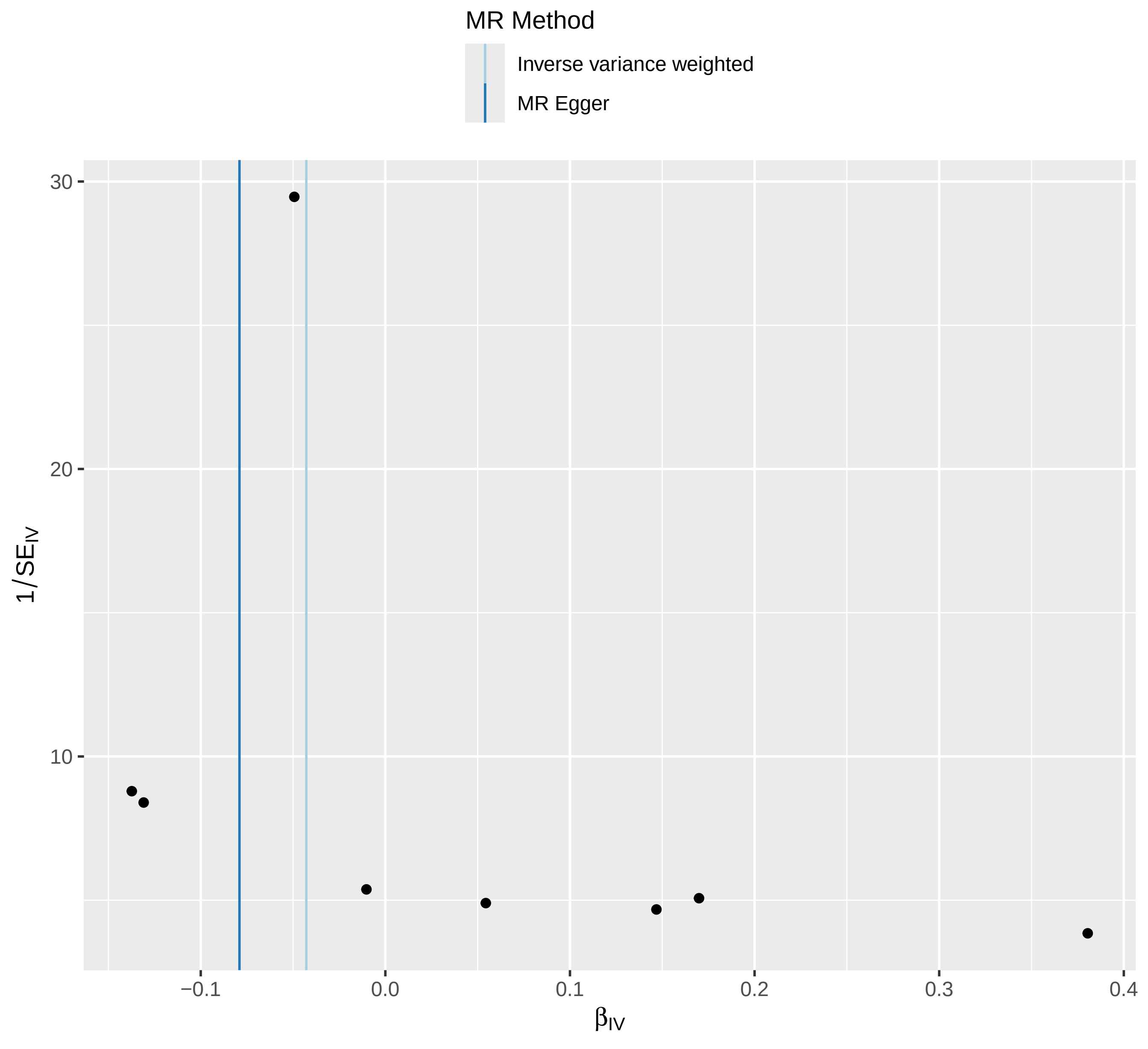
MR Method
Inverse variance weighted
MR Egger
30
20
10
1/SE$_{IV}$
−0.1
0.0
0.1
0.2
0.3
0.4
β$_{IV}$

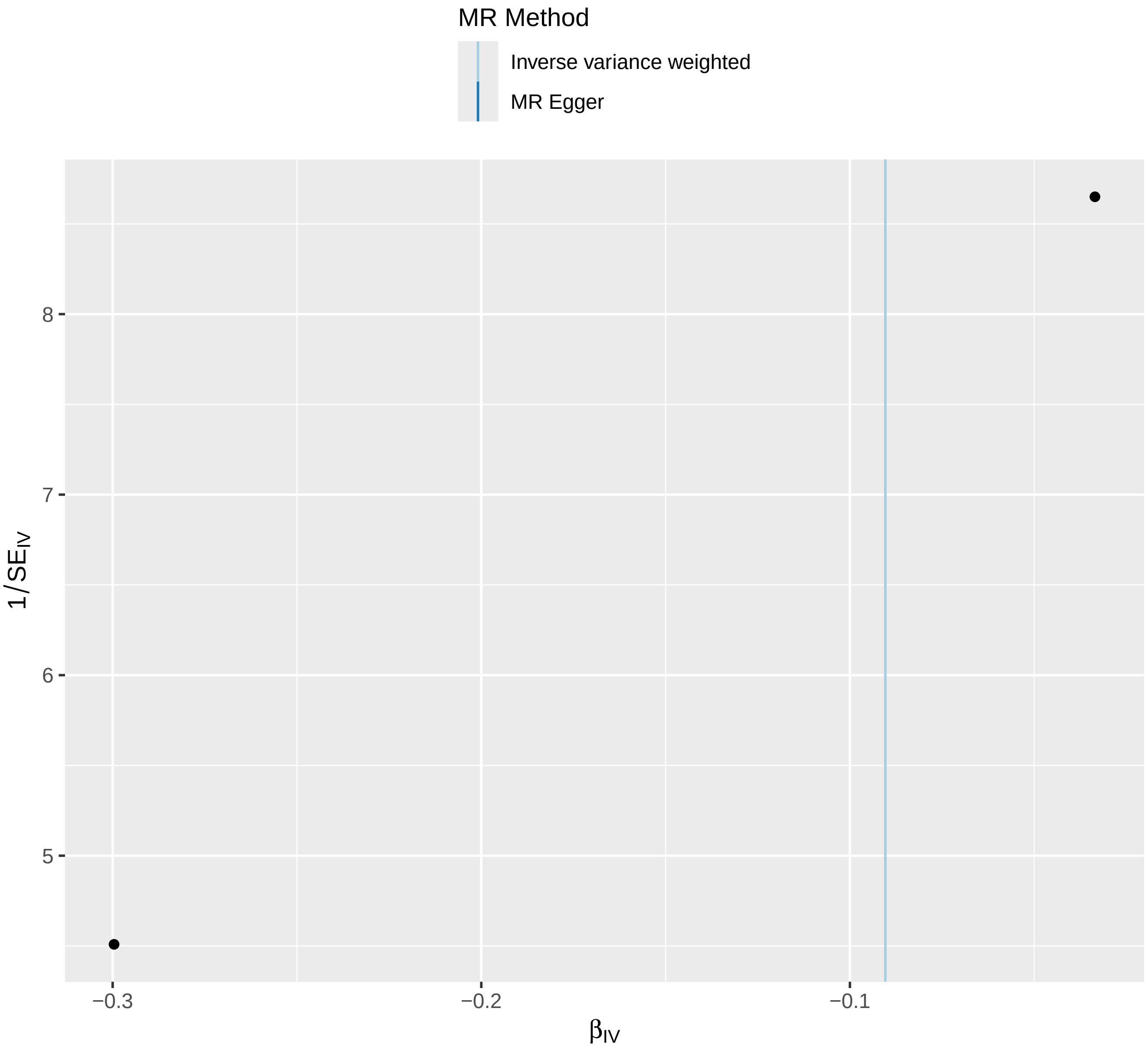
MR Method
Inverse variance weighted
MR Egger
8
7
6
5
$1/SE_{IV}$
−0.3
−0.2
−0.1
$\beta_{IV}$

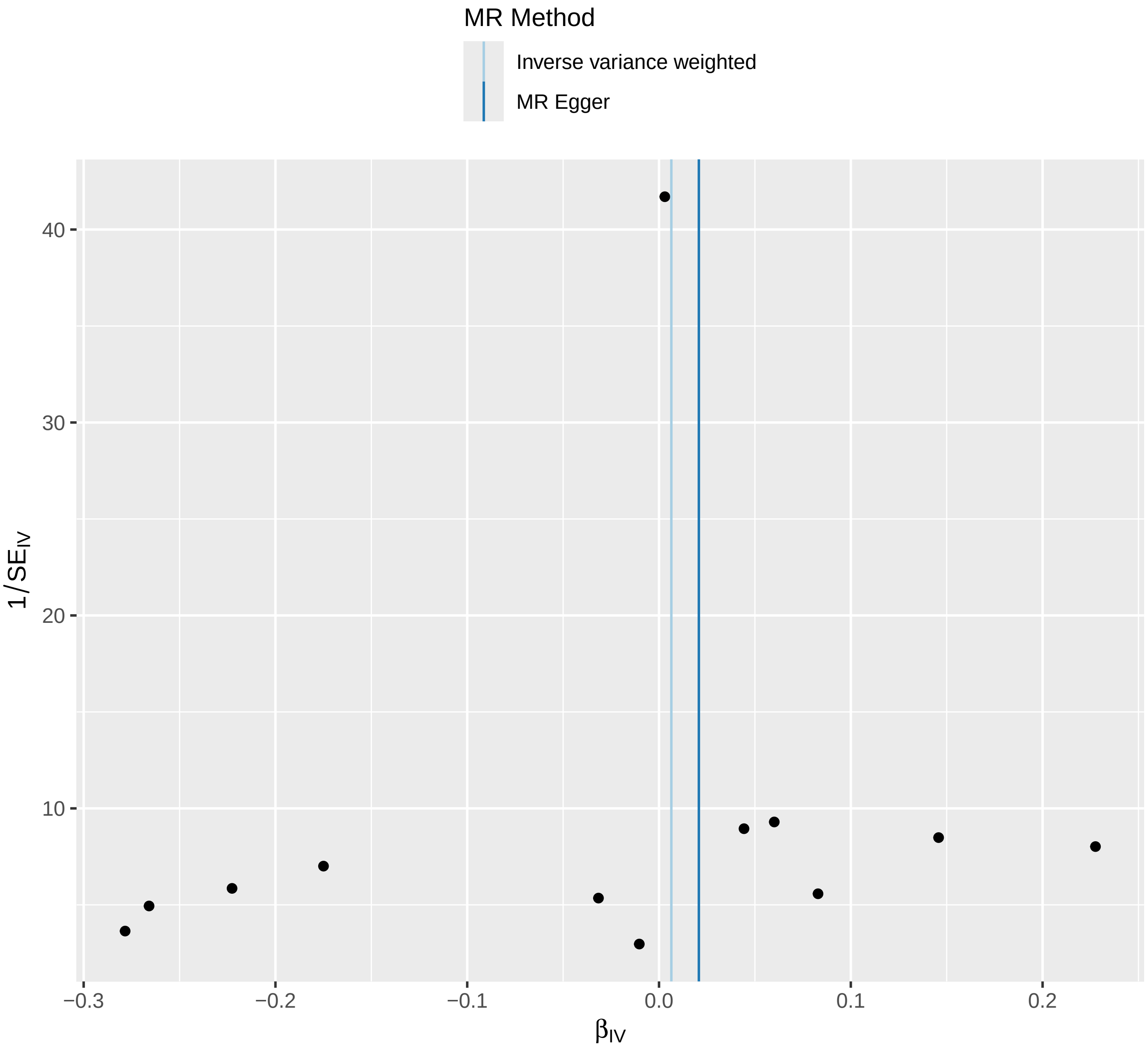
MR Method
Inverse variance weighted
MR Egger
40
30
20
10
1/SE_IV
−0.3
−0.2
−0.1
0.0
0.1
0.2
β_IV

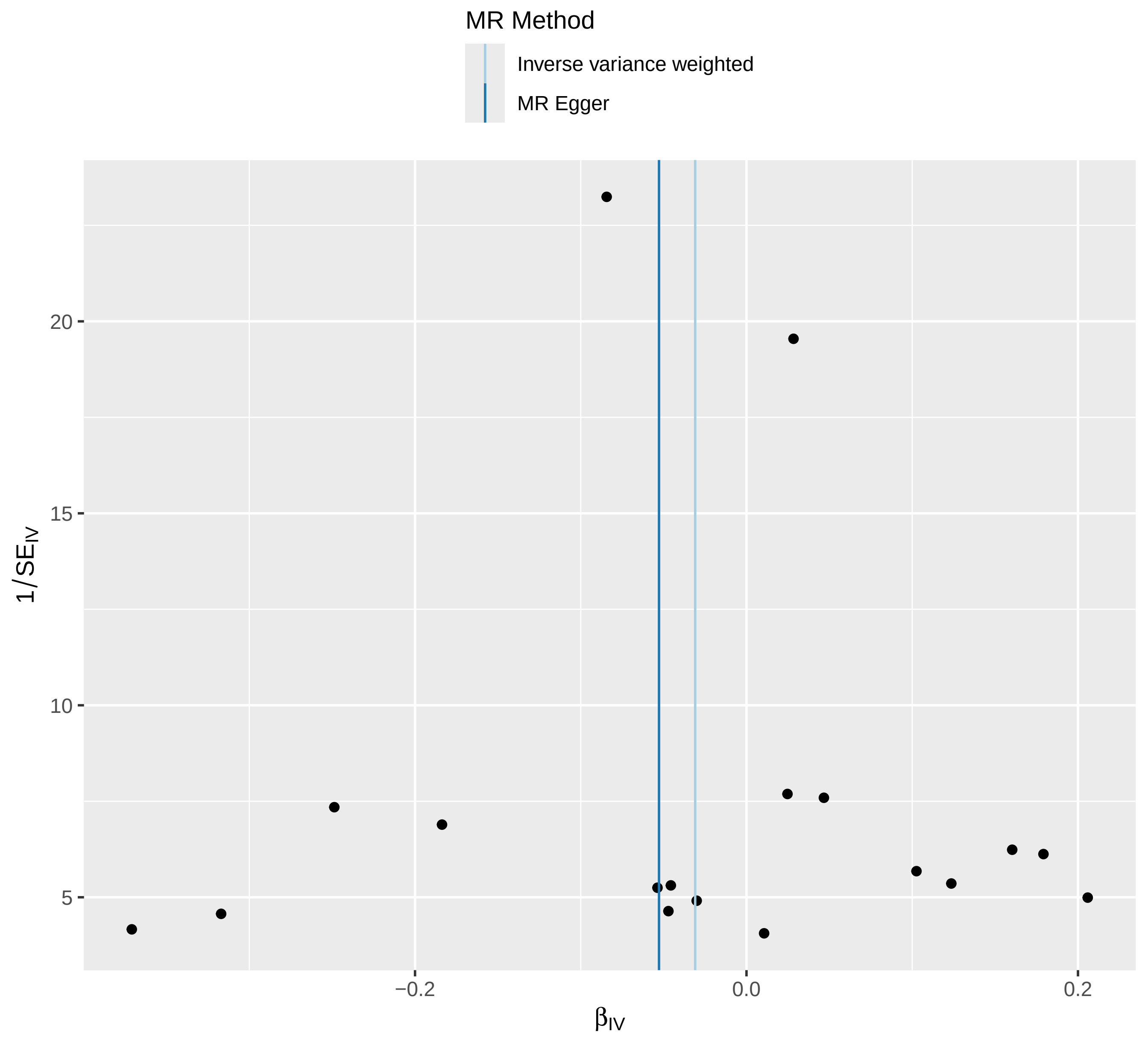
MR Method
Inverse variance weighted
MR Egger
20
15
10
5
1/SE_IV
−0.2
0.0
0.2
β_IV

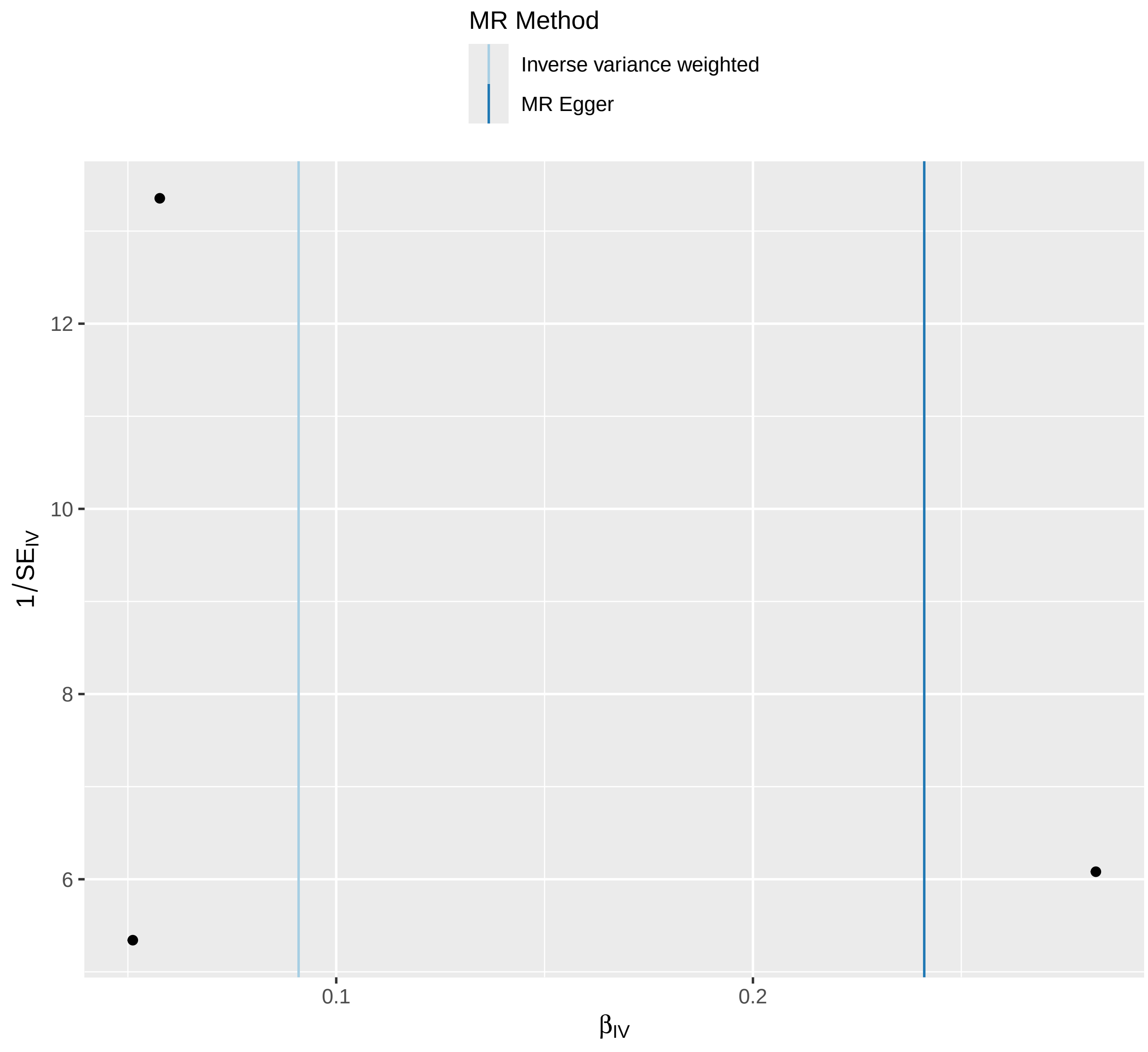

MR Method
Inverse variance weighted
MR Egger
12
10
8
6
0.1
0.2
$1/SE_{IV}$
$\beta_{IV}$

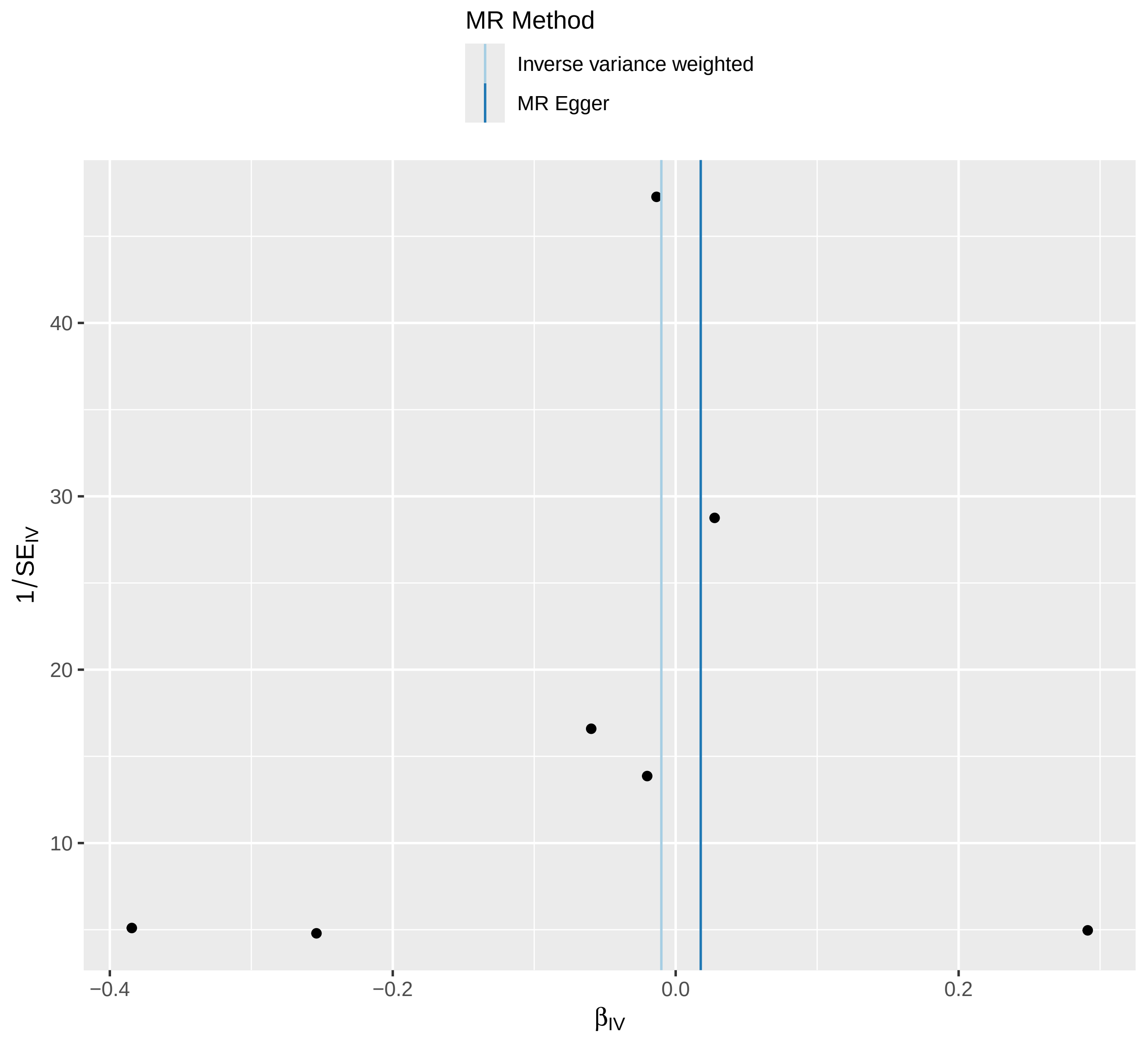
MR Method
Inverse variance weighted
MR Egger
40
30
20
10
−0.4
−0.2
0.0
0.2
$1/SE_{IV}$
$\beta_{IV}$

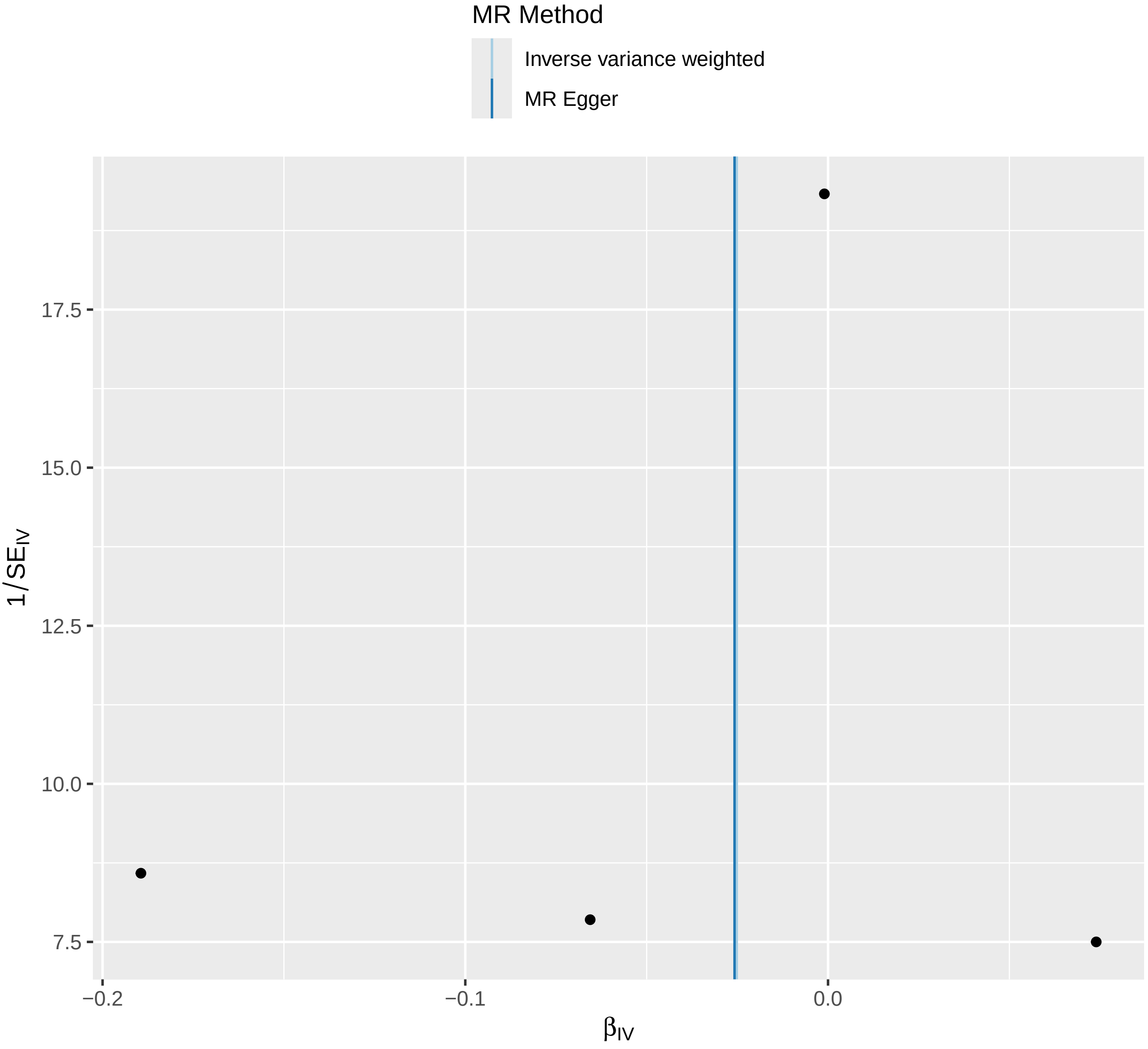

MR Method
Inverse variance weighted
MR Egger
17.5
15.0
12.5
10.0
7.5
1/SE$_{IV}$
−0.2
−0.1
0.0
β$_{IV}$

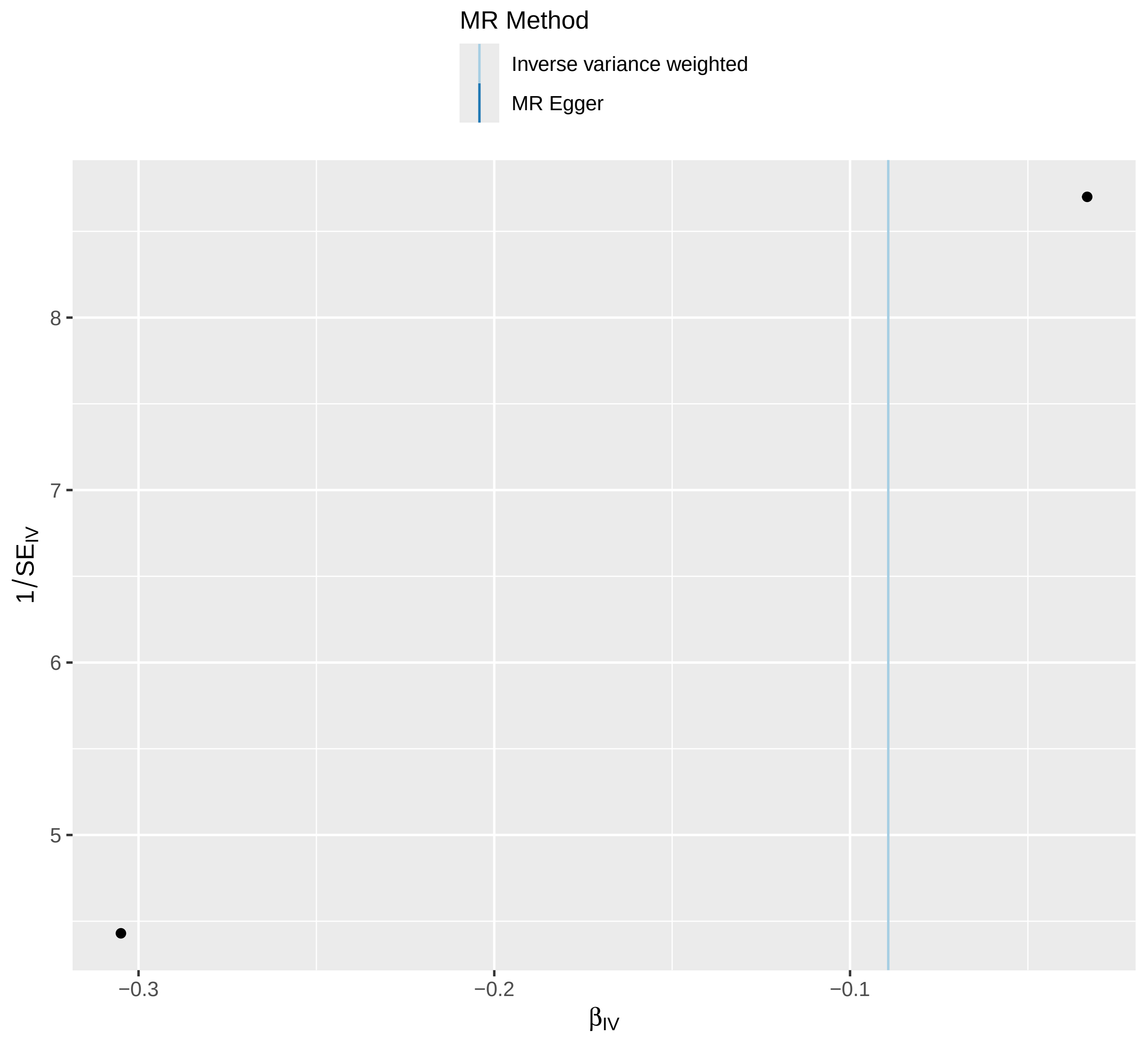
MR Method
Inverse variance weighted
MR Egger
8
7
6
5
1/SE$_{IV}$
−0.3
−0.2
−0.1
β$_{IV}$

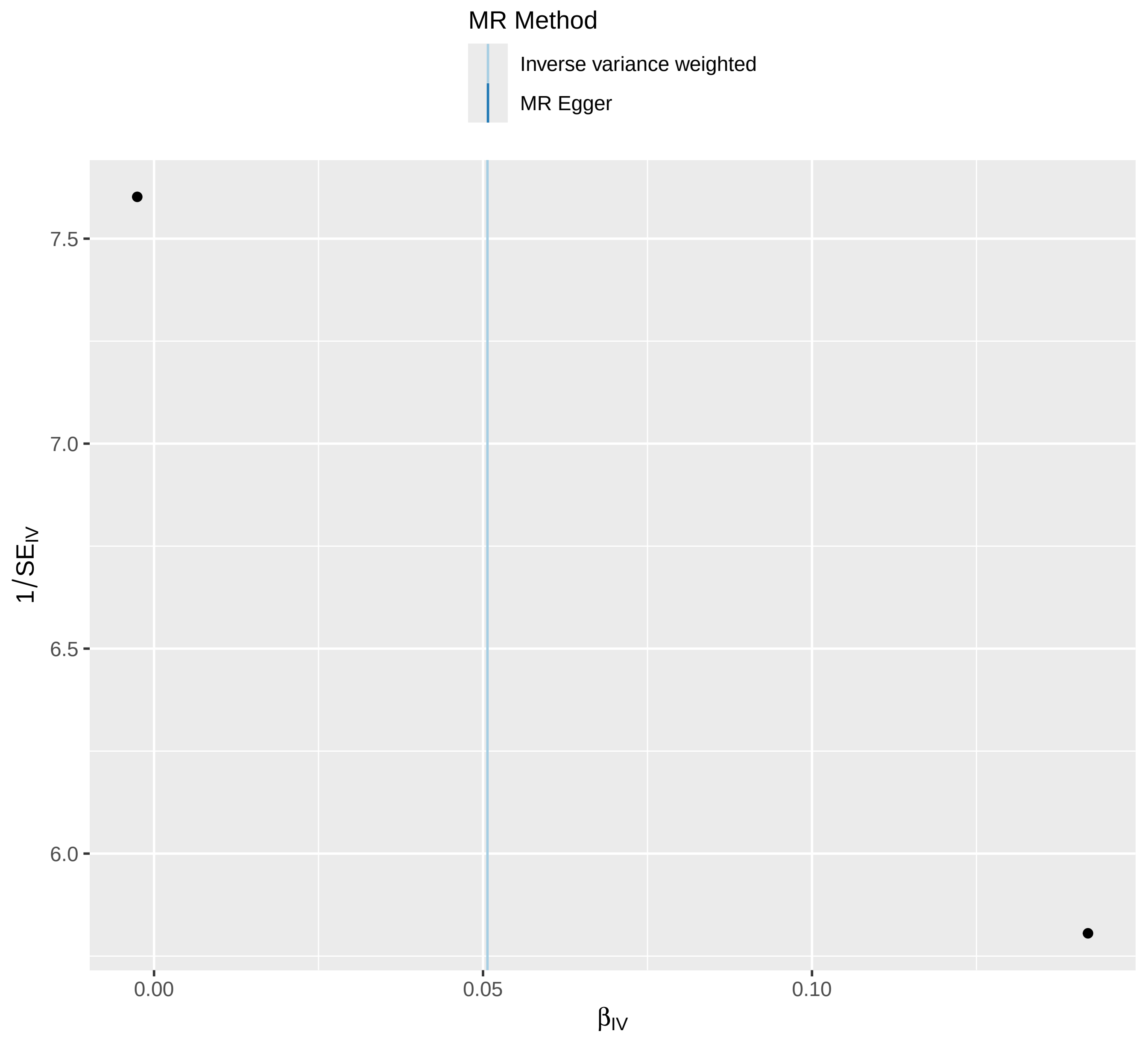
MR Method
Inverse variance weighted
MR Egger
7.5
7.0
6.5
6.0
1/SE_IV
0.00
0.05
0.10
β_IV

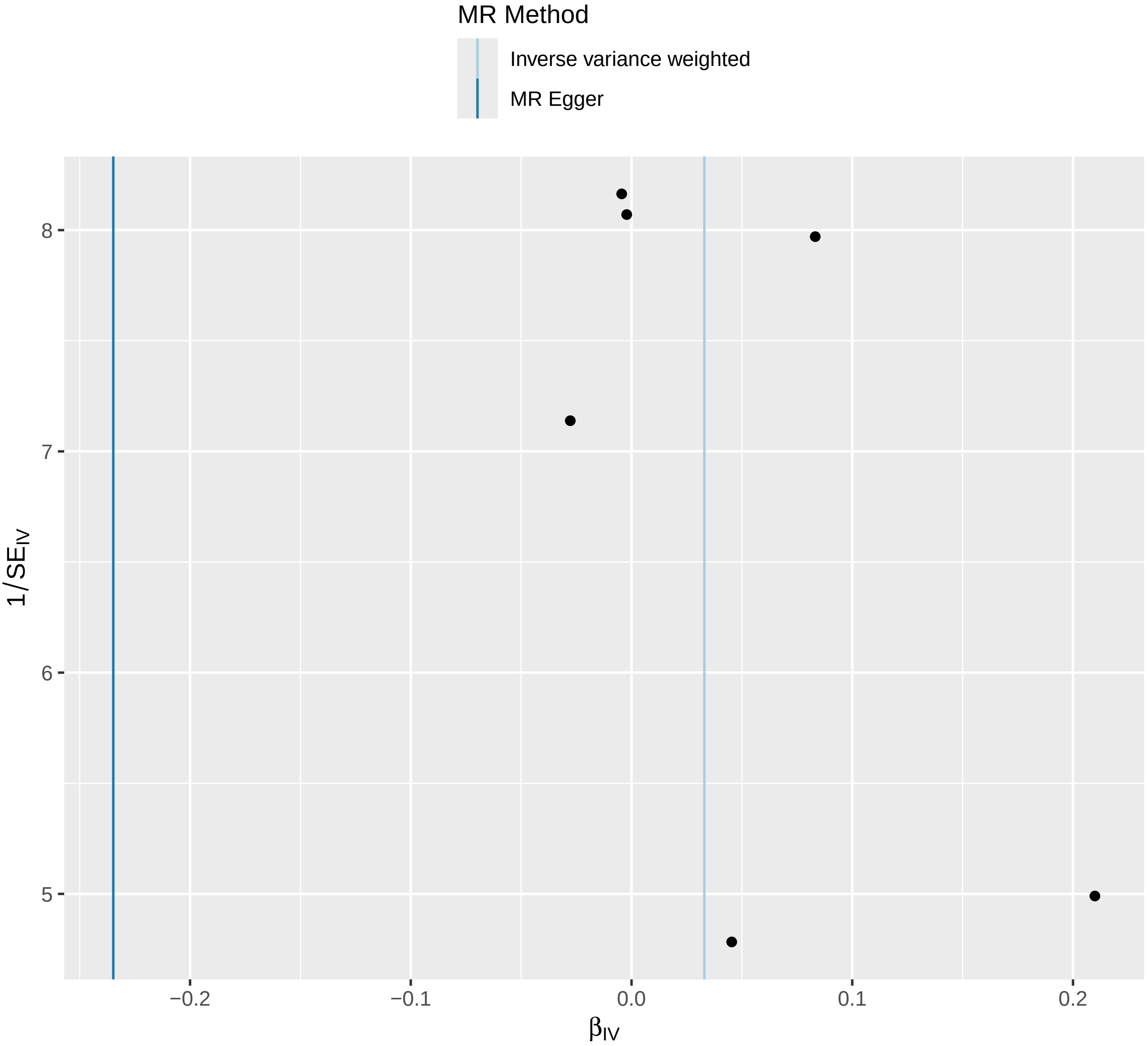
MR Method
Inverse variance weighted
MR Egger
8
7
6
5
1/SE$_{IV}$
−0.2
−0.1
0.0
0.1
0.2
β$_{IV}$

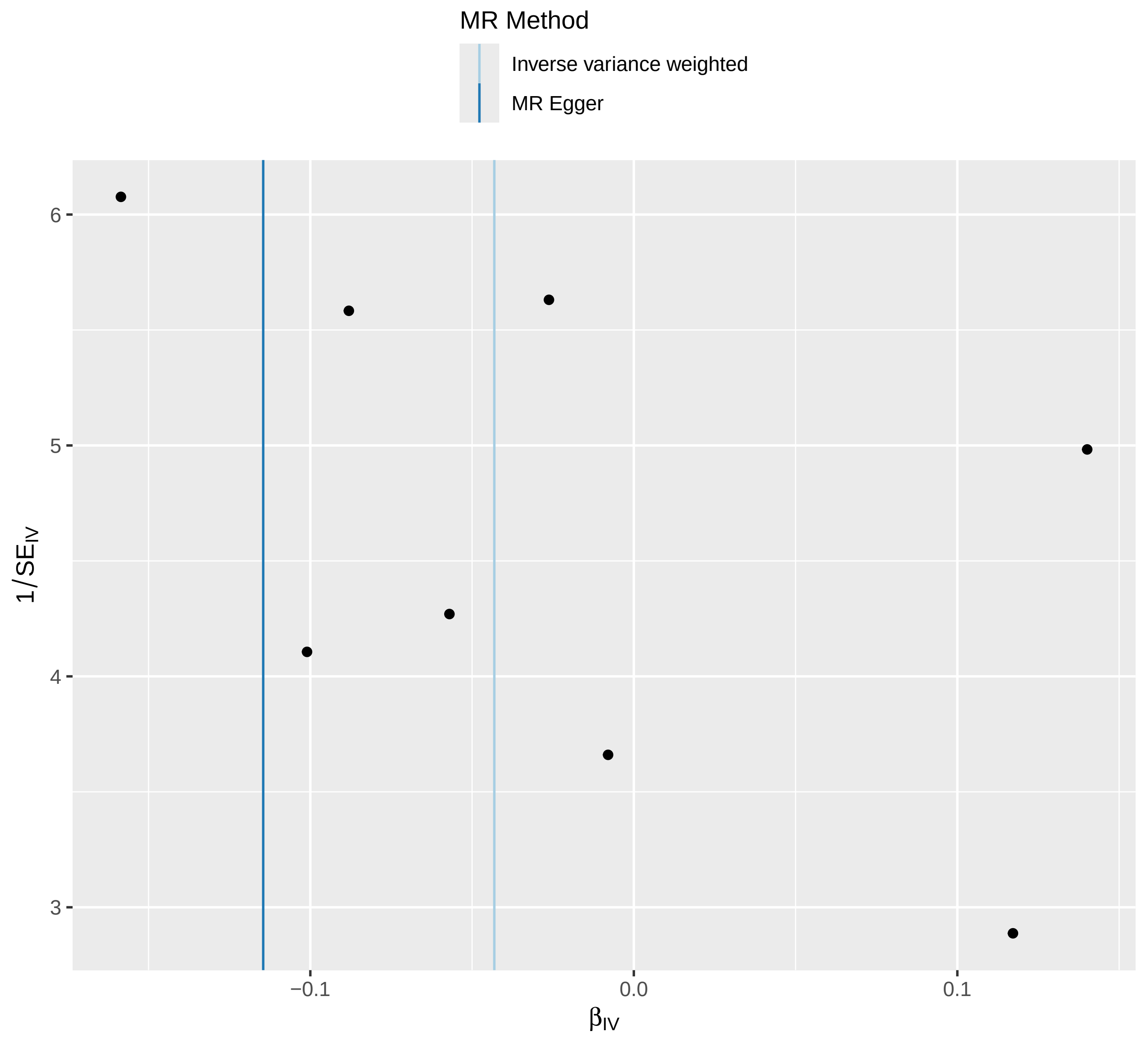

MR Method
Inverse variance weighted
MR Egger
6
5
4
3
1/SE_IV
−0.1
0.0
0.1
β_IV

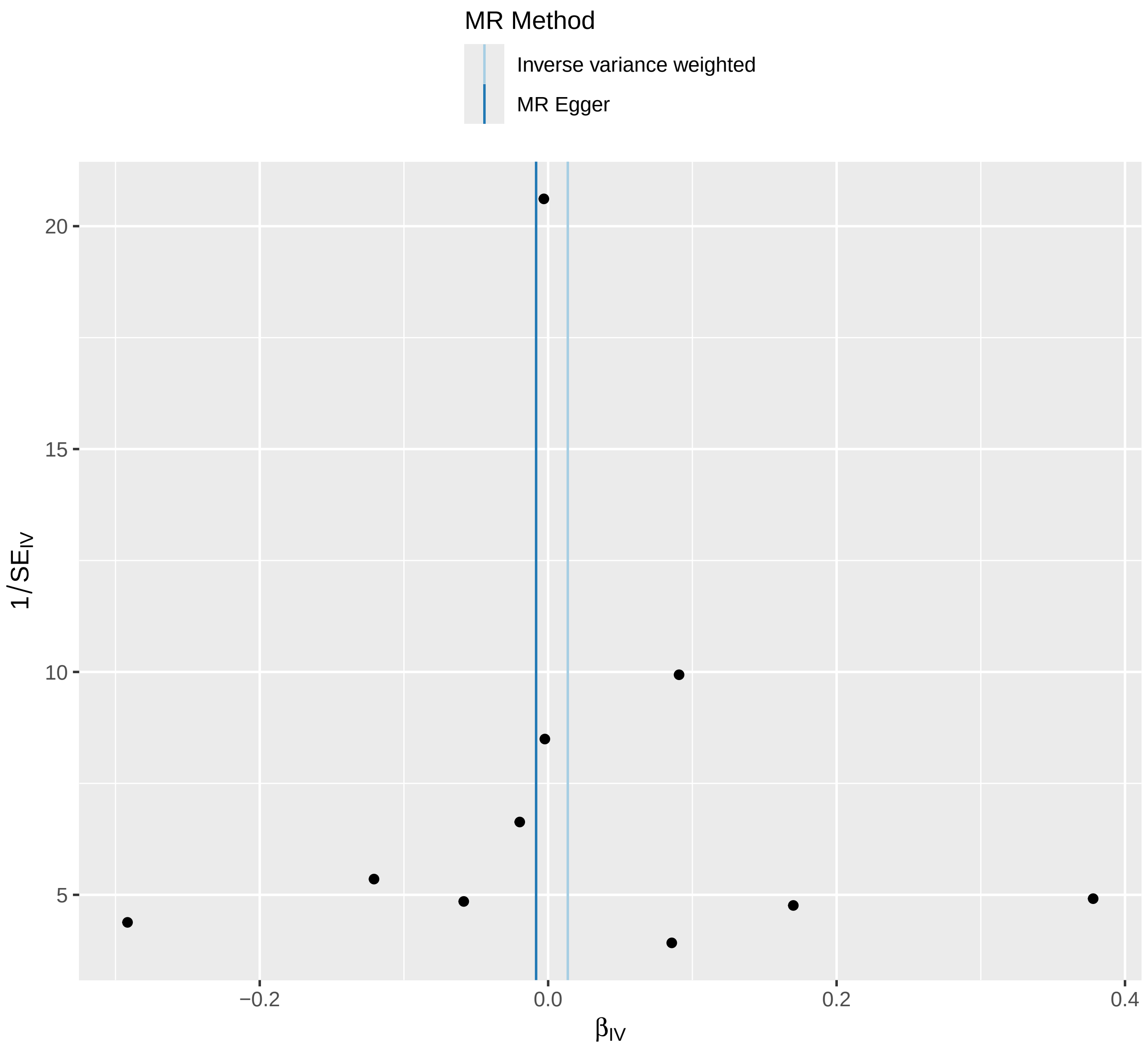
MR Method
Inverse variance weighted
MR Egger
20
15
10
5
$1/SE_{IV}$
−0.2
0.0
0.2
0.4
$\beta_{IV}$

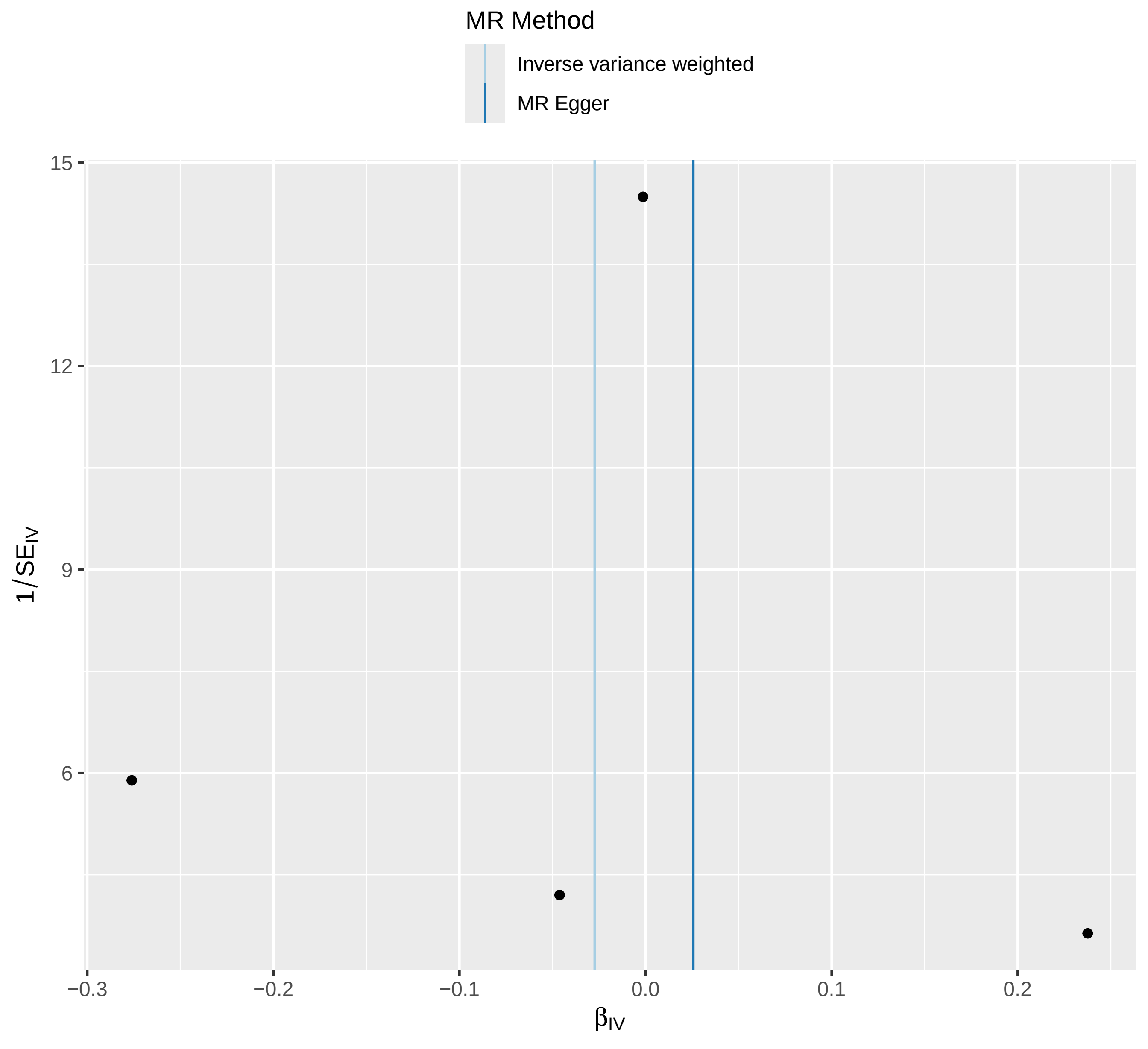
MR Method
Inverse variance weighted
MR Egger
15
12
9
6
1/SE$_{IV}$
−0.3
−0.2
−0.1
0.0
0.1
0.2
β$_{IV}$

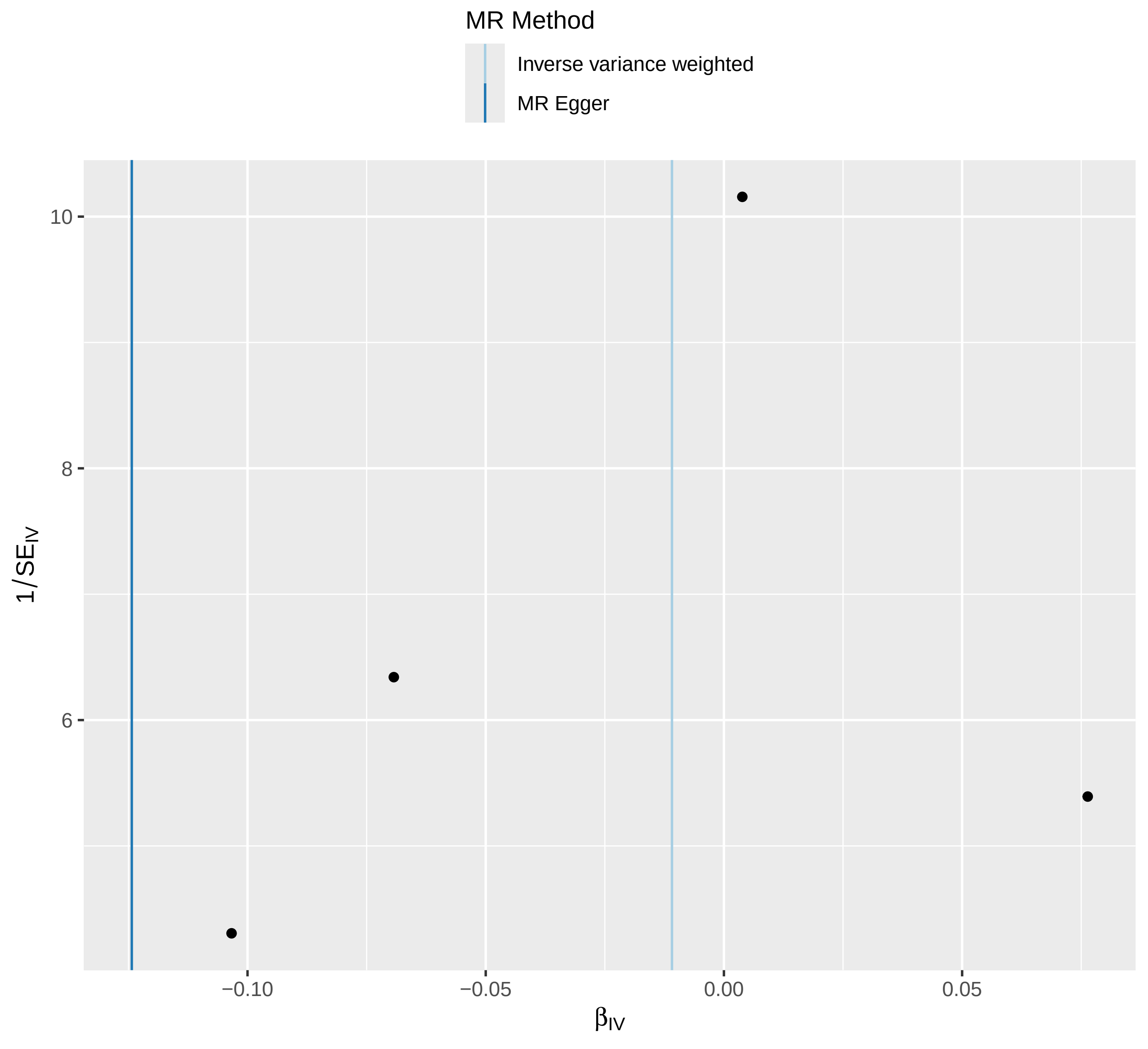
MR Method
Inverse variance weighted
MR Egger
10
8
6
1/SE$_{IV}$
−0.10
−0.05
0.00
0.05
β$_{IV}$

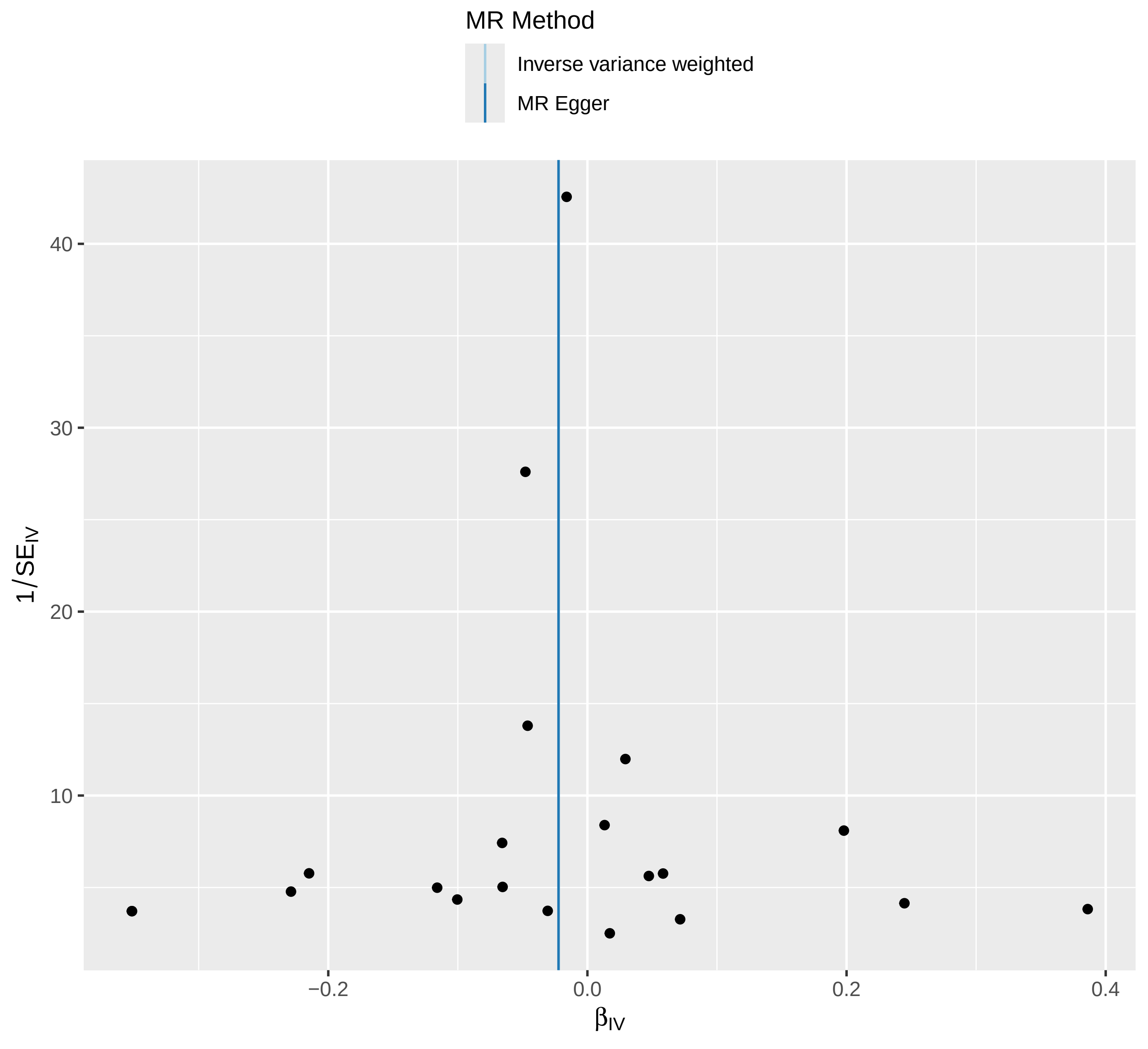

MR Method
Inverse variance weighted
MR Egger
40
30
20
10
1/SE_IV
−0.2
0.0
0.2
0.4
β_IV

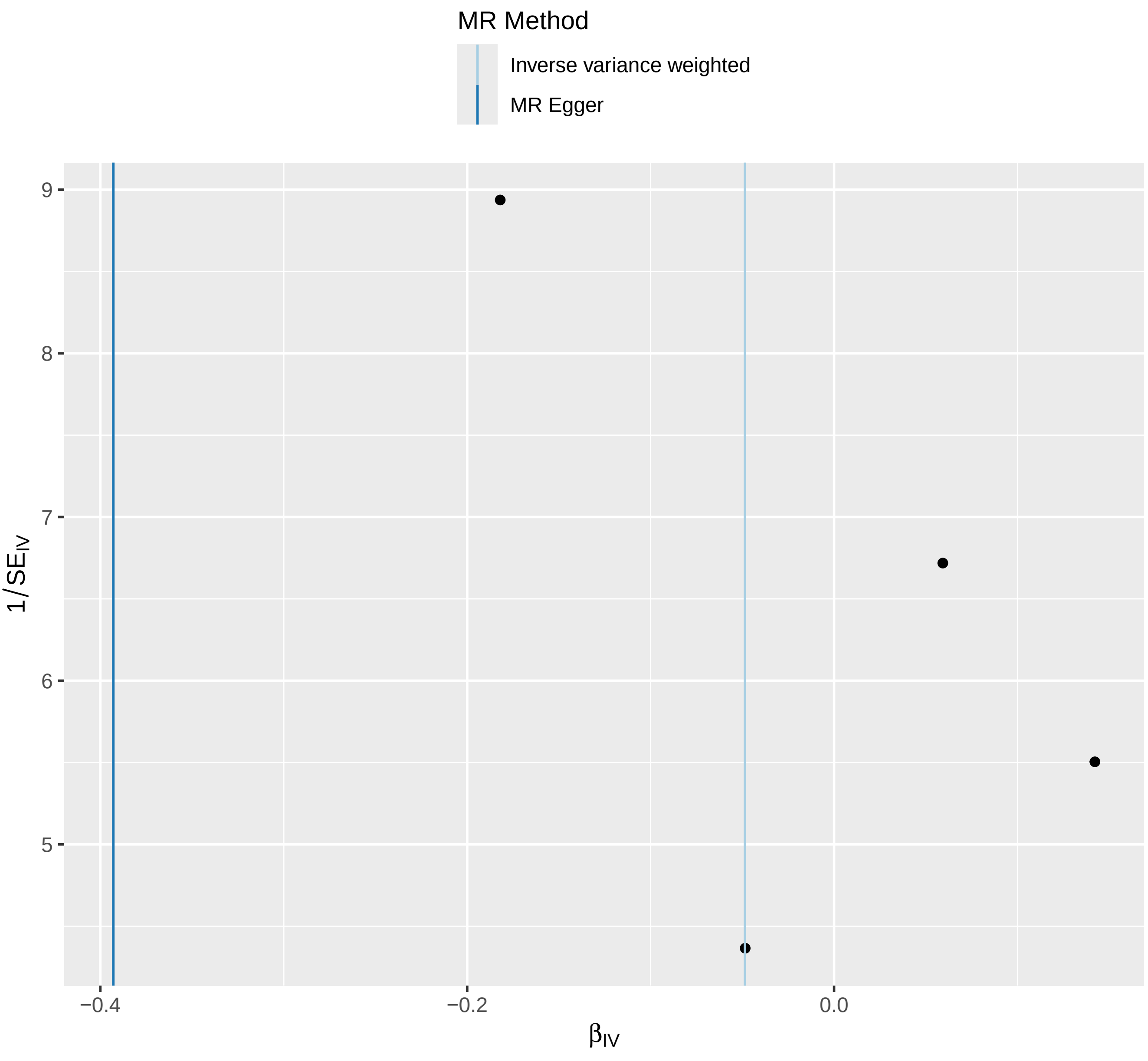

MR Method
Inverse variance weighted
MR Egger
9
8
7
6
5
1/SE_IV
−0.4
−0.2
0.0
β_IV

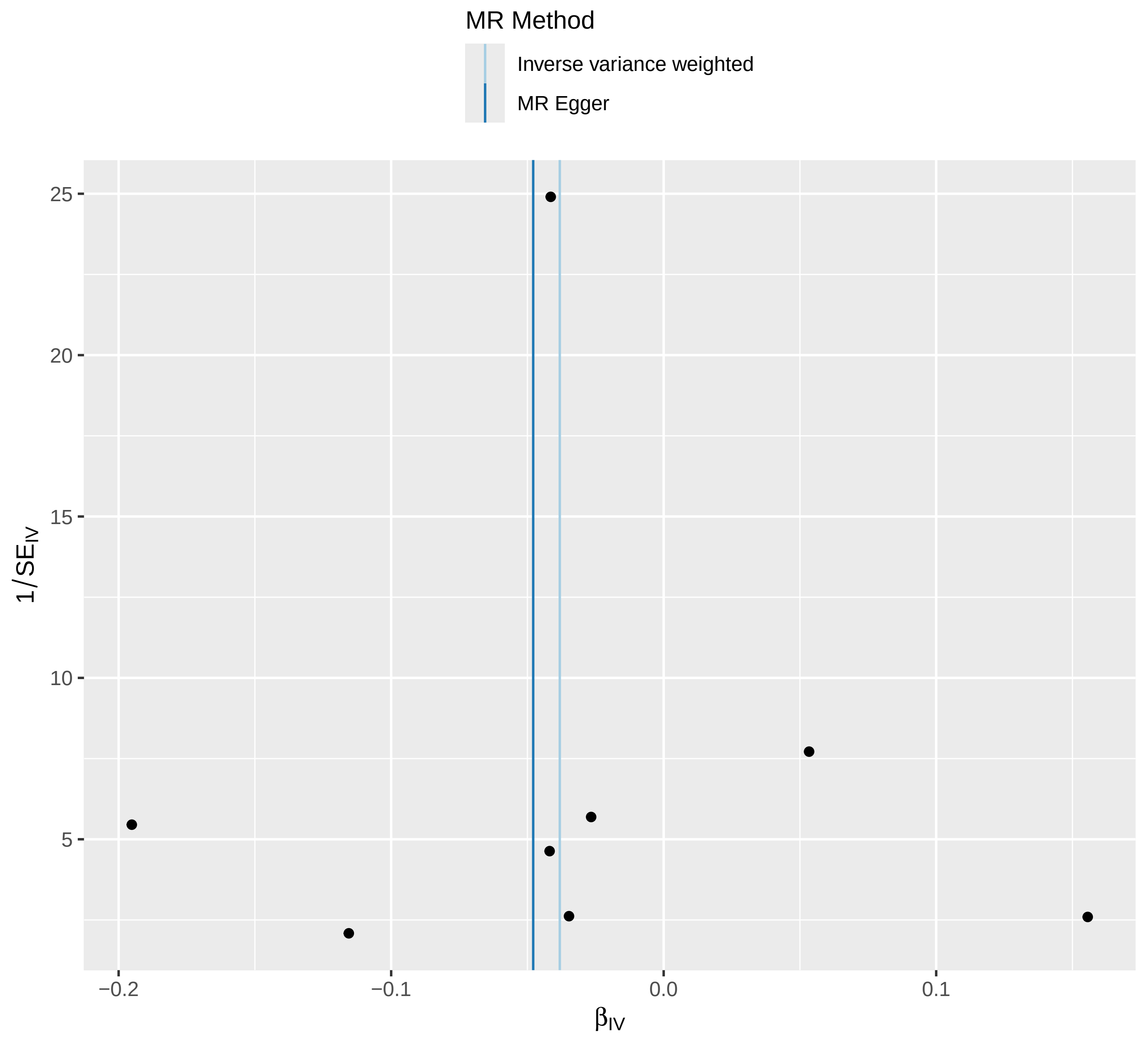
MR Method
Inverse variance weighted
MR Egger
25
20
15
10
5
1/SE_IV
−0.2
−0.1
0.0
0.1
β_IV

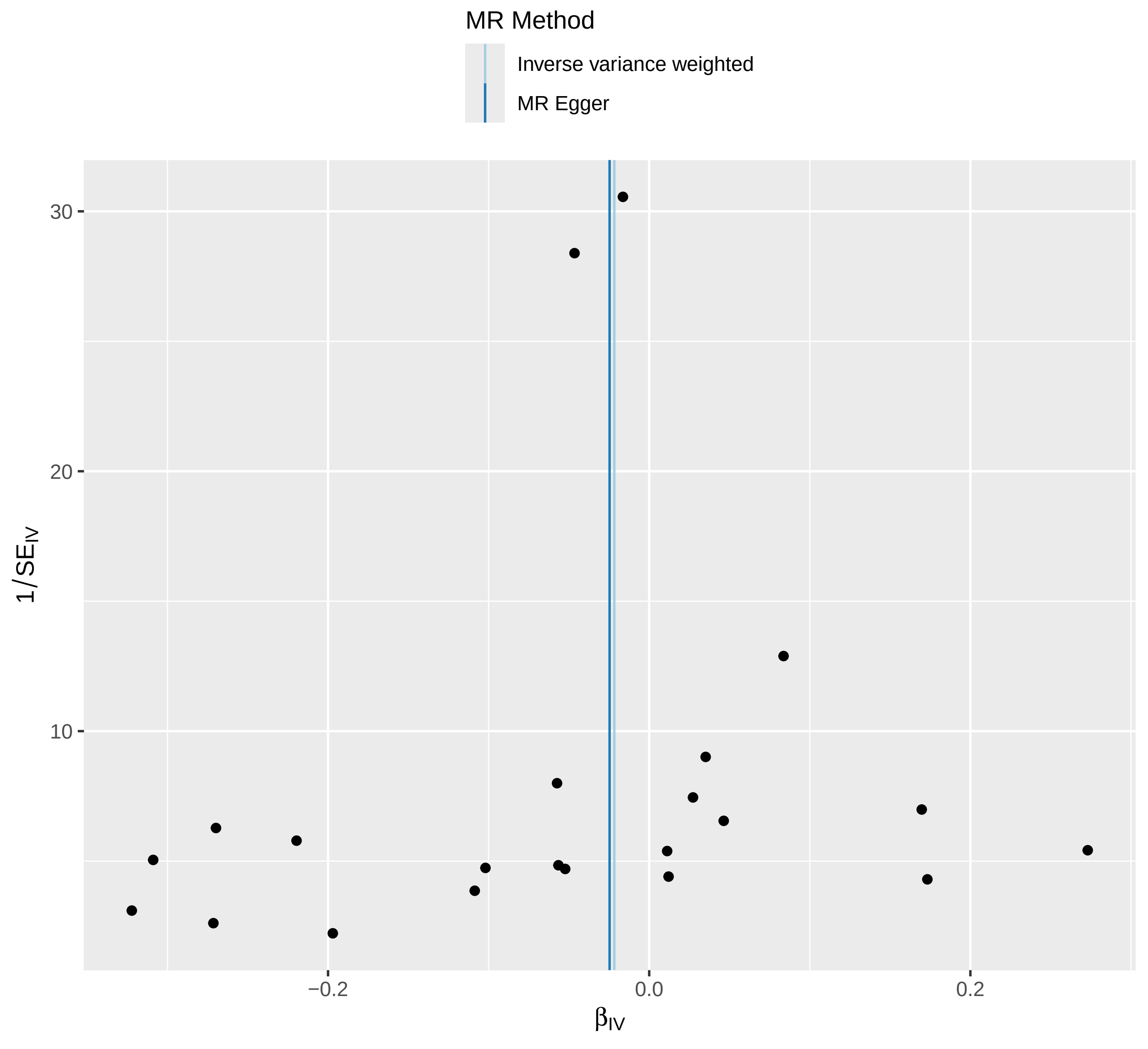
MR Method
Inverse variance weighted
MR Egger
30
20
10
1/SE$_{IV}$
−0.2
0.0
0.2
β$_{IV}$

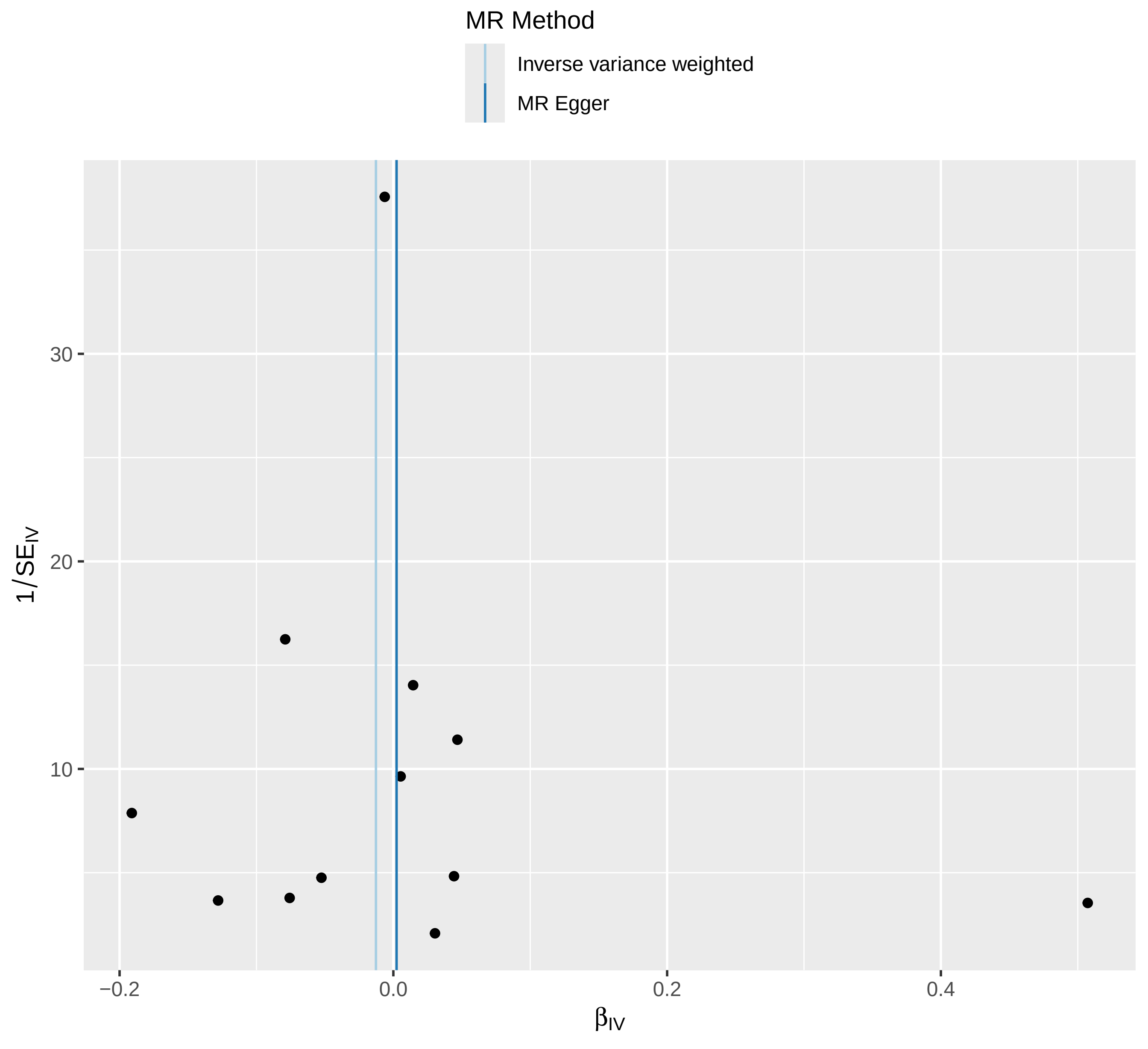
MR Method
Inverse variance weighted
MR Egger
1/SE$_{IV}$
30
20
10
−0.2
0.0
0.2
0.4
β$_{IV}$

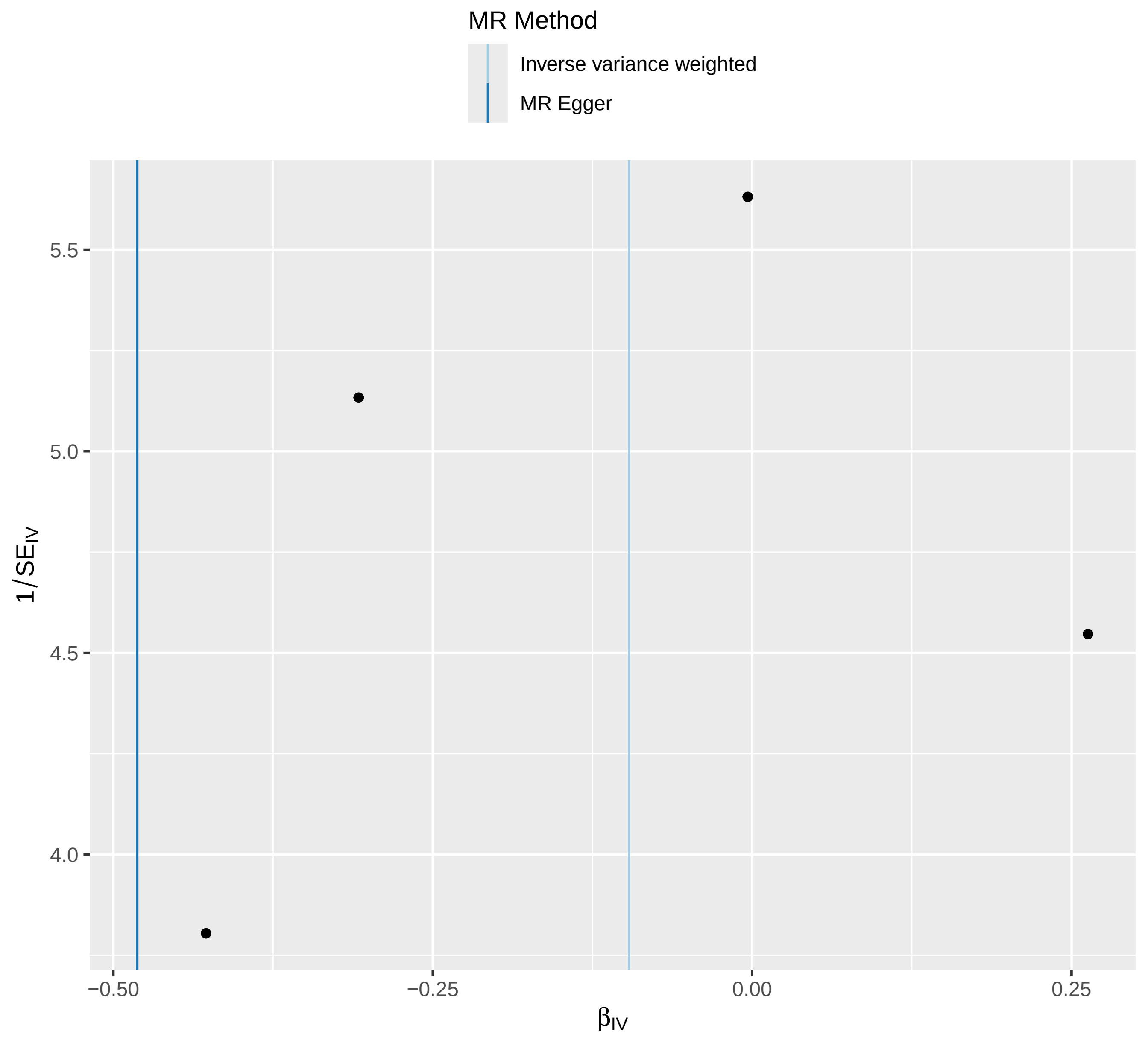
MR Method
Inverse variance weighted
MR Egger
5.5
5.0
4.5
4.0
1/SE_IV
−0.50
−0.25
0.00
0.25
β_IV

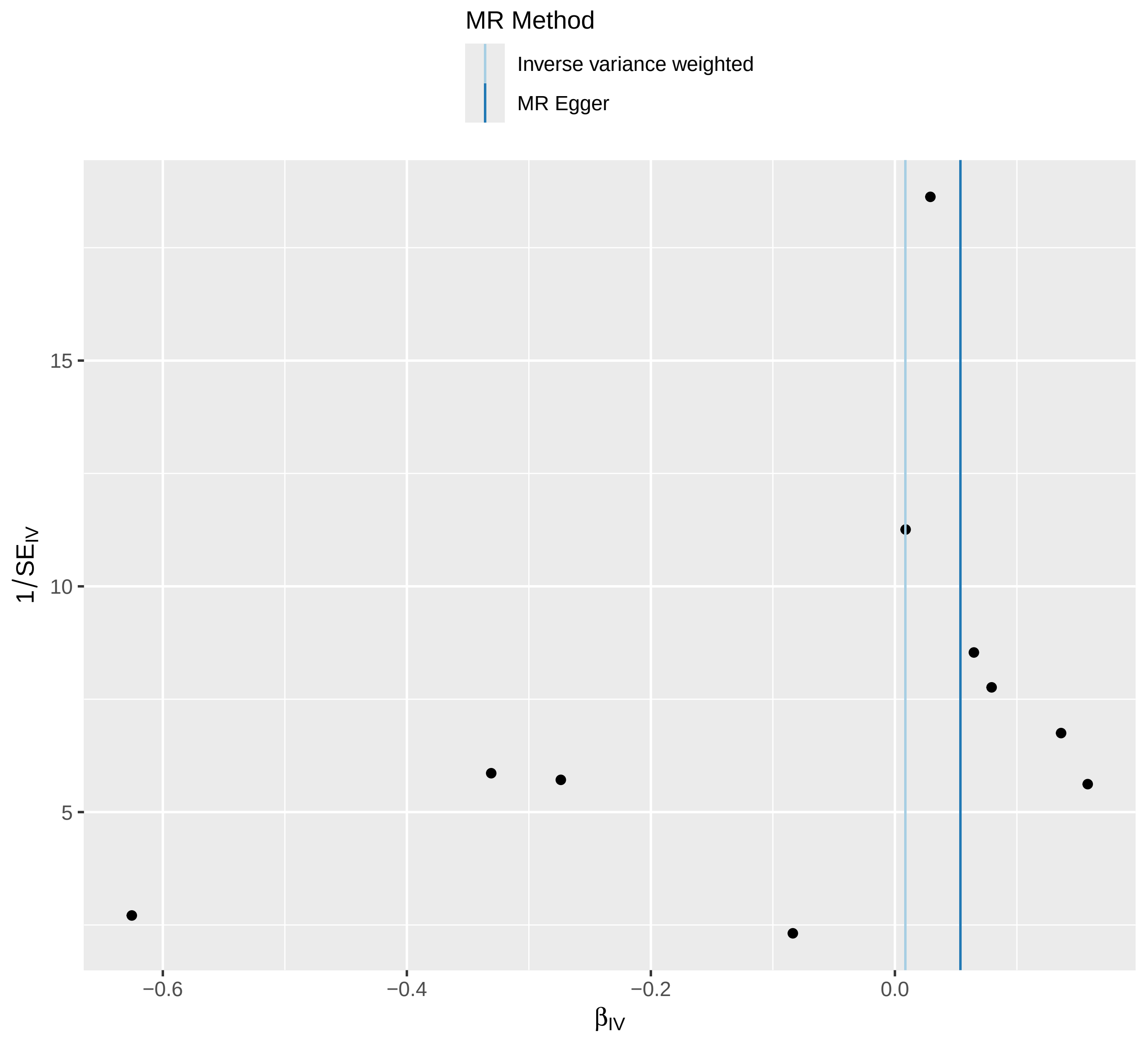

MR Method
Inverse variance weighted
MR Egger
1/SE_IV
15
10
5
−0.6
−0.4
−0.2
0.0
β_IV

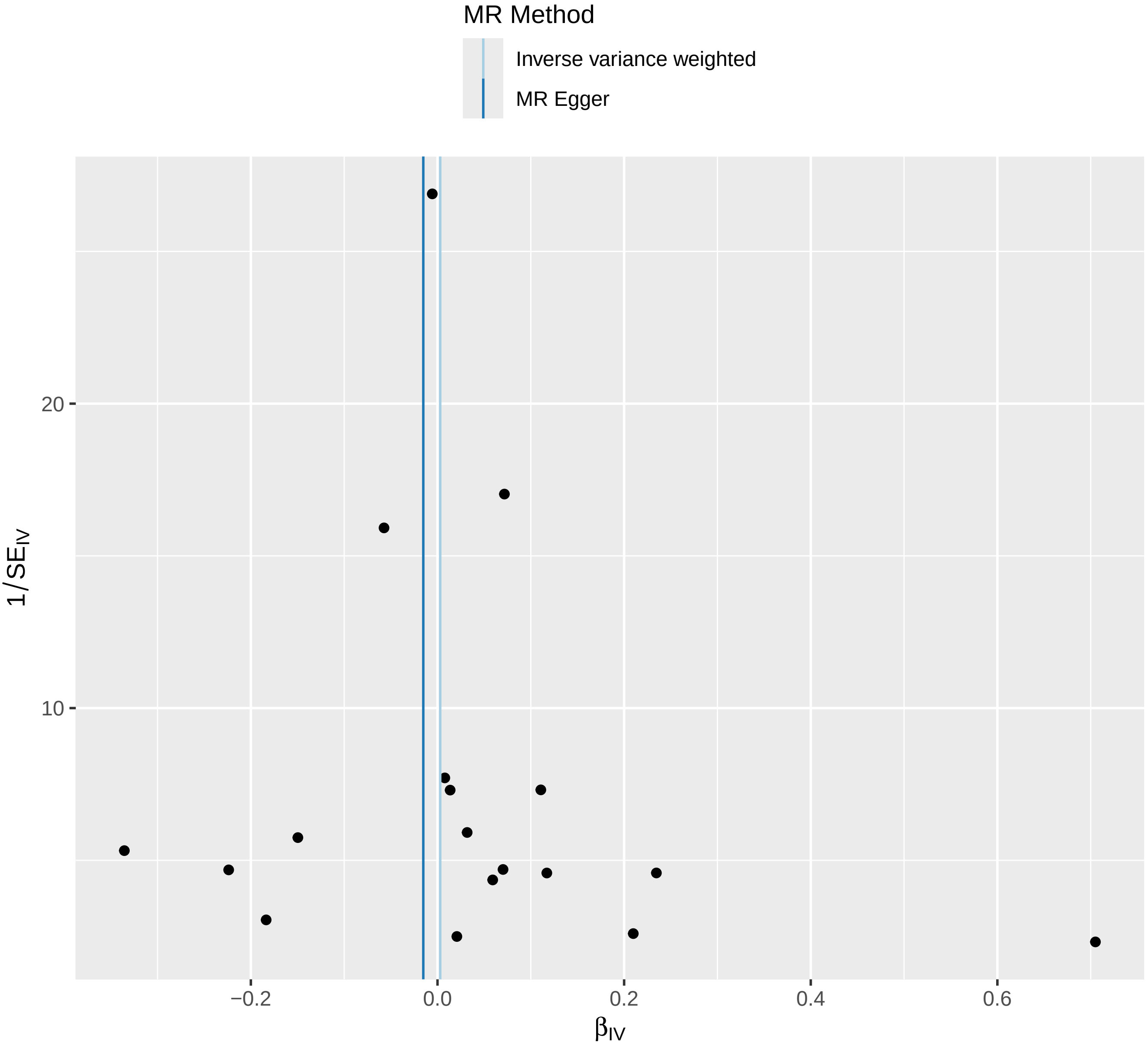
MR Method
Inverse variance weighted
MR Egger
20
10
−0.2
0.0
0.2
0.4
0.6
1/SE_IV
β_IV

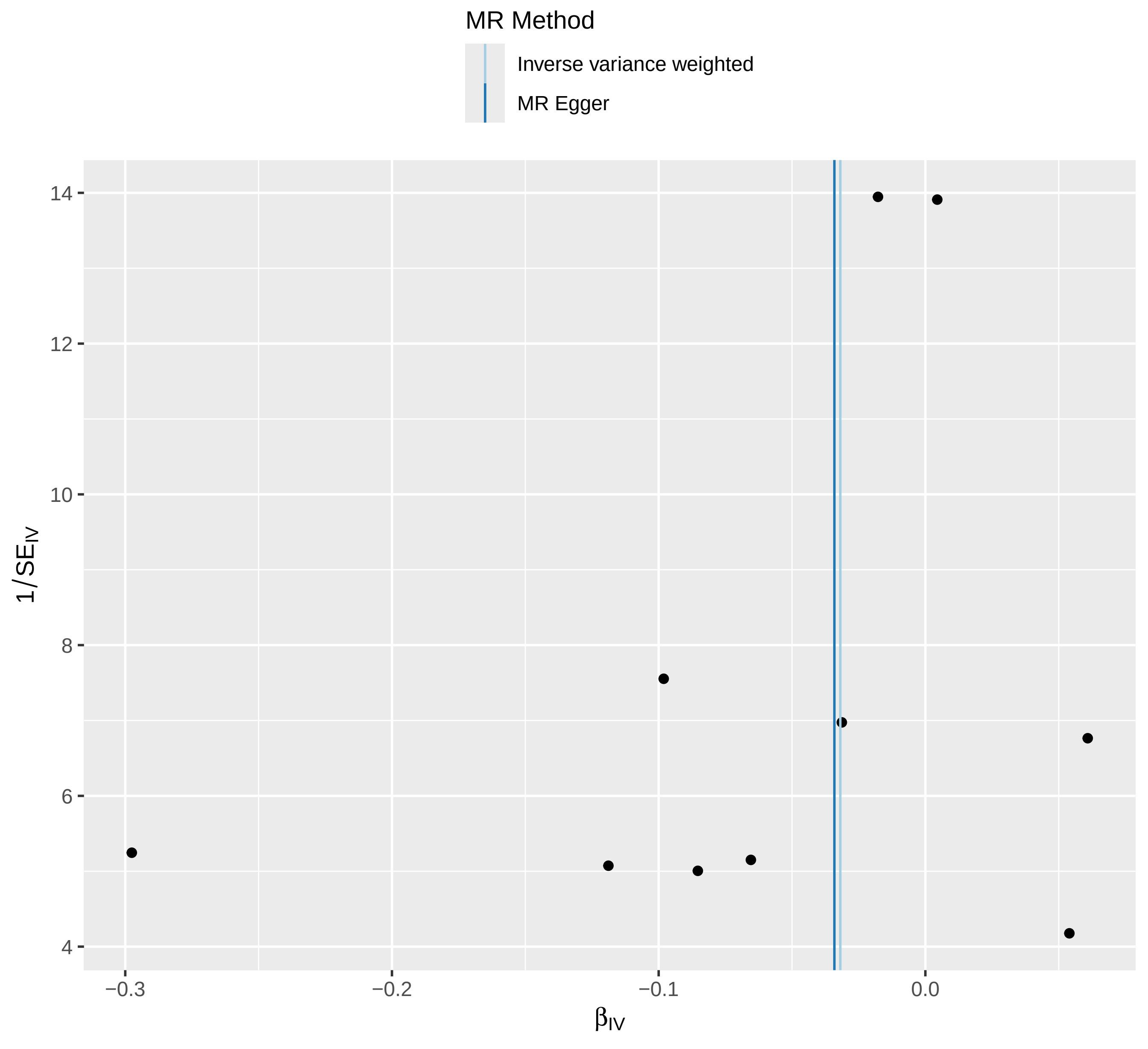

MR Method
Inverse variance weighted
MR Egger
14
12
10
8
6
4
1/SE$_{IV}$
−0.3
−0.2
−0.1
0.0
β$_{IV}$

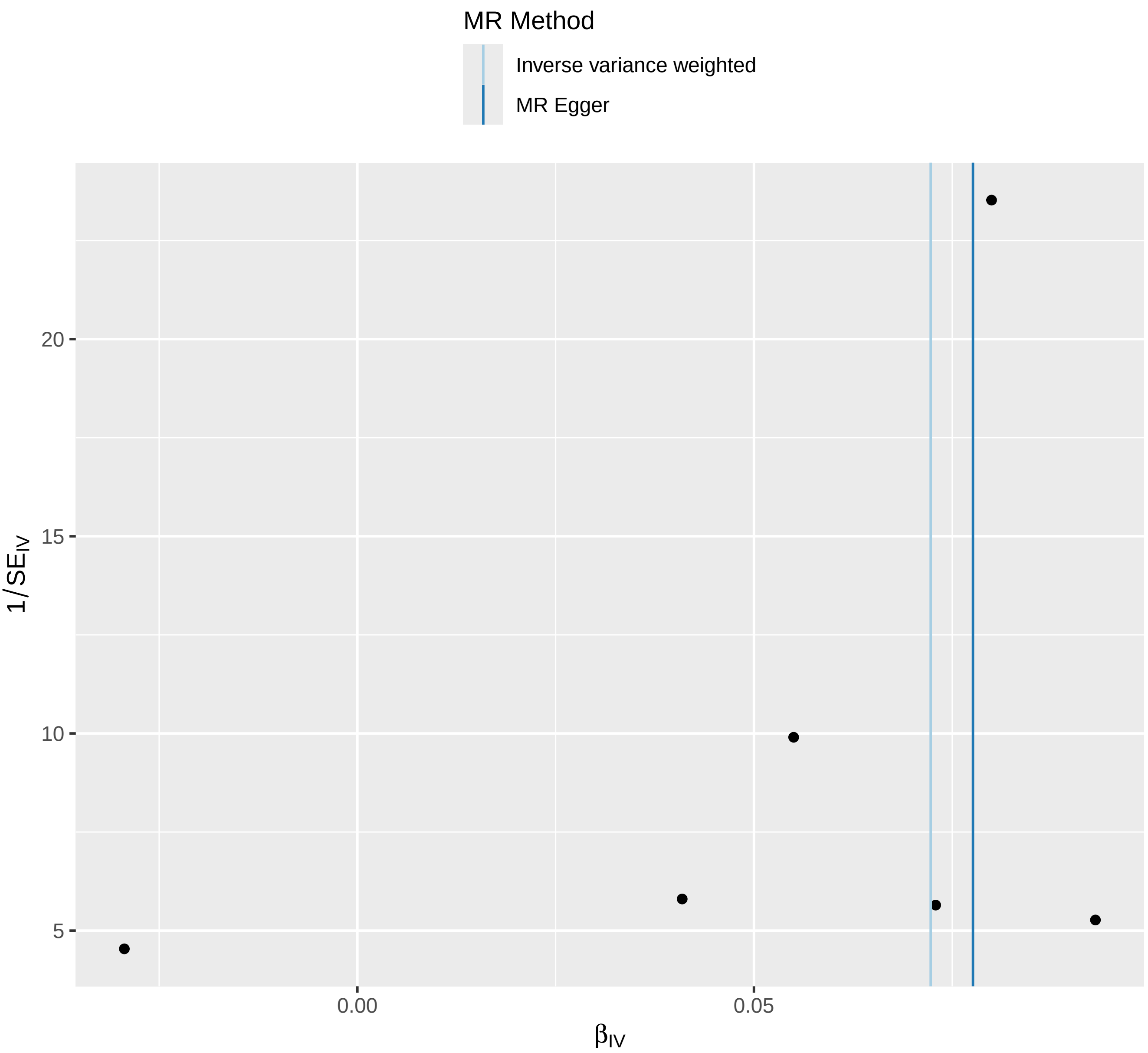

MR Method
Inverse variance weighted
MR Egger
20
15
10
5
1/SE_IV
0.00
0.05
β_IV

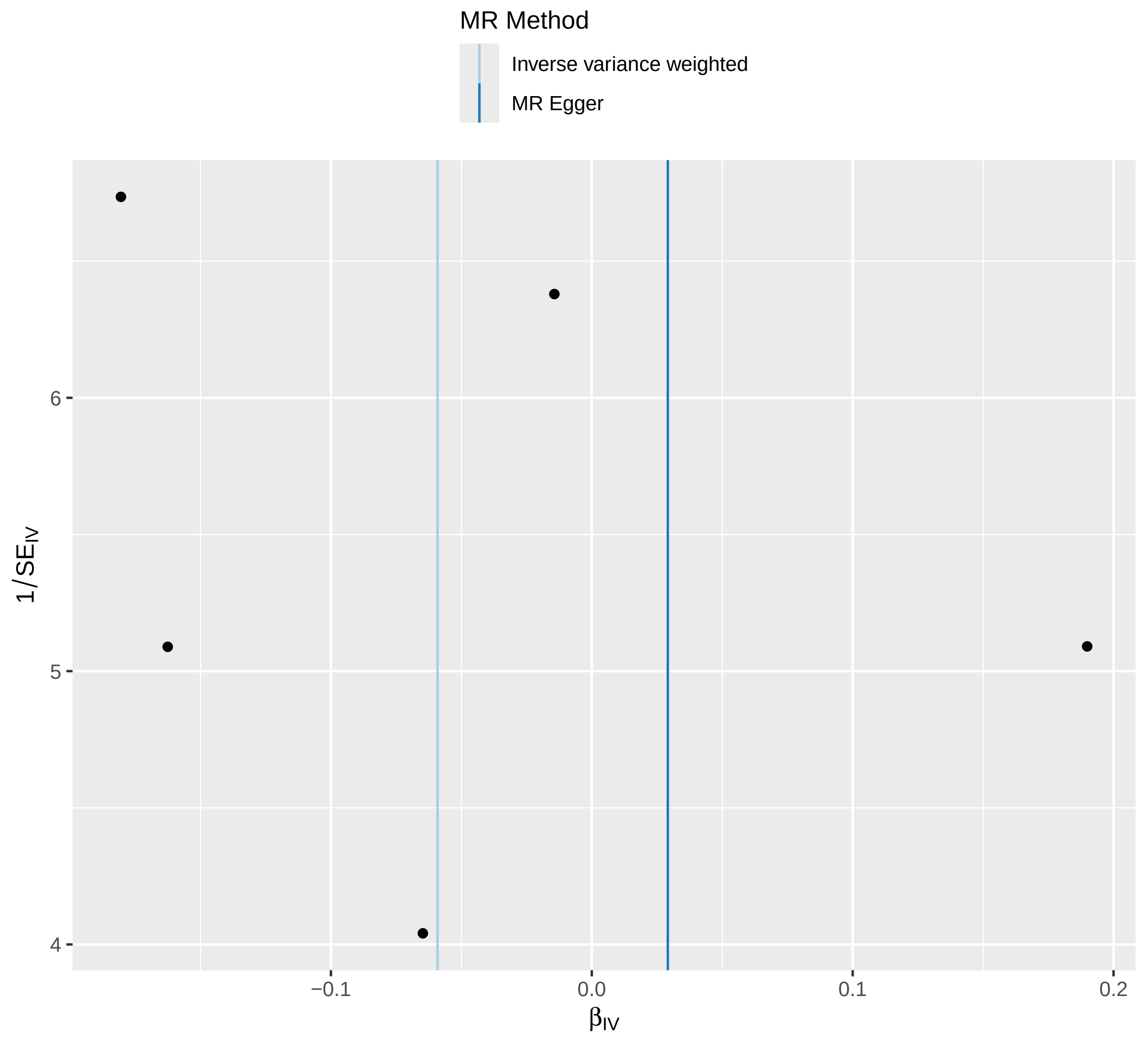
MR Method
Inverse variance weighted
MR Egger
1/SE$_{IV}$
6
5
4
−0.1
0.0
0.1
0.2
β$_{IV}$

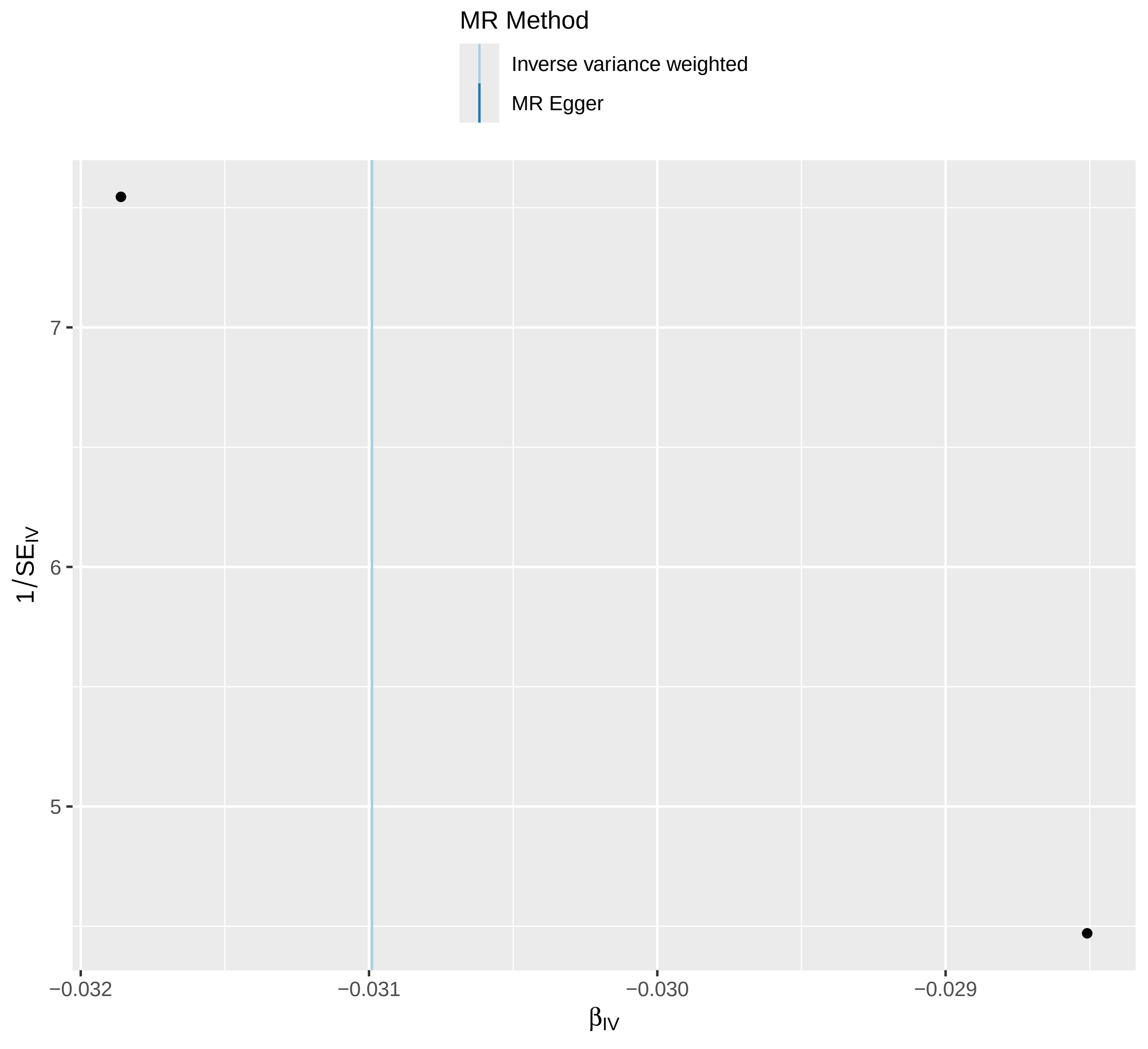
MR Method
Inverse variance weighted
MR Egger
7
6
5
1/SE_IV
−0.032
−0.031
−0.030
−0.029
β_IV

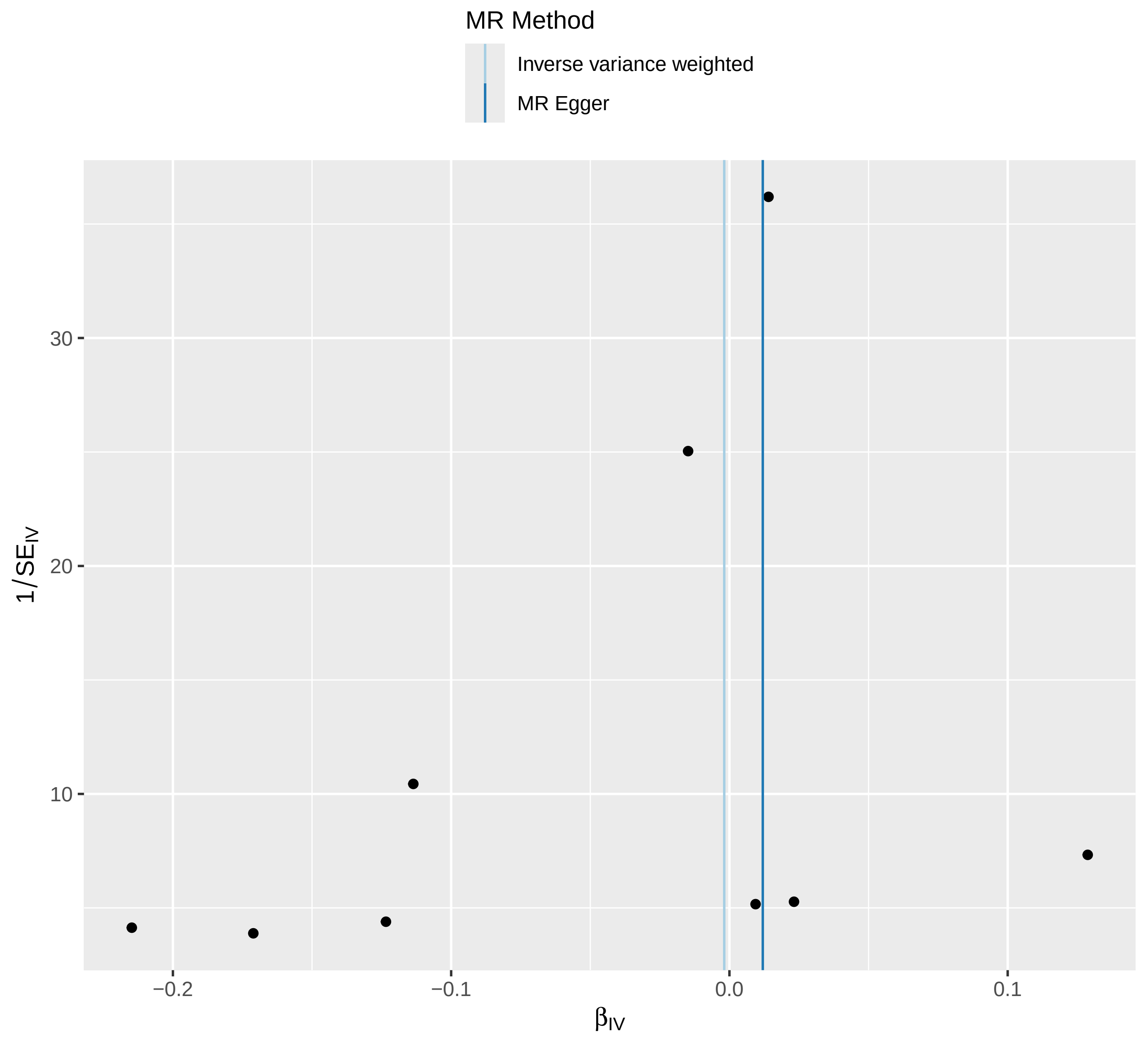

MR Method
Inverse variance weighted
MR Egger
30
20
10
1/$SE_{IV}$
−0.2
−0.1
0.0
0.1
$\beta_{IV}$

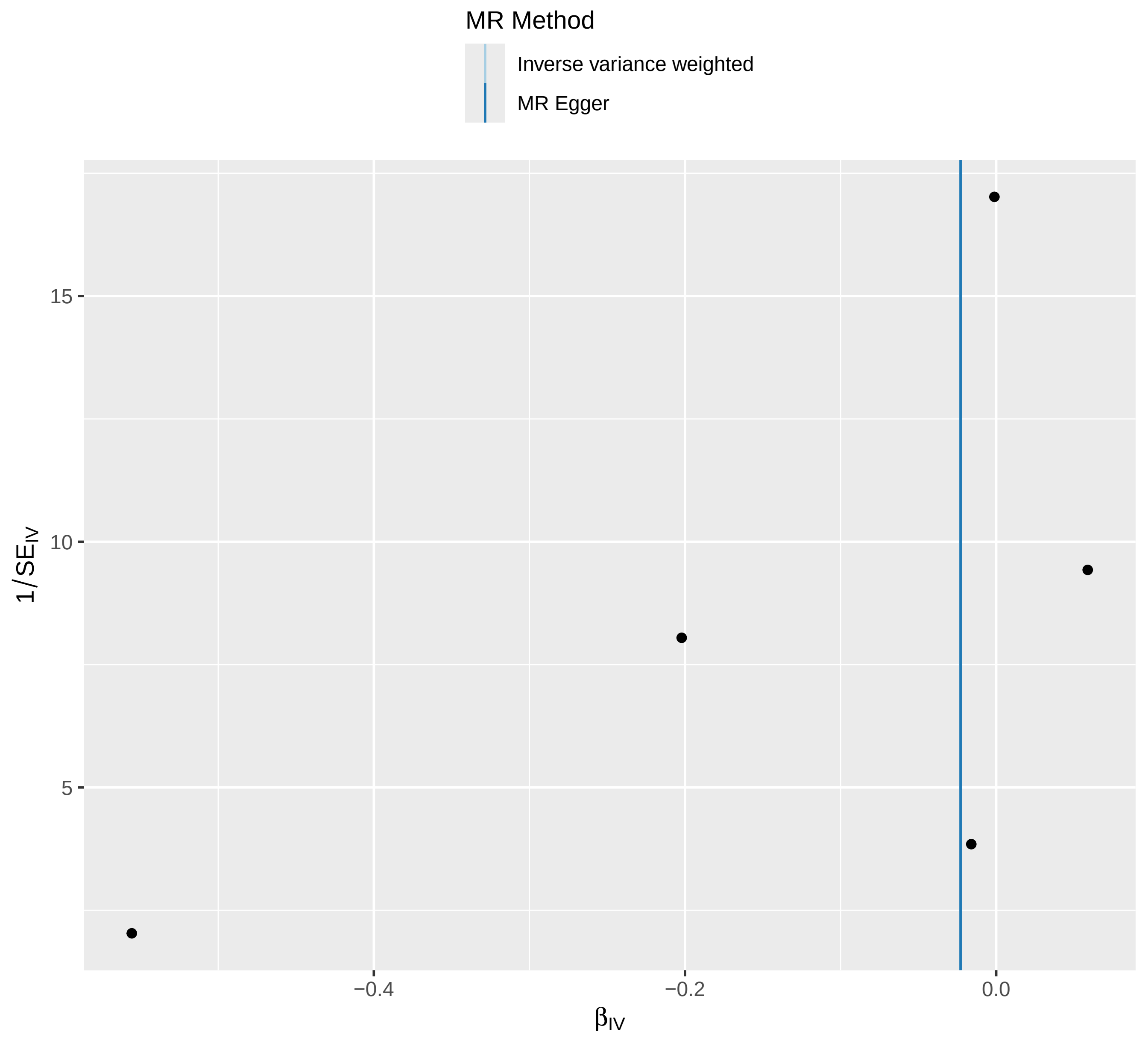

MR Method
Inverse variance weighted
MR Egger
15
10
5
1/SE$_{IV}$
−0.4
−0.2
0.0
β$_{IV}$

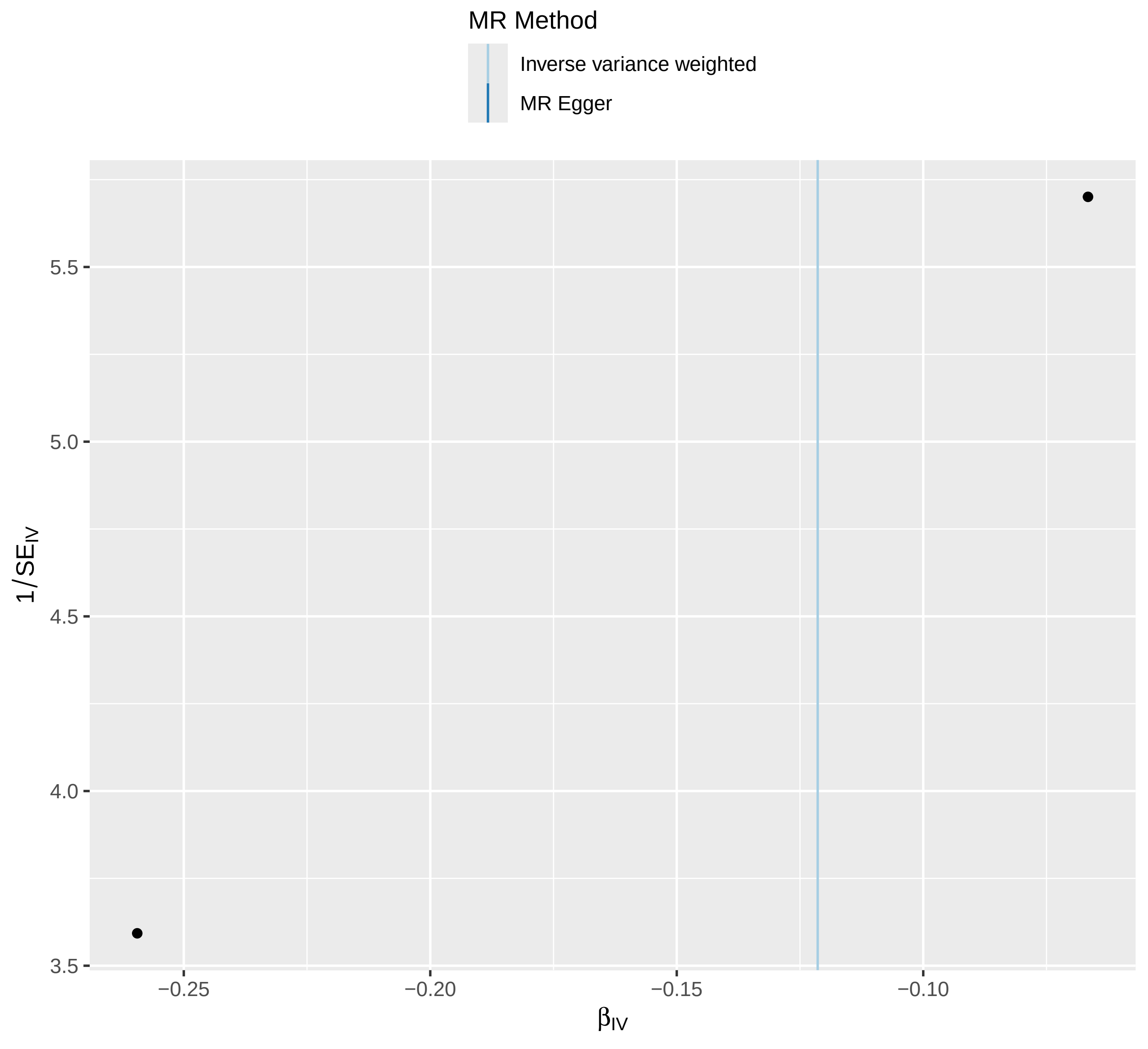
MR Method
Inverse variance weighted
MR Egger
1/SE_IV
5.5
5.0
4.5
4.0
3.5
−0.25
−0.20
−0.15
−0.10
β_IV

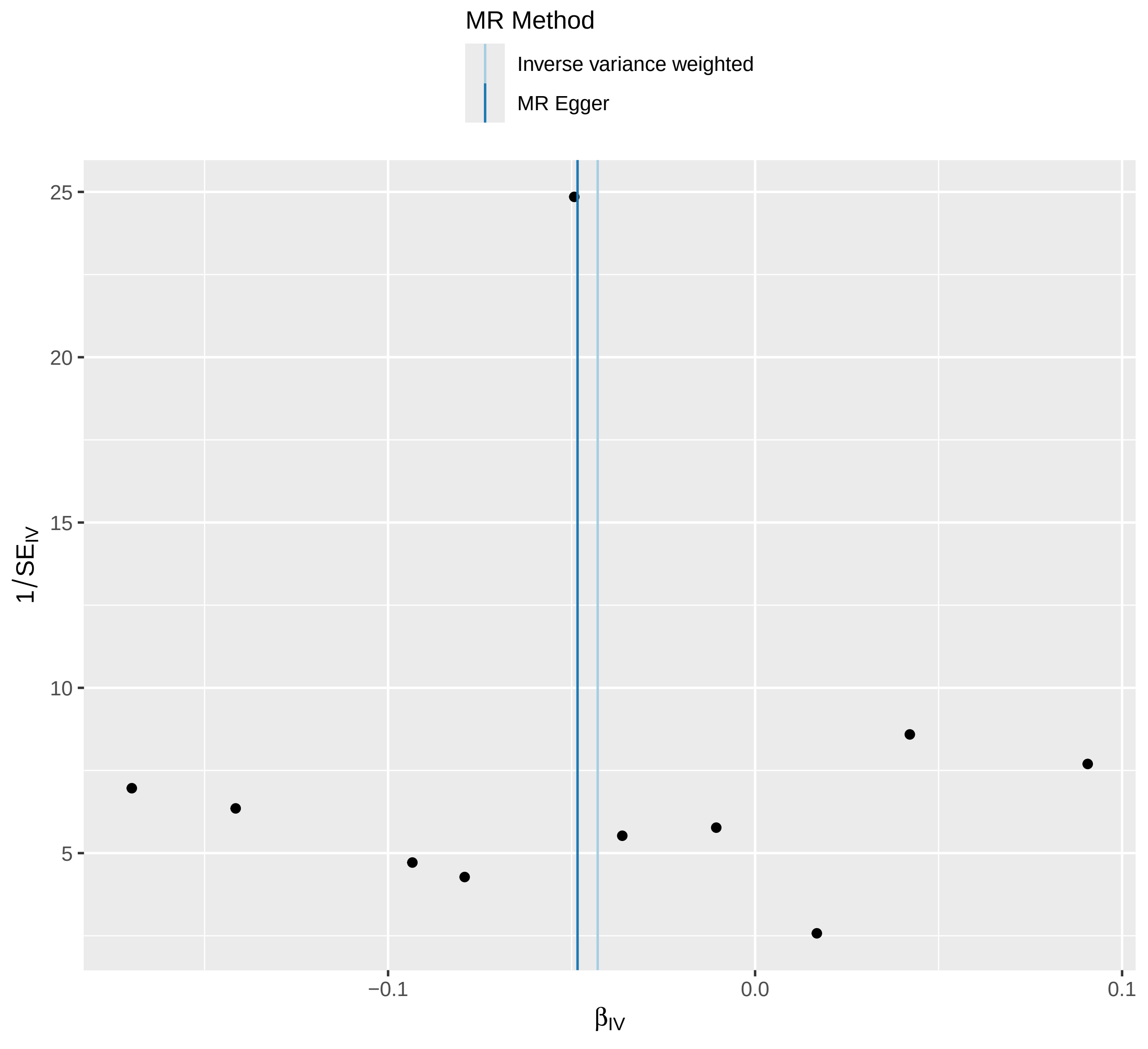

MR Method
Inverse variance weighted
MR Egger
25
20
15
10
5
1/SE_IV
−0.1
0.0
0.1
β_IV

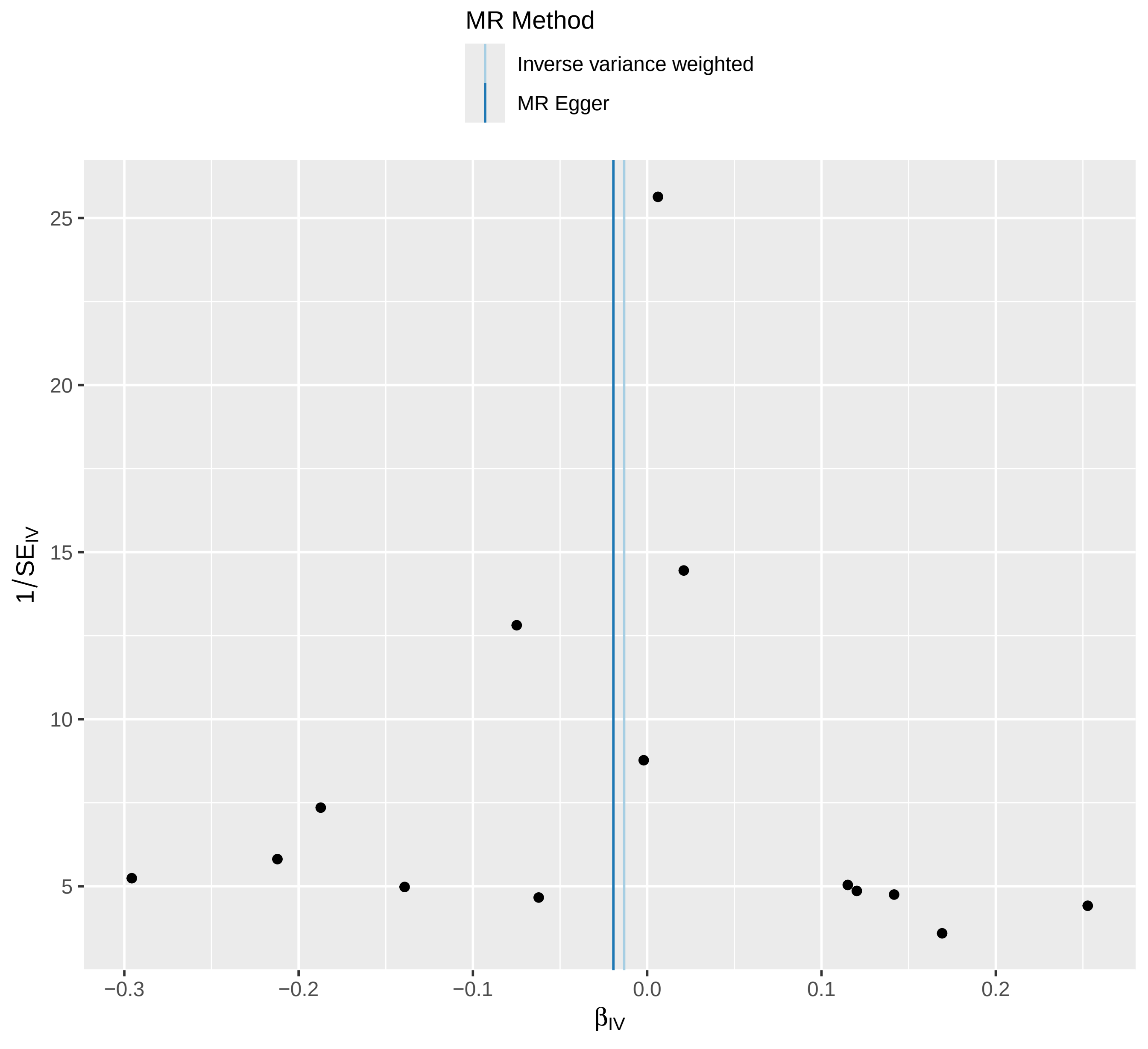

MR Method
Inverse variance weighted
MR Egger
25
20
15
10
5
1/SE$_{IV}$
−0.3
−0.2
−0.1
0.0
0.1
0.2
β$_{IV}$

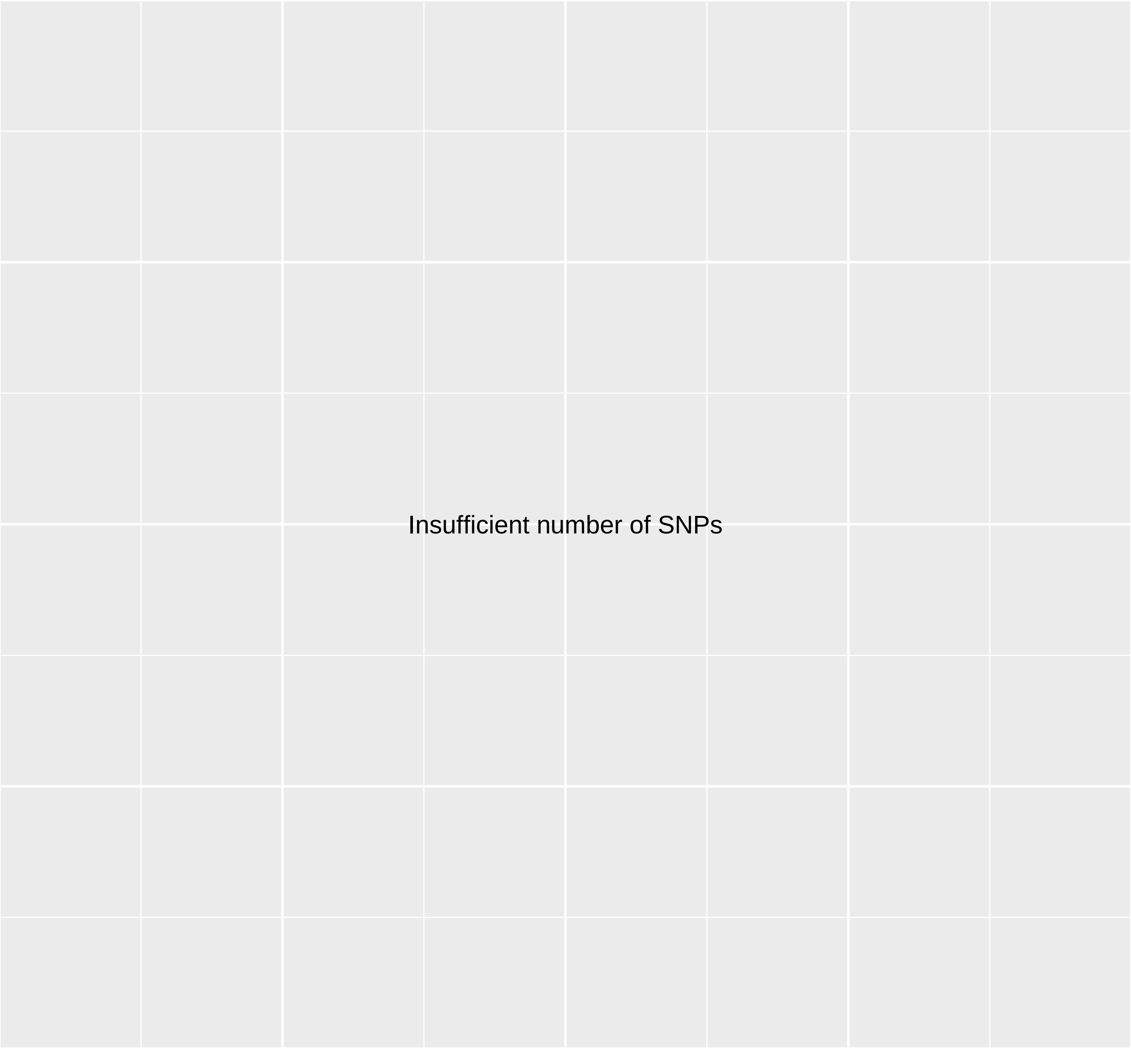
Insufficient number of SNPs

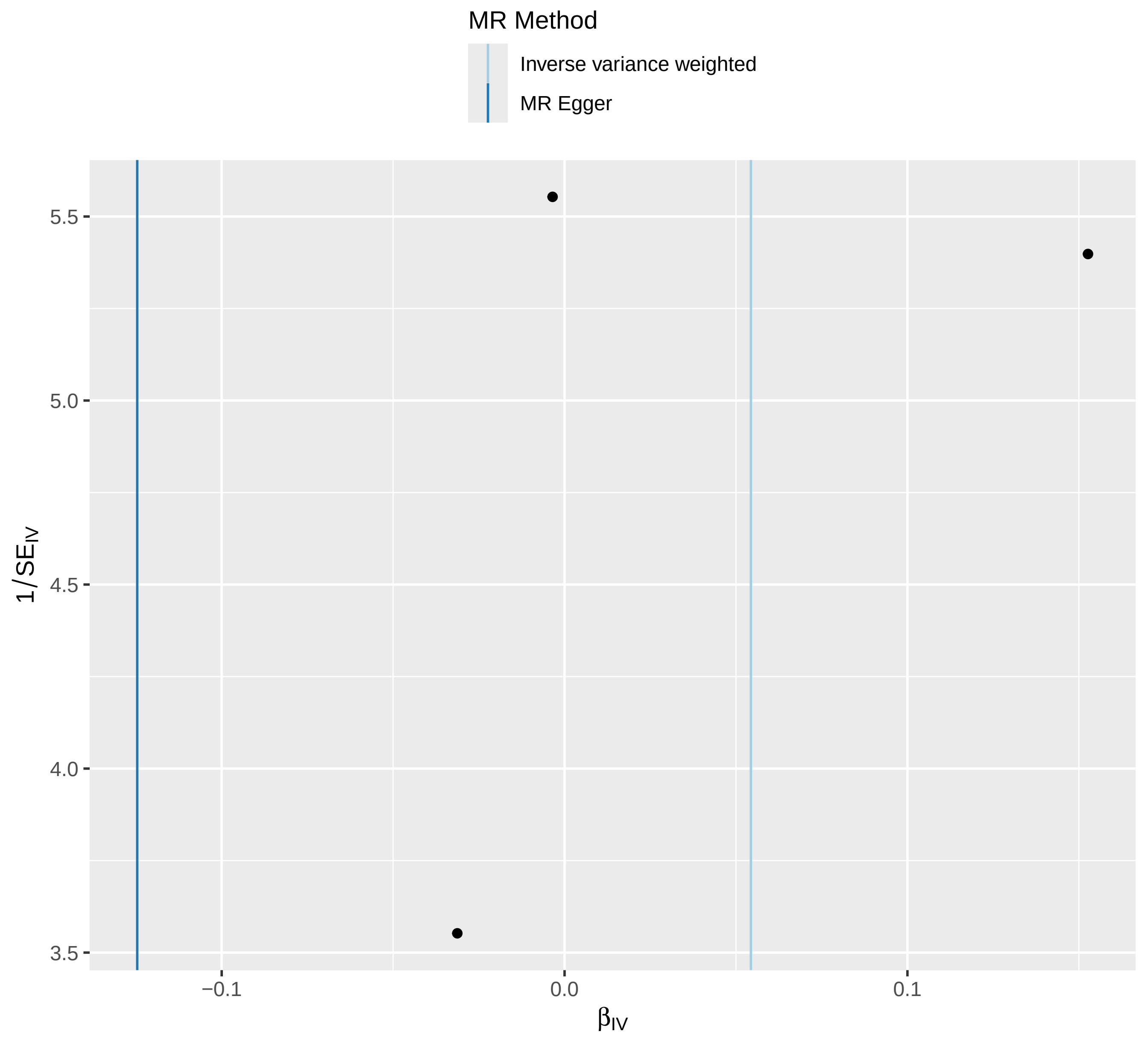
MR Method
Inverse variance weighted
MR Egger
5.5
5.0
4.5
4.0
3.5
1/SE_IV
−0.1
0.0
0.1
β_IV

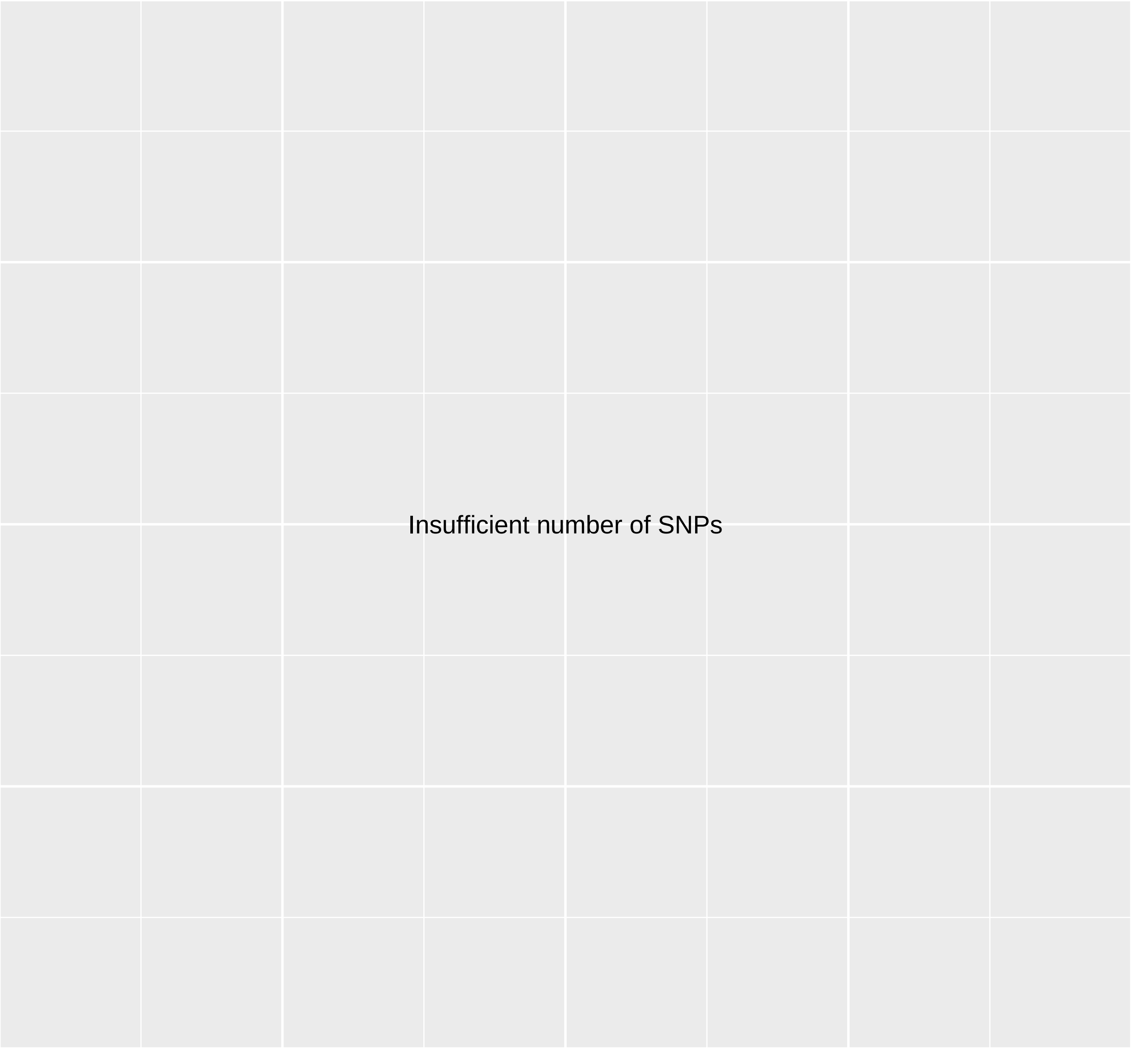
Insufficient number of SNPs

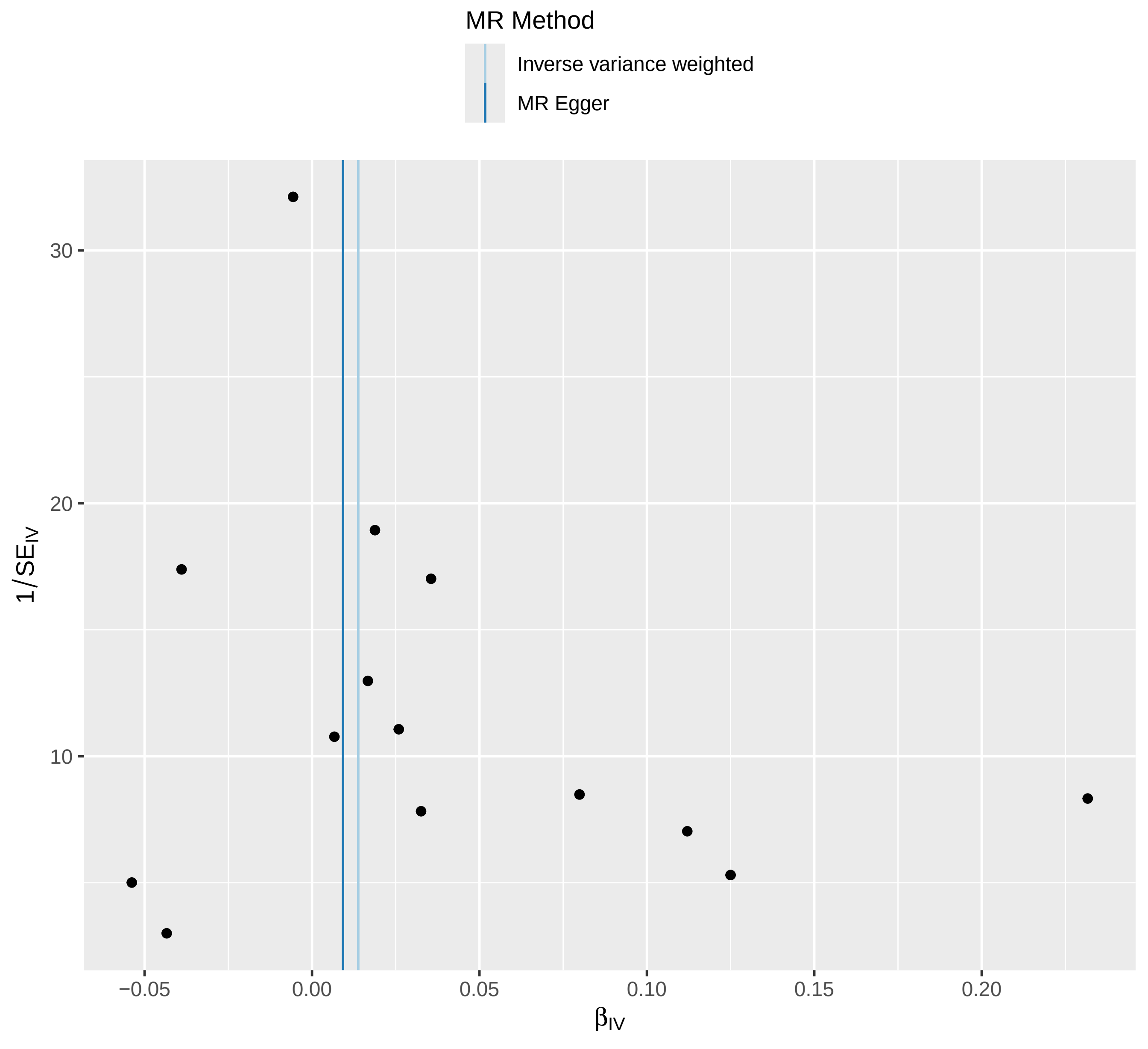
MR Method
Inverse variance weighted
MR Egger
30
20
10
1/SE$_{IV}$
−0.05
0.00
0.05
0.10
0.15
0.20
β$_{IV}$

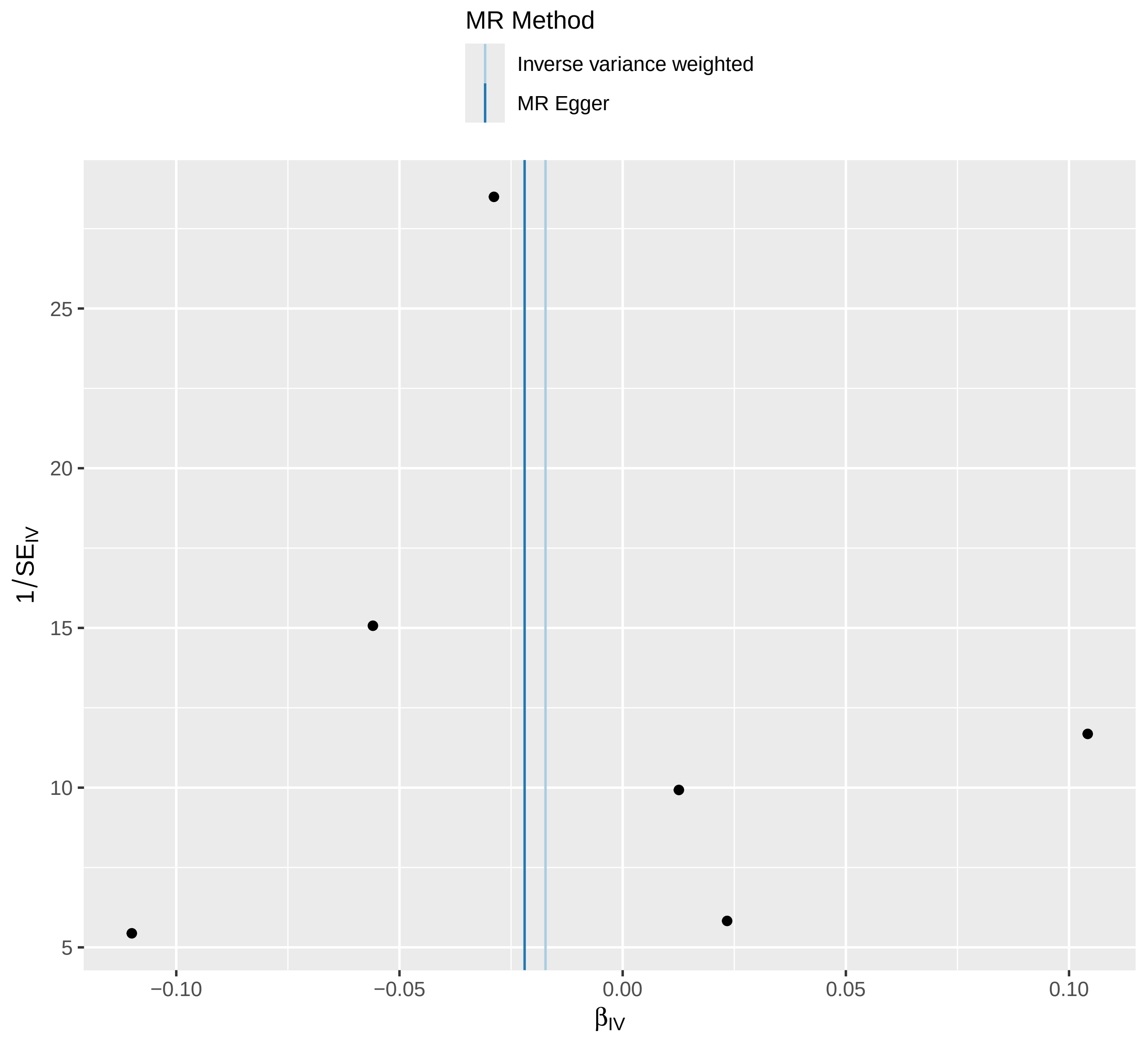
MR Method
Inverse variance weighted
MR Egger
1/SE_IV
25
20
15
10
5
−0.10
−0.05
0.00
0.05
0.10
β_IV

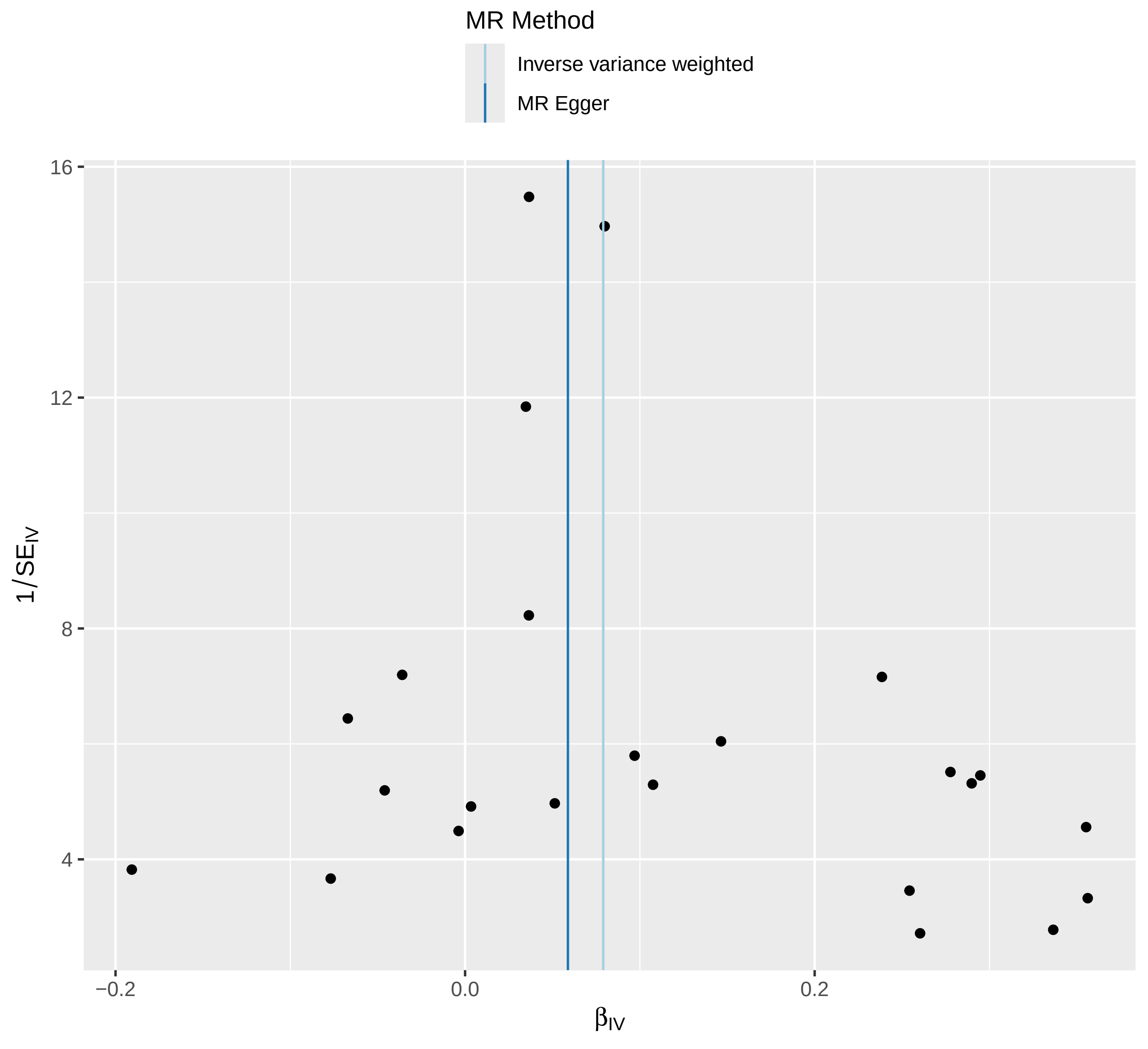
MR Method
Inverse variance weighted
MR Egger
16
12
8
4
1/SE_IV
−0.2
0.0
0.2
β_IV

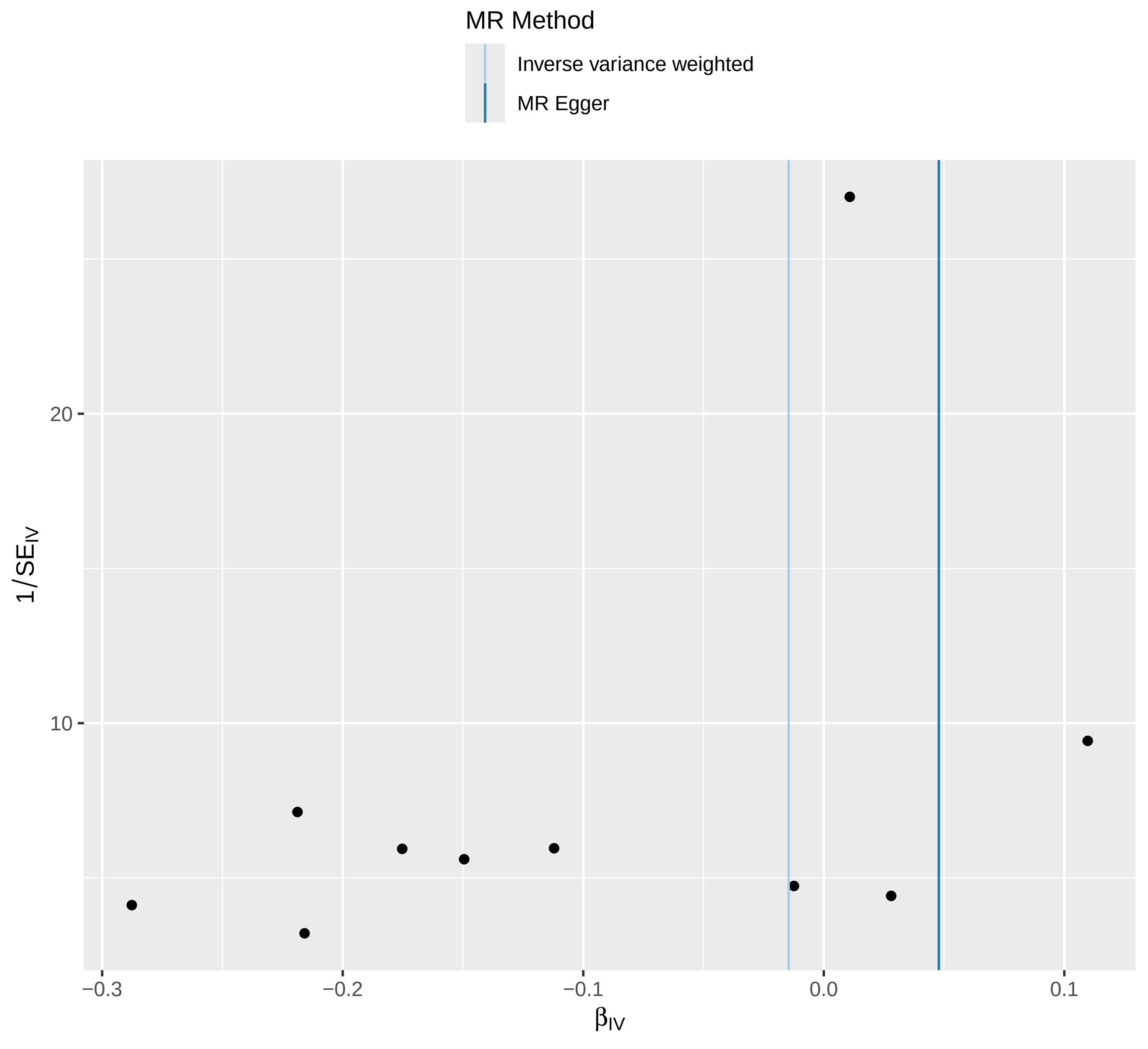
MR Method
Inverse variance weighted
MR Egger
20
10
$1/SE_{IV}$
−0.3
−0.2
−0.1
0.0
0.1
$\beta_{IV}$

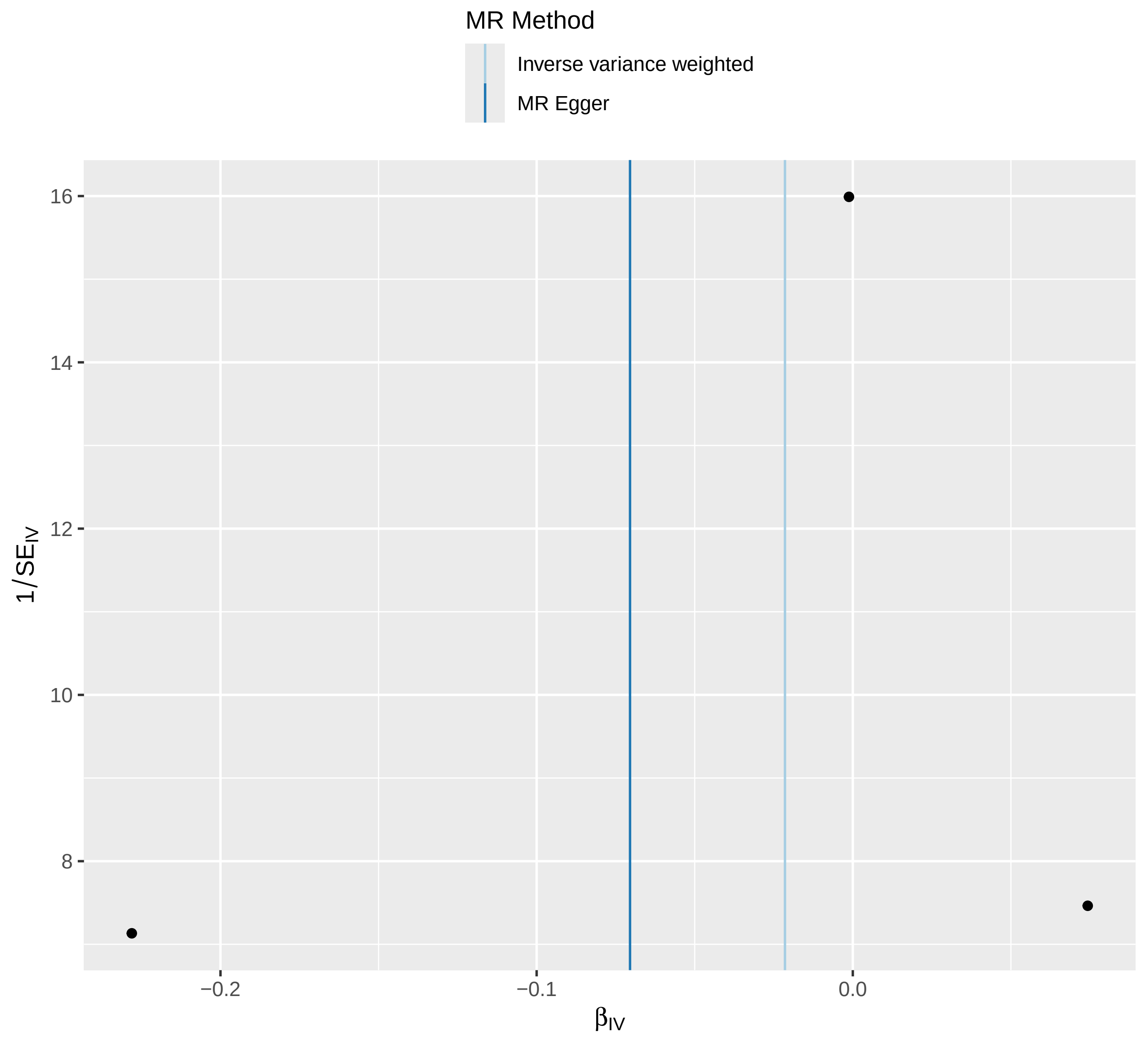

MR Method
Inverse variance weighted
MR Egger
16
14
12
10
8
1/SE_IV
−0.2
−0.1
0.0
β_IV

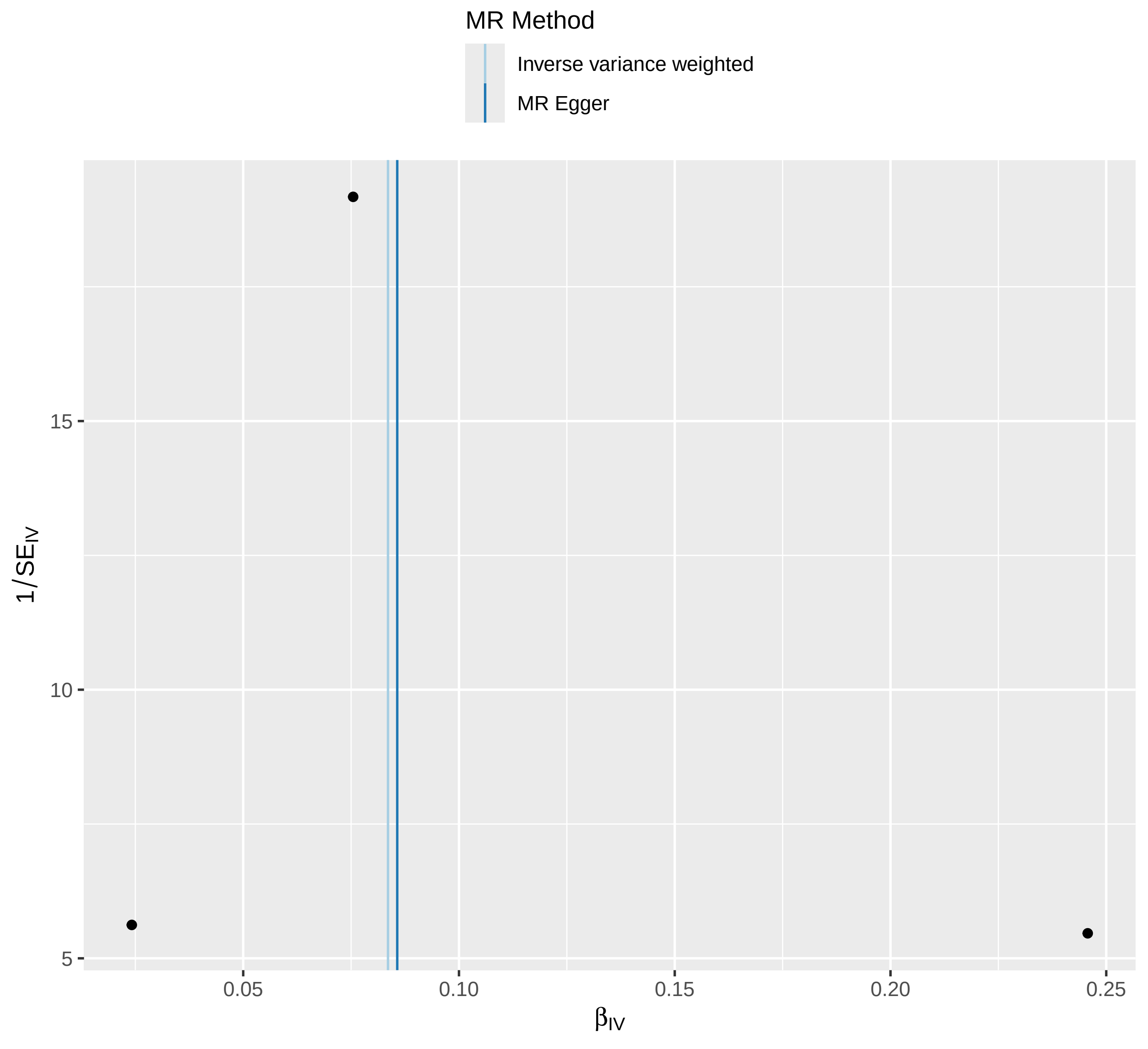

MR Method
Inverse variance weighted
MR Egger
15
10
5
1/SE_IV
0.05
0.10
0.15
0.20
0.25
β_IV

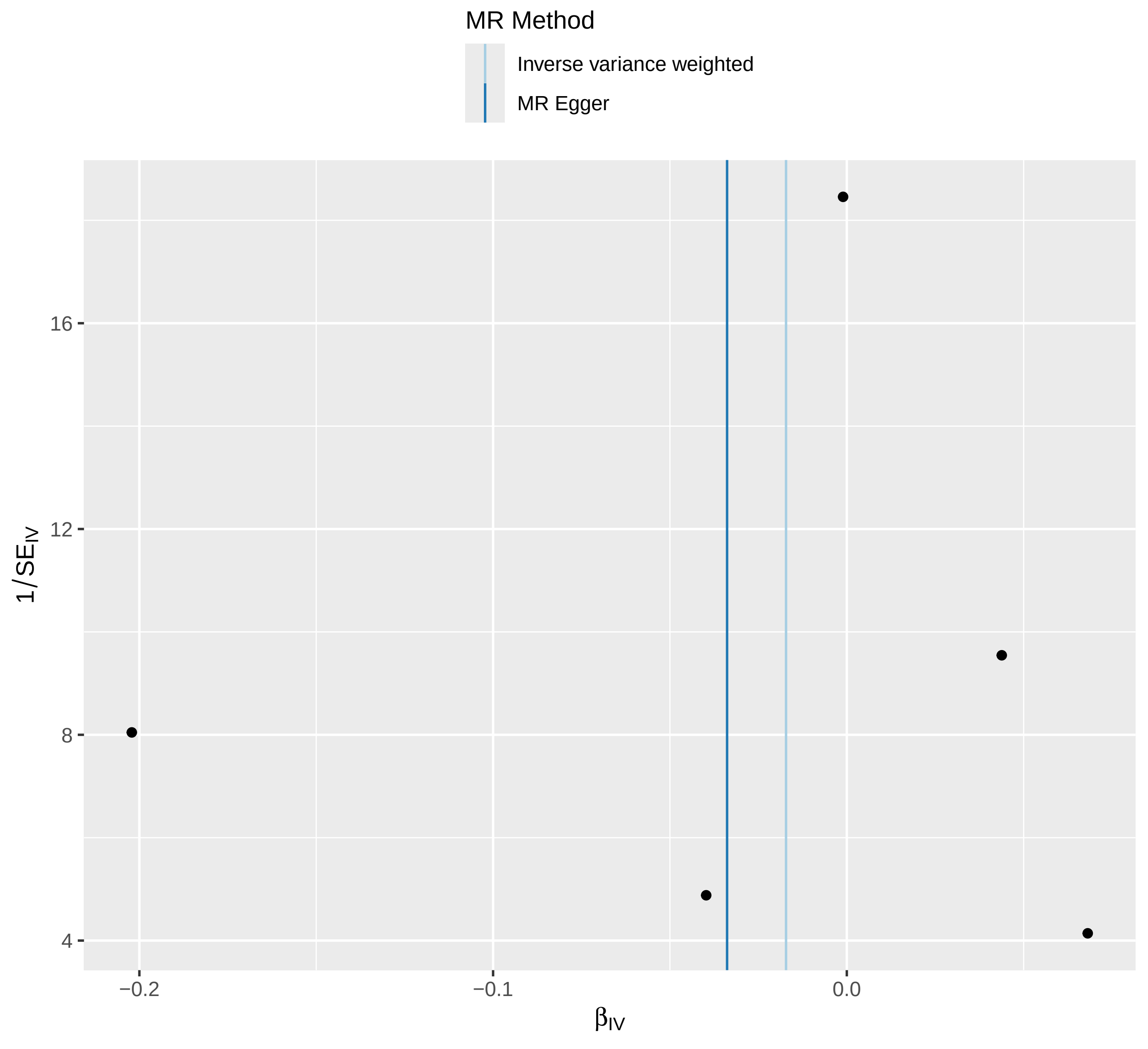

MR Method
Inverse variance weighted
MR Egger
16
12
8
4
1/SE$_{IV}$
−0.2
−0.1
0.0
β$_{IV}$

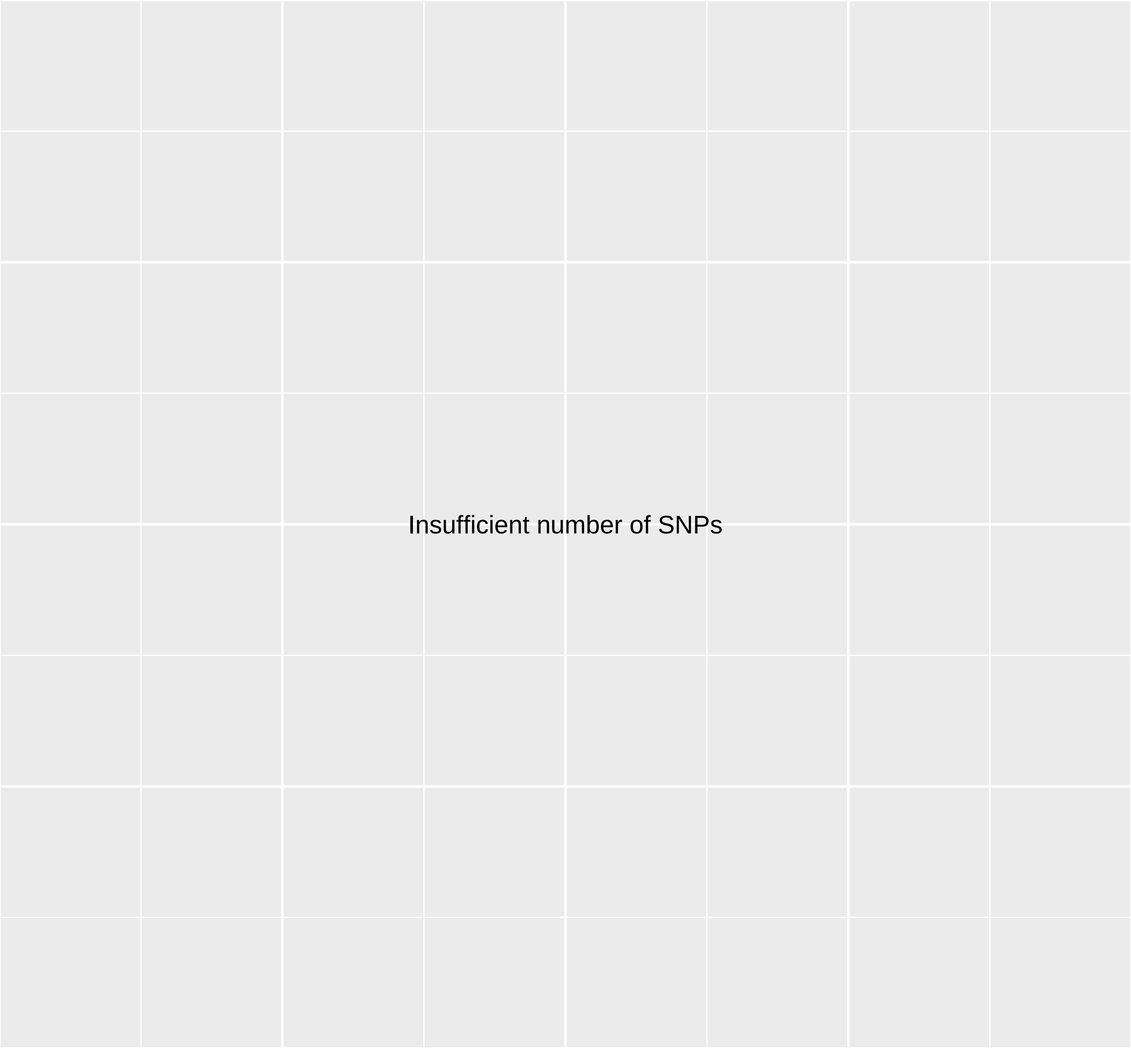
Insufficient number of SNPs

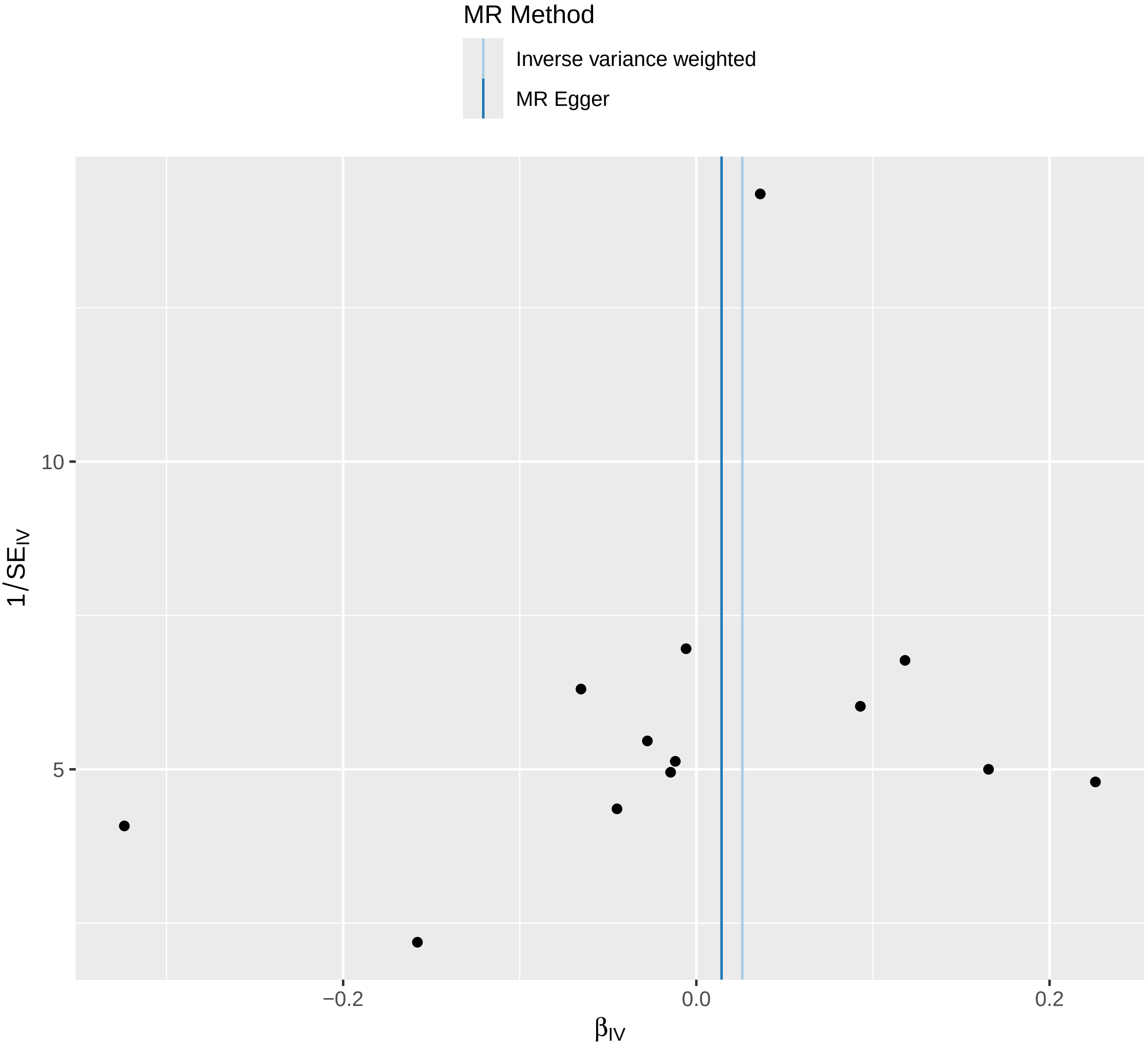
MR Method
Inverse variance weighted
MR Egger
10
5
1/SE_IV
−0.2
0.0
0.2
β_IV

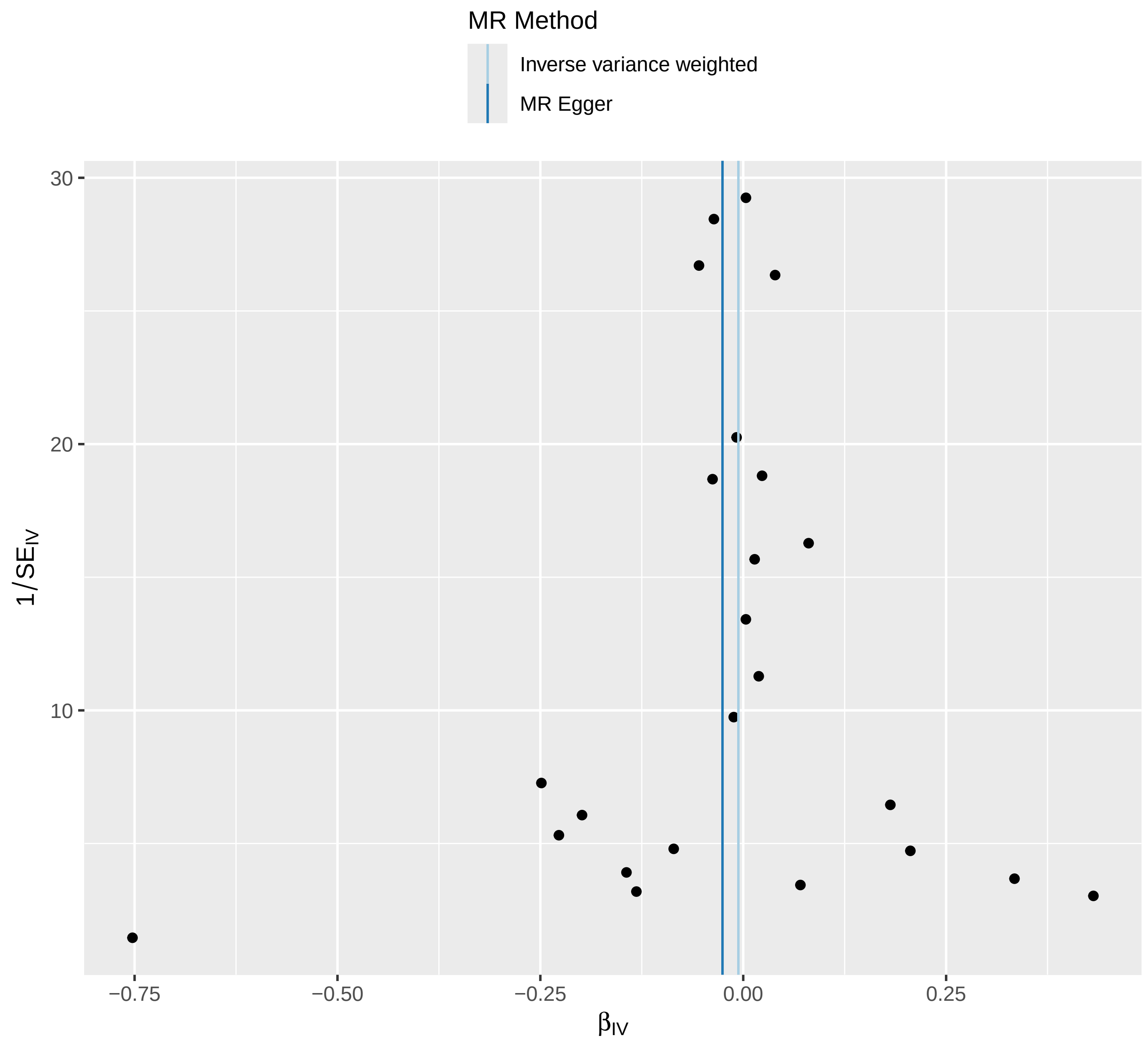
MR Method
Inverse variance weighted
MR Egger
30
20
10
1/SE_IV
−0.75
−0.50
−0.25
0.00
0.25
β_IV

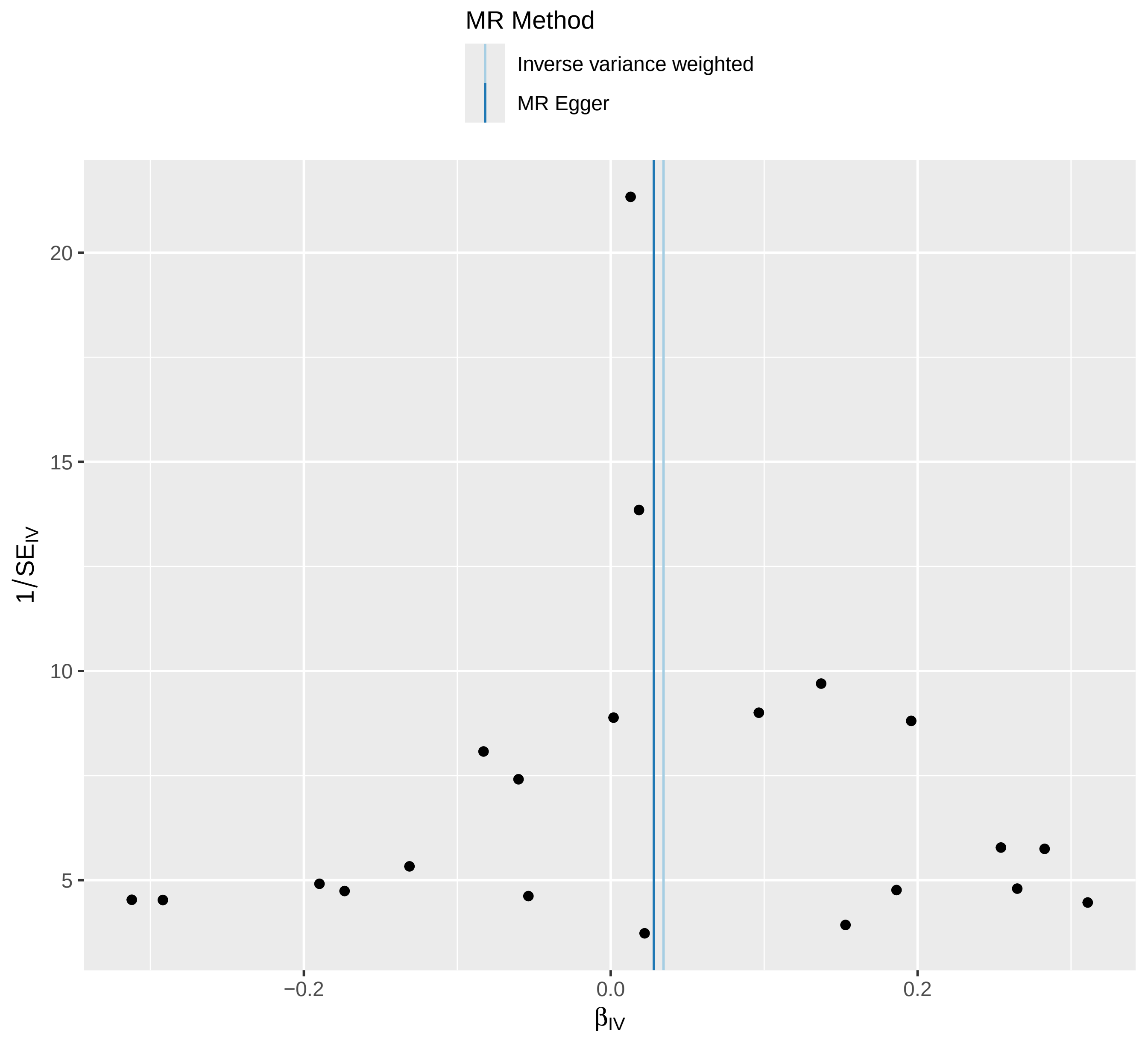

MR Method
Inverse variance weighted
MR Egger
20
15
10
5
1/SE$_{IV}$
−0.2
0.0
0.2
β$_{IV}$

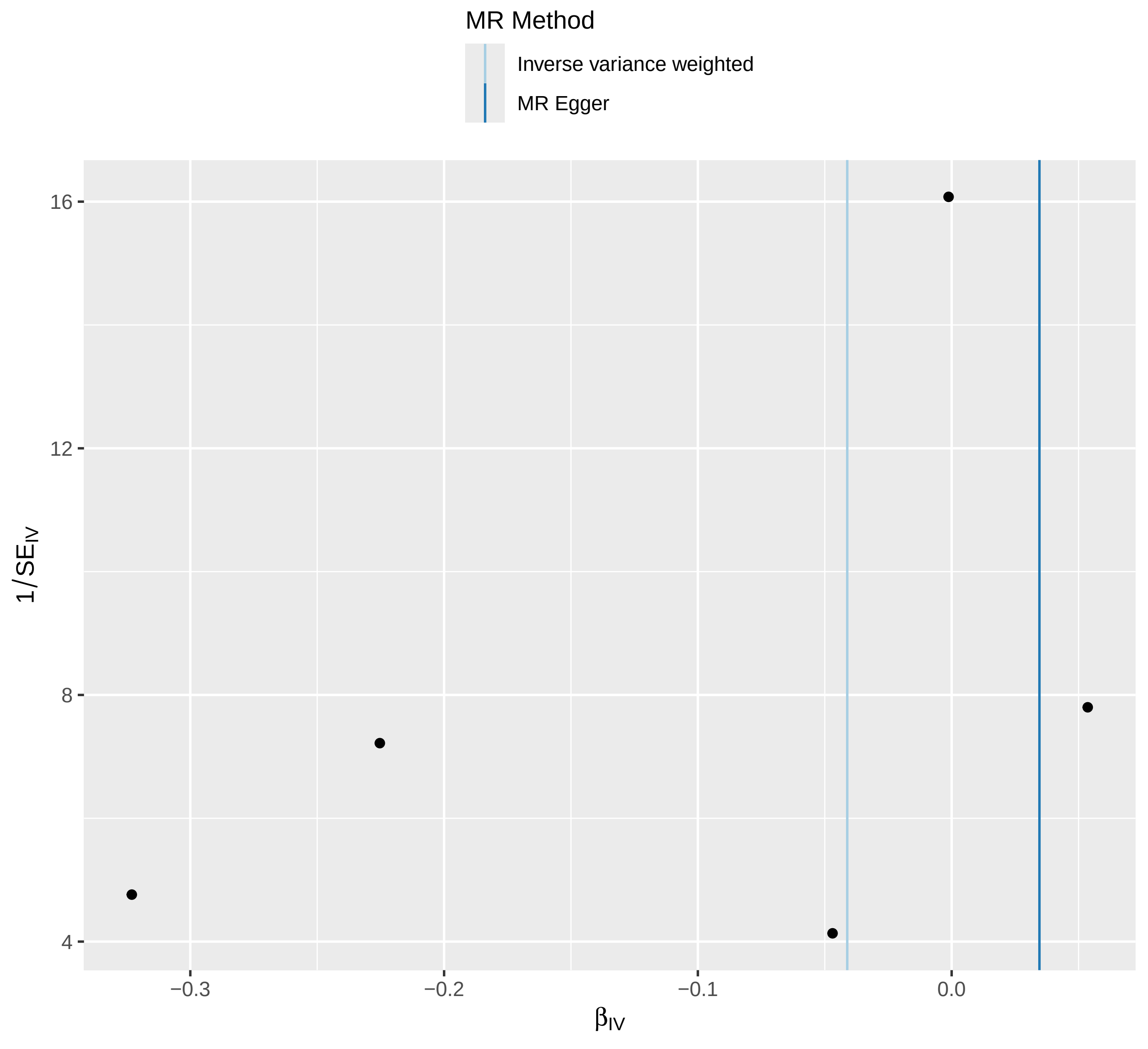

MR Method
Inverse variance weighted
MR Egger
16
12
8
4
−0.3
−0.2
−0.1
0.0
1/SE_IV
β_IV

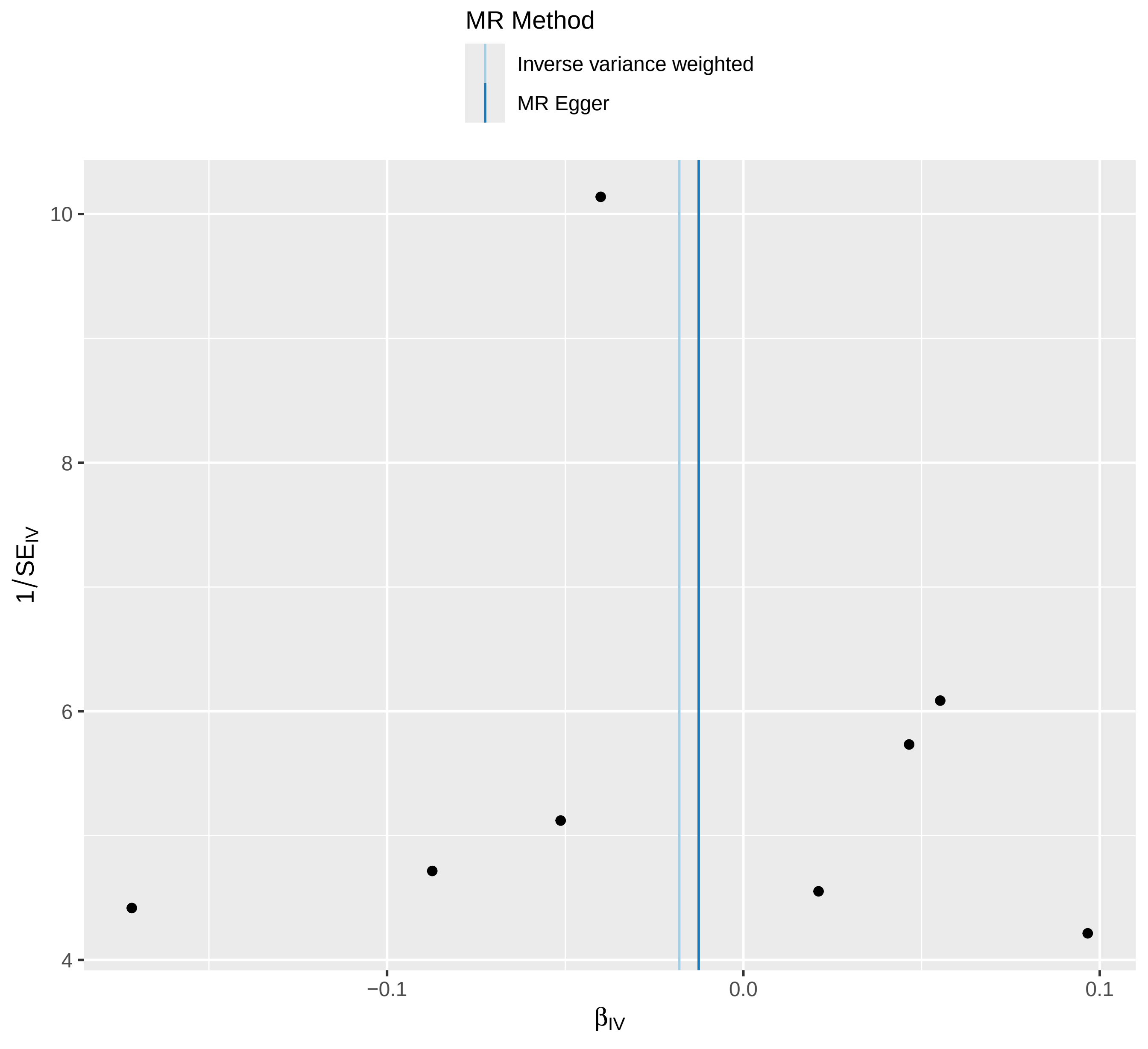

MR Method
Inverse variance weighted
MR Egger
10
8
6
4
1/SE$_{IV}$
−0.1
0.0
0.1
β$_{IV}$

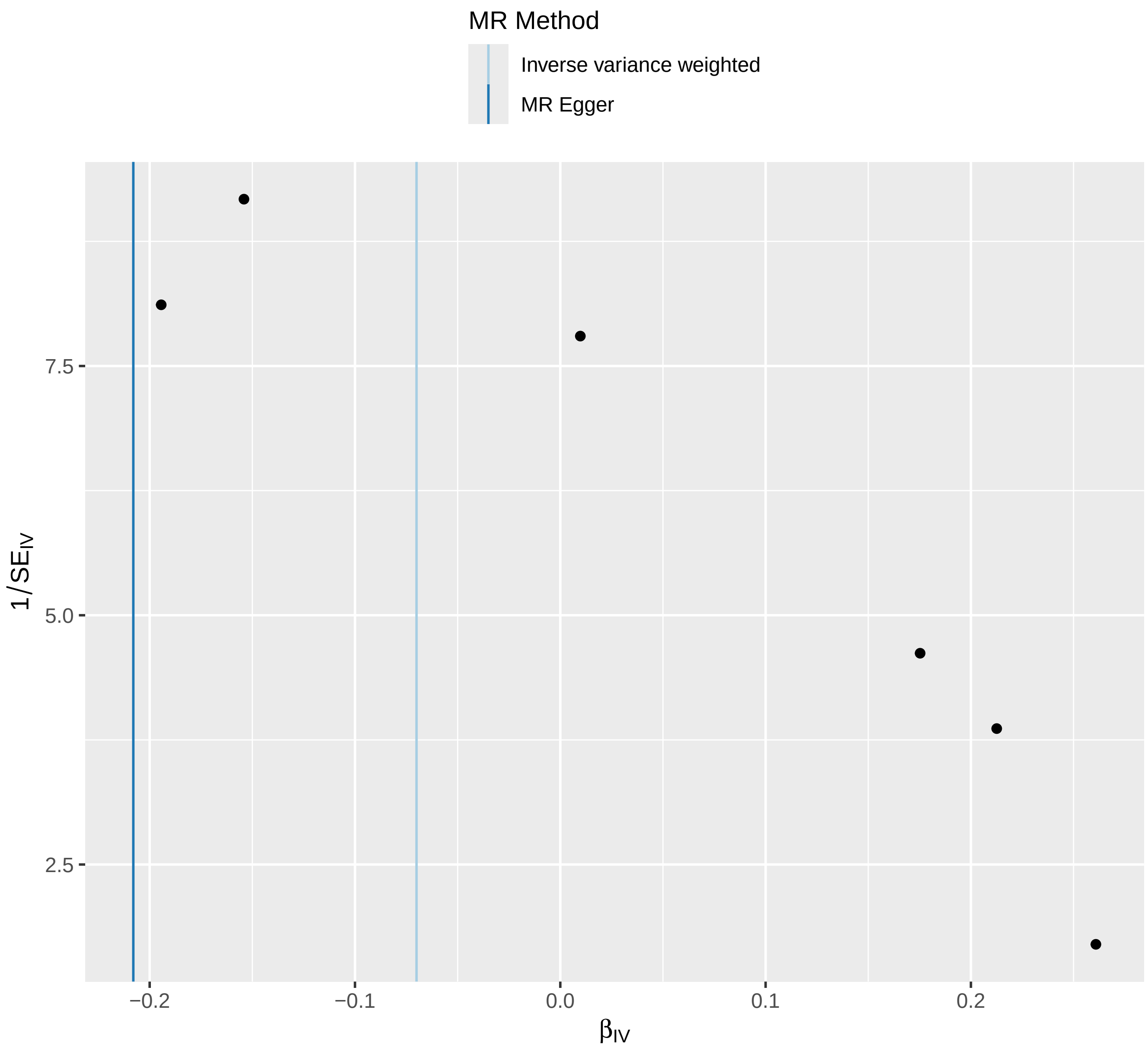
MR Method
Inverse variance weighted
MR Egger
7.5
5.0
2.5
1/SE$_{IV}$
−0.2
−0.1
0.0
0.1
0.2
β$_{IV}$

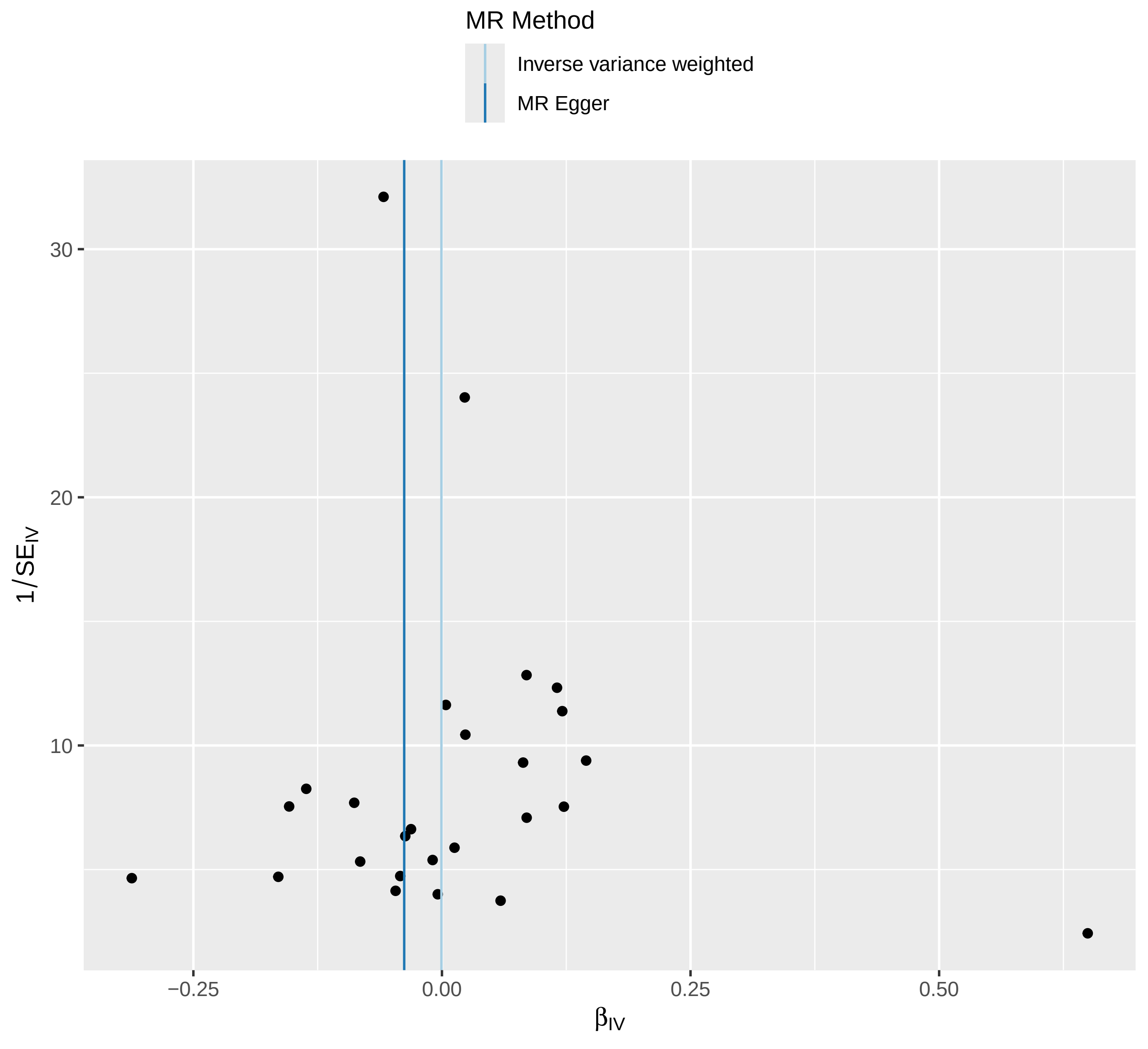
MR Method
Inverse variance weighted
MR Egger
30
20
10
1/$SE_{IV}$
−0.25
0.00
0.25
0.50
$\beta_{IV}$

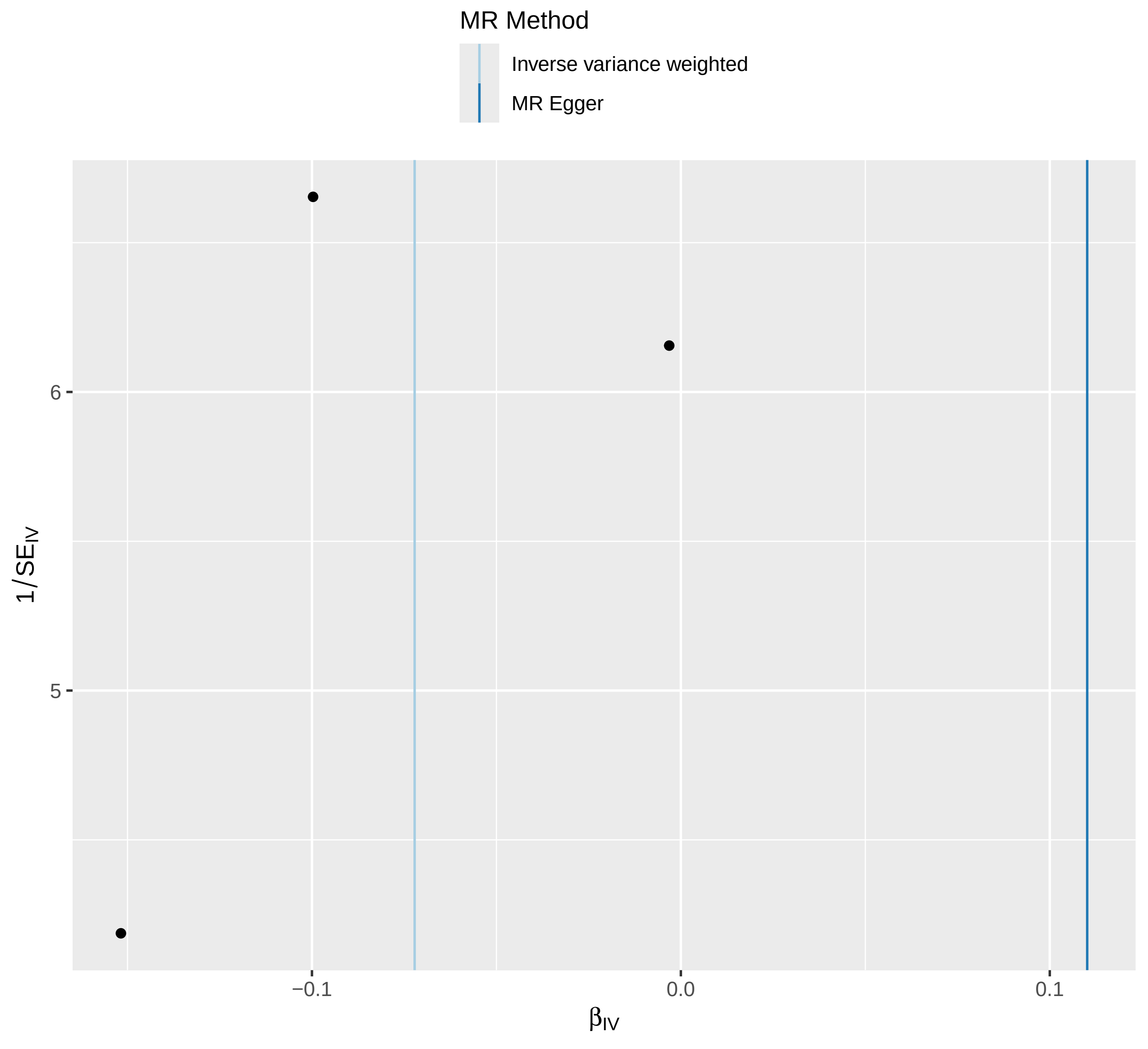
MR Method
Inverse variance weighted
MR Egger
6
5
1/SE_IV
−0.1
0.0
0.1
β_IV

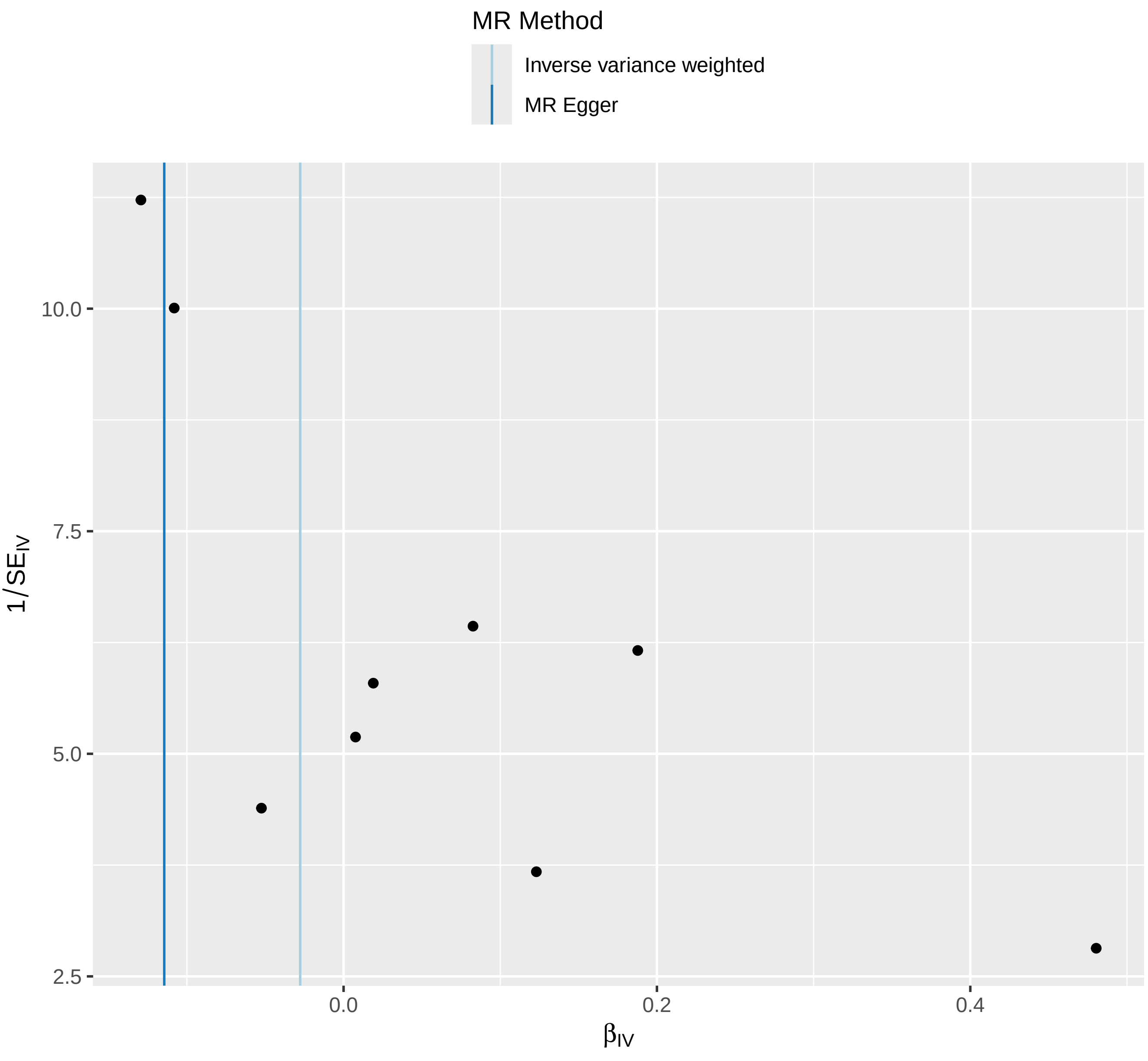
MR Method
Inverse variance weighted
MR Egger
10.0
7.5
5.0
2.5
0.0
0.2
0.4
$1/SE_{IV}$
$\beta_{IV}$

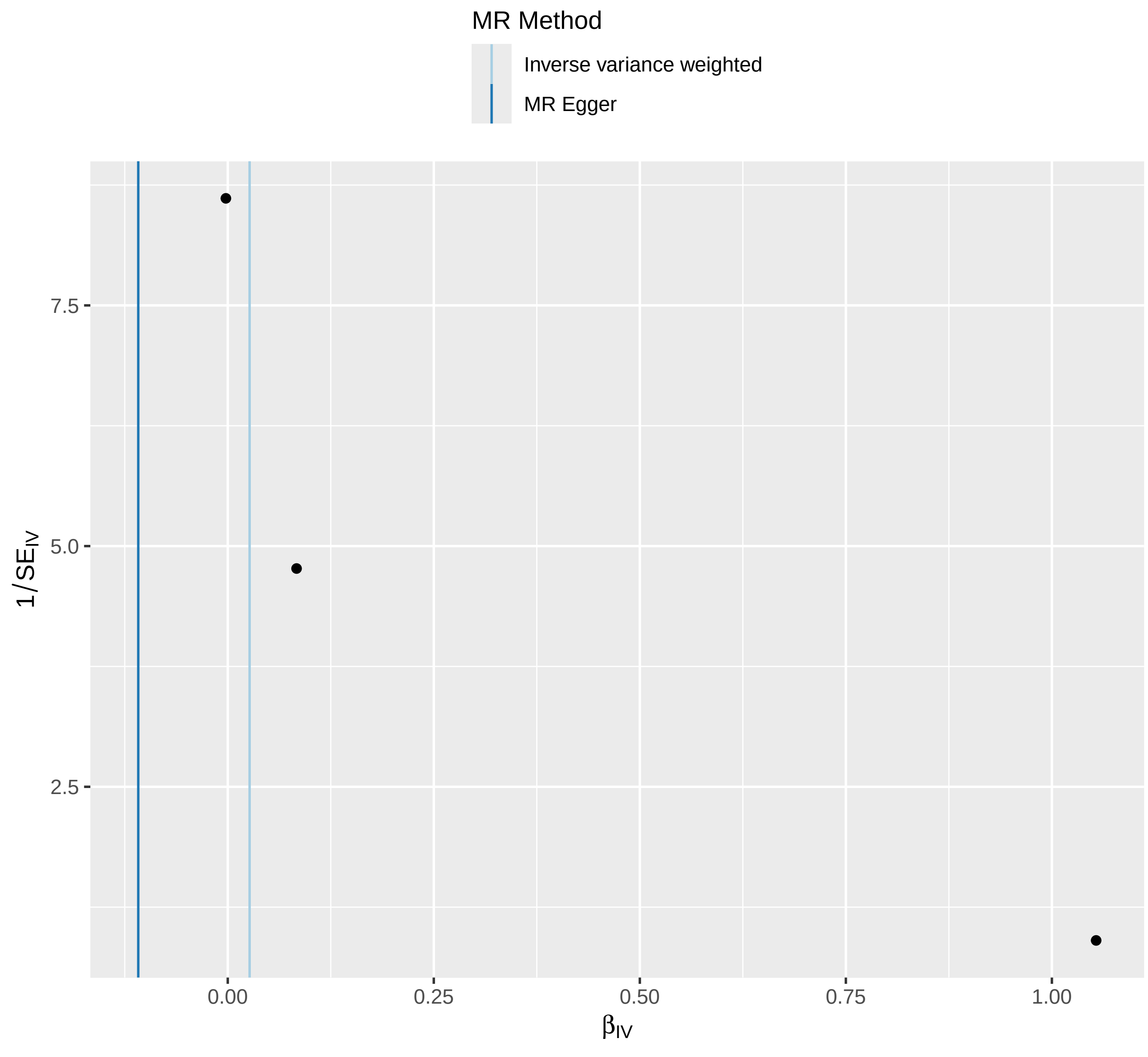
MR Method
Inverse variance weighted
MR Egger
7.5
5.0
2.5
1/SE$_{IV}$
0.00
0.25
0.50
0.75
1.00
$\beta_{IV}$

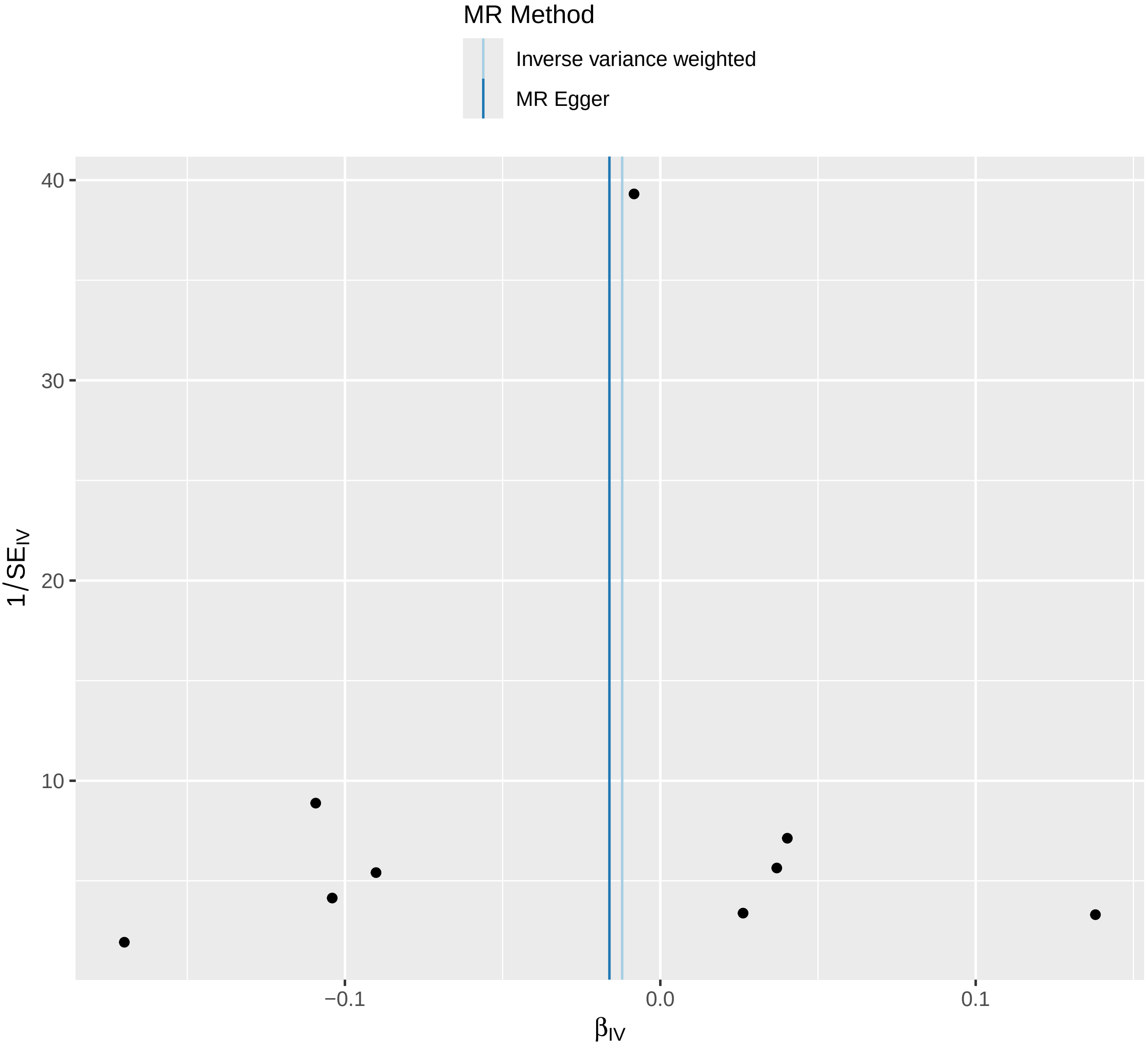
MR Method
Inverse variance weighted
MR Egger
40
30
20
10
1/SE$_{IV}$
−0.1
0.0
0.1
β$_{IV}$

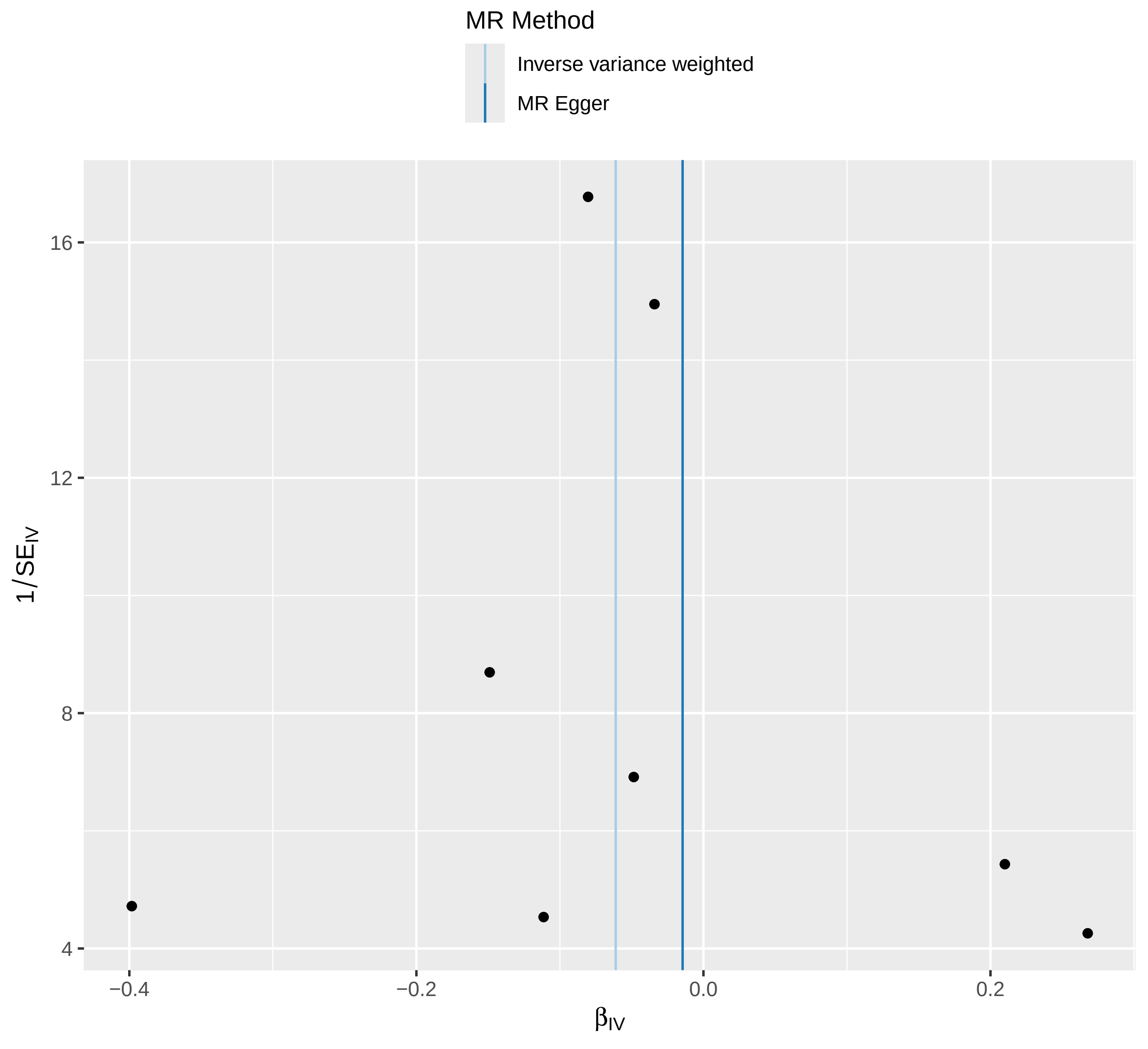
MR Method
Inverse variance weighted
MR Egger
16
12
8
4
1/SE$_{IV}$
−0.4
−0.2
0.0
0.2
β$_{IV}$

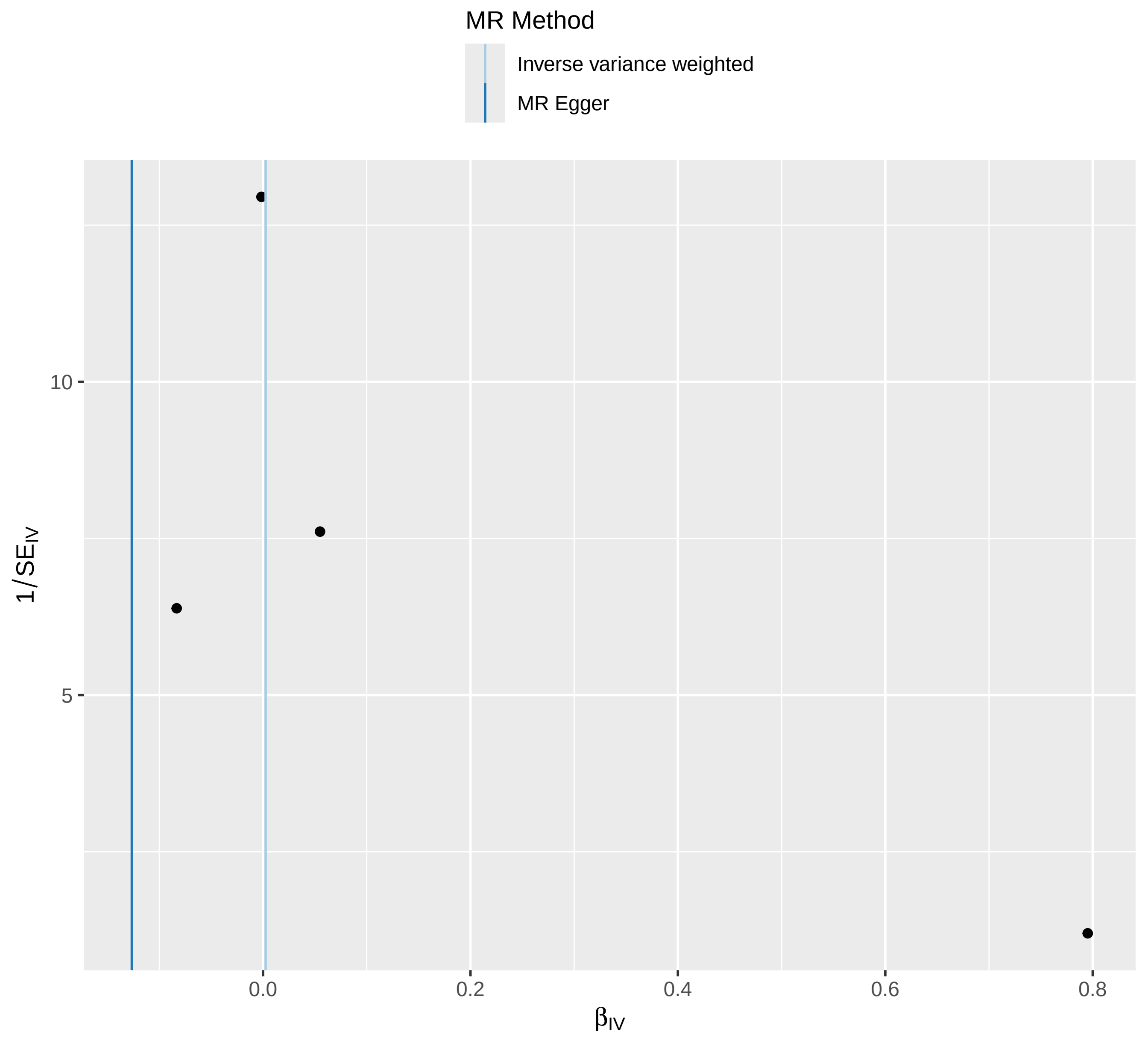
MR Method
Inverse variance weighted
MR Egger
10
5
1/SE$_{IV}$
0.0
0.2
0.4
0.6
0.8
β$_{IV}$

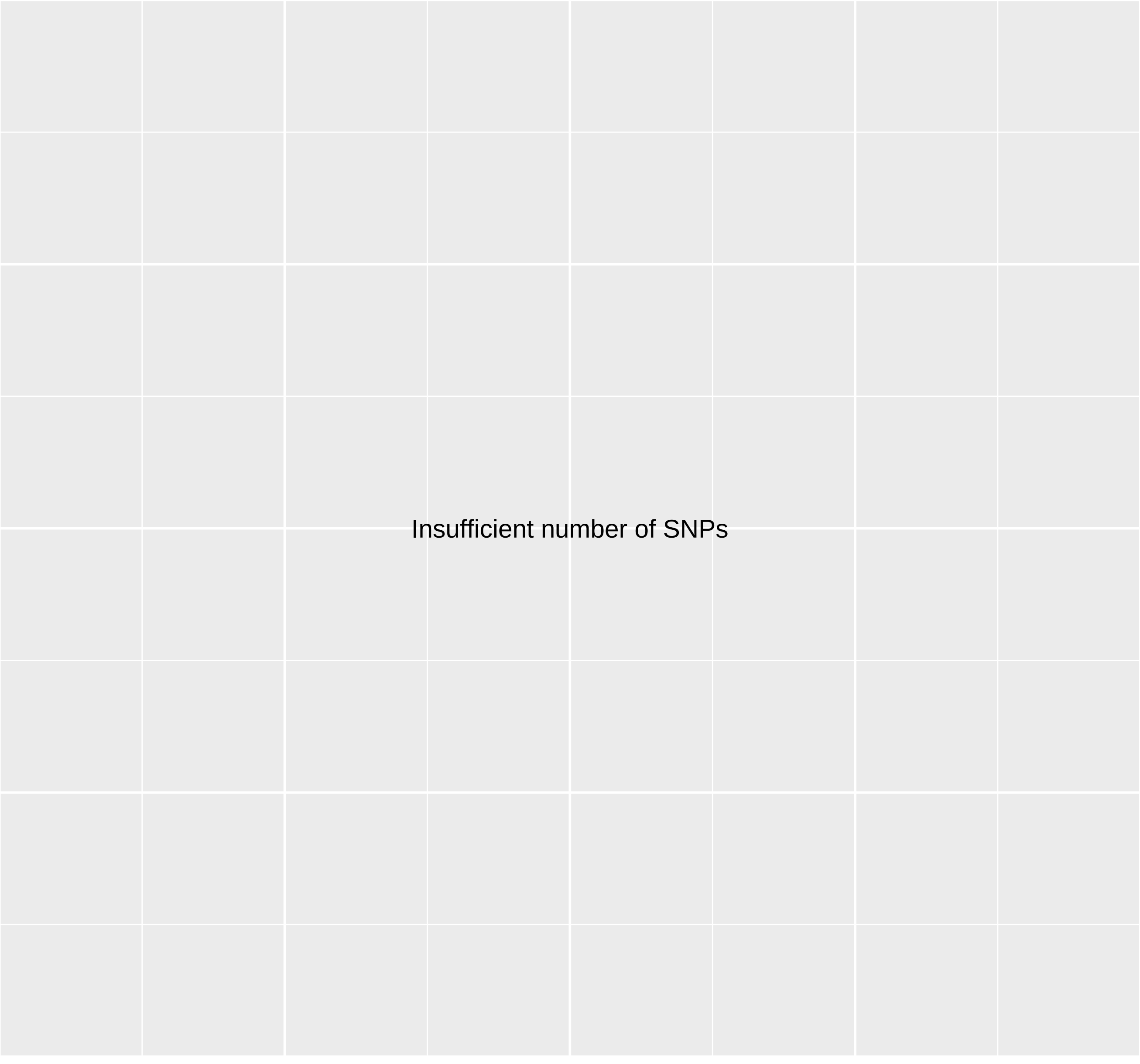
Insufficient number of SNPs

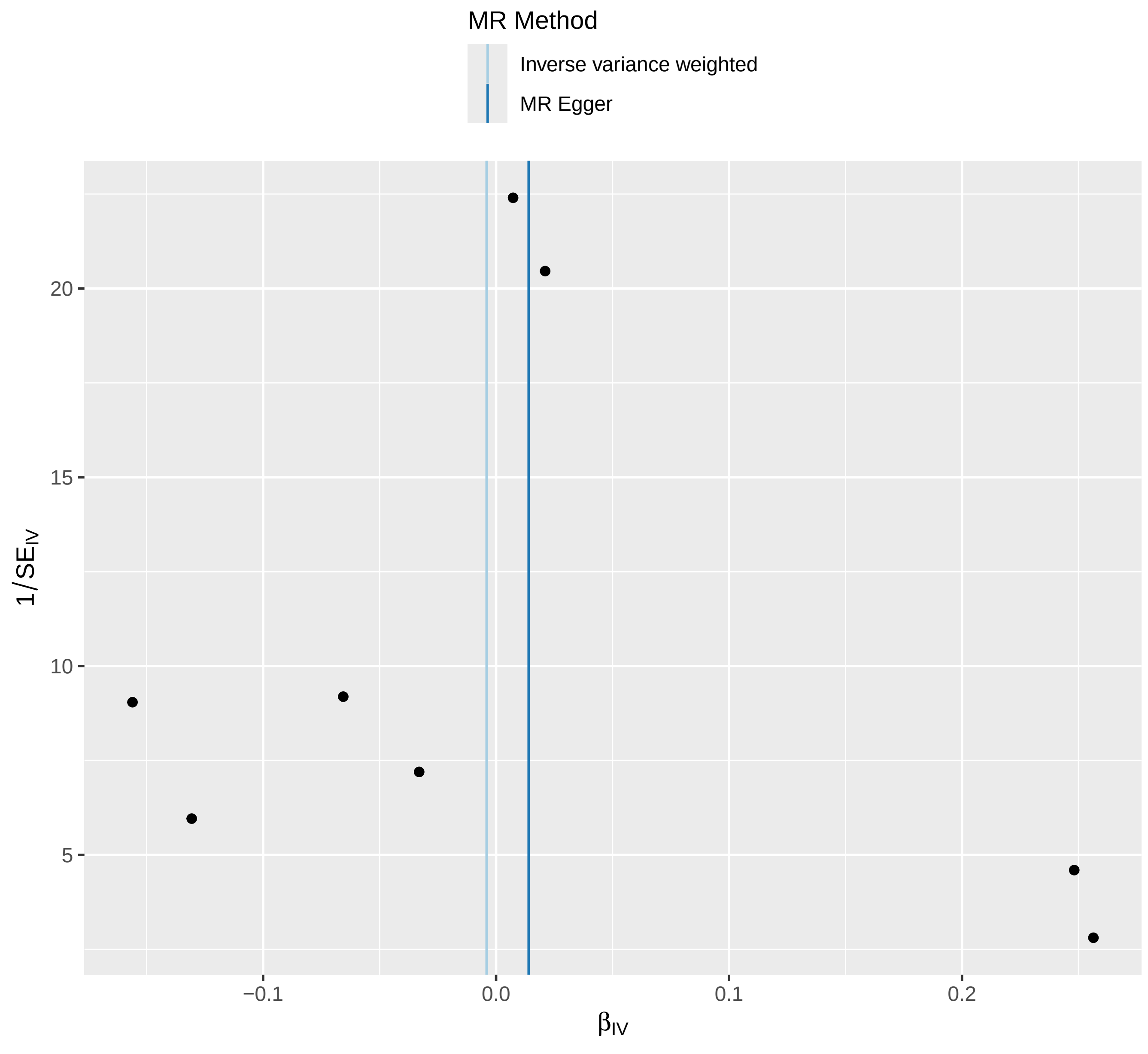
MR Method
Inverse variance weighted
MR Egger
20
15
10
5
1/SE_IV
−0.1
0.0
0.1
0.2
β_IV

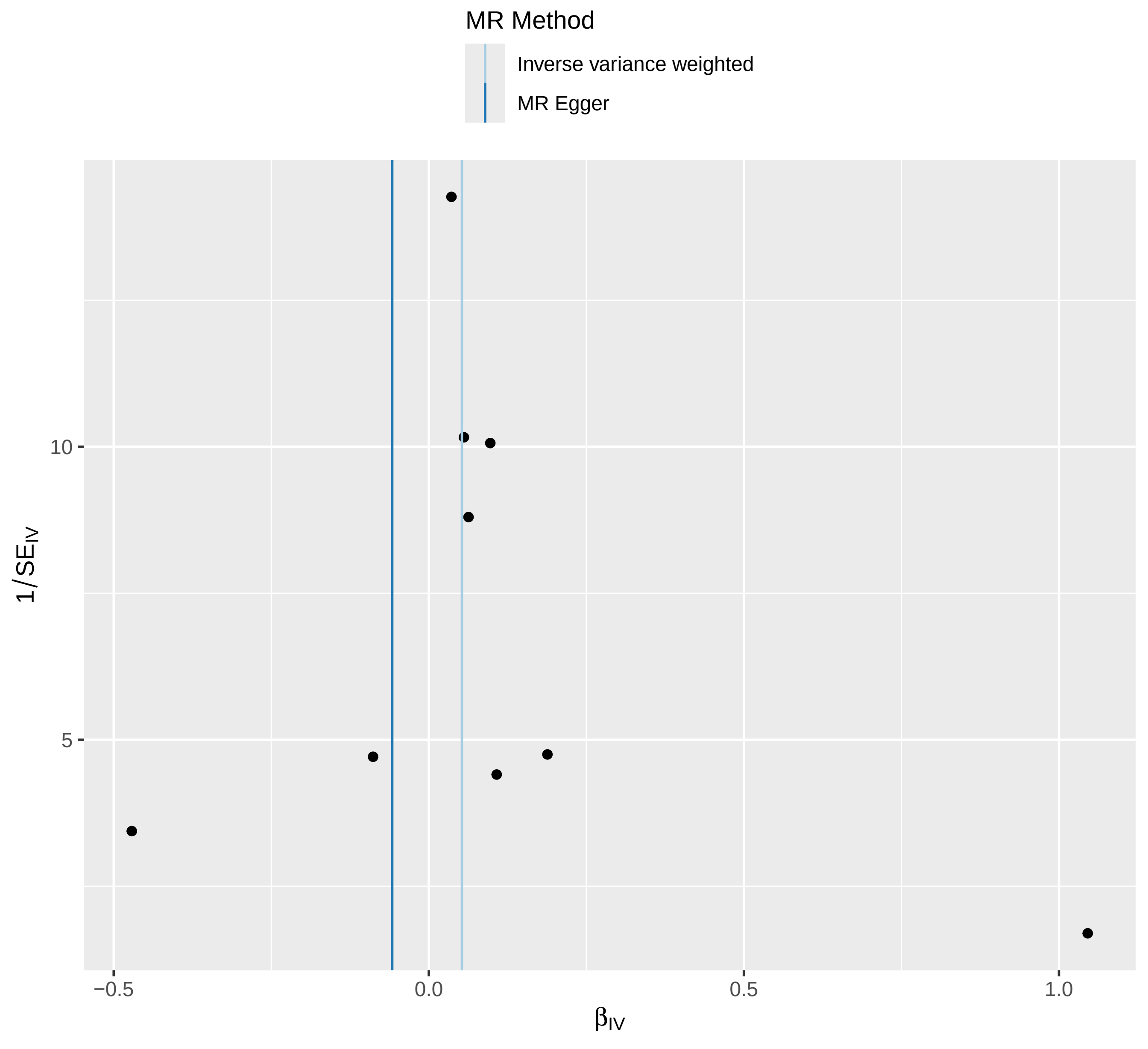

MR Method
Inverse variance weighted
MR Egger
10
5
1/SE$_{IV}$
−0.5
0.0
0.5
1.0
β$_{IV}$

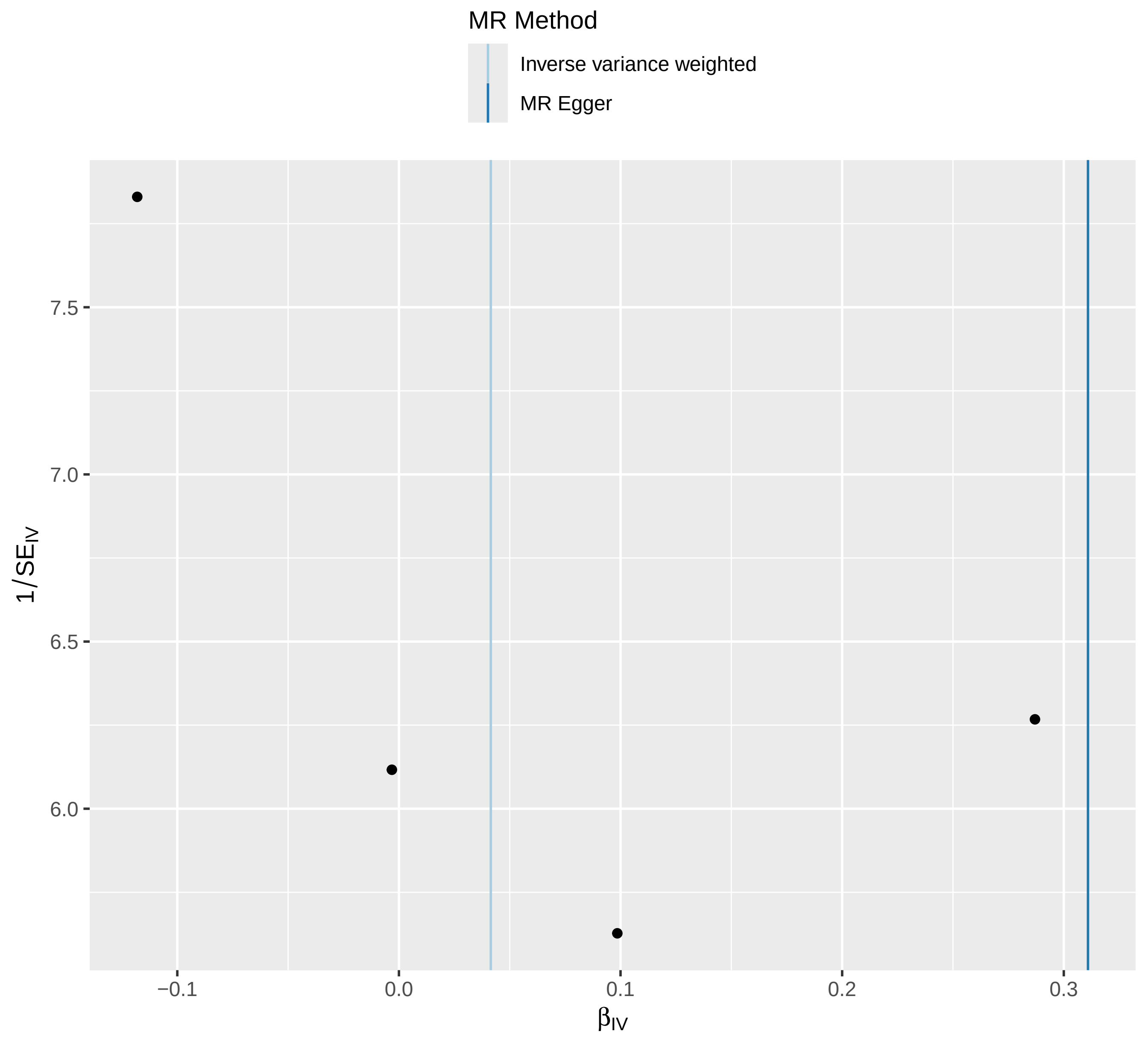
MR Method
Inverse variance weighted
MR Egger
7.5
7.0
6.5
6.0
1/SE$_{IV}$
−0.1
0.0
0.1
0.2
0.3
β$_{IV}$

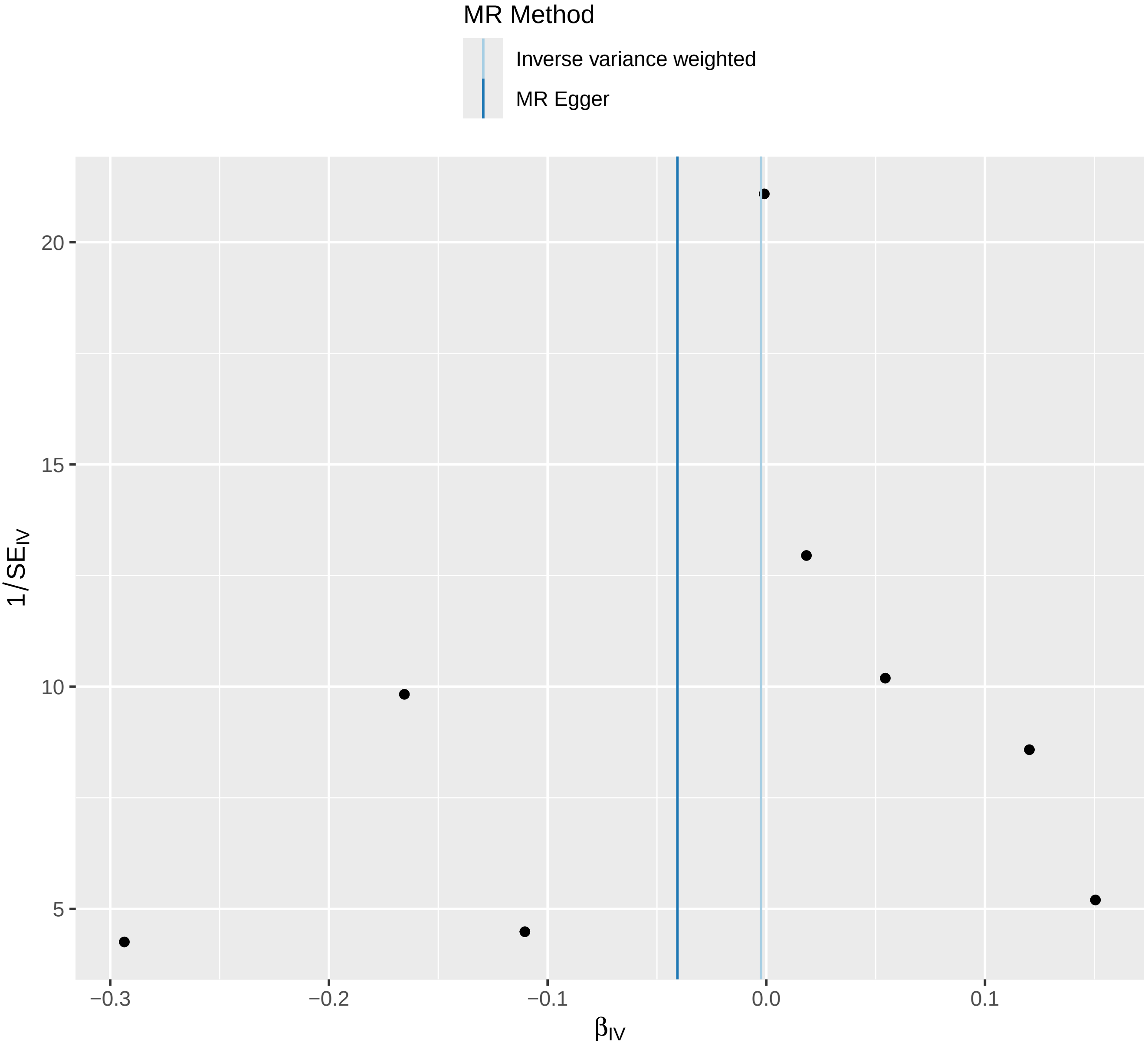
MR Method
Inverse variance weighted
MR Egger
20
15
10
5
1/SE_IV
−0.3
−0.2
−0.1
0.0
0.1
β_IV

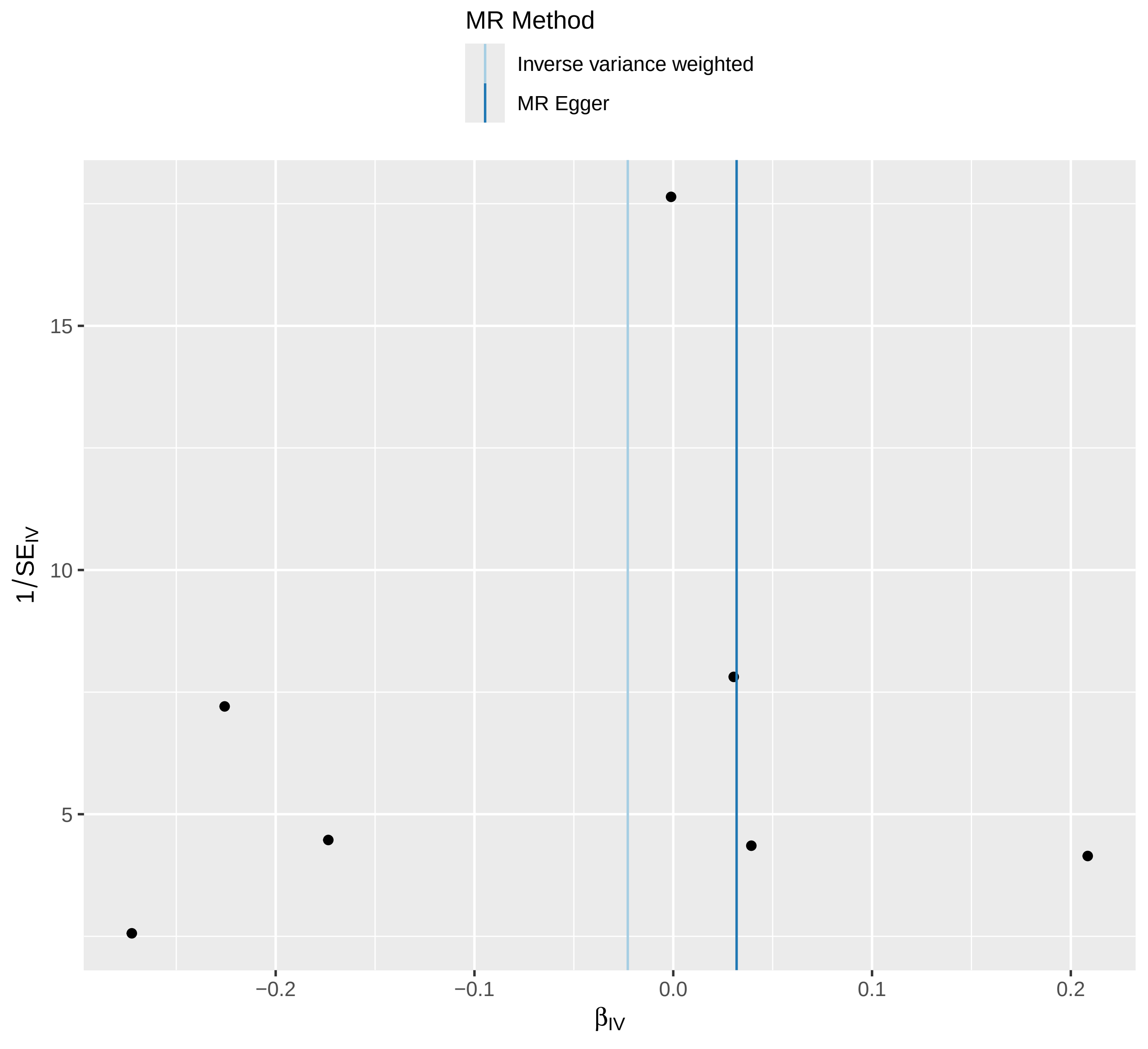
MR Method
Inverse variance weighted
MR Egger
15
10
5
$1/SE_{IV}$
−0.2
−0.1
0.0
0.1
0.2
$\beta_{IV}$

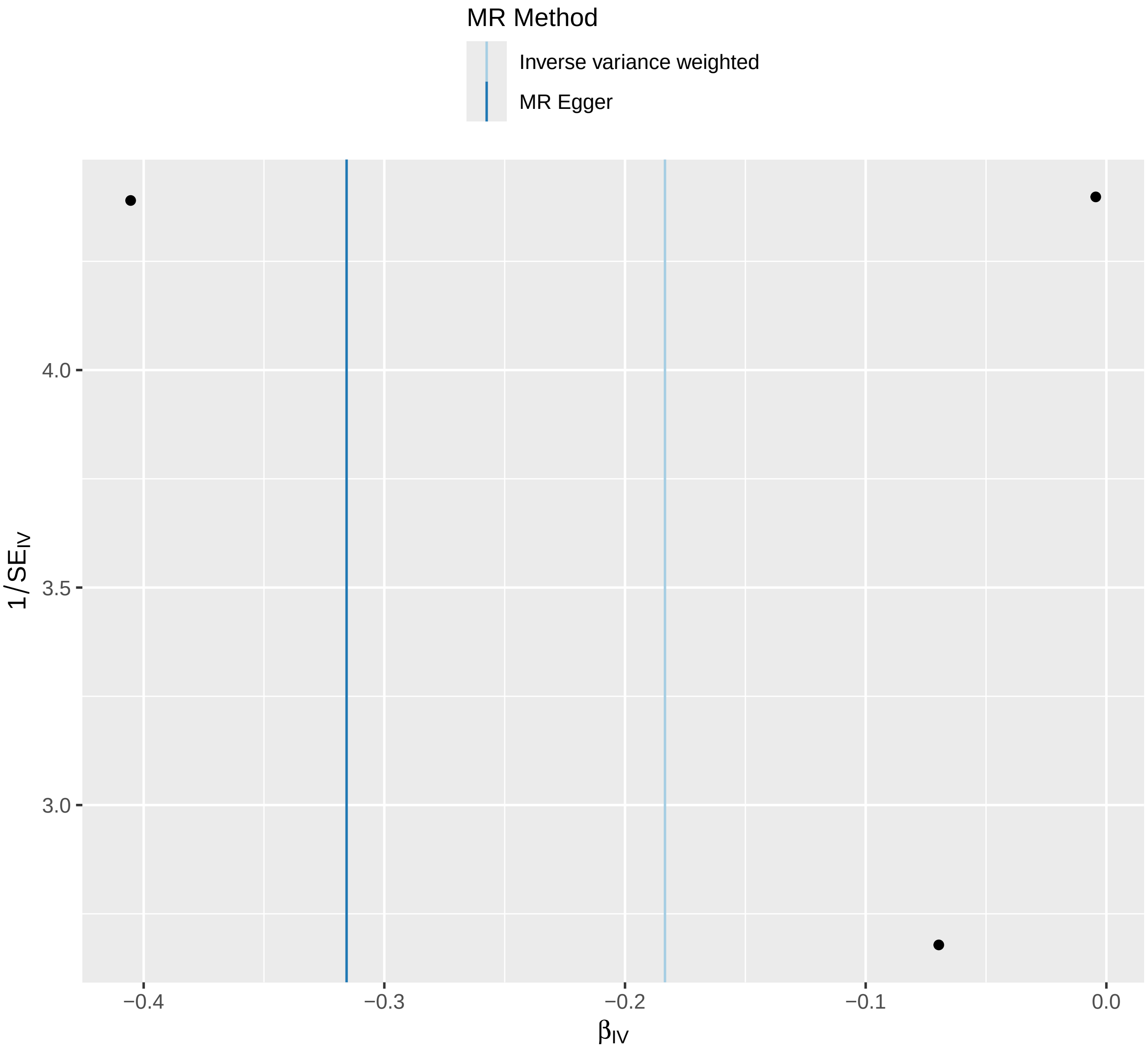

MR Method
Inverse variance weighted
MR Egger
4.0
3.5
3.0
1/SE_IV
−0.4
−0.3
−0.2
−0.1
0.0
β_IV

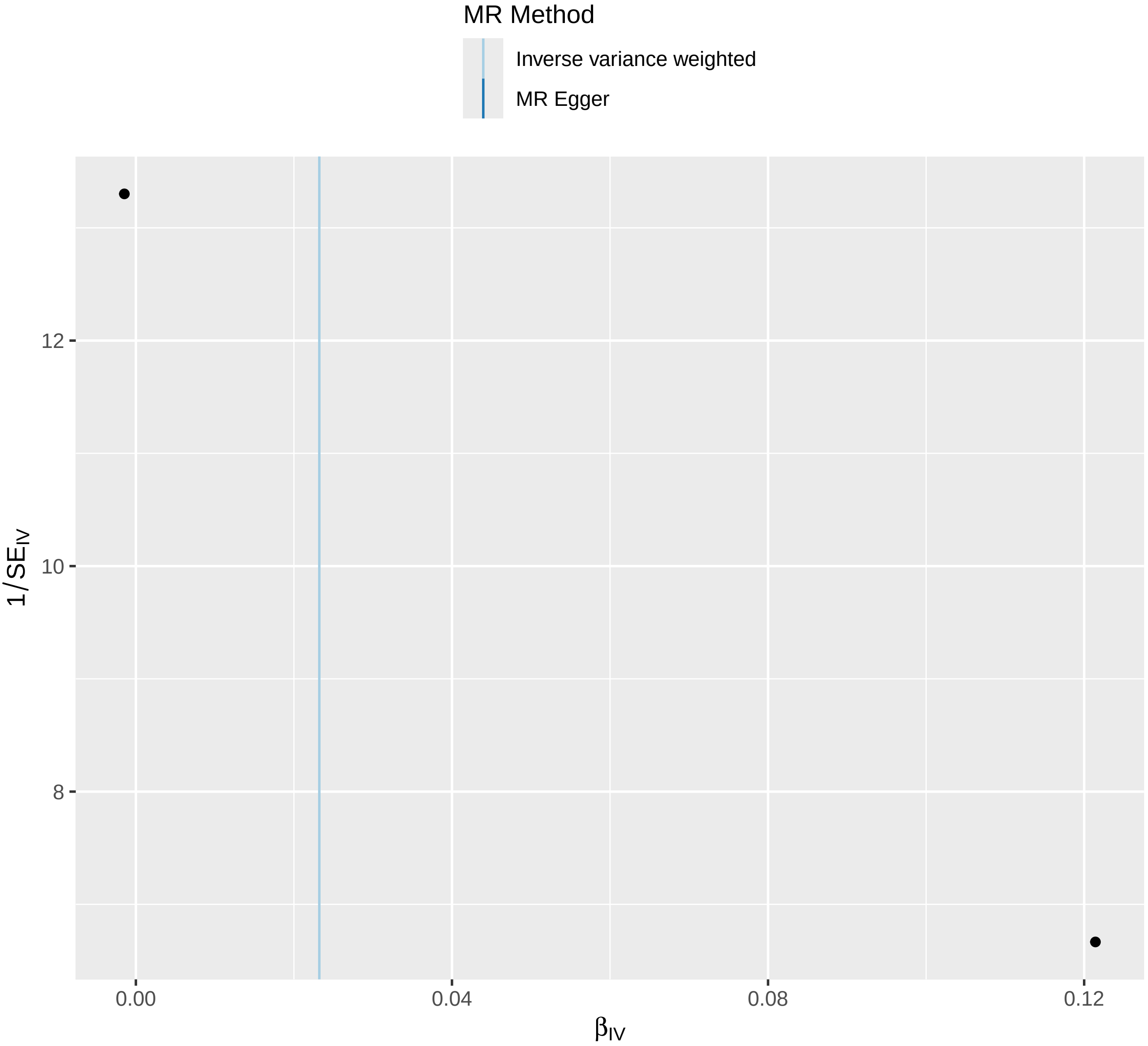
MR Method
Inverse variance weighted
MR Egger
1/SE_IV
12
10
8
0.00
0.04
0.08
0.12
β_IV

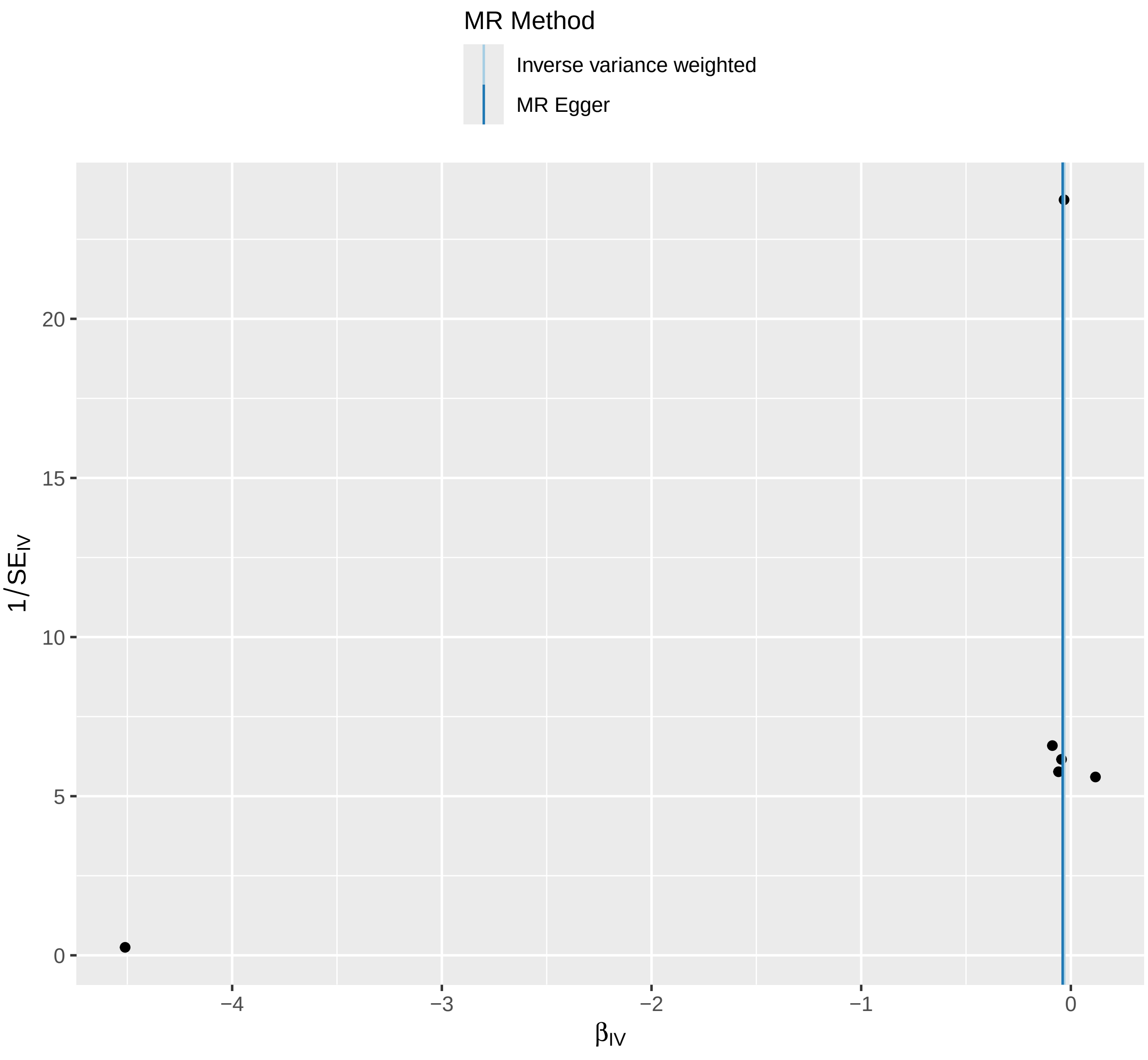
MR Method
Inverse variance weighted
MR Egger
1/SE_IV
β_IV
20
15
10
5
0
−4
−3
−2
−1
0

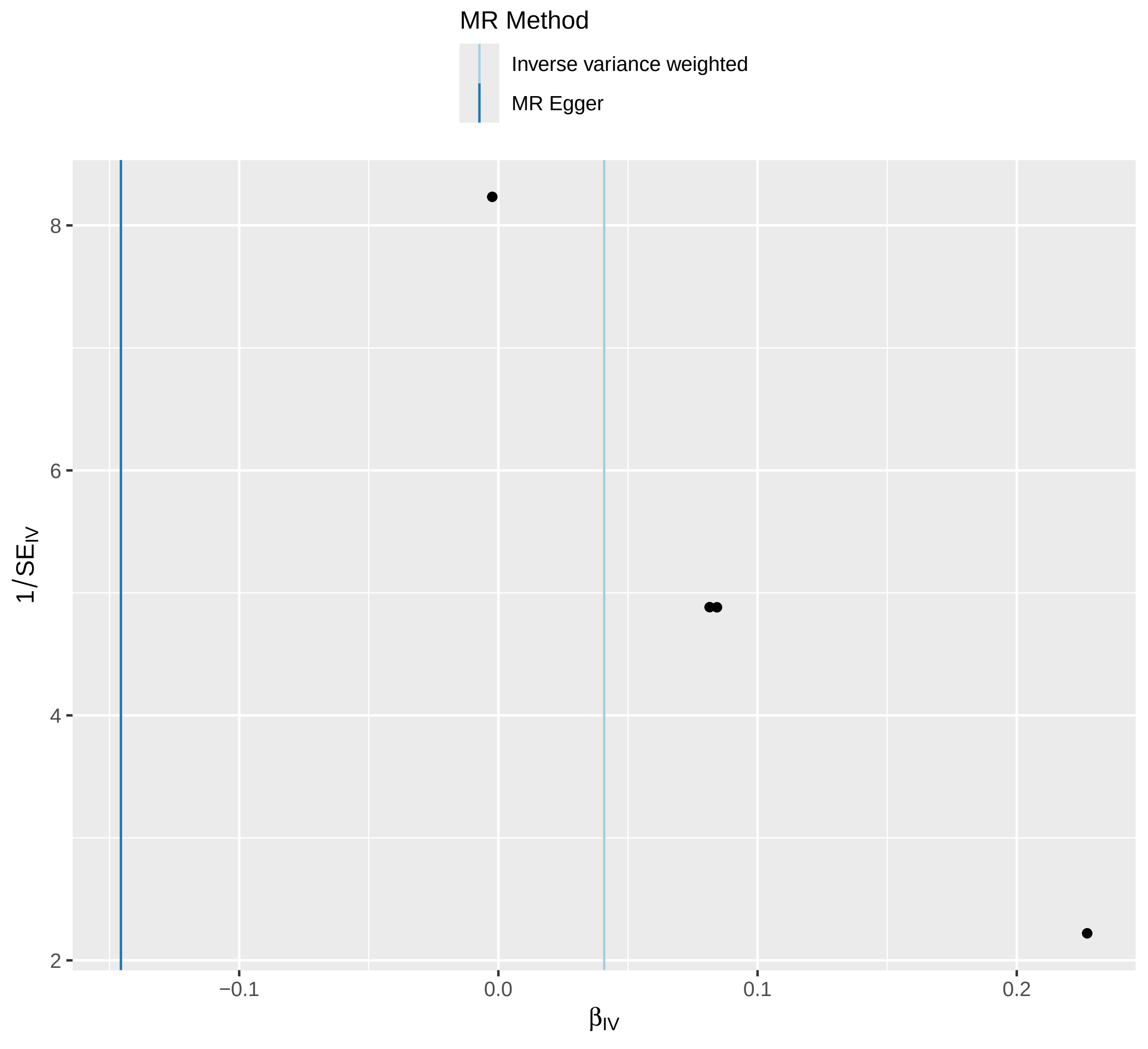
MR Method
Inverse variance weighted
MR Egger
1/SE_IV
8
6
4
2
−0.1
0.0
0.1
0.2
β_IV

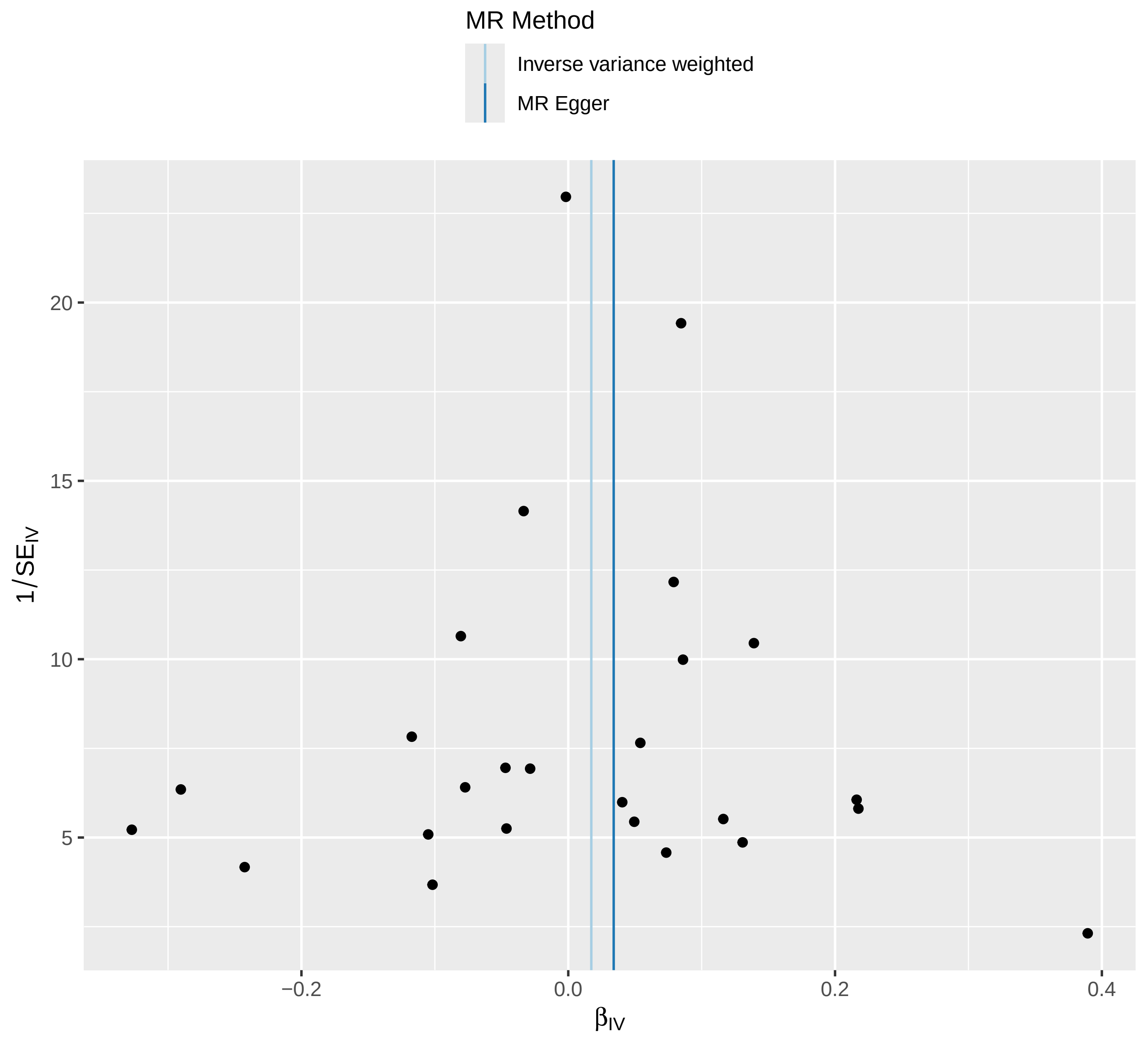
MR Method
Inverse variance weighted
MR Egger
20
15
10
5
1/SE_IV
−0.2
0.0
0.2
0.4
β_IV

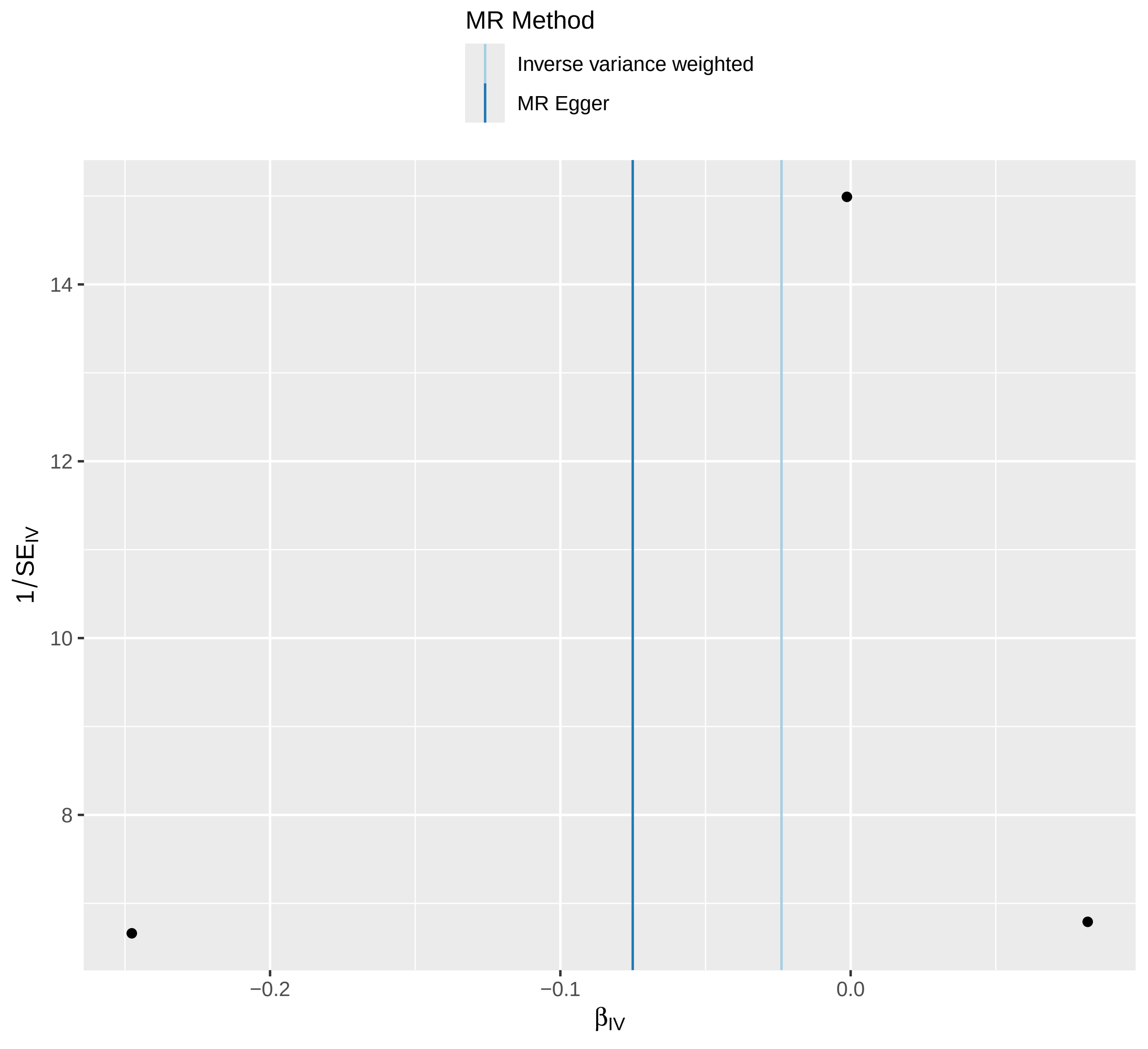

MR Method
Inverse variance weighted
MR Egger
14
12
10
8
1/SE_IV
−0.2
−0.1
0.0
β_IV

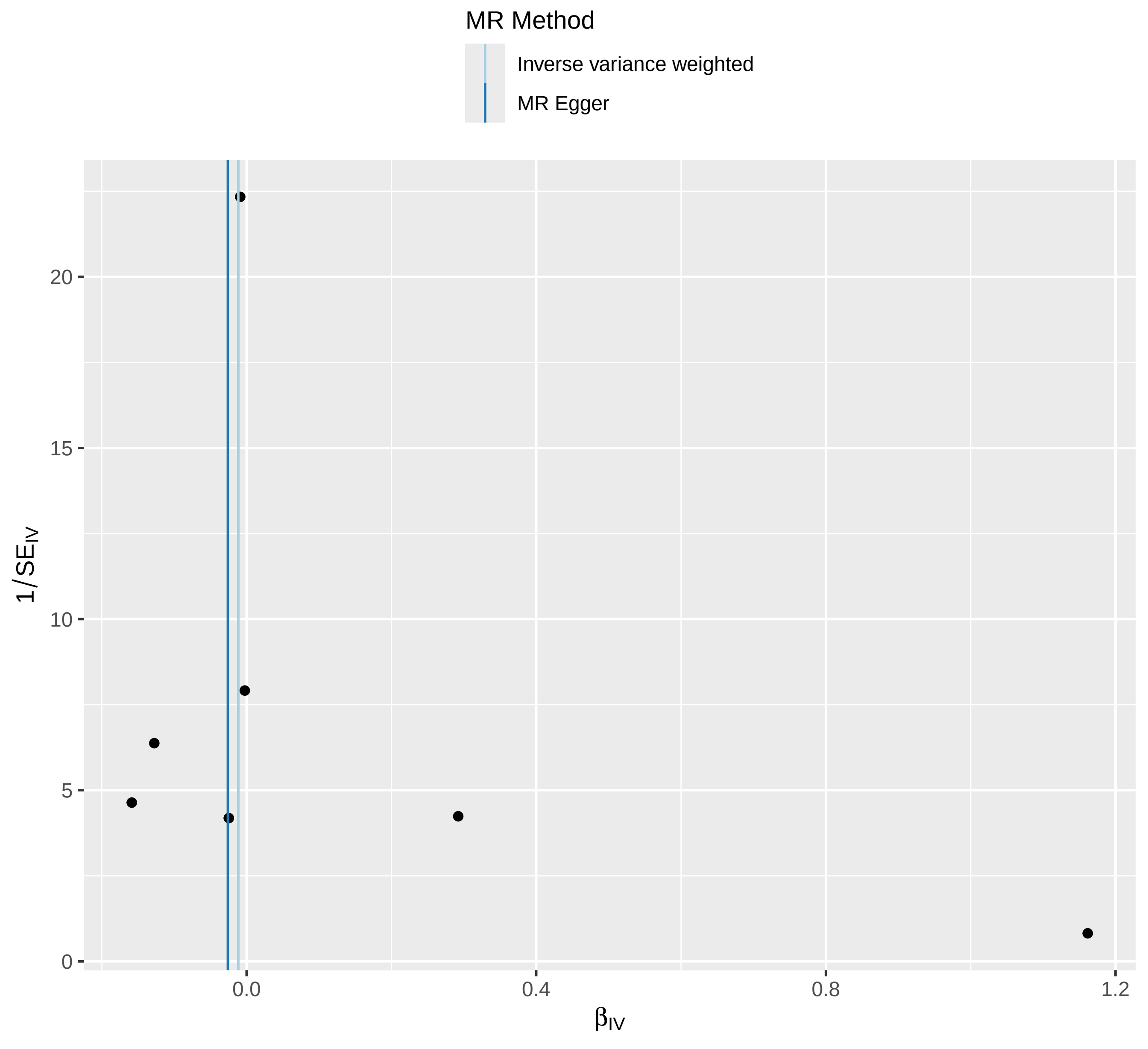
MR Method
Inverse variance weighted
MR Egger
20
15
10
5
0
1/SE_IV
0.0
0.4
0.8
1.2
β_IV

Insufficient number of SNPs

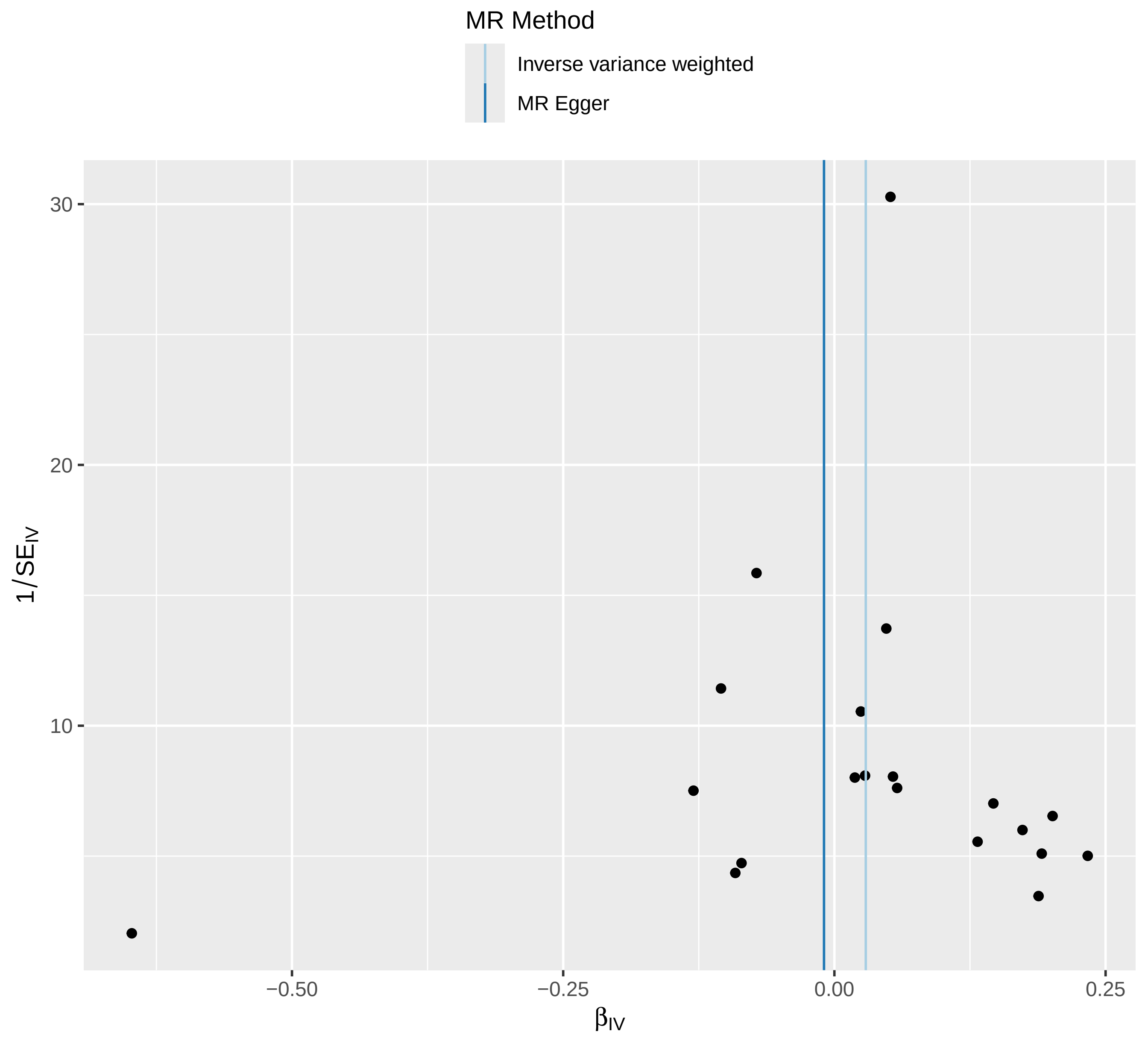

MR Method
Inverse variance weighted
MR Egger
30
20
10
1/SE_IV
−0.50
−0.25
0.00
0.25
β_IV

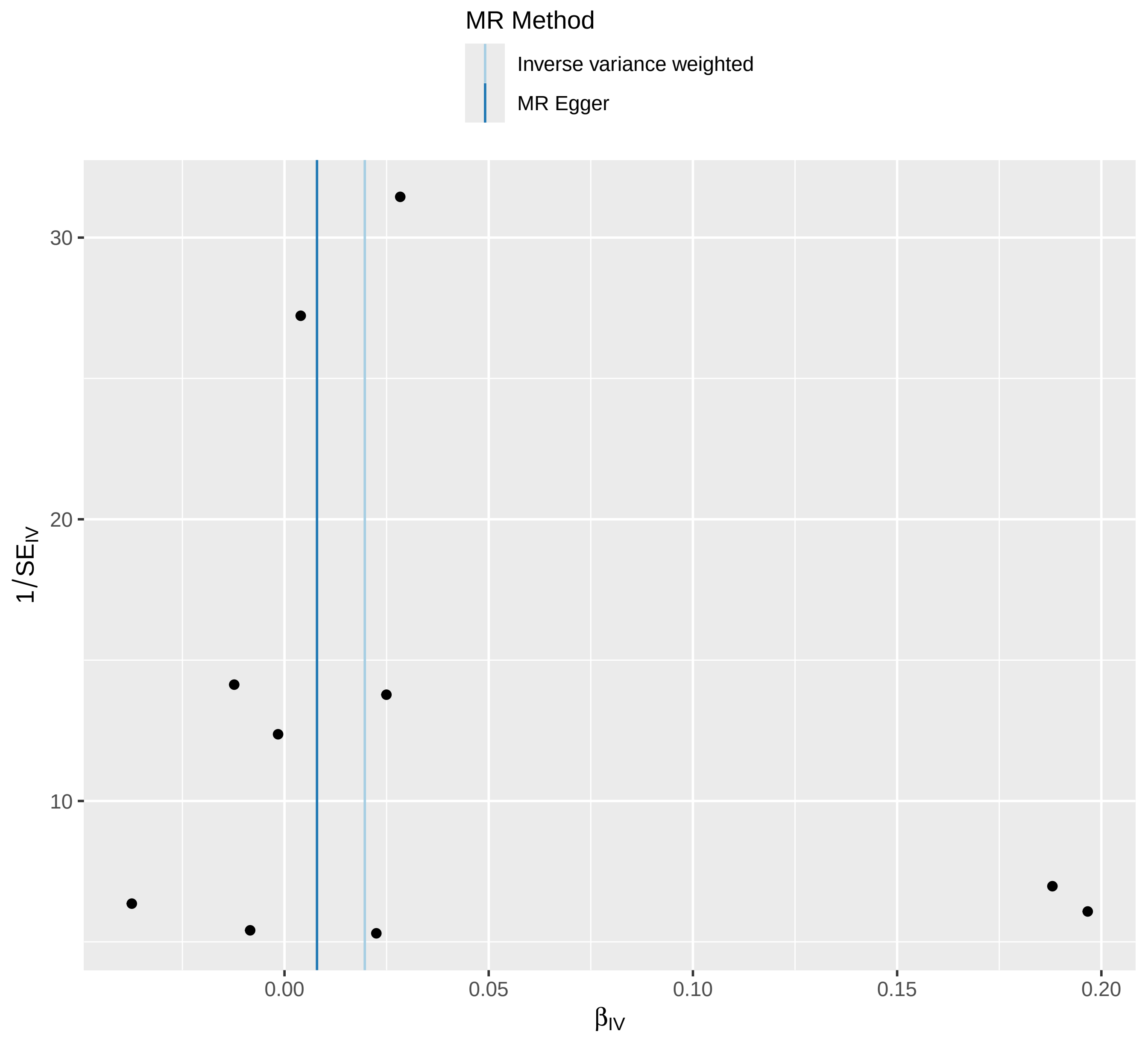

MR Method
Inverse variance weighted
MR Egger
30
20
10
1/SE$_{IV}$
0.00
0.05
0.10
0.15
0.20
β$_{IV}$

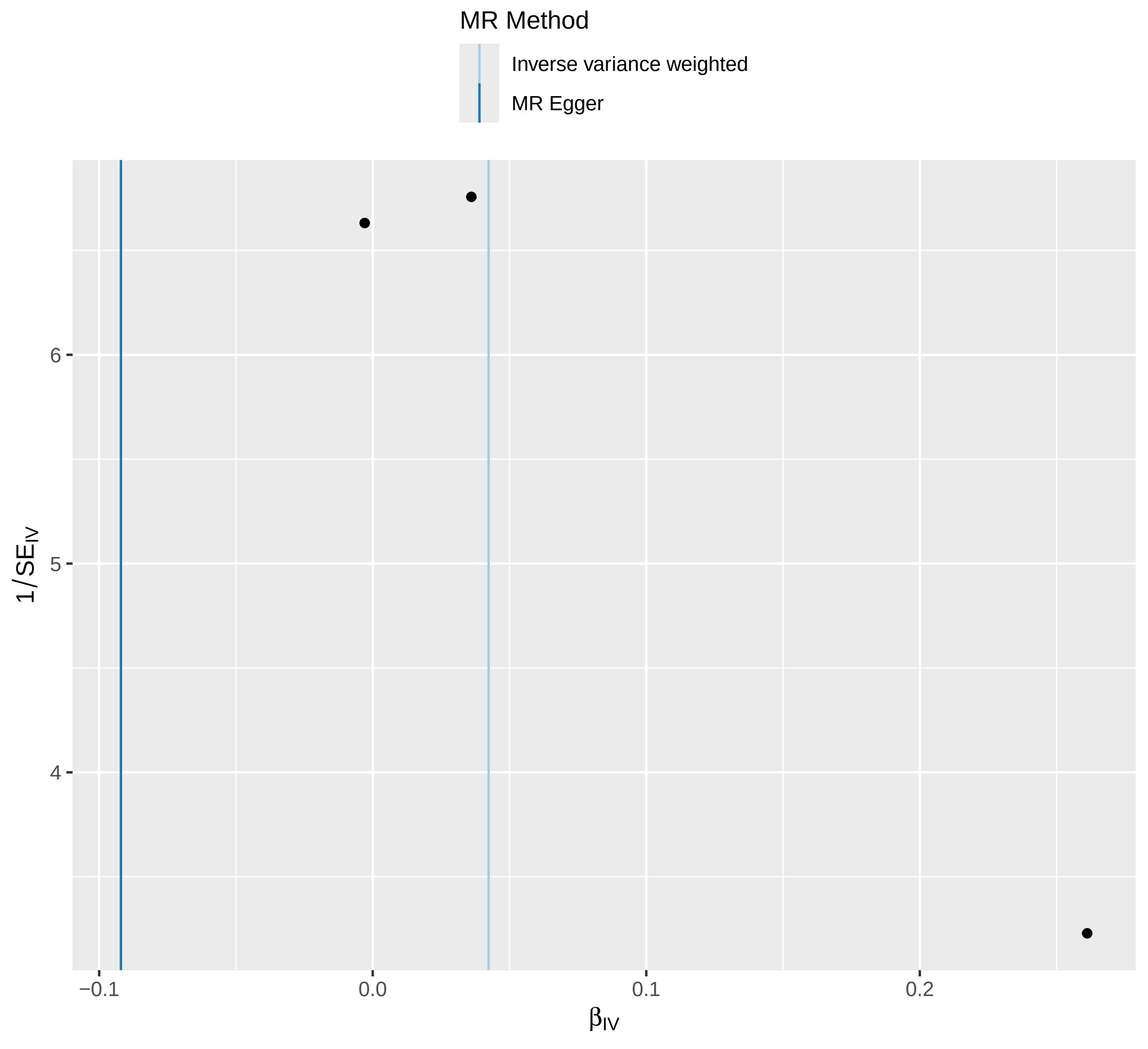
MR Method
Inverse variance weighted
MR Egger
1/SE_IV
β_IV
6
5
4
−0.1
0.0
0.1
0.2

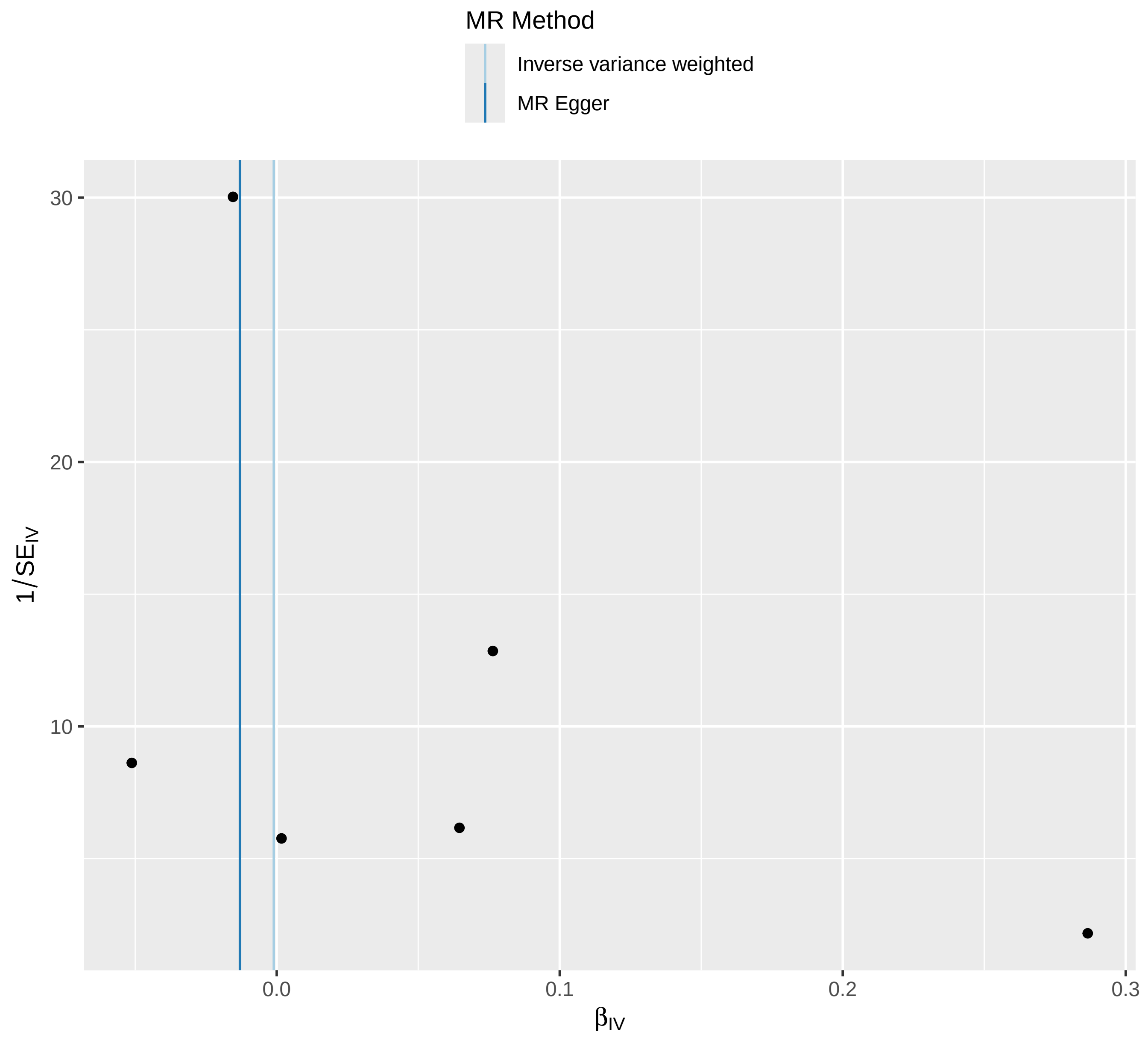
MR Method
Inverse variance weighted
MR Egger
30
20
10
1/SE_IV
0.0
0.1
0.2
0.3
β_IV

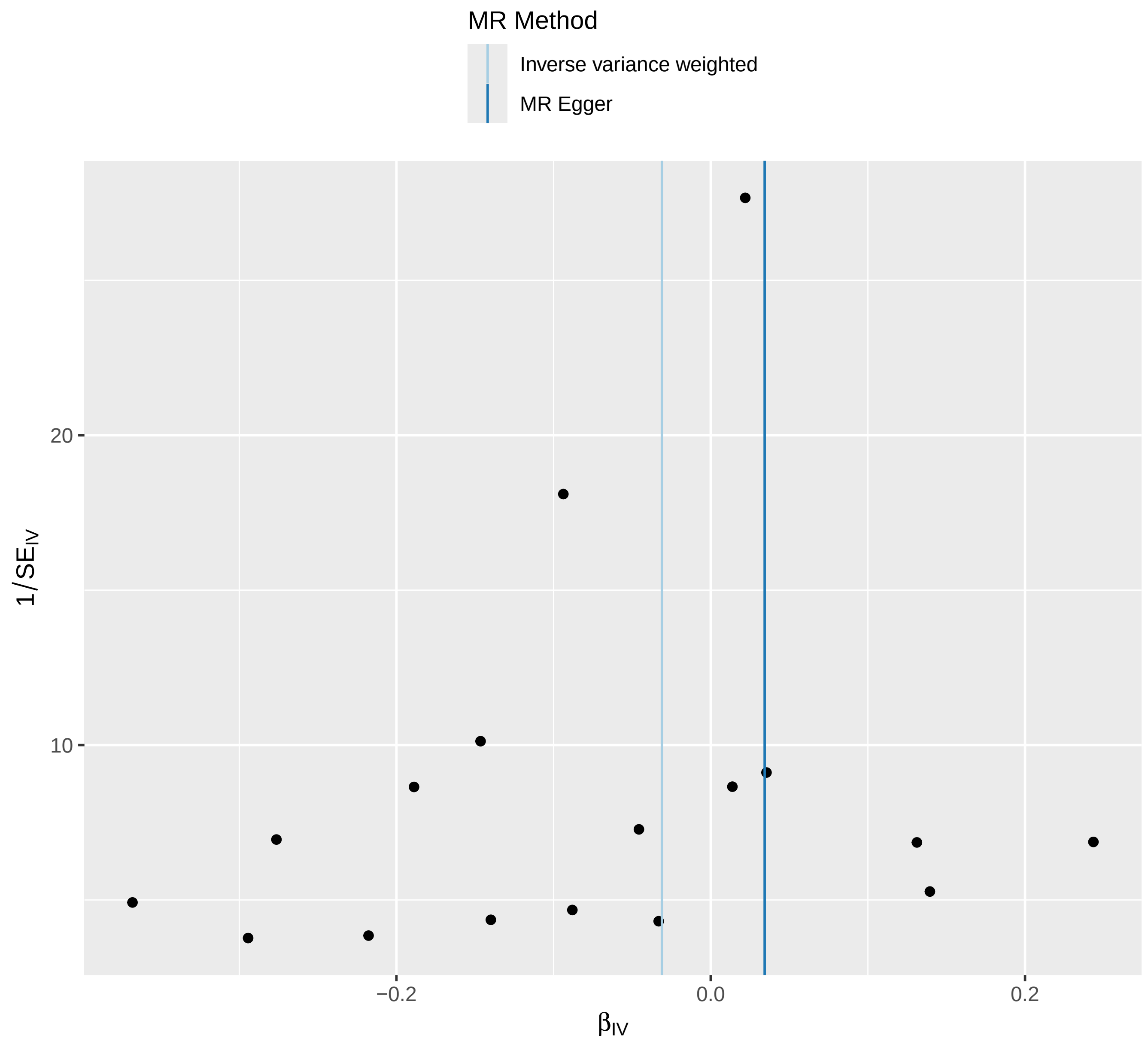
MR Method
Inverse variance weighted
MR Egger
20
10
1/SE$_{IV}$
−0.2
0.0
0.2
β$_{IV}$

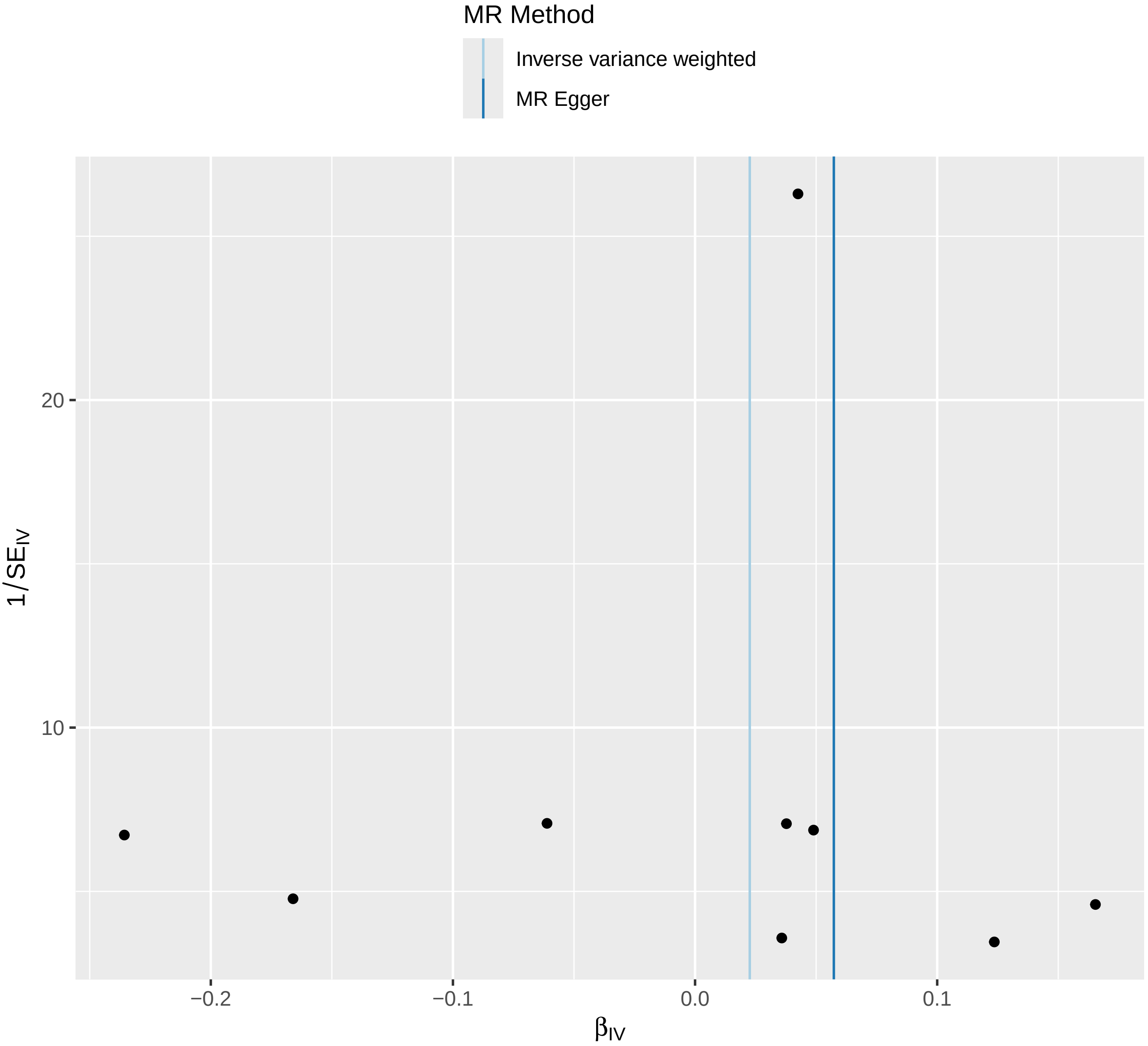
MR Method
Inverse variance weighted
MR Egger
20
10
1/SE$_{IV}$
−0.2
−0.1
0.0
0.1
β$_{IV}$

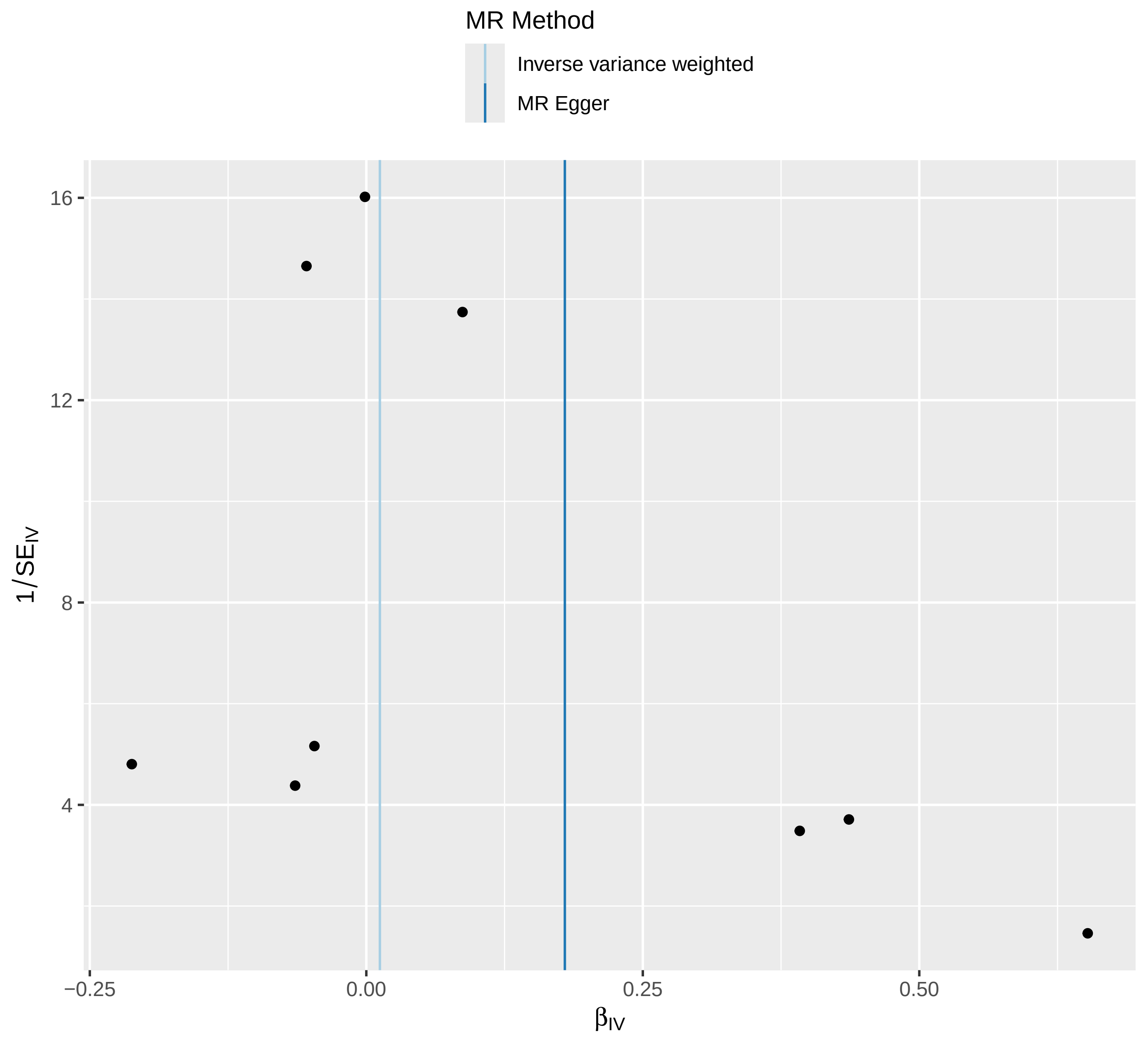

MR Method
Inverse variance weighted
MR Egger
16
12
8
4
1/SE$_{IV}$
−0.25
0.00
0.25
0.50
β$_{IV}$

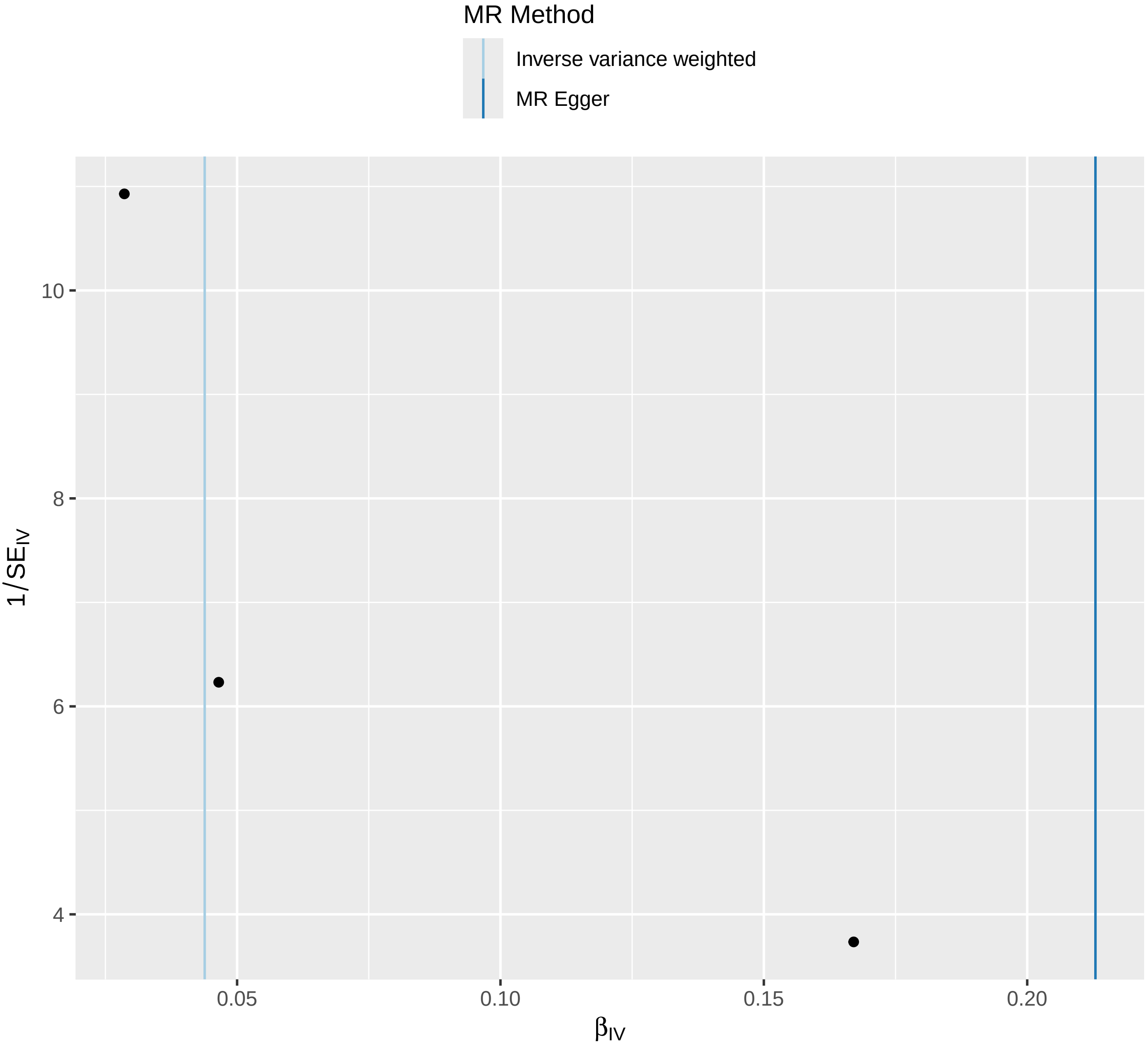
MR Method
Inverse variance weighted
MR Egger
10
8
6
4
0.05
0.10
0.15
0.20
$1/SE_{IV}$
$\beta_{IV}$

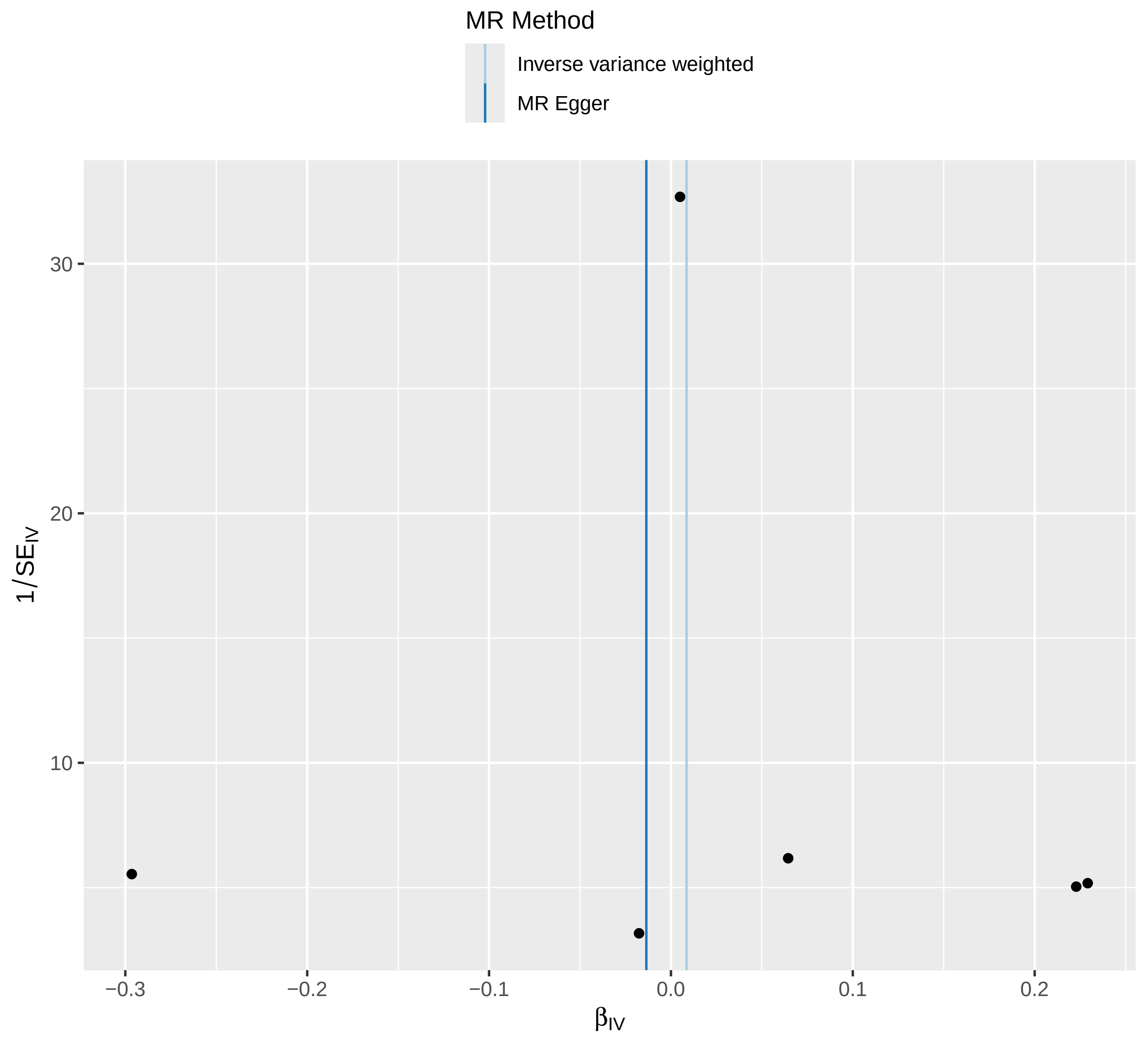
MR Method
Inverse variance weighted
MR Egger
30
20
10
1/SE_IV
−0.3
−0.2
−0.1
0.0
0.1
0.2
β_IV

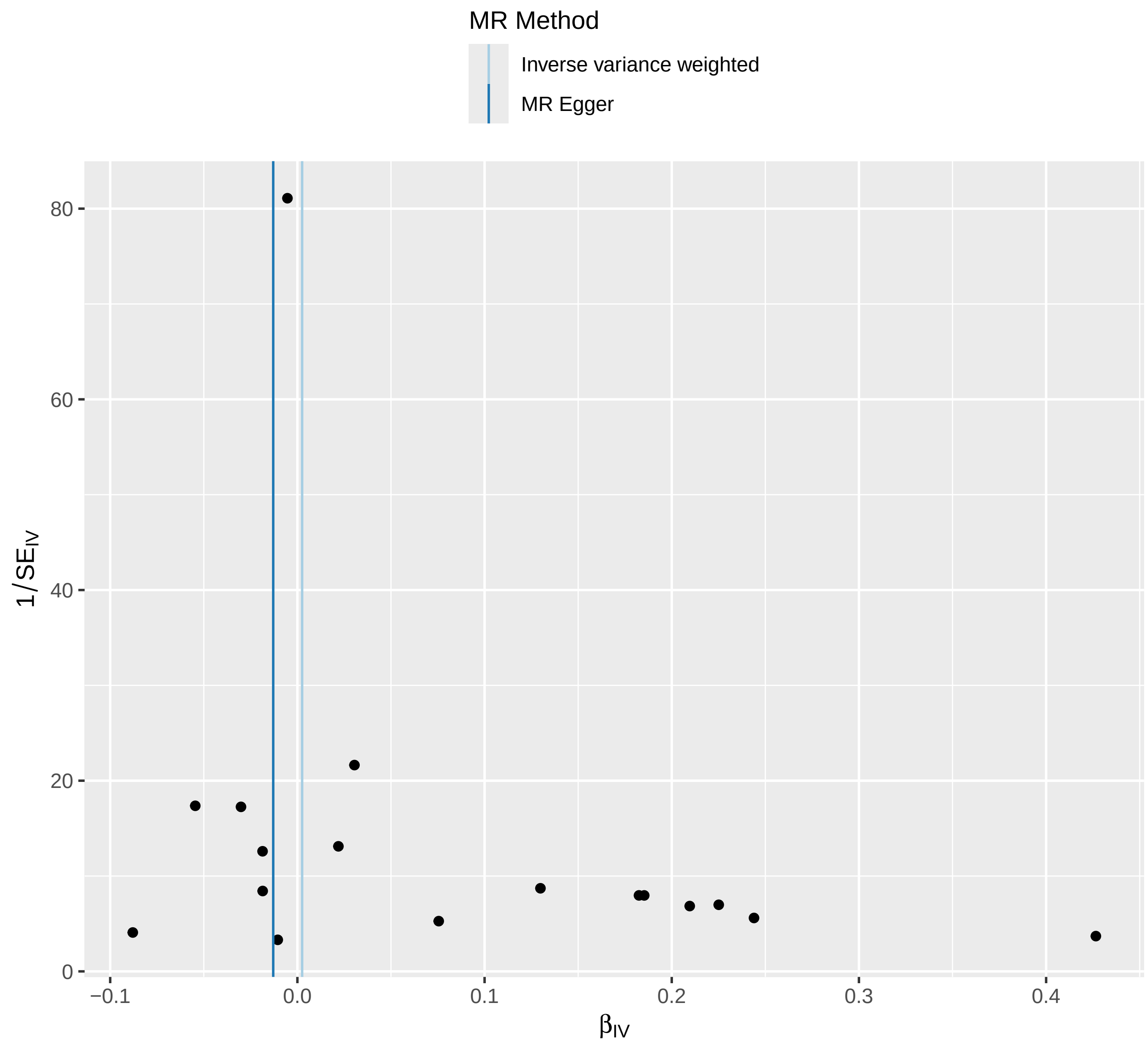
MR Method
Inverse variance weighted
MR Egger
80
60
40
20
0
−0.1
0.0
0.1
0.2
0.3
0.4
$1/SE_{IV}$
$\beta_{IV}$

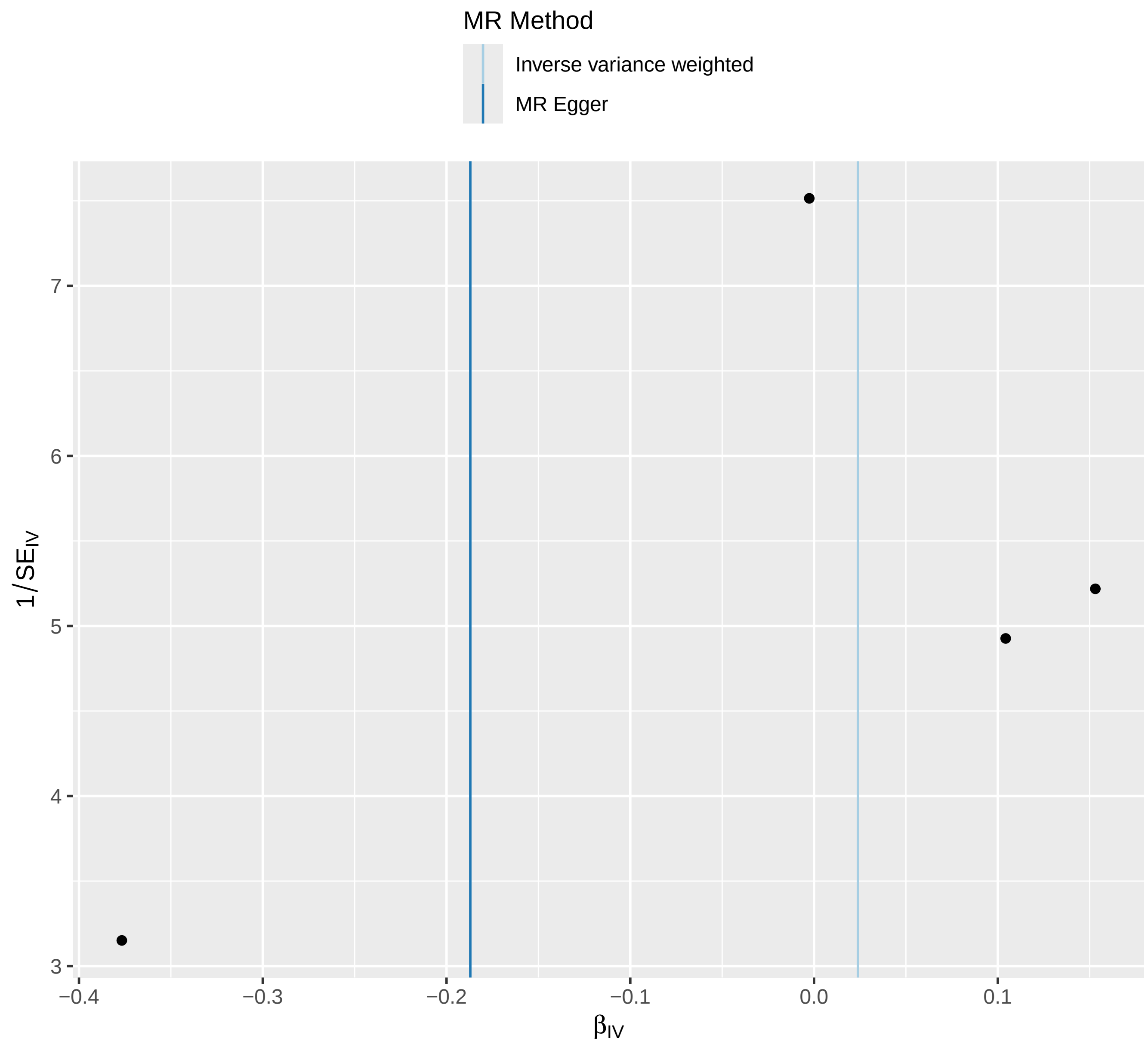
MR Method
Inverse variance weighted
MR Egger
7
6
5
4
3
$1/SE_{IV}$
−0.4
−0.3
−0.2
−0.1
0.0
0.1
$\beta_{IV}$

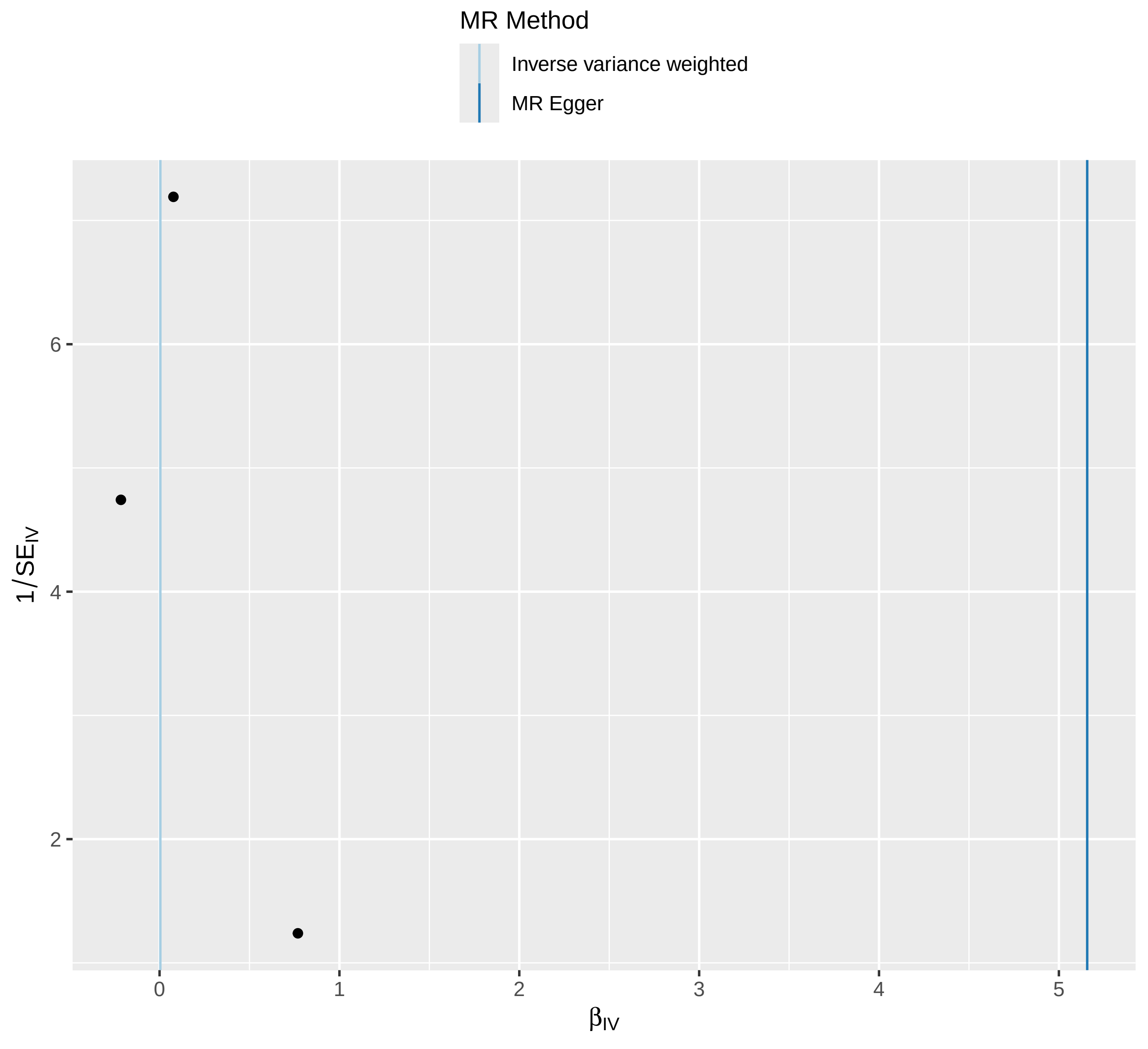

MR Method
Inverse variance weighted
MR Egger
6
4
2
1/SE_IV
0
1
2
3
4
5
β_IV

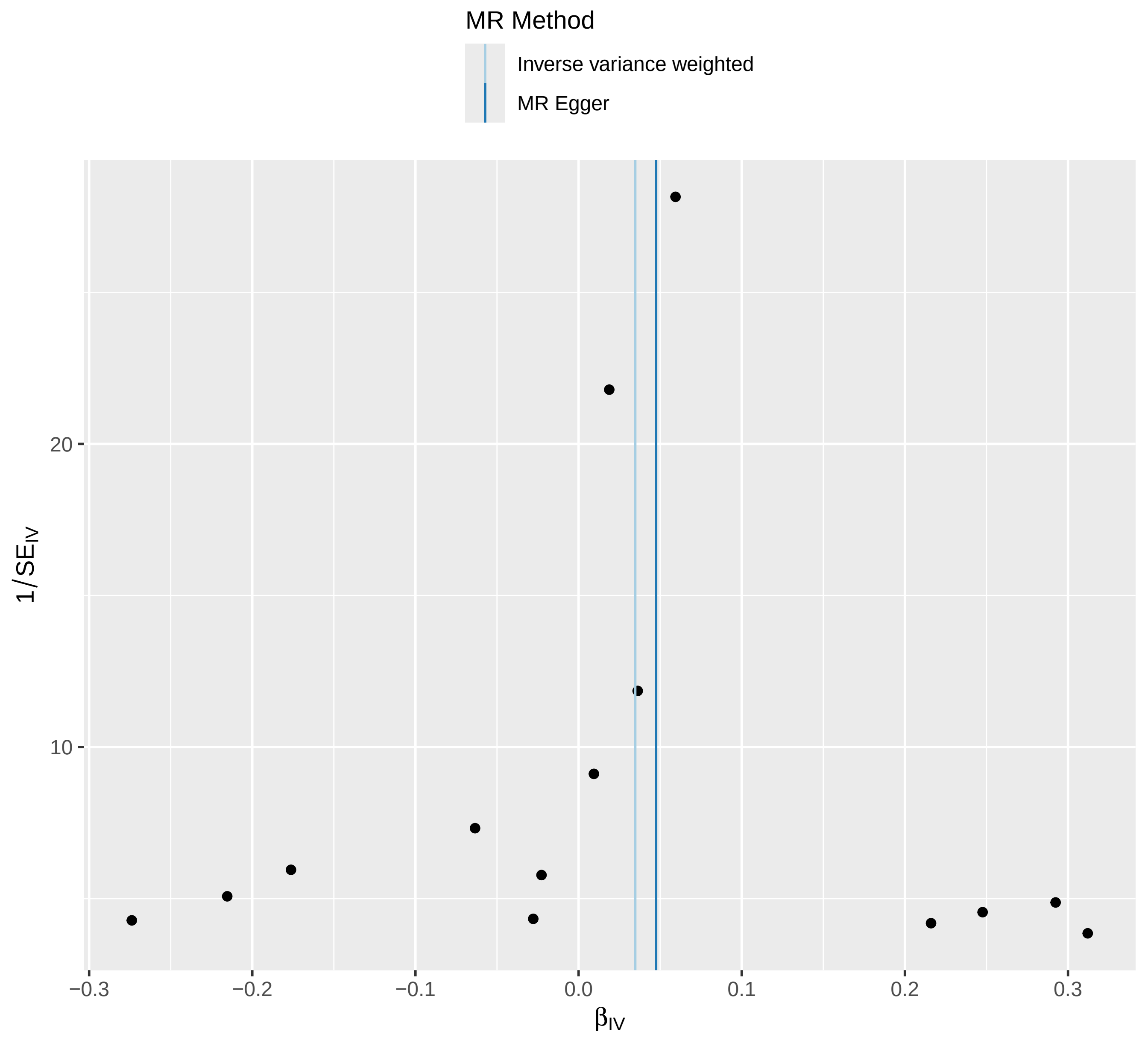
MR Method
Inverse variance weighted
MR Egger
20
10
1/SE_IV
−0.3
−0.2
−0.1
0.0
0.1
0.2
0.3
β_IV

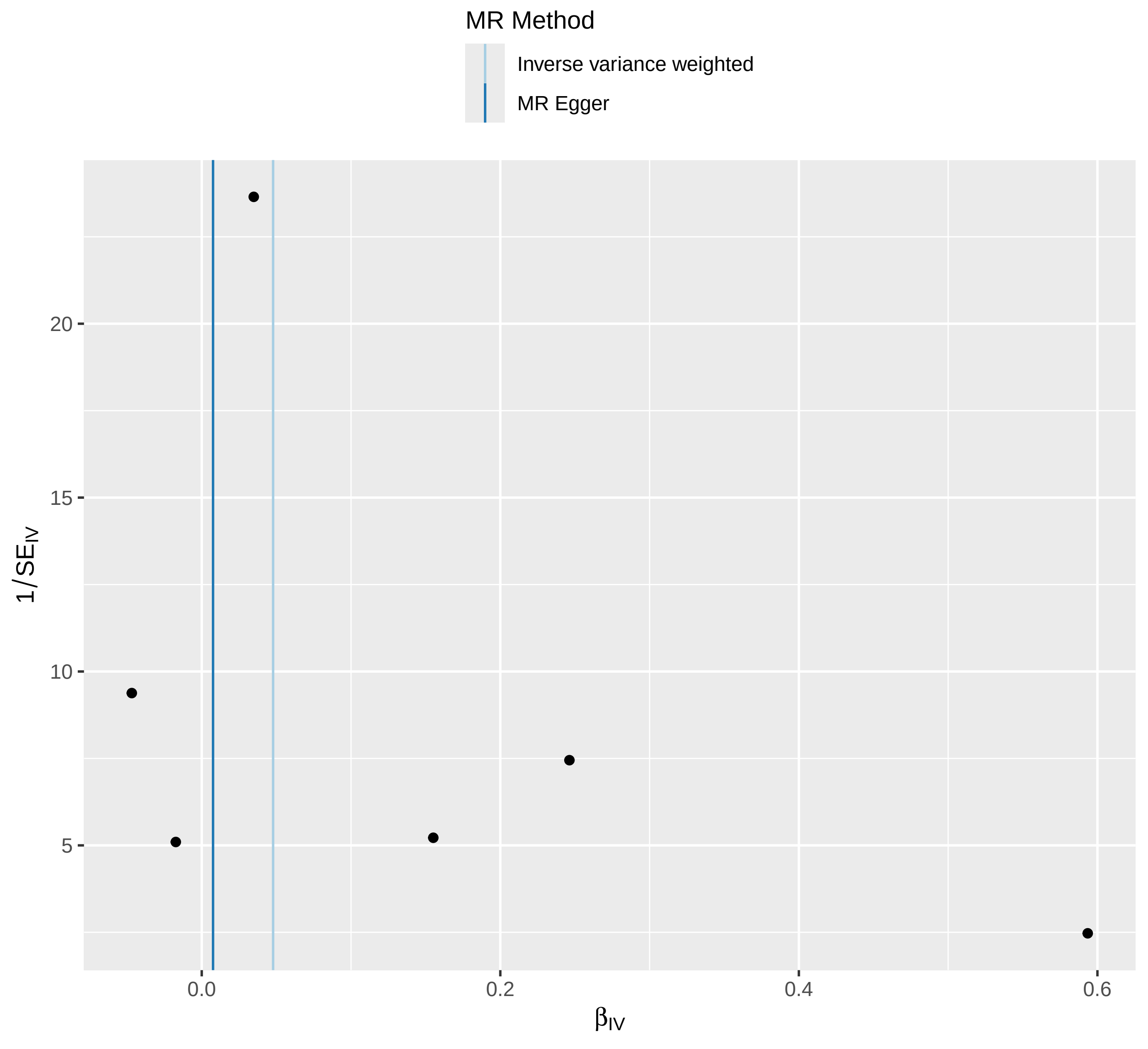

MR Method
Inverse variance weighted
MR Egger
1/SE_IV
β_IV
20
15
10
5
0.0
0.2
0.4
0.6

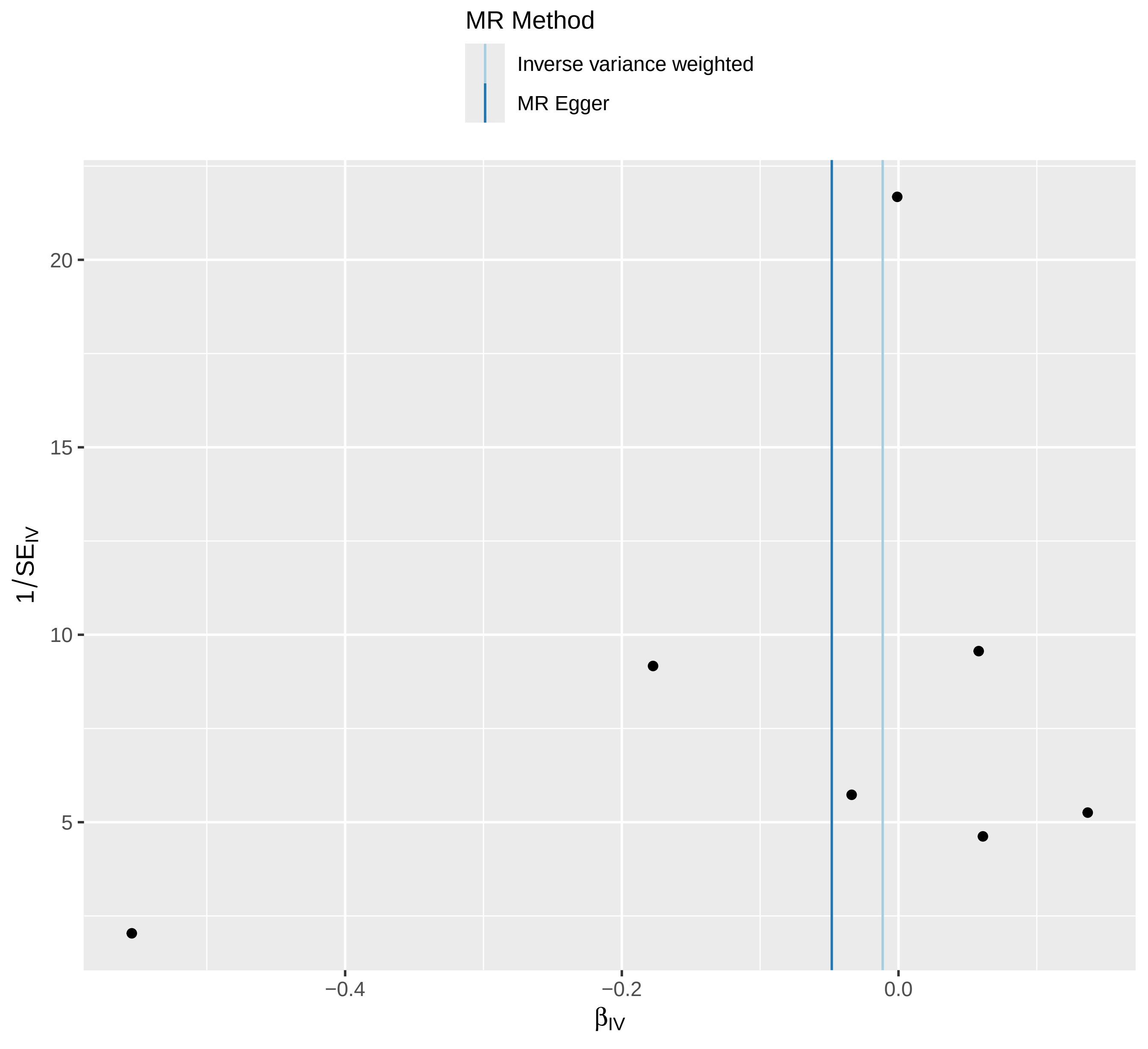
MR Method
Inverse variance weighted
MR Egger
1/SE$_{IV}$
20
15
10
5
−0.4
−0.2
0.0
β$_{IV}$

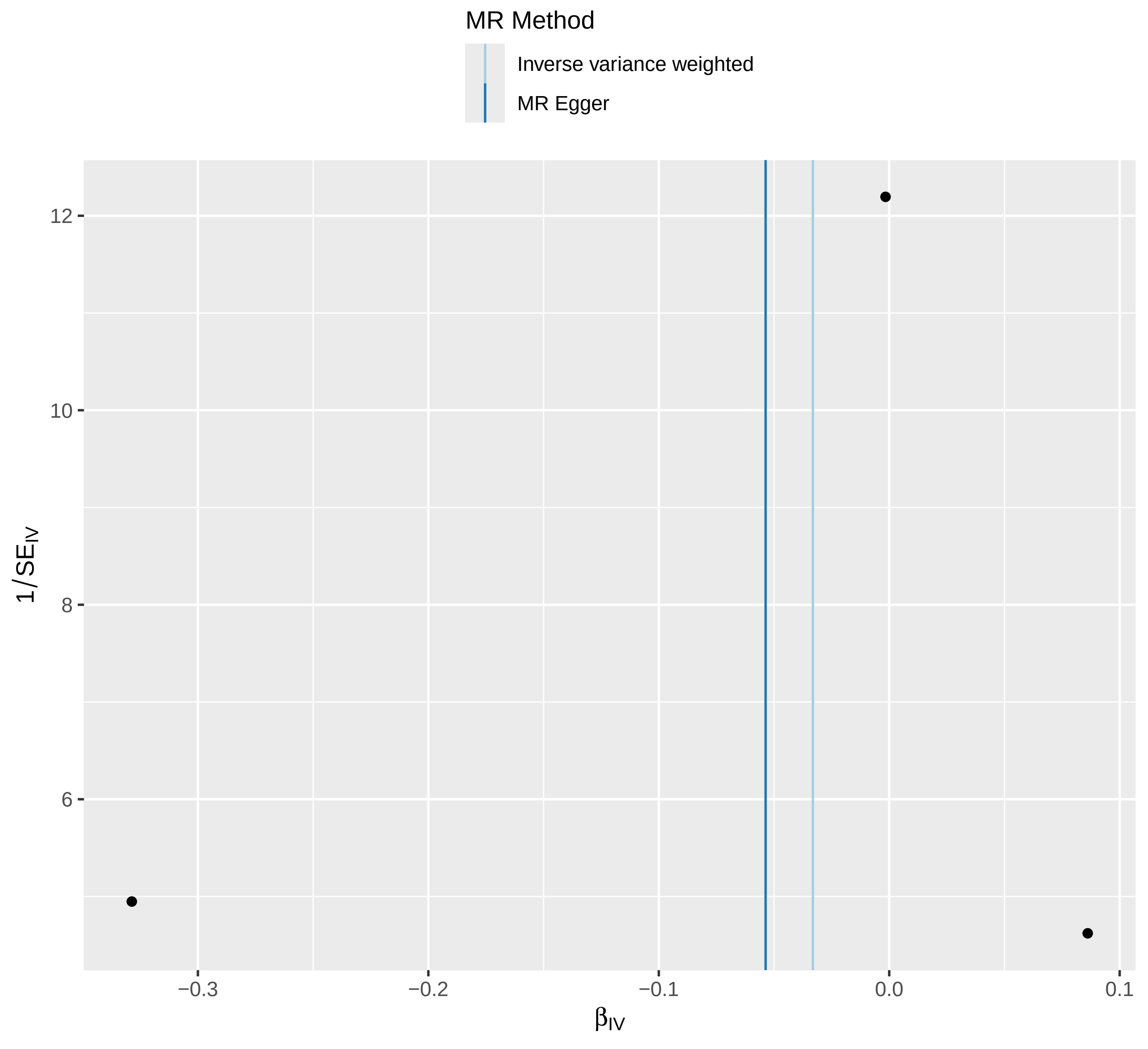
MR Method
Inverse variance weighted
MR Egger
12
10
8
6
1/SE$_{IV}$
−0.3
−0.2
−0.1
0.0
0.1
β$_{IV}$

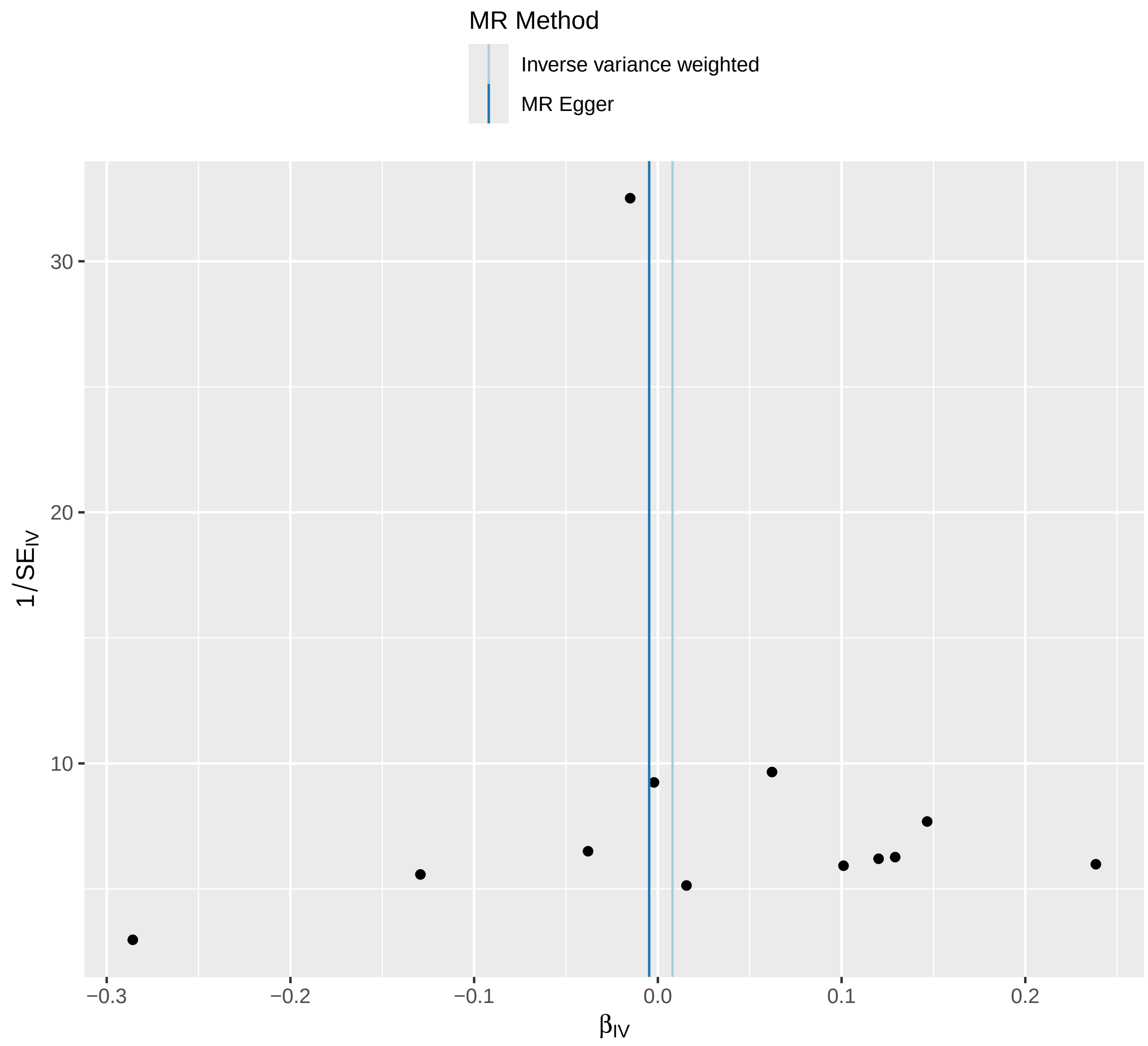

MR Method
Inverse variance weighted
MR Egger
30
20
10
1/SE$_{IV}$
−0.3
−0.2
−0.1
0.0
0.1
0.2
β$_{IV}$

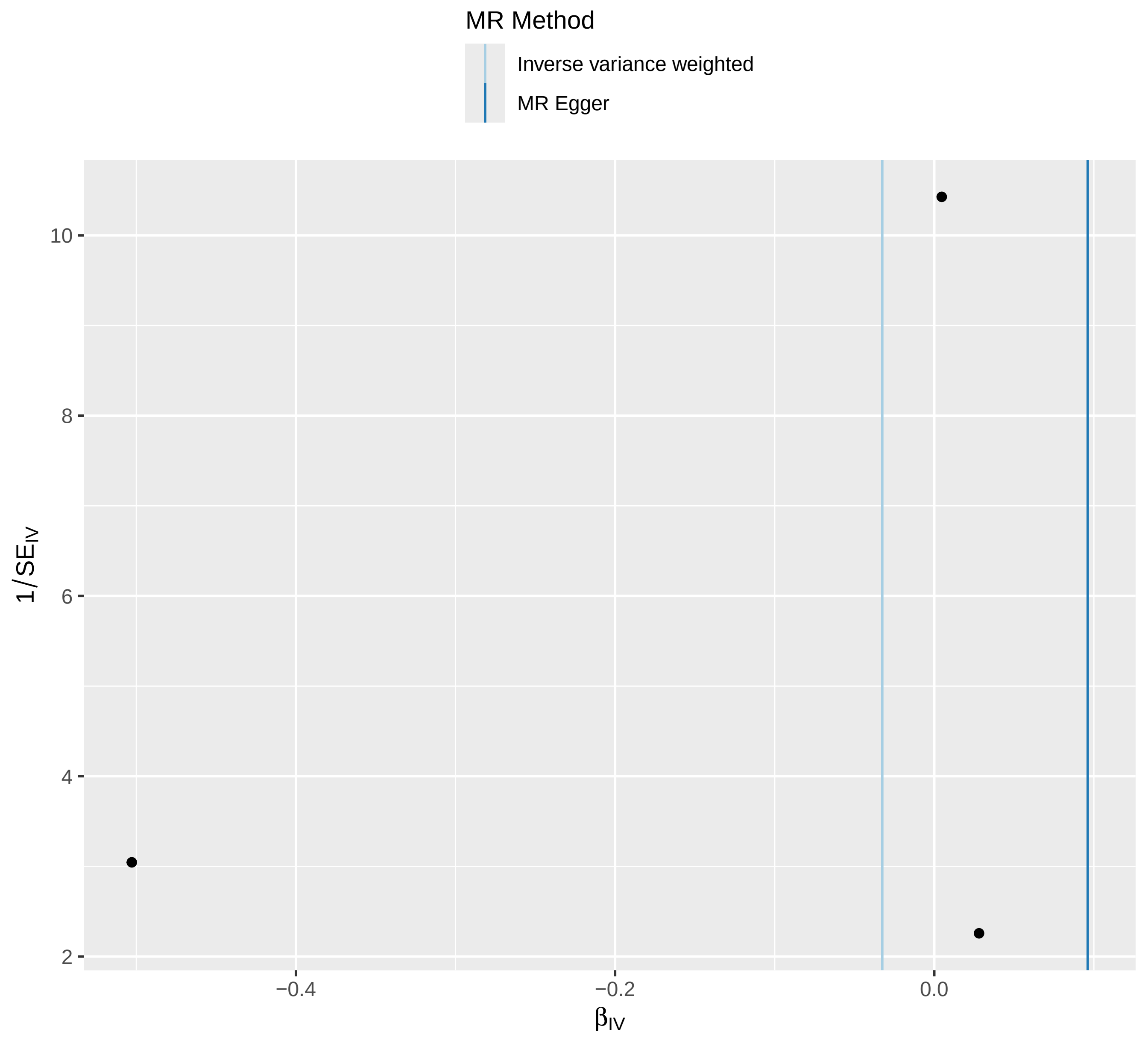
MR Method
Inverse variance weighted
MR Egger
10
8
6
4
2
1/SE_IV
−0.4
−0.2
0.0
β_IV

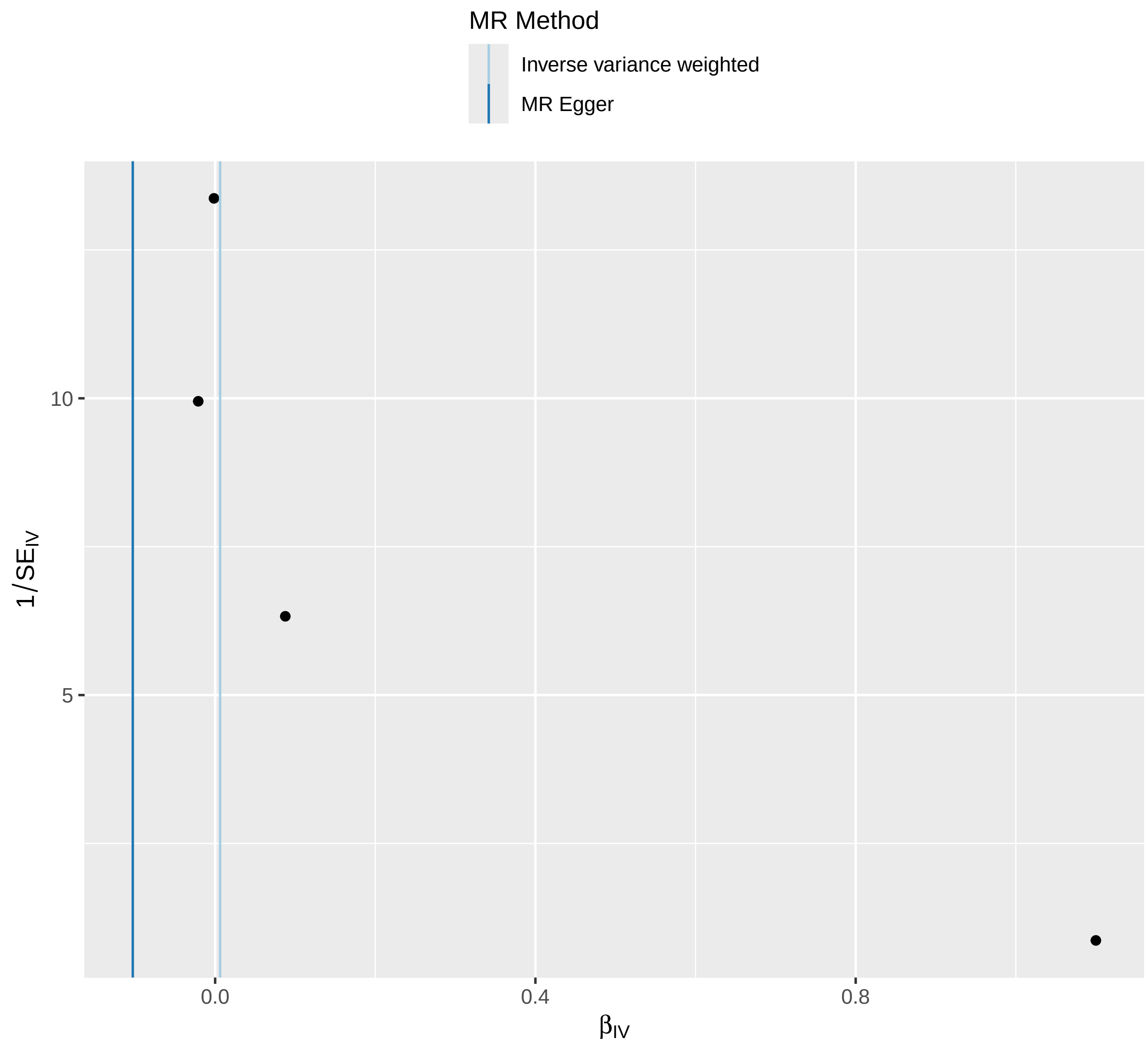
MR Method
Inverse variance weighted
MR Egger
10
5
1/SE$_{IV}$
0.0
0.4
0.8
β$_{IV}$

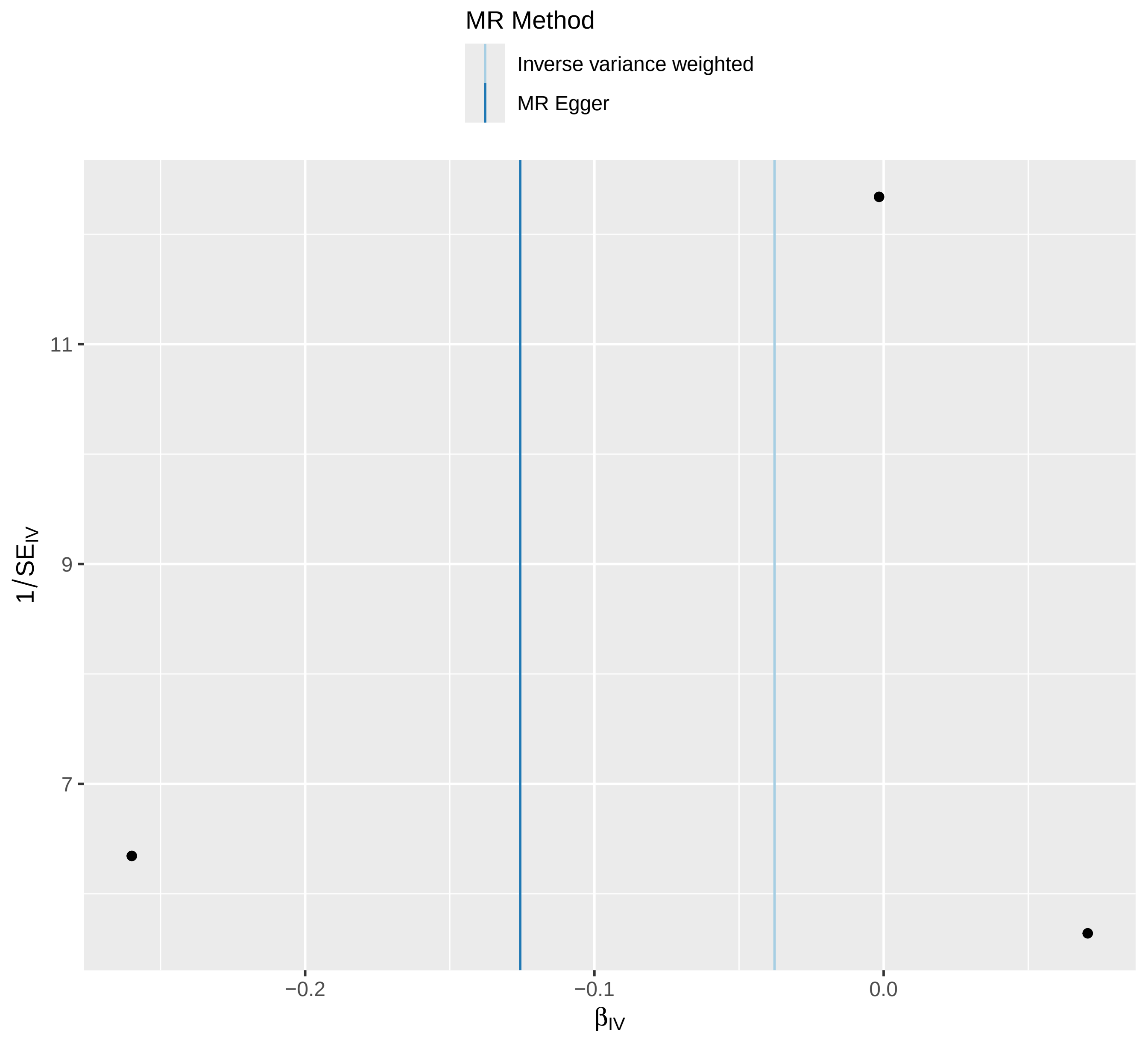

MR Method
Inverse variance weighted
MR Egger
11
9
7
1/SE$_{IV}$
−0.2
−0.1
0.0
β$_{IV}$

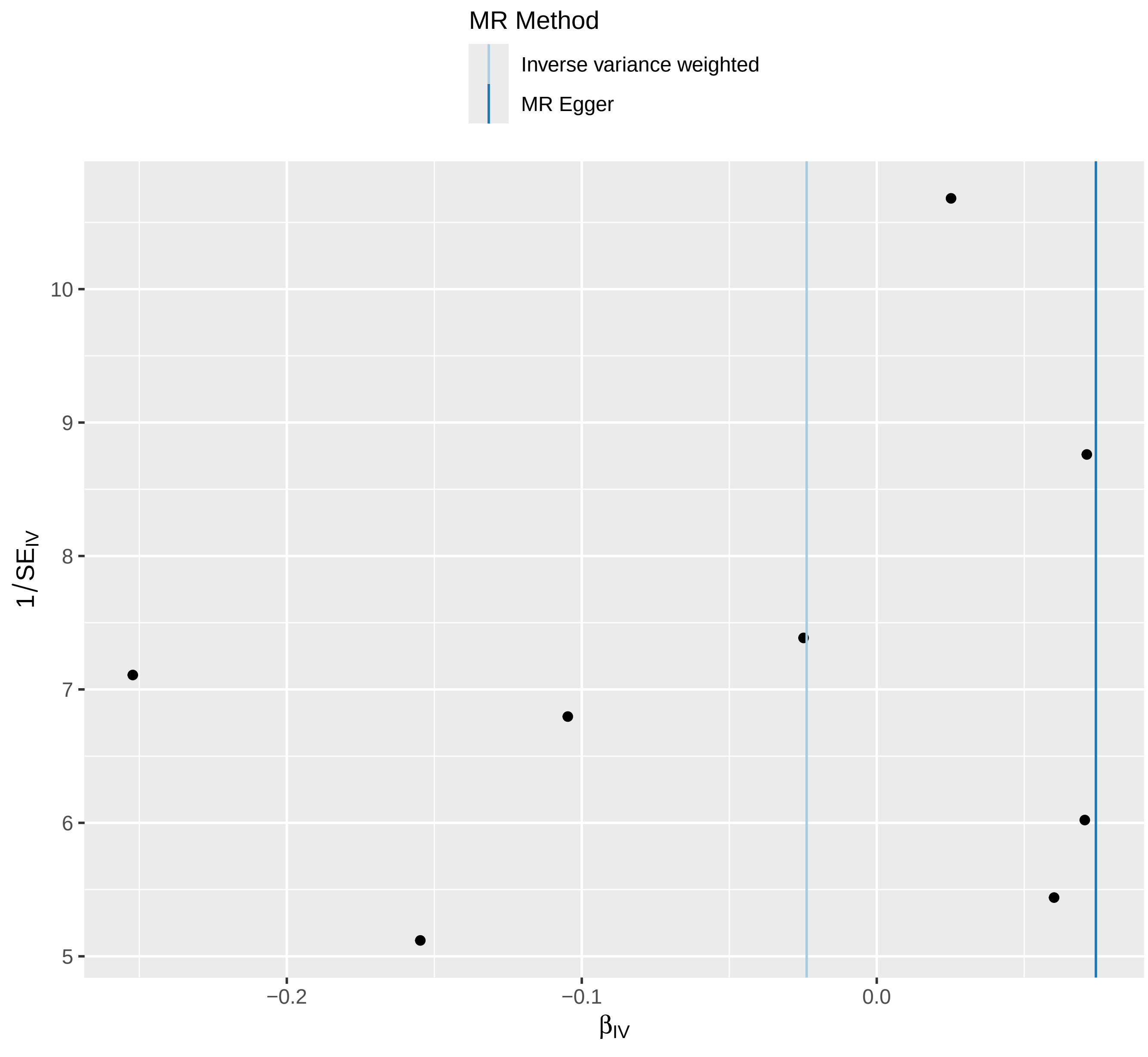

MR Method
Inverse variance weighted
MR Egger
10
9
8
7
6
5
1/SE_IV
−0.2
−0.1
0.0
β_IV

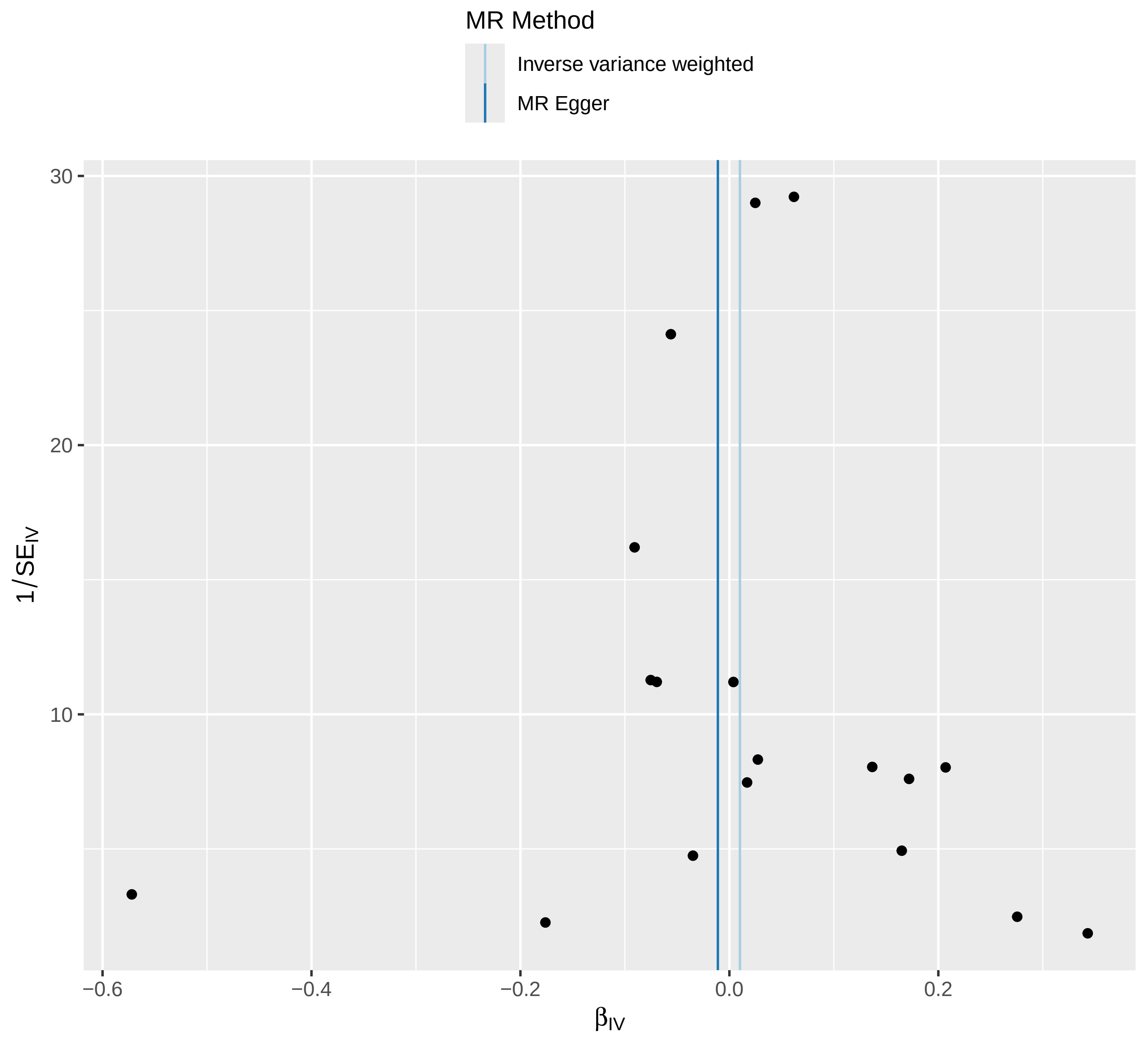
MR Method
Inverse variance weighted
MR Egger
30
20
10
1/SE_IV
−0.6
−0.4
−0.2
0.0
0.2
β_IV

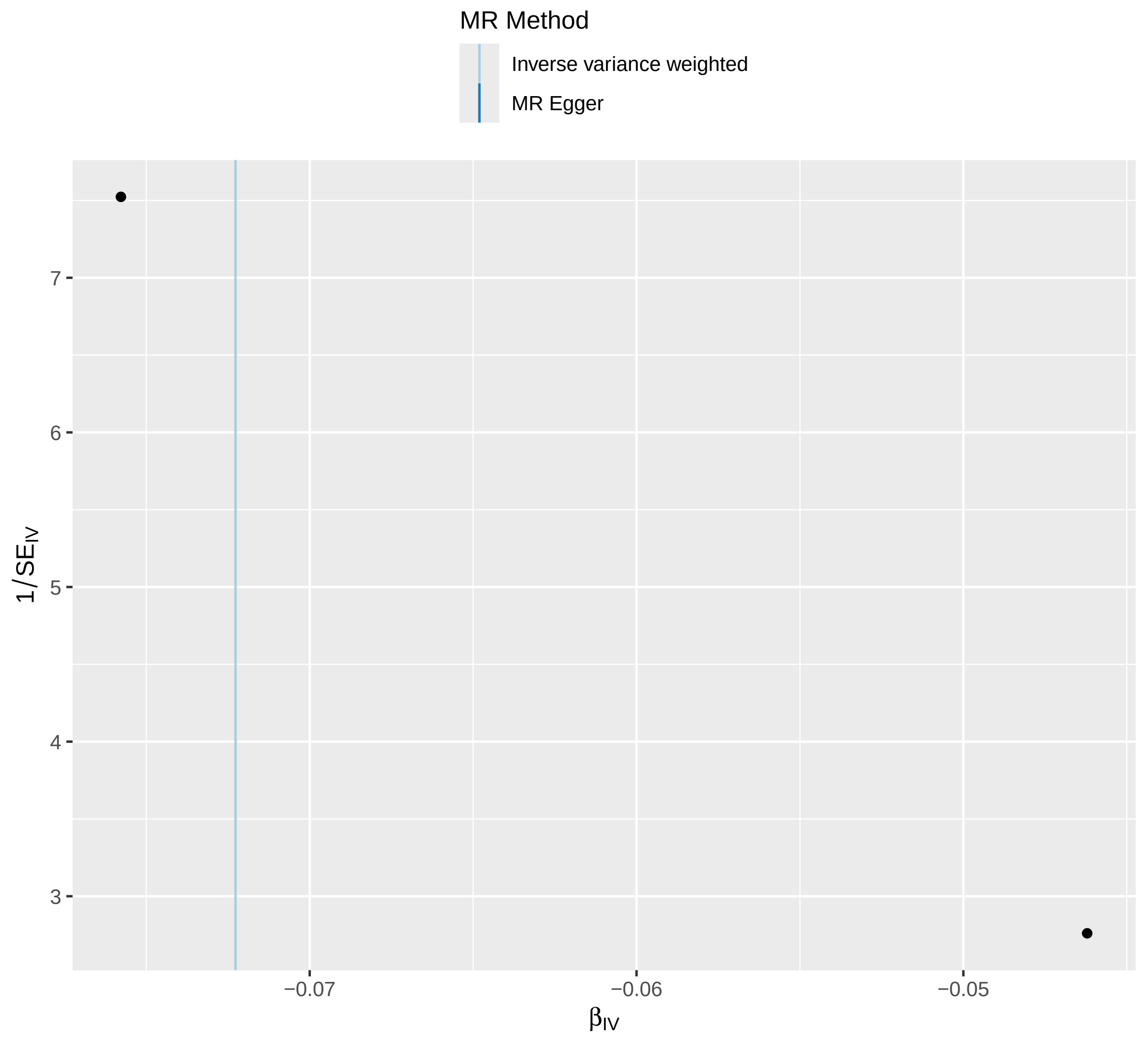
MR Method
Inverse variance weighted
MR Egger
7
6
5
4
3
1/SE$_{IV}$
−0.07
−0.06
−0.05
β$_{IV}$

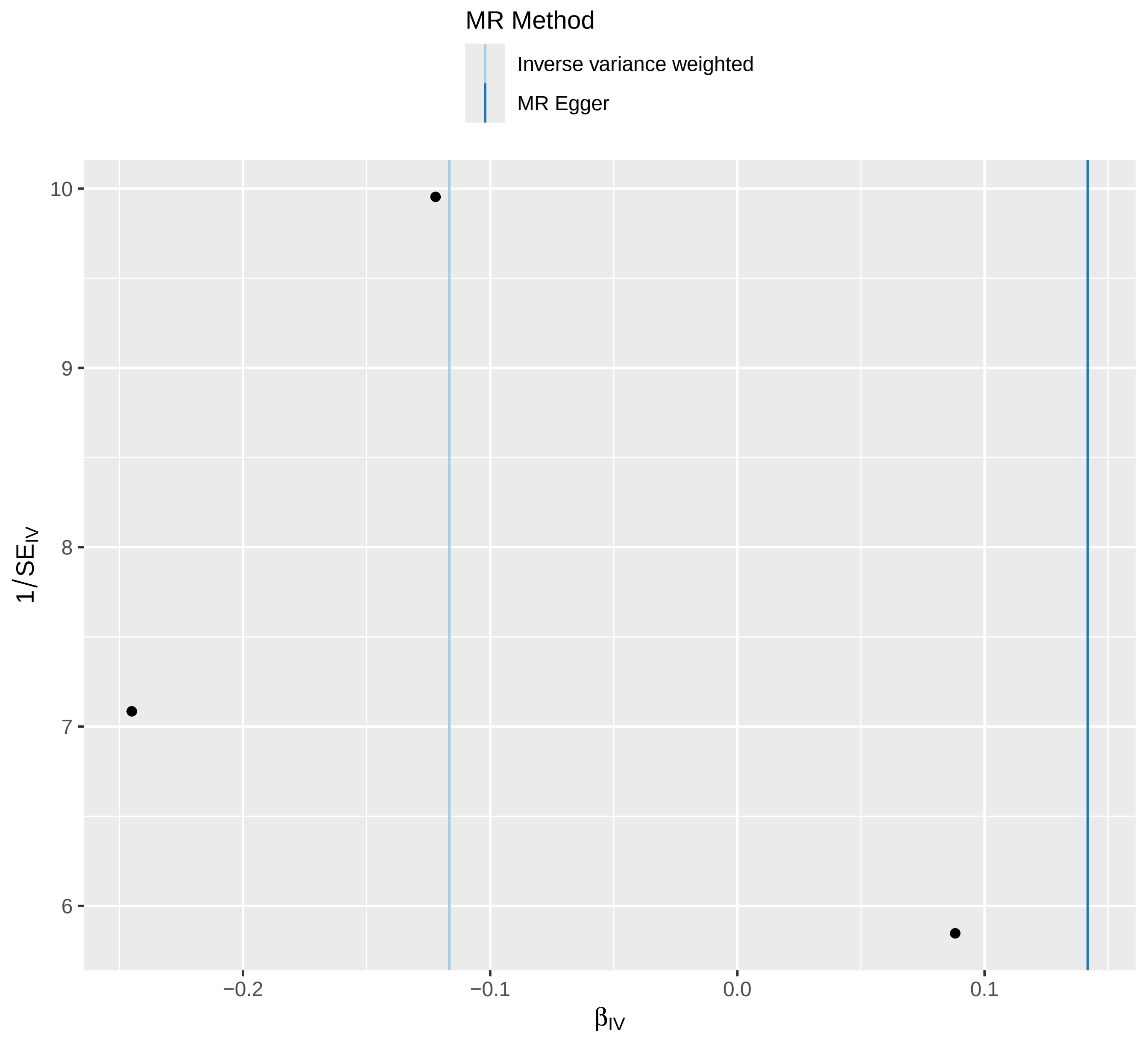
MR Method
Inverse variance weighted
MR Egger
10
9
8
7
6
1/SE_IV
−0.2
−0.1
0.0
0.1
β_IV

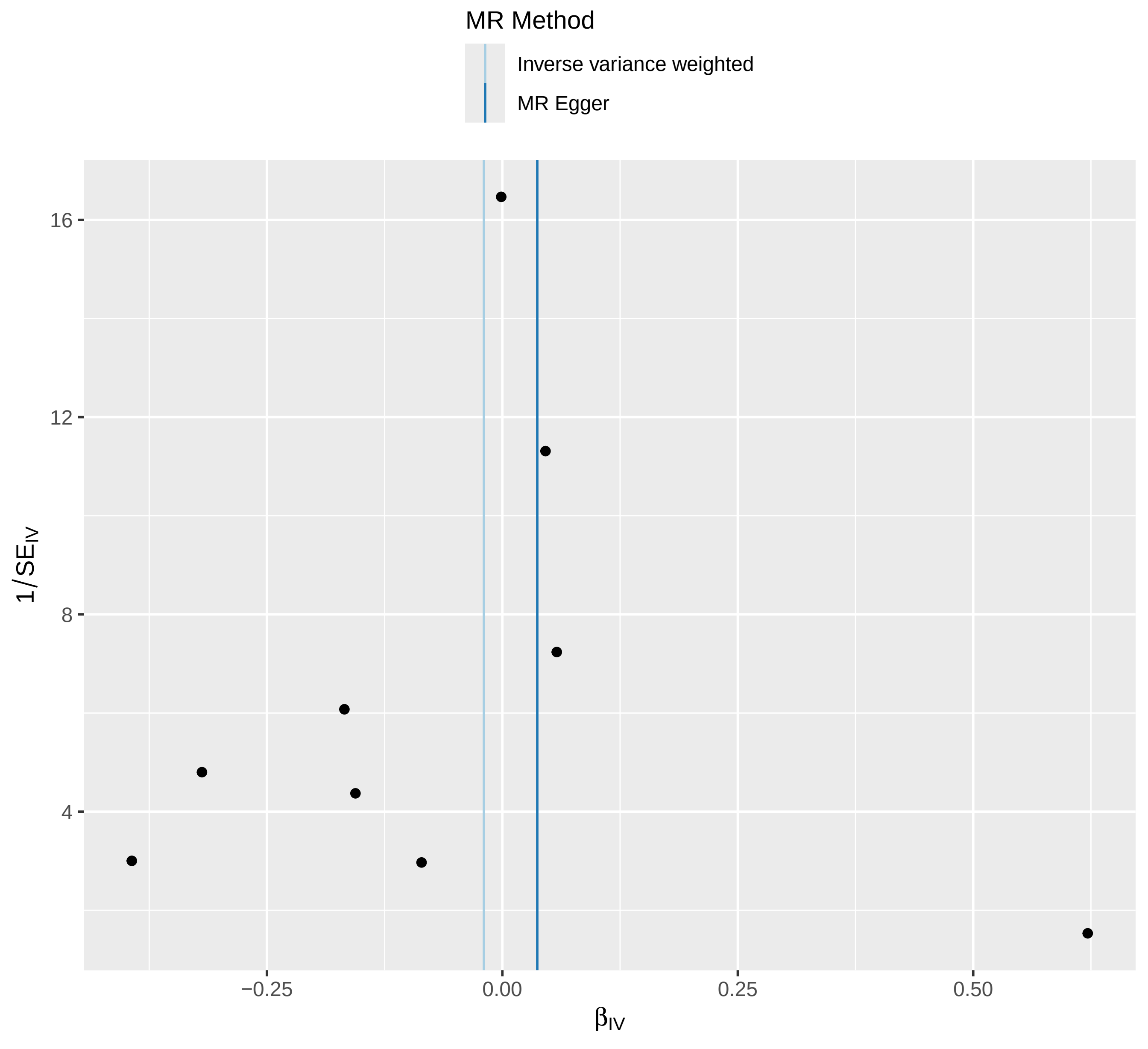

MR Method
Inverse variance weighted
MR Egger
16
12
8
4
$1/SE_{IV}$
−0.25
0.00
0.25
0.50
$\beta_{IV}$

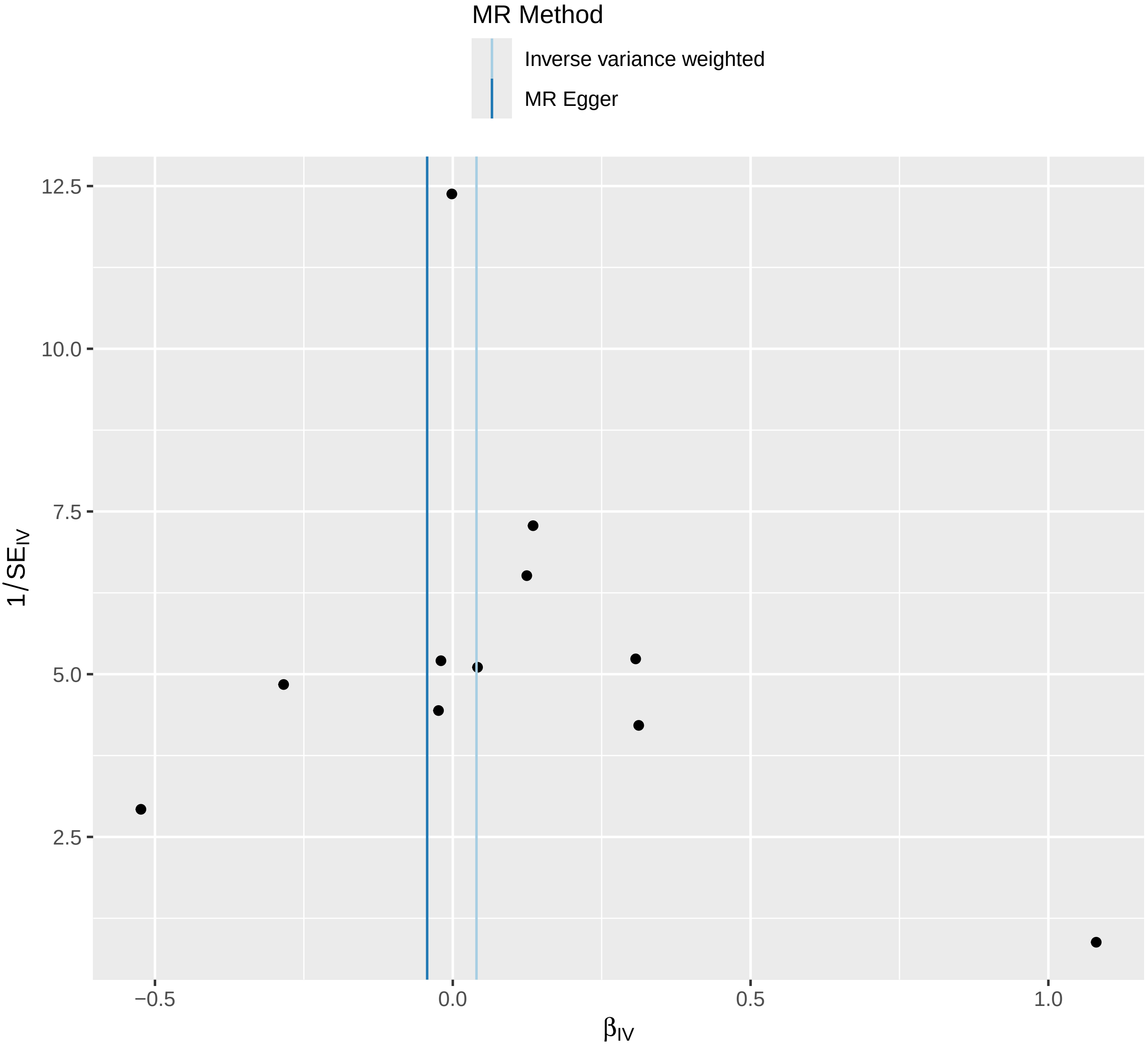
MR Method
Inverse variance weighted
MR Egger
12.5
10.0
7.5
5.0
2.5
−0.5
0.0
0.5
1.0
$1/SE_{IV}$
$\beta_{IV}$

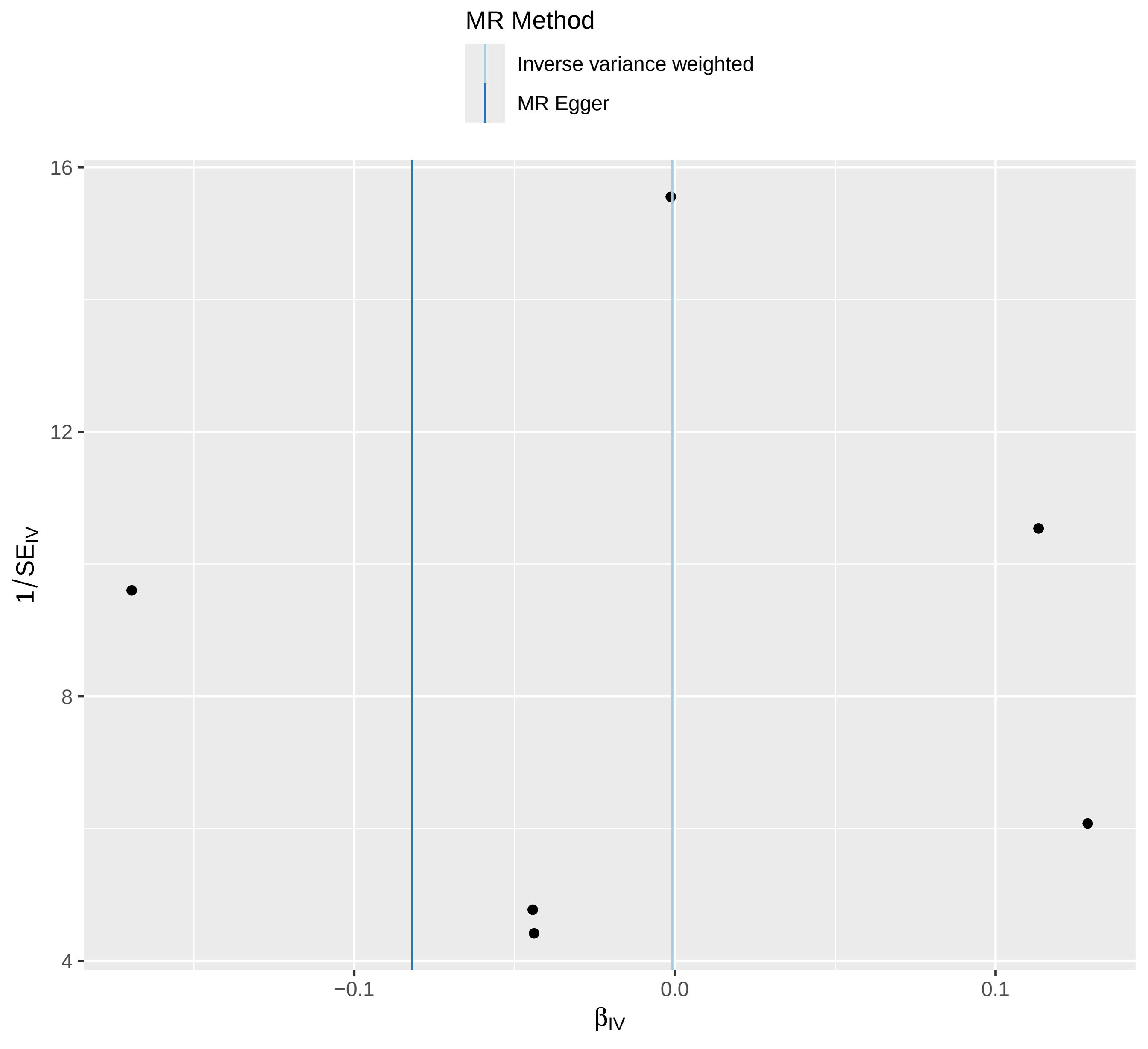

MR Method
Inverse variance weighted
MR Egger
16
12
8
4
1/SE_IV
−0.1
0.0
0.1
β_IV

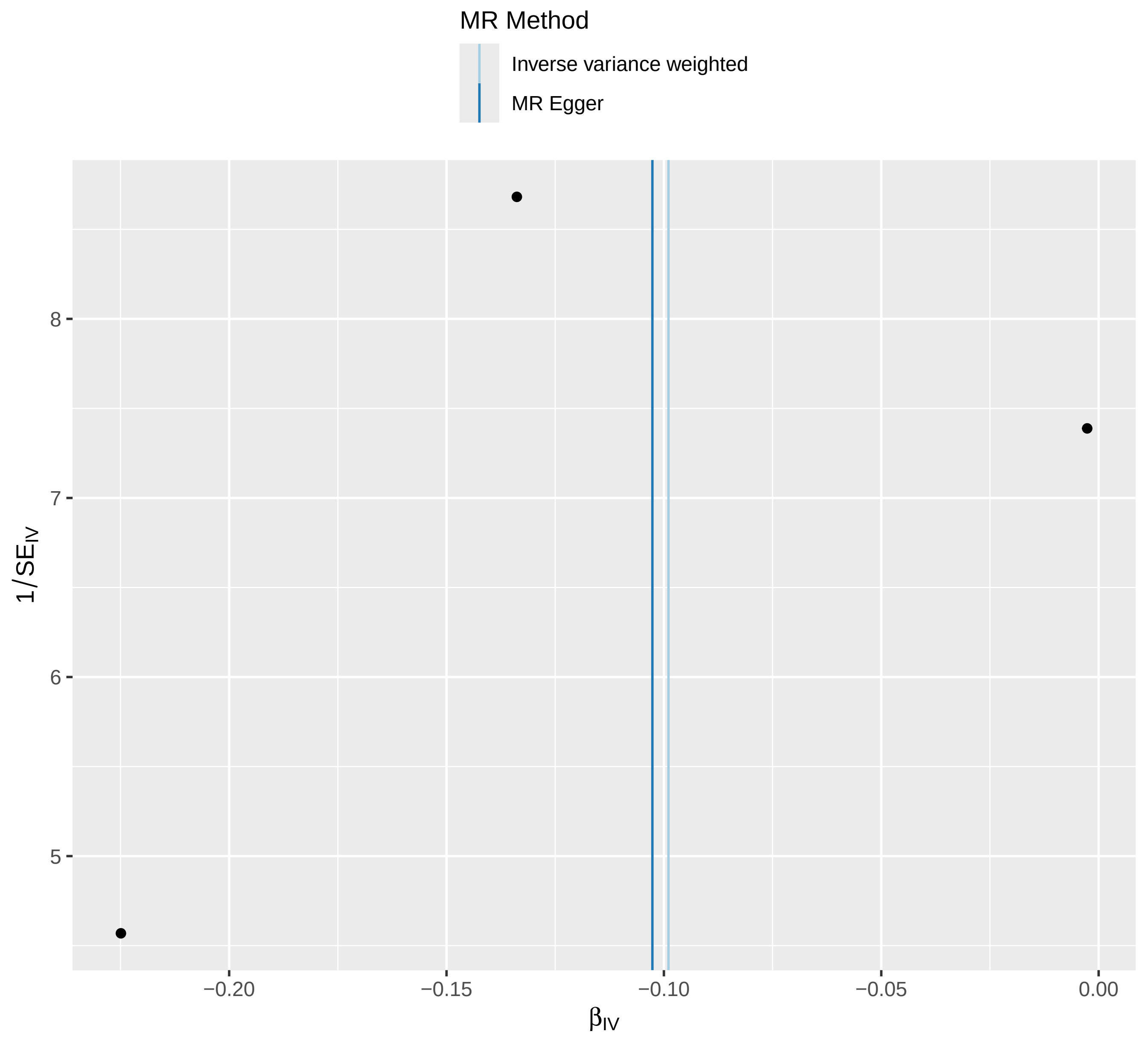
MR Method
Inverse variance weighted
MR Egger
8
7
6
5
1/SE_IV
−0.20
−0.15
−0.10
−0.05
0.00
β_IV

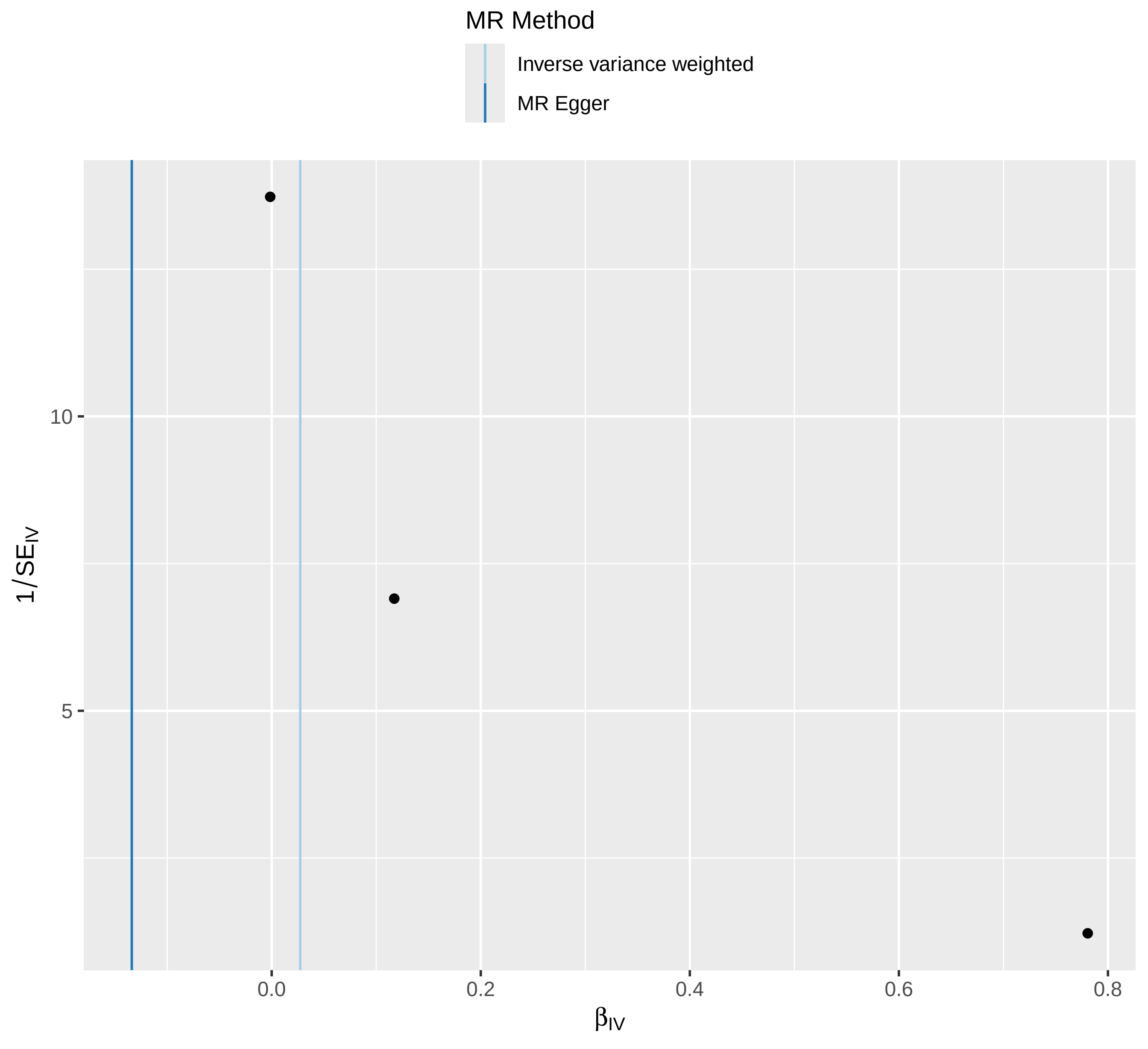
MR Method
Inverse variance weighted
MR Egger
1/SE_IV
10
5
0.0
0.2
0.4
0.6
0.8
β_IV

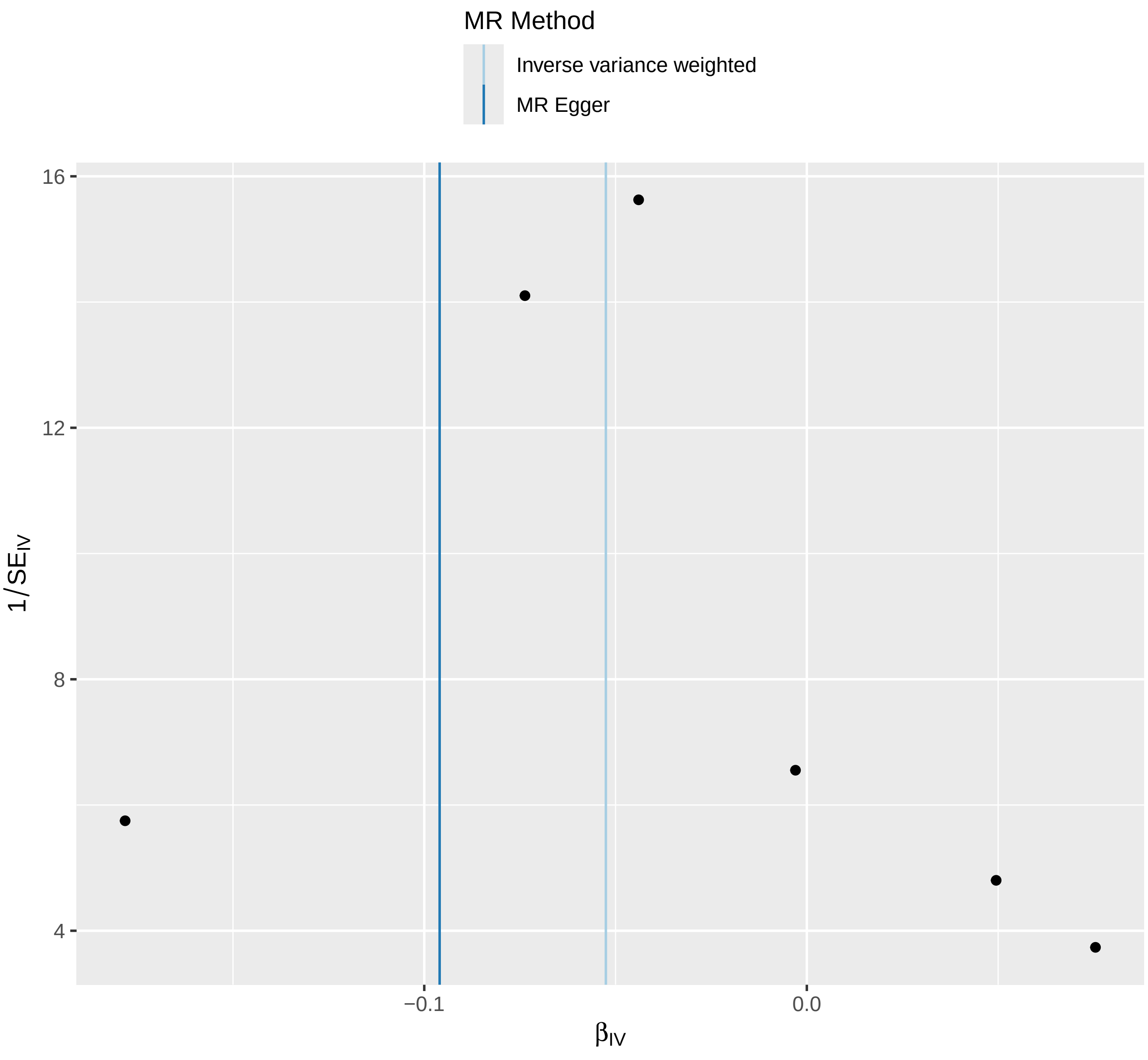
MR Method
Inverse variance weighted
MR Egger
16
12
8
4
1/SE$_{IV}$
−0.1
0.0
β$_{IV}$

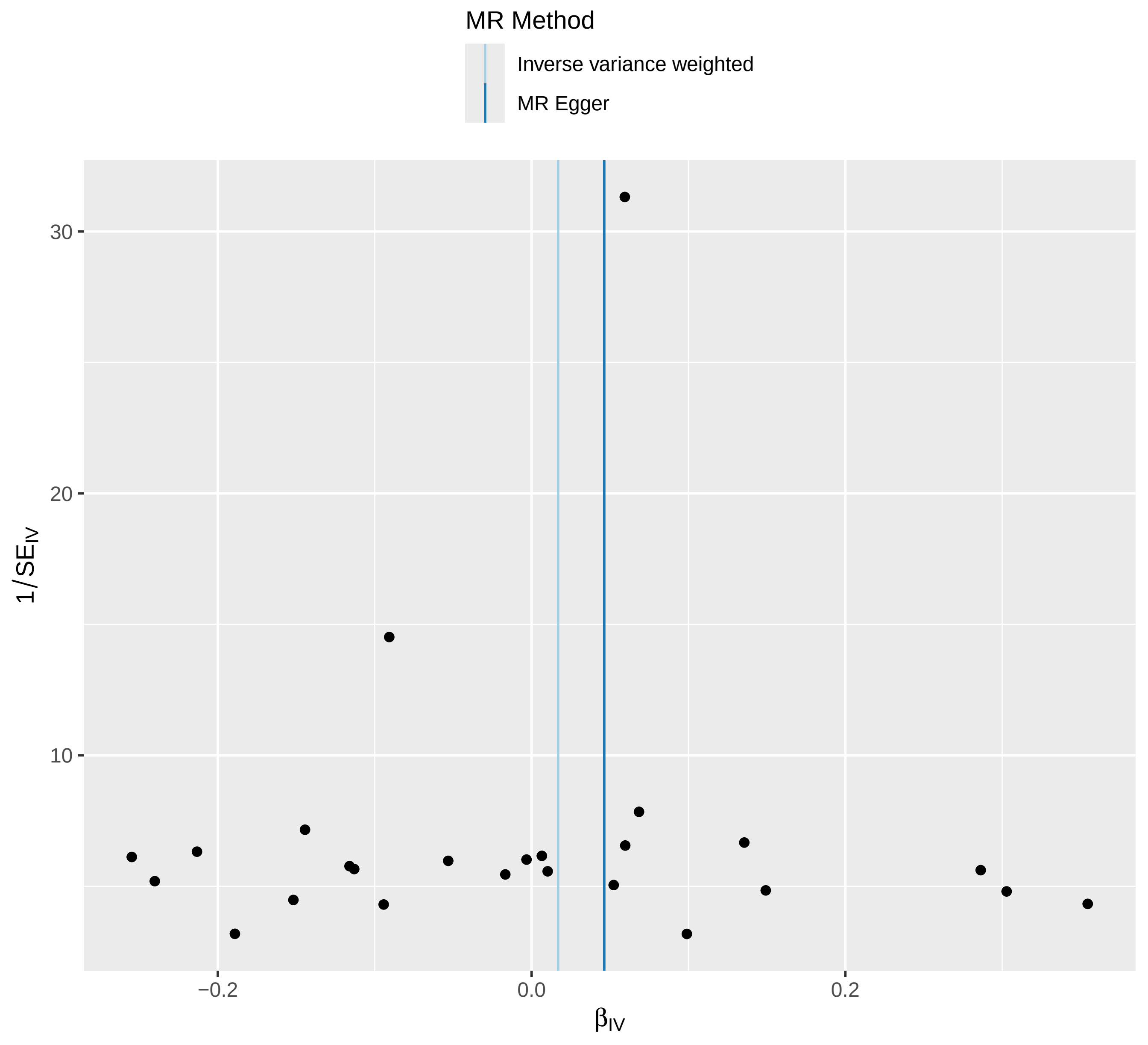
MR Method
Inverse variance weighted
MR Egger
30
20
10
1/SE$_{IV}$
−0.2
0.0
0.2
β$_{IV}$

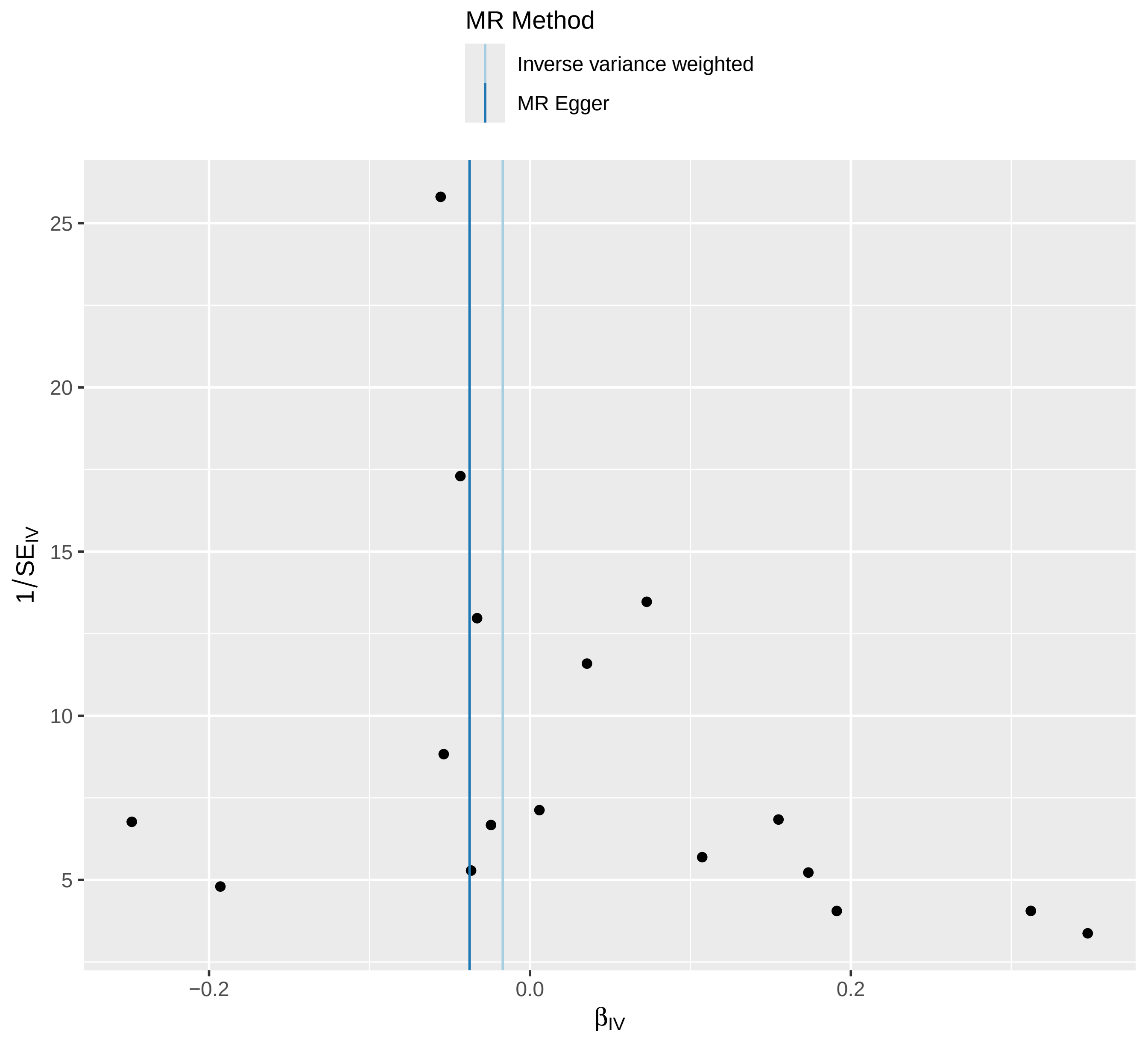
MR Method
Inverse variance weighted
MR Egger
25
20
15
10
5
$1/SE_{IV}$
−0.2
0.0
0.2
$\beta_{IV}$

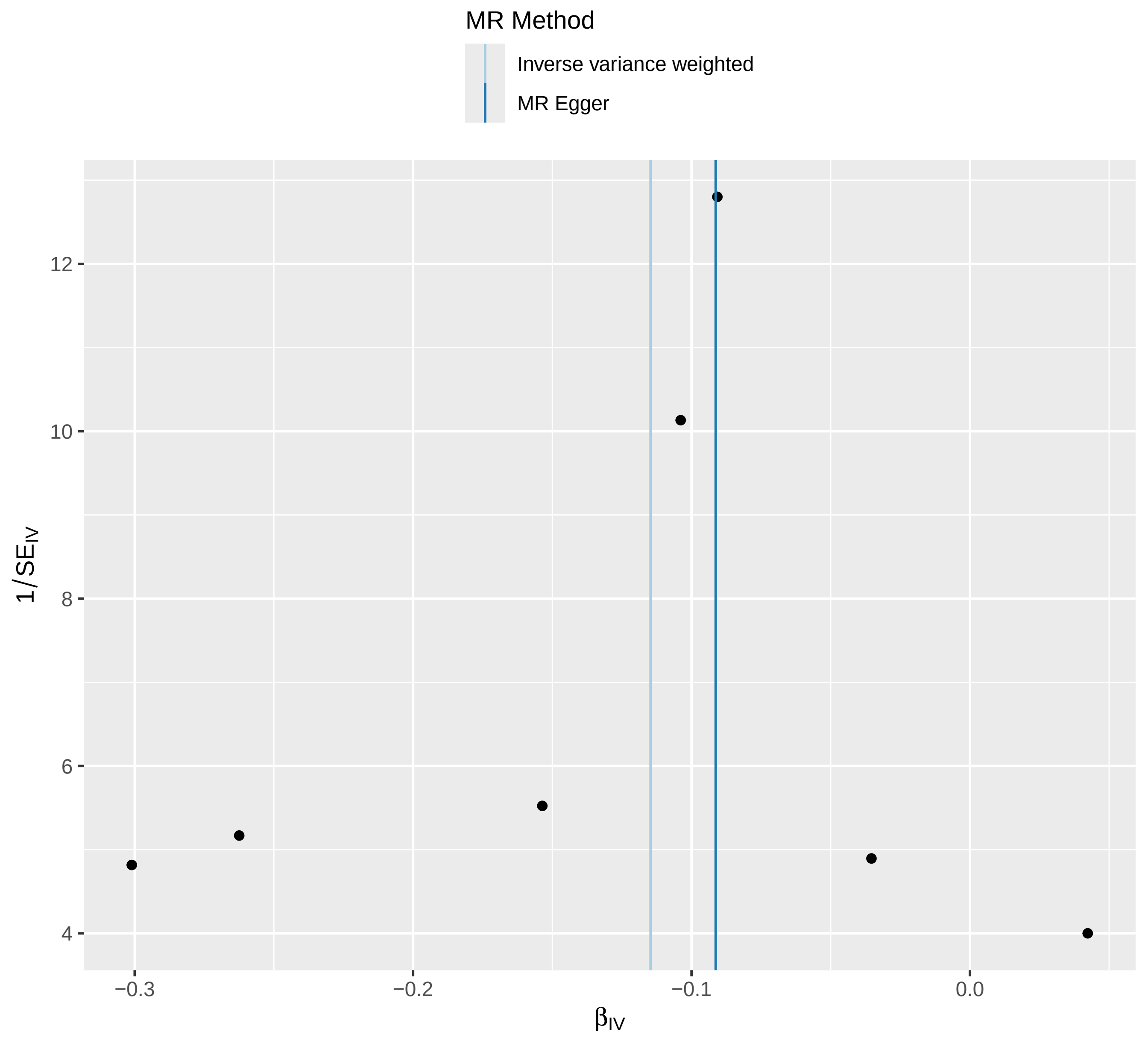

MR Method
Inverse variance weighted
MR Egger
1/SE$_{IV}$
4
6
8
10
12
−0.3
−0.2
−0.1
0.0
β$_{IV}$

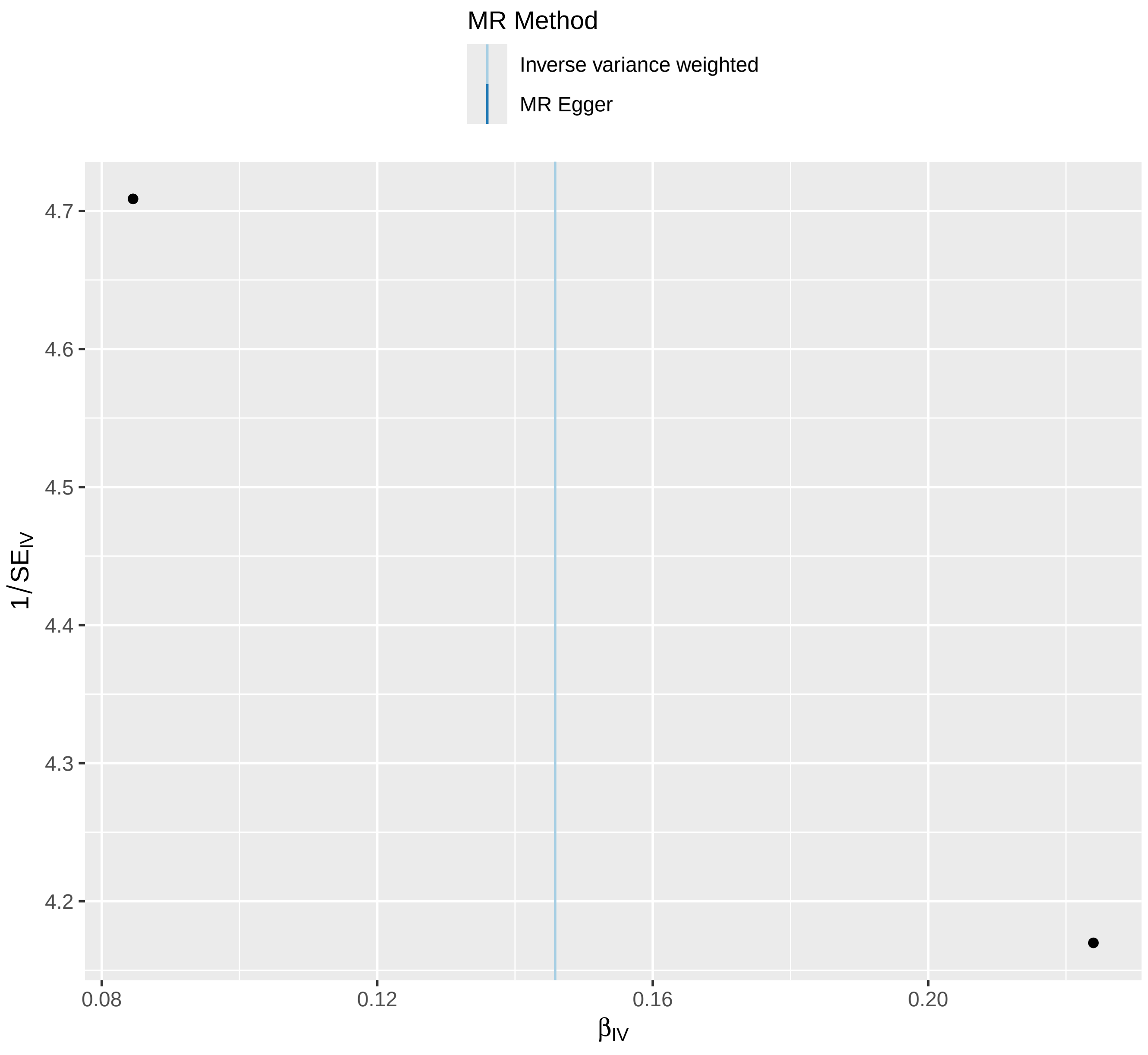
MR Method
Inverse variance weighted
MR Egger
4.7
4.6
4.5
4.4
4.3
4.2
$1/SE_{IV}$
0.08
0.12
0.16
0.20
$\beta_{IV}$

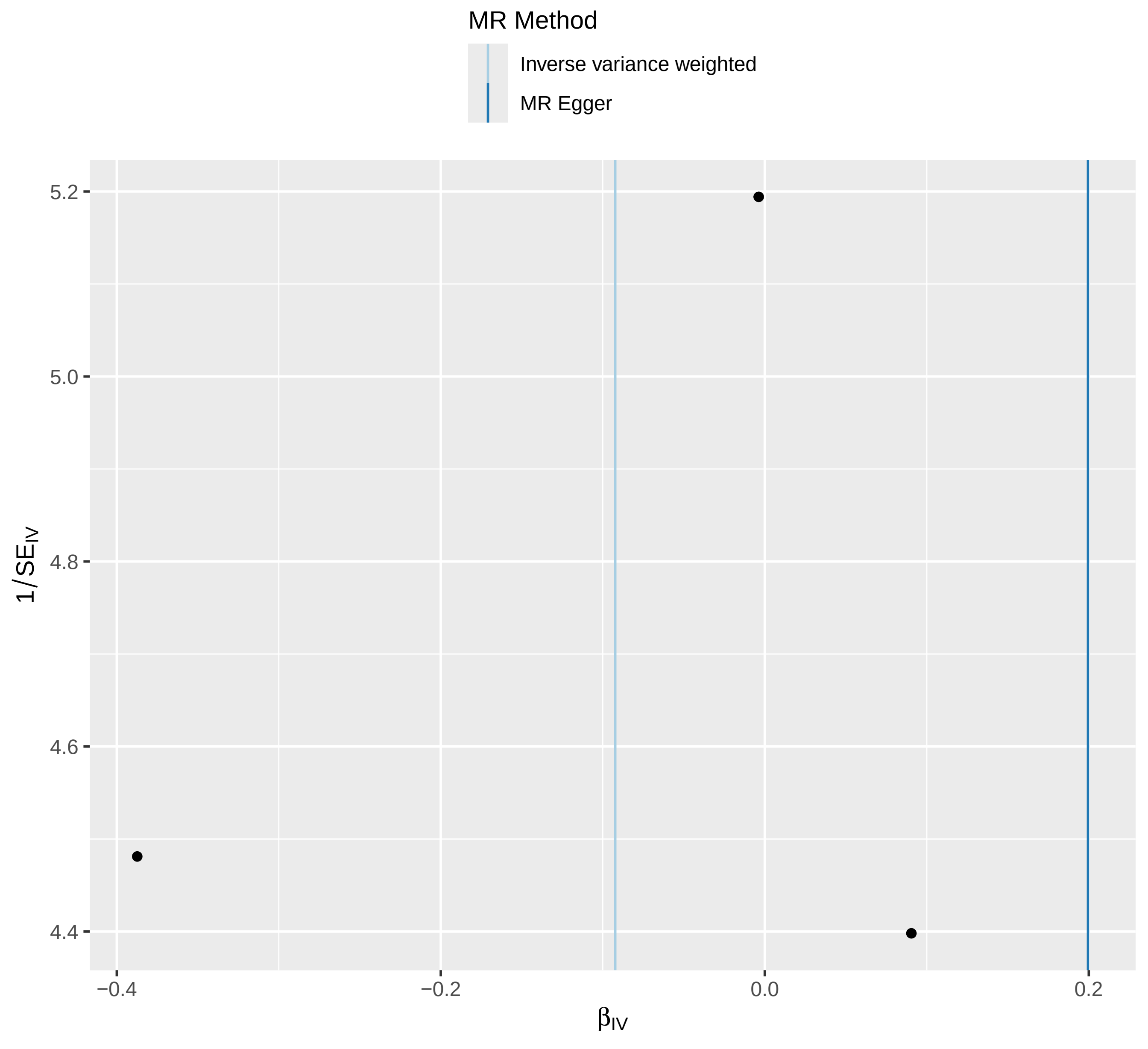

MR Method
Inverse variance weighted
MR Egger
5.2
5.0
4.8
4.6
4.4
$1/SE_{IV}$
−0.4
−0.2
0.0
0.2
$\beta_{IV}$

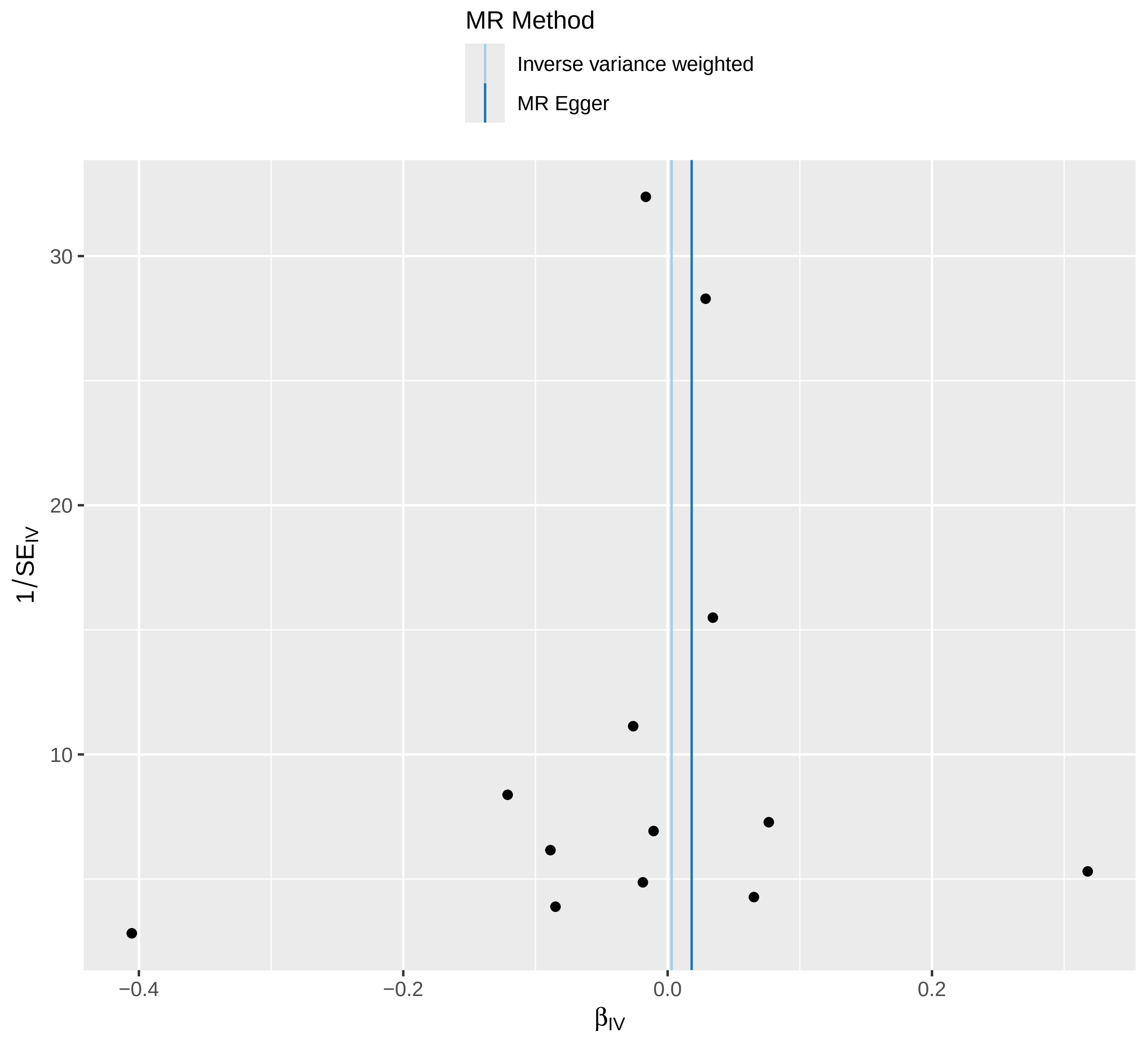

MR Method
Inverse variance weighted
MR Egger
30
20
10
1/SE_IV
−0.4
−0.2
0.0
0.2
β_IV

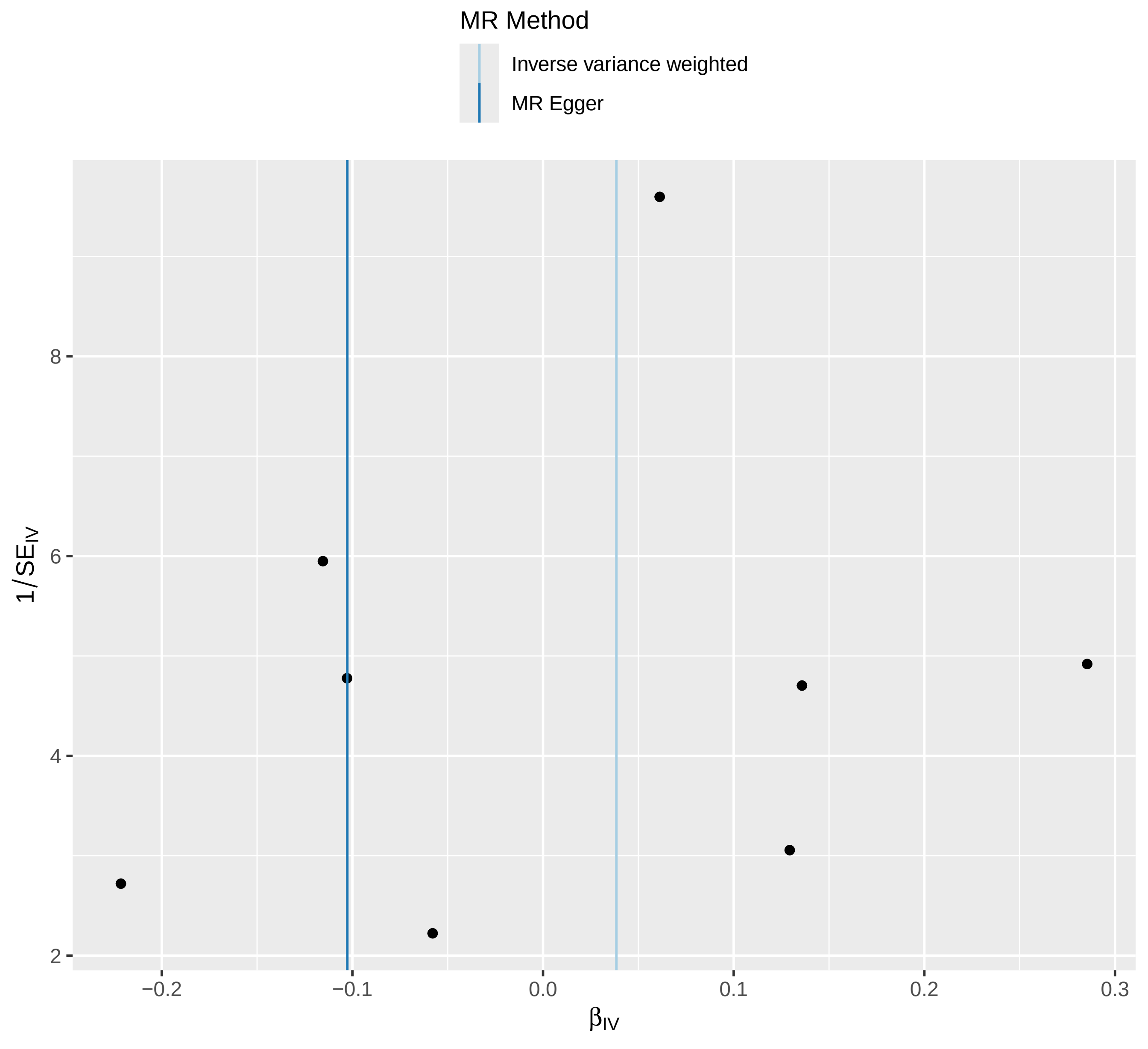

MR Method
Inverse variance weighted
MR Egger
8
6
4
2
1/SE_IV
−0.2
−0.1
0.0
0.1
0.2
0.3
β_IV

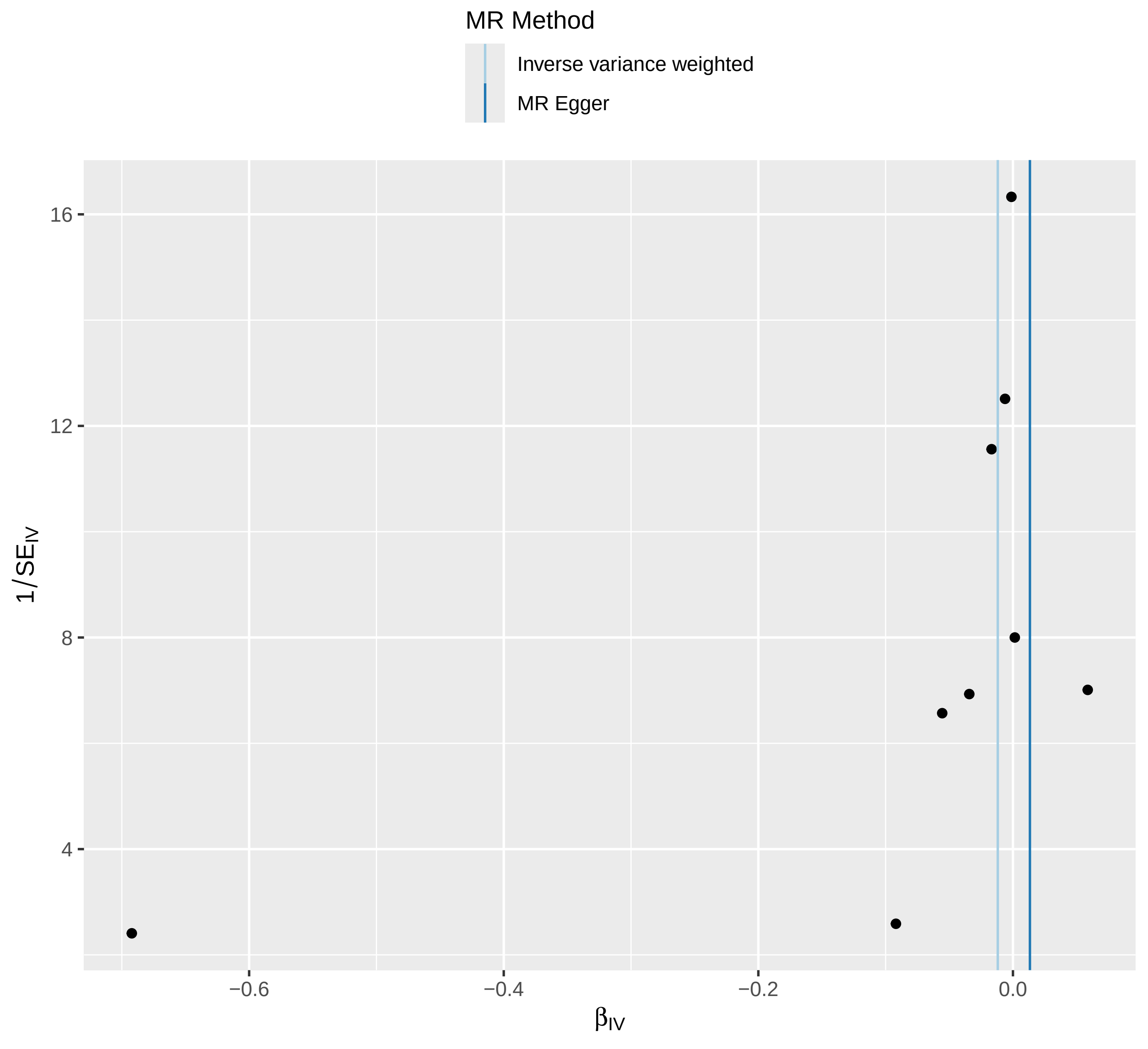
MR Method
Inverse variance weighted
MR Egger
16
12
8
4
−0.6
−0.4
−0.2
0.0
1/SE_IV
β_IV

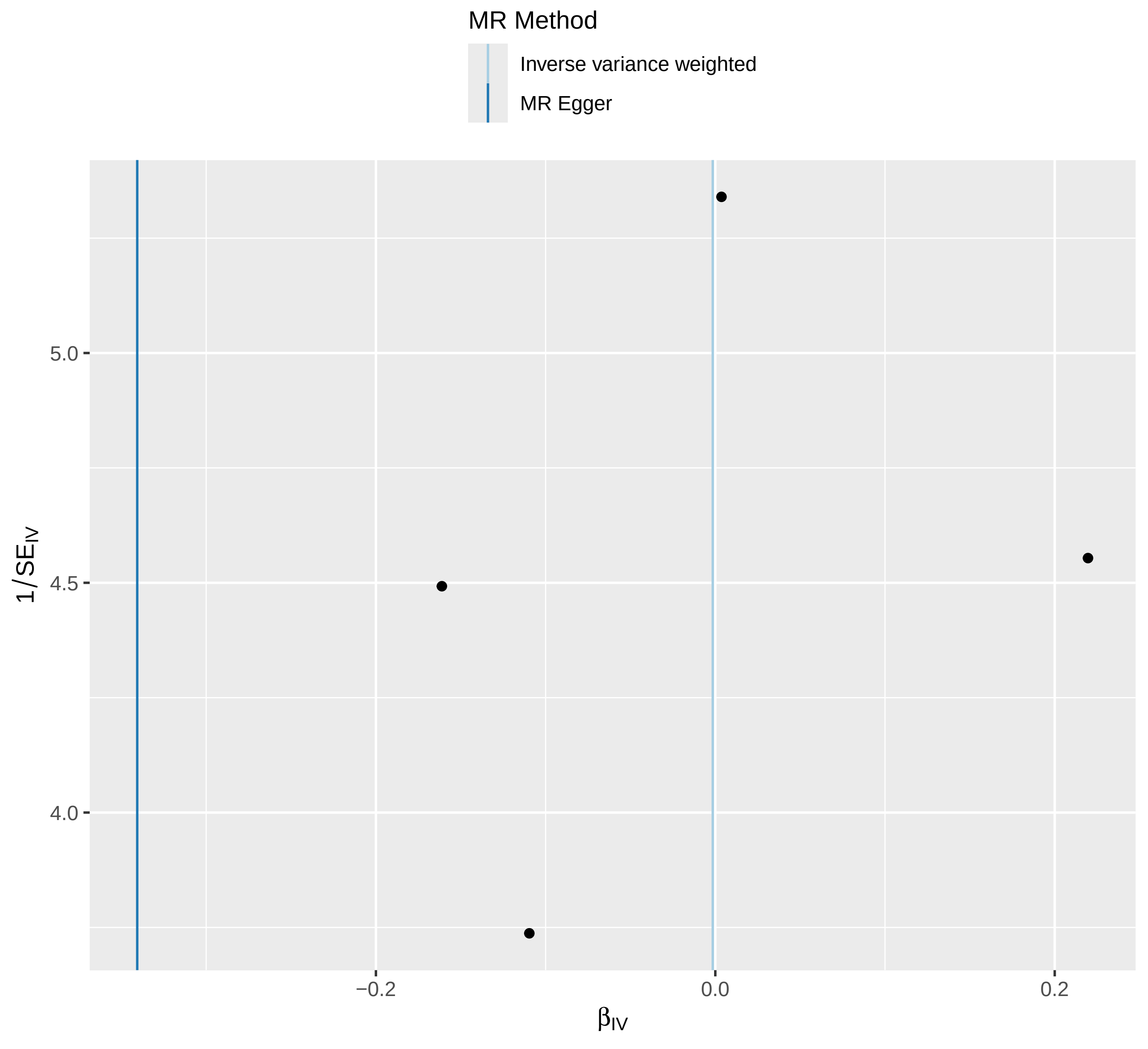
MR Method
Inverse variance weighted
MR Egger
5.0
4.5
4.0
1/SE$_{IV}$
−0.2
0.0
0.2
β$_{IV}$

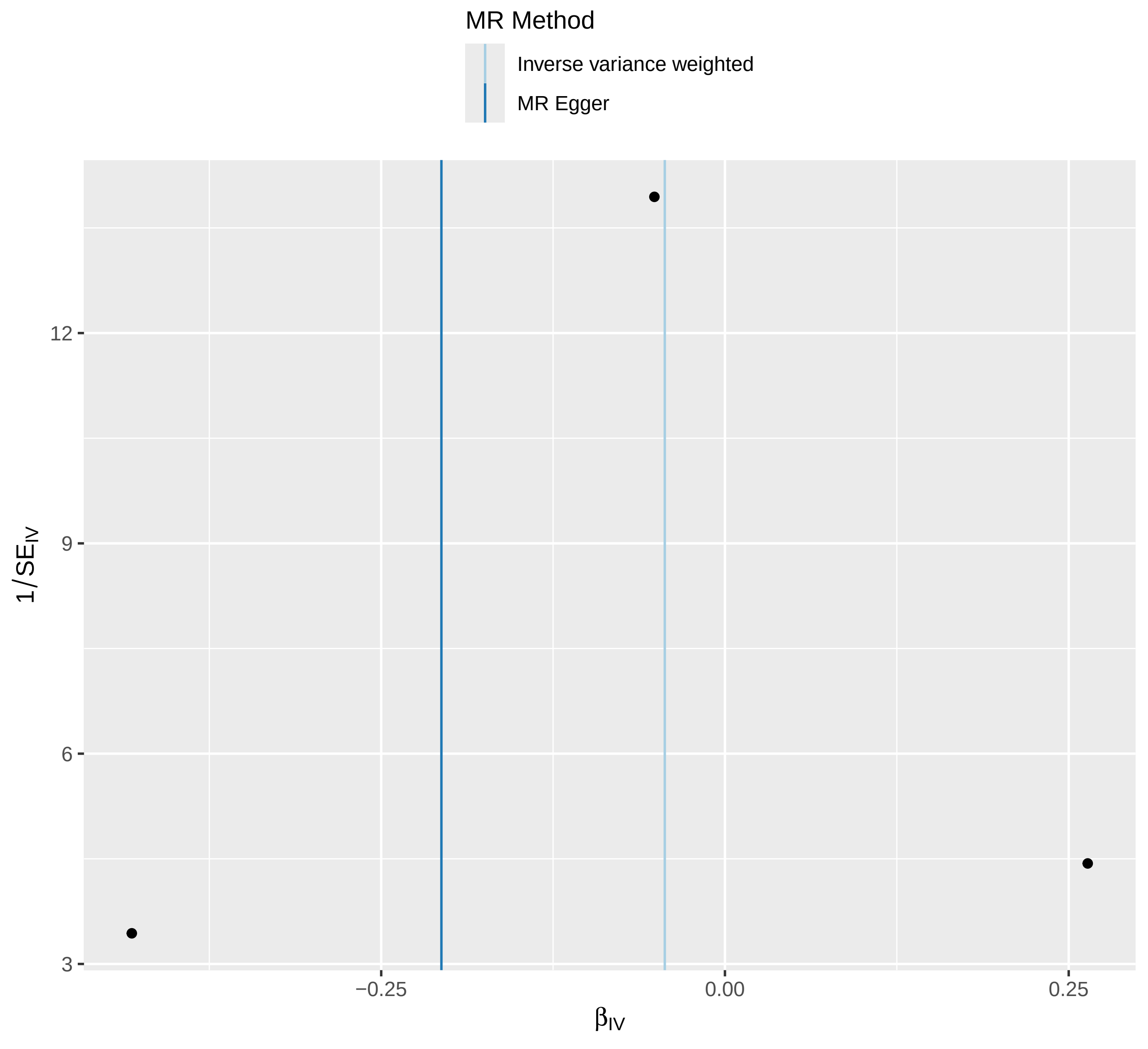
MR Method
Inverse variance weighted
MR Egger
12
9
6
3
1/SE$_{IV}$
−0.25
0.00
0.25
β$_{IV}$

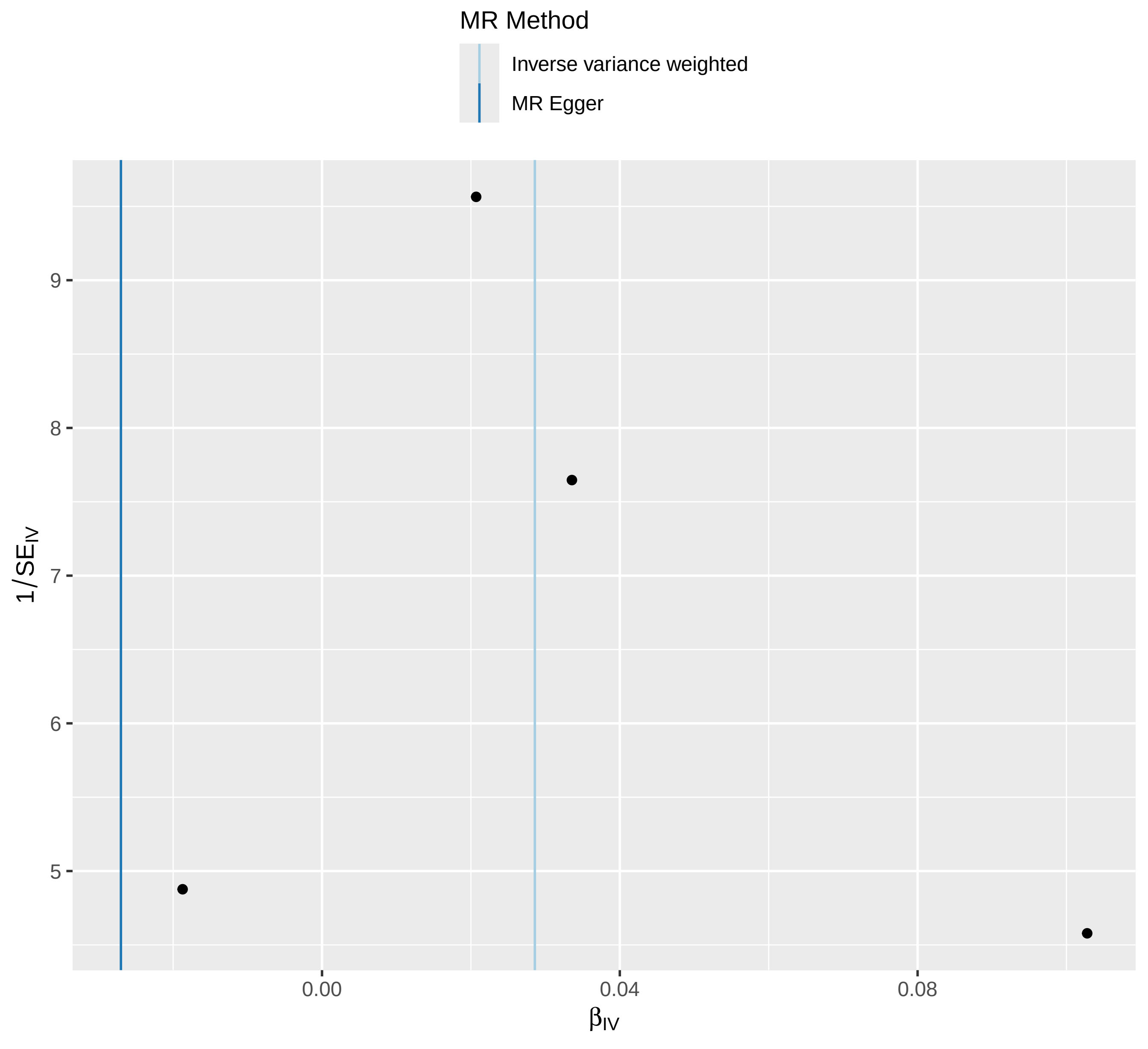
MR Method
Inverse variance weighted
MR Egger
9
8
7
6
5
1/SE_IV
0.00
0.04
0.08
β_IV

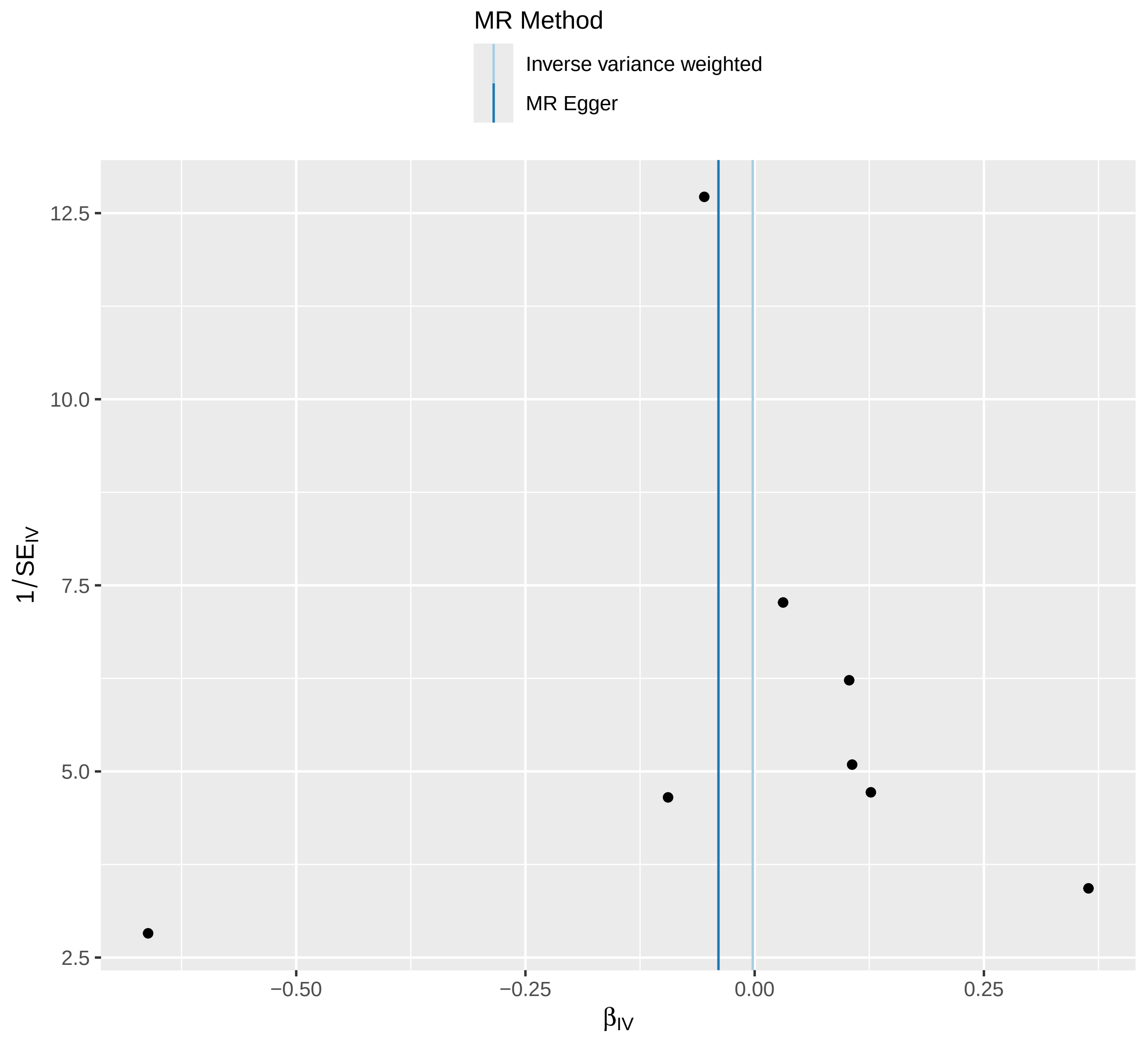
MR Method
Inverse variance weighted
MR Egger
12.5
10.0
7.5
5.0
2.5
1/SE_IV
−0.50
−0.25
0.00
0.25
β_IV

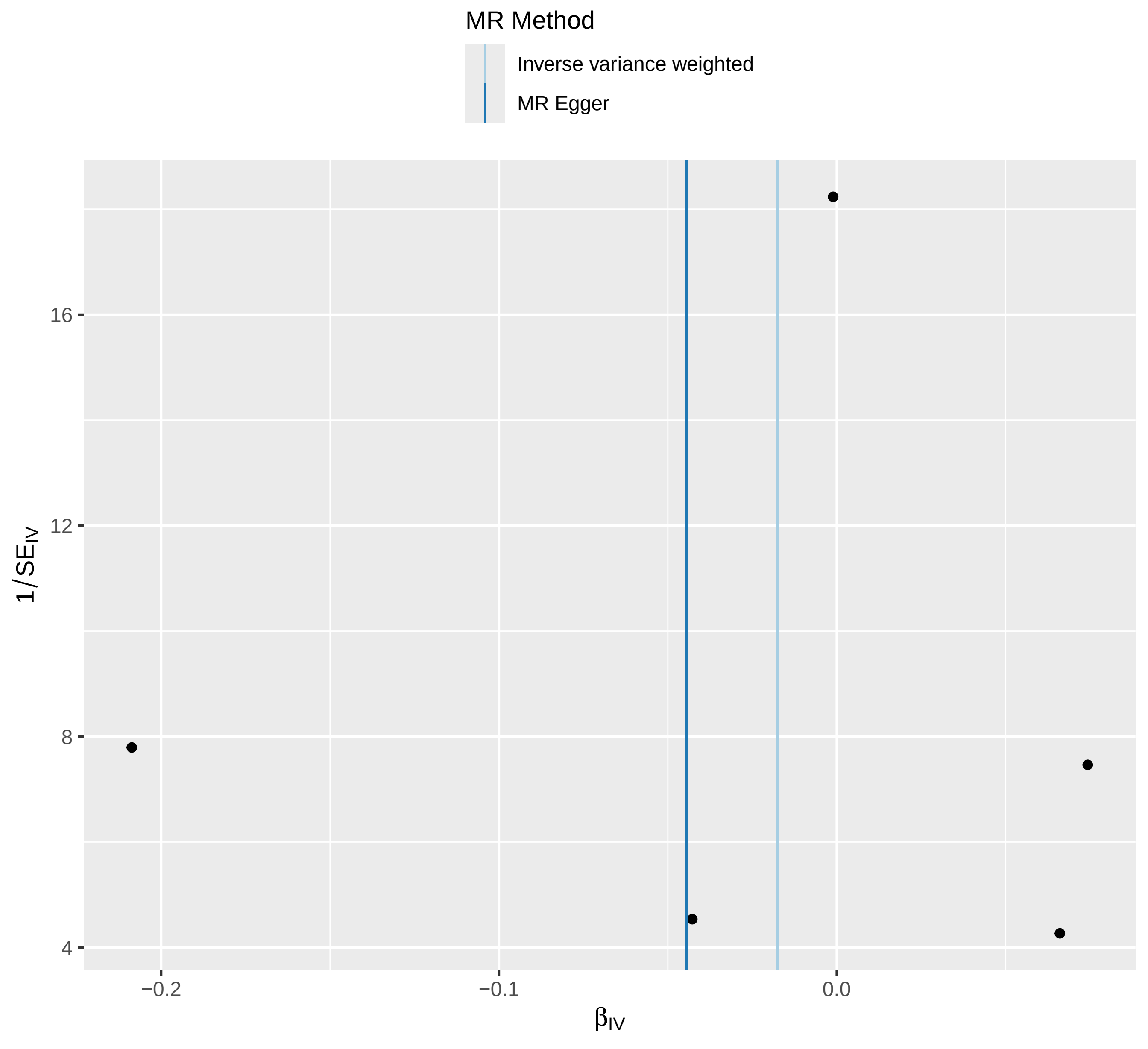
MR Method
Inverse variance weighted
MR Egger
16
12
8
4
1/SE$_{IV}$
−0.2
−0.1
0.0
β$_{IV}$

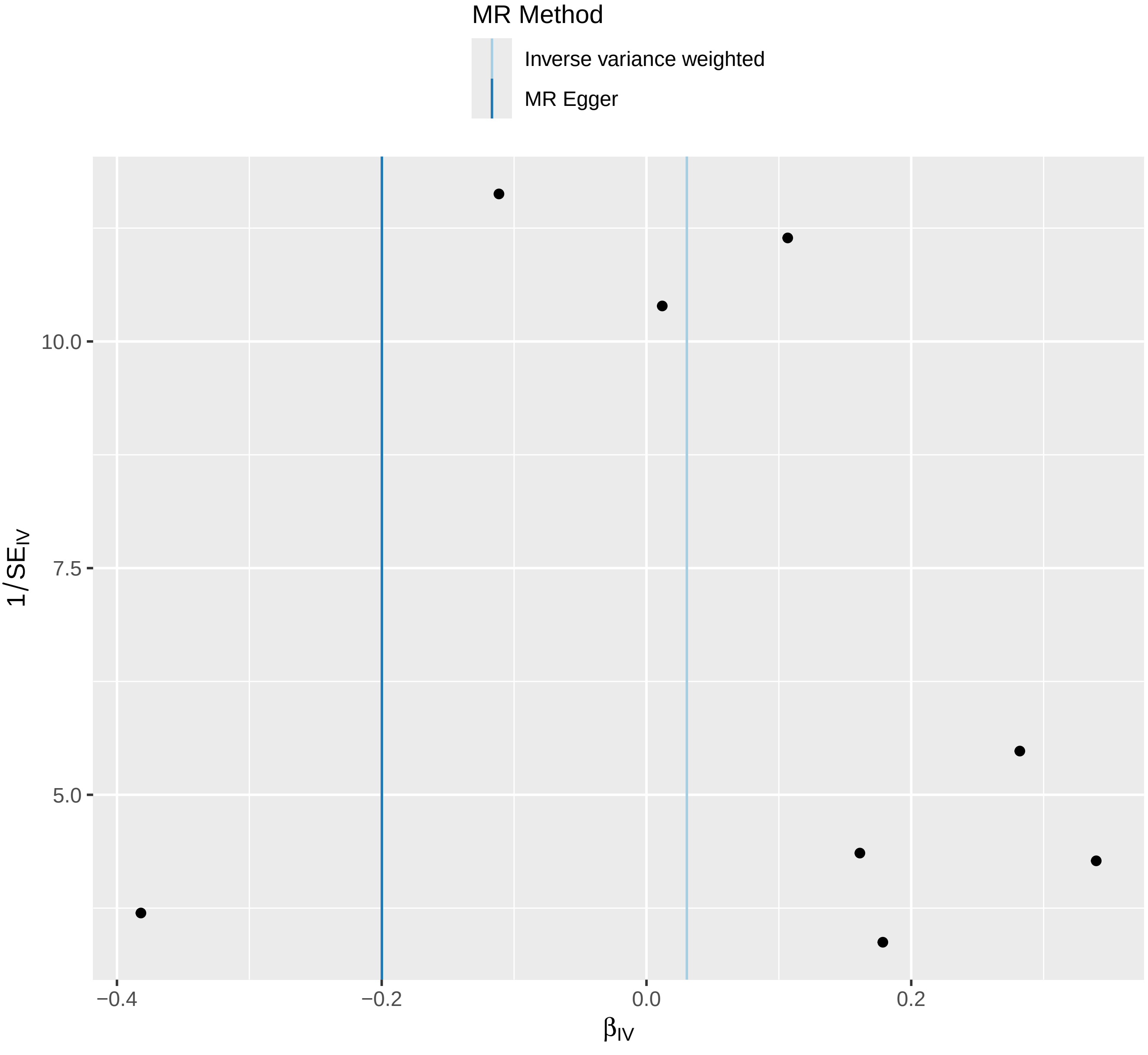
MR Method
Inverse variance weighted
MR Egger
10.0
7.5
5.0
1/SE_IV
−0.4
−0.2
0.0
0.2
β_IV

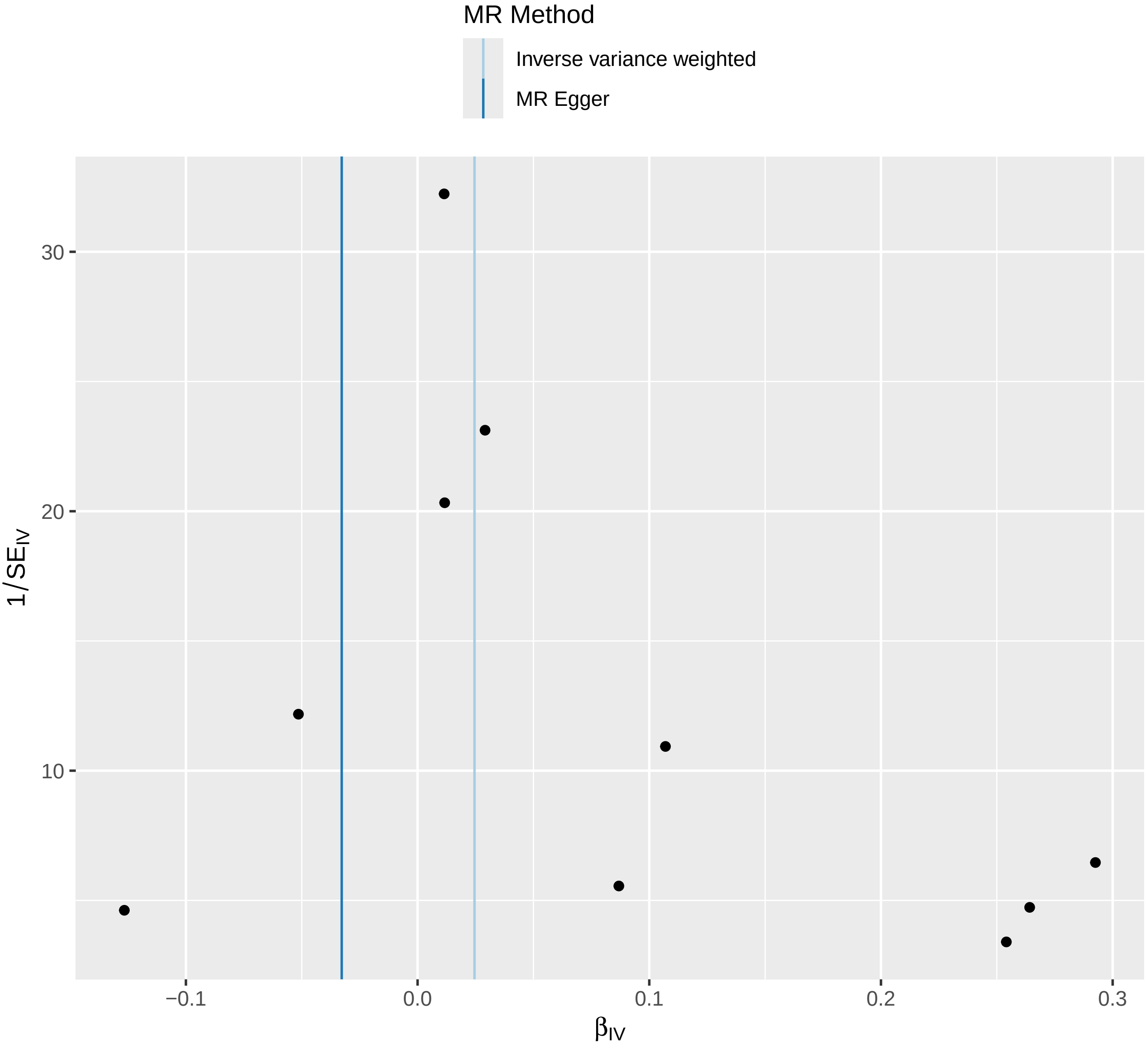

MR Method
Inverse variance weighted
MR Egger
30
20
10
1/SE_IV
−0.1
0.0
0.1
0.2
0.3
β_IV

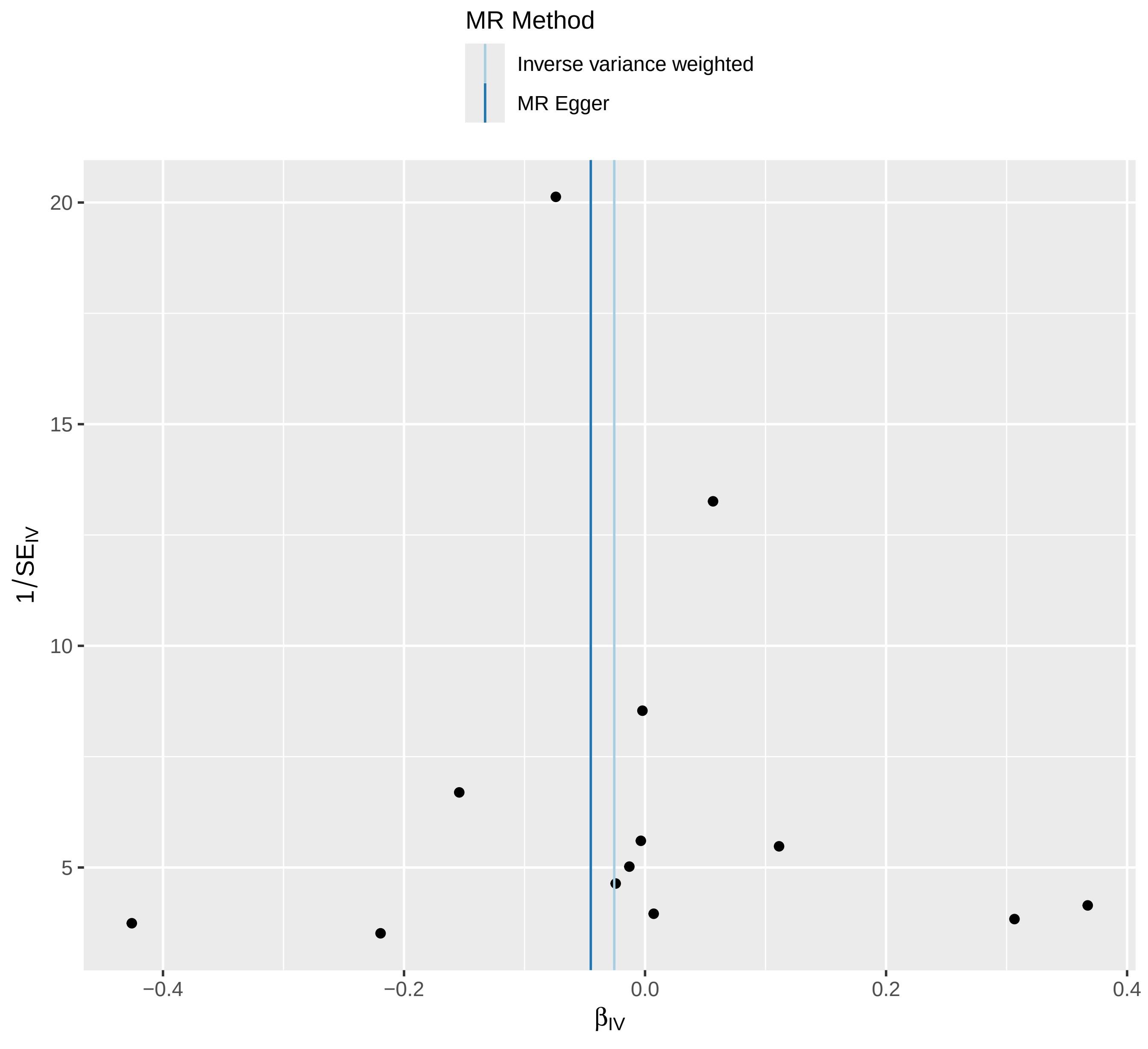

MR Method
Inverse variance weighted
MR Egger
20
15
10
5
1/SE$_{IV}$
−0.4
−0.2
0.0
0.2
0.4
β$_{IV}$

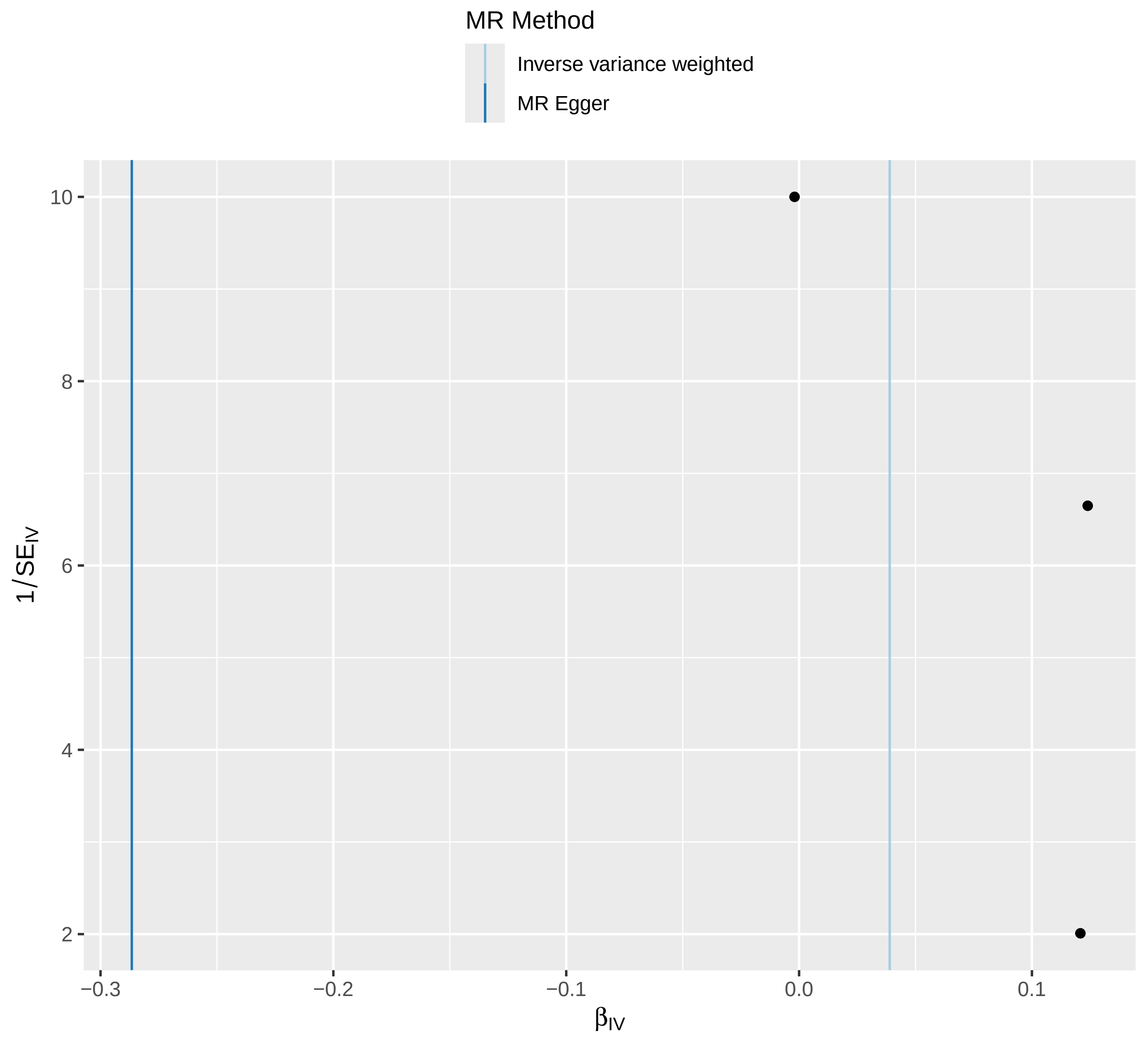
MR Method
Inverse variance weighted
MR Egger
10
8
6
4
2
1/SE_IV
−0.3
−0.2
−0.1
0.0
0.1
β_IV

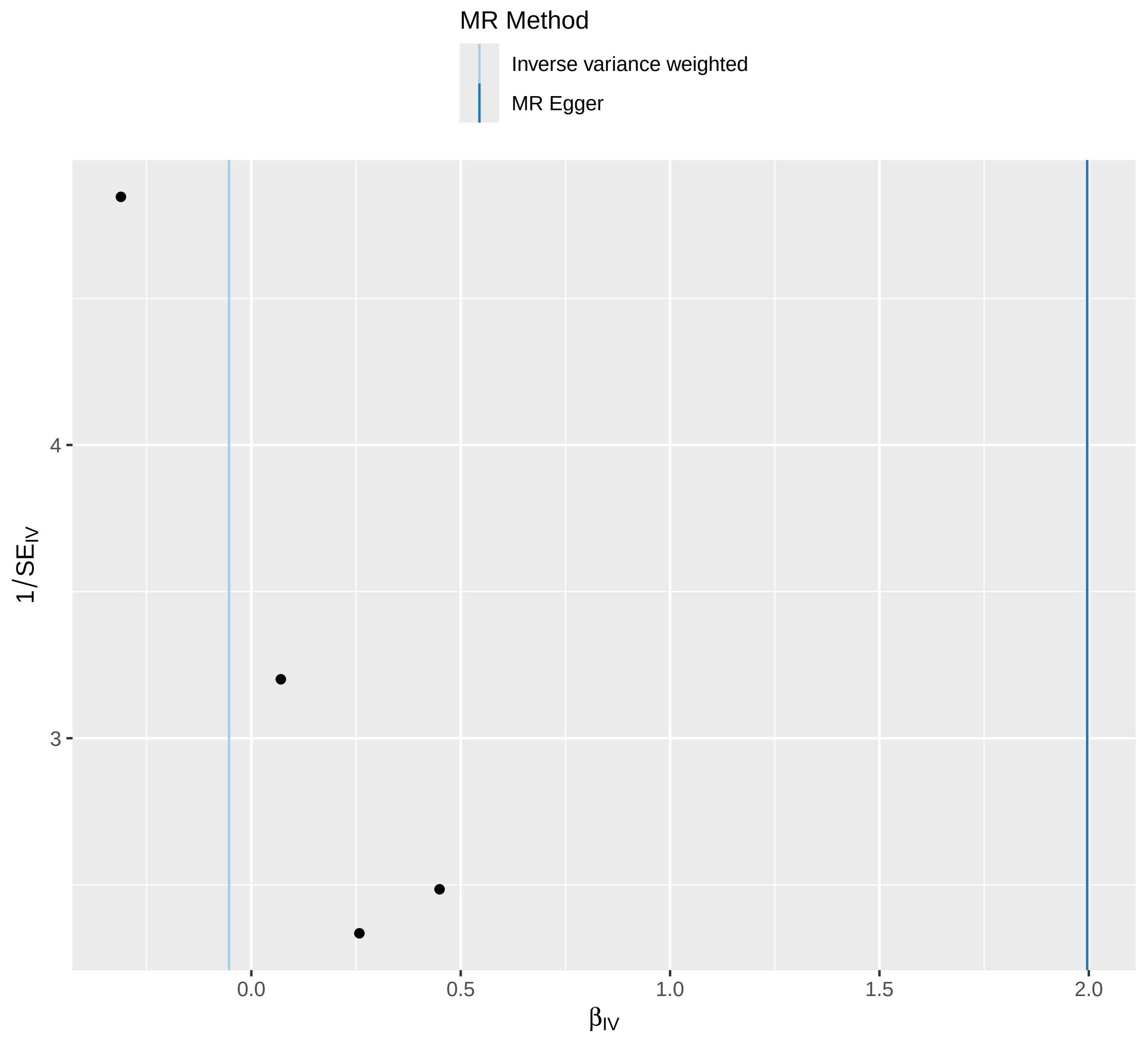

MR Method
Inverse variance weighted
MR Egger
4
3
1/SE_IV
0.0
0.5
1.0
1.5
2.0
β_IV

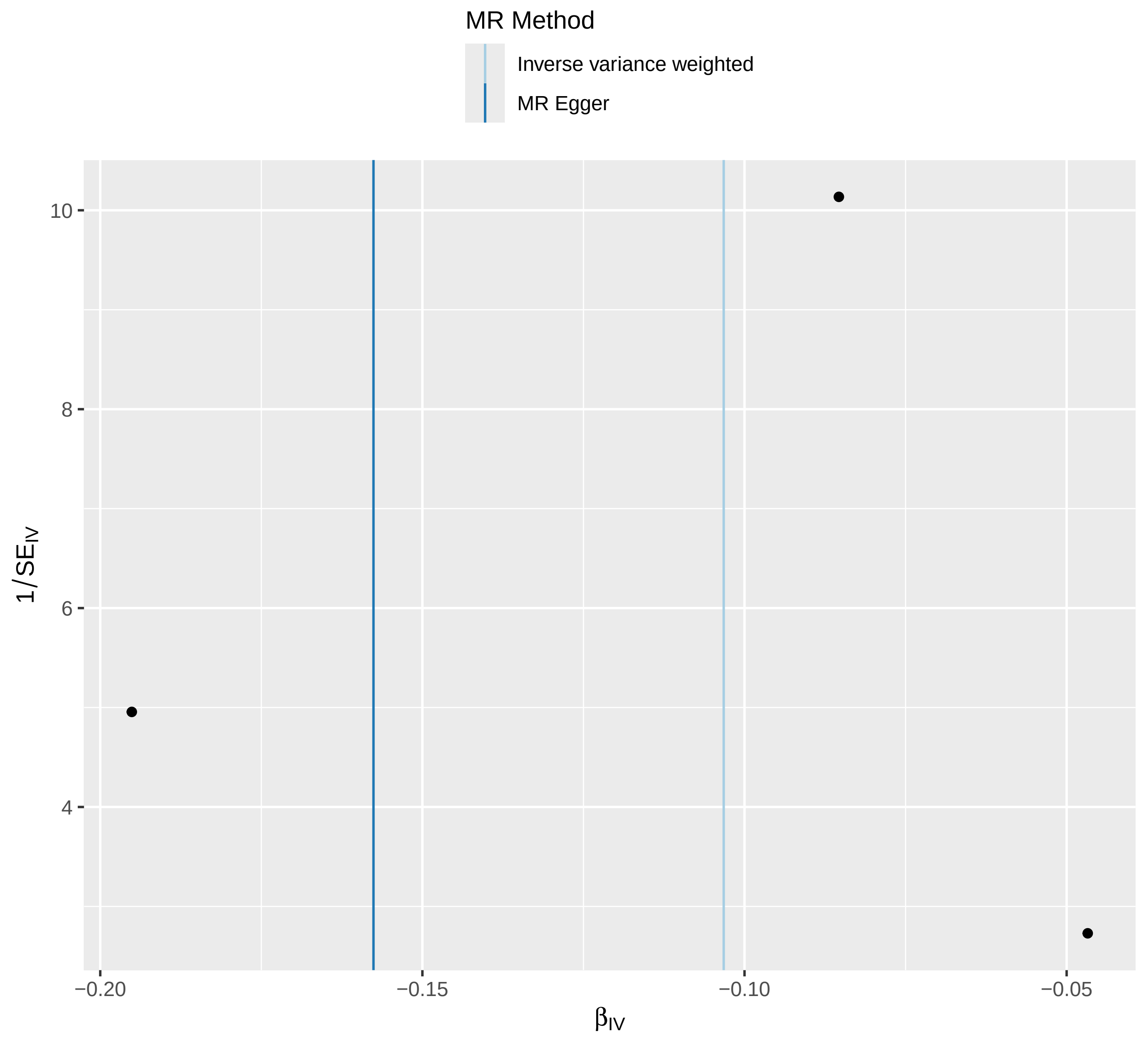

MR Method
Inverse variance weighted
MR Egger
10
8
6
4
1/SE_IV
−0.20
−0.15
−0.10
−0.05
β_IV

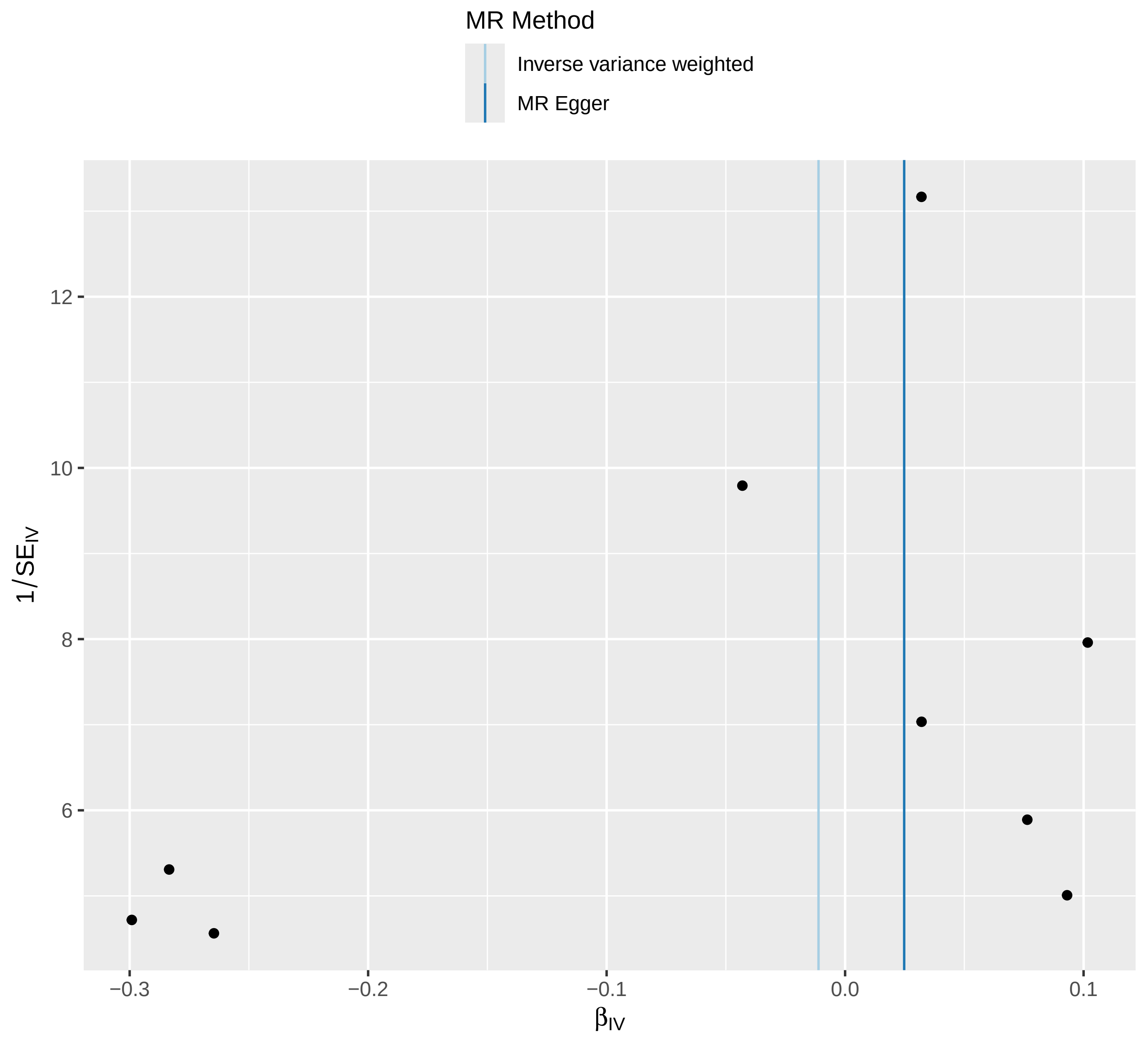
MR Method
Inverse variance weighted
MR Egger
1/SE$_{IV}$
12
10
8
6
−0.3
−0.2
−0.1
0.0
0.1
β$_{IV}$

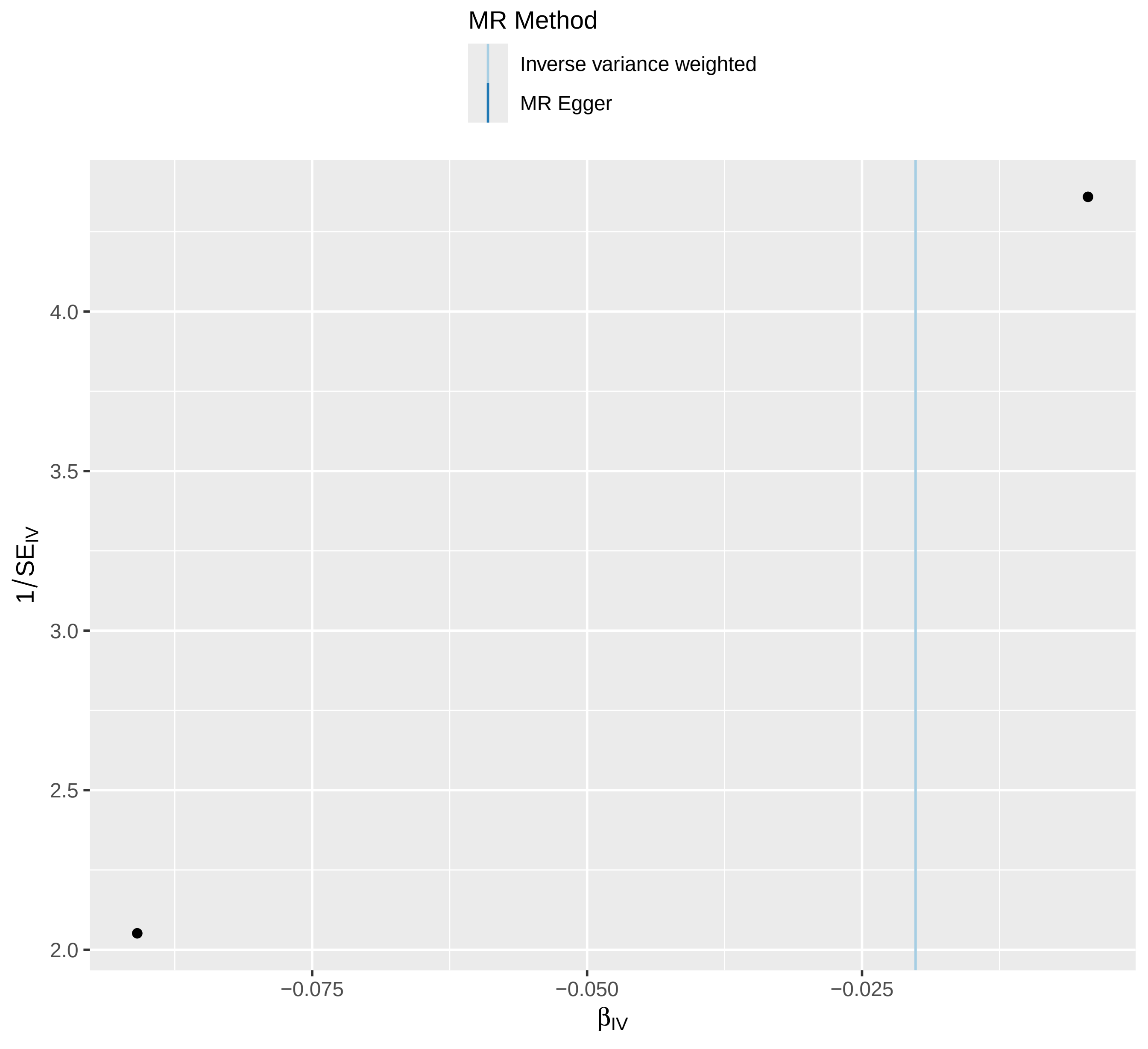

MR Method
Inverse variance weighted
MR Egger
1/SE$_{IV}$
4.0
3.5
3.0
2.5
2.0
−0.075
−0.050
−0.025
β$_{IV}$

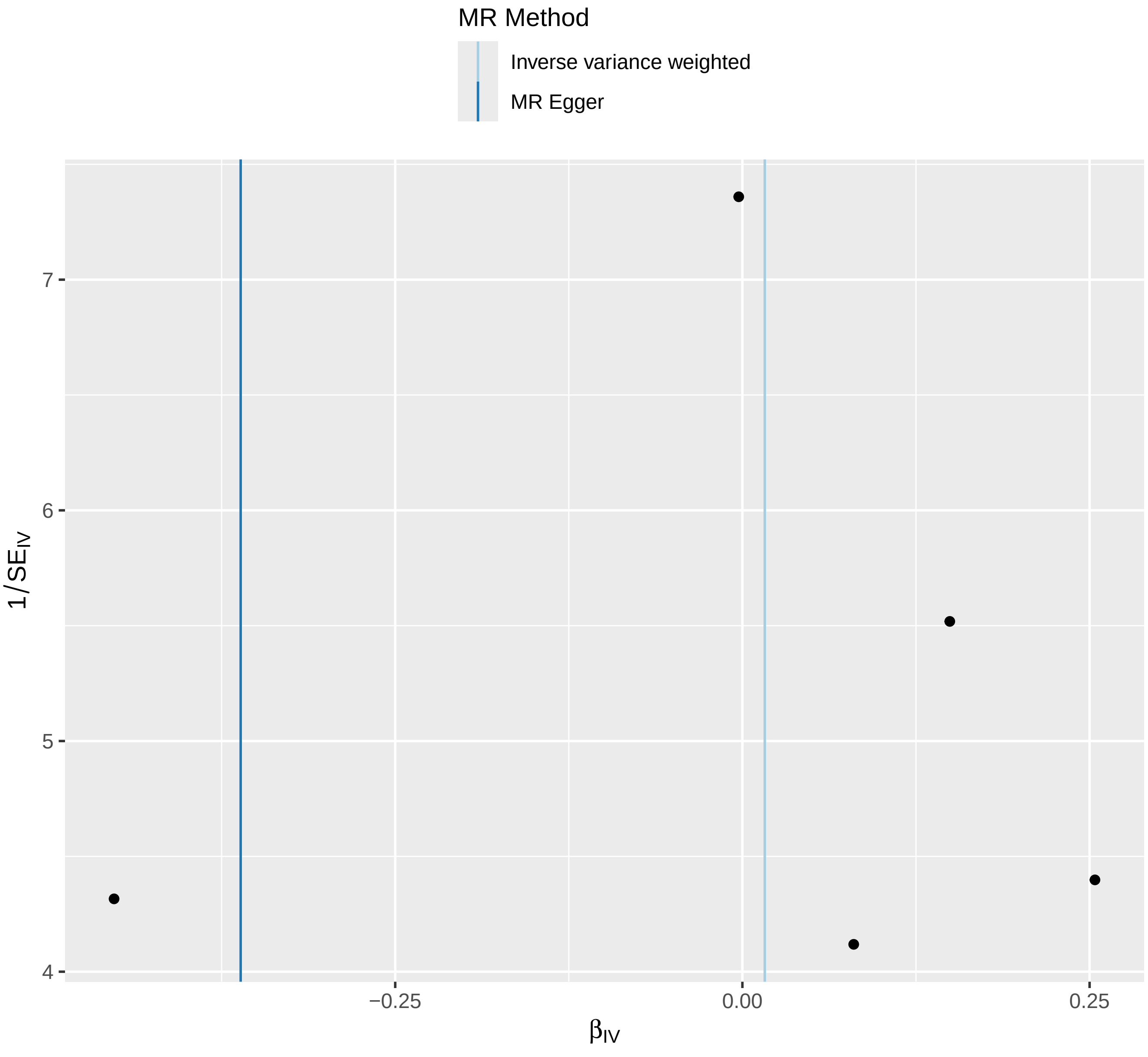
MR Method
Inverse variance weighted
MR Egger
7
6
5
4
1/SE_IV
−0.25
0.00
0.25
β_IV

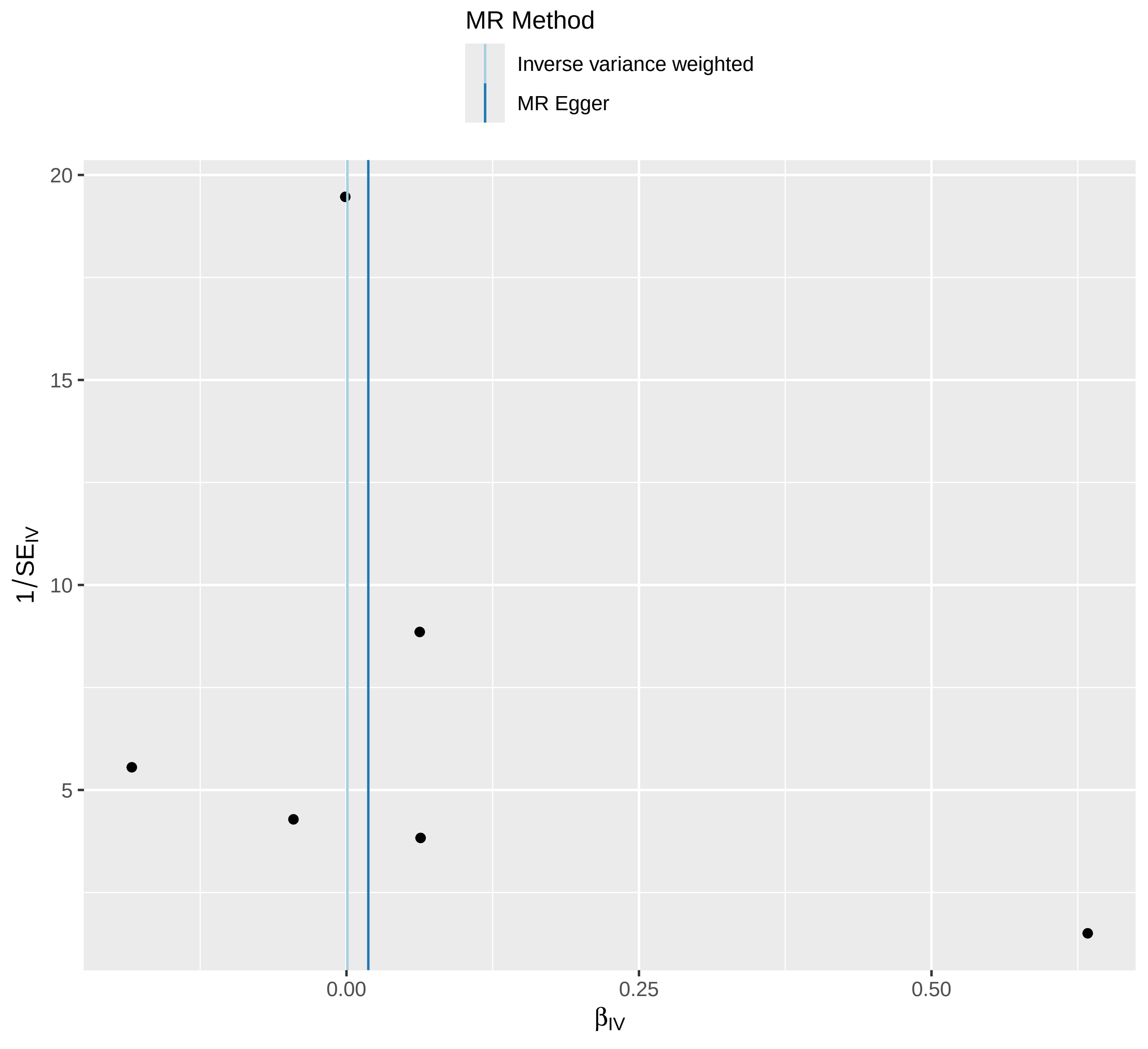

MR Method
Inverse variance weighted
MR Egger
20
15
10
5
1/SE_IV
0.00
0.25
0.50
β_IV

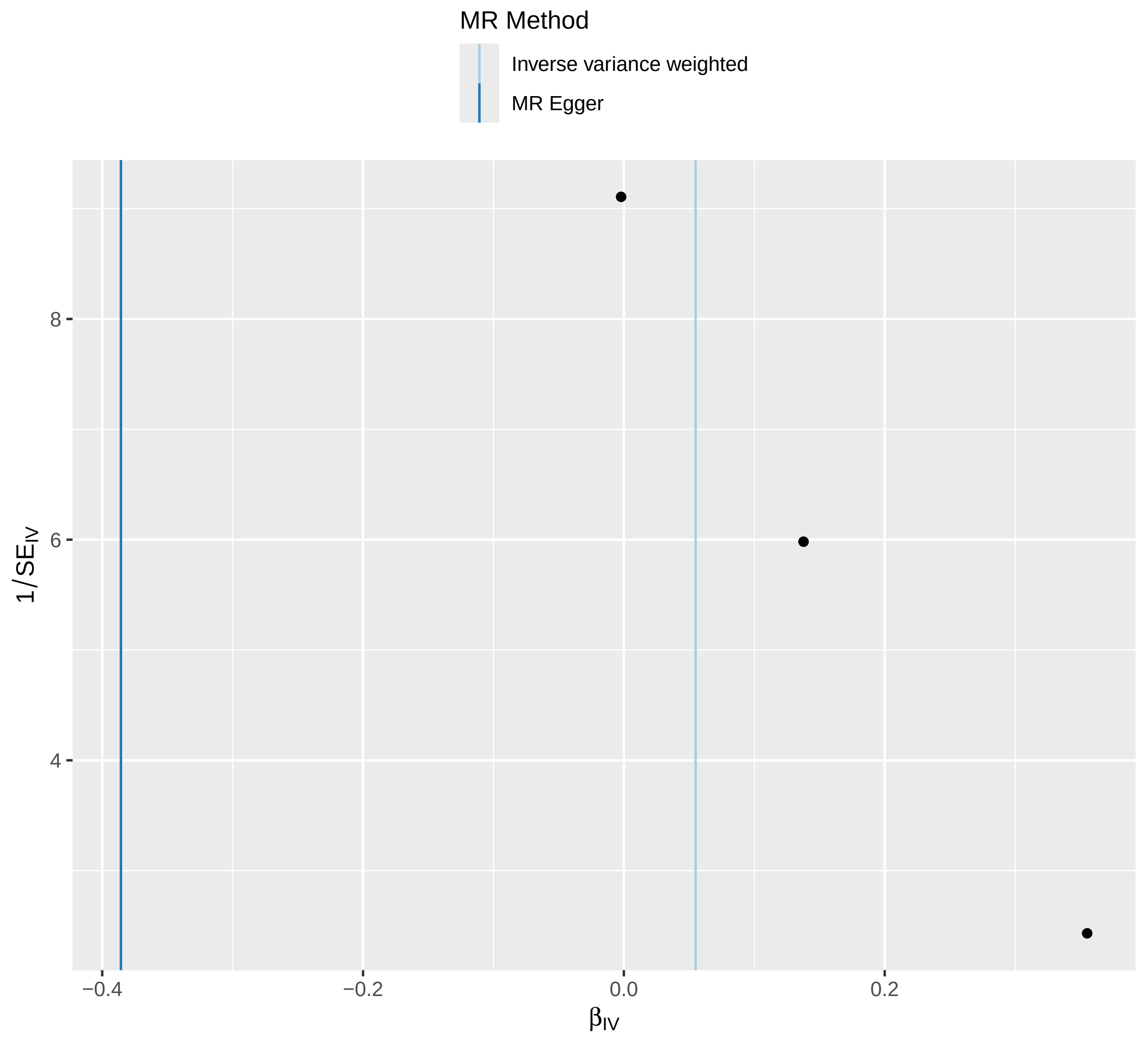
MR Method
Inverse variance weighted
MR Egger
8
6
4
1/SE$_{IV}$
−0.4
−0.2
0.0
0.2
β$_{IV}$

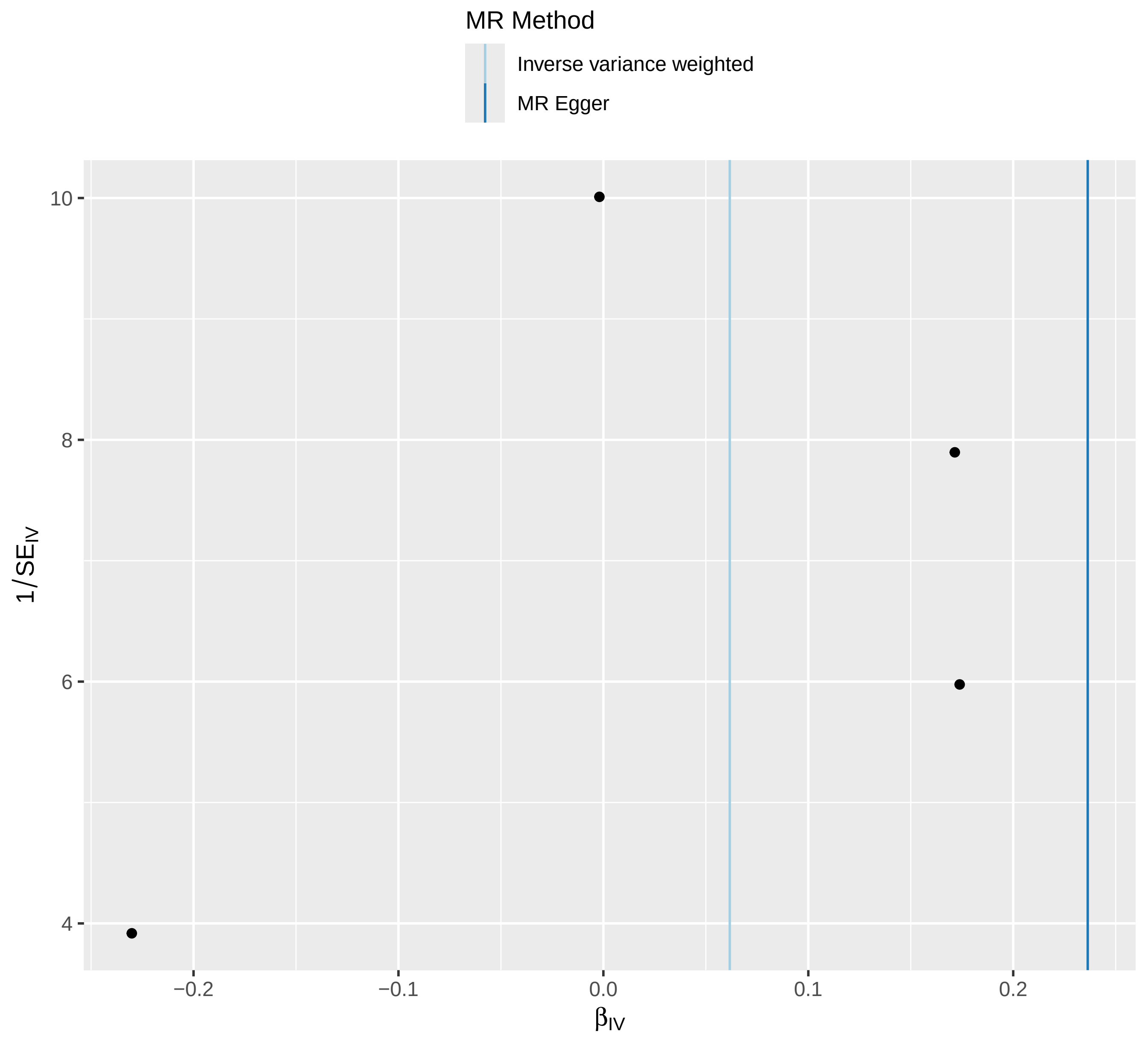

MR Method
Inverse variance weighted
MR Egger
10
8
6
4
1/SE$_{IV}$
−0.2
−0.1
0.0
0.1
0.2
β$_{IV}$

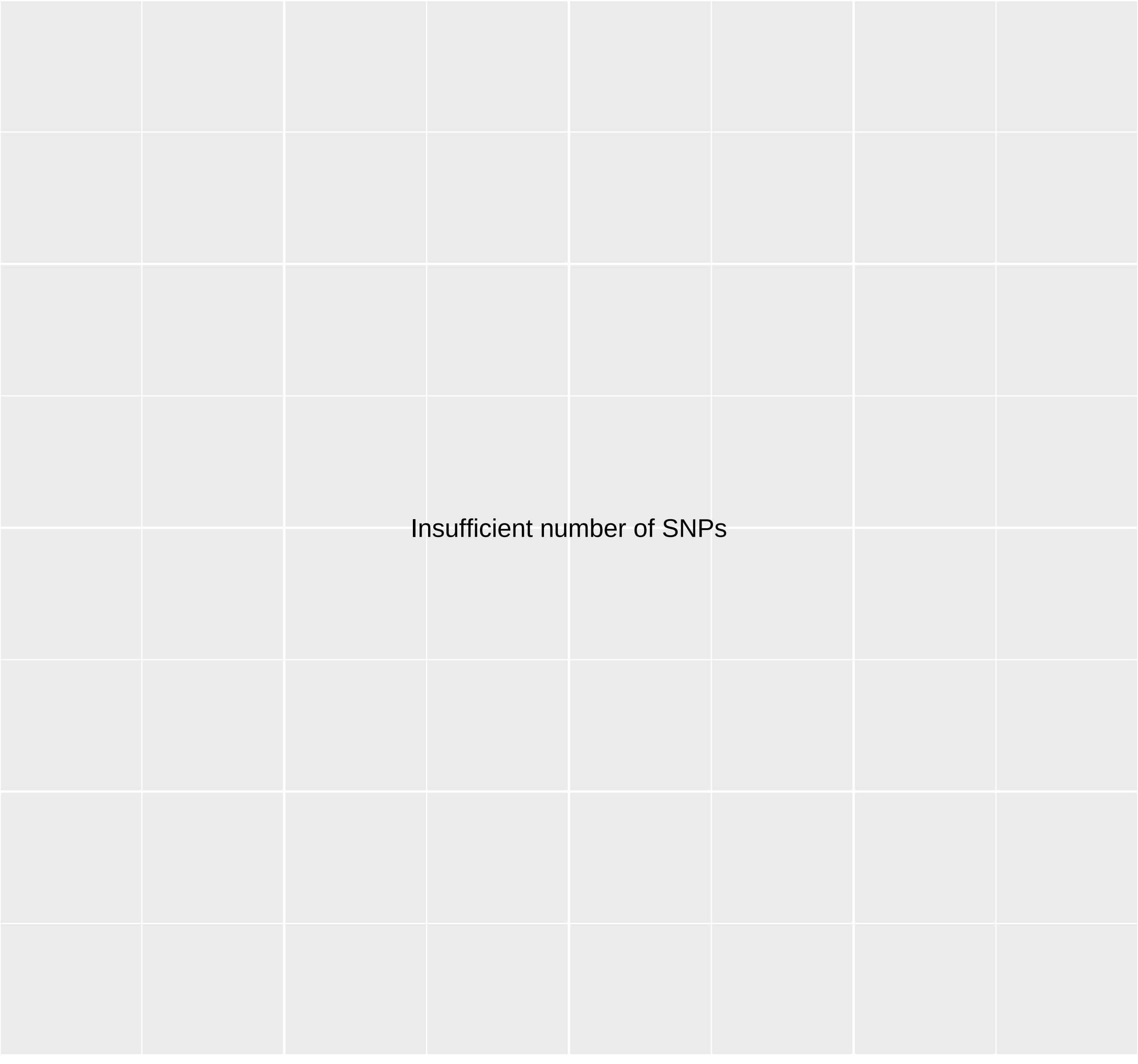
Insufficient number of SNPs

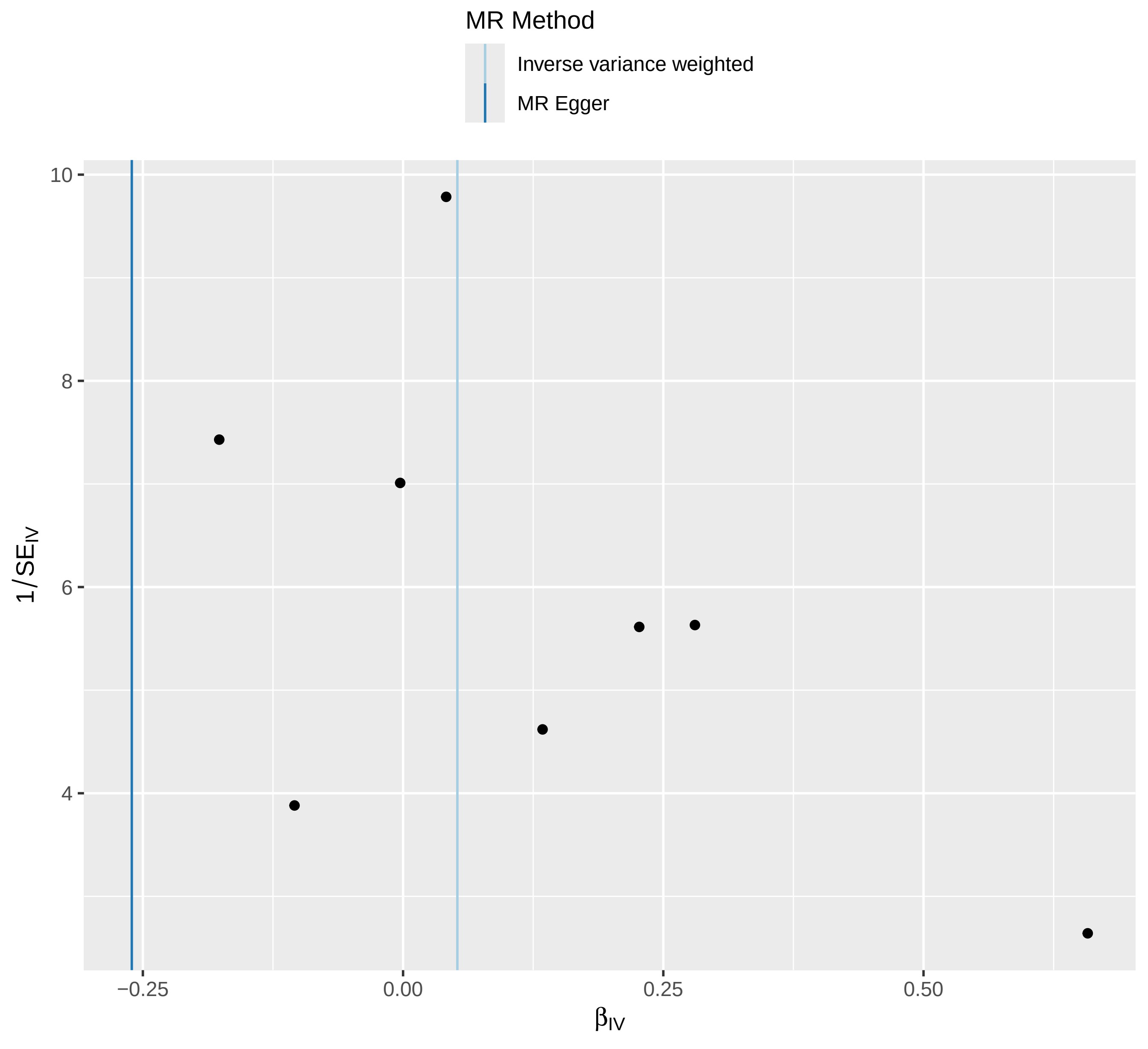

MR Method
Inverse variance weighted
MR Egger
10
8
6
4
1/SE_IV
−0.25
0.00
0.25
0.50
β_IV

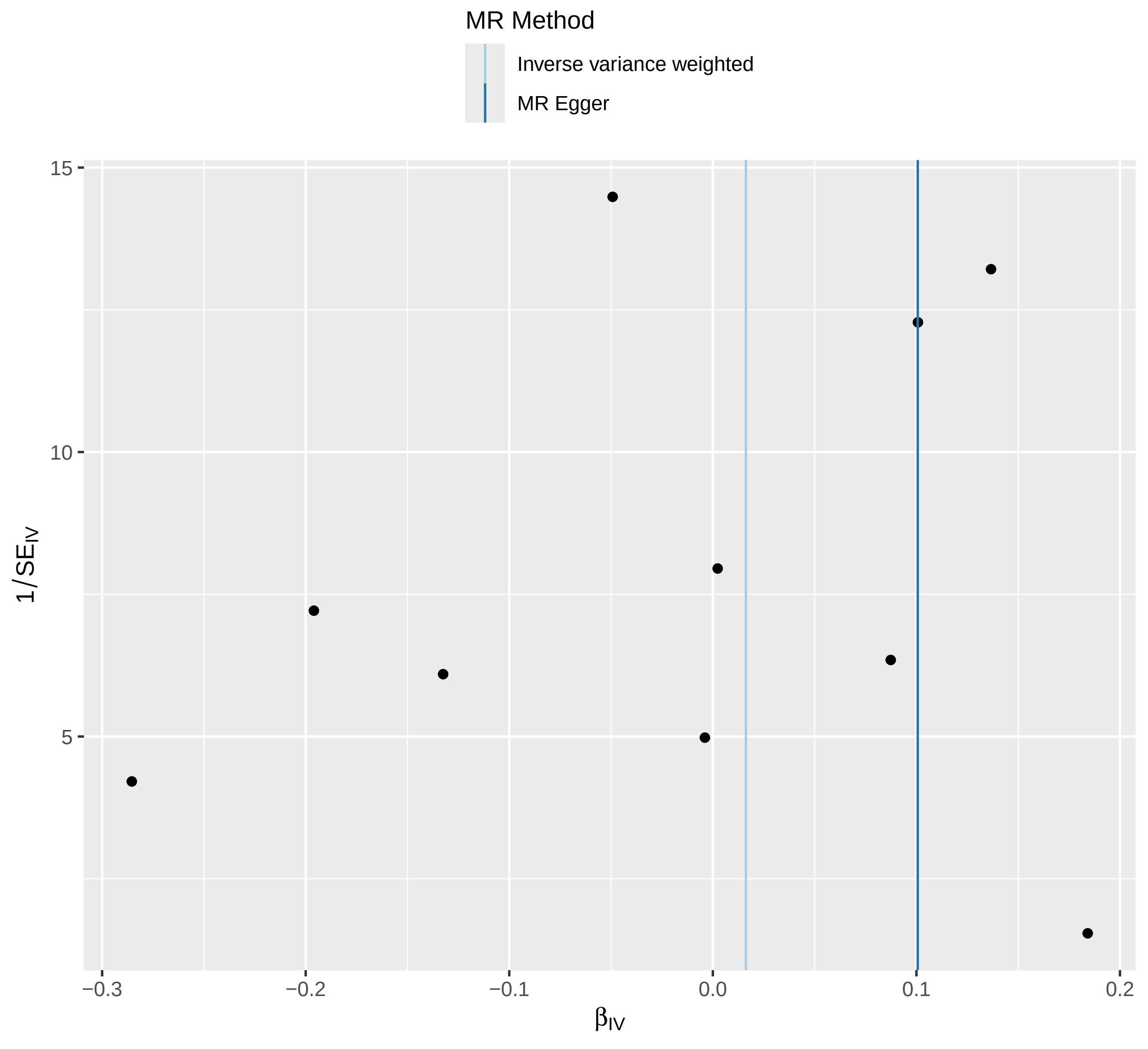
MR Method
Inverse variance weighted
MR Egger
15
10
5
1/SE_IV
−0.3
−0.2
−0.1
0.0
0.1
0.2
β_IV

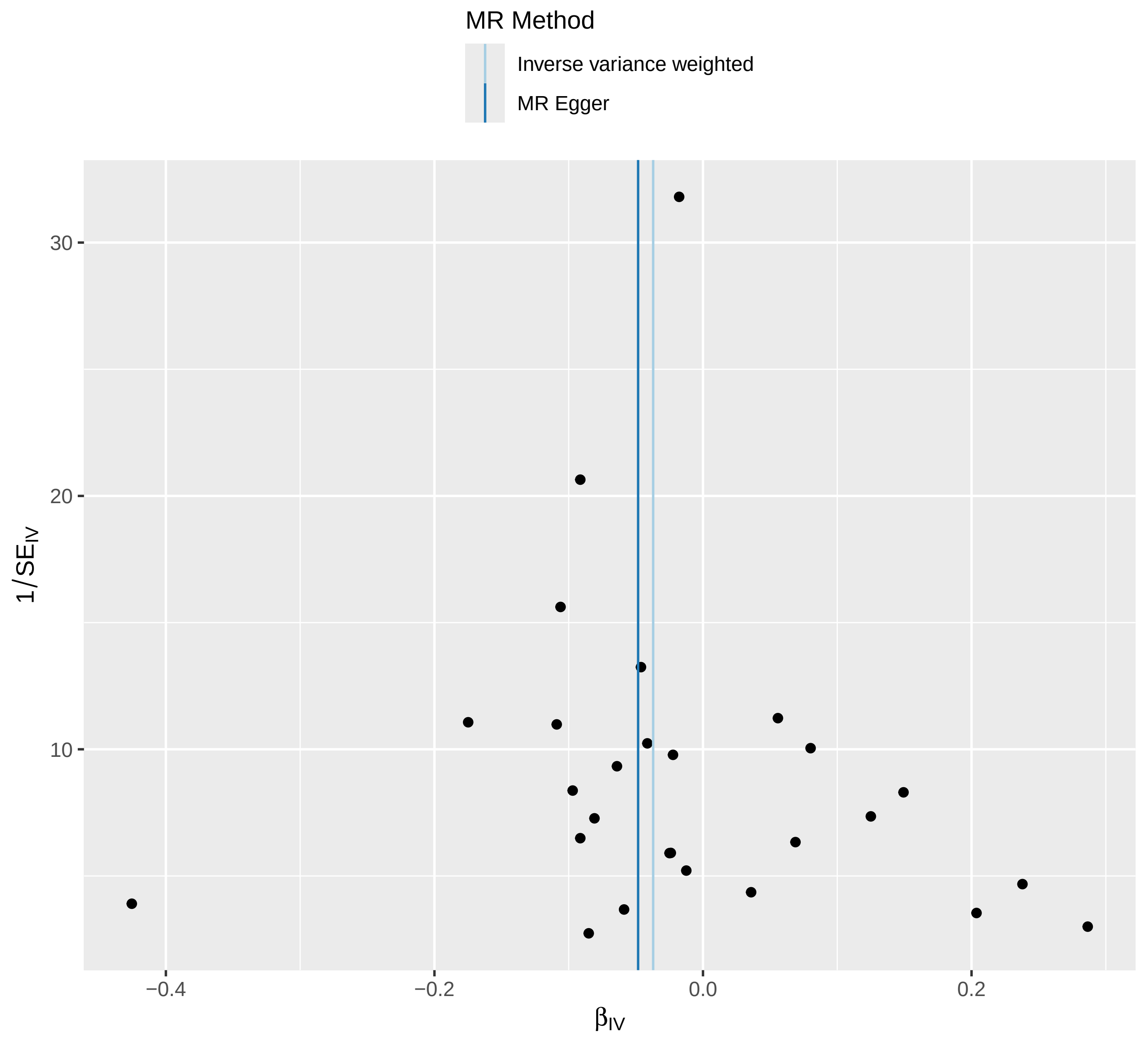

MR Method
Inverse variance weighted
MR Egger
30
20
10
$1/SE_{IV}$
−0.4
−0.2
0.0
0.2
$\beta_{IV}$

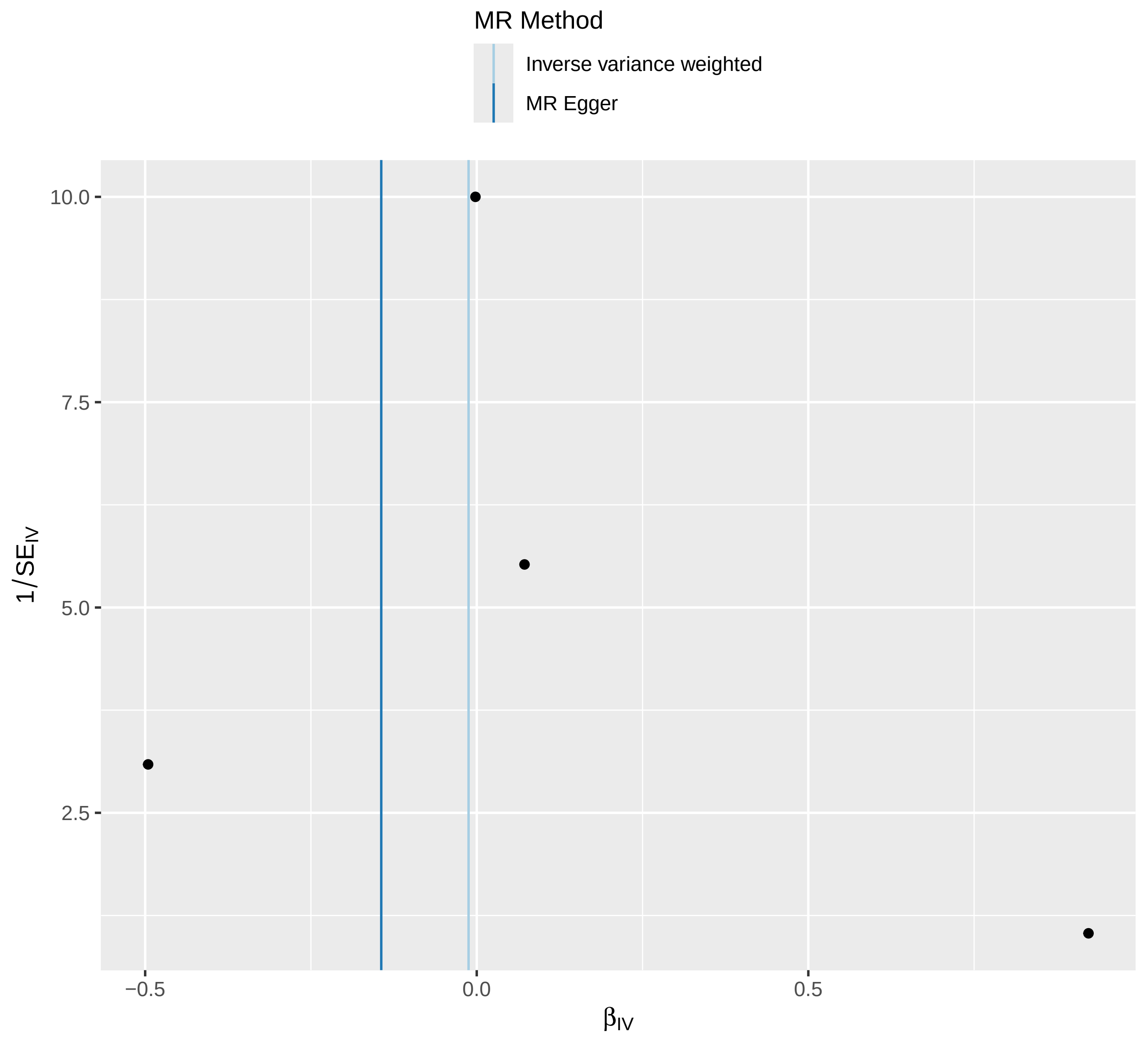
MR Method
Inverse variance weighted
MR Egger
10.0
7.5
5.0
2.5
1/SE_IV
−0.5
0.0
0.5
β_IV

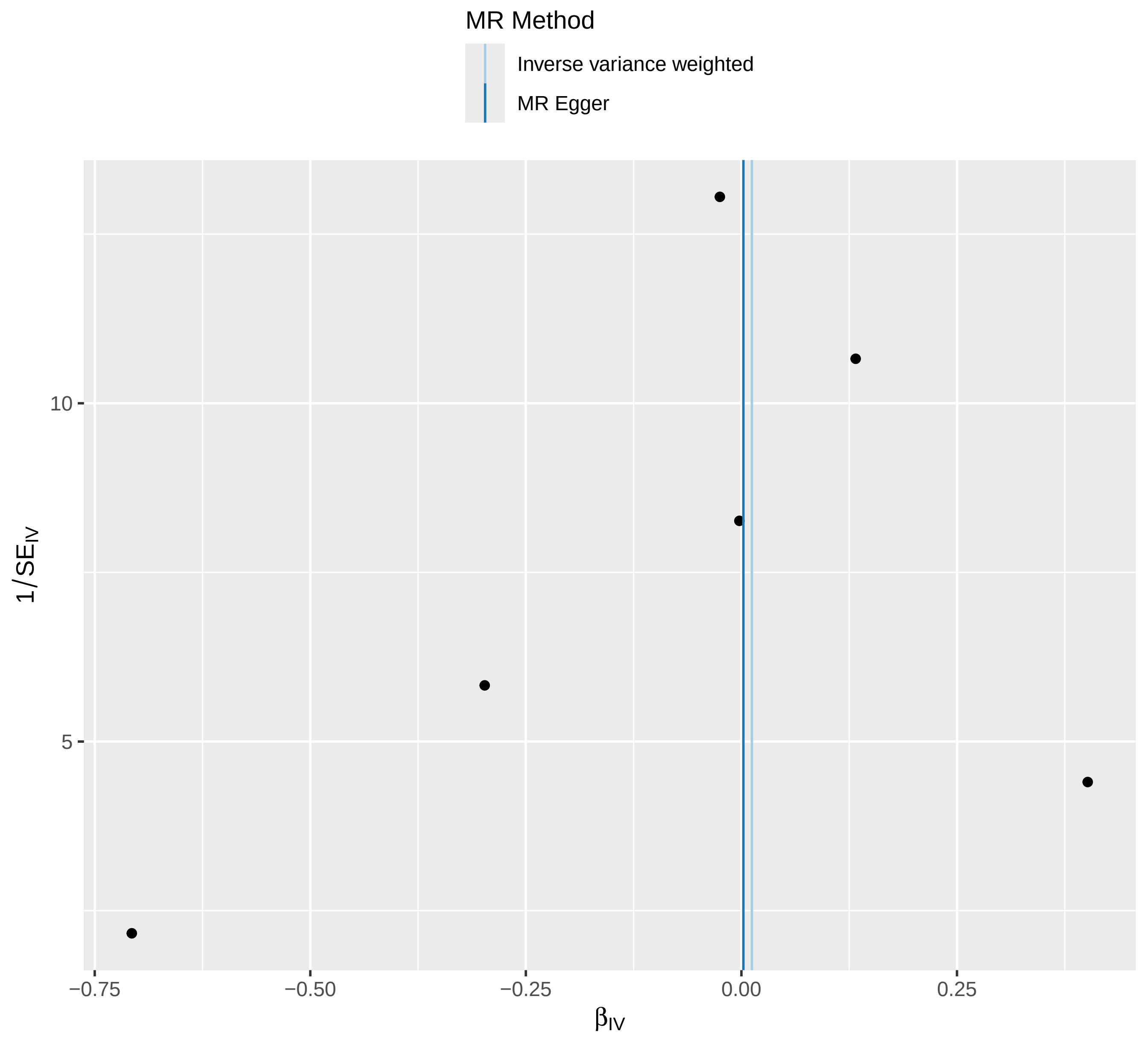
MR Method
Inverse variance weighted
MR Egger
10
5
1/SE$_{IV}$
−0.75
−0.50
−0.25
0.00
0.25
β$_{IV}$

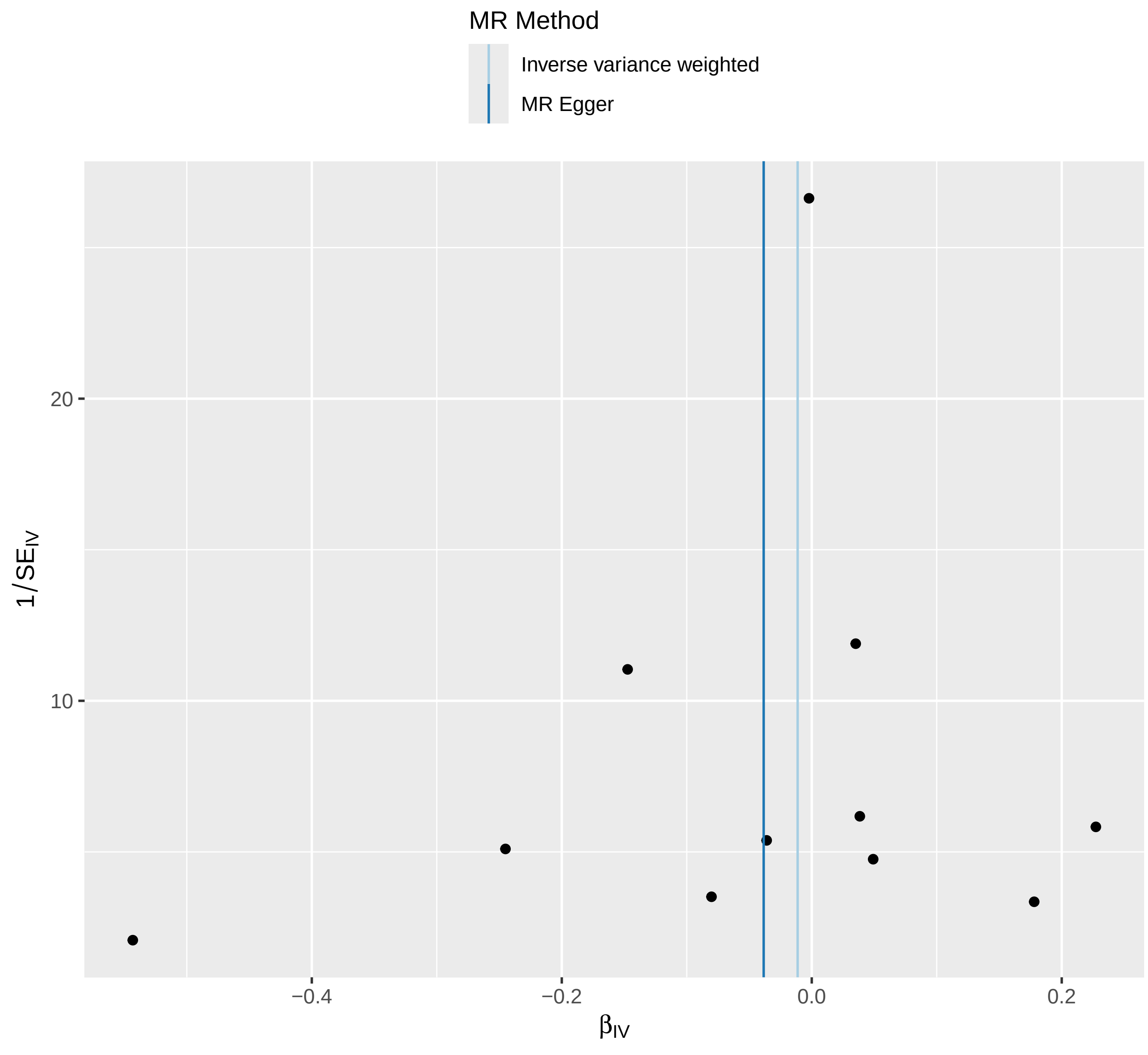
MR Method
Inverse variance weighted
MR Egger
20
10
$1/SE_{IV}$
−0.4
−0.2
0.0
0.2
$\beta_{IV}$

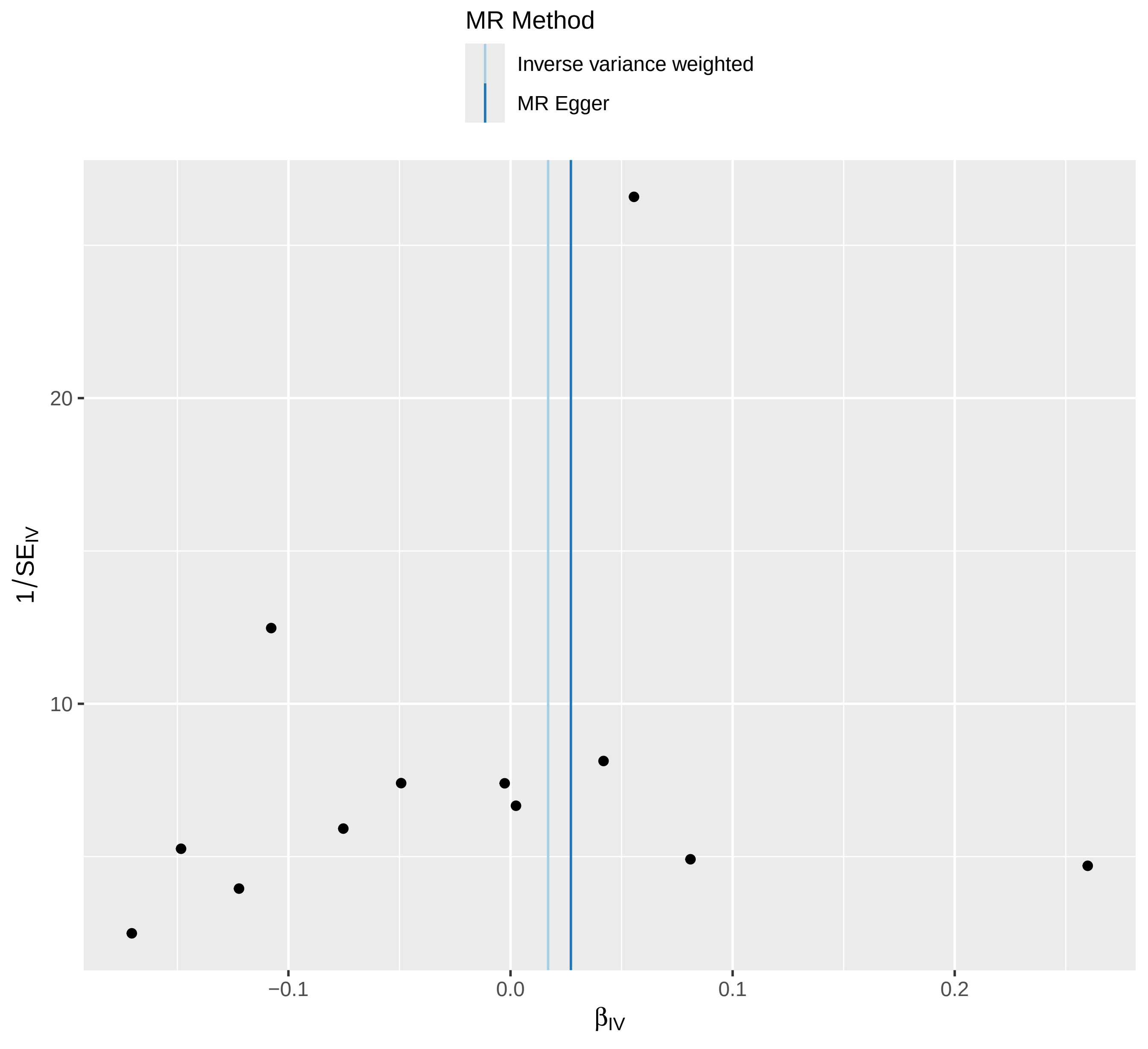
MR Method
Inverse variance weighted
MR Egger
20
10
$1/SE_{IV}$
−0.1
0.0
0.1
0.2
$\beta_{IV}$

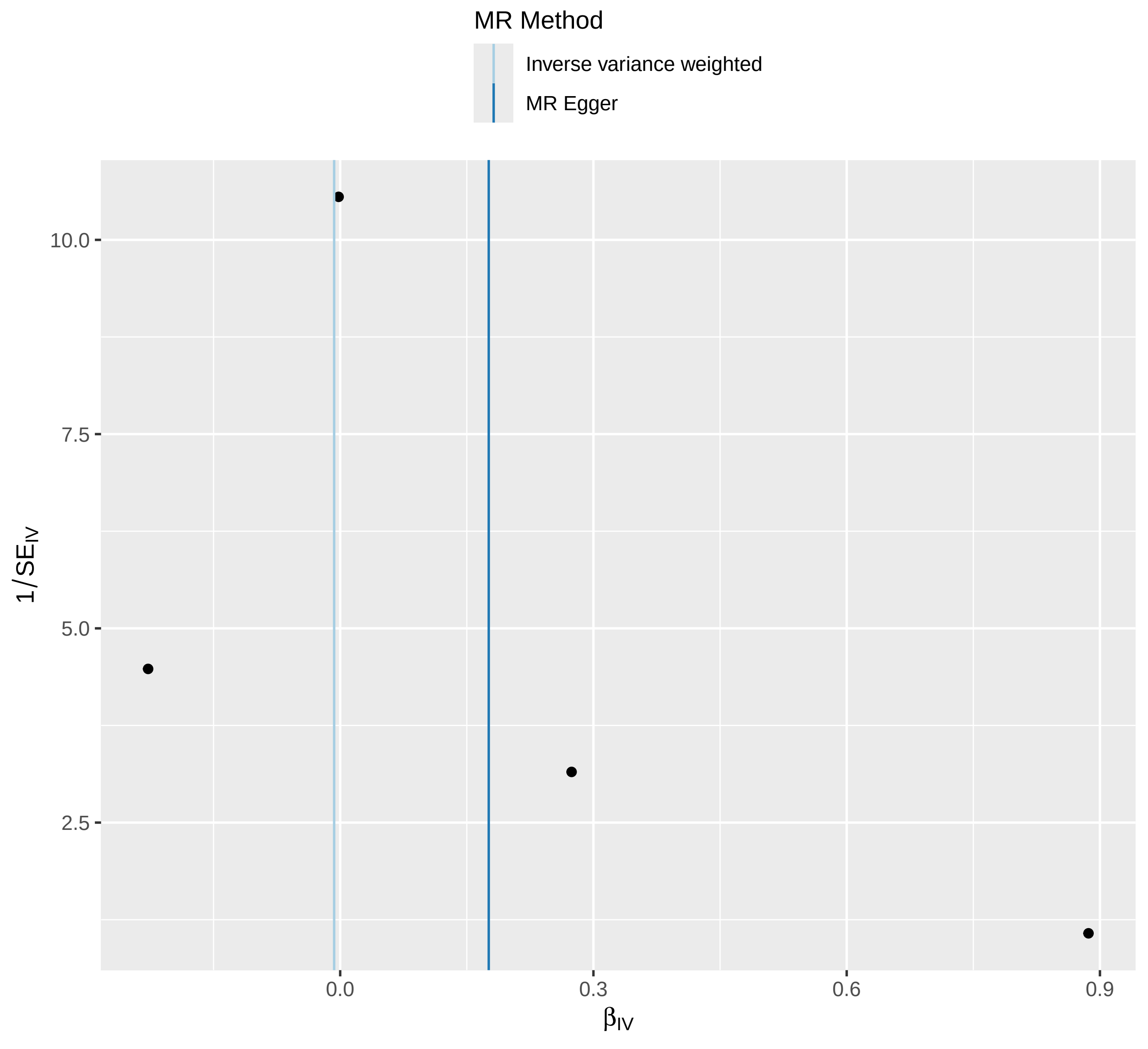

MR Method
Inverse variance weighted
MR Egger
10.0
7.5
5.0
2.5
1/SE_IV
0.0
0.3
0.6
0.9
β_IV

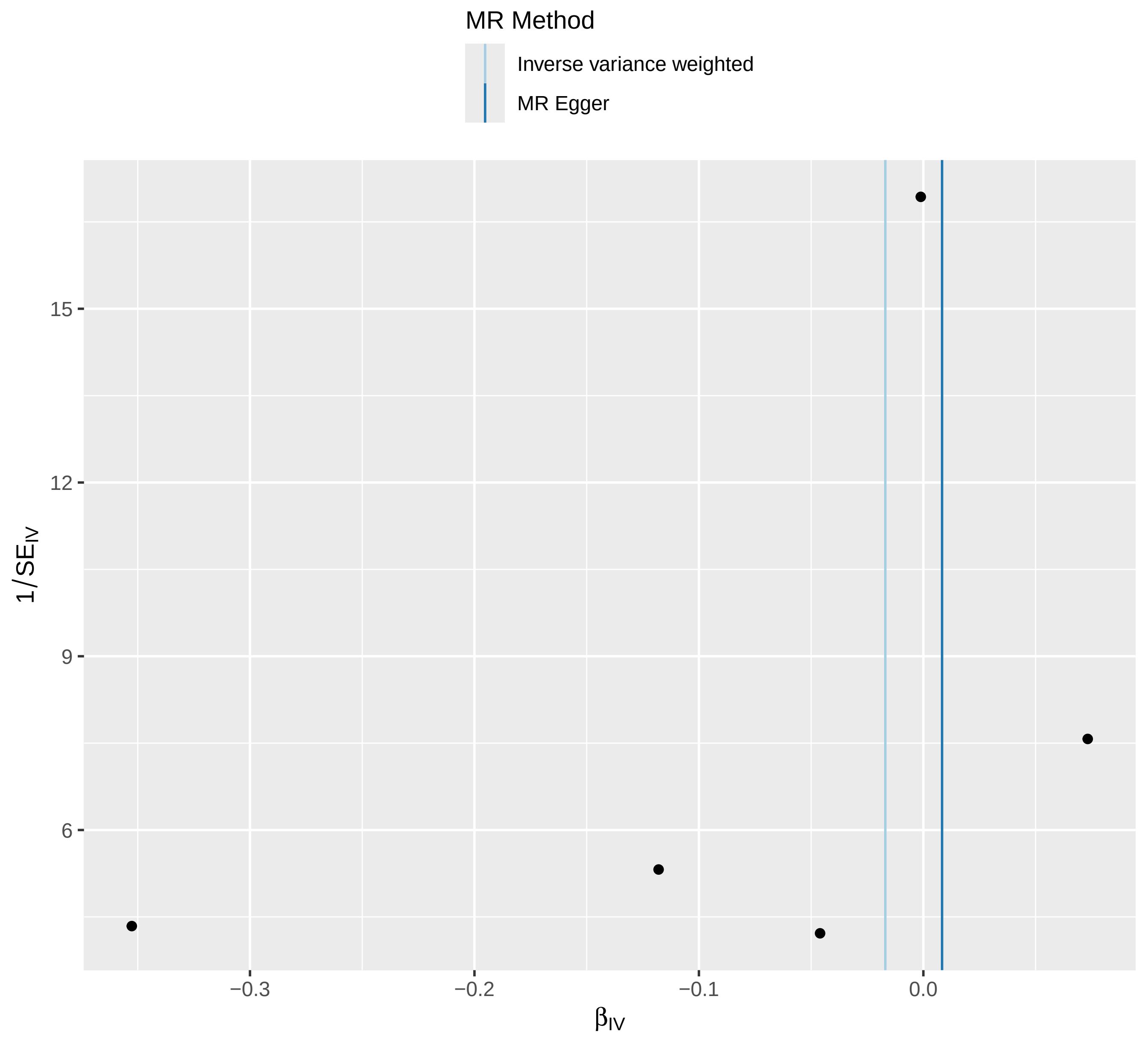
MR Method
Inverse variance weighted
MR Egger
15
12
9
6
$1/SE_{IV}$
−0.3
−0.2
−0.1
0.0
$\beta_{IV}$

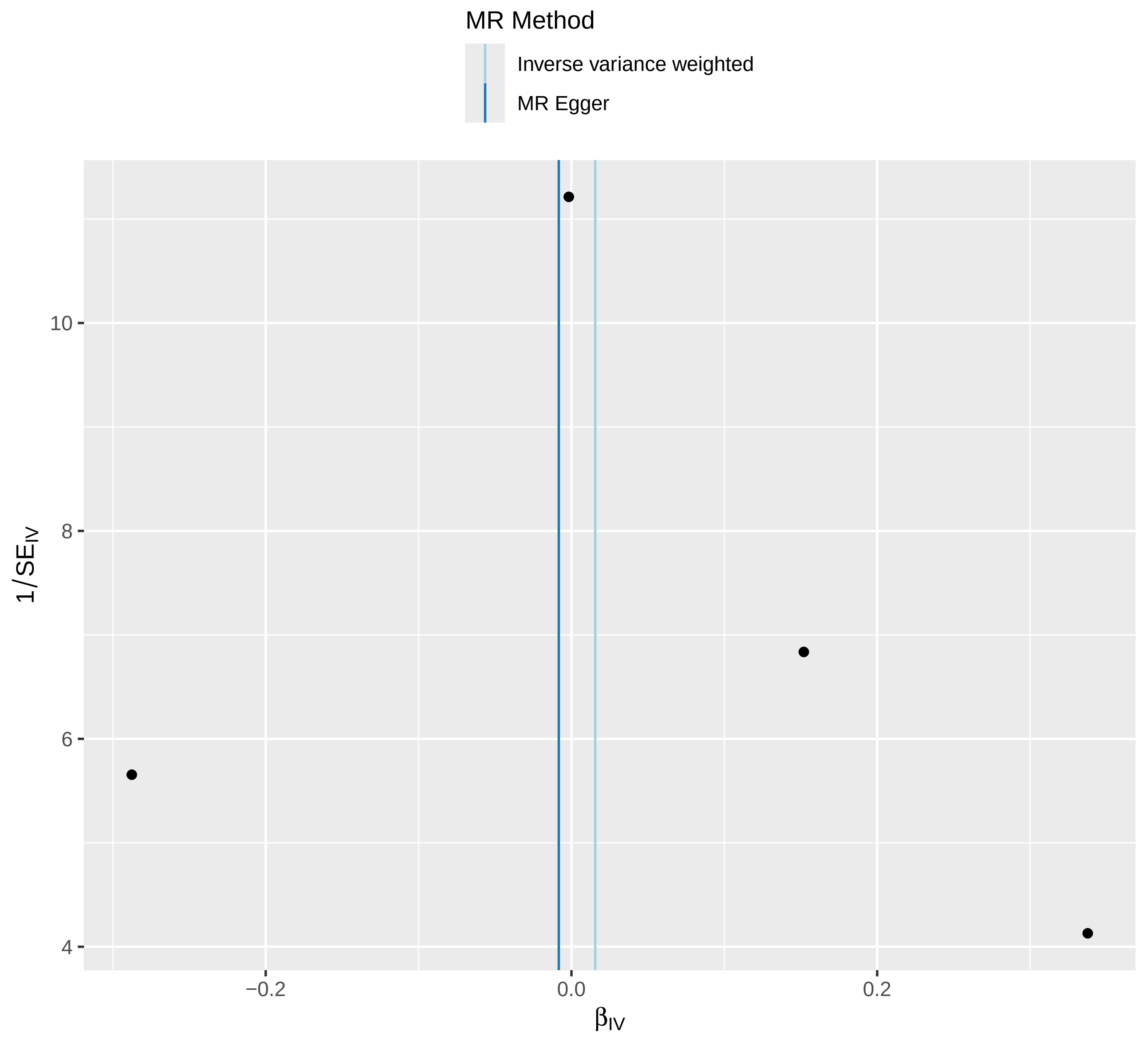

MR Method
Inverse variance weighted
MR Egger
10
8
6
4
1/SE$_{IV}$
−0.2
0.0
0.2
β$_{IV}$

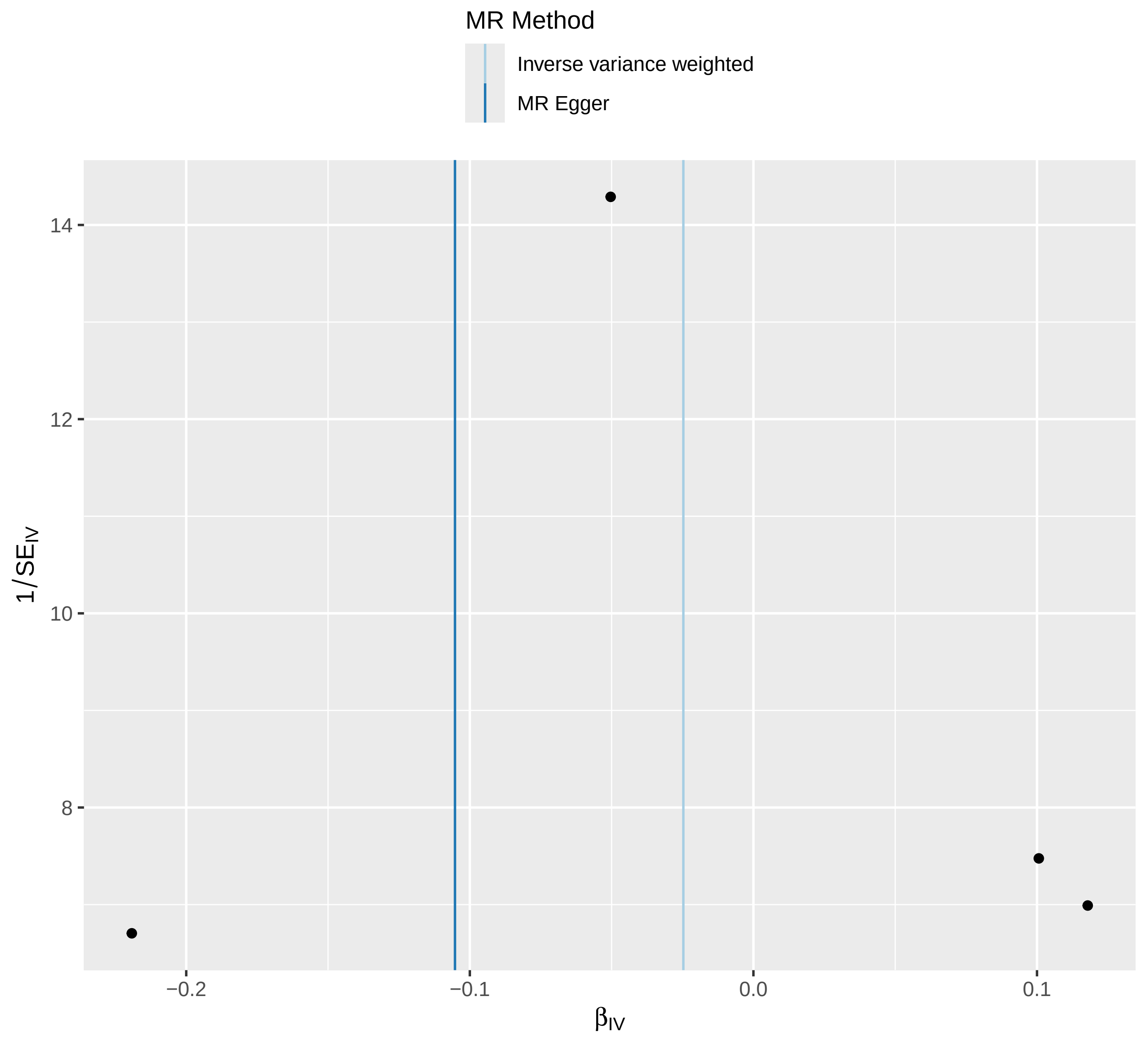
MR Method
Inverse variance weighted
MR Egger
14
12
10
8
1/SE$_{IV}$
−0.2
−0.1
0.0
0.1
β$_{IV}$

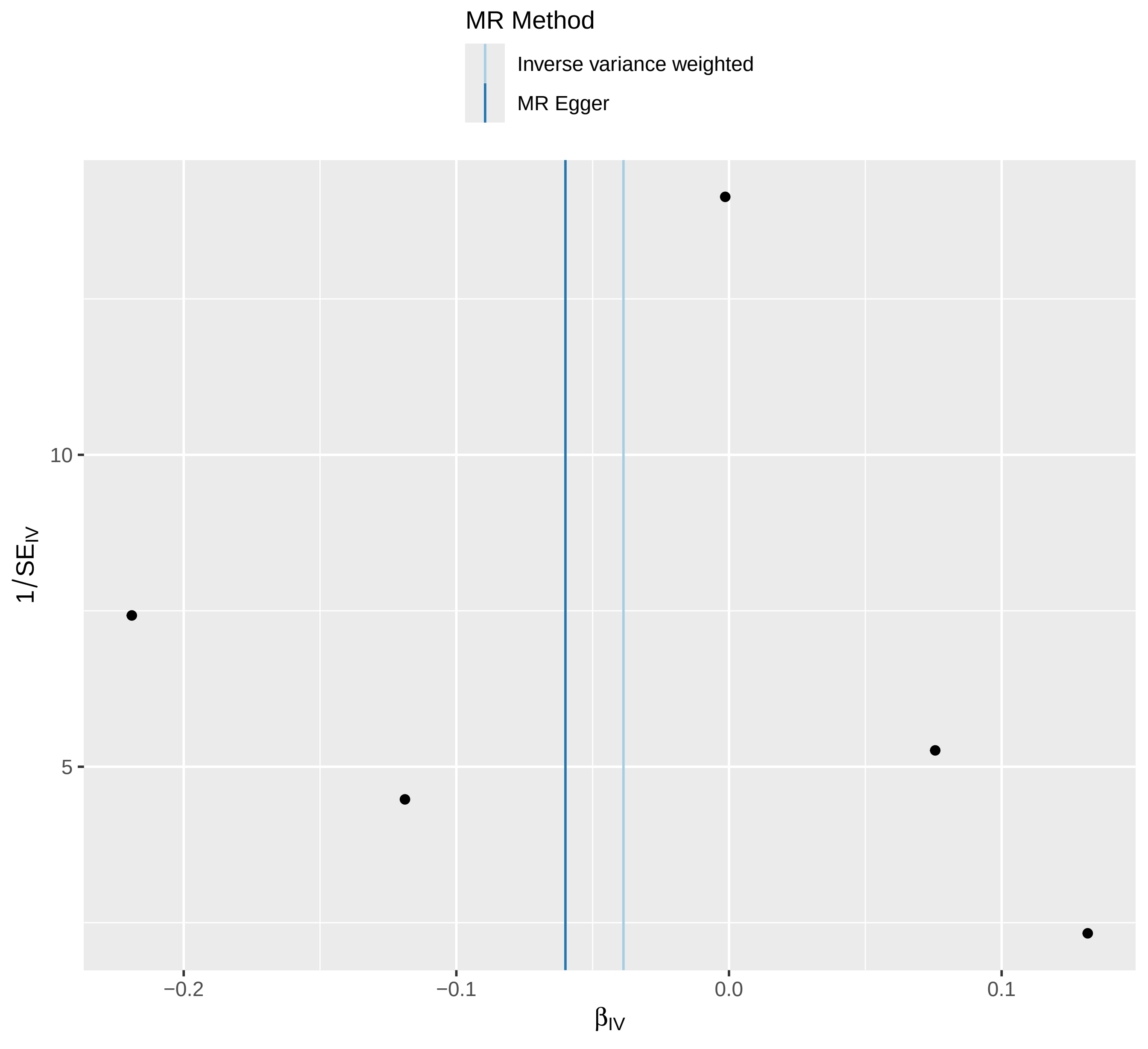
MR Method
Inverse variance weighted
MR Egger
10
5
1/SE$_{IV}$
−0.2
−0.1
0.0
0.1
β$_{IV}$

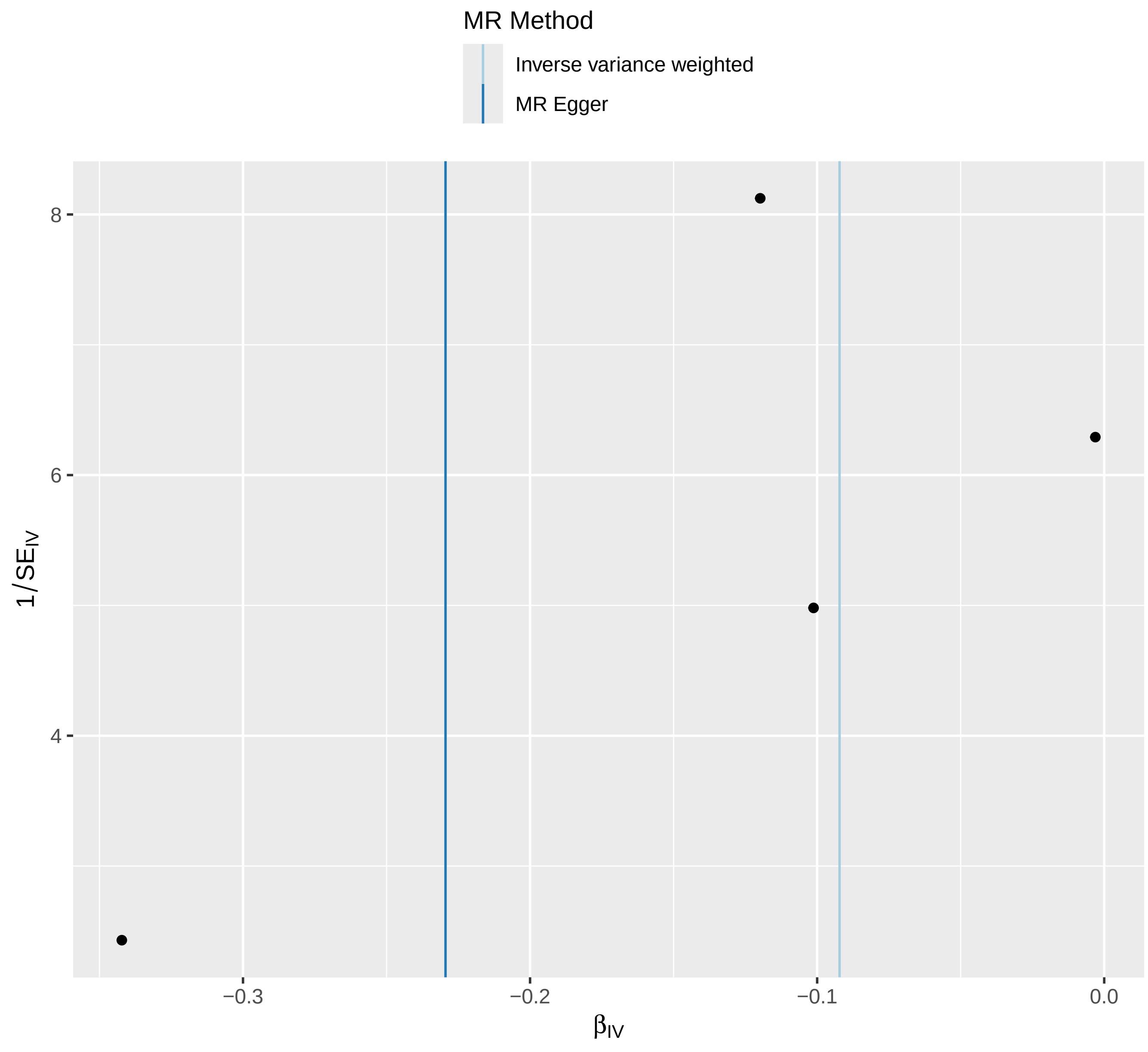
MR Method
Inverse variance weighted
MR Egger
8
6
4
1/SE$_{IV}$
−0.3
−0.2
−0.1
0.0
β$_{IV}$

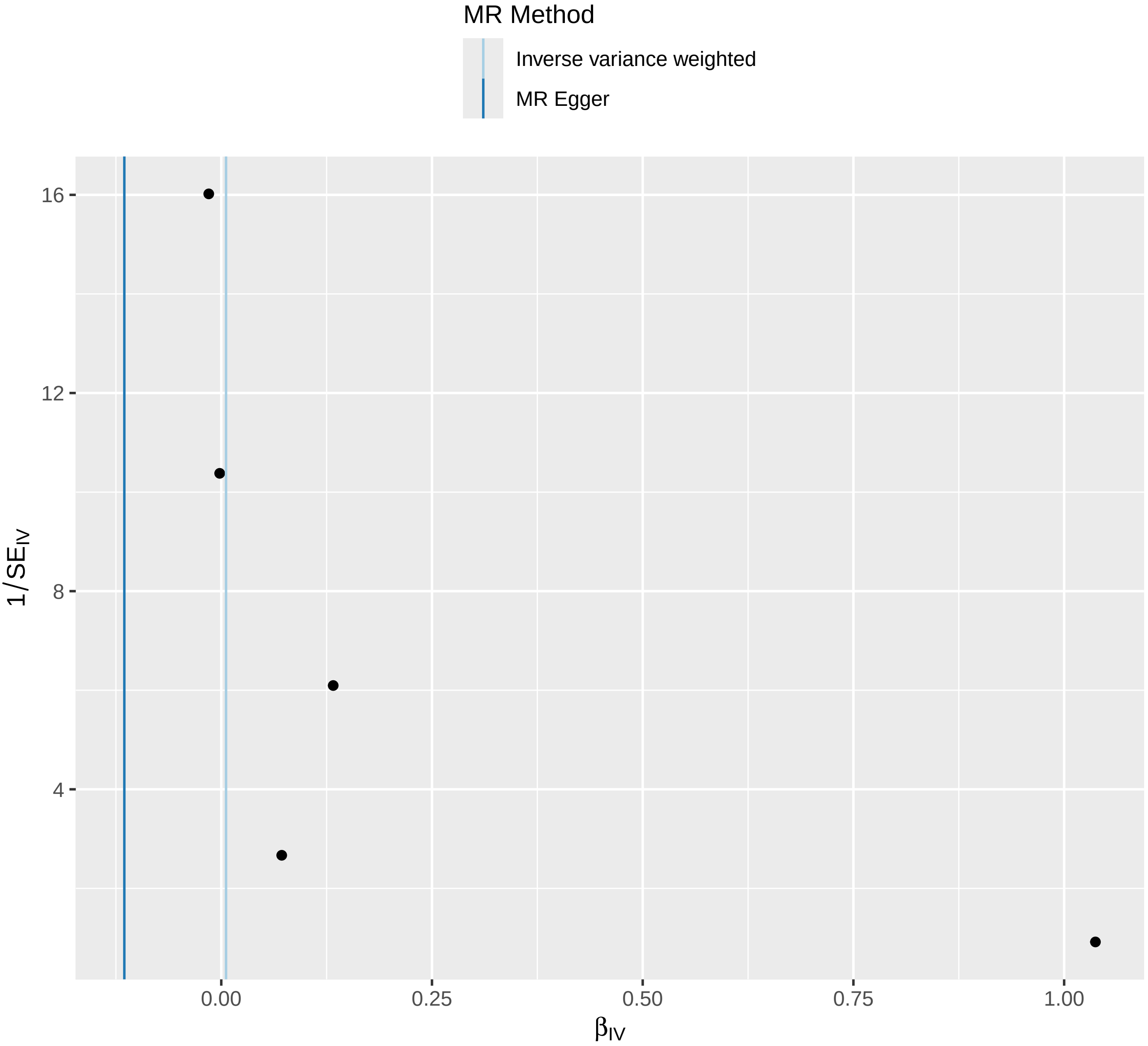
MR Method
Inverse variance weighted
MR Egger
16
12
8
4
1/SE$_{IV}$
0.00
0.25
0.50
0.75
1.00
β$_{IV}$

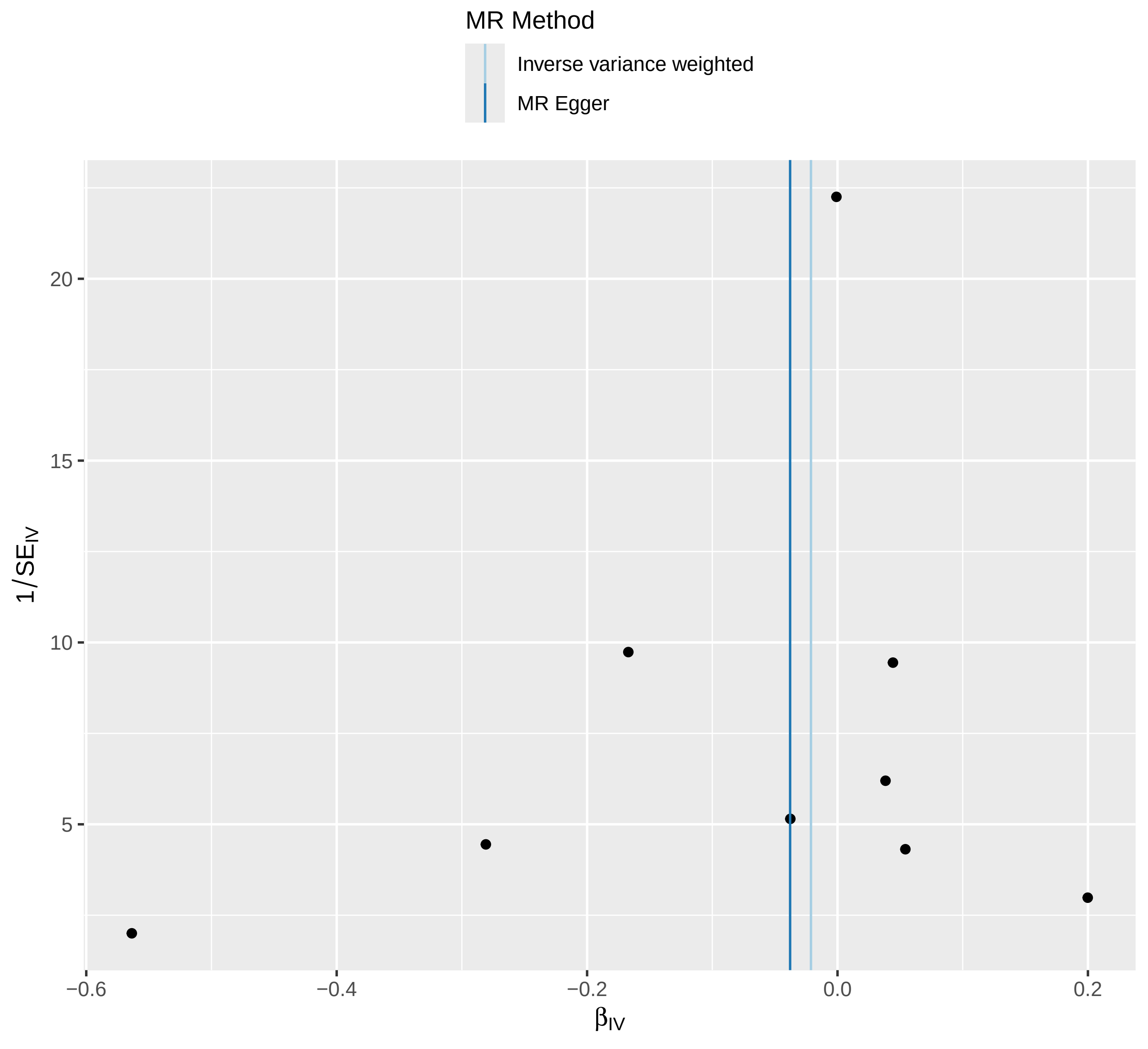
MR Method
Inverse variance weighted
MR Egger
20
15
10
5
1/SE_IV
−0.6
−0.4
−0.2
0.0
0.2
β_IV

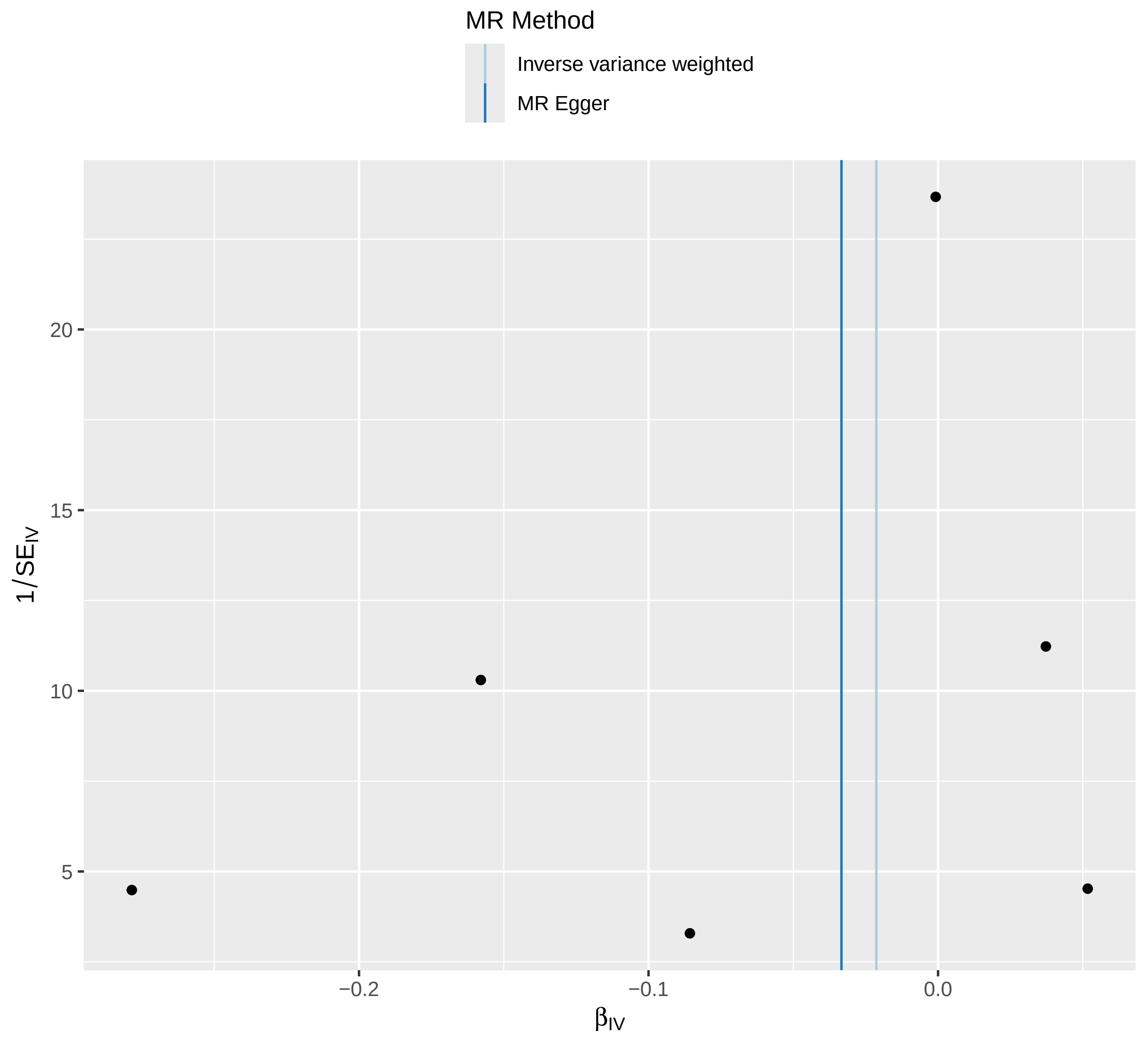

MR Method
Inverse variance weighted
MR Egger
20
15
10
5
1/SE$_{IV}$
−0.2
−0.1
0.0
β$_{IV}$

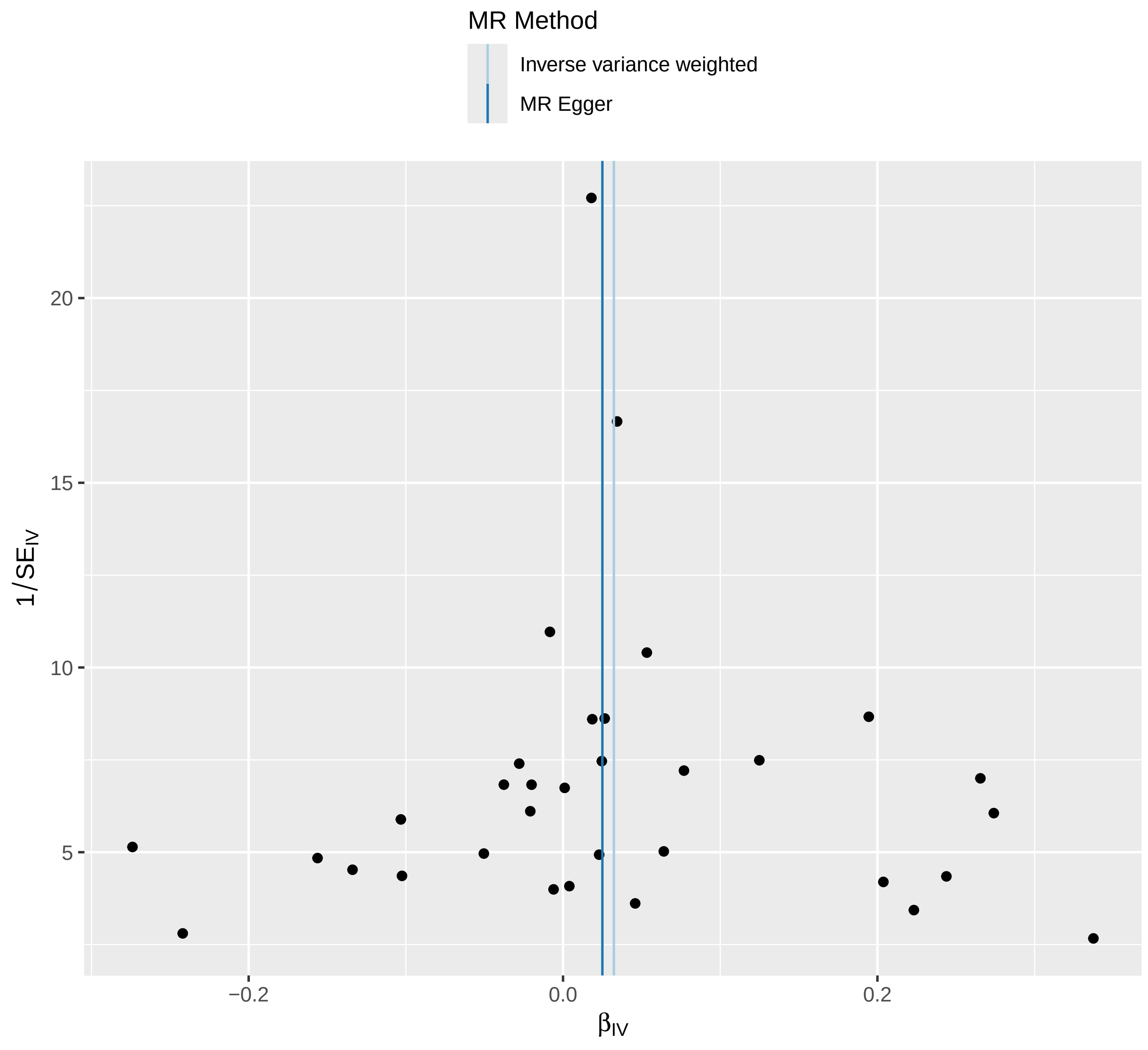
MR Method
Inverse variance weighted
MR Egger
20
15
10
5
1/SE_IV
−0.2
0.0
0.2
β_IV

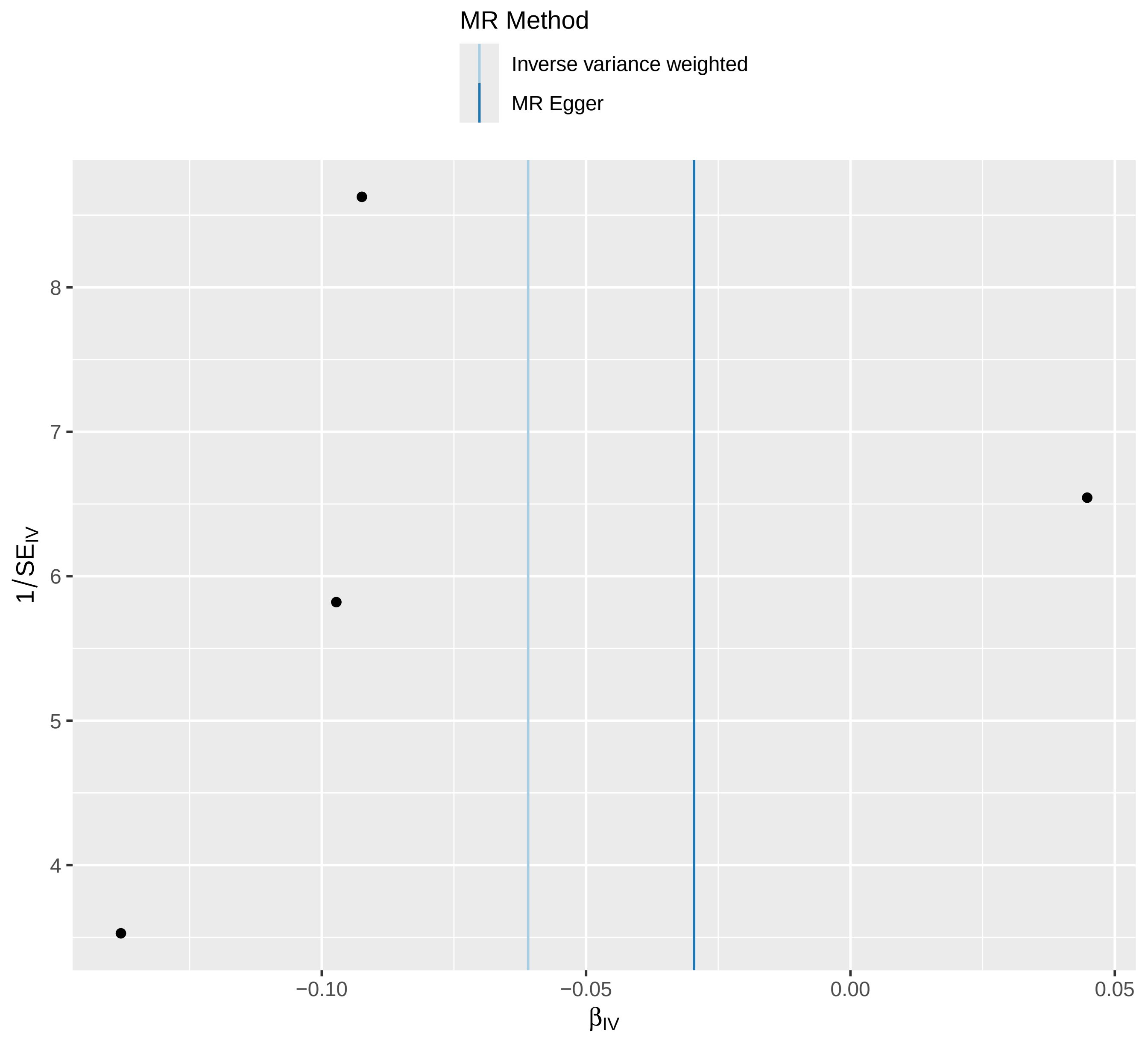
MR Method
Inverse variance weighted
MR Egger
8
7
6
5
4
$1/SE_{IV}$
−0.10
−0.05
0.00
0.05
$\beta_{IV}$

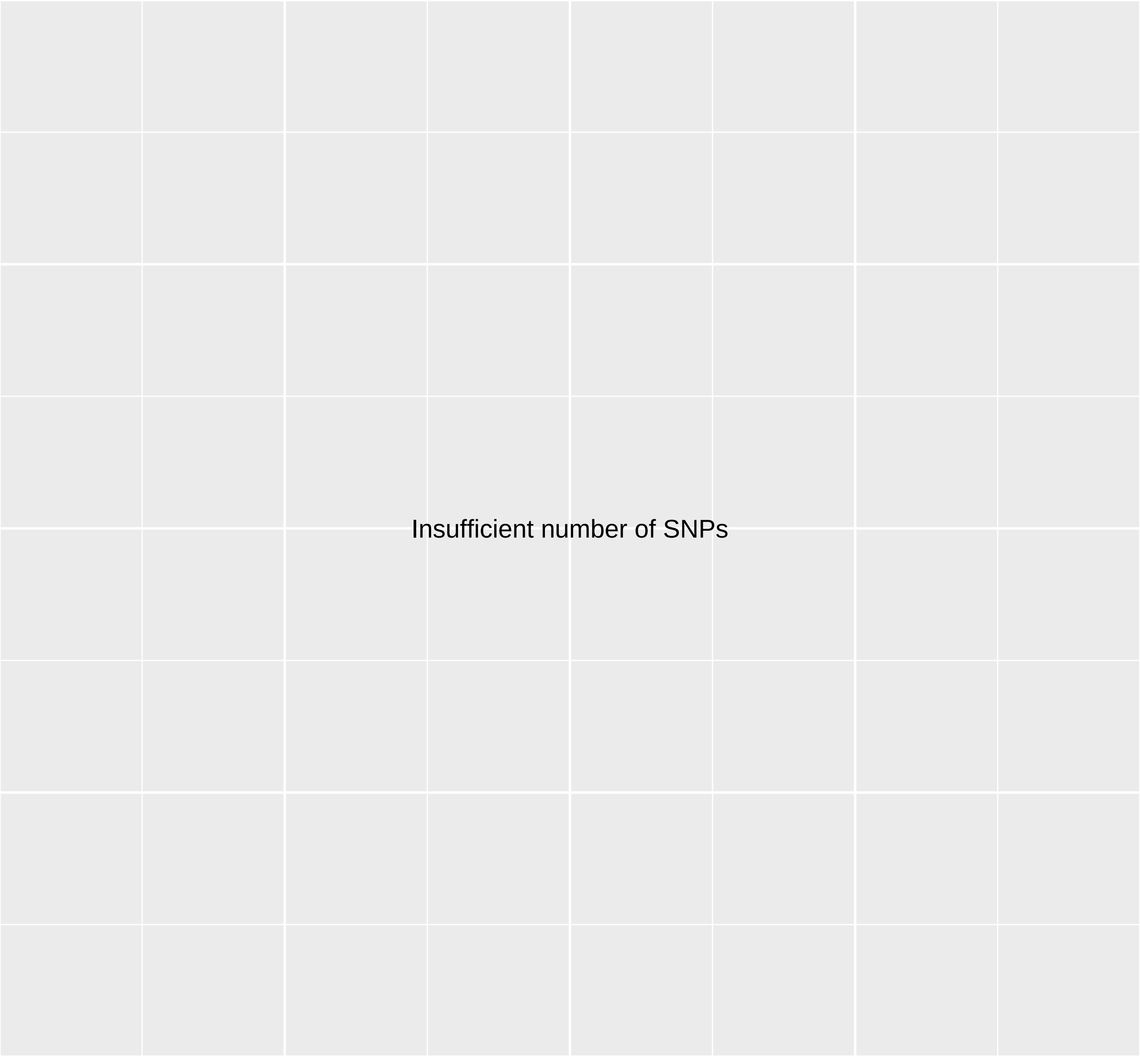
Insufficient number of SNPs

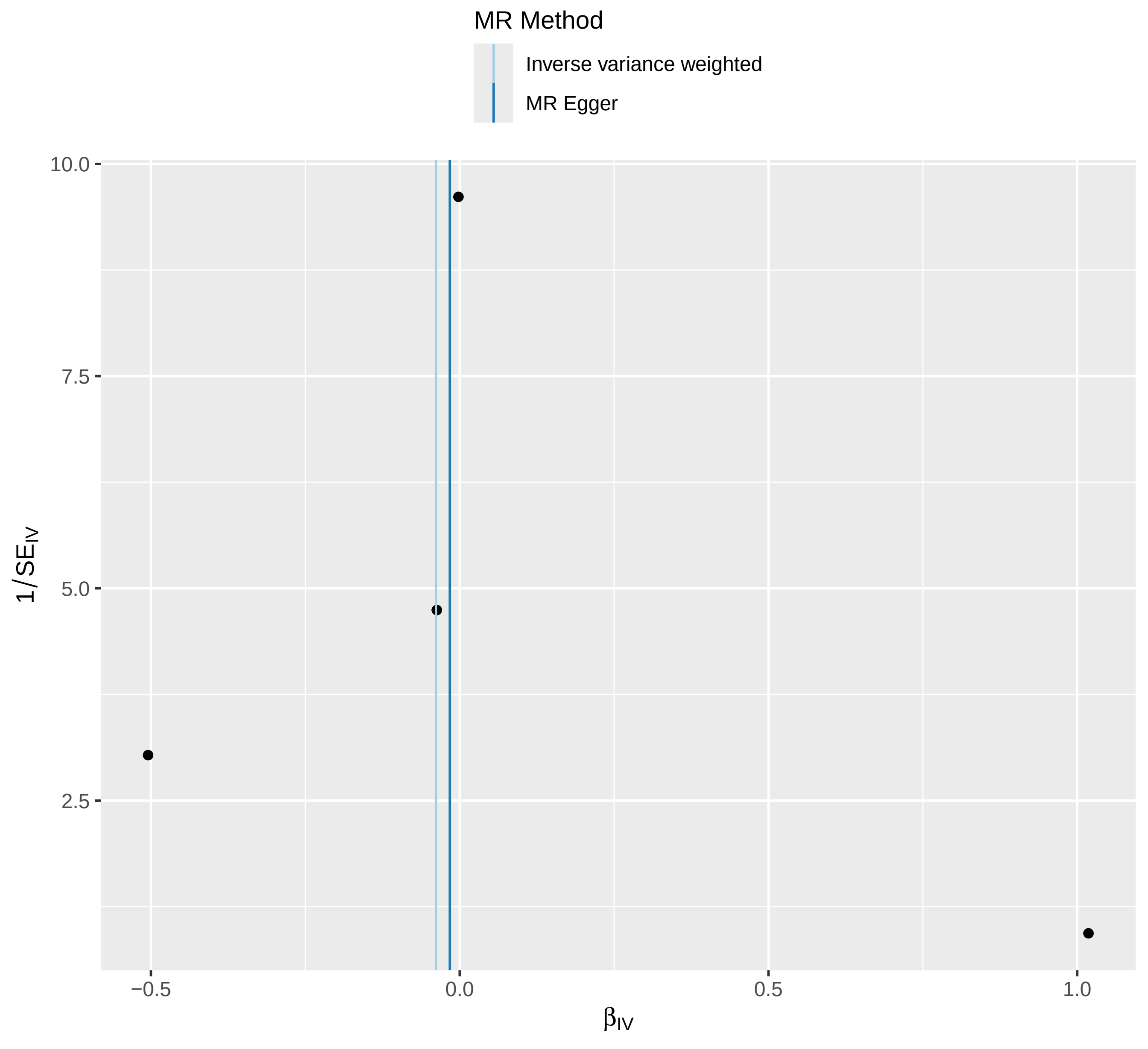
MR Method
Inverse variance weighted
MR Egger
10.0
7.5
5.0
2.5
1/SE_IV
−0.5
0.0
0.5
1.0
β_IV

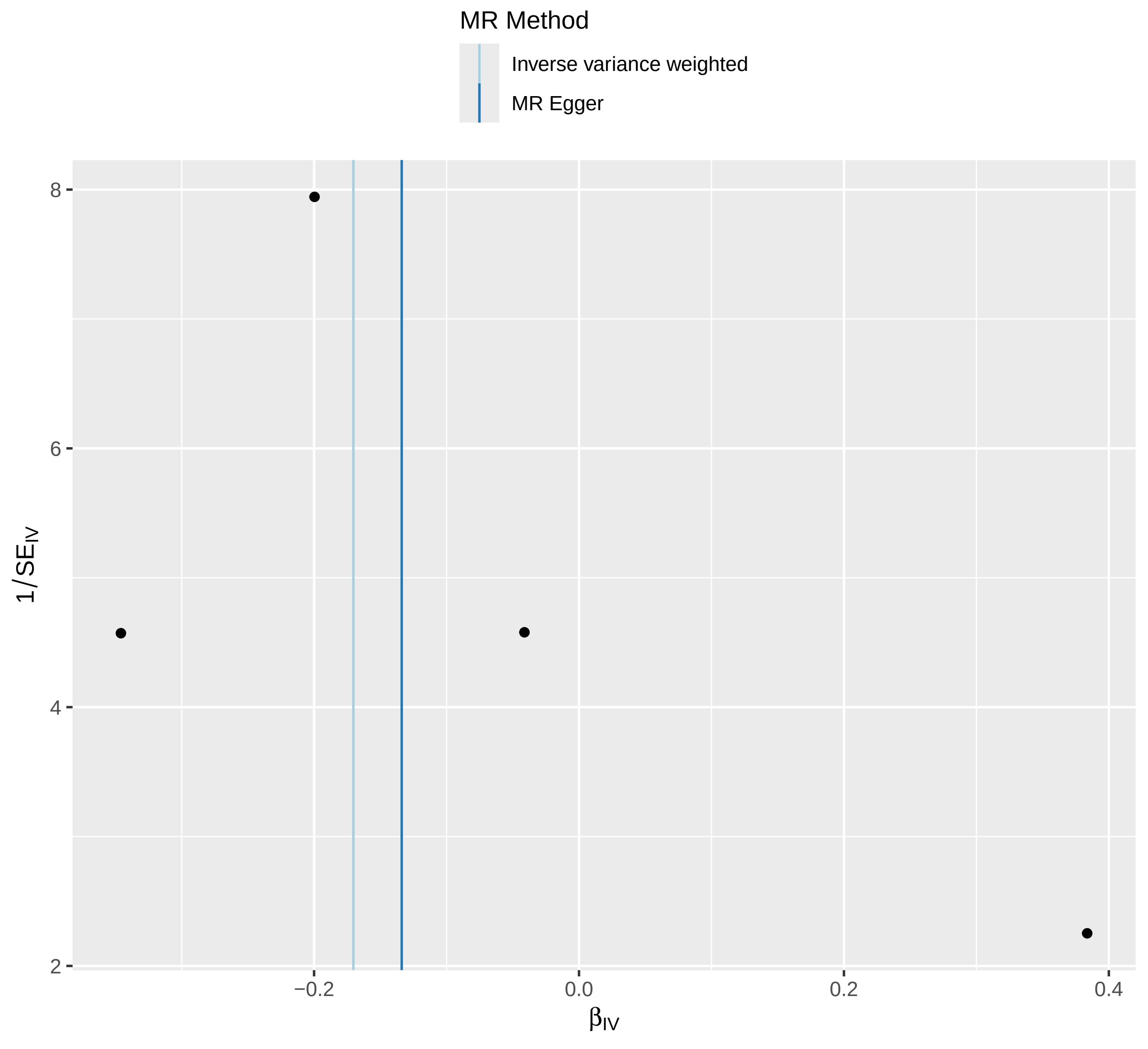
MR Method
Inverse variance weighted
MR Egger
8
6
4
2
−0.2
0.0
0.2
0.4
$1/SE_{IV}$
$\beta_{IV}$

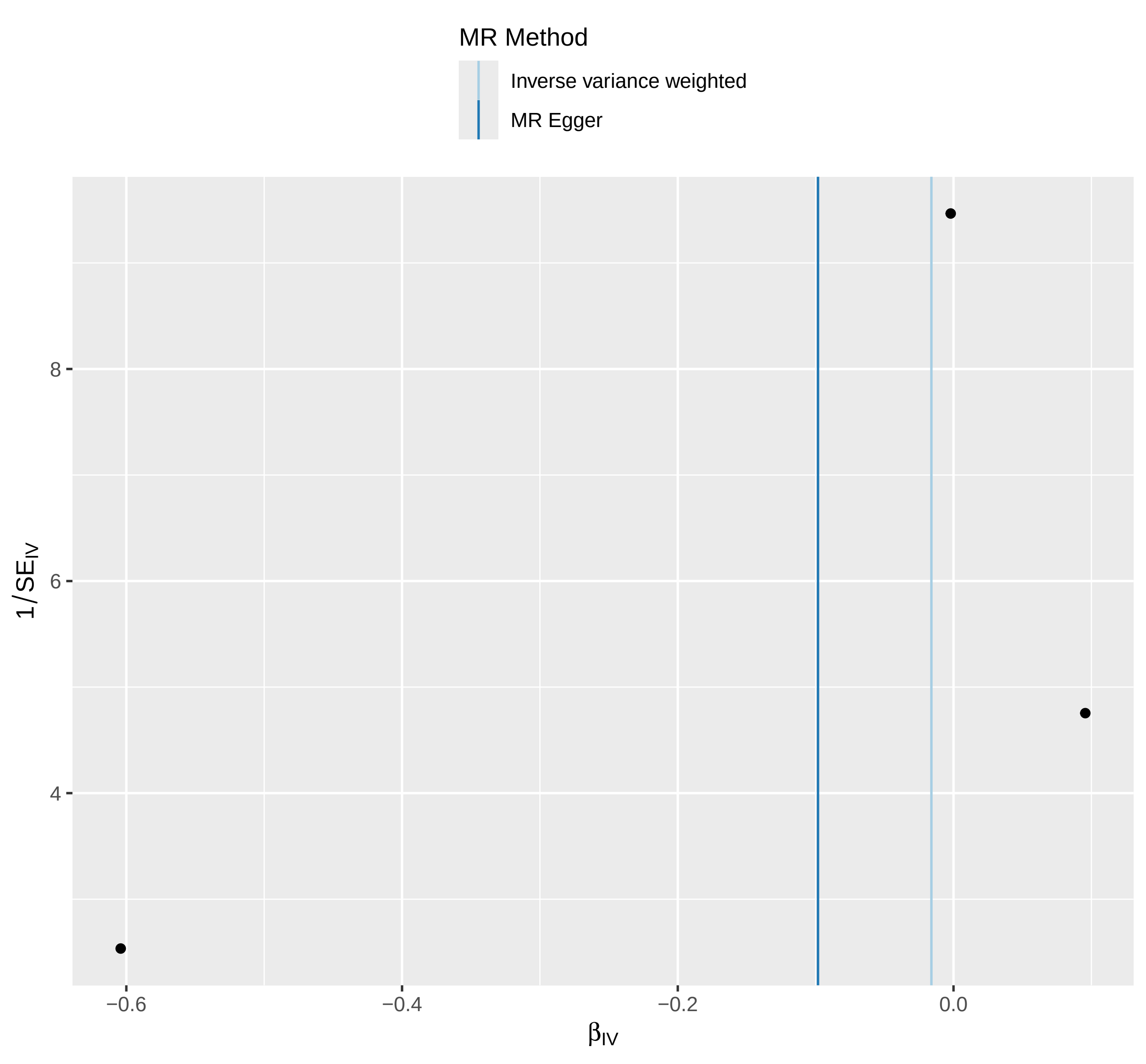

MR Method
Inverse variance weighted
MR Egger
8
6
4
1/SE$_{IV}$
−0.6
−0.4
−0.2
0.0
β$_{IV}$

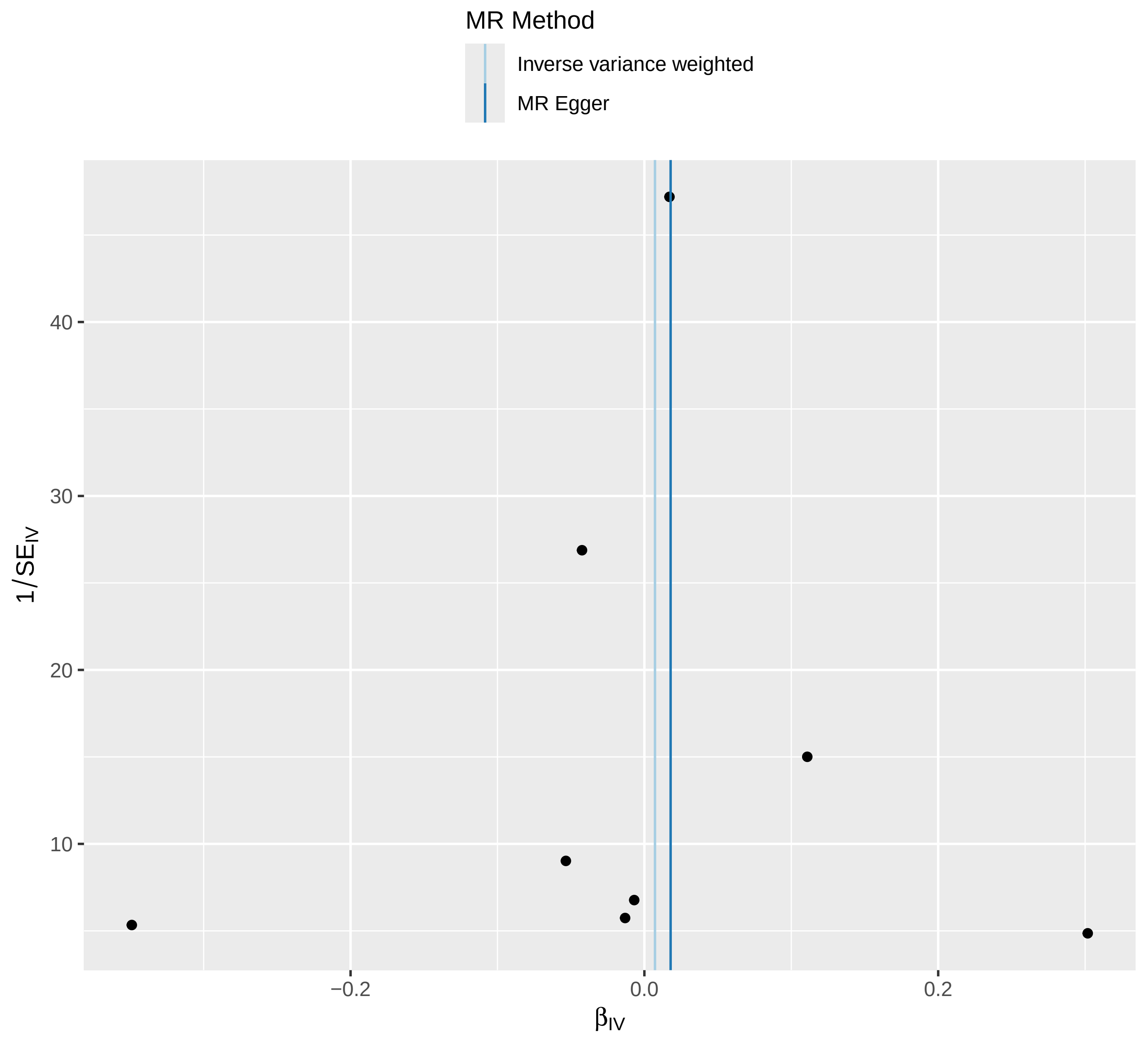
MR Method
Inverse variance weighted
MR Egger
1/SE$_{IV}$
40
30
20
10
−0.2
0.0
0.2
β$_{IV}$

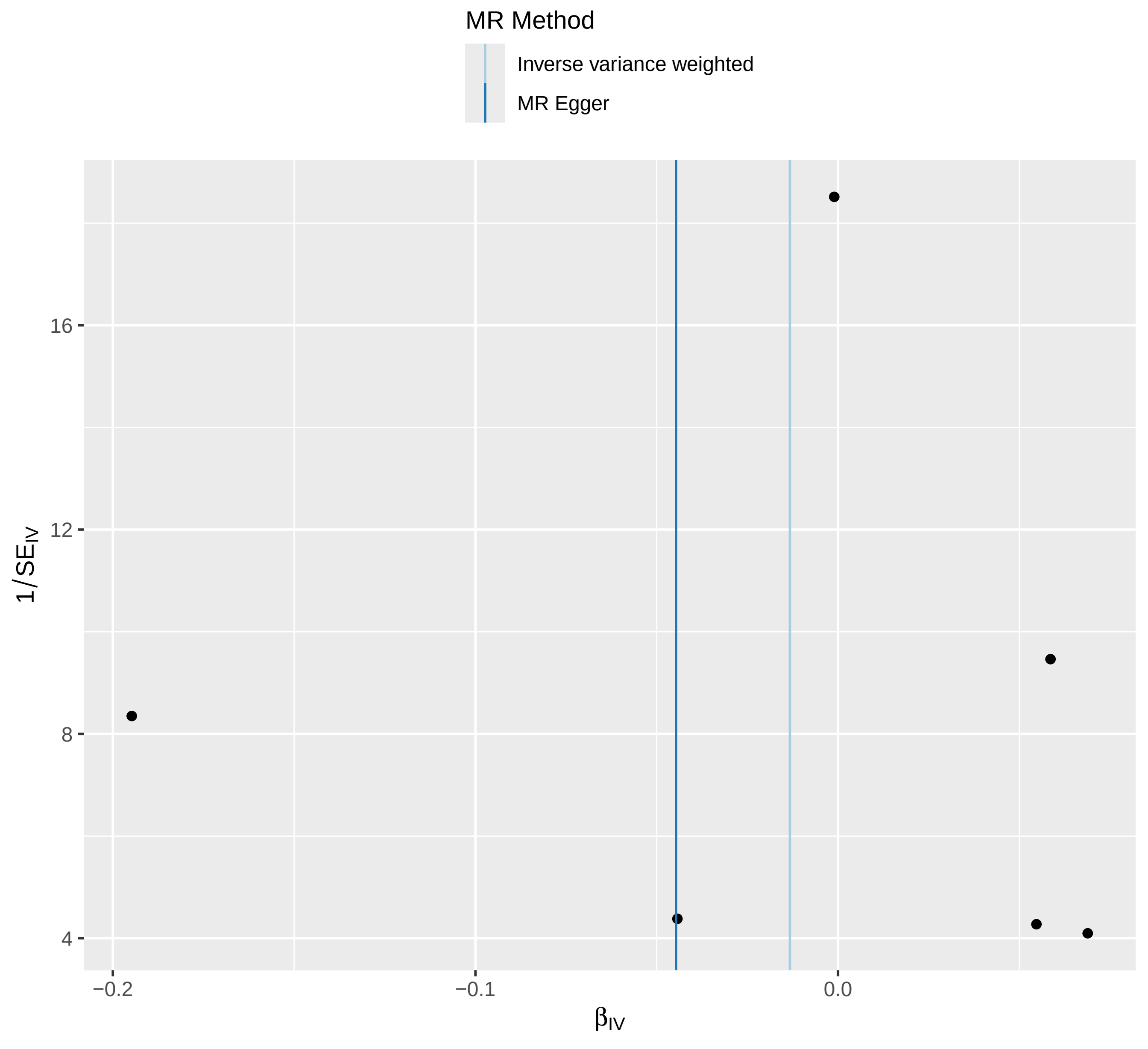
MR Method
Inverse variance weighted
MR Egger
16
12
8
4
1/SE_IV
−0.2
−0.1
0.0
β_IV

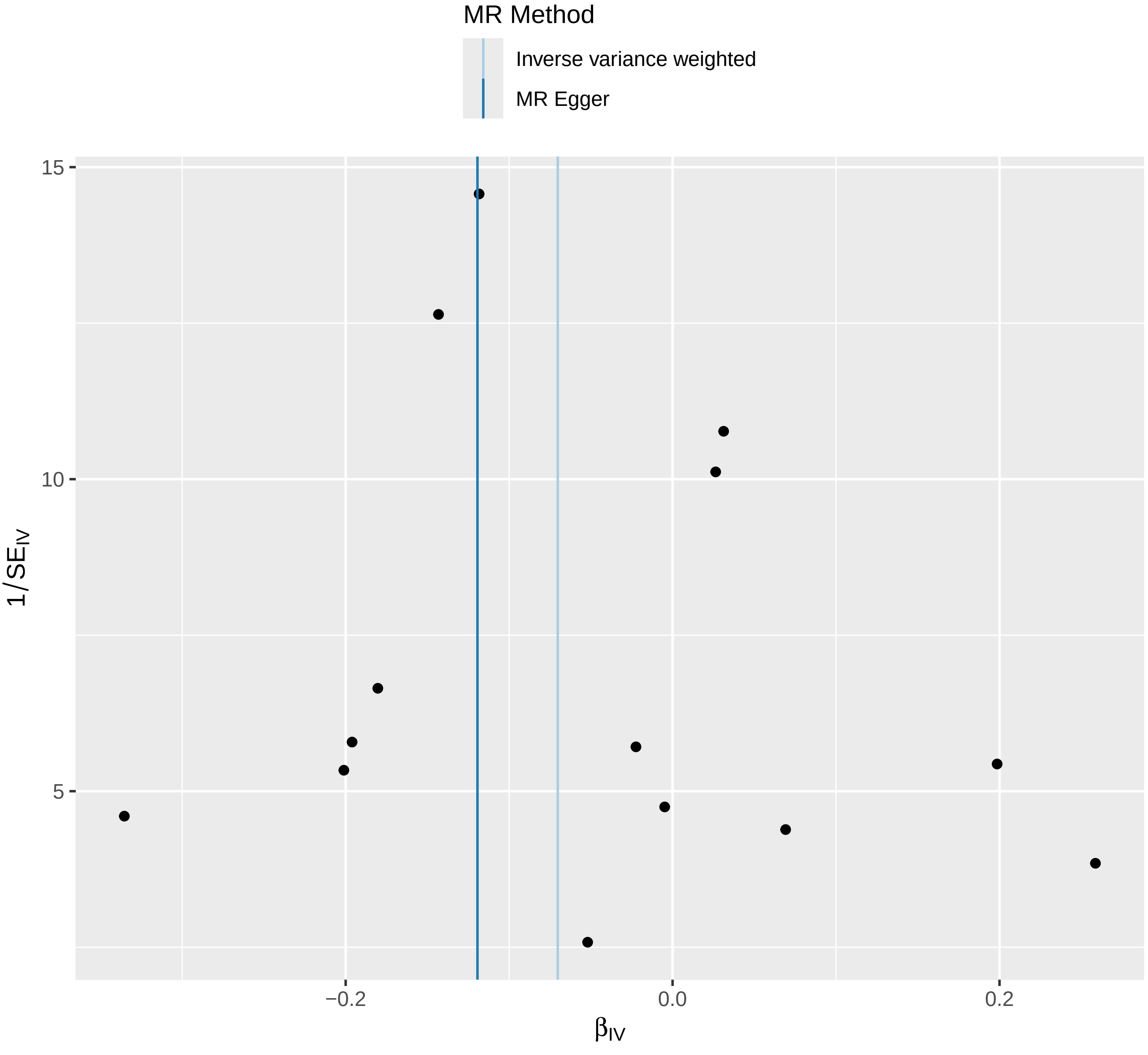

MR Method
Inverse variance weighted
MR Egger
15
10
5
1/SE_IV
−0.2
0.0
0.2
β_IV

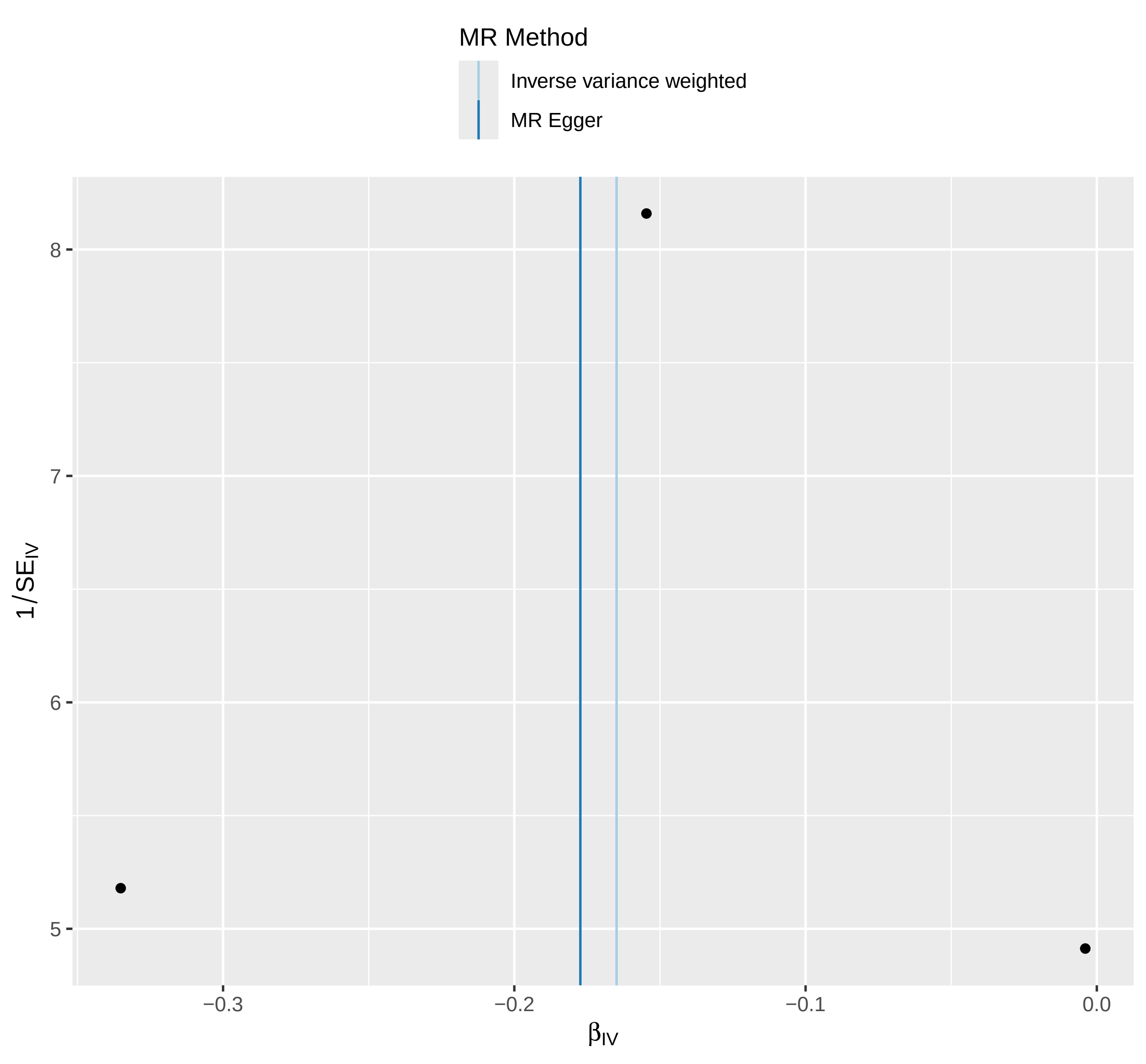

MR Method
Inverse variance weighted
MR Egger
8
7
6
5
1/SE$_{IV}$
−0.3
−0.2
−0.1
0.0
β$_{IV}$

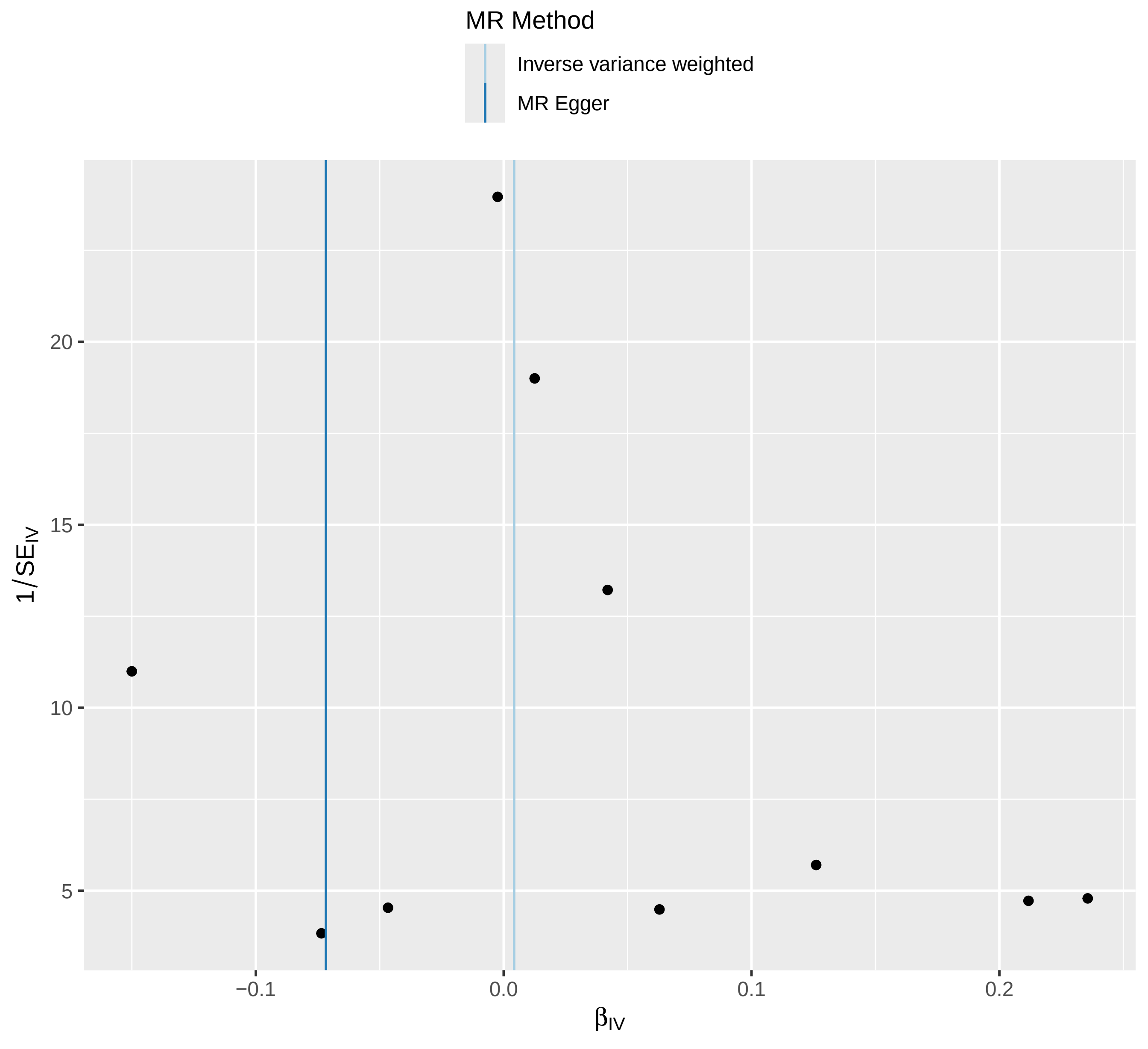
MR Method
Inverse variance weighted
MR Egger
1/SE_IV
20
15
10
5
−0.1
0.0
0.1
0.2
β_IV

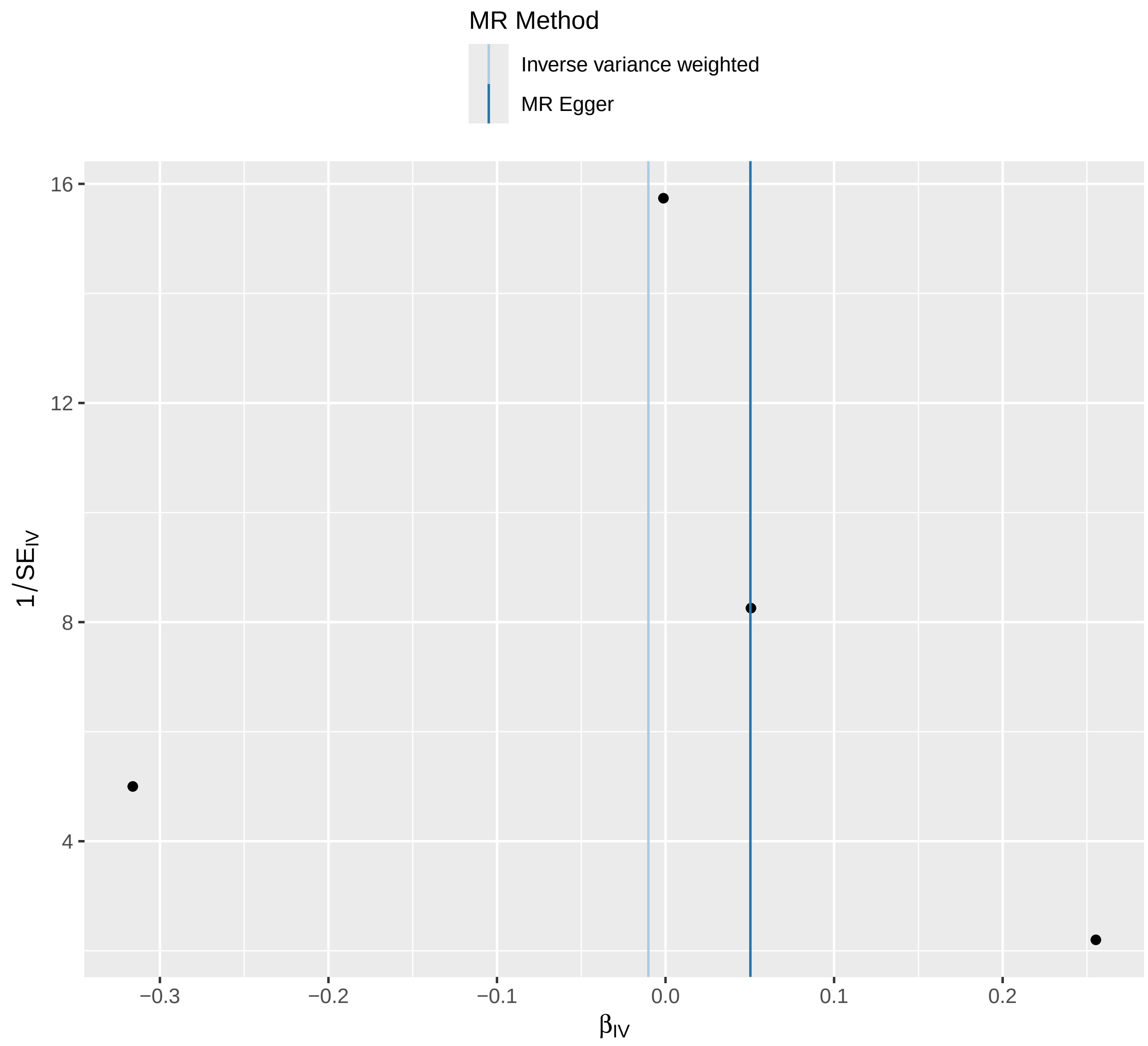
MR Method
Inverse variance weighted
MR Egger
16
12
8
4
1/SE$_{IV}$
−0.3
−0.2
−0.1
0.0
0.1
0.2
β$_{IV}$

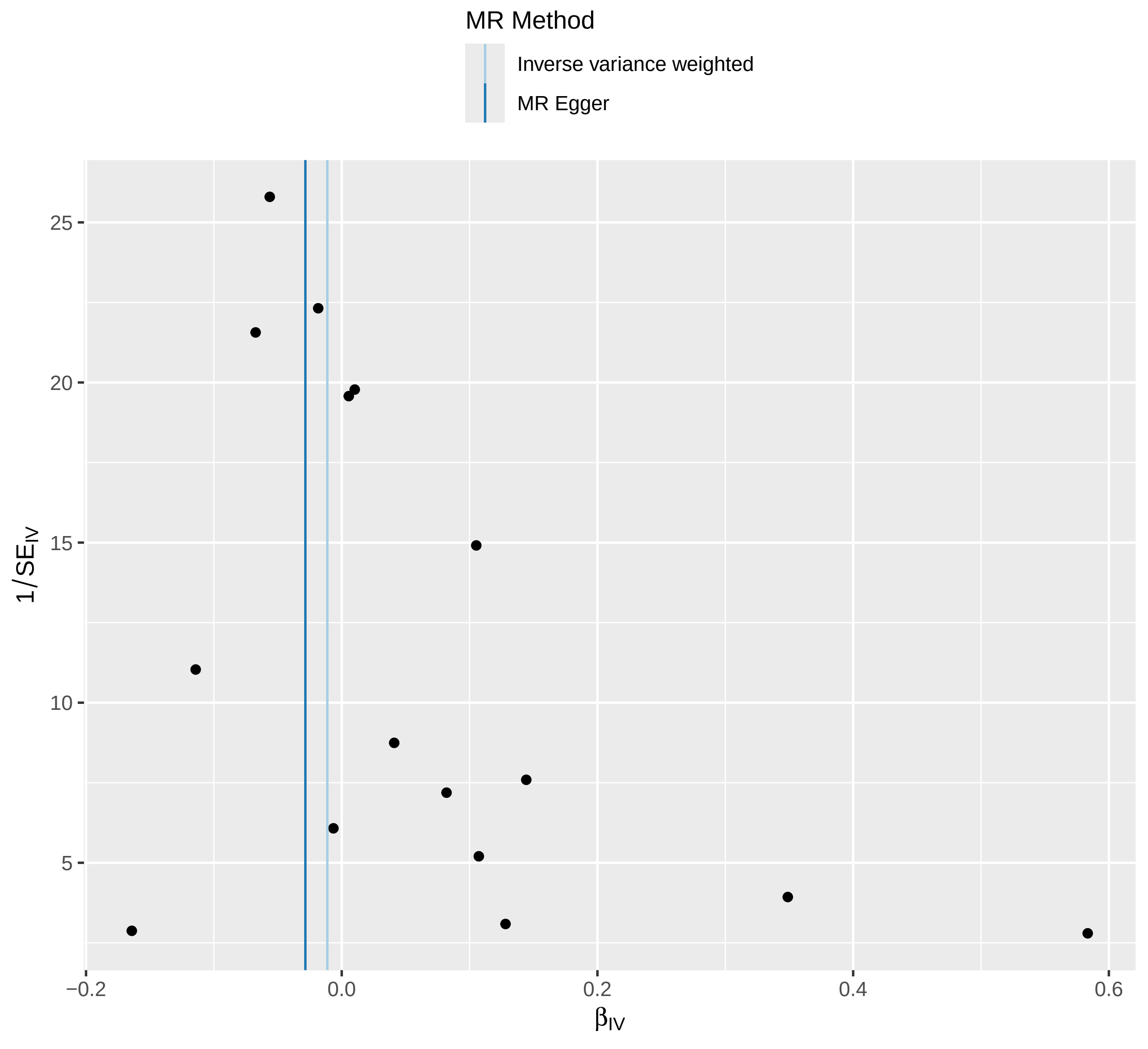
MR Method
Inverse variance weighted
MR Egger
25
20
15
10
5
1/SE_IV
−0.2
0.0
0.2
0.4
0.6
β_IV

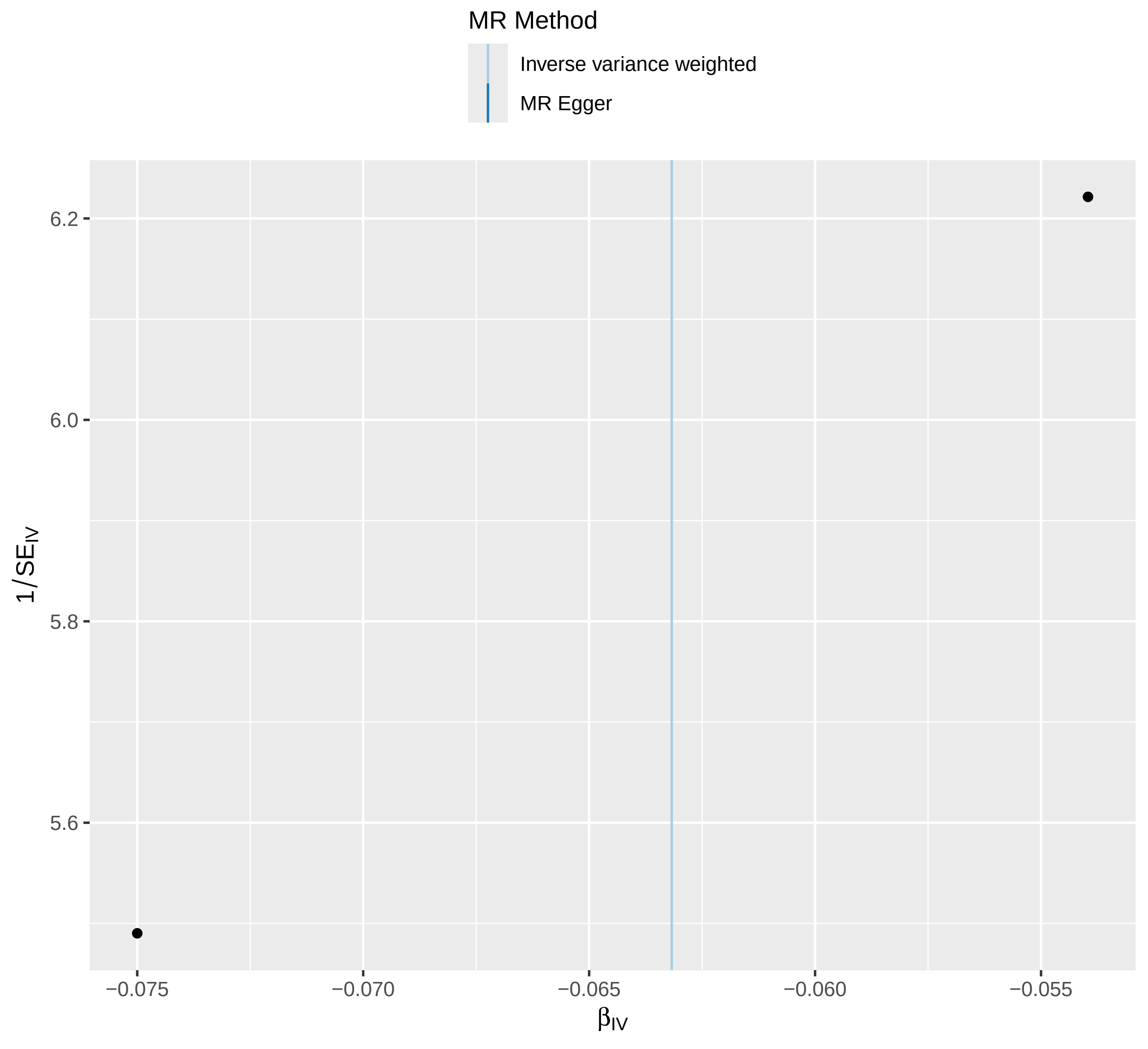
MR Method
Inverse variance weighted
MR Egger
6.2
6.0
5.8
5.6
1/SE_IV
−0.075
−0.070
−0.065
−0.060
−0.055
β_IV

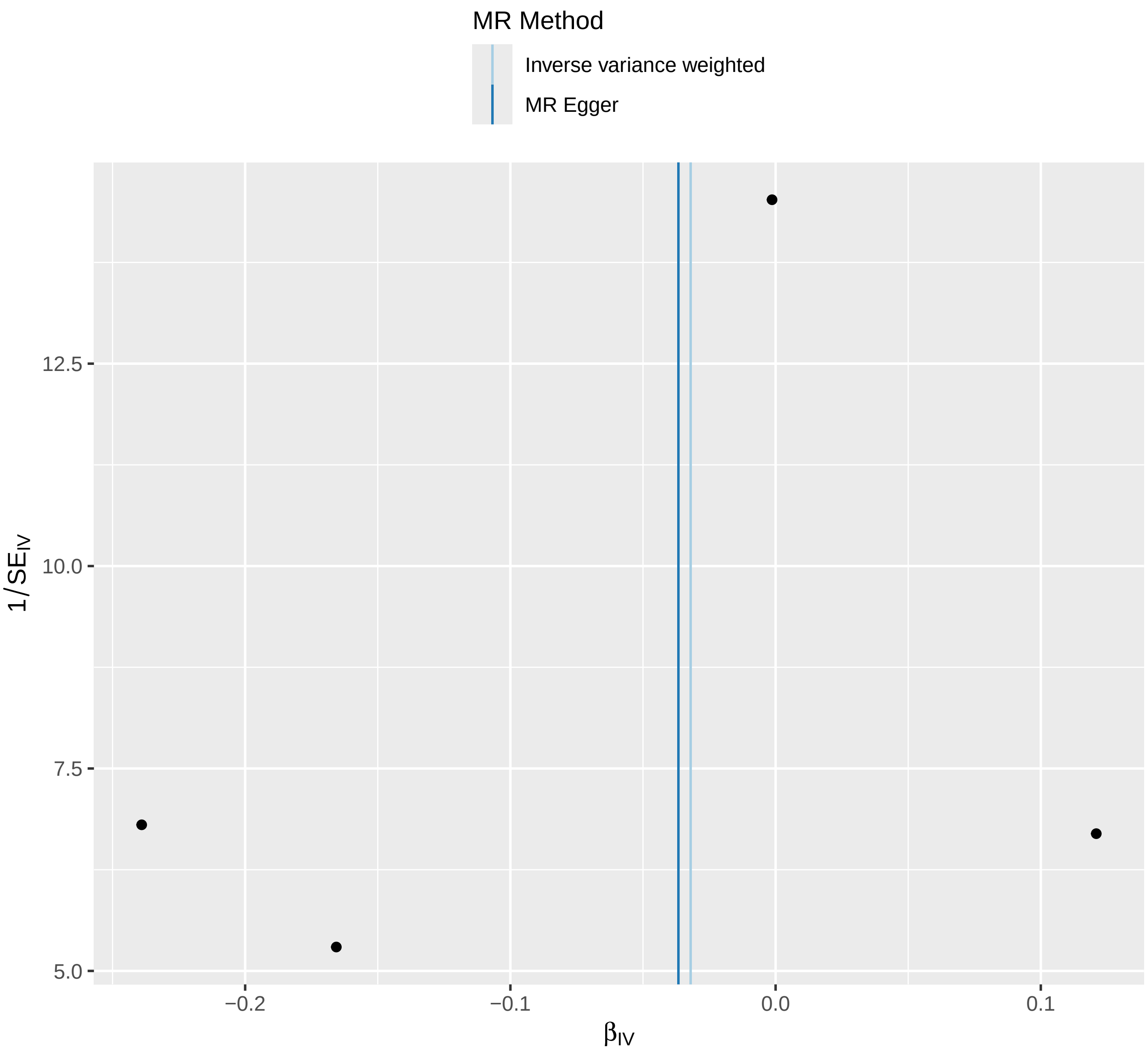
MR Method
Inverse variance weighted
MR Egger
1/SE_IV
12.5
10.0
7.5
5.0
−0.2
−0.1
0.0
0.1
β_IV

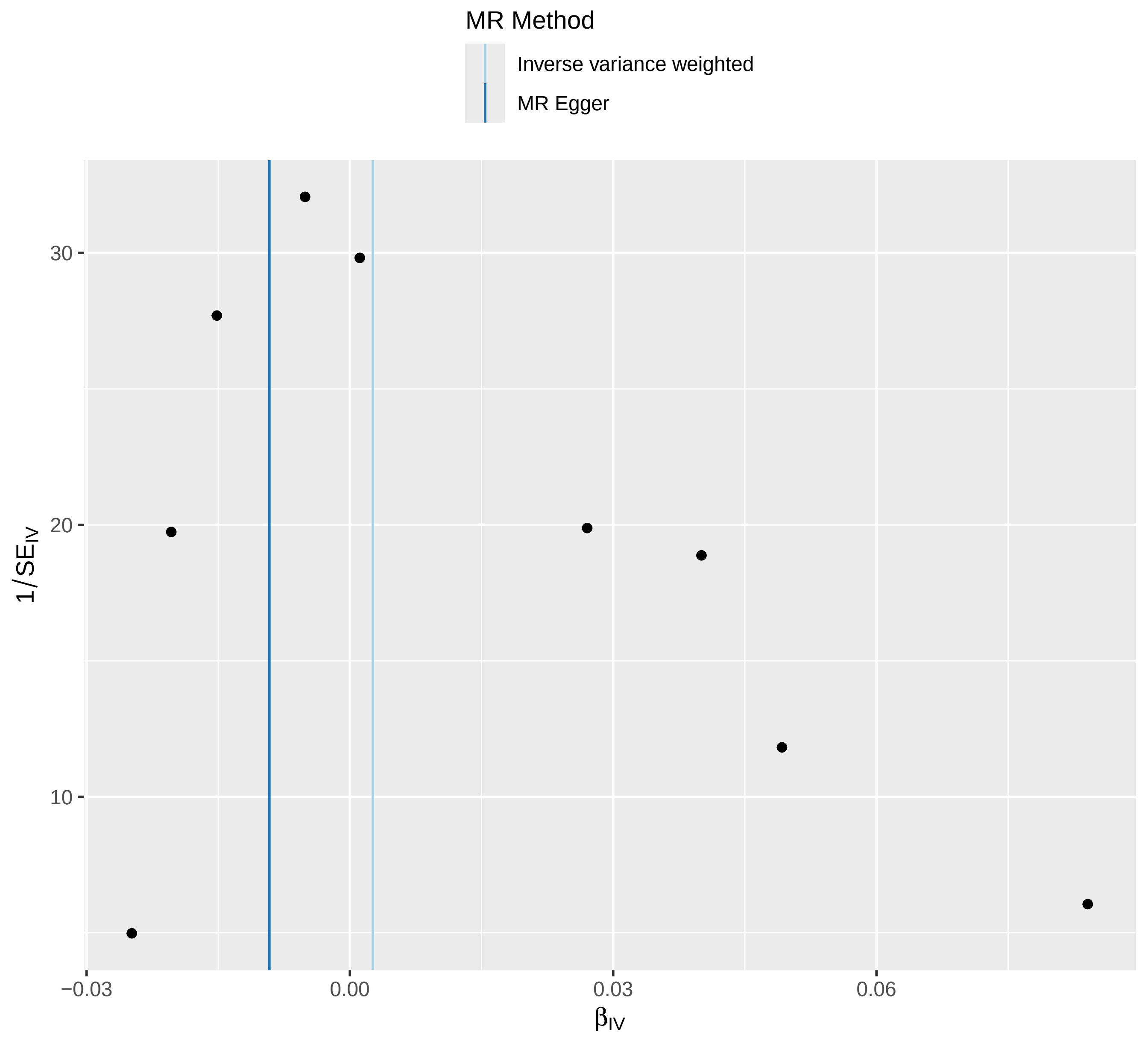
MR Method
Inverse variance weighted
MR Egger
30
20
10
1/SE$_{IV}$
−0.03
0.00
0.03
0.06
β$_{IV}$

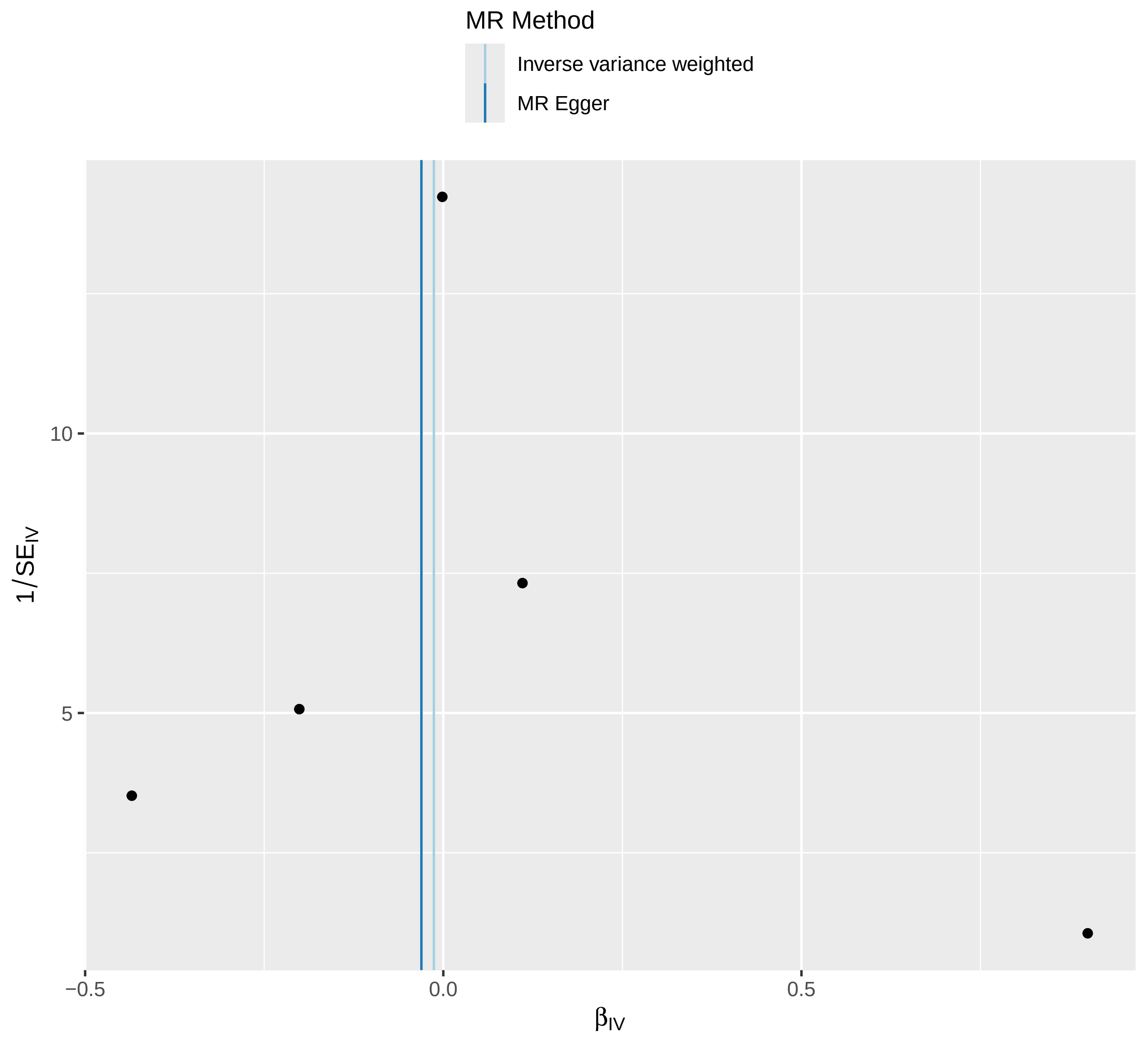
MR Method
Inverse variance weighted
MR Egger
10
5
1/SE_IV
−0.5
0.0
0.5
β_IV

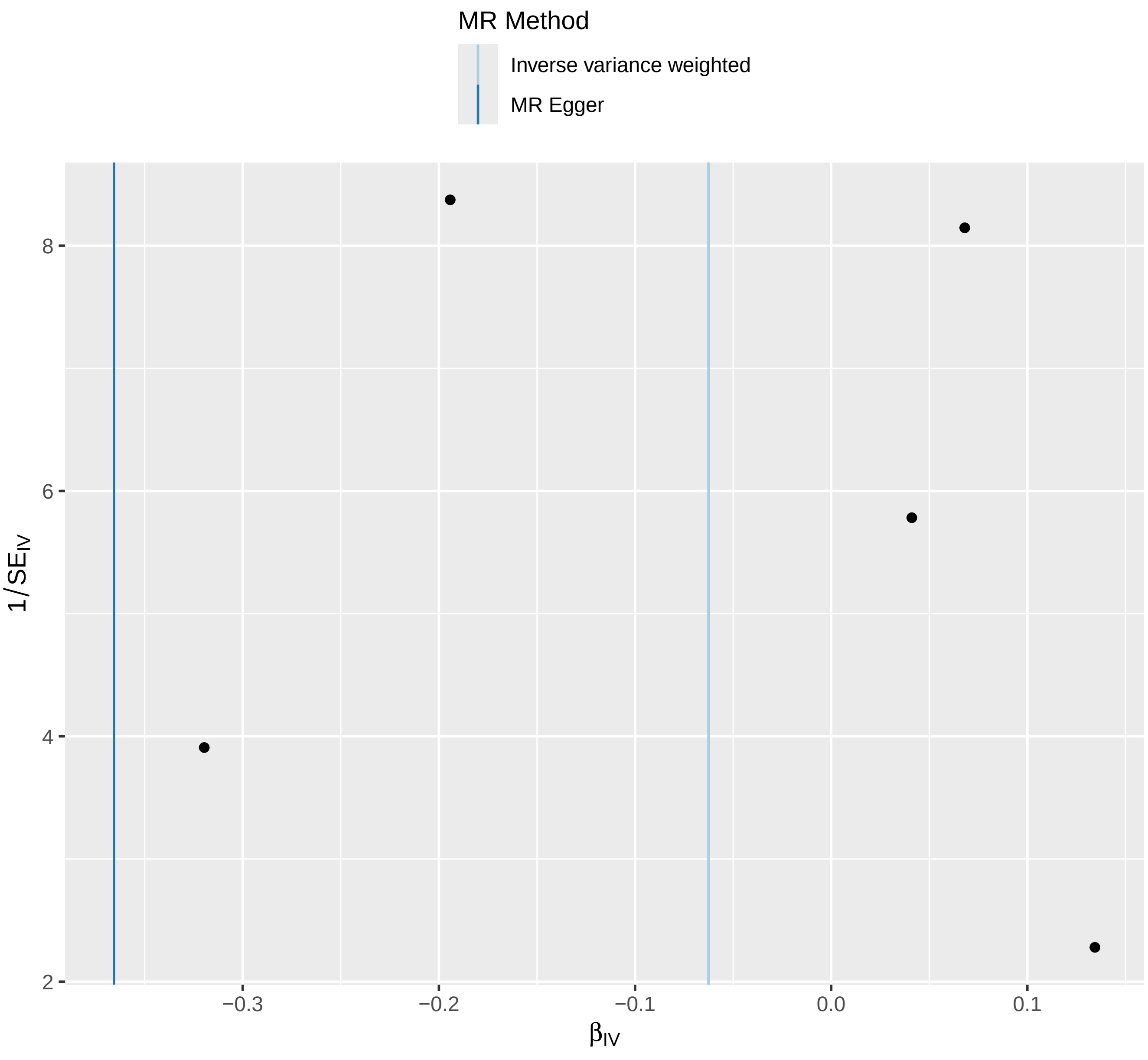
MR Method
Inverse variance weighted
MR Egger
8
6
4
2
1/SE$_{IV}$
−0.3
−0.2
−0.1
0.0
0.1
β$_{IV}$

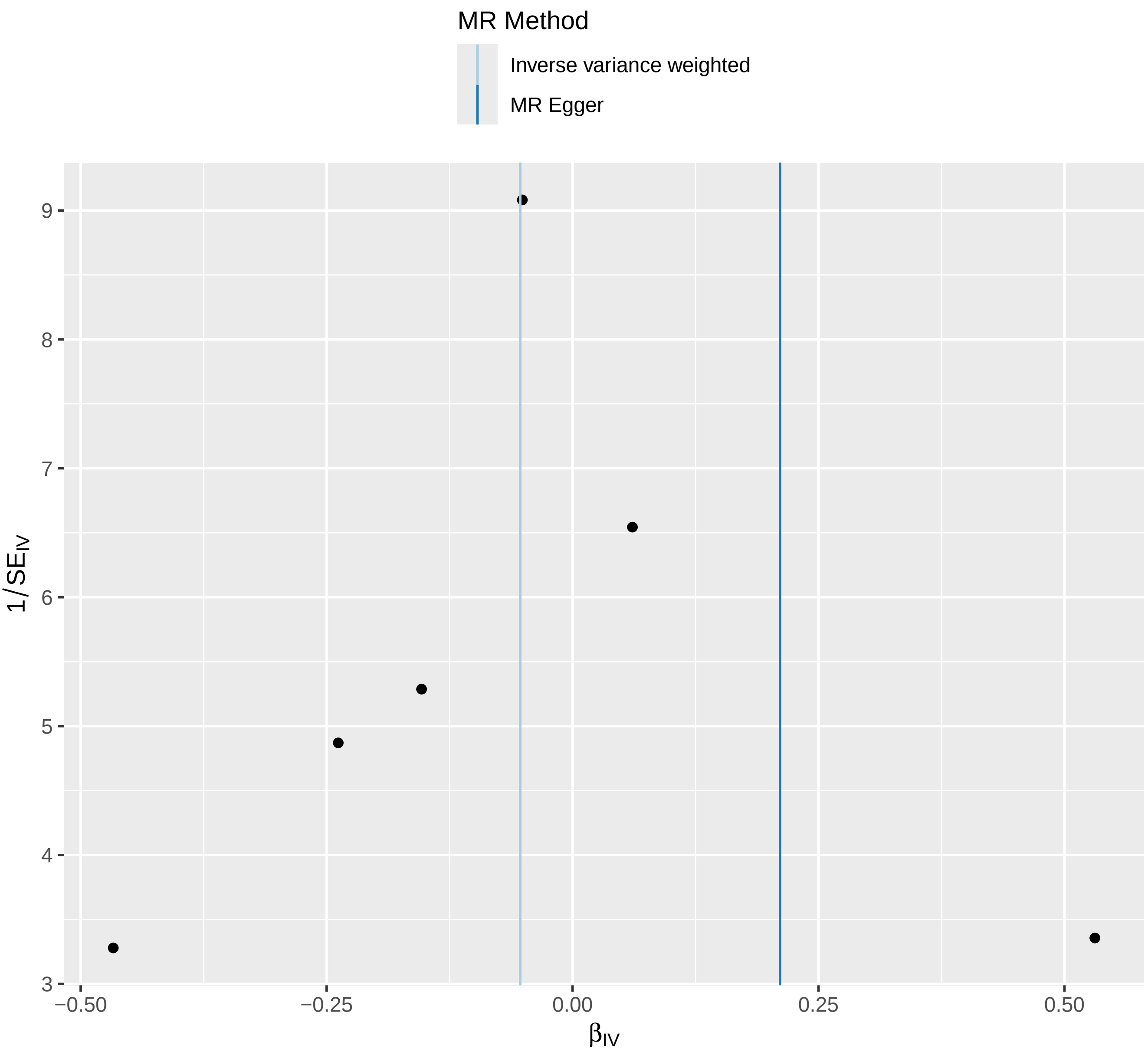
MR Method
Inverse variance weighted
MR Egger
1/SE_IV
9
8
7
6
5
4
3
−0.50
−0.25
0.00
0.25
0.50
β_IV

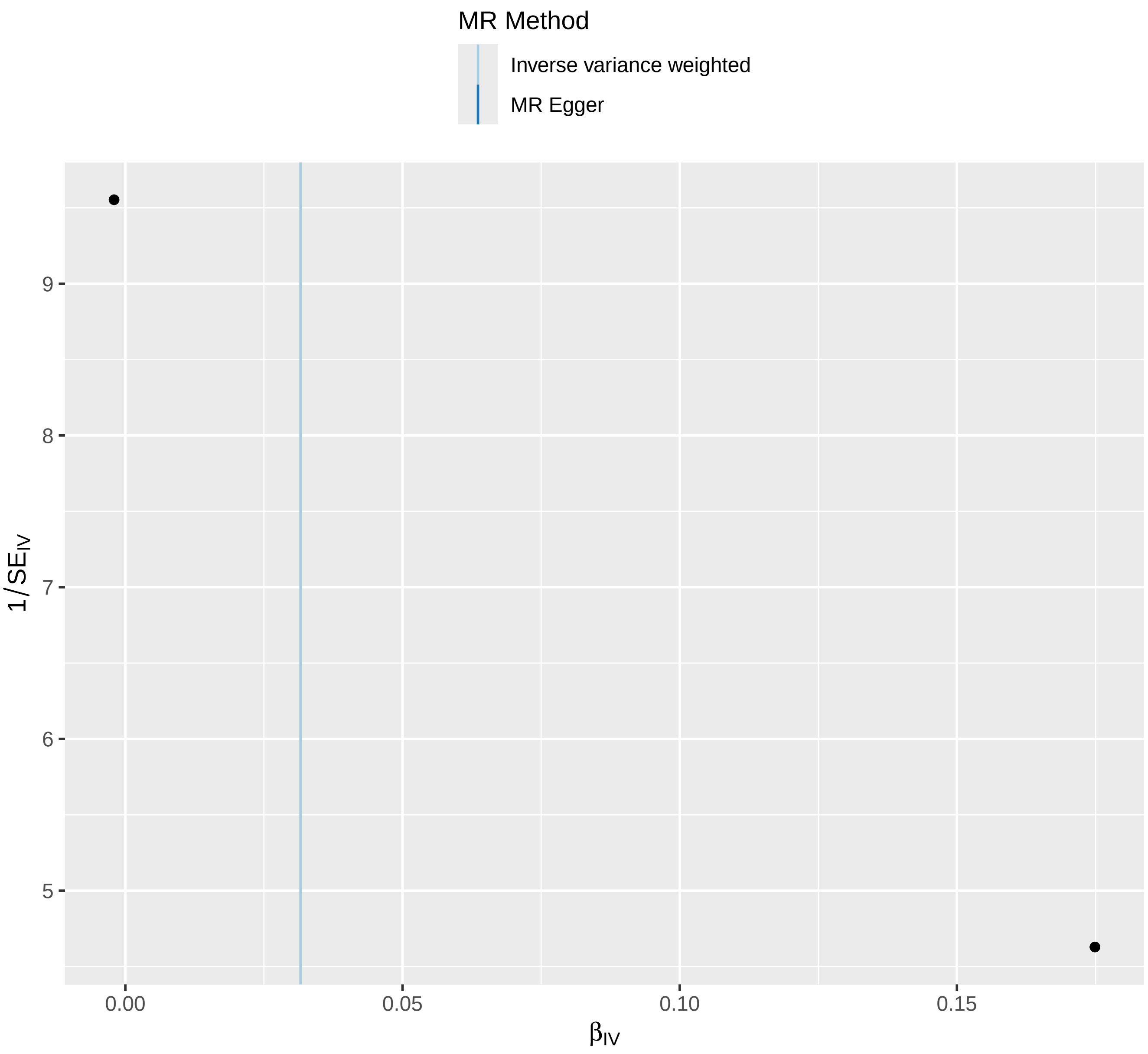
MR Method
Inverse variance weighted
MR Egger
1/SE_IV
9
8
7
6
5
0.00
0.05
0.10
0.15
β_IV

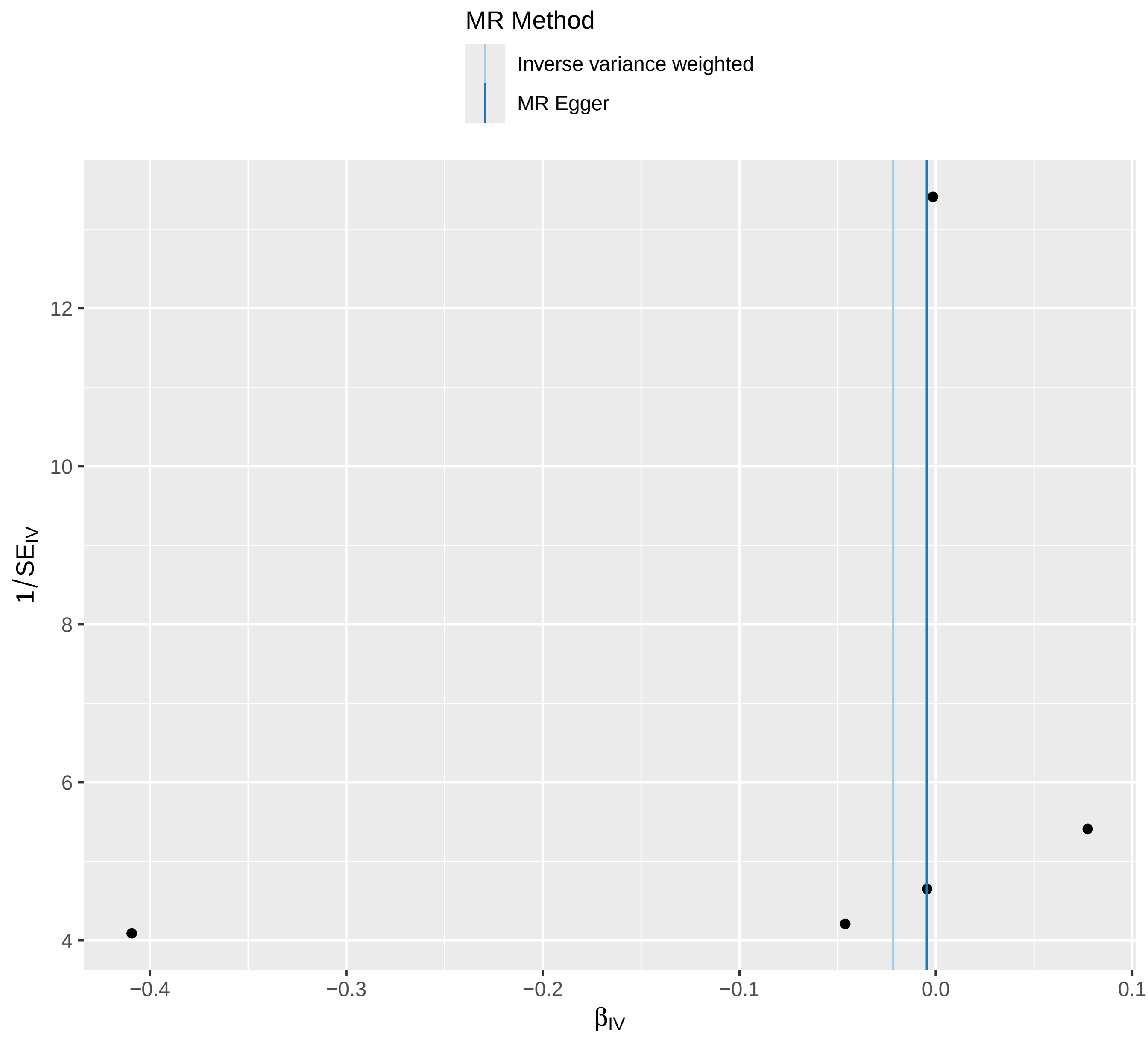

MR Method
Inverse variance weighted
MR Egger
12
10
8
6
4
1/SE$_{IV}$
−0.4
−0.3
−0.2
−0.1
0.0
0.1
β$_{IV}$

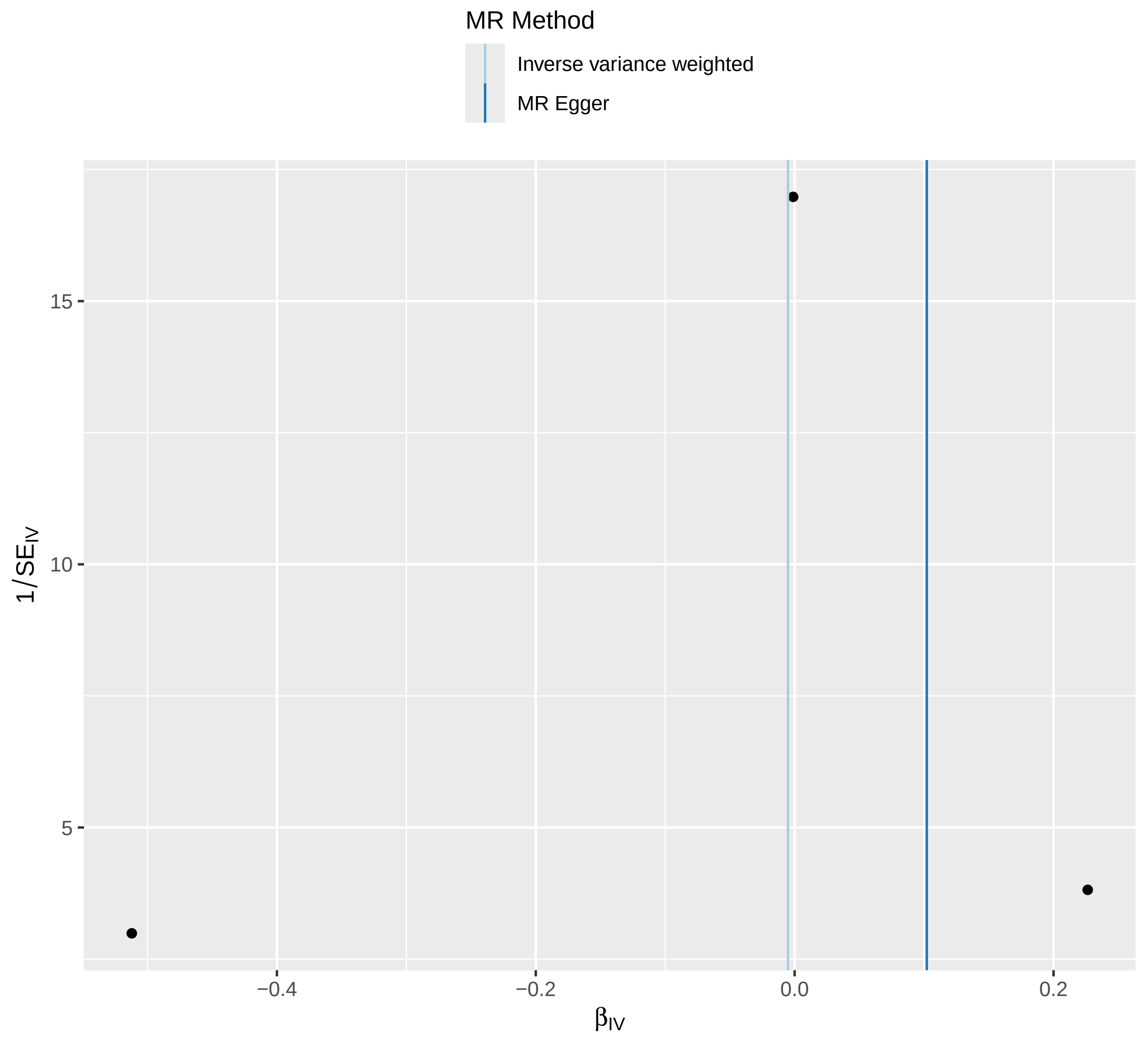
MR Method
Inverse variance weighted
MR Egger
15
10
5
−0.4
−0.2
0.0
0.2
1/SE$_{IV}$
β$_{IV}$

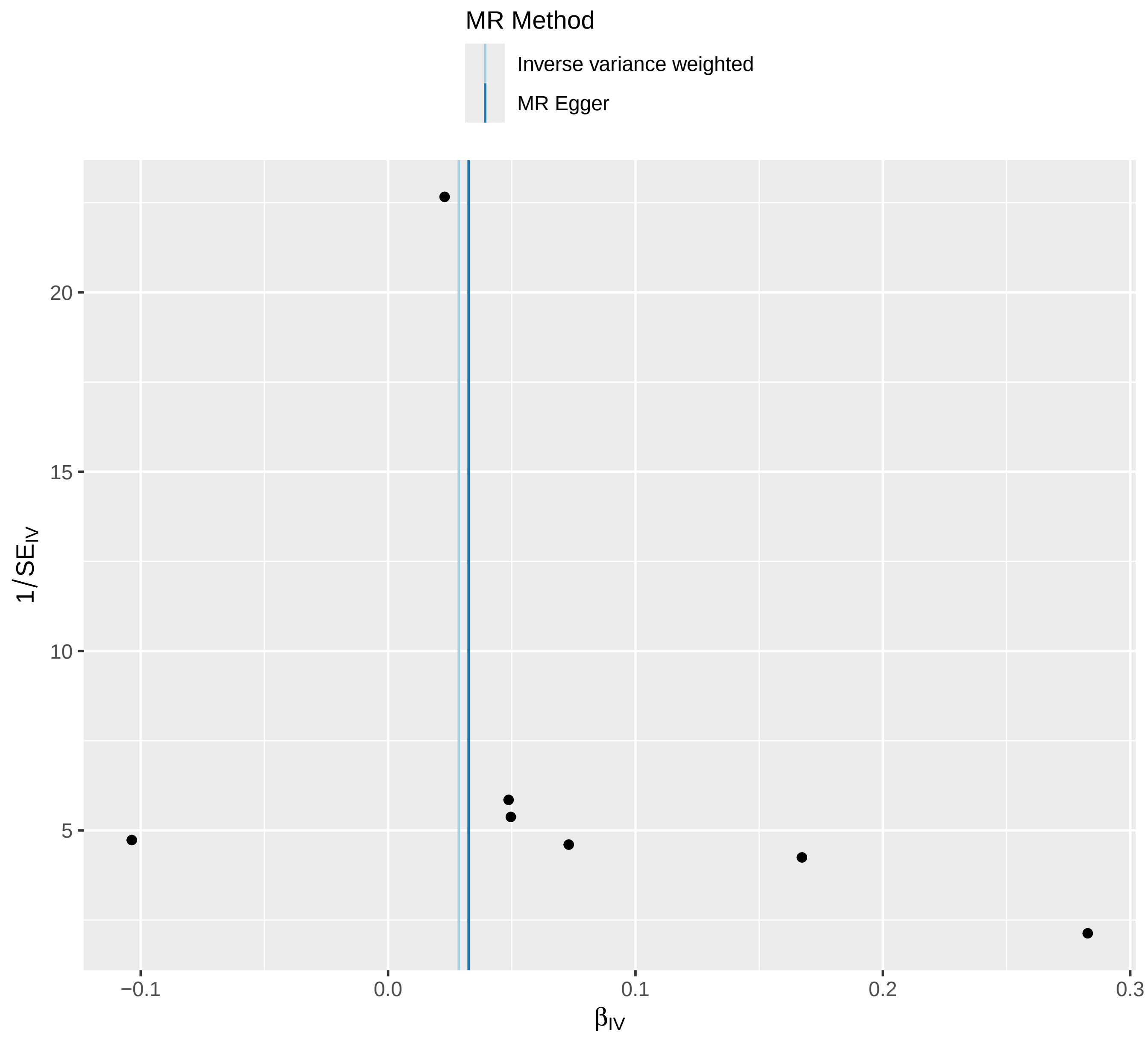
MR Method
Inverse variance weighted
MR Egger
20
15
10
5
1/SE_IV
−0.1
0.0
0.1
0.2
0.3
β_IV

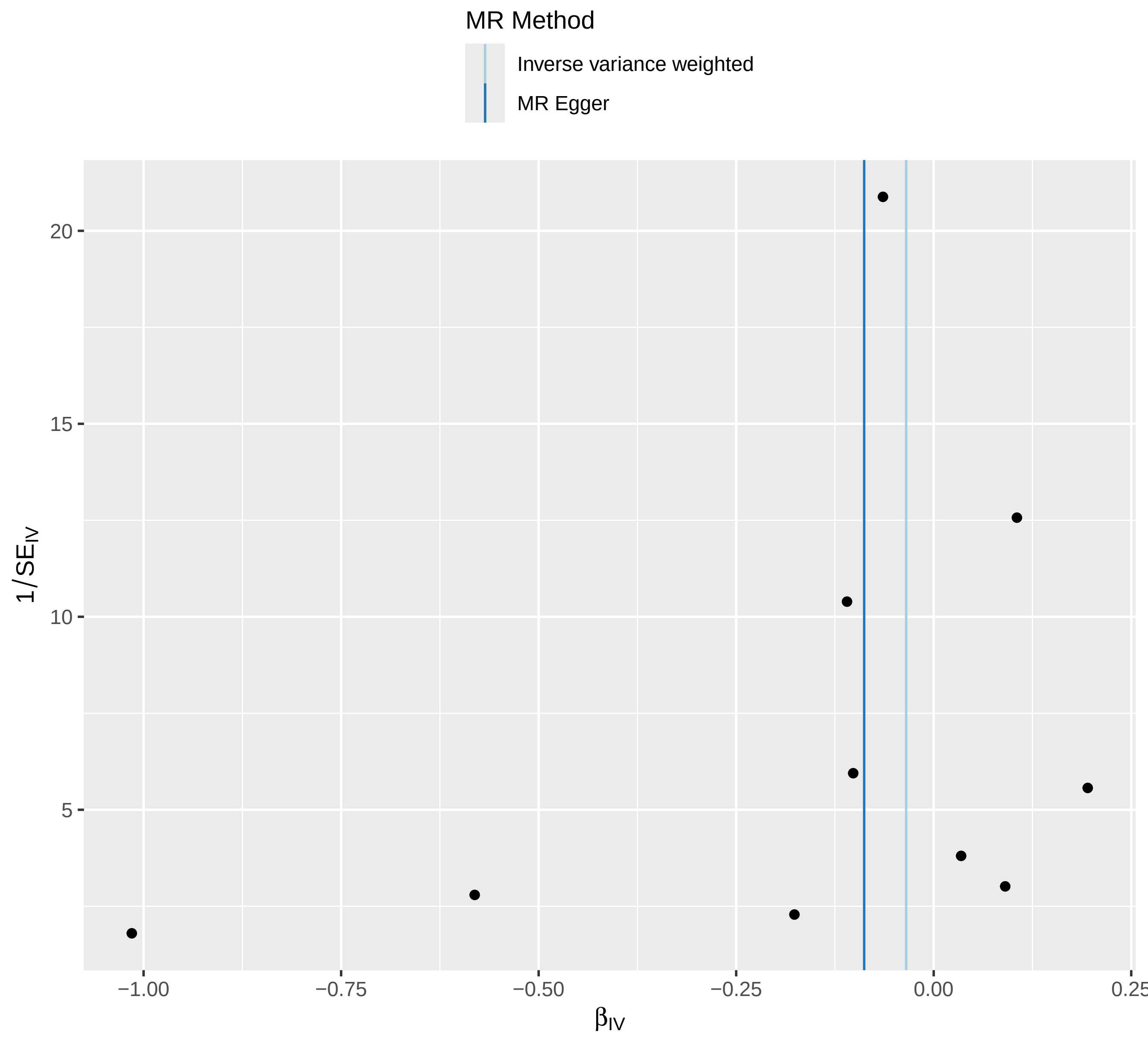
MR Method
Inverse variance weighted
MR Egger
20
15
10
5
1/SE_IV
−1.00
−0.75
−0.50
−0.25
0.00
0.25
β_IV

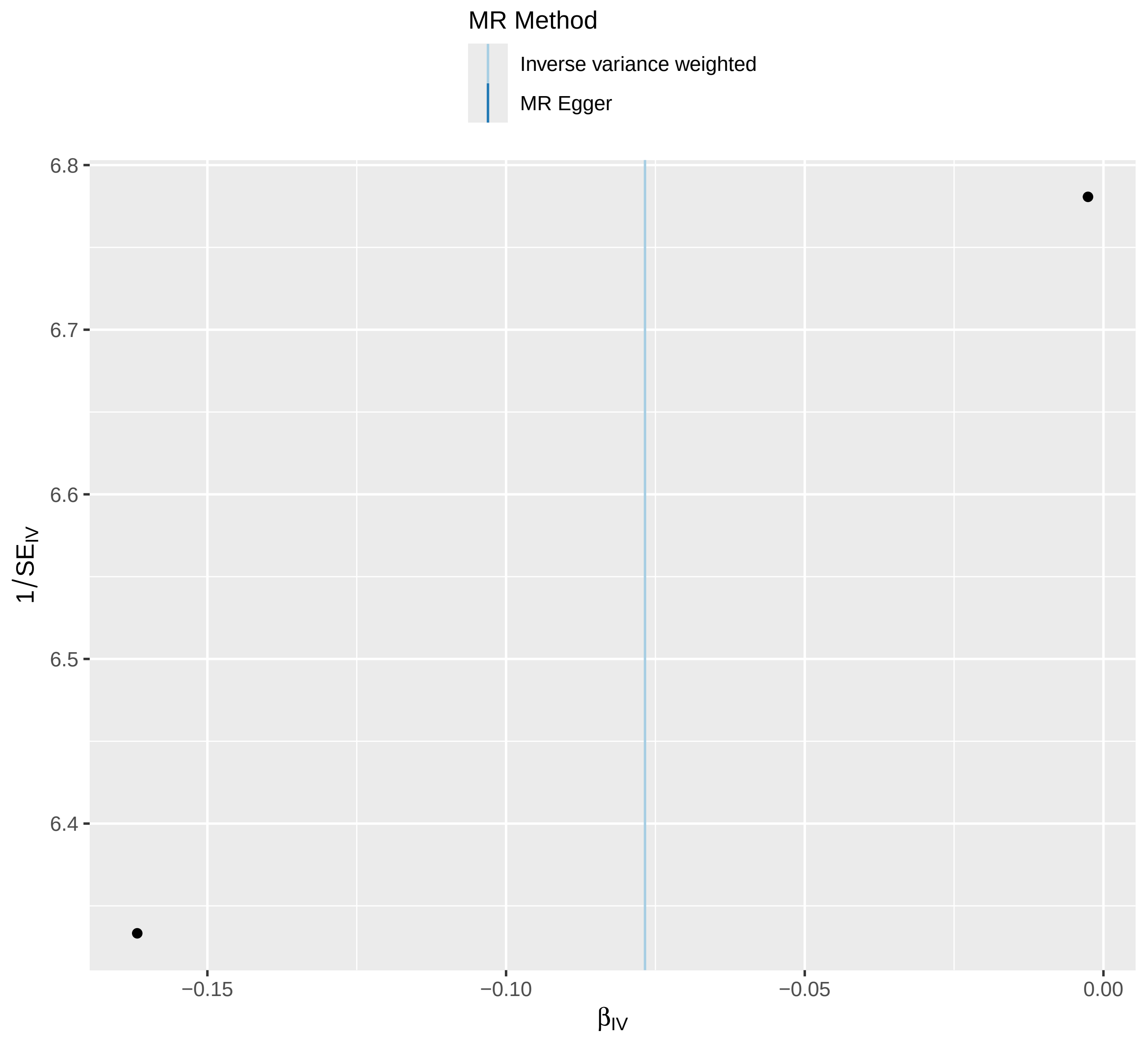
MR Method
Inverse variance weighted
MR Egger
6.8
6.7
6.6
6.5
6.4
1/SE$_{IV}$
−0.15
−0.10
−0.05
0.00
β$_{IV}$

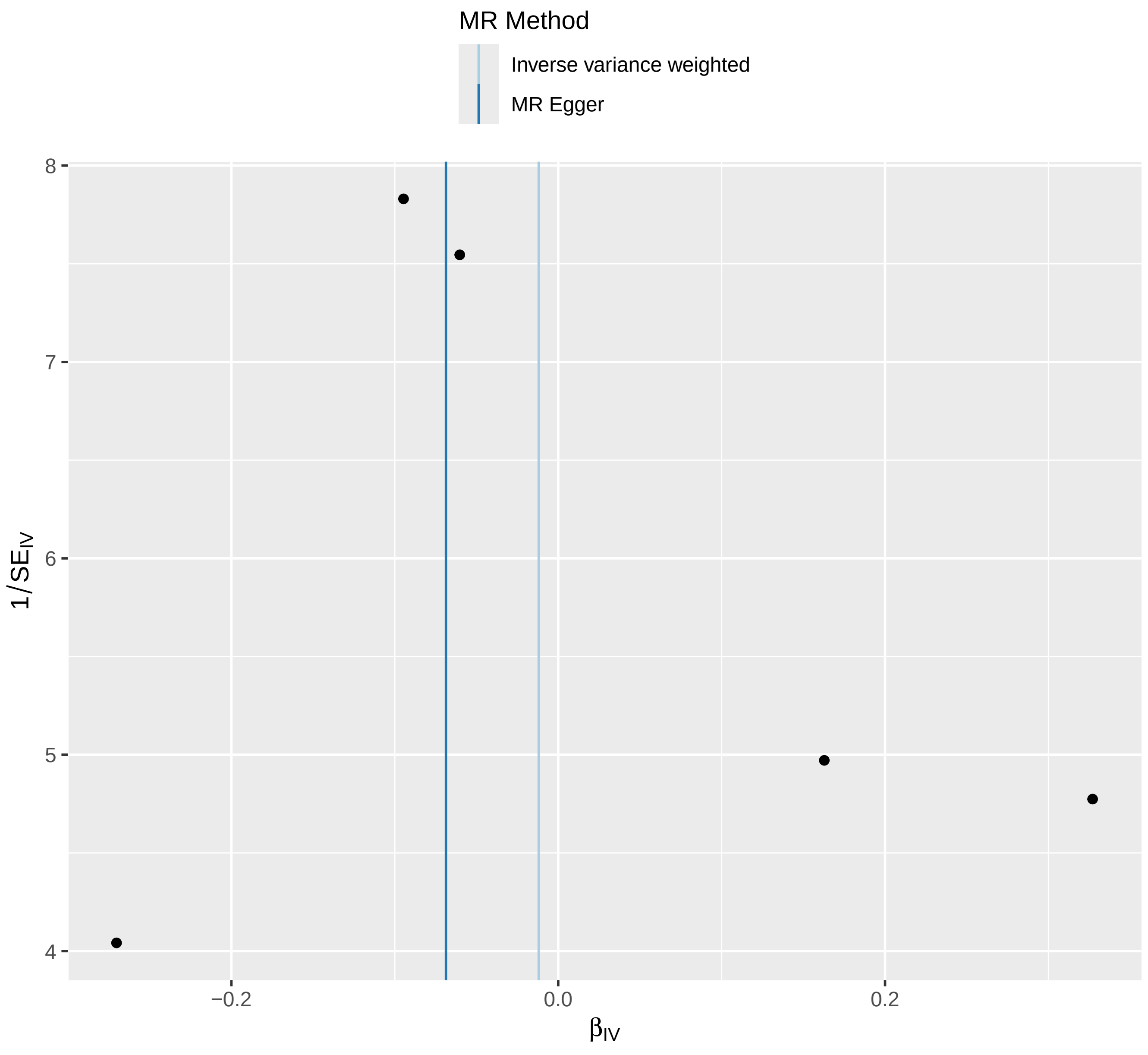

MR Method
Inverse variance weighted
MR Egger
8
7
6
5
4
1/SE$_{IV}$
−0.2
0.0
0.2
β$_{IV}$

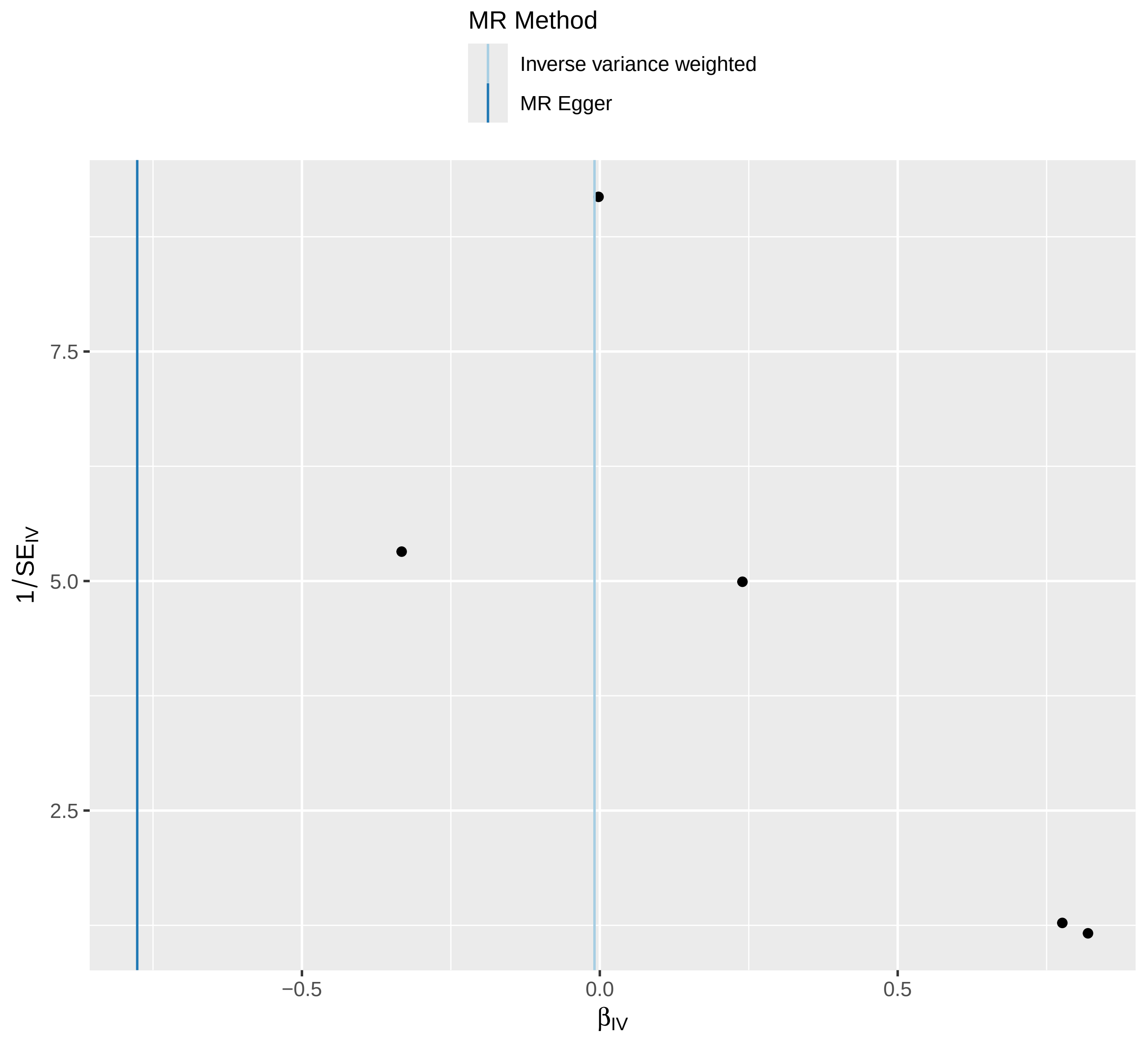
MR Method
Inverse variance weighted
MR Egger
7.5
5.0
2.5
1/SE$_{IV}$
−0.5
0.0
0.5
β$_{IV}$

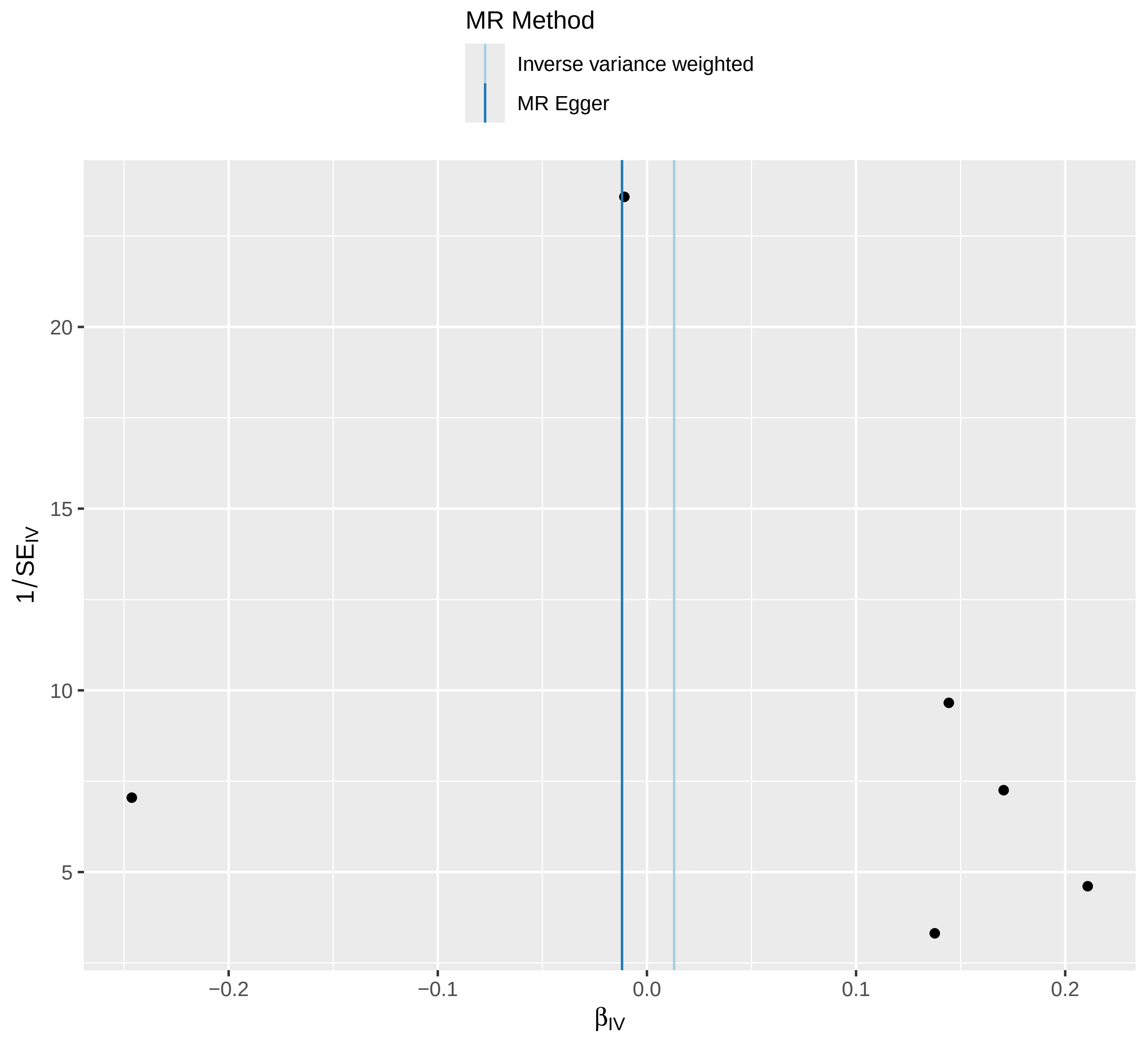
MR Method
Inverse variance weighted
MR Egger
20
15
10
5
1/SE_IV
−0.2
−0.1
0.0
0.1
0.2
β_IV

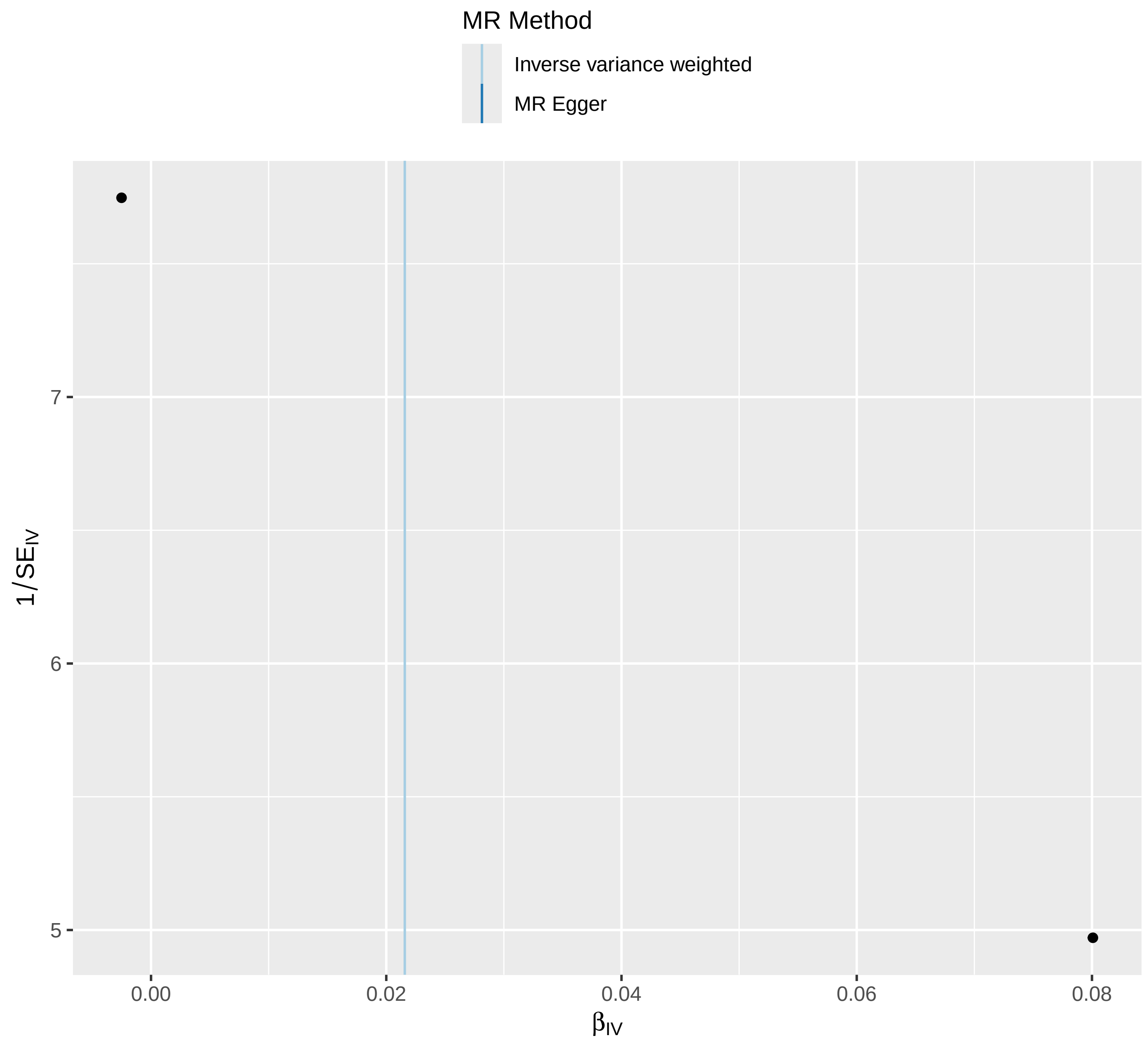

MR Method
Inverse variance weighted
MR Egger
7
6
5
1/SE_IV
0.00
0.02
0.04
0.06
0.08
β_IV

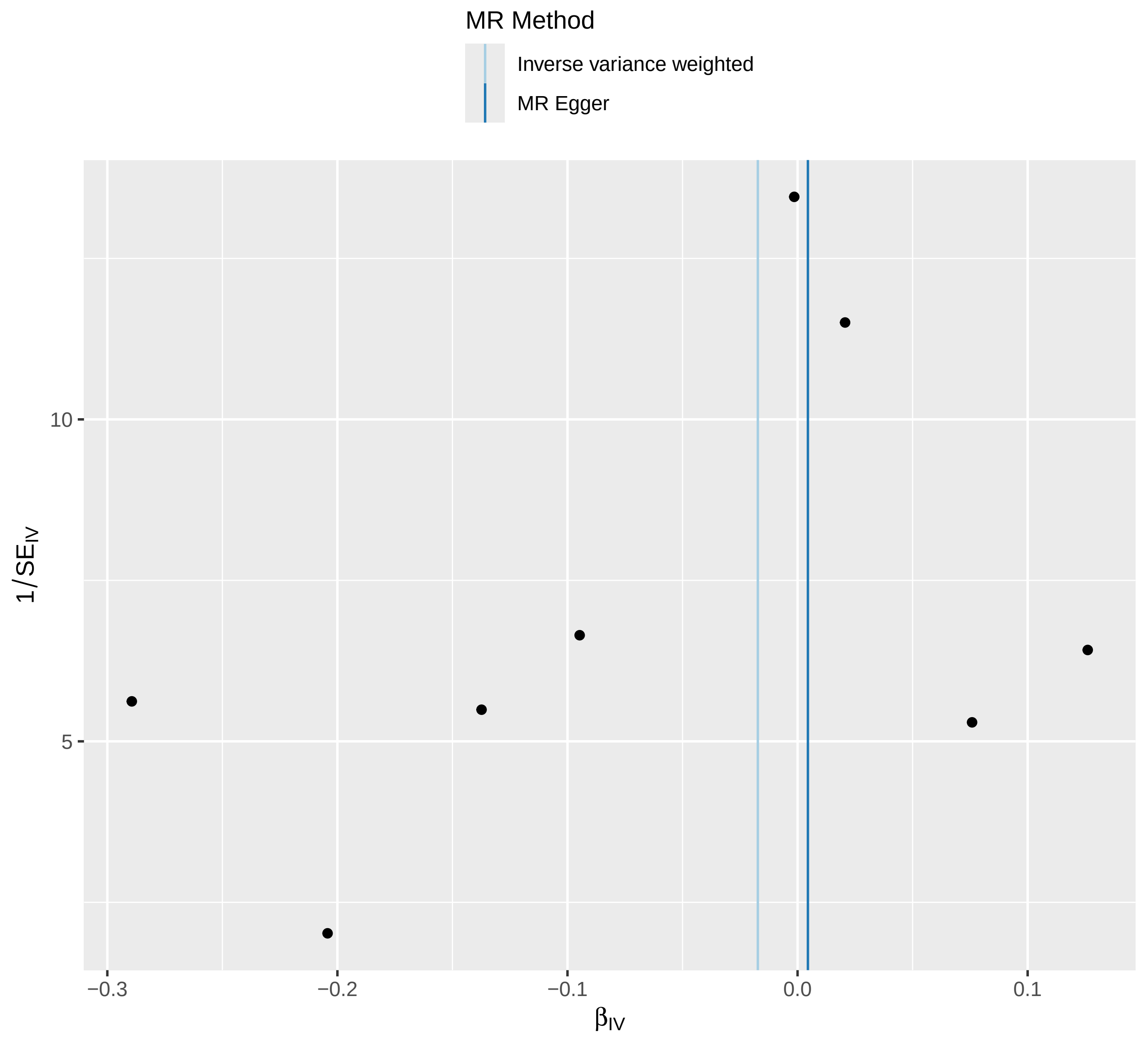
MR Method
Inverse variance weighted
MR Egger
10
5
1/SE_IV
−0.3
−0.2
−0.1
0.0
0.1
β_IV

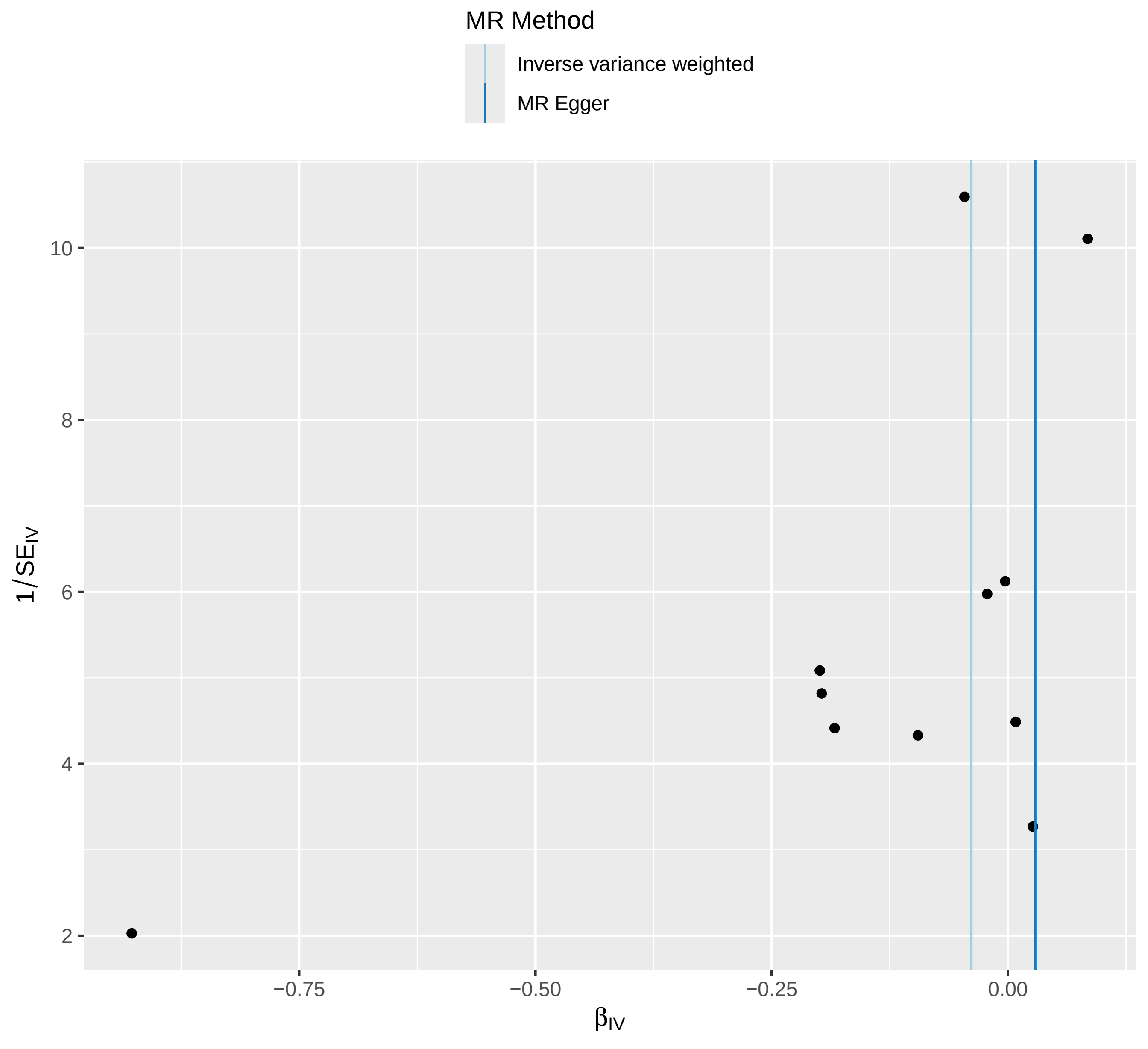
MR Method
Inverse variance weighted
MR Egger
1/SE_IV
10
8
6
4
2
−0.75
−0.50
−0.25
0.00
β_IV

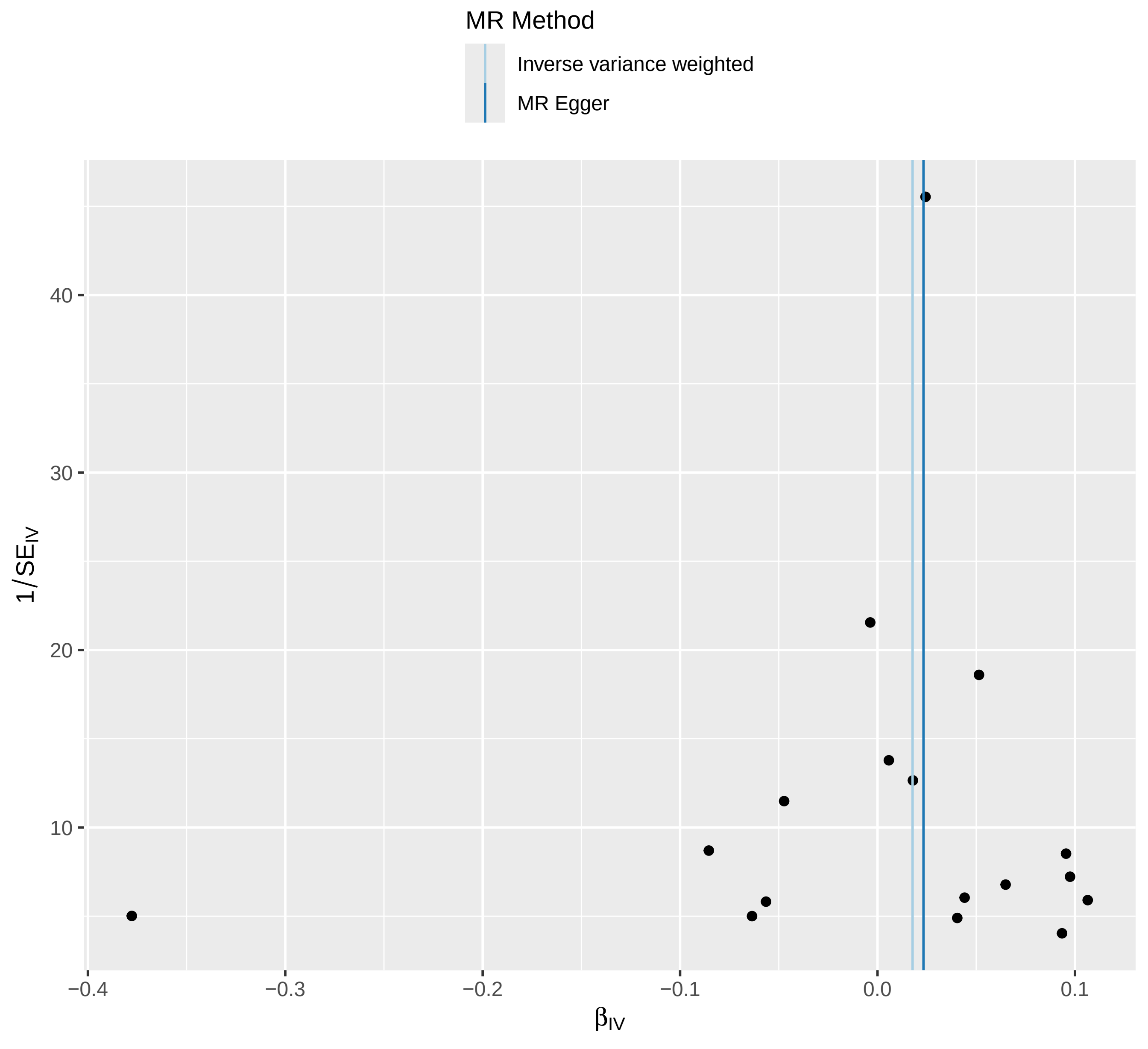

MR Method
Inverse variance weighted
MR Egger
40
30
20
10
1/SE_IV
−0.4
−0.3
−0.2
−0.1
0.0
0.1
β_IV

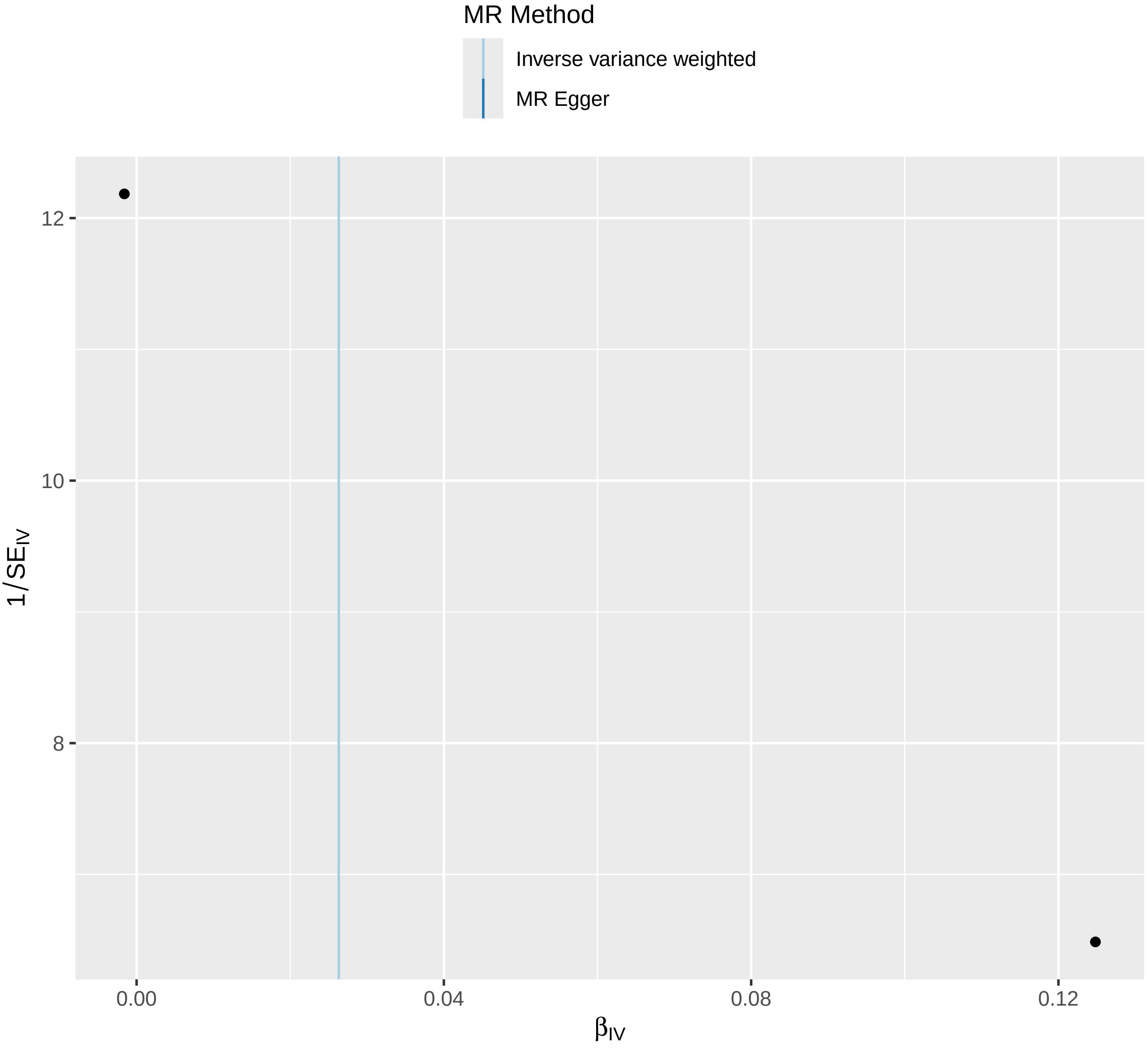

MR Method
Inverse variance weighted
MR Egger
12
10
8
$1/SE_{IV}$
0.00
0.04
0.08
0.12
$\beta_{IV}$

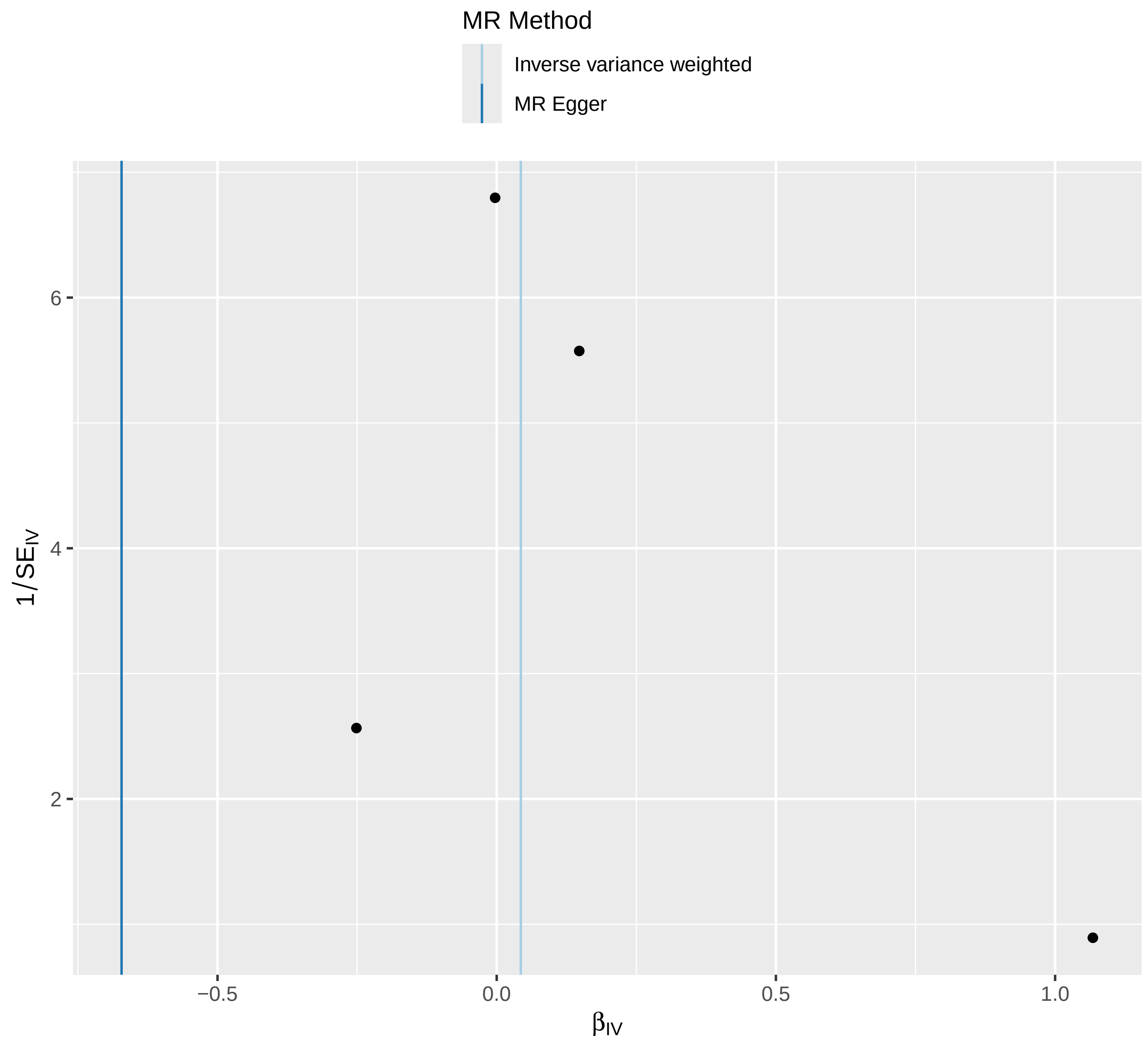
MR Method
Inverse variance weighted
MR Egger
6
4
2
1/SE_IV
−0.5
0.0
0.5
1.0
β_IV

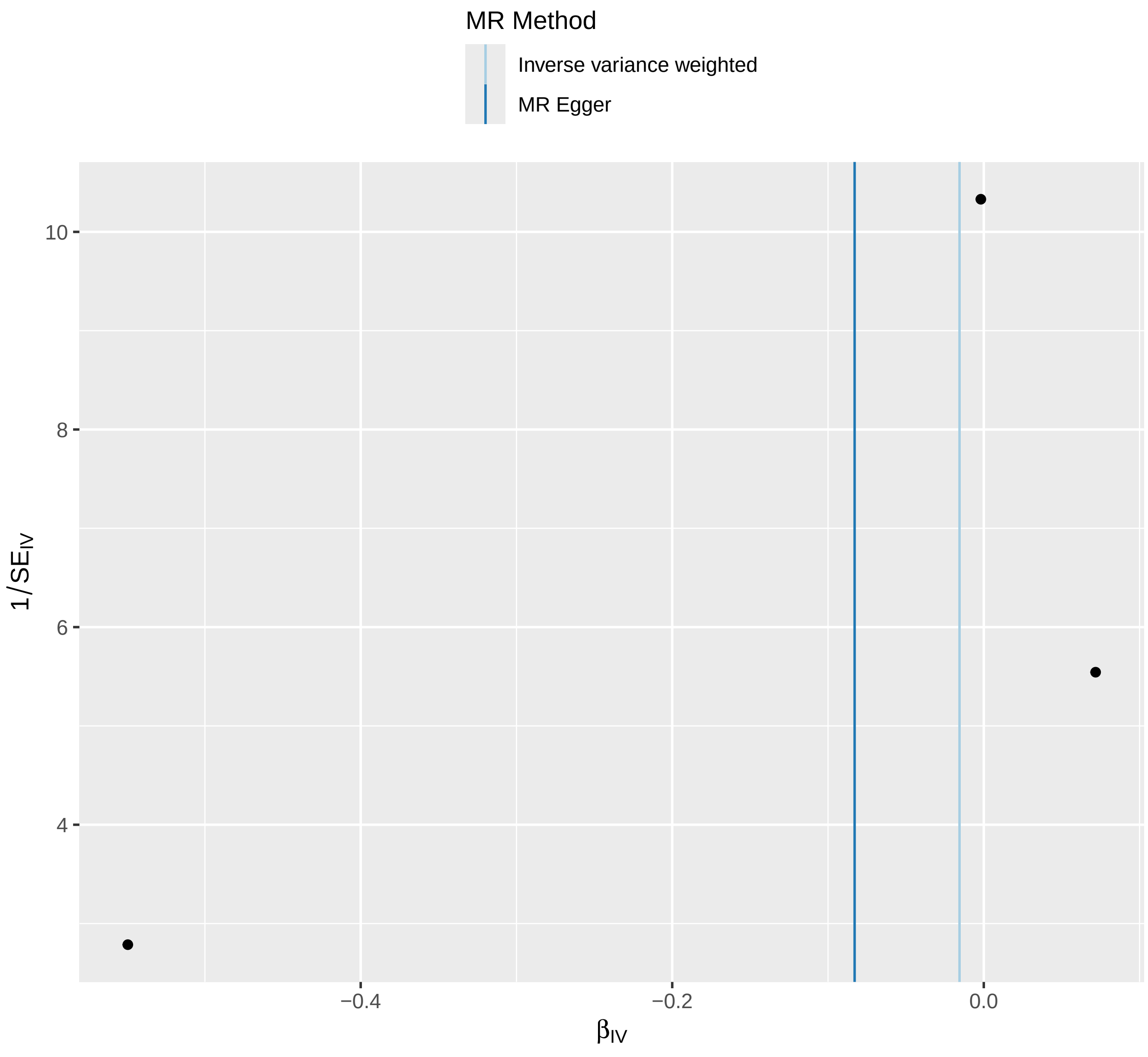
MR Method
Inverse variance weighted
MR Egger
10
8
6
4
1/SE$_{IV}$
−0.4
−0.2
0.0
β$_{IV}$

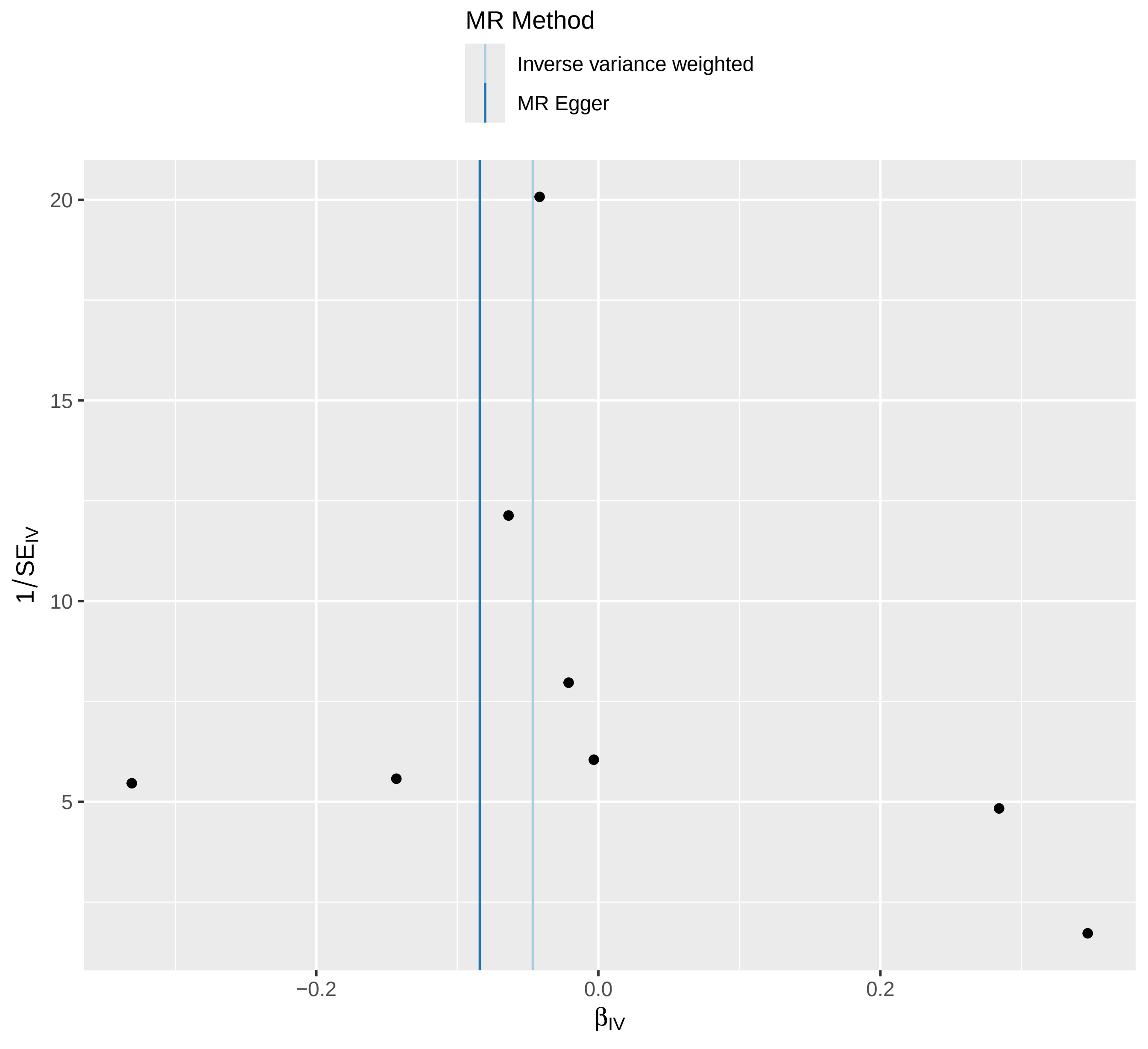
MR Method
Inverse variance weighted
MR Egger
20
15
10
5
1/SE_IV
−0.2
0.0
0.2
β_IV

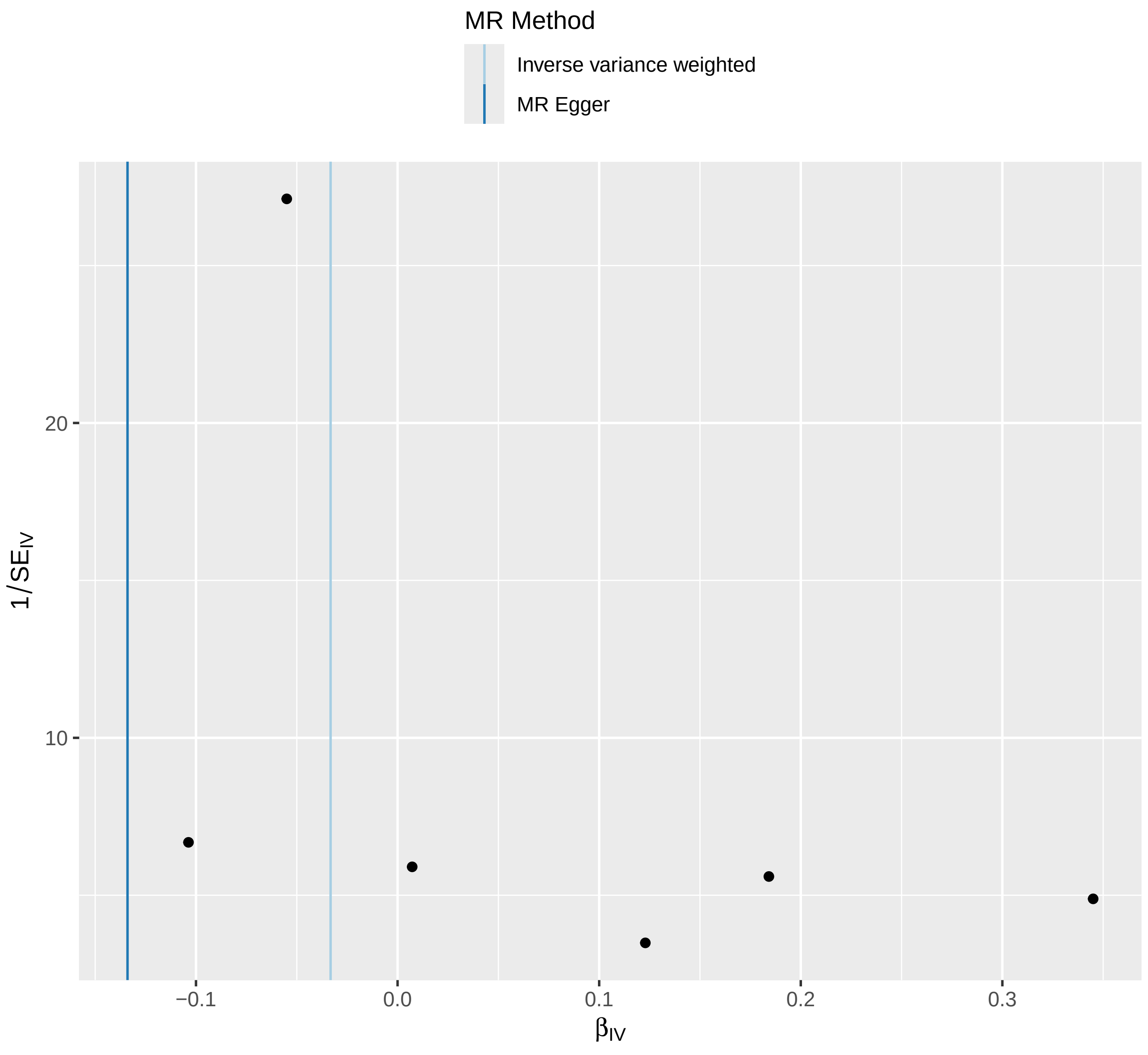

MR Method
Inverse variance weighted
MR Egger
20
10
1/SE_IV
−0.1
0.0
0.1
0.2
0.3
β_IV

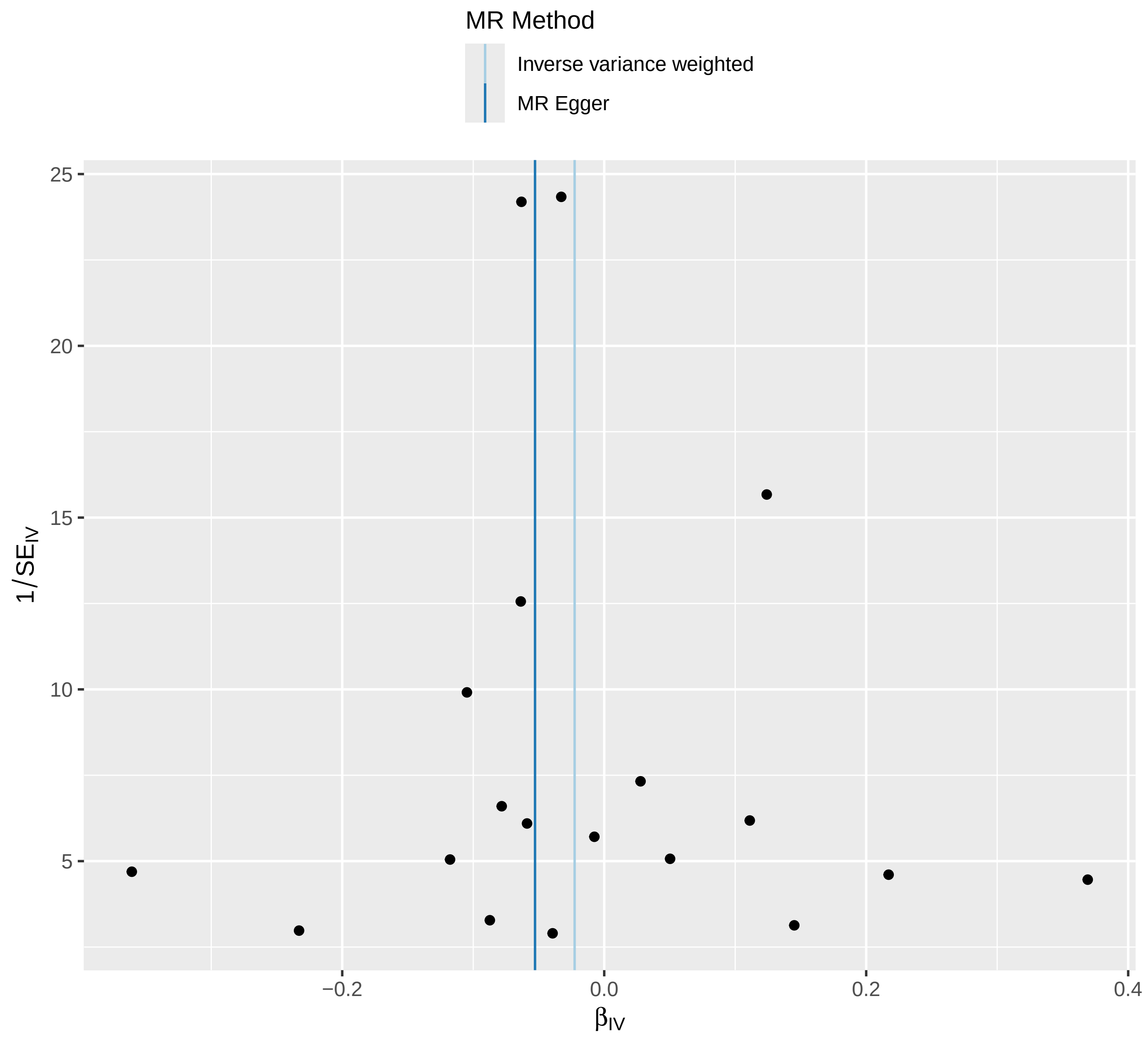
MR Method
Inverse variance weighted
MR Egger
25
20
15
10
5
1/SE_IV
−0.2
0.0
0.2
0.4
β_IV

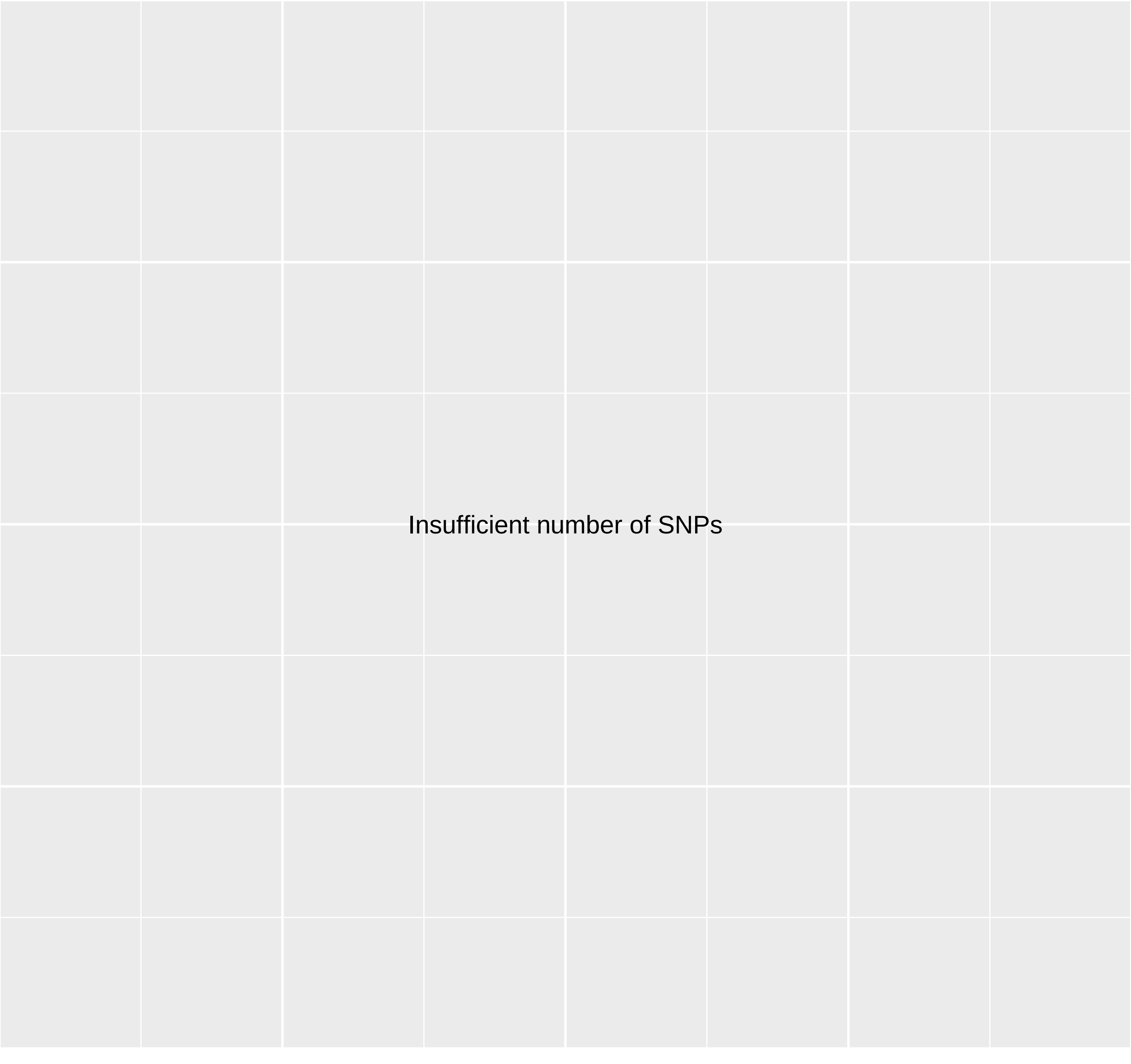
Insufficient number of SNPs

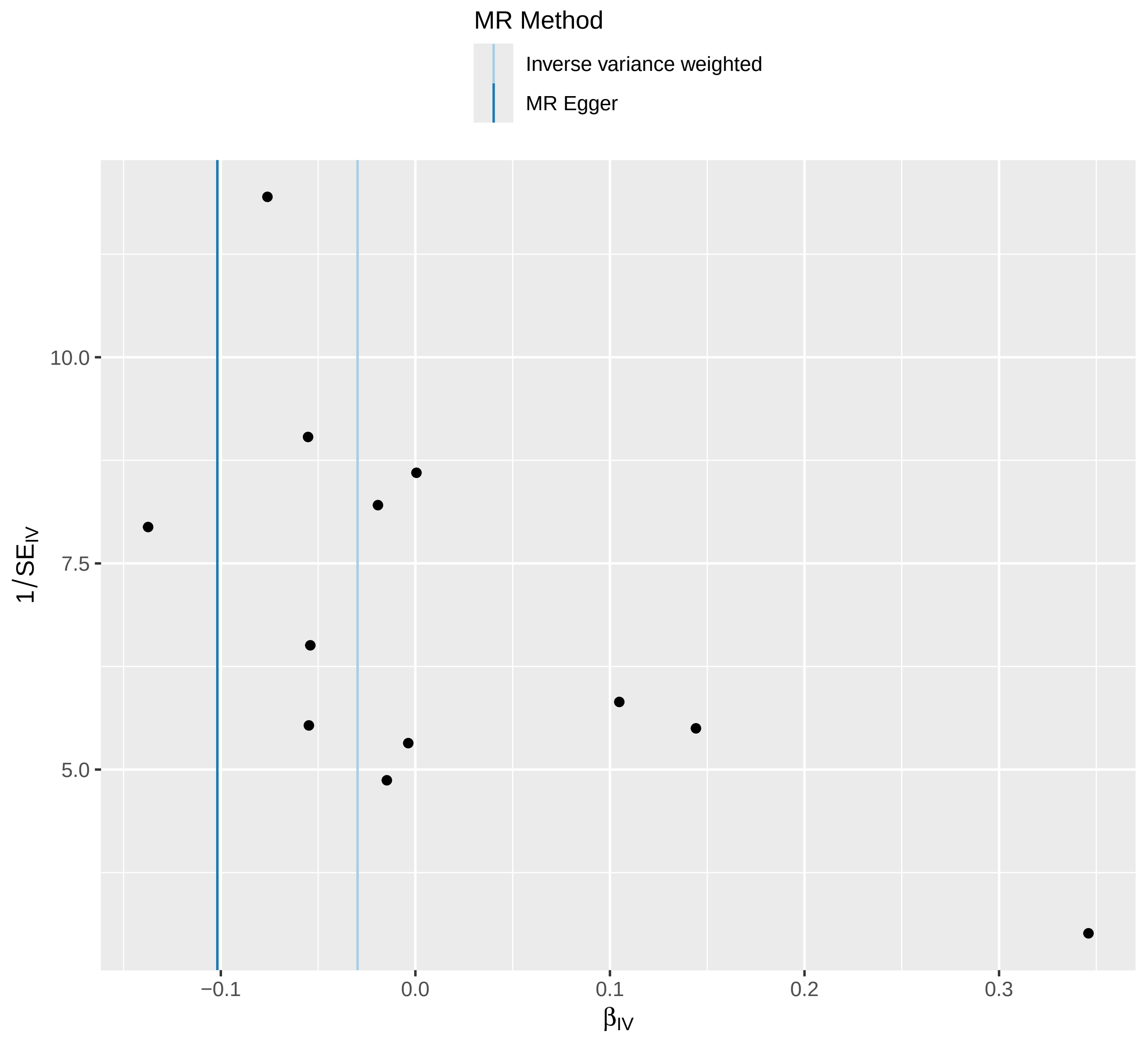
MR Method
Inverse variance weighted
MR Egger
10.0
7.5
5.0
1/SE_IV
−0.1
0.0
0.1
0.2
0.3
β_IV

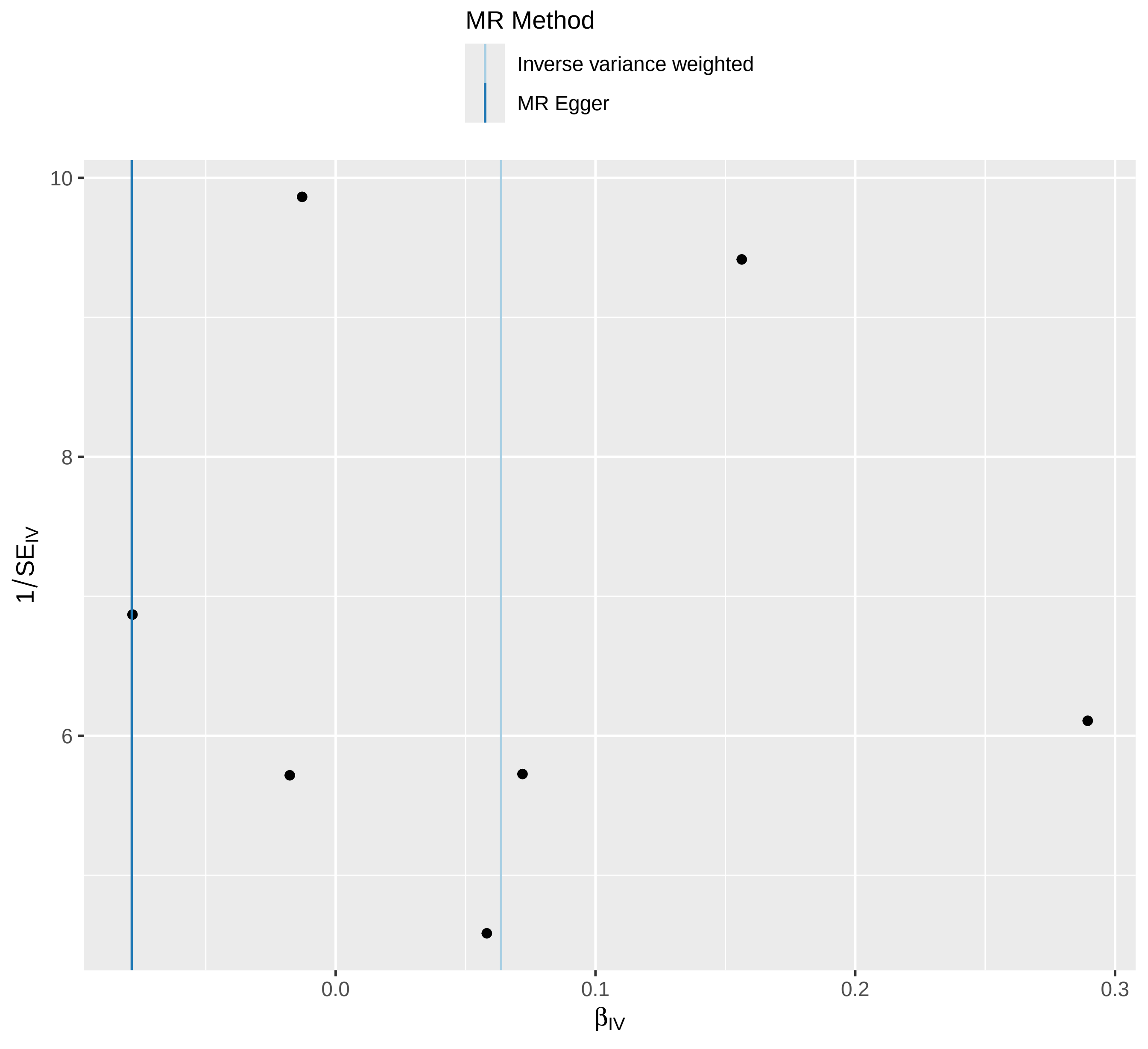
MR Method
Inverse variance weighted
MR Egger
10
8
6
1/SE$_{IV}$
0.0
0.1
0.2
0.3
β$_{IV}$

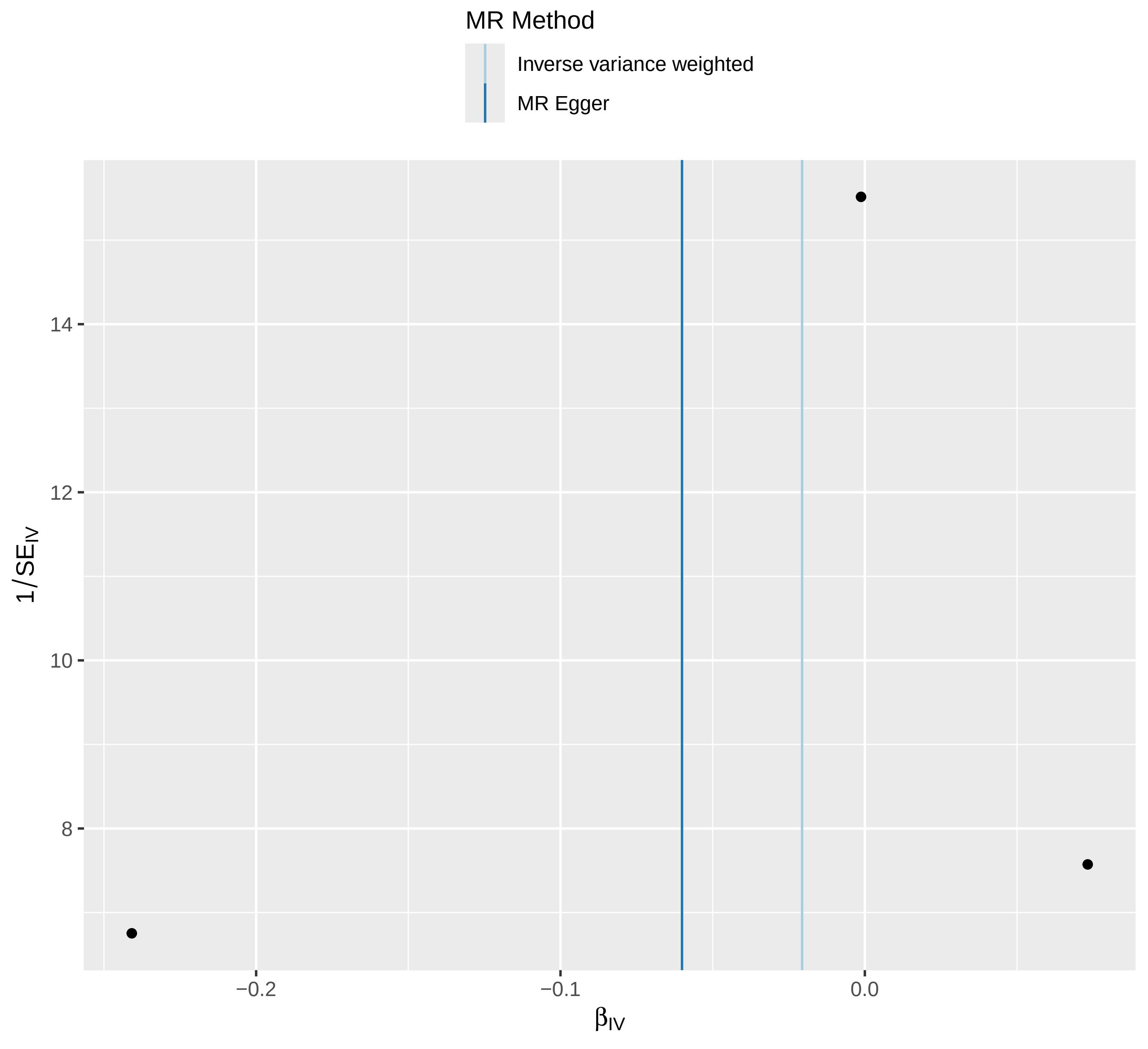
MR Method
Inverse variance weighted
MR Egger
14
12
10
8
1/SE_IV
−0.2
−0.1
0.0
β_IV

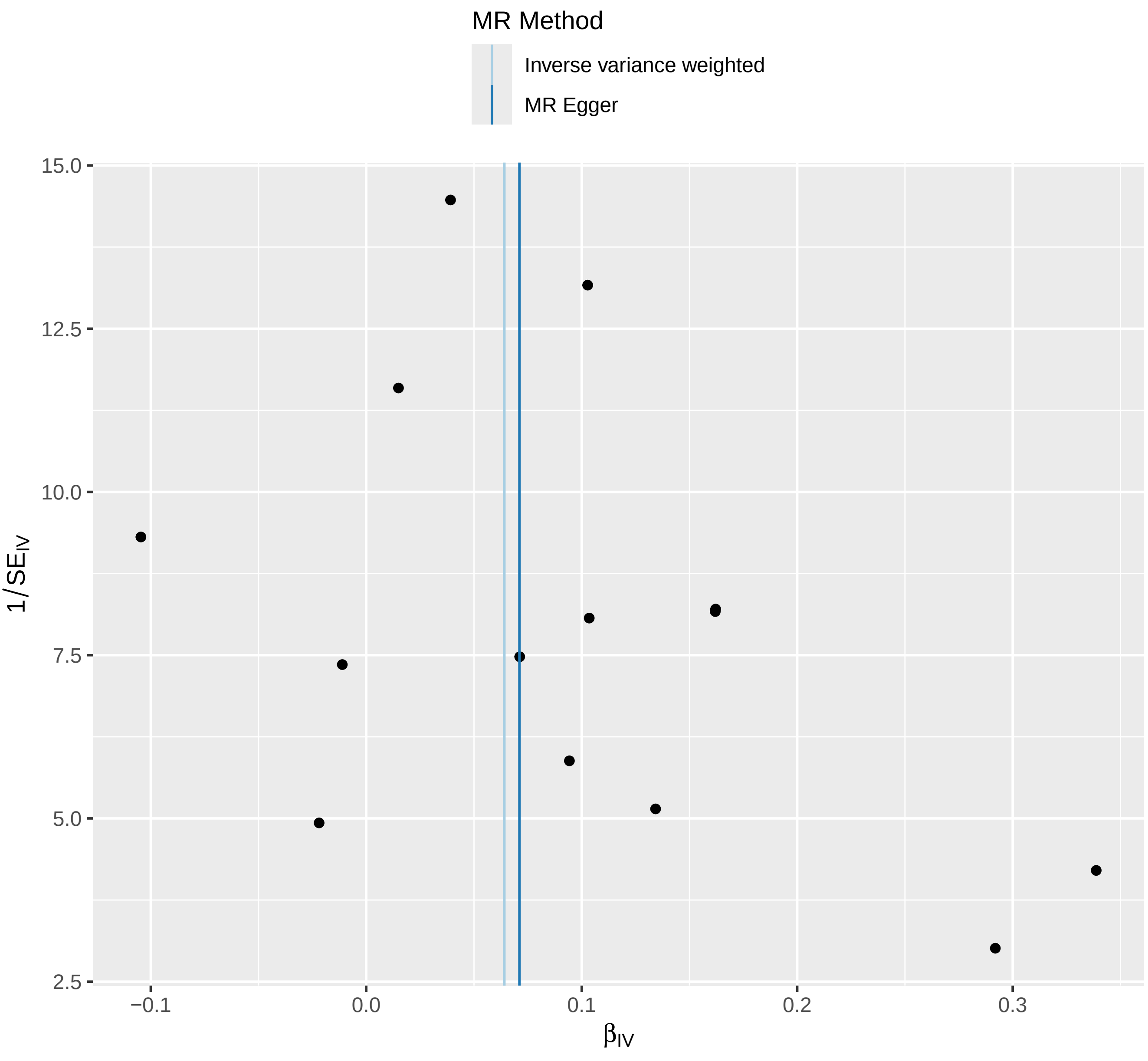
MR Method
Inverse variance weighted
MR Egger
15.0
12.5
10.0
7.5
5.0
2.5
1/SE_IV
−0.1
0.0
0.1
0.2
0.3
β_IV

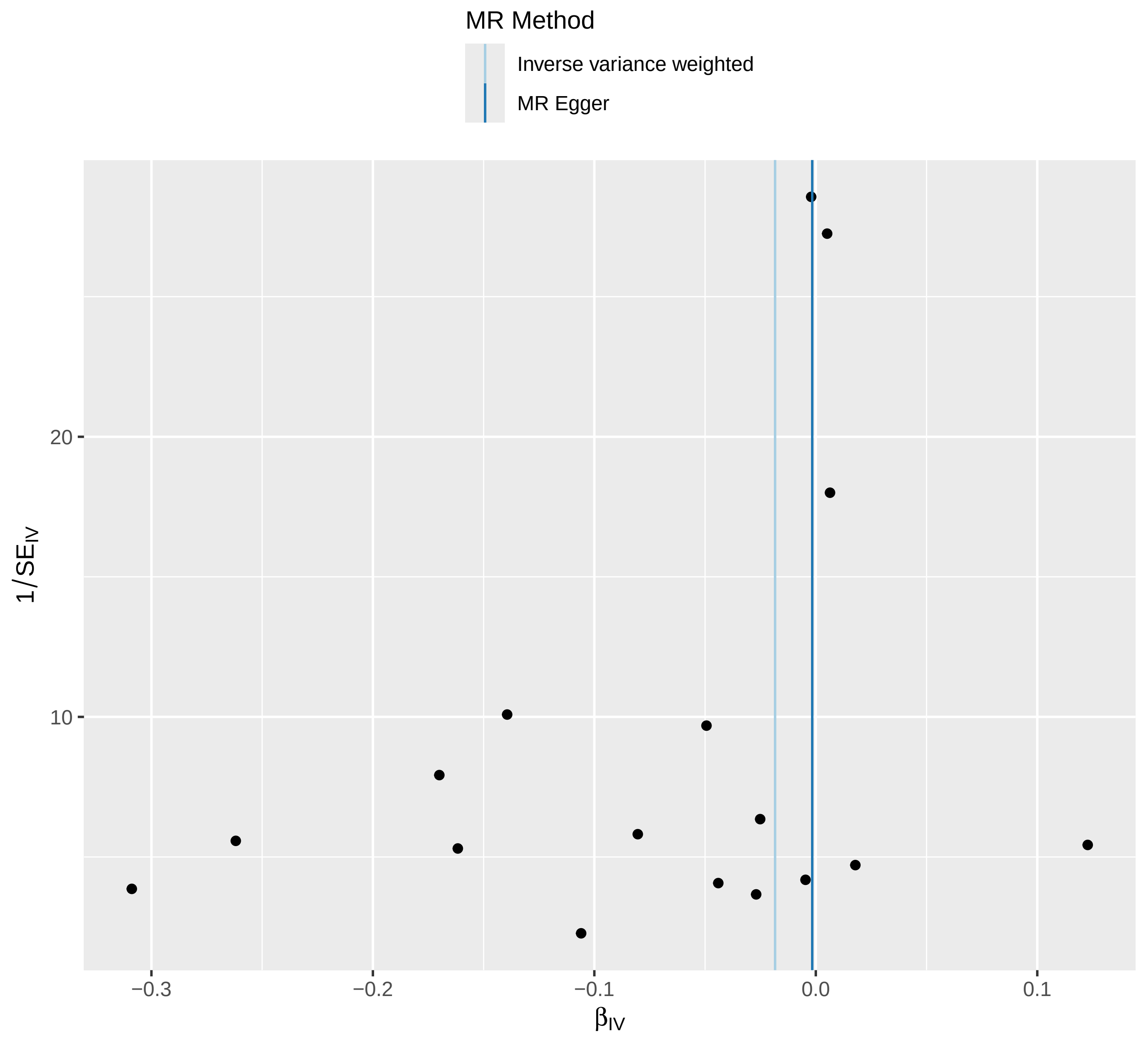
MR Method
Inverse variance weighted
MR Egger
20
10
1/SE$_{IV}$
−0.3
−0.2
−0.1
0.0
0.1
β$_{IV}$

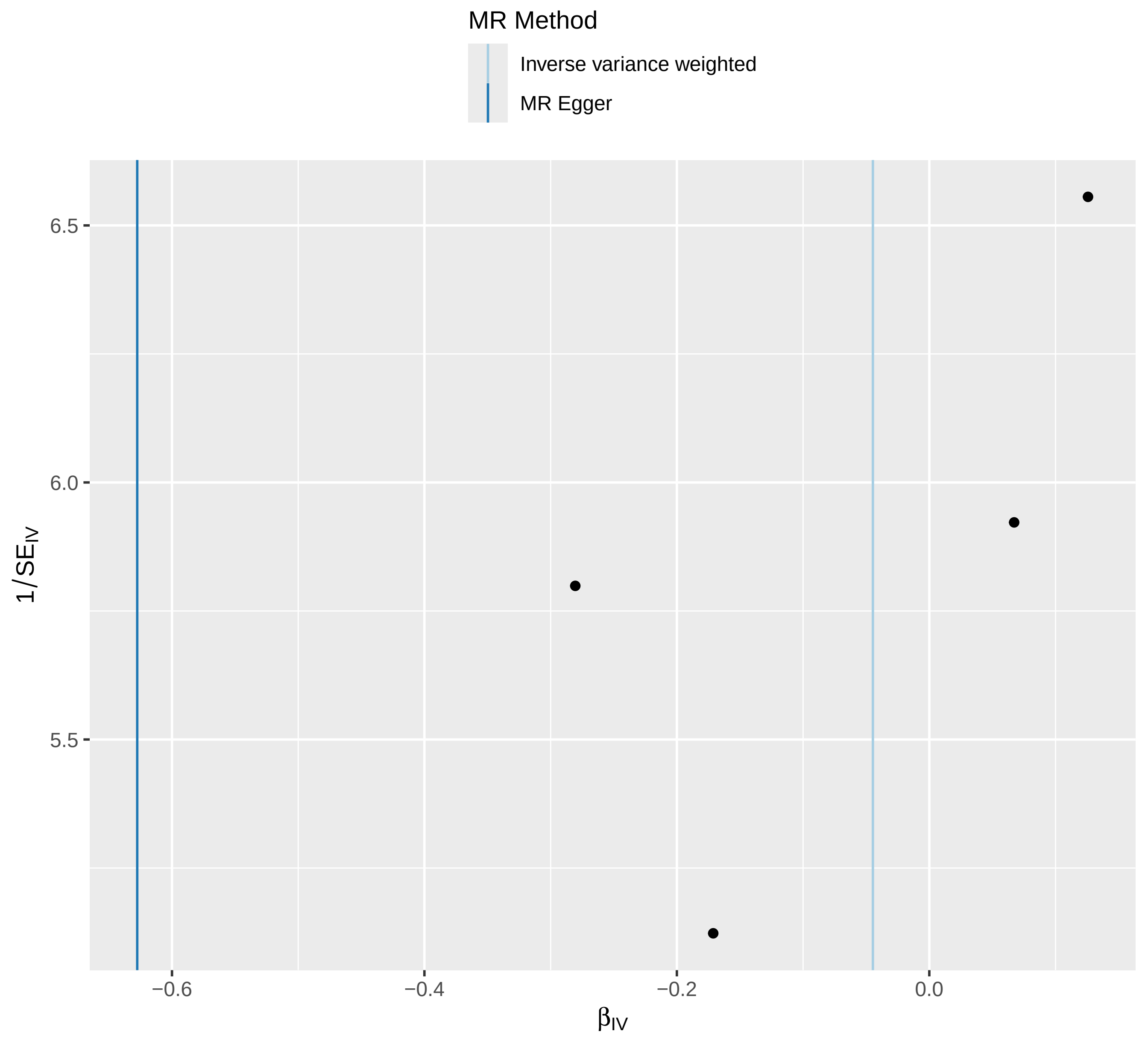
MR Method
Inverse variance weighted
MR Egger
6.5
6.0
5.5
1/SE_IV
−0.6
−0.4
−0.2
0.0
β_IV

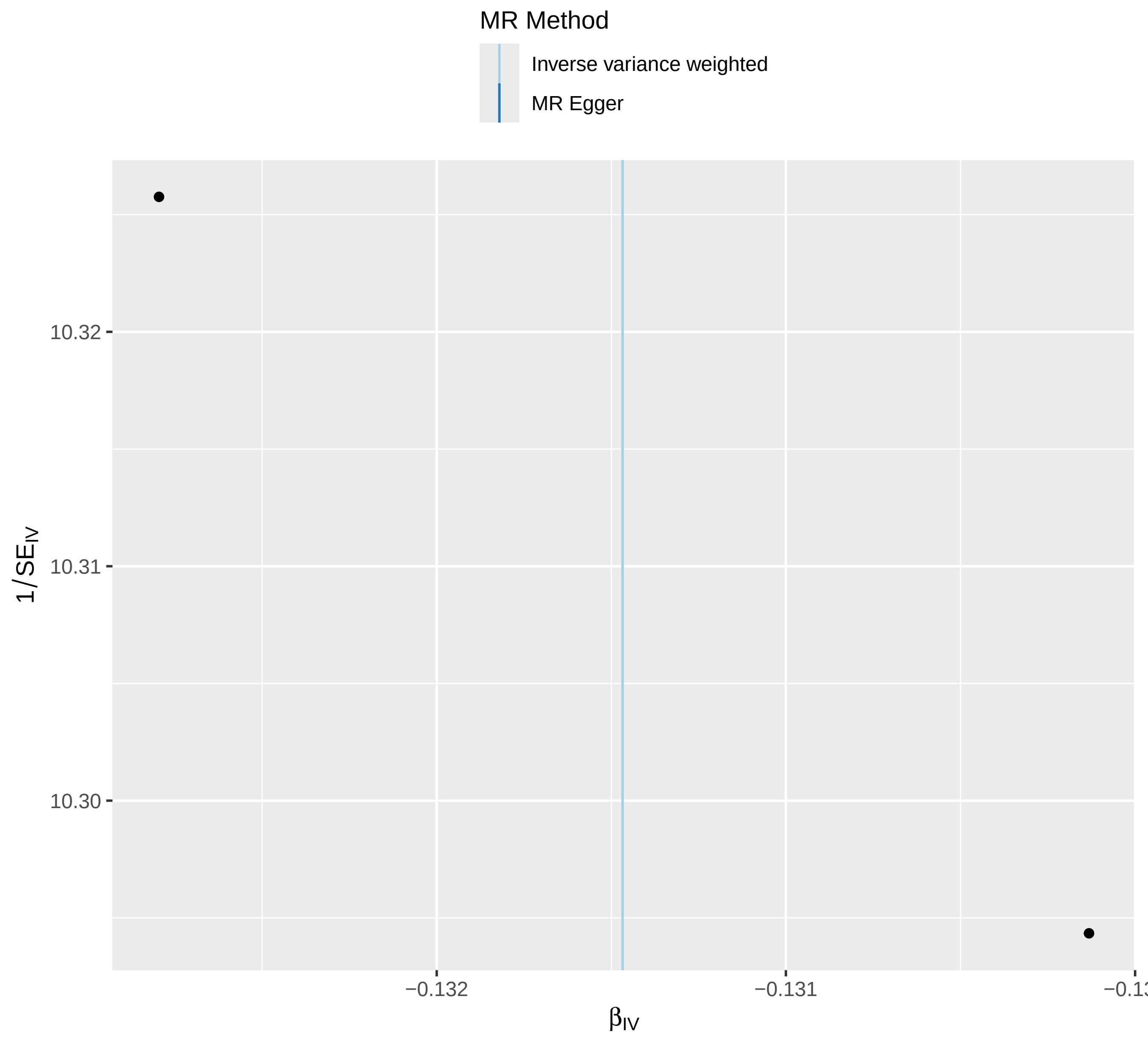
MR Method
Inverse variance weighted
MR Egger
10.32
10.31
10.30
1/SE_IV
−0.132
−0.131
−0.13
β_IV

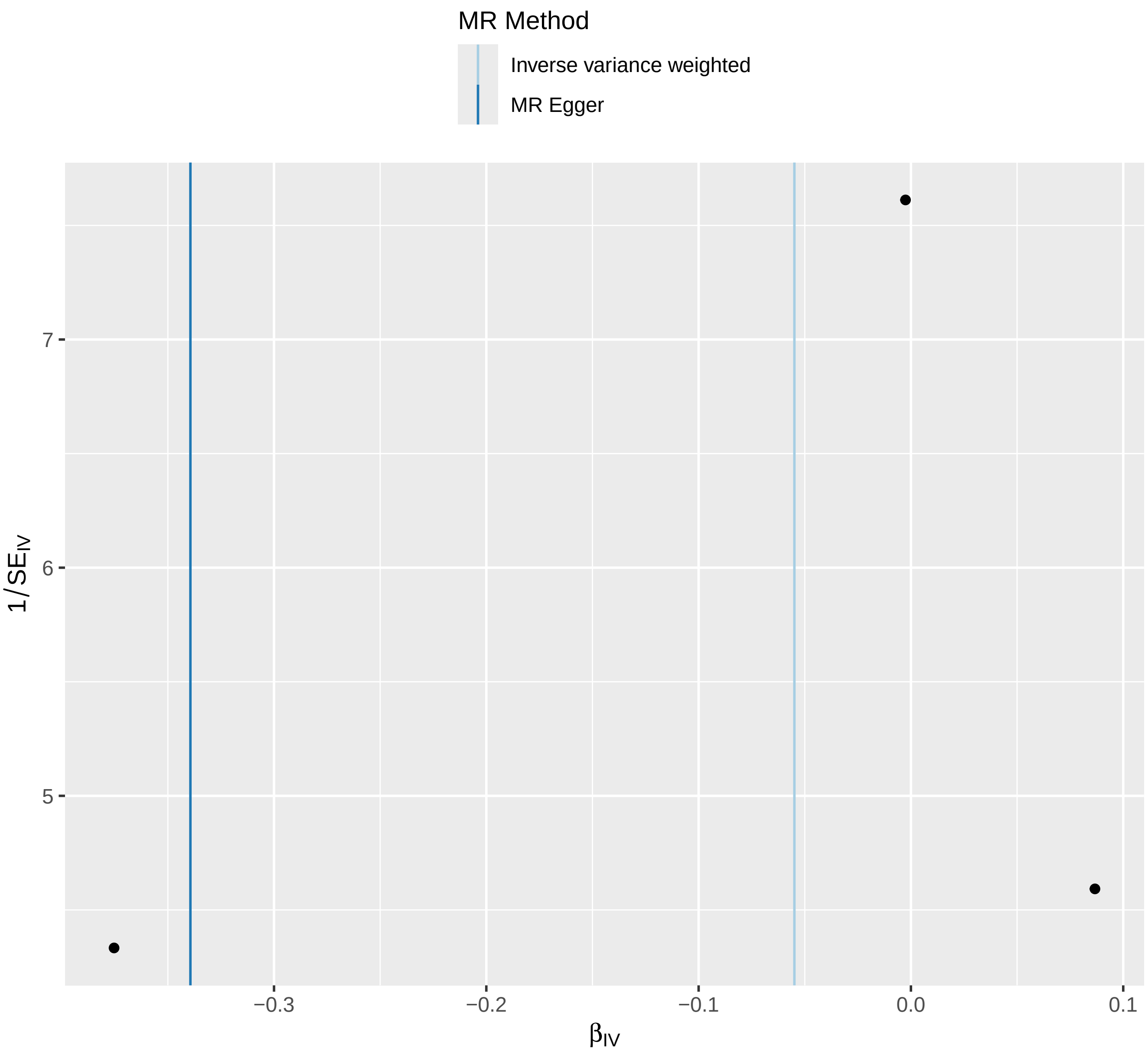

MR Method
Inverse variance weighted
MR Egger
7
6
5
1/SE_IV
−0.3
−0.2
−0.1
0.0
0.1
β_IV

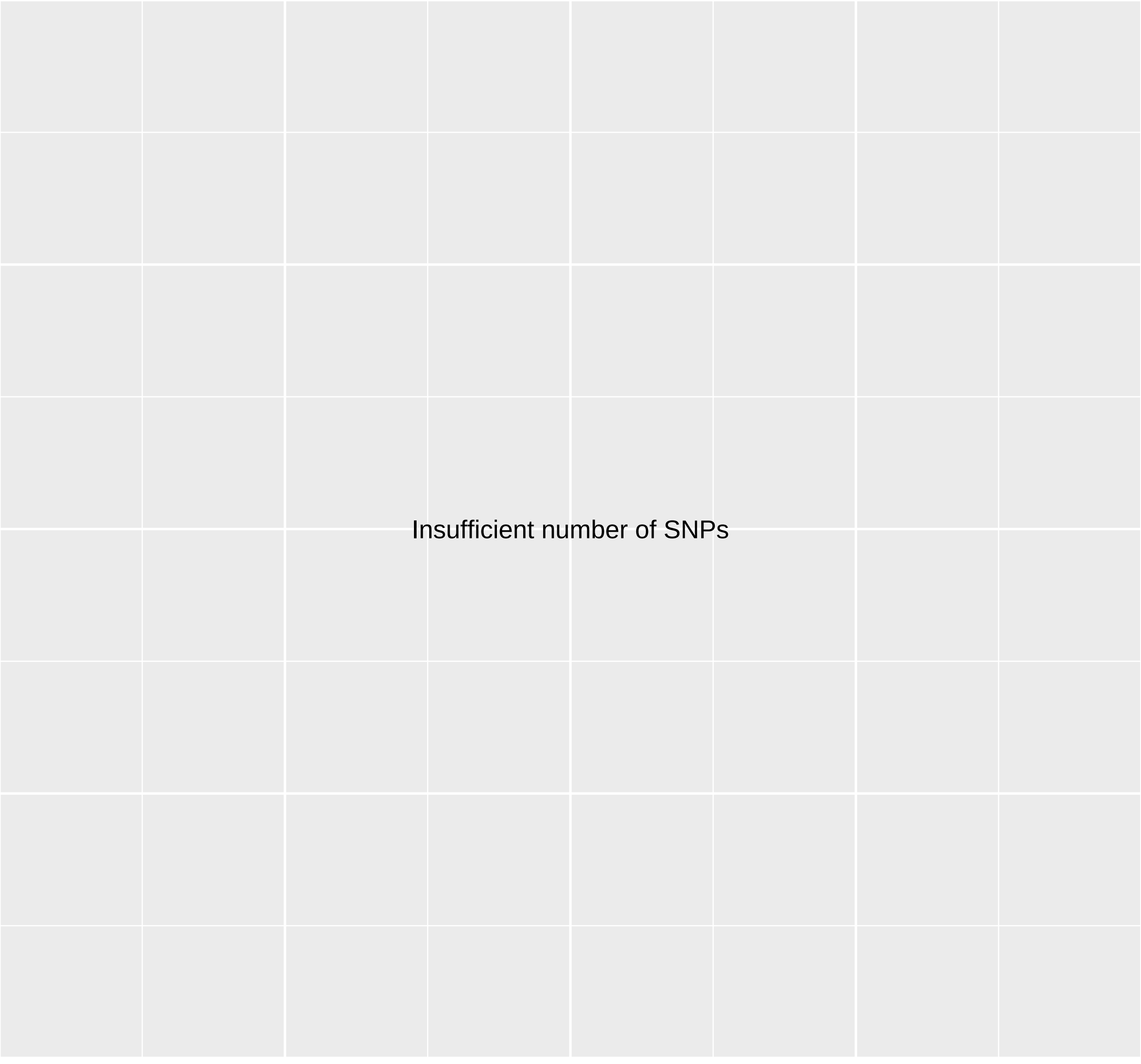
Insufficient number of SNPs

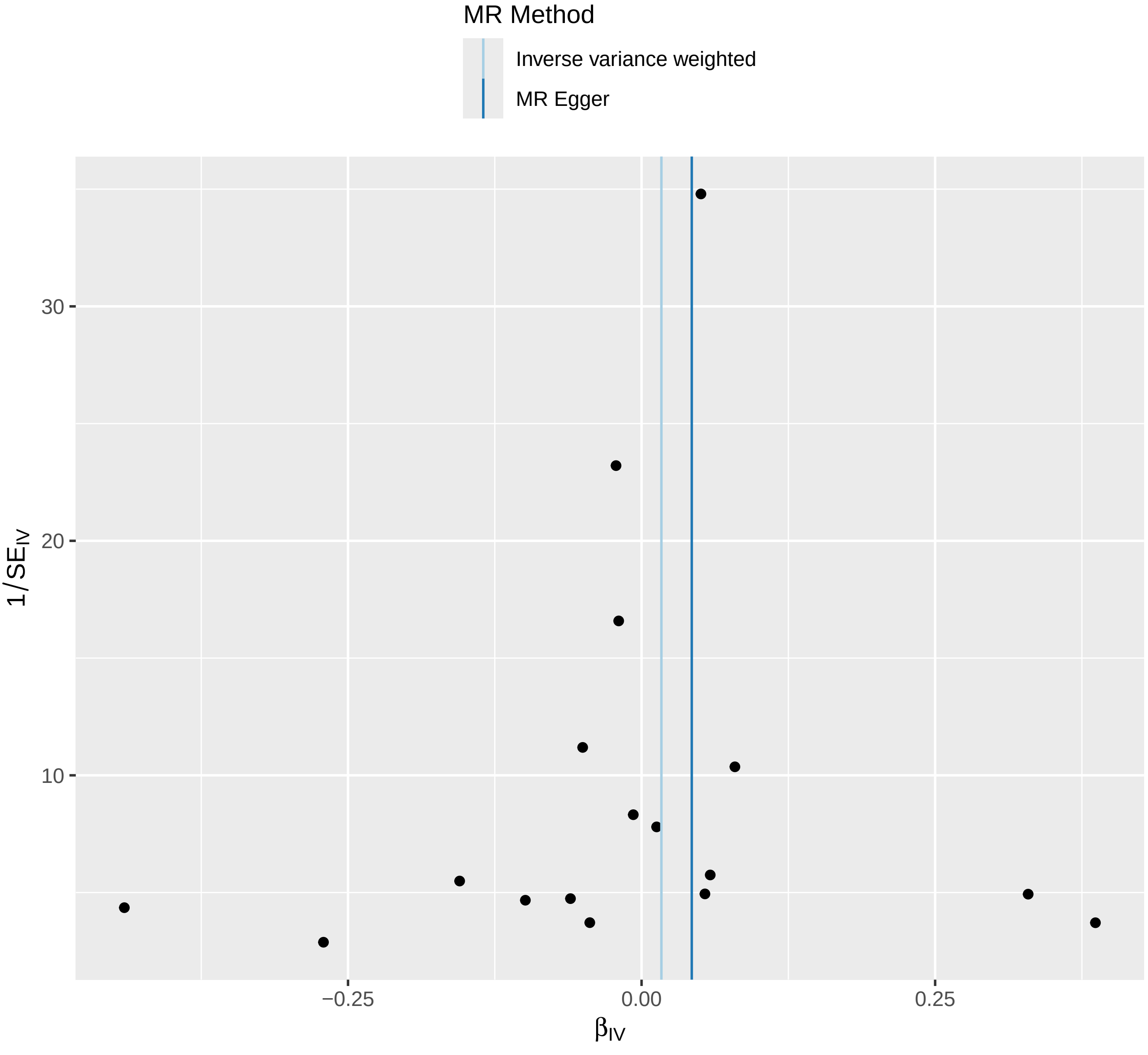
MR Method
Inverse variance weighted
MR Egger
30
20
10
1/SE$_{IV}$
−0.25
0.00
0.25
β$_{IV}$

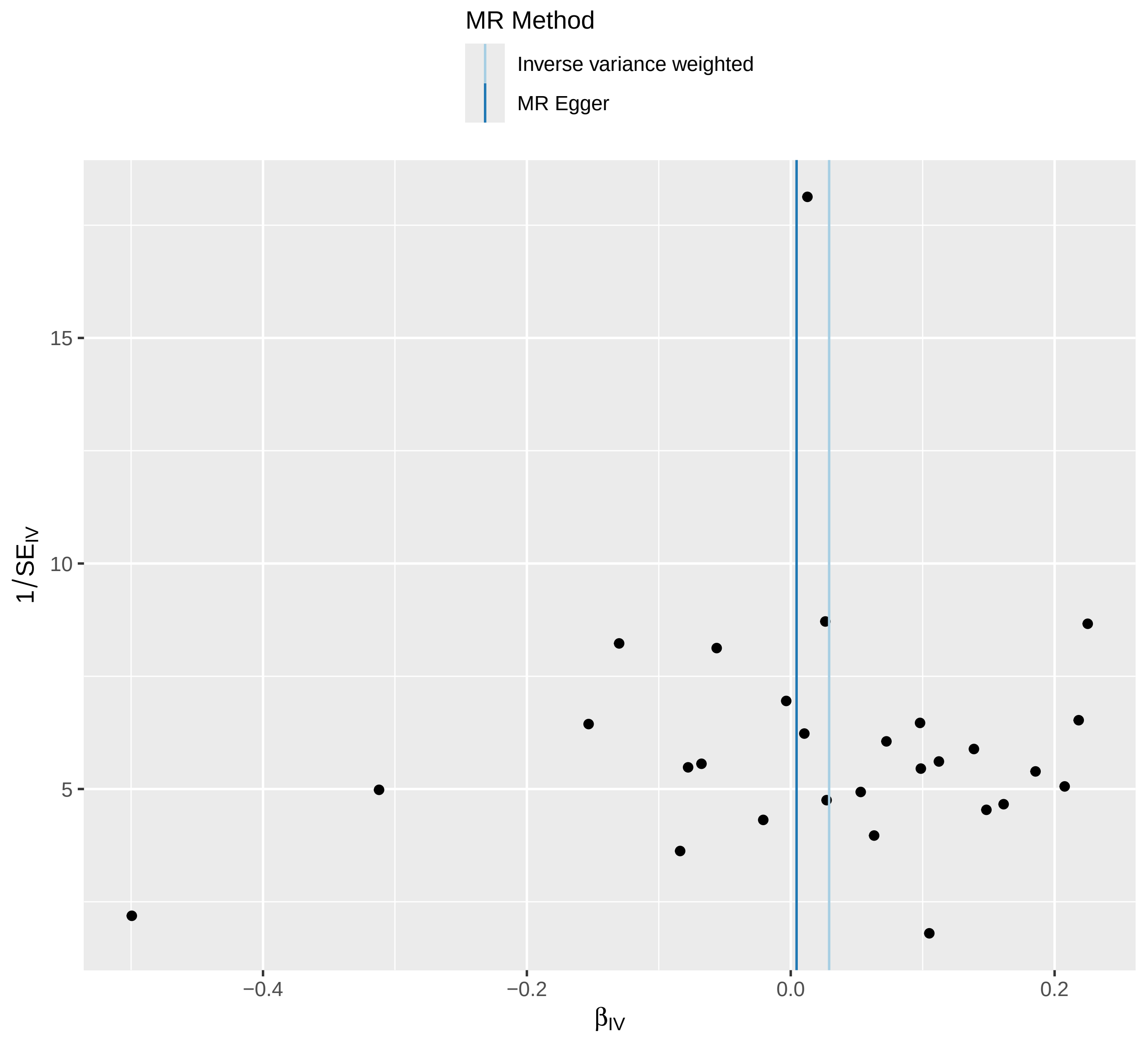
MR Method
Inverse variance weighted
MR Egger
15
10
5
1/SE_IV
−0.4
−0.2
0.0
0.2
β_IV

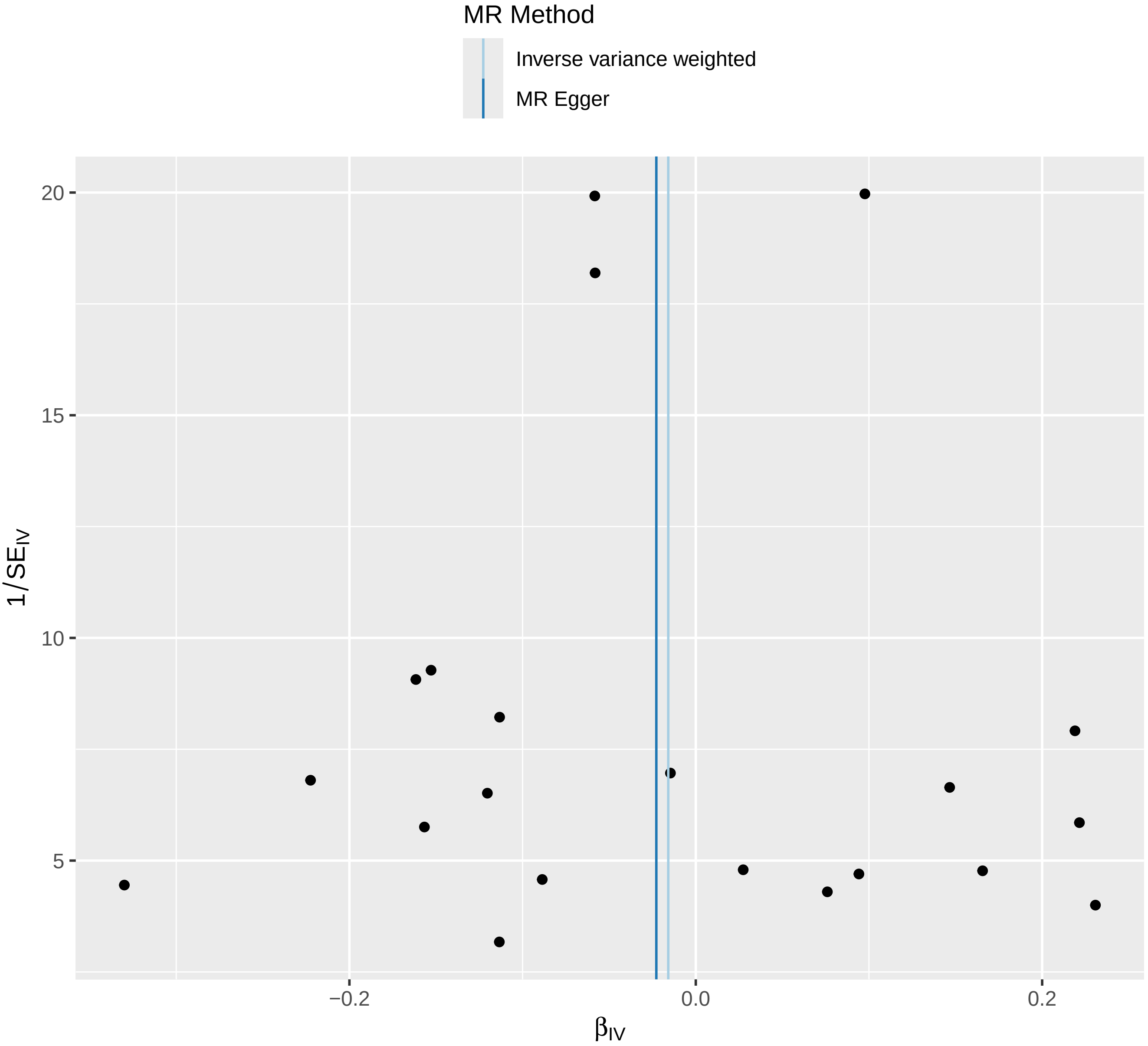

MR Method
Inverse variance weighted
MR Egger
20
15
10
5
1/SE_IV
−0.2
0.0
0.2
β_IV

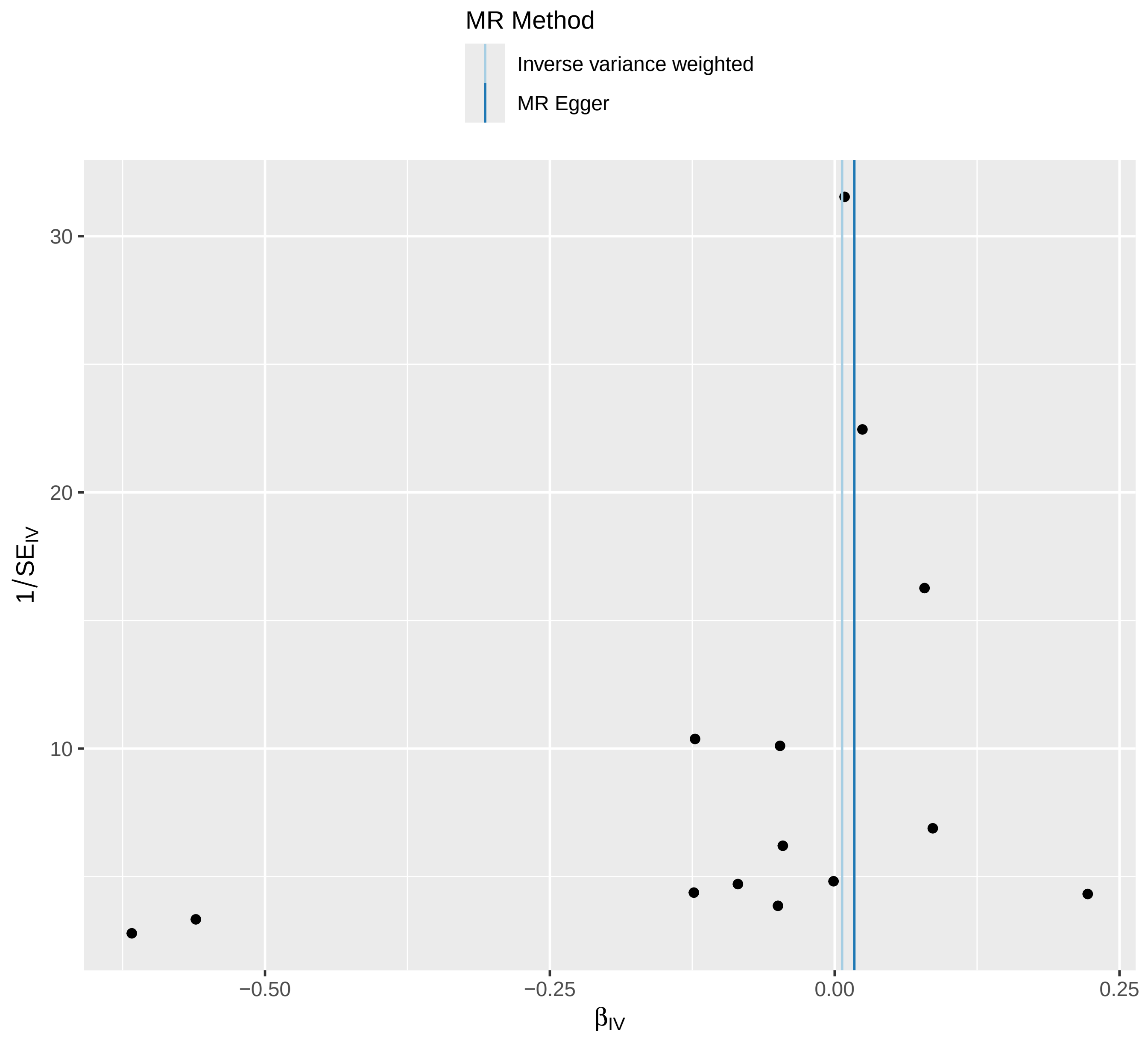

MR Method
Inverse variance weighted
MR Egger
30
20
10
1/SE$_{IV}$
−0.50
−0.25
0.00
0.25
β$_{IV}$

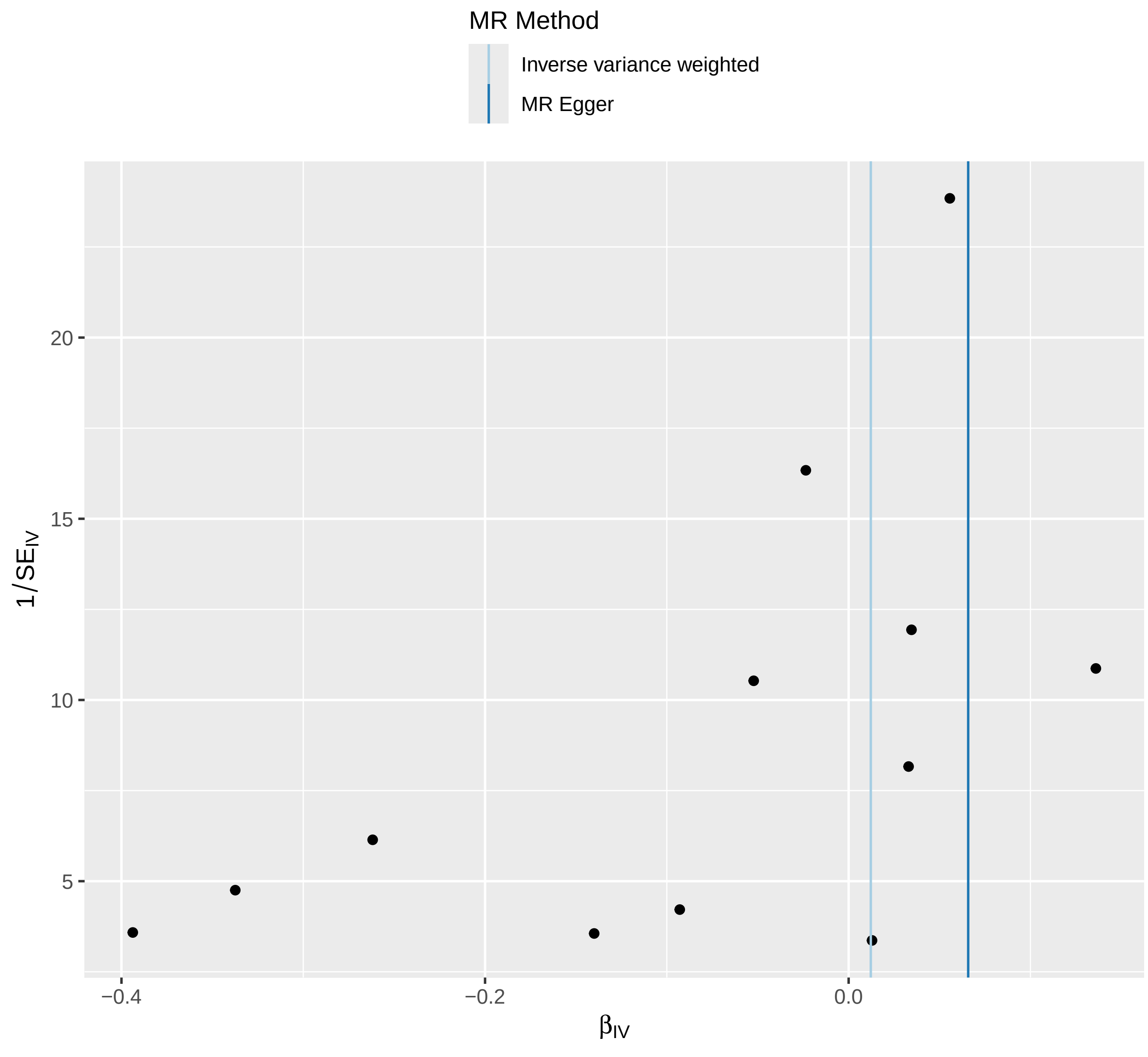
MR Method
Inverse variance weighted
MR Egger
20
15
10
5
1/SE$_{IV}$
−0.4
−0.2
0.0
β$_{IV}$

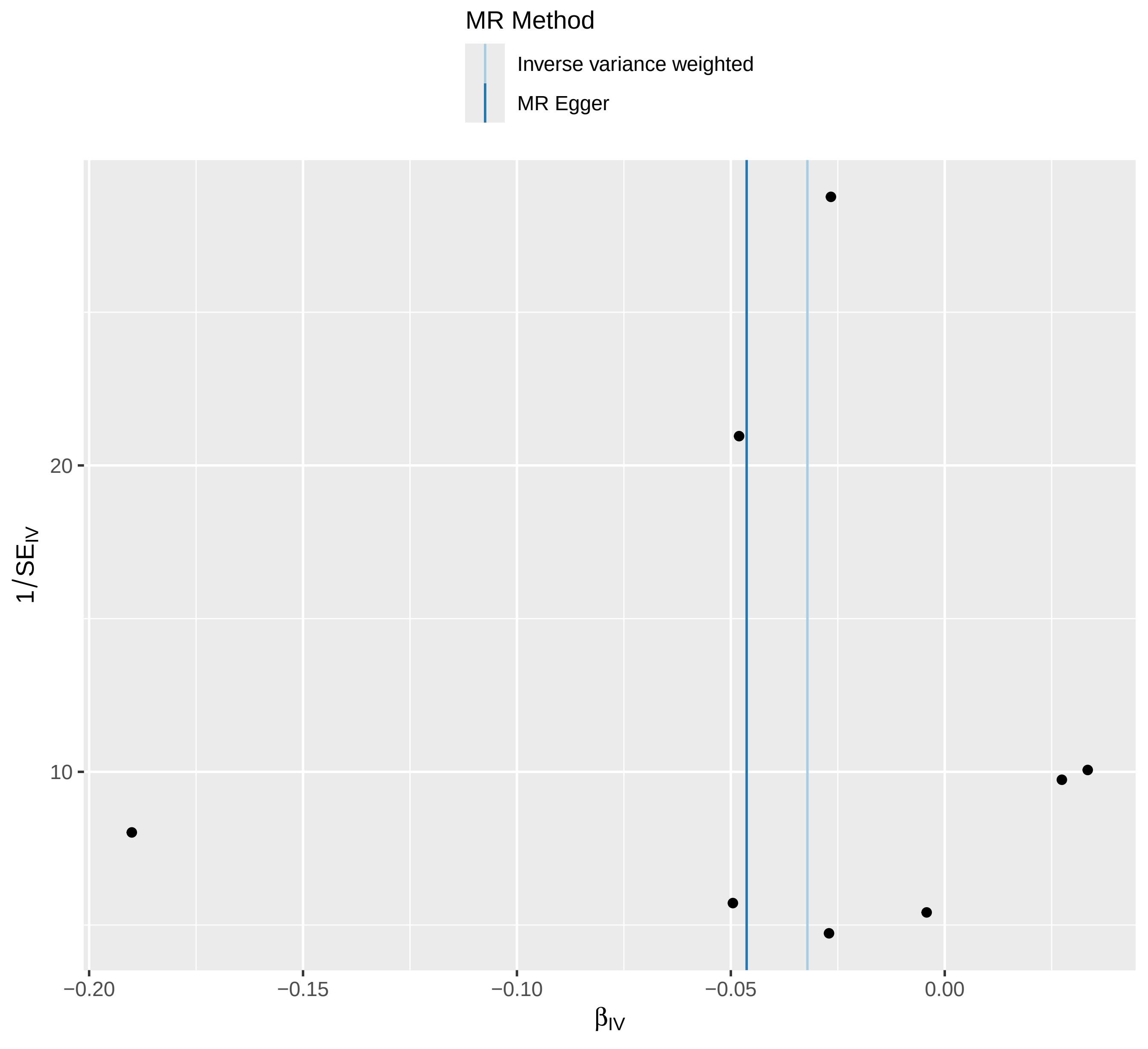

MR Method
Inverse variance weighted
MR Egger
20
10
1/SE$_{IV}$
−0.20
−0.15
−0.10
−0.05
0.00
β$_{IV}$

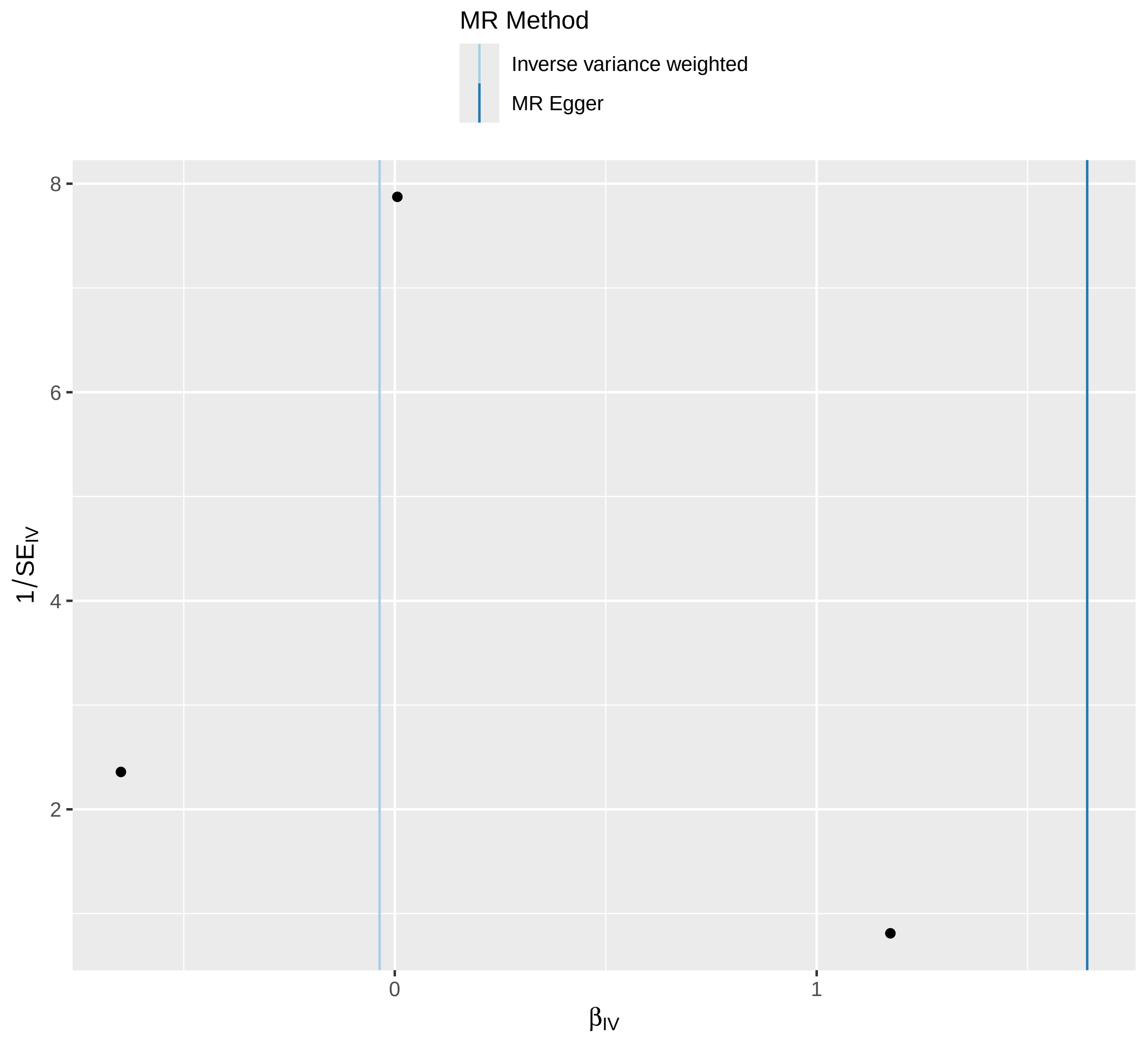
MR Method
Inverse variance weighted
MR Egger
8
6
4
2
0
1
1/SE_IV
β_IV

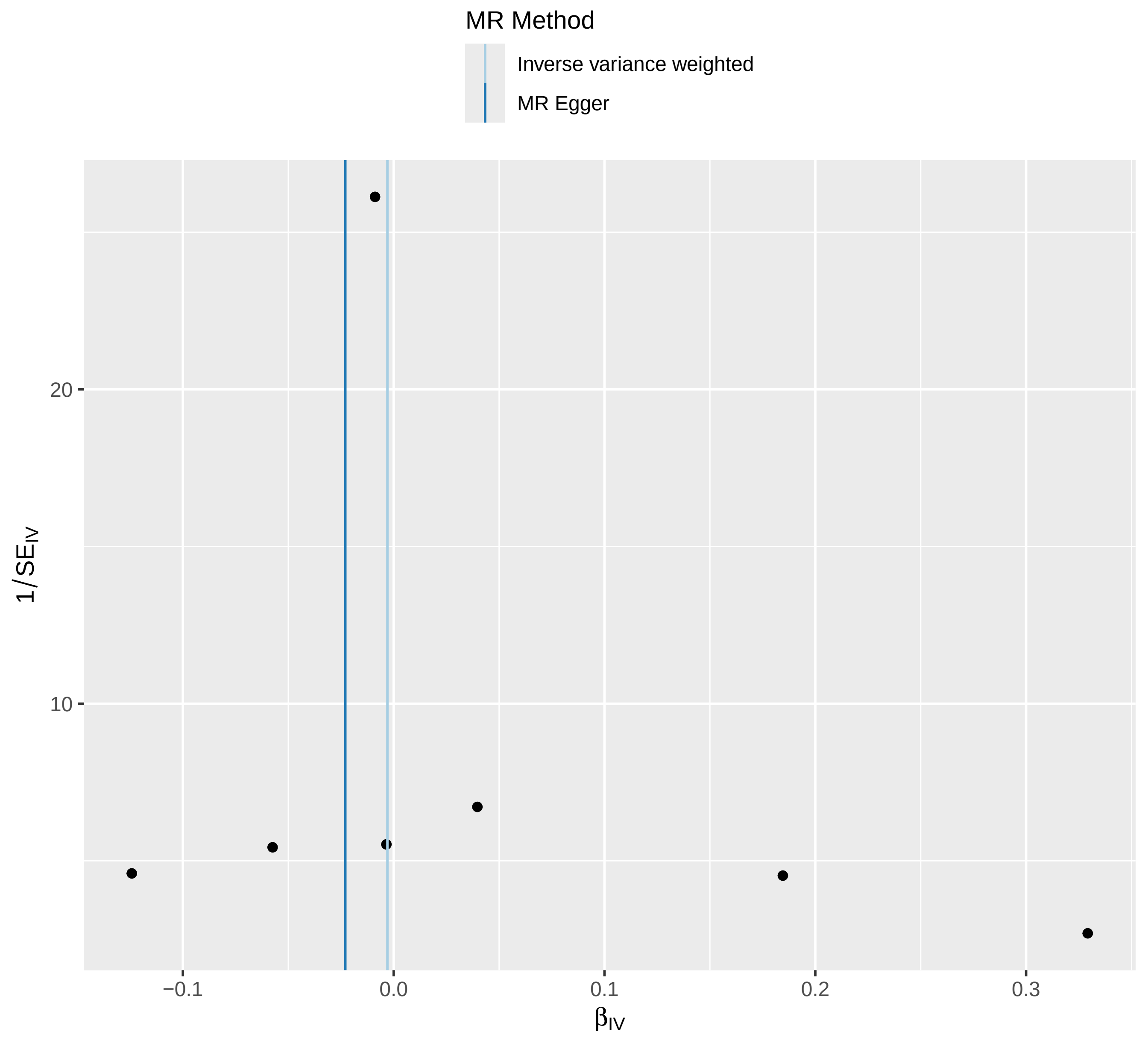

MR Method
Inverse variance weighted
MR Egger
20
10
1/SE$_{IV}$
−0.1
0.0
0.1
0.2
0.3
β$_{IV}$

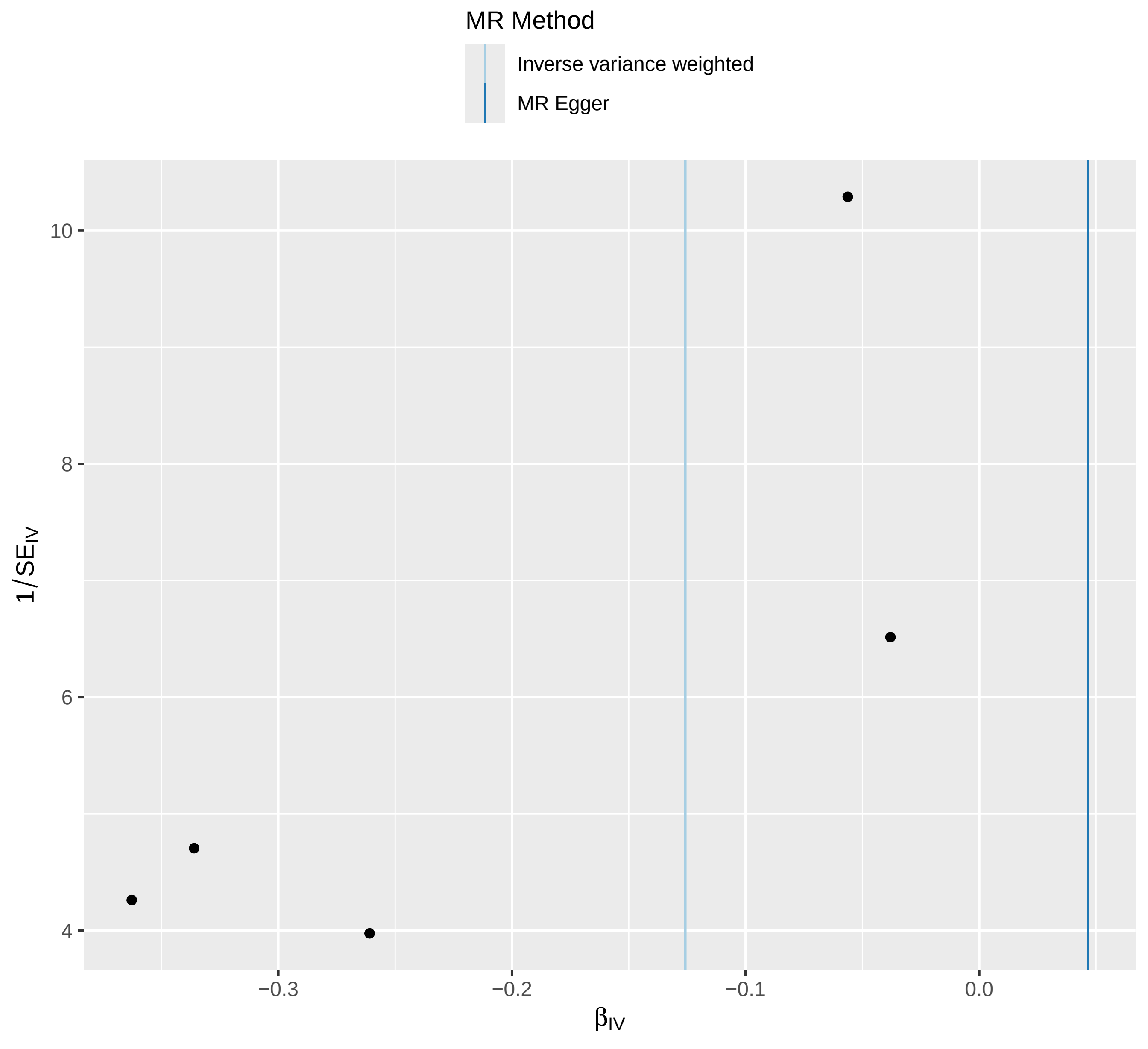
MR Method
Inverse variance weighted
MR Egger
1/SE_IV
10
8
6
4
−0.3
−0.2
−0.1
0.0
β_IV

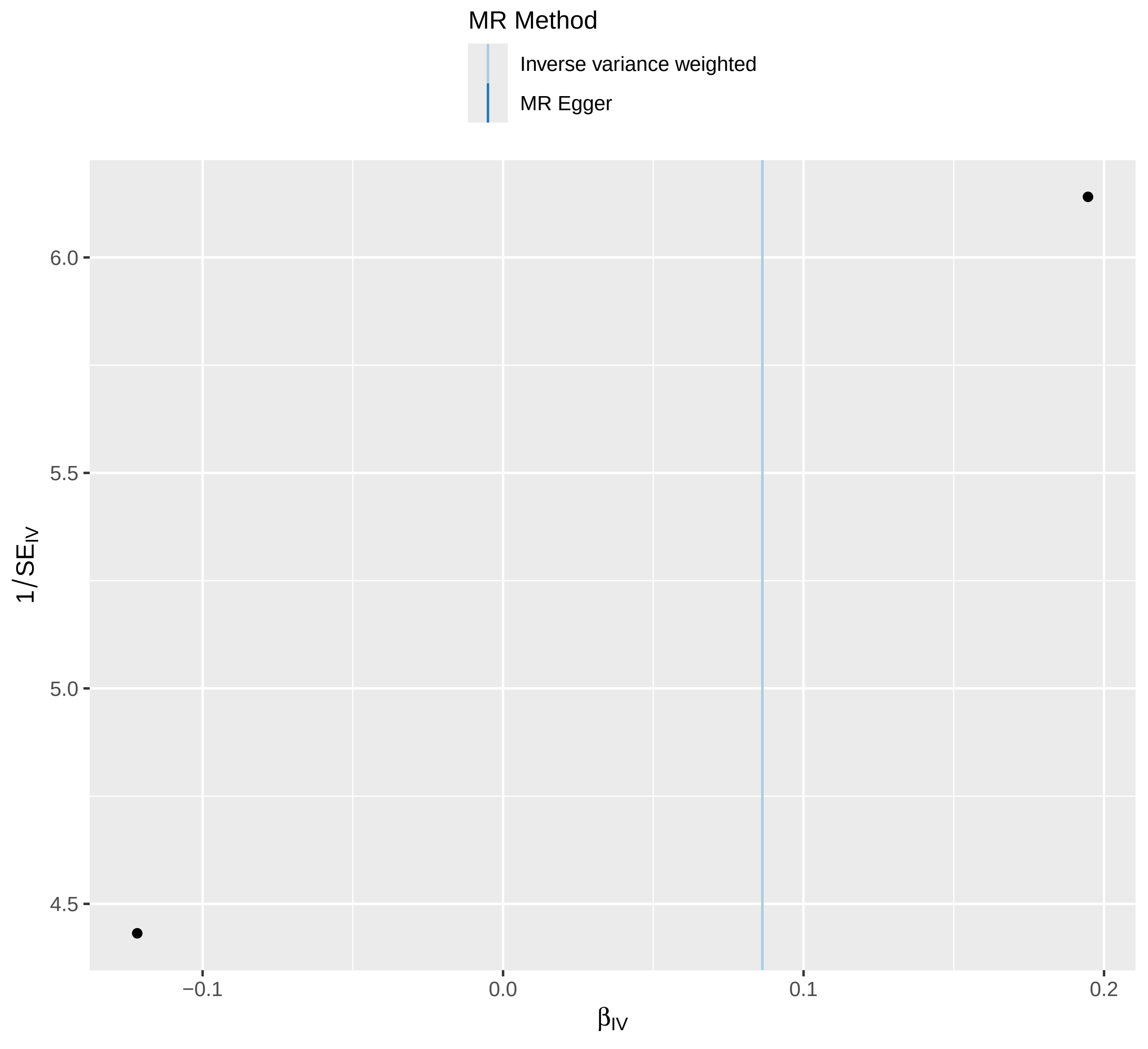

MR Method
Inverse variance weighted
MR Egger
6.0
5.5
5.0
4.5
1/SE_IV
−0.1
0.0
0.1
0.2
β_IV

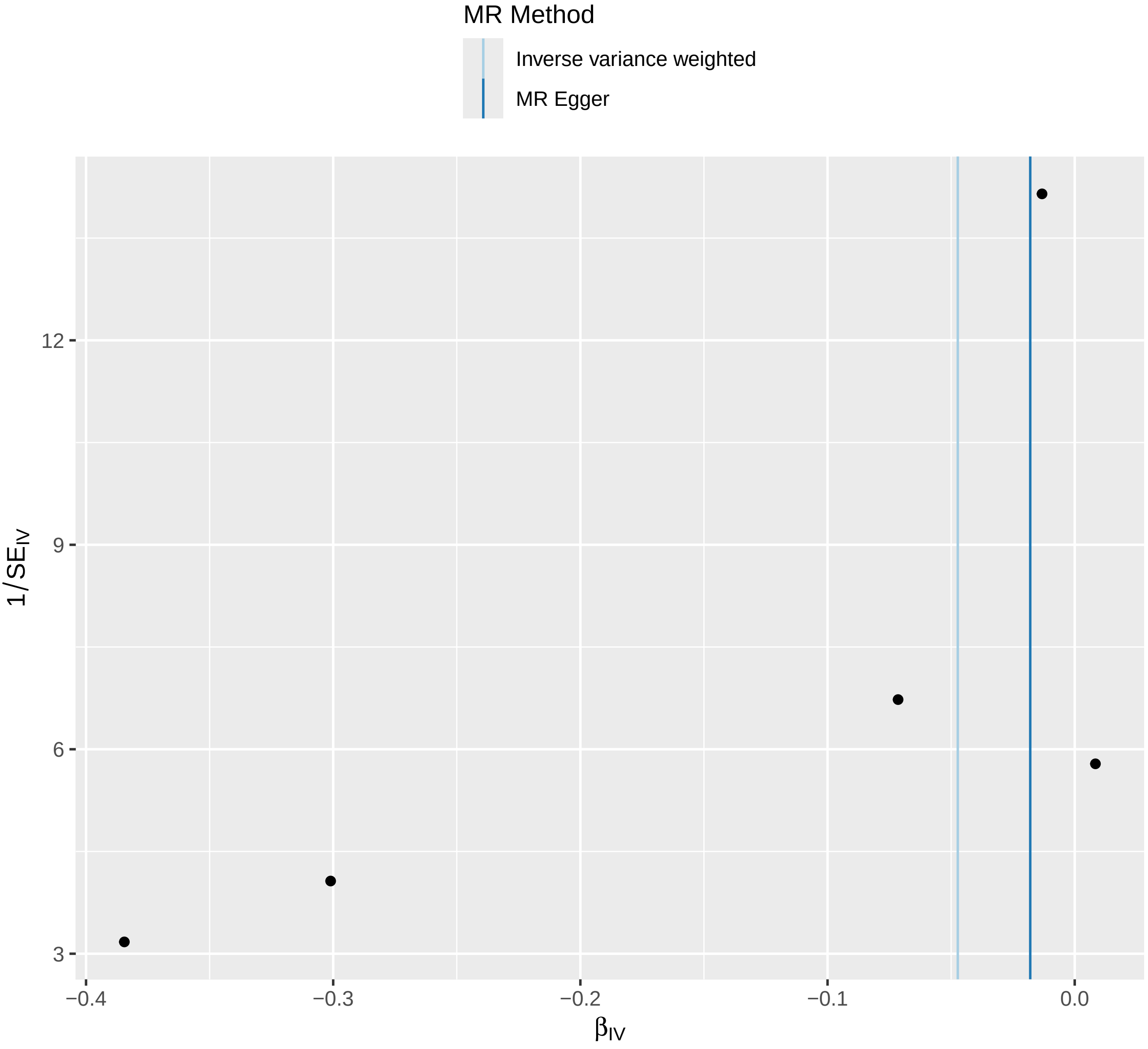
MR Method
Inverse variance weighted
MR Egger
1/SE$_{IV}$
3
6
9
12
−0.4
−0.3
−0.2
−0.1
0.0
β$_{IV}$

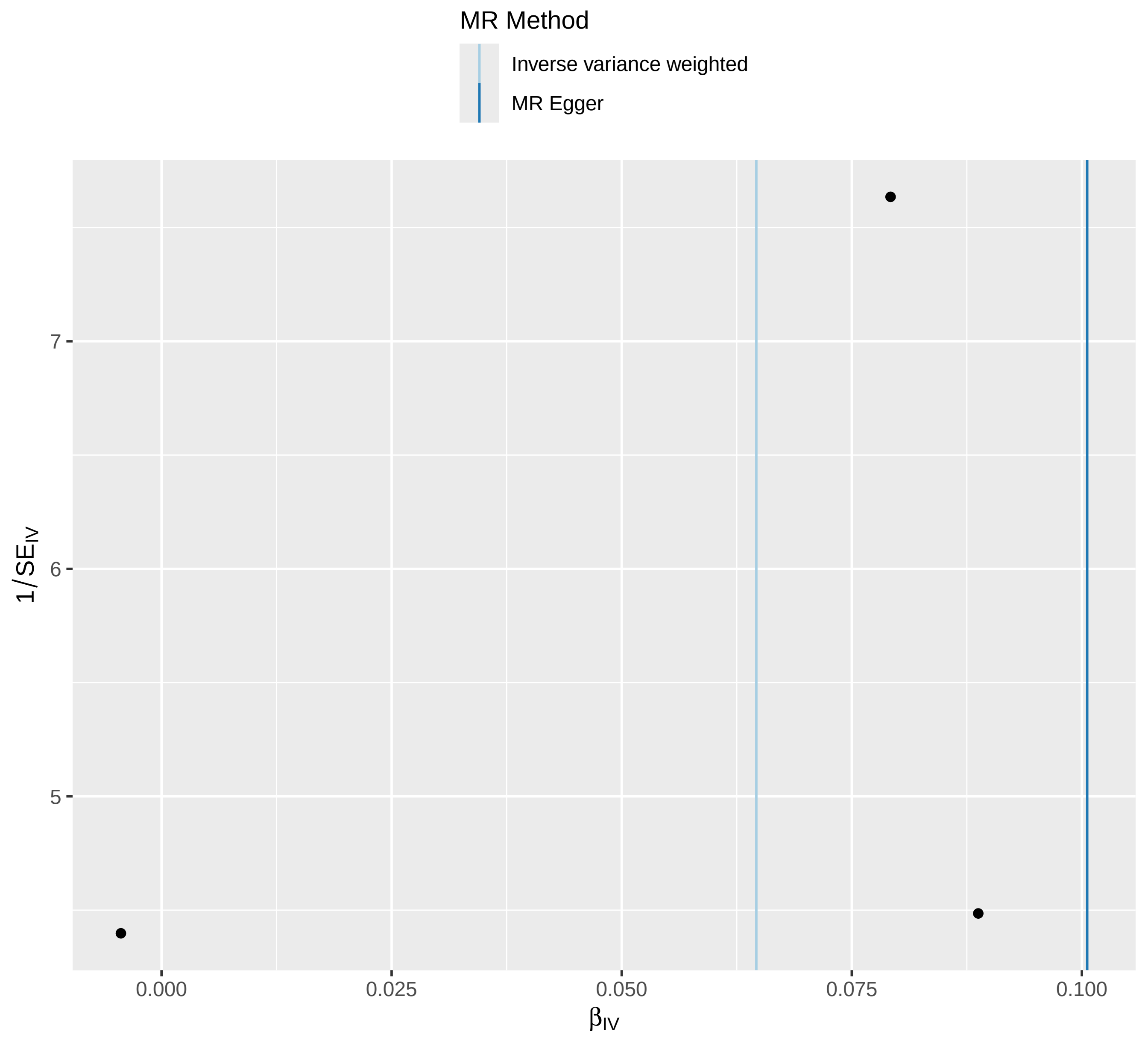

MR Method
Inverse variance weighted
MR Egger
7
6
5
1/SE$_{IV}$
0.000
0.025
0.050
0.075
0.100
β$_{IV}$

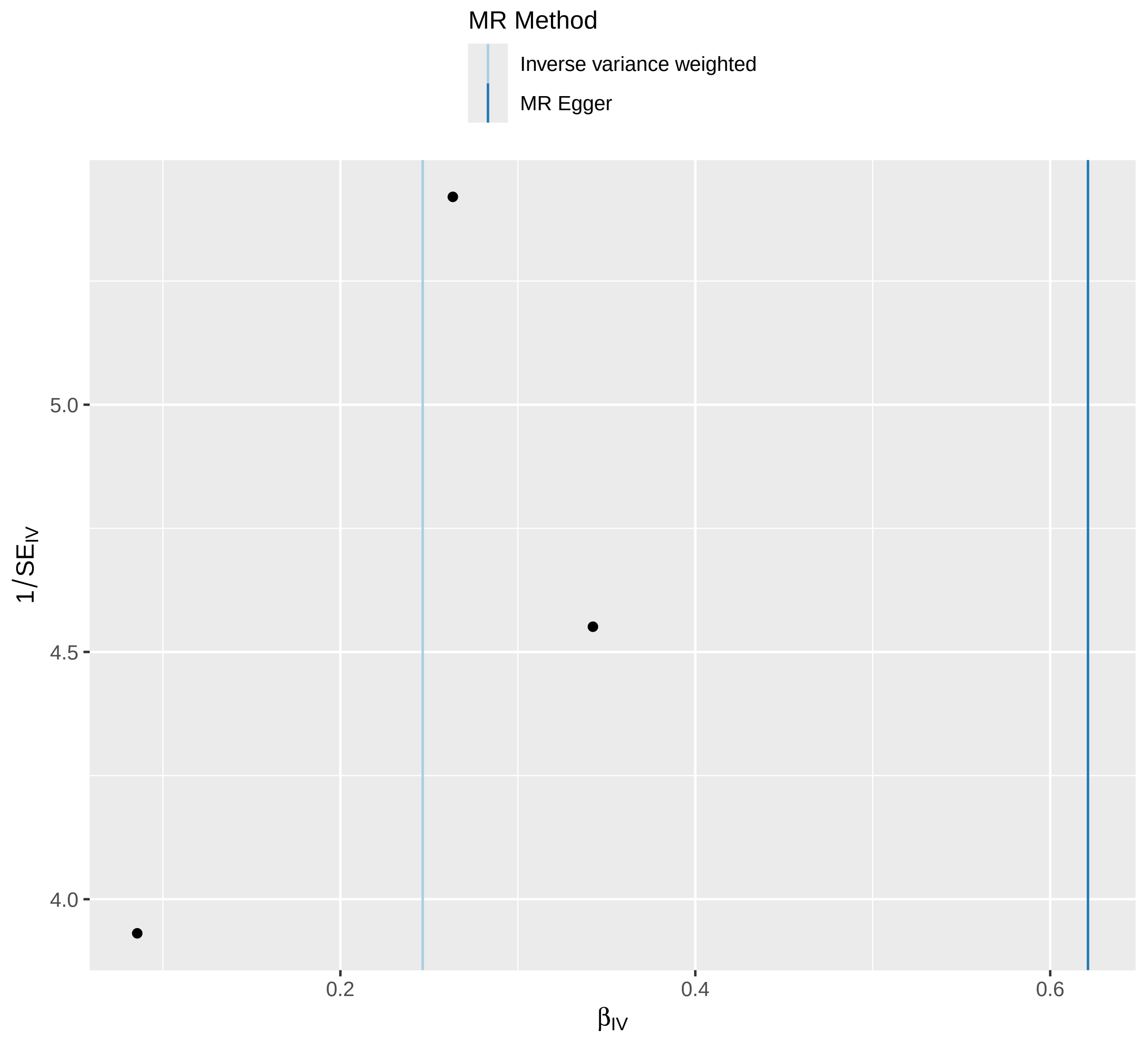
MR Method
Inverse variance weighted
MR Egger
5.0
4.5
4.0
1/SE_IV
0.2
0.4
0.6
β_IV

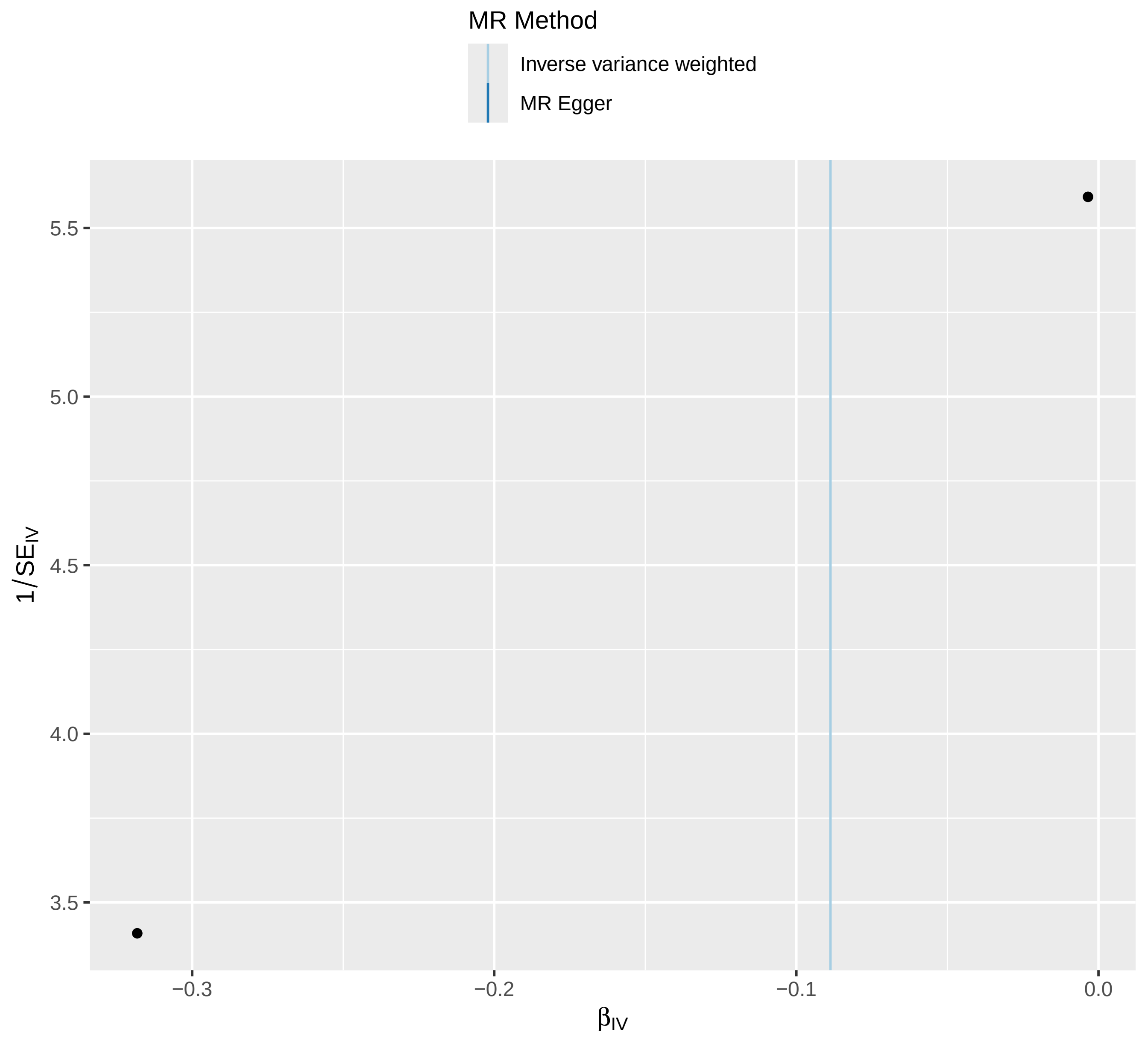
MR Method
Inverse variance weighted
MR Egger
5.5
5.0
4.5
4.0
3.5
$1/SE_{IV}$
−0.3
−0.2
−0.1
0.0
$\beta_{IV}$

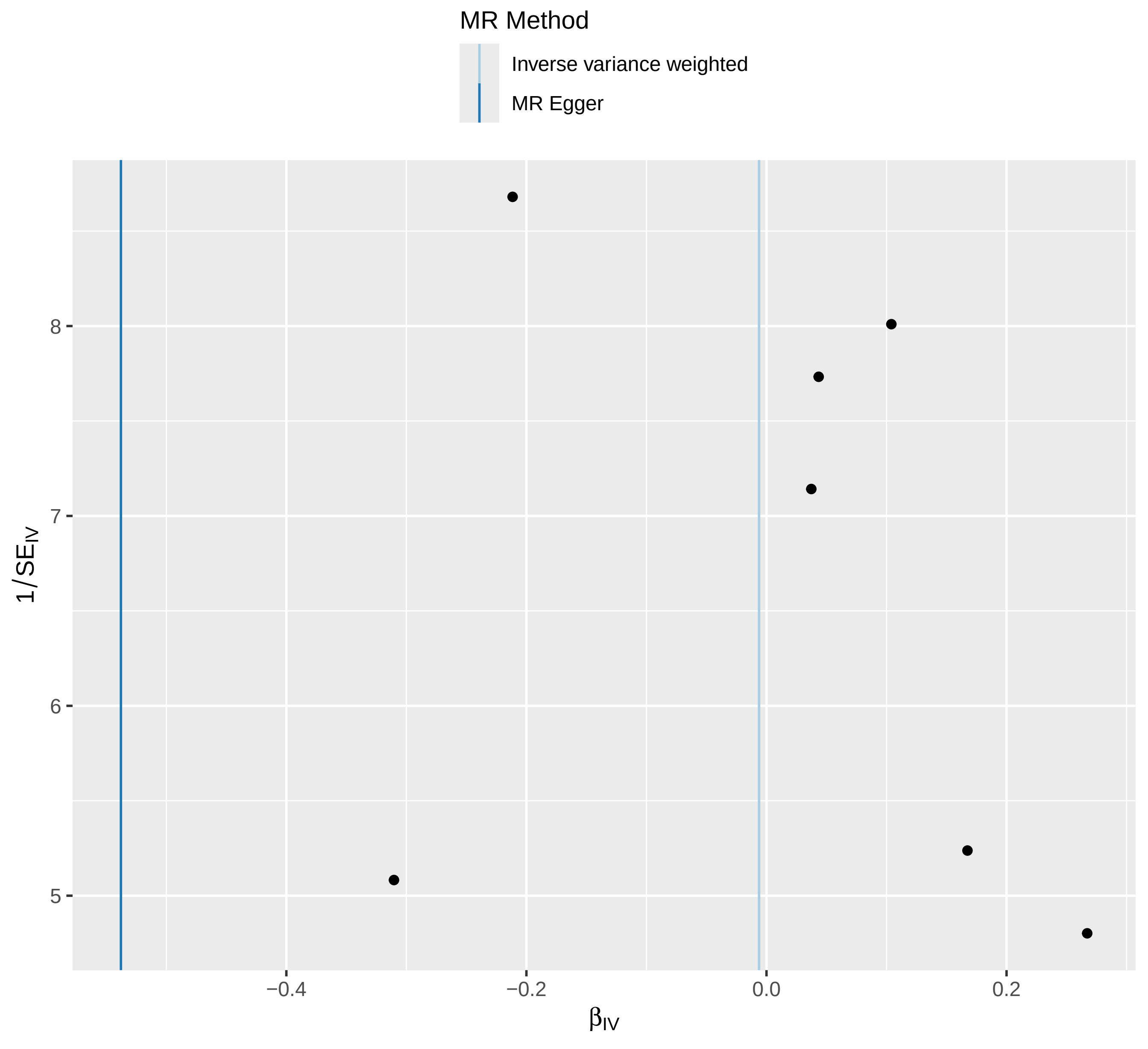
MR Method
Inverse variance weighted
MR Egger
8
7
6
5
$1/SE_{IV}$
−0.4
−0.2
0.0
0.2
$\beta_{IV}$

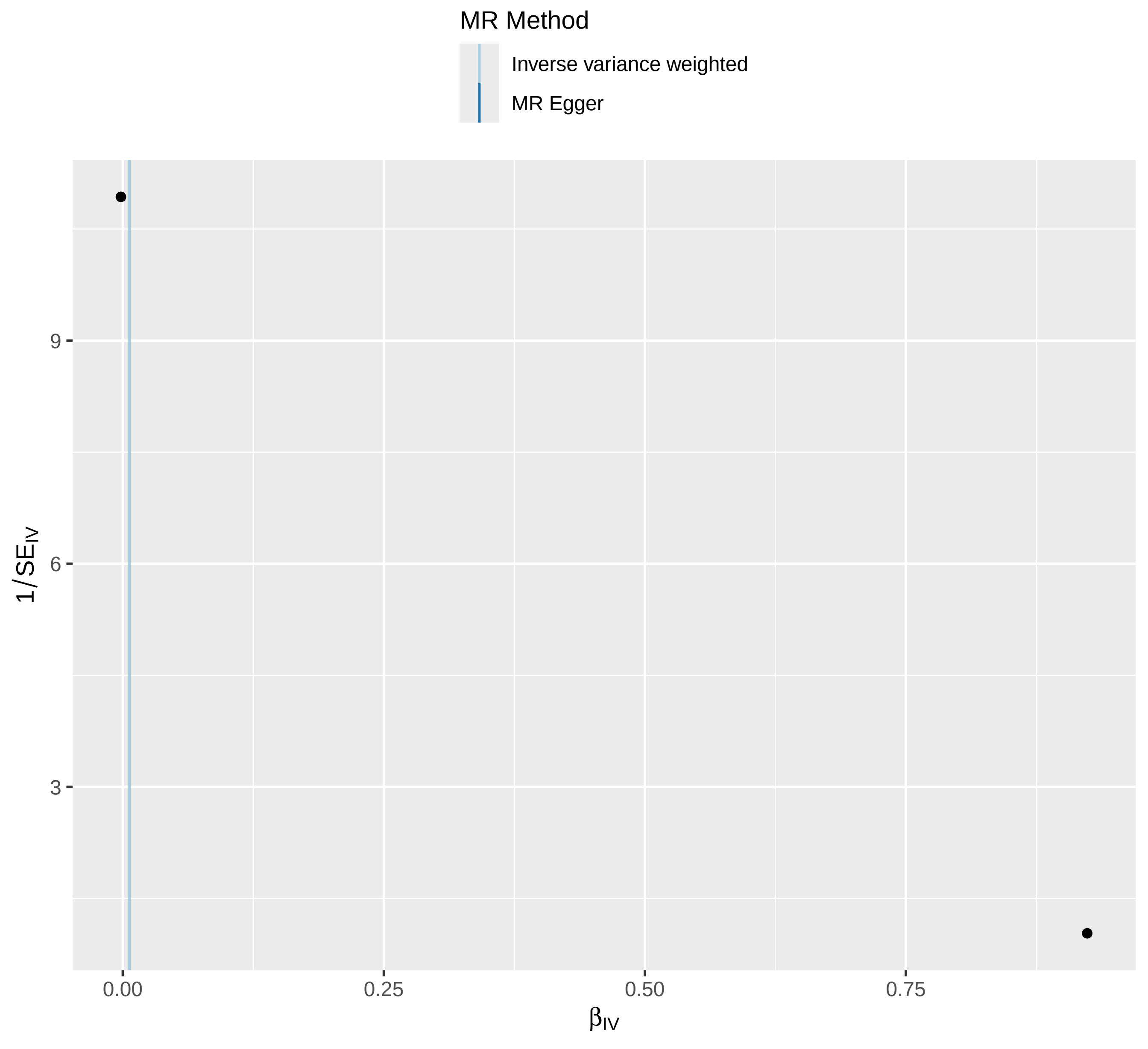
MR Method
Inverse variance weighted
MR Egger
9
6
3
1/SE$_{IV}$
0.00
0.25
0.50
0.75
$\beta_{IV}$

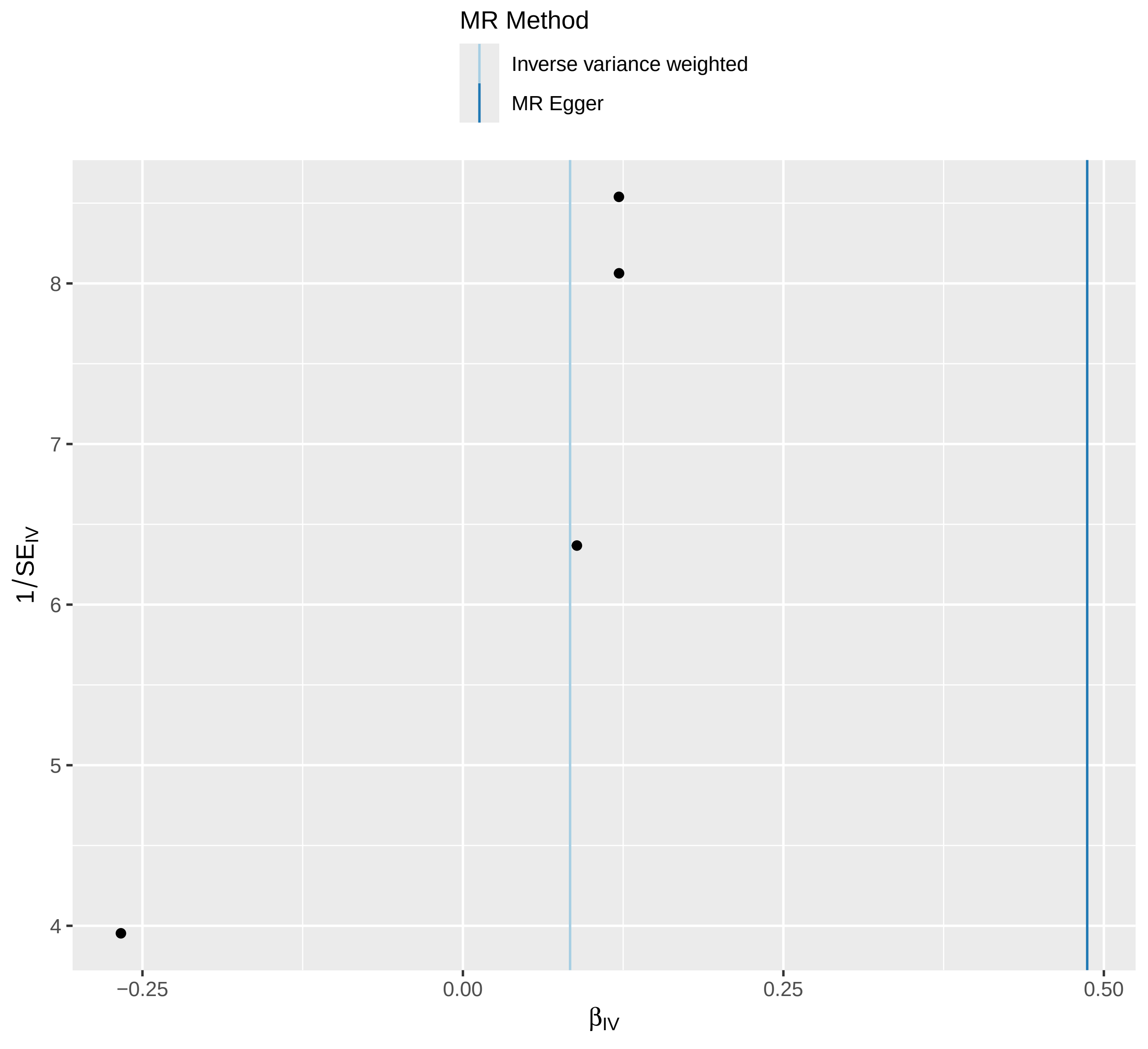
MR Method
Inverse variance weighted
MR Egger
1/SE_IV
8
7
6
5
4
−0.25
0.00
0.25
0.50
β_IV

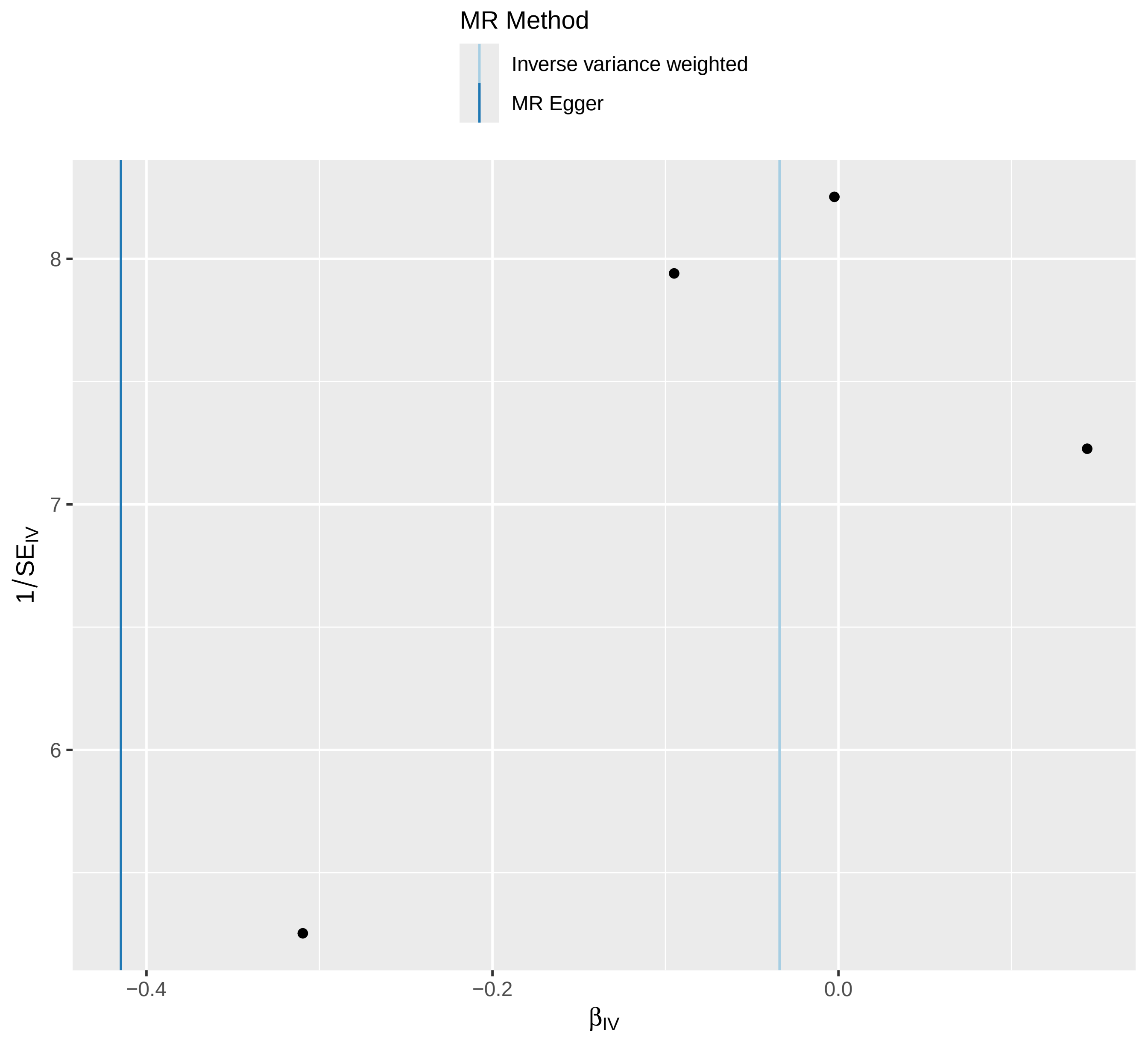
MR Method
Inverse variance weighted
MR Egger
8
7
6
1/SE_IV
−0.4
−0.2
0.0
β_IV

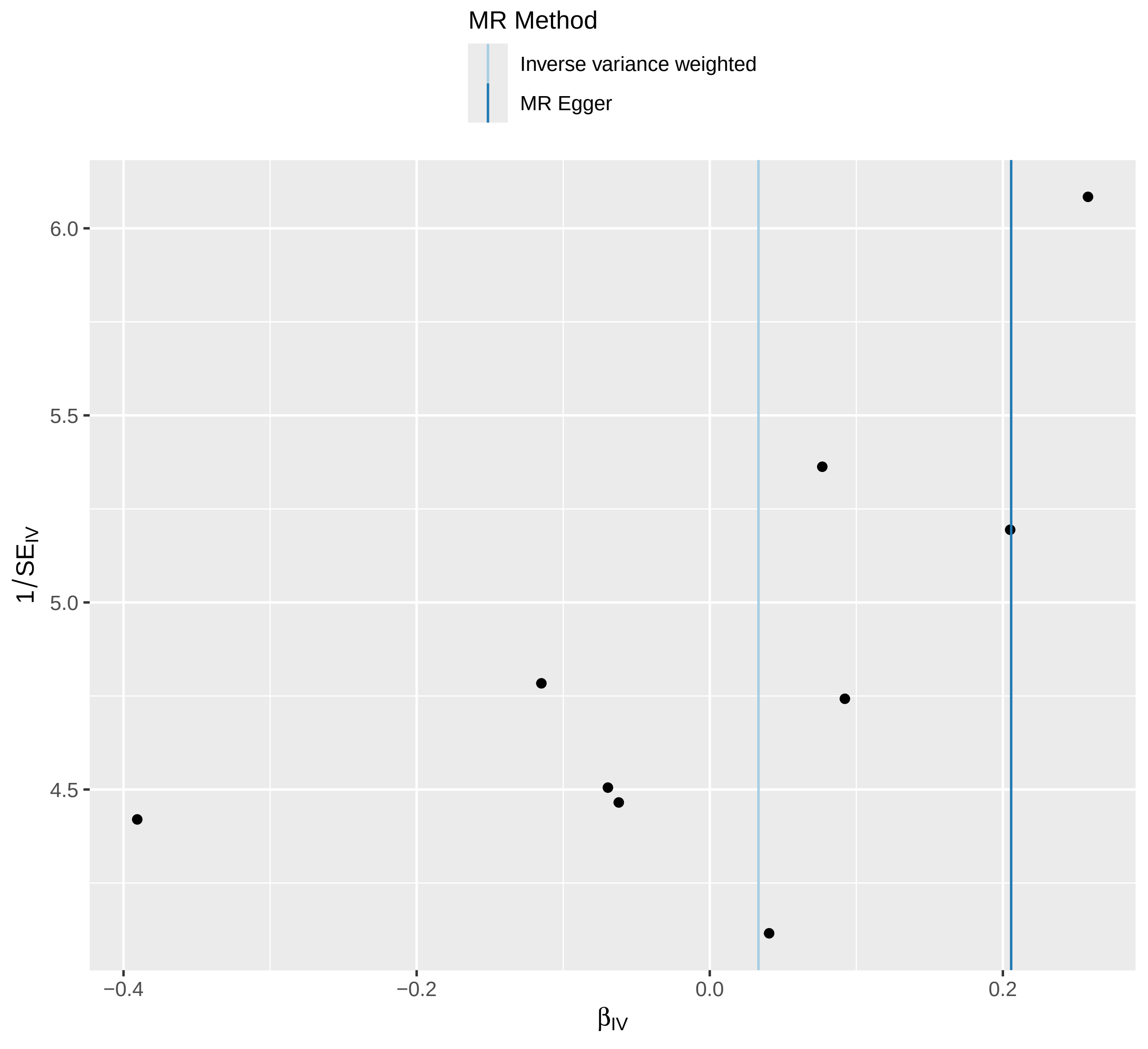

MR Method
Inverse variance weighted
MR Egger
6.0
5.5
5.0
4.5
1/SE_IV
−0.4
−0.2
0.0
0.2
β_IV

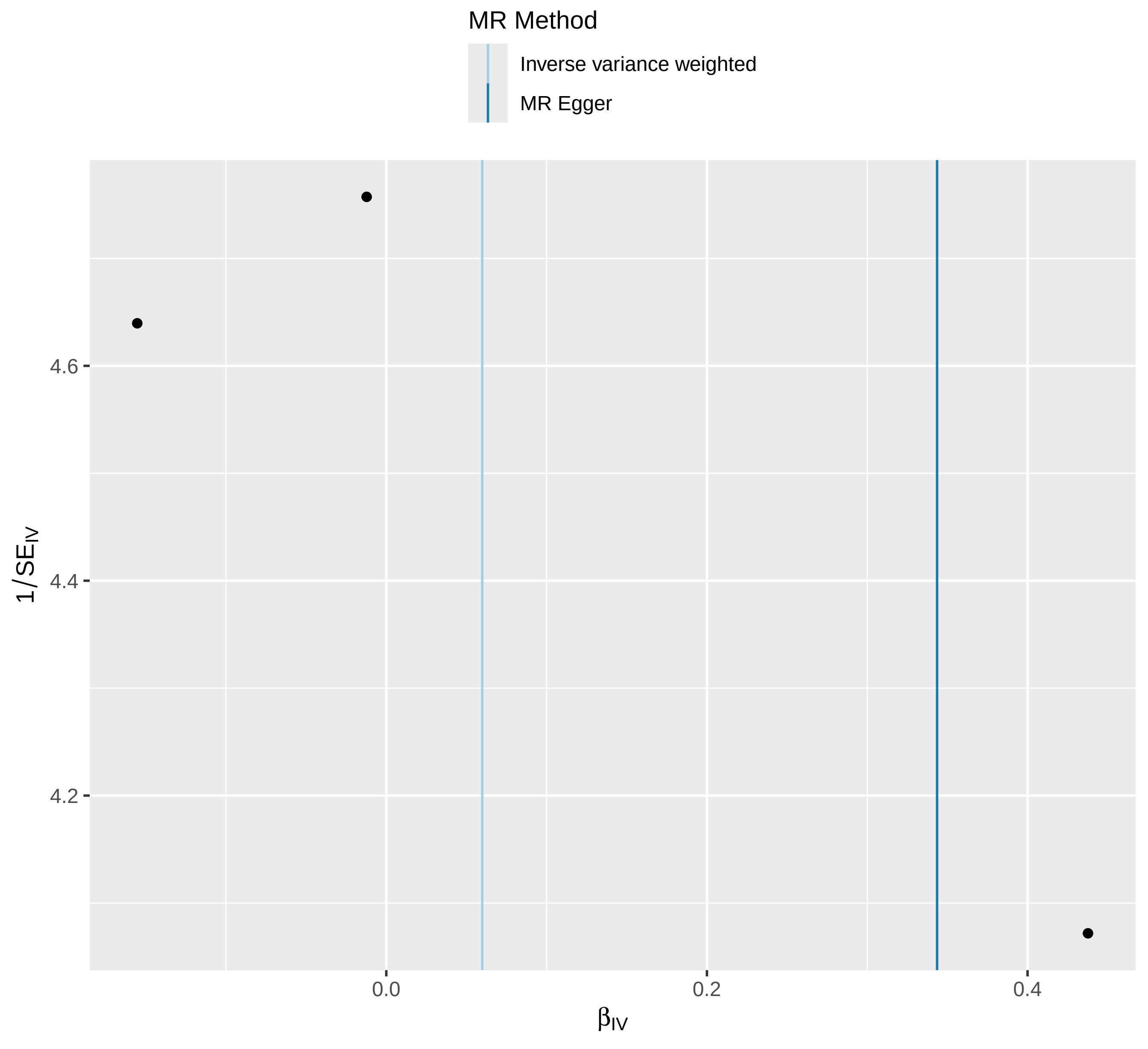

MR Method
Inverse variance weighted
MR Egger
4.6
4.4
4.2
1/SE_IV
0.0
0.2
0.4
β_IV

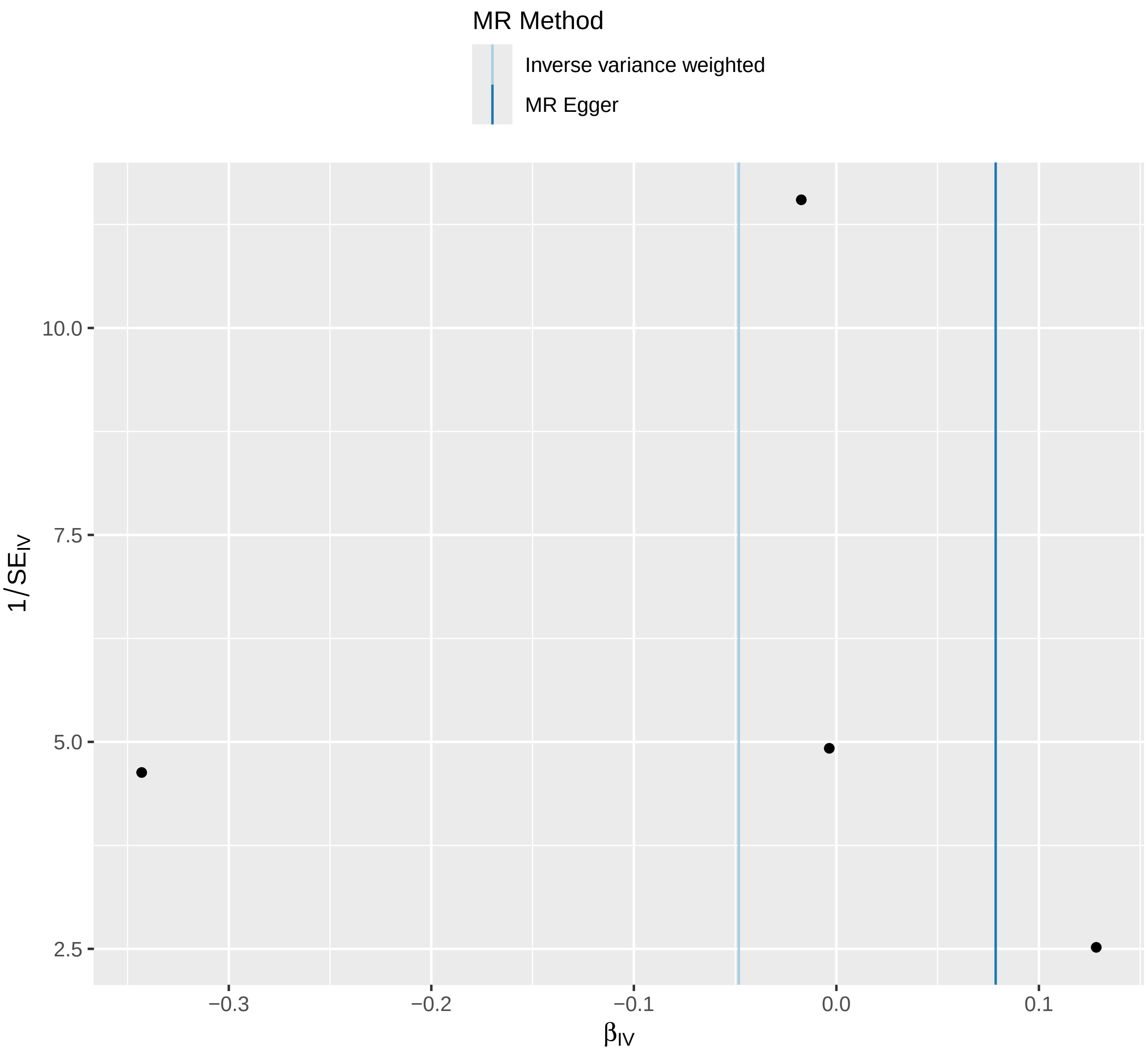
MR Method
Inverse variance weighted
MR Egger
10.0
7.5
5.0
2.5
1/SE_IV
−0.3
−0.2
−0.1
0.0
0.1
β_IV

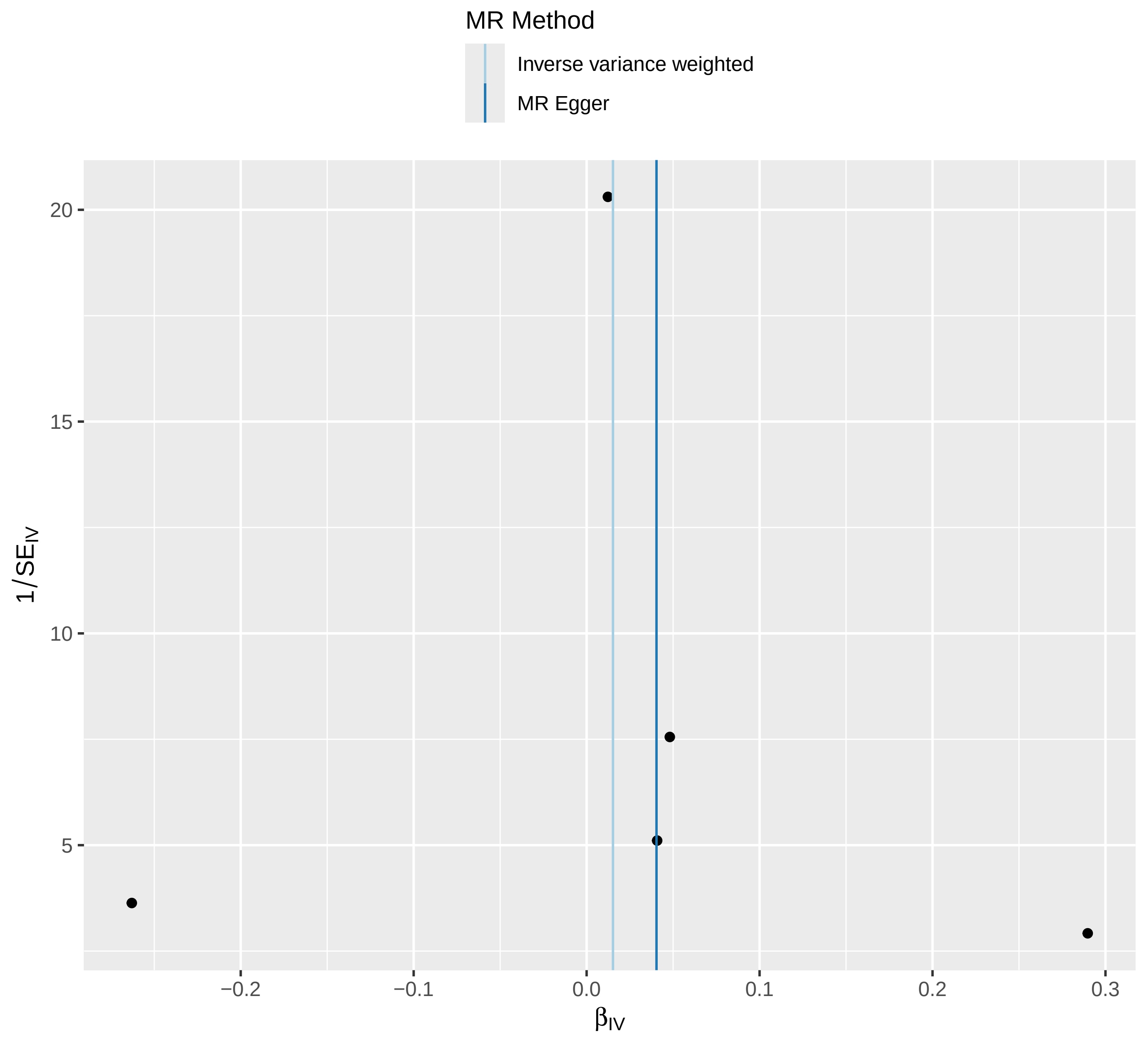
MR Method
Inverse variance weighted
MR Egger
20
15
10
5
1/SE$_{IV}$
−0.2
−0.1
0.0
0.1
0.2
0.3
β$_{IV}$

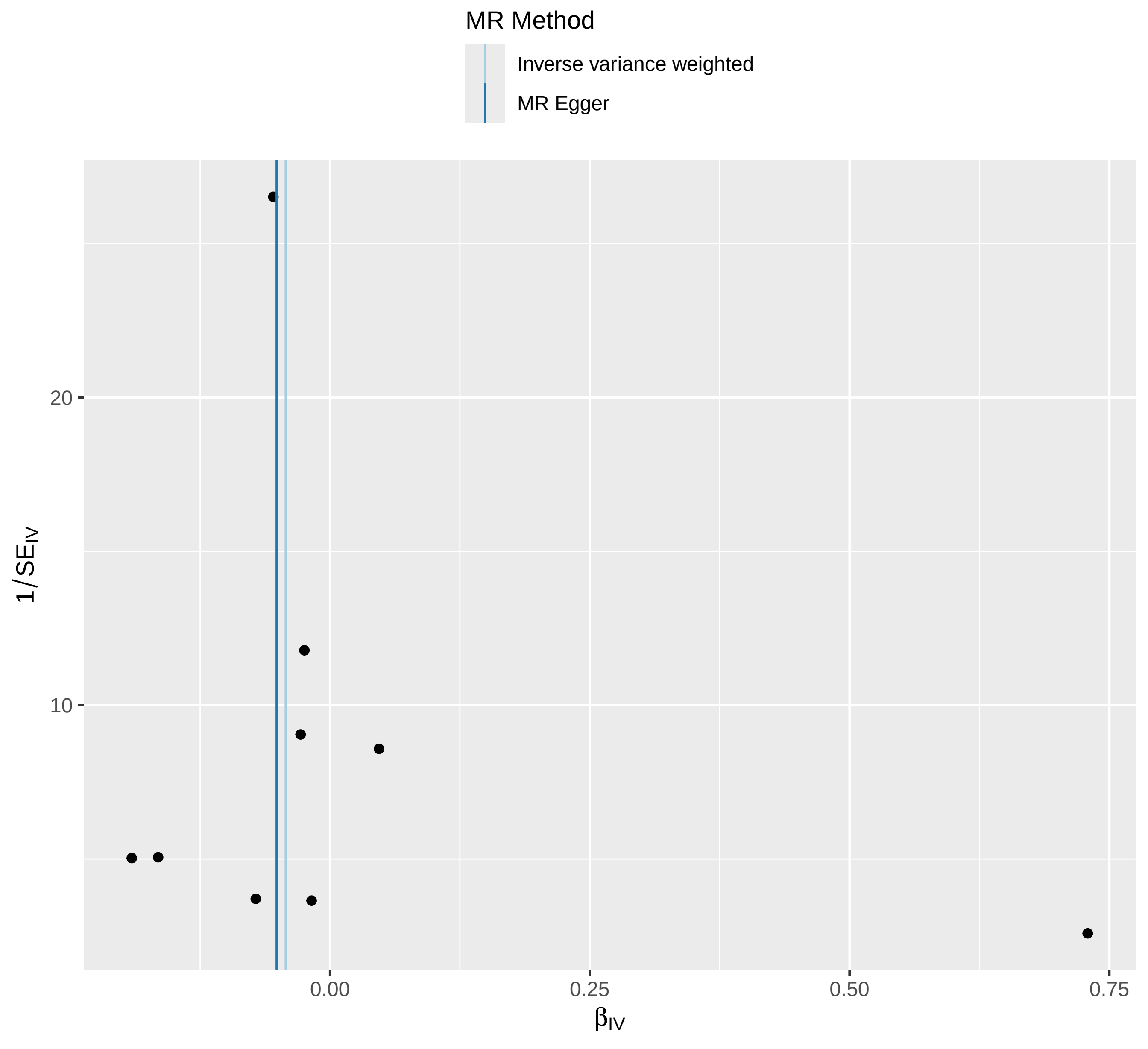

MR Method
Inverse variance weighted
MR Egger
20
10
1/SE_IV
0.00
0.25
0.50
0.75
β_IV

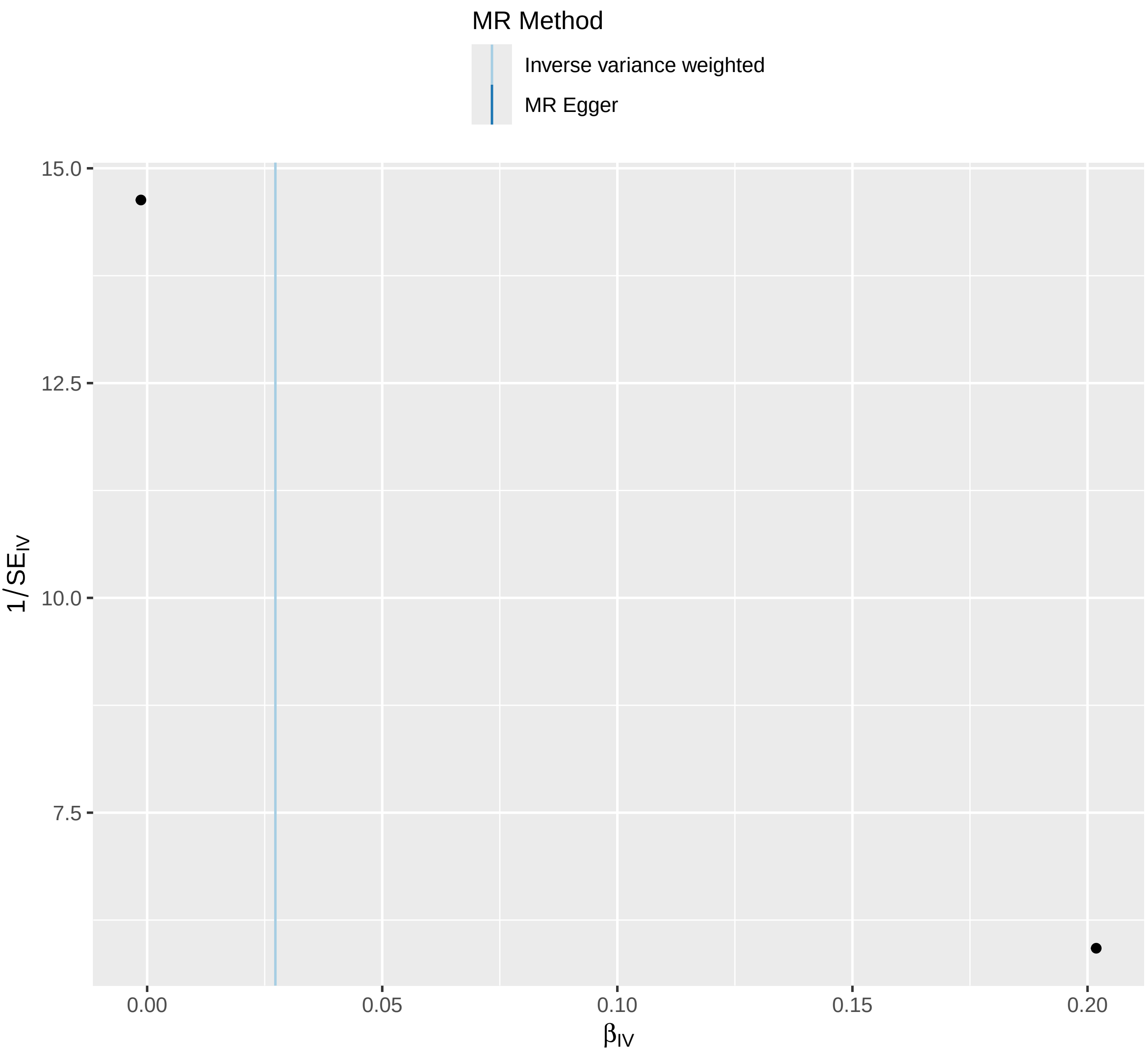
MR Method
Inverse variance weighted
MR Egger
15.0
12.5
10.0
7.5
1/SE_IV
0.00
0.05
0.10
0.15
0.20
β_IV

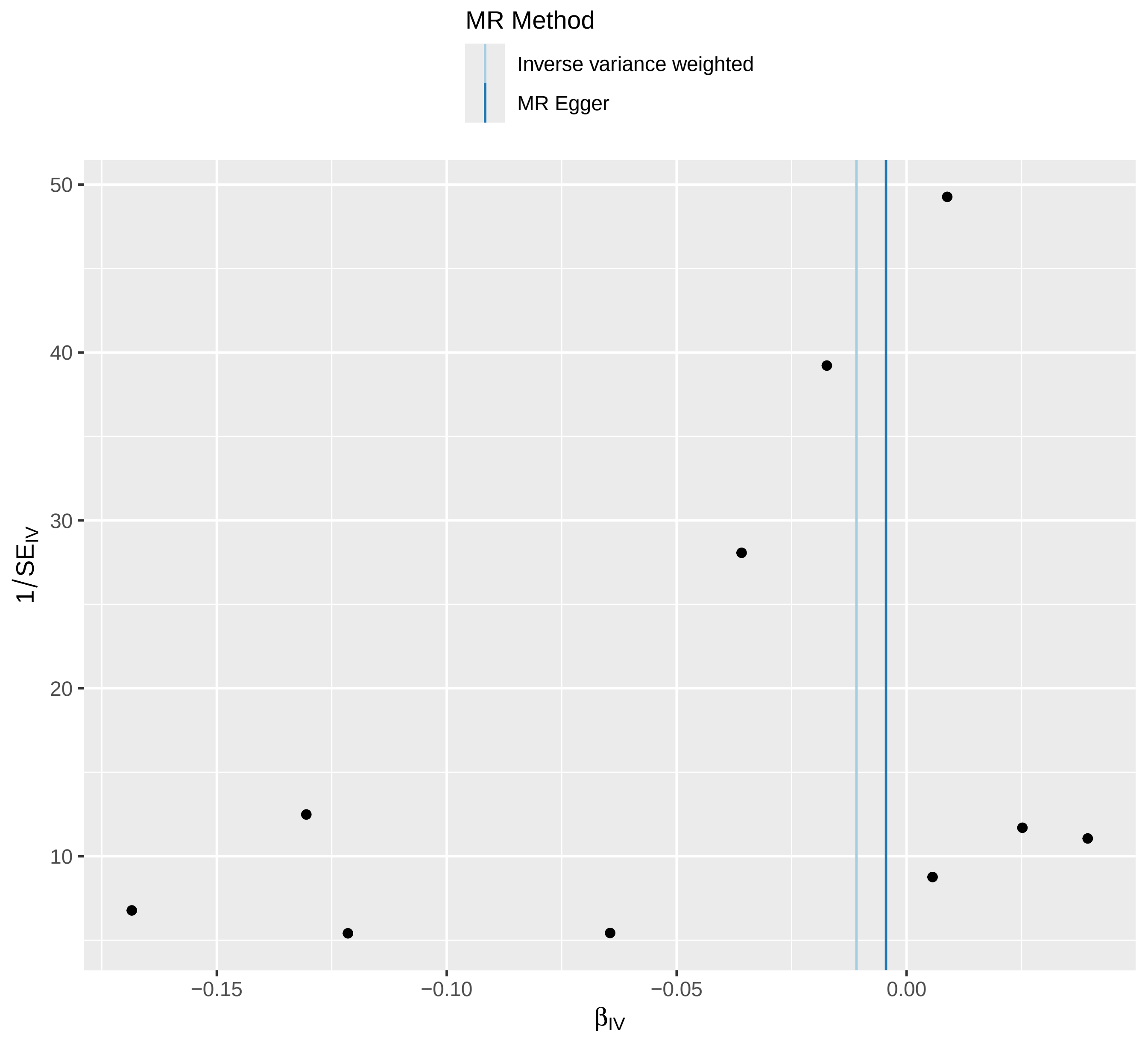
MR Method
Inverse variance weighted
MR Egger
50
40
30
20
10
1/SE_IV
−0.15
−0.10
−0.05
0.00
β_IV

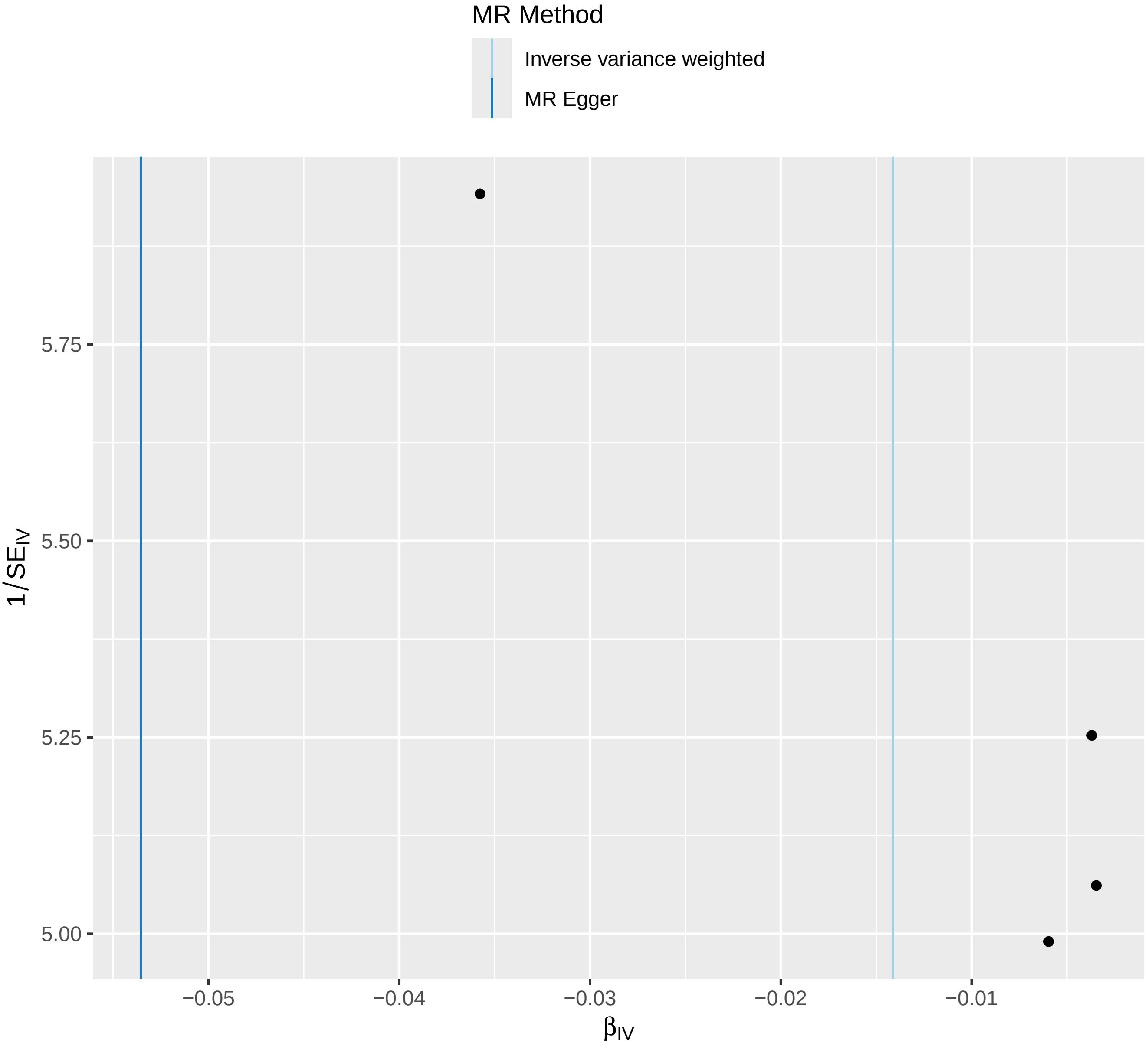
MR Method
Inverse variance weighted
MR Egger
5.75
5.50
5.25
5.00
1/SE_IV
−0.05
−0.04
−0.03
−0.02
−0.01
β_IV

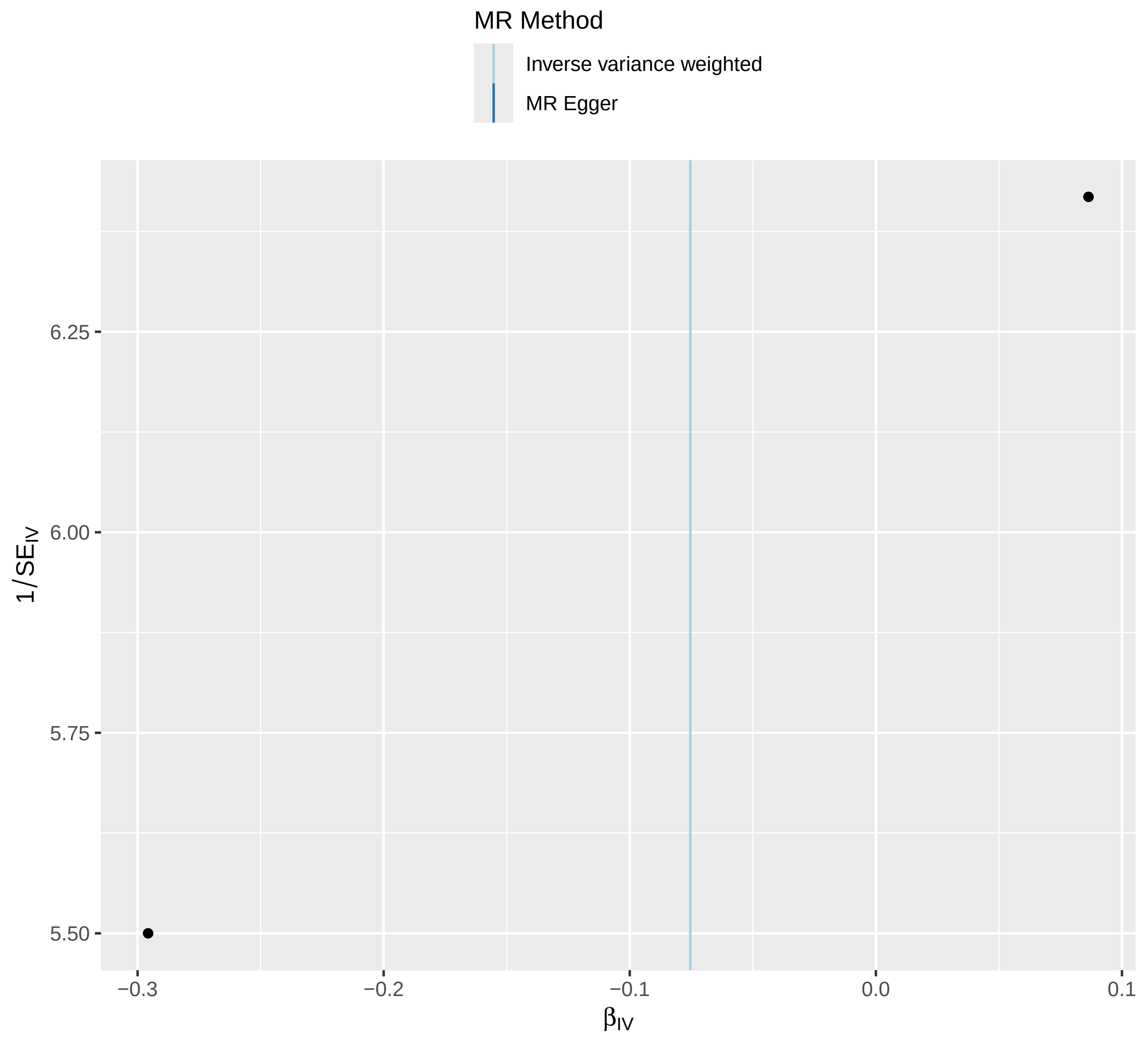

MR Method
Inverse variance weighted
MR Egger
6.25
6.00
5.75
5.50
1/SE_IV
−0.3
−0.2
−0.1
0.0
0.1
β_IV

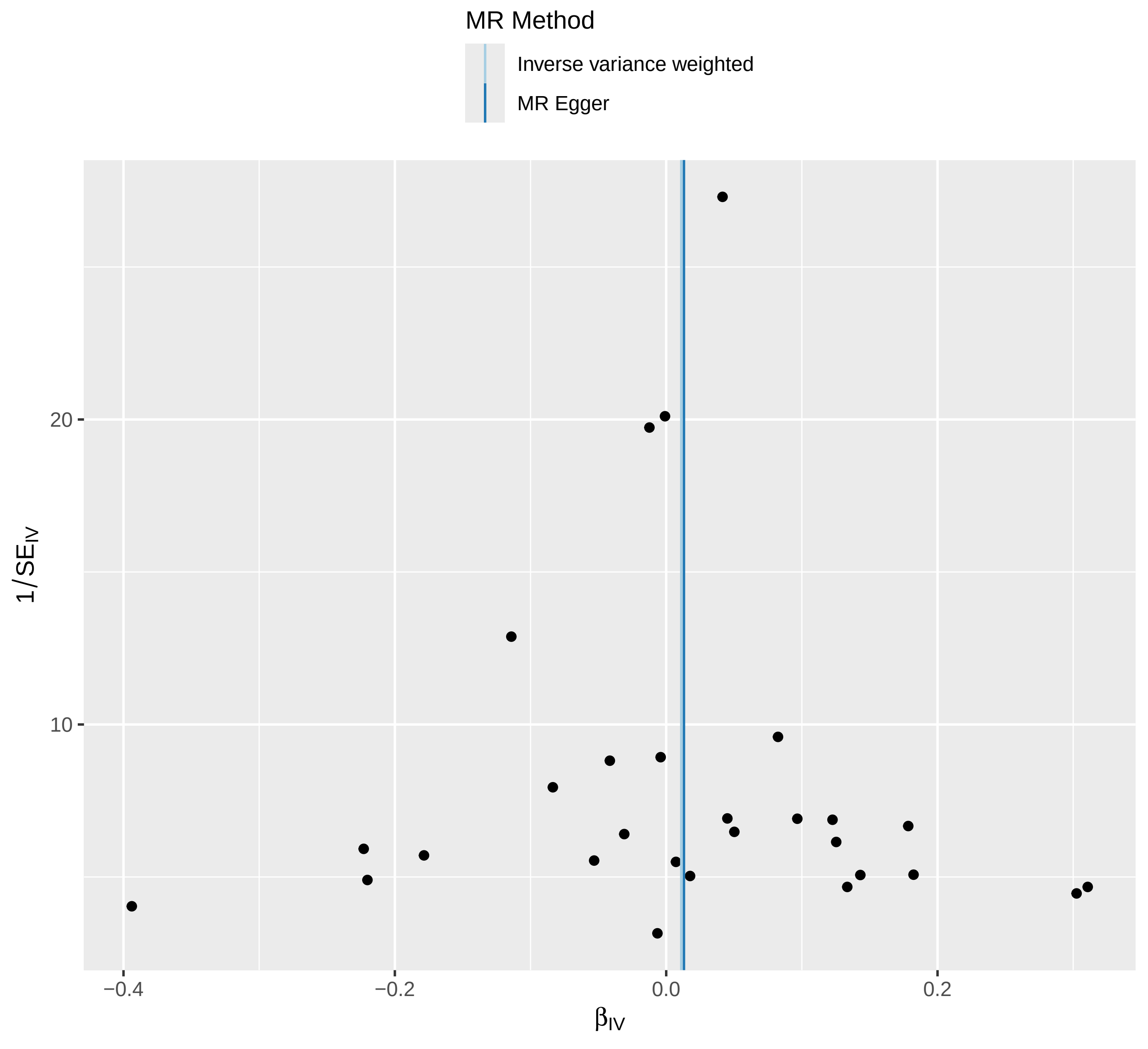
MR Method
Inverse variance weighted
MR Egger
20
10
1/SE_IV
−0.4
−0.2
0.0
0.2
β_IV

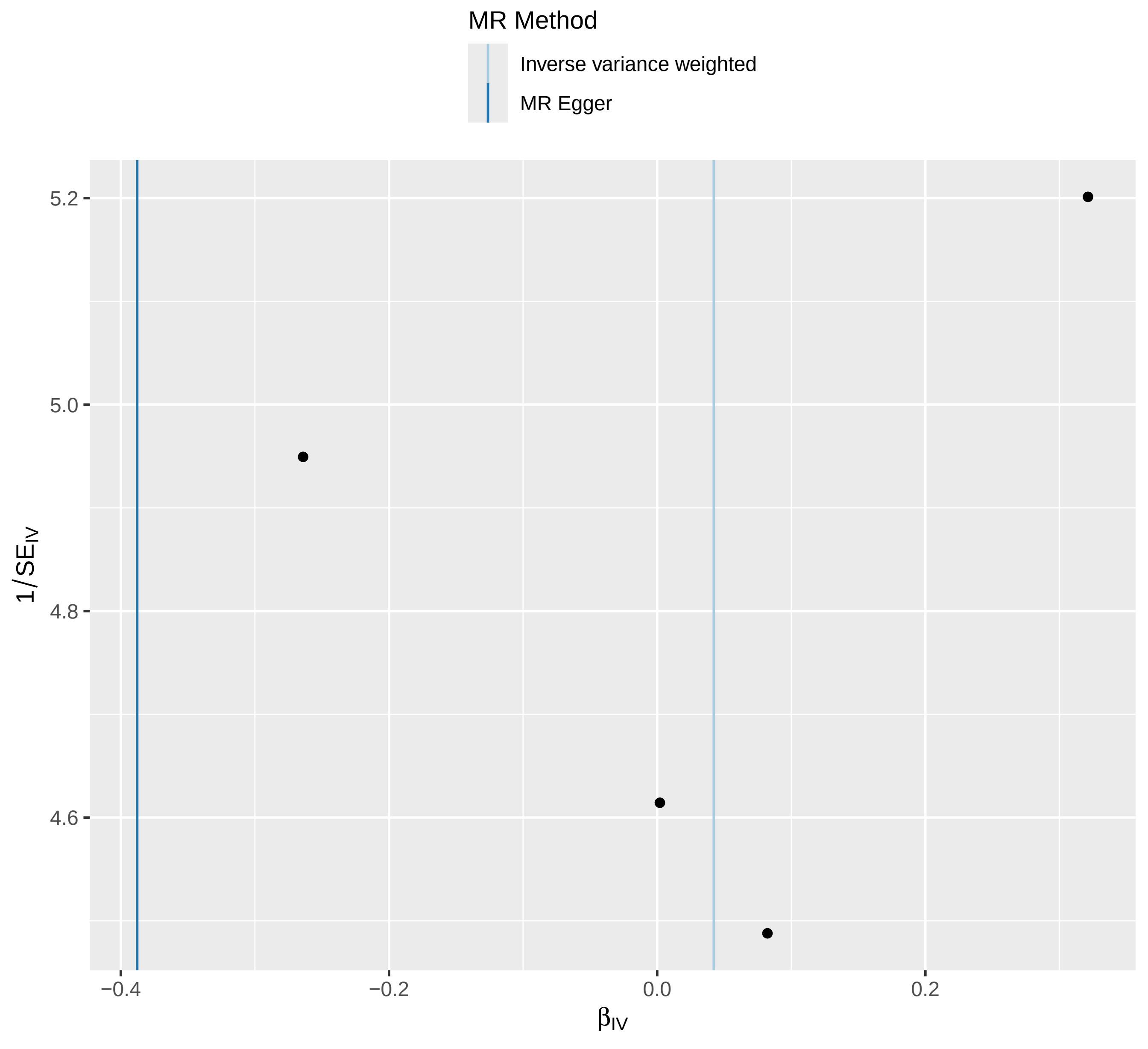

MR Method
Inverse variance weighted
MR Egger
5.2
5.0
4.8
4.6
1/SE_IV
−0.4
−0.2
0.0
0.2
β_IV

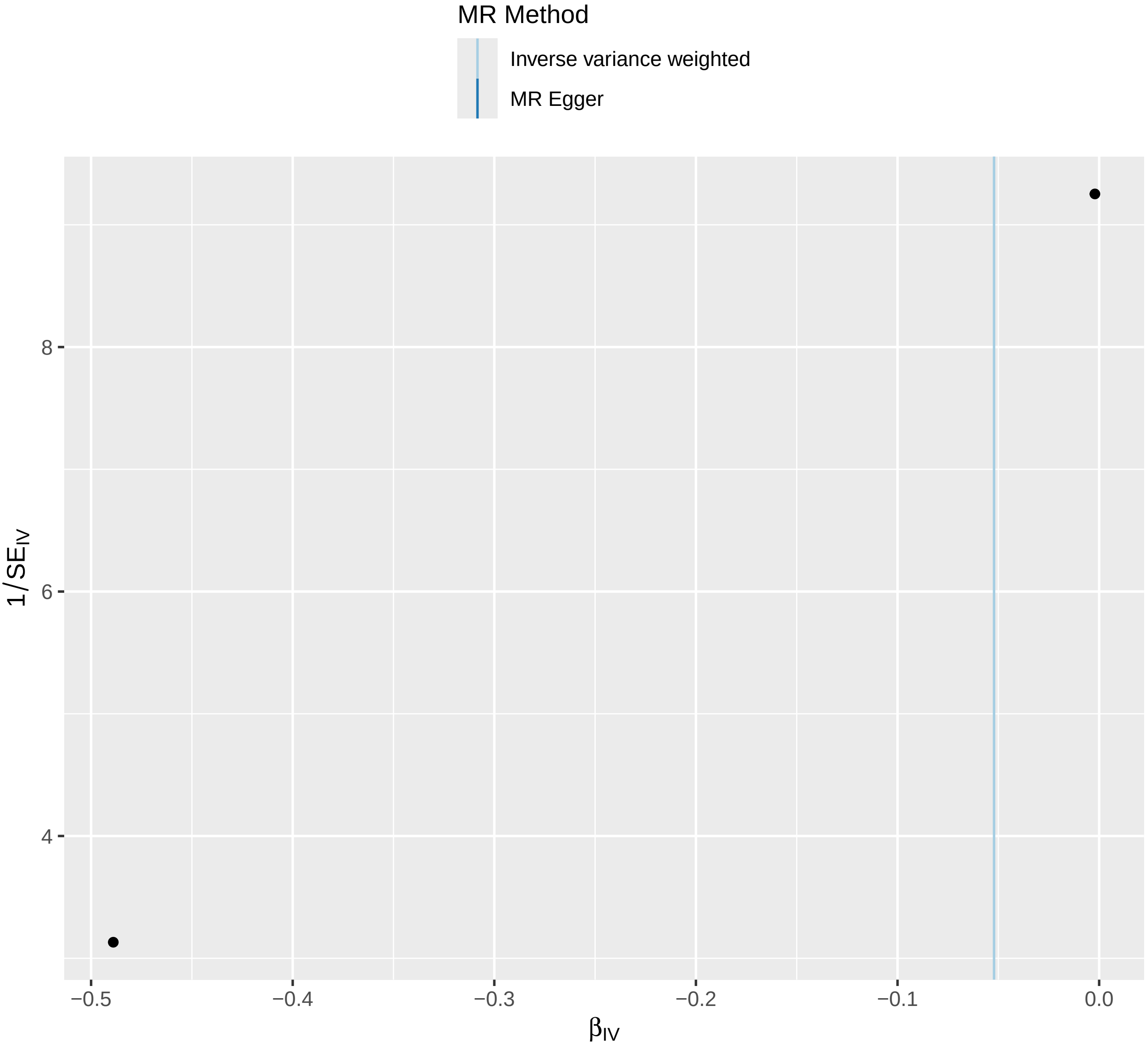

MR Method
Inverse variance weighted
MR Egger
8
6
4
1/SE$_{IV}$
−0.5
−0.4
−0.3
−0.2
−0.1
0.0
β$_{IV}$

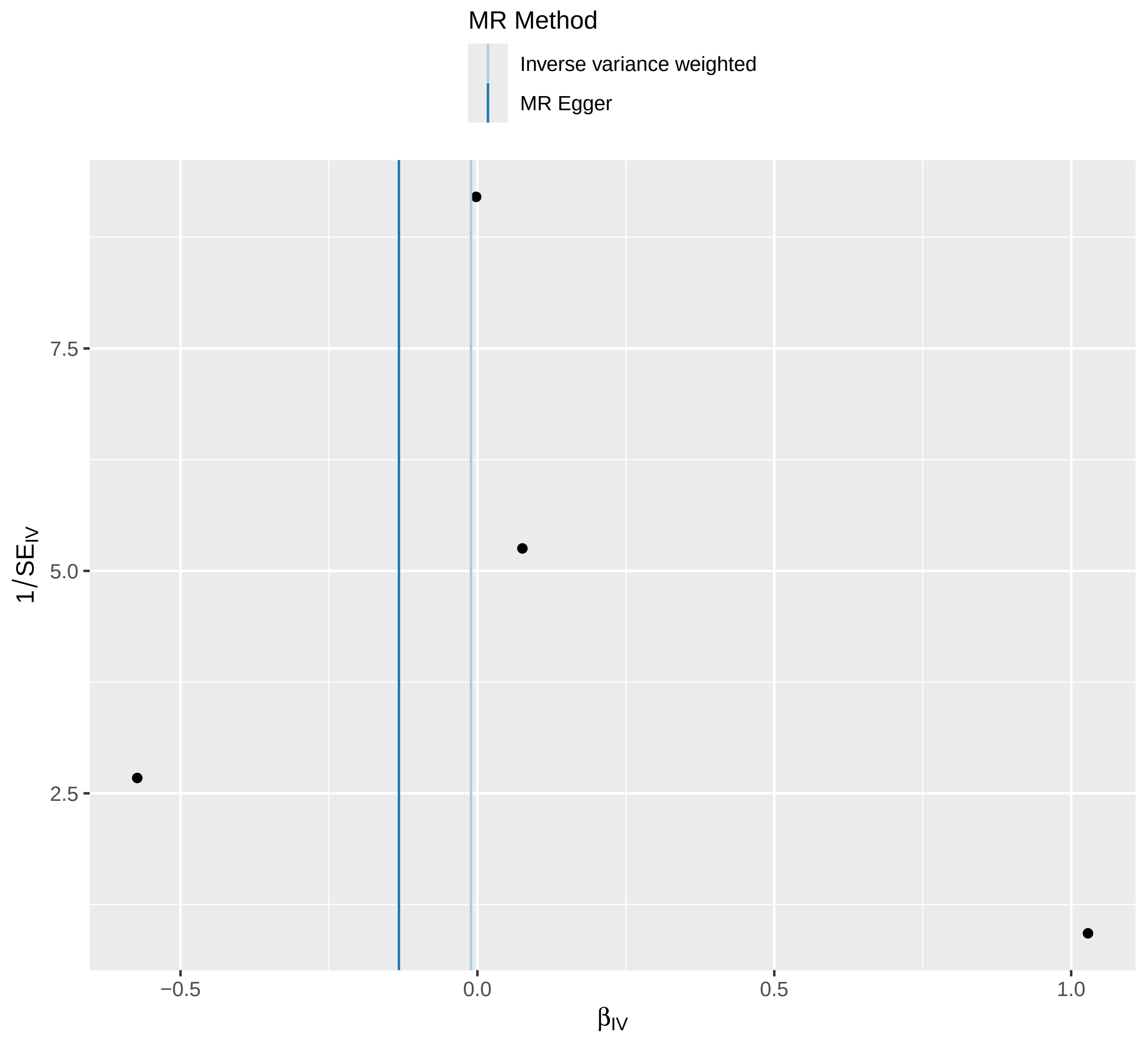
MR Method
Inverse variance weighted
MR Egger
7.5
5.0
2.5
$1/SE_{IV}$
−0.5
0.0
0.5
1.0
$\beta_{IV}$

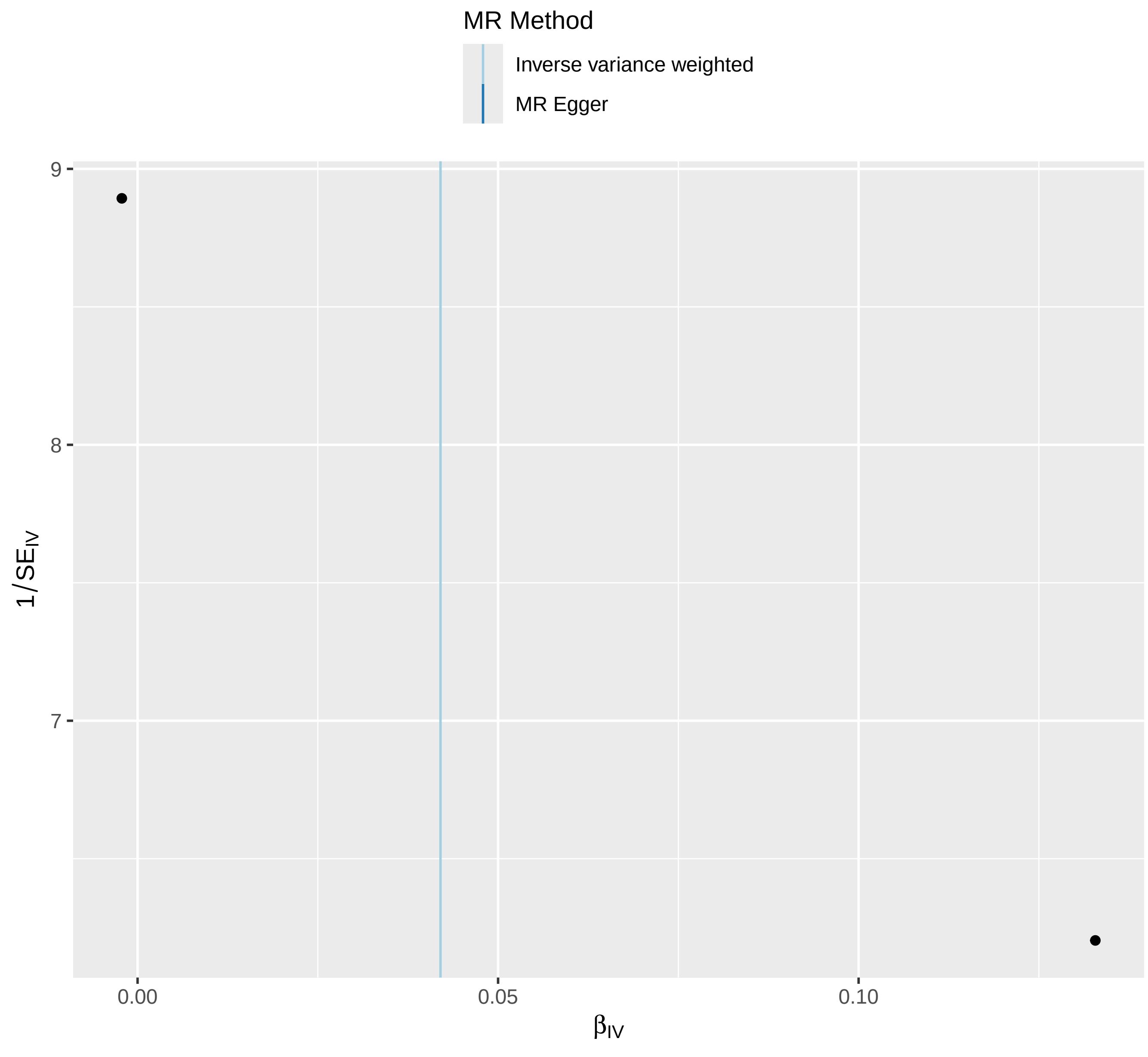

MR Method
Inverse variance weighted
MR Egger
9
8
7
1/SE$_{IV}$
0.00
0.05
0.10
β$_{IV}$

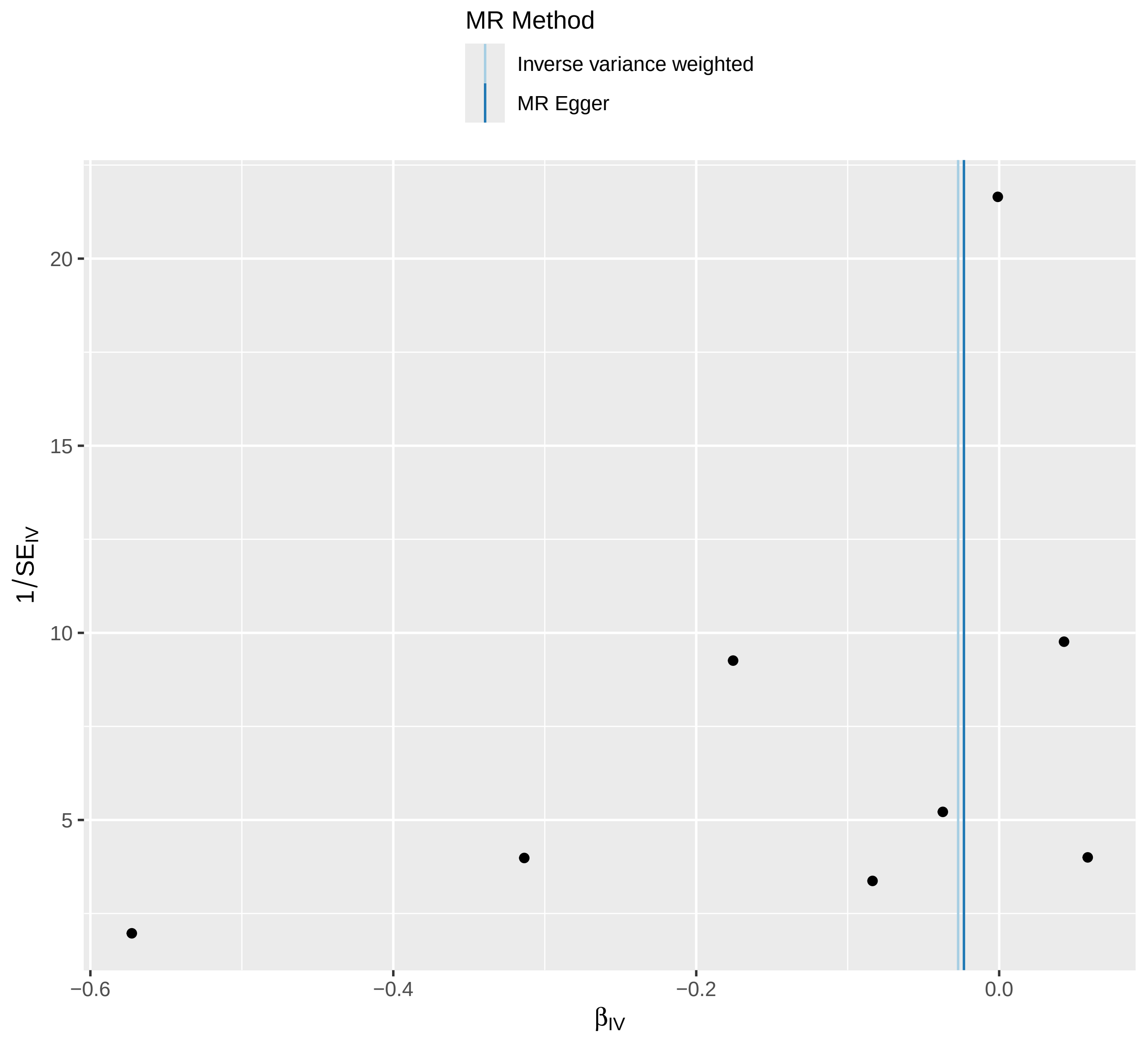
MR Method
Inverse variance weighted
MR Egger
20
15
10
5
$1/SE_{IV}$
−0.6
−0.4
−0.2
0.0
$\beta_{IV}$

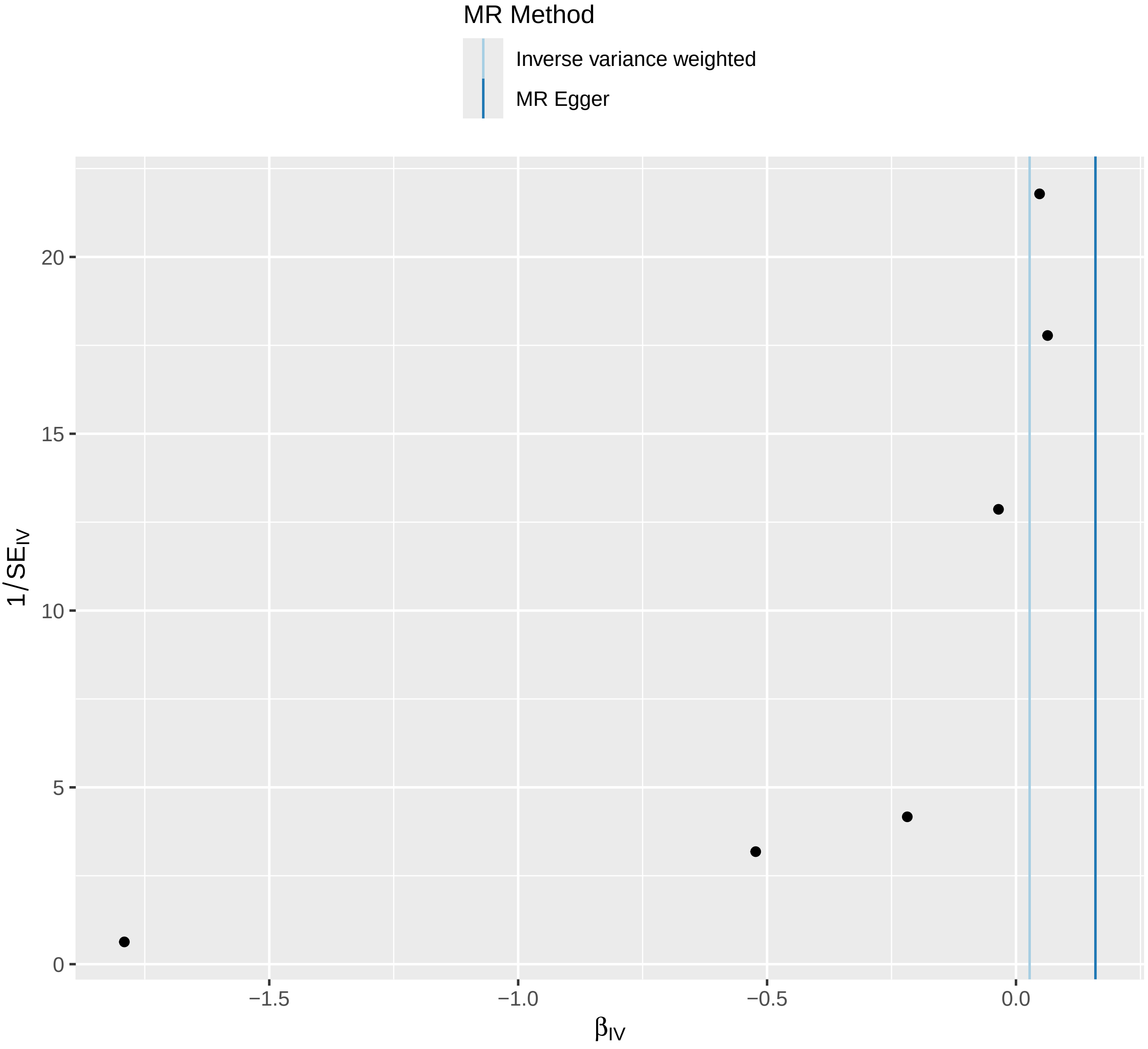

MR Method
Inverse variance weighted
MR Egger
20
15
10
5
0
−1.5
−1.0
−0.5
0.0
$1/SE_{IV}$
$\beta_{IV}$

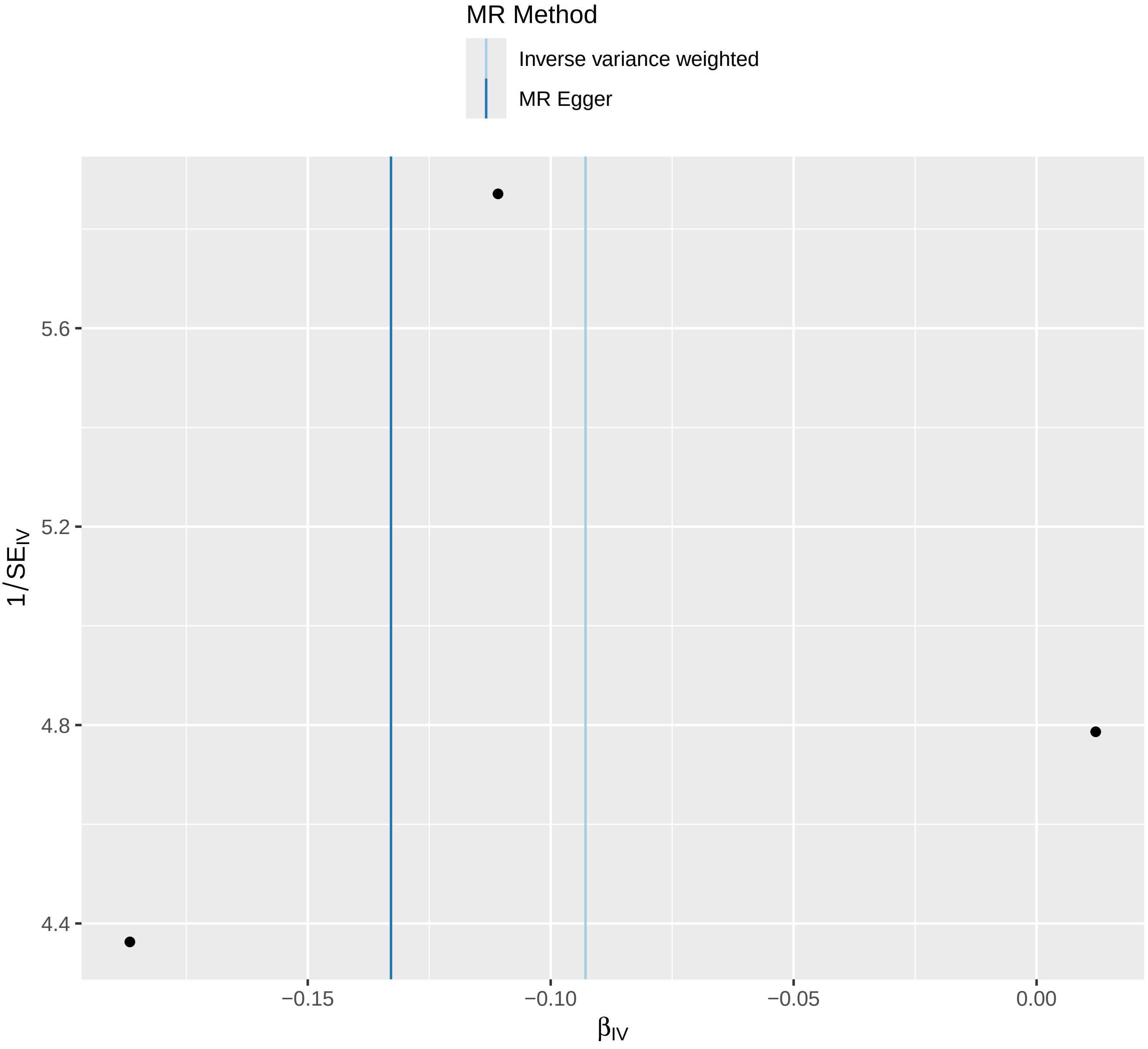
MR Method
Inverse variance weighted
MR Egger
5.6
5.2
4.8
4.4
1/SE_IV
−0.15
−0.10
−0.05
0.00
β_IV

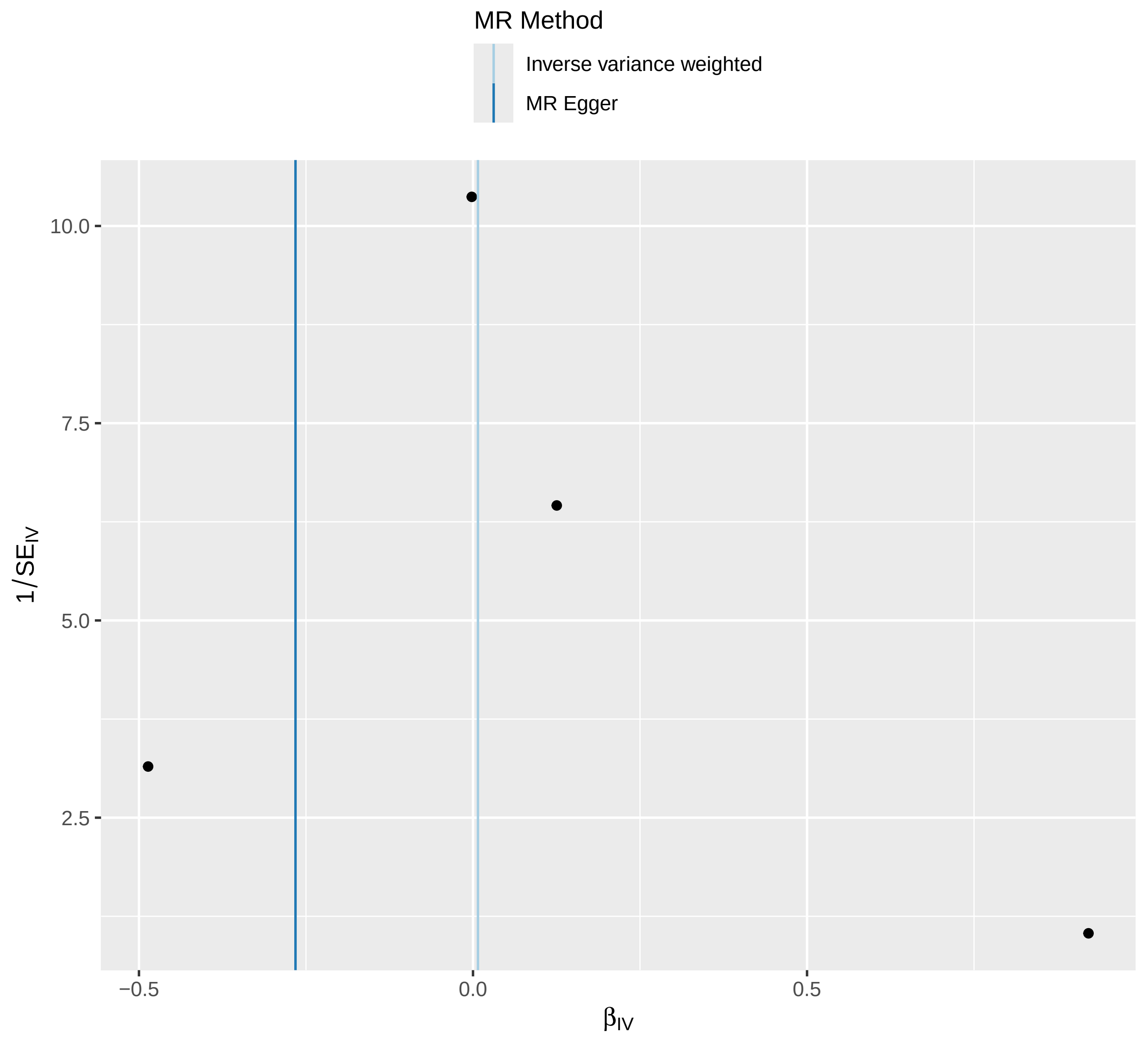
MR Method
Inverse variance weighted
MR Egger
10.0
7.5
5.0
2.5
1/SE_IV
−0.5
0.0
0.5
β_IV

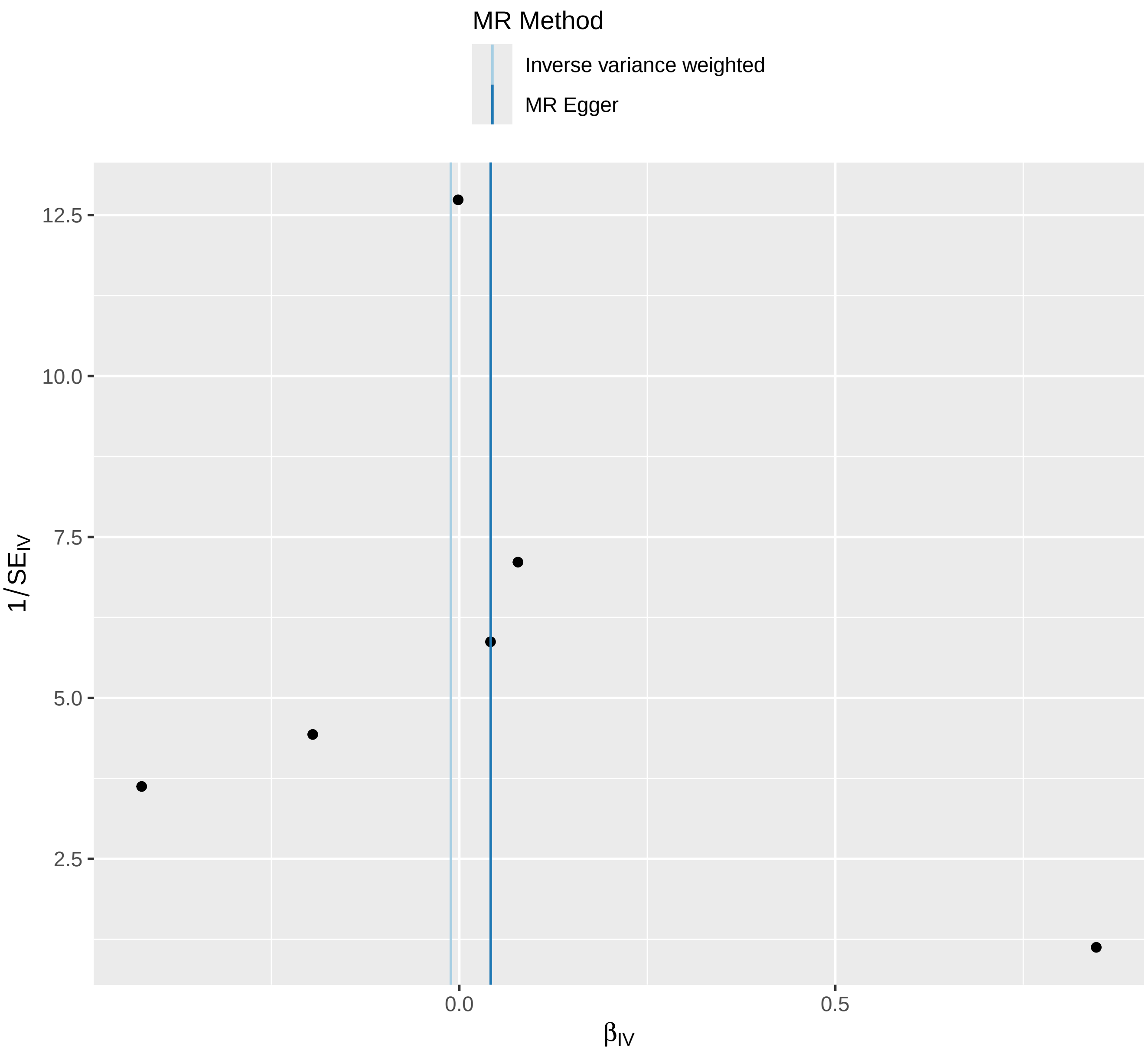

MR Method
Inverse variance weighted
MR Egger
12.5
10.0
7.5
5.0
2.5
1/SE_IV
0.0
0.5
β_IV

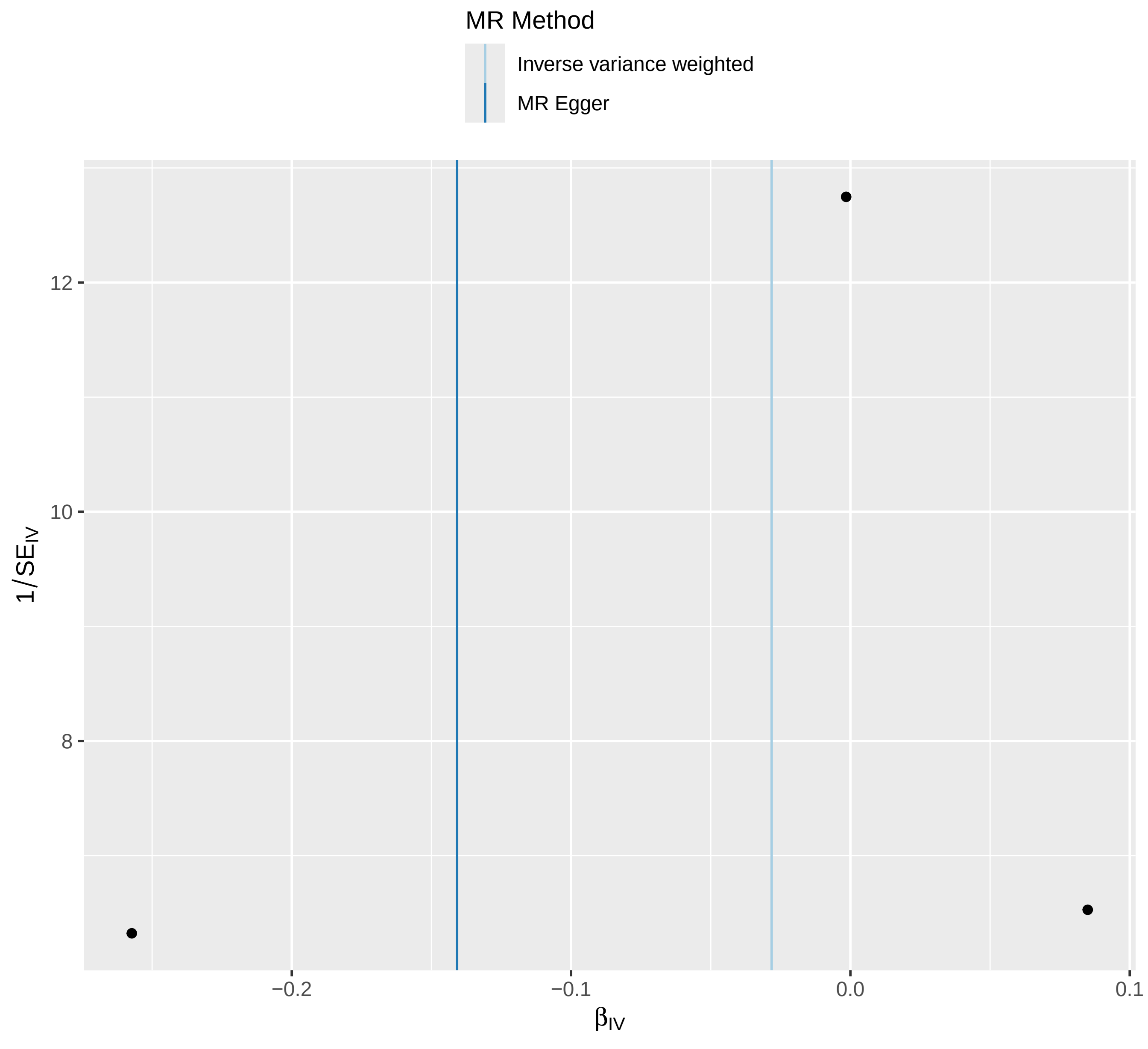

MR Method
Inverse variance weighted
MR Egger
12
10
8
1/SE$_{IV}$
−0.2
−0.1
0.0
0.1
β$_{IV}$

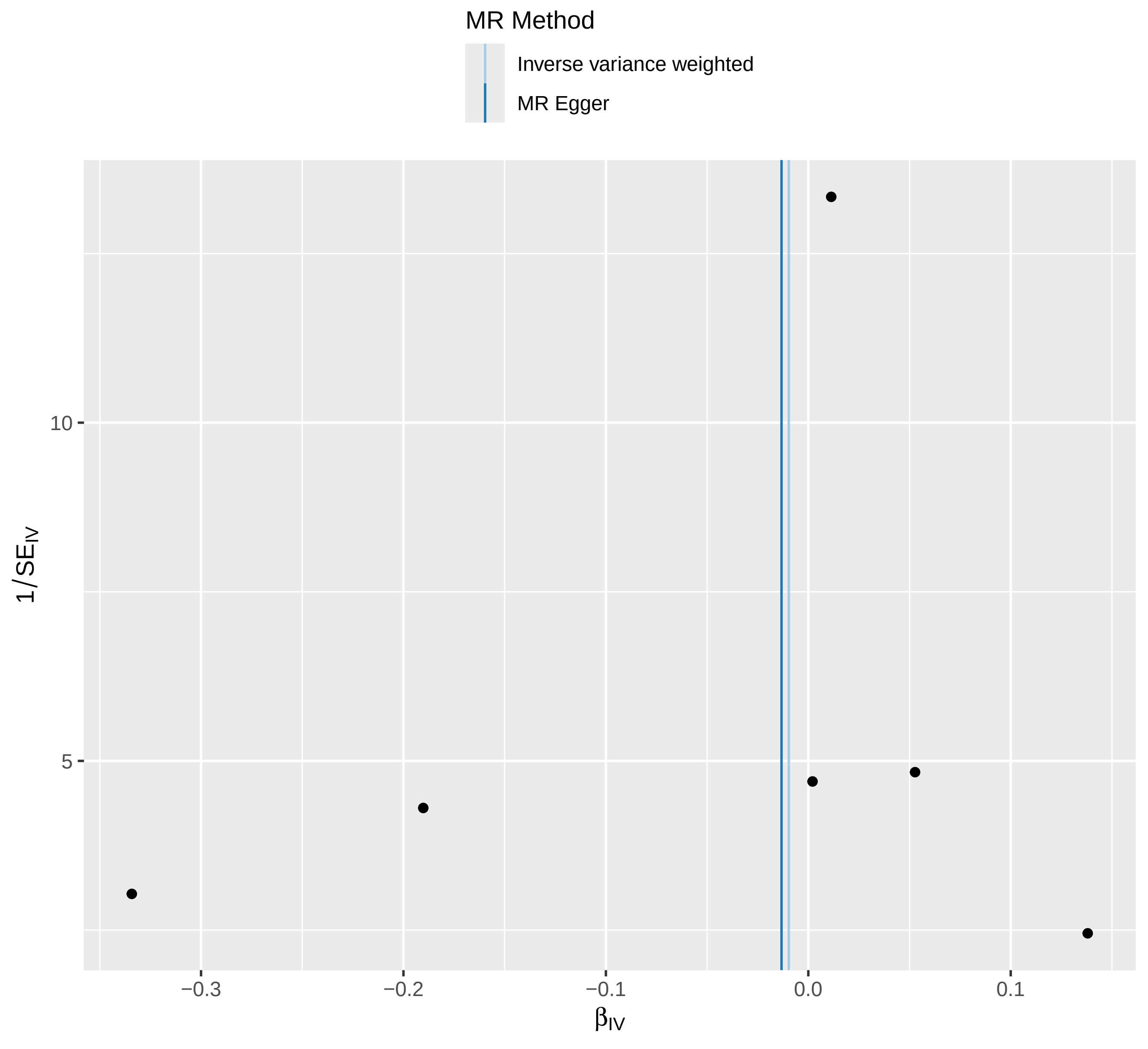
MR Method
Inverse variance weighted
MR Egger
10
5
1/SE$_{IV}$
−0.3
−0.2
−0.1
0.0
0.1
β$_{IV}$

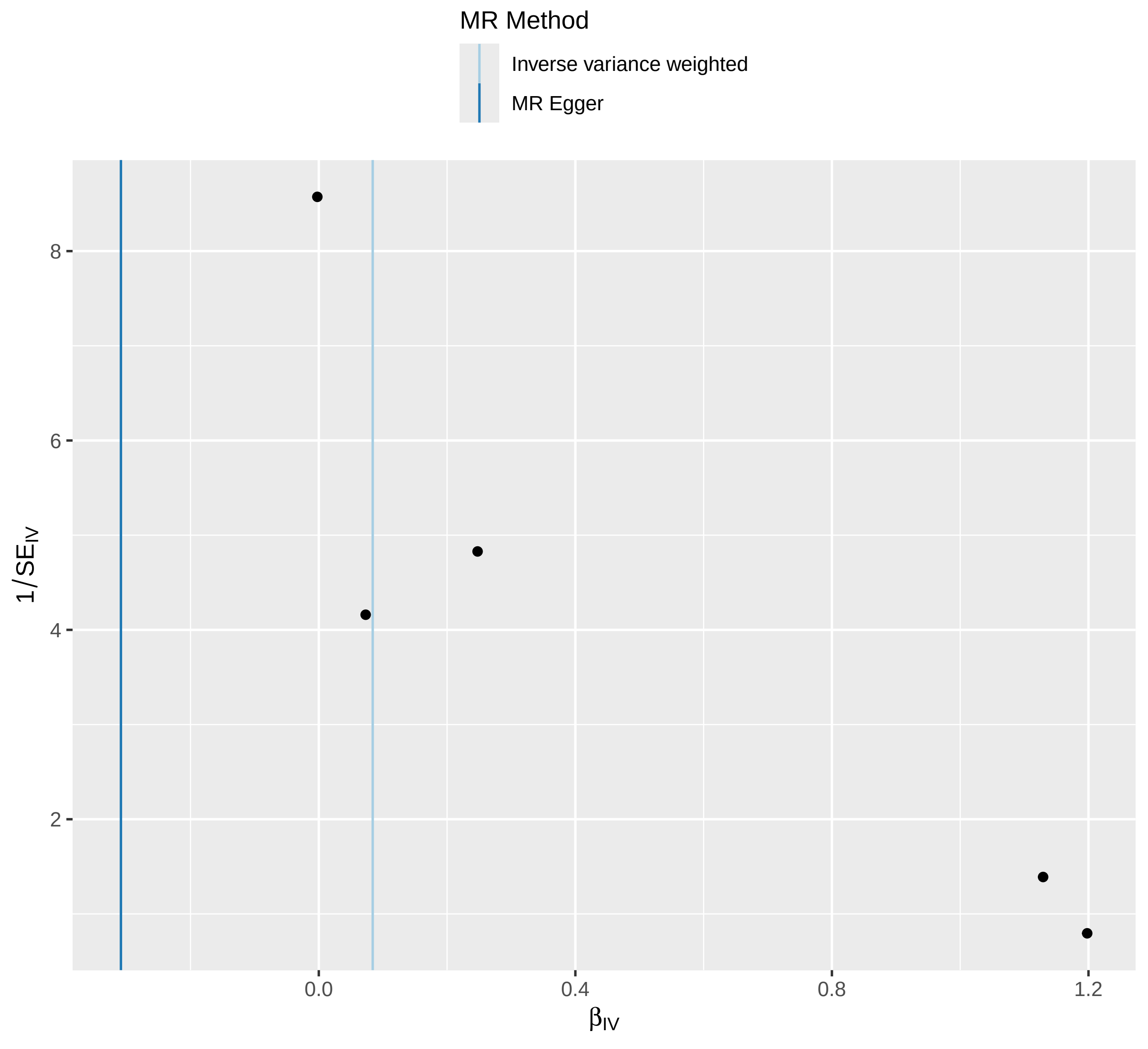

MR Method
Inverse variance weighted
MR Egger
8
6
4
2
1/SE_IV
0.0
0.4
0.8
1.2
β_IV

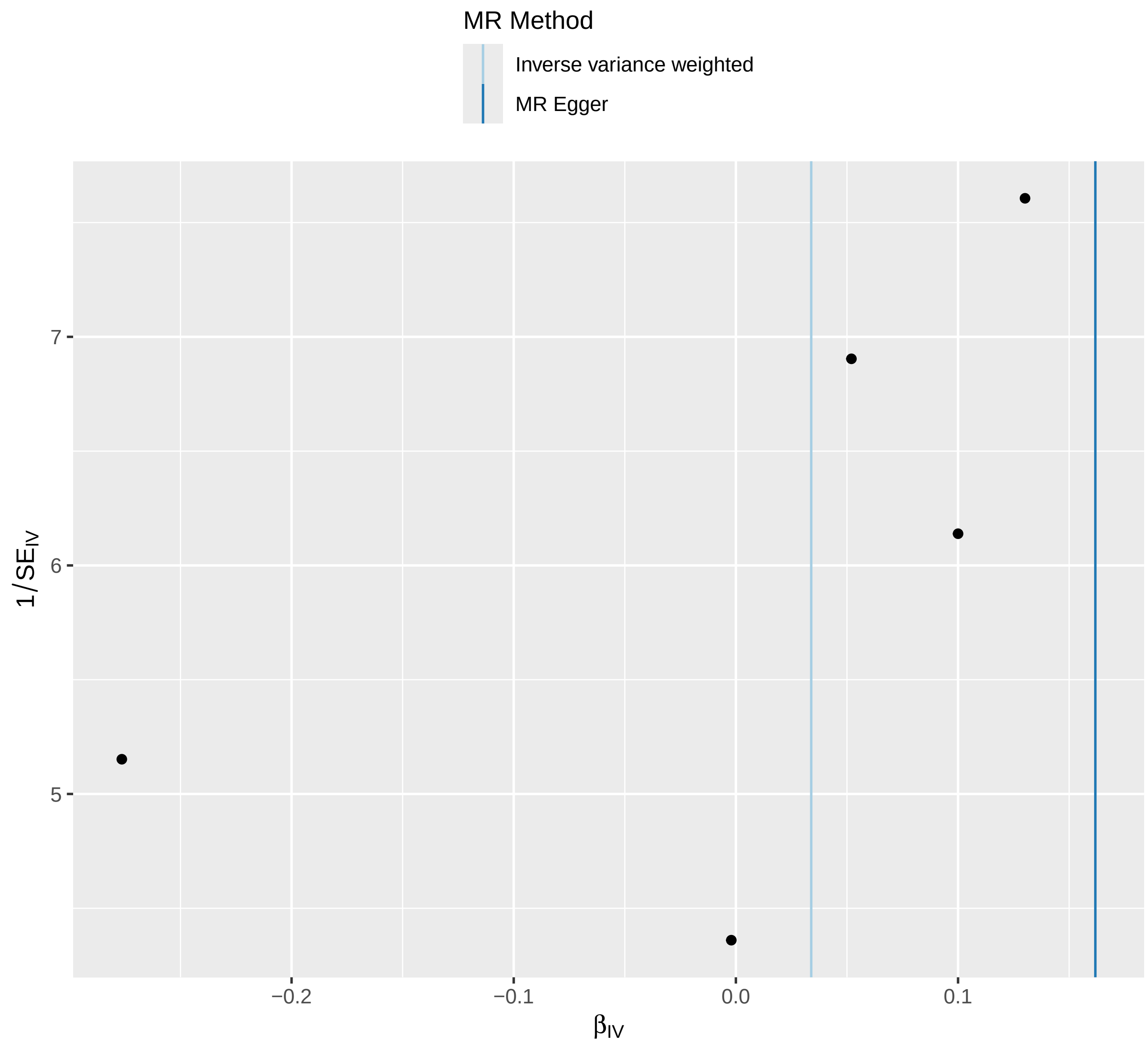
MR Method
Inverse variance weighted
MR Egger
7
6
5
1/SE_IV
−0.2
−0.1
0.0
0.1
β_IV

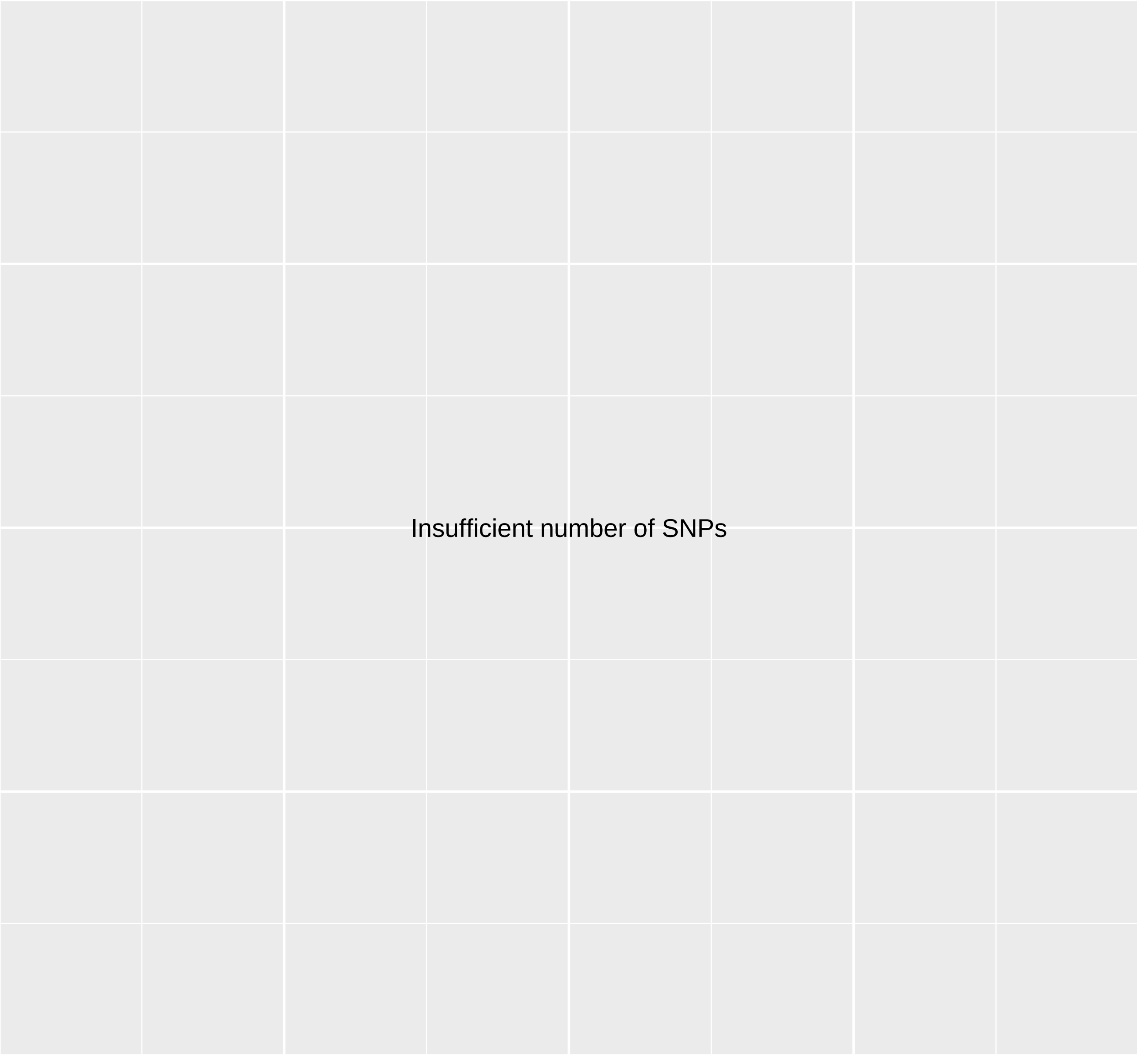
Insufficient number of SNPs

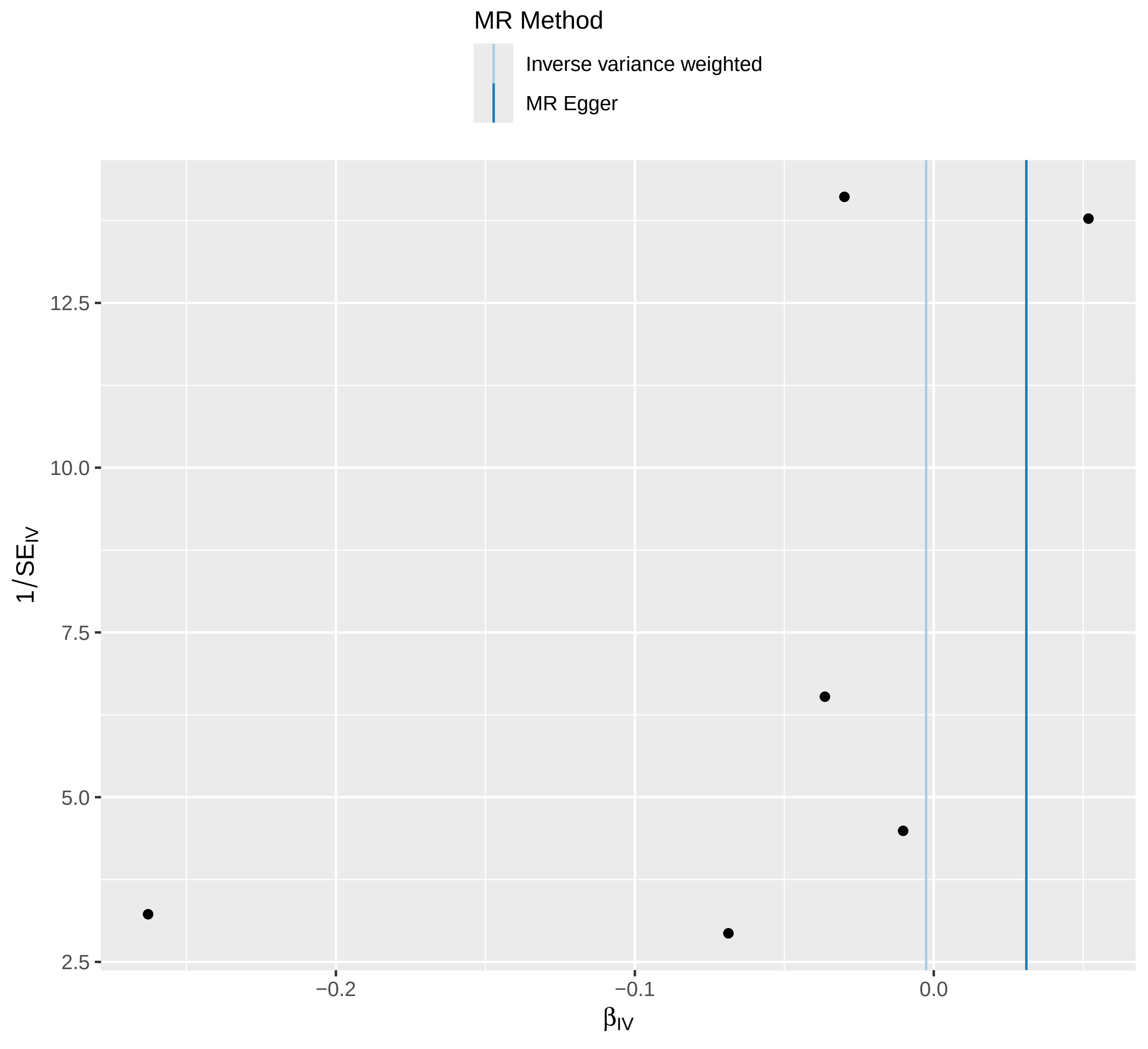
MR Method
Inverse variance weighted
MR Egger
1/SE$_{IV}$
12.5
10.0
7.5
5.0
2.5
−0.2
−0.1
0.0
β$_{IV}$

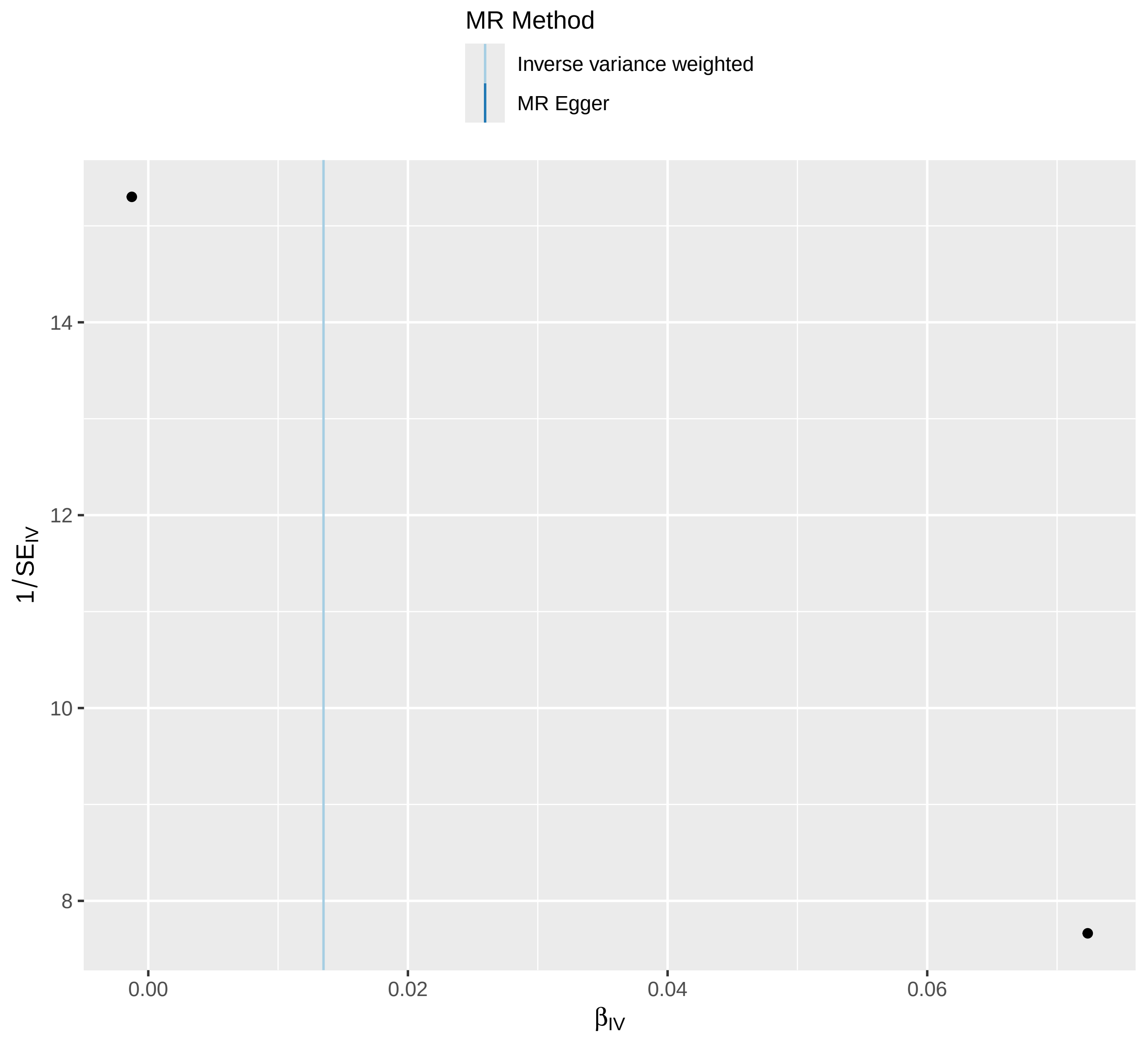
MR Method
Inverse variance weighted
MR Egger
14
12
10
8
0.00
0.02
0.04
0.06
$\beta_{IV}$
$1/SE_{IV}$

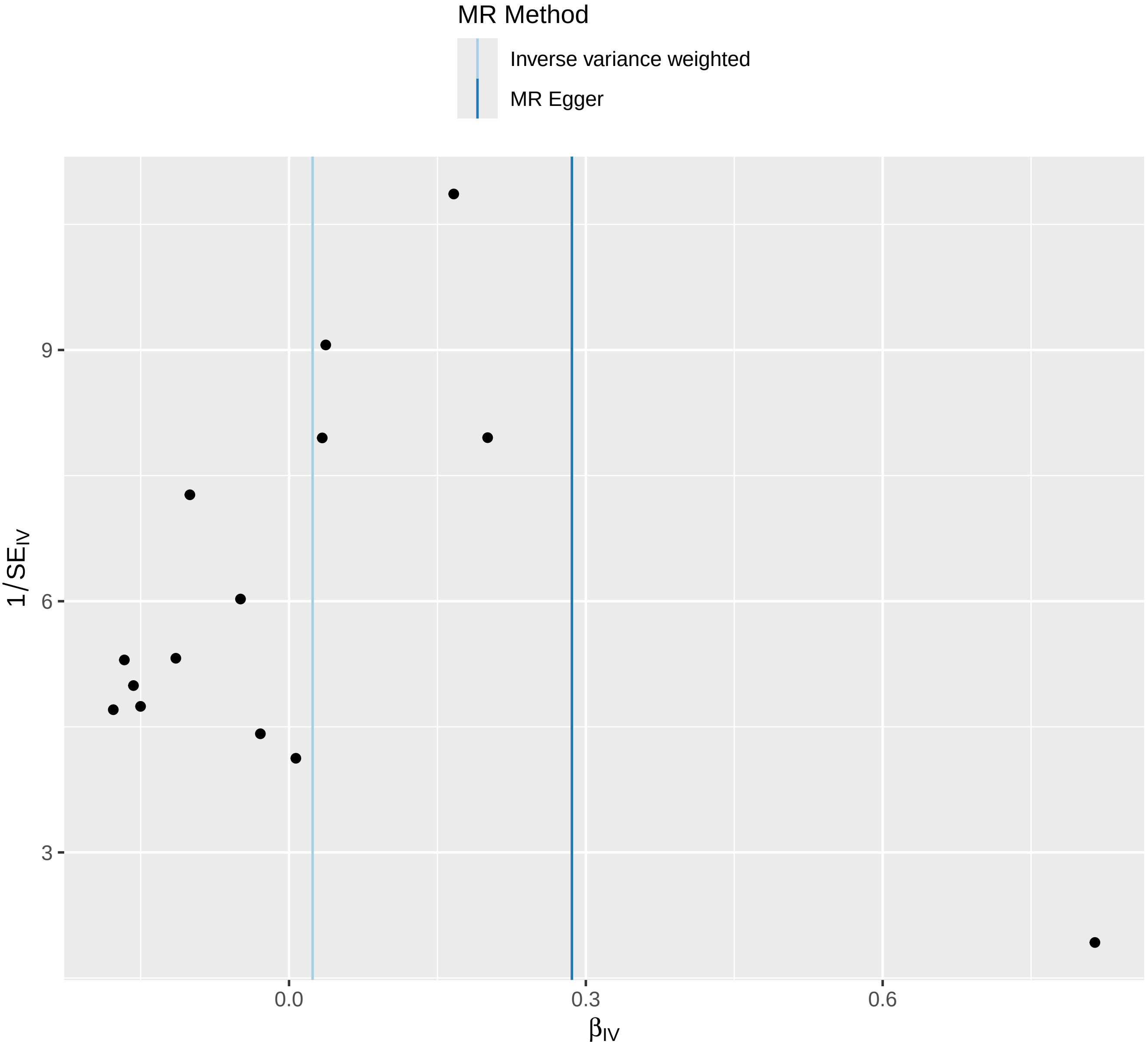

MR Method
Inverse variance weighted
MR Egger
9
6
3
$1/SE_{IV}$
0.0
0.3
0.6
$\beta_{IV}$

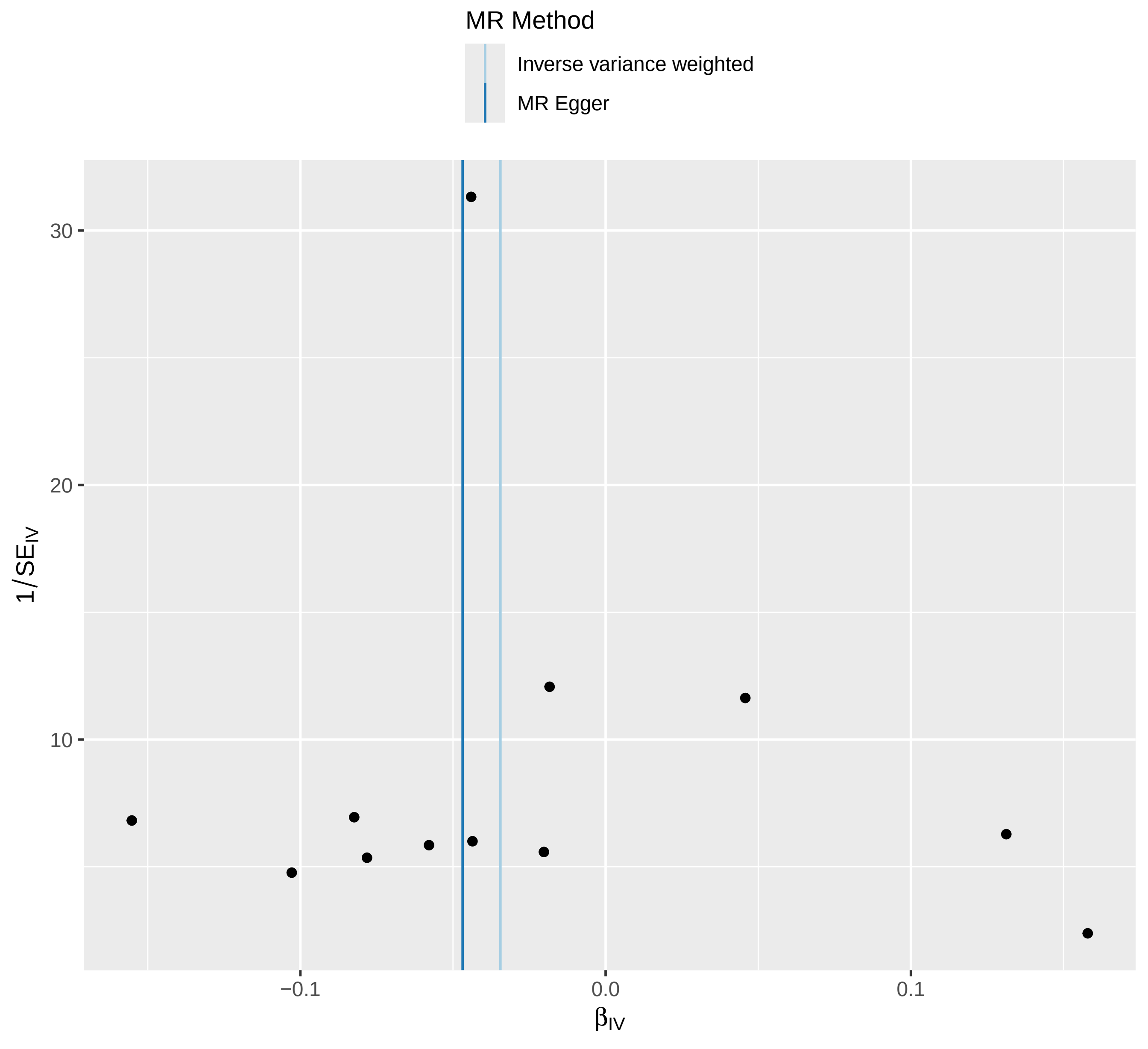

MR Method
Inverse variance weighted
MR Egger
30
20
10
1/SE_IV
−0.1
0.0
0.1
β_IV

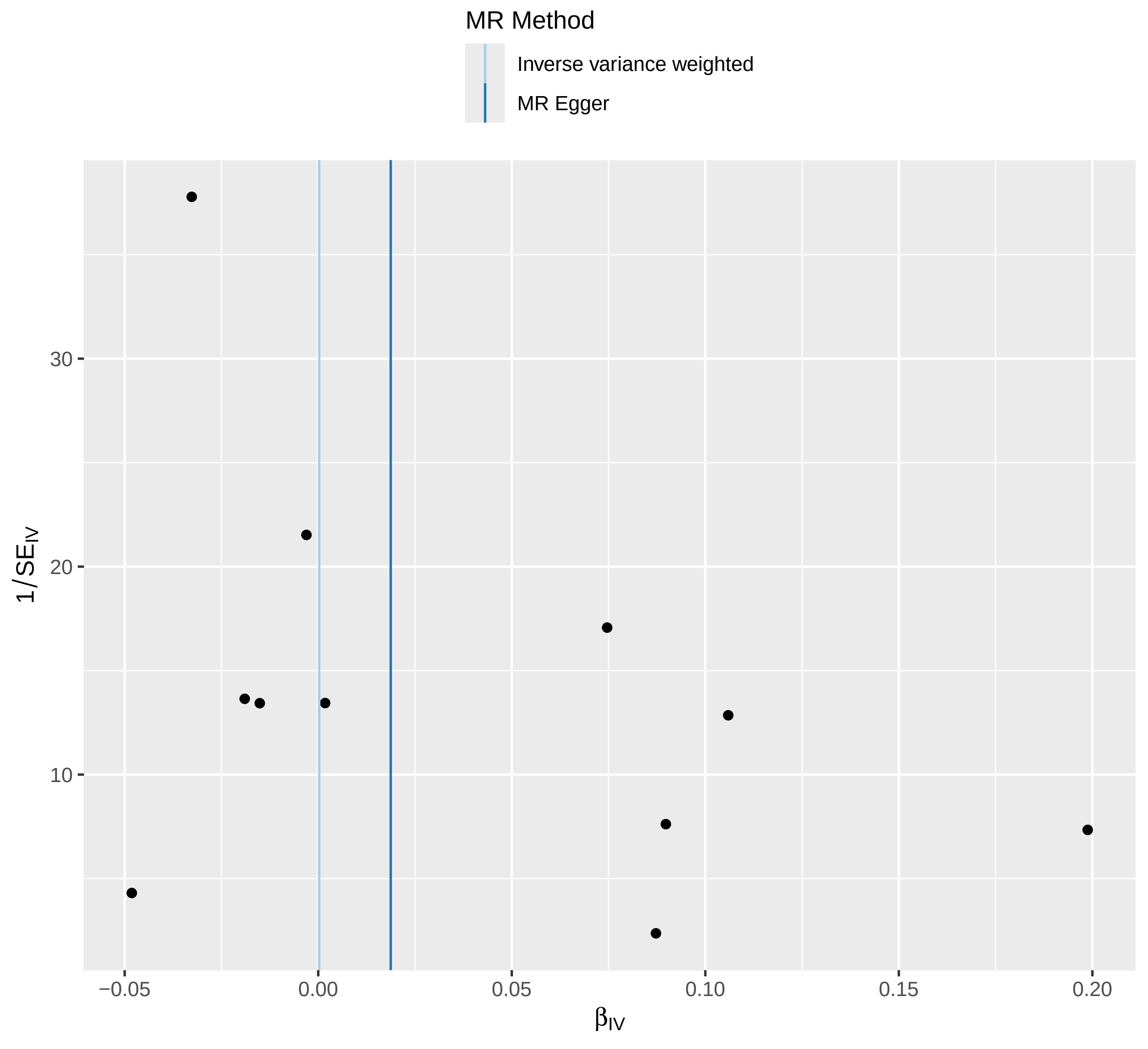
MR Method
Inverse variance weighted
MR Egger
30
20
10
1/$SE_{IV}$
−0.05
0.00
0.05
0.10
0.15
0.20
$\beta_{IV}$

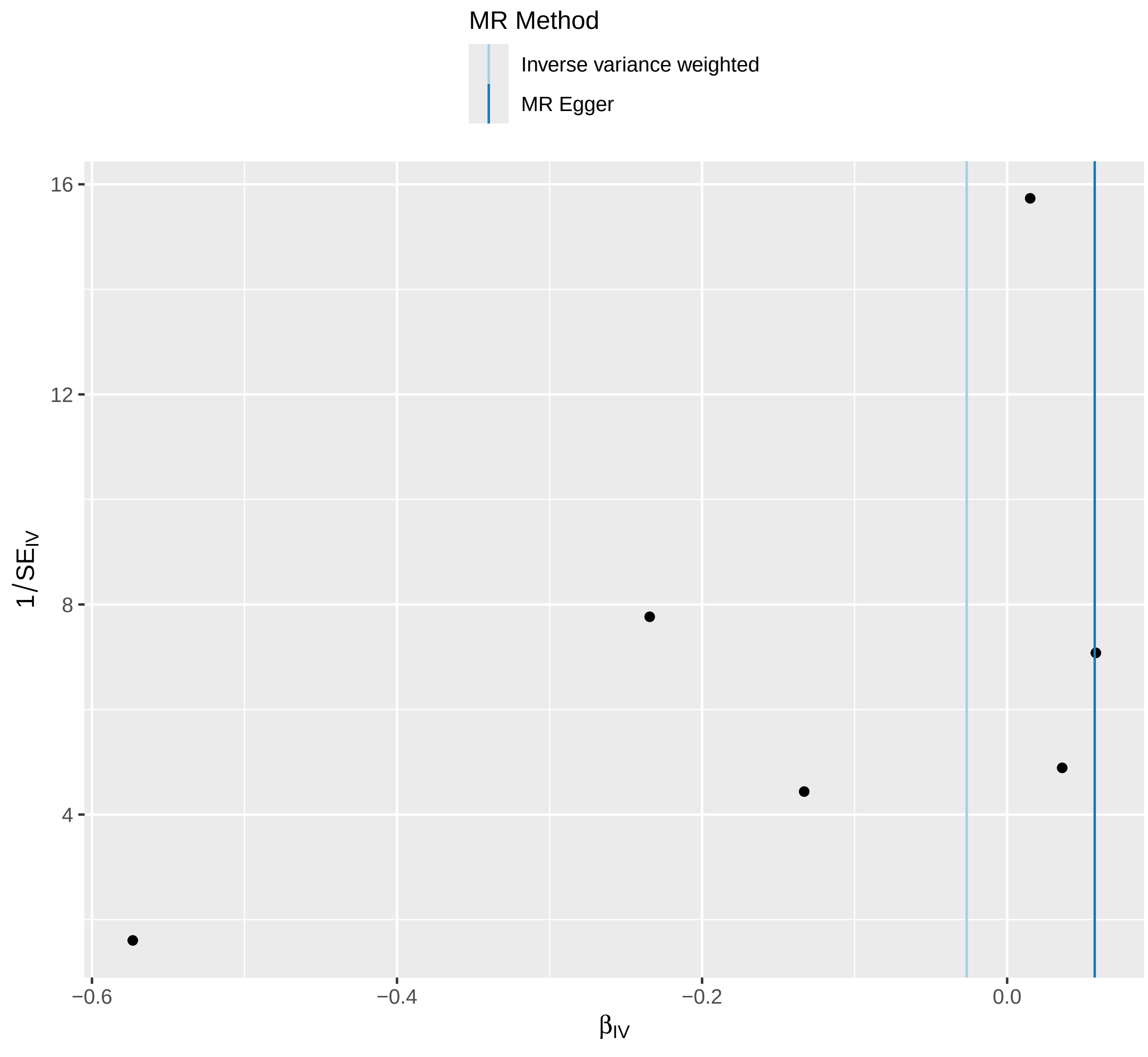
MR Method
Inverse variance weighted
MR Egger
16
12
8
4
1/SE$_{IV}$
−0.6
−0.4
−0.2
0.0
β$_{IV}$

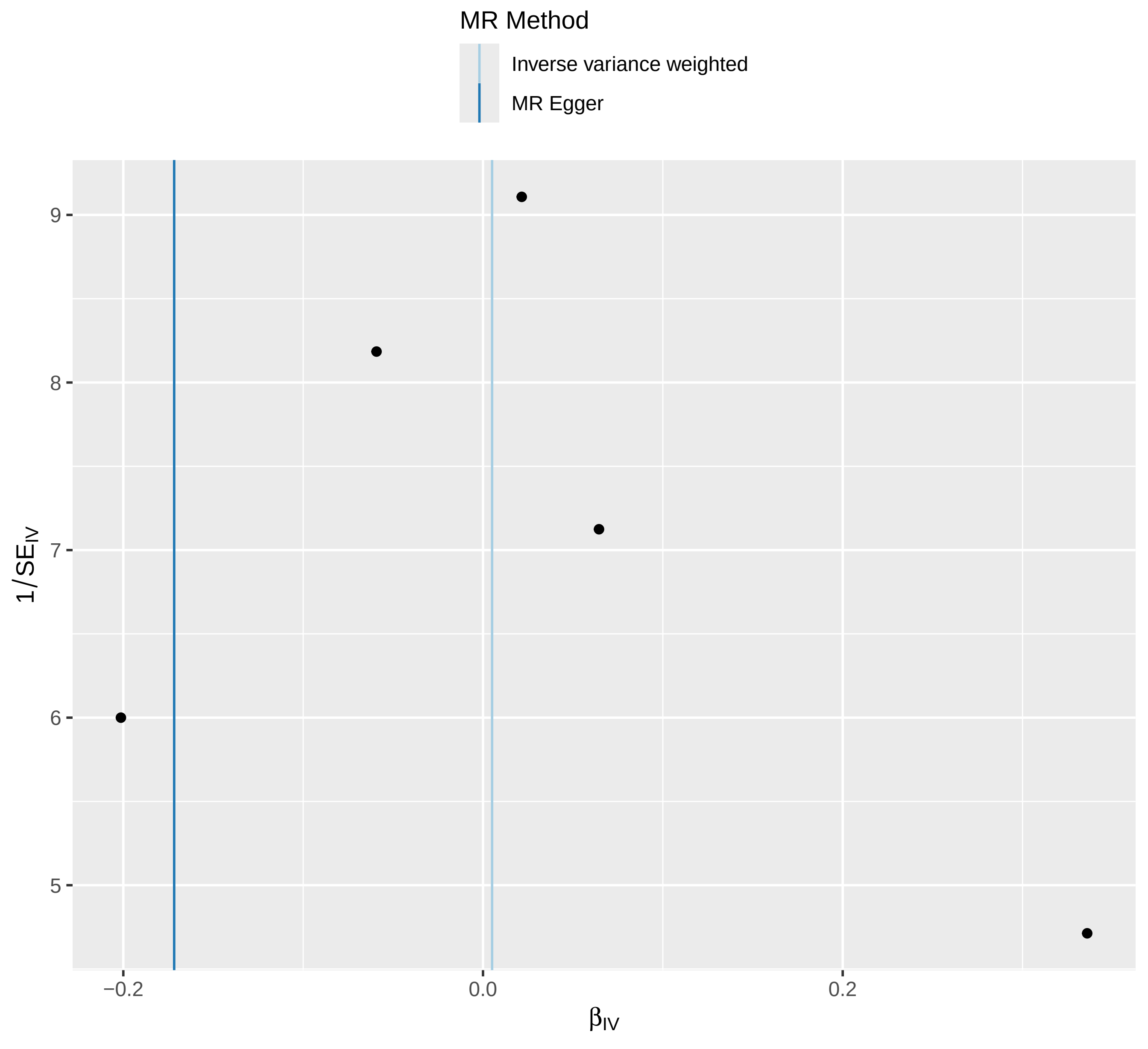
MR Method
Inverse variance weighted
MR Egger
9
8
7
6
5
1/SE$_{IV}$
−0.2
0.0
0.2
β$_{IV}$

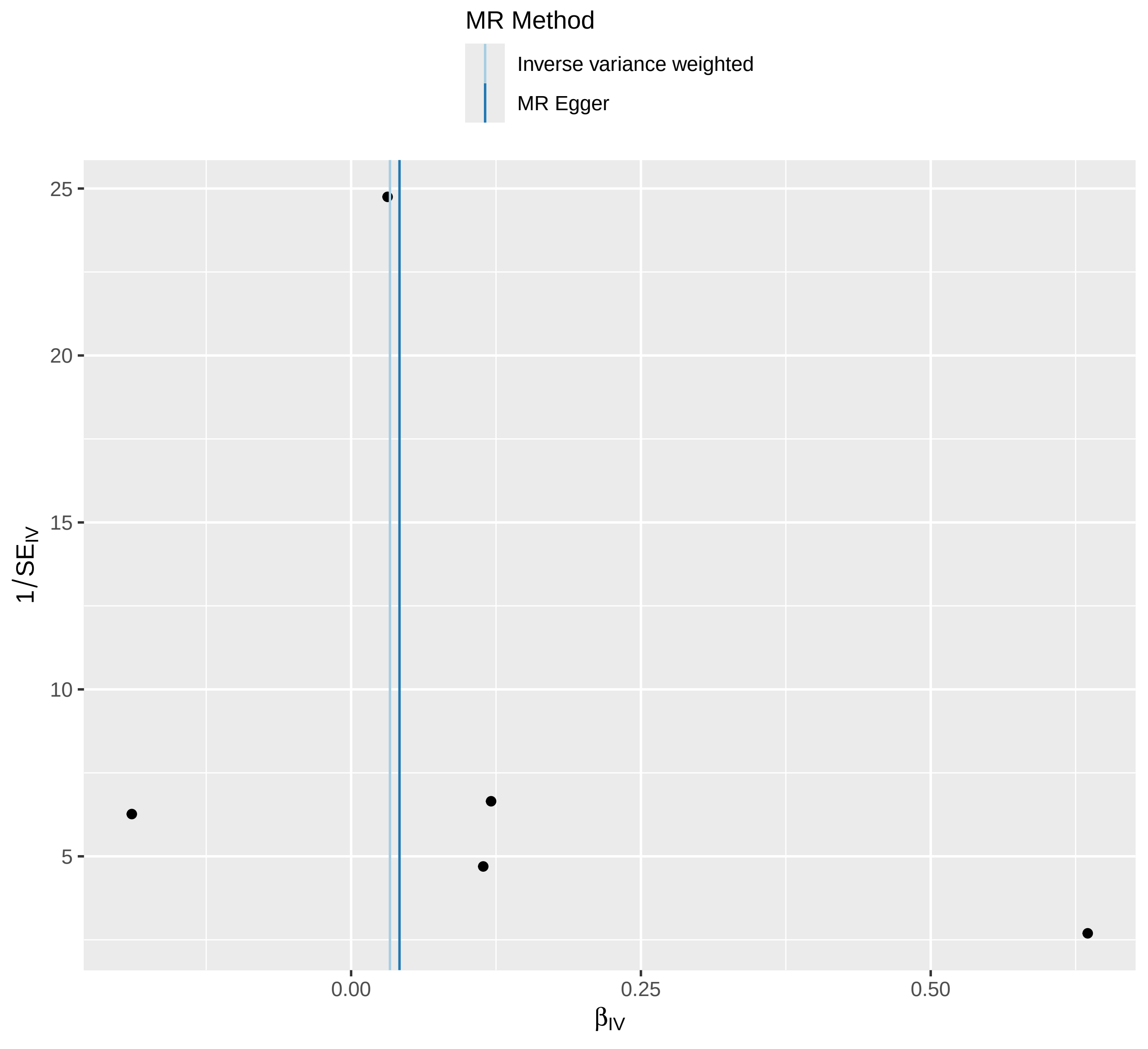
MR Method
Inverse variance weighted
MR Egger
25
20
15
10
5
0.00
0.25
0.50
$1/SE_{IV}$
$\beta_{IV}$

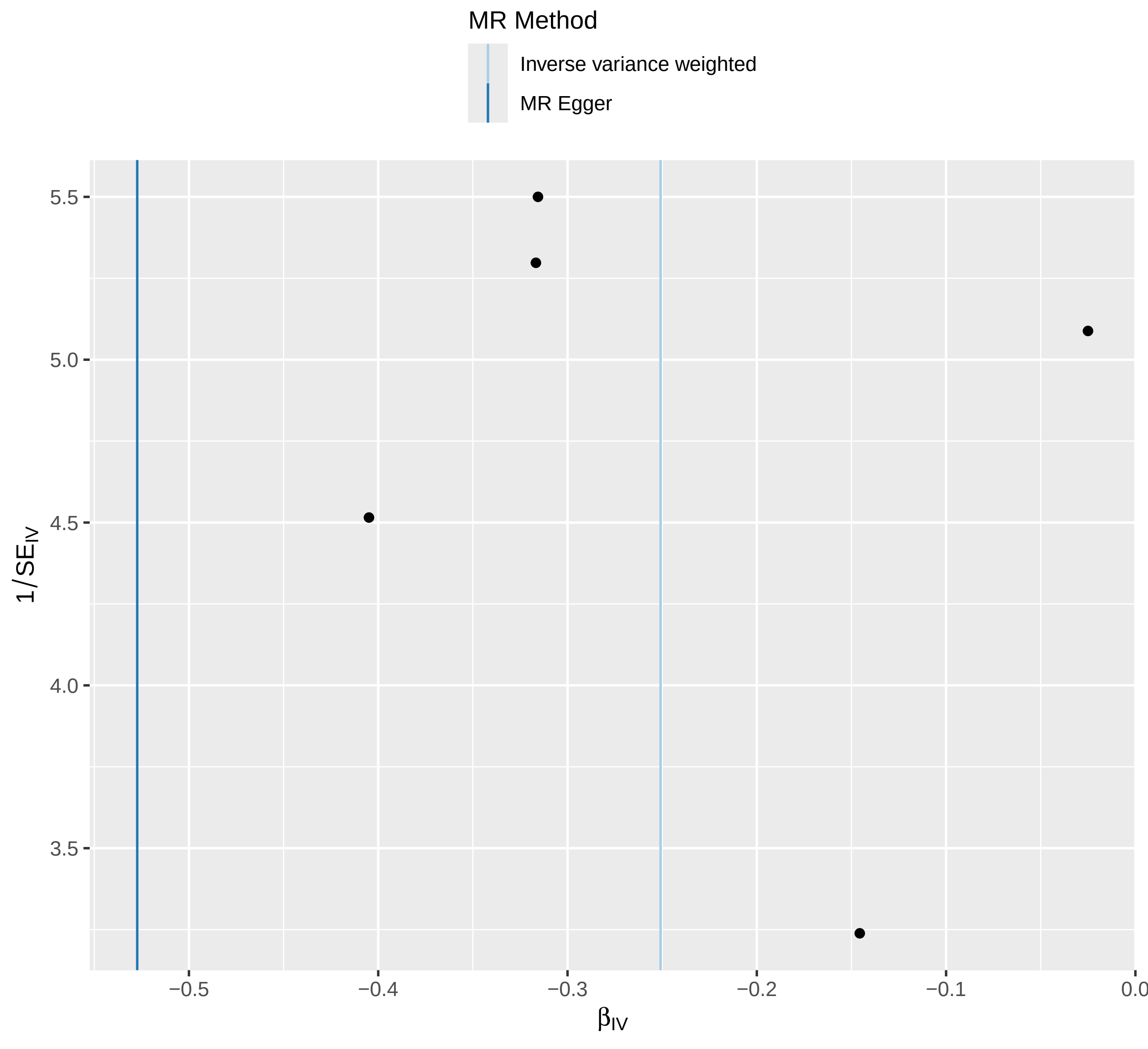
MR Method
Inverse variance weighted
MR Egger
5.5
5.0
4.5
4.0
3.5
1/SE$_{IV}$
−0.5
−0.4
−0.3
−0.2
−0.1
0.0
β$_{IV}$

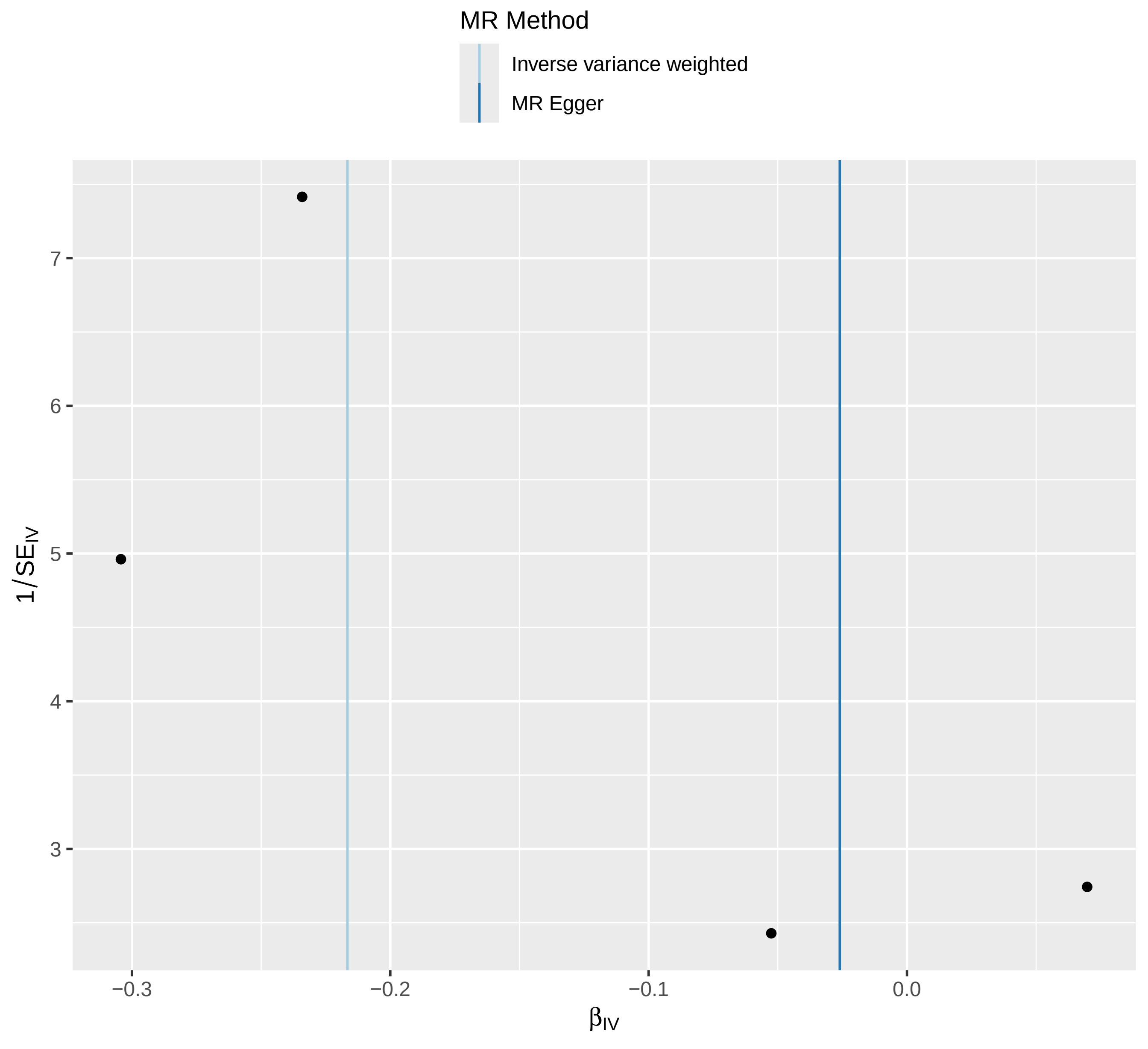
MR Method
Inverse variance weighted
MR Egger
7
6
5
4
3
1/SE_IV
−0.3
−0.2
−0.1
0.0
β_IV

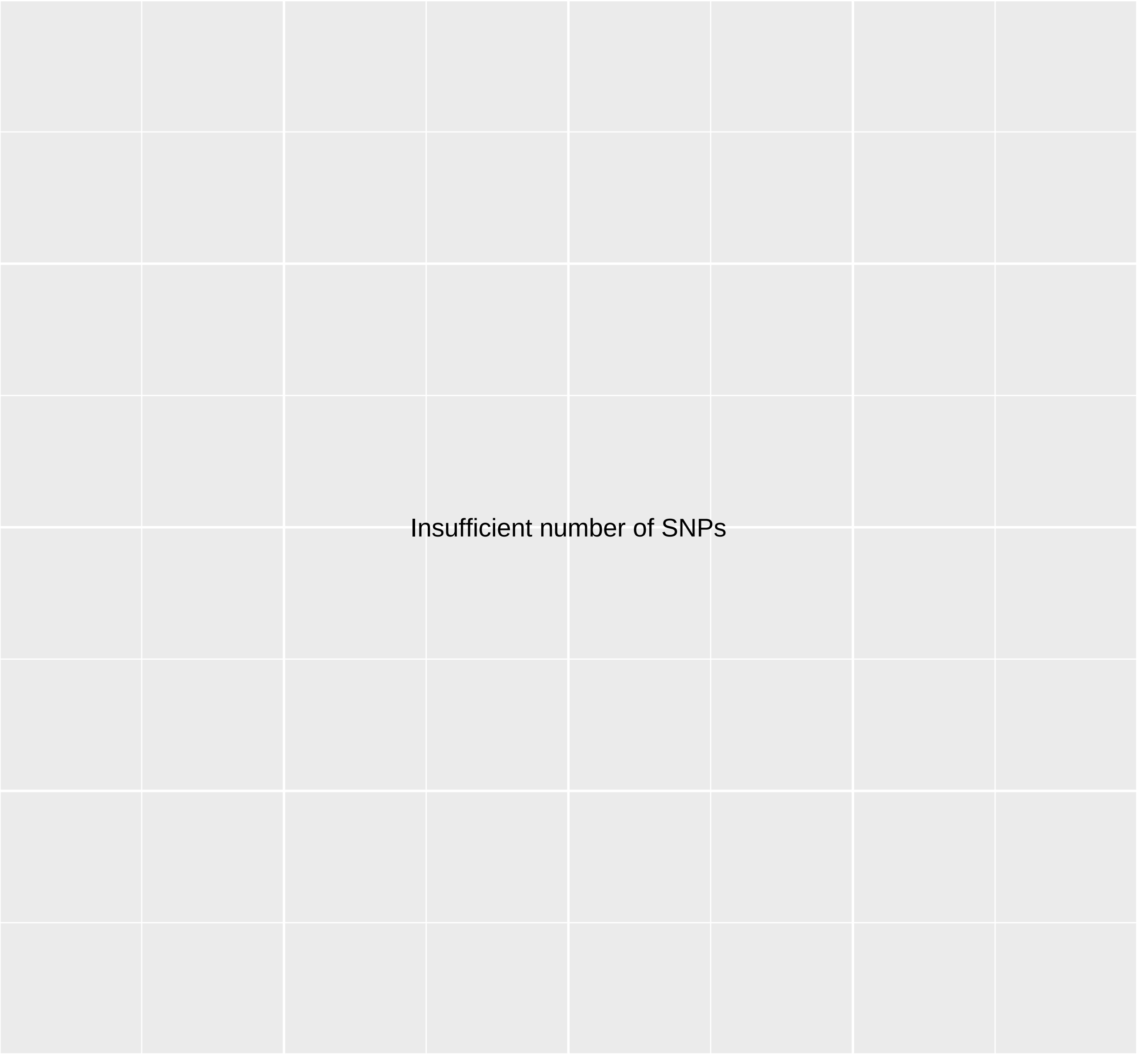
Insufficient number of SNPs

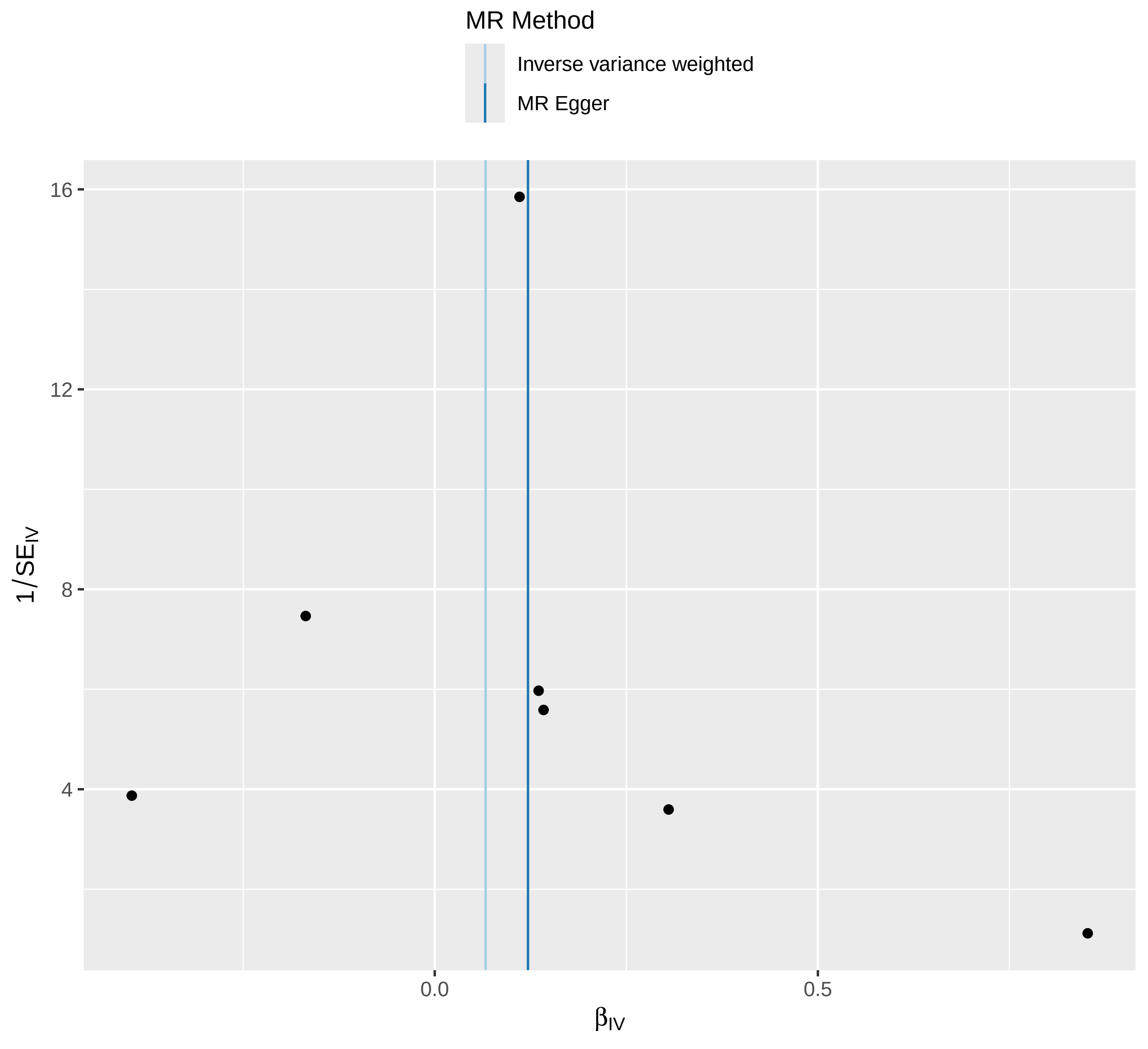
MR Method
Inverse variance weighted
MR Egger
16
12
8
4
1/SE_IV
0.0
0.5
β_IV

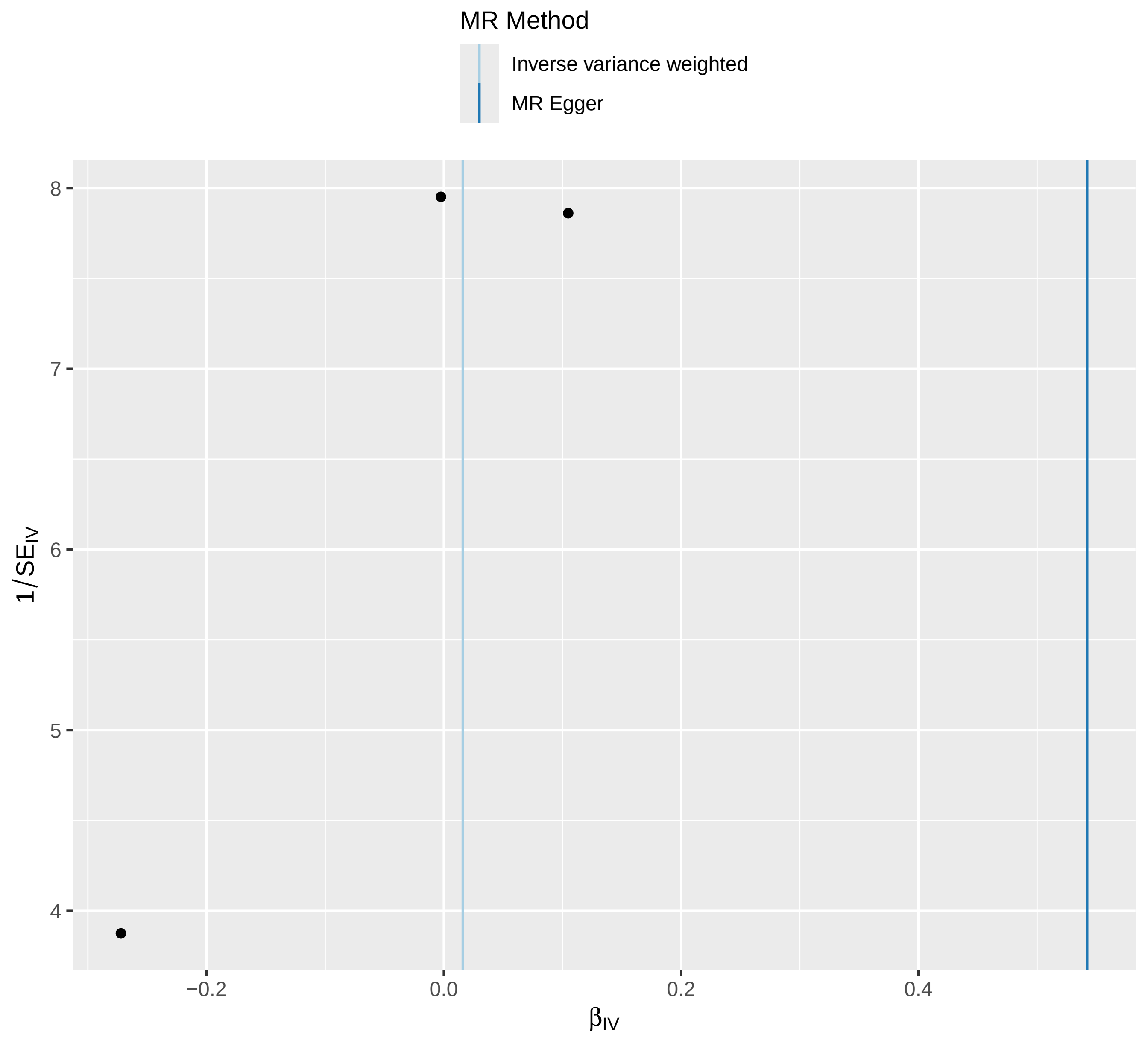
MR Method
Inverse variance weighted
MR Egger
8
7
6
5
4
1/SE_IV
−0.2
0.0
0.2
0.4
β_IV

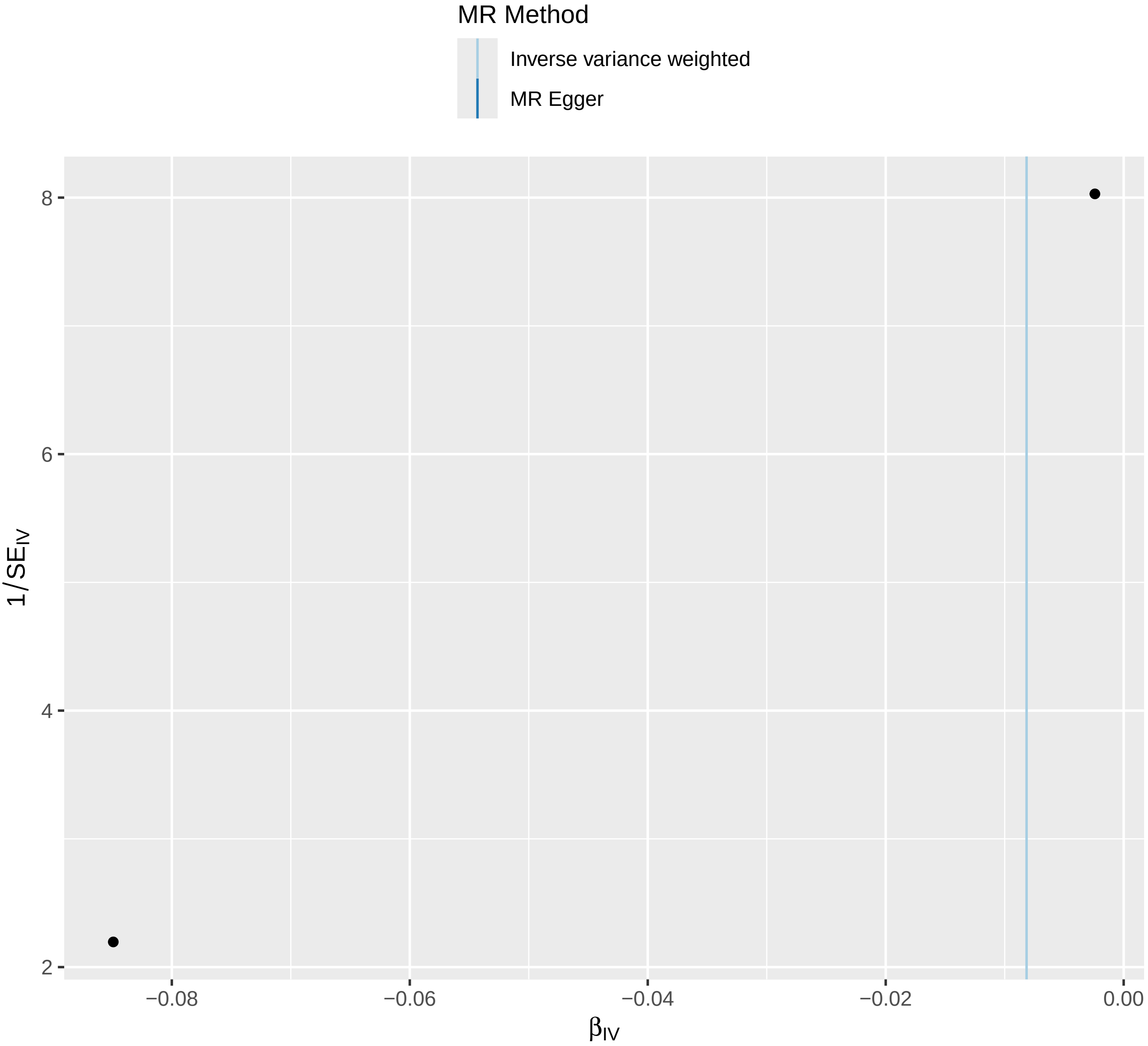

MR Method
Inverse variance weighted
MR Egger
8
6
4
2
1/SE$_{IV}$
−0.08
−0.06
−0.04
−0.02
0.00
β$_{IV}$

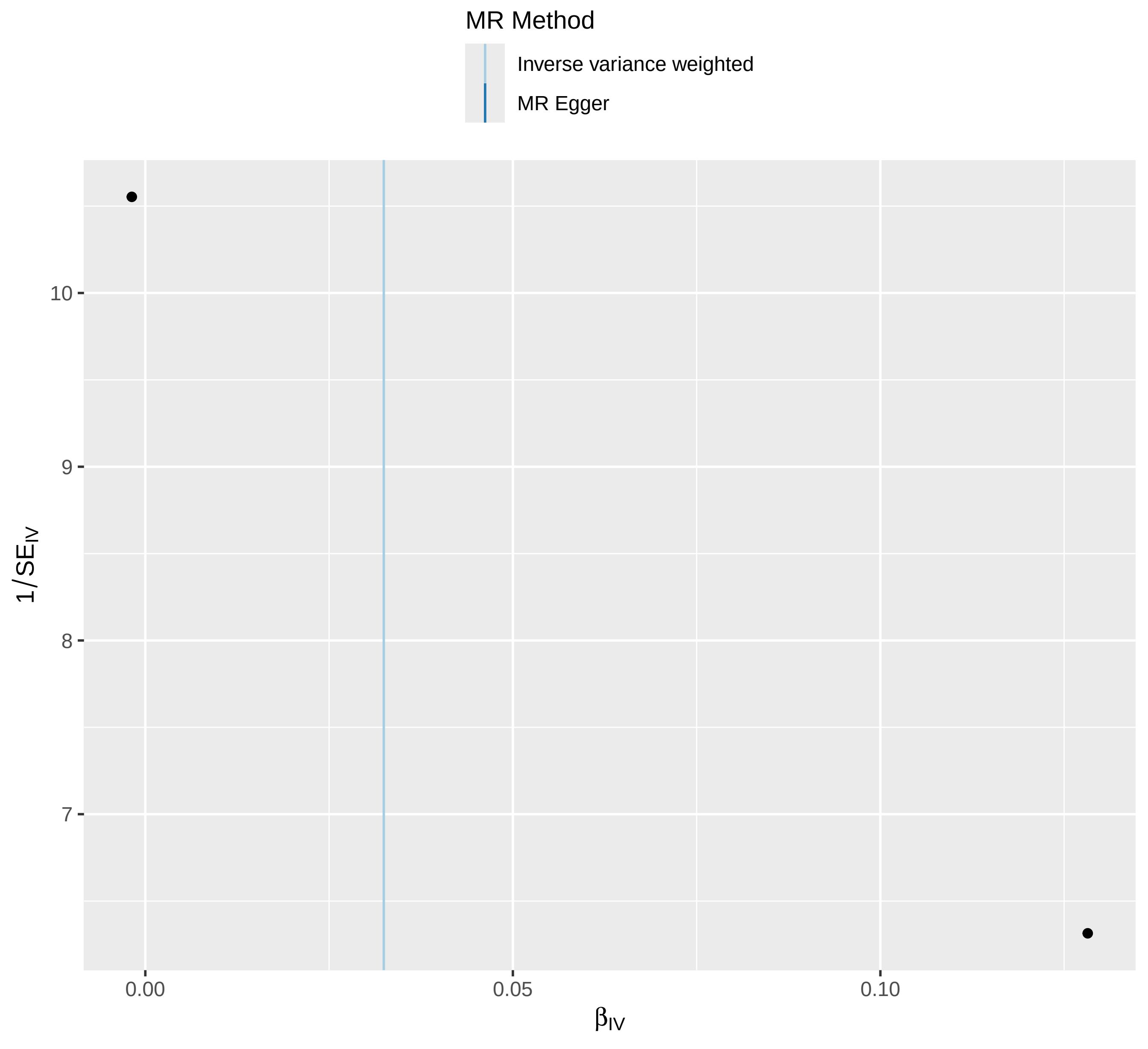
MR Method
Inverse variance weighted
MR Egger
10
9
8
7
$1/SE_{IV}$
0.00
0.05
0.10
$\beta_{IV}$

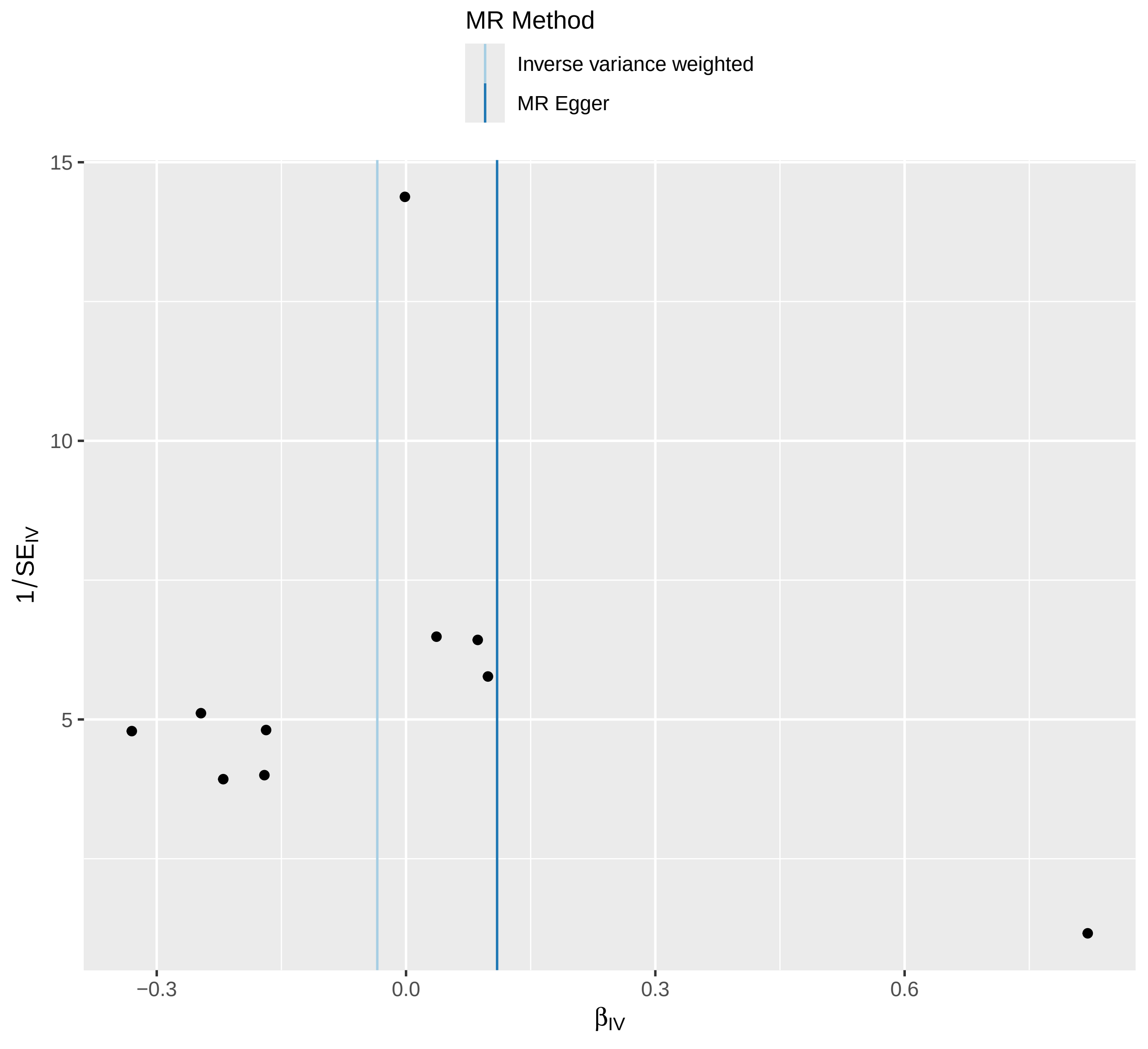
MR Method
Inverse variance weighted
MR Egger
15
10
5
1/SE_IV
−0.3
0.0
0.3
0.6
β_IV

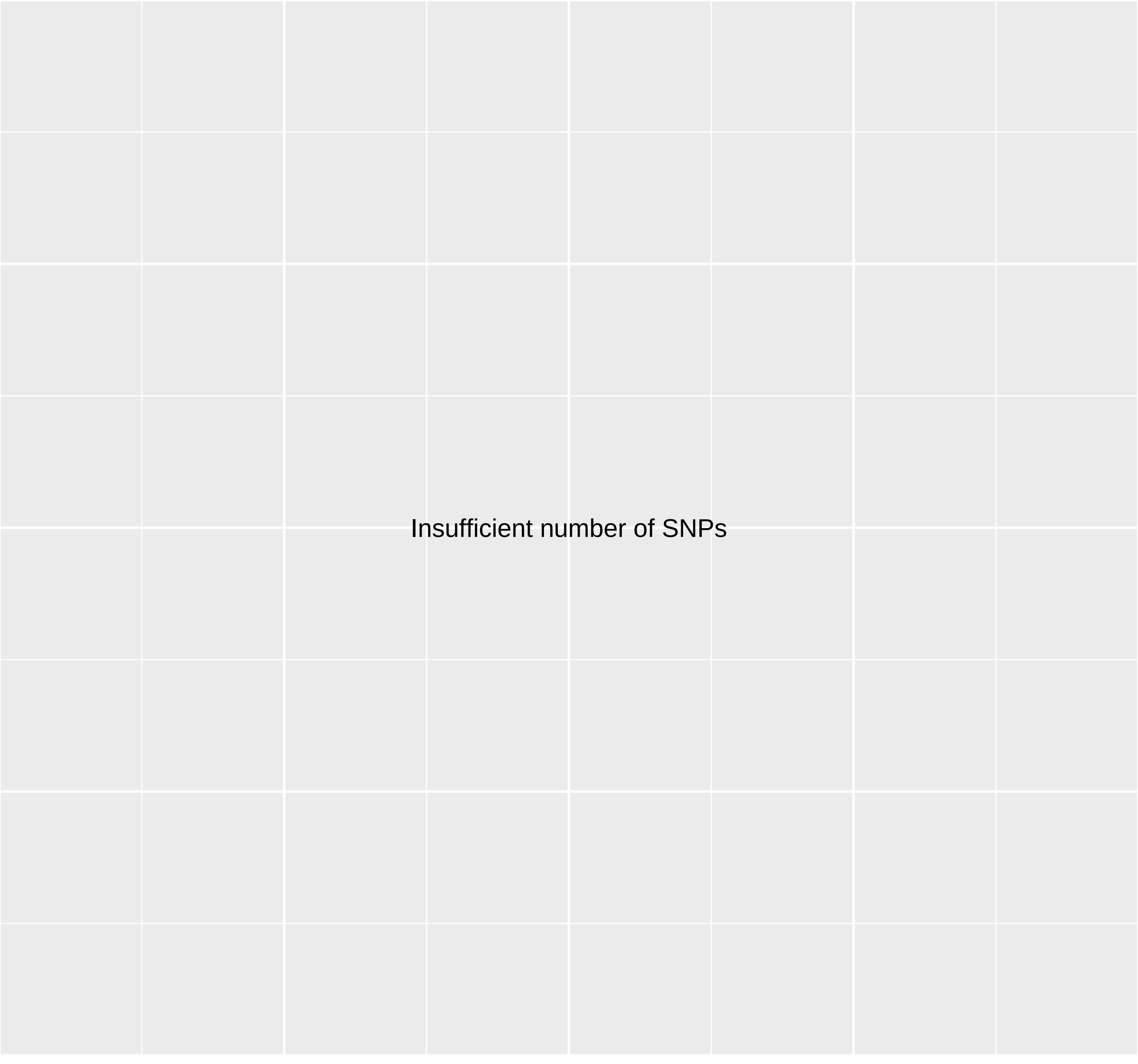
Insufficient number of SNPs

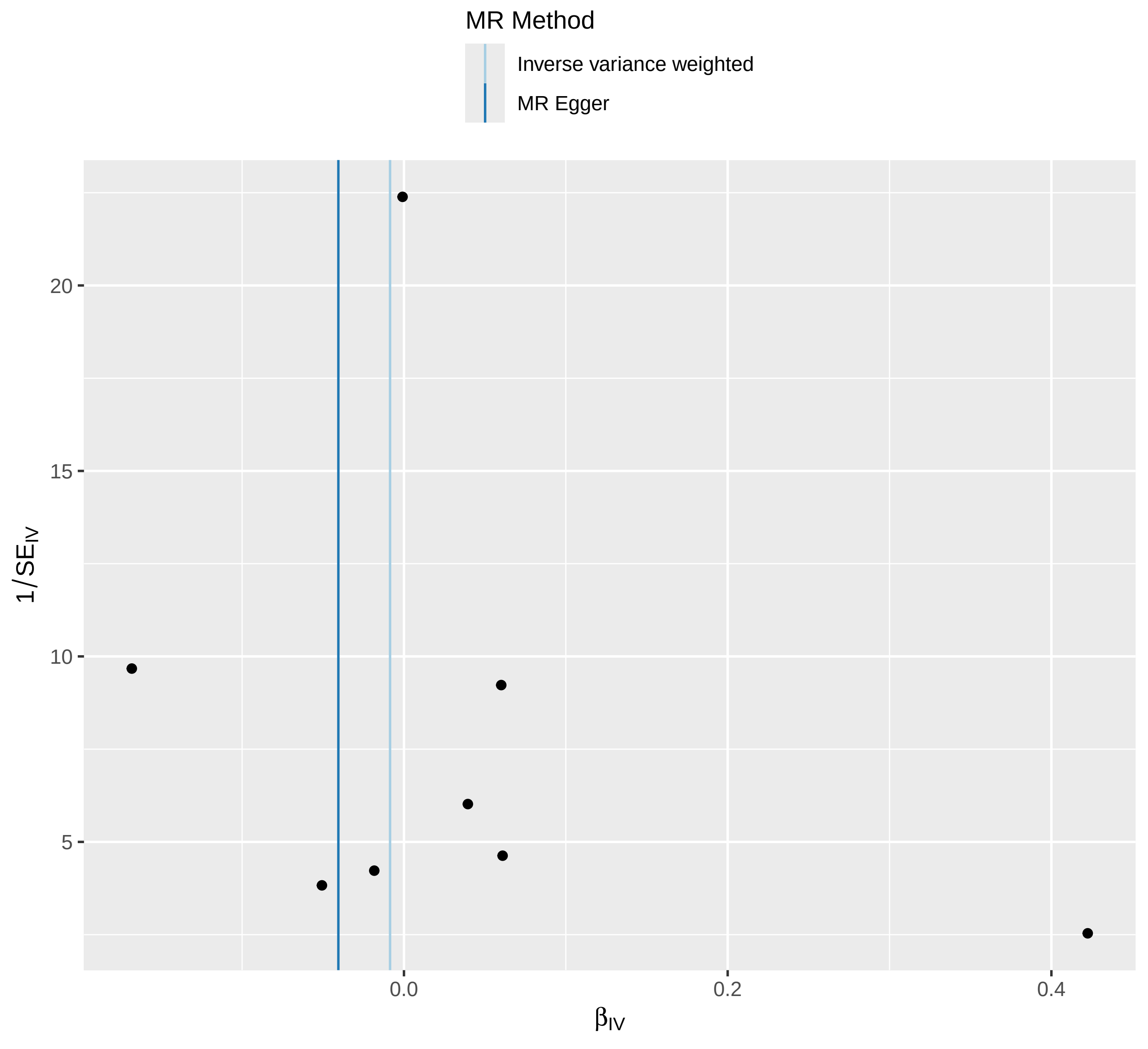
MR Method
Inverse variance weighted
MR Egger
20
15
10
5
1/SE_IV
0.0
0.2
0.4
β_IV

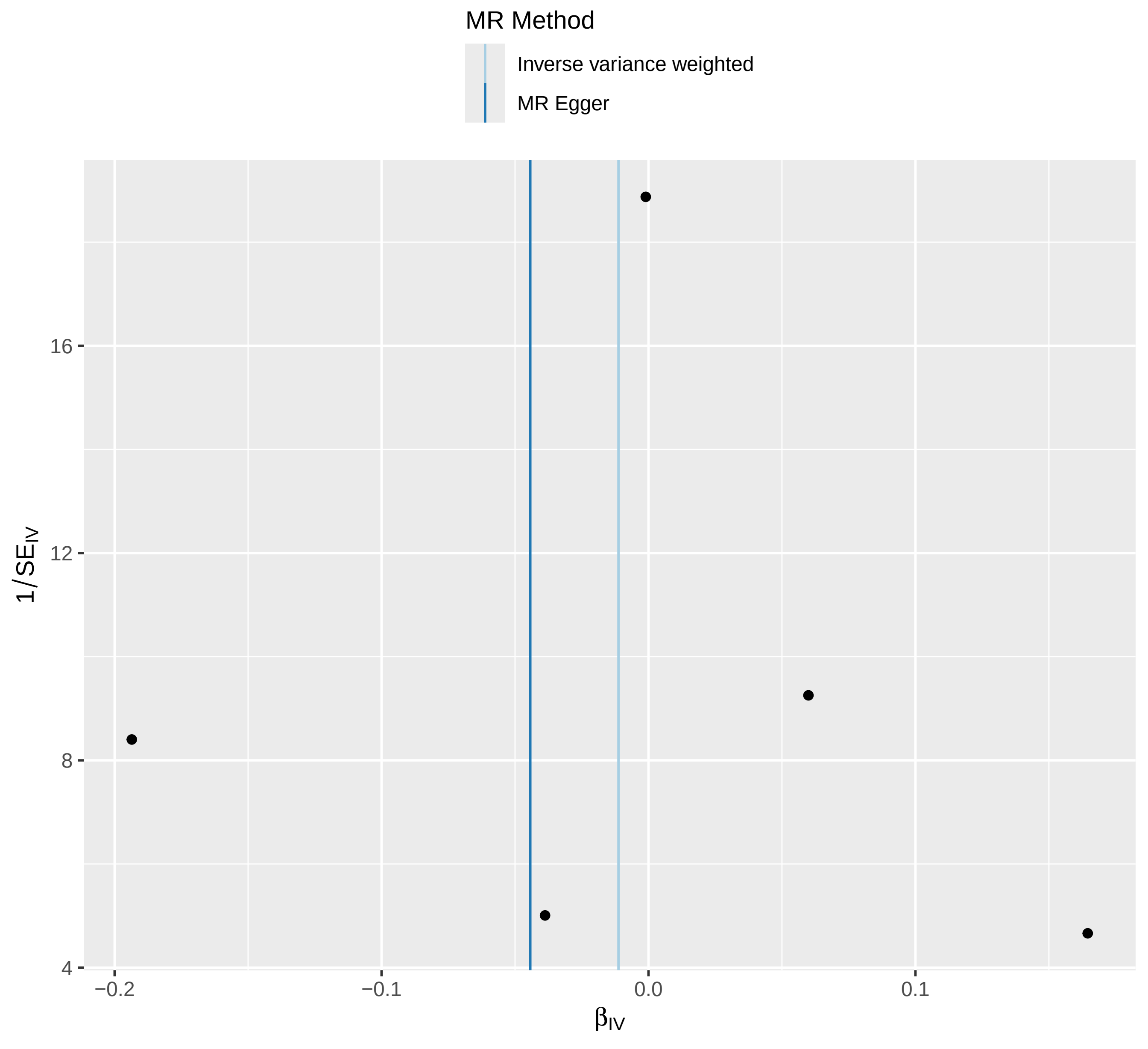
MR Method
Inverse variance weighted
MR Egger
16
12
8
4
−0.2
−0.1
0.0
0.1
1/SE_IV
β_IV

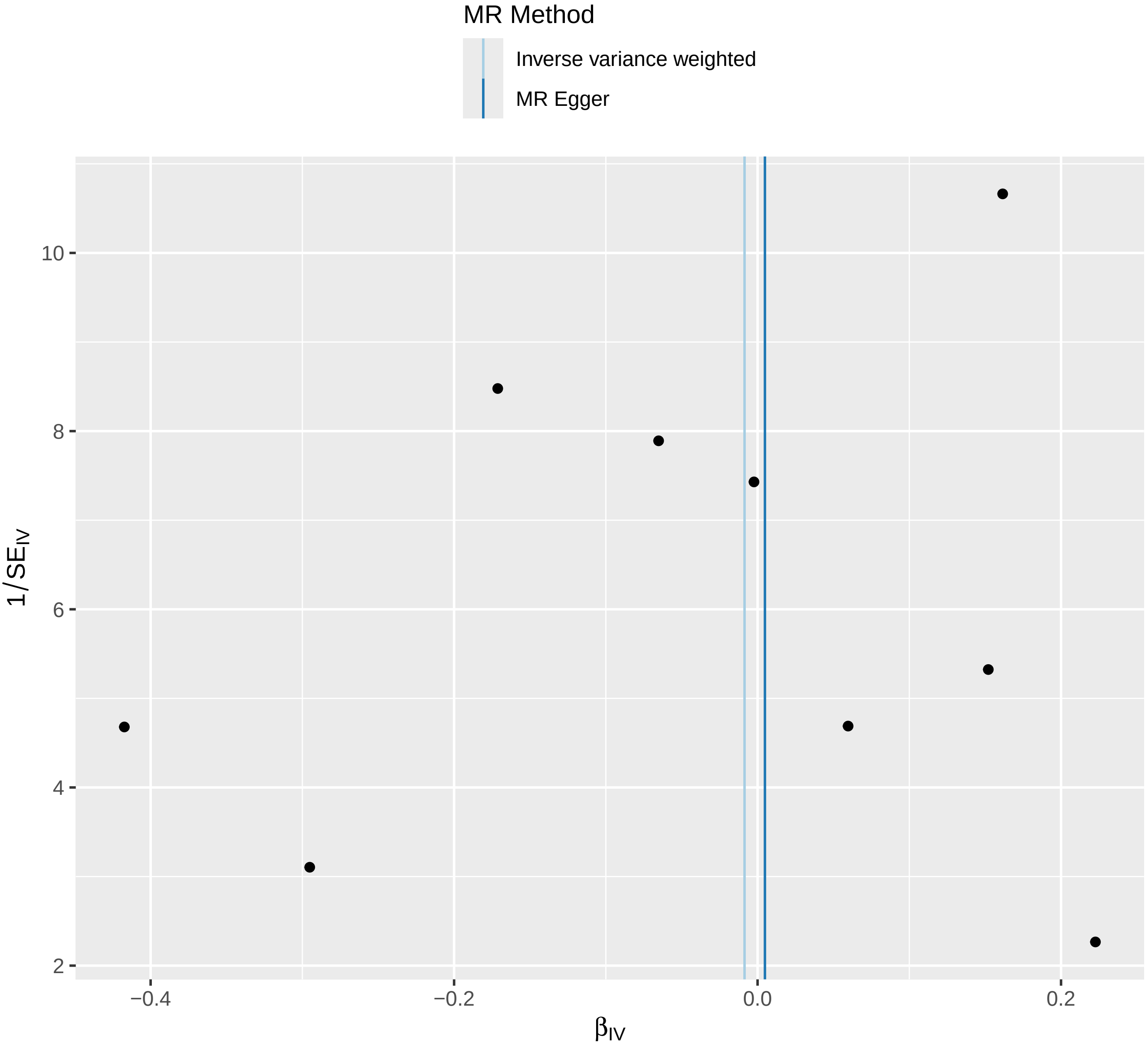

MR Method
Inverse variance weighted
MR Egger
10
8
6
4
2
1/SE$_{IV}$
−0.4
−0.2
0.0
0.2
β$_{IV}$

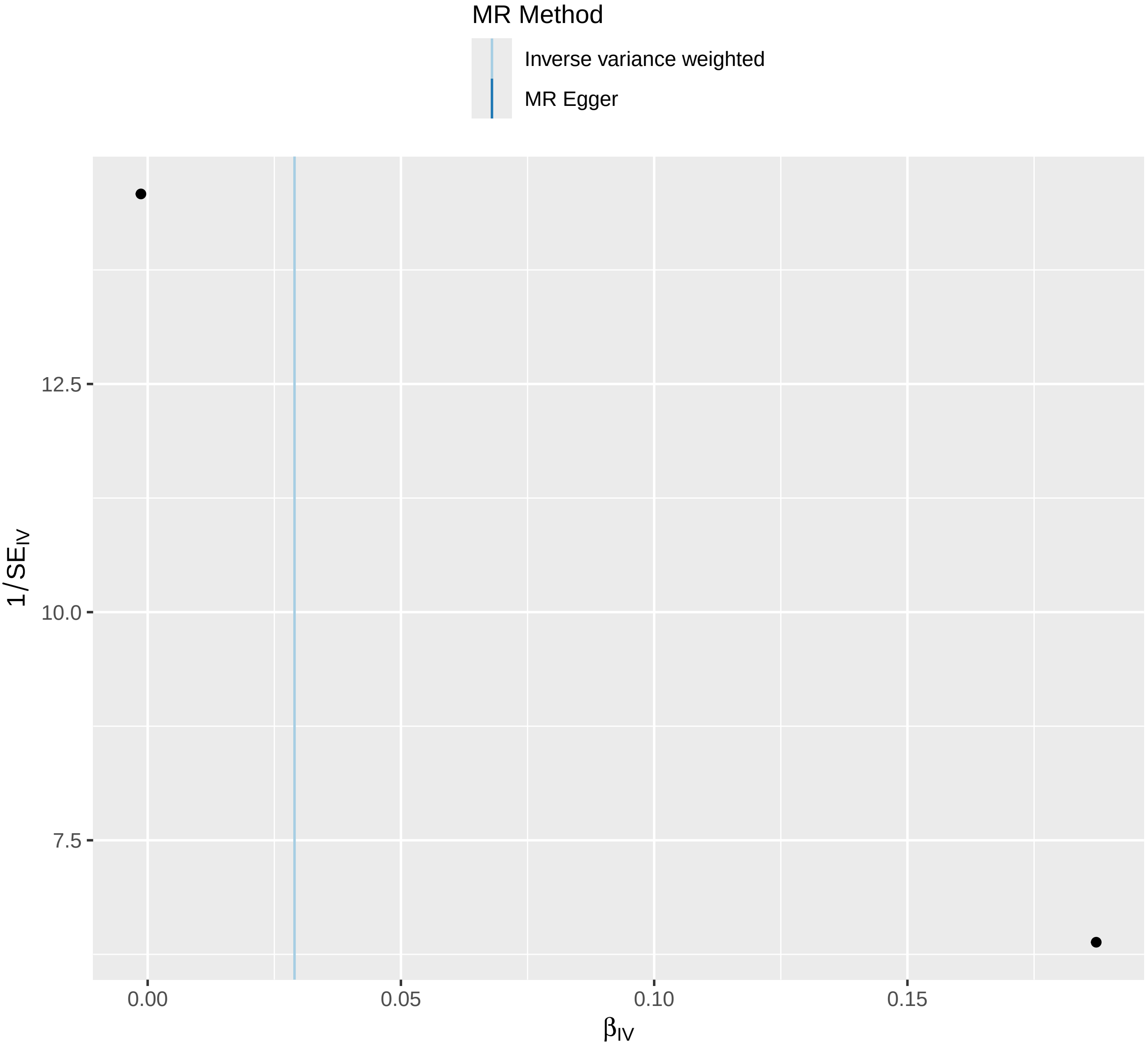
MR Method
Inverse variance weighted
MR Egger
12.5
10.0
7.5
1/SE$_{IV}$
0.00
0.05
0.10
0.15
β$_{IV}$

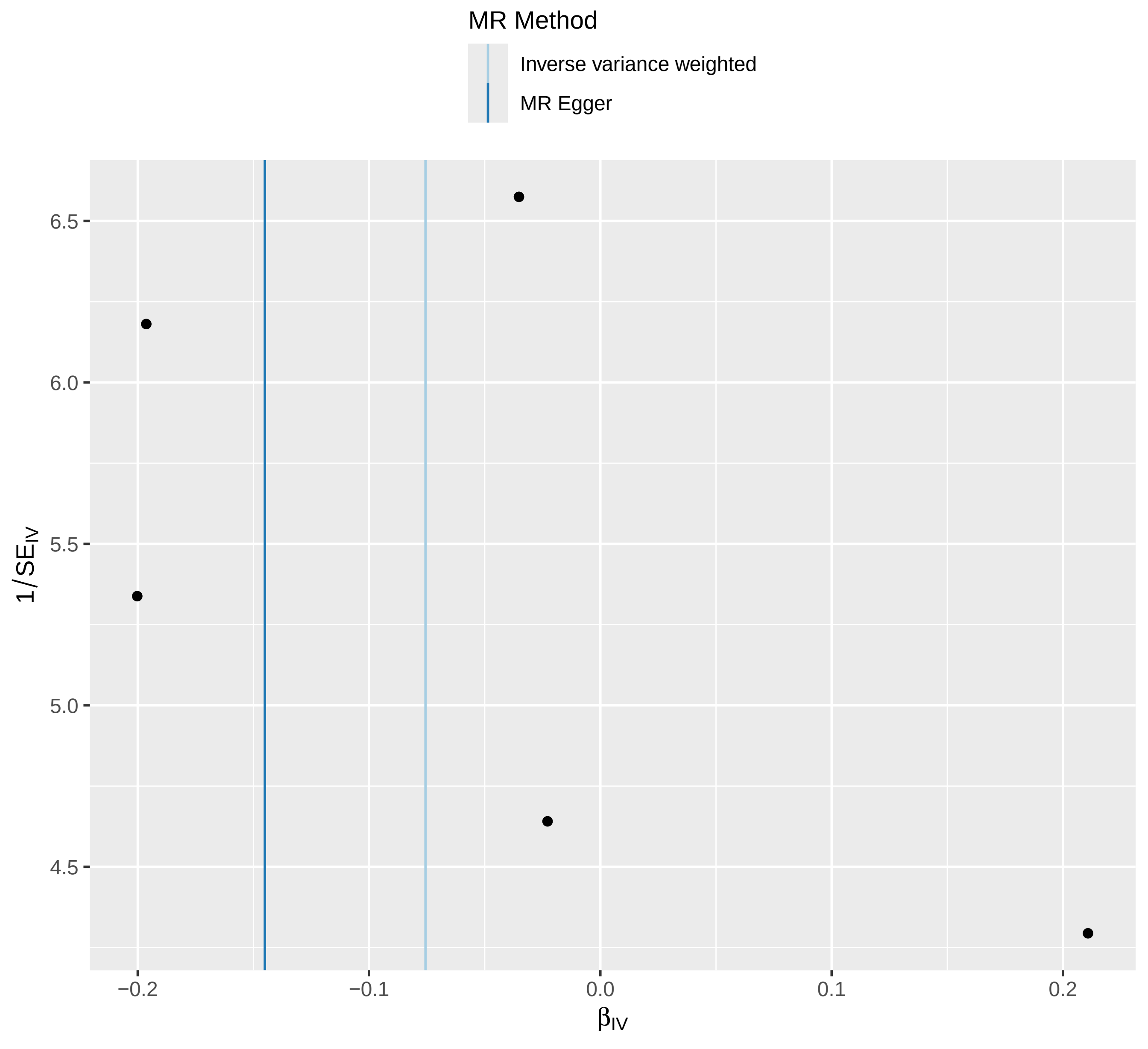

MR Method
Inverse variance weighted
MR Egger
6.5
6.0
5.5
5.0
4.5
1/SE$_{IV}$
−0.2
−0.1
0.0
0.1
0.2
$\beta_{IV}$

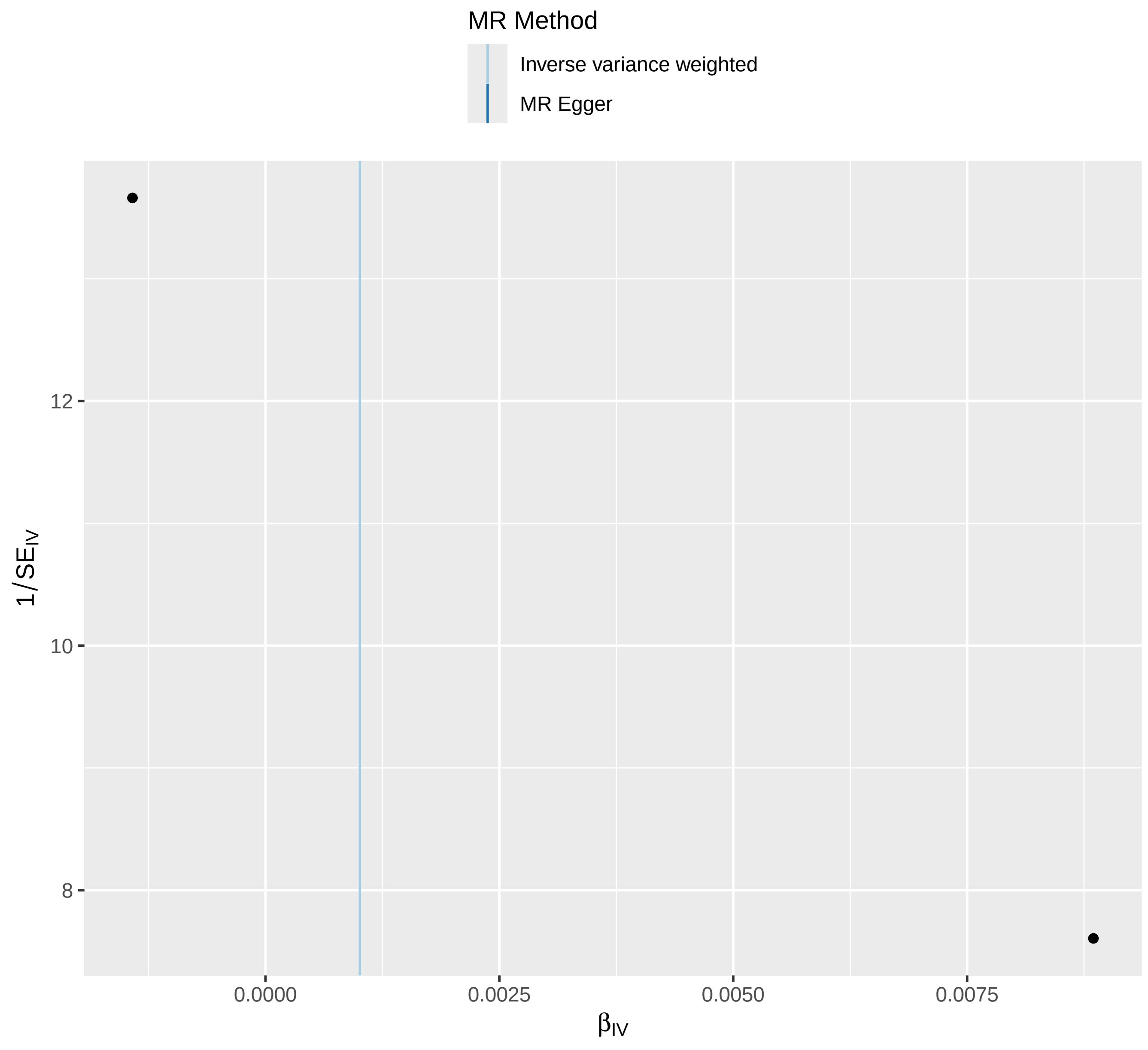
MR Method
Inverse variance weighted
MR Egger
12
10
8
0.0000
0.0025
0.0050
0.0075
1/SE$_{IV}$
β$_{IV}$

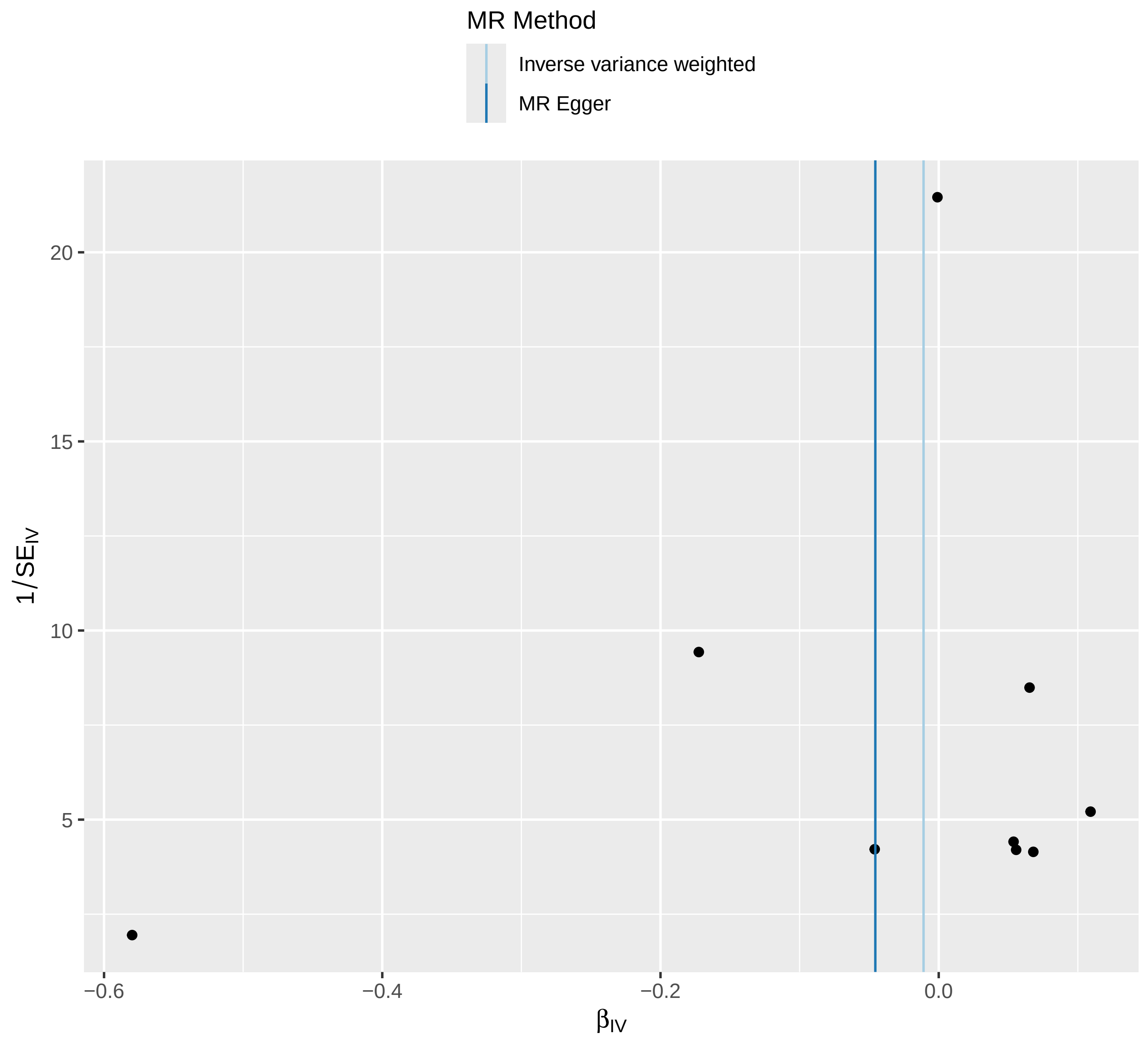
MR Method
Inverse variance weighted
MR Egger
20
15
10
5
1/SE$_{IV}$
−0.6
−0.4
−0.2
0.0
β$_{IV}$

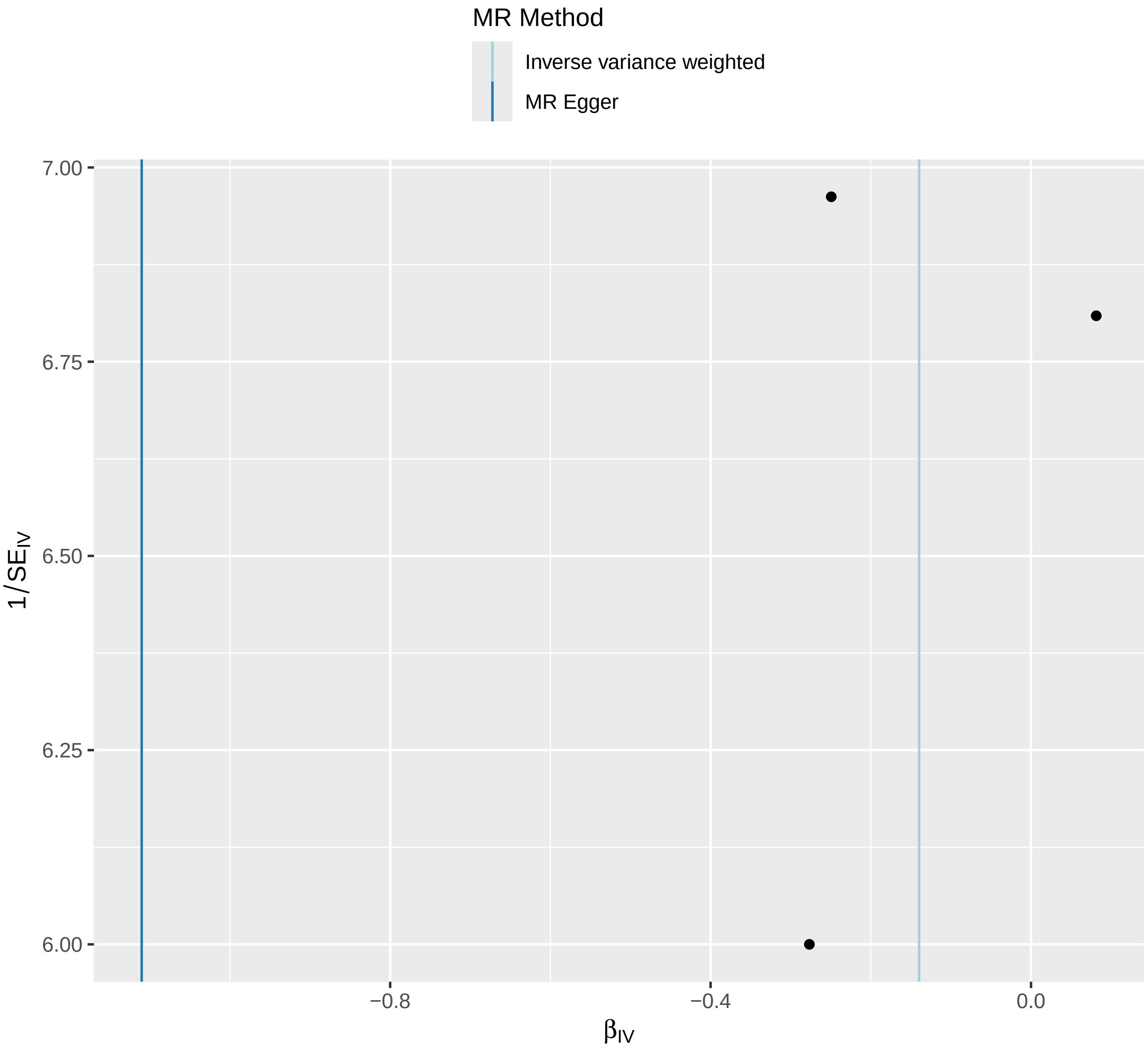
MR Method
Inverse variance weighted
MR Egger
7.00
6.75
6.50
6.25
6.00
1/SE_IV
−0.8
−0.4
0.0
β_IV

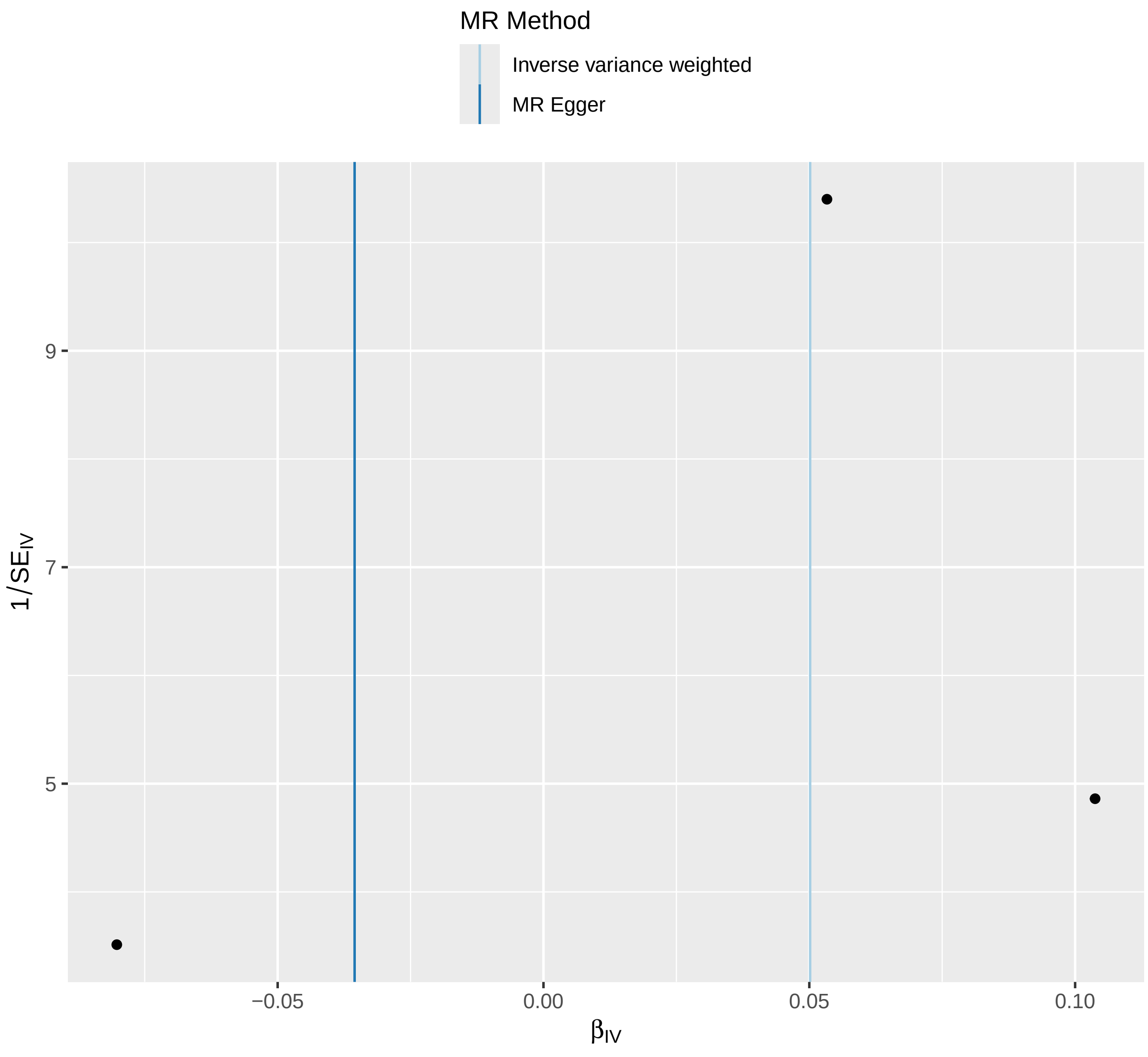
MR Method
Inverse variance weighted
MR Egger
9
7
5
$1/SE_{IV}$
−0.05
0.00
0.05
0.10
$\beta_{IV}$

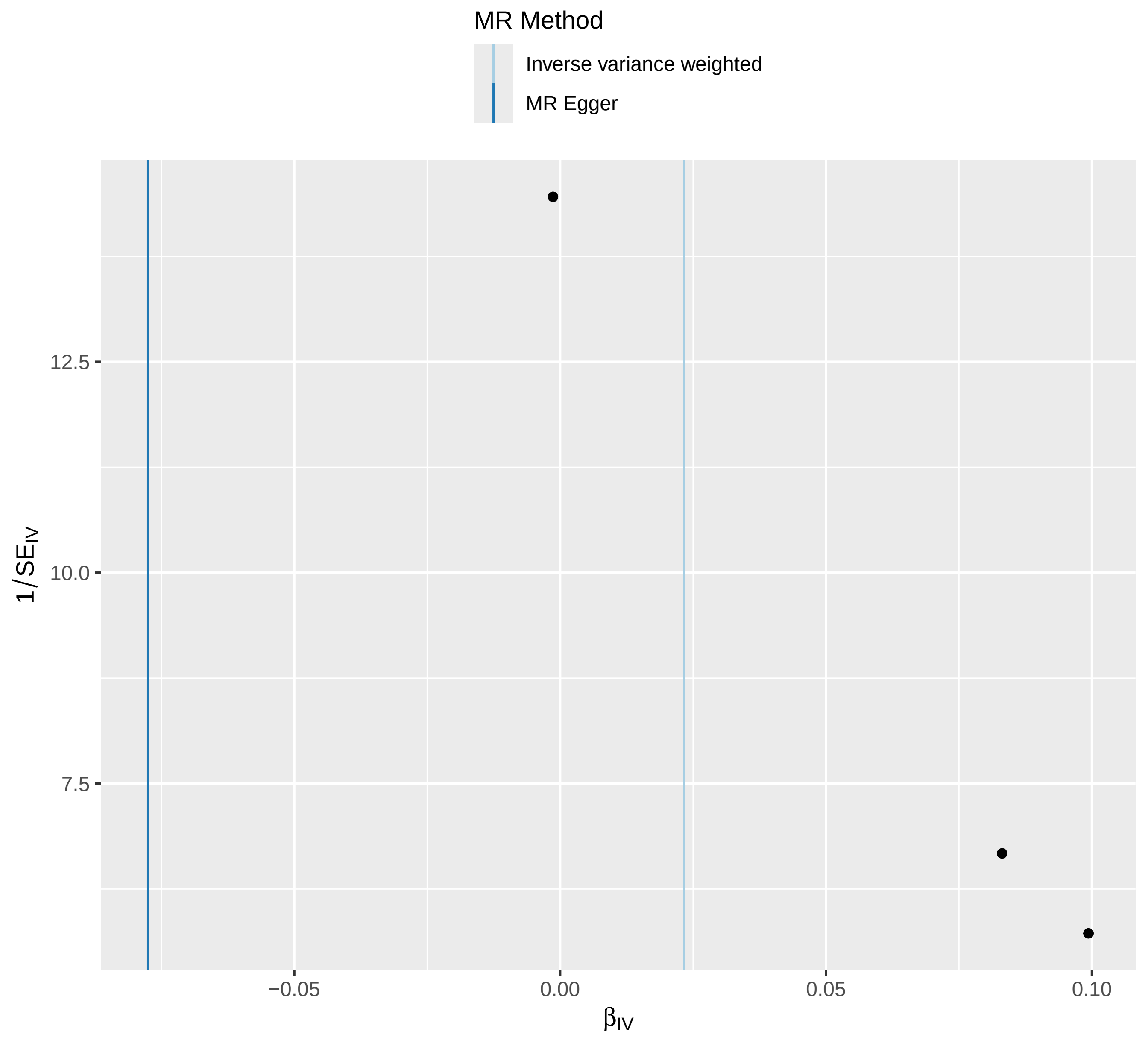

MR Method
Inverse variance weighted
MR Egger
12.5
10.0
7.5
1/SE_IV
−0.05
0.00
0.05
0.10
β_IV

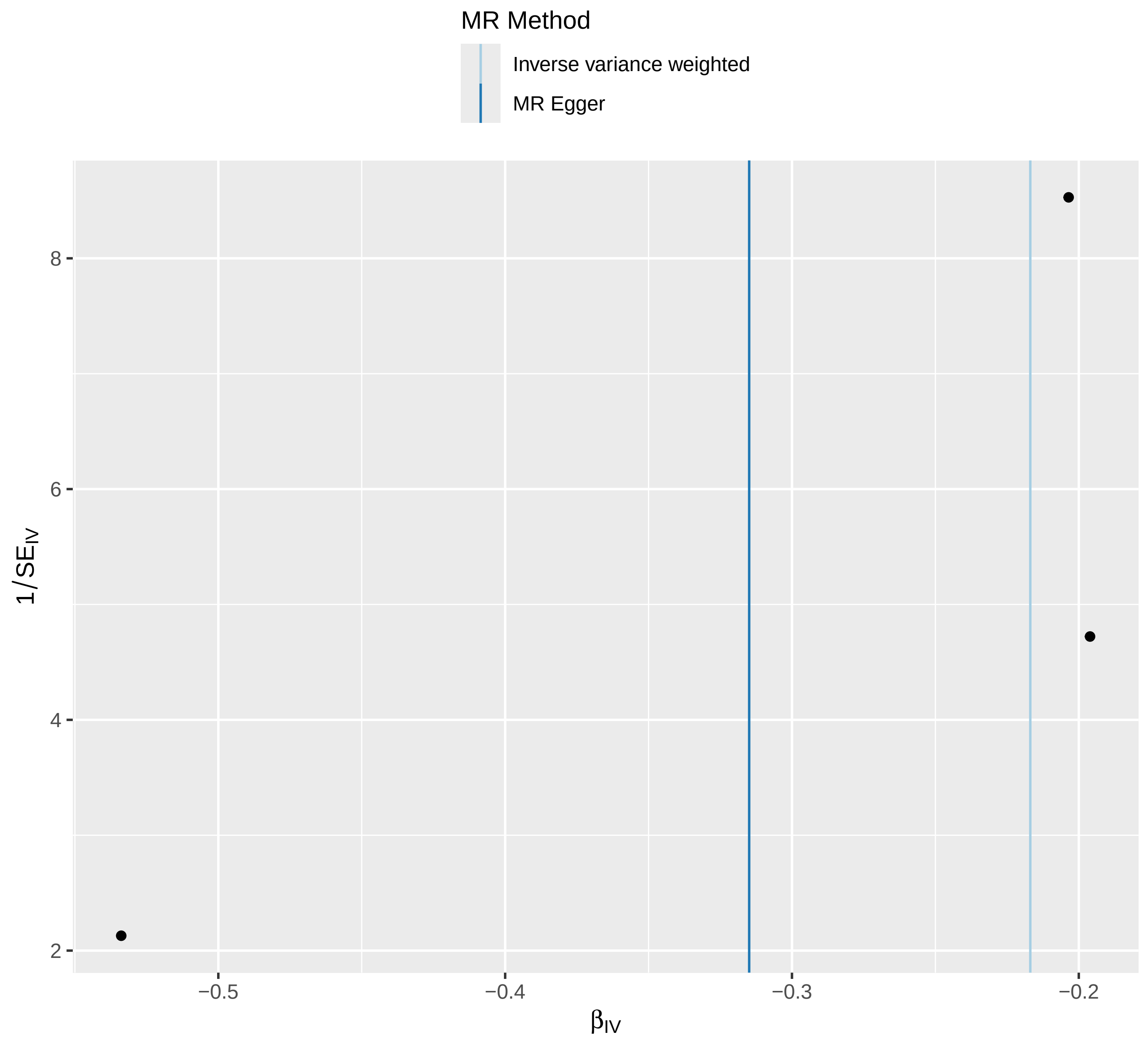
MR Method
Inverse variance weighted
MR Egger
8
6
4
2
−0.5
−0.4
−0.3
−0.2
$1/SE_{IV}$
$\beta_{IV}$

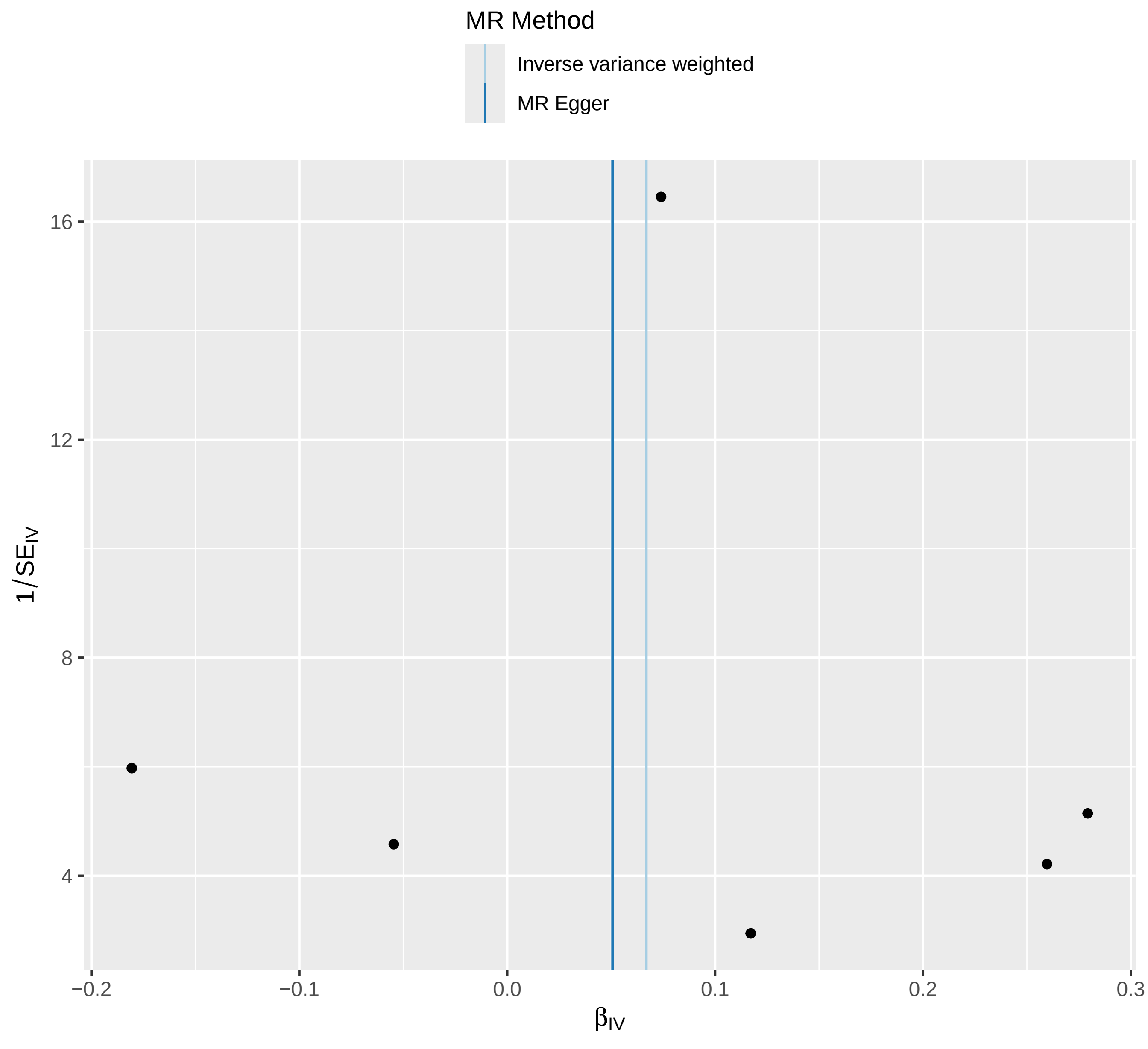

MR Method
Inverse variance weighted
MR Egger
16
12
8
4
1/SE_IV
−0.2
−0.1
0.0
0.1
0.2
0.3
β_IV

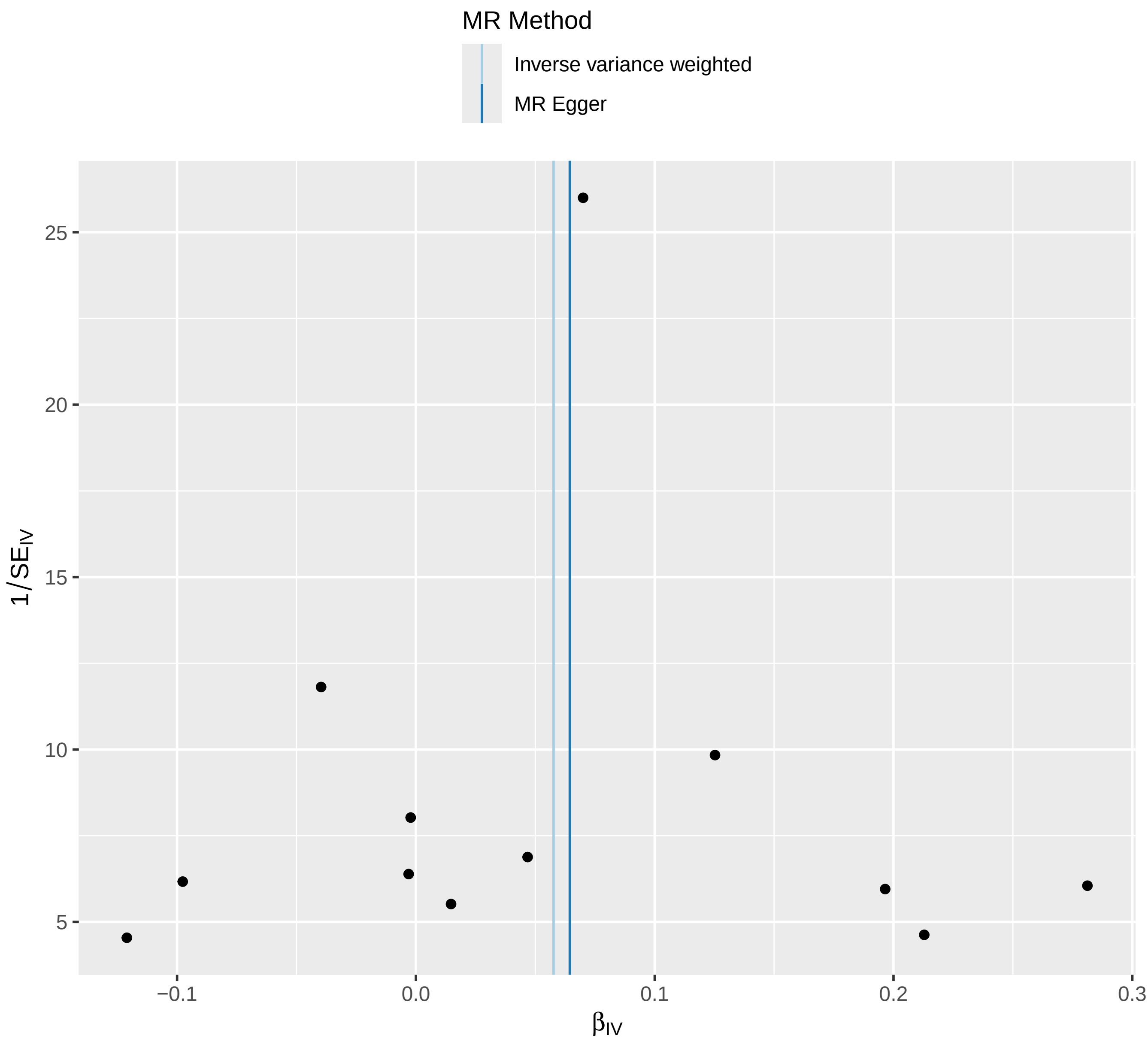
MR Method
Inverse variance weighted
MR Egger
25
20
15
10
5
1/SE_IV
−0.1
0.0
0.1
0.2
0.3
β_IV

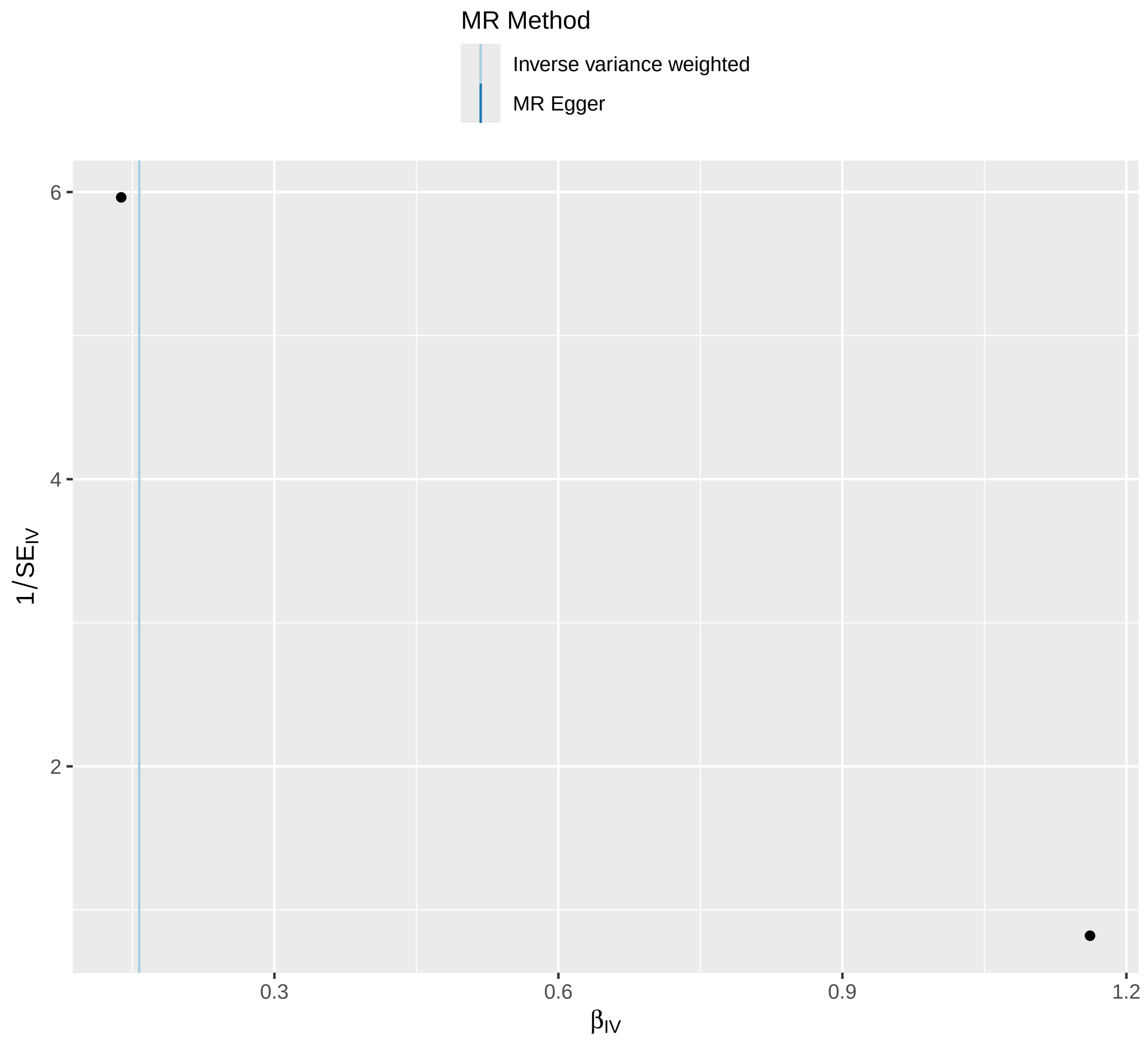

MR Method
Inverse variance weighted
MR Egger
6
4
2
1/SE$_{IV}$
0.3
0.6
0.9
1.2
β$_{IV}$

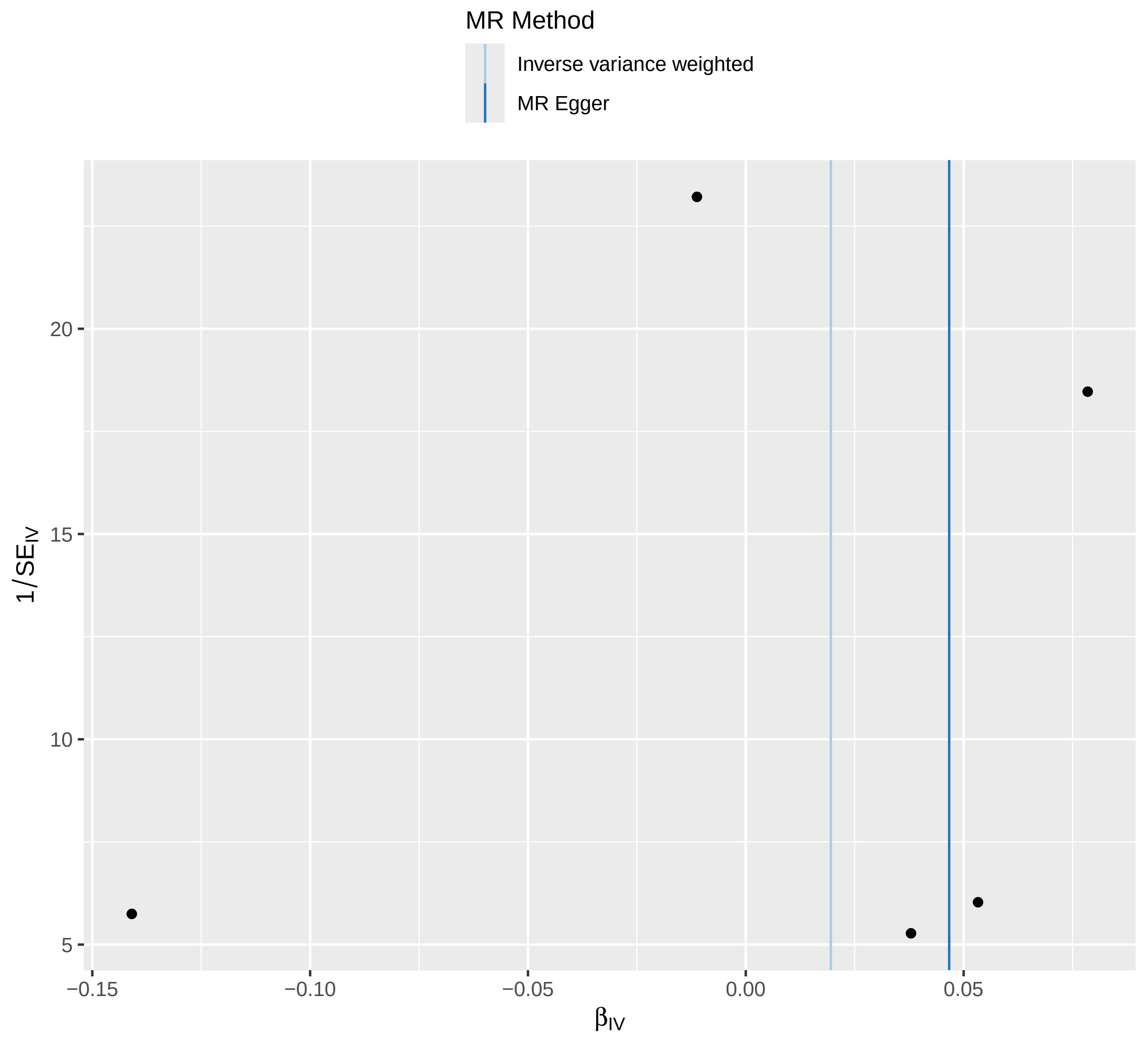

MR Method
Inverse variance weighted
MR Egger
20
15
10
5
$1/SE_{IV}$
−0.15
−0.10
−0.05
0.00
0.05
$\beta_{IV}$

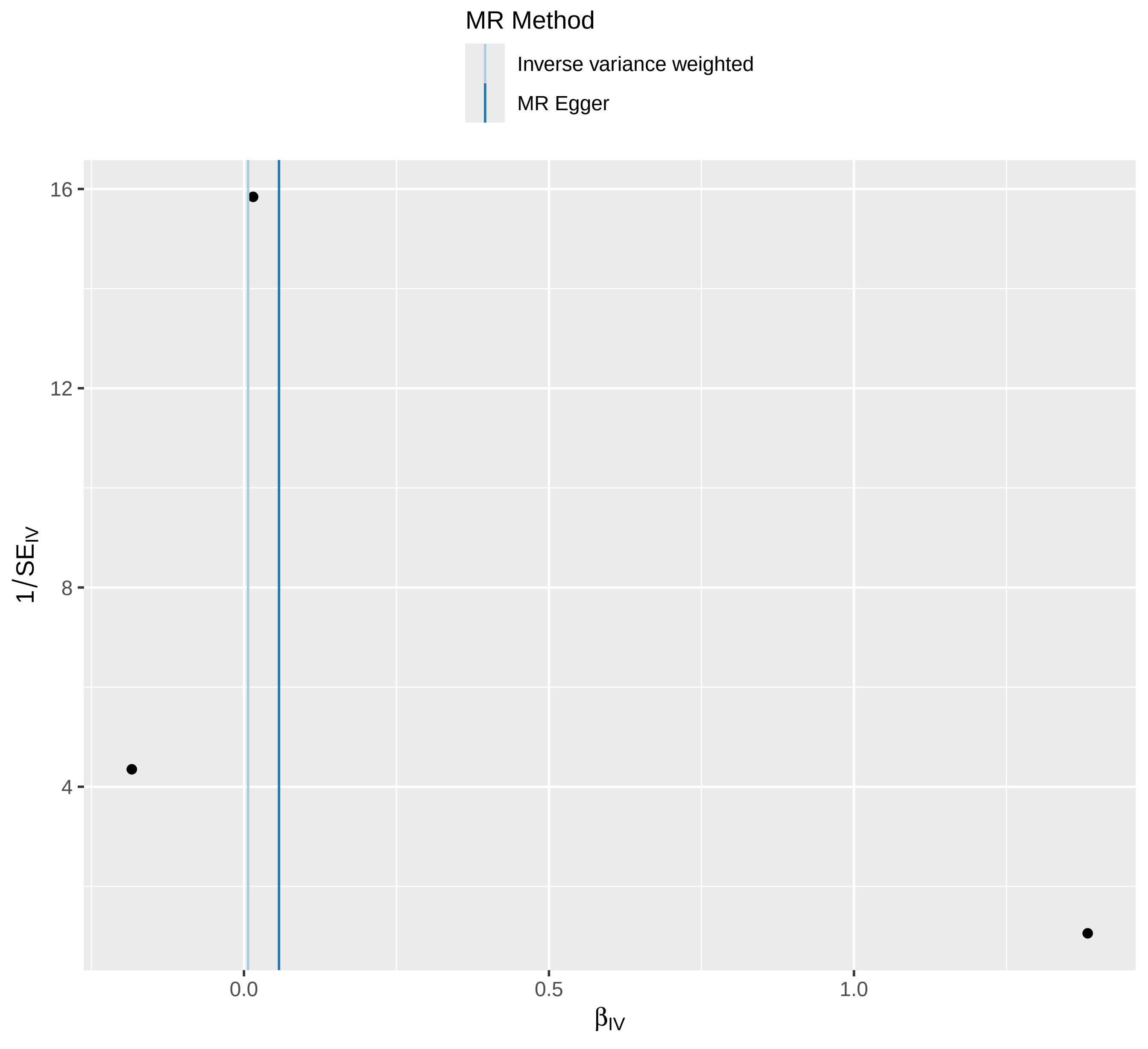
MR Method
Inverse variance weighted
MR Egger
16
12
8
4
1/SE_IV
0.0
0.5
1.0
β_IV

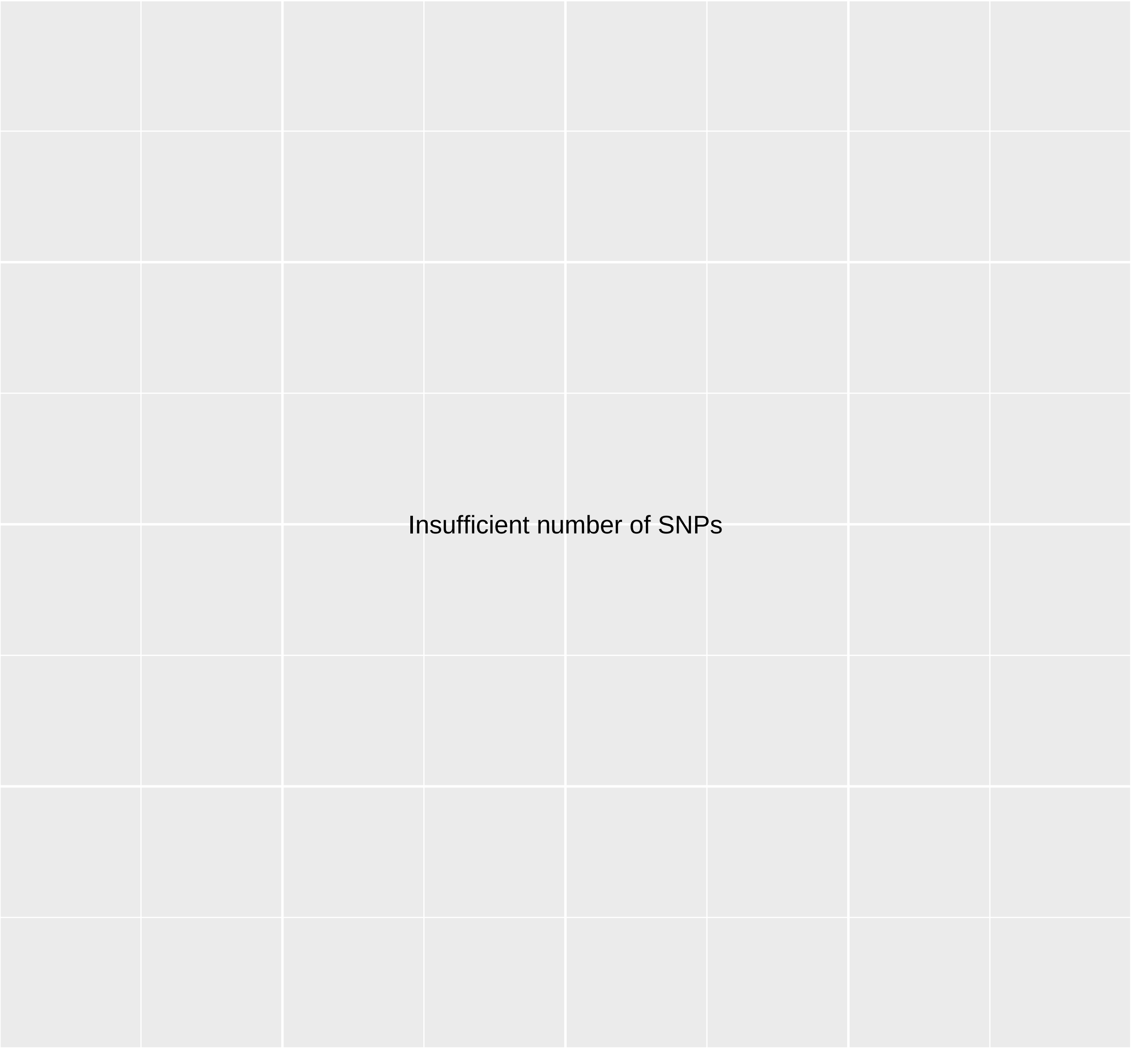
Insufficient number of SNPs

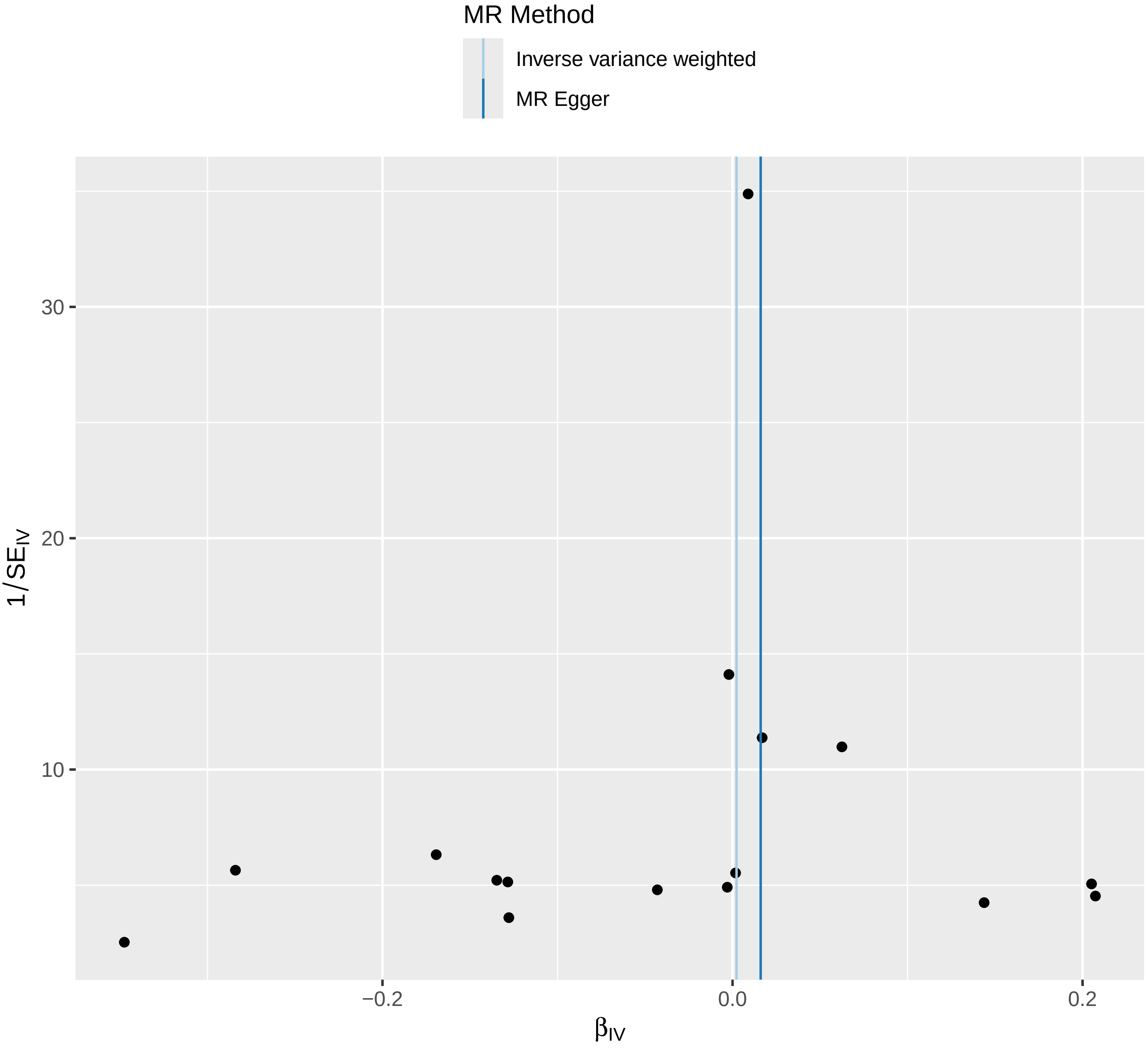

MR Method
Inverse variance weighted
MR Egger
30
20
10
1/SE_IV
−0.2
0.0
0.2
β_IV

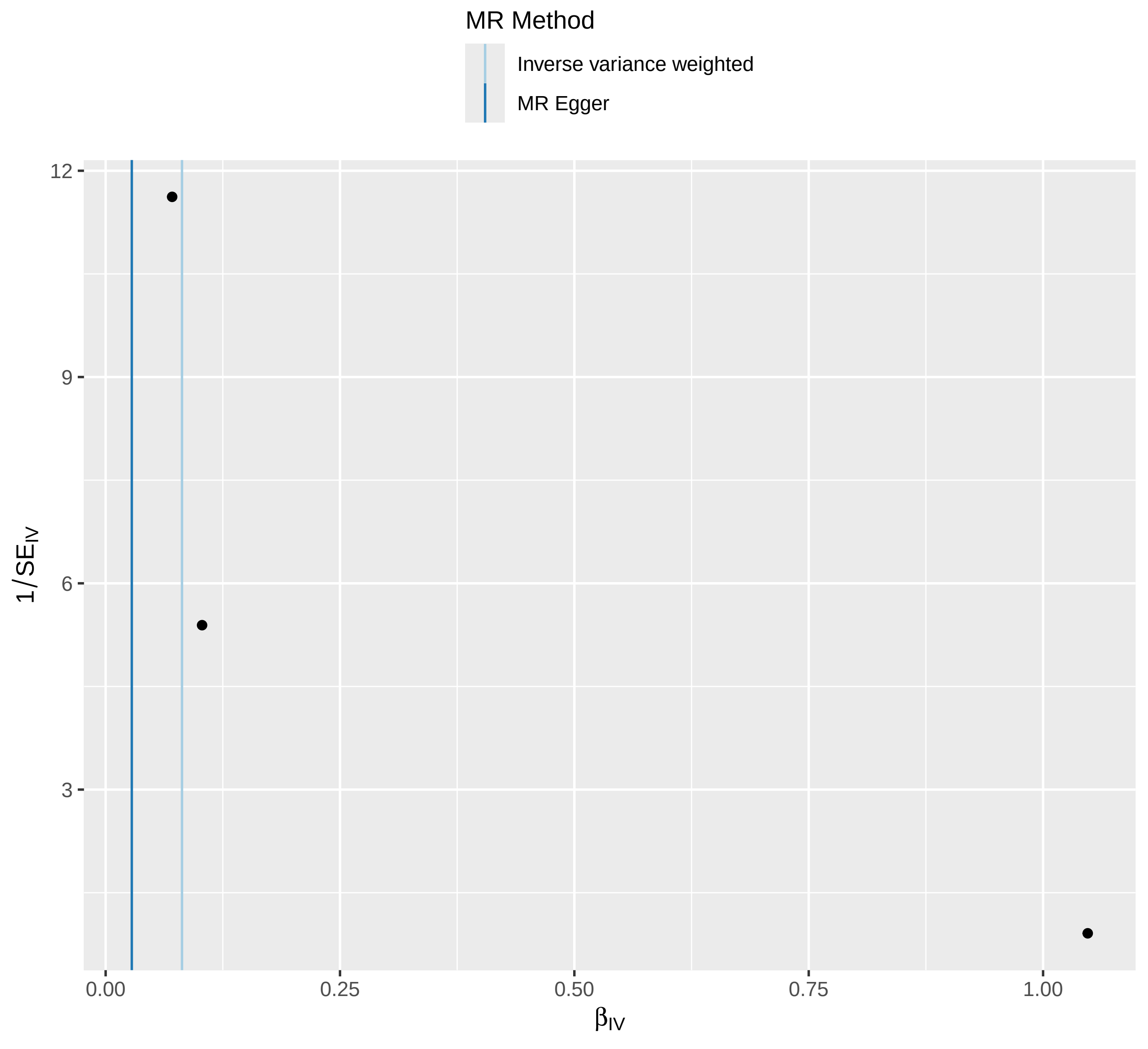

MR Method
Inverse variance weighted
MR Egger
12
9
6
3
1/SE_IV
0.00
0.25
0.50
0.75
1.00
β_IV

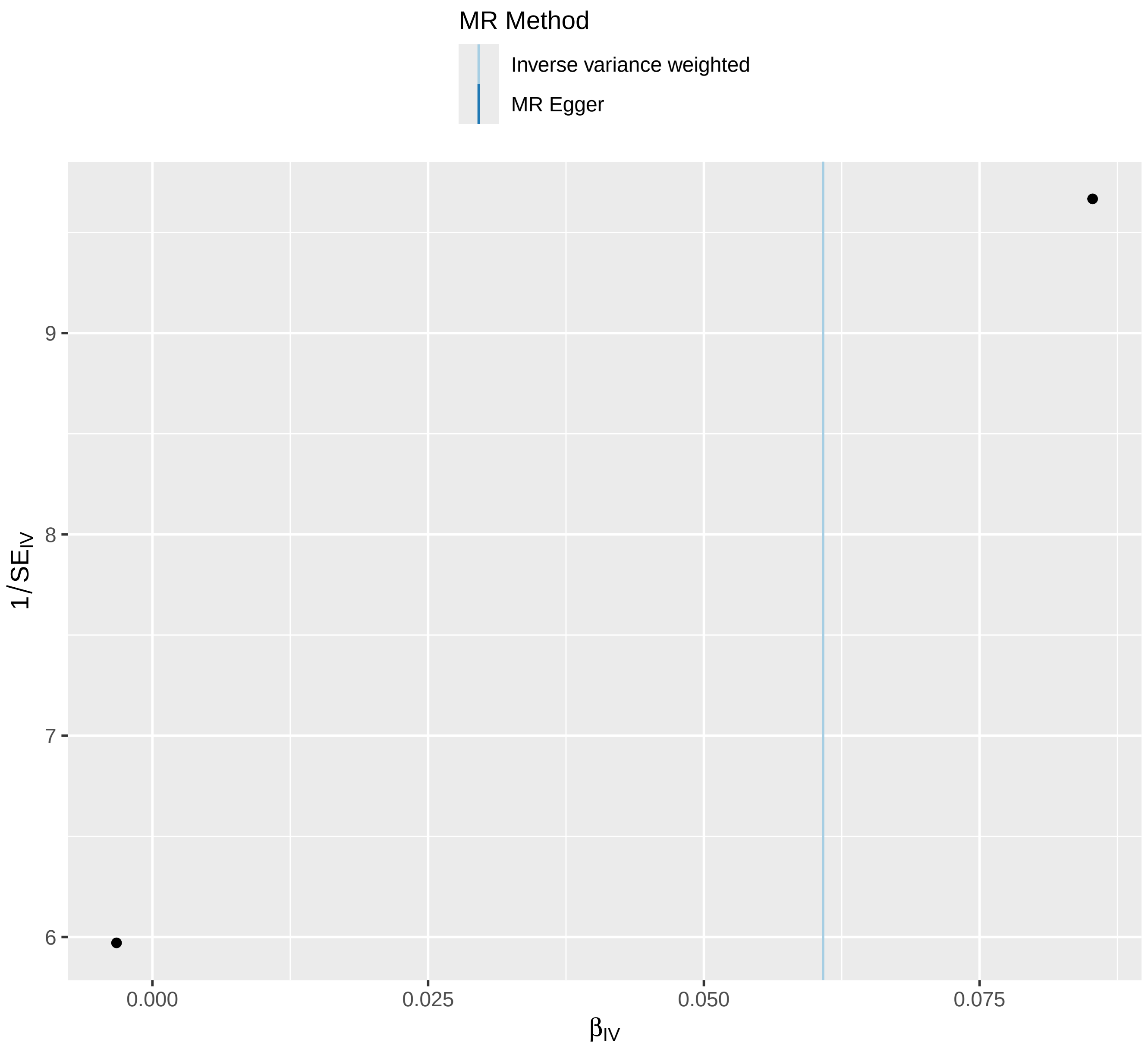

MR Method
Inverse variance weighted
MR Egger
9
8
7
6
1/SE$_{IV}$
0.000
0.025
0.050
0.075
β$_{IV}$

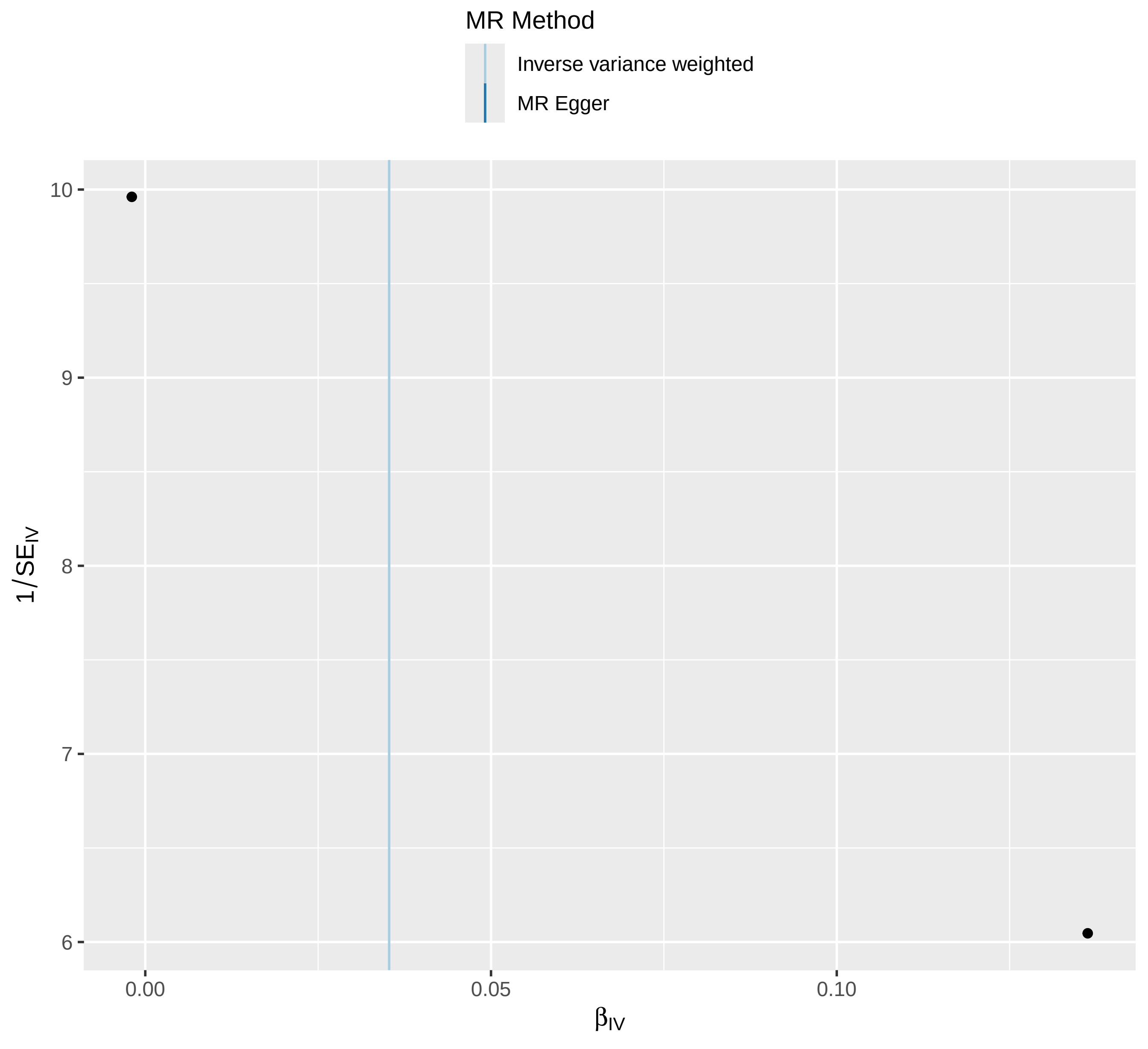
MR Method
Inverse variance weighted
MR Egger
10
9
8
7
6
1/SE_IV
0.00
0.05
0.10
β_IV

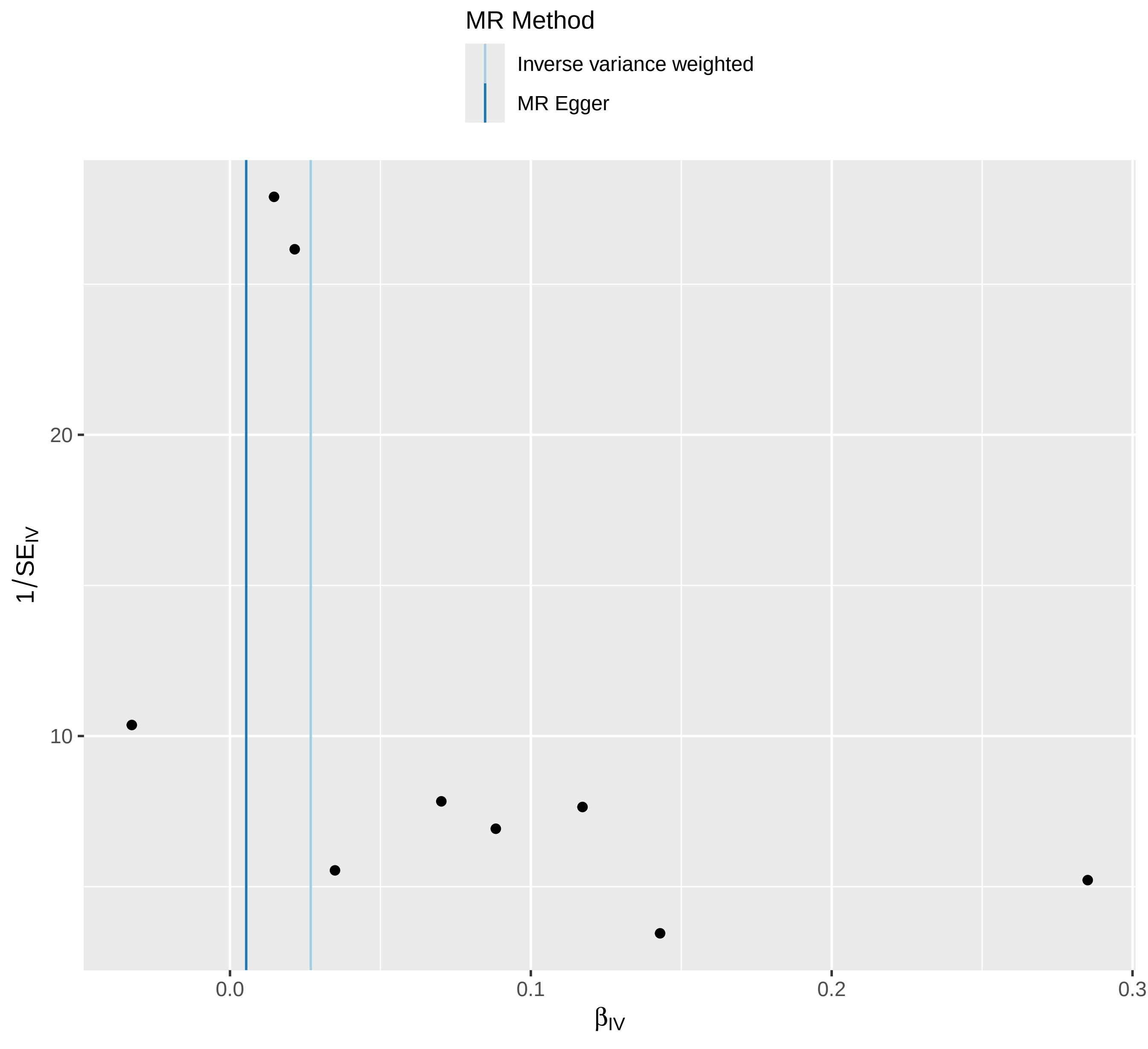

MR Method
Inverse variance weighted
MR Egger
20
10
$1/SE_{IV}$
0.0
0.1
0.2
0.3
$\beta_{IV}$

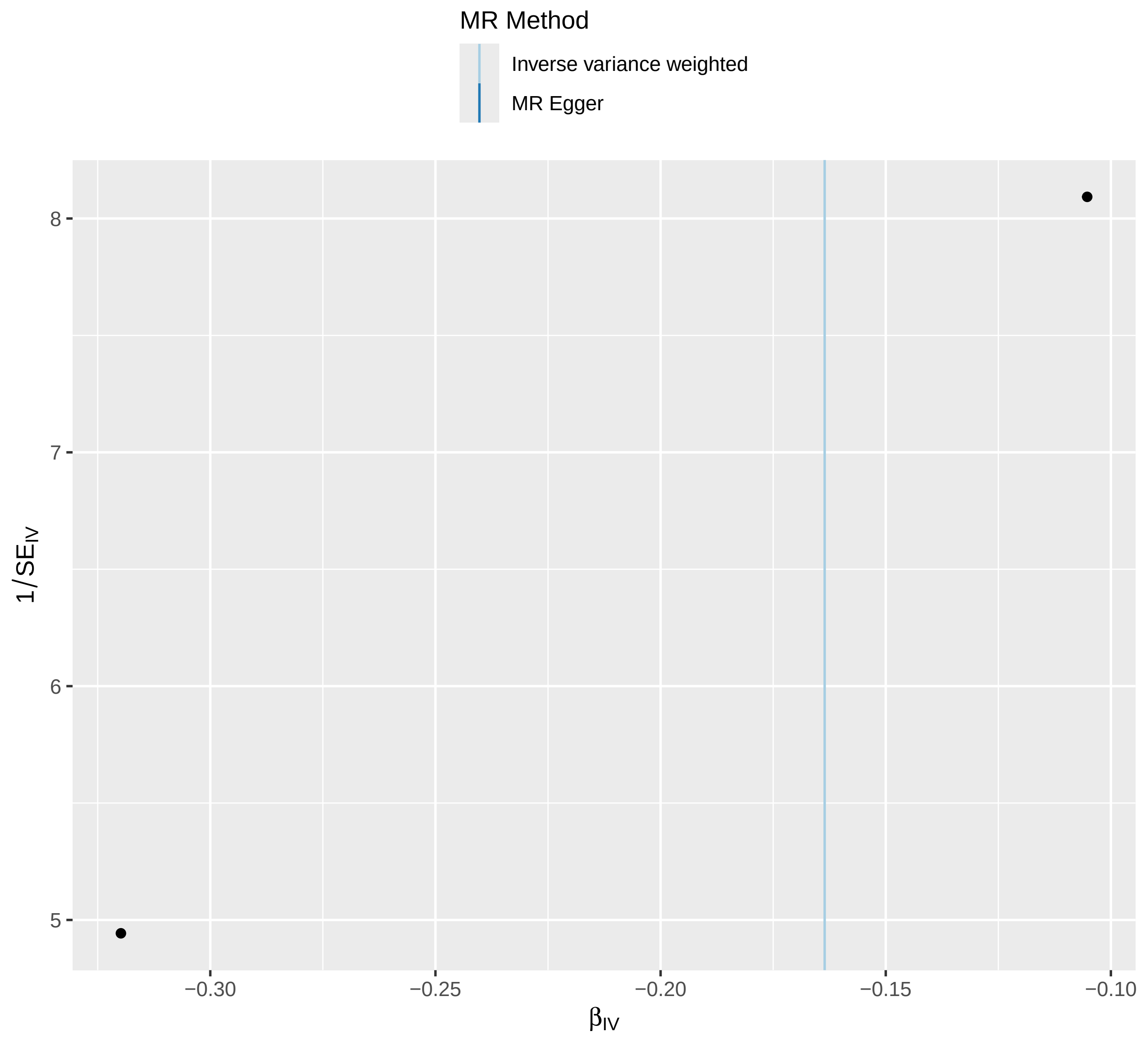
MR Method
Inverse variance weighted
MR Egger
8
7
6
5
1/SE_IV
−0.30
−0.25
−0.20
−0.15
−0.10
β_IV

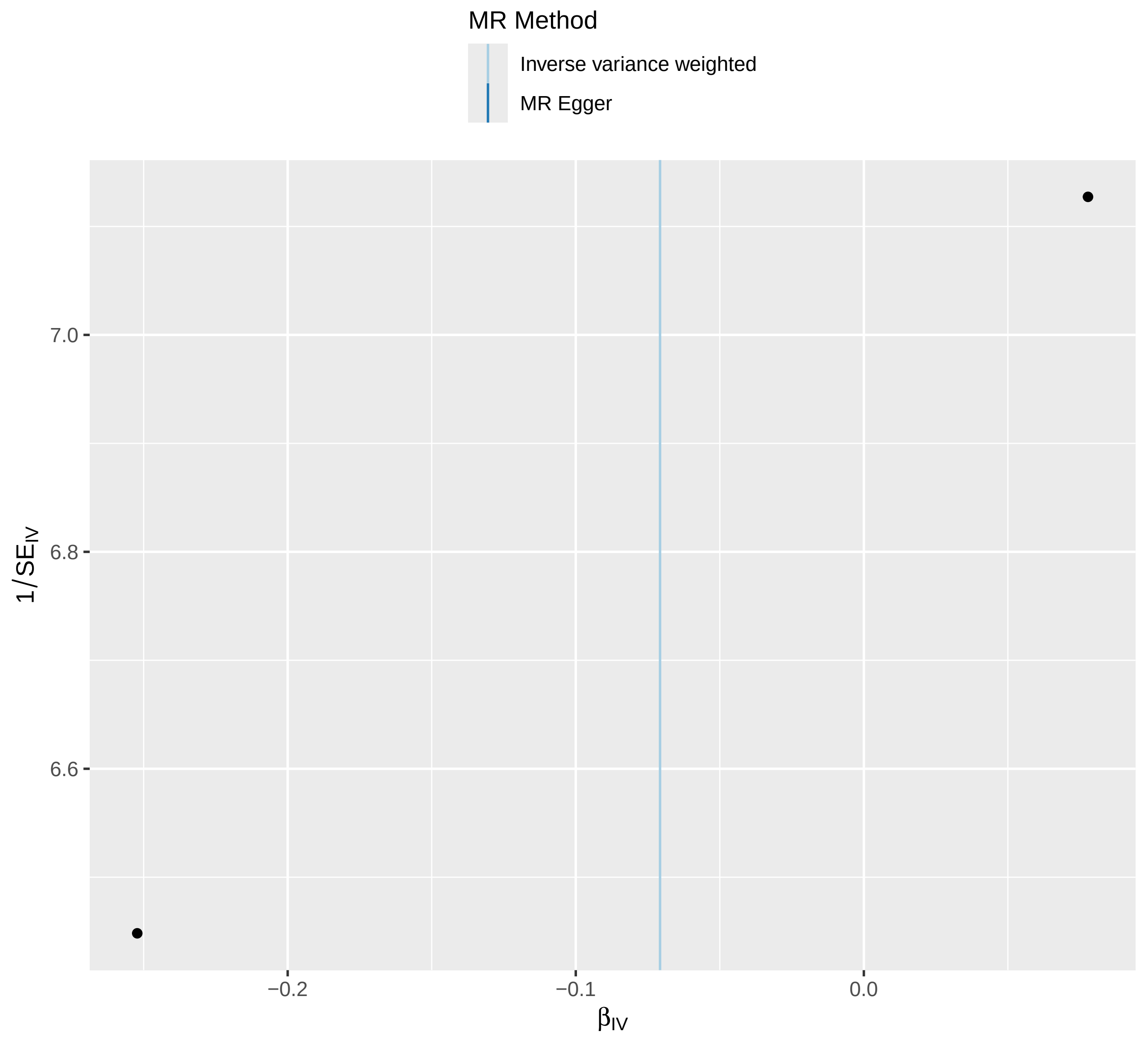

MR Method
Inverse variance weighted
MR Egger
7.0
6.8
6.6
1/SE_IV
−0.2
−0.1
0.0
β_IV

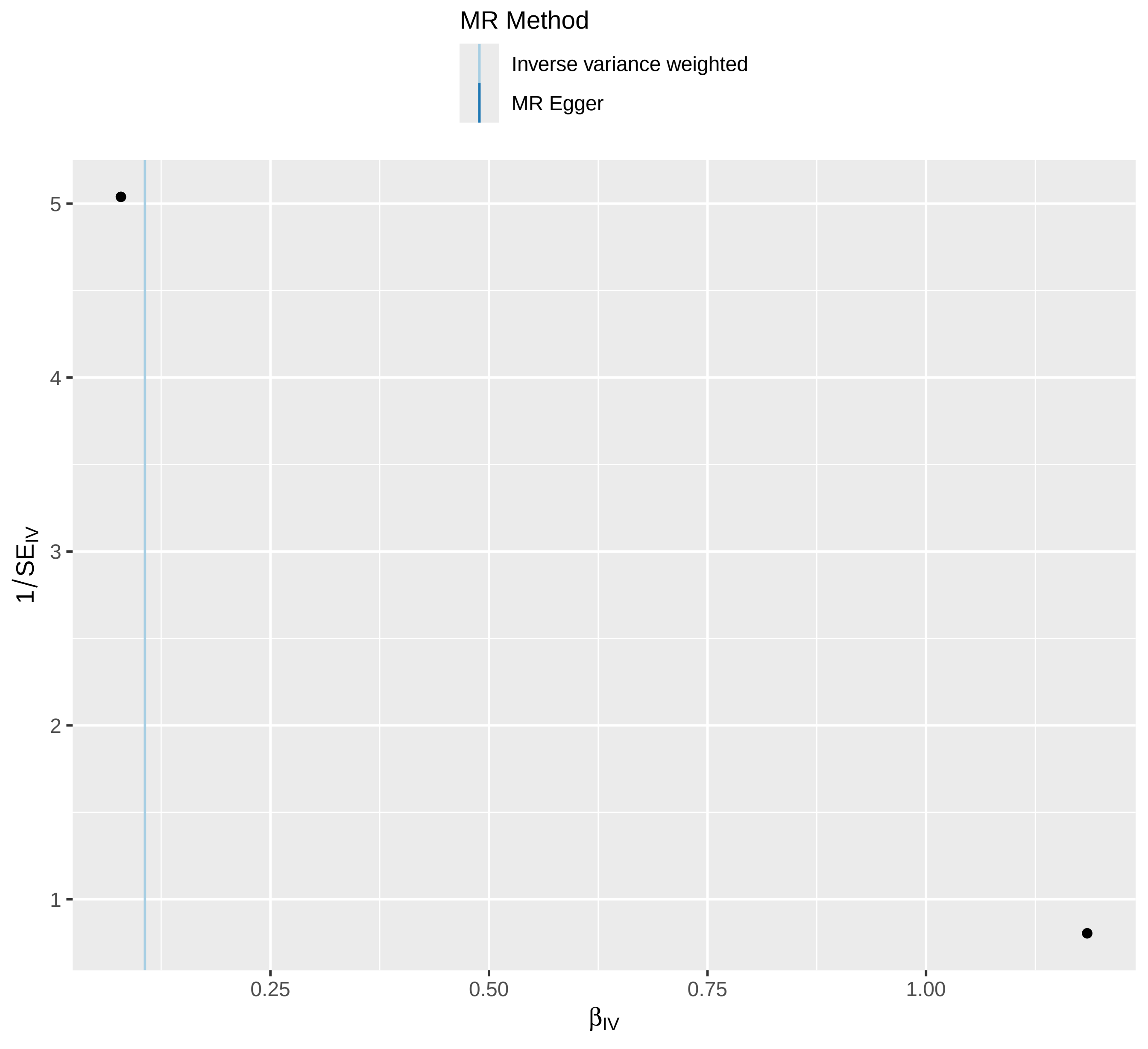
MR Method
Inverse variance weighted
MR Egger
1/SE$_{IV}$
5
4
3
2
1
0.25
0.50
0.75
1.00
β$_{IV}$

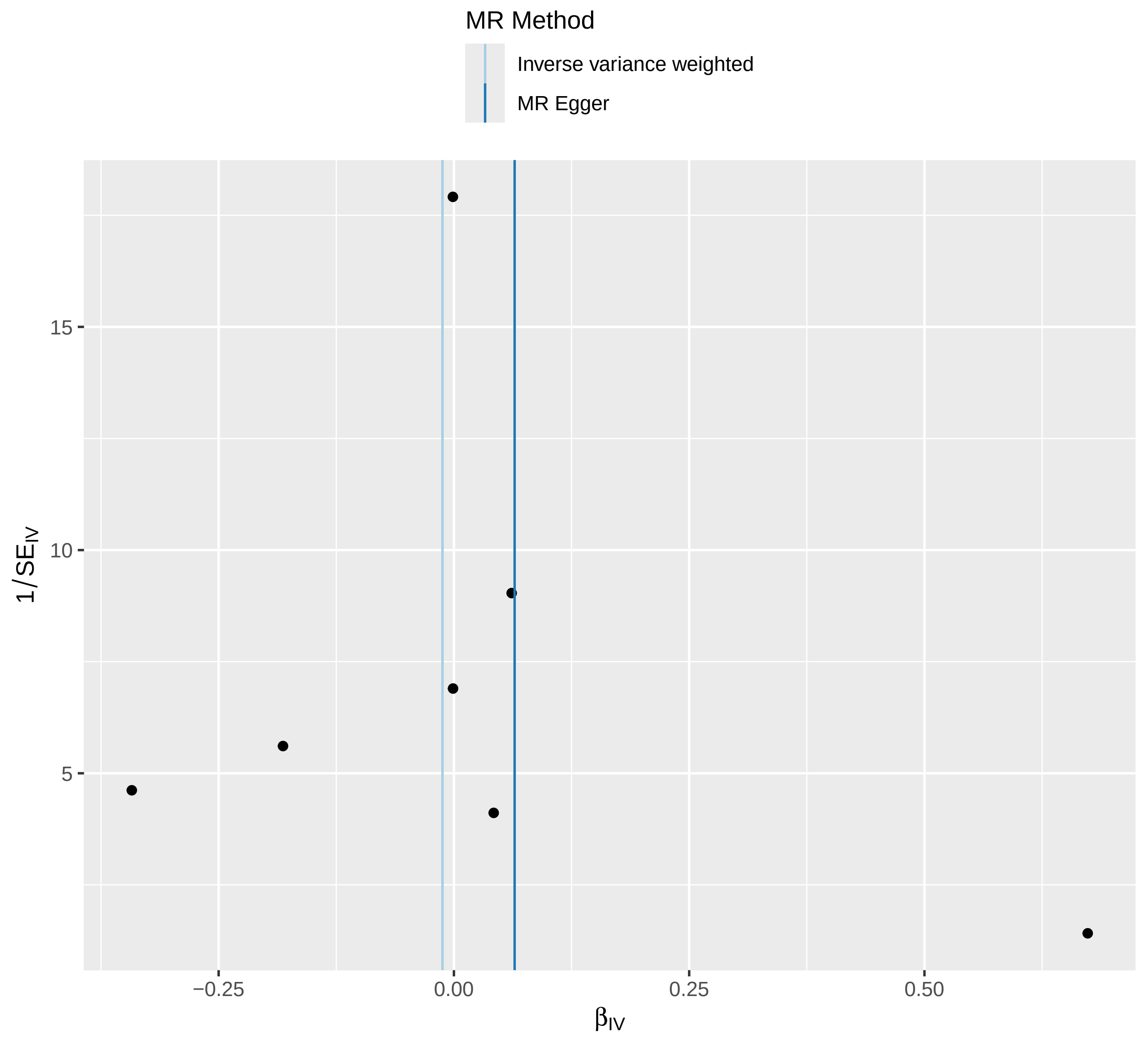
MR Method
Inverse variance weighted
MR Egger
15
10
5
1/SE_IV
−0.25
0.00
0.25
0.50
β_IV

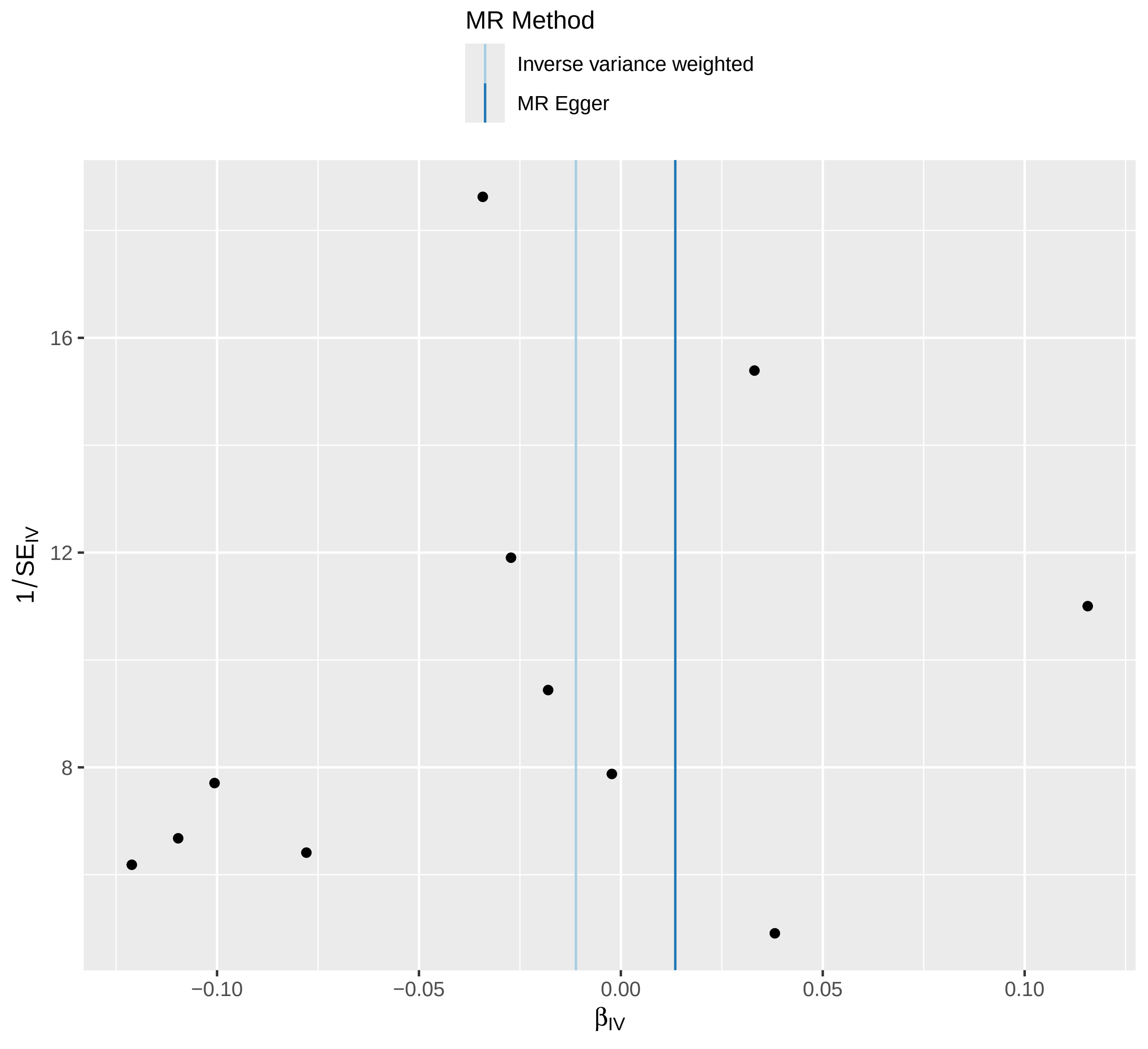

MR Method
Inverse variance weighted
MR Egger
16
12
8
1/SE$_{IV}$
−0.10
−0.05
0.00
0.05
0.10
$\beta_{IV}$

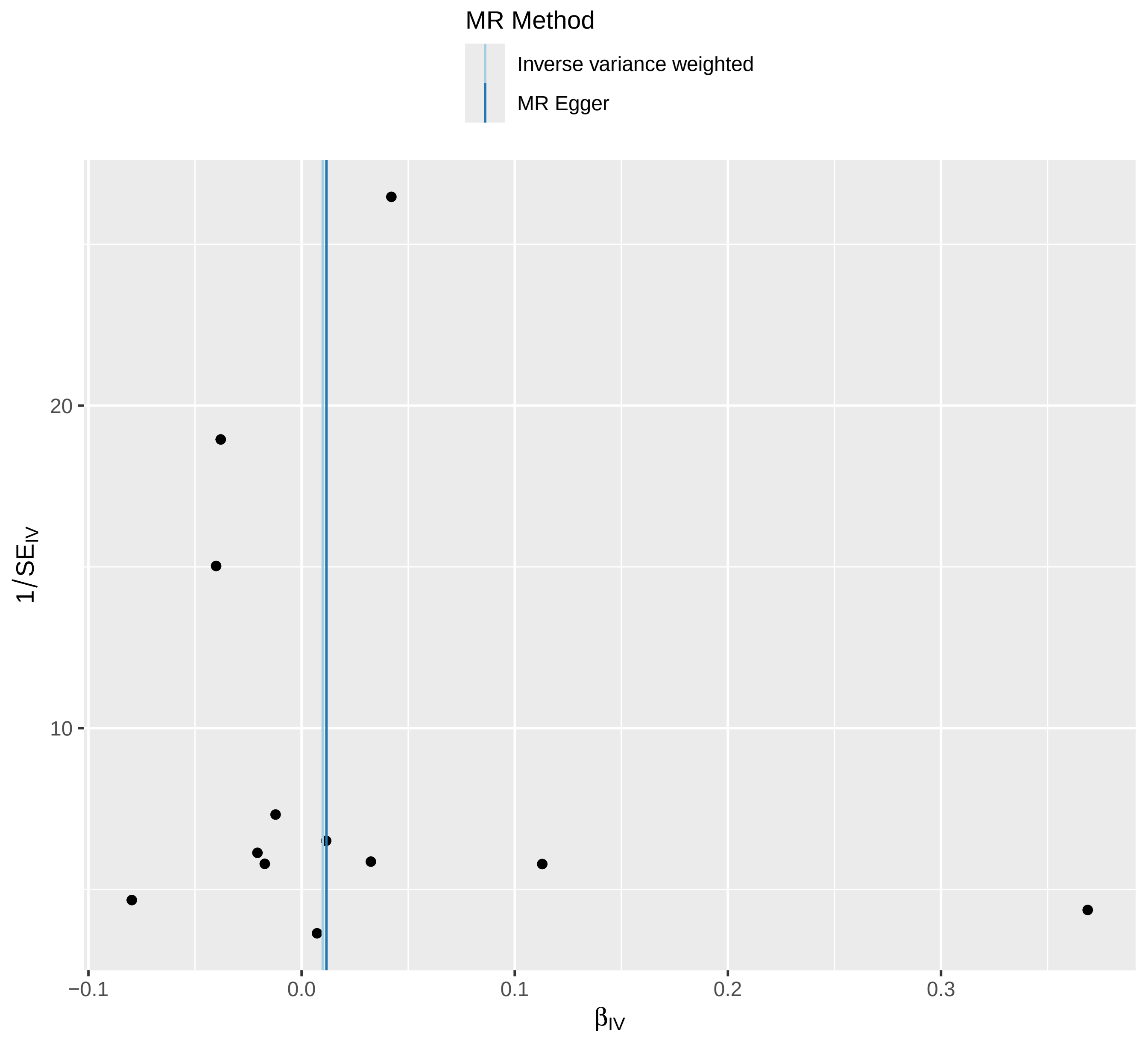
MR Method
Inverse variance weighted
MR Egger
20
10
1/SE$_{IV}$
−0.1
0.0
0.1
0.2
0.3
β$_{IV}$

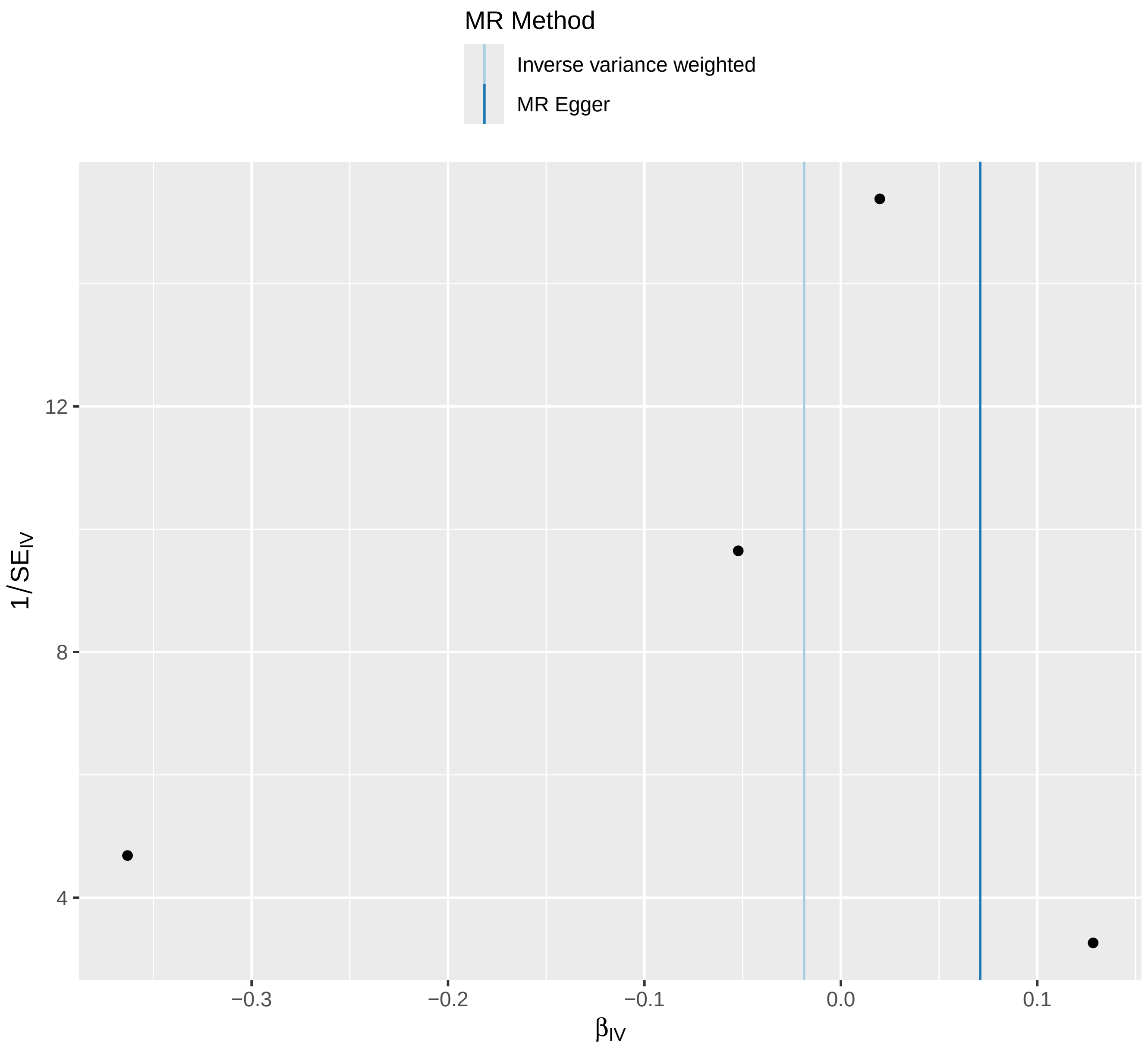
MR Method
Inverse variance weighted
MR Egger
1/SE$_{IV}$
12
8
4
−0.3
−0.2
−0.1
0.0
0.1
β$_{IV}$

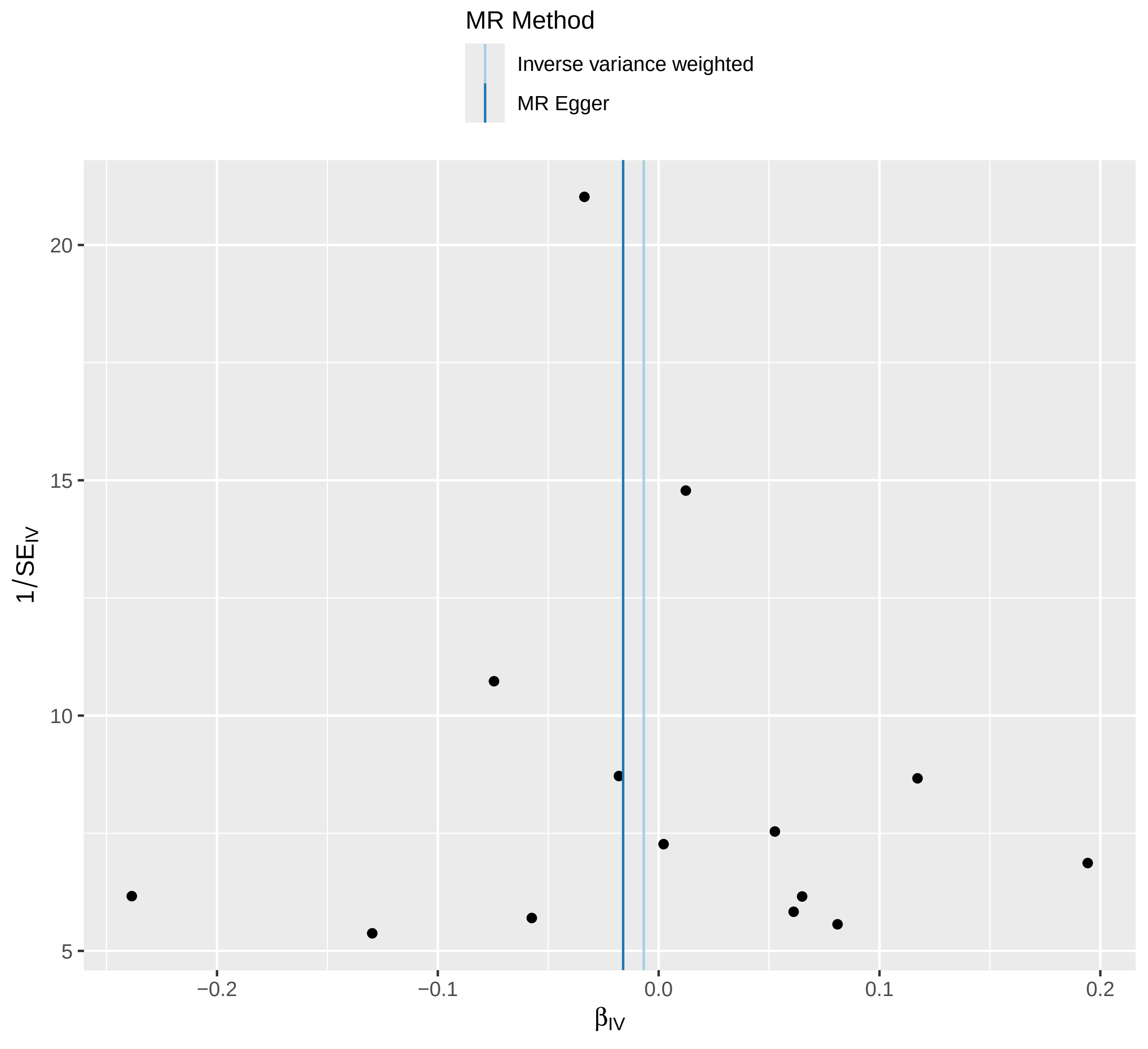
MR Method
Inverse variance weighted
MR Egger
20
15
10
5
1/SE_IV
−0.2
−0.1
0.0
0.1
0.2
β_IV

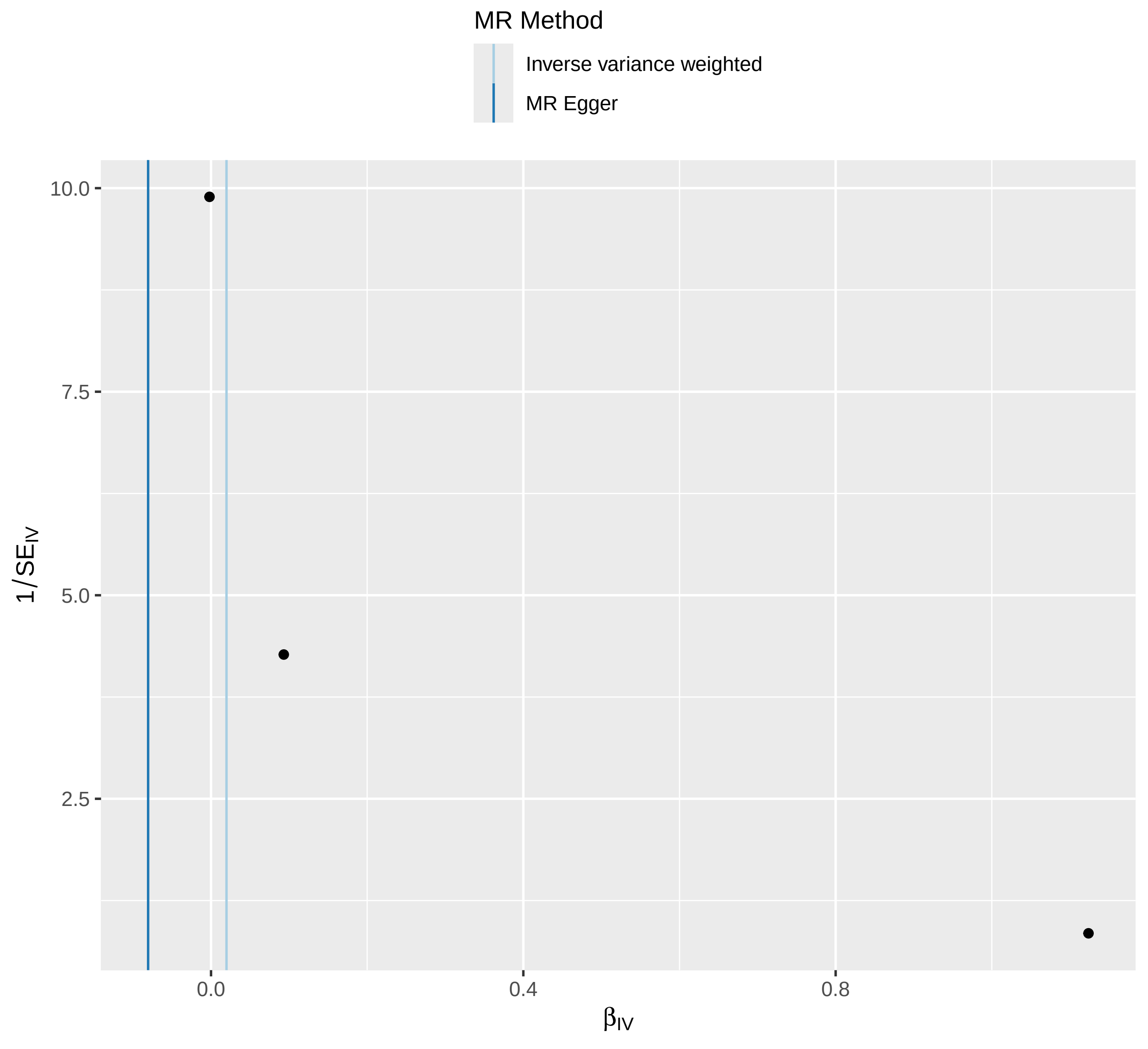

MR Method
Inverse variance weighted
MR Egger
10.0
7.5
5.0
2.5
1/SE_IV
0.0
0.4
0.8
β_IV

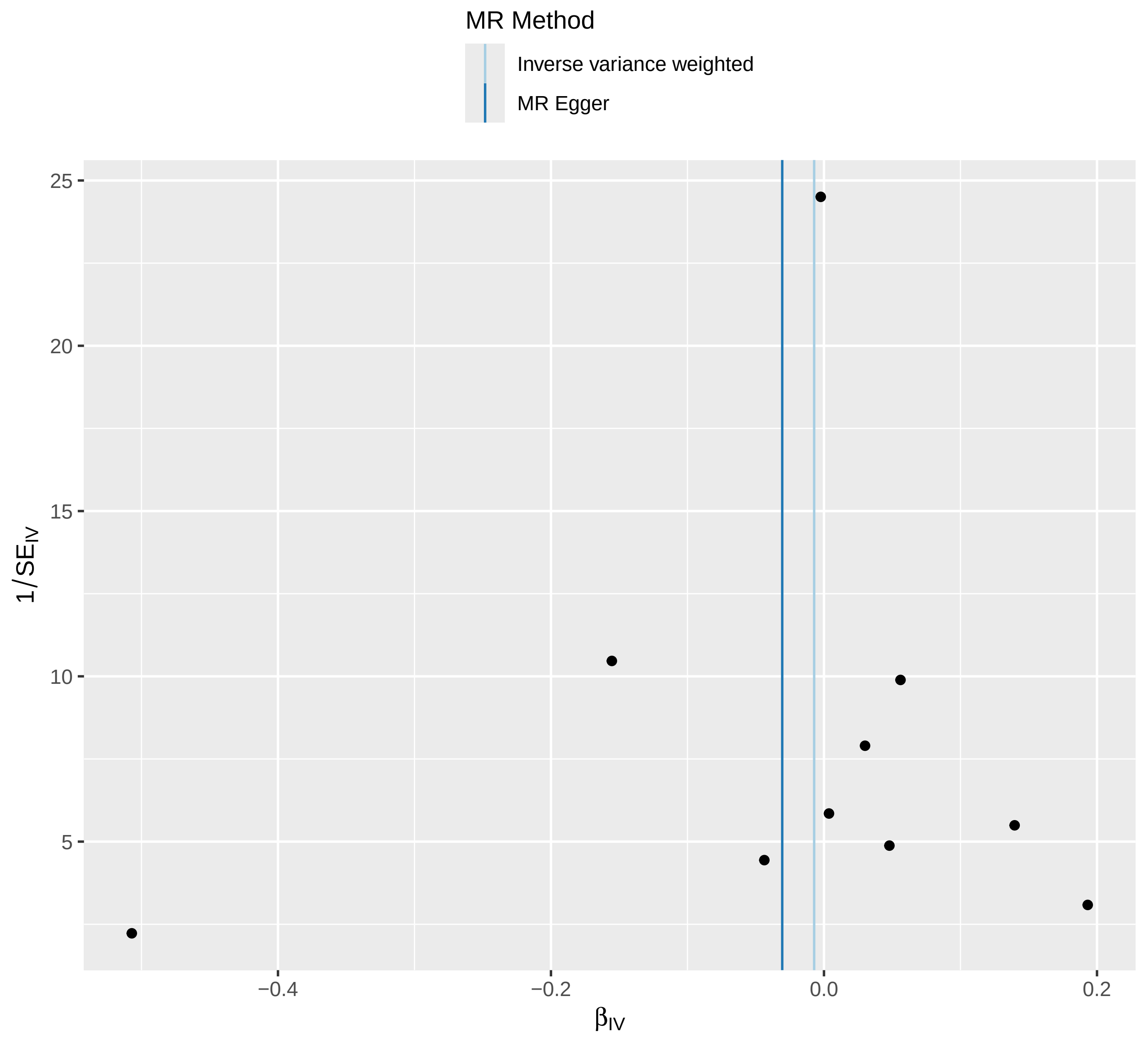

MR Method
Inverse variance weighted
MR Egger
25
20
15
10
5
1/SE_IV
−0.4
−0.2
0.0
0.2
β_IV

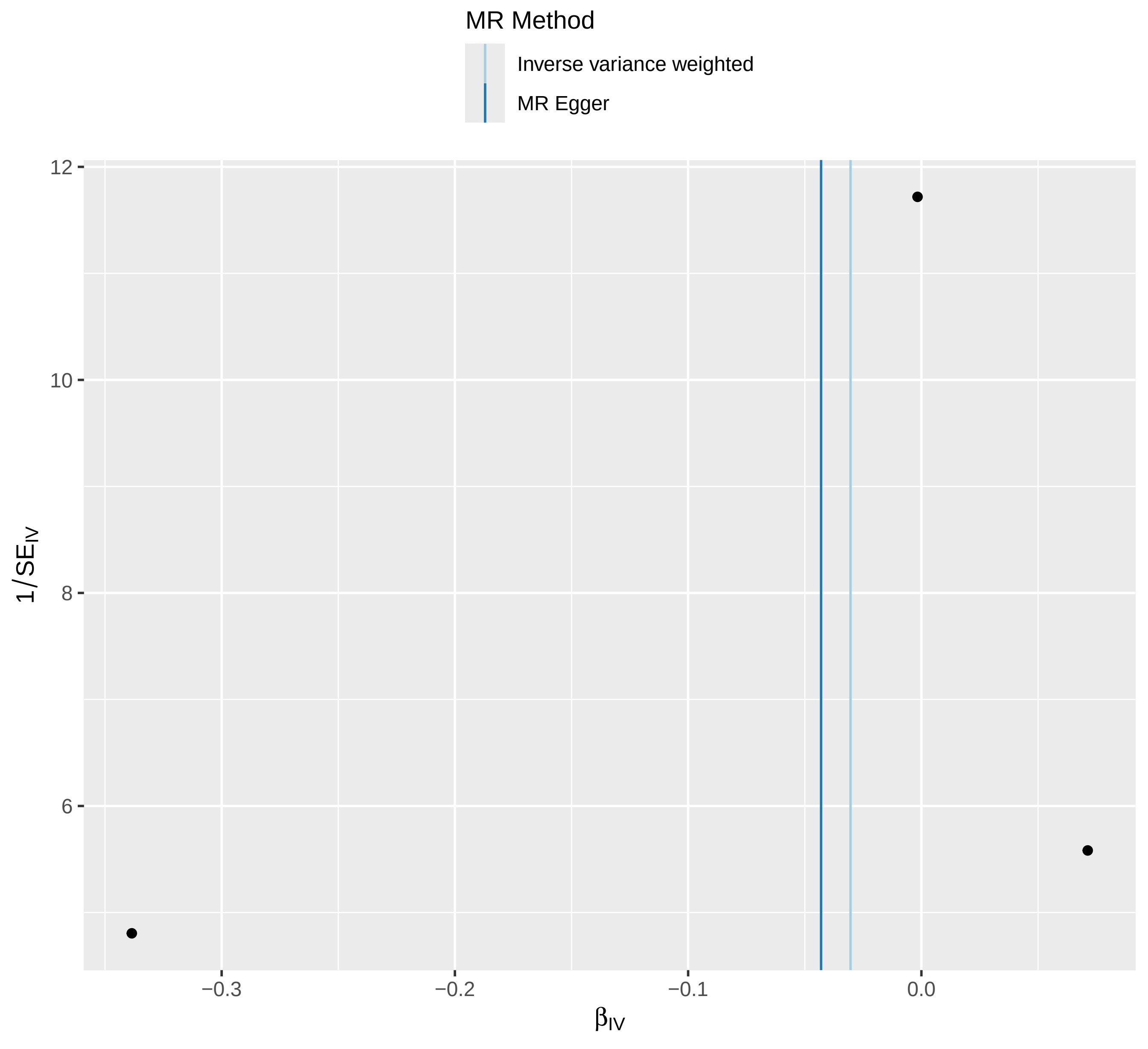
MR Method
Inverse variance weighted
MR Egger
12
10
8
6
1/SE$_{IV}$
−0.3
−0.2
−0.1
0.0
β$_{IV}$

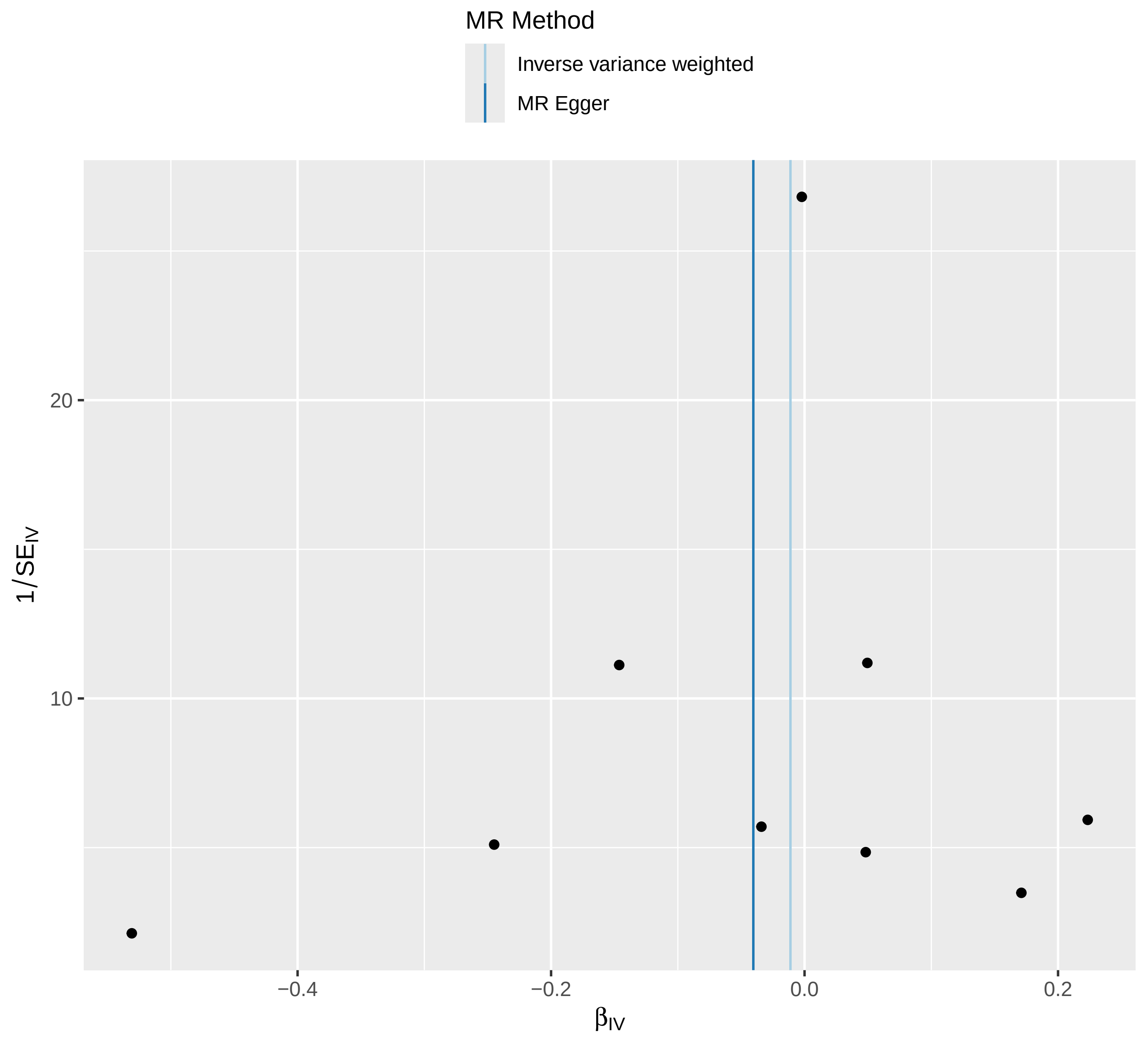

MR Method
Inverse variance weighted
MR Egger
20
10
1/SE_IV
−0.4
−0.2
0.0
0.2
β_IV

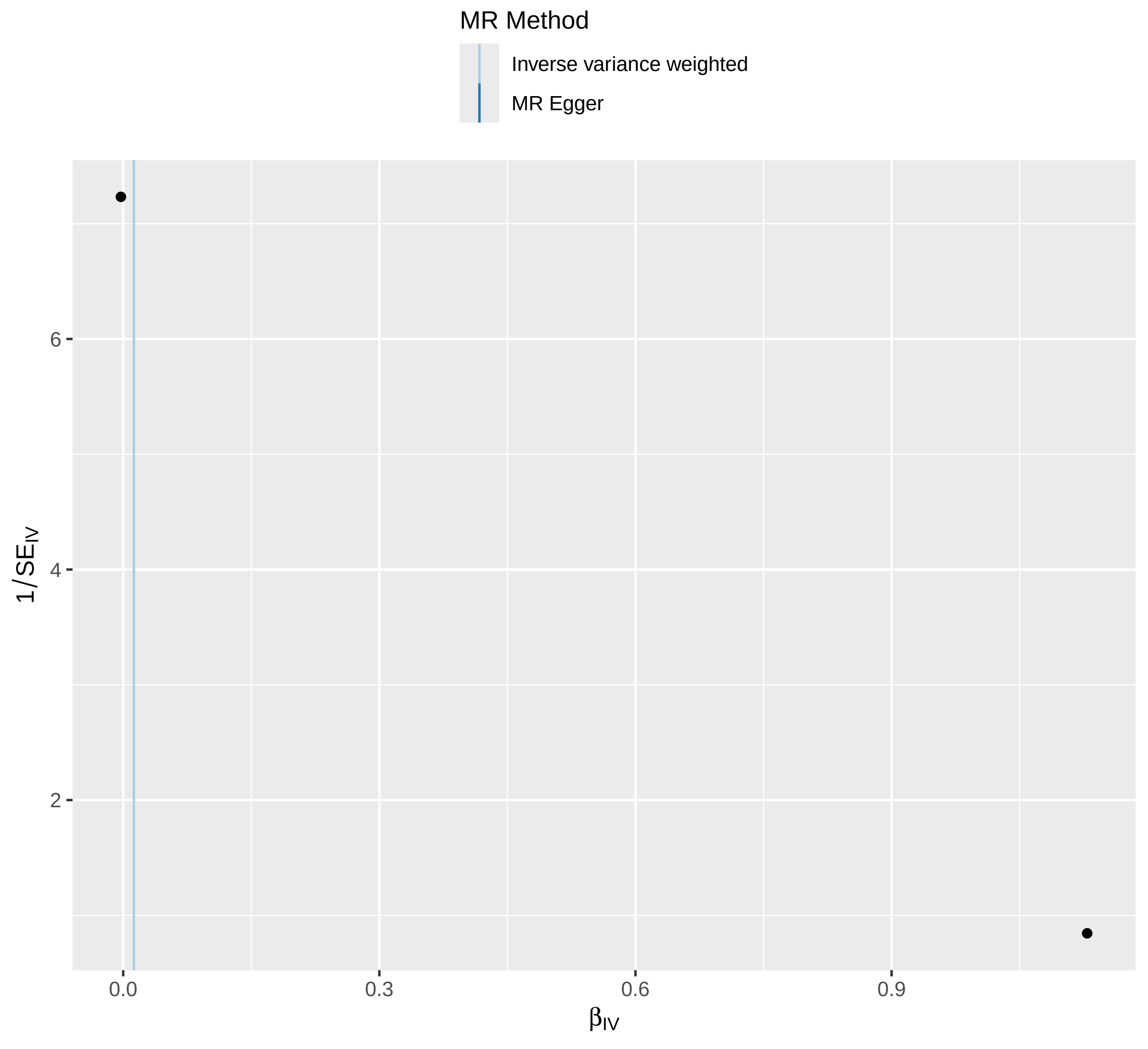
MR Method
Inverse variance weighted
MR Egger
6
4
2
1/SE_IV
0.0
0.3
0.6
0.9
β_IV

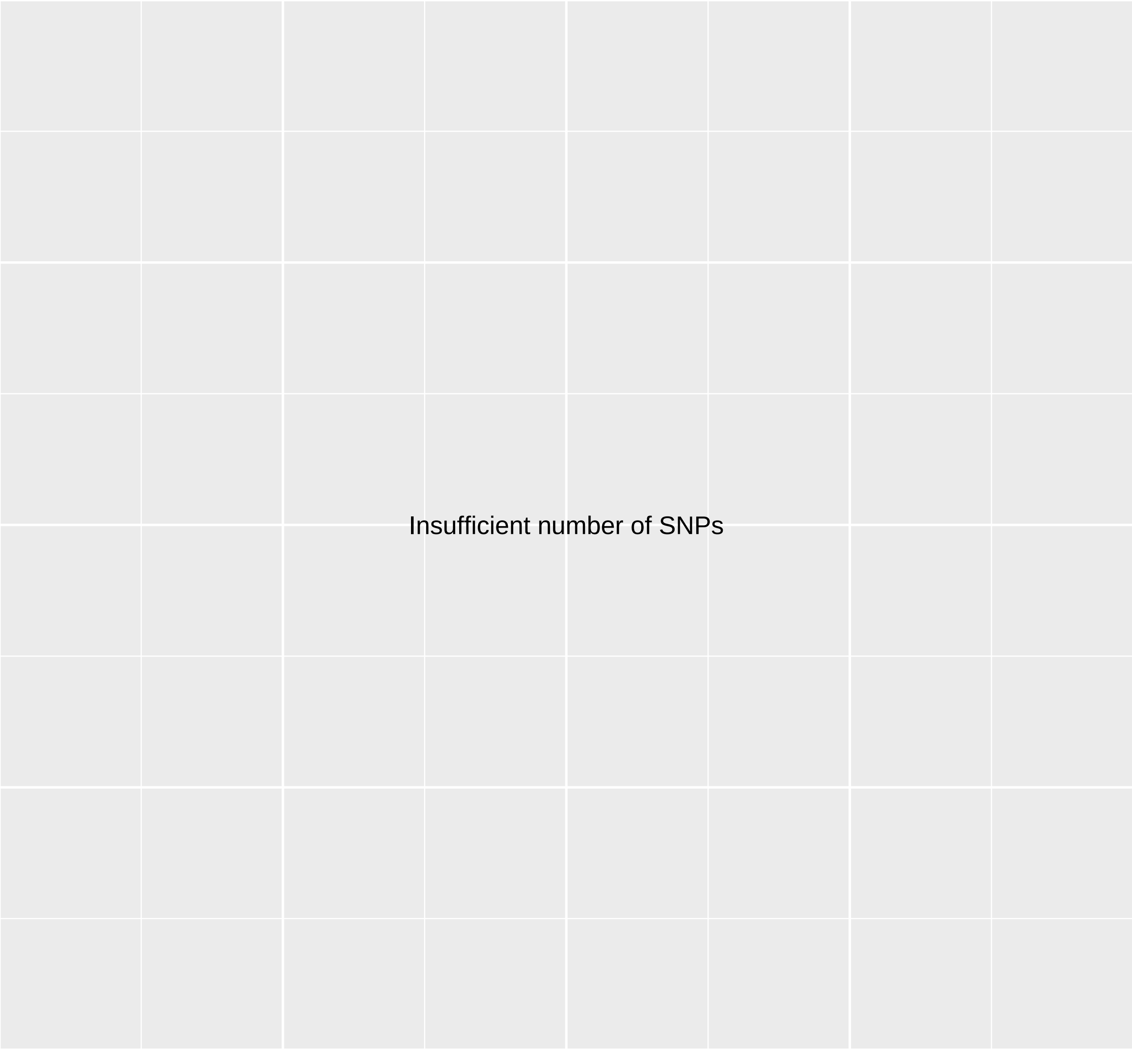
Insufficient number of SNPs

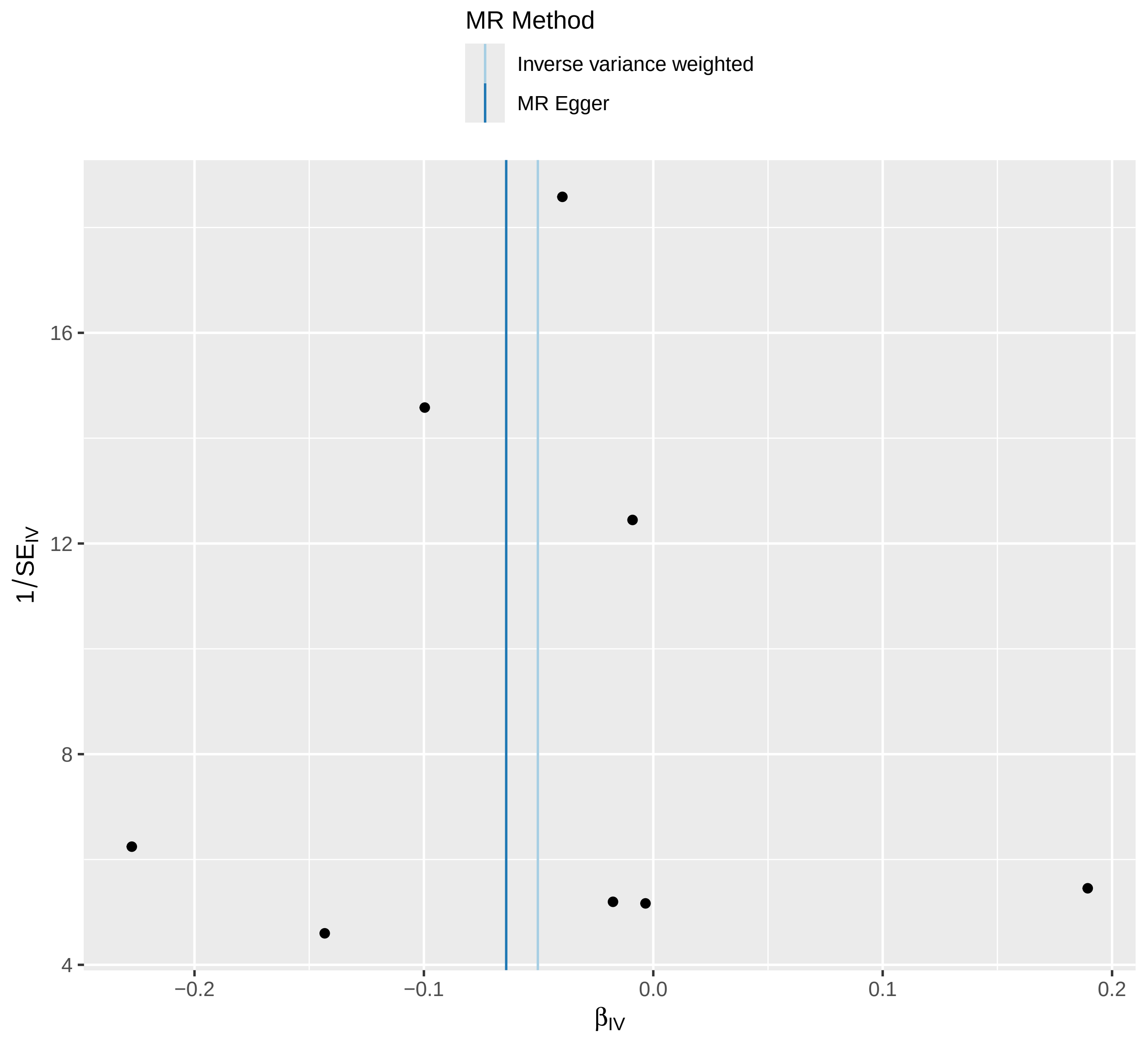

MR Method
Inverse variance weighted
MR Egger
16
12
8
4
−0.2
−0.1
0.0
0.1
0.2
$1/SE_{IV}$
$\beta_{IV}$

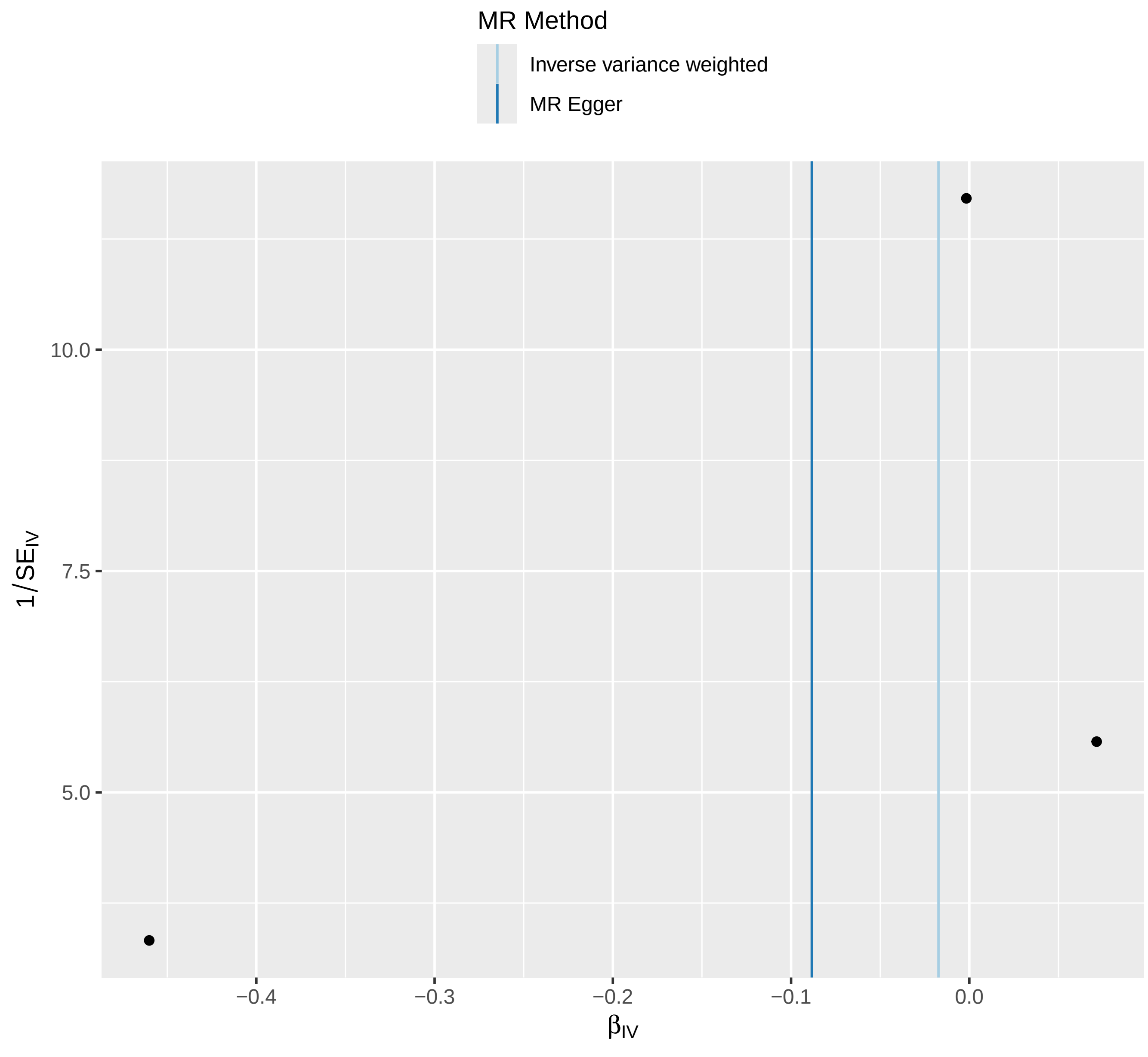

MR Method
Inverse variance weighted
MR Egger
10.0
7.5
5.0
1/SE$_{IV}$
−0.4
−0.3
−0.2
−0.1
0.0
β$_{IV}$

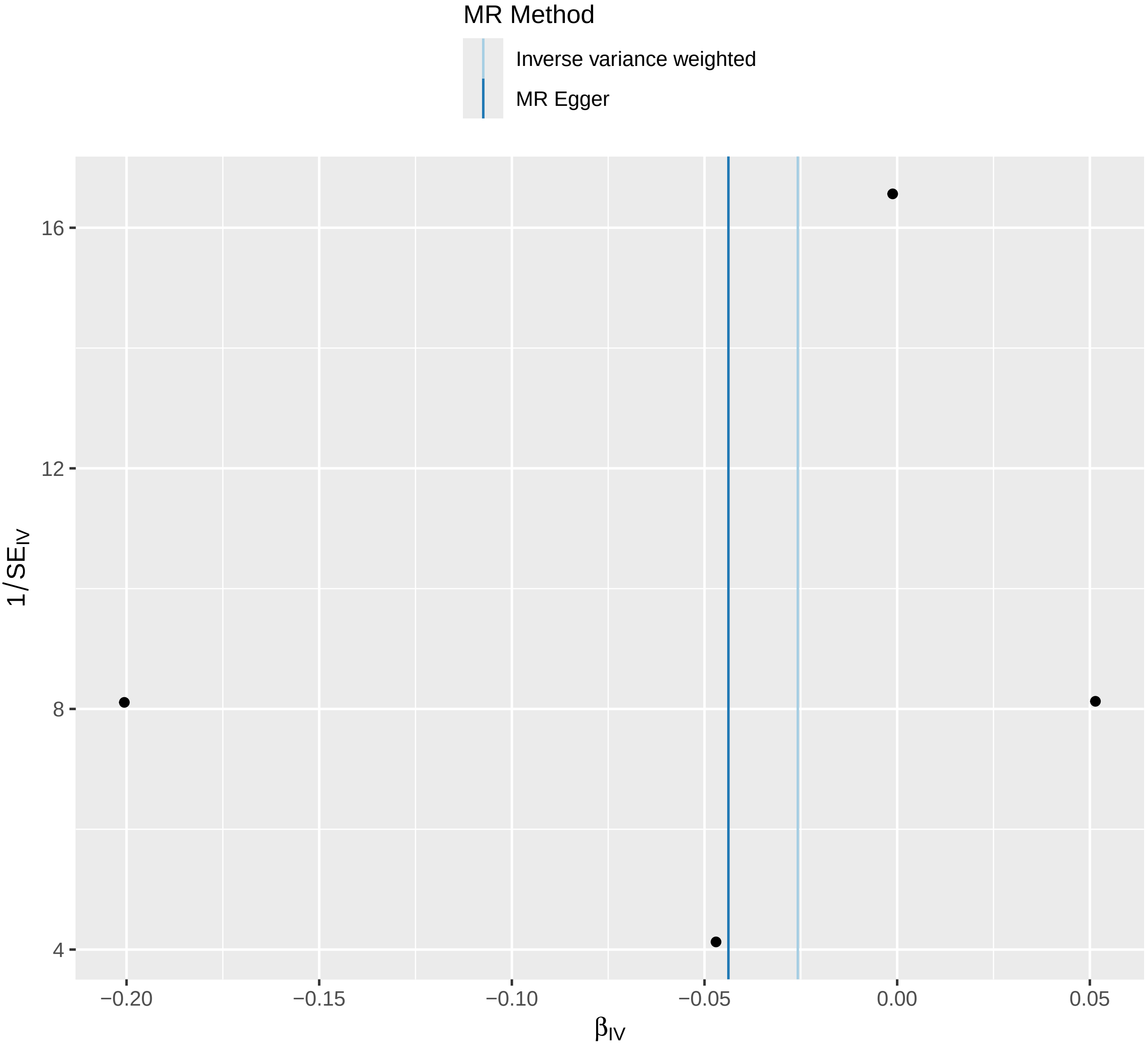

MR Method
Inverse variance weighted
MR Egger
16
12
8
4
1/SE_IV
−0.20
−0.15
−0.10
−0.05
0.00
0.05
β_IV

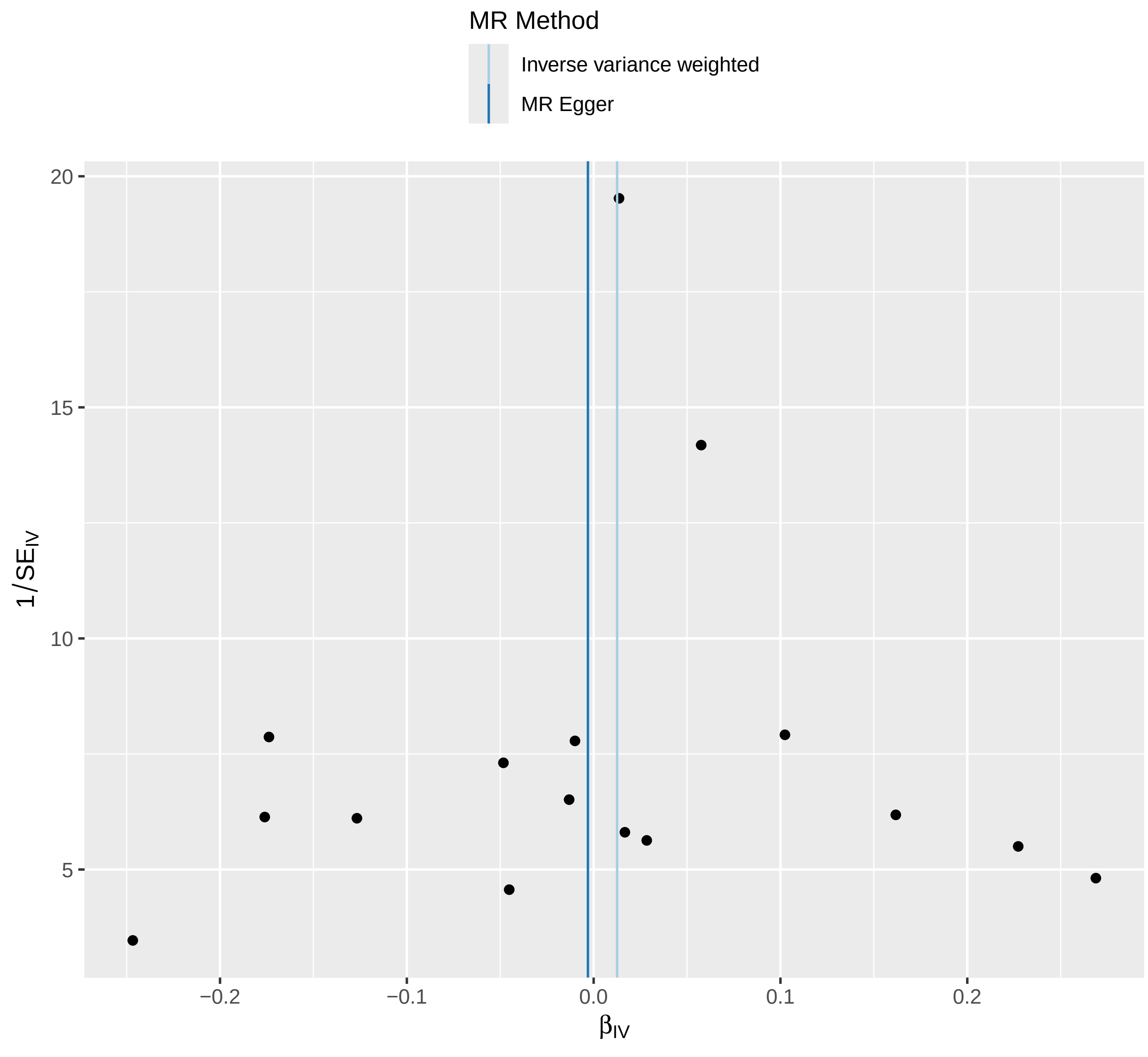
MR Method
Inverse variance weighted
MR Egger
20
15
10
5
−0.2
−0.1
0.0
0.1
0.2
$1/SE_{IV}$
$\beta_{IV}$

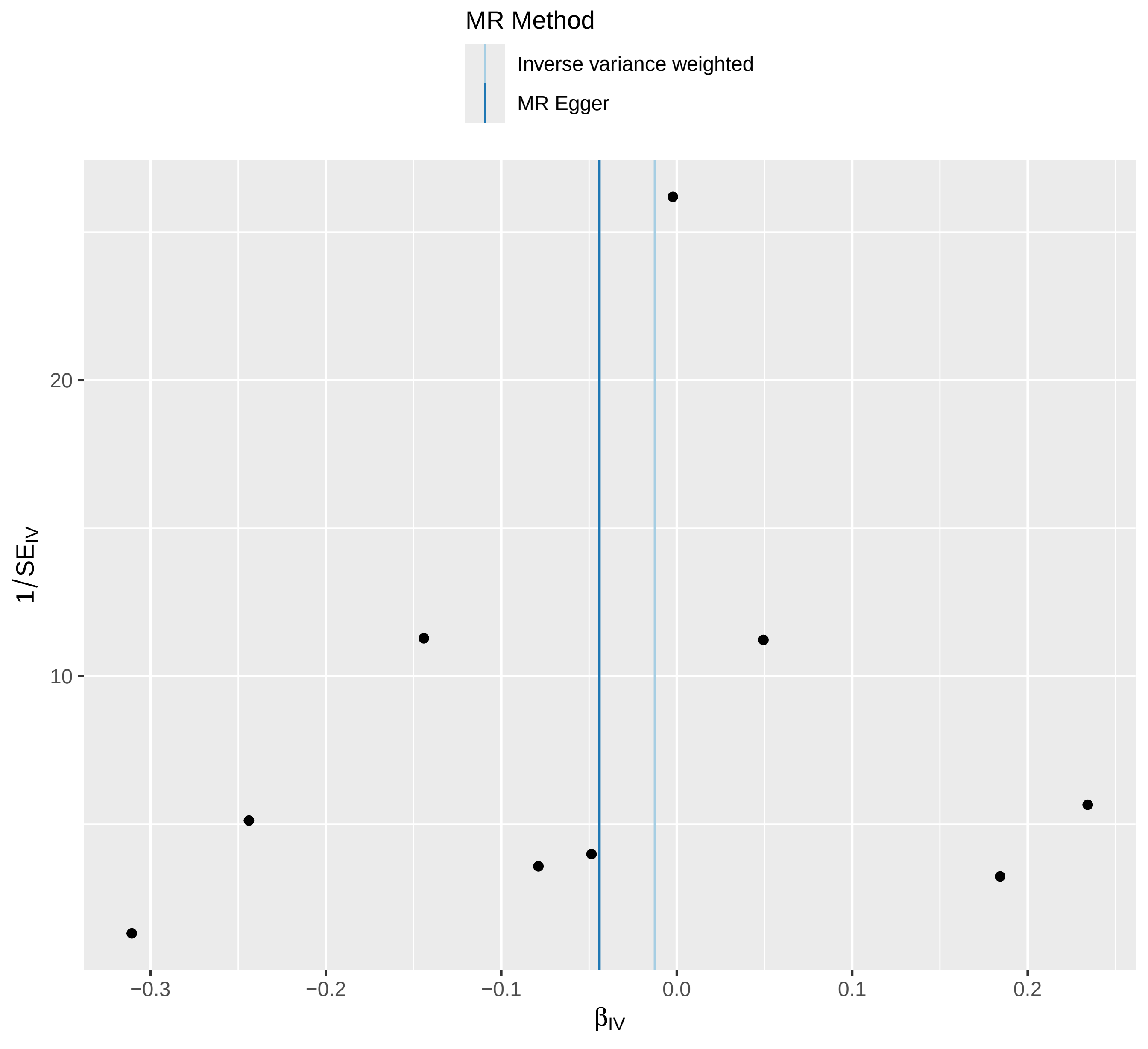
MR Method
Inverse variance weighted
MR Egger
20
10
1/SE$_{IV}$
−0.3
−0.2
−0.1
0.0
0.1
0.2
β$_{IV}$

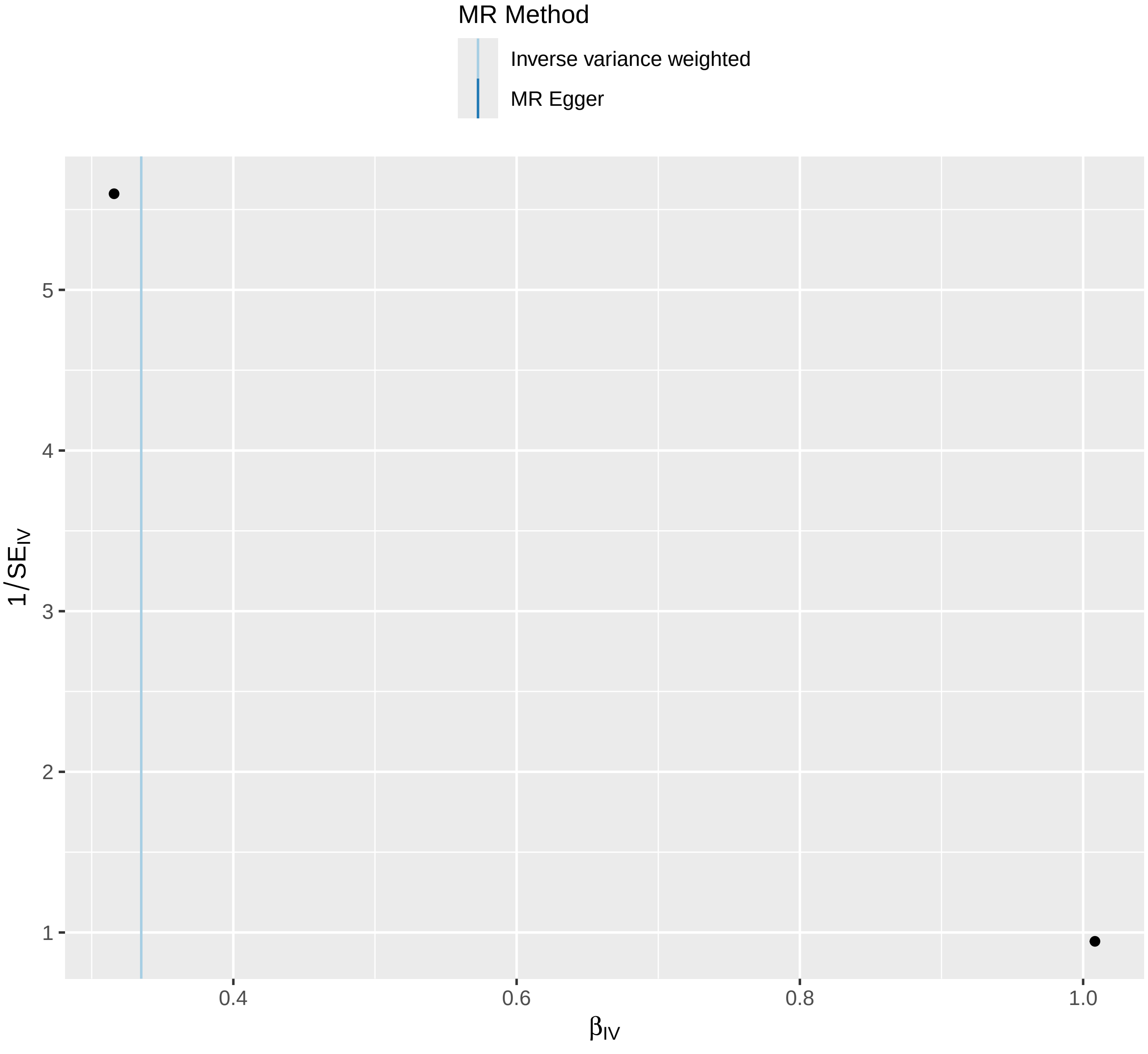
MR Method
Inverse variance weighted
MR Egger
1/SE_IV
5
4
3
2
1
0.4
0.6
0.8
1.0
β_IV

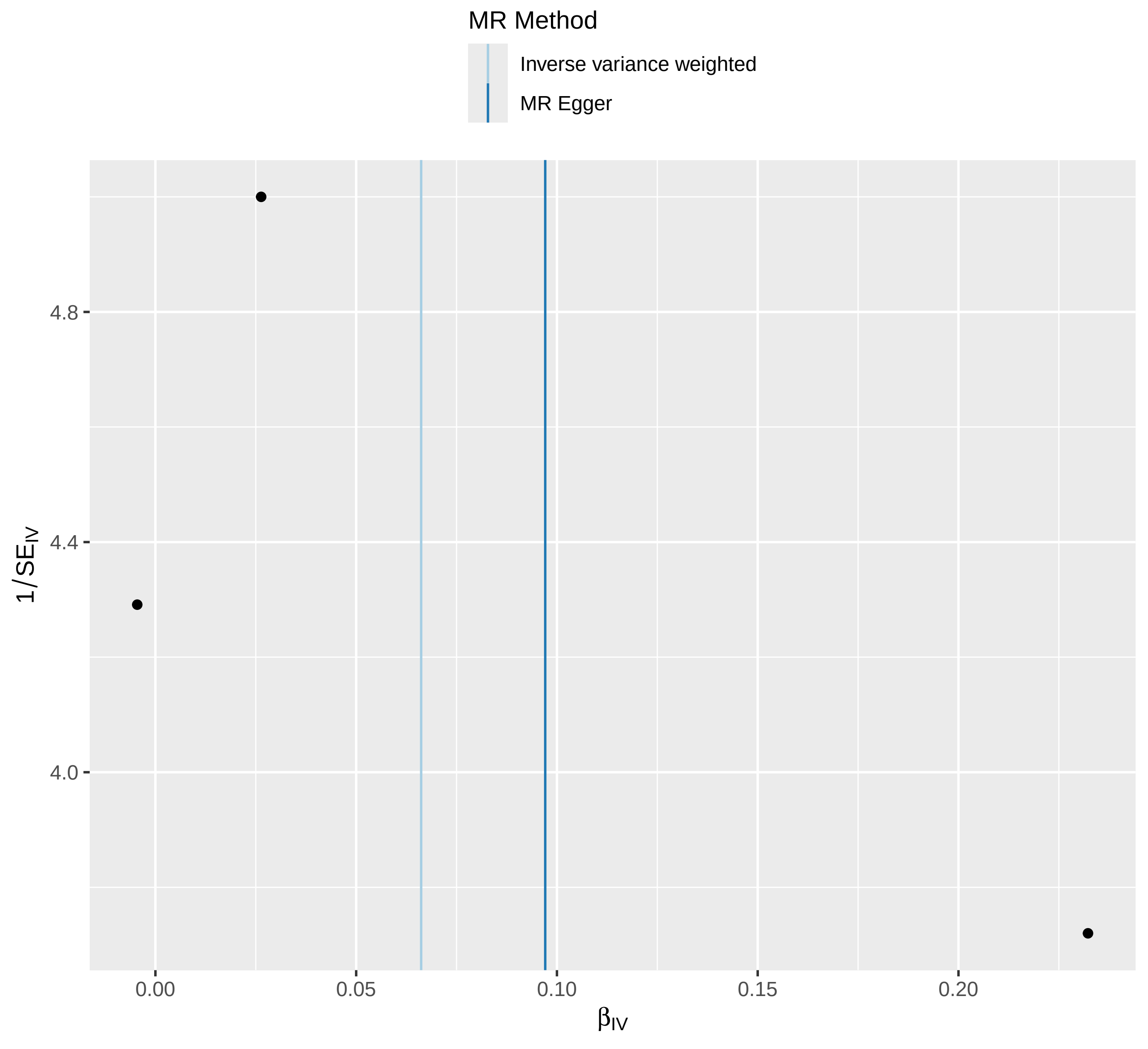

MR Method
Inverse variance weighted
MR Egger
4.8
4.4
4.0
1/SE$_{IV}$
0.00
0.05
0.10
0.15
0.20
β$_{IV}$

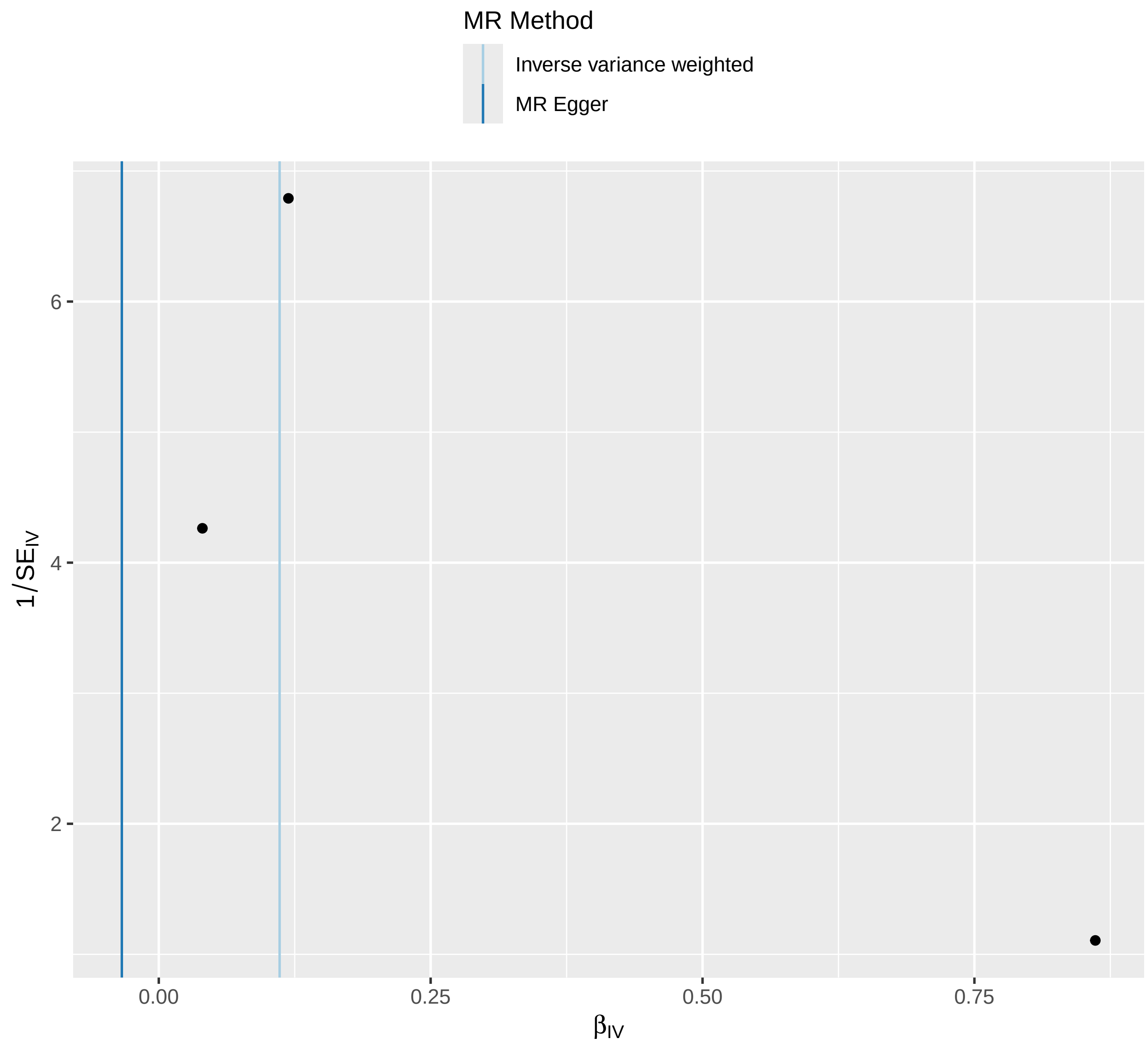
MR Method
Inverse variance weighted
MR Egger
6
4
2
1/SE_IV
0.00
0.25
0.50
0.75
β_IV

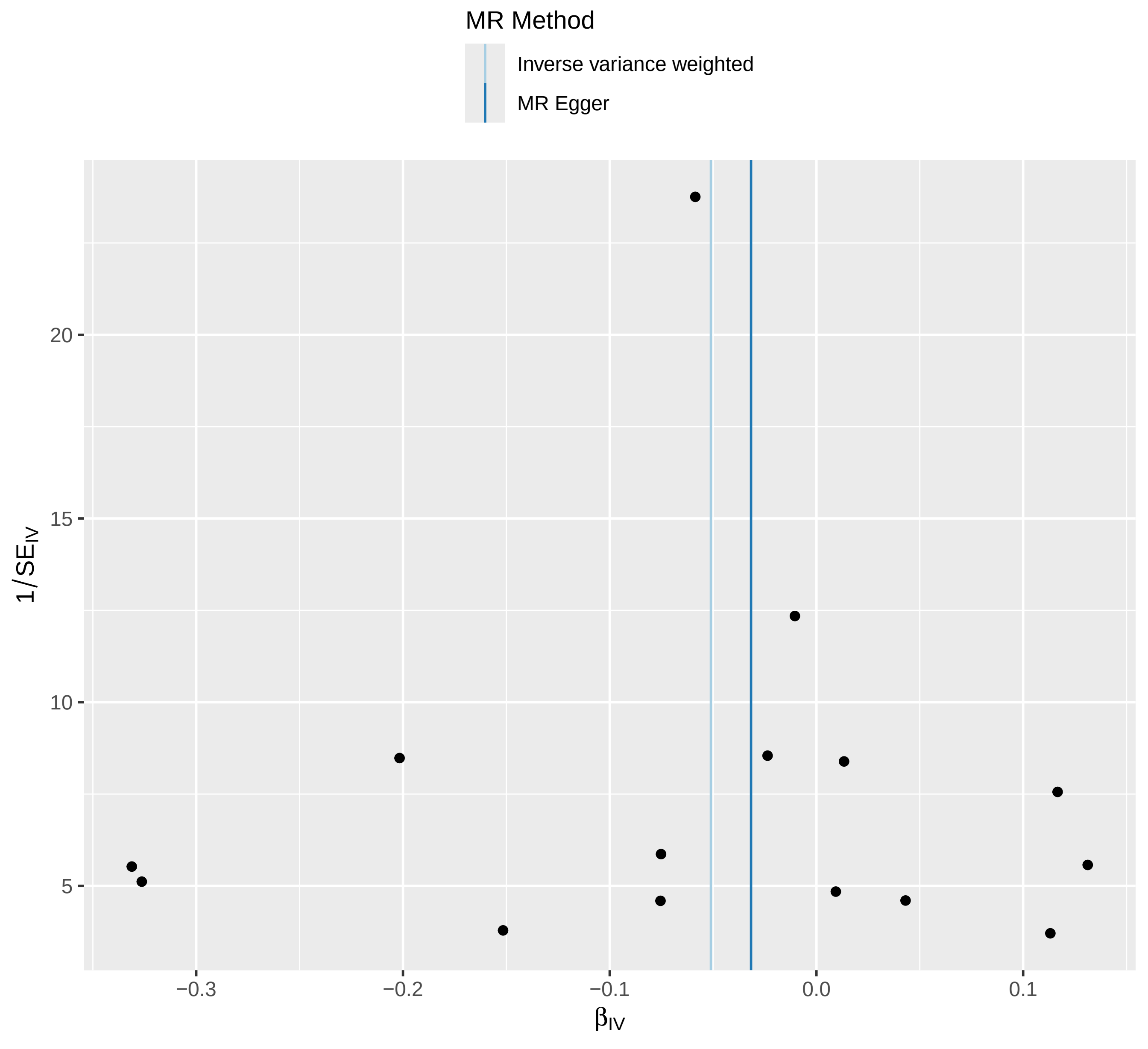
MR Method
Inverse variance weighted
MR Egger
20
15
10
5
$1/SE_{IV}$
−0.3
−0.2
−0.1
0.0
0.1
$\beta_{IV}$

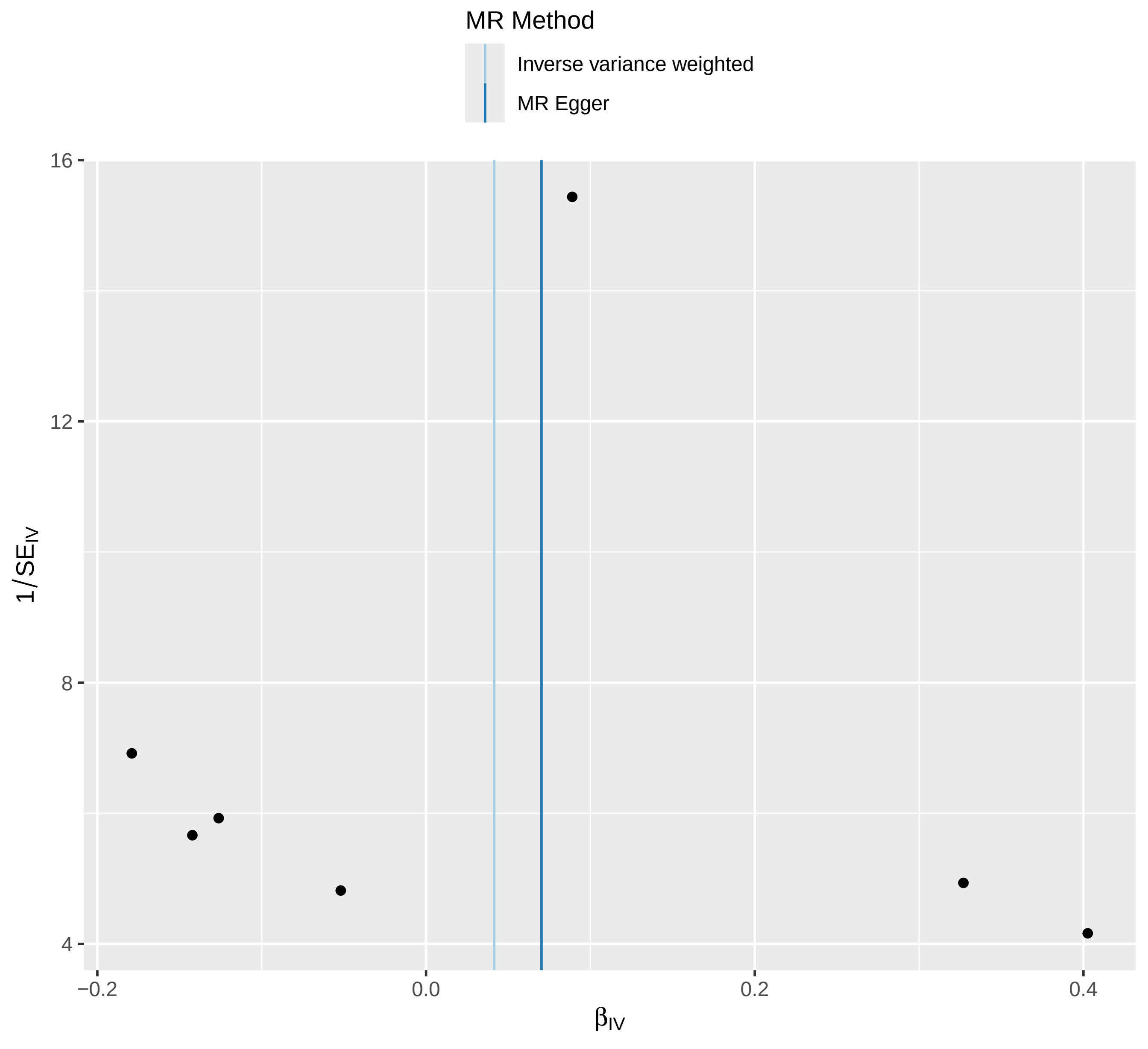
MR Method
Inverse variance weighted
MR Egger
16
12
8
4
$1/SE_{IV}$
−0.2
0.0
0.2
0.4
$\beta_{IV}$

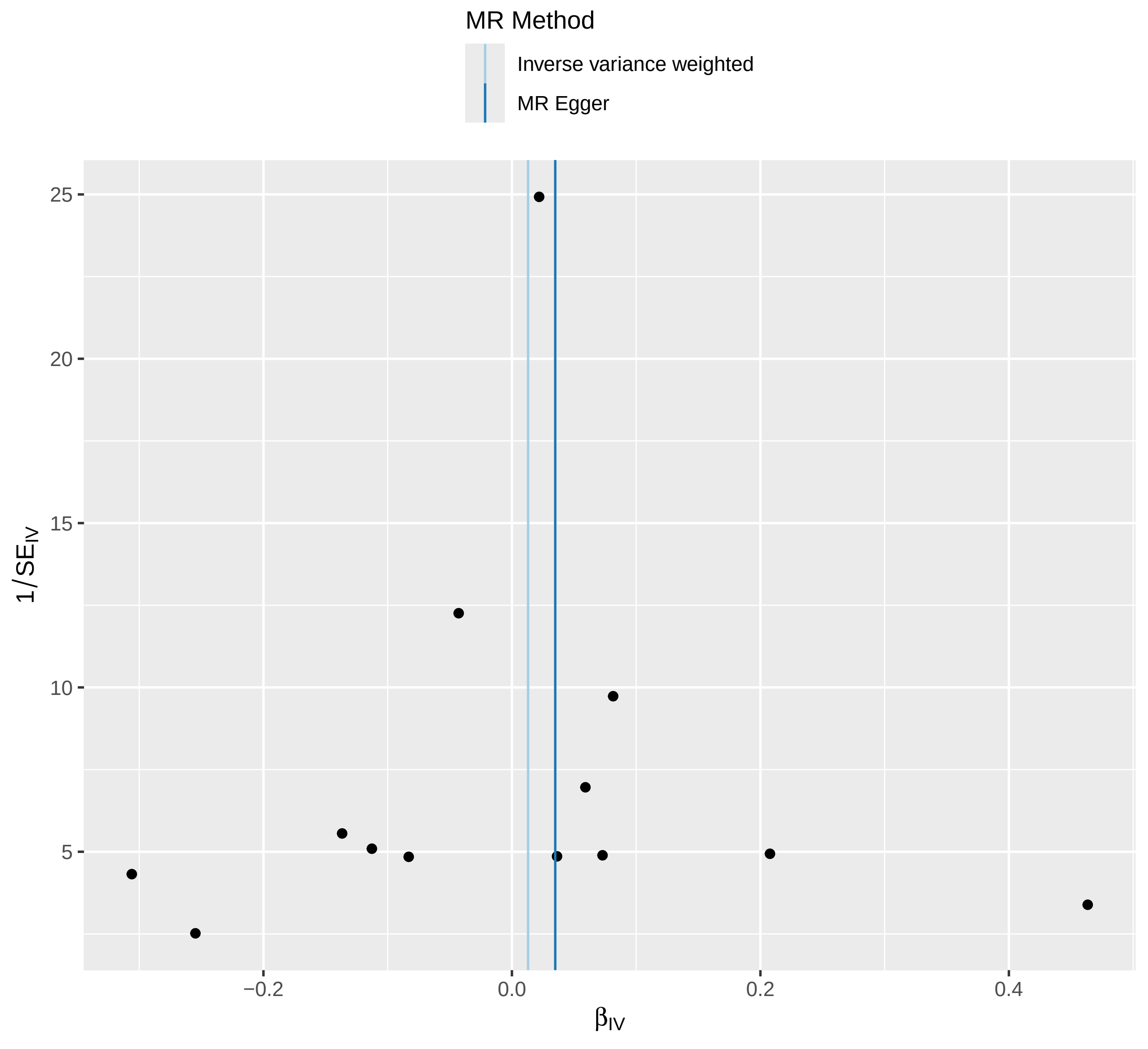

MR Method
Inverse variance weighted
MR Egger
25
20
15
10
5
$1/SE_{IV}$
−0.2
0.0
0.2
0.4
$\beta_{IV}$

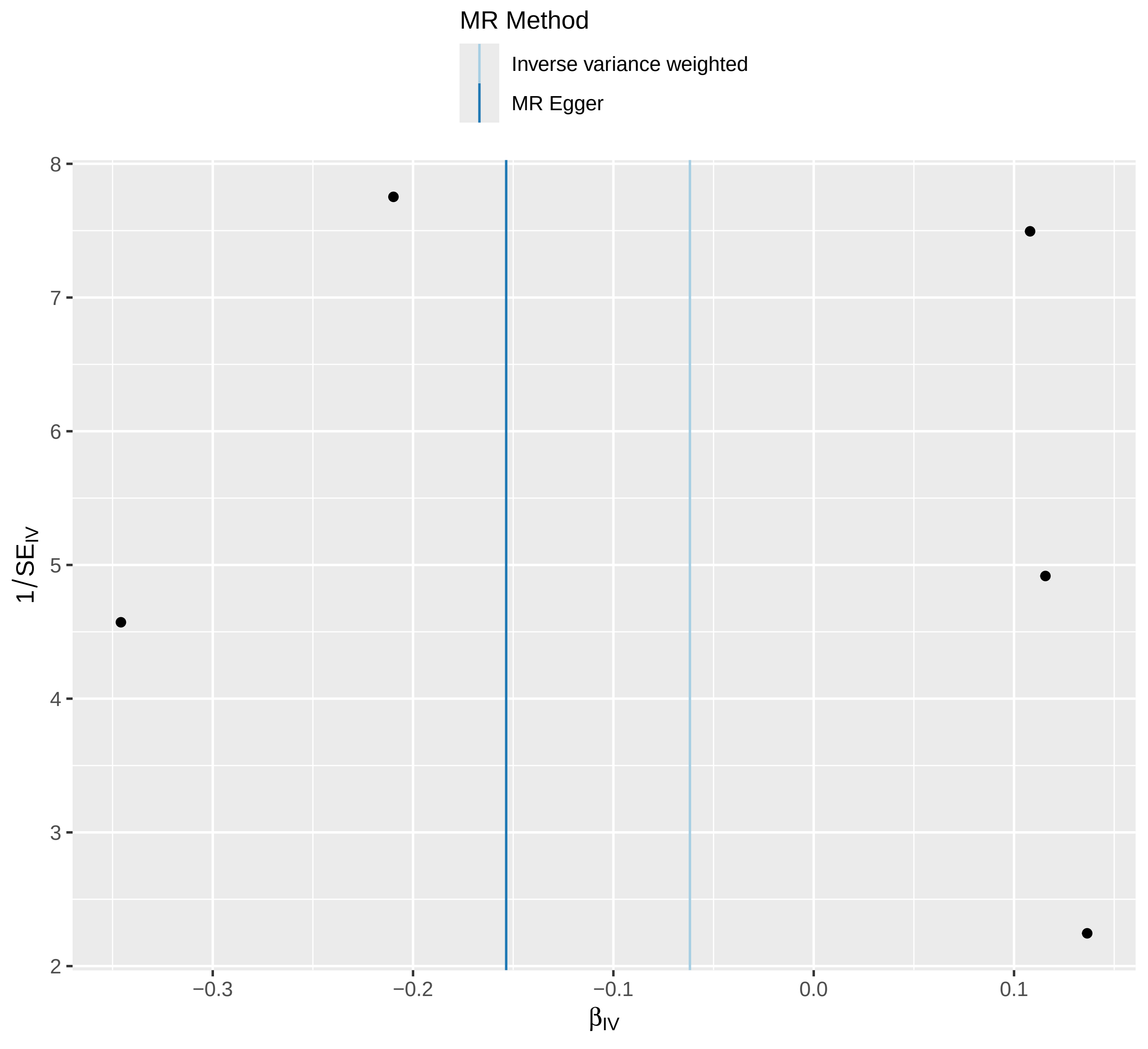
MR Method
Inverse variance weighted
MR Egger
1/SE_IV
β_IV
8
7
6
5
4
3
2
−0.3
−0.2
−0.1
0.0
0.1

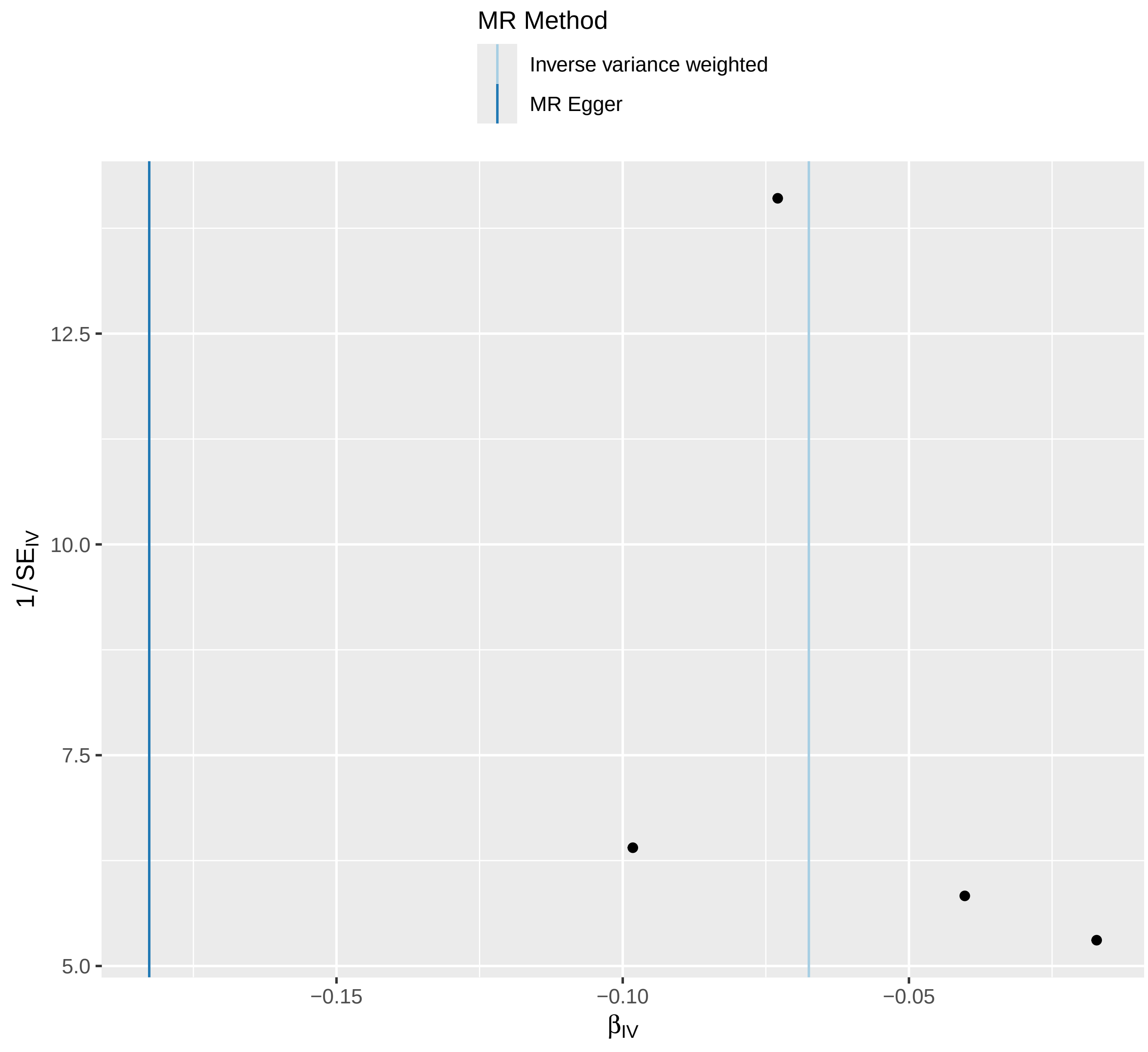
MR Method
Inverse variance weighted
MR Egger
12.5
10.0
7.5
5.0
−0.15
−0.10
−0.05
$1/SE_{IV}$
$\beta_{IV}$

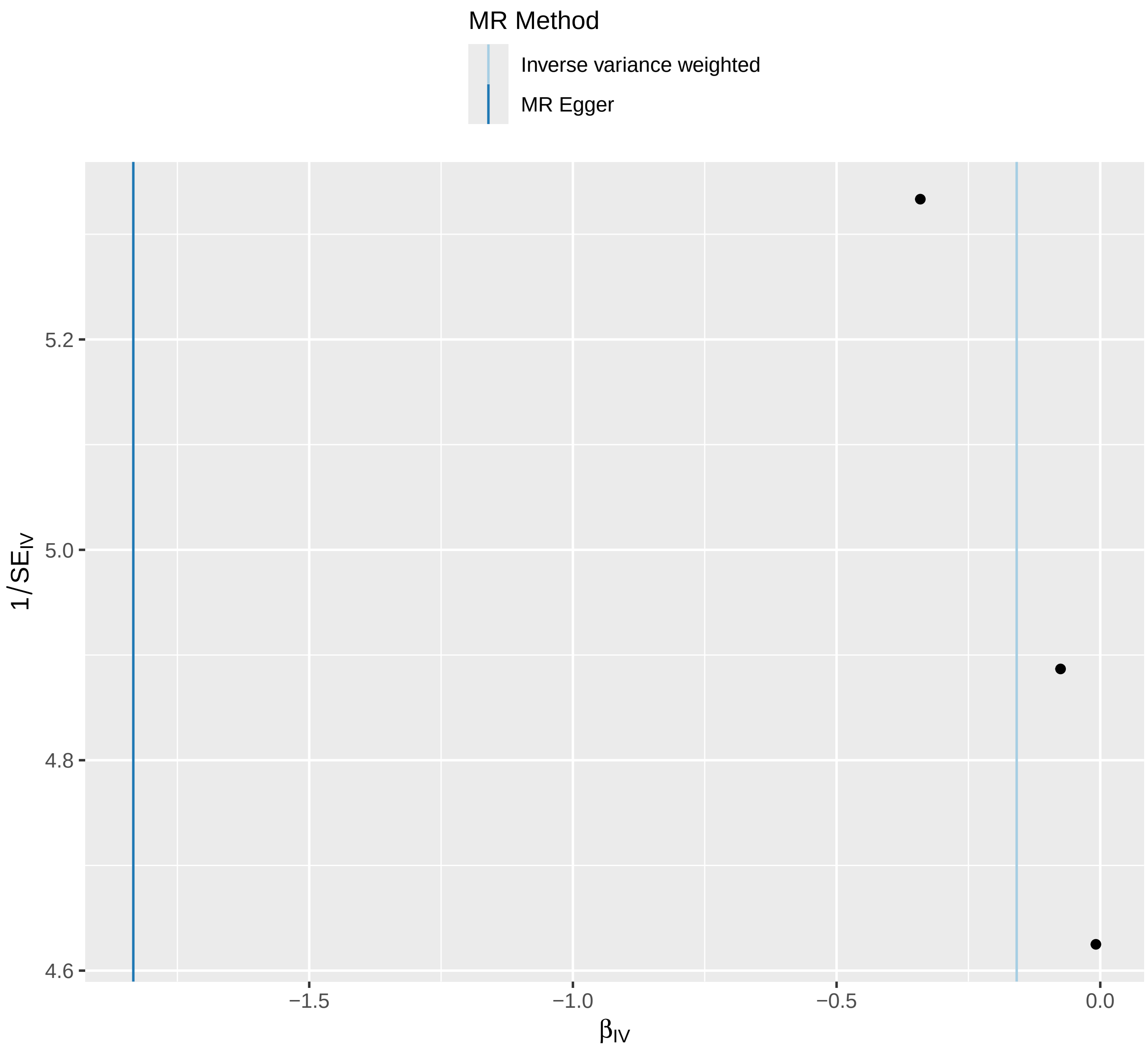

MR Method
Inverse variance weighted
MR Egger
5.2
5.0
4.8
4.6
1/SE$_{IV}$
−1.5
−1.0
−0.5
0.0
β$_{IV}$

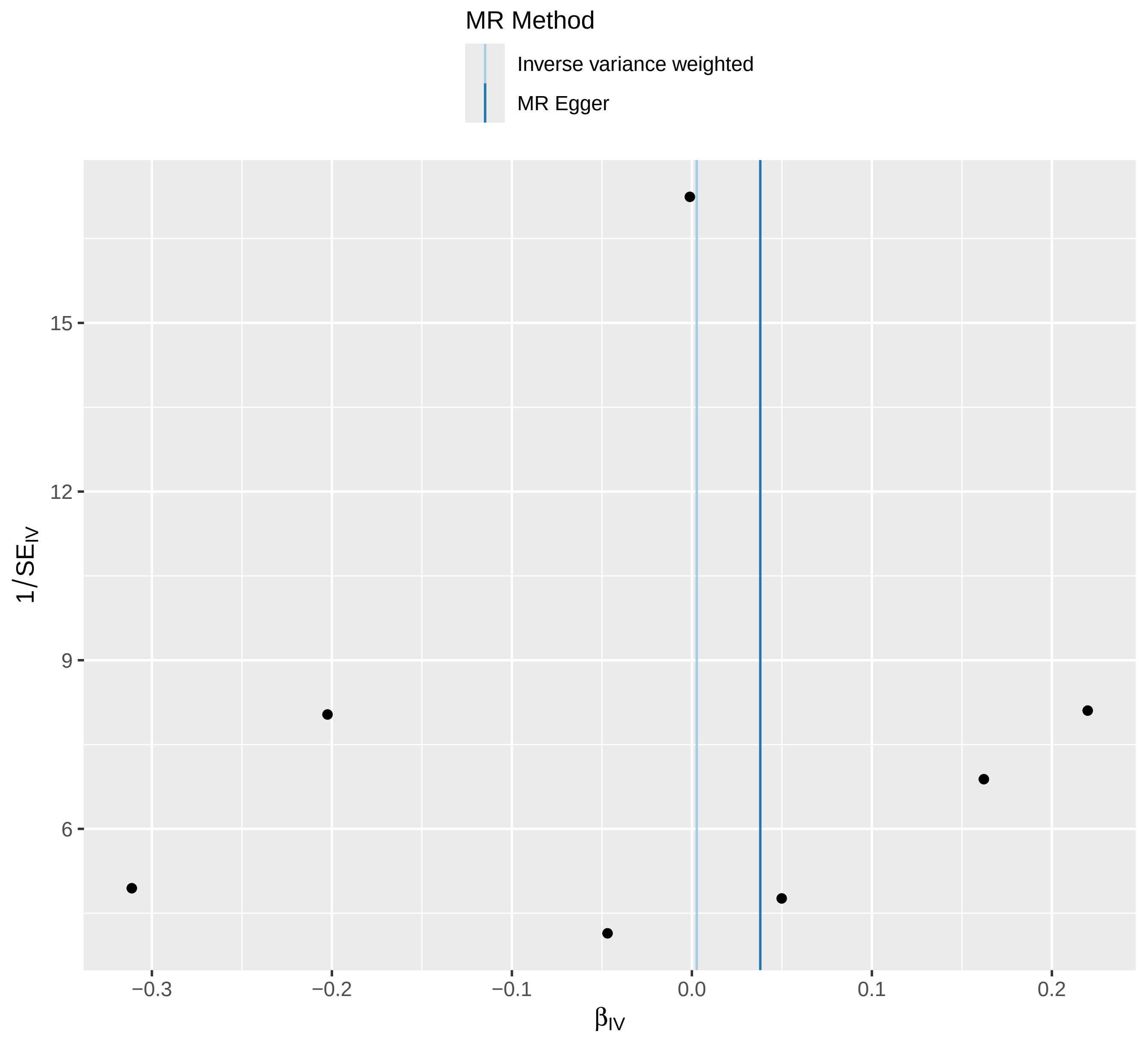

MR Method
Inverse variance weighted
MR Egger
15
12
9
6
$1/SE_{IV}$
−0.3
−0.2
−0.1
0.0
0.1
0.2
$\beta_{IV}$

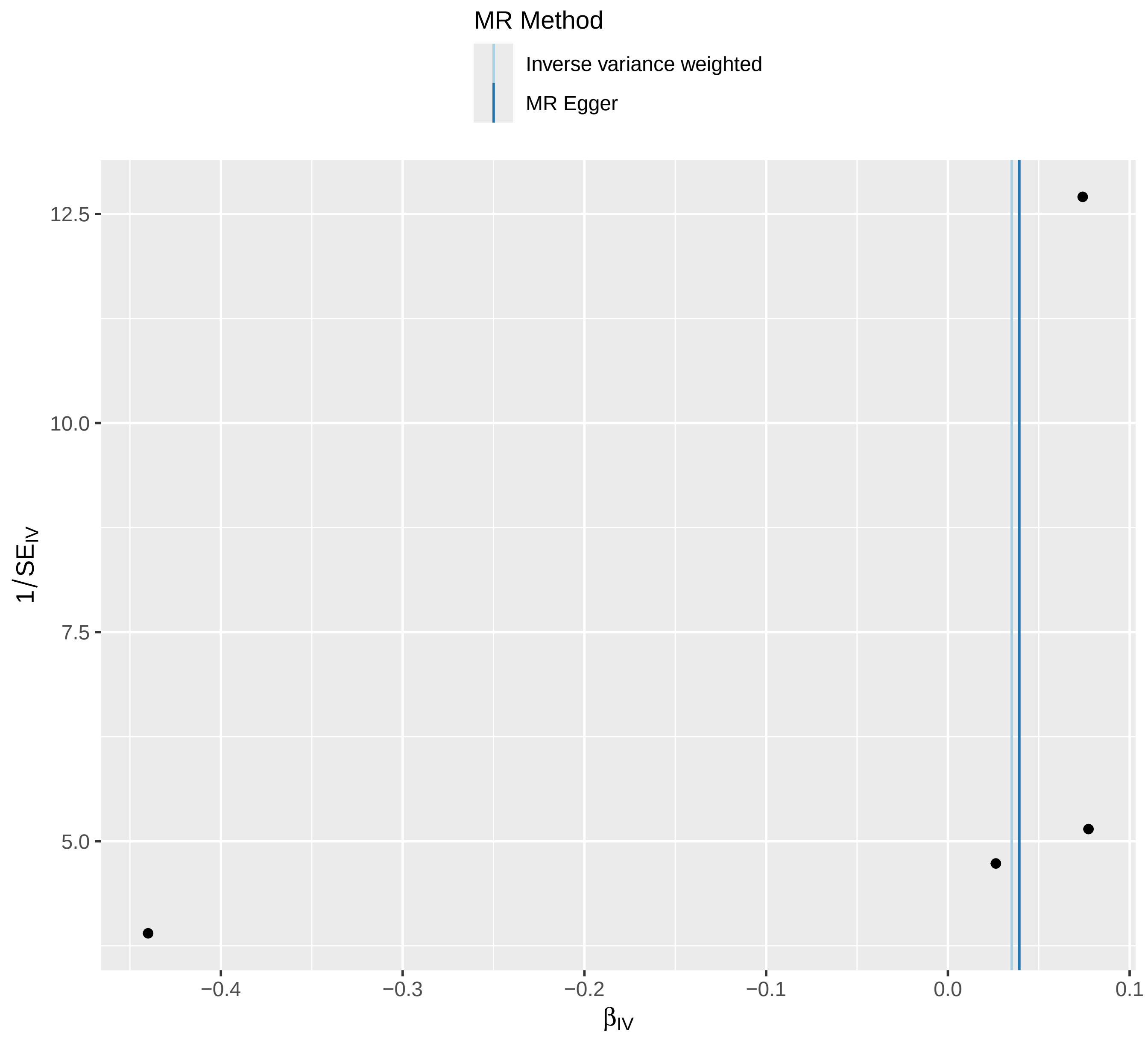

MR Method
Inverse variance weighted
MR Egger
12.5
10.0
7.5
5.0
1/SE_IV
−0.4
−0.3
−0.2
−0.1
0.0
0.1
β_IV

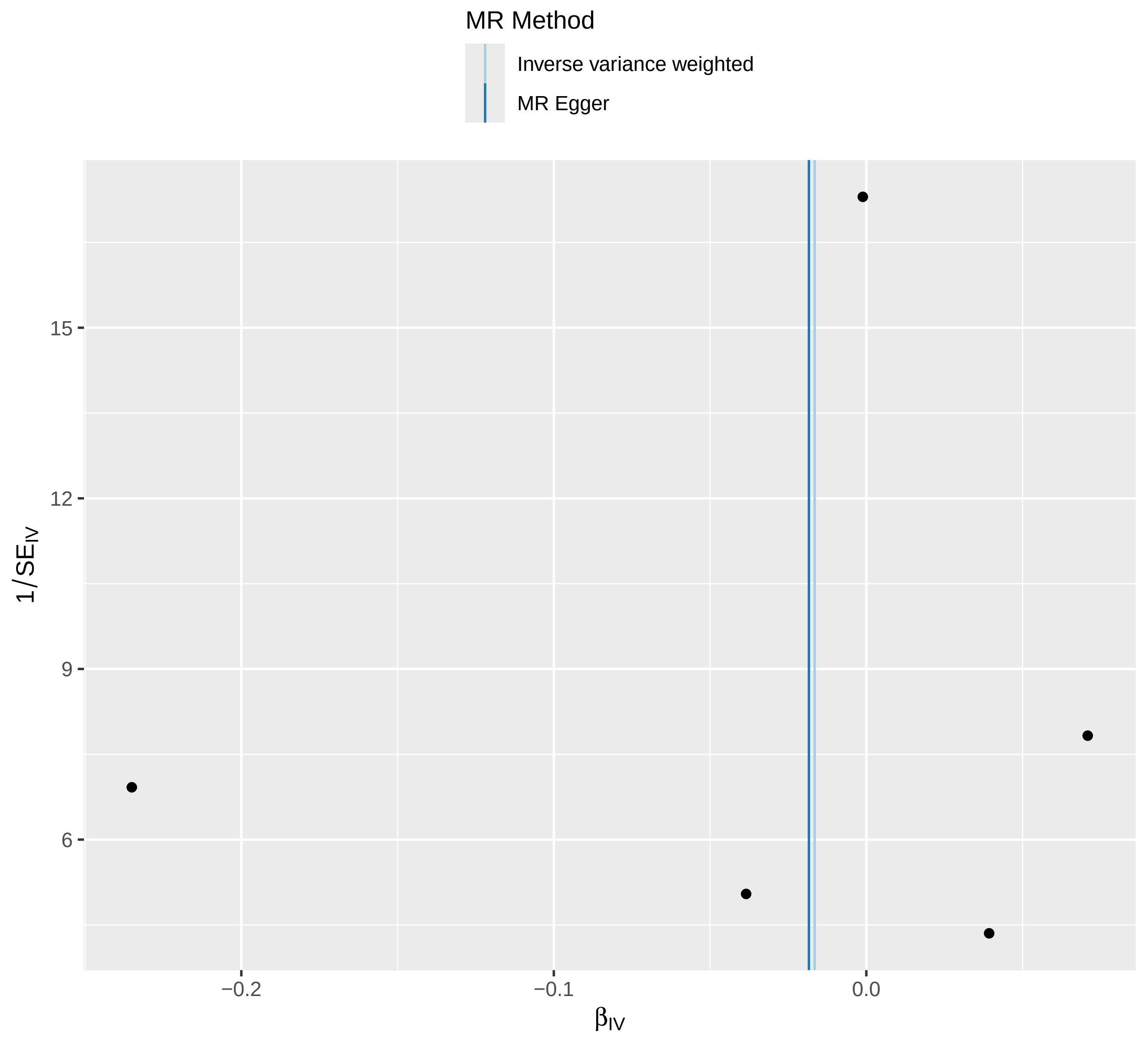
MR Method
Inverse variance weighted
MR Egger
15
12
9
6
1/SE$_{IV}$
−0.2
−0.1
0.0
β$_{IV}$

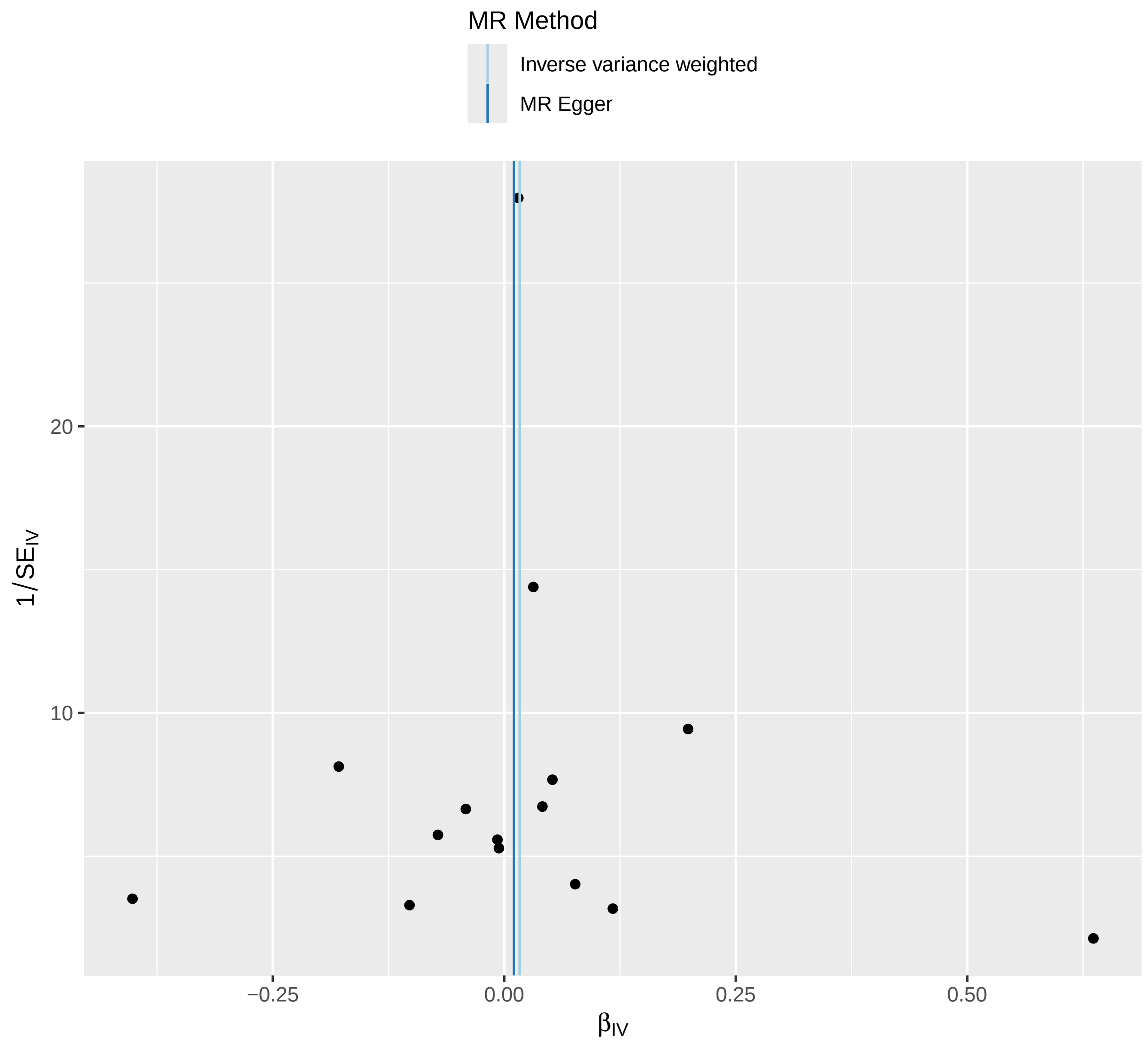
MR Method
Inverse variance weighted
MR Egger
20
10
$1/SE_{IV}$
−0.25
0.00
0.25
0.50
$\beta_{IV}$

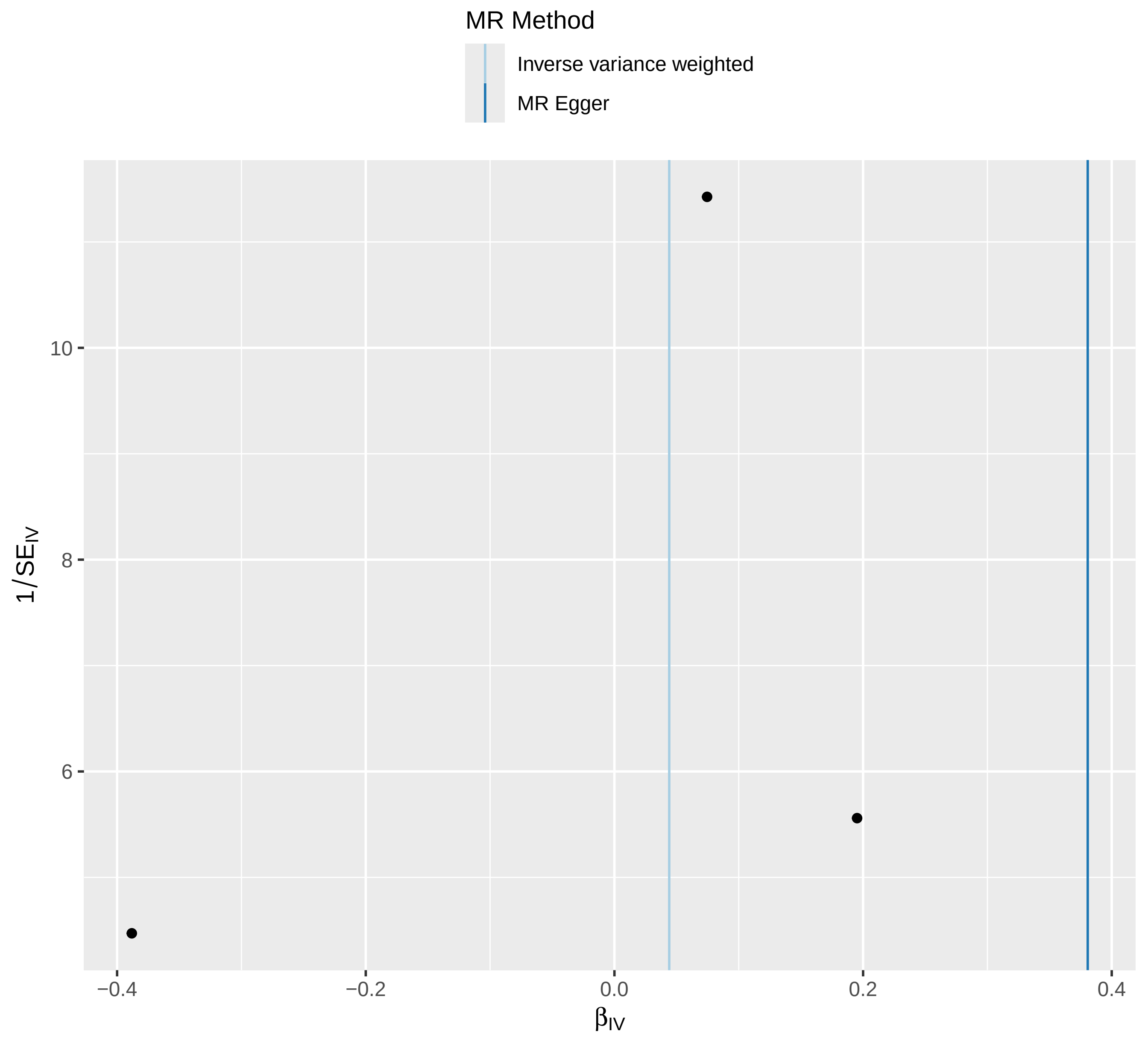
MR Method
Inverse variance weighted
MR Egger
10
8
6
1/SE_IV
−0.4
−0.2
0.0
0.2
0.4
β_IV

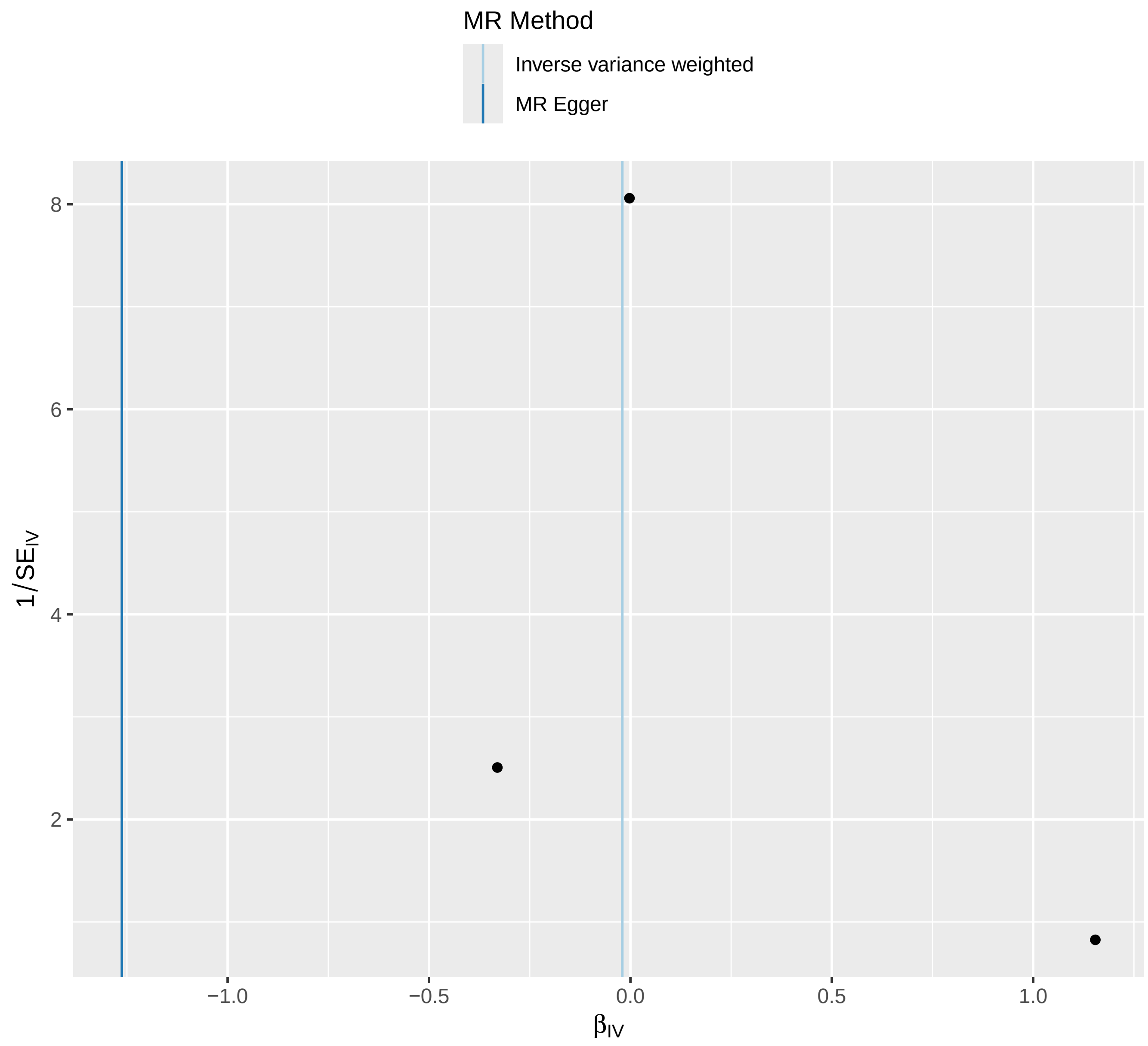
MR Method
Inverse variance weighted
MR Egger
1/SE_IV
8
6
4
2
−1.0
−0.5
0.0
0.5
1.0
β_IV

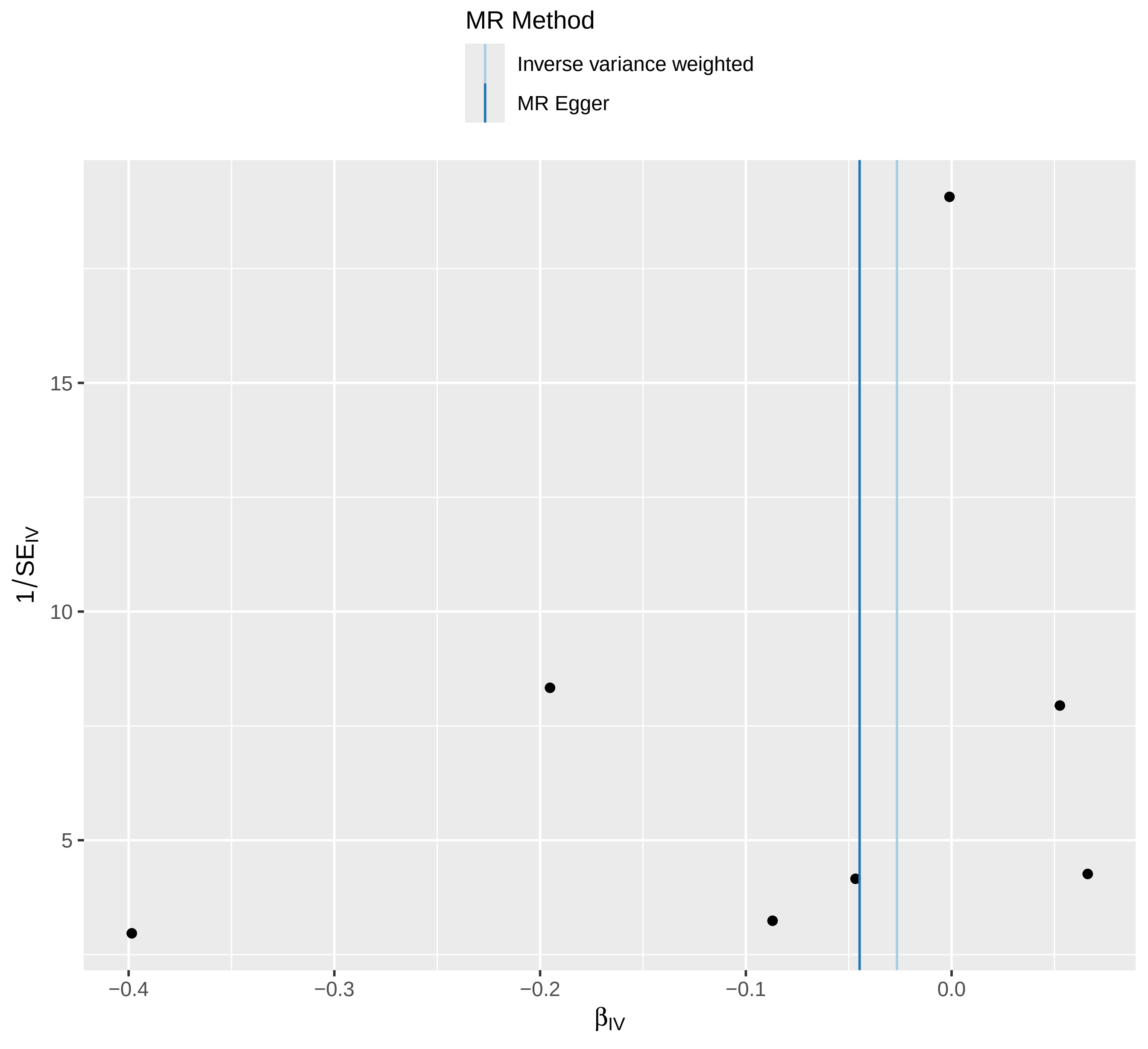

MR Method
Inverse variance weighted
MR Egger
1/SE_IV
15
10
5
−0.4
−0.3
−0.2
−0.1
0.0
β_IV

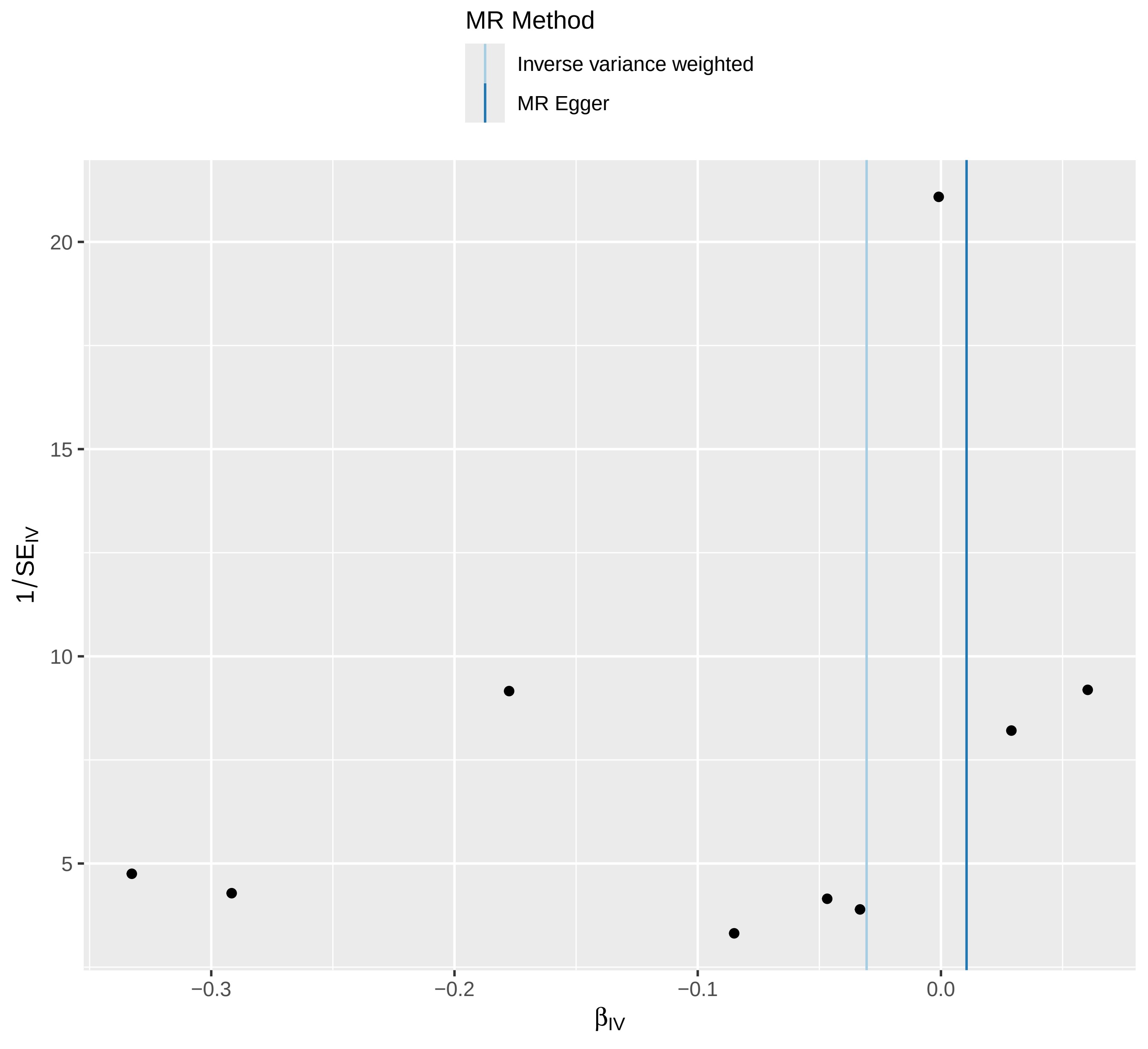
MR Method
Inverse variance weighted
MR Egger
20
15
10
5
1/SE_IV
−0.3
−0.2
−0.1
0.0
β_IV

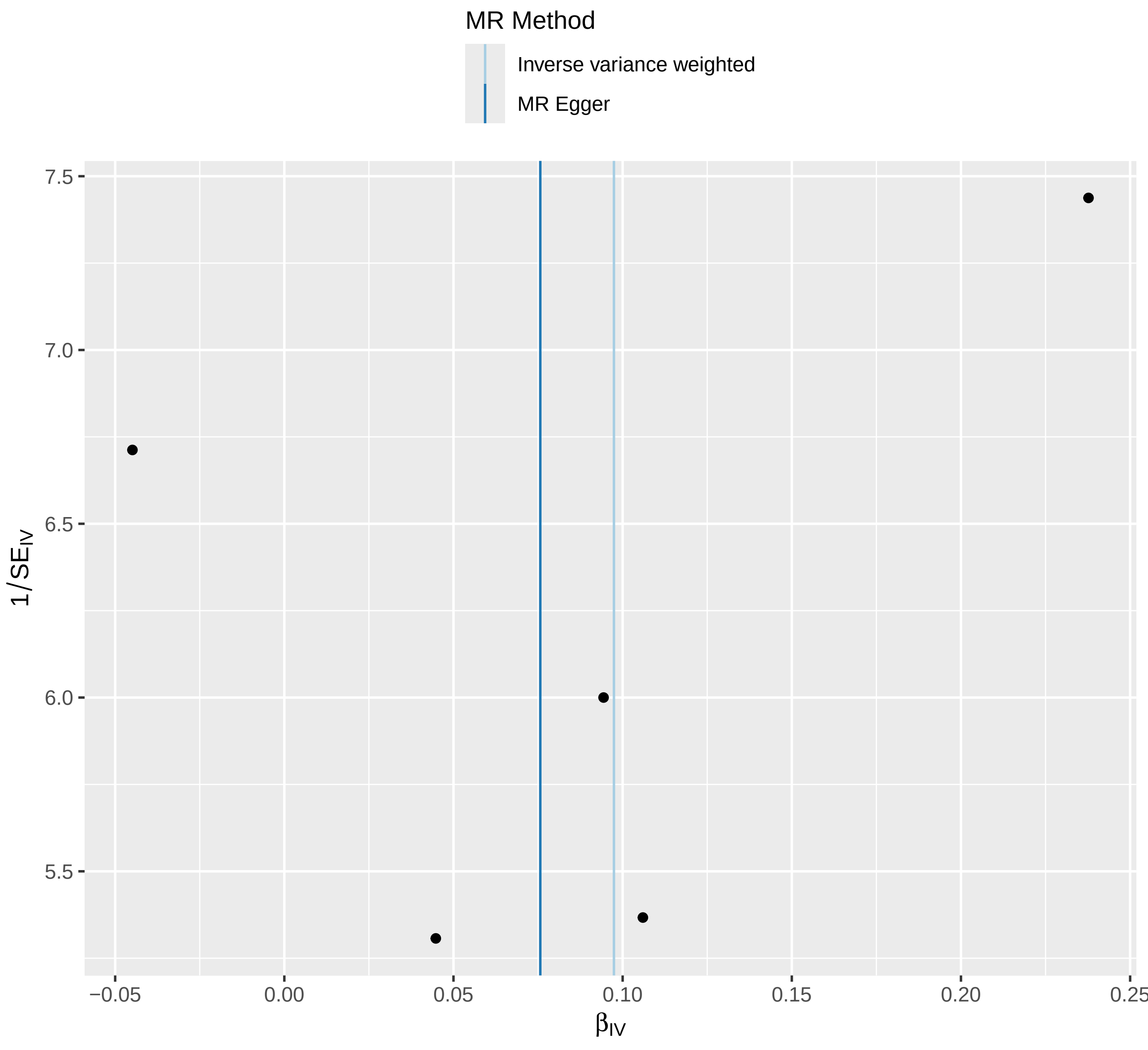
MR Method
Inverse variance weighted
MR Egger
7.5
7.0
6.5
6.0
5.5
1/SE$_{IV}$
−0.05
0.00
0.05
0.10
0.15
0.20
0.25
β$_{IV}$

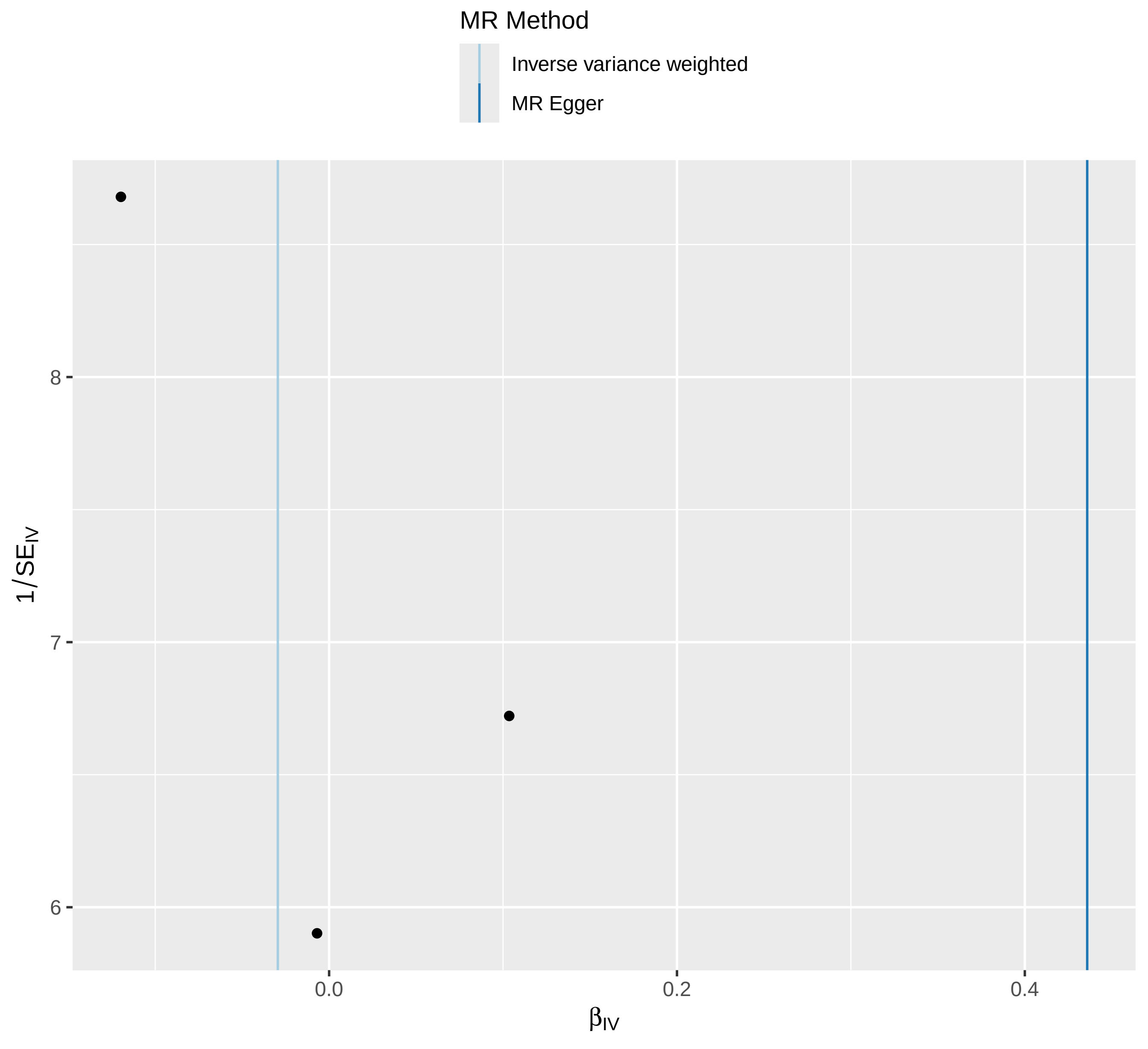

MR Method
Inverse variance weighted
MR Egger
1/SE_IV
8
7
6
0.0
0.2
0.4
β_IV

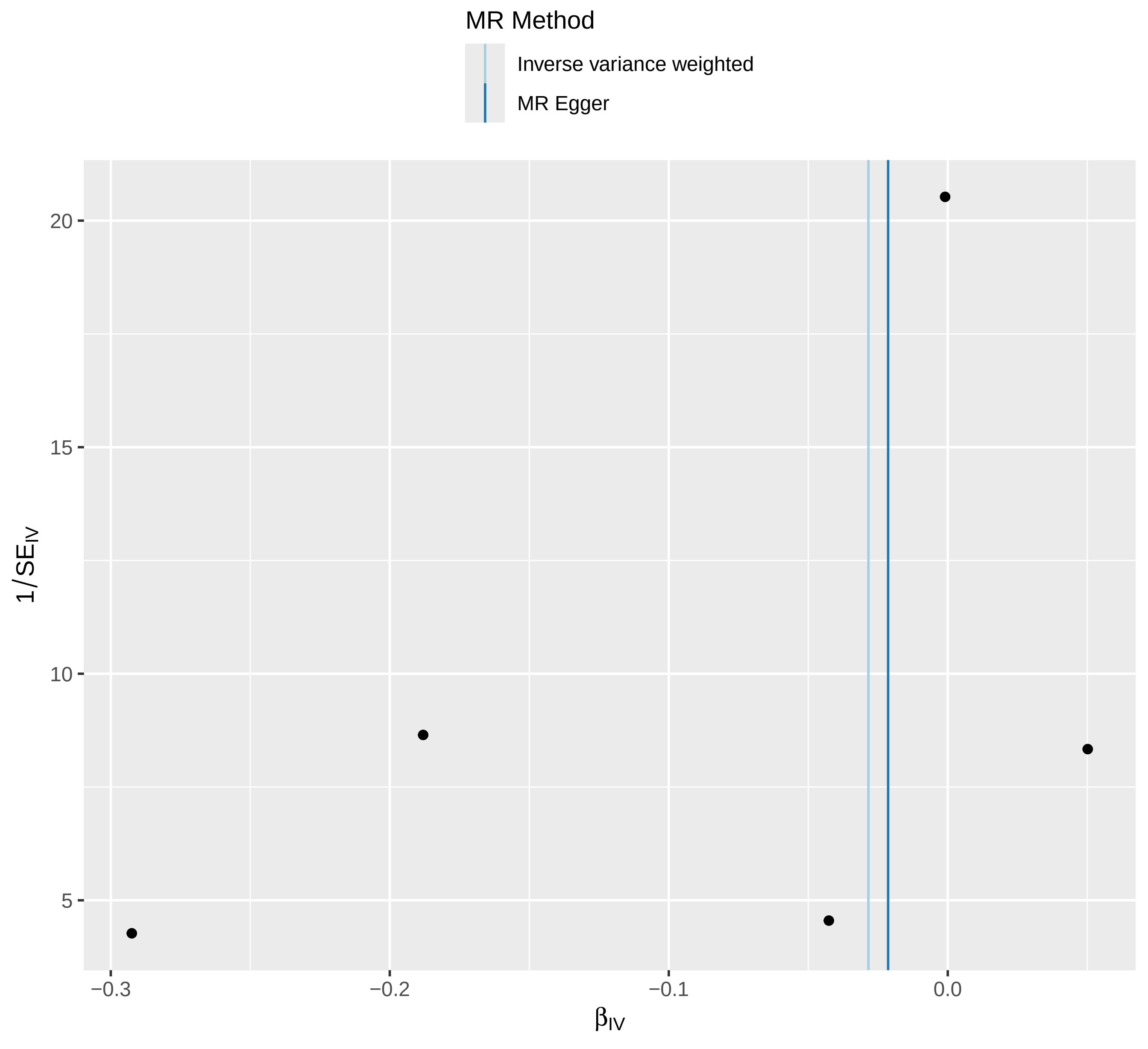
MR Method
Inverse variance weighted
MR Egger
20
15
10
5
1/SE_IV
−0.3
−0.2
−0.1
0.0
β_IV

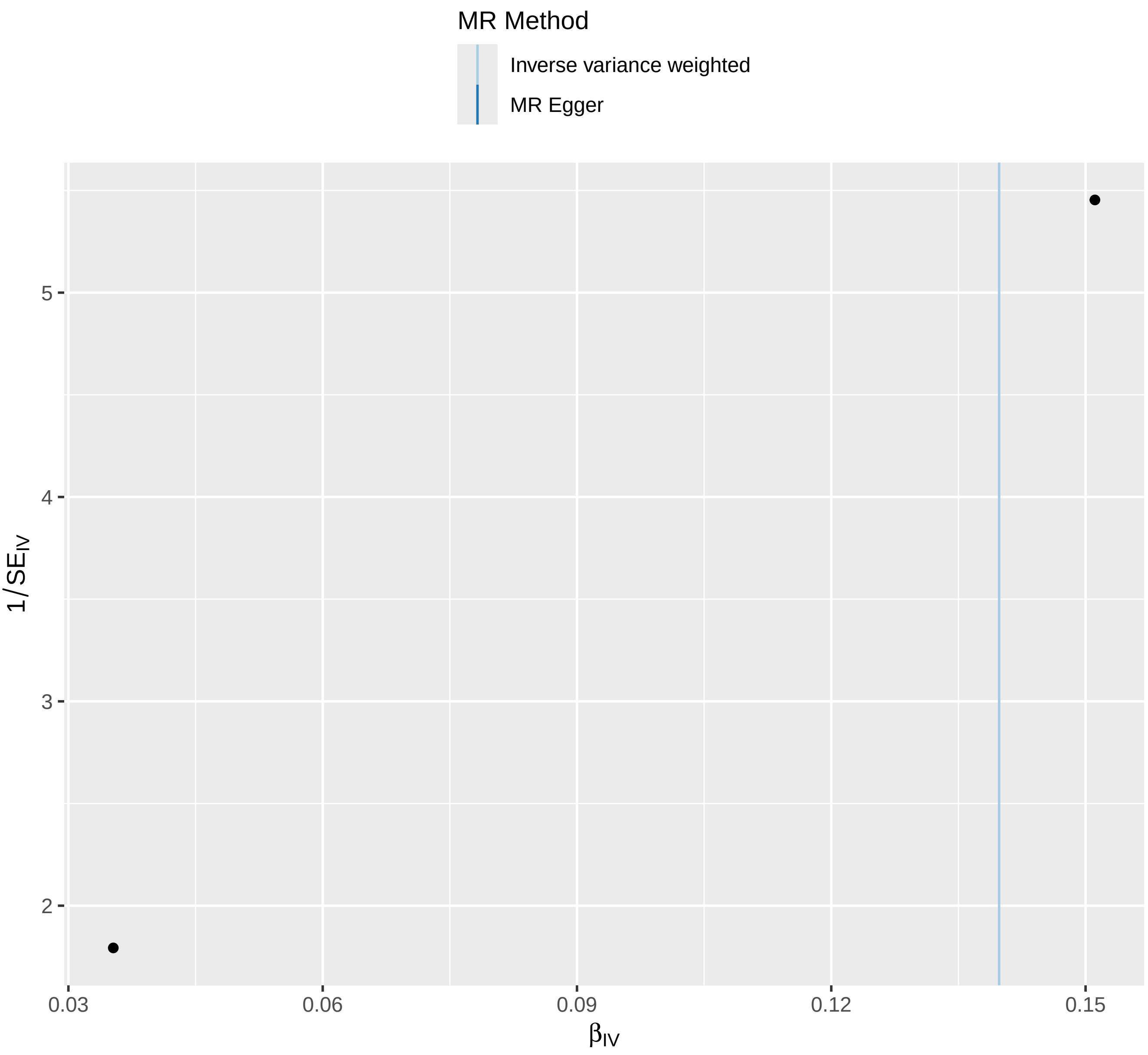
MR Method
Inverse variance weighted
MR Egger
5
4
3
2
0.03
0.06
0.09
0.12
0.15
$1/SE_{IV}$
$\beta_{IV}$

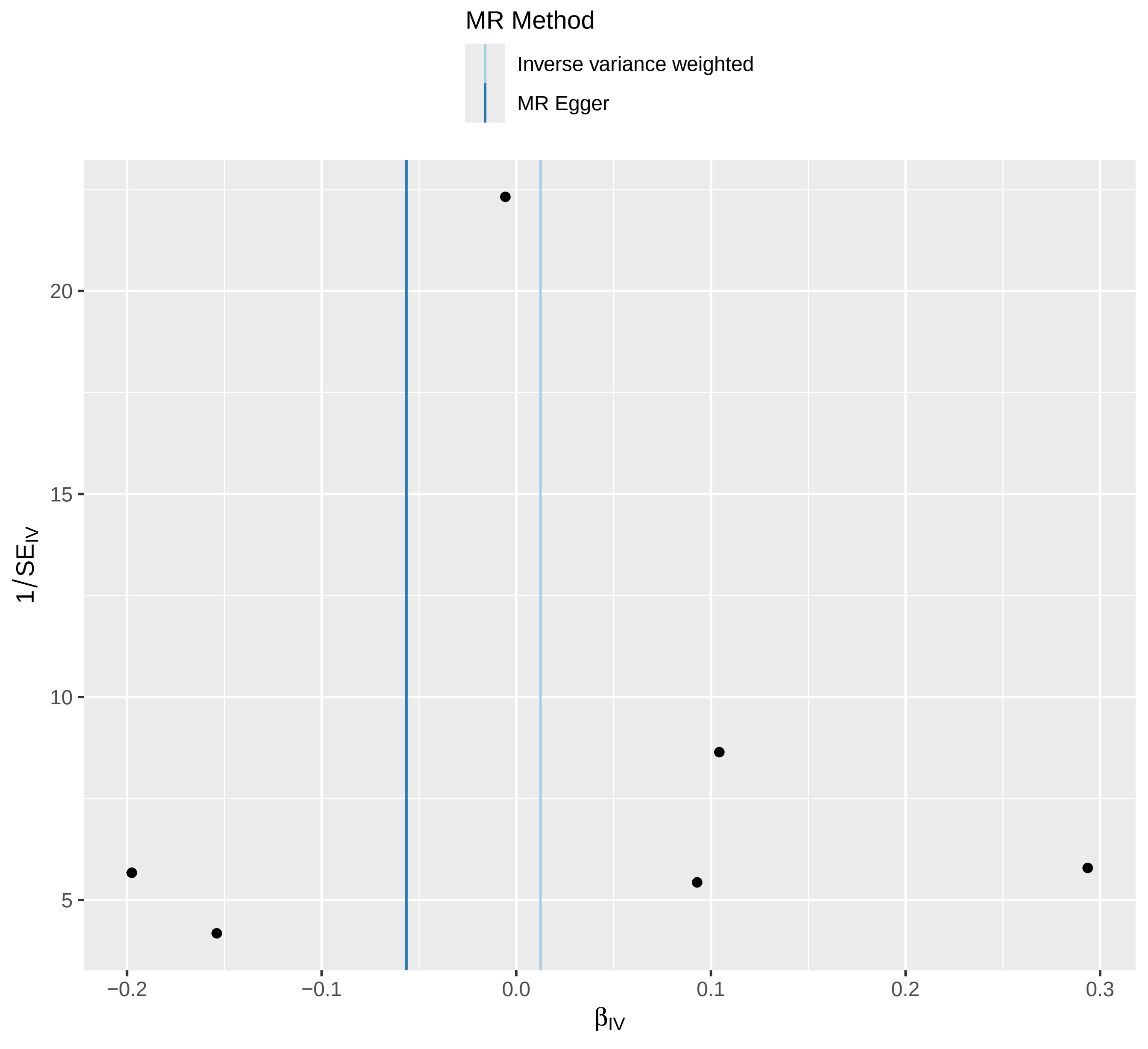
MR Method
Inverse variance weighted
MR Egger
20
15
10
5
$1/SE_{IV}$
−0.2
−0.1
0.0
0.1
0.2
0.3
$\beta_{IV}$

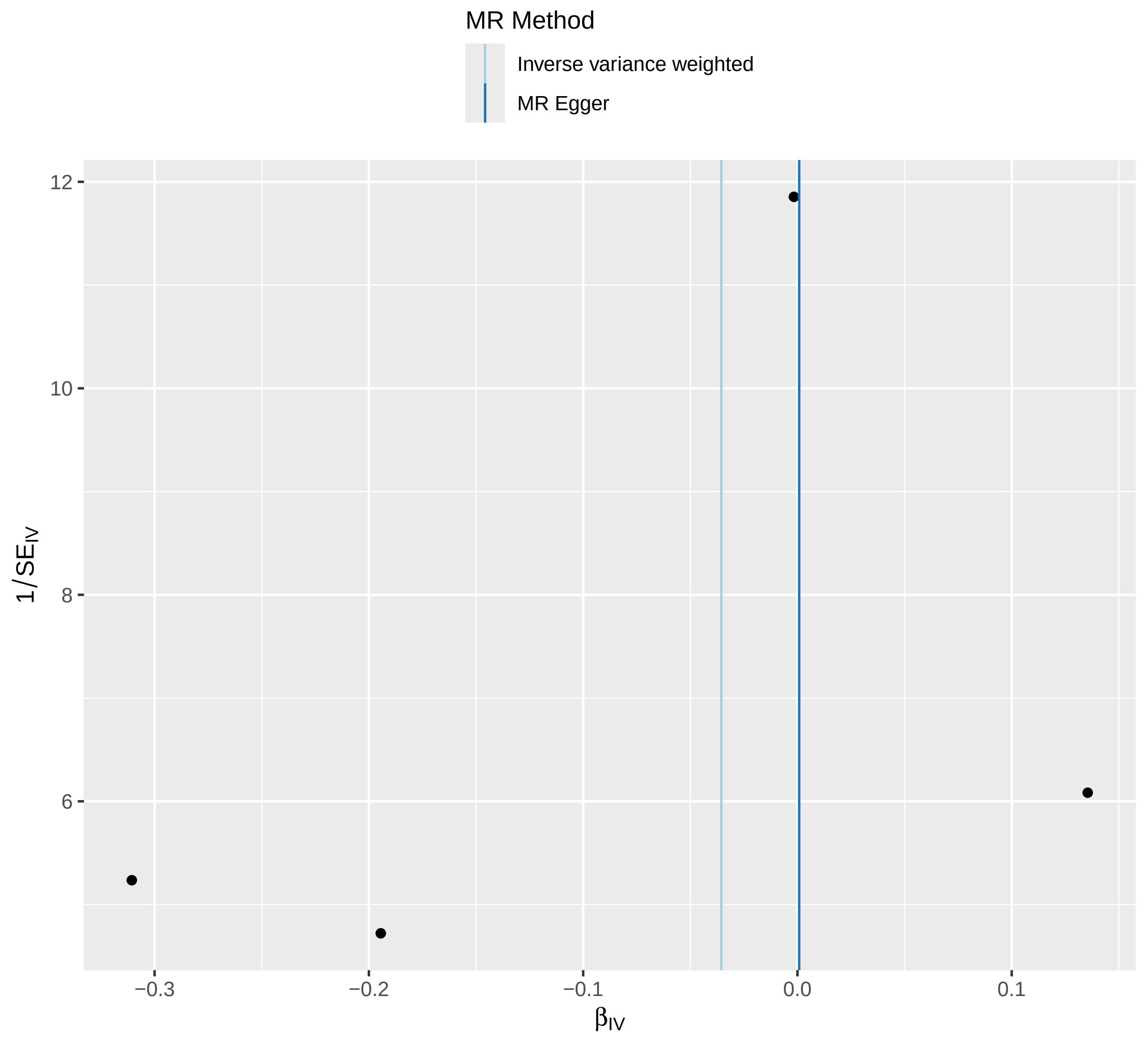
MR Method
Inverse variance weighted
MR Egger
12
10
8
6
1/SE_IV
−0.3
−0.2
−0.1
0.0
0.1
β_IV

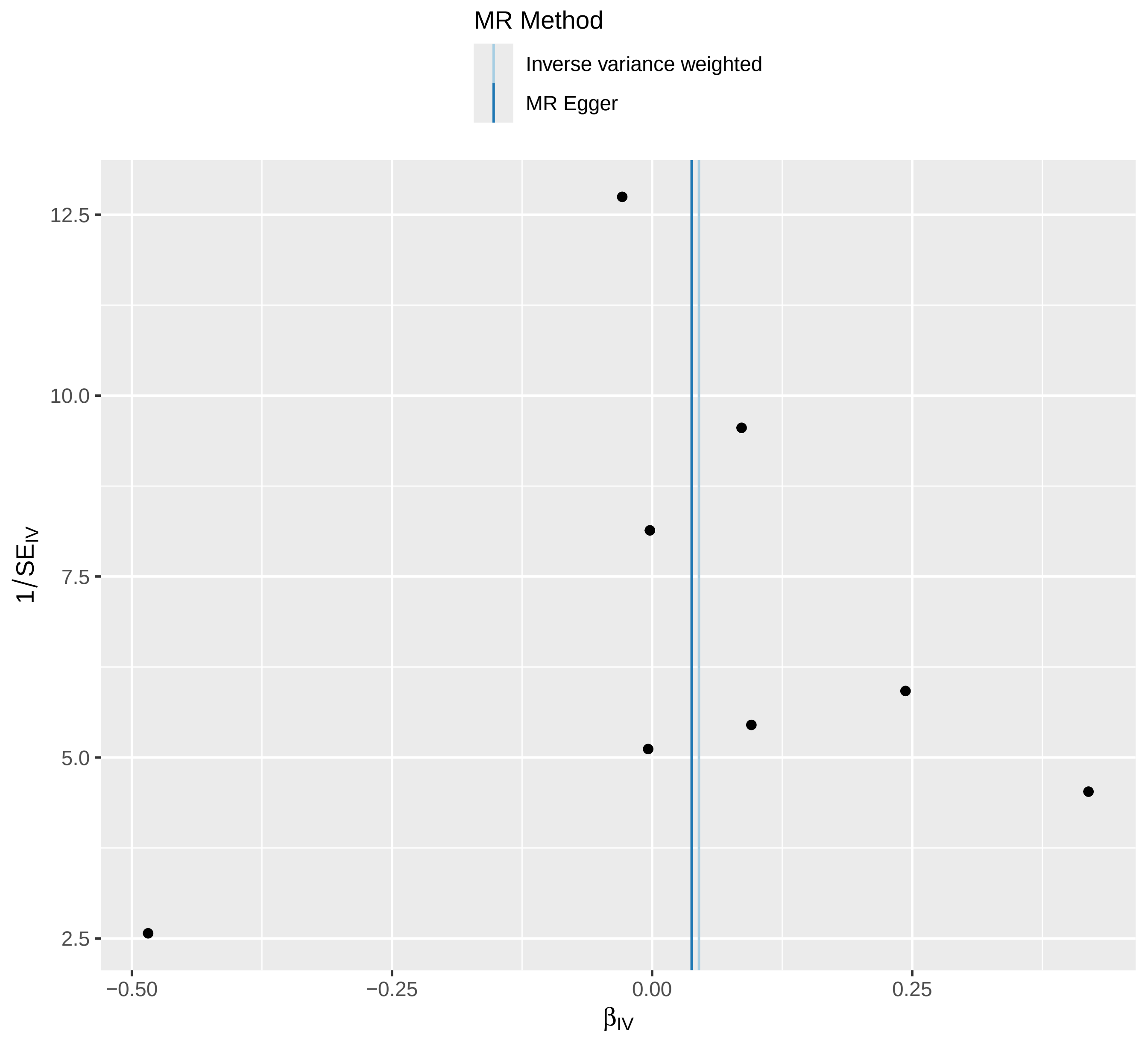
MR Method
Inverse variance weighted
MR Egger
12.5
10.0
7.5
5.0
2.5
1/SE$_{IV}$
−0.50
−0.25
0.00
0.25
β$_{IV}$

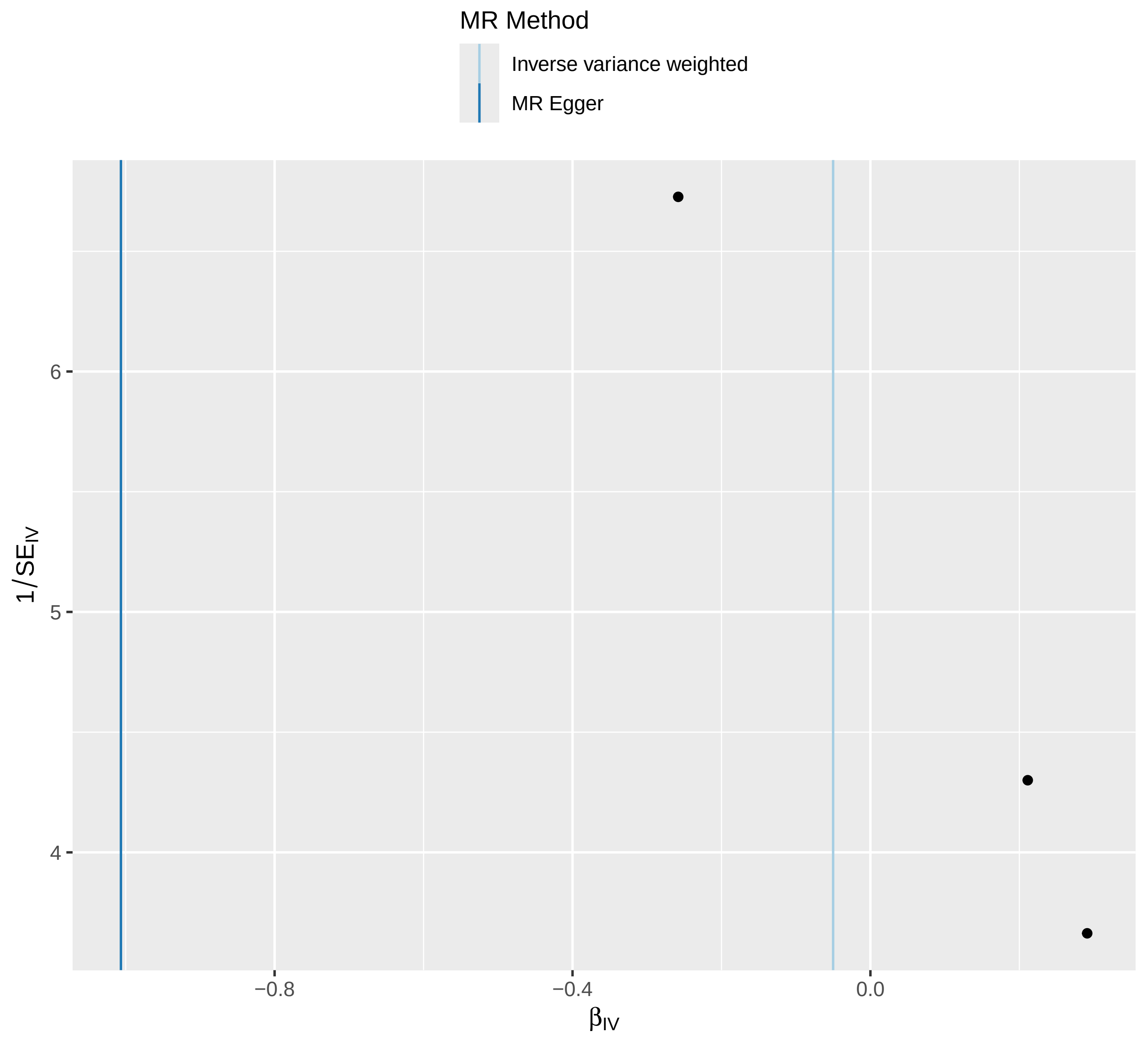

MR Method
Inverse variance weighted
MR Egger
6
5
4
1/SE_IV
−0.8
−0.4
0.0
β_IV

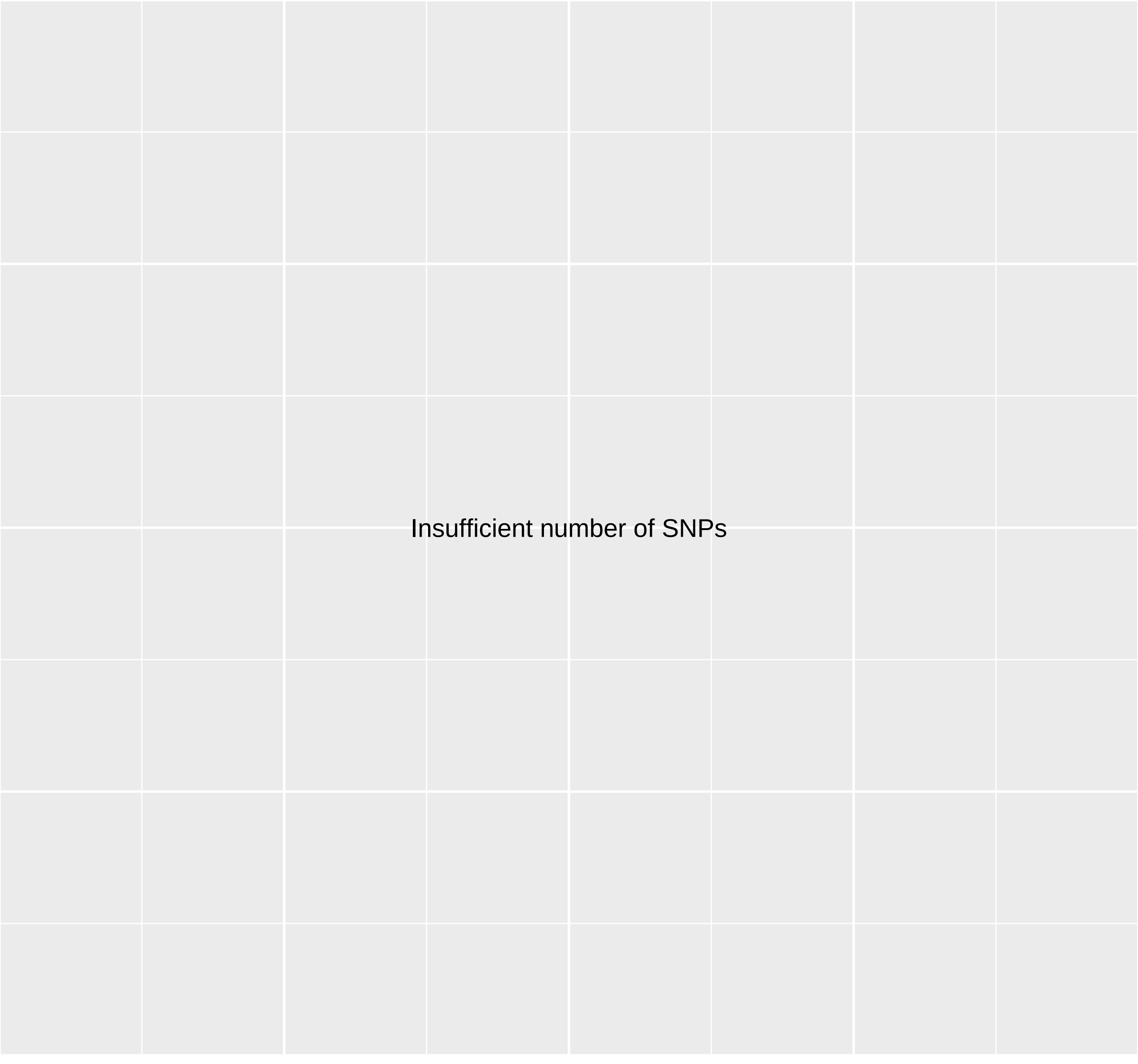
Insufficient number of SNPs

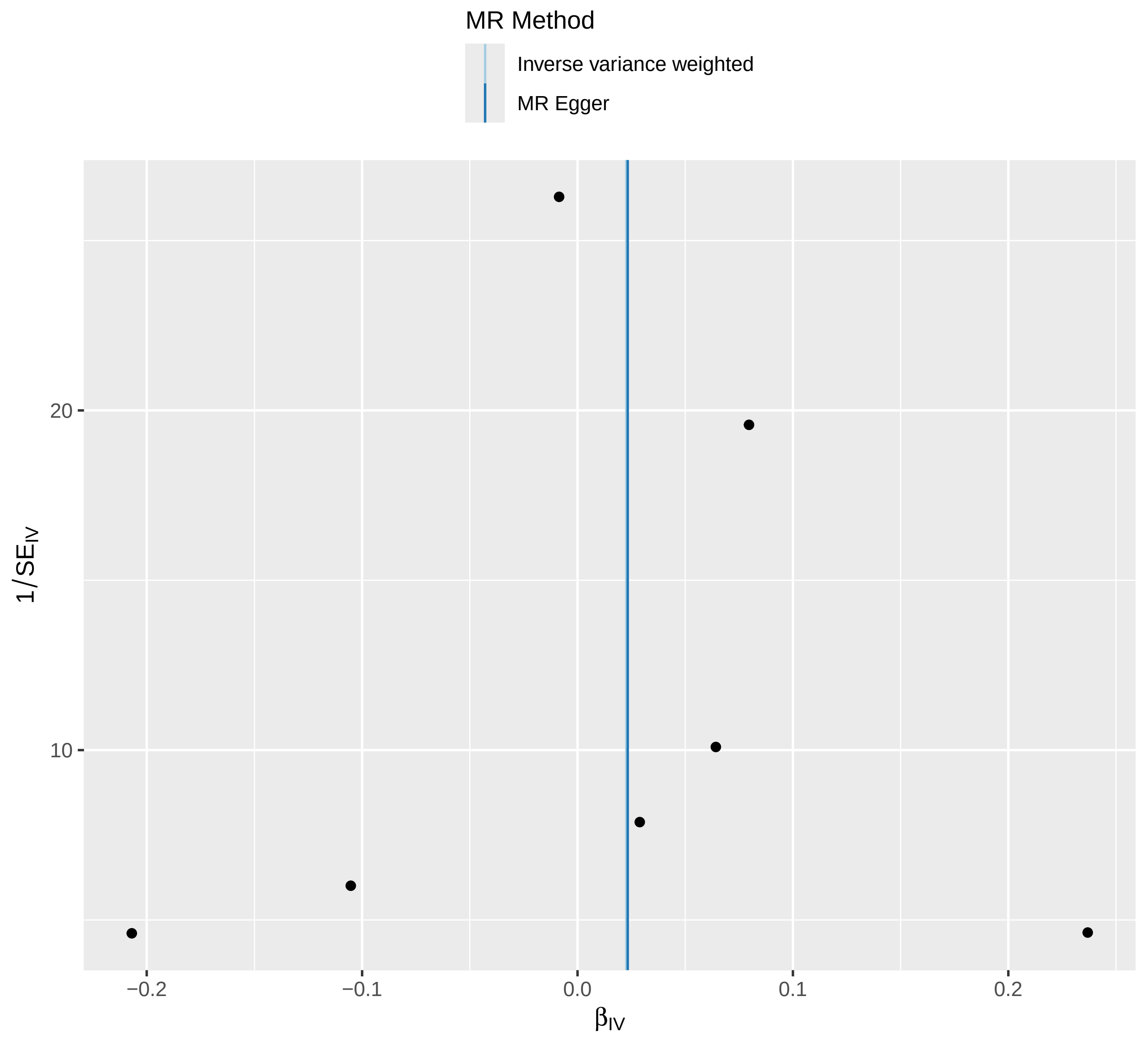
MR Method
Inverse variance weighted
MR Egger
20
10
1/SE_IV
−0.2
−0.1
0.0
0.1
0.2
β_IV

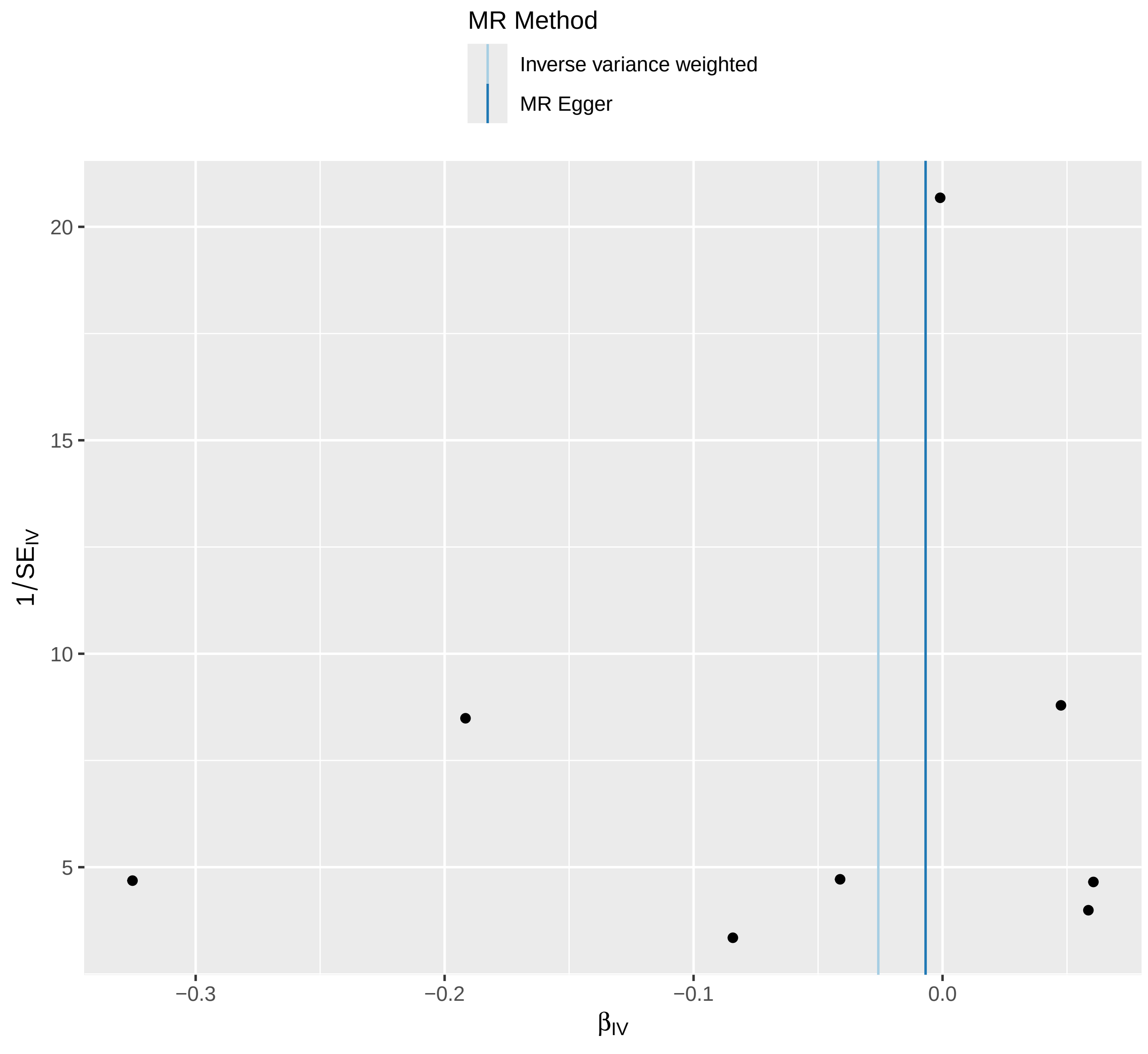
MR Method
Inverse variance weighted
MR Egger
20
15
10
5
1/SE$_{IV}$
−0.3
−0.2
−0.1
0.0
β$_{IV}$

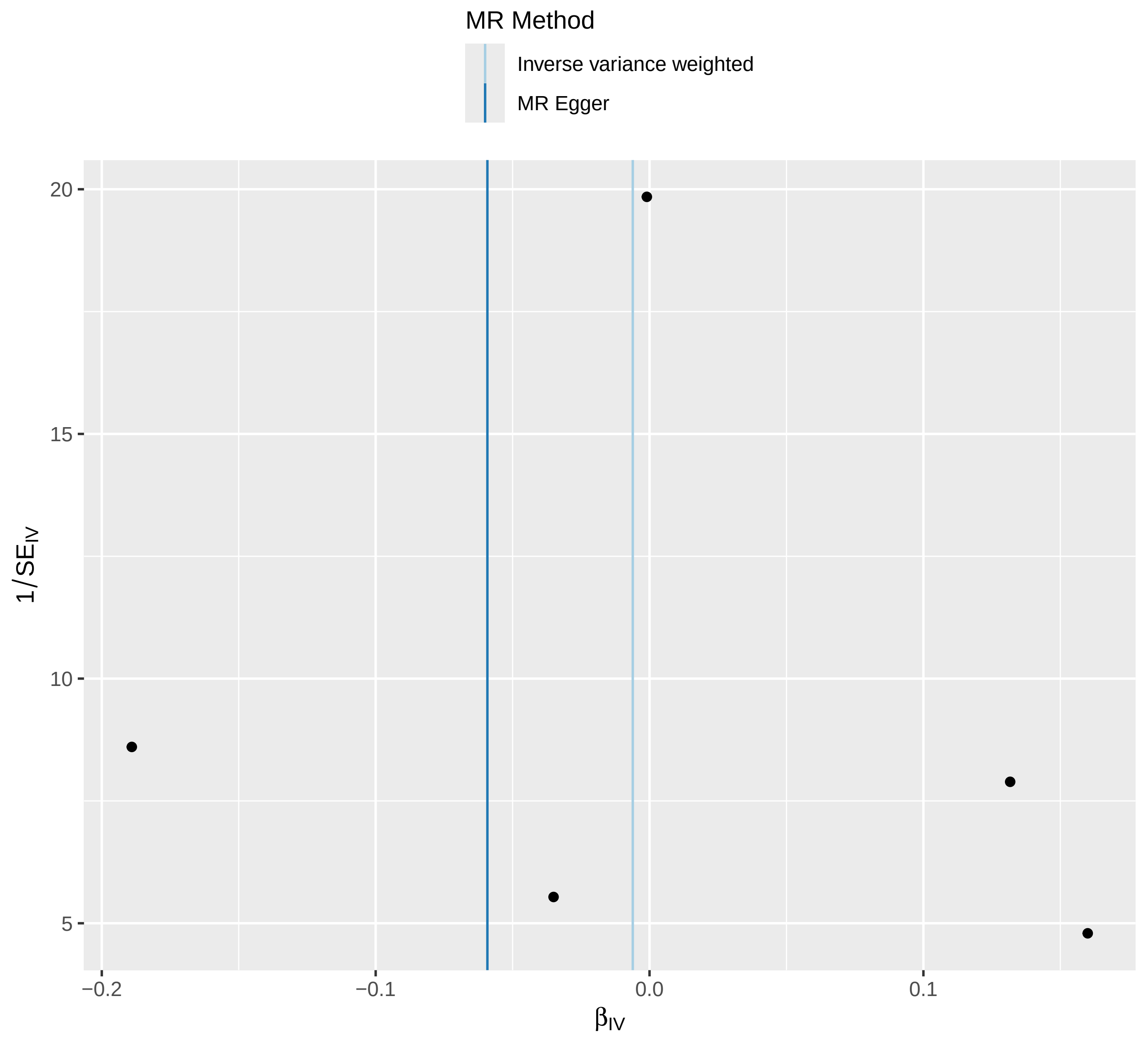
MR Method
Inverse variance weighted
MR Egger
20
15
10
5
$1/SE_{IV}$
−0.2
−0.1
0.0
0.1
$\beta_{IV}$

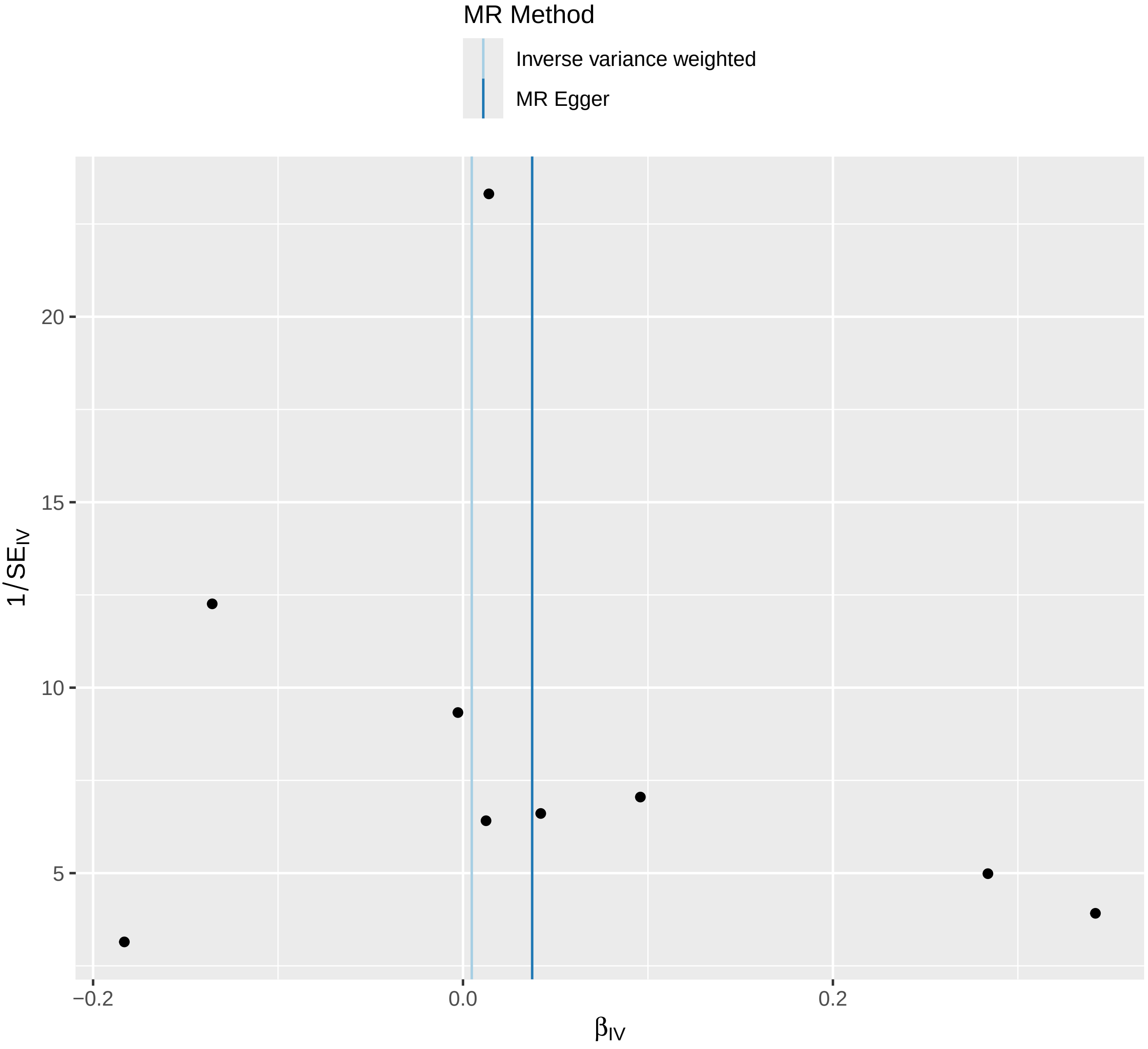

MR Method
Inverse variance weighted
MR Egger
20
15
10
5
1/SE$_{IV}$
−0.2
0.0
0.2
β$_{IV}$

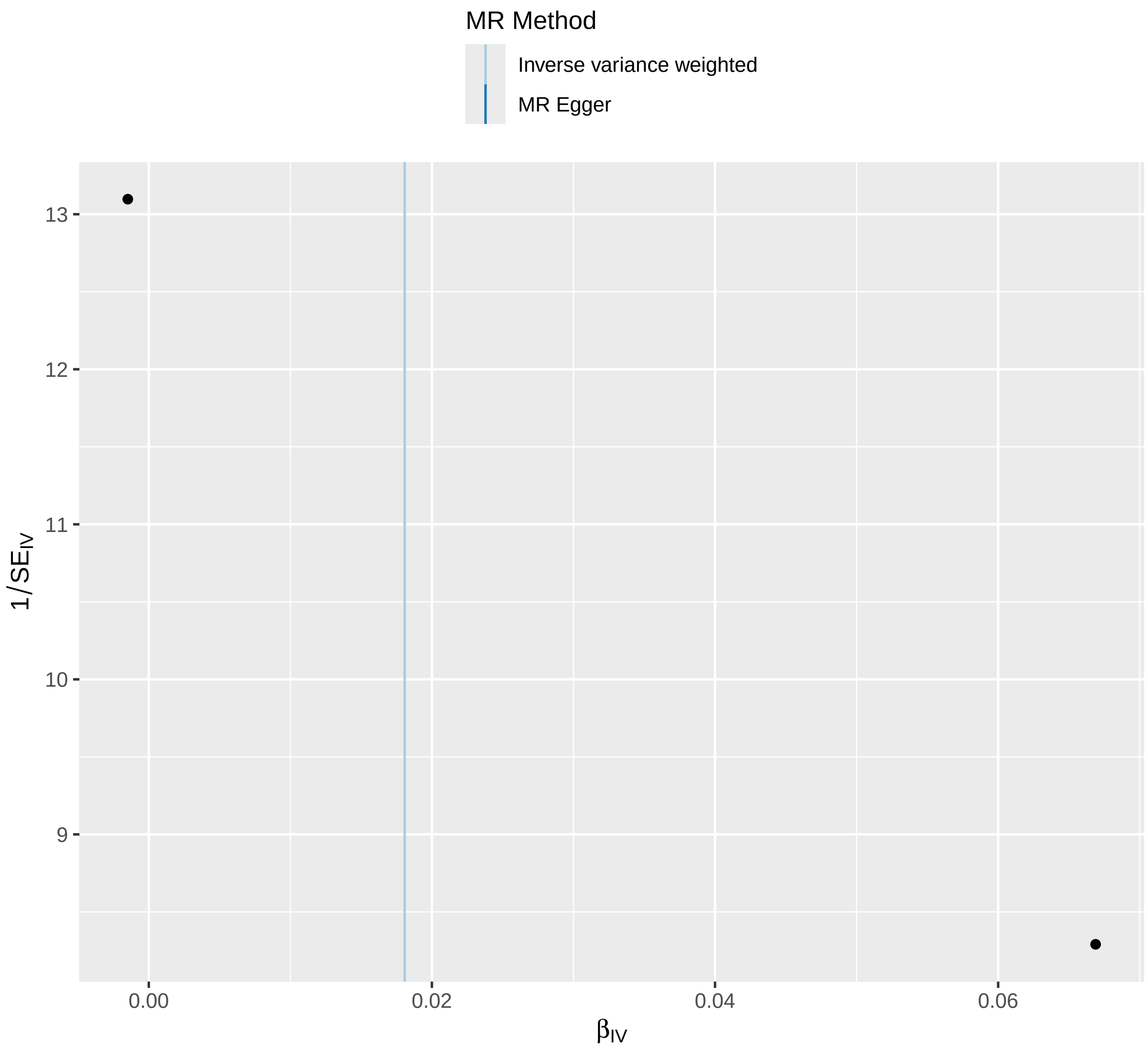

MR Method
Inverse variance weighted
MR Egger
13
12
11
10
9
1/SE_IV
0.00
0.02
0.04
0.06
β_IV

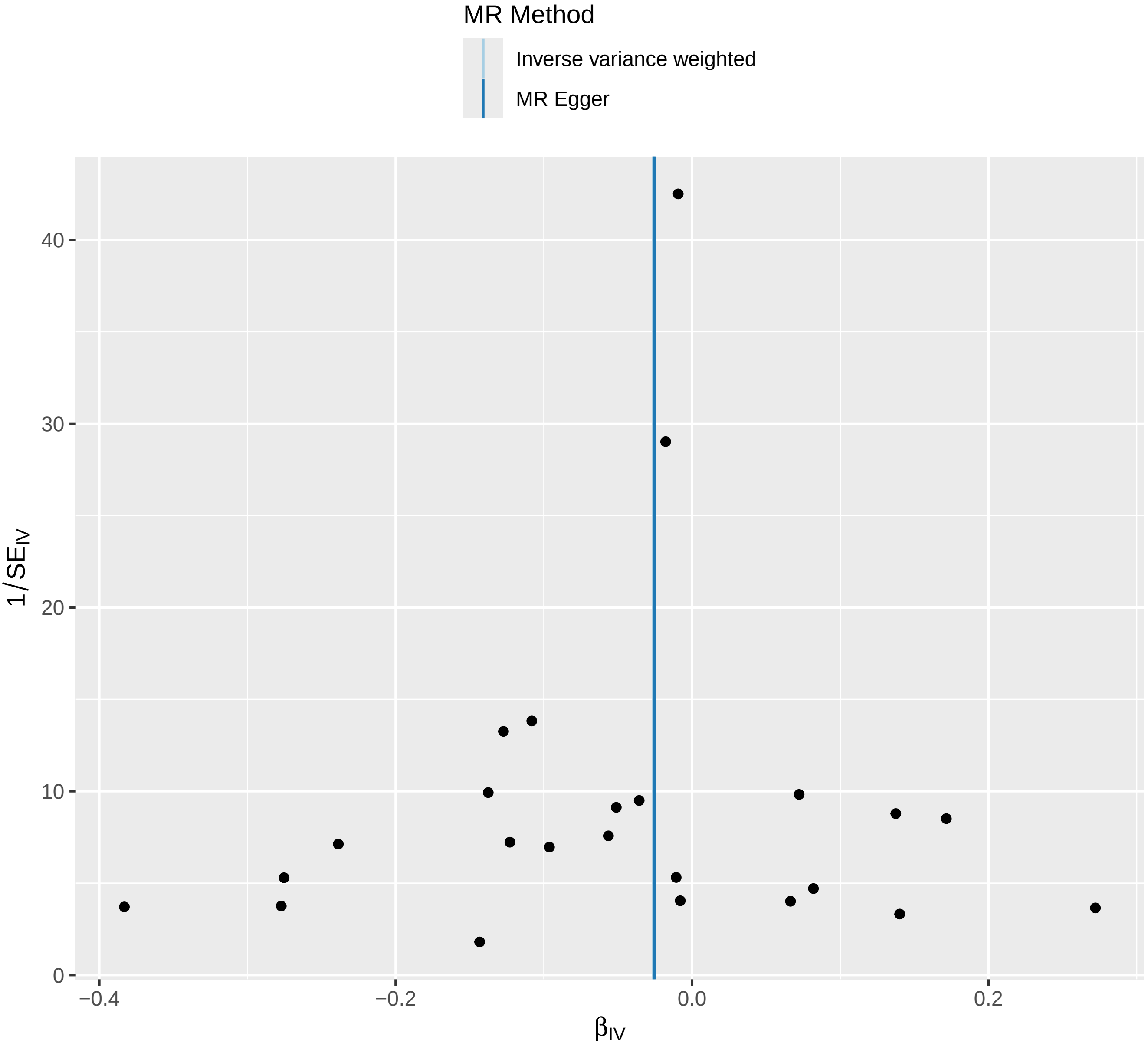
MR Method
Inverse variance weighted
MR Egger
40
30
20
10
0
−0.4
−0.2
0.0
0.2
$1/SE_{IV}$
$\beta_{IV}$

Insufficient number of SNPs

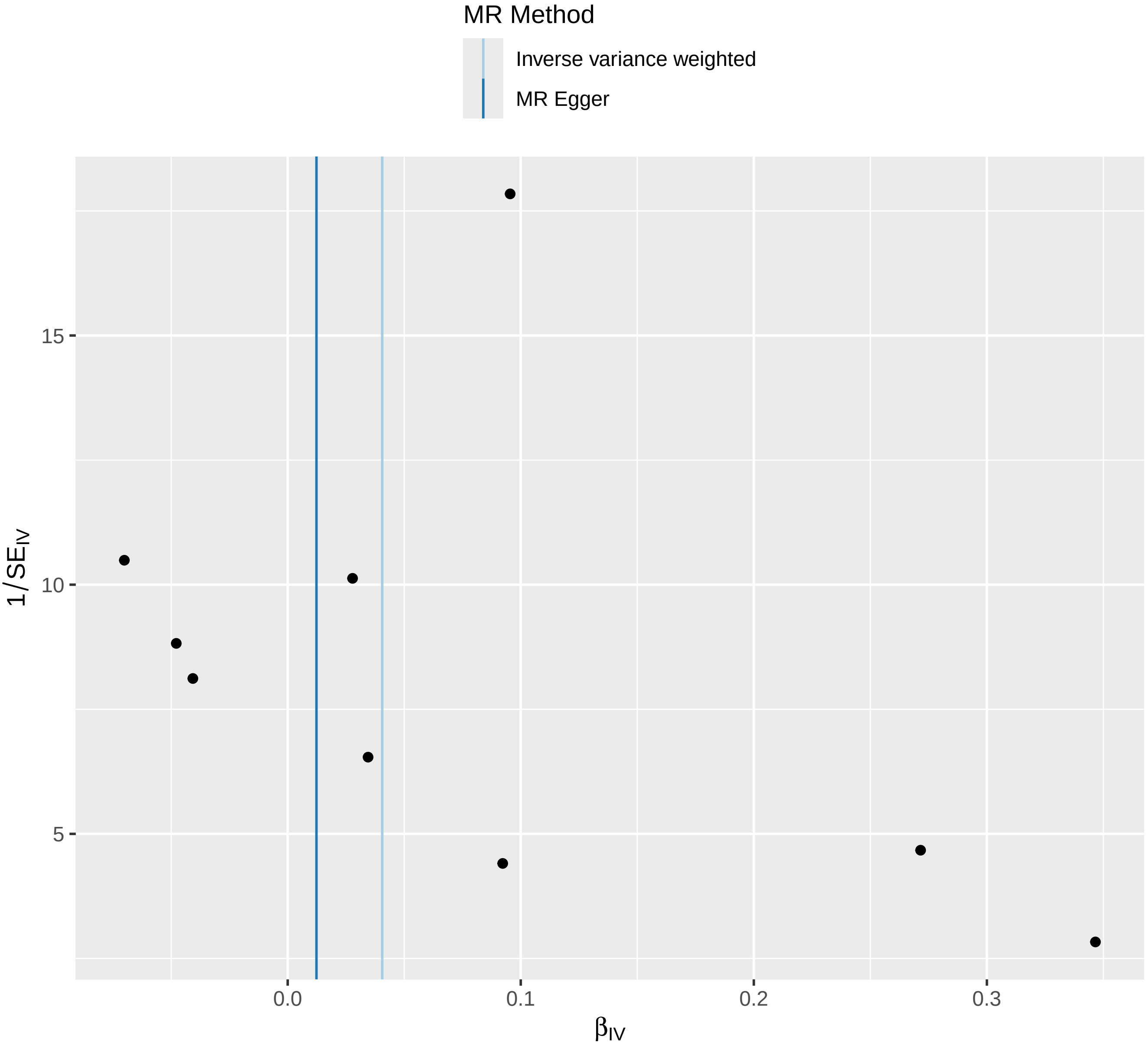

MR Method
Inverse variance weighted
MR Egger
15
10
5
$1/SE_{IV}$
0.0
0.1
0.2
0.3
$\beta_{IV}$

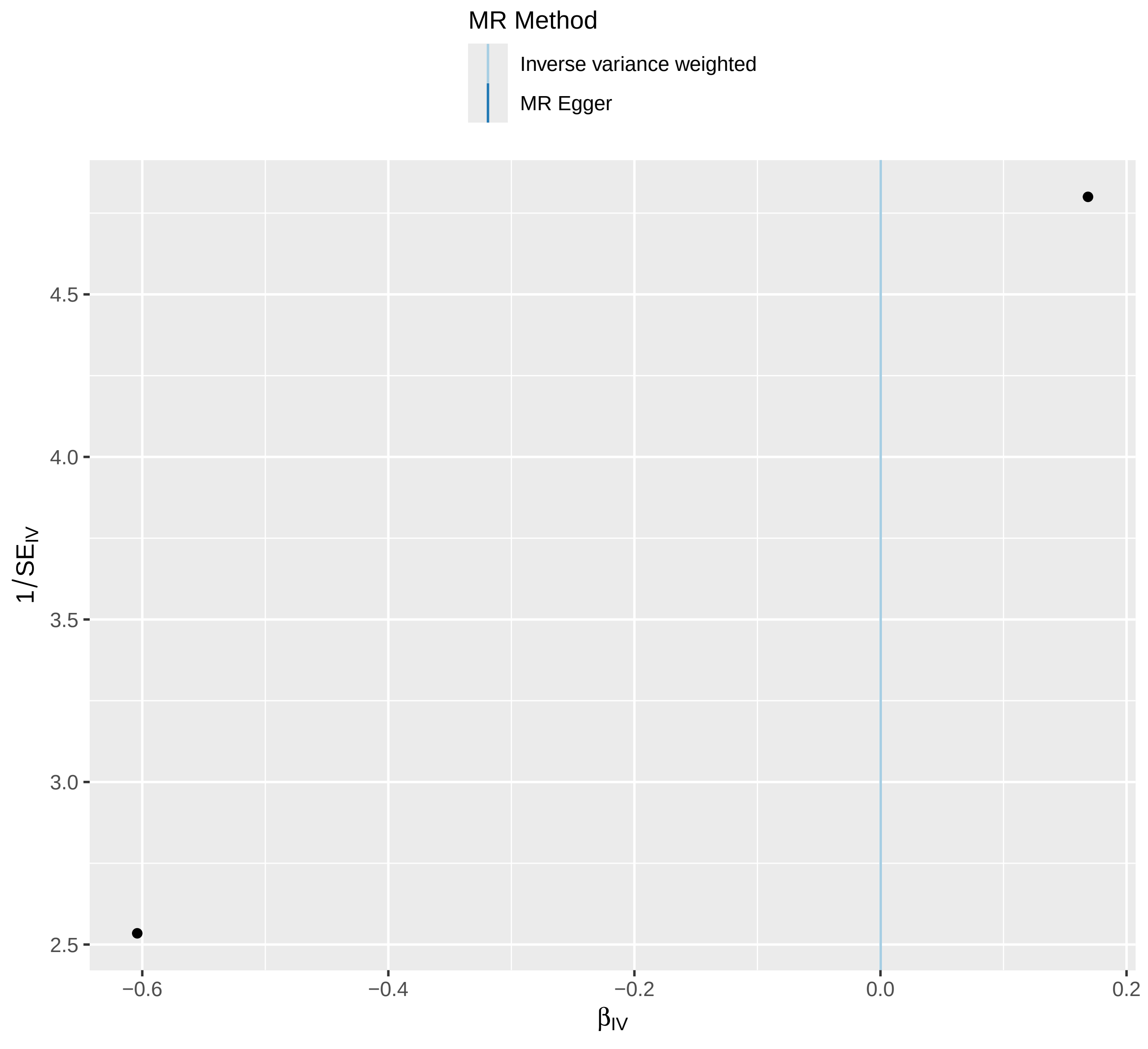
MR Method
Inverse variance weighted
MR Egger
4.5
4.0
3.5
3.0
2.5
1/SE_IV
−0.6
−0.4
−0.2
0.0
0.2
β_IV

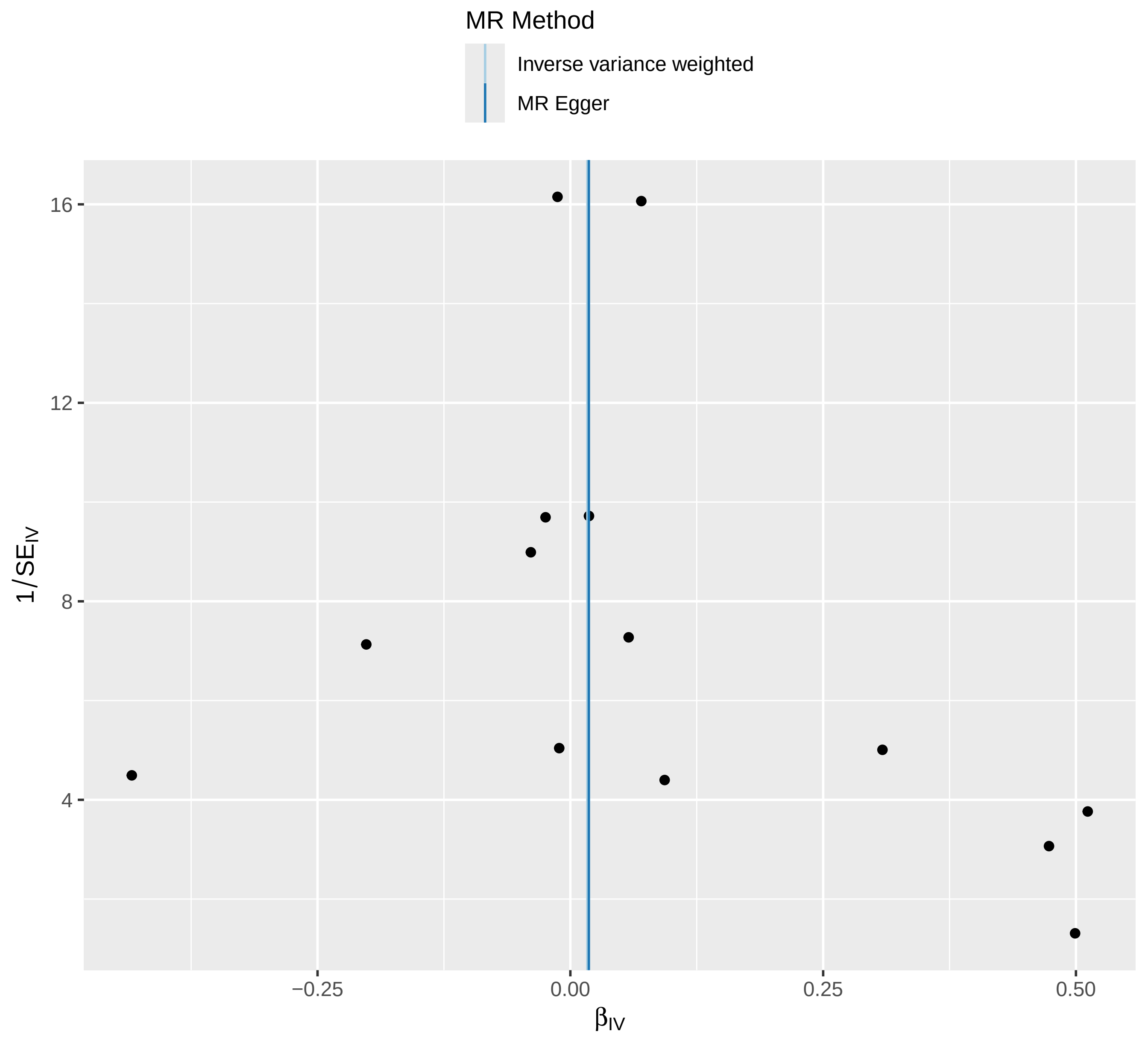
MR Method
Inverse variance weighted
MR Egger
16
12
8
4
$1/SE_{IV}$
−0.25
0.00
0.25
0.50
$\beta_{IV}$

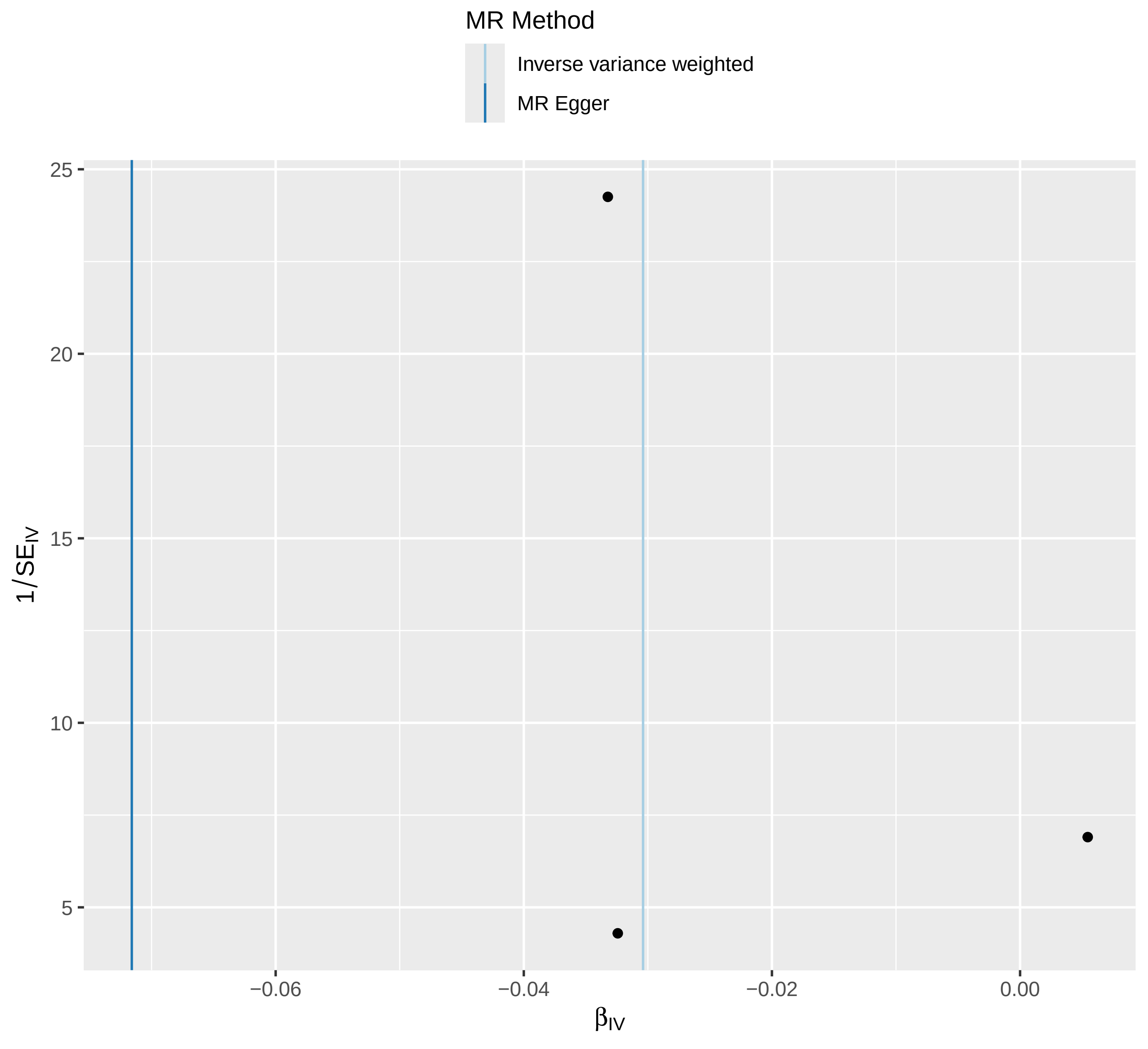
MR Method
Inverse variance weighted
MR Egger
25
20
15
10
5
1/SE_IV
−0.06
−0.04
−0.02
0.00
β_IV

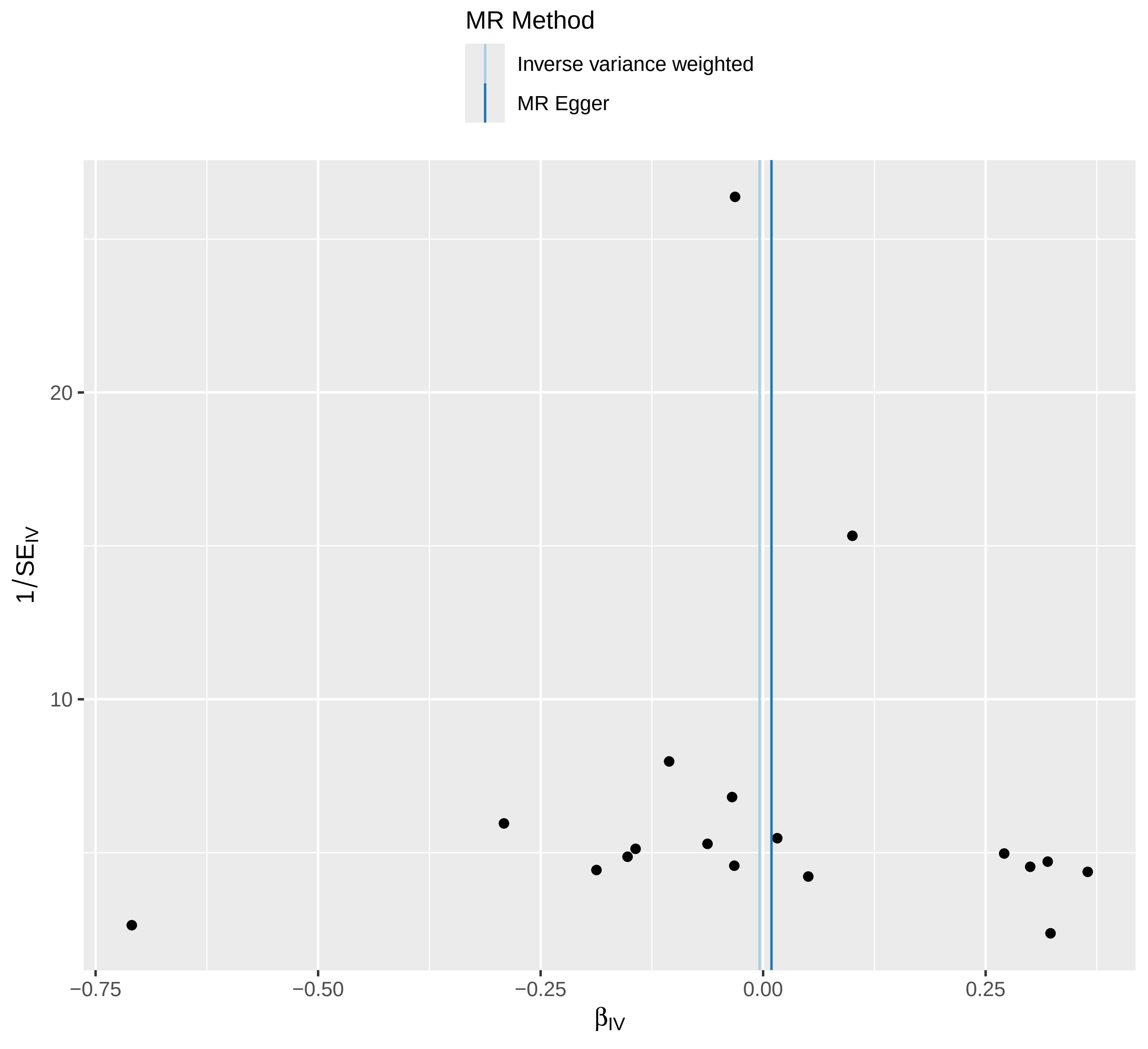
MR Method
Inverse variance weighted
MR Egger
1/SE$_{IV}$
20
10
−0.75
−0.50
−0.25
0.00
0.25
β$_{IV}$

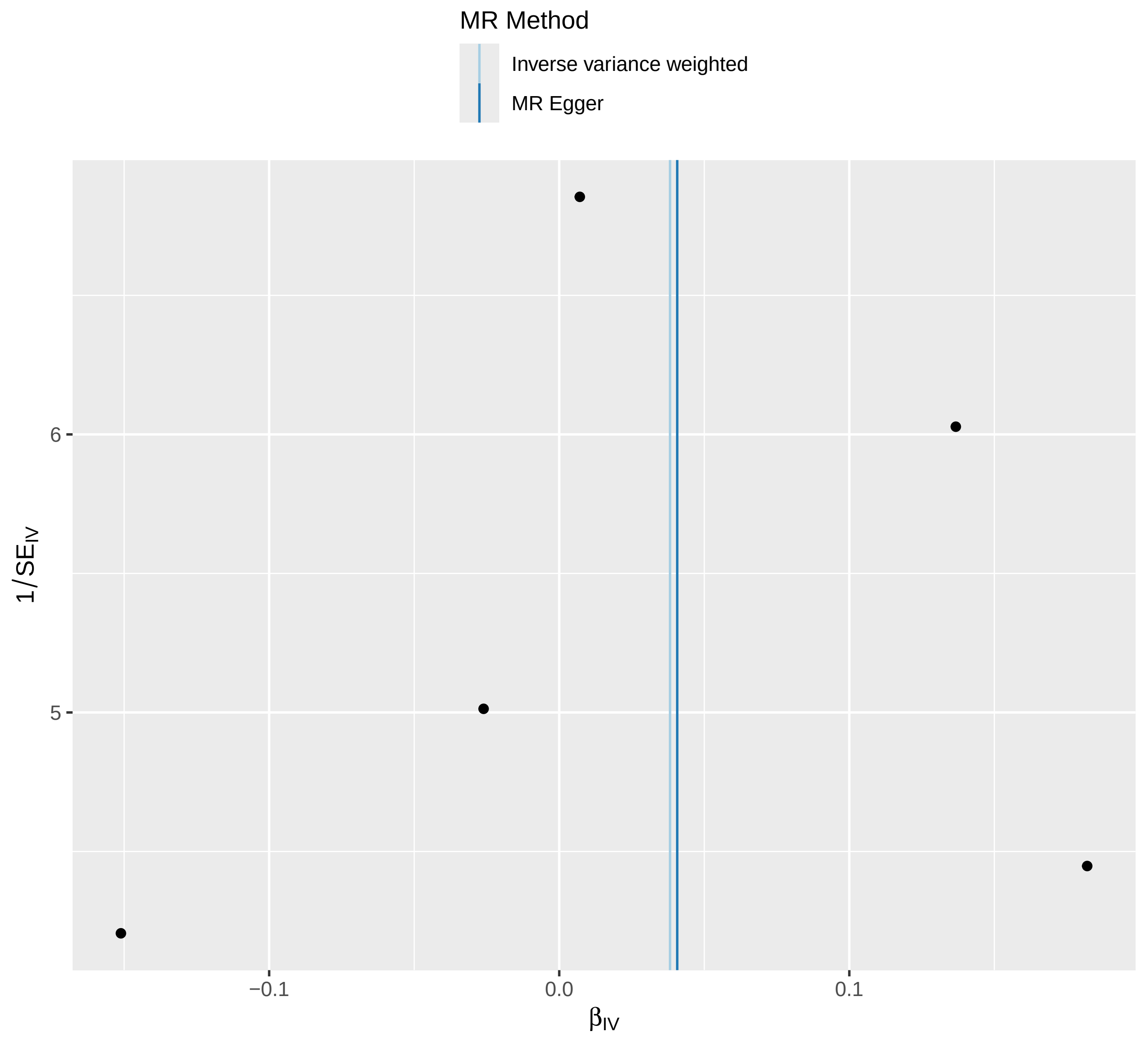

MR Method
Inverse variance weighted
MR Egger
6
5
1/SE_IV
−0.1
0.0
0.1
β_IV

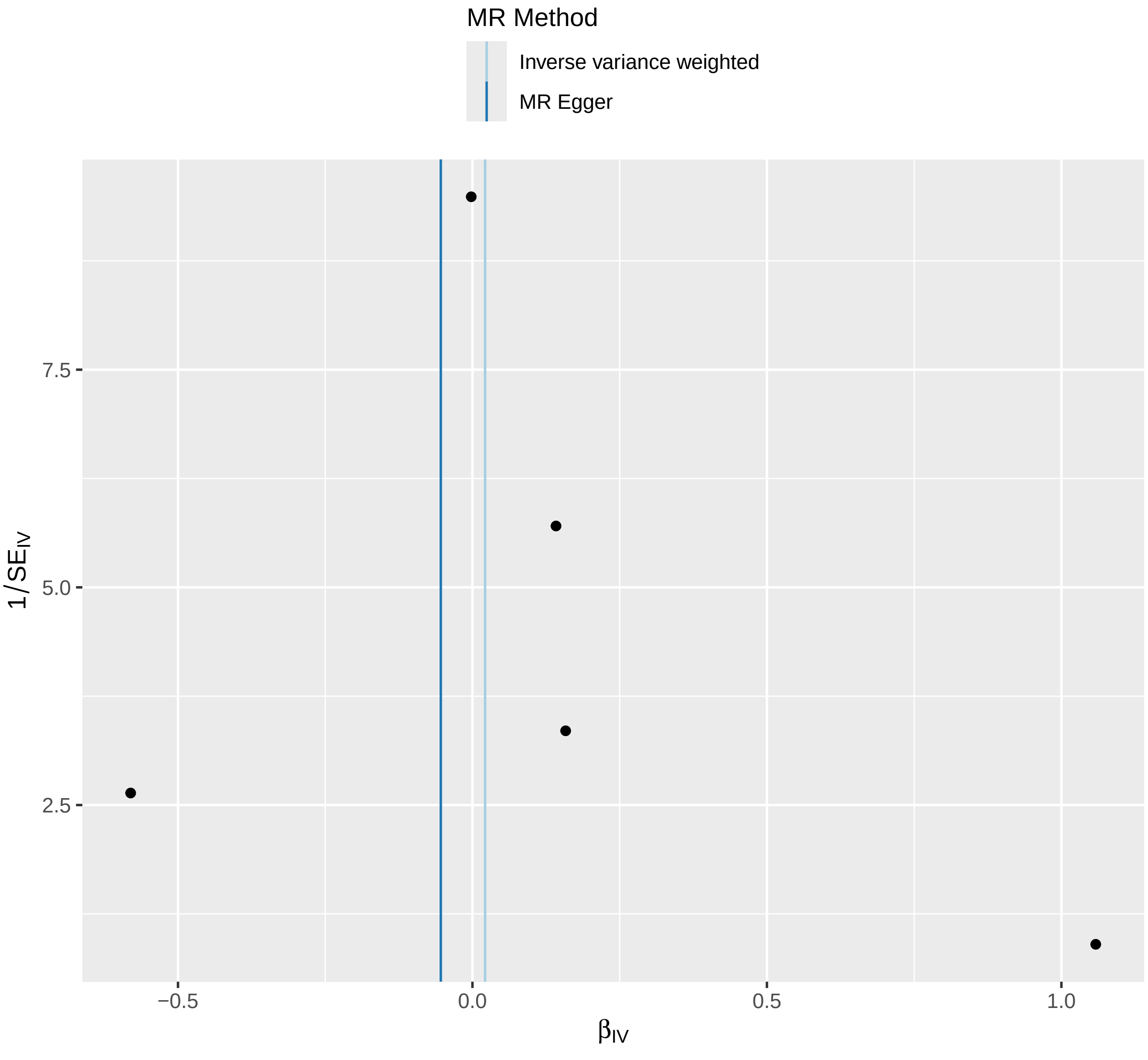

MR Method
Inverse variance weighted
MR Egger
7.5
5.0
2.5
1/SE$_{IV}$
−0.5
0.0
0.5
1.0
β$_{IV}$

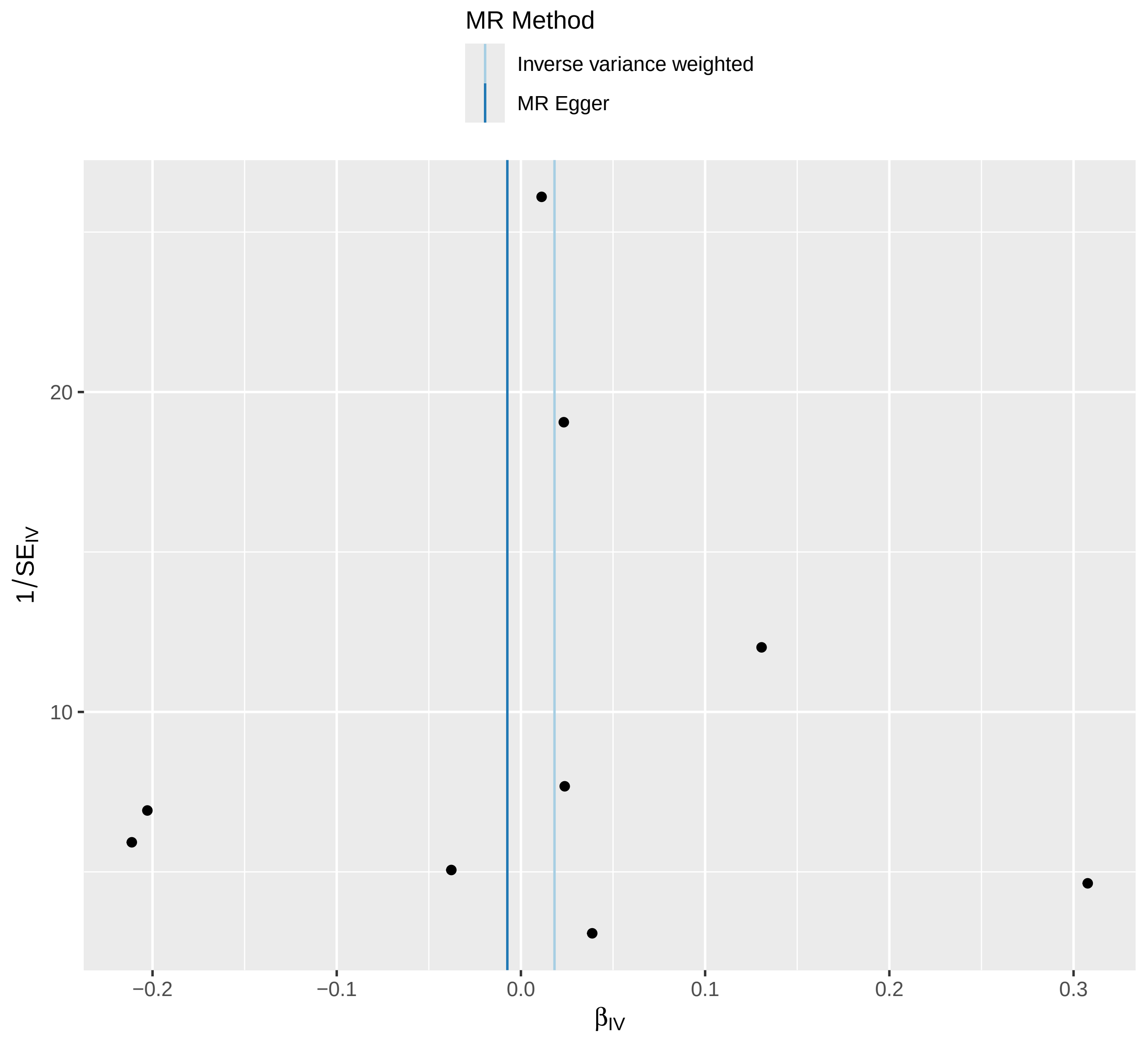

MR Method
Inverse variance weighted
MR Egger
20
10
1/SE$_{IV}$
−0.2
−0.1
0.0
0.1
0.2
0.3
β$_{IV}$

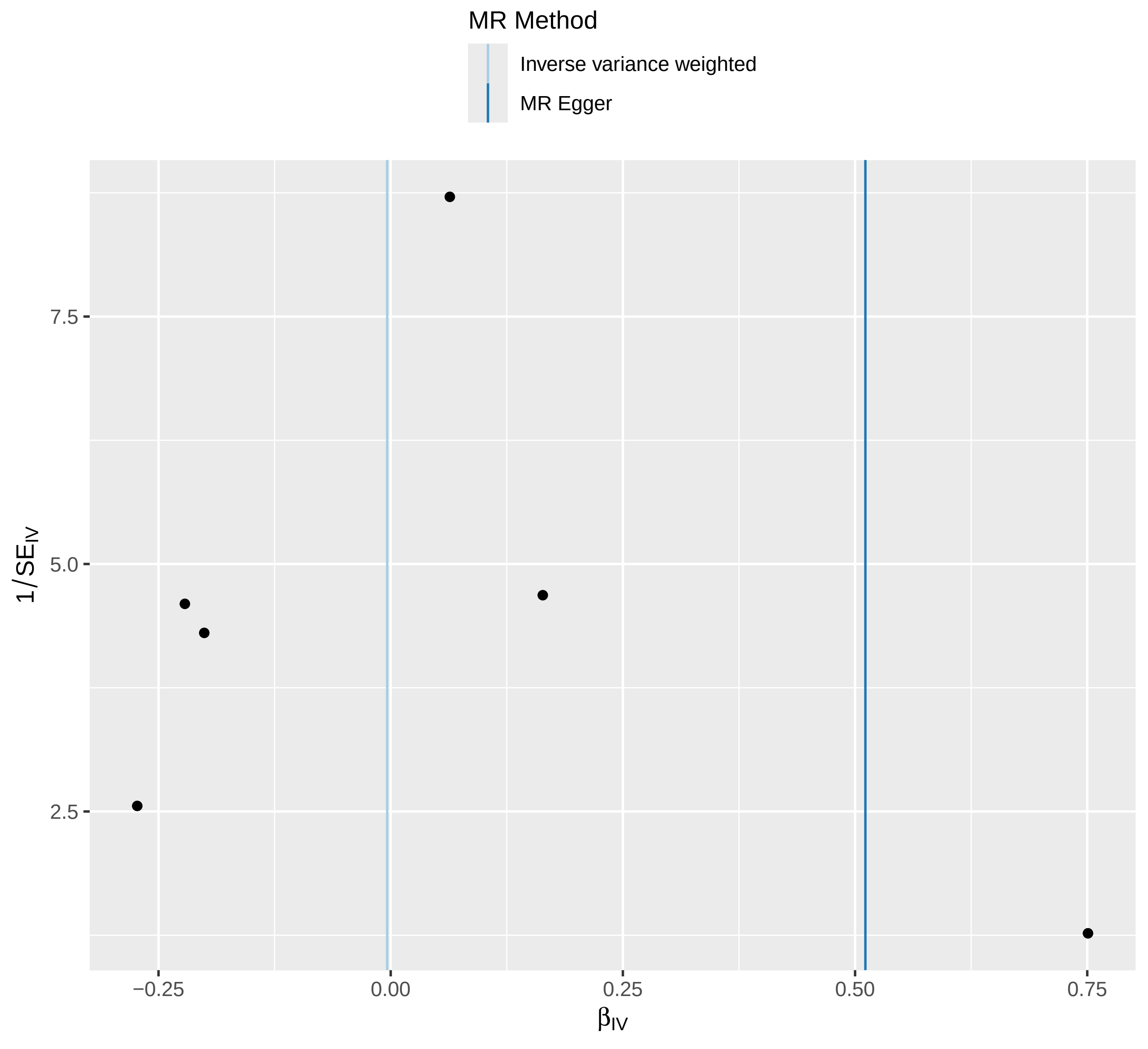
MR Method
Inverse variance weighted
MR Egger
7.5
5.0
2.5
1/SE$_{IV}$
−0.25
0.00
0.25
0.50
0.75
β$_{IV}$

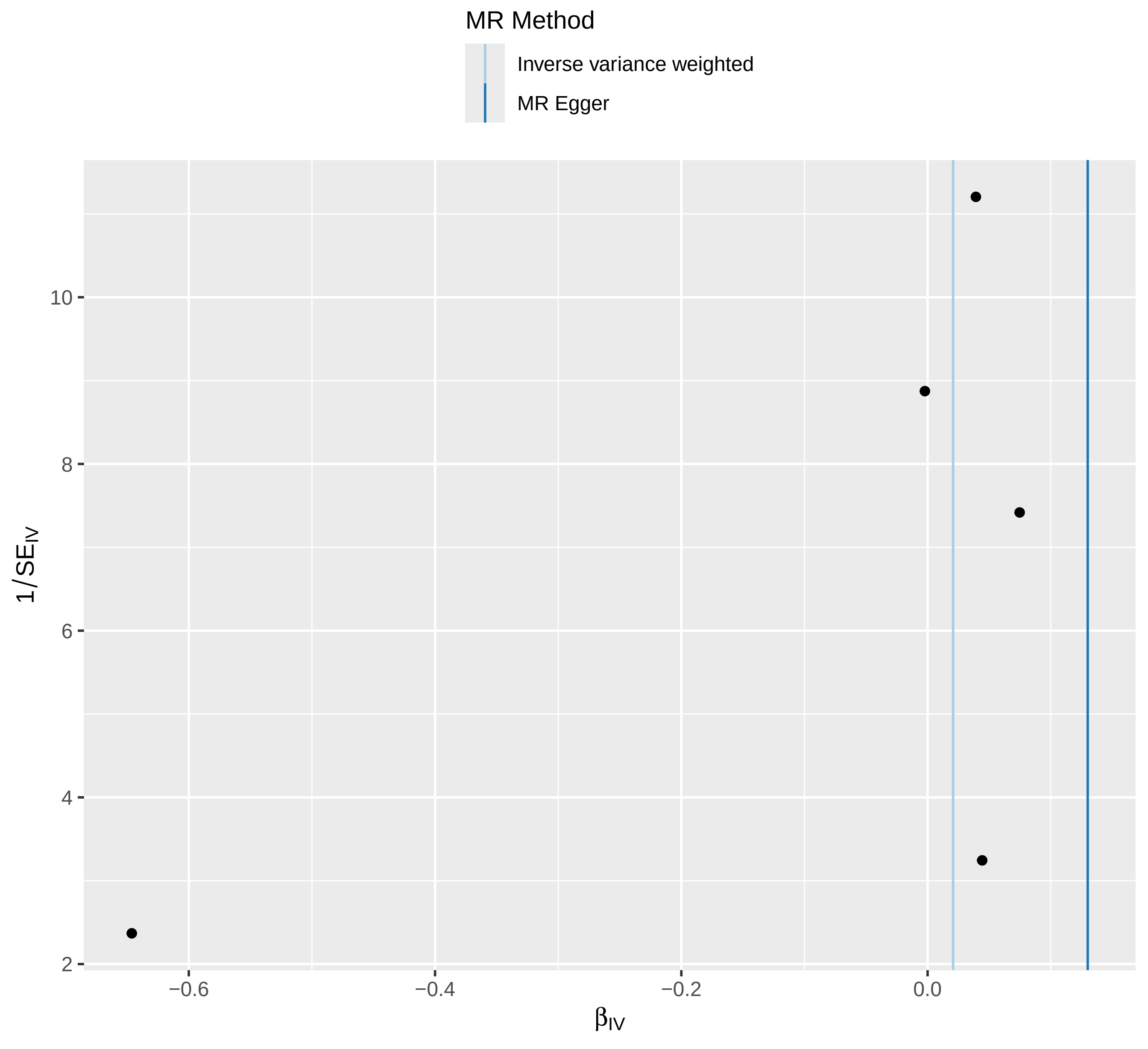

MR Method
Inverse variance weighted
MR Egger
1/SE$_{IV}$
10
8
6
4
2
−0.6
−0.4
−0.2
0.0
β$_{IV}$

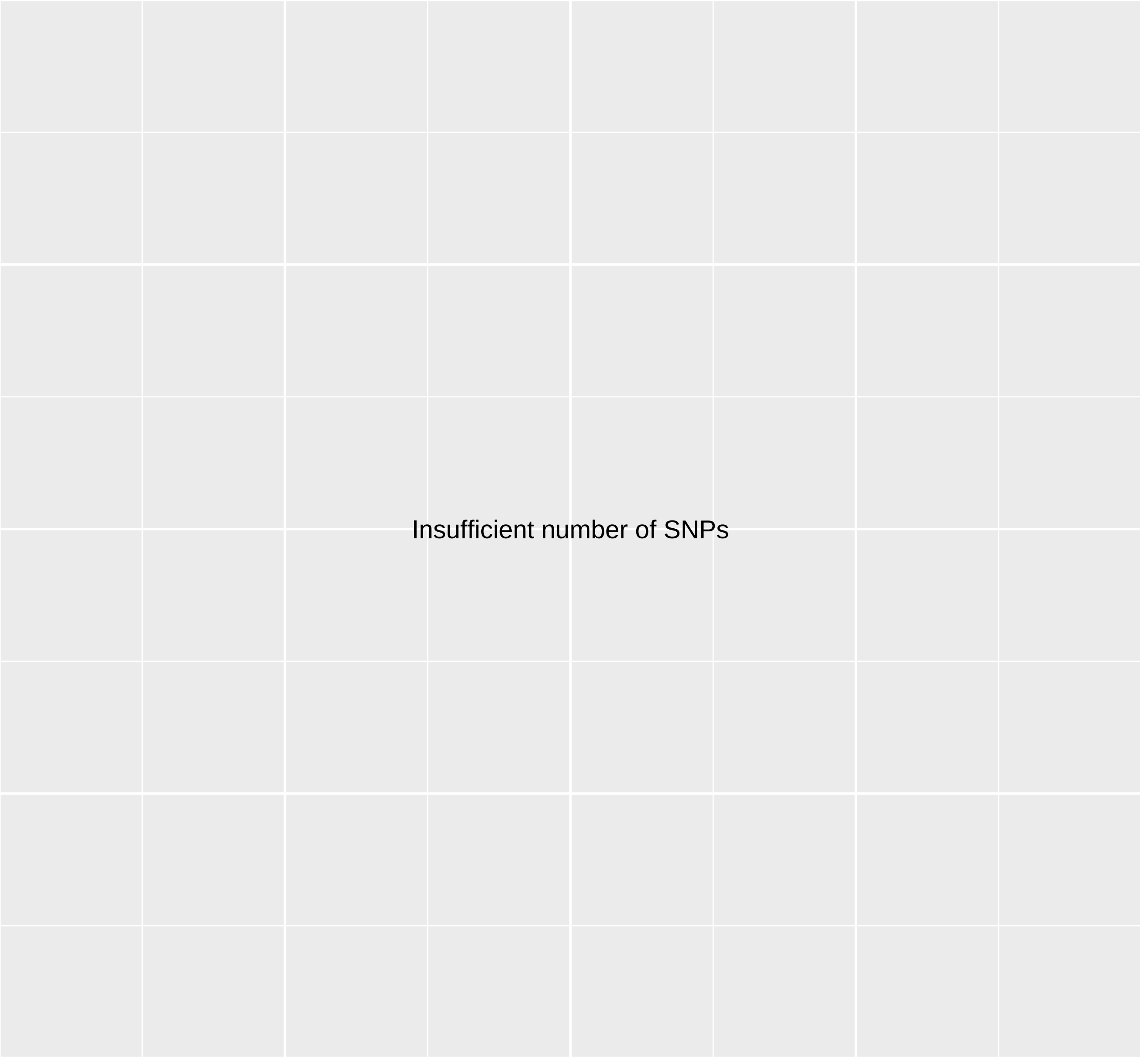
Insufficient number of SNPs

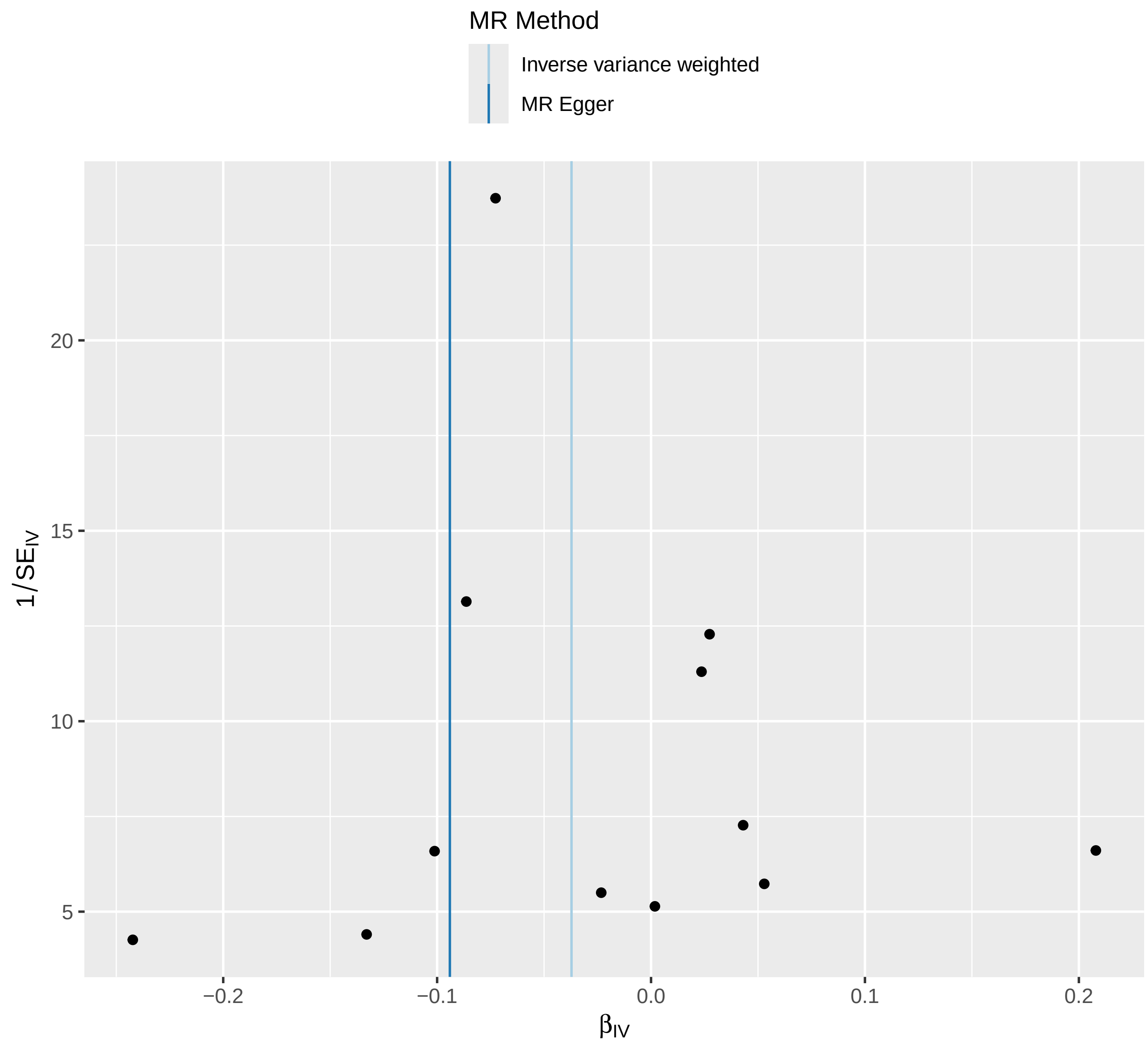
MR Method
Inverse variance weighted
MR Egger
1/$SE_{IV}$
20
15
10
5
−0.2
−0.1
0.0
0.1
0.2
$\beta_{IV}$

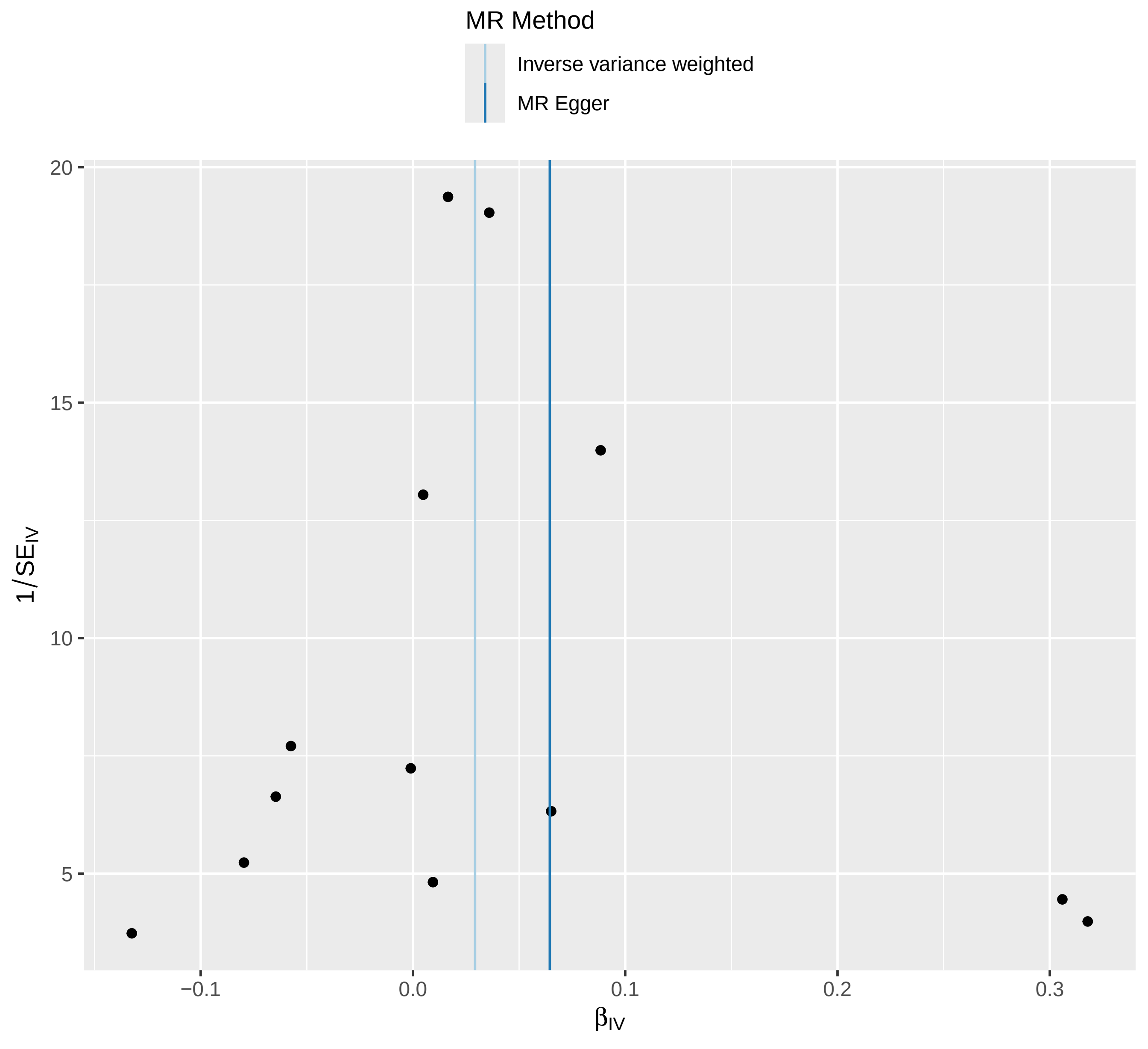

MR Method
Inverse variance weighted
MR Egger
20
15
10
5
$1/SE_{IV}$
−0.1
0.0
0.1
0.2
0.3
$\beta_{IV}$

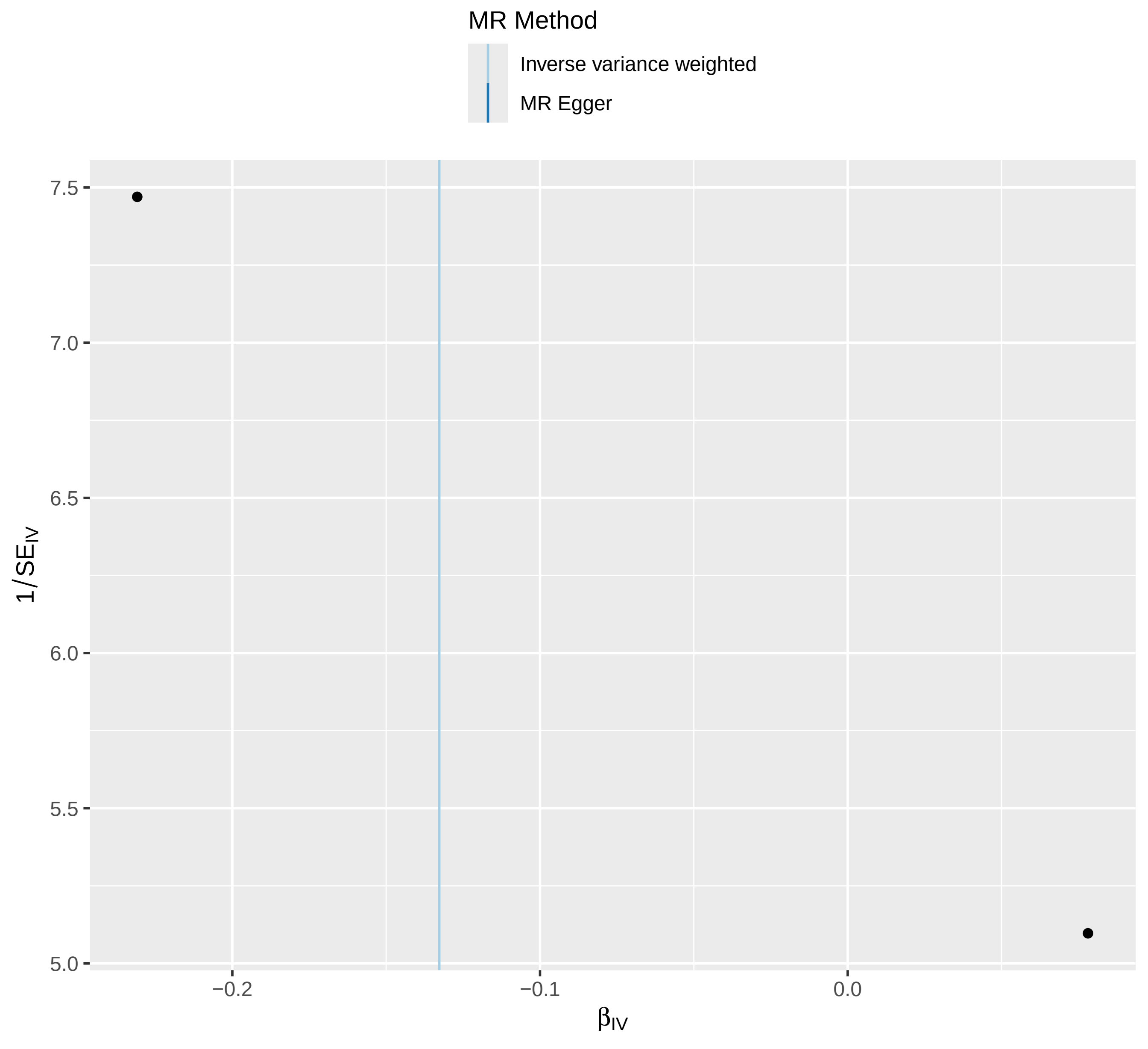
MR Method
Inverse variance weighted
MR Egger
7.5
7.0
6.5
6.0
5.5
5.0
1/SE_IV
−0.2
−0.1
0.0
β_IV

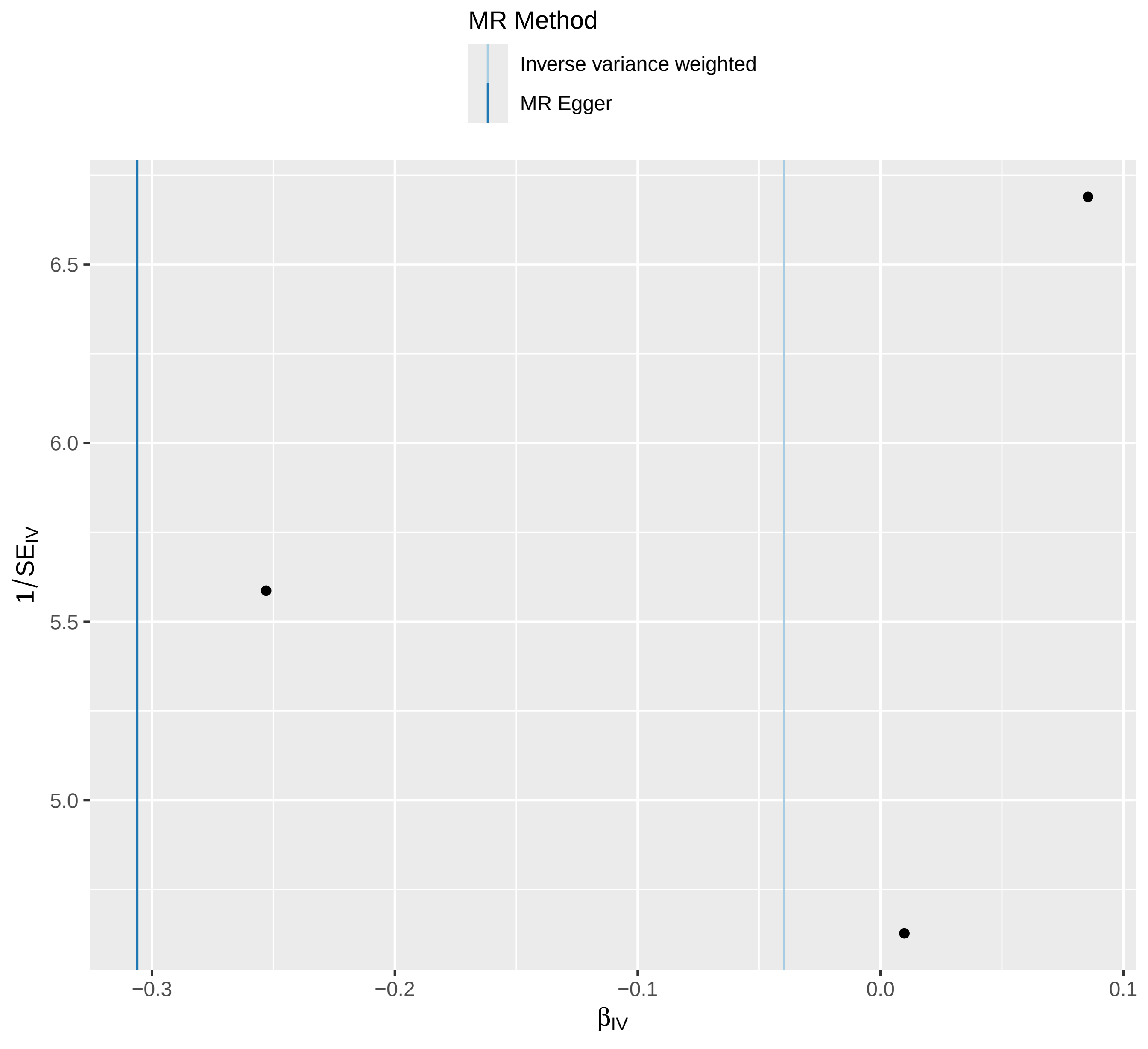

MR Method
Inverse variance weighted
MR Egger
6.5
6.0
5.5
5.0
1/SE_IV
−0.3
−0.2
−0.1
0.0
0.1
β_IV

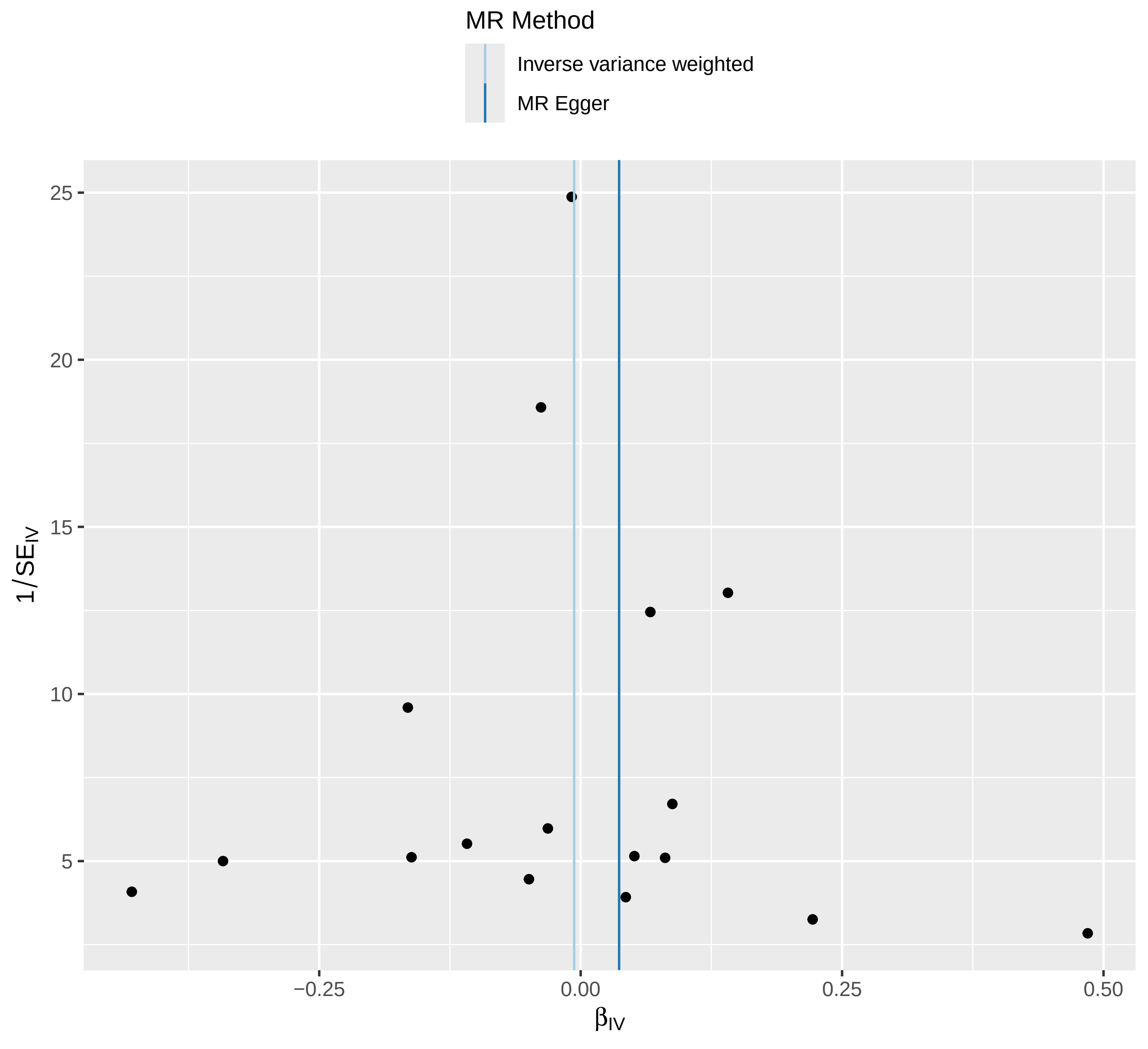
MR Method
Inverse variance weighted
MR Egger
25
20
15
10
5
1/SE$_{IV}$
−0.25
0.00
0.25
0.50
β$_{IV}$

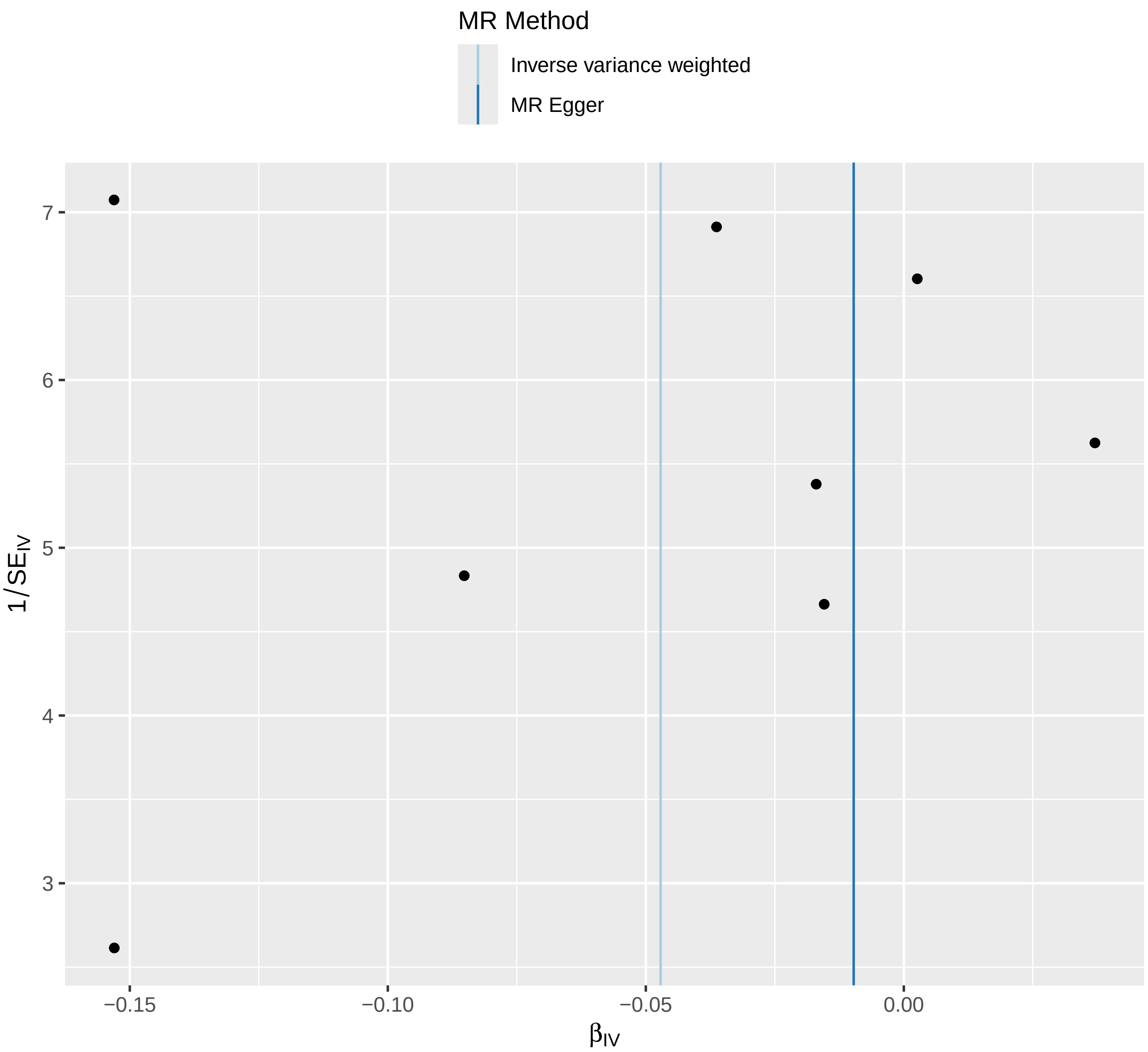
MR Method
Inverse variance weighted
MR Egger
1/SE_IV
7
6
5
4
3
−0.15
−0.10
−0.05
0.00
β_IV

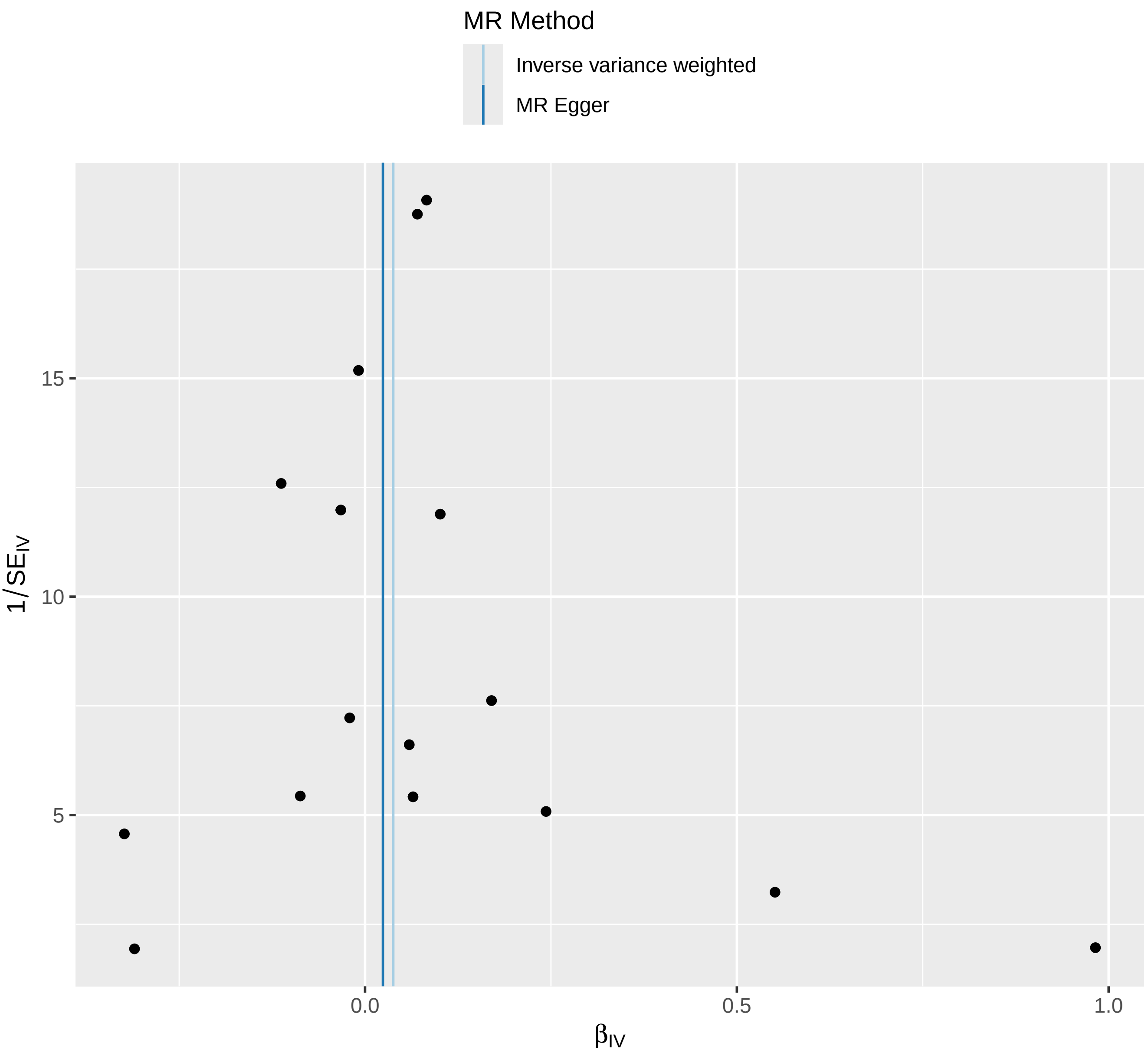
MR Method
Inverse variance weighted
MR Egger
15
10
5
1/SE$_{IV}$
0.0
0.5
1.0
β$_{IV}$

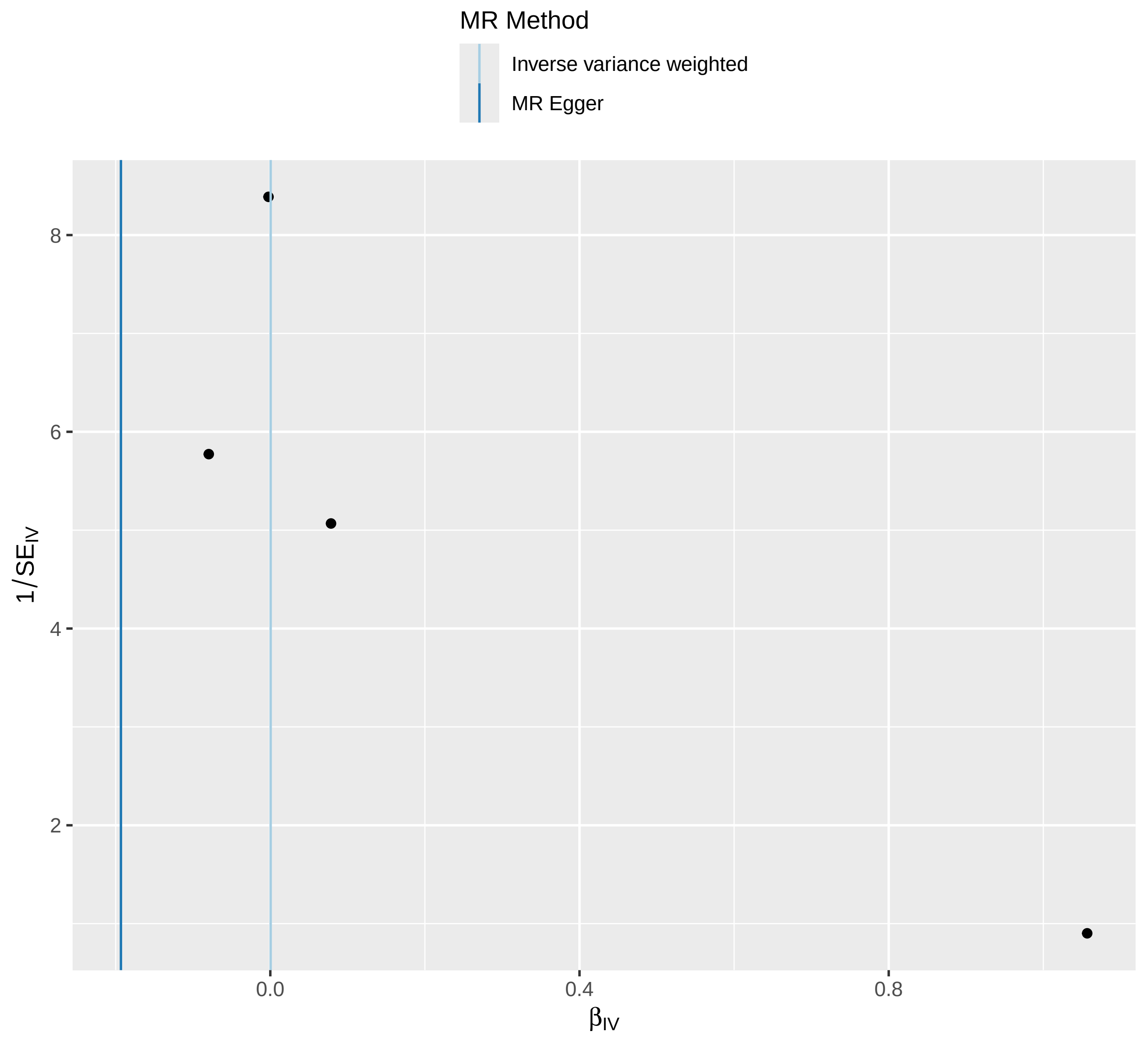
MR Method
Inverse variance weighted
MR Egger
8
6
4
2
$1/SE_{IV}$
0.0
0.4
0.8
$\beta_{IV}$

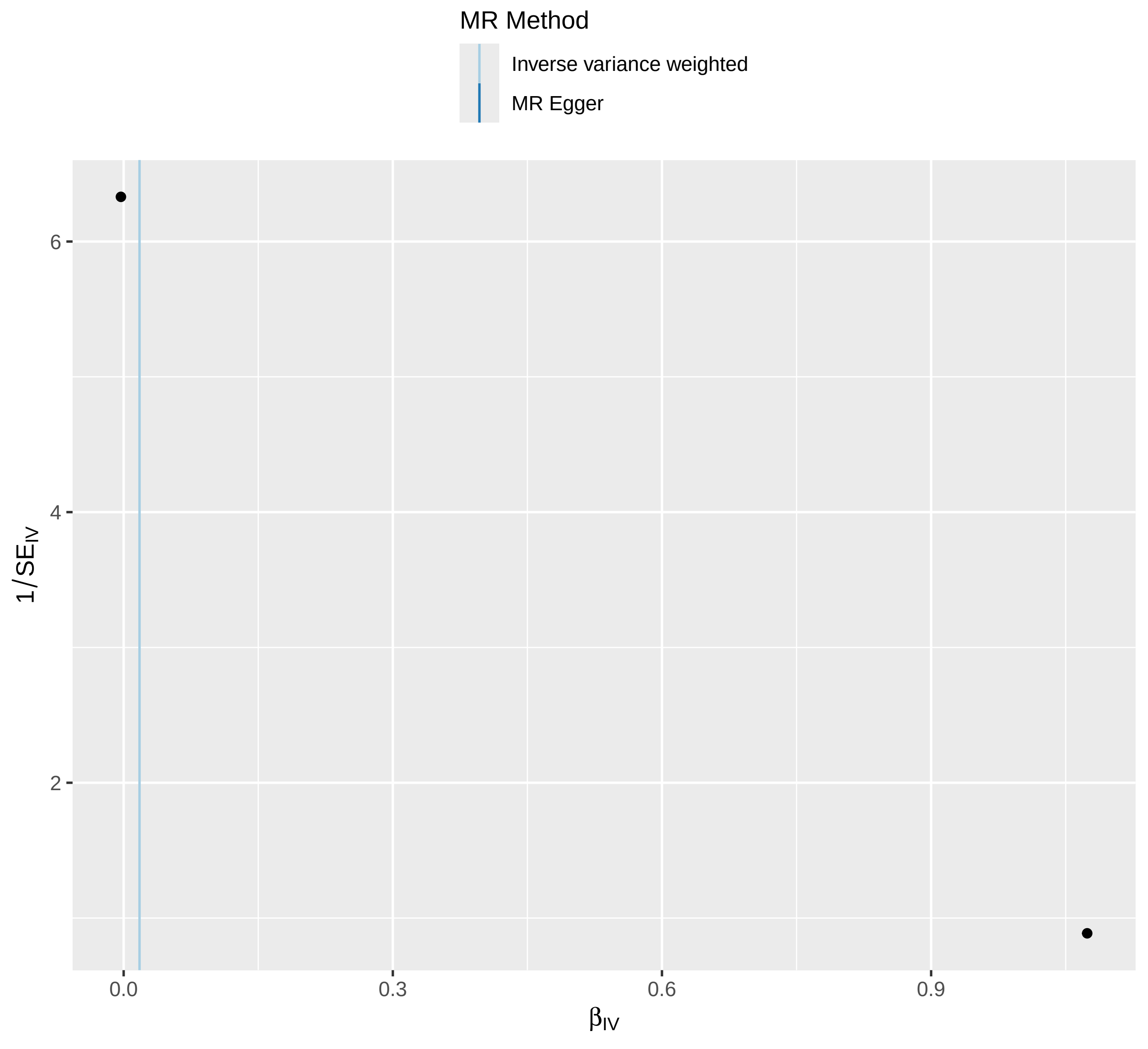
MR Method
Inverse variance weighted
MR Egger
6
4
2
0.0
0.3
0.6
0.9
$1/SE_{IV}$
$\beta_{IV}$

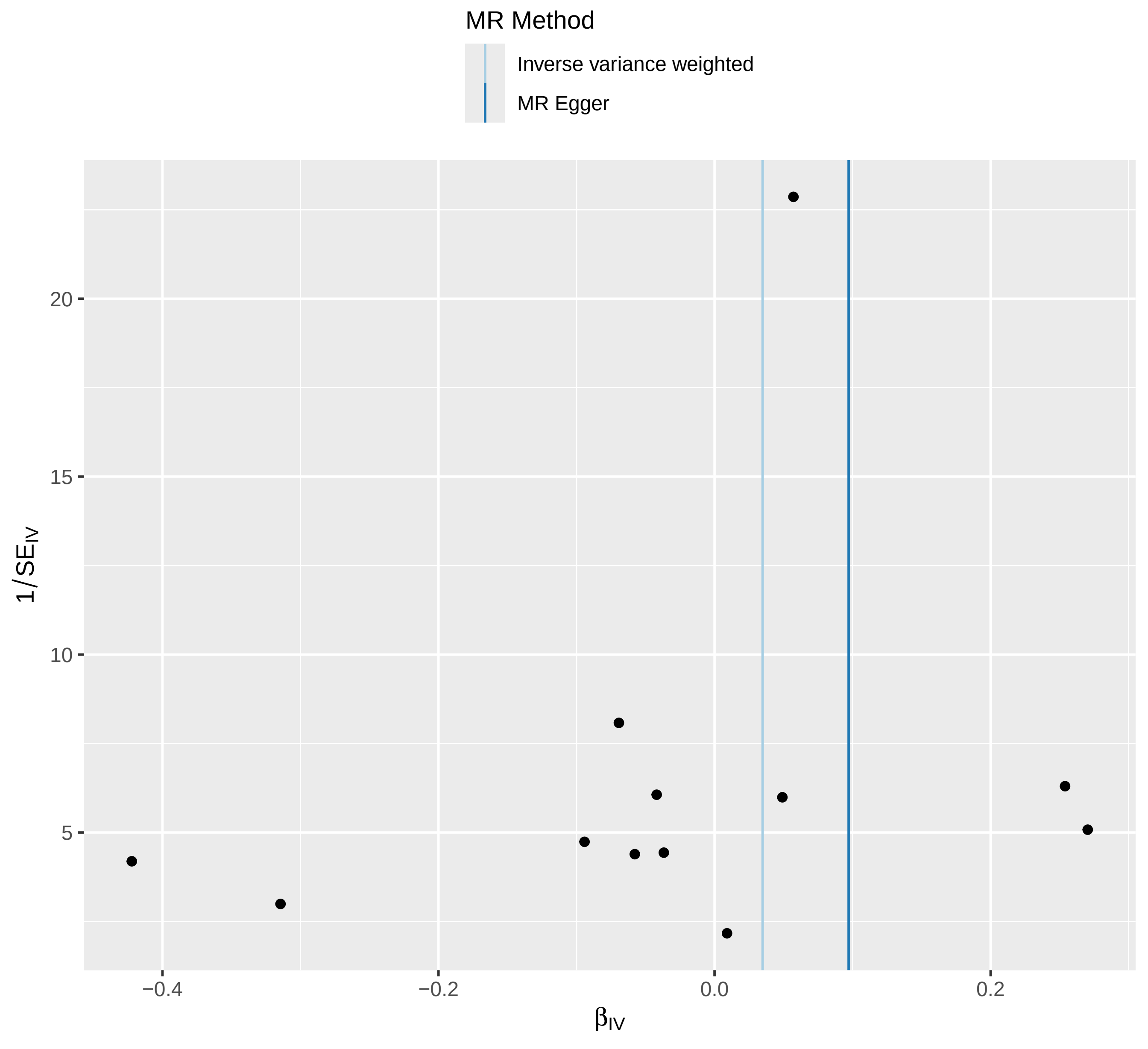

MR Method
Inverse variance weighted
MR Egger
20
15
10
5
1/SE_IV
−0.4
−0.2
0.0
0.2
β_IV

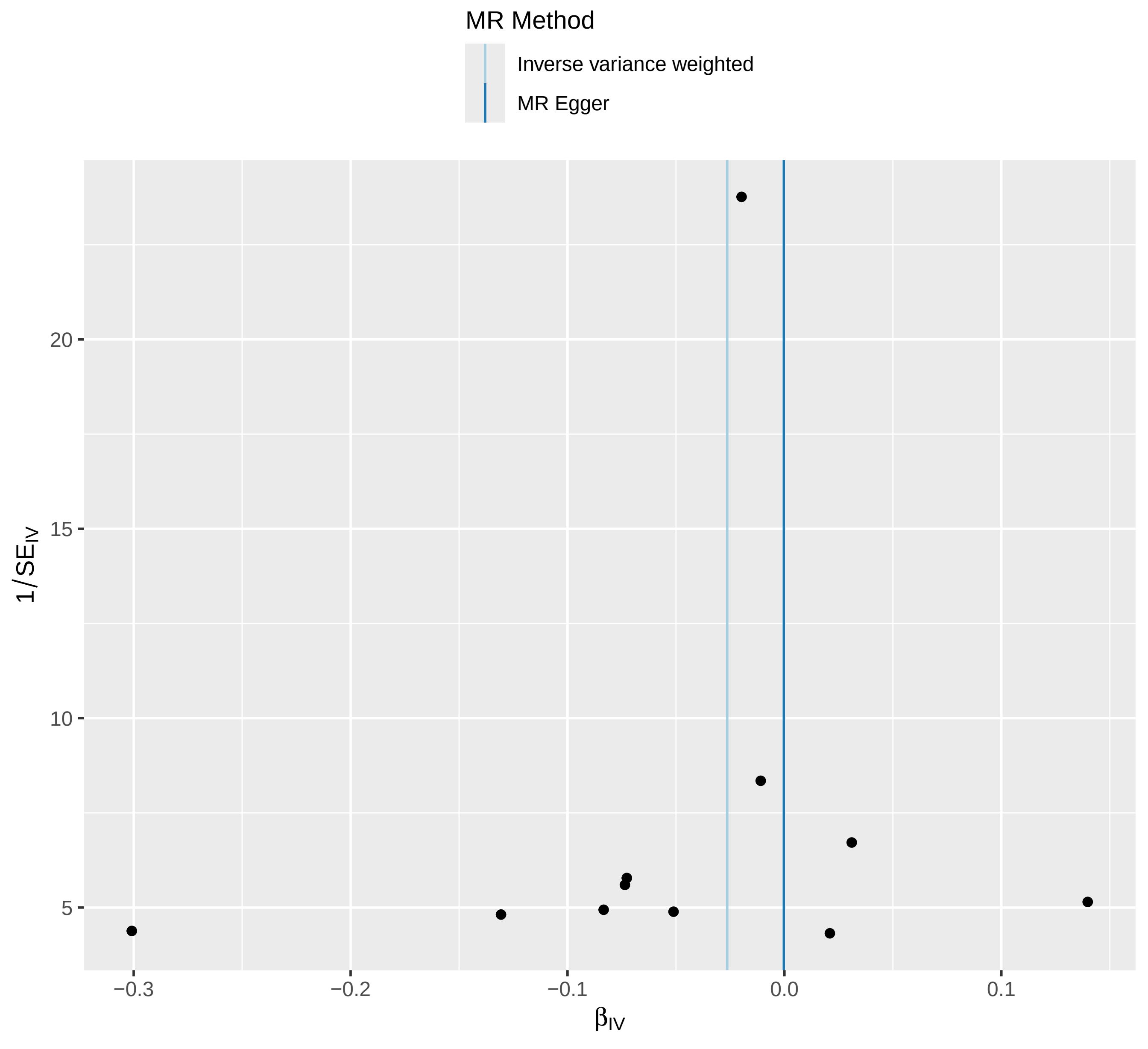
MR Method
Inverse variance weighted
MR Egger
1/SE$_{IV}$
20
15
10
5
−0.3
−0.2
−0.1
0.0
0.1
β$_{IV}$

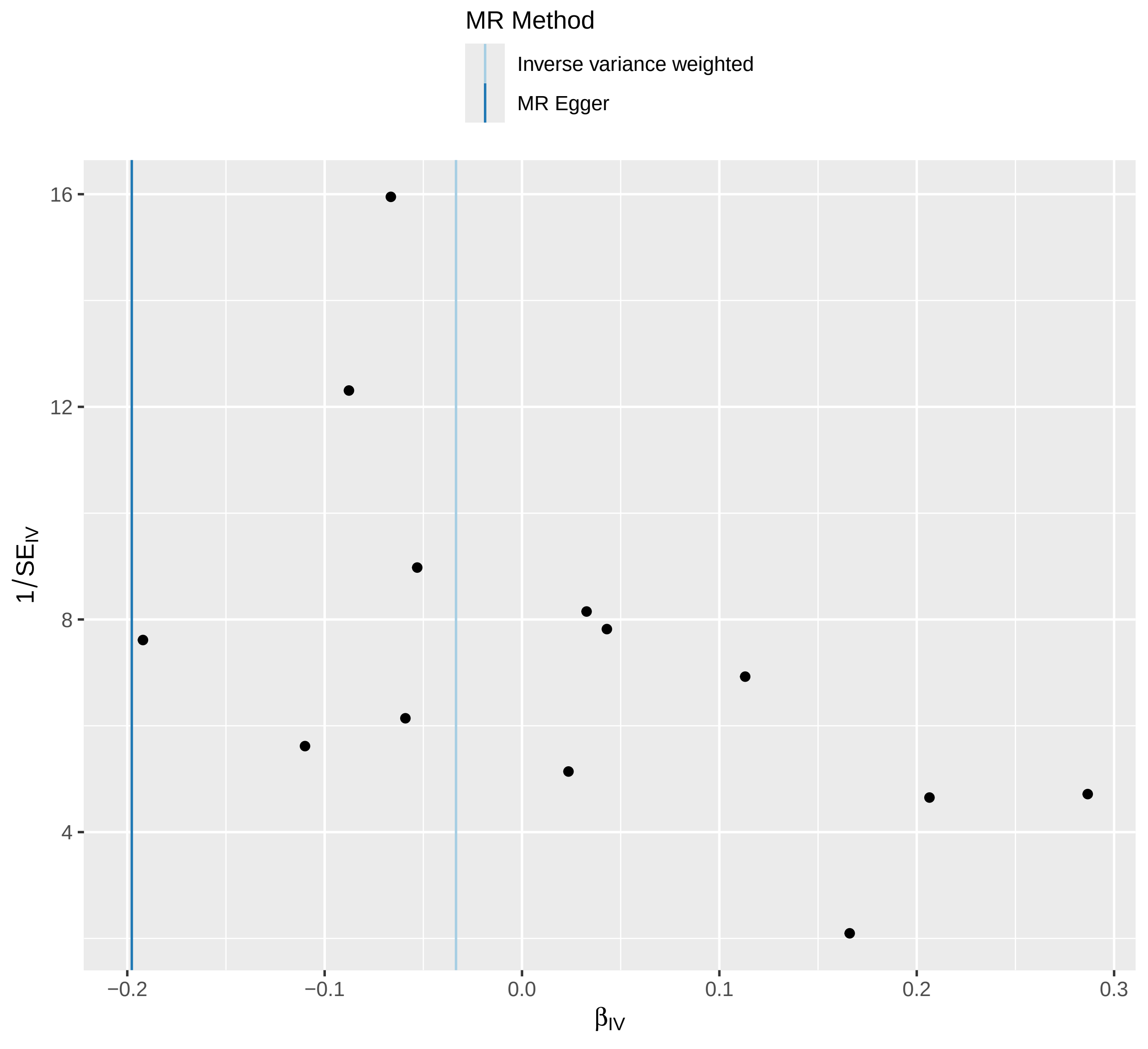
MR Method
Inverse variance weighted
MR Egger
16
12
8
4
1/SE$_{IV}$
−0.2
−0.1
0.0
0.1
0.2
0.3
β$_{IV}$

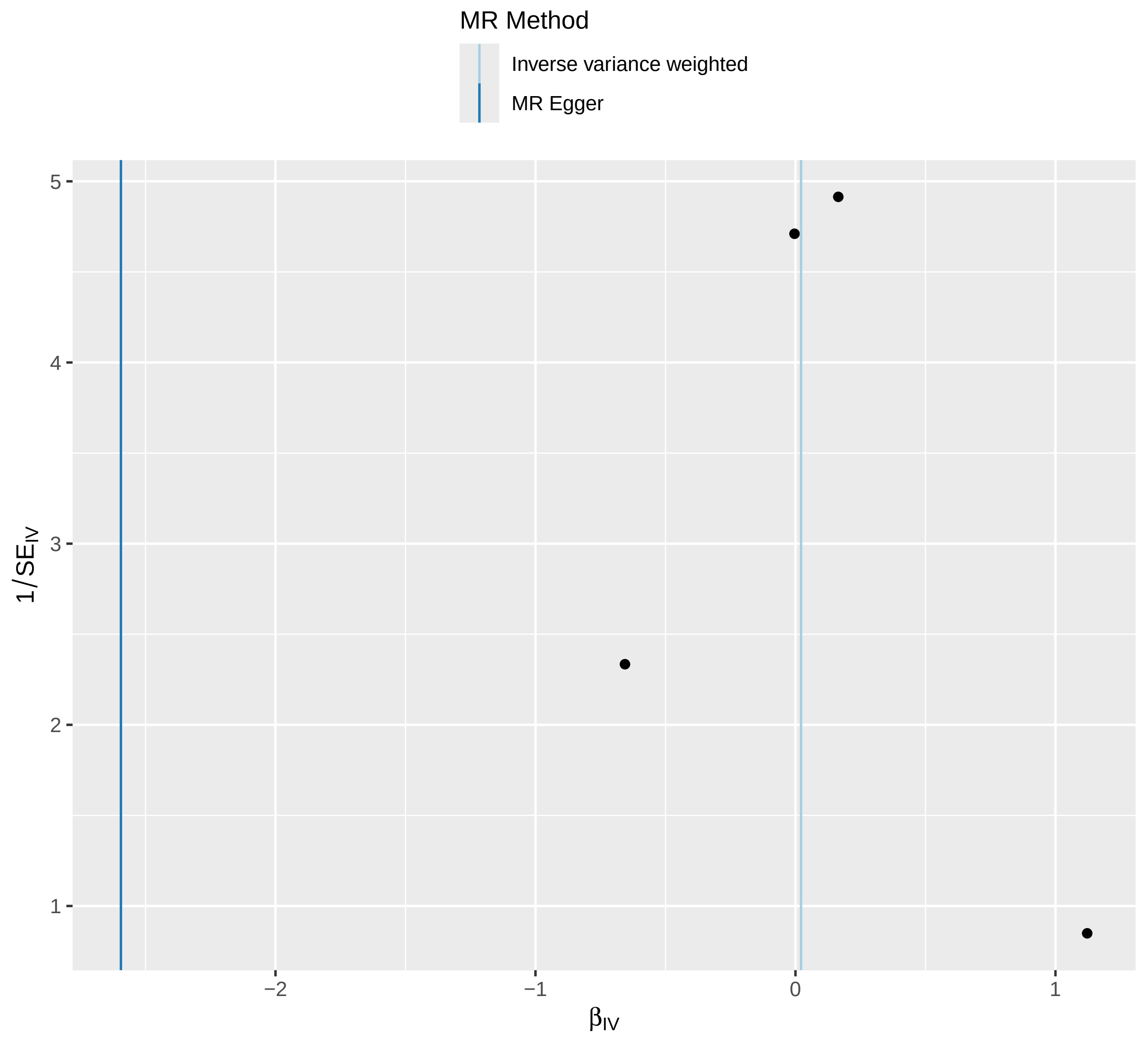
MR Method
Inverse variance weighted
MR Egger
1/SE$_{IV}$
5
4
3
2
1
−2
−1
0
1
β$_{IV}$

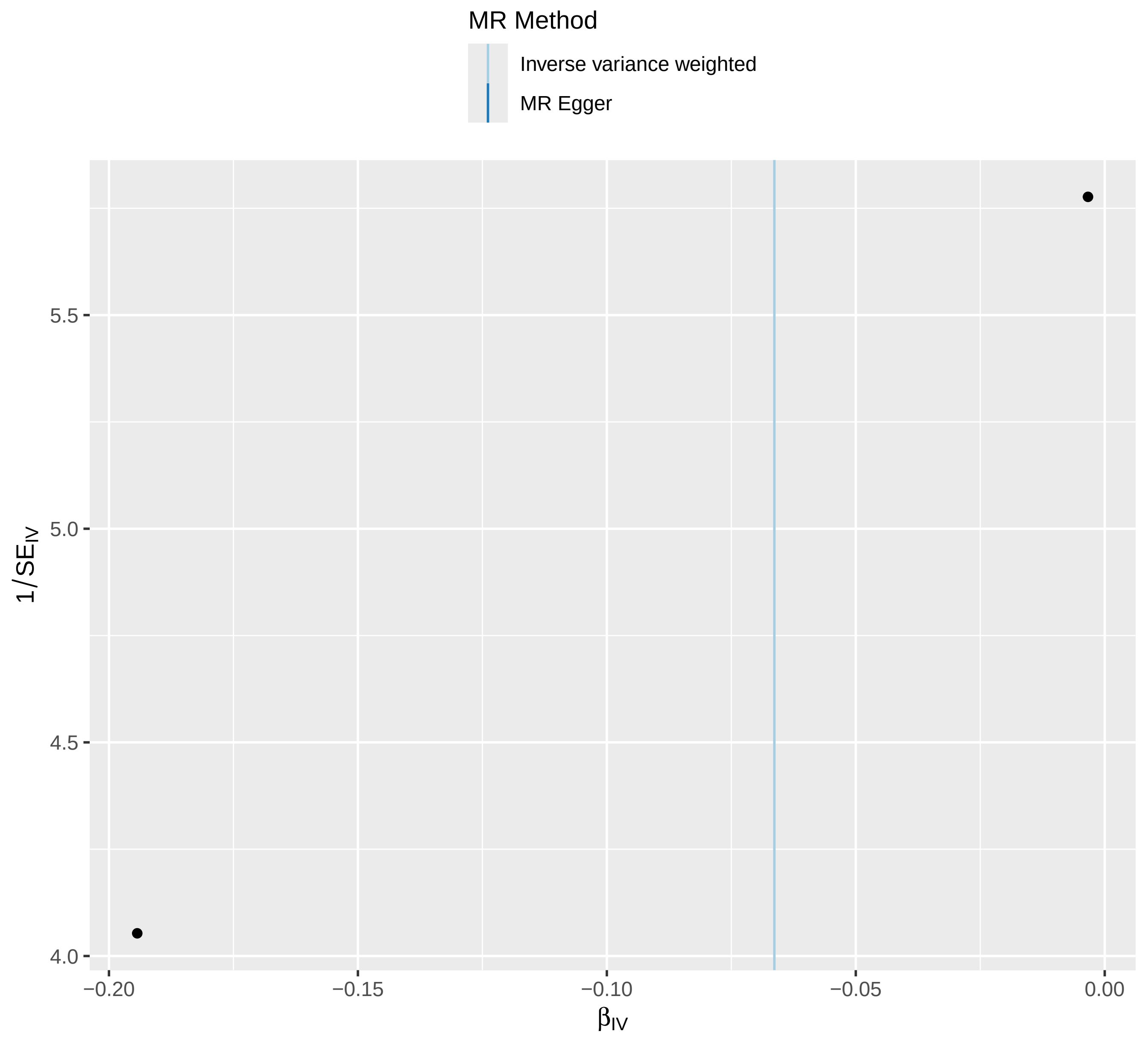

MR Method
Inverse variance weighted
MR Egger
5.5
5.0
4.5
4.0
1/SE$_{IV}$
−0.20
−0.15
−0.10
−0.05
0.00
β$_{IV}$

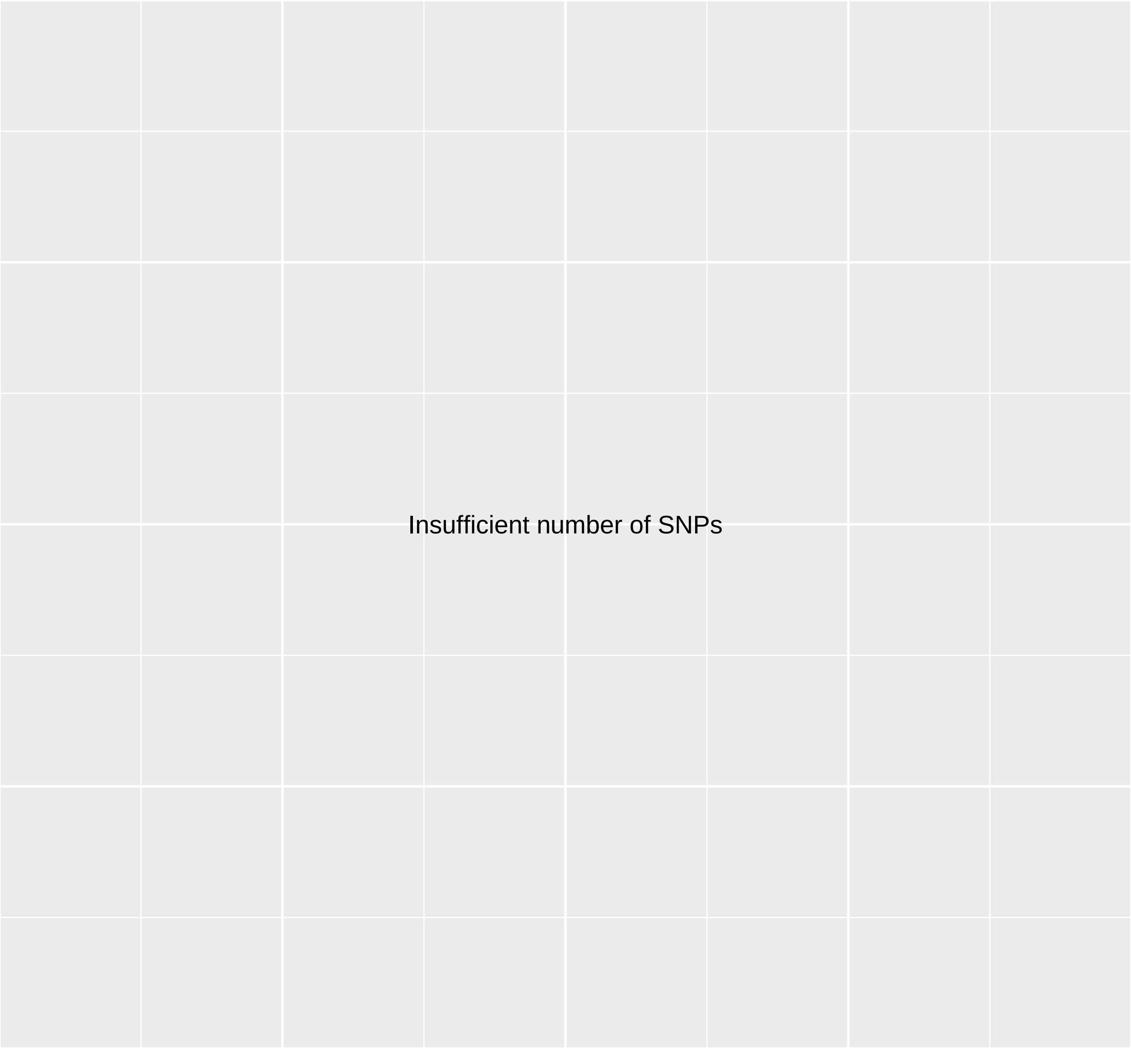
Insufficient number of SNPs

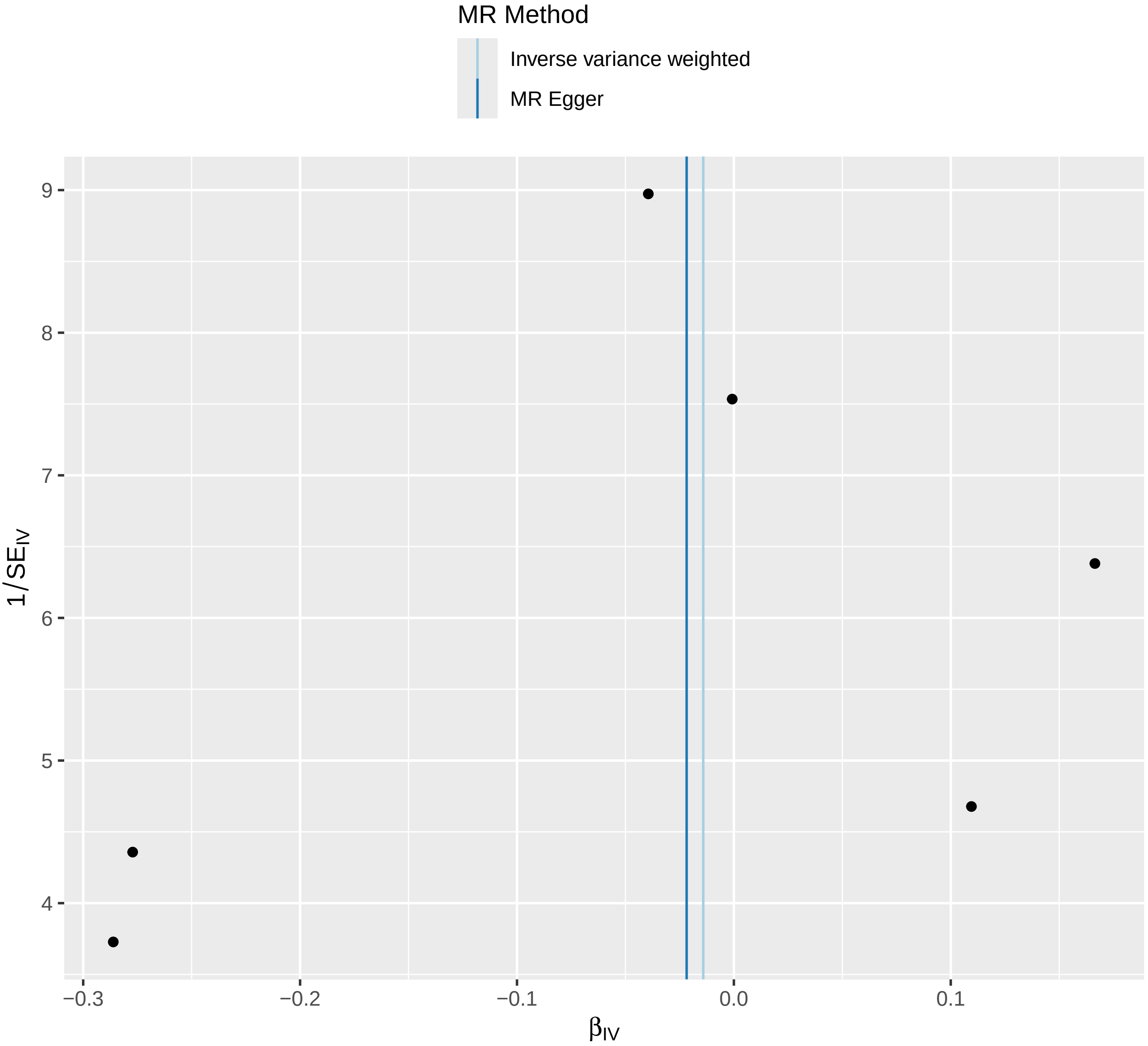
MR Method
Inverse variance weighted
MR Egger
9
8
7
6
5
4
1/SE_IV
−0.3
−0.2
−0.1
0.0
0.1
β_IV

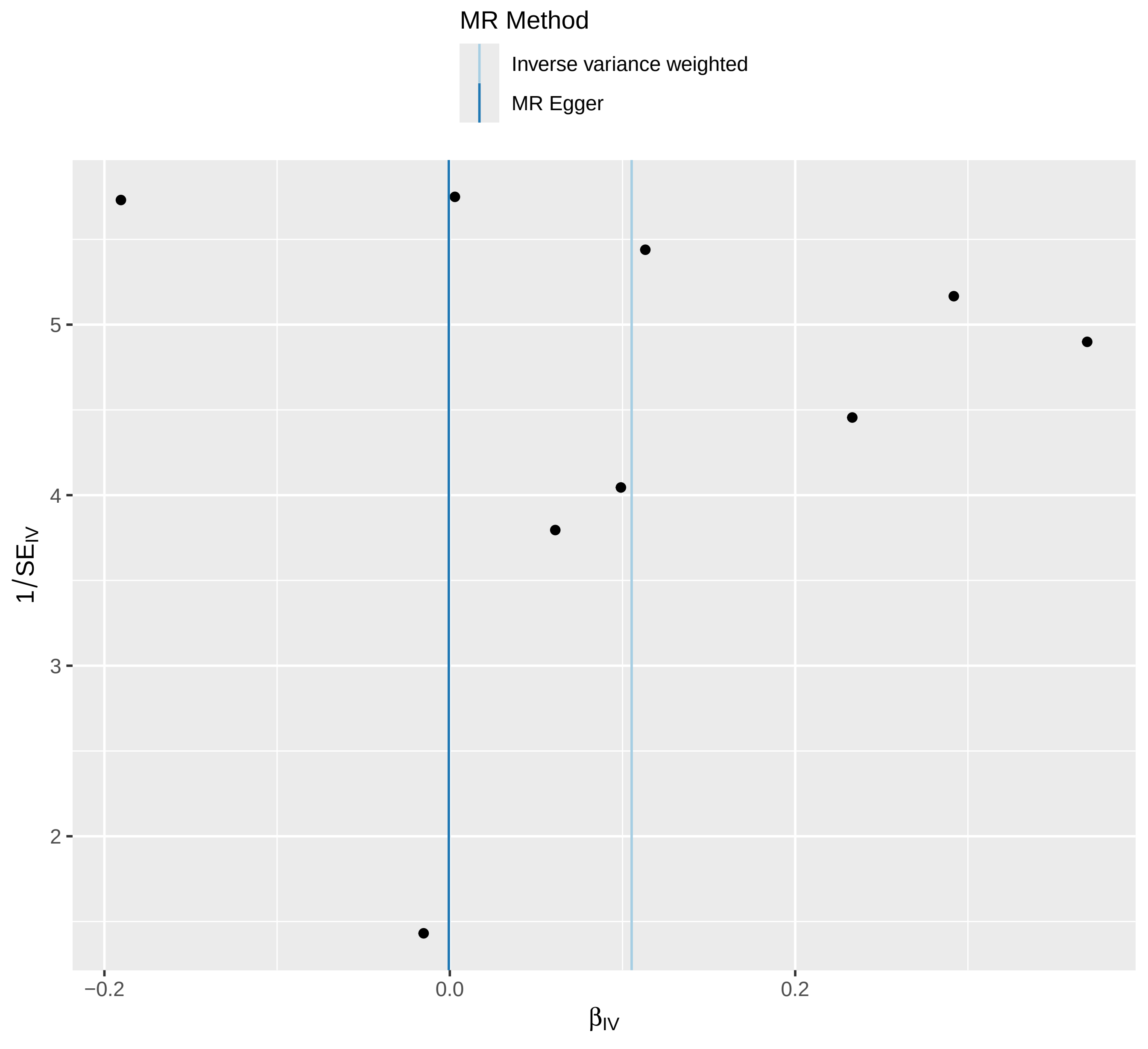
MR Method
Inverse variance weighted
MR Egger
5
4
3
2
$1/SE_{IV}$
−0.2
0.0
0.2
$\beta_{IV}$

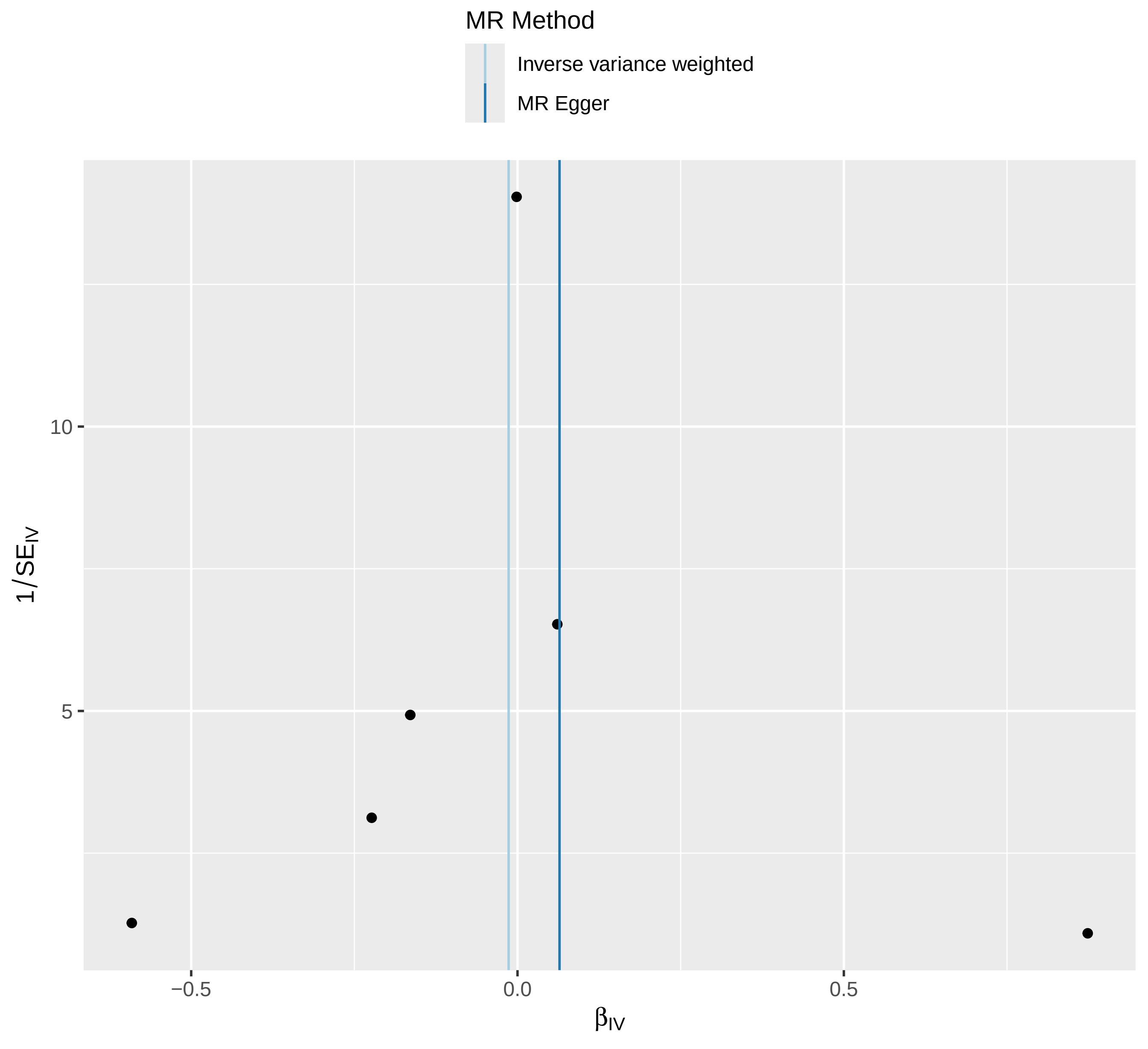

MR Method
Inverse variance weighted
MR Egger
10
5
1/SE$_{IV}$
−0.5
0.0
0.5
β$_{IV}$

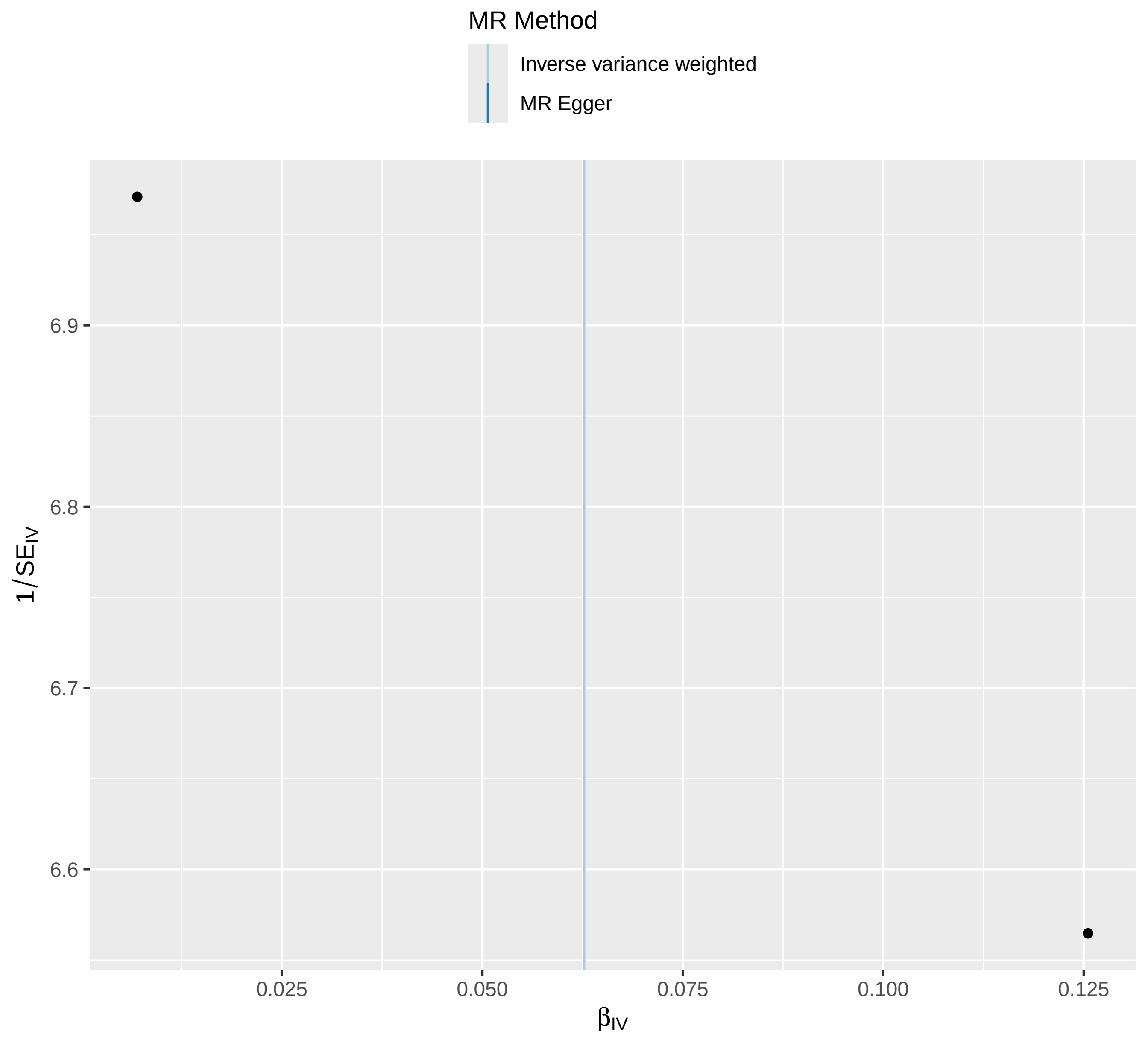

MR Method
Inverse variance weighted
MR Egger
6.9
6.8
6.7
6.6
1/SE_IV
0.025
0.050
0.075
0.100
0.125
β_IV

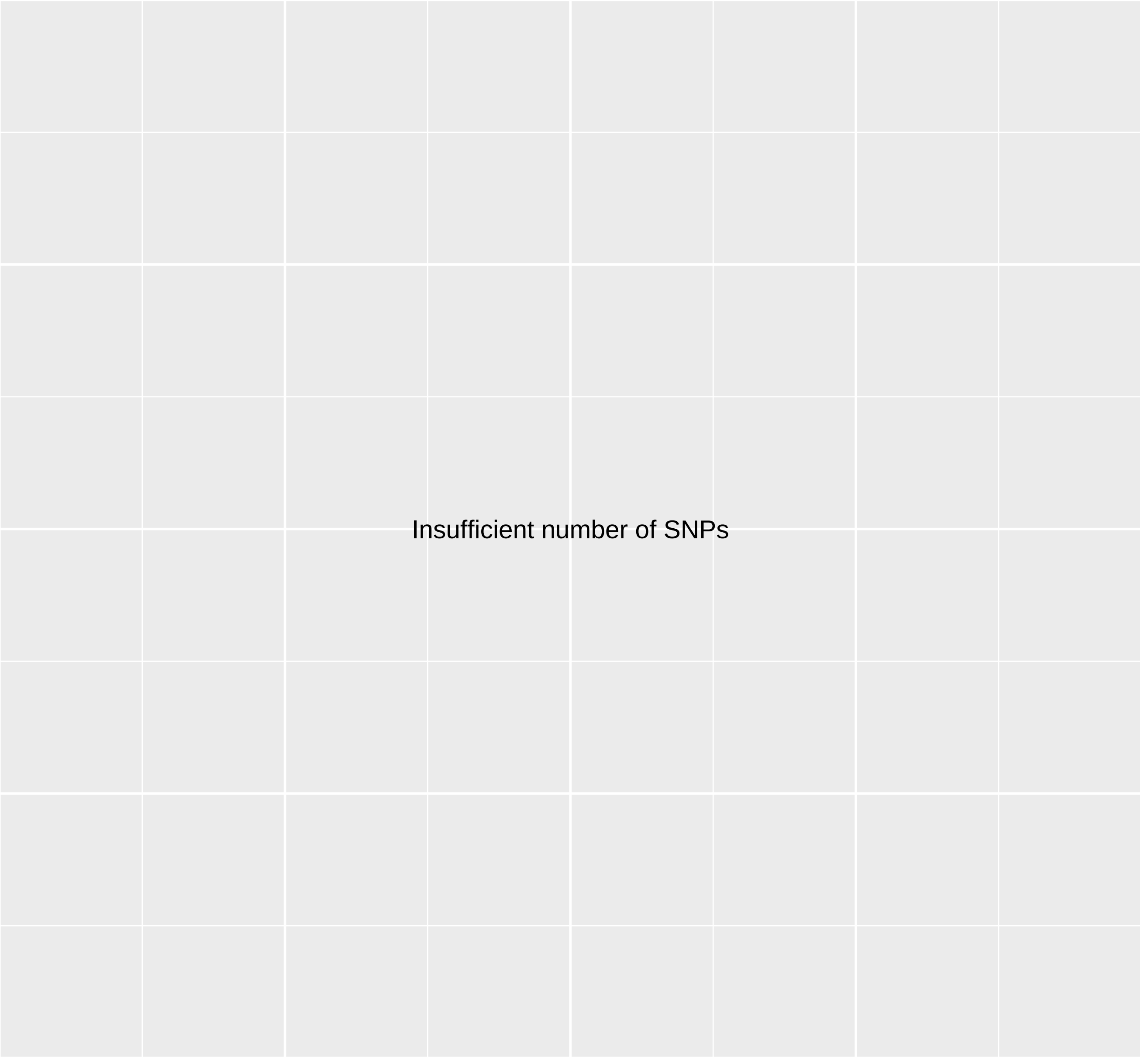
Insufficient number of SNPs

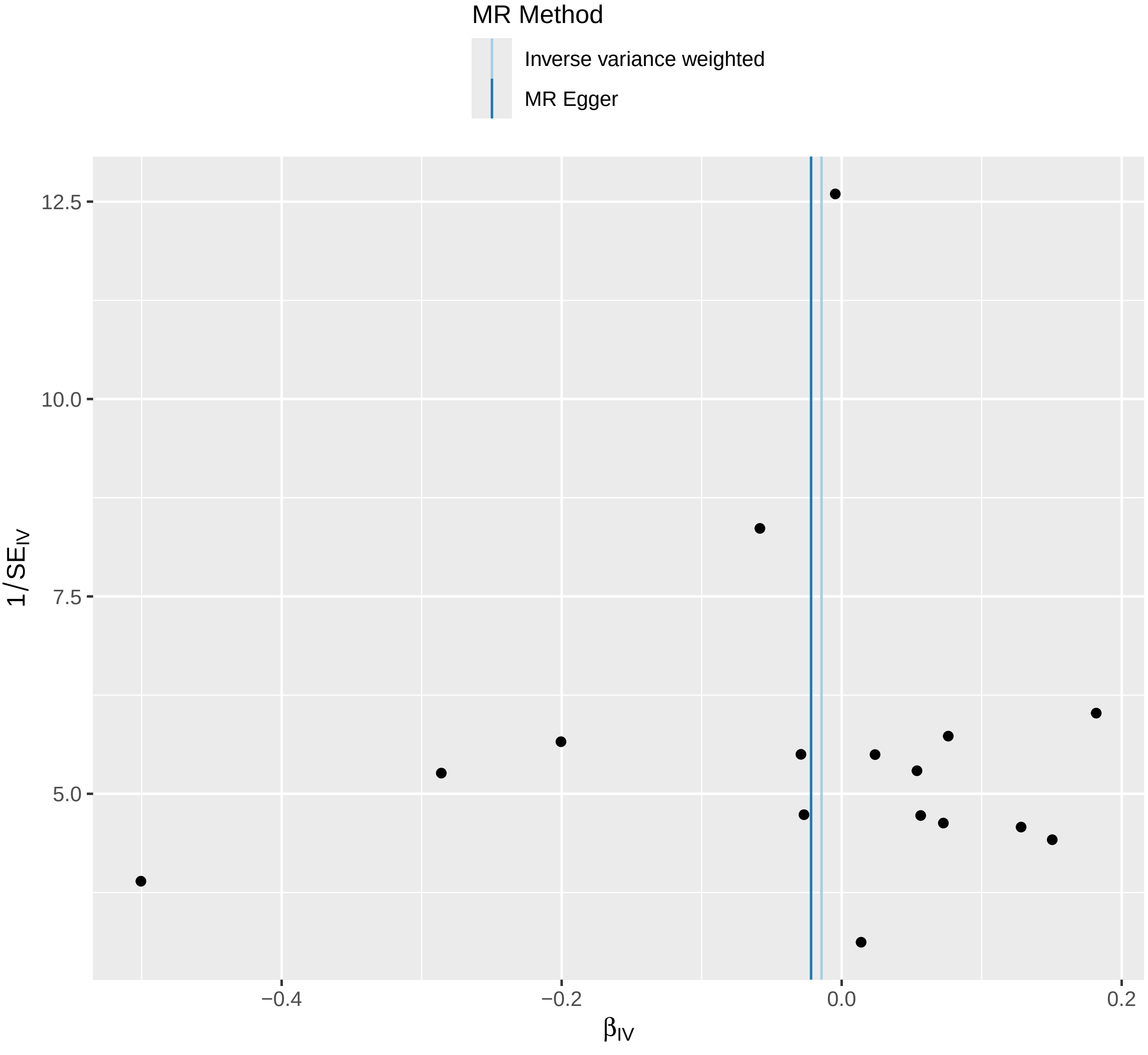

MR Method
Inverse variance weighted
MR Egger
12.5
10.0
7.5
5.0
1/SE$_{IV}$
−0.4
−0.2
0.0
0.2
β$_{IV}$

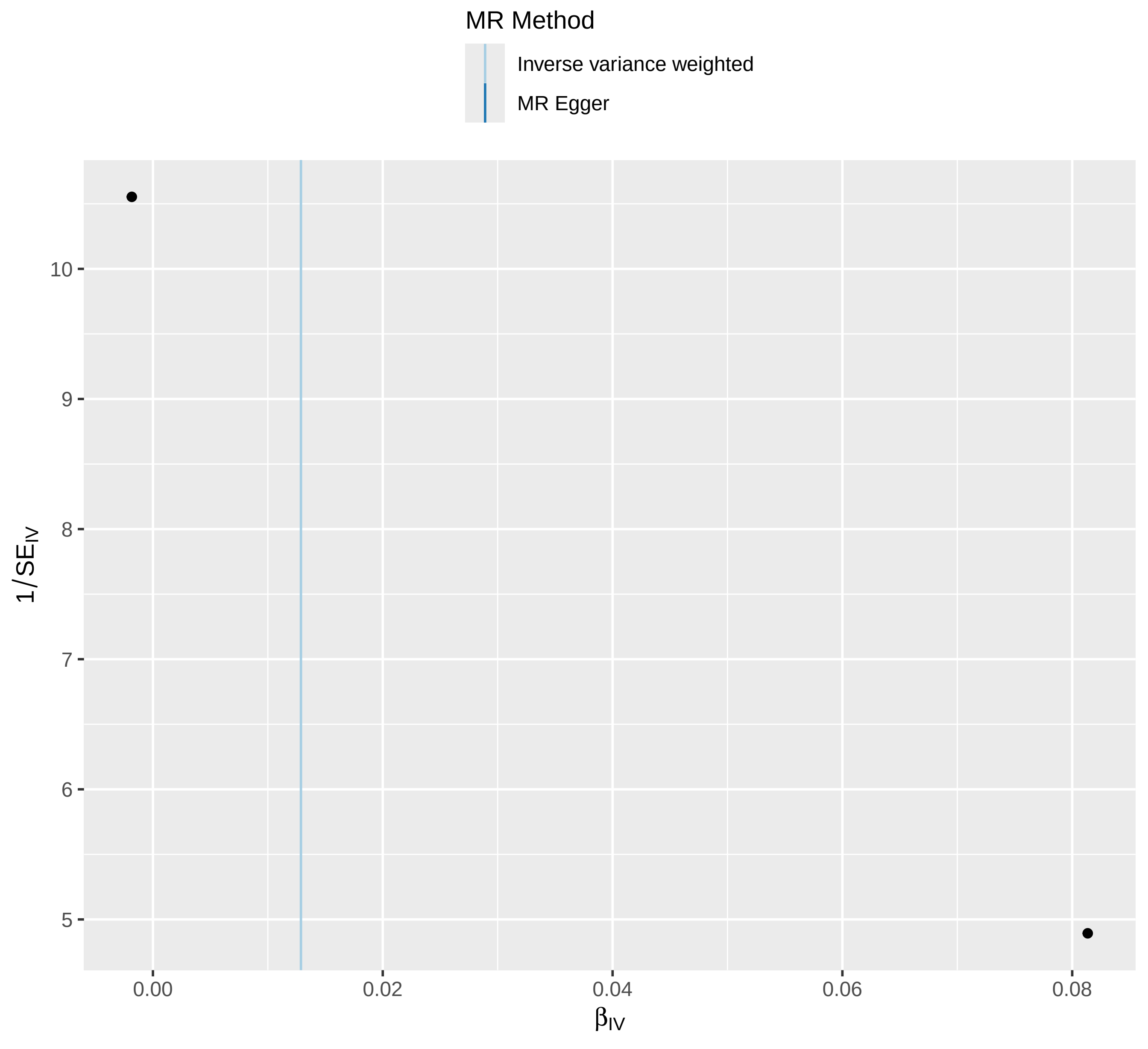

MR Method
Inverse variance weighted
MR Egger
1/SE_IV
10
9
8
7
6
5
0.00
0.02
0.04
0.06
0.08
β_IV

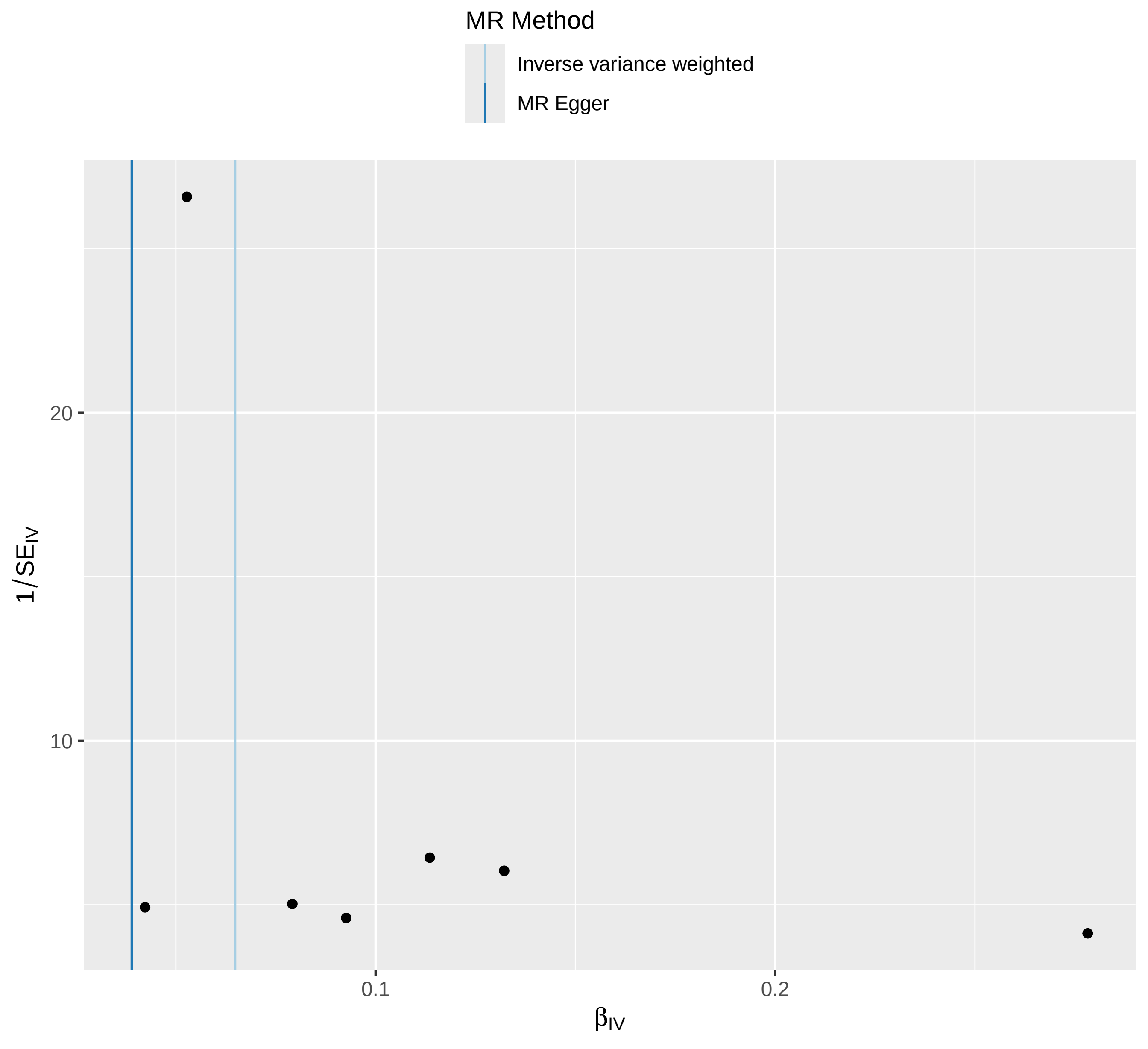
MR Method
Inverse variance weighted
MR Egger
20
10
1/SE$_{IV}$
0.1
0.2
β$_{IV}$

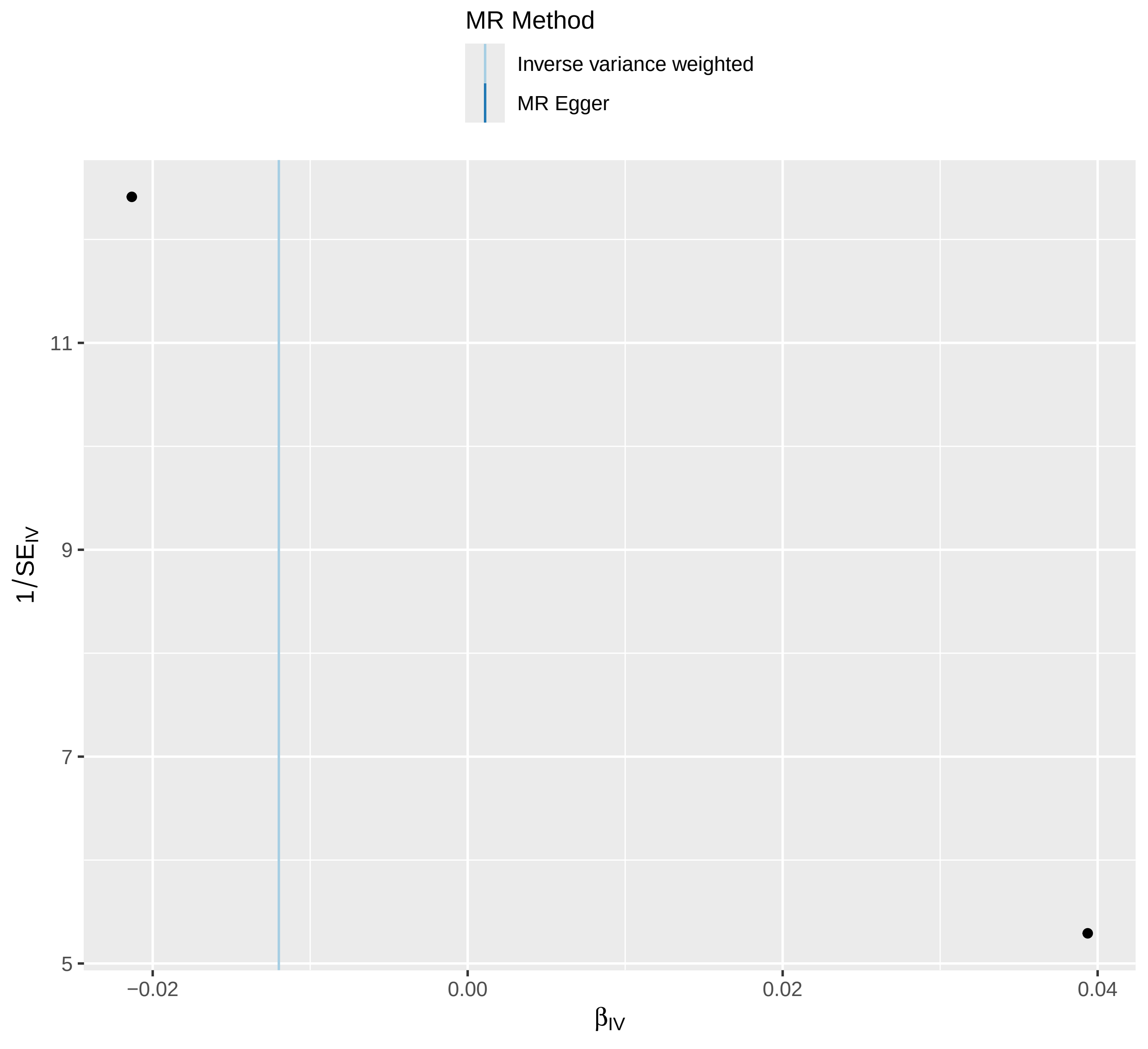

MR Method
Inverse variance weighted
MR Egger
11
9
7
5
1/SE_IV
−0.02
0.00
0.02
0.04
β_IV

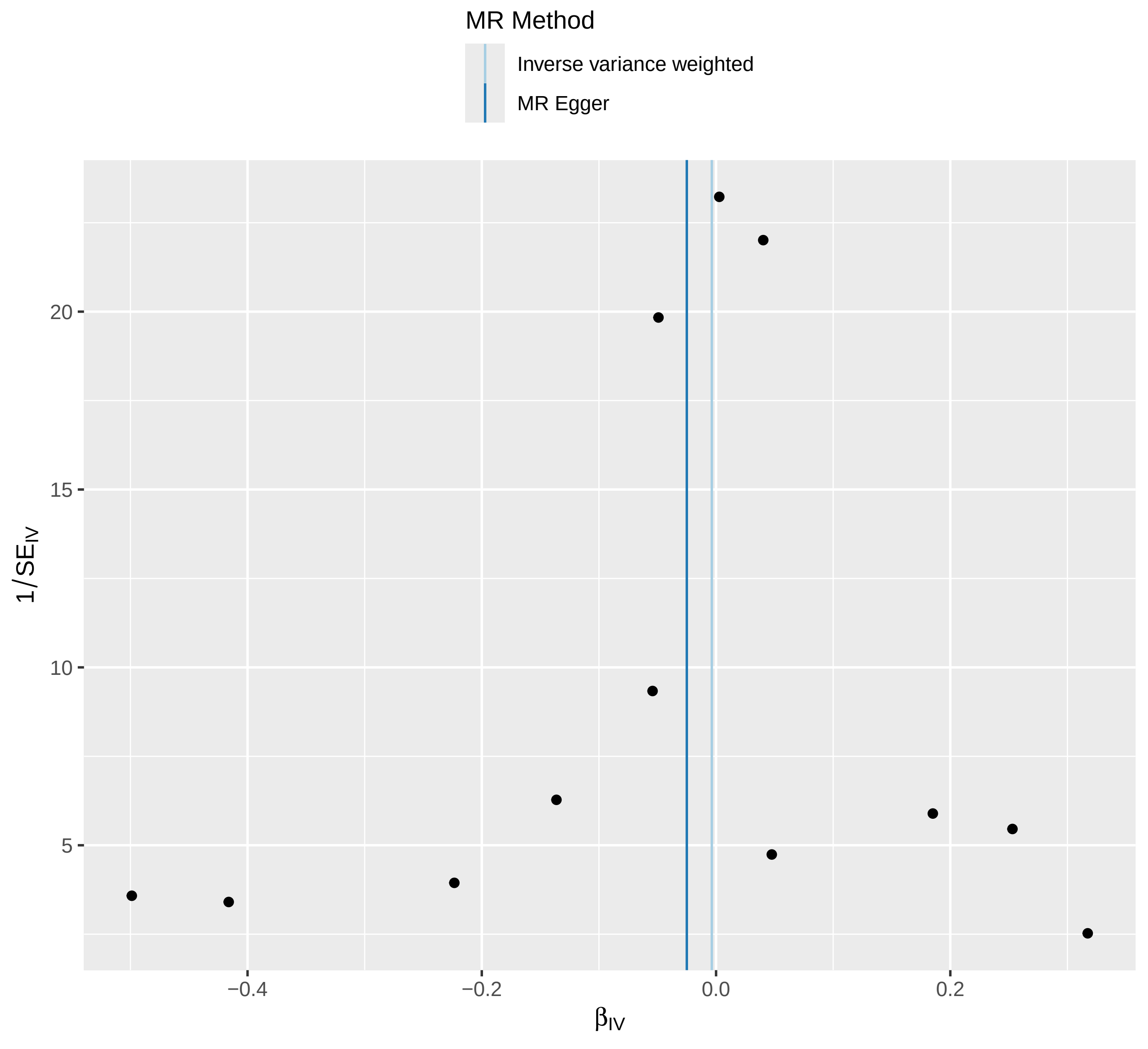

MR Method
Inverse variance weighted
MR Egger
20
15
10
5
$1/SE_{IV}$
−0.4
−0.2
0.0
0.2
$\beta_{IV}$

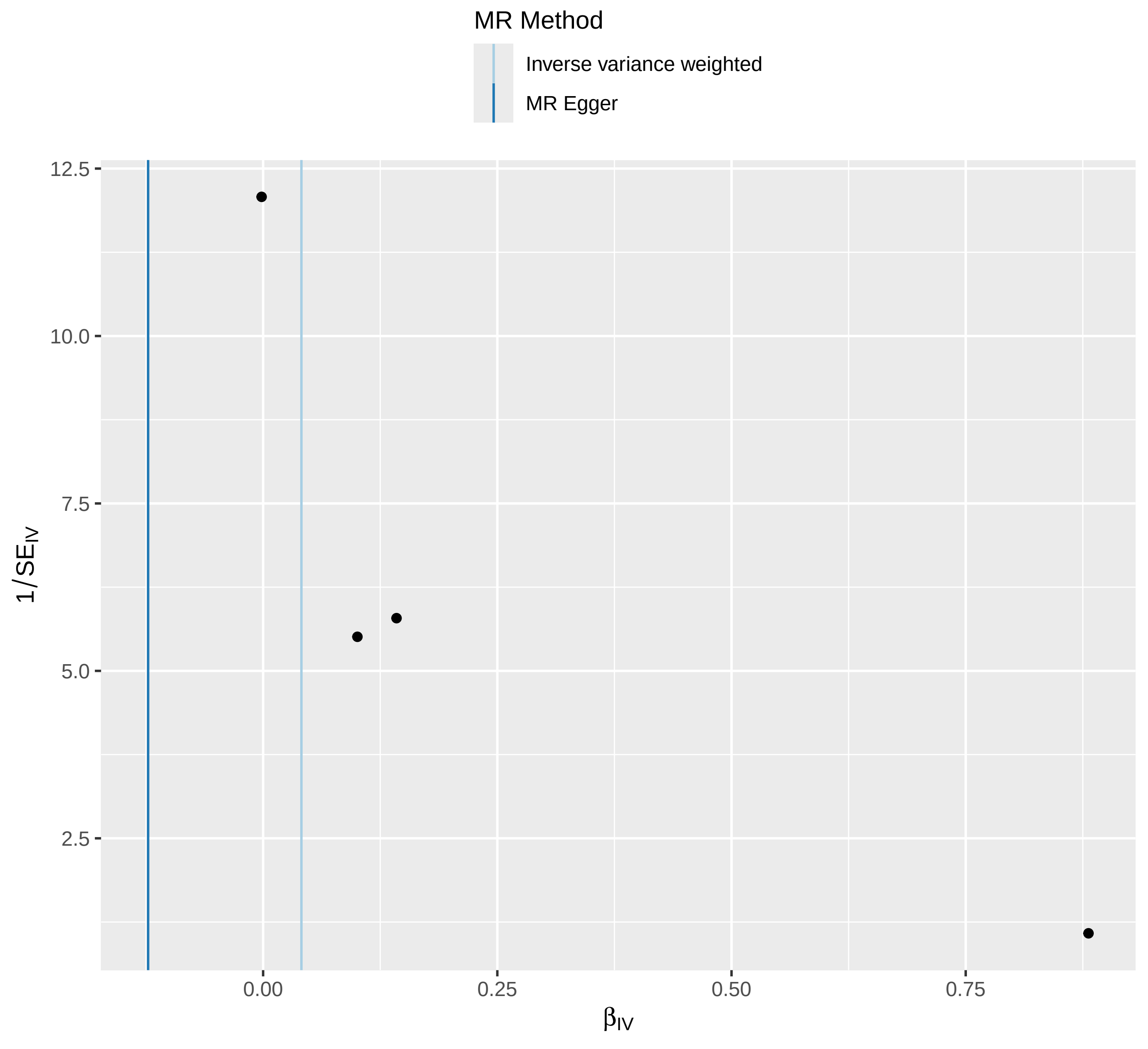
MR Method
Inverse variance weighted
MR Egger
12.5
10.0
7.5
5.0
2.5
$1/SE_{IV}$
0.00
0.25
0.50
0.75
$\beta_{IV}$

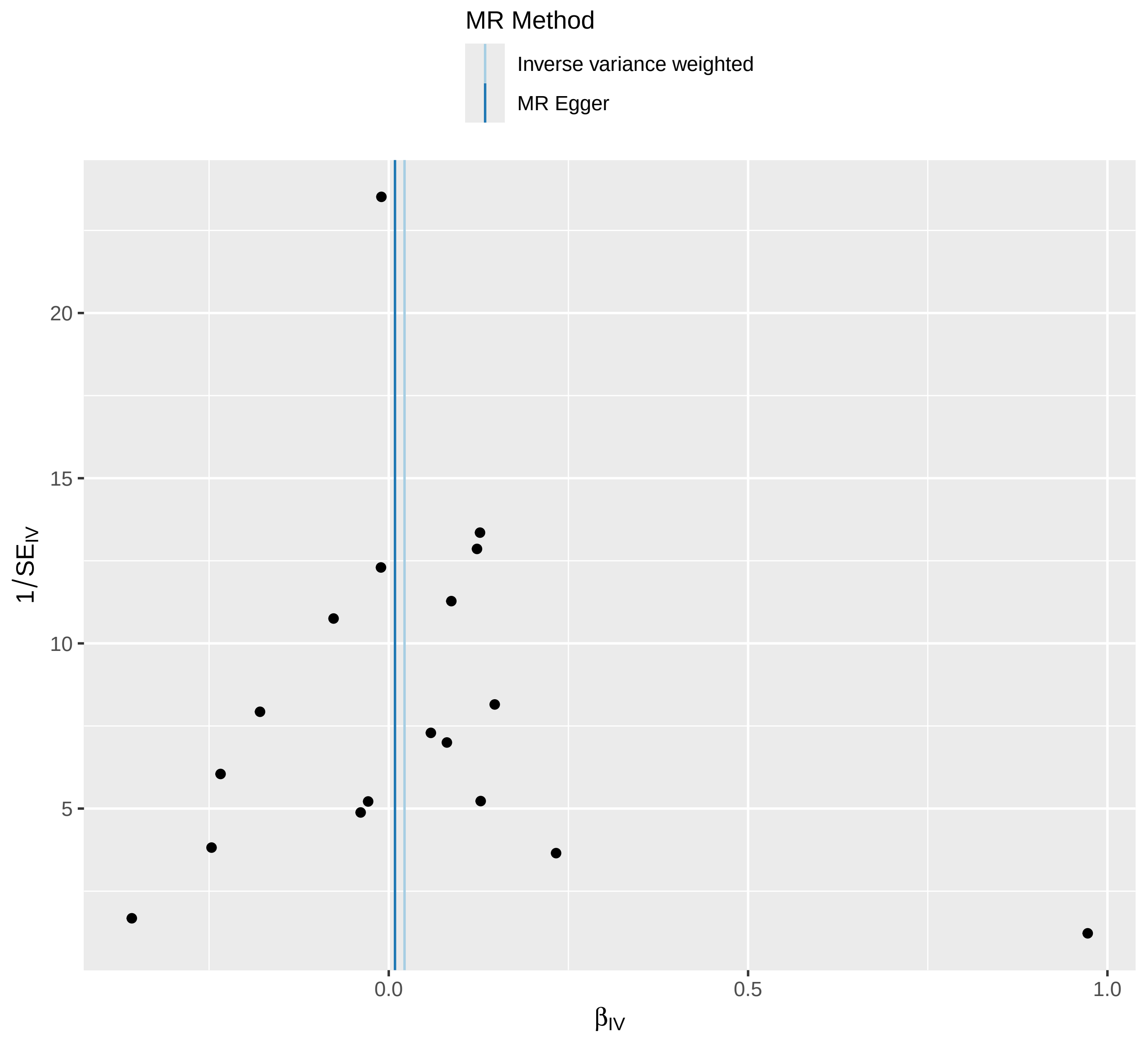
MR Method
Inverse variance weighted
MR Egger
20
15
10
5
1/SE_IV
0.0
0.5
1.0
β_IV

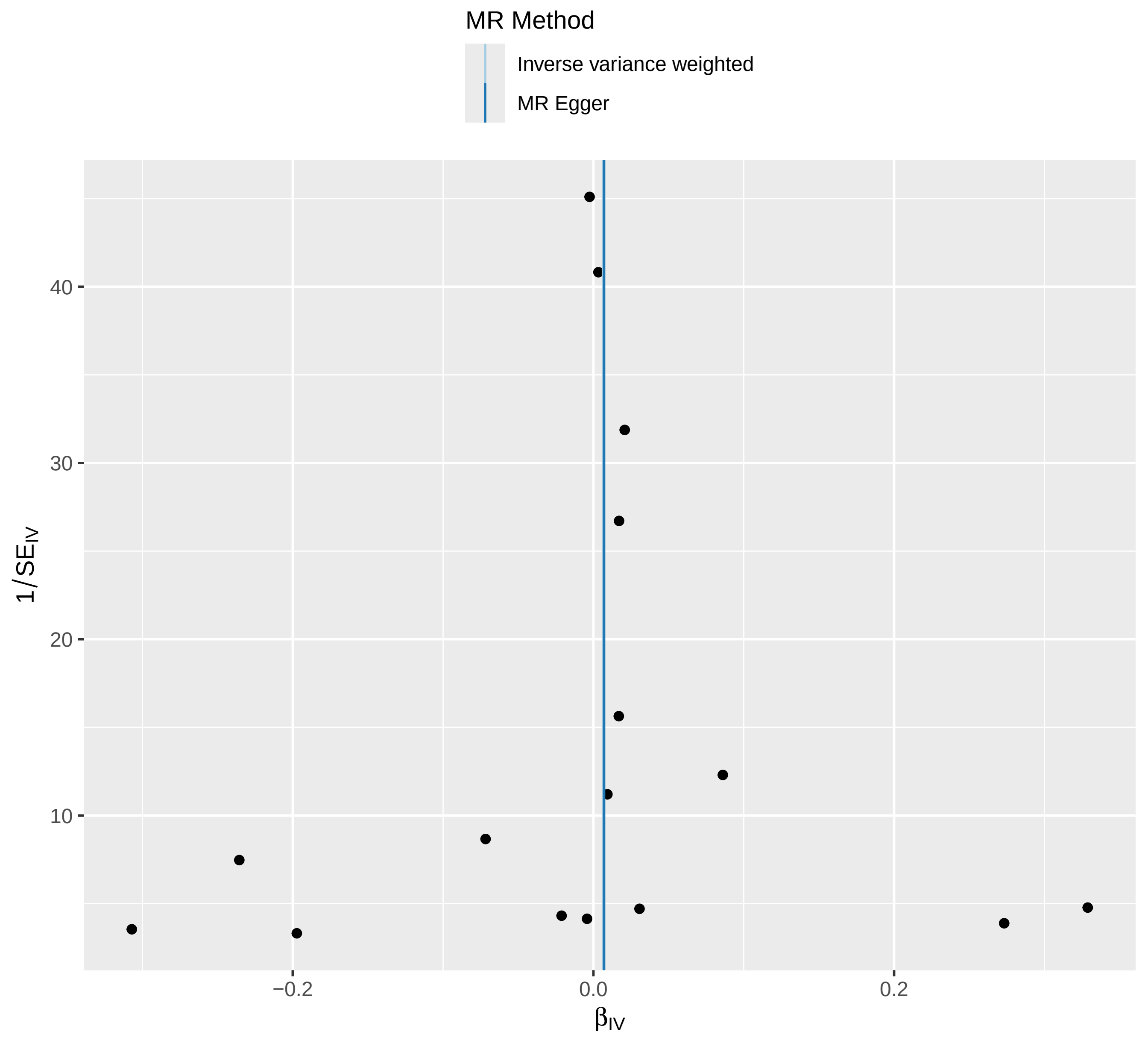

MR Method
Inverse variance weighted
MR Egger
40
30
20
10
1/SE_IV
−0.2
0.0
0.2
β_IV

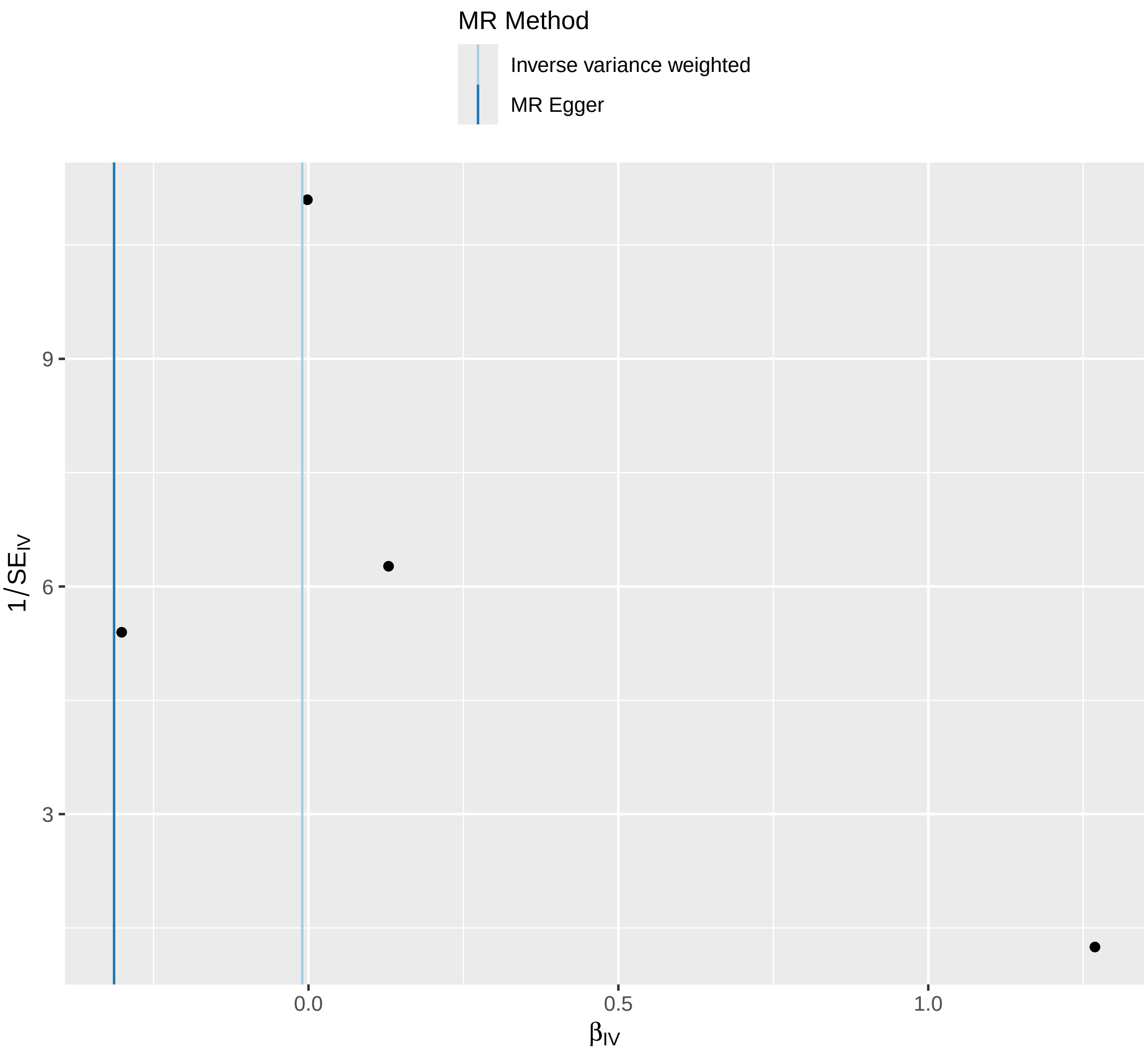
MR Method
Inverse variance weighted
MR Egger
9
6
3
0.0
0.5
1.0
1/SE_IV
β_IV

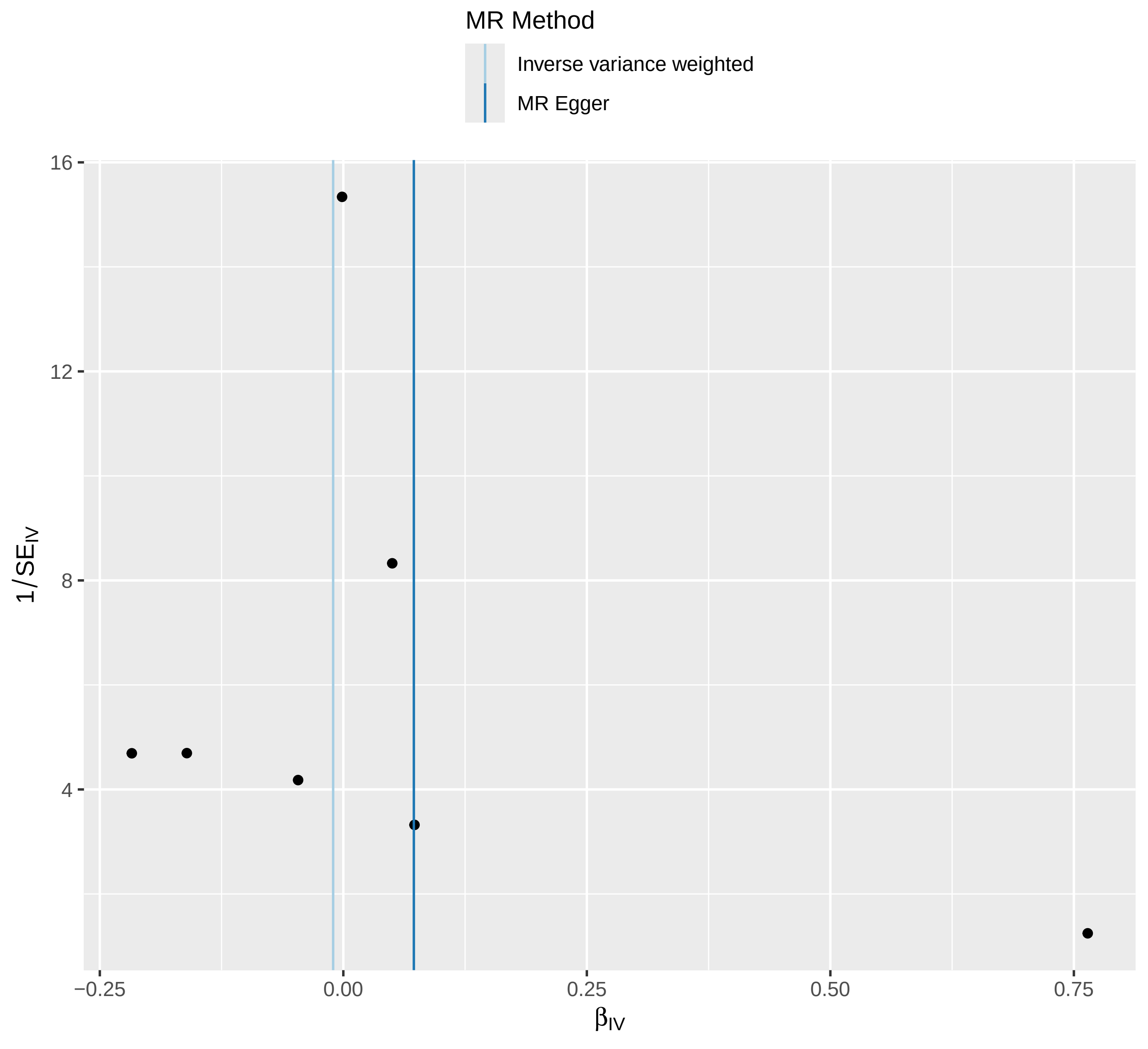
MR Method
Inverse variance weighted
MR Egger
16
12
8
4
1/SE$_{IV}$
−0.25
0.00
0.25
0.50
0.75
β$_{IV}$

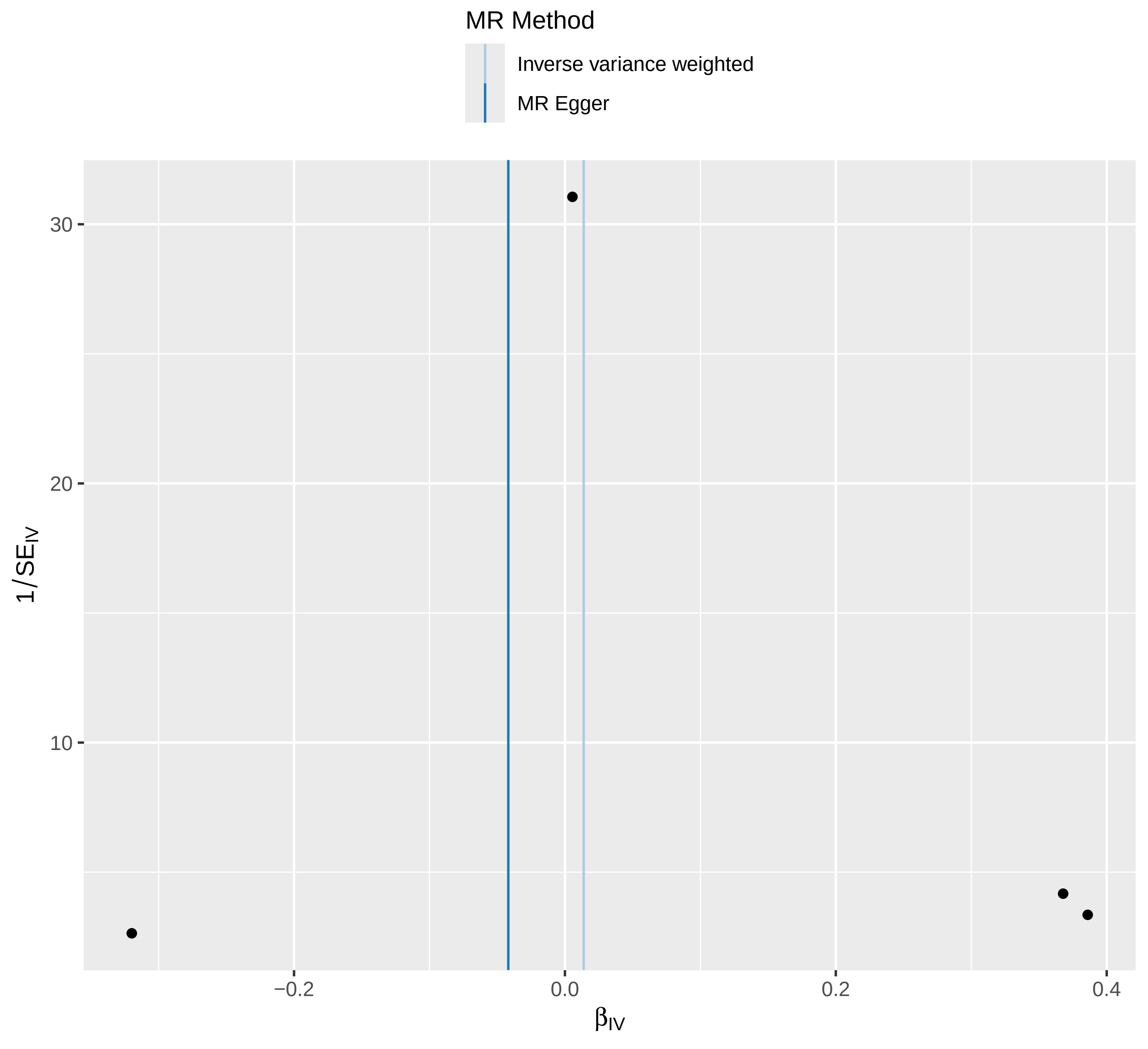

MR Method
Inverse variance weighted
MR Egger
30
20
10
1/SE_IV
−0.2
0.0
0.2
0.4
β_IV

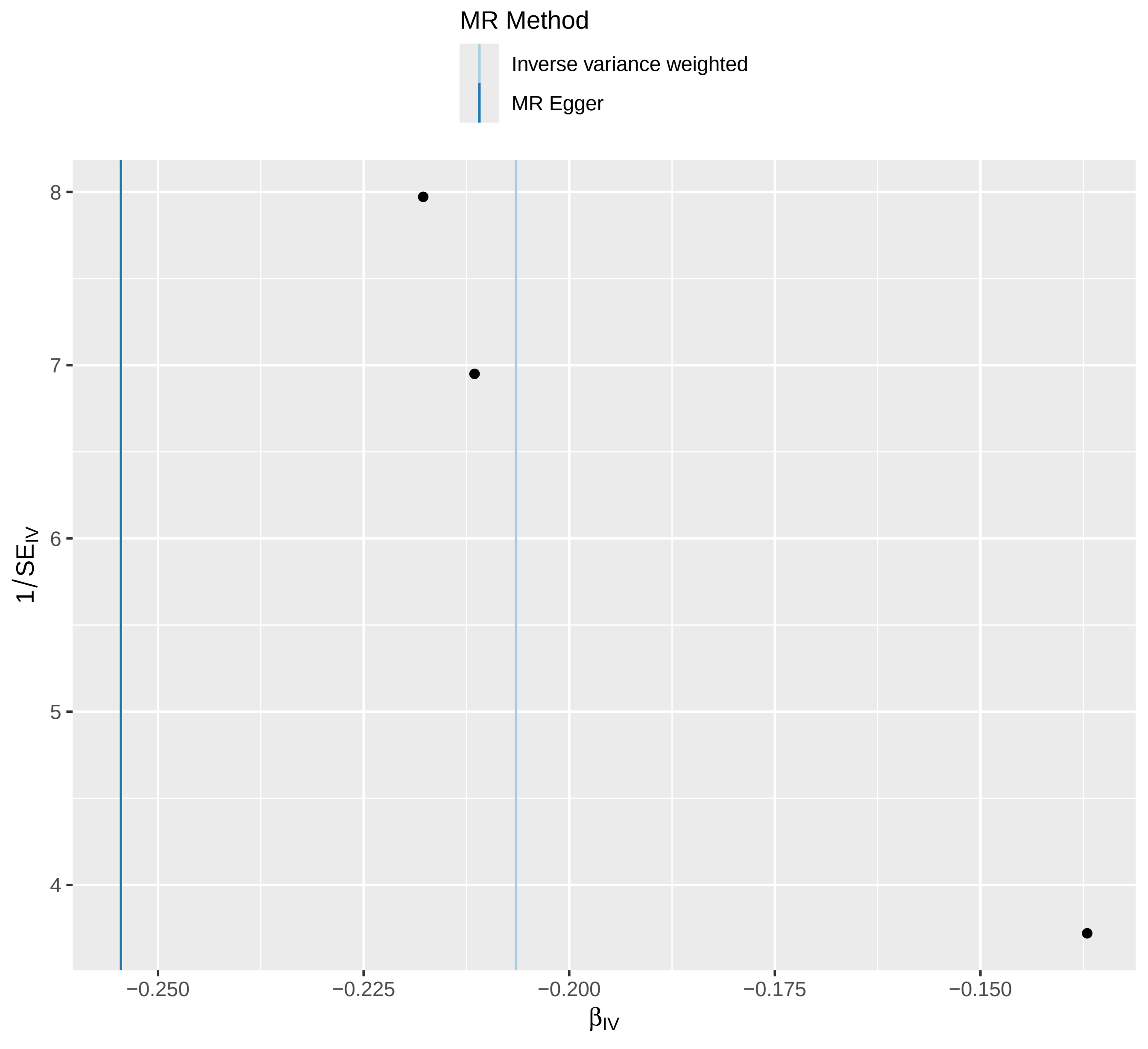
MR Method
Inverse variance weighted
MR Egger
1/SE$_{IV}$
8
7
6
5
4
−0.250
−0.225
−0.200
−0.175
−0.150
β$_{IV}$

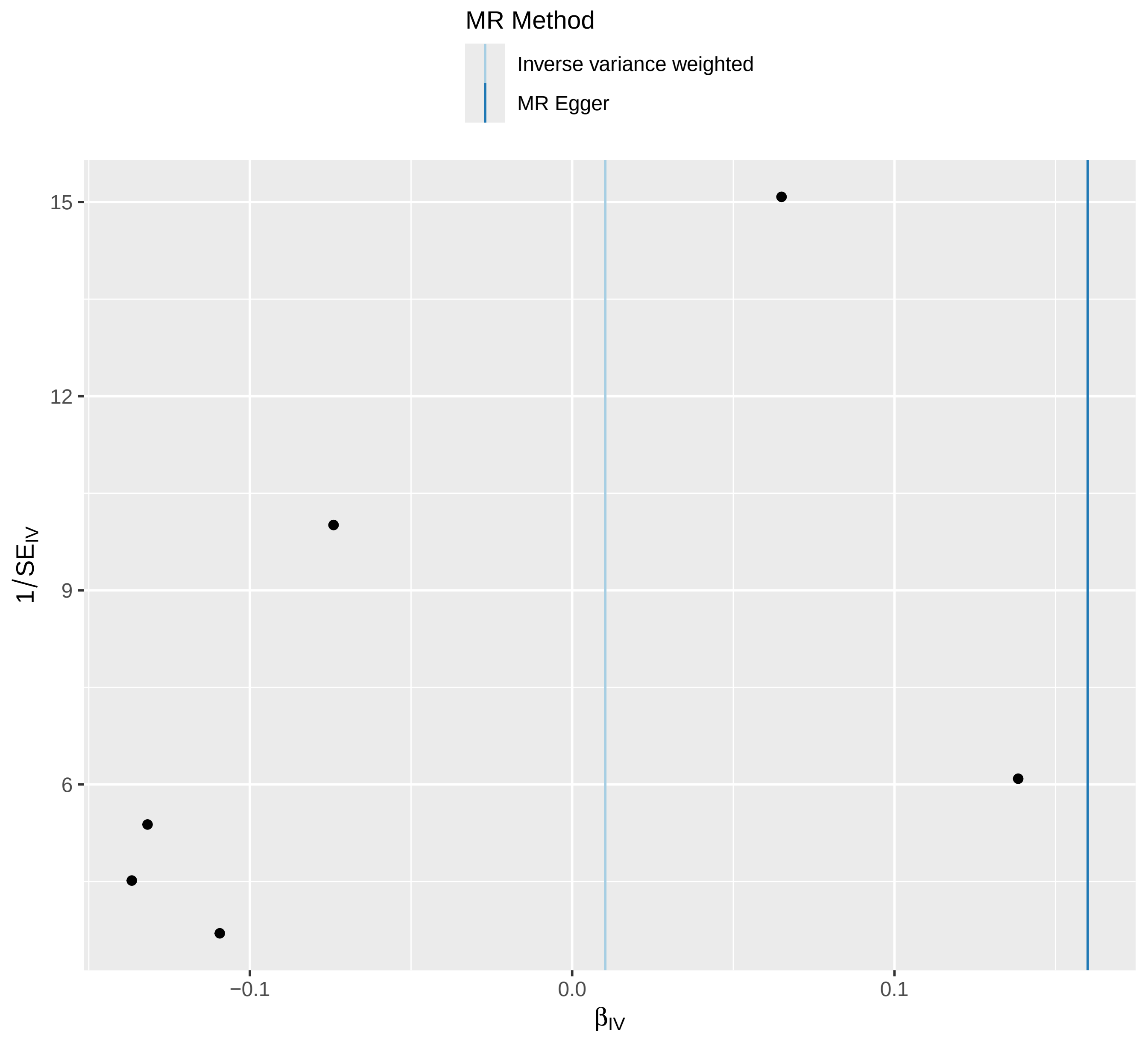
MR Method
Inverse variance weighted
MR Egger
15
12
9
6
1/SE_IV
−0.1
0.0
0.1
β_IV

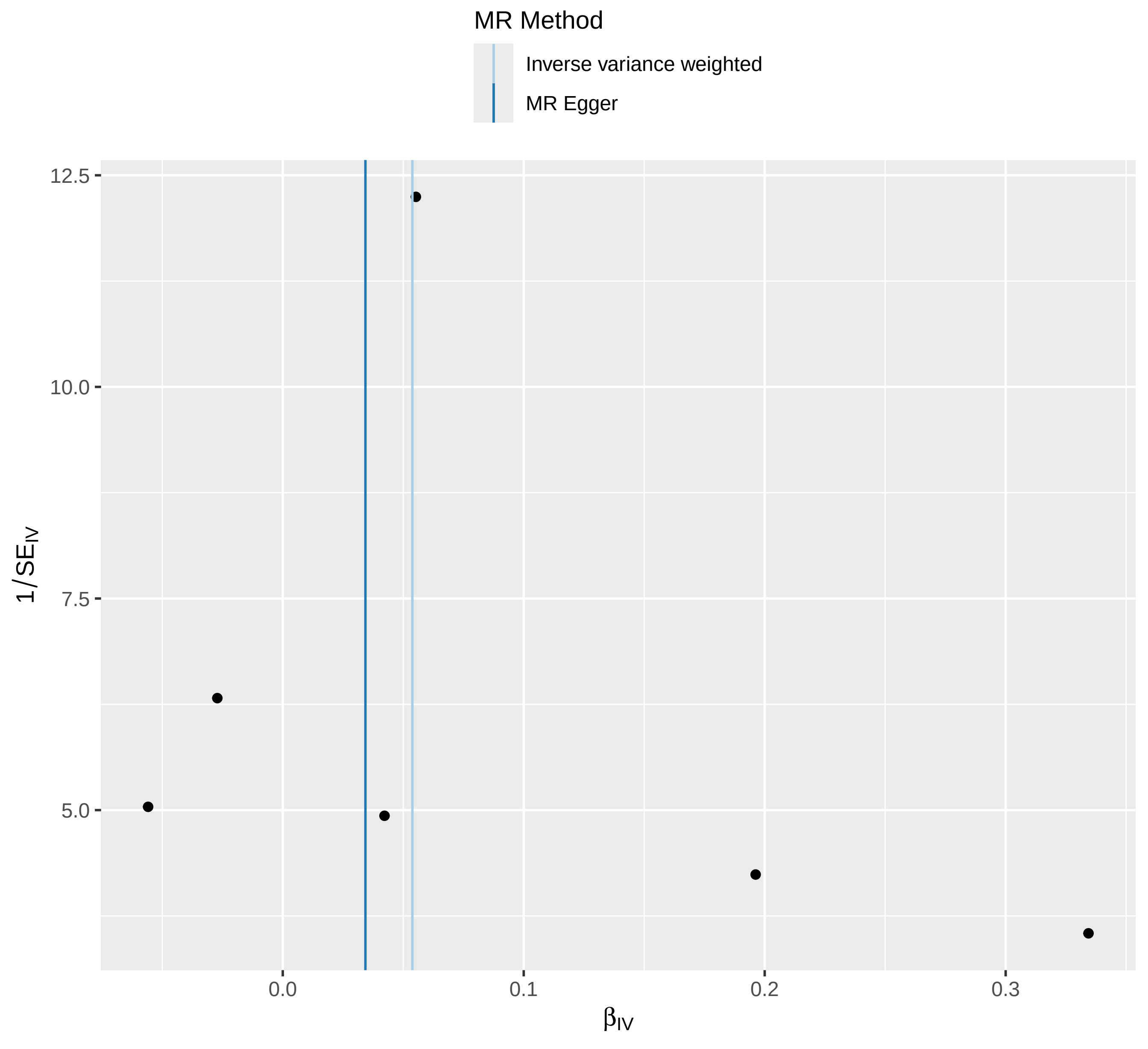

MR Method
Inverse variance weighted
MR Egger
12.5
10.0
7.5
5.0
1/SE_IV
0.0
0.1
0.2
0.3
β_IV

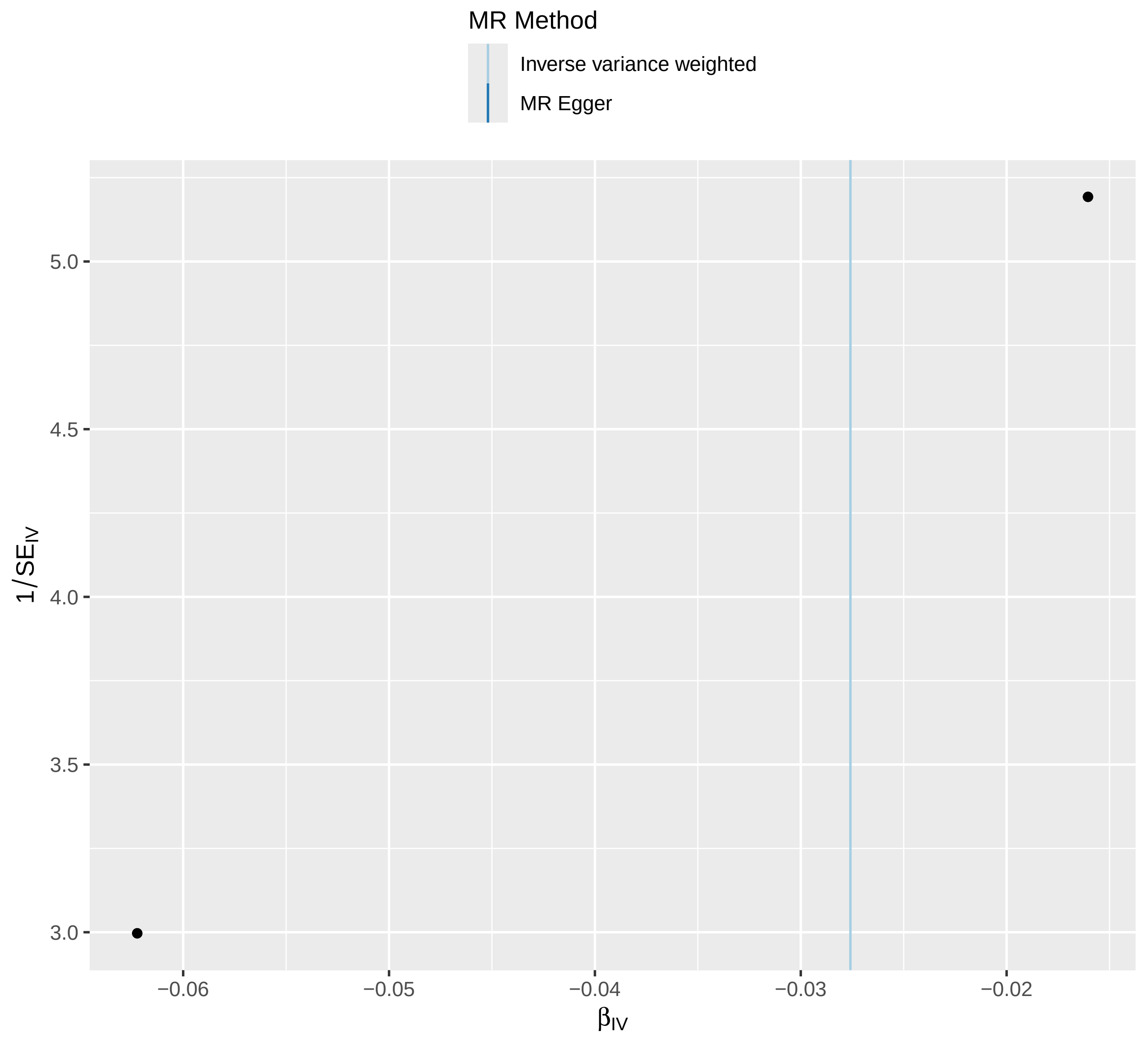
MR Method
Inverse variance weighted
MR Egger
1/SE_IV
5.0
4.5
4.0
3.5
3.0
−0.06
−0.05
−0.04
−0.03
−0.02
β_IV

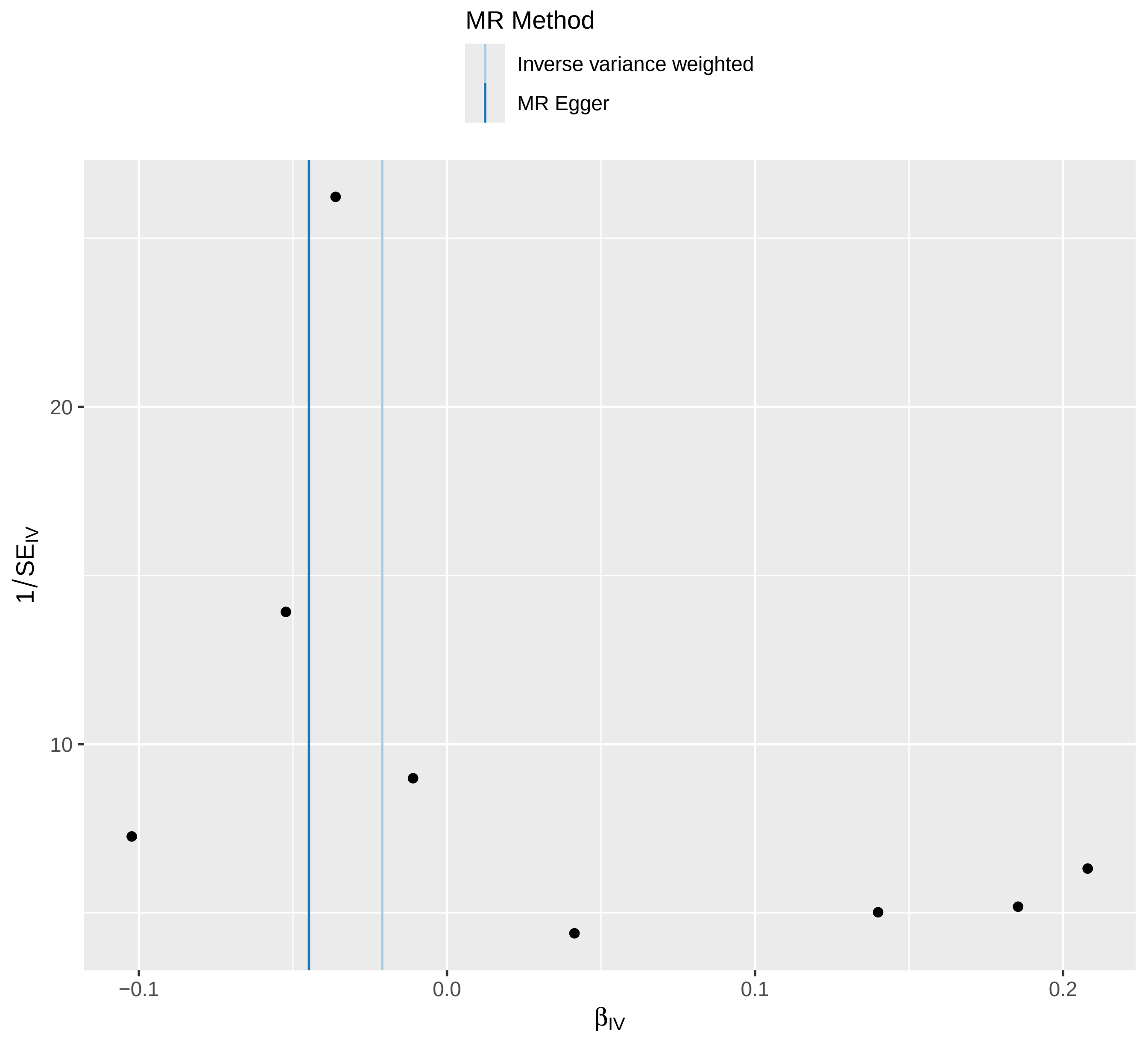
MR Method
Inverse variance weighted
MR Egger
20
10
$1/SE_{IV}$
−0.1
0.0
0.1
0.2
$\beta_{IV}$

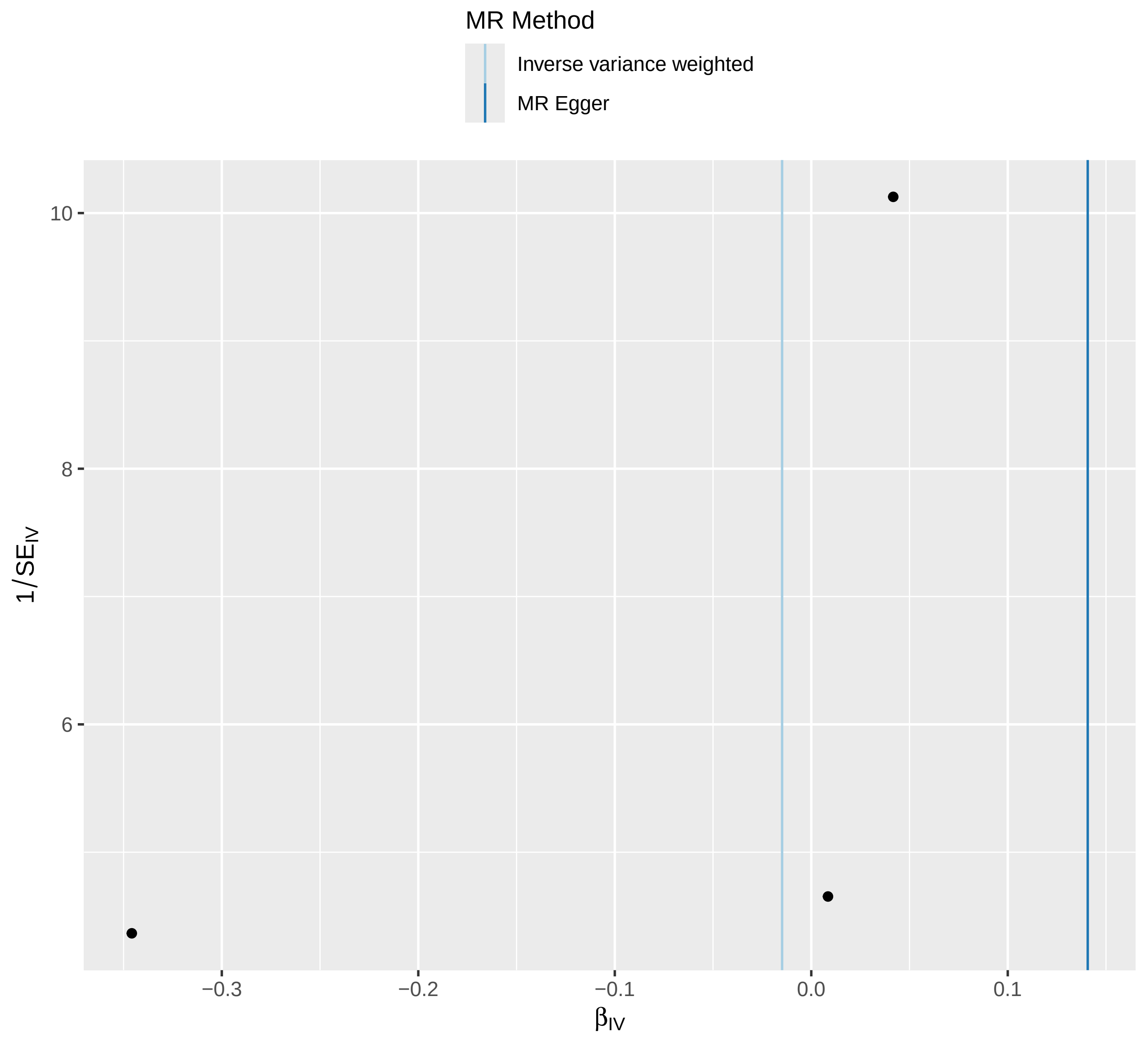
MR Method
Inverse variance weighted
MR Egger
10
8
6
1/SE$_{IV}$
−0.3
−0.2
−0.1
0.0
0.1
β$_{IV}$

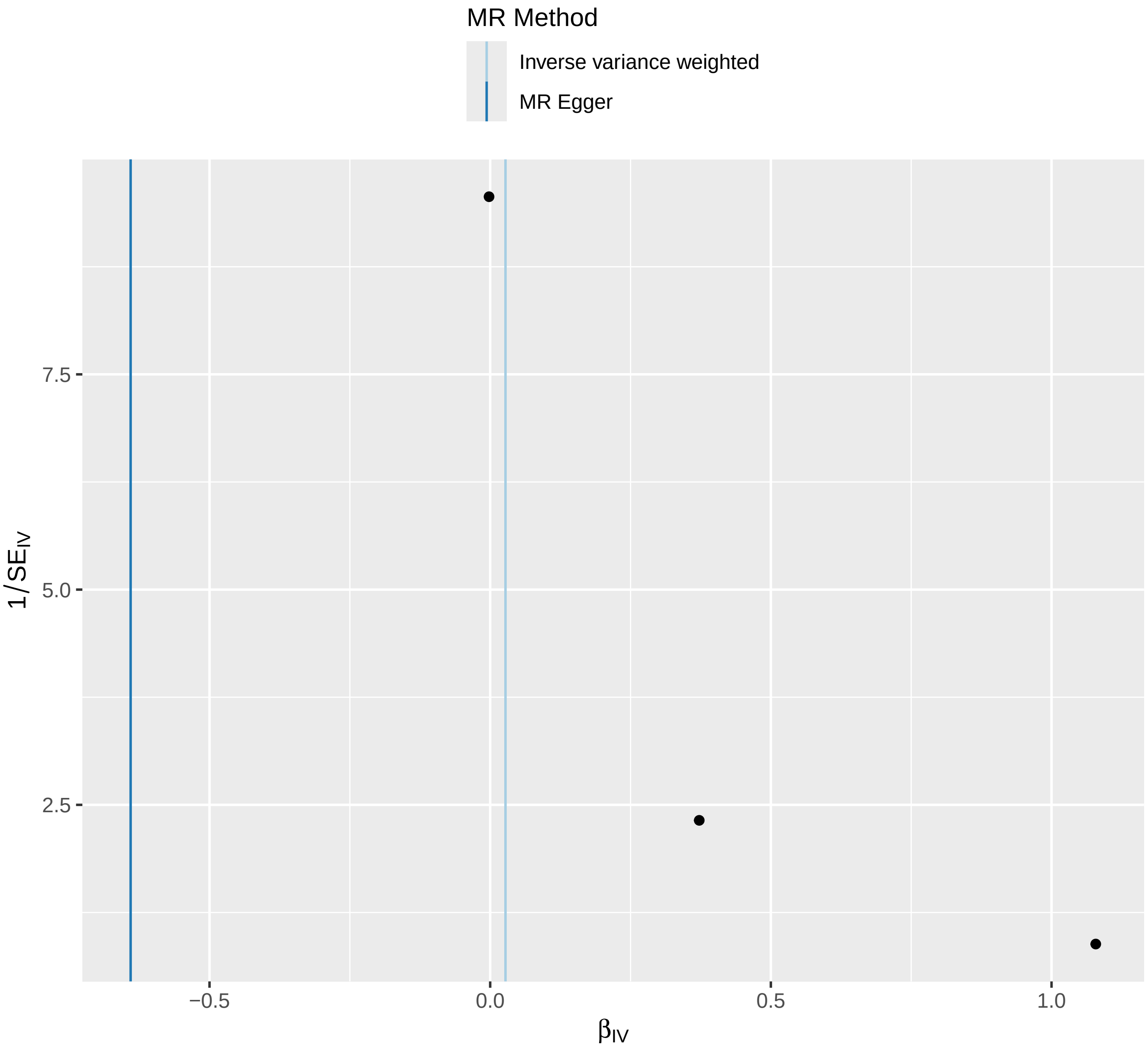
MR Method
Inverse variance weighted
MR Egger
7.5
5.0
2.5
1/SE_IV
−0.5
0.0
0.5
1.0
β_IV

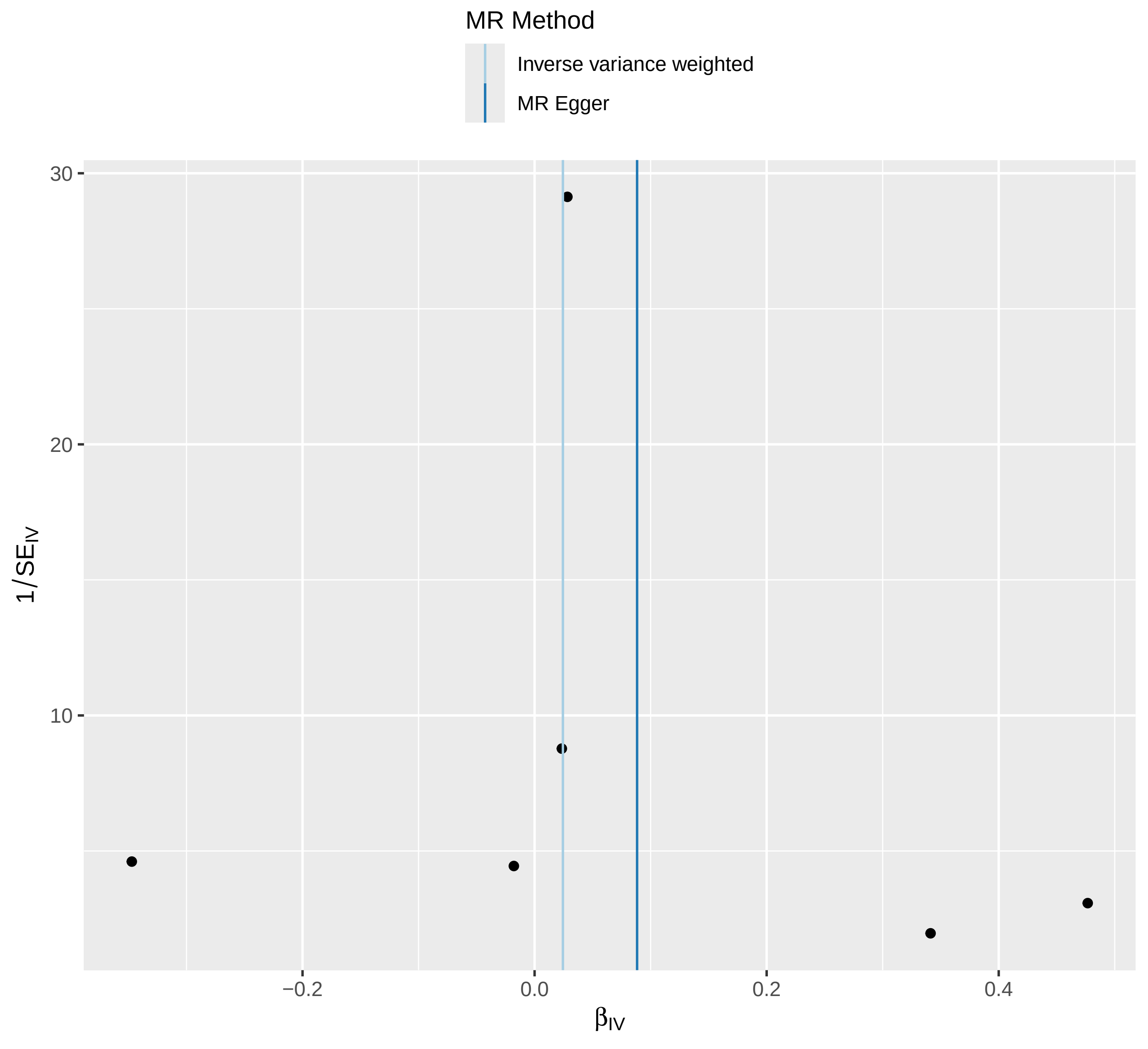
MR Method
Inverse variance weighted
MR Egger
30
20
10
1/SE_IV
−0.2
0.0
0.2
0.4
β_IV

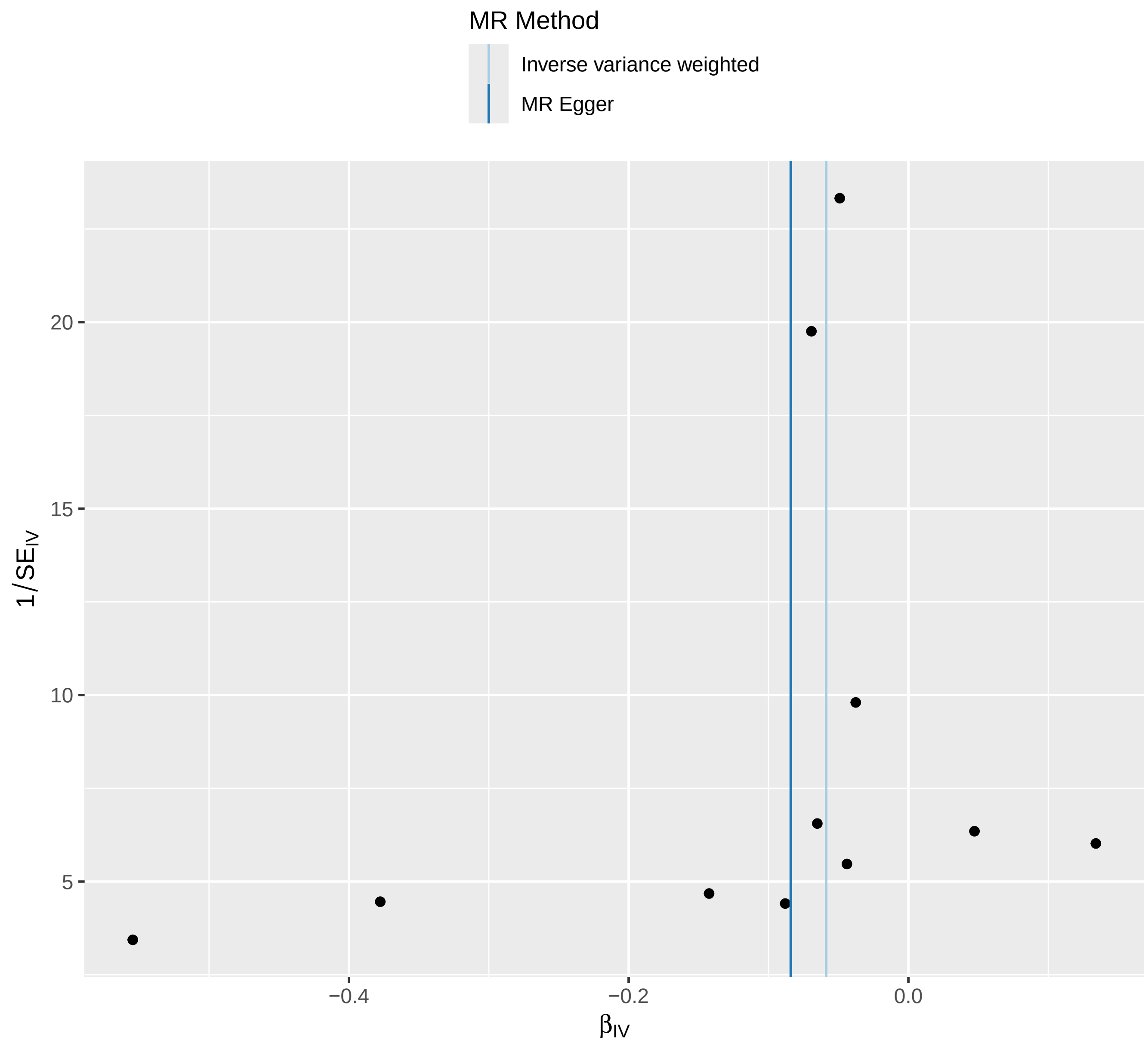
MR Method
Inverse variance weighted
MR Egger
20
15
10
5
1/SE_IV
−0.4
−0.2
0.0
β_IV

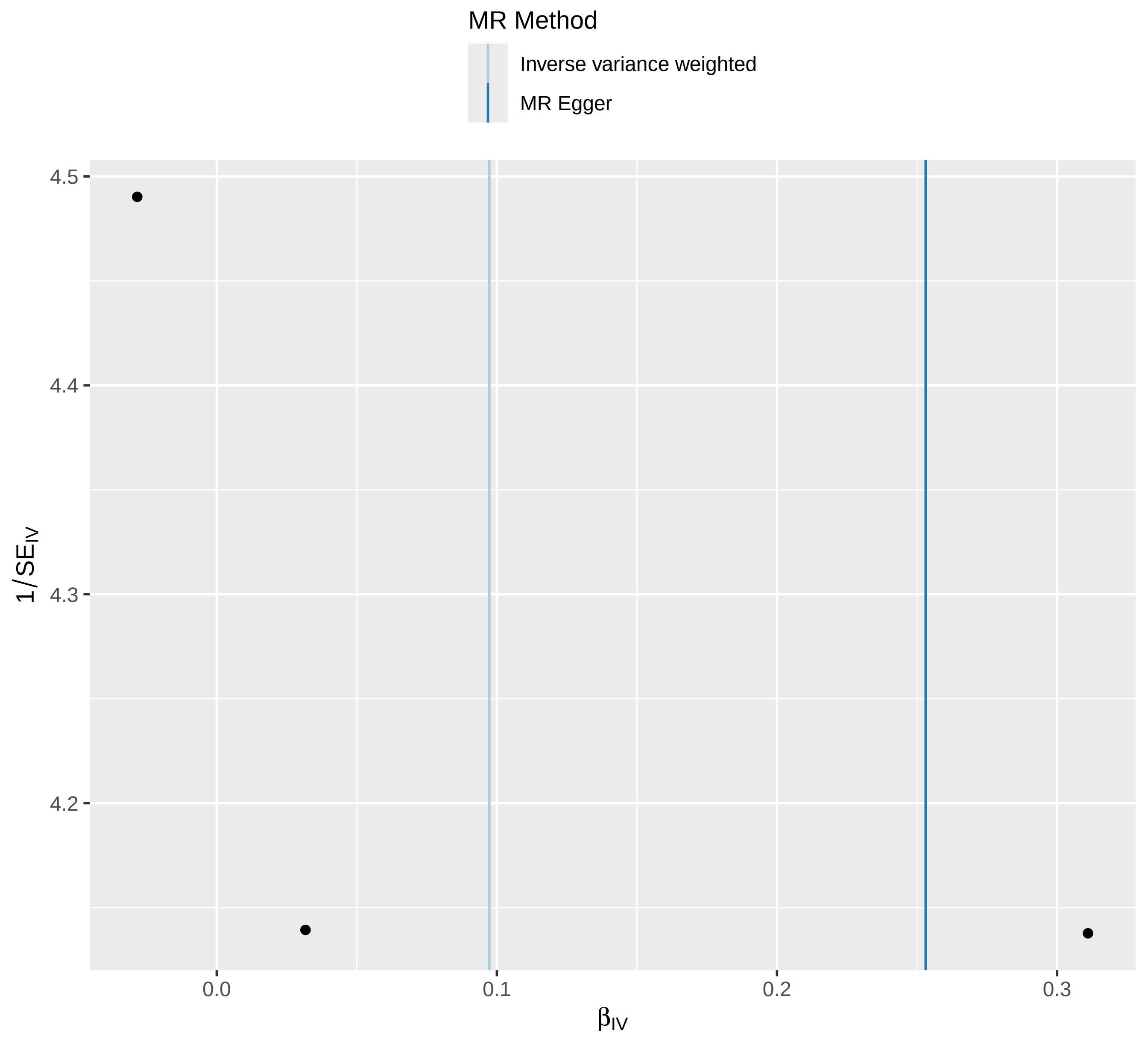

MR Method
Inverse variance weighted
MR Egger
4.5
4.4
4.3
4.2
$1/SE_{IV}$
0.0
0.1
0.2
0.3
$\beta_{IV}$

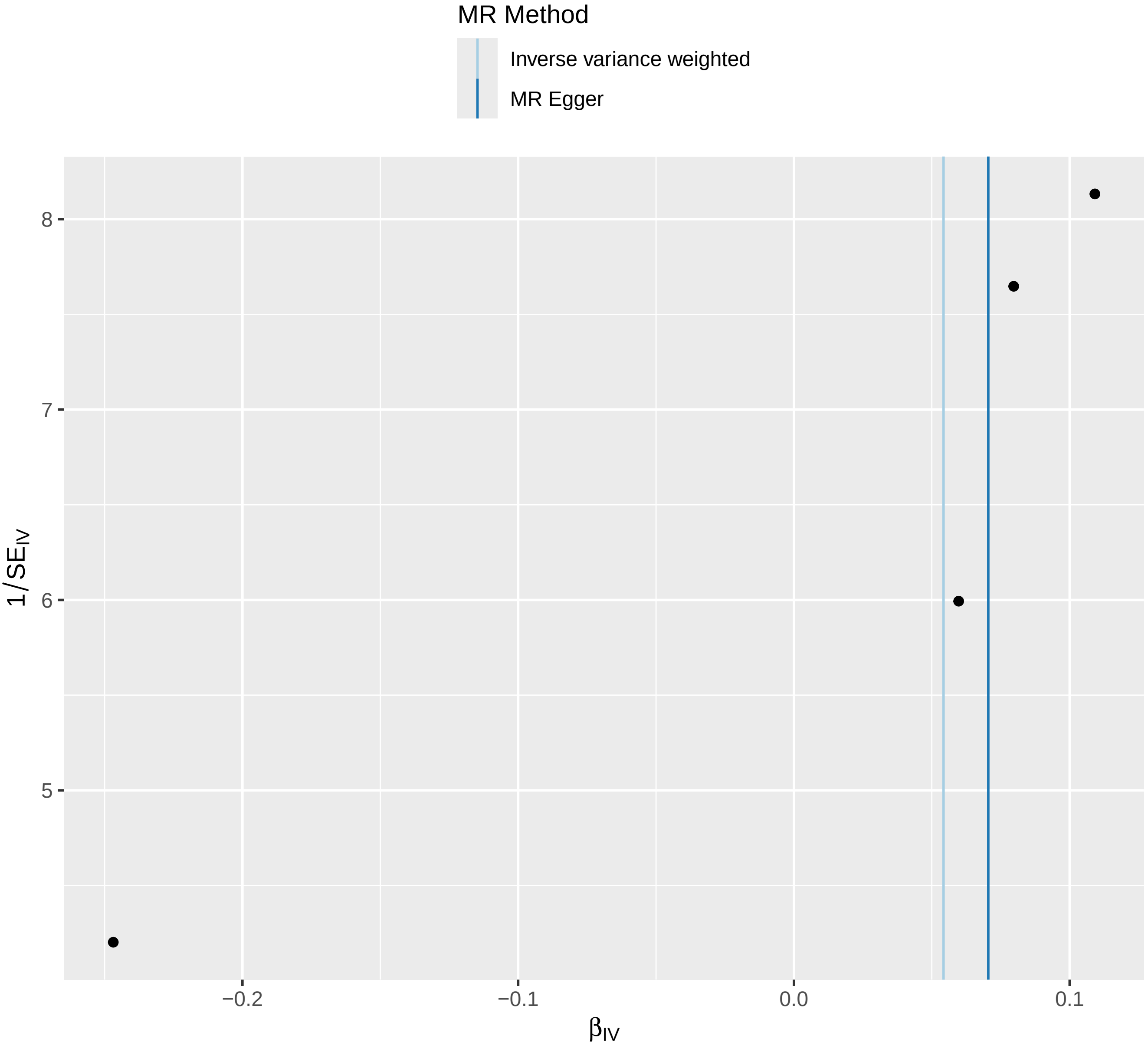
MR Method
Inverse variance weighted
MR Egger
8
7
6
5
1/SE_IV
−0.2
−0.1
0.0
0.1
β_IV

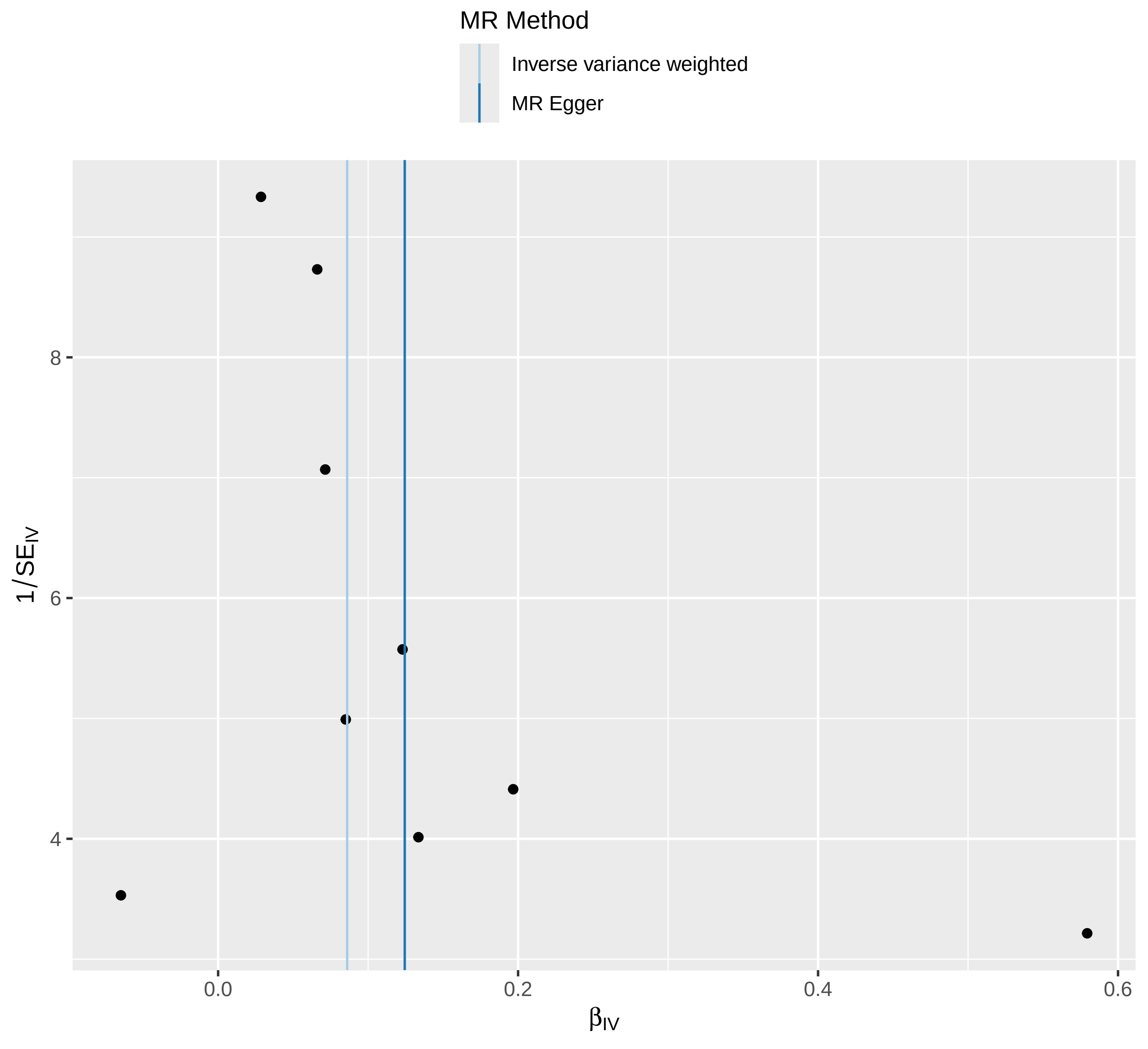
MR Method
Inverse variance weighted
MR Egger
8
6
4
0.0
0.2
0.4
0.6
1/SE_IV
β_IV

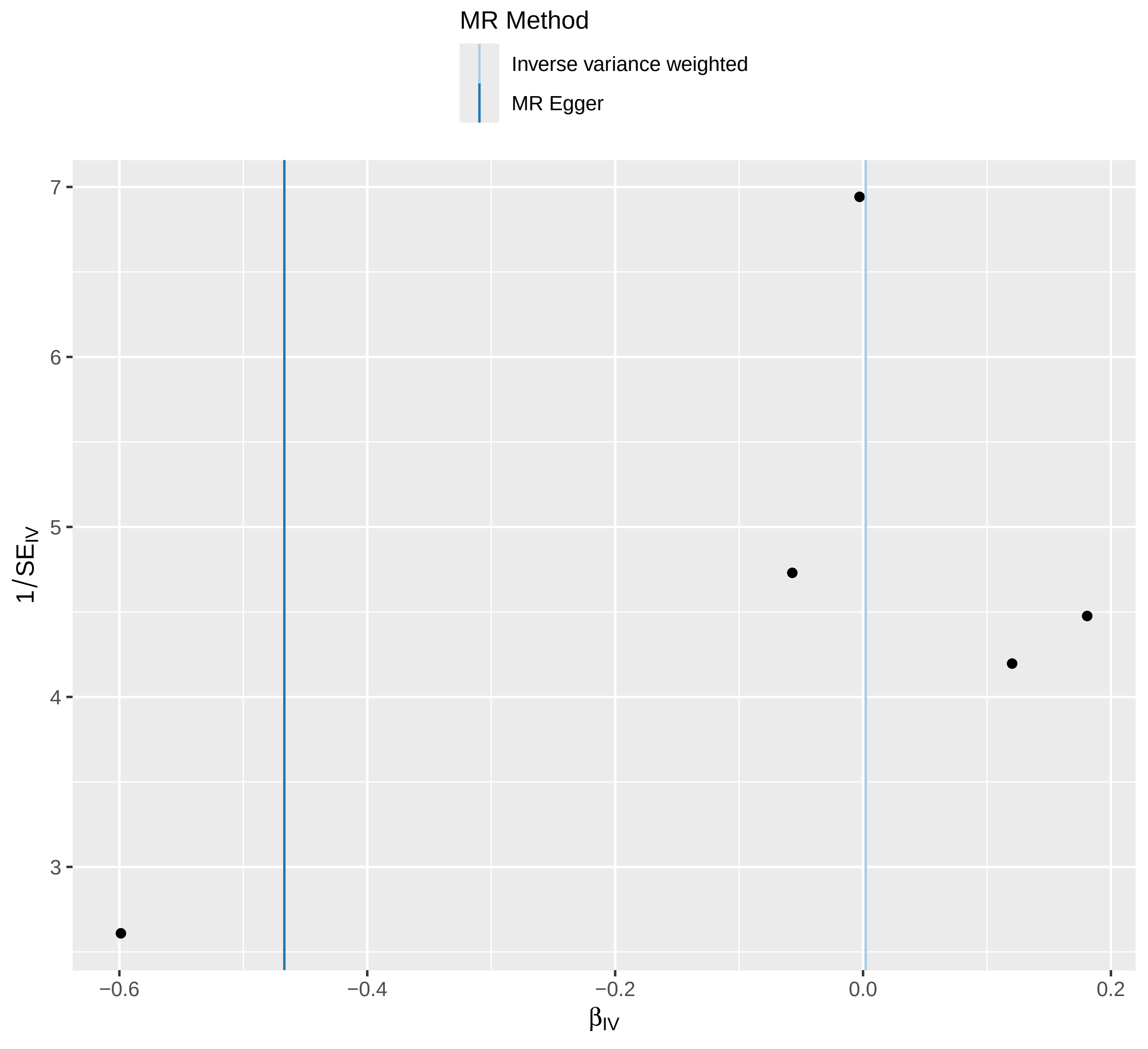

MR Method
Inverse variance weighted
MR Egger
7
6
5
4
3
1/SE_IV
−0.6
−0.4
−0.2
0.0
0.2
β_IV

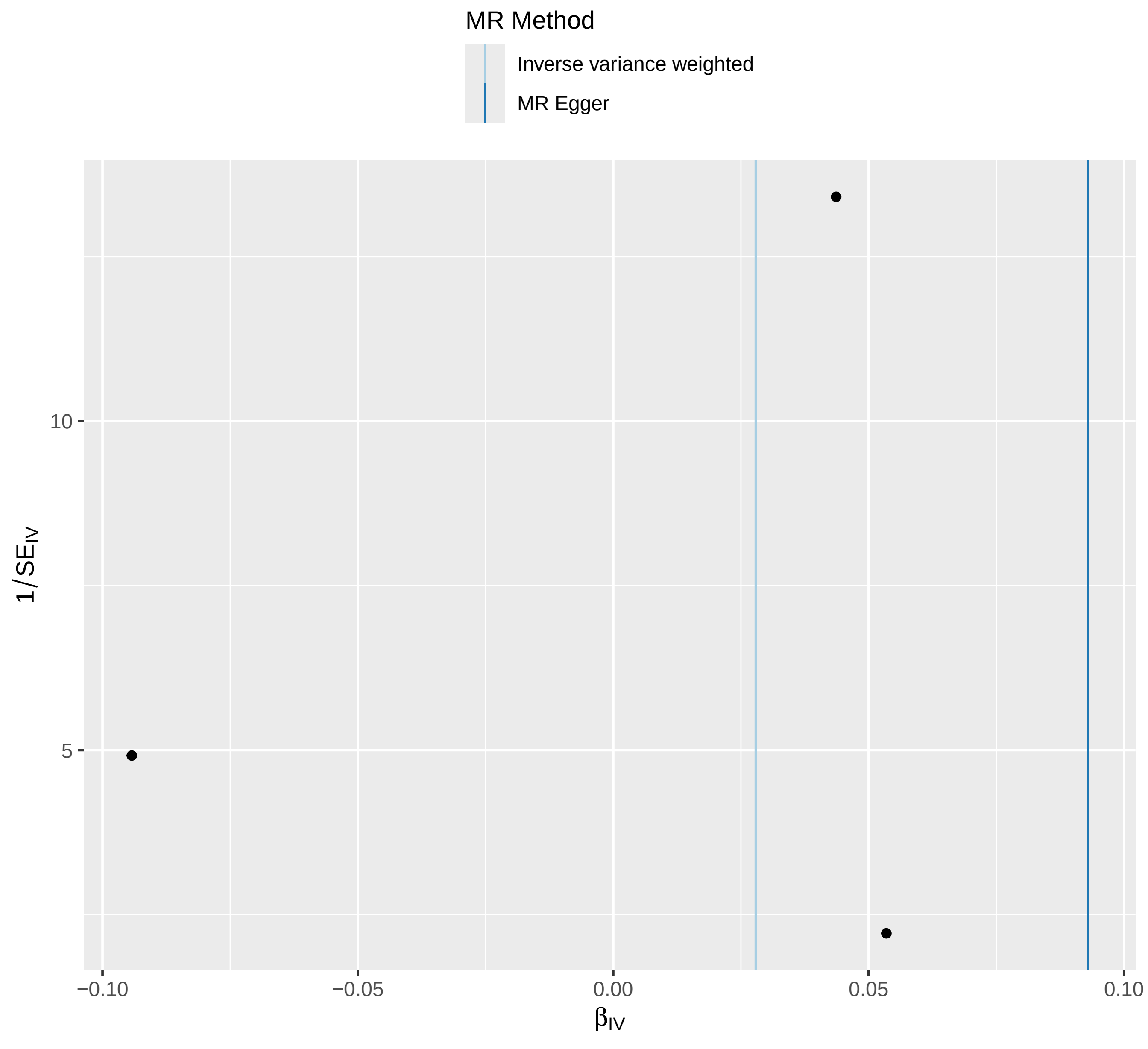
MR Method
Inverse variance weighted
MR Egger
10
5
1/SE_IV
−0.10
−0.05
0.00
0.05
0.10
β_IV

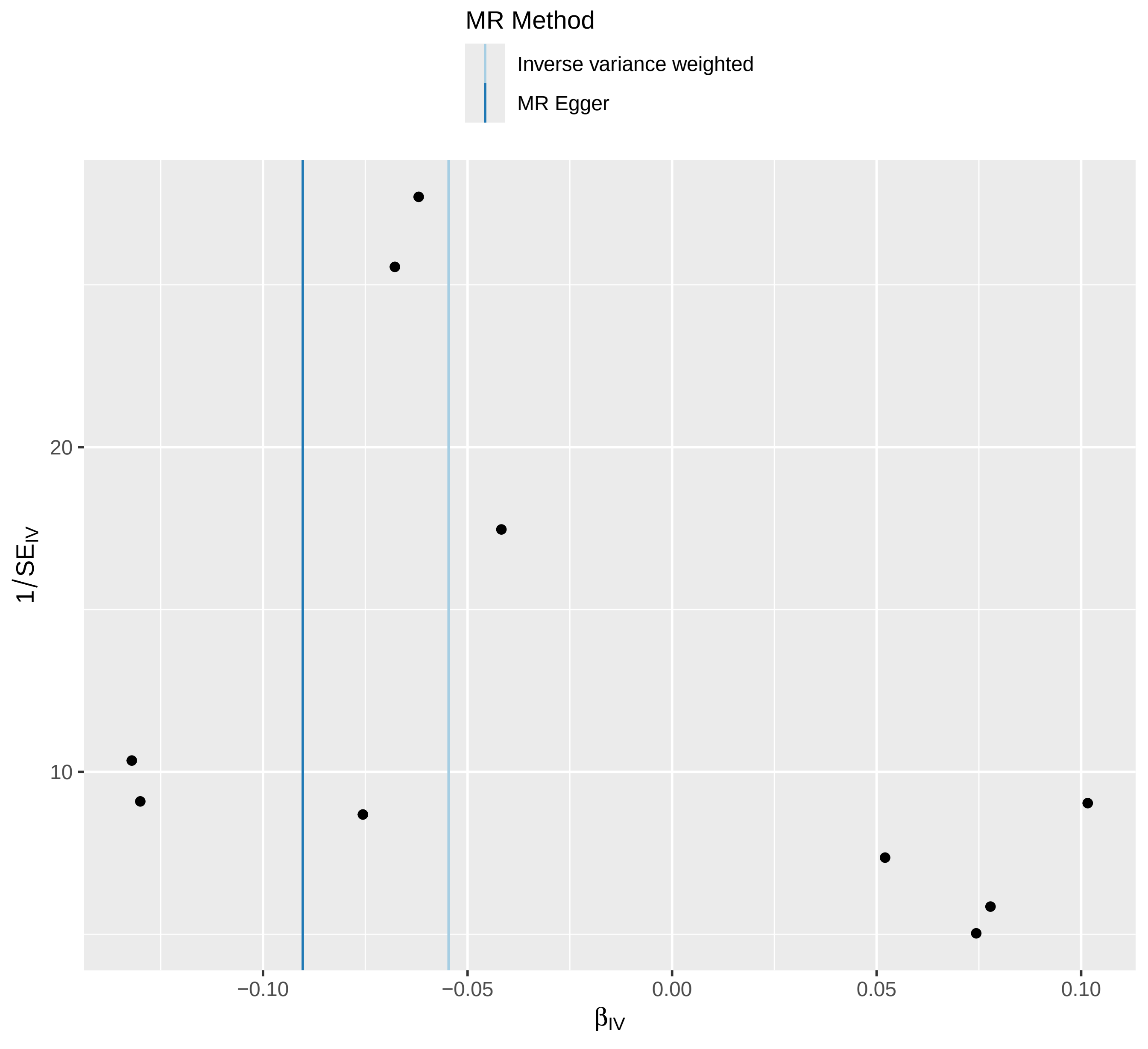
MR Method
Inverse variance weighted
MR Egger
20
10
$1/SE_{IV}$
−0.10
−0.05
0.00
0.05
0.10
$\beta_{IV}$

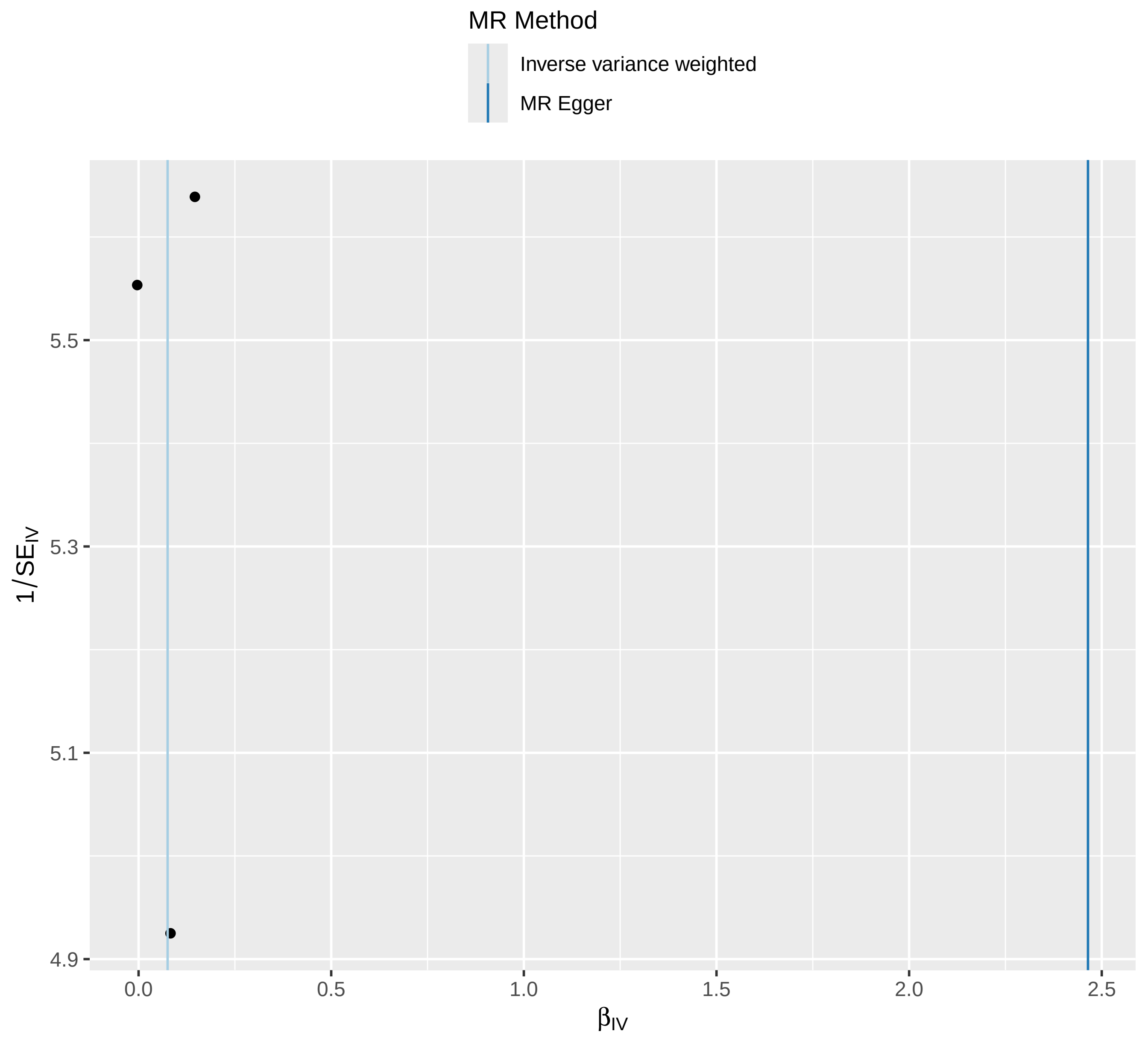

MR Method
Inverse variance weighted
MR Egger
5.5
5.3
5.1
4.9
1/SE_IV
0.0
0.5
1.0
1.5
2.0
2.5
β_IV

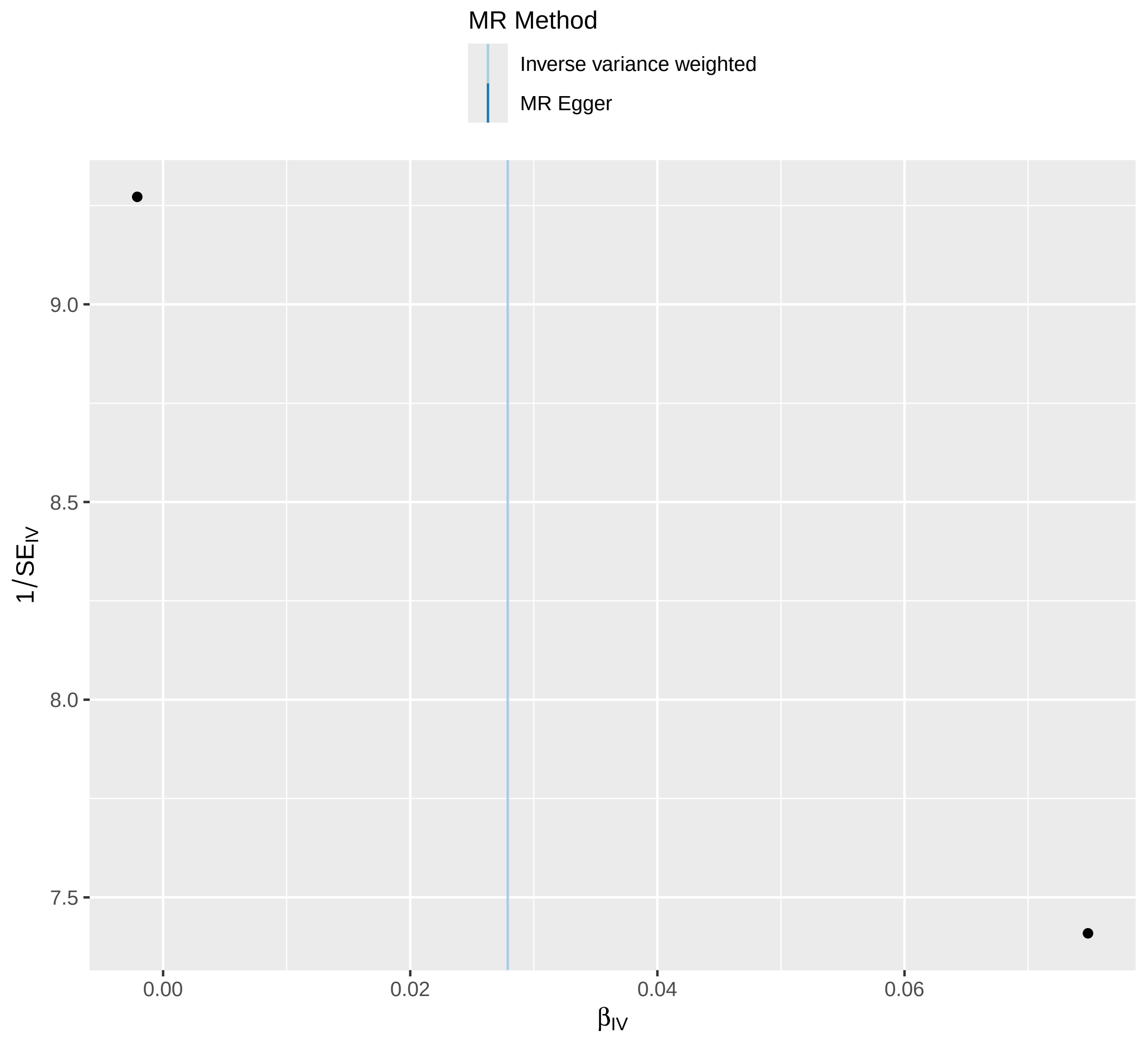

MR Method
Inverse variance weighted
MR Egger
9.0
8.5
8.0
7.5
1/SE_IV
0.00
0.02
0.04
0.06
β_IV

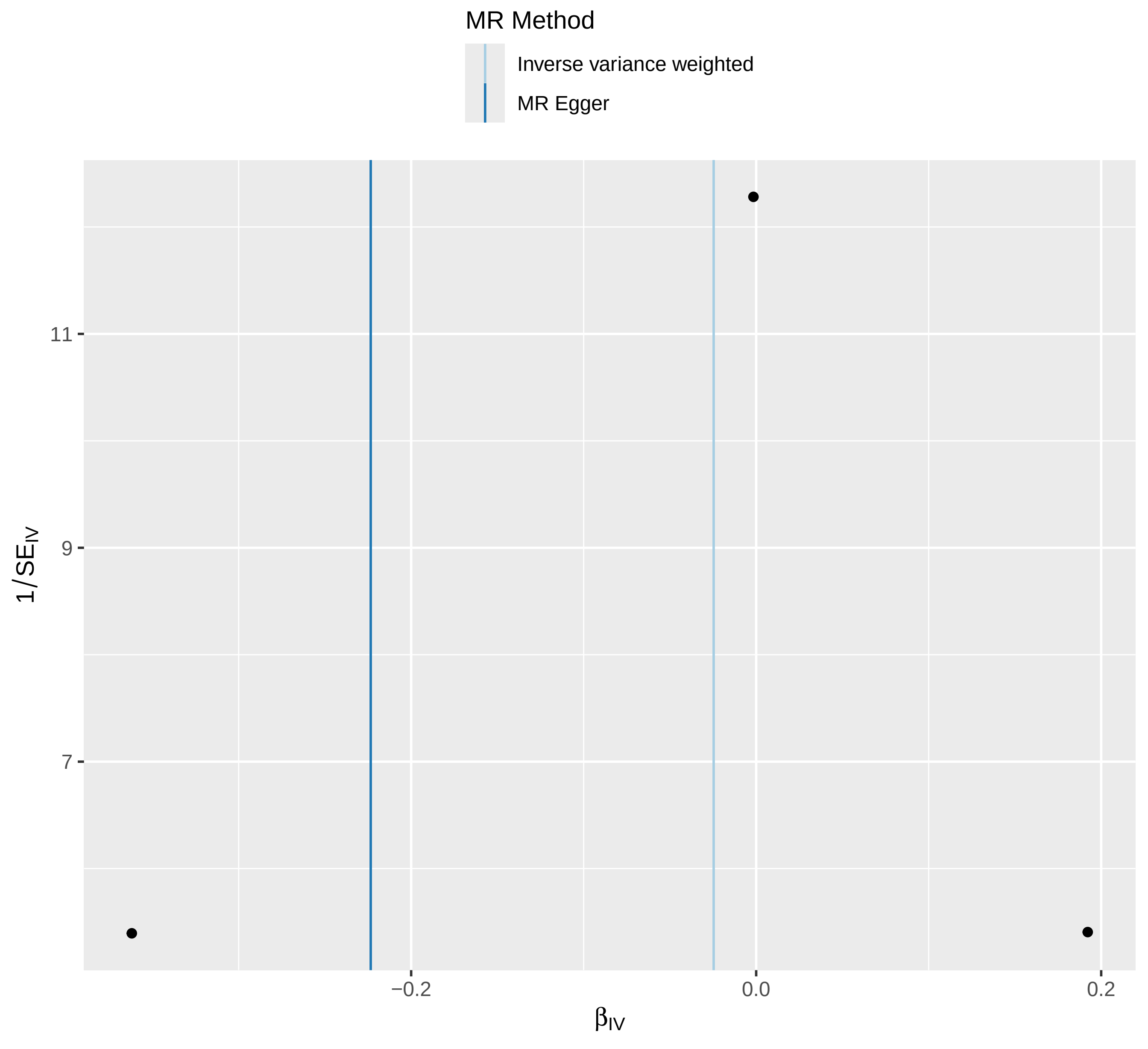

MR Method
Inverse variance weighted
MR Egger
11
9
7
1/SE$_{IV}$
−0.2
0.0
0.2
β$_{IV}$

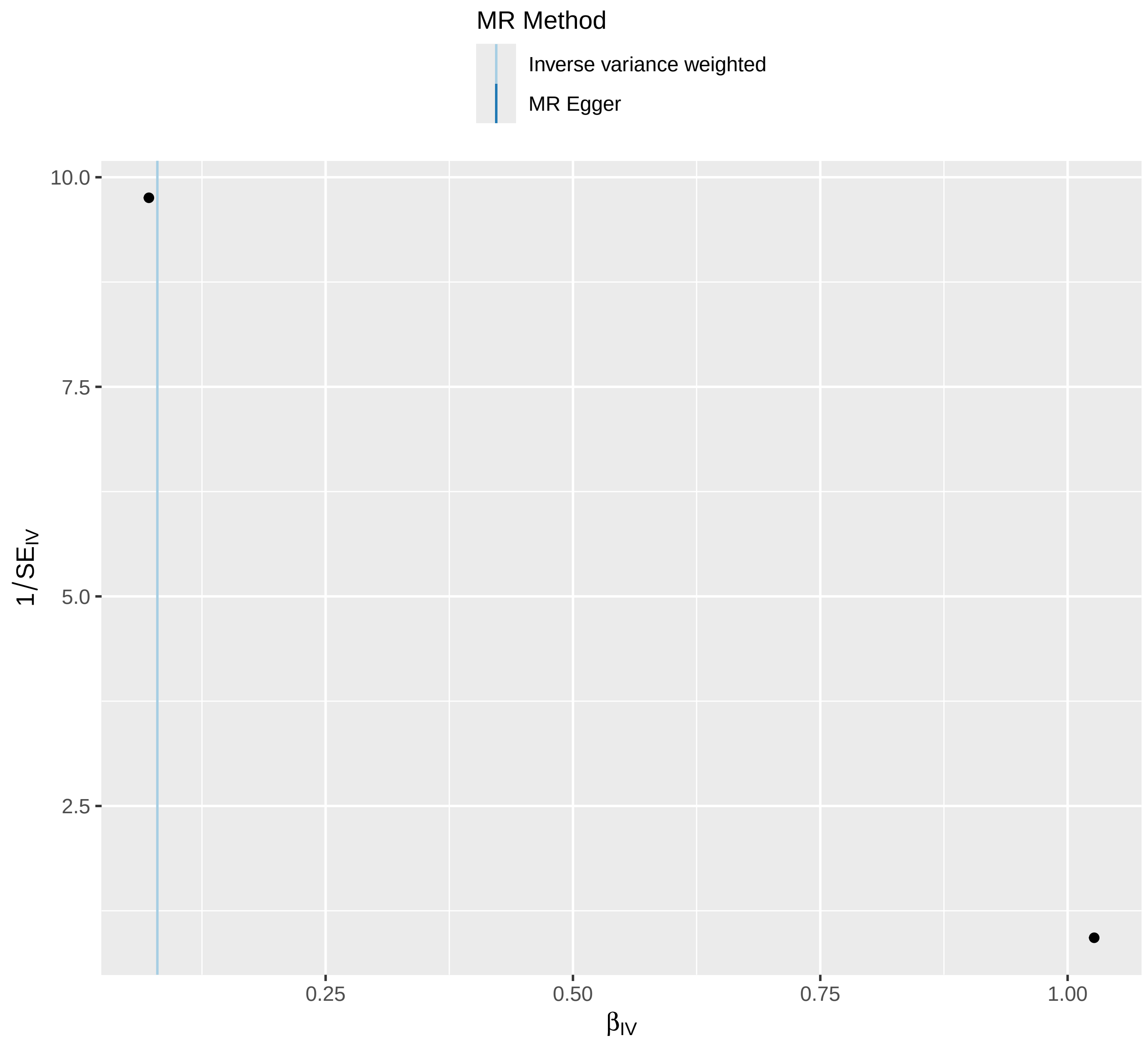

MR Method
Inverse variance weighted
MR Egger
10.0
7.5
5.0
2.5
$1/SE_{IV}$
0.25
0.50
0.75
1.00
$\beta_{IV}$

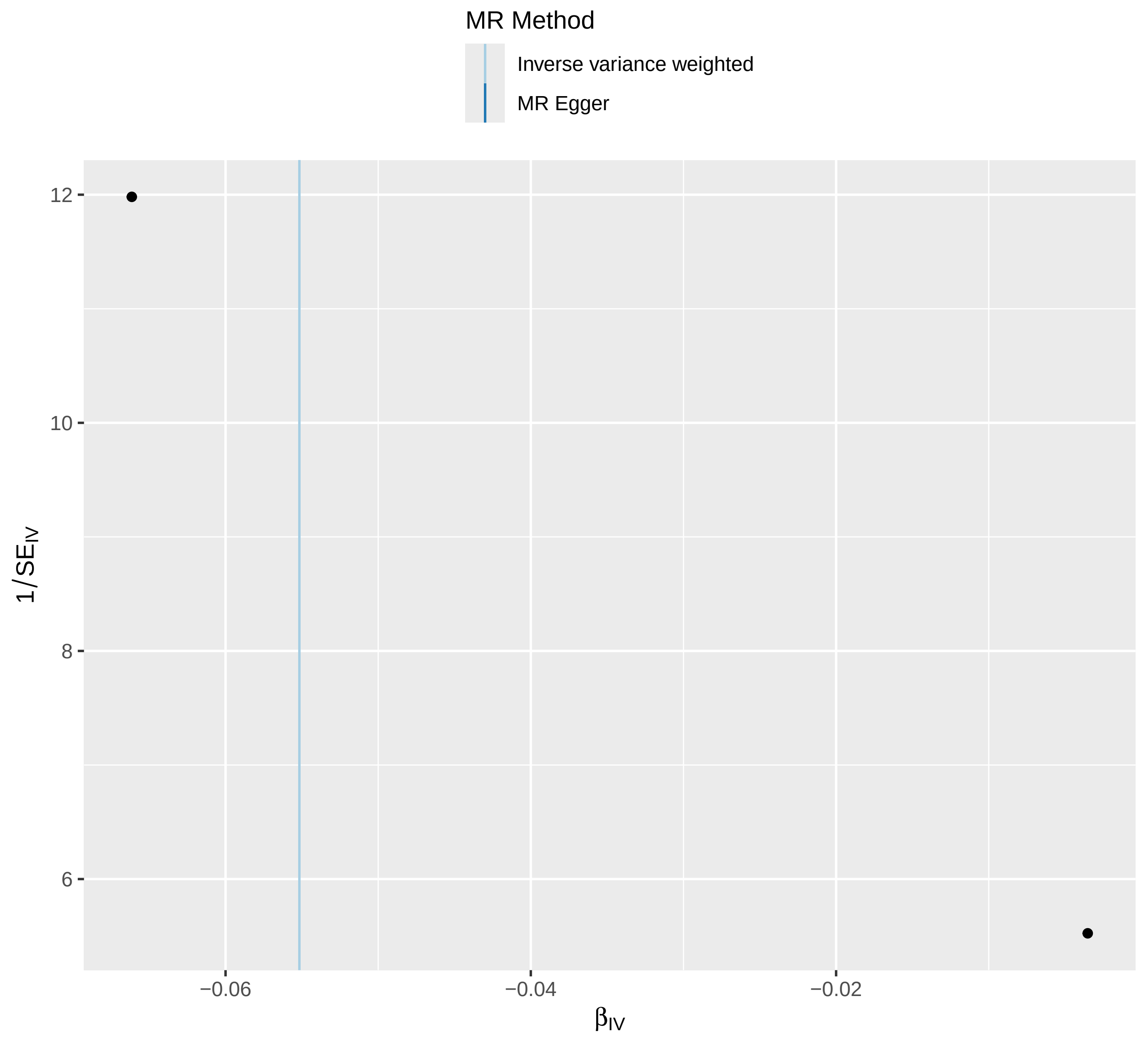

MR Method
Inverse variance weighted
MR Egger
12
10
8
6
1/SE$_{IV}$
−0.06
−0.04
−0.02
β$_{IV}$

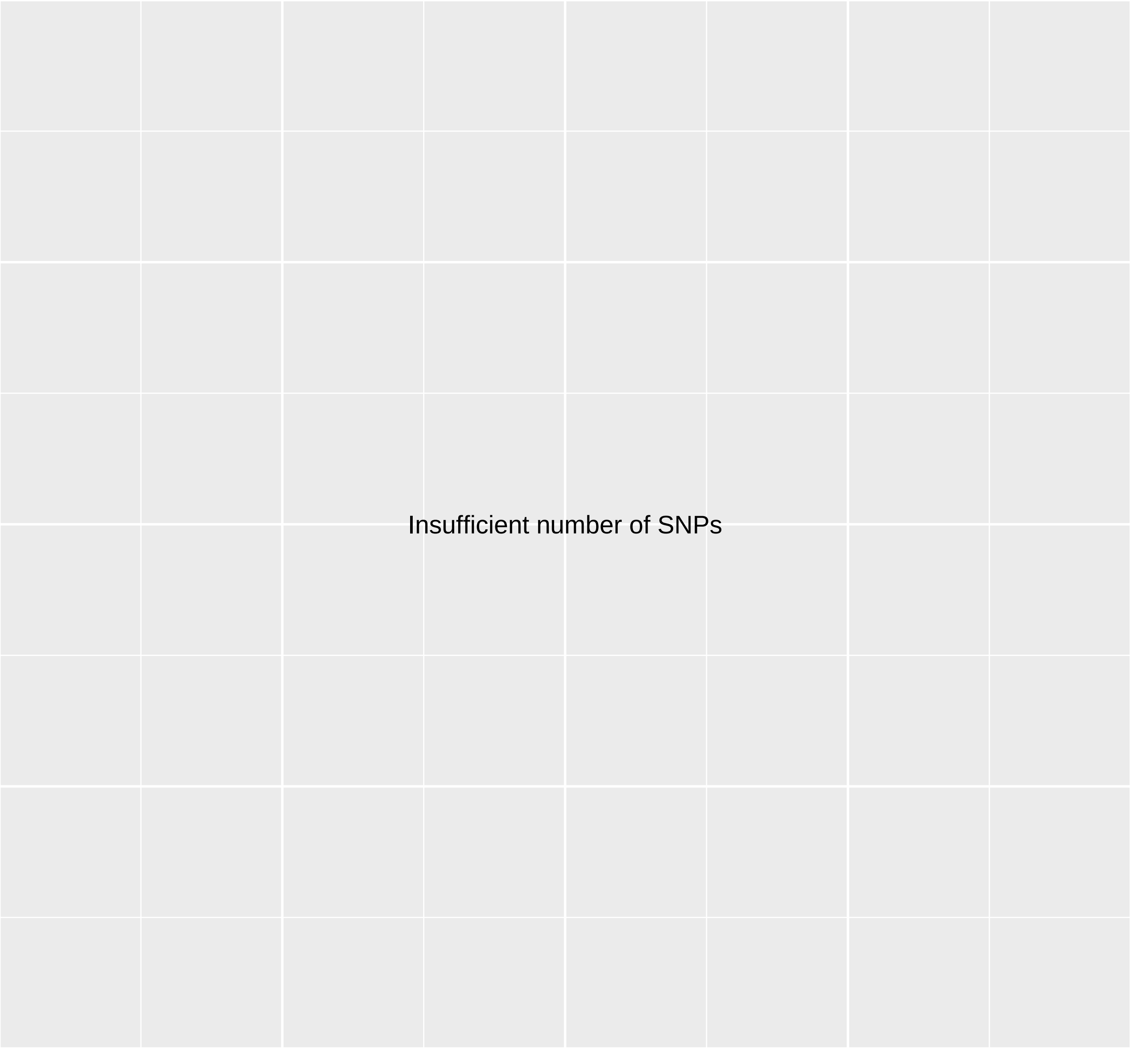
Insufficient number of SNPs

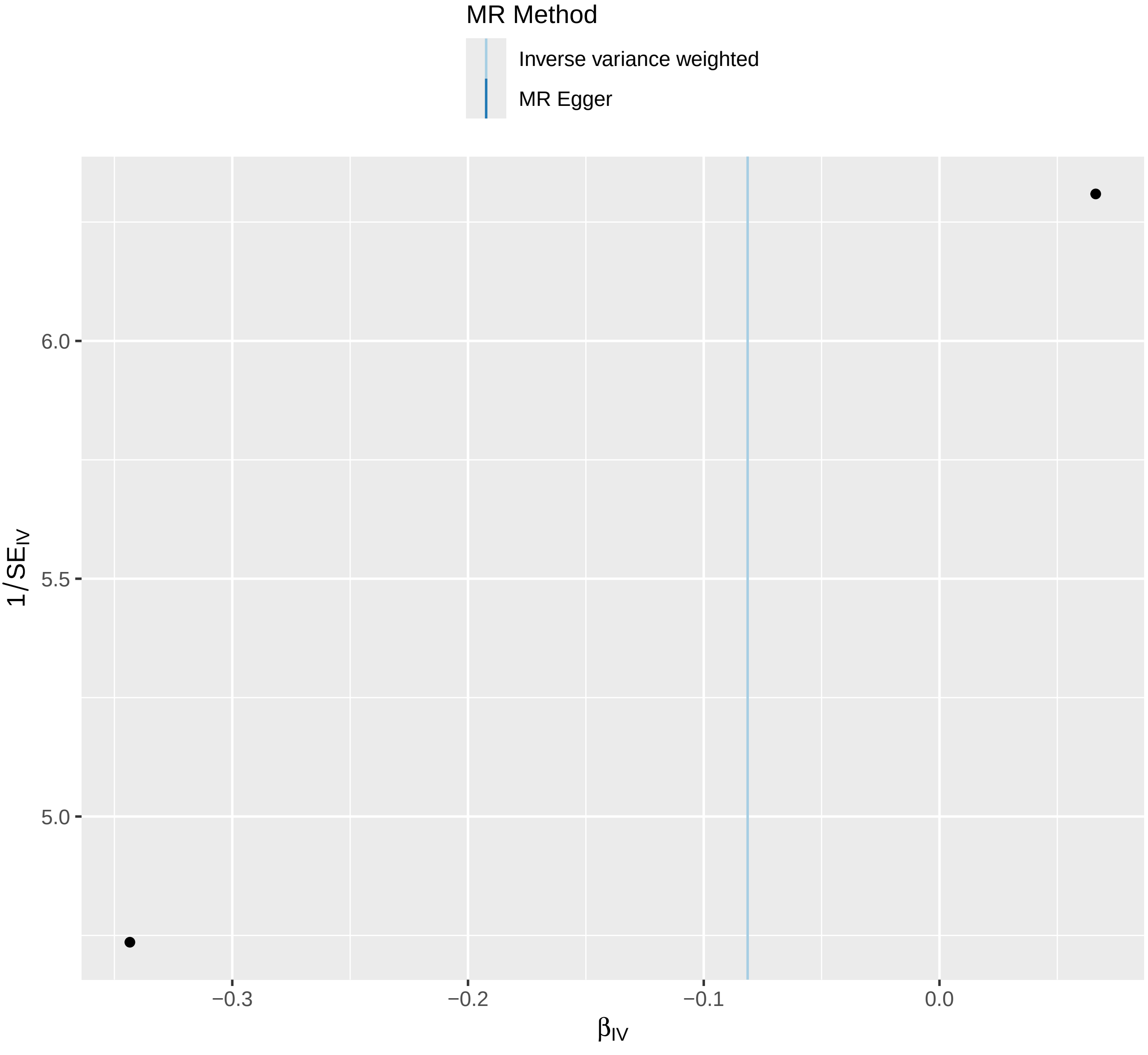
MR Method
Inverse variance weighted
MR Egger
6.0
5.5
5.0
1/SE_IV
−0.3
−0.2
−0.1
0.0
β_IV

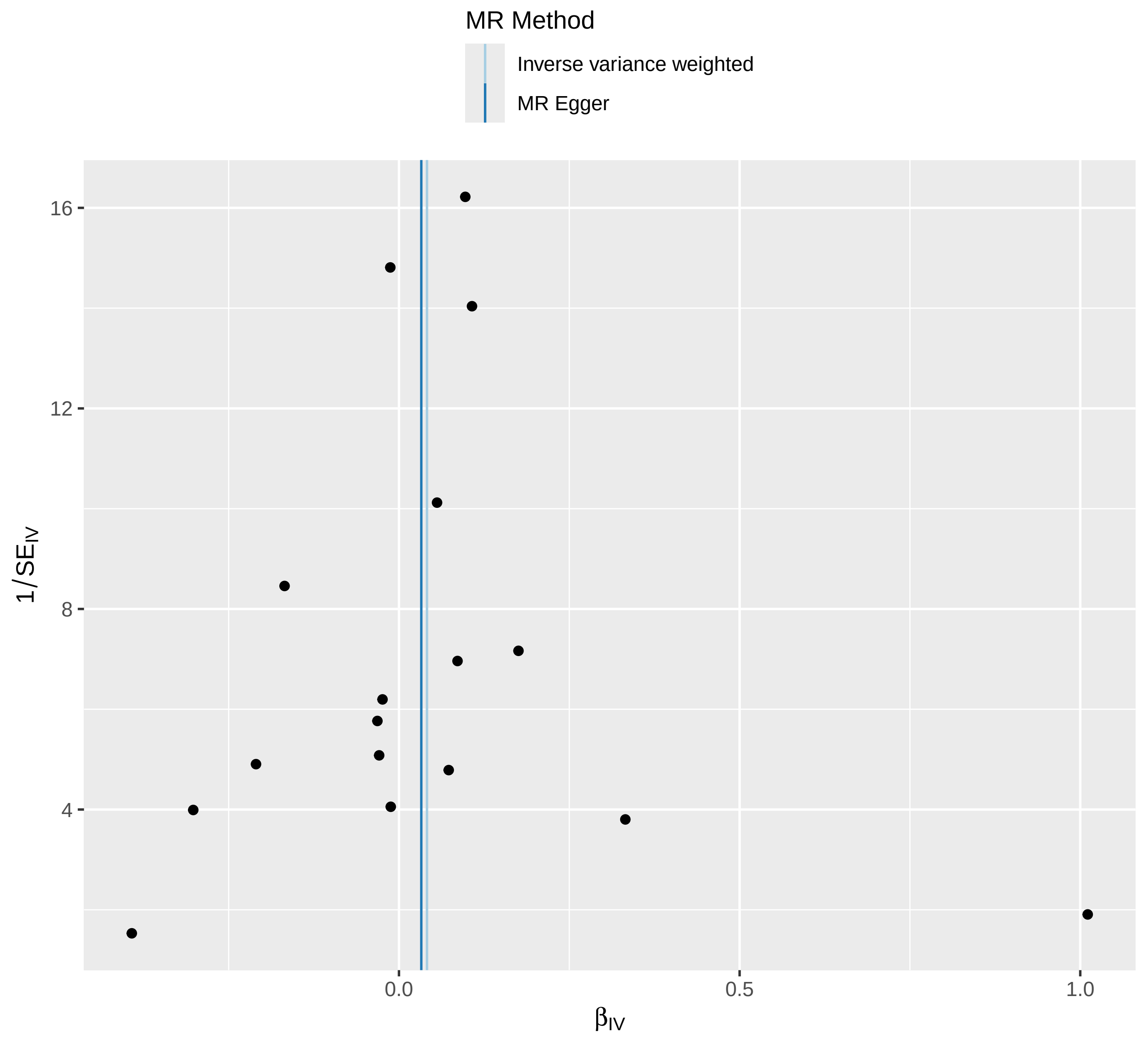

MR Method
Inverse variance weighted
MR Egger
16
12
8
4
1/SE$_{IV}$
0.0
0.5
1.0
β$_{IV}$

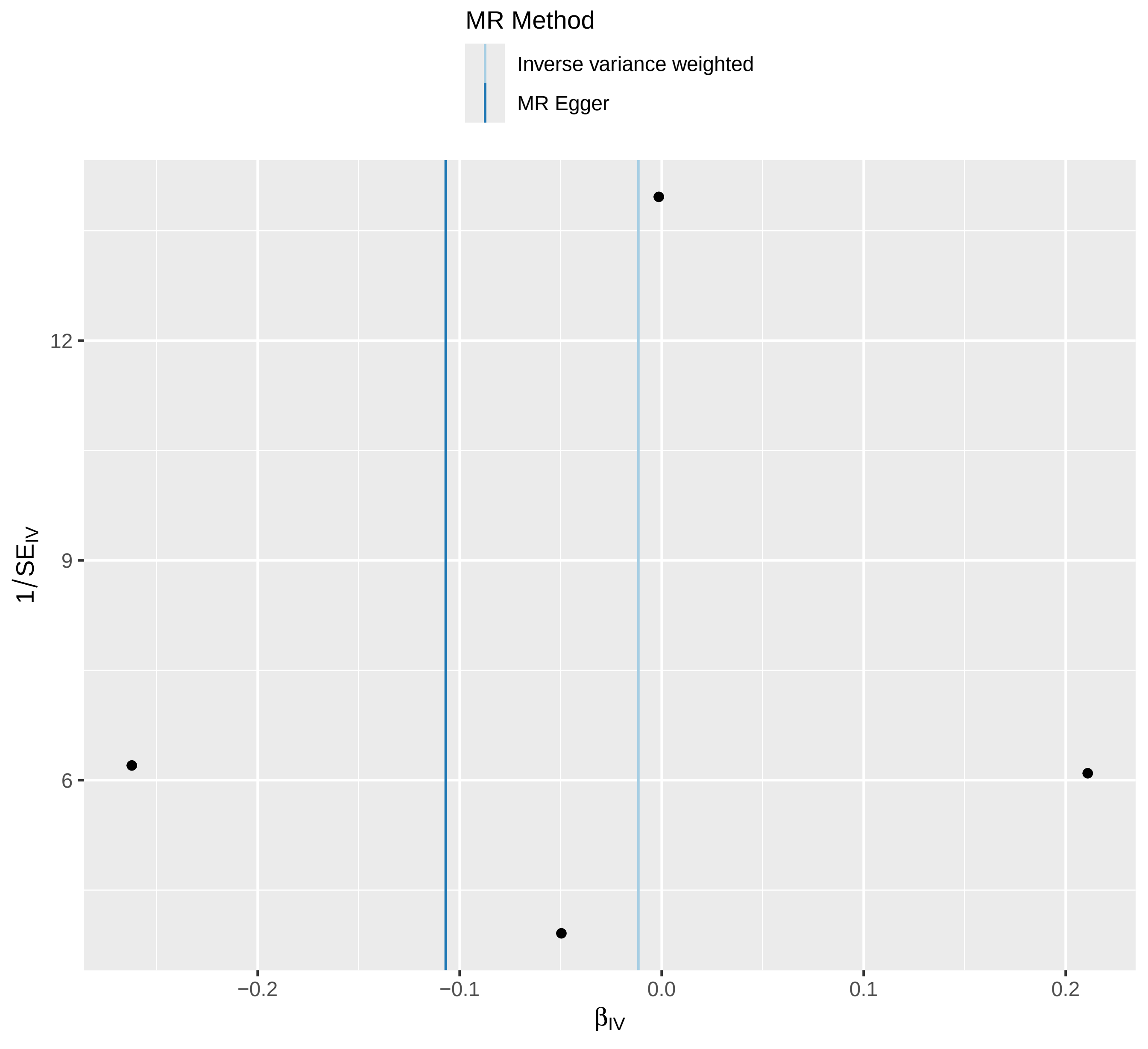
MR Method
Inverse variance weighted
MR Egger
1/SE_IV
12
9
6
−0.2
−0.1
0.0
0.1
0.2
β_IV

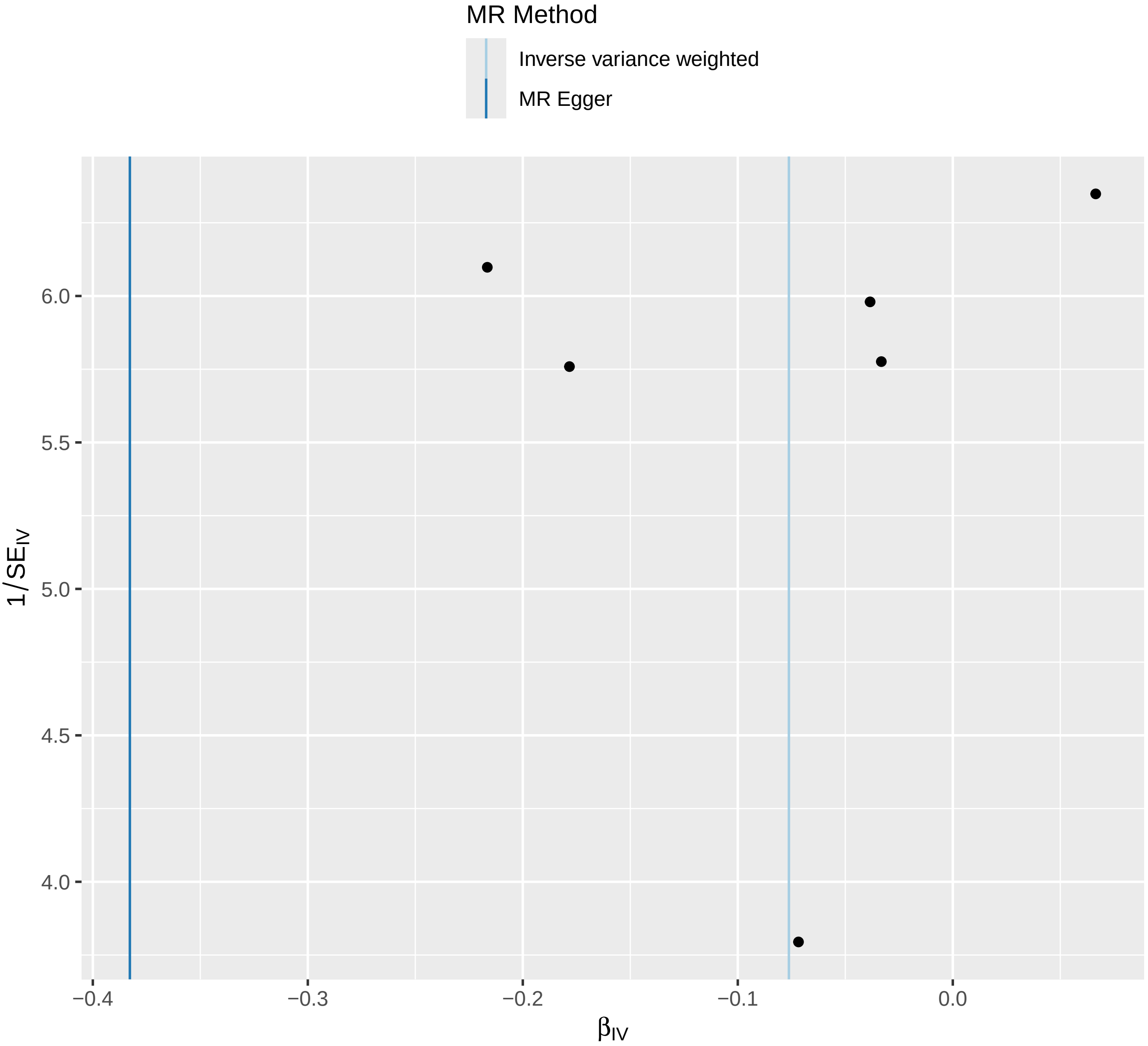
MR Method
Inverse variance weighted
MR Egger
6.0
5.5
5.0
4.5
4.0
1/SE$_{IV}$
−0.4
−0.3
−0.2
−0.1
0.0
β$_{IV}$

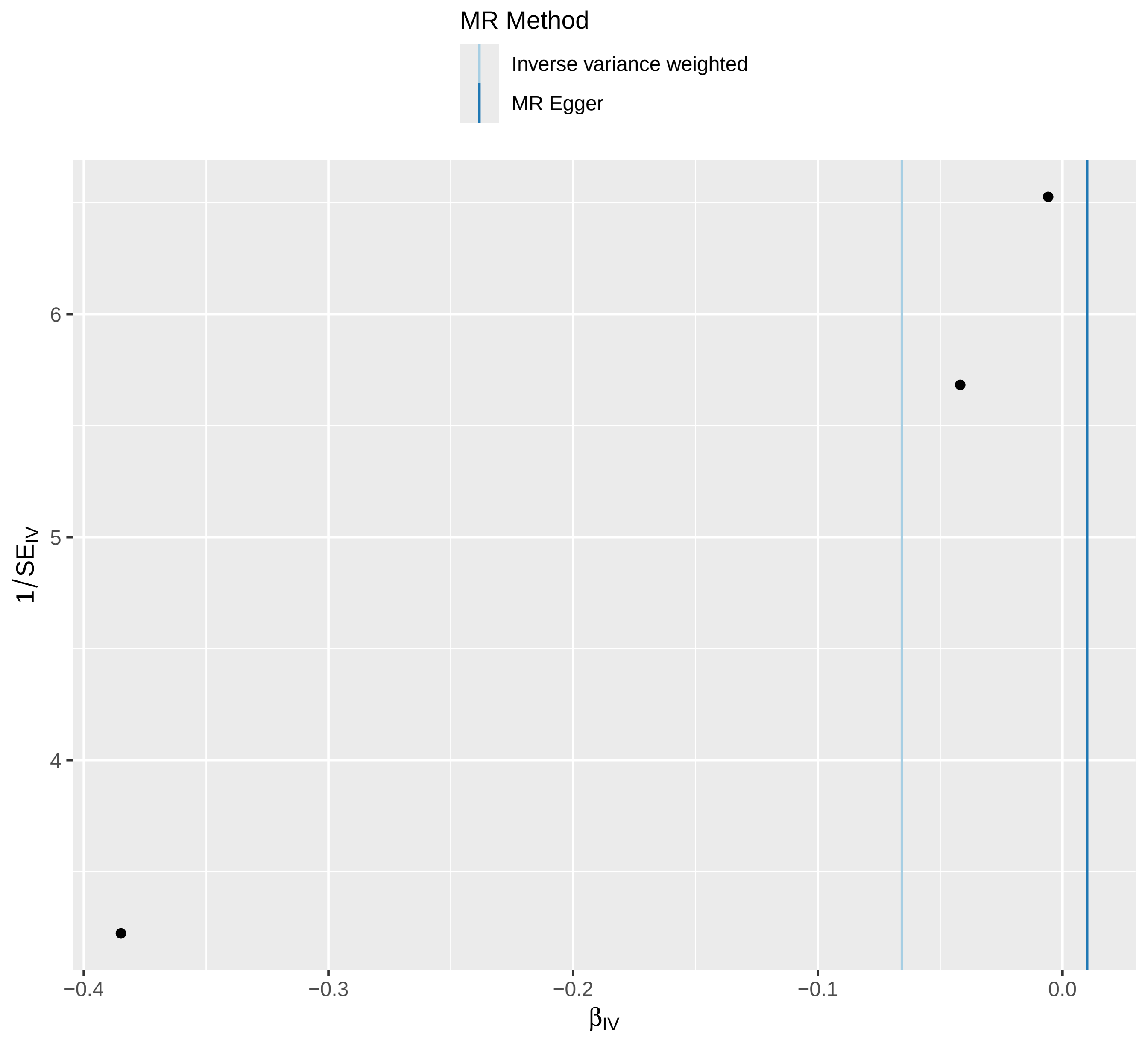

MR Method
Inverse variance weighted
MR Egger
6
5
4
1/SE_IV
−0.4
−0.3
−0.2
−0.1
0.0
β_IV

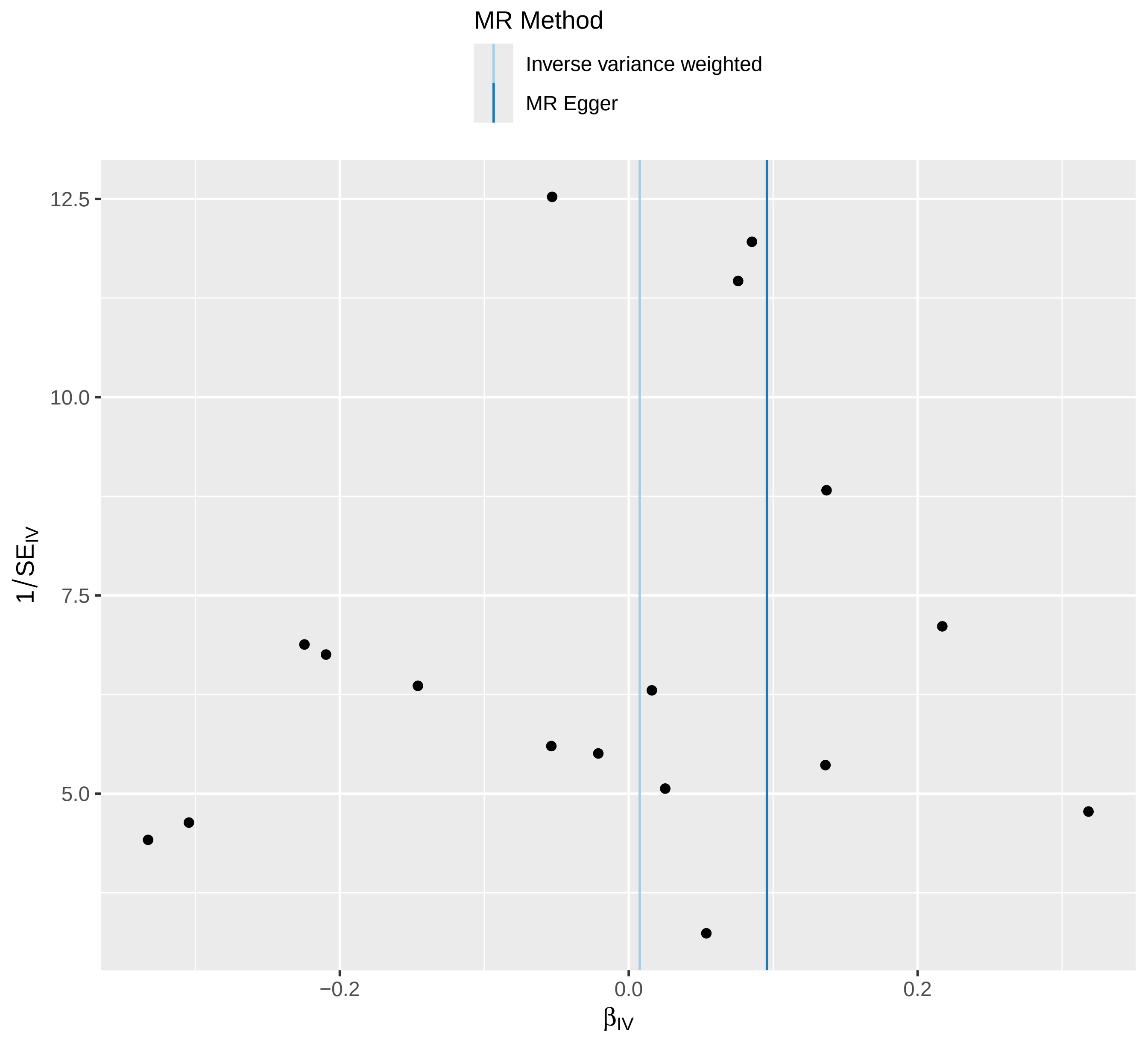

MR Method
Inverse variance weighted
MR Egger
12.5
10.0
7.5
5.0
$1/SE_{IV}$
−0.2
0.0
0.2
$\beta_{IV}$

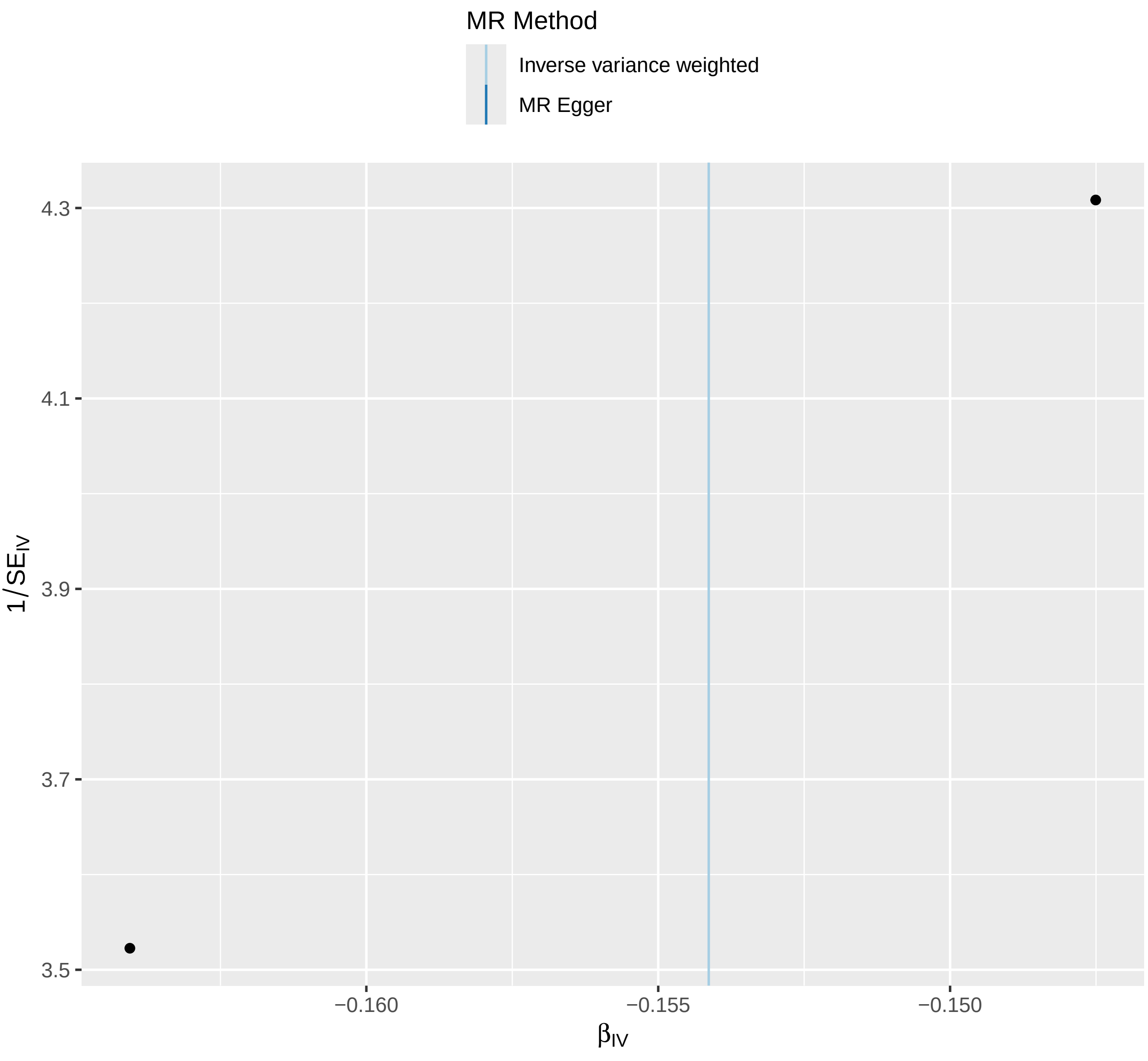

MR Method
Inverse variance weighted
MR Egger
4.3
4.1
3.9
3.7
3.5
1/SE$_{IV}$
−0.160
−0.155
−0.150
β$_{IV}$

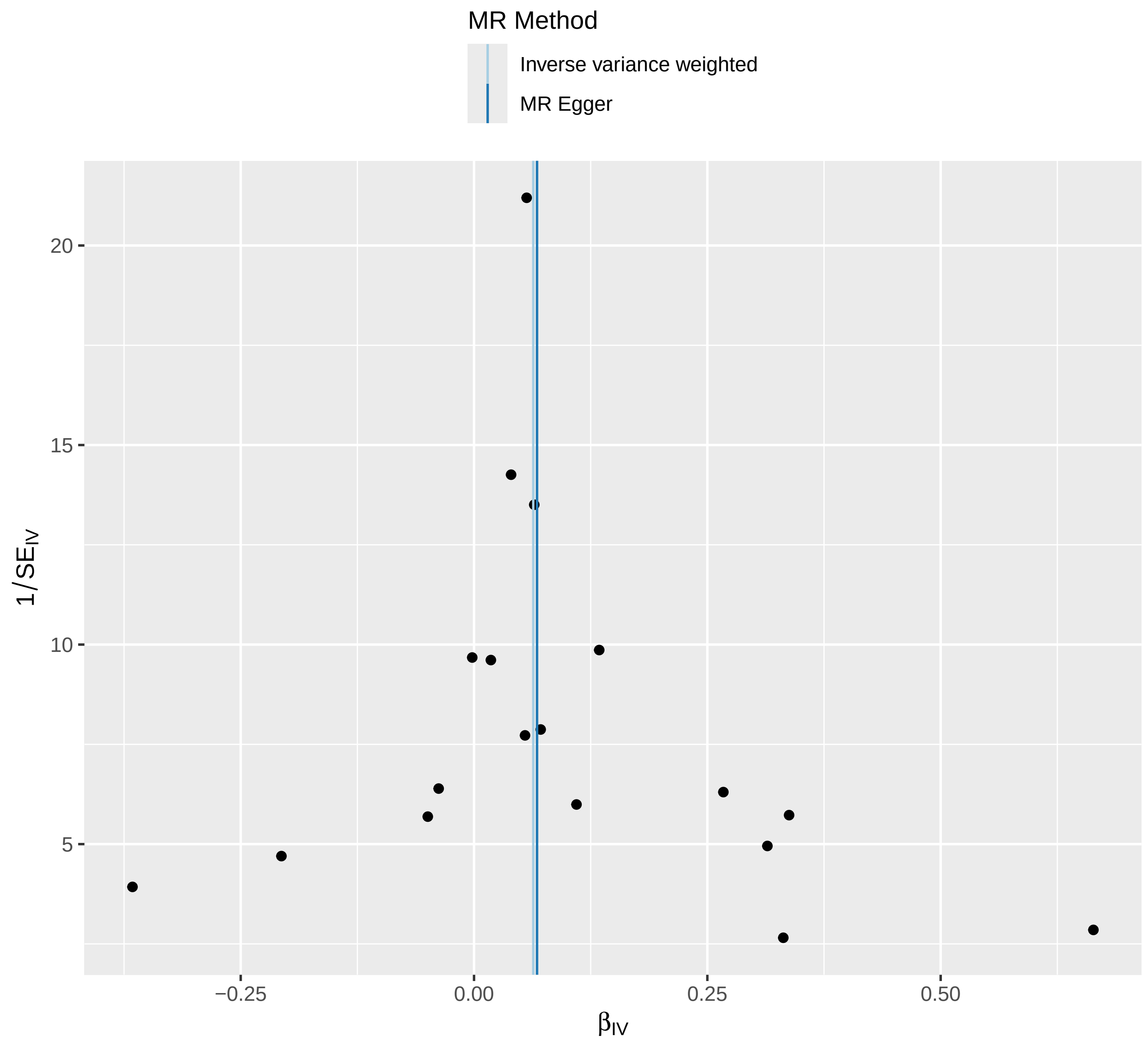

MR Method
Inverse variance weighted
MR Egger
20
15
10
5
1/SE_IV
−0.25
0.00
0.25
0.50
β_IV

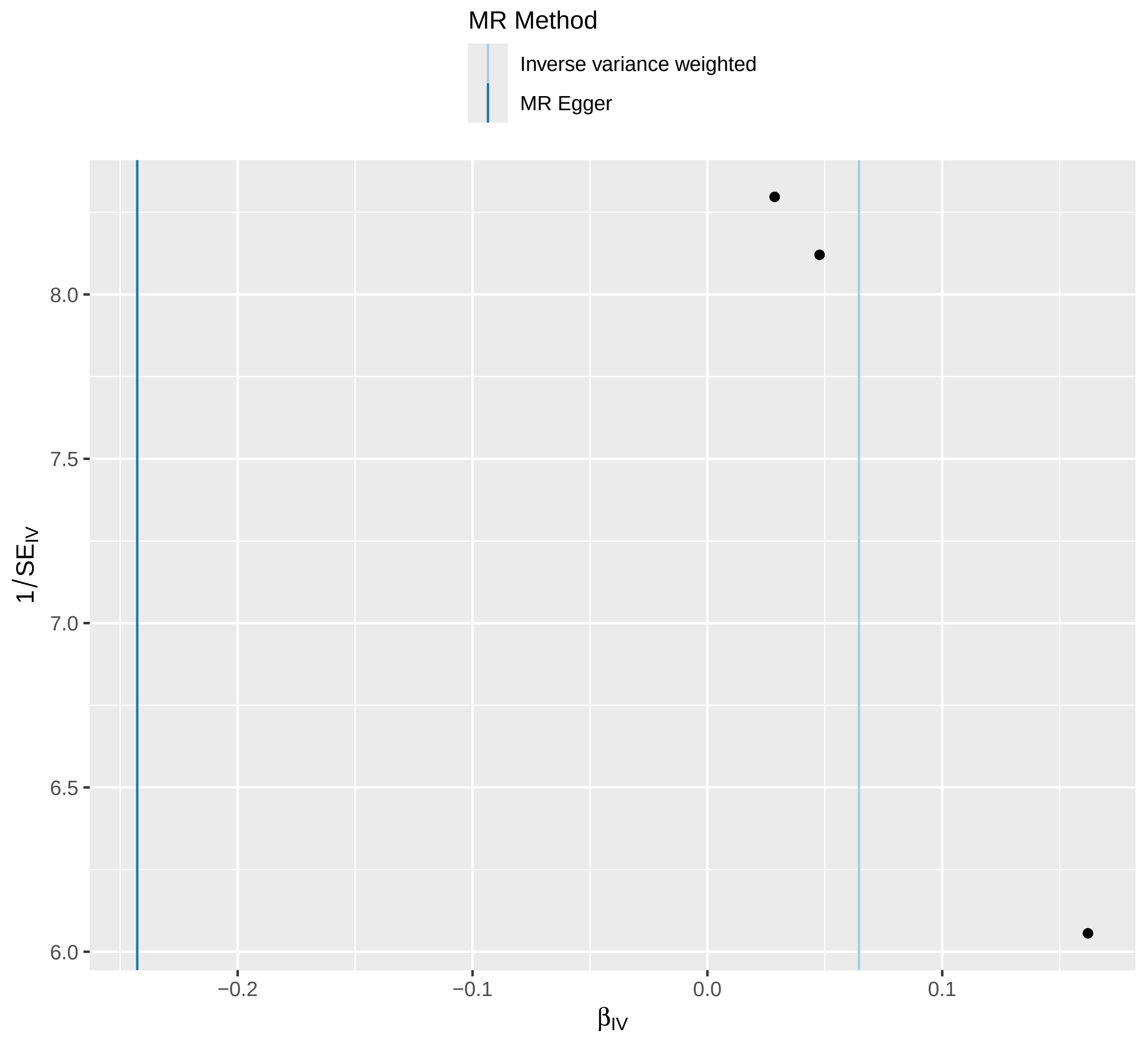
MR Method
Inverse variance weighted
MR Egger
8.0
7.5
7.0
6.5
6.0
1/SE$_{IV}$
−0.2
−0.1
0.0
0.1
β$_{IV}$

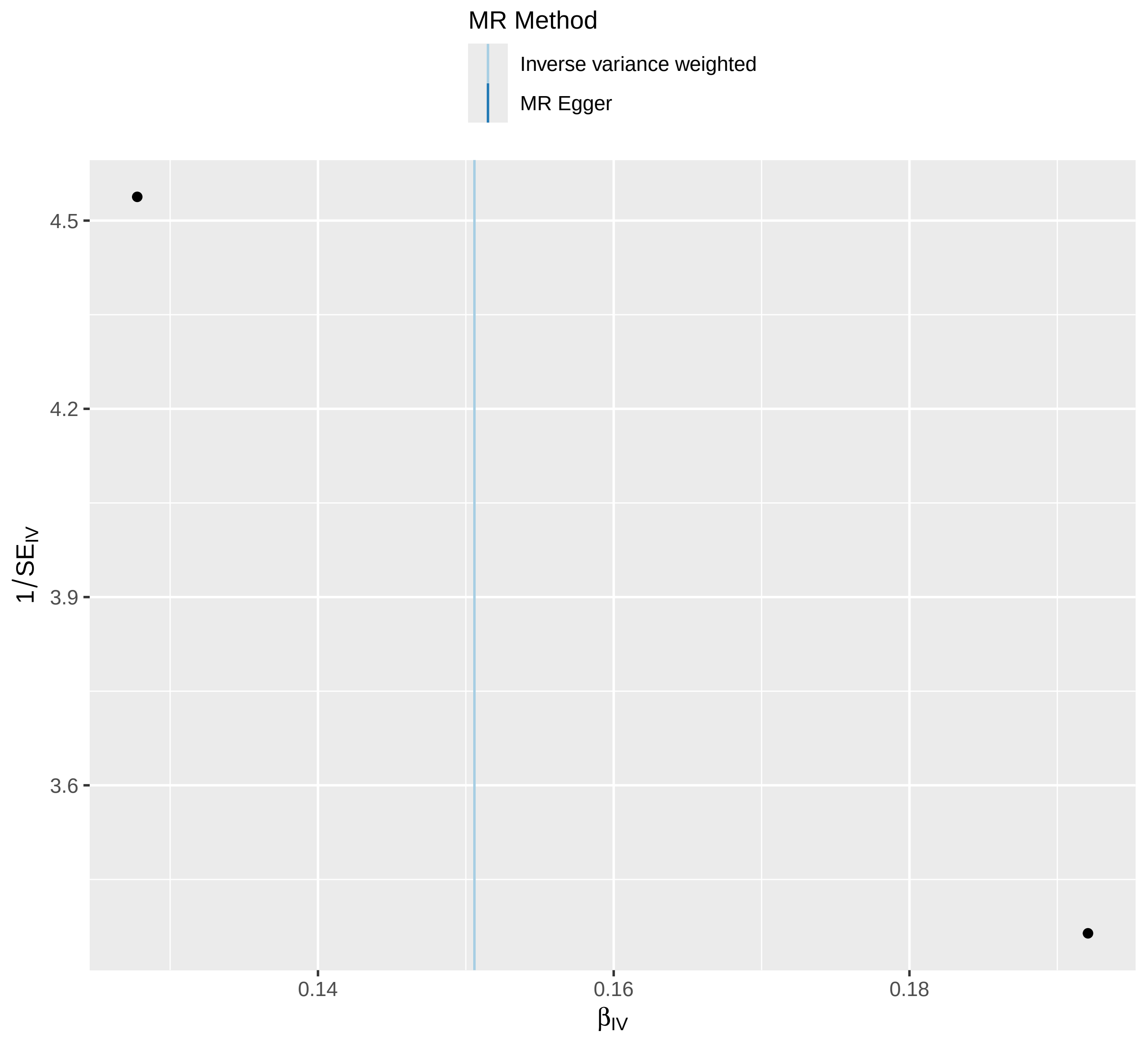
MR Method
Inverse variance weighted
MR Egger
4.5
4.2
3.9
3.6
1/SE_IV
0.14
0.16
0.18
β_IV

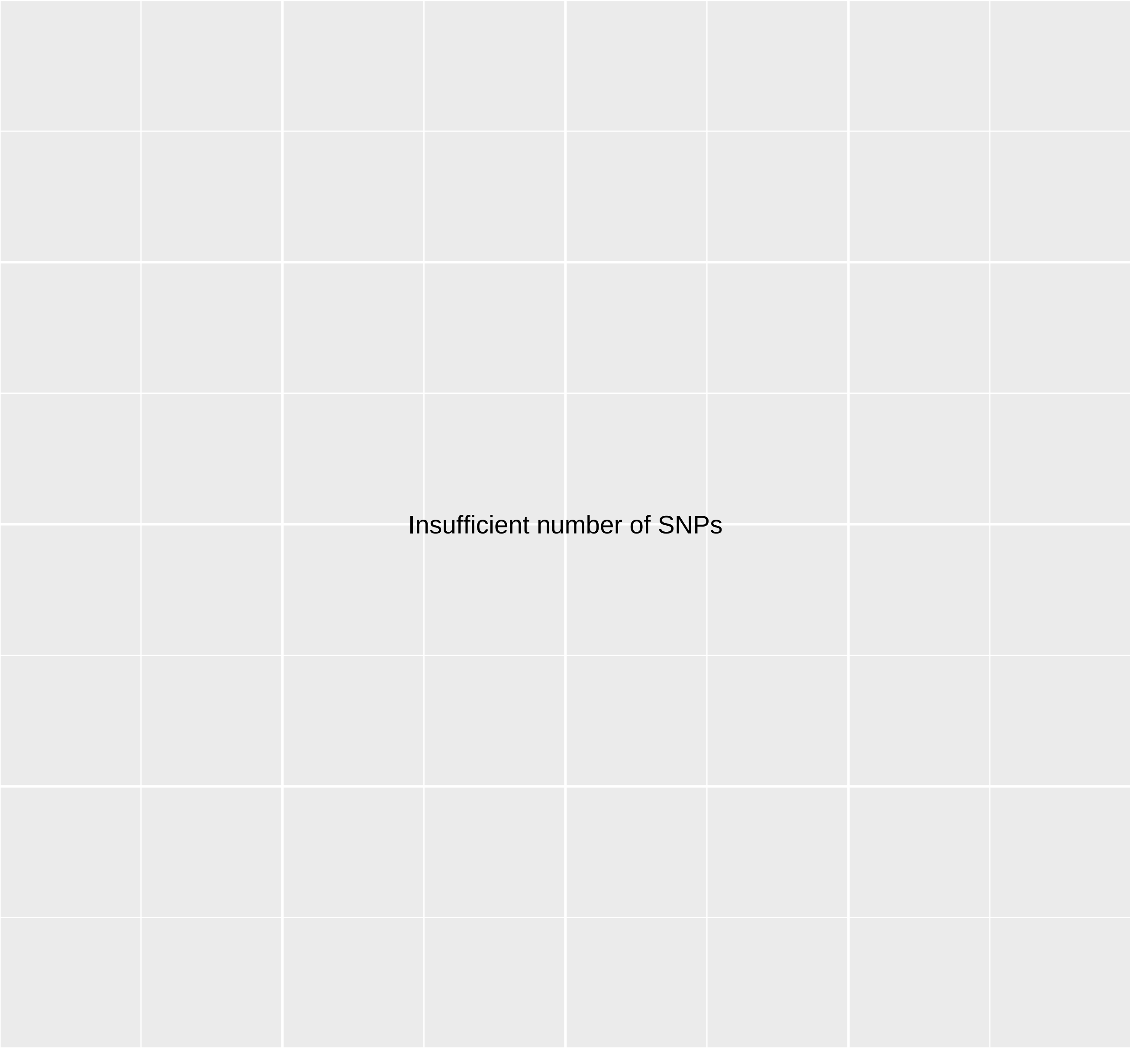
Insufficient number of SNPs

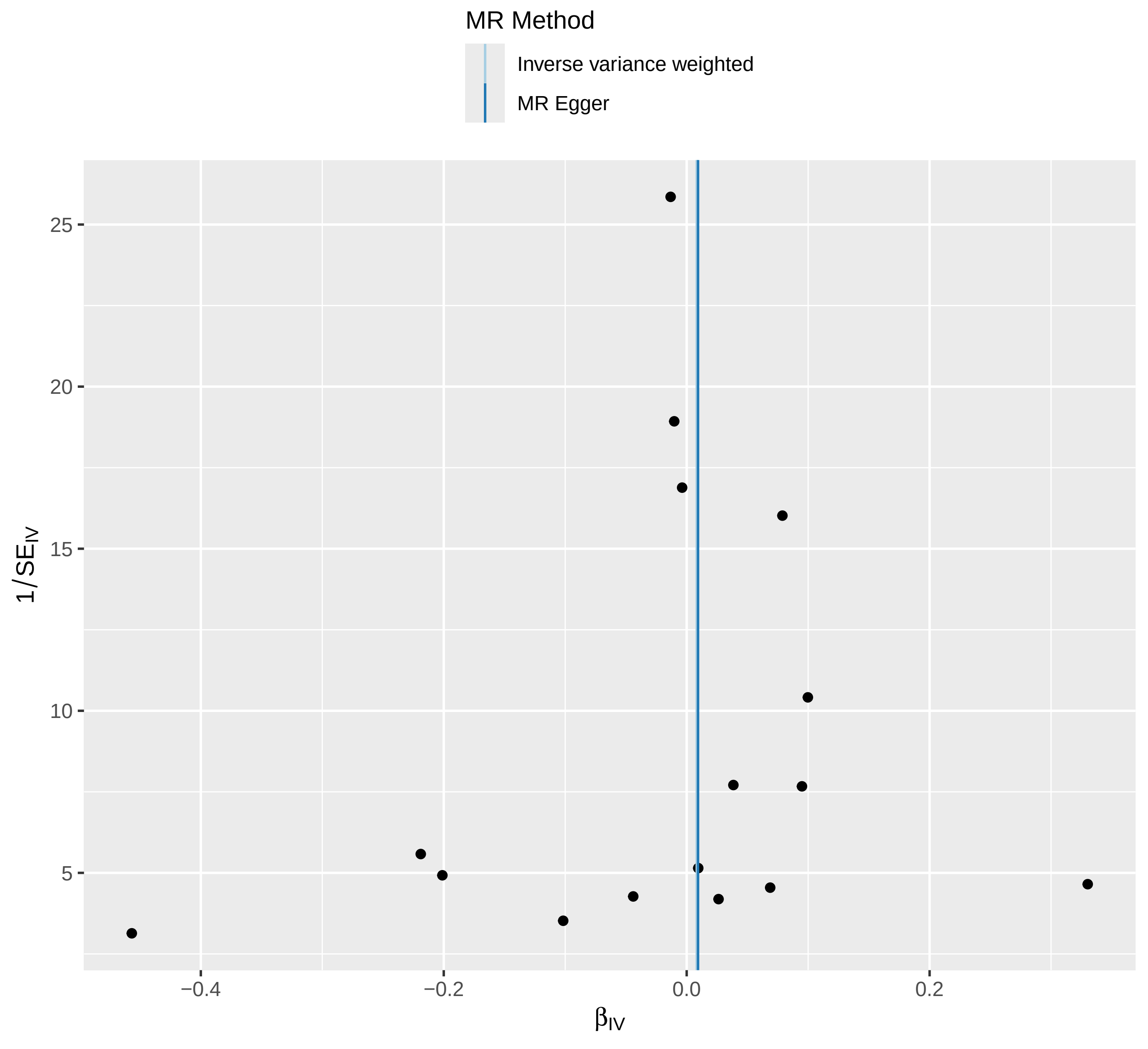
MR Method
Inverse variance weighted
MR Egger
25
20
15
10
5
1/SE$_{IV}$
−0.4
−0.2
0.0
0.2
β$_{IV}$

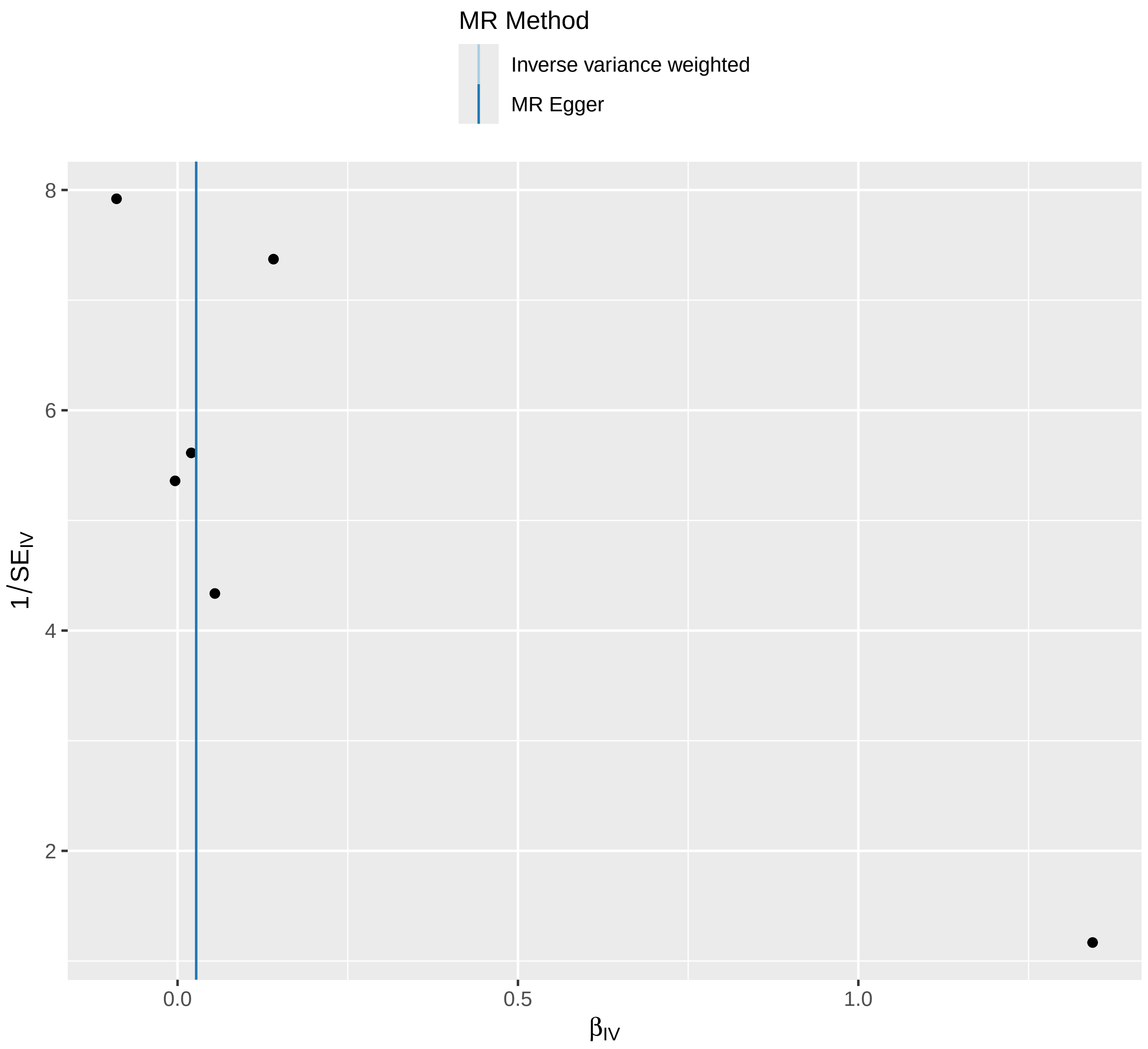
MR Method
Inverse variance weighted
MR Egger
1/SE_IV
β_IV
8
6
4
2
0.0
0.5
1.0

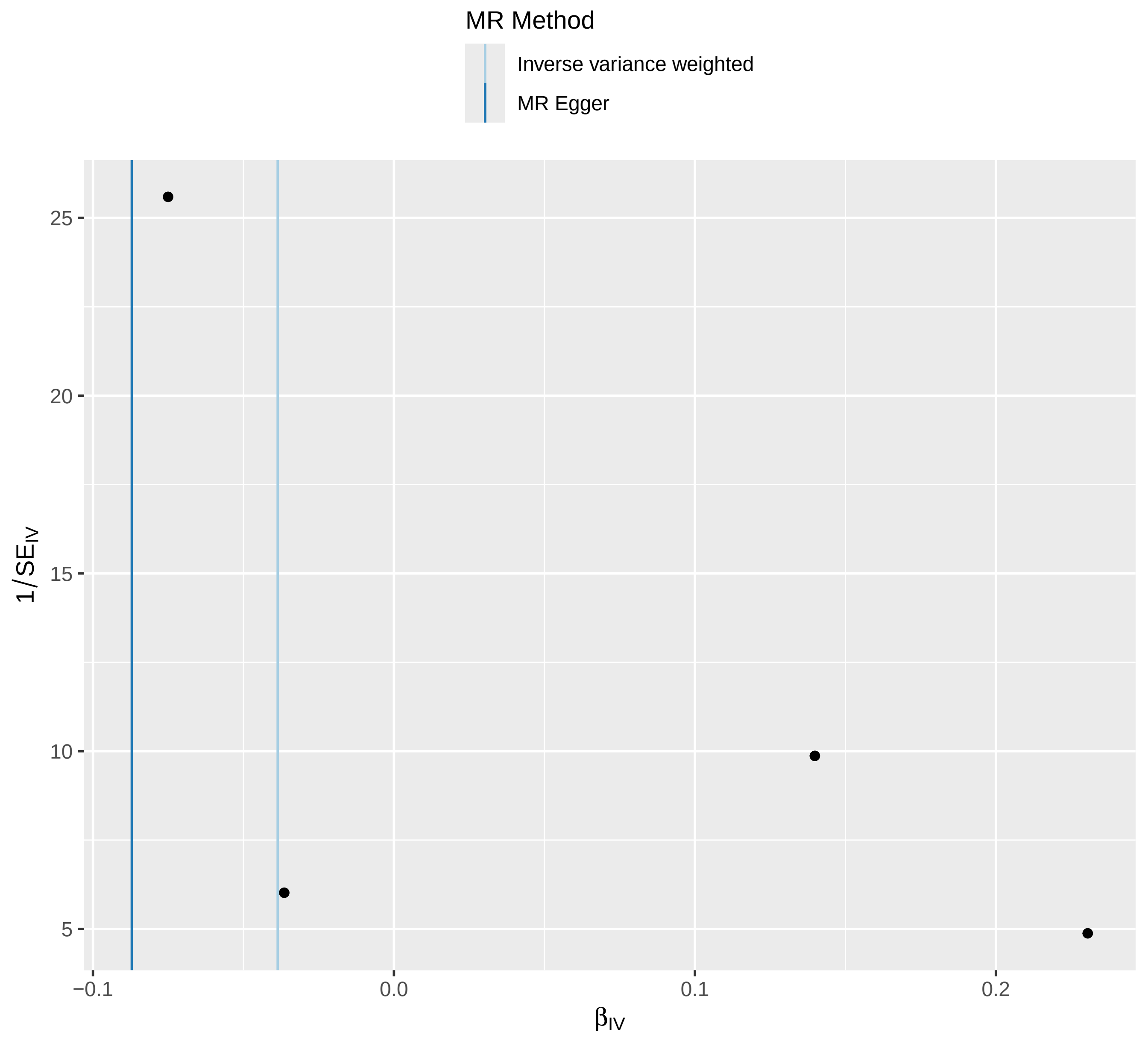

MR Method
Inverse variance weighted
MR Egger
25
20
15
10
5
1/SE_IV
−0.1
0.0
0.1
0.2
β_IV

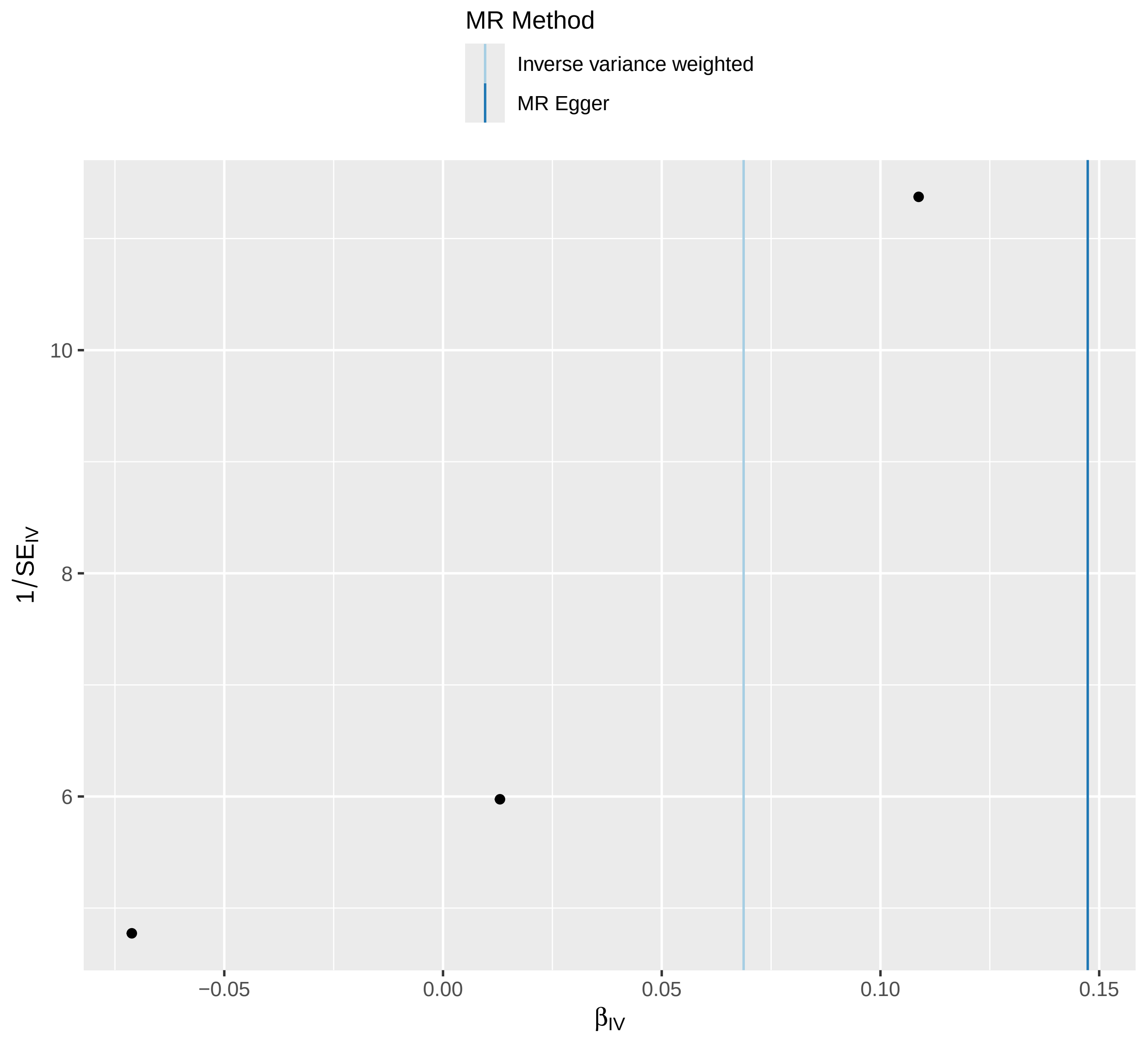

MR Method
Inverse variance weighted
MR Egger
10
8
6
−0.05
0.00
0.05
0.10
0.15
$1/SE_{IV}$
$\beta_{IV}$

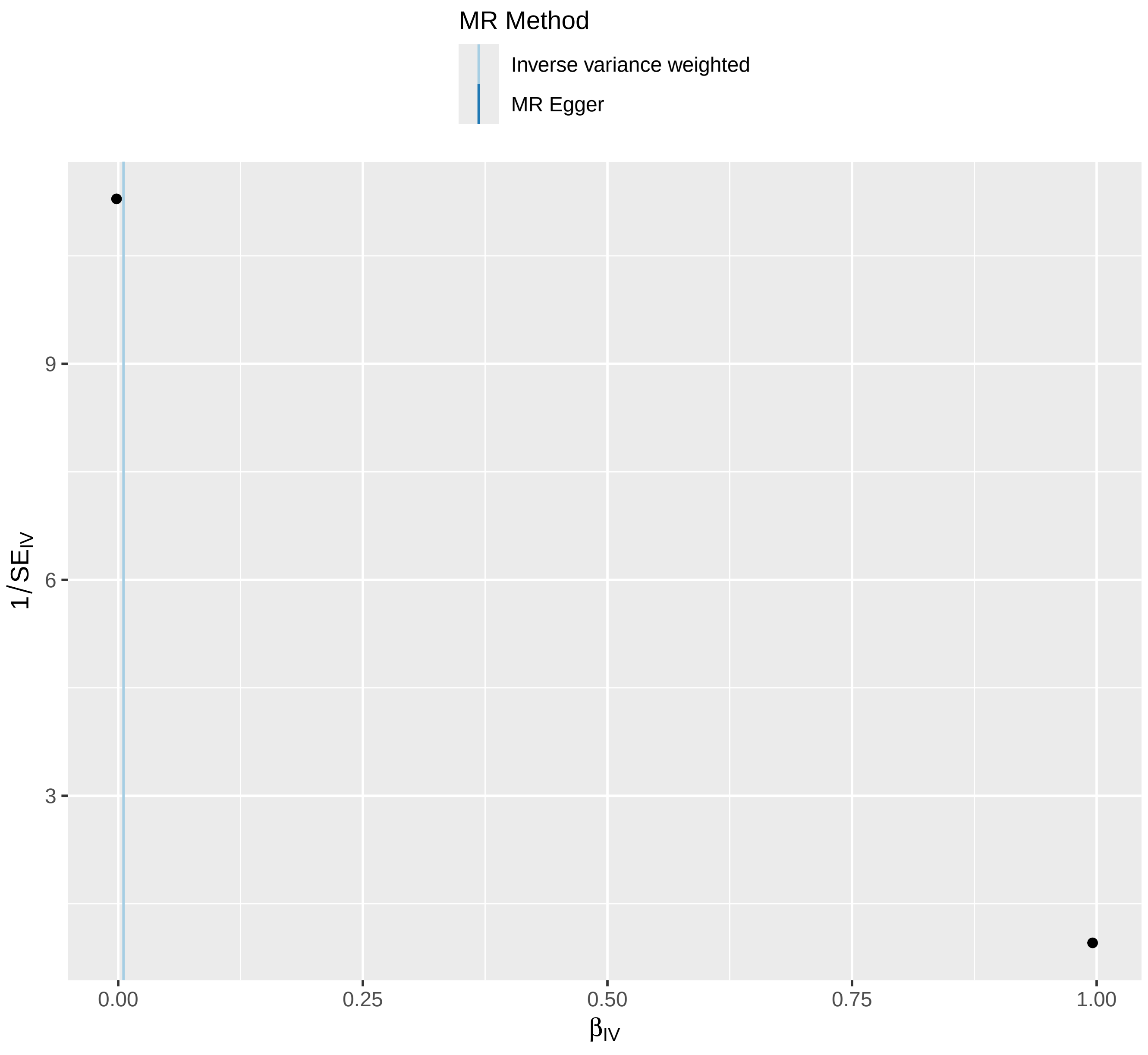
MR Method
Inverse variance weighted
MR Egger
9
6
3
0.00
0.25
0.50
0.75
1.00
$1/SE_{IV}$
$\beta_{IV}$

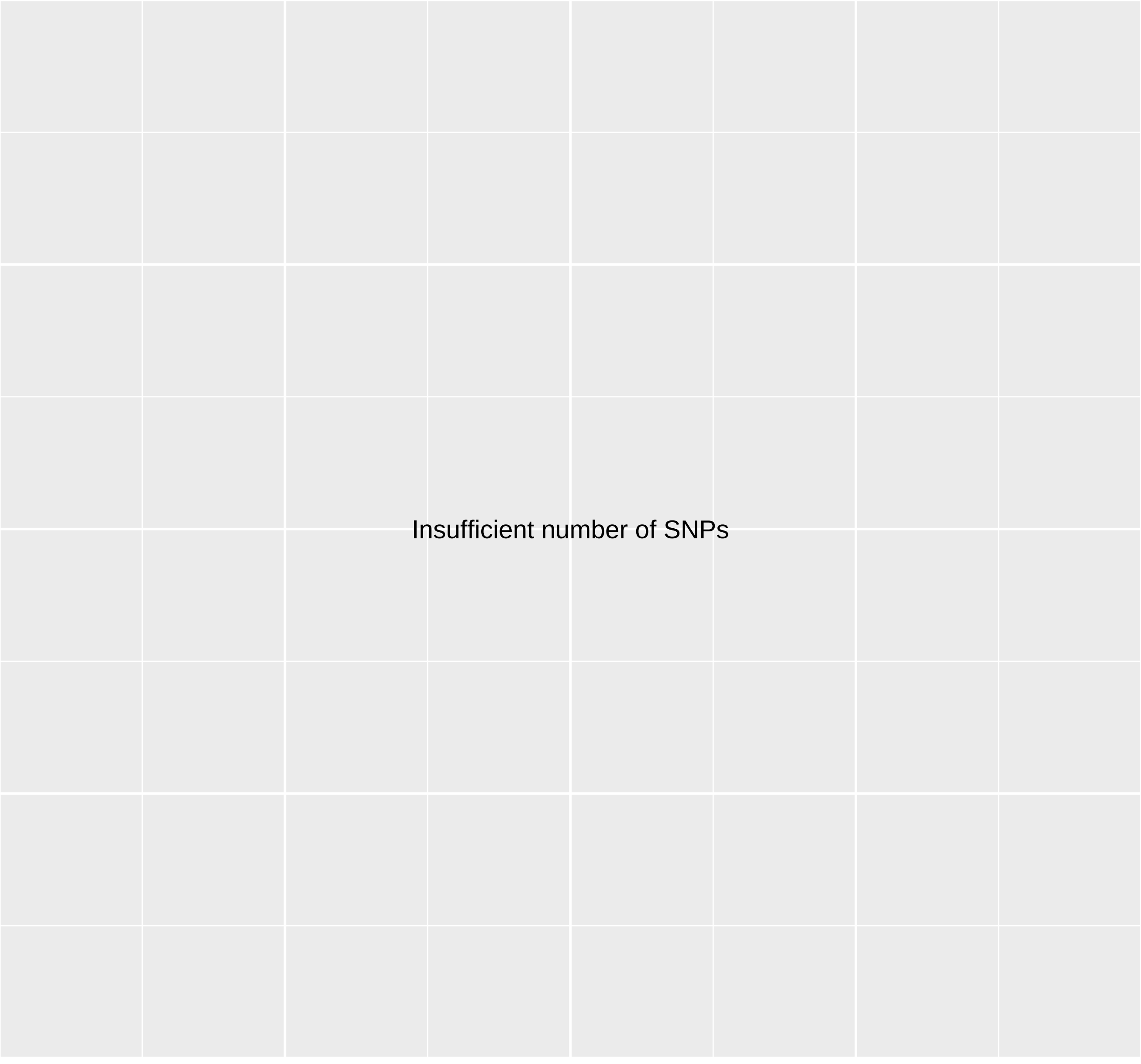
Insufficient number of SNPs

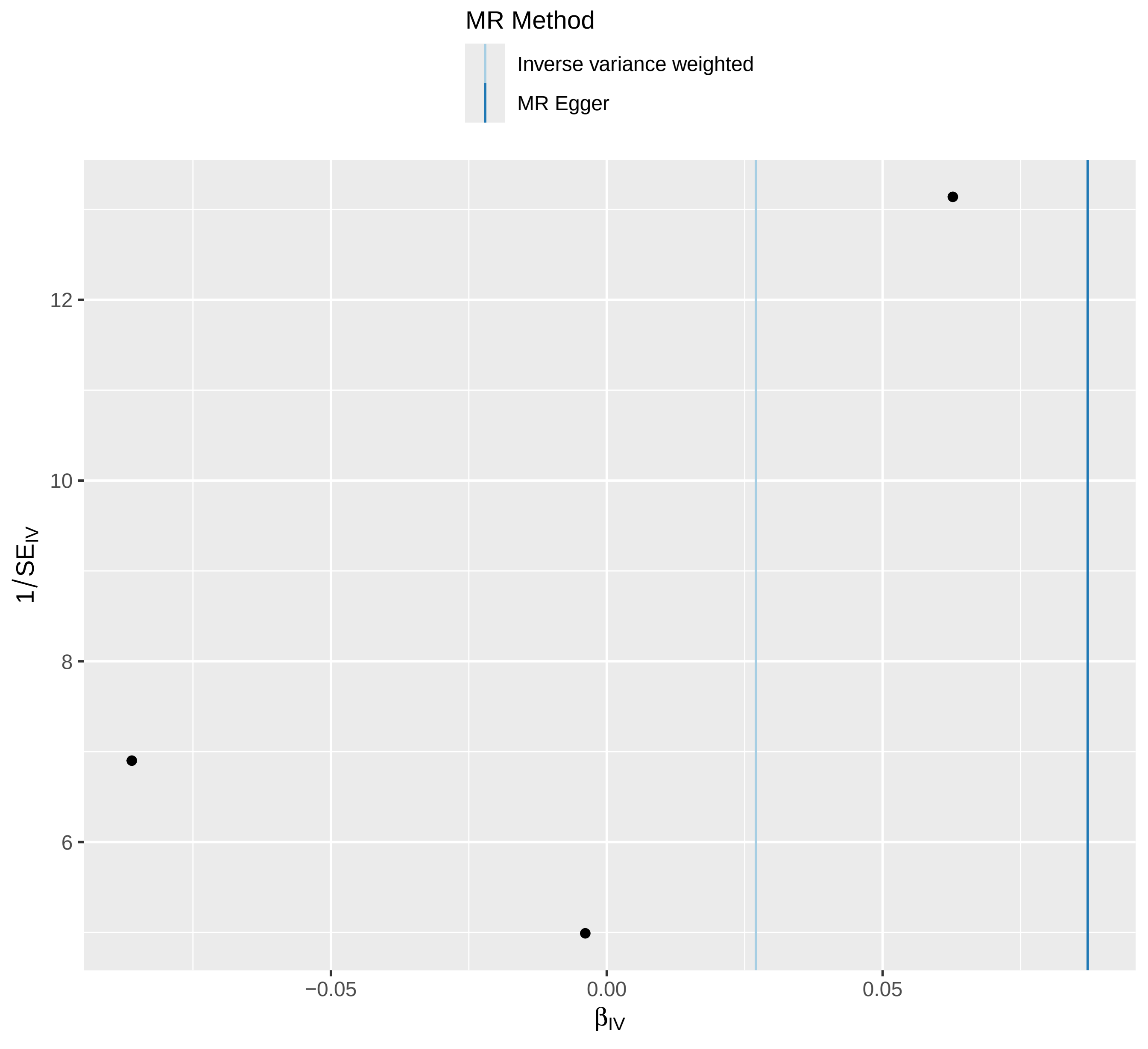

MR Method
Inverse variance weighted
MR Egger
12
10
8
6
1/SE_IV
−0.05
0.00
0.05
β_IV

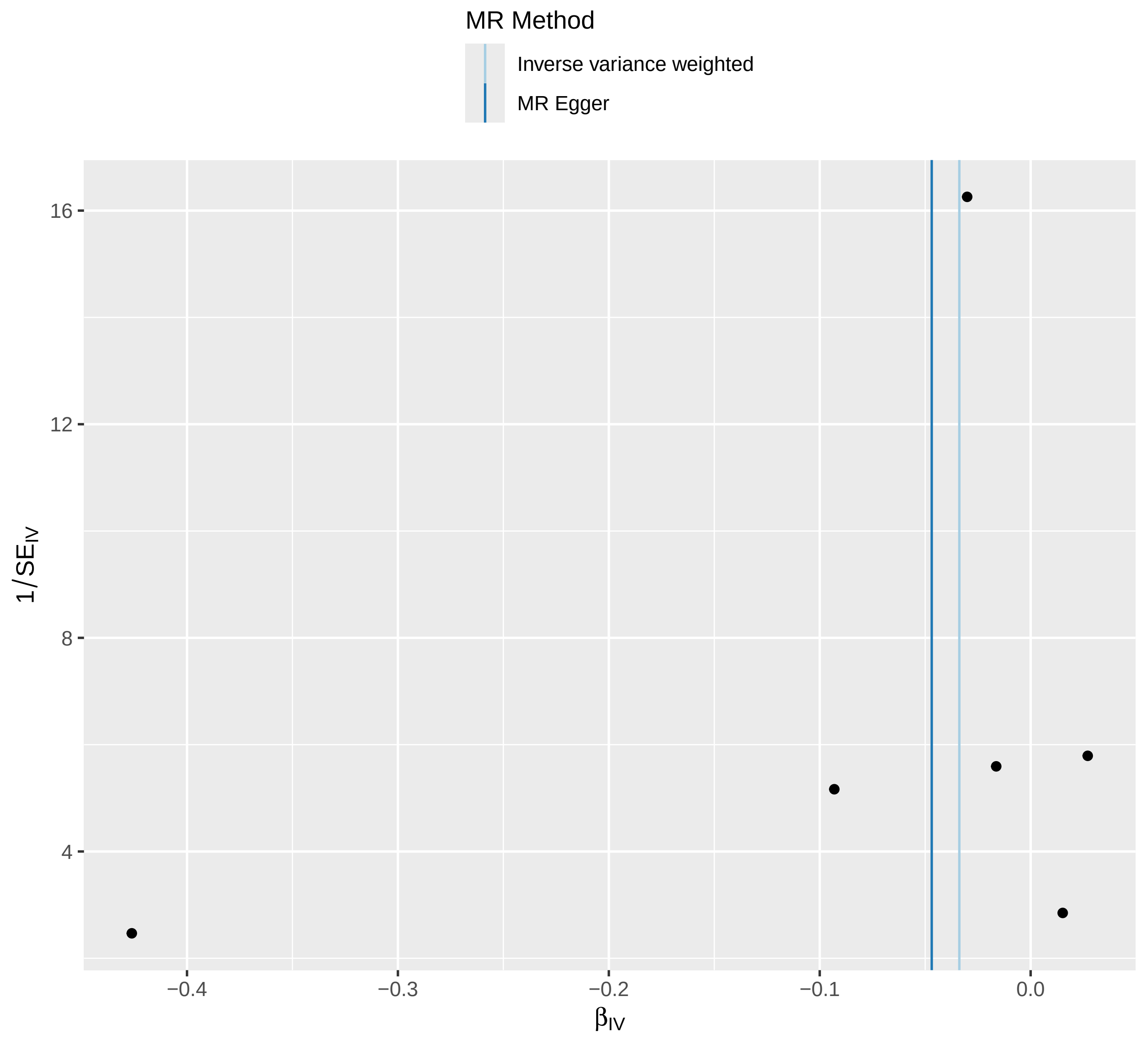
MR Method
Inverse variance weighted
MR Egger
16
12
8
4
1/SE$_{IV}$
−0.4
−0.3
−0.2
−0.1
0.0
β$_{IV}$

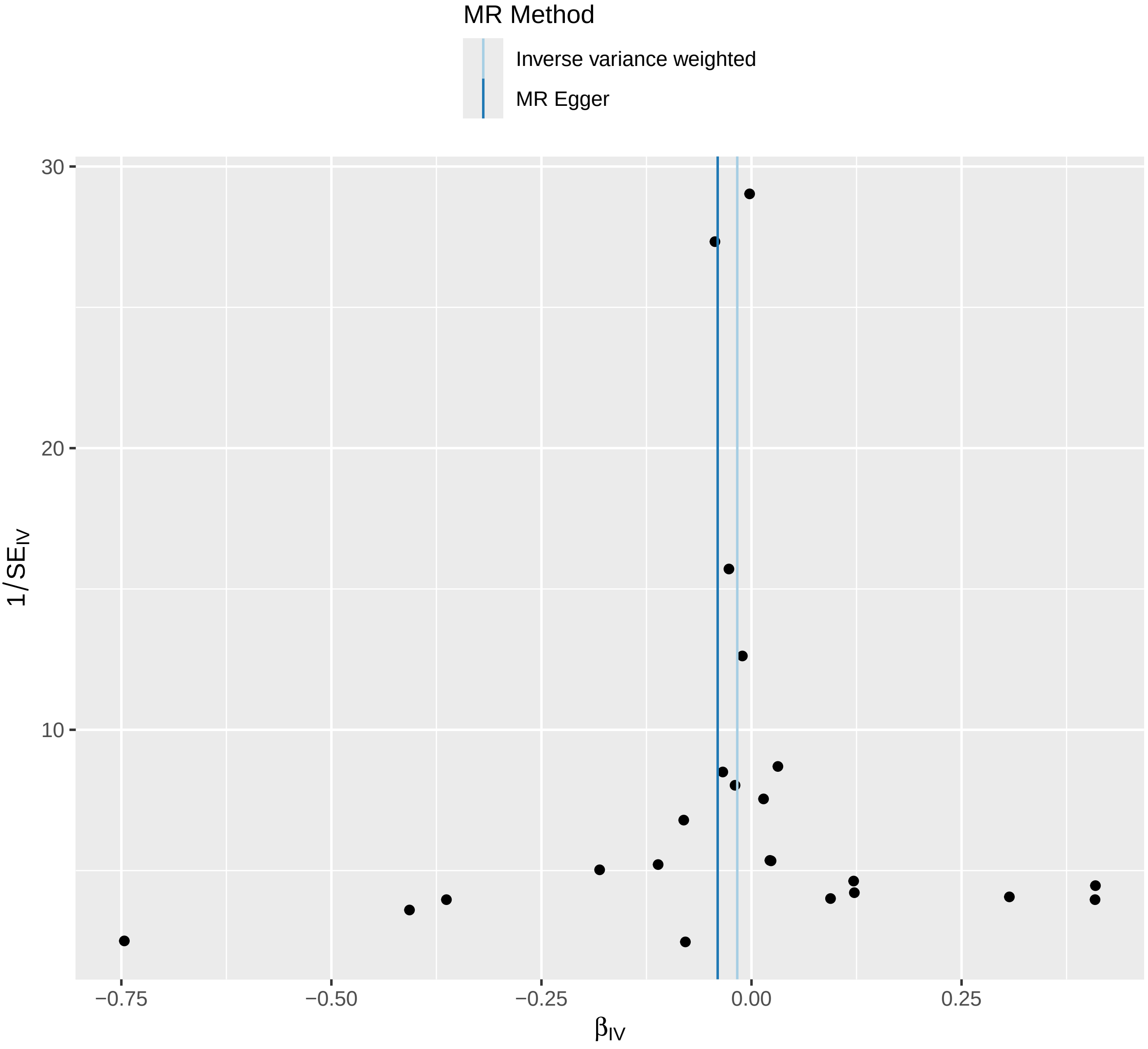
MR Method
Inverse variance weighted
MR Egger
30
20
10
1/SE$_{IV}$
−0.75
−0.50
−0.25
0.00
0.25
β$_{IV}$

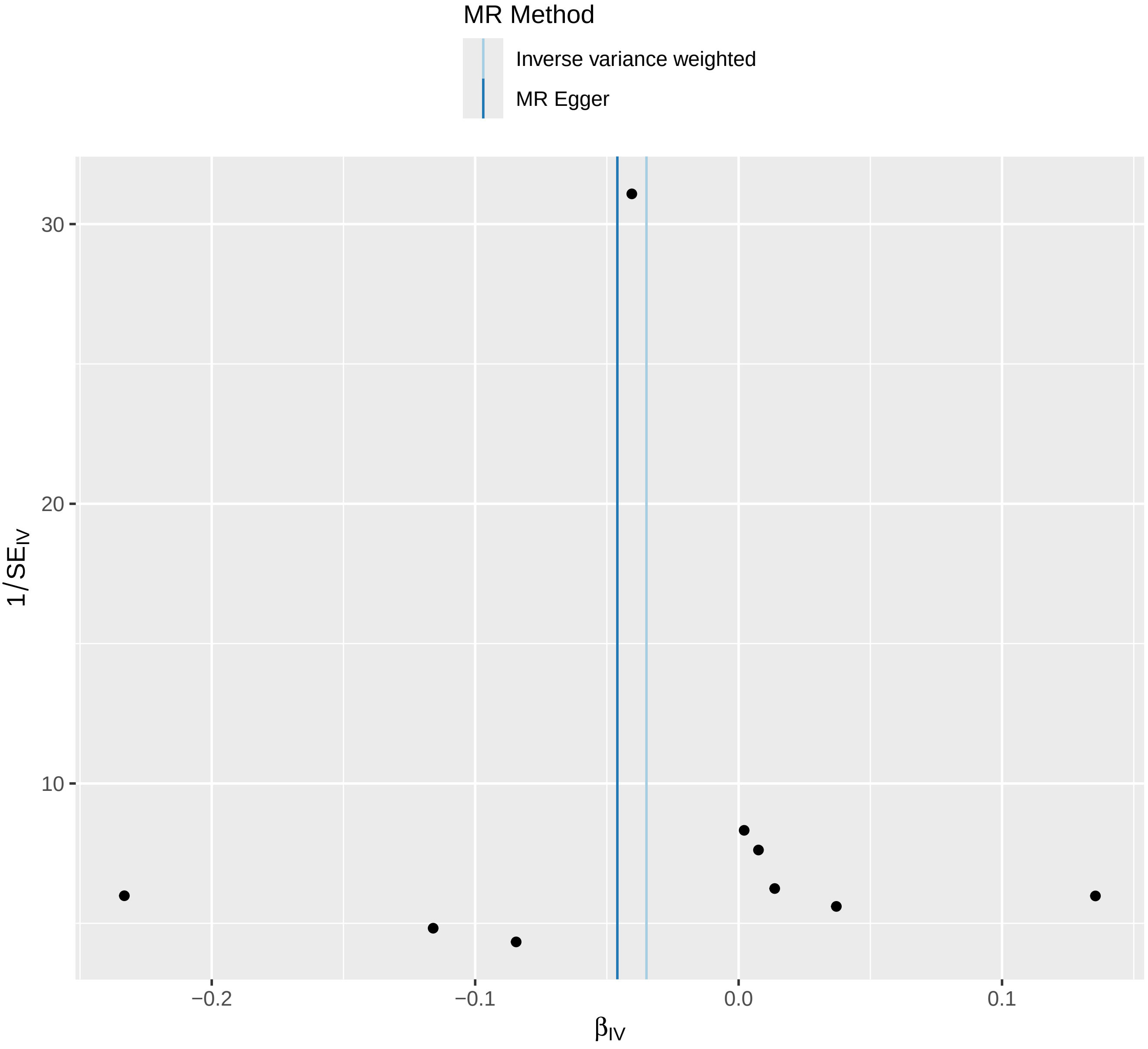
MR Method
Inverse variance weighted
MR Egger
30
20
10
1/SE$_{IV}$
−0.2
−0.1
0.0
0.1
β$_{IV}$

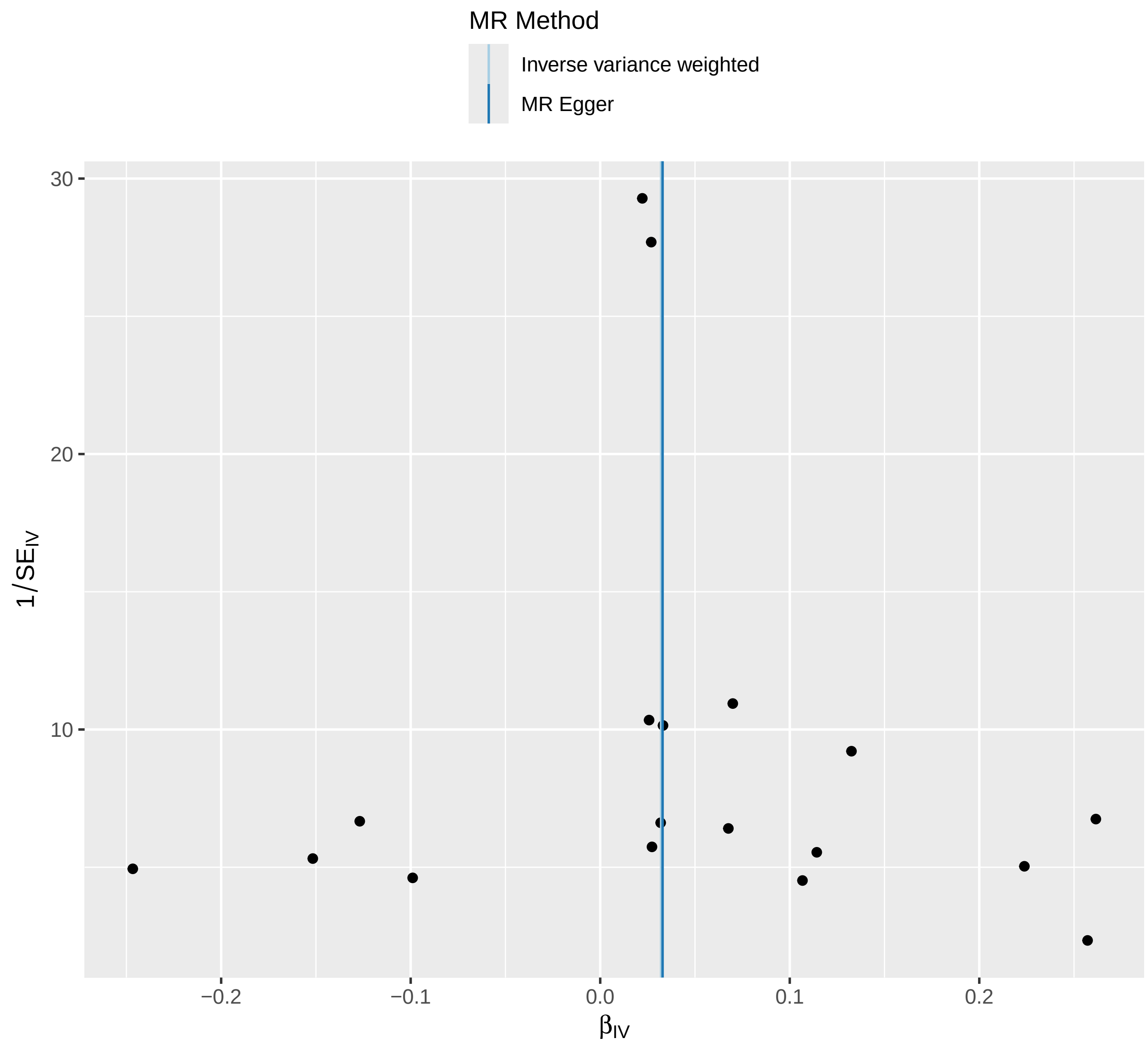
MR Method
Inverse variance weighted
MR Egger
30
20
10
$1/SE_{IV}$
−0.2
−0.1
0.0
0.1
0.2
$\beta_{IV}$

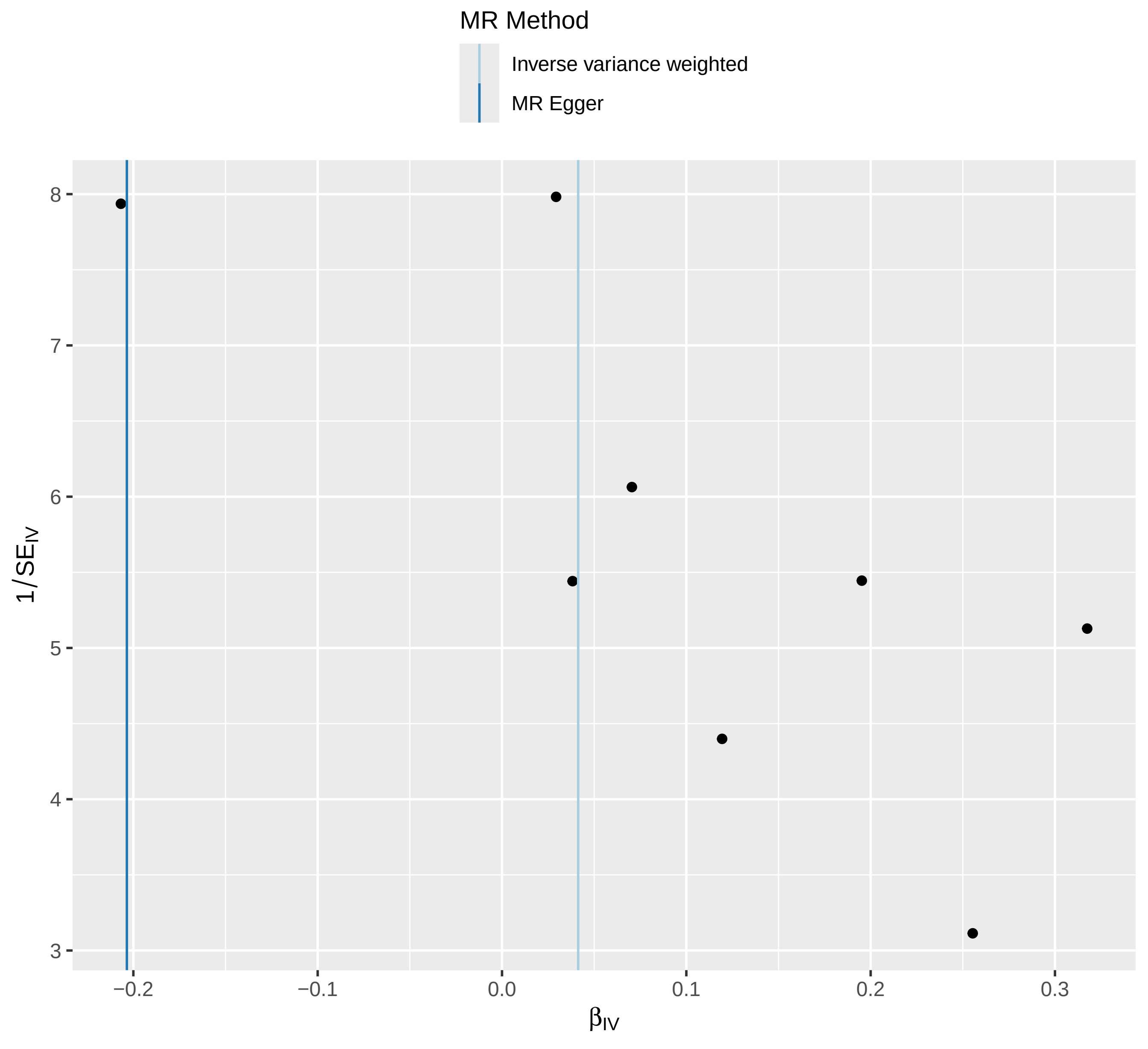
MR Method
Inverse variance weighted
MR Egger
8
7
6
5
4
3
1/SE_IV
−0.2
−0.1
0.0
0.1
0.2
0.3
β_IV

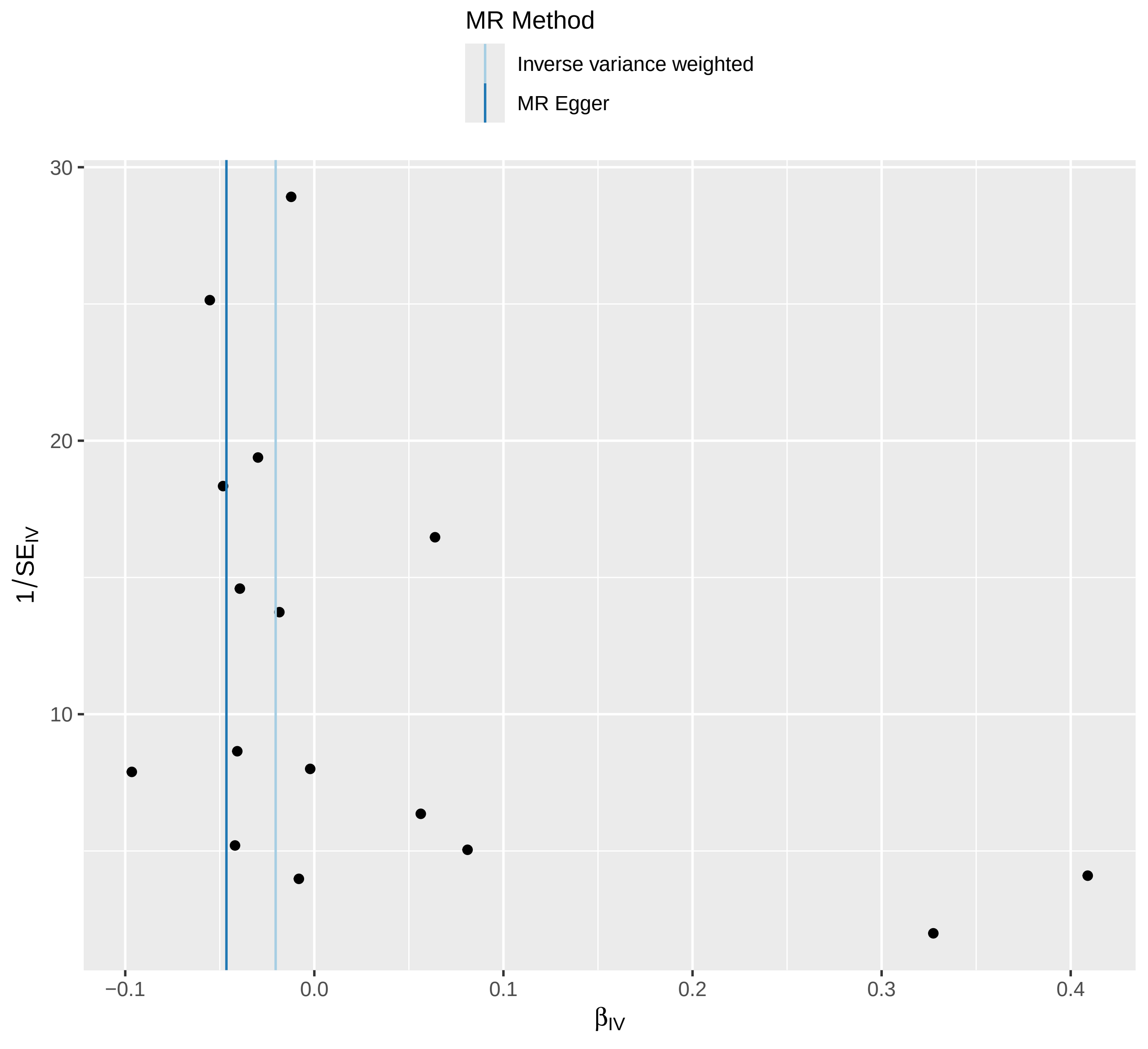
MR Method
Inverse variance weighted
MR Egger
30
20
10
1/SE$_{IV}$
−0.1
0.0
0.1
0.2
0.3
0.4
β$_{IV}$

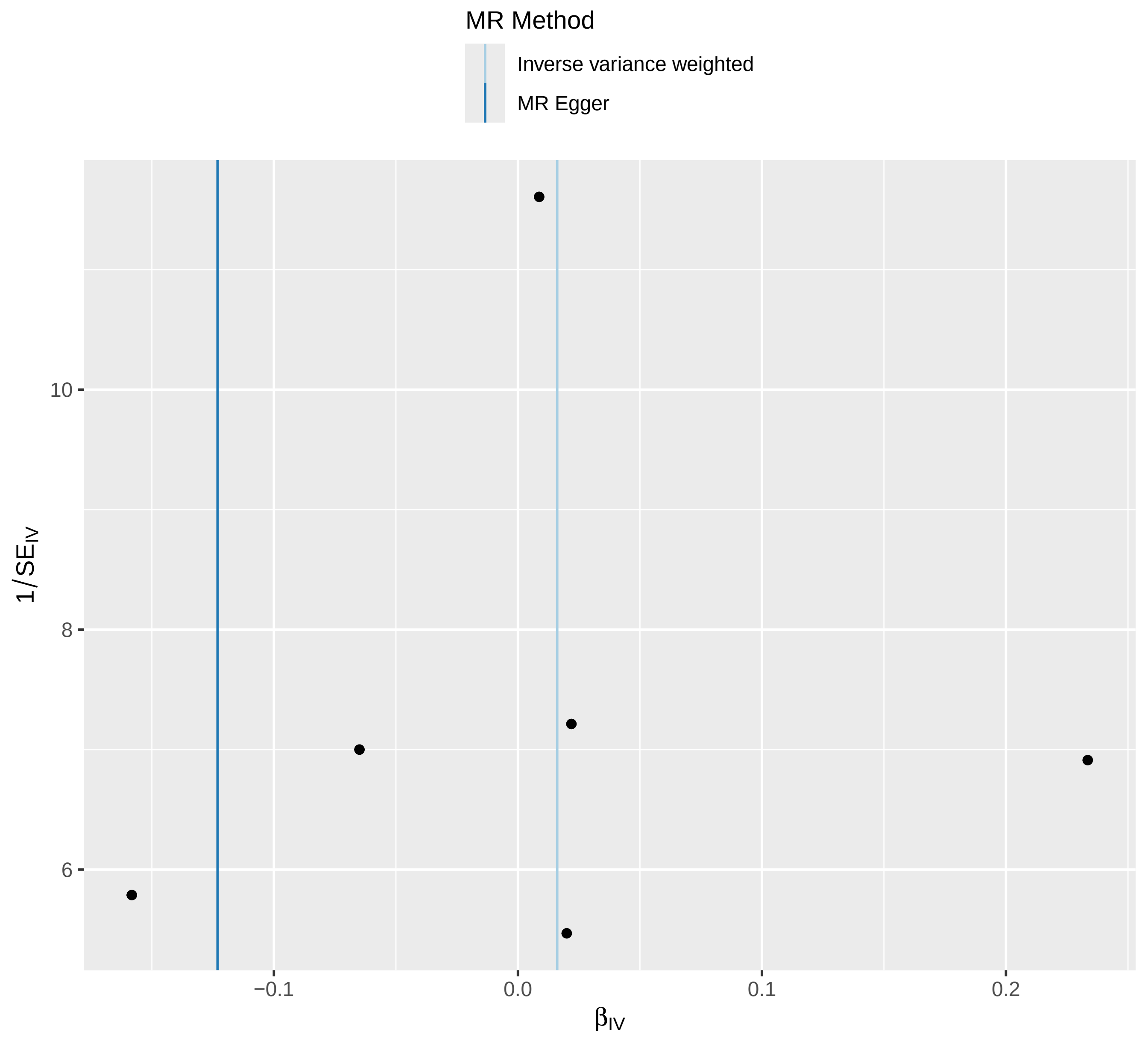
MR Method
Inverse variance weighted
MR Egger
1/SE$_{IV}$
10
8
6
−0.1
0.0
0.1
0.2
β$_{IV}$

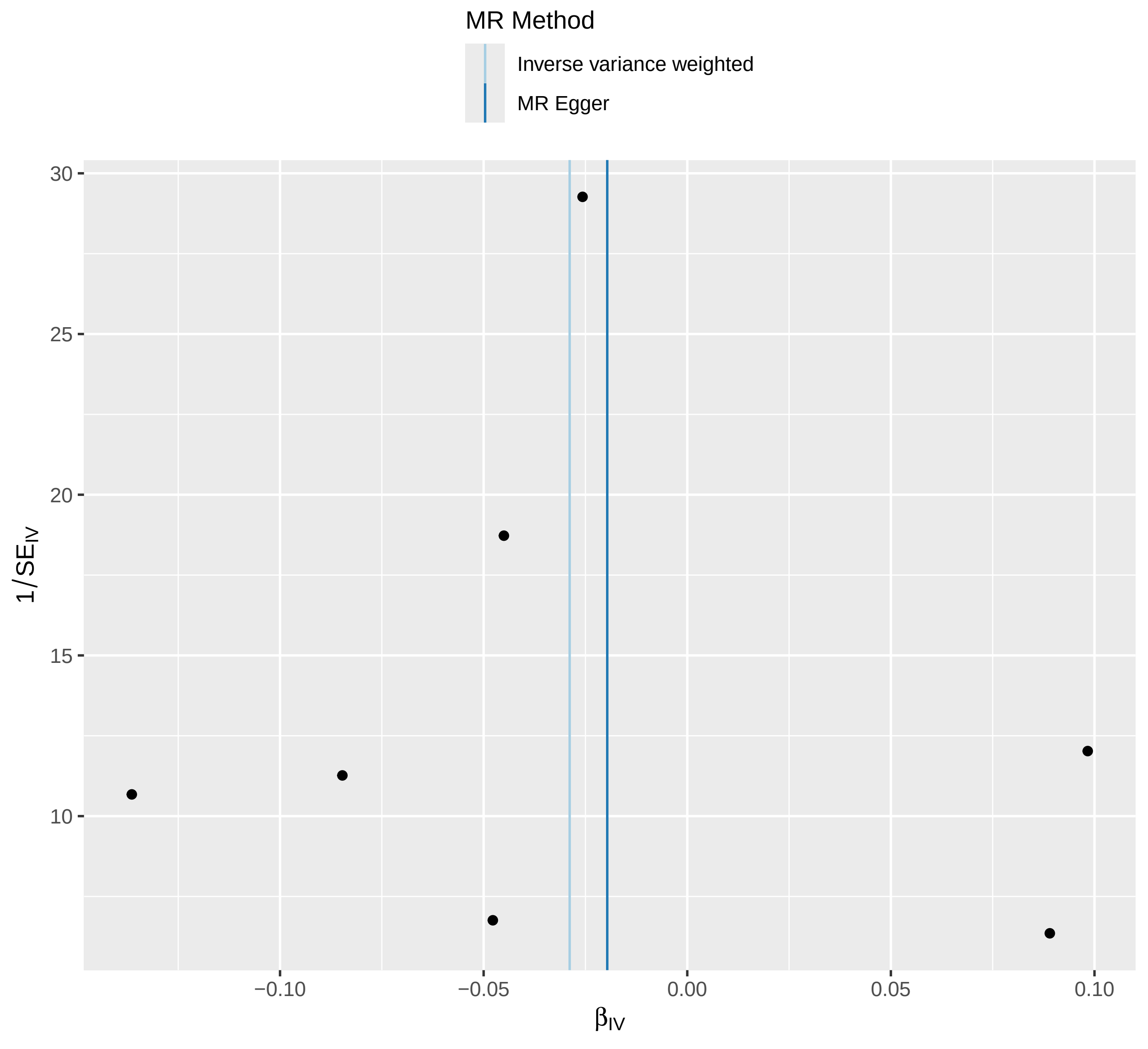

MR Method
Inverse variance weighted
MR Egger
30
25
20
15
10
1/SE$_{IV}$
−0.10
−0.05
0.00
0.05
0.10
β$_{IV}$

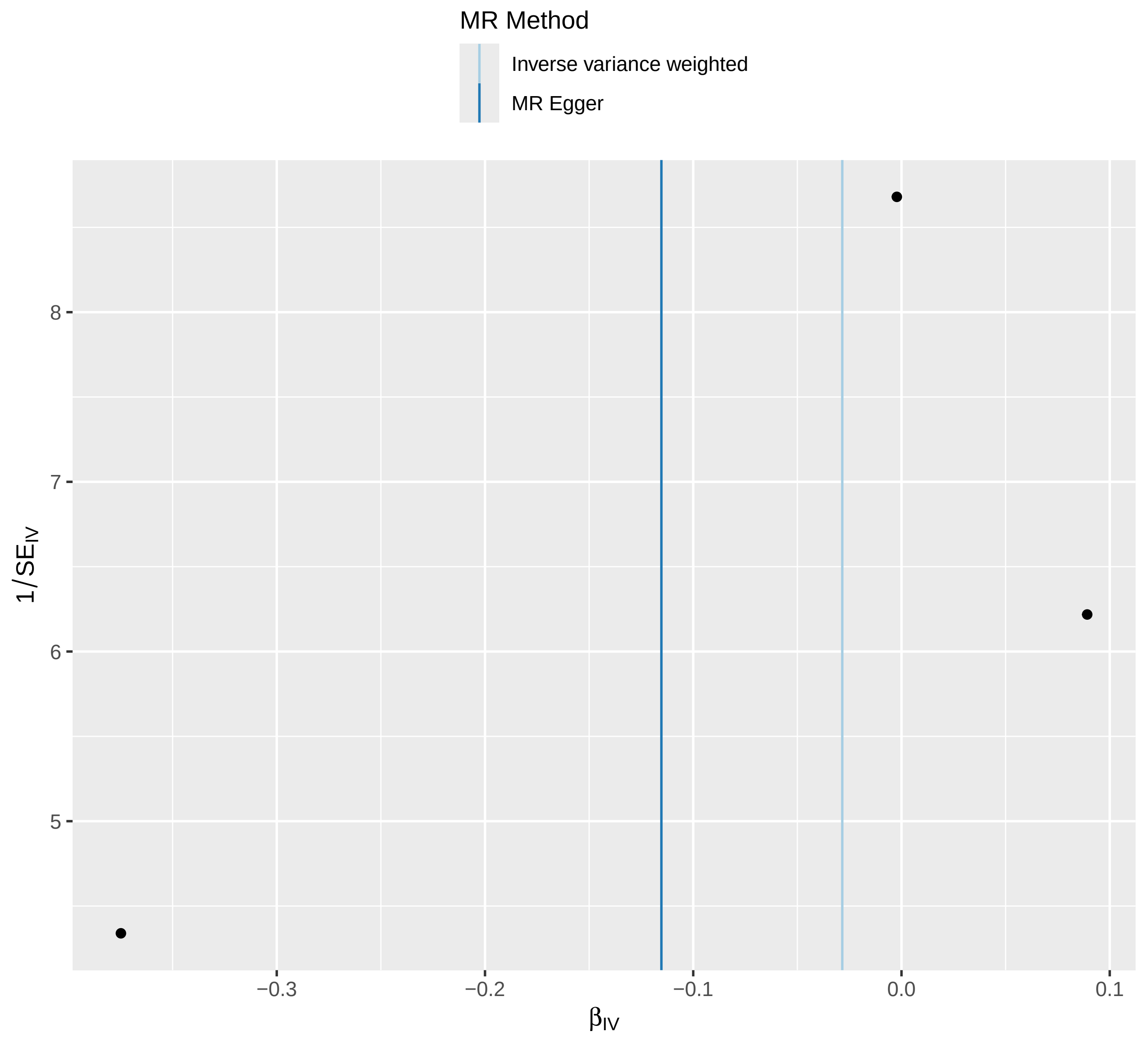
MR Method
Inverse variance weighted
MR Egger
8
7
6
5
1/SE_IV
−0.3
−0.2
−0.1
0.0
0.1
β_IV

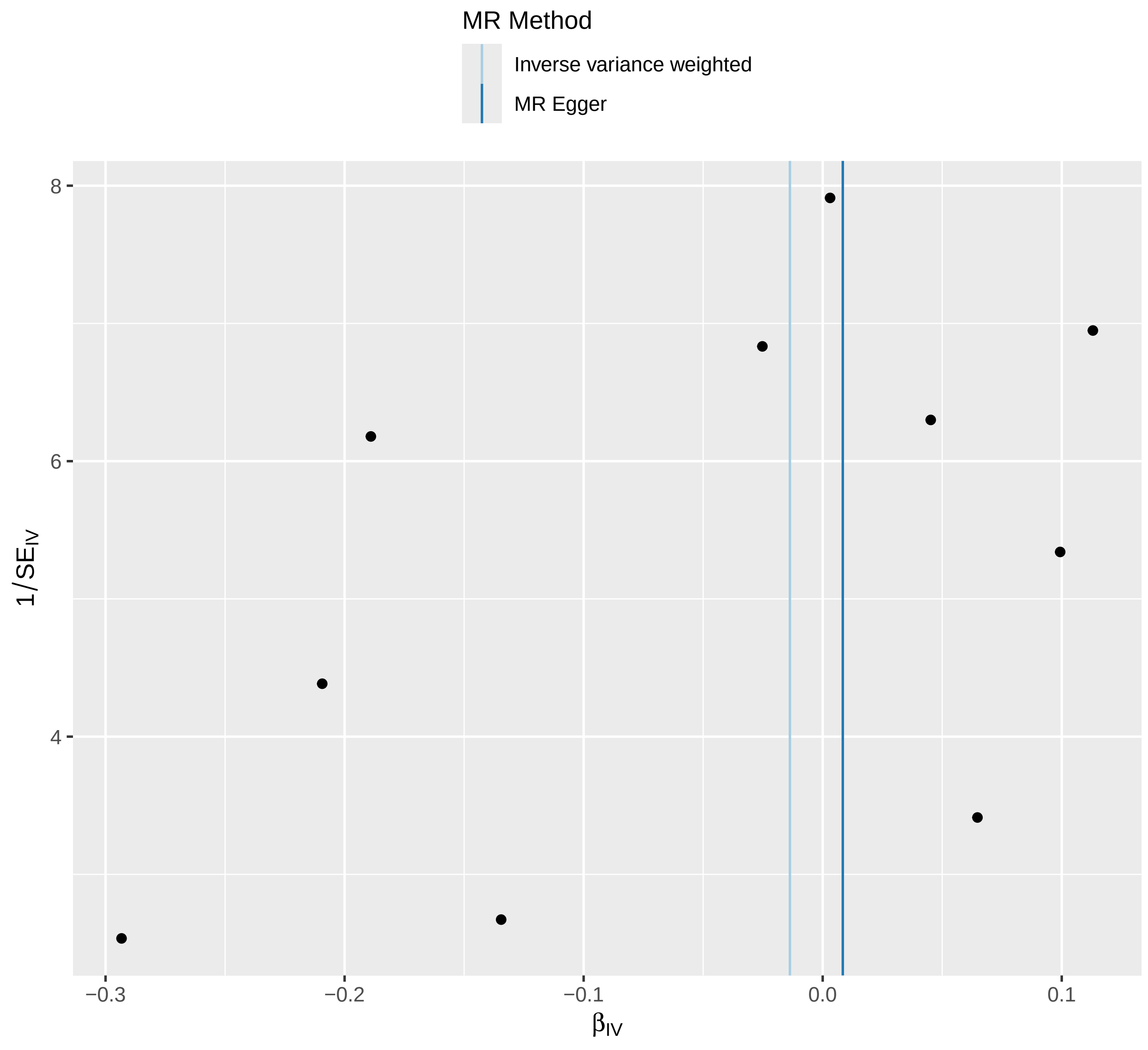
MR Method
Inverse variance weighted
MR Egger
8
6
4
1/SE$_{IV}$
−0.3
−0.2
−0.1
0.0
0.1
β$_{IV}$

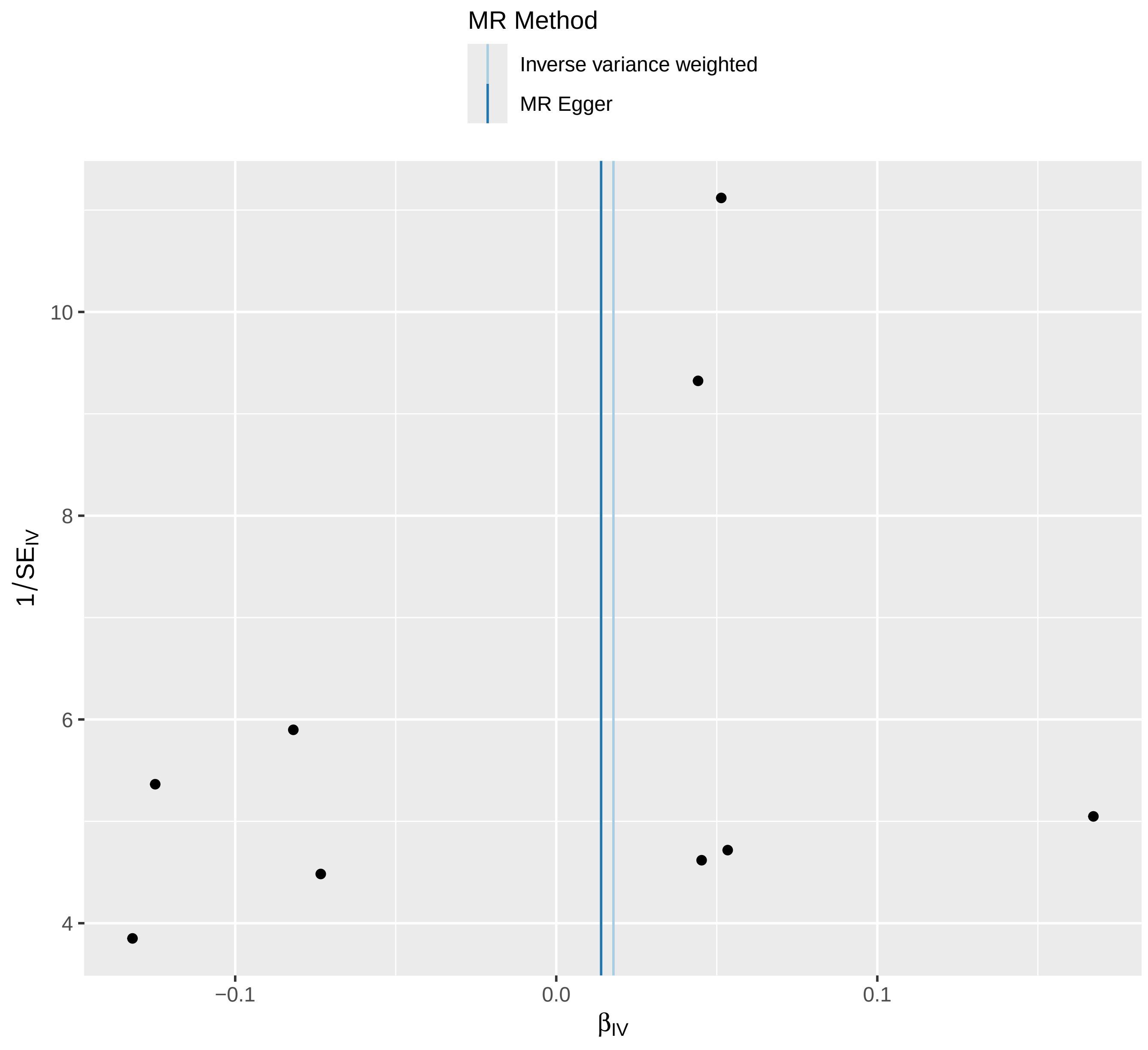
MR Method
Inverse variance weighted
MR Egger
10
8
6
4
1/SE_IV
−0.1
0.0
0.1
β_IV

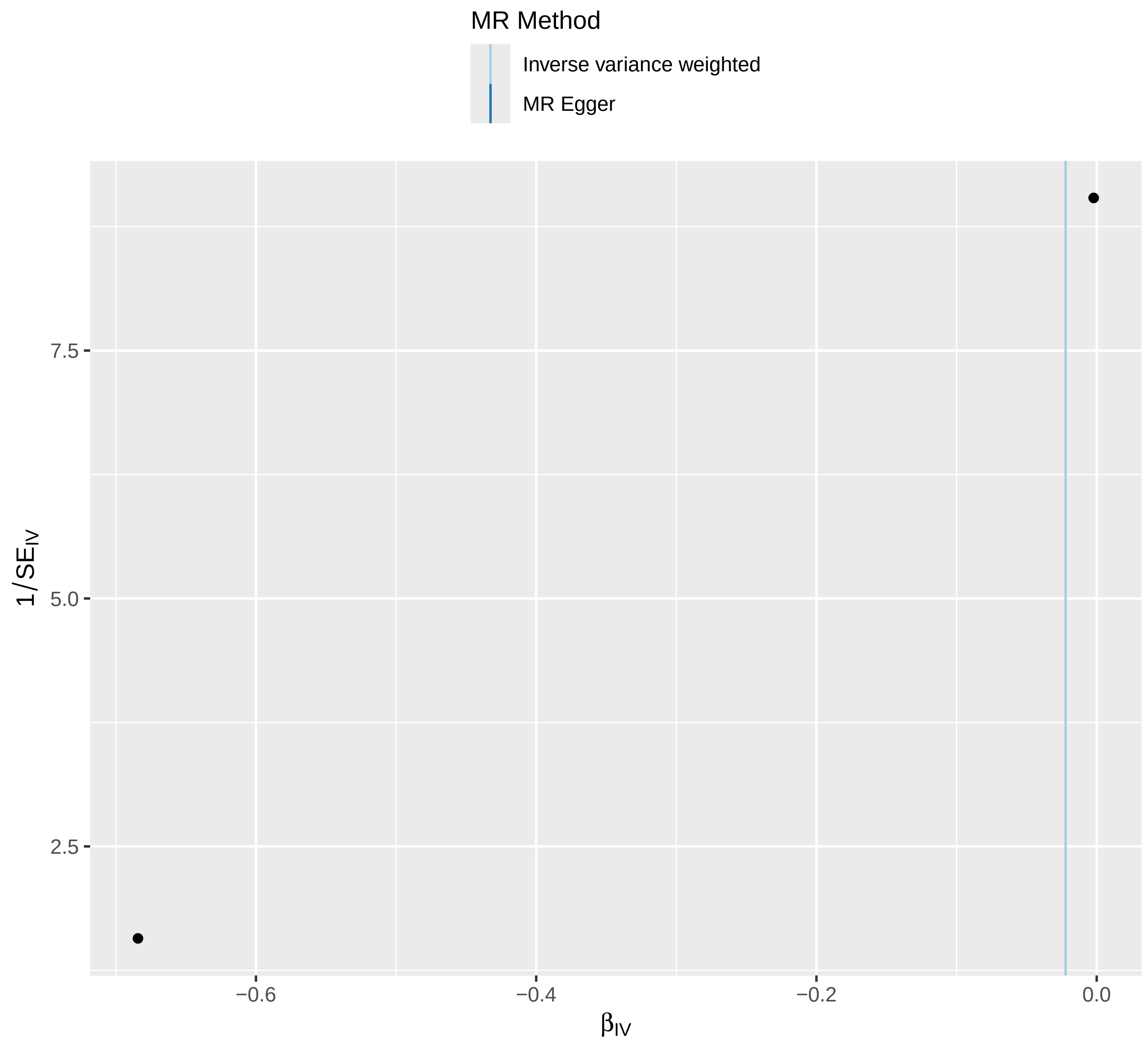

MR Method
Inverse variance weighted
MR Egger
7.5
5.0
2.5
1/SE$_{IV}$
−0.6
−0.4
−0.2
0.0
β$_{IV}$

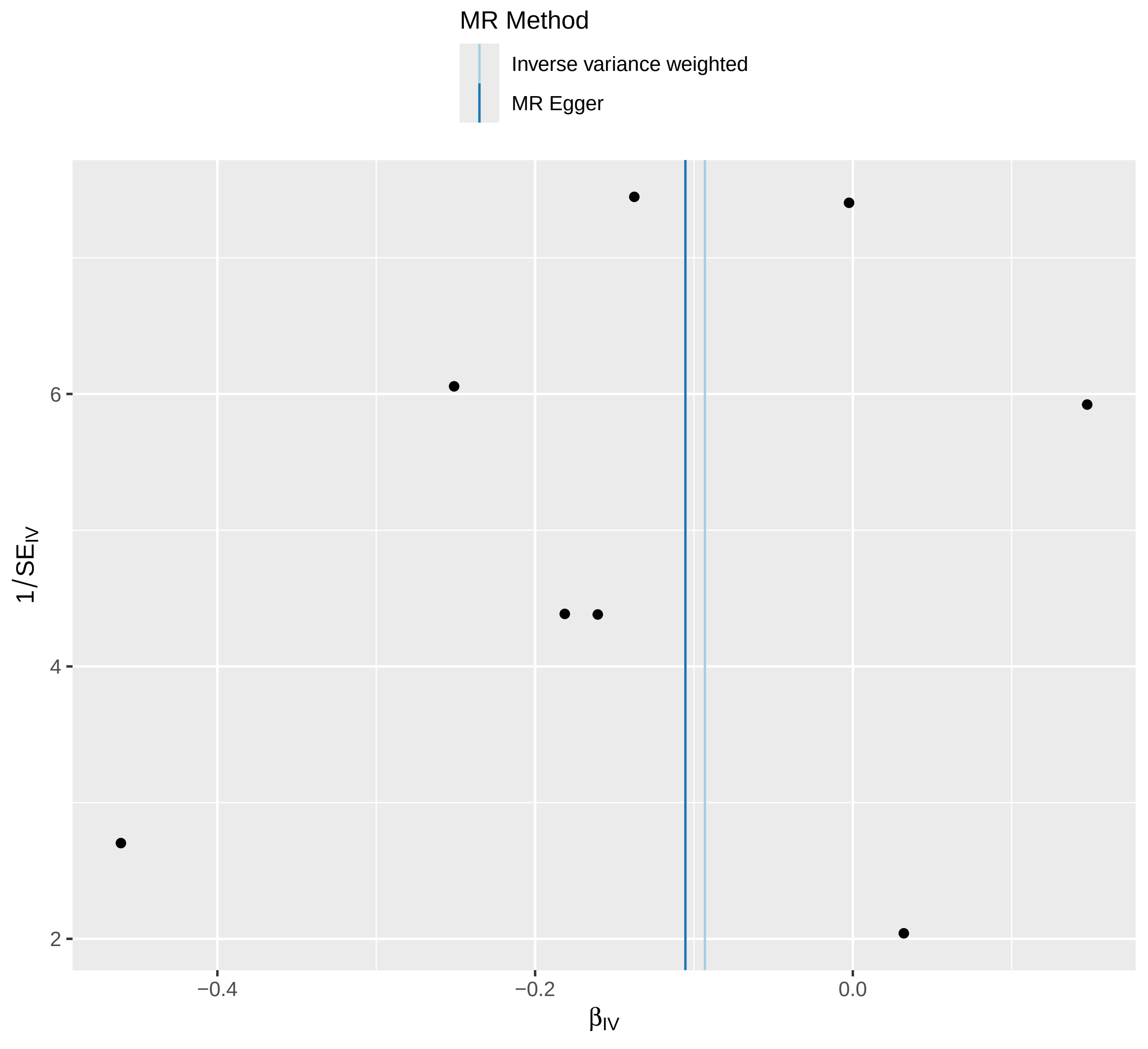

MR Method
Inverse variance weighted
MR Egger
6
4
2
1/SE$_{IV}$
−0.4
−0.2
0.0
β$_{IV}$

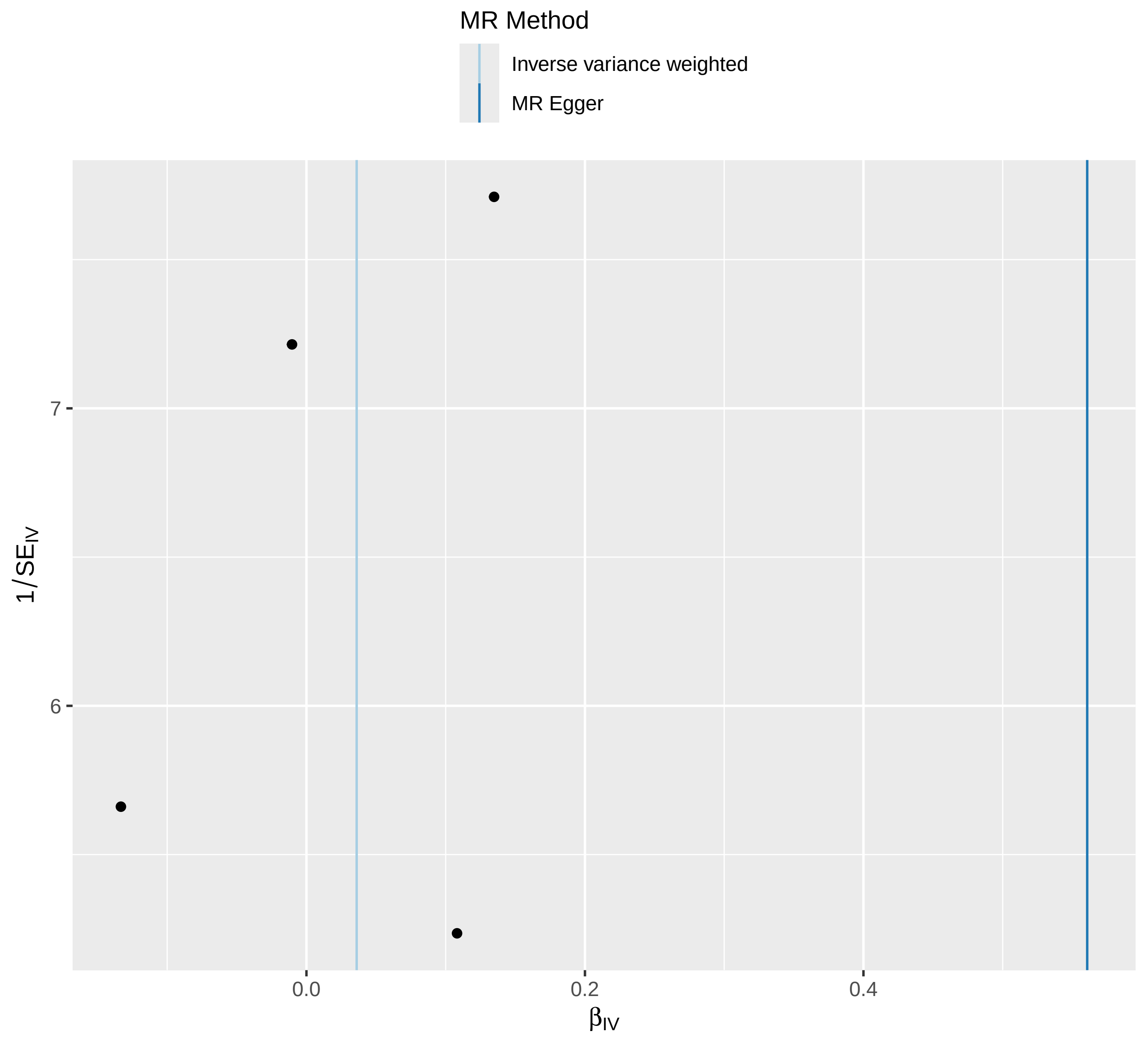
MR Method
Inverse variance weighted
MR Egger
1/SE_IV
7
6
0.0
0.2
0.4
β_IV

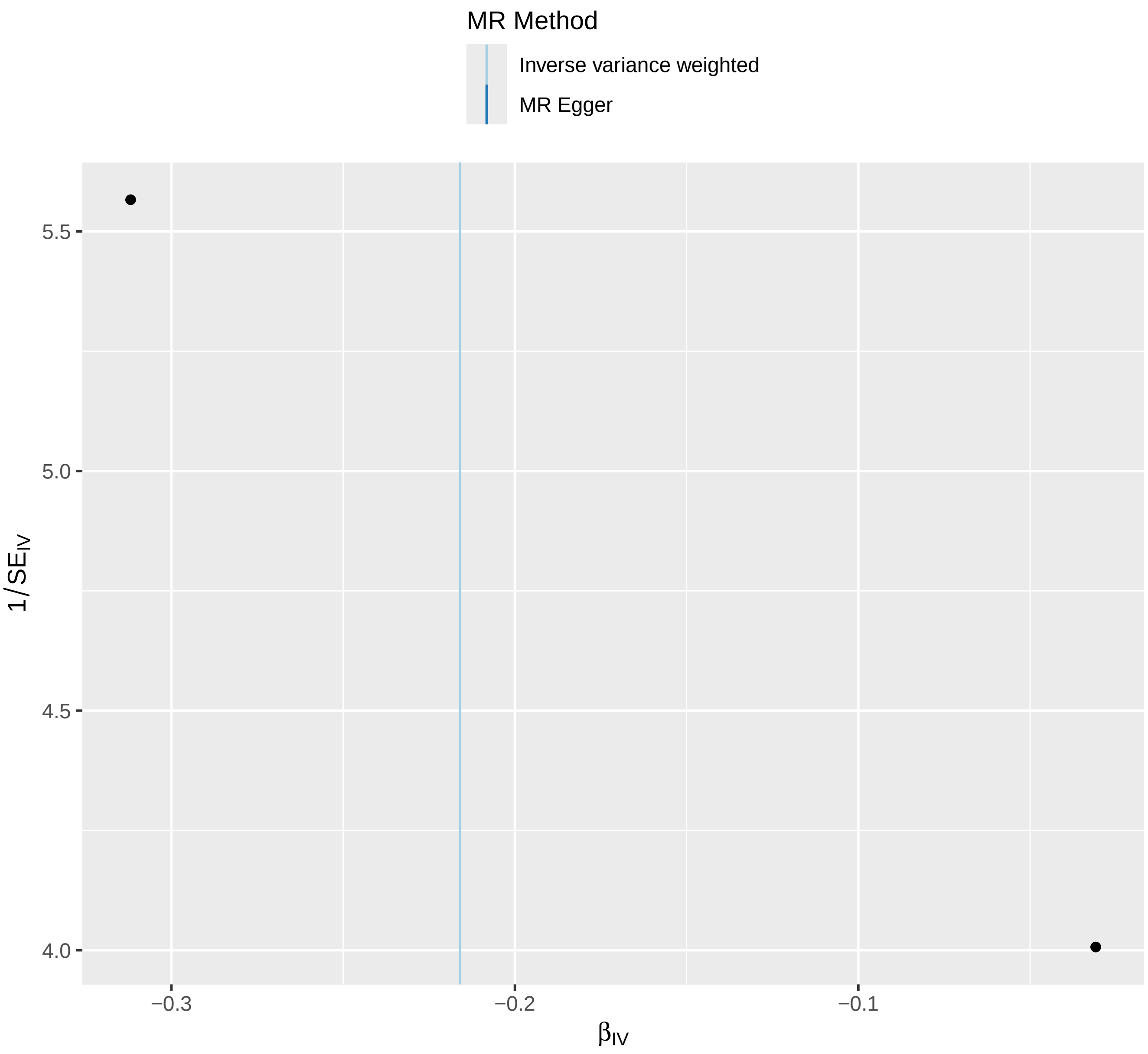
MR Method
Inverse variance weighted
MR Egger
1/SE_IV
5.5
5.0
4.5
4.0
β_IV
−0.3
−0.2
−0.1

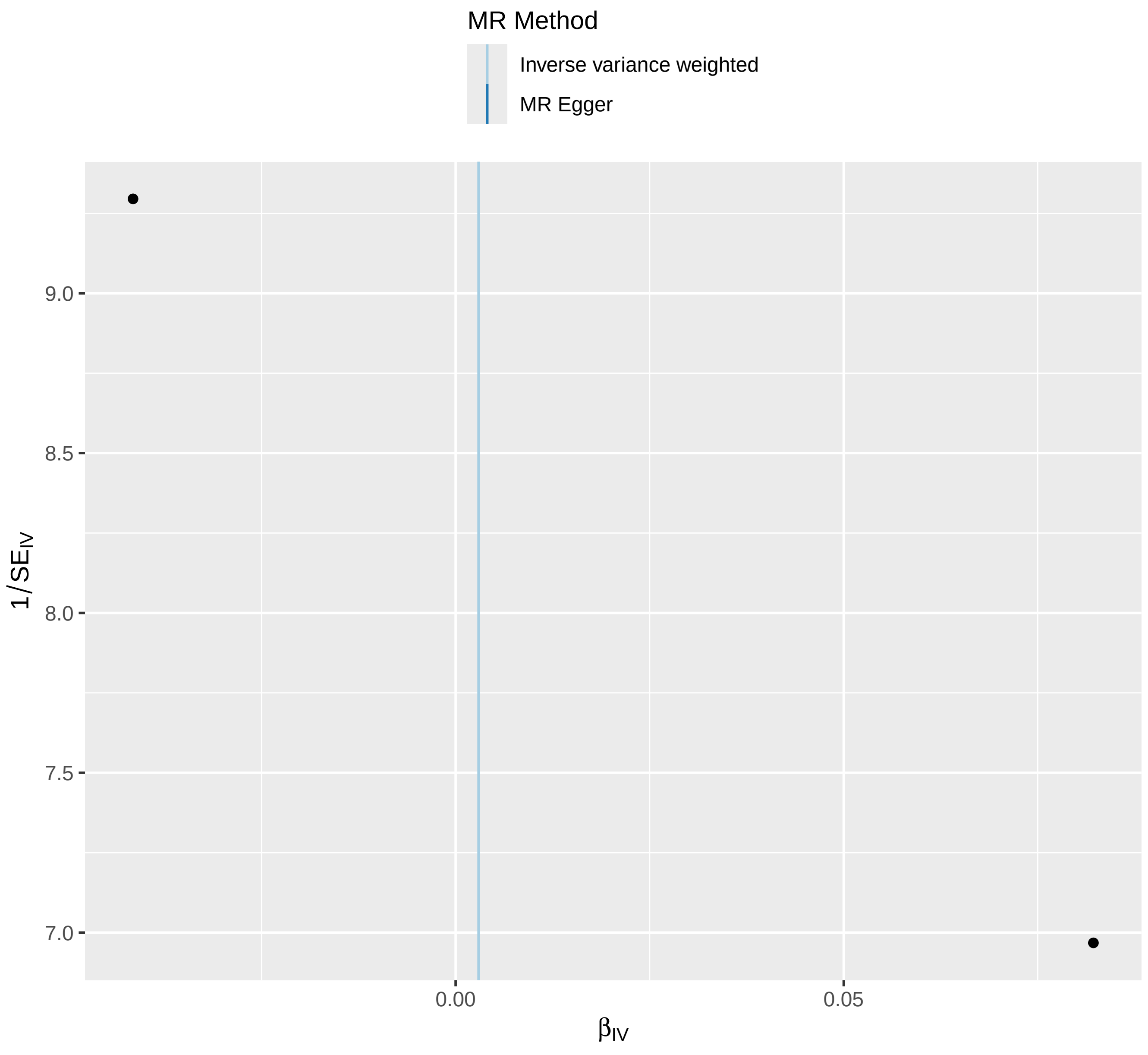

MR Method
Inverse variance weighted
MR Egger
9.0
8.5
8.0
7.5
7.0
1/SE_IV
0.00
0.05
β_IV

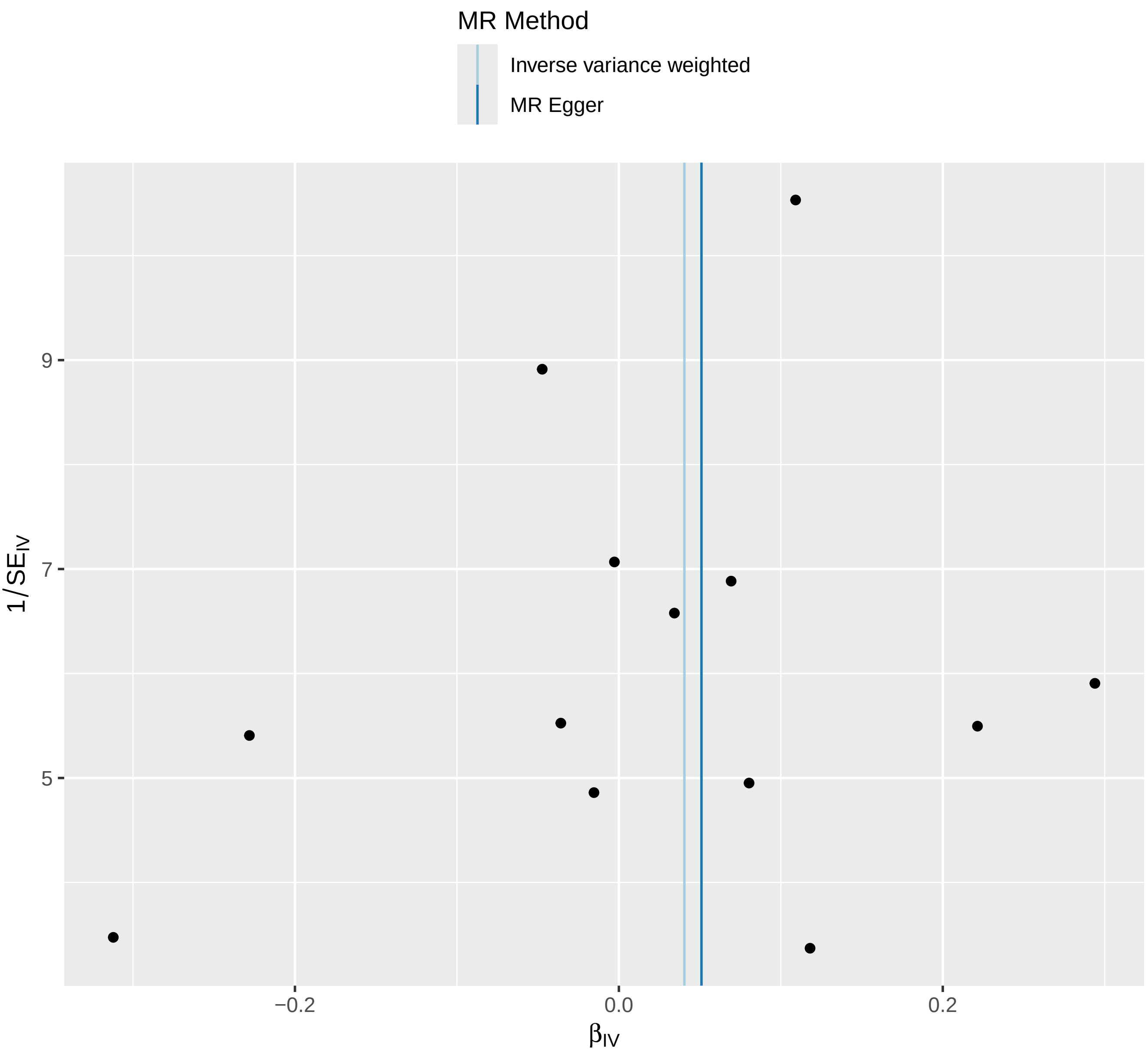
MR Method
Inverse variance weighted
MR Egger
9
7
5
1/SE_IV
−0.2
0.0
0.2
β_IV

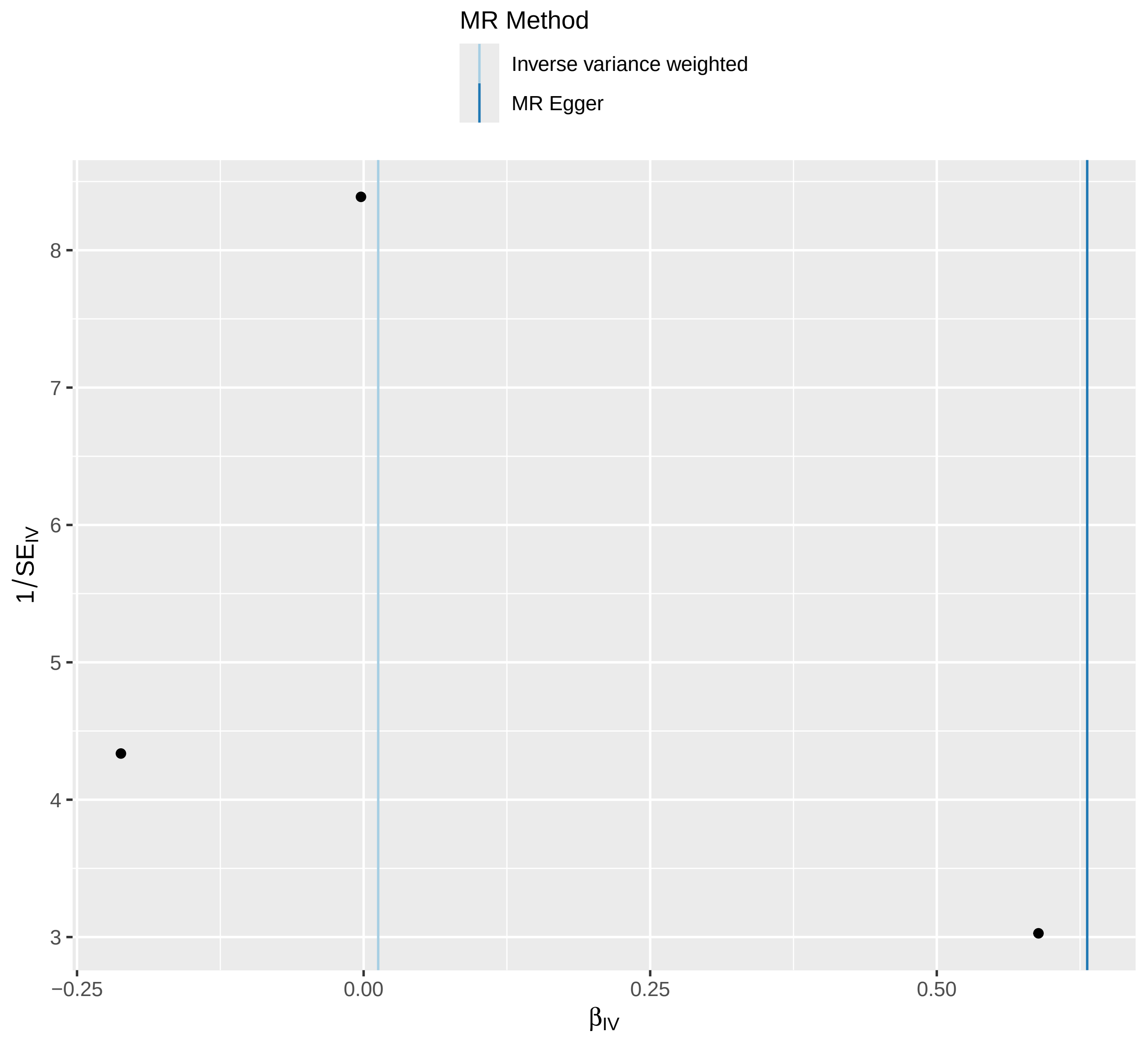
MR Method
Inverse variance weighted
MR Egger
8
7
6
5
4
3
1/SE_IV
−0.25
0.00
0.25
0.50
β_IV

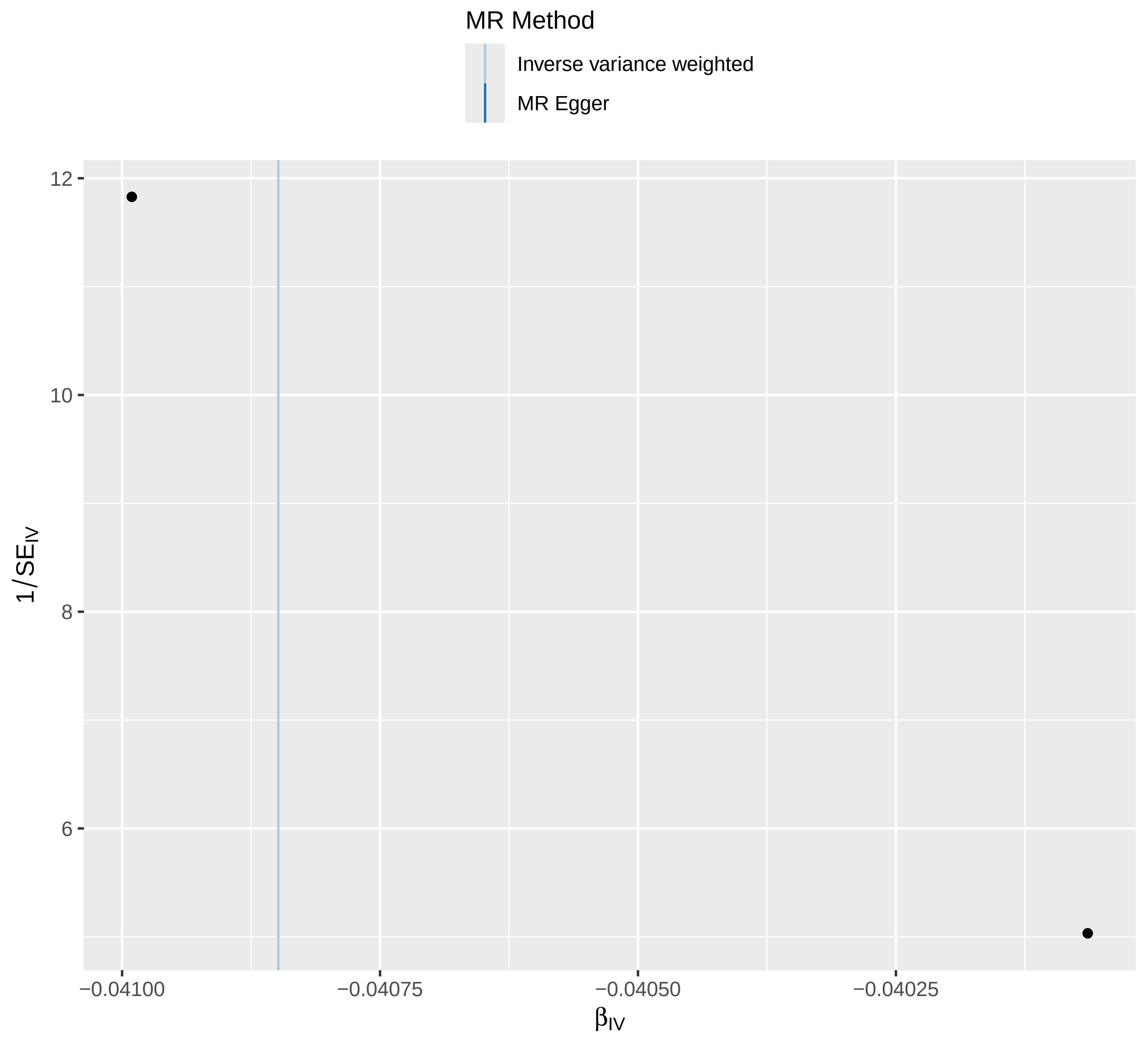

MR Method
Inverse variance weighted
MR Egger
12
10
8
6
1/SE$_{IV}$
−0.04100
−0.04075
−0.04050
−0.04025
β$_{IV}$

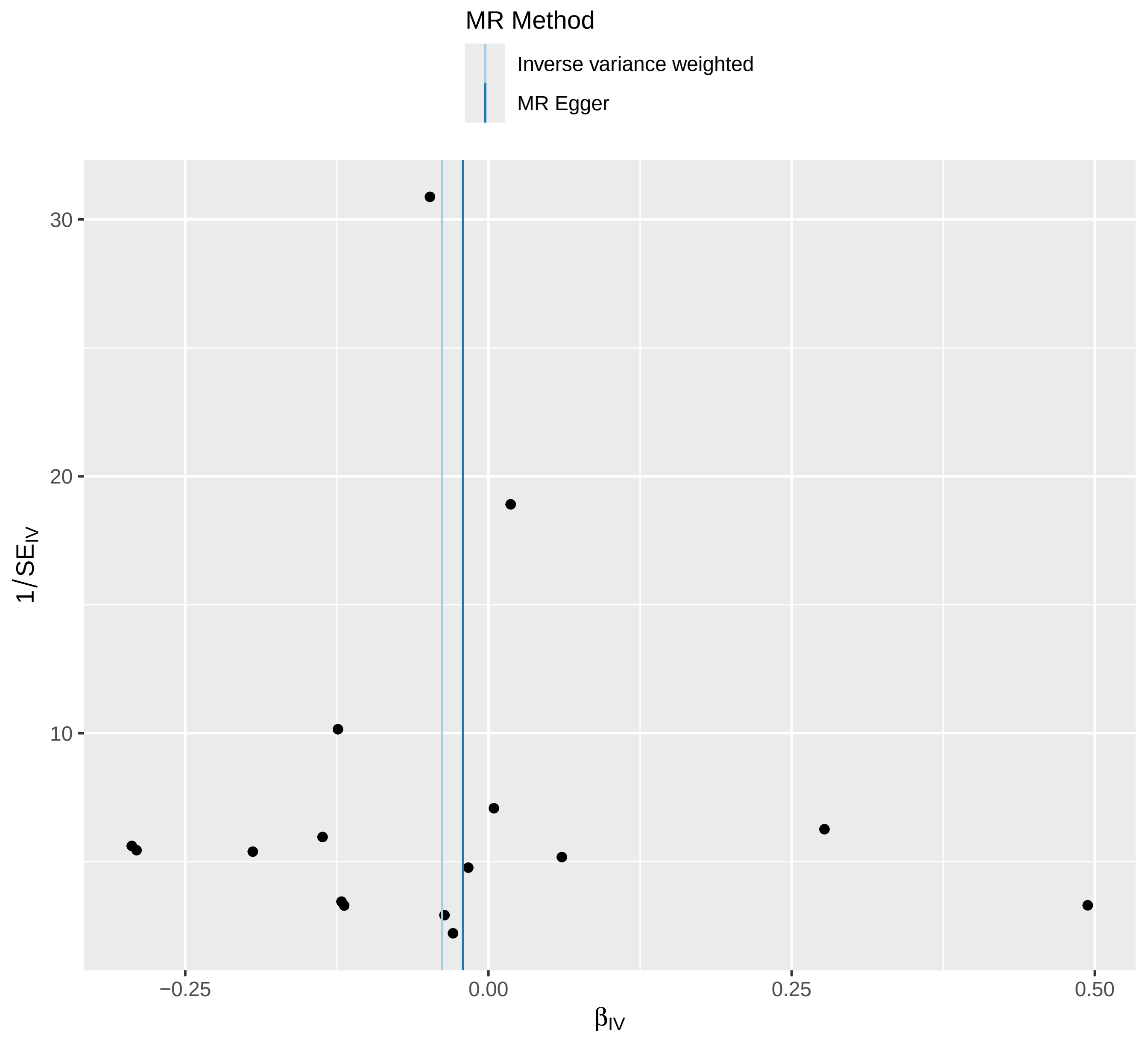
MR Method
Inverse variance weighted
MR Egger
30
20
10
1/SE$_{IV}$
−0.25
0.00
0.25
0.50
β$_{IV}$

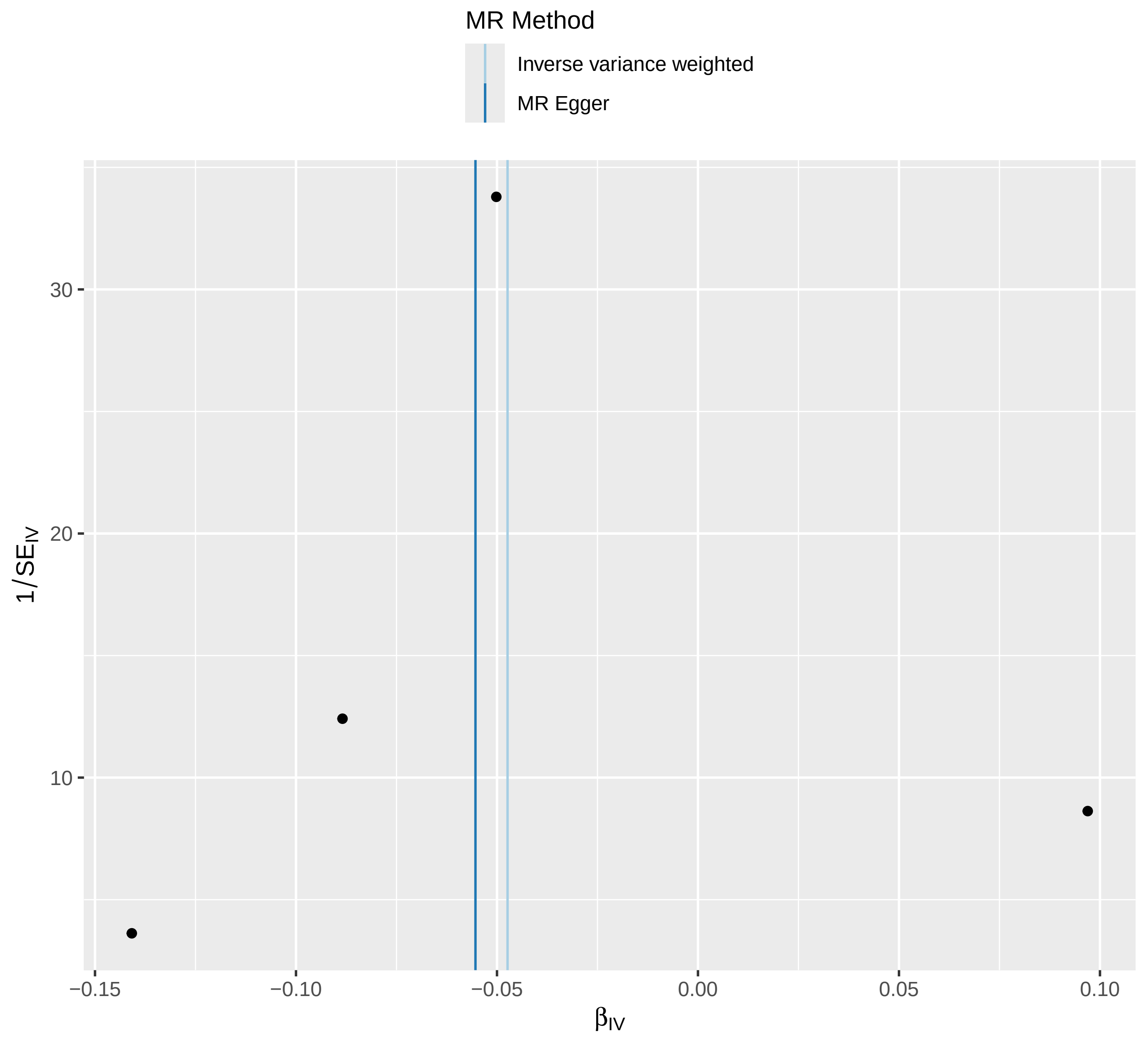
MR Method
Inverse variance weighted
MR Egger
30
20
10
1/SE$_{IV}$
−0.15
−0.10
−0.05
0.00
0.05
0.10
β$_{IV}$

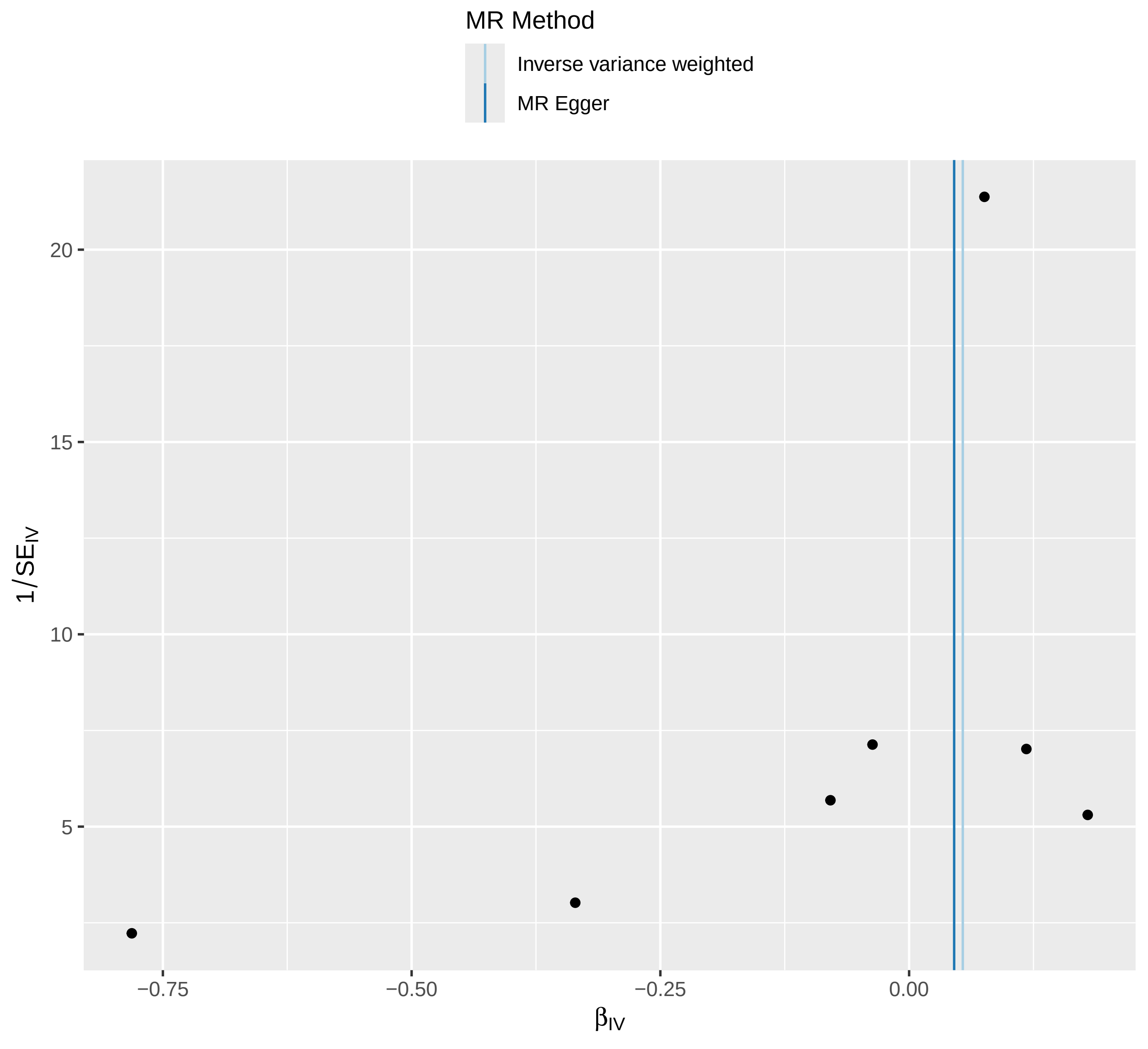
MR Method
Inverse variance weighted
MR Egger
20
15
10
5
1/SE$_{IV}$
−0.75
−0.50
−0.25
0.00
β$_{IV}$

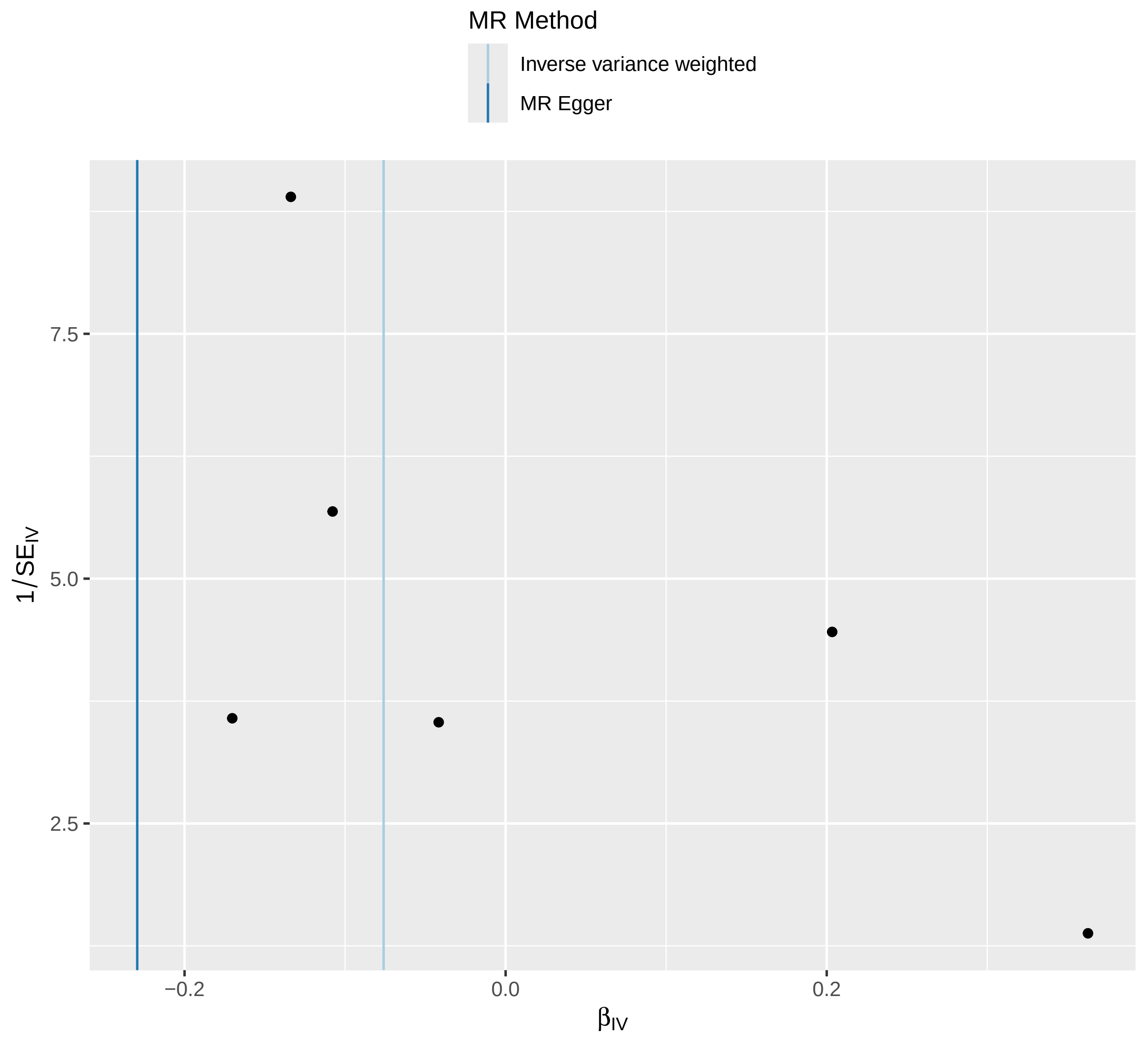
MR Method
Inverse variance weighted
MR Egger
7.5
5.0
2.5
1/SE_IV
−0.2
0.0
0.2
β_IV

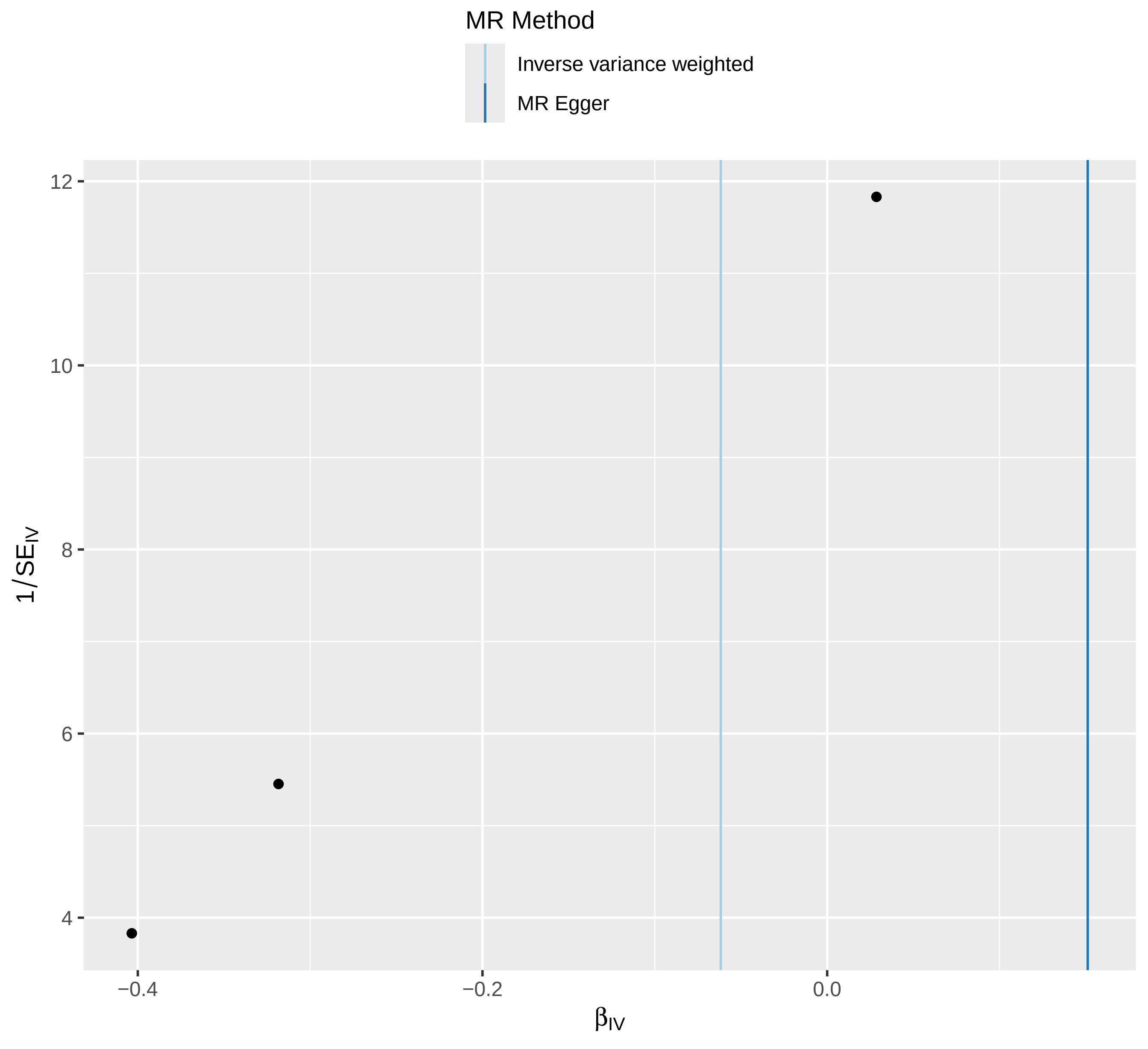
MR Method
Inverse variance weighted
MR Egger
12
10
8
6
4
1/SE_IV
−0.4
−0.2
0.0
β_IV

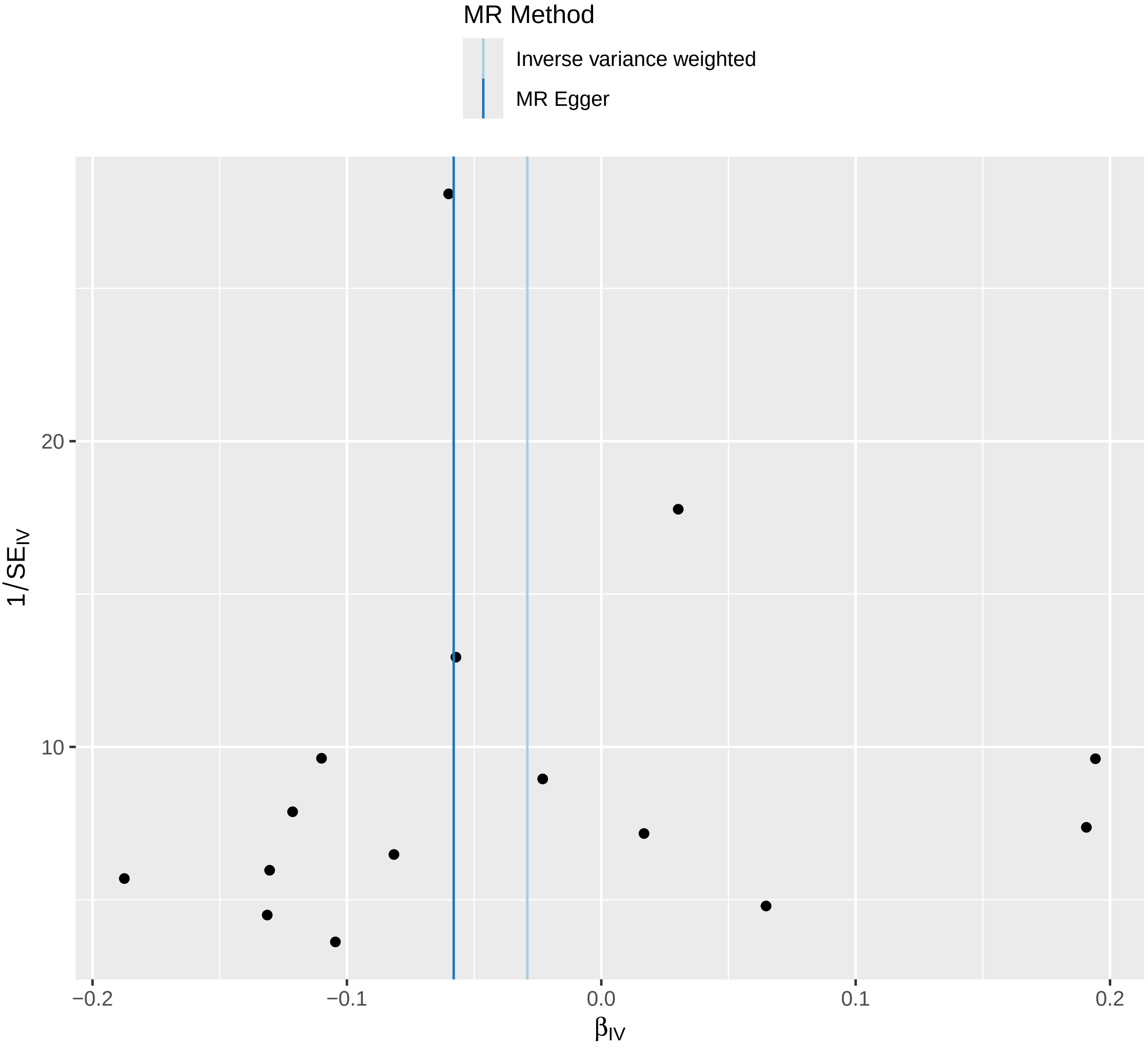
MR Method
Inverse variance weighted
MR Egger
20
10
1/SE$_{IV}$
−0.2
−0.1
0.0
0.1
0.2
β$_{IV}$

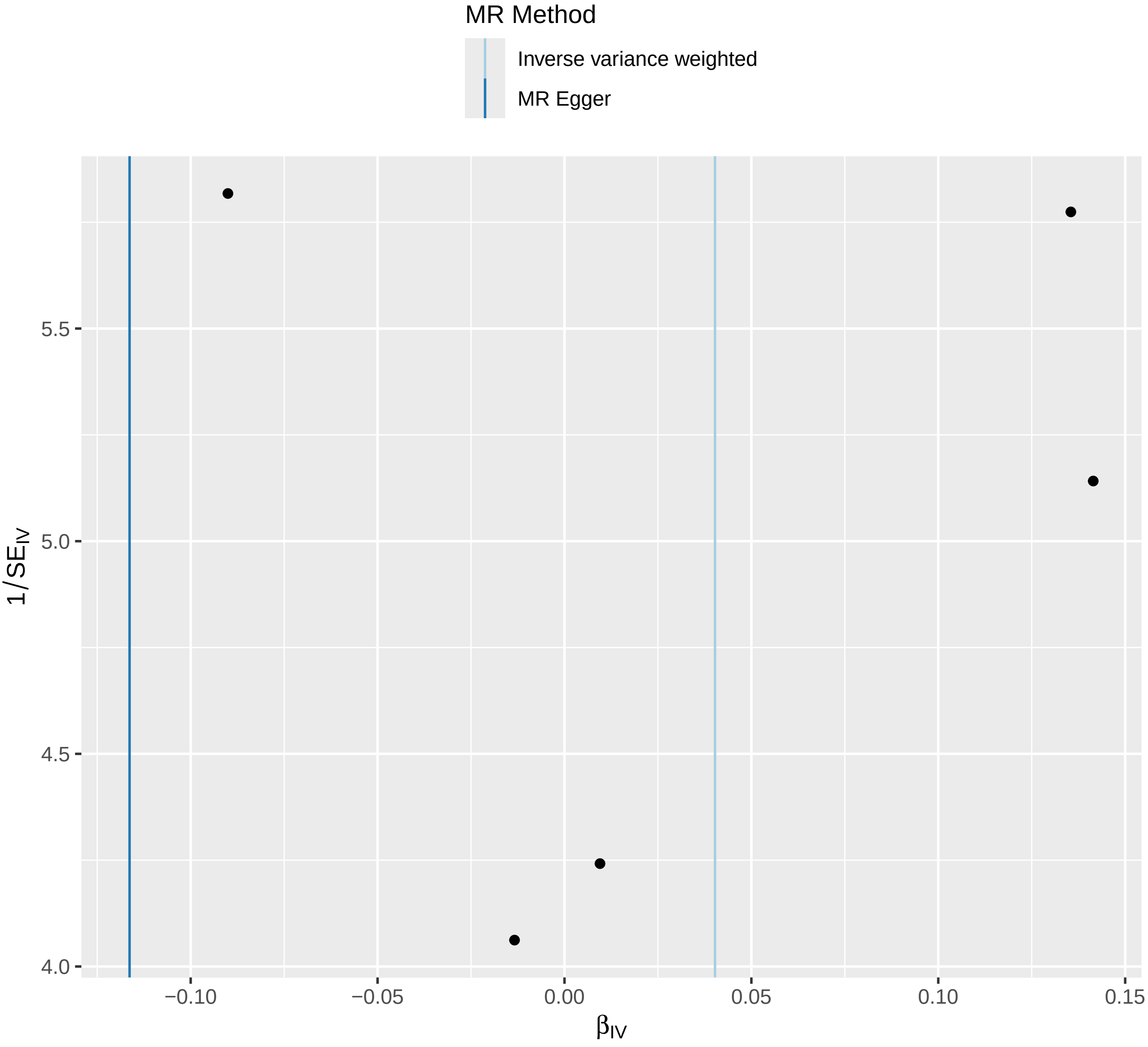

MR Method
Inverse variance weighted
MR Egger
1/SE$_{IV}$
β$_{IV}$
5.5
5.0
4.5
4.0
−0.10
−0.05
0.00
0.05
0.10
0.15

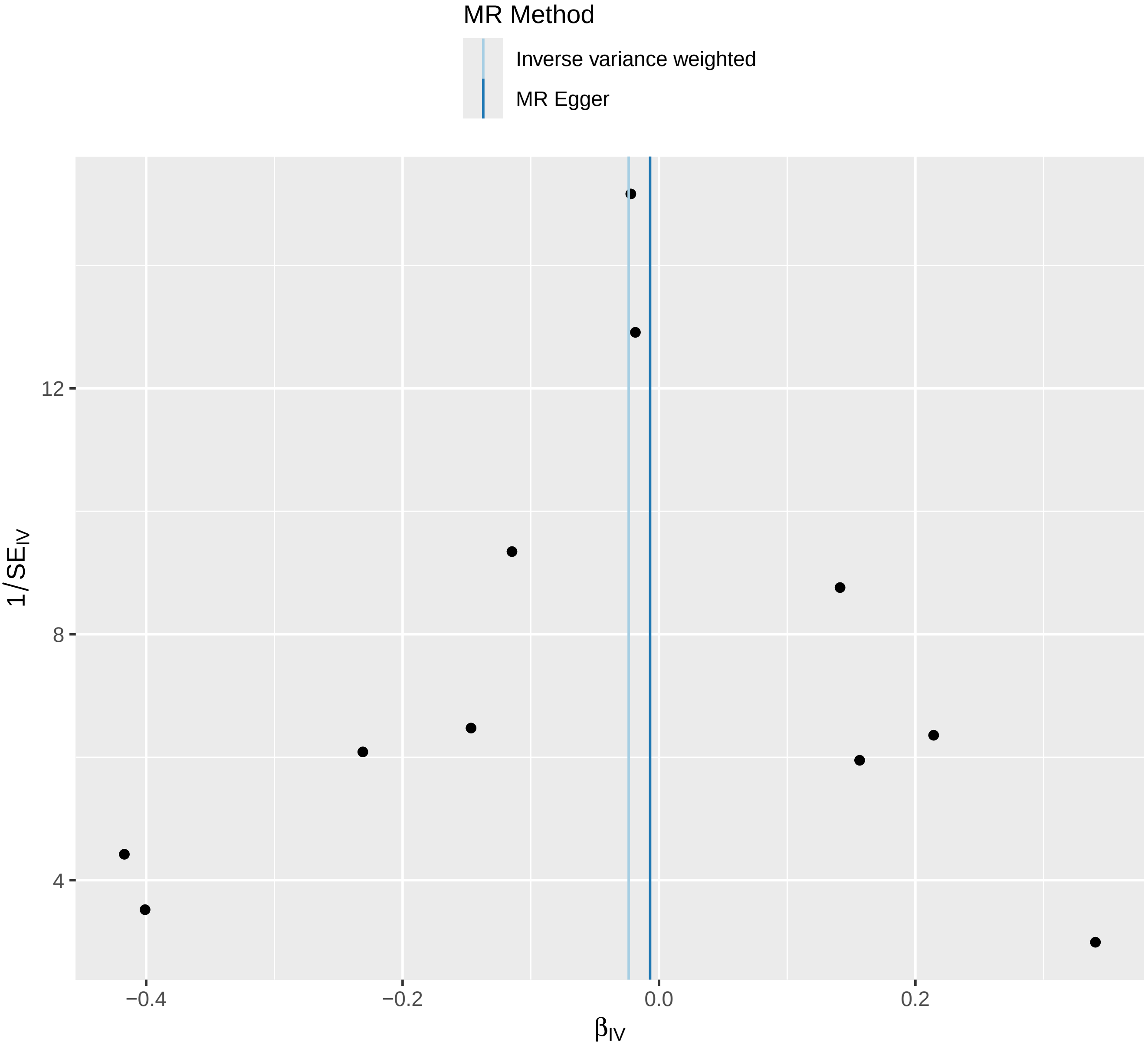

MR Method
Inverse variance weighted
MR Egger
12
8
4
1/SE_IV
−0.4
−0.2
0.0
0.2
β_IV

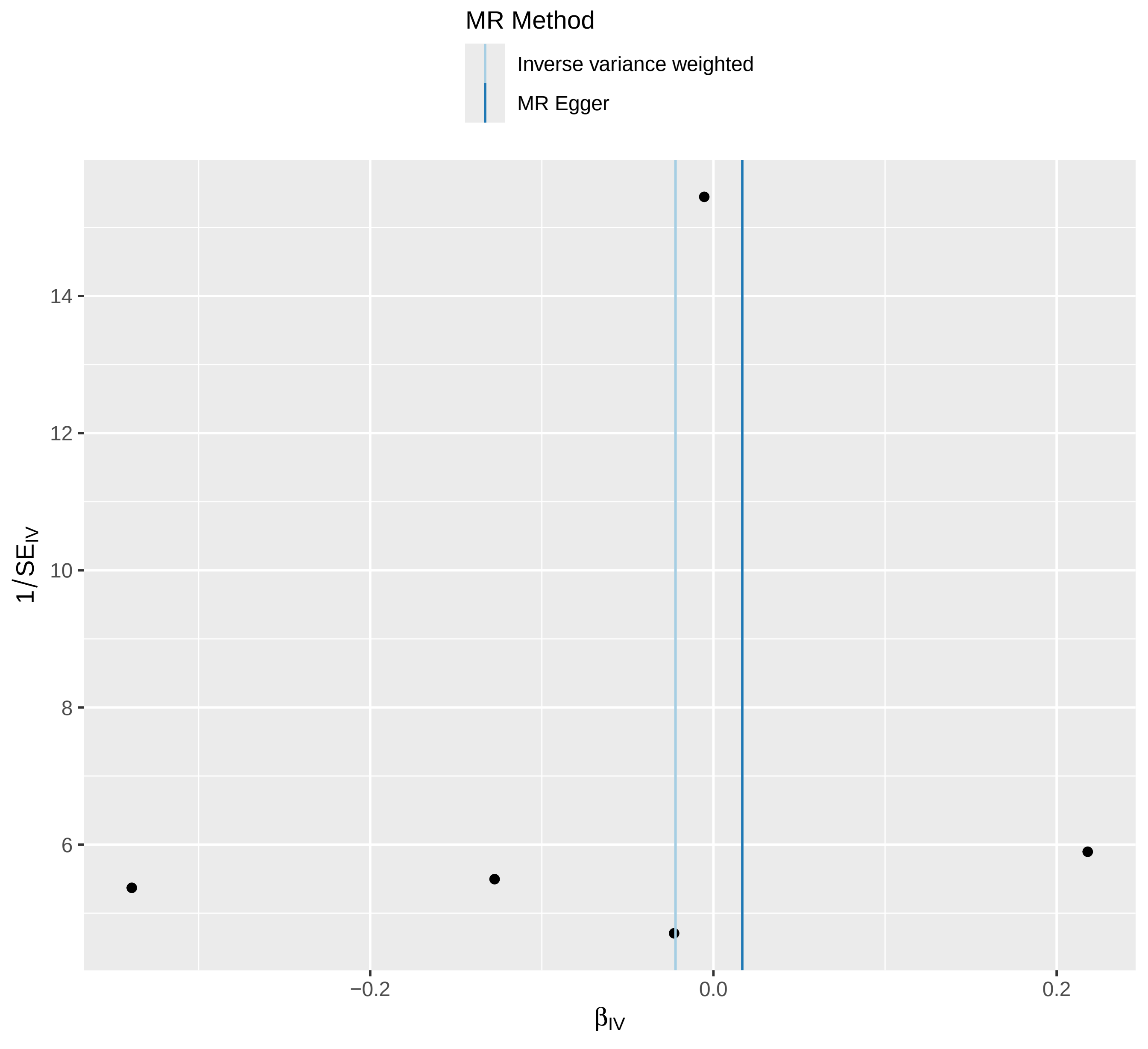

MR Method
Inverse variance weighted
MR Egger
14
12
10
8
6
1/SE_IV
−0.2
0.0
0.2
β_IV

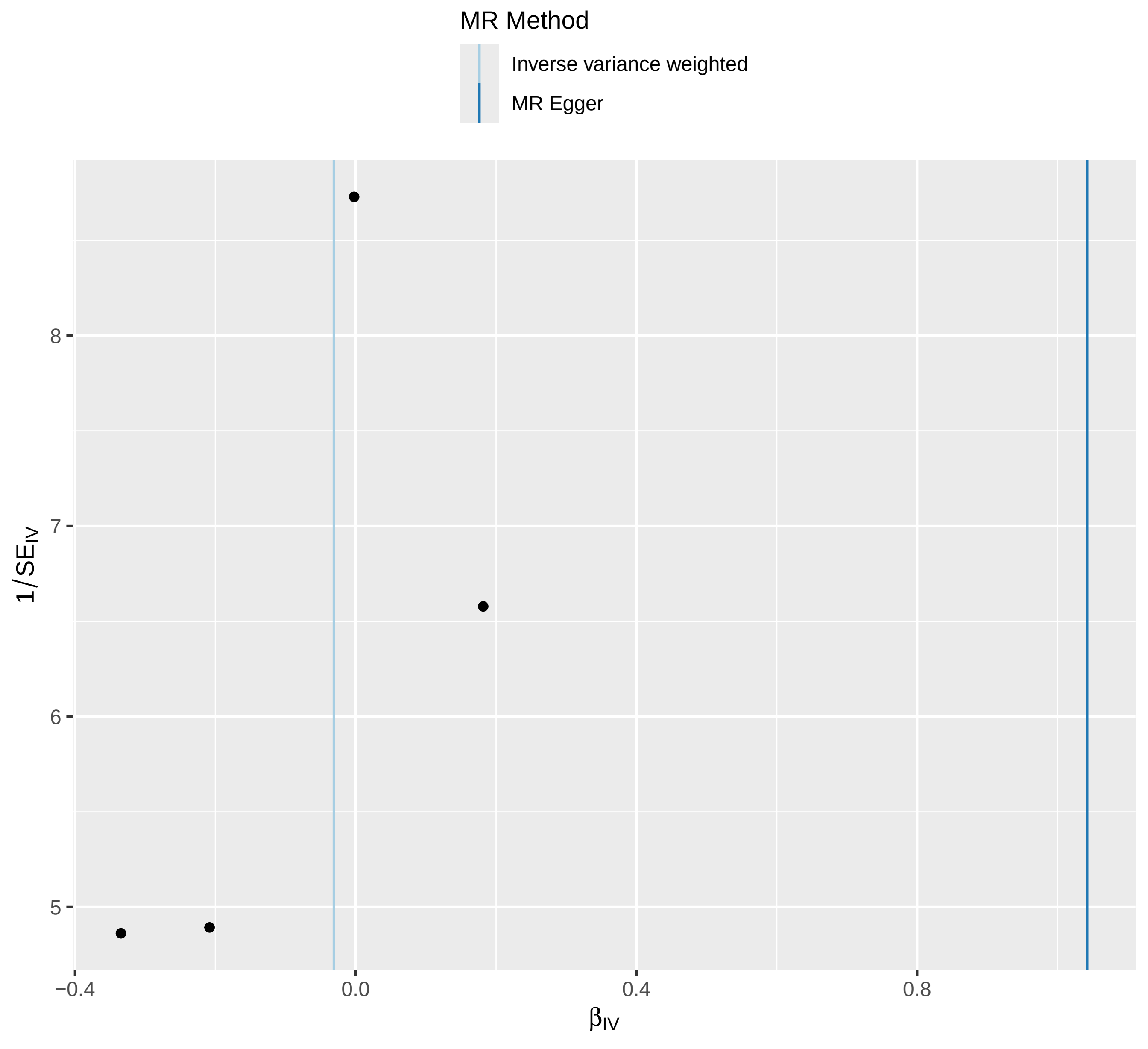
MR Method
Inverse variance weighted
MR Egger
8
7
6
5
1/SE$_{IV}$
−0.4
0.0
0.4
0.8
β$_{IV}$

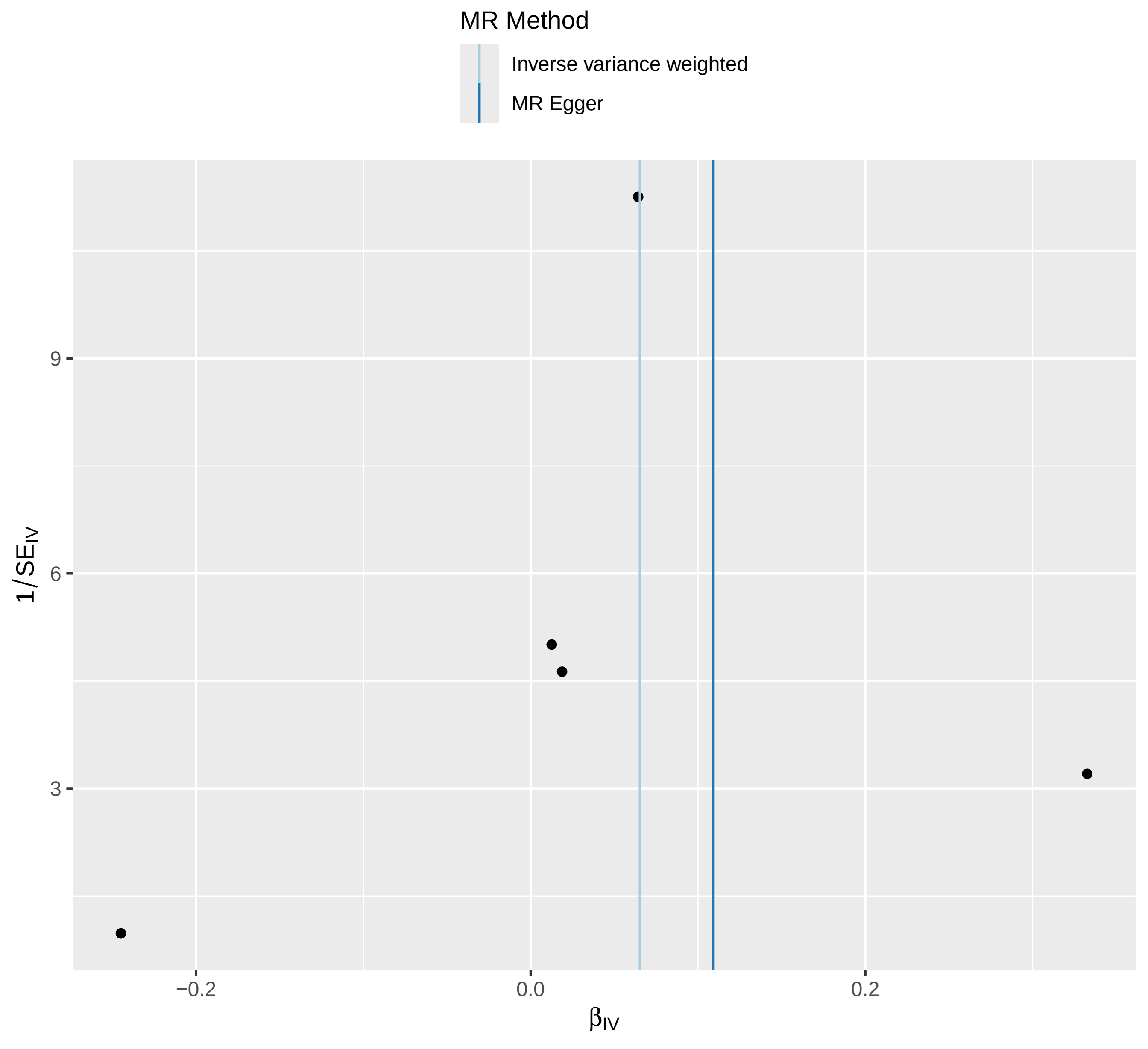
MR Method
Inverse variance weighted
MR Egger
9
6
3
−0.2
0.0
0.2
1/SE_IV
β_IV

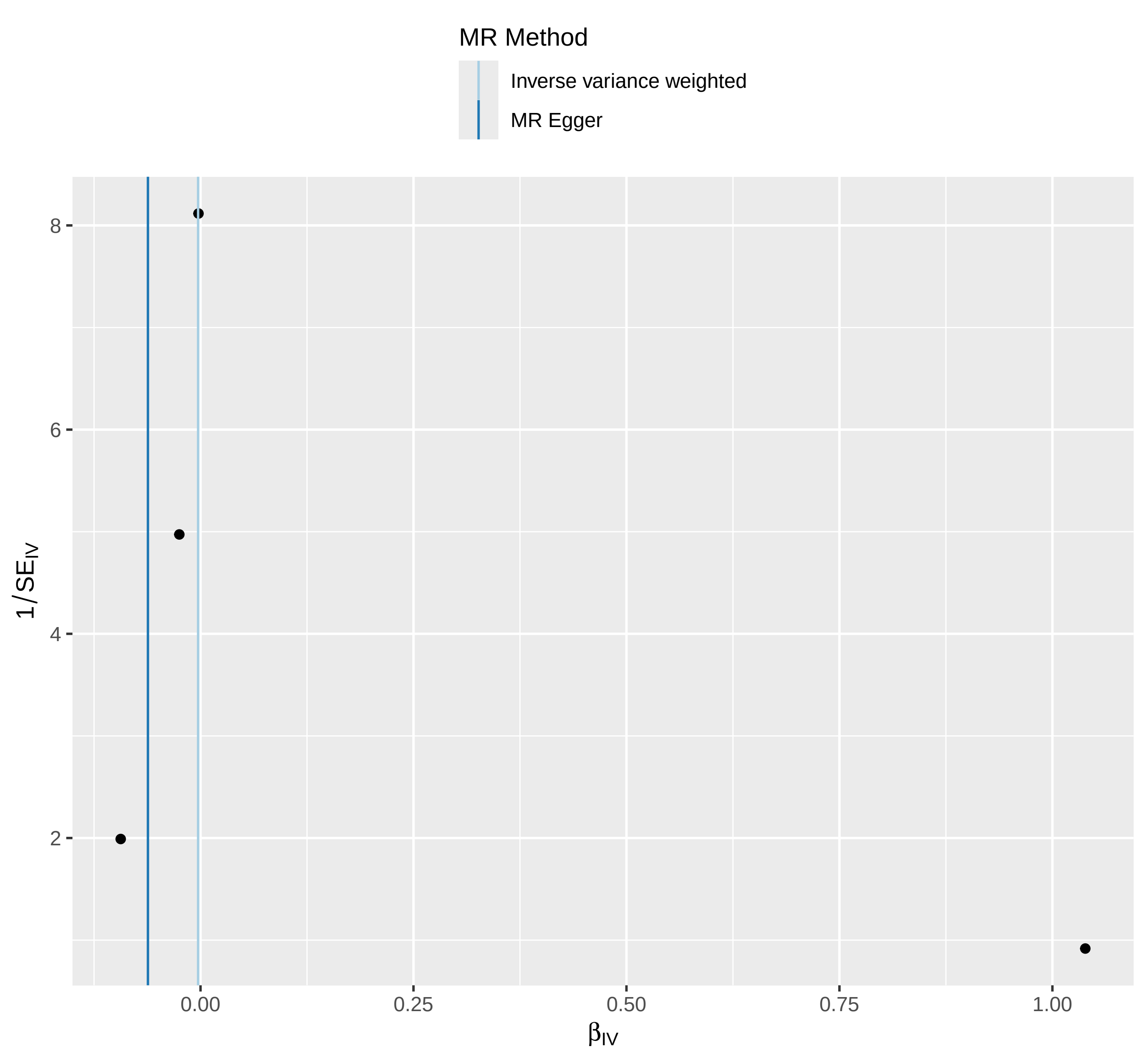

MR Method
Inverse variance weighted
MR Egger
8
6
4
2
1/SE_IV
0.00
0.25
0.50
0.75
1.00
β_IV

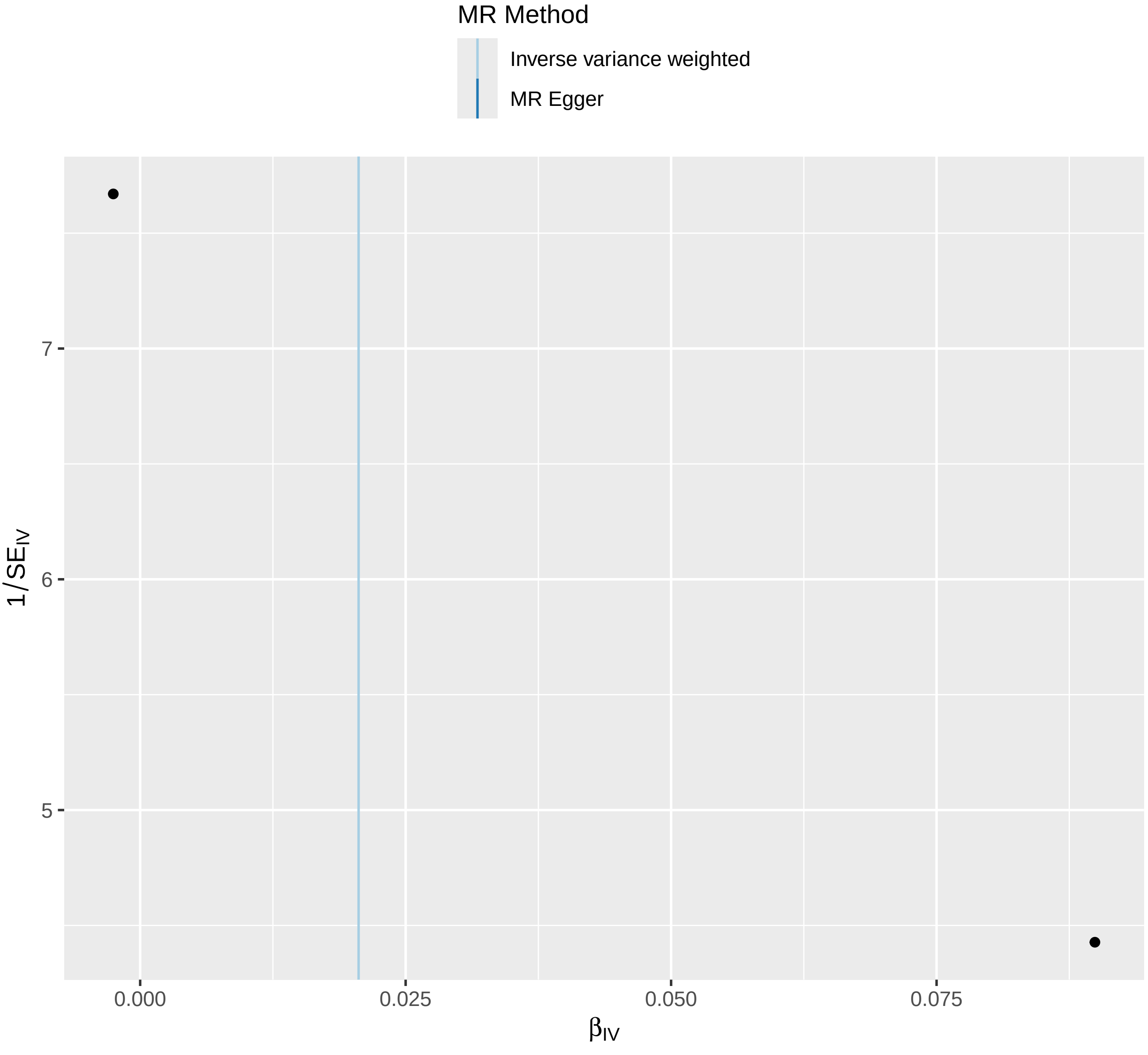

MR Method
Inverse variance weighted
MR Egger
1/SE_IV
7
6
5
0.000
0.025
0.050
0.075
β_IV

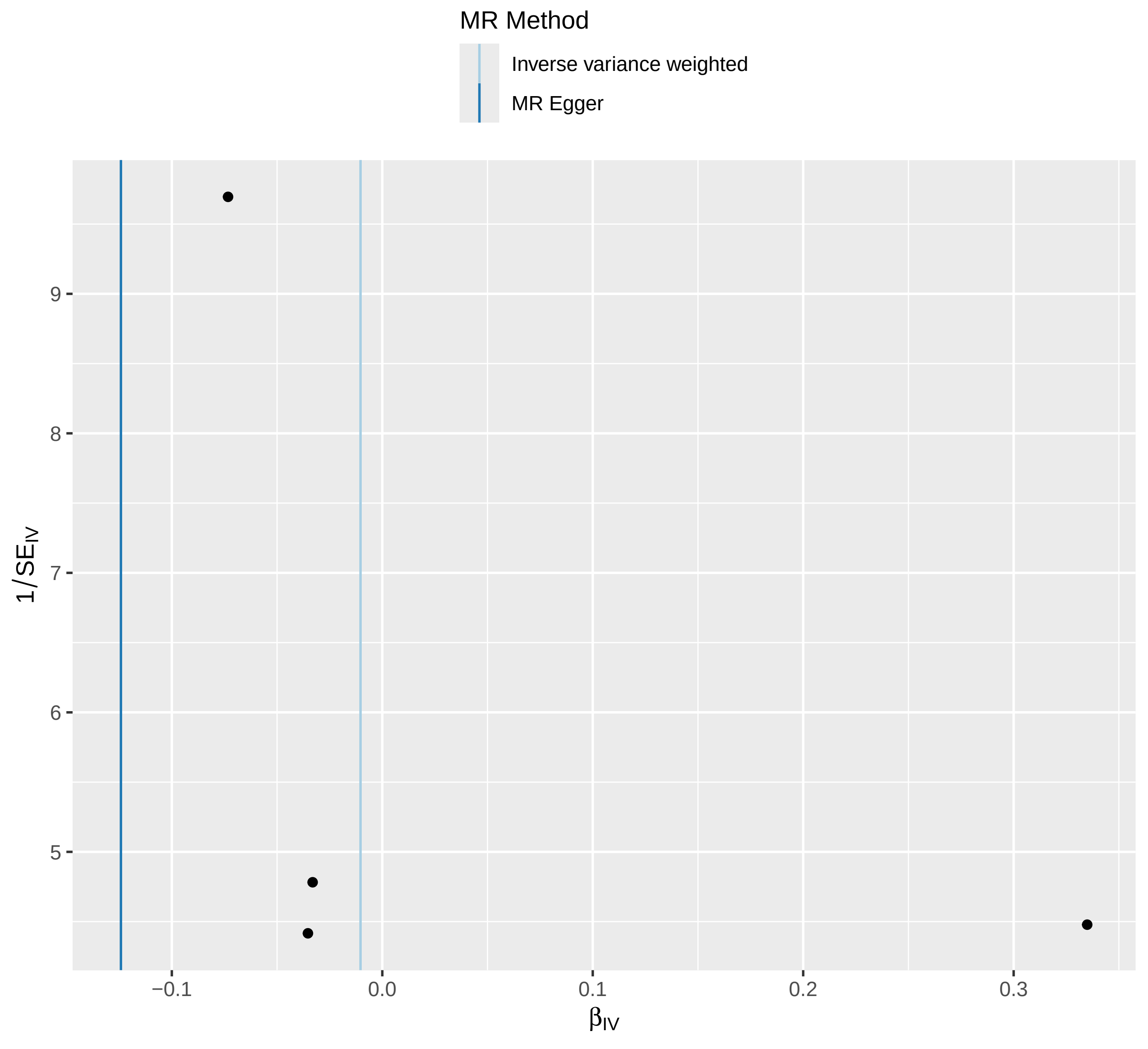

MR Method
Inverse variance weighted
MR Egger
9
8
7
6
5
1/SE_IV
−0.1
0.0
0.1
0.2
0.3
β_IV

Insufficient number of SNPs

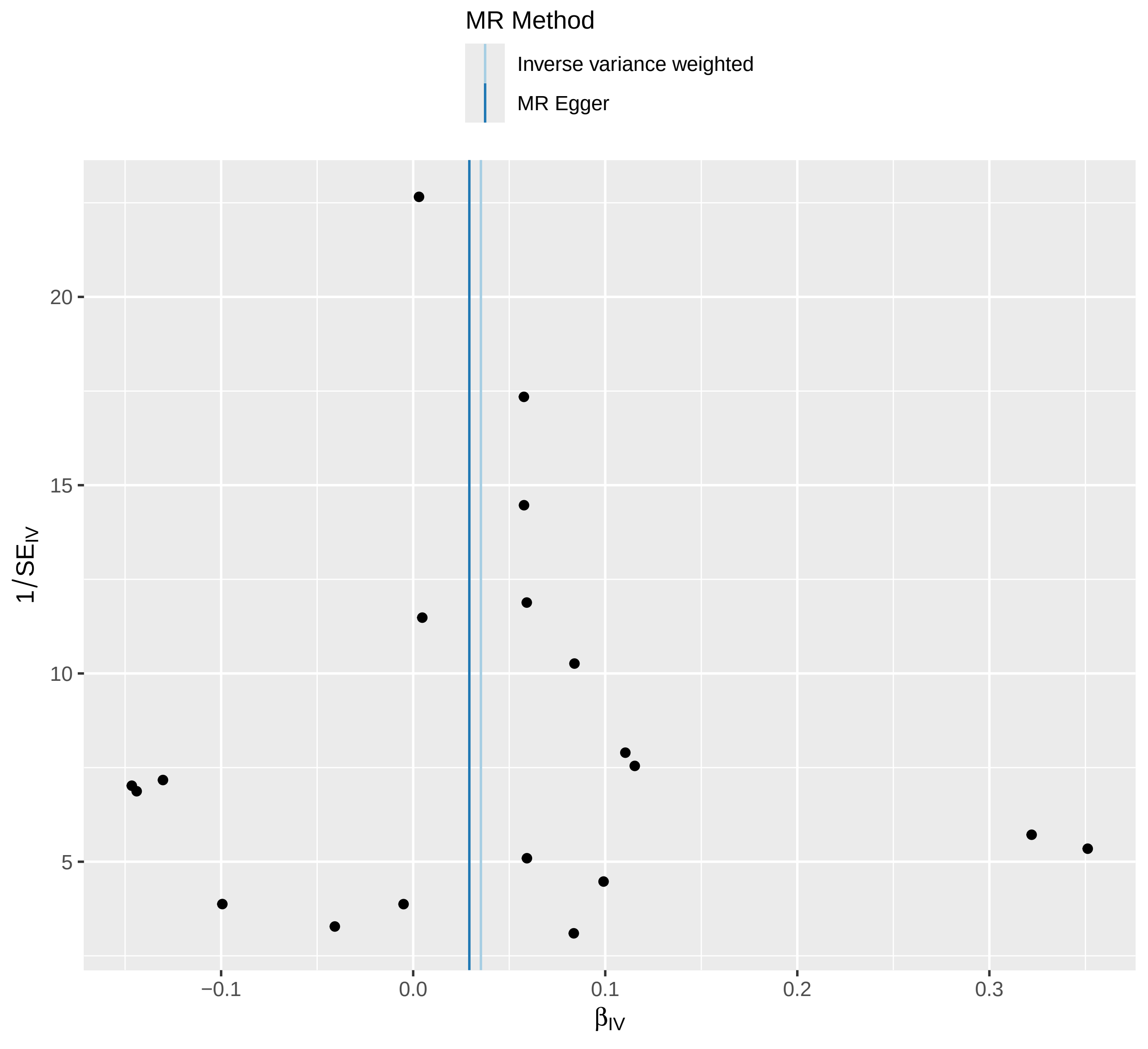
MR Method
Inverse variance weighted
MR Egger
20
15
10
5
1/SE_IV
−0.1
0.0
0.1
0.2
0.3
β_IV

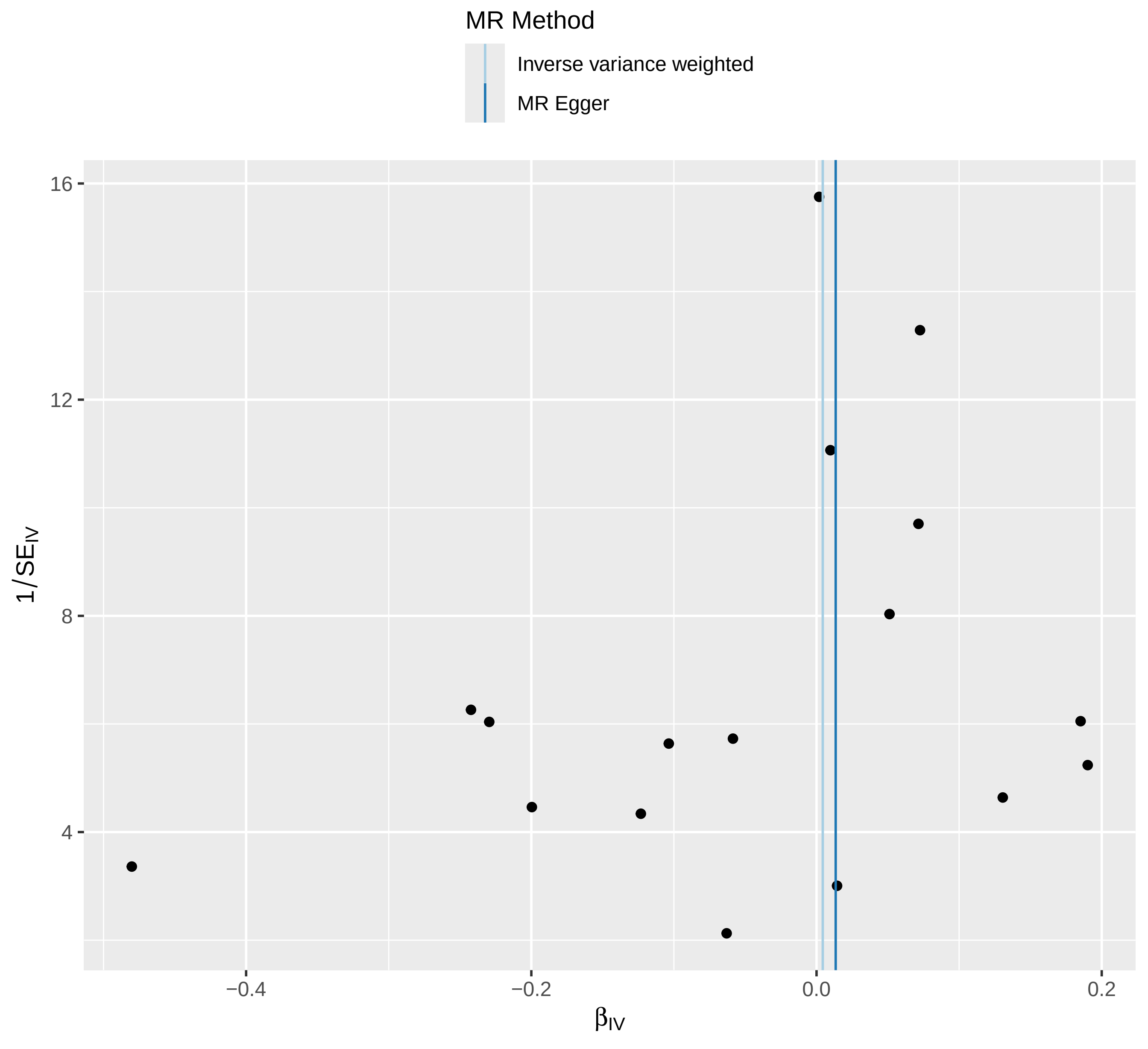
MR Method
Inverse variance weighted
MR Egger
1/SE_IV
16
12
8
4
−0.4
−0.2
0.0
0.2
β_IV

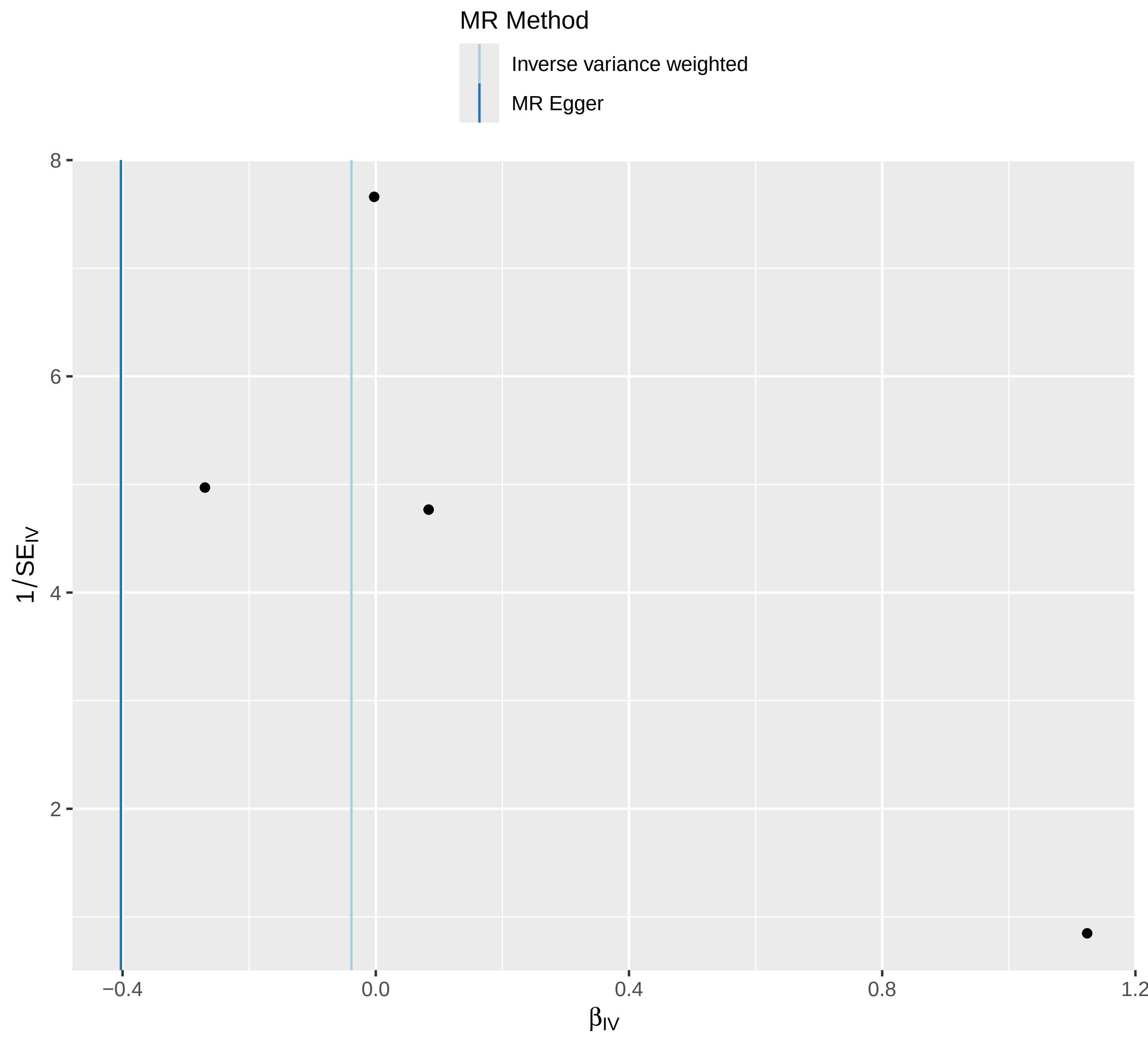
MR Method
Inverse variance weighted
MR Egger
8
6
4
2
1/SE_IV
−0.4
0.0
0.4
0.8
1.2
β_IV

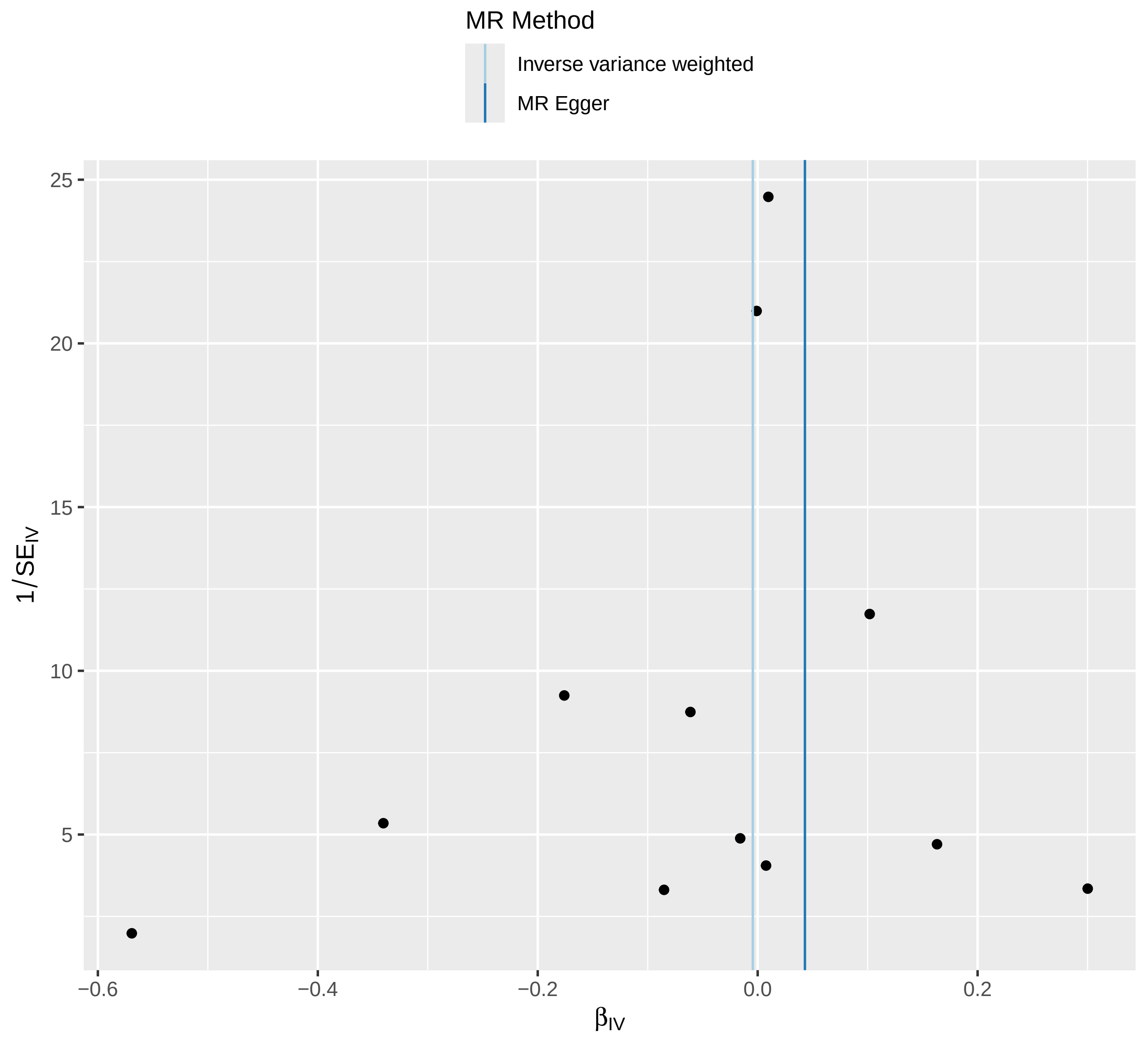

MR Method
Inverse variance weighted
MR Egger
25
20
15
10
5
1/SE$_{IV}$
−0.6
−0.4
−0.2
0.0
0.2
β$_{IV}$

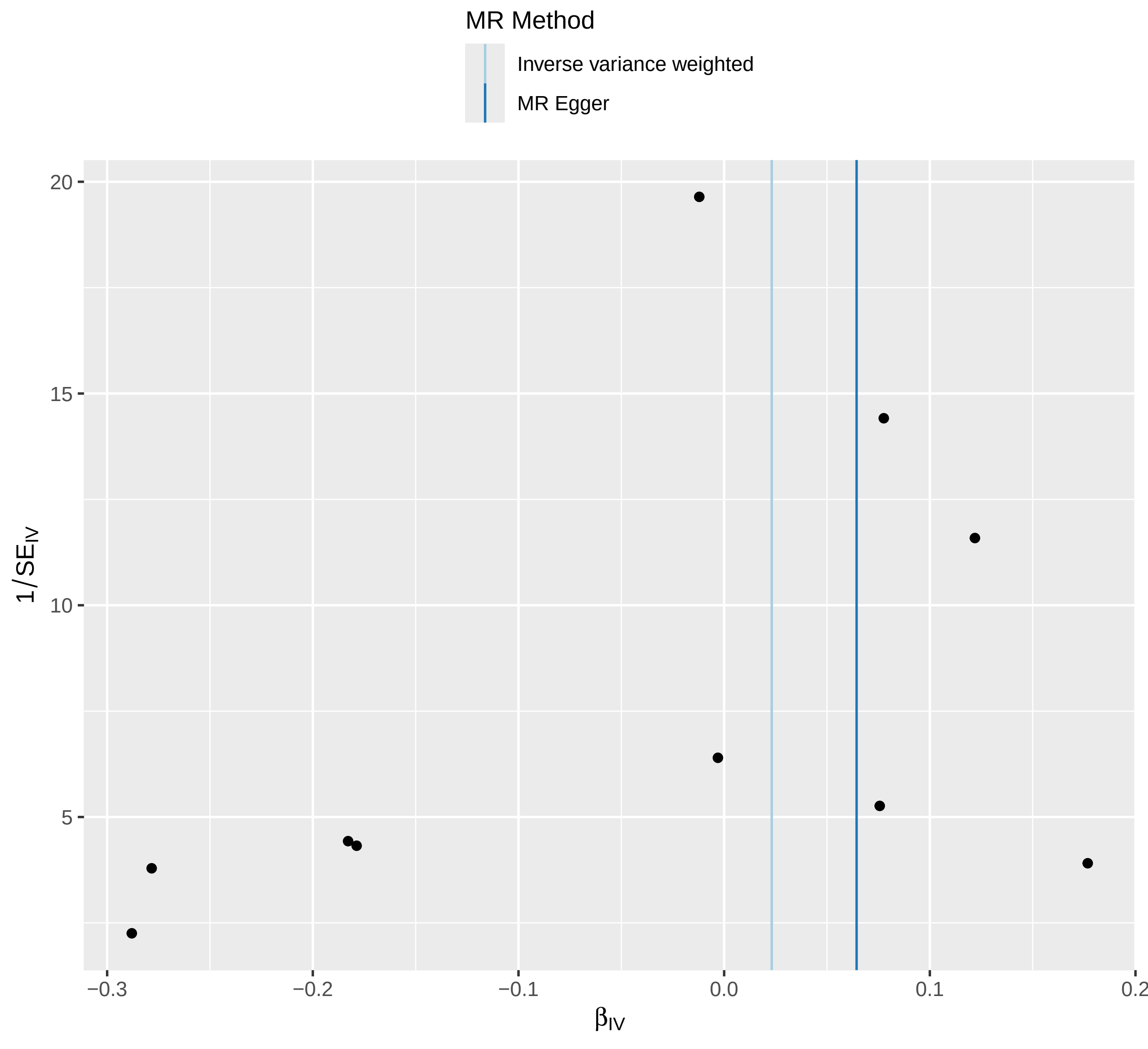

MR Method
Inverse variance weighted
MR Egger
20
15
10
5
1/SE_IV
−0.3
−0.2
−0.1
0.0
0.1
0.2
β_IV

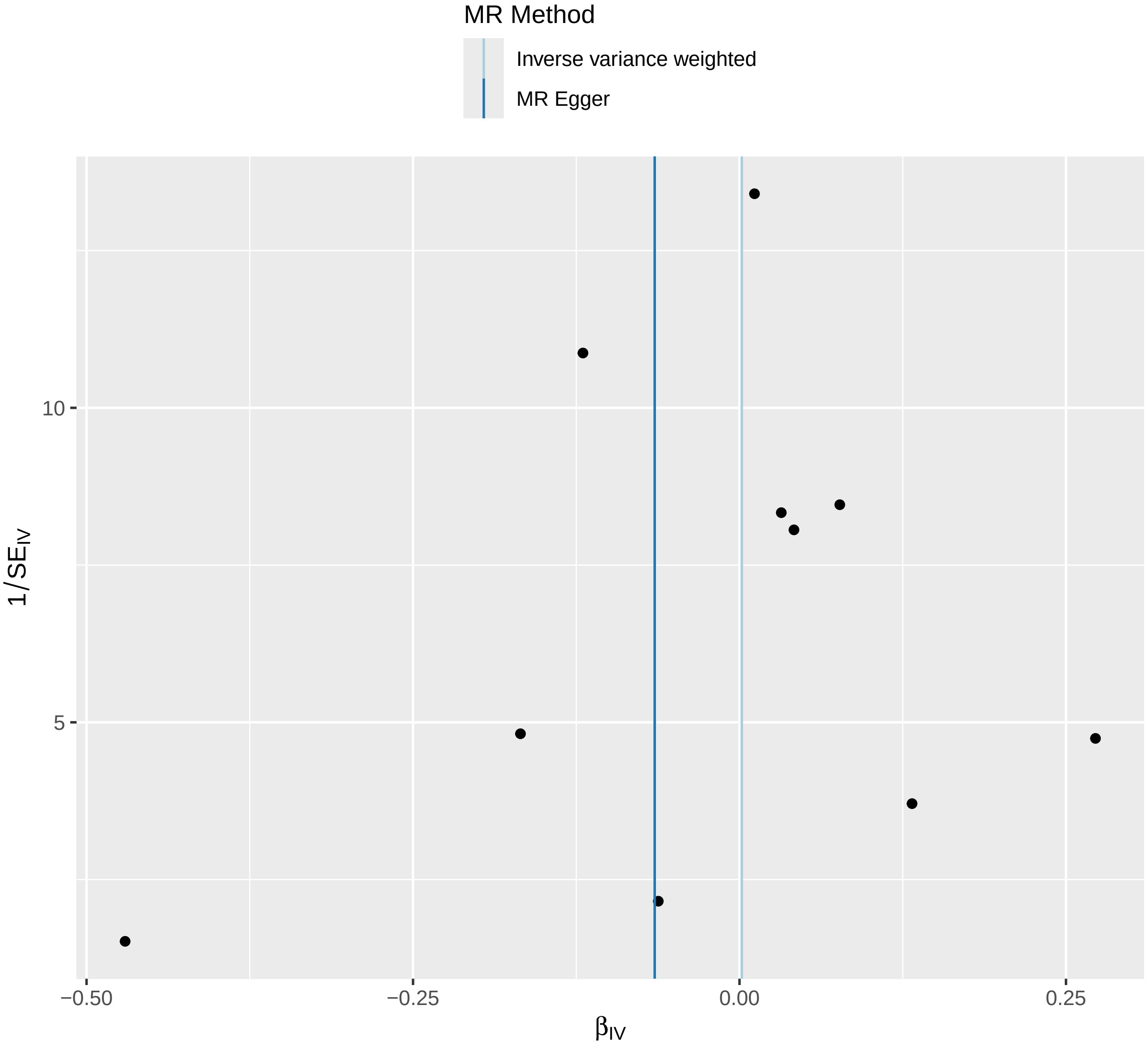
MR Method
Inverse variance weighted
MR Egger
10
5
1/SE$_{IV}$
−0.50
−0.25
0.00
0.25
β$_{IV}$

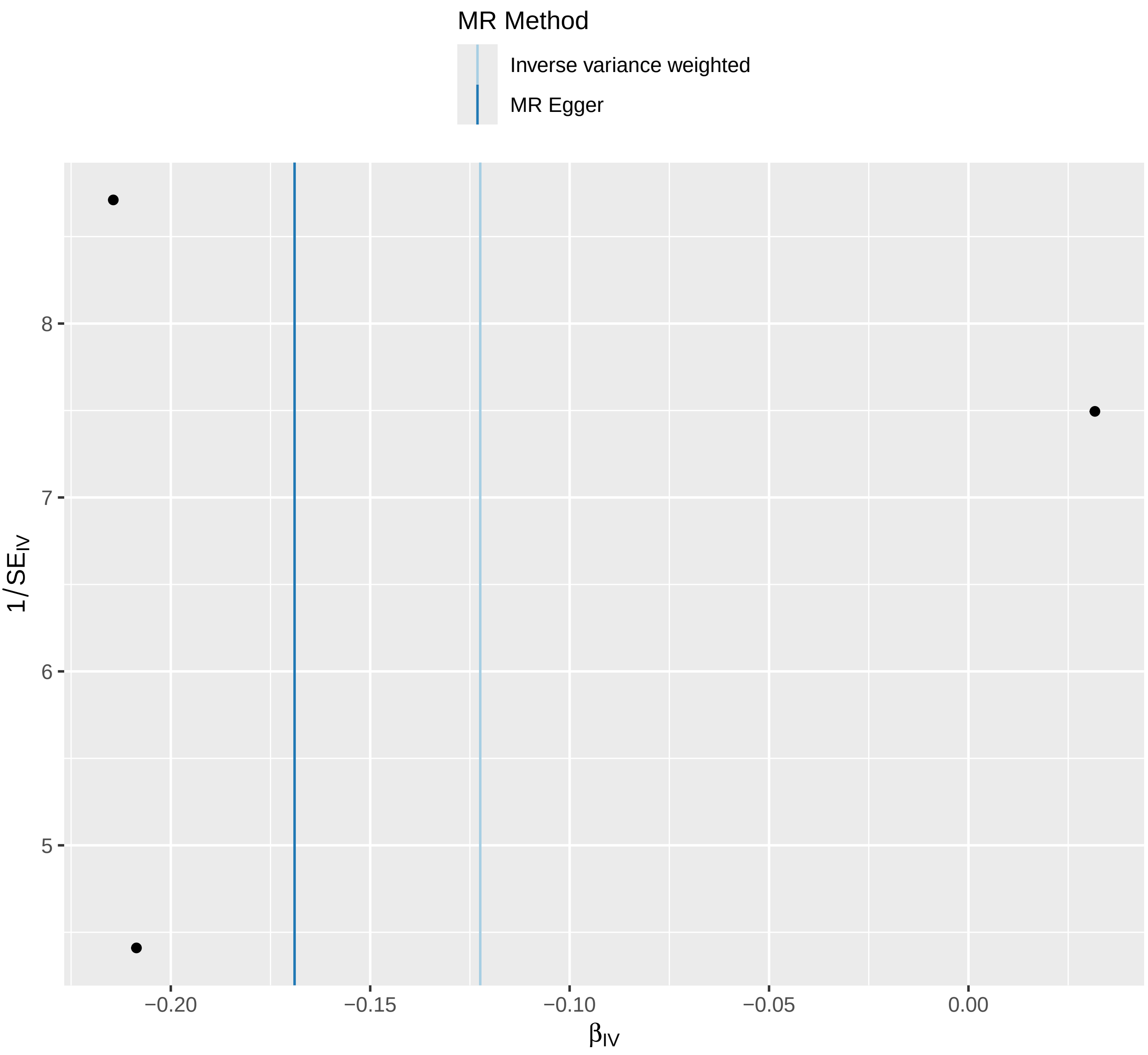
MR Method
Inverse variance weighted
MR Egger
8
7
6
5
$1/SE_{IV}$
−0.20
−0.15
−0.10
−0.05
0.00
$\beta_{IV}$

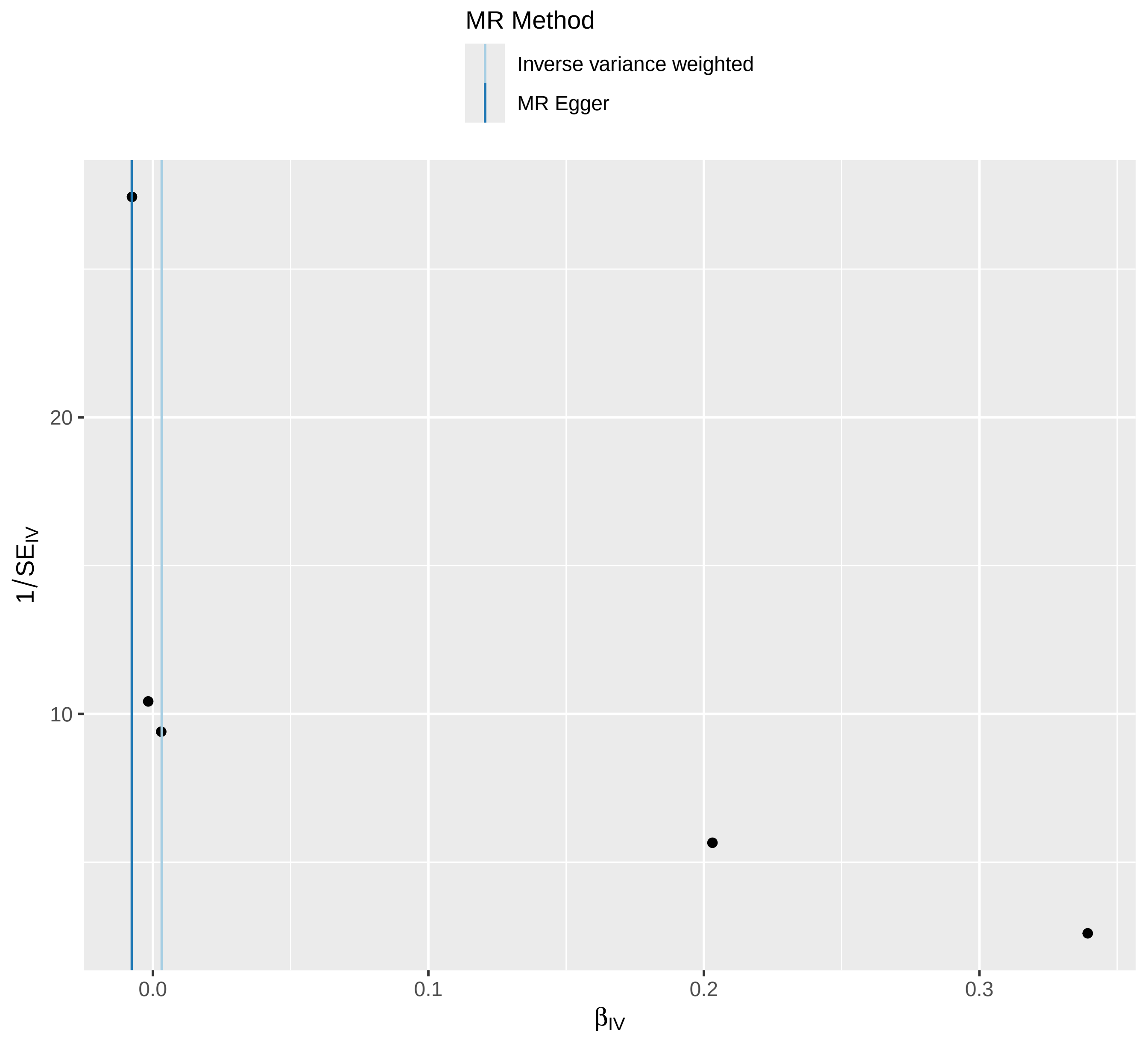

MR Method
Inverse variance weighted
MR Egger
20
10
1/SE$_{IV}$
0.0
0.1
0.2
0.3
β$_{IV}$

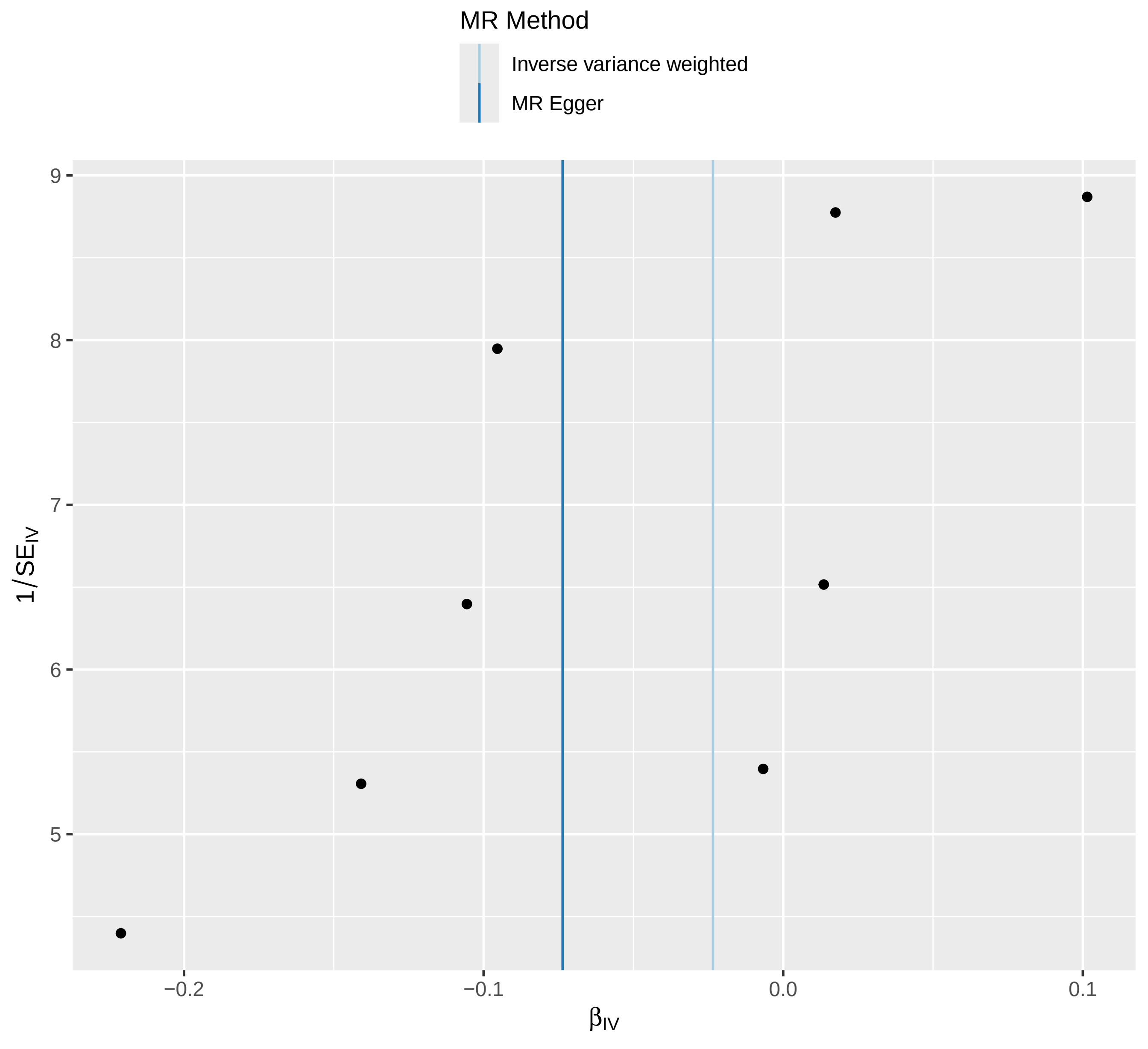

MR Method
Inverse variance weighted
MR Egger
9
8
7
6
5
1/SE_IV
−0.2
−0.1
0.0
0.1
β_IV

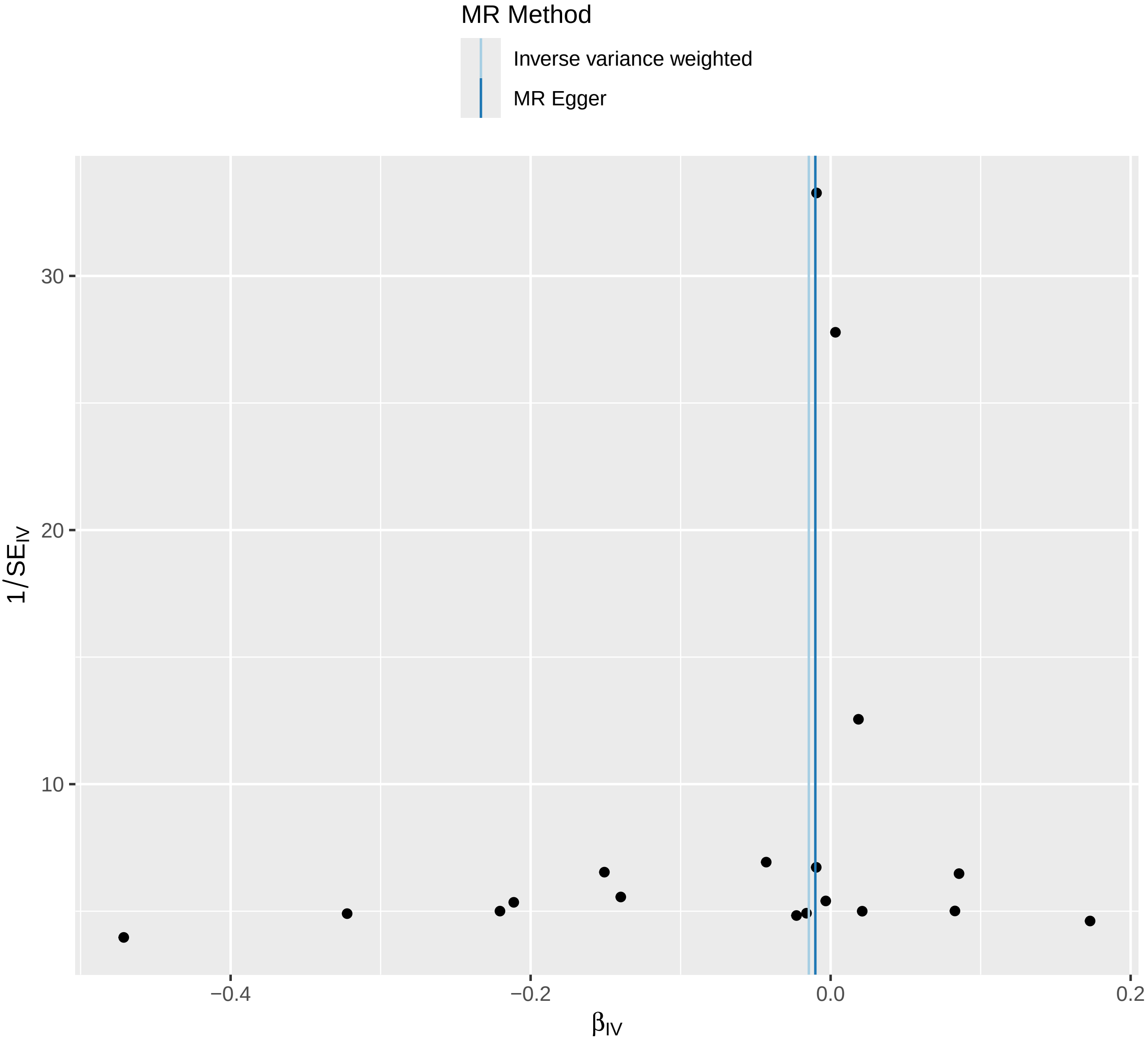

MR Method
Inverse variance weighted
MR Egger
30
20
10
1/SE$_{IV}$
−0.4
−0.2
0.0
0.2
β$_{IV}$

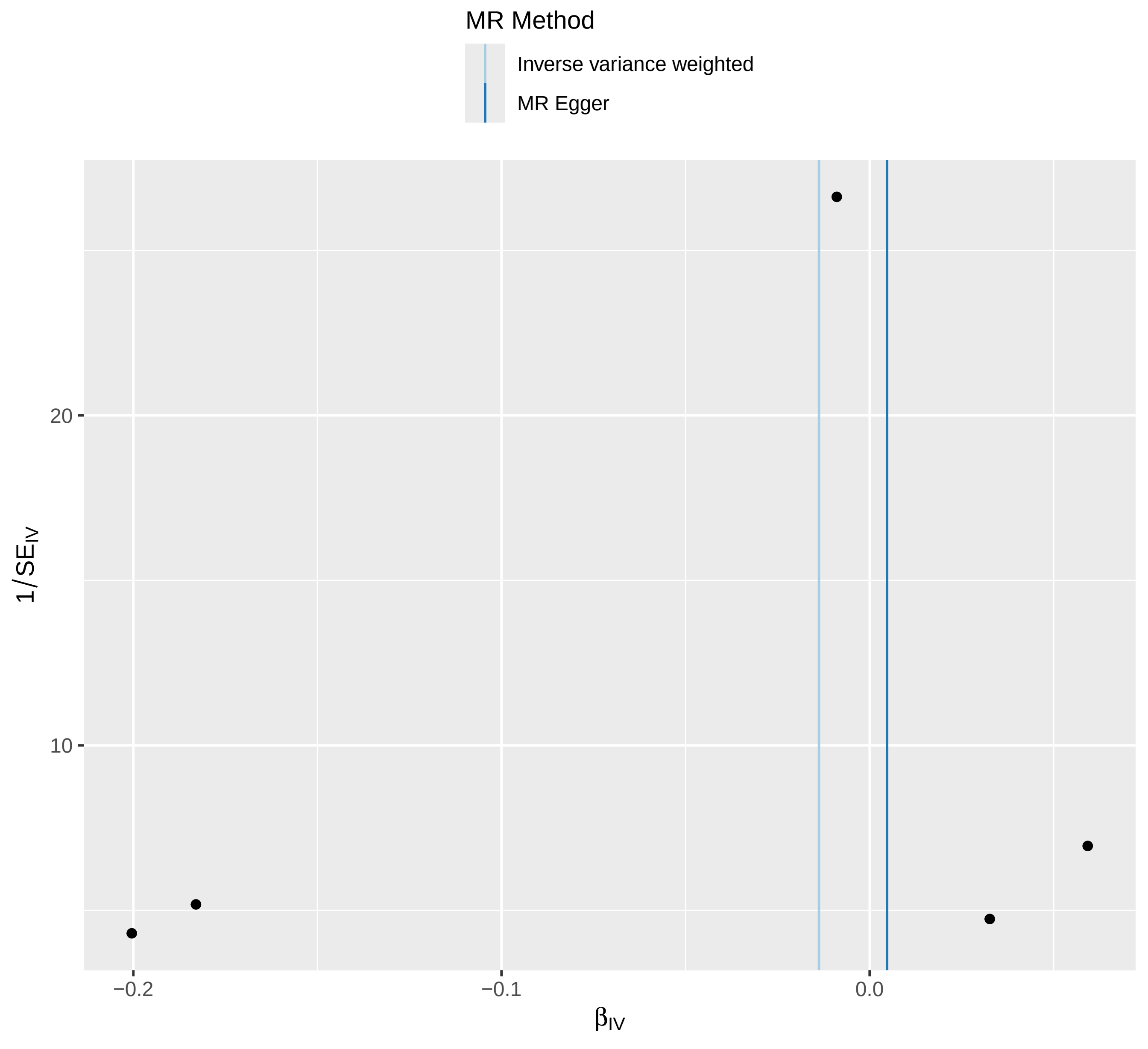

MR Method
Inverse variance weighted
MR Egger
20
10
1/SE$_{IV}$
−0.2
−0.1
0.0
β$_{IV}$

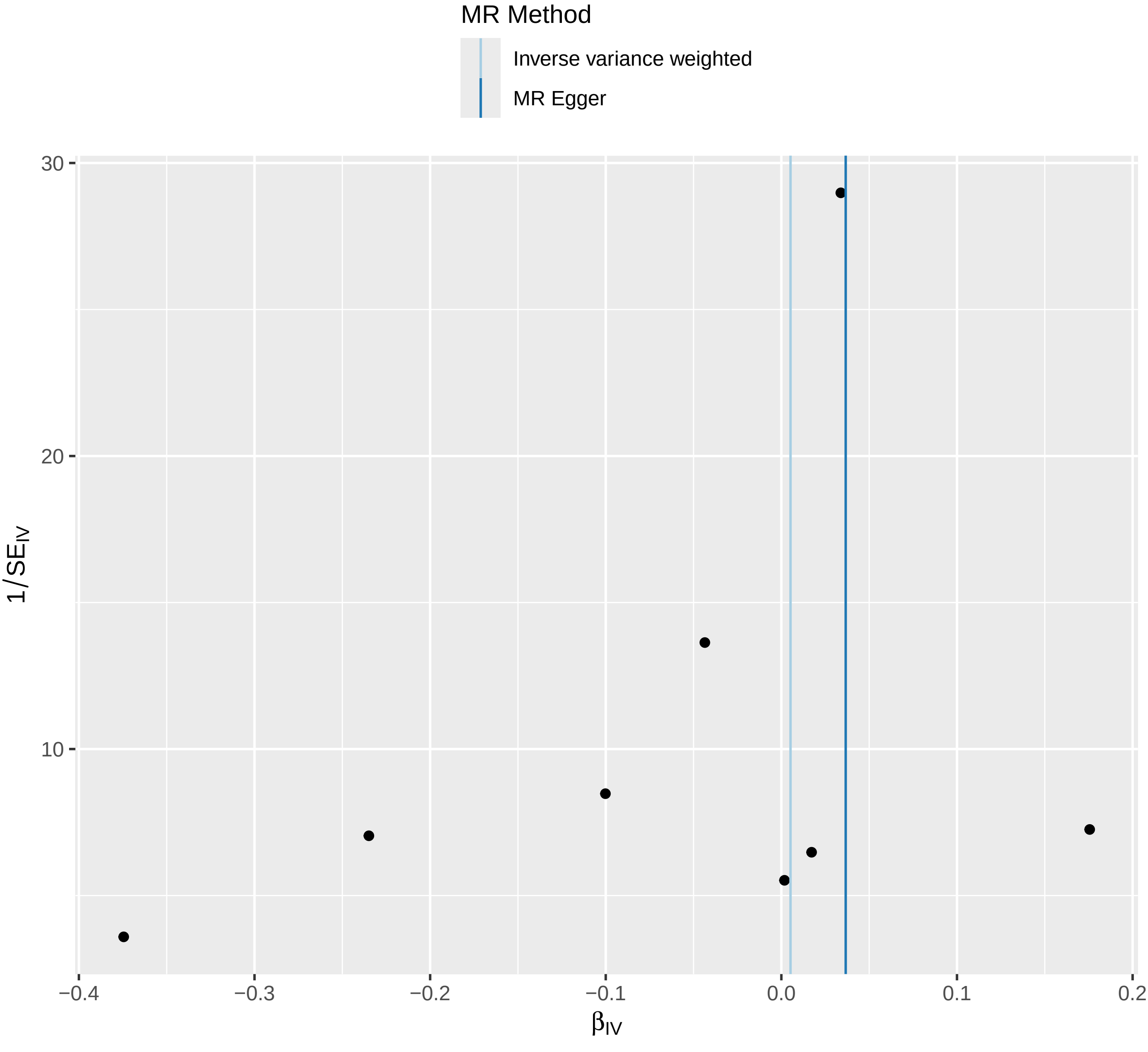

MR Method
Inverse variance weighted
MR Egger
30
20
10
1/SE_IV
−0.4
−0.3
−0.2
−0.1
0.0
0.1
0.2
β_IV

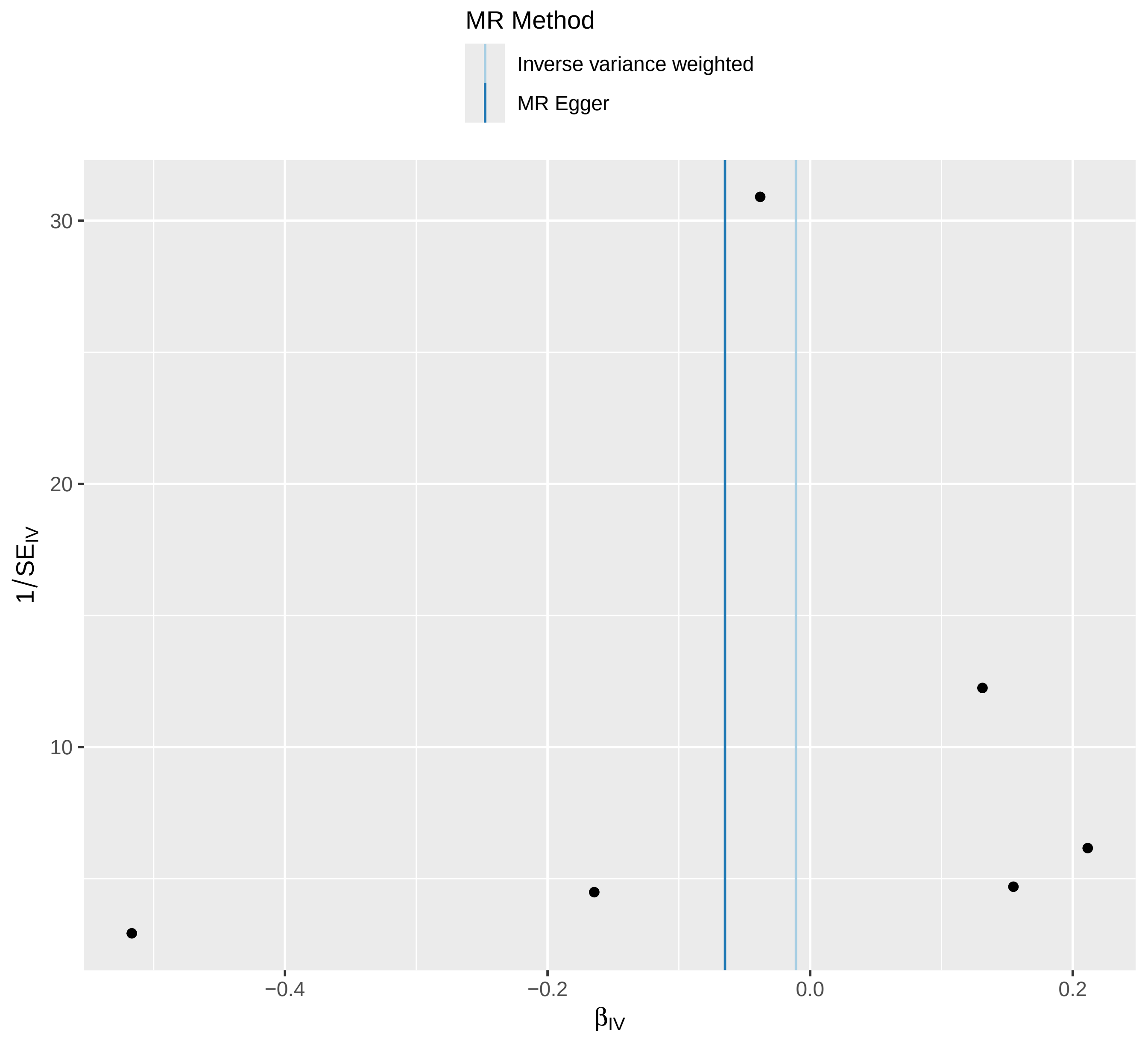

MR Method
Inverse variance weighted
MR Egger
30
20
10
1/SE_IV
−0.4
−0.2
0.0
0.2
β_IV

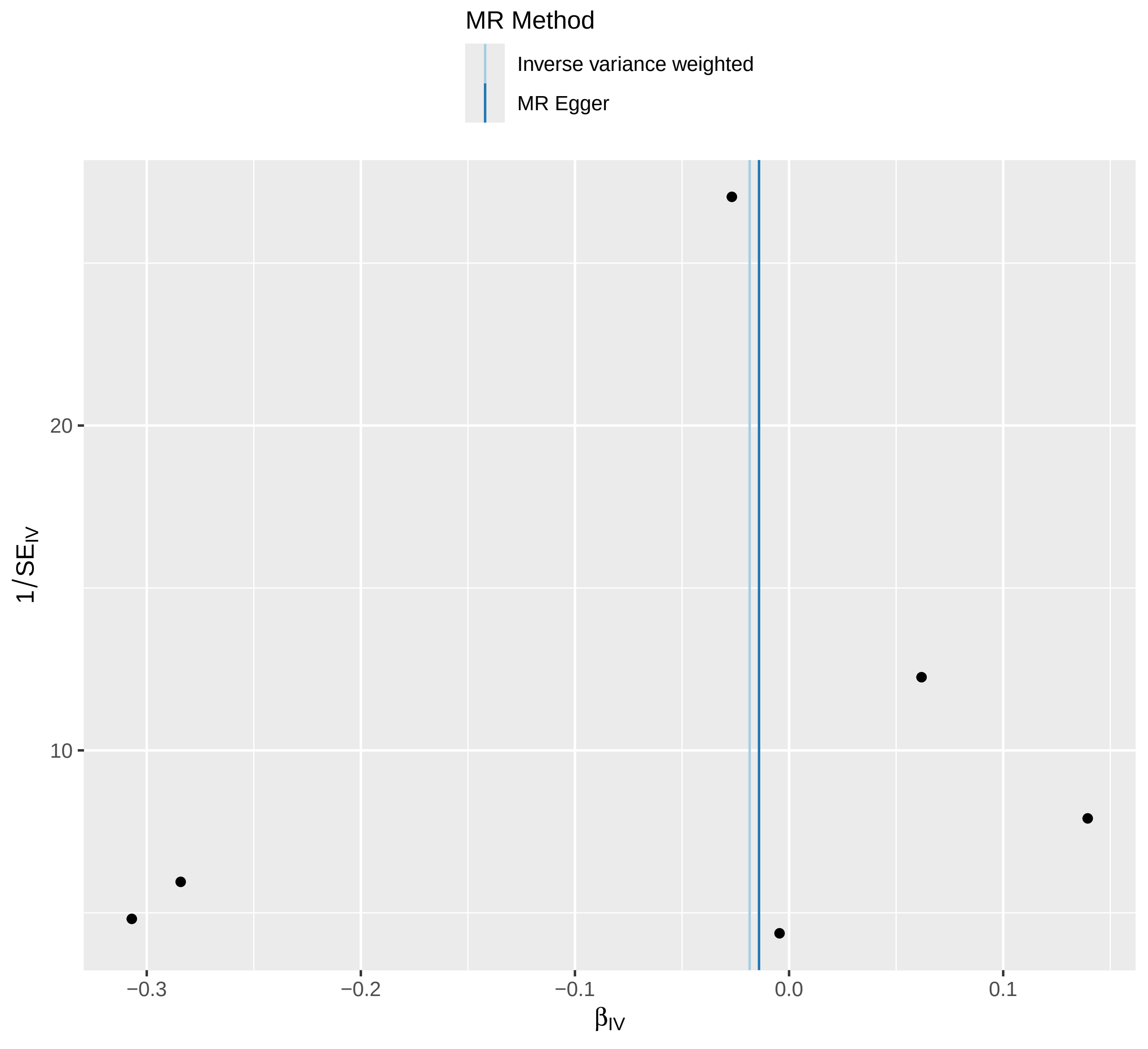

MR Method
Inverse variance weighted
MR Egger
20
10
1/SE$_{IV}$
−0.3
−0.2
−0.1
0.0
0.1
β$_{IV}$

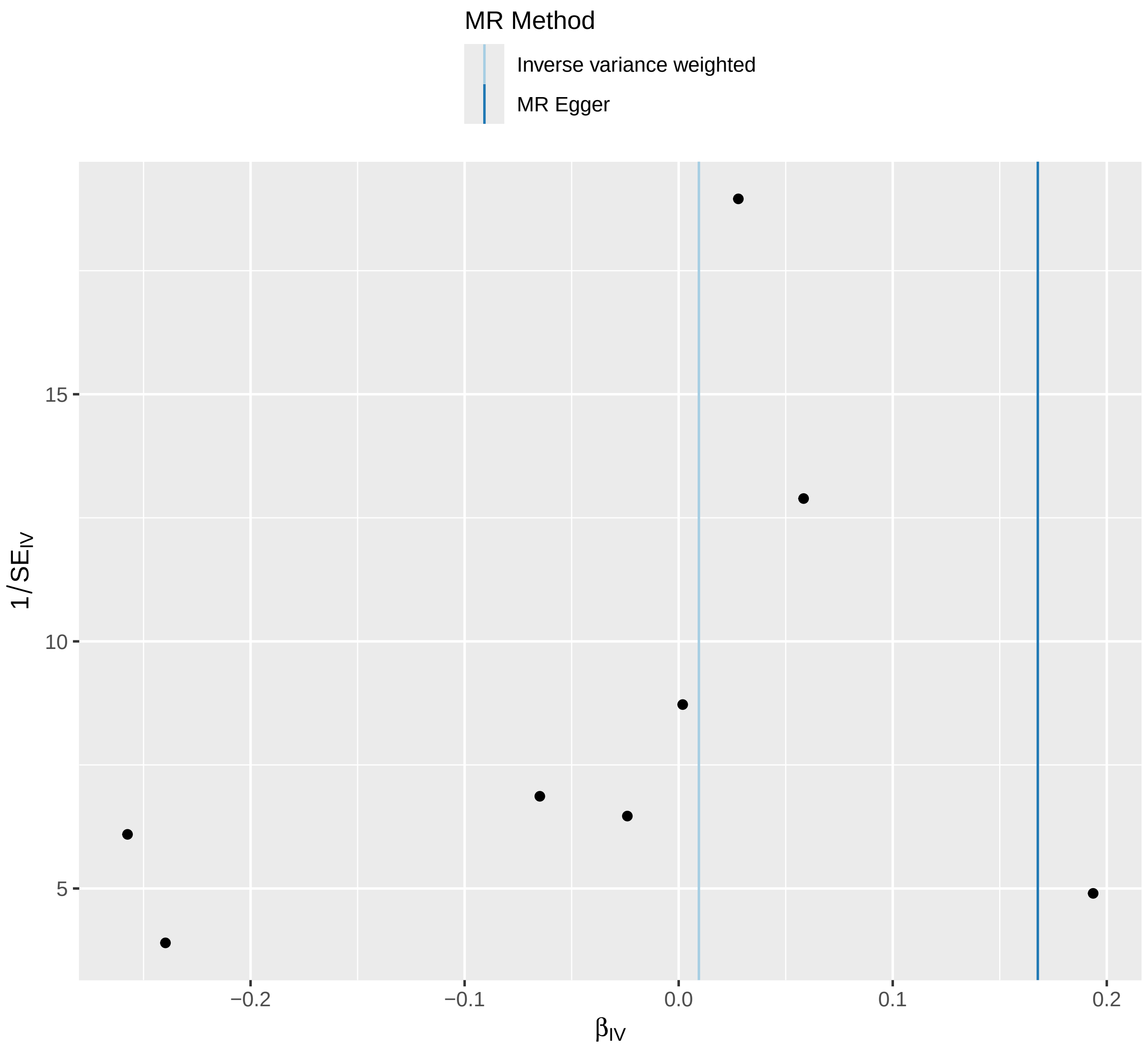
MR Method
Inverse variance weighted
MR Egger
15
10
5
1/SE_IV
−0.2
−0.1
0.0
0.1
0.2
β_IV

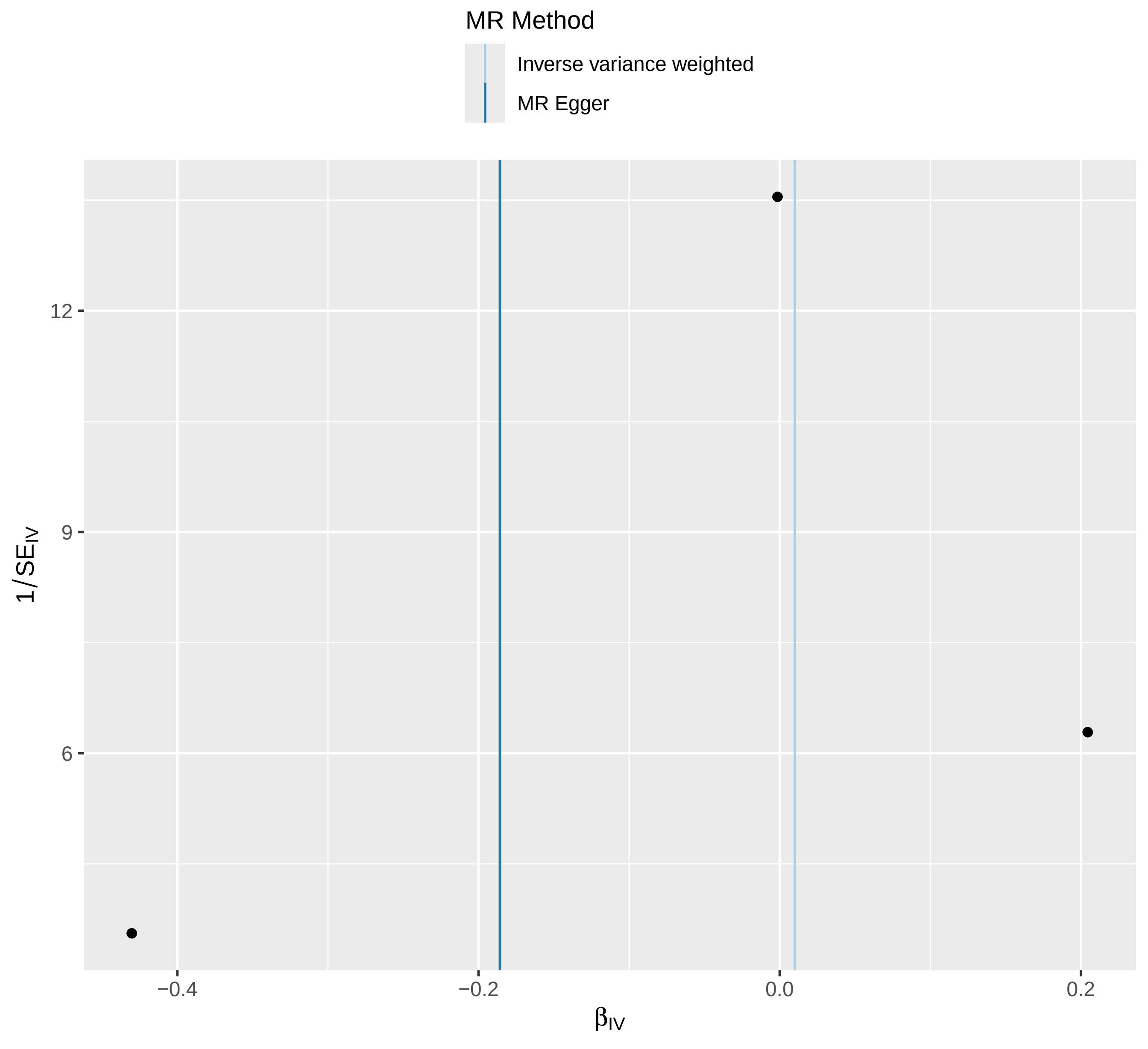

MR Method
Inverse variance weighted
MR Egger
12
9
6
1/SE$_{IV}$
−0.4
−0.2
0.0
0.2
β$_{IV}$

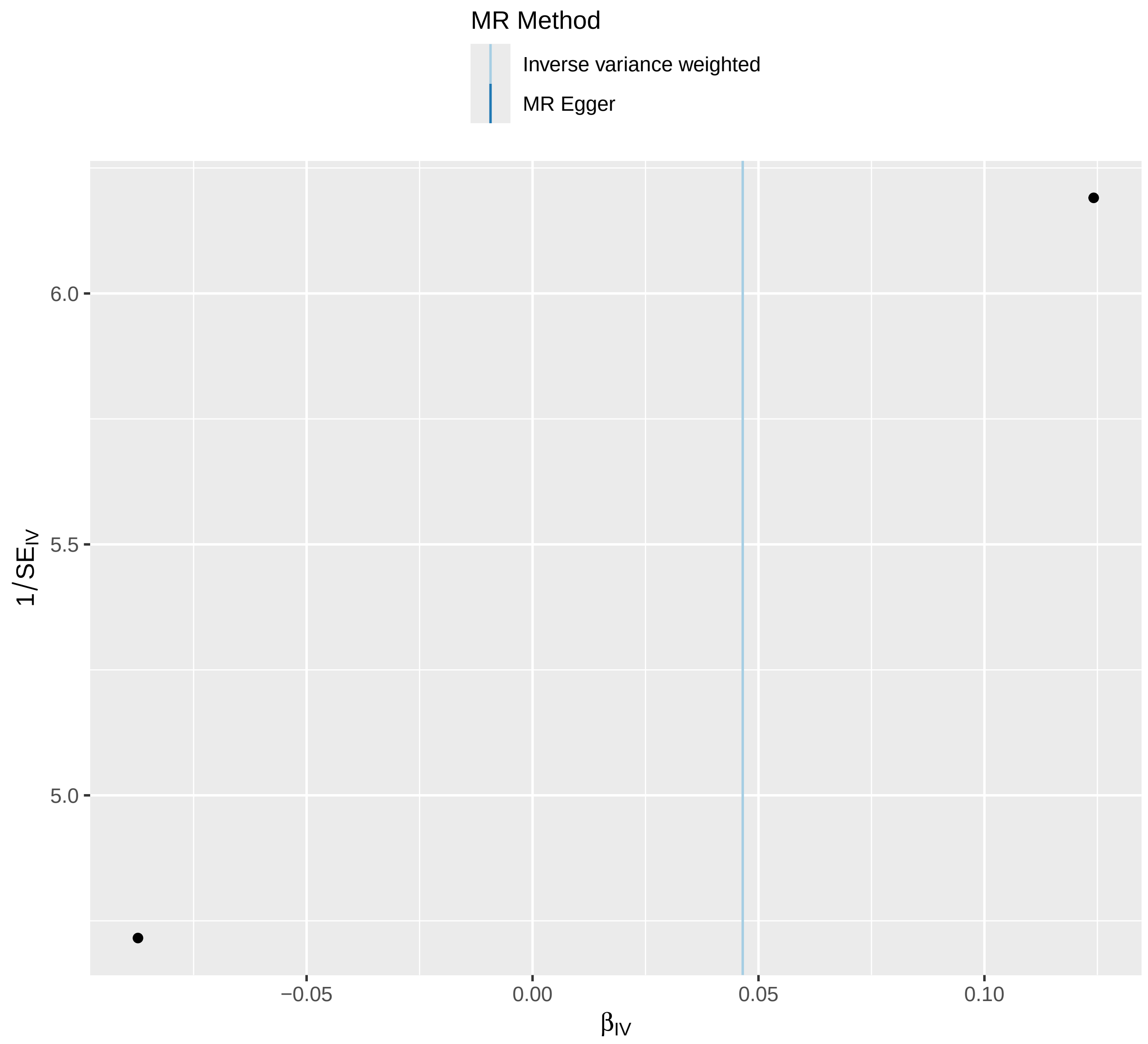
MR Method
Inverse variance weighted
MR Egger
6.0
5.5
5.0
1/SE_IV
−0.05
0.00
0.05
0.10
β_IV

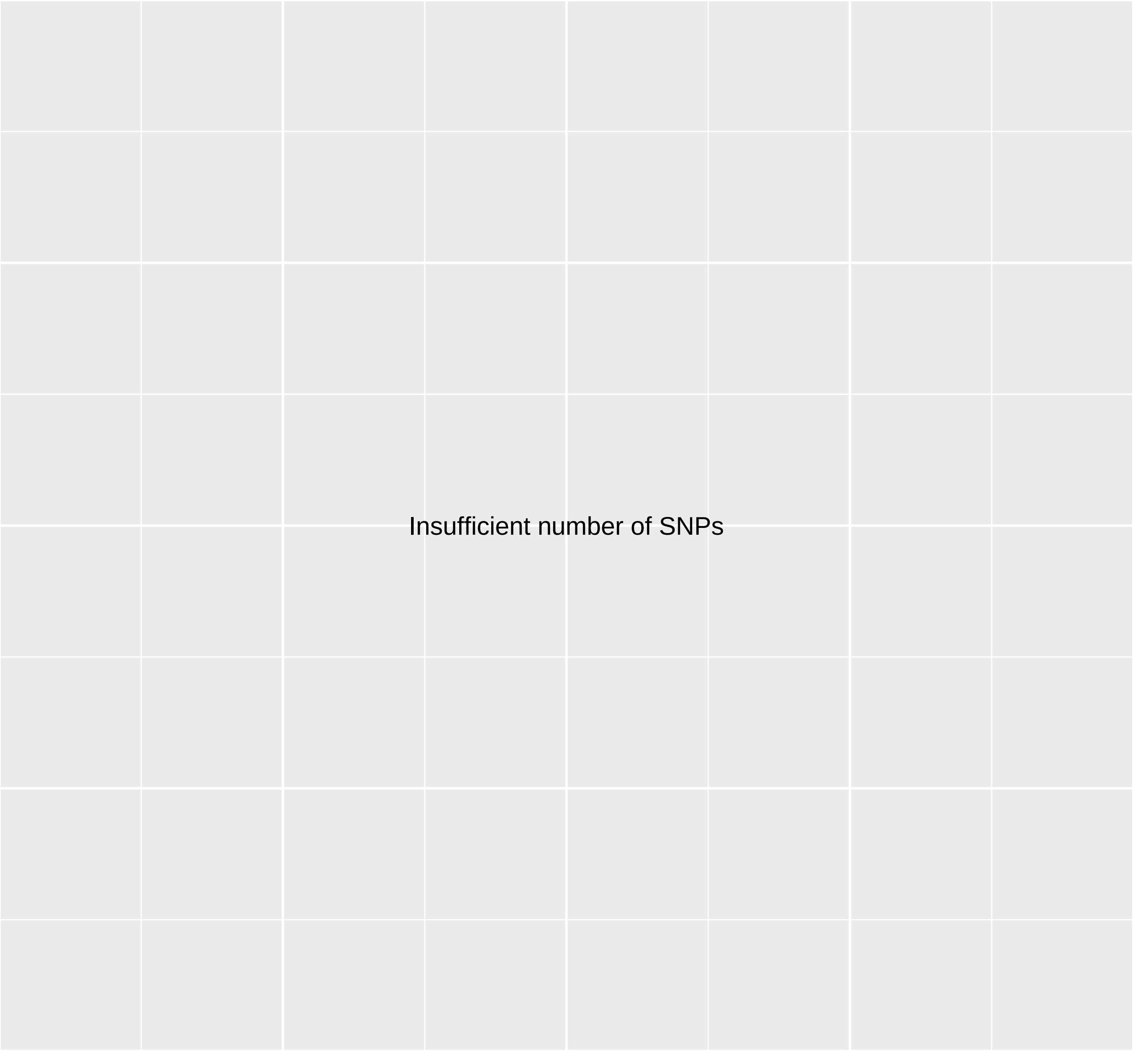
Insufficient number of SNPs

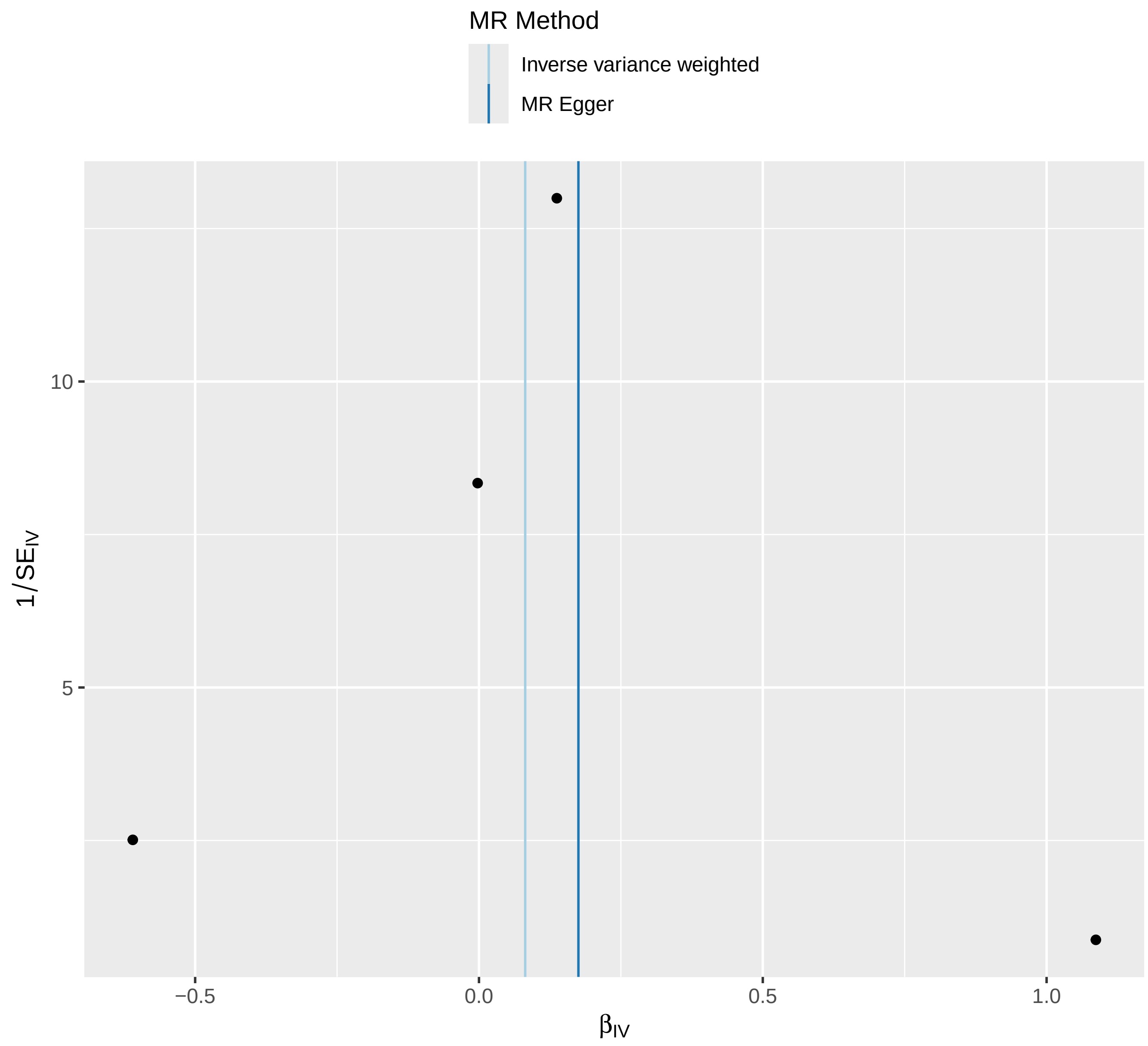

MR Method
Inverse variance weighted
MR Egger
10
5
1/SE$_{IV}$
−0.5
0.0
0.5
1.0
β$_{IV}$

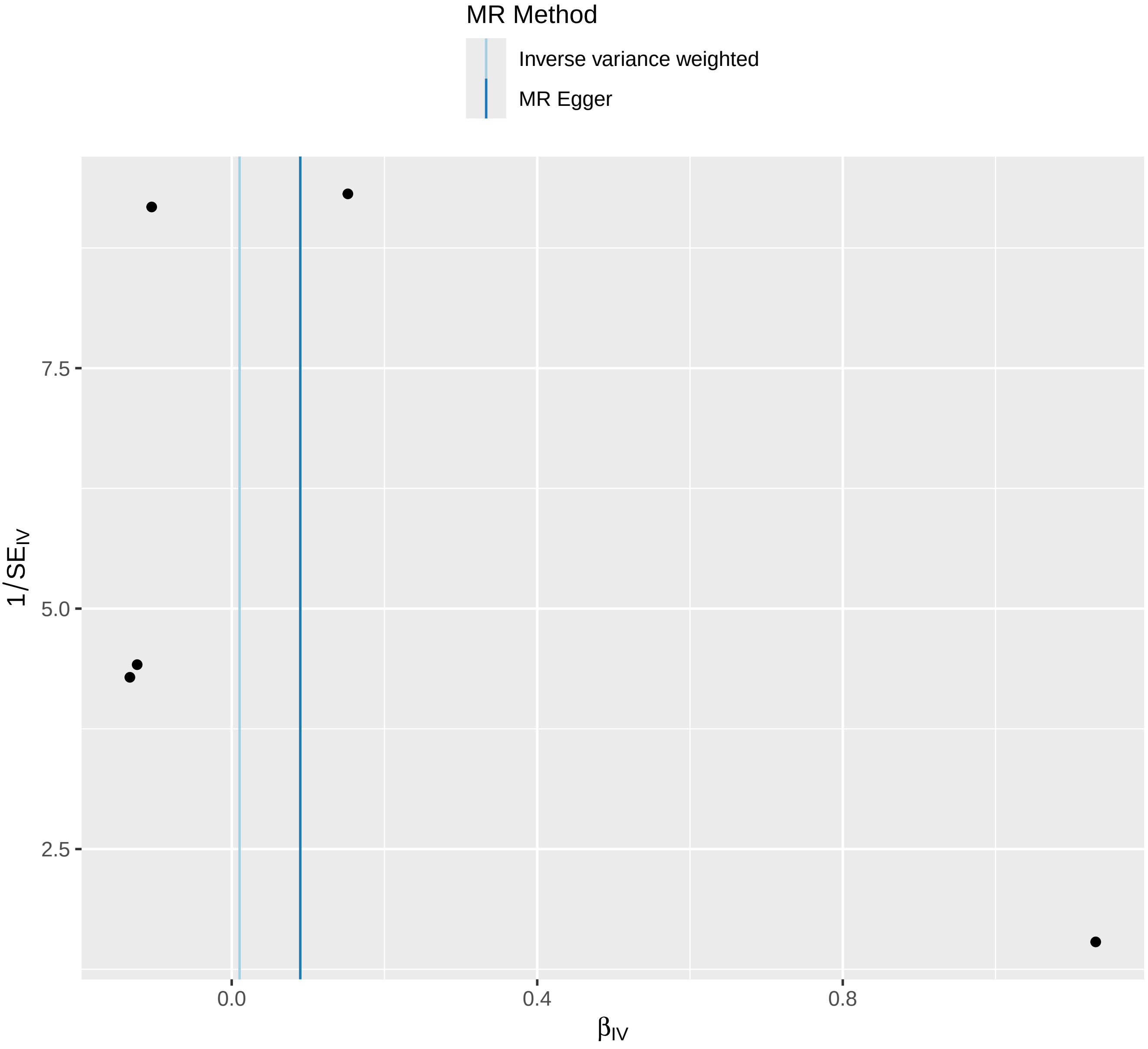
MR Method
Inverse variance weighted
MR Egger
7.5
5.0
2.5
1/SE_IV
0.0
0.4
0.8
β_IV

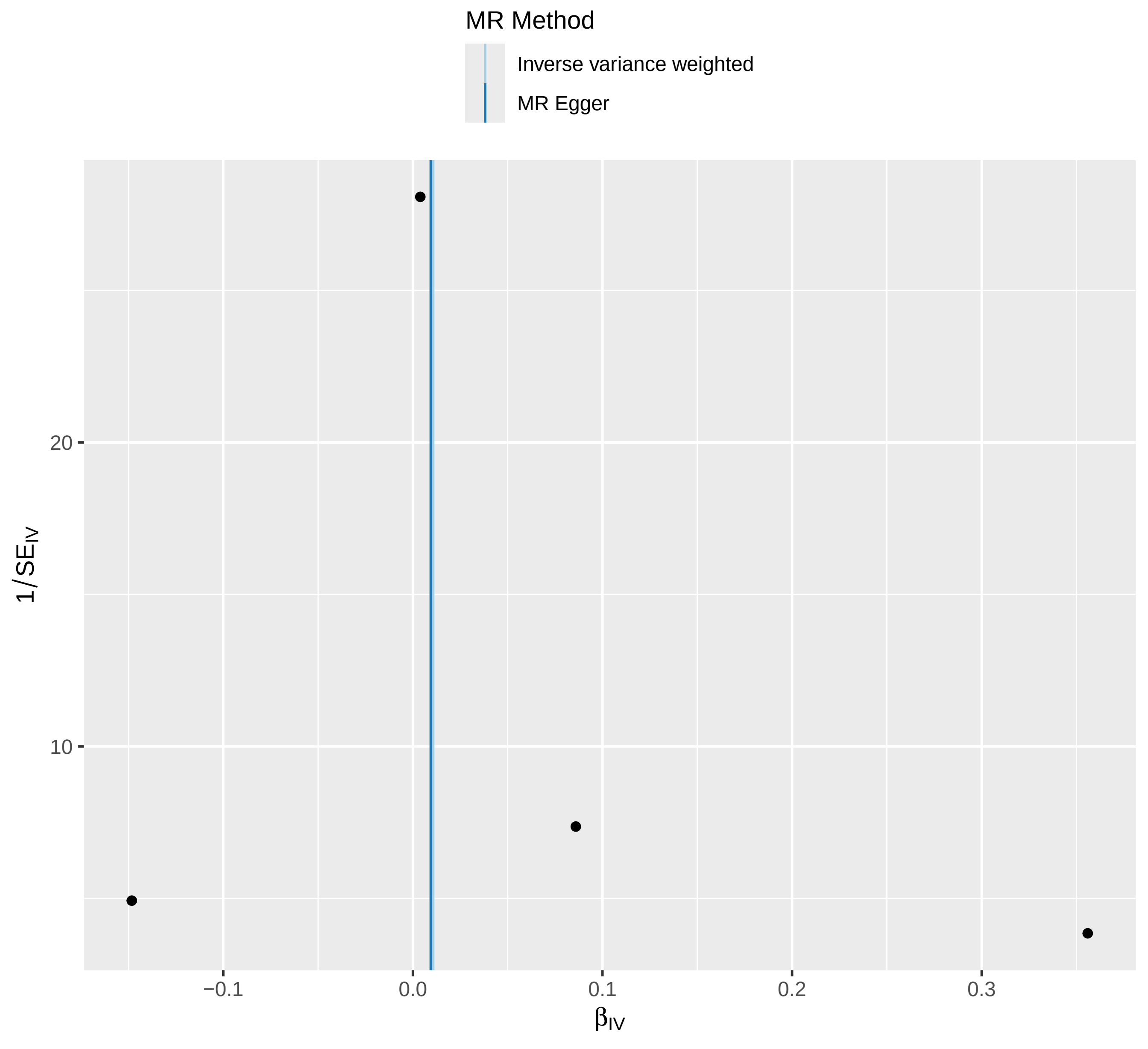
MR Method
Inverse variance weighted
MR Egger
20
10
1/SE$_{IV}$
−0.1
0.0
0.1
0.2
0.3
β$_{IV}$

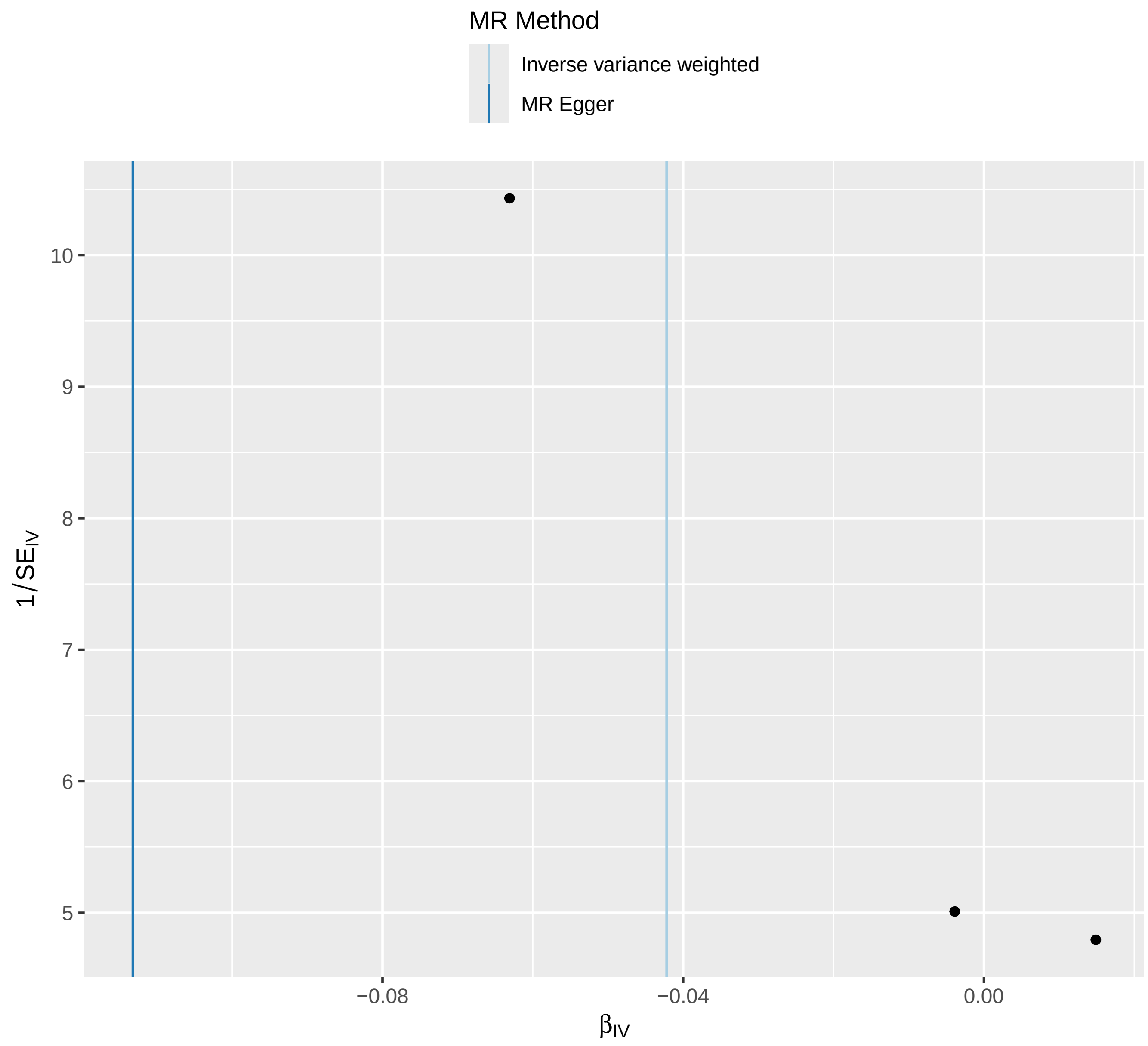
MR Method
Inverse variance weighted
MR Egger
1/$SE_{IV}$
10
9
8
7
6
5
−0.08
−0.04
0.00
$\beta_{IV}$

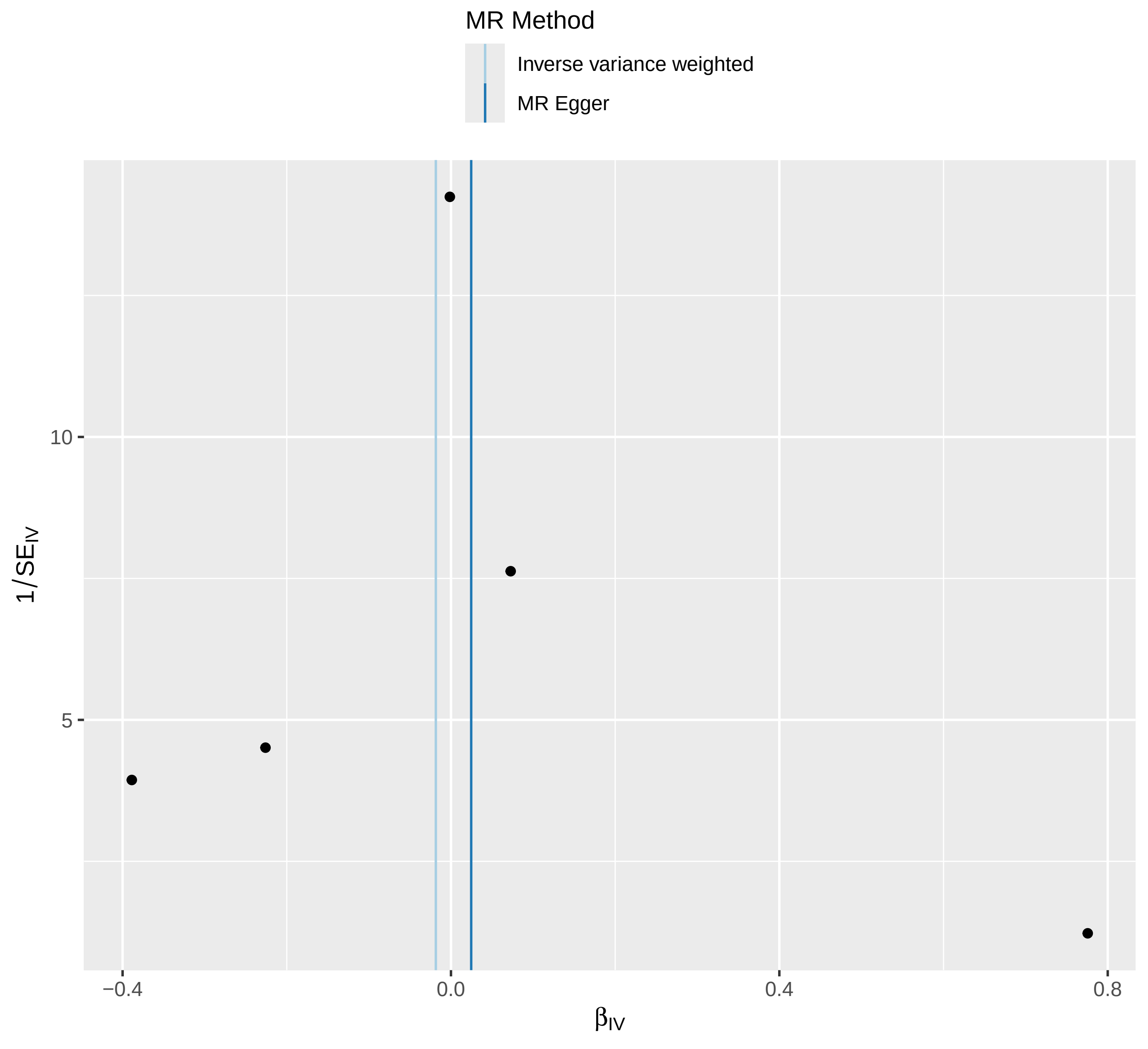

MR Method
Inverse variance weighted
MR Egger
10
5
1/SE$_{IV}$
−0.4
0.0
0.4
0.8
β$_{IV}$

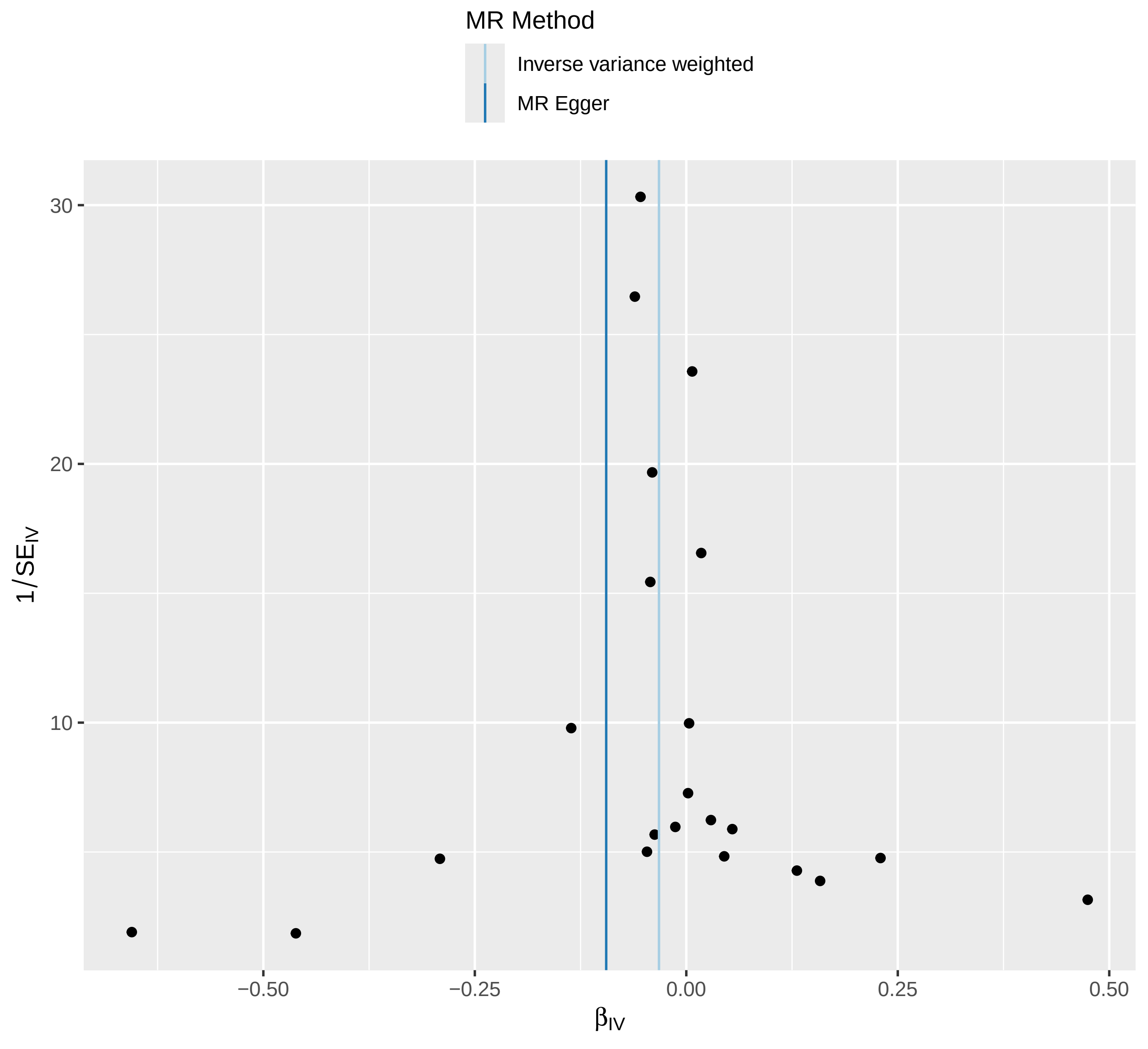

MR Method
Inverse variance weighted
MR Egger
30
20
10
1/SE$_{IV}$
−0.50
−0.25
0.00
0.25
0.50
β$_{IV}$

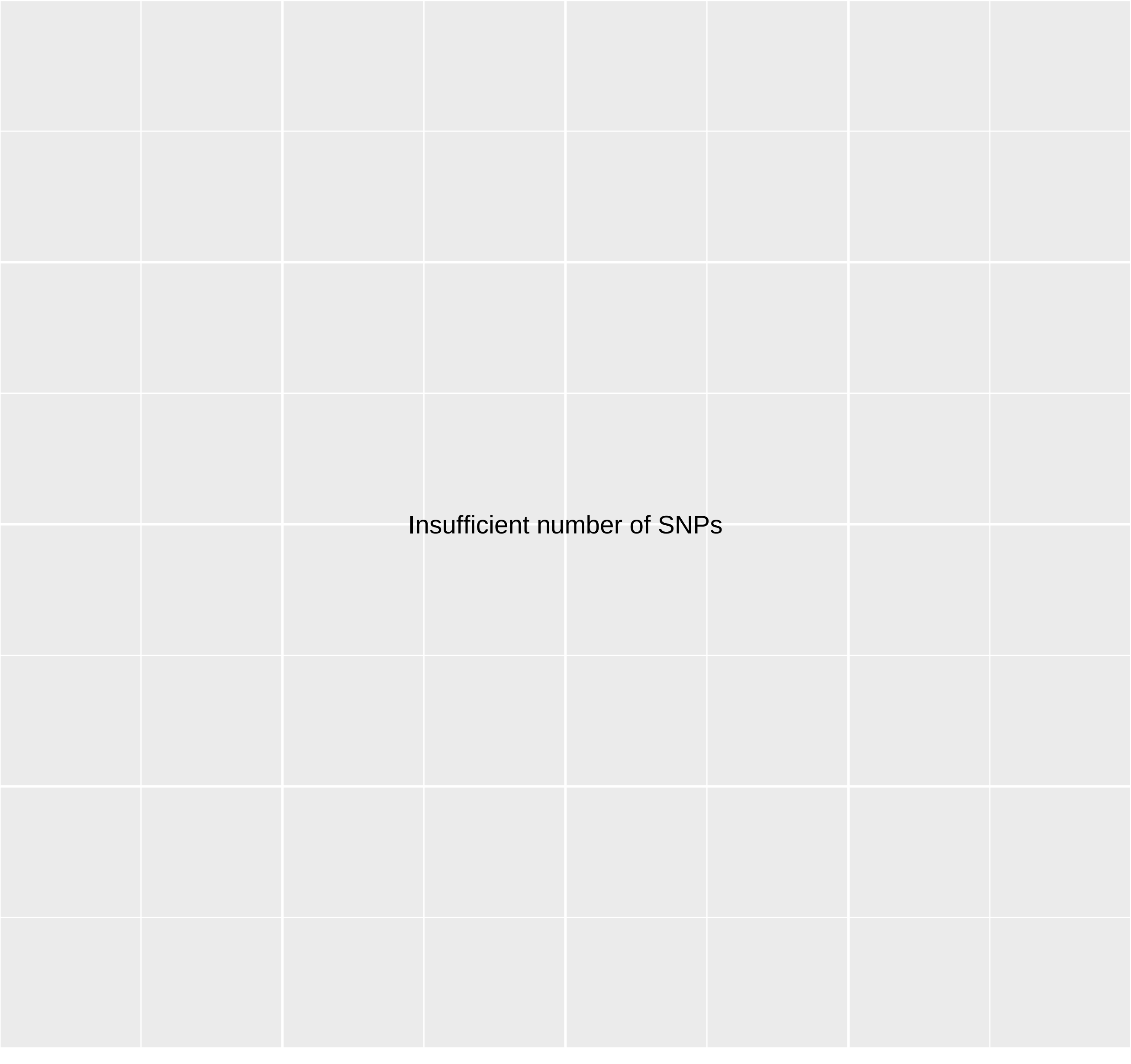
Insufficient number of SNPs

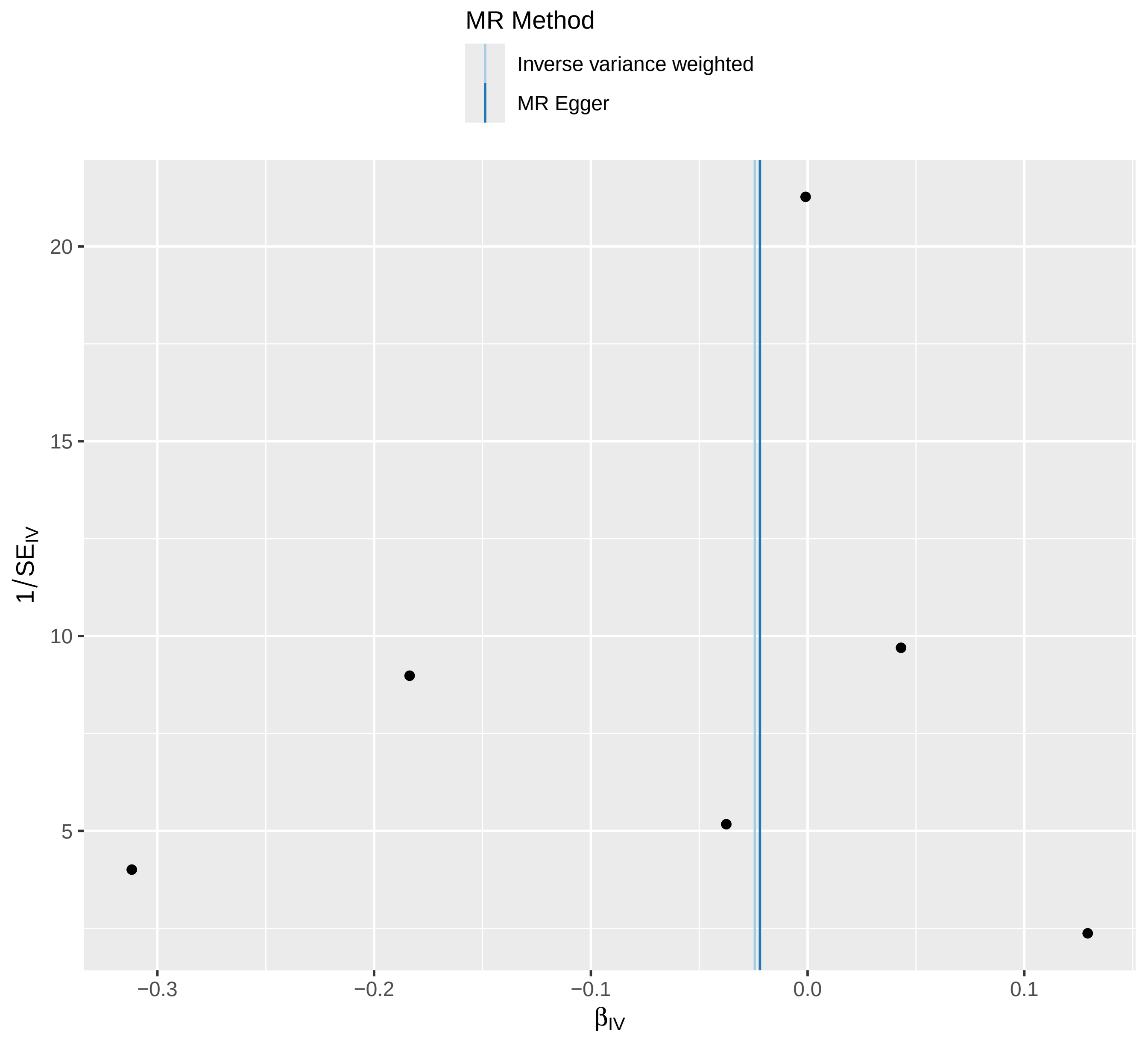

MR Method
Inverse variance weighted
MR Egger
20
15
10
5
1/SE_IV
−0.3
−0.2
−0.1
0.0
0.1
β_IV

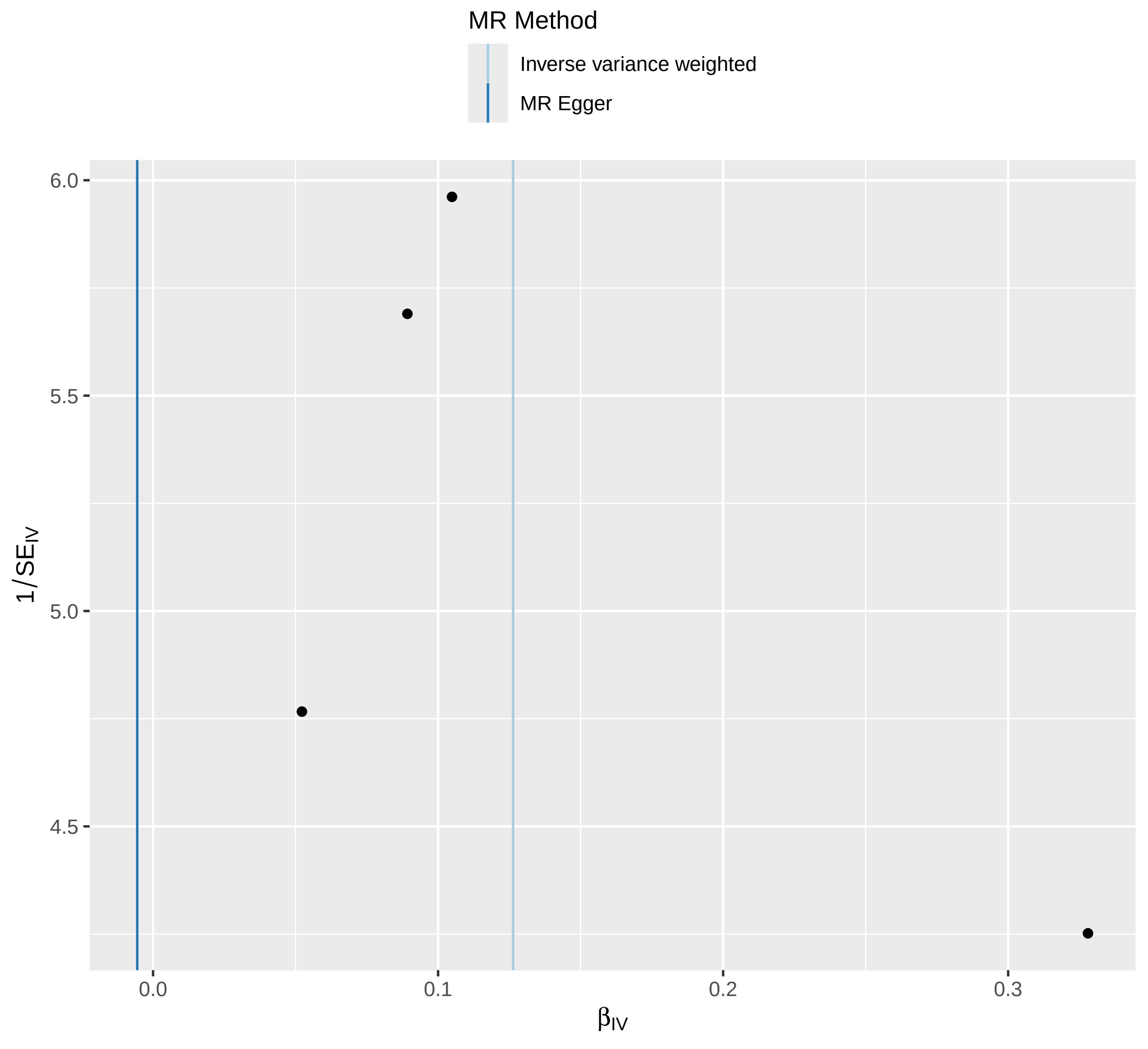
MR Method
Inverse variance weighted
MR Egger
6.0
5.5
5.0
4.5
1/SE_IV
0.0
0.1
0.2
0.3
β_IV

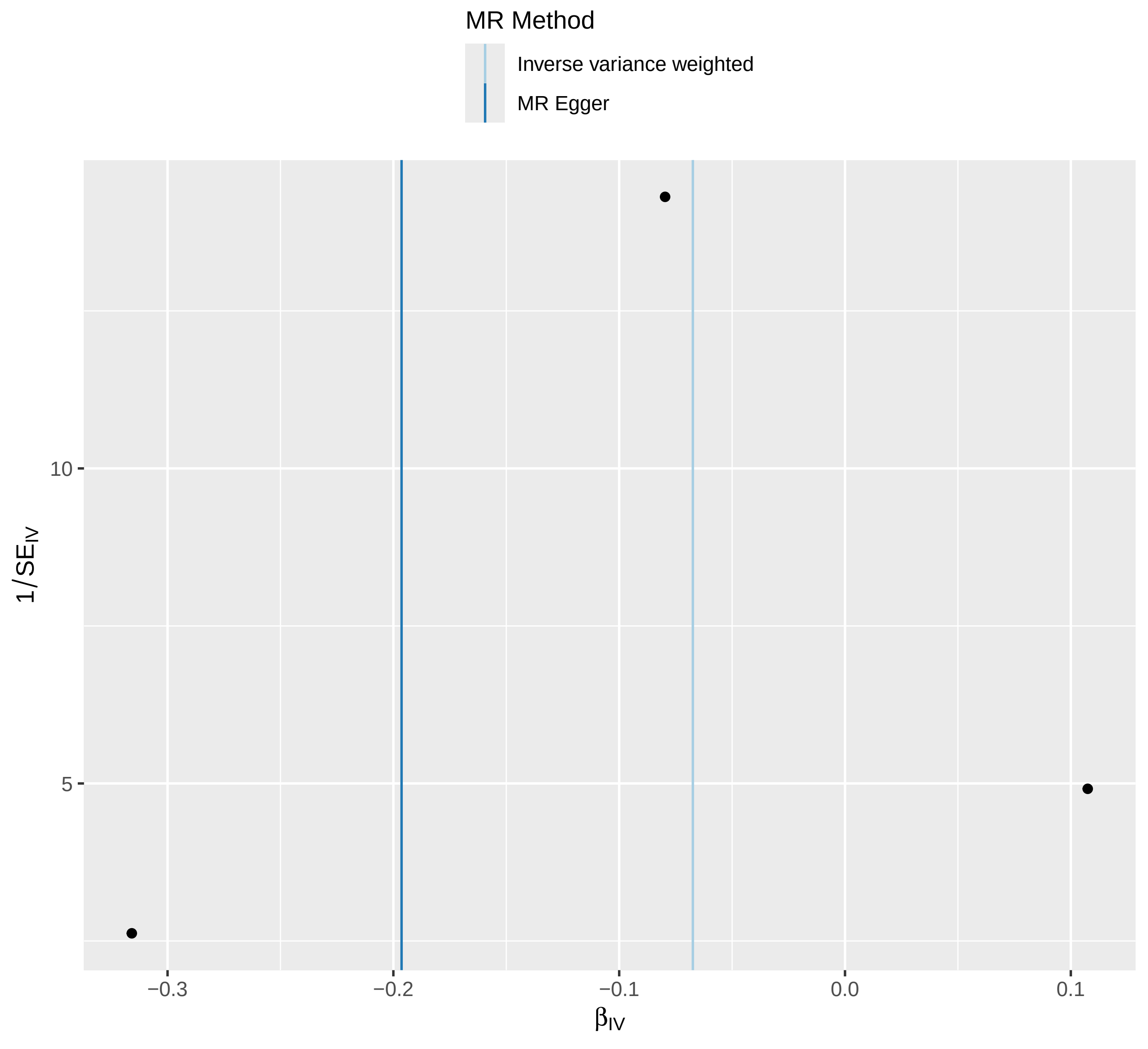

MR Method
Inverse variance weighted
MR Egger
10
5
1/SE_IV
−0.3
−0.2
−0.1
0.0
0.1
β_IV

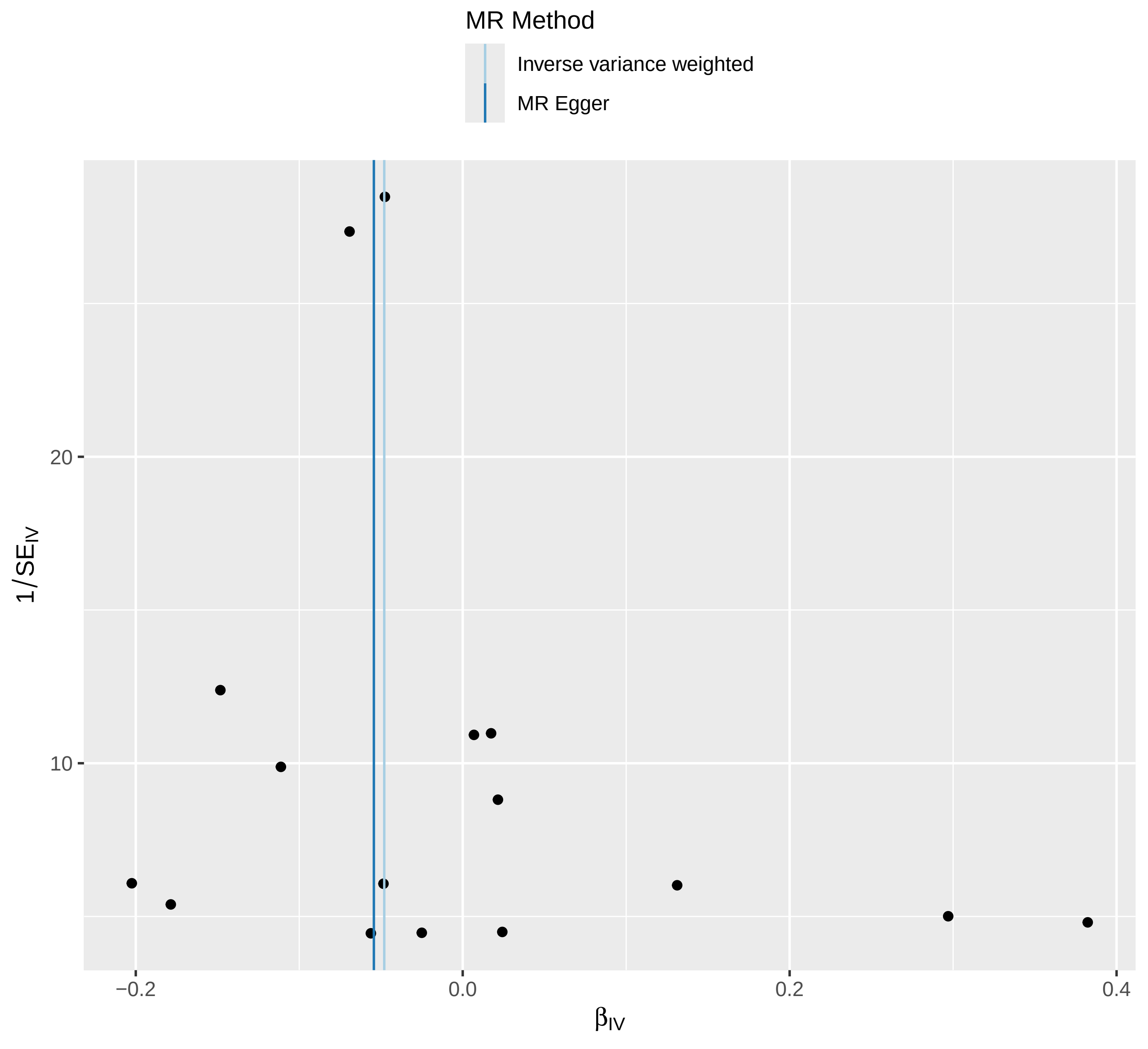
MR Method
Inverse variance weighted
MR Egger
20
10
1/SE$_{IV}$
−0.2
0.0
0.2
0.4
β$_{IV}$

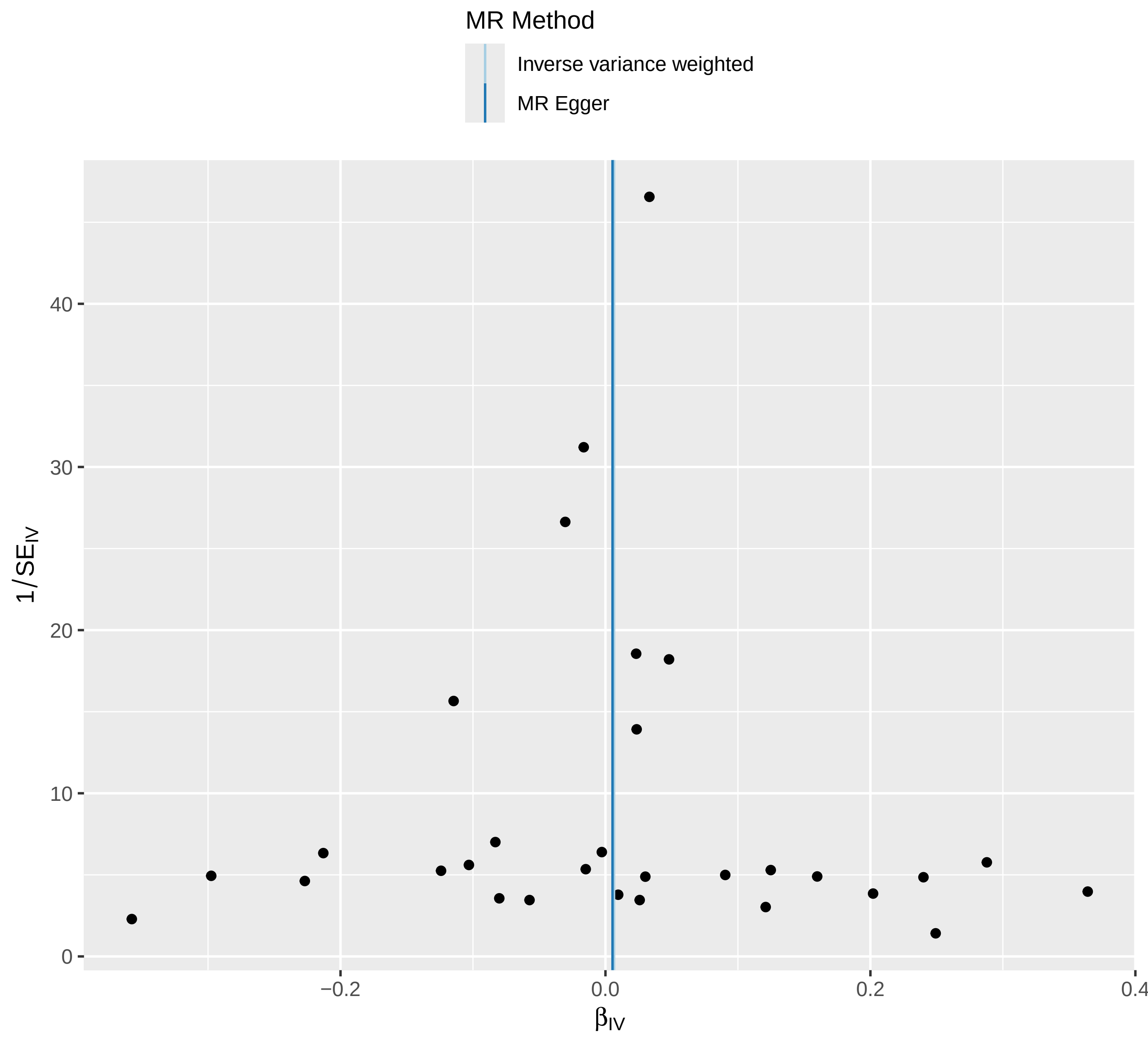

MR Method
Inverse variance weighted
MR Egger
40
30
20
10
0
1/SE$_{IV}$
−0.2
0.0
0.2
0.4
β$_{IV}$

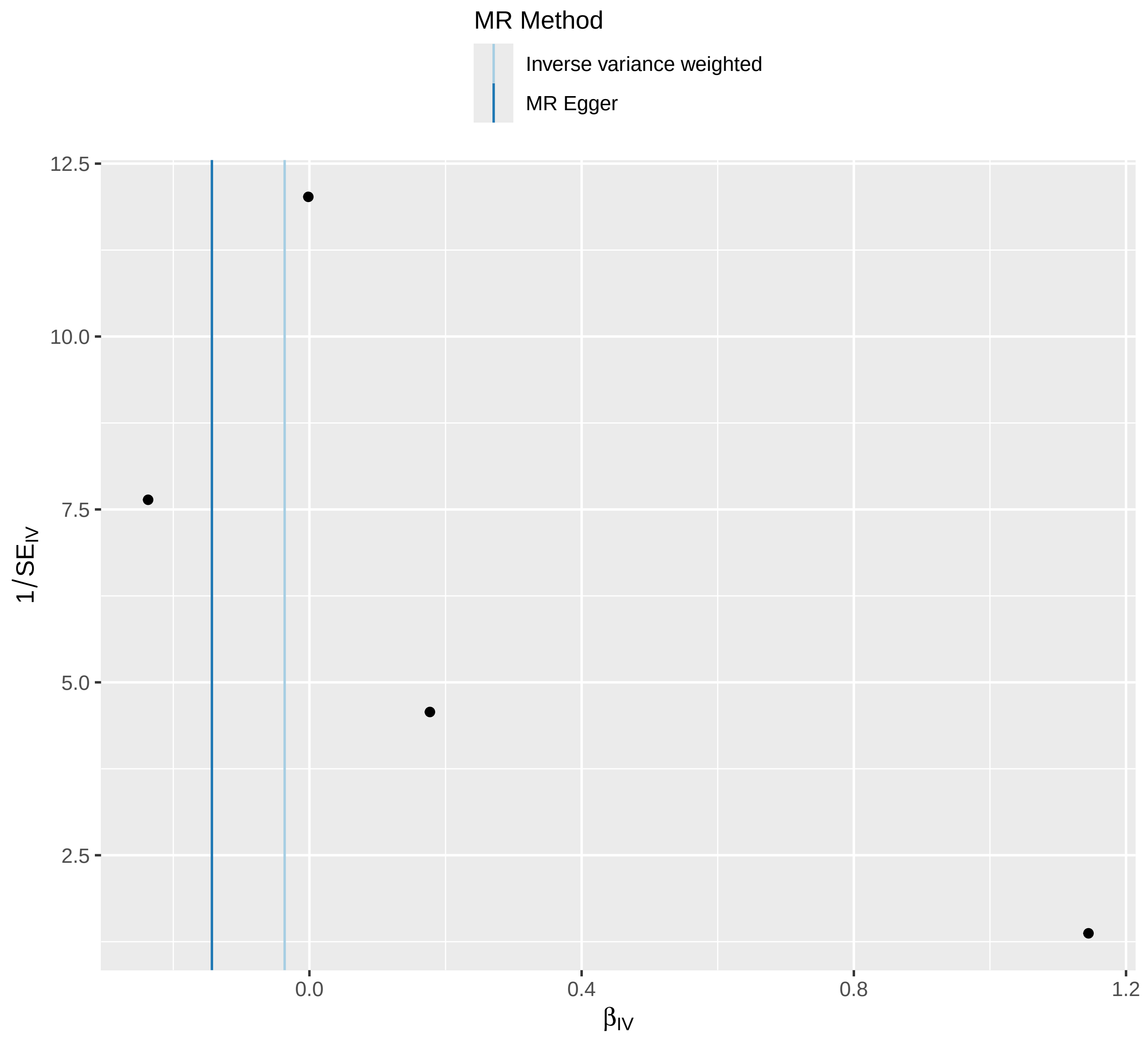

MR Method
Inverse variance weighted
MR Egger
12.5
10.0
7.5
5.0
2.5
1/SE_IV
0.0
0.4
0.8
1.2
β_IV

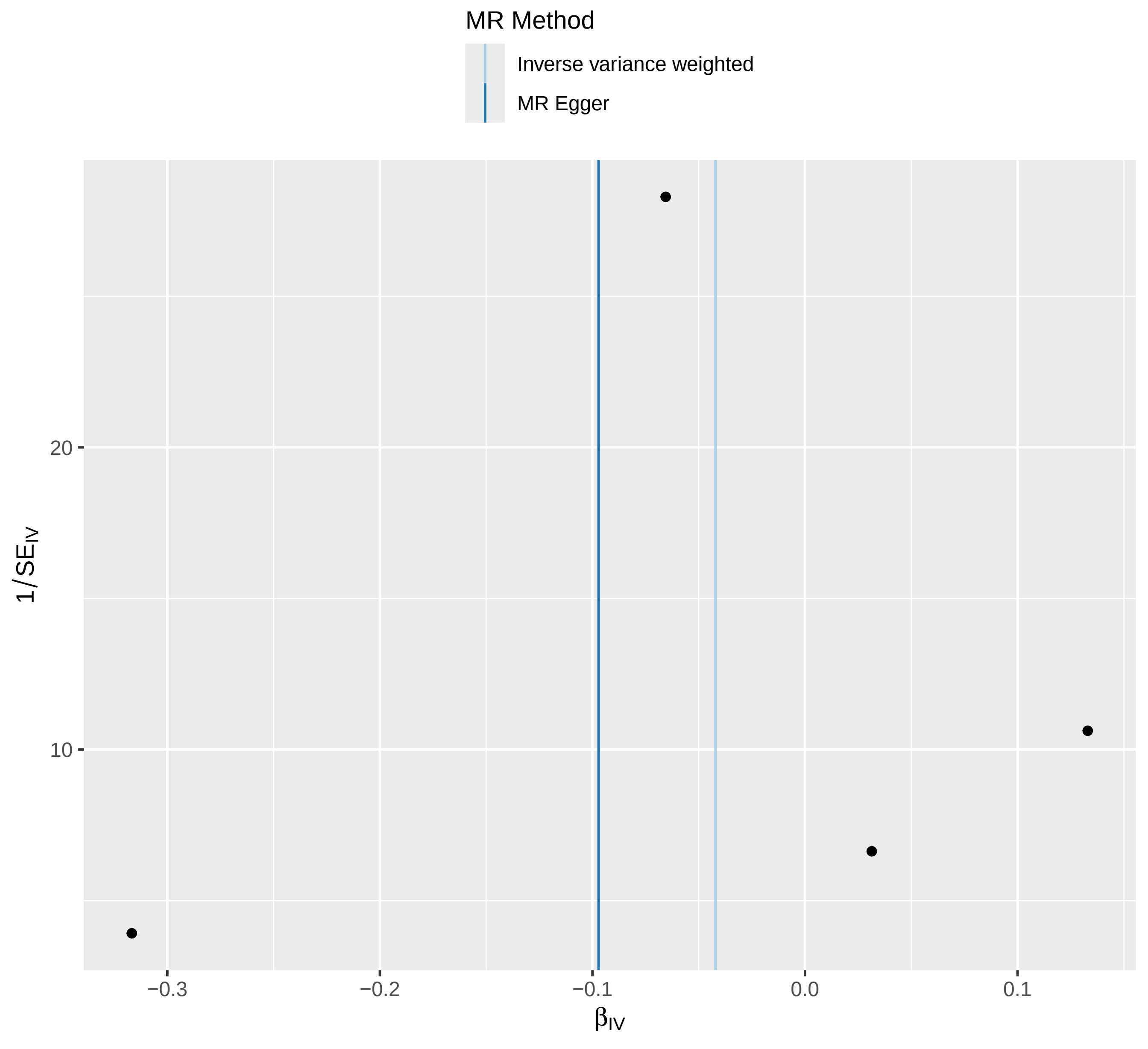

MR Method
Inverse variance weighted
MR Egger
20
10
$1/SE_{IV}$
−0.3
−0.2
−0.1
0.0
0.1
$\beta_{IV}$

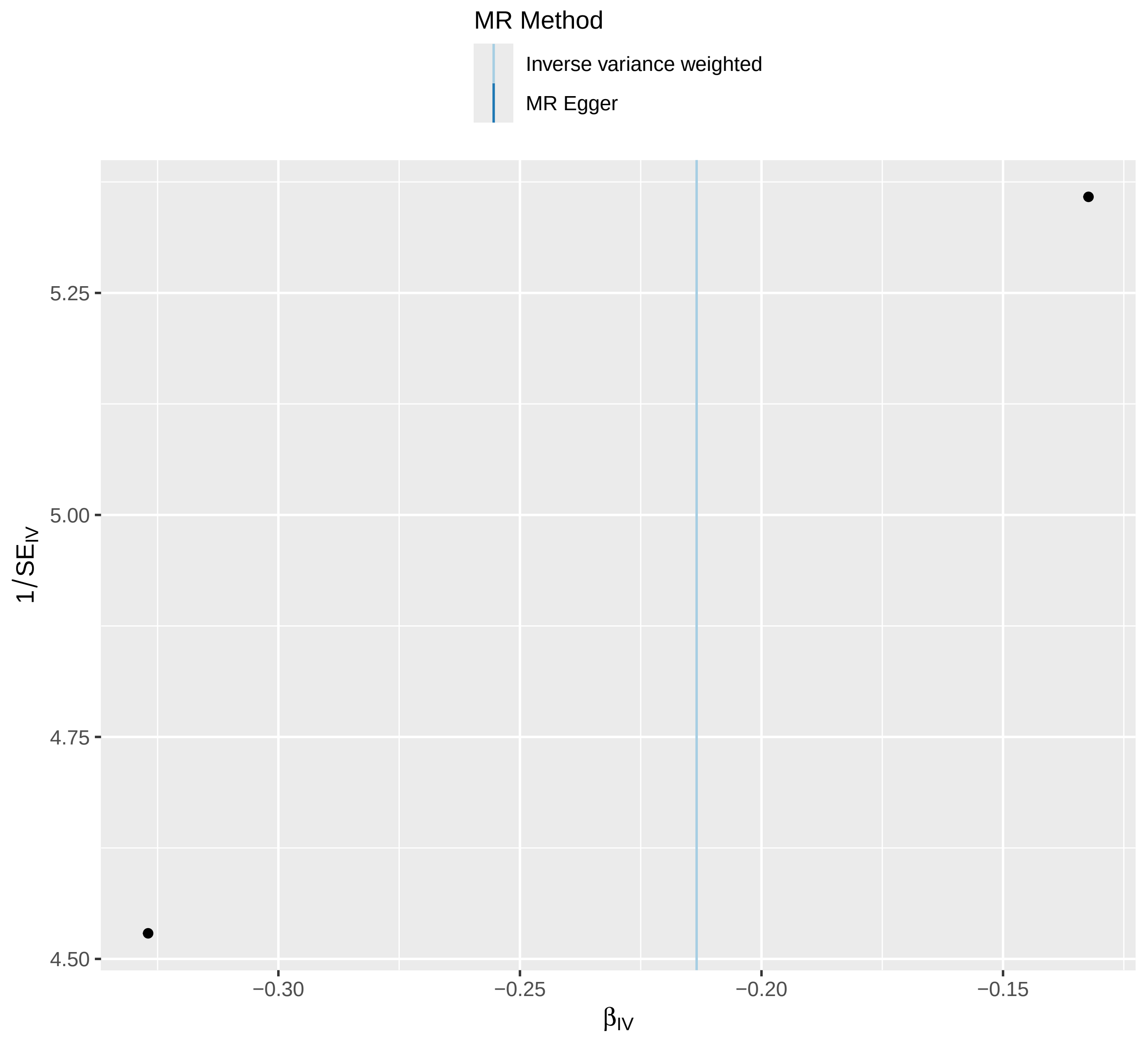

MR Method
Inverse variance weighted
MR Egger
5.25
5.00
4.75
4.50
$1/SE_{IV}$
−0.30
−0.25
−0.20
−0.15
$\beta_{IV}$

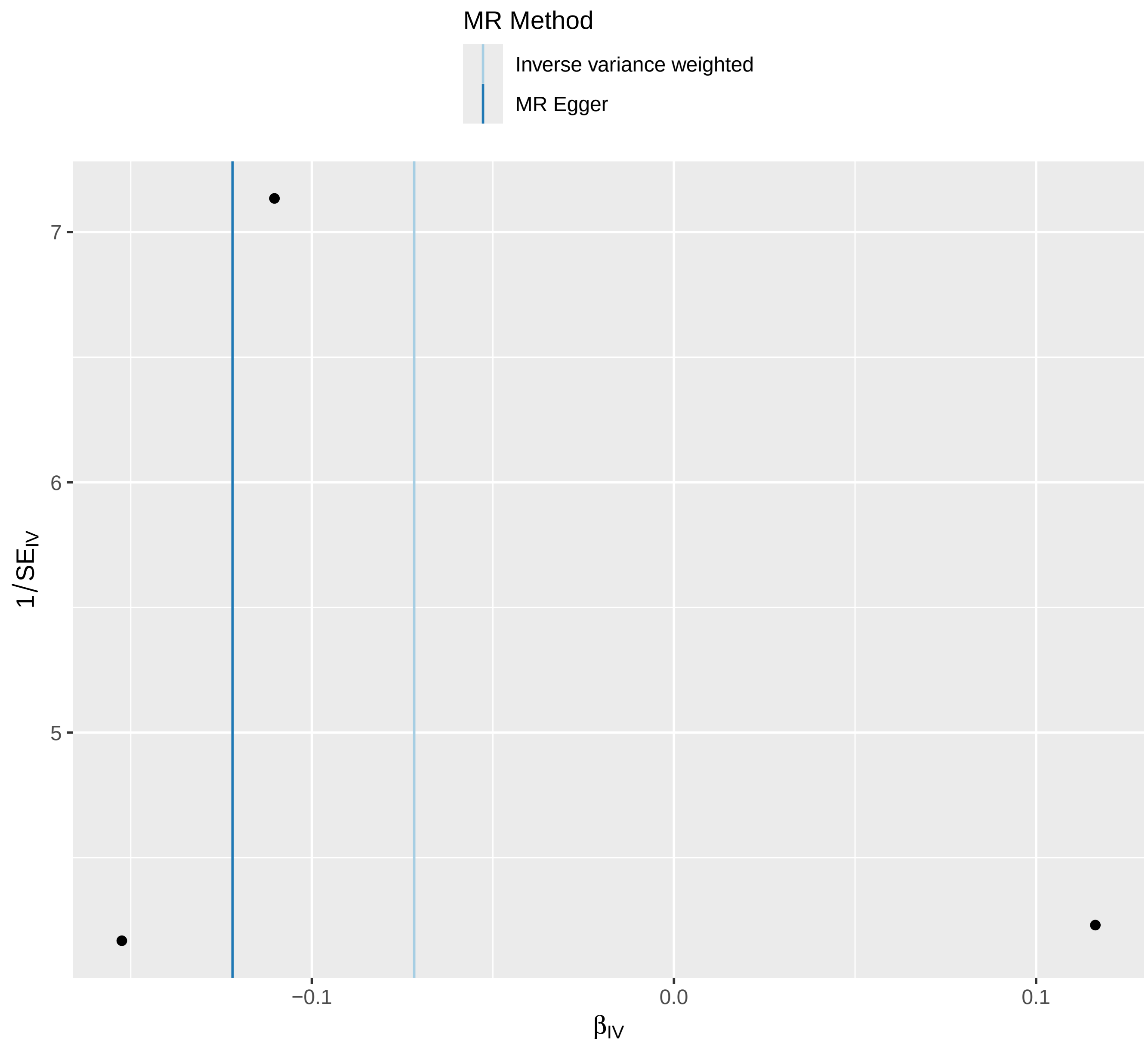

MR Method
Inverse variance weighted
MR Egger
7
6
5
1/SE$_{IV}$
−0.1
0.0
0.1
β$_{IV}$

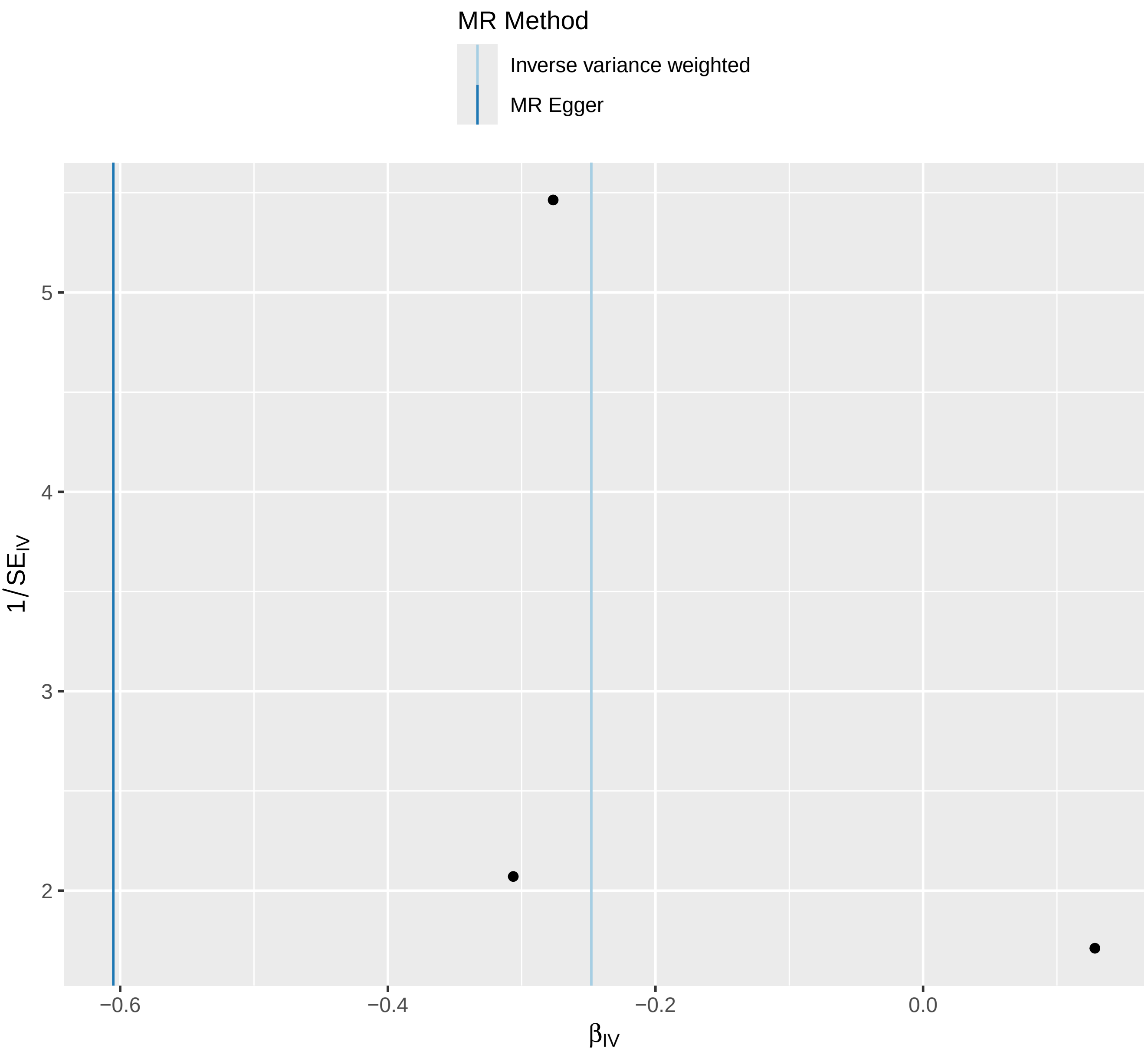

MR Method
Inverse variance weighted
MR Egger
5
4
3
2
1/SE$_{IV}$
−0.6
−0.4
−0.2
0.0
β$_{IV}$

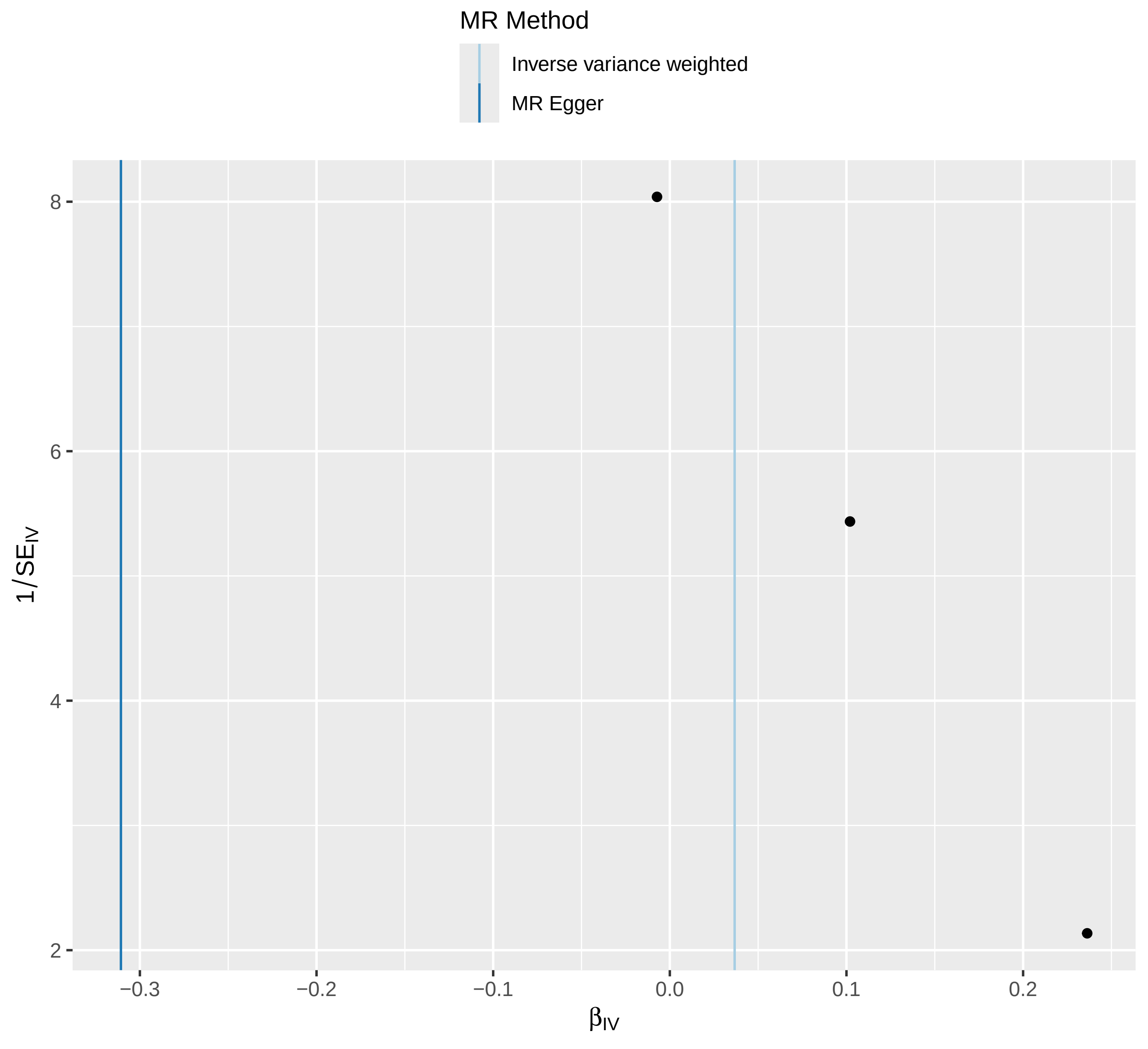
MR Method
Inverse variance weighted
MR Egger
8
6
4
2
1/SE$_{IV}$
−0.3
−0.2
−0.1
0.0
0.1
0.2
β$_{IV}$

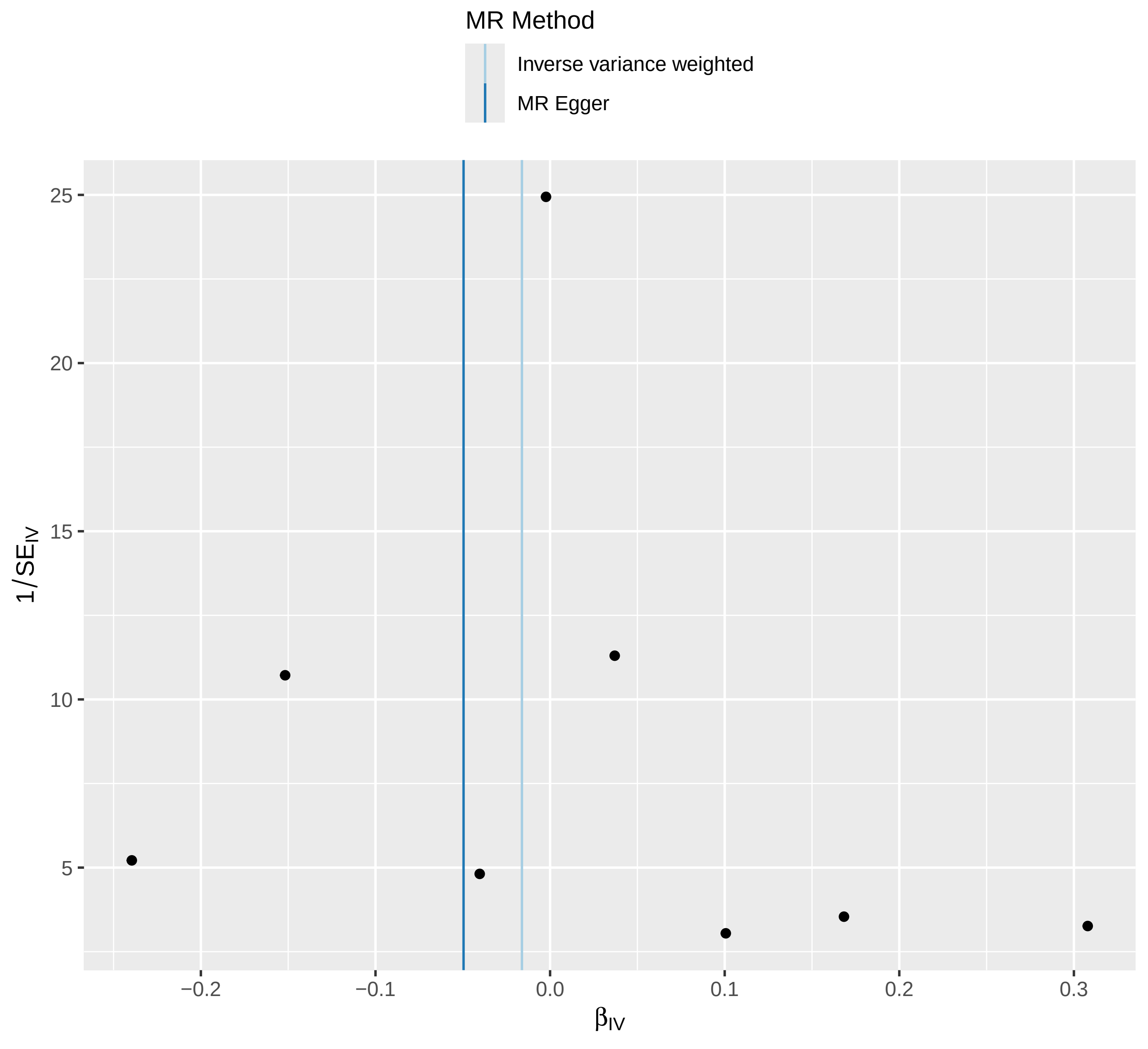

MR Method
Inverse variance weighted
MR Egger
25
20
15
10
5
1/SE_IV
−0.2
−0.1
0.0
0.1
0.2
0.3
β_IV

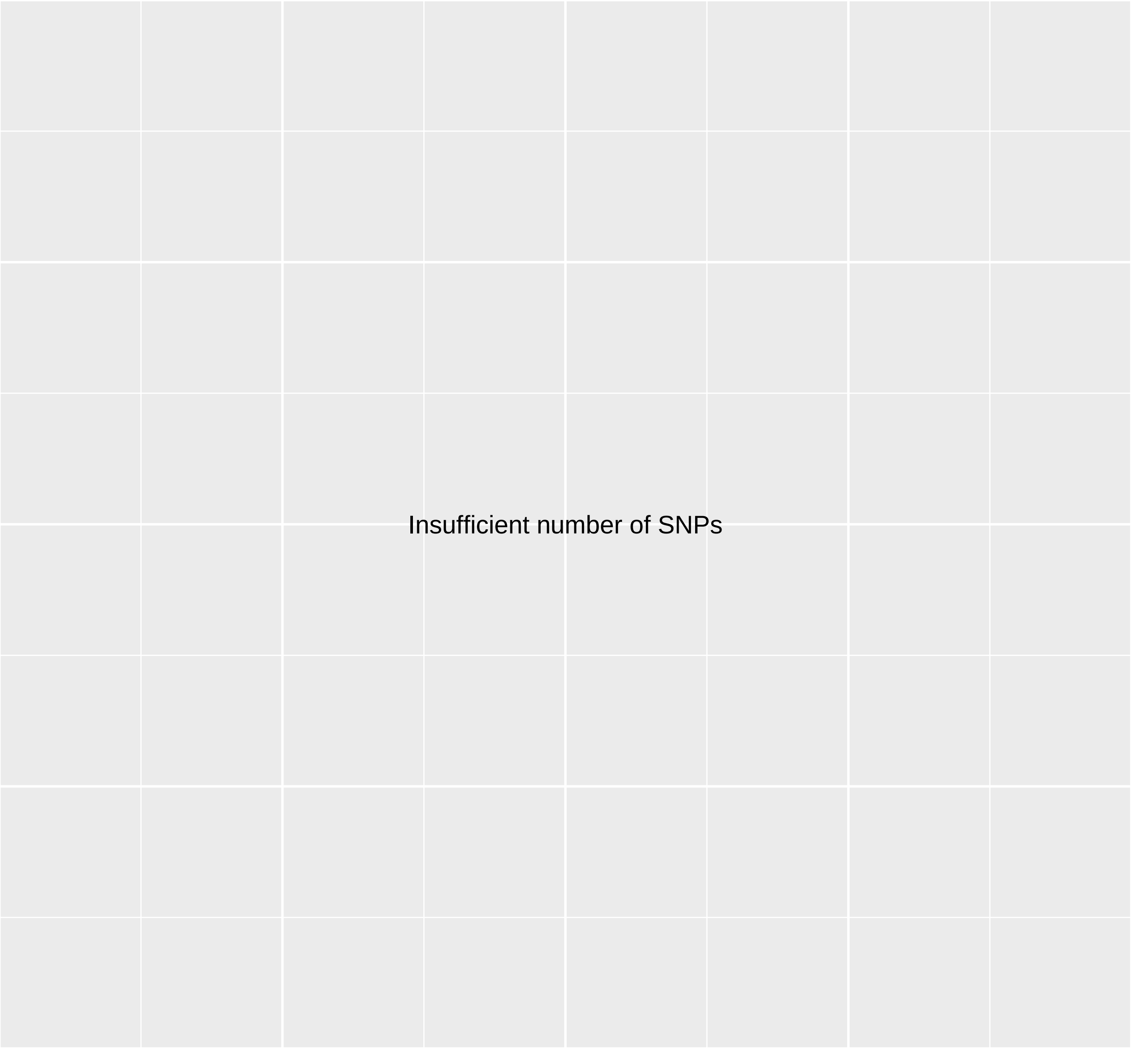
Insufficient number of SNPs

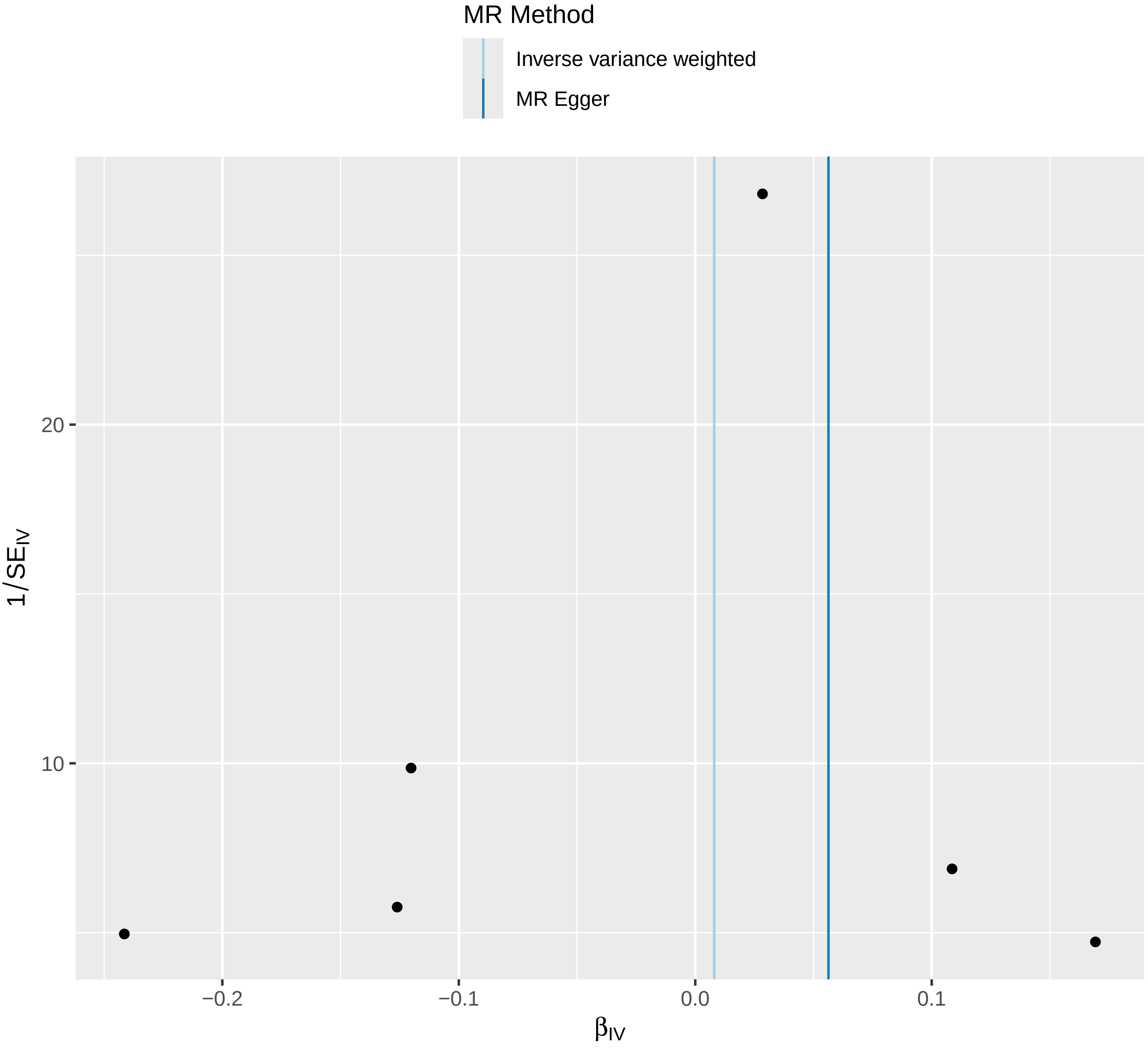
MR Method
Inverse variance weighted
MR Egger
20
10
1/SE_IV
−0.2
−0.1
0.0
0.1
β_IV

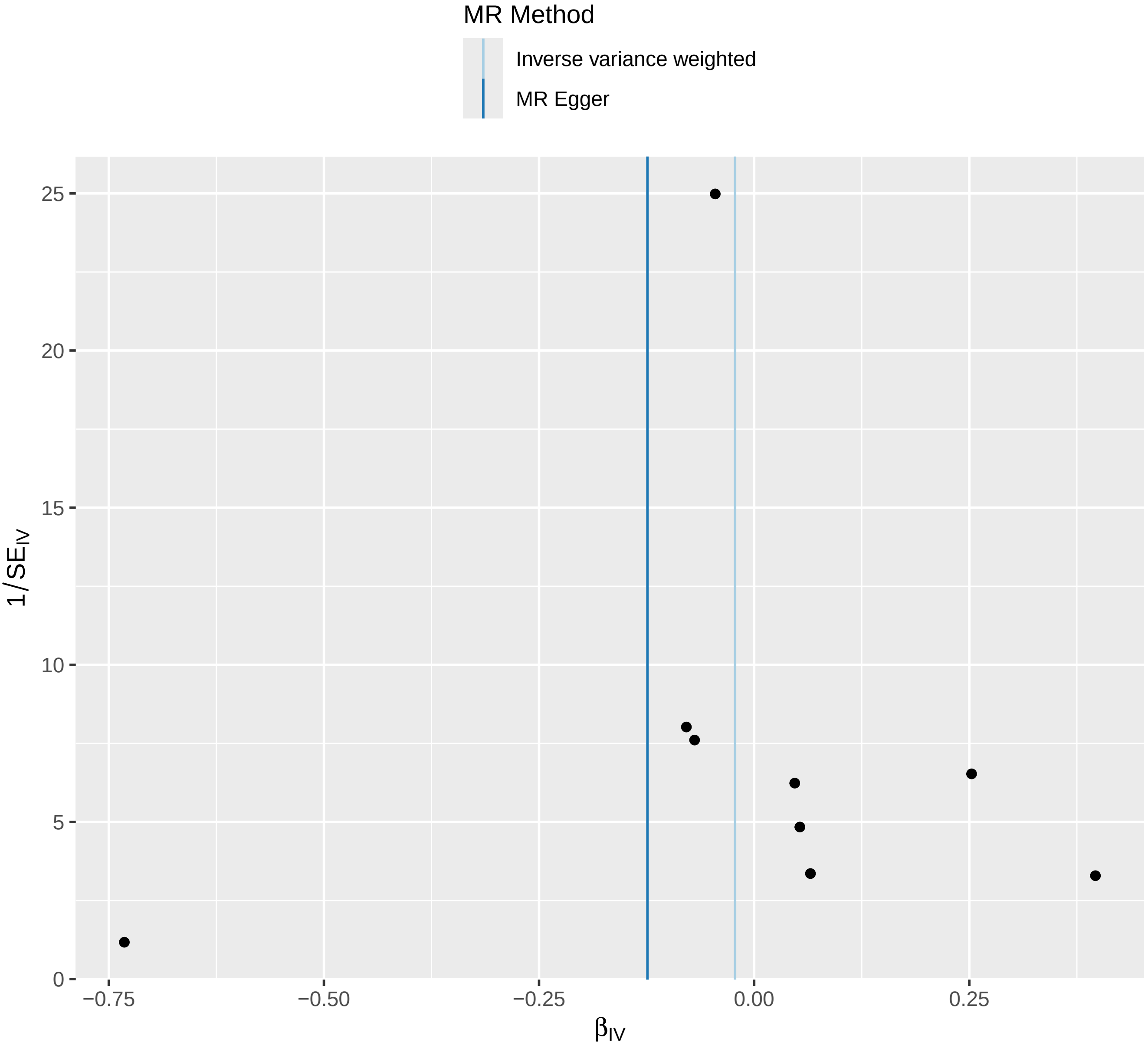
MR Method
Inverse variance weighted
MR Egger
25
20
15
10
5
0
1/SE_IV
−0.75
−0.50
−0.25
0.00
0.25
β_IV

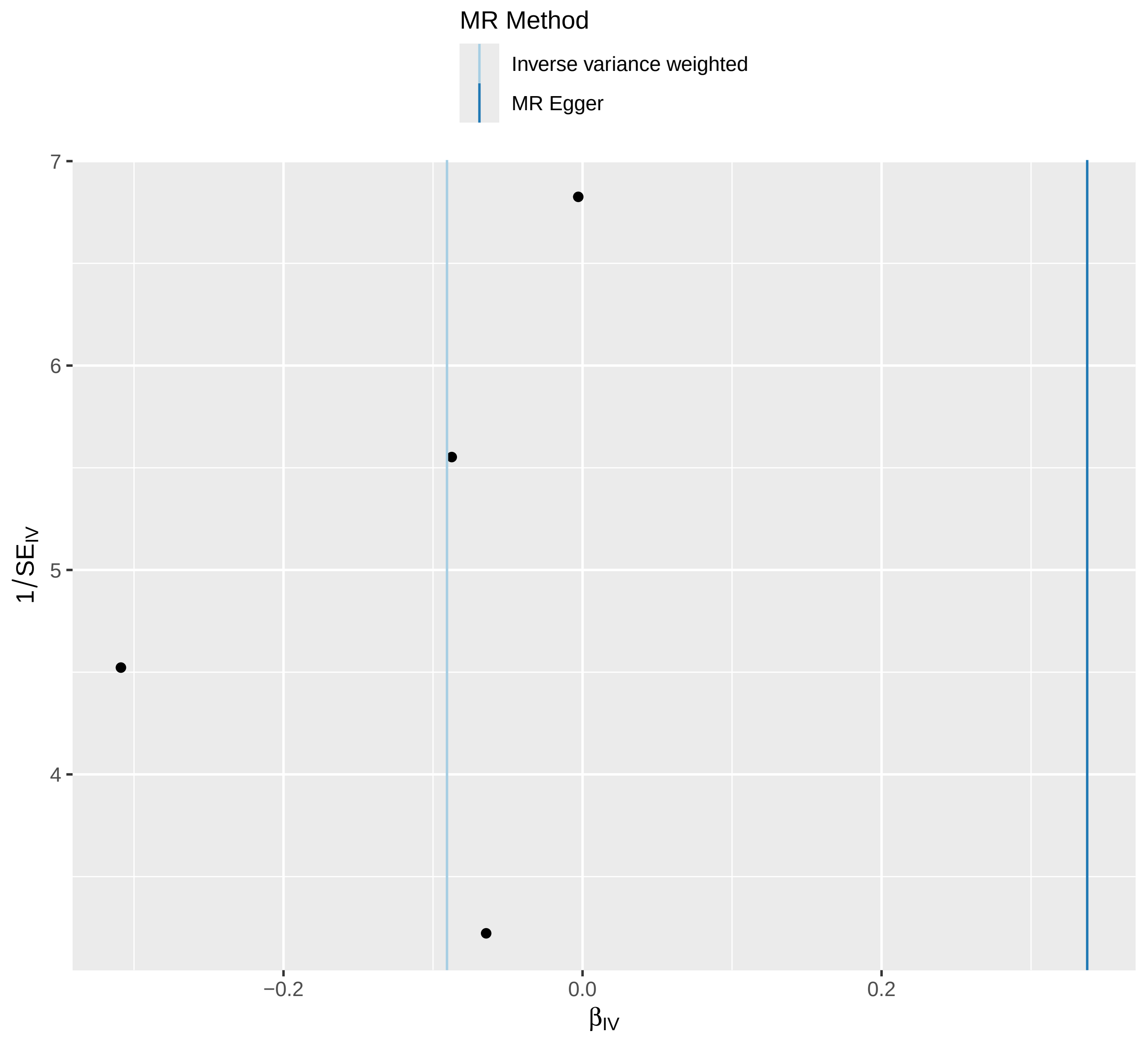
MR Method
Inverse variance weighted
MR Egger
7
6
5
4
1/SE_IV
−0.2
0.0
0.2
β_IV

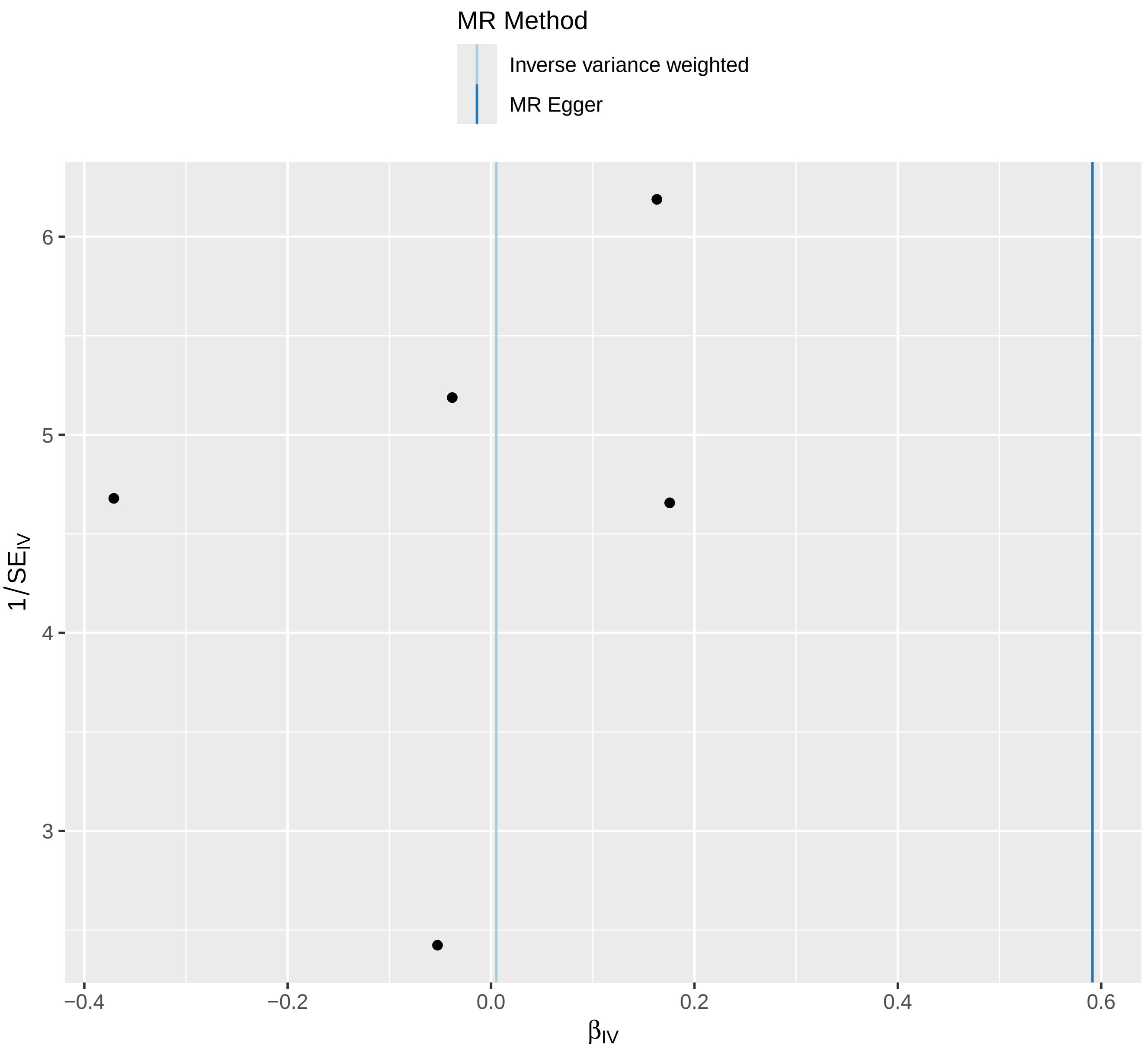

MR Method
Inverse variance weighted
MR Egger
6
5
4
3
1/SE_IV
−0.4
−0.2
0.0
0.2
0.4
0.6
β_IV

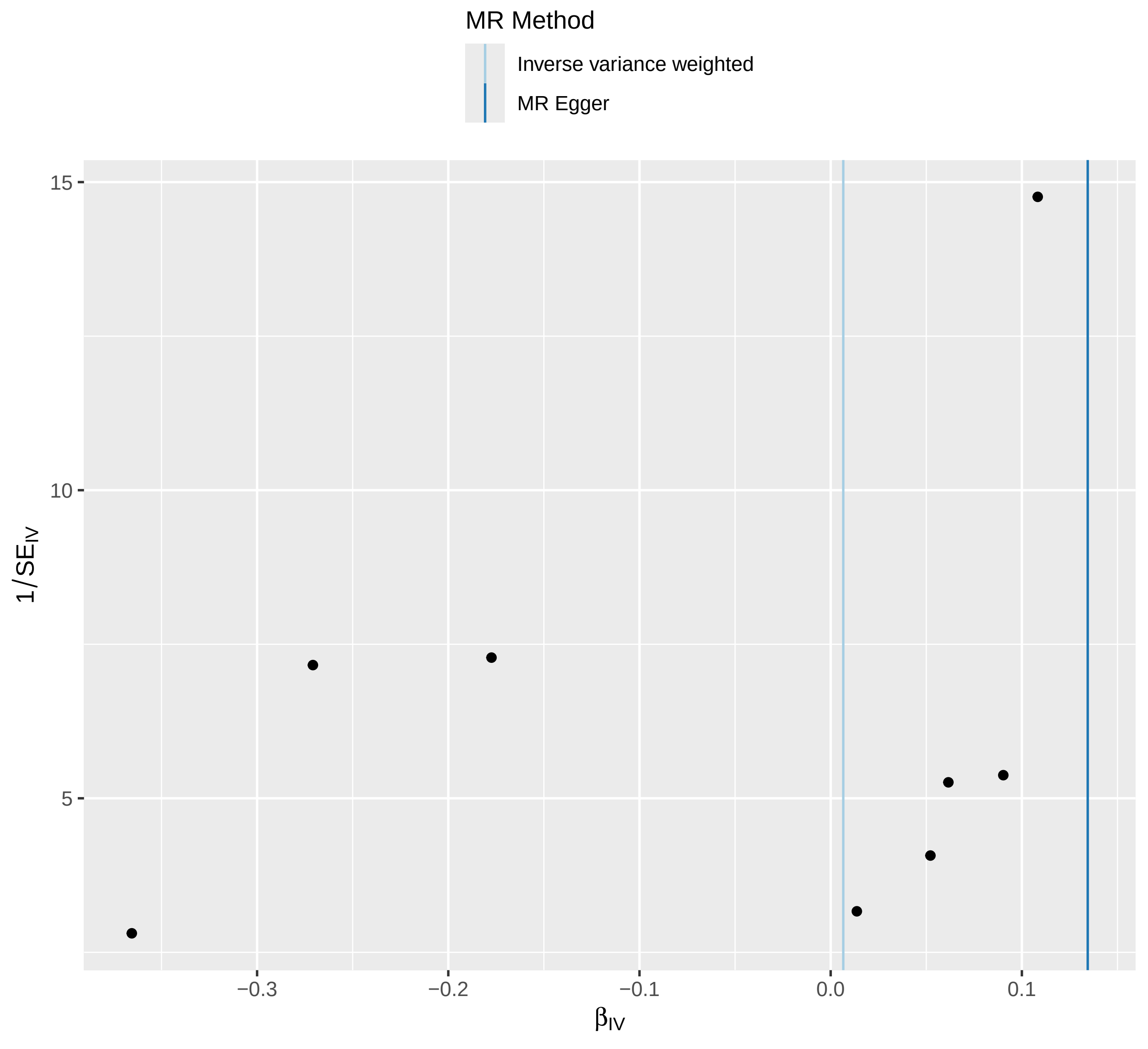

MR Method
Inverse variance weighted
MR Egger
15
10
5
−0.3
−0.2
−0.1
0.0
0.1
$1/SE_{IV}$
$\beta_{IV}$

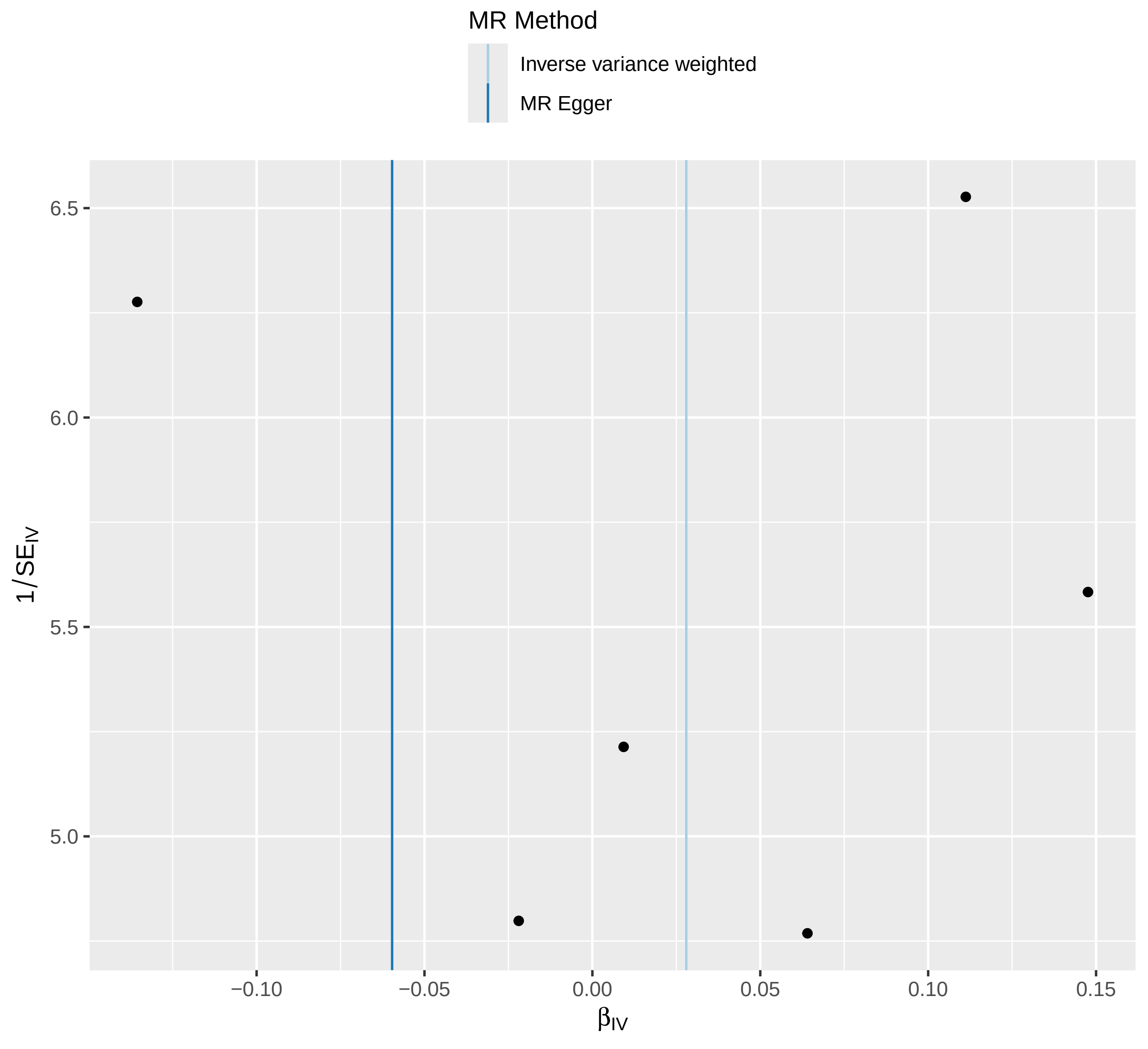
MR Method
Inverse variance weighted
MR Egger
6.5
6.0
5.5
5.0
1/SE$_{IV}$
−0.10
−0.05
0.00
0.05
0.10
0.15
β$_{IV}$

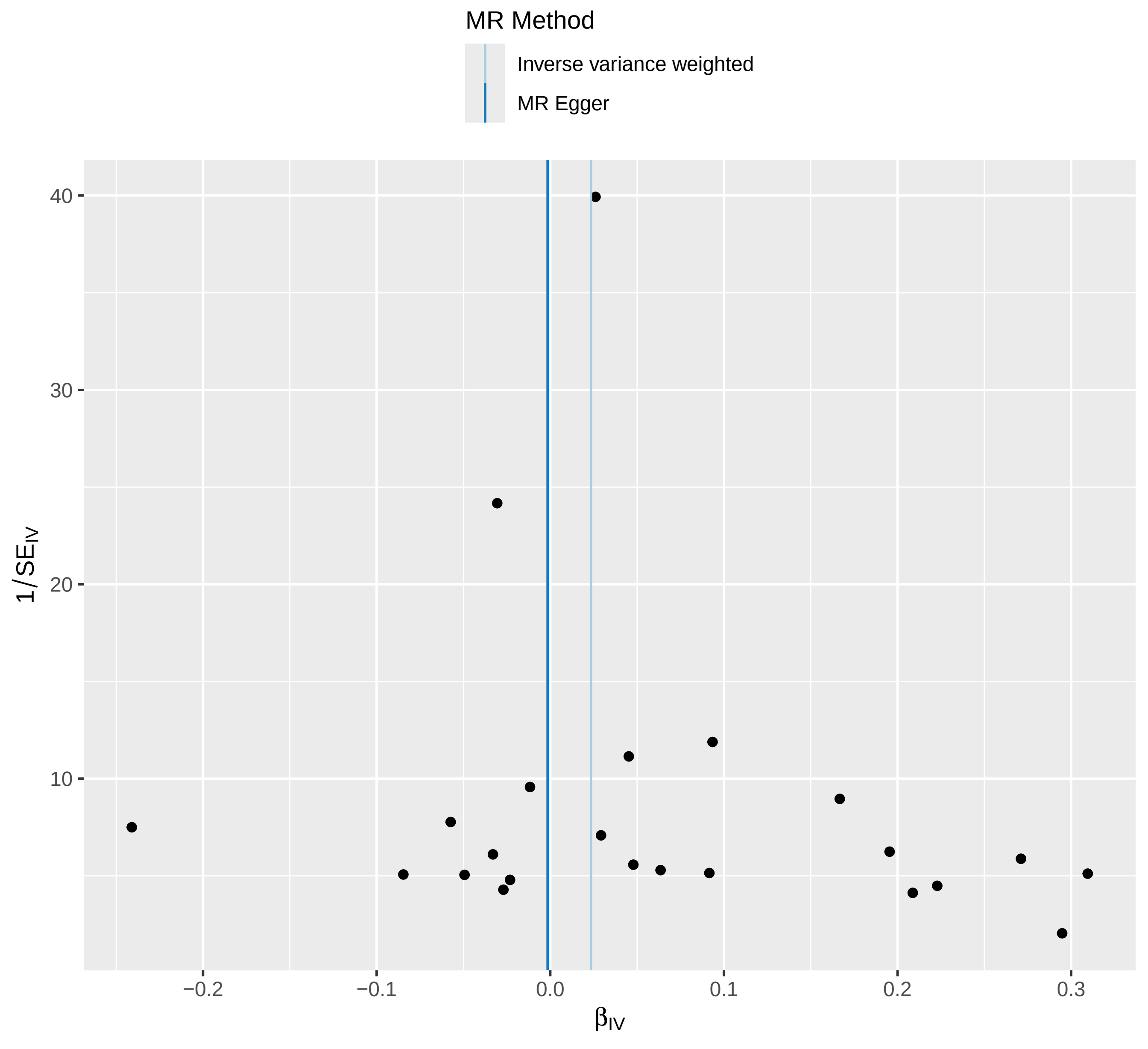

MR Method
Inverse variance weighted
MR Egger
40
30
20
10
1/SE_IV
−0.2
−0.1
0.0
0.1
0.2
0.3
β_IV

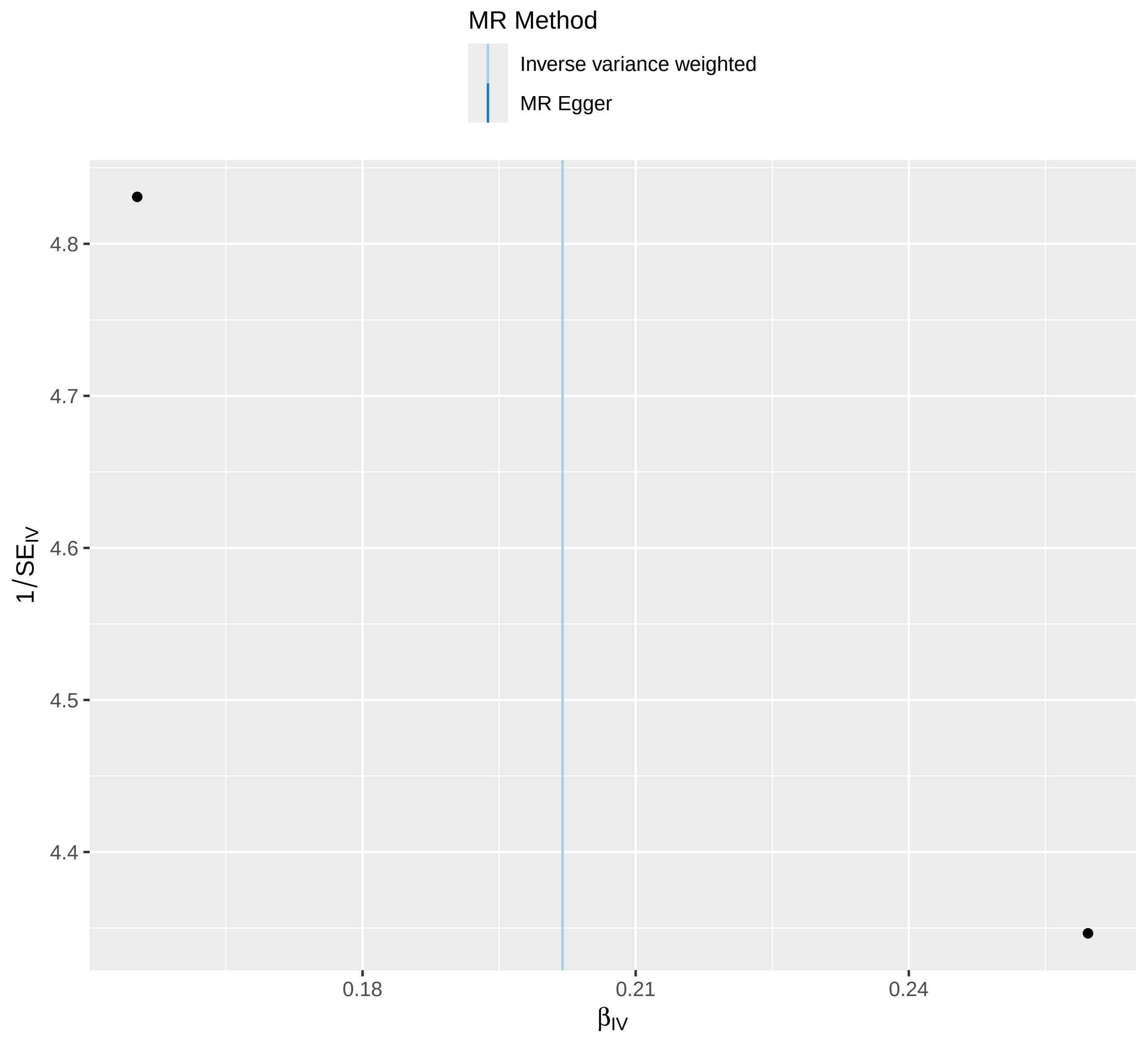
MR Method
Inverse variance weighted
MR Egger
4.8
4.7
4.6
4.5
4.4
1/SE_IV
0.18
0.21
0.24
β_IV

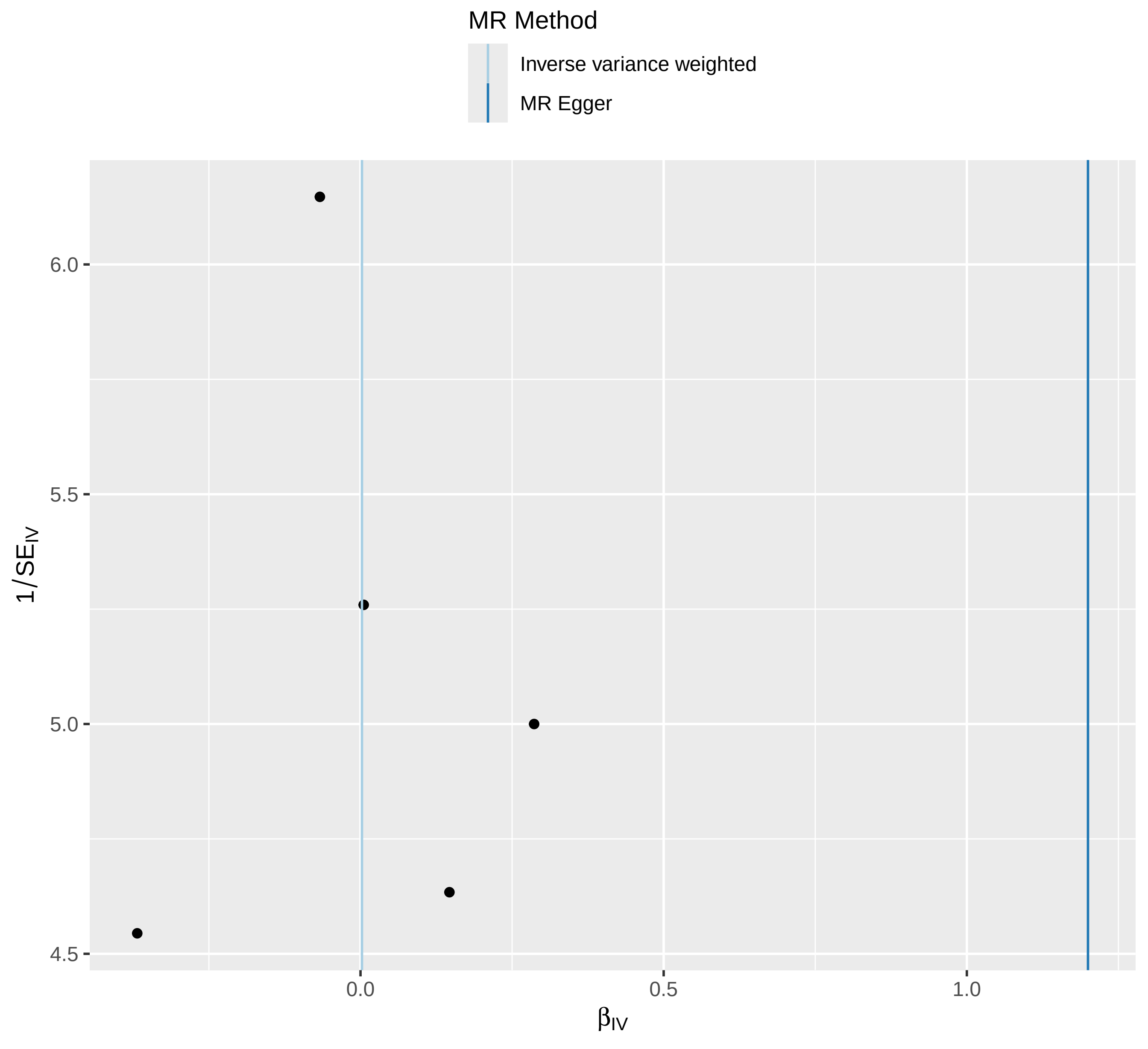
MR Method
Inverse variance weighted
MR Egger
6.0
5.5
5.0
4.5
$1/SE_{IV}$
0.0
0.5
1.0
$\beta_{IV}$

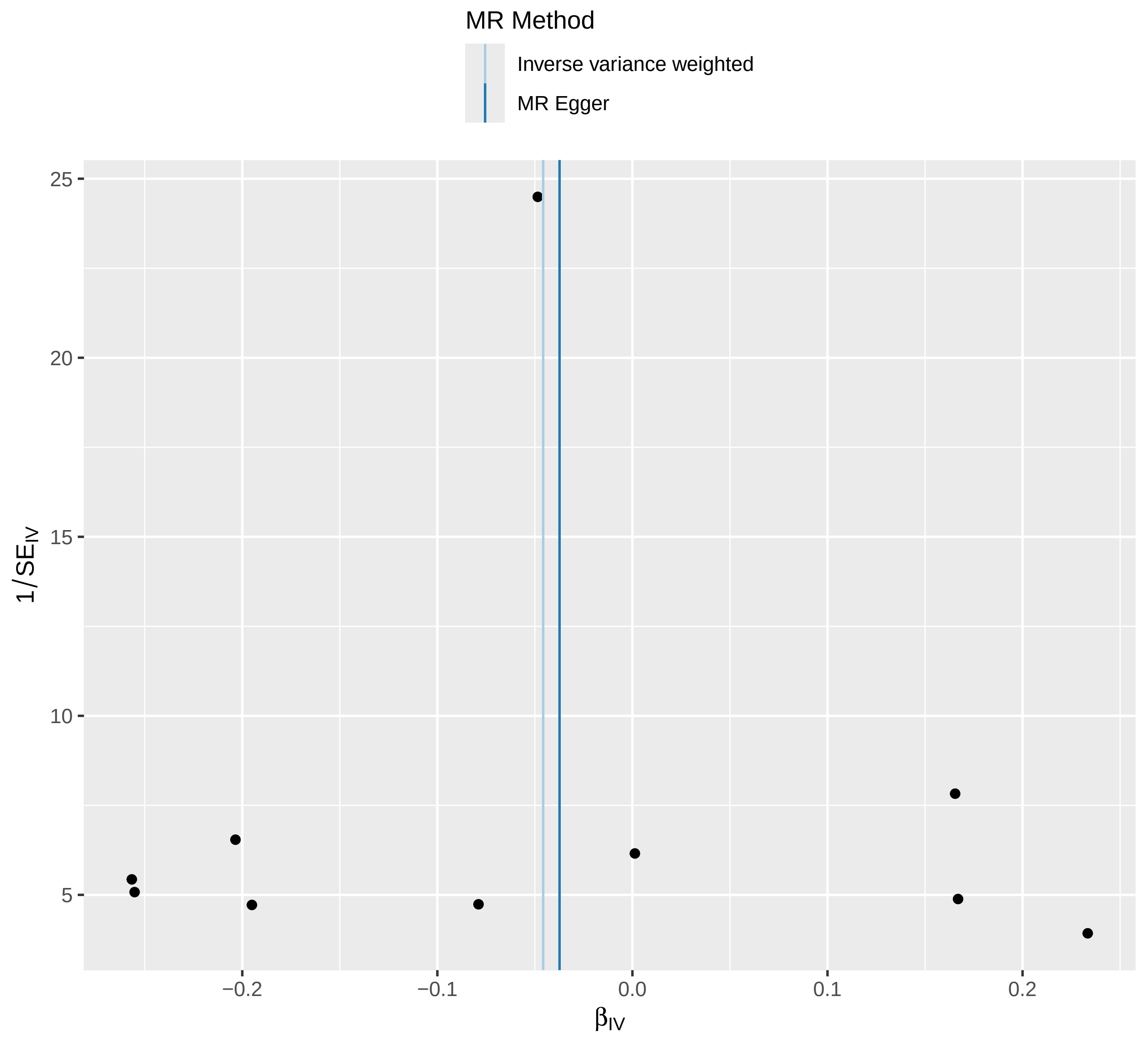

MR Method
Inverse variance weighted
MR Egger
25
20
15
10
5
1/SE_IV
−0.2
−0.1
0.0
0.1
0.2
β_IV

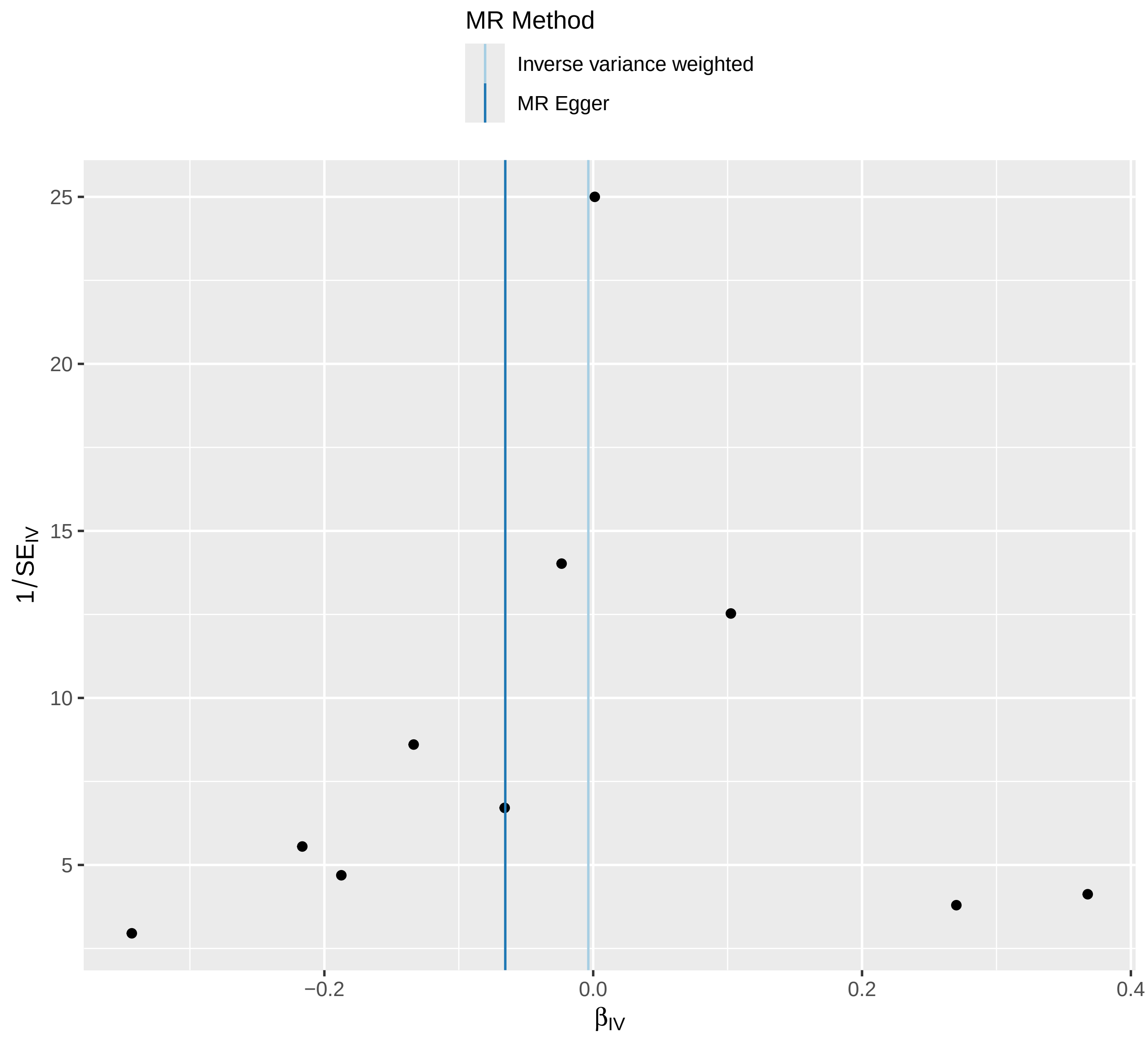
MR Method
Inverse variance weighted
MR Egger
25
20
15
10
5
1/SE_IV
−0.2
0.0
0.2
0.4
β_IV

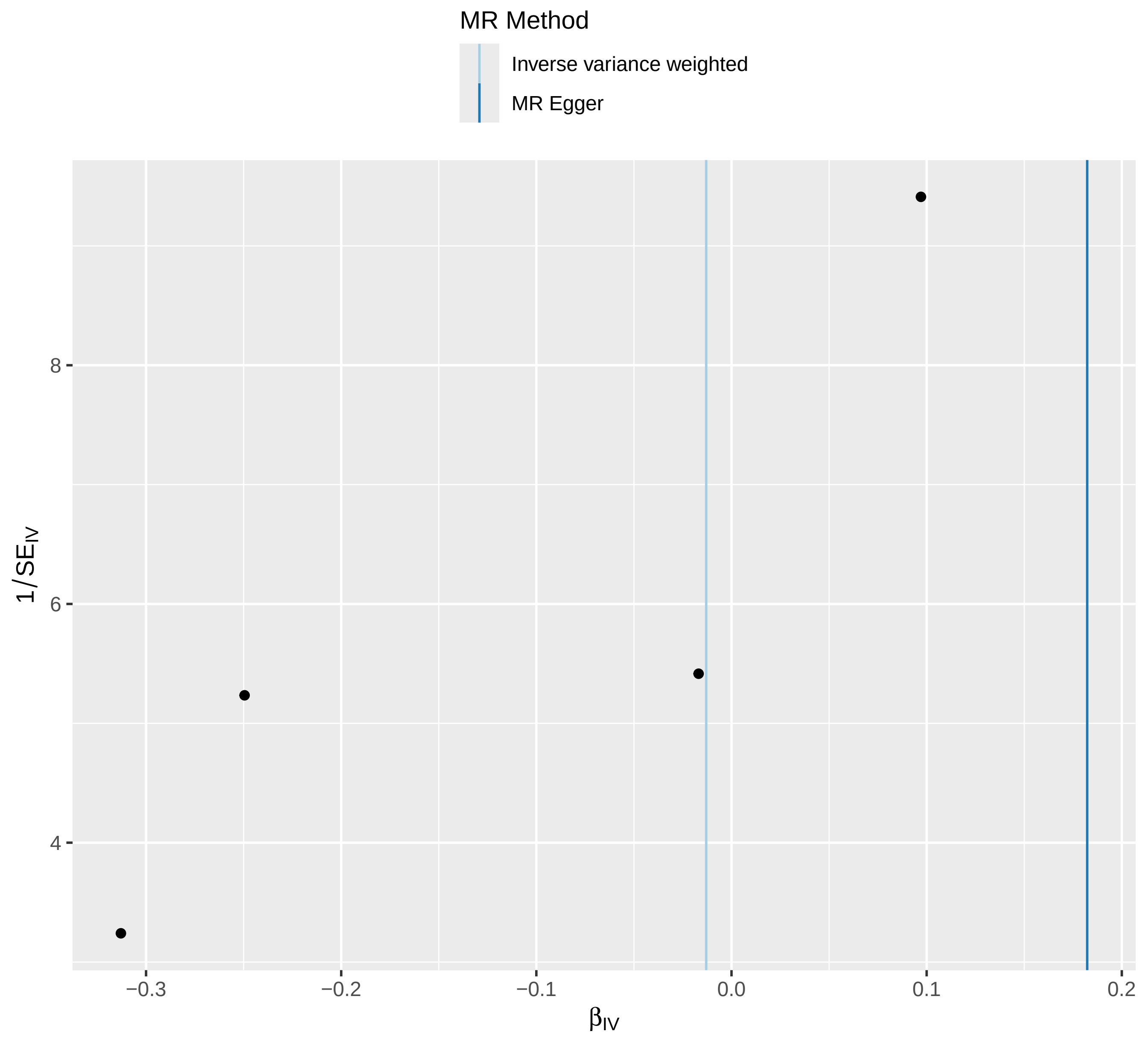

MR Method
Inverse variance weighted
MR Egger
8
6
4
1/SE$_{IV}$
−0.3
−0.2
−0.1
0.0
0.1
0.2
β$_{IV}$

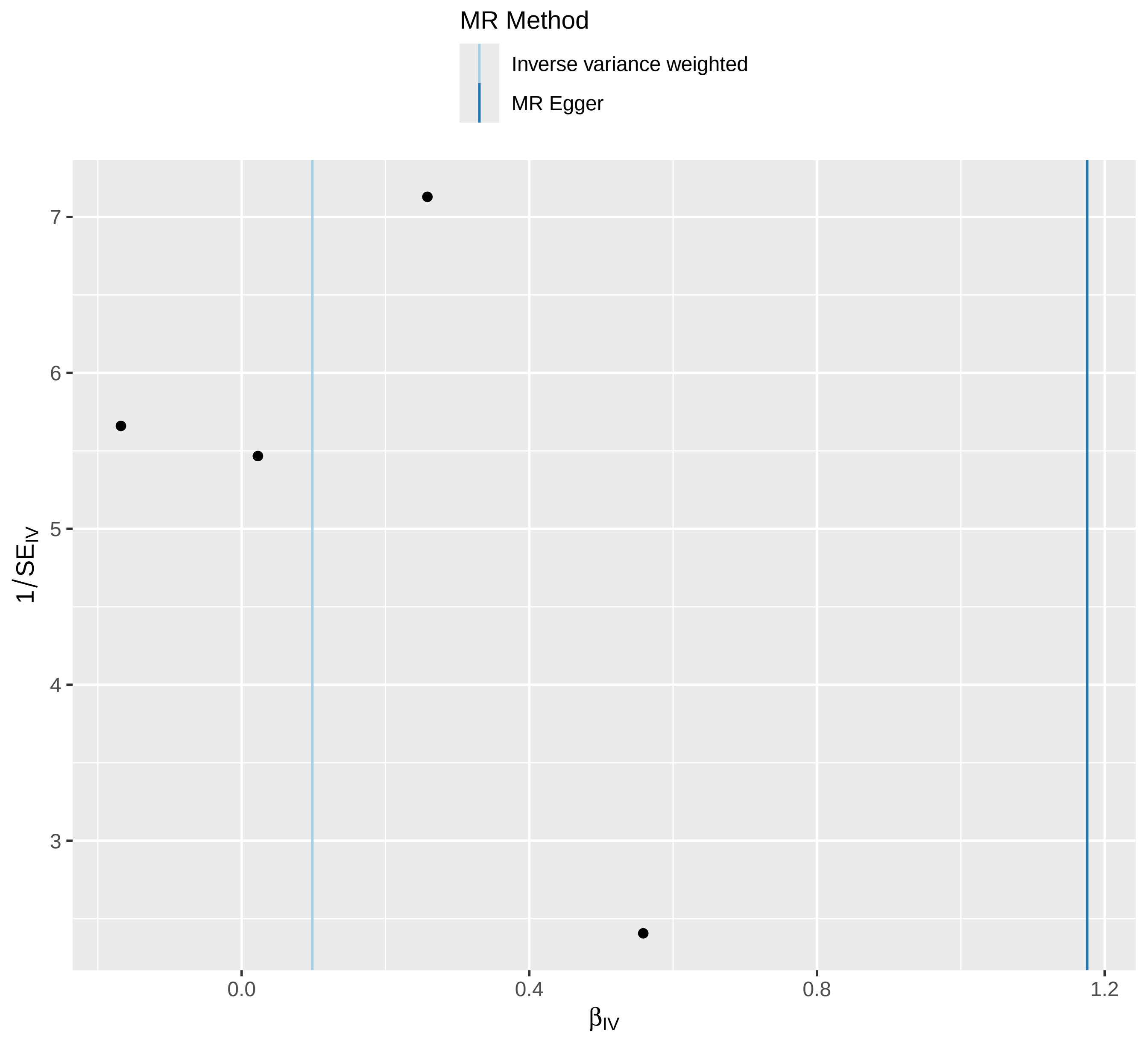
MR Method
Inverse variance weighted
MR Egger
7
6
5
4
3
1/SE$_{IV}$
0.0
0.4
0.8
1.2
β$_{IV}$

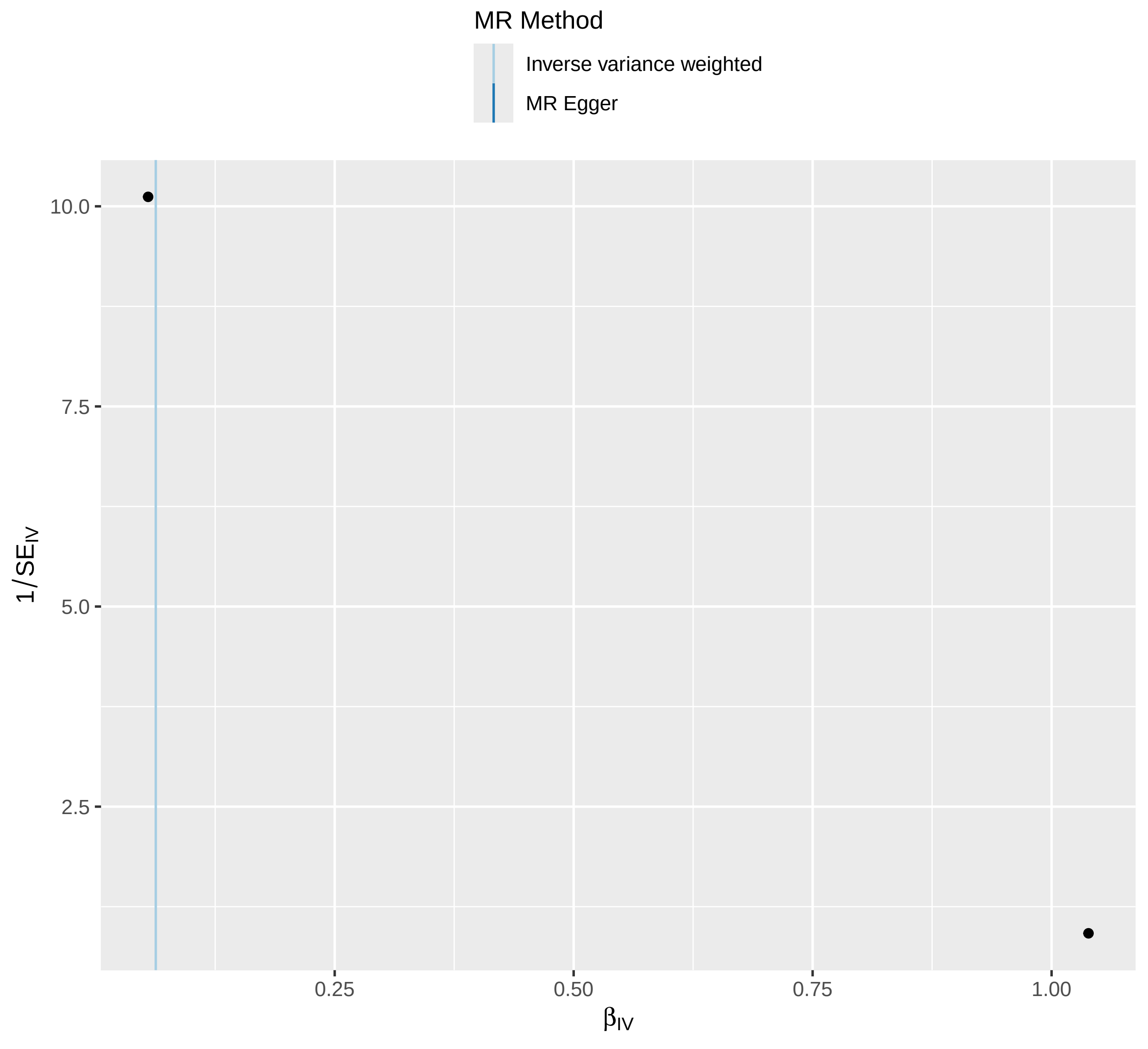

MR Method
Inverse variance weighted
MR Egger
10.0
7.5
5.0
2.5
1/SE_IV
0.25
0.50
0.75
1.00
β_IV

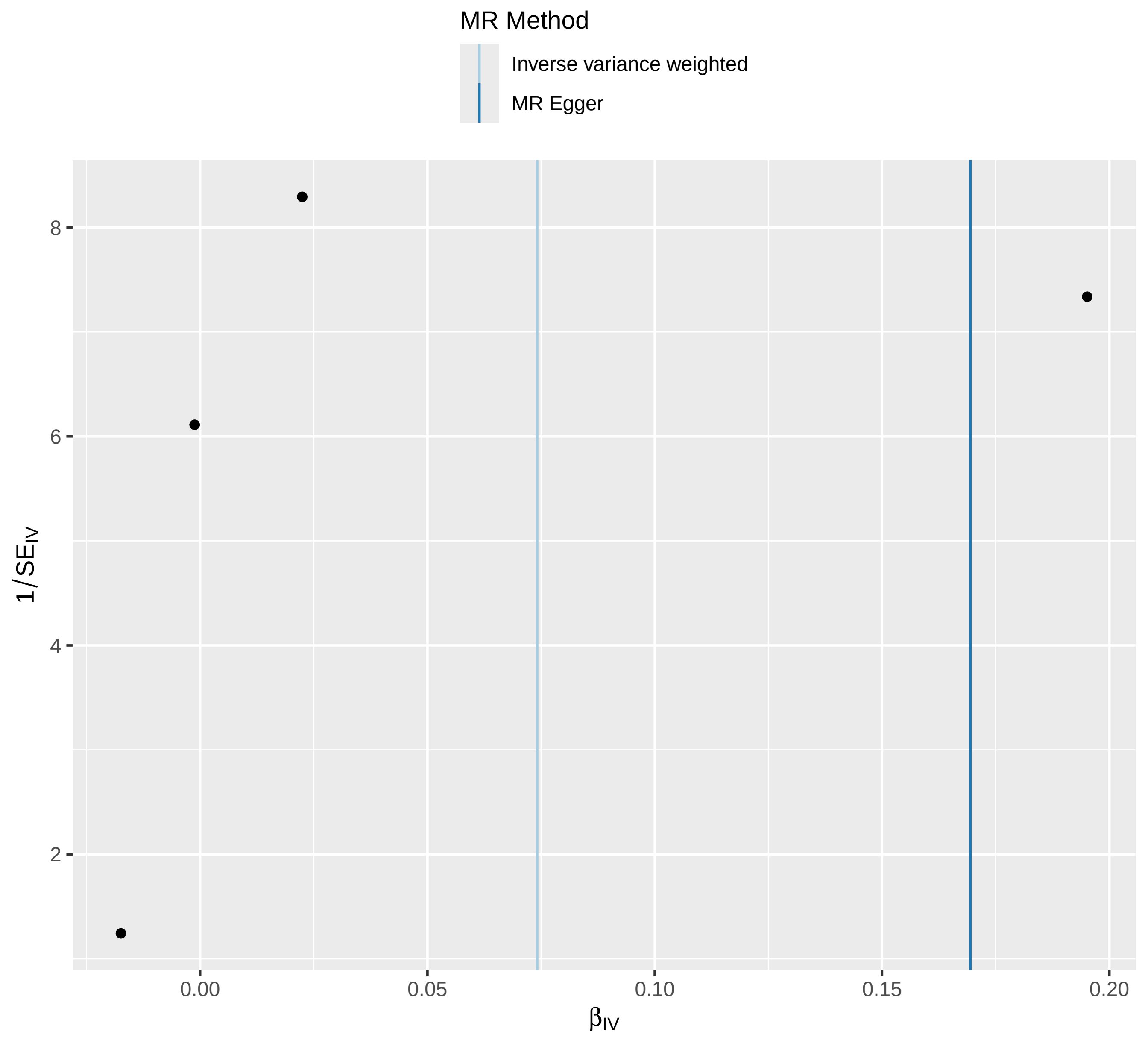
MR Method
Inverse variance weighted
MR Egger
8
6
4
2
1/SE_IV
0.00
0.05
0.10
0.15
0.20
β_IV

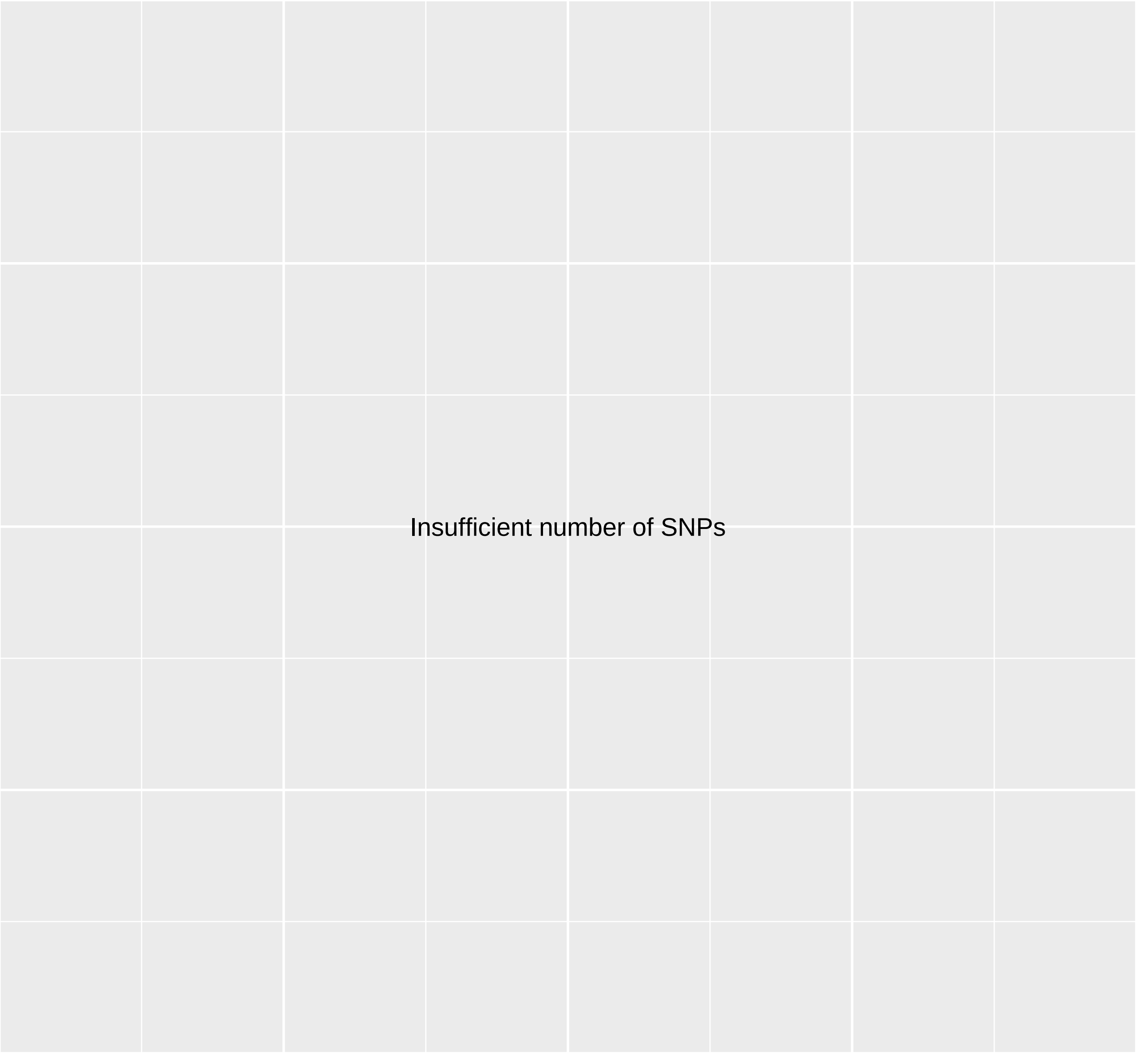
Insufficient number of SNPs

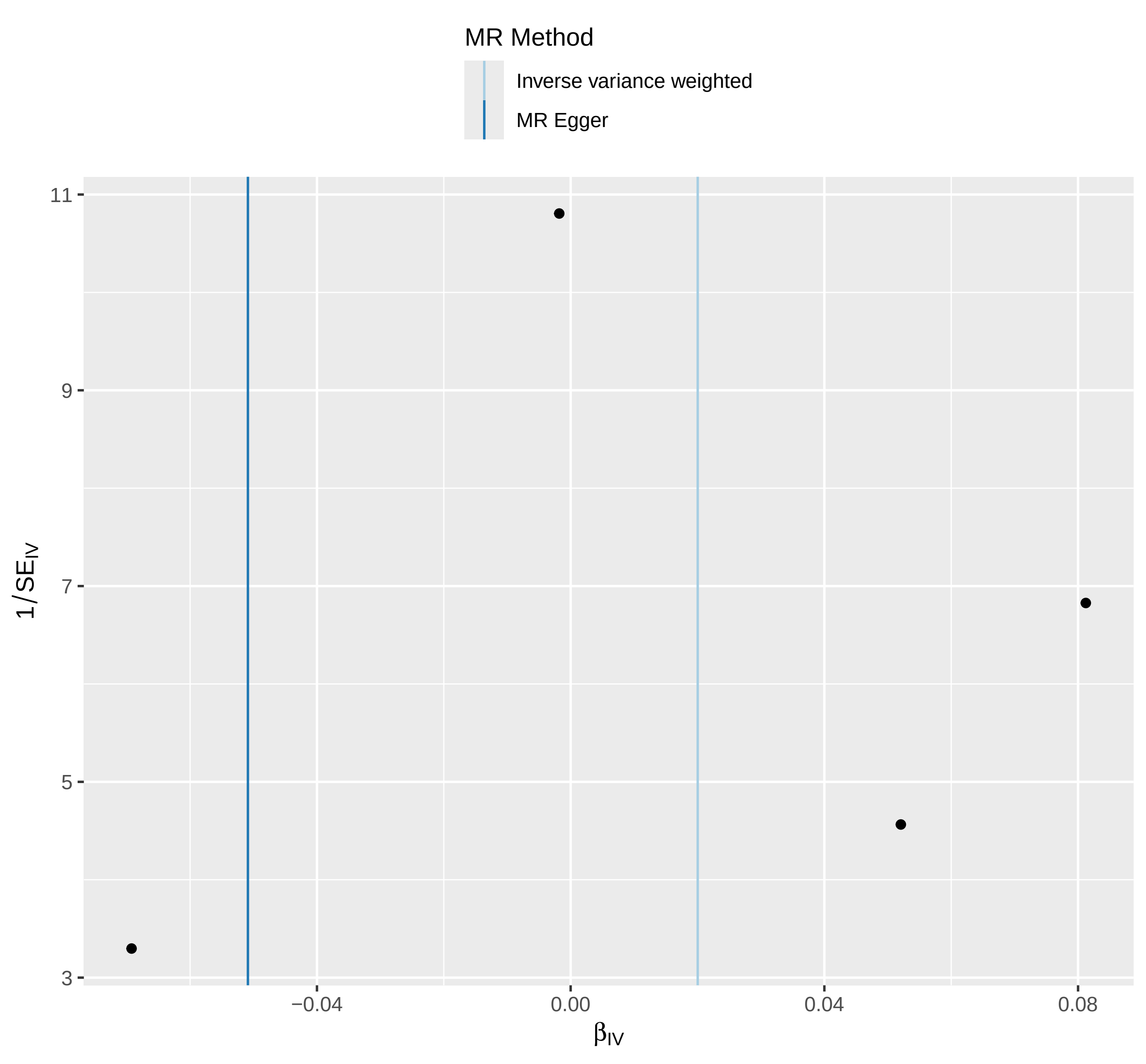
MR Method
Inverse variance weighted
MR Egger
11
9
7
5
3
1/SE_IV
−0.04
0.00
0.04
0.08
β_IV

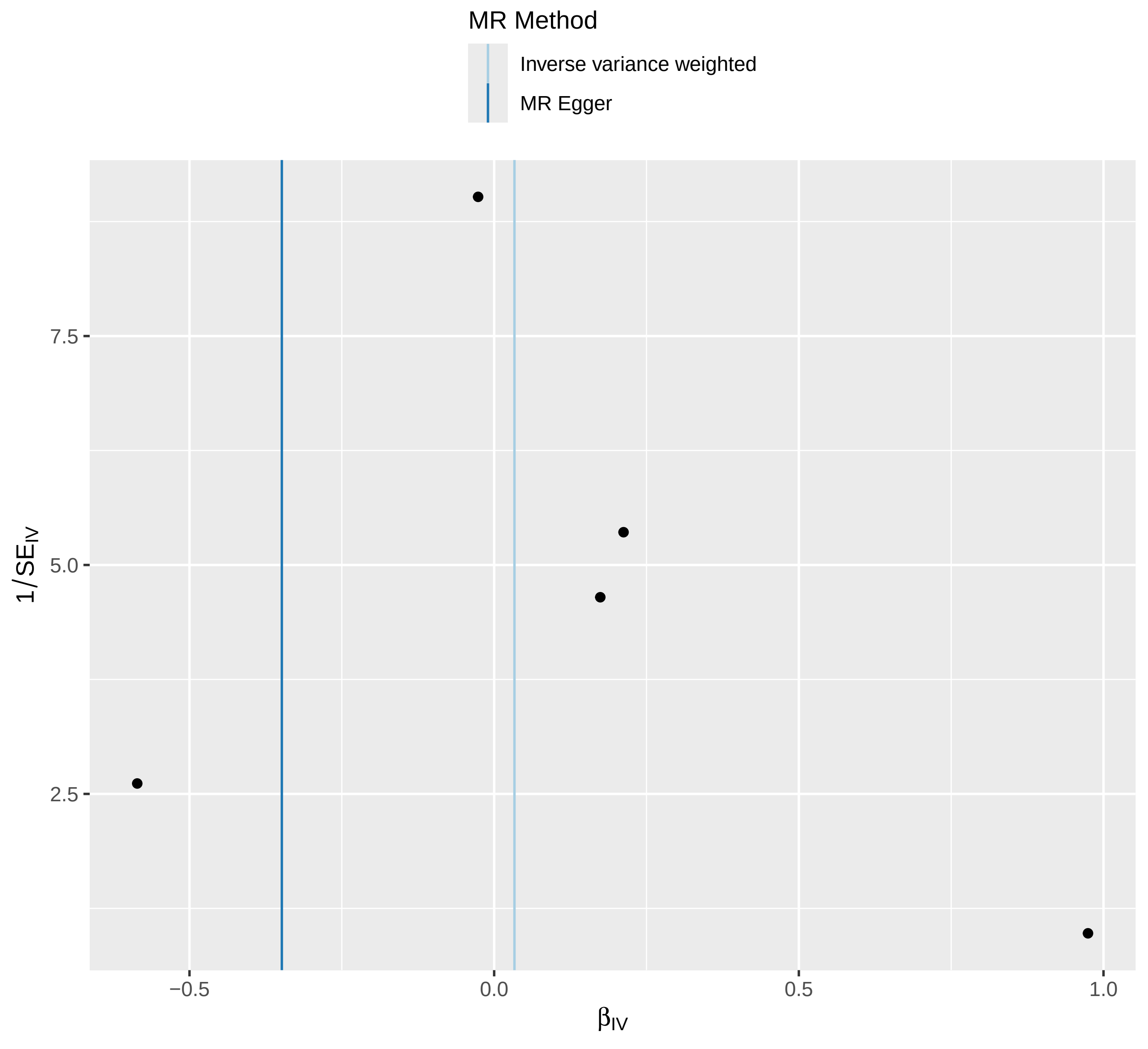
MR Method
Inverse variance weighted
MR Egger
7.5
5.0
2.5
$1/SE_{IV}$
−0.5
0.0
0.5
1.0
$\beta_{IV}$

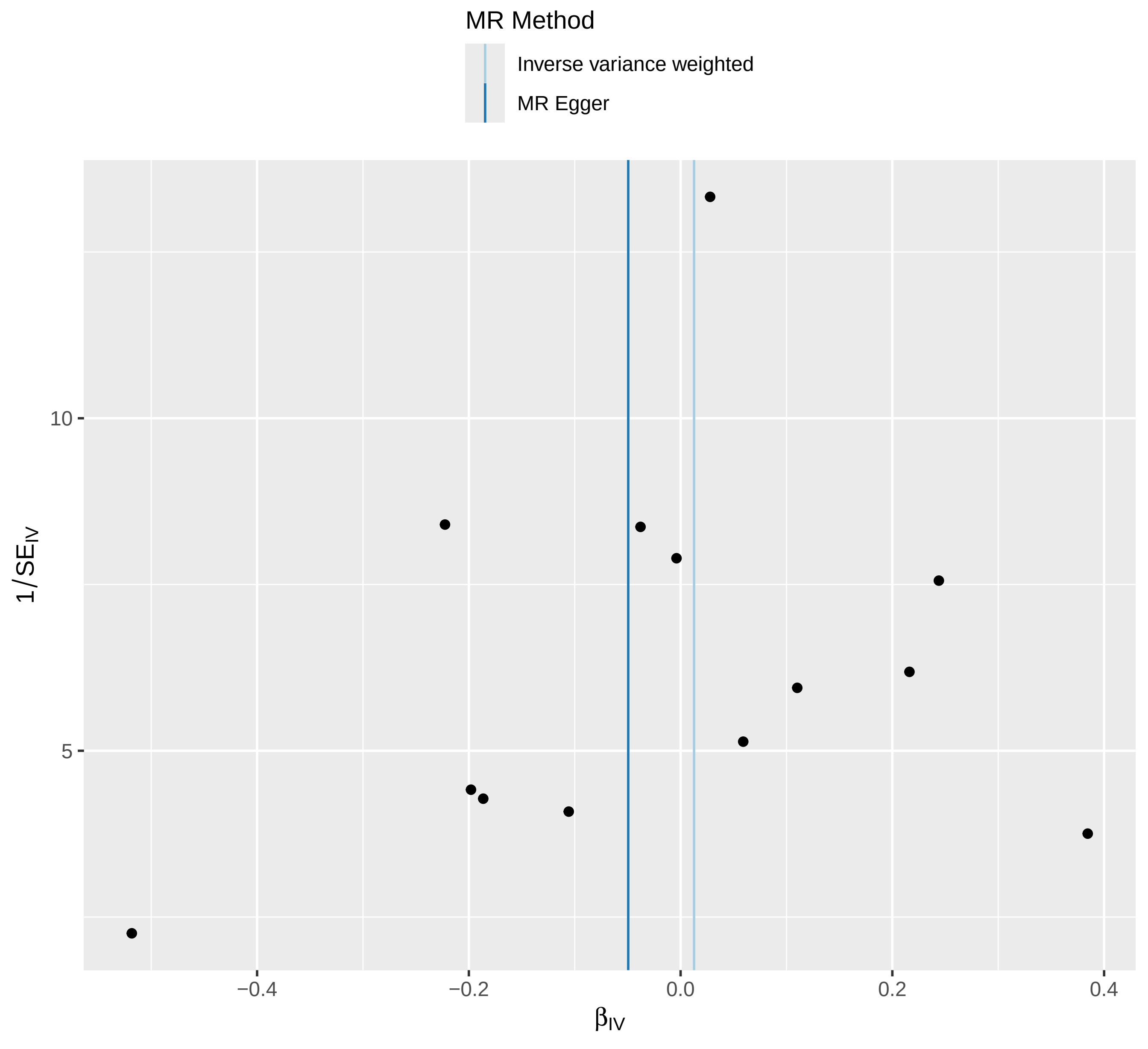
MR Method
Inverse variance weighted
MR Egger
10
5
1/SE$_{IV}$
−0.4
−0.2
0.0
0.2
0.4
β$_{IV}$

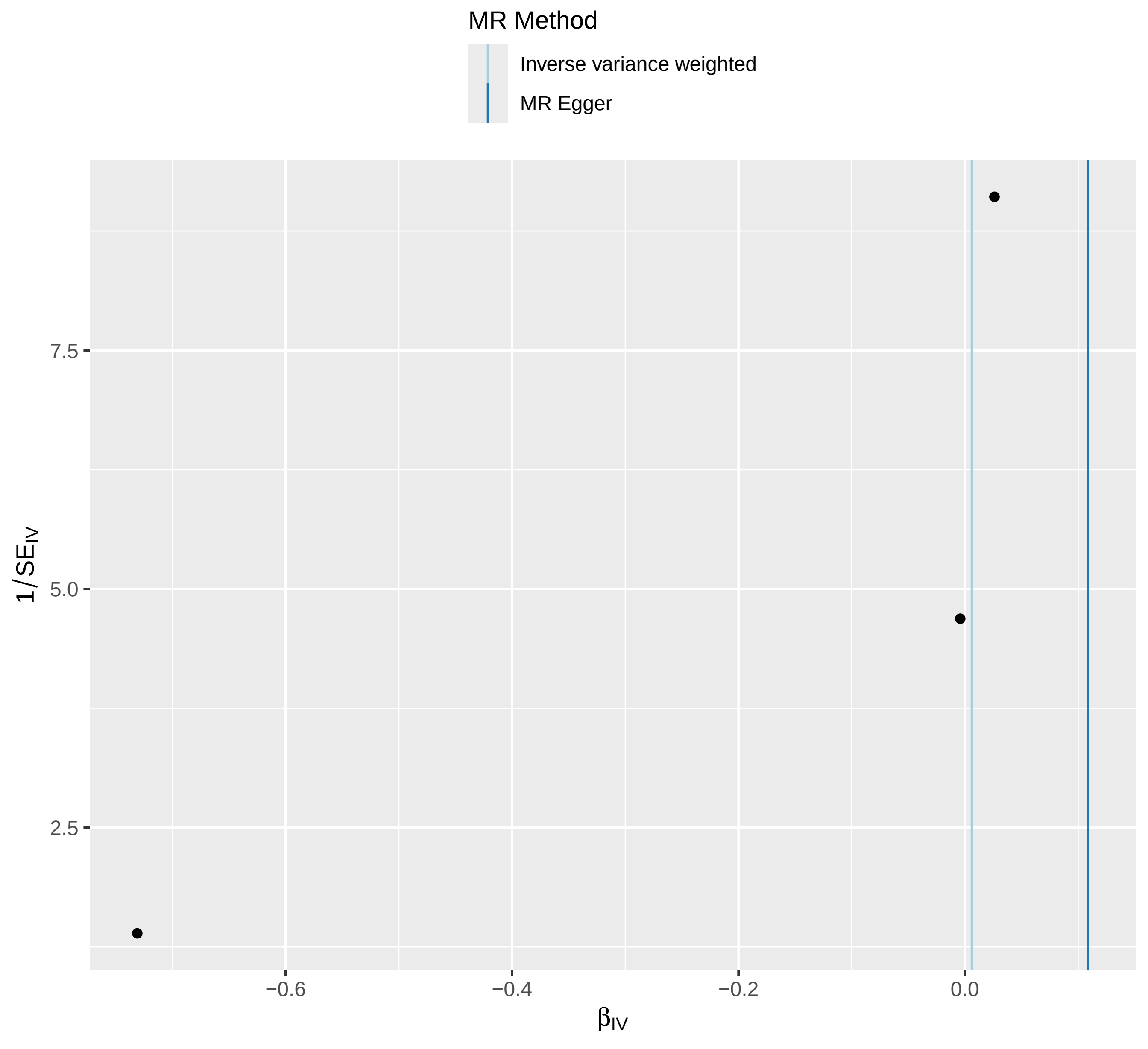
MR Method
Inverse variance weighted
MR Egger
1/SE$_{IV}$
7.5
5.0
2.5
−0.6
−0.4
−0.2
0.0
β$_{IV}$

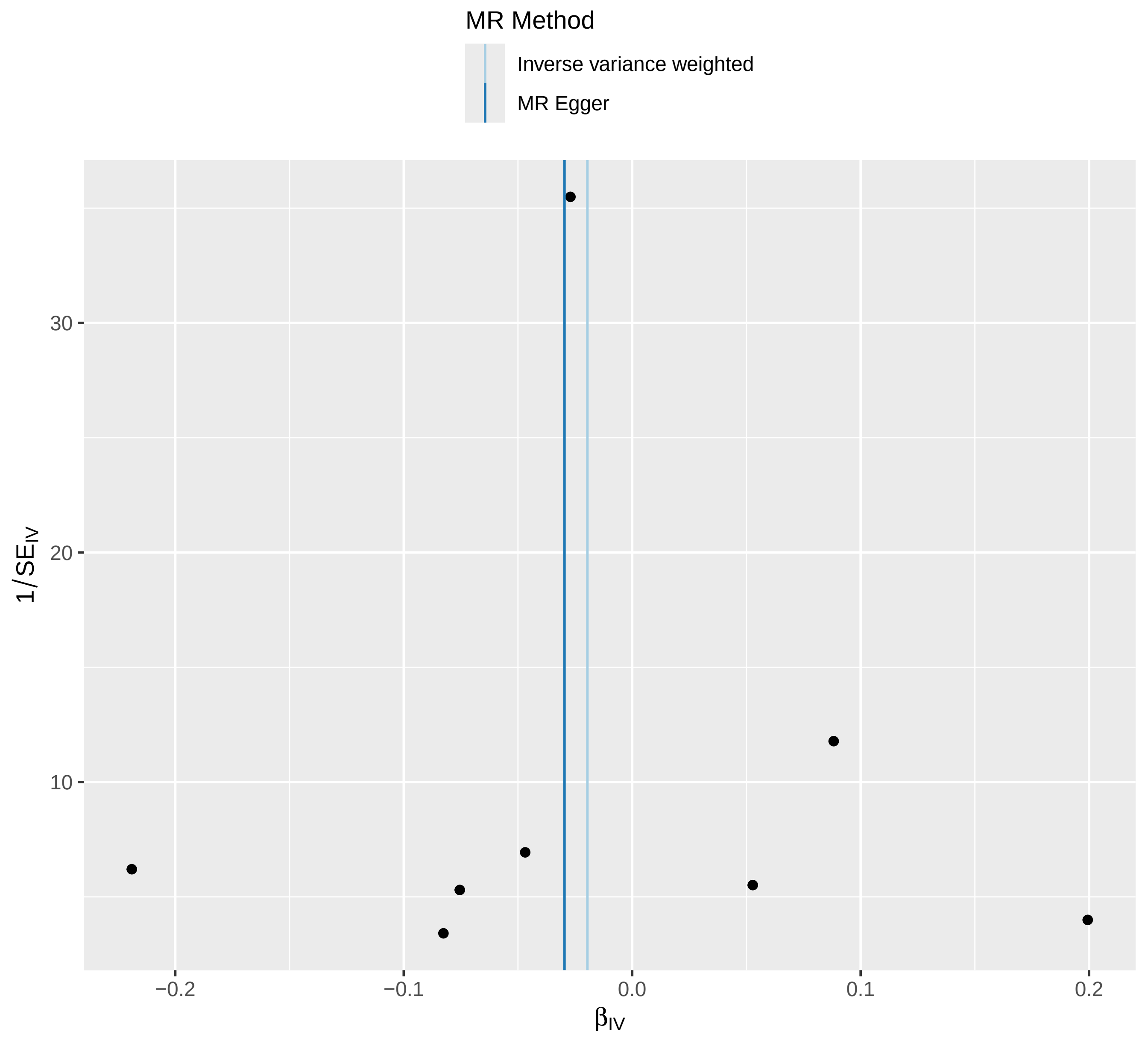
MR Method
Inverse variance weighted
MR Egger
30
20
10
1/SE$_{IV}$
−0.2
−0.1
0.0
0.1
0.2
β$_{IV}$

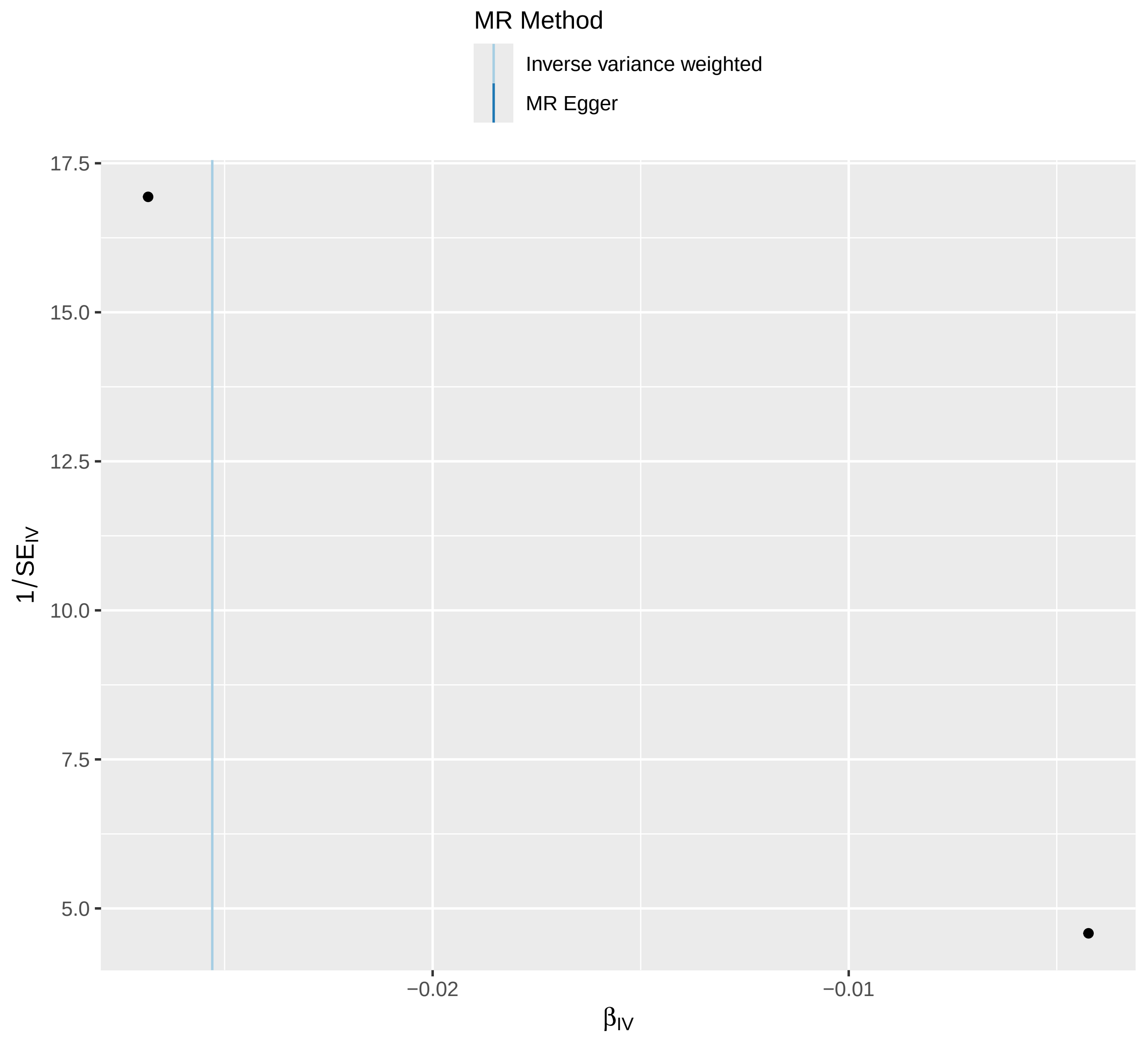
MR Method
Inverse variance weighted
MR Egger
17.5
15.0
12.5
10.0
7.5
5.0
1/SE_IV
−0.02
−0.01
β_IV

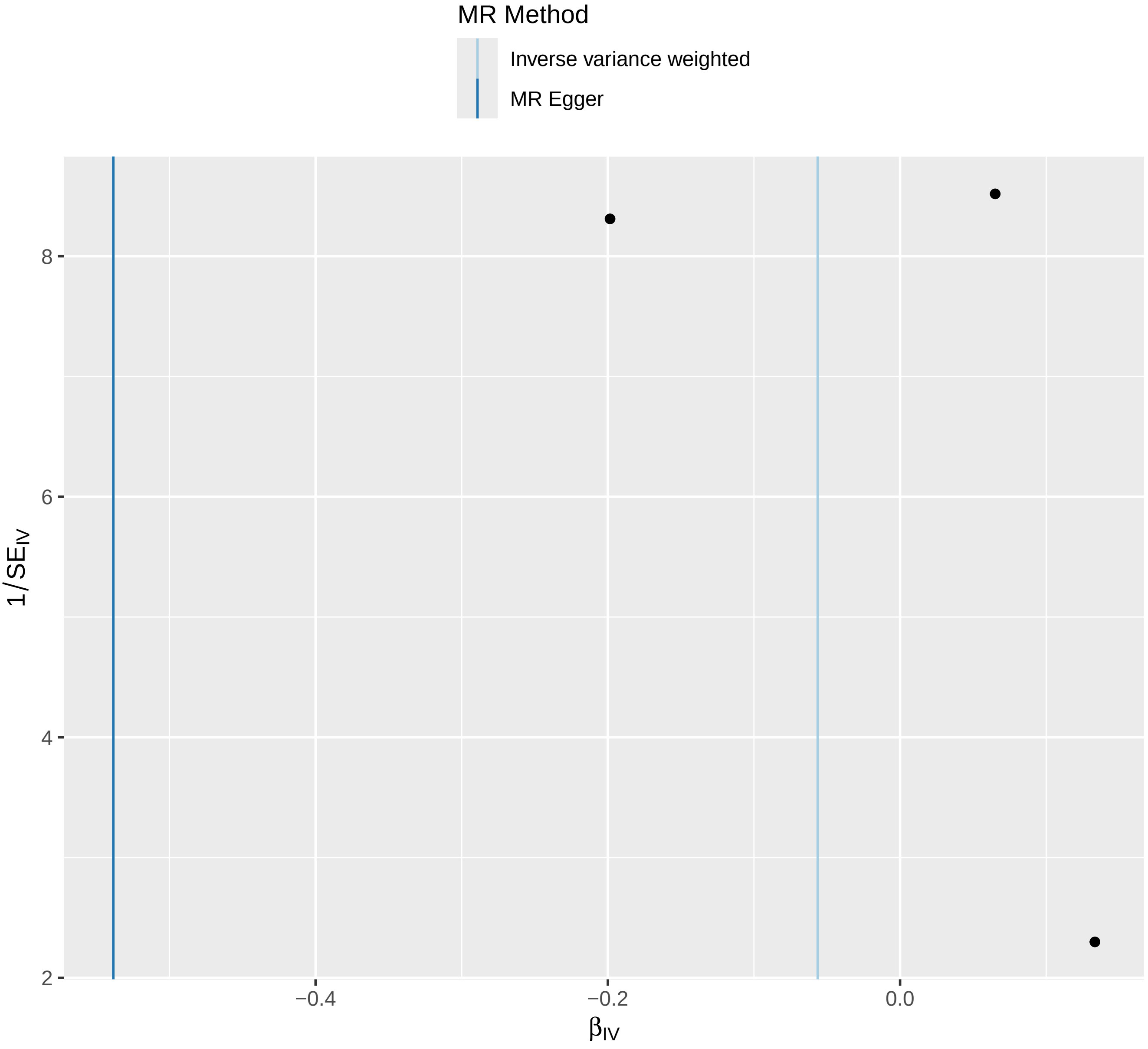
MR Method
Inverse variance weighted
MR Egger
8
6
4
2
1/SE$_{IV}$
−0.4
−0.2
0.0
β$_{IV}$

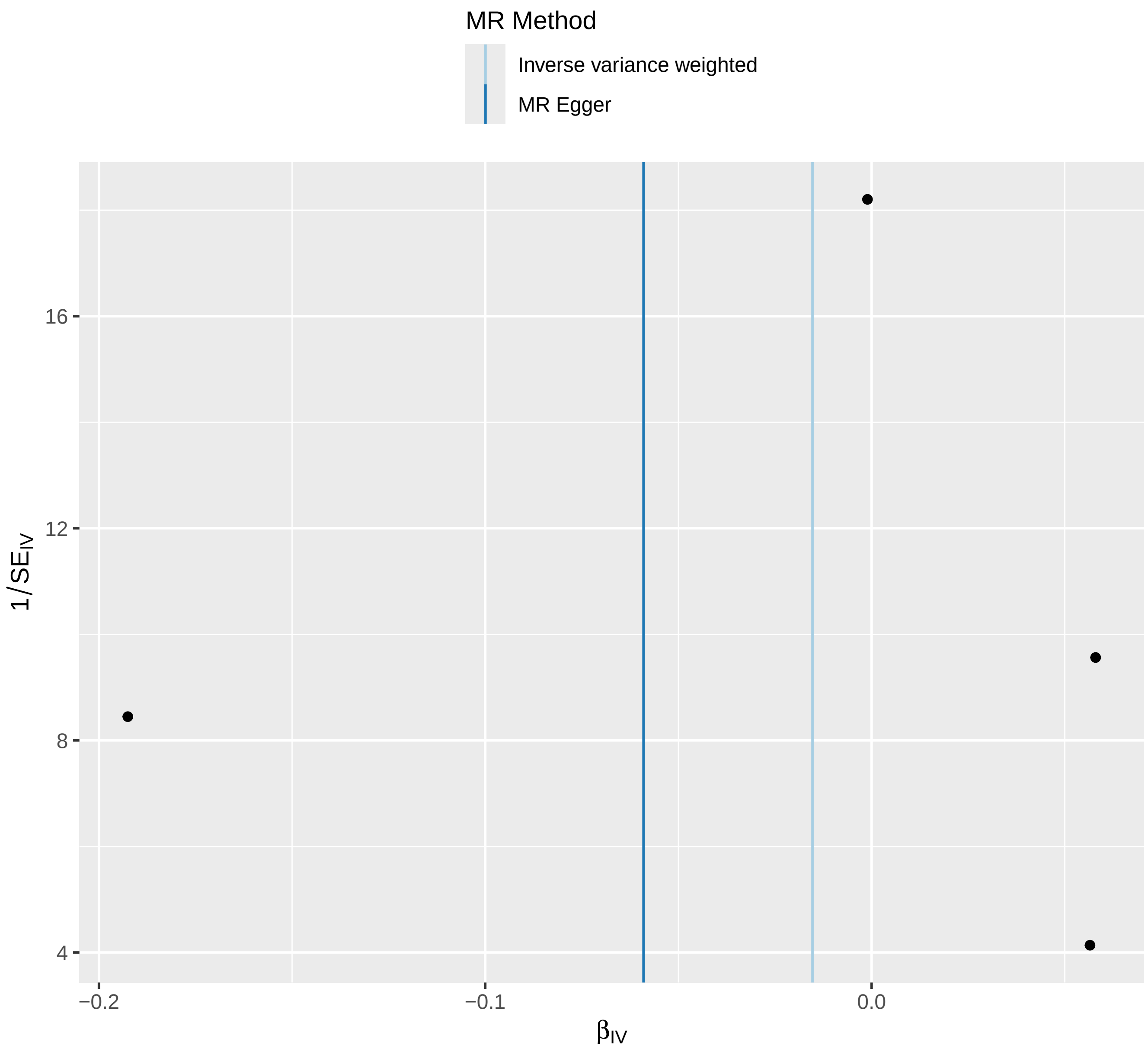
MR Method
Inverse variance weighted
MR Egger
16
12
8
4
1/SE$_{IV}$
−0.2
−0.1
0.0
β$_{IV}$

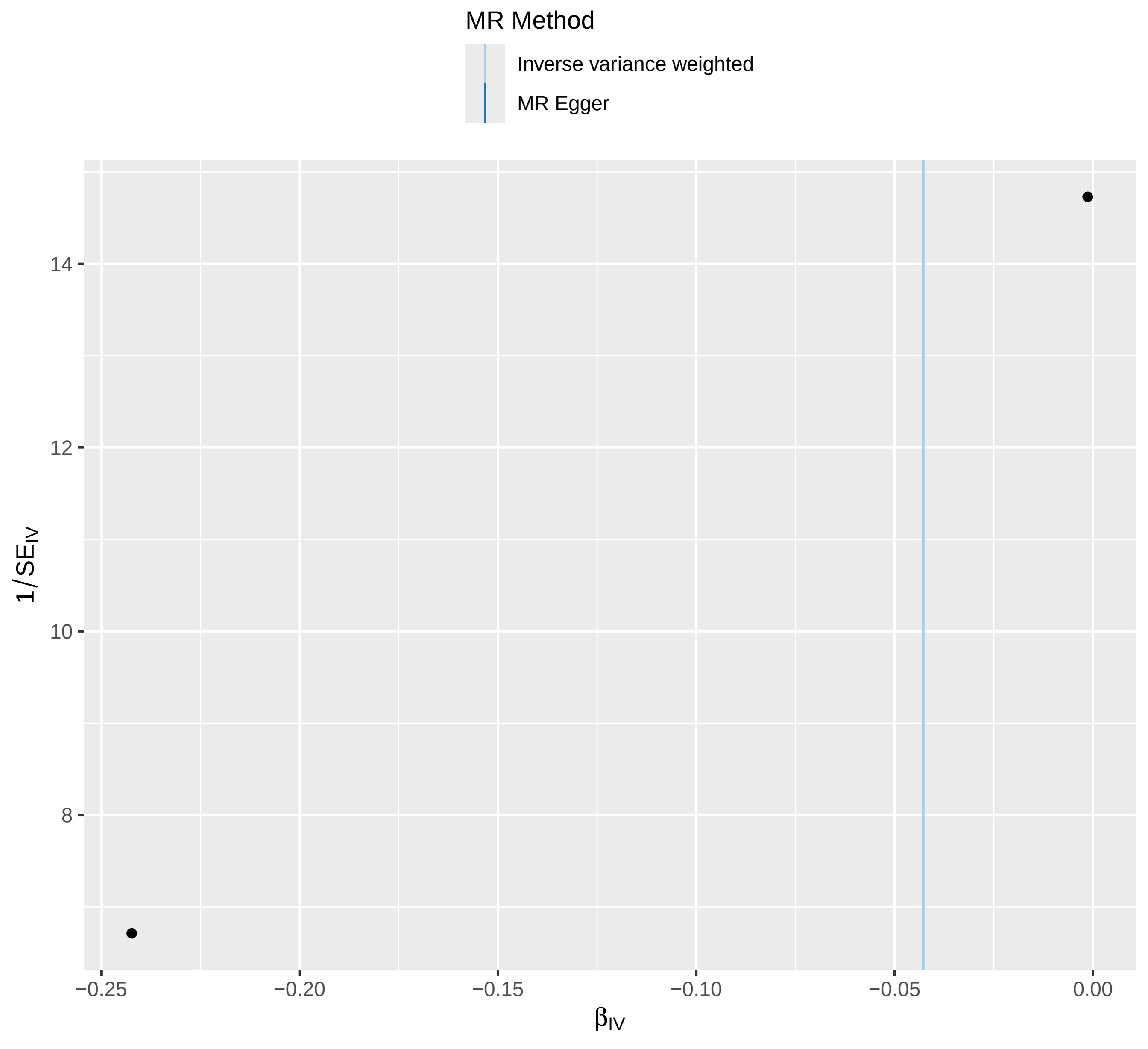

MR Method
Inverse variance weighted
MR Egger
14
12
10
8
1/SE_IV
−0.25
−0.20
−0.15
−0.10
−0.05
0.00
β_IV

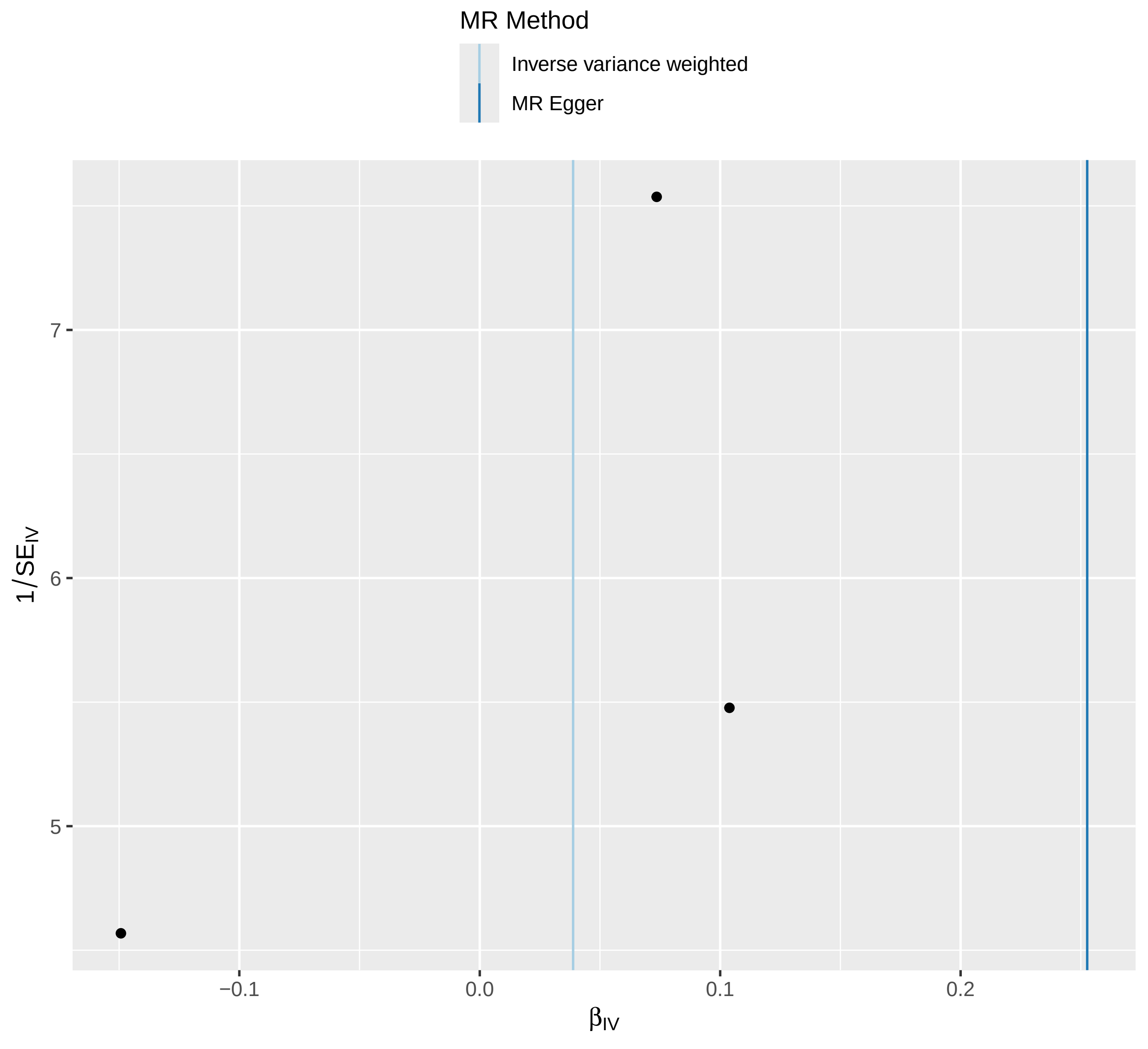
MR Method
Inverse variance weighted
MR Egger
7
6
5
1/SE_IV
−0.1
0.0
0.1
0.2
β_IV

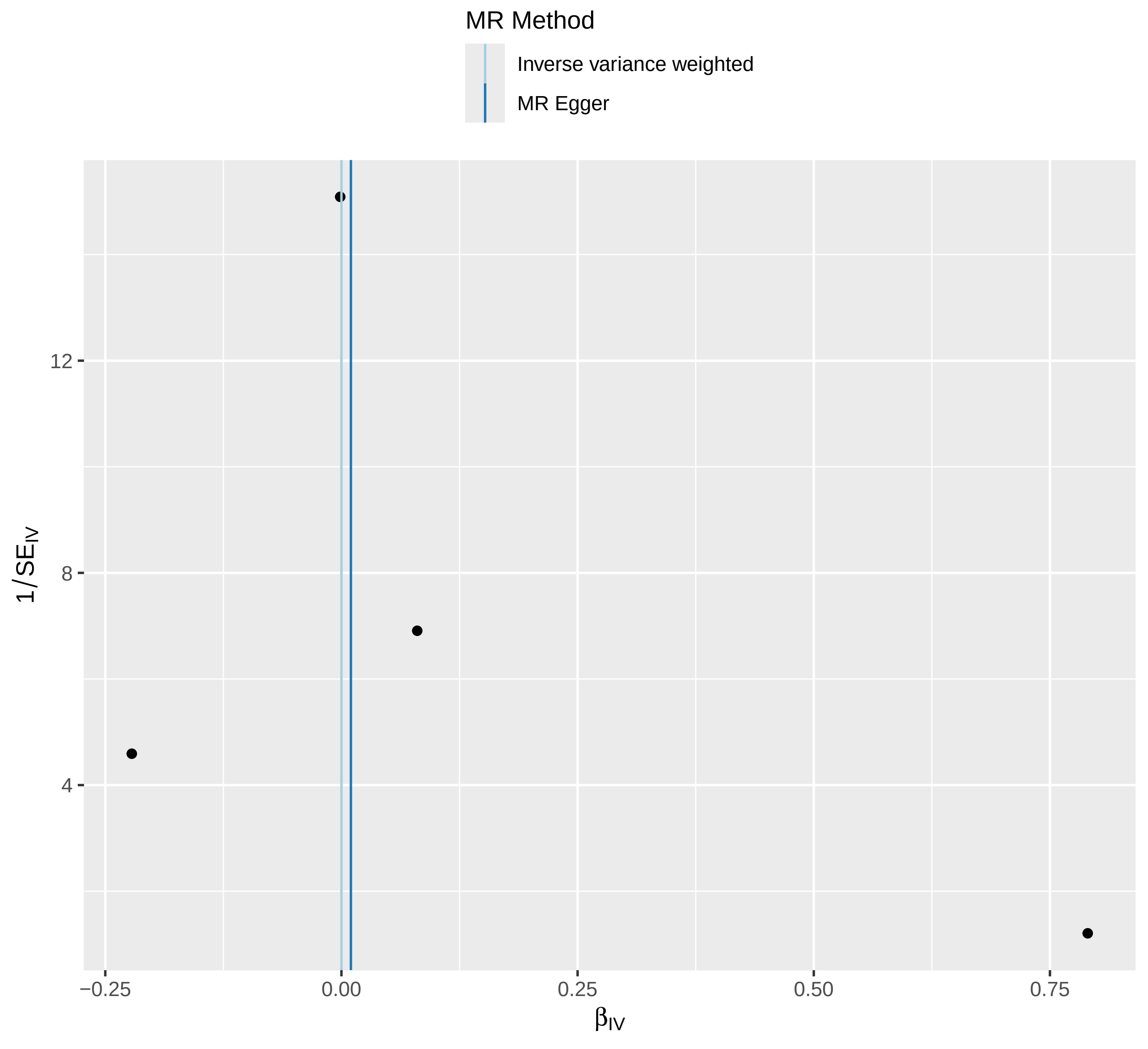

MR Method
Inverse variance weighted
MR Egger
1/SE$_{IV}$
12
8
4
−0.25
0.00
0.25
0.50
0.75
β$_{IV}$

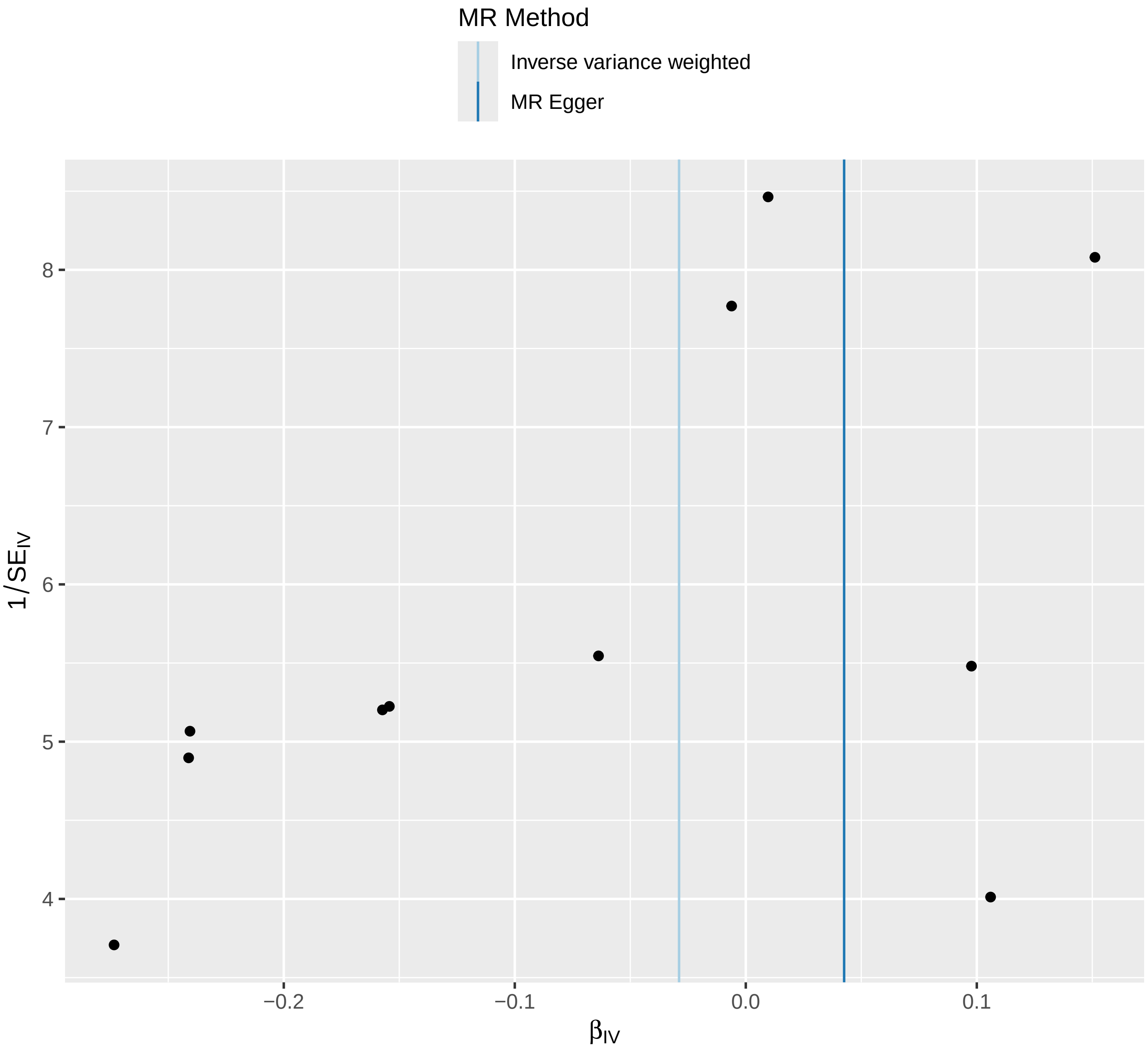
MR Method
Inverse variance weighted
MR Egger
8
7
6
5
4
$1/SE_{IV}$
−0.2
−0.1
0.0
0.1
$\beta_{IV}$

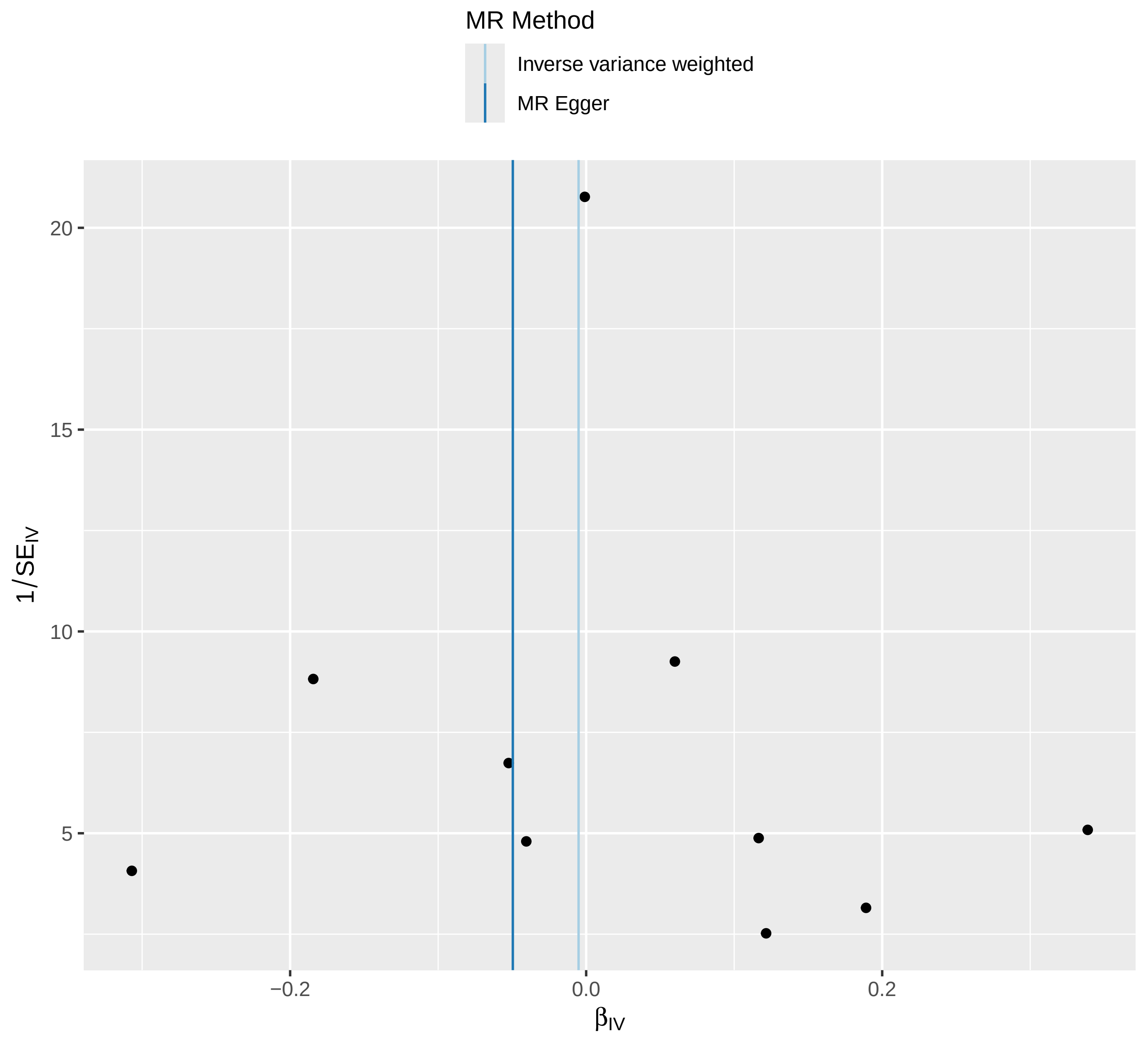

MR Method
Inverse variance weighted
MR Egger
20
15
10
5
1/SE_IV
−0.2
0.0
0.2
β_IV

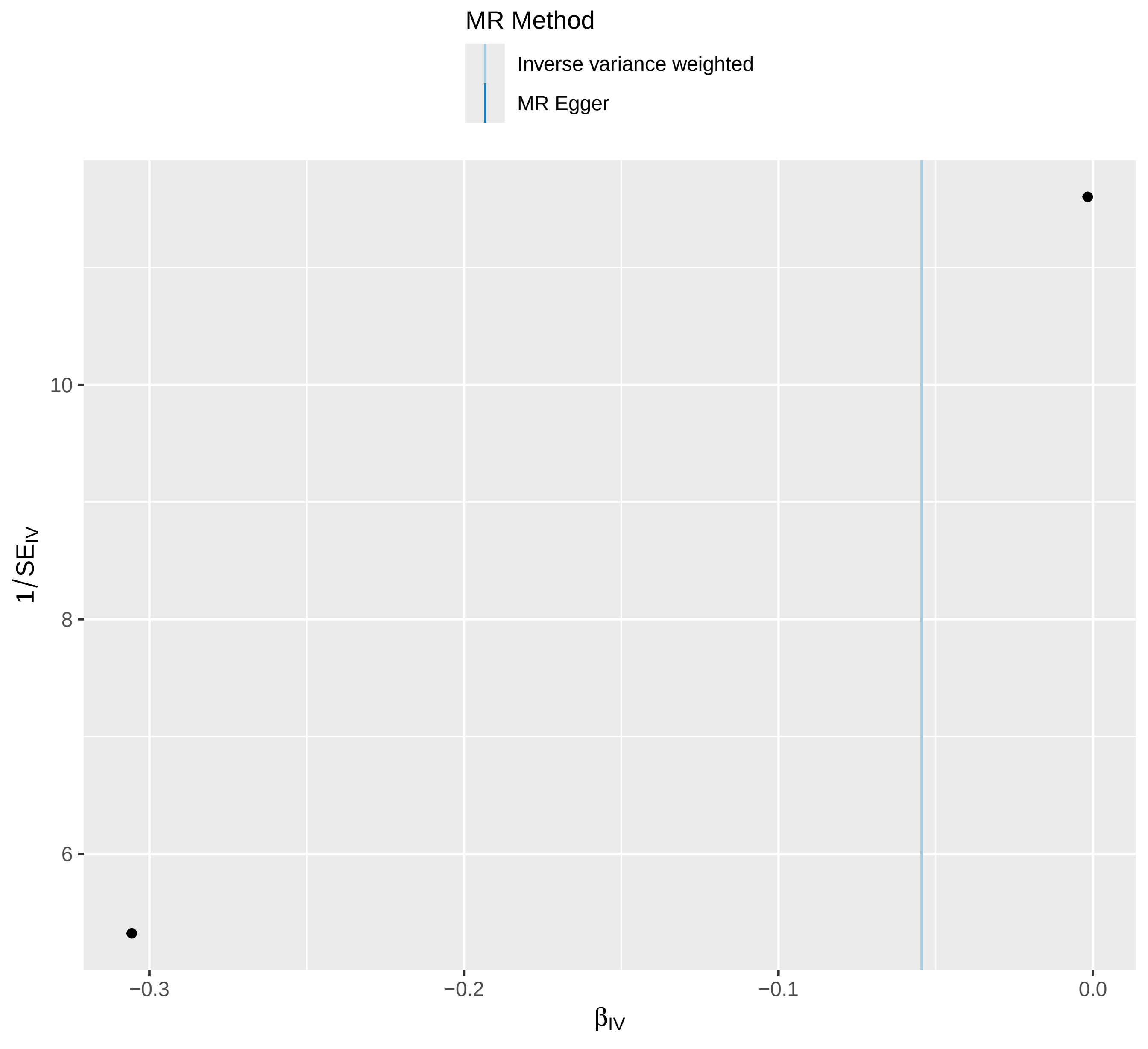
MR Method
Inverse variance weighted
MR Egger
10
8
6
1/SE_IV
−0.3
−0.2
−0.1
0.0
β_IV

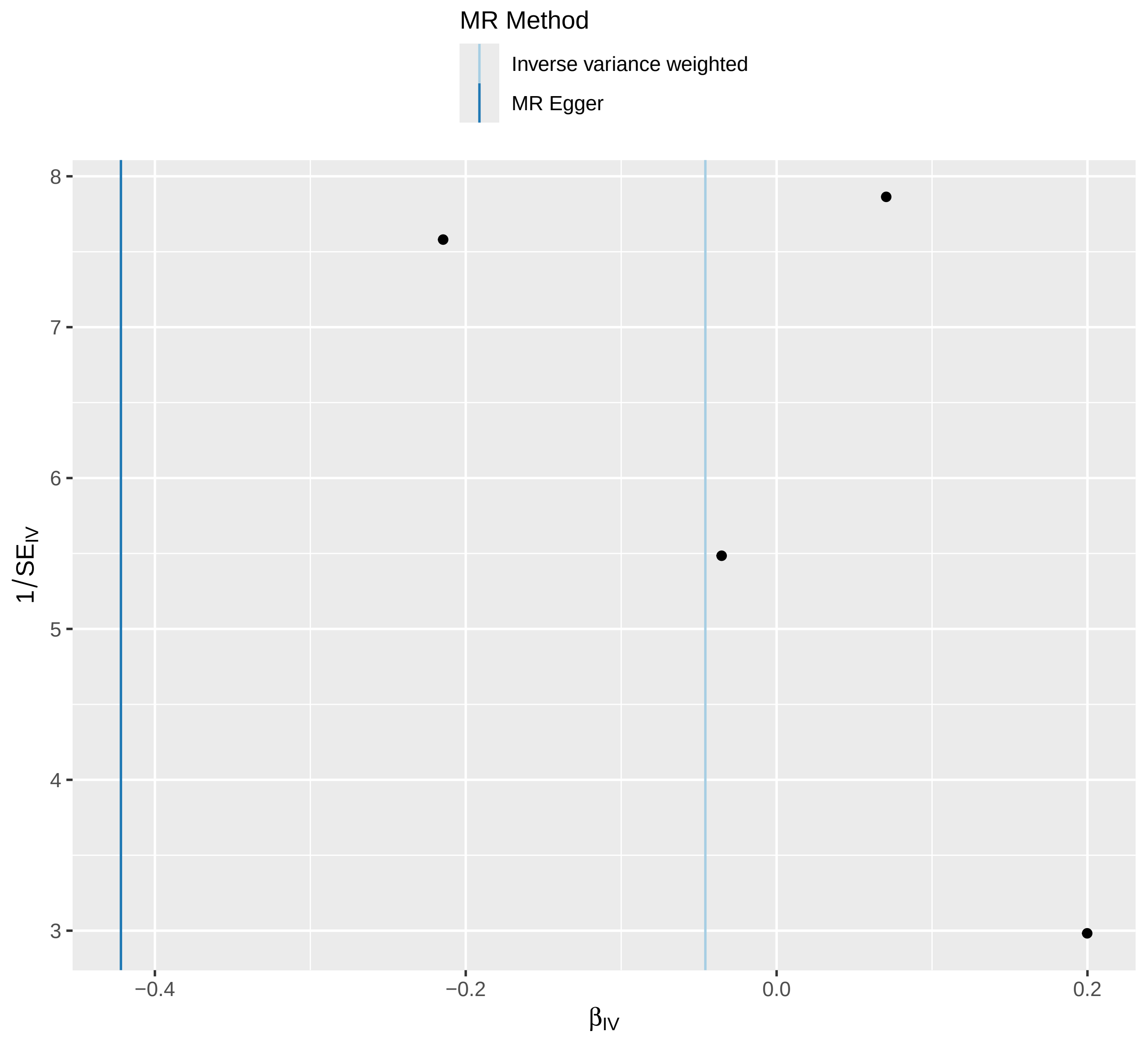
MR Method
Inverse variance weighted
MR Egger
1/SE_IV
β_IV
8
7
6
5
4
3
−0.4
−0.2
0.0
0.2

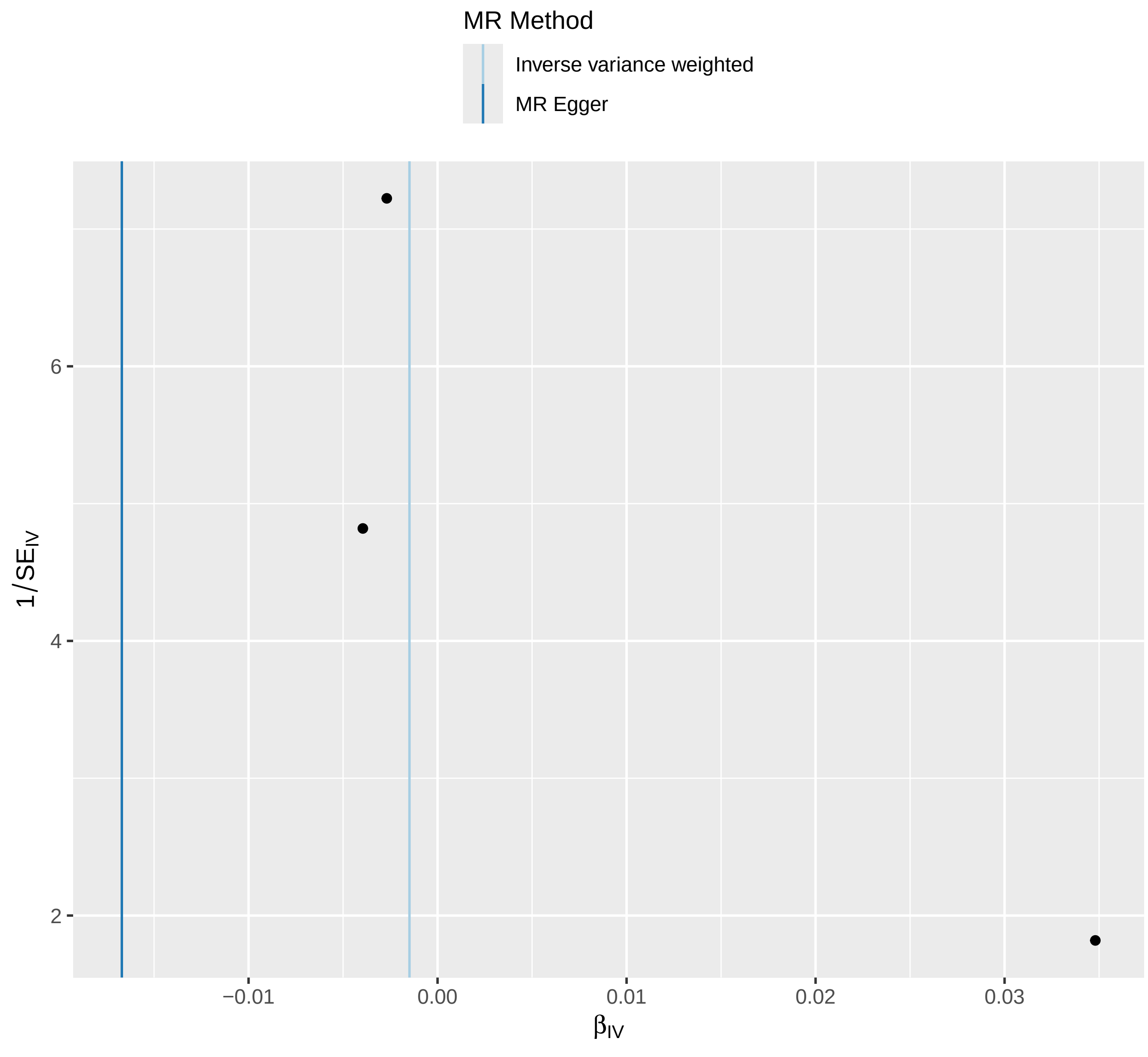
MR Method
Inverse variance weighted
MR Egger
6
4
2
1/SE_IV
−0.01
0.00
0.01
0.02
0.03
β_IV

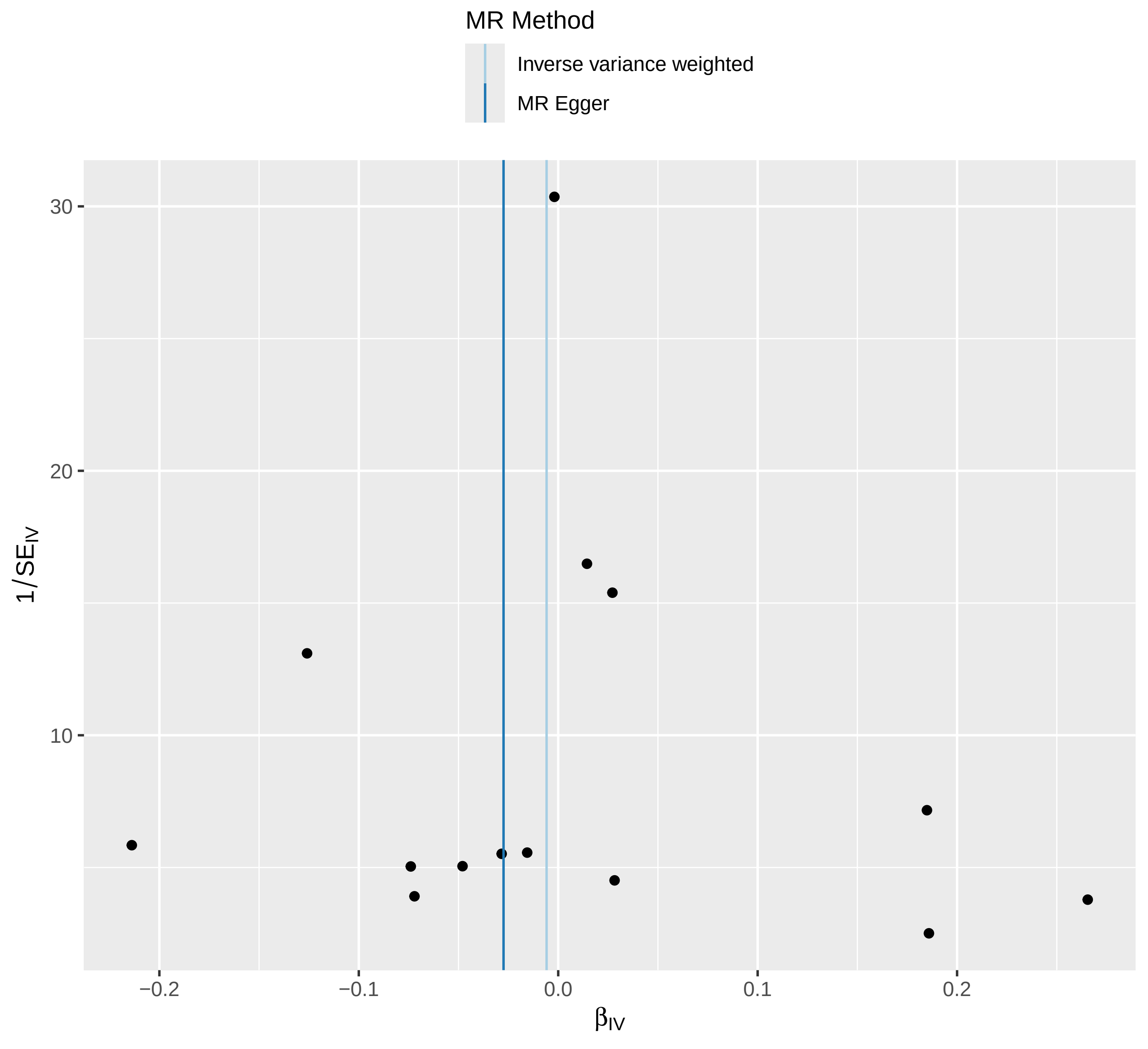

MR Method
Inverse variance weighted
MR Egger
30
20
10
1/SE_IV
−0.2
−0.1
0.0
0.1
0.2
β_IV

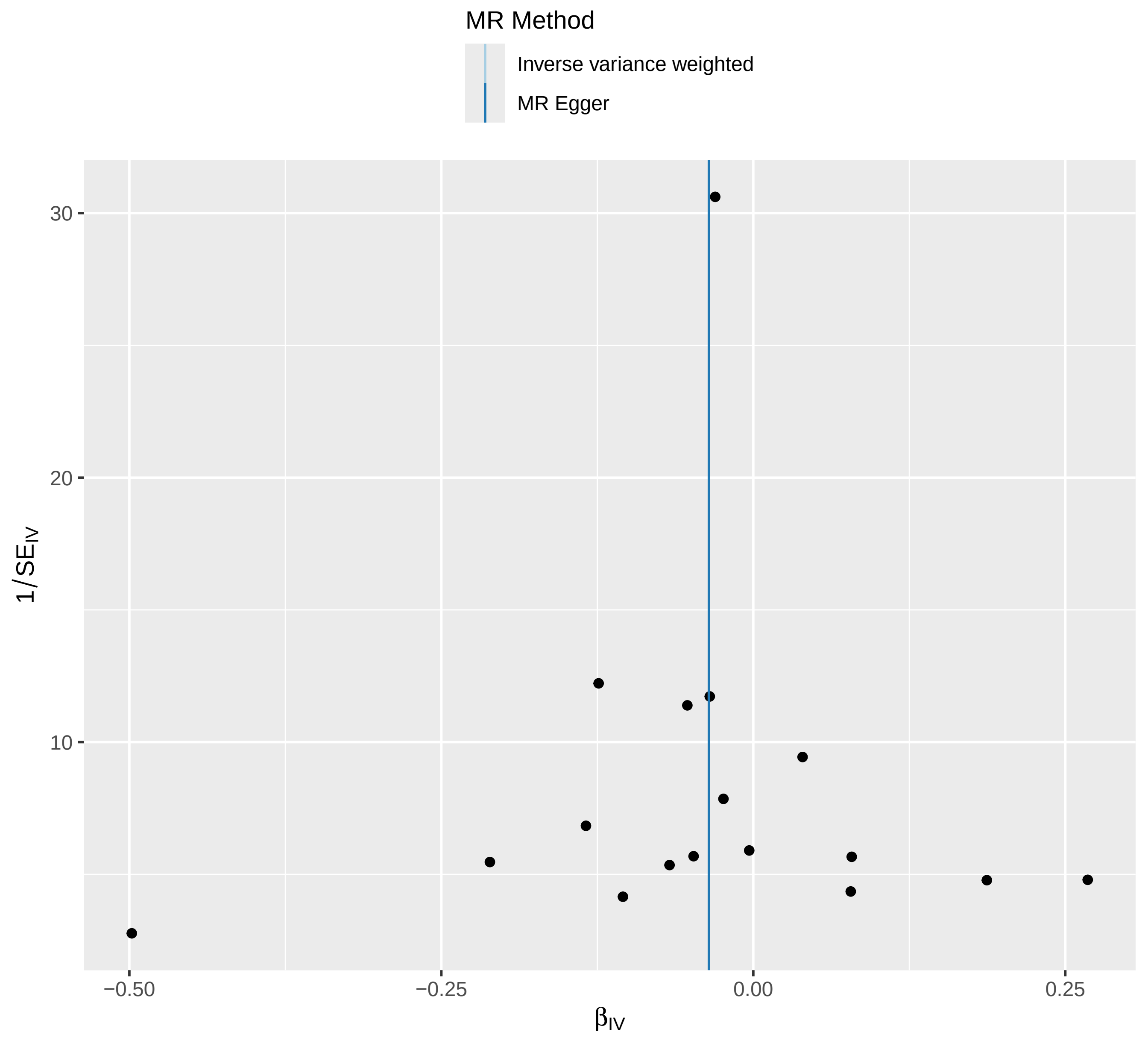
MR Method
Inverse variance weighted
MR Egger
30
20
10
1/SE$_{IV}$
−0.50
−0.25
0.00
0.25
β$_{IV}$

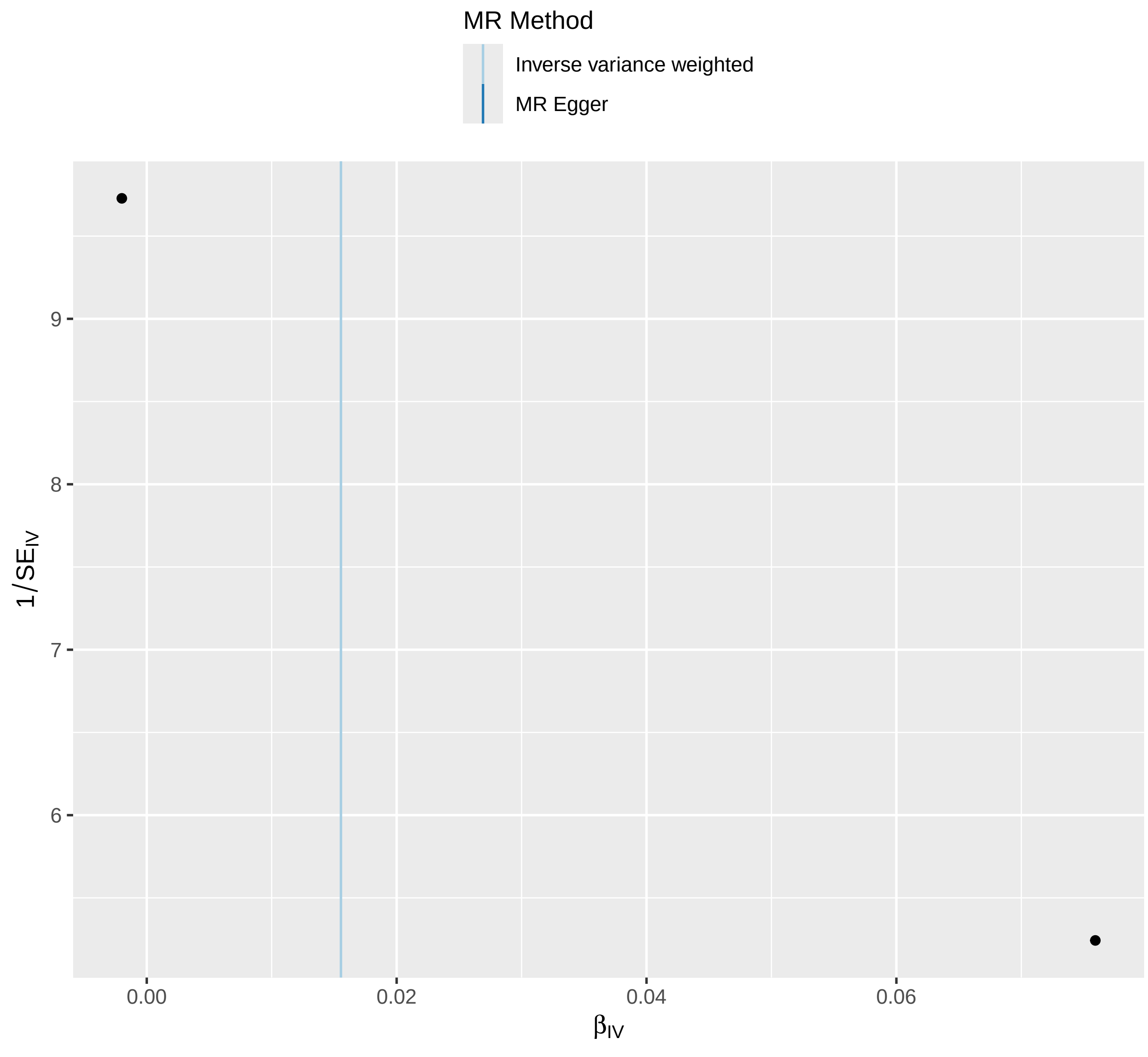
MR Method
Inverse variance weighted
MR Egger
9
8
7
6
1/SE_IV
0.00
0.02
0.04
0.06
β_IV

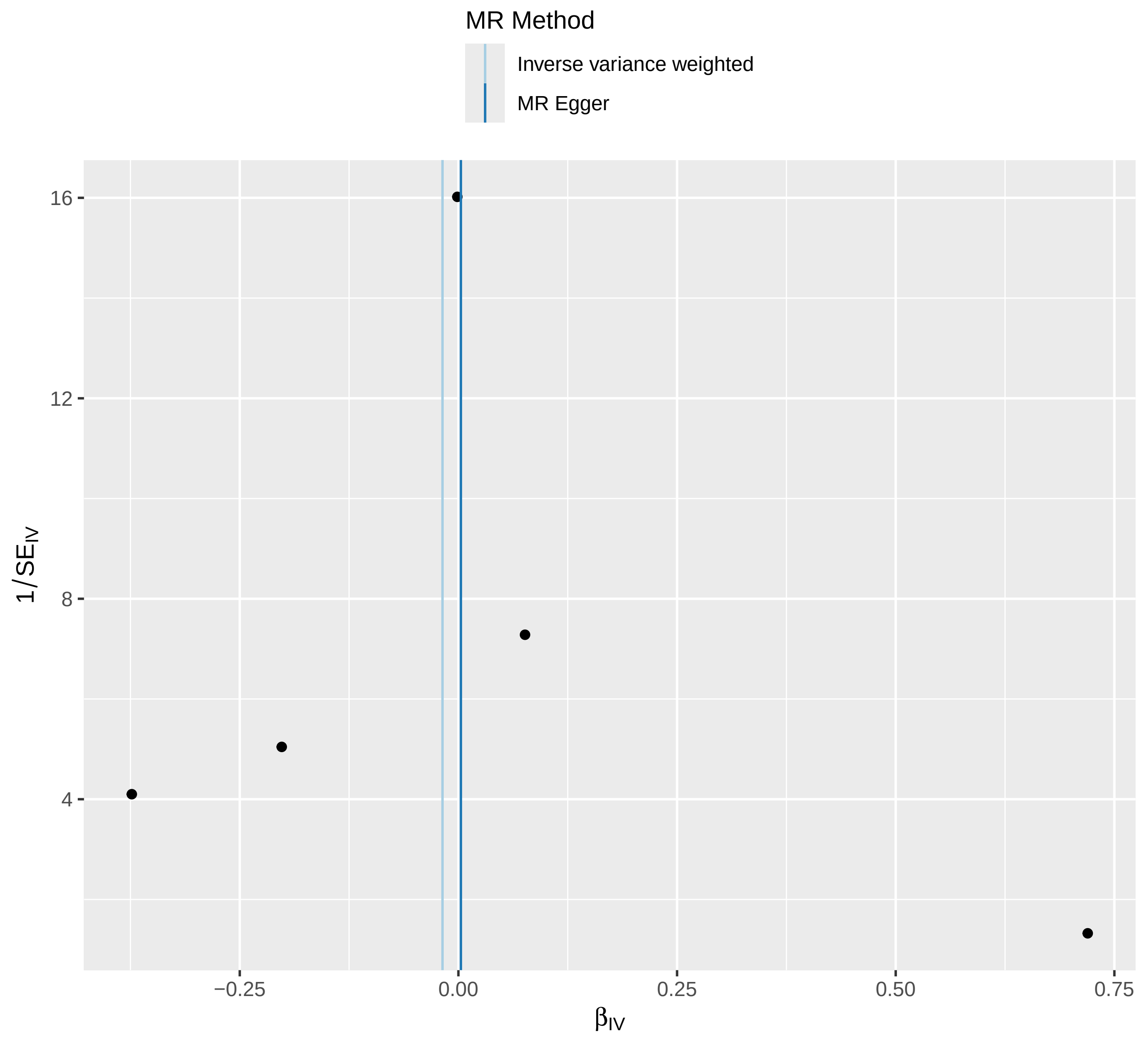
MR Method
Inverse variance weighted
MR Egger
16
12
8
4
1/SE_IV
−0.25
0.00
0.25
0.50
0.75
β_IV

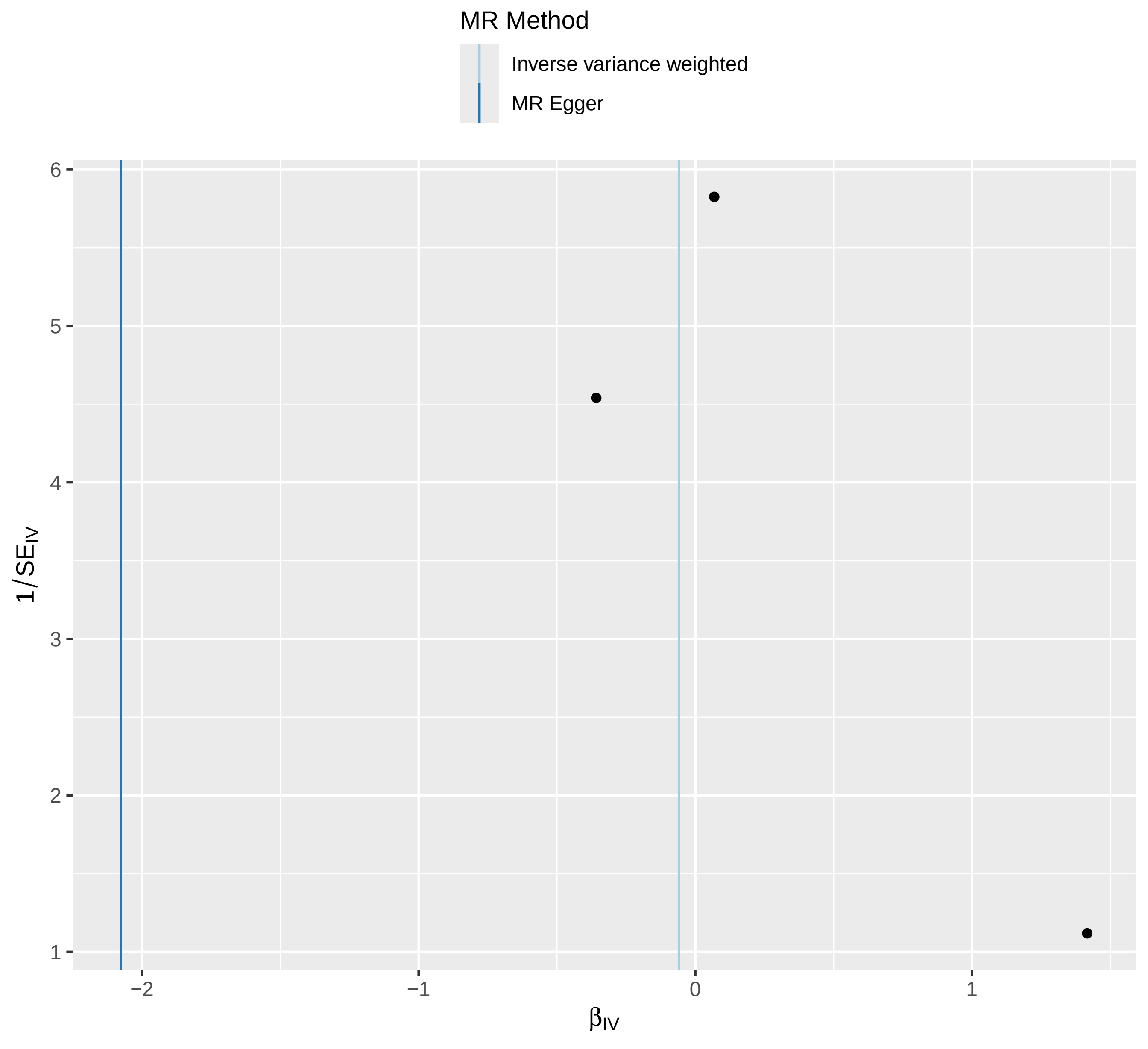
MR Method
Inverse variance weighted
MR Egger
1/SE$_{IV}$
6
5
4
3
2
1
−2
−1
0
1
β$_{IV}$

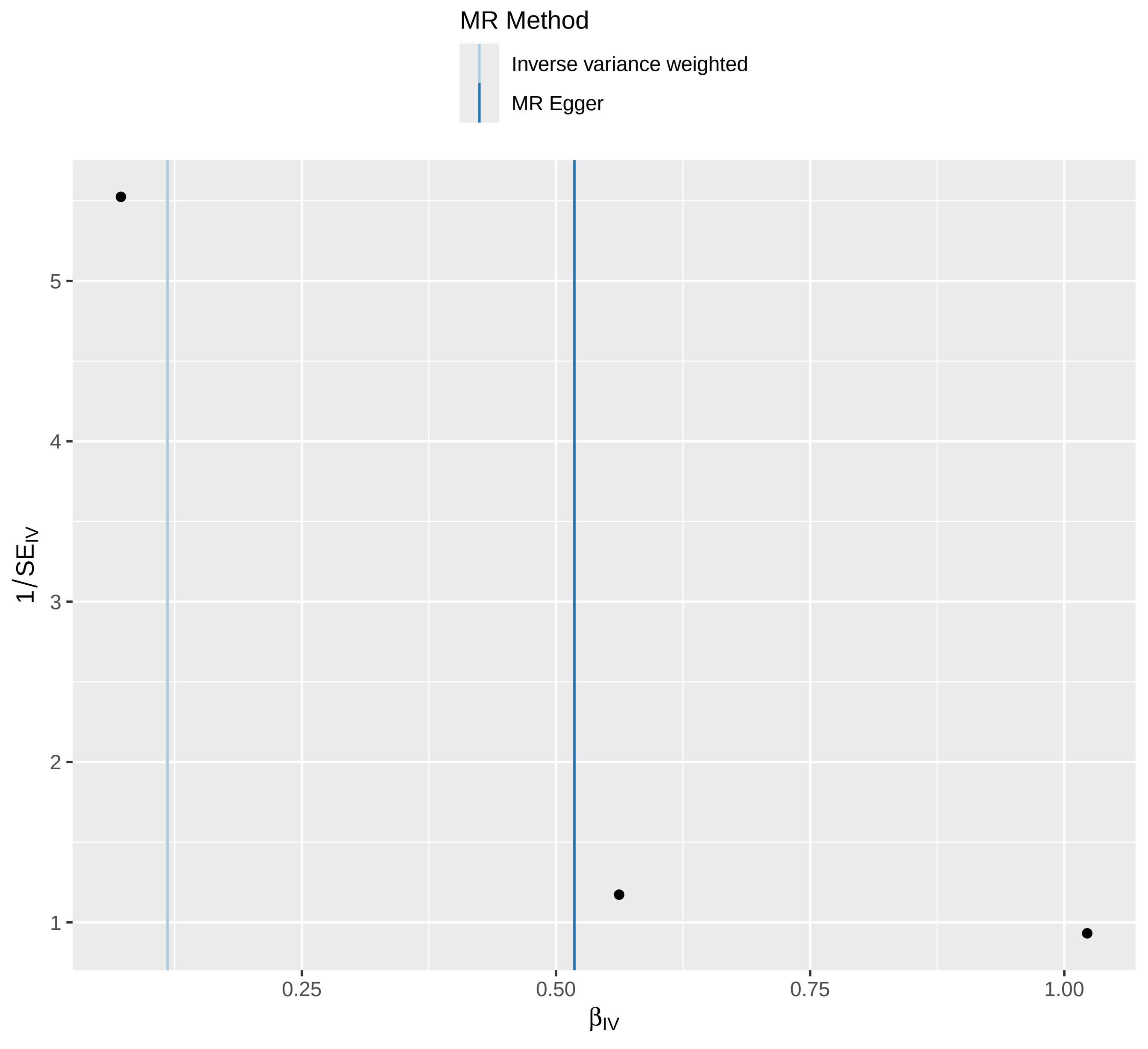

MR Method
Inverse variance weighted
MR Egger
5
4
3
2
1
1/SE_IV
0.25
0.50
0.75
1.00
β_IV

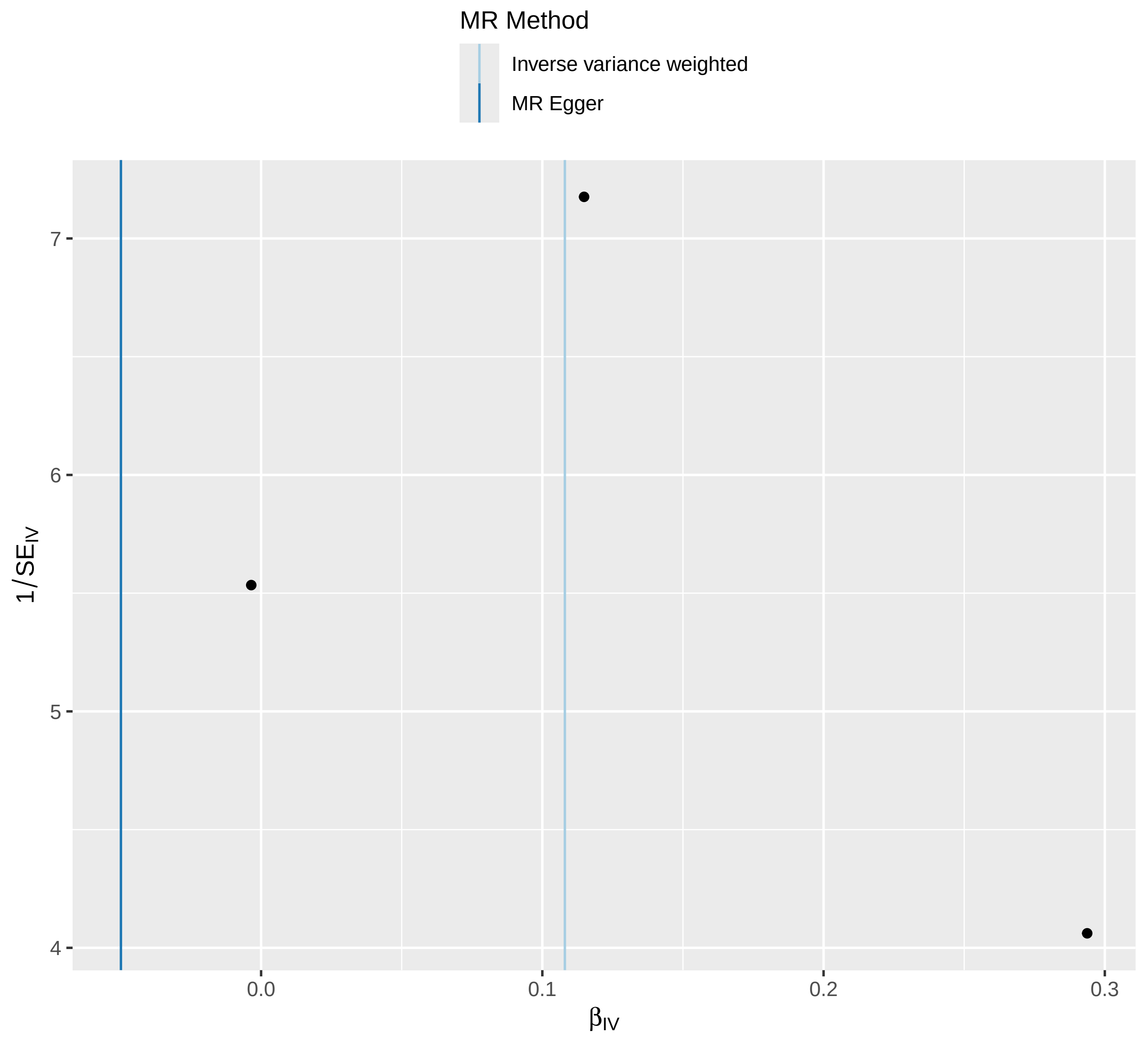

MR Method
Inverse variance weighted
MR Egger
7
6
5
4
1/SE$_{IV}$
0.0
0.1
0.2
0.3
β$_{IV}$

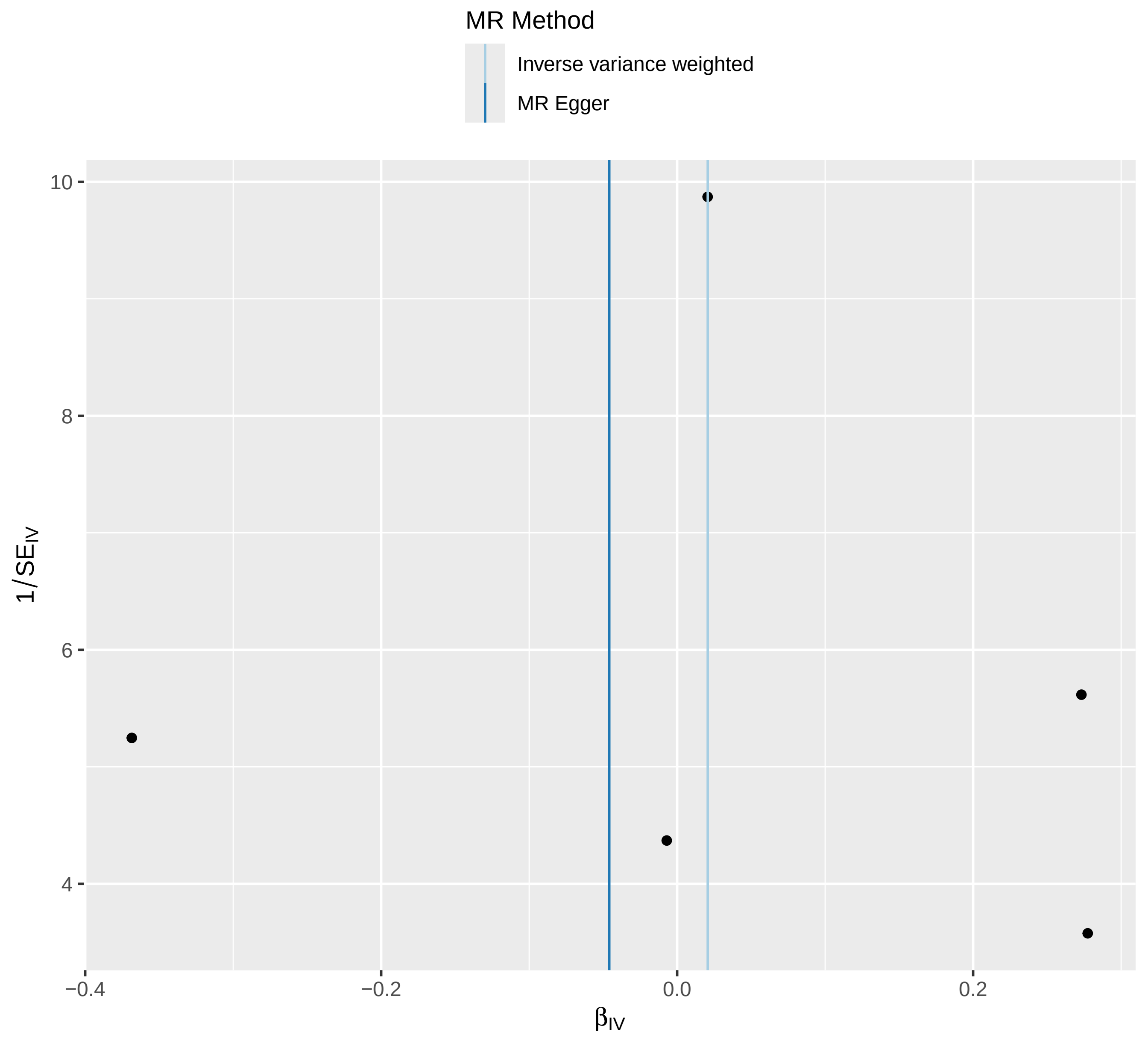
MR Method
Inverse variance weighted
MR Egger
10
8
6
4
1/SE$_{IV}$
−0.4
−0.2
0.0
0.2
β$_{IV}$

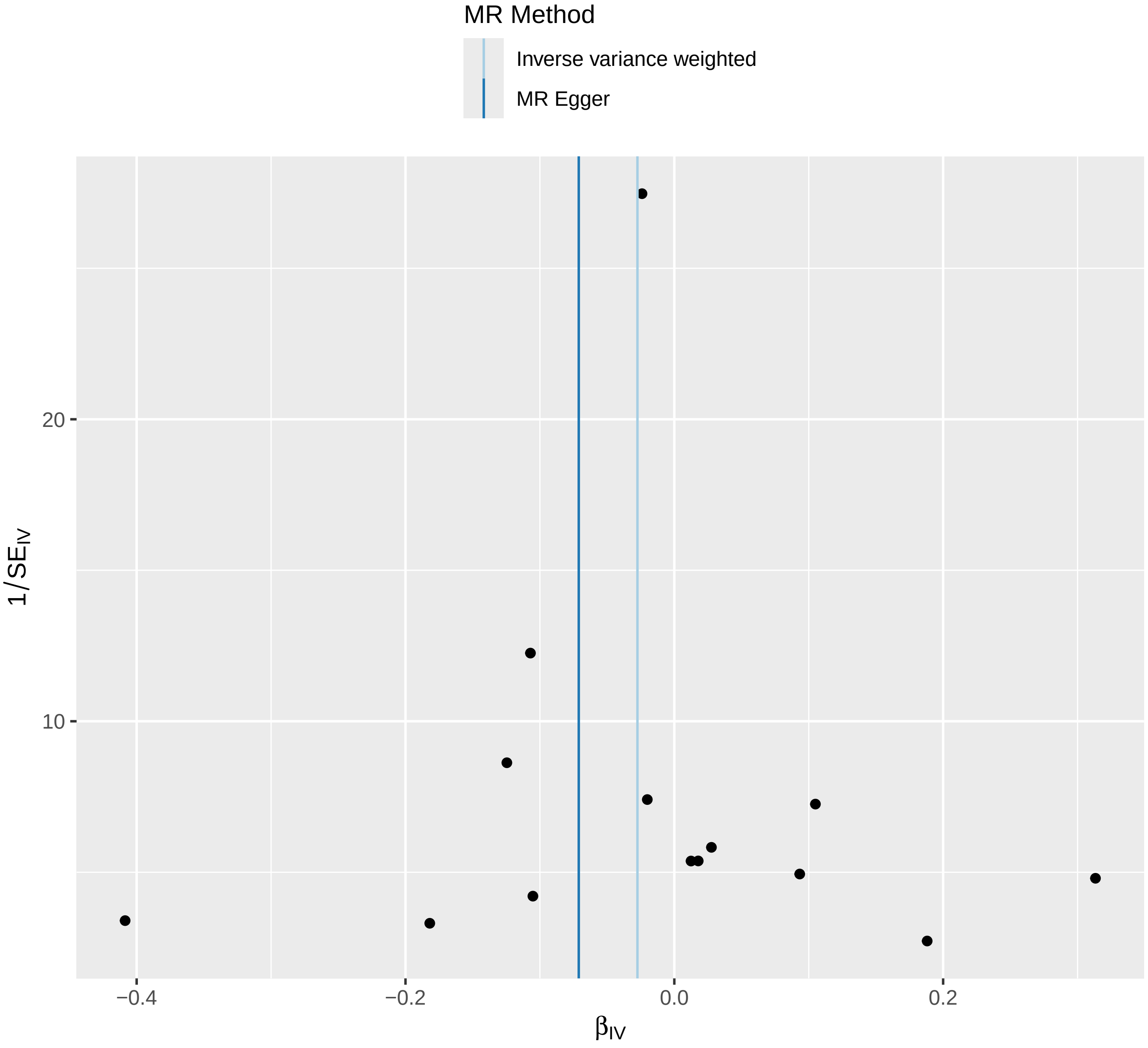
MR Method
Inverse variance weighted
MR Egger
20
10
1/SE$_{IV}$
−0.4
−0.2
0.0
0.2
β$_{IV}$

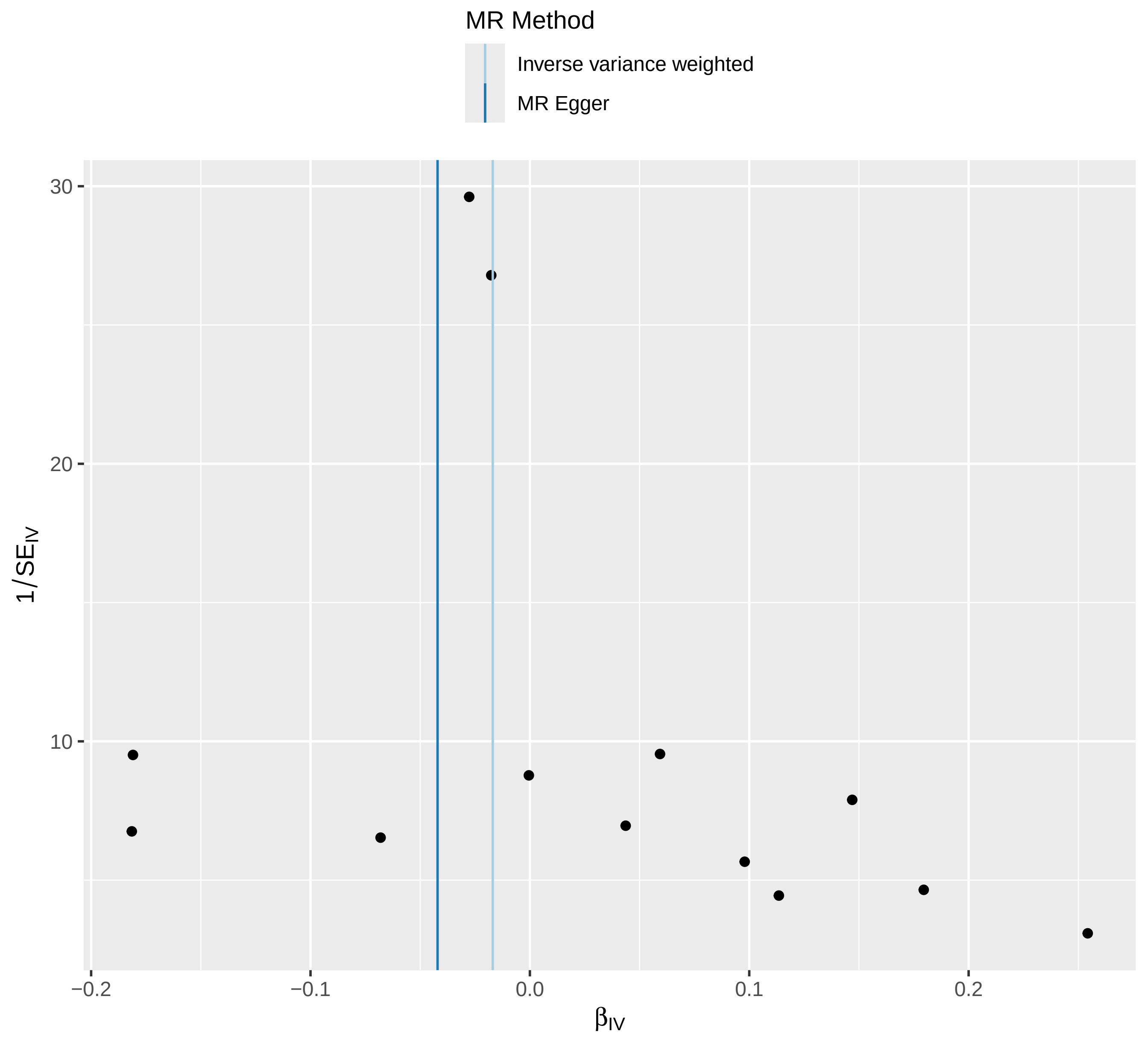
MR Method
Inverse variance weighted
MR Egger
30
20
10
1/SE$_{IV}$
−0.2
−0.1
0.0
0.1
0.2
β$_{IV}$

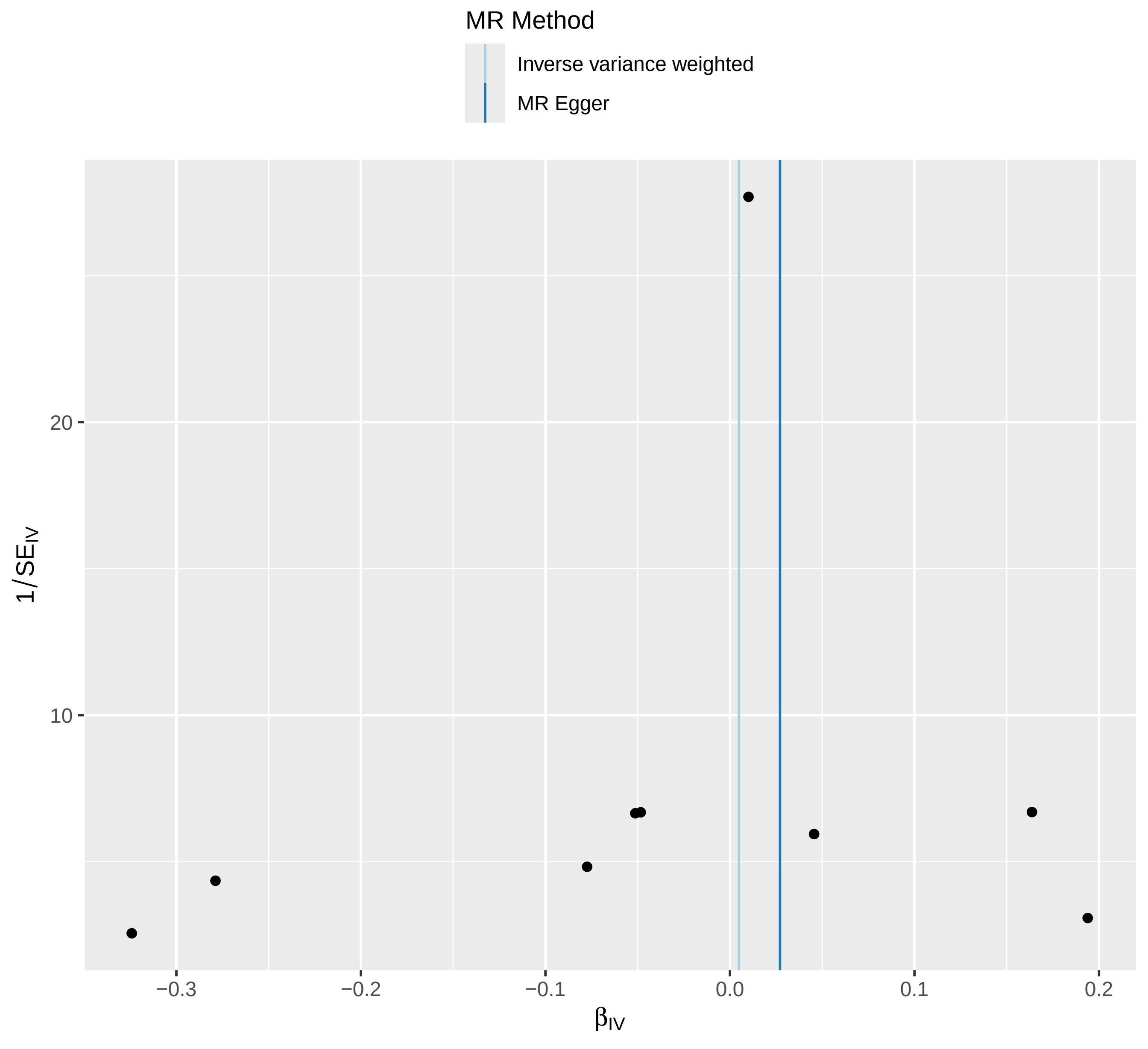
MR Method
Inverse variance weighted
MR Egger
20
10
1/SE$_{IV}$
−0.3
−0.2
−0.1
0.0
0.1
0.2
β$_{IV}$

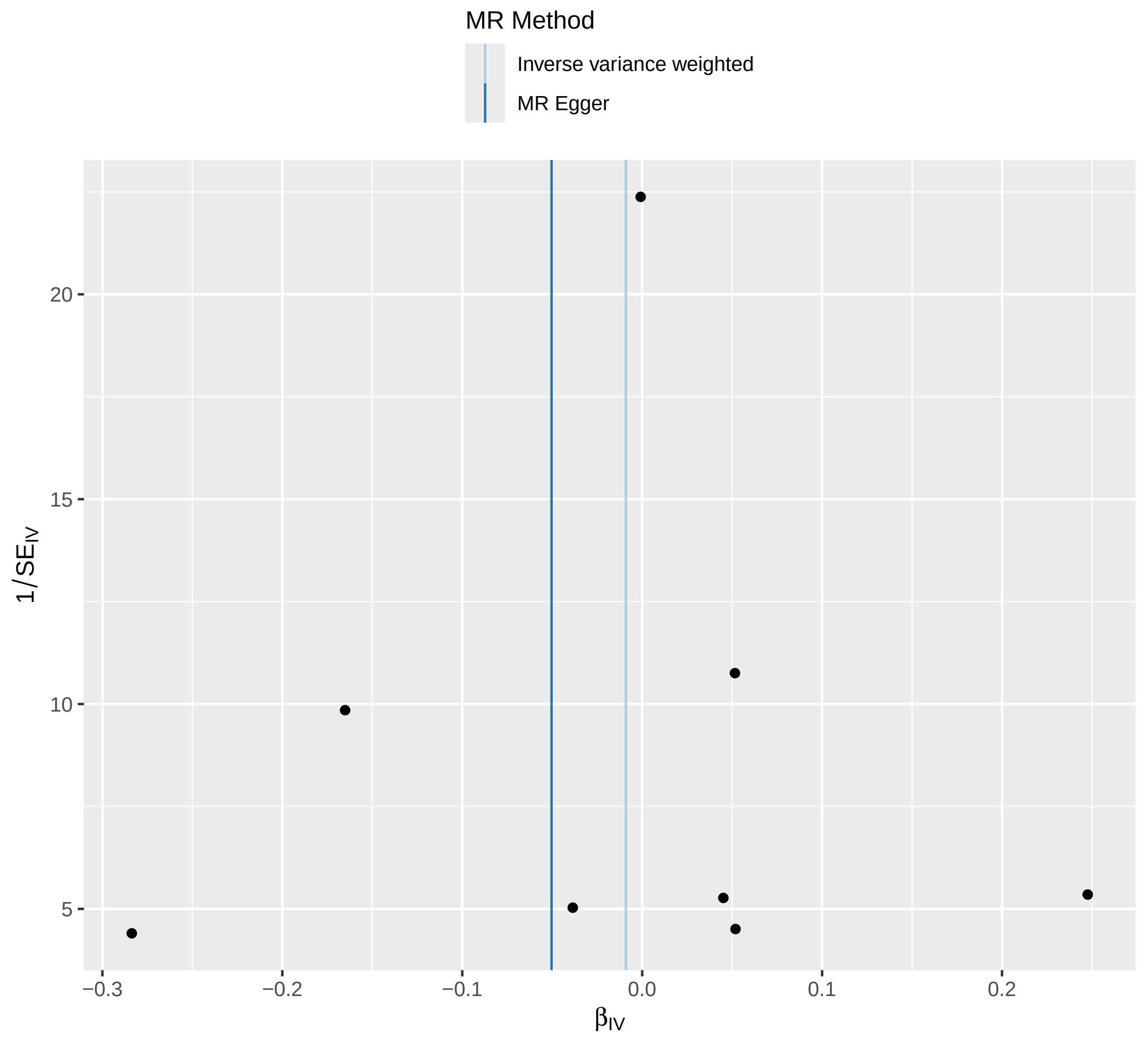
MR Method
Inverse variance weighted
MR Egger
20
15
10
5
1/SE$_{IV}$
−0.3
−0.2
−0.1
0.0
0.1
0.2
β$_{IV}$

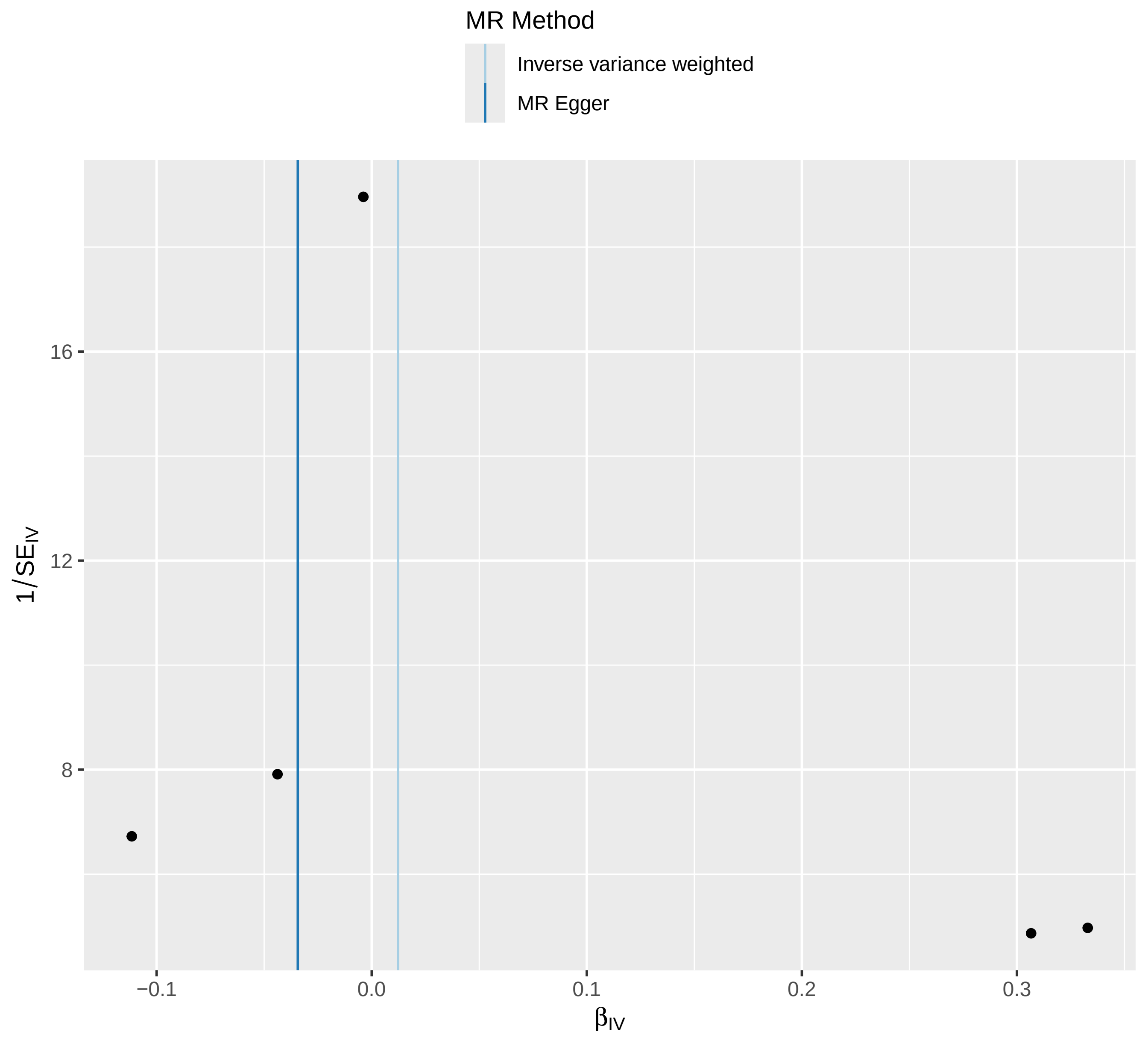
MR Method
Inverse variance weighted
MR Egger
16
12
8
1/SE$_{IV}$
−0.1
0.0
0.1
0.2
0.3
β$_{IV}$

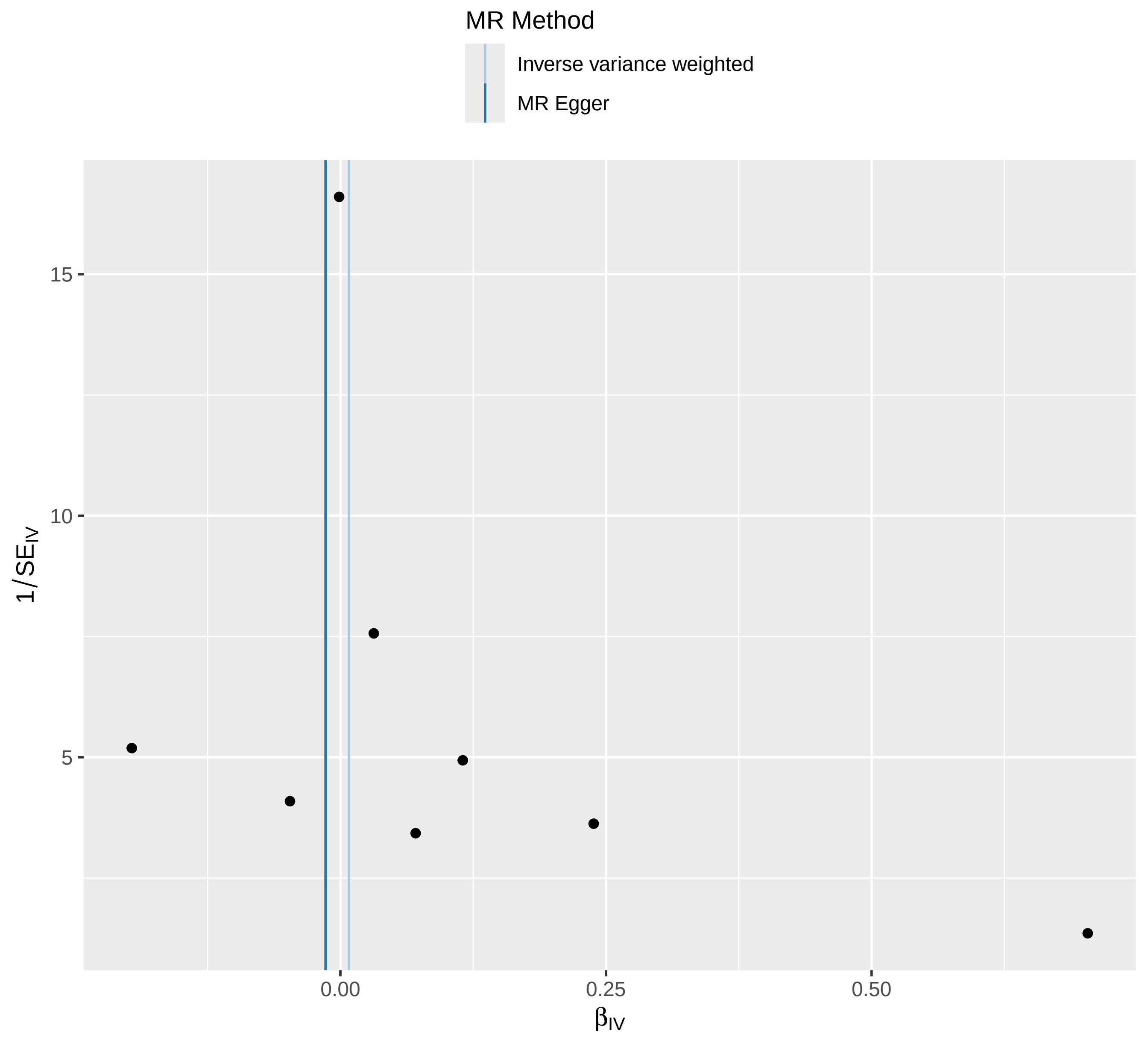

MR Method
Inverse variance weighted
MR Egger
1/SE$_{IV}$
15
10
5
0.00
0.25
0.50
β$_{IV}$

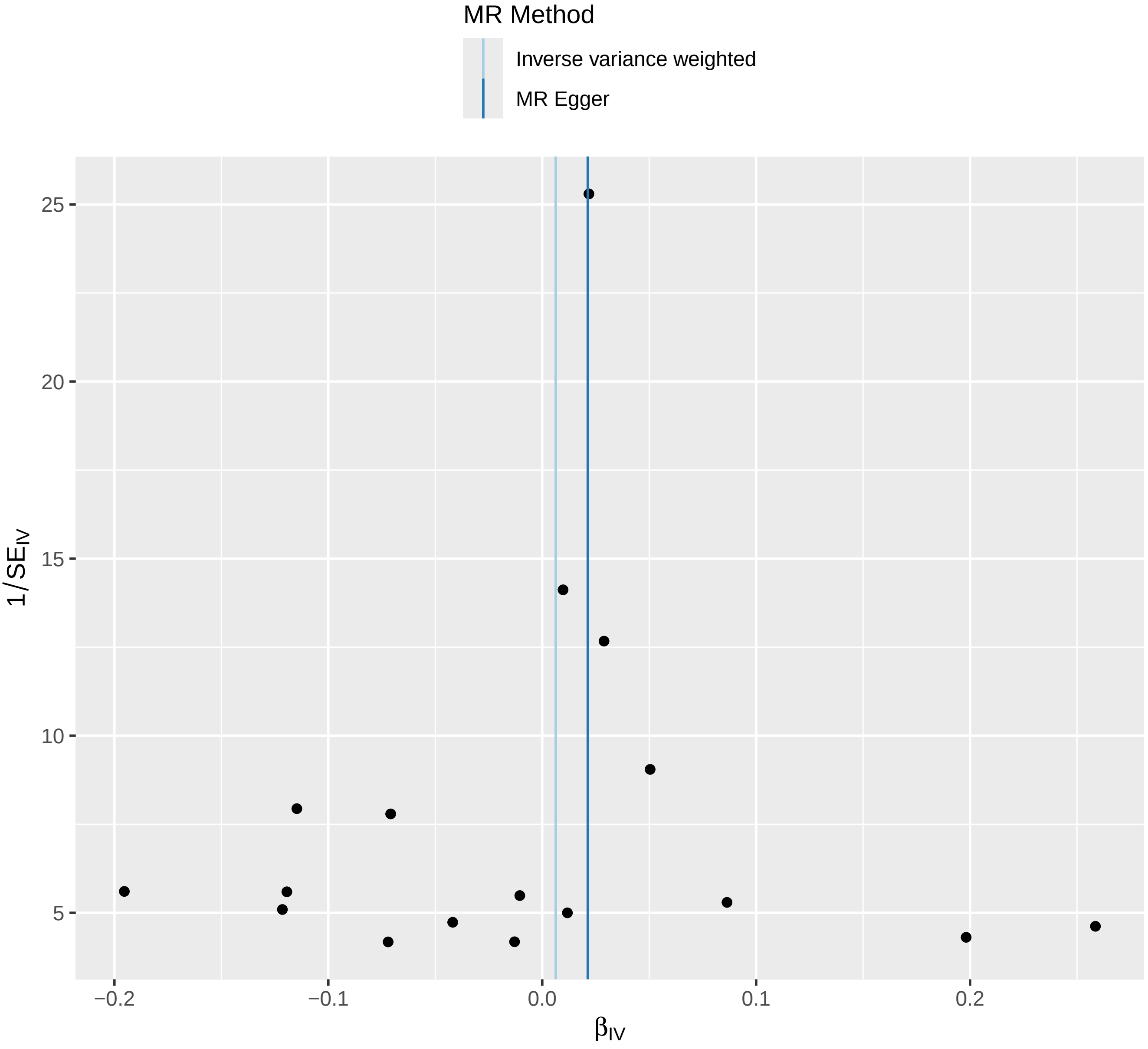
MR Method
Inverse variance weighted
MR Egger
25
20
15
10
5
1/SE$_{IV}$
−0.2
−0.1
0.0
0.1
0.2
β$_{IV}$

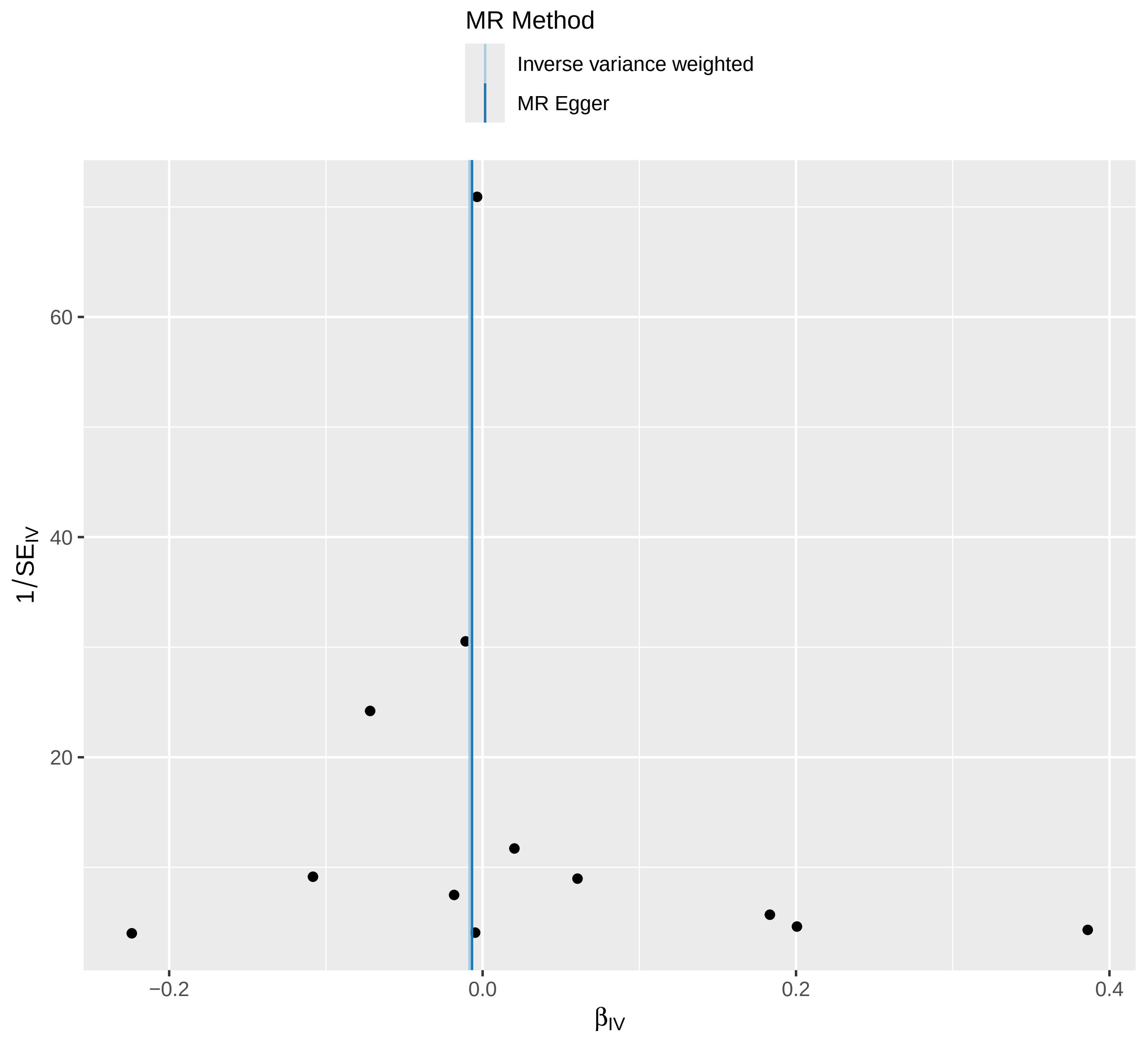

MR Method
Inverse variance weighted
MR Egger
60
40
20
1/SE$_{IV}$
−0.2
0.0
0.2
0.4
$\beta_{IV}$

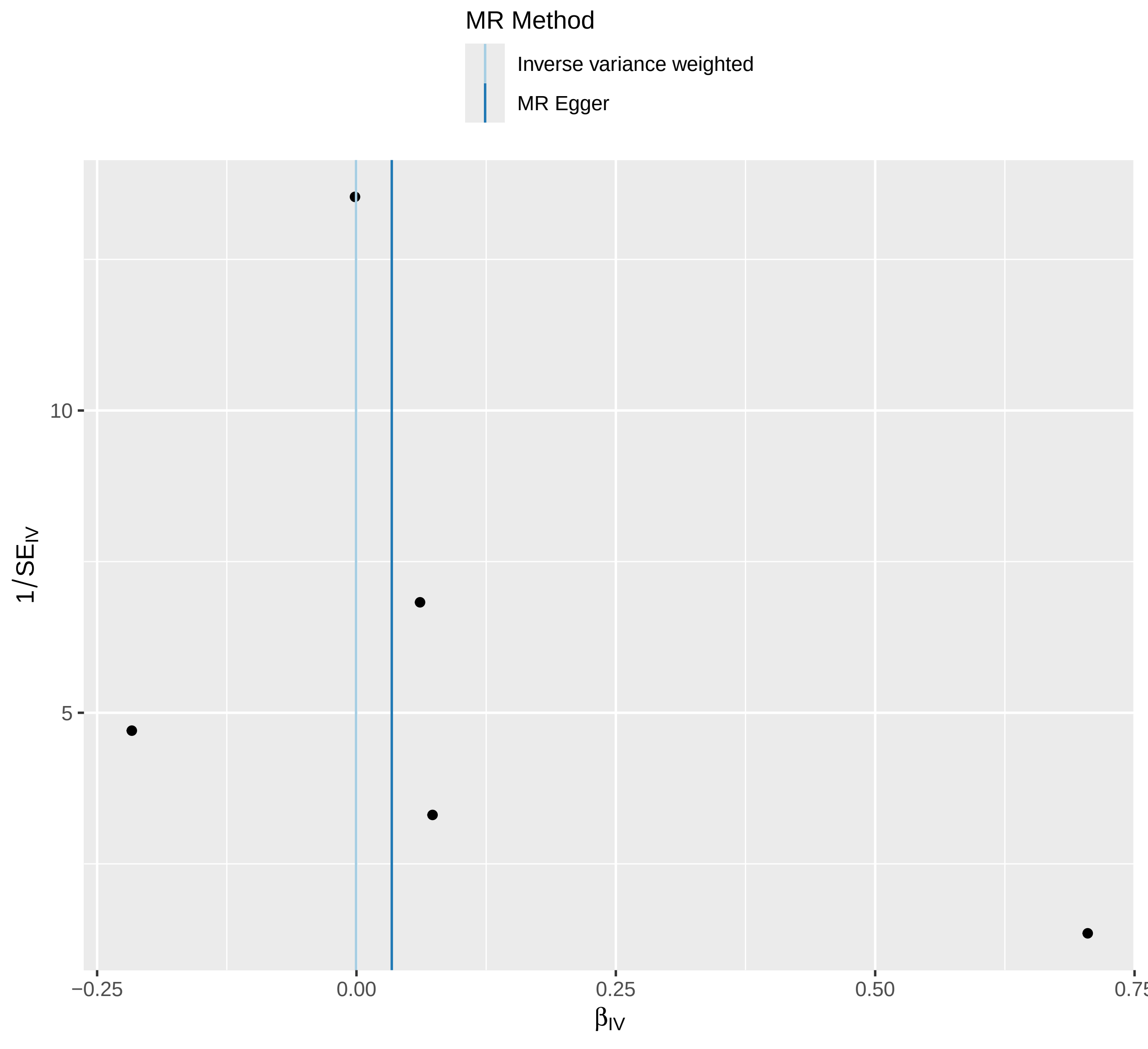
MR Method
Inverse variance weighted
MR Egger
10
5
$1/SE_{IV}$
−0.25
0.00
0.25
0.50
0.75
$\beta_{IV}$

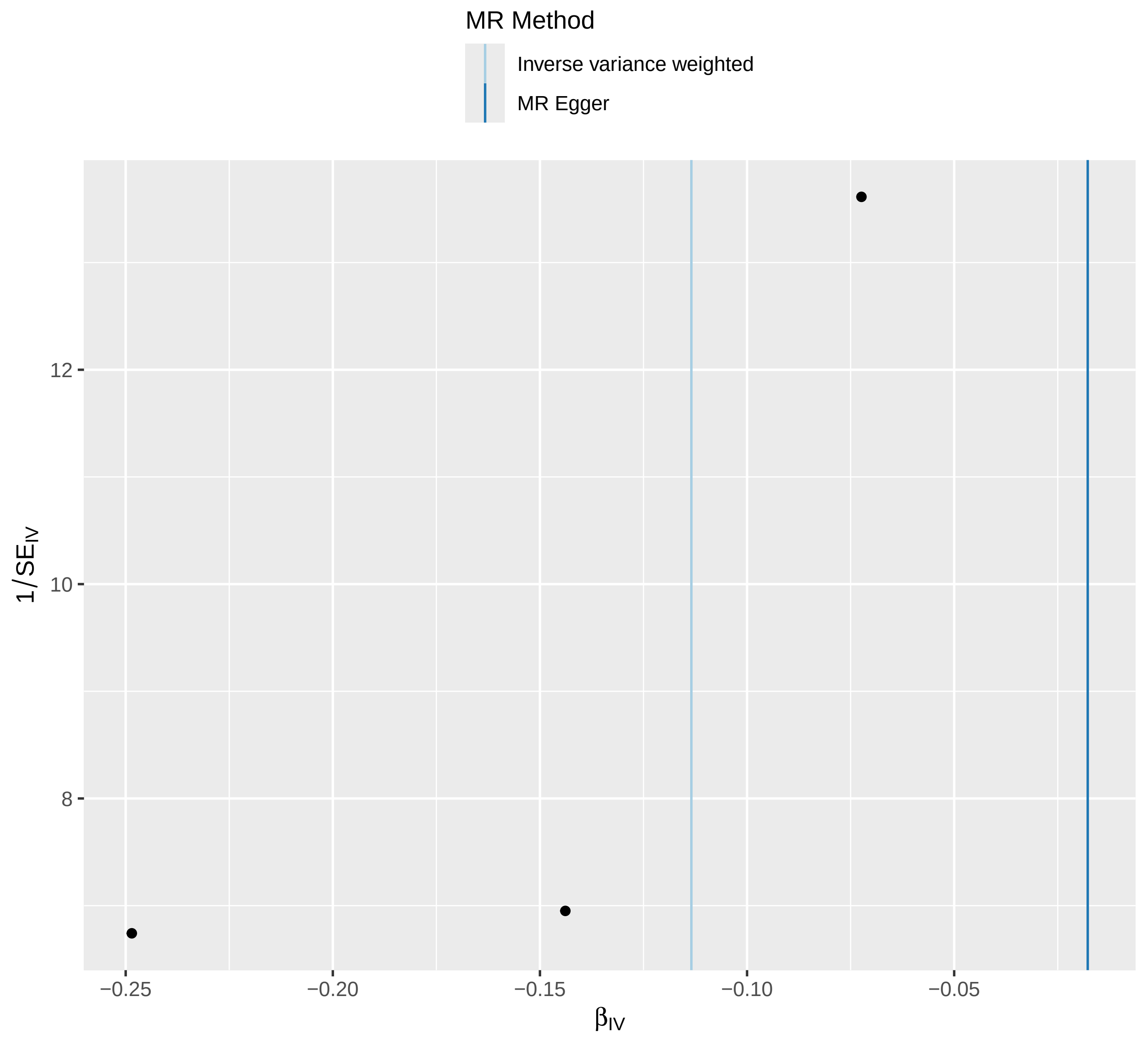

MR Method
Inverse variance weighted
MR Egger
12
10
8
1/SE_IV
−0.25
−0.20
−0.15
−0.10
−0.05
β_IV

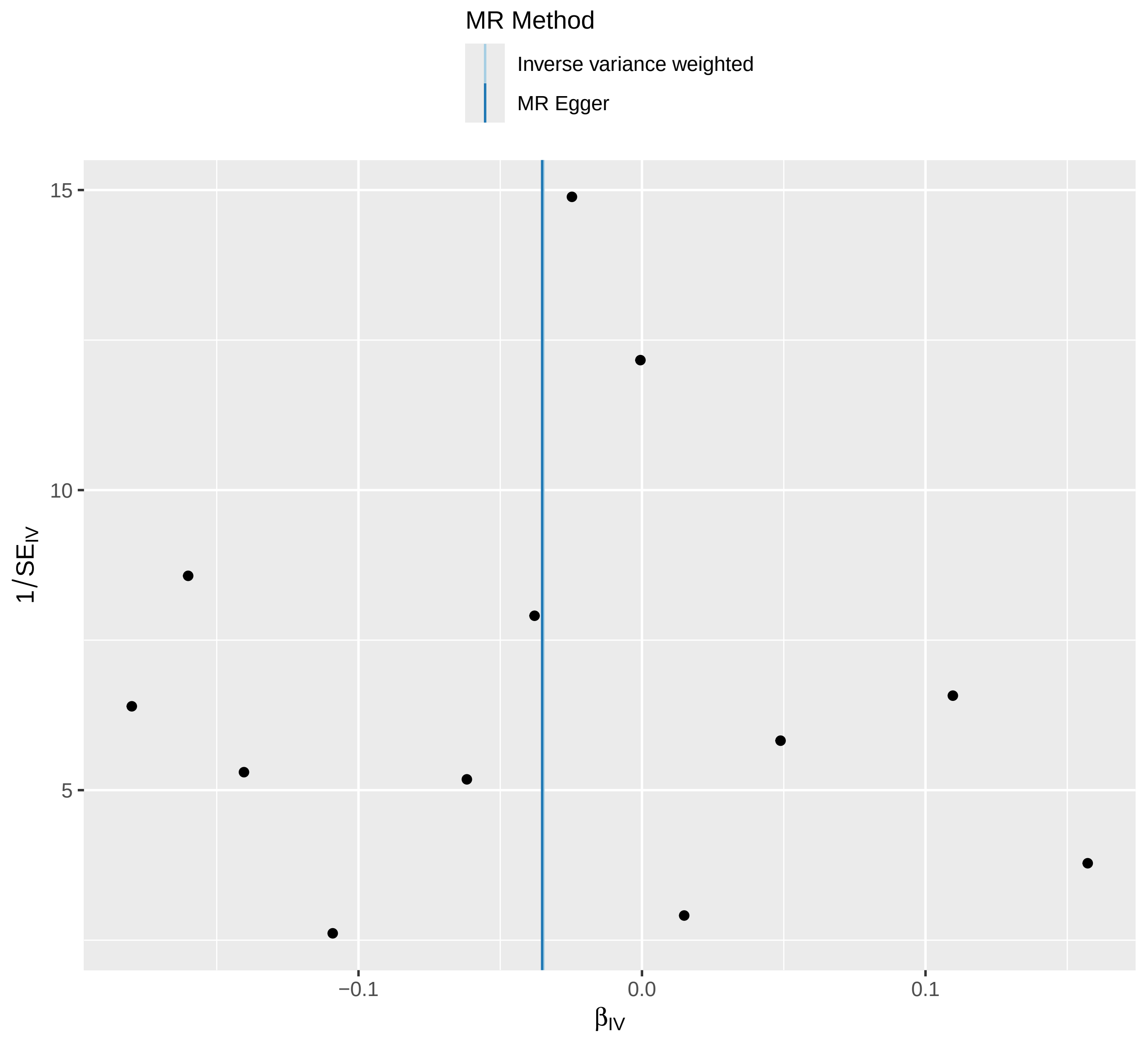

MR Method
Inverse variance weighted
MR Egger
15
10
5
1/SE$_{IV}$
−0.1
0.0
0.1
β$_{IV}$

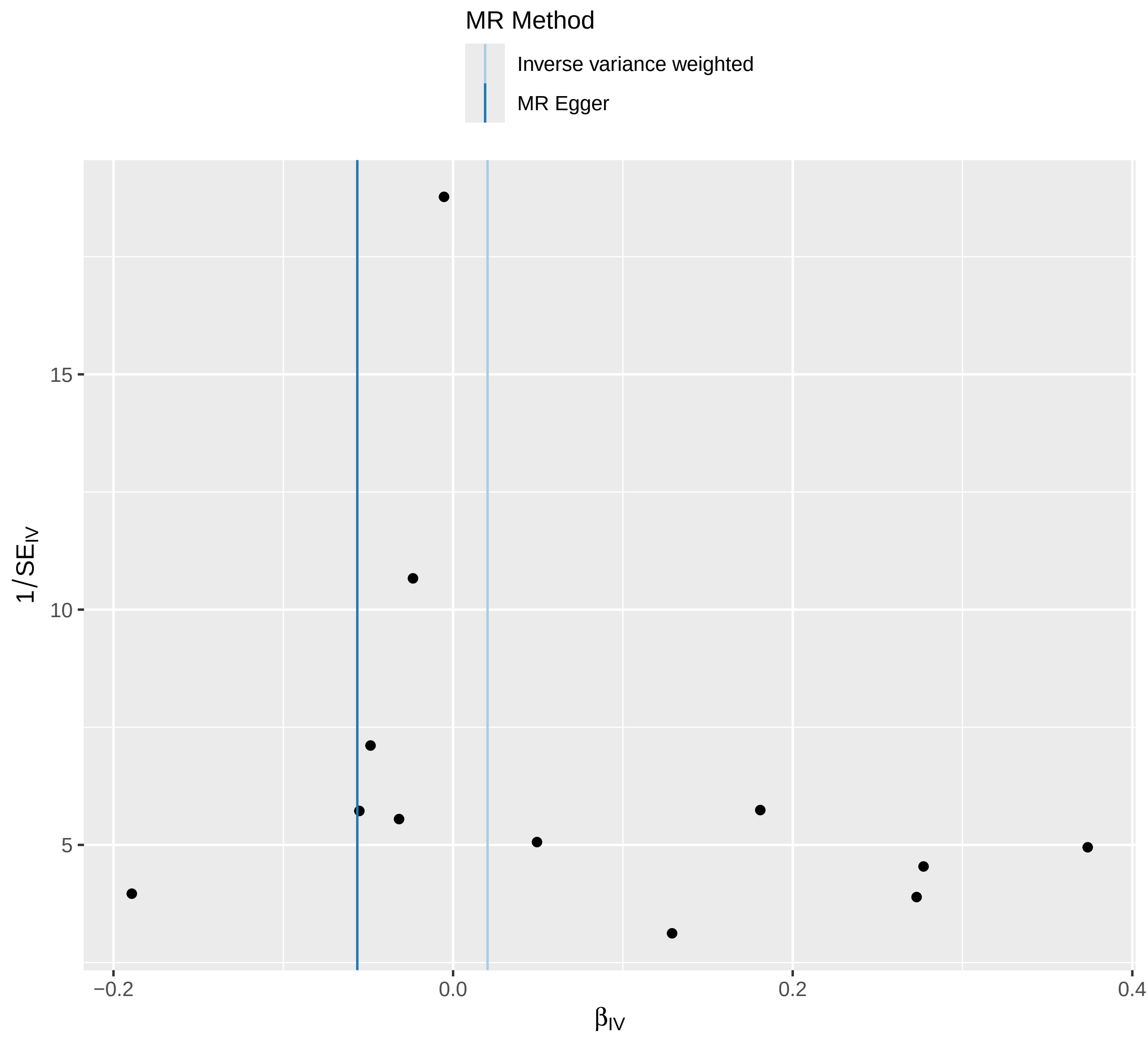

MR Method
Inverse variance weighted
MR Egger
15
10
5
1/SE$_{IV}$
−0.2
0.0
0.2
0.4
β$_{IV}$

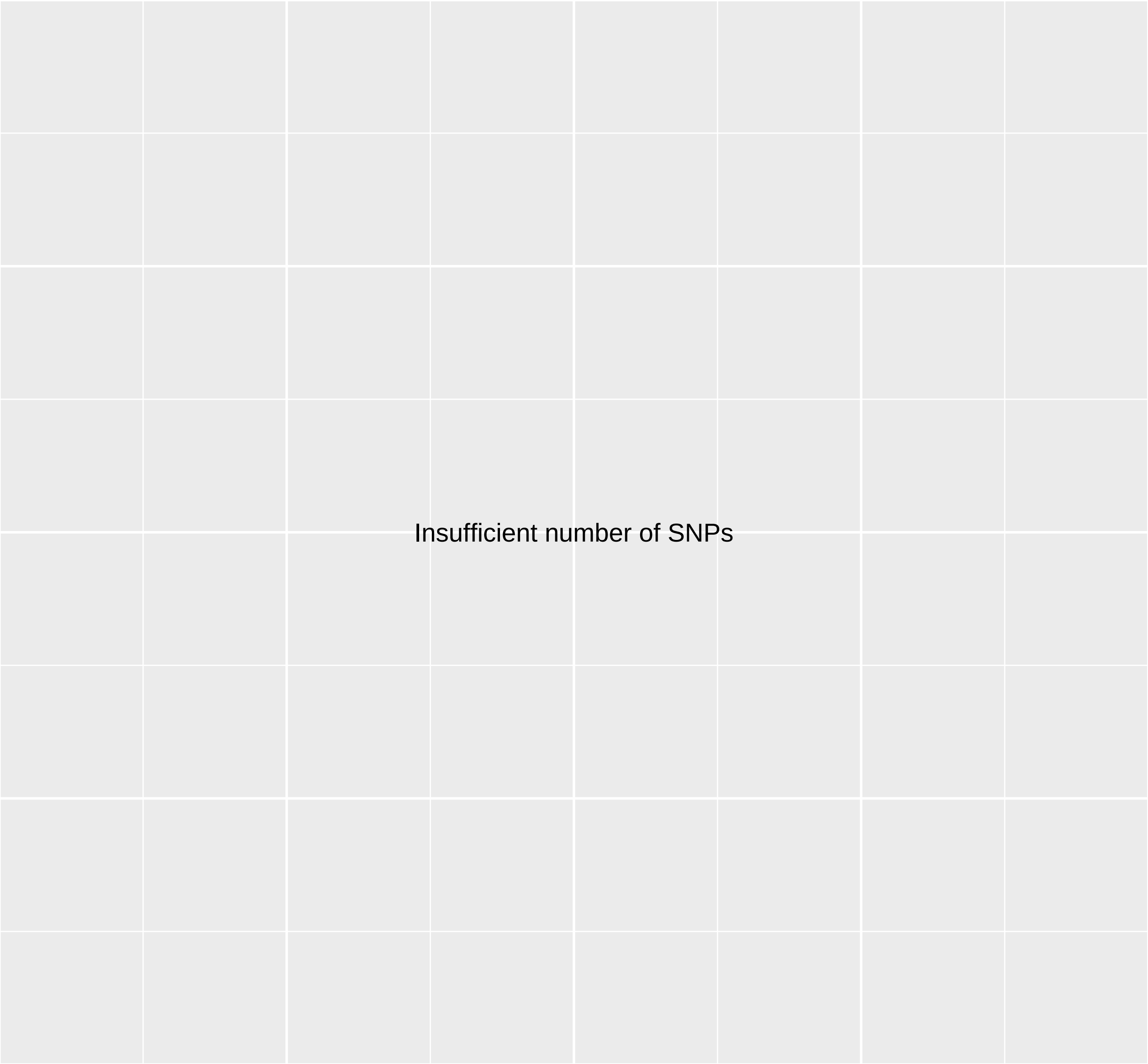
Insufficient number of SNPs

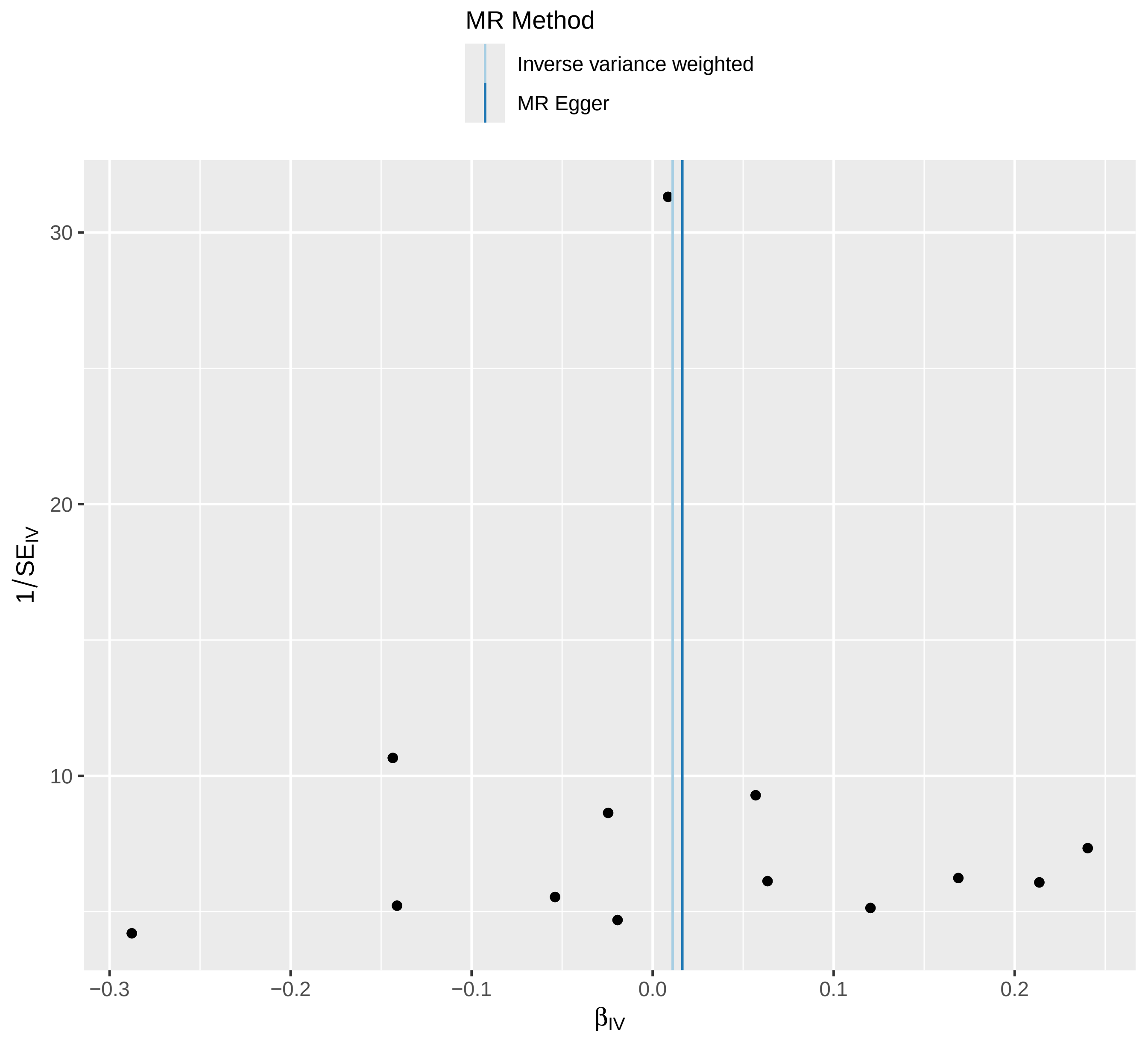
MR Method
Inverse variance weighted
MR Egger
30
20
10
1/SE$_{IV}$
−0.3
−0.2
−0.1
0.0
0.1
0.2
β$_{IV}$

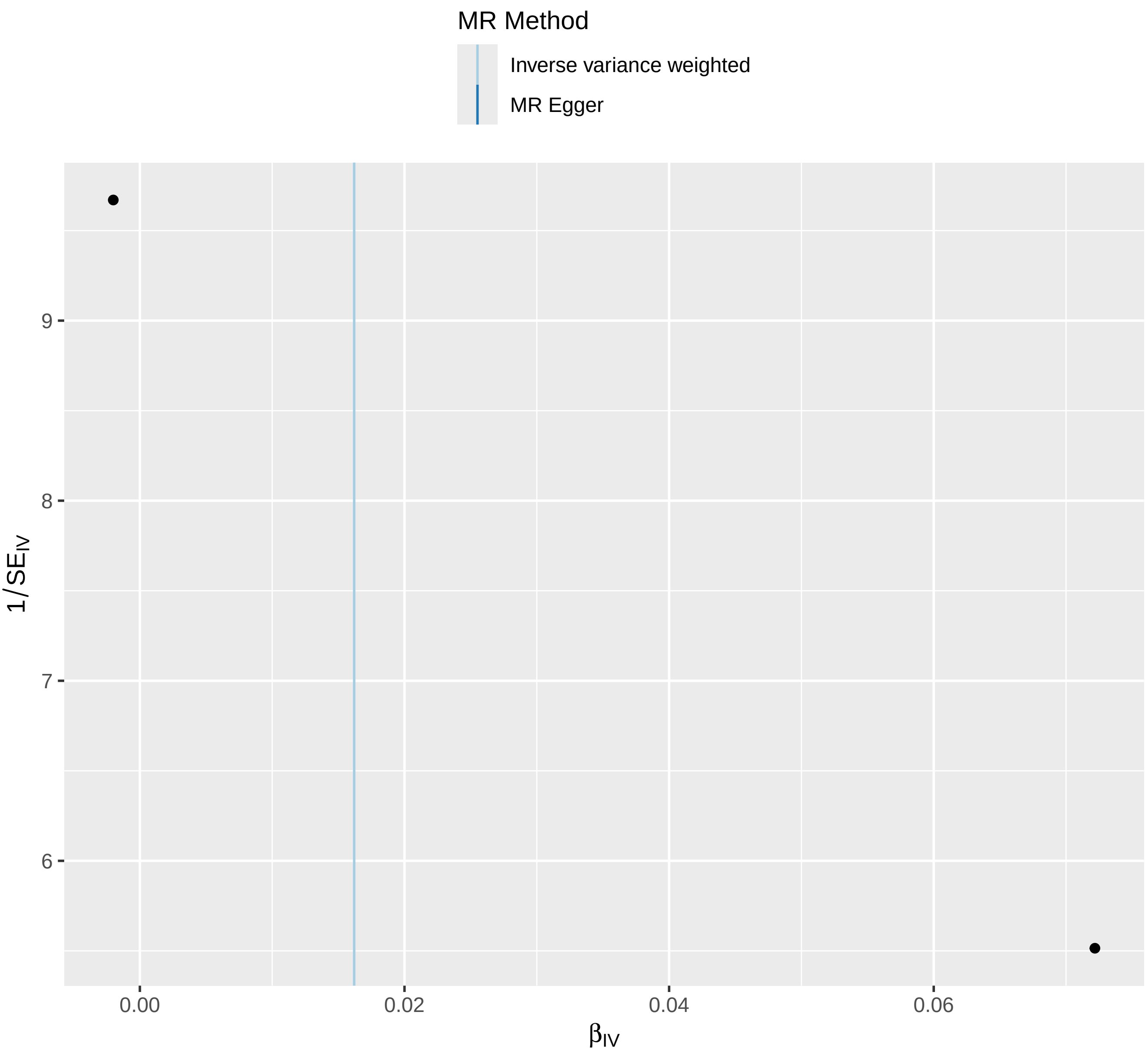
MR Method
Inverse variance weighted
MR Egger
9
8
7
6
1/SE_IV
0.00
0.02
0.04
0.06
β_IV

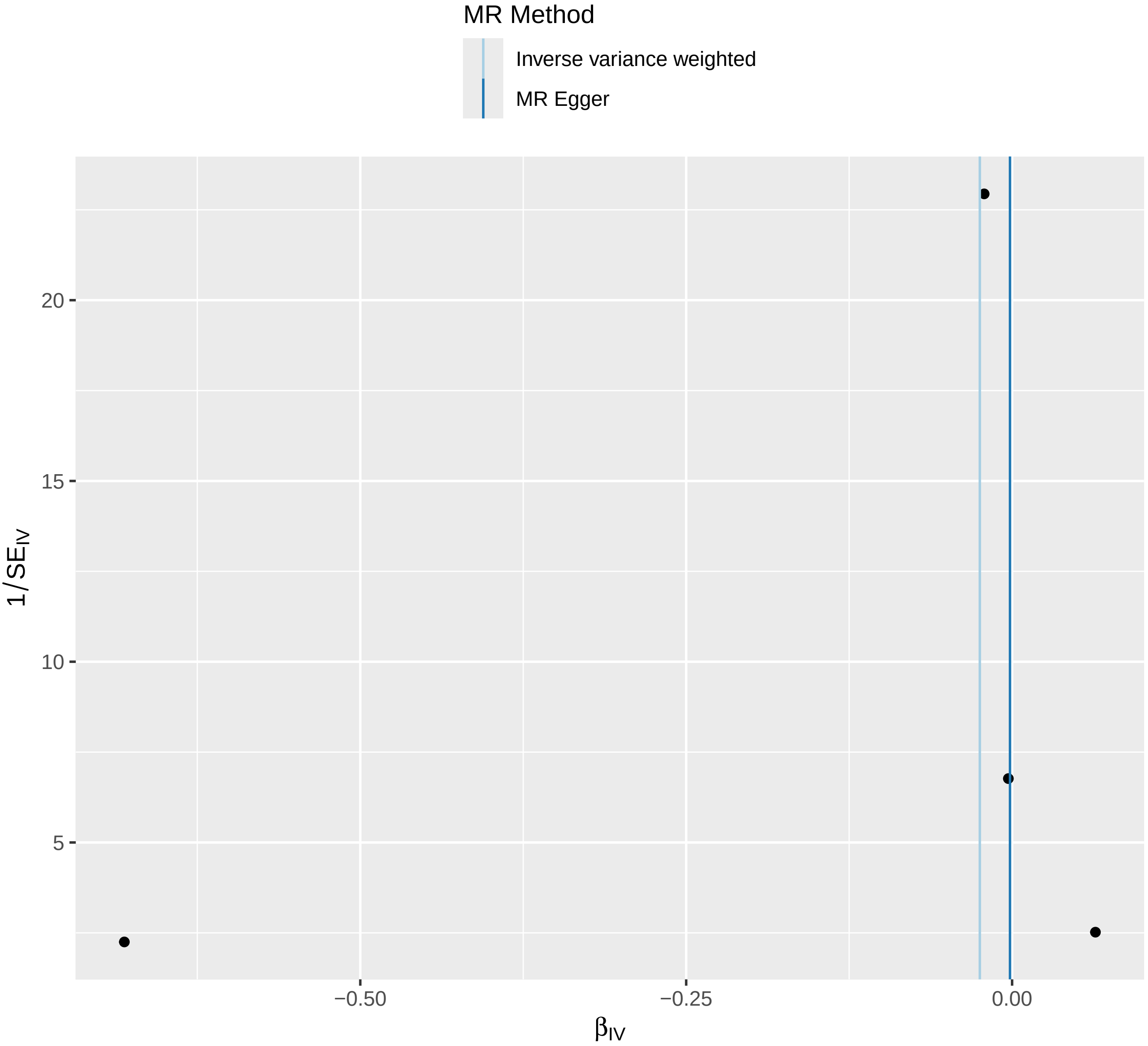

MR Method
Inverse variance weighted
MR Egger
20
15
10
5
1/SE_IV
−0.50
−0.25
0.00
β_IV

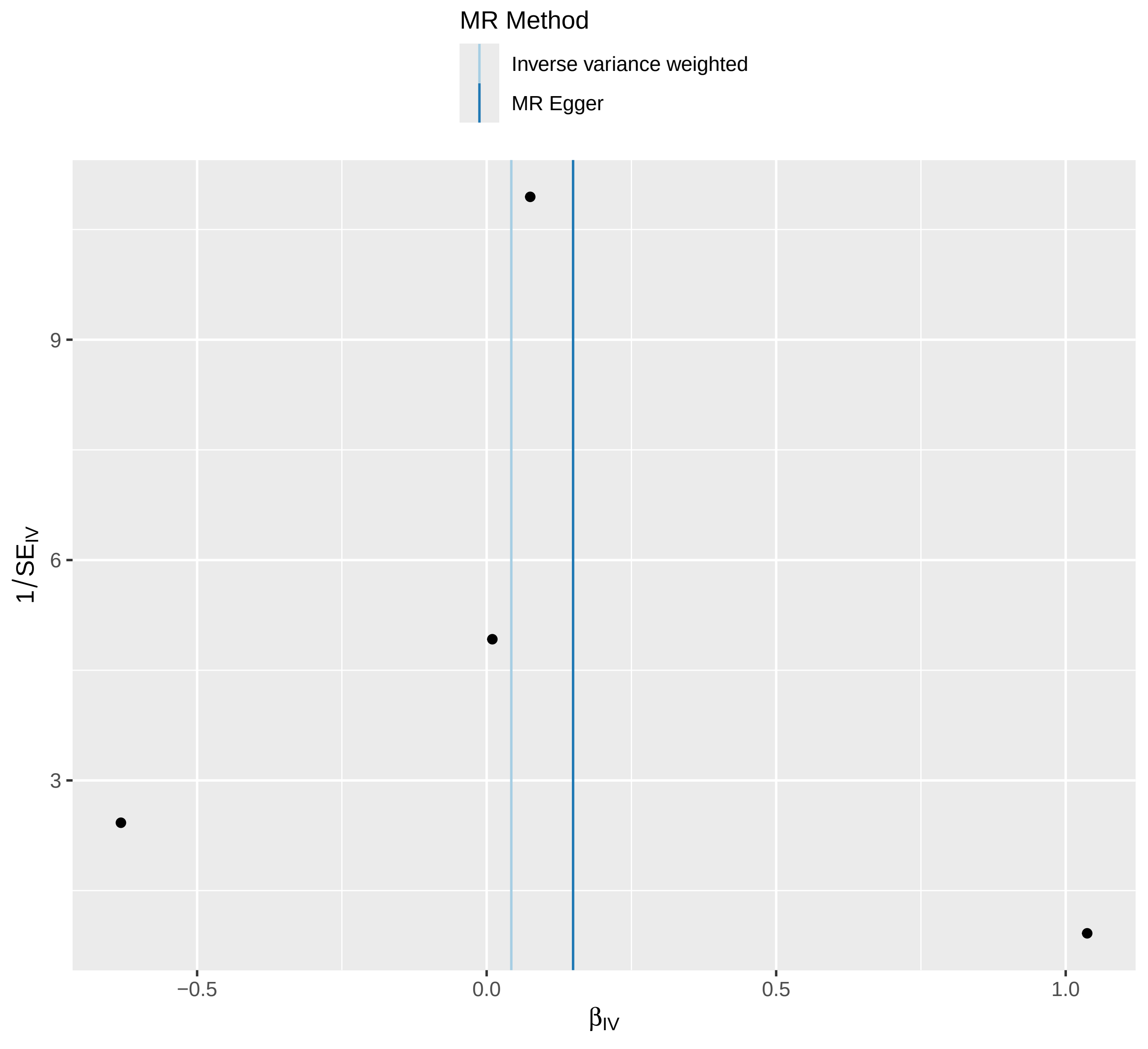

MR Method
Inverse variance weighted
MR Egger
9
6
3
1/SE_IV
−0.5
0.0
0.5
1.0
β_IV

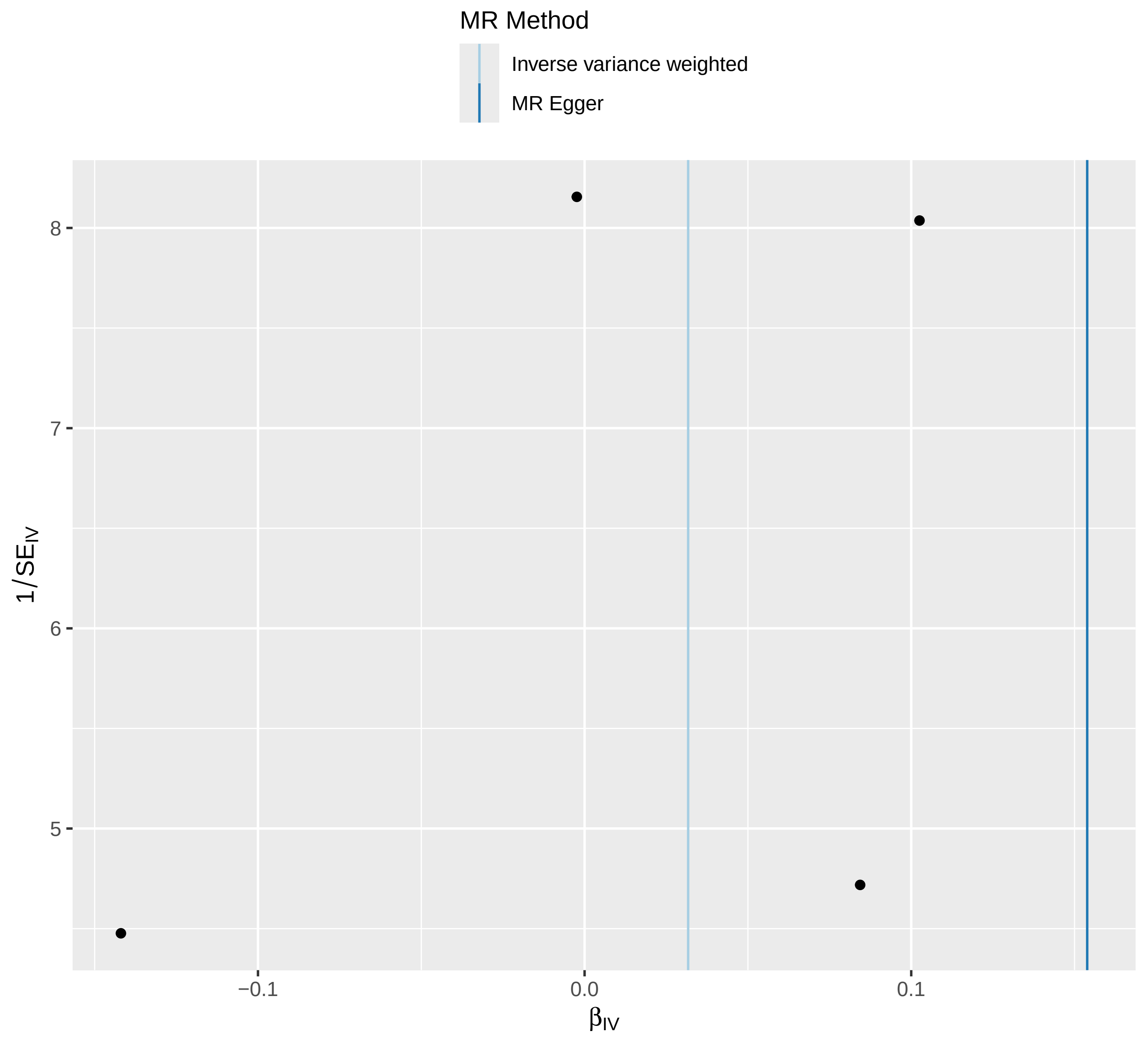
MR Method
Inverse variance weighted
MR Egger
8
7
6
5
1/SE$_{IV}$
−0.1
0.0
0.1
β$_{IV}$

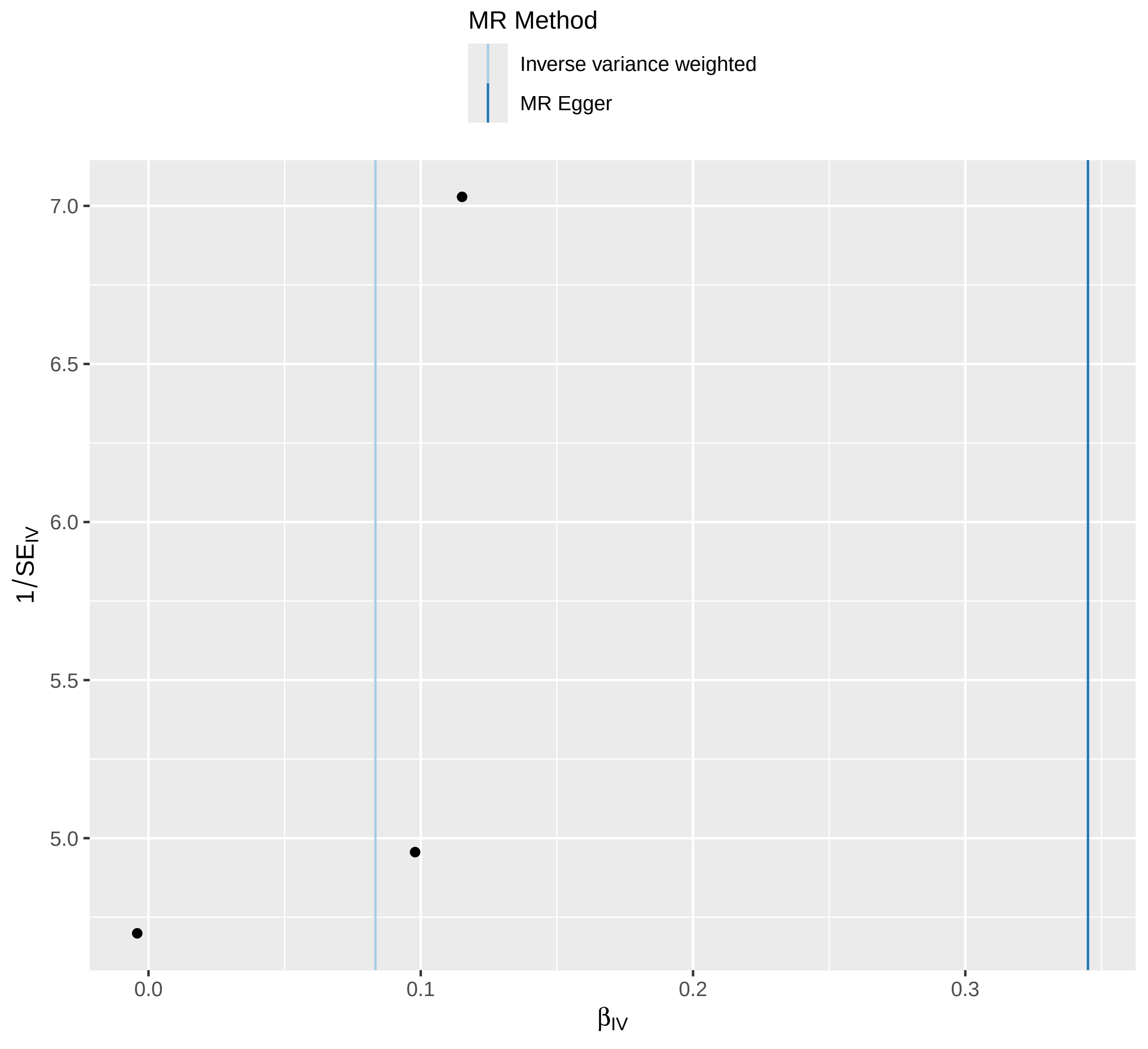
MR Method
Inverse variance weighted
MR Egger
7.0
6.5
6.0
5.5
5.0
1/SE_IV
0.0
0.1
0.2
0.3
β_IV

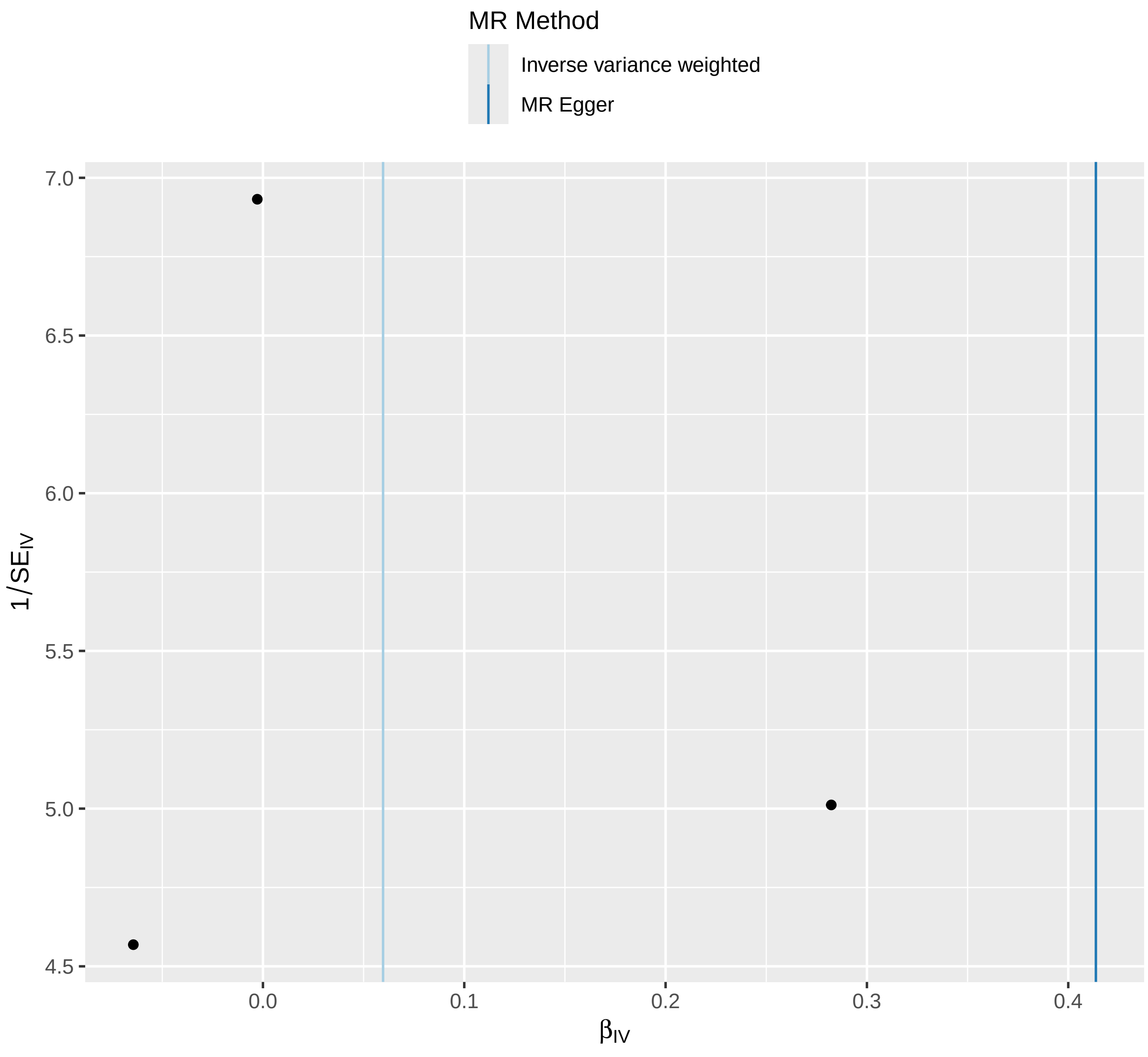

MR Method
Inverse variance weighted
MR Egger
7.0
6.5
6.0
5.5
5.0
4.5
1/SE_IV
0.0
0.1
0.2
0.3
0.4
β_IV

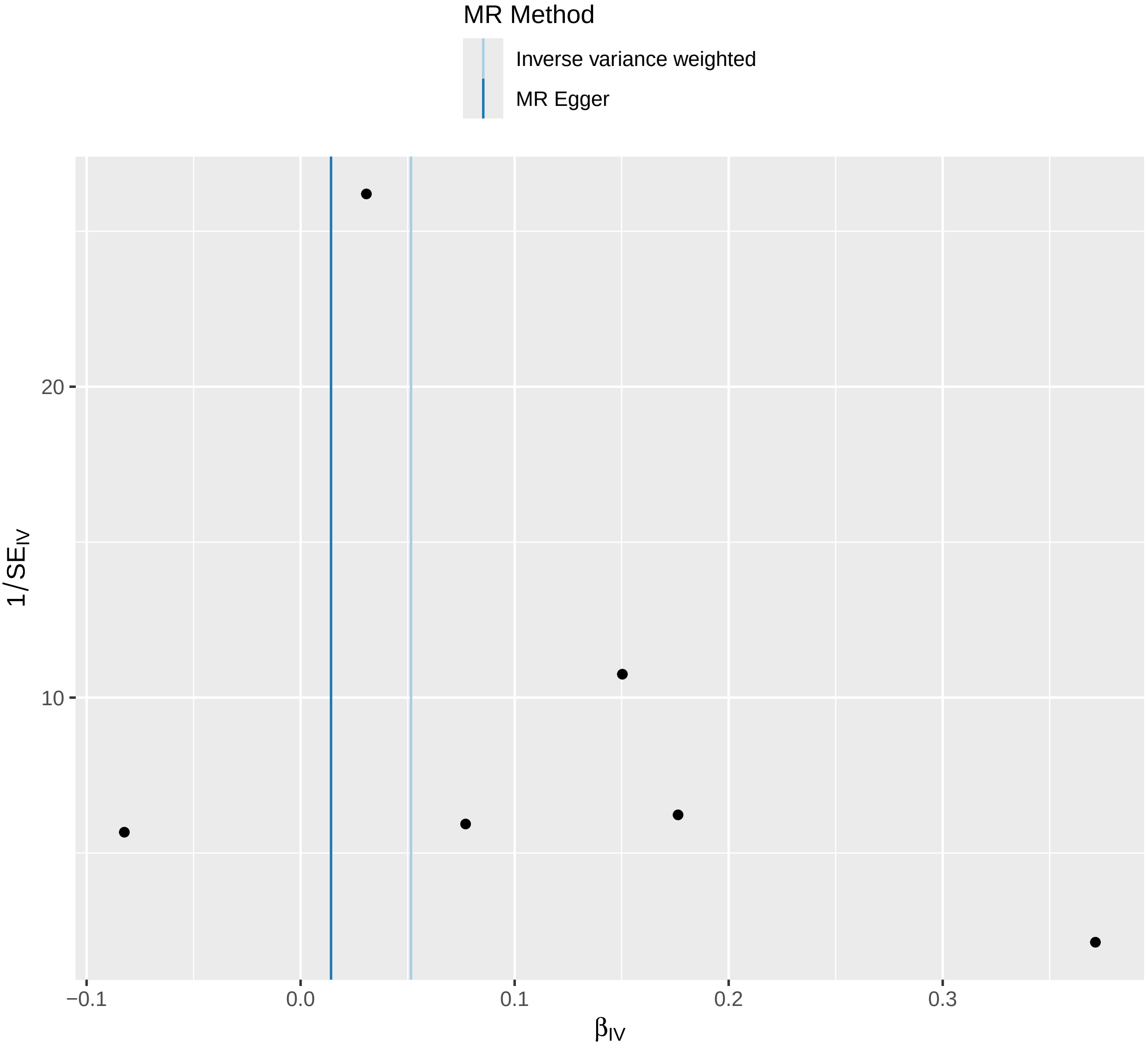
MR Method
Inverse variance weighted
MR Egger
20
10
1/SE_IV
−0.1
0.0
0.1
0.2
0.3
β_IV

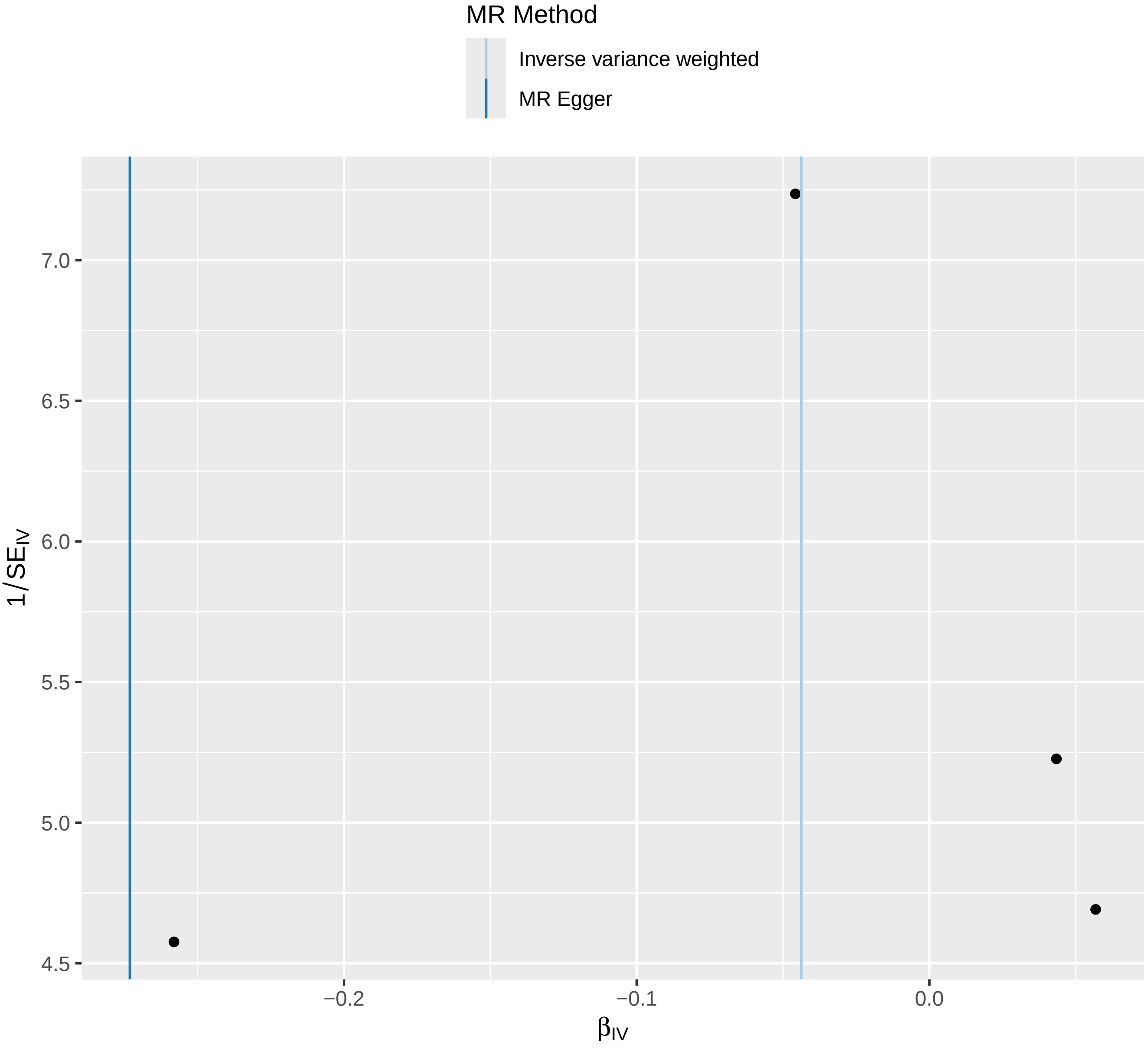

MR Method
Inverse variance weighted
MR Egger
7.0
6.5
6.0
5.5
5.0
4.5
1/SE$_{IV}$
−0.2
−0.1
0.0
β$_{IV}$

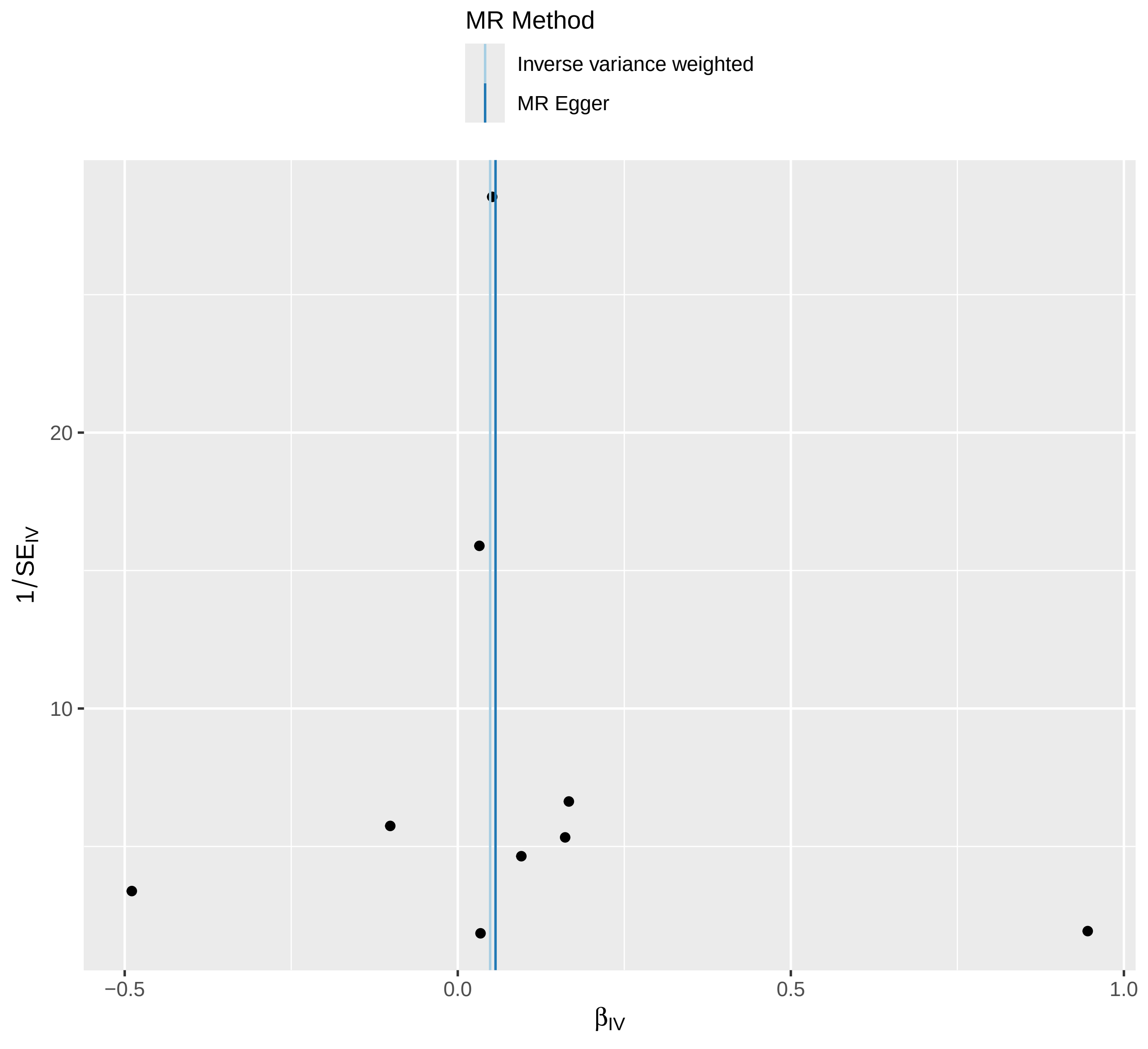

MR Method
Inverse variance weighted
MR Egger
1/SE_IV
20
10
−0.5
0.0
0.5
1.0
β_IV

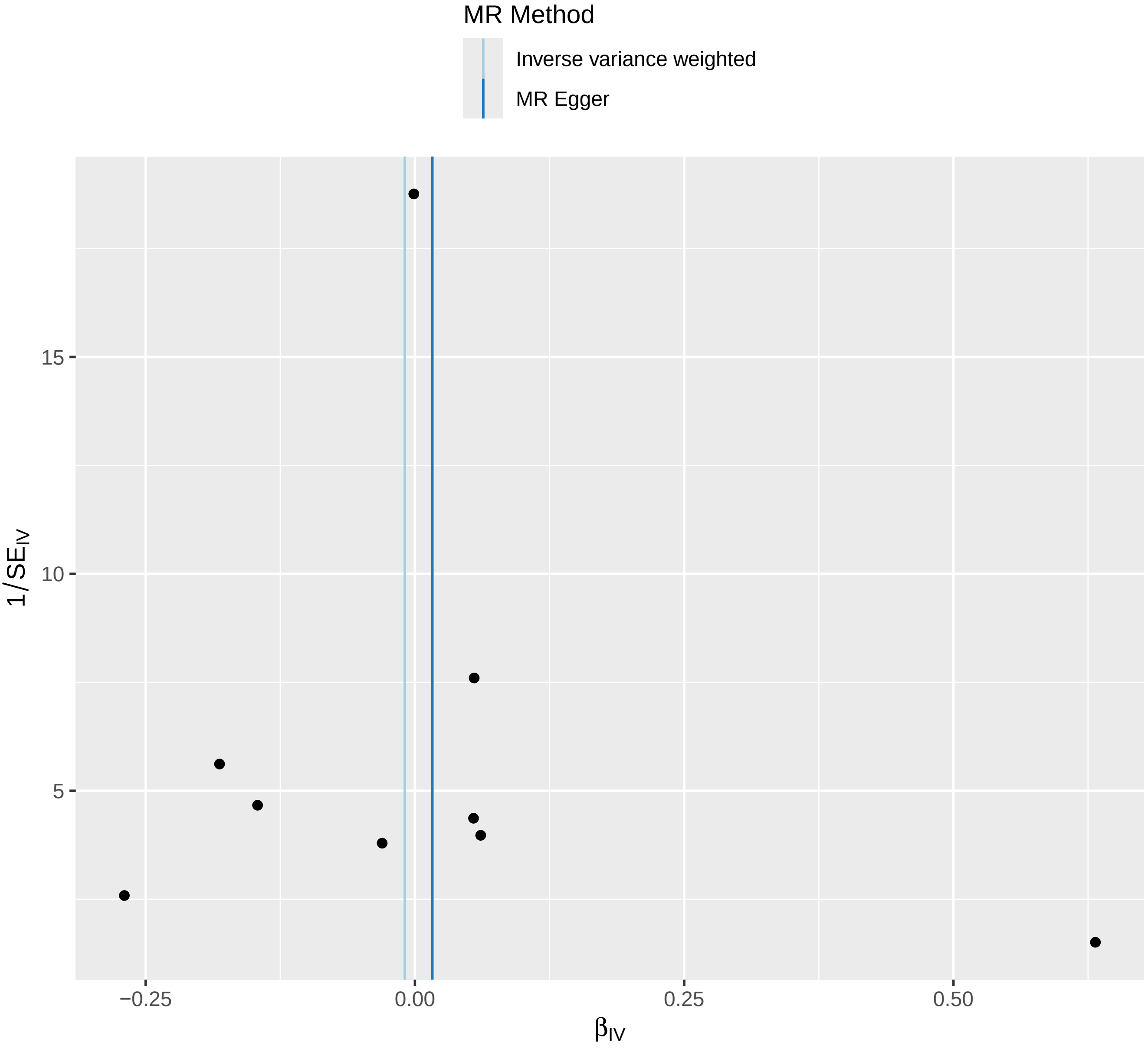
MR Method
Inverse variance weighted
MR Egger
15
10
5
1/SE$_{IV}$
−0.25
0.00
0.25
0.50
β$_{IV}$

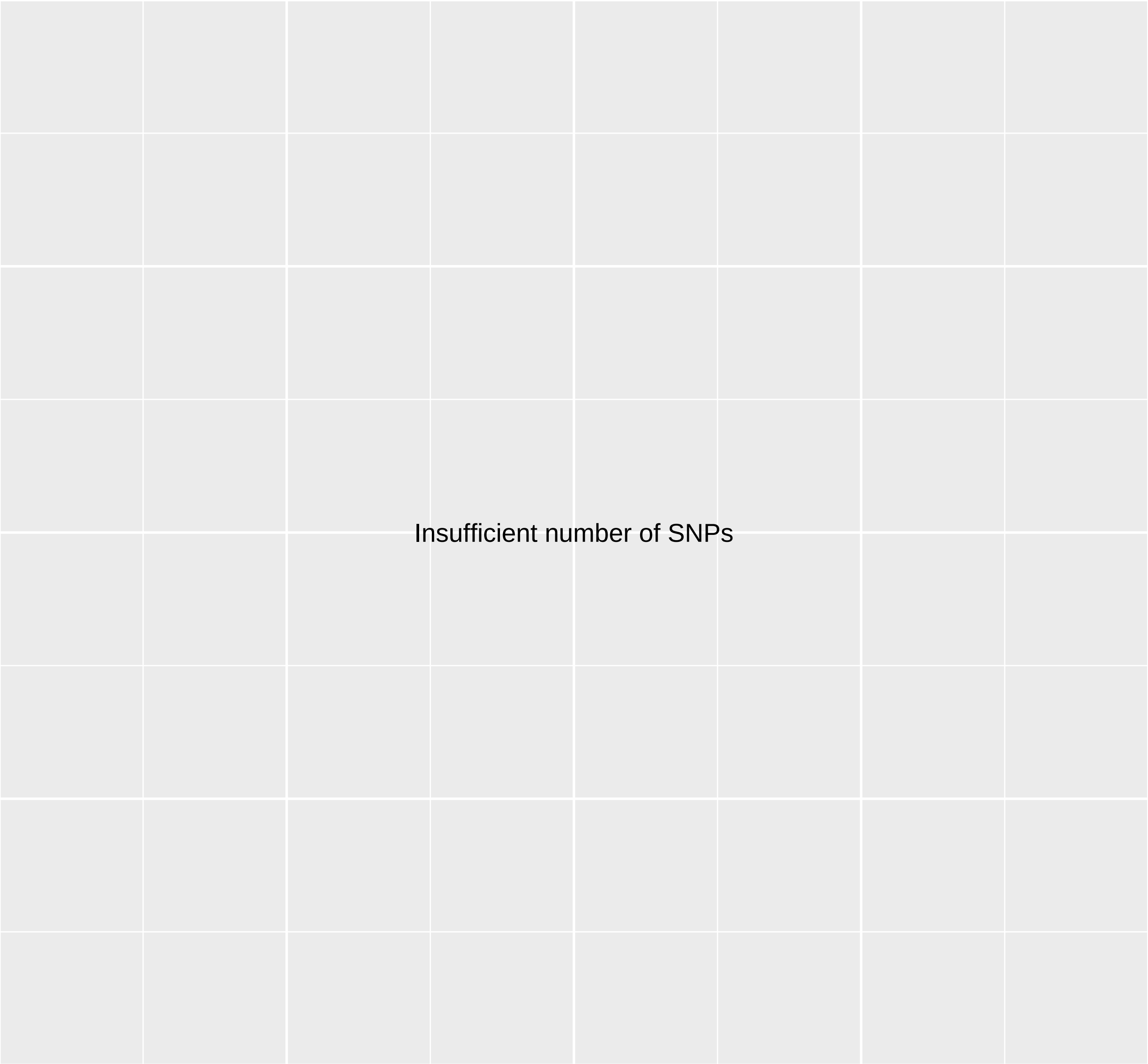
Insufficient number of SNPs

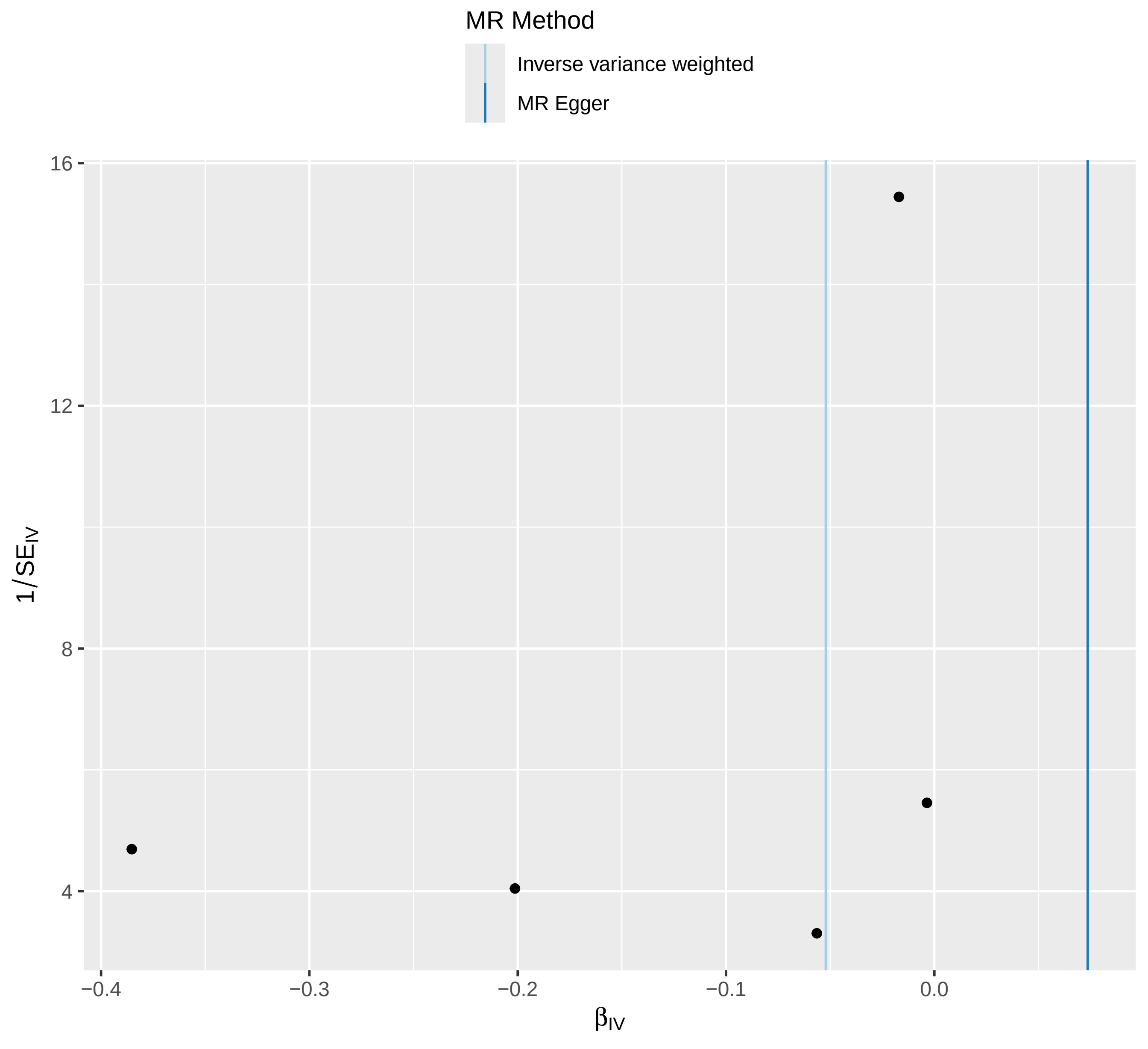

MR Method
Inverse variance weighted
MR Egger
16
12
8
4
1/SE_IV
−0.4
−0.3
−0.2
−0.1
0.0
β_IV

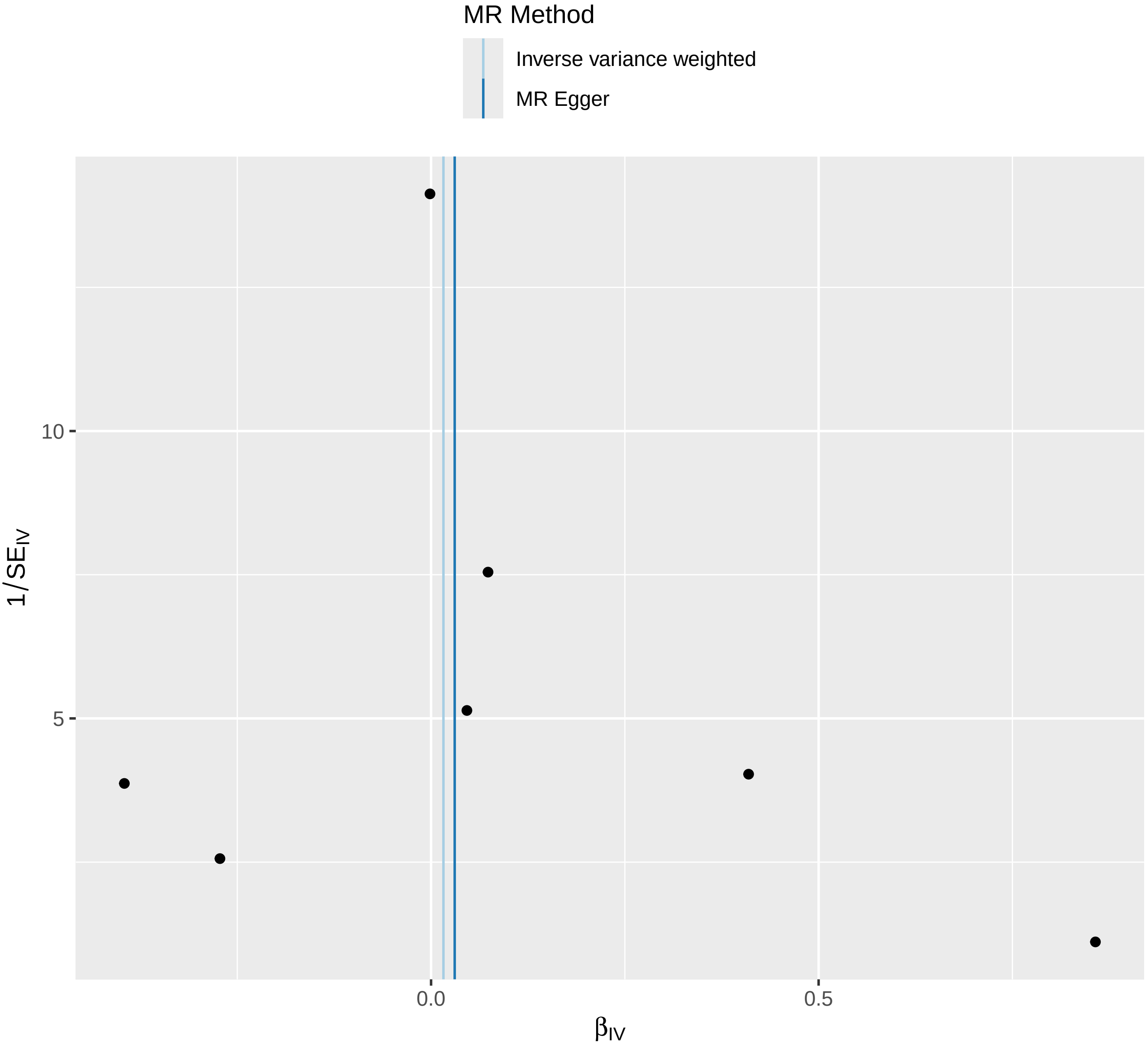
MR Method
Inverse variance weighted
MR Egger
10
5
1/SE_IV
0.0
0.5
β_IV

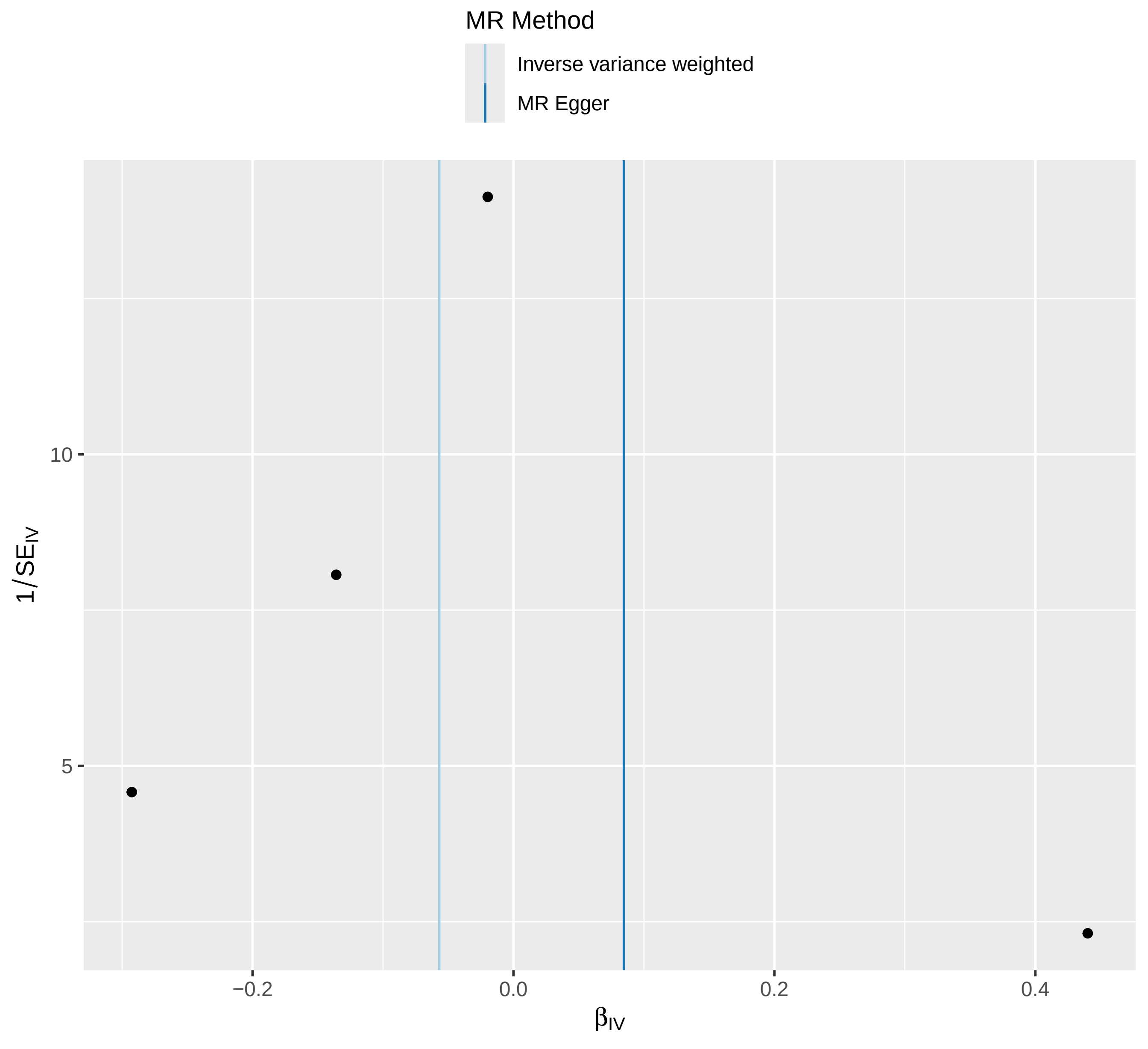

MR Method
Inverse variance weighted
MR Egger
10
5
1/SE_IV
−0.2
0.0
0.2
0.4
β_IV

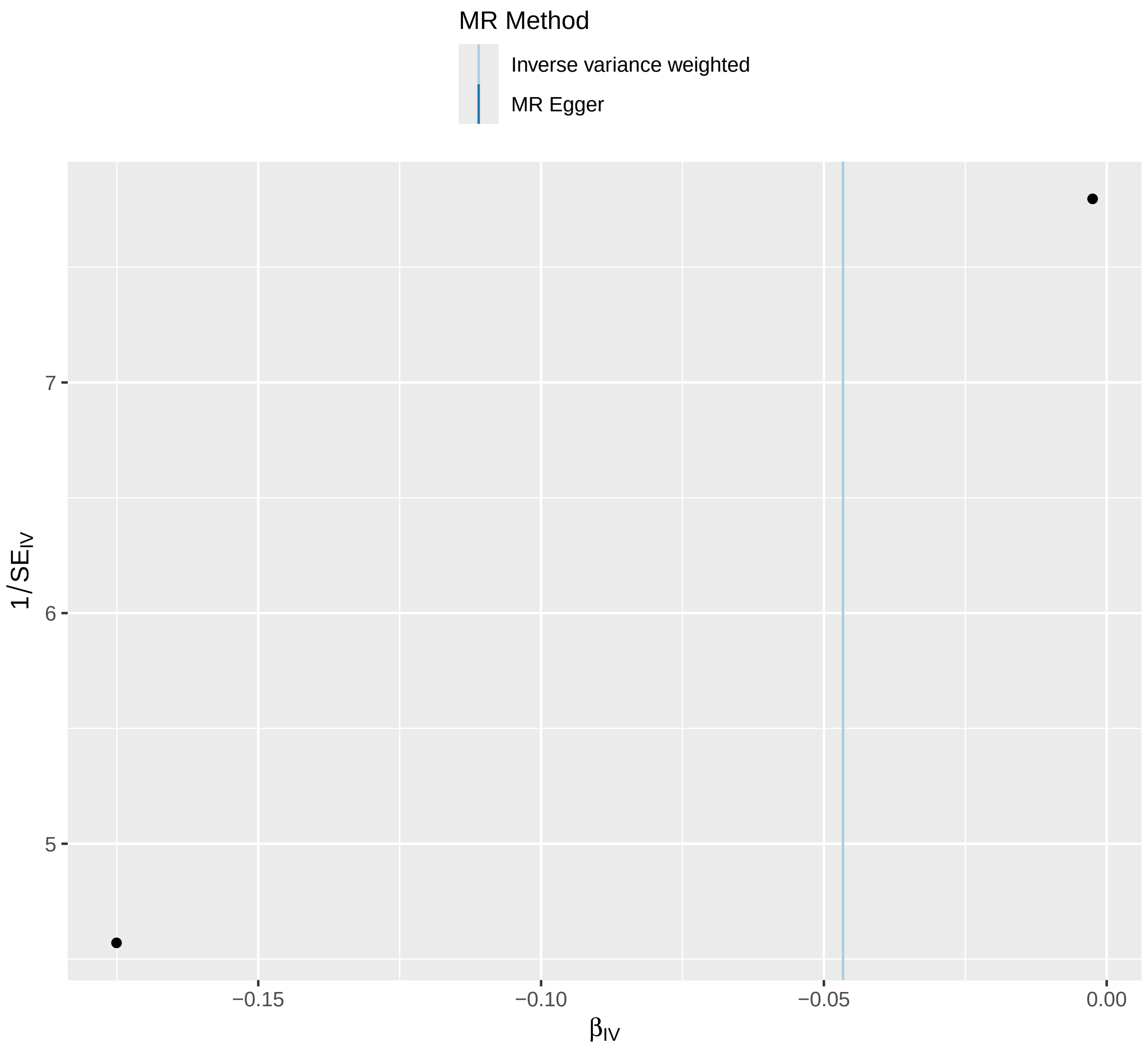

MR Method
Inverse variance weighted
MR Egger
7
6
5
1/SE_IV
−0.15
−0.10
−0.05
0.00
β_IV

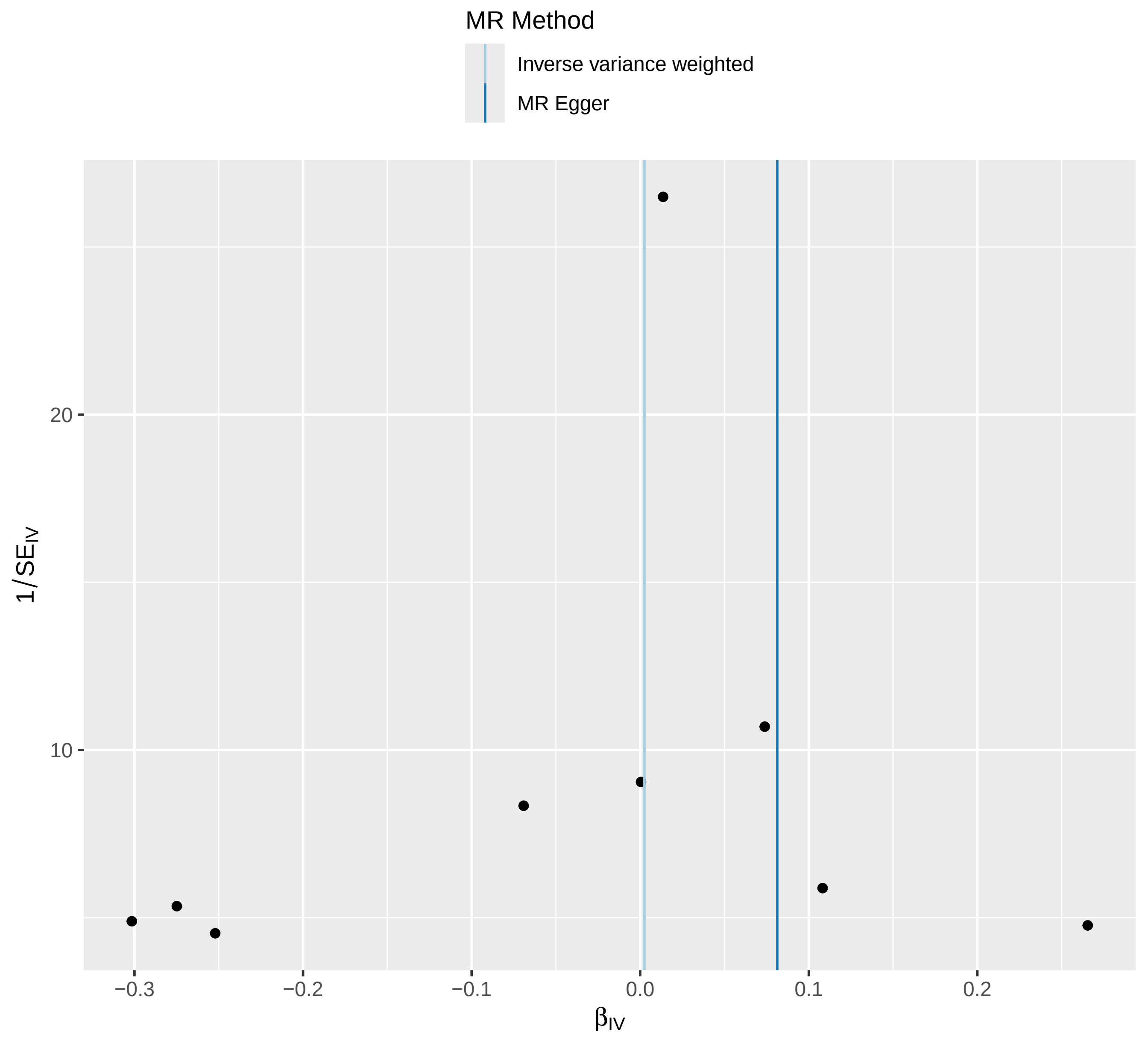
MR Method
Inverse variance weighted
MR Egger
20
10
$1/SE_{IV}$
−0.3
−0.2
−0.1
0.0
0.1
0.2
$\beta_{IV}$

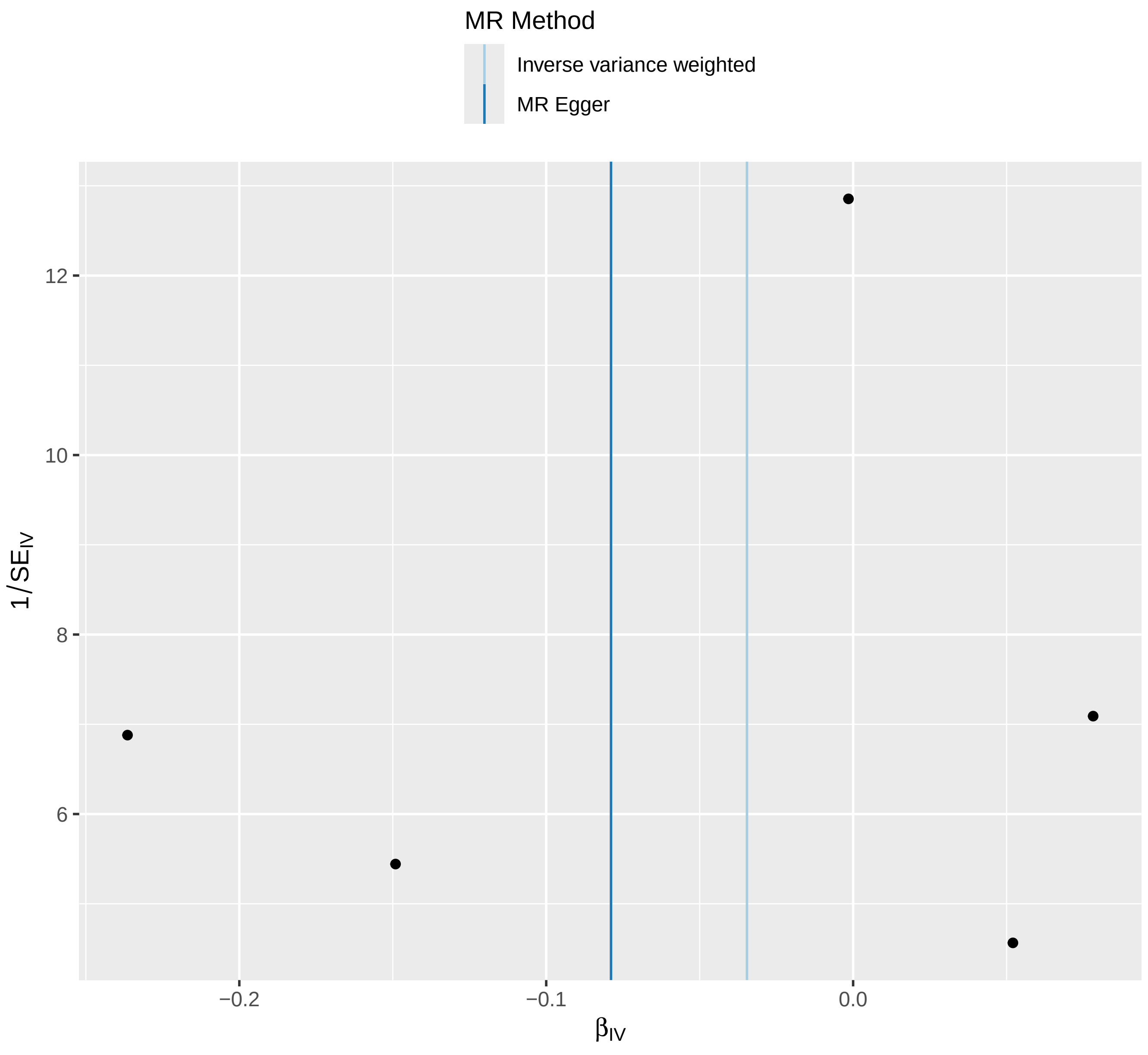
MR Method
Inverse variance weighted
MR Egger
12
10
8
6
1/SE$_{IV}$
−0.2
−0.1
0.0
β$_{IV}$

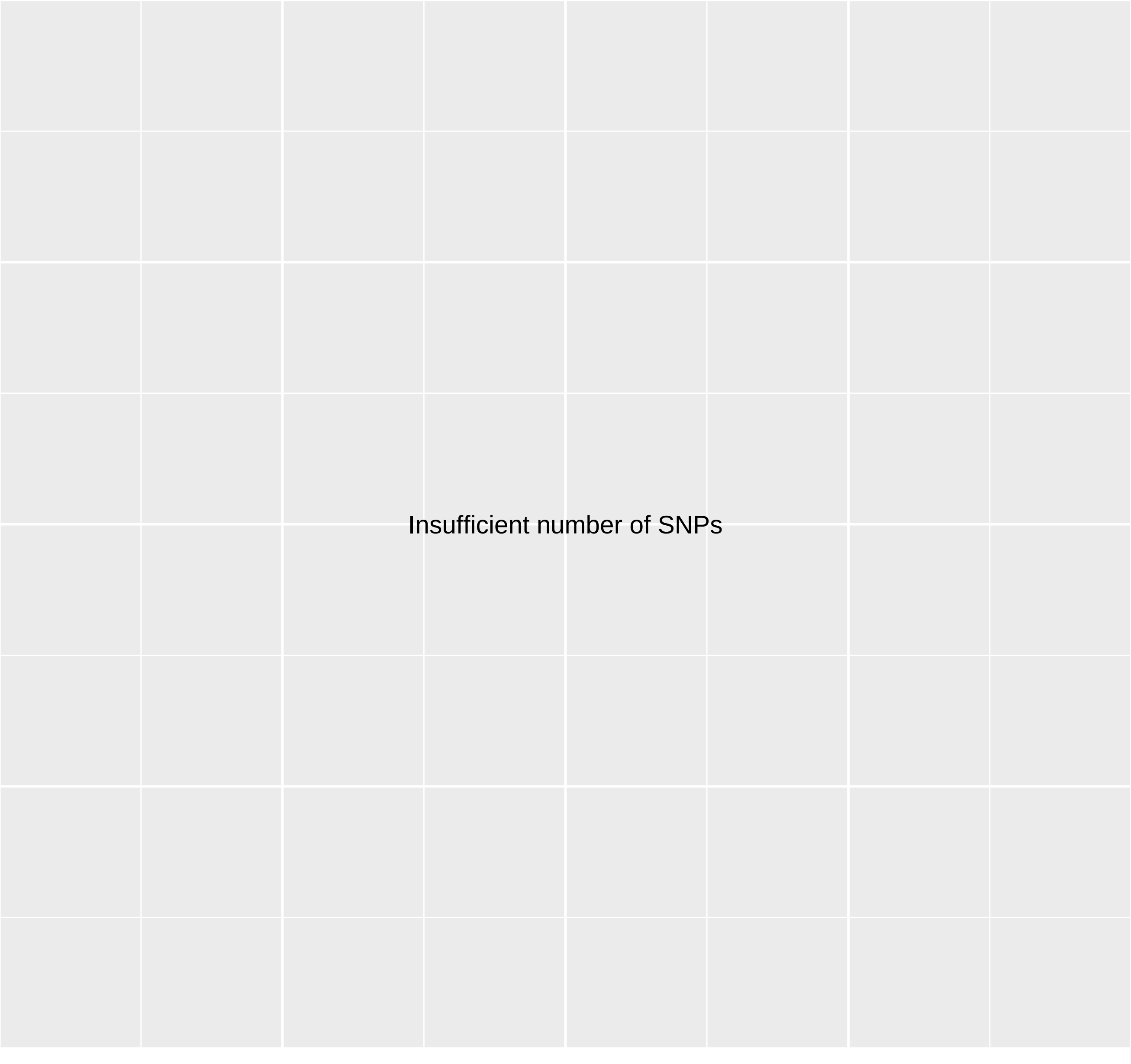
Insufficient number of SNPs

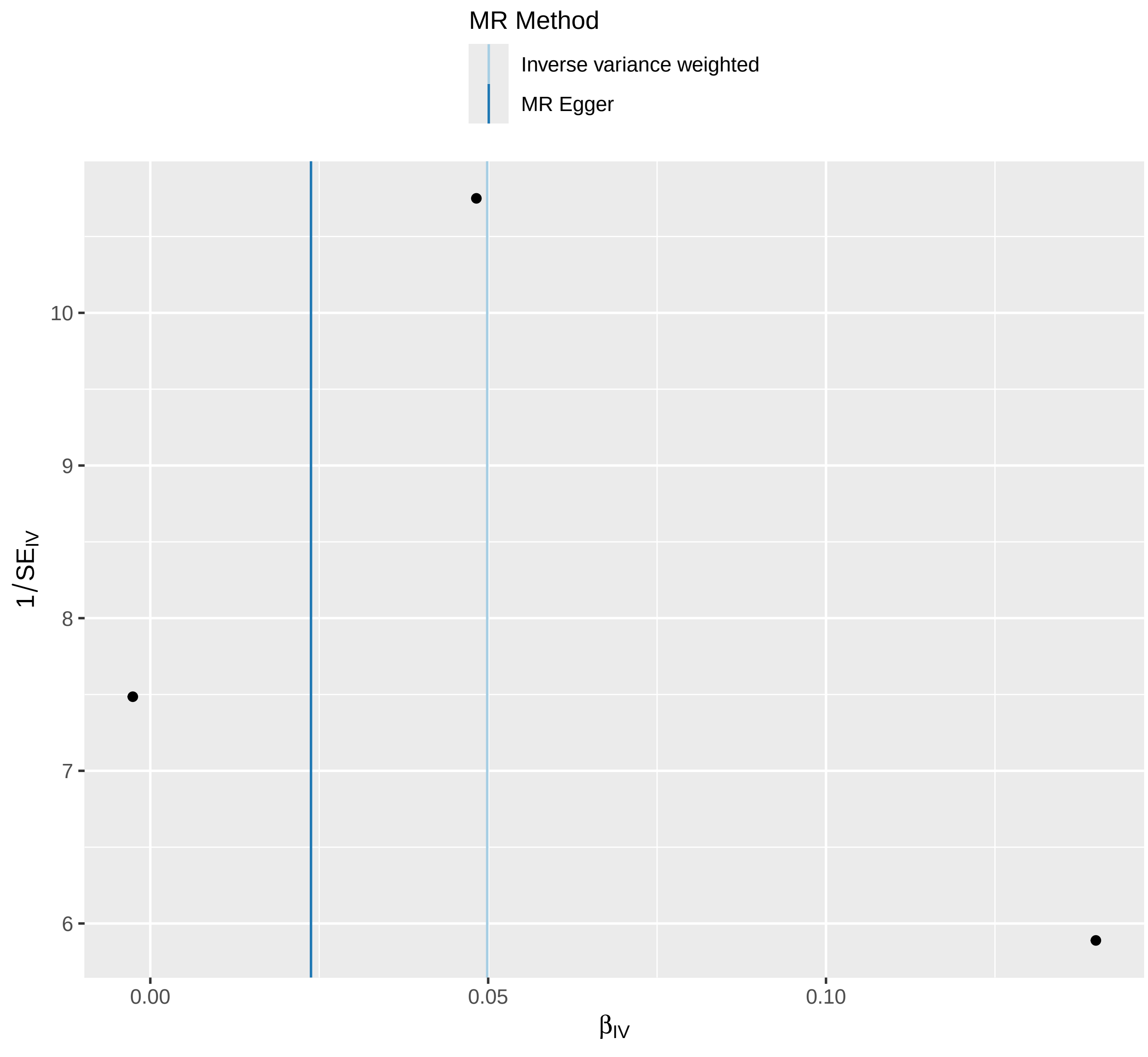
MR Method
Inverse variance weighted
MR Egger
1/SE_IV
10
9
8
7
6
0.00
0.05
0.10
β_IV

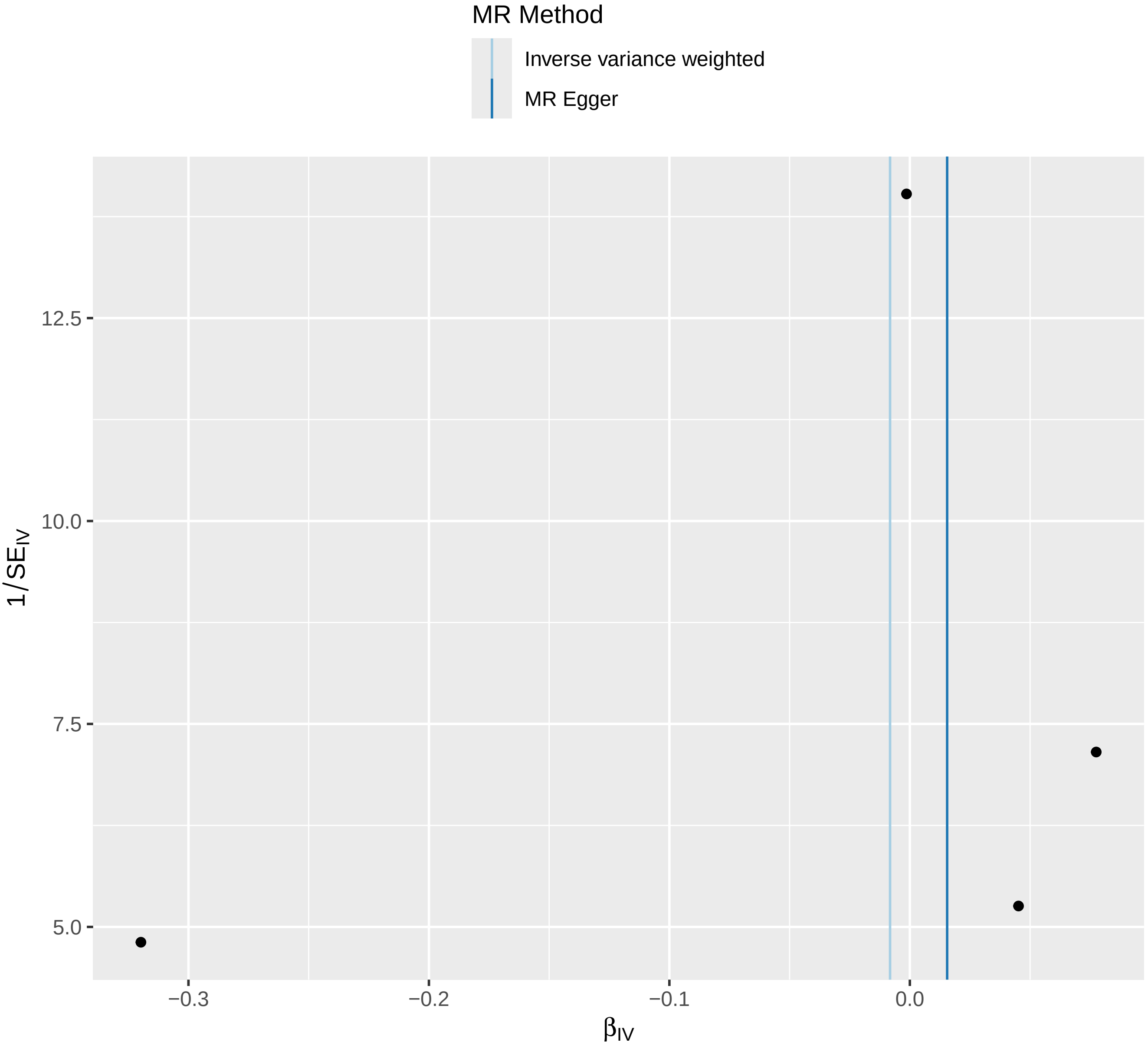

MR Method
Inverse variance weighted
MR Egger
12.5
10.0
7.5
5.0
1/SE$_{IV}$
−0.3
−0.2
−0.1
0.0
β$_{IV}$

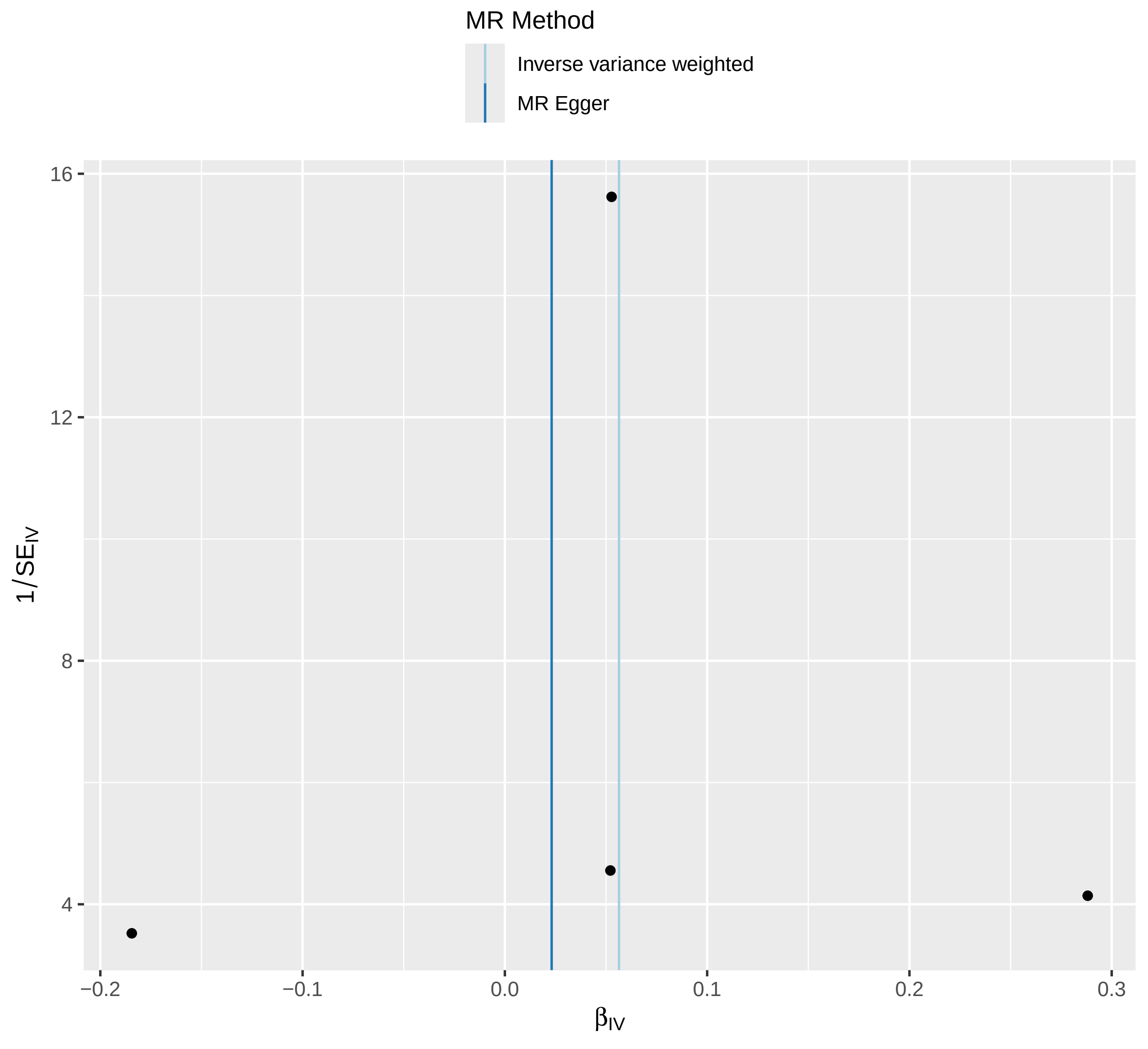

MR Method
Inverse variance weighted
MR Egger
16
12
8
4
$1/SE_{IV}$
−0.2
−0.1
0.0
0.1
0.2
0.3
$\beta_{IV}$

Insufficient number of SNPs

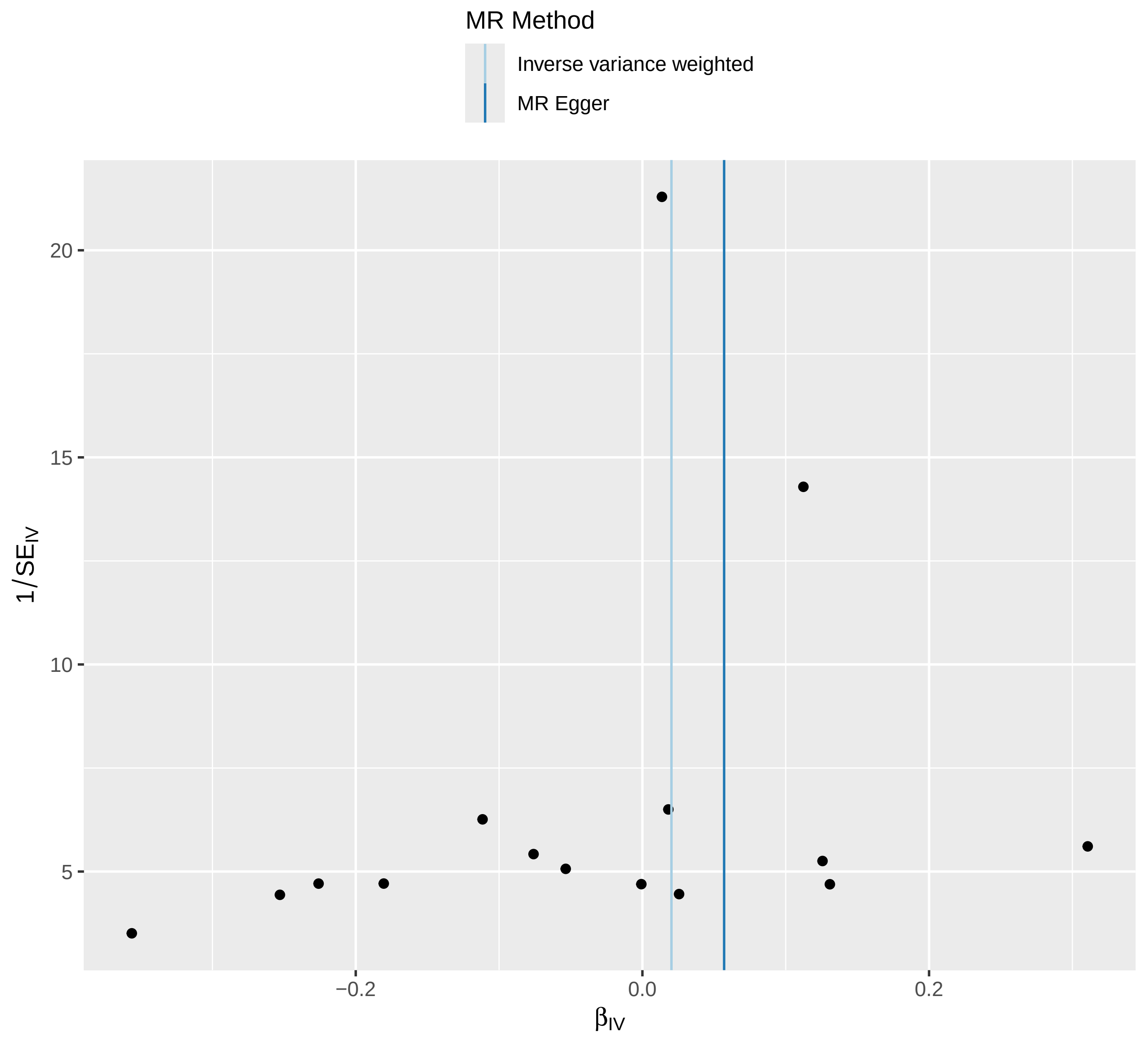
MR Method
Inverse variance weighted
MR Egger
20
15
10
5
1/SE_IV
−0.2
0.0
0.2
β_IV

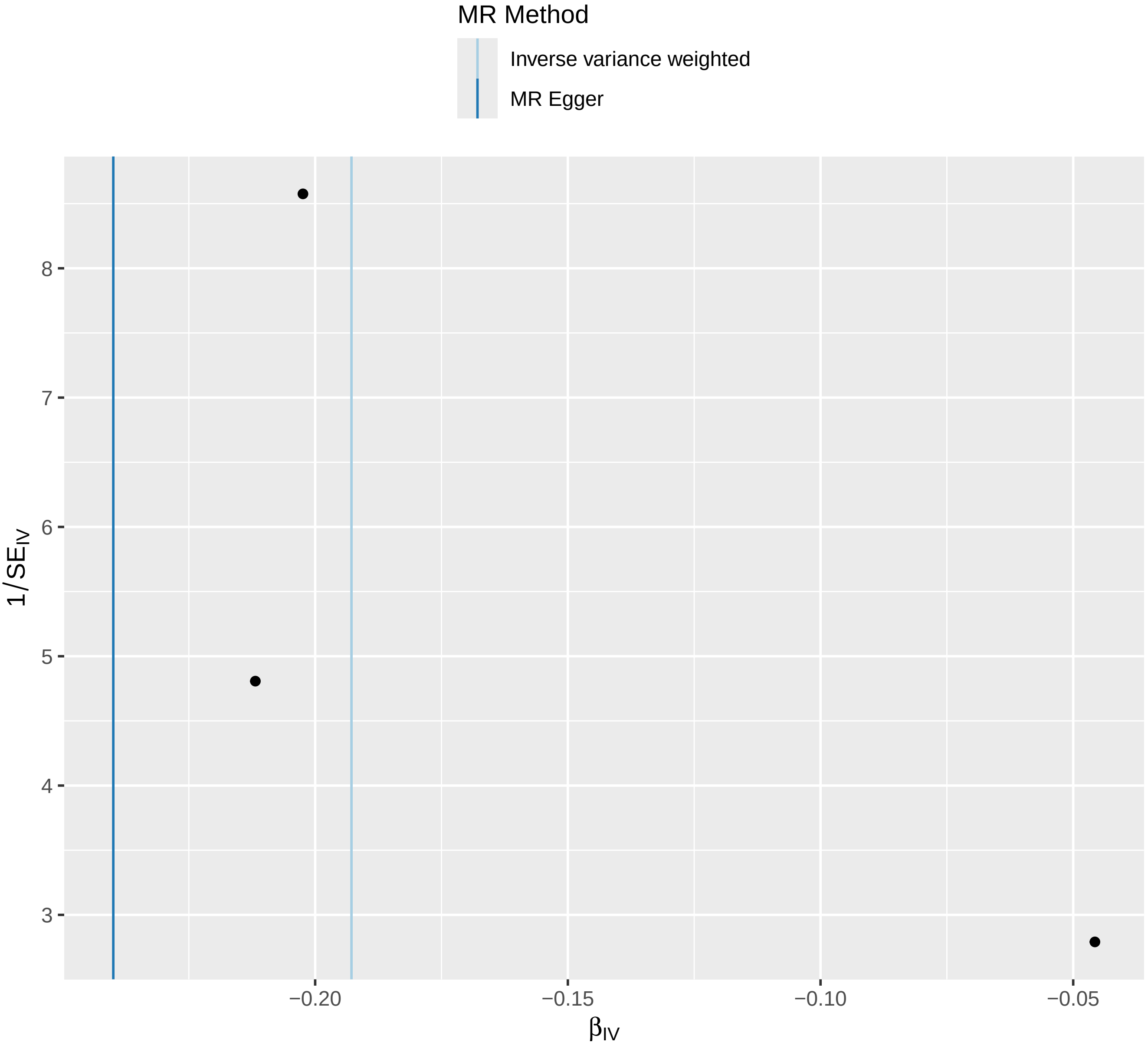
MR Method
Inverse variance weighted
MR Egger
8
7
6
5
4
3
1/SE$_{IV}$
−0.20
−0.15
−0.10
−0.05
β$_{IV}$

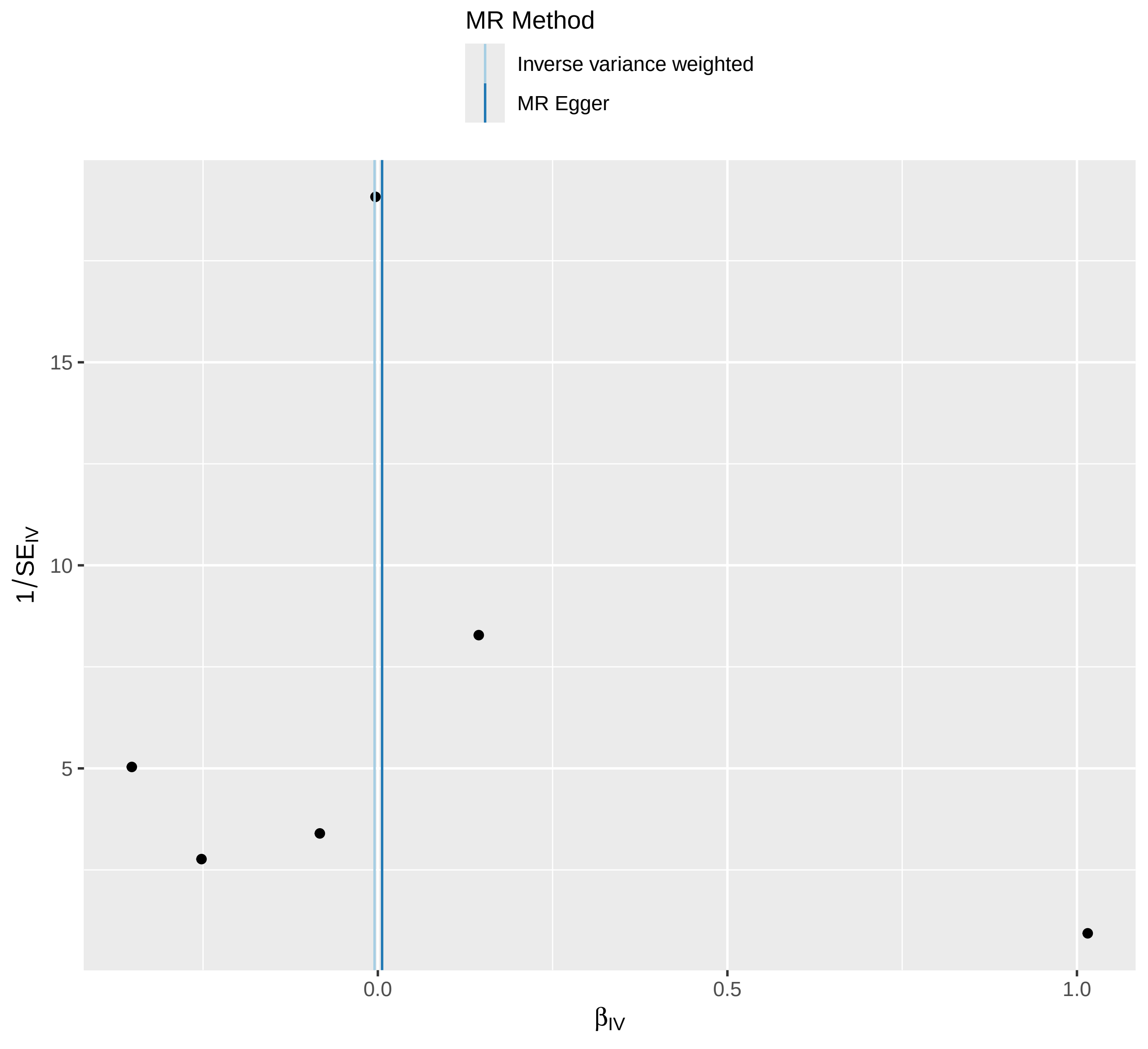
MR Method
Inverse variance weighted
MR Egger
15
10
5
0.0
0.5
1.0
1/SE$_{IV}$
β$_{IV}$

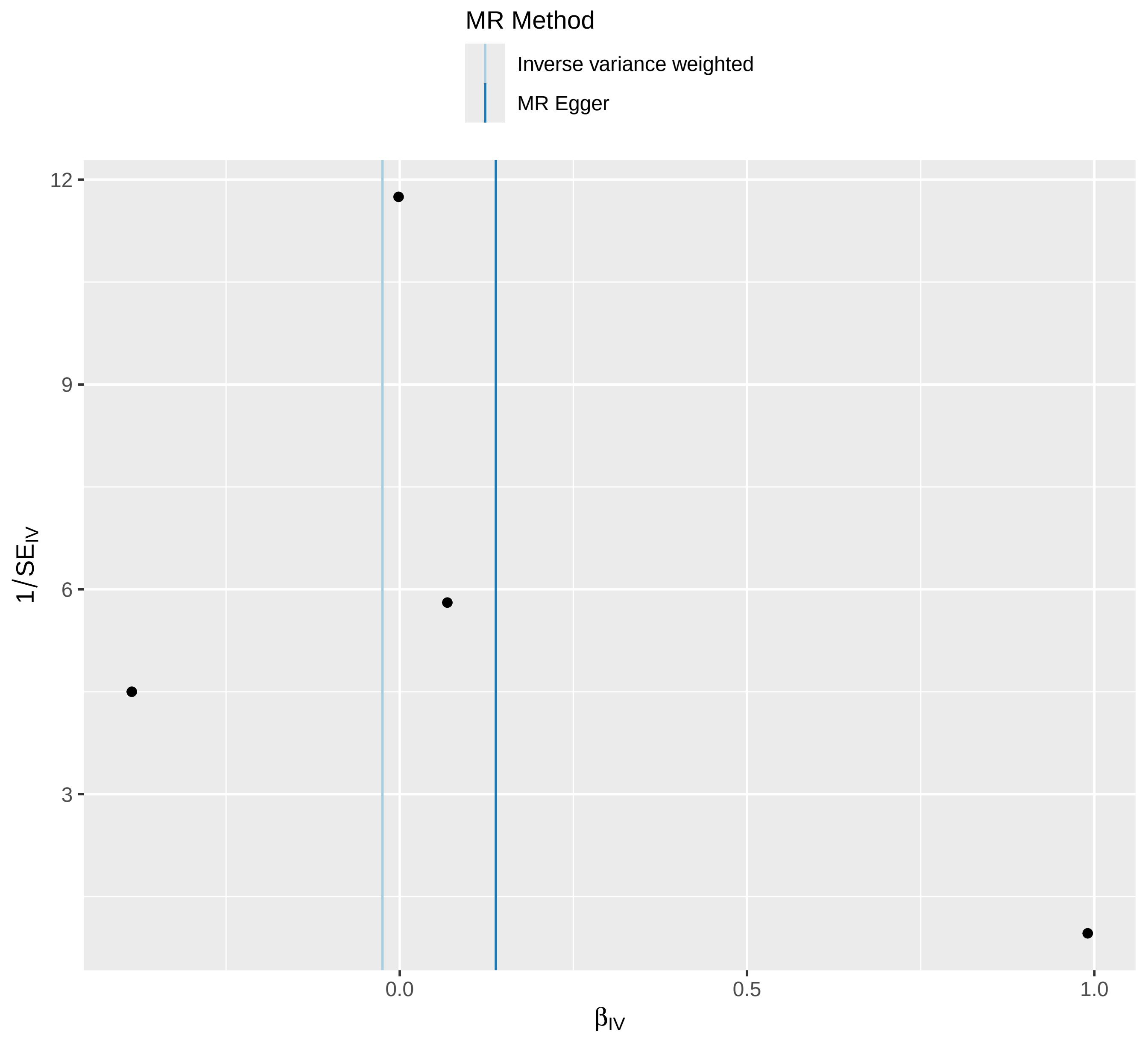
MR Method
Inverse variance weighted
MR Egger
12
9
6
3
$1/SE_{IV}$
0.0
0.5
1.0
$\beta_{IV}$

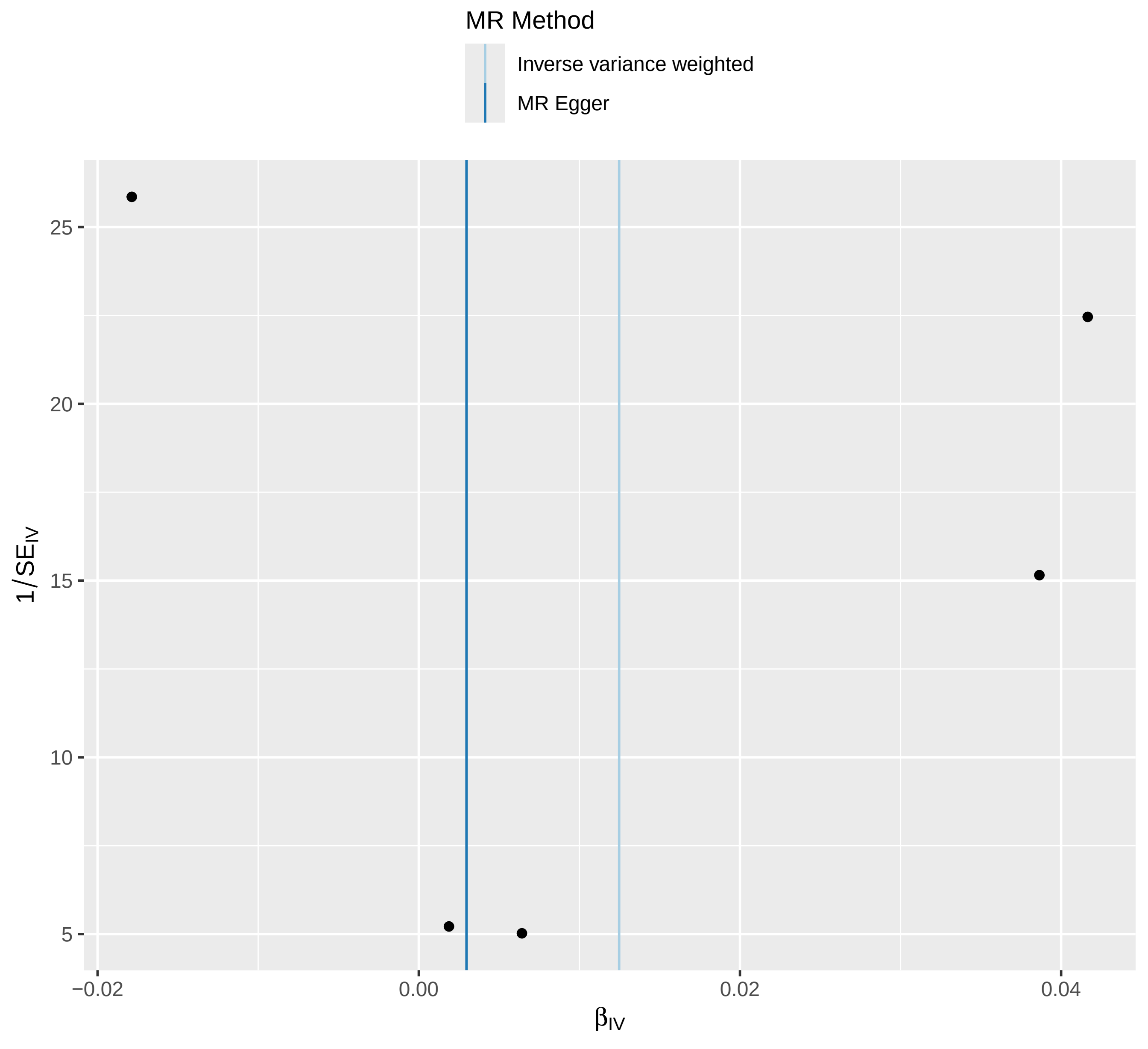

MR Method
Inverse variance weighted
MR Egger
1/$SE_{IV}$
25
20
15
10
5
−0.02
0.00
0.02
0.04
$\beta_{IV}$

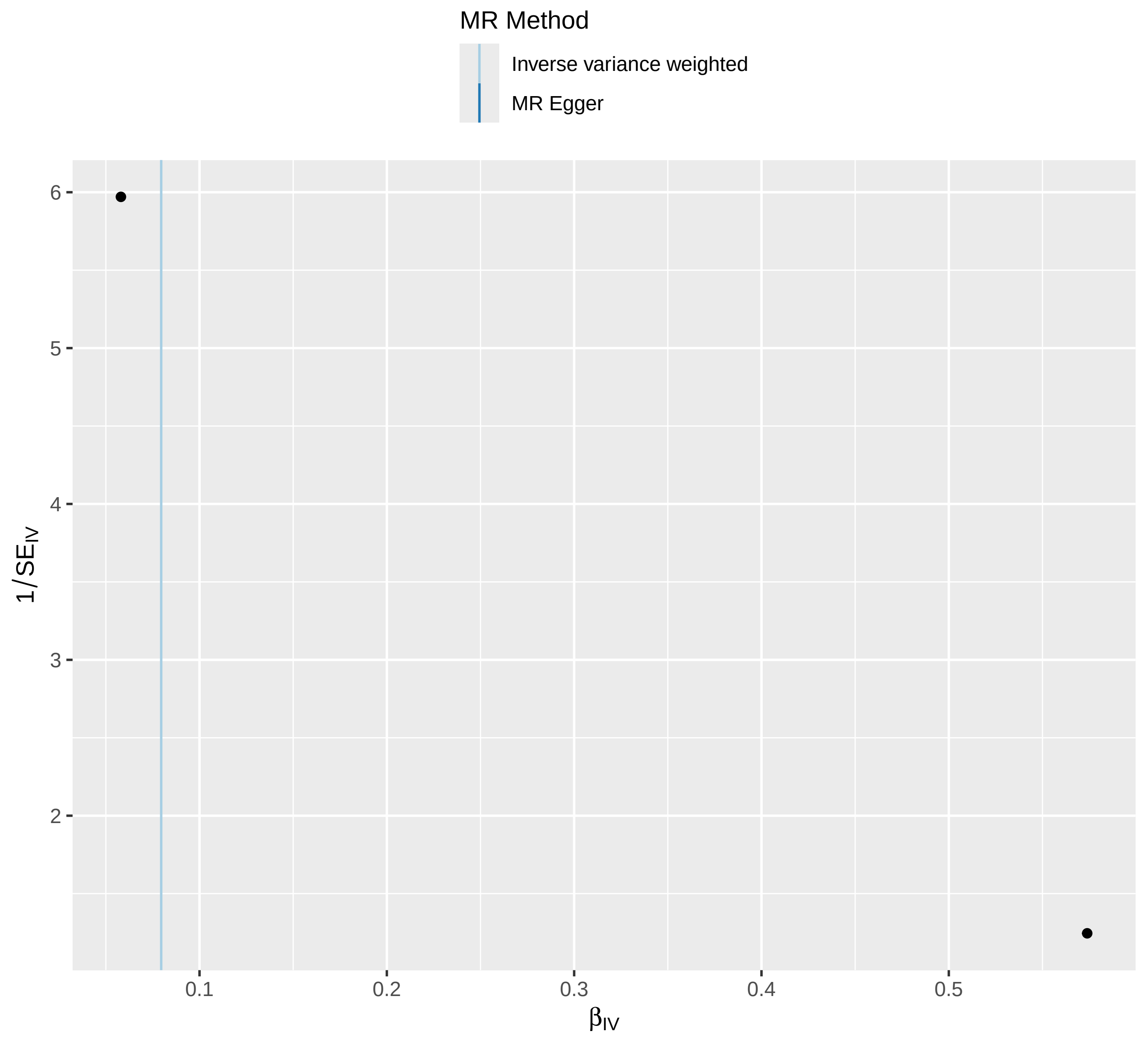

MR Method
Inverse variance weighted
MR Egger
6
5
4
3
2
1/SE$_{IV}$
0.1
0.2
0.3
0.4
0.5
β$_{IV}$

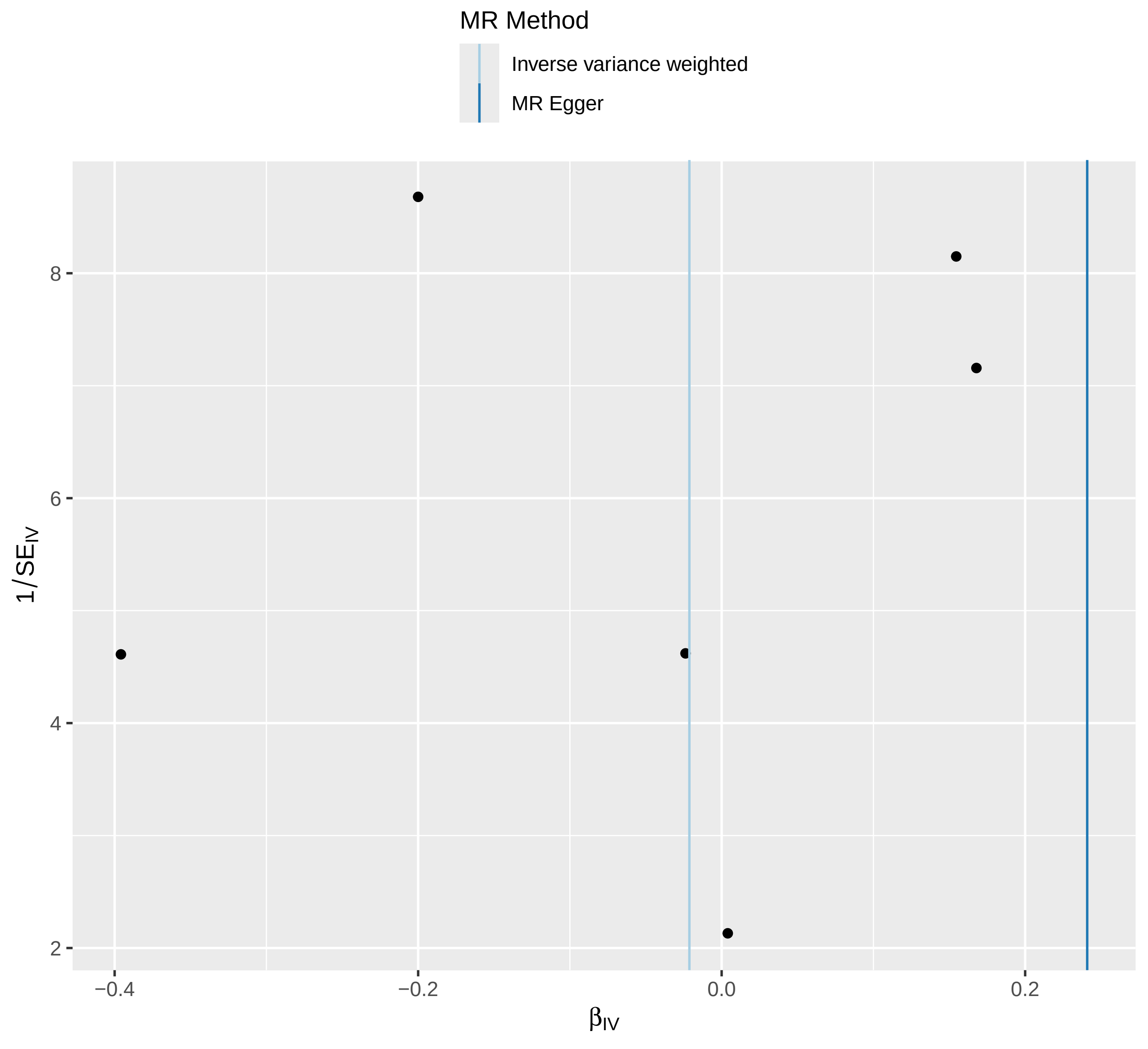
MR Method
Inverse variance weighted
MR Egger
8
6
4
2
1/SE_IV
−0.4
−0.2
0.0
0.2
β_IV

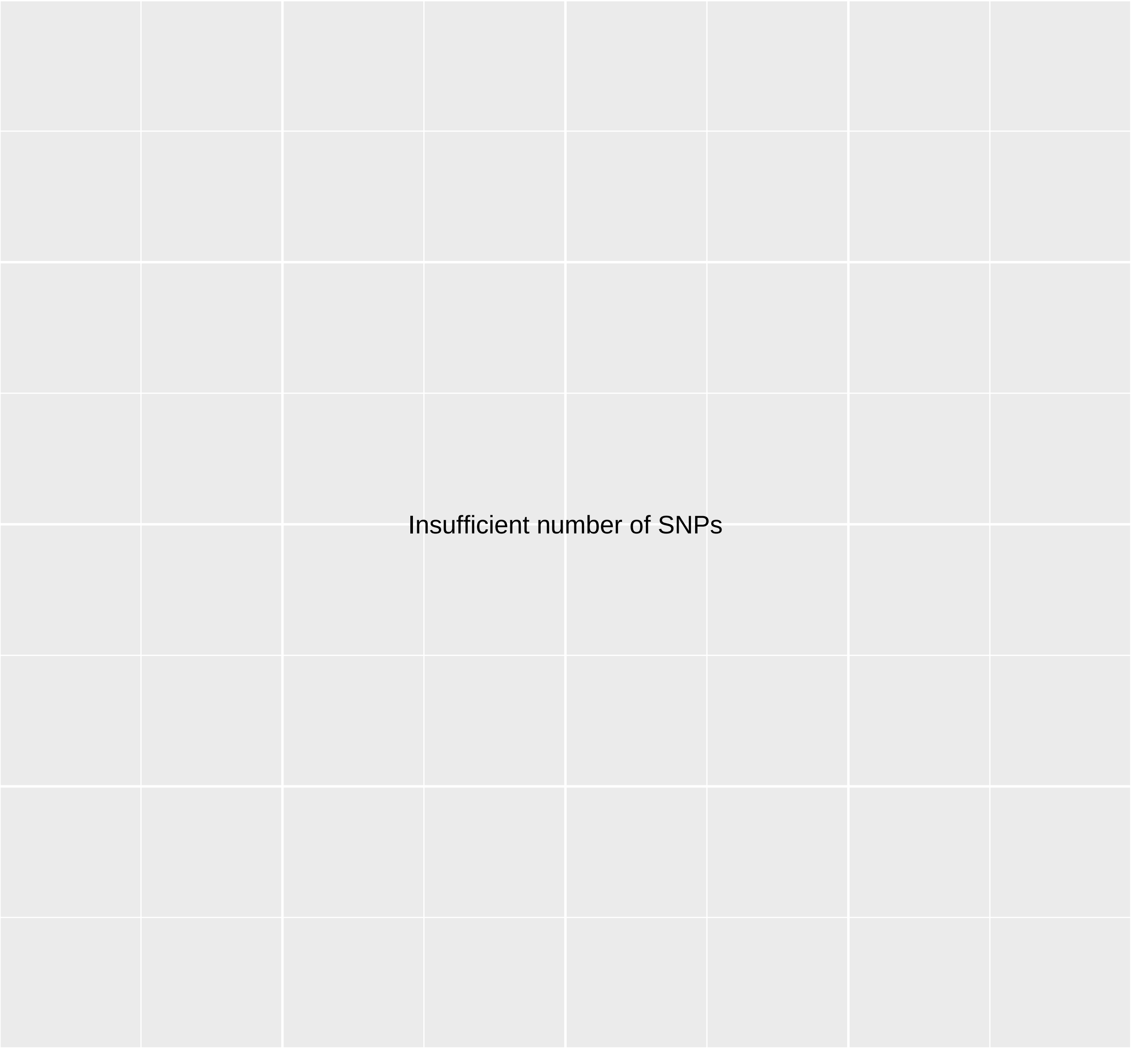
Insufficient number of SNPs

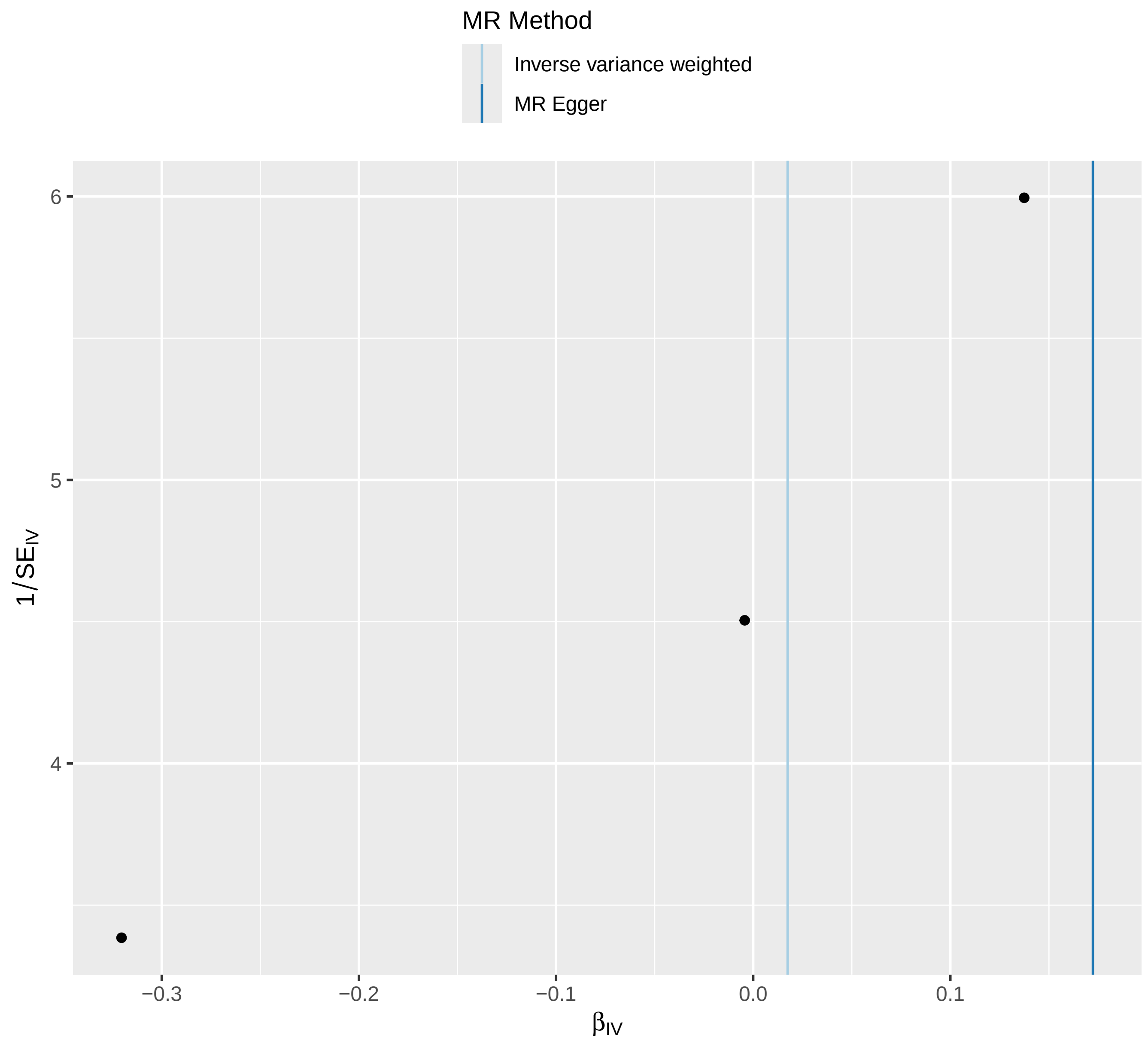

MR Method
Inverse variance weighted
MR Egger
6
5
4
1/SE_IV
−0.3
−0.2
−0.1
0.0
0.1
β_IV

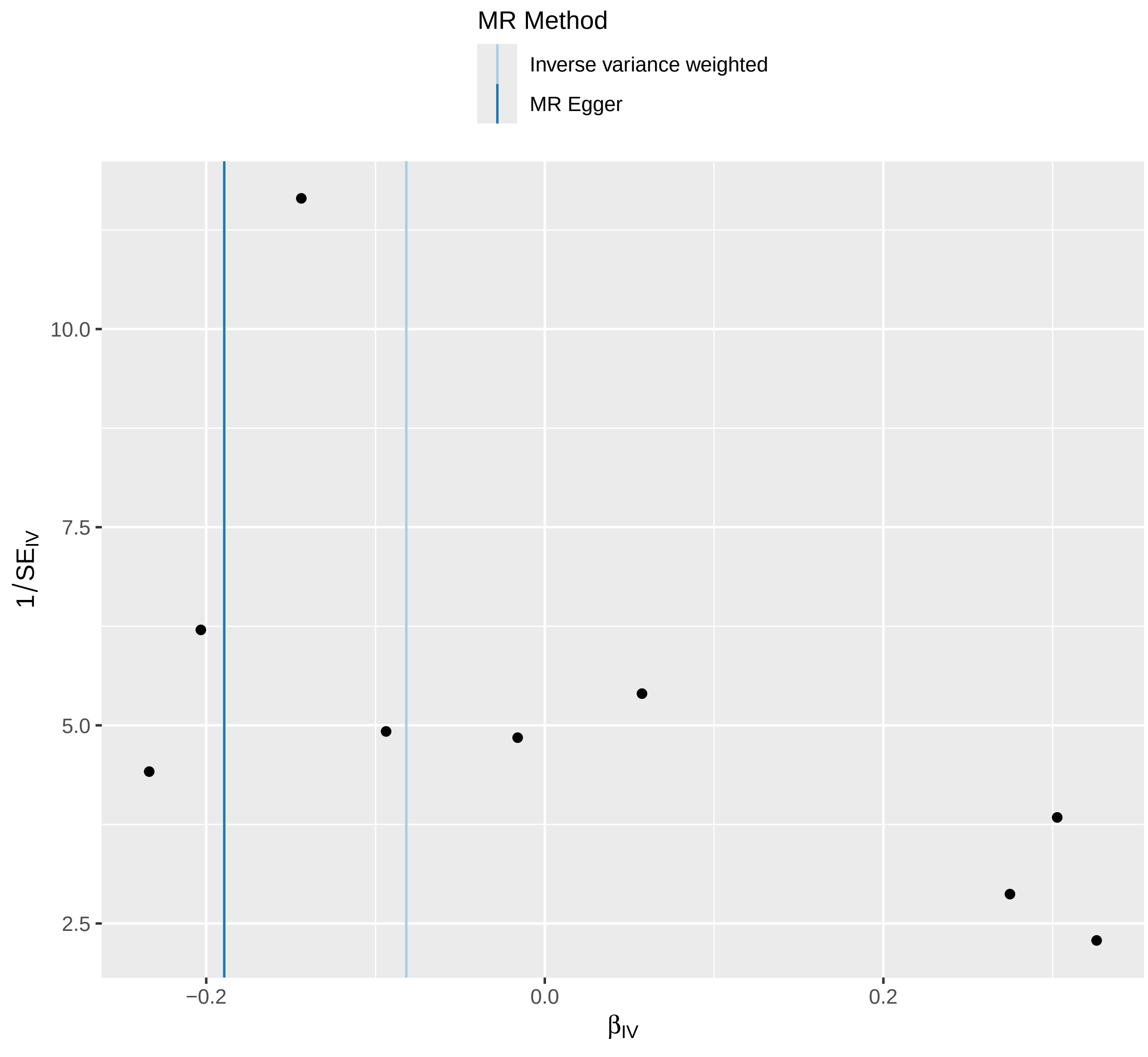
MR Method
Inverse variance weighted
MR Egger
10.0
7.5
5.0
2.5
1/SE_IV
−0.2
0.0
0.2
β_IV

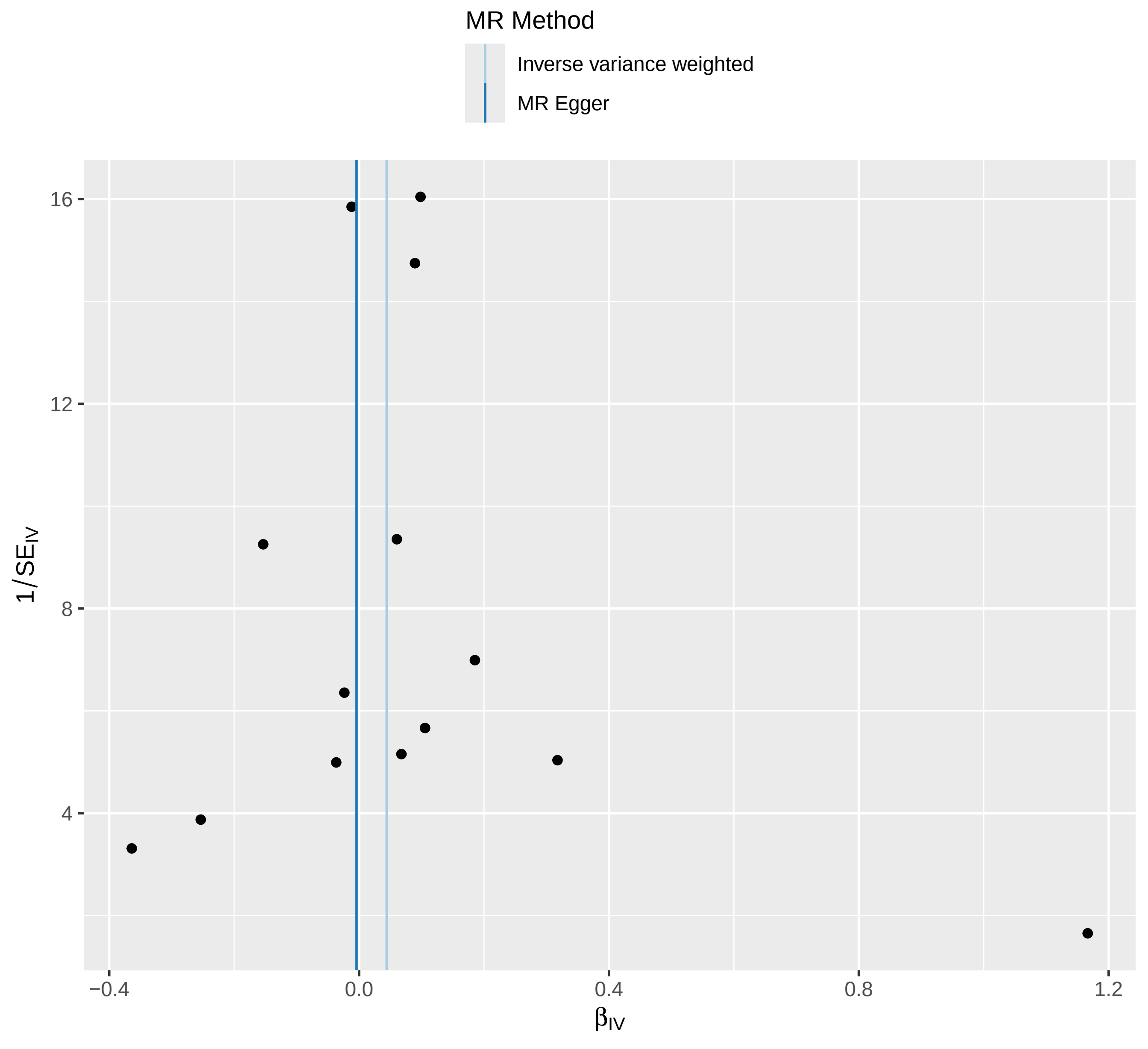

MR Method
Inverse variance weighted
MR Egger
16
12
8
4
$1/SE_{IV}$
−0.4
0.0
0.4
0.8
1.2
$\beta_{IV}$

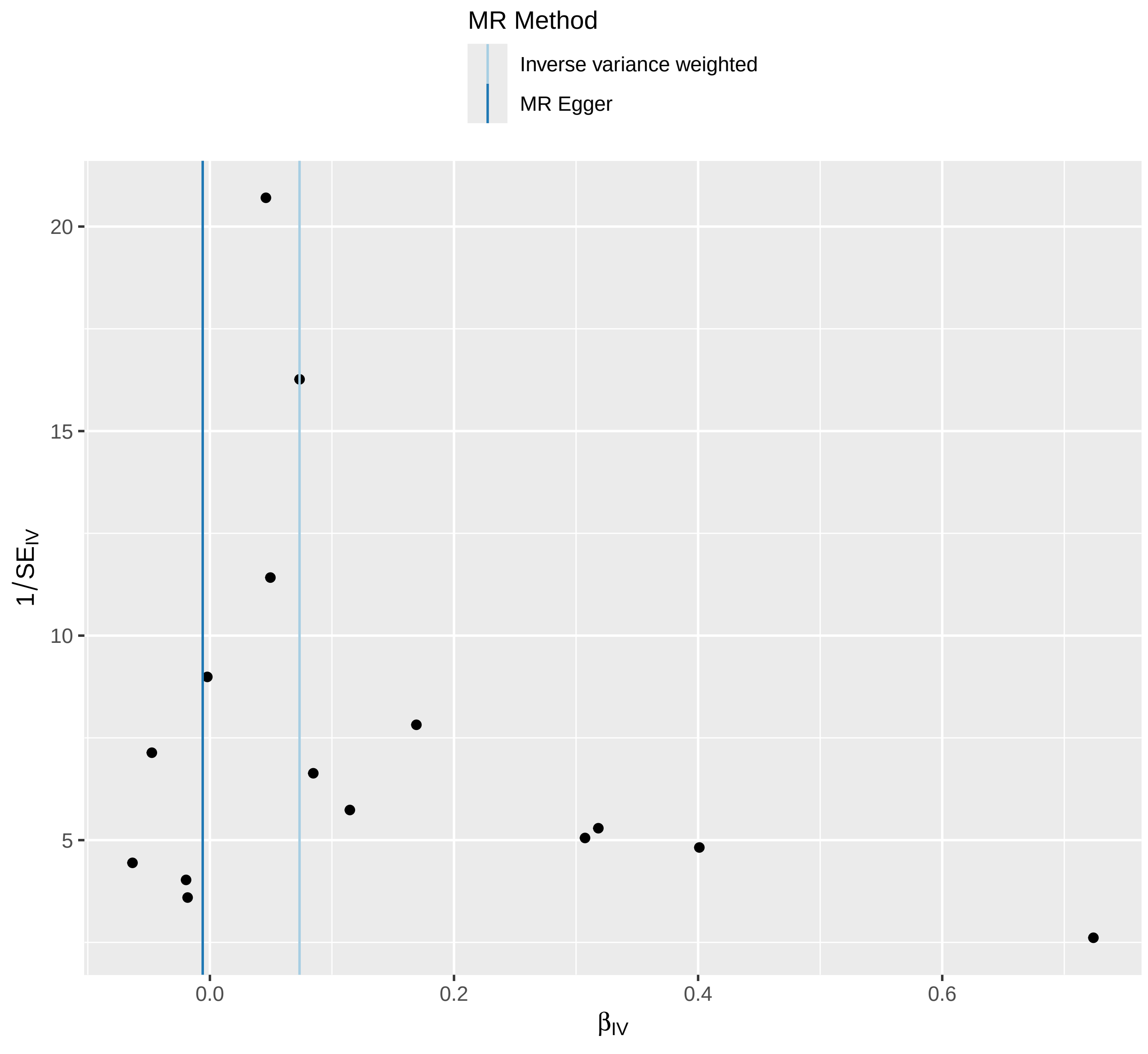
MR Method
Inverse variance weighted
MR Egger
20
15
10
5
0.0
0.2
0.4
0.6
1/SE_IV
β_IV

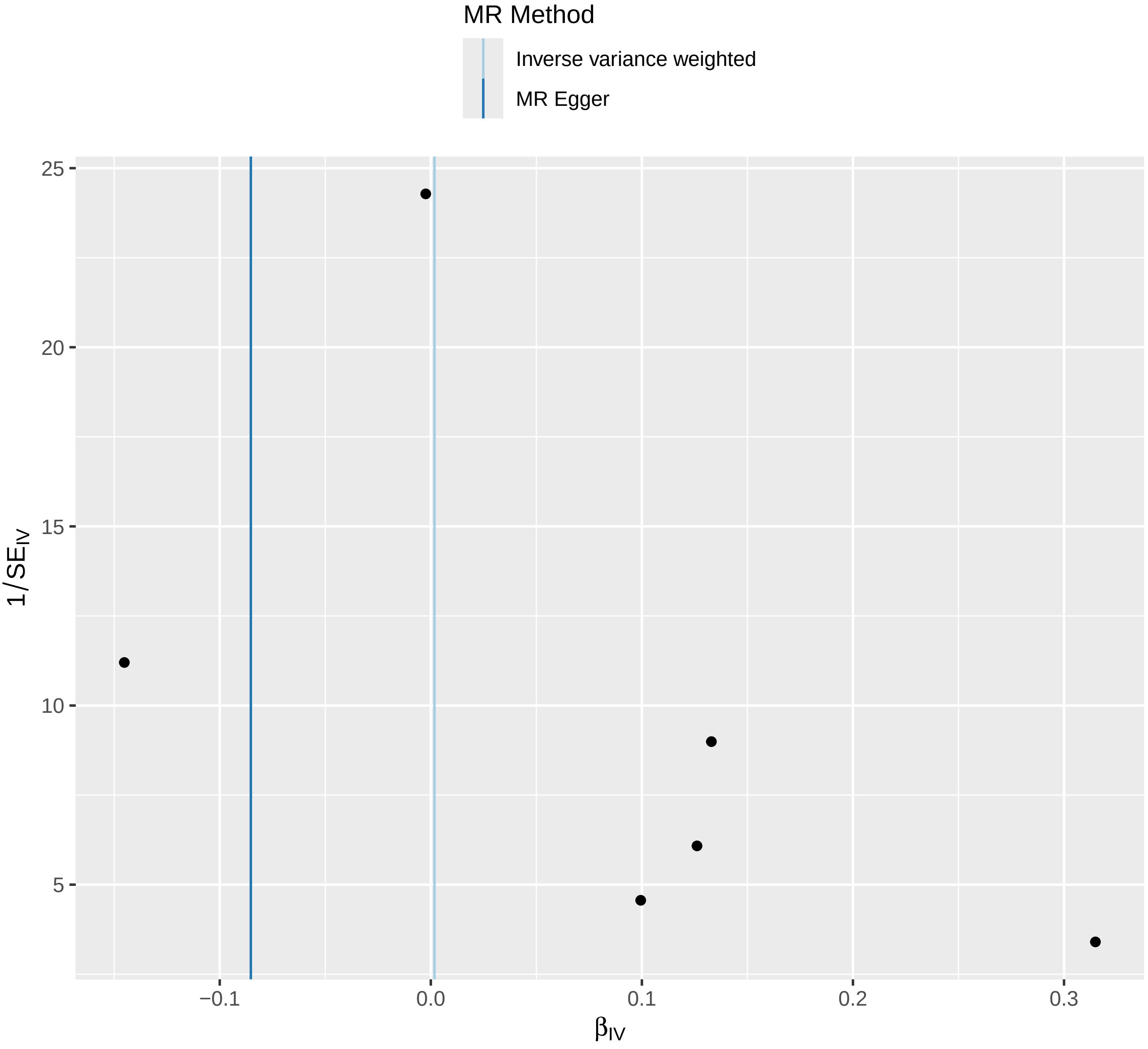

MR Method
Inverse variance weighted
MR Egger
25
20
15
10
5
1/SE$_{IV}$
−0.1
0.0
0.1
0.2
0.3
β$_{IV}$

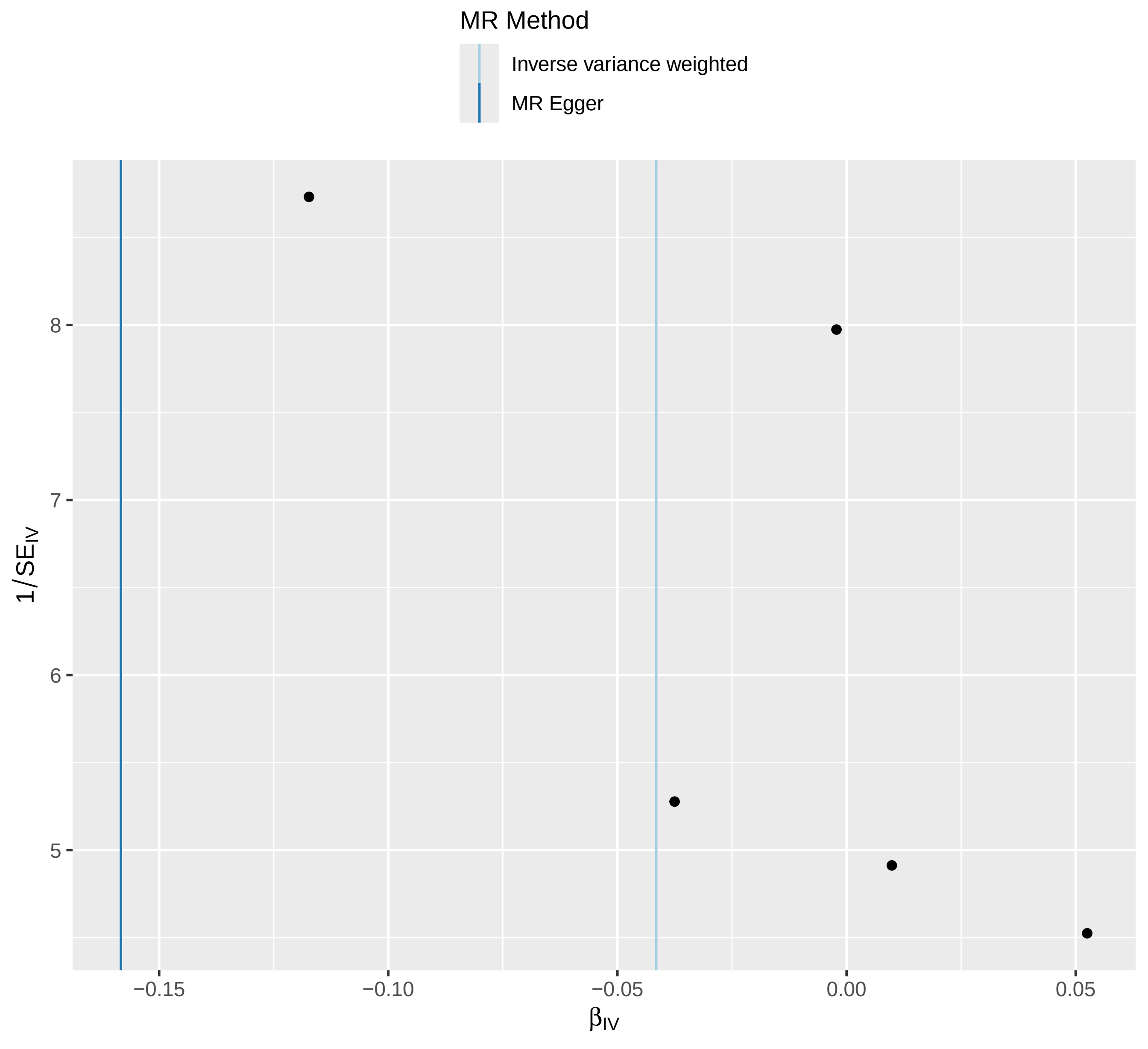

MR Method
Inverse variance weighted
MR Egger
8
7
6
5
$1/SE_{IV}$
−0.15
−0.10
−0.05
0.00
0.05
$\beta_{IV}$

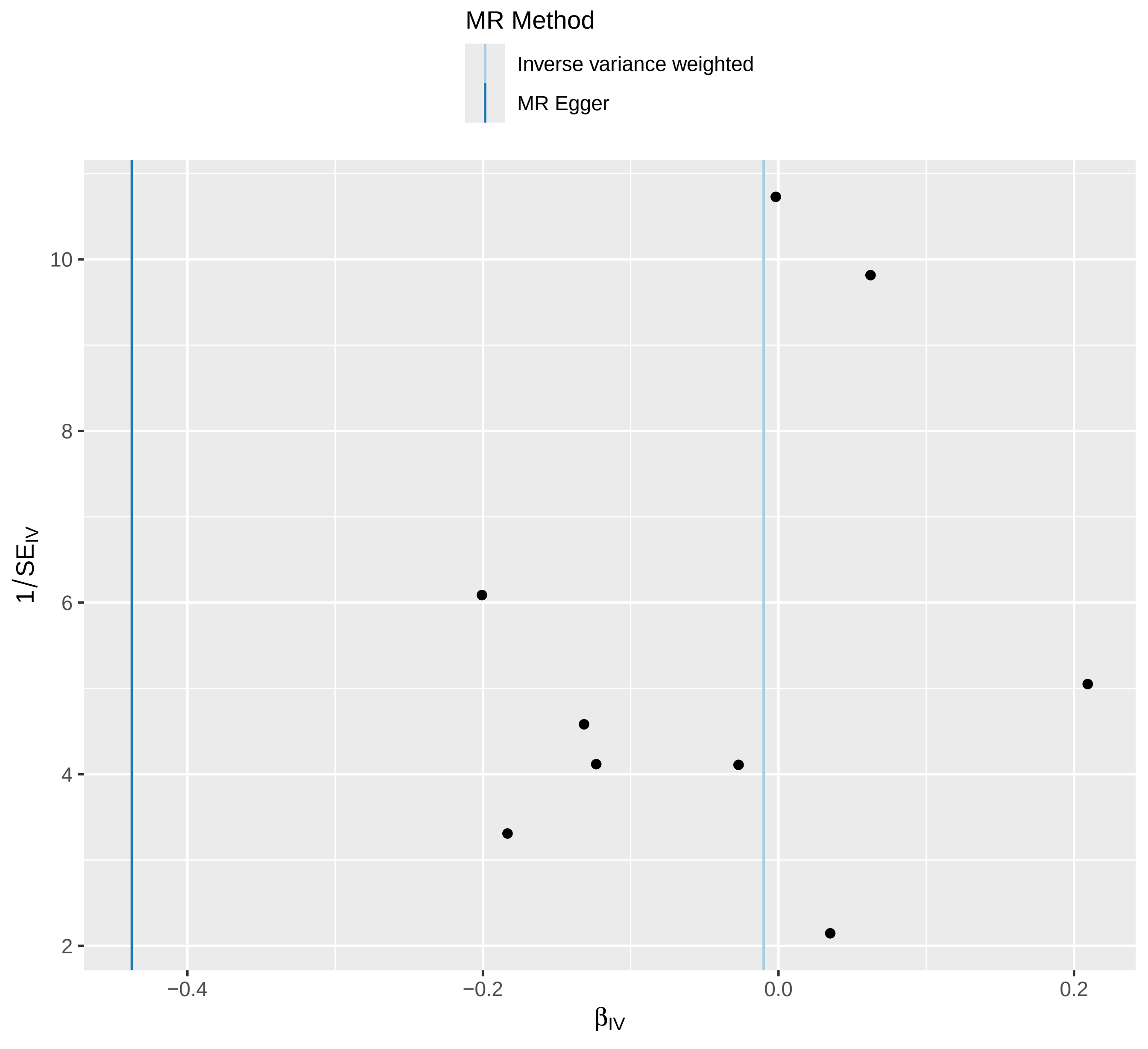

MR Method
Inverse variance weighted
MR Egger
10
8
6
4
2
1/SE$_{IV}$
−0.4
−0.2
0.0
0.2
β$_{IV}$

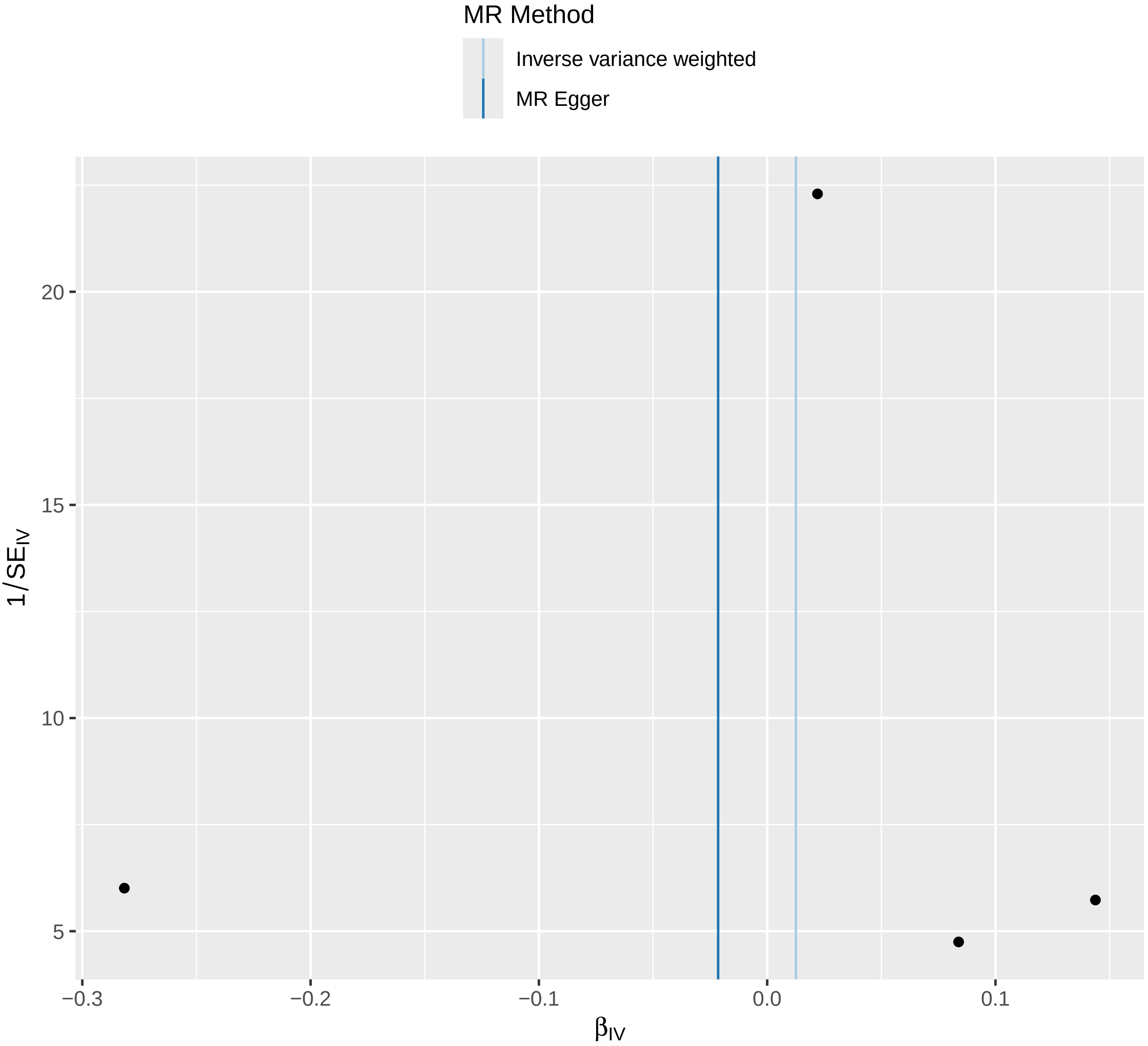

MR Method
Inverse variance weighted
MR Egger
20
15
10
5
1/SE$_{IV}$
−0.3
−0.2
−0.1
0.0
0.1
β$_{IV}$

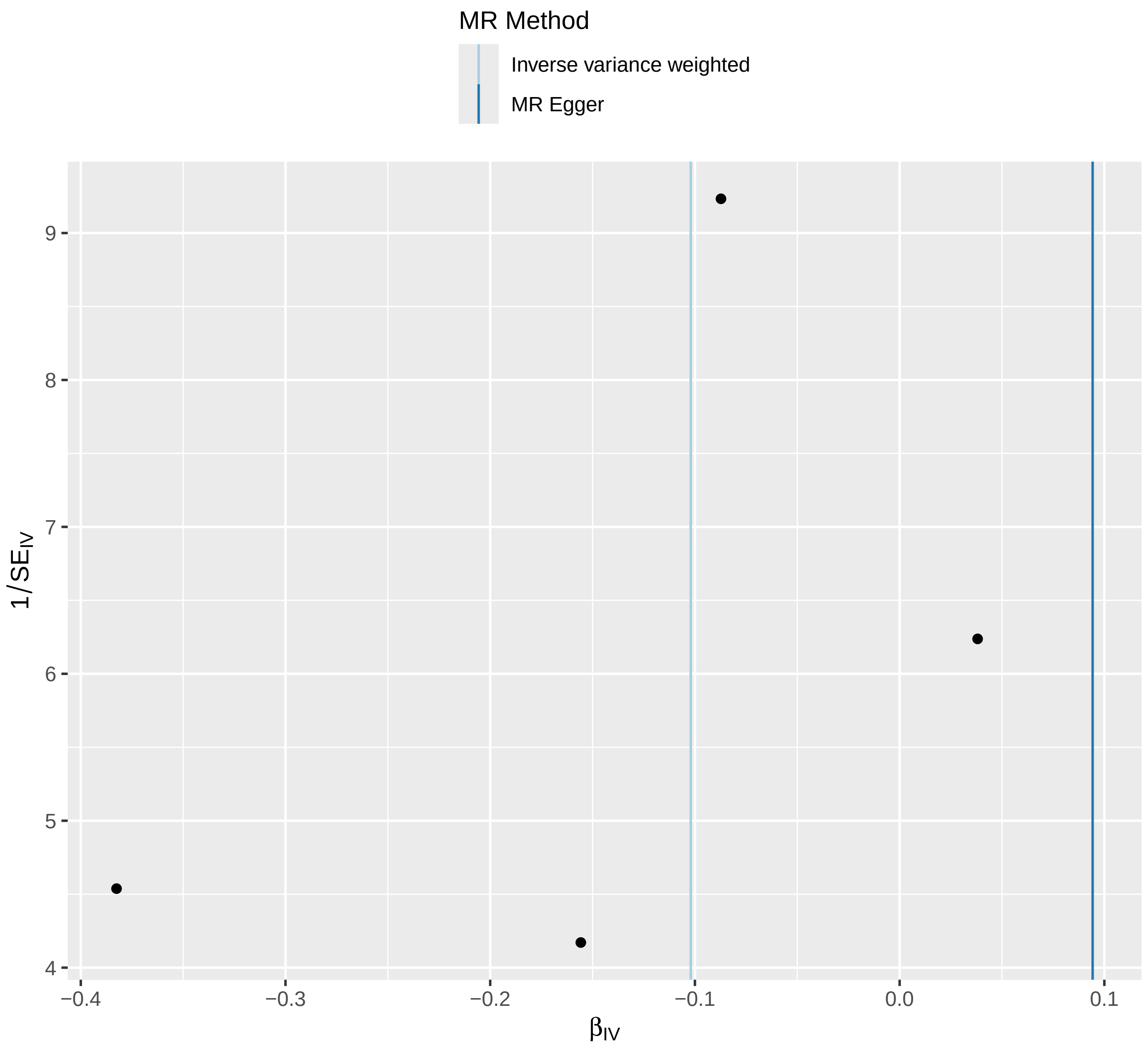
MR Method
Inverse variance weighted
MR Egger
9
8
7
6
5
4
1/SE_IV
−0.4
−0.3
−0.2
−0.1
0.0
0.1
β_IV

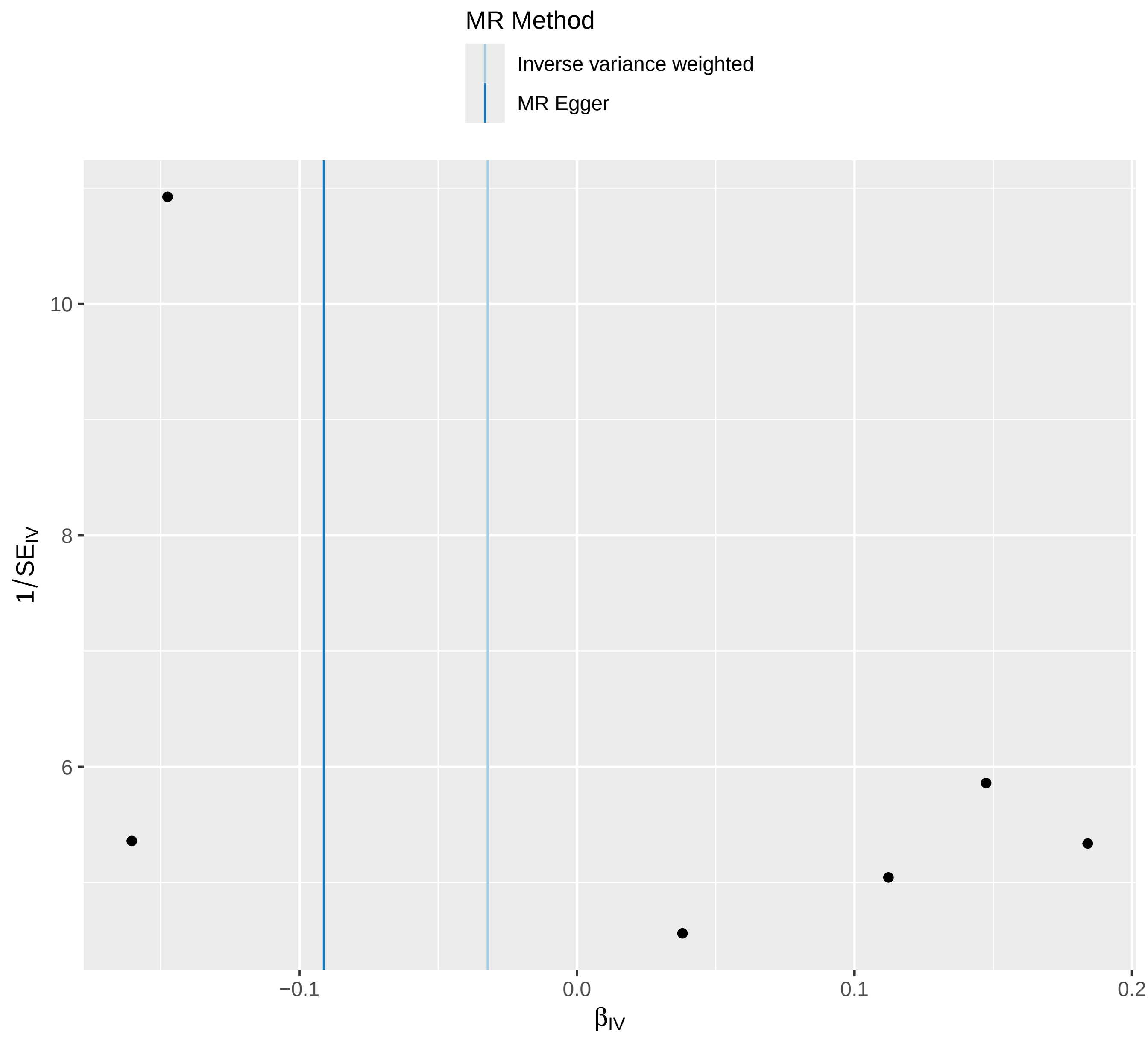

MR Method
Inverse variance weighted
MR Egger
10
8
6
1/SE_IV
−0.1
0.0
0.1
0.2
β_IV

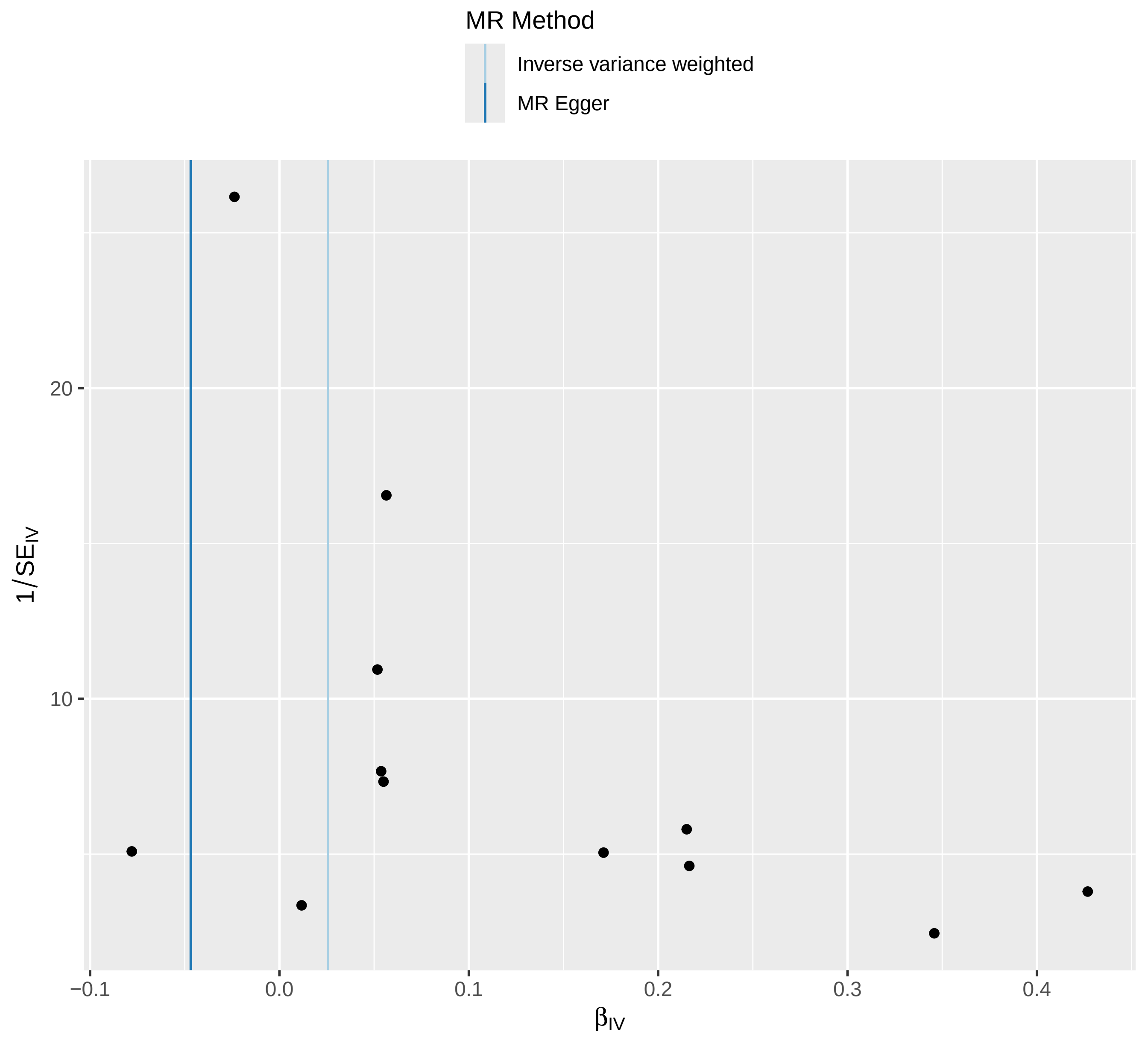

MR Method
Inverse variance weighted
MR Egger
20
10
1/SE_IV
−0.1
0.0
0.1
0.2
0.3
0.4
β_IV

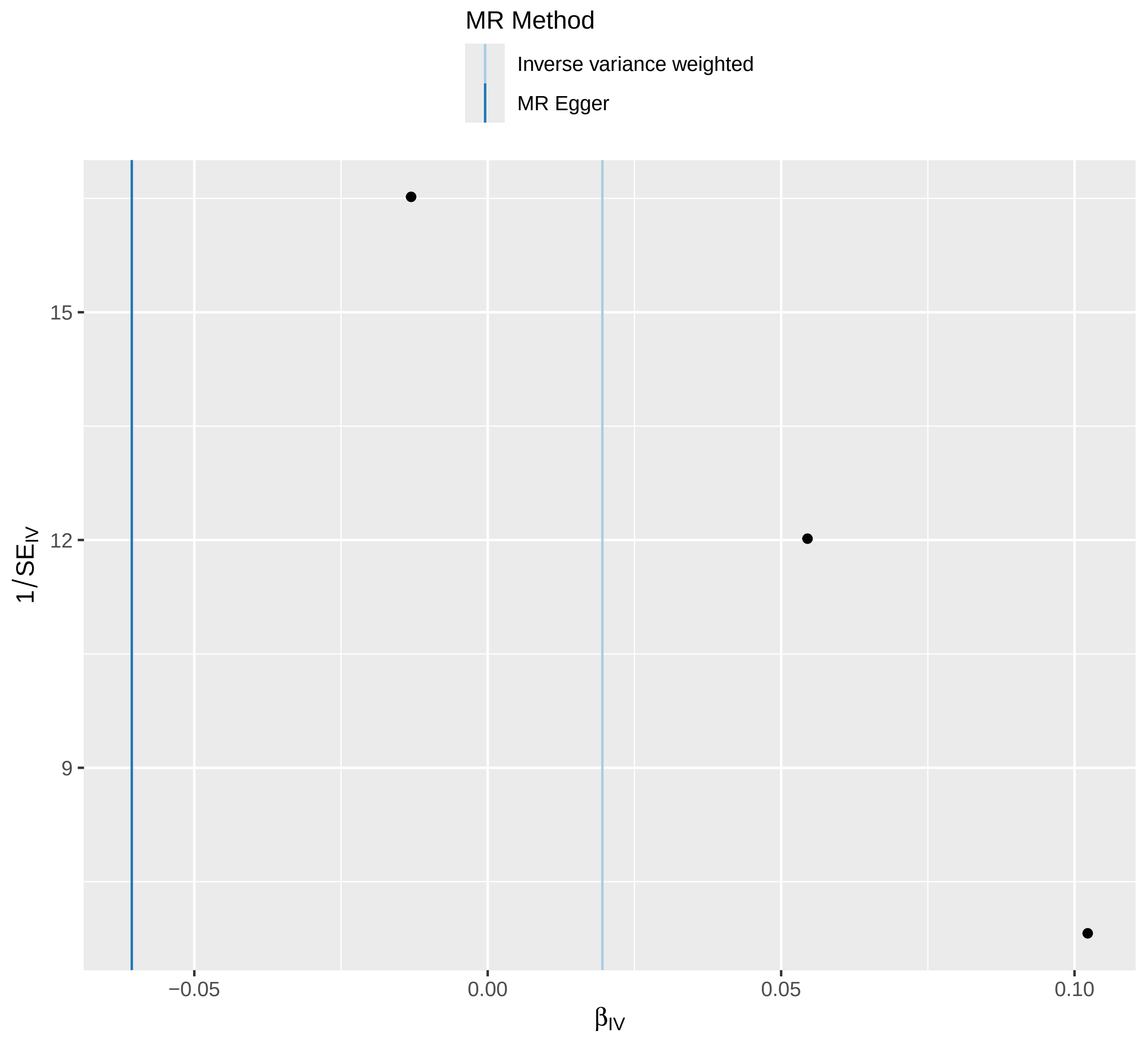
MR Method
Inverse variance weighted
MR Egger
15
12
9
1/SE_IV
−0.05
0.00
0.05
0.10
β_IV

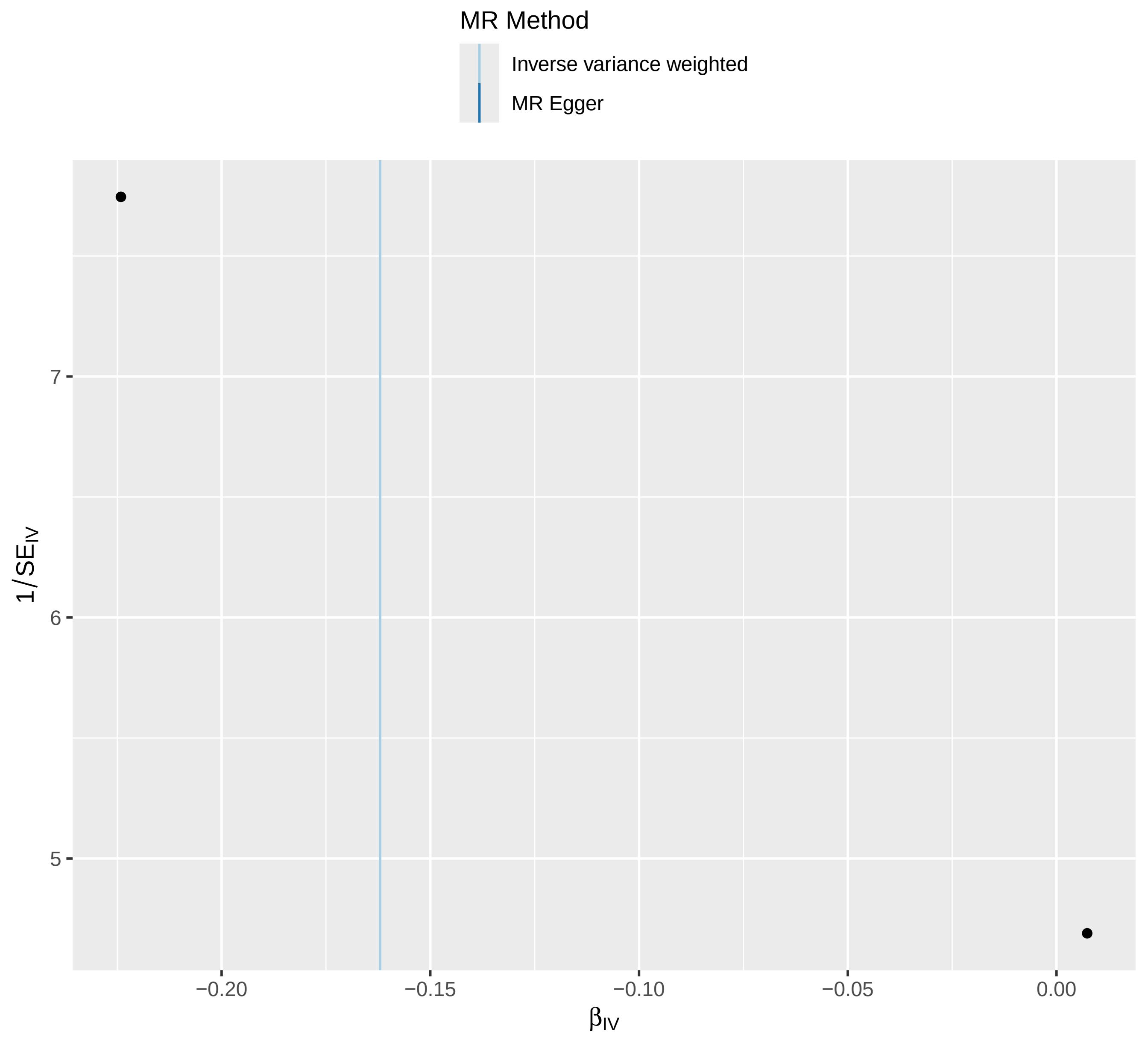
MR Method
Inverse variance weighted
MR Egger
1/SE_IV
7
6
5
−0.20
−0.15
−0.10
−0.05
0.00
β_IV

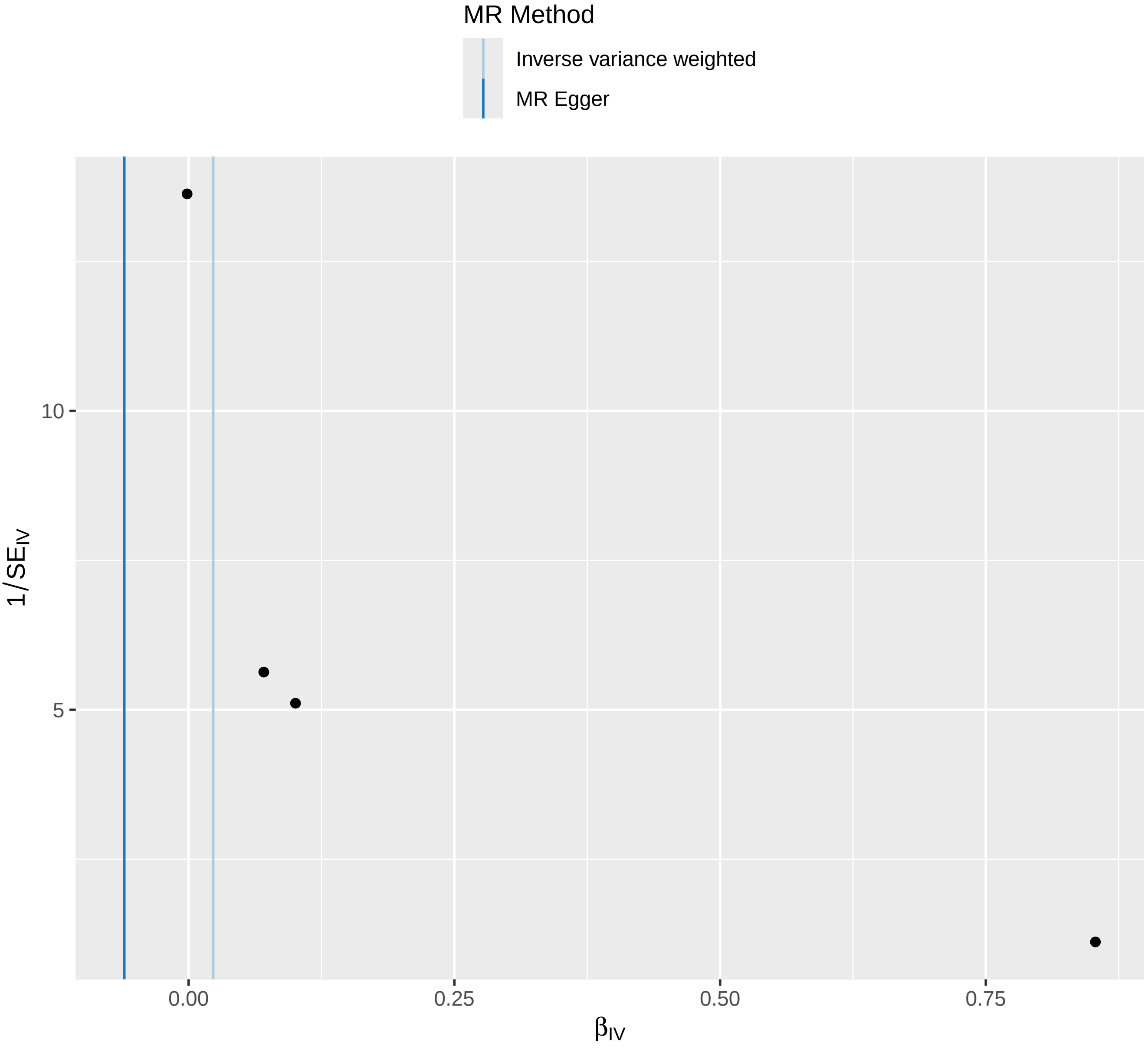
MR Method
Inverse variance weighted
MR Egger
10
5
1/SE_IV
0.00
0.25
0.50
0.75
β_IV

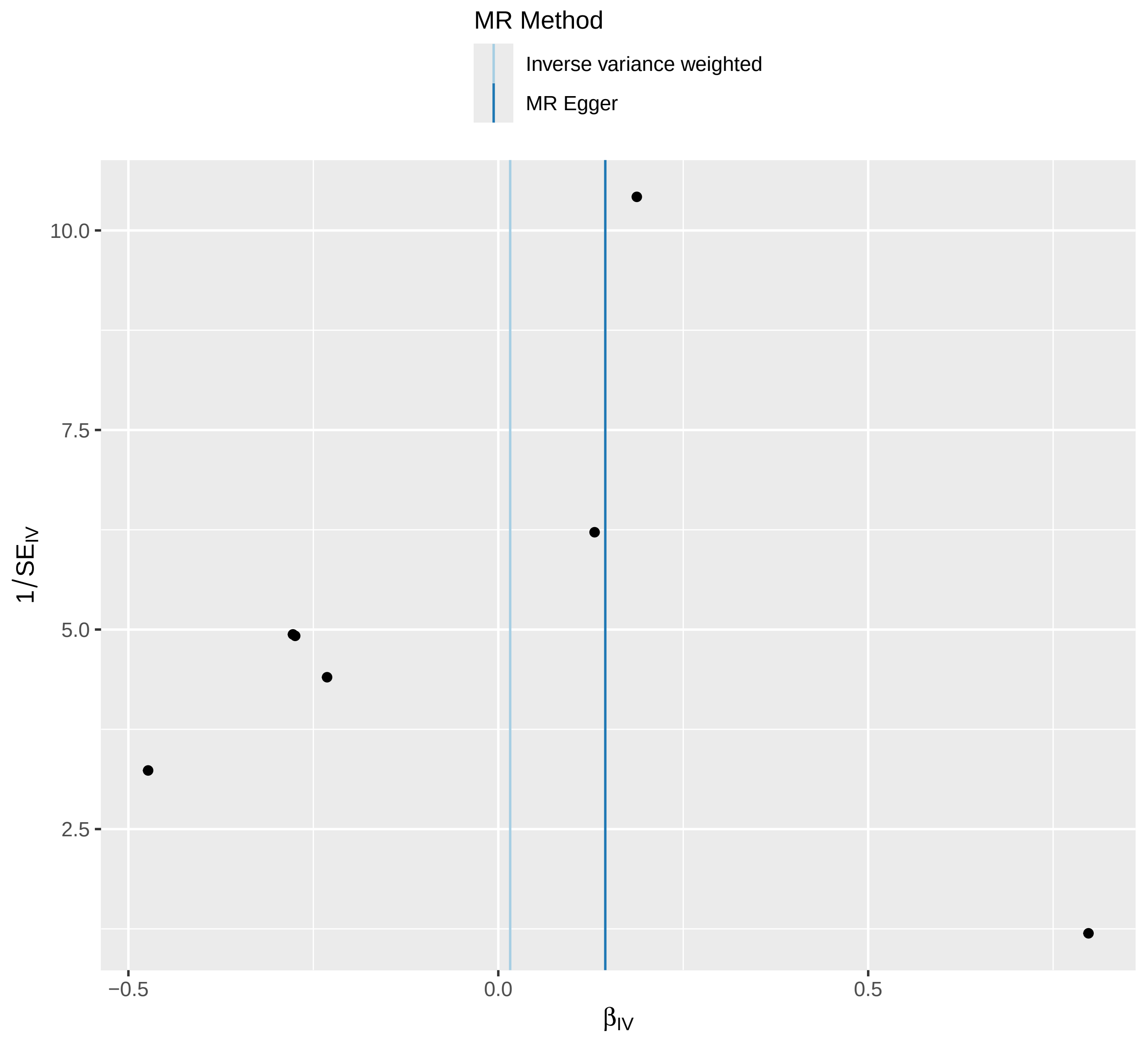

MR Method
Inverse variance weighted
MR Egger
10.0
7.5
5.0
2.5
−0.5
0.0
0.5
$1/SE_{IV}$
$\beta_{IV}$

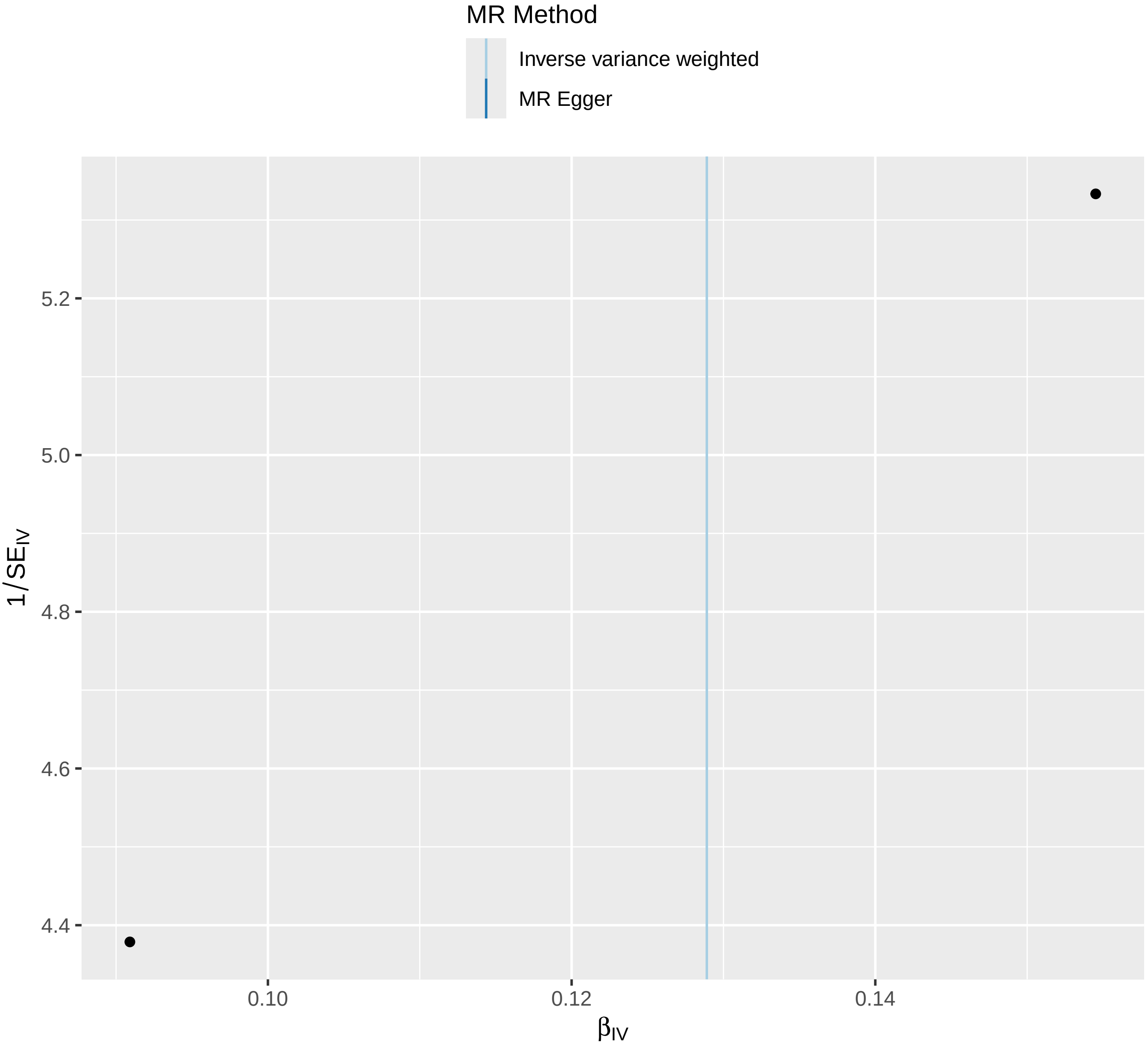

MR Method
Inverse variance weighted
MR Egger
5.2
5.0
4.8
4.6
4.4
0.10
0.12
0.14
$1/SE_{IV}$
$\beta_{IV}$

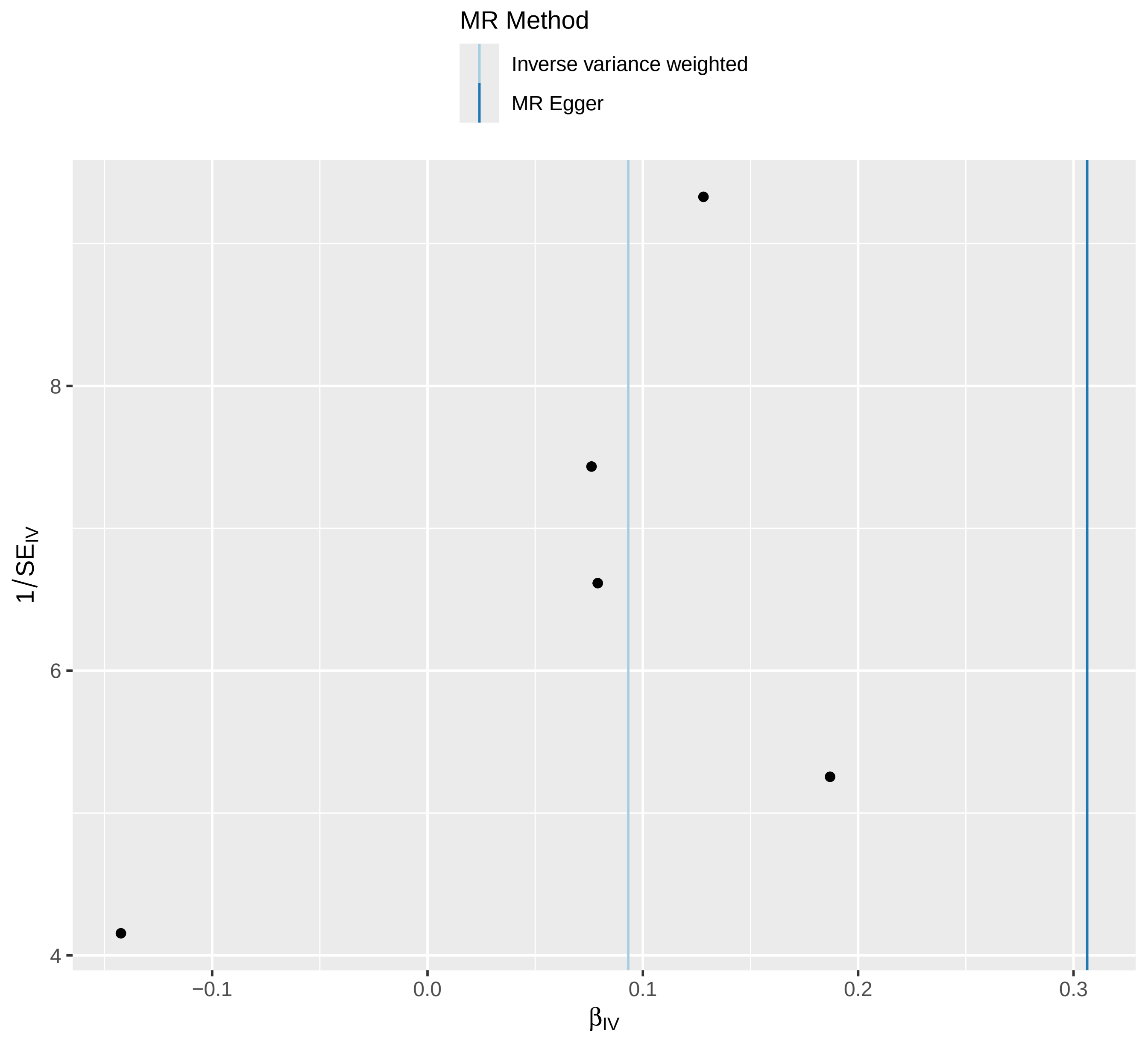

MR Method
Inverse variance weighted
MR Egger
8
6
4
1/SE_IV
−0.1
0.0
0.1
0.2
0.3
β_IV

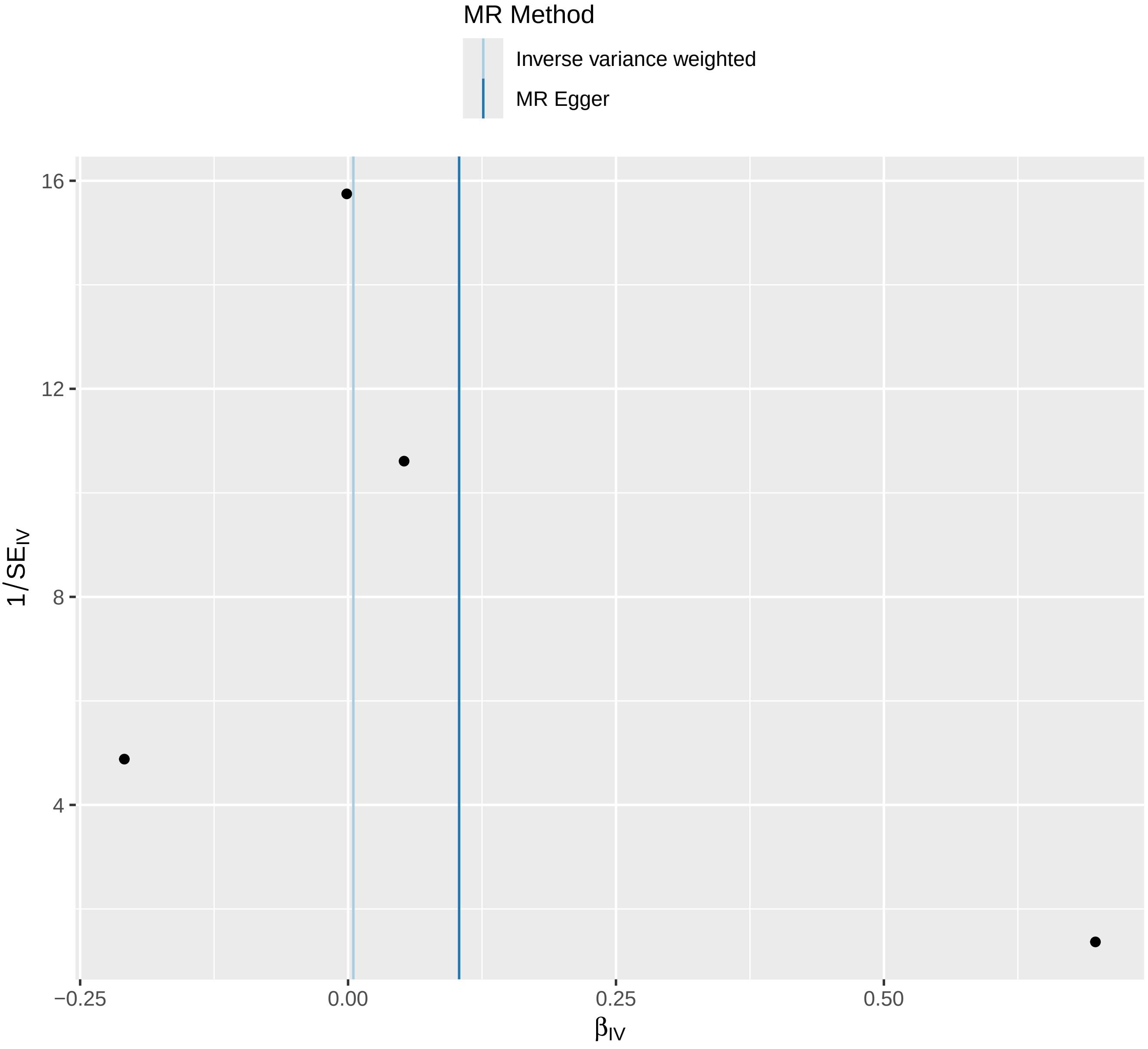

MR Method
Inverse variance weighted
MR Egger
16
12
8
4
1/SE_IV
−0.25
0.00
0.25
0.50
β_IV

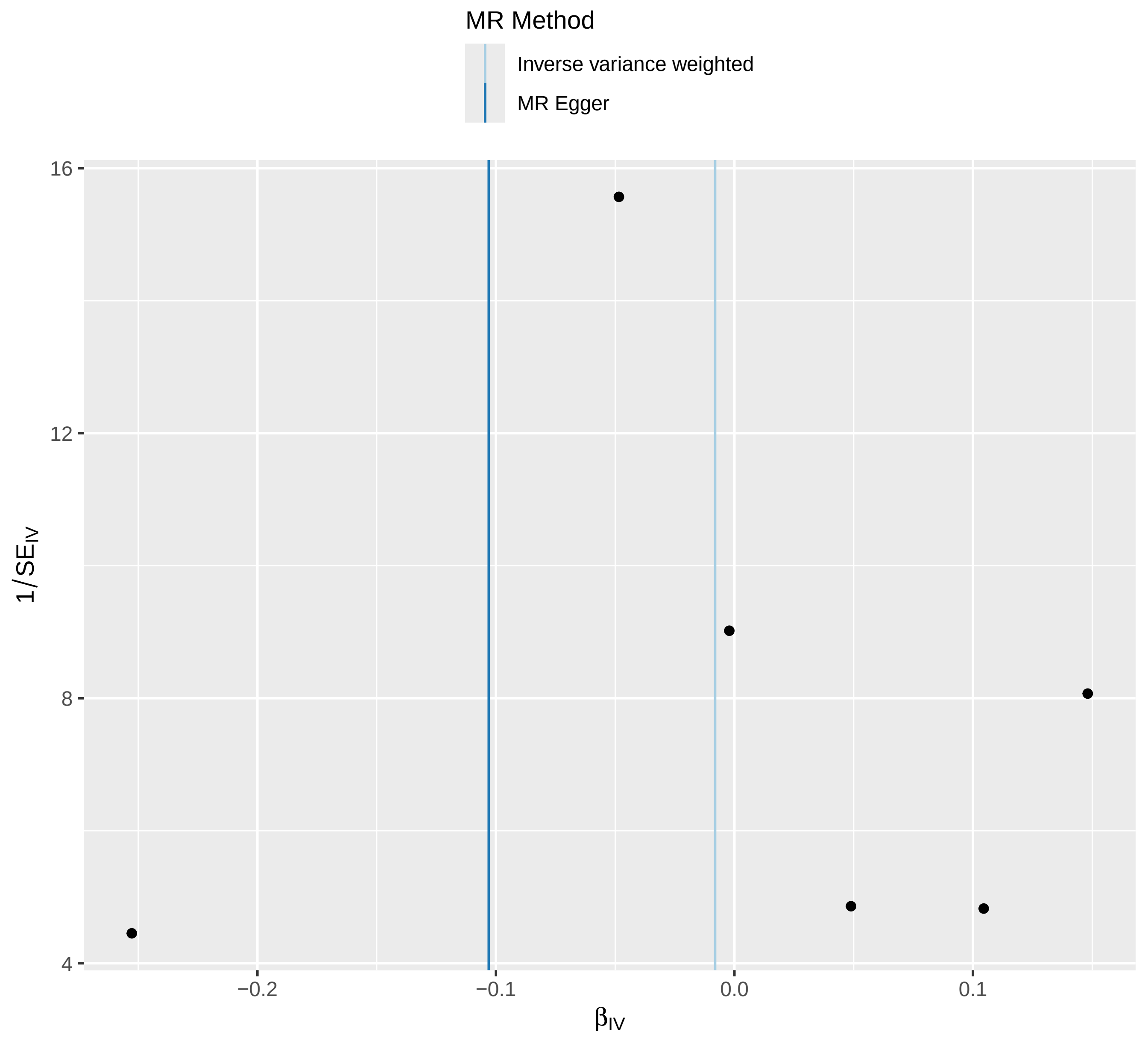

MR Method
Inverse variance weighted
MR Egger
16
12
8
4
−0.2
−0.1
0.0
0.1
$1/SE_{IV}$
$\beta_{IV}$

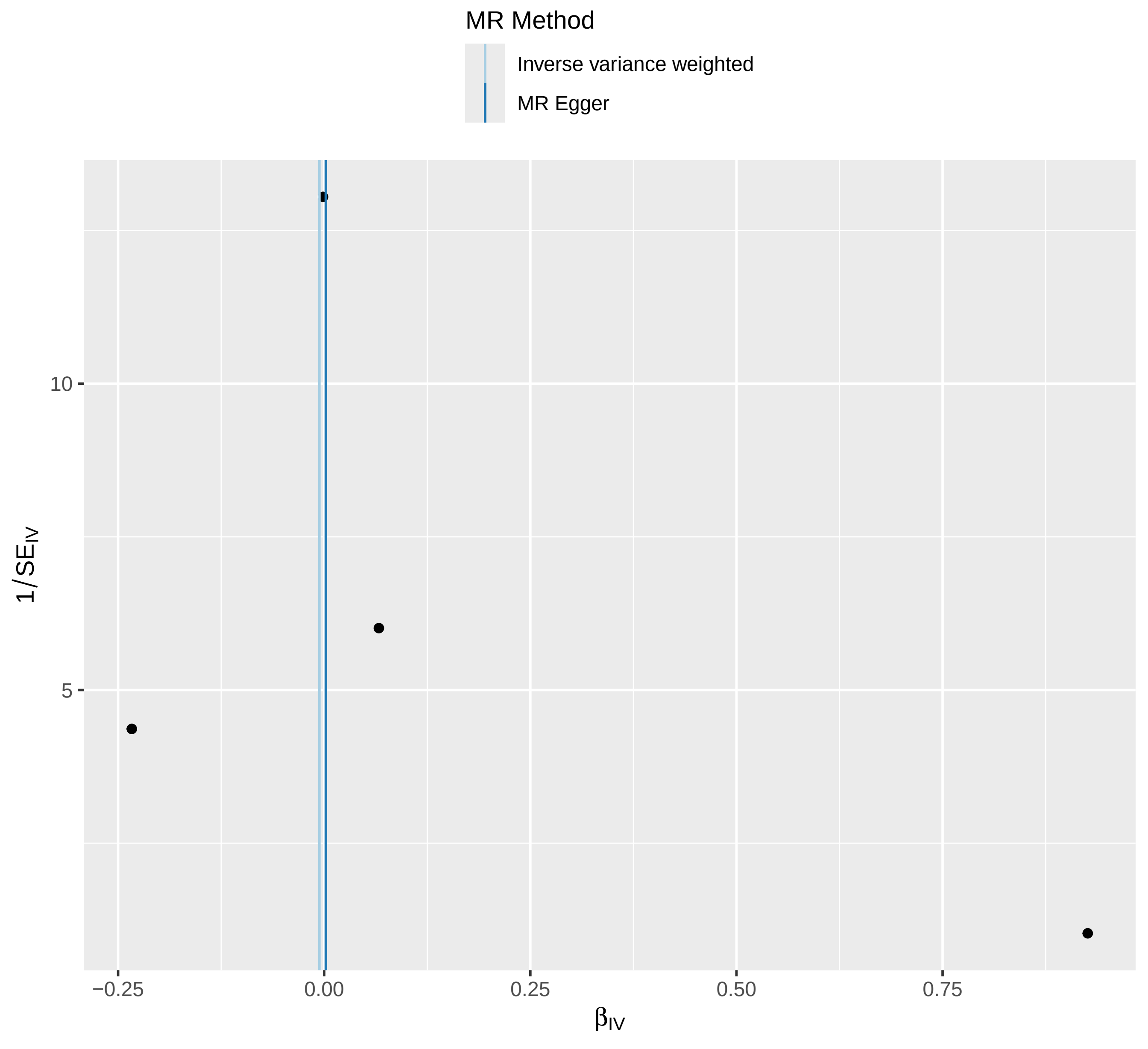
MR Method
Inverse variance weighted
MR Egger
10
5
1/SE_IV
−0.25
0.00
0.25
0.50
0.75
β_IV

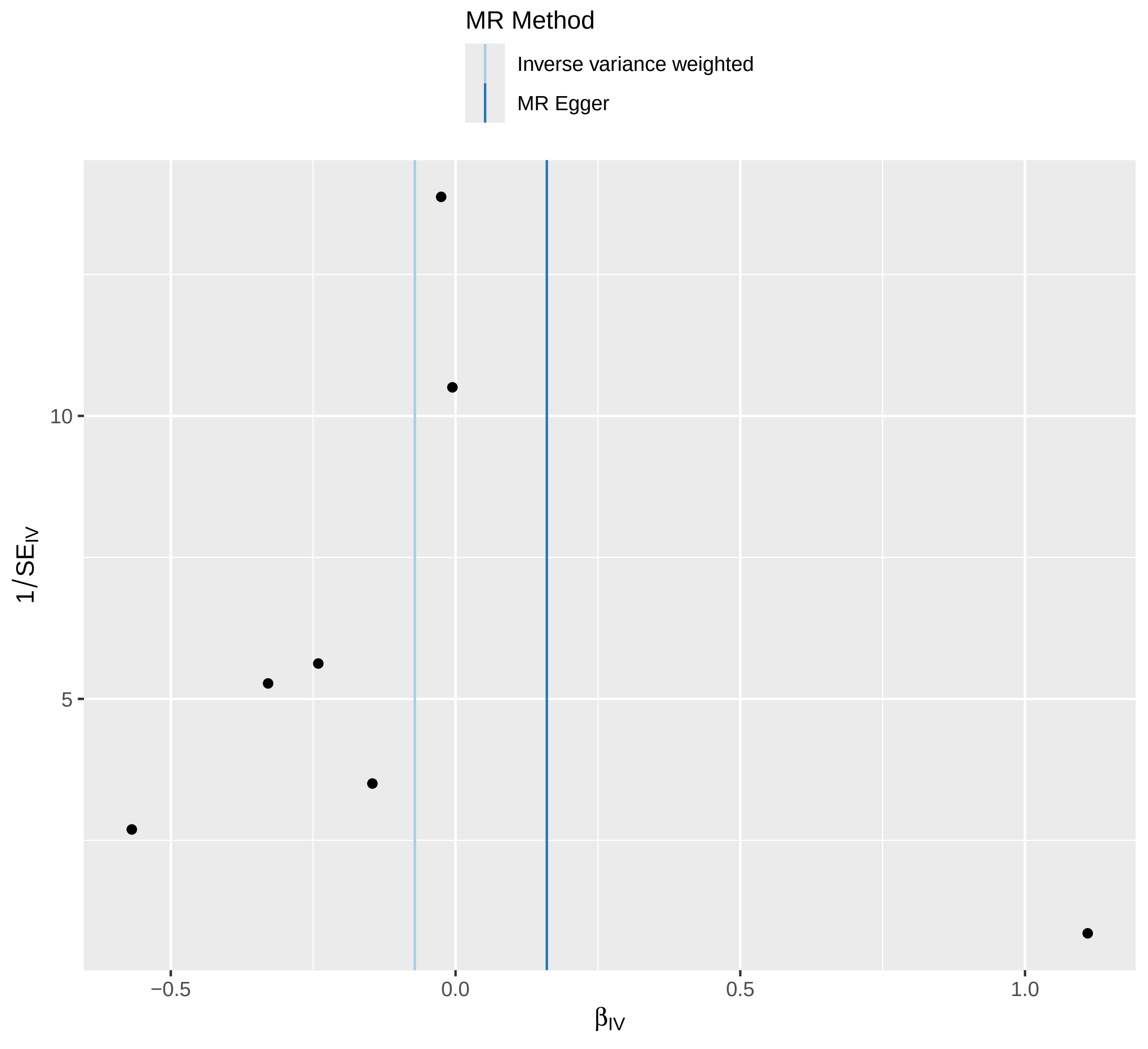

MR Method
Inverse variance weighted
MR Egger
10
5
1/SE$_{IV}$
−0.5
0.0
0.5
1.0
β$_{IV}$

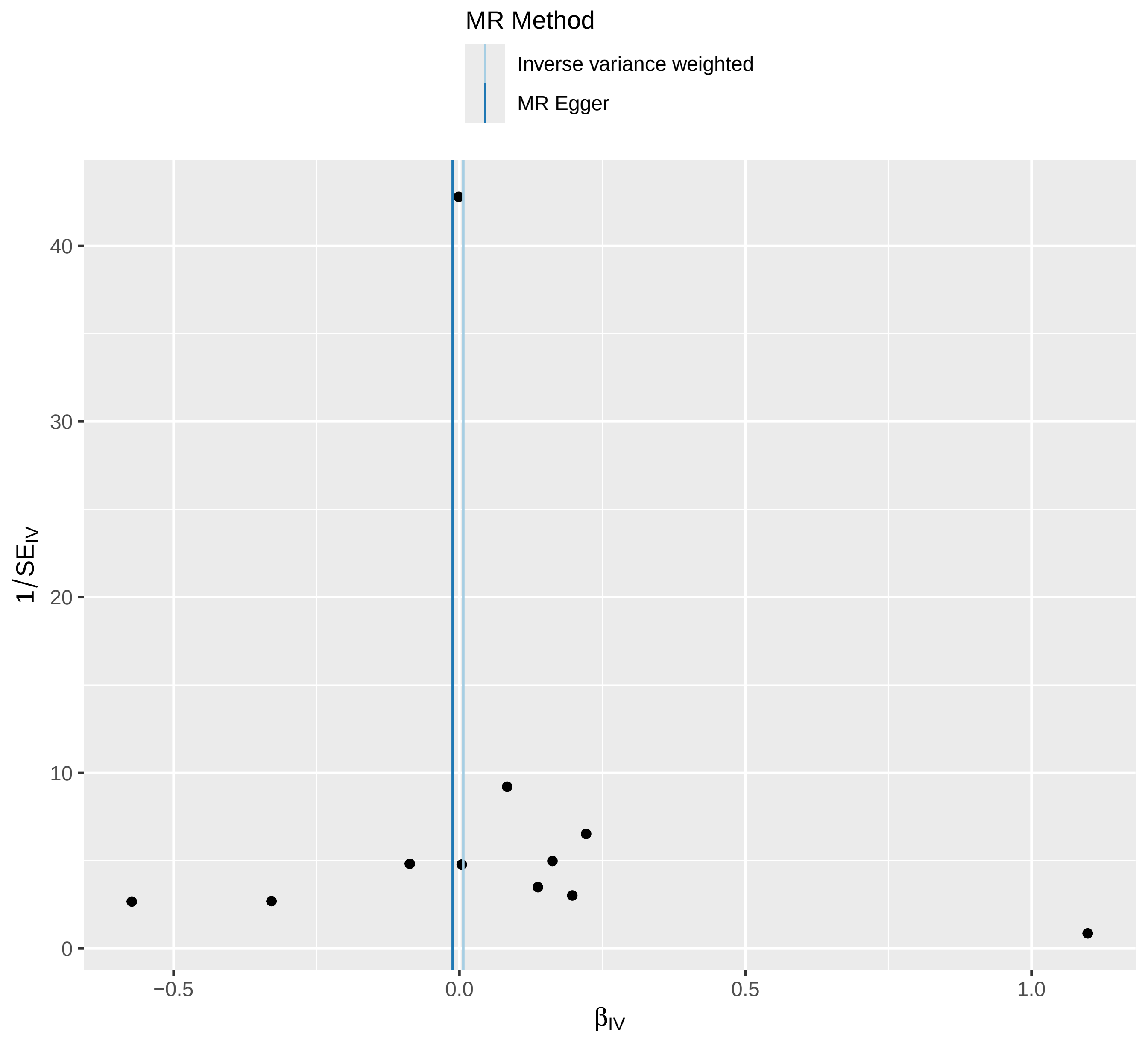

MR Method
Inverse variance weighted
MR Egger
40
30
20
10
0
1/SE_IV
−0.5
0.0
0.5
1.0
β_IV

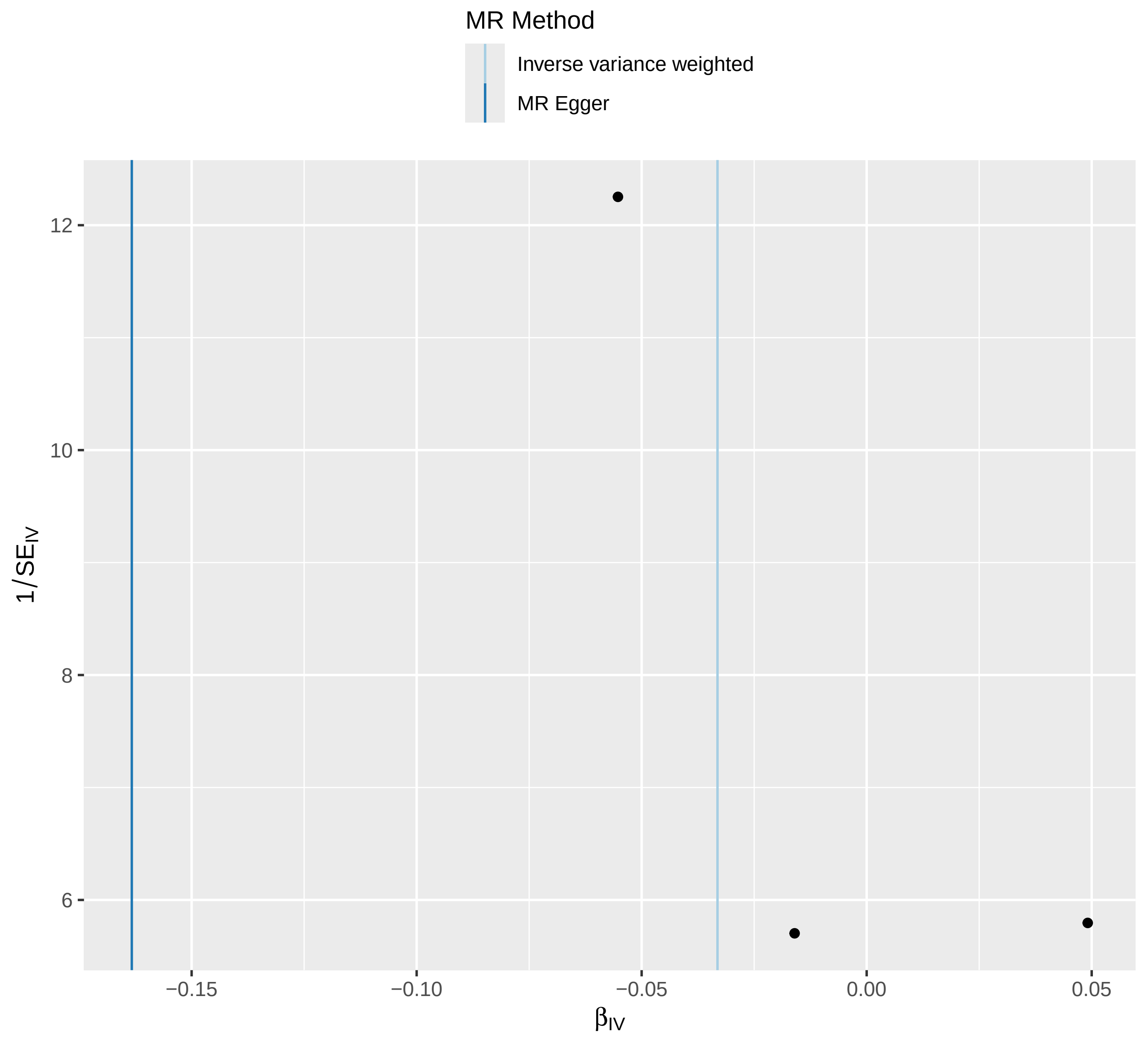

MR Method
Inverse variance weighted
MR Egger
12
10
8
6
1/SE_IV
−0.15
−0.10
−0.05
0.00
0.05
β_IV

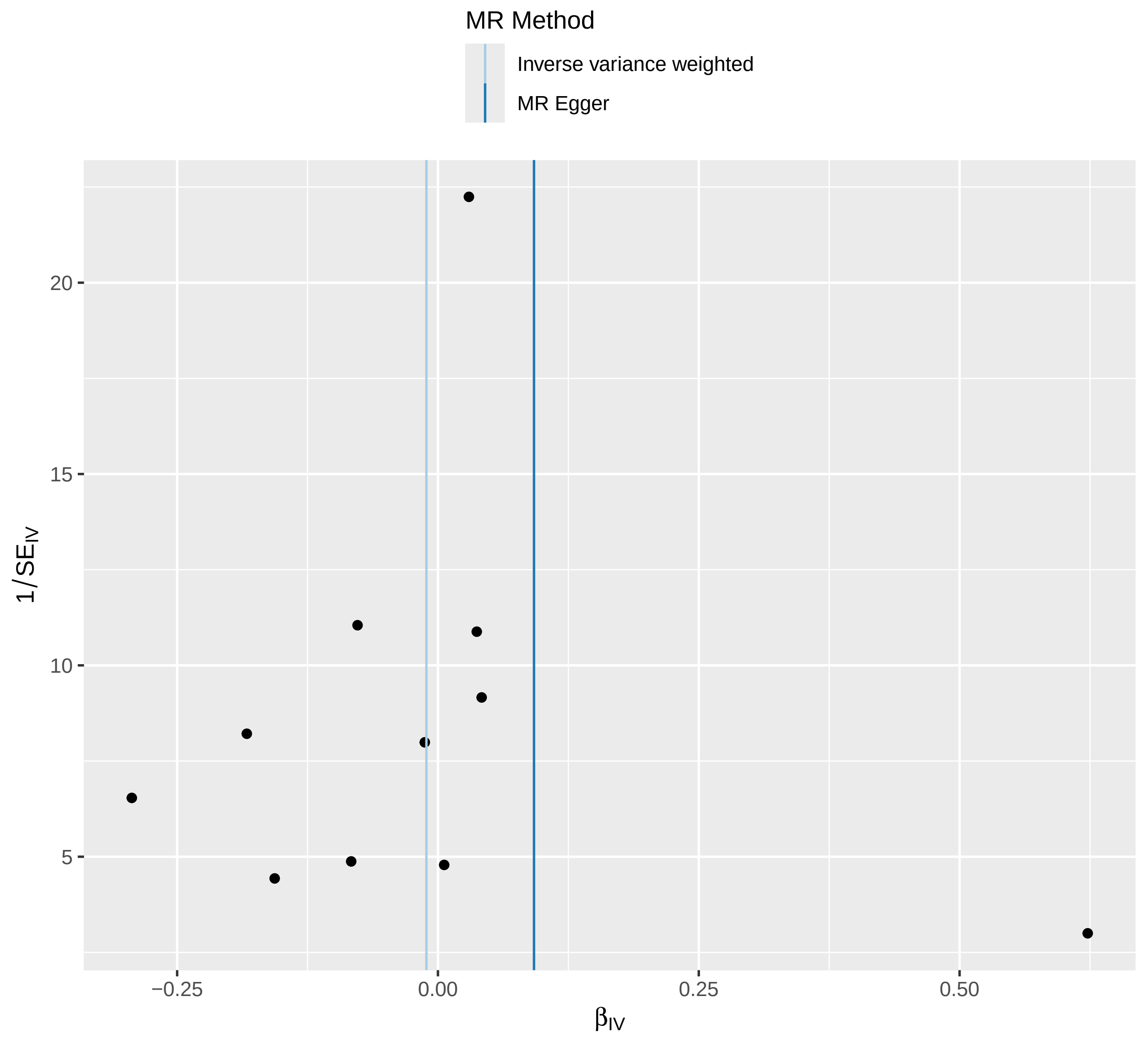

MR Method
Inverse variance weighted
MR Egger
20
15
10
5
1/SE$_{IV}$
−0.25
0.00
0.25
0.50
β$_{IV}$

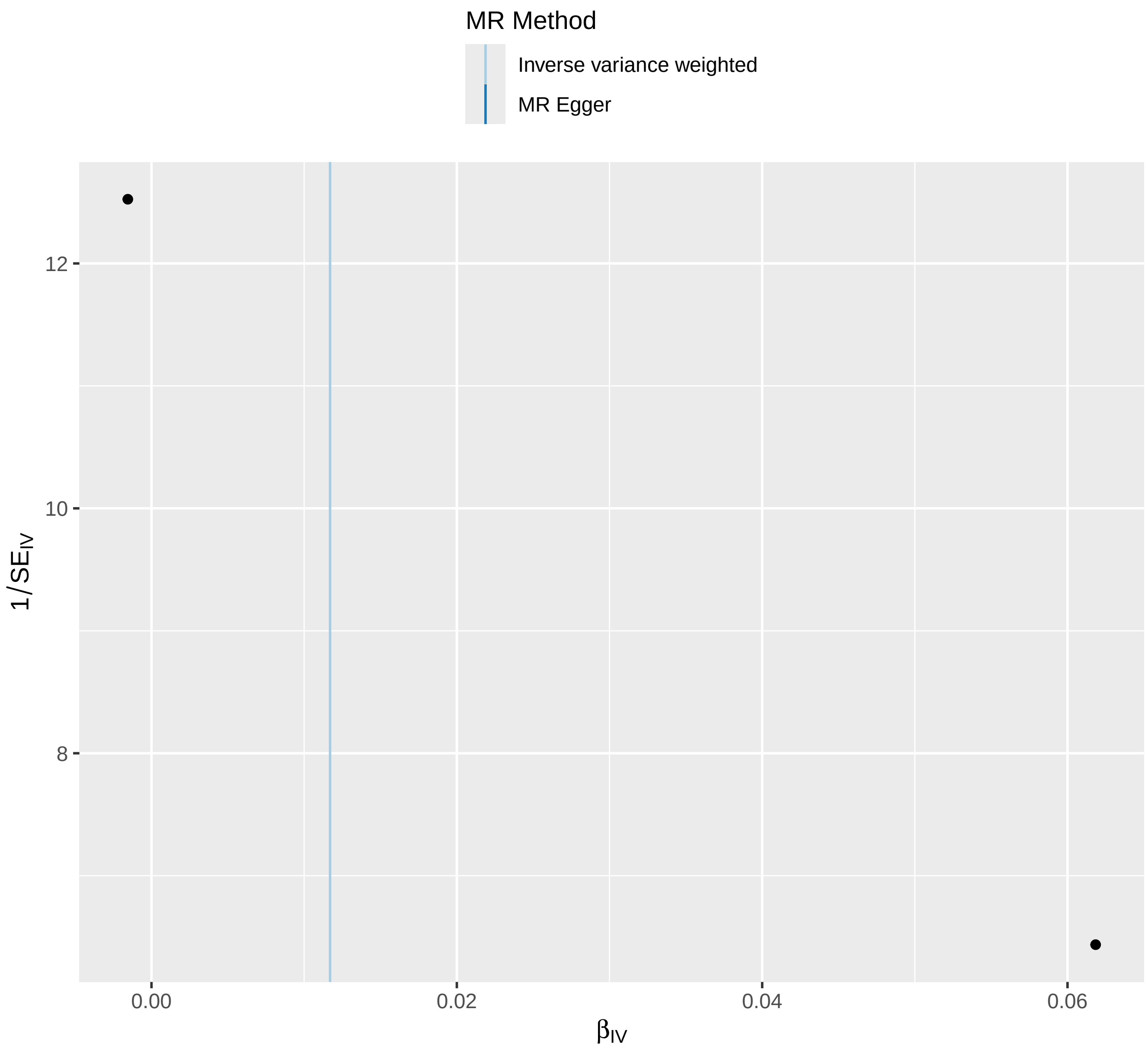

MR Method
Inverse variance weighted
MR Egger
12
10
8
1/SE_IV
0.00
0.02
0.04
0.06
β_IV

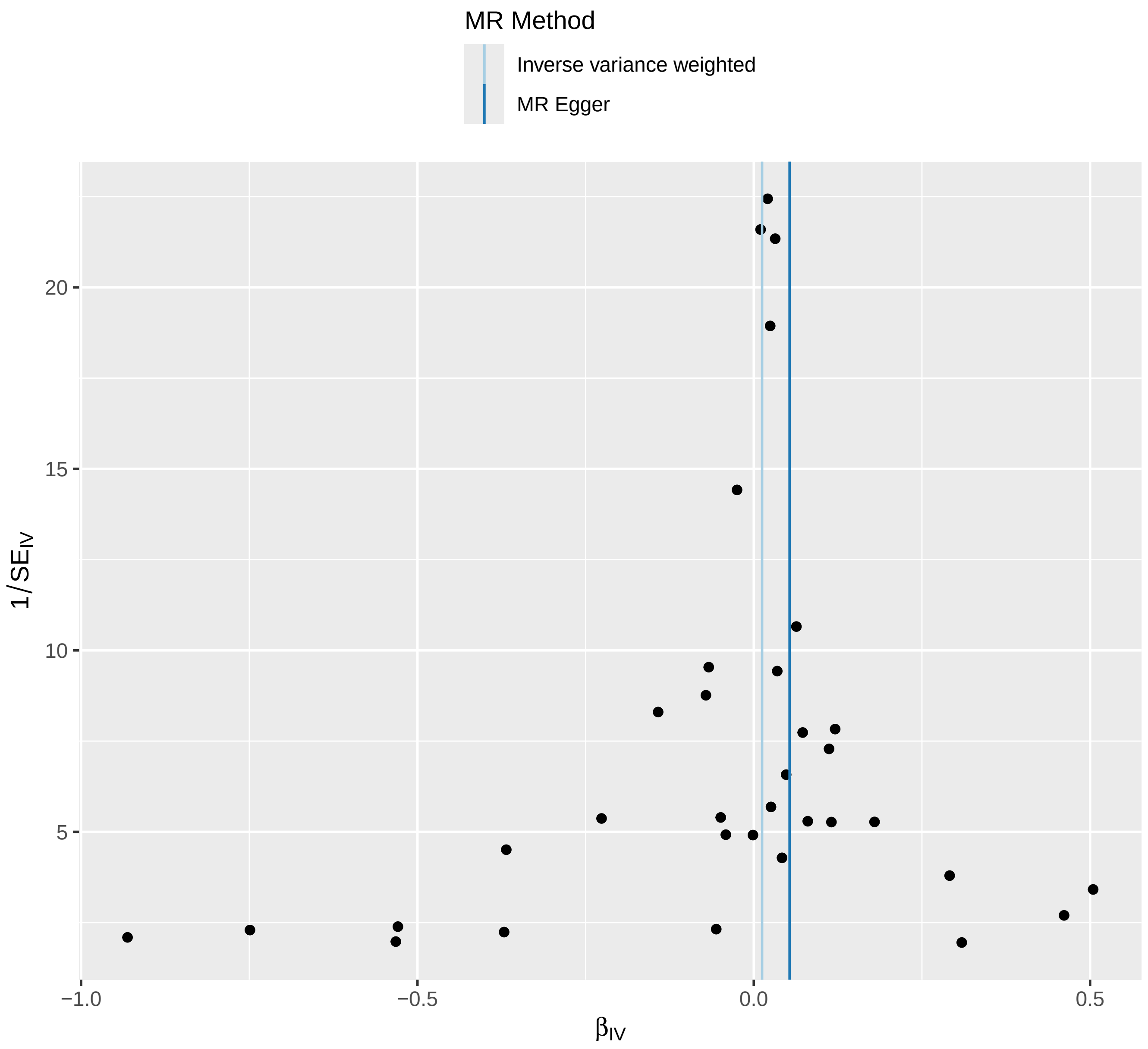

MR Method
Inverse variance weighted
MR Egger
20
15
10
5
1/SE_IV
−1.0
−0.5
0.0
0.5
β_IV

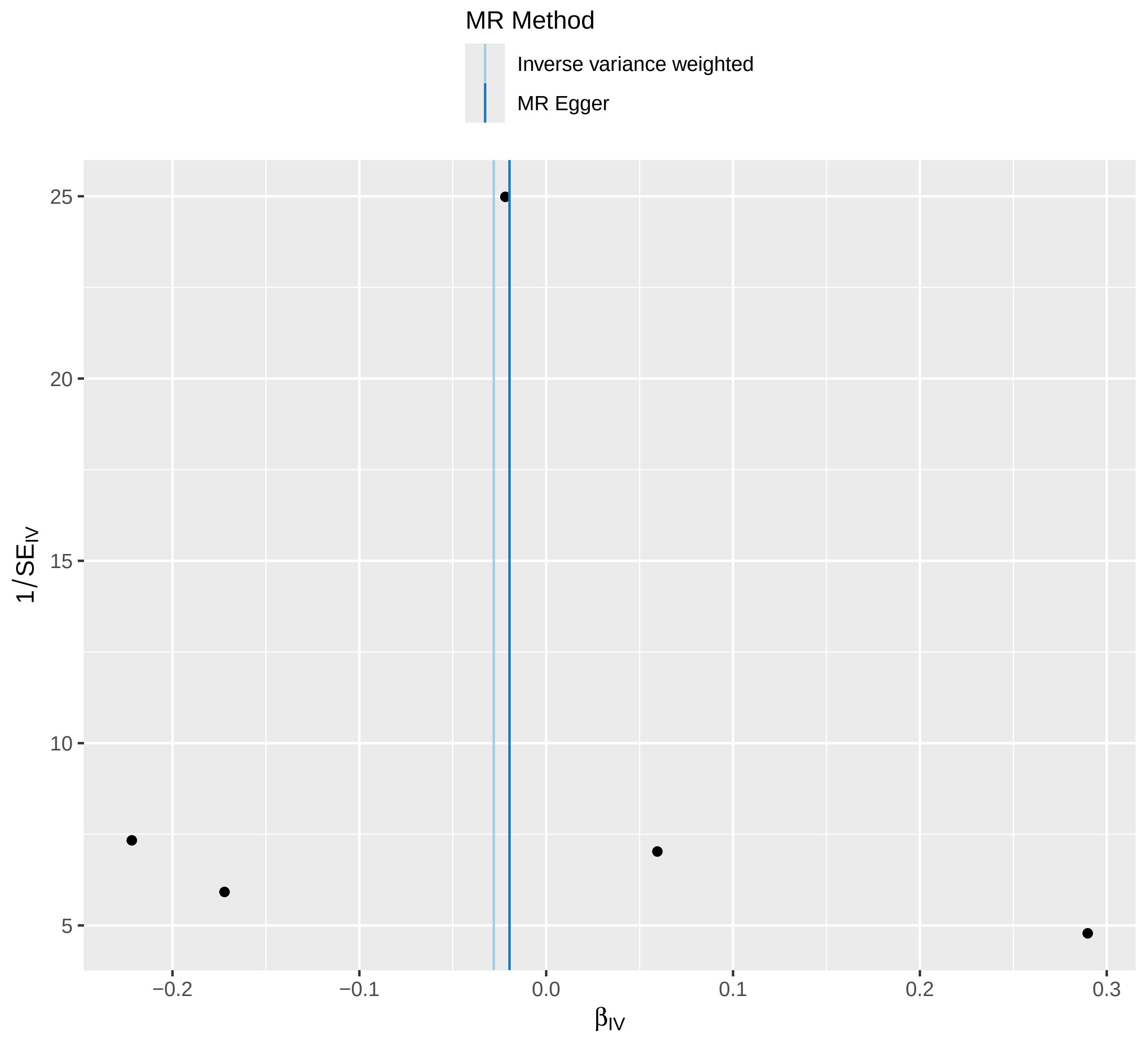
MR Method
Inverse variance weighted
MR Egger
25
20
15
10
5
1/SE_IV
−0.2
−0.1
0.0
0.1
0.2
0.3
β_IV

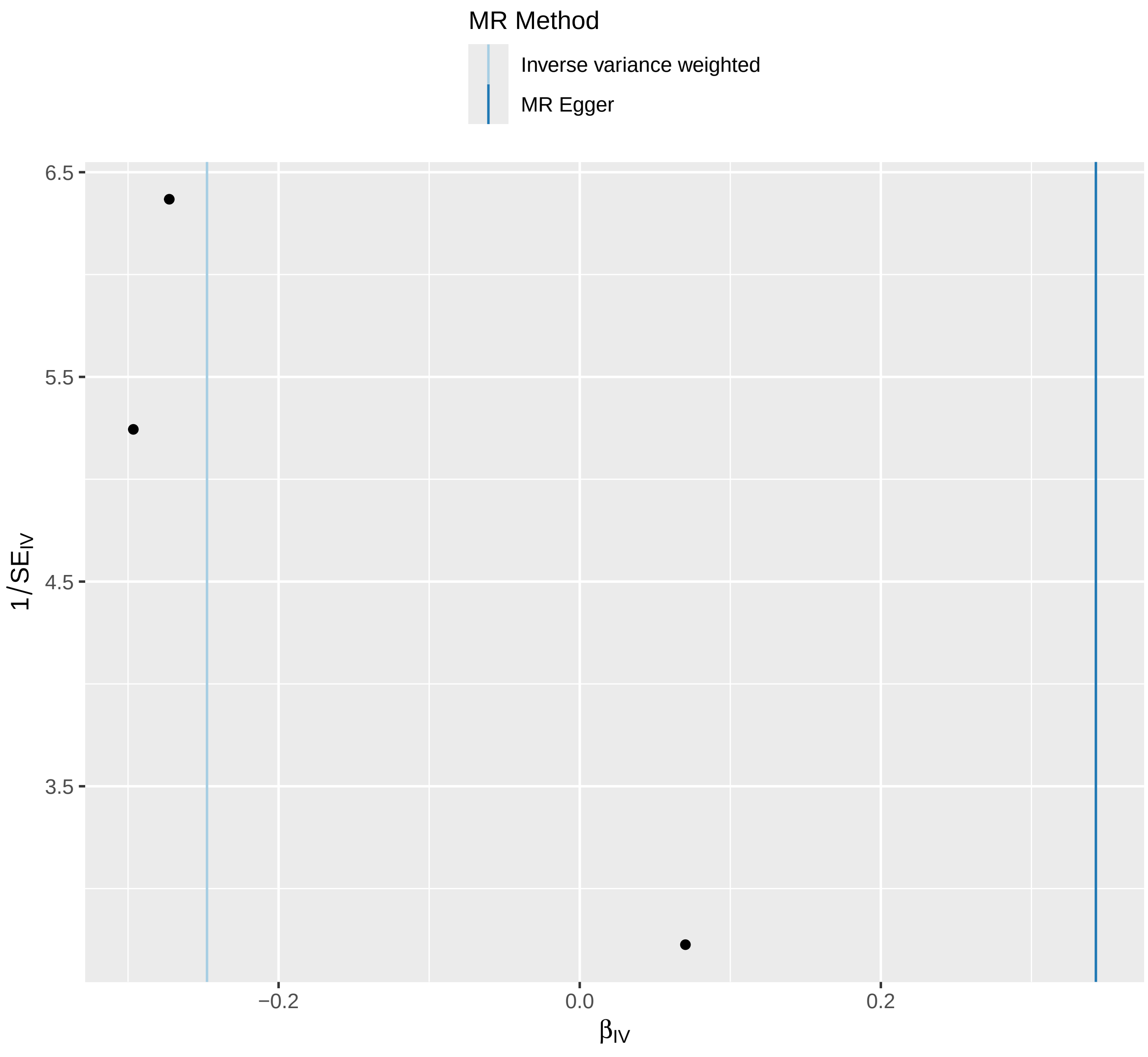
MR Method
Inverse variance weighted
MR Egger
6.5
5.5
4.5
3.5
1/SE_IV
−0.2
0.0
0.2
β_IV

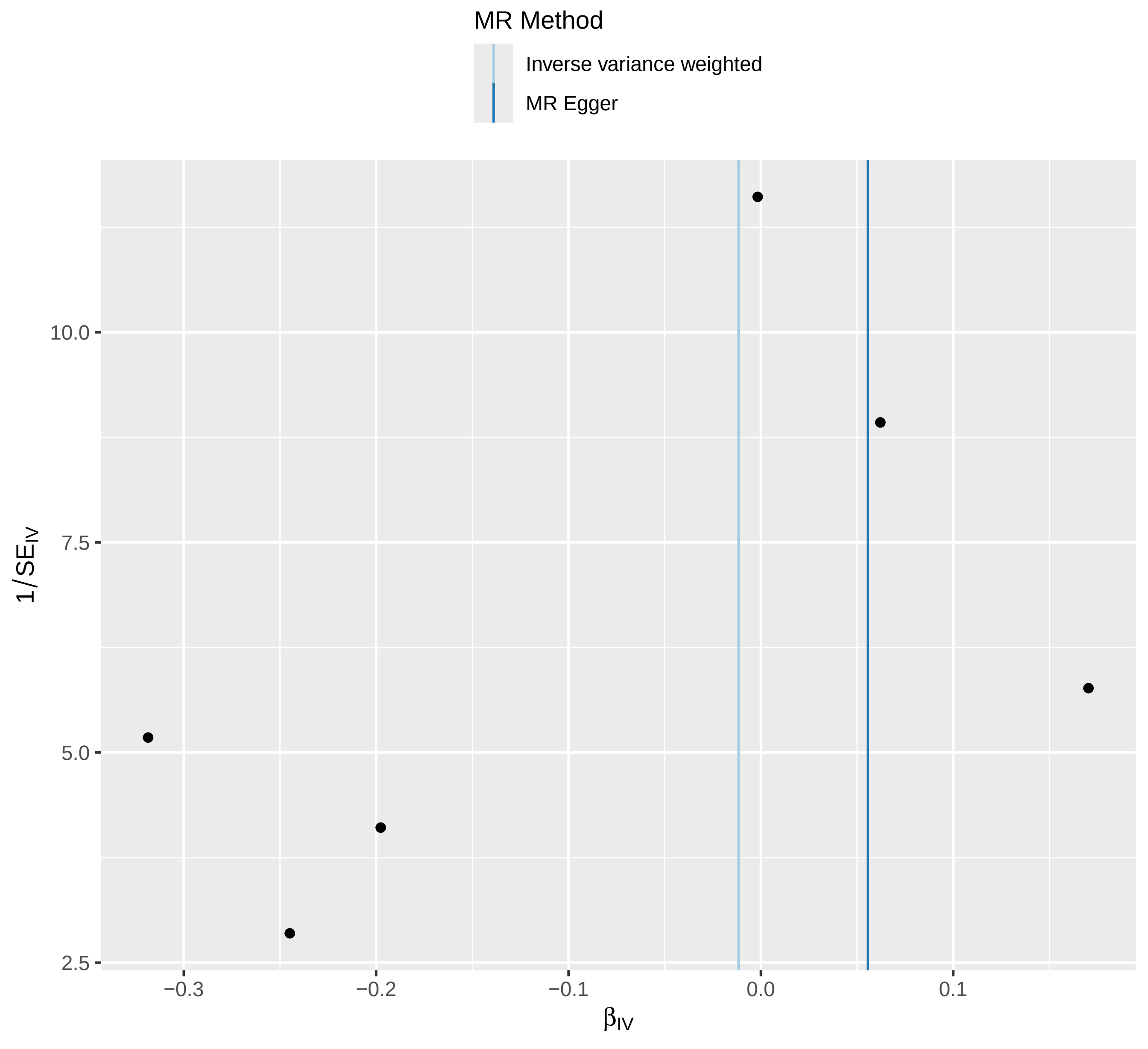
MR Method
Inverse variance weighted
MR Egger
10.0
7.5
5.0
2.5
1/SE_IV
−0.3
−0.2
−0.1
0.0
0.1
β_IV

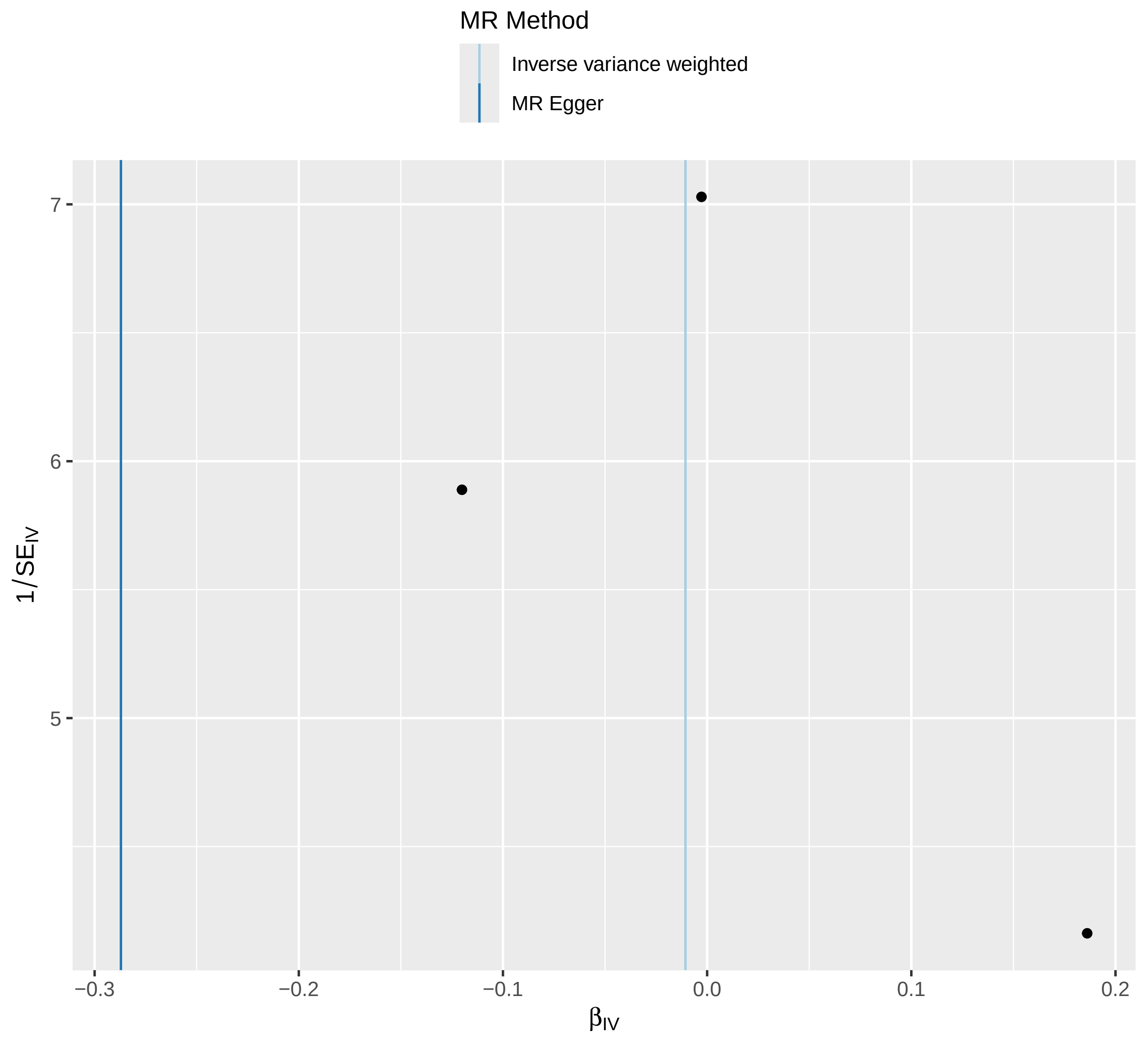

MR Method
Inverse variance weighted
MR Egger
7
6
5
1/SE$_{IV}$
−0.3
−0.2
−0.1
0.0
0.1
0.2
β$_{IV}$

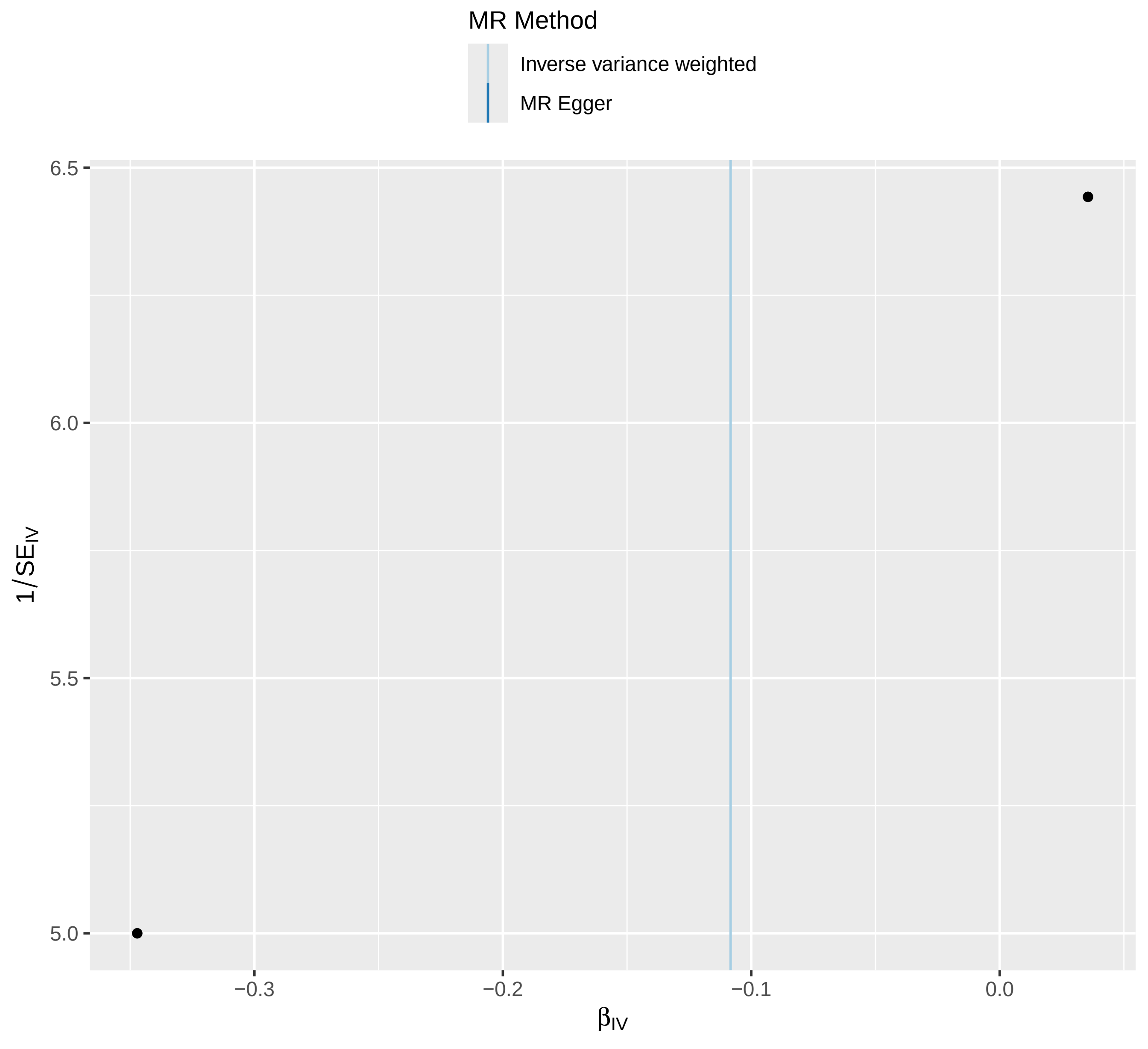
MR Method
Inverse variance weighted
MR Egger
6.5
6.0
5.5
5.0
1/SE_IV
−0.3
−0.2
−0.1
0.0
β_IV

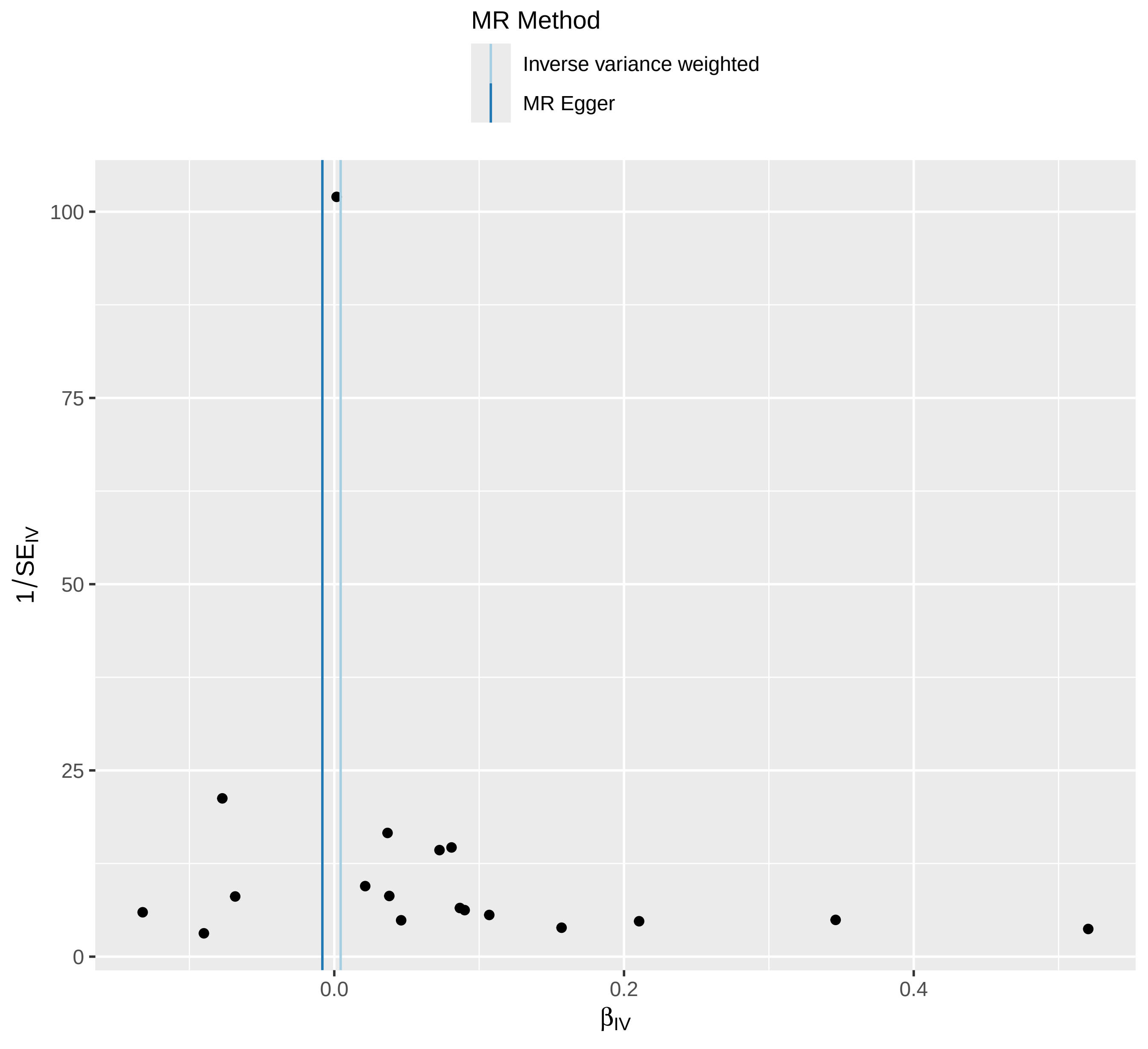
MR Method
Inverse variance weighted
MR Egger
100
75
50
25
0
1/SE$_{IV}$
0.0
0.2
0.4
β$_{IV}$

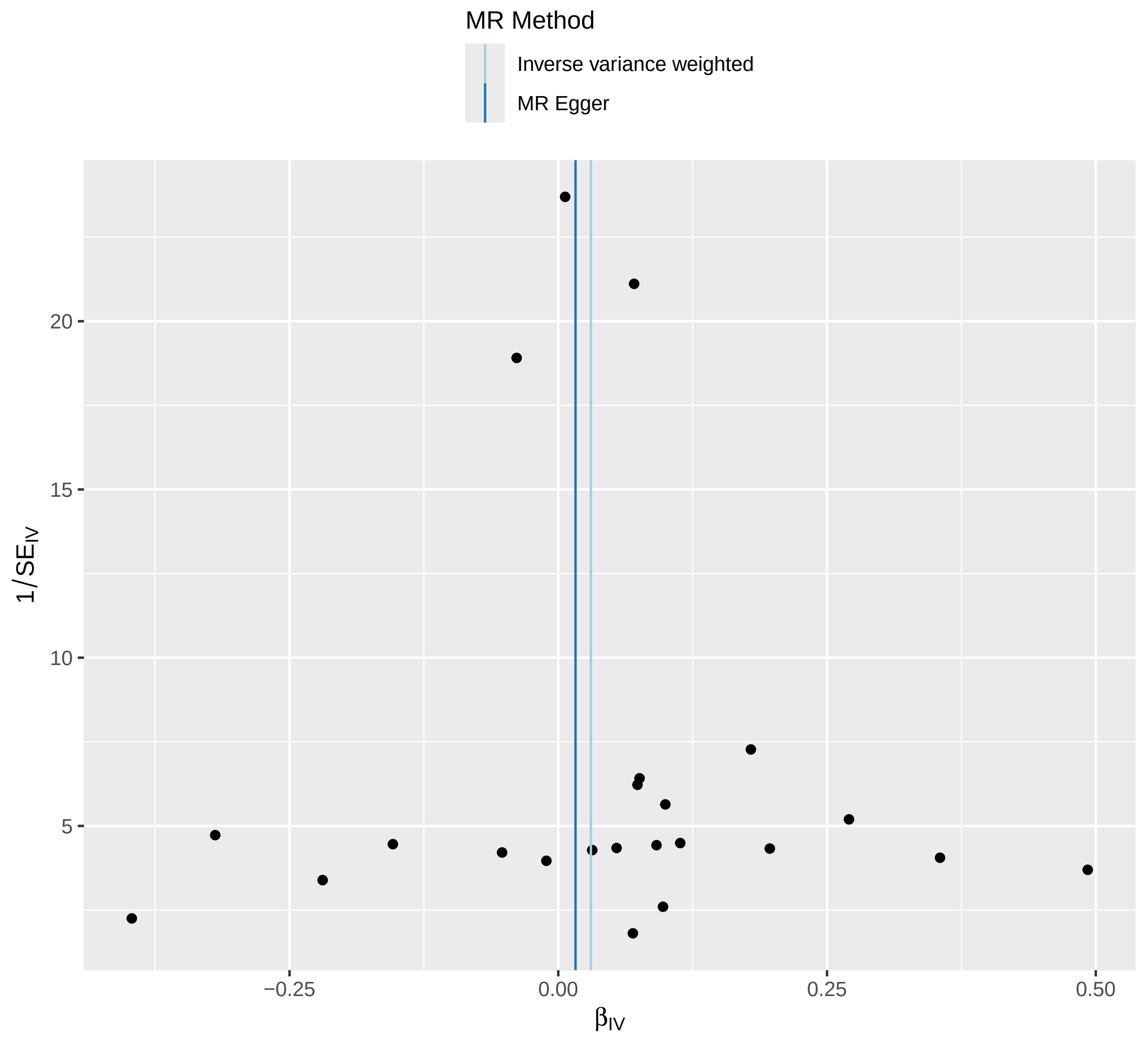

MR Method
Inverse variance weighted
MR Egger
20
15
10
5
1/SE$_{IV}$
−0.25
0.00
0.25
0.50
β$_{IV}$

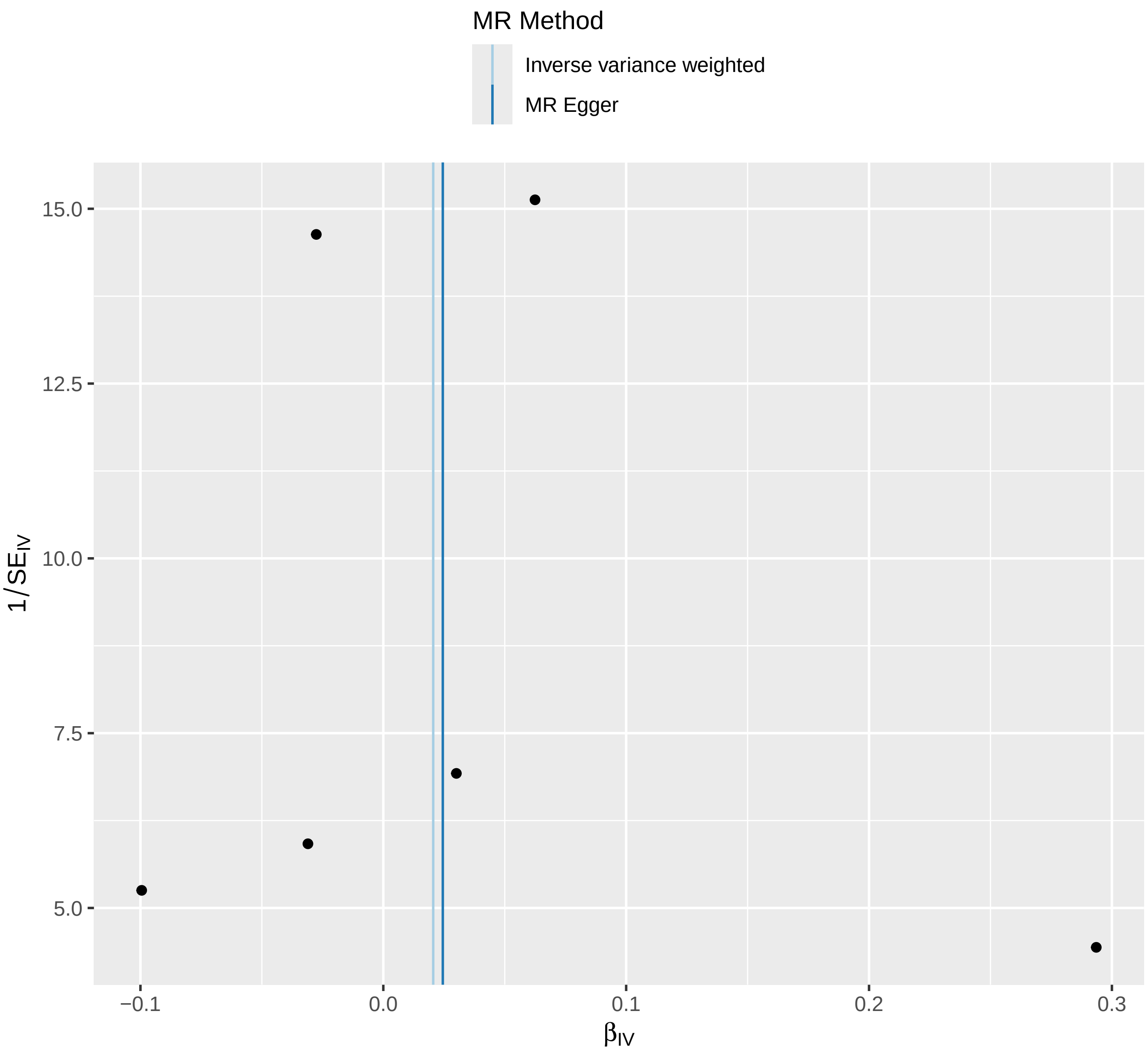
MR Method
Inverse variance weighted
MR Egger
15.0
12.5
10.0
7.5
5.0
1/SE_IV
−0.1
0.0
0.1
0.2
0.3
β_IV

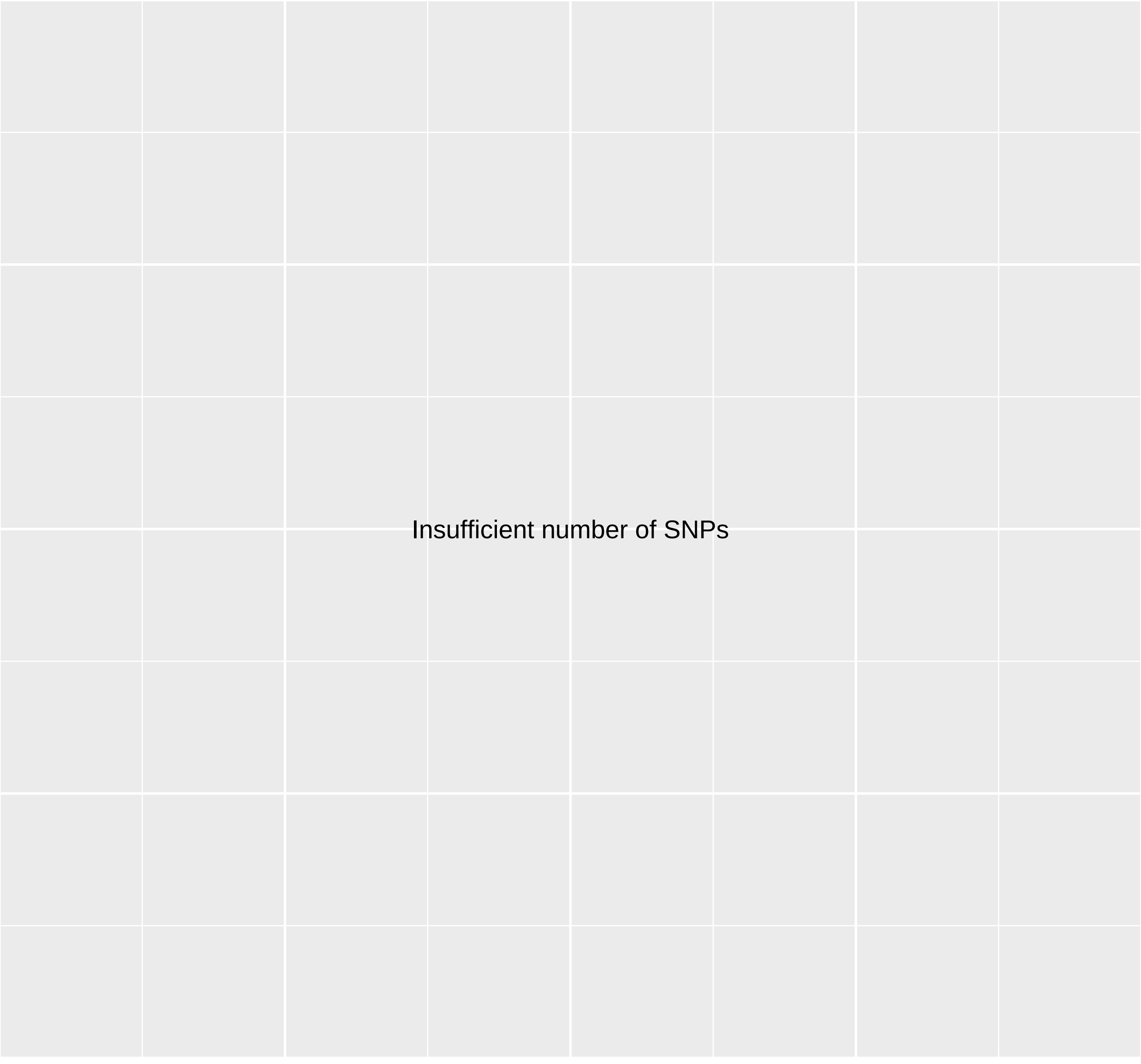
Insufficient number of SNPs

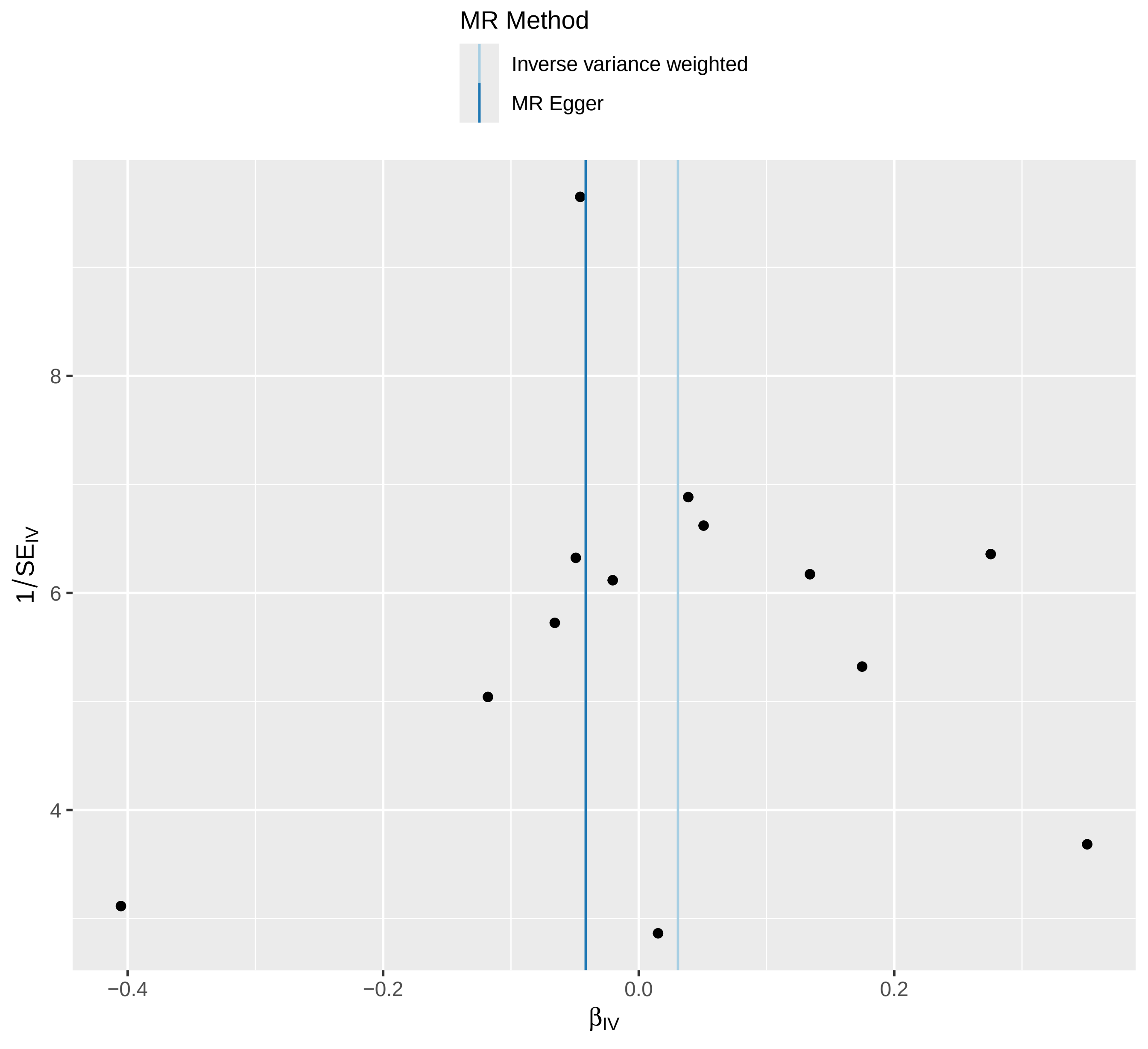

MR Method
Inverse variance weighted
MR Egger
1/SE_IV
8
6
4
−0.4
−0.2
0.0
0.2
β_IV

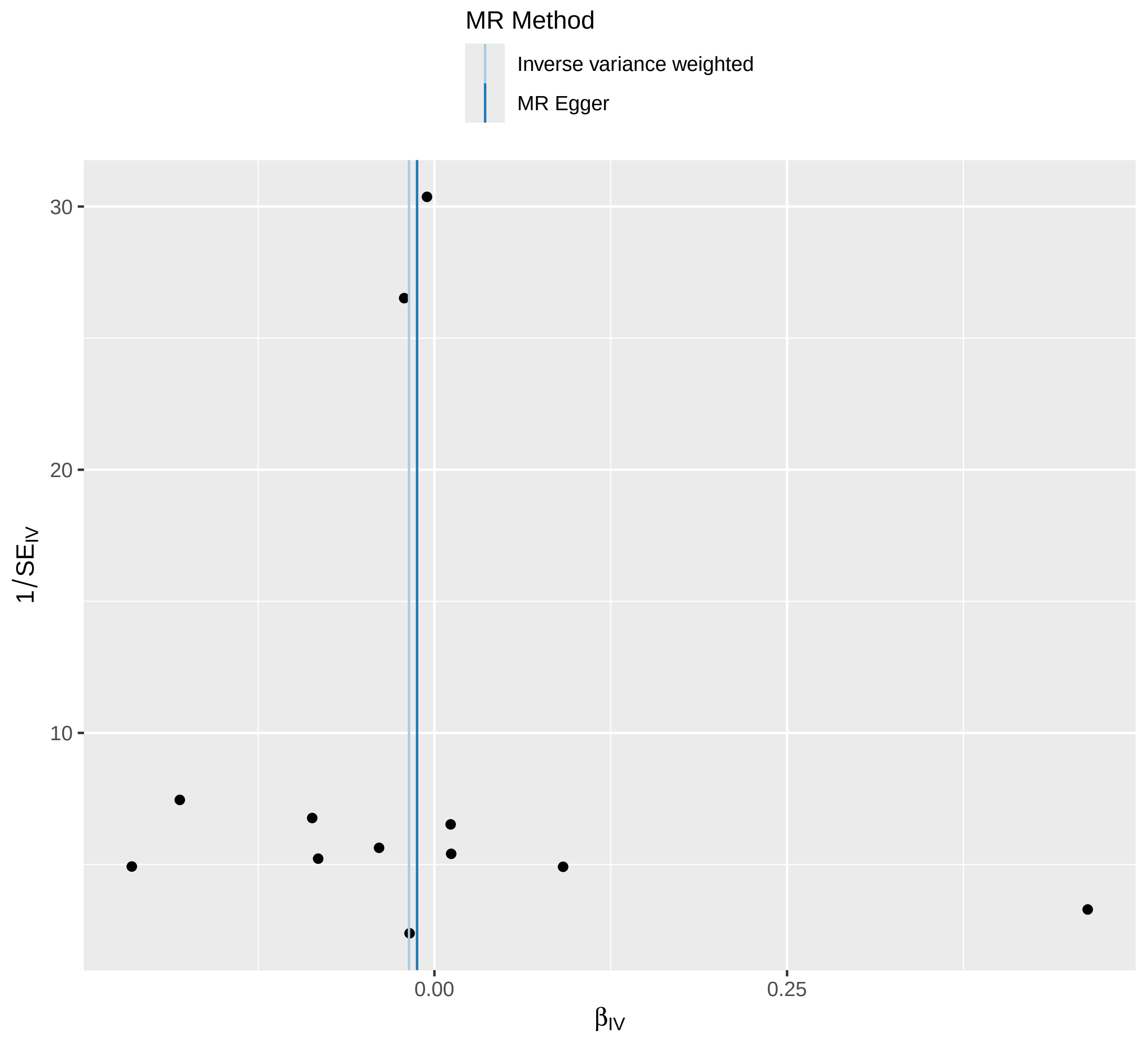
MR Method
Inverse variance weighted
MR Egger
30
20
10
1/SE_IV
0.00
0.25
β_IV

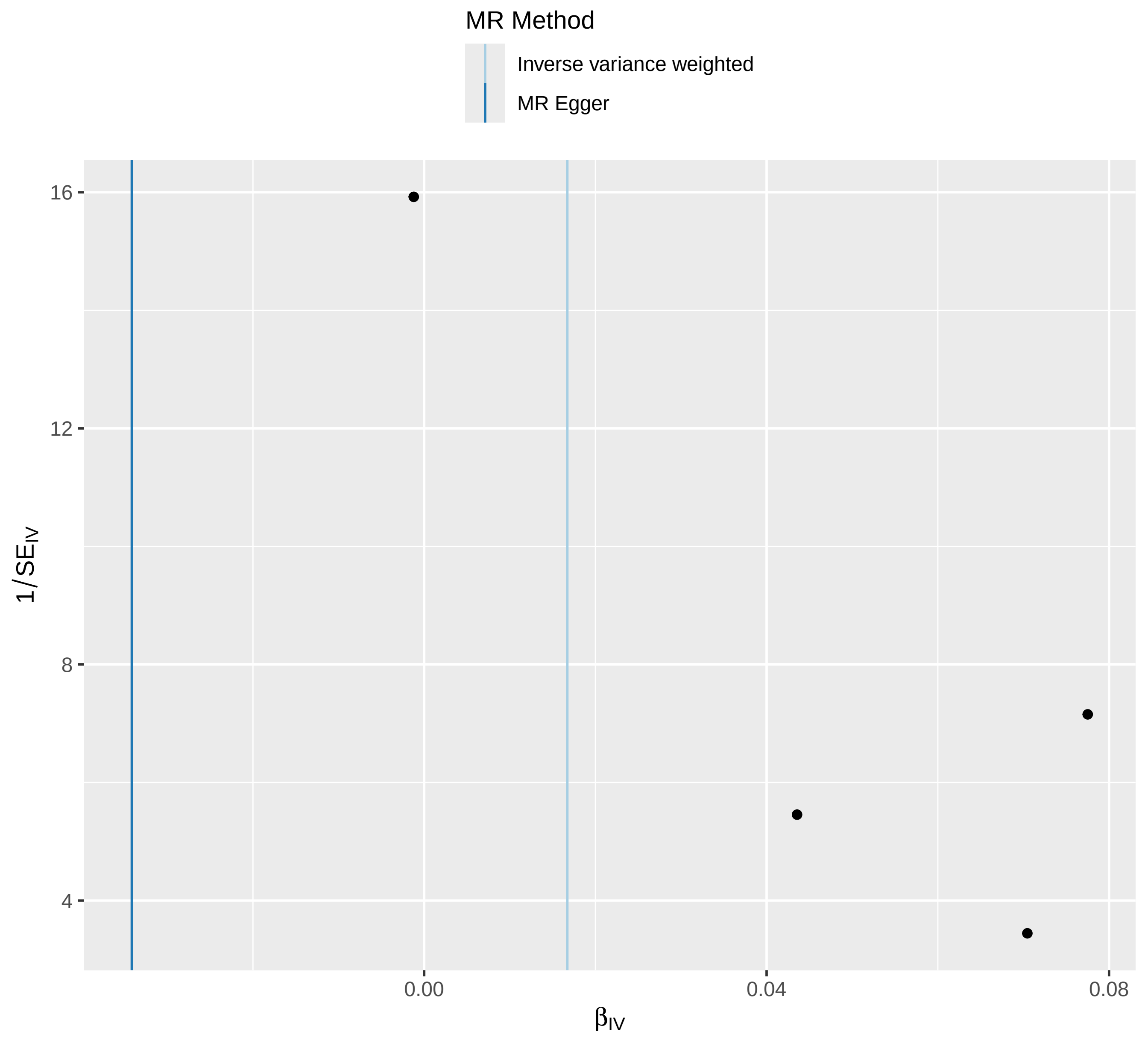
MR Method
Inverse variance weighted
MR Egger
16
12
8
4
1/SE_IV
0.00
0.04
0.08
β_IV

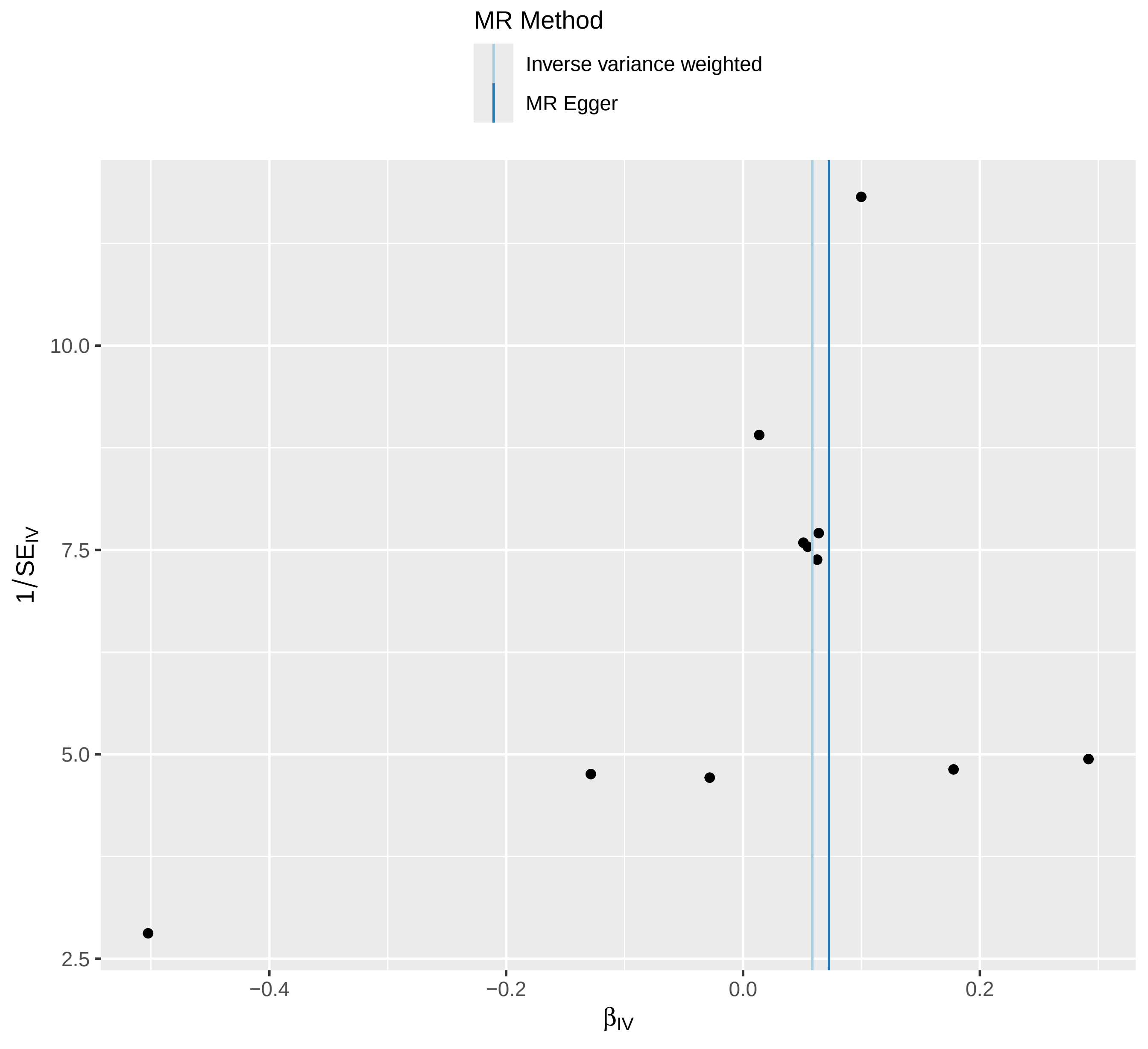
MR Method
Inverse variance weighted
MR Egger
10.0
7.5
5.0
2.5
1/SE_IV
−0.4
−0.2
0.0
0.2
β_IV

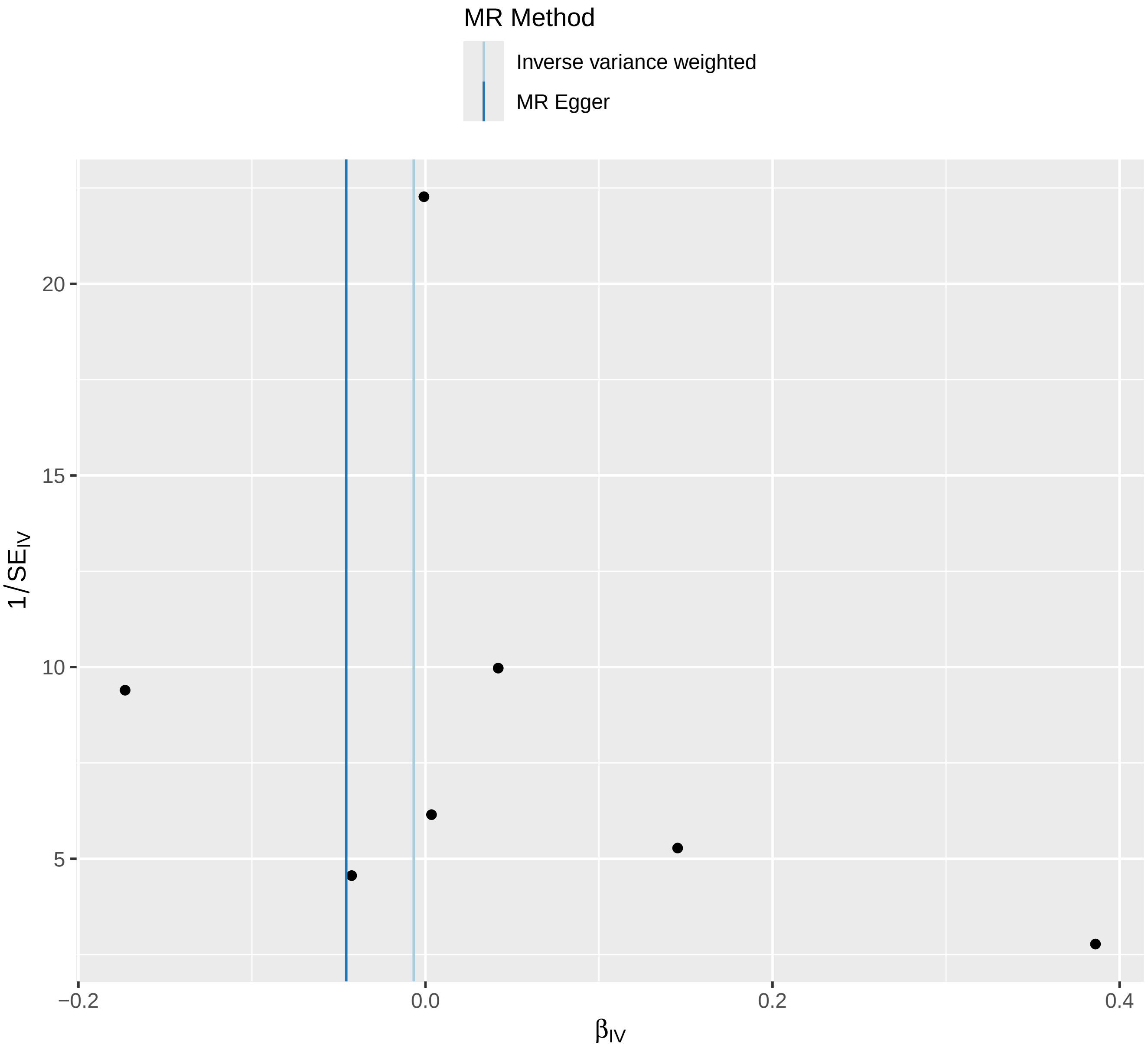
MR Method
Inverse variance weighted
MR Egger
20
15
10
5
1/SE_IV
−0.2
0.0
0.2
0.4
β_IV

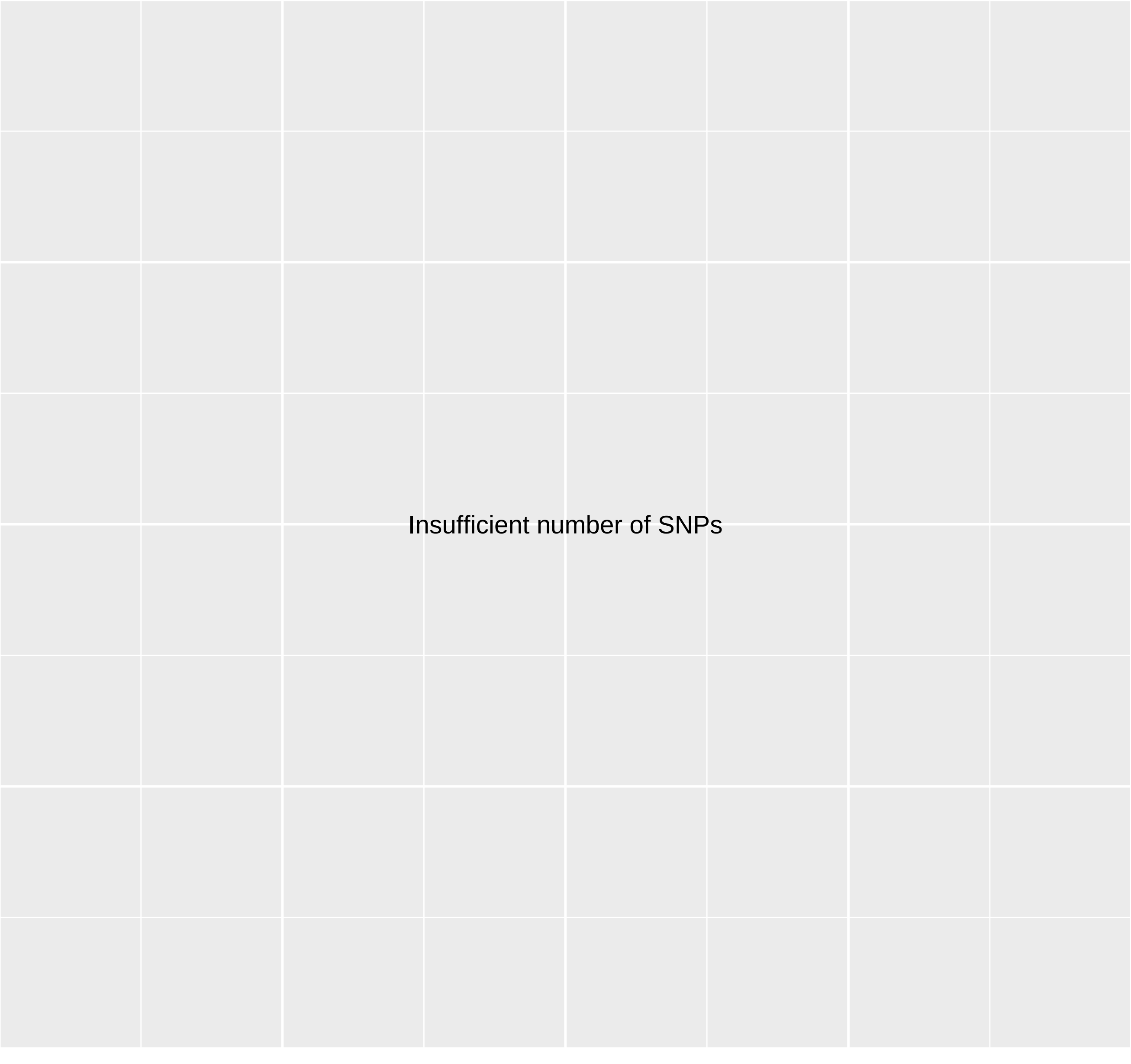
Insufficient number of SNPs

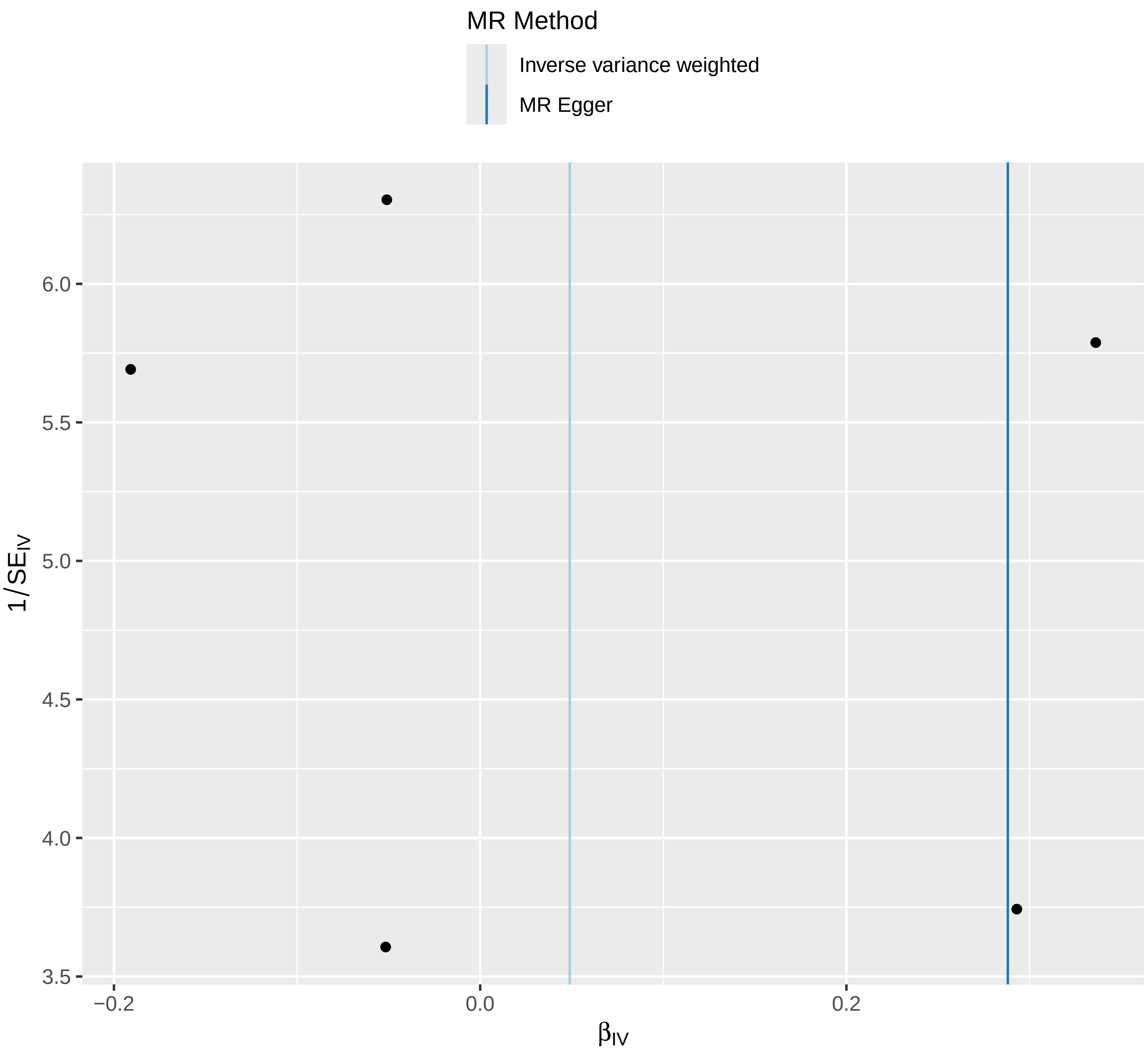
MR Method
Inverse variance weighted
MR Egger
6.0
5.5
5.0
4.5
4.0
3.5
1/SE_IV
−0.2
0.0
0.2
β_IV

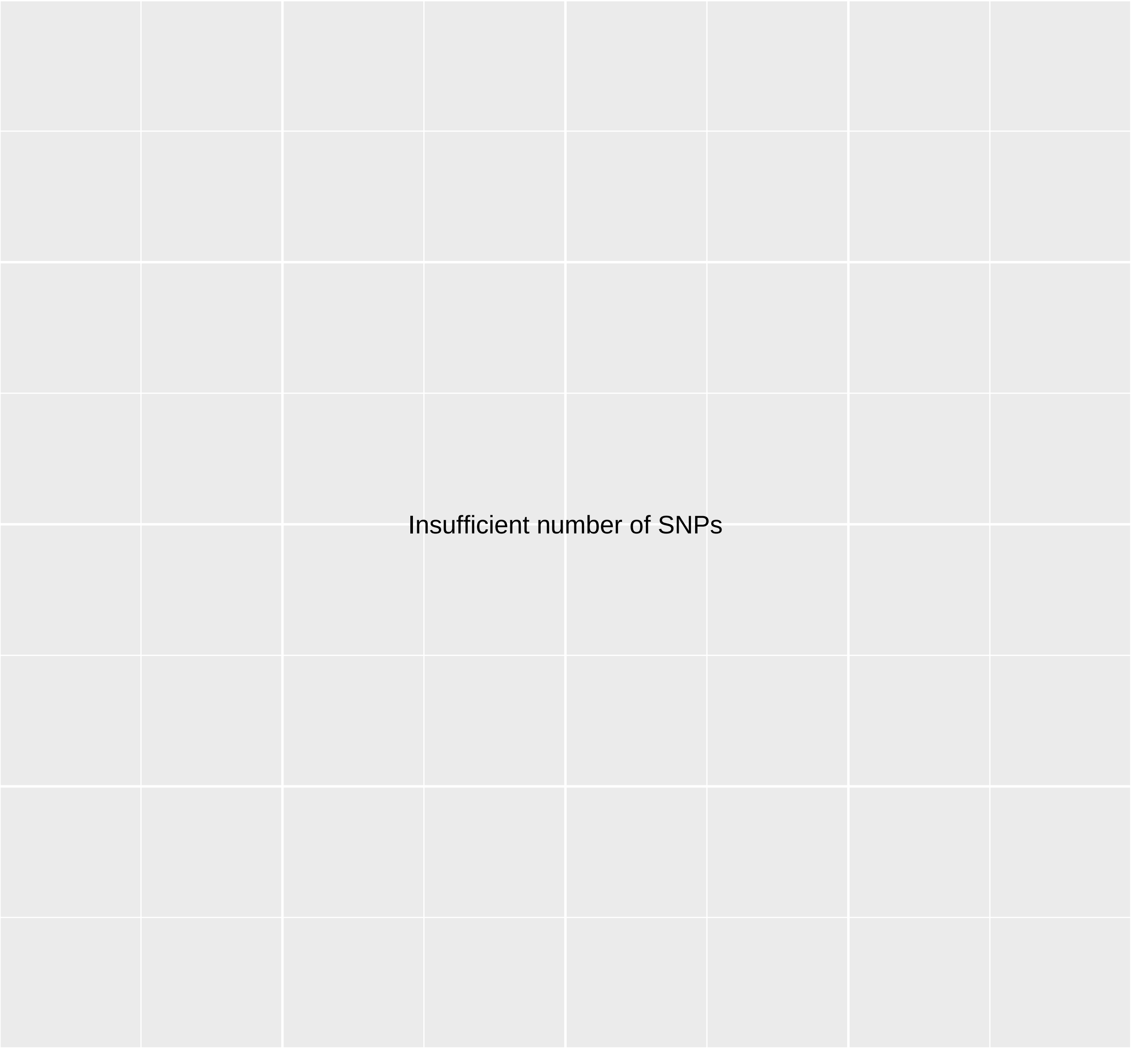
Insufficient number of SNPs

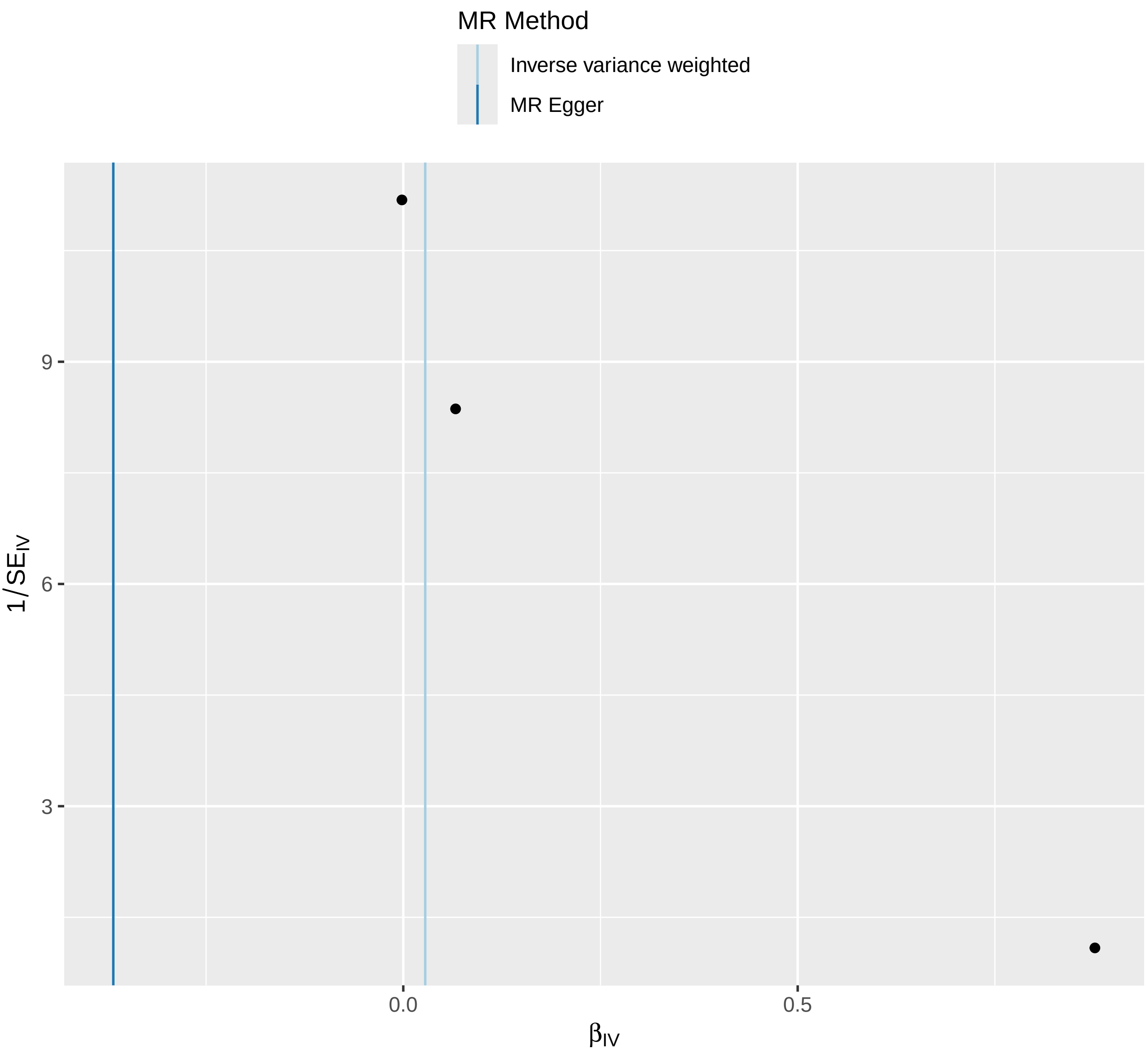
MR Method
Inverse variance weighted
MR Egger
9
6
3
$1/SE_{IV}$
0.0
0.5
$\beta_{IV}$

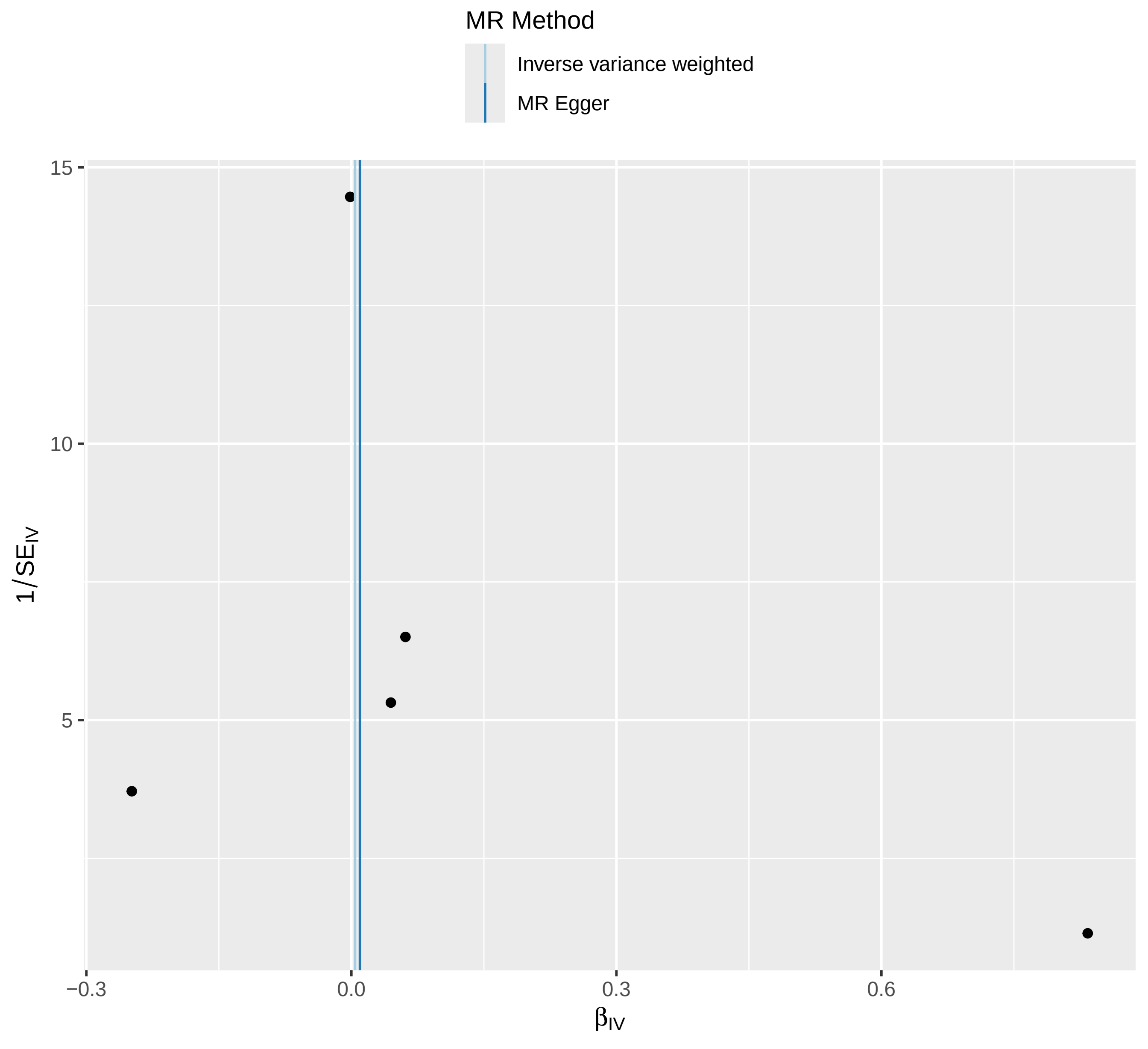

MR Method
Inverse variance weighted
MR Egger
15
10
5
$1/SE_{IV}$
−0.3
0.0
0.3
0.6
$\beta_{IV}$

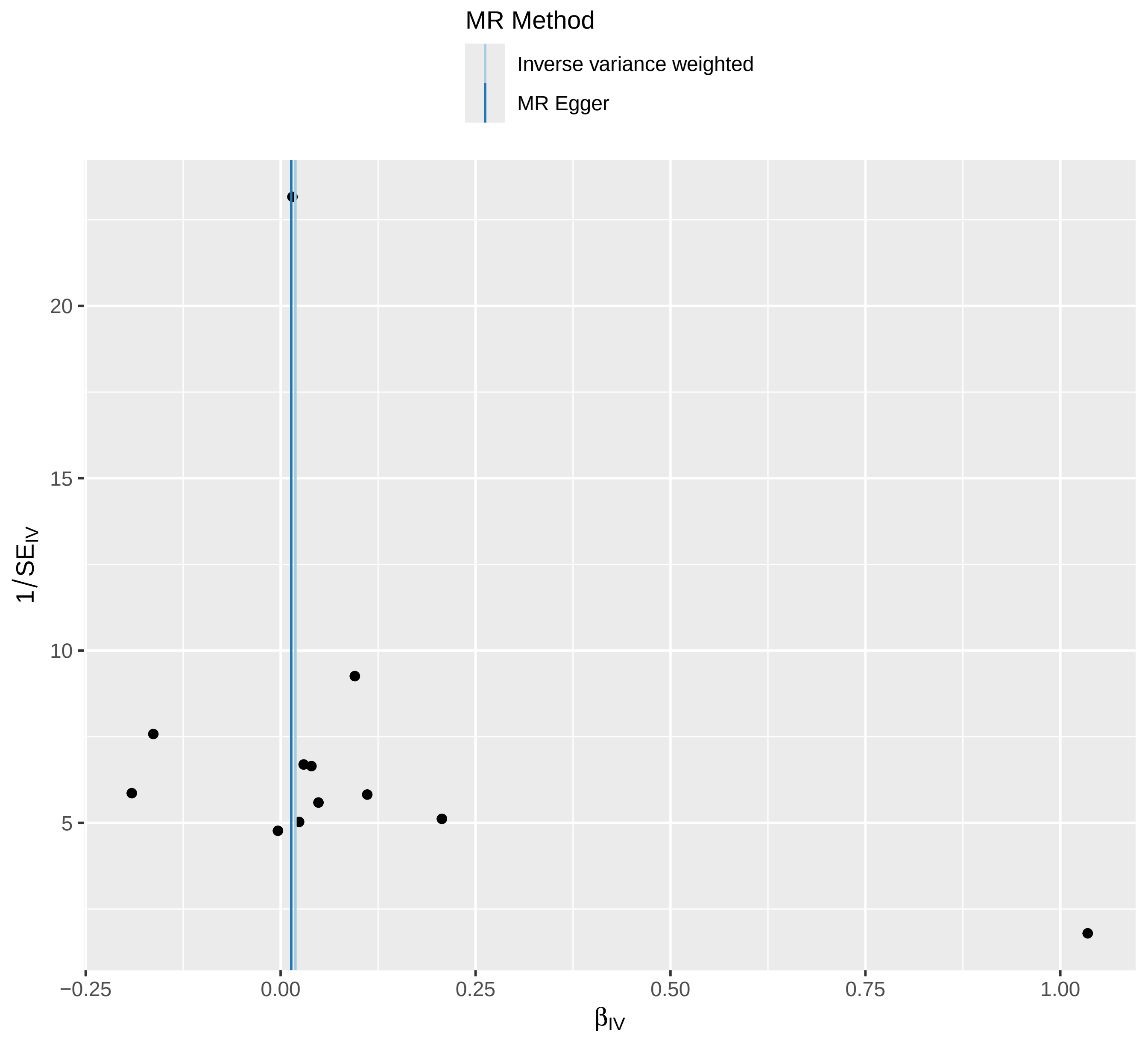

MR Method
Inverse variance weighted
MR Egger
20
15
10
5
$1/SE_{IV}$
−0.25
0.00
0.25
0.50
0.75
1.00
$\beta_{IV}$

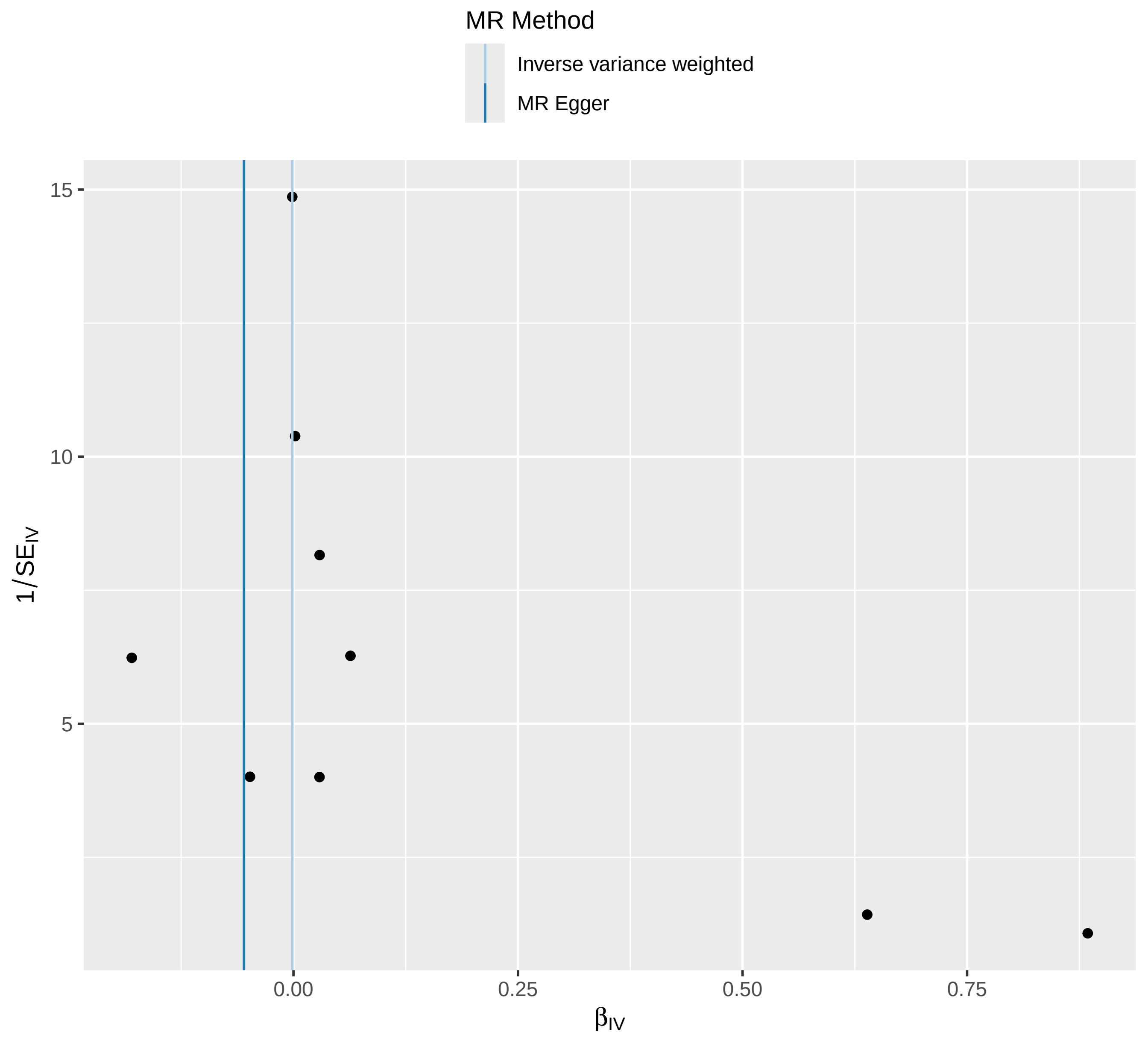
MR Method
Inverse variance weighted
MR Egger
15
10
5
$1/SE_{IV}$
0.00
0.25
0.50
0.75
$\beta_{IV}$

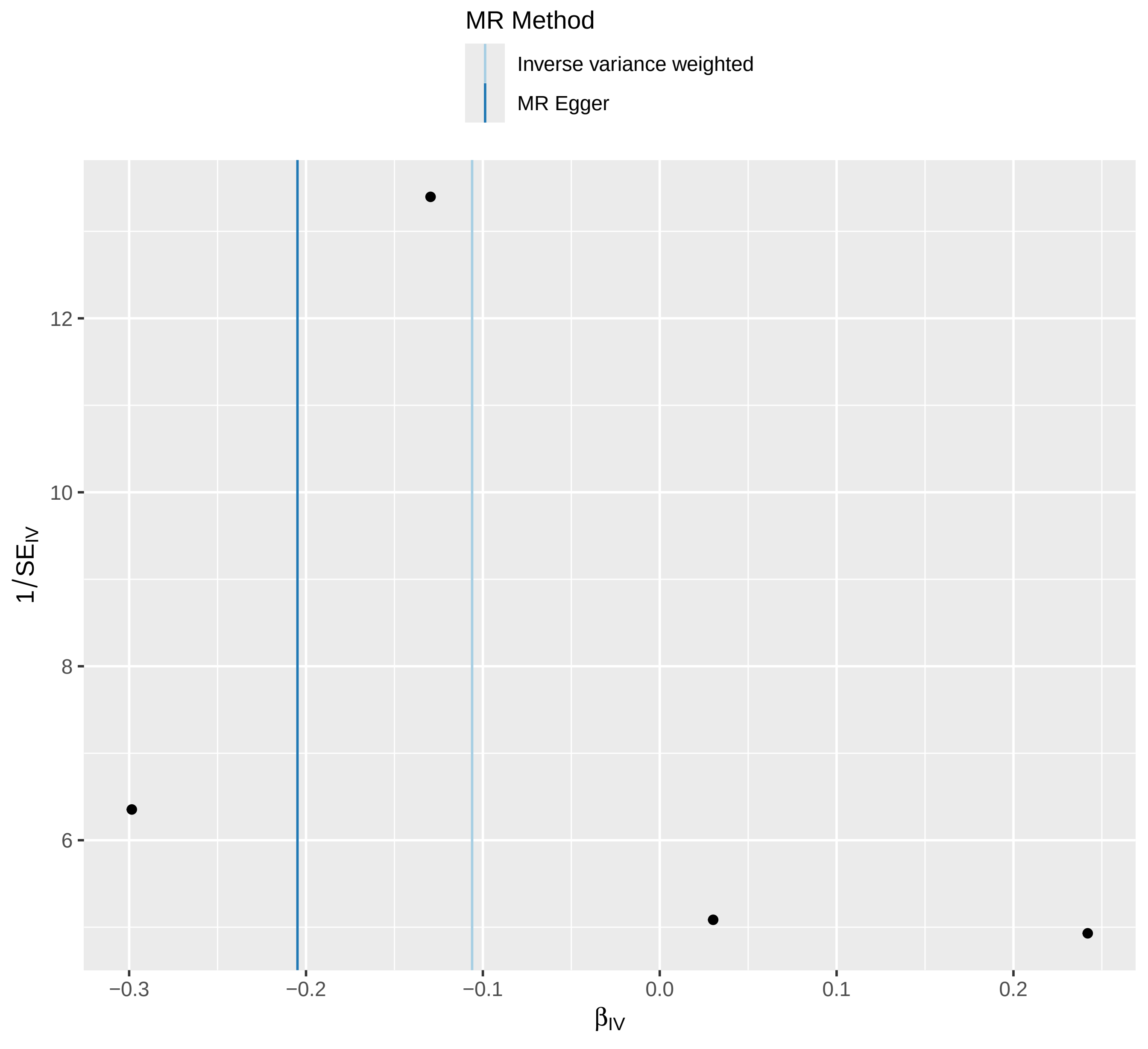
MR Method
Inverse variance weighted
MR Egger
12
10
8
6
1/SE$_{IV}$
−0.3
−0.2
−0.1
0.0
0.1
0.2
β$_{IV}$

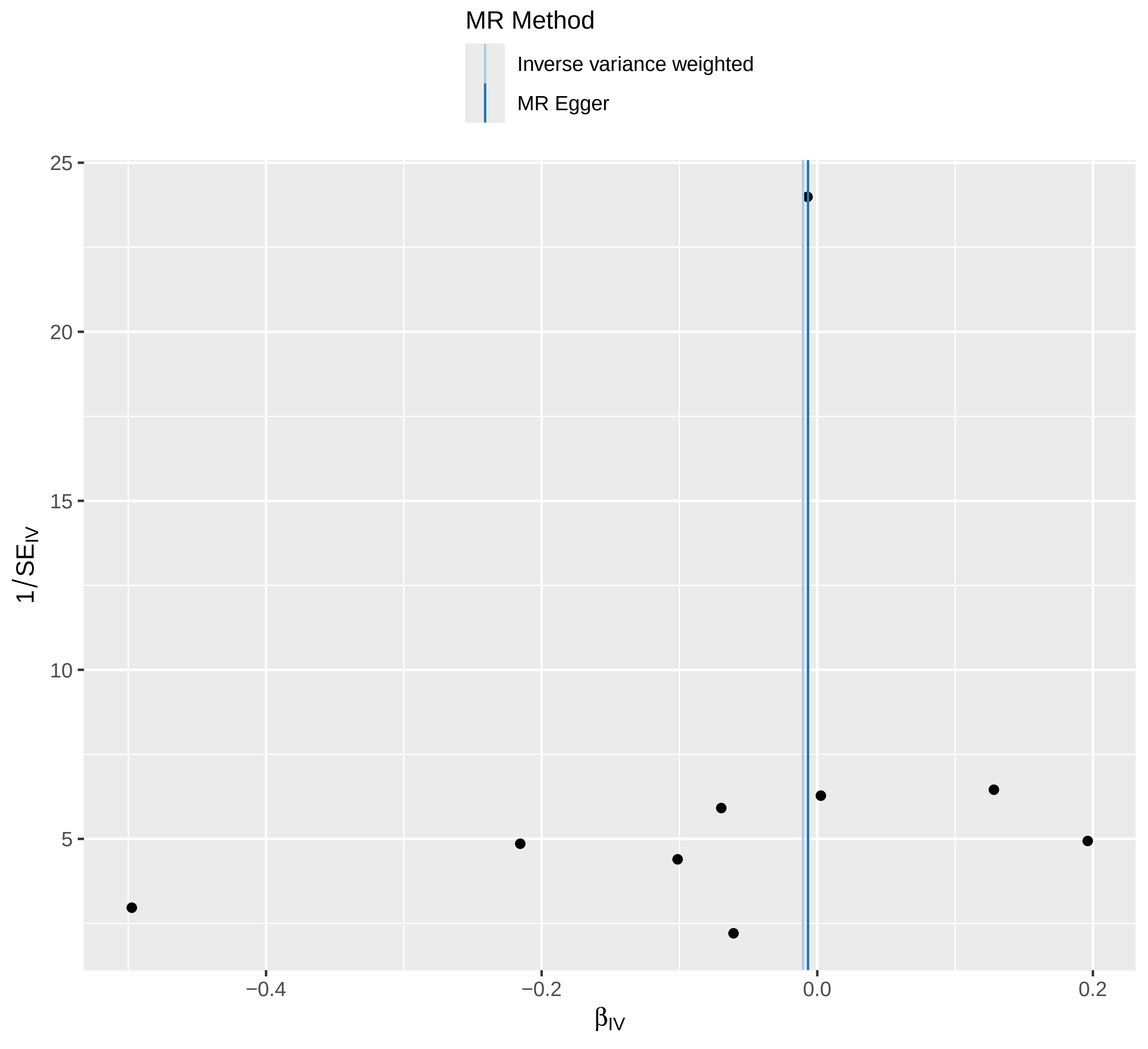

MR Method
Inverse variance weighted
MR Egger
25
20
15
10
5
1/SE_IV
−0.4
−0.2
0.0
0.2
β_IV

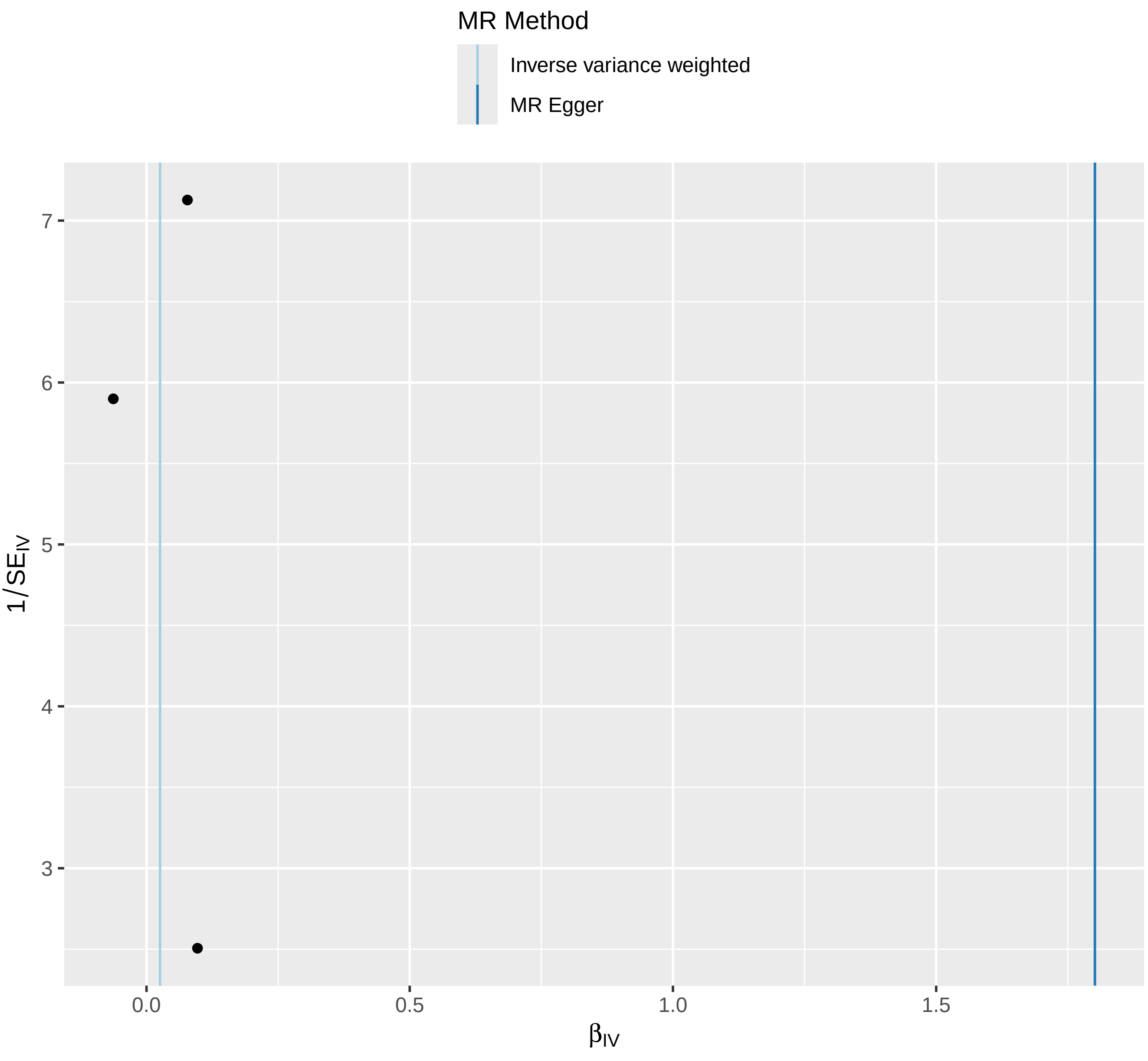
MR Method
Inverse variance weighted
MR Egger
1/SE_IV
7
6
5
4
3
0.0
0.5
1.0
1.5
β_IV

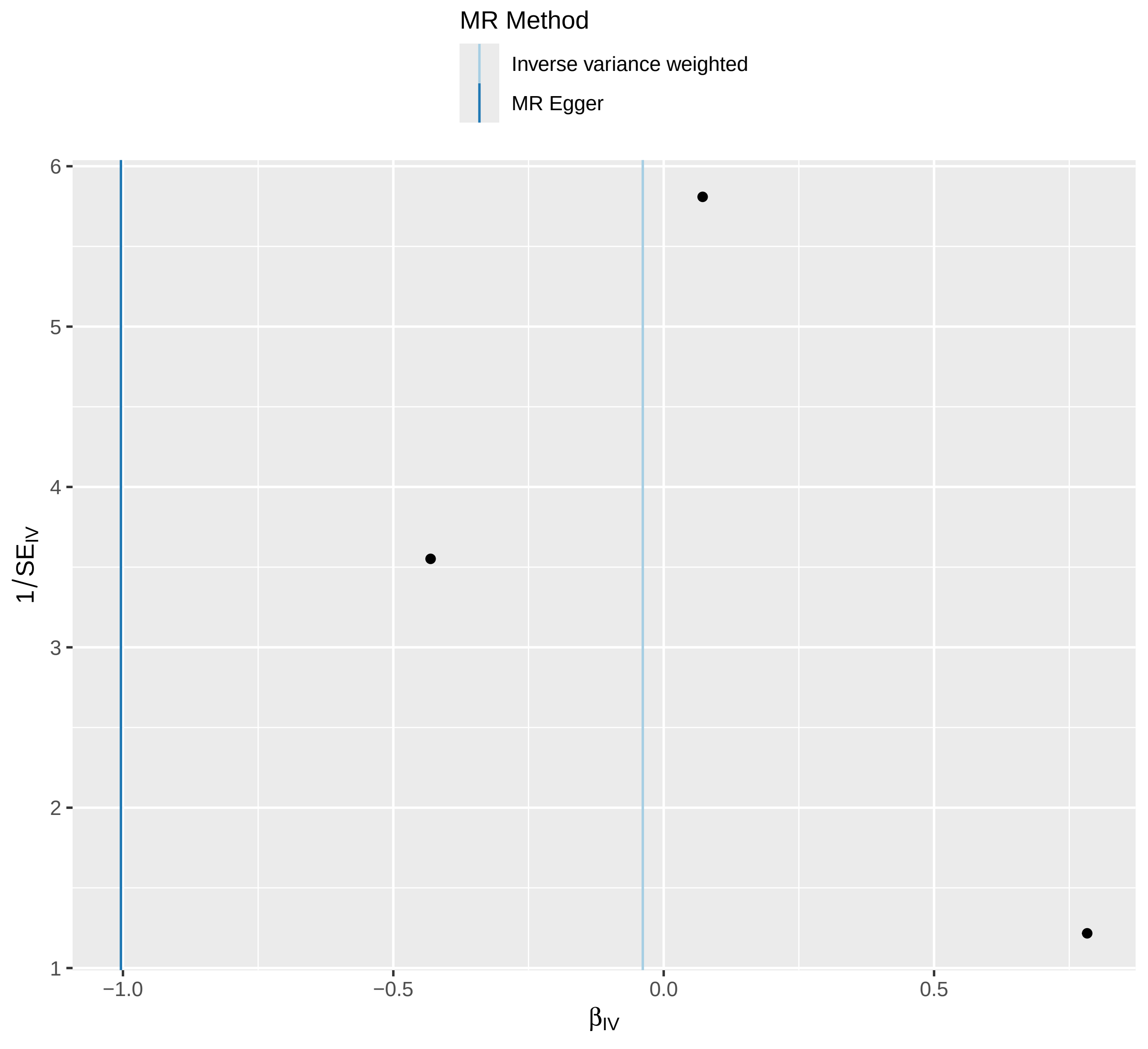

MR Method
Inverse variance weighted
MR Egger
1/SE_IV
β_IV
6
5
4
3
2
1
−1.0
−0.5
0.0
0.5

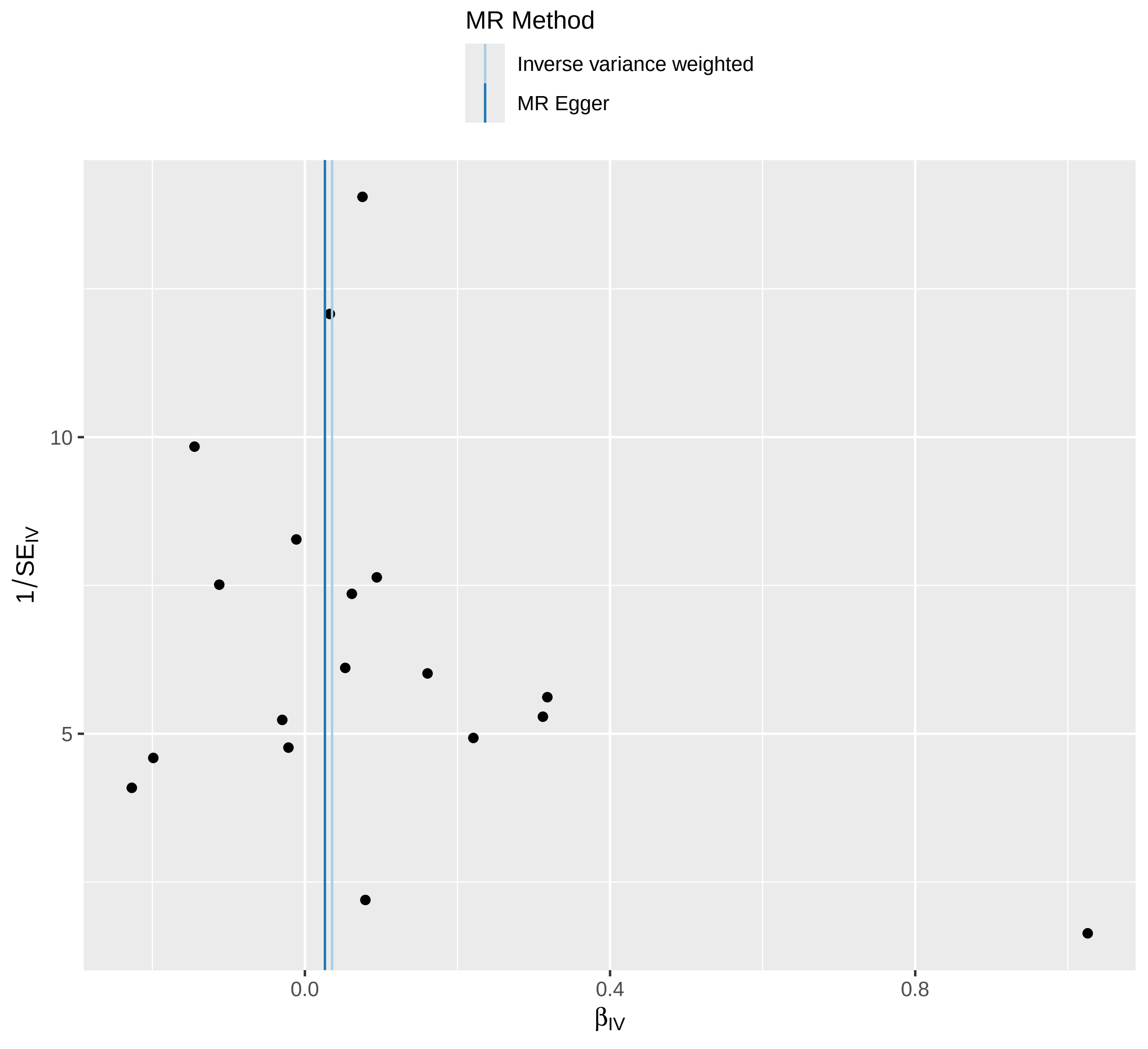
MR Method
Inverse variance weighted
MR Egger
10
5
1/SE_IV
0.0
0.4
0.8
β_IV

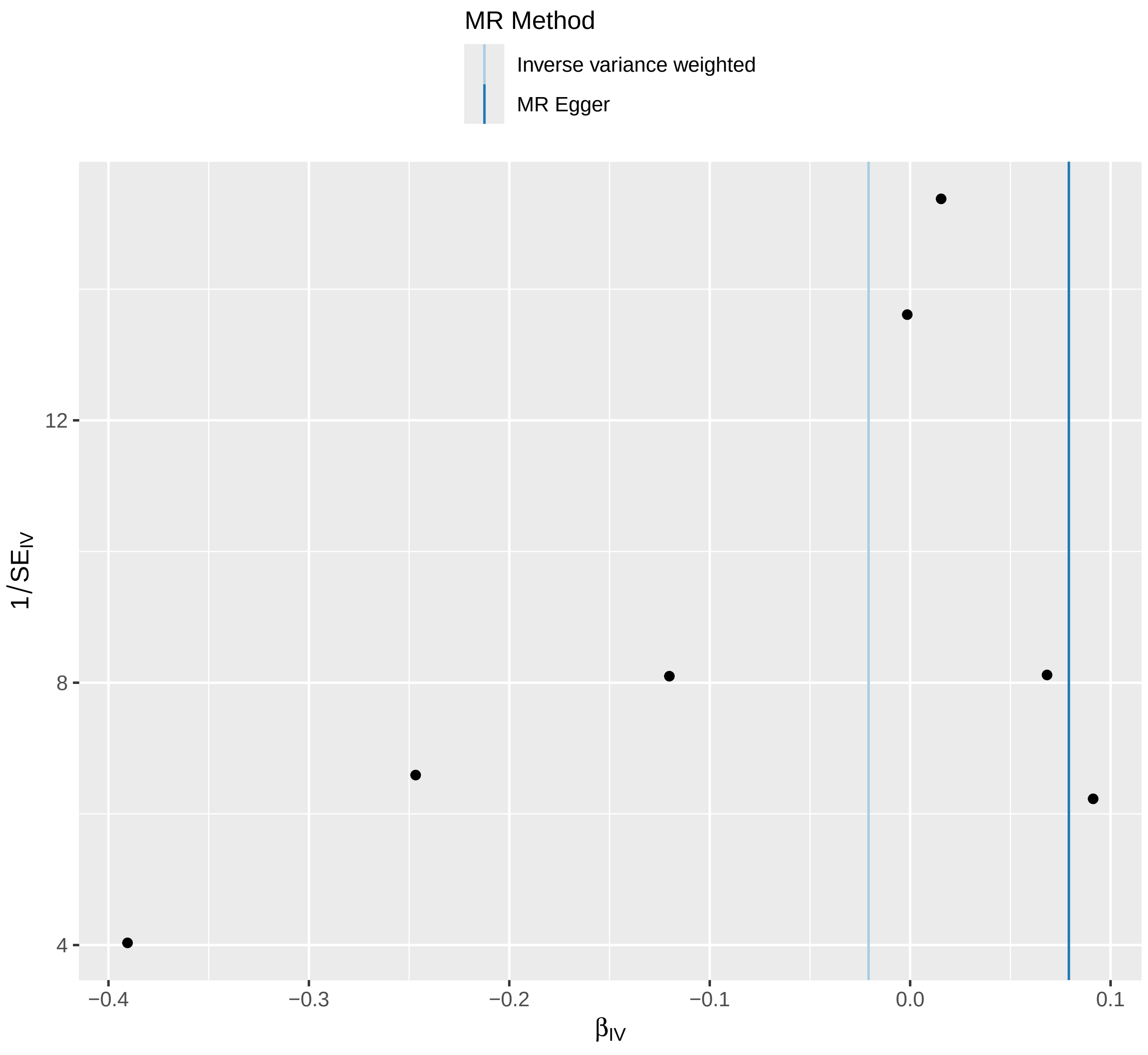

MR Method
Inverse variance weighted
MR Egger
12
8
4
1/SE_IV
−0.4
−0.3
−0.2
−0.1
0.0
0.1
β_IV

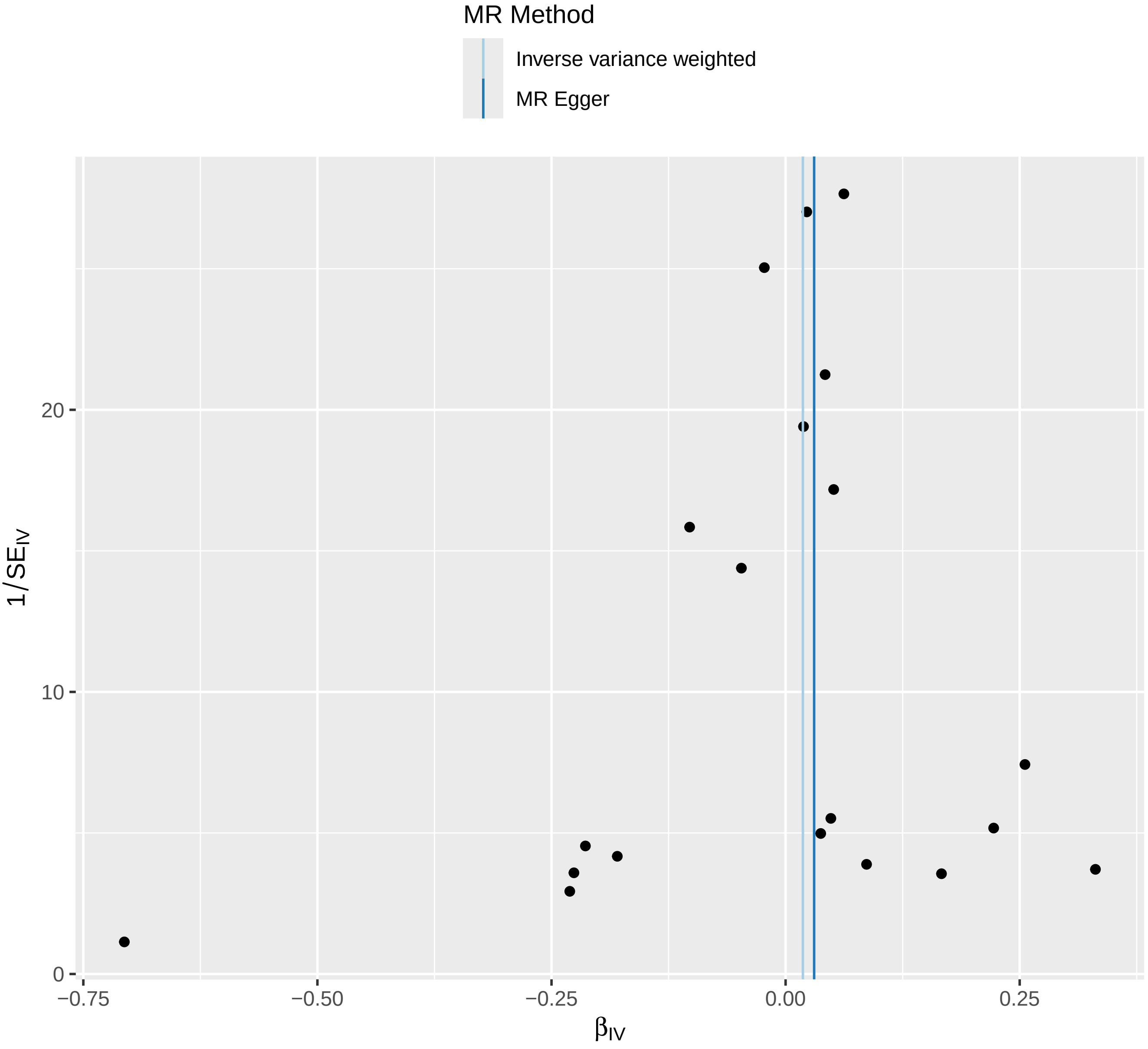

MR Method
Inverse variance weighted
MR Egger
20
10
0
1/SE$_{IV}$
−0.75
−0.50
−0.25
0.00
0.25
β$_{IV}$

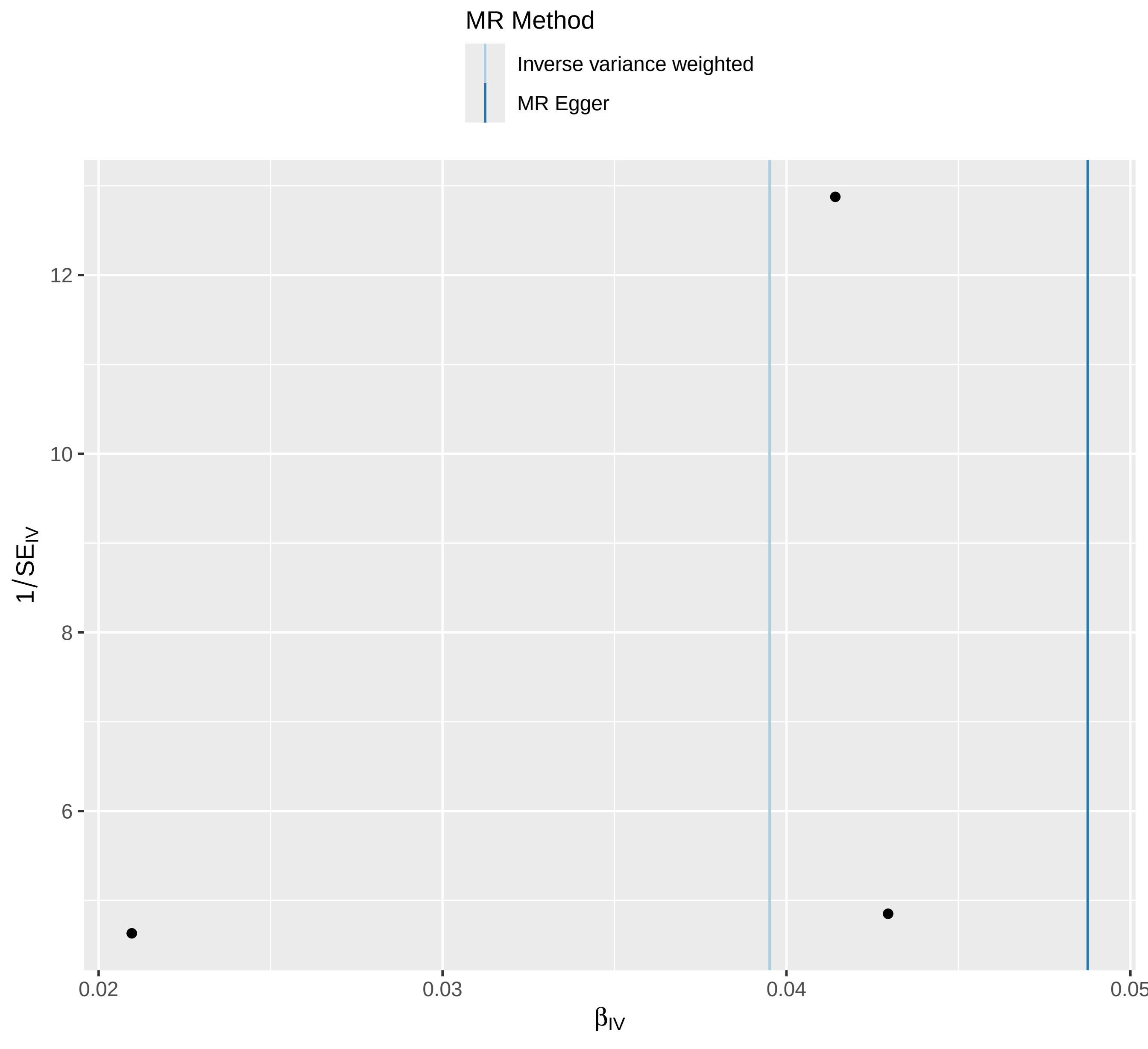

MR Method
Inverse variance weighted
MR Egger
12
10
8
6
1/SE$_{IV}$
0.02
0.03
0.04
0.05
β$_{IV}$

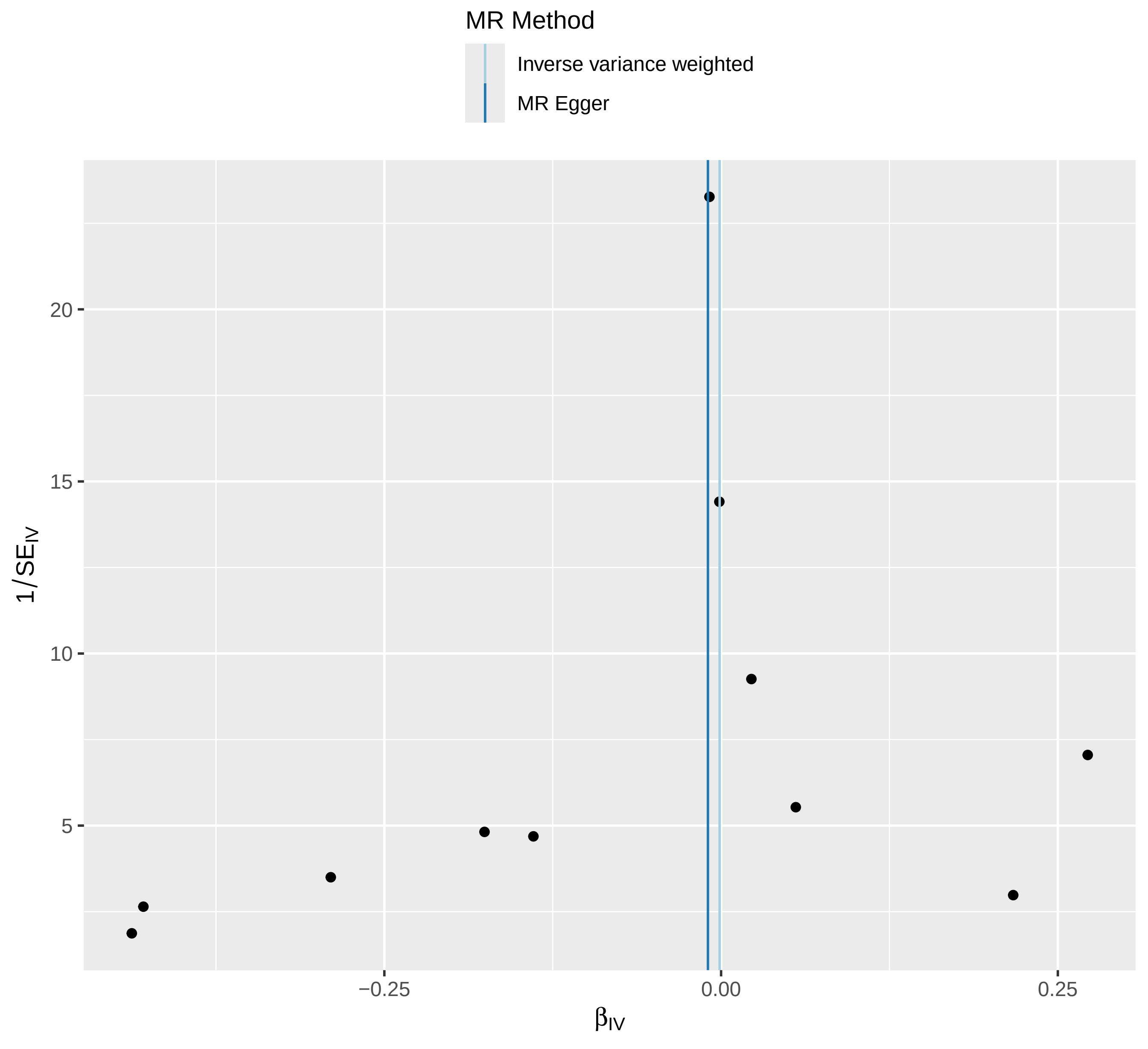

MR Method
Inverse variance weighted
MR Egger
20
15
10
5
1/SE_IV
−0.25
0.00
0.25
β_IV

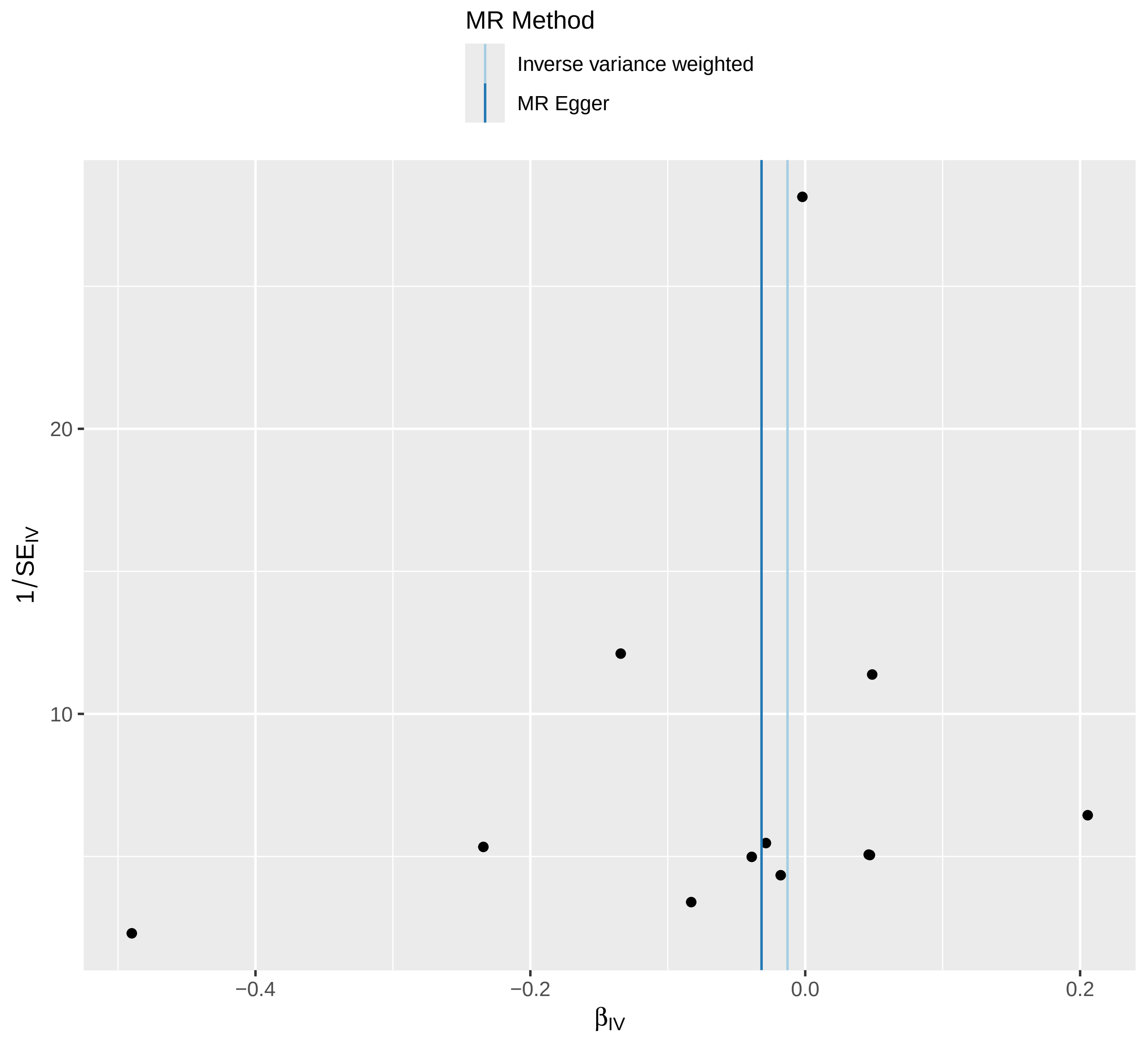
MR Method
Inverse variance weighted
MR Egger
20
10
1/SE$_{IV}$
−0.4
−0.2
0.0
0.2
β$_{IV}$

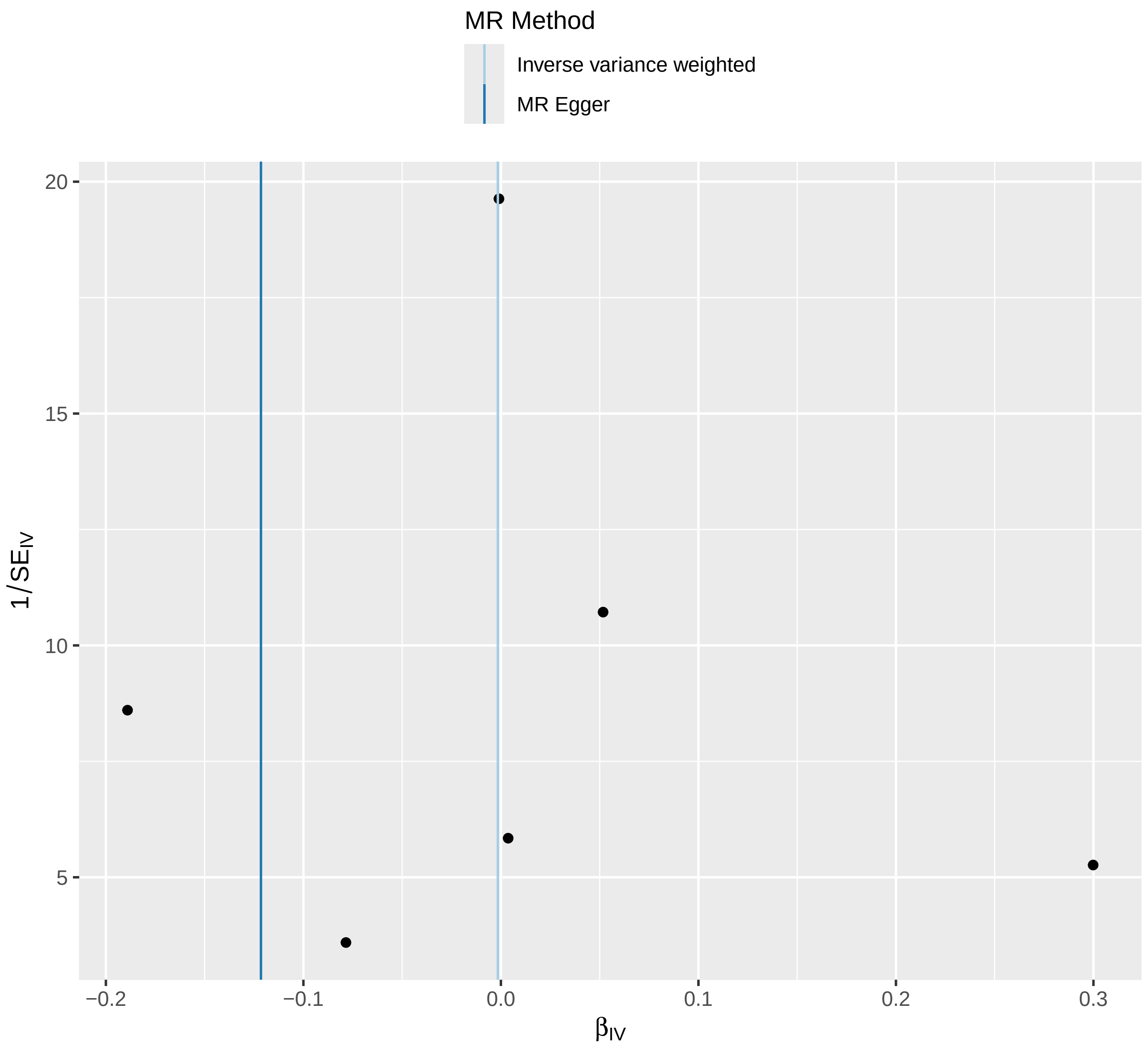
MR Method
Inverse variance weighted
MR Egger
20
15
10
5
1/SE_IV
−0.2
−0.1
0.0
0.1
0.2
0.3
β_IV

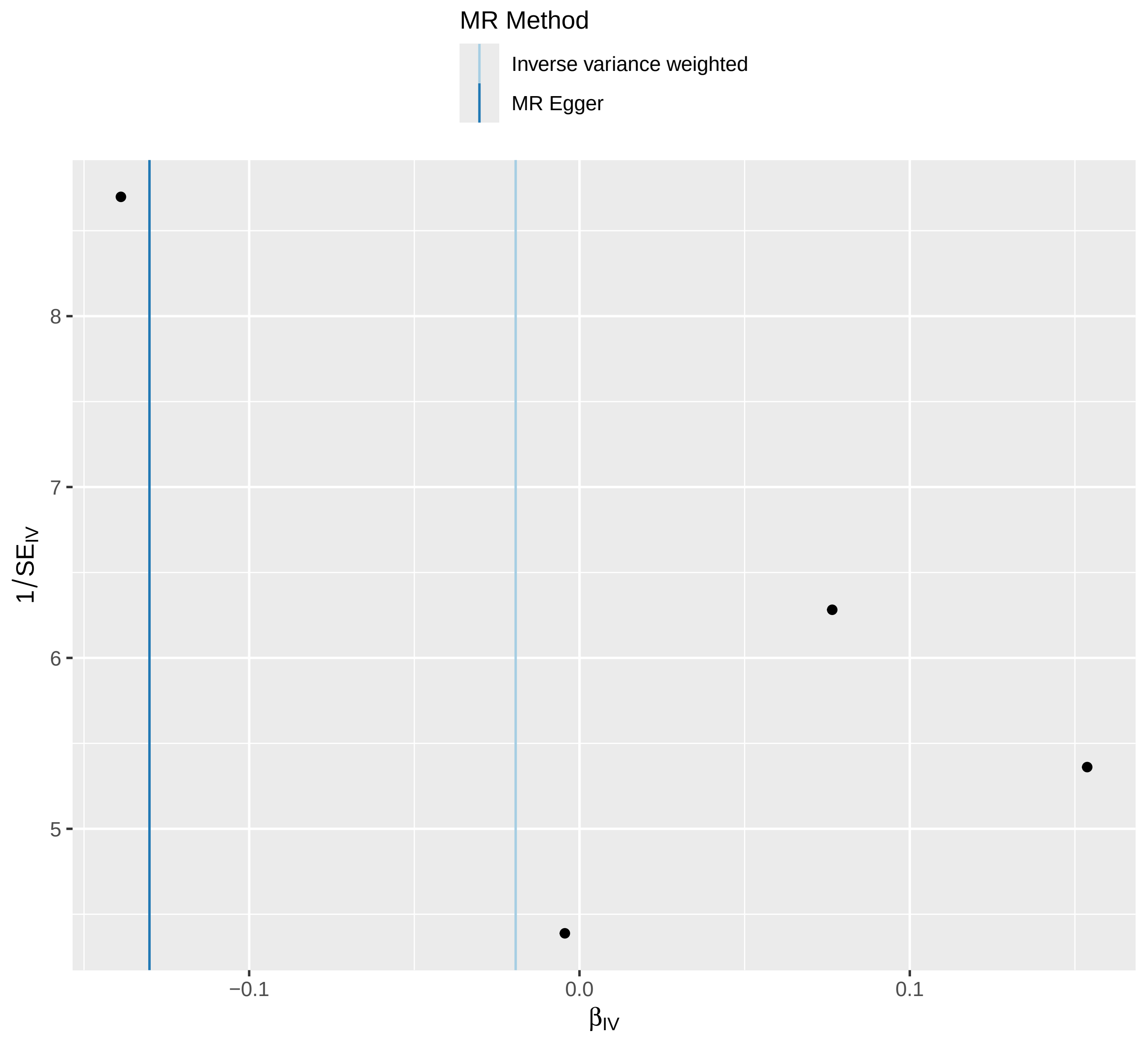
MR Method
Inverse variance weighted
MR Egger
8
7
6
5
1/SE_IV
−0.1
0.0
0.1
β_IV

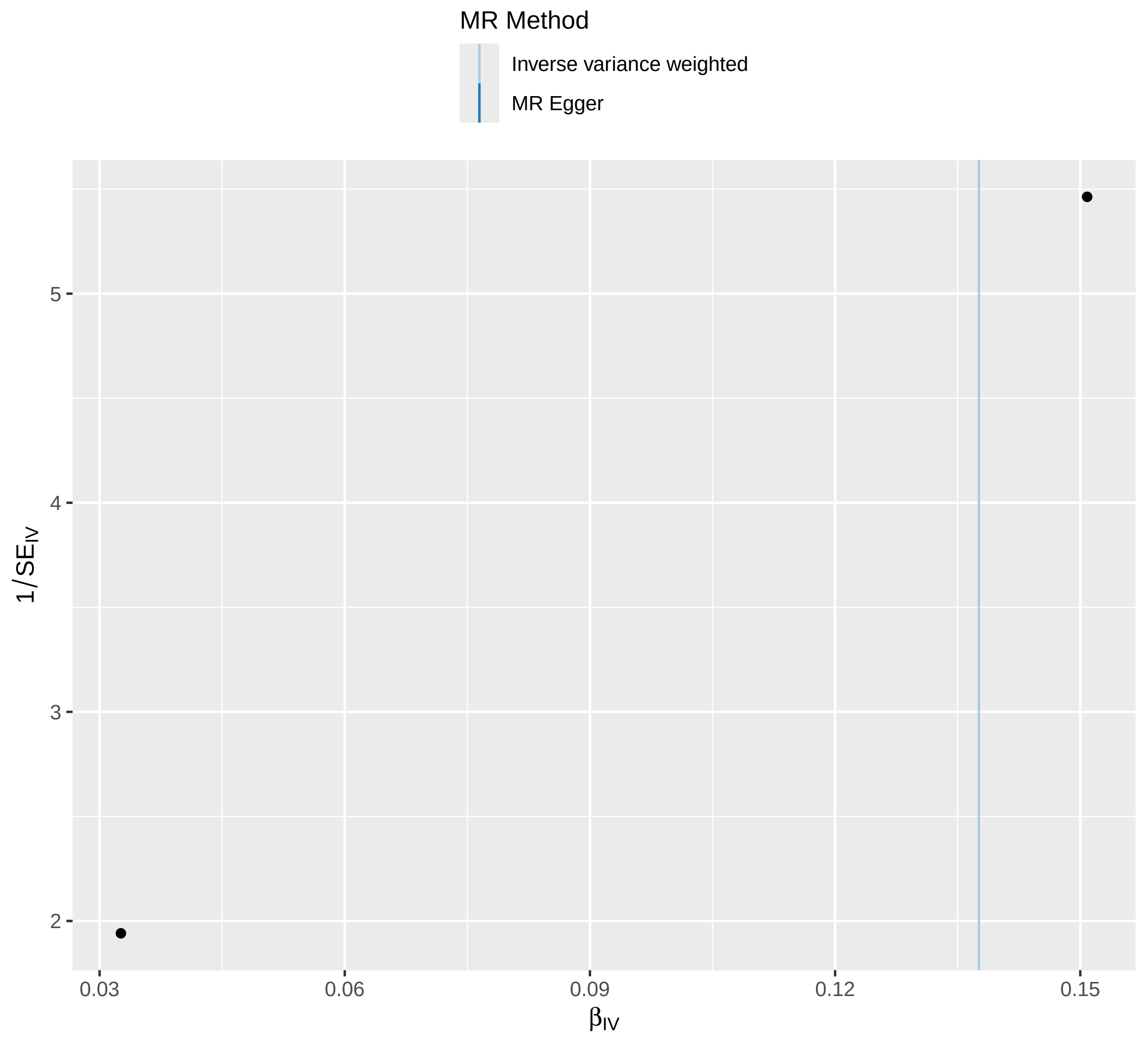
MR Method
Inverse variance weighted
MR Egger
5
4
3
2
$1/SE_{IV}$
0.03
0.06
0.09
0.12
0.15
$\beta_{IV}$

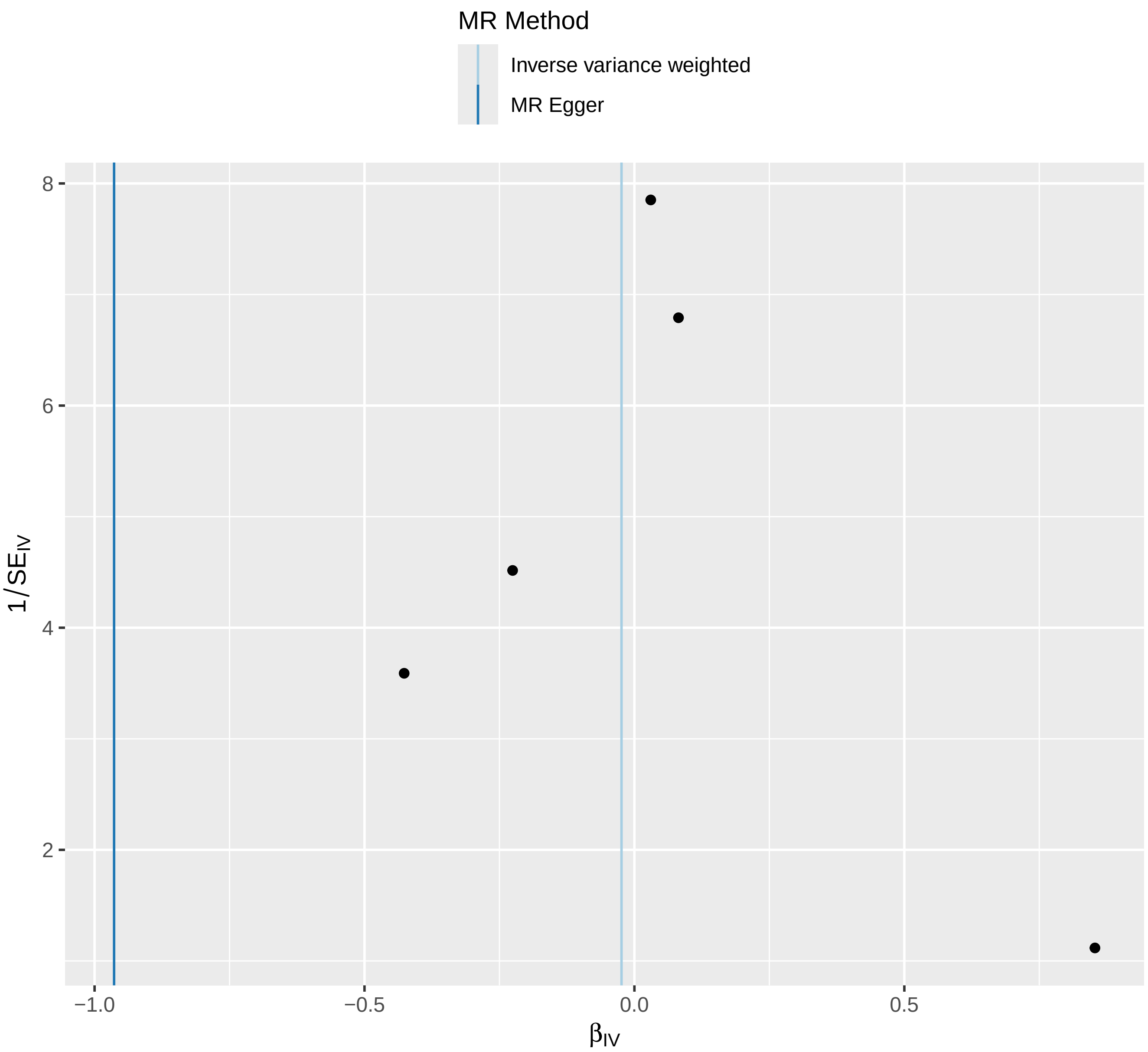

MR Method
Inverse variance weighted
MR Egger
1/SE_IV
β_IV
8
6
4
2
−1.0
−0.5
0.0
0.5

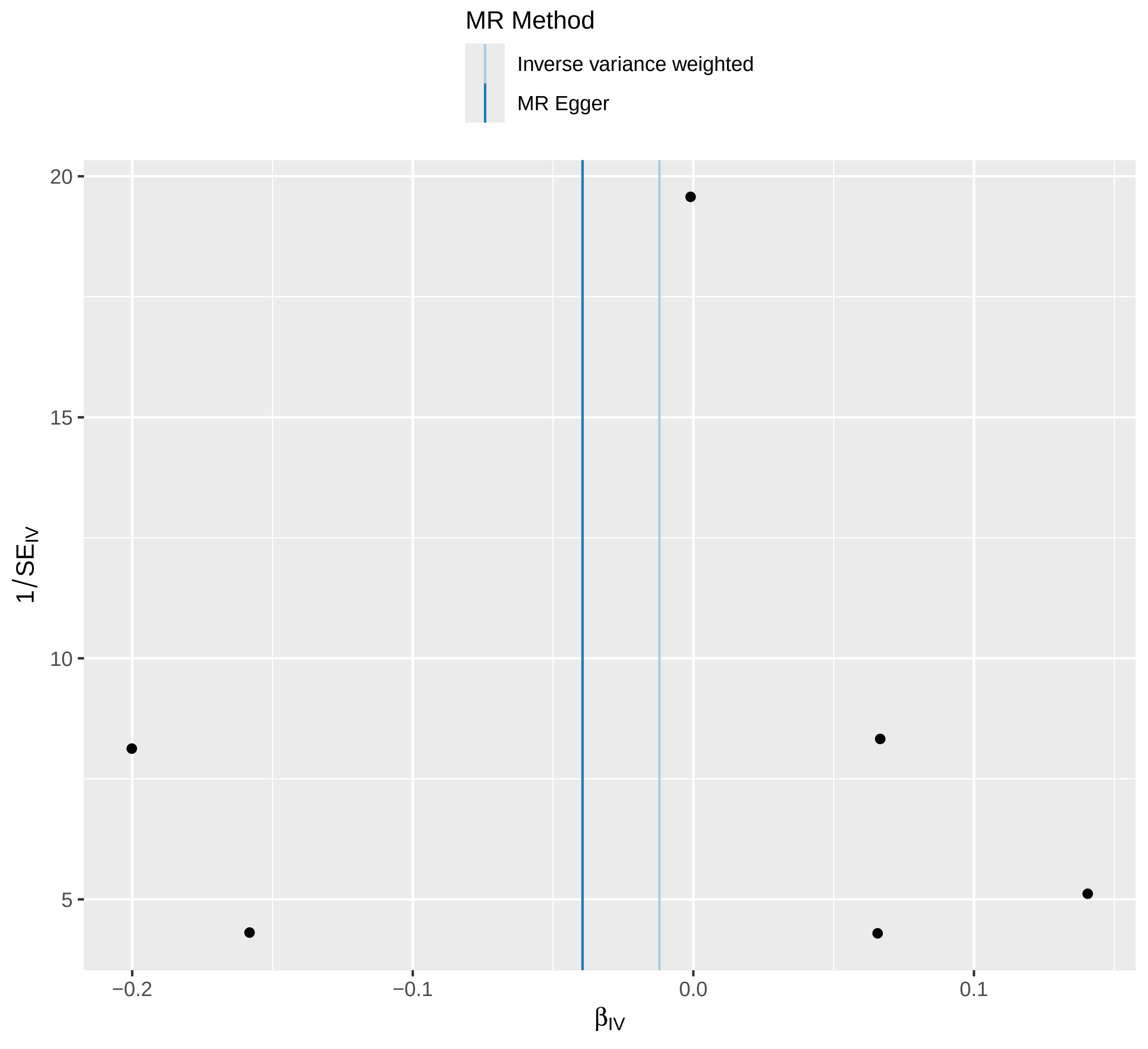
MR Method
Inverse variance weighted
MR Egger
20
15
10
5
$1/SE_{IV}$
−0.2
−0.1
0.0
0.1
$\beta_{IV}$

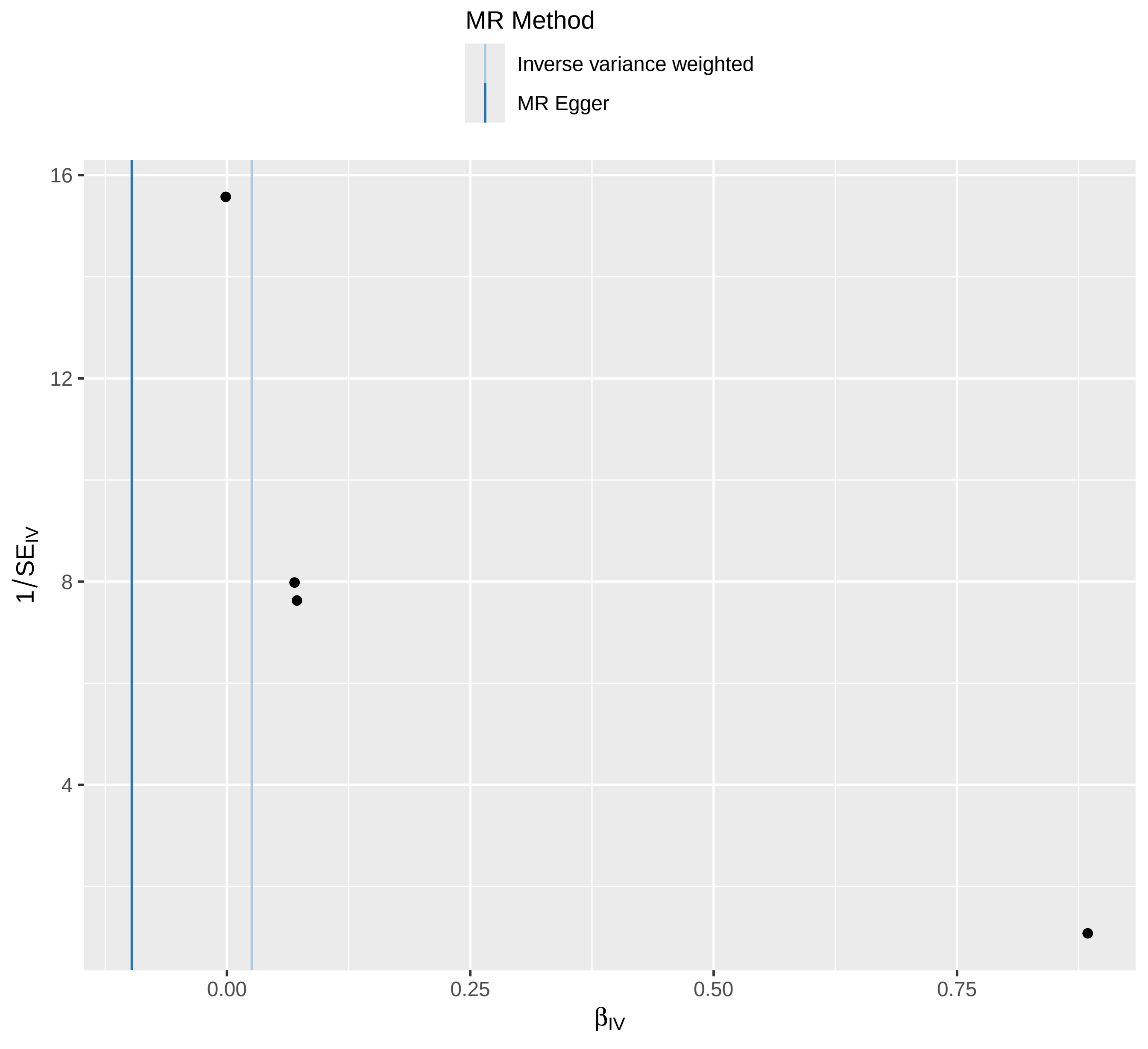
MR Method
Inverse variance weighted
MR Egger
16
12
8
4
1/SE_IV
0.00
0.25
0.50
0.75
β_IV

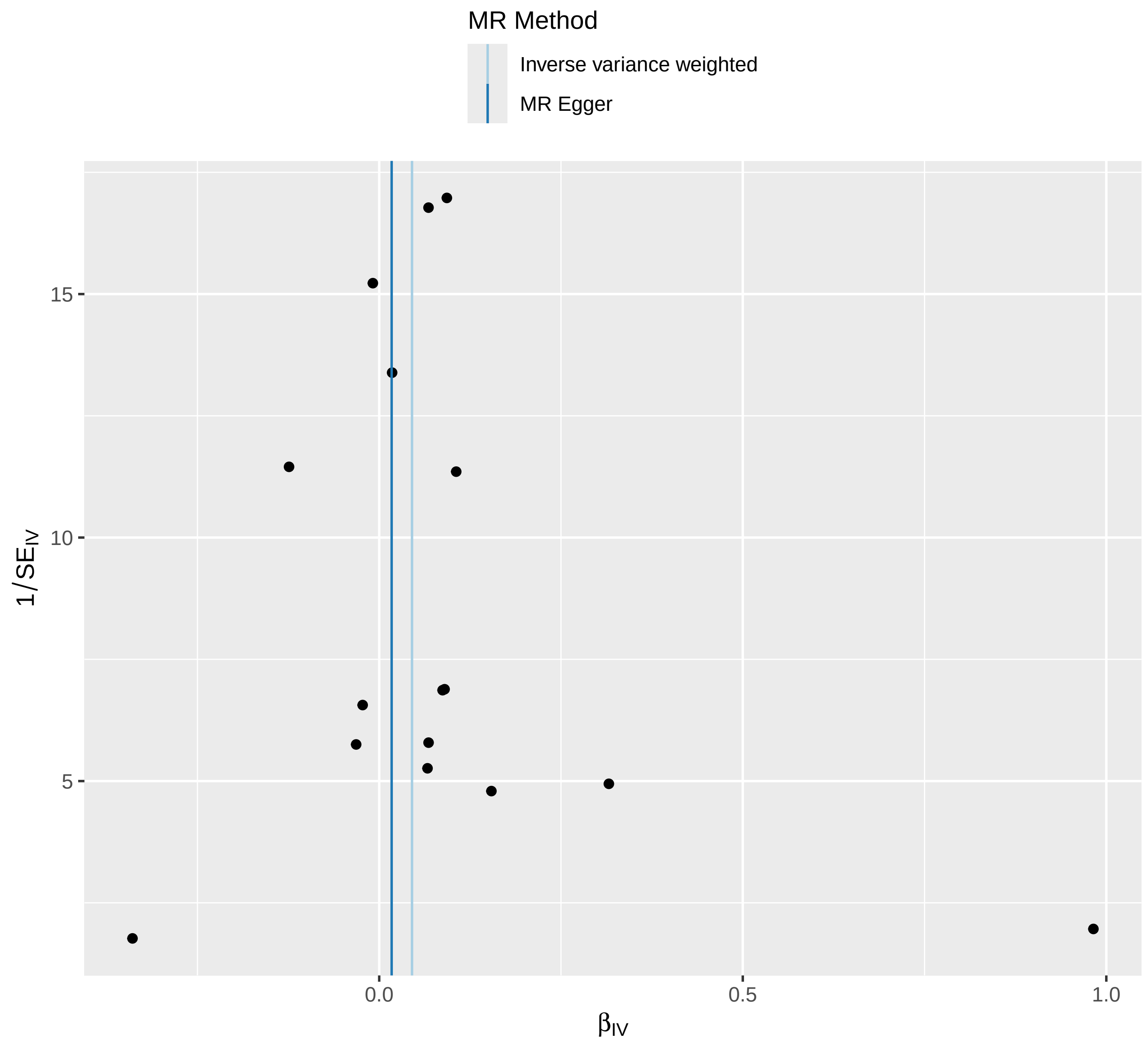
MR Method
Inverse variance weighted
MR Egger
15
10
5
1/SE$_{IV}$
0.0
0.5
1.0
β$_{IV}$

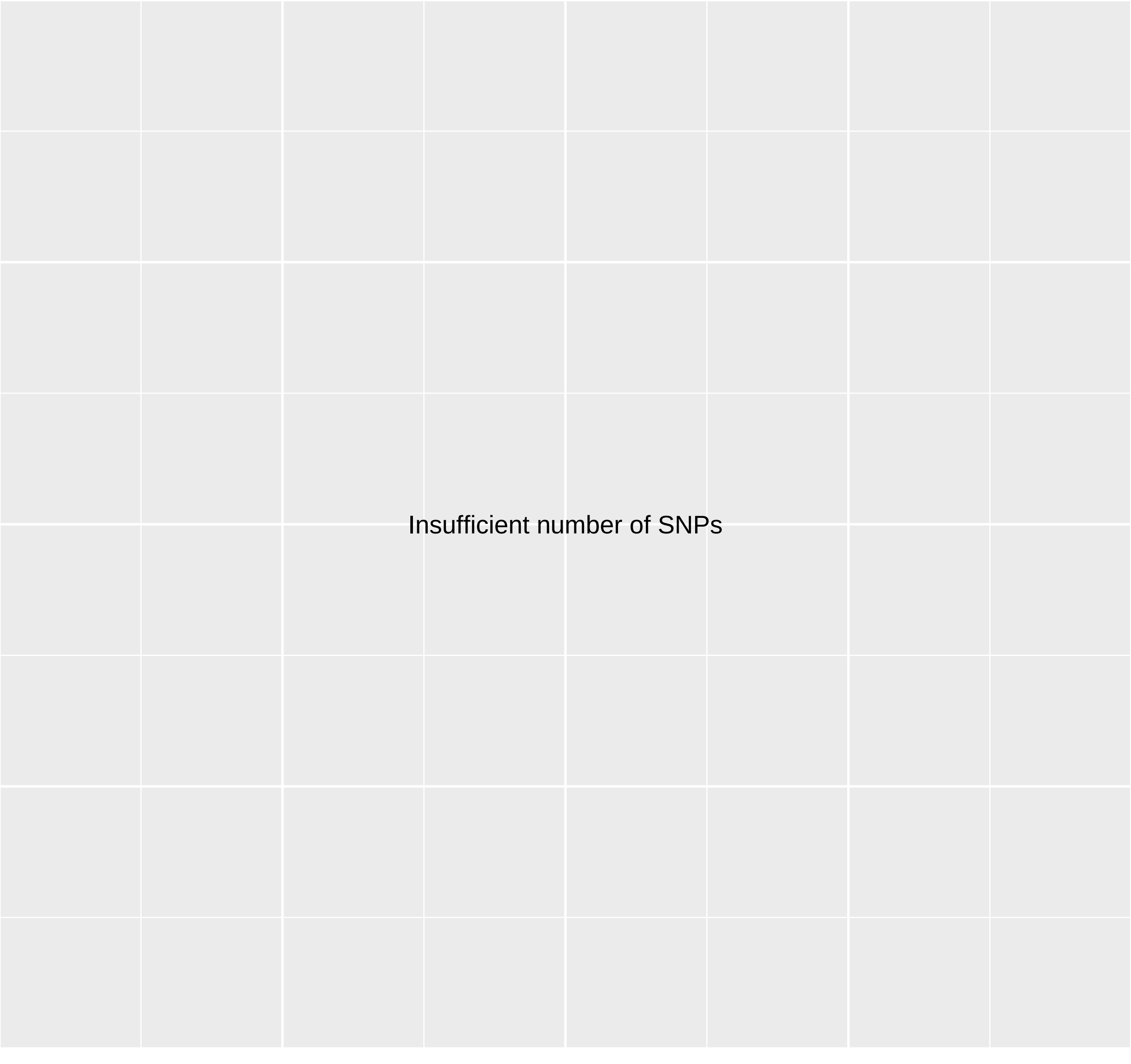
Insufficient number of SNPs

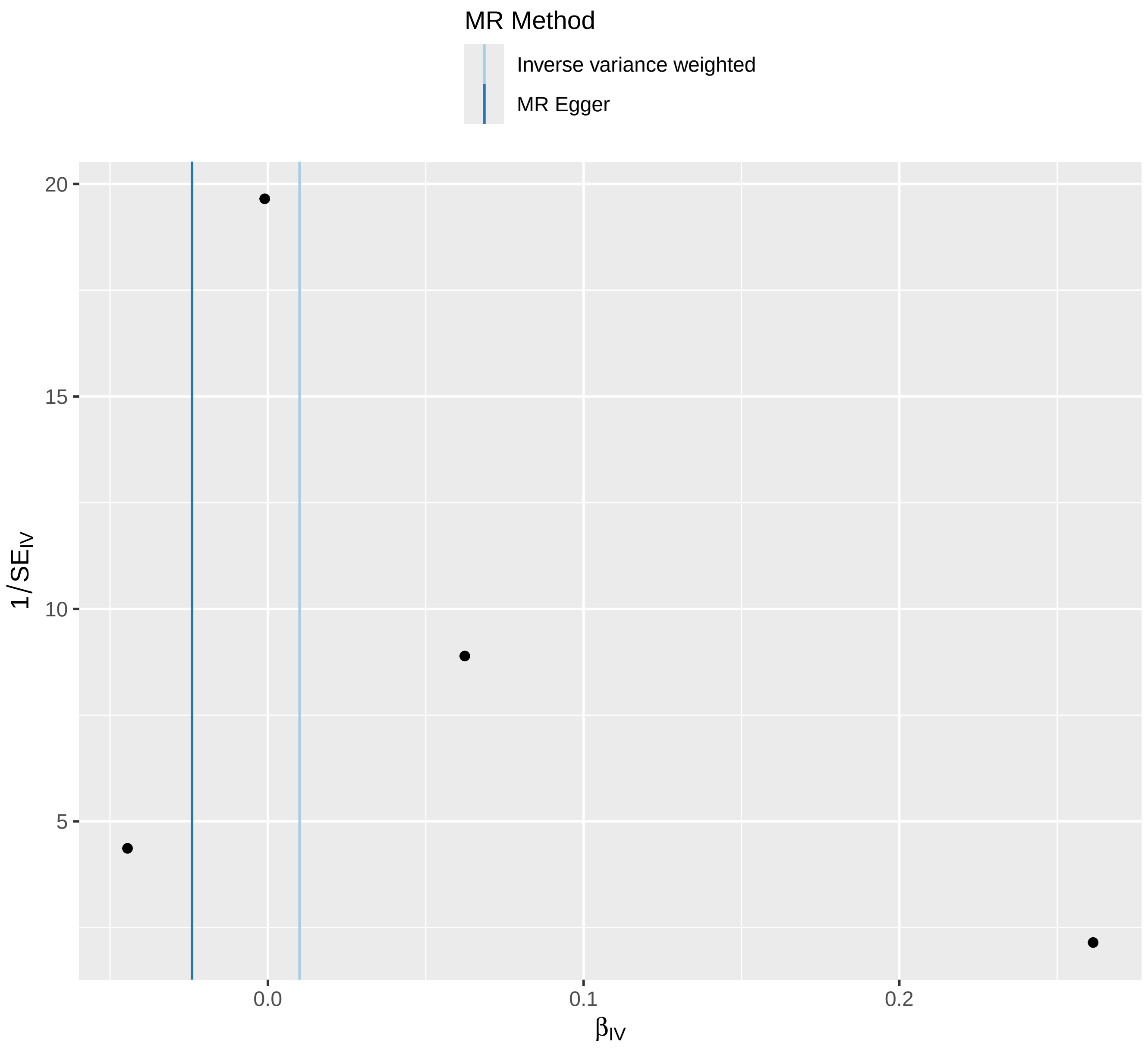
MR Method
Inverse variance weighted
MR Egger
20
15
10
5
0.0
0.1
0.2
$\beta_{IV}$
$1/SE_{IV}$

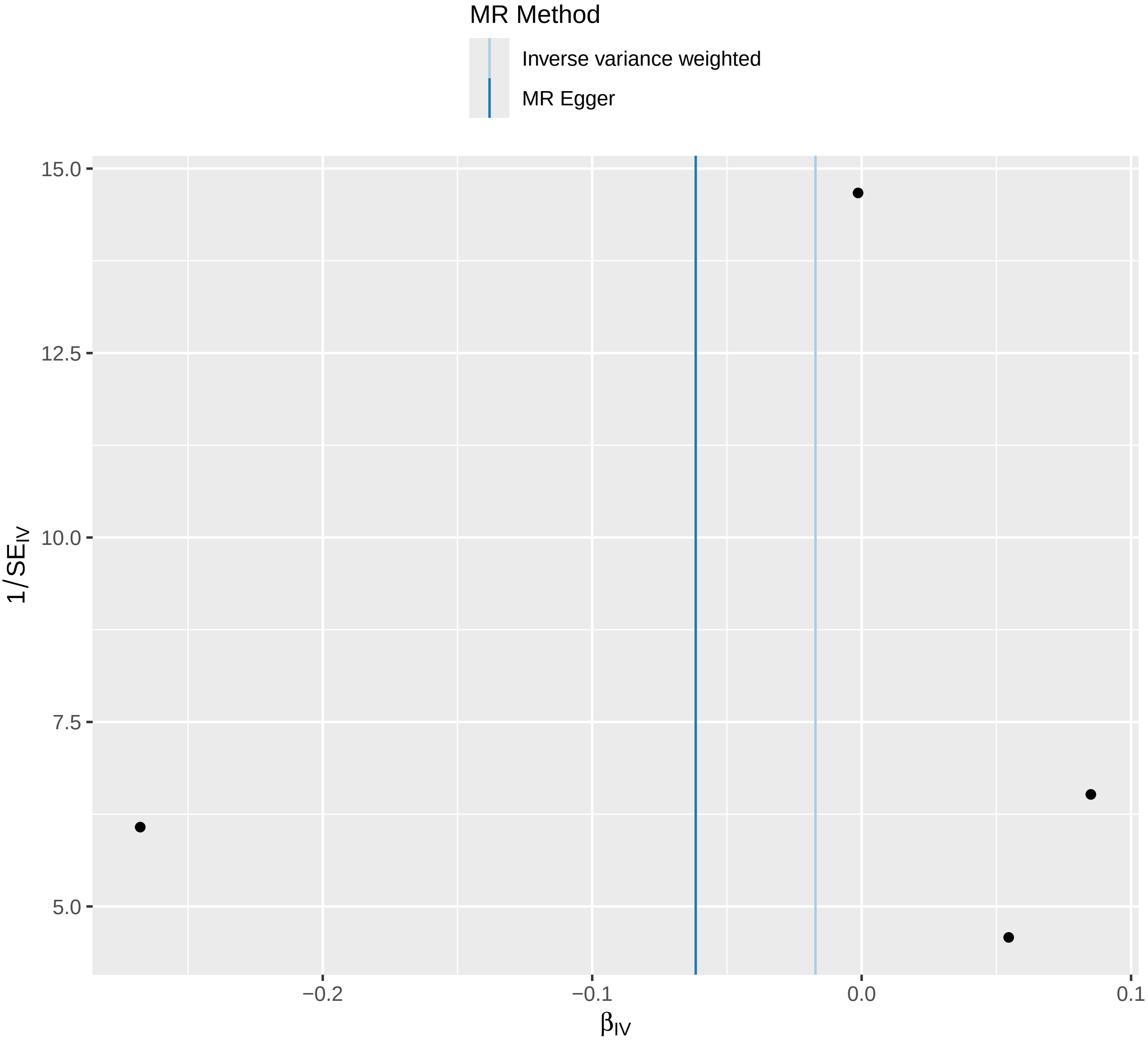

MR Method
Inverse variance weighted
MR Egger
15.0
12.5
10.0
7.5
5.0
1/SE$_{IV}$
−0.2
−0.1
0.0
0.1
β$_{IV}$

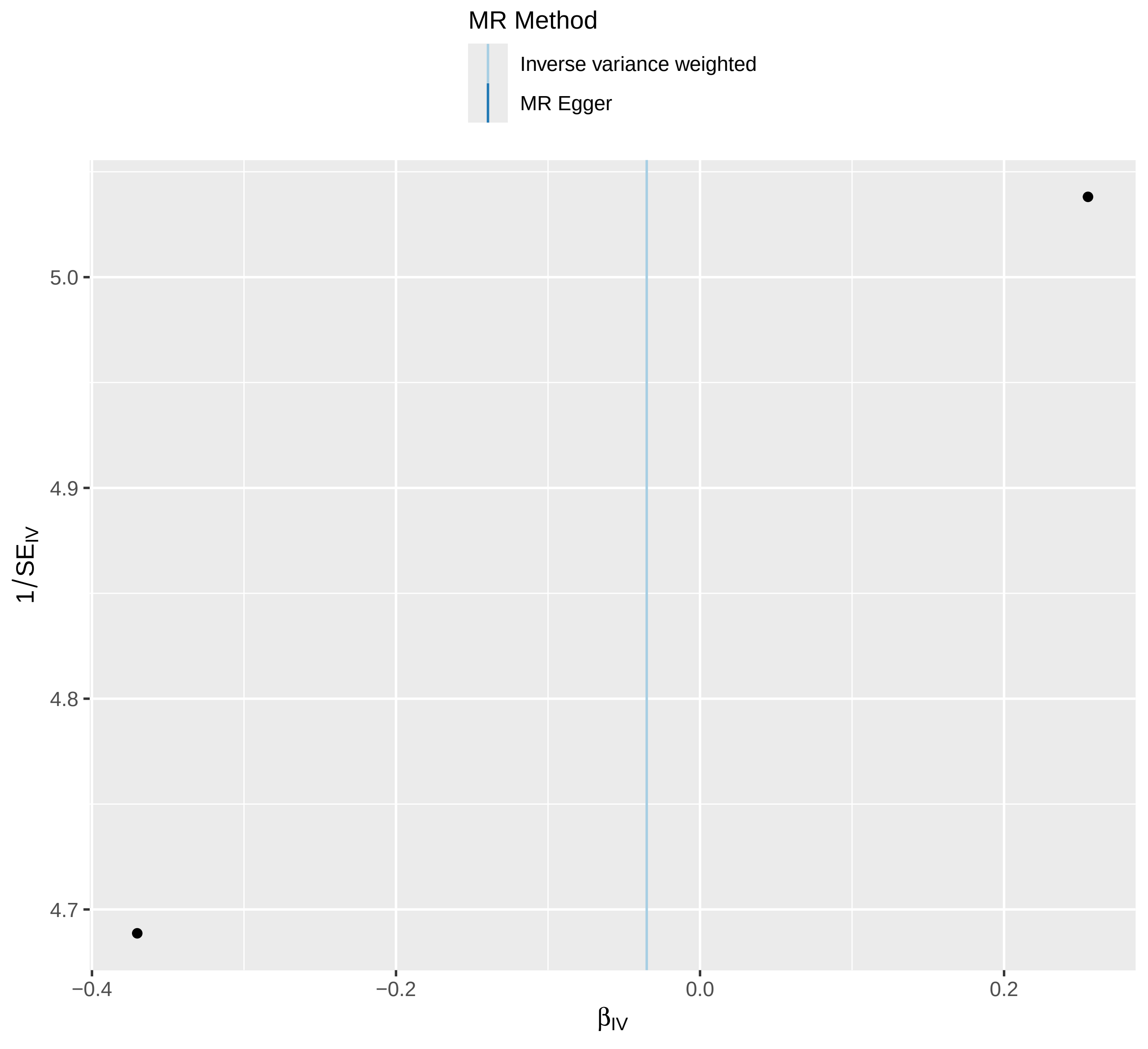
MR Method
Inverse variance weighted
MR Egger
5.0
4.9
4.8
4.7
1/SE$_{IV}$
−0.4
−0.2
0.0
0.2
β$_{IV}$

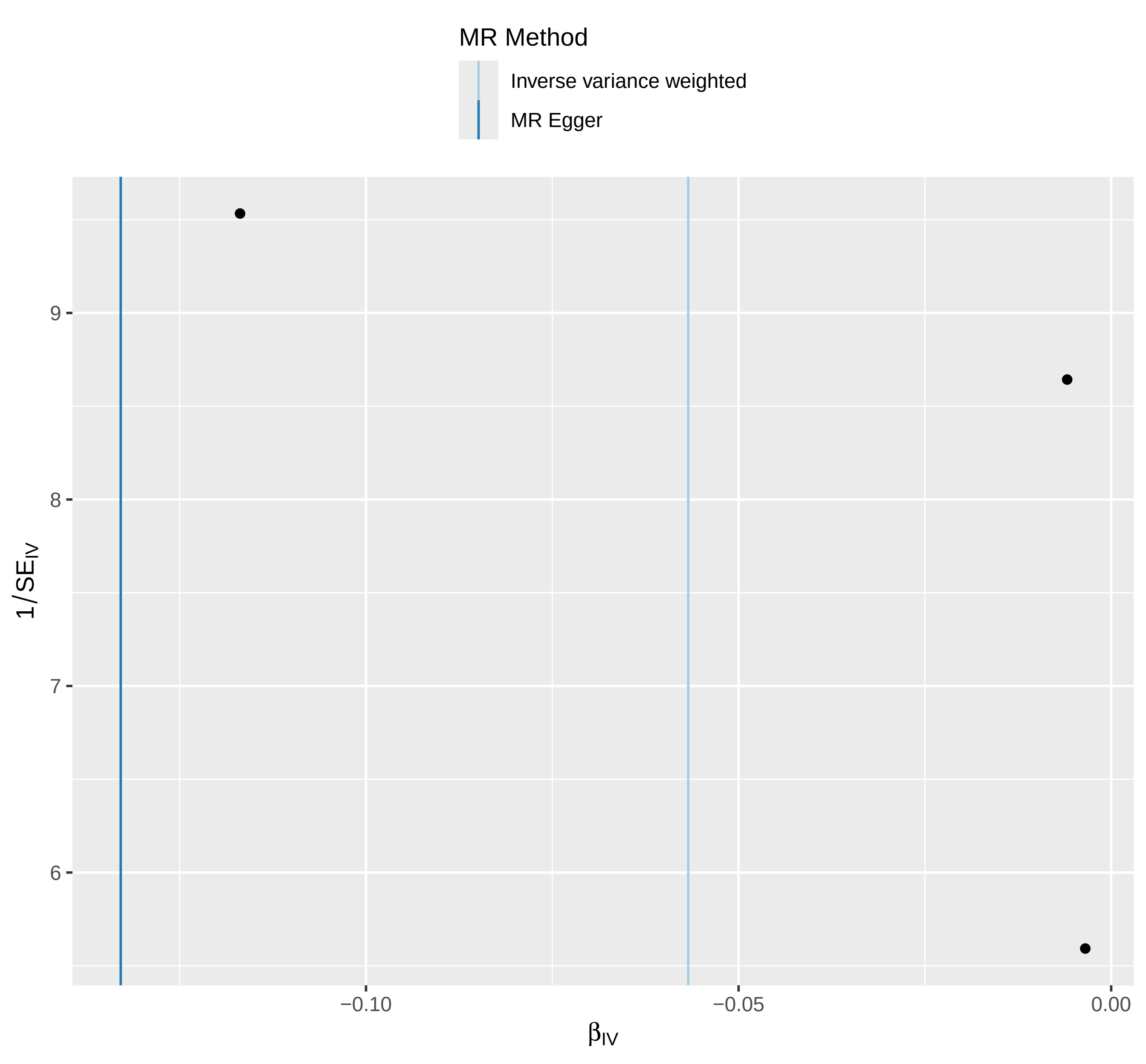

MR Method
Inverse variance weighted
MR Egger
9
8
7
6
1/SE_IV
−0.10
−0.05
0.00
β_IV

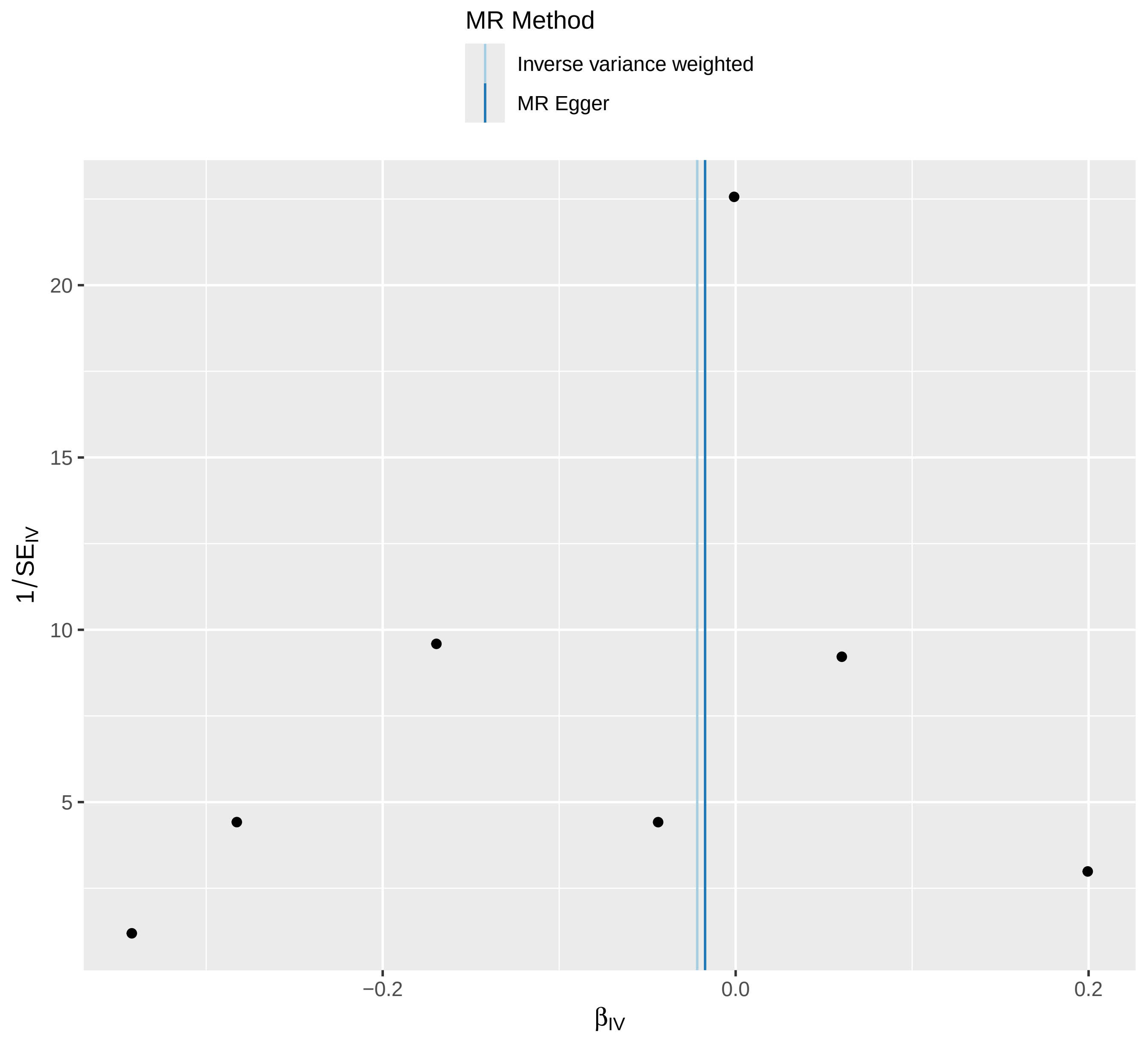

MR Method
Inverse variance weighted
MR Egger
1/SE_IV
20
15
10
5
−0.2
0.0
0.2
β_IV

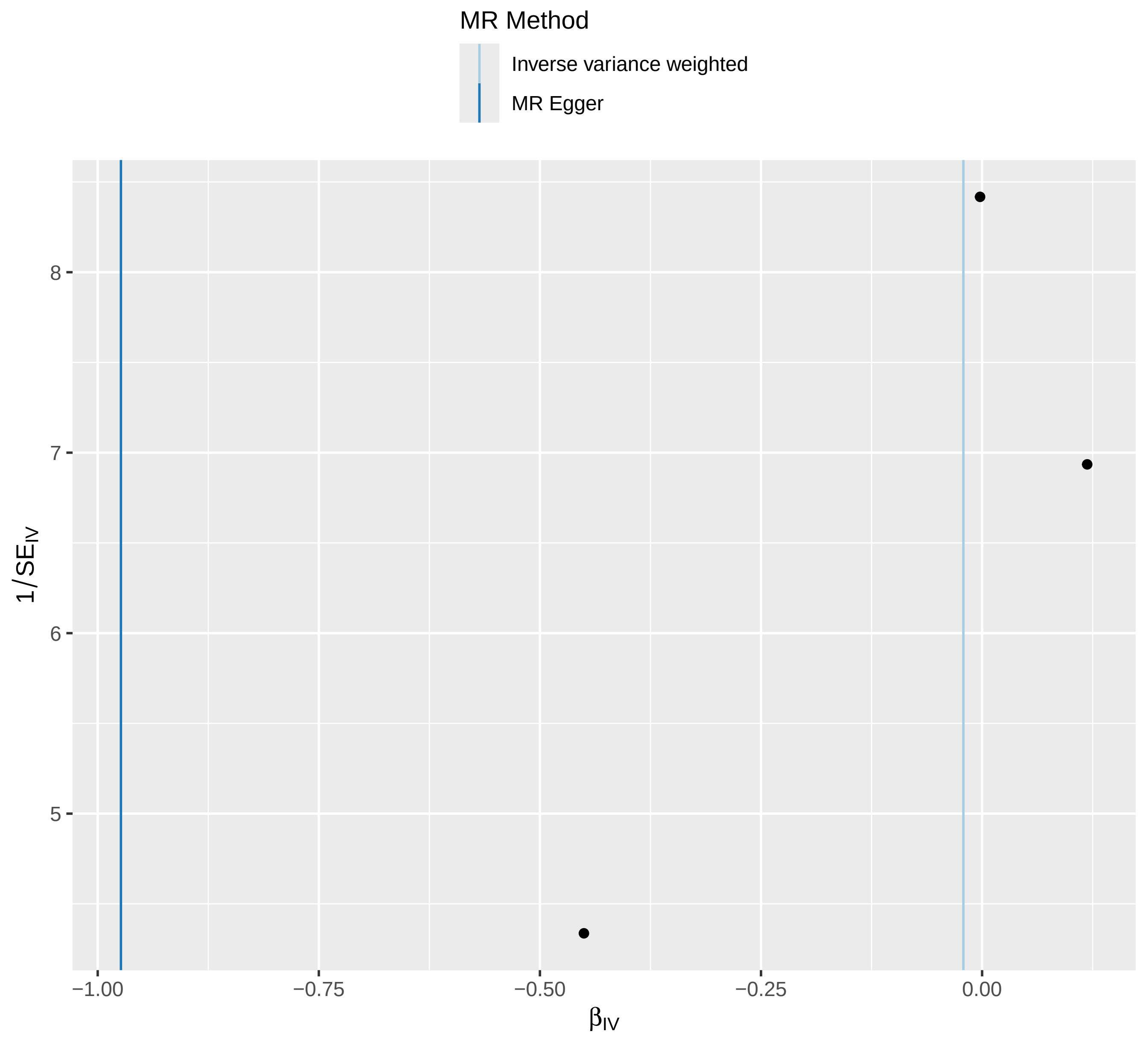
MR Method
Inverse variance weighted
MR Egger
8
7
6
5
1/SE$_{IV}$
−1.00
−0.75
−0.50
−0.25
0.00
β$_{IV}$

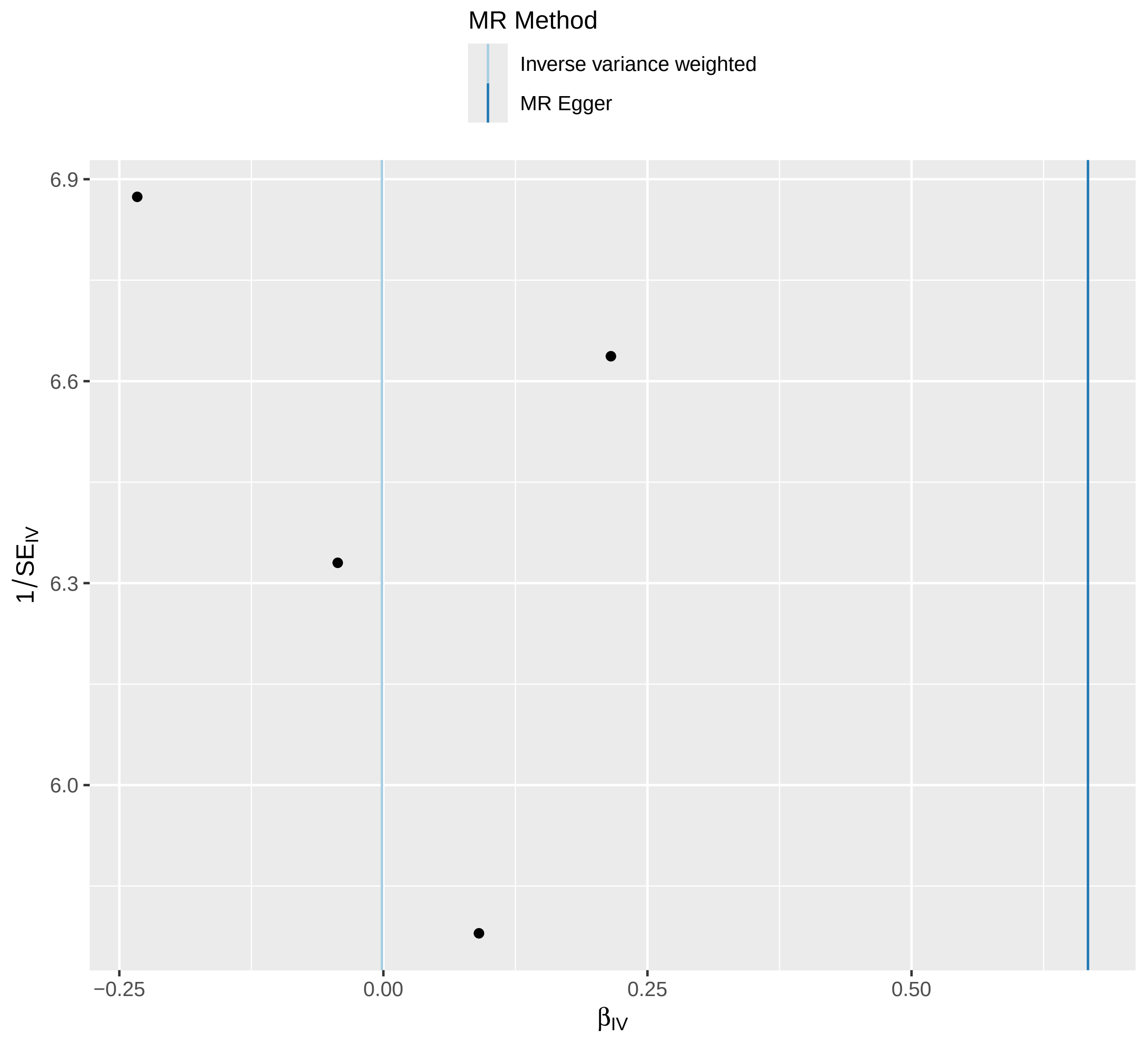
MR Method
Inverse variance weighted
MR Egger
6.9
6.6
6.3
6.0
$1/SE_{IV}$
−0.25
0.00
0.25
0.50
$\beta_{IV}$

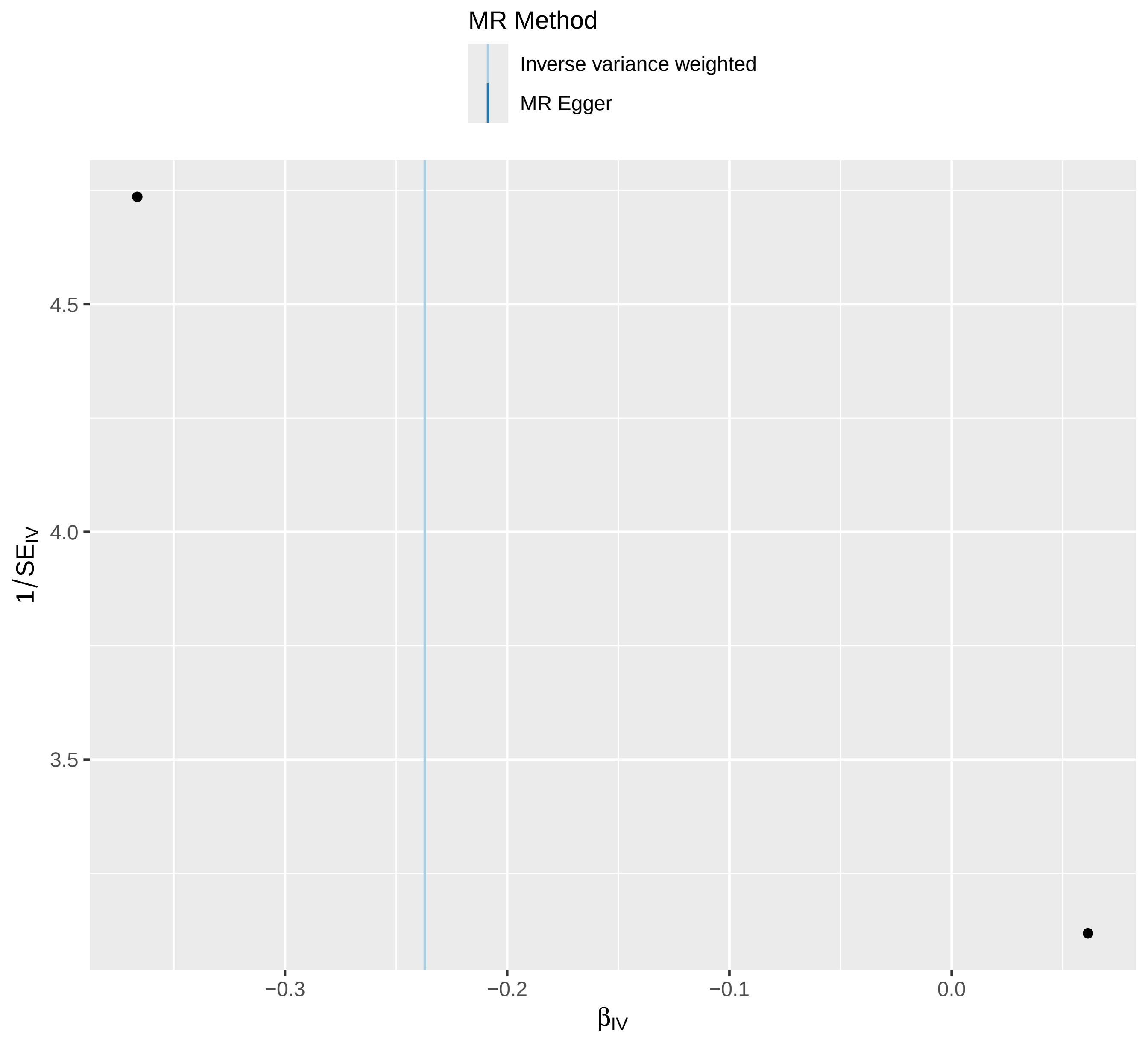
MR Method
Inverse variance weighted
MR Egger
4.5
4.0
3.5
1/SE_IV
−0.3
−0.2
−0.1
0.0
β_IV

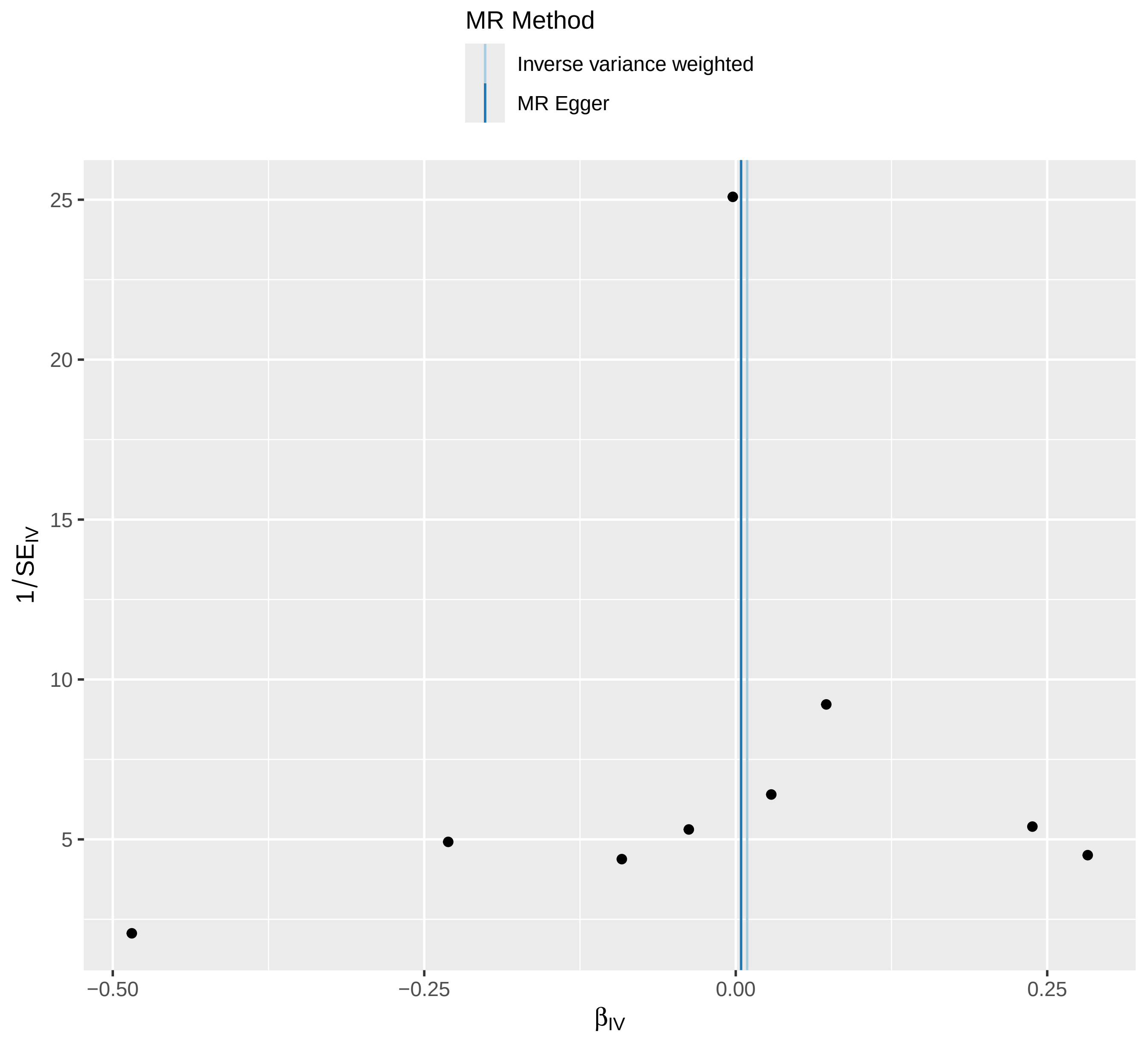
MR Method
Inverse variance weighted
MR Egger
1/SE$_{IV}$
β$_{IV}$
25
20
15
10
5
−0.50
−0.25
0.00
0.25

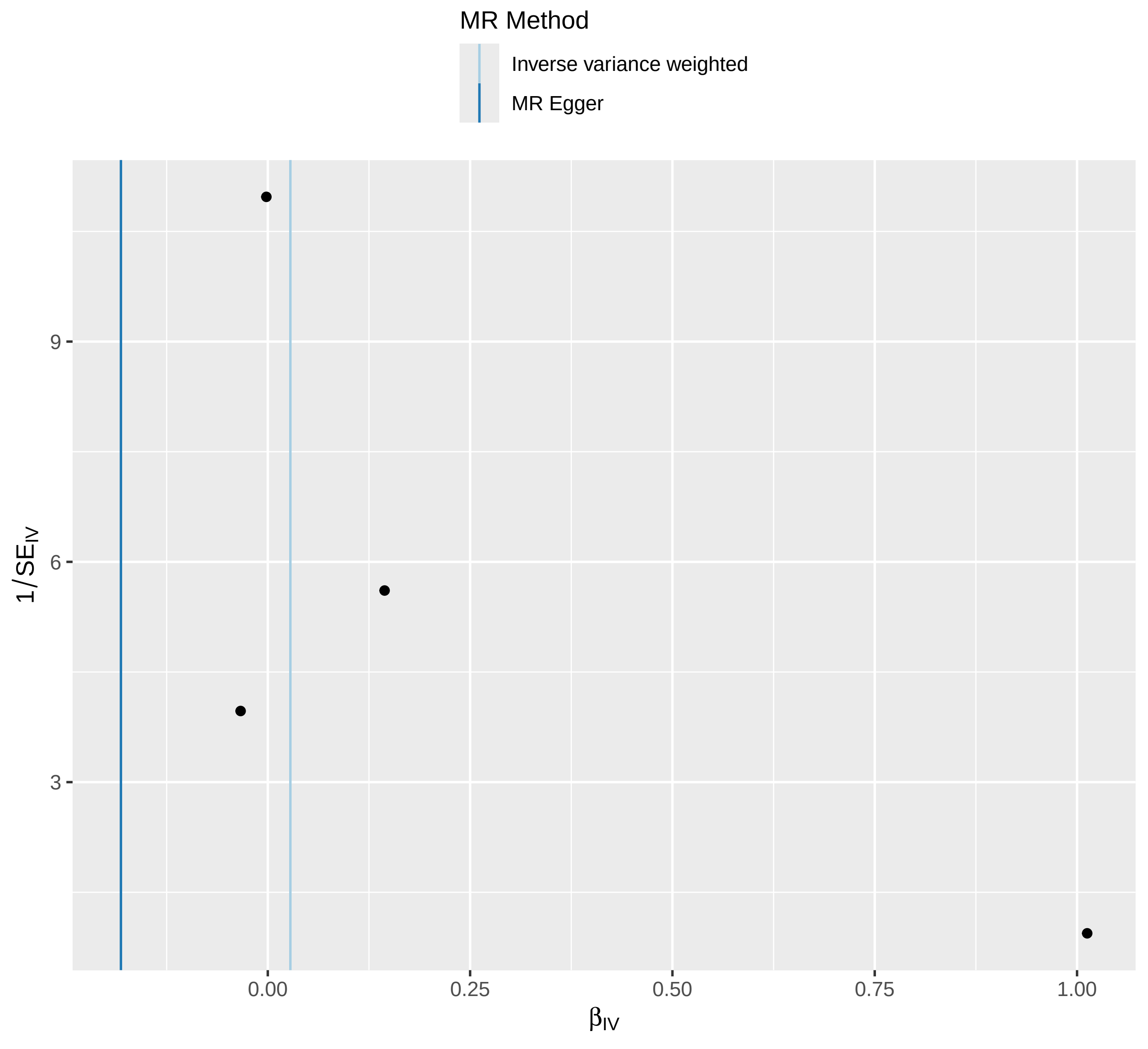

MR Method
Inverse variance weighted
MR Egger
9
6
3
1/SE_IV
0.00
0.25
0.50
0.75
1.00
β_IV

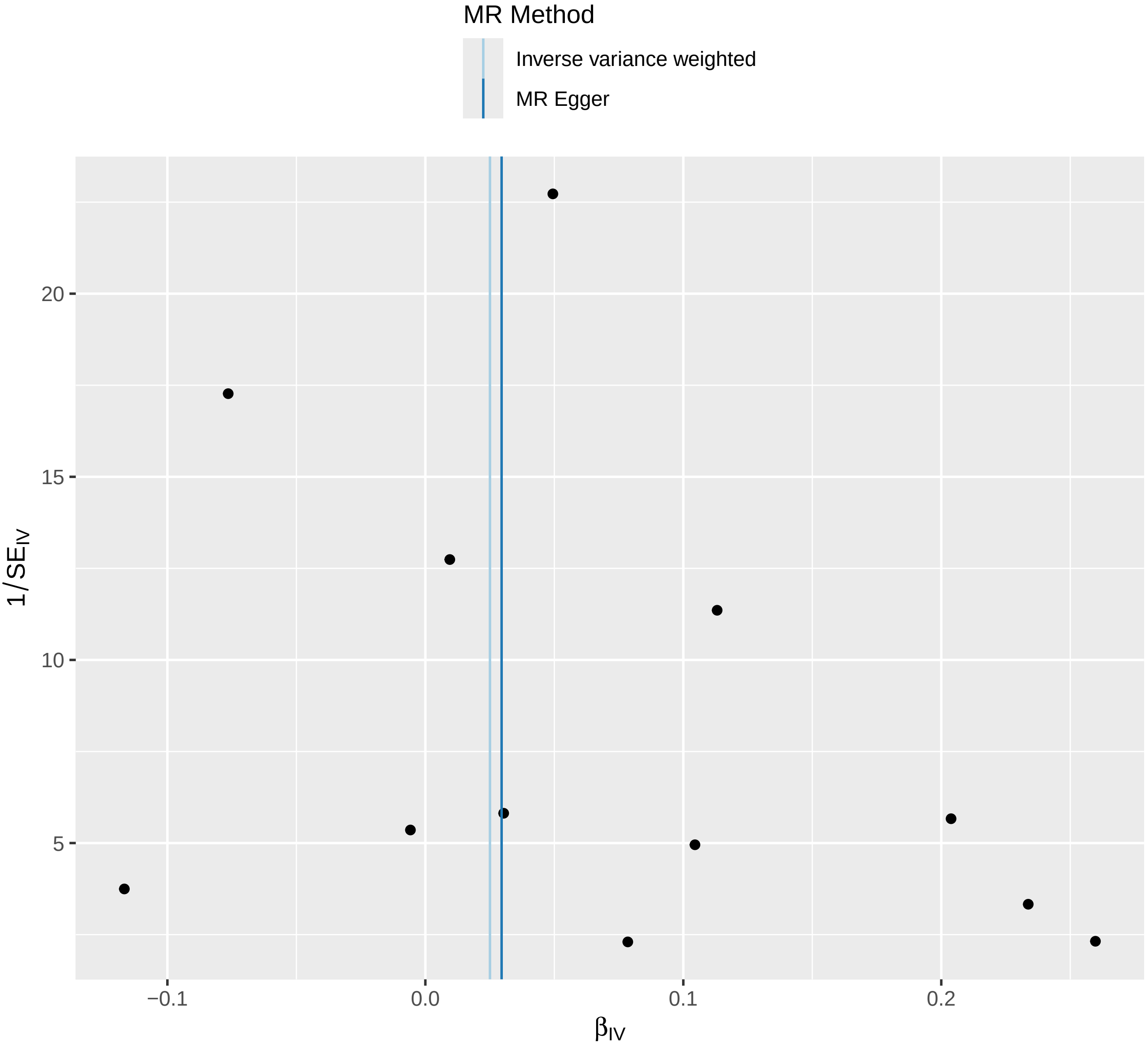
MR Method
Inverse variance weighted
MR Egger
20
15
10
5
1/SE_IV
−0.1
0.0
0.1
0.2
β_IV

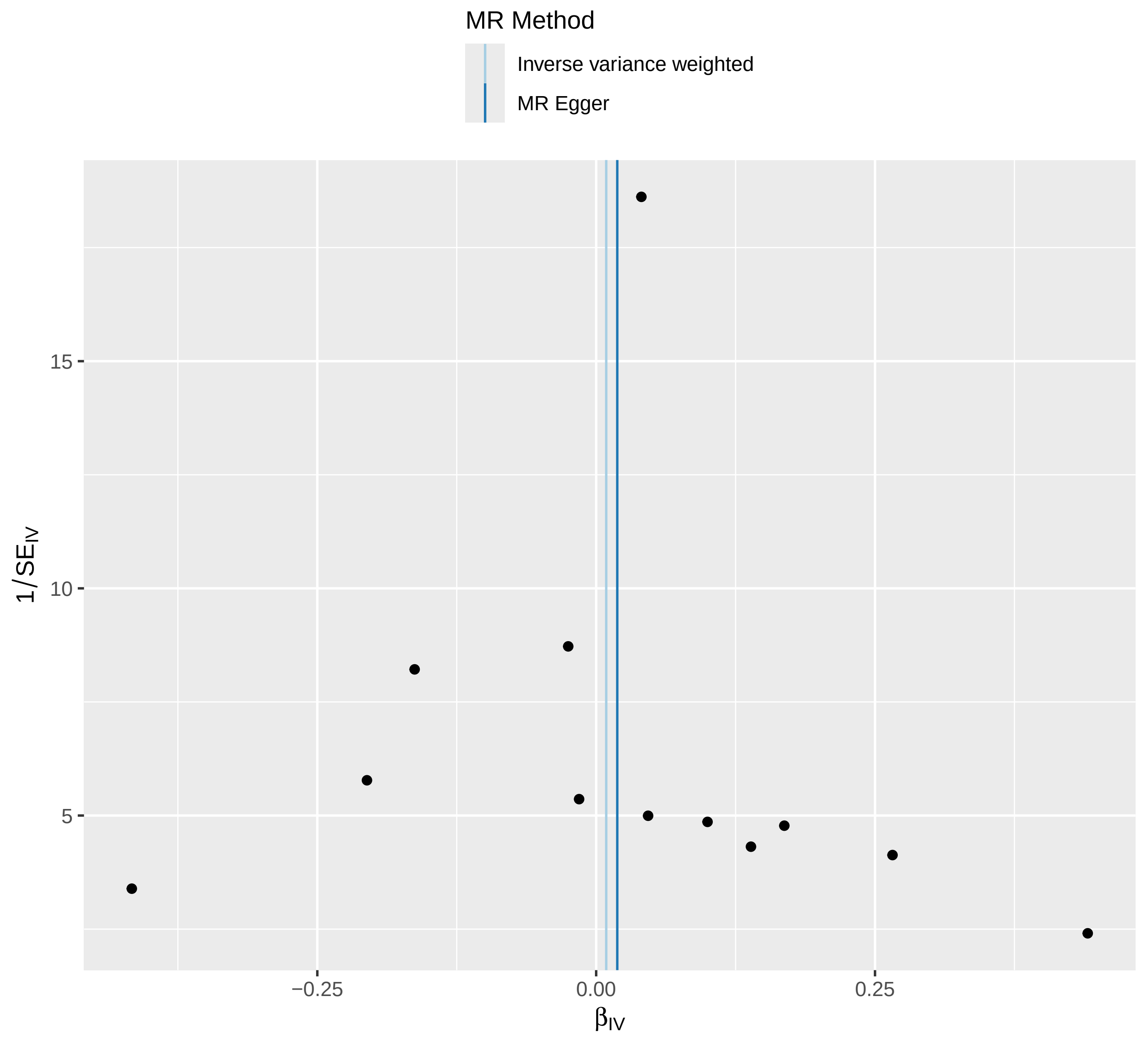
MR Method
Inverse variance weighted
MR Egger
1/SE_IV
15
10
5
−0.25
0.00
0.25
β_IV

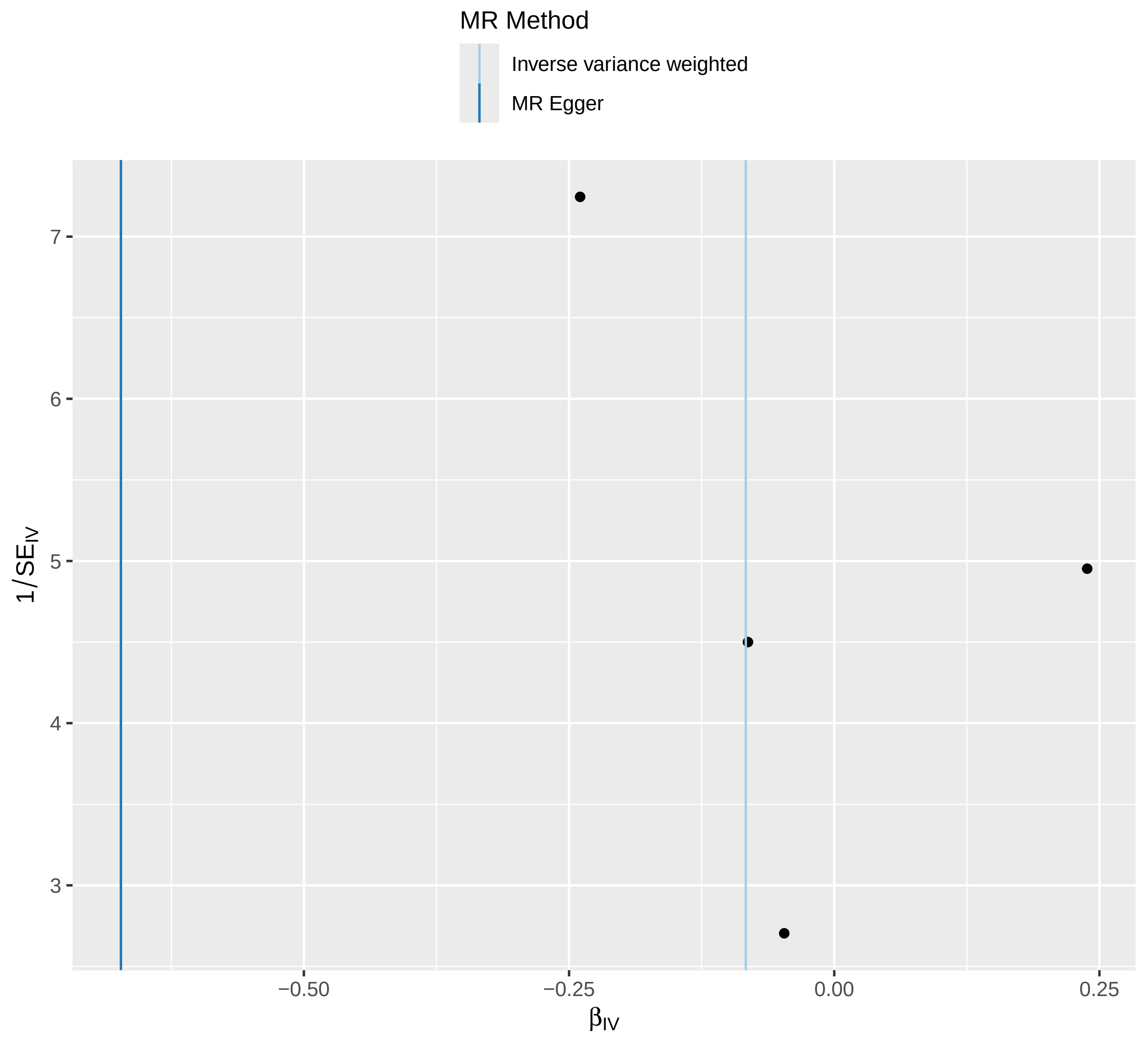

MR Method
Inverse variance weighted
MR Egger
1/SE$_{IV}$
7
6
5
4
3
−0.50
−0.25
0.00
0.25
β$_{IV}$

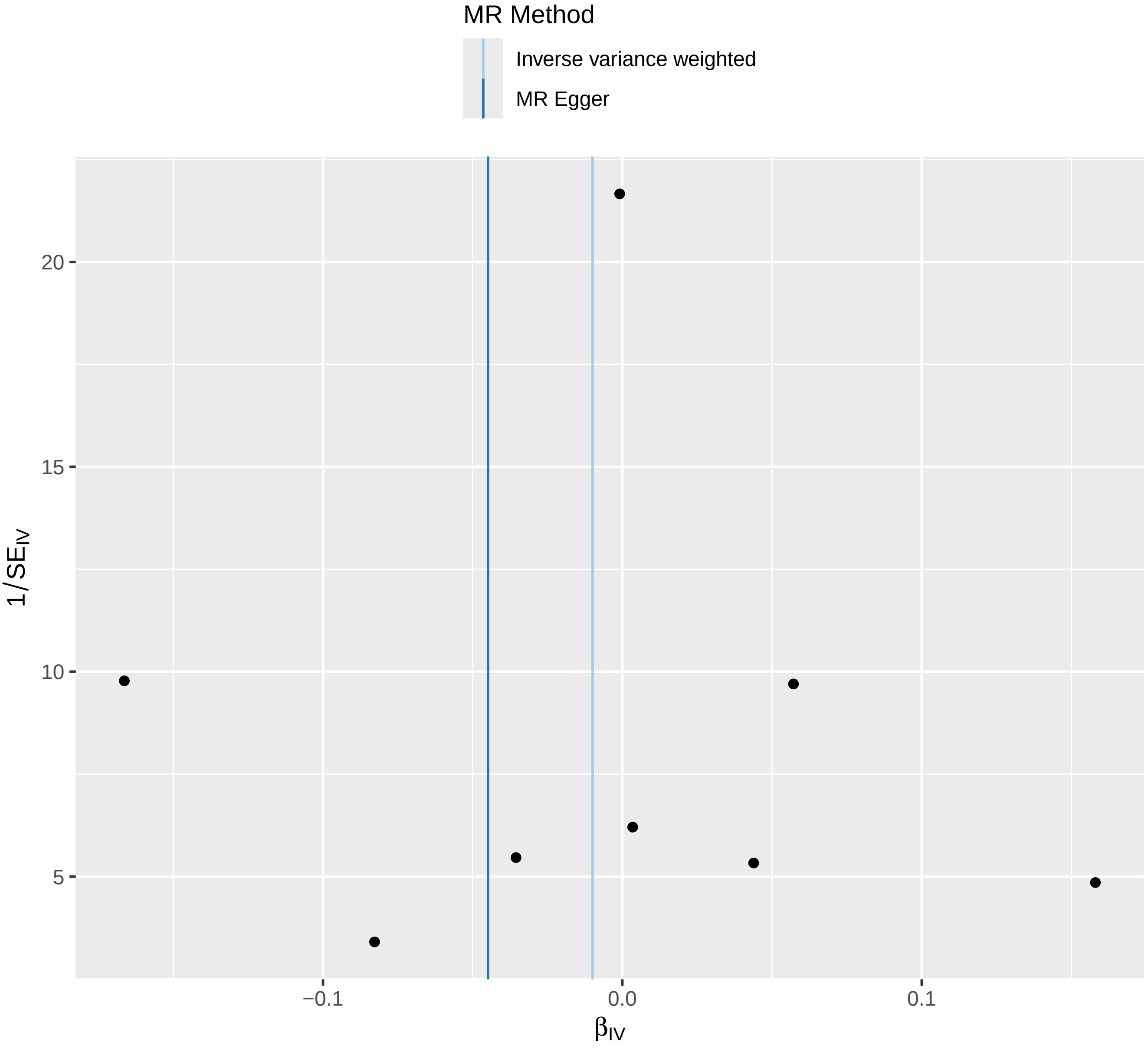
MR Method
Inverse variance weighted
MR Egger
20
15
10
5
1/SE$_{IV}$
−0.1
0.0
0.1
β$_{IV}$

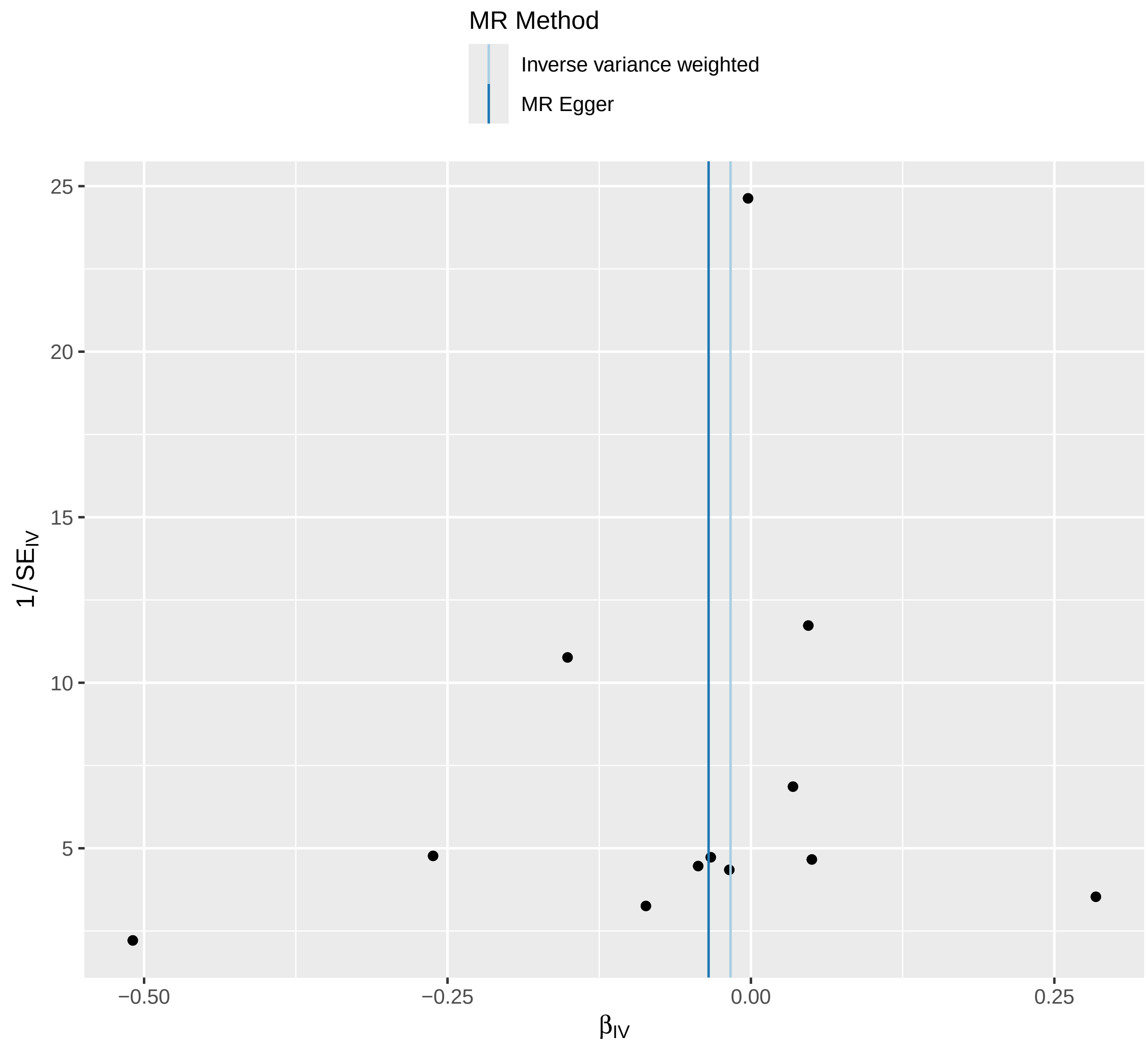

MR Method
Inverse variance weighted
MR Egger
25
20
15
10
5
$1/SE_{IV}$
−0.50
−0.25
0.00
0.25
$\beta_{IV}$

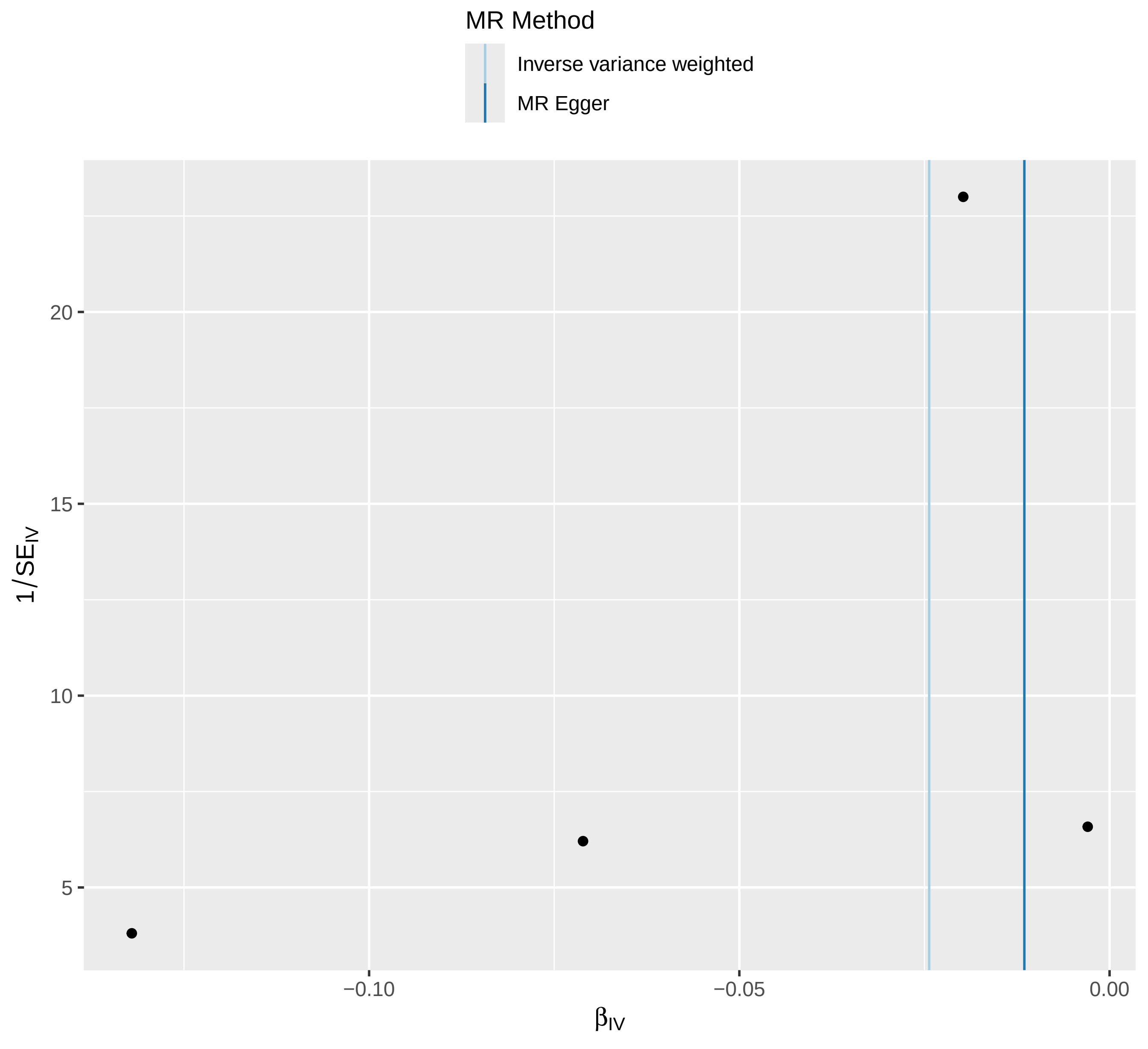
MR Method
Inverse variance weighted
MR Egger
20
15
10
5
1/SE_IV
−0.10
−0.05
0.00
β_IV

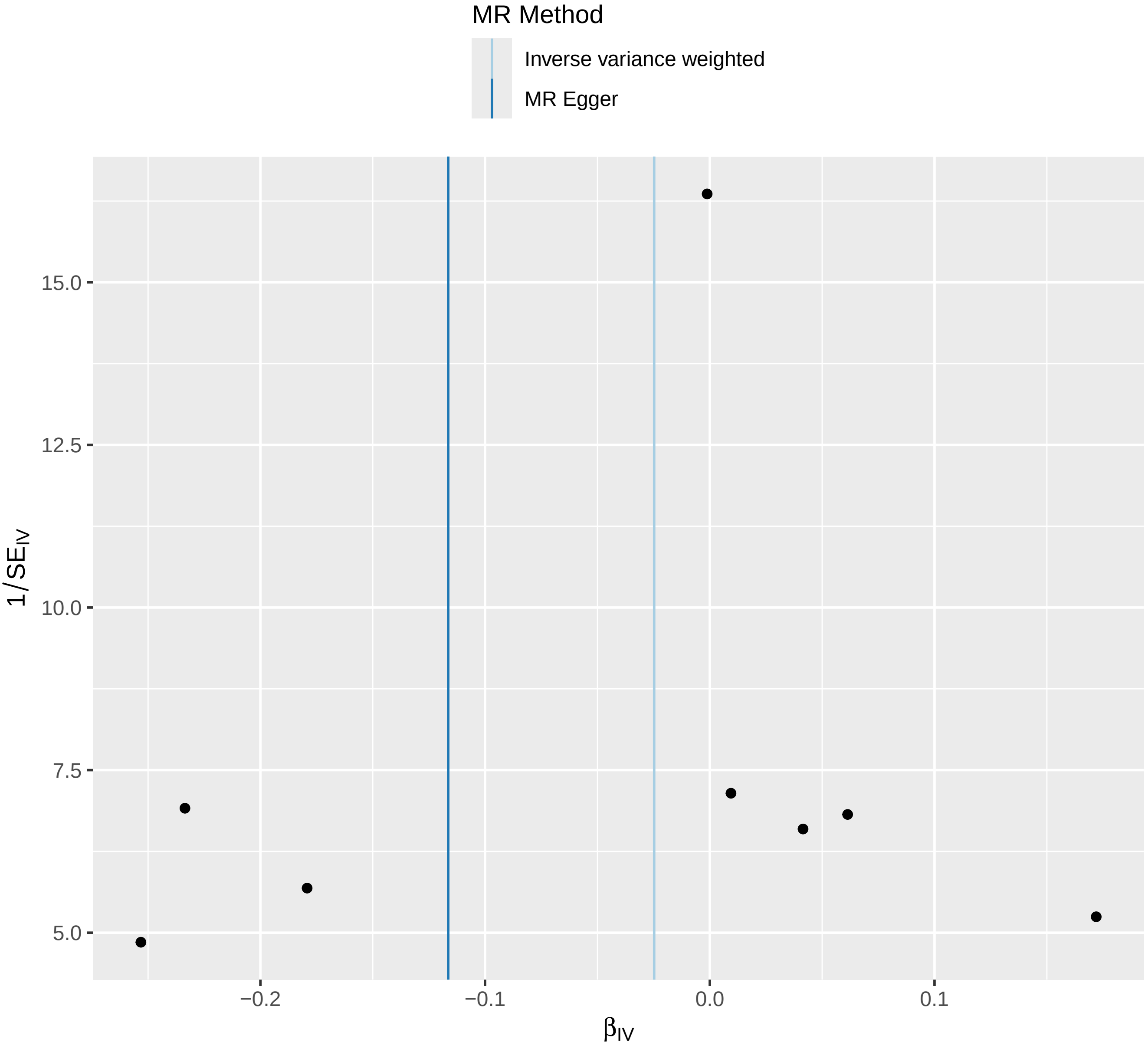

MR Method
Inverse variance weighted
MR Egger
15.0
12.5
10.0
7.5
5.0
1/SE_IV
−0.2
−0.1
0.0
0.1
β_IV

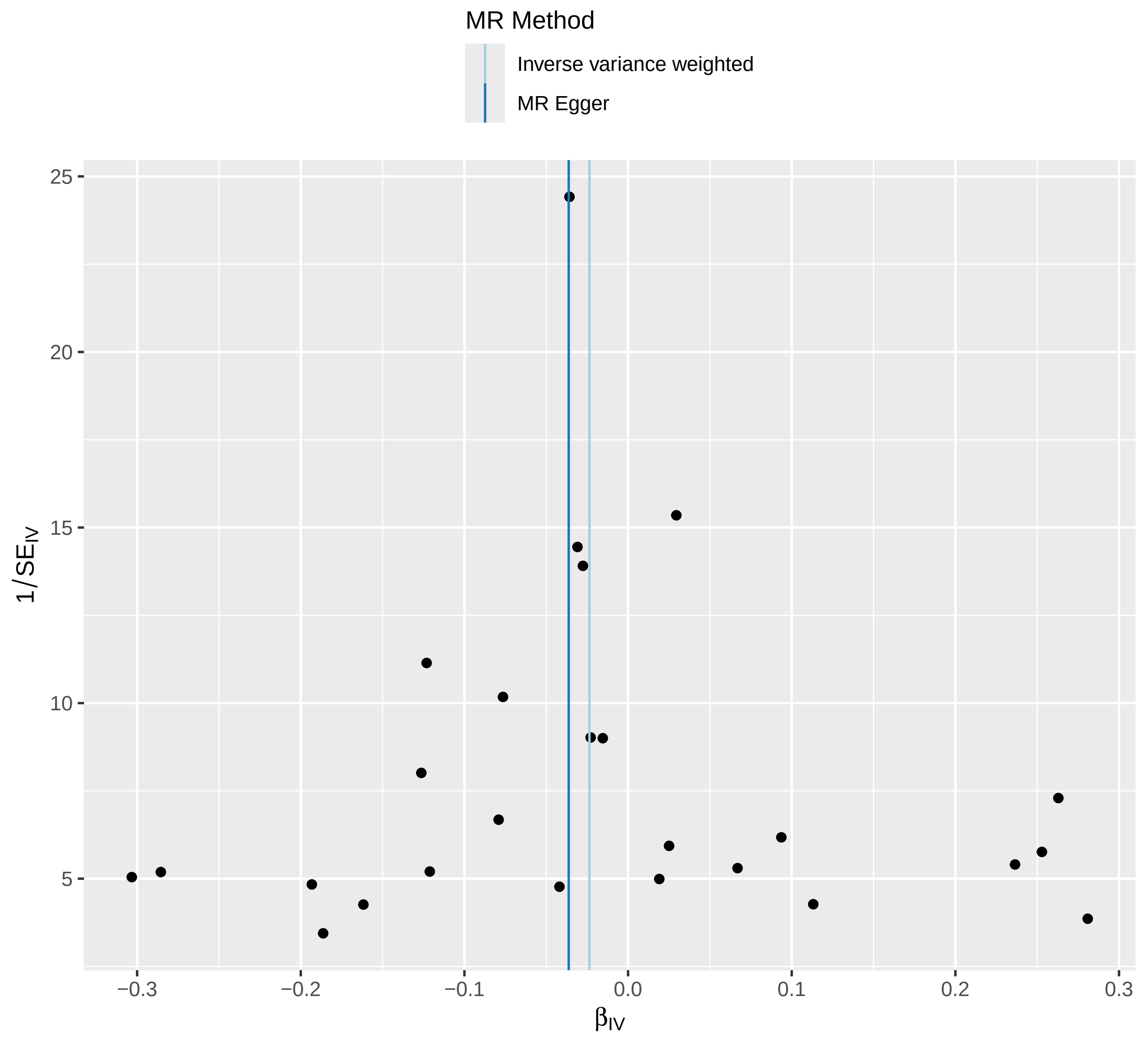
MR Method
Inverse variance weighted
MR Egger
1/$SE_{IV}$
$\beta_{IV}$
25
20
15
10
5
−0.3
−0.2
−0.1
0.0
0.1
0.2
0.3

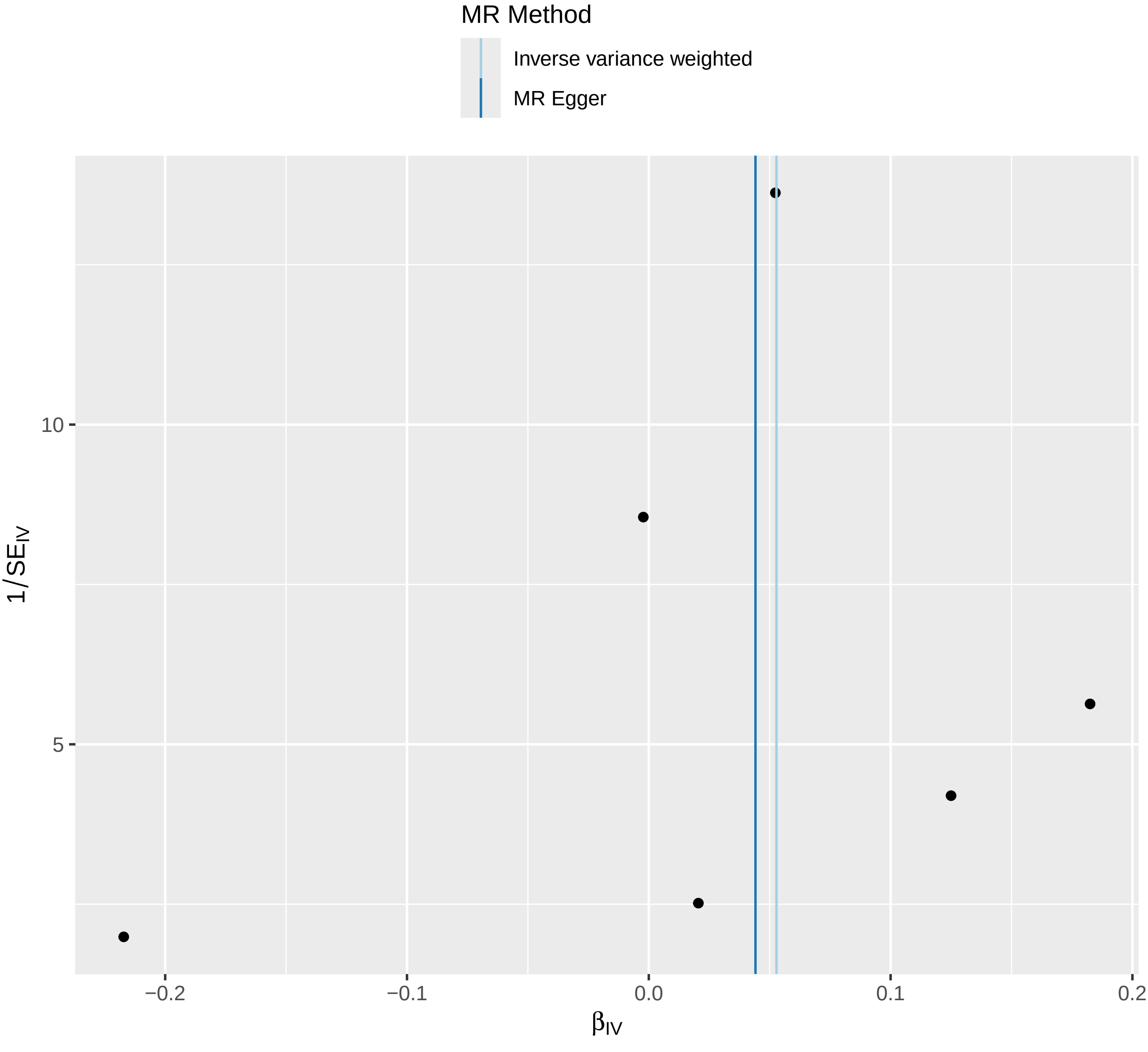

MR Method
Inverse variance weighted
MR Egger
10
5
1/SE_IV
−0.2
−0.1
0.0
0.1
0.2
β_IV

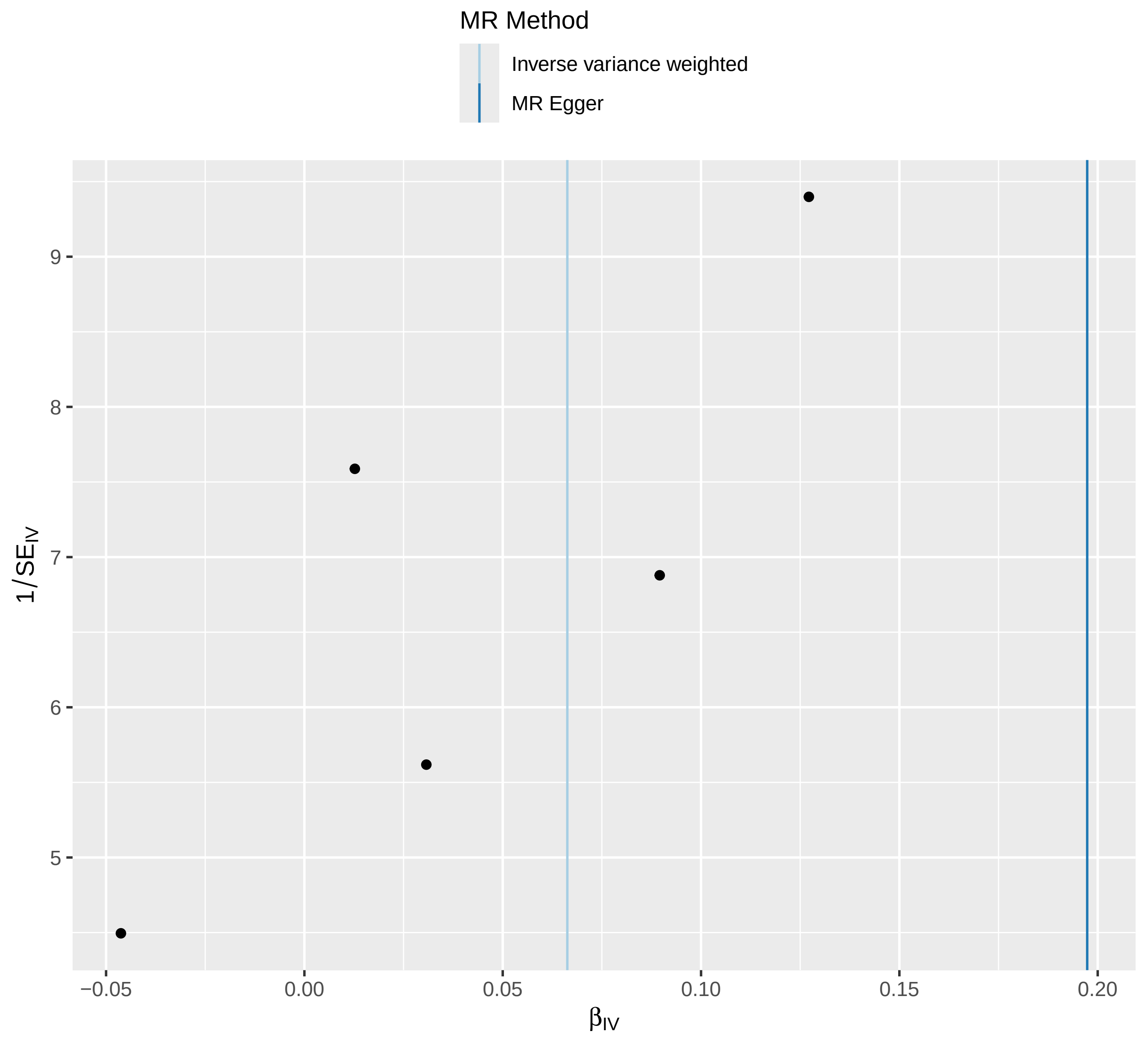

MR Method
Inverse variance weighted
MR Egger
9
8
7
6
5
1/SE_IV
−0.05
0.00
0.05
0.10
0.15
0.20
β_IV

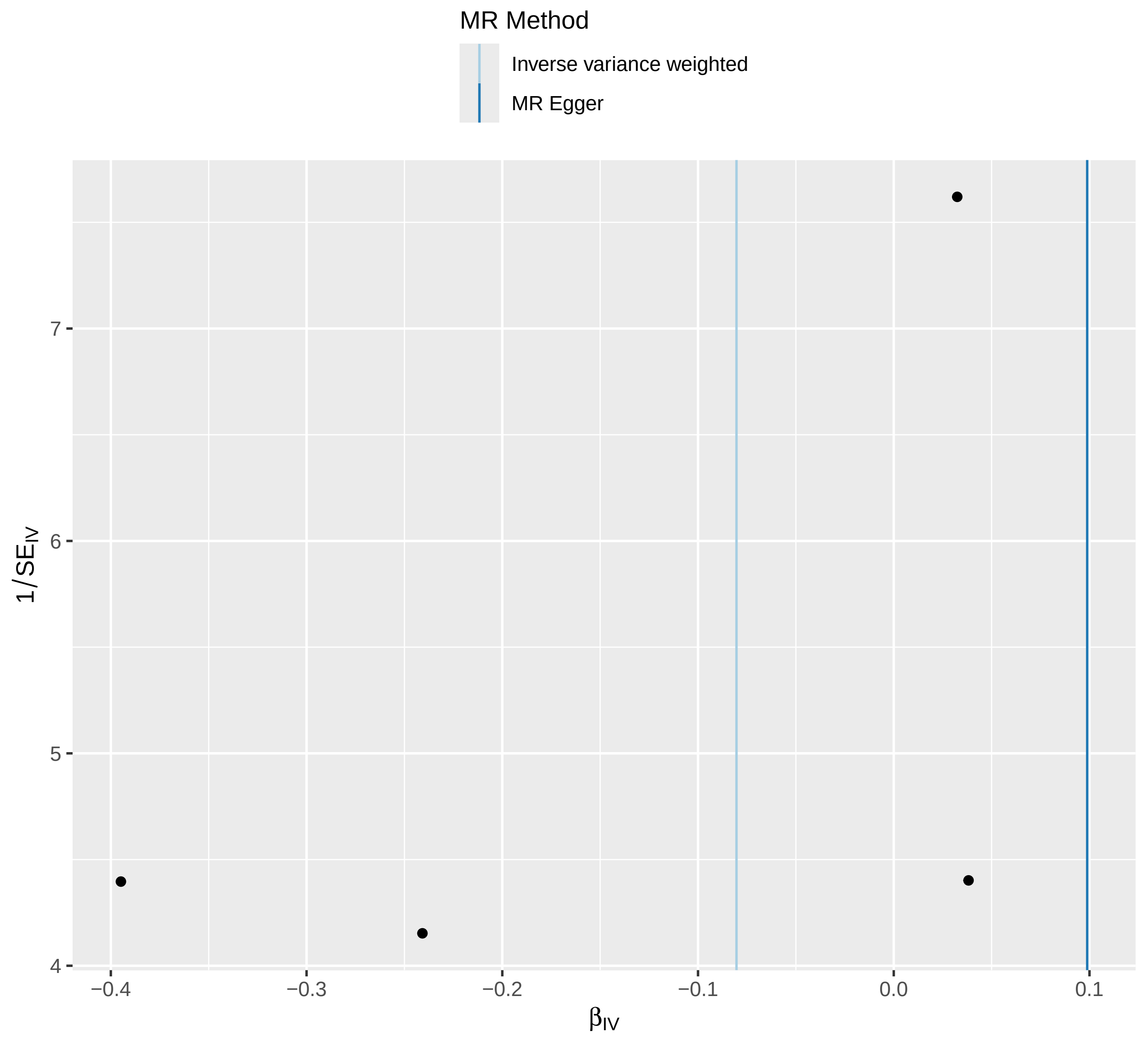
MR Method
Inverse variance weighted
MR Egger
1/SE_IV
7
6
5
4
−0.4
−0.3
−0.2
−0.1
0.0
0.1
β_IV

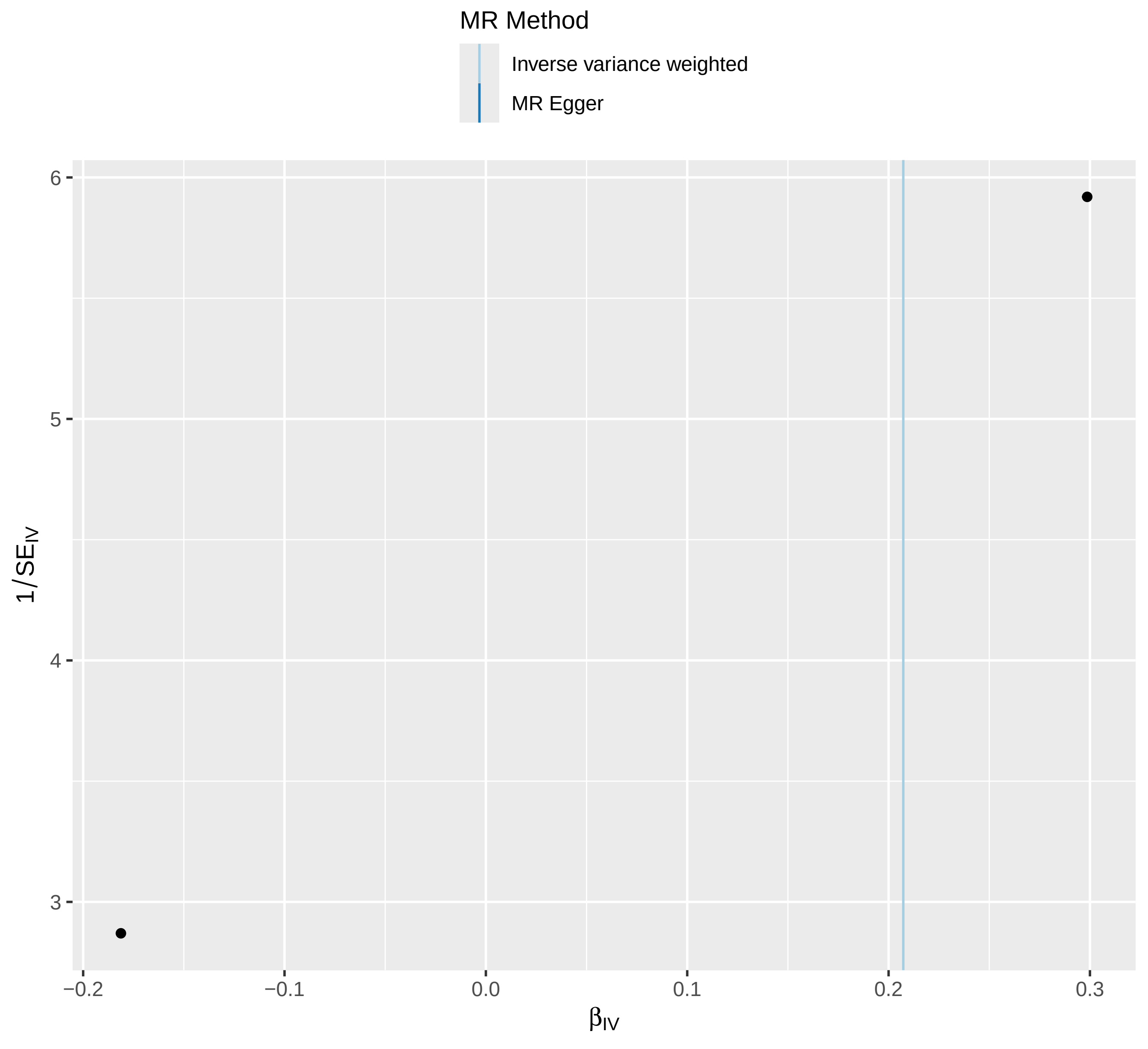
MR Method
Inverse variance weighted
MR Egger
1/SE_IV
β_IV
6
5
4
3
−0.2
−0.1
0.0
0.1
0.2
0.3

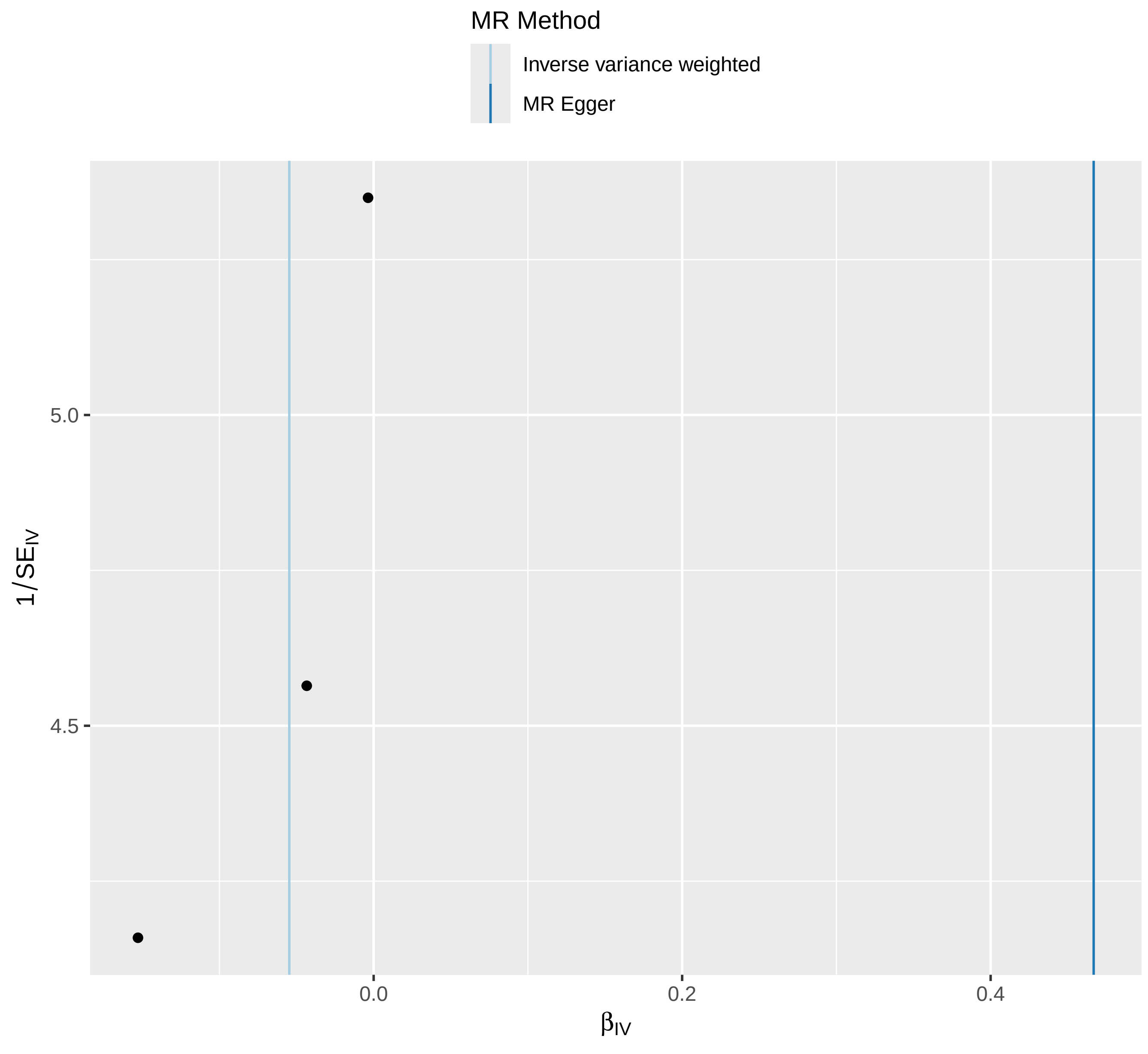
MR Method
Inverse variance weighted
MR Egger
5.0
4.5
1/SE_IV
0.0
0.2
0.4
β_IV

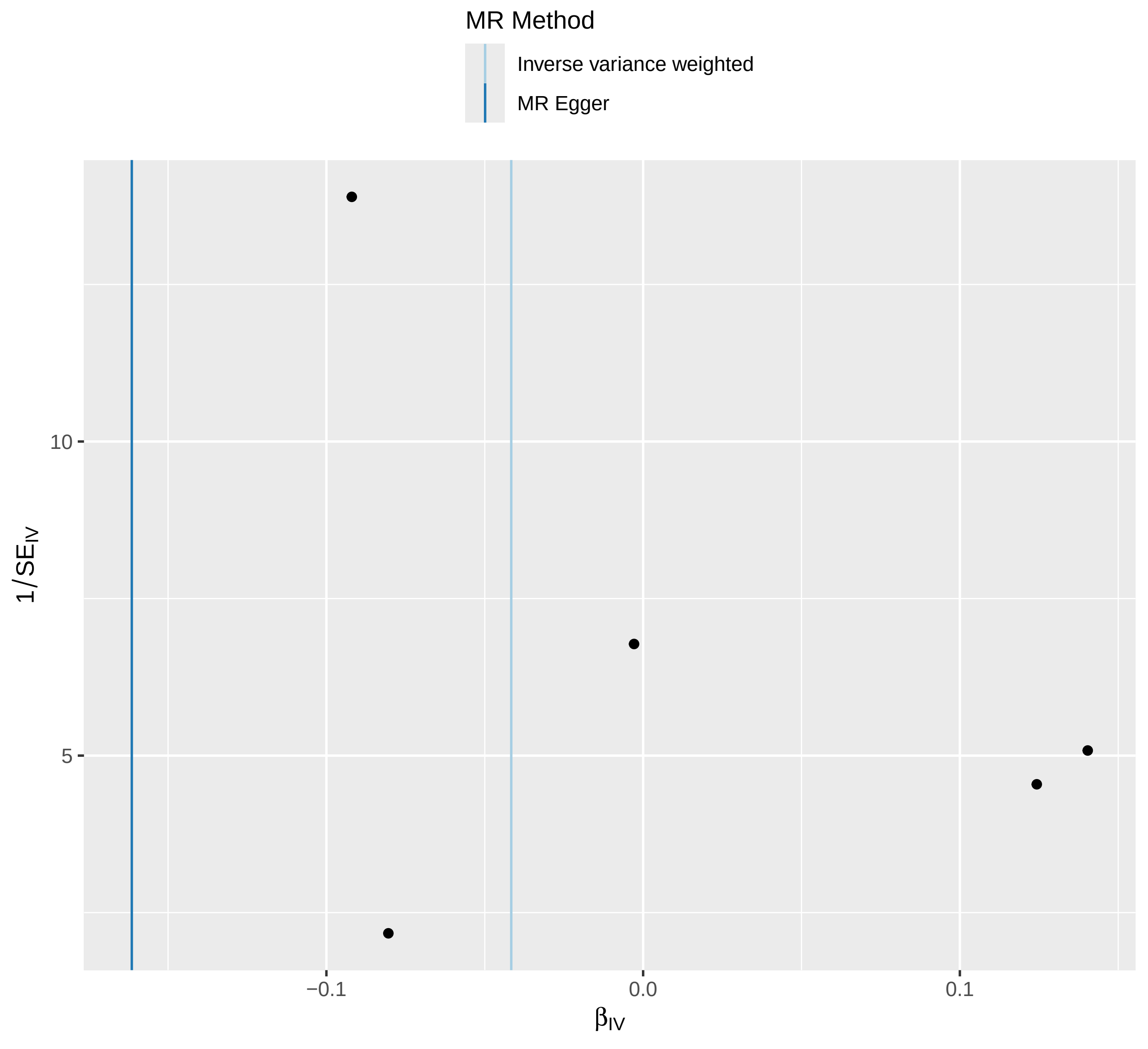
MR Method
Inverse variance weighted
MR Egger
10
5
1/SE$_{IV}$
−0.1
0.0
0.1
β$_{IV}$

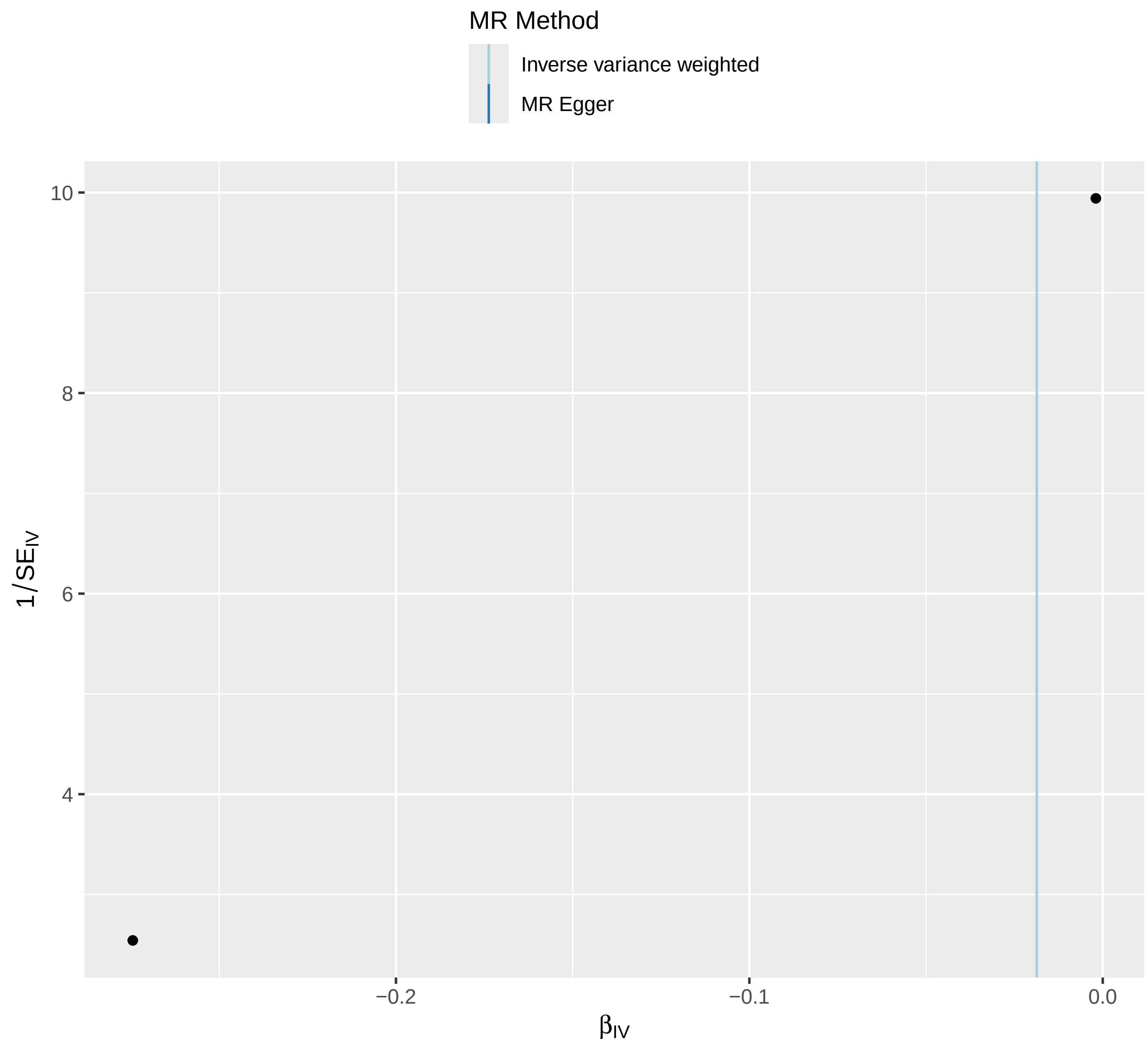
MR Method
Inverse variance weighted
MR Egger
10
8
6
4
1/SE_IV
−0.2
−0.1
0.0
β_IV

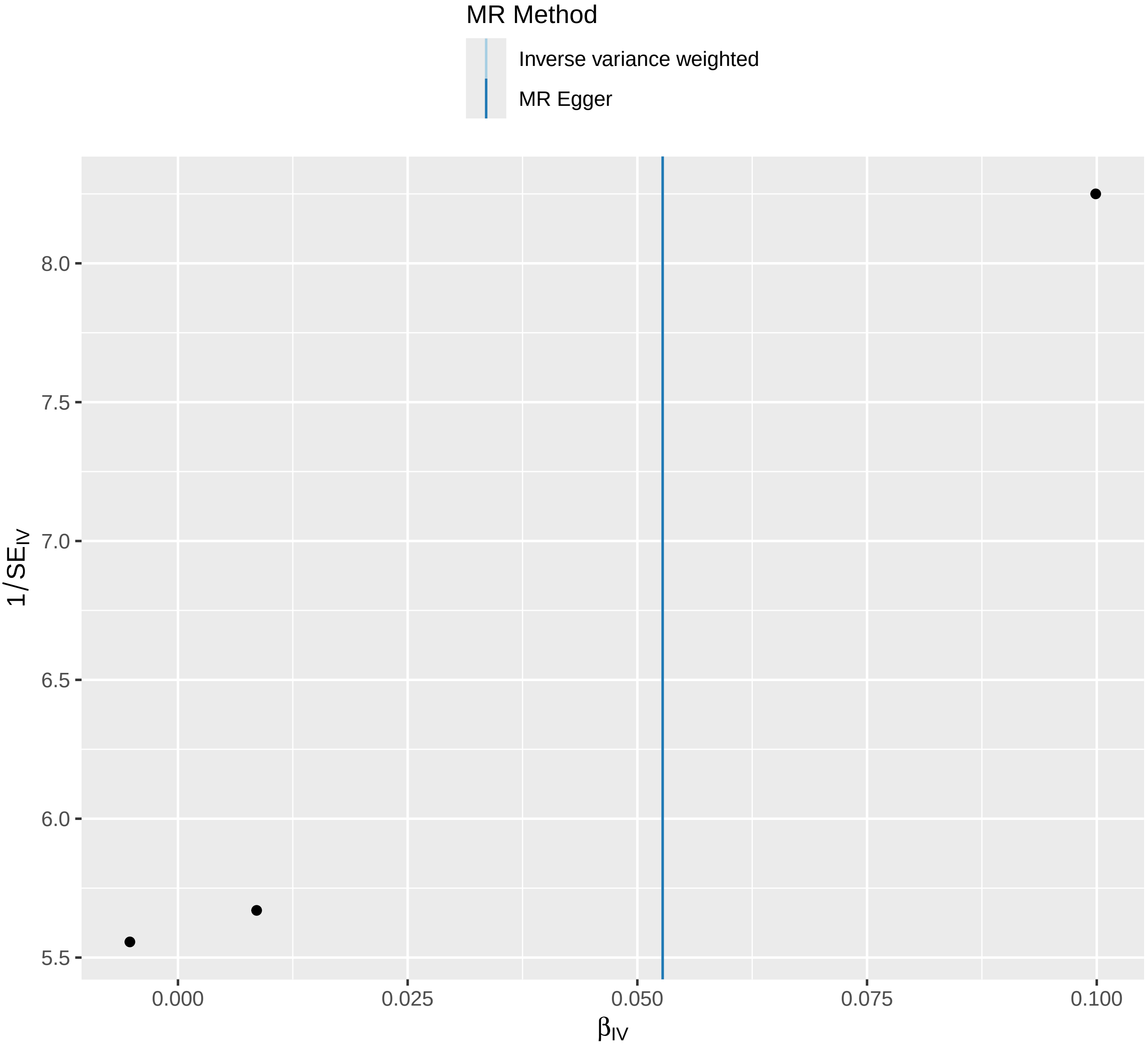

MR Method
Inverse variance weighted
MR Egger
8.0
7.5
7.0
6.5
6.0
5.5
1/SE_IV
0.000
0.025
0.050
0.075
0.100
β_IV

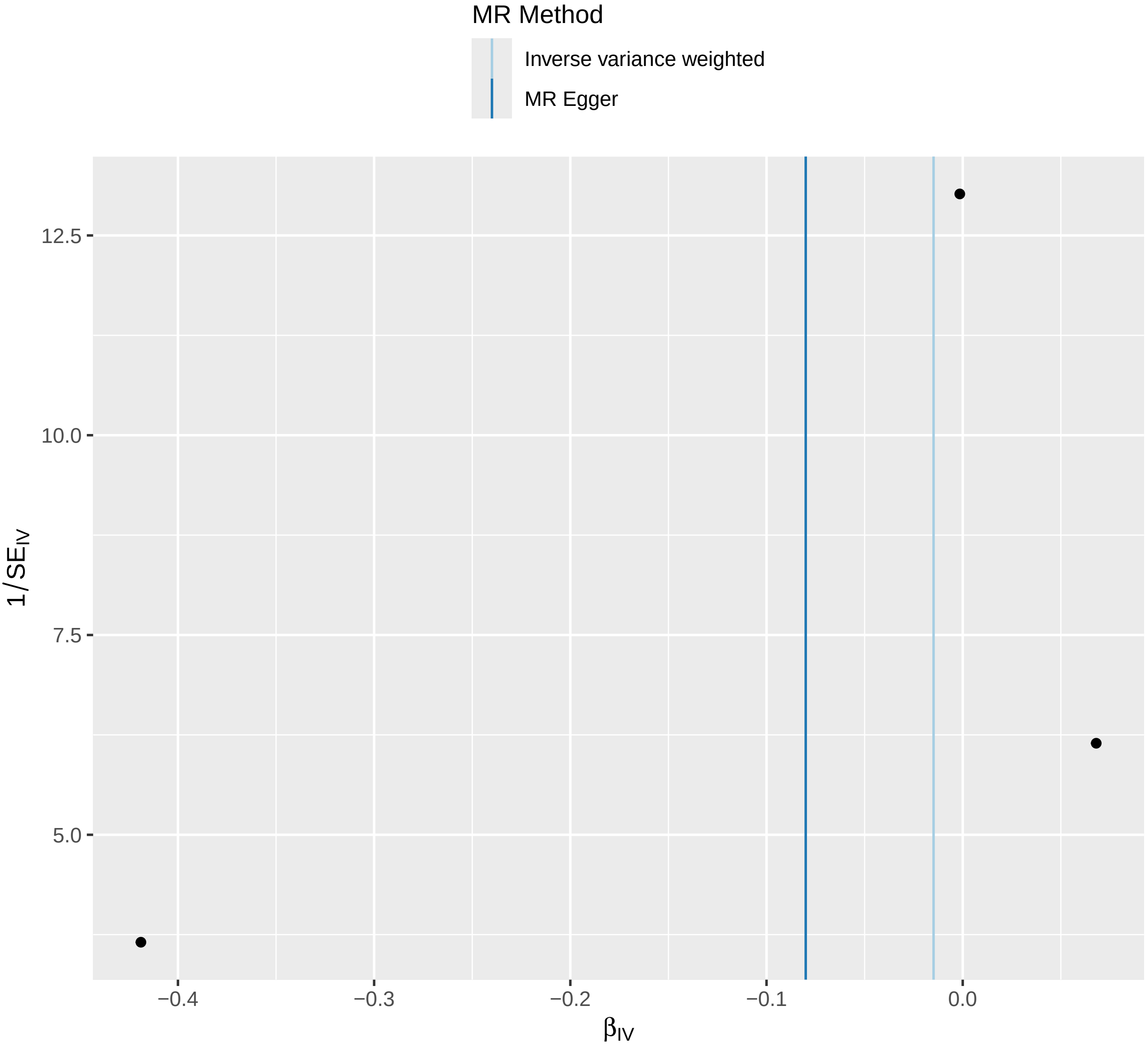
MR Method
Inverse variance weighted
MR Egger
12.5
10.0
7.5
5.0
1/SE_IV
−0.4
−0.3
−0.2
−0.1
0.0
β_IV

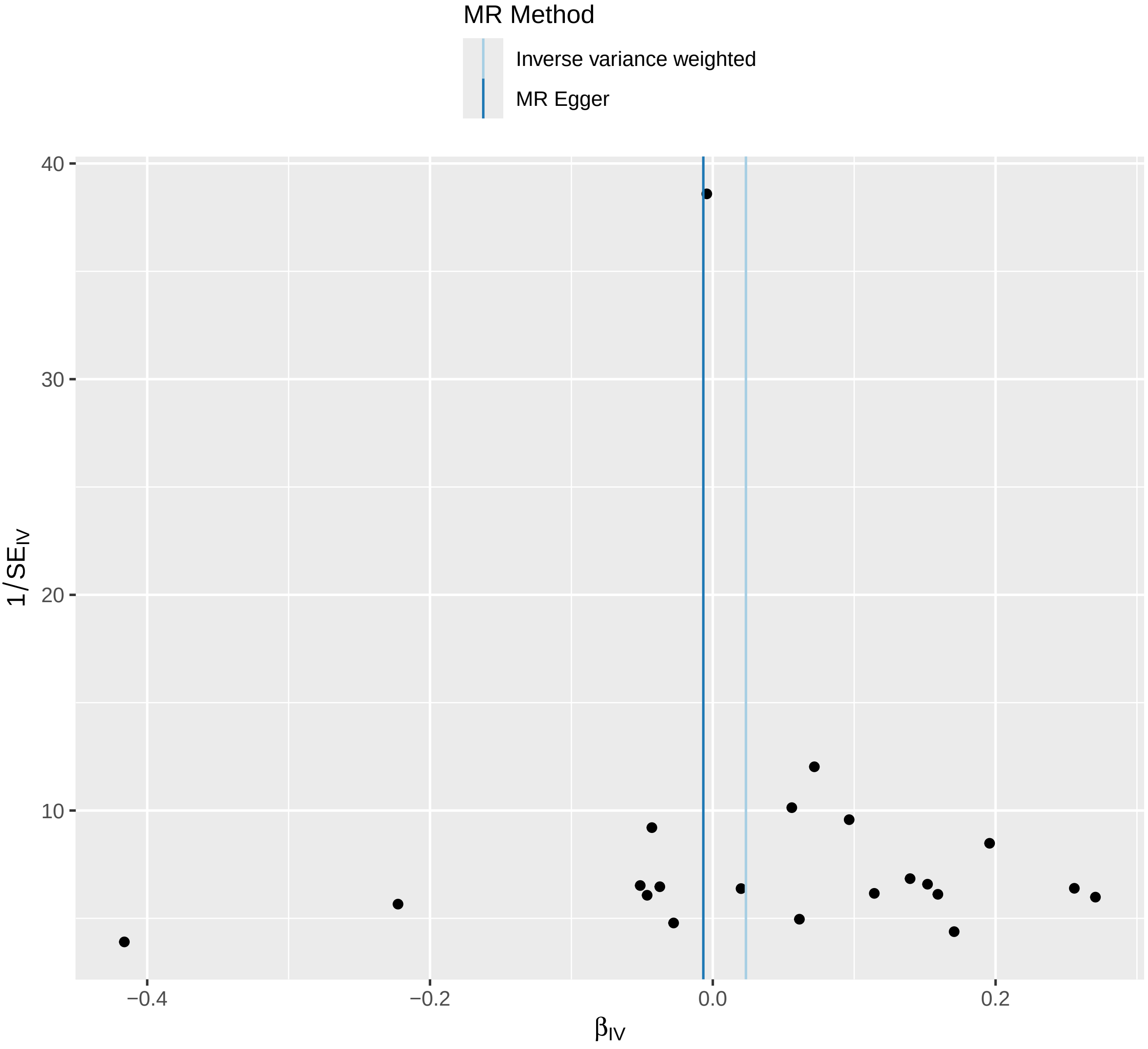

MR Method
Inverse variance weighted
MR Egger
40
30
20
10
1/SE$_{IV}$
−0.4
−0.2
0.0
0.2
β$_{IV}$

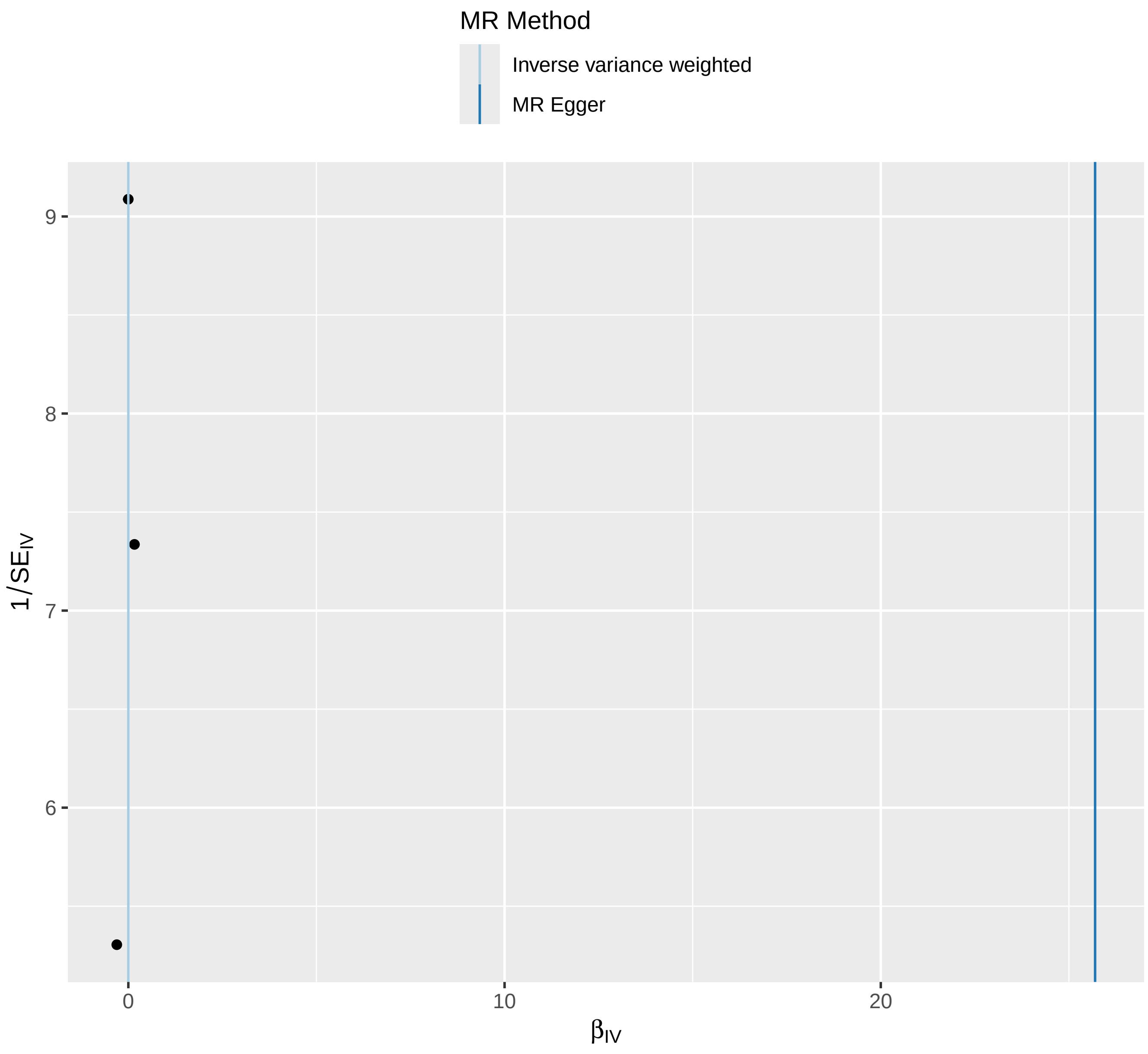

MR Method
Inverse variance weighted
MR Egger
9
8
7
6
1/SE_IV
0
10
20
β_IV

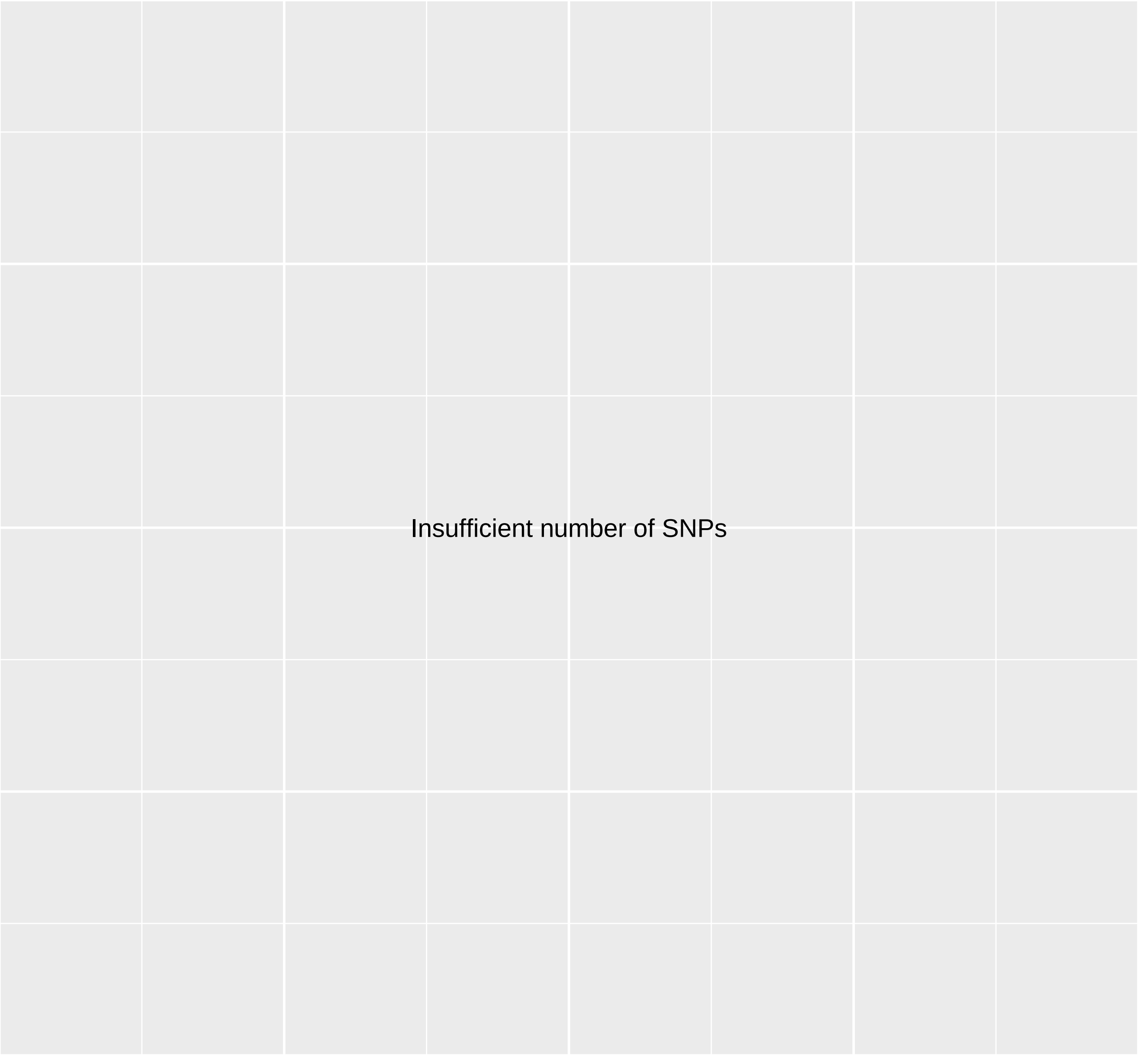
Insufficient number of SNPs

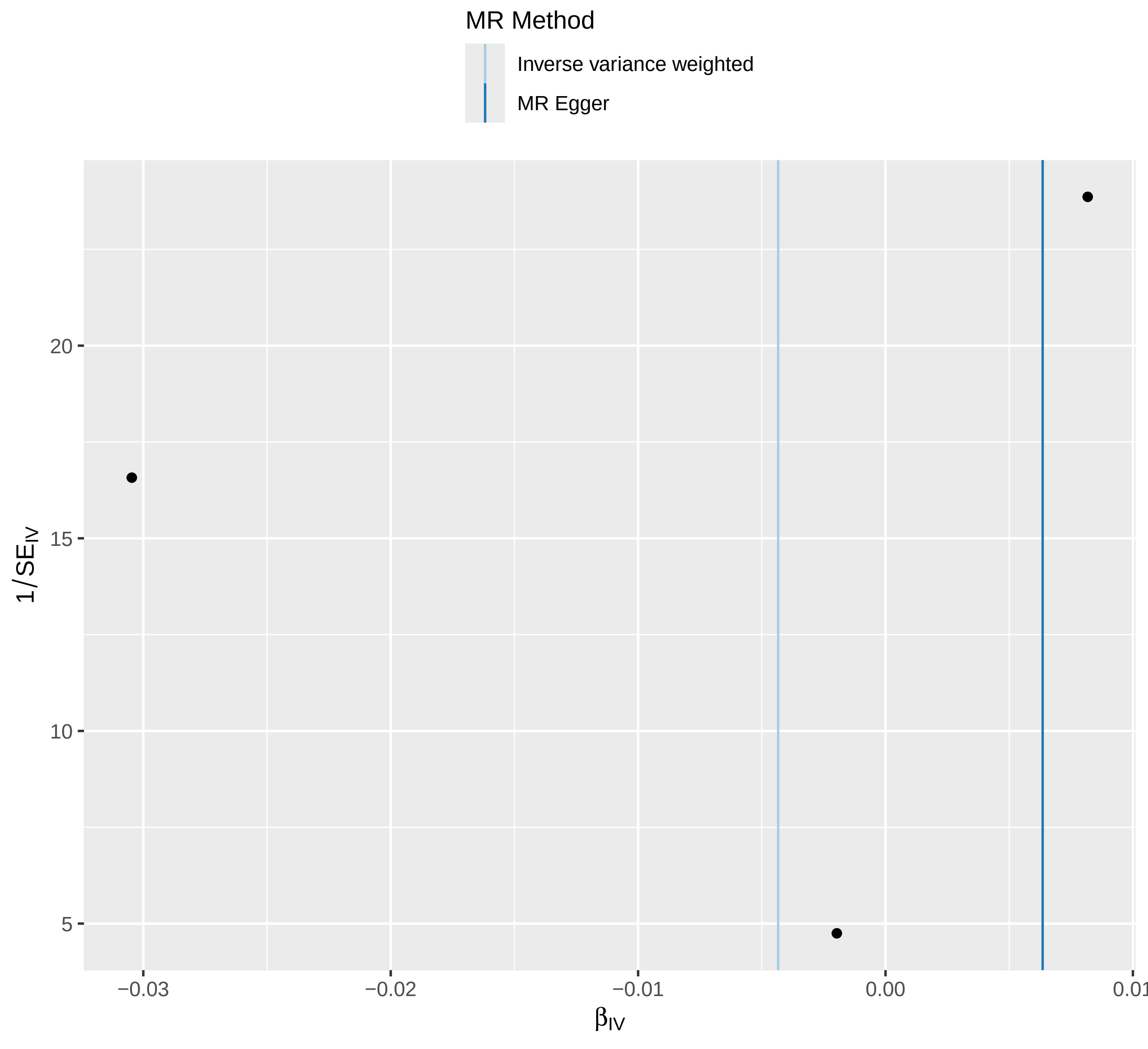
MR Method
Inverse variance weighted
MR Egger
20
15
10
5
1/SE_IV
−0.03
−0.02
−0.01
0.00
0.01
β_IV

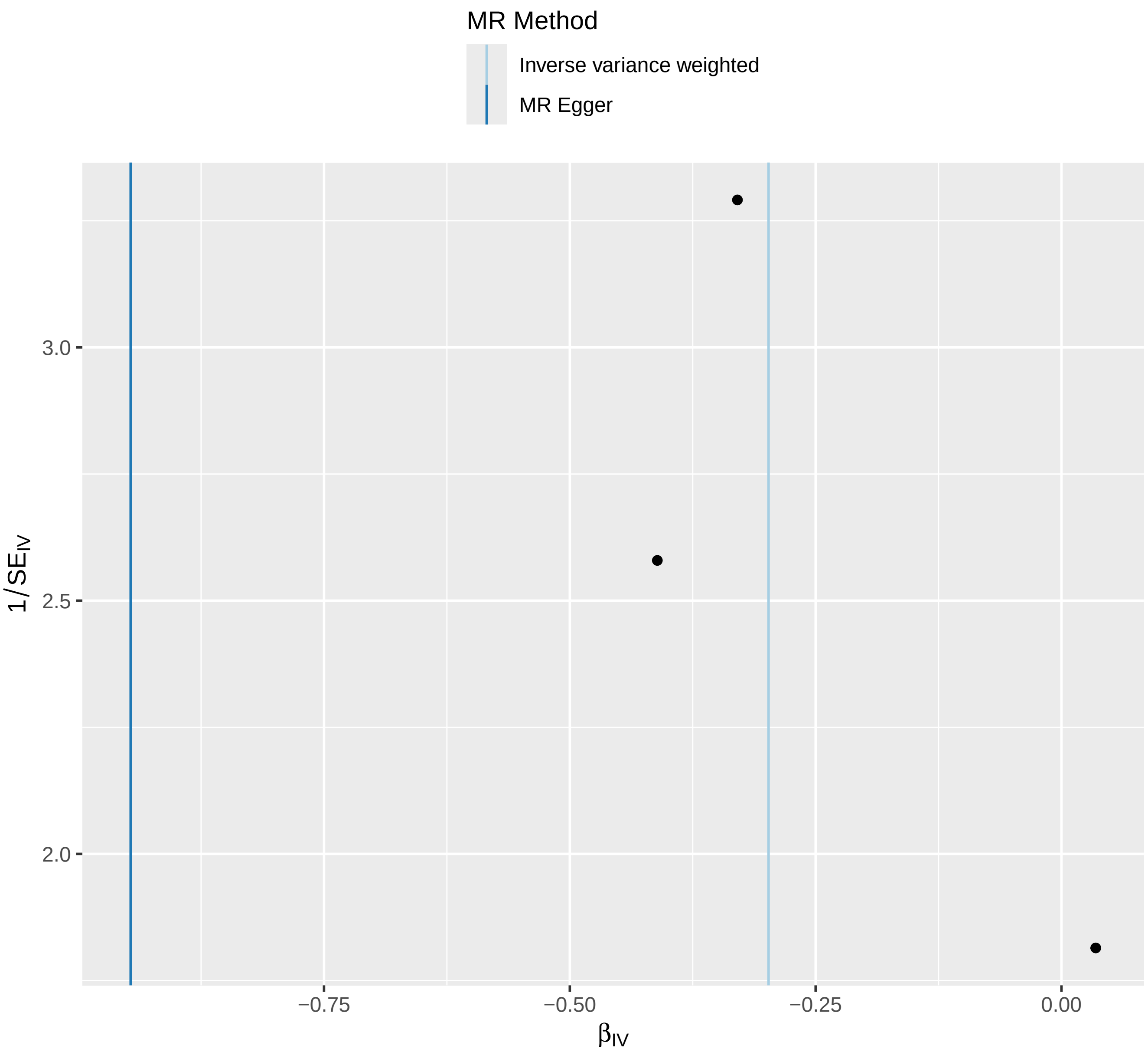
MR Method
Inverse variance weighted
MR Egger
3.0
2.5
2.0
1/$SE_{IV}$
−0.75
−0.50
−0.25
0.00
$\beta_{IV}$

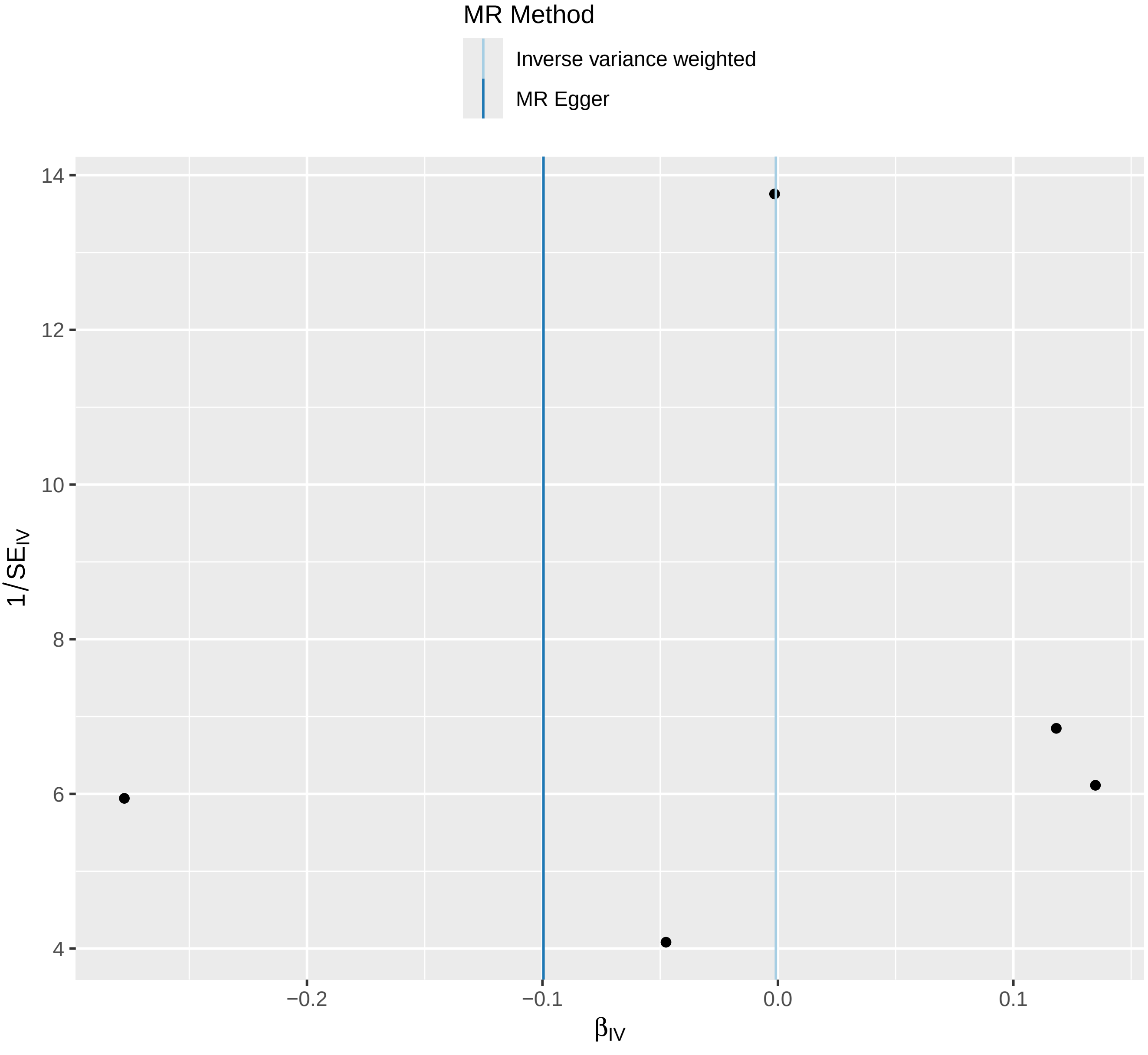

MR Method
Inverse variance weighted
MR Egger
14
12
10
8
6
4
1/SE$_{IV}$
−0.2
−0.1
0.0
0.1
β$_{IV}$

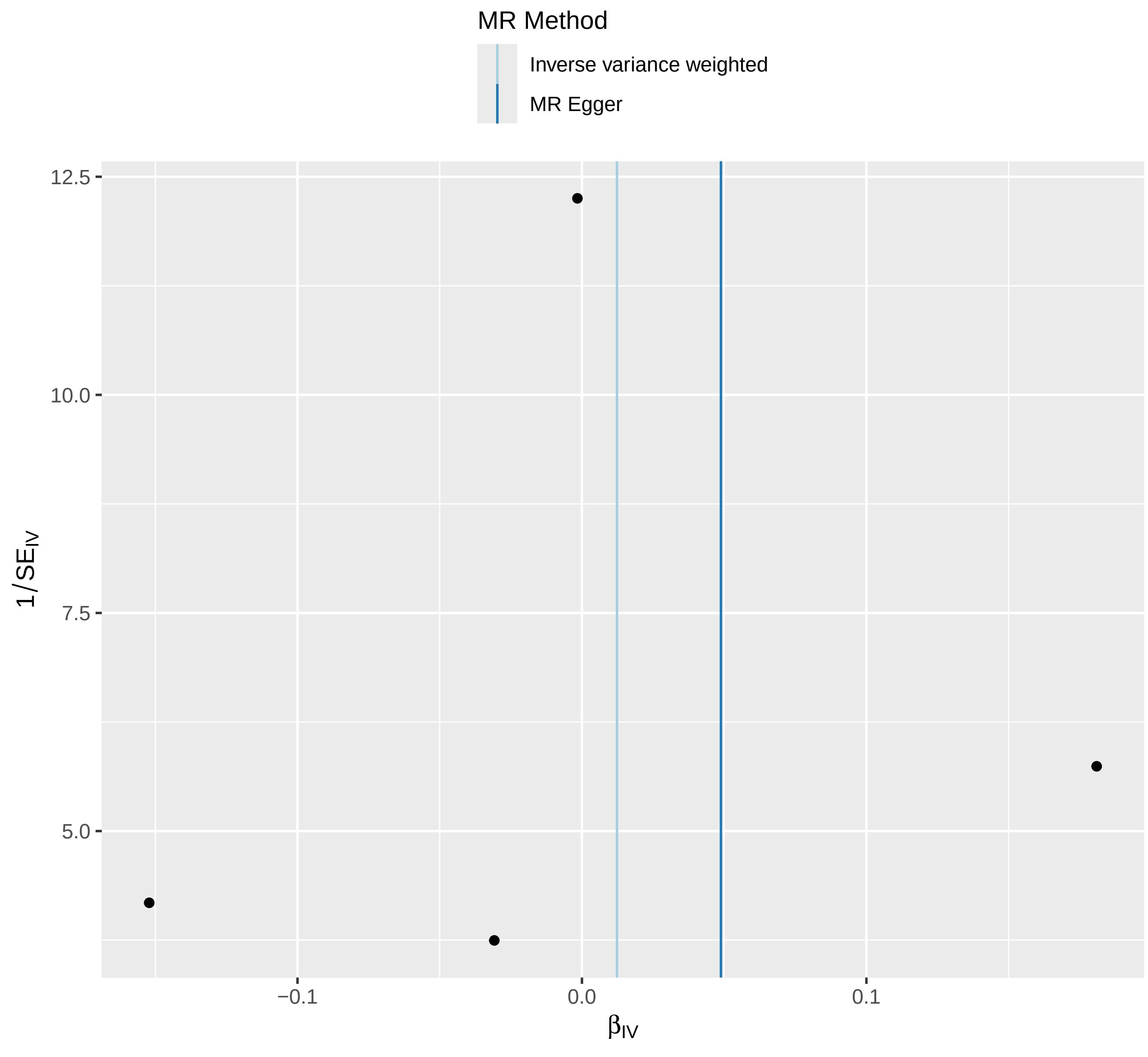
MR Method
Inverse variance weighted
MR Egger
12.5
10.0
7.5
5.0
1/SE$_{IV}$
−0.1
0.0
0.1
β$_{IV}$

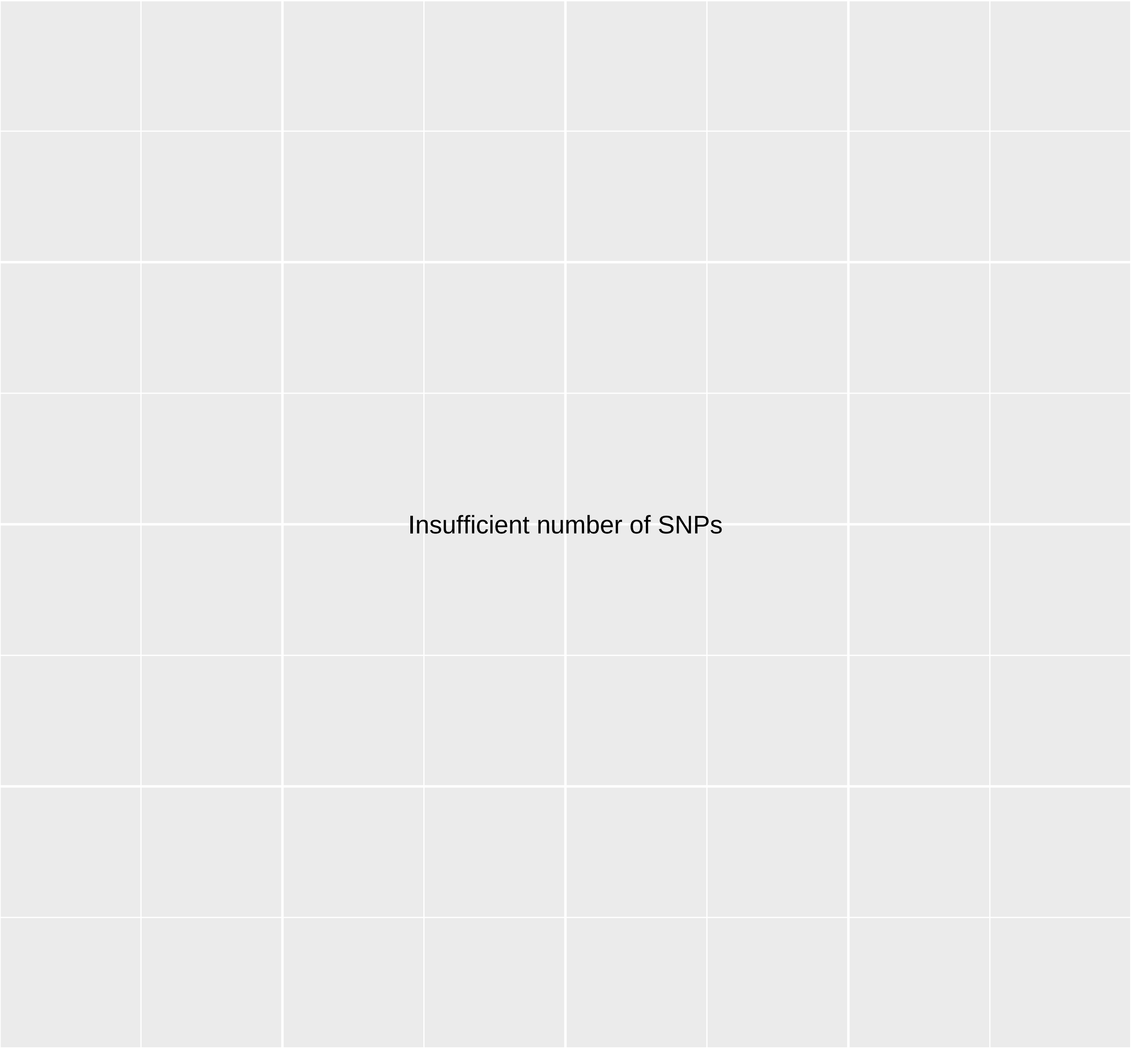
Insufficient number of SNPs

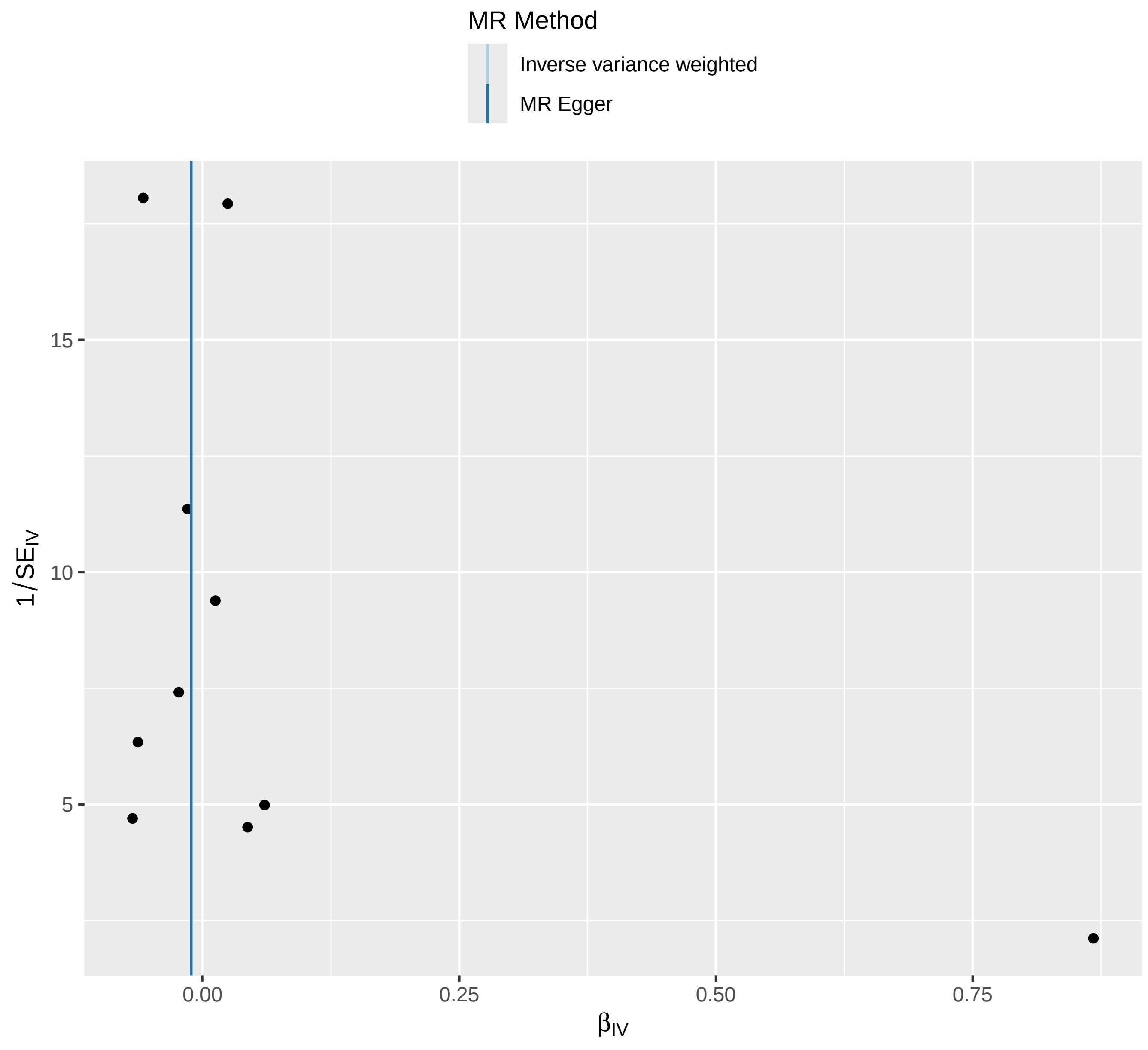
MR Method
Inverse variance weighted
MR Egger
1/SE_IV
15
10
5
0.00
0.25
0.50
0.75
β_IV

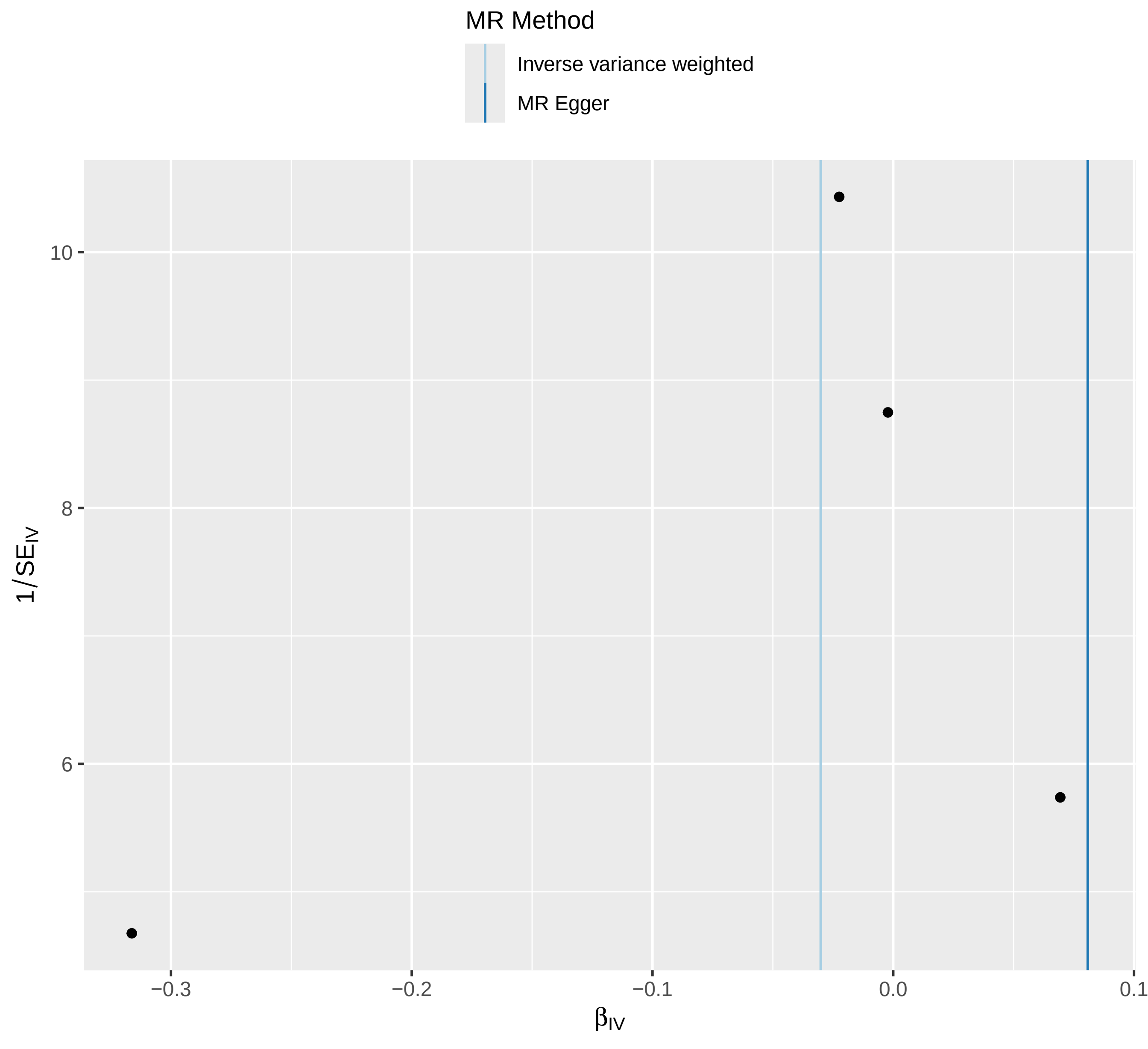
MR Method
Inverse variance weighted
MR Egger
10
8
6
$1/SE_{IV}$
−0.3
−0.2
−0.1
0.0
0.1
$\beta_{IV}$

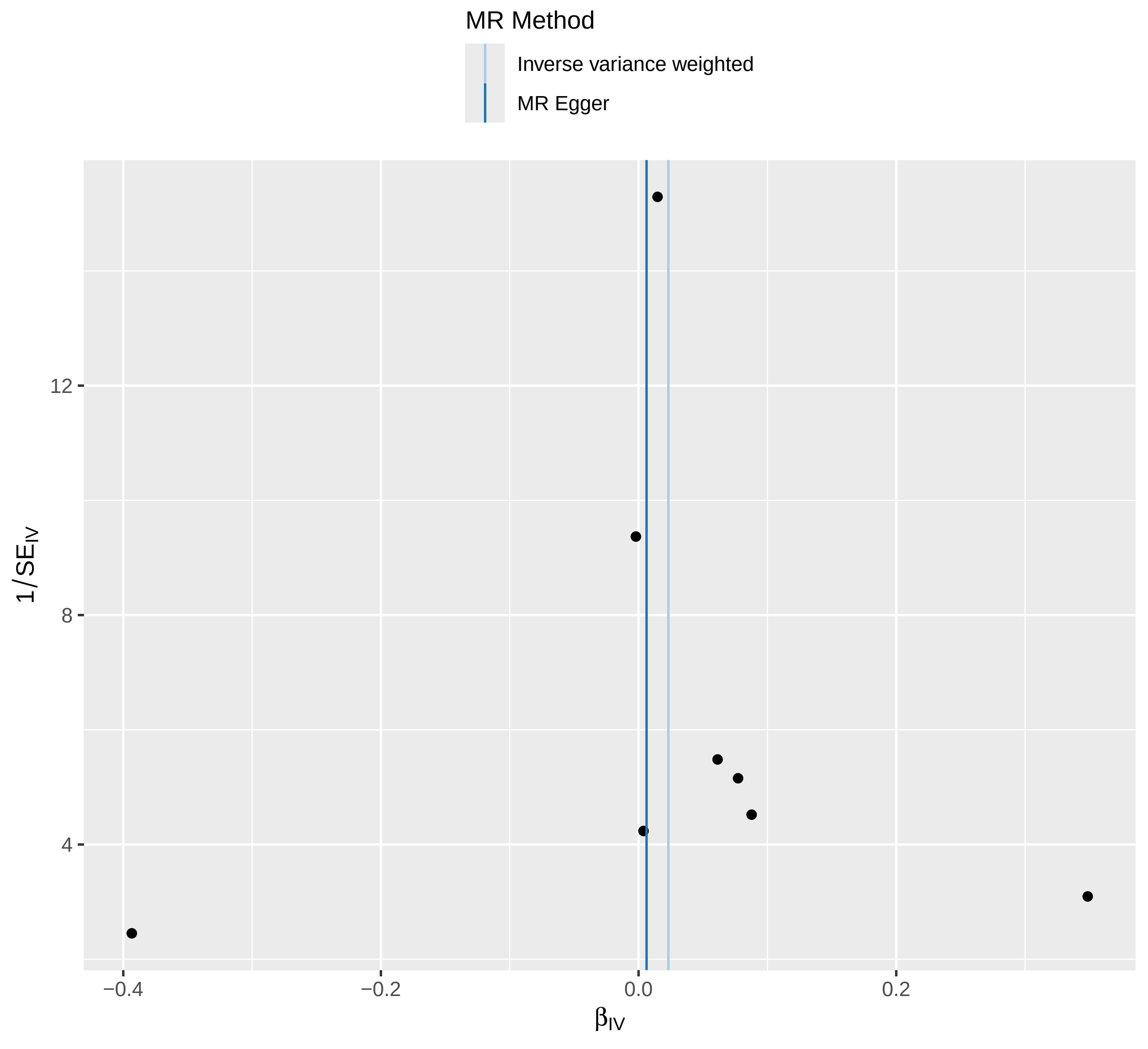

MR Method
Inverse variance weighted
MR Egger
12
8
4
1/SE$_{IV}$
−0.4
−0.2
0.0
0.2
β$_{IV}$

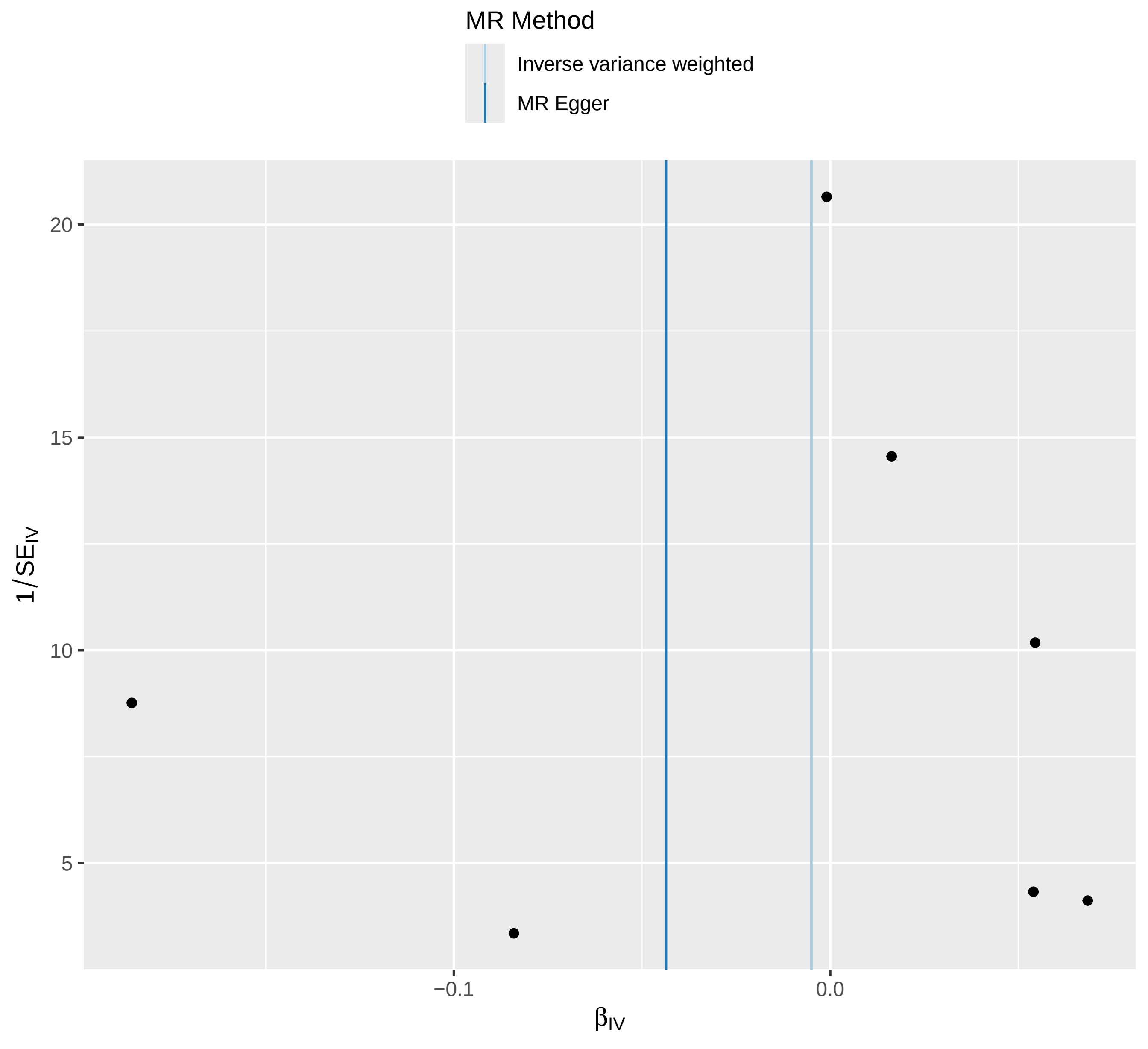
MR Method
Inverse variance weighted
MR Egger
20
15
10
5
1/SE_IV
−0.1
0.0
β_IV

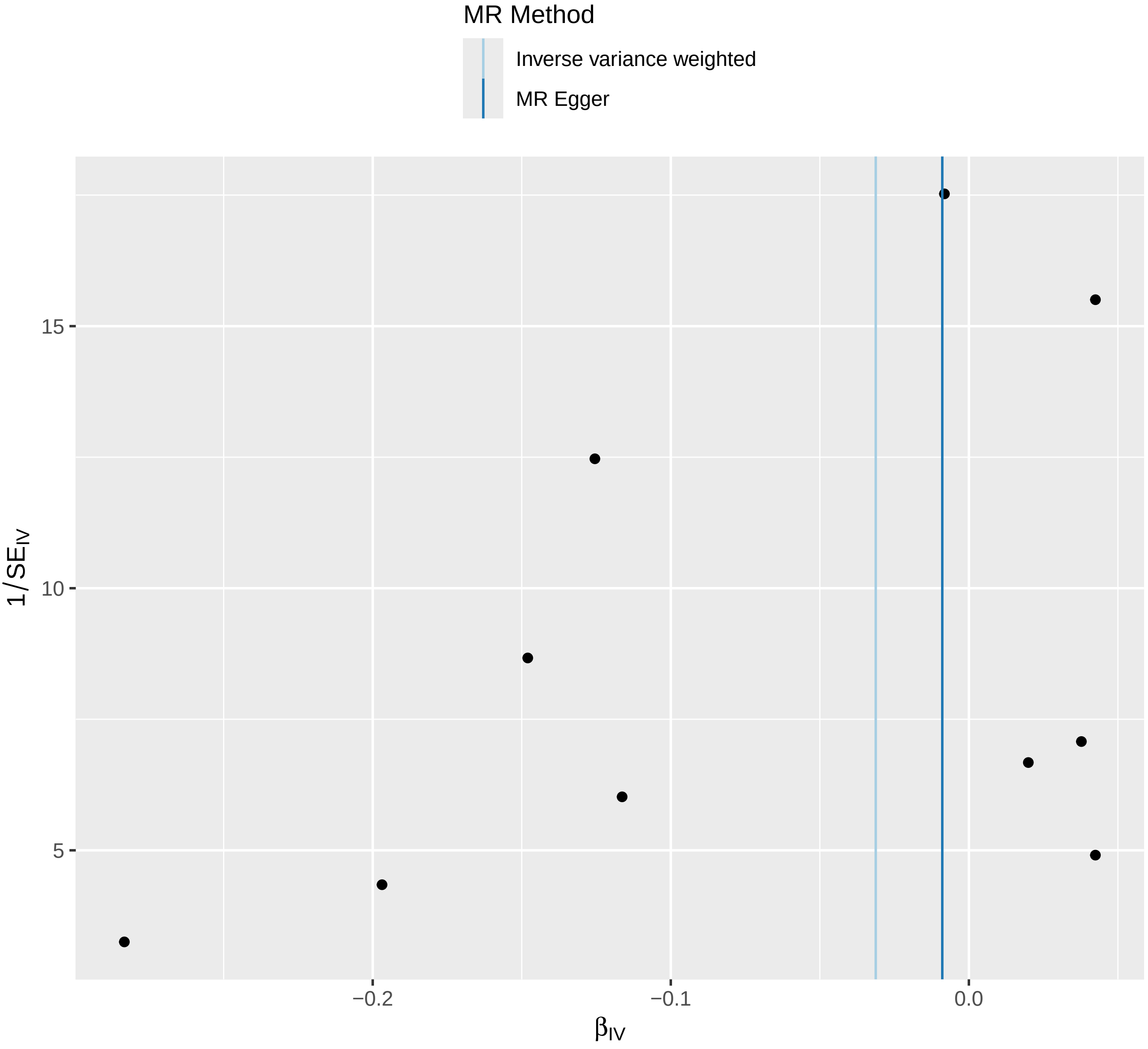

MR Method
Inverse variance weighted
MR Egger
15
10
5
1/SE$_{IV}$
−0.2
−0.1
0.0
β$_{IV}$

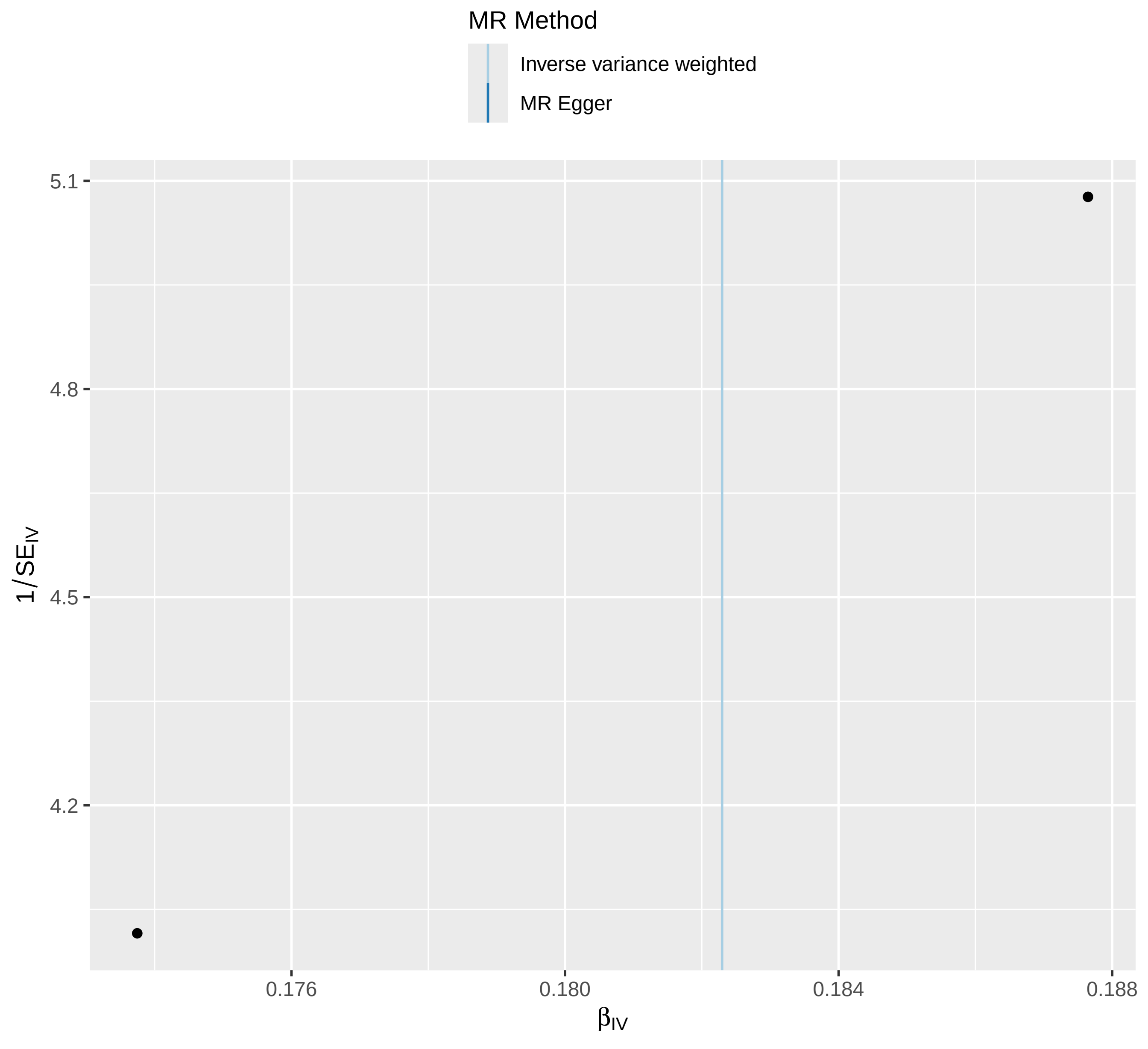
MR Method
Inverse variance weighted
MR Egger
5.1
4.8
4.5
4.2
1/SE_IV
0.176
0.180
0.184
0.188
β_IV

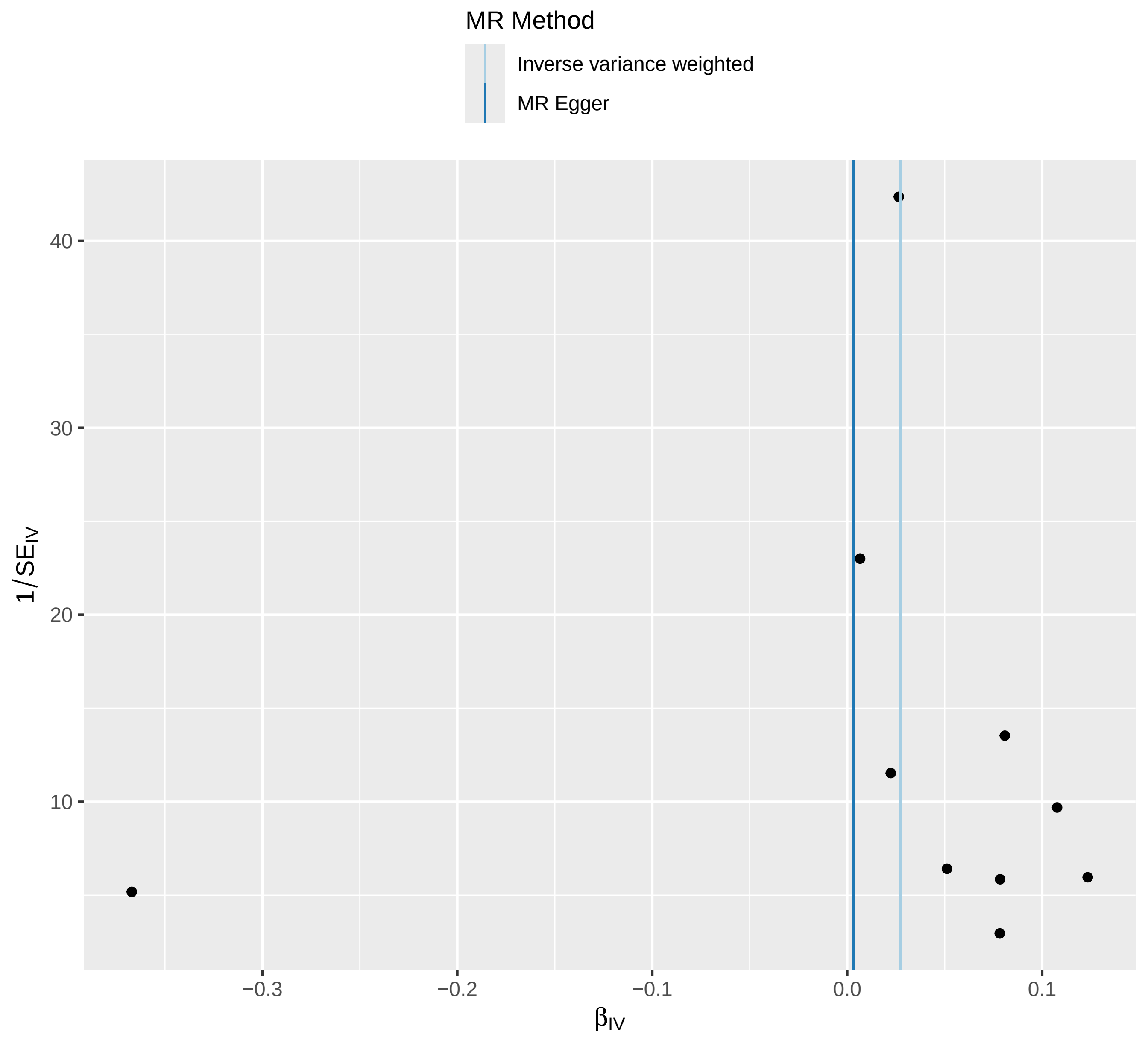

MR Method
Inverse variance weighted
MR Egger
40
30
20
10
1/SE_IV
−0.3
−0.2
−0.1
0.0
0.1
β_IV

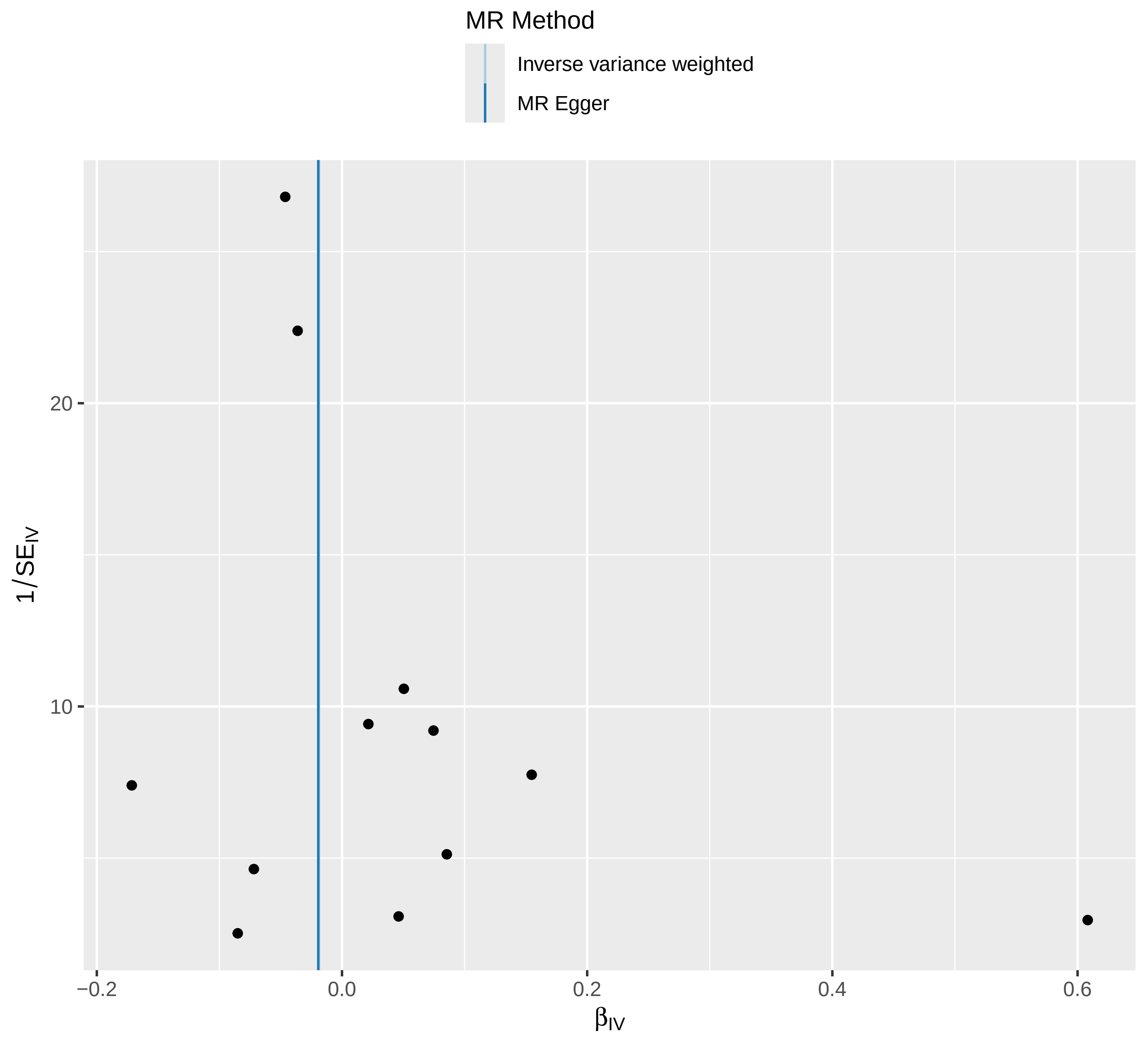
MR Method
Inverse variance weighted
MR Egger
20
10
$1/SE_{IV}$
−0.2
0.0
0.2
0.4
0.6
$\beta_{IV}$

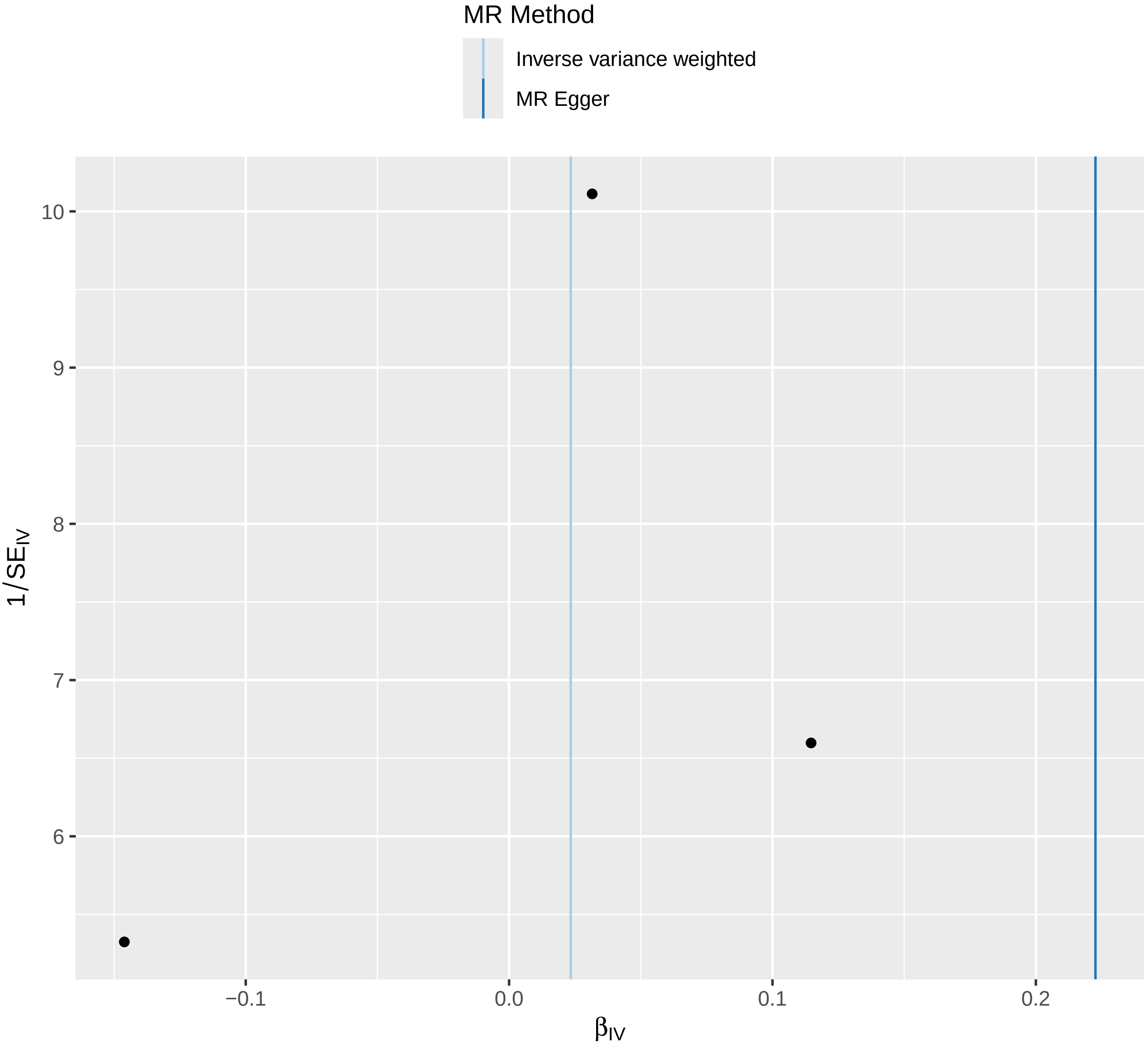

MR Method
Inverse variance weighted
MR Egger
10
9
8
7
6
1/SE_IV
−0.1
0.0
0.1
0.2
β_IV

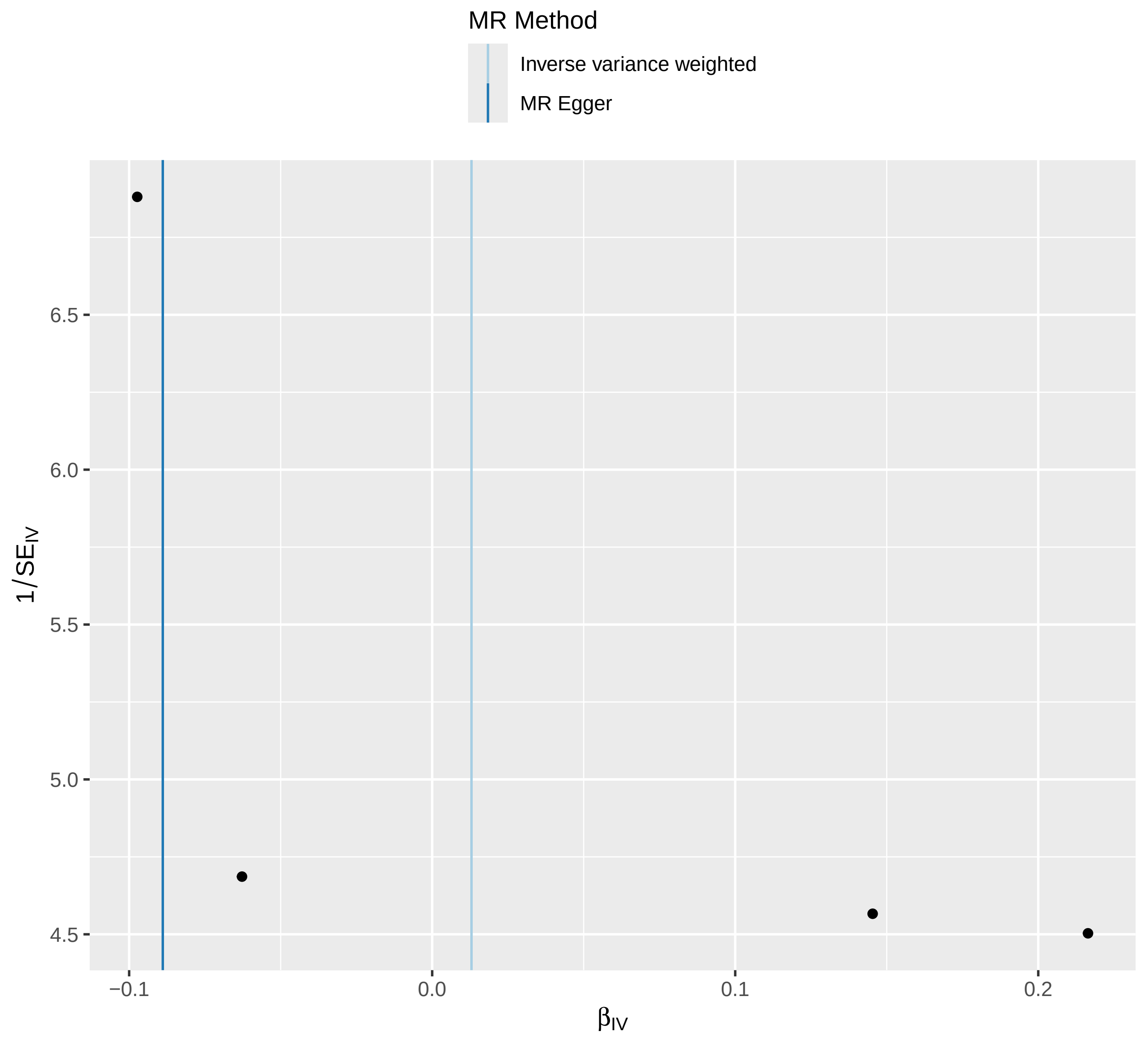
MR Method
Inverse variance weighted
MR Egger
1/SE$_{IV}$
6.5
6.0
5.5
5.0
4.5
−0.1
0.0
0.1
0.2
β$_{IV}$

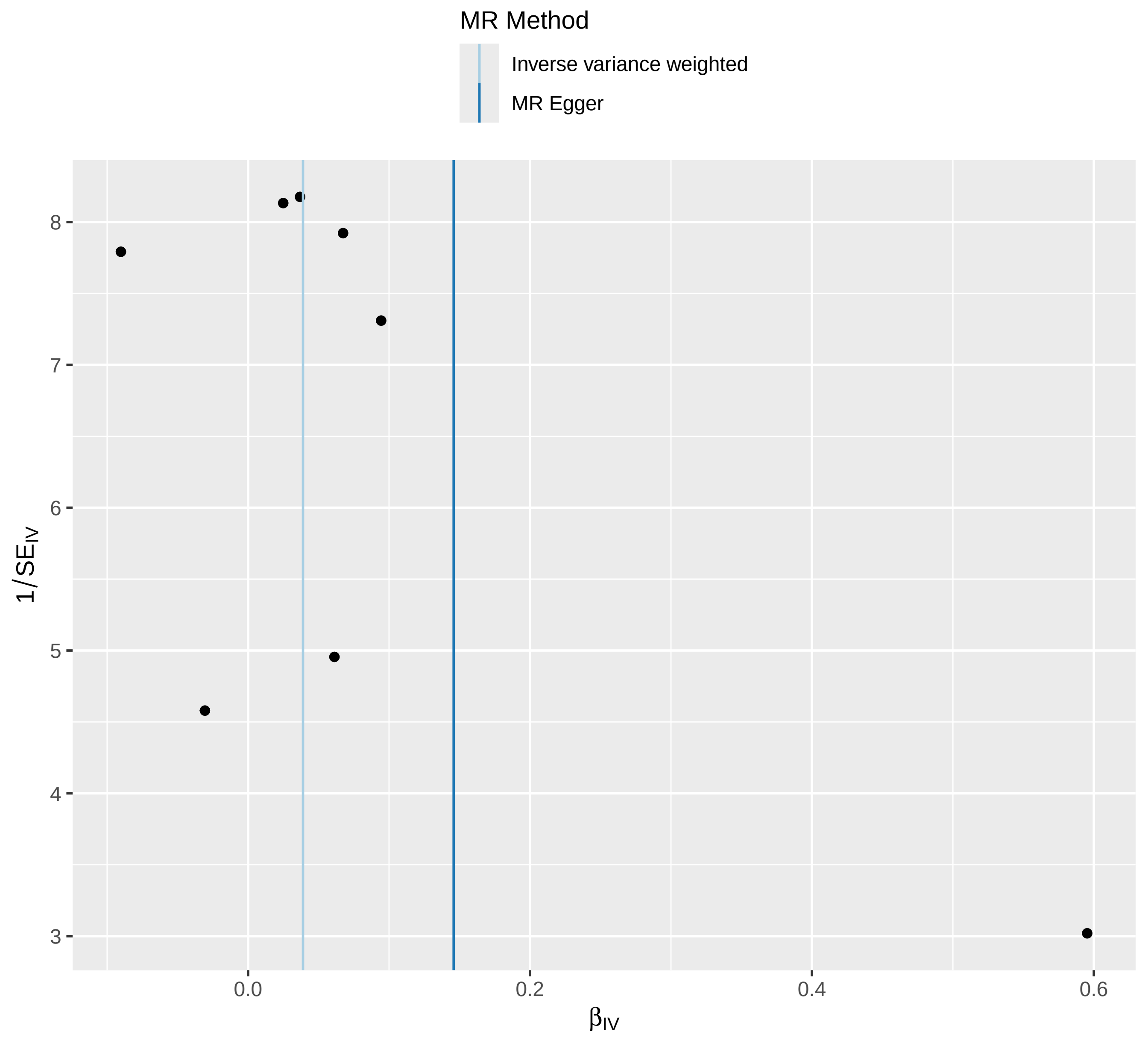

MR Method
Inverse variance weighted
MR Egger
1/SE_IV
8
7
6
5
4
3
0.0
0.2
0.4
0.6
β_IV

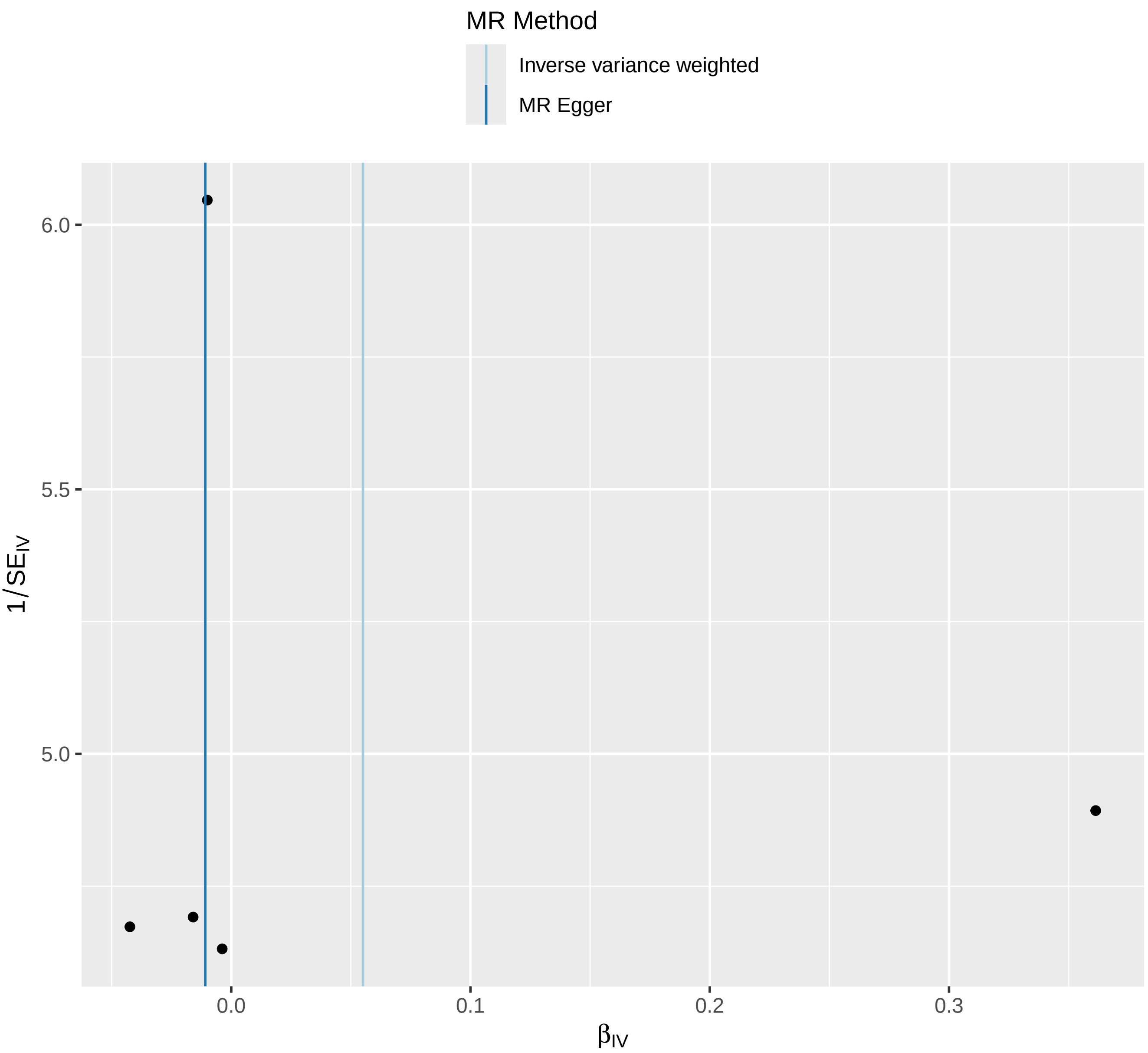

MR Method
Inverse variance weighted
MR Egger
6.0
5.5
5.0
1/SE_IV
0.0
0.1
0.2
0.3
β_IV

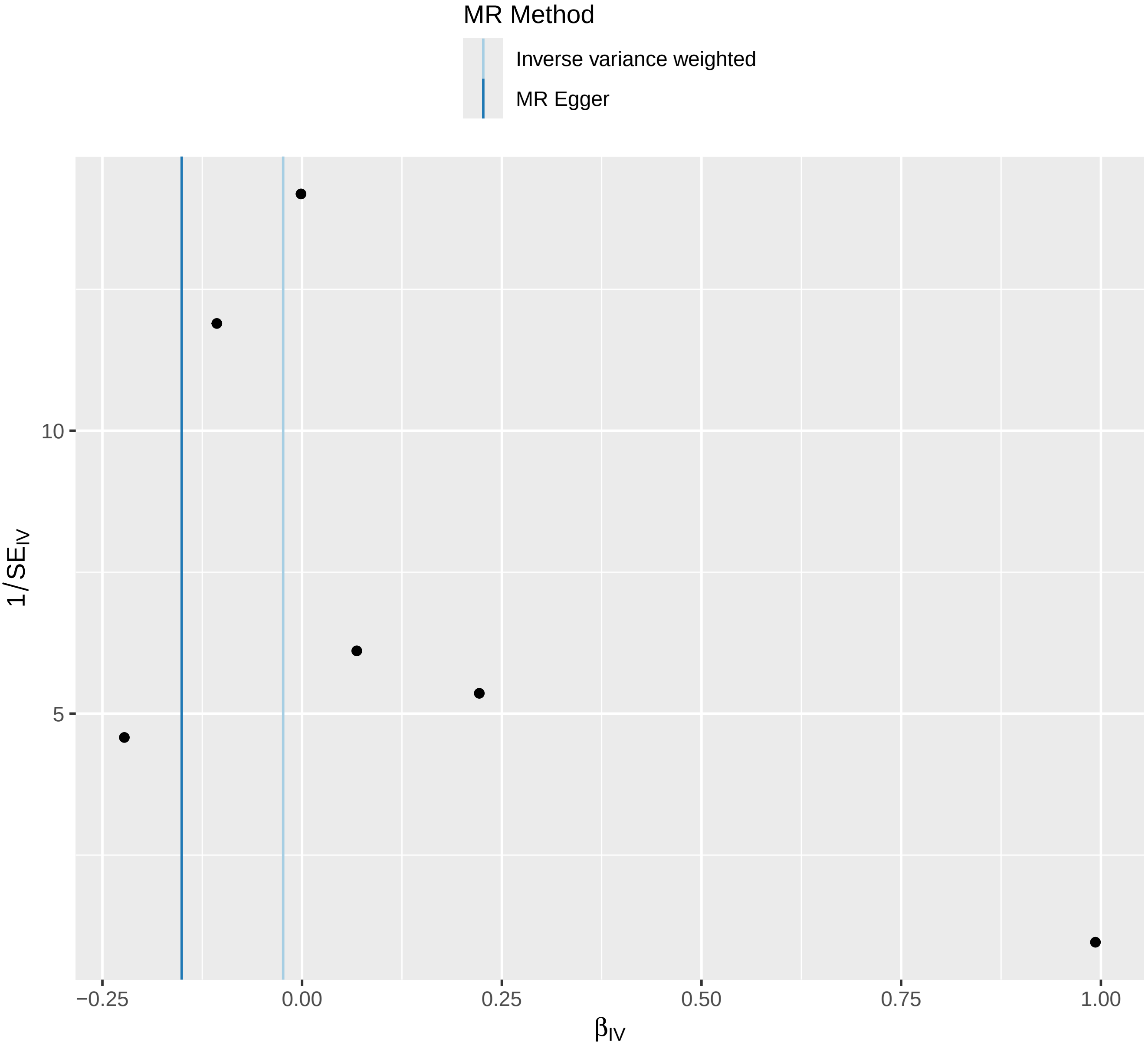

MR Method
Inverse variance weighted
MR Egger
10
5
1/SE$_{IV}$
−0.25
0.00
0.25
0.50
0.75
1.00
β$_{IV}$

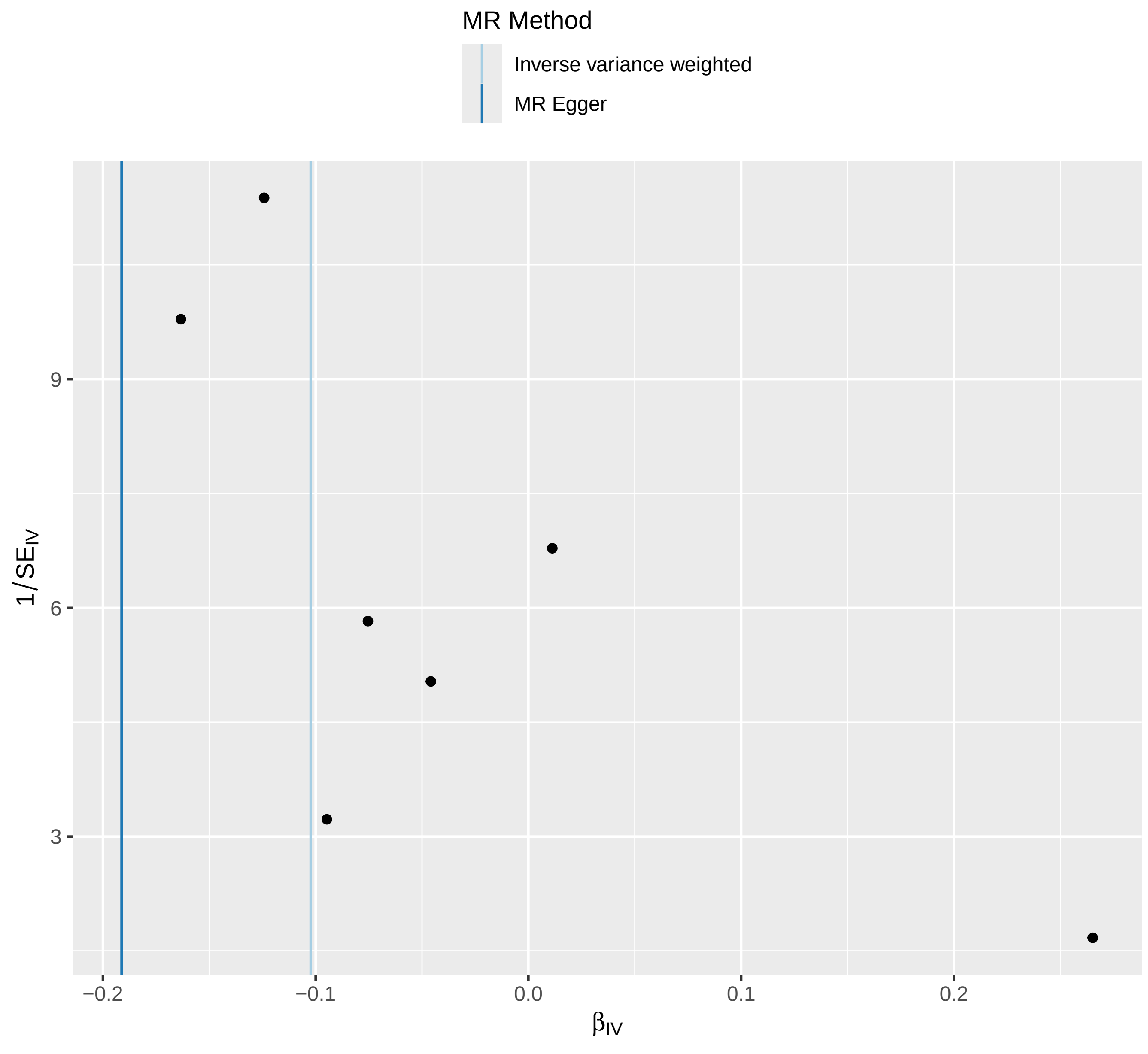
MR Method
Inverse variance weighted
MR Egger
1/SE_IV
9
6
3
−0.2
−0.1
0.0
0.1
0.2
β_IV

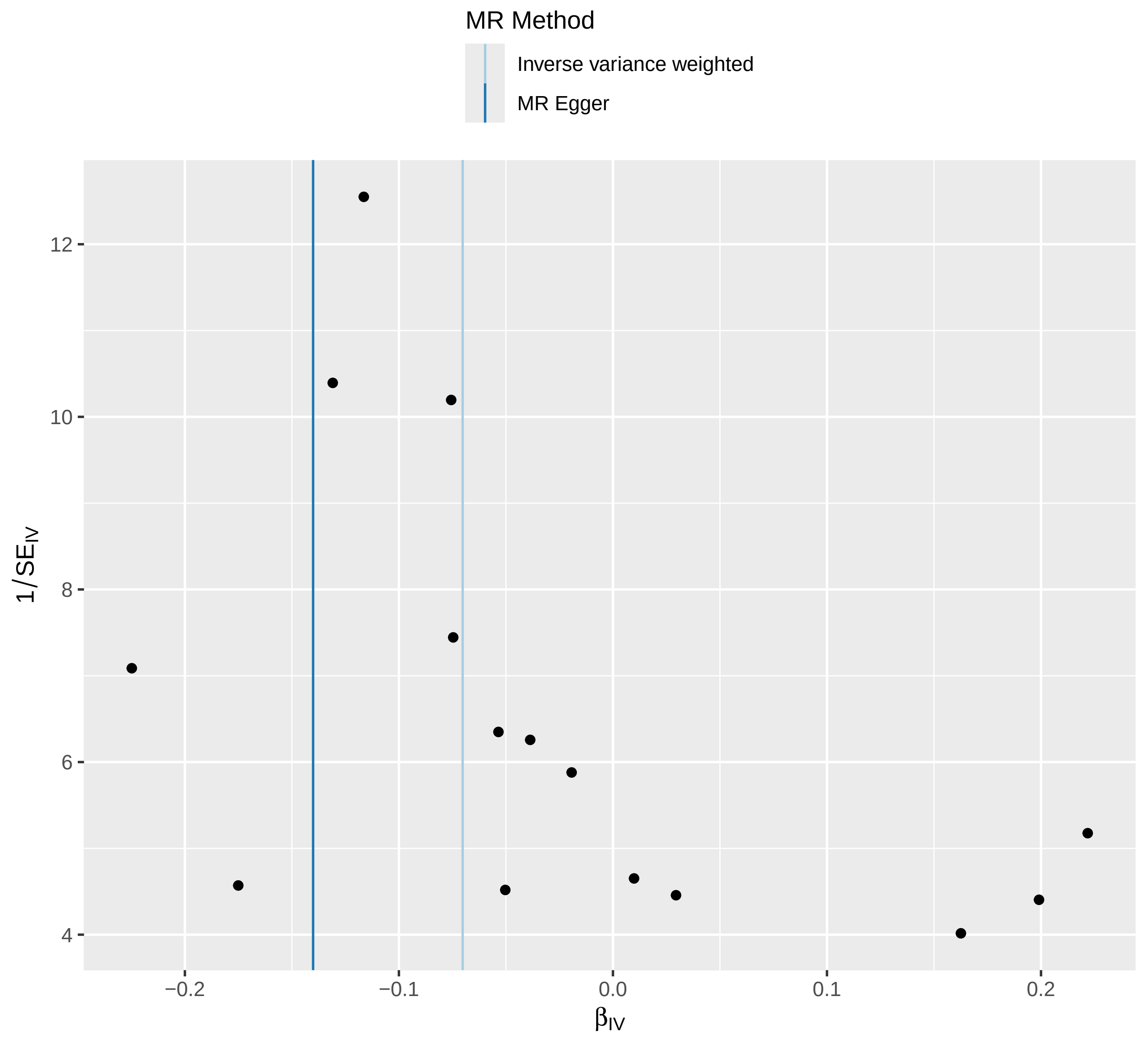
MR Method
Inverse variance weighted
MR Egger
12
10
8
6
4
1/SE$_{IV}$
−0.2
−0.1
0.0
0.1
0.2
β$_{IV}$

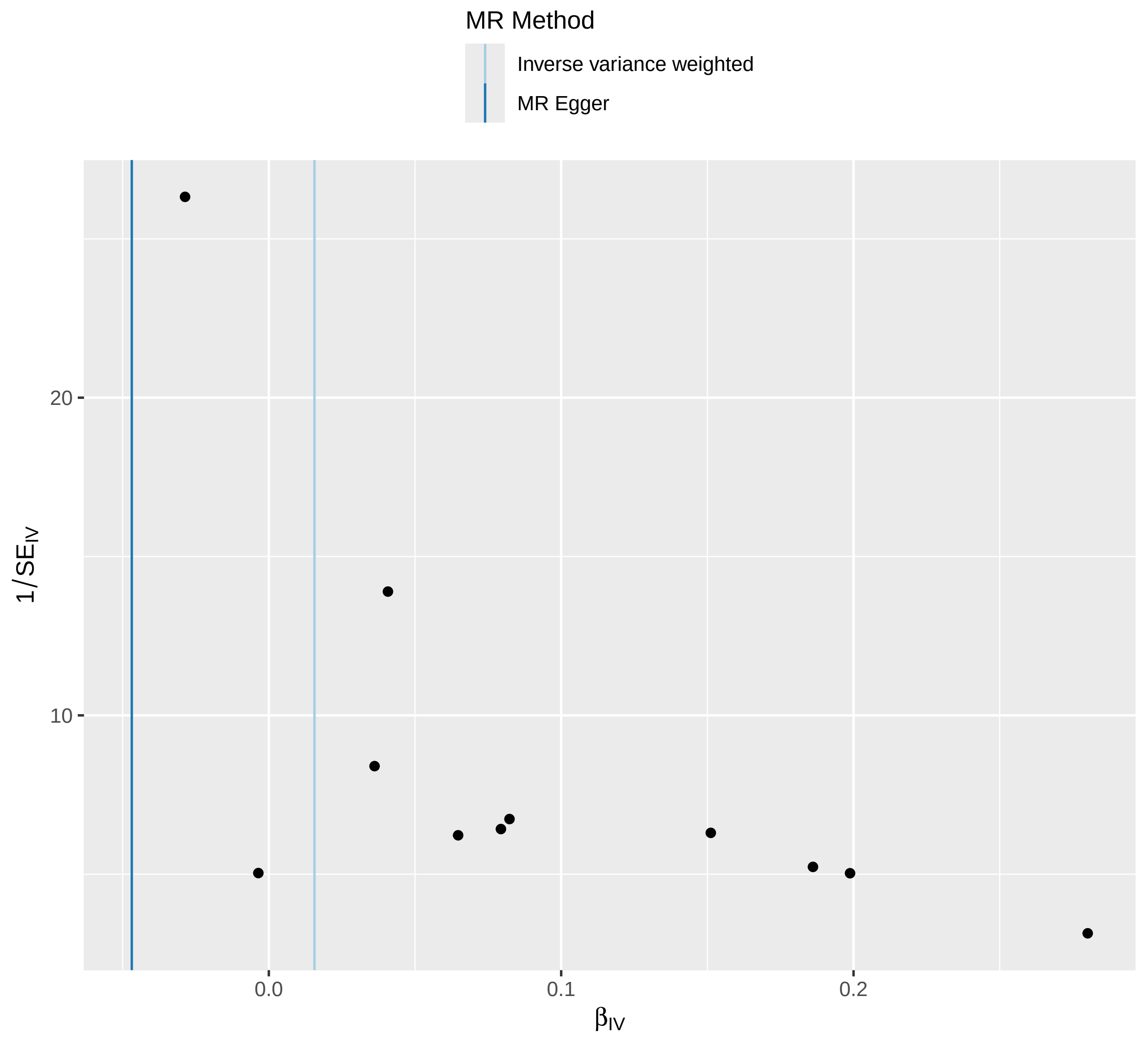
MR Method
Inverse variance weighted
MR Egger
20
10
1/SE_IV
0.0
0.1
0.2
β_IV

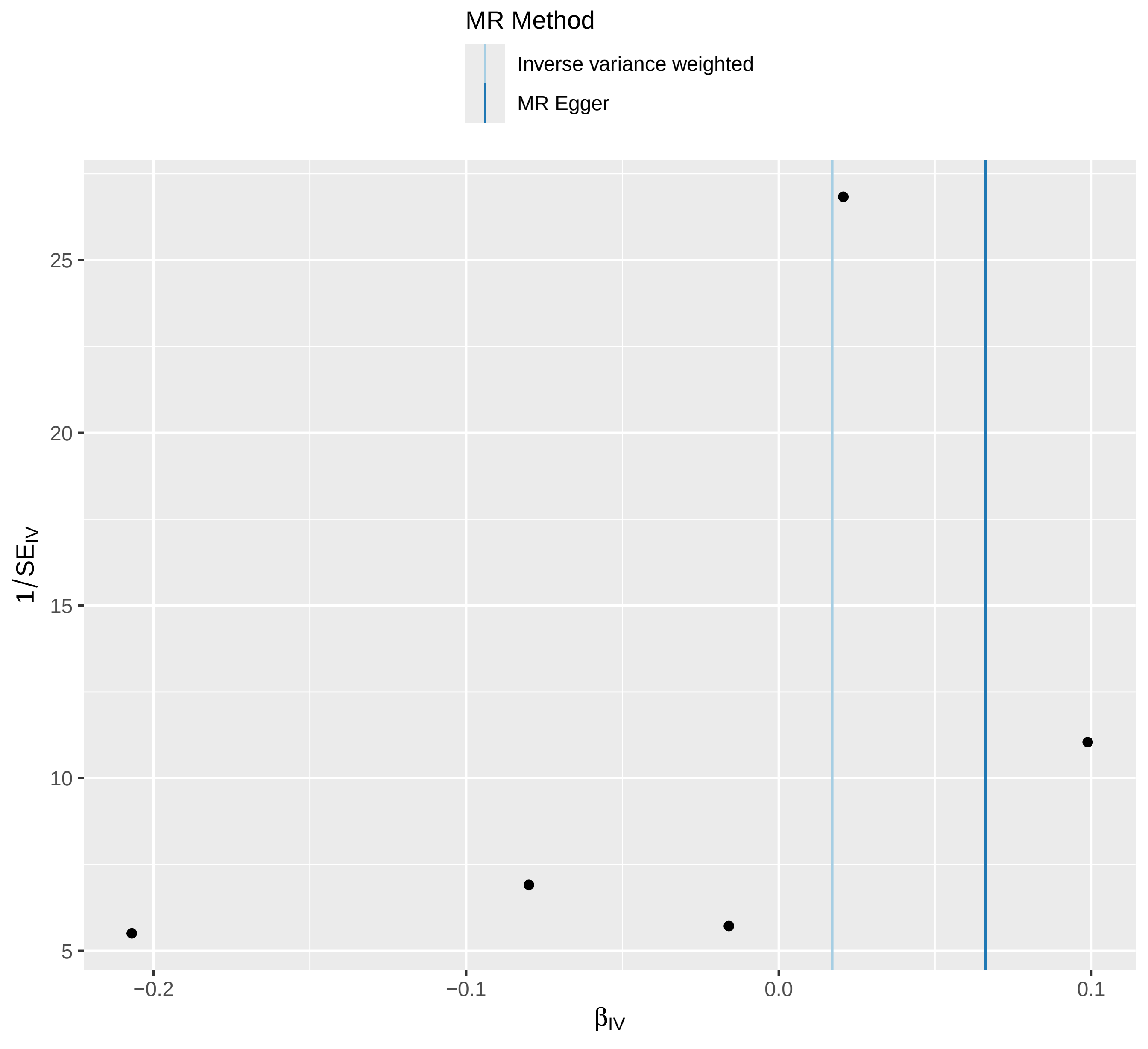
MR Method
Inverse variance weighted
MR Egger
1/SE_IV
25
20
15
10
5
−0.2
−0.1
0.0
0.1
β_IV

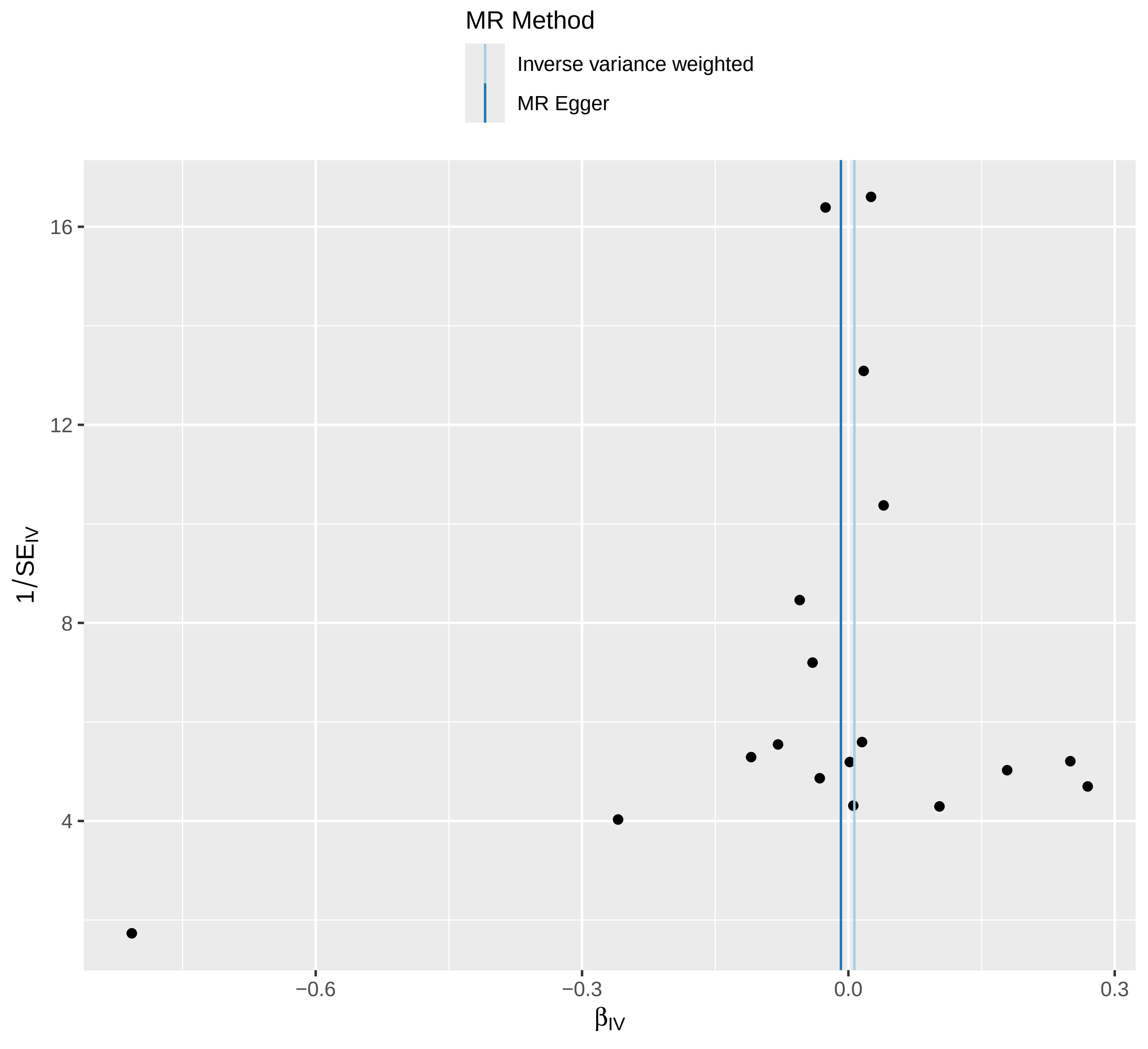

MR Method
Inverse variance weighted
MR Egger
16
12
8
4
1/SE$_{IV}$
−0.6
−0.3
0.0
0.3
β$_{IV}$

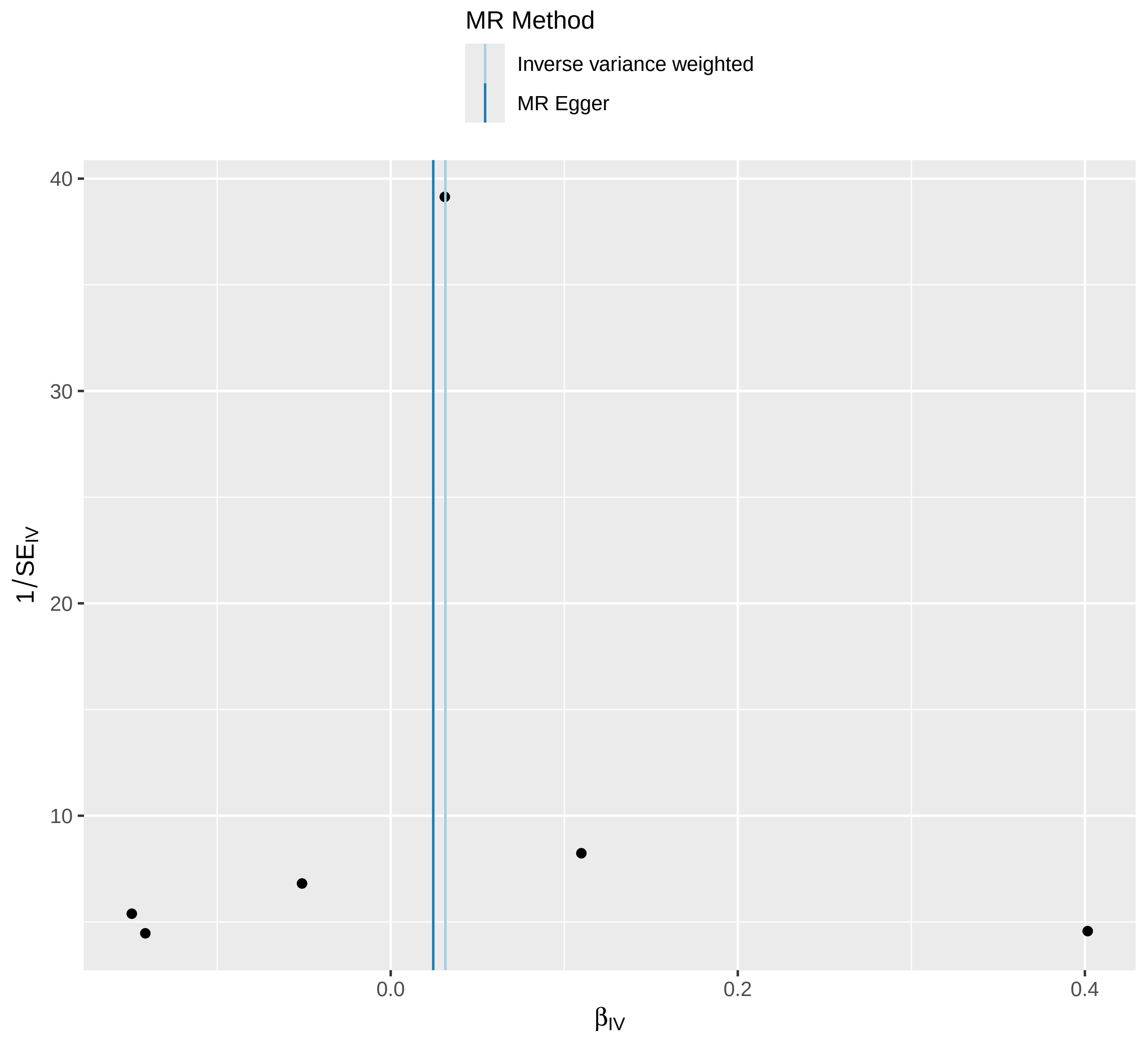

MR Method
Inverse variance weighted
MR Egger
40
30
20
10
1/SE$_{IV}$
0.0
0.2
0.4
β$_{IV}$

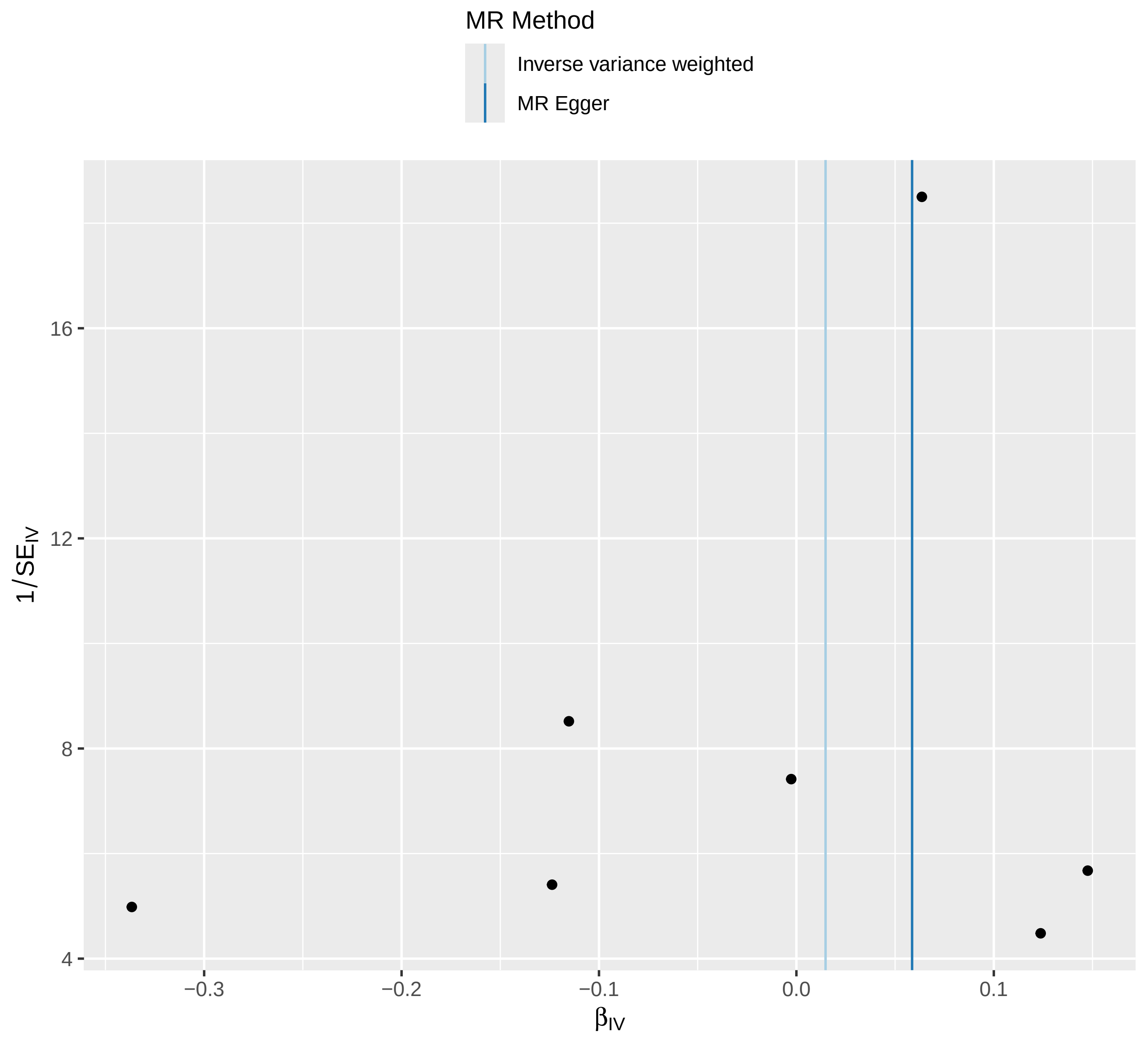

MR Method
Inverse variance weighted
MR Egger
16
12
8
4
1/SE_IV
−0.3
−0.2
−0.1
0.0
0.1
β_IV

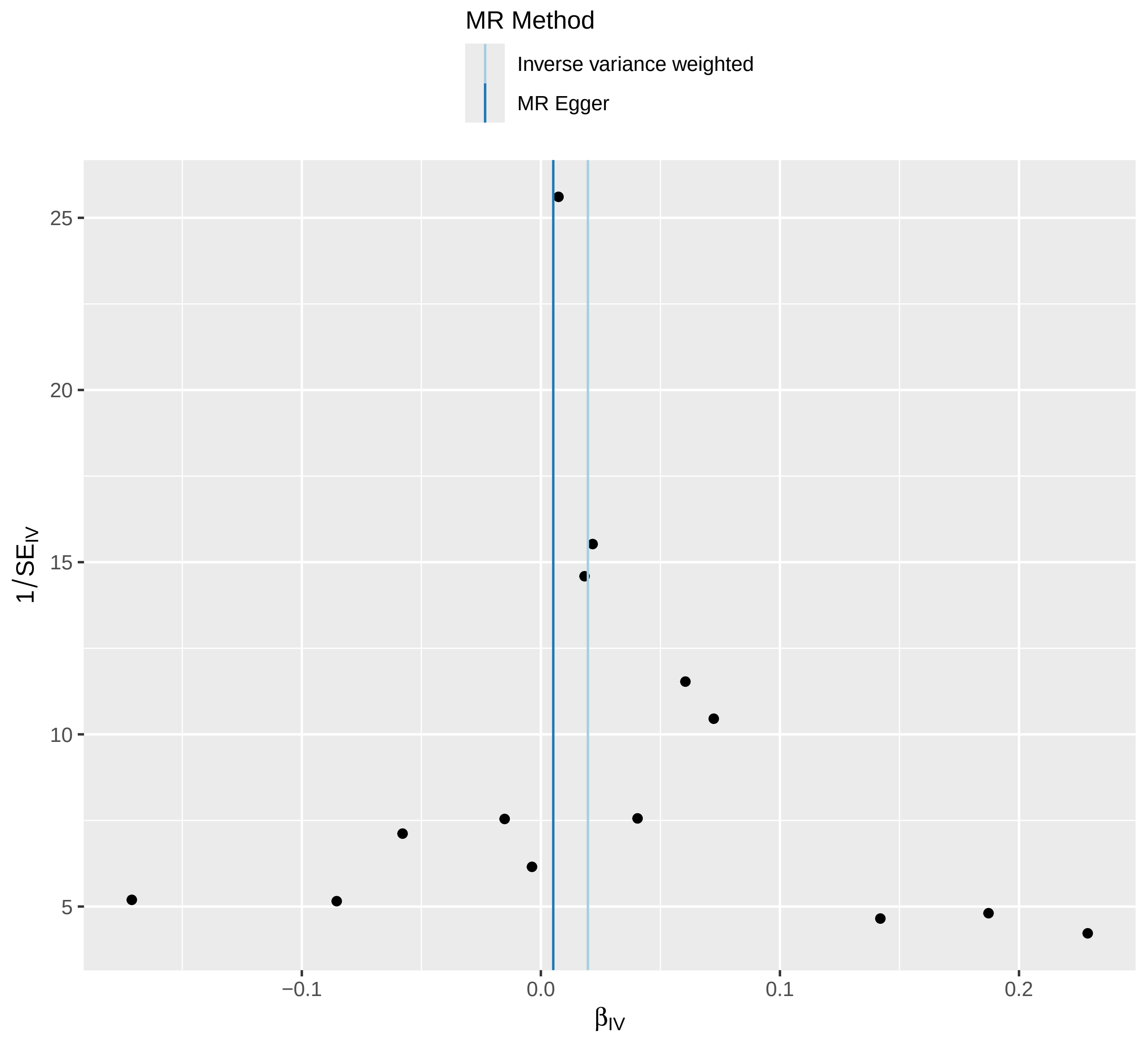
MR Method
Inverse variance weighted
MR Egger
25
20
15
10
5
1/SE$_{IV}$
−0.1
0.0
0.1
0.2
$\beta_{IV}$

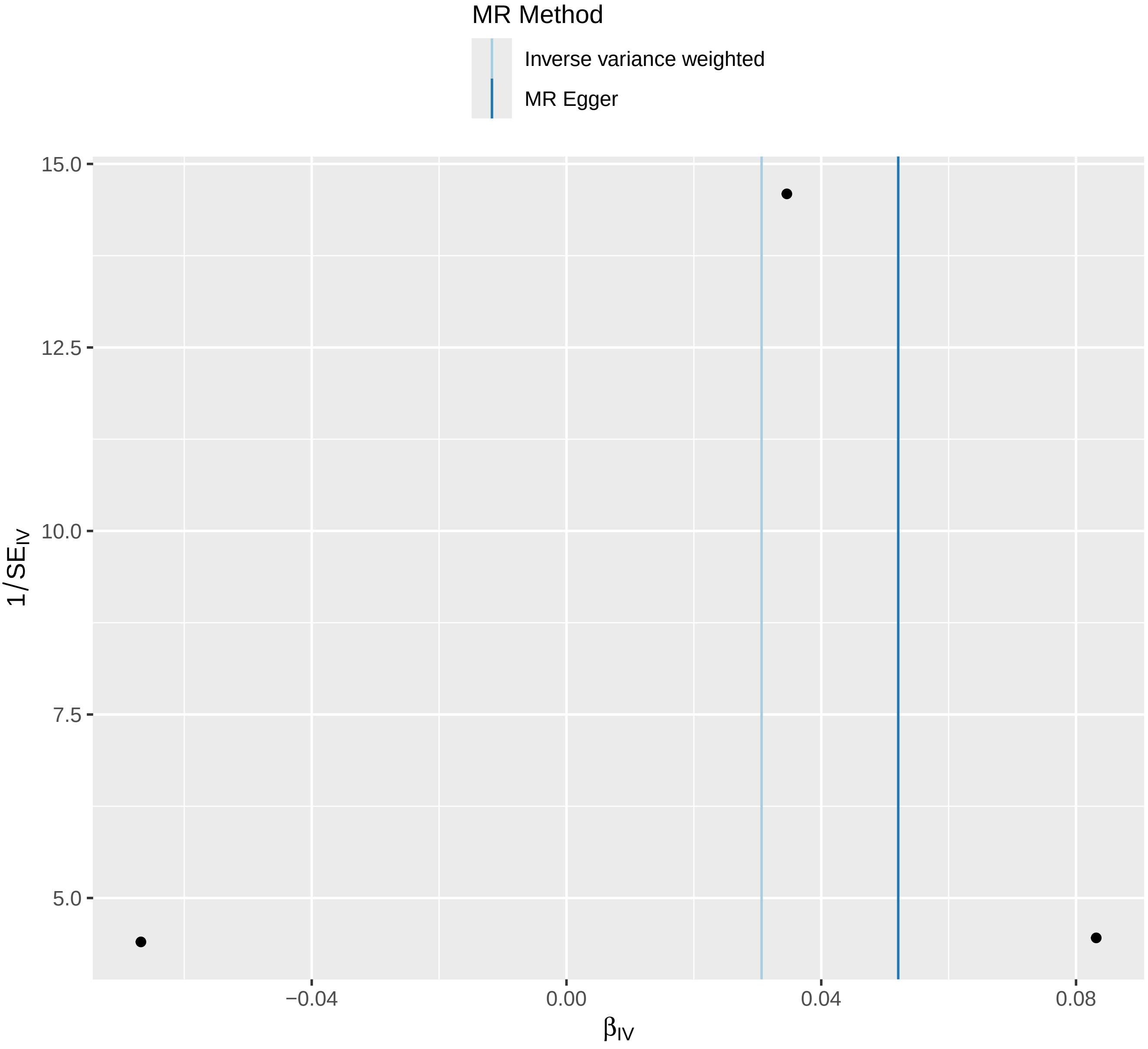
MR Method
Inverse variance weighted
MR Egger
15.0
12.5
10.0
7.5
5.0
1/SE$_{IV}$
−0.04
0.00
0.04
0.08
β$_{IV}$

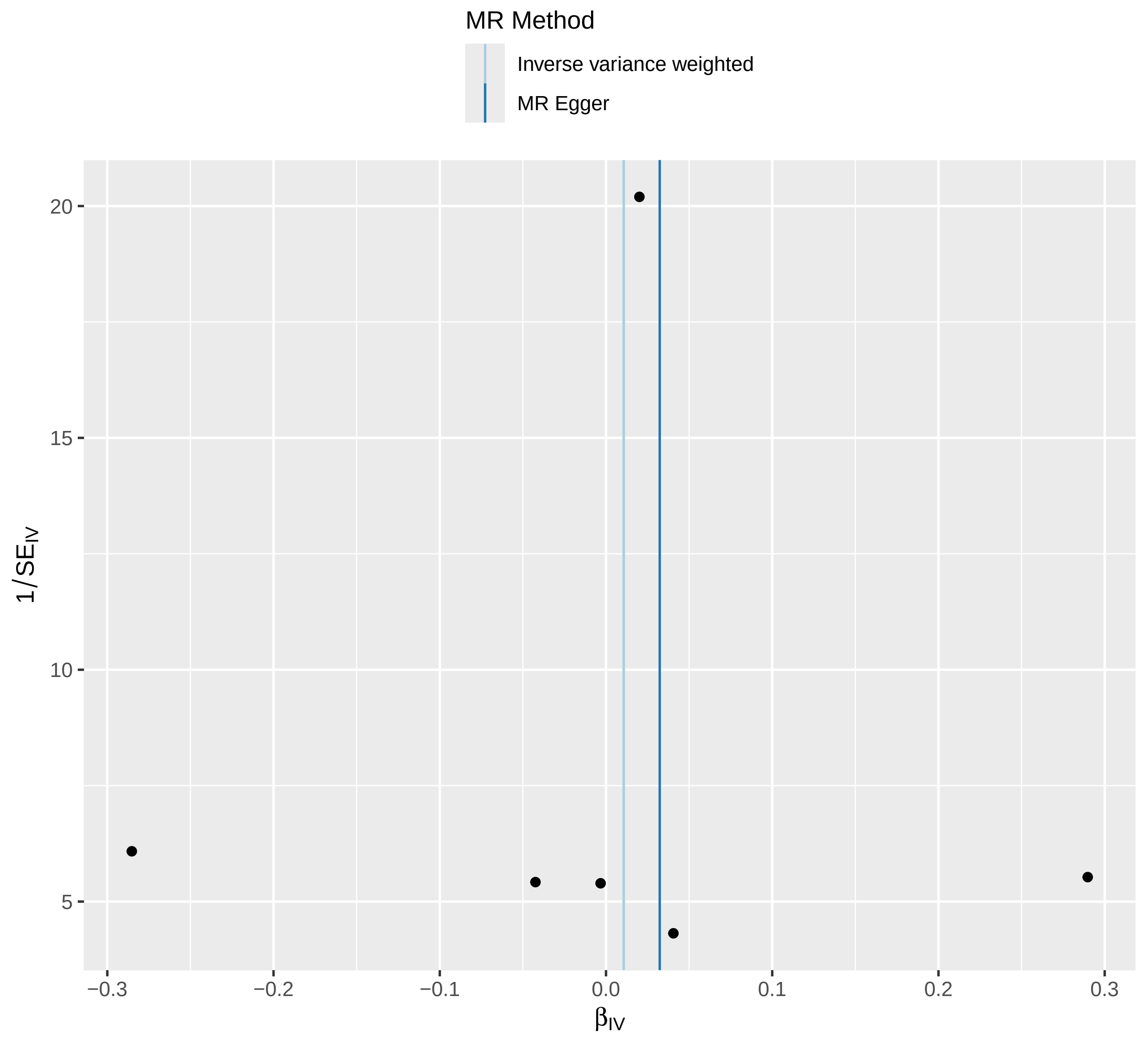
MR Method
Inverse variance weighted
MR Egger
20
15
10
5
1/SE_IV
−0.3
−0.2
−0.1
0.0
0.1
0.2
0.3
β_IV

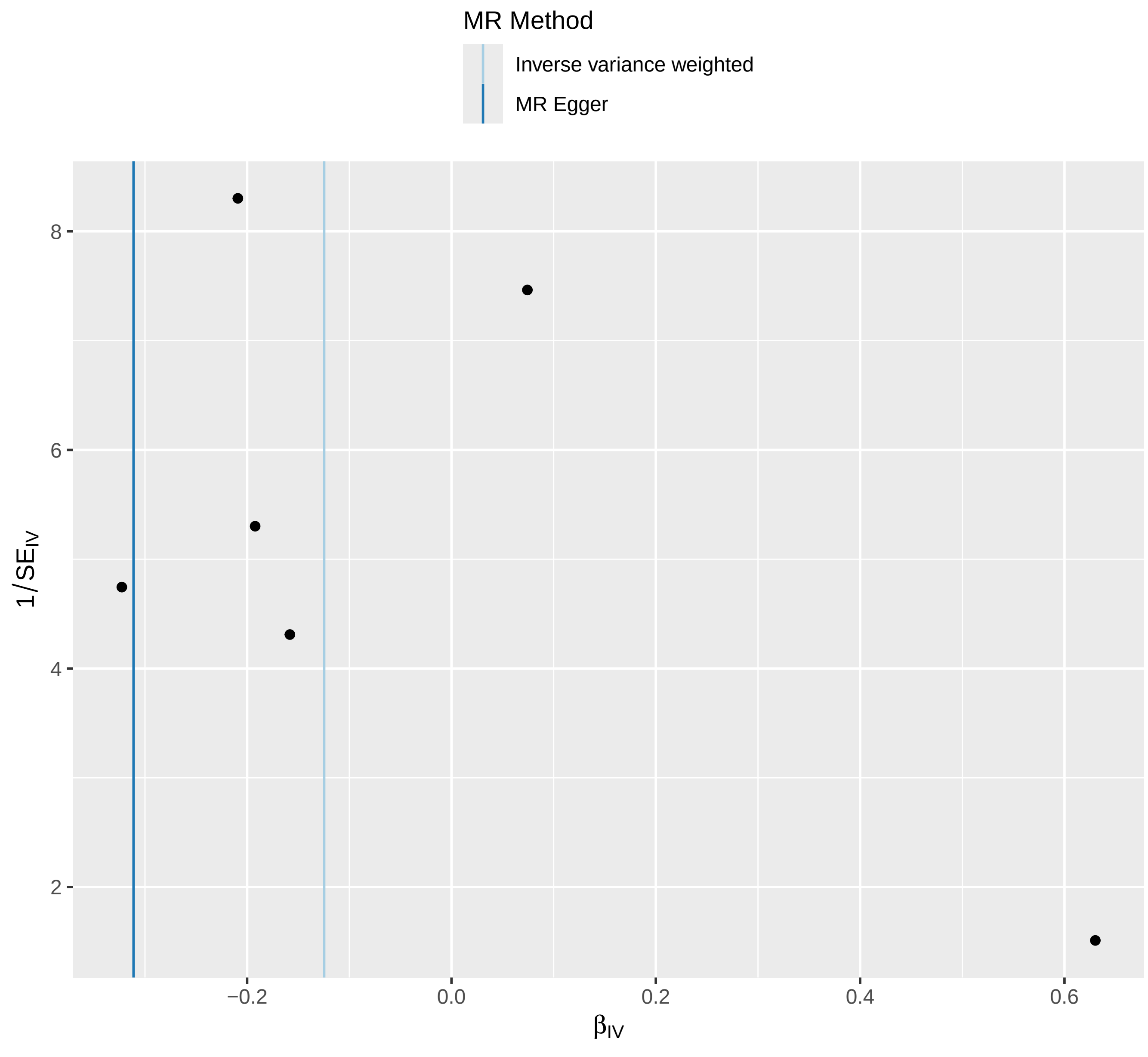

MR Method
Inverse variance weighted
MR Egger
8
6
4
2
1/SE_IV
−0.2
0.0
0.2
0.4
0.6
β_IV

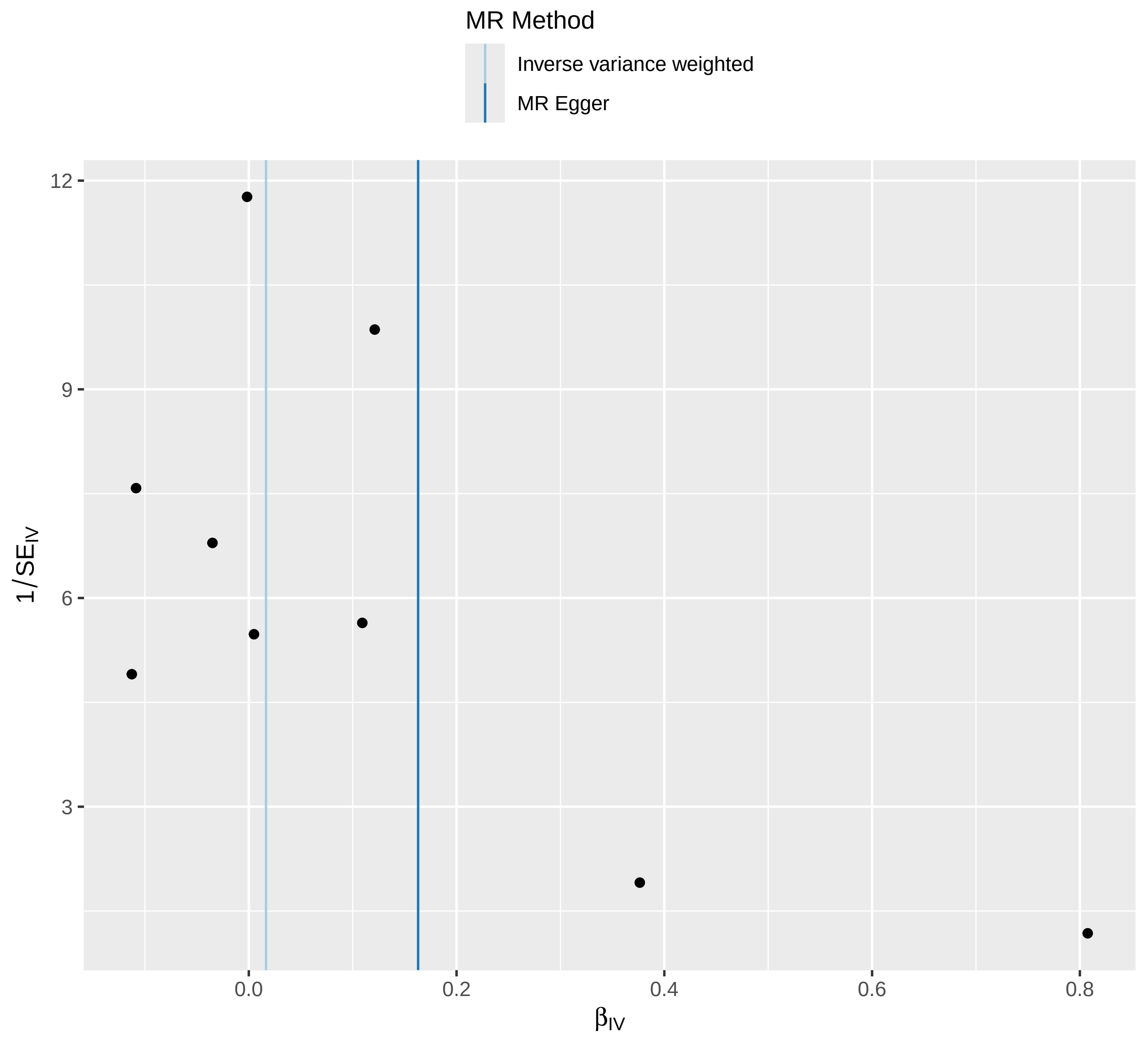
MR Method
Inverse variance weighted
MR Egger
12
9
6
3
1/SE_IV
0.0
0.2
0.4
0.6
0.8
β_IV

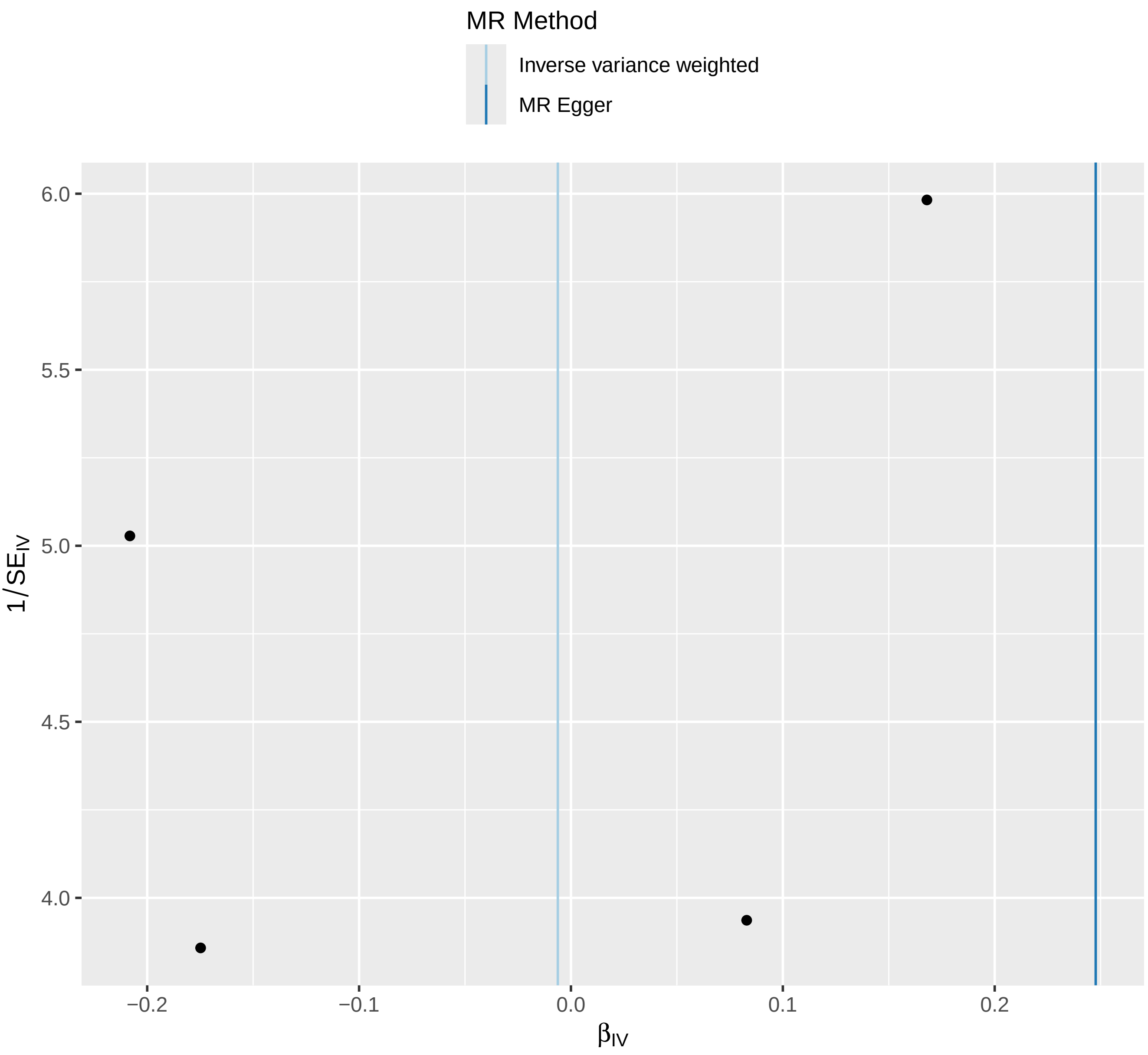
MR Method
Inverse variance weighted
MR Egger
6.0
5.5
5.0
4.5
4.0
1/SE_IV
−0.2
−0.1
0.0
0.1
0.2
β_IV

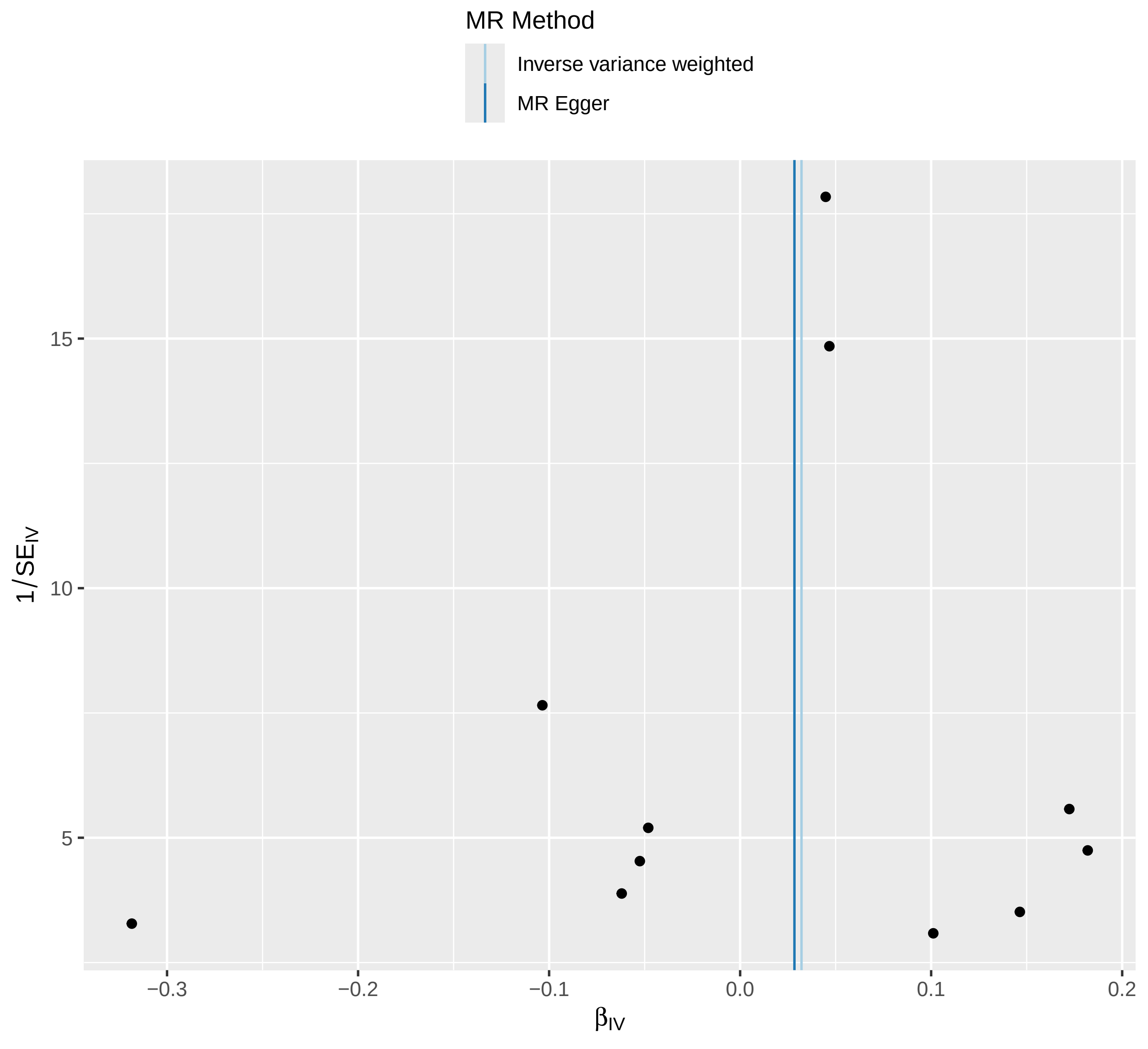
MR Method
Inverse variance weighted
MR Egger
15
10
5
1/SE_IV
−0.3
−0.2
−0.1
0.0
0.1
0.2
β_IV

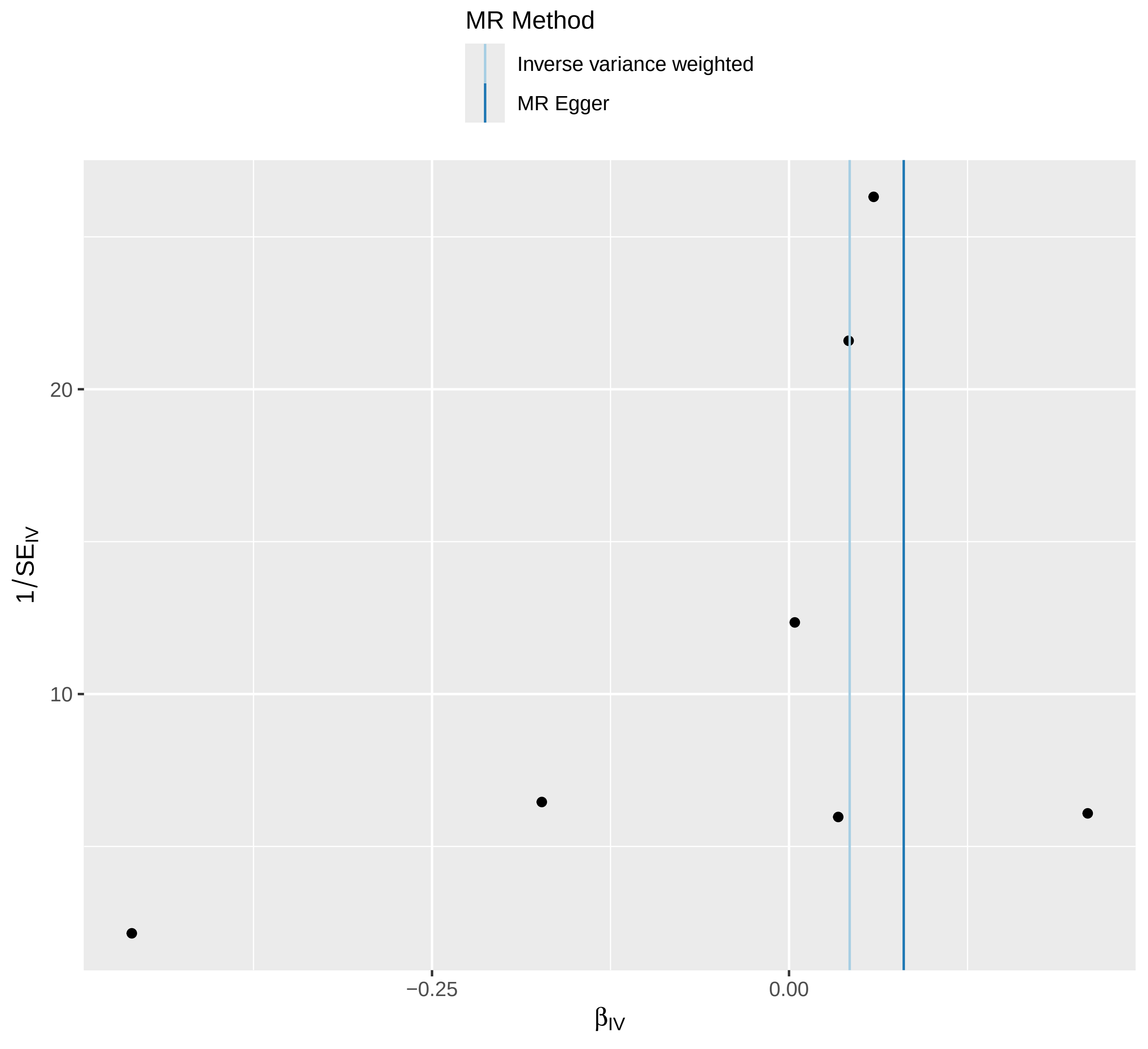
MR Method
Inverse variance weighted
MR Egger
20
10
1/SE$_{IV}$
−0.25
0.00
$\beta_{IV}$

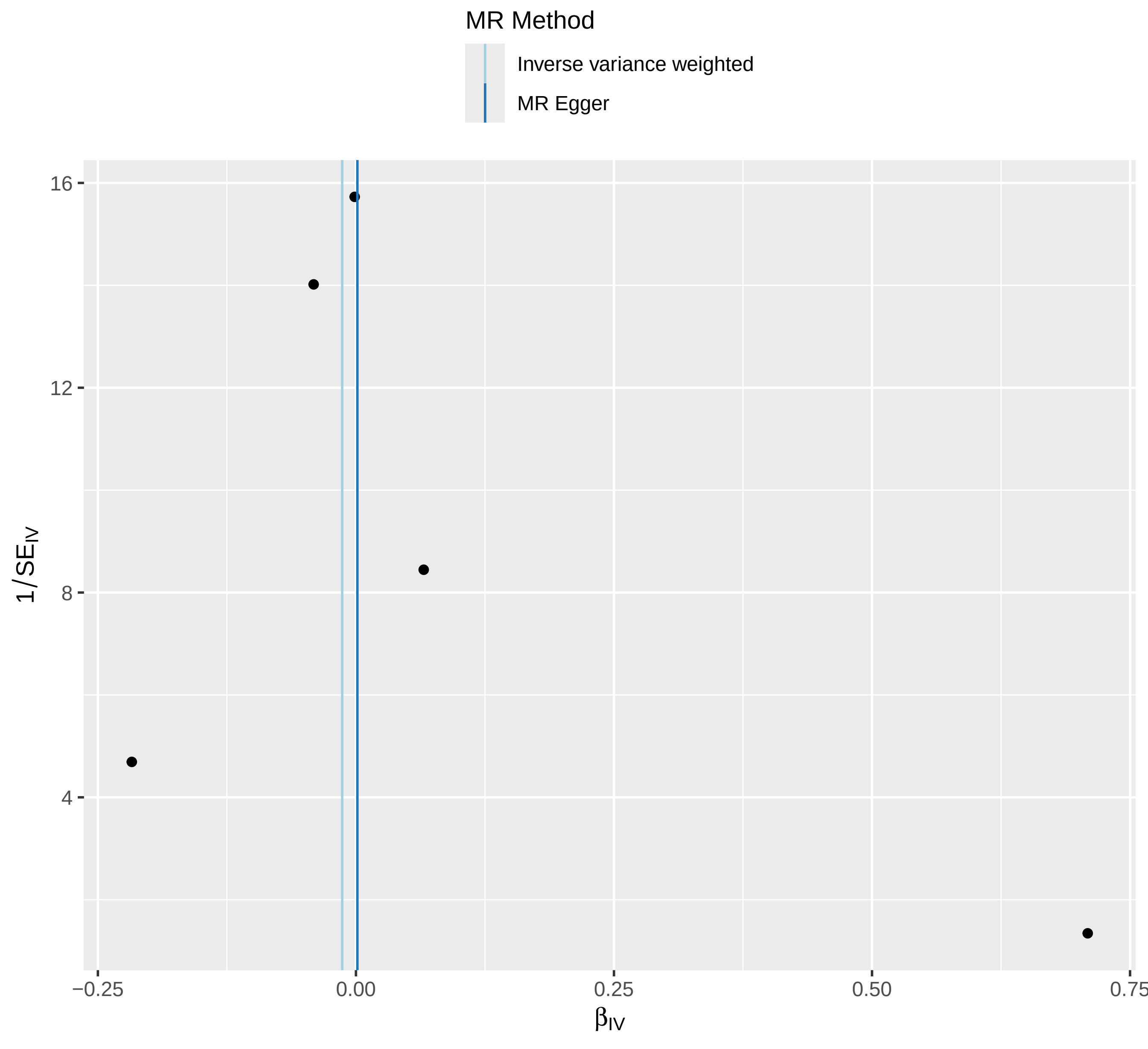

MR Method
Inverse variance weighted
MR Egger
16
12
8
4
$1/SE_{IV}$
−0.25
0.00
0.25
0.50
0.75
$\beta_{IV}$

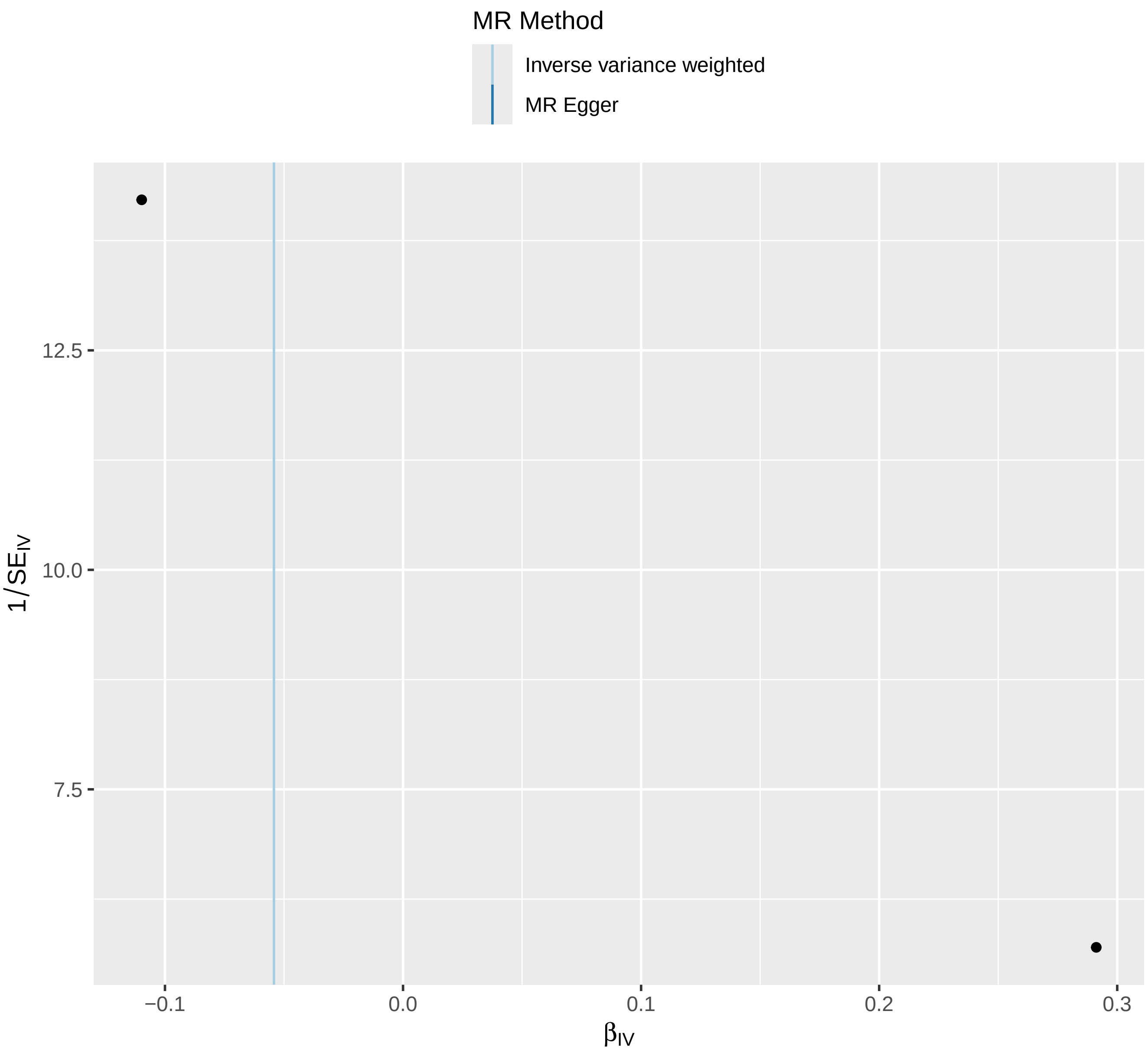
MR Method
Inverse variance weighted
MR Egger
12.5
10.0
7.5
1/SE$_{IV}$
−0.1
0.0
0.1
0.2
0.3
β$_{IV}$

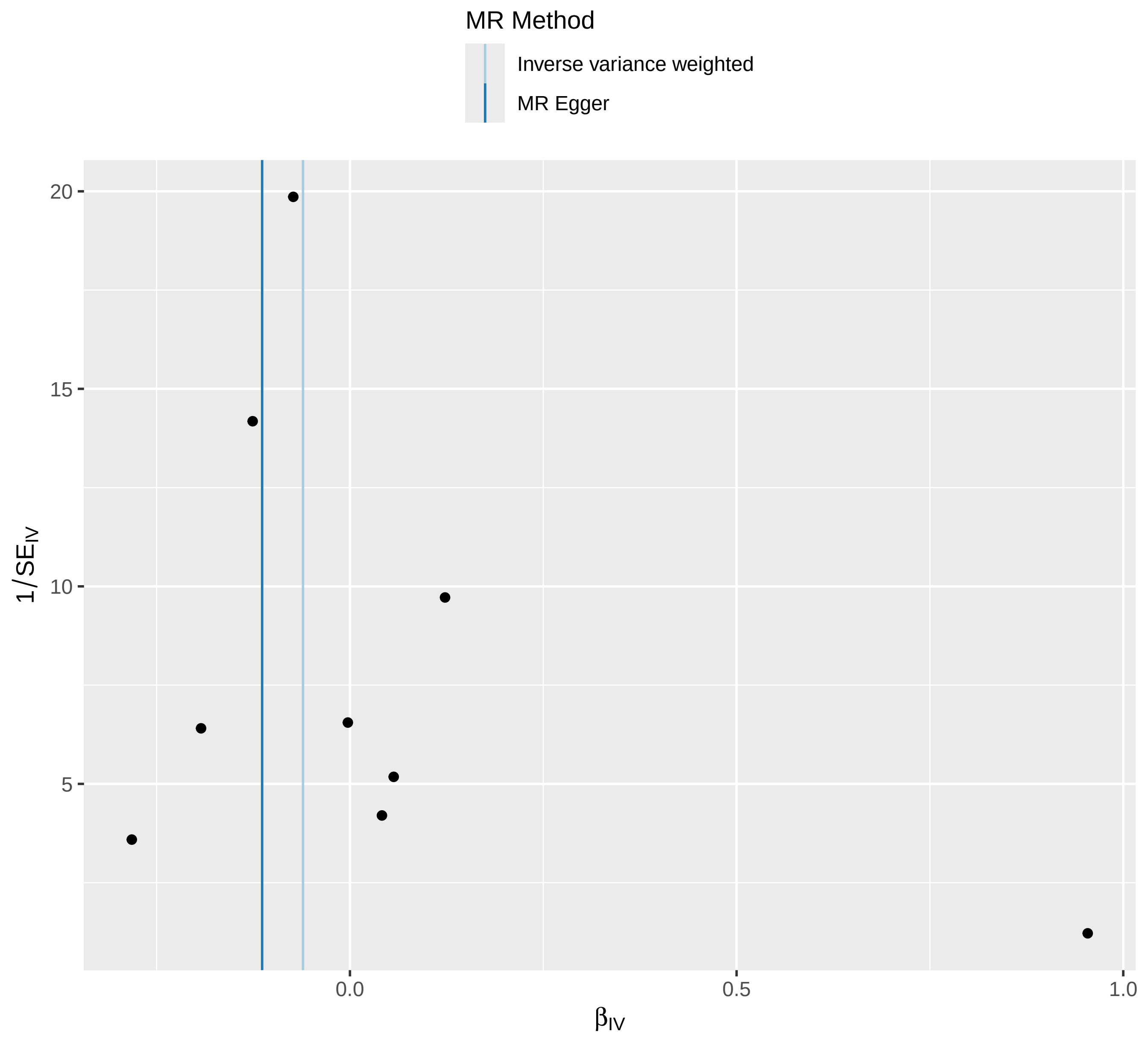
MR Method
Inverse variance weighted
MR Egger
20
15
10
5
0.0
0.5
1.0
1/SE_IV
β_IV

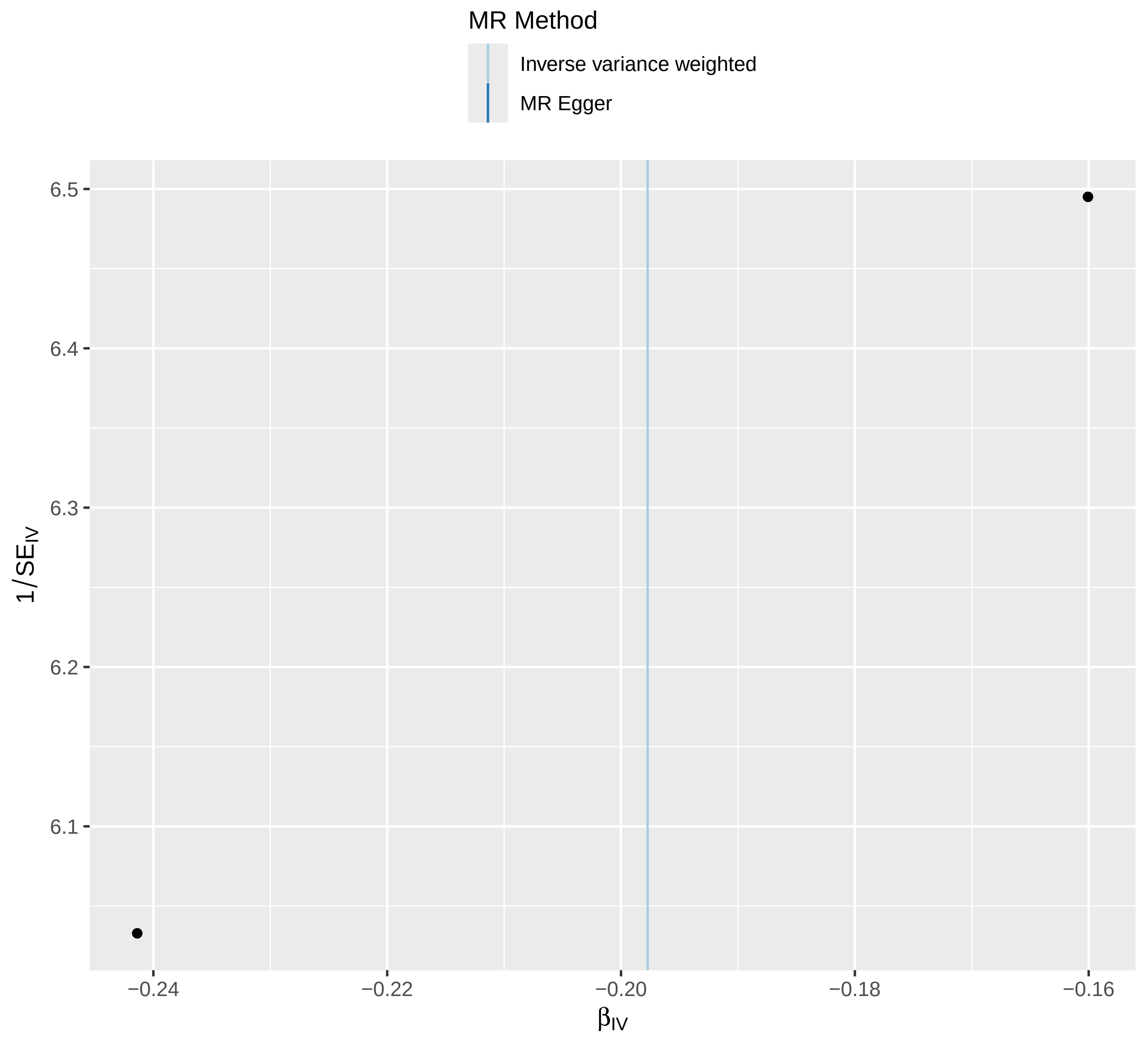
MR Method
Inverse variance weighted
MR Egger
6.5
6.4
6.3
6.2
6.1
1/SE_IV
−0.24
−0.22
−0.20
−0.18
−0.16
β_IV

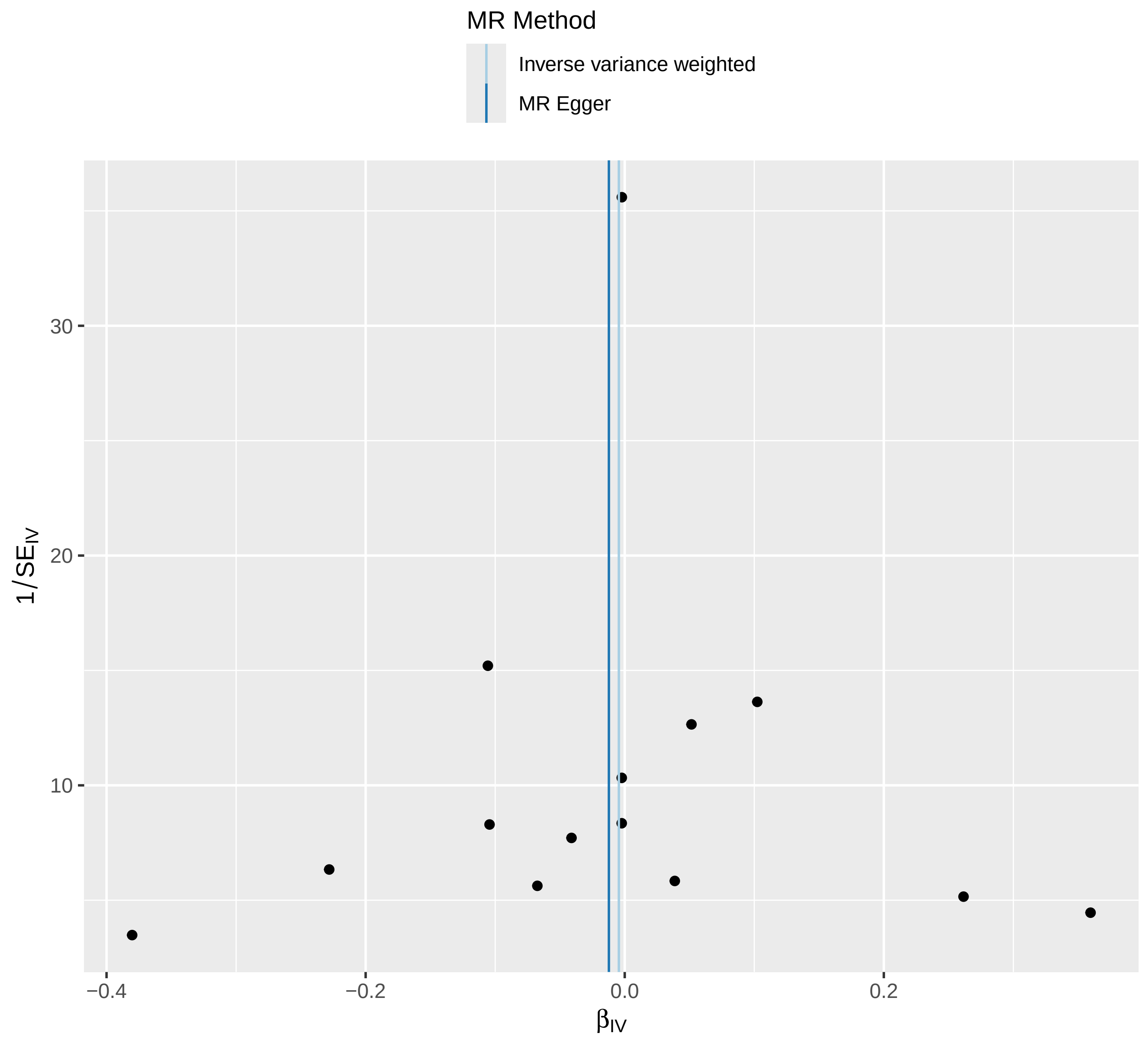
MR Method
Inverse variance weighted
MR Egger
30
20
10
1/SE_IV
−0.4
−0.2
0.0
0.2
β_IV

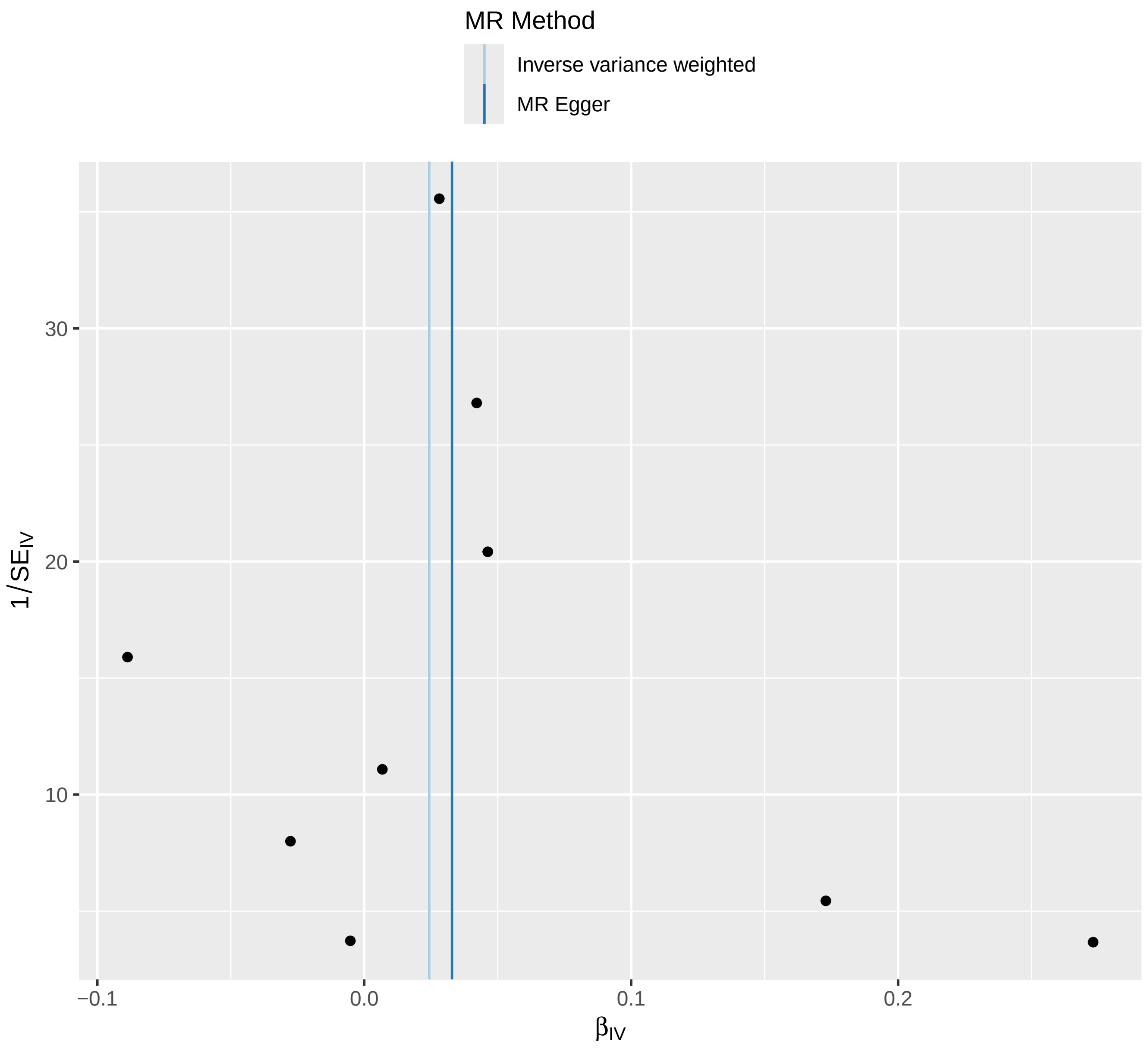
MR Method
Inverse variance weighted
MR Egger
30
20
10
1/SE_IV
−0.1
0.0
0.1
0.2
β_IV

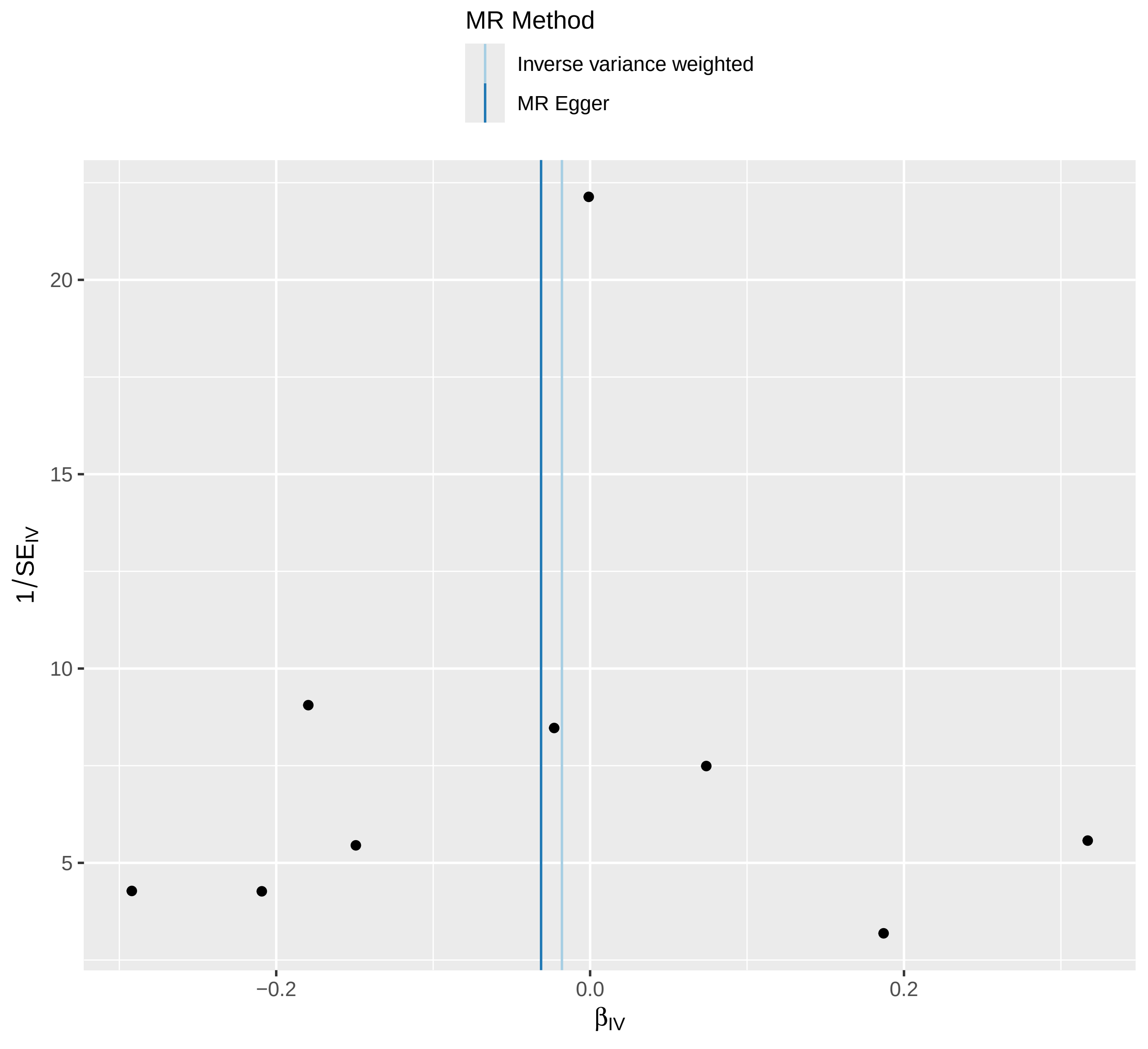

MR Method
Inverse variance weighted
MR Egger
20
15
10
5
1/SE_IV
−0.2
0.0
0.2
β_IV

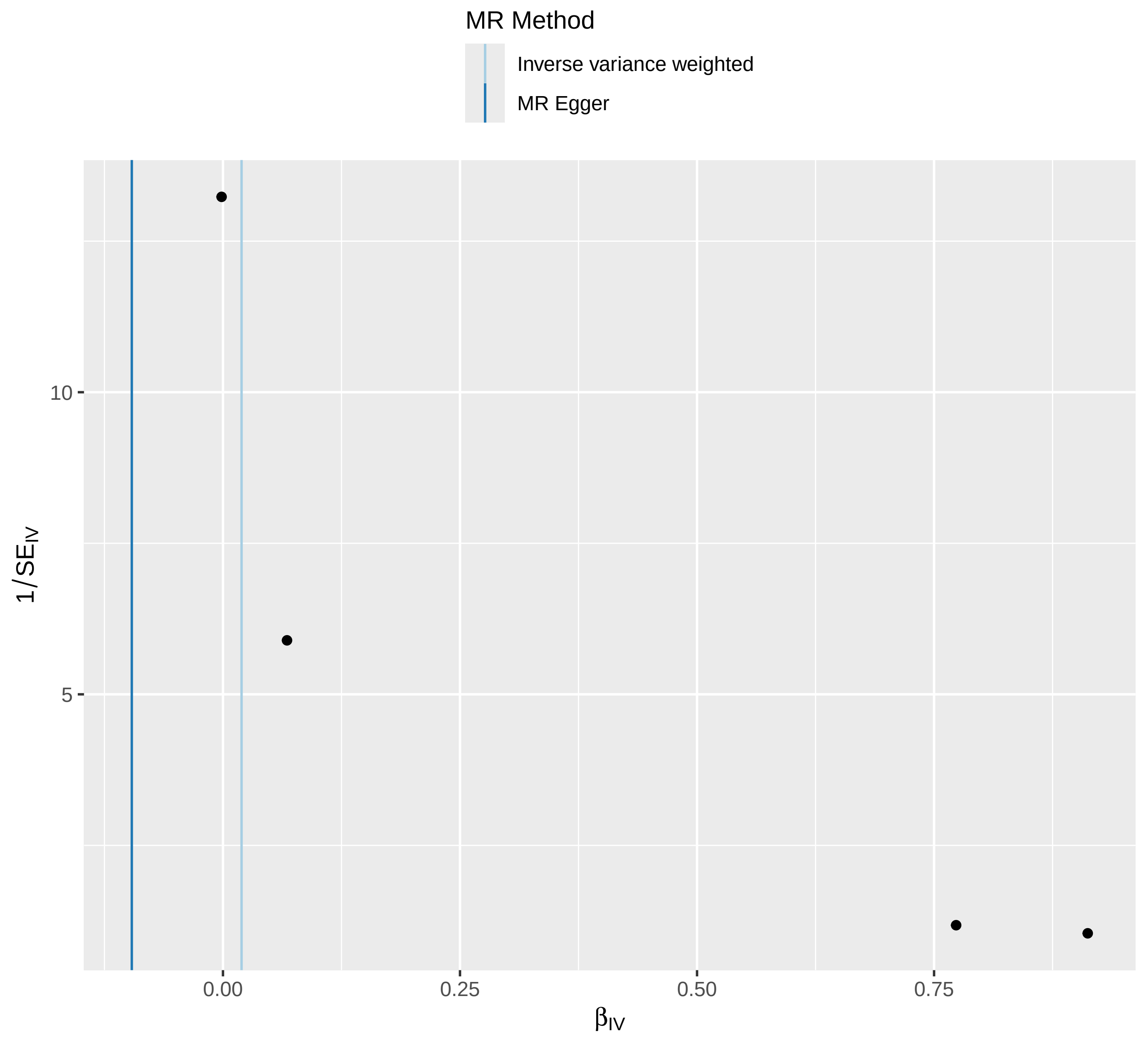

MR Method
Inverse variance weighted
MR Egger
10
5
1/SE_IV
0.00
0.25
0.50
0.75
β_IV

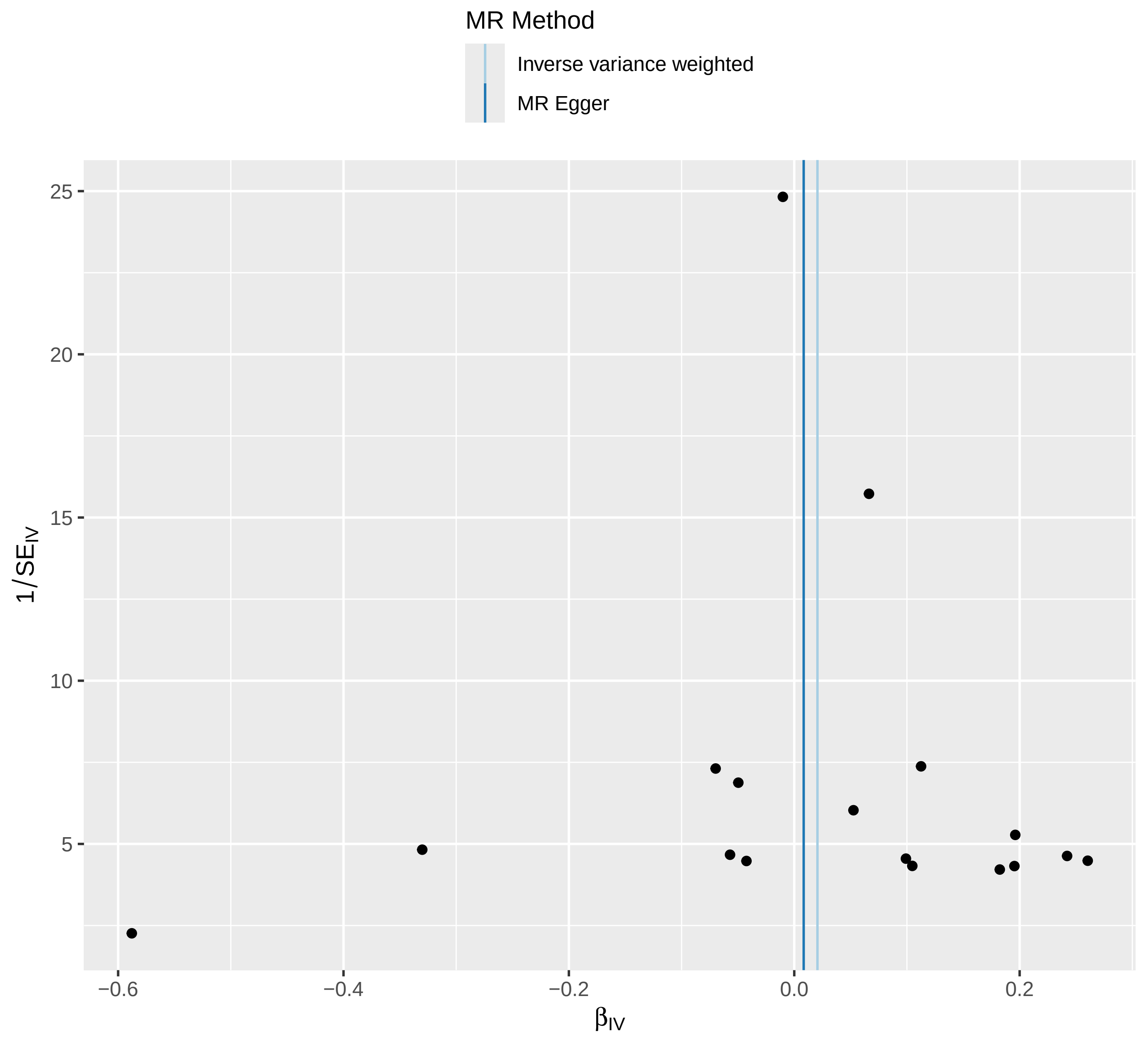
MR Method
Inverse variance weighted
MR Egger
25
20
15
10
5
1/SE$_{IV}$
−0.6
−0.4
−0.2
0.0
0.2
β$_{IV}$

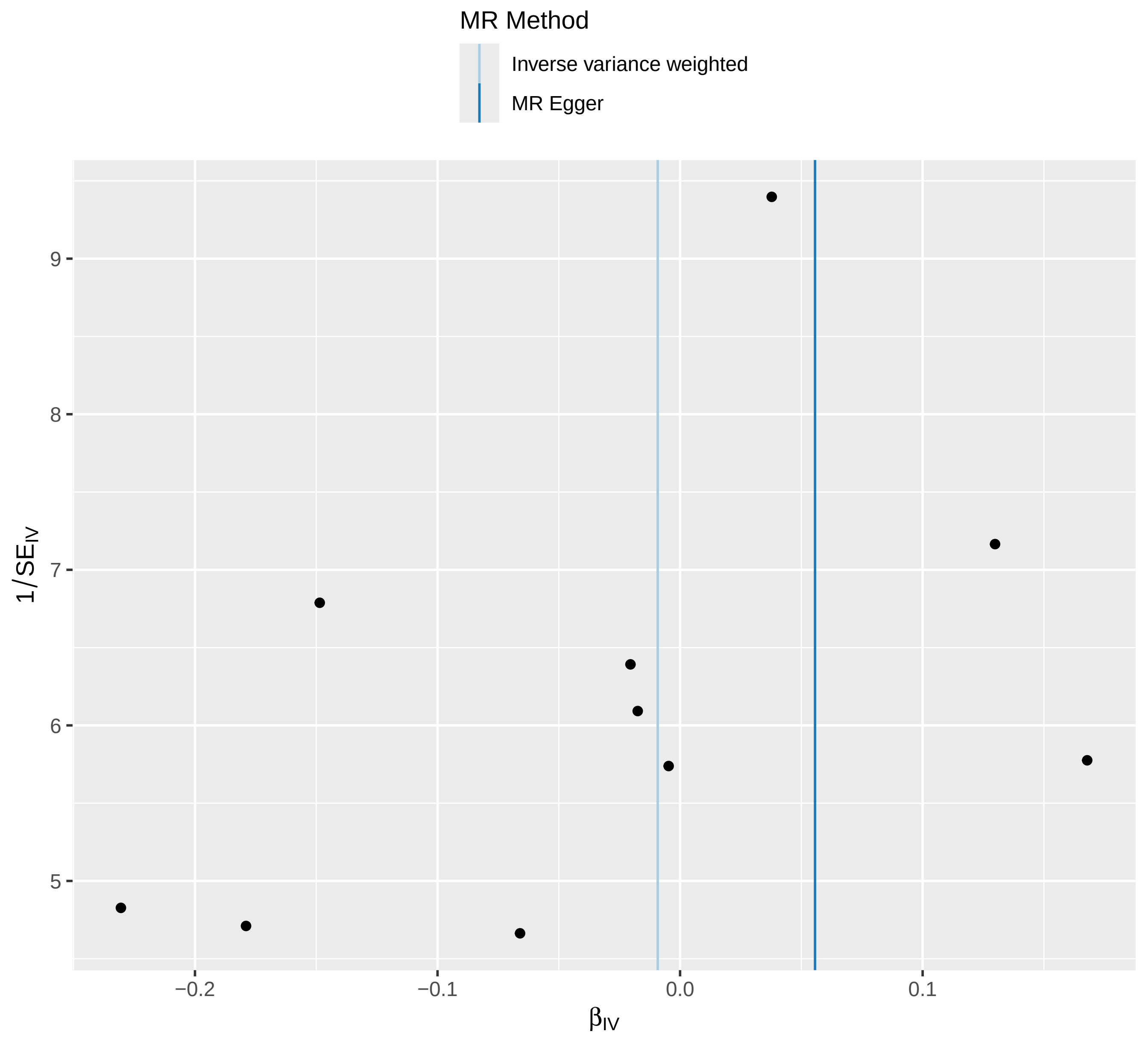
MR Method
Inverse variance weighted
MR Egger
9
8
7
6
5
1/SE_IV
−0.2
−0.1
0.0
0.1
β_IV

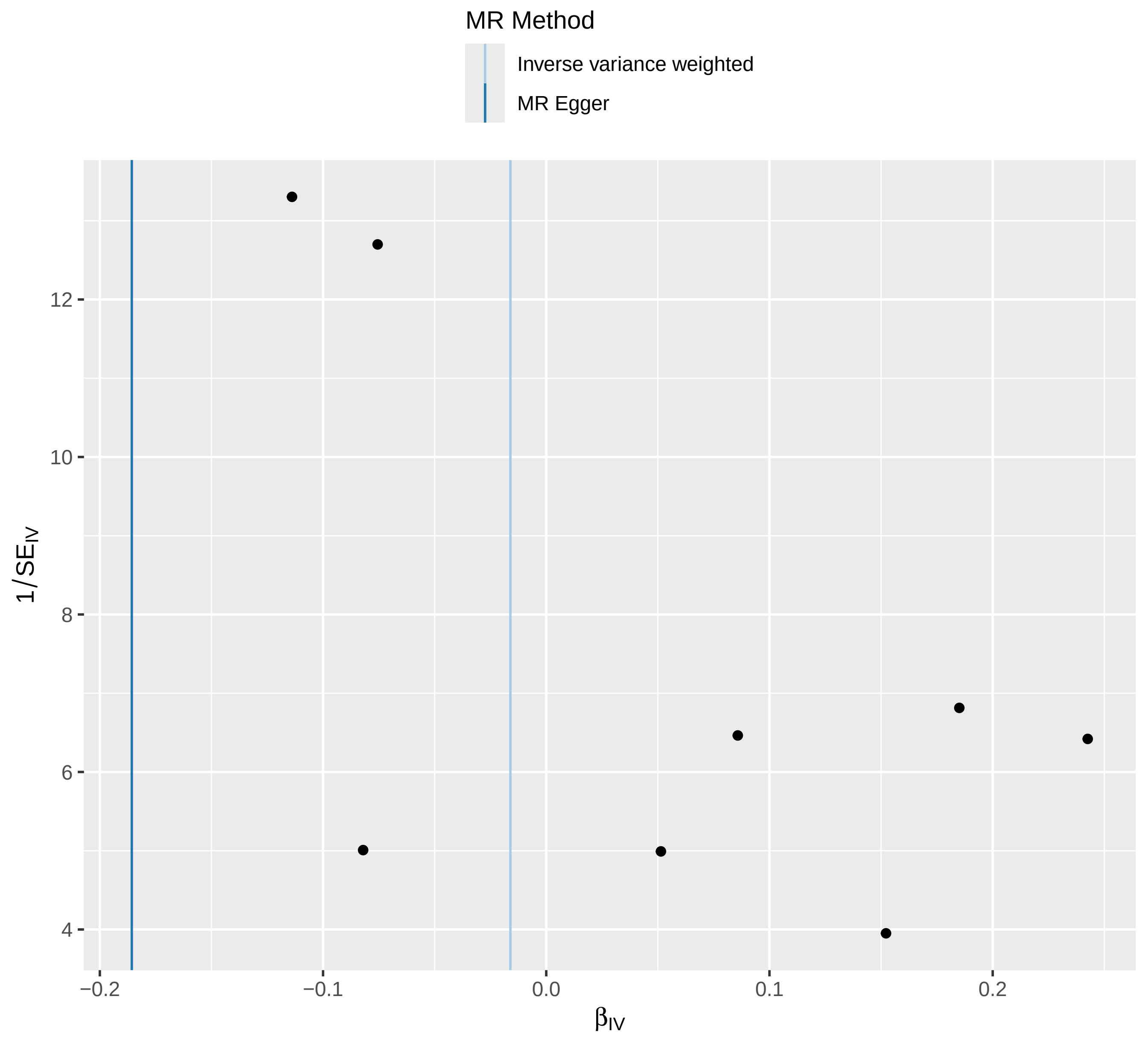
MR Method
Inverse variance weighted
MR Egger
12
10
8
6
4
1/SE$_{IV}$
−0.2
−0.1
0.0
0.1
0.2
β$_{IV}$

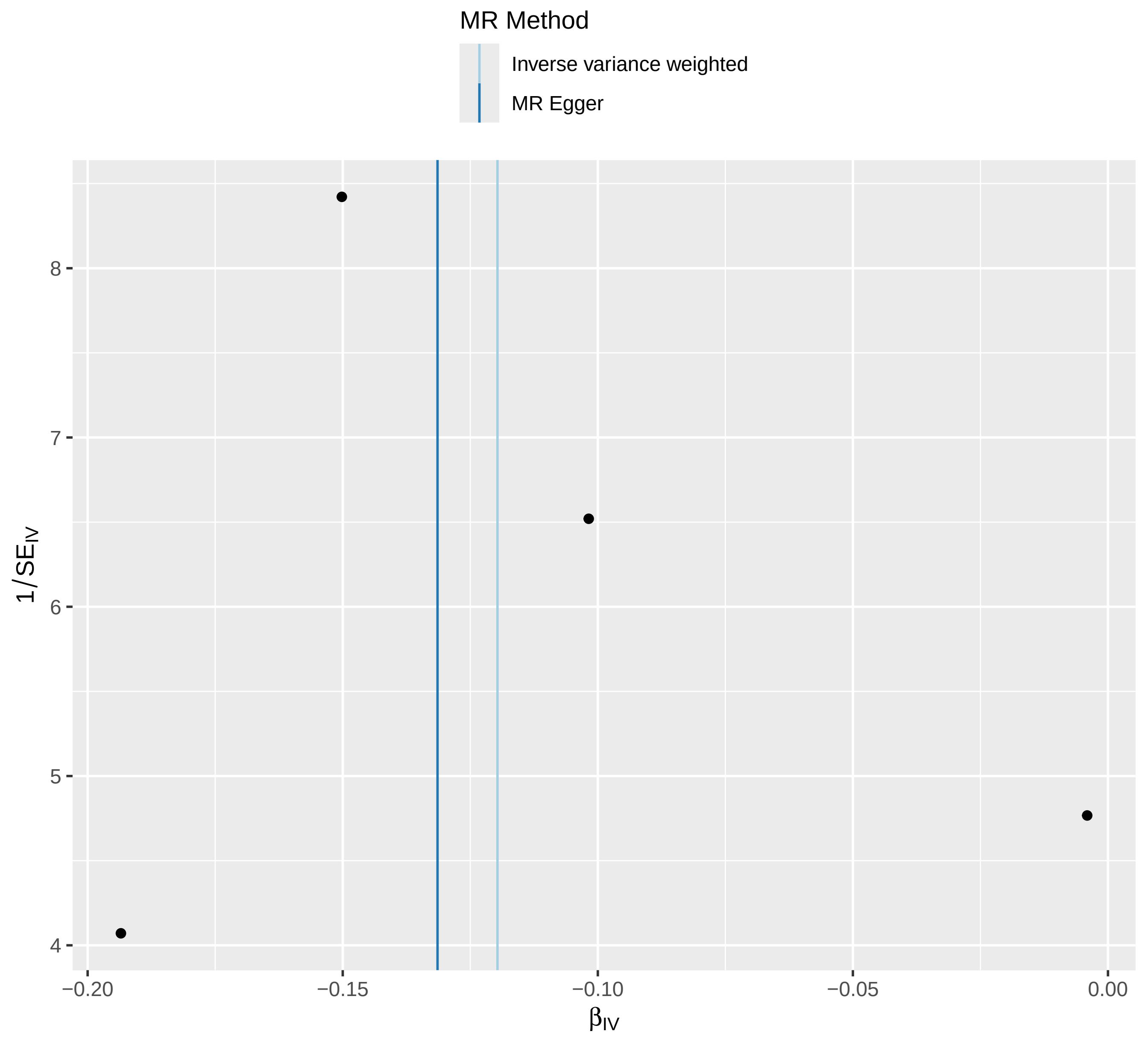

MR Method
Inverse variance weighted
MR Egger
8
7
6
5
4
1/SE_IV
−0.20
−0.15
−0.10
−0.05
0.00
β_IV

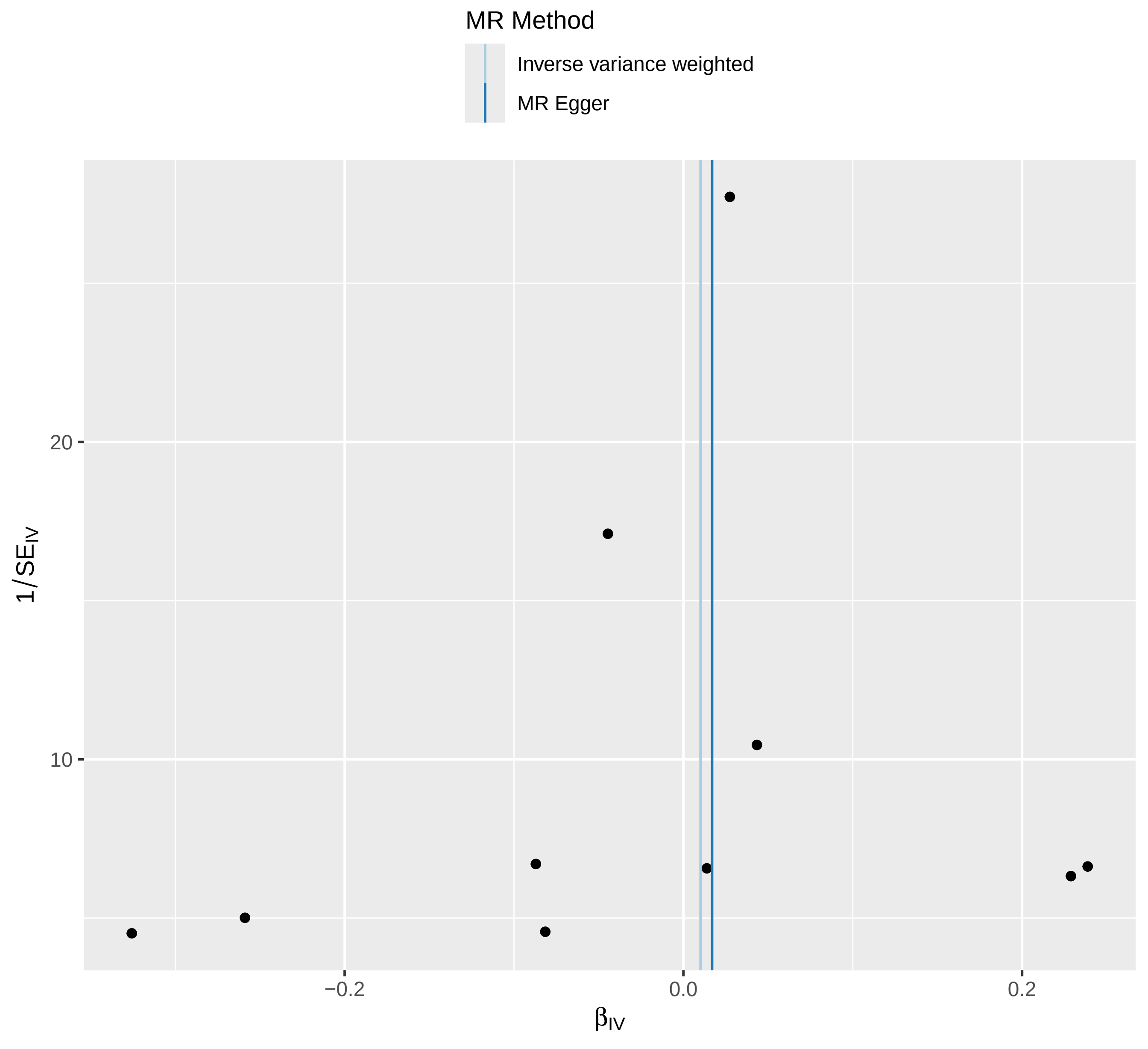

MR Method
Inverse variance weighted
MR Egger
20
10
1/SE$_{IV}$
−0.2
0.0
0.2
β$_{IV}$

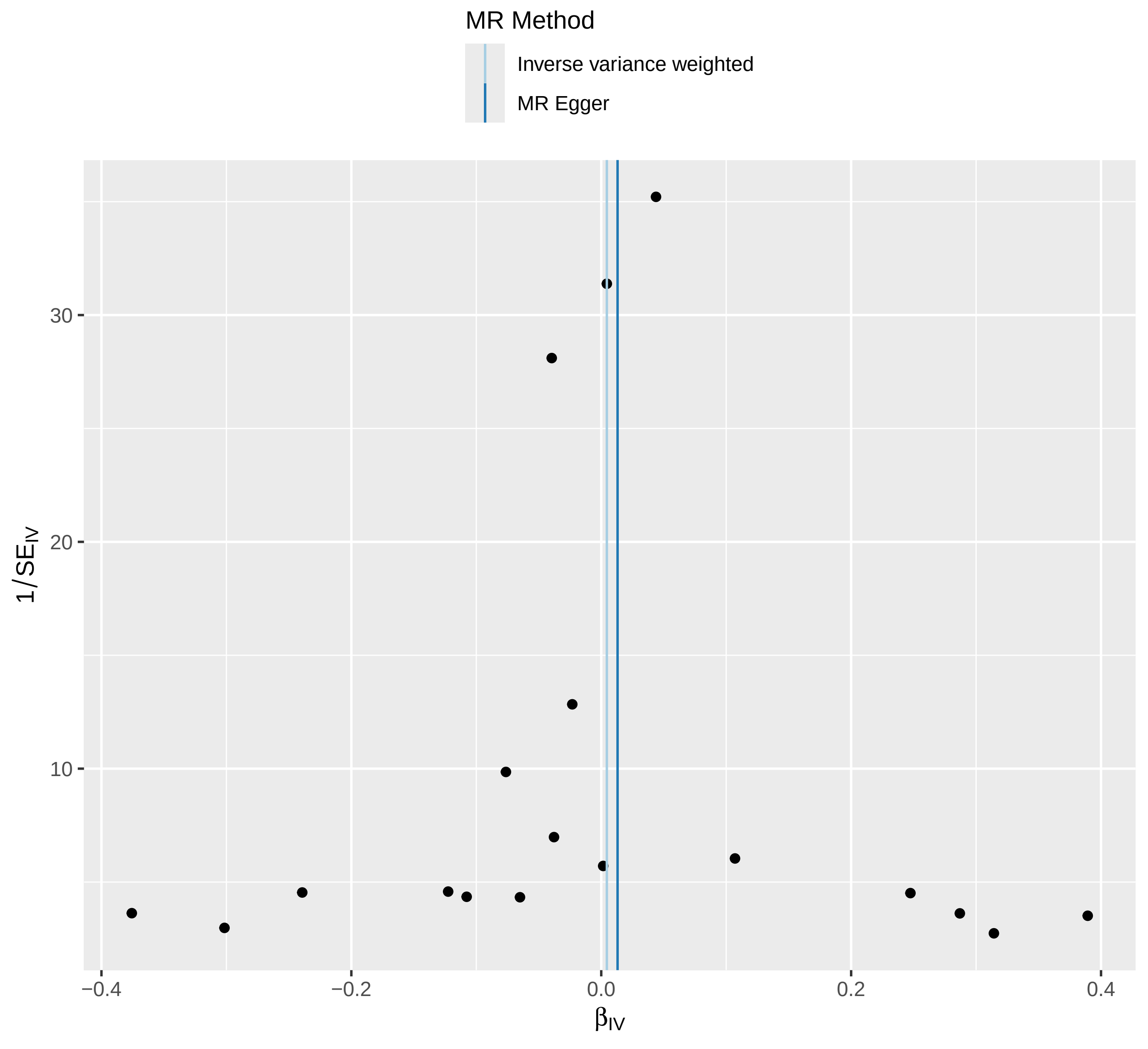
MR Method
Inverse variance weighted
MR Egger
30
20
10
1/SE$_{IV}$
−0.4
−0.2
0.0
0.2
0.4
β$_{IV}$

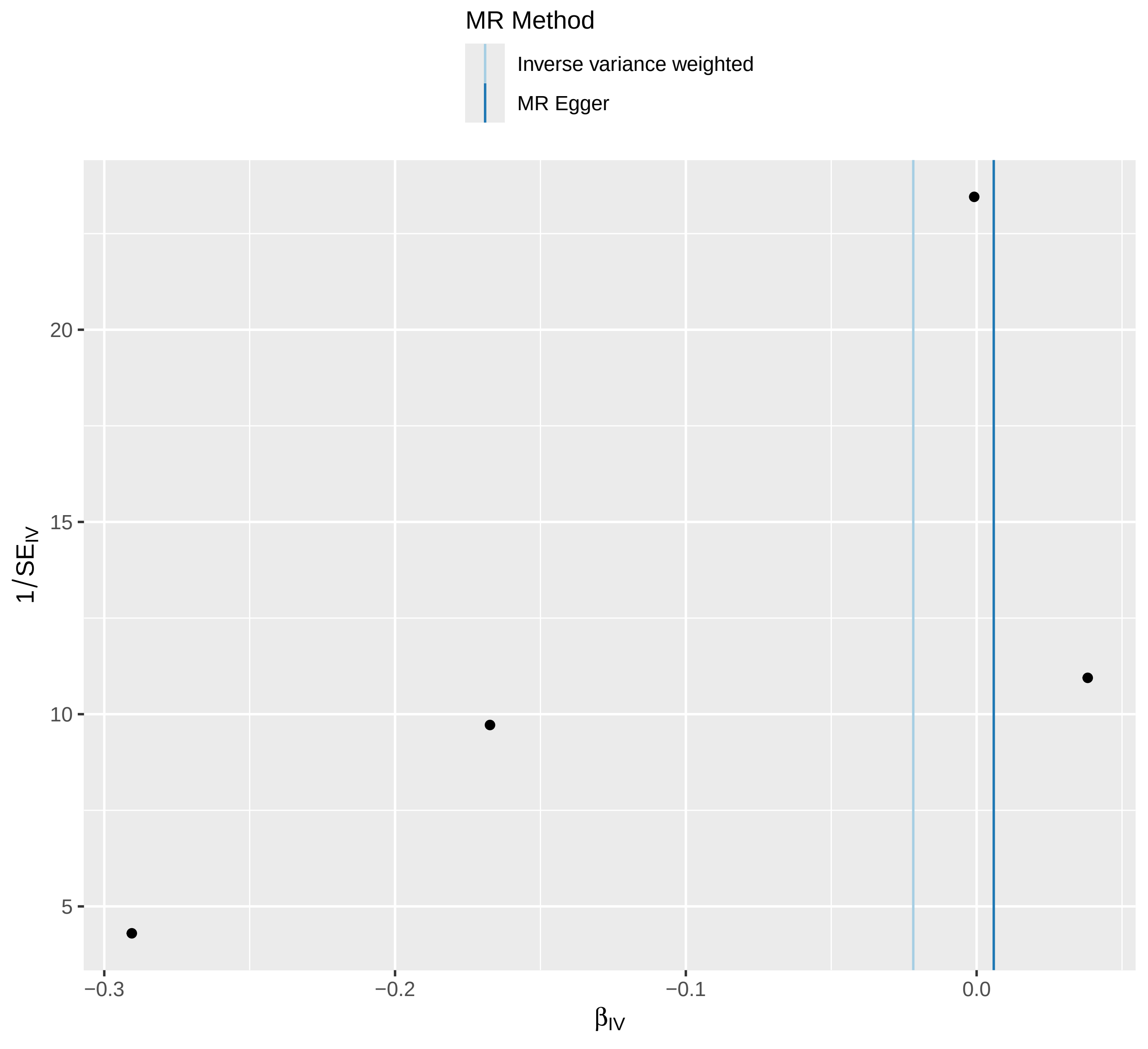

MR Method
Inverse variance weighted
MR Egger
20
15
10
5
1/SE$_{IV}$
−0.3
−0.2
−0.1
0.0
β$_{IV}$

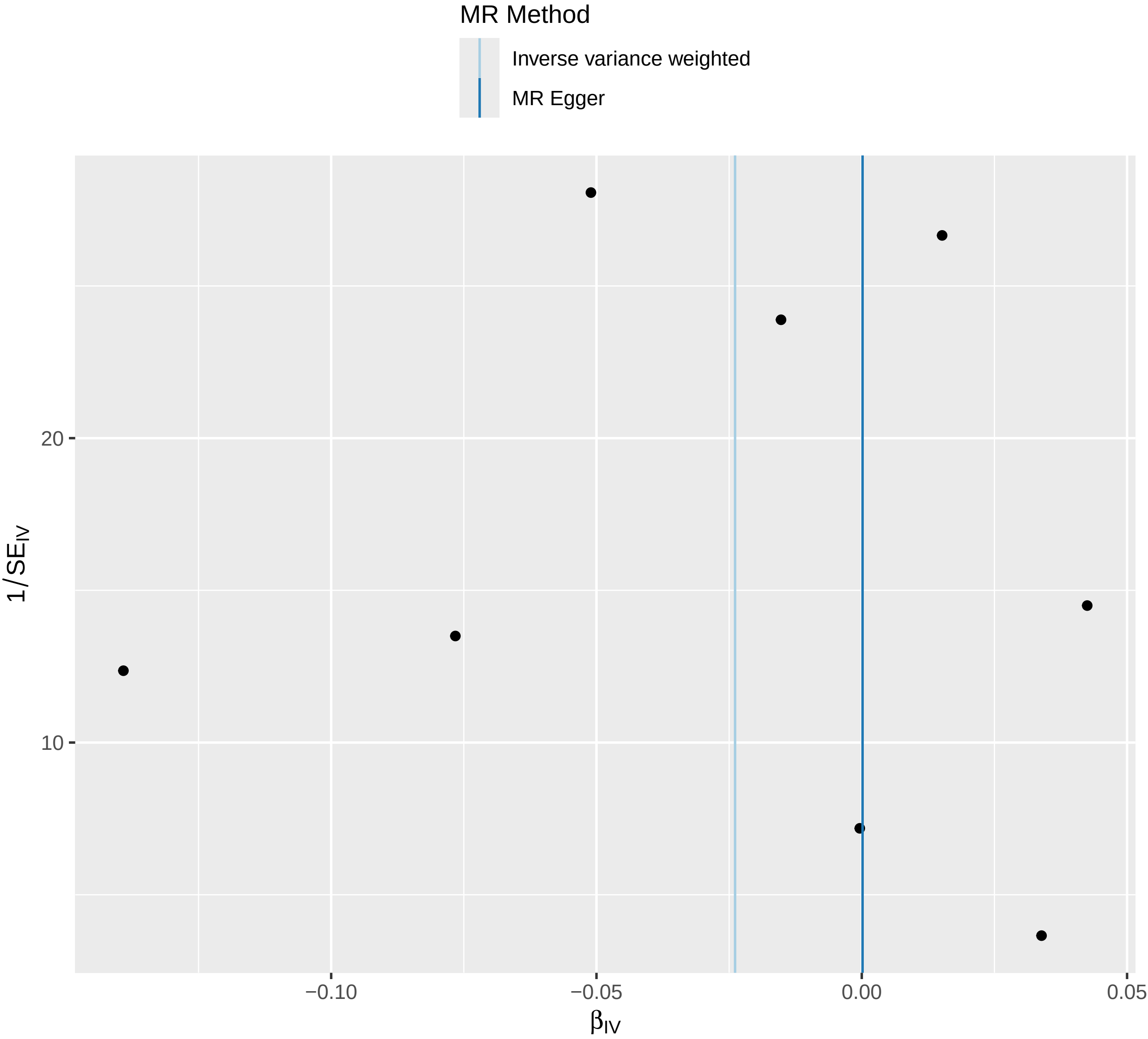
MR Method
Inverse variance weighted
MR Egger
20
10
1/SE$_{IV}$
−0.10
−0.05
0.00
0.05
β$_{IV}$

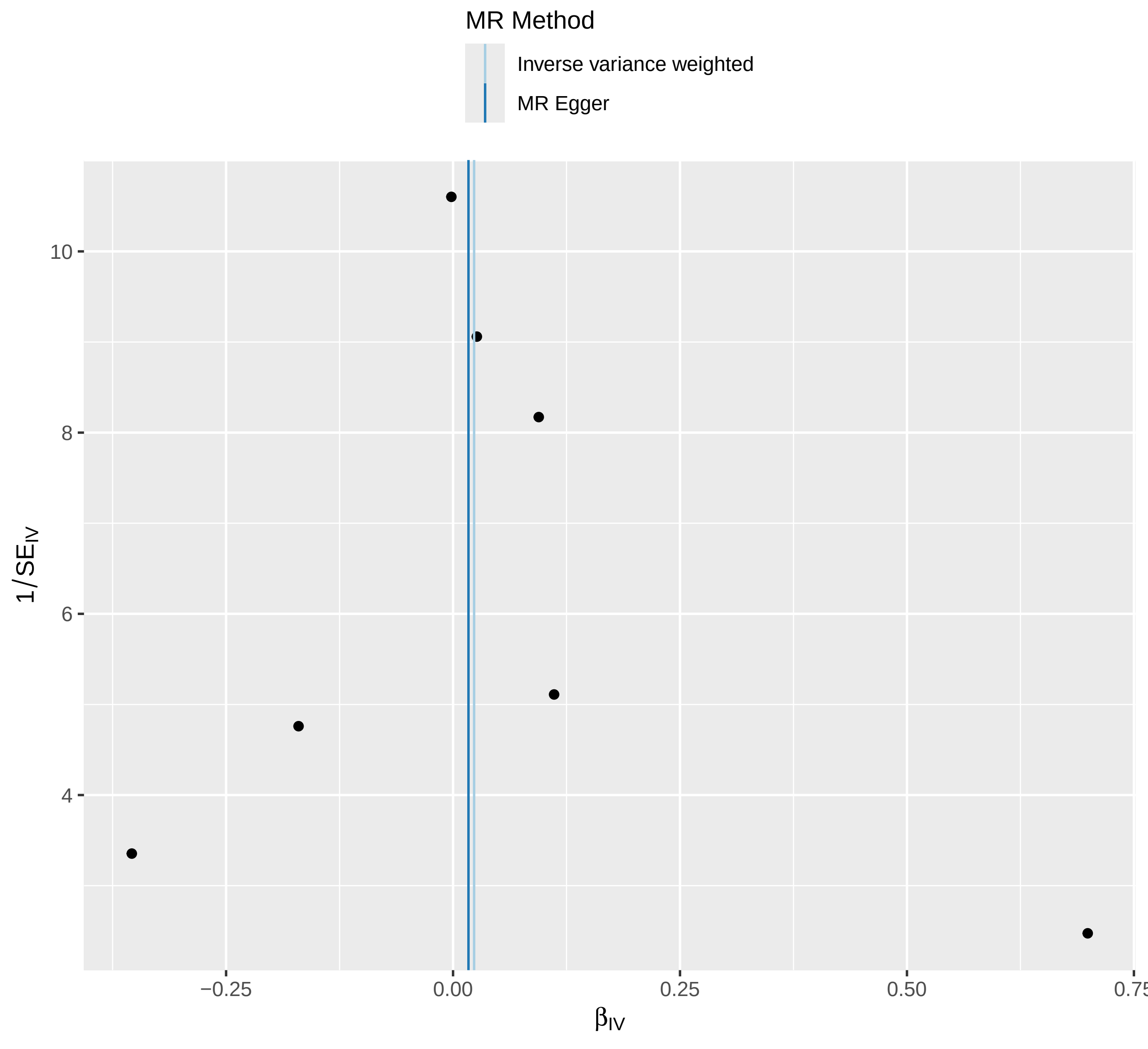
MR Method
Inverse variance weighted
MR Egger
10
8
6
4
$1/SE_{IV}$
−0.25
0.00
0.25
0.50
0.75
$\beta_{IV}$

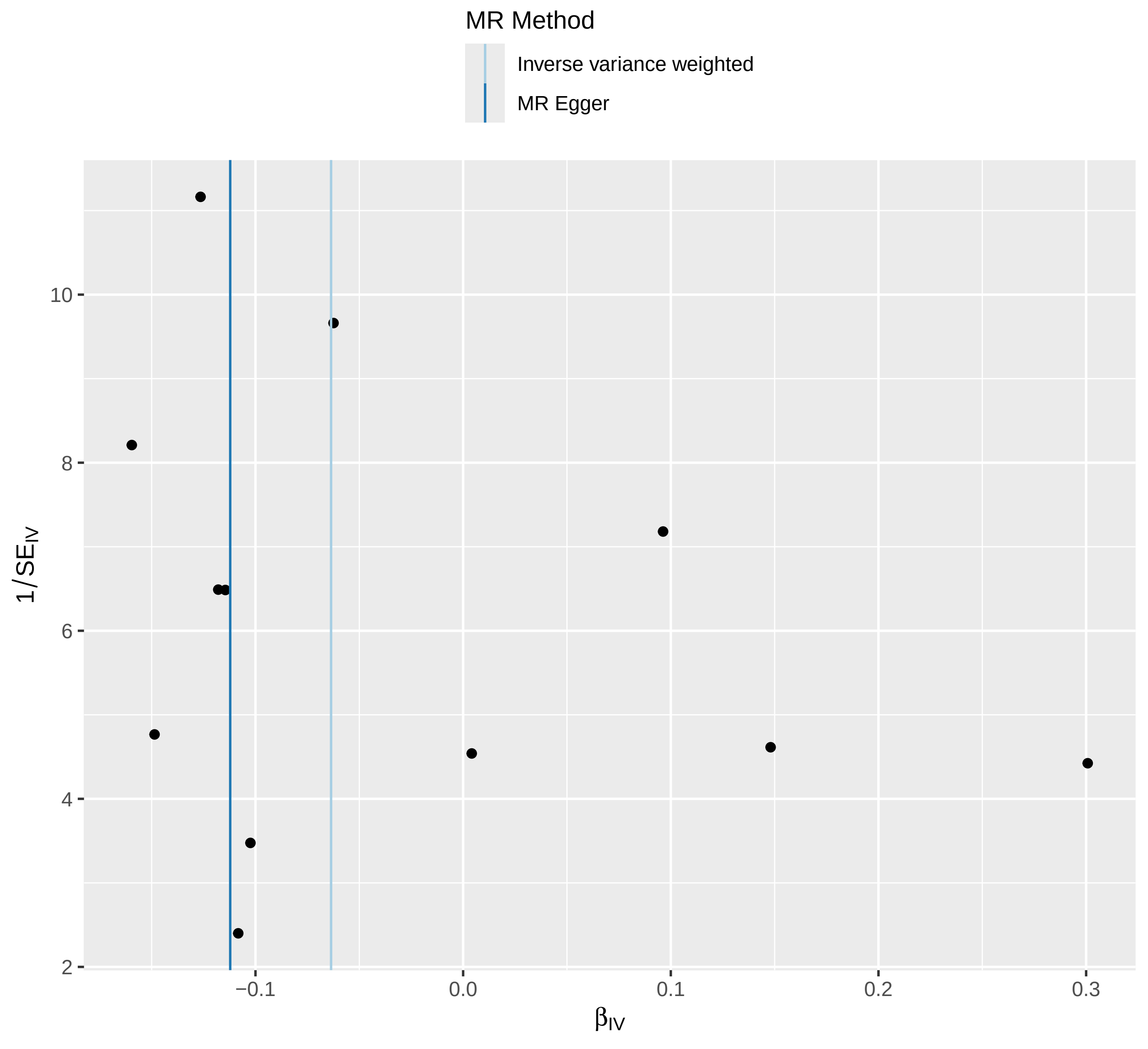

MR Method
Inverse variance weighted
MR Egger
10
8
6
4
2
1/SE_IV
−0.1
0.0
0.1
0.2
0.3
β_IV

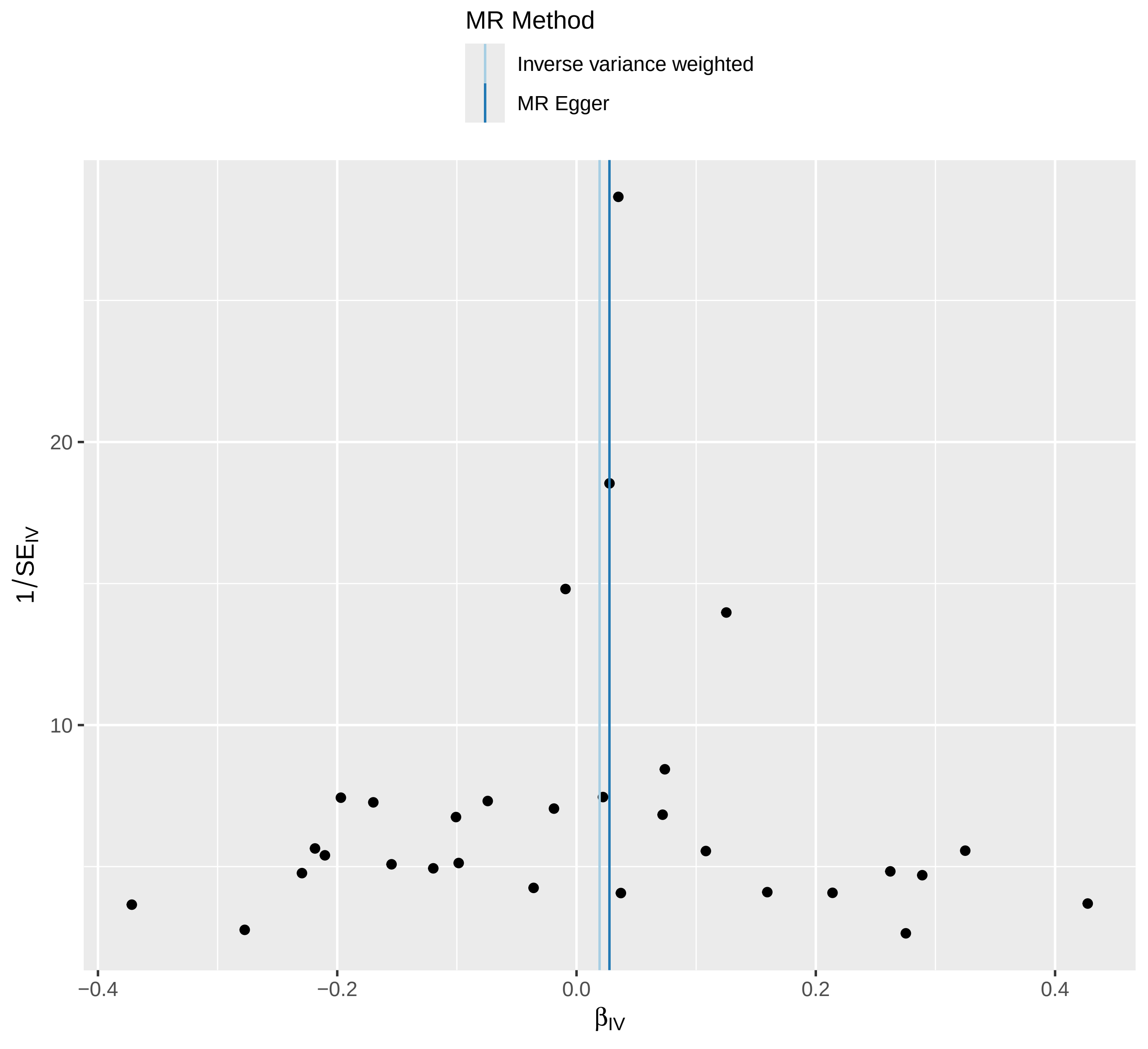
MR Method
Inverse variance weighted
MR Egger
20
10
1/SE$_{IV}$
−0.4
−0.2
0.0
0.2
0.4
β$_{IV}$

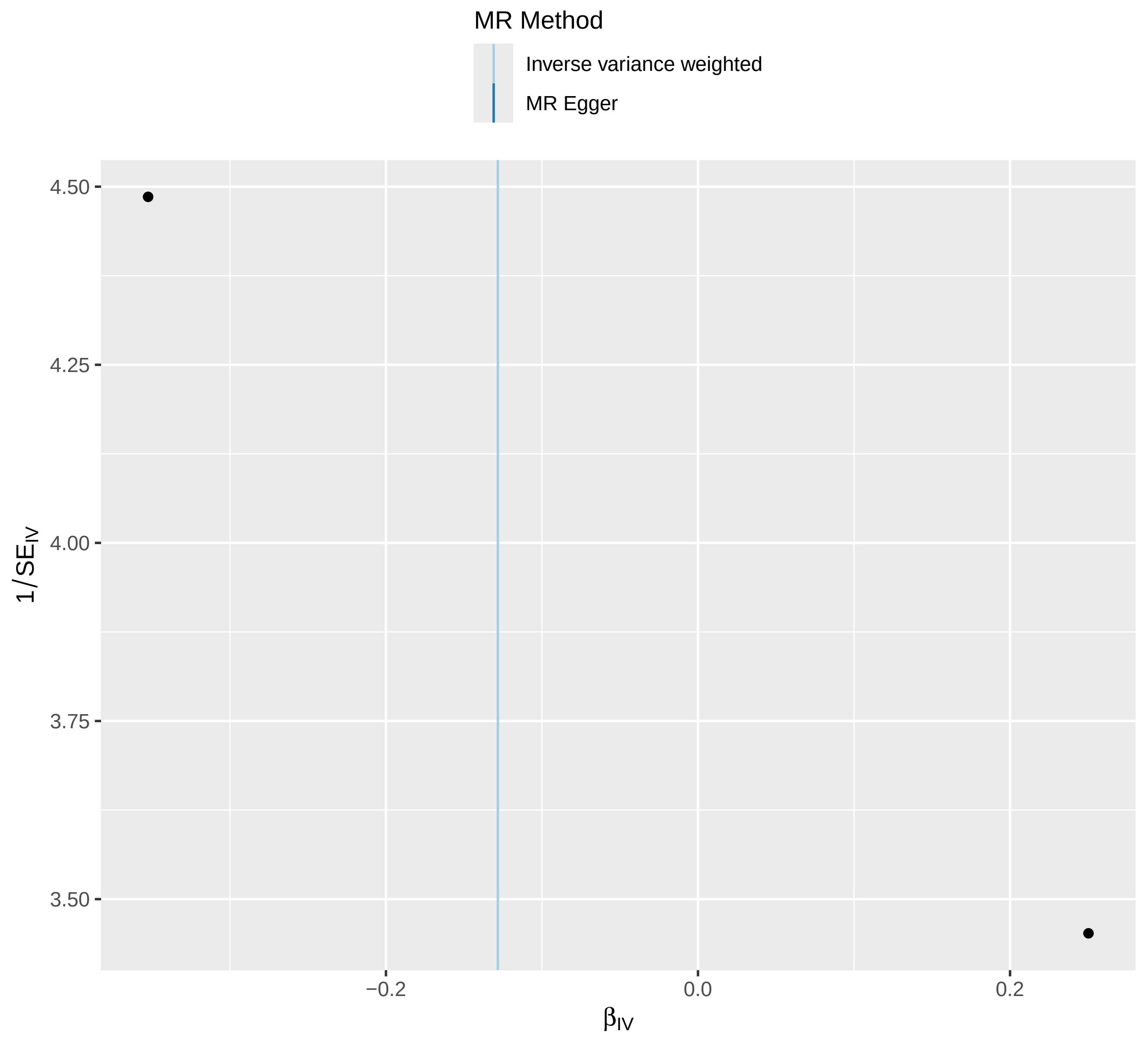

MR Method
Inverse variance weighted
MR Egger
4.50
4.25
4.00
3.75
3.50
1/SE_IV
−0.2
0.0
0.2
β_IV

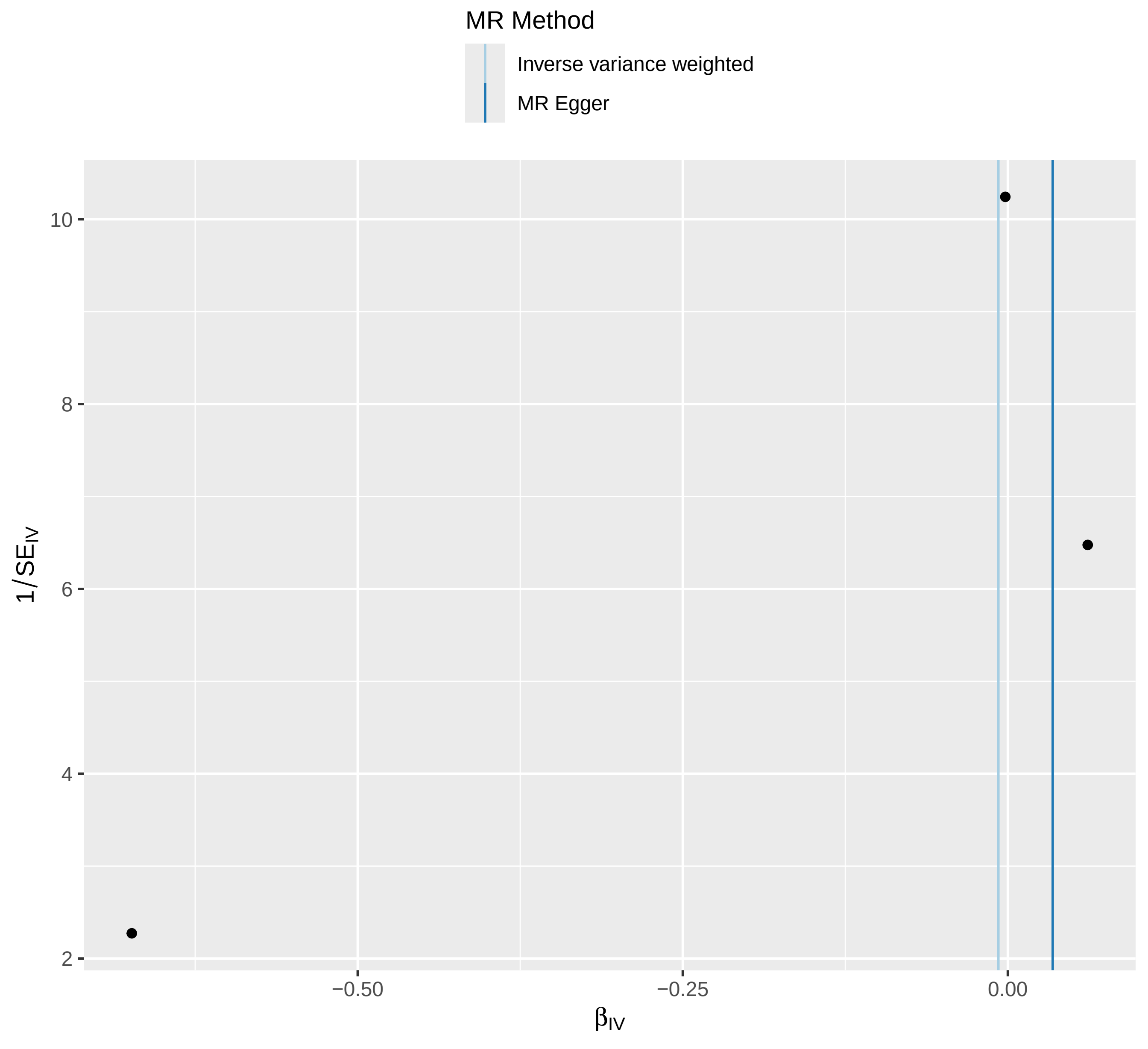

MR Method
Inverse variance weighted
MR Egger
10
8
6
4
2
1/SE_IV
−0.50
−0.25
0.00
β_IV

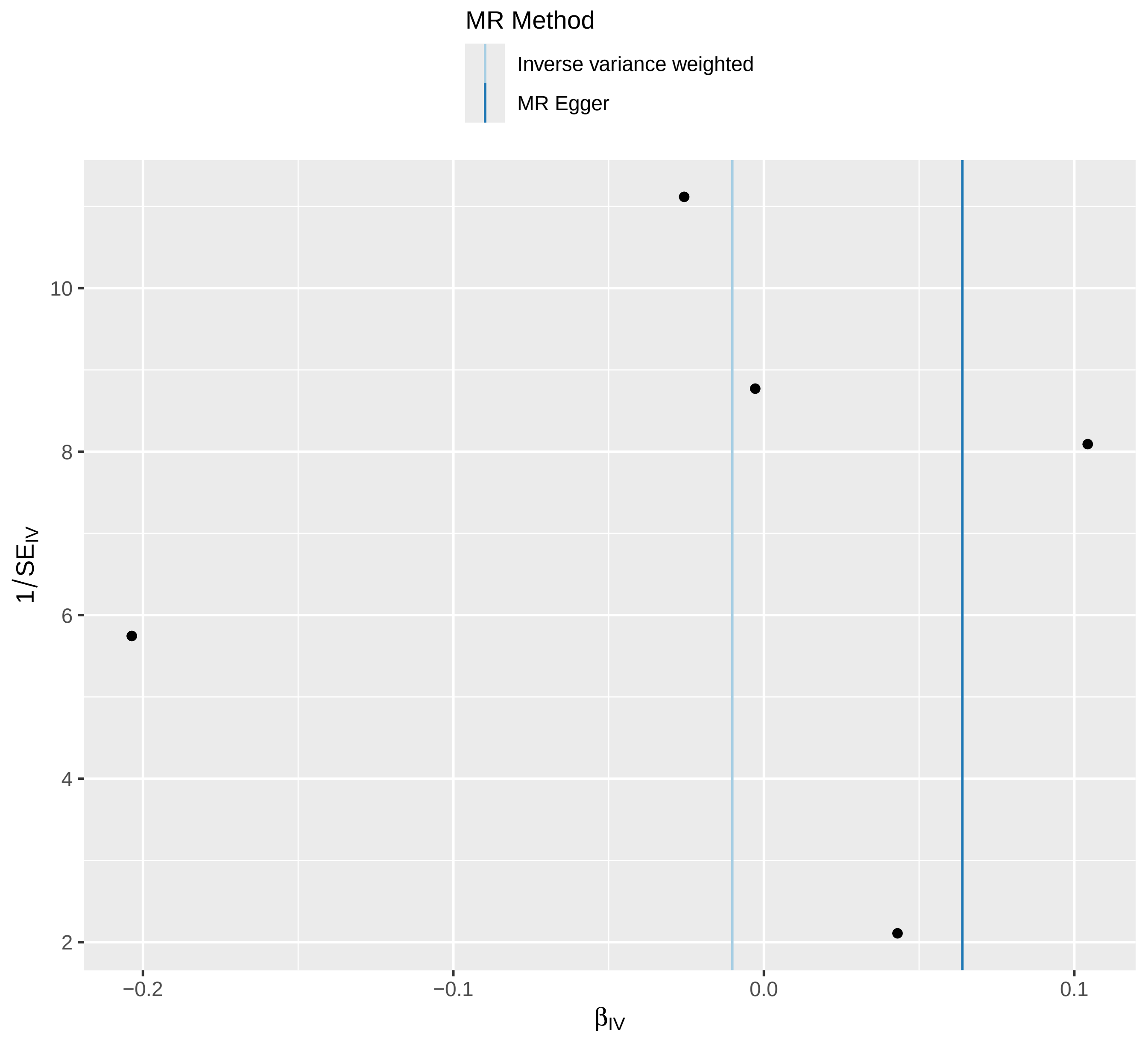
MR Method
Inverse variance weighted
MR Egger
1/SE_IV
10
8
6
4
2
−0.2
−0.1
0.0
0.1
β_IV

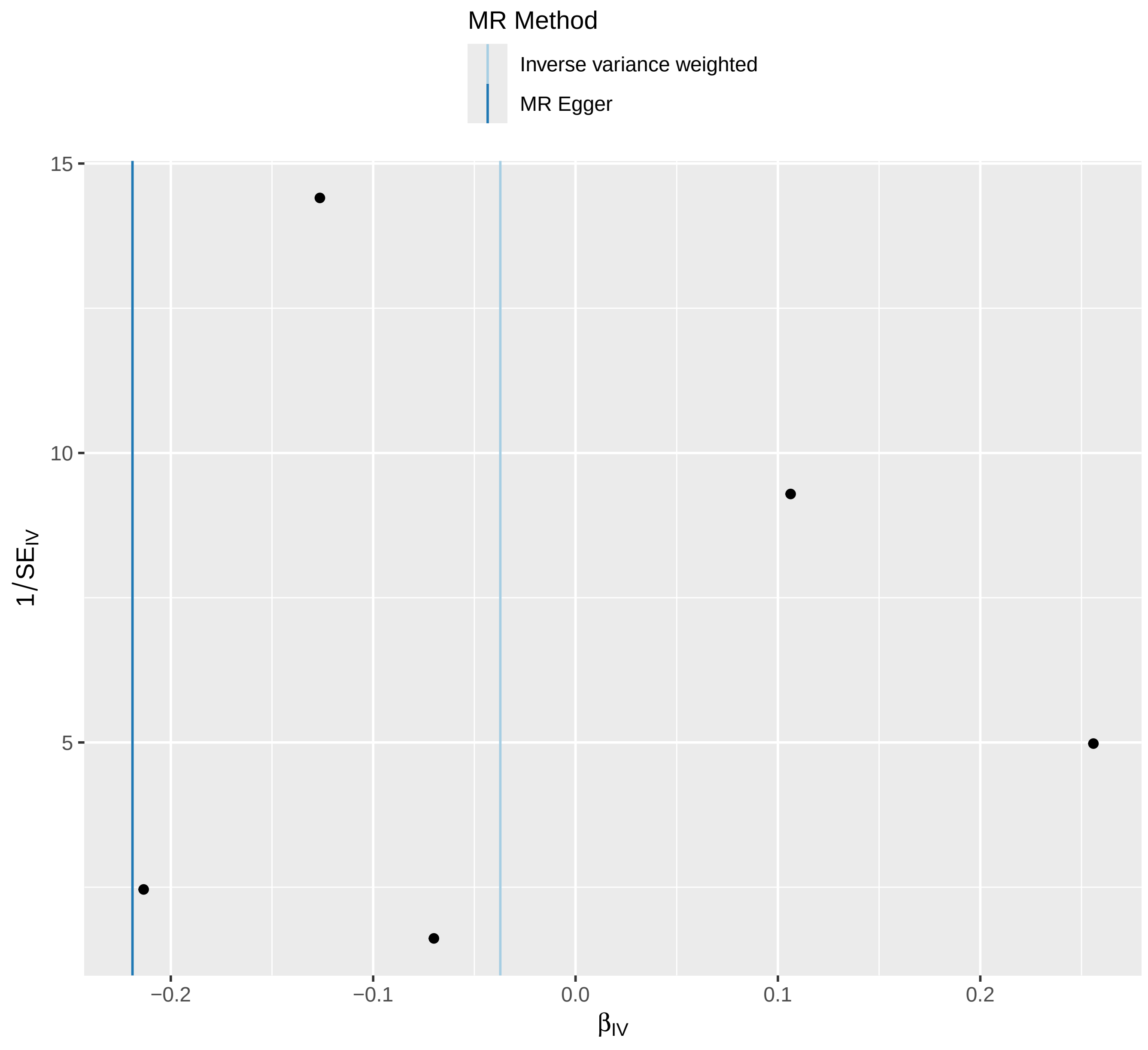
MR Method
Inverse variance weighted
MR Egger
15
10
5
1/SE$_{IV}$
−0.2
−0.1
0.0
0.1
0.2
β$_{IV}$

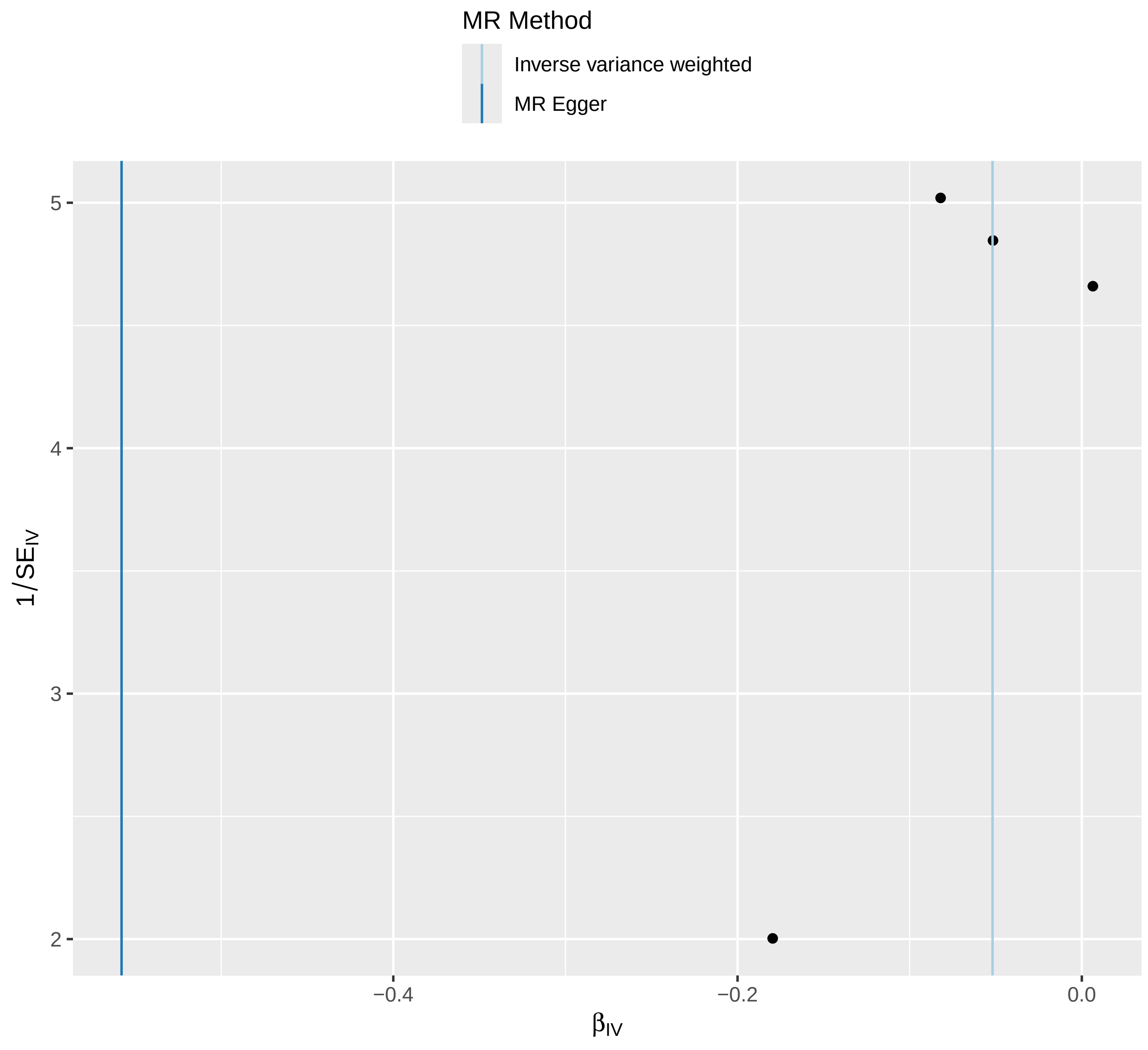

MR Method
Inverse variance weighted
MR Egger
5
4
3
2
1/SE_IV
−0.4
−0.2
0.0
β_IV

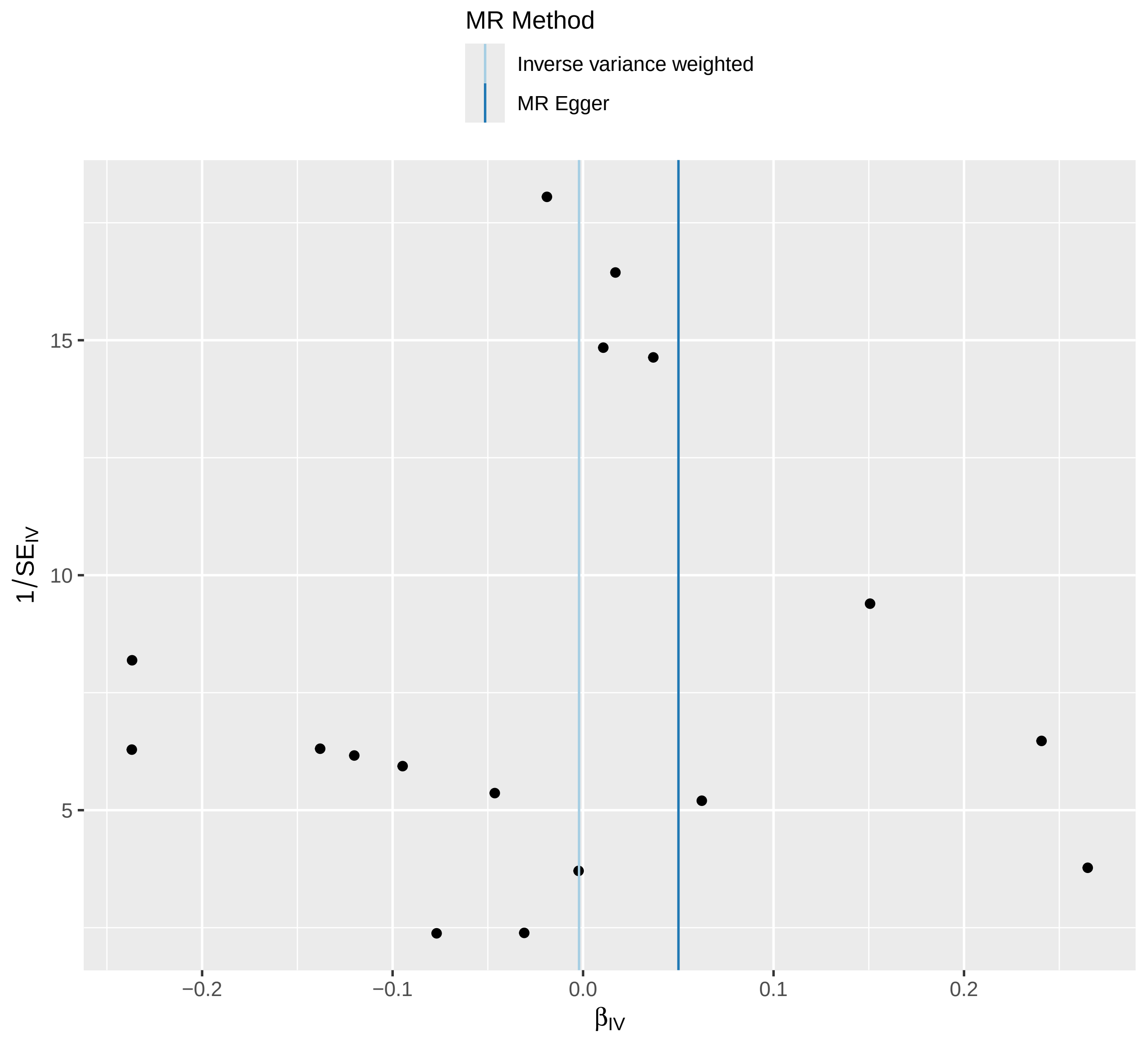

MR Method
Inverse variance weighted
MR Egger
15
10
5
$1/SE_{IV}$
−0.2
−0.1
0.0
0.1
0.2
$\beta_{IV}$

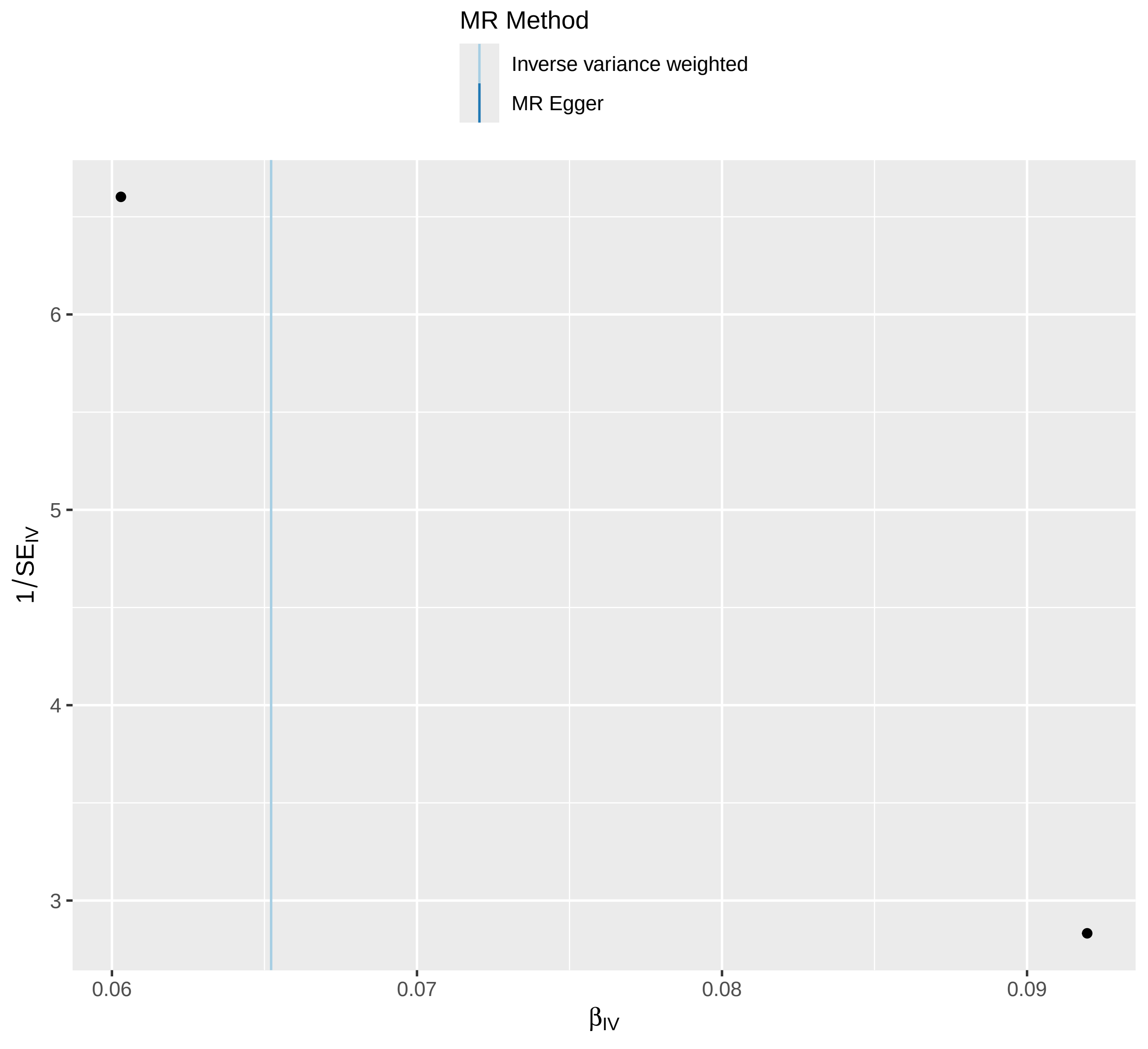

MR Method
Inverse variance weighted
MR Egger
6
5
4
3
1/SE$_{IV}$
0.06
0.07
0.08
0.09
β$_{IV}$

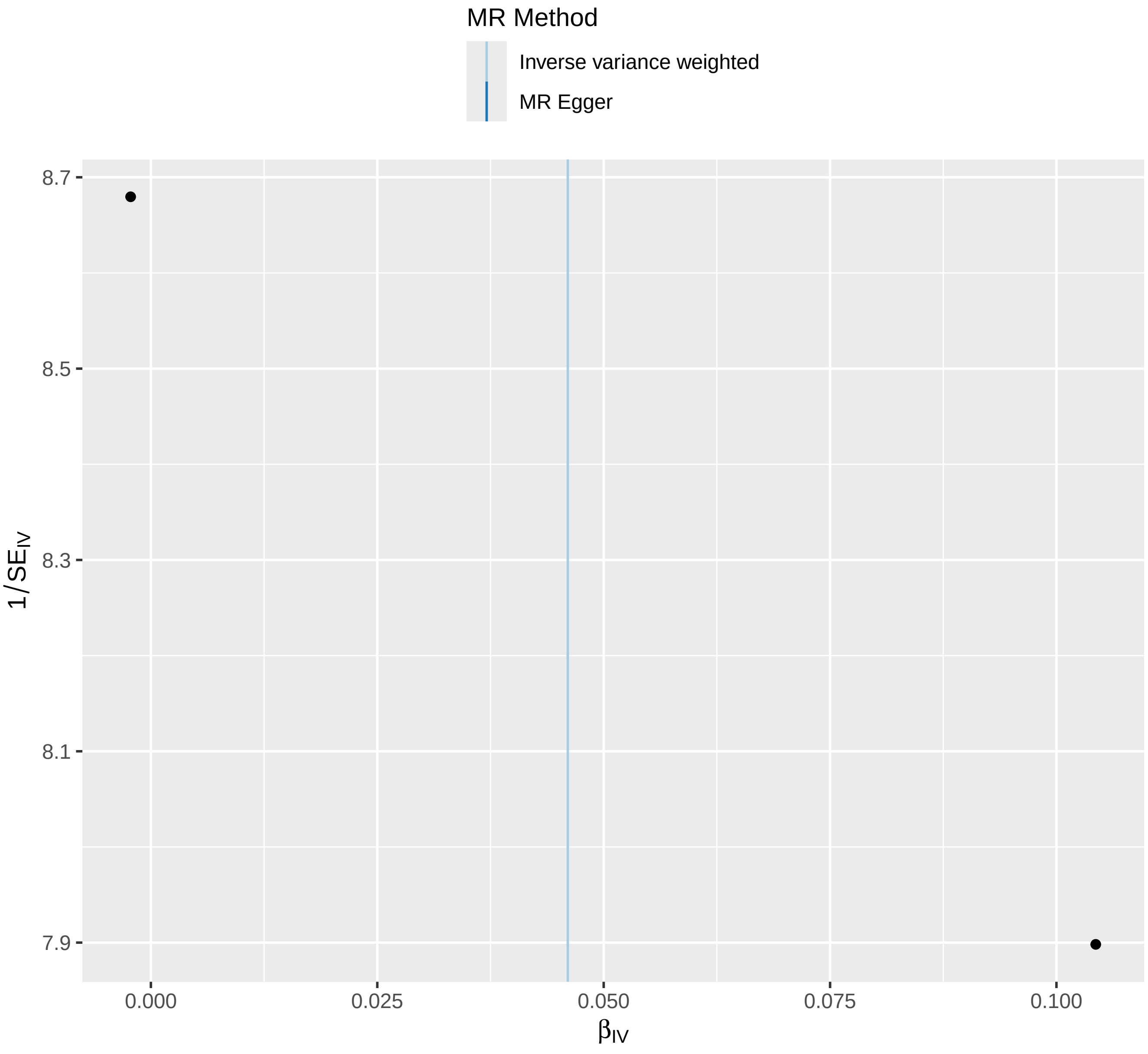

MR Method
Inverse variance weighted
MR Egger
8.7
8.5
8.3
8.1
7.9
$1/SE_{IV}$
0.000
0.025
0.050
0.075
0.100
$\beta_{IV}$

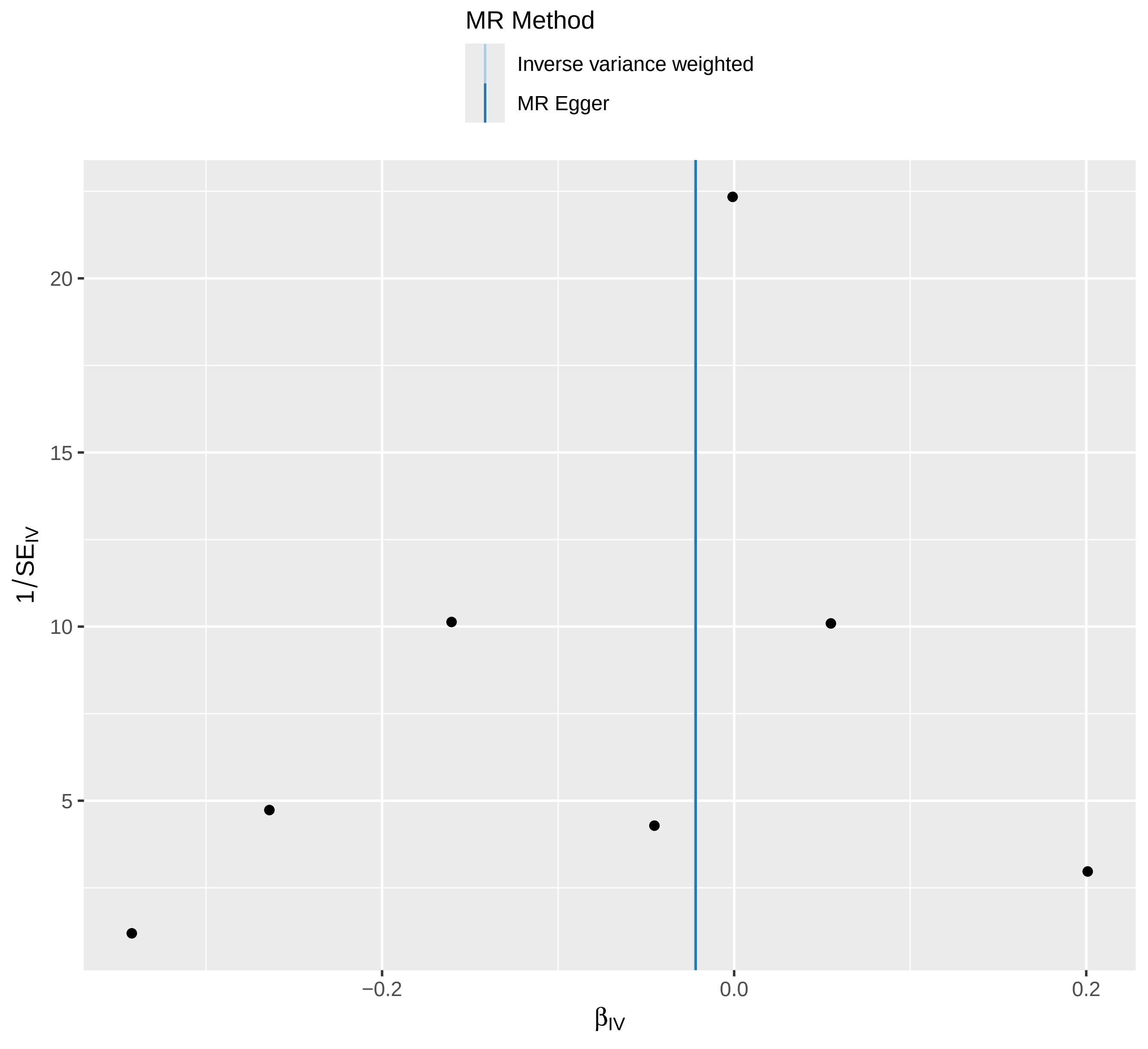

MR Method
Inverse variance weighted
MR Egger
20
15
10
5
$1/SE_{IV}$
−0.2
0.0
0.2
$\beta_{IV}$

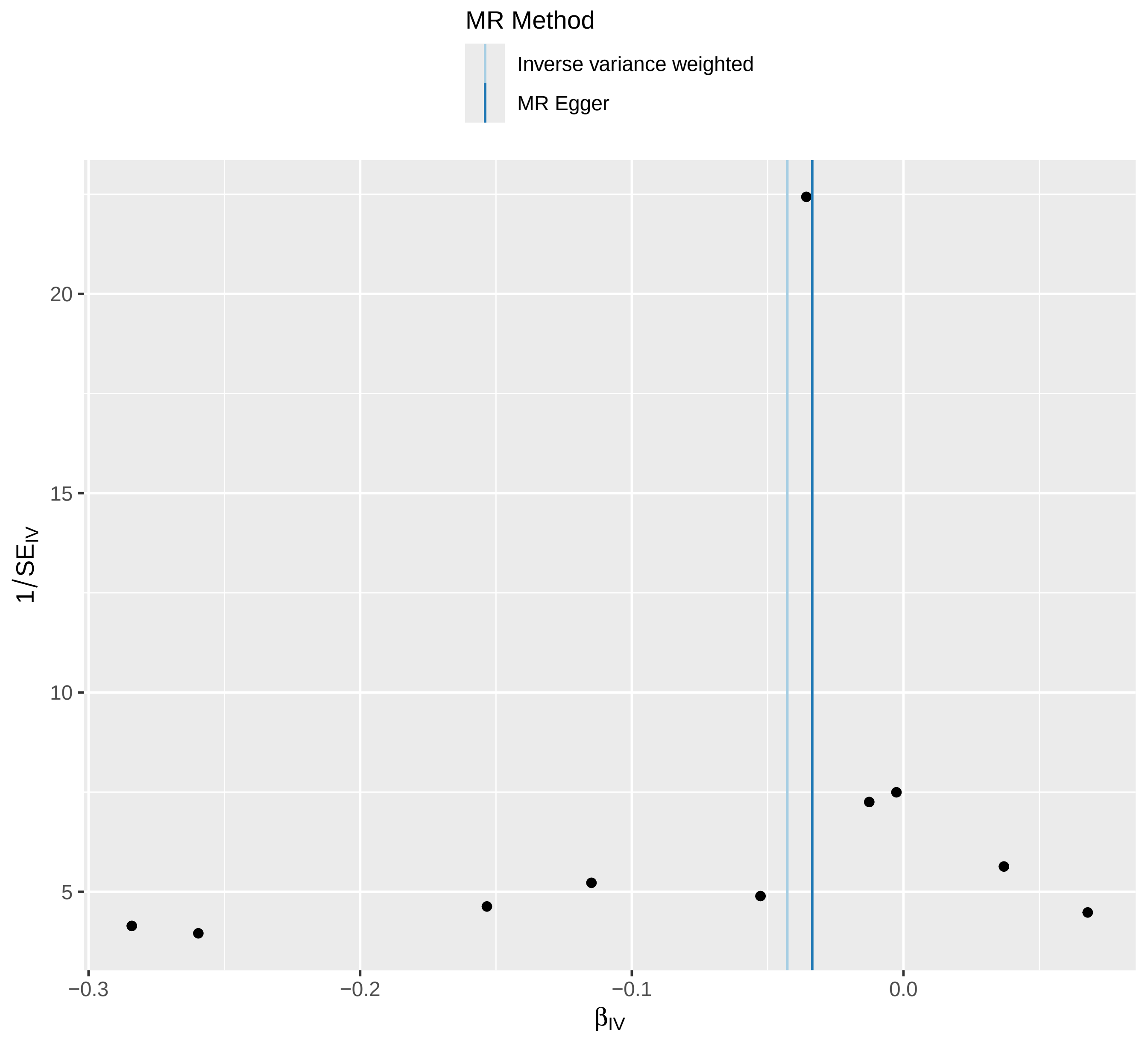
MR Method
Inverse variance weighted
MR Egger
20
15
10
5
1/SE_IV
−0.3
−0.2
−0.1
0.0
β_IV

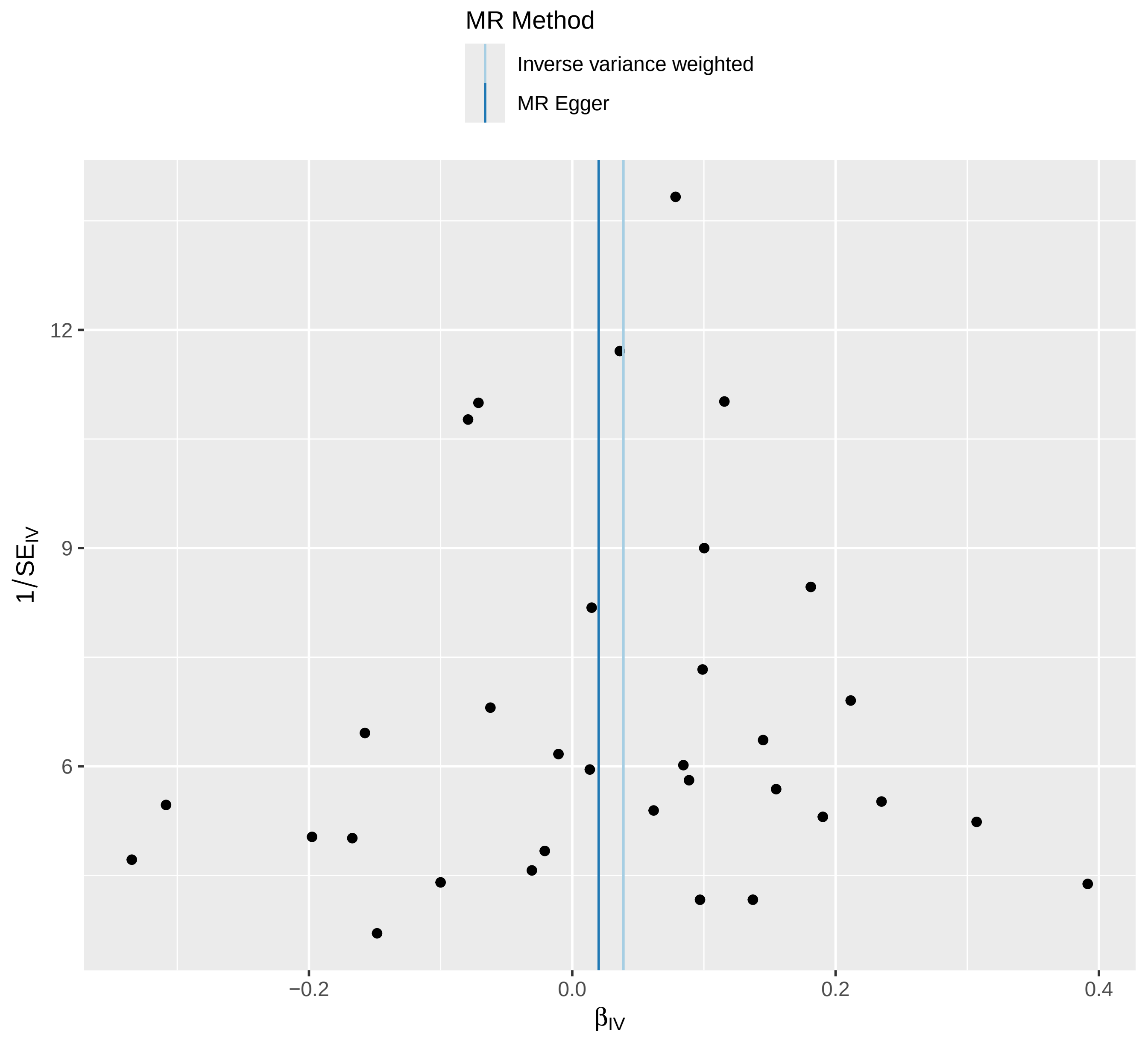

MR Method
Inverse variance weighted
MR Egger
12
9
6
1/SE_IV
−0.2
0.0
0.2
0.4
β_IV

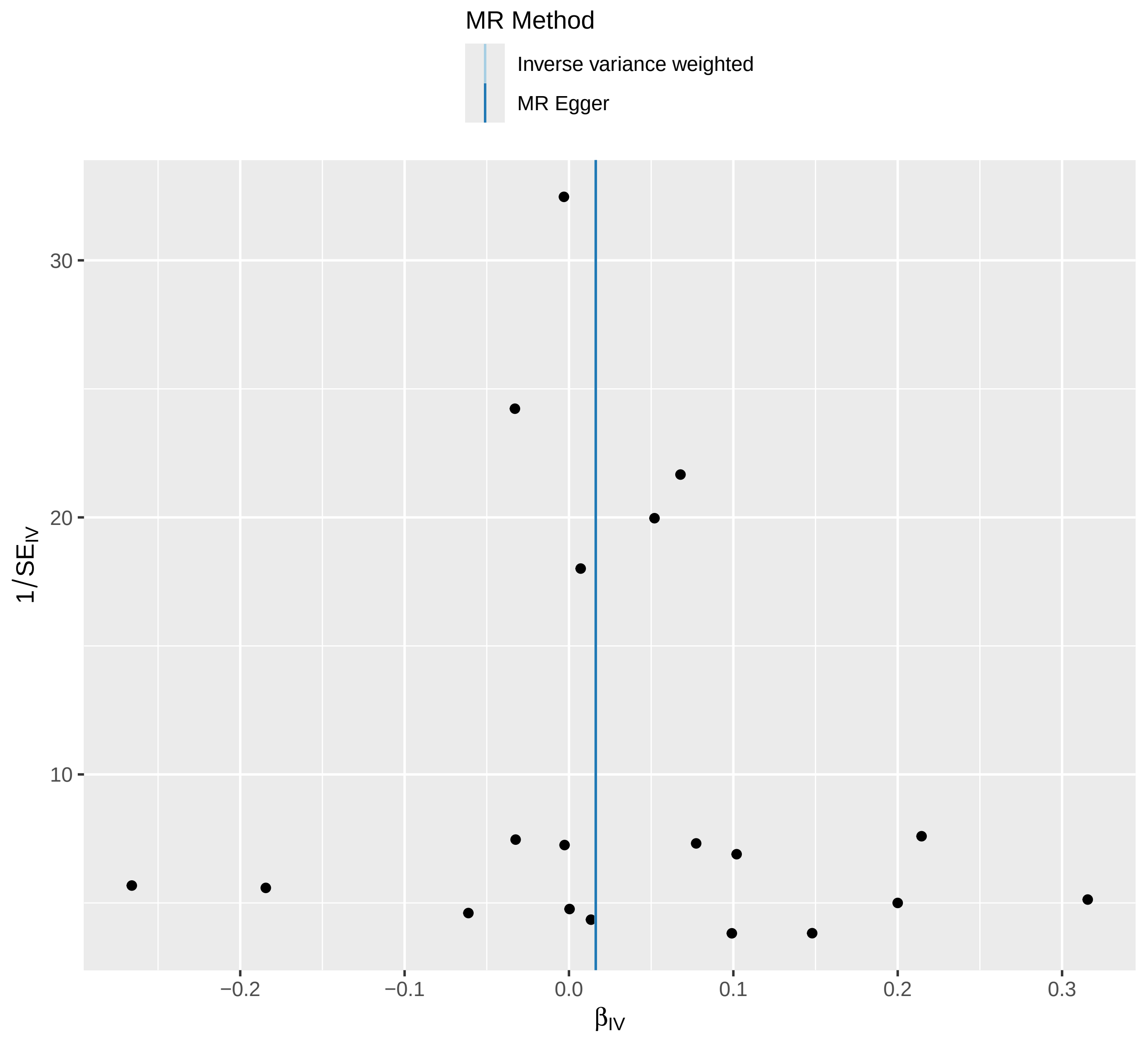
MR Method
Inverse variance weighted
MR Egger
30
20
10
1/SE$_{IV}$
−0.2
−0.1
0.0
0.1
0.2
0.3
β$_{IV}$

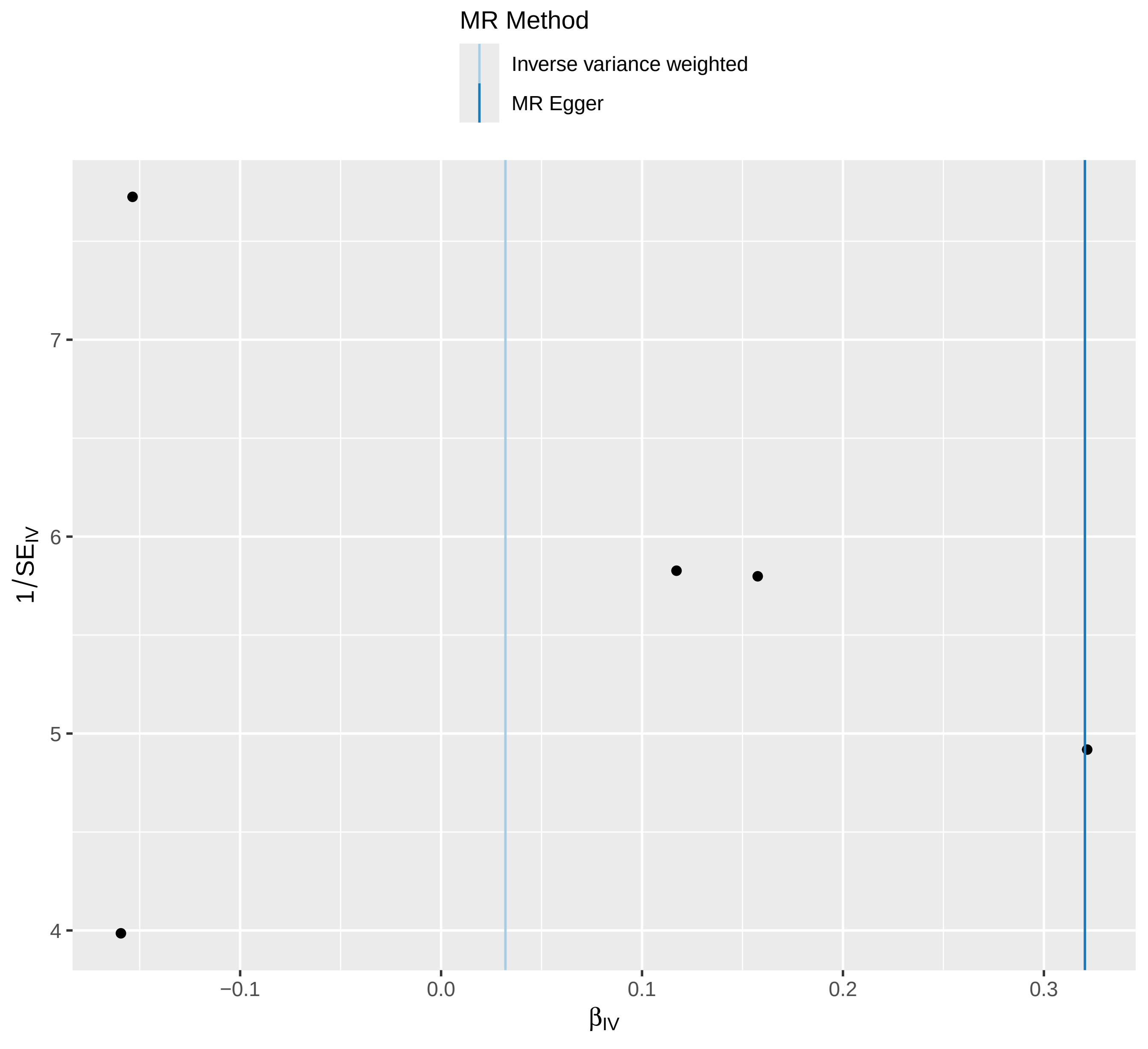

MR Method
Inverse variance weighted
MR Egger
7
6
5
4
1/SE_IV
−0.1
0.0
0.1
0.2
0.3
β_IV

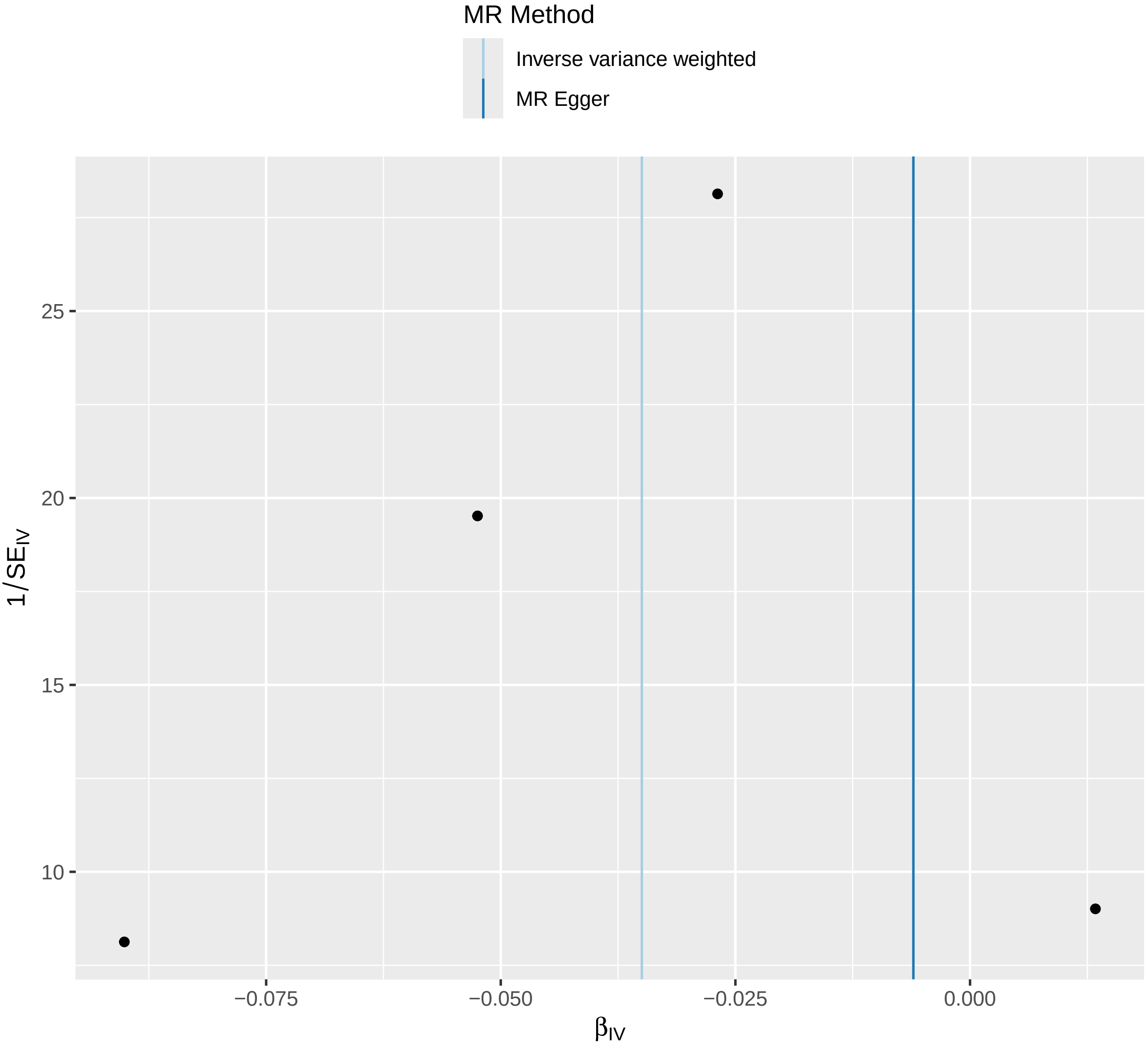
MR Method
Inverse variance weighted
MR Egger
25
20
15
10
1/SE$_{IV}$
−0.075
−0.050
−0.025
0.000
β$_{IV}$

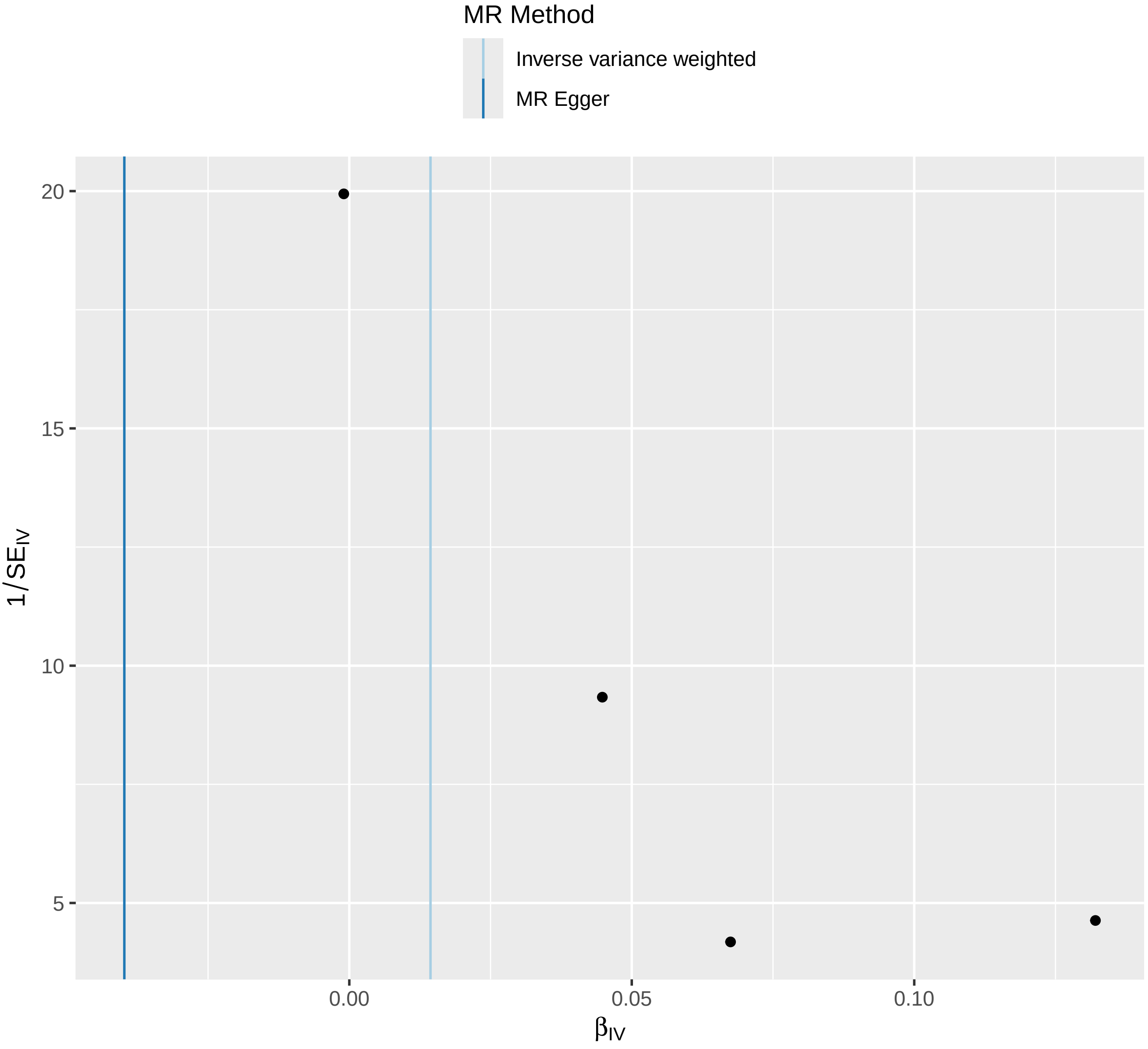
MR Method
Inverse variance weighted
MR Egger
20
15
10
5
1/SE_IV
0.00
0.05
0.10
β_IV

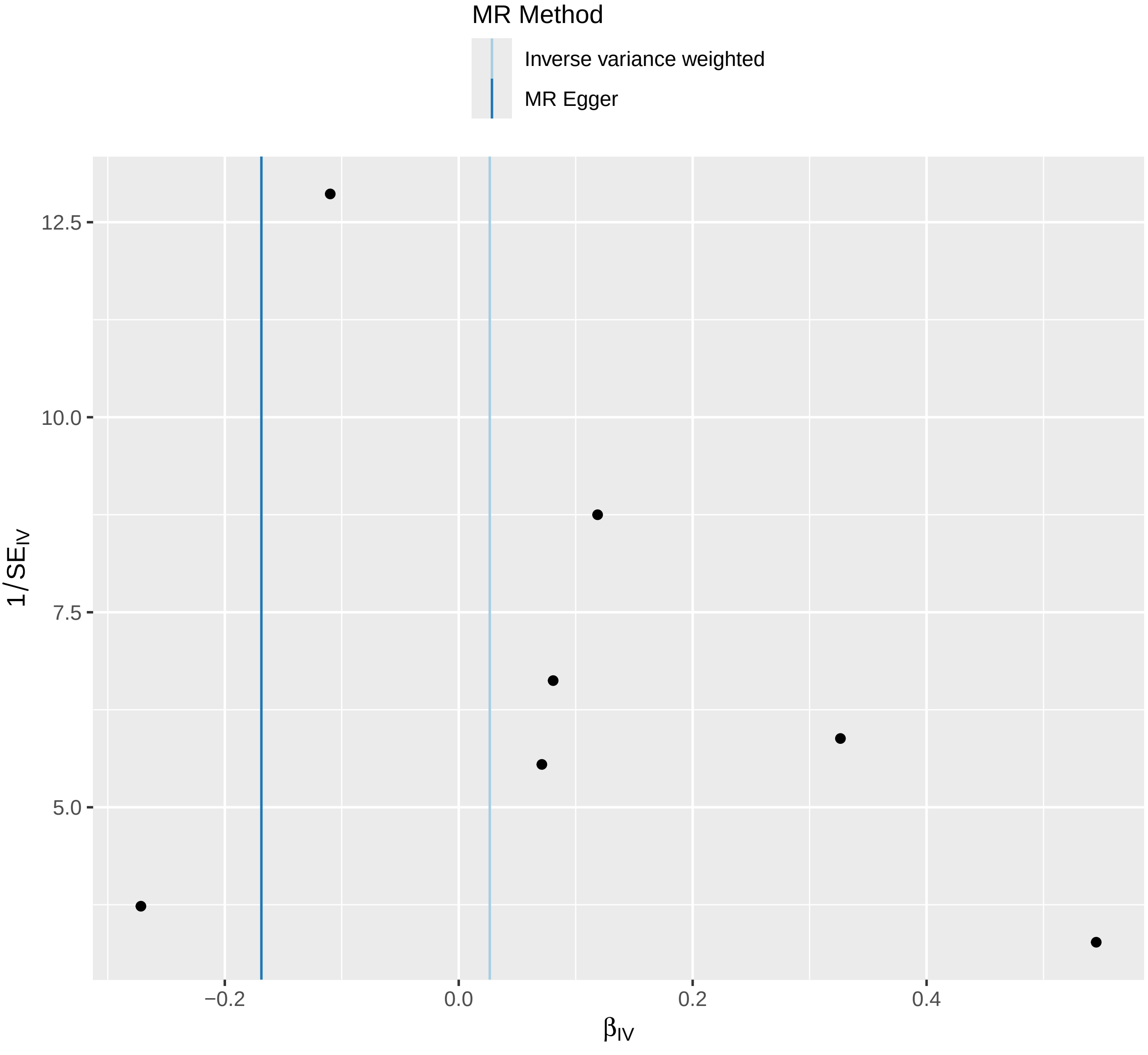

MR Method
Inverse variance weighted
MR Egger
12.5
10.0
7.5
5.0
1/SE_IV
−0.2
0.0
0.2
0.4
β_IV

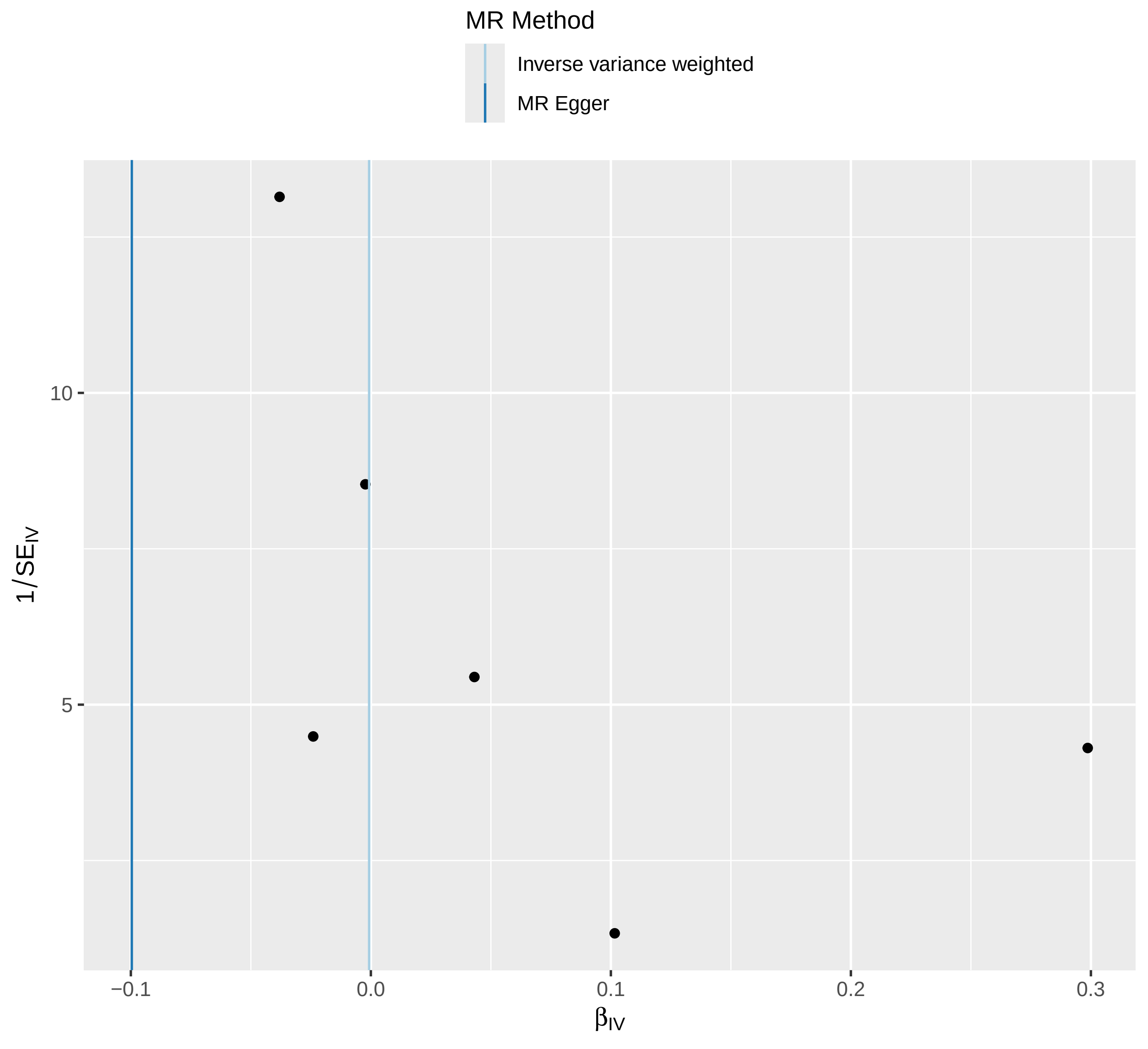

MR Method
Inverse variance weighted
MR Egger
1/SE$_{IV}$
10
5
−0.1
0.0
0.1
0.2
0.3
β$_{IV}$

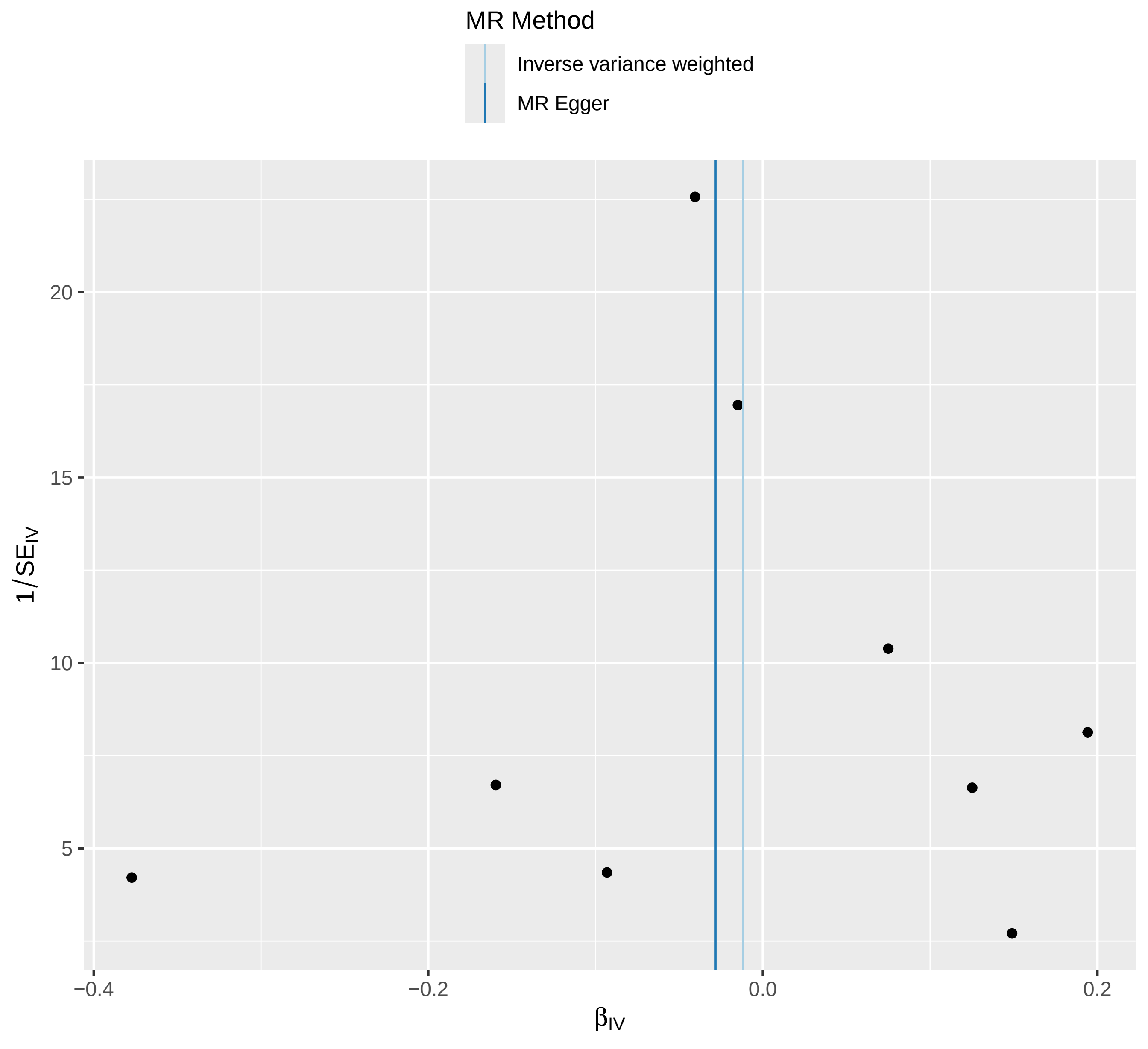
MR Method
Inverse variance weighted
MR Egger
1/SE$_{IV}$
20
15
10
5
−0.4
−0.2
0.0
0.2
β$_{IV}$

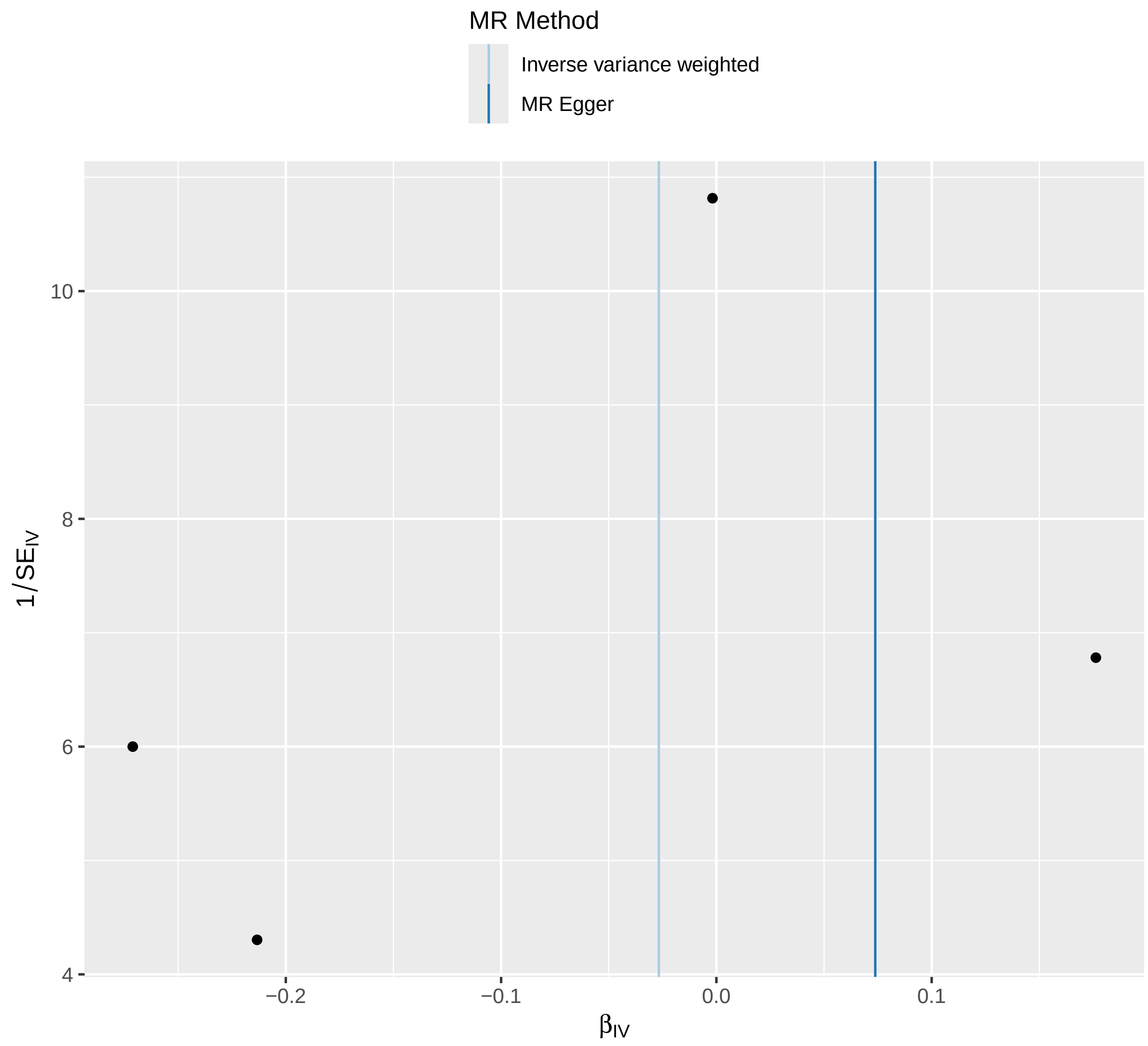

MR Method
Inverse variance weighted
MR Egger
10
8
6
4
1/SE$_{IV}$
−0.2
−0.1
0.0
0.1
β$_{IV}$

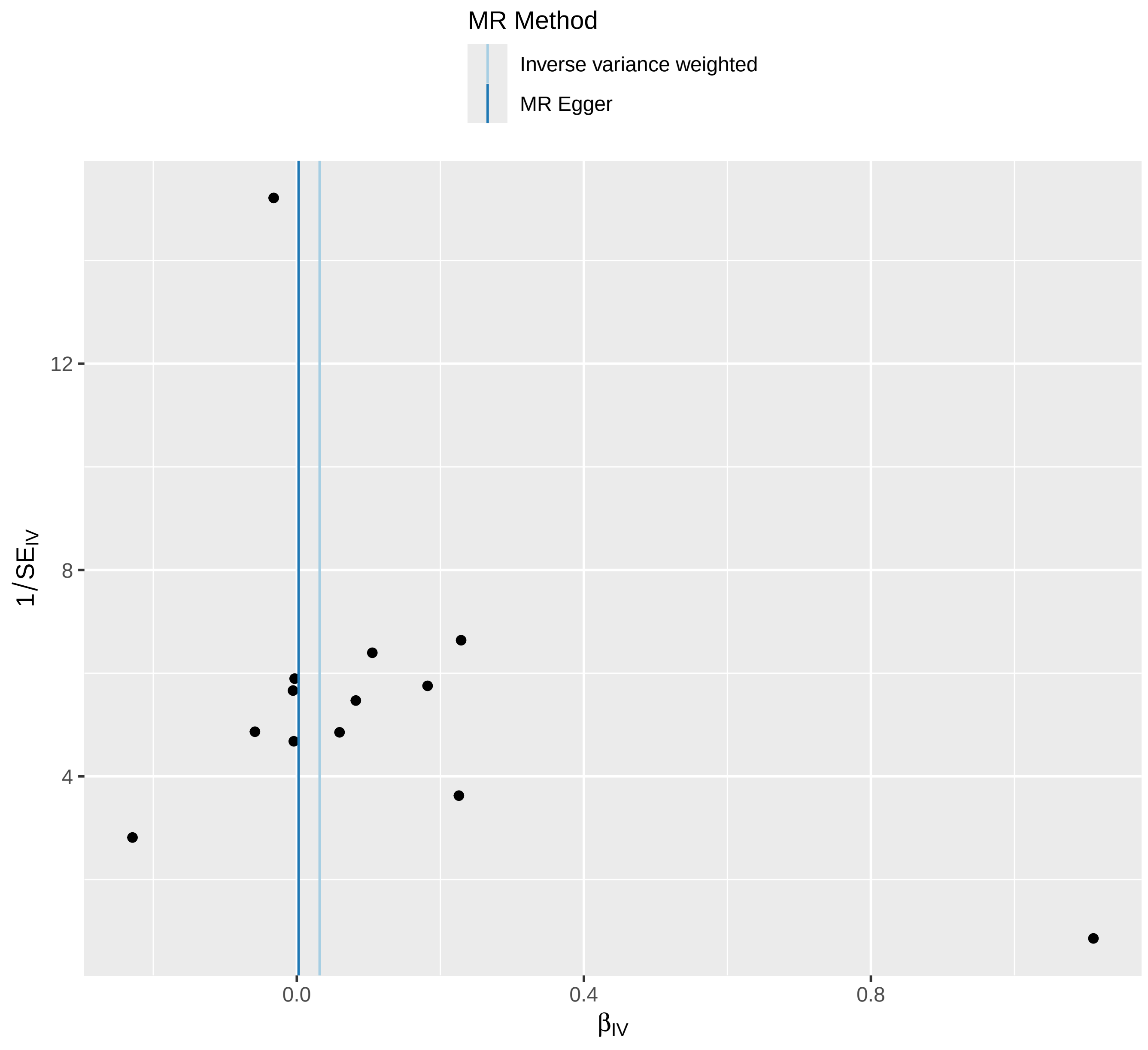

MR Method
Inverse variance weighted
MR Egger
12
8
4
0.0
0.4
0.8
$1/SE_{IV}$
$\beta_{IV}$

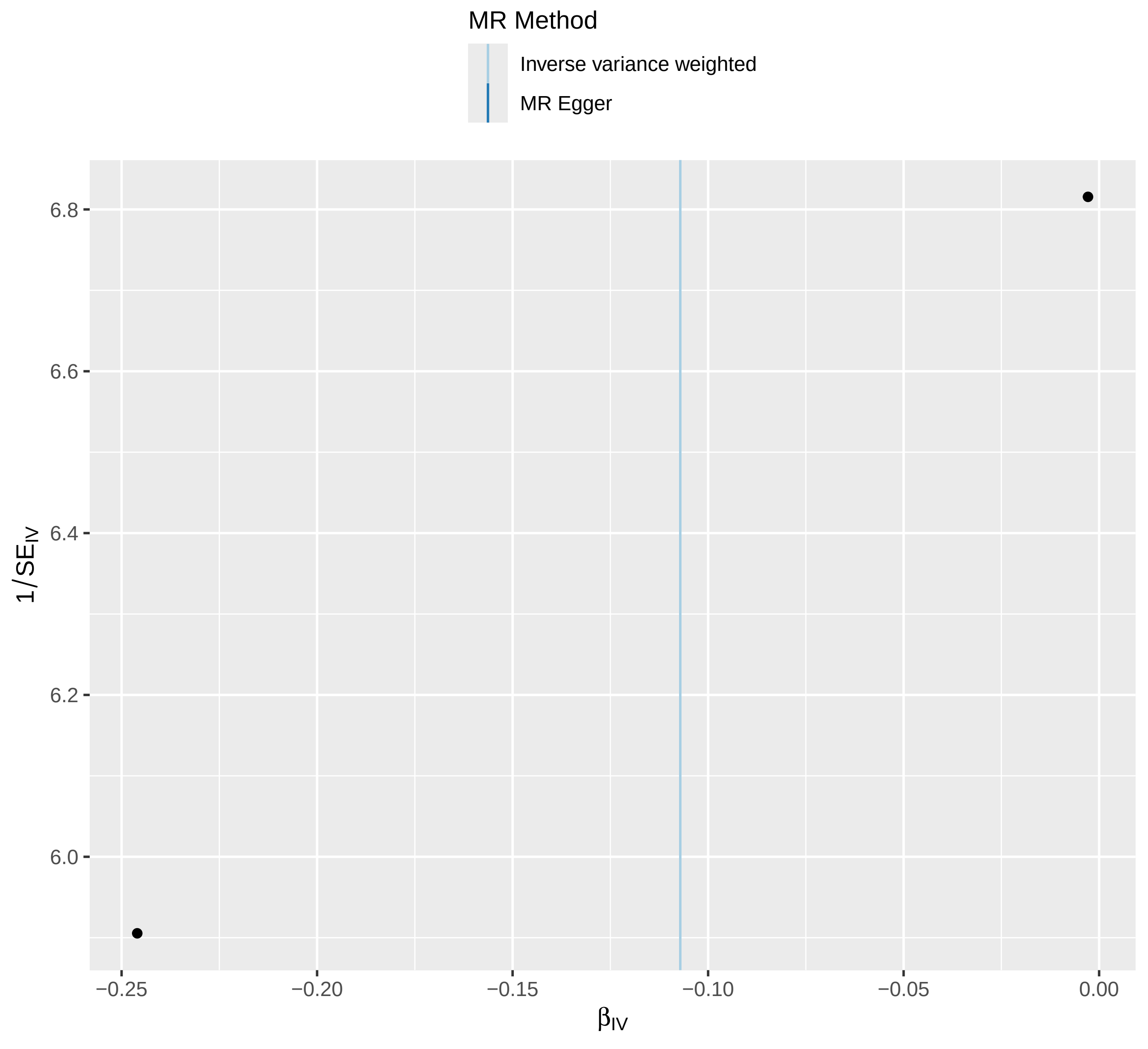
MR Method
Inverse variance weighted
MR Egger
1/SE$_{IV}$
6.8
6.6
6.4
6.2
6.0
−0.25
−0.20
−0.15
−0.10
−0.05
0.00
β$_{IV}$

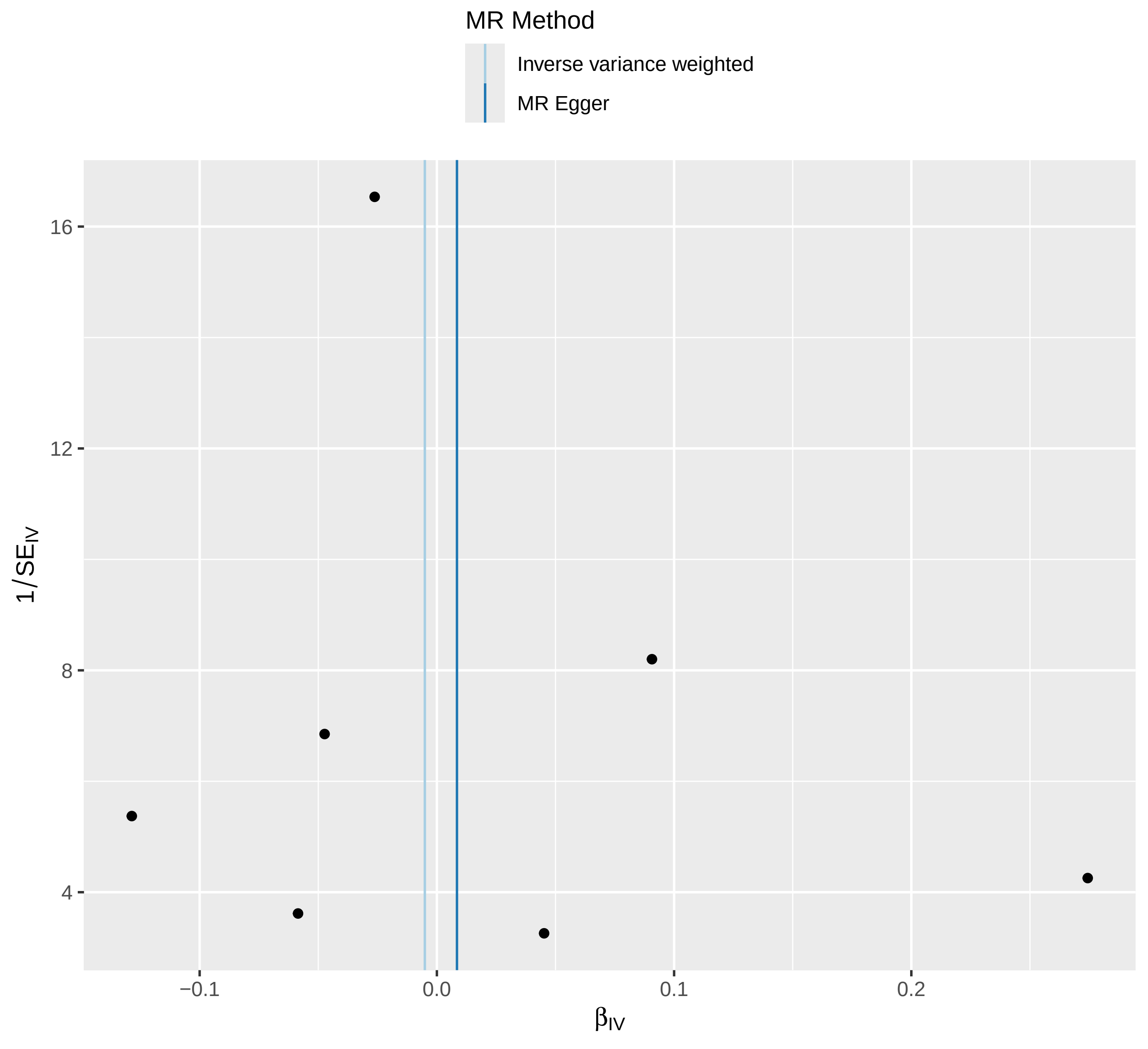

MR Method
Inverse variance weighted
MR Egger
16
12
8
4
1/SE_IV
−0.1
0.0
0.1
0.2
β_IV

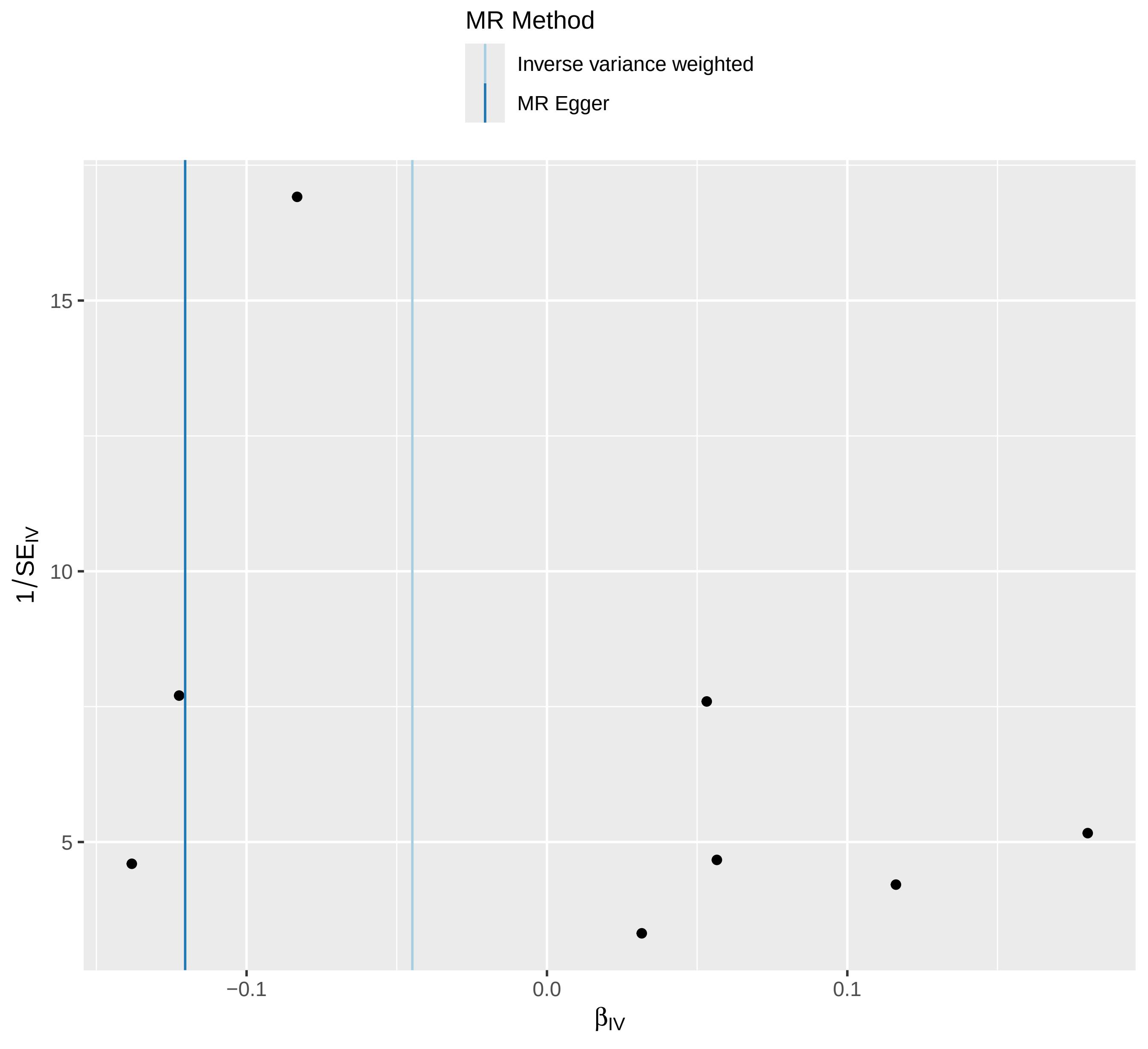
MR Method
Inverse variance weighted
MR Egger
15
10
5
1/SE_IV
−0.1
0.0
0.1
β_IV

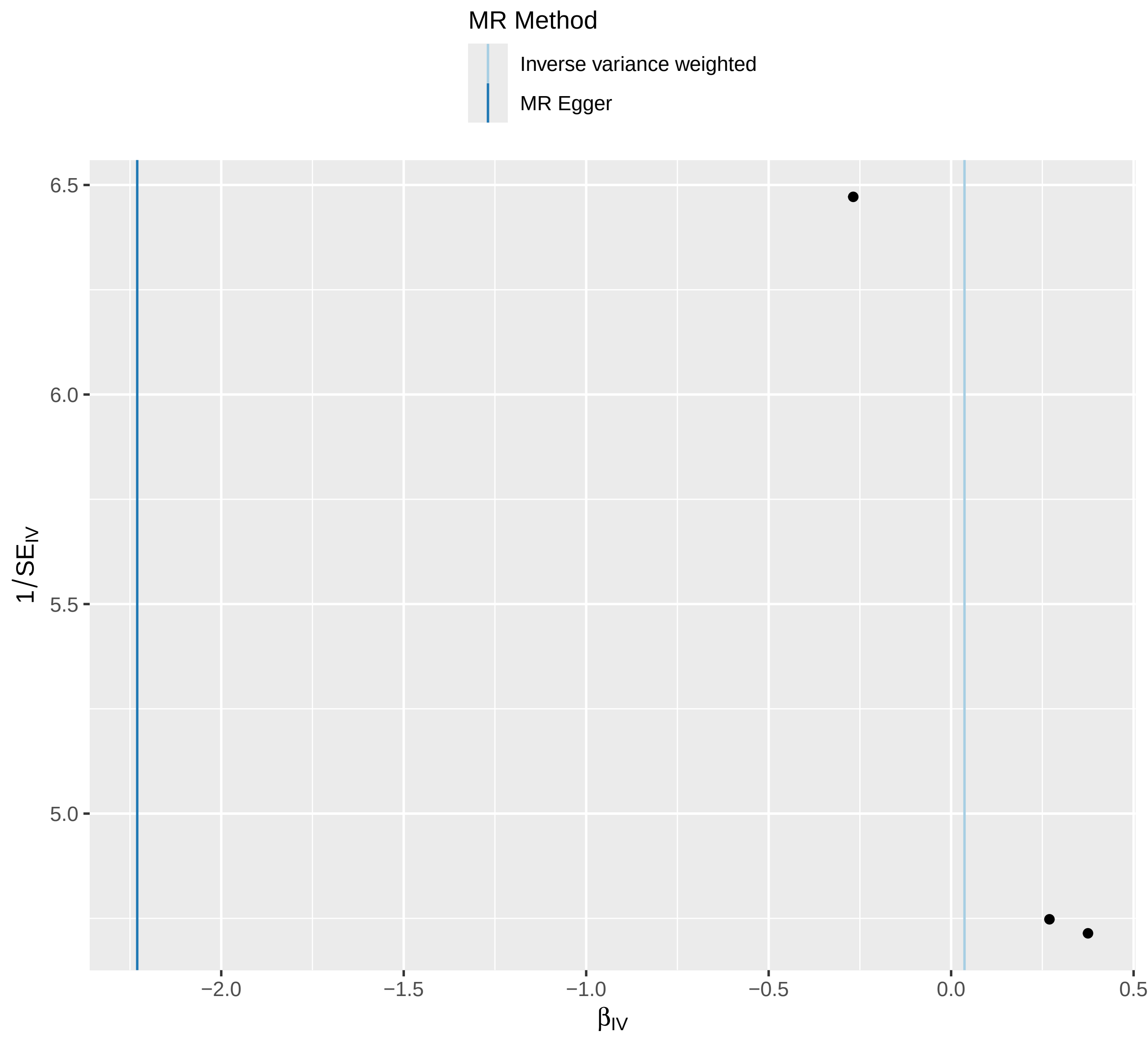
MR Method
Inverse variance weighted
MR Egger
6.5
6.0
5.5
5.0
$1/SE_{IV}$
−2.0
−1.5
−1.0
−0.5
0.0
0.5
$\beta_{IV}$

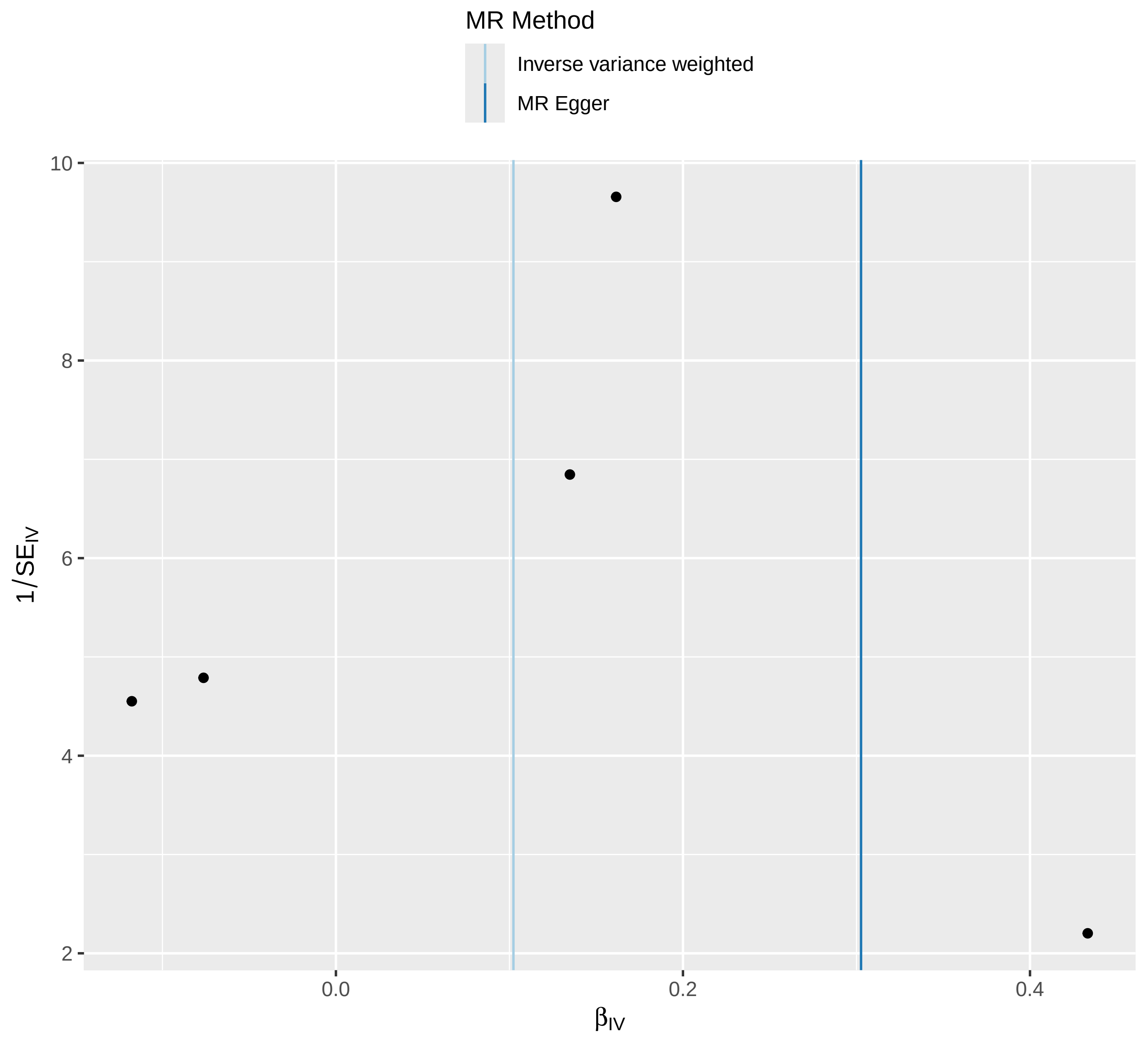

MR Method
Inverse variance weighted
MR Egger
10
8
6
4
2
1/SE_IV
0.0
0.2
0.4
β_IV

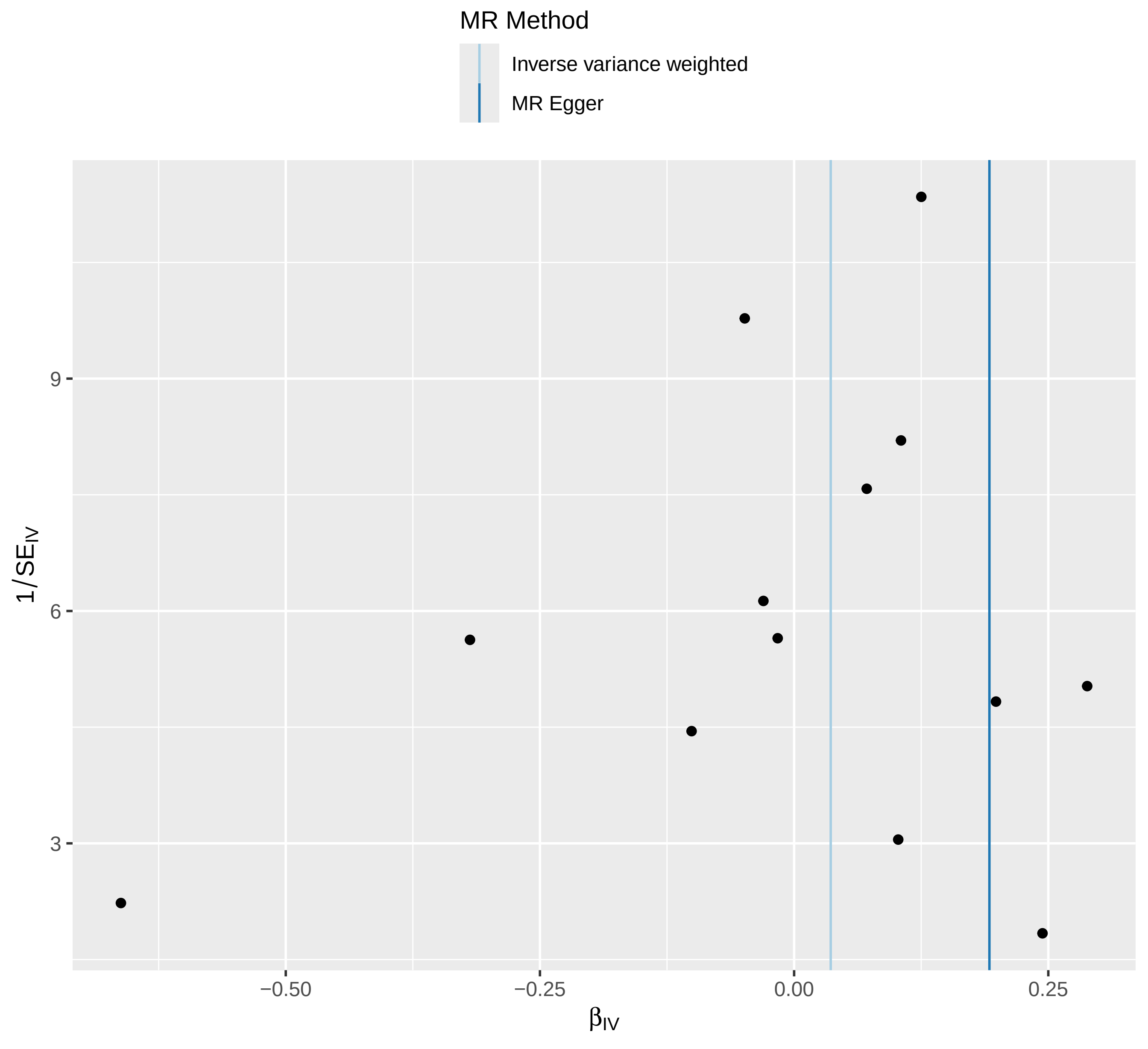
MR Method
Inverse variance weighted
MR Egger
9
6
3
1/SE_IV
−0.50
−0.25
0.00
0.25
β_IV

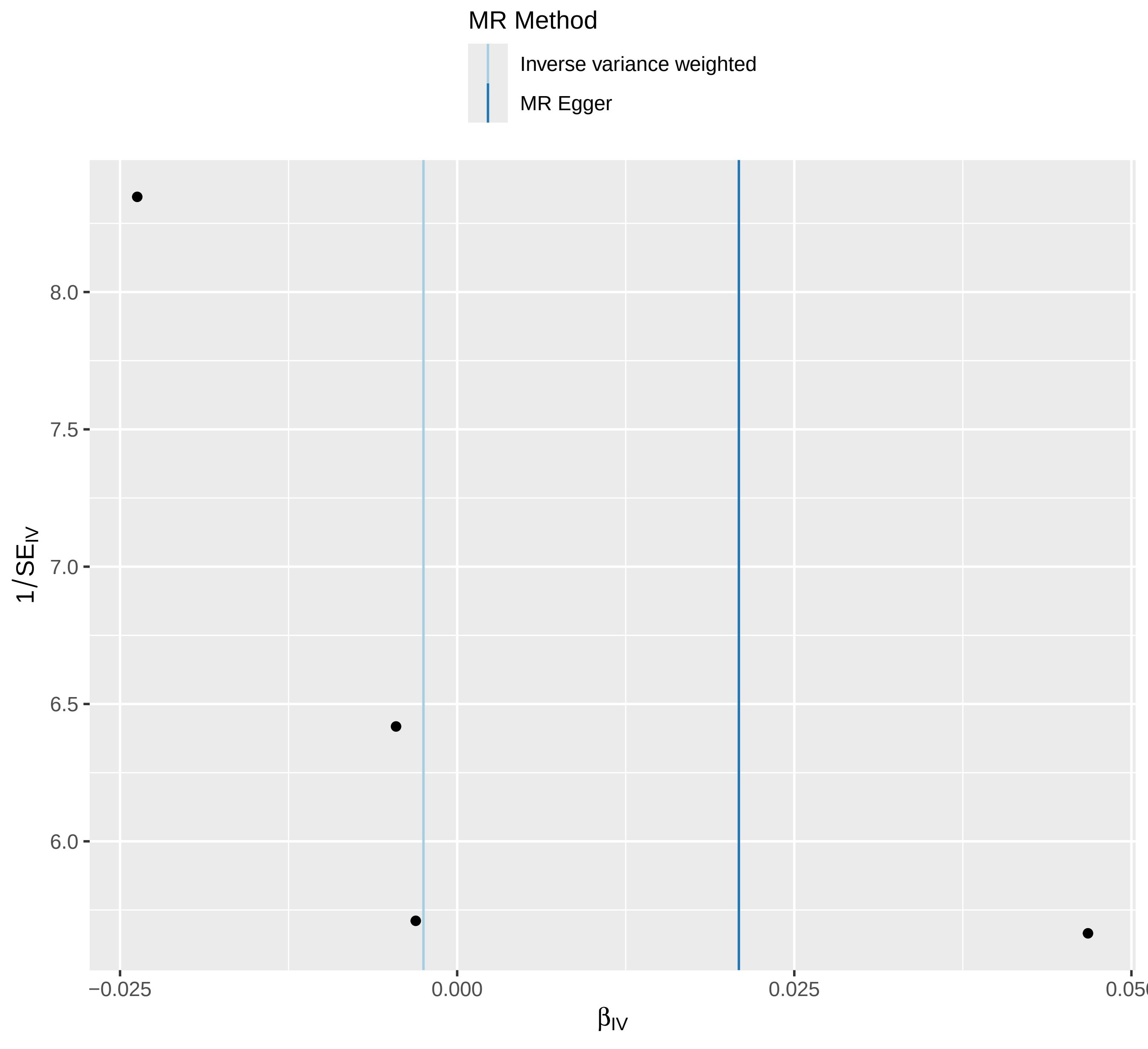

MR Method
Inverse variance weighted
MR Egger
8.0
7.5
7.0
6.5
6.0
1/SE$_{IV}$
−0.025
0.000
0.025
0.050
β$_{IV}$

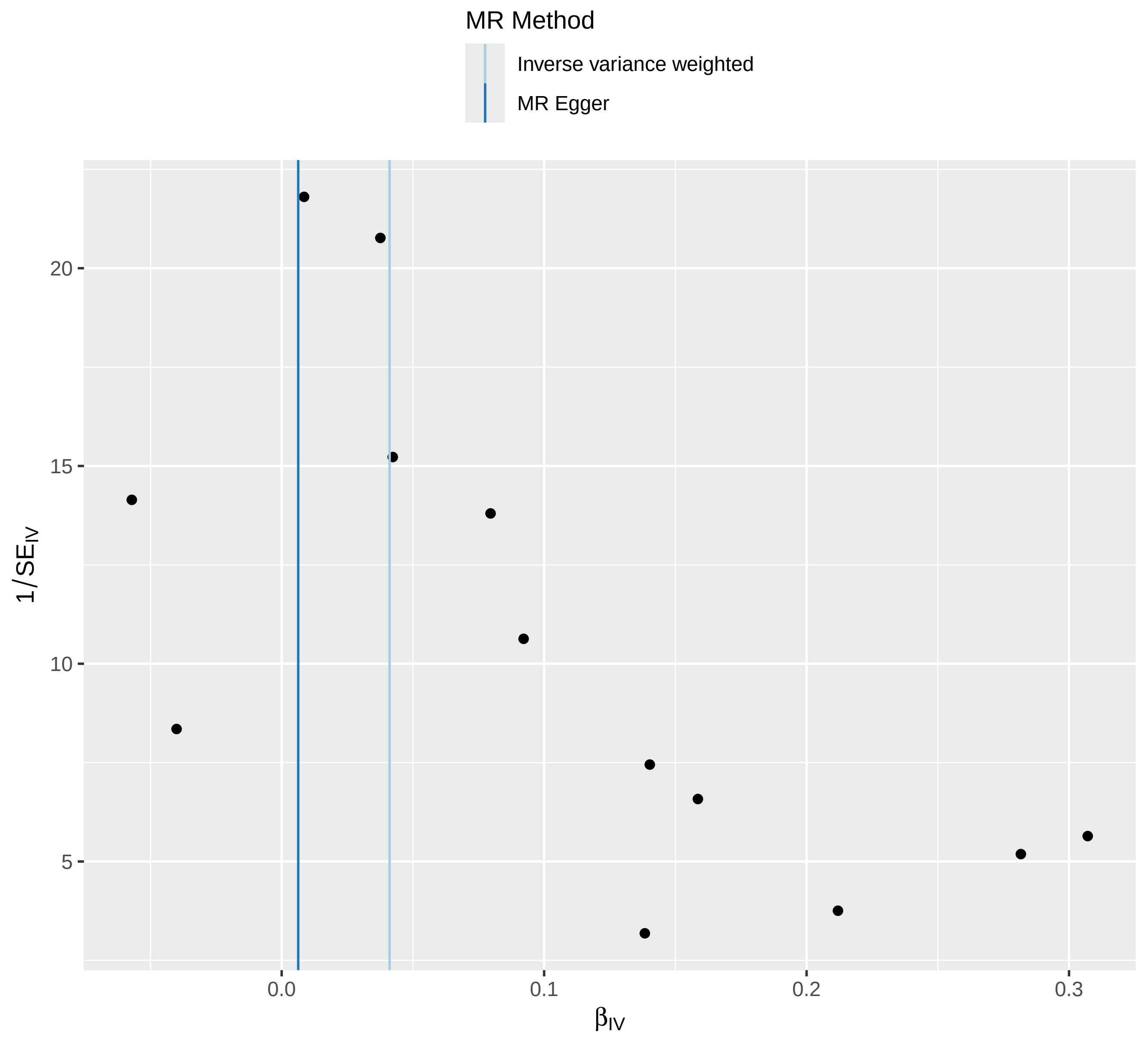

MR Method
Inverse variance weighted
MR Egger
20
15
10
5
0.0
0.1
0.2
0.3
1/SE_IV
β_IV

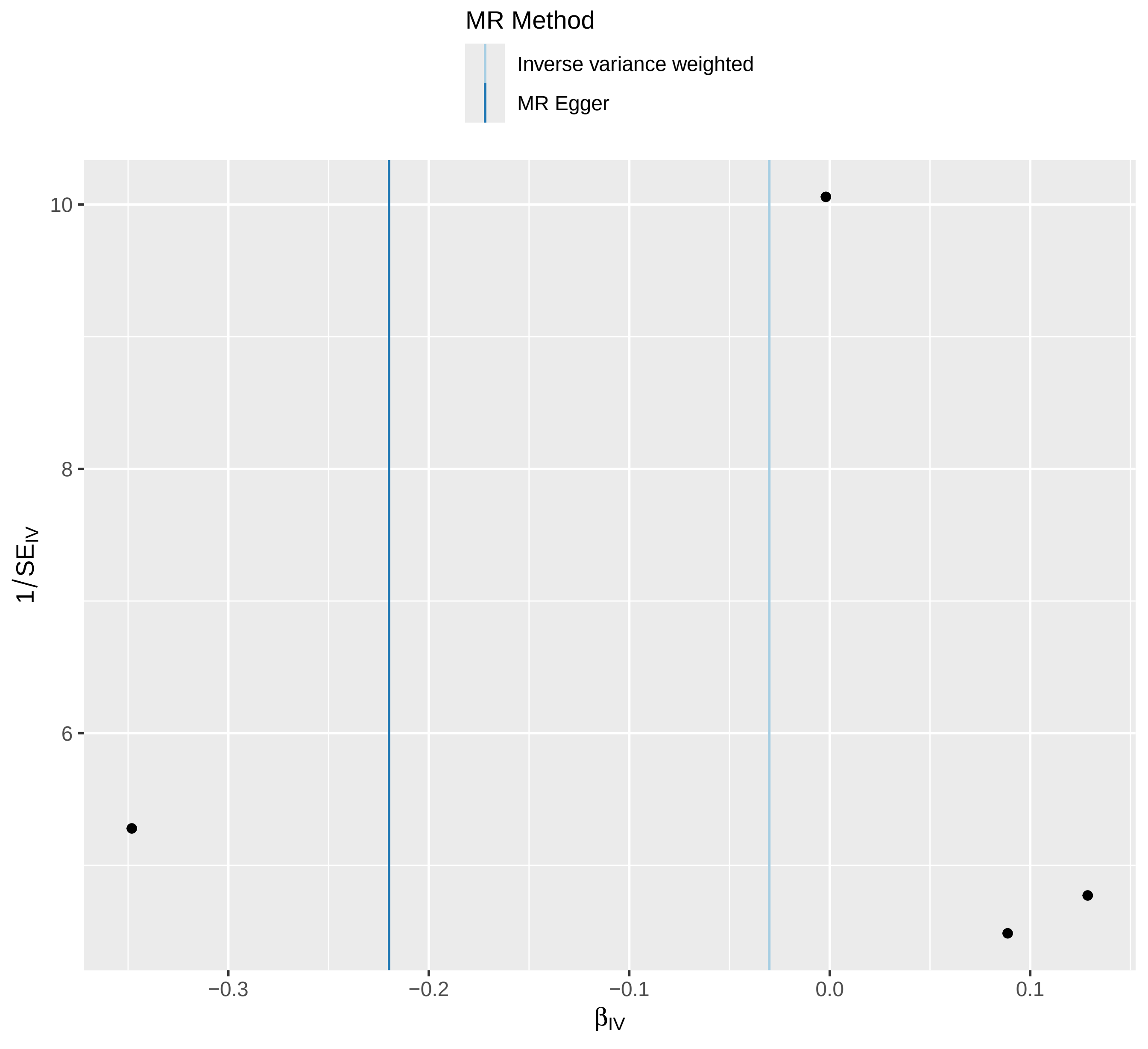
MR Method
Inverse variance weighted
MR Egger
10
8
6
1/SE$_{IV}$
−0.3
−0.2
−0.1
0.0
0.1
β$_{IV}$

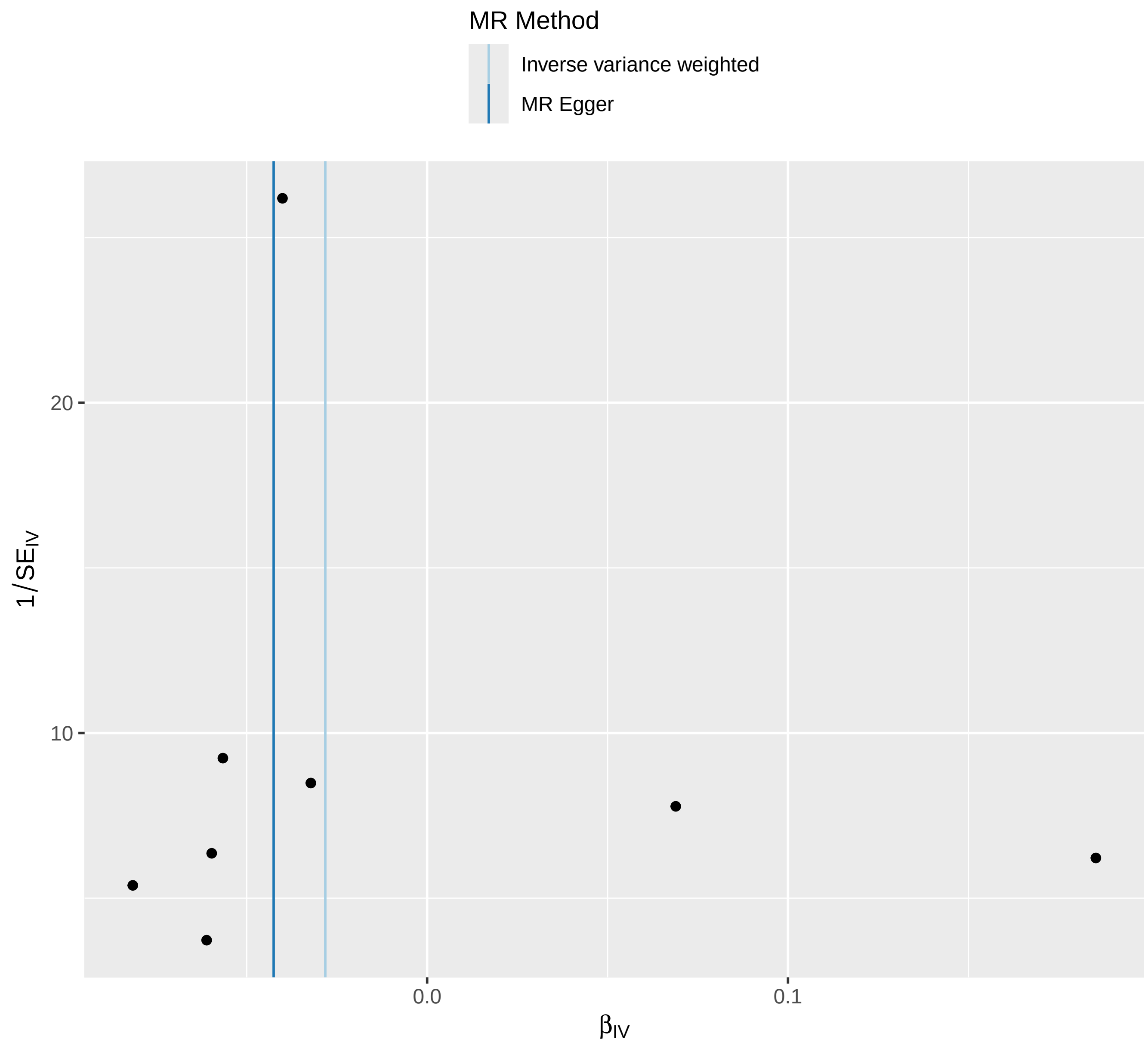
MR Method
Inverse variance weighted
MR Egger
20
10
0.0
0.1
1/SE_IV
β_IV

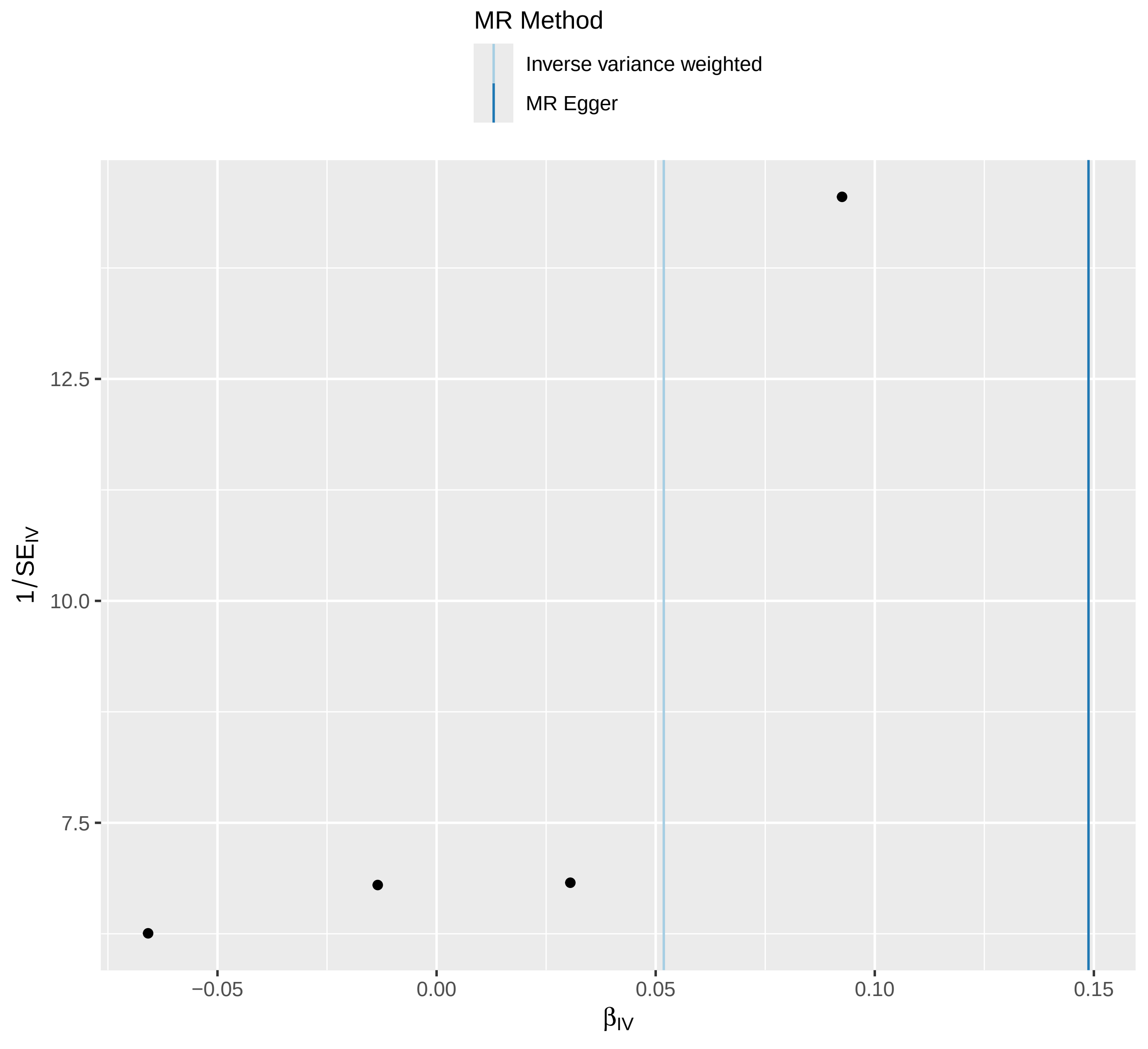

MR Method
Inverse variance weighted
MR Egger
12.5
10.0
7.5
1/SE$_{IV}$
−0.05
0.00
0.05
0.10
0.15
β$_{IV}$

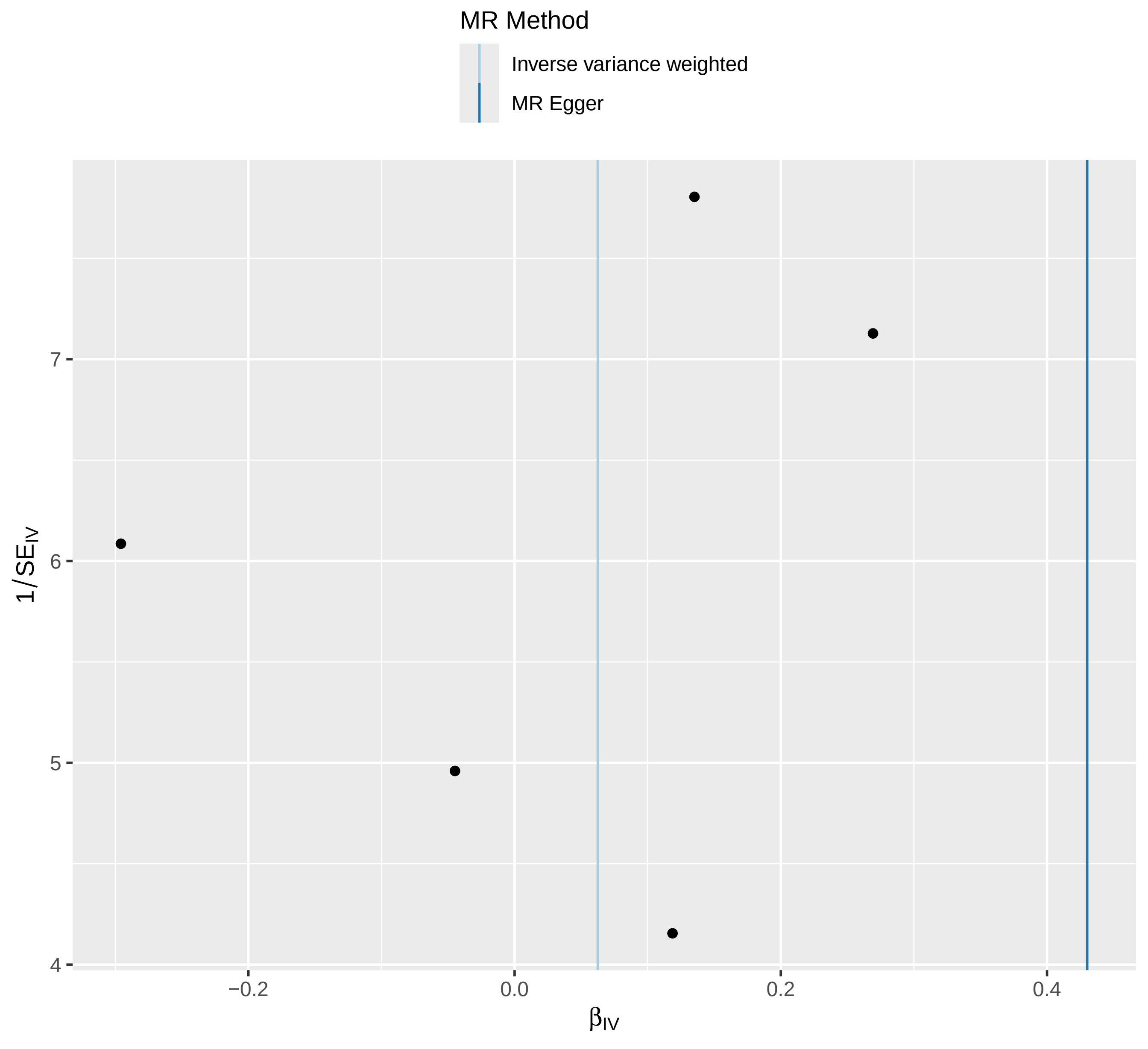
MR Method
Inverse variance weighted
MR Egger
1/SE$_{IV}$
4
5
6
7
−0.2
0.0
0.2
0.4
β$_{IV}$

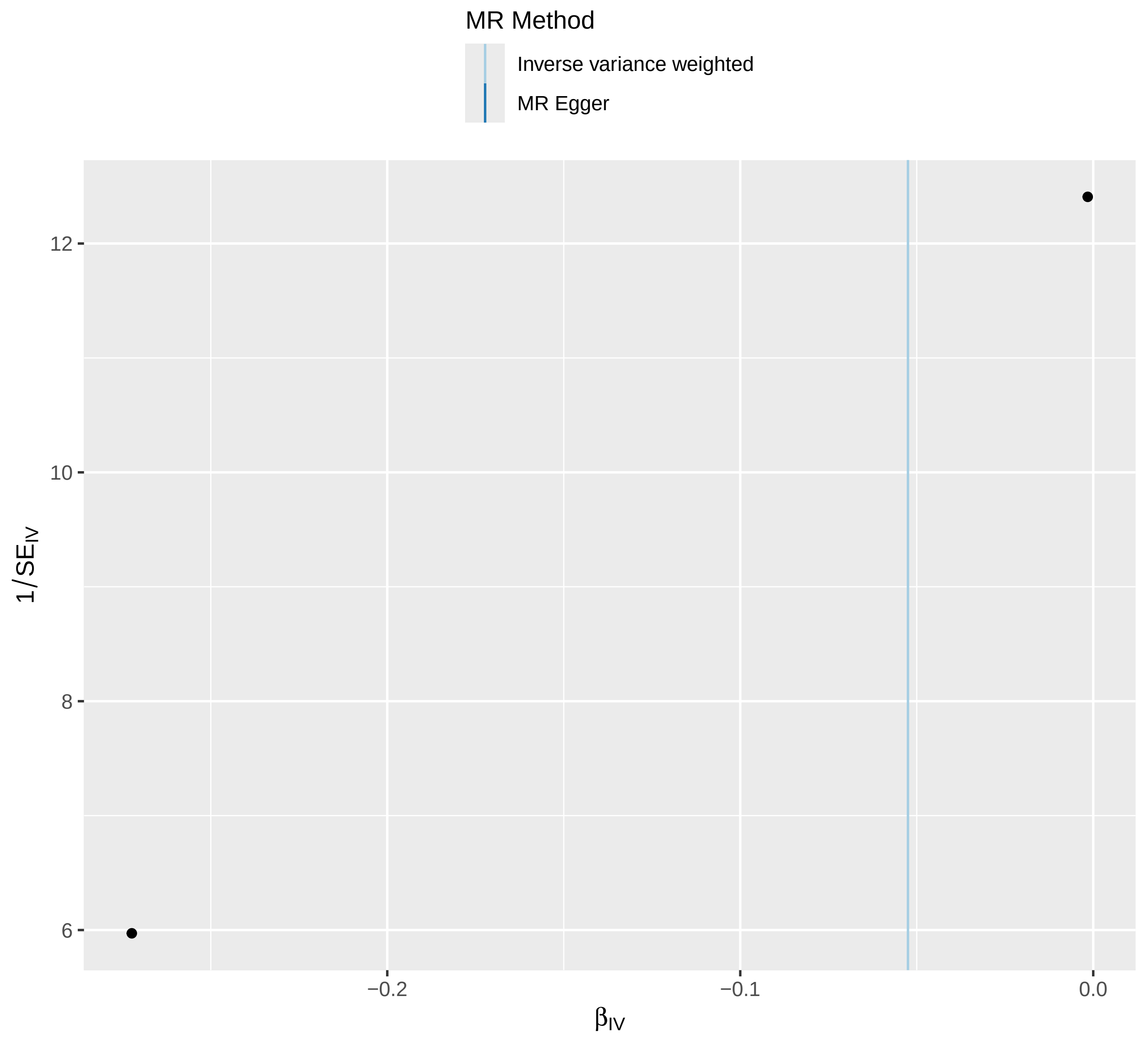
MR Method
Inverse variance weighted
MR Egger
12
10
8
6
1/SE_IV
−0.2
−0.1
0.0
β_IV

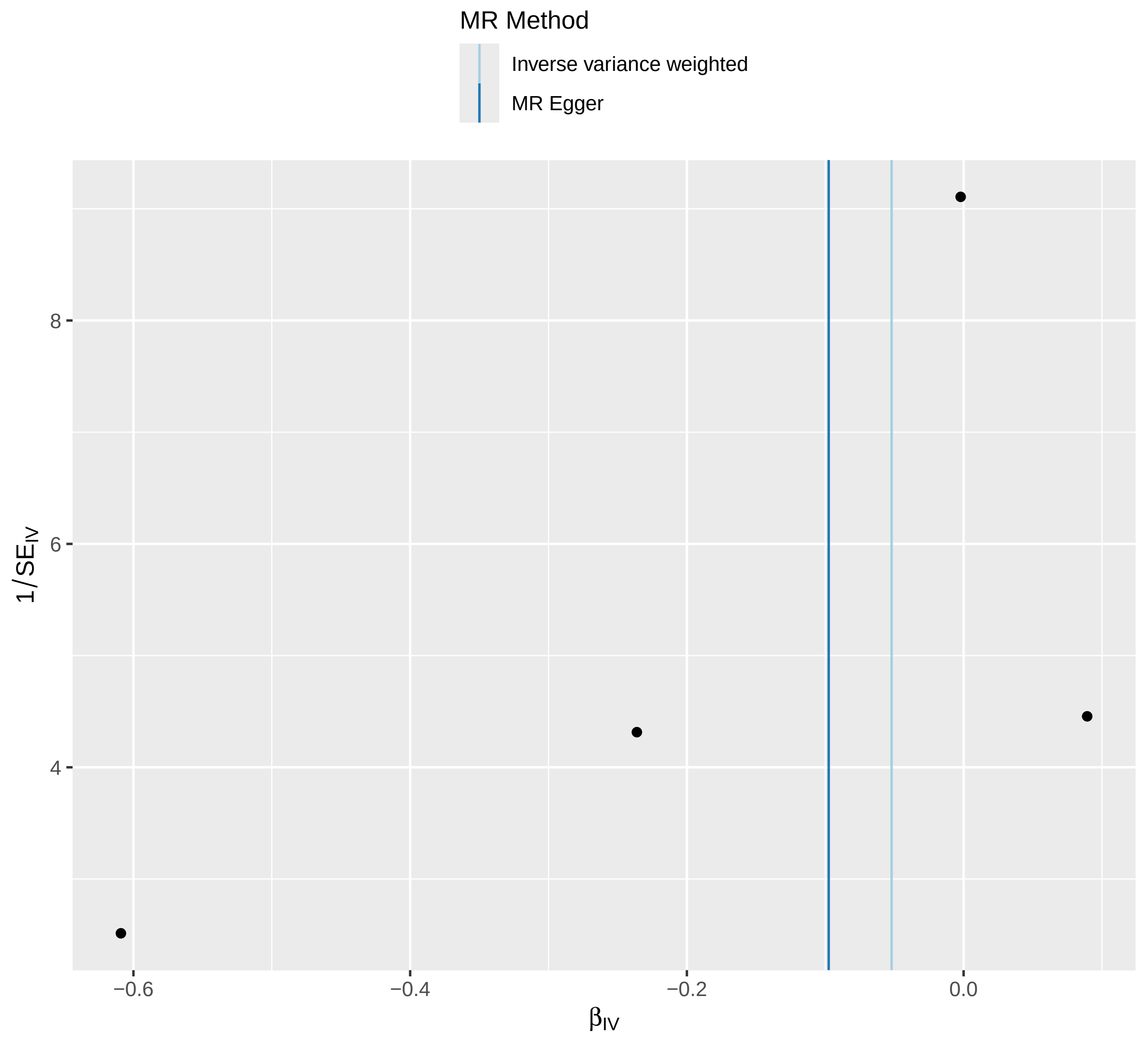

MR Method
Inverse variance weighted
MR Egger
8
6
4
$1/SE_{IV}$
−0.6
−0.4
−0.2
0.0
$\beta_{IV}$

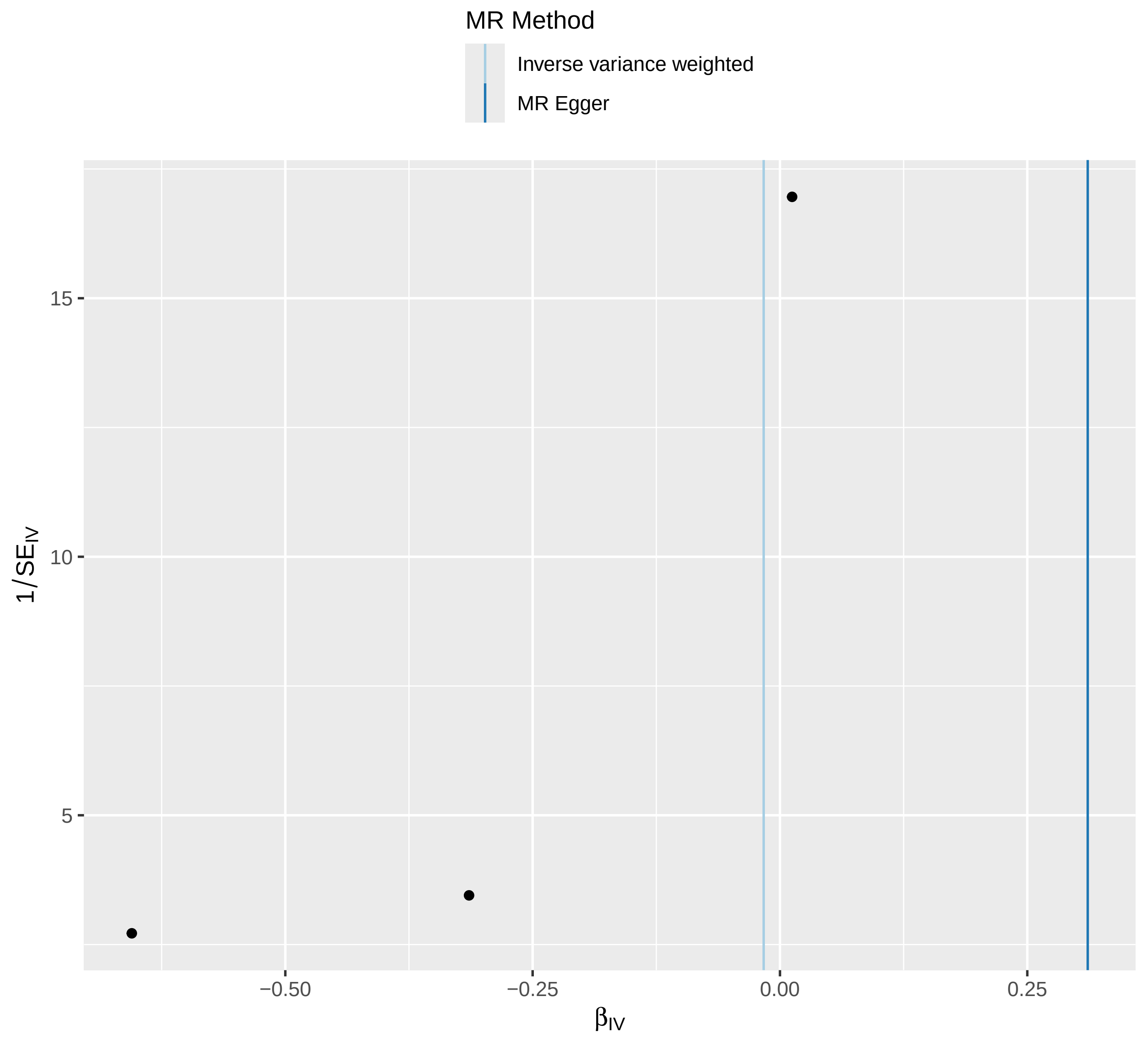

MR Method
Inverse variance weighted
MR Egger
15
10
5
1/SE_IV
−0.50
−0.25
0.00
0.25
β_IV

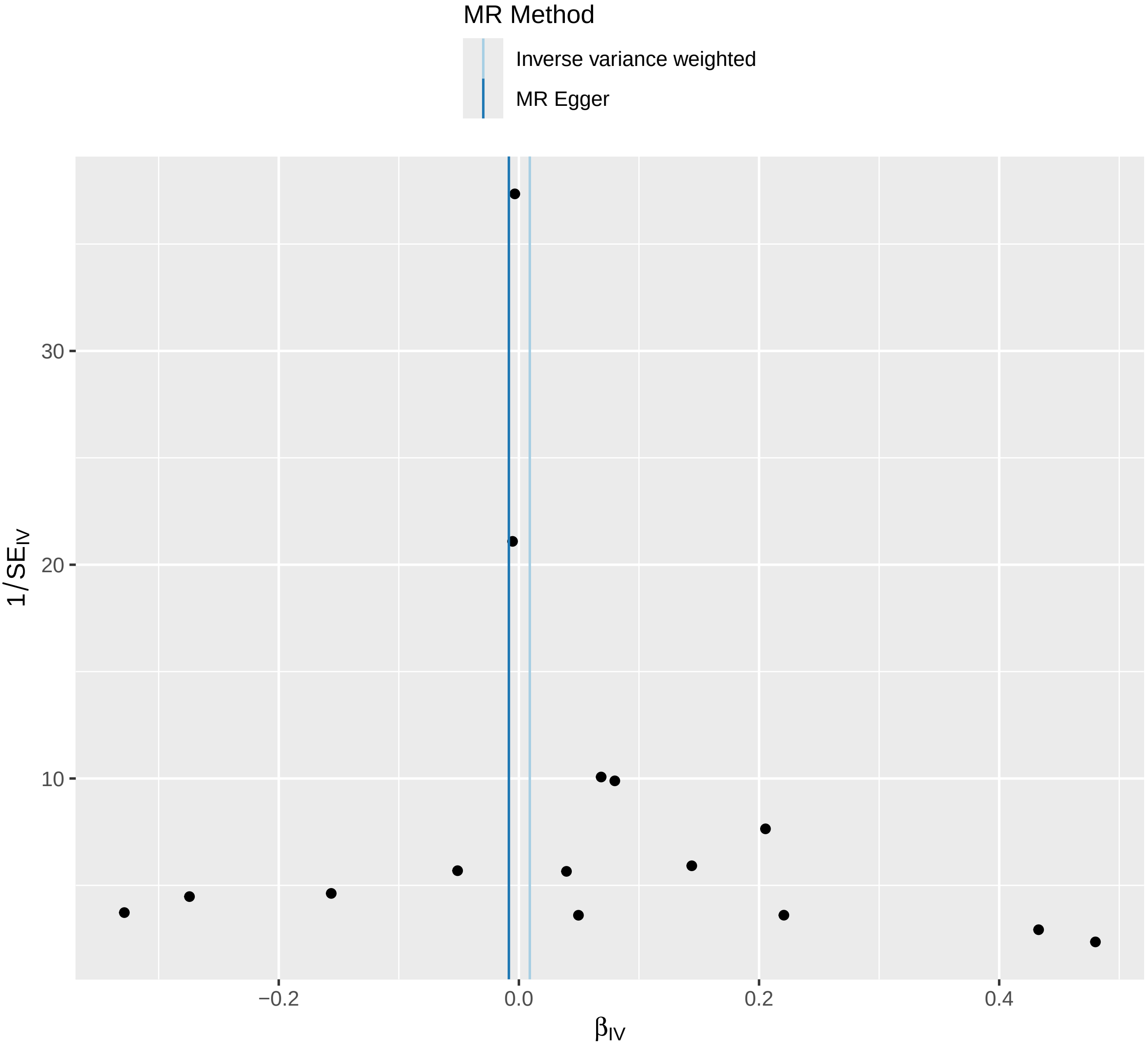
MR Method
Inverse variance weighted
MR Egger
30
20
10
1/SE$_{IV}$
−0.2
0.0
0.2
0.4
β$_{IV}$

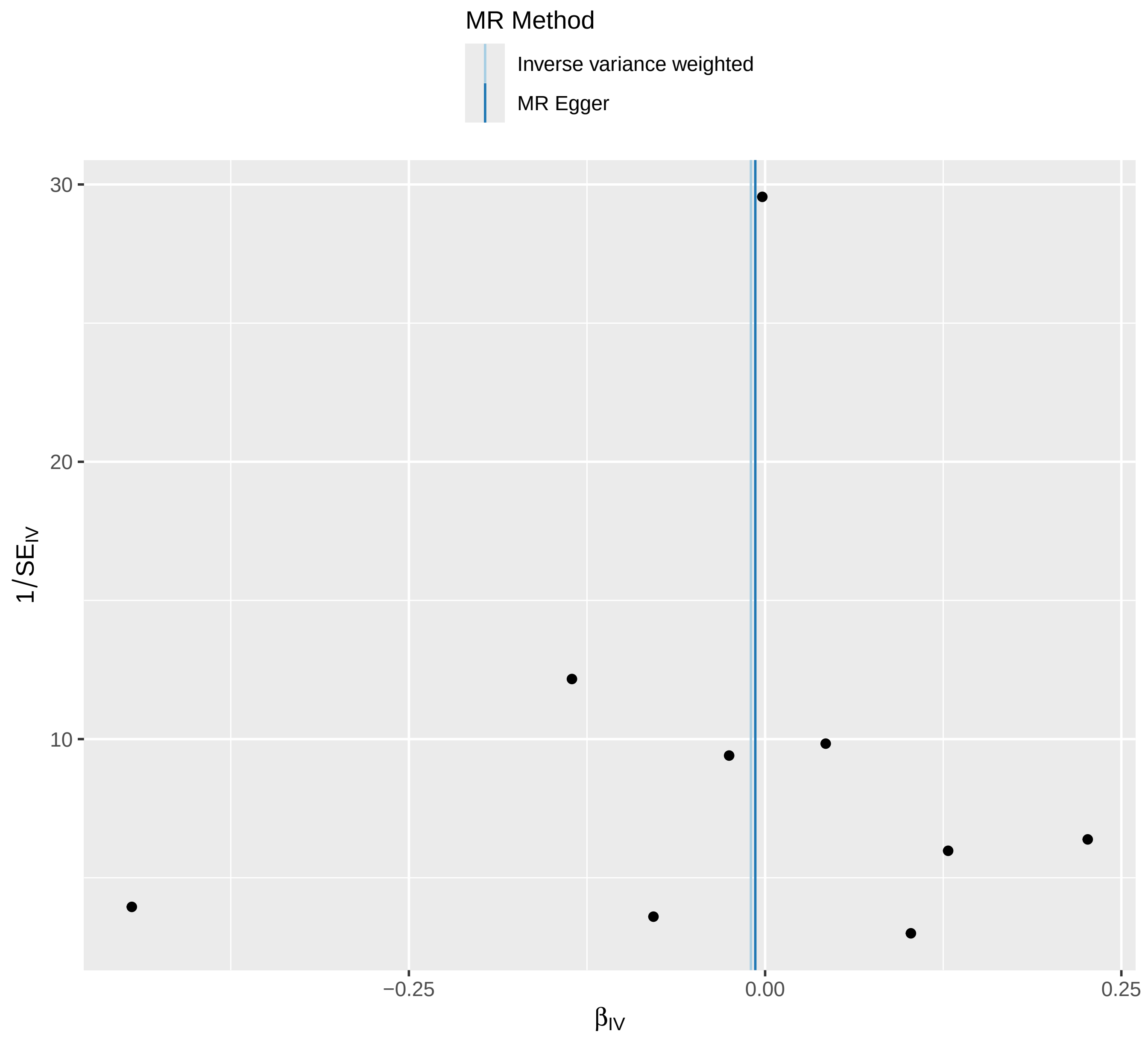

MR Method
Inverse variance weighted
MR Egger
30
20
10
1/SE$_{IV}$
−0.25
0.00
0.25
β$_{IV}$

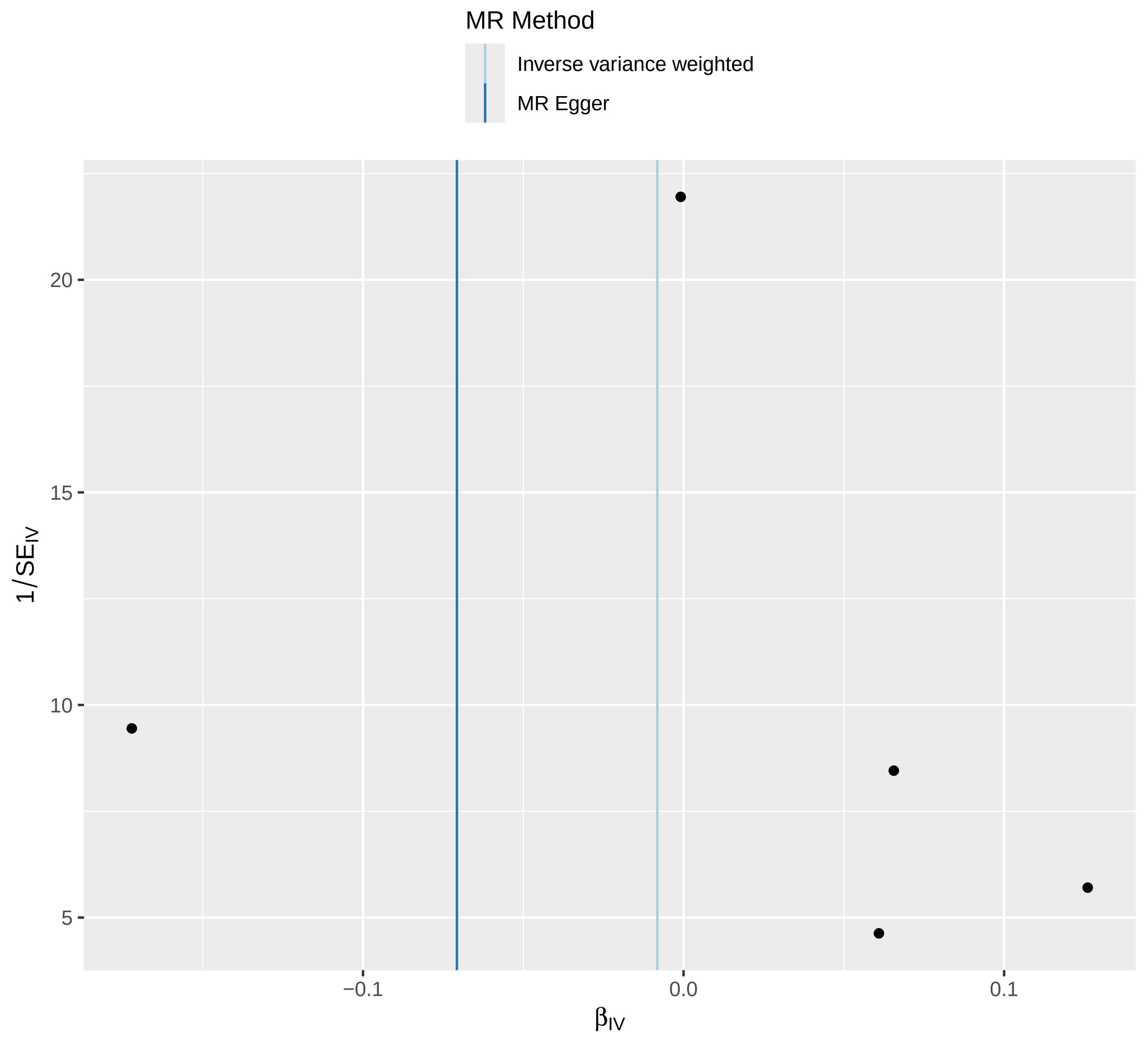

MR Method
Inverse variance weighted
MR Egger
20
15
10
5
1/SE$_{IV}$
−0.1
0.0
0.1
β$_{IV}$

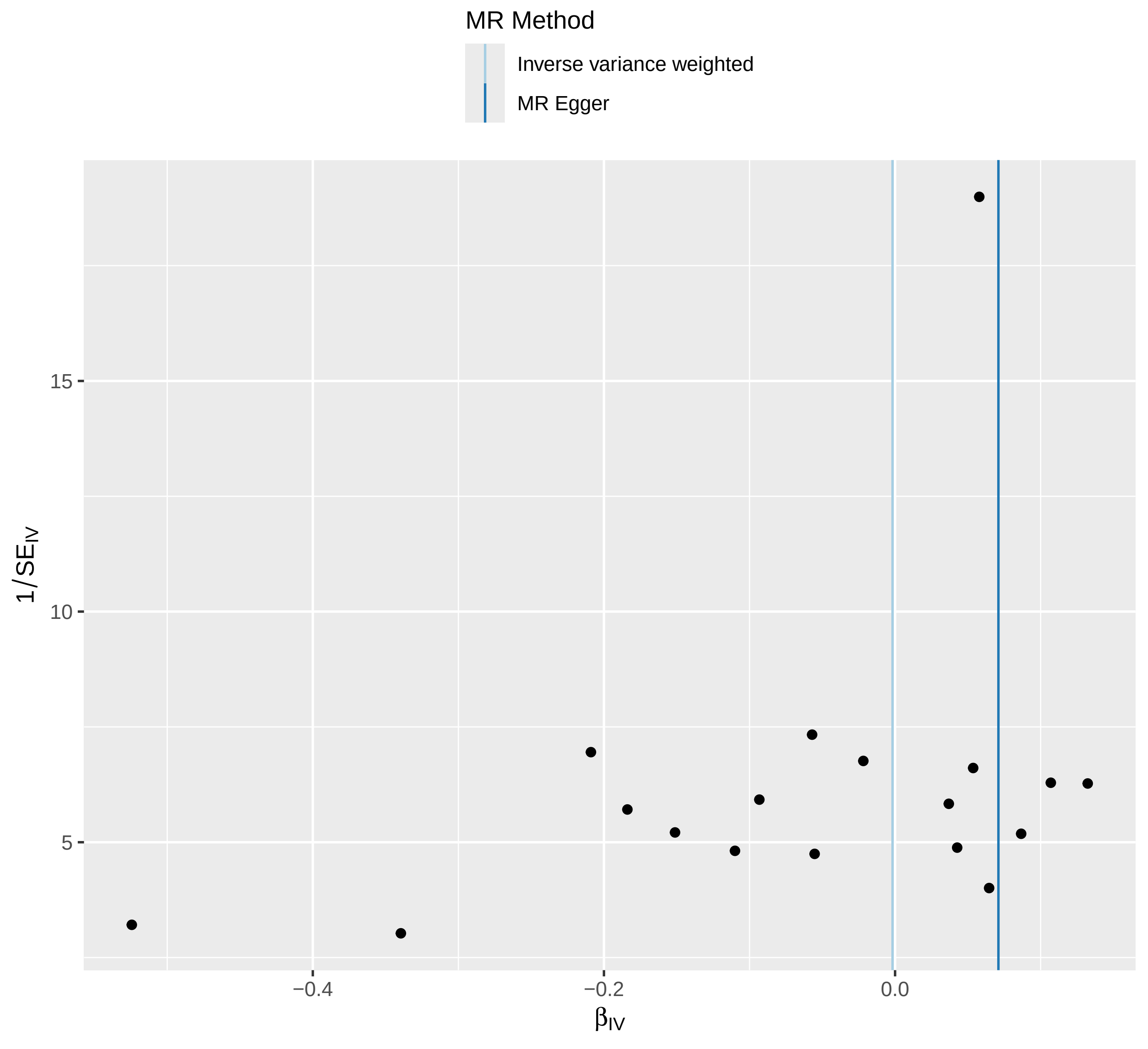

MR Method
Inverse variance weighted
MR Egger
15
10
5
1/SE$_{IV}$
−0.4
−0.2
0.0
β$_{IV}$

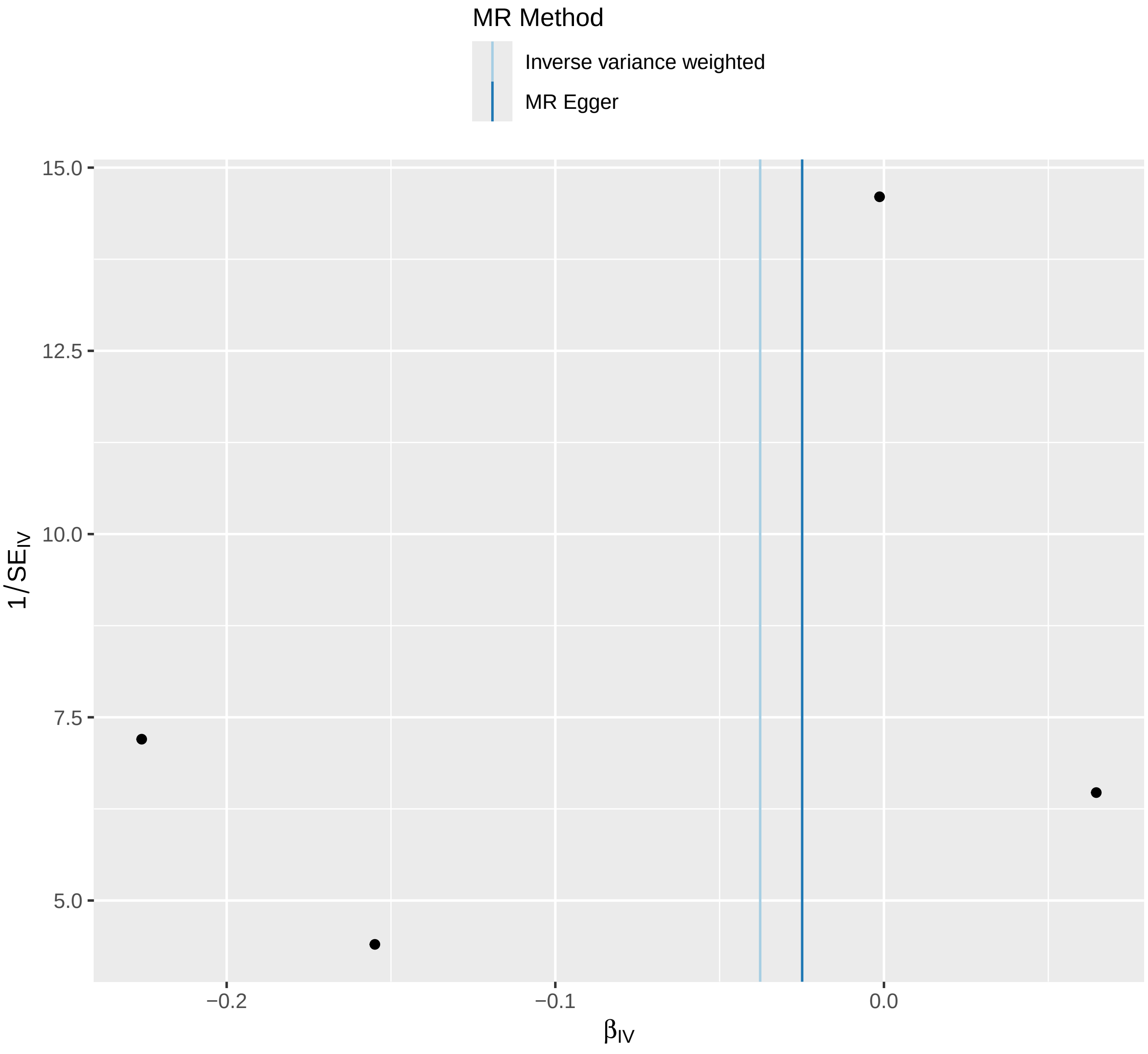
MR Method
Inverse variance weighted
MR Egger
15.0
12.5
10.0
7.5
5.0
1/SE$_{IV}$
−0.2
−0.1
0.0
β$_{IV}$

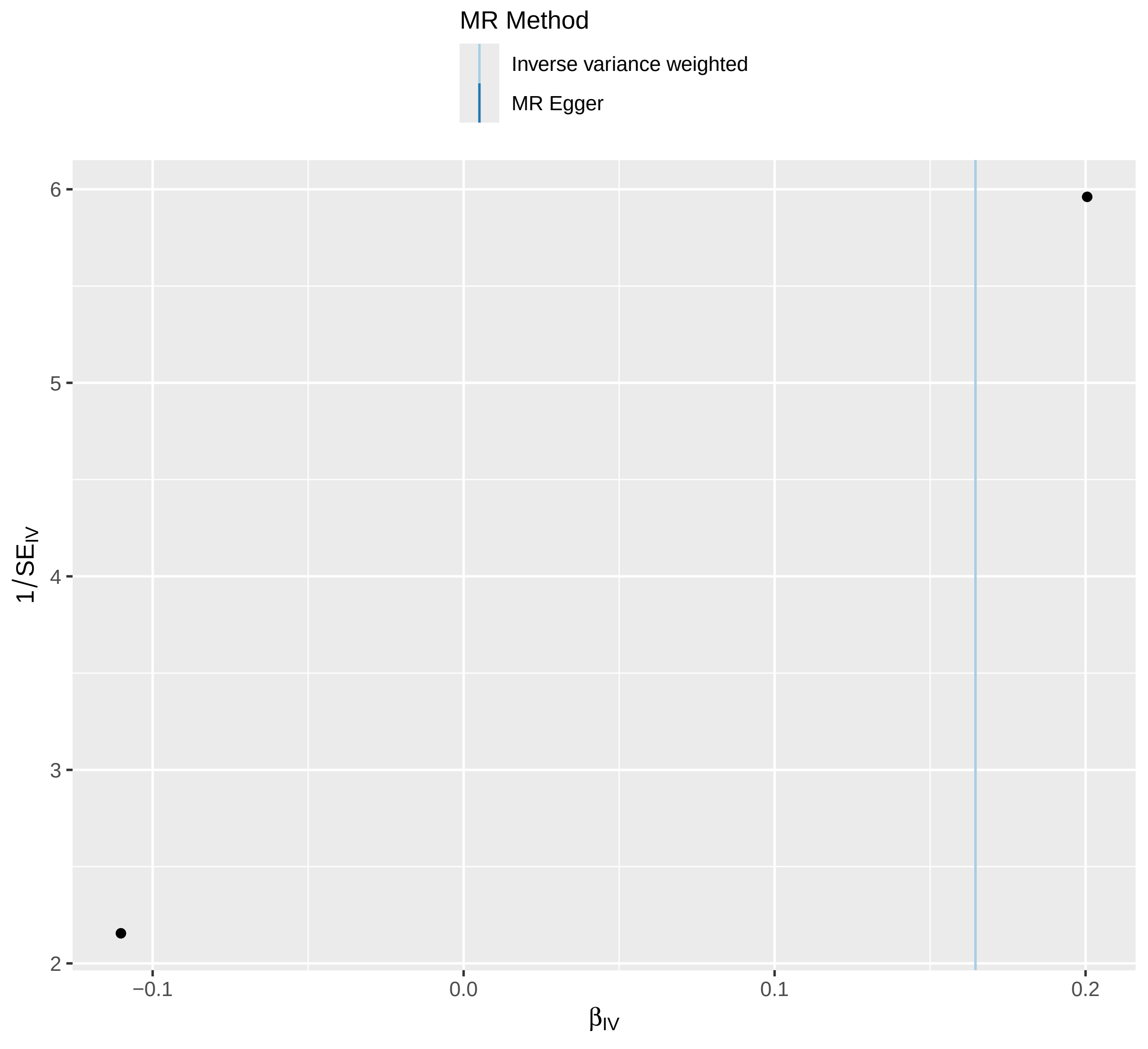

MR Method
Inverse variance weighted
MR Egger
6
5
4
3
2
1/SE_IV
−0.1
0.0
0.1
0.2
β_IV

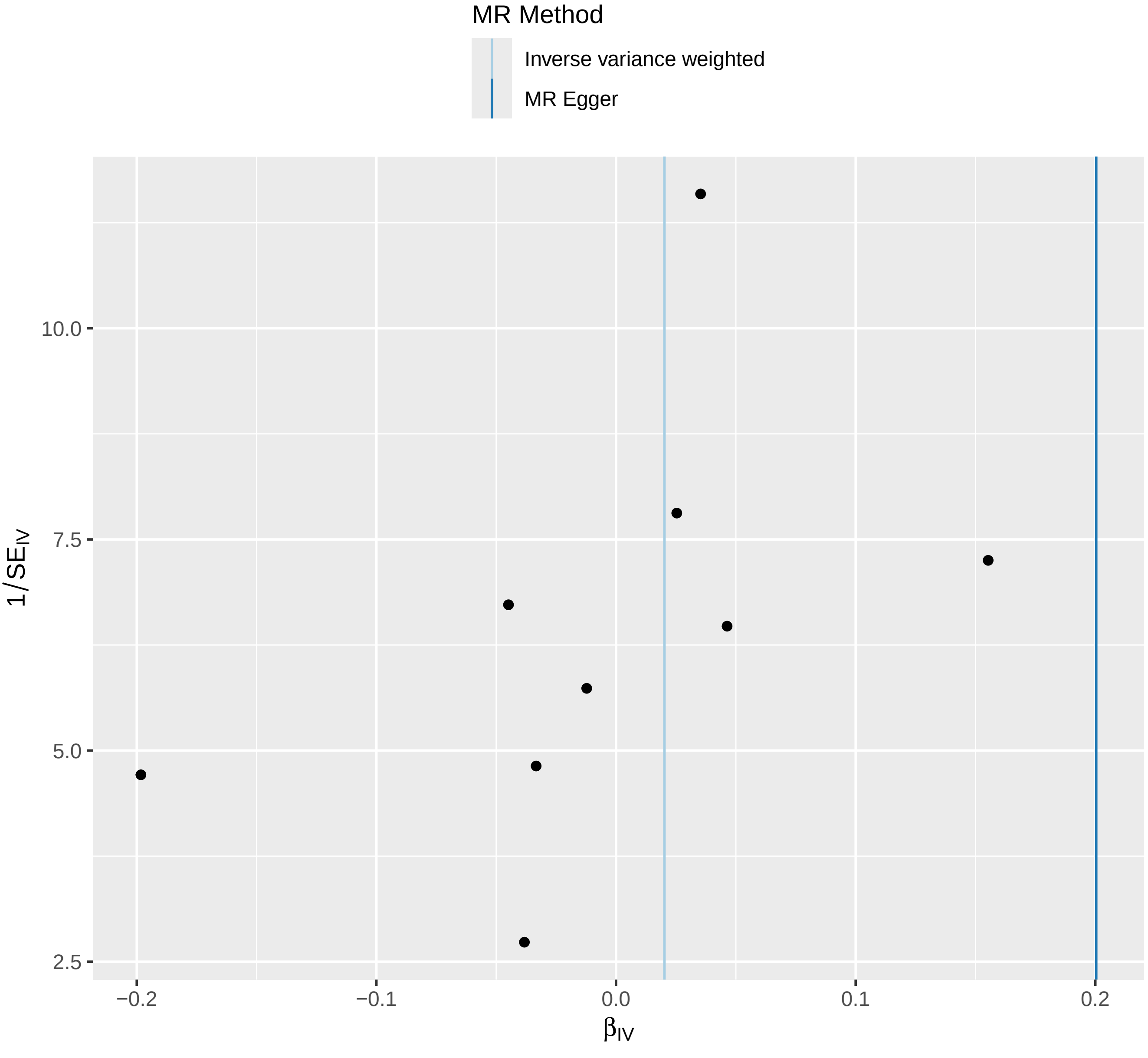
MR Method
Inverse variance weighted
MR Egger
1/SE_IV
10.0
7.5
5.0
2.5
−0.2
−0.1
0.0
0.1
0.2
β_IV

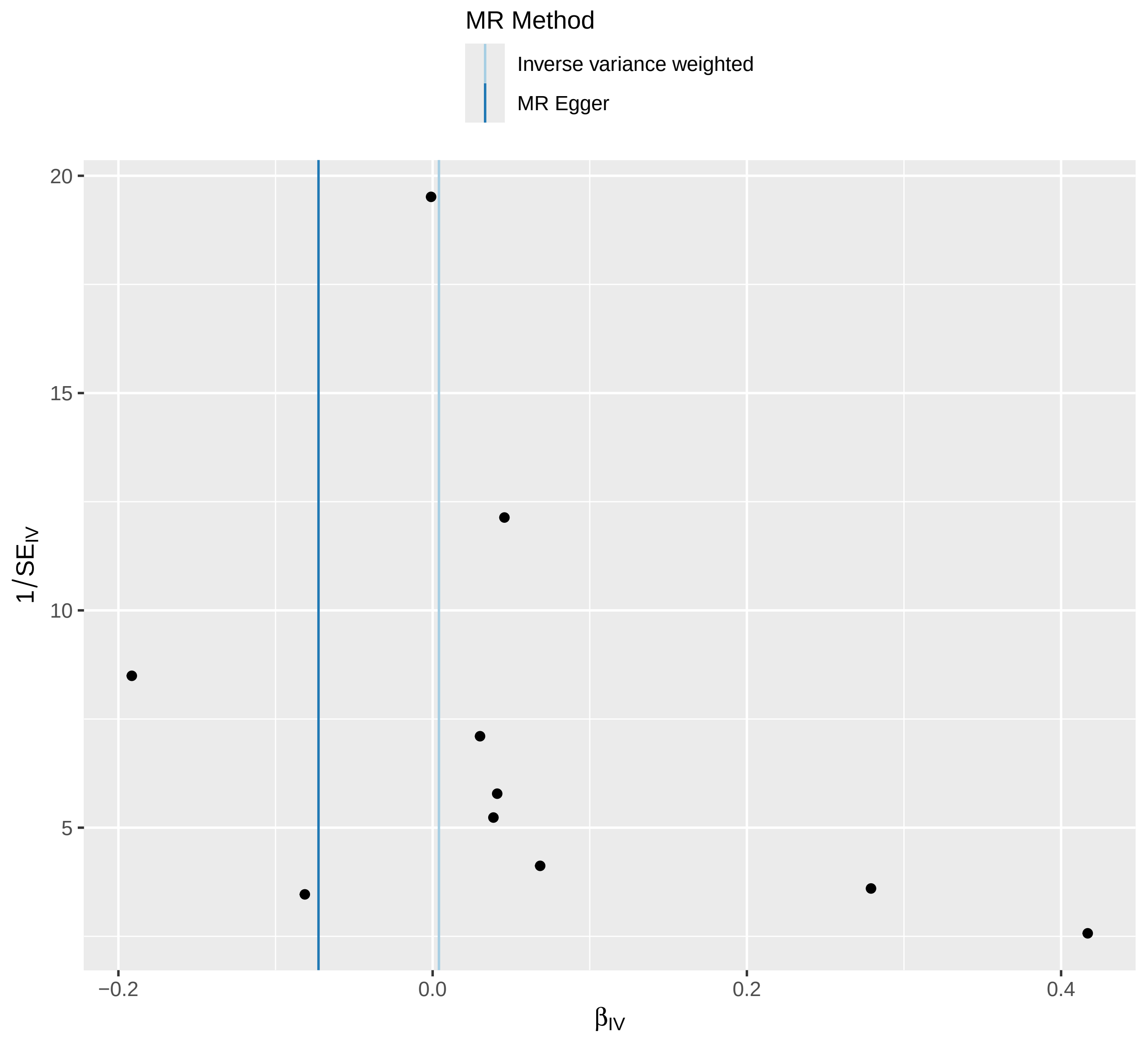

MR Method
Inverse variance weighted
MR Egger
20
15
10
5
−0.2
0.0
0.2
0.4
1/SE$_{IV}$
β$_{IV}$

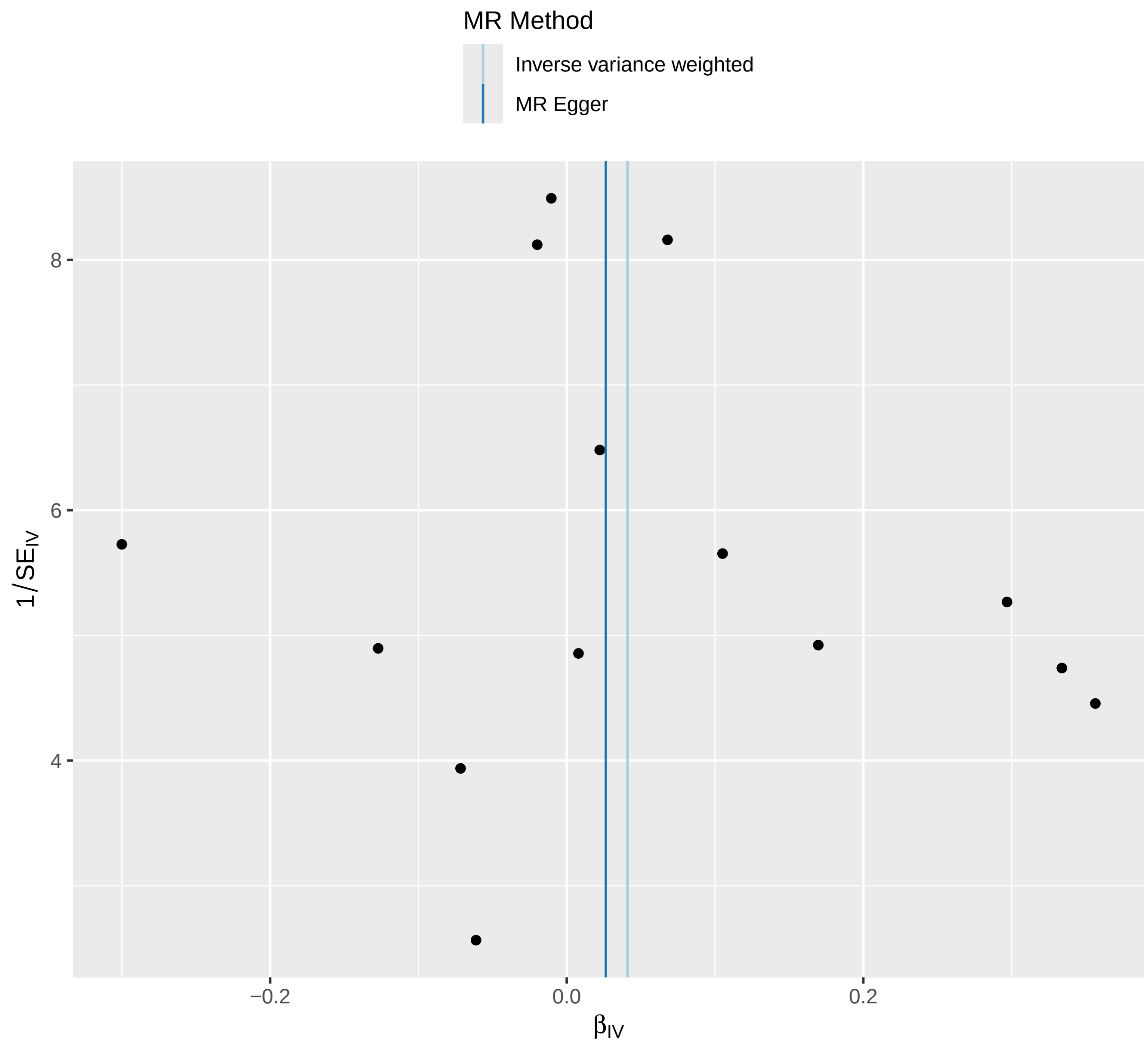

MR Method
Inverse variance weighted
MR Egger
8
6
4
1/SE$_{IV}$
−0.2
0.0
0.2
β$_{IV}$

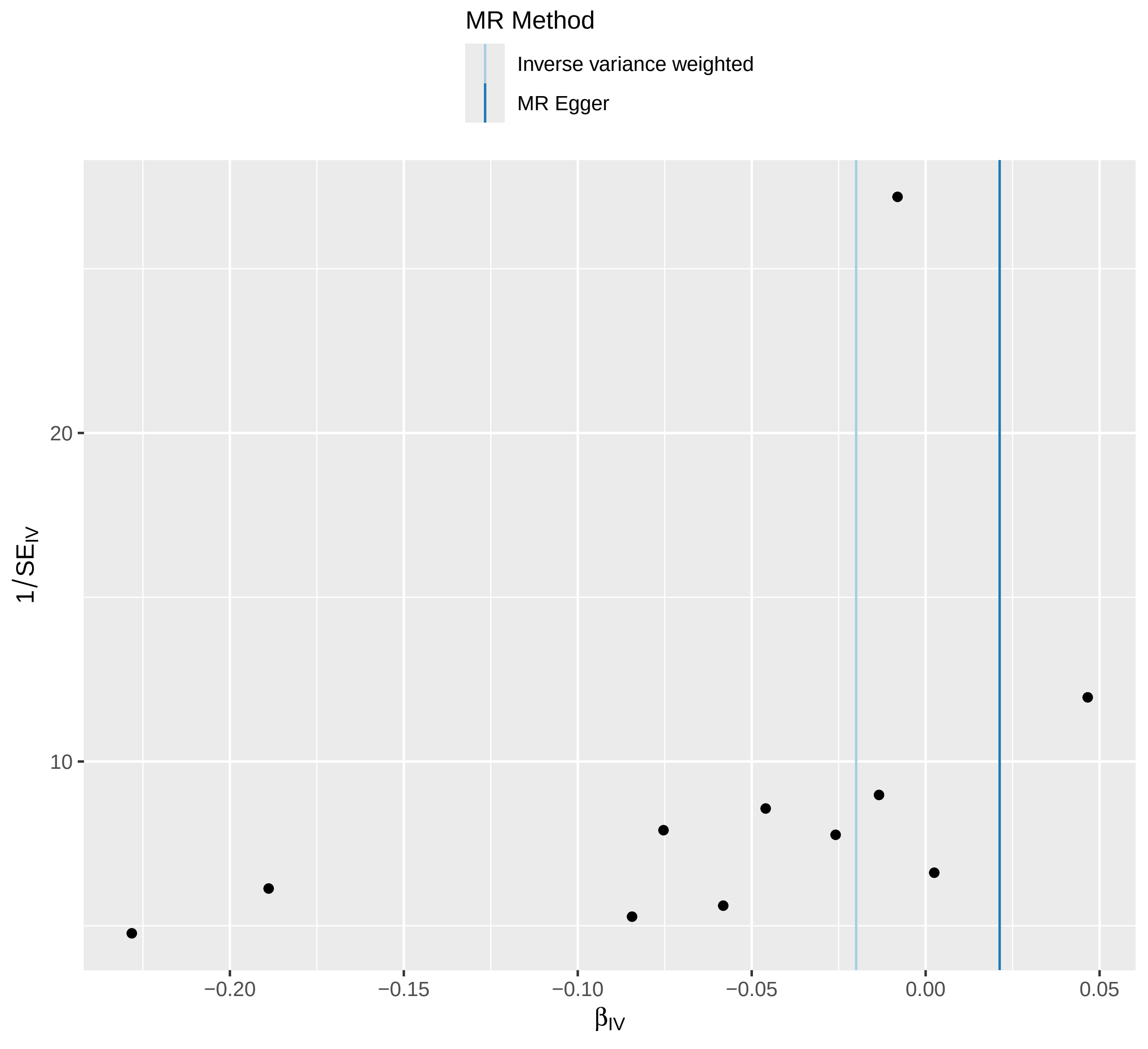
MR Method
Inverse variance weighted
MR Egger
20
10
1/SE$_{IV}$
−0.20
−0.15
−0.10
−0.05
0.00
0.05
β$_{IV}$

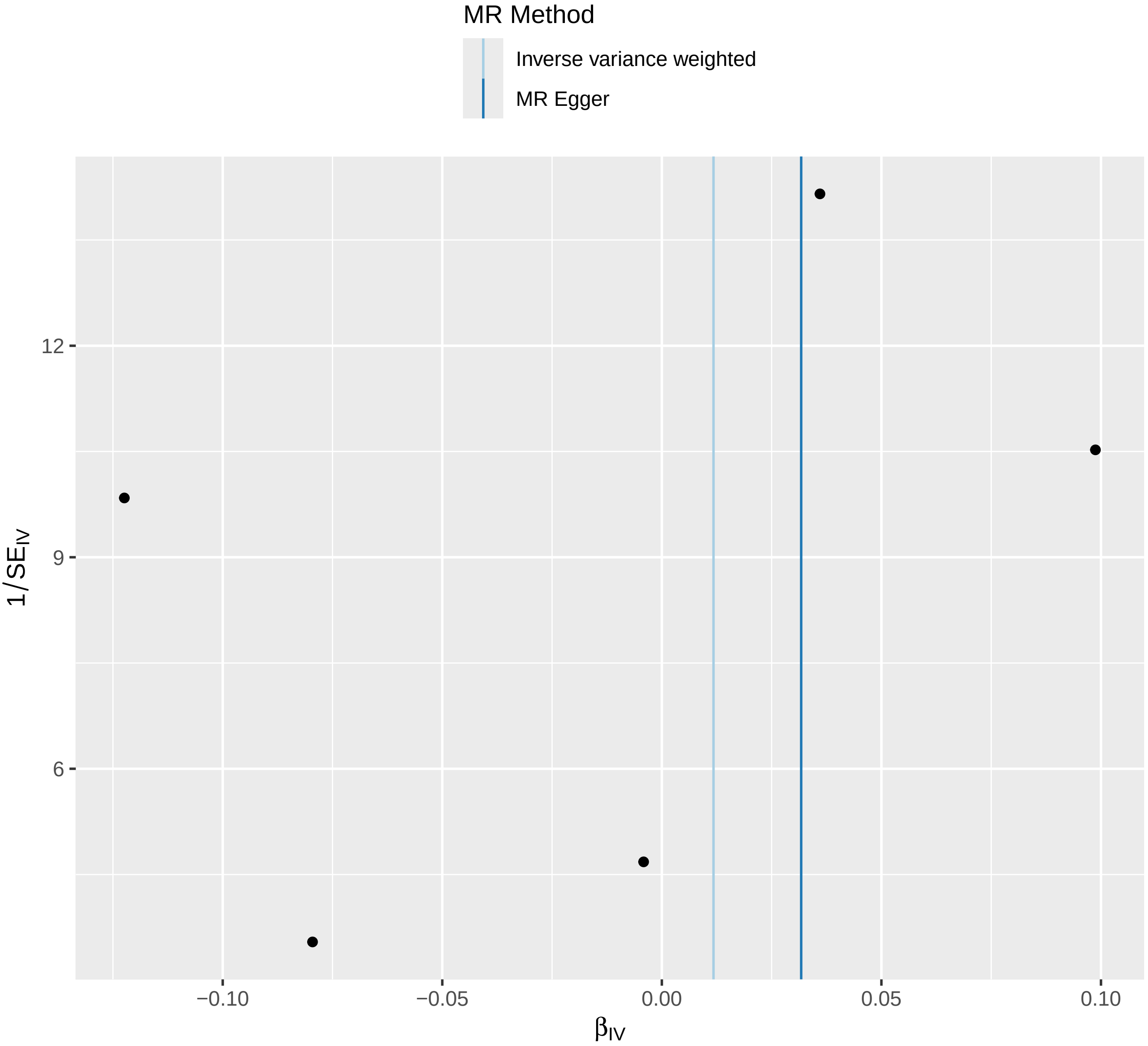

MR Method
Inverse variance weighted
MR Egger
12
9
6
$1/SE_{IV}$
−0.10
−0.05
0.00
0.05
0.10
$\beta_{IV}$

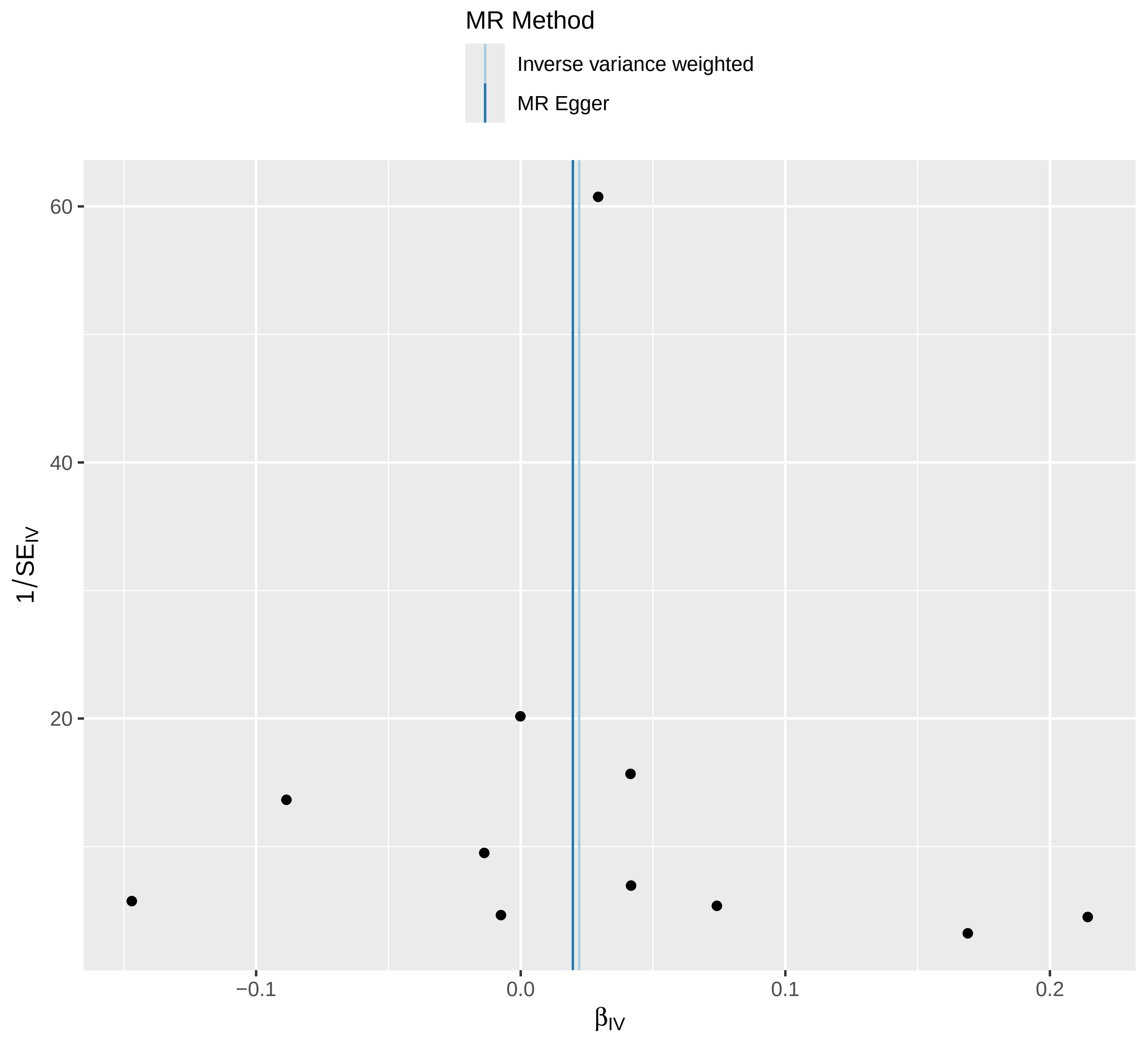
MR Method
Inverse variance weighted
MR Egger
60
40
20
1/SE$_{IV}$
−0.1
0.0
0.1
0.2
β$_{IV}$

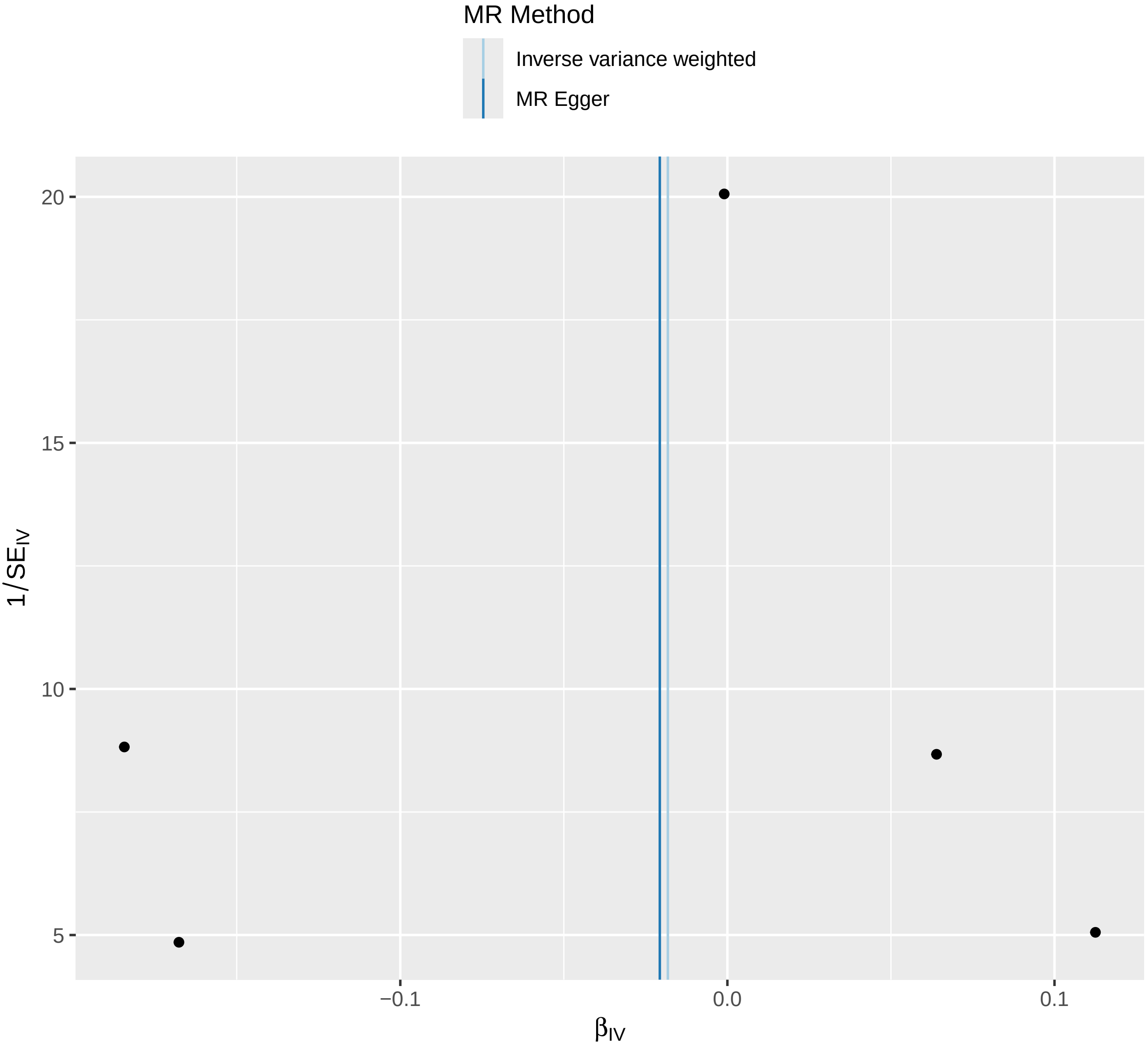
MR Method
Inverse variance weighted
MR Egger
20
15
10
5
1/SE_IV
−0.1
0.0
0.1
β_IV

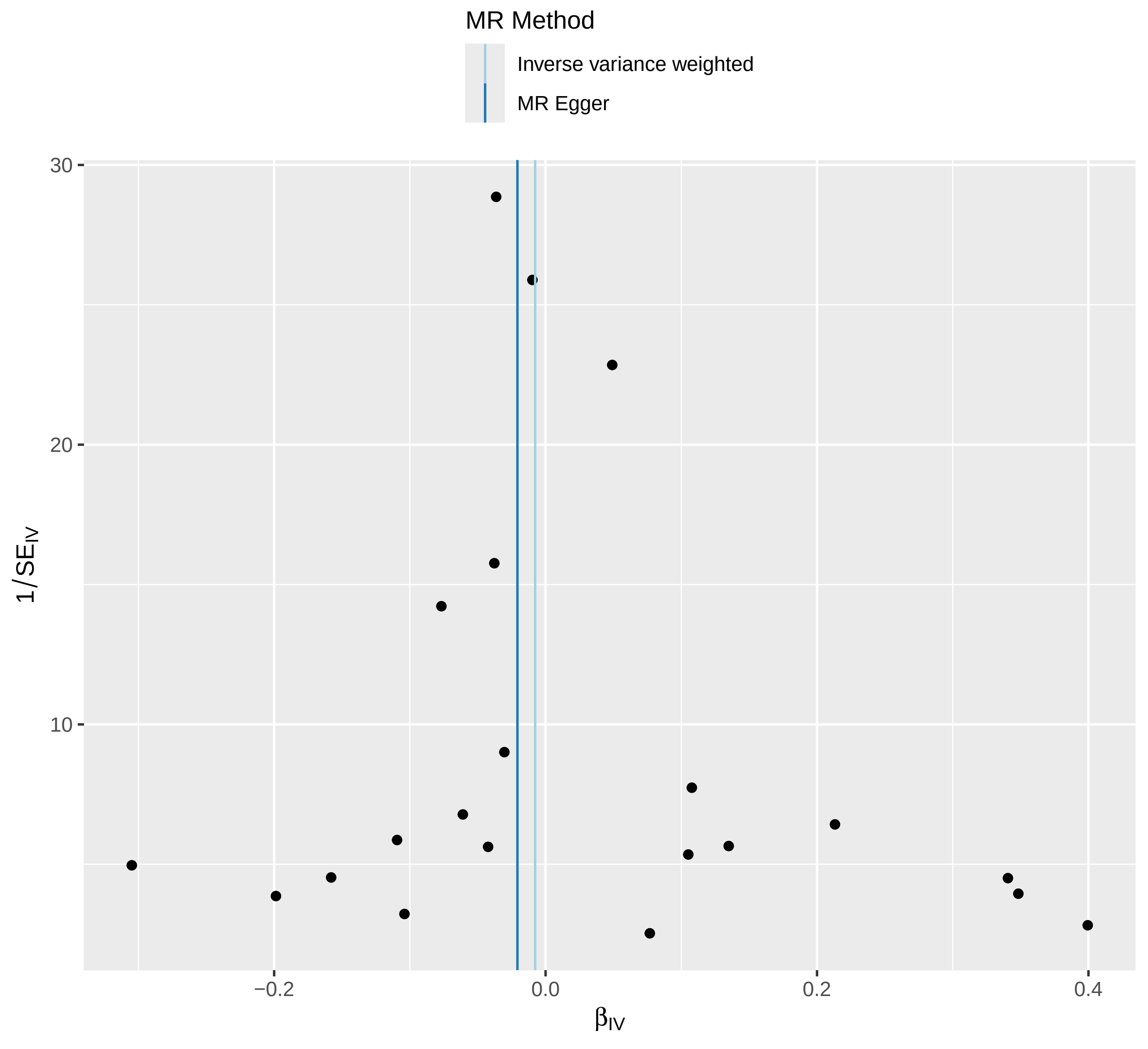
MR Method
Inverse variance weighted
MR Egger
30
20
10
1/SE_IV
−0.2
0.0
0.2
0.4
β_IV

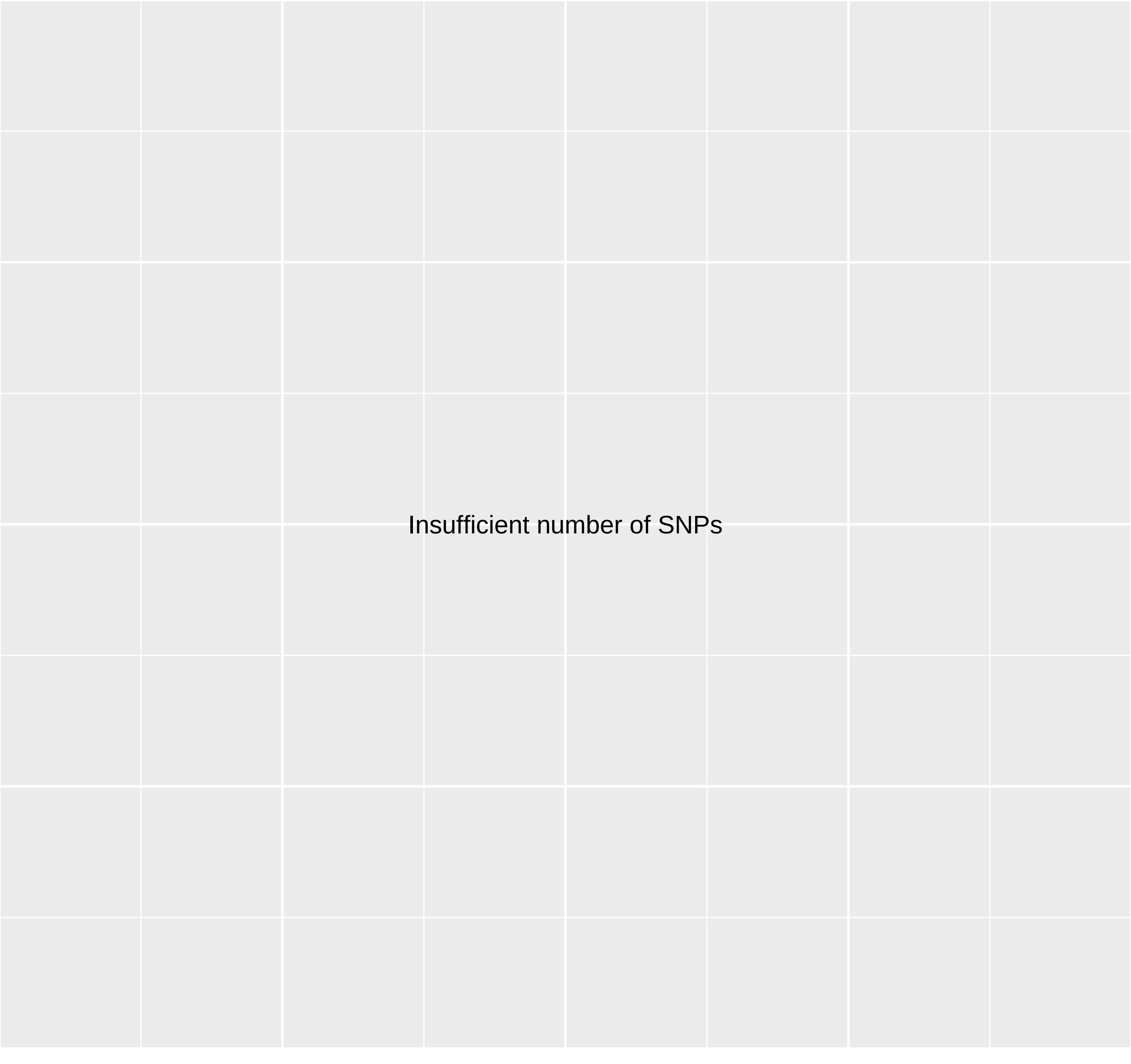
Insufficient number of SNPs

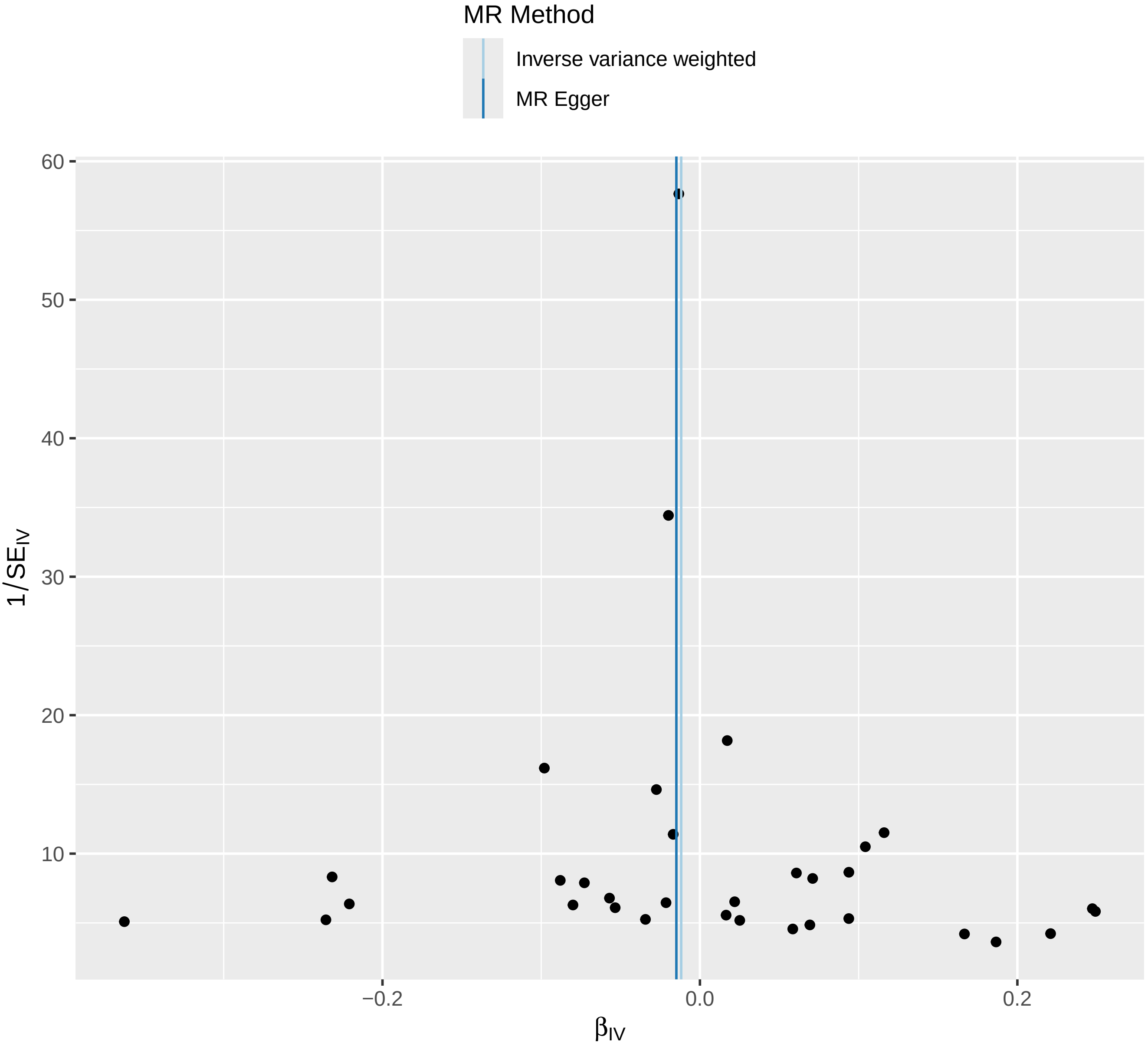
MR Method
Inverse variance weighted
MR Egger
60
50
40
30
20
10
1/SE$_{IV}$
−0.2
0.0
0.2
β$_{IV}$

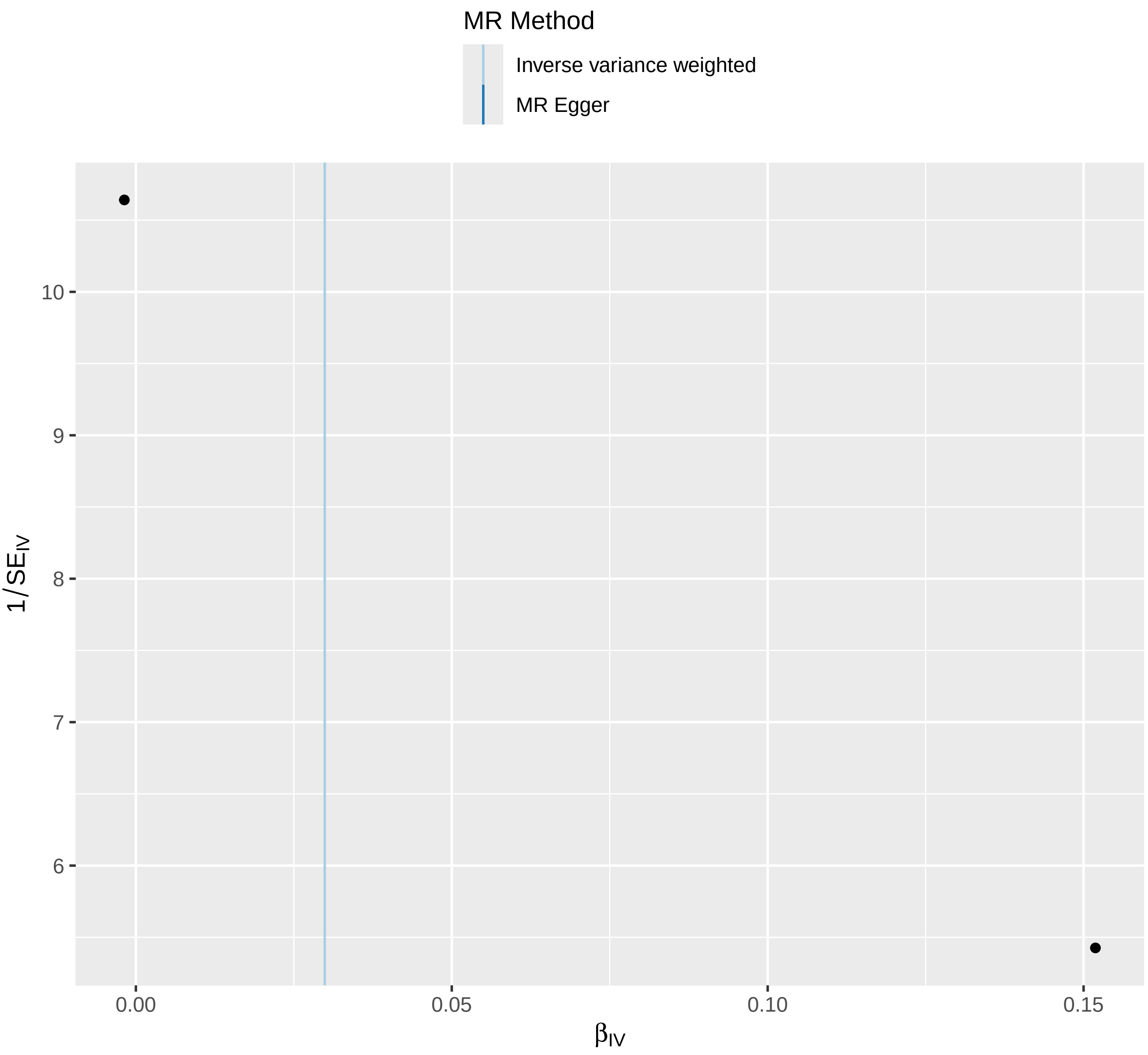

MR Method
Inverse variance weighted
MR Egger
10
9
8
7
6
0.00
0.05
0.10
0.15
1/SE_IV
β_IV

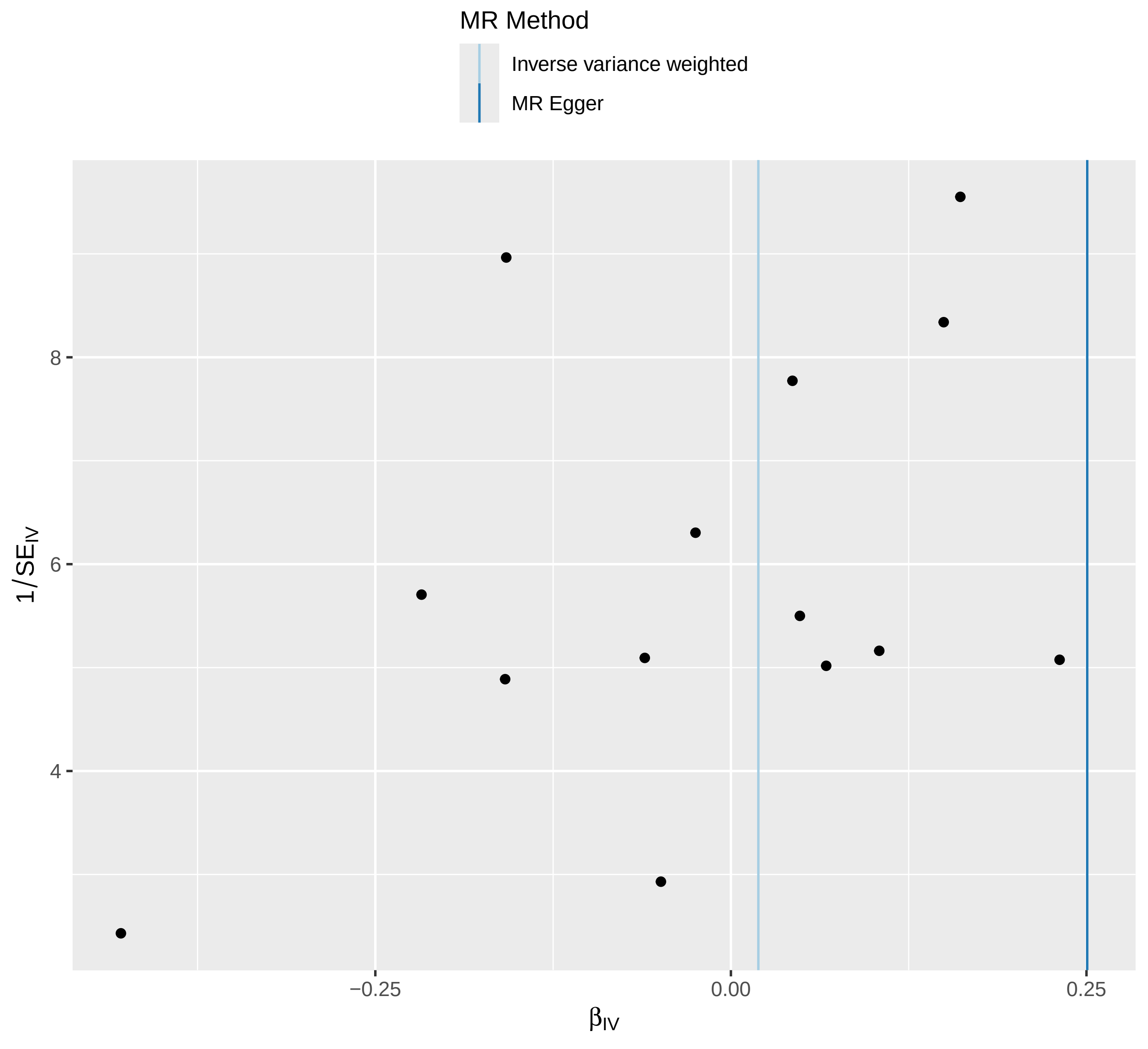

MR Method
Inverse variance weighted
MR Egger
8
6
4
1/SE$_{IV}$
−0.25
0.00
0.25
β$_{IV}$

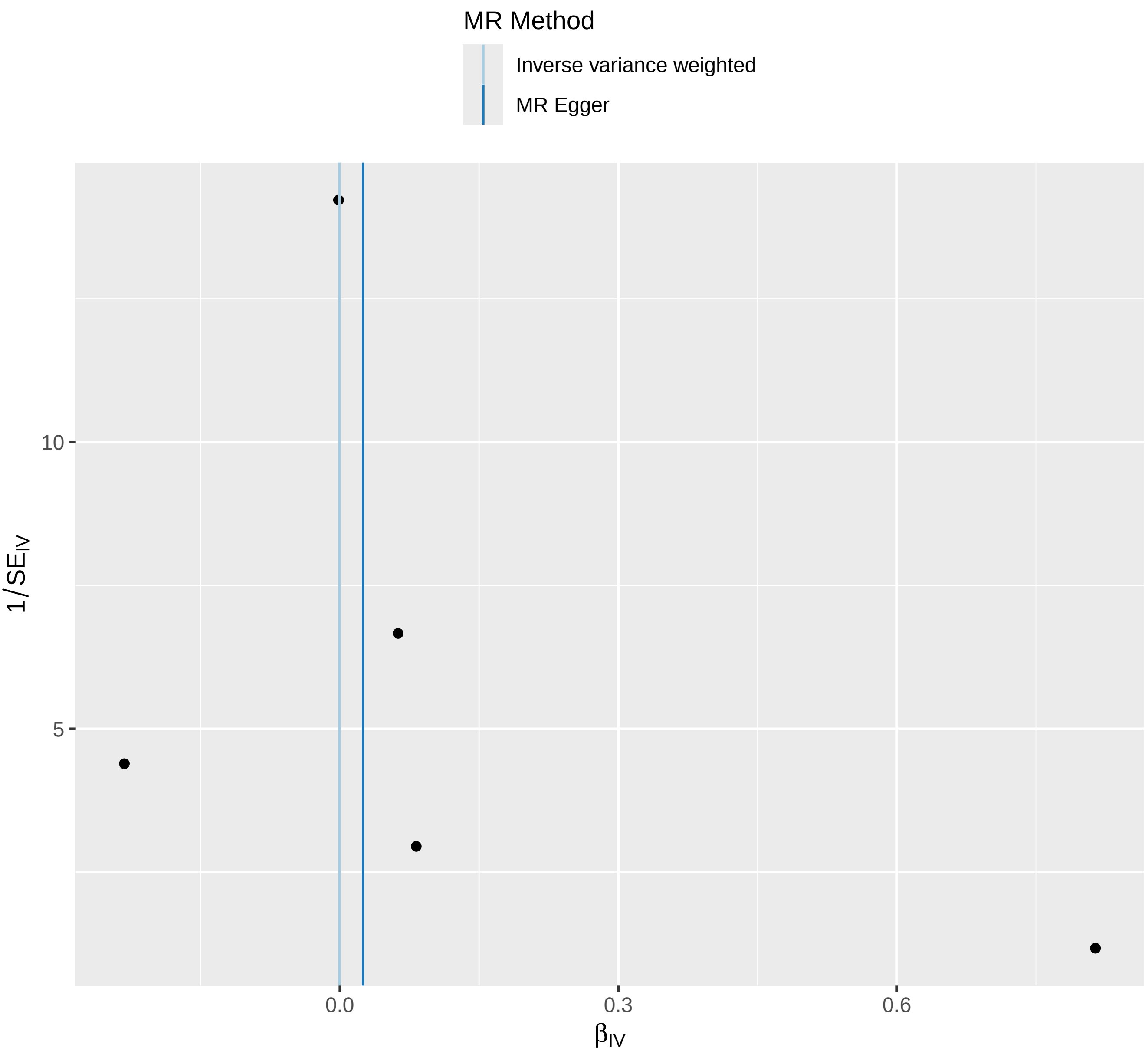

MR Method
Inverse variance weighted
MR Egger
10
5
0.0
0.3
0.6
$1/SE_{IV}$
$\beta_{IV}$

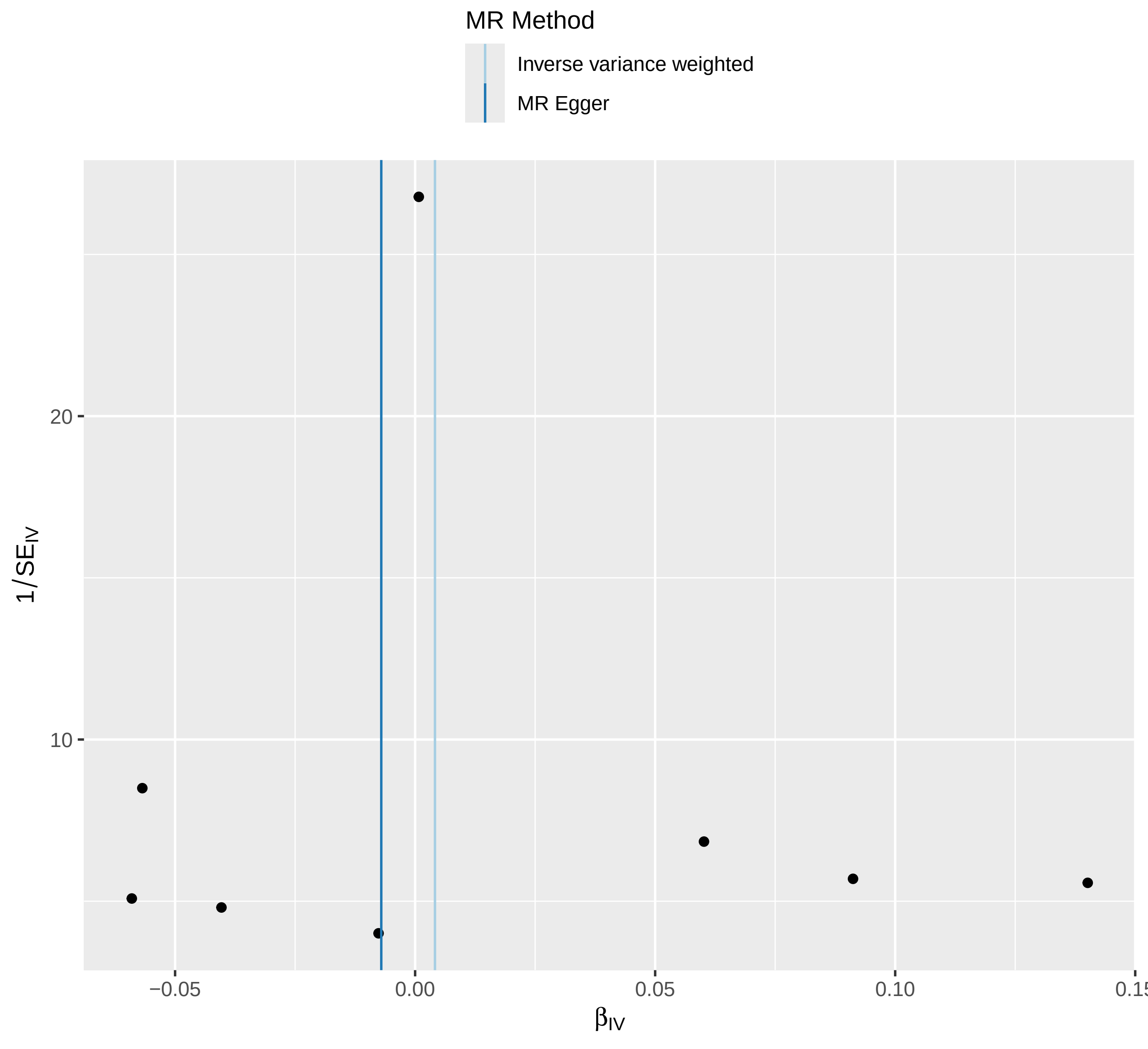
MR Method
Inverse variance weighted
MR Egger
20
10
1/SE_IV
−0.05
0.00
0.05
0.10
0.15
β_IV

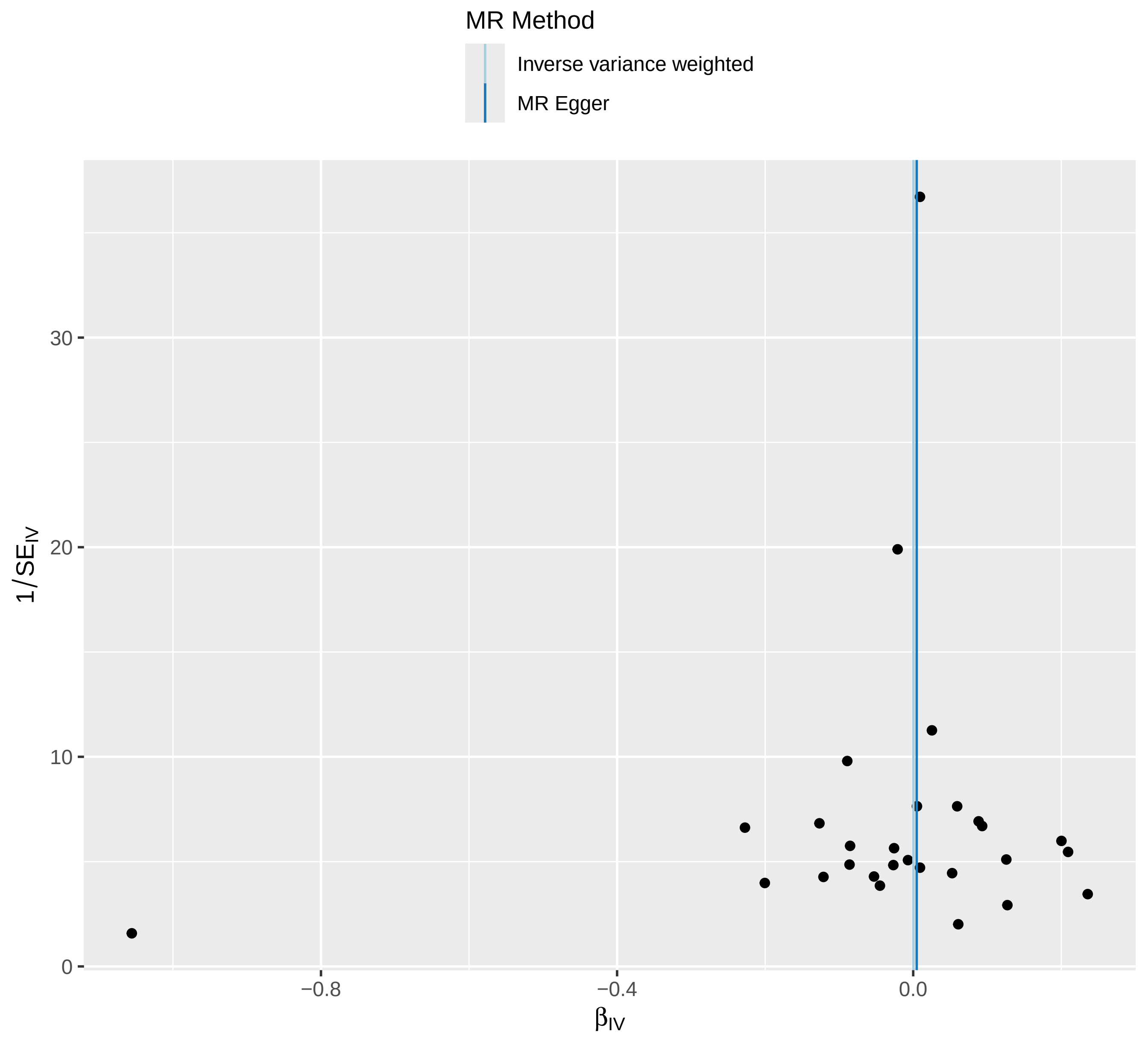

MR Method
Inverse variance weighted
MR Egger
1/SE_IV
30
20
10
0
−0.8
−0.4
0.0
β_IV

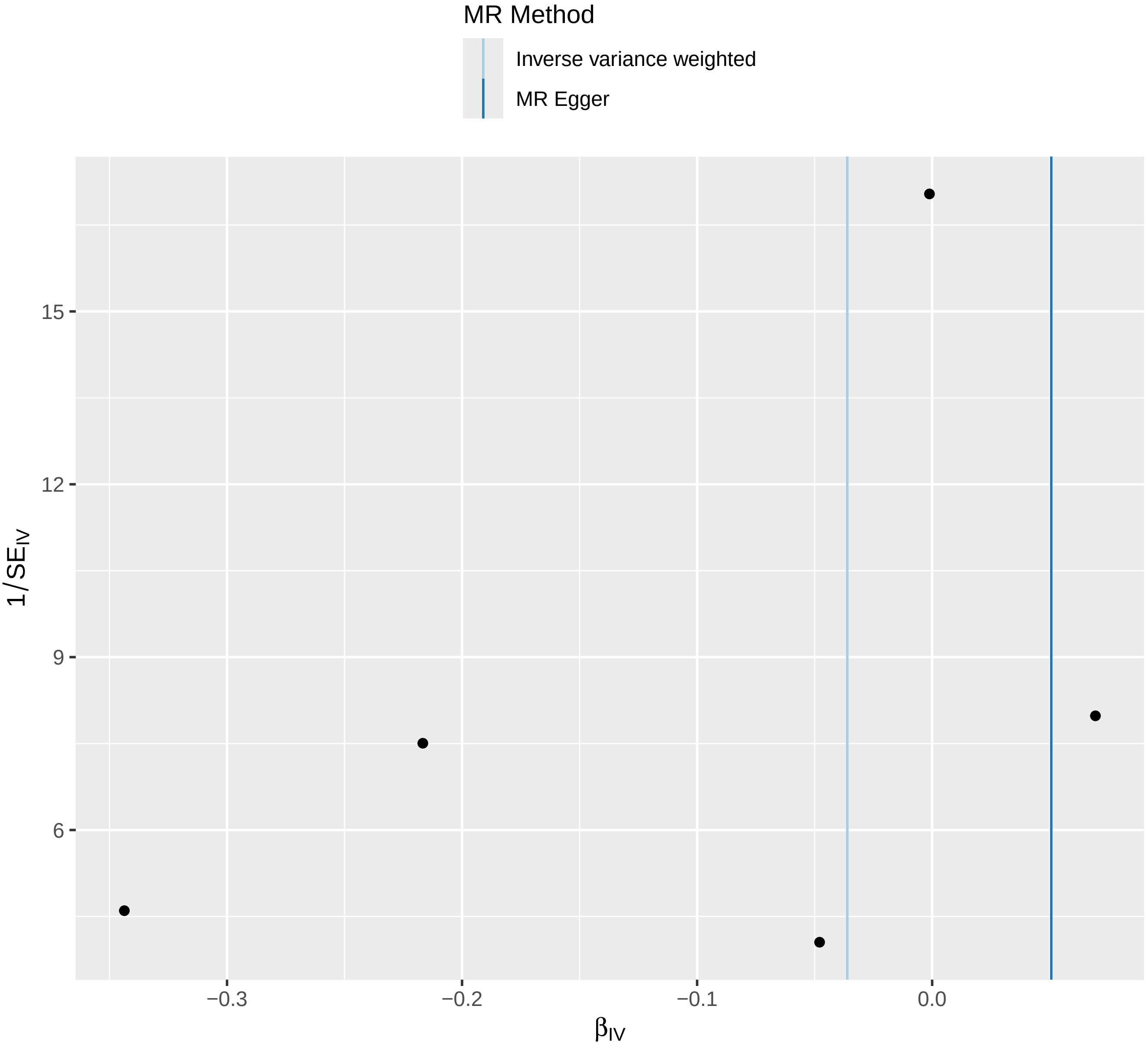
MR Method
Inverse variance weighted
MR Egger
15
12
9
6
$1/SE_{IV}$
−0.3
−0.2
−0.1
0.0
$\beta_{IV}$

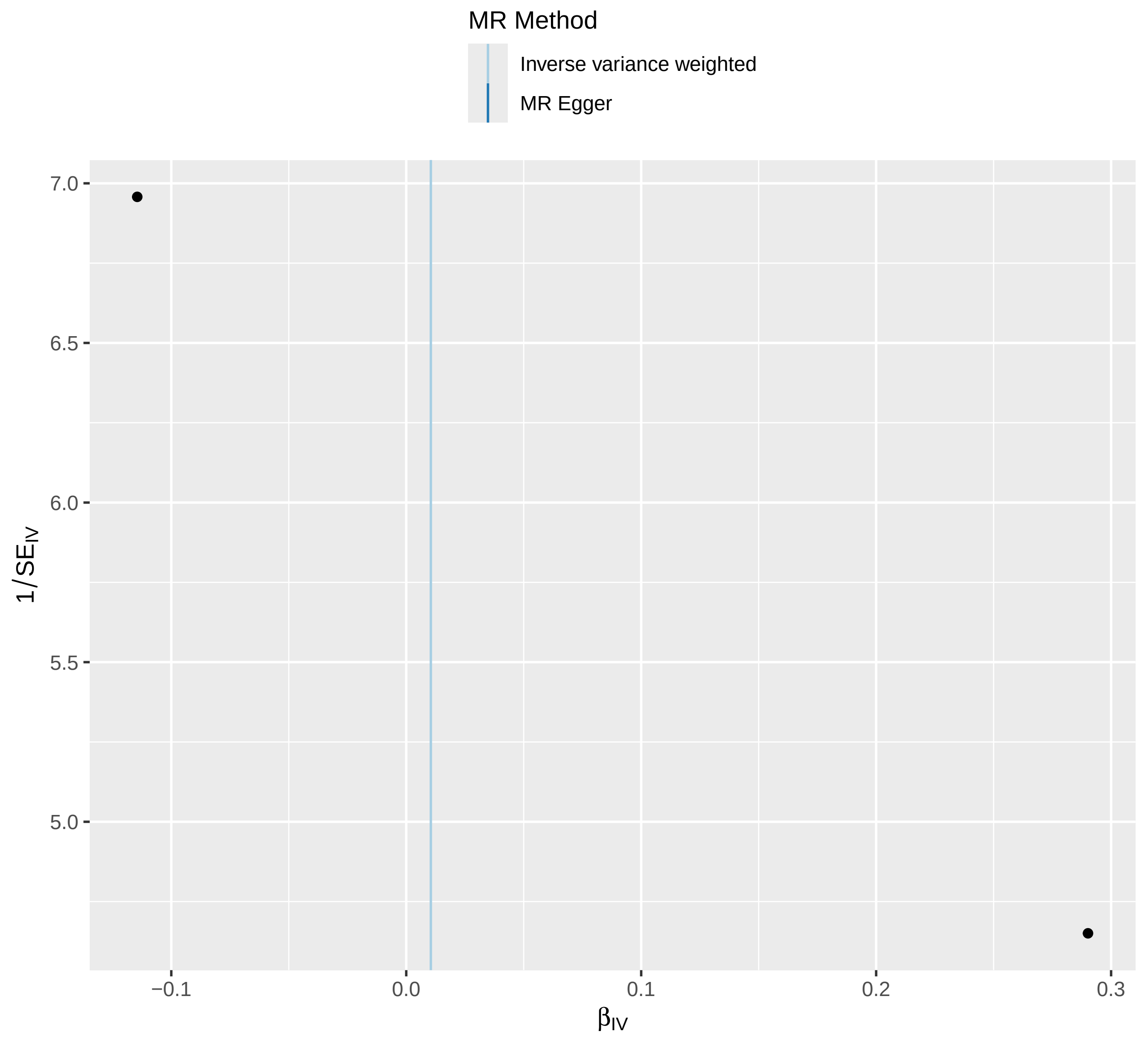

MR Method
Inverse variance weighted
MR Egger
7.0
6.5
6.0
5.5
5.0
1/SE_IV
−0.1
0.0
0.1
0.2
0.3
β_IV

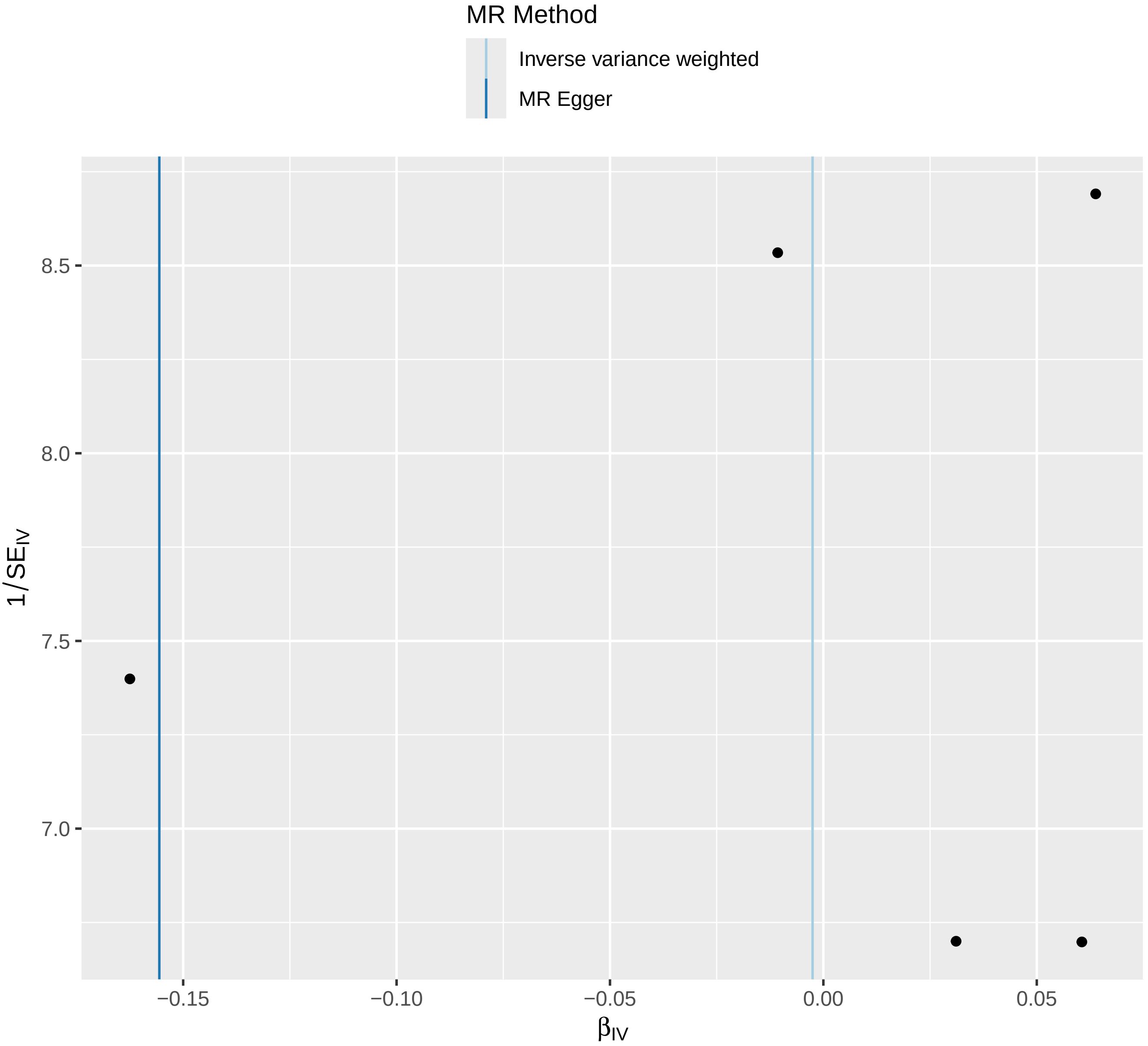
MR Method
Inverse variance weighted
MR Egger
8.5
8.0
7.5
7.0
1/SE$_{IV}$
−0.15
−0.10
−0.05
0.00
0.05
β$_{IV}$

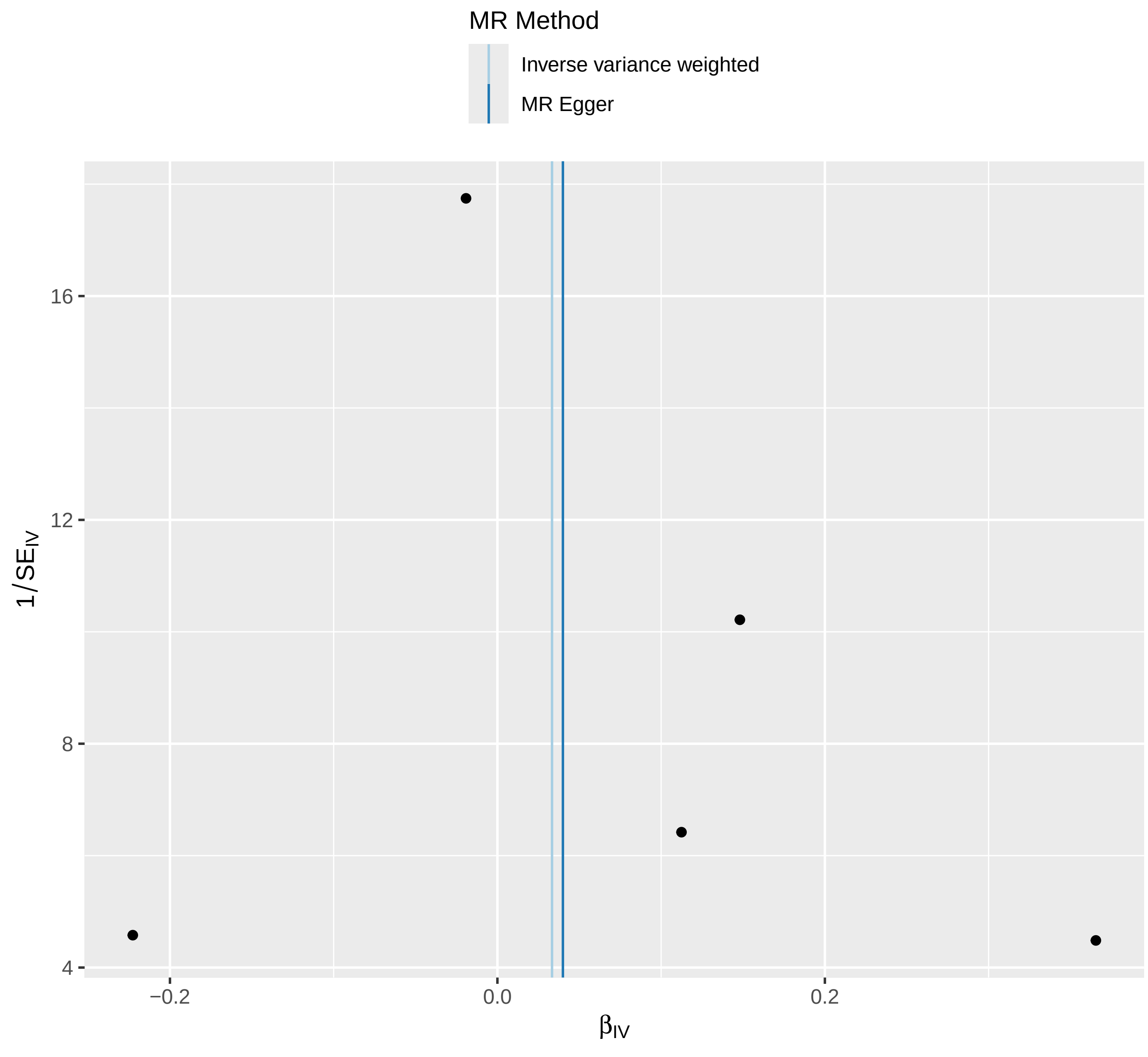

MR Method
Inverse variance weighted
MR Egger
16
12
8
4
1/SE$_{IV}$
−0.2
0.0
0.2
β$_{IV}$

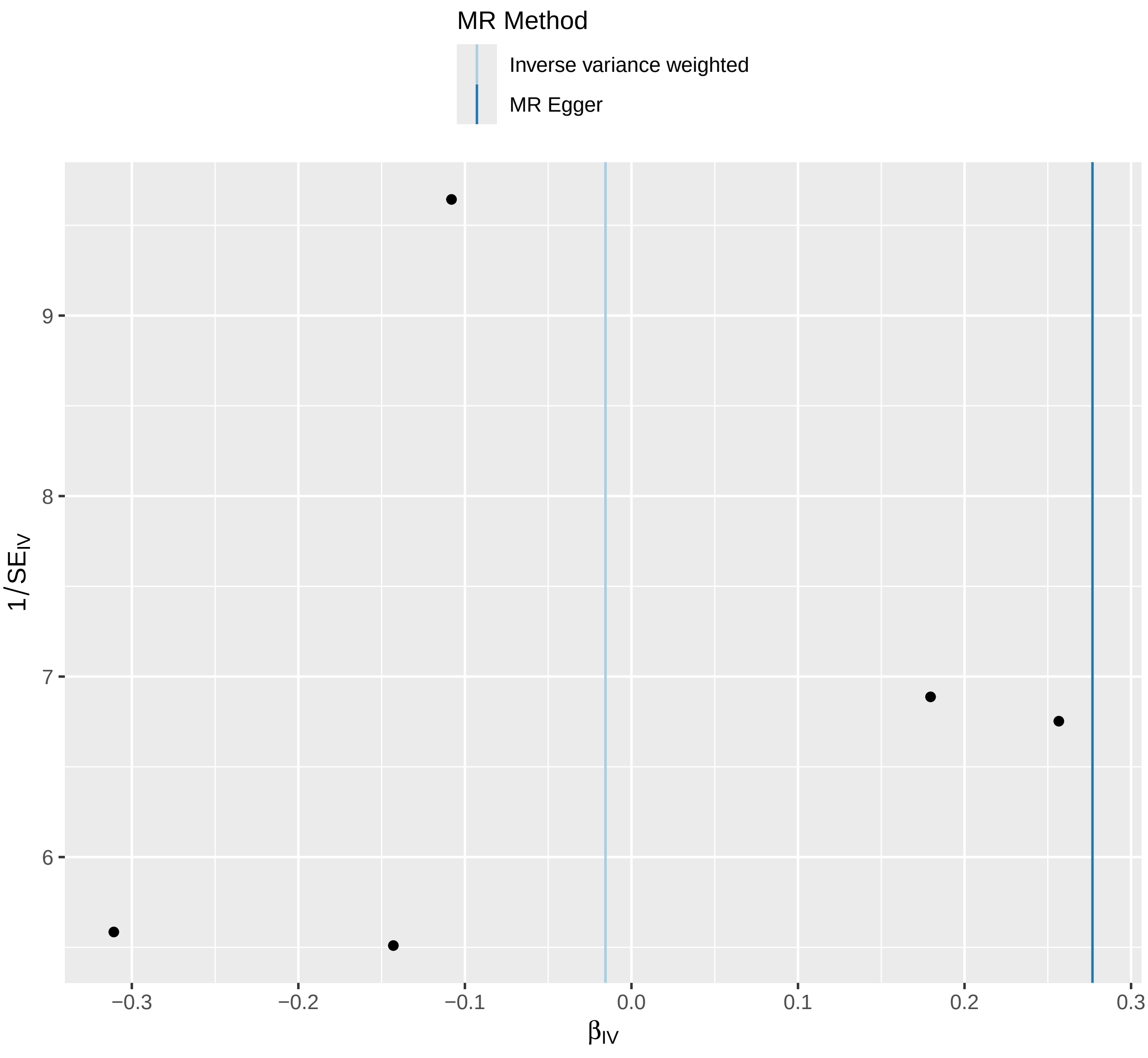
MR Method
Inverse variance weighted
MR Egger
1/SE_IV
9
8
7
6
−0.3
−0.2
−0.1
0.0
0.1
0.2
0.3
β_IV

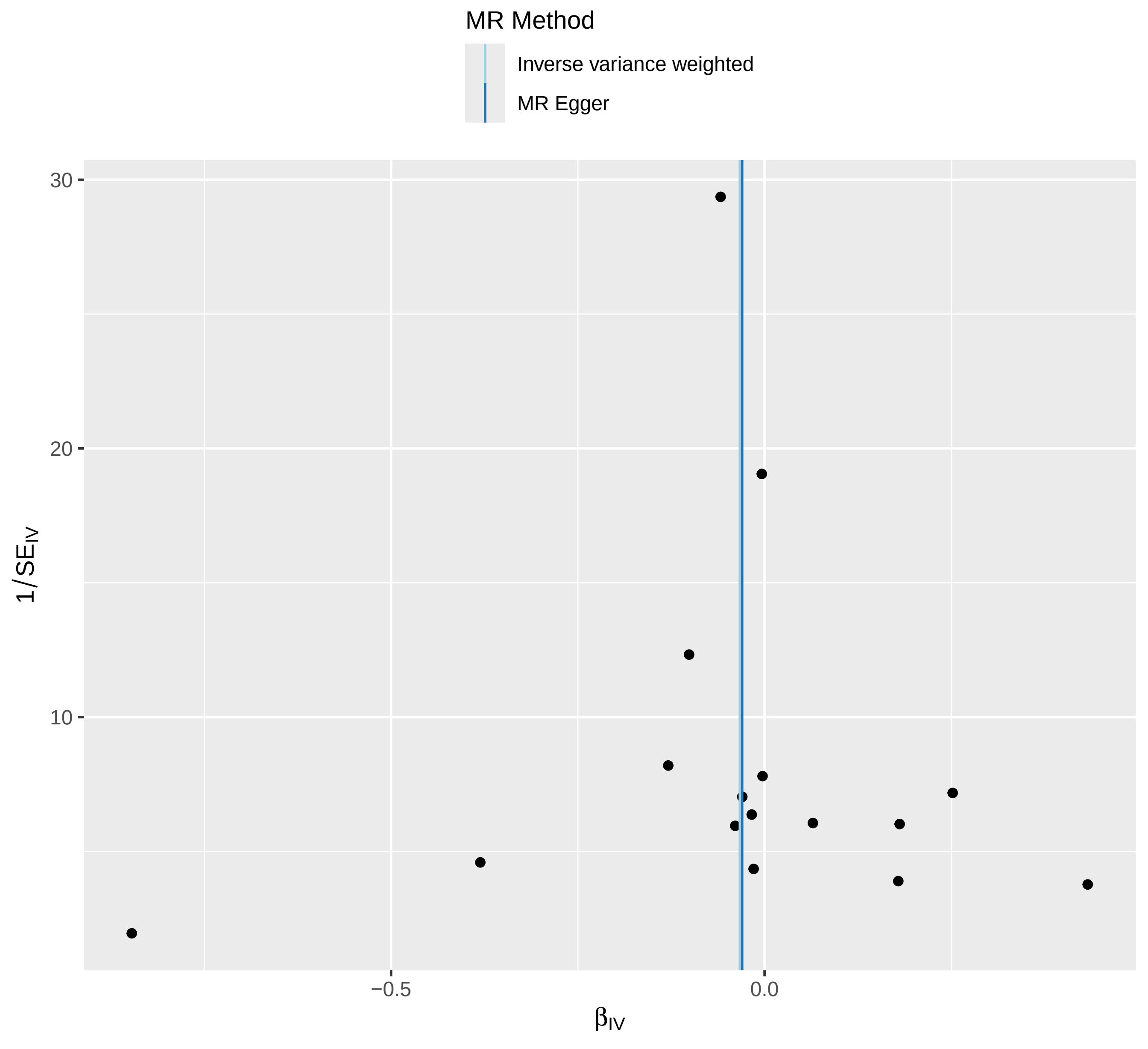

MR Method
Inverse variance weighted
MR Egger
30
20
10
1/SE$_{IV}$
−0.5
0.0
β$_{IV}$

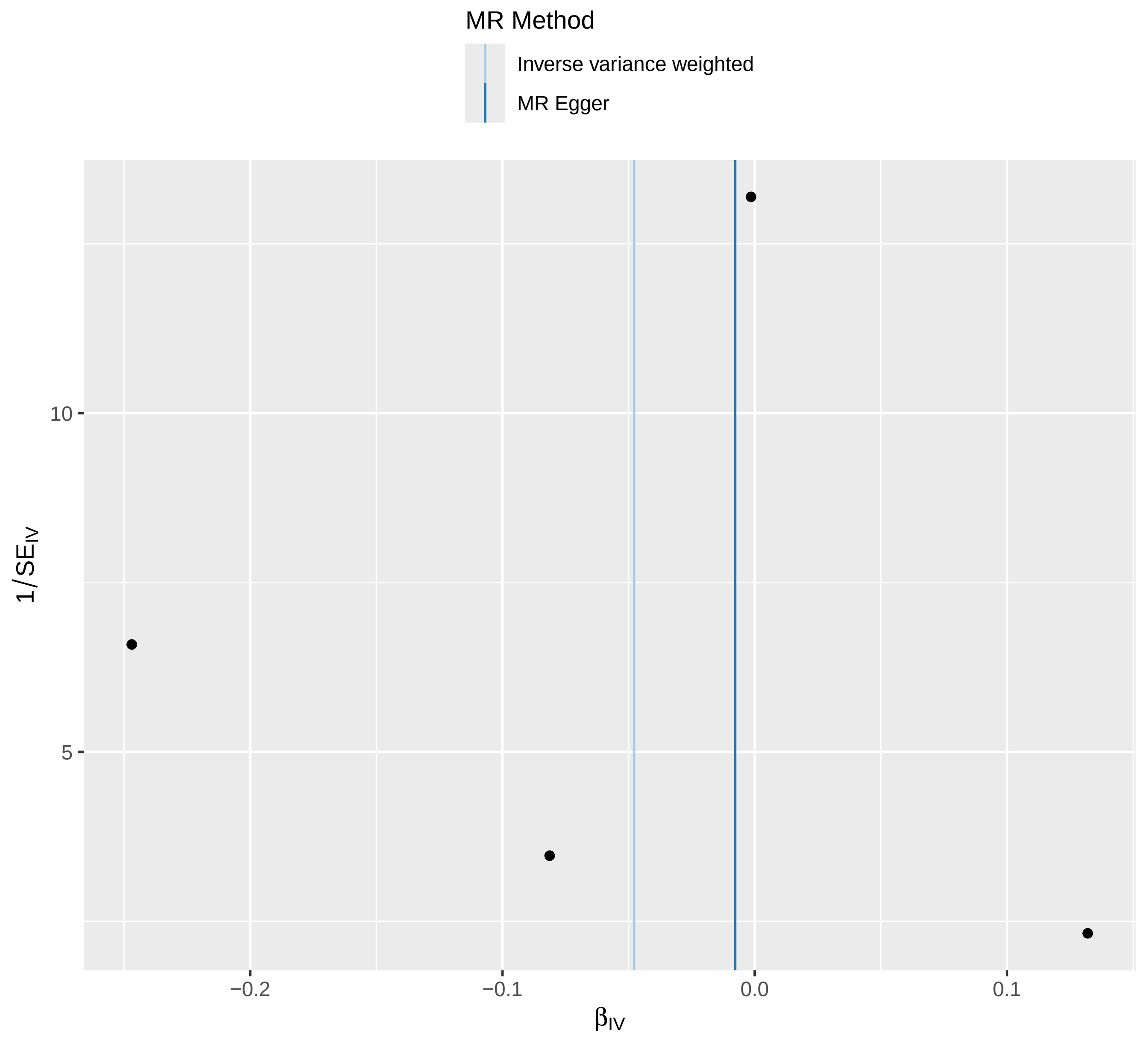
MR Method
Inverse variance weighted
MR Egger
10
5
$1/SE_{IV}$
−0.2
−0.1
0.0
0.1
$\beta_{IV}$

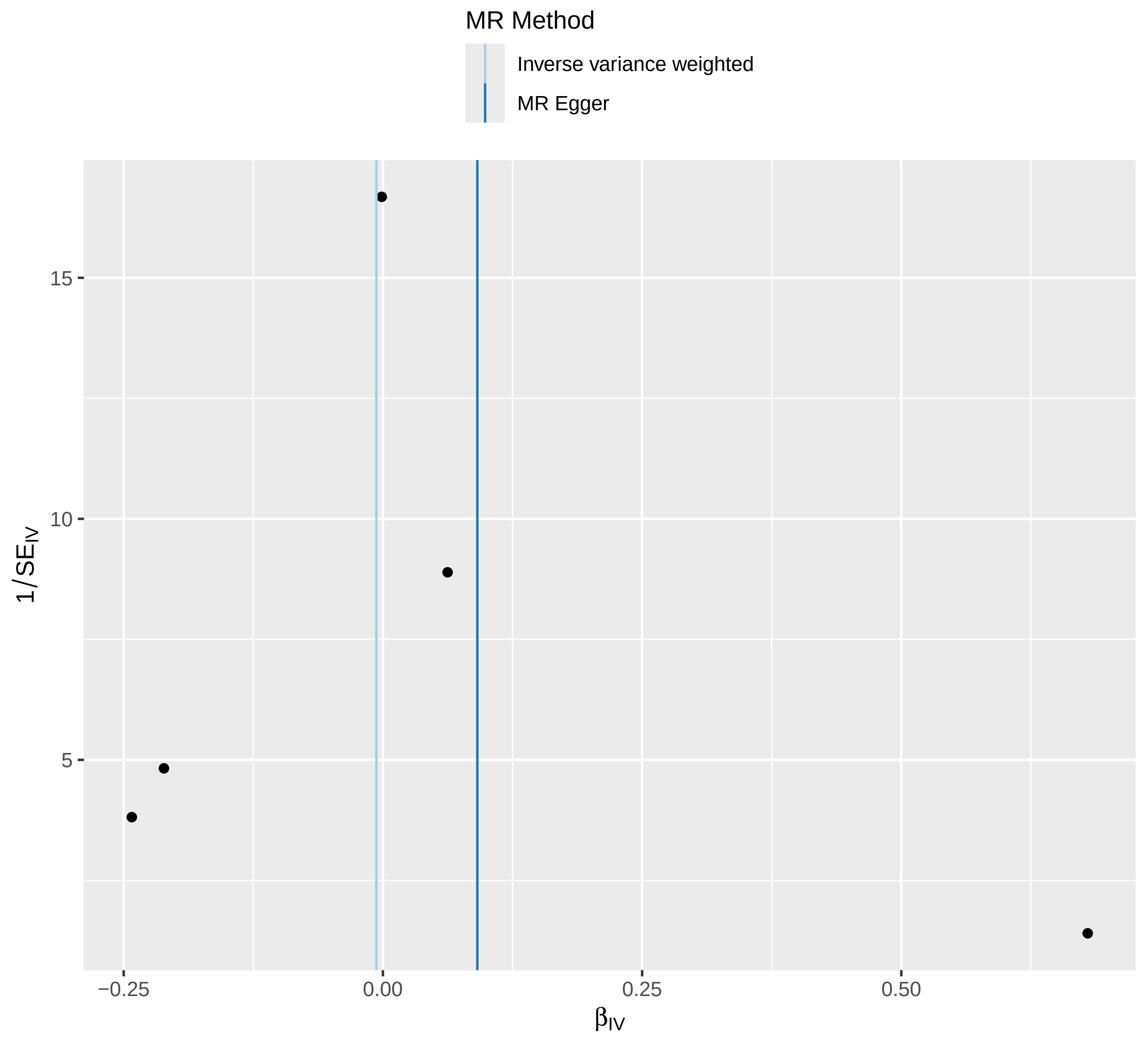

MR Method
Inverse variance weighted
MR Egger
15
10
5
1/SE_IV
−0.25
0.00
0.25
0.50
β_IV

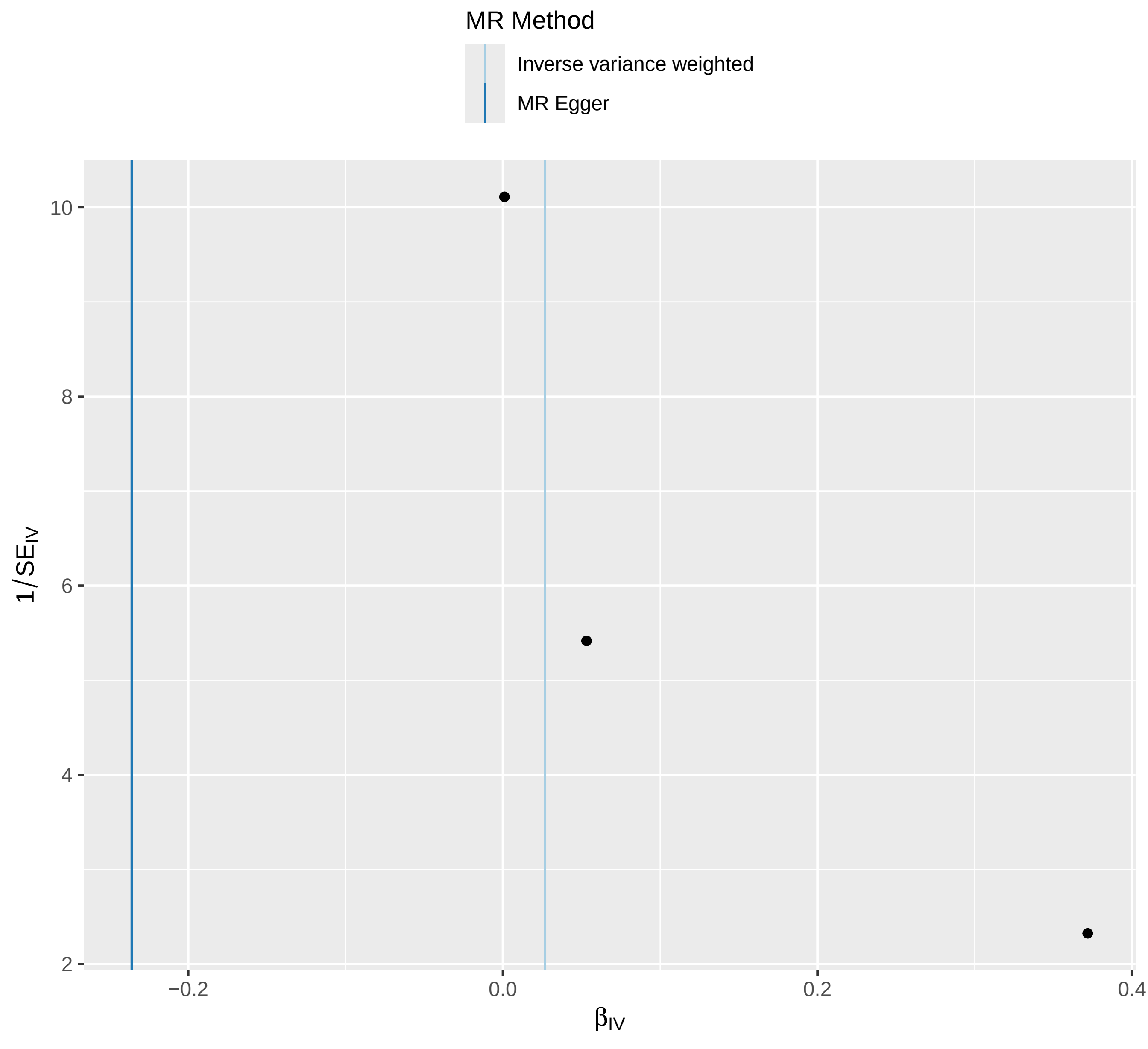
MR Method
Inverse variance weighted
MR Egger
10
8
6
4
2
1/SE_IV
−0.2
0.0
0.2
0.4
β_IV

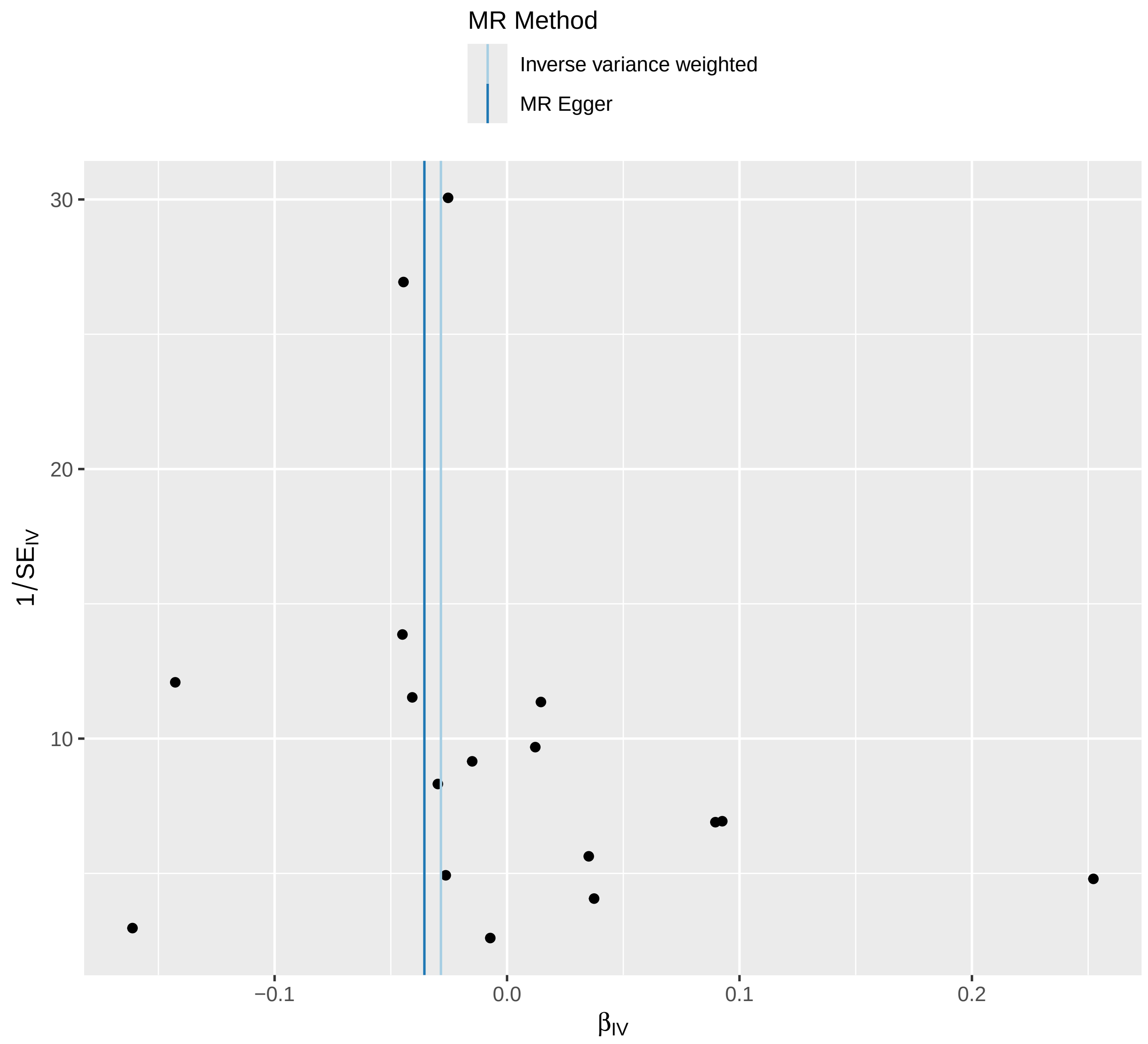
MR Method
Inverse variance weighted
MR Egger
30
20
10
1/SE_IV
−0.1
0.0
0.1
0.2
β_IV

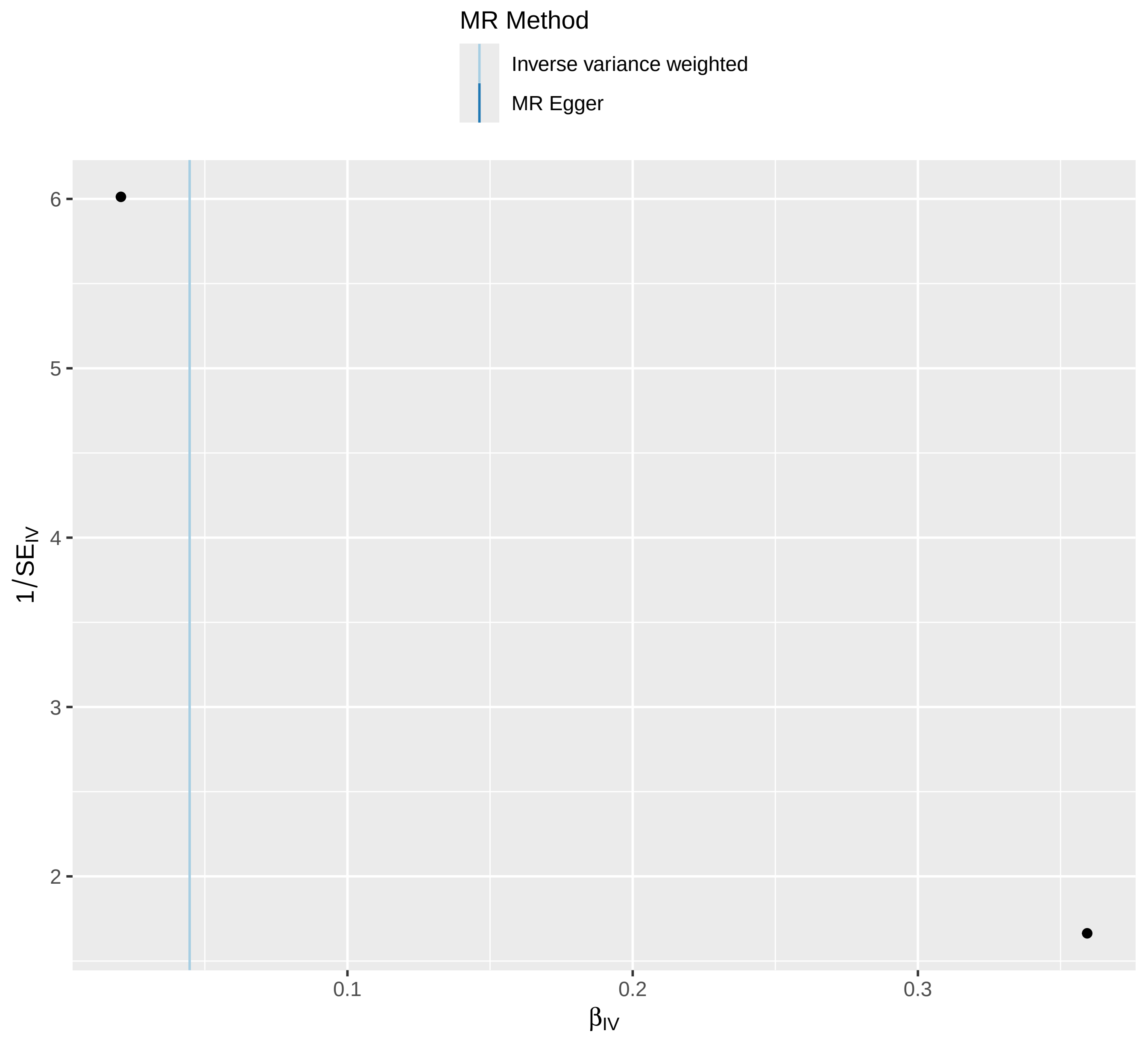
MR Method
Inverse variance weighted
MR Egger
6
5
4
3
2
0.1
0.2
0.3
$\beta_{IV}$
$1/SE_{IV}$

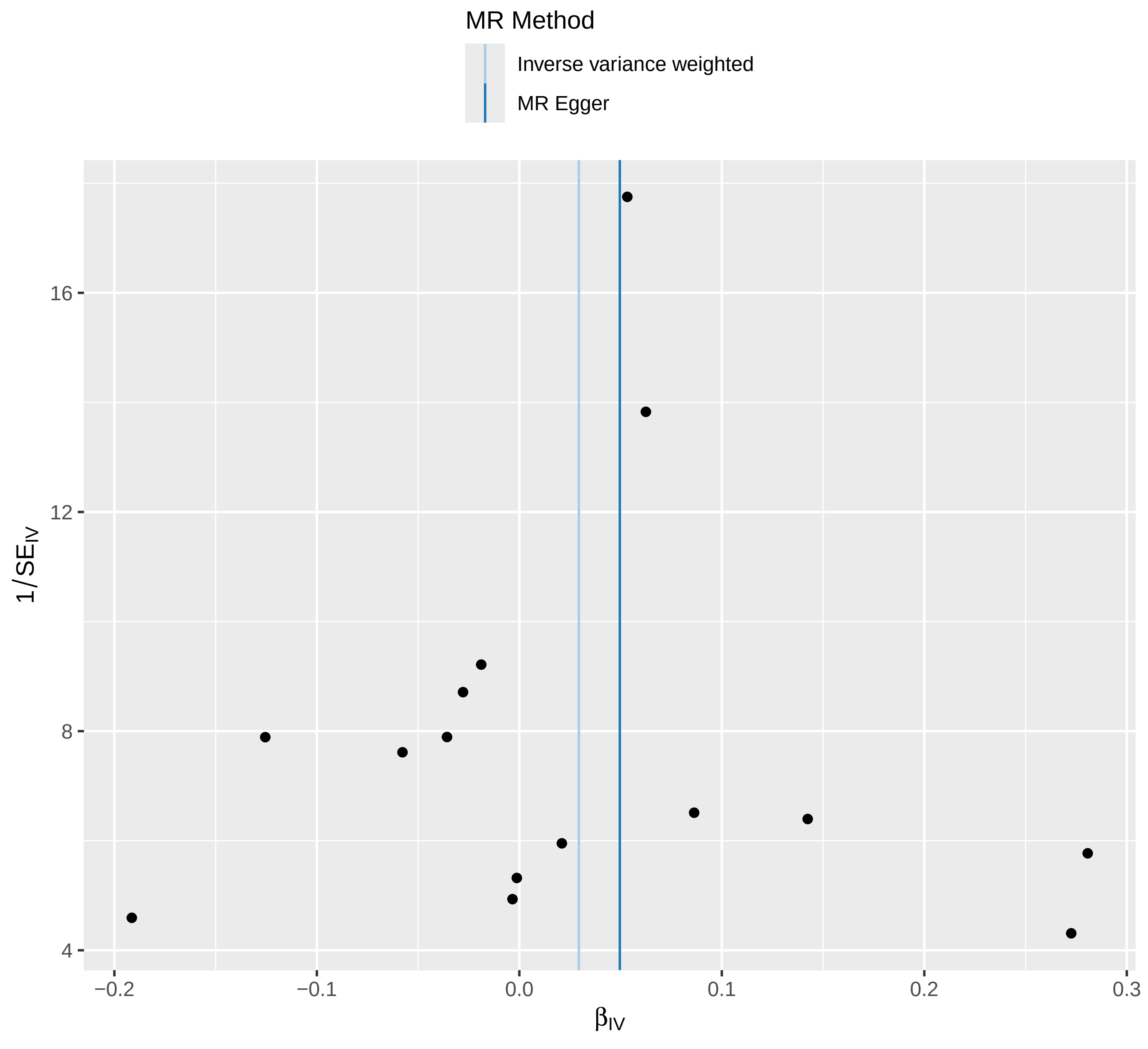

MR Method
Inverse variance weighted
MR Egger
16
12
8
4
1/SE$_{IV}$
−0.2
−0.1
0.0
0.1
0.2
0.3
β$_{IV}$

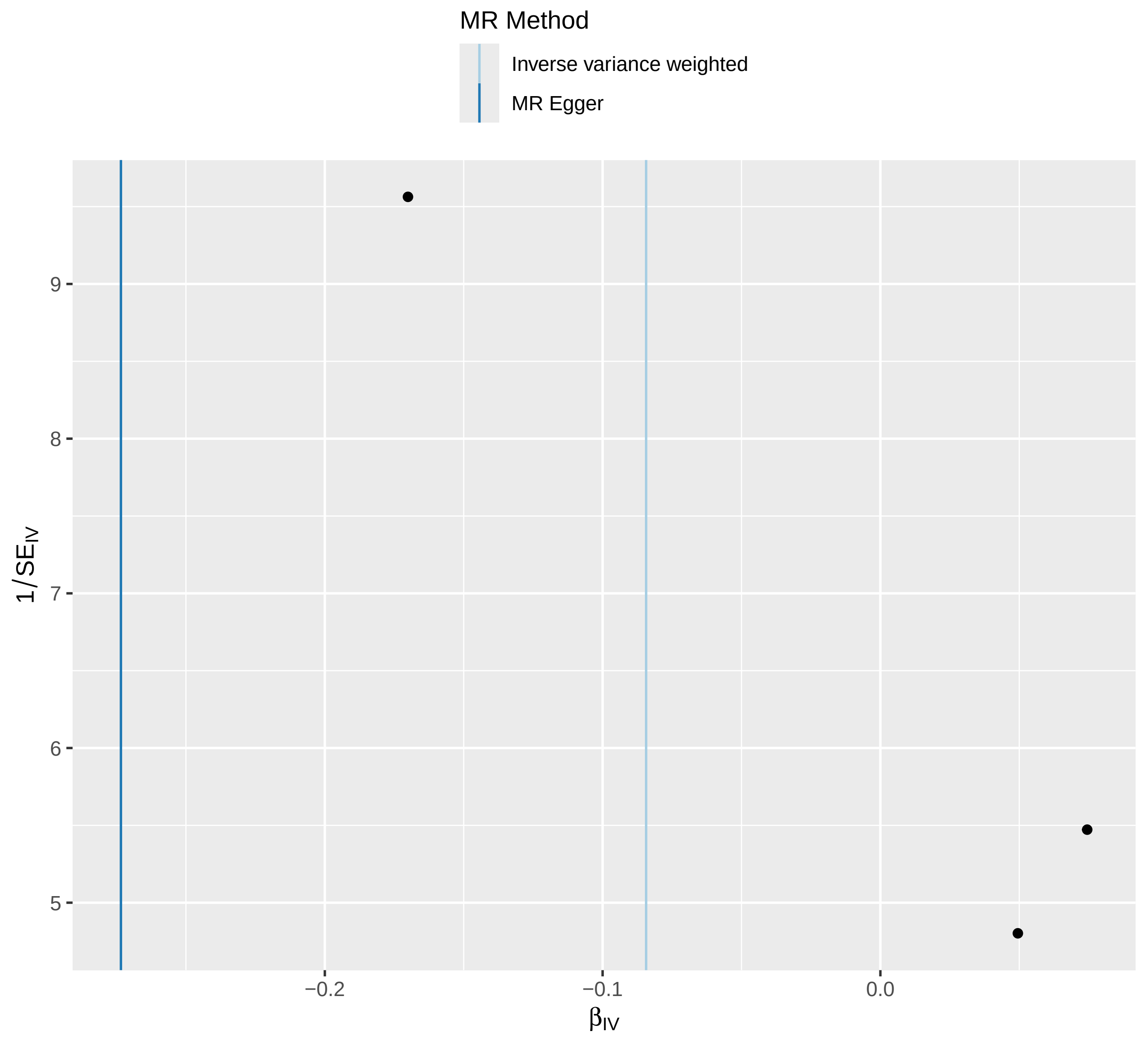
MR Method
Inverse variance weighted
MR Egger
9
8
7
6
5
1/SE$_{IV}$
−0.2
−0.1
0.0
β$_{IV}$

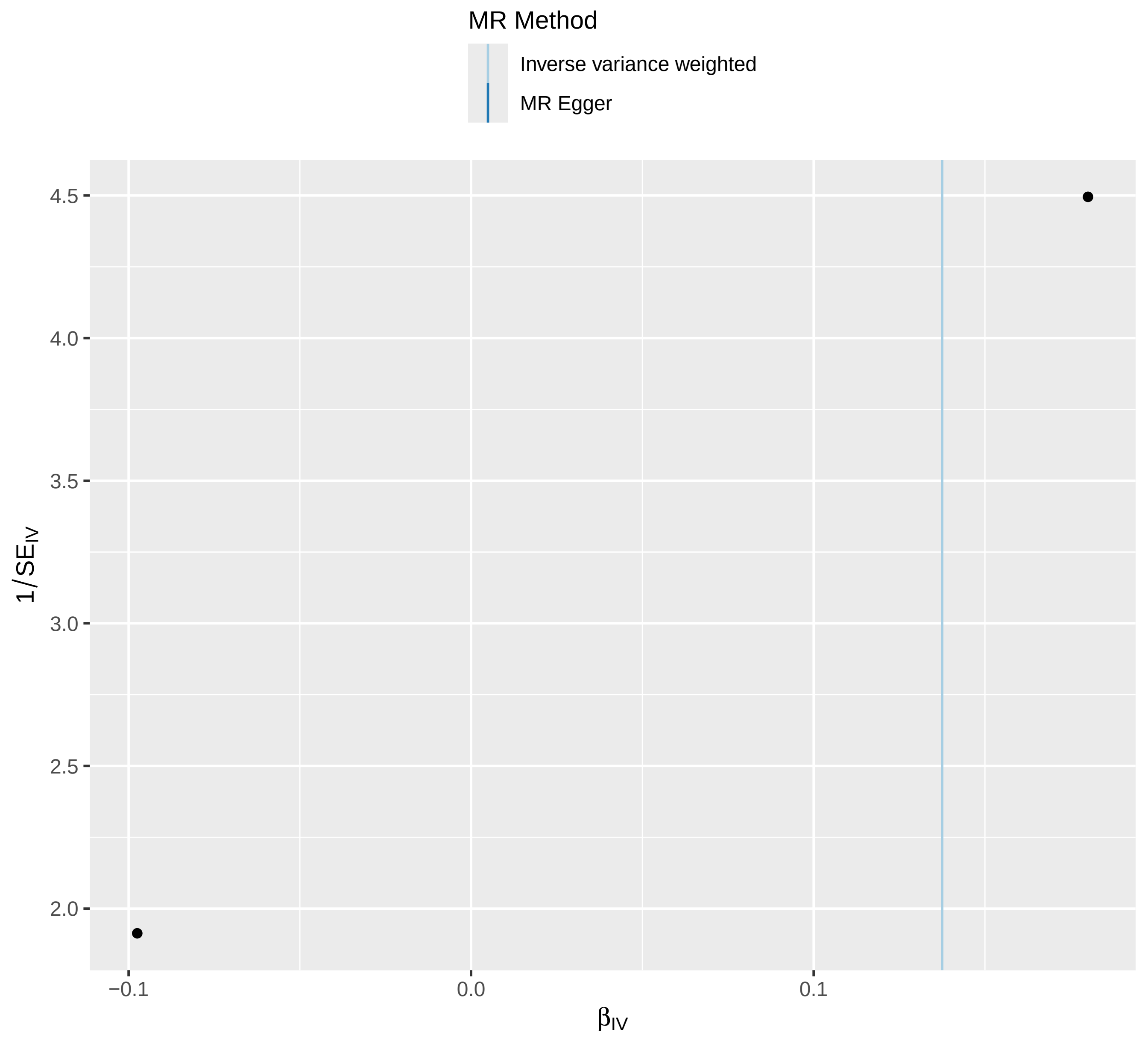
MR Method
Inverse variance weighted
MR Egger
4.5
4.0
3.5
3.0
2.5
2.0
$1/SE_{IV}$
−0.1
0.0
0.1
$\beta_{IV}$

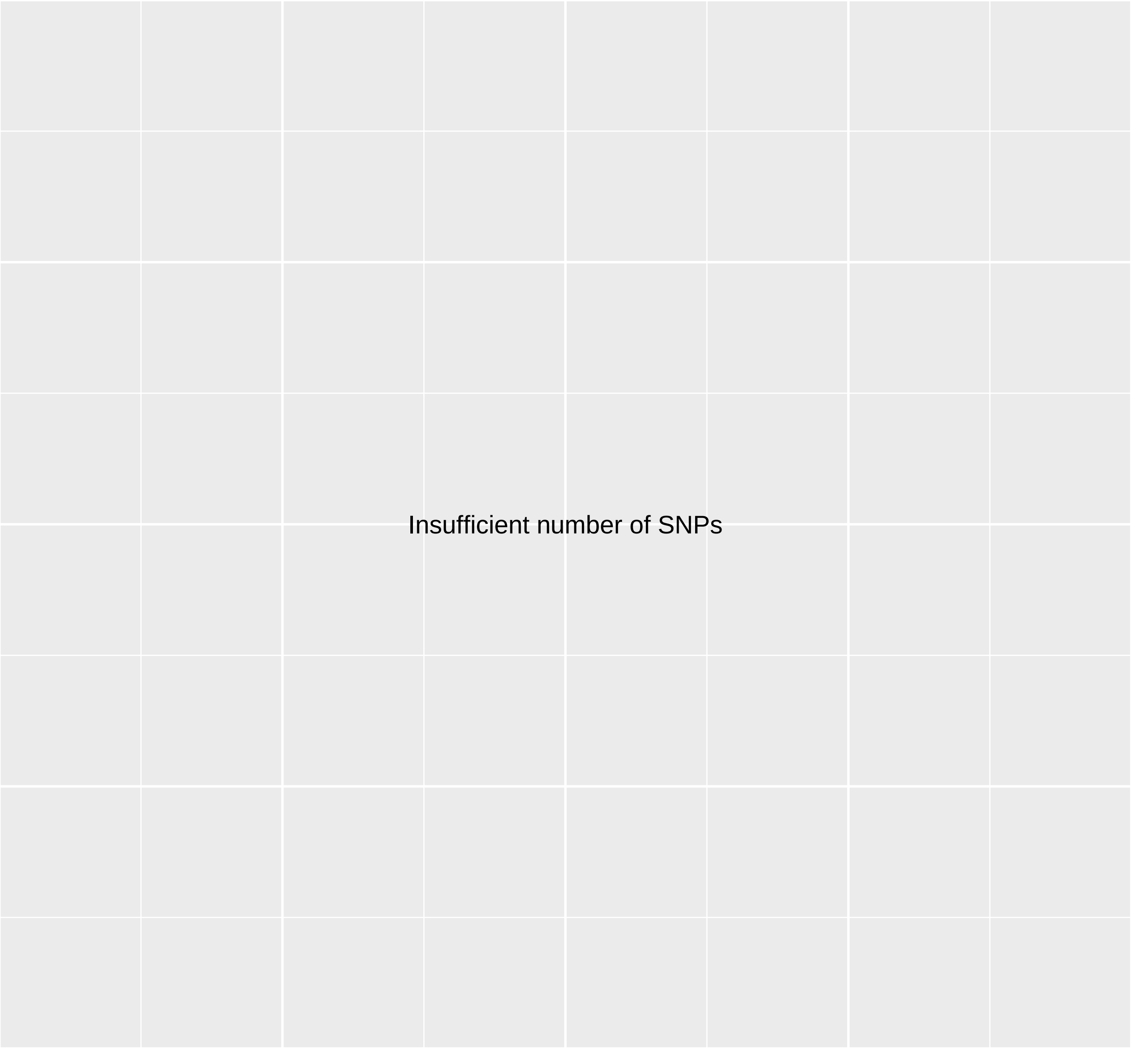
Insufficient number of SNPs

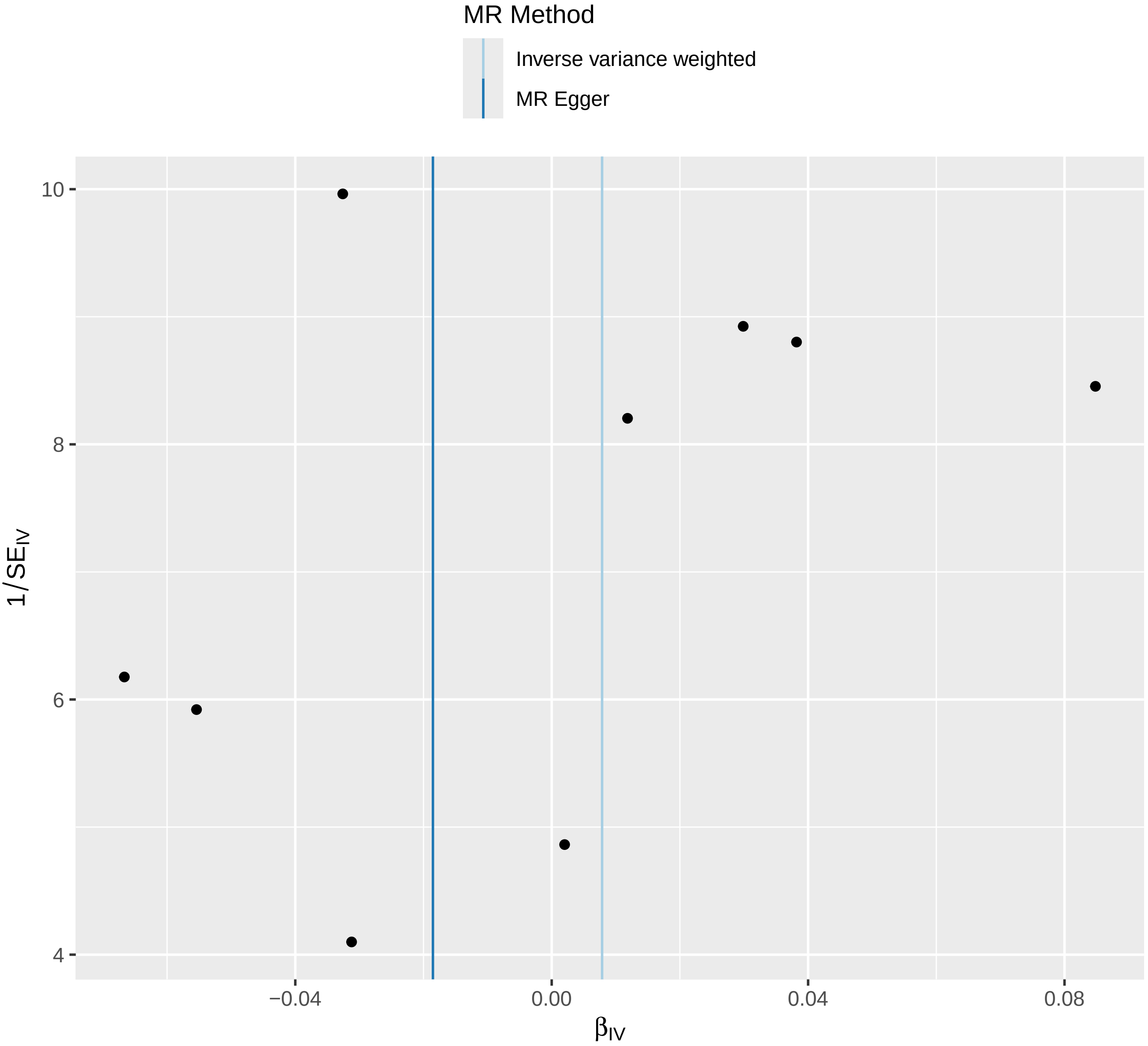

MR Method
Inverse variance weighted
MR Egger
10
8
6
4
$1/SE_{IV}$
−0.04
0.00
0.04
0.08
$\beta_{IV}$

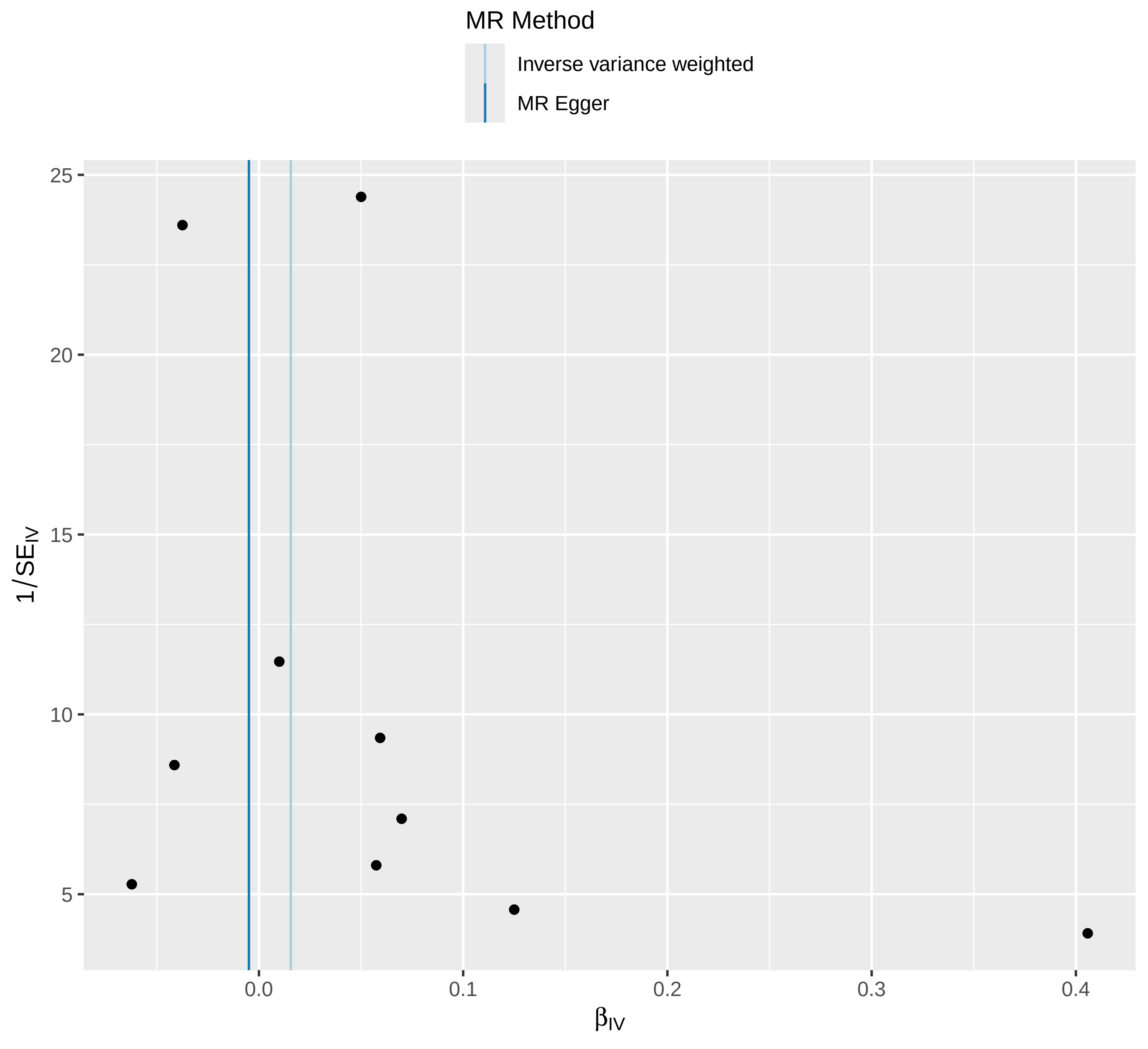
MR Method
Inverse variance weighted
MR Egger
25
20
15
10
5
1/SE_IV
0.0
0.1
0.2
0.3
0.4
β_IV

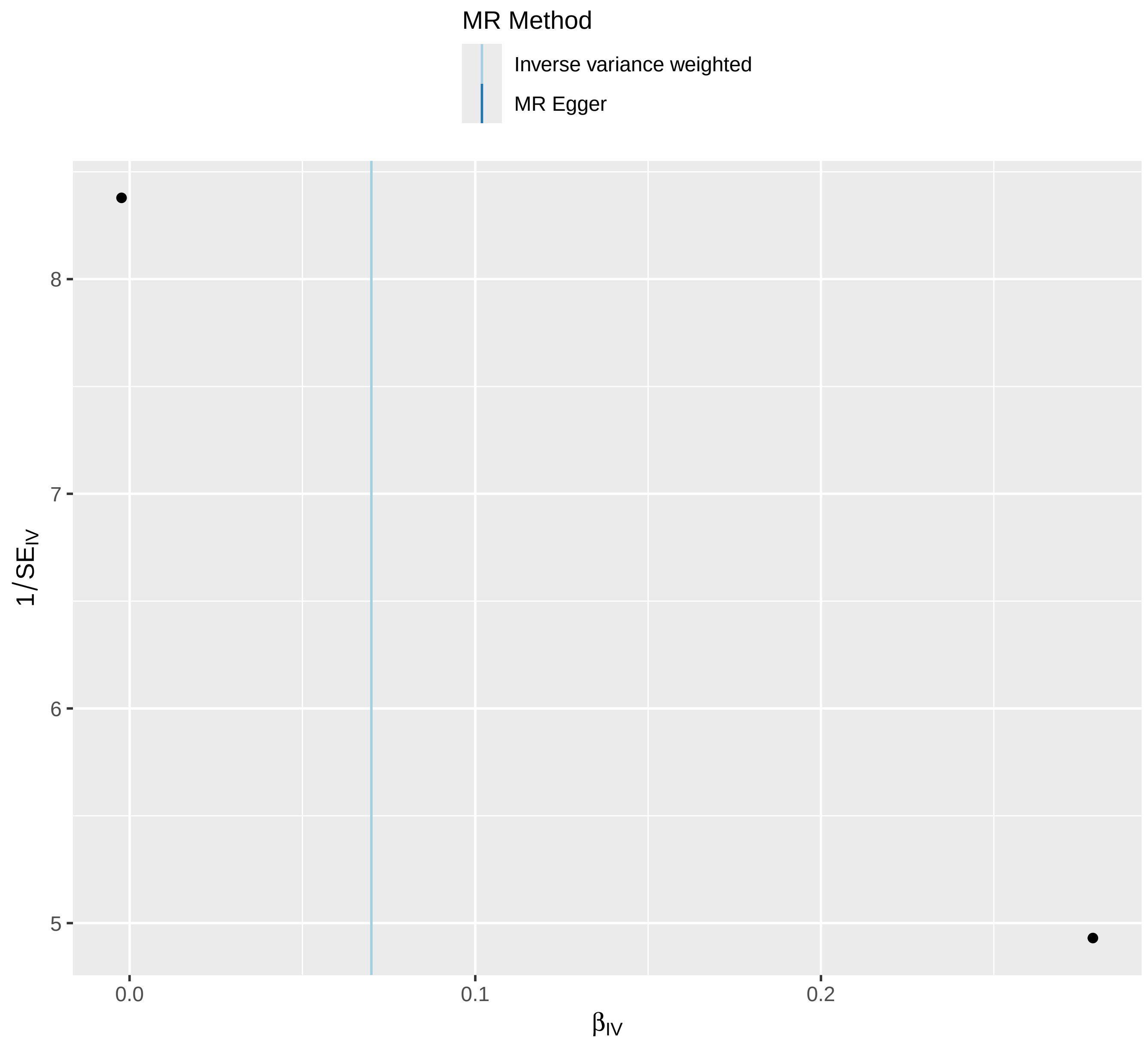

MR Method
Inverse variance weighted
MR Egger
8
7
6
5
1/SE_IV
0.0
0.1
0.2
β_IV

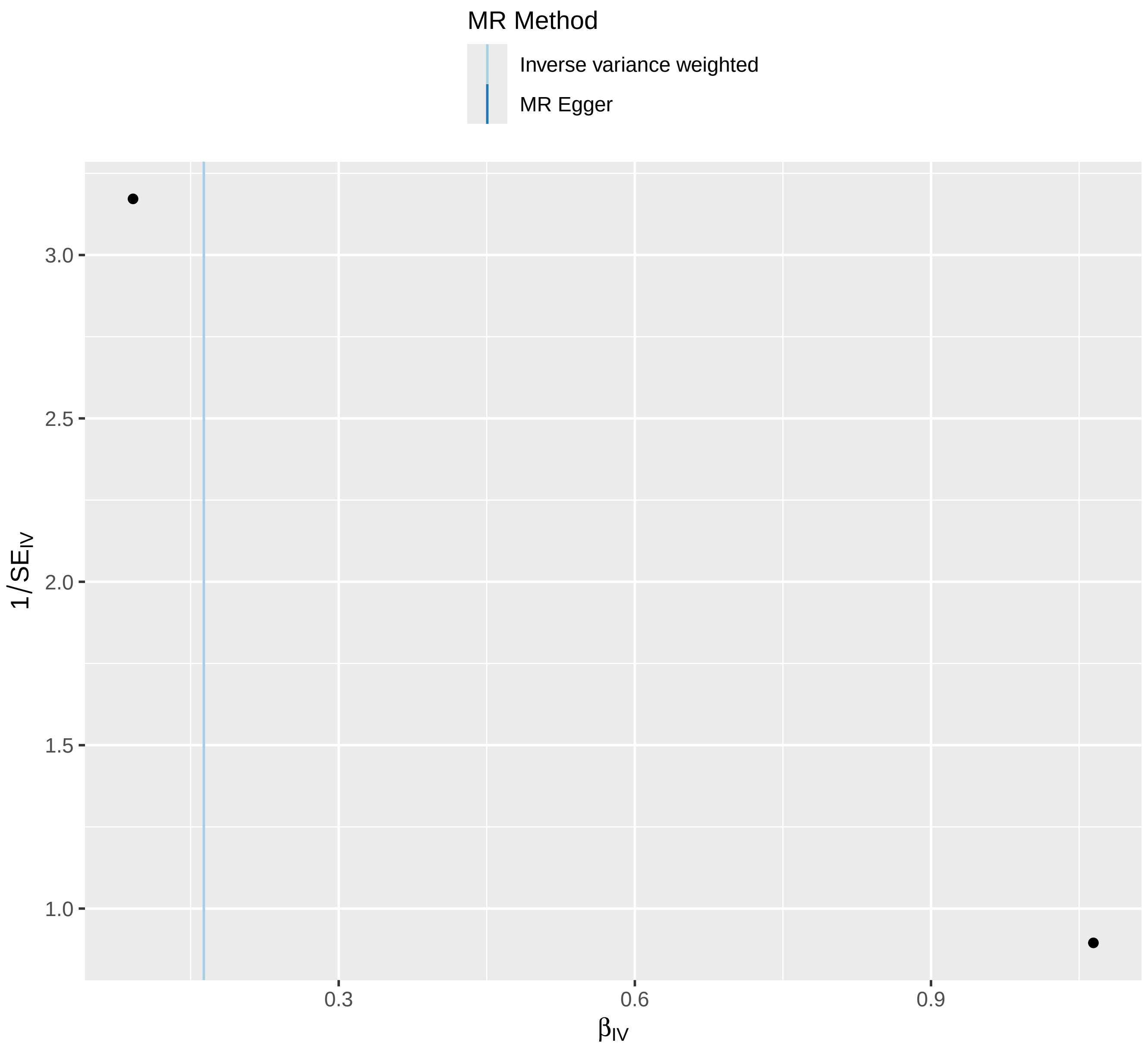

MR Method
Inverse variance weighted
MR Egger
3.0
2.5
2.0
1.5
1.0
1/SE_IV
0.3
0.6
0.9
β_IV

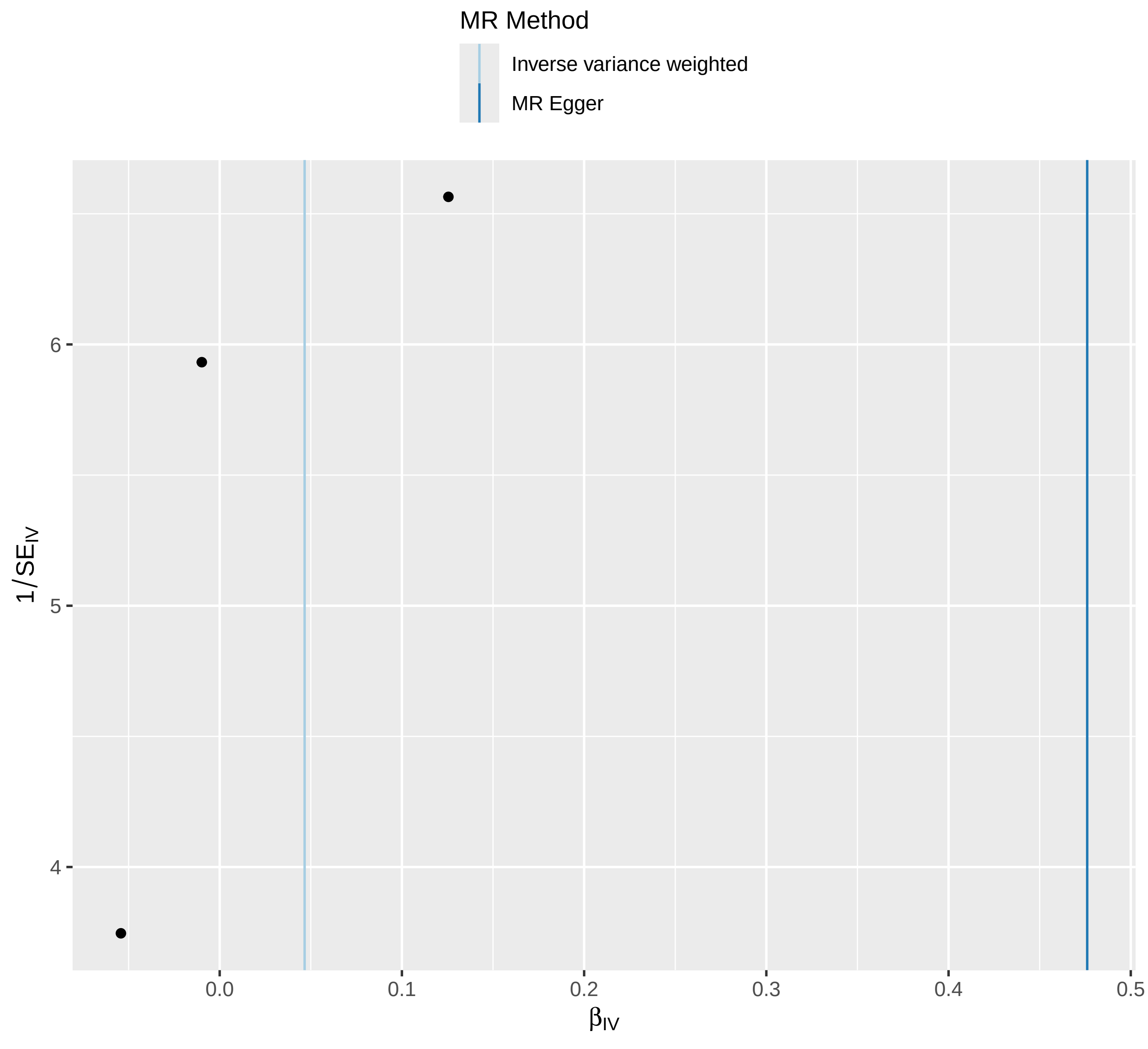

MR Method
Inverse variance weighted
MR Egger
1/SE_IV
6
5
4
0.0
0.1
0.2
0.3
0.4
0.5
β_IV

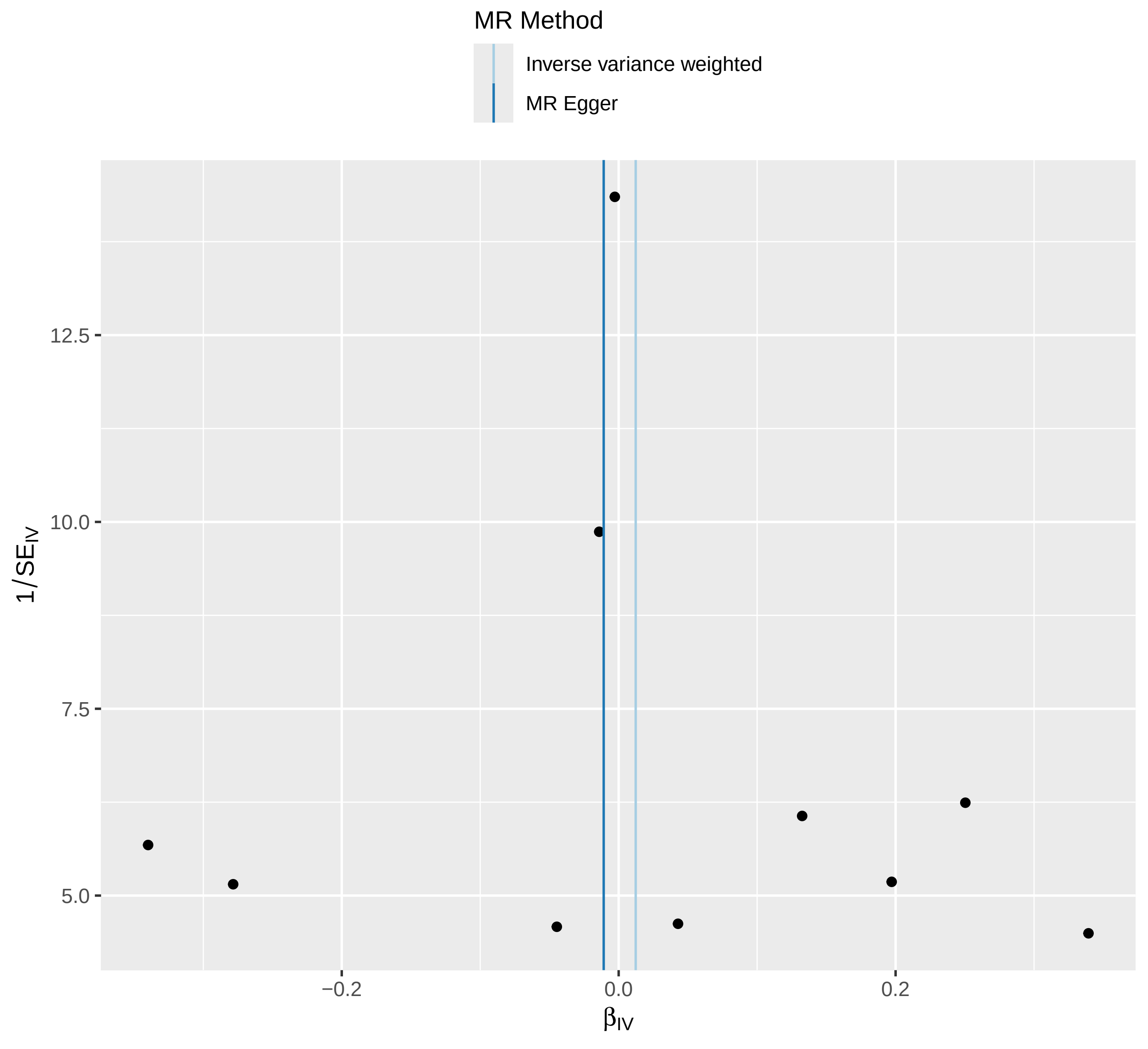

MR Method
Inverse variance weighted
MR Egger
12.5
10.0
7.5
5.0
1/SE$_{IV}$
−0.2
0.0
0.2
β$_{IV}$

Insufficient number of SNPs

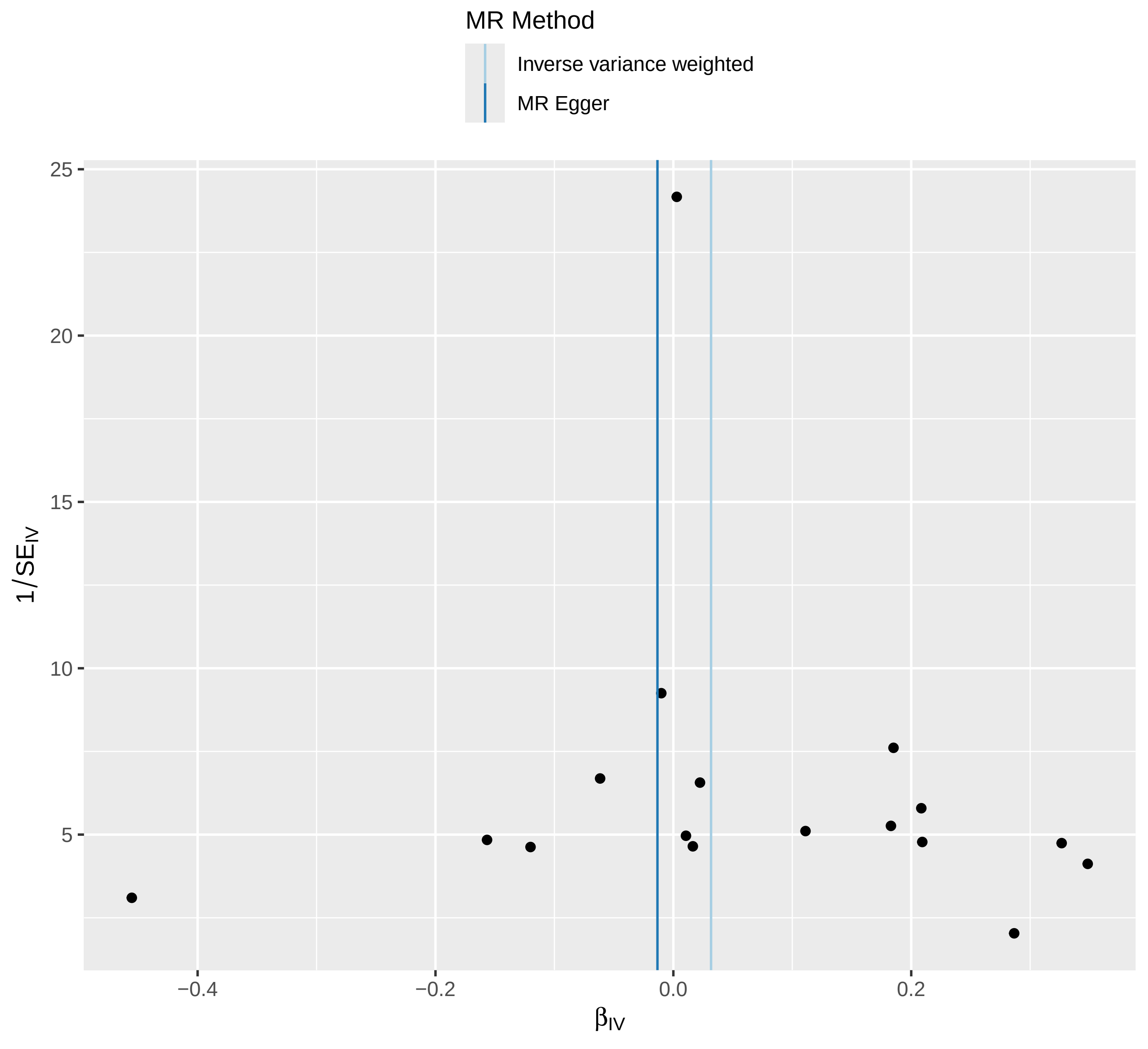

MR Method
Inverse variance weighted
MR Egger
25
20
15
10
5
1/SE_IV
−0.4
−0.2
0.0
0.2
β_IV

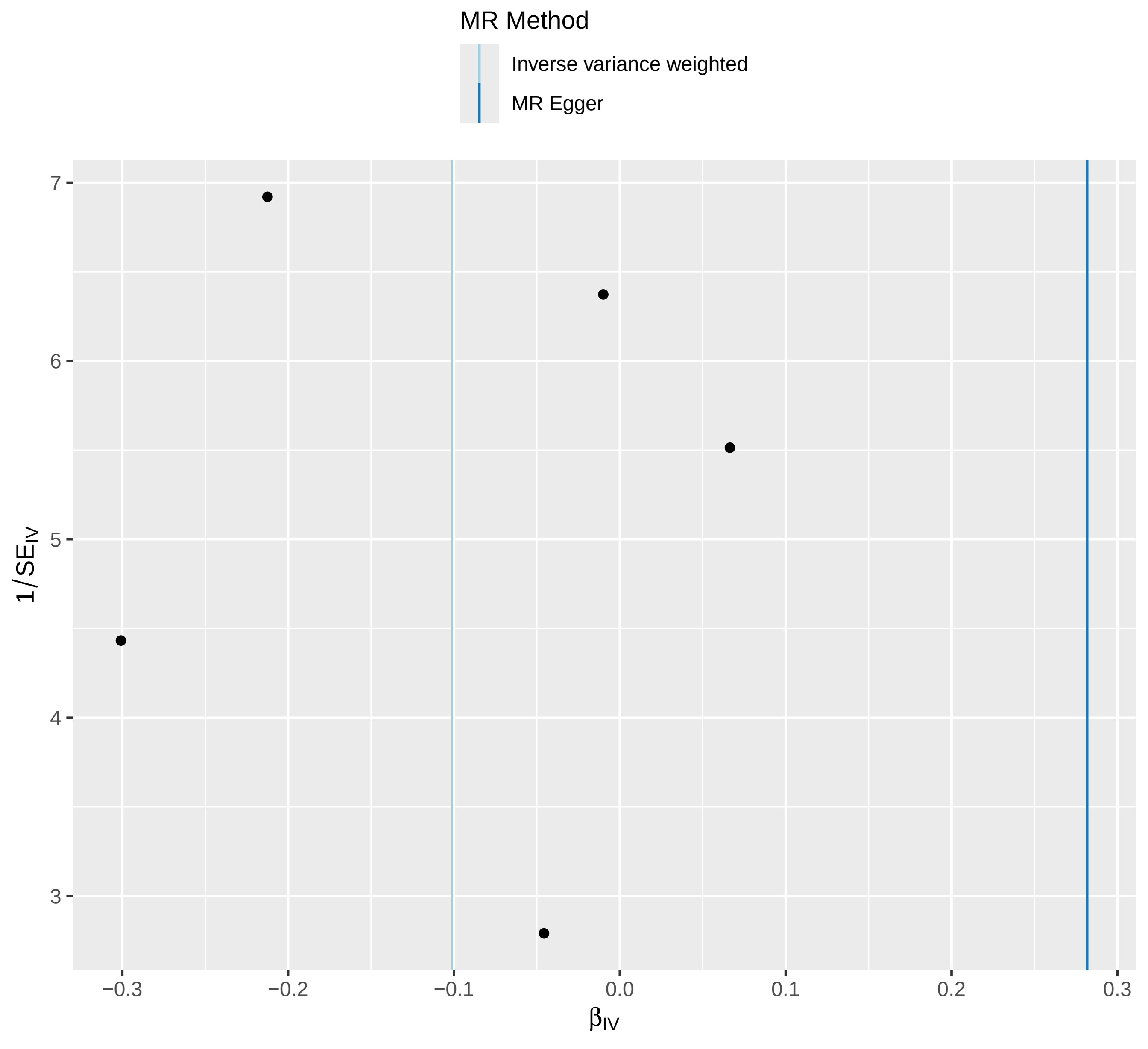

MR Method
Inverse variance weighted
MR Egger
7
6
5
4
3
1/SE$_{IV}$
−0.3
−0.2
−0.1
0.0
0.1
0.2
0.3
β$_{IV}$

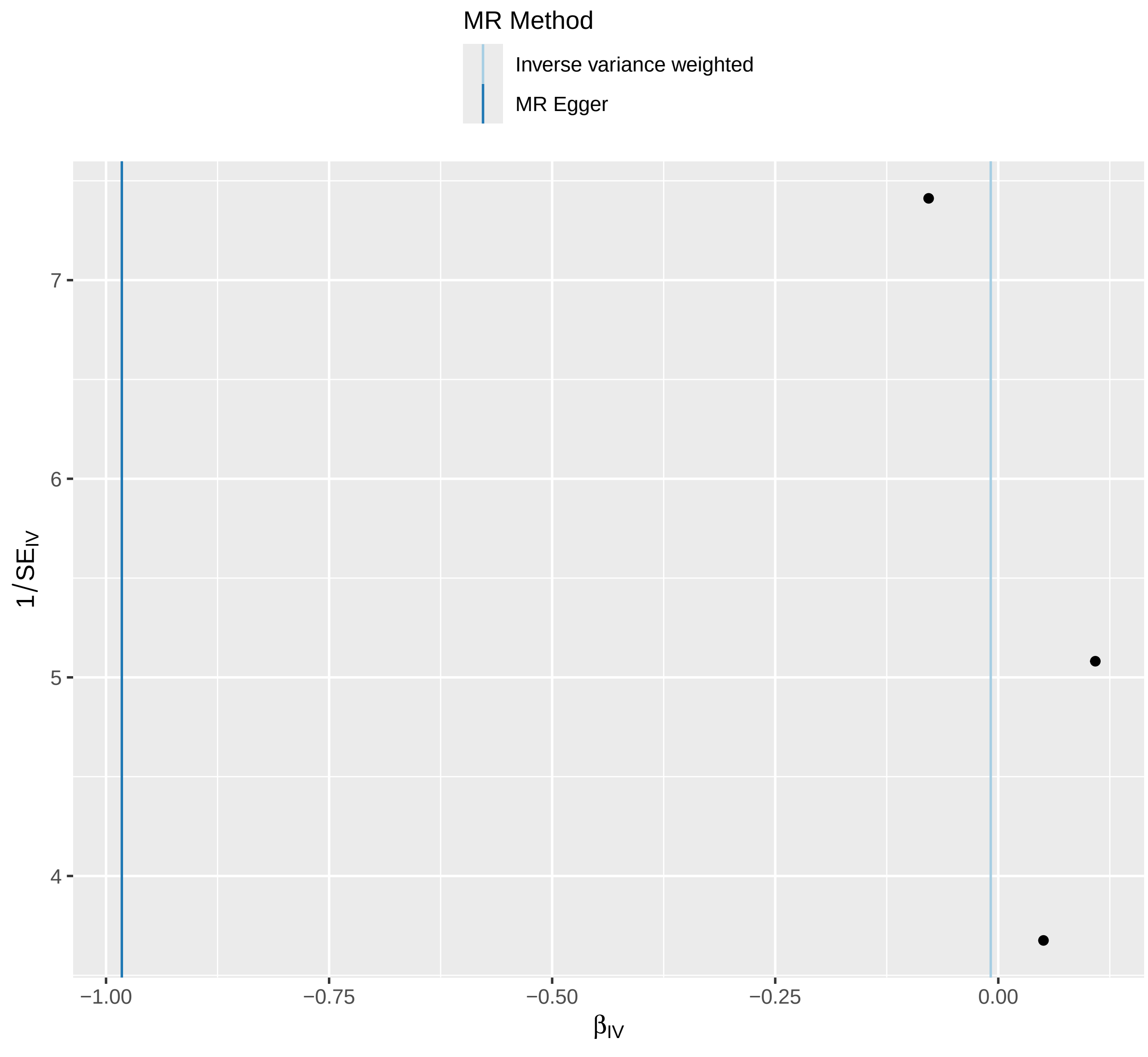

MR Method
Inverse variance weighted
MR Egger
7
6
5
4
1/SE_IV
−1.00
−0.75
−0.50
−0.25
0.00
β_IV

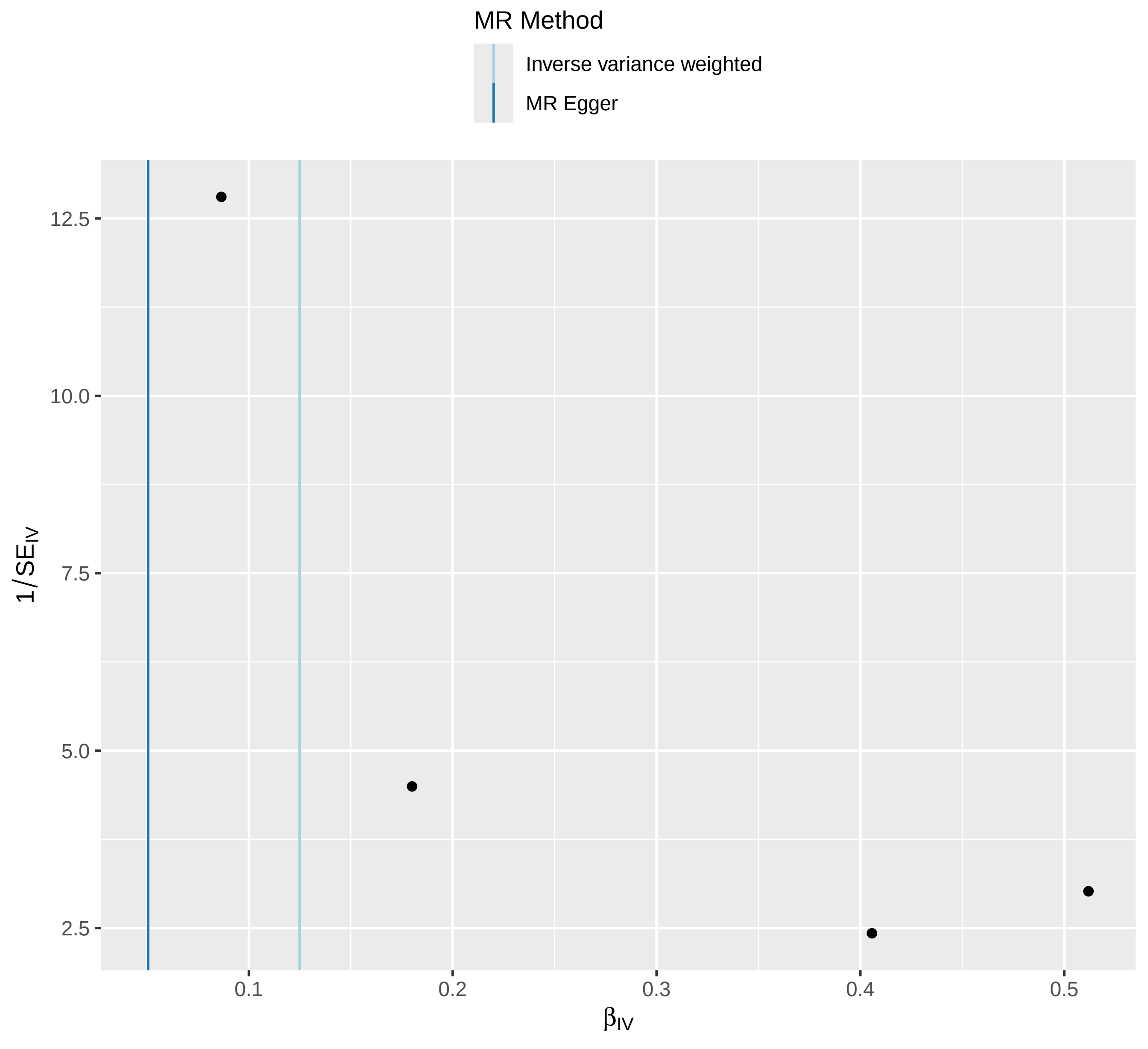
MR Method
Inverse variance weighted
MR Egger
12.5
10.0
7.5
5.0
2.5
$1/SE_{IV}$
0.1
0.2
0.3
0.4
0.5
$\beta_{IV}$

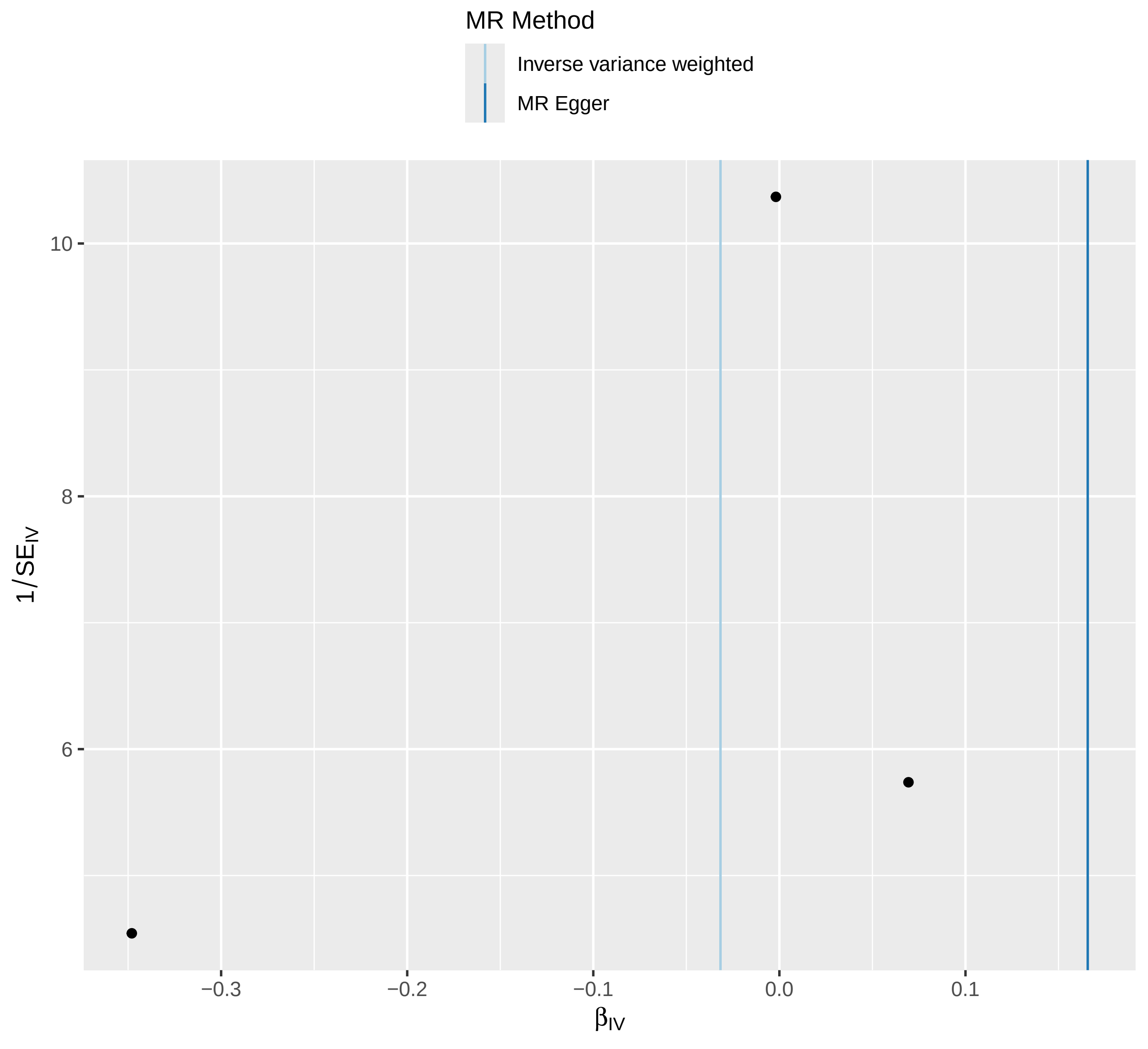

MR Method
Inverse variance weighted
MR Egger
10
8
6
$1/SE_{IV}$
−0.3
−0.2
−0.1
0.0
0.1
$\beta_{IV}$

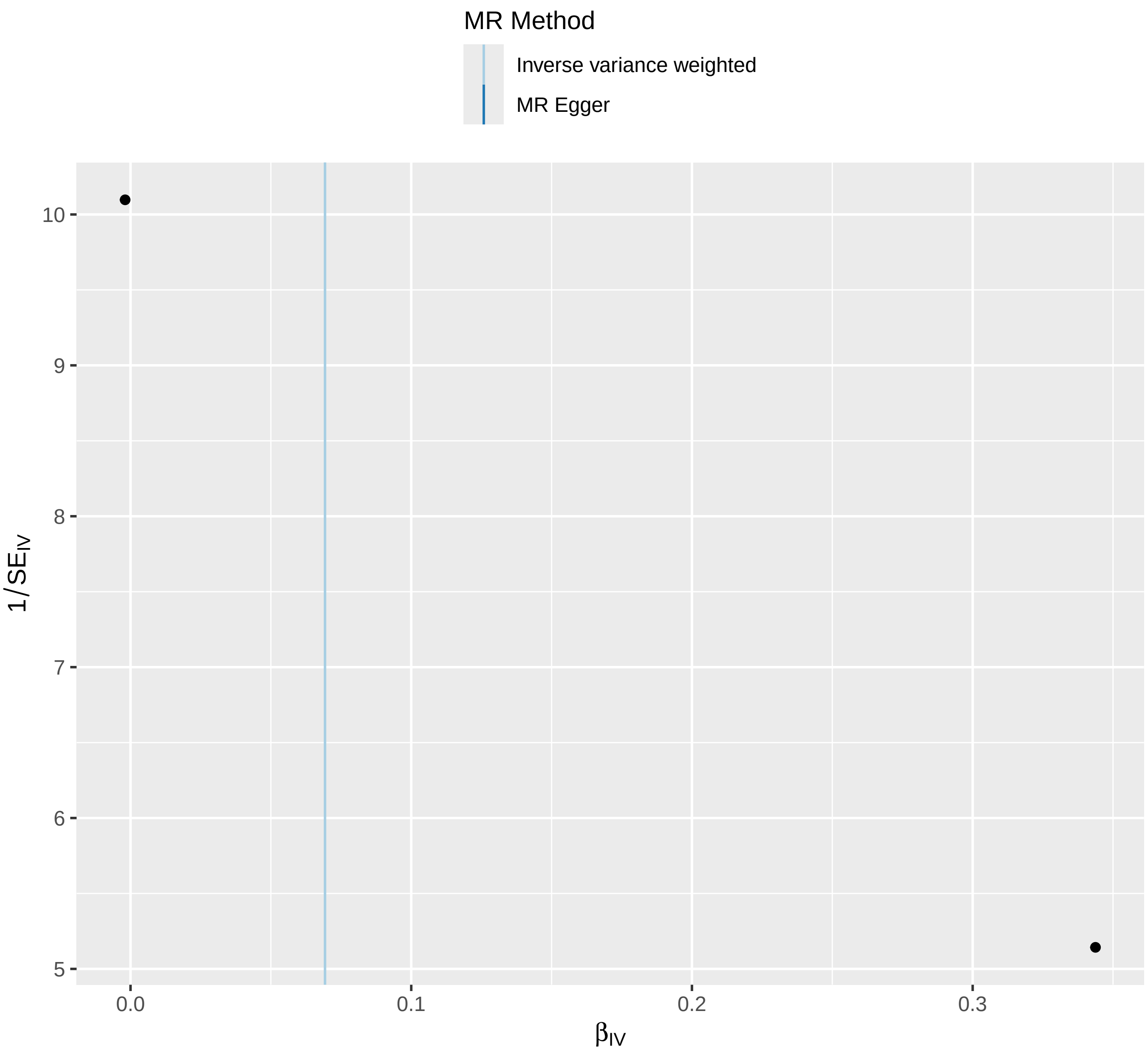

MR Method
Inverse variance weighted
MR Egger
10
9
8
7
6
5
0.0
0.1
0.2
0.3
1/SE_IV
β_IV

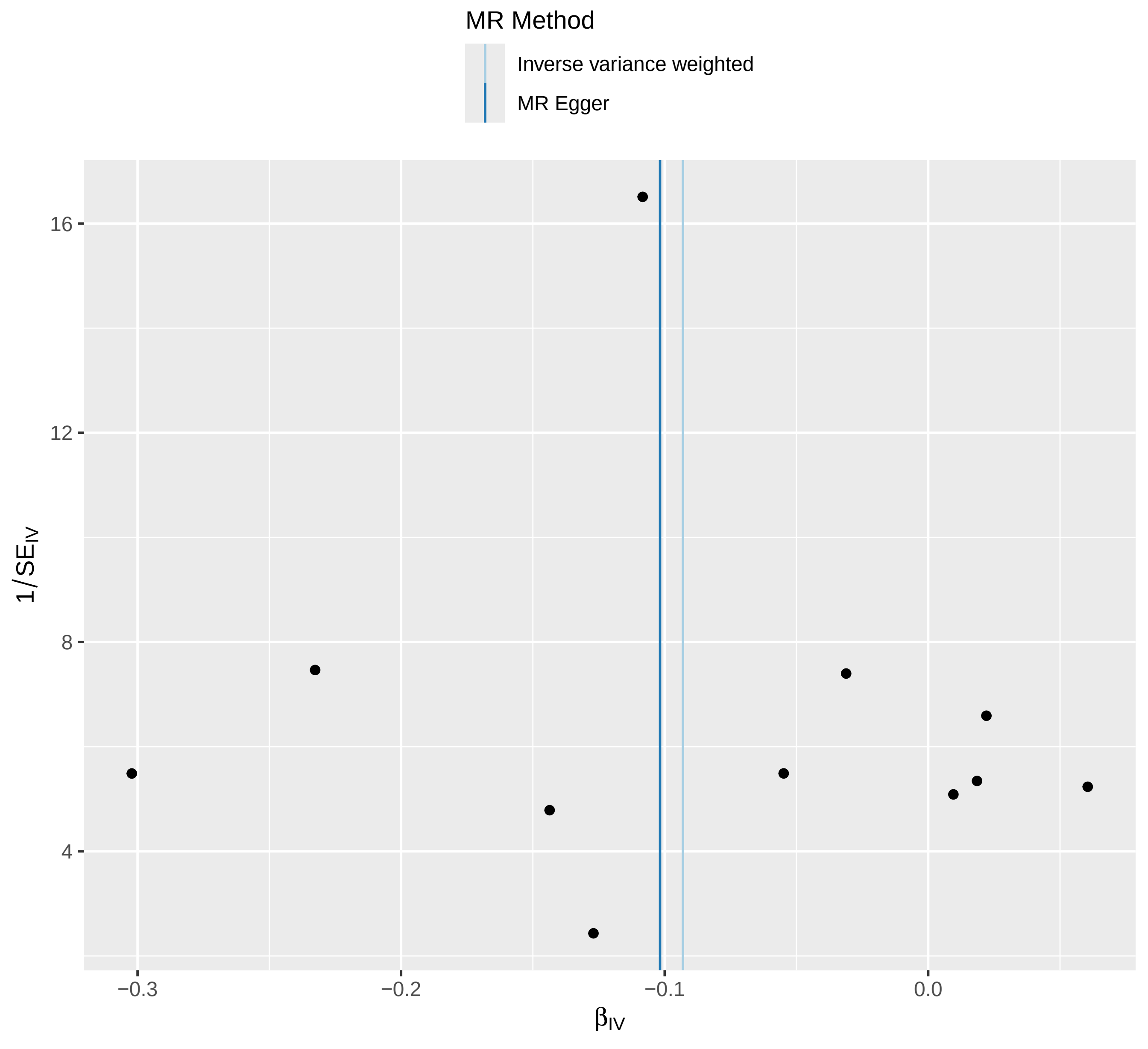
MR Method
Inverse variance weighted
MR Egger
16
12
8
4
1/SE_IV
−0.3
−0.2
−0.1
0.0
β_IV

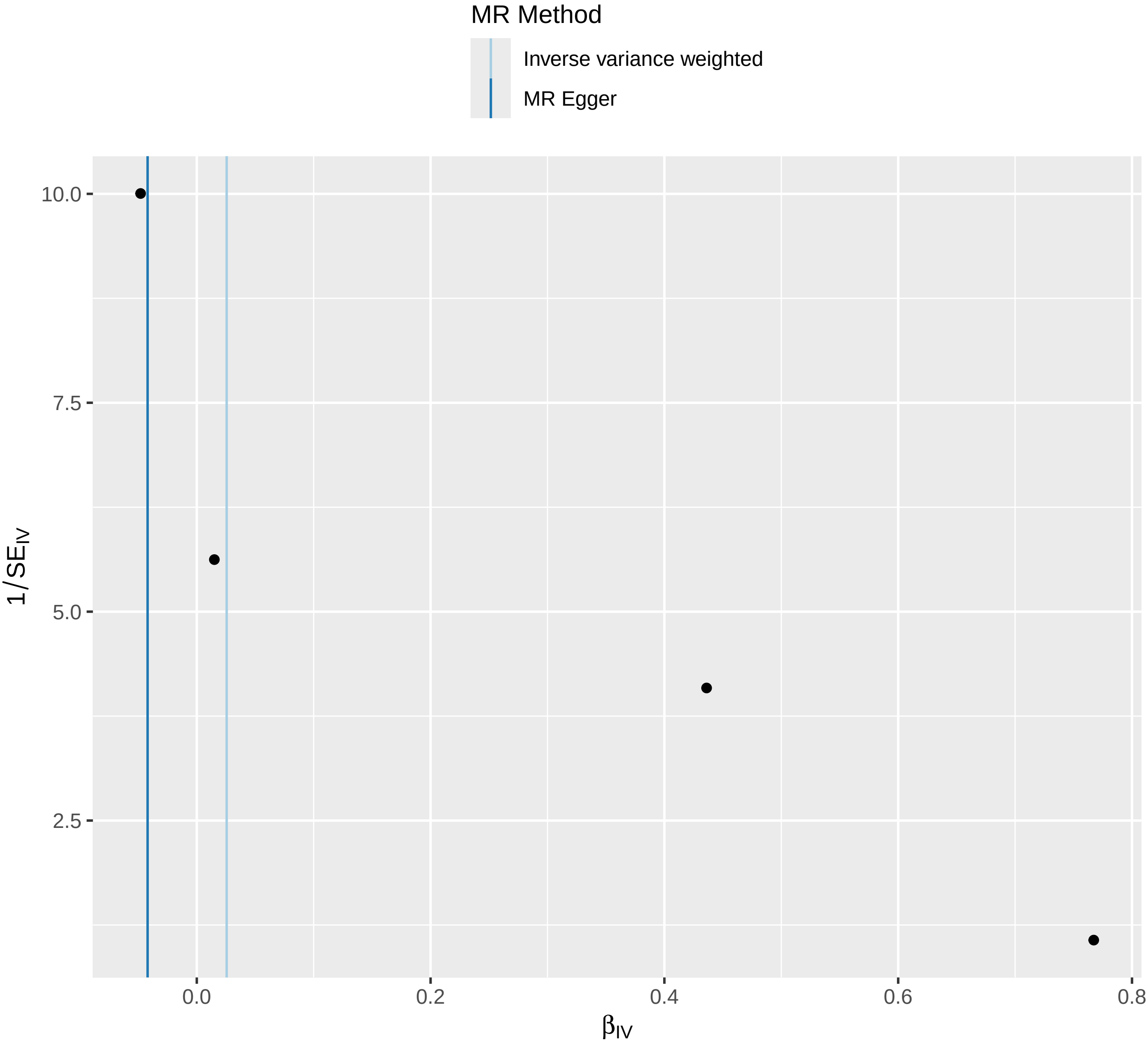
MR Method
Inverse variance weighted
MR Egger
10.0
7.5
5.0
2.5
1/SE_IV
0.0
0.2
0.4
0.6
0.8
β_IV

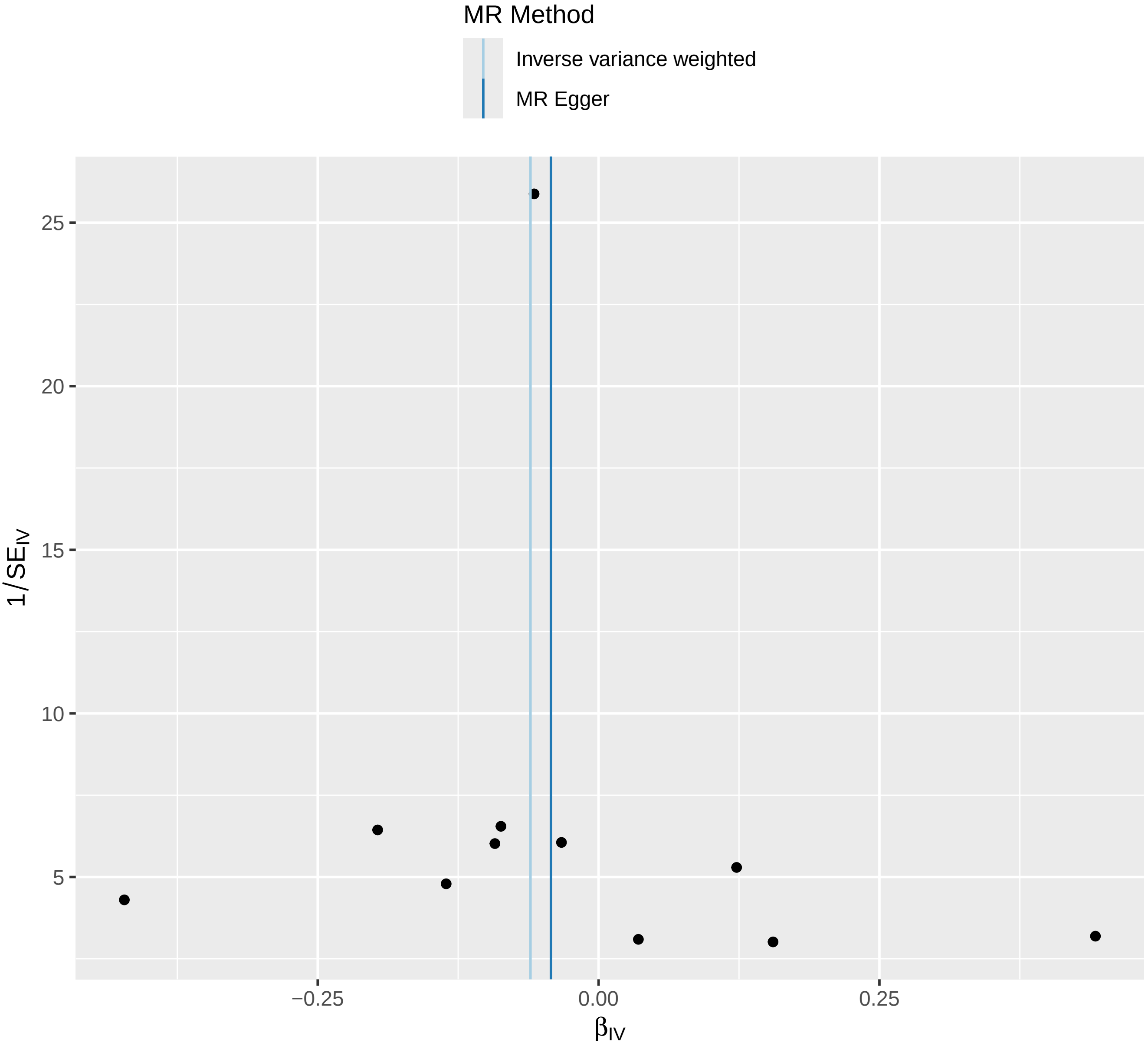

MR Method
Inverse variance weighted
MR Egger
25
20
15
10
5
1/SE_IV
−0.25
0.00
0.25
β_IV

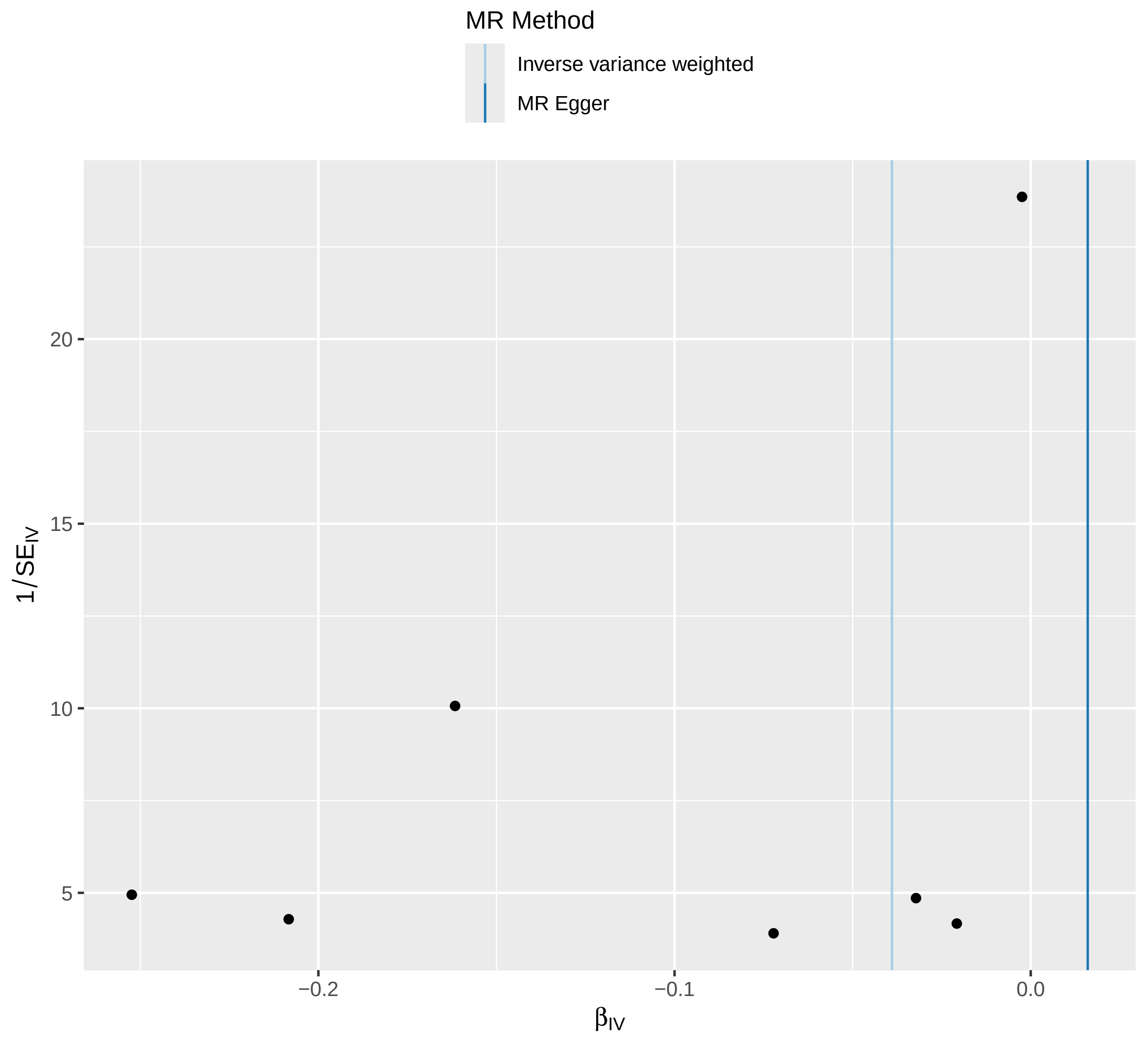

MR Method
Inverse variance weighted
MR Egger
1/$SE_{IV}$
20
15
10
5
−0.2
−0.1
0.0
$\beta_{IV}$

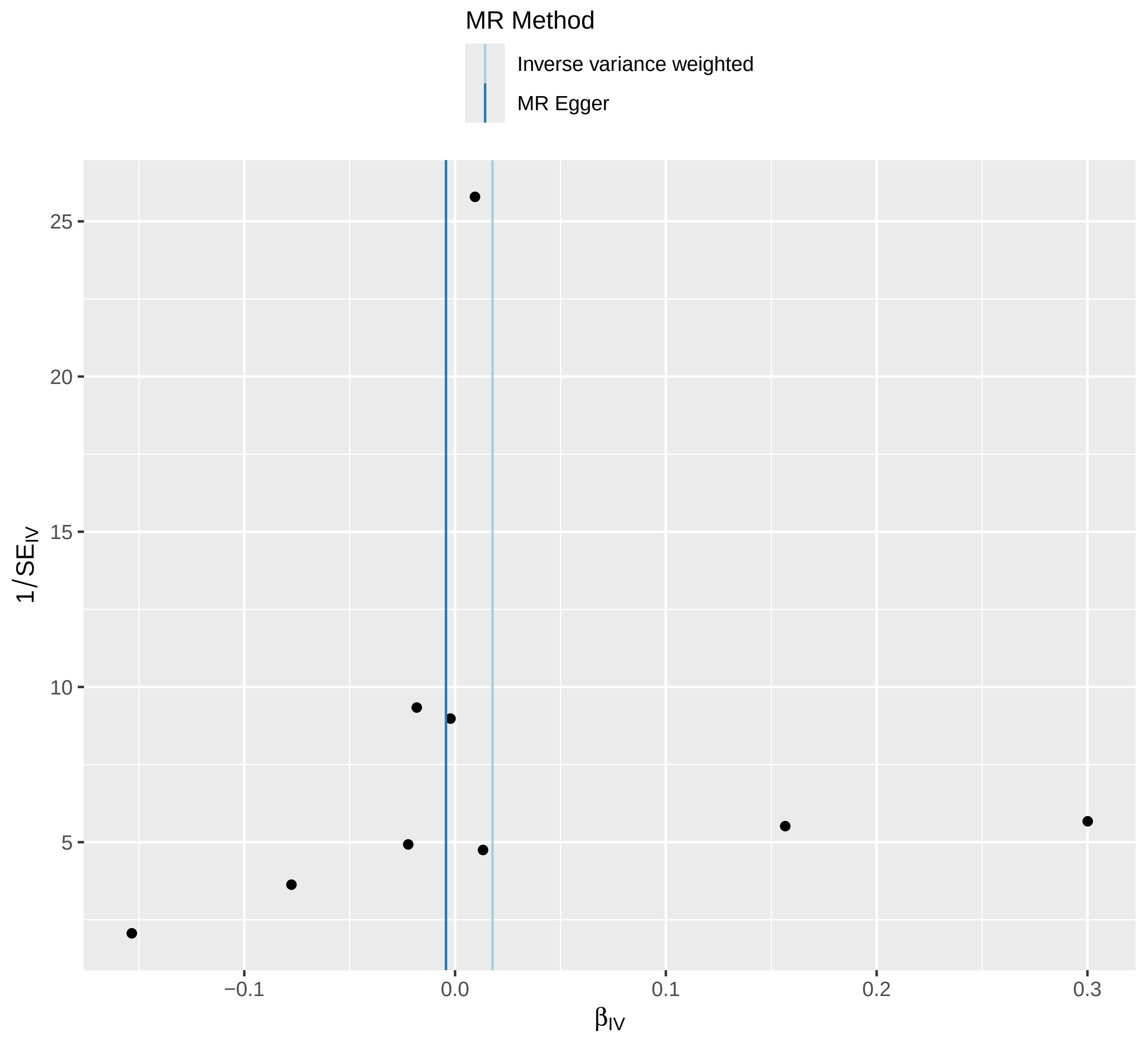
MR Method
Inverse variance weighted
MR Egger
25
20
15
10
5
1/SE_IV
−0.1
0.0
0.1
0.2
0.3
β_IV

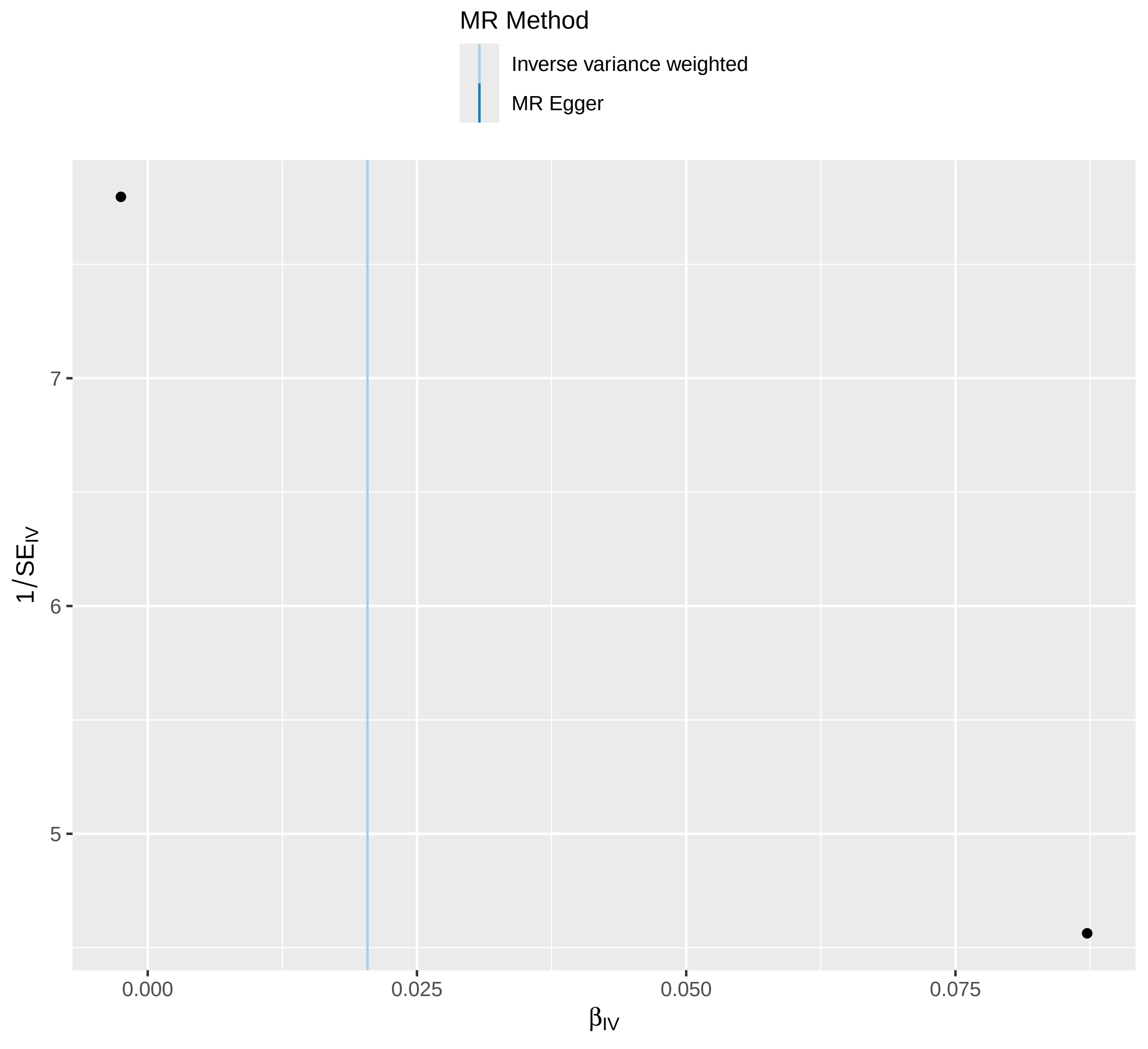
MR Method
Inverse variance weighted
MR Egger
7
6
5
1/SE_IV
0.000
0.025
0.050
0.075
β_IV

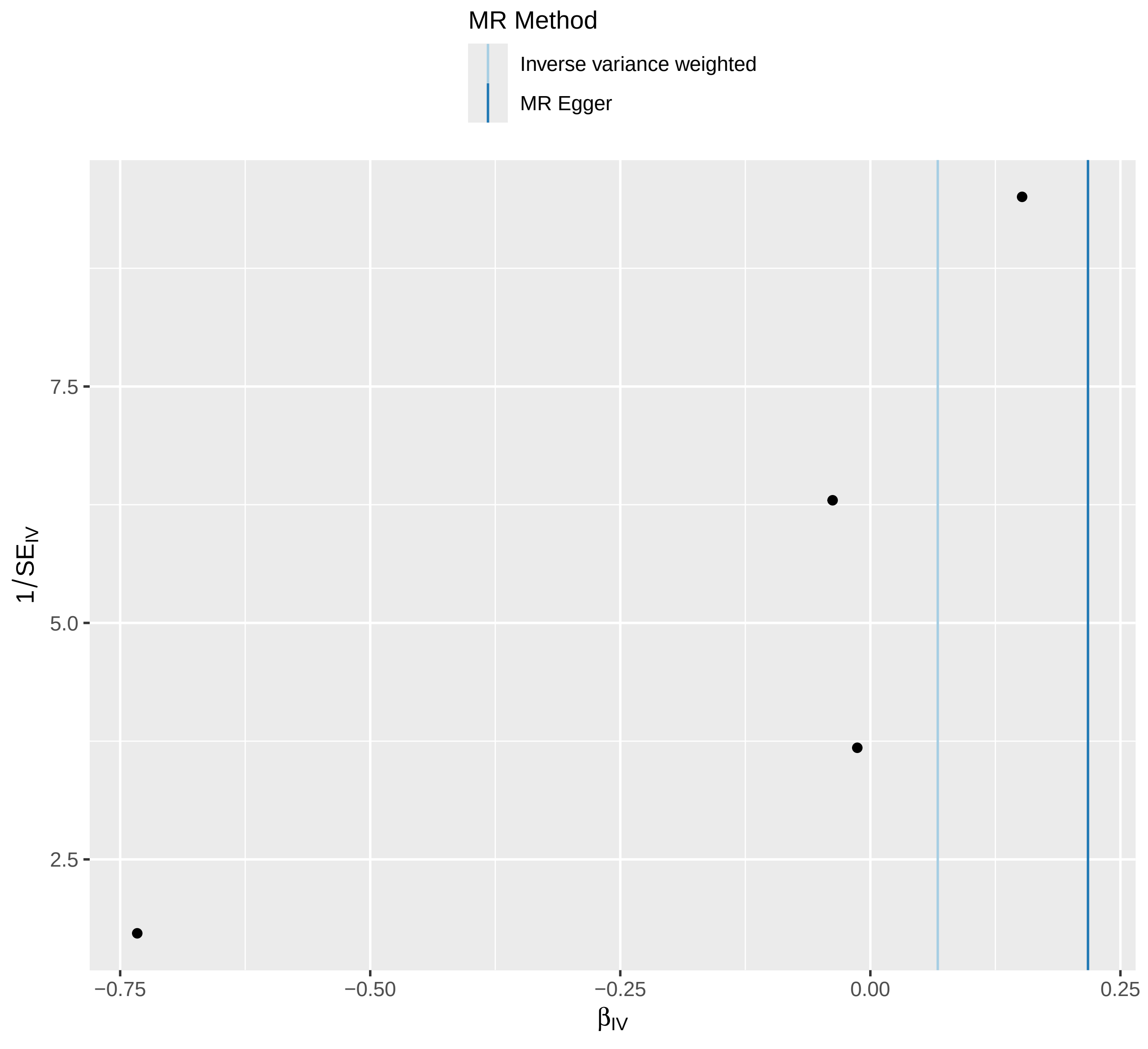
MR Method
Inverse variance weighted
MR Egger
7.5
5.0
2.5
1/SE_IV
−0.75
−0.50
−0.25
0.00
0.25
β_IV

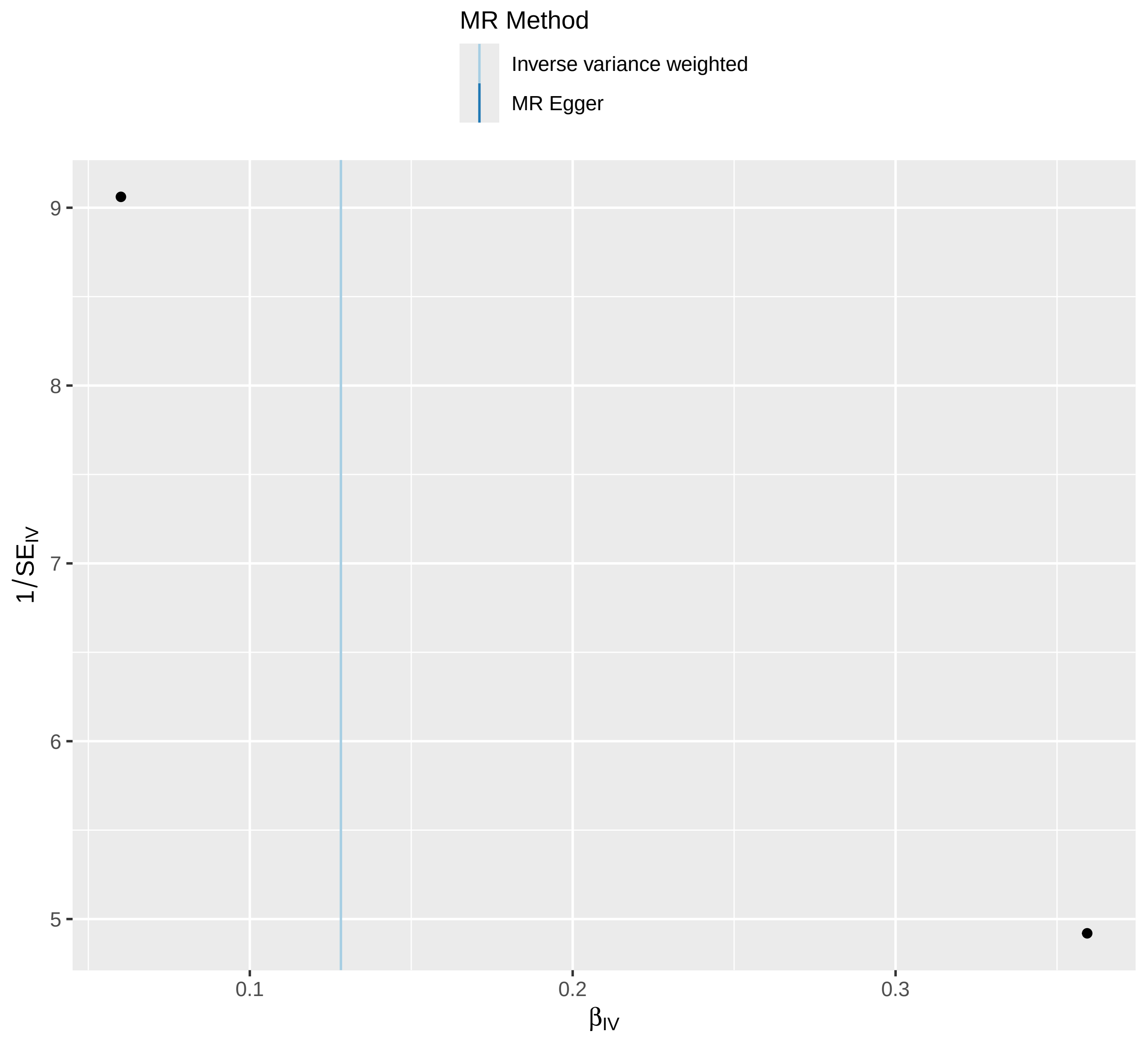

MR Method
Inverse variance weighted
MR Egger
9
8
7
6
5
1/SE$_{IV}$
0.1
0.2
0.3
$\beta_{IV}$

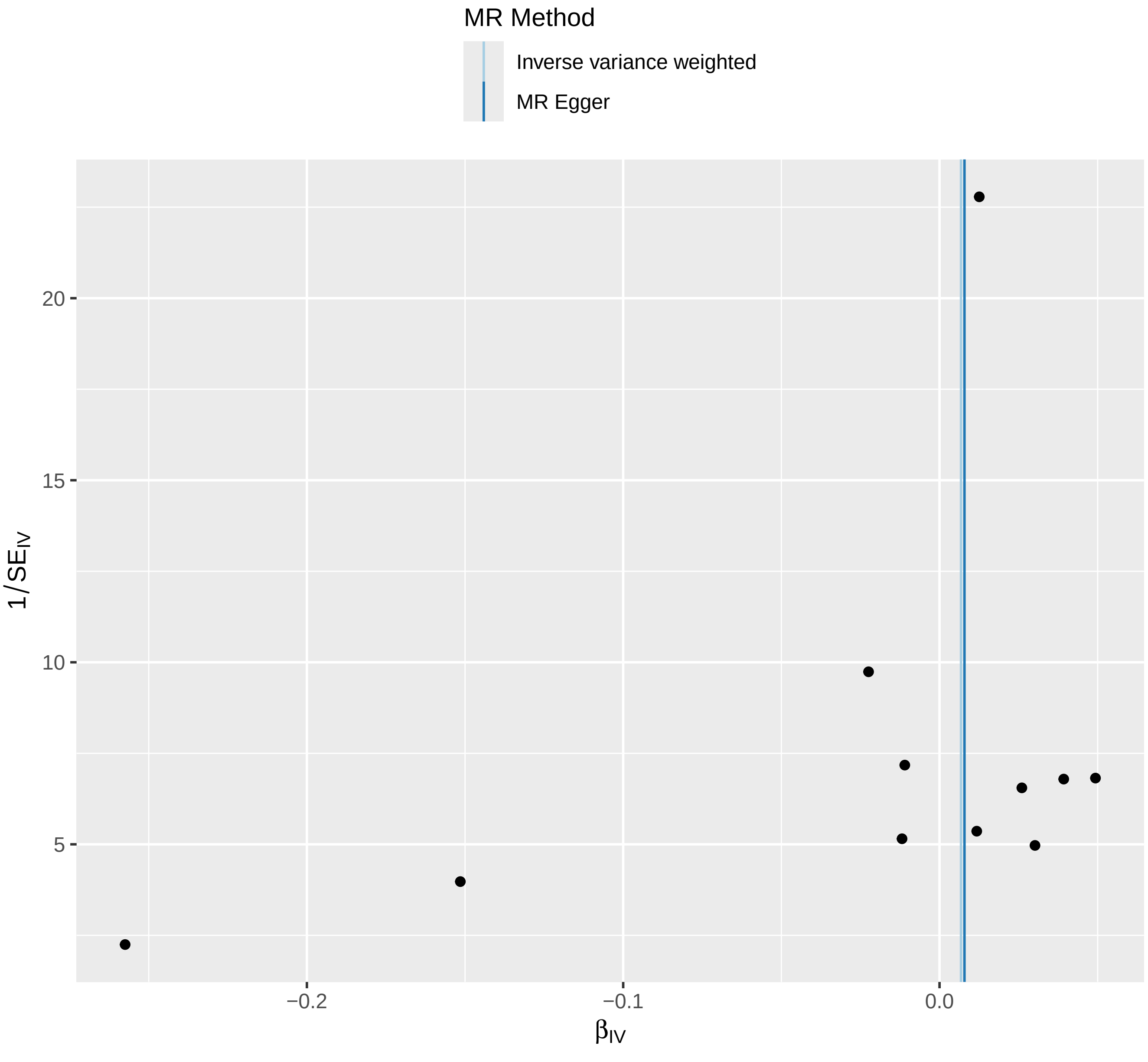

MR Method
Inverse variance weighted
MR Egger
20
15
10
5
1/SE_IV
−0.2
−0.1
0.0
β_IV

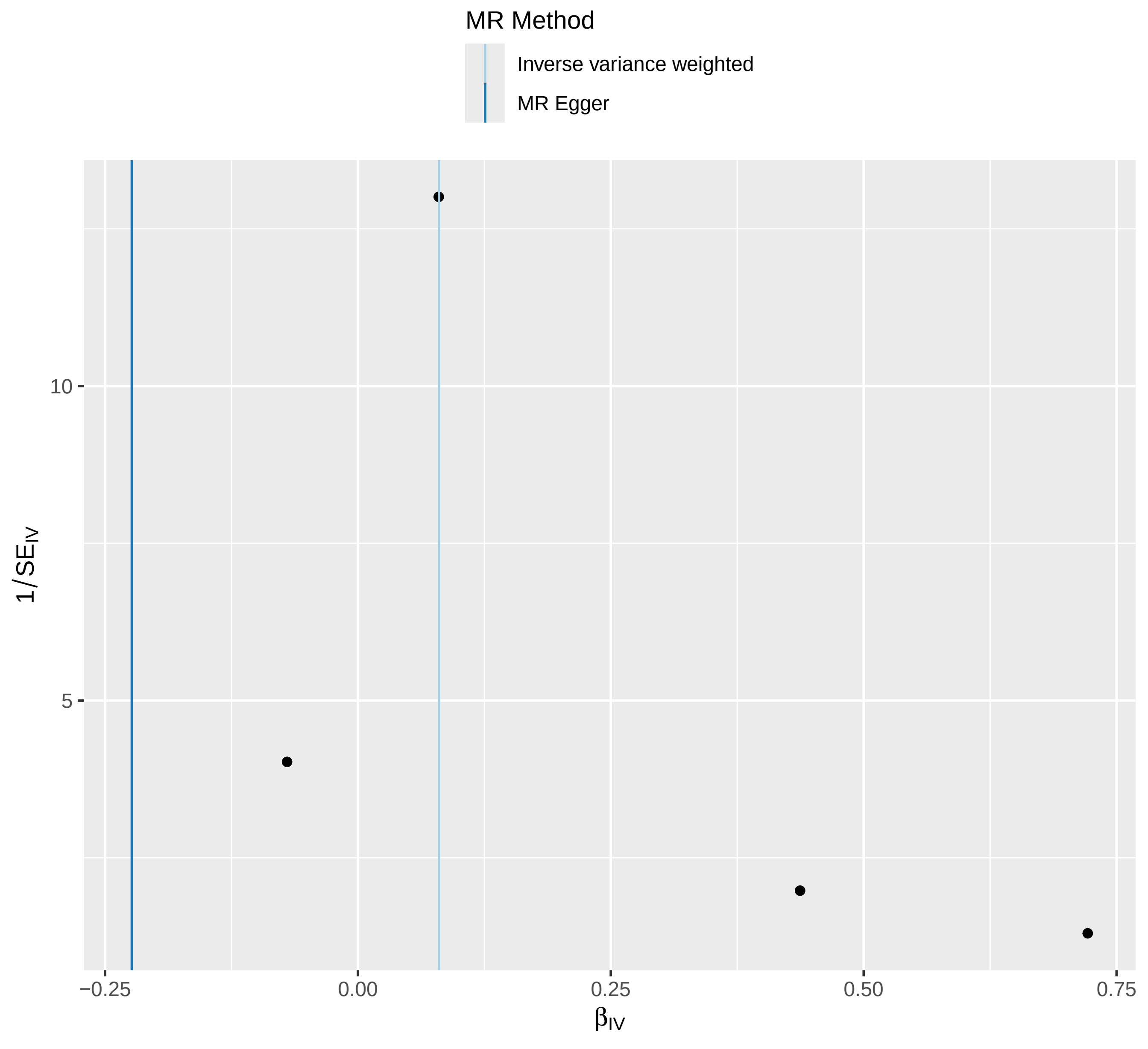
MR Method
Inverse variance weighted
MR Egger
10
5
1/SE_IV
−0.25
0.00
0.25
0.50
0.75
β_IV

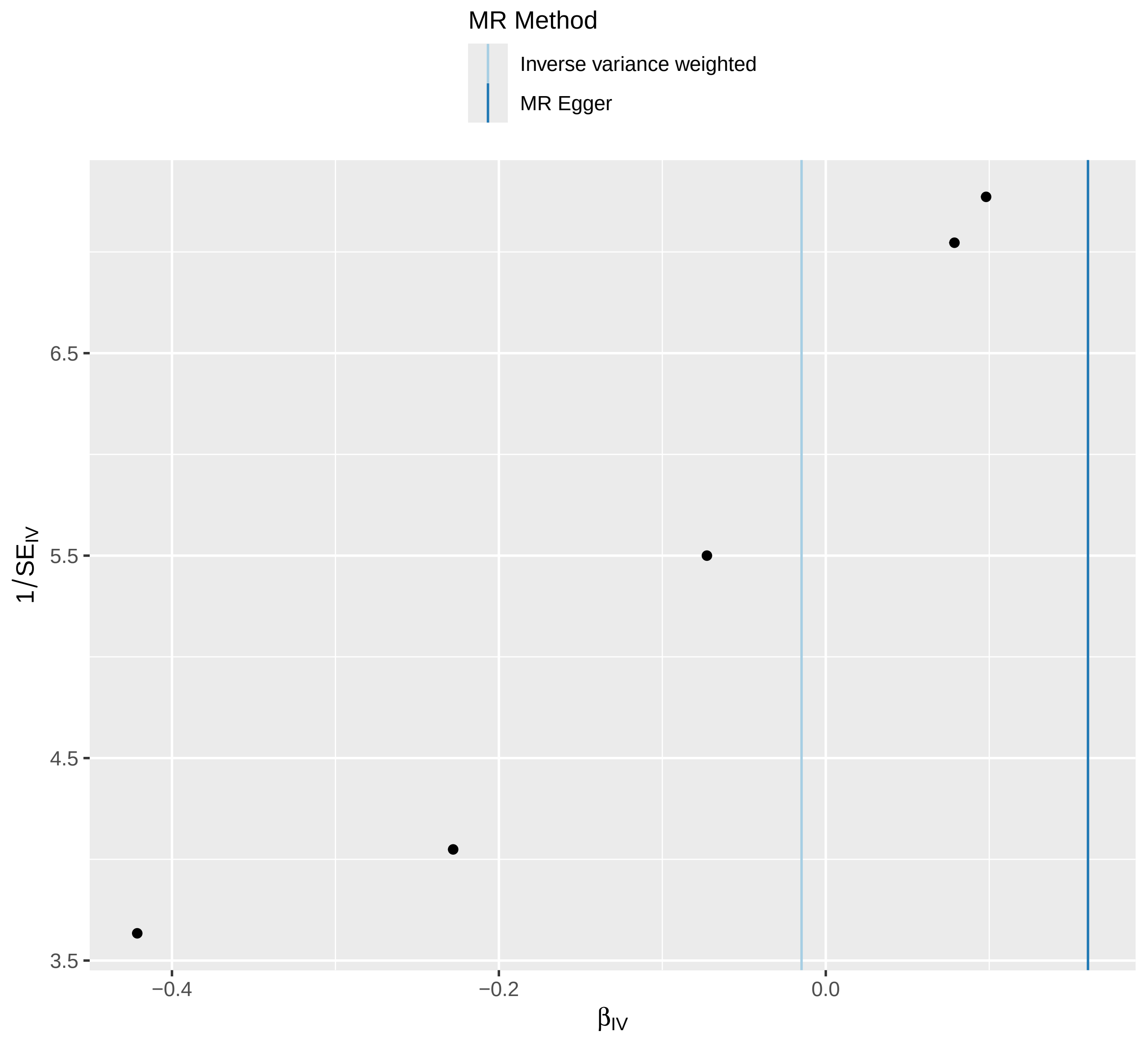

MR Method
Inverse variance weighted
MR Egger
6.5
5.5
4.5
3.5
1/SE_IV
−0.4
−0.2
0.0
β_IV

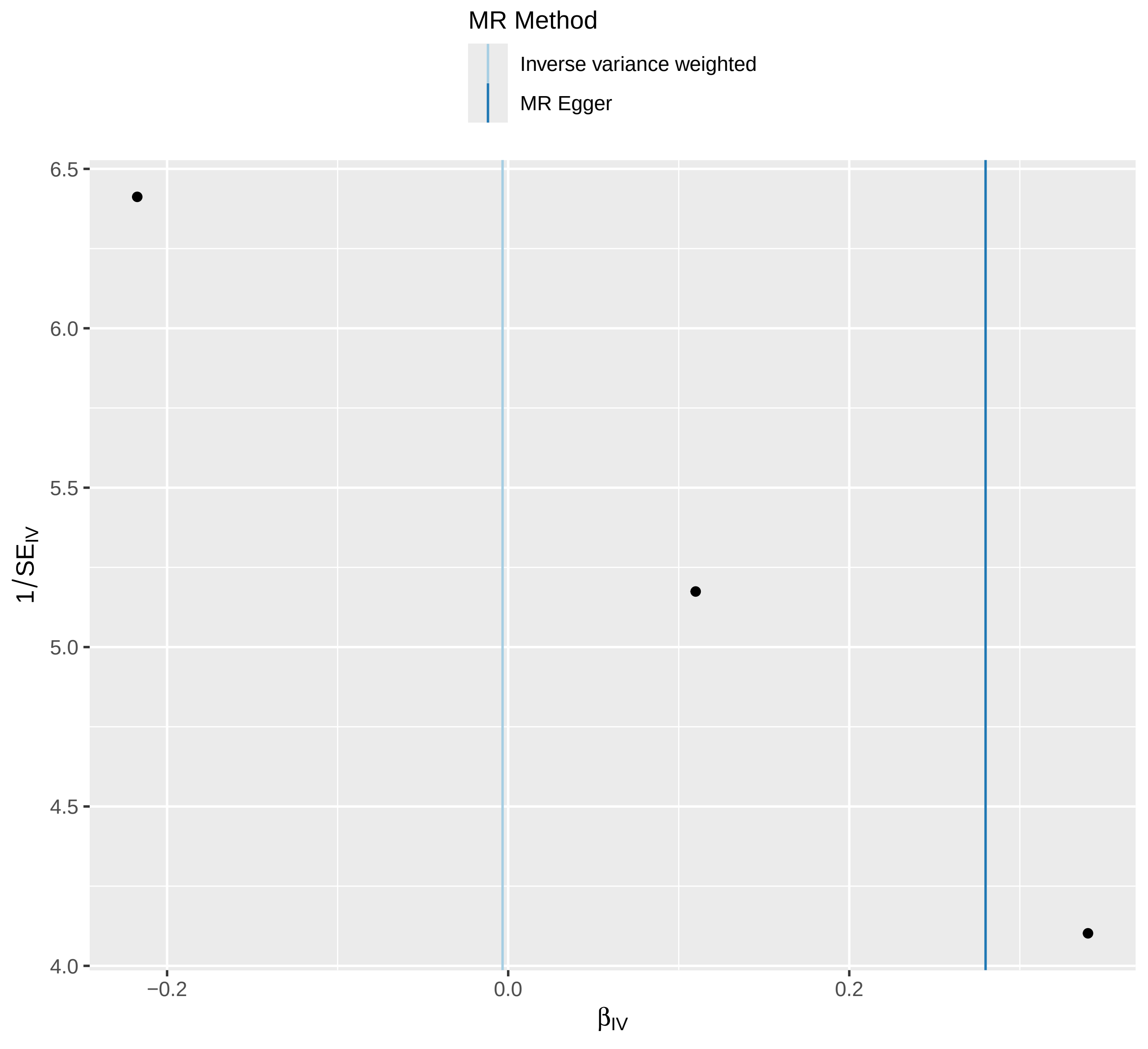
MR Method
Inverse variance weighted
MR Egger
6.5
6.0
5.5
5.0
4.5
4.0
1/SE_IV
−0.2
0.0
0.2
β_IV

Insufficient number of SNPs

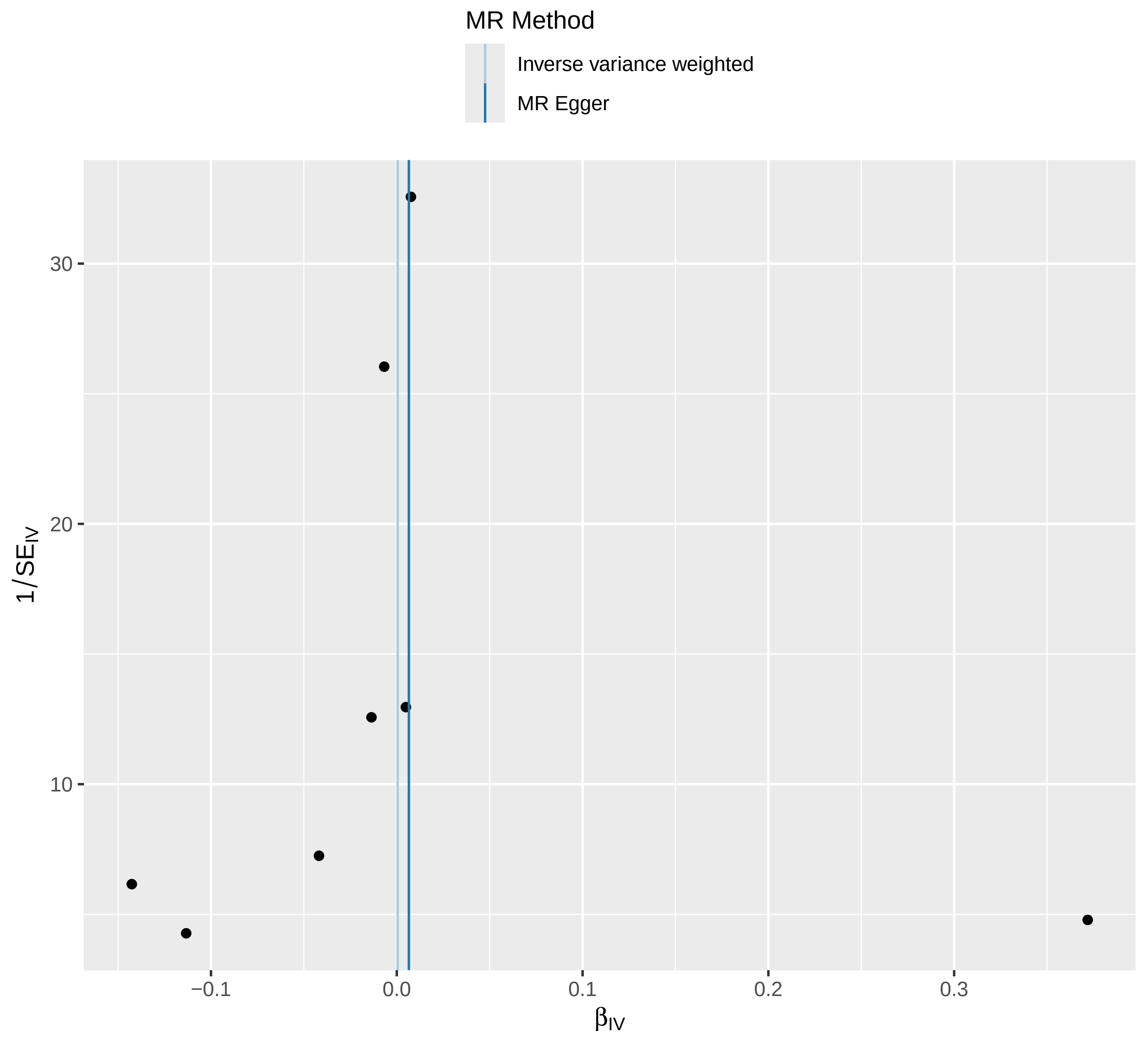

MR Method
Inverse variance weighted
MR Egger
30
20
10
1/SE$_{IV}$
−0.1
0.0
0.1
0.2
0.3
β$_{IV}$

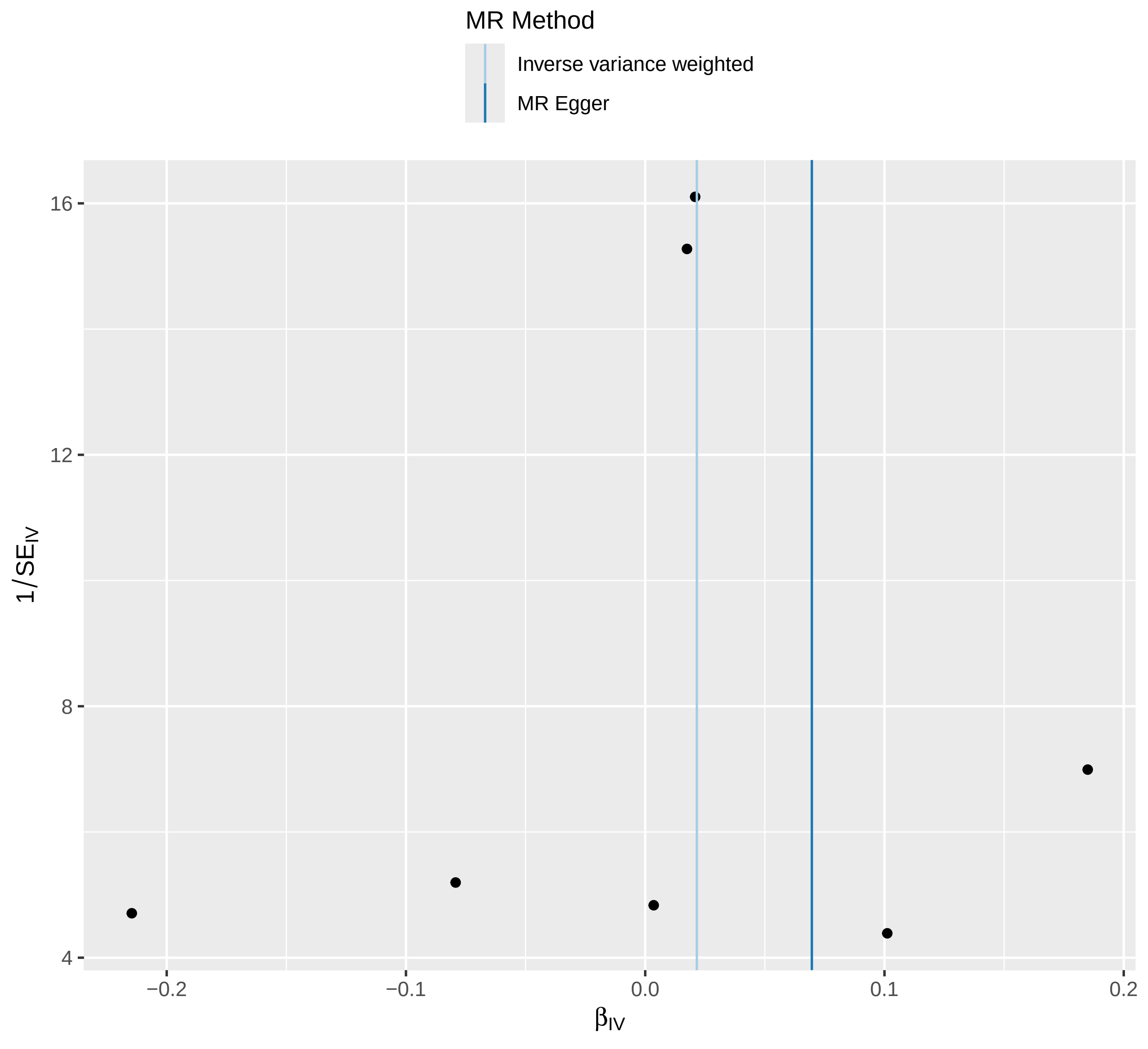
MR Method
Inverse variance weighted
MR Egger
16
12
8
4
−0.2
−0.1
0.0
0.1
0.2
$1/SE_{IV}$
$\beta_{IV}$

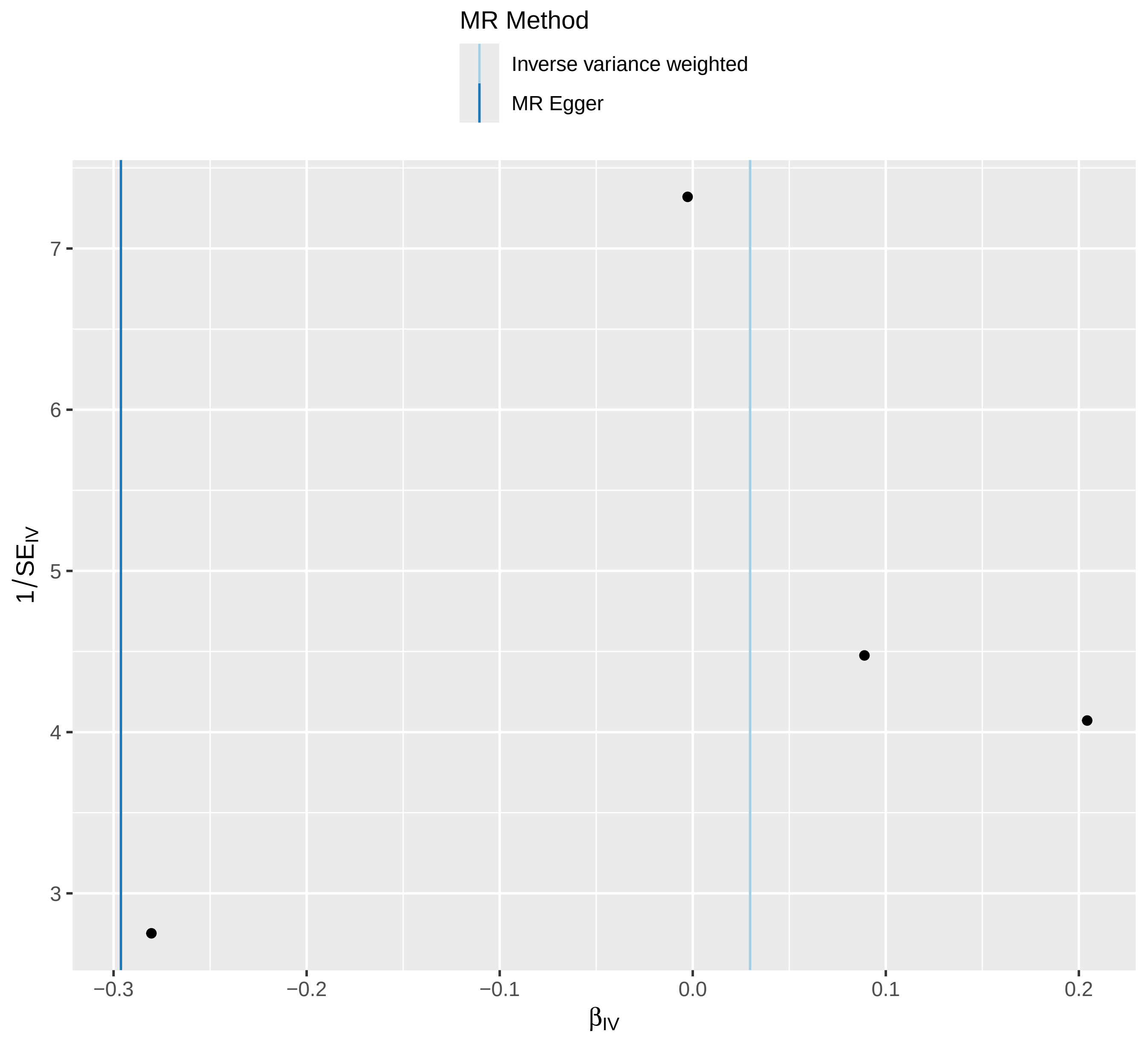
MR Method
Inverse variance weighted
MR Egger
7
6
5
4
3
1/SE_IV
−0.3
−0.2
−0.1
0.0
0.1
0.2
β_IV

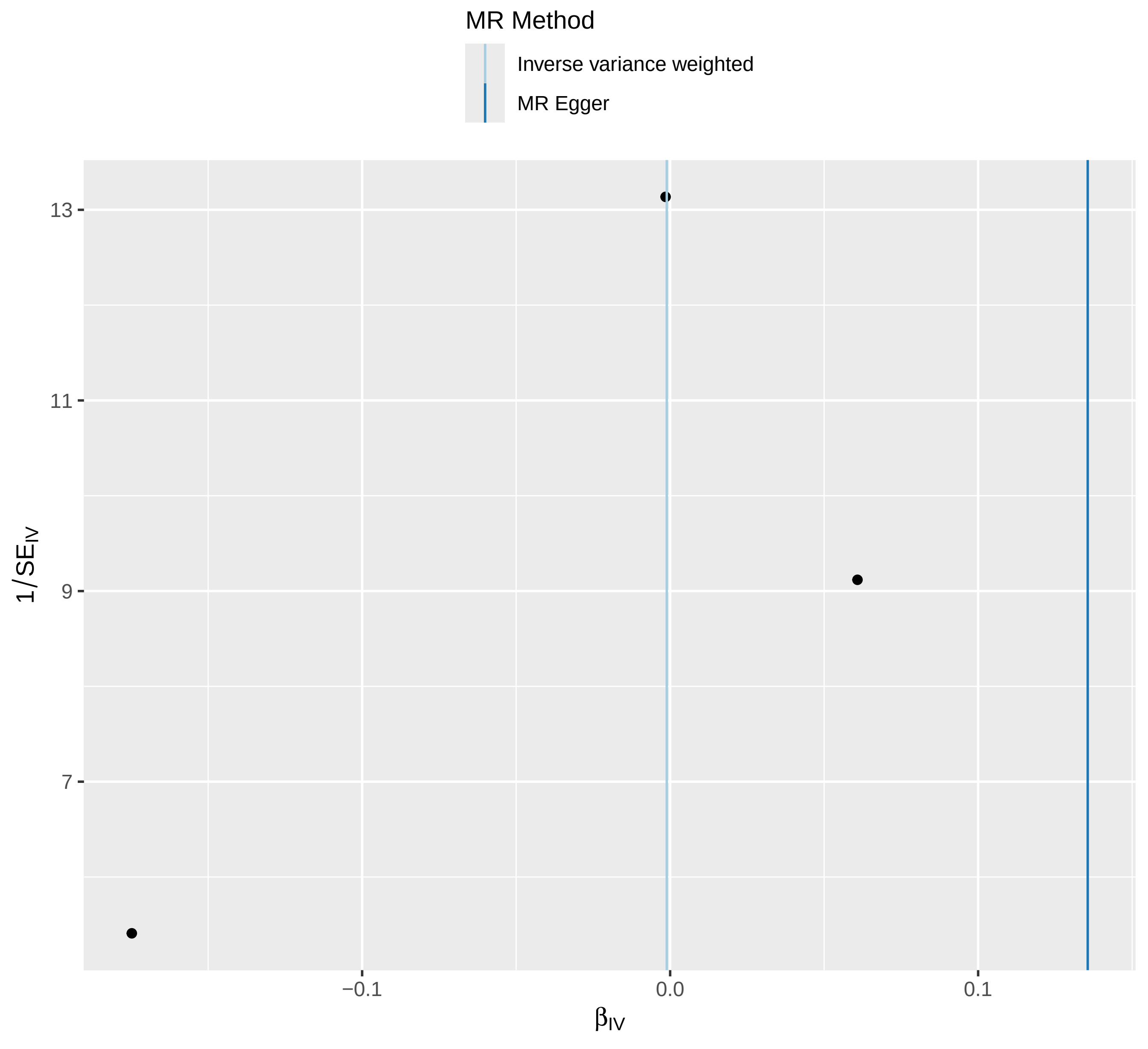

MR Method
Inverse variance weighted
MR Egger
13
11
9
7
1/SE_IV
−0.1
0.0
0.1
β_IV

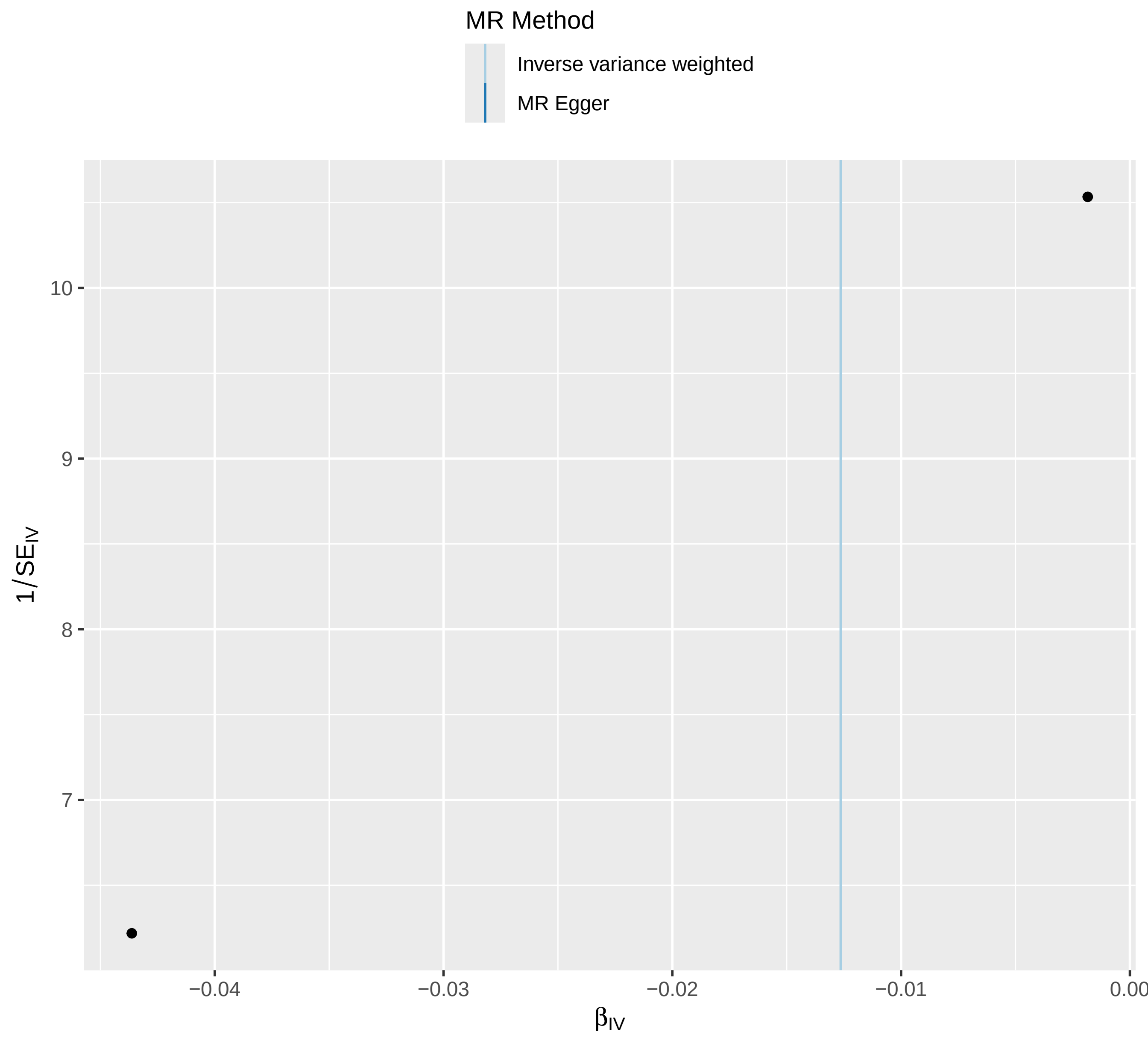
MR Method
Inverse variance weighted
MR Egger
10
9
8
7
1/SE$_{IV}$
−0.04
−0.03
−0.02
−0.01
0.00
β$_{IV}$

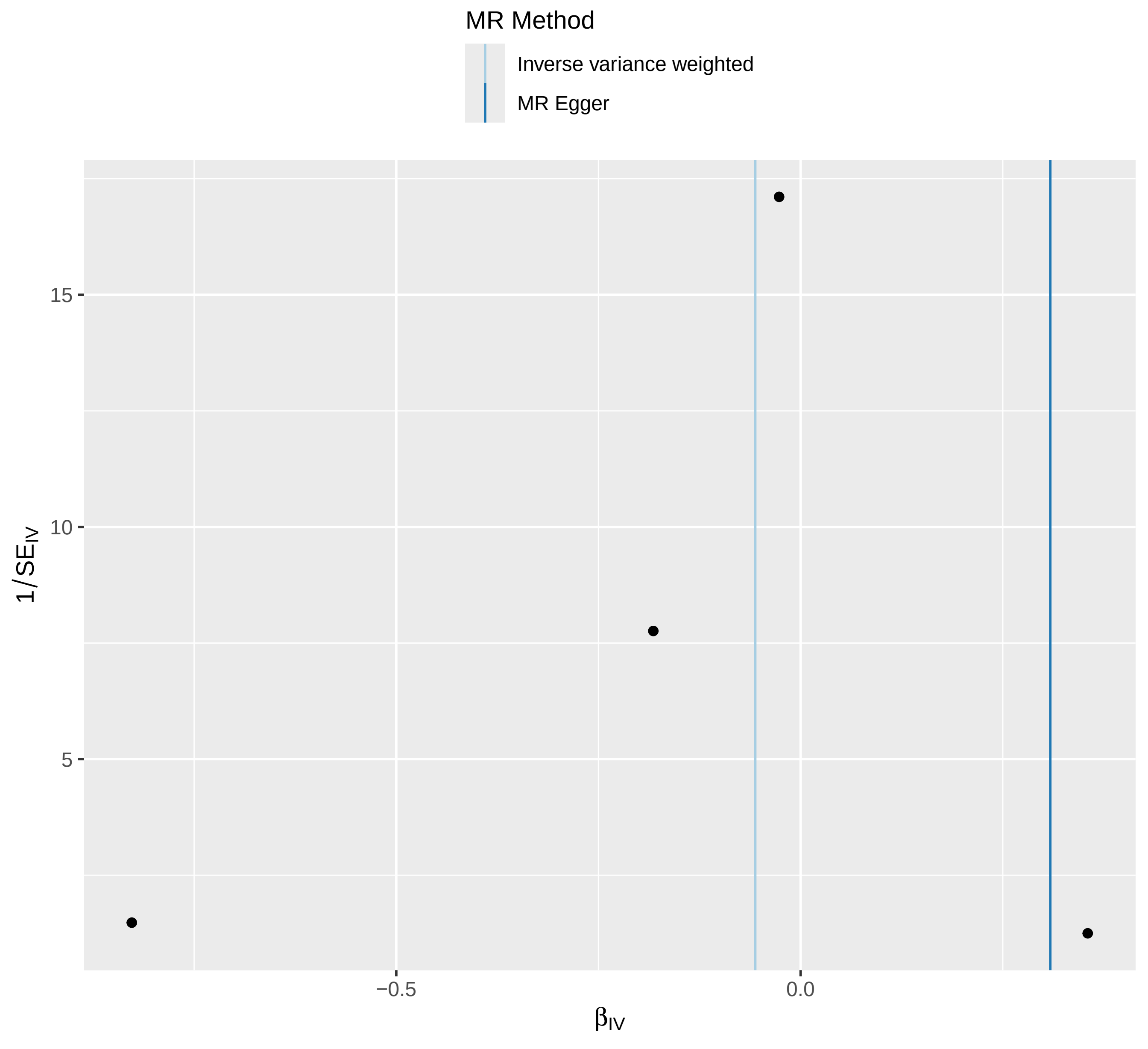
MR Method
Inverse variance weighted
MR Egger
15
10
5
1/SE_IV
−0.5
0.0
β_IV

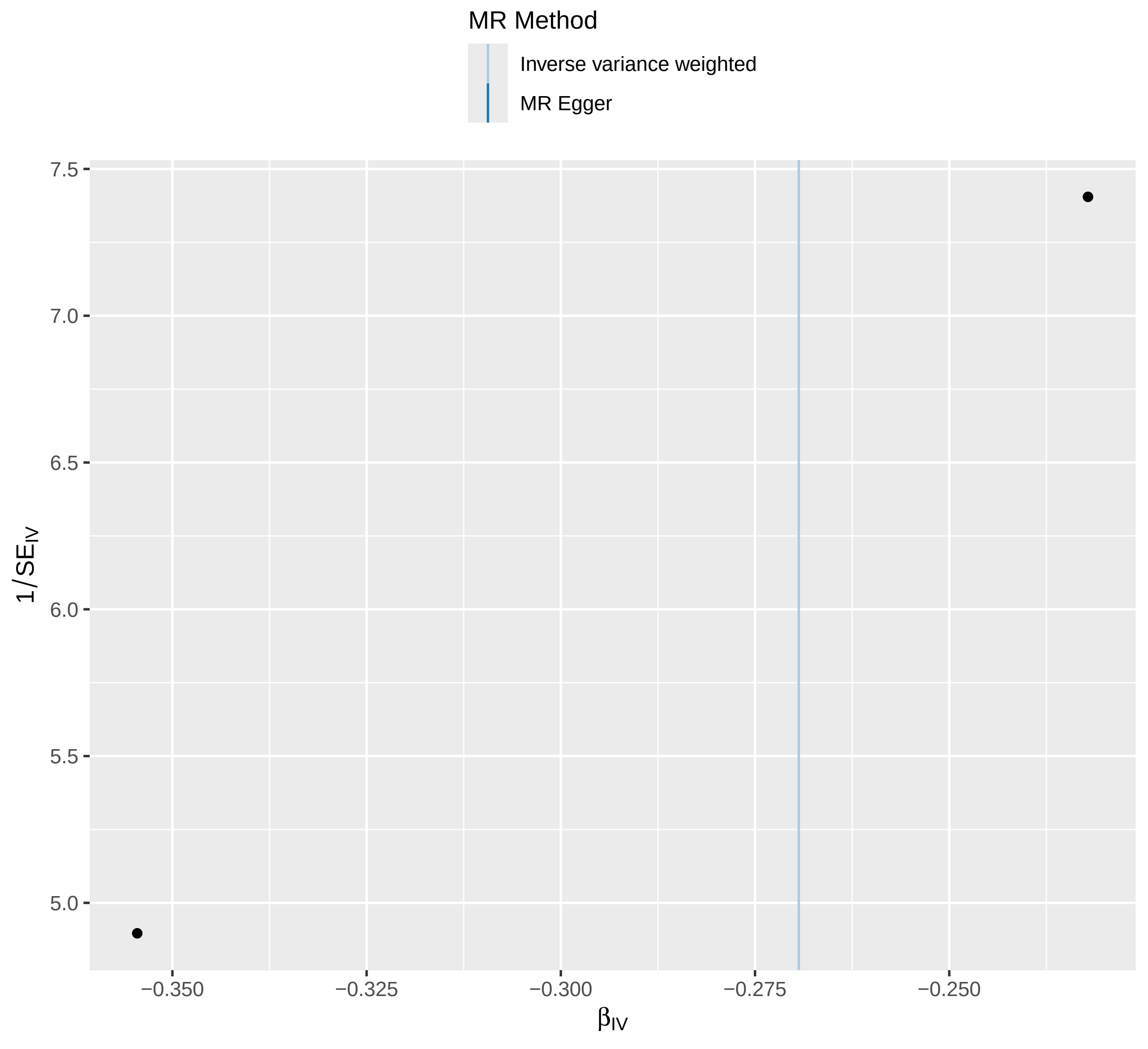
MR Method
Inverse variance weighted
MR Egger
7.5
7.0
6.5
6.0
5.5
5.0
1/SE_IV
−0.350
−0.325
−0.300
−0.275
−0.250
β_IV

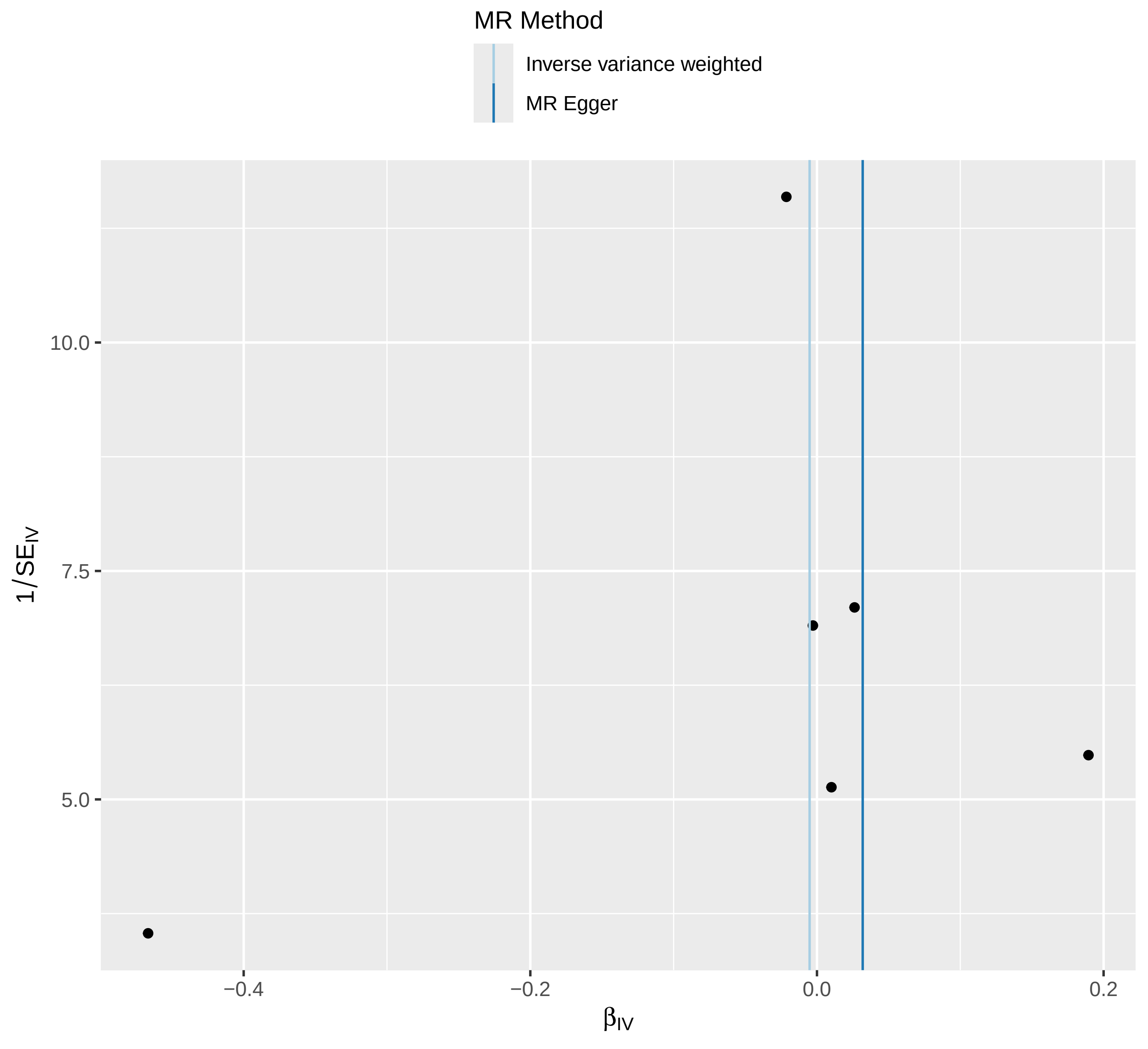

MR Method
Inverse variance weighted
MR Egger
10.0
7.5
5.0
1/SE_IV
−0.4
−0.2
0.0
0.2
β_IV

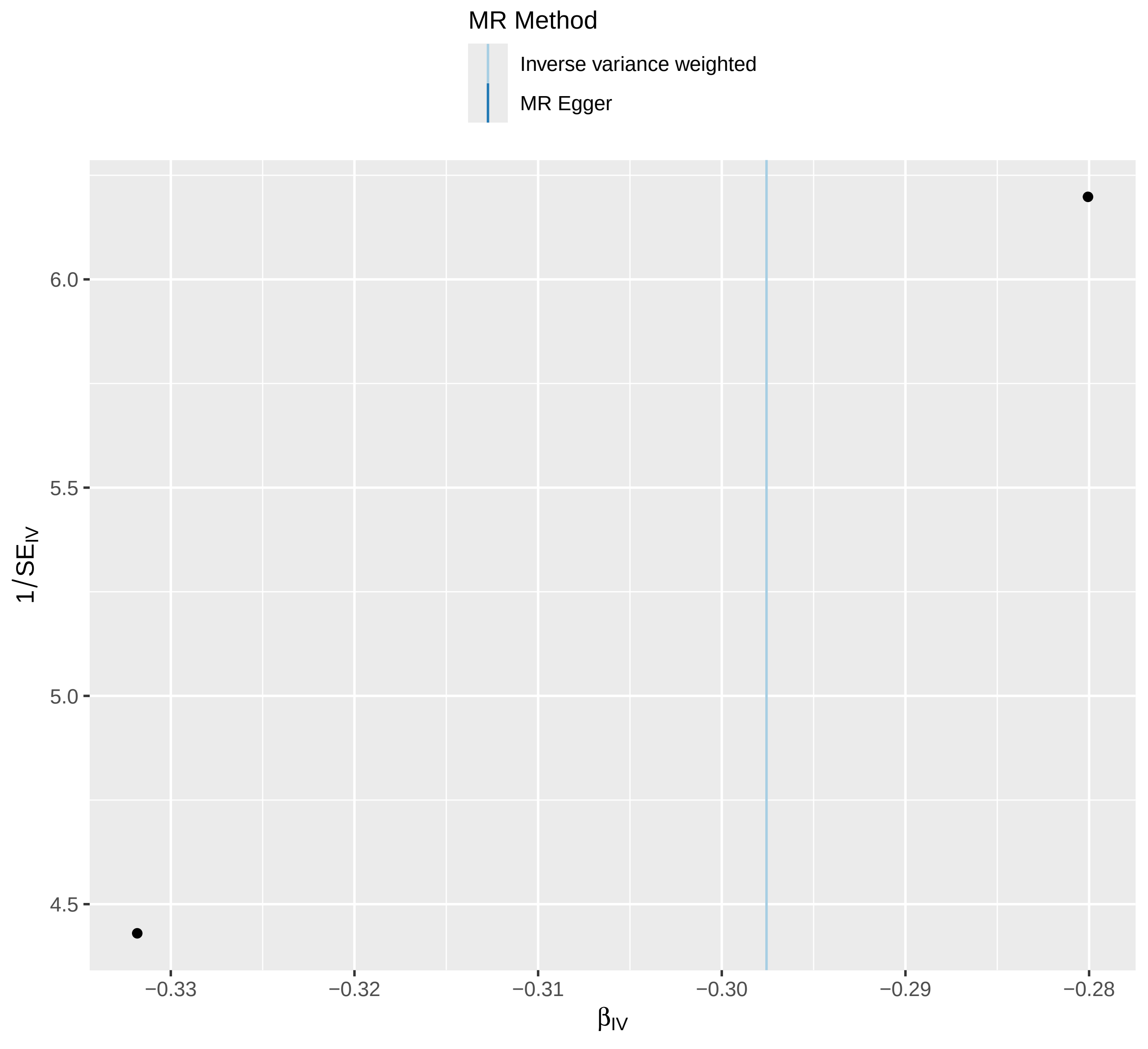

MR Method
Inverse variance weighted
MR Egger
6.0
5.5
5.0
4.5
1/SE_IV
−0.33
−0.32
−0.31
−0.30
−0.29
−0.28
β_IV

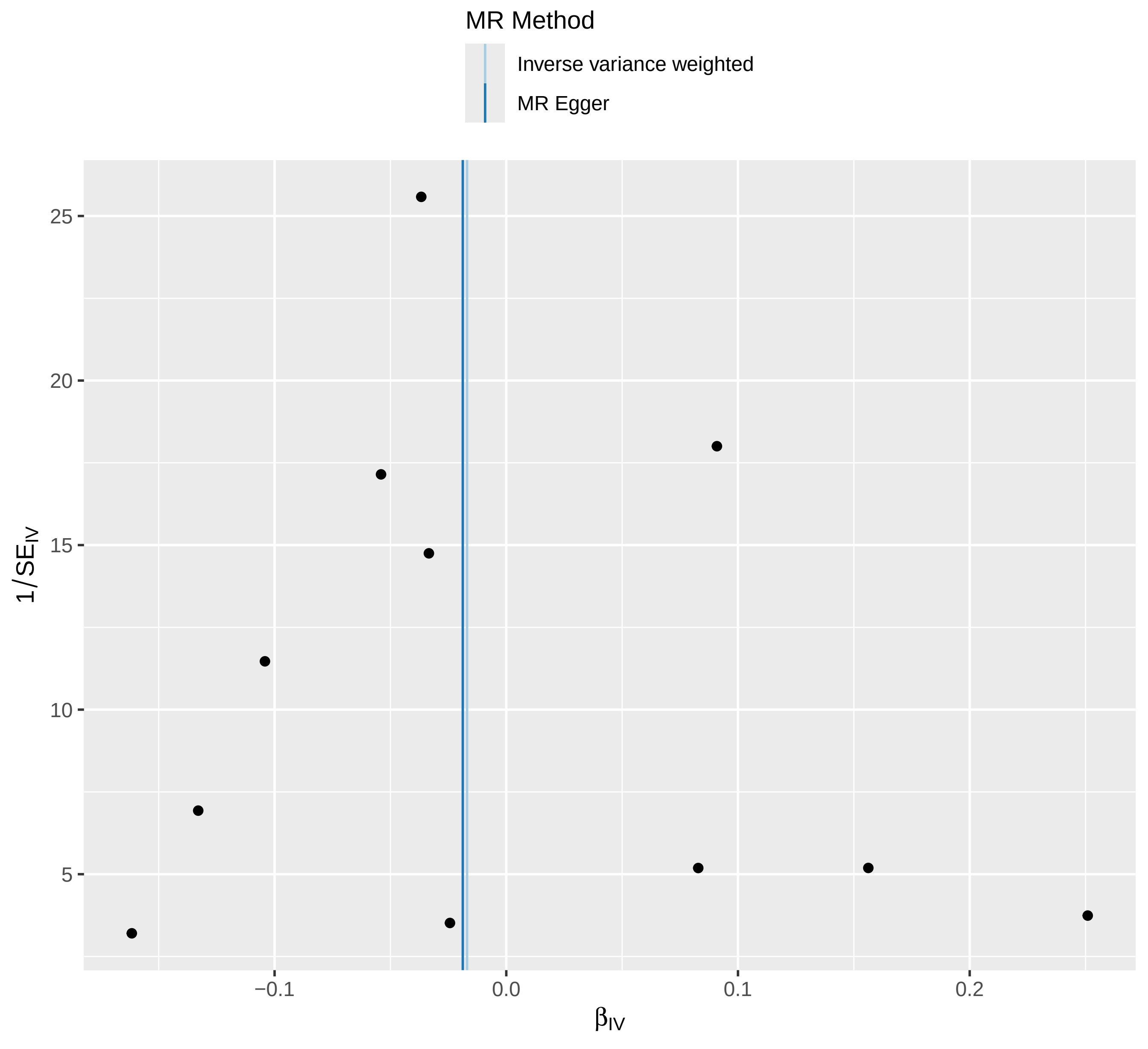

MR Method
Inverse variance weighted
MR Egger
25
20
15
10
5
1/SE$_{IV}$
−0.1
0.0
0.1
0.2
β$_{IV}$

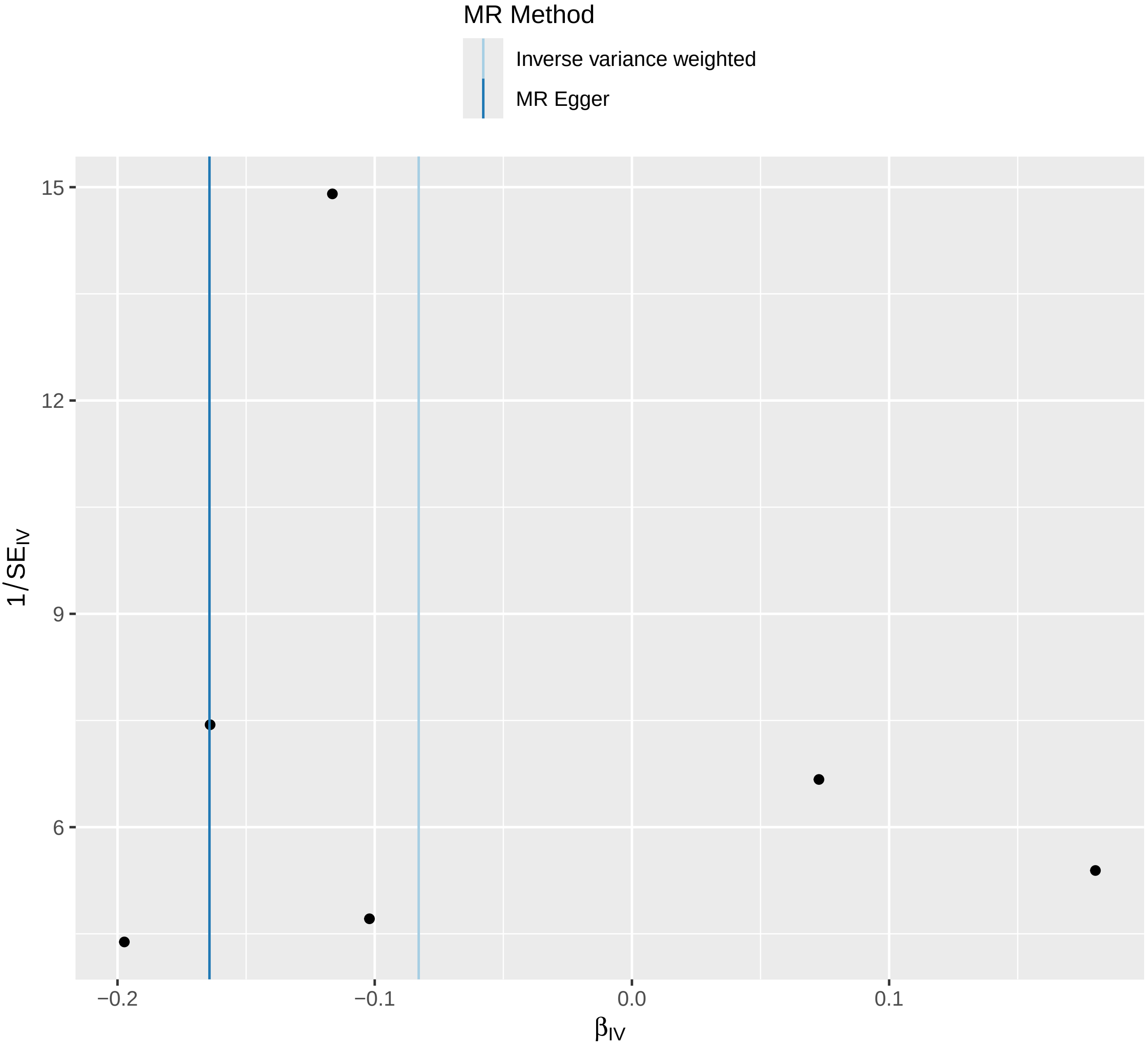
MR Method
Inverse variance weighted
MR Egger
15
12
9
6
1/SE_IV
−0.2
−0.1
0.0
0.1
β_IV

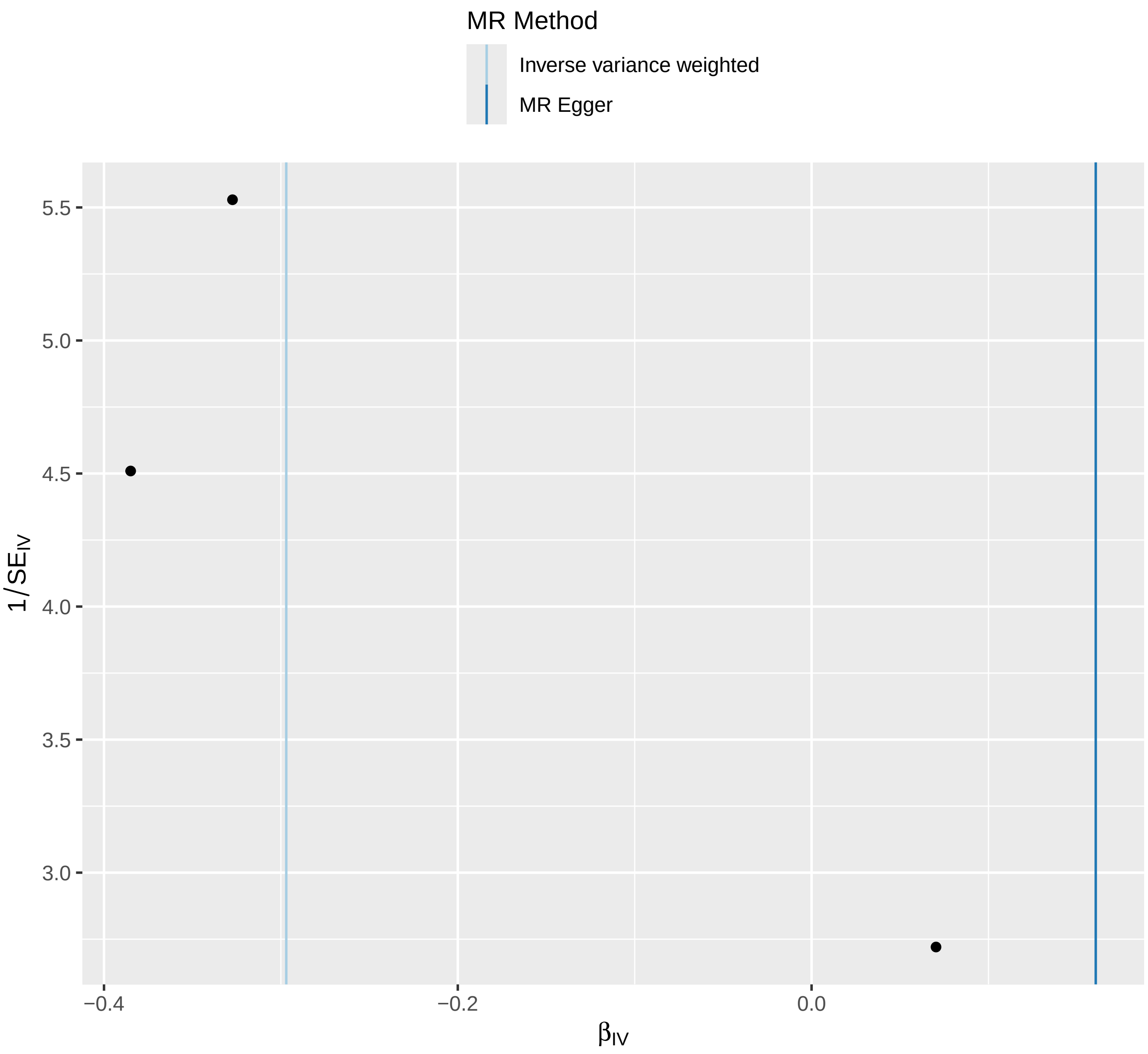
MR Method
Inverse variance weighted
MR Egger
5.5
5.0
4.5
4.0
3.5
3.0
1/SE_IV
−0.4
−0.2
0.0
β_IV

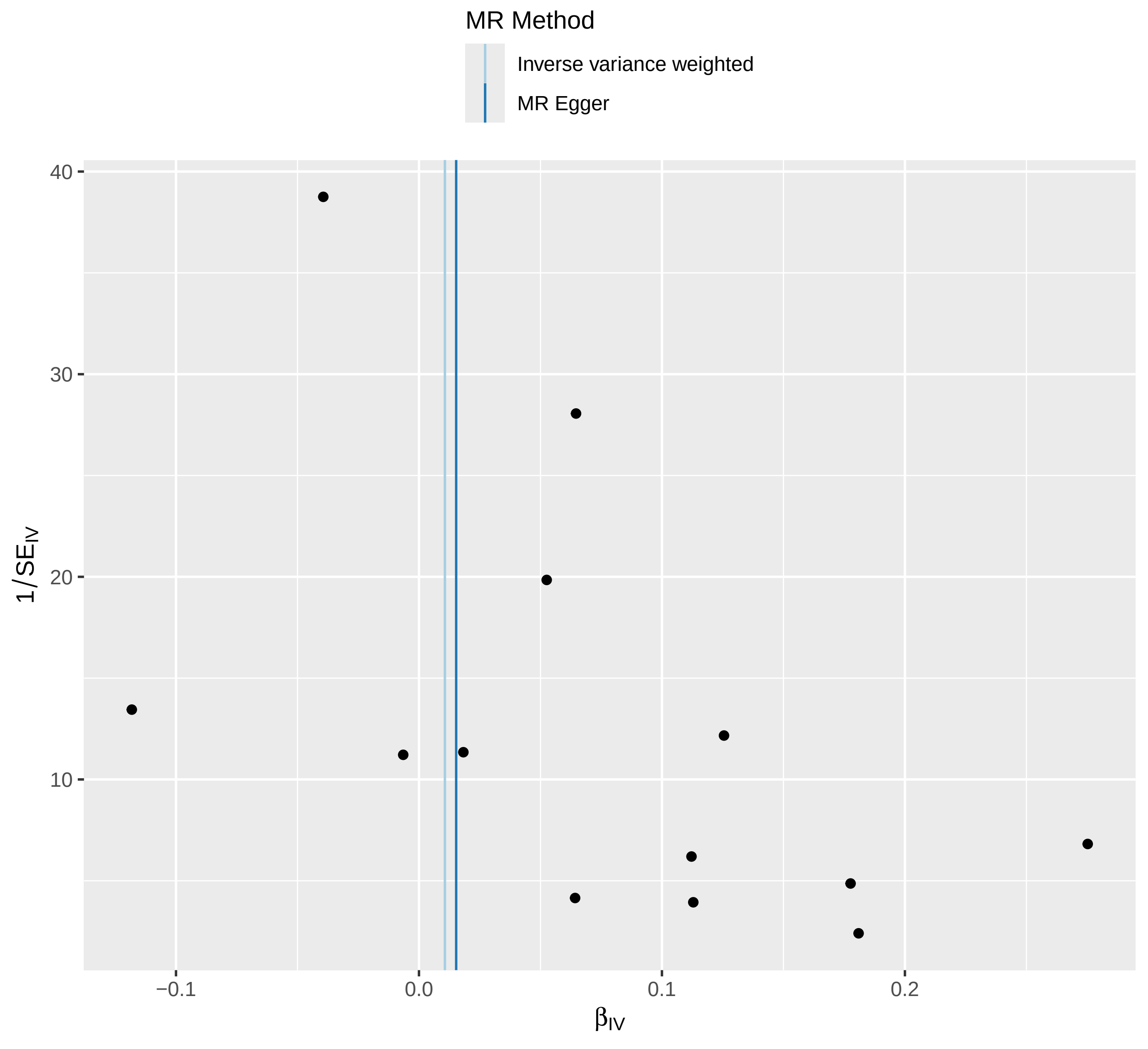

MR Method
Inverse variance weighted
MR Egger
40
30
20
10
$1/SE_{IV}$
−0.1
0.0
0.1
0.2
$\beta_{IV}$

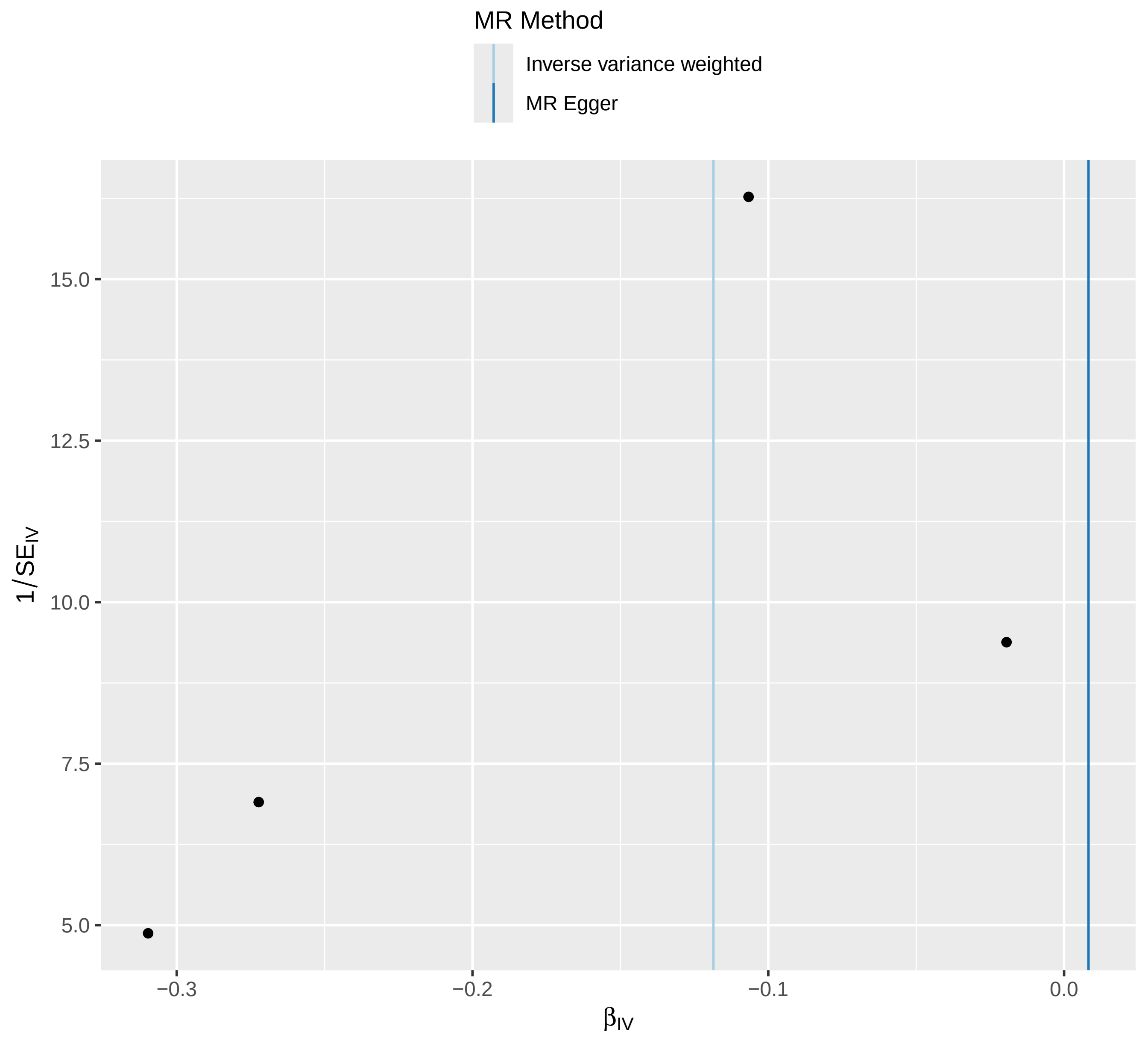
MR Method
Inverse variance weighted
MR Egger
15.0
12.5
10.0
7.5
5.0
$1/SE_{IV}$
−0.3
−0.2
−0.1
0.0
$\beta_{IV}$

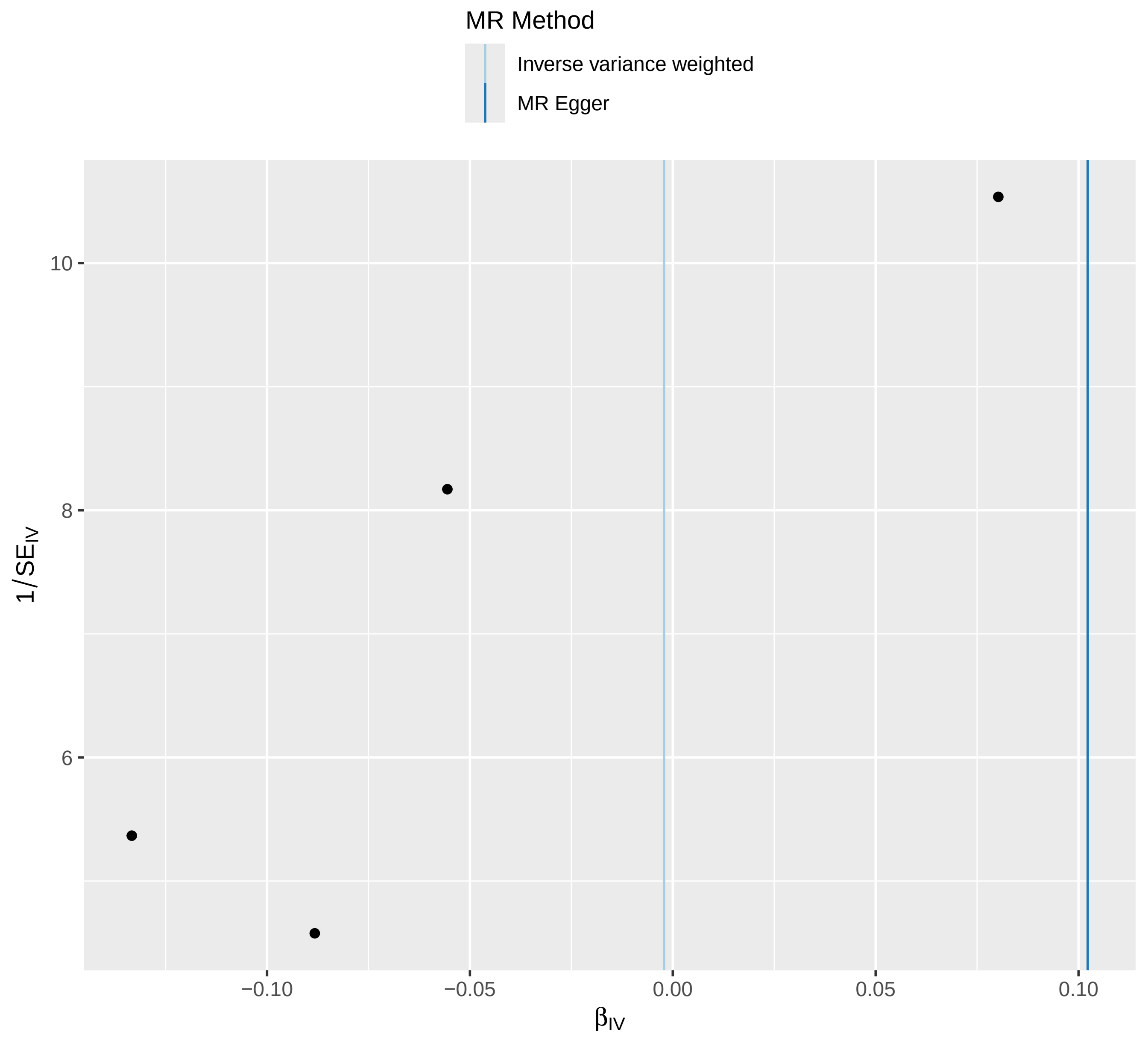
MR Method
Inverse variance weighted
MR Egger
10
8
6
$1/SE_{IV}$
−0.10
−0.05
0.00
0.05
0.10
$\beta_{IV}$

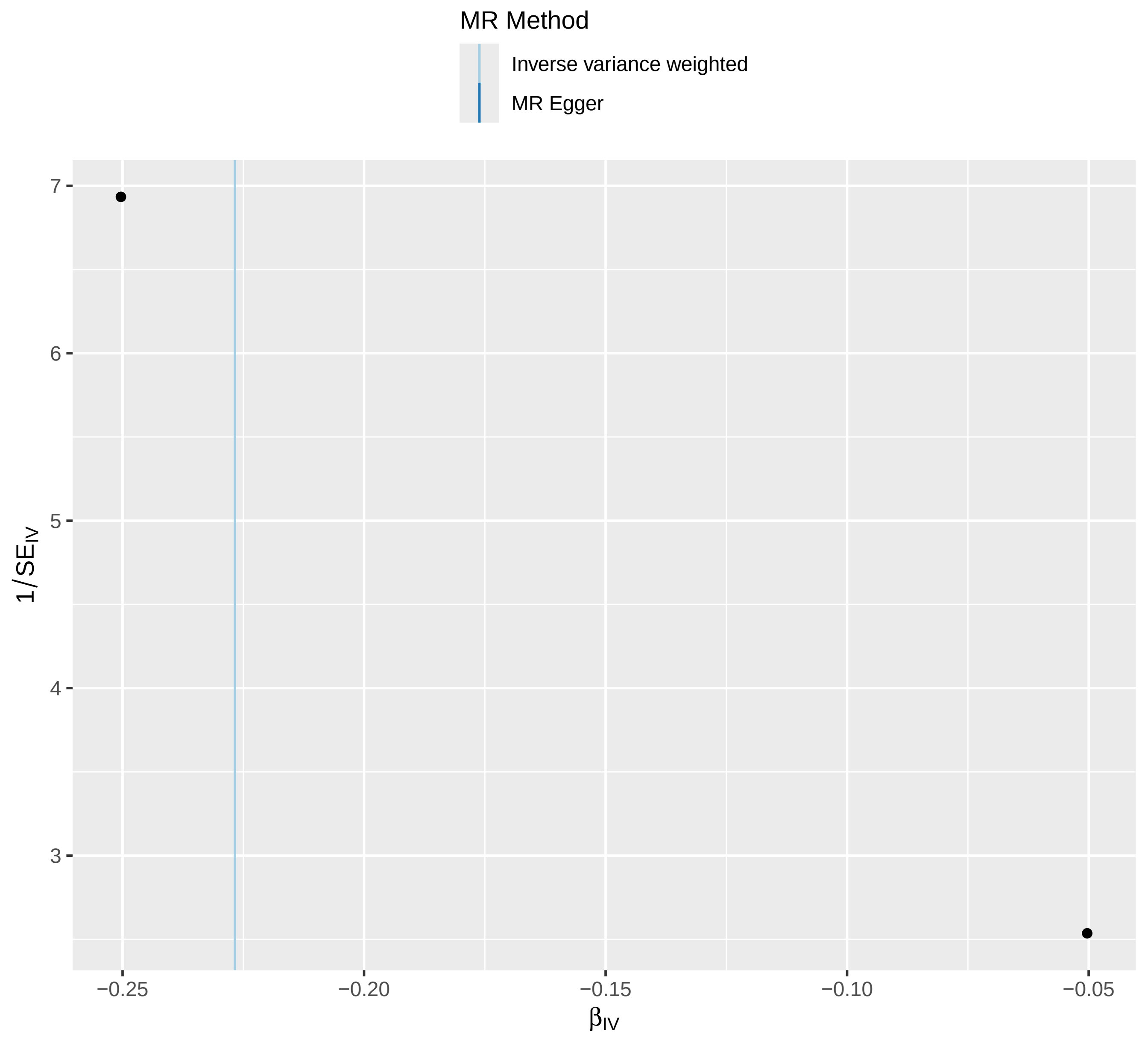

MR Method
Inverse variance weighted
MR Egger
7
6
5
4
3
1/SE$_{IV}$
−0.25
−0.20
−0.15
−0.10
−0.05
β$_{IV}$

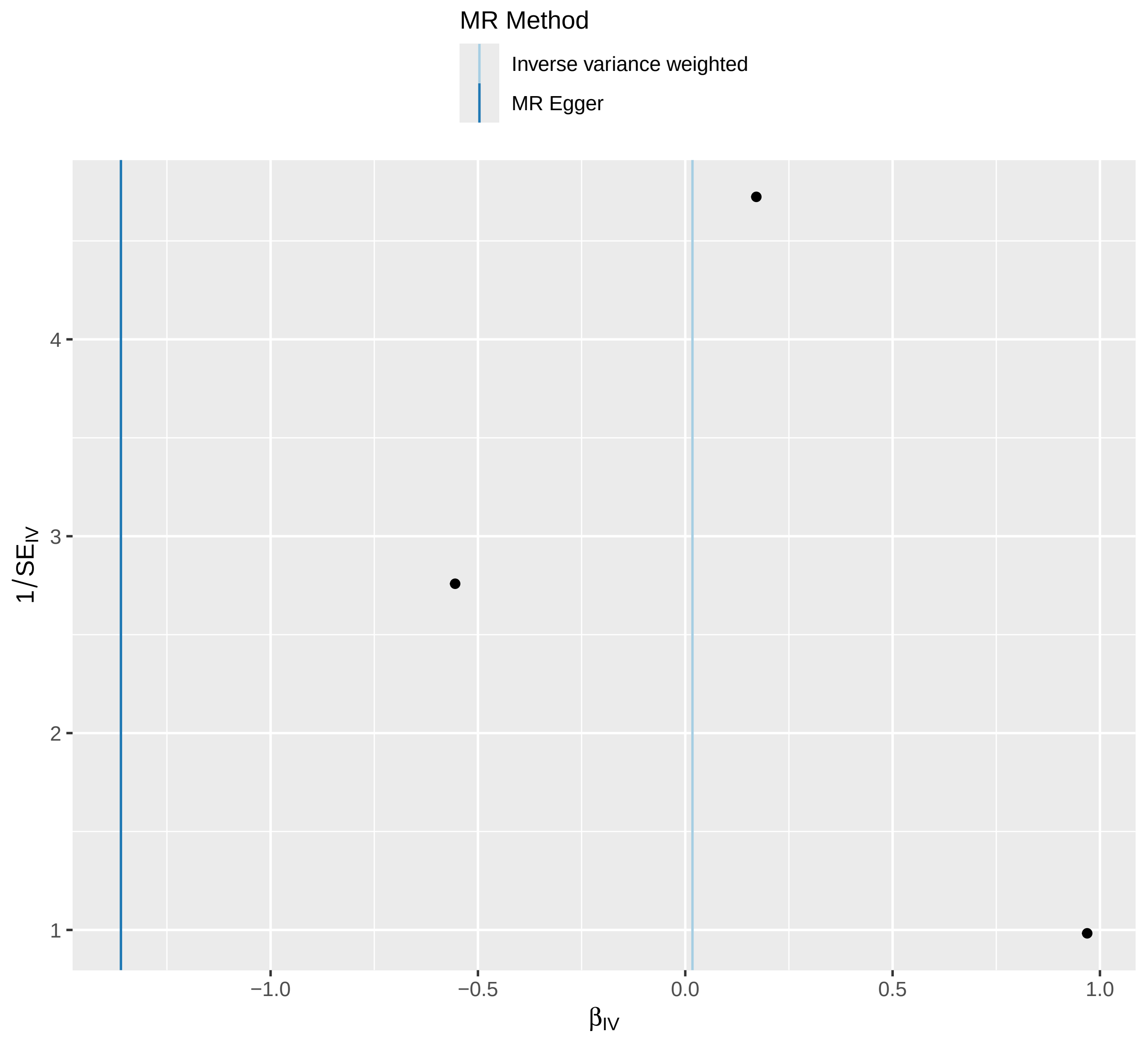
MR Method
Inverse variance weighted
MR Egger
1/SE$_{IV}$
4
3
2
1
−1.0
−0.5
0.0
0.5
1.0
β$_{IV}$

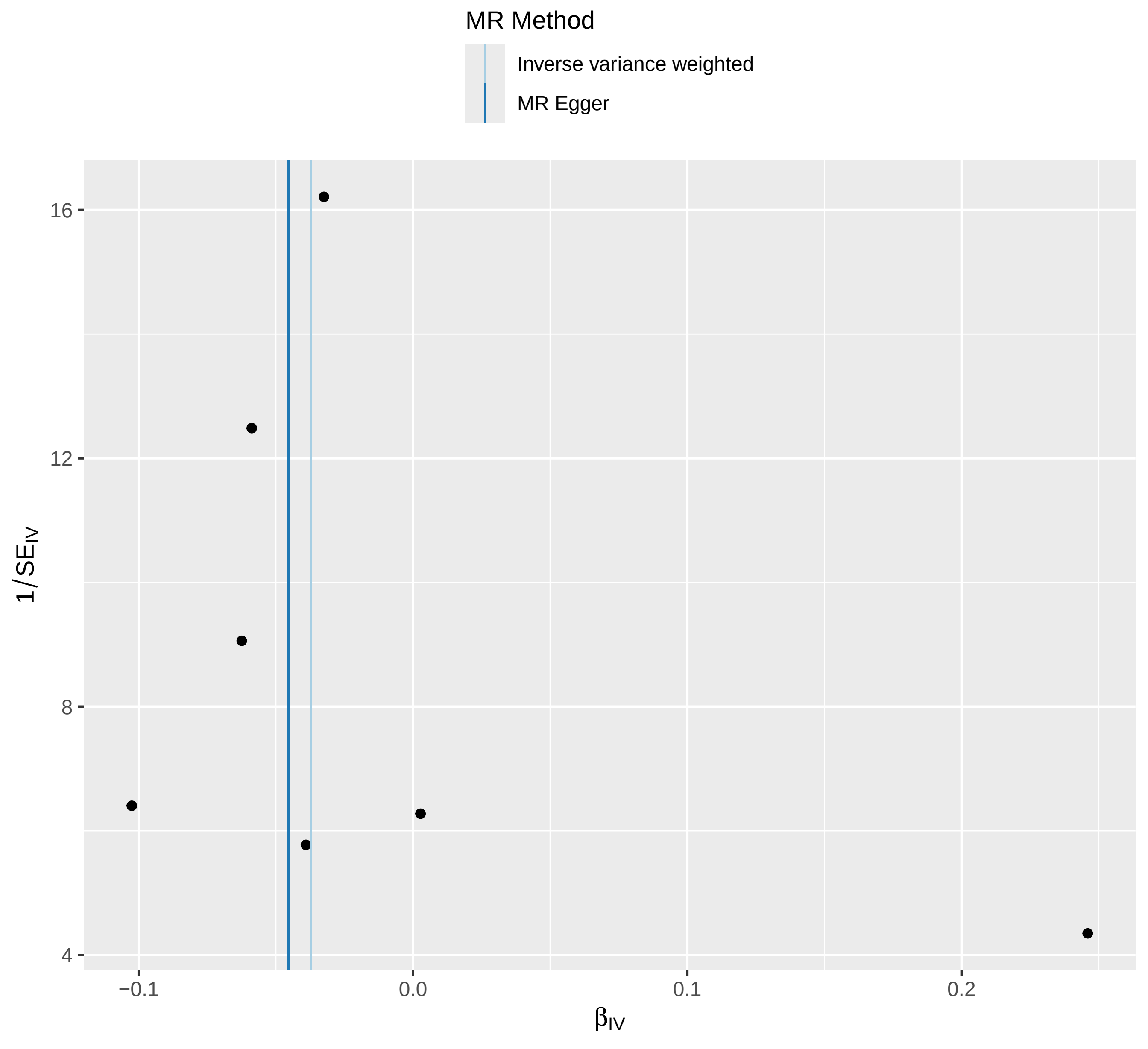

MR Method
Inverse variance weighted
MR Egger
16
12
8
4
$1/SE_{IV}$
−0.1
0.0
0.1
0.2
$\beta_{IV}$

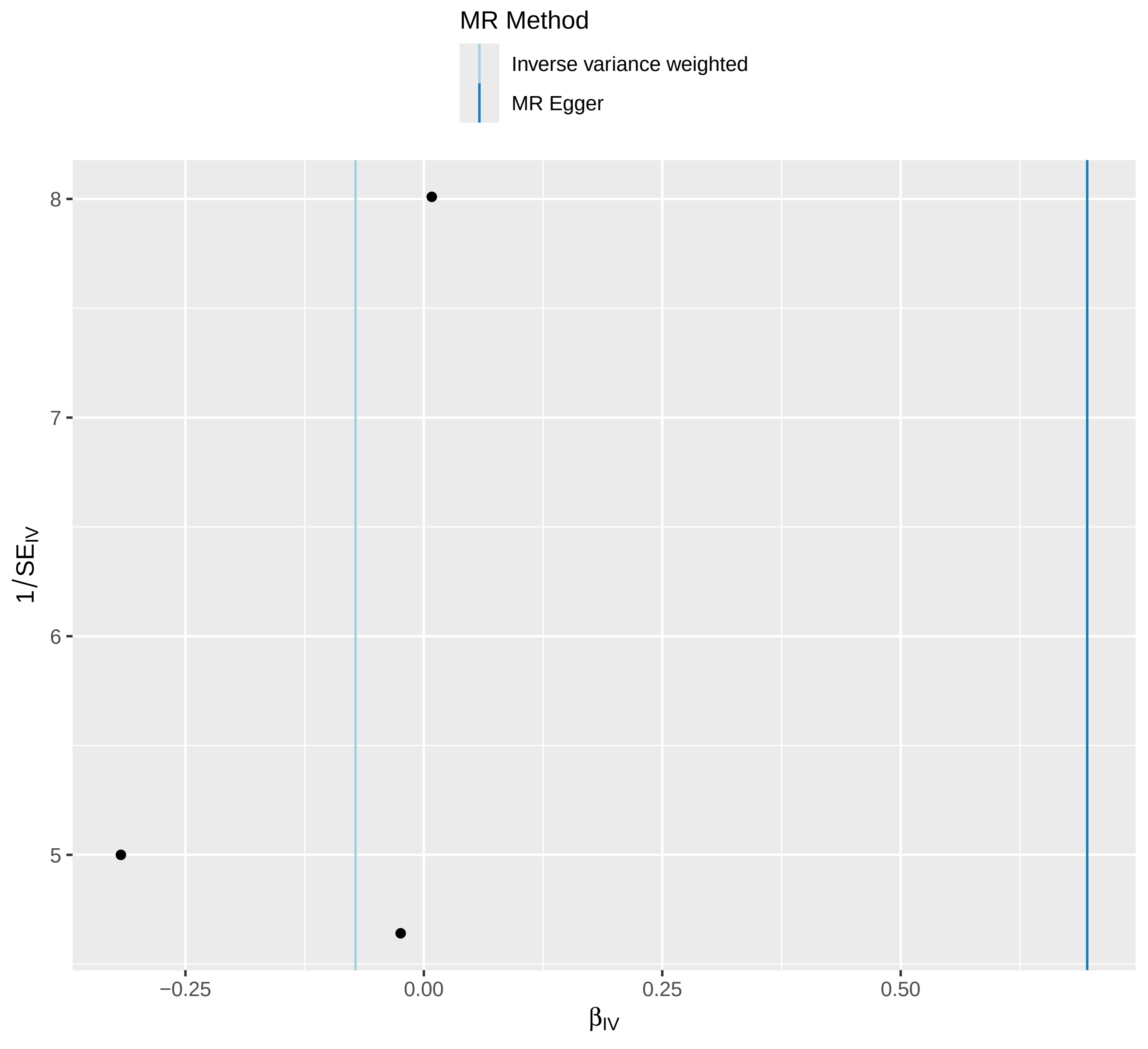
MR Method
Inverse variance weighted
MR Egger
8
7
6
5
1/SE$_{IV}$
−0.25
0.00
0.25
0.50
β$_{IV}$

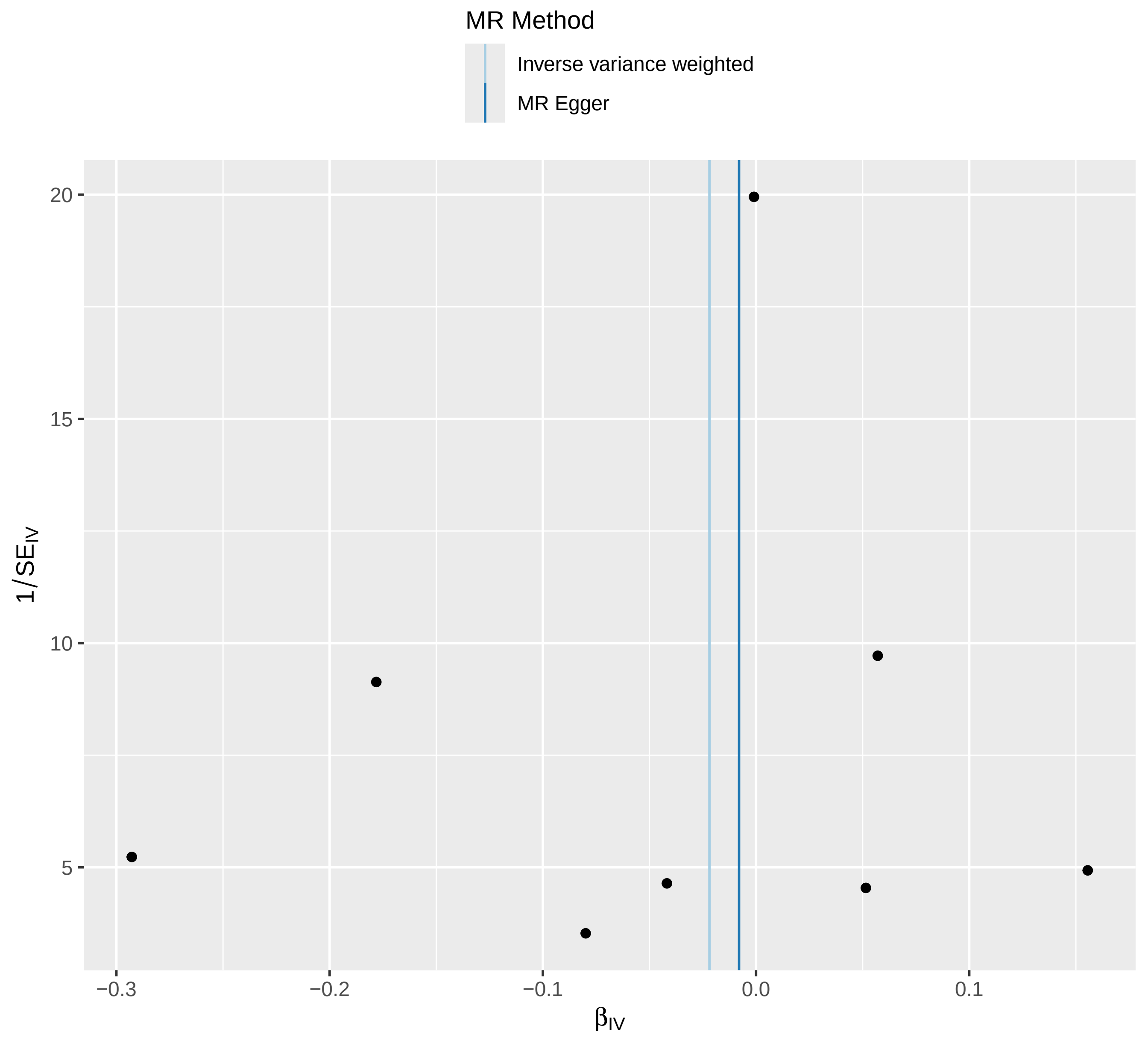

MR Method
Inverse variance weighted
MR Egger
20
15
10
5
1/SE_IV
−0.3
−0.2
−0.1
0.0
0.1
β_IV

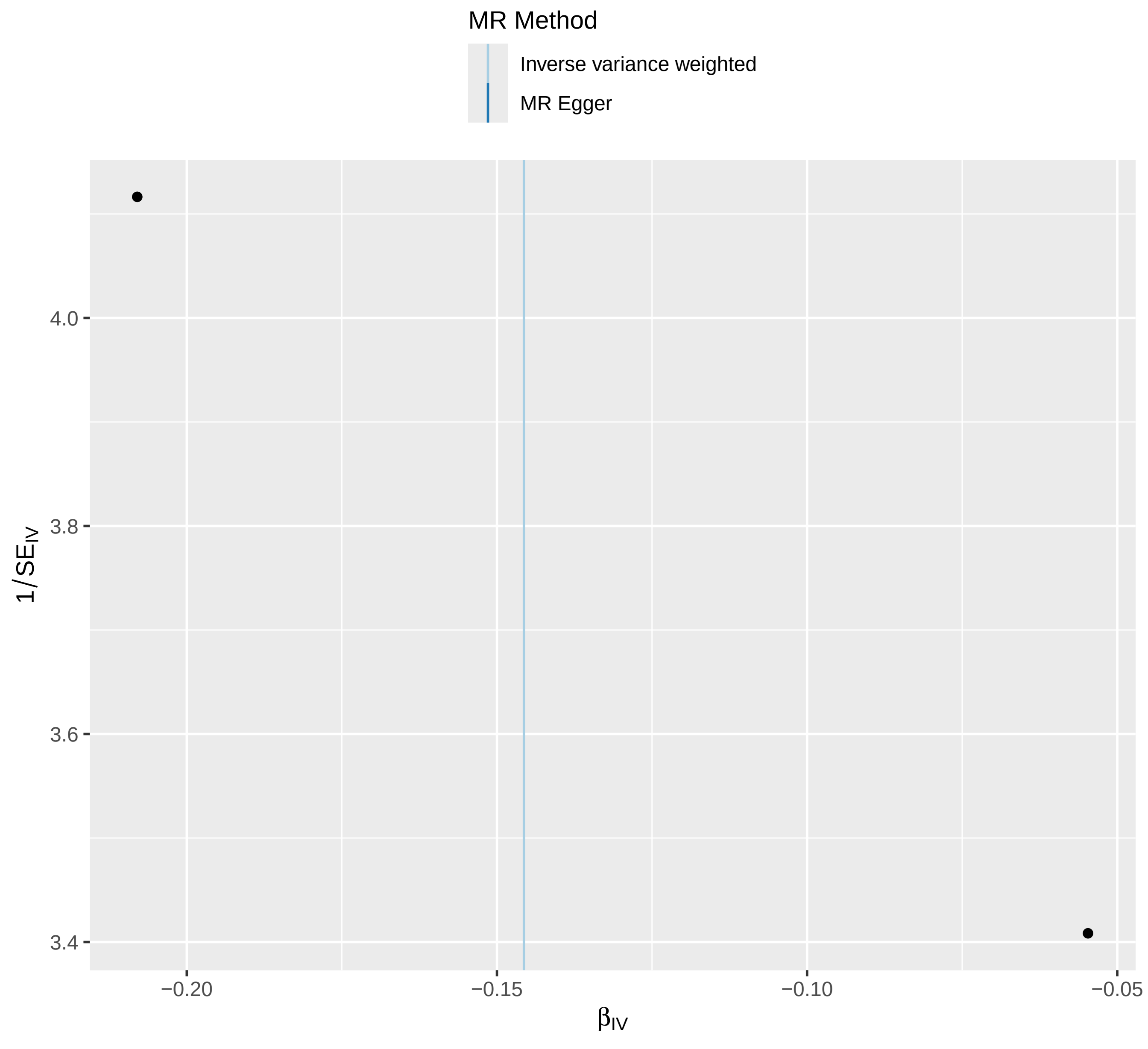
MR Method
Inverse variance weighted
MR Egger
4.0
3.8
3.6
3.4
$1/SE_{IV}$
−0.20
−0.15
−0.10
−0.05
$\beta_{IV}$

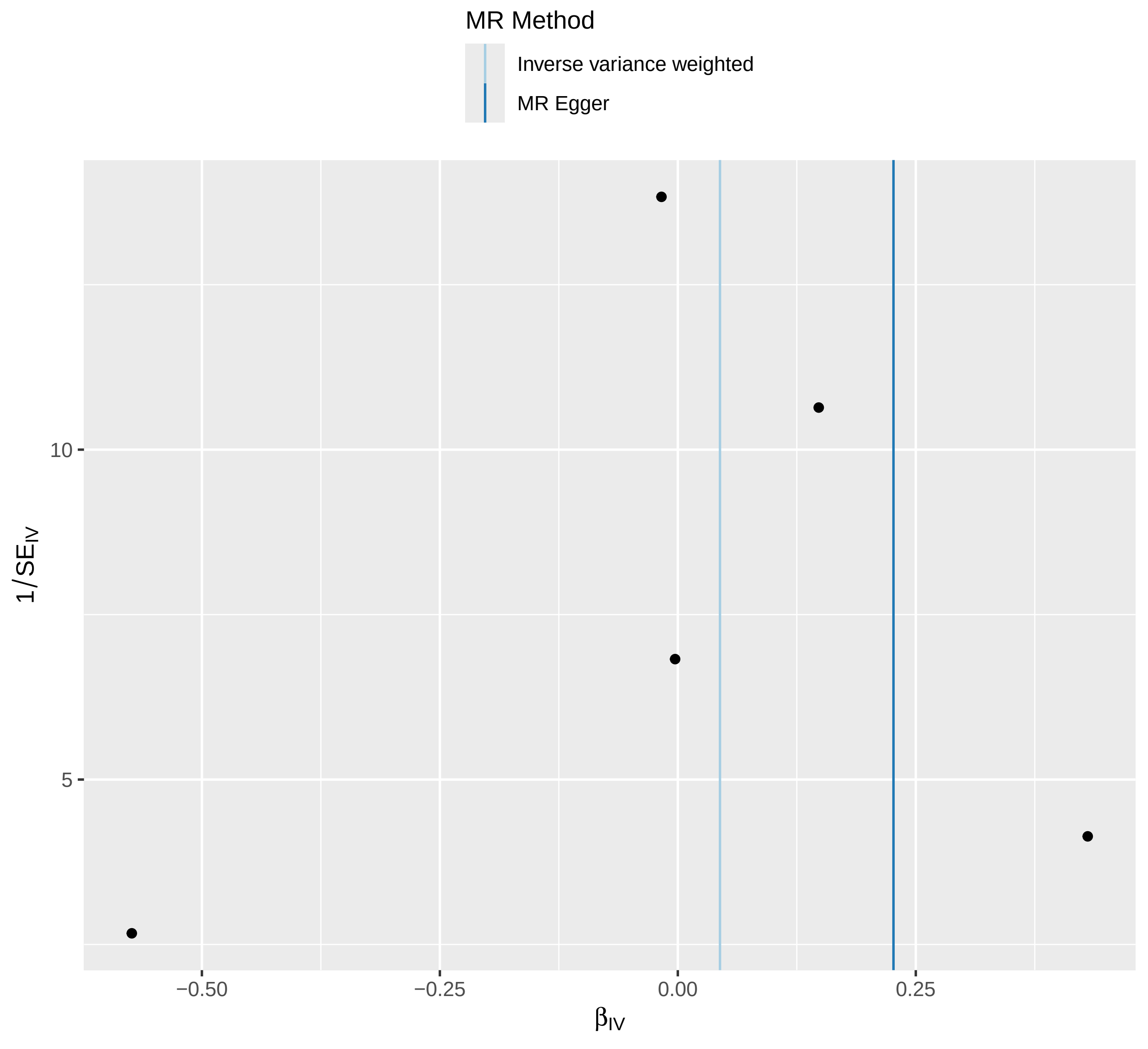

MR Method
Inverse variance weighted
MR Egger
10
5
1/SE_IV
−0.50
−0.25
0.00
0.25
β_IV

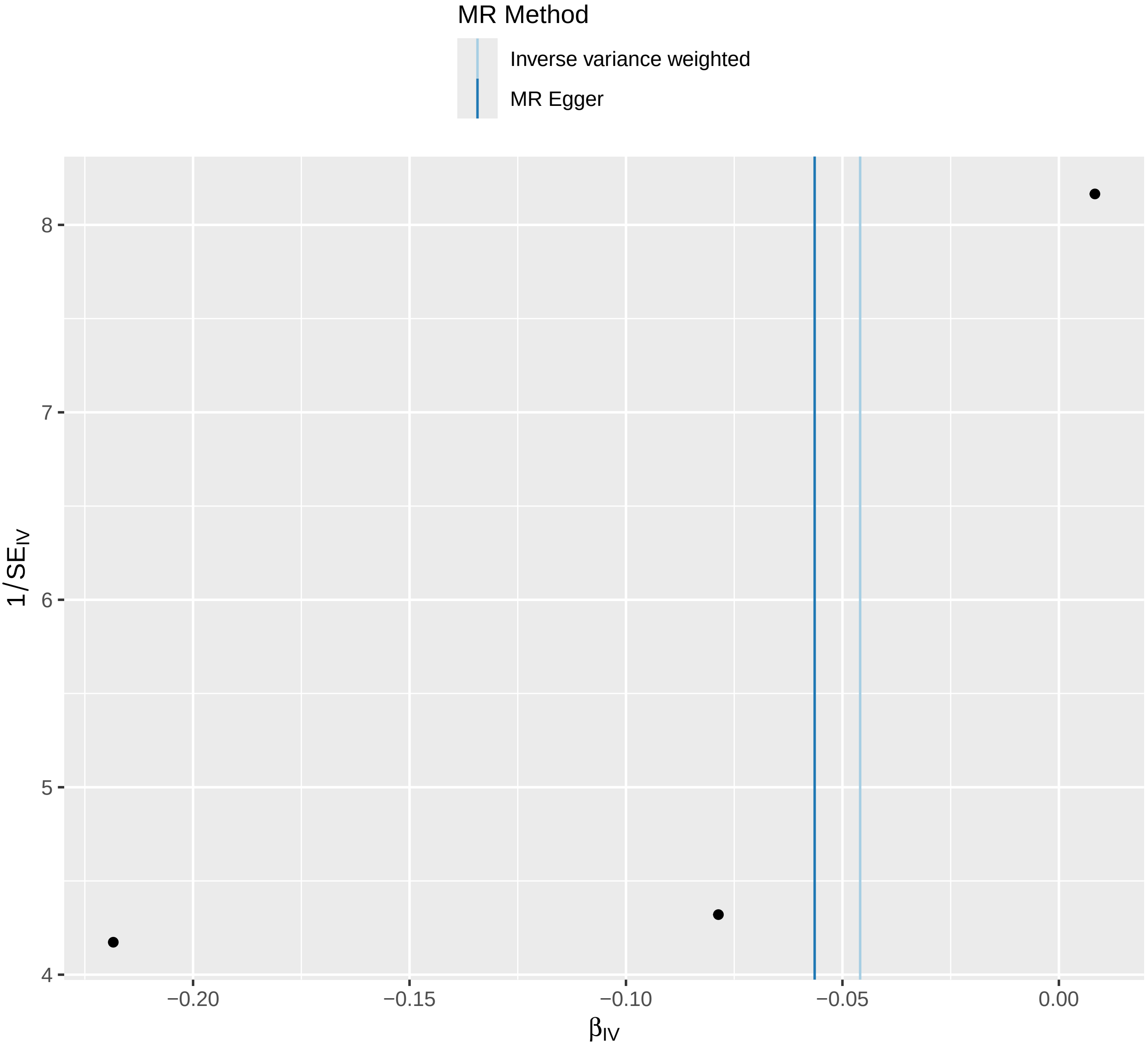

MR Method
Inverse variance weighted
MR Egger
8
7
6
5
4
1/SE_IV
−0.20
−0.15
−0.10
−0.05
0.00
β_IV

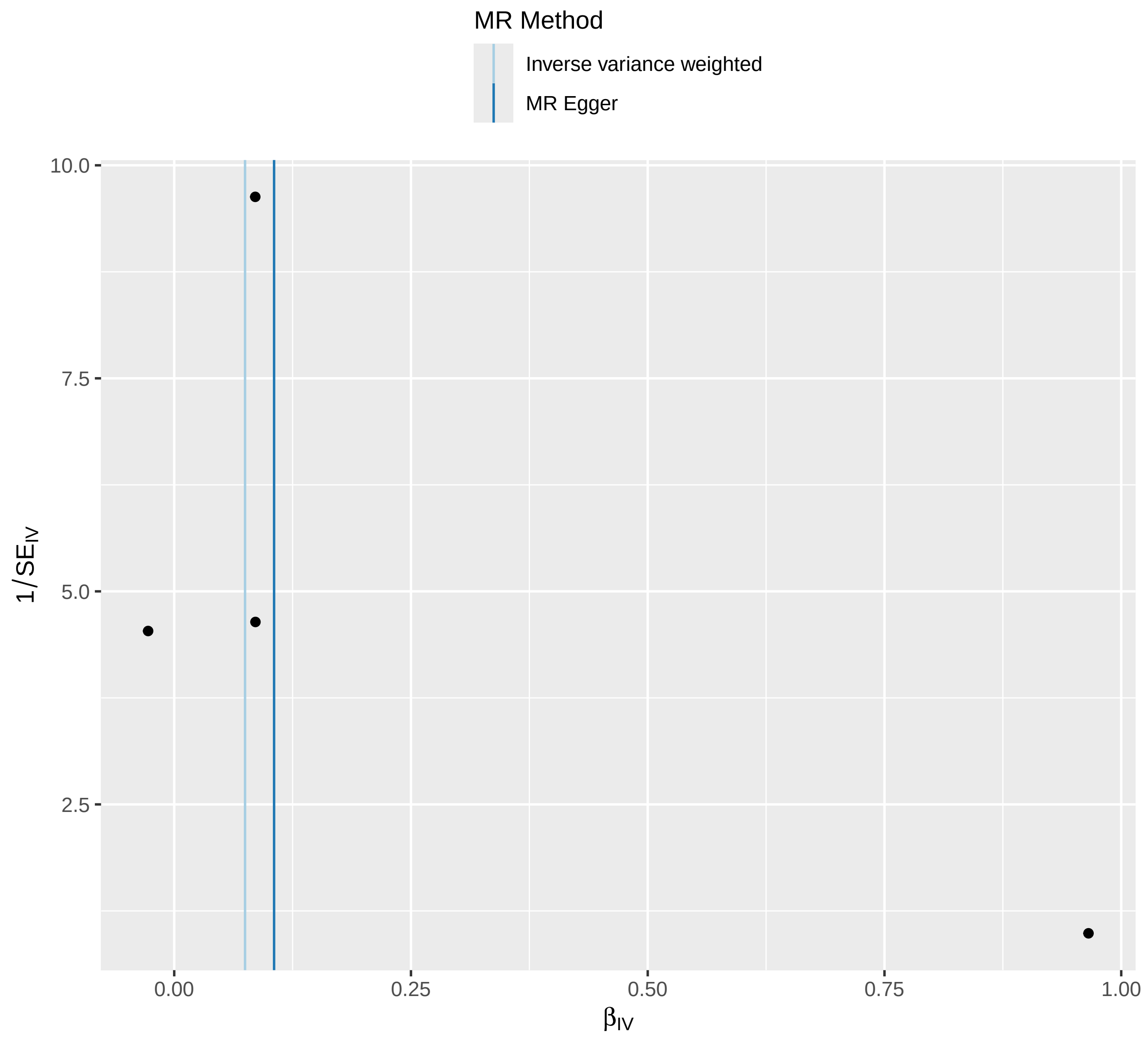
MR Method
Inverse variance weighted
MR Egger
10.0
7.5
5.0
2.5
1/SE_IV
0.00
0.25
0.50
0.75
1.00
β_IV

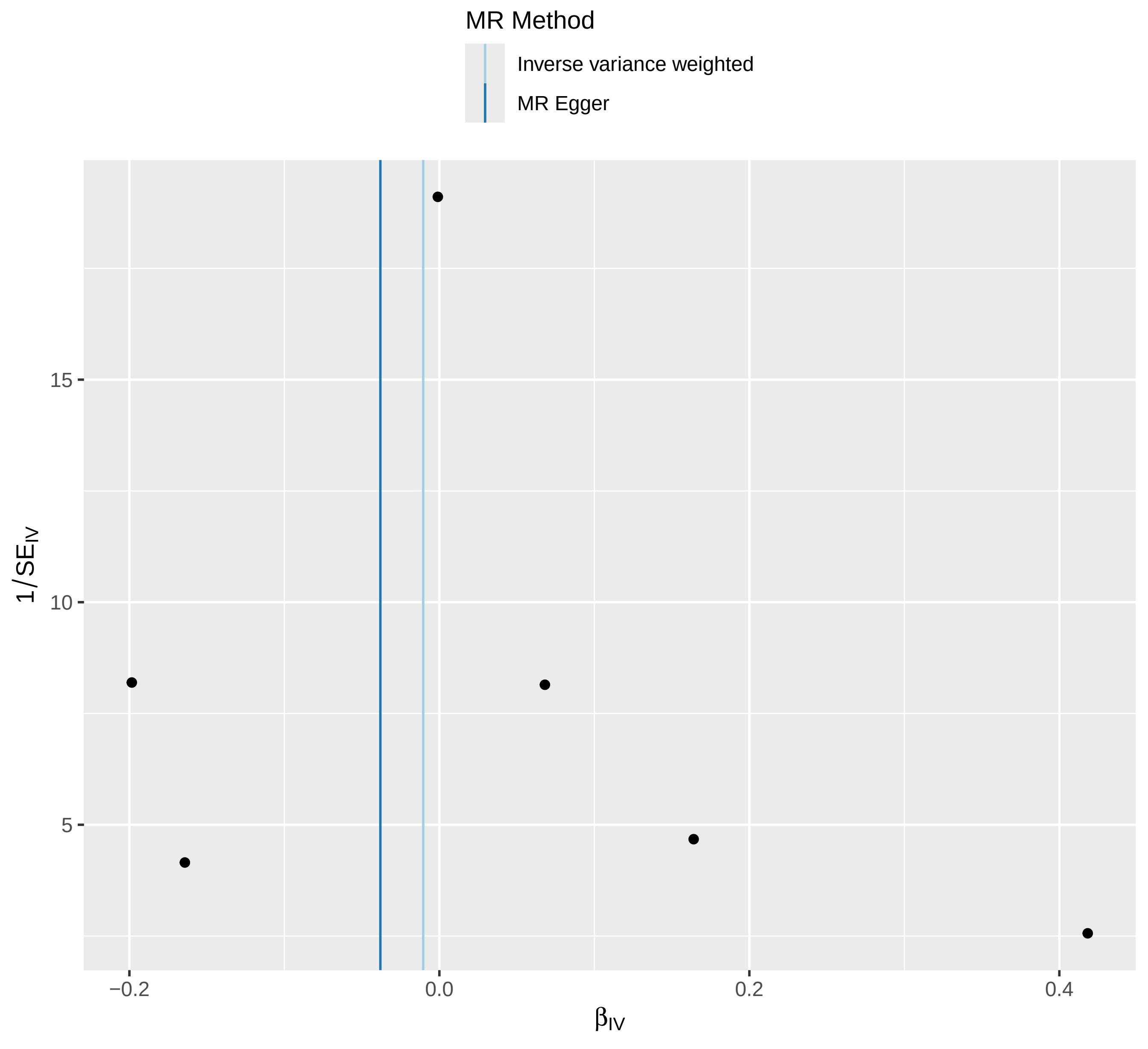
MR Method
Inverse variance weighted
MR Egger
1/SE_IV
β_IV
15
10
5
−0.2
0.0
0.2
0.4

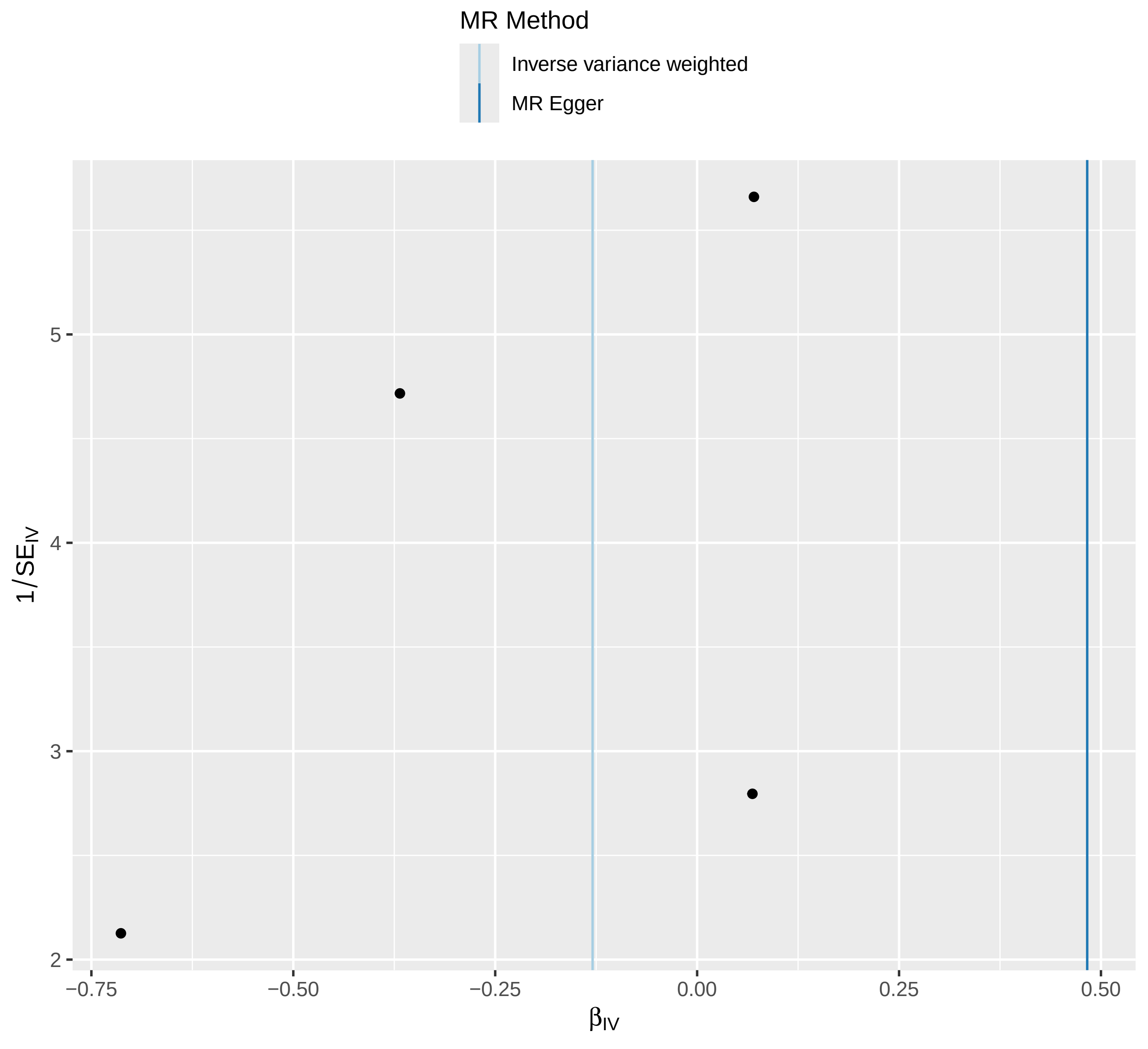
MR Method
Inverse variance weighted
MR Egger
$1/SE_{IV}$
5
4
3
2
−0.75
−0.50
−0.25
0.00
0.25
0.50
$\beta_{IV}$

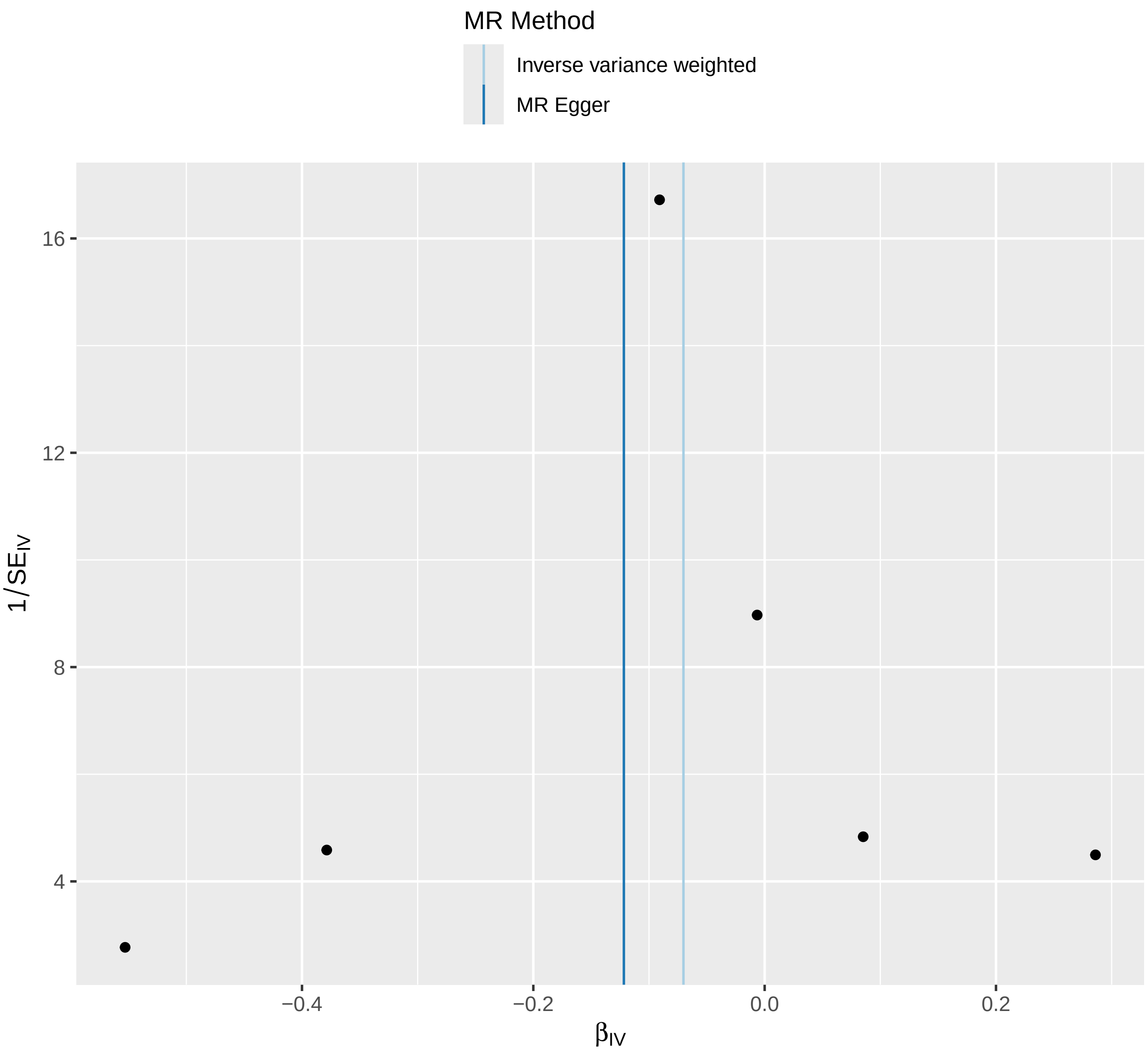
MR Method
Inverse variance weighted
MR Egger
16
12
8
4
1/SE$_{IV}$
−0.4
−0.2
0.0
0.2
β$_{IV}$

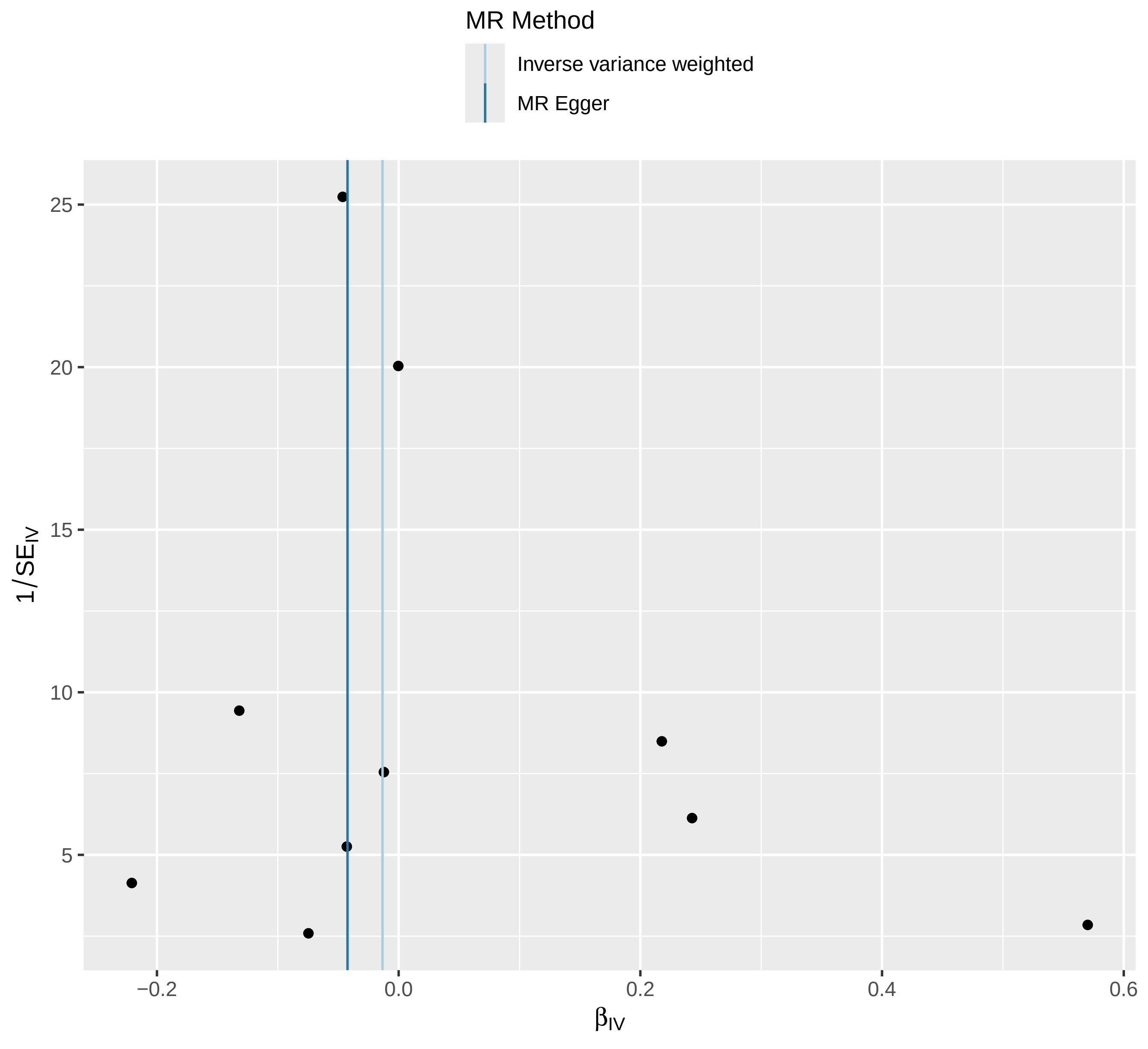
MR Method
Inverse variance weighted
MR Egger
25
20
15
10
5
1/SE_IV
−0.2
0.0
0.2
0.4
0.6
β_IV

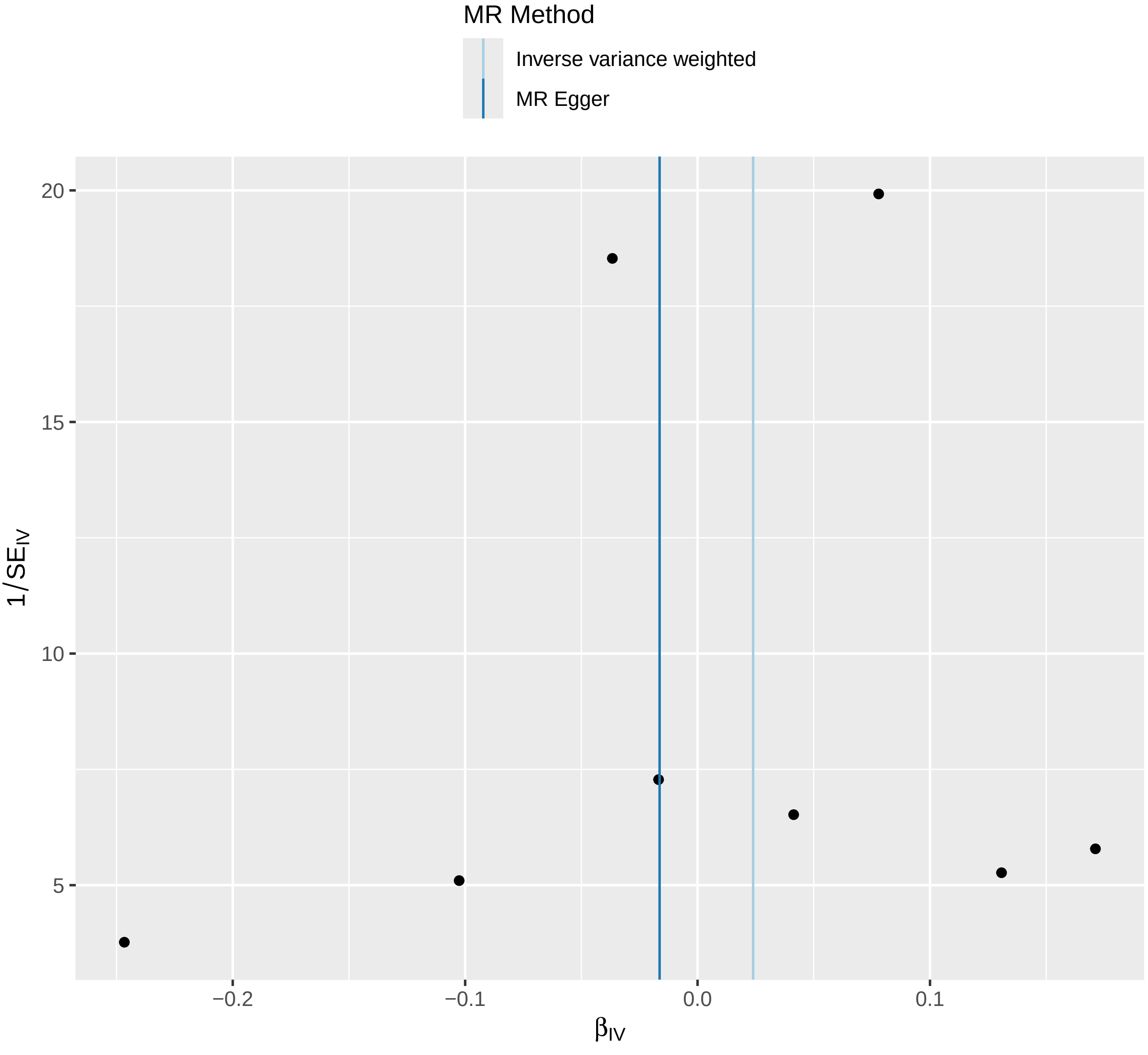

MR Method
Inverse variance weighted
MR Egger
20
15
10
5
1/SE_IV
−0.2
−0.1
0.0
0.1
β_IV

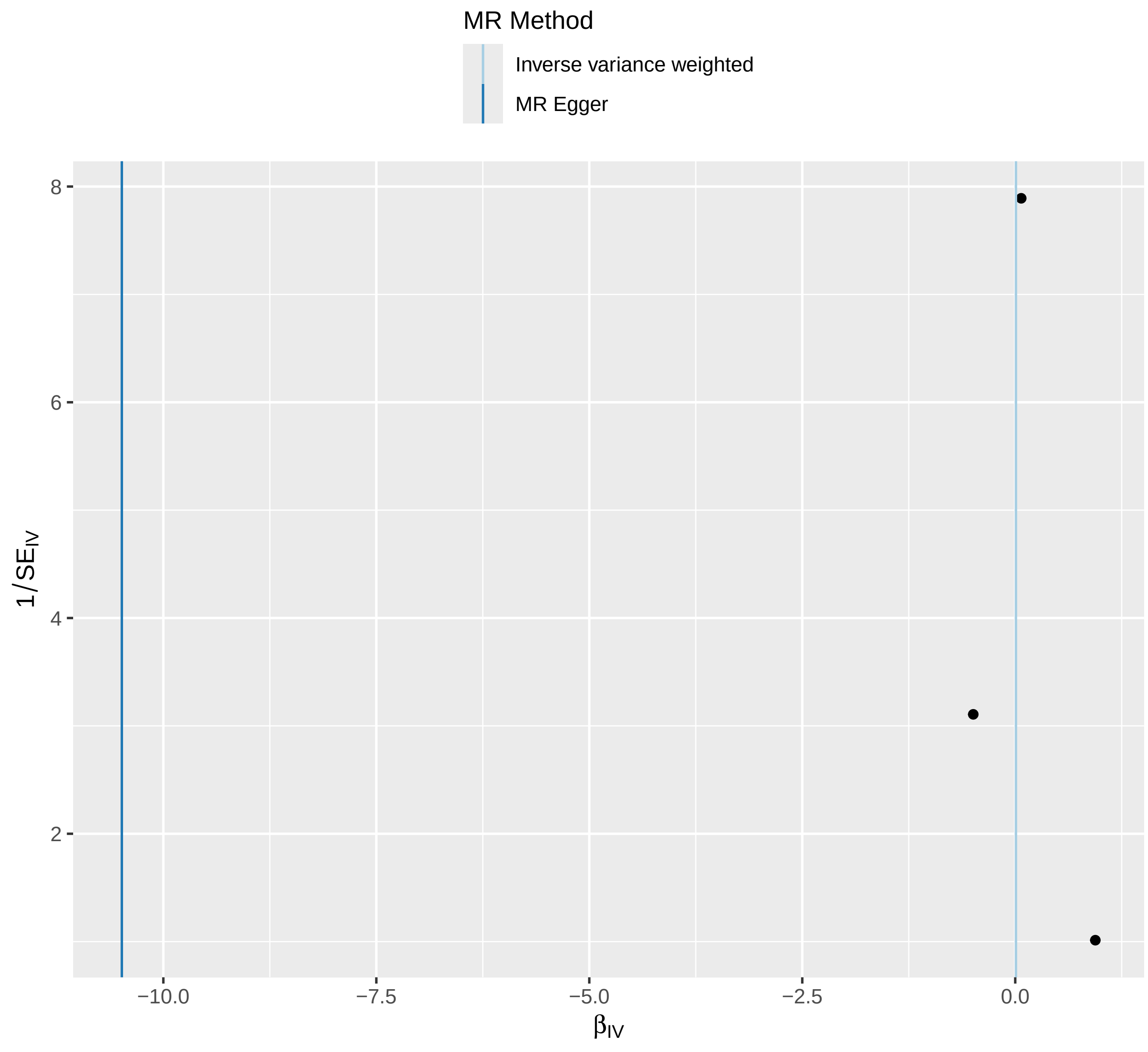

MR Method
Inverse variance weighted
MR Egger
8
6
4
2
1/SE_IV
−10.0
−7.5
−5.0
−2.5
0.0
β_IV

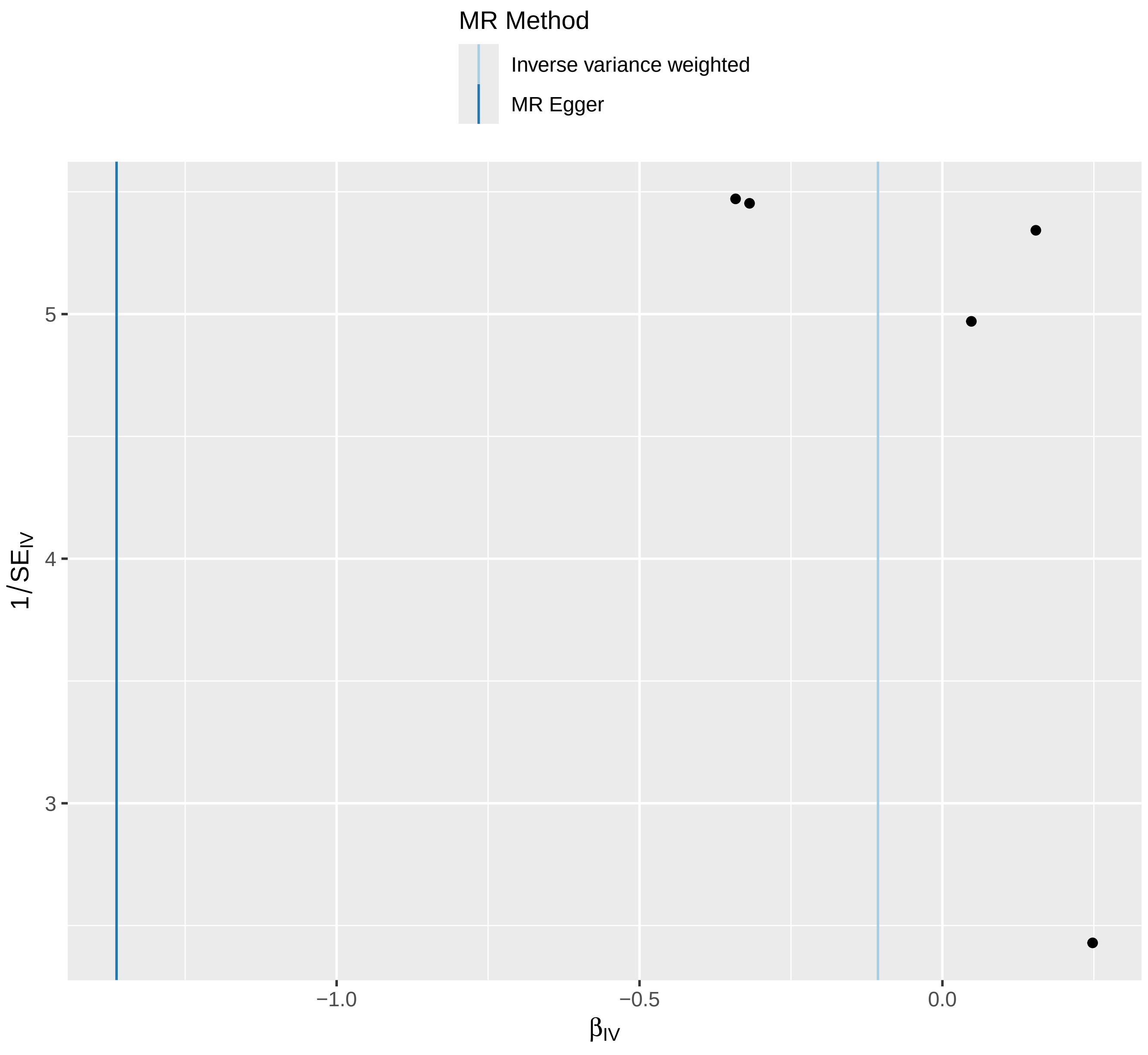
MR Method
Inverse variance weighted
MR Egger
5
4
3
1/SE_IV
−1.0
−0.5
0.0
β_IV

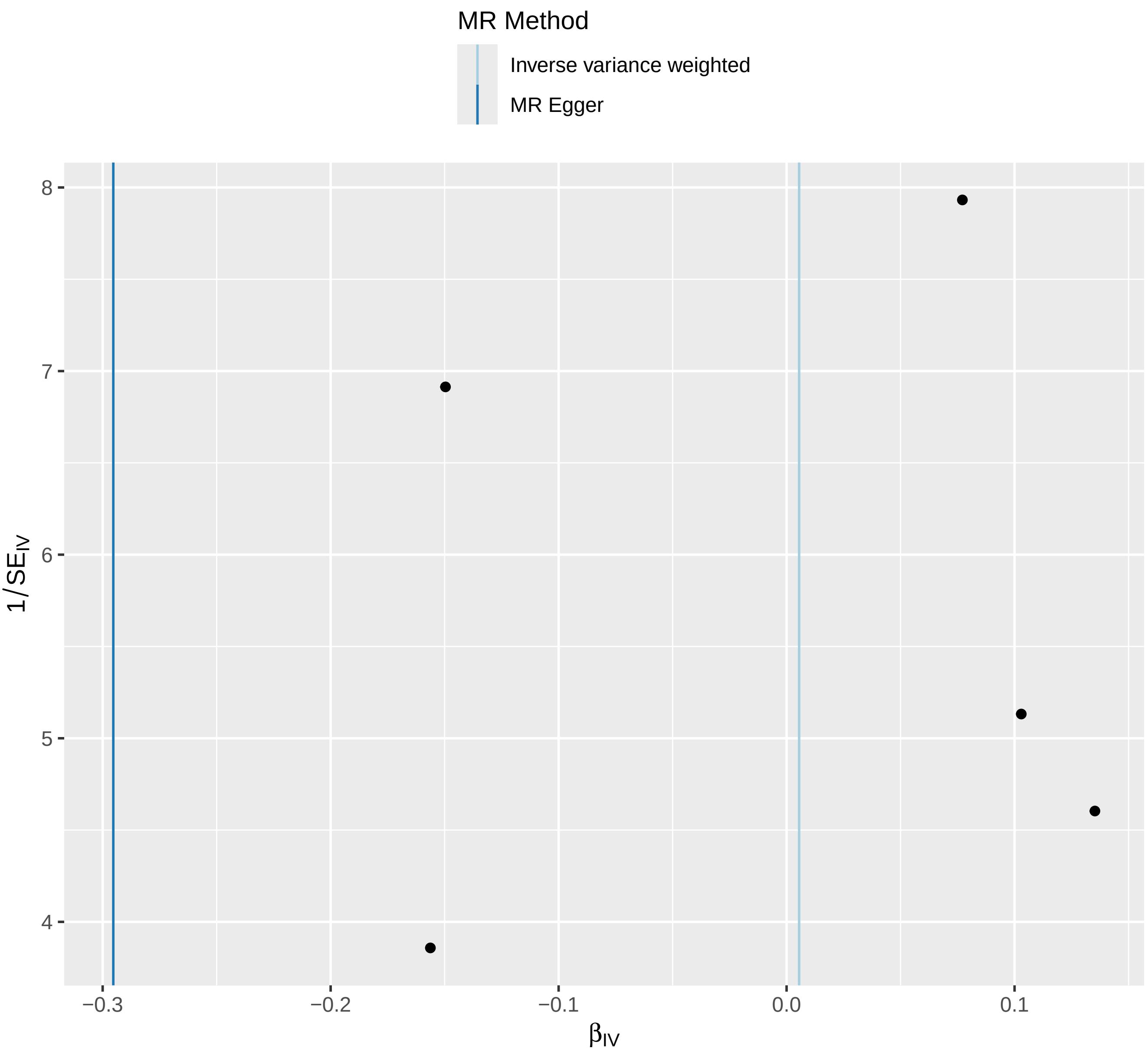

MR Method
Inverse variance weighted
MR Egger
1/$SE_{IV}$
8
7
6
5
4
−0.3
−0.2
−0.1
0.0
0.1
$\beta_{IV}$

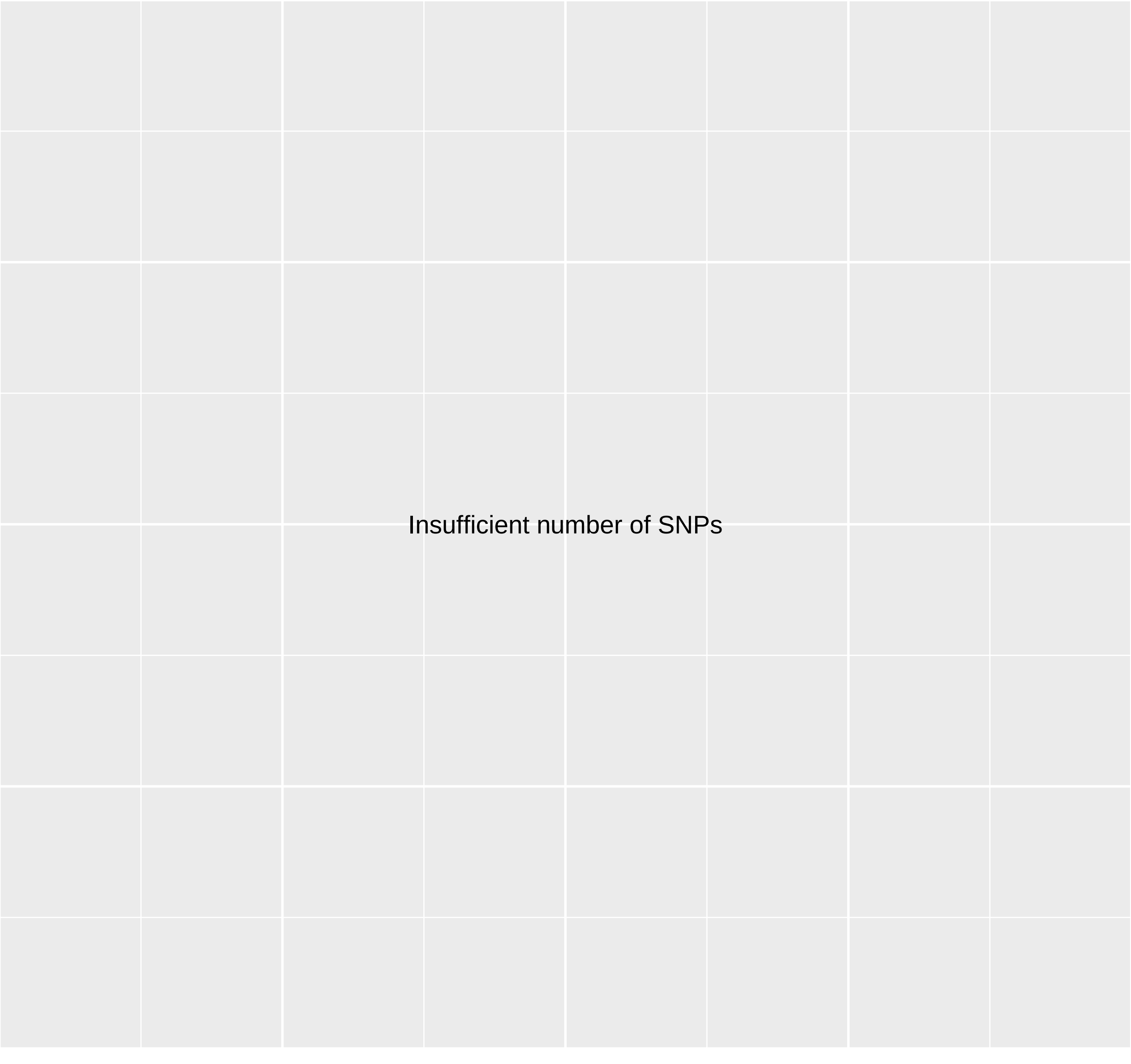
Insufficient number of SNPs

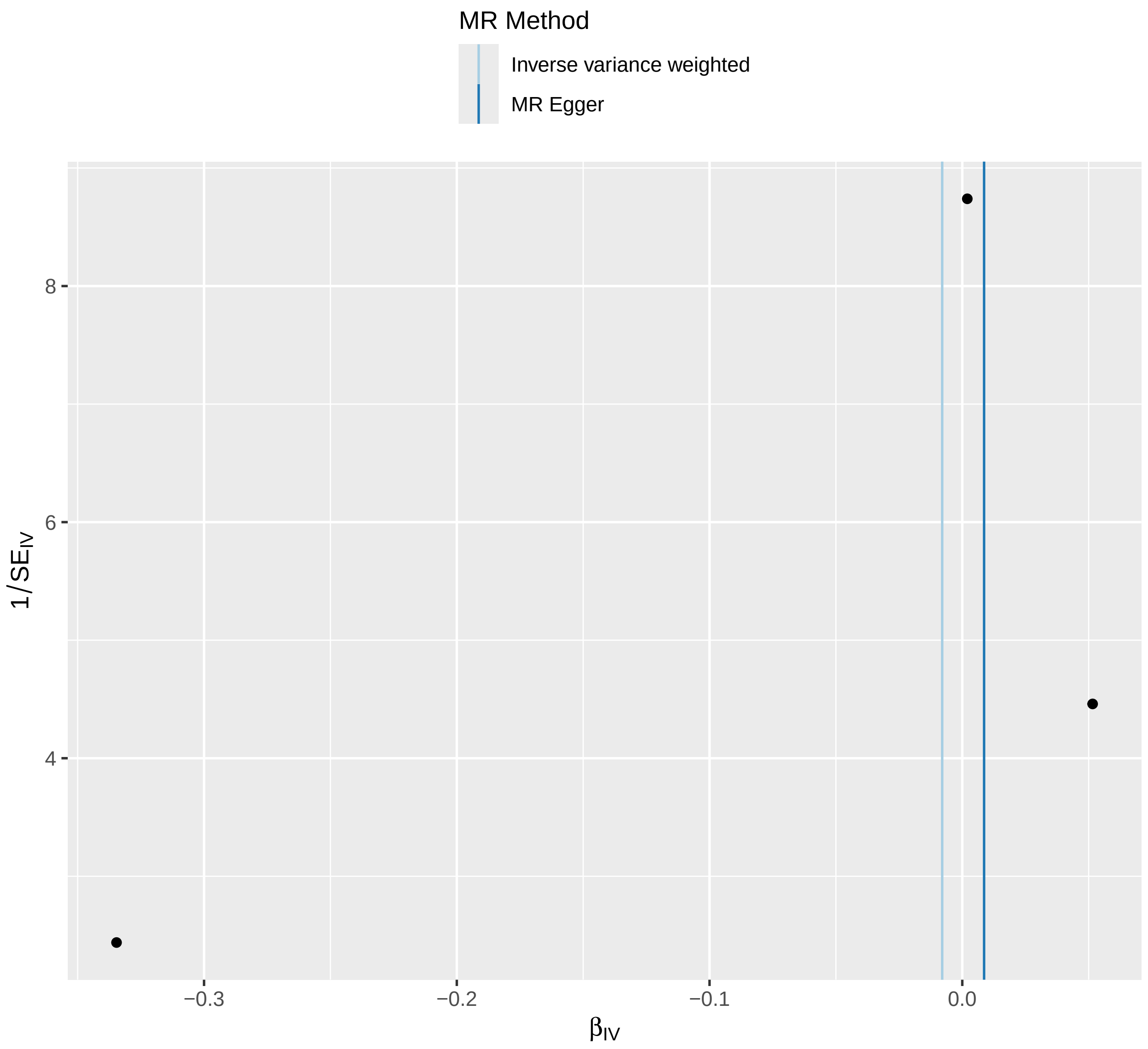
MR Method
Inverse variance weighted
MR Egger
8
6
4
1/SE_IV
−0.3
−0.2
−0.1
0.0
β_IV

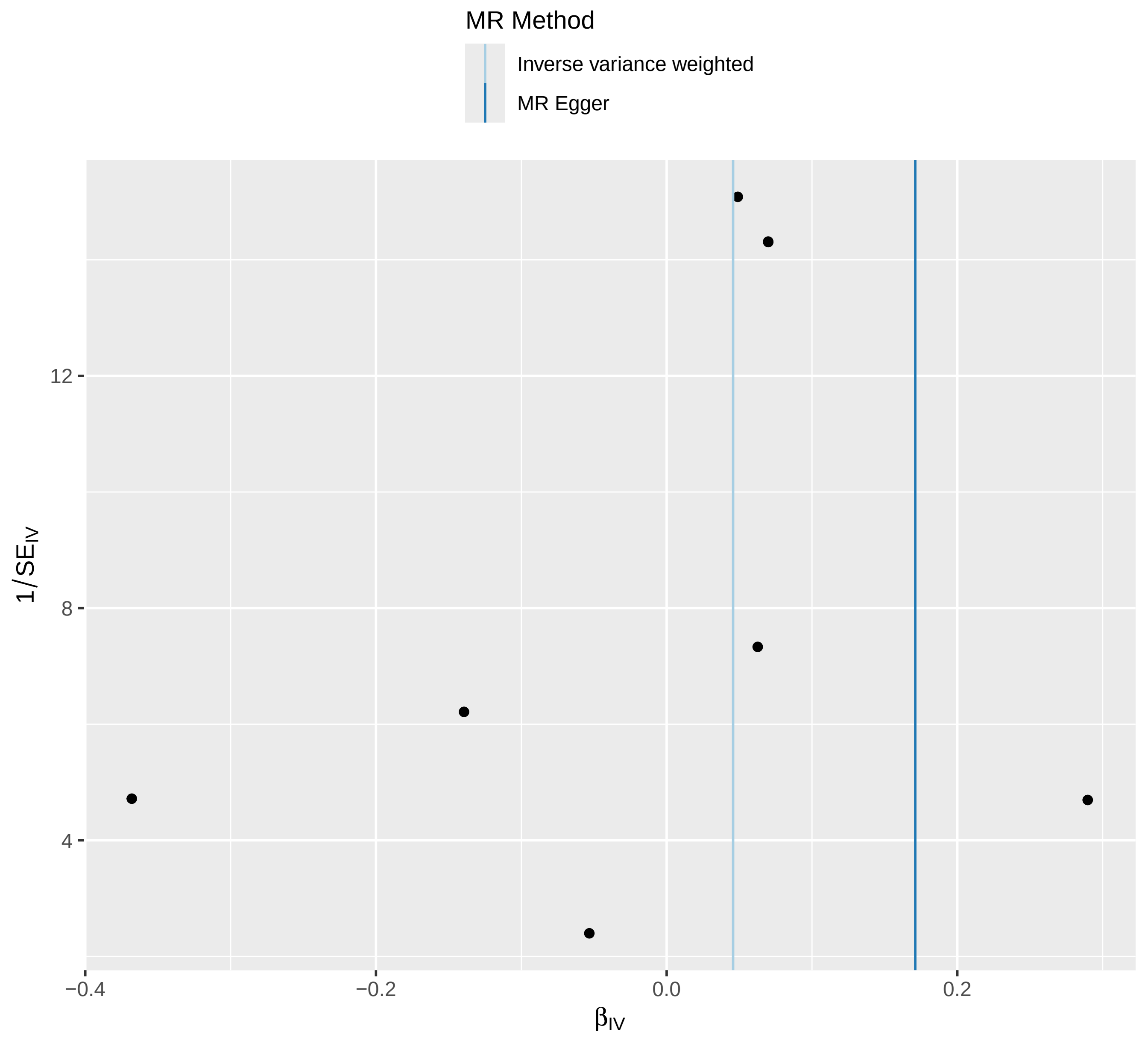
MR Method
Inverse variance weighted
MR Egger
12
8
4
−0.4
−0.2
0.0
0.2
1/SE_IV
β_IV

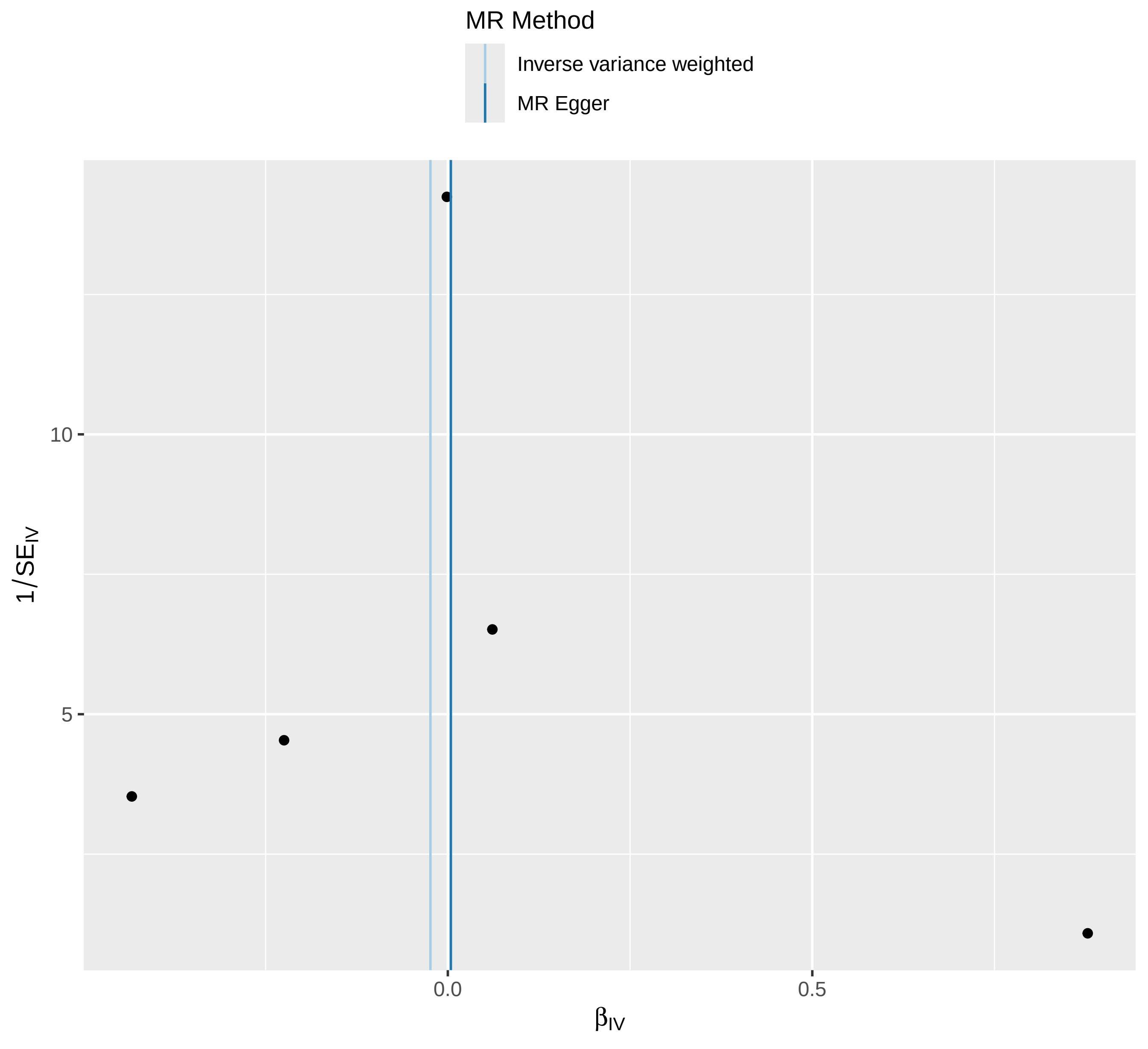
MR Method
Inverse variance weighted
MR Egger
10
5
1/SE_IV
0.0
0.5
β_IV

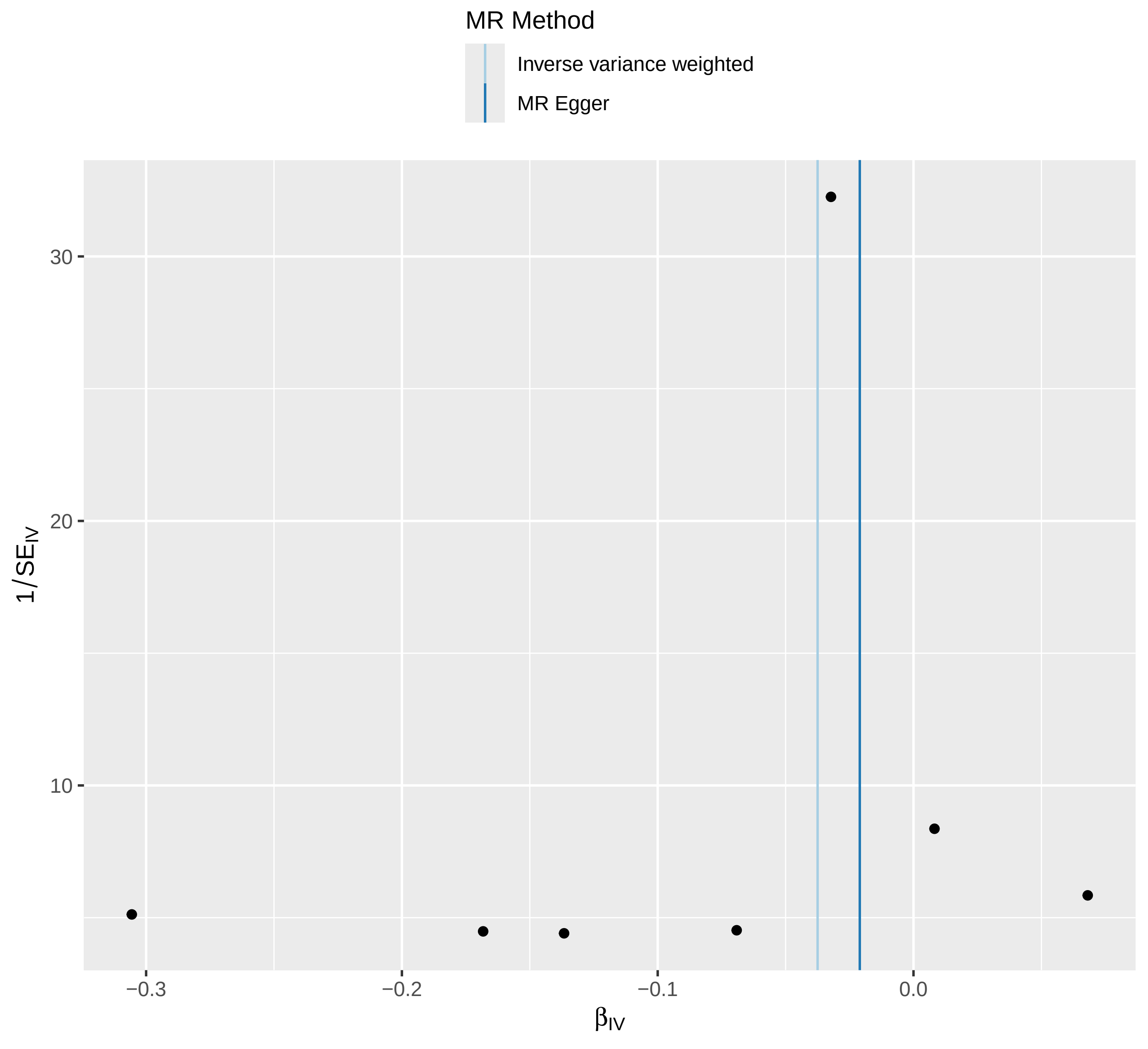

MR Method
Inverse variance weighted
MR Egger
30
20
10
−0.3
−0.2
−0.1
0.0
$1/SE_{IV}$
$\beta_{IV}$

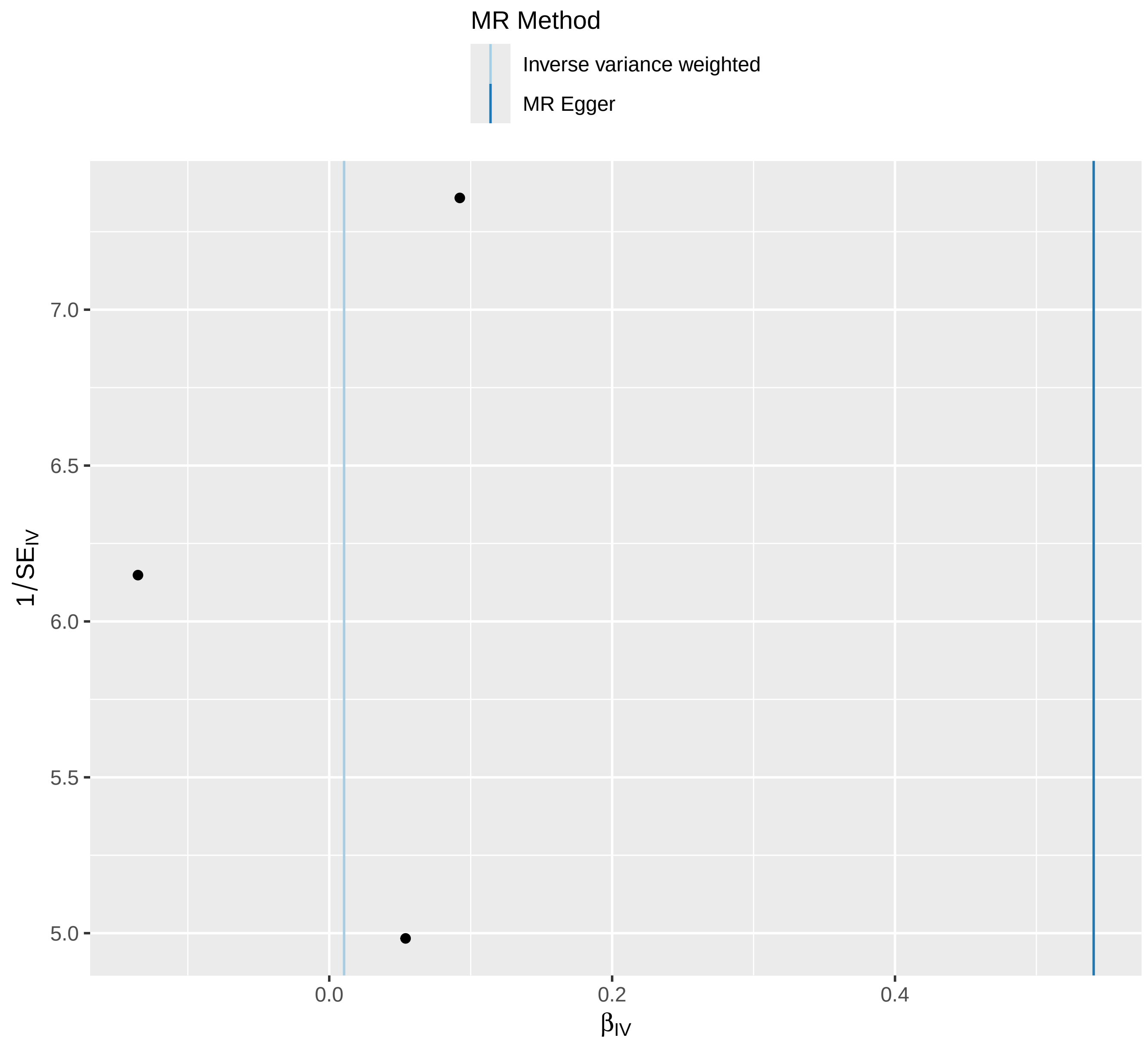

MR Method
Inverse variance weighted
MR Egger
7.0
6.5
6.0
5.5
5.0
1/SE_IV
0.0
0.2
0.4
β_IV

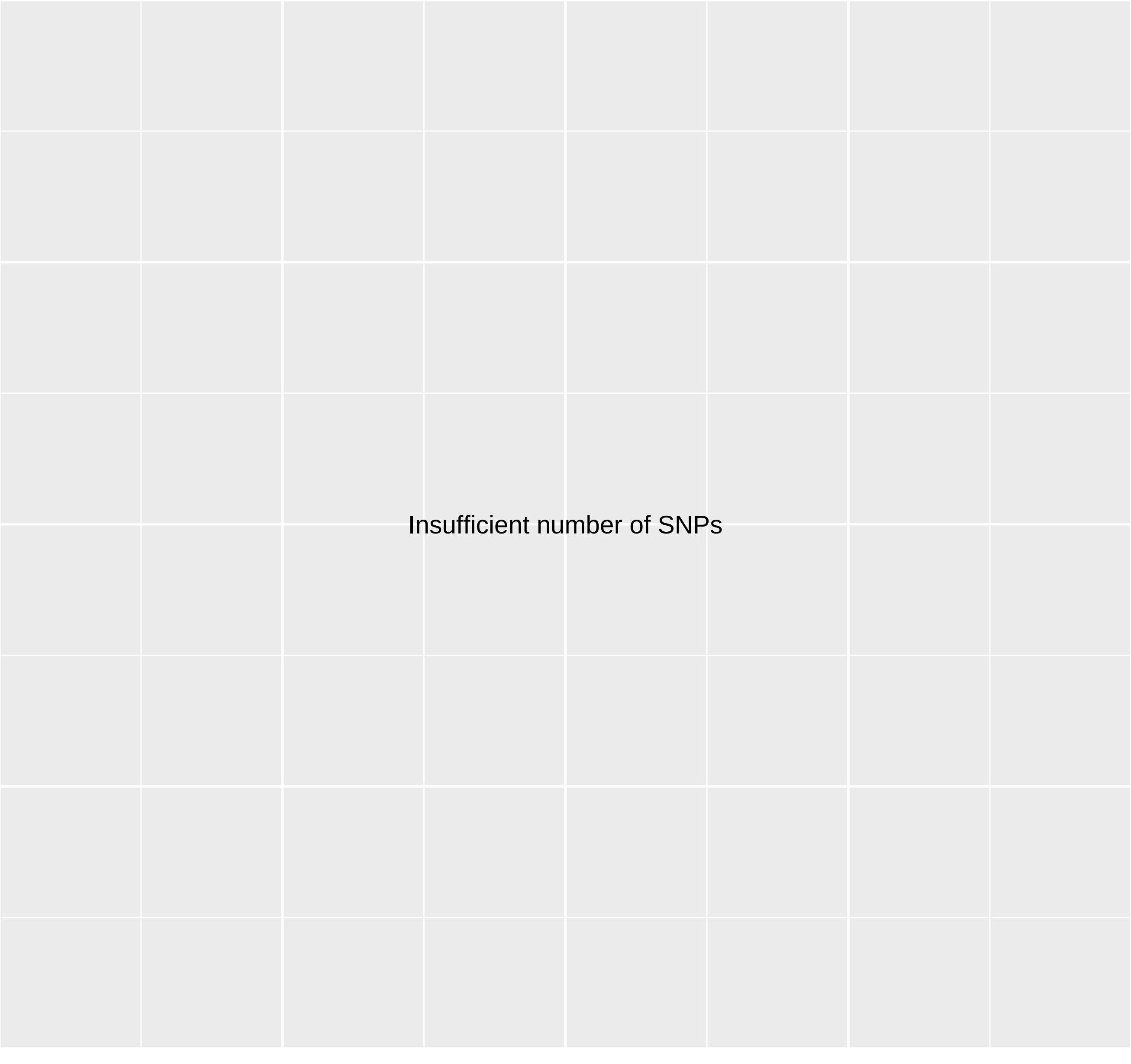
Insufficient number of SNPs

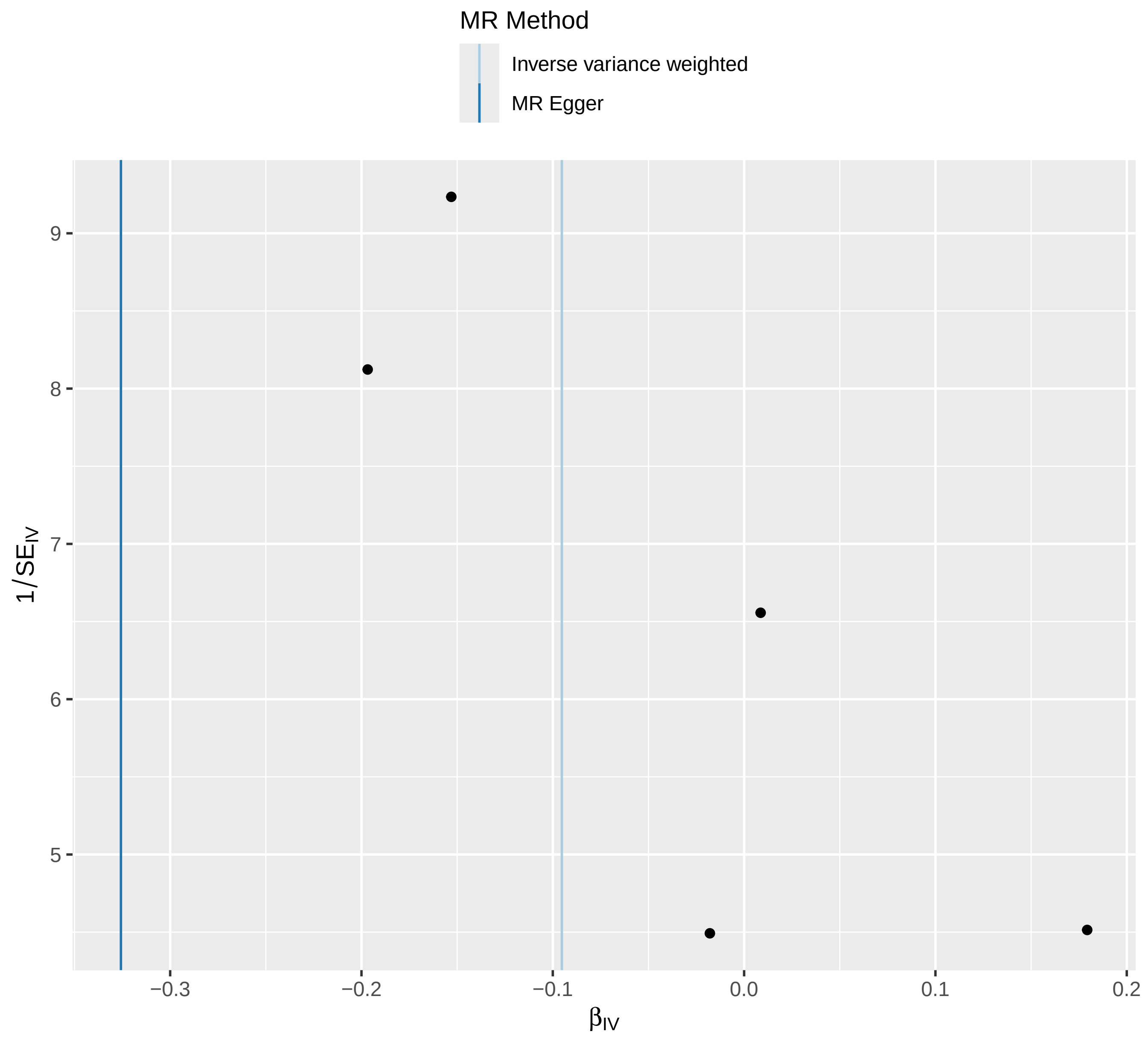

MR Method
Inverse variance weighted
MR Egger
9
8
7
6
5
1/SE_IV
−0.3
−0.2
−0.1
0.0
0.1
0.2
β_IV

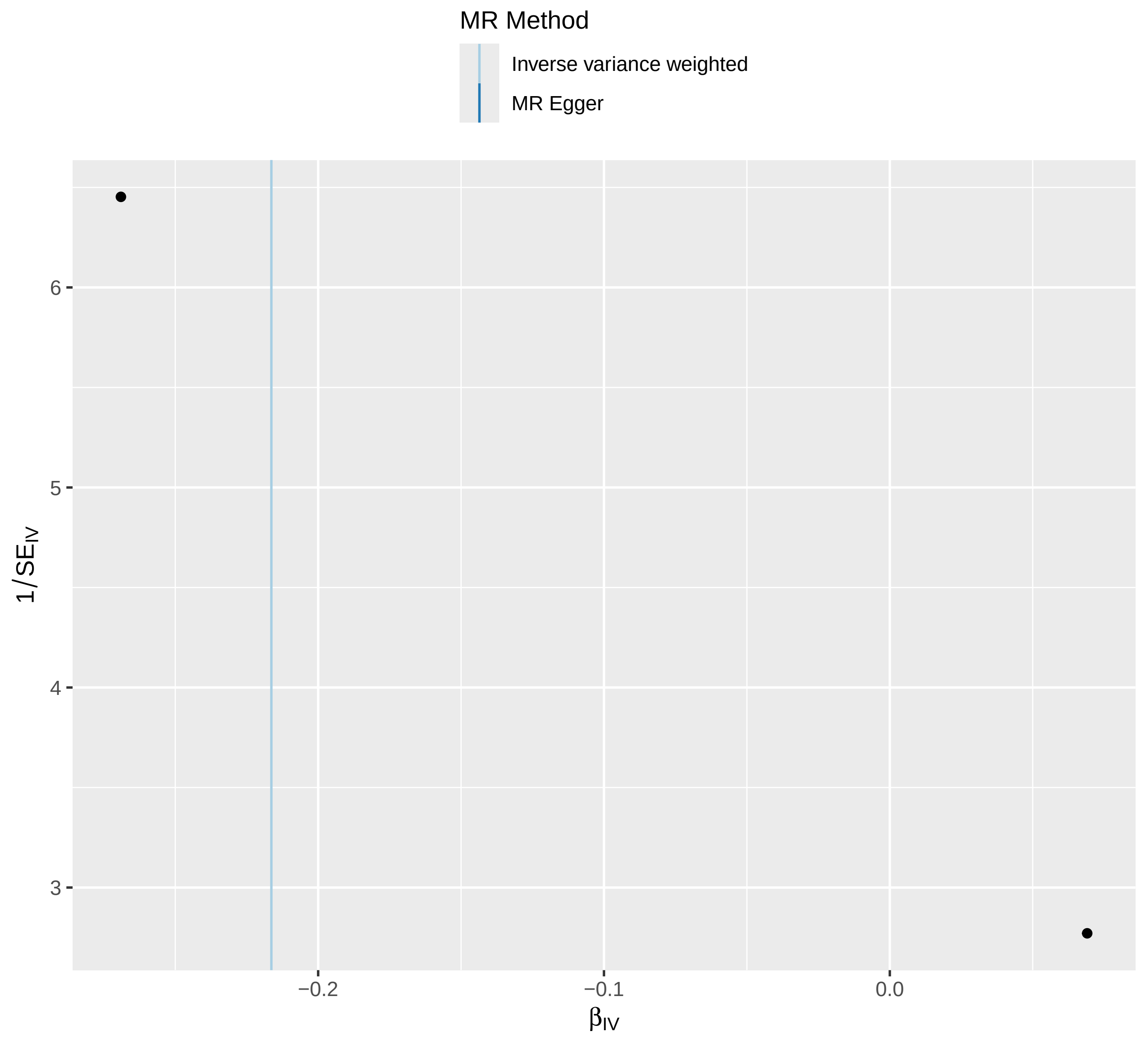
MR Method
Inverse variance weighted
MR Egger
6
5
4
3
1/SE$_{IV}$
−0.2
−0.1
0.0
β$_{IV}$

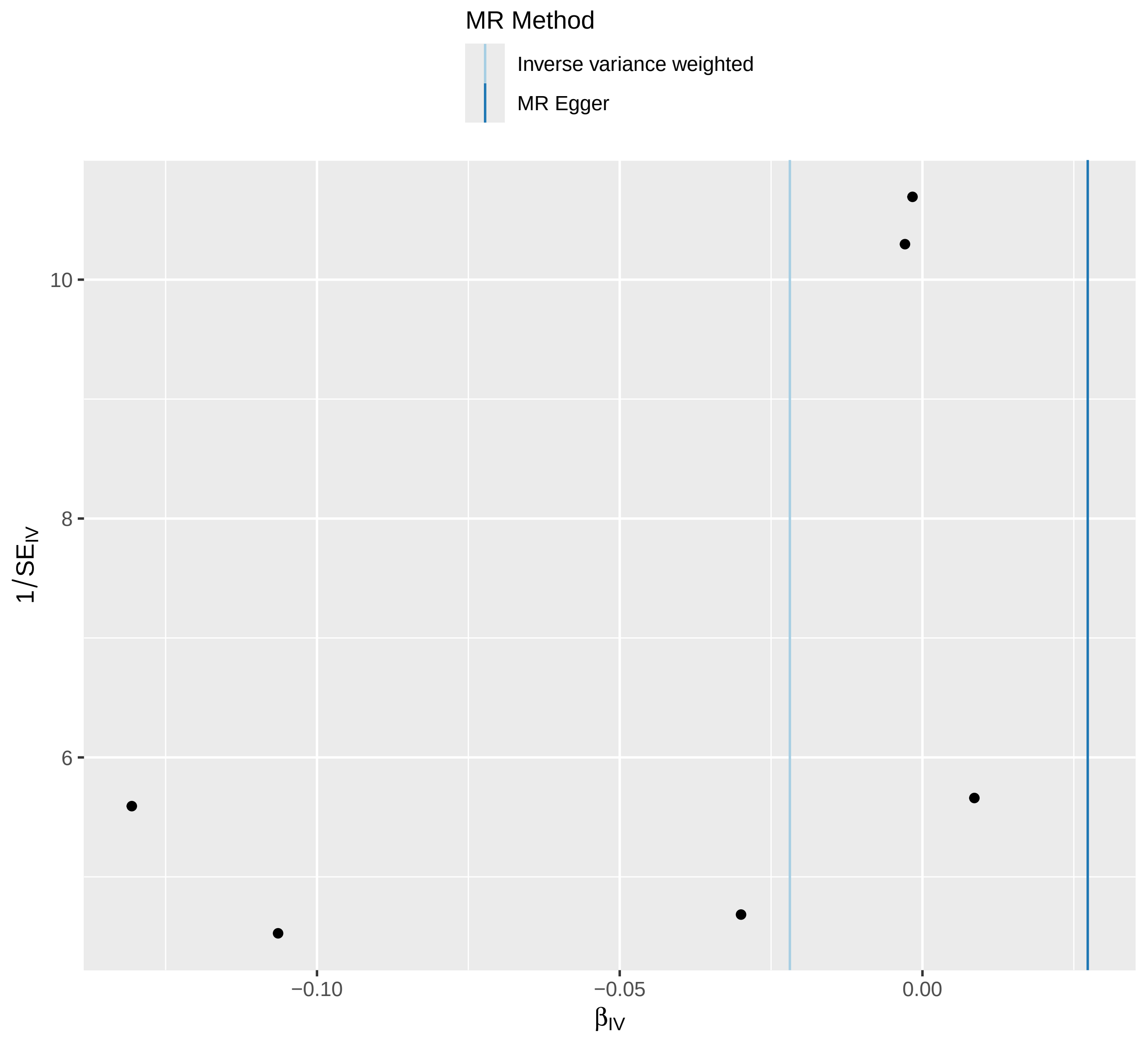

MR Method
Inverse variance weighted
MR Egger
10
8
6
1/SE$_{IV}$
−0.10
−0.05
0.00
β$_{IV}$

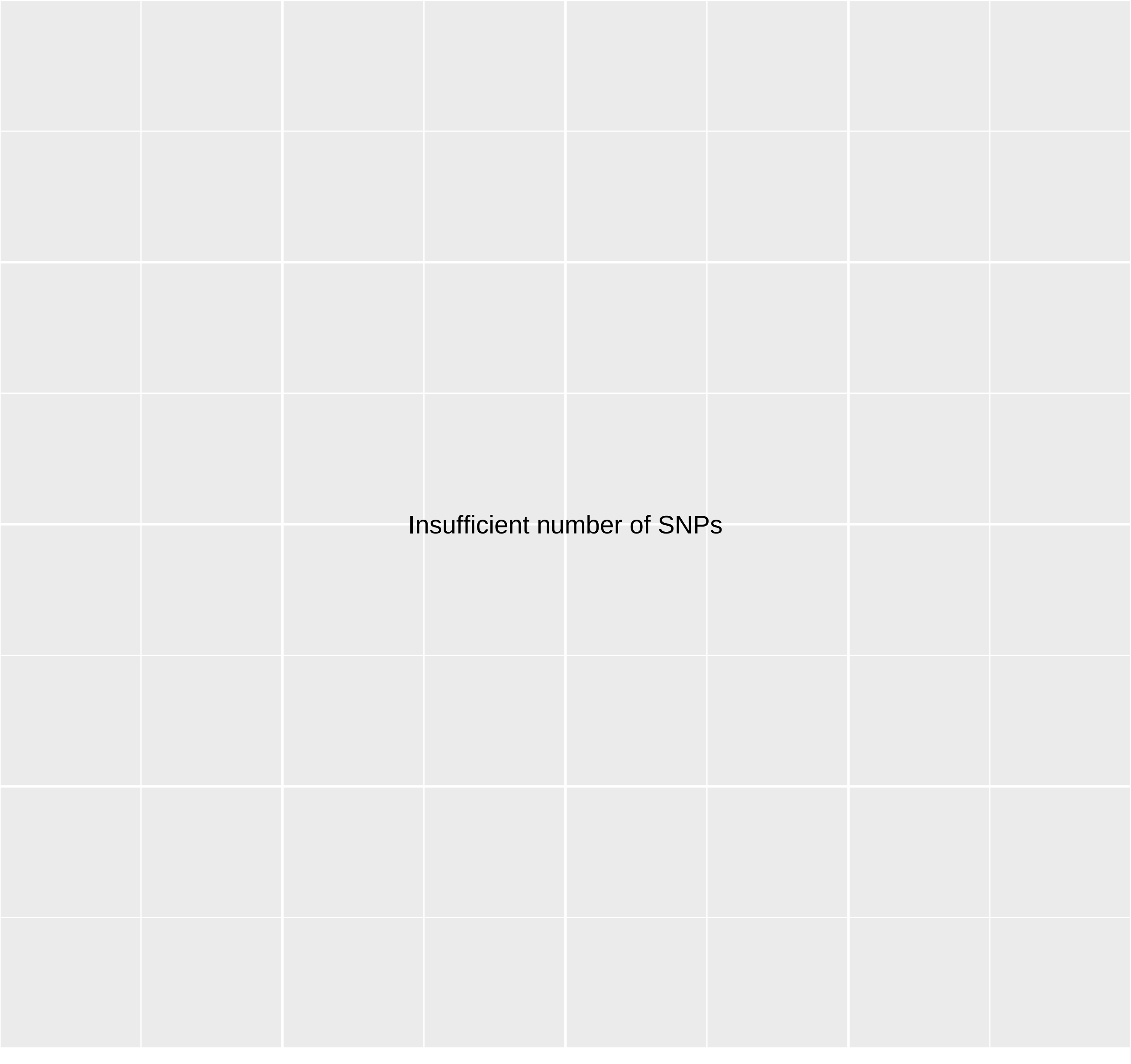
Insufficient number of SNPs

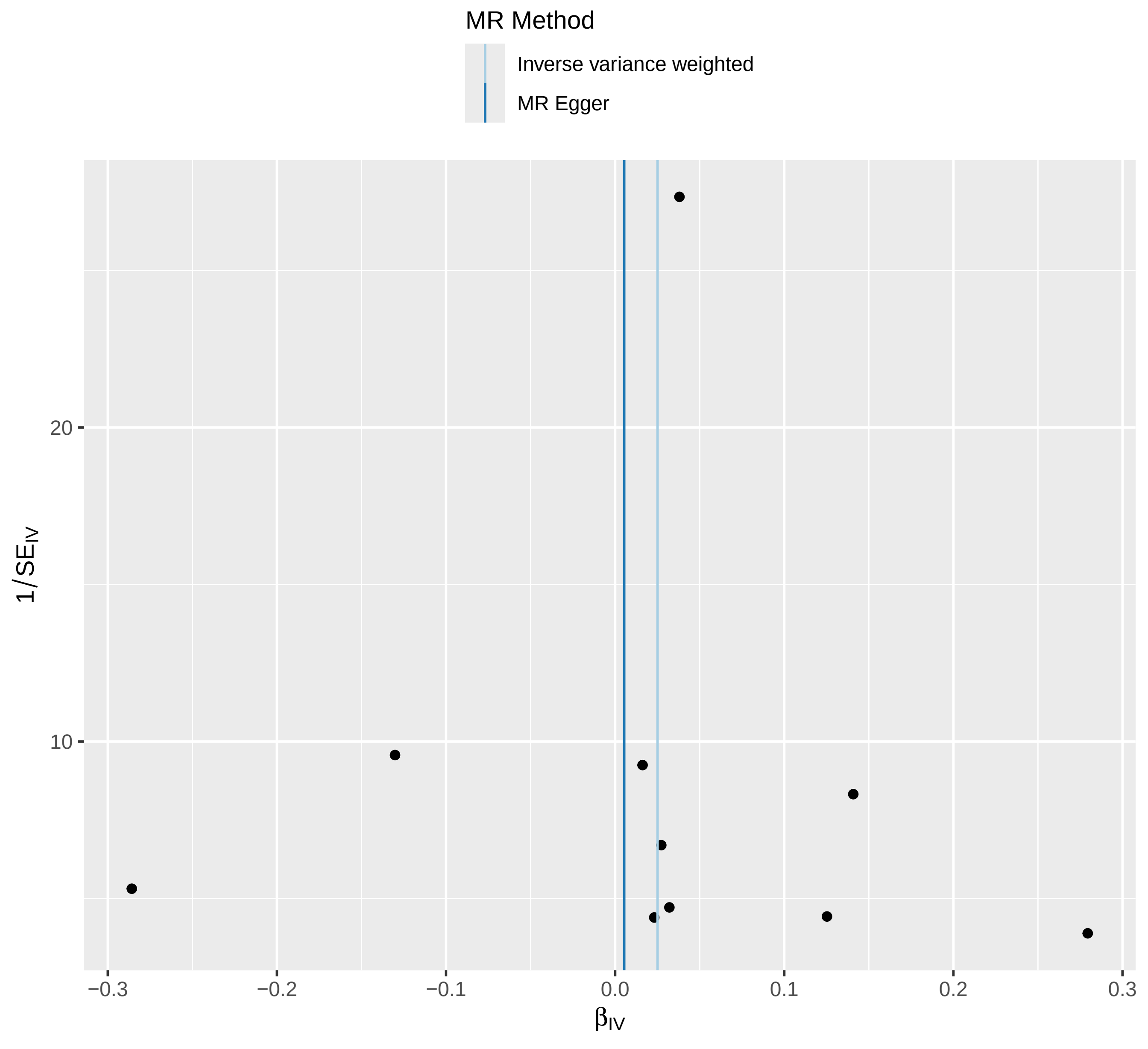
MR Method
Inverse variance weighted
MR Egger
1/SE_IV
20
10
−0.3
−0.2
−0.1
0.0
0.1
0.2
0.3
β_IV

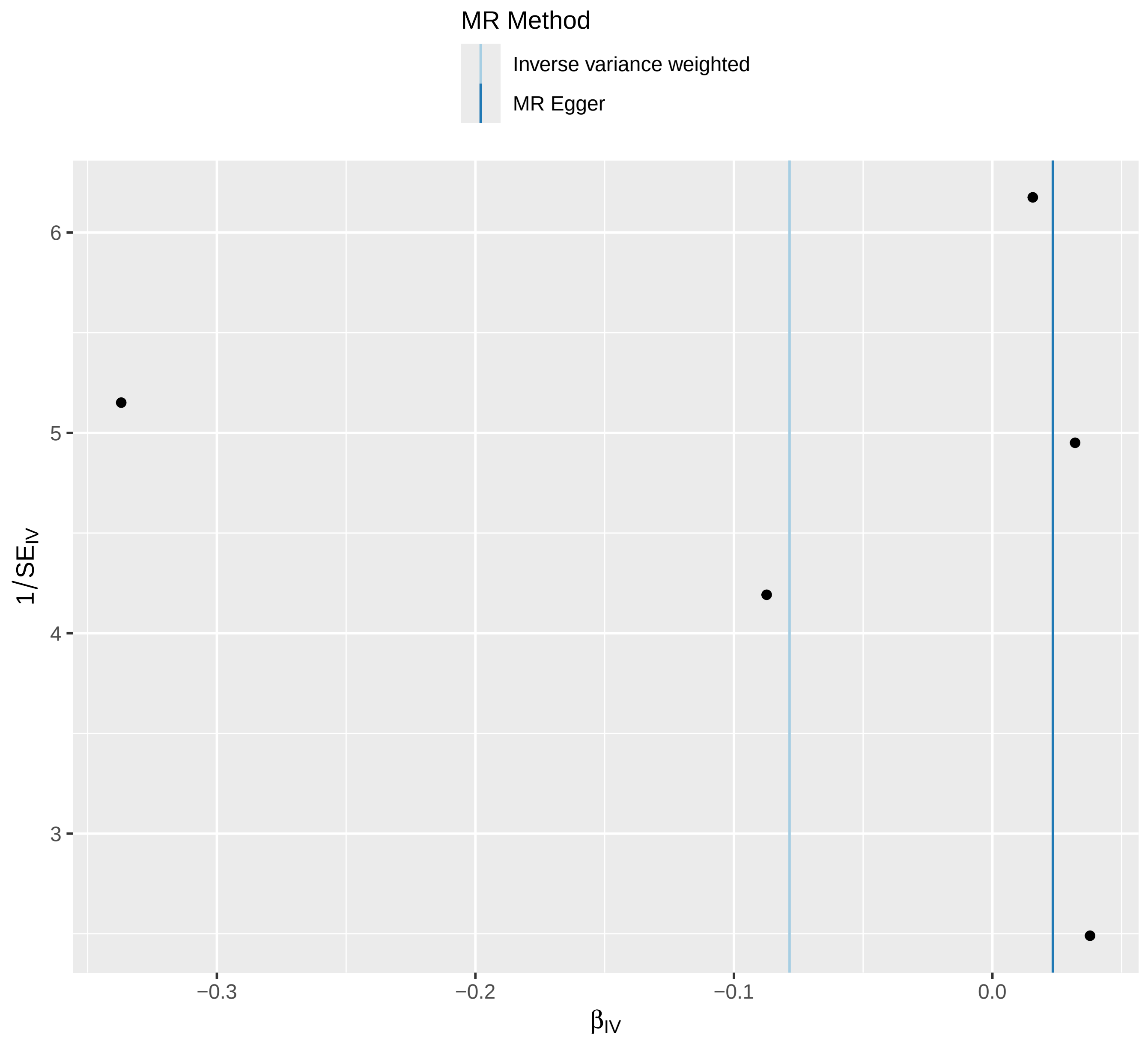

MR Method
Inverse variance weighted
MR Egger
6
5
4
3
1/SE_IV
−0.3
−0.2
−0.1
0.0
β_IV

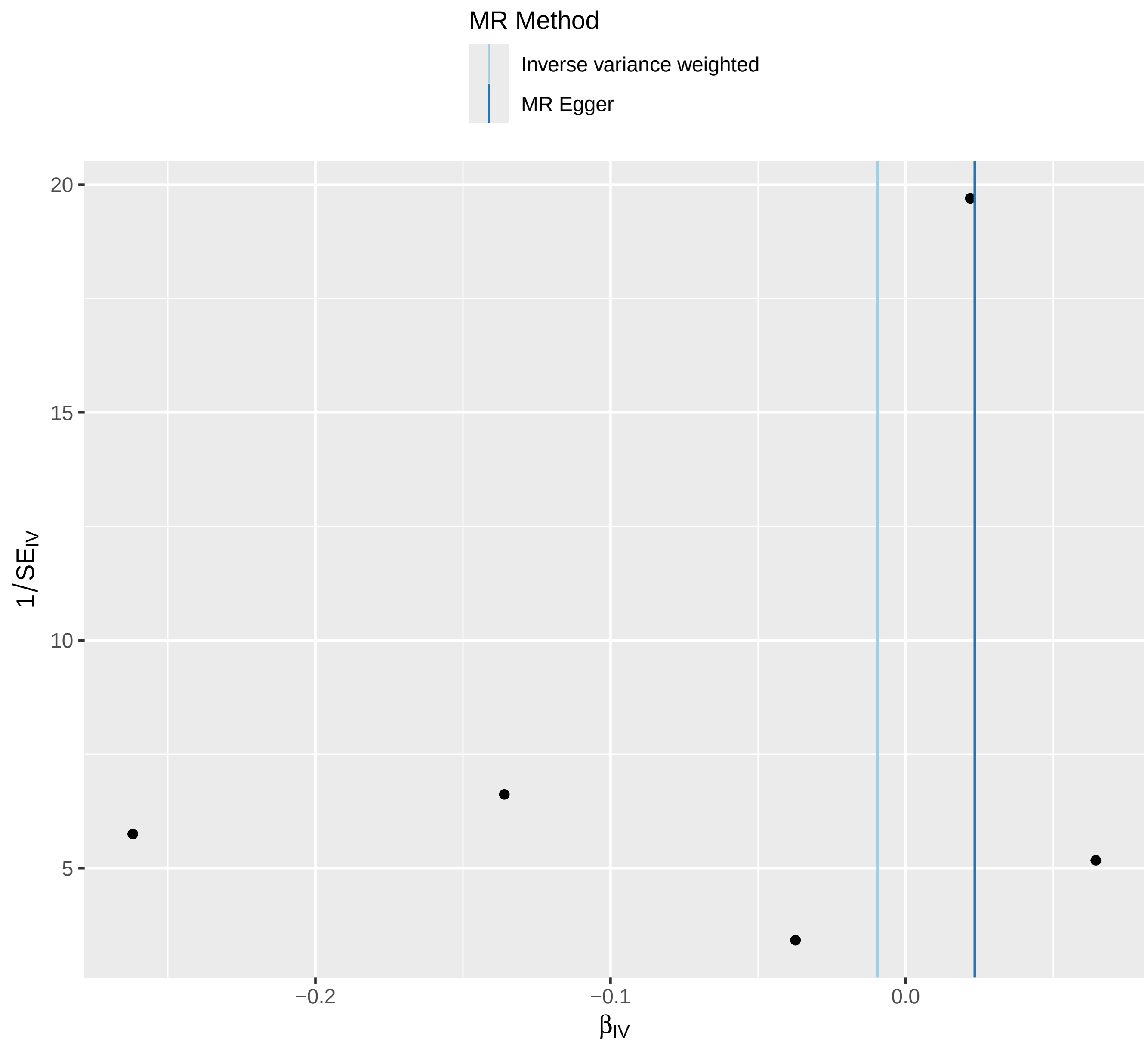

MR Method
Inverse variance weighted
MR Egger
20
15
10
5
1/SE$_{IV}$
−0.2
−0.1
0.0
β$_{IV}$

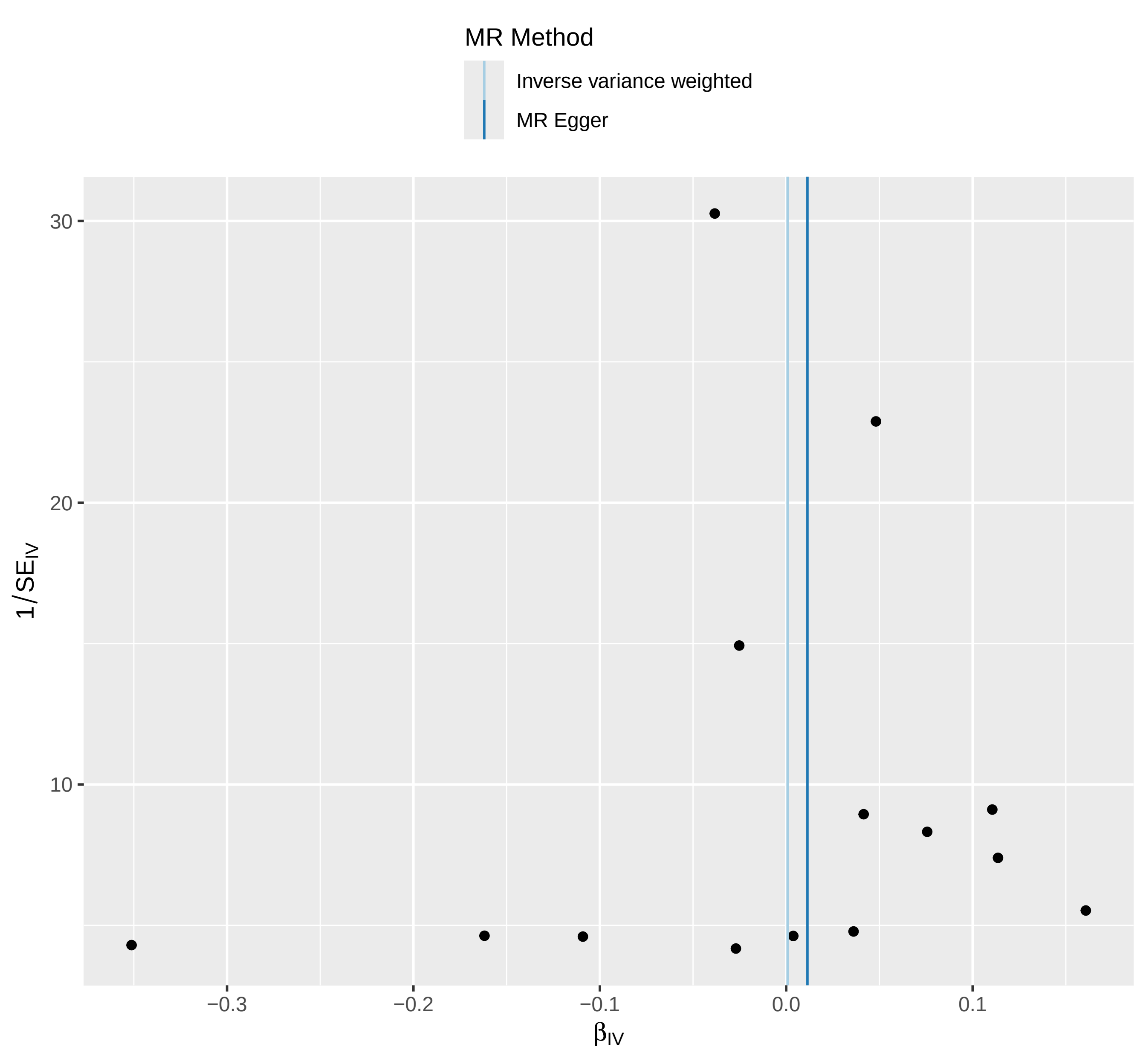

MR Method
Inverse variance weighted
MR Egger
1/SE_IV
30
20
10
−0.3
−0.2
−0.1
0.0
0.1
β_IV

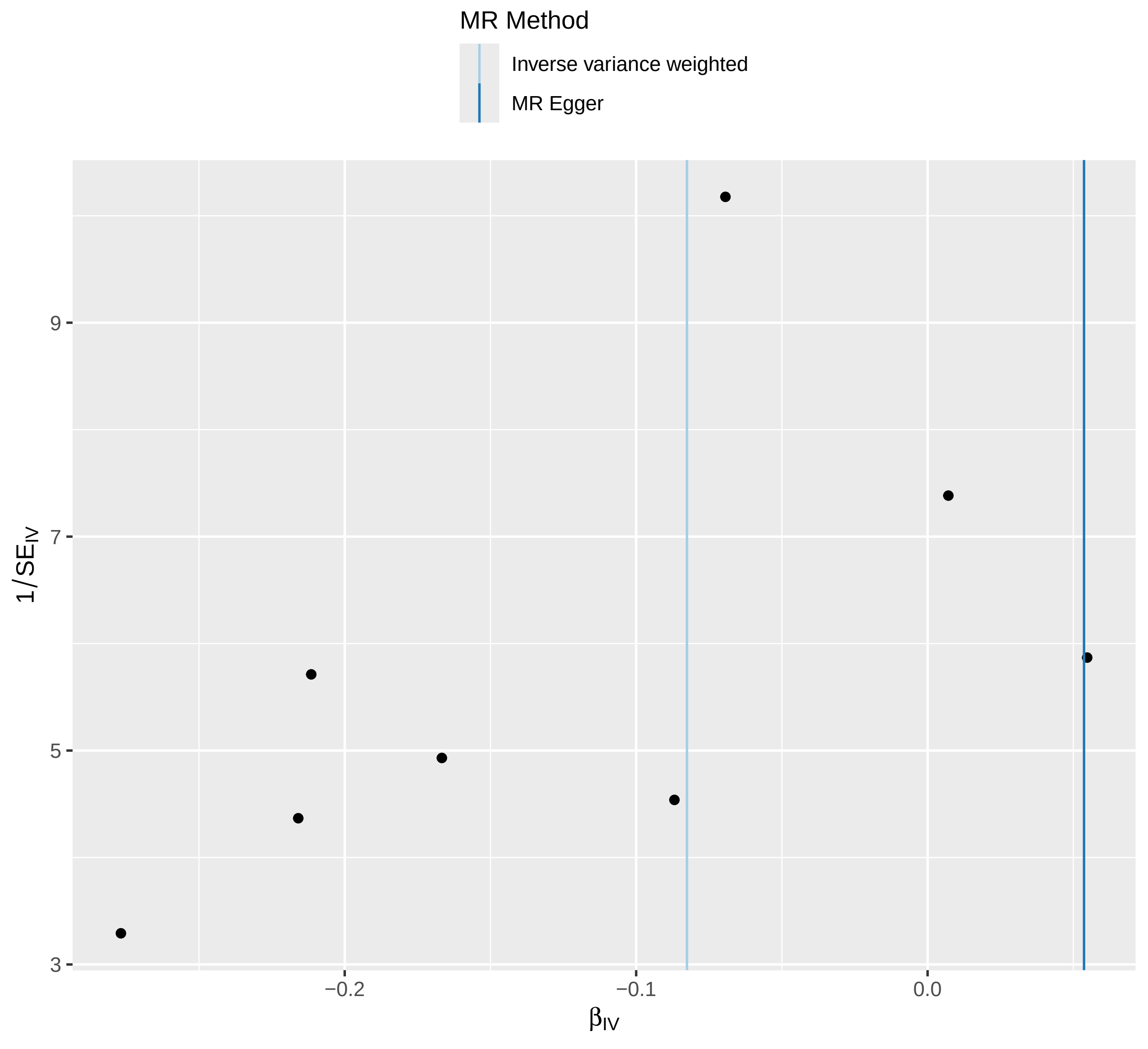
MR Method
Inverse variance weighted
MR Egger
9
7
5
3
1/SE$_{IV}$
−0.2
−0.1
0.0
β$_{IV}$

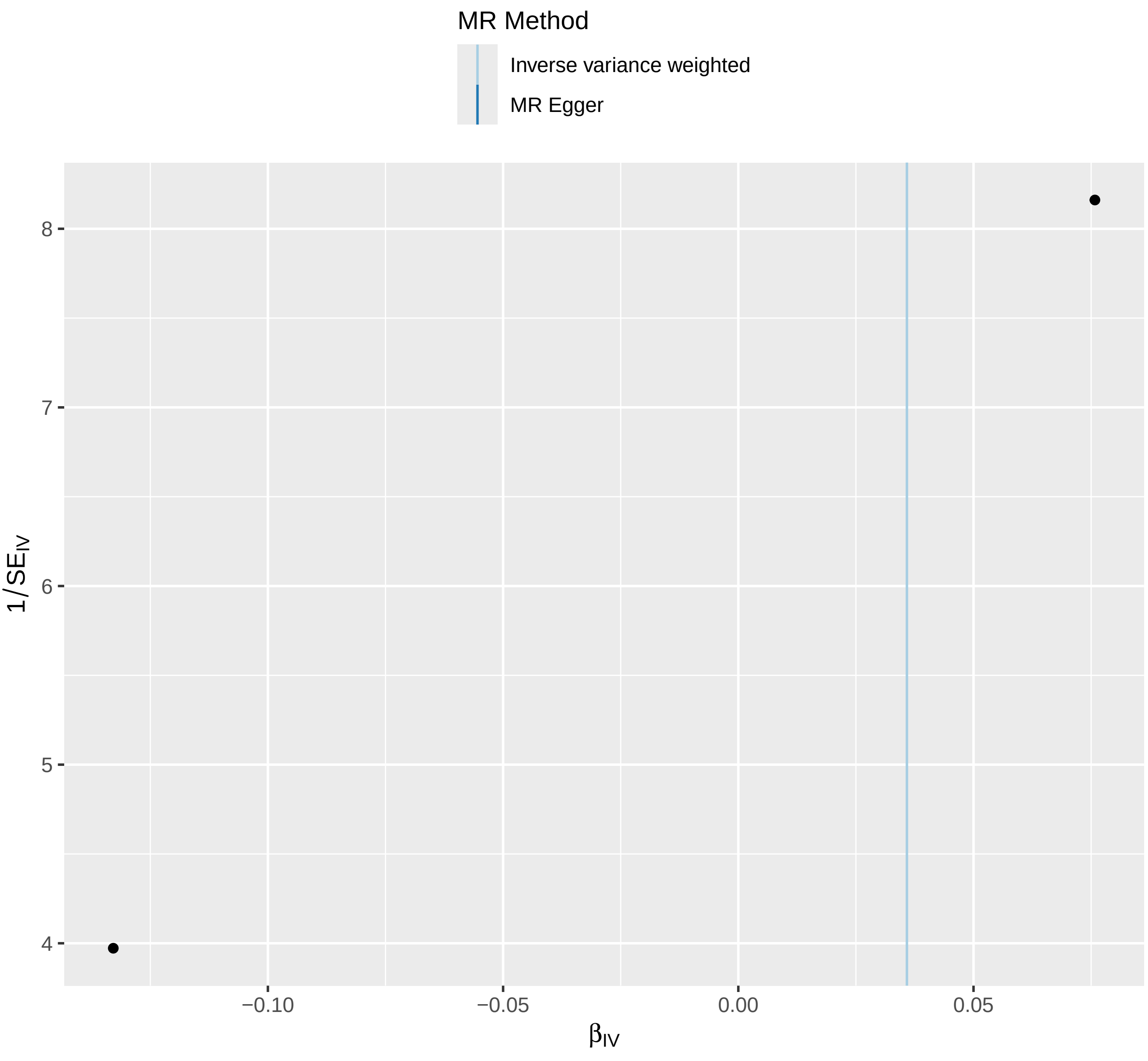

MR Method
Inverse variance weighted
MR Egger
8
7
6
5
4
1/SE$_{IV}$
−0.10
−0.05
0.00
0.05
β$_{IV}$

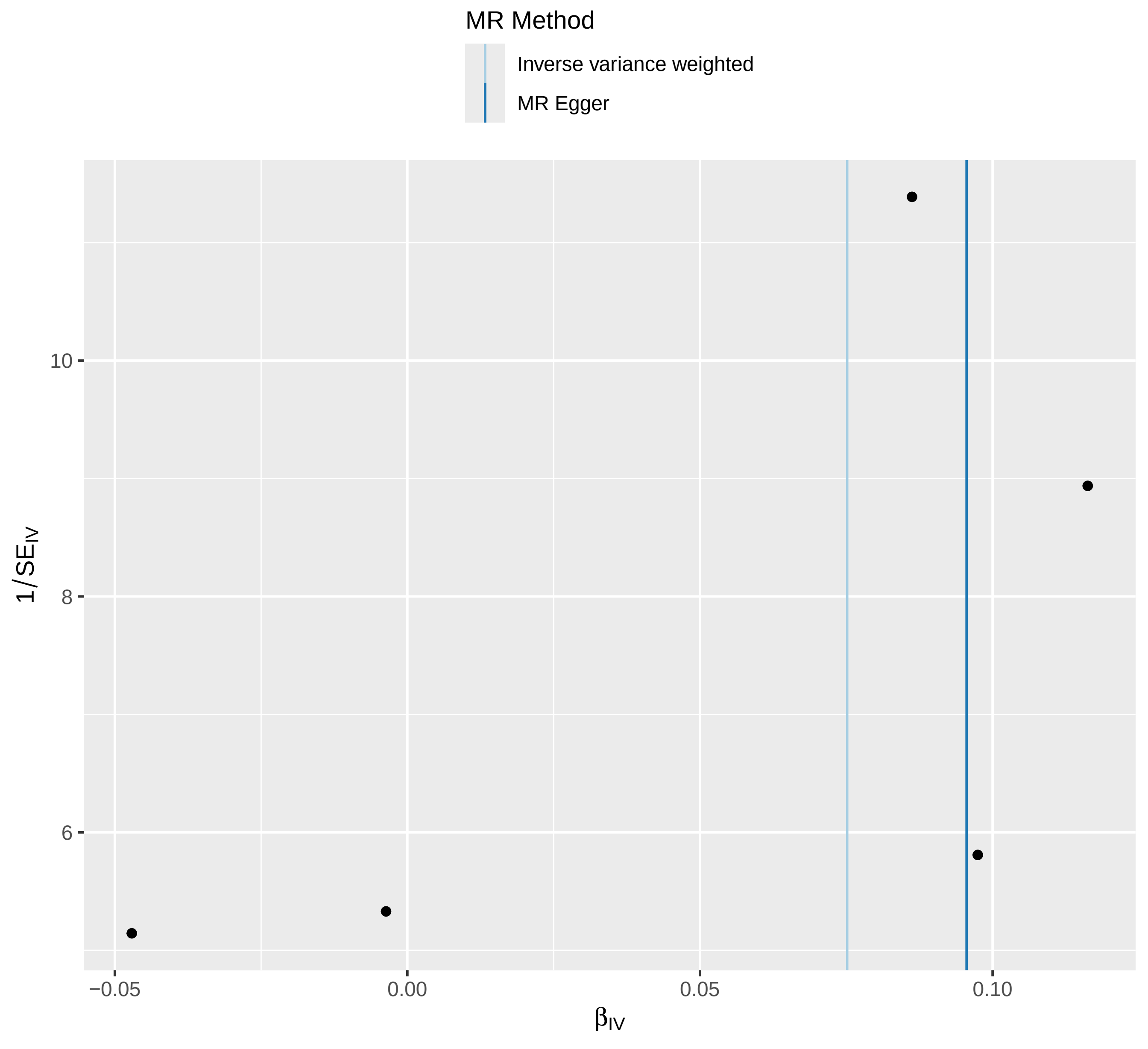
MR Method
Inverse variance weighted
MR Egger
1/SE$_{IV}$
10
8
6
−0.05
0.00
0.05
0.10
β$_{IV}$

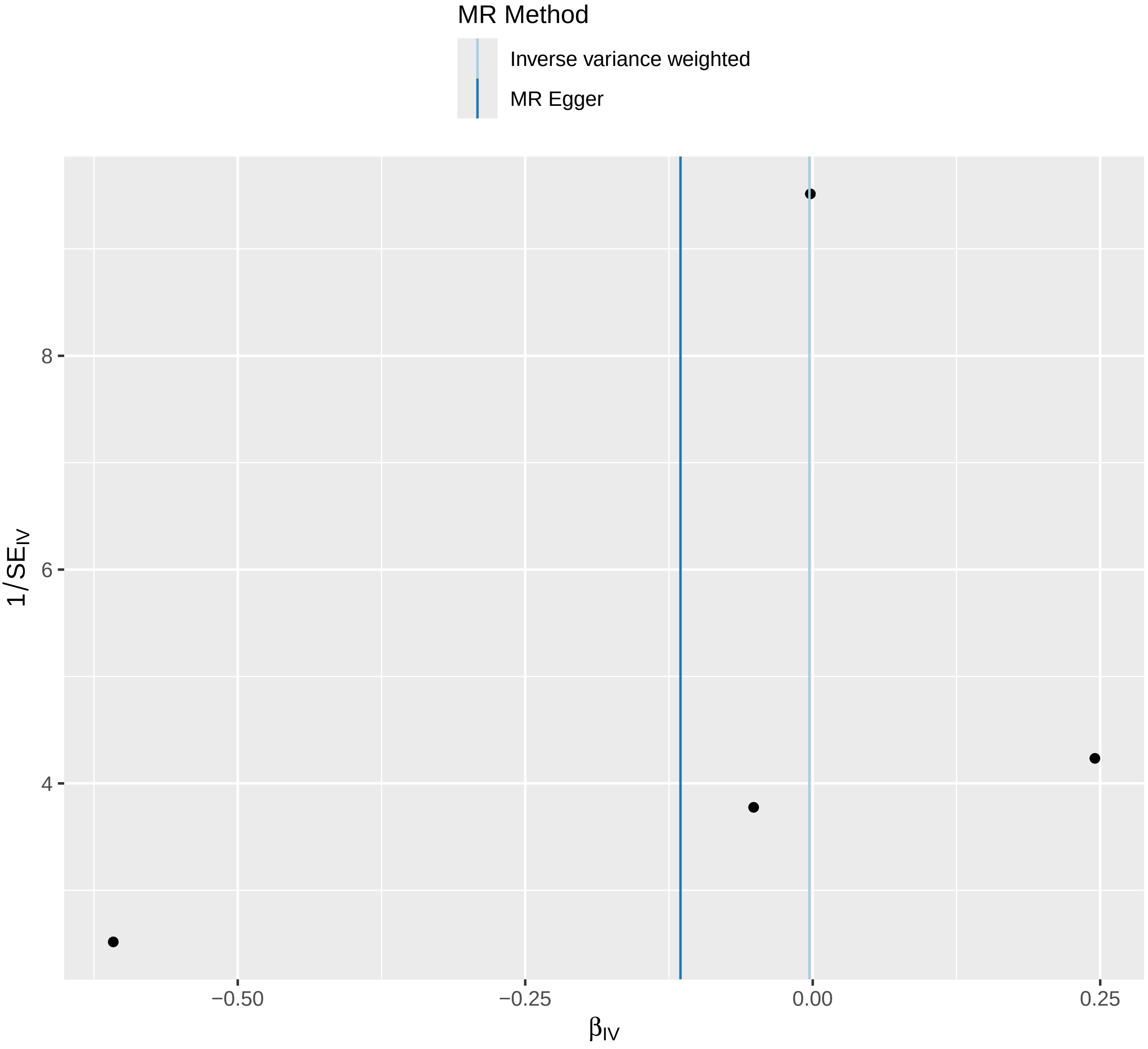
MR Method
Inverse variance weighted
MR Egger
8
6
4
−0.50
−0.25
0.00
0.25
$1/SE_{IV}$
$\beta_{IV}$

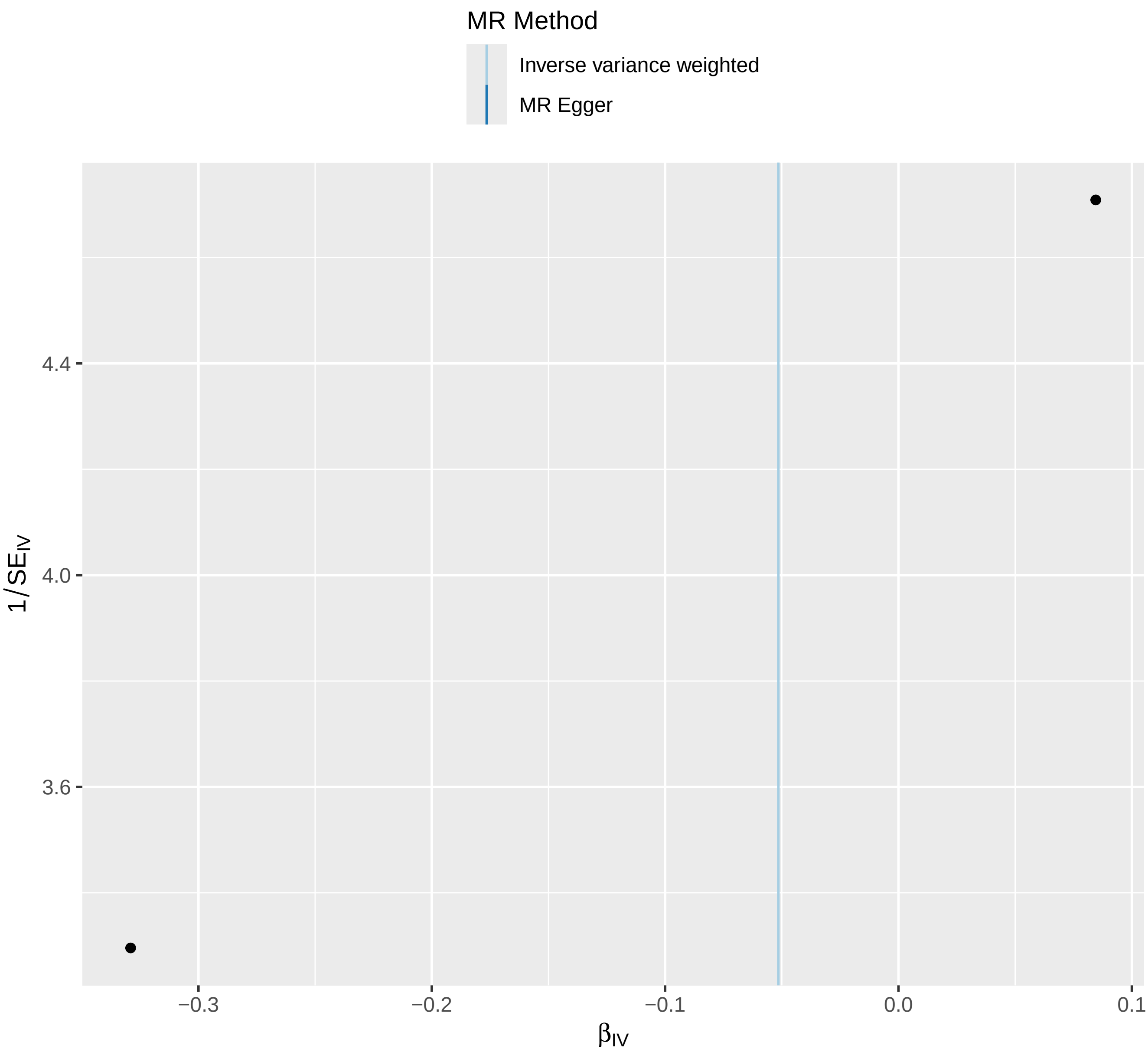
MR Method
Inverse variance weighted
MR Egger
4.4
4.0
3.6
1/SE$_{IV}$
−0.3
−0.2
−0.1
0.0
0.1
β$_{IV}$

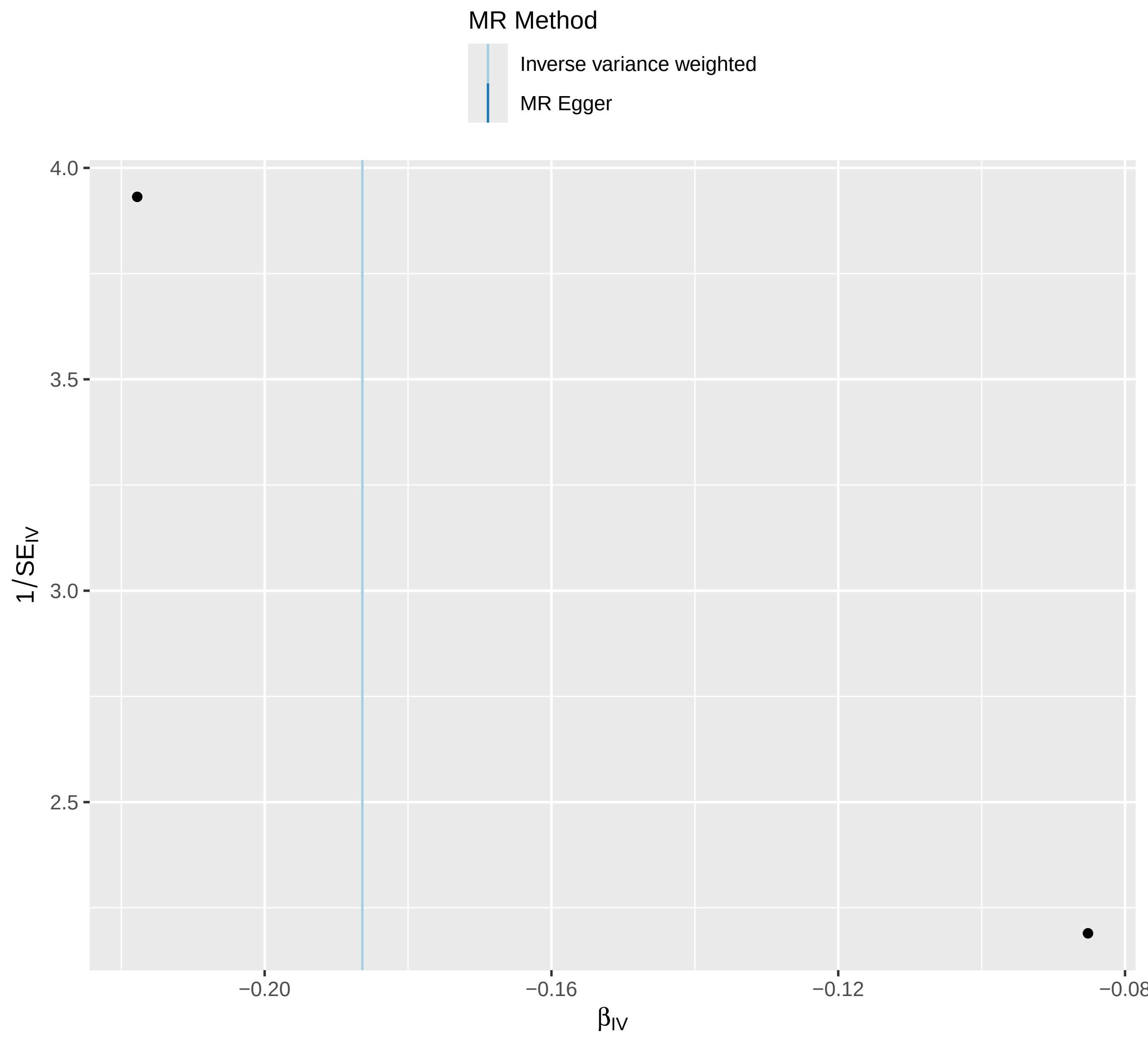
MR Method
Inverse variance weighted
MR Egger
4.0
3.5
3.0
2.5
$1/SE_{IV}$
−0.20
−0.16
−0.12
−0.08
$\beta_{IV}$

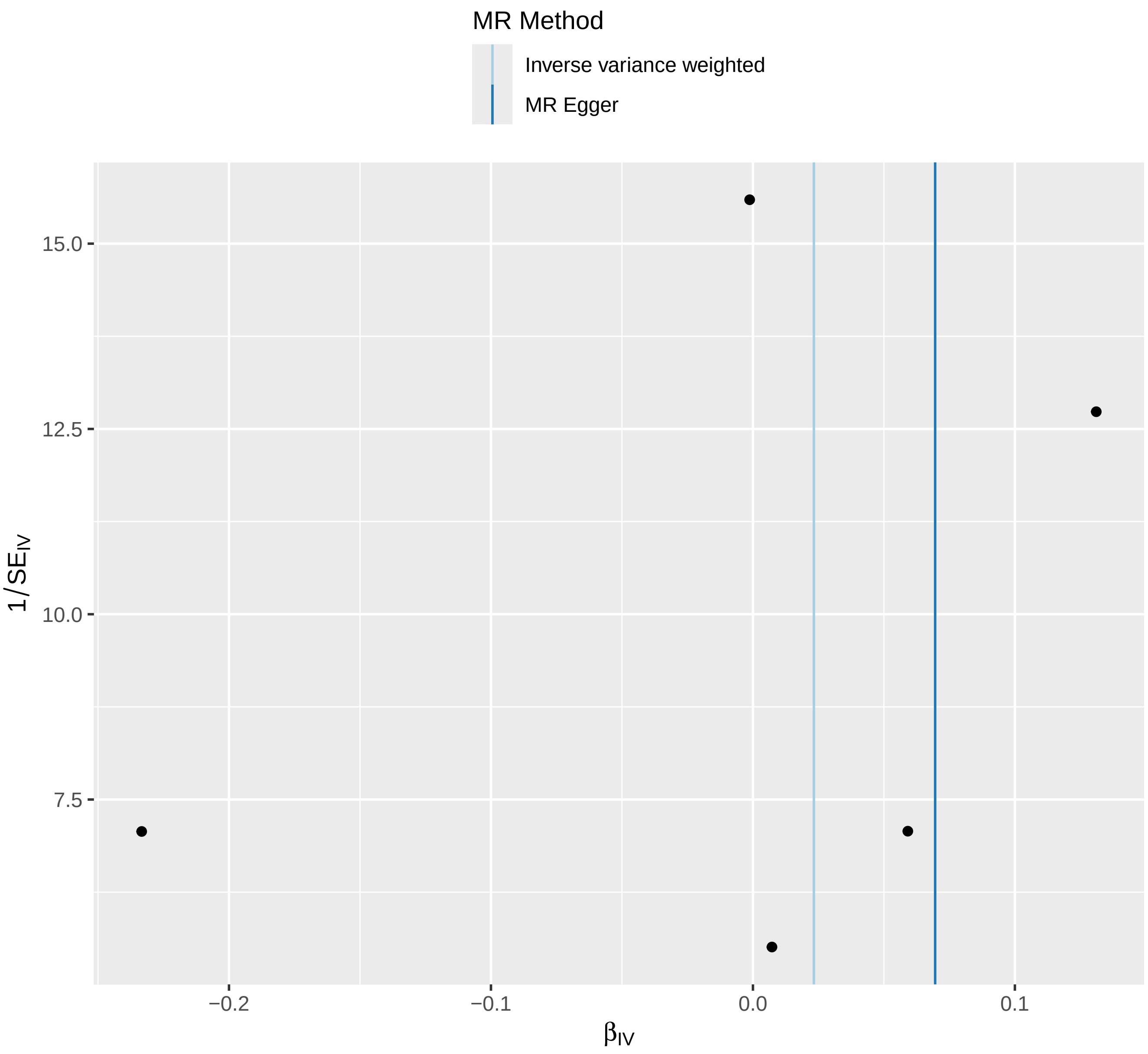
MR Method
Inverse variance weighted
MR Egger
15.0
12.5
10.0
7.5
1/SE_IV
−0.2
−0.1
0.0
0.1
β_IV

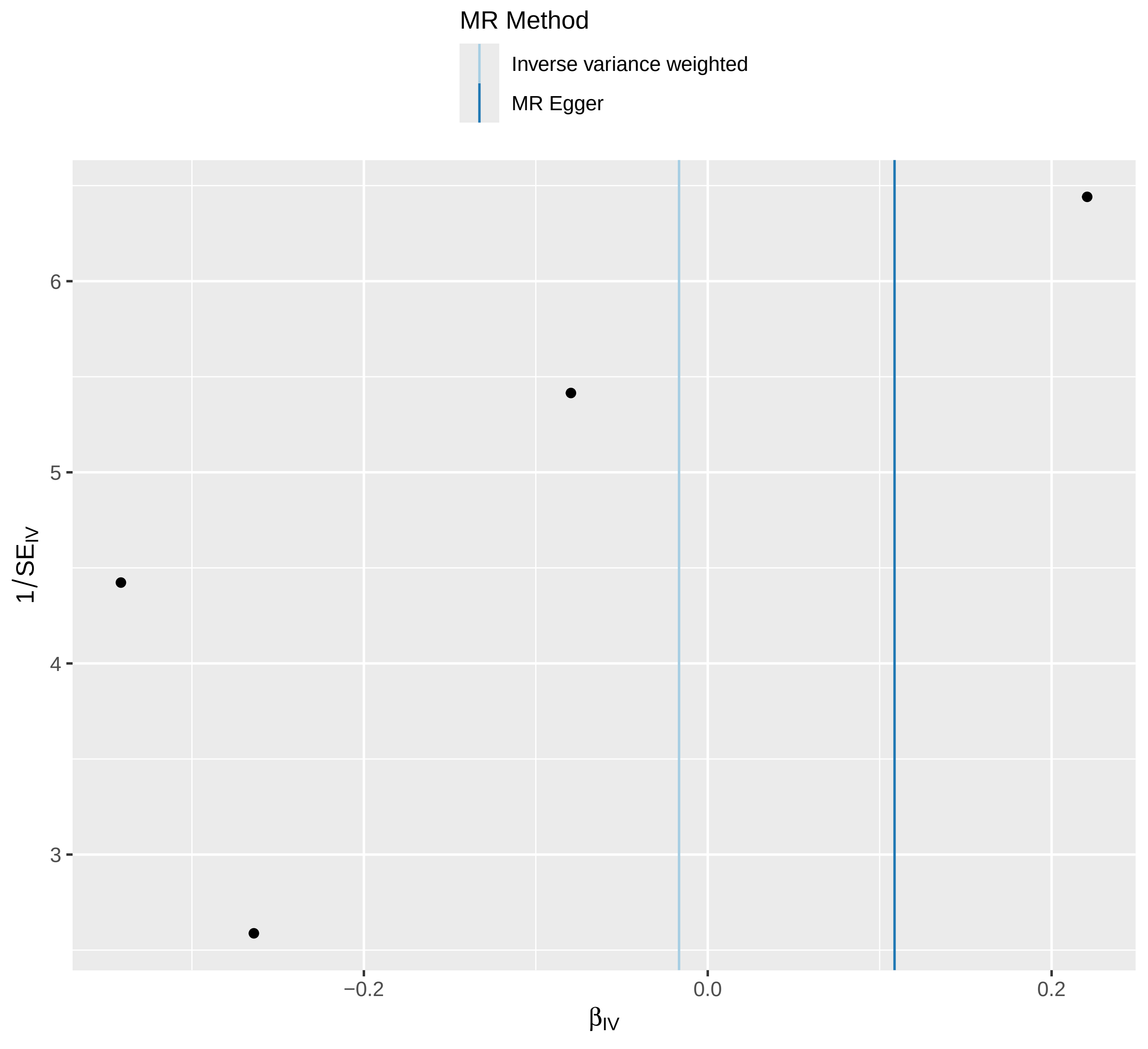
MR Method
Inverse variance weighted
MR Egger
1/SE$_{IV}$
6
5
4
3
−0.2
0.0
0.2
β$_{IV}$

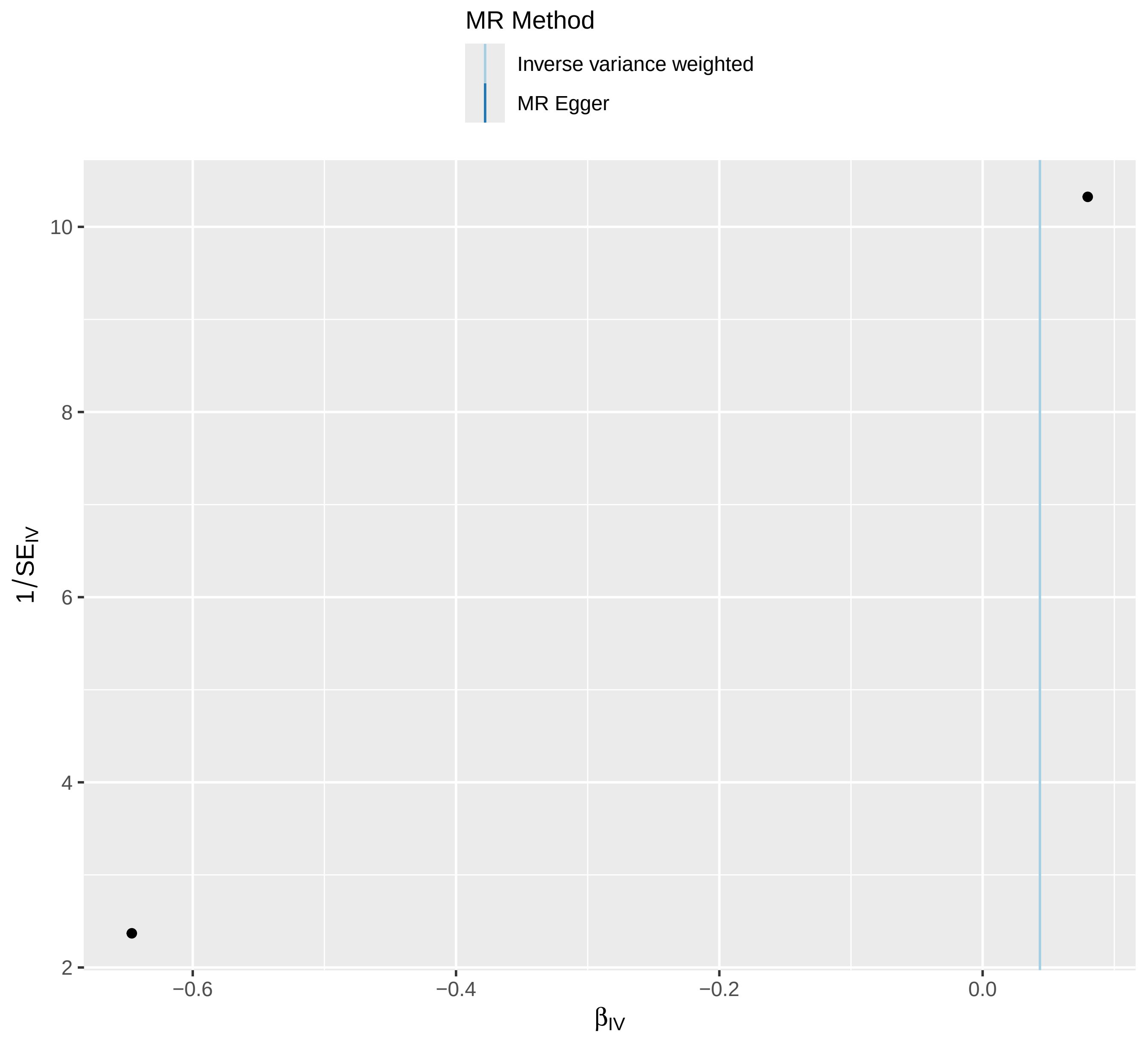

MR Method
Inverse variance weighted
MR Egger
10
8
6
4
2
1/SE$_{IV}$
−0.6
−0.4
−0.2
0.0
β$_{IV}$

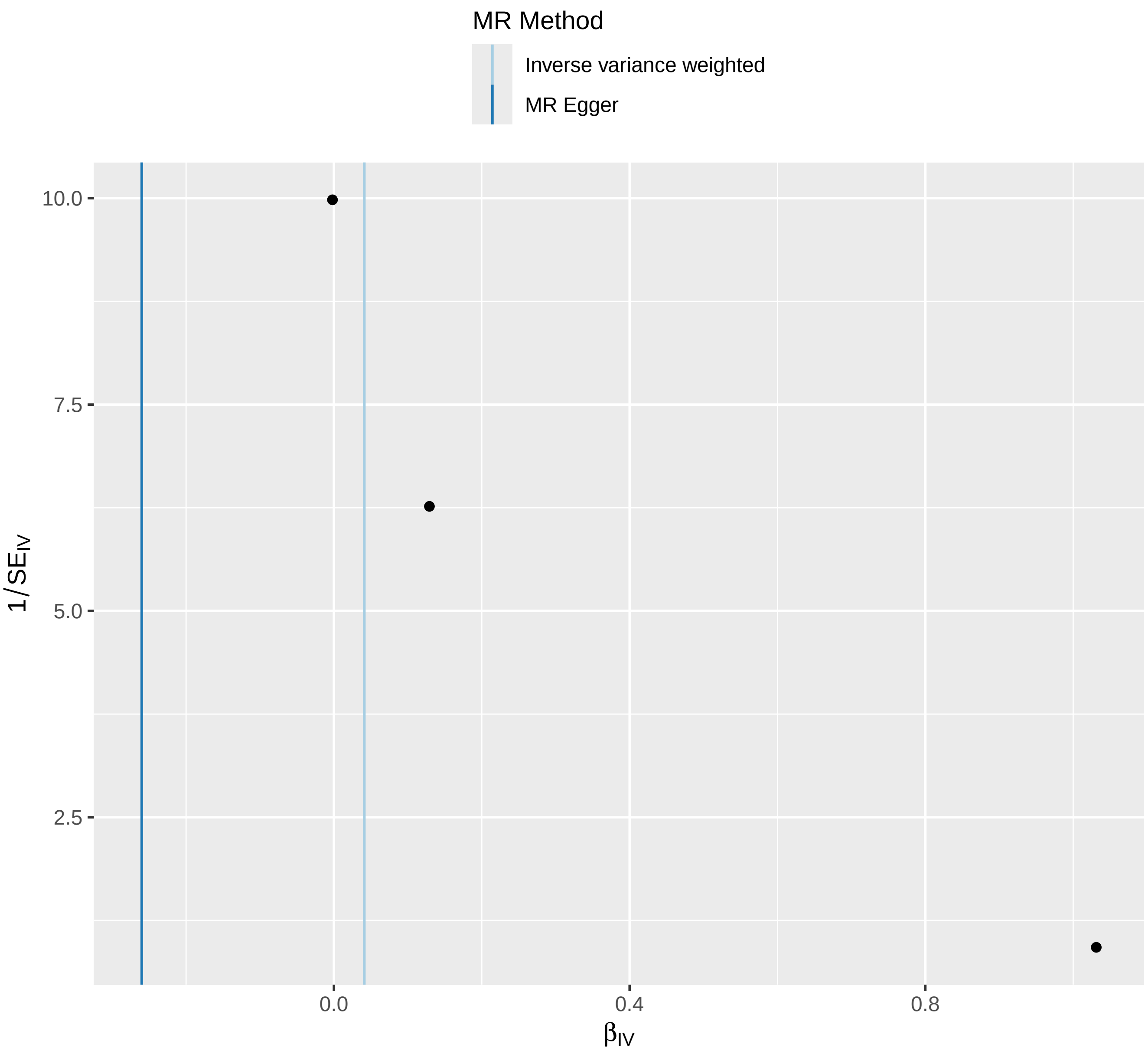

MR Method
Inverse variance weighted
MR Egger
10.0
7.5
5.0
2.5
0.0
0.4
0.8
$1/SE_{IV}$
$\beta_{IV}$

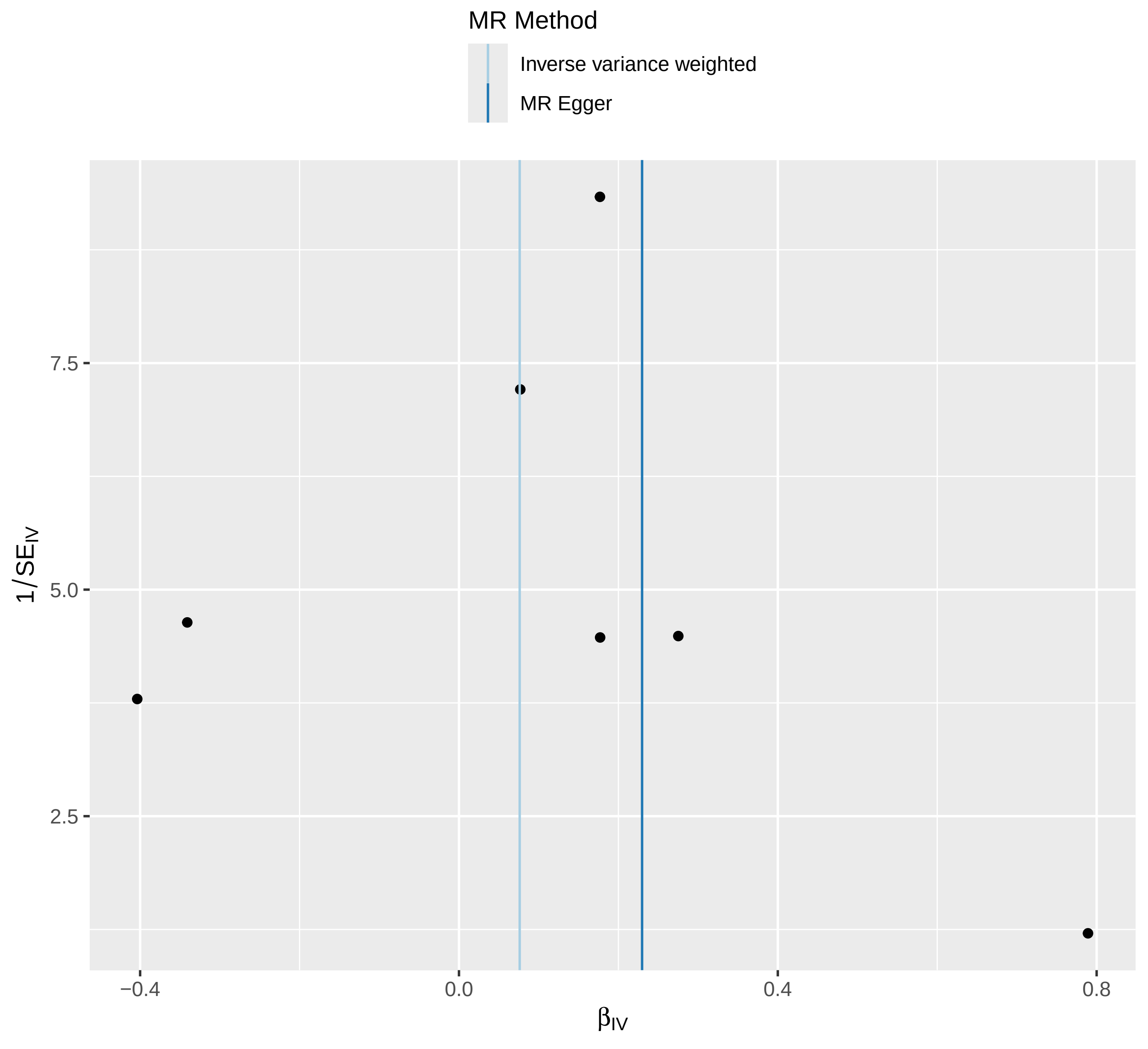
MR Method
Inverse variance weighted
MR Egger
7.5
5.0
2.5
1/SE_IV
−0.4
0.0
0.4
0.8
β_IV

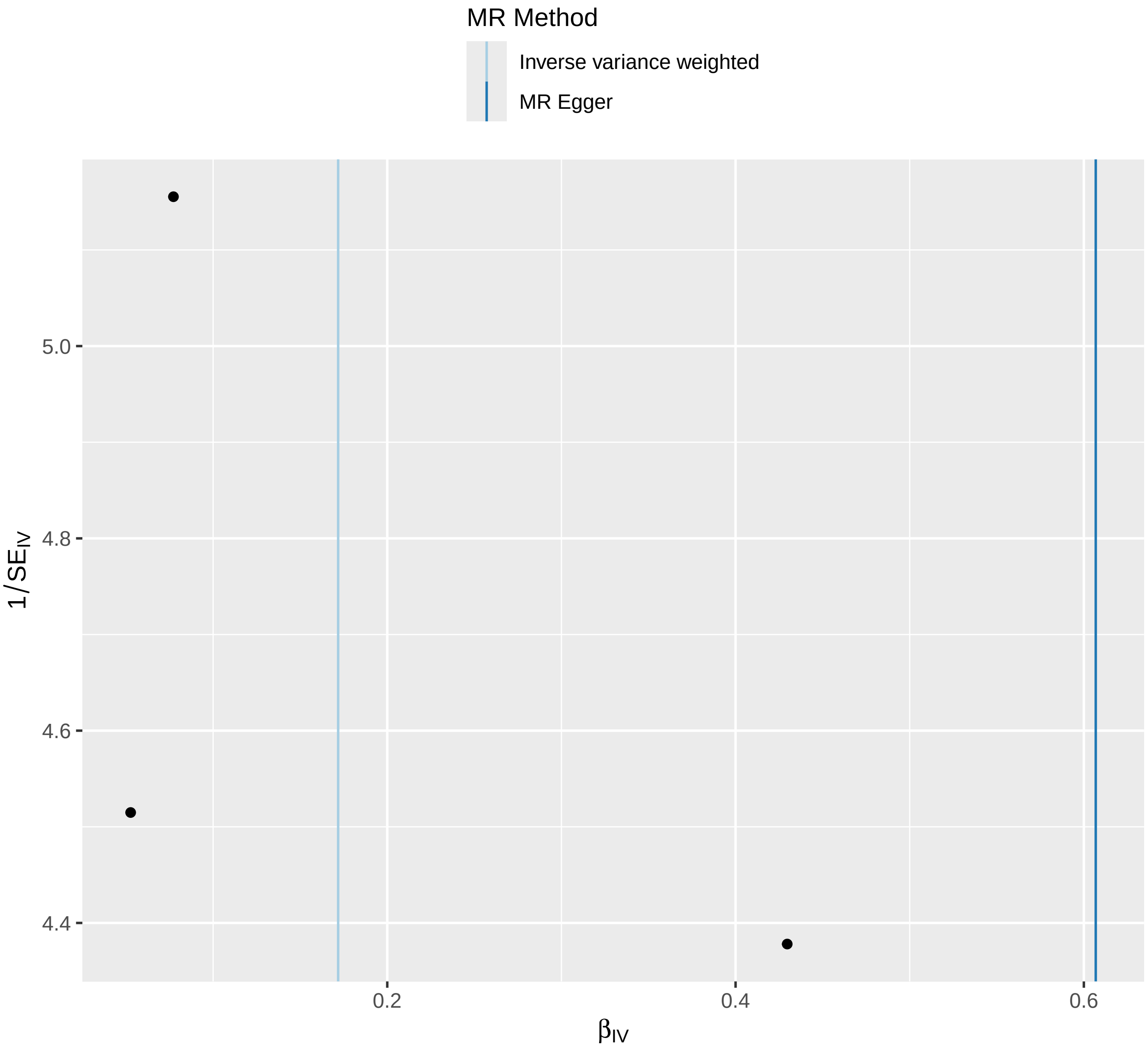

MR Method
Inverse variance weighted
MR Egger
5.0
4.8
4.6
4.4
$1/SE_{IV}$
0.2
0.4
0.6
$\beta_{IV}$

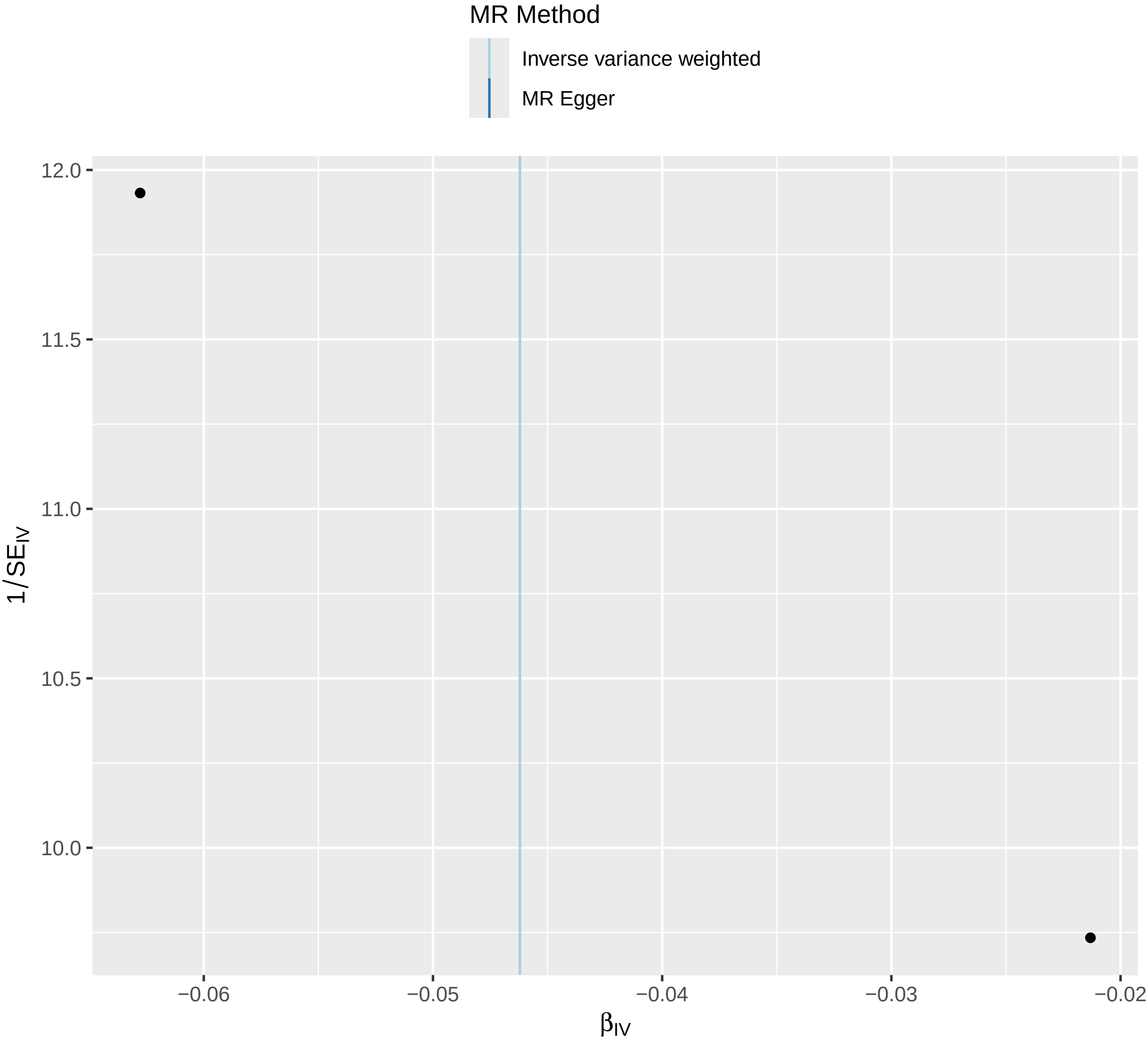
MR Method
Inverse variance weighted
MR Egger
12.0
11.5
11.0
10.5
10.0
$1/SE_{IV}$
−0.06
−0.05
−0.04
−0.03
−0.02
$\beta_{IV}$

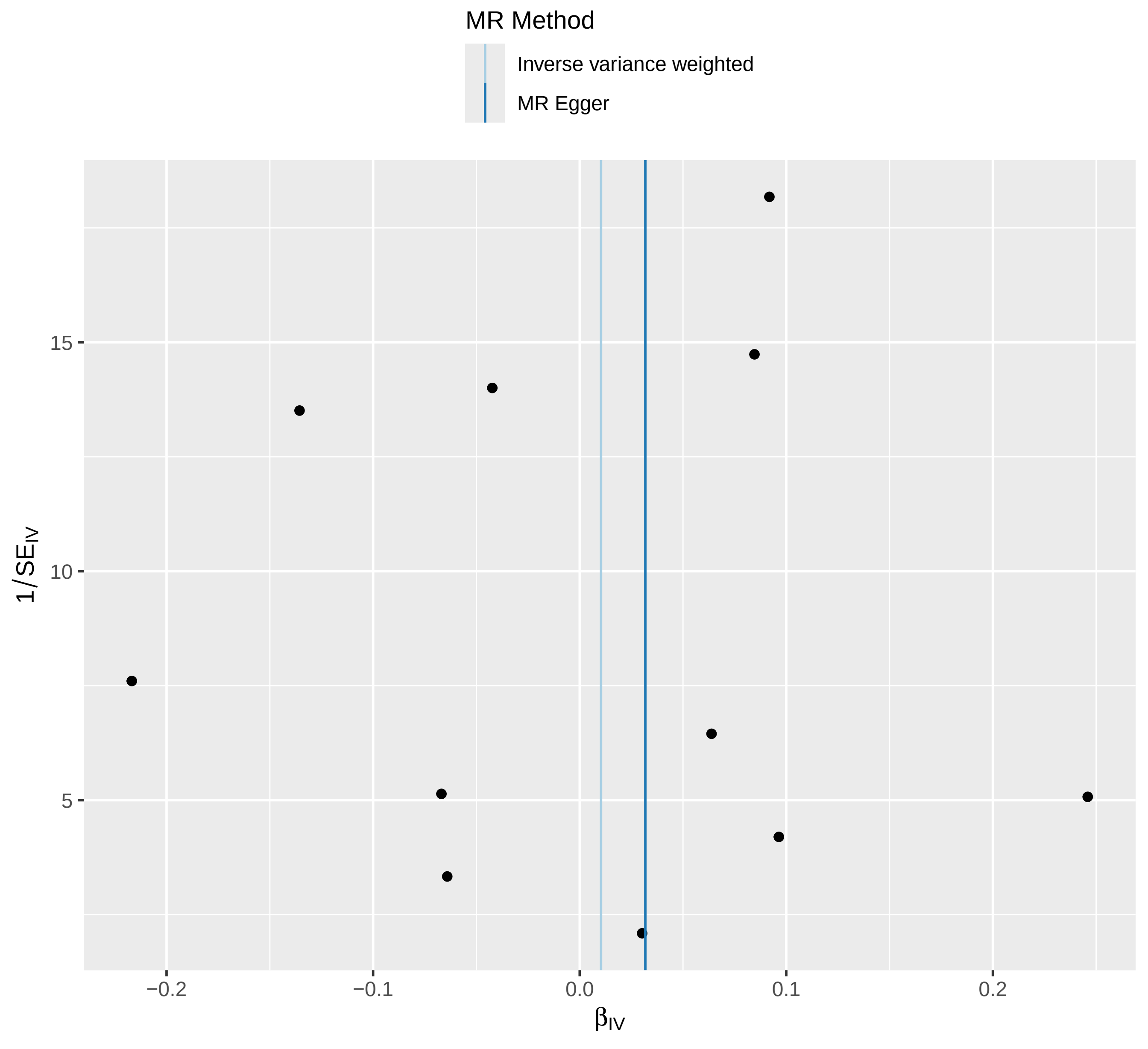

MR Method
Inverse variance weighted
MR Egger
15
10
5
1/SE_IV
−0.2
−0.1
0.0
0.1
0.2
β_IV

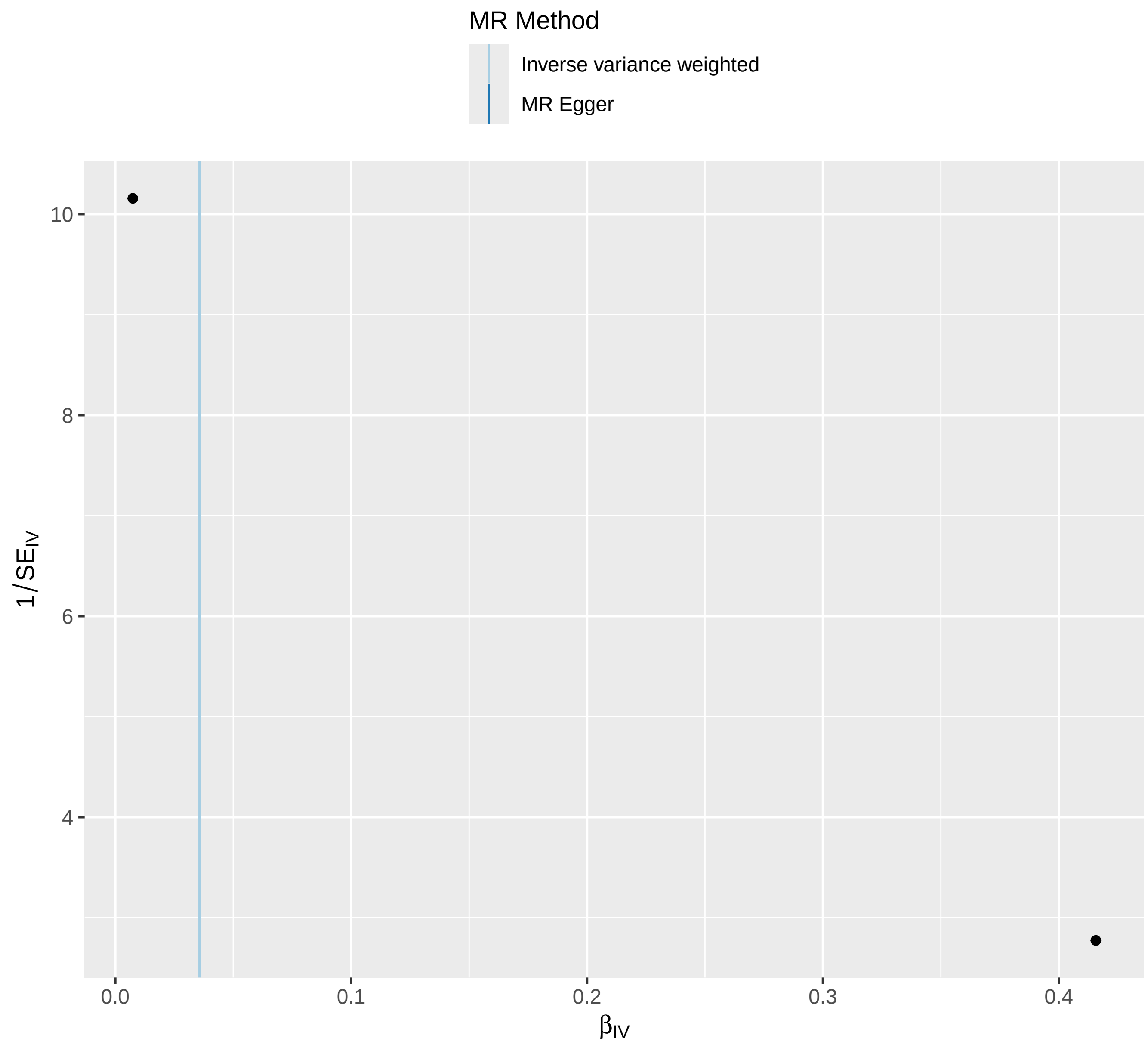

MR Method
Inverse variance weighted
MR Egger
10
8
6
4
0.0
0.1
0.2
0.3
0.4
1/SE_IV
β_IV

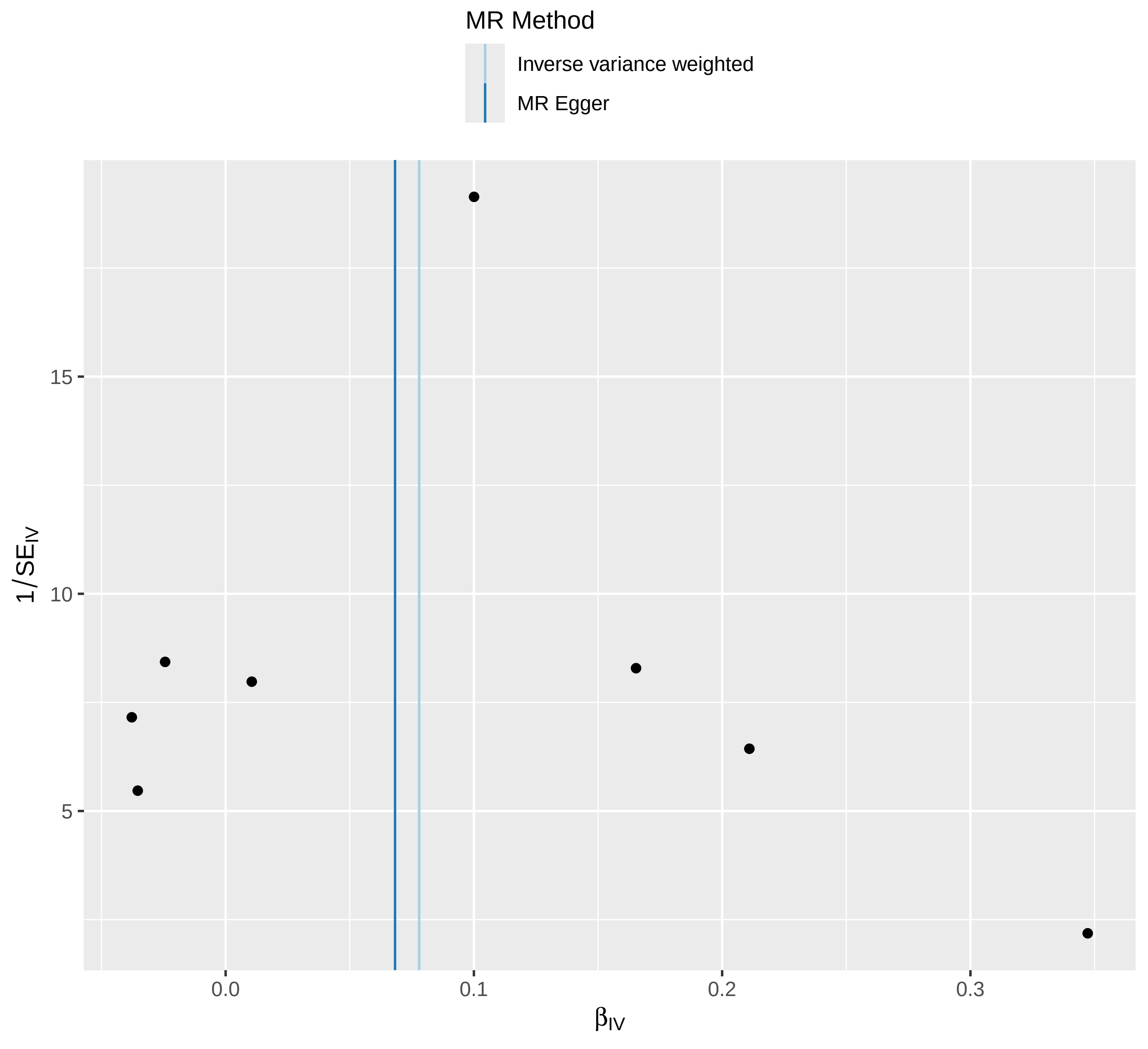

MR Method
Inverse variance weighted
MR Egger
15
10
5
0.0
0.1
0.2
0.3
1/SE_IV
β_IV

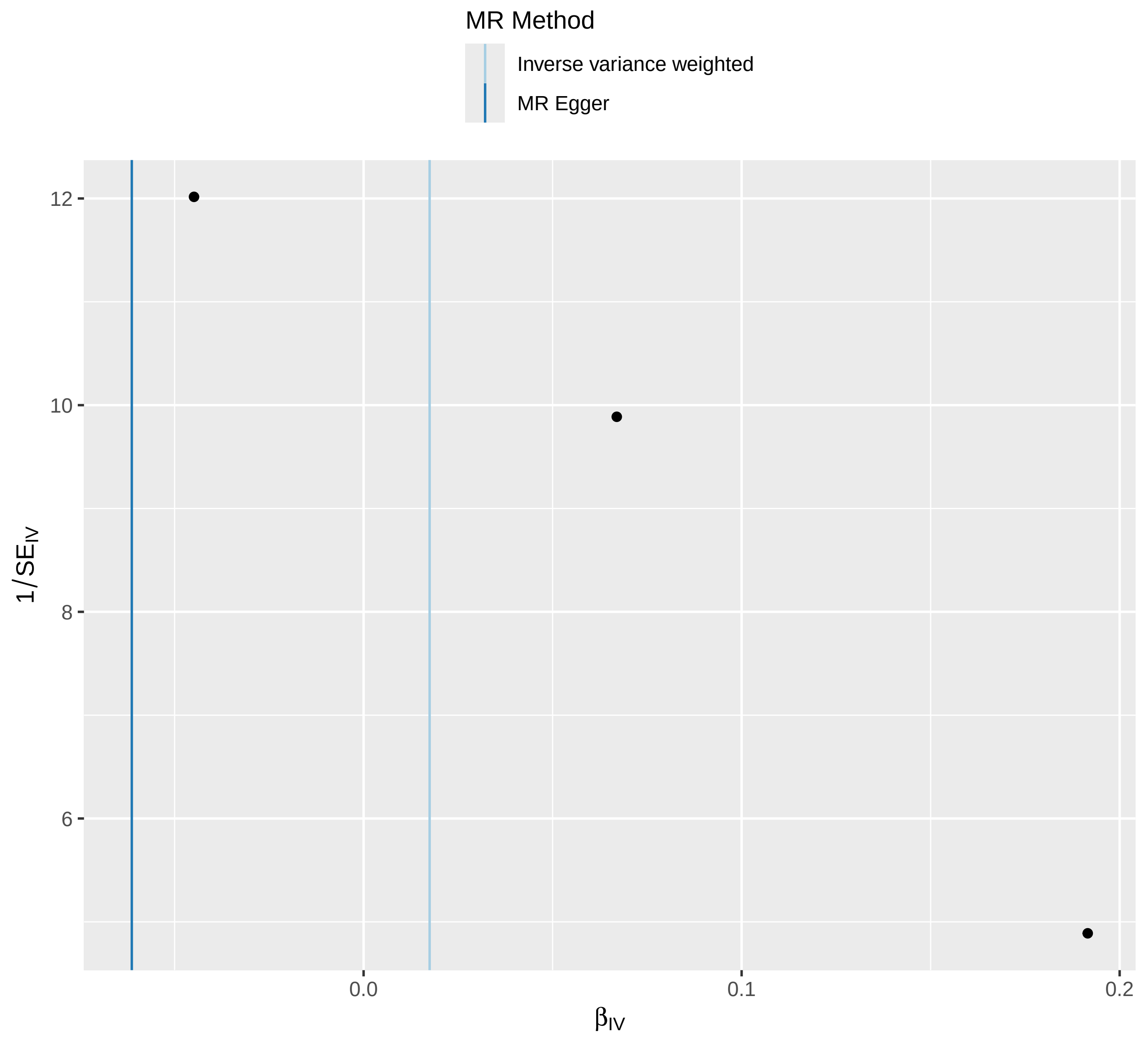
MR Method
Inverse variance weighted
MR Egger
12
10
8
6
1/SE_IV
0.0
0.1
0.2
β_IV

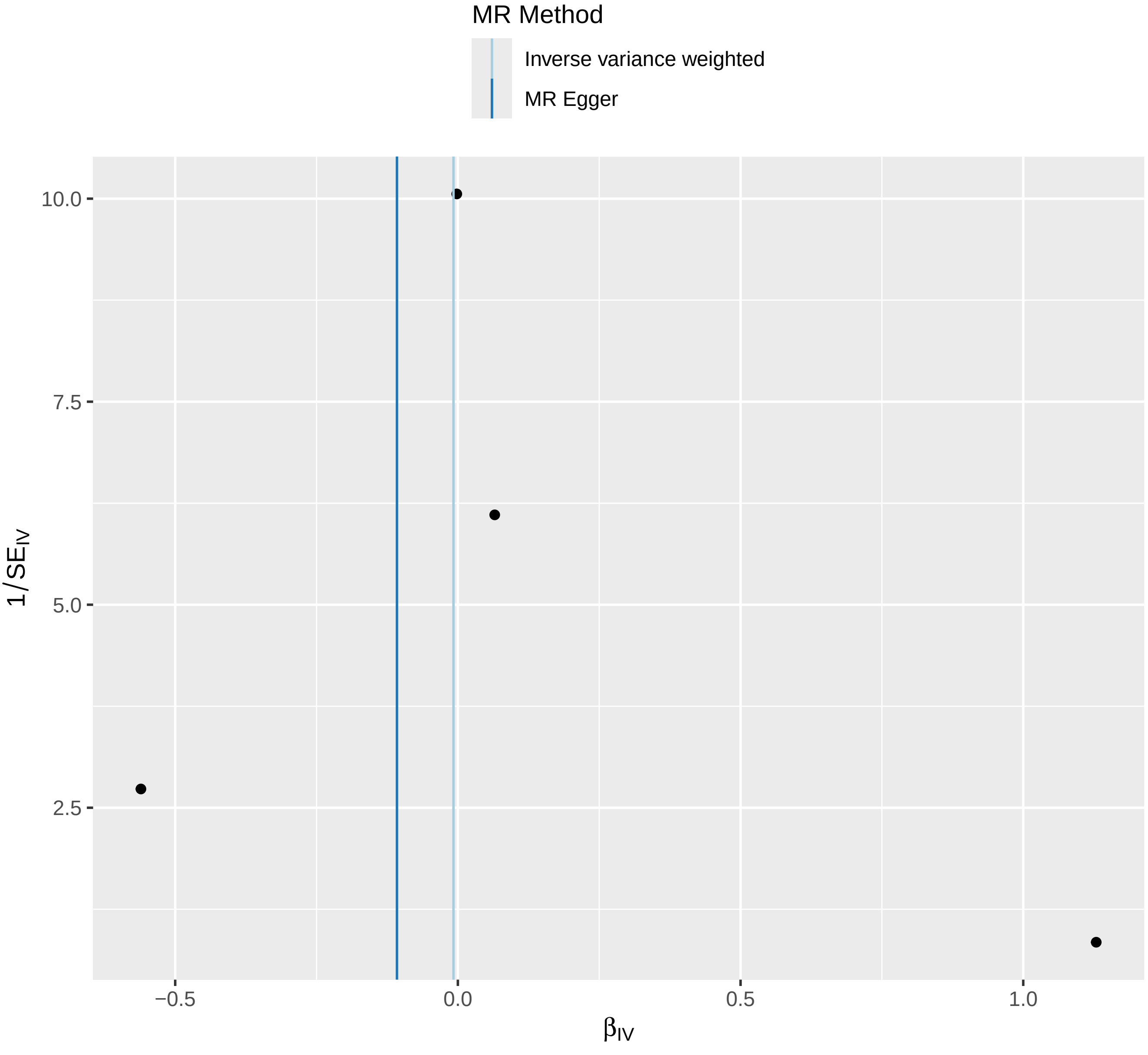

MR Method
Inverse variance weighted
MR Egger
10.0
7.5
5.0
2.5
1/SE_IV
−0.5
0.0
0.5
1.0
β_IV

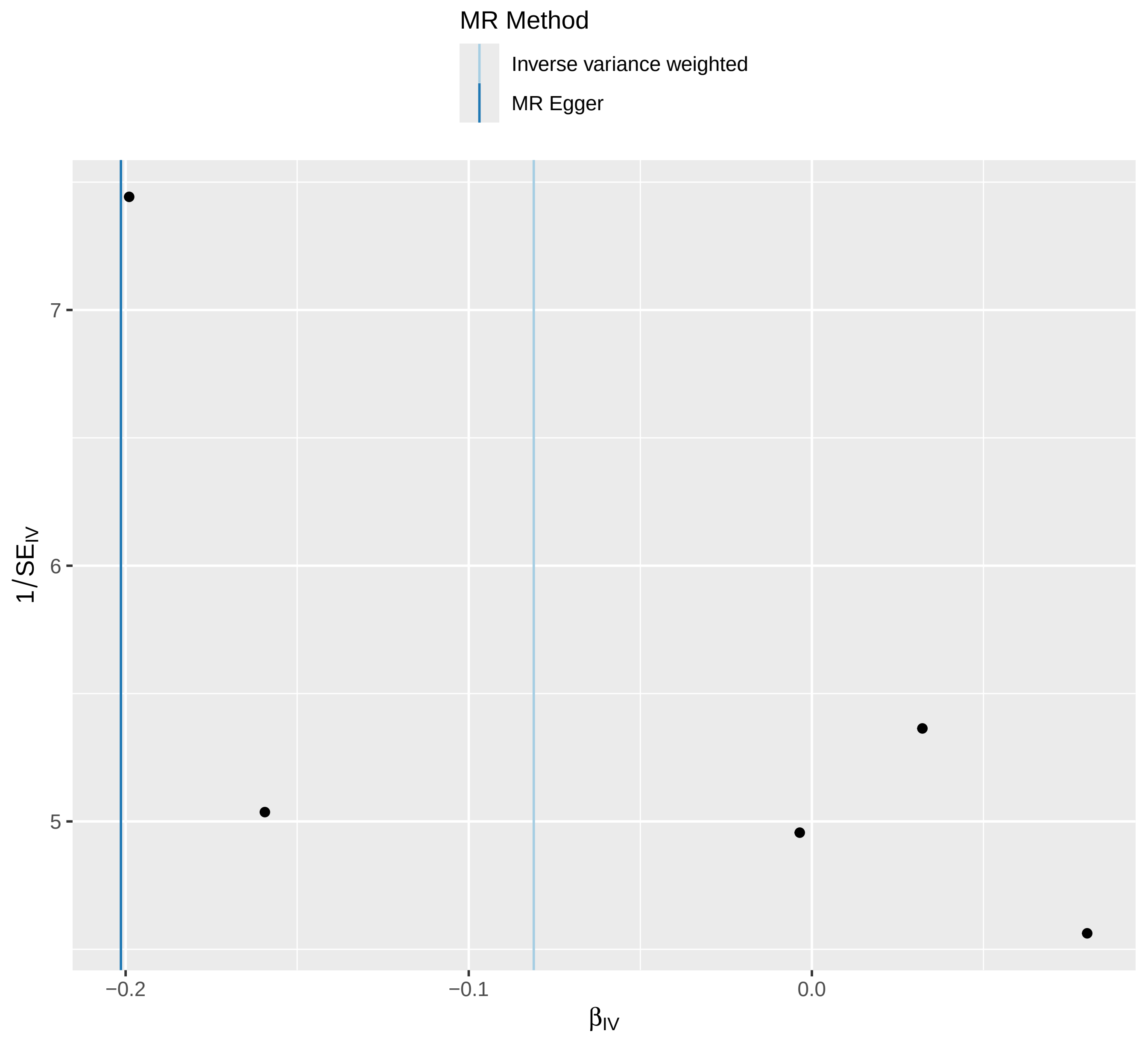
MR Method
Inverse variance weighted
MR Egger
7
6
5
1/SE$_{IV}$
−0.2
−0.1
0.0
β$_{IV}$

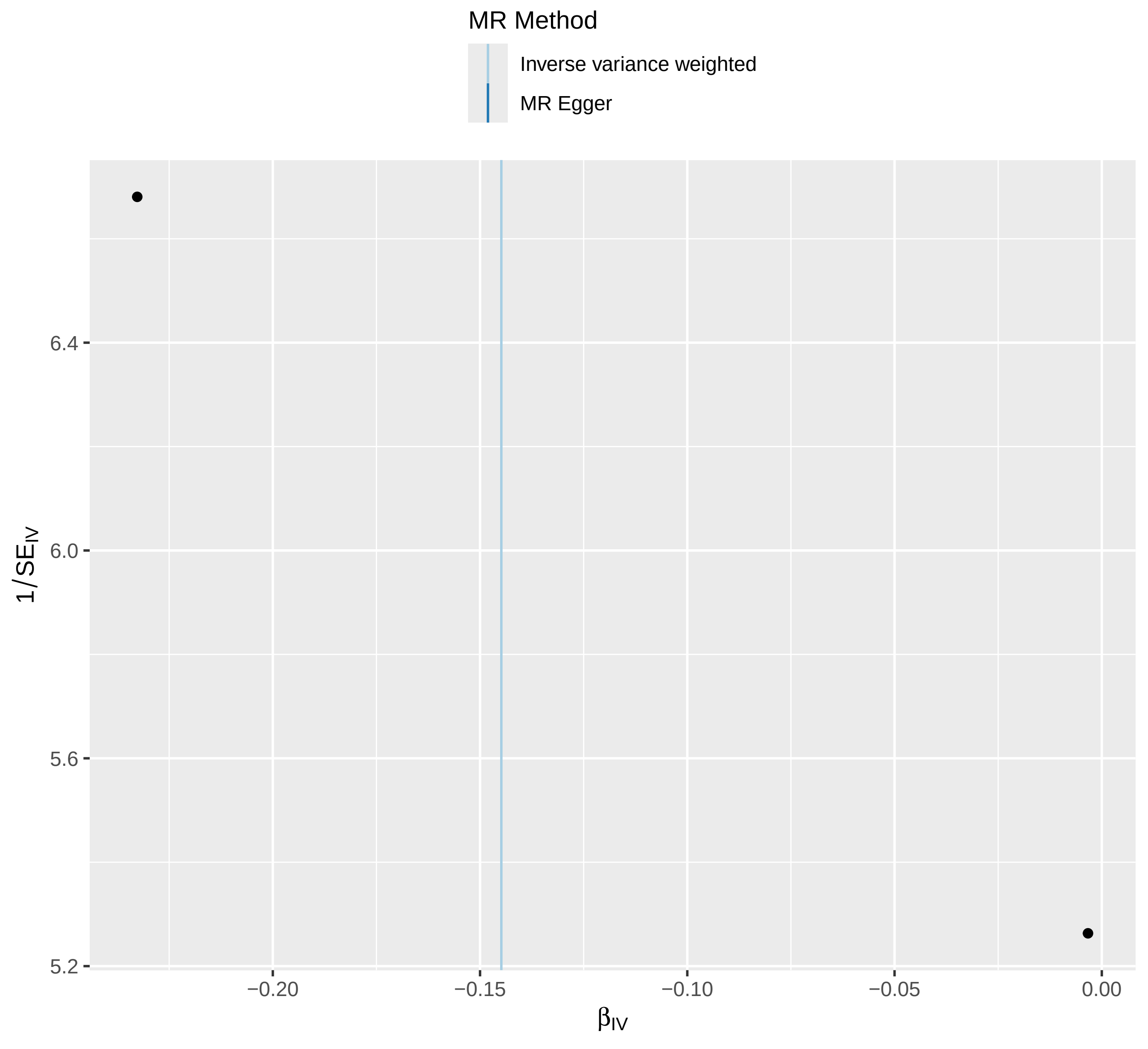
MR Method
Inverse variance weighted
MR Egger
6.4
6.0
5.6
5.2
1/SE_IV
−0.20
−0.15
−0.10
−0.05
0.00
β_IV

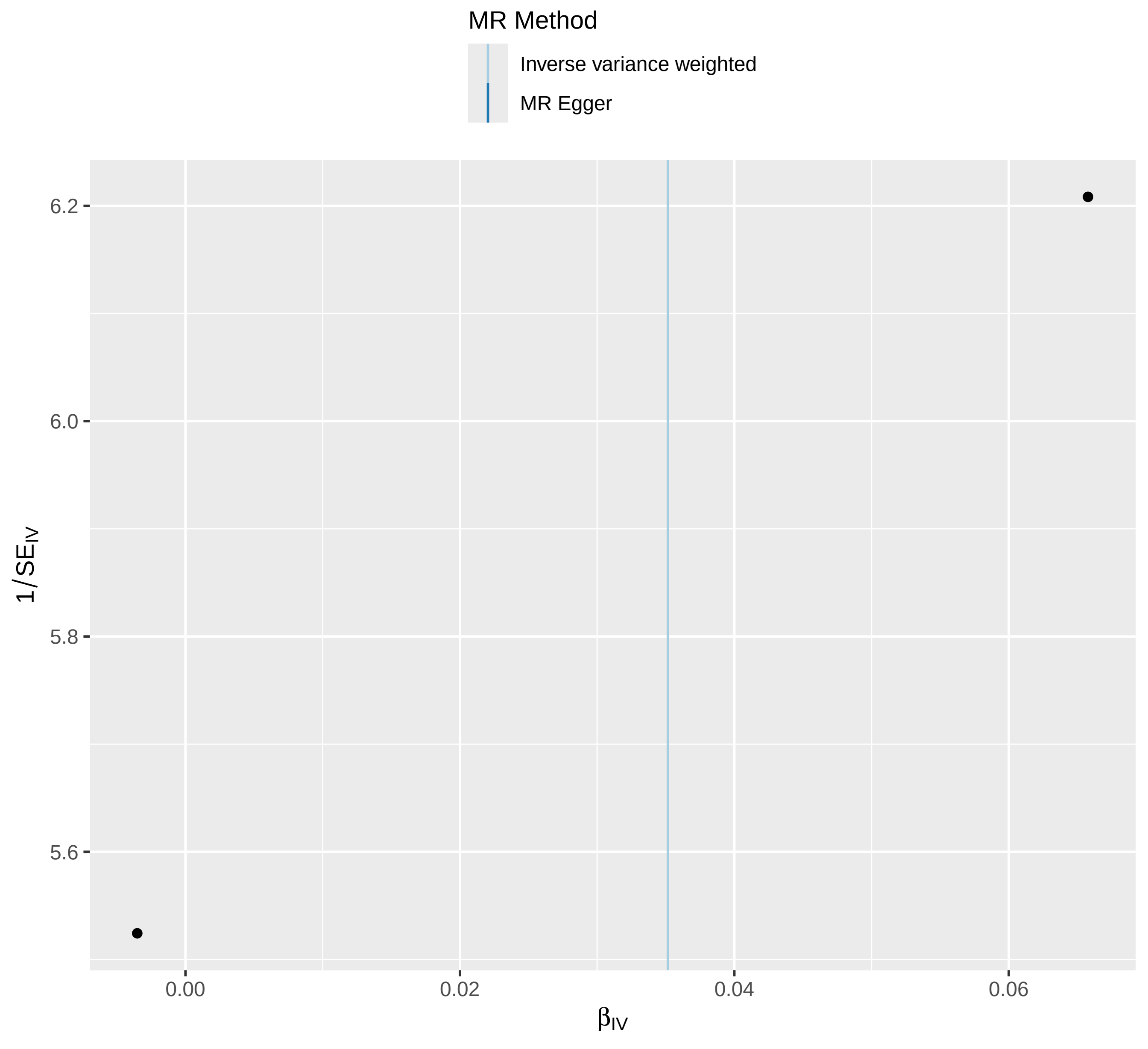
MR Method
Inverse variance weighted
MR Egger
6.2
6.0
5.8
5.6
1/SE_IV
0.00
0.02
0.04
0.06
β_IV

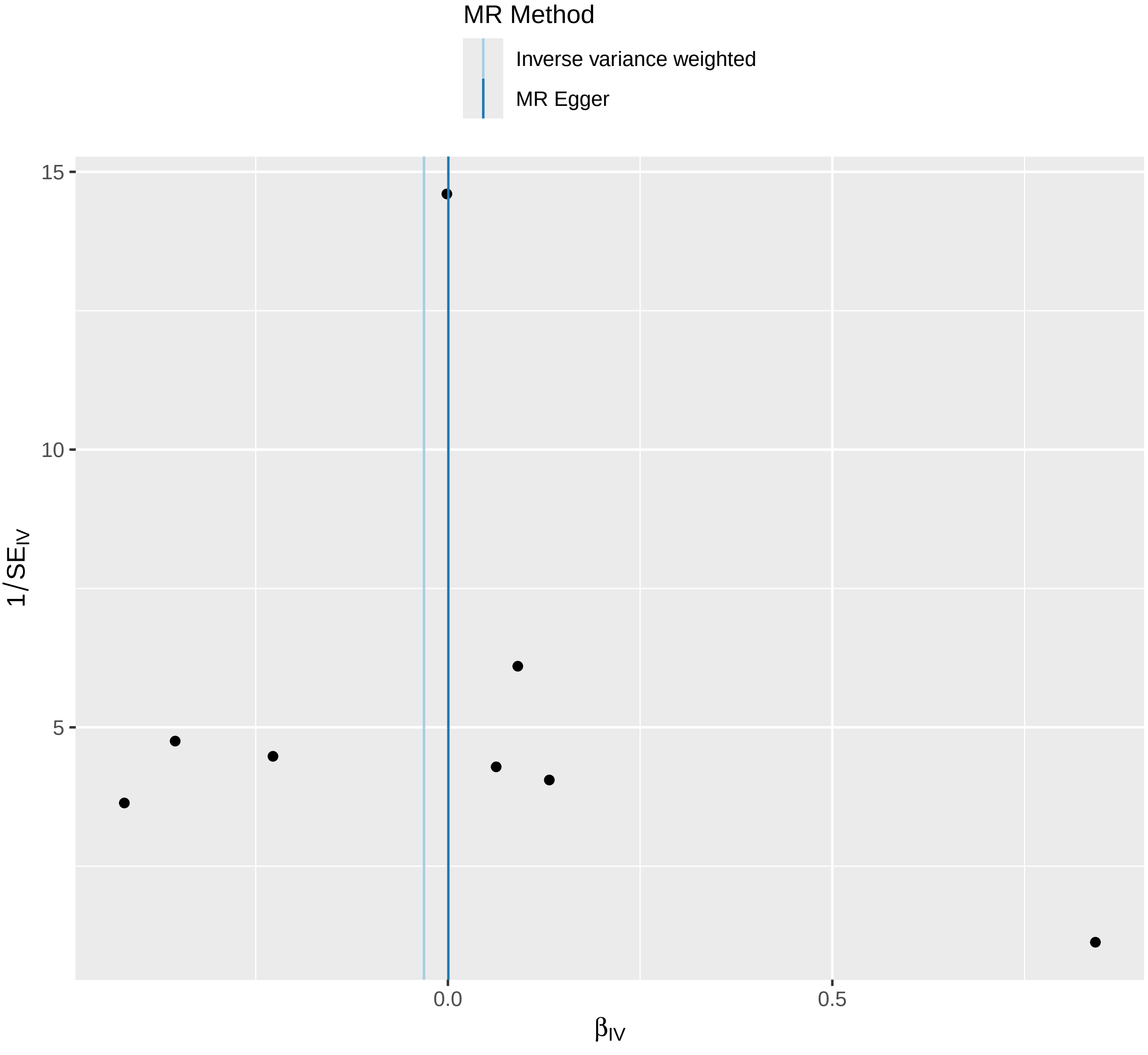

MR Method
Inverse variance weighted
MR Egger
15
10
5
0.0
0.5
1/SE$_{IV}$
β$_{IV}$

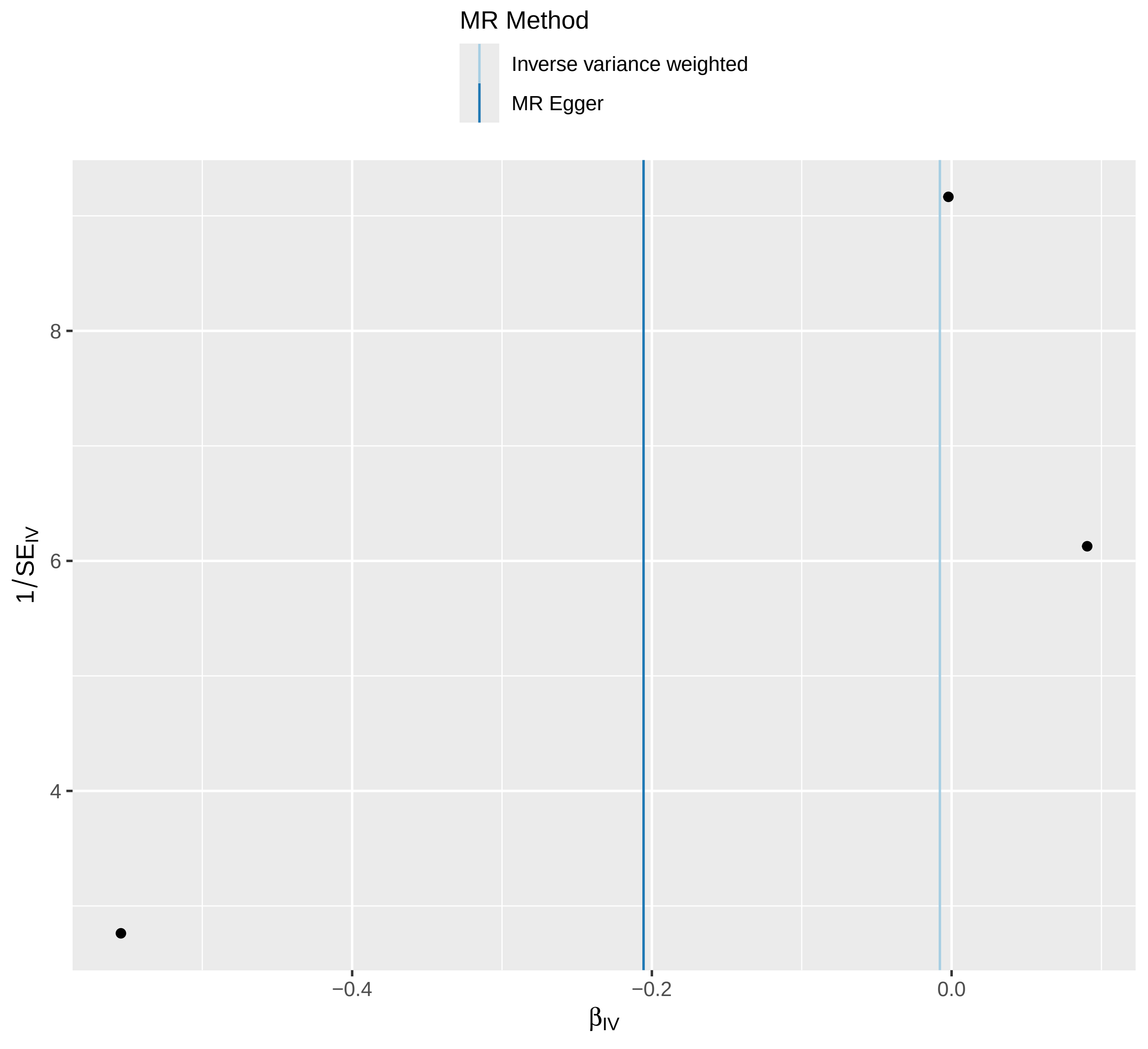

MR Method
Inverse variance weighted
MR Egger
8
6
4
1/SE$_{IV}$
−0.4
−0.2
0.0
β$_{IV}$

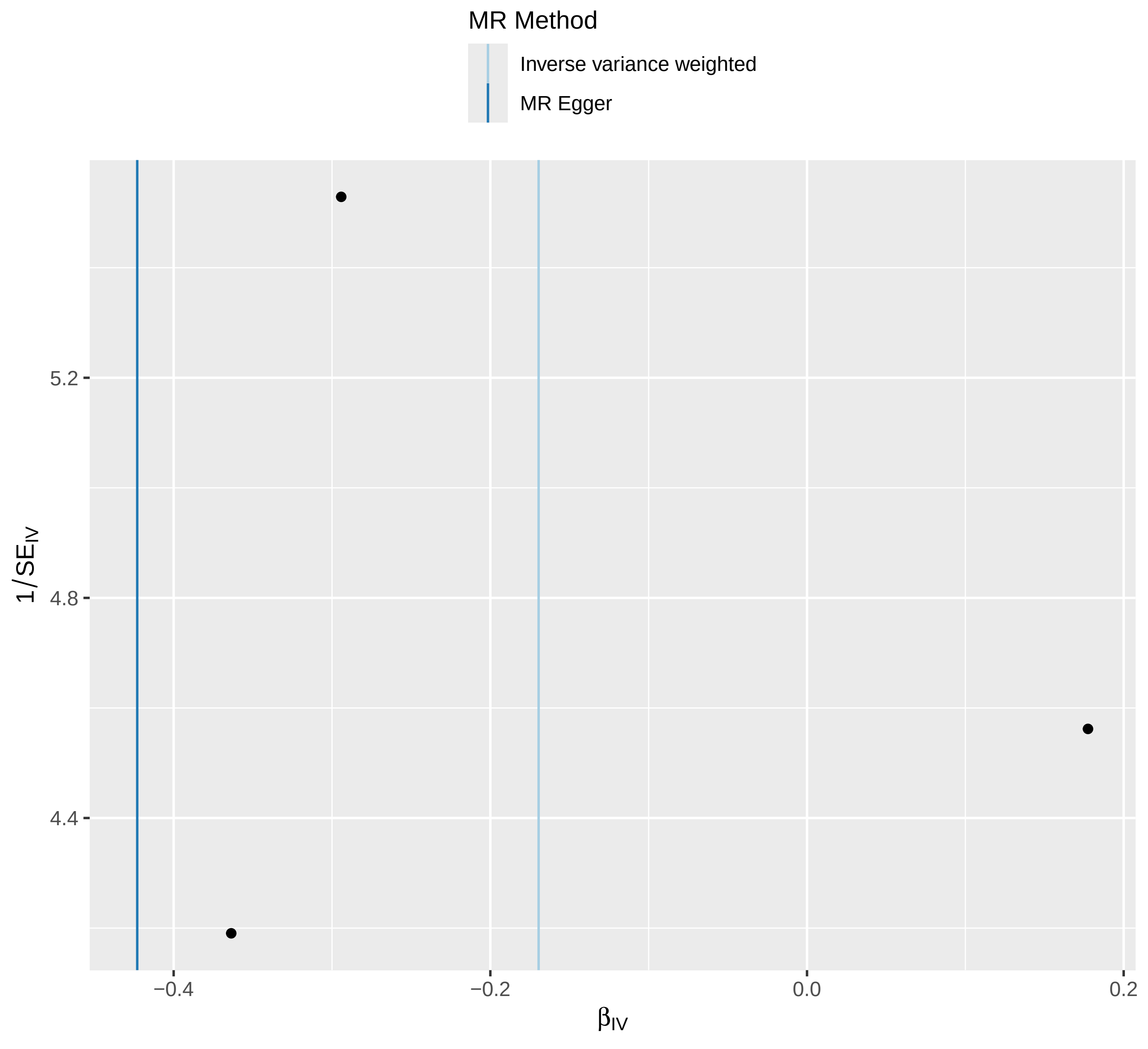
MR Method
Inverse variance weighted
MR Egger
5.2
4.8
4.4
1/SE_IV
−0.4
−0.2
0.0
0.2
β_IV

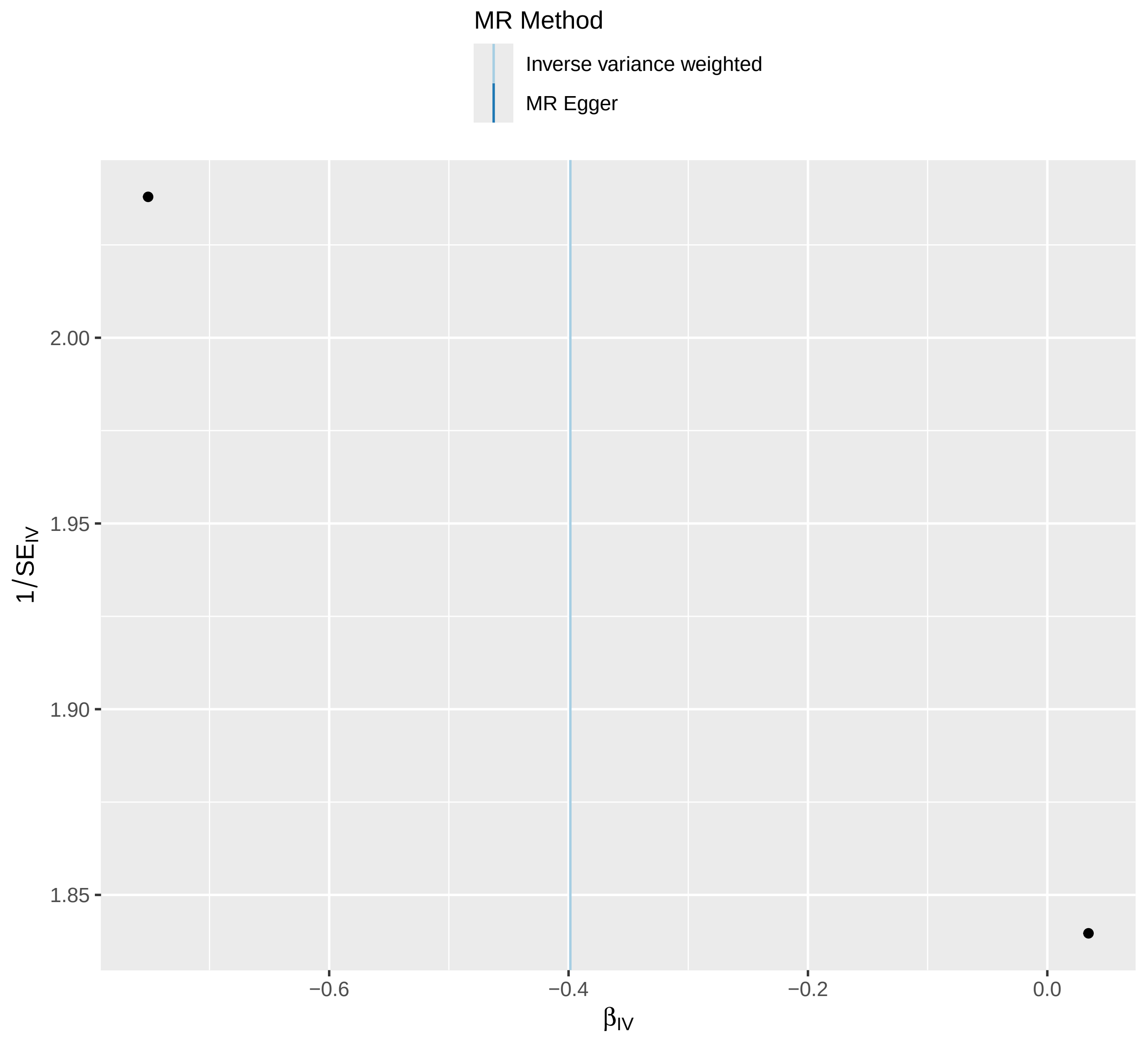

MR Method
Inverse variance weighted
MR Egger
2.00
1.95
1.90
1.85
1/SE_IV
−0.6
−0.4
−0.2
0.0
β_IV

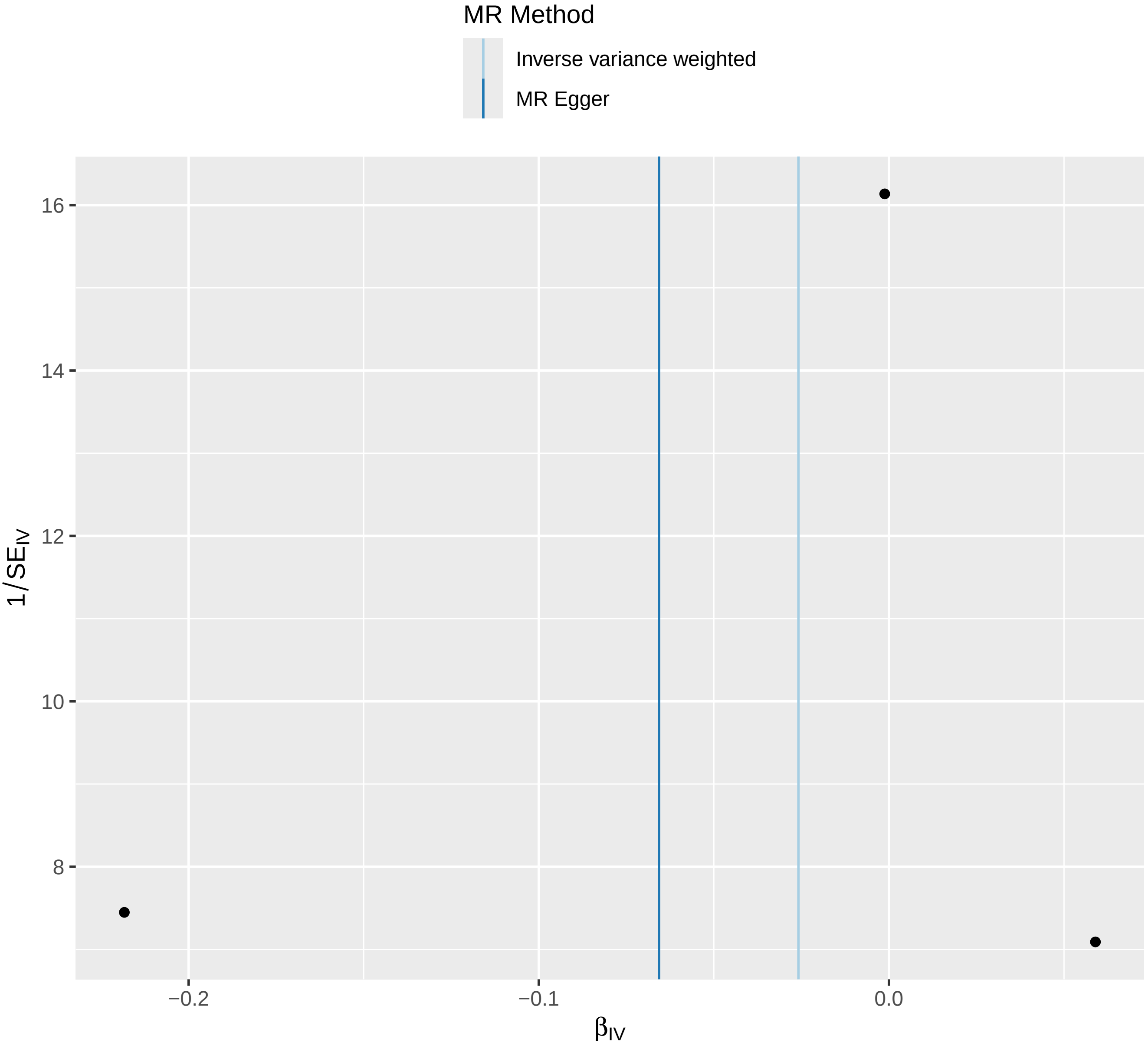

MR Method
Inverse variance weighted
MR Egger
16
14
12
10
8
1/SE$_{IV}$
−0.2
−0.1
0.0
β$_{IV}$

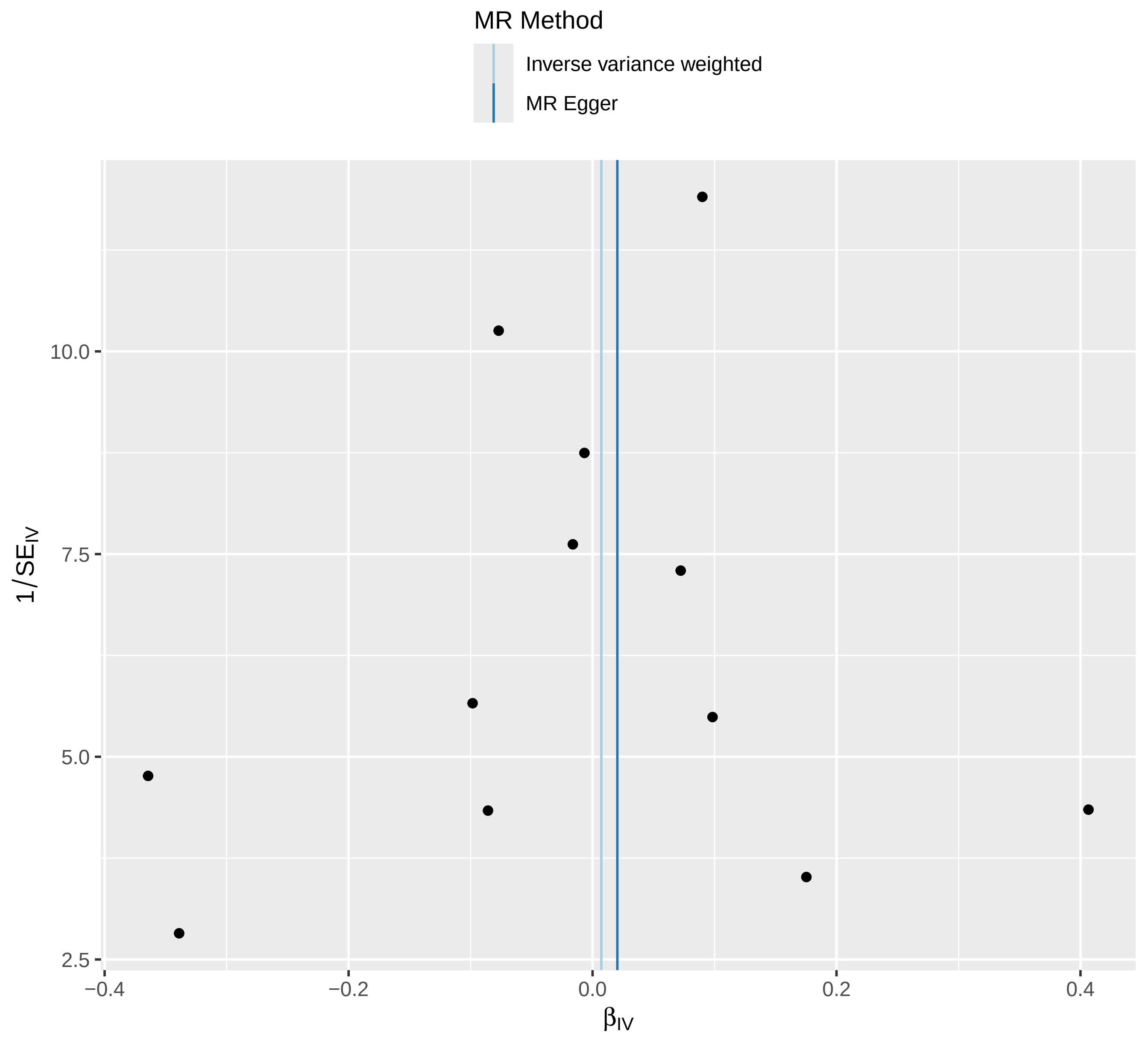
MR Method
Inverse variance weighted
MR Egger
1/SE_IV
10.0
7.5
5.0
2.5
−0.4
−0.2
0.0
0.2
0.4
β_IV

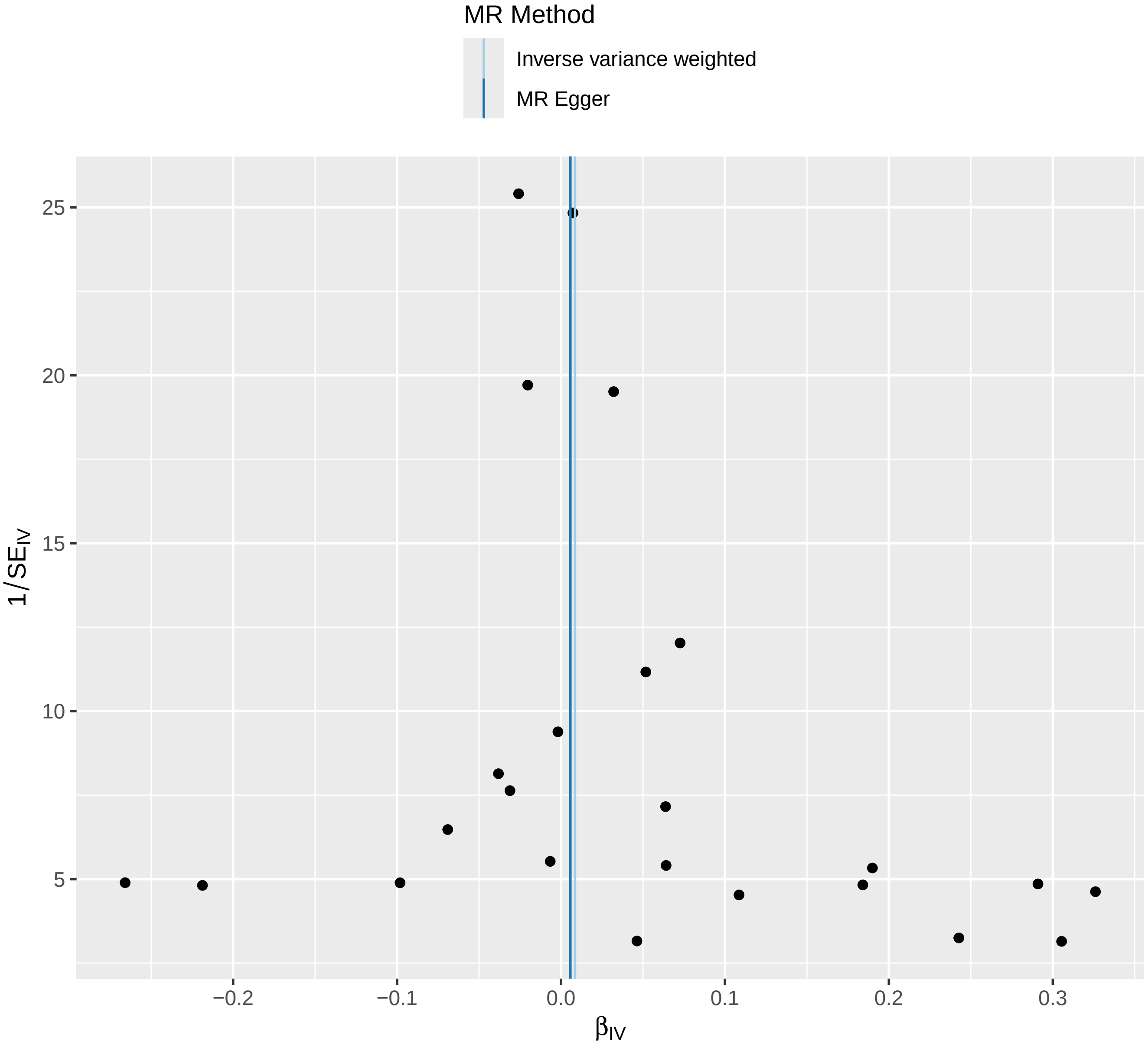

MR Method
Inverse variance weighted
MR Egger
25
20
15
10
5
$1/SE_{IV}$
−0.2
−0.1
0.0
0.1
0.2
0.3
$\beta_{IV}$

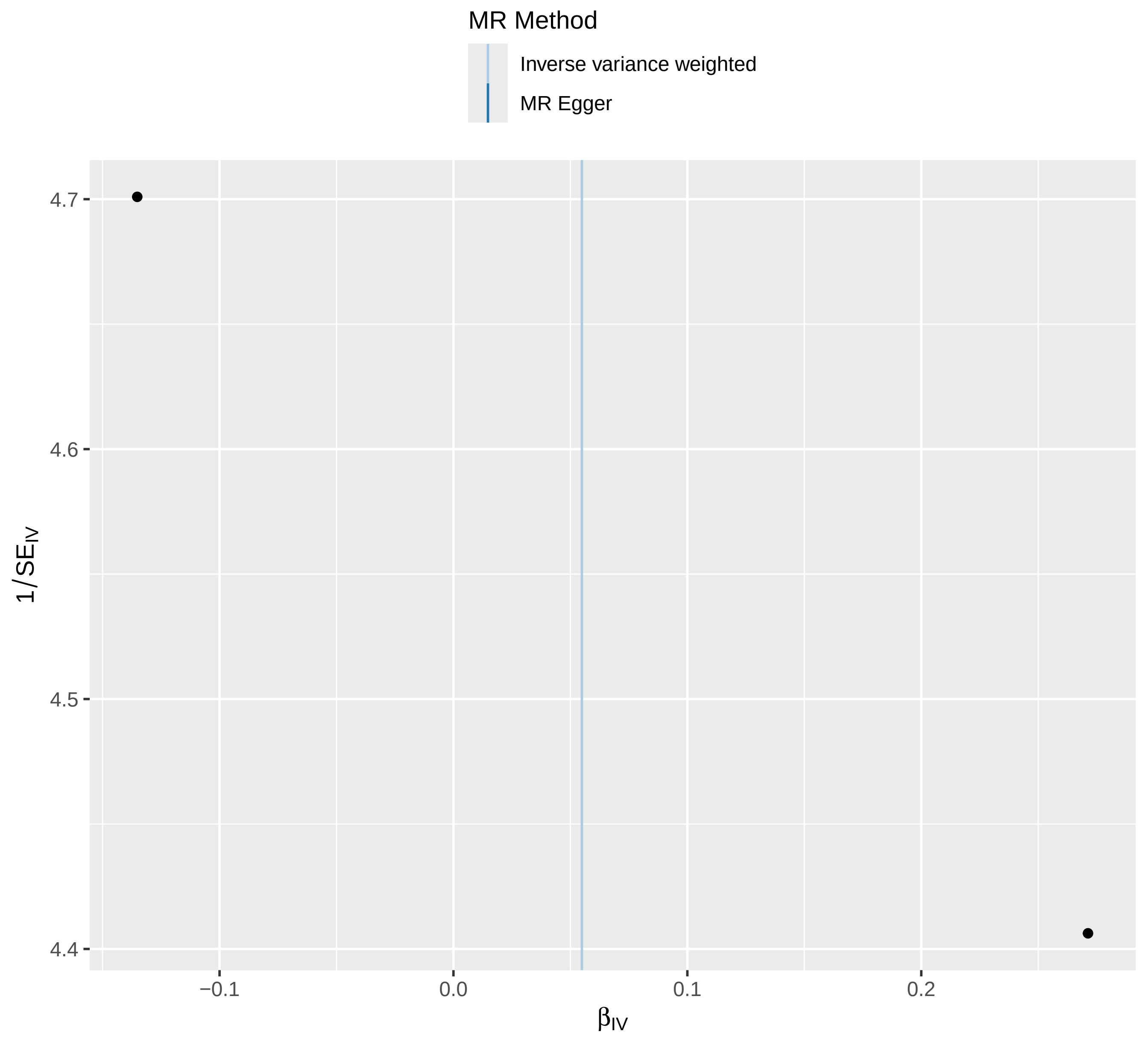

MR Method
Inverse variance weighted
MR Egger
1/SE_IV
4.7
4.6
4.5
4.4
−0.1
0.0
0.1
0.2
β_IV

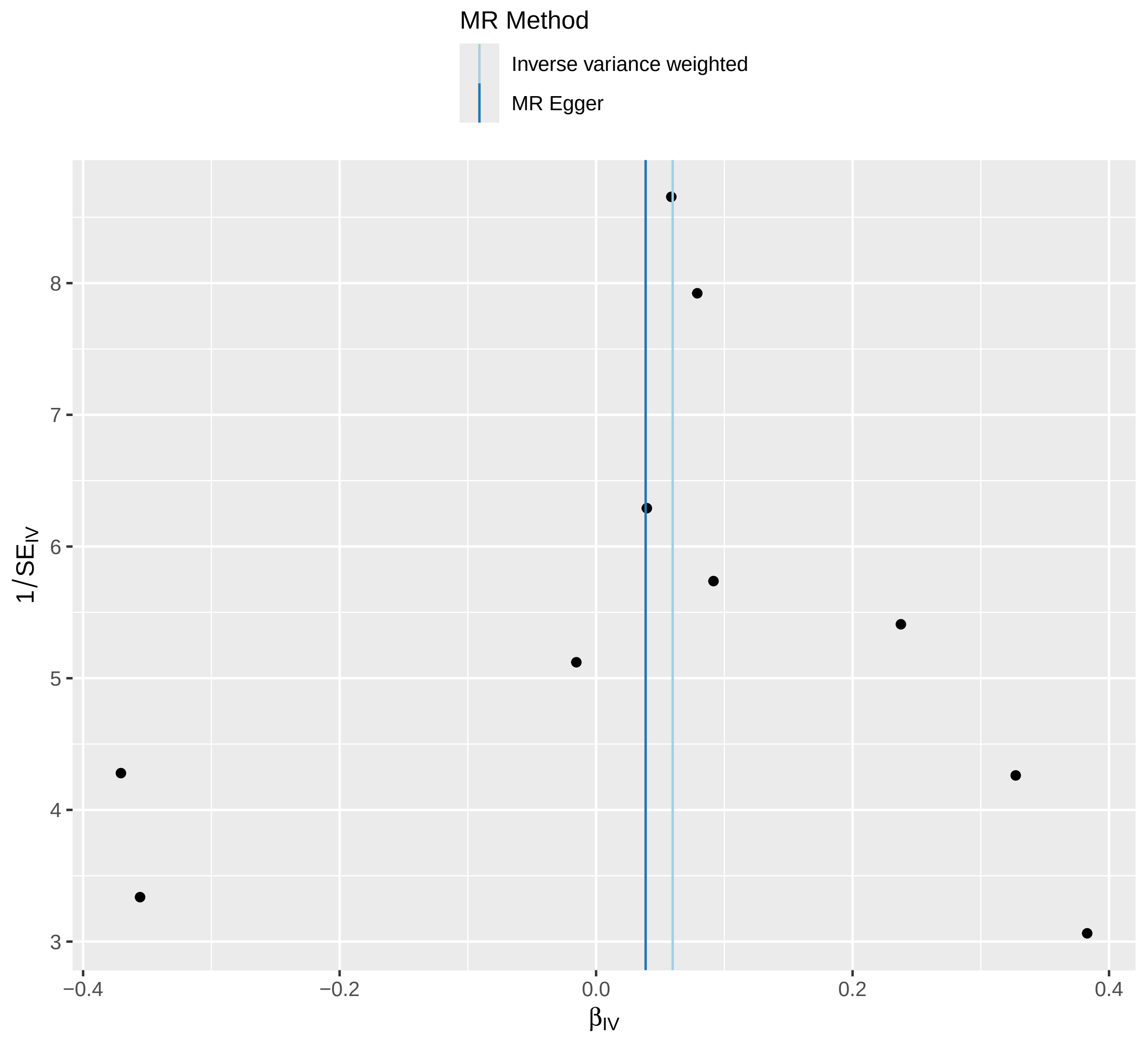
MR Method
Inverse variance weighted
MR Egger
8
7
6
5
4
3
$1/SE_{IV}$
−0.4
−0.2
0.0
0.2
0.4
$\beta_{IV}$

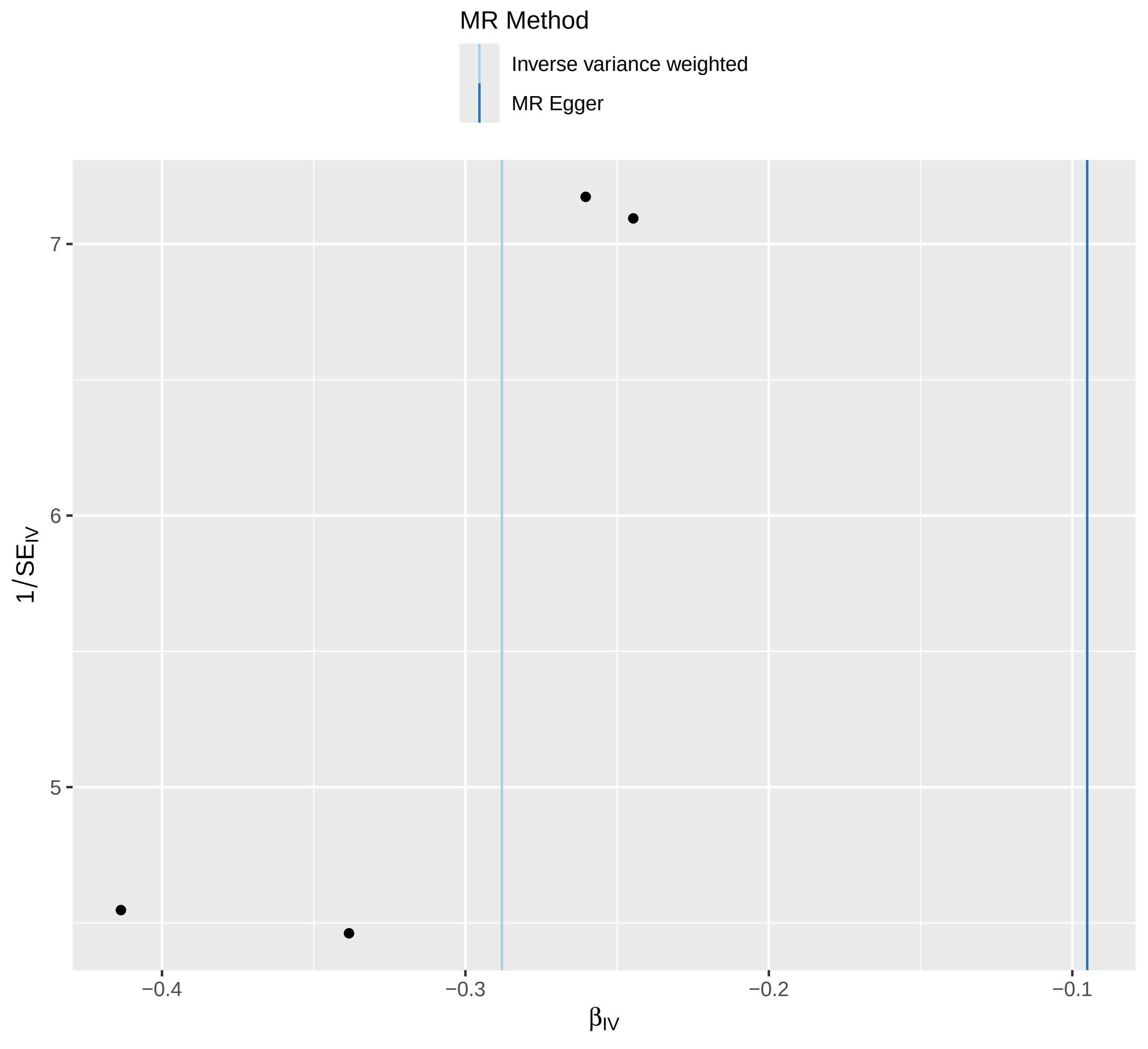
MR Method
Inverse variance weighted
MR Egger
7
6
5
1/SE_IV
−0.4
−0.3
−0.2
−0.1
β_IV

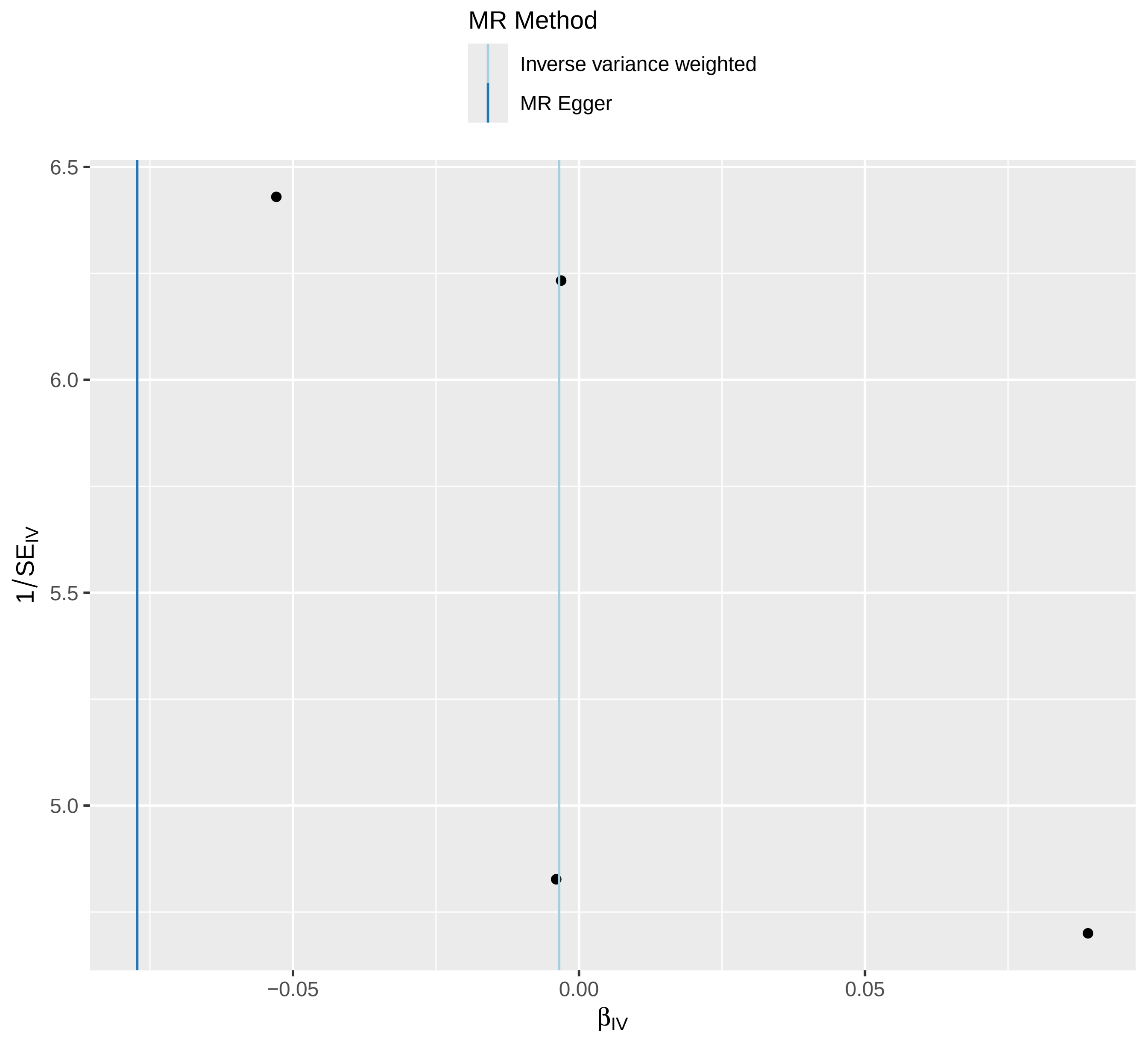
MR Method
Inverse variance weighted
MR Egger
6.5
6.0
5.5
5.0
1/SE_IV
−0.05
0.00
0.05
β_IV

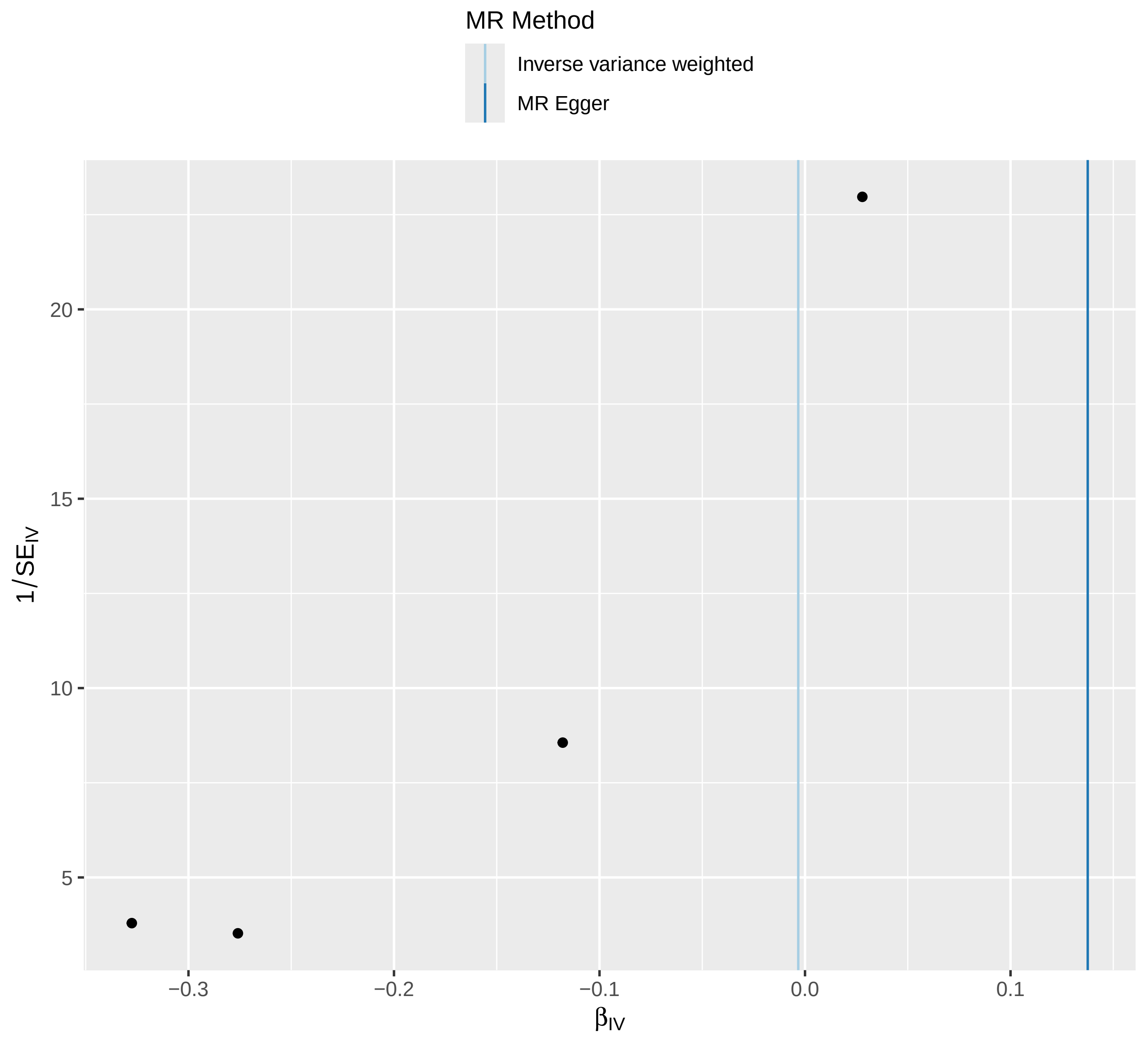

MR Method
Inverse variance weighted
MR Egger
20
15
10
5
1/SE$_{IV}$
−0.3
−0.2
−0.1
0.0
0.1
β$_{IV}$

Insufficient number of SNPs

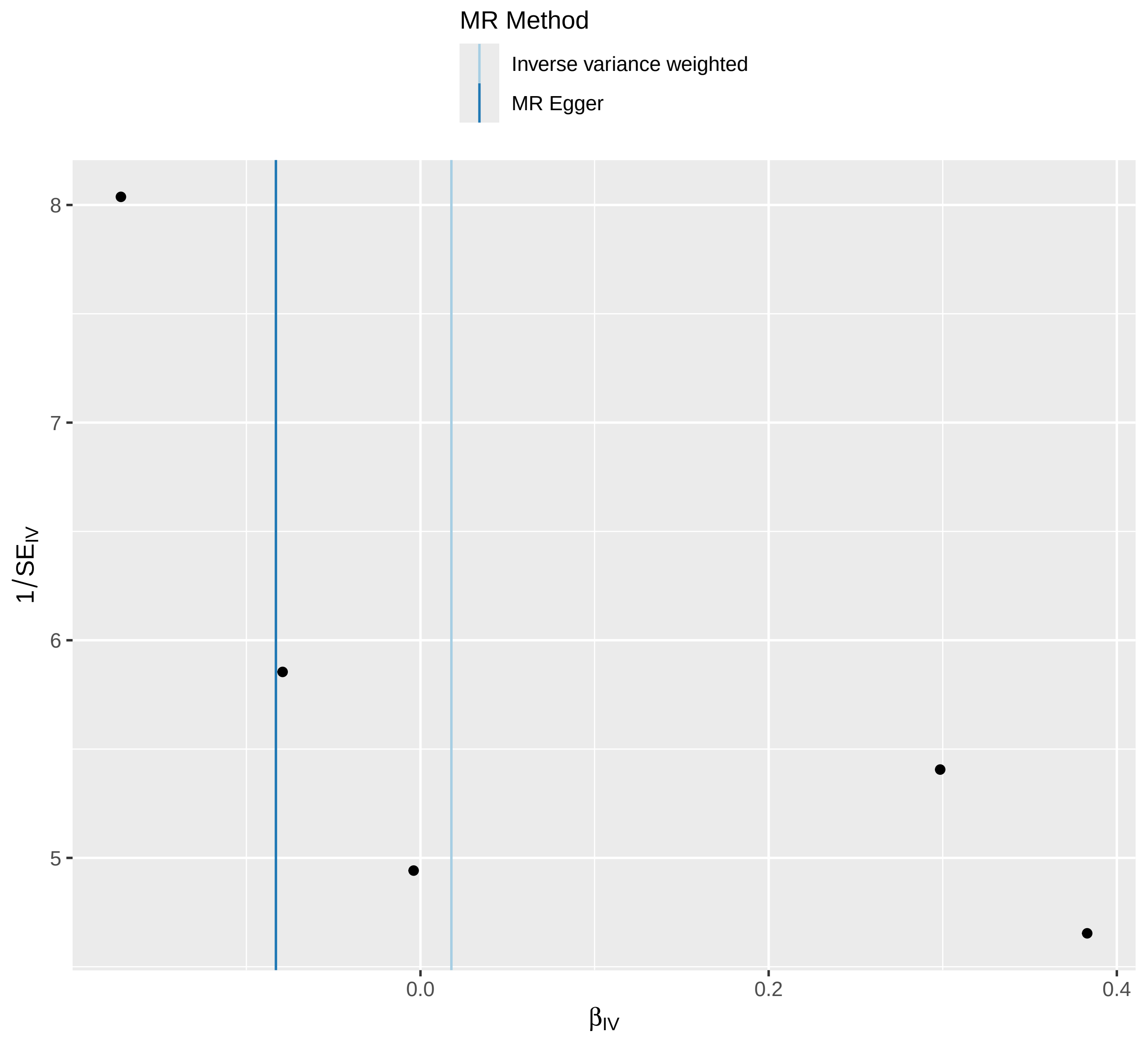
MR Method
Inverse variance weighted
MR Egger
8
7
6
5
1/SE_IV
0.0
0.2
0.4
β_IV

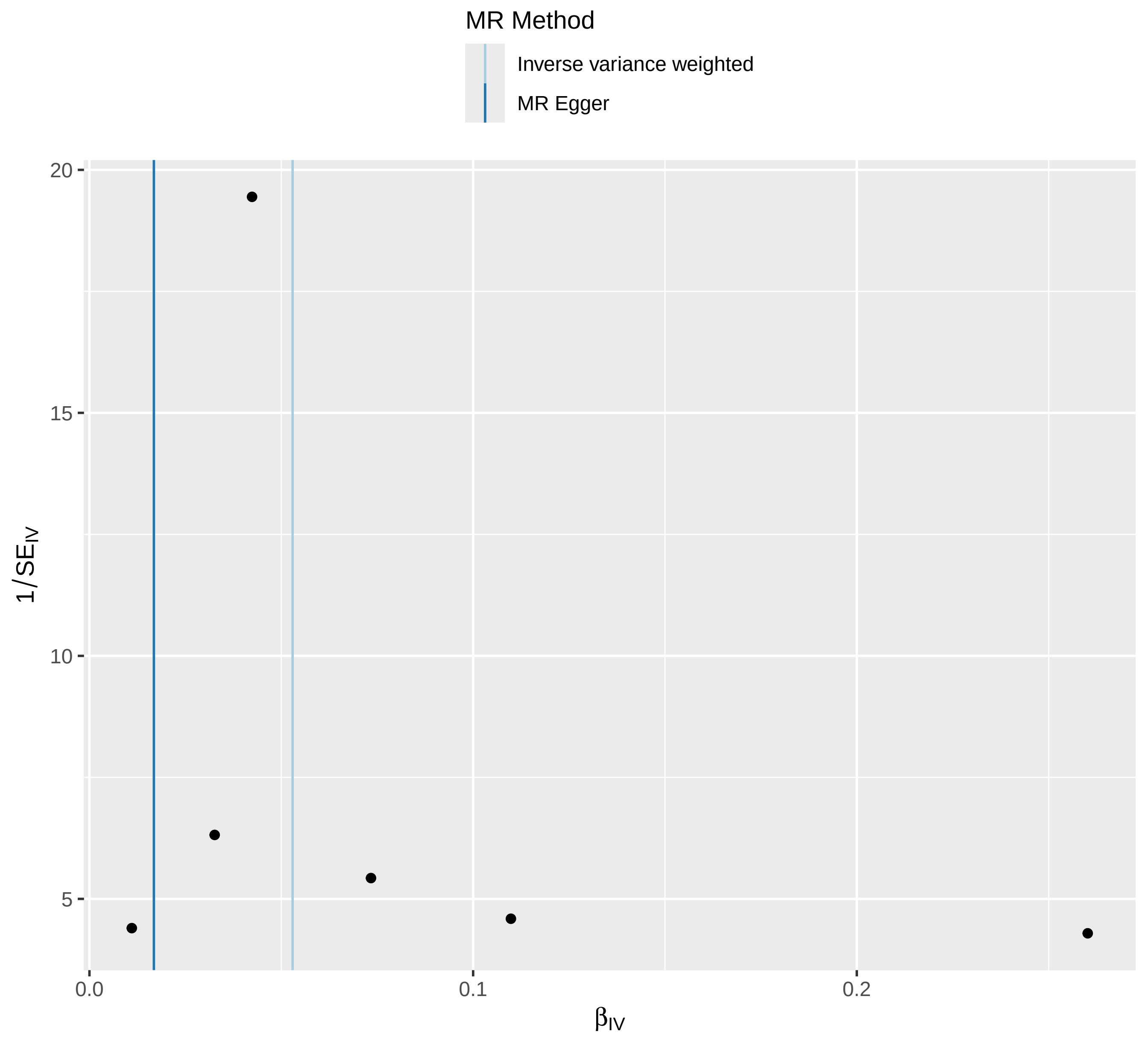

MR Method
Inverse variance weighted
MR Egger
20
15
10
5
1/SE_IV
0.0
0.1
0.2
β_IV

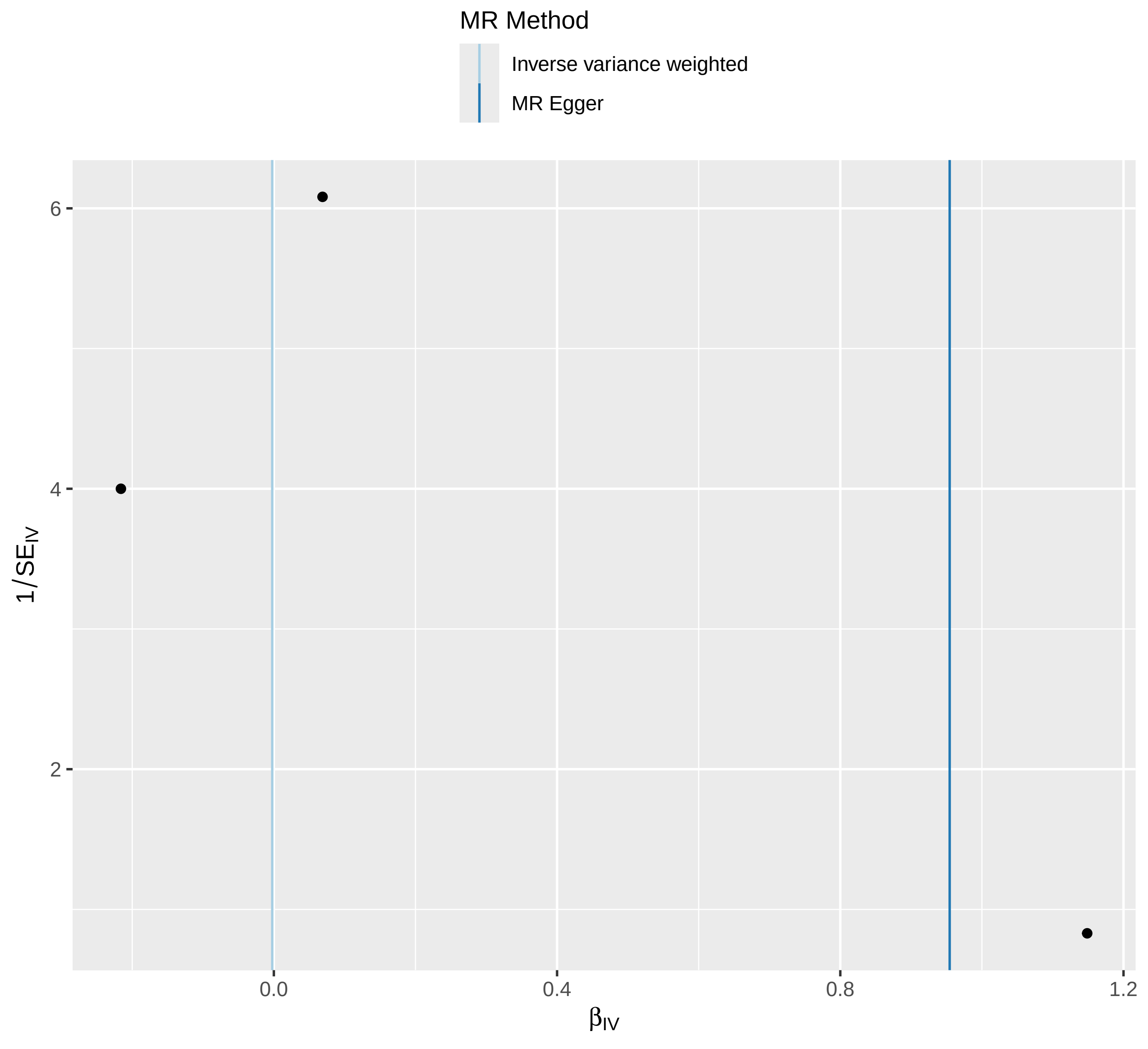

MR Method
Inverse variance weighted
MR Egger
6
4
2
1/SE_IV
0.0
0.4
0.8
1.2
β_IV

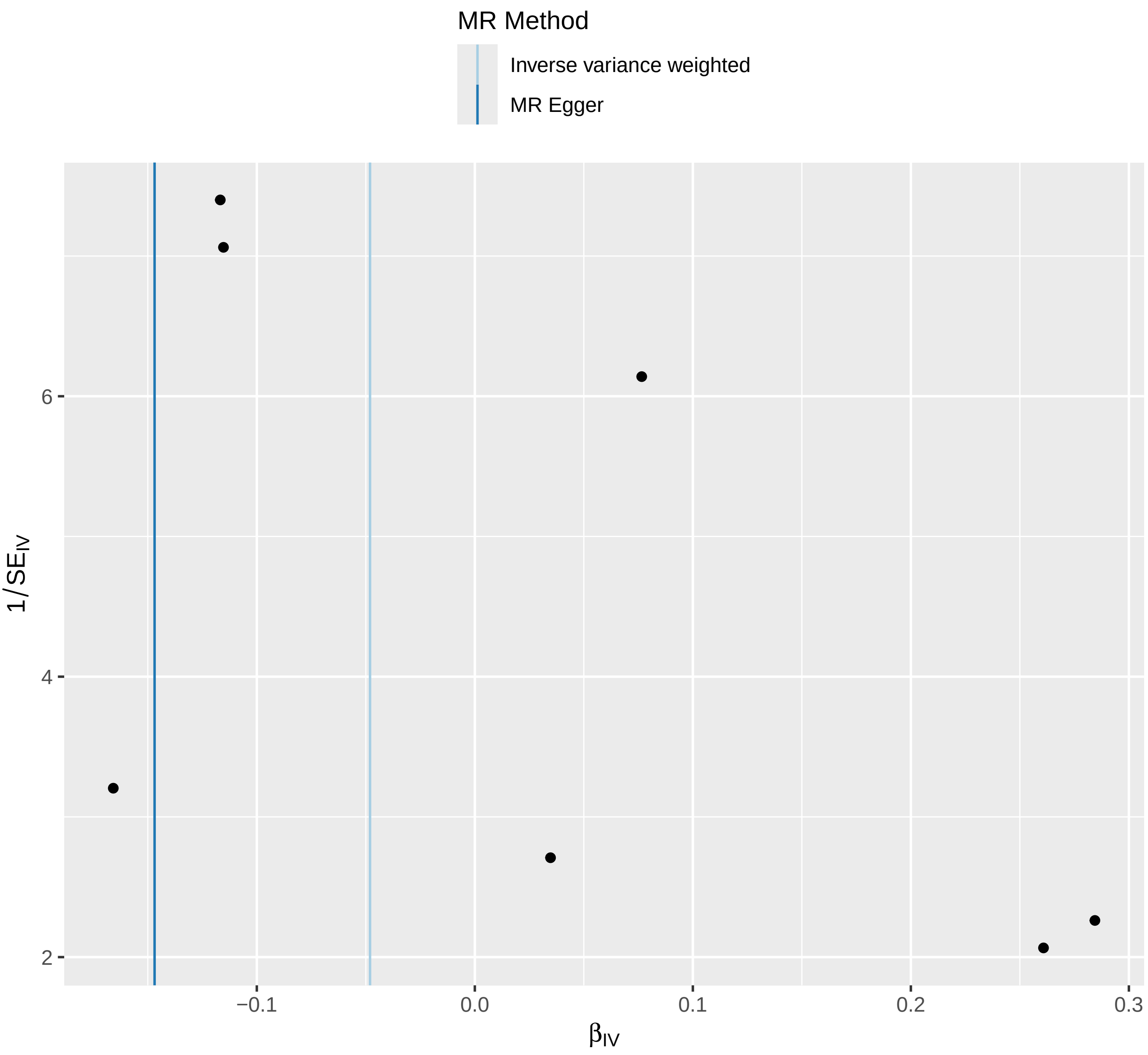
MR Method
Inverse variance weighted
MR Egger
6
4
2
$1/SE_{IV}$
−0.1
0.0
0.1
0.2
0.3
$\beta_{IV}$

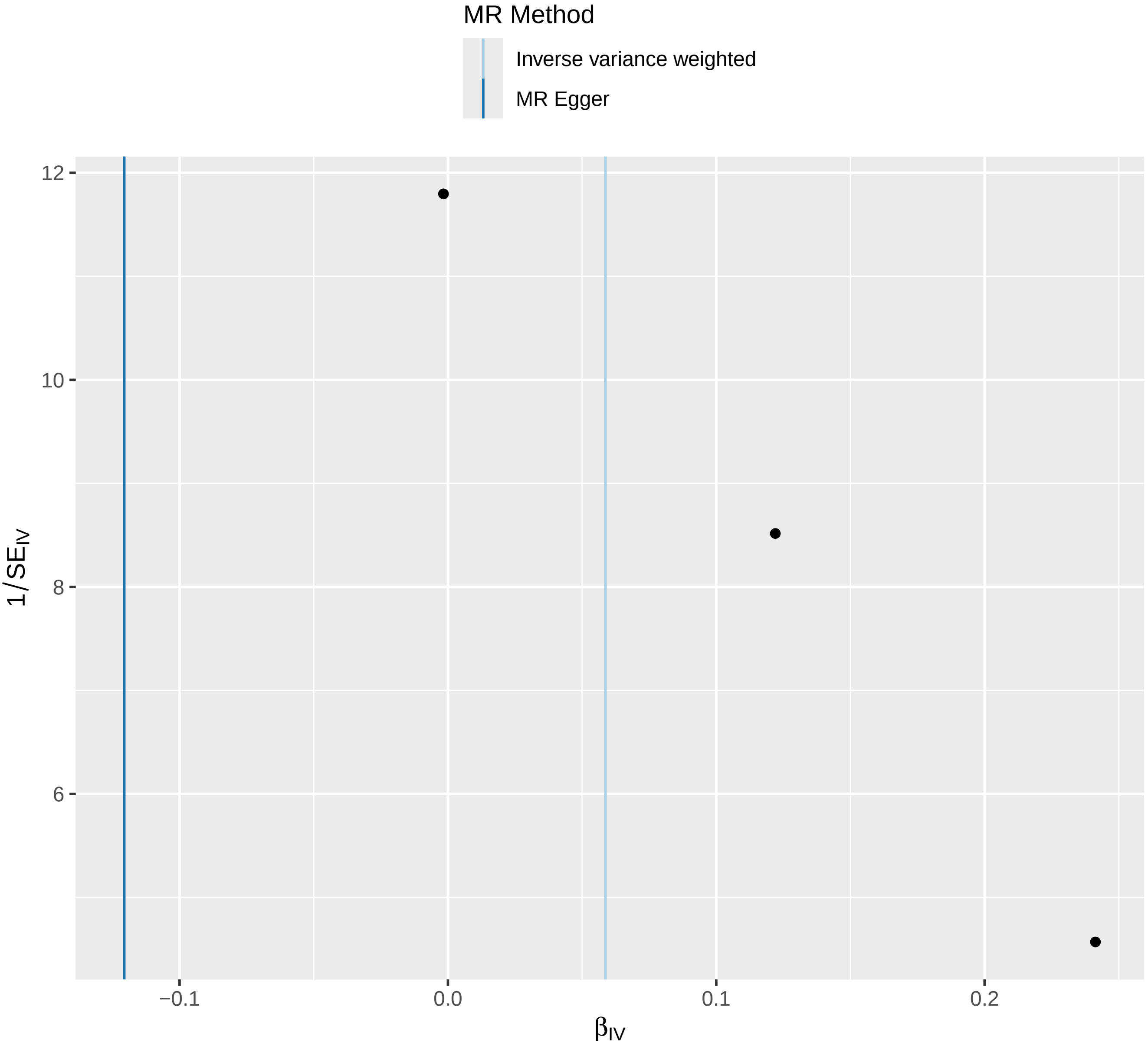

MR Method
Inverse variance weighted
MR Egger
12
10
8
6
1/SE$_{IV}$
−0.1
0.0
0.1
0.2
β$_{IV}$

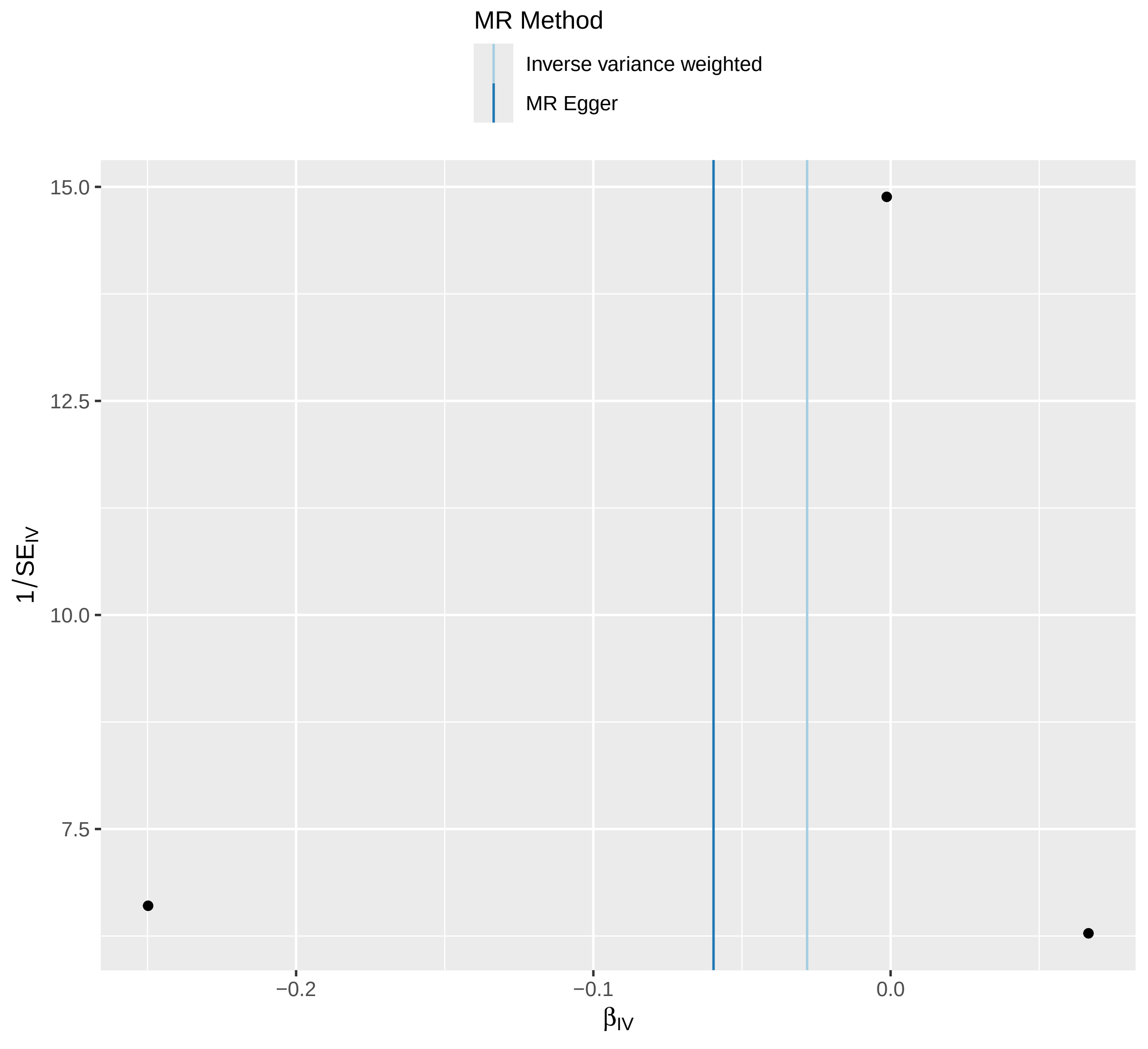
MR Method
Inverse variance weighted
MR Egger
15.0
12.5
10.0
7.5
1/SE$_{IV}$
−0.2
−0.1
0.0
$\beta_{IV}$

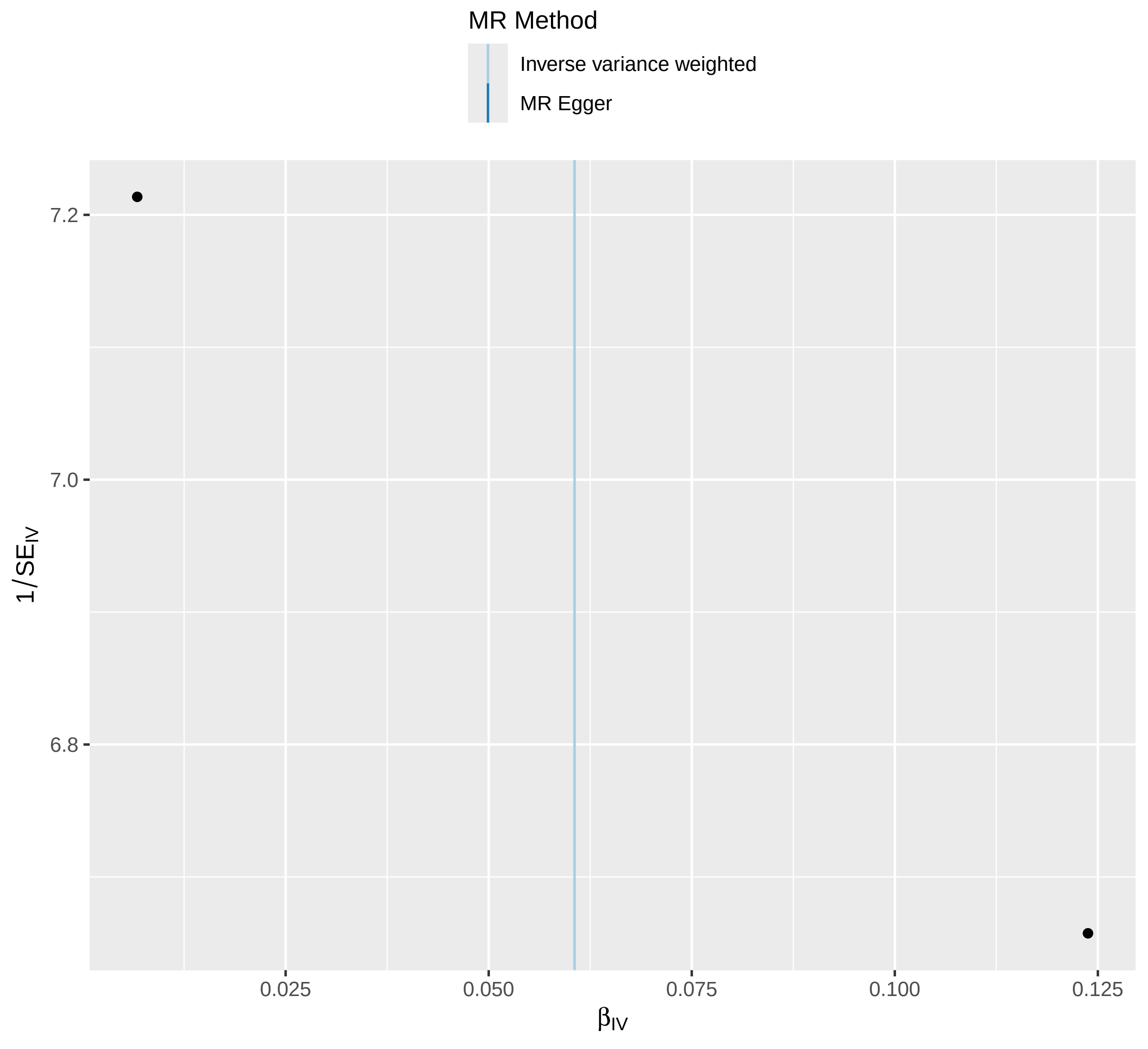
MR Method
Inverse variance weighted
MR Egger
7.2
7.0
6.8
1/SE_IV
0.025
0.050
0.075
0.100
0.125
β_IV

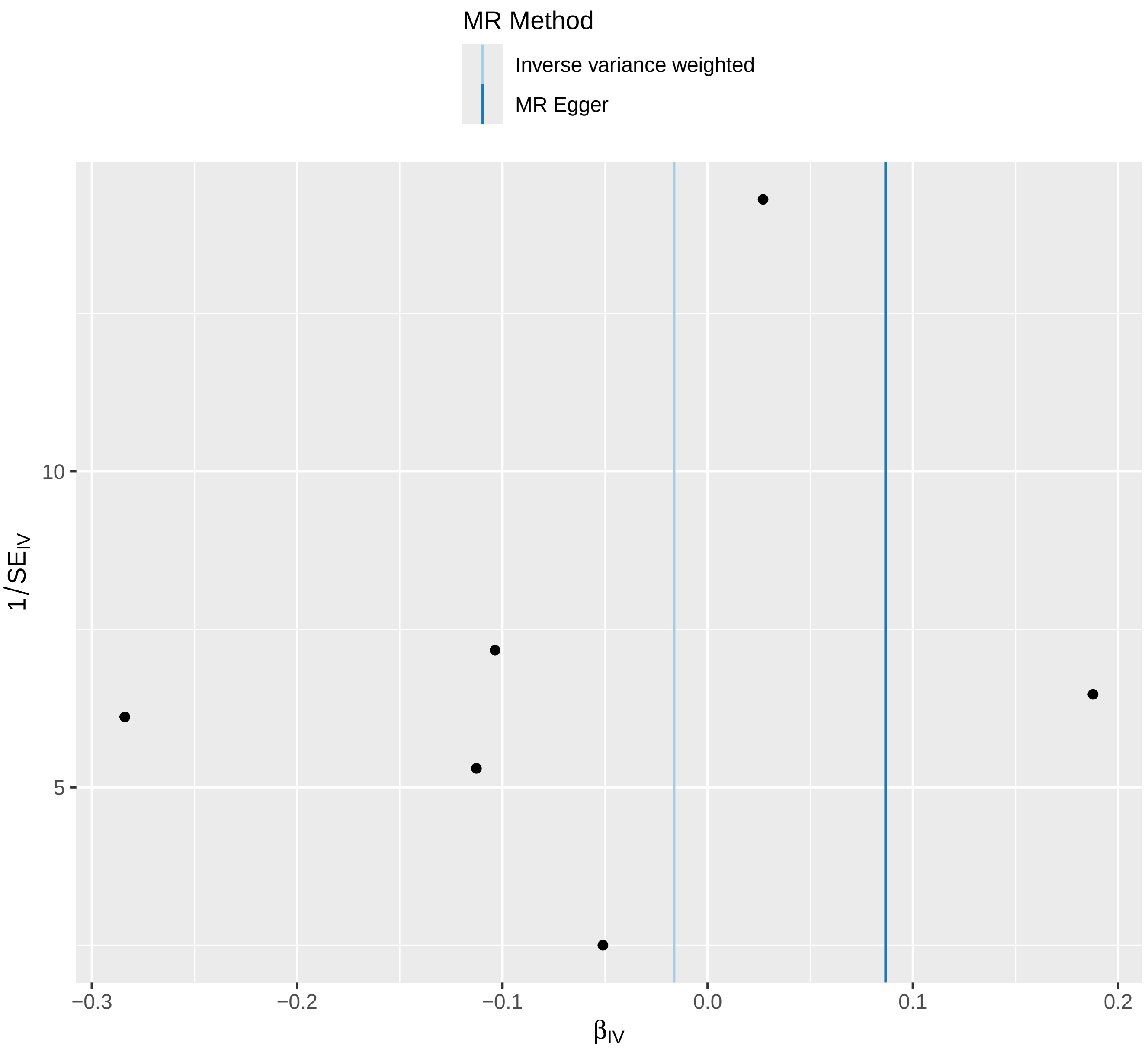
MR Method
Inverse variance weighted
MR Egger
10
5
1/SE_IV
−0.3
−0.2
−0.1
0.0
0.1
0.2
β_IV

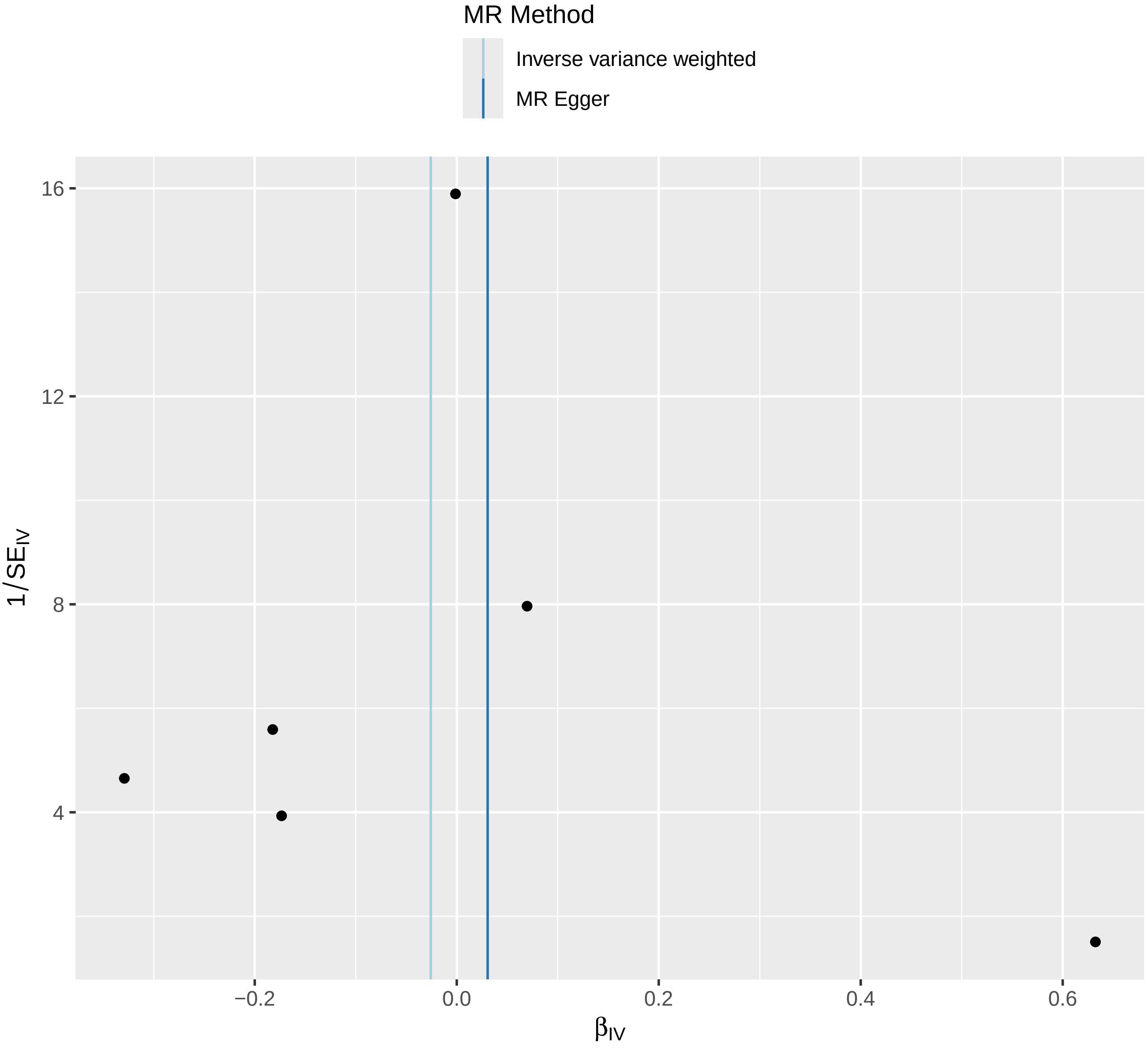
MR Method
Inverse variance weighted
MR Egger
16
12
8
4
1/SE_IV
−0.2
0.0
0.2
0.4
0.6
β_IV

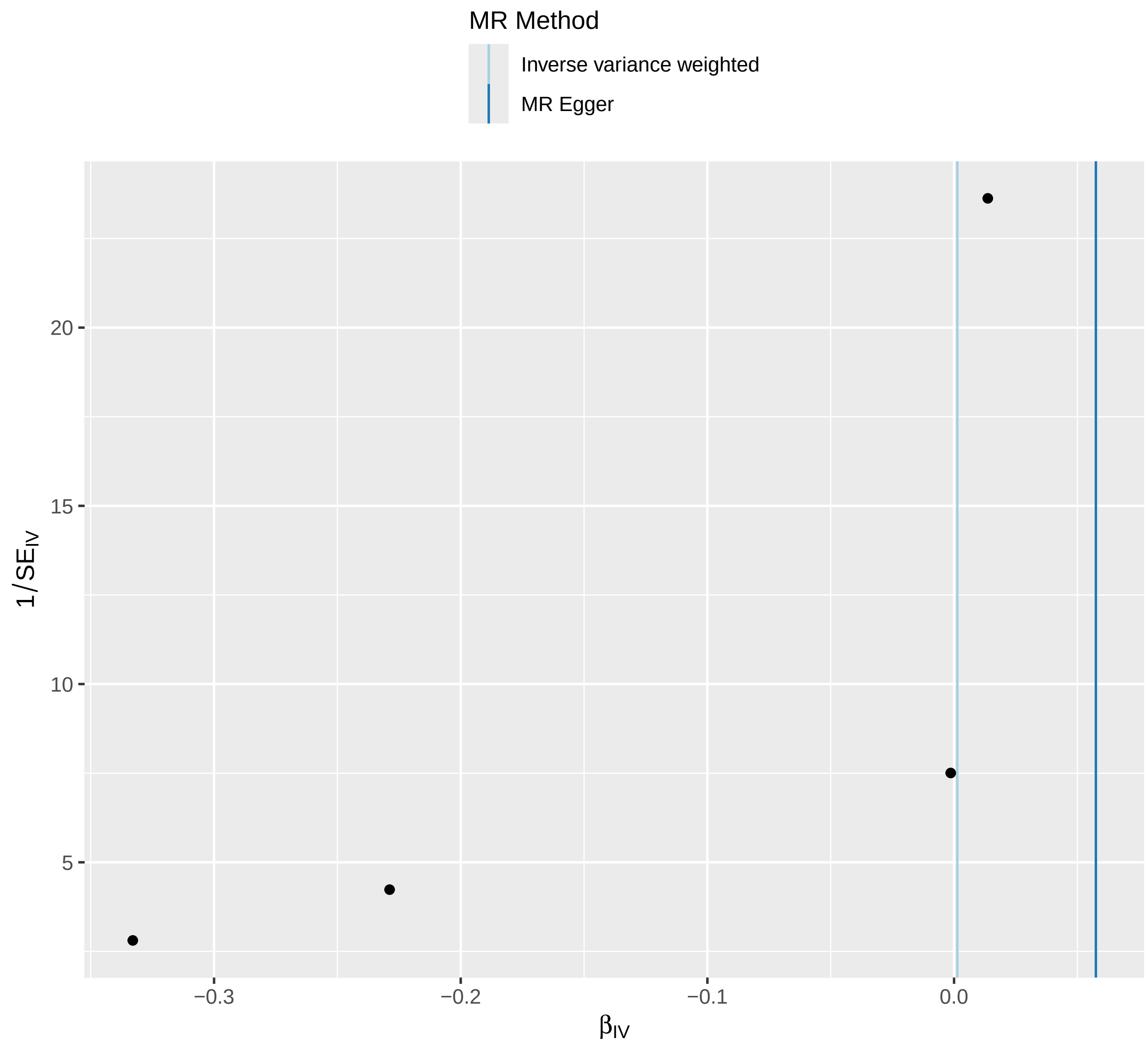

MR Method
Inverse variance weighted
MR Egger
20
15
10
5
1/SE_IV
−0.3
−0.2
−0.1
0.0
β_IV

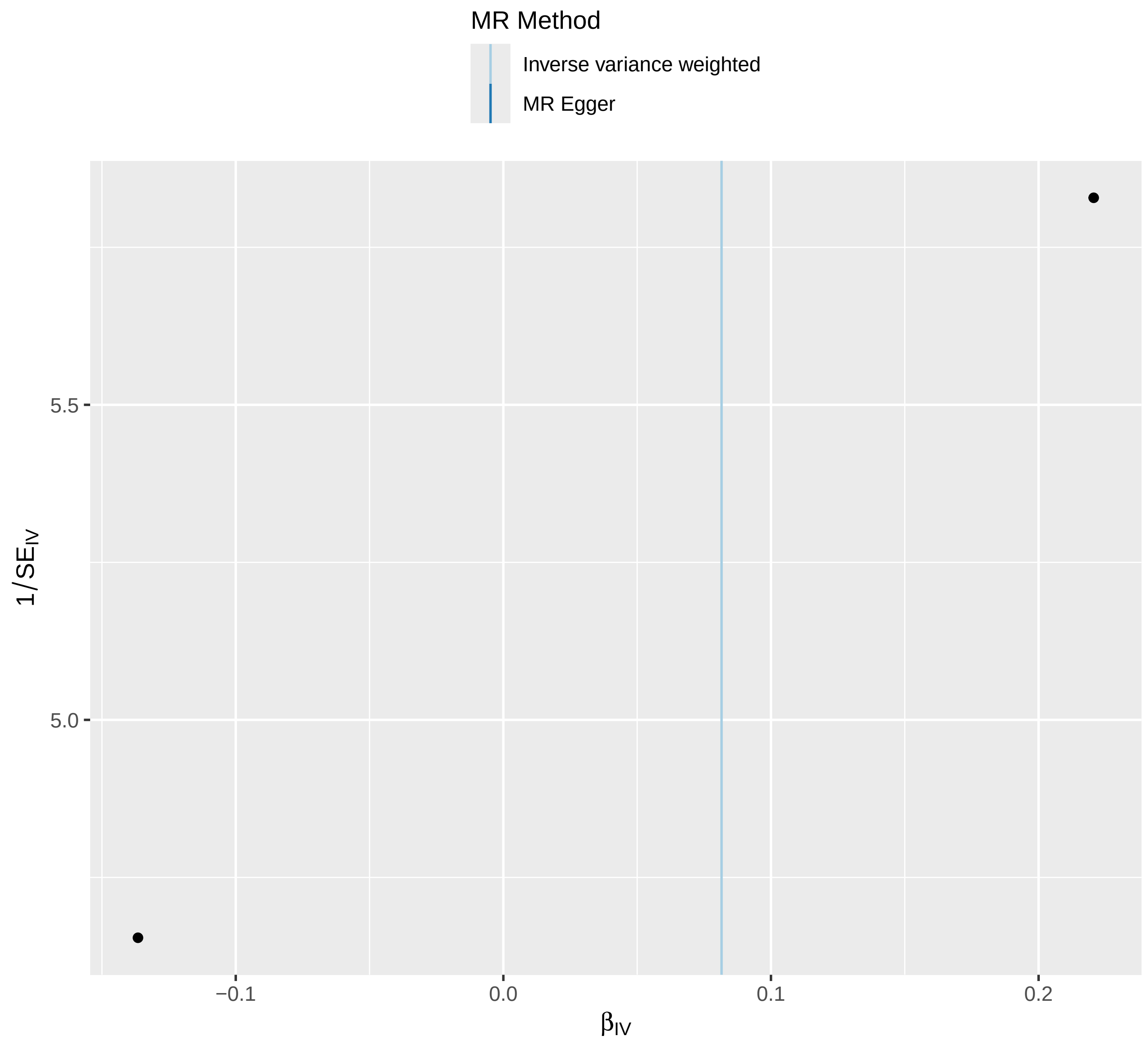
MR Method
Inverse variance weighted
MR Egger
5.5
5.0
1/SE$_{IV}$
−0.1
0.0
0.1
0.2
β$_{IV}$

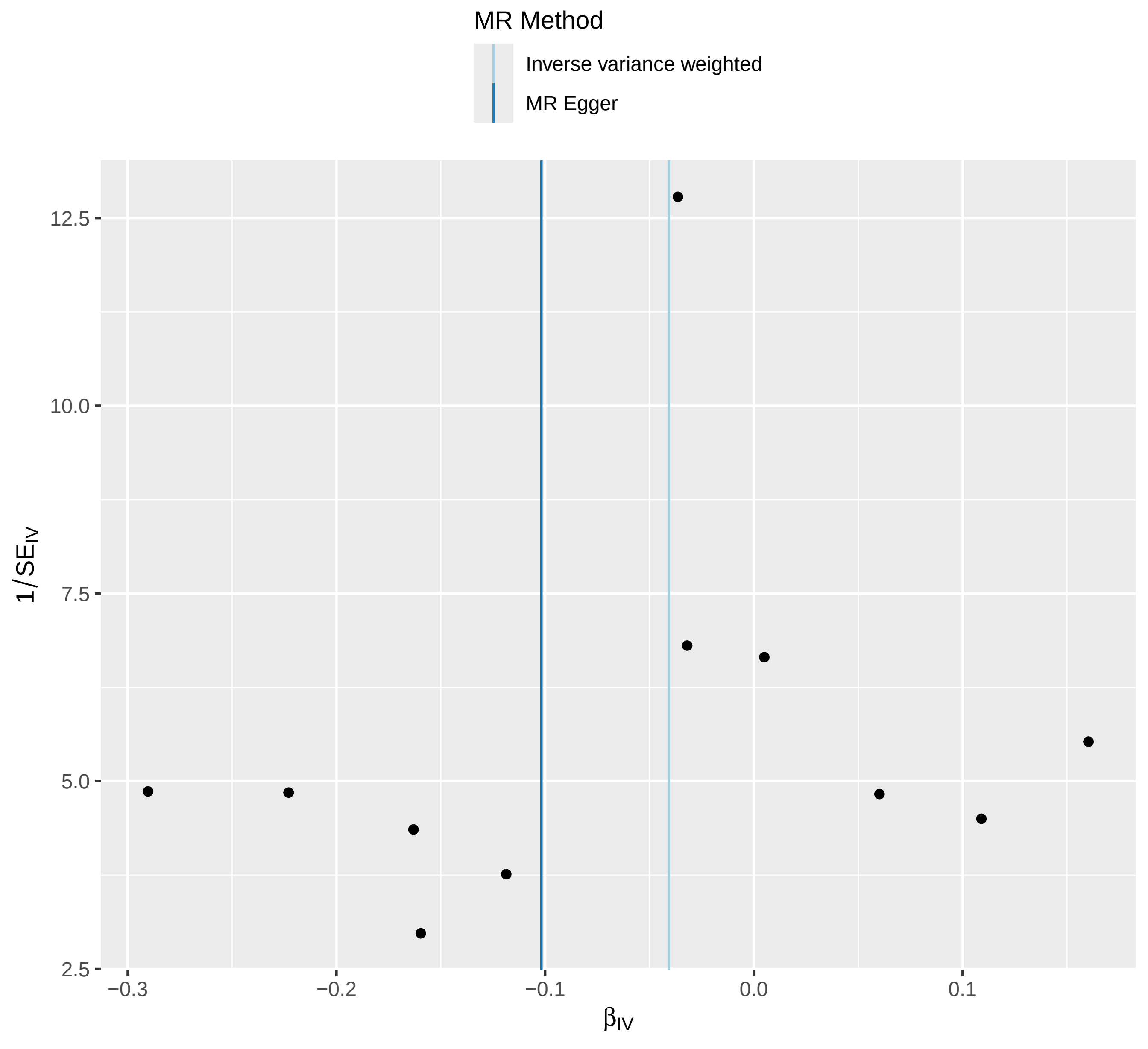

MR Method
Inverse variance weighted
MR Egger
12.5
10.0
7.5
5.0
2.5
1/SE_IV
−0.3
−0.2
−0.1
0.0
0.1
β_IV

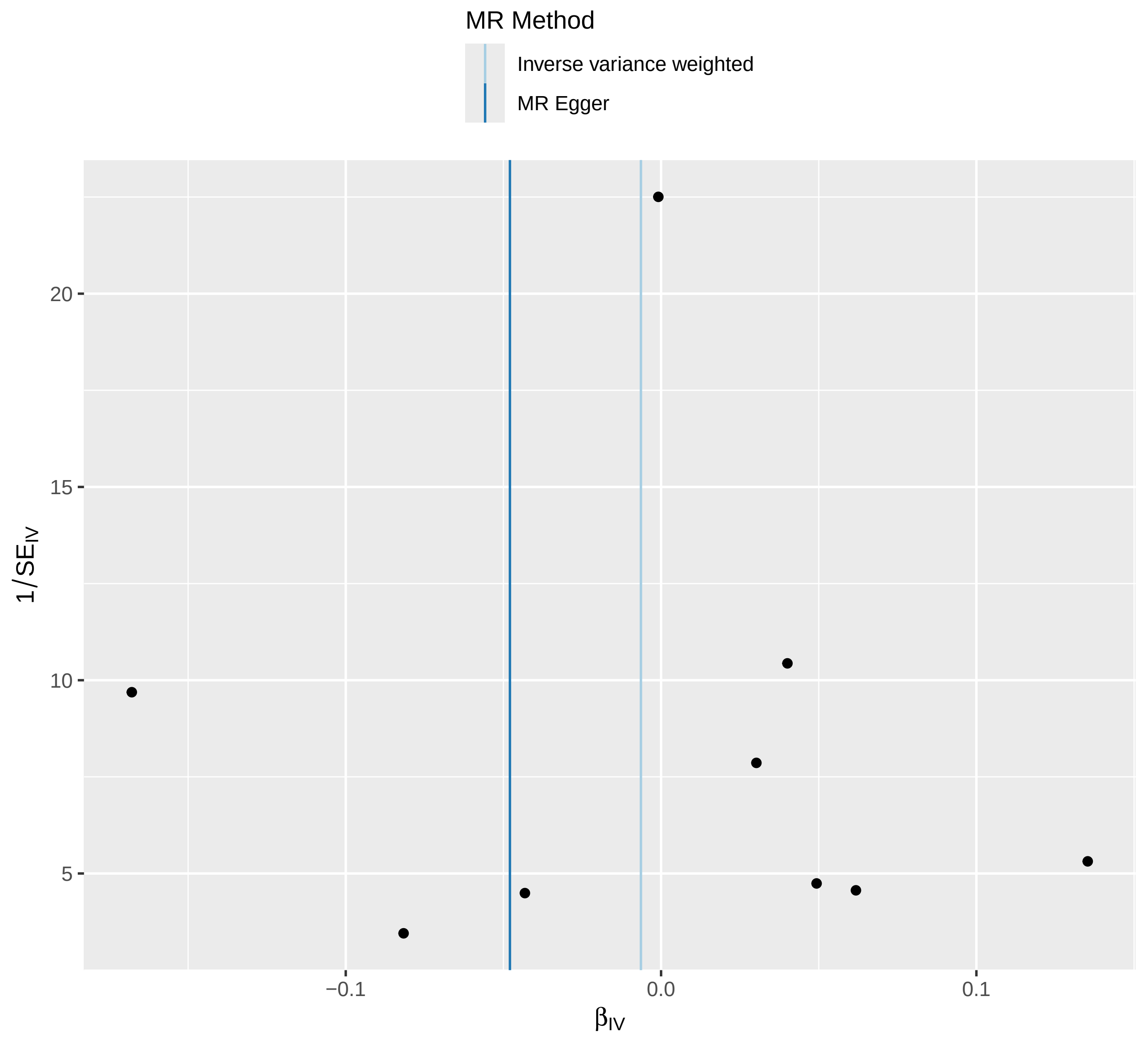
MR Method
Inverse variance weighted
MR Egger
20
15
10
5
1/SE$_{IV}$
−0.1
0.0
0.1
β$_{IV}$

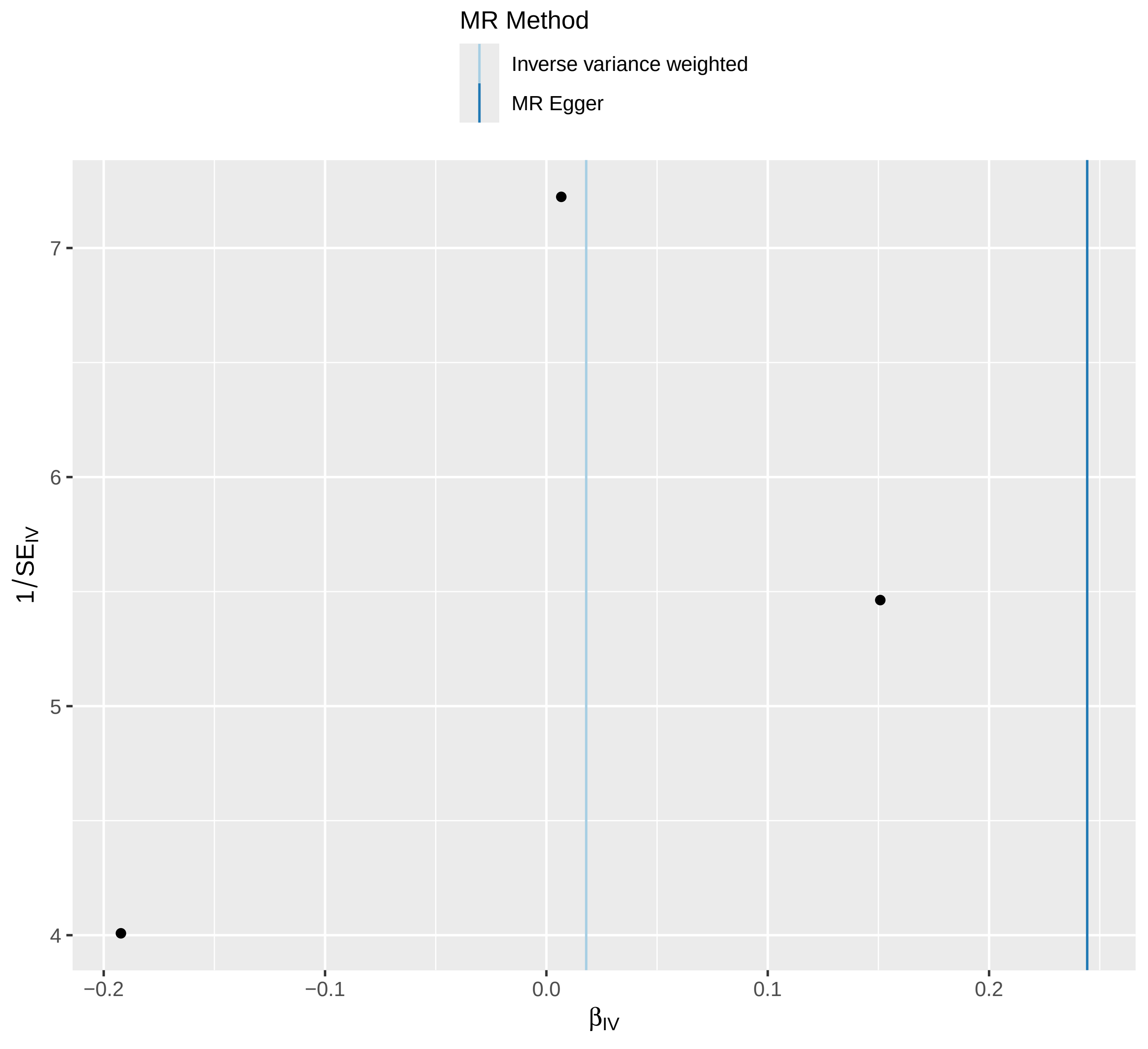
MR Method
Inverse variance weighted
MR Egger
1/SE_IV
7
6
5
4
−0.2
−0.1
0.0
0.1
0.2
β_IV

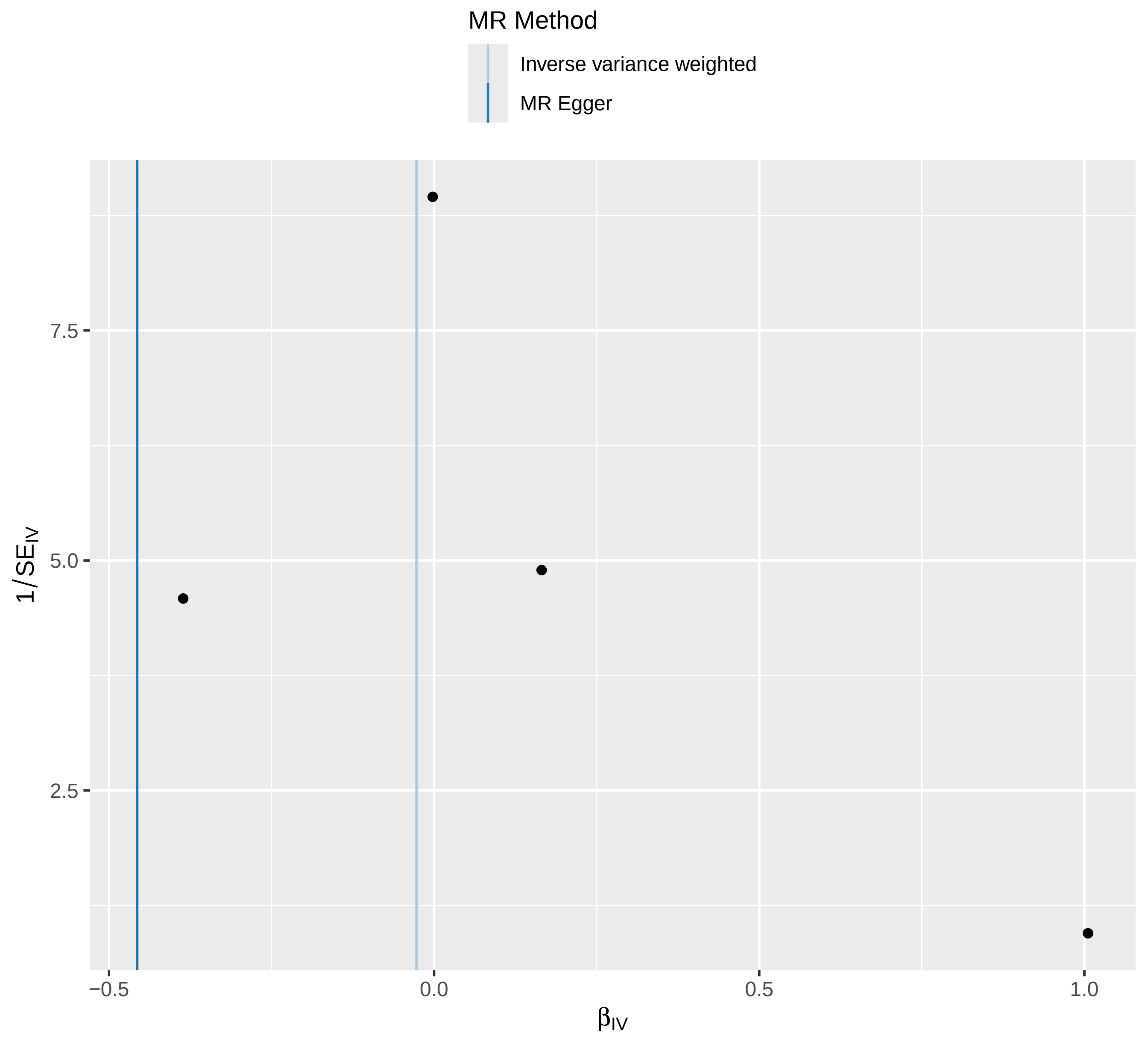

MR Method
Inverse variance weighted
MR Egger
7.5
5.0
2.5
$1/SE_{IV}$
−0.5
0.0
0.5
1.0
$\beta_{IV}$

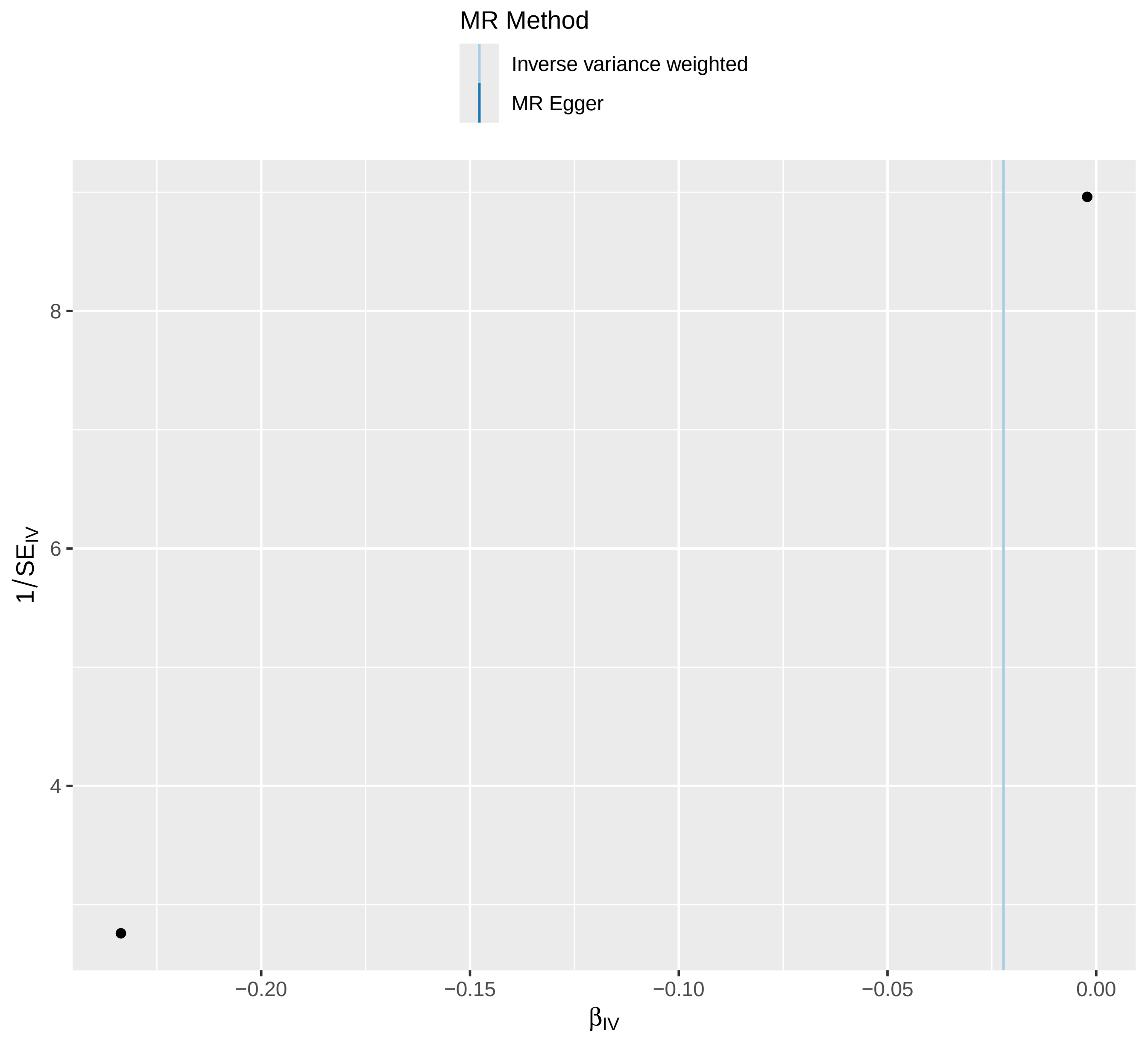

MR Method
Inverse variance weighted
MR Egger
8
6
4
1/SE_IV
−0.20
−0.15
−0.10
−0.05
0.00
β_IV

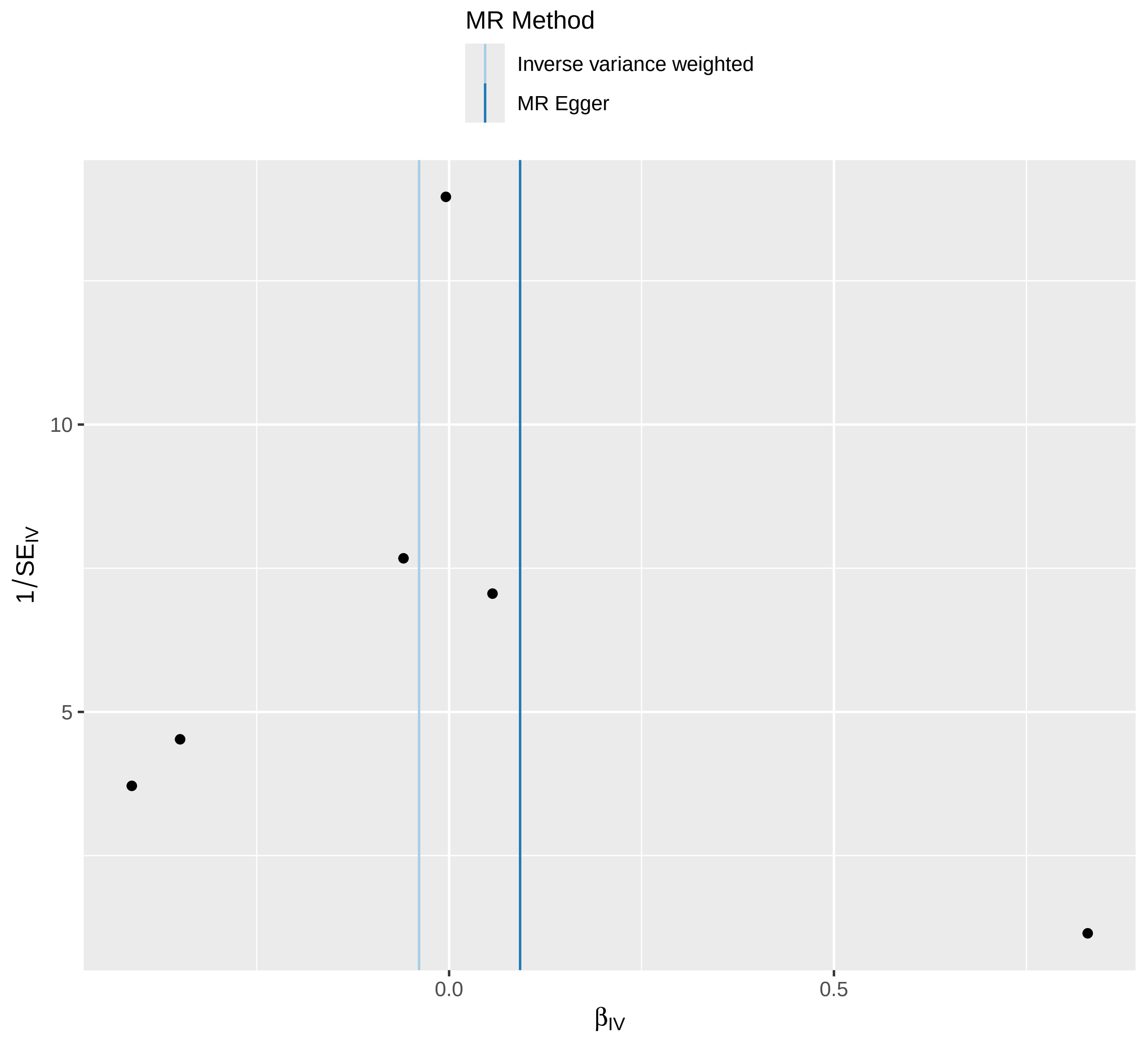

MR Method
Inverse variance weighted
MR Egger
10
5
0.0
0.5
1/SE$_{IV}$
β$_{IV}$

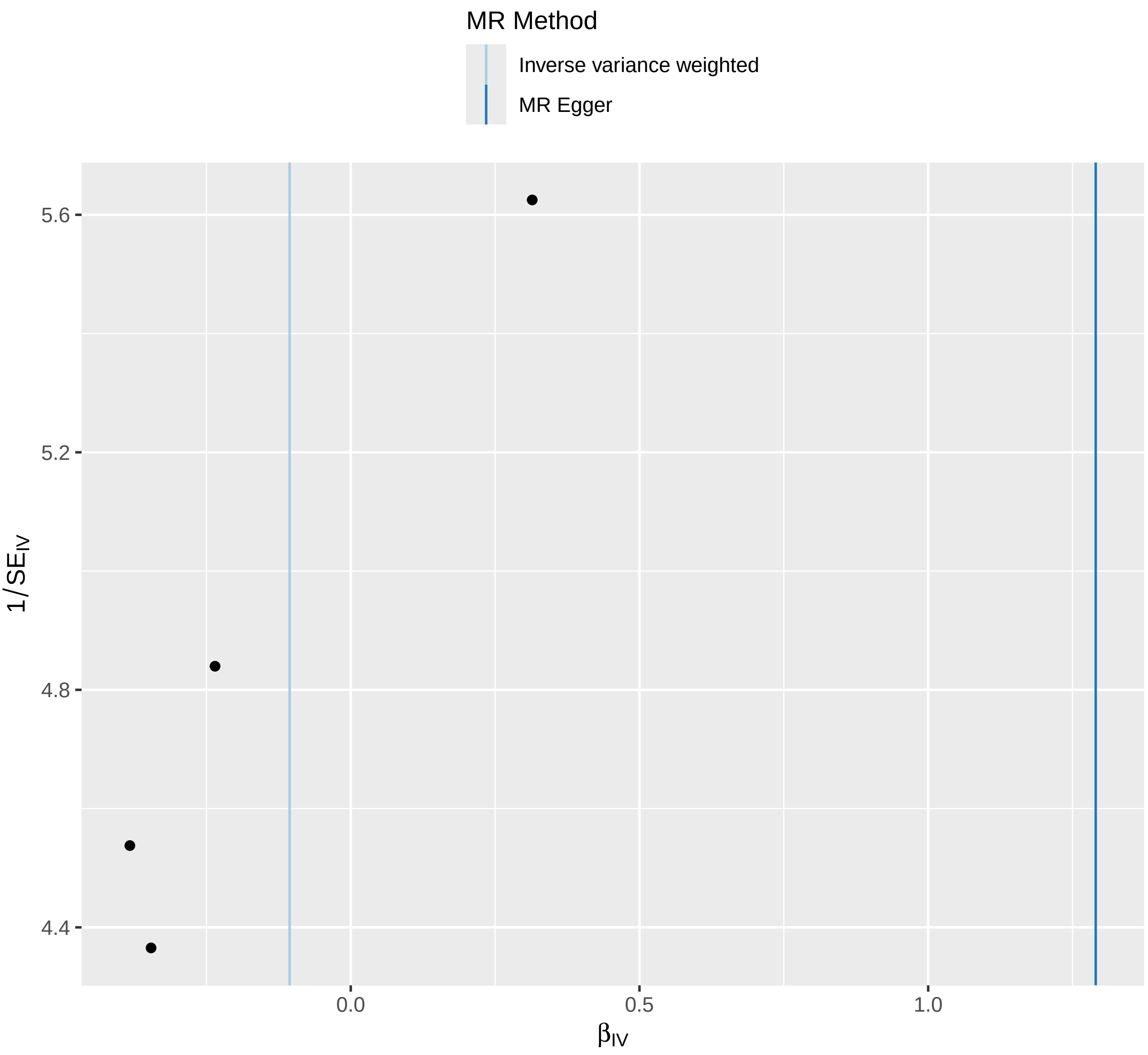
MR Method
Inverse variance weighted
MR Egger
5.6
5.2
4.8
4.4
$1/SE_{IV}$
0.0
0.5
1.0
$\beta_{IV}$

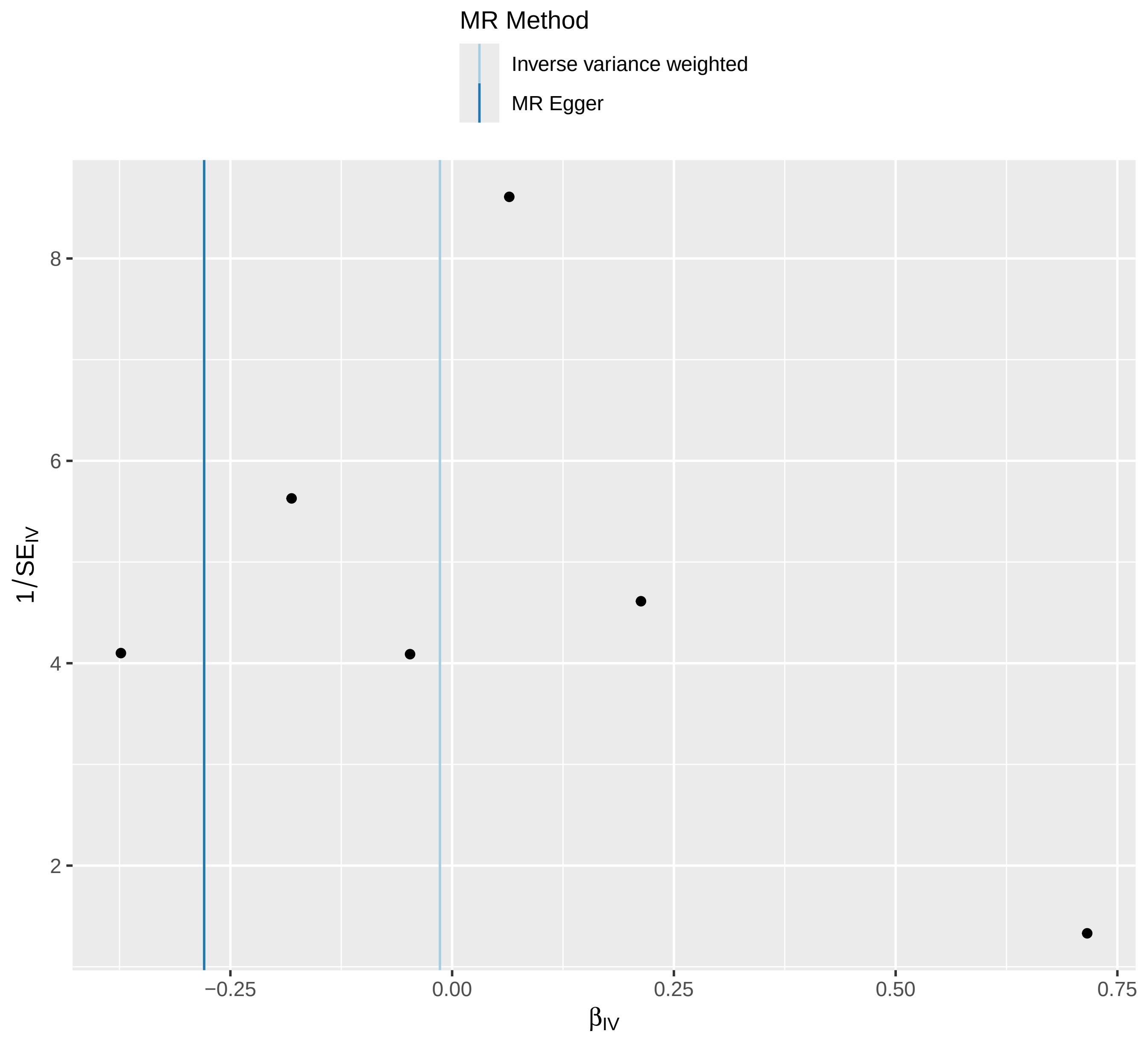

MR Method
Inverse variance weighted
MR Egger
8
6
4
2
1/SE_IV
−0.25
0.00
0.25
0.50
0.75
β_IV

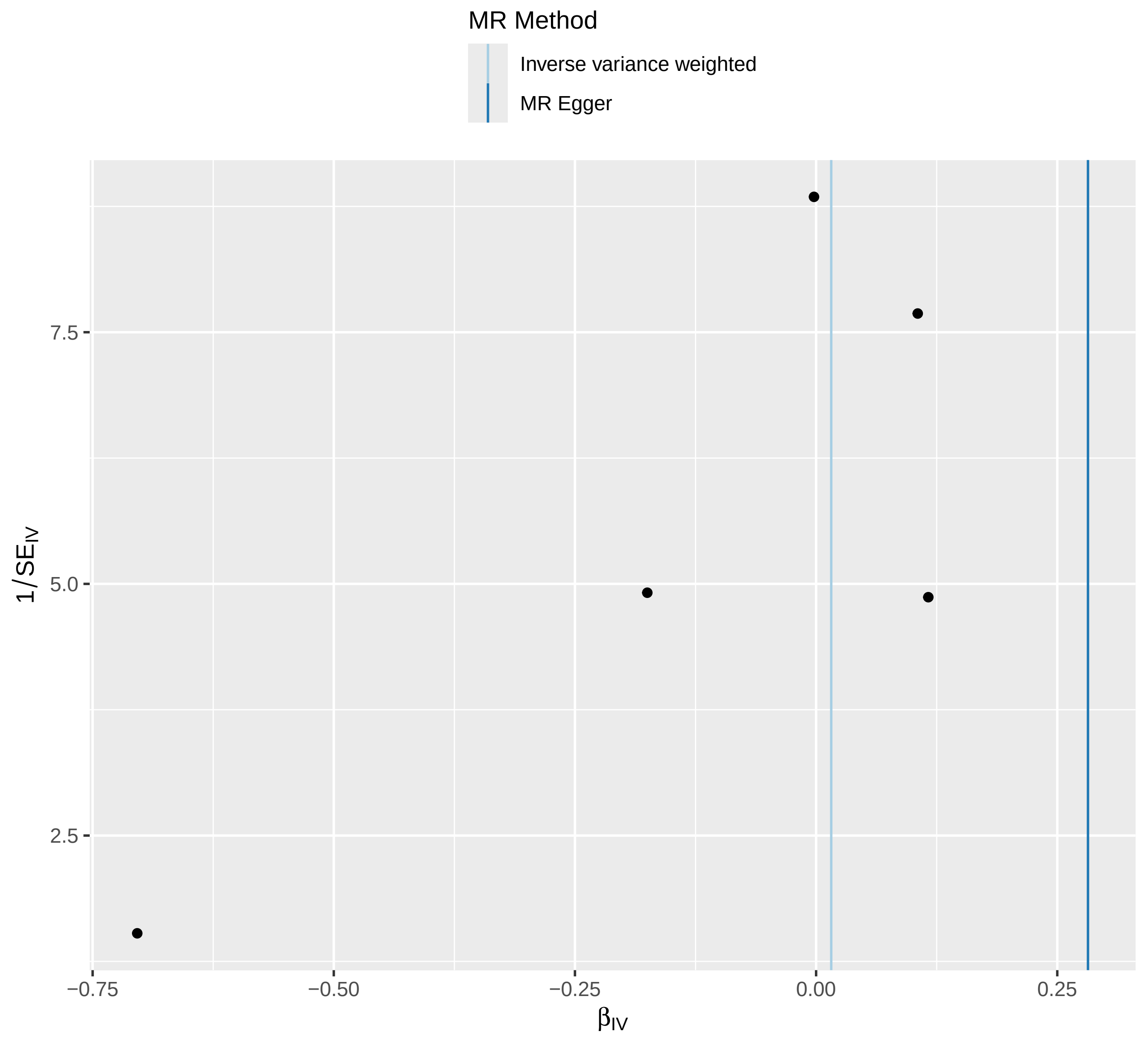
MR Method
Inverse variance weighted
MR Egger
1/$SE_{IV}$
7.5
5.0
2.5
−0.75
−0.50
−0.25
0.00
0.25
$\beta_{IV}$

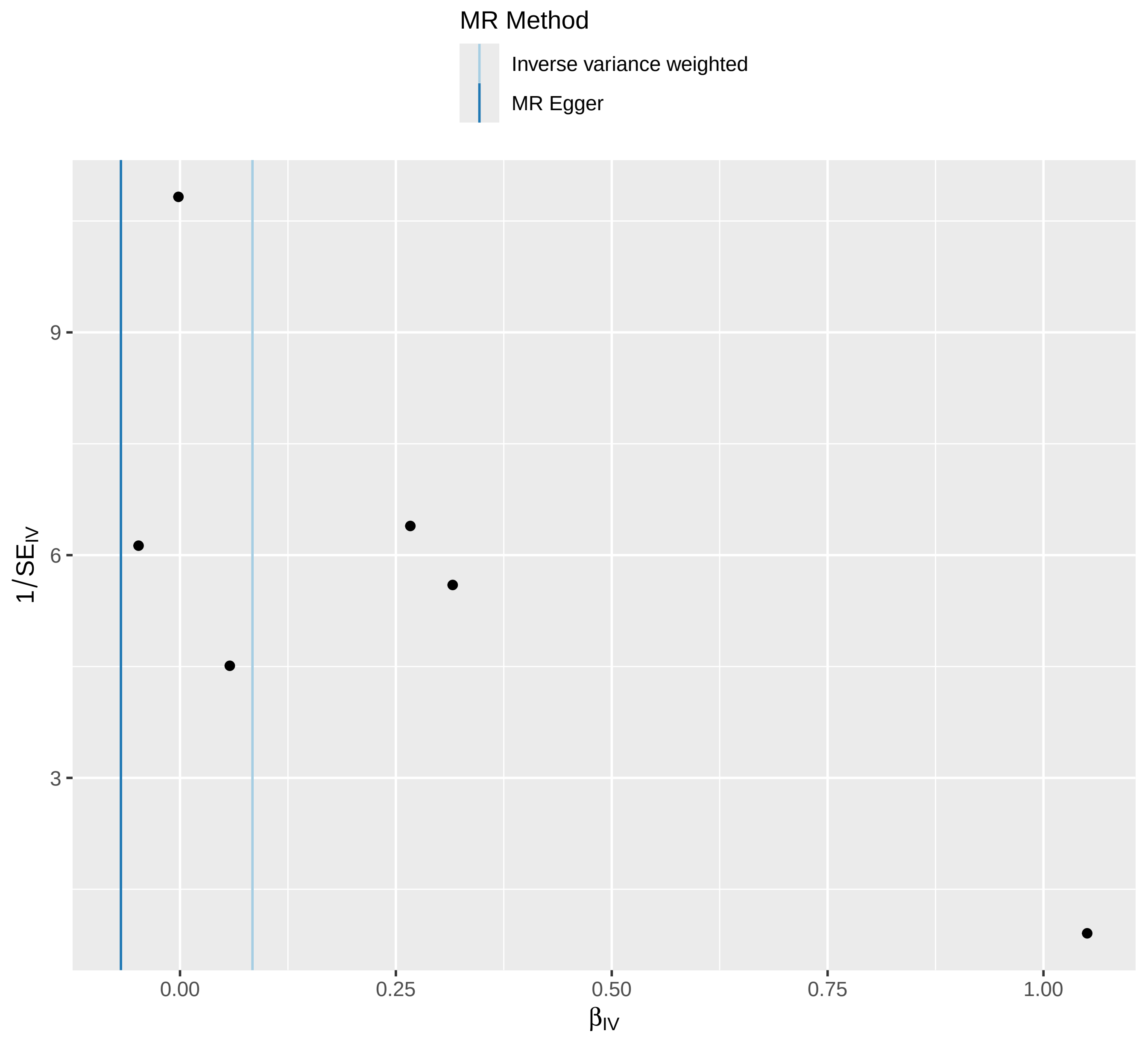
MR Method
Inverse variance weighted
MR Egger
1/SE_IV
9
6
3
0.00
0.25
0.50
0.75
1.00
β_IV

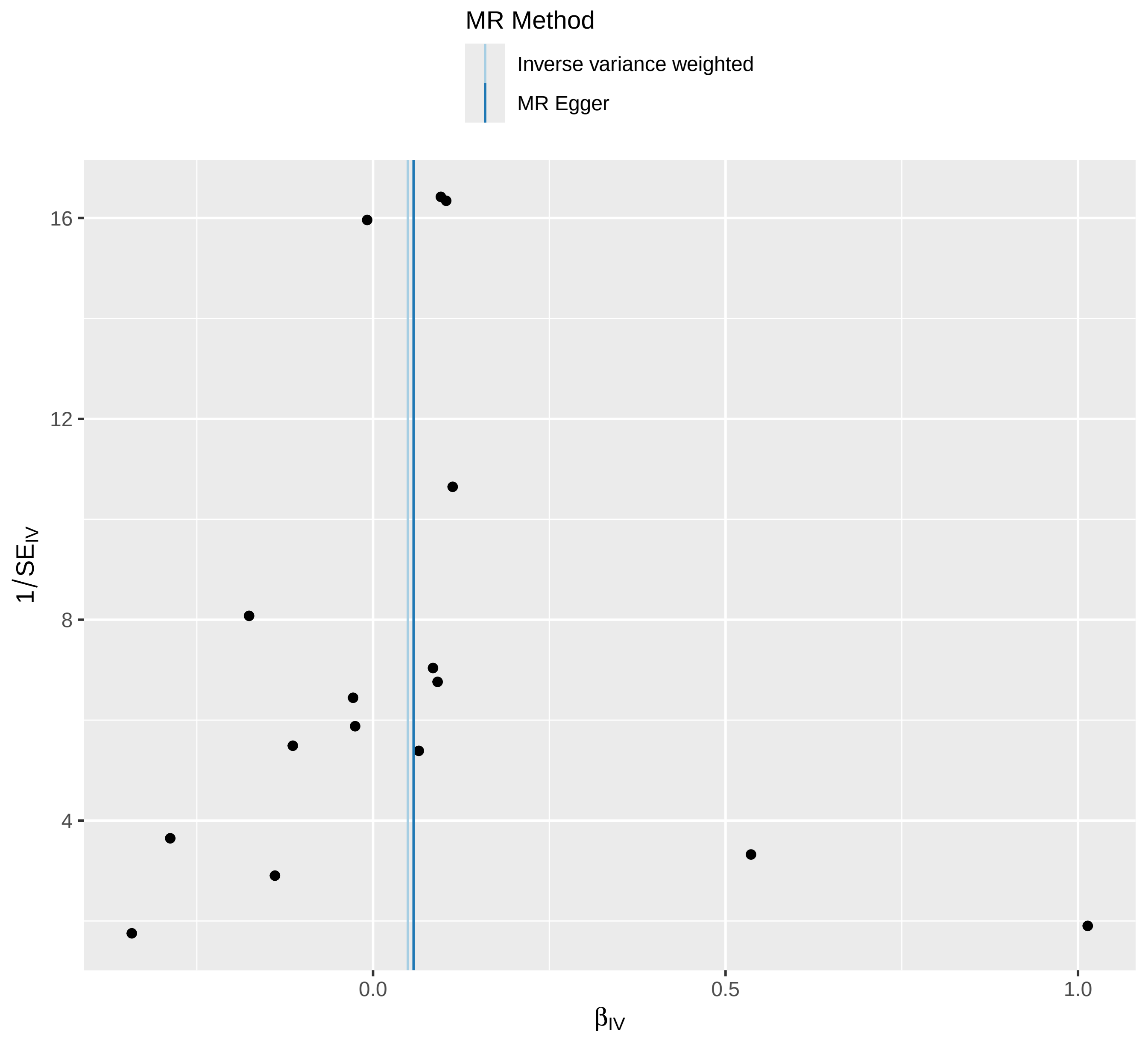

MR Method
Inverse variance weighted
MR Egger
16
12
8
4
$1/SE_{IV}$
0.0
0.5
1.0
$\beta_{IV}$

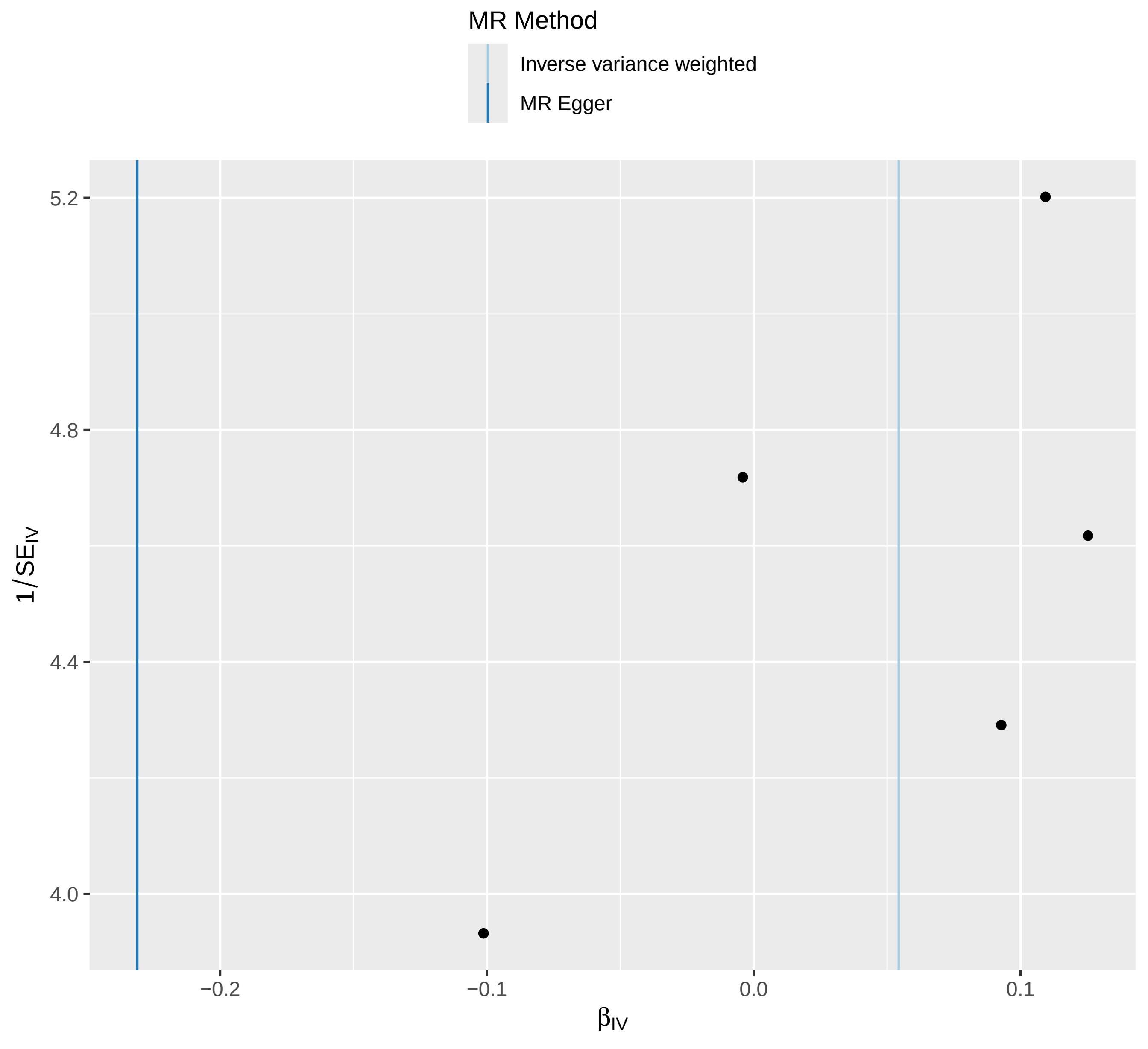
MR Method
Inverse variance weighted
MR Egger
5.2
4.8
4.4
4.0
1/SE_IV
−0.2
−0.1
0.0
0.1
β_IV

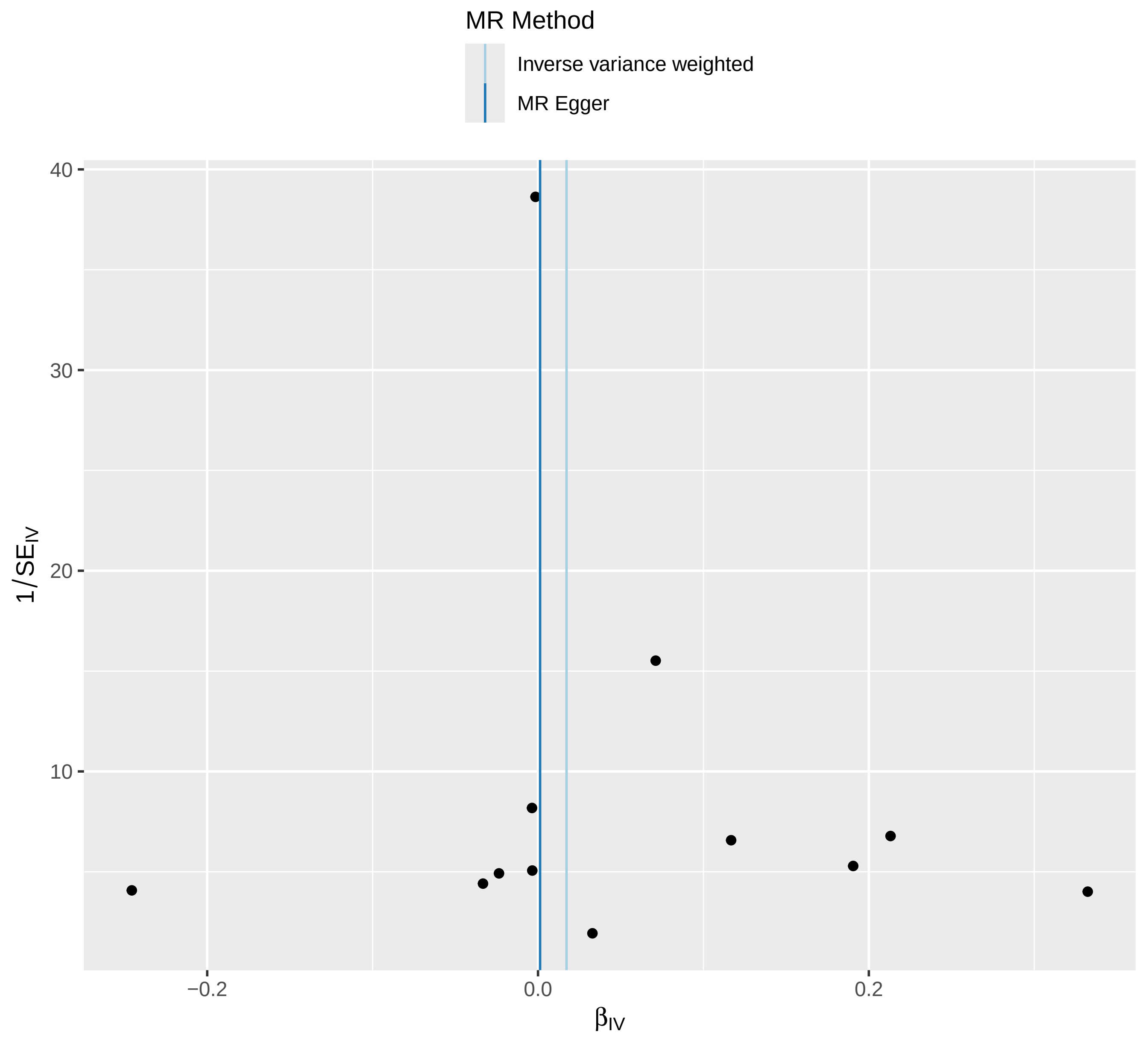

MR Method
Inverse variance weighted
MR Egger
40
30
20
10
1/SE$_{IV}$
−0.2
0.0
0.2
β$_{IV}$

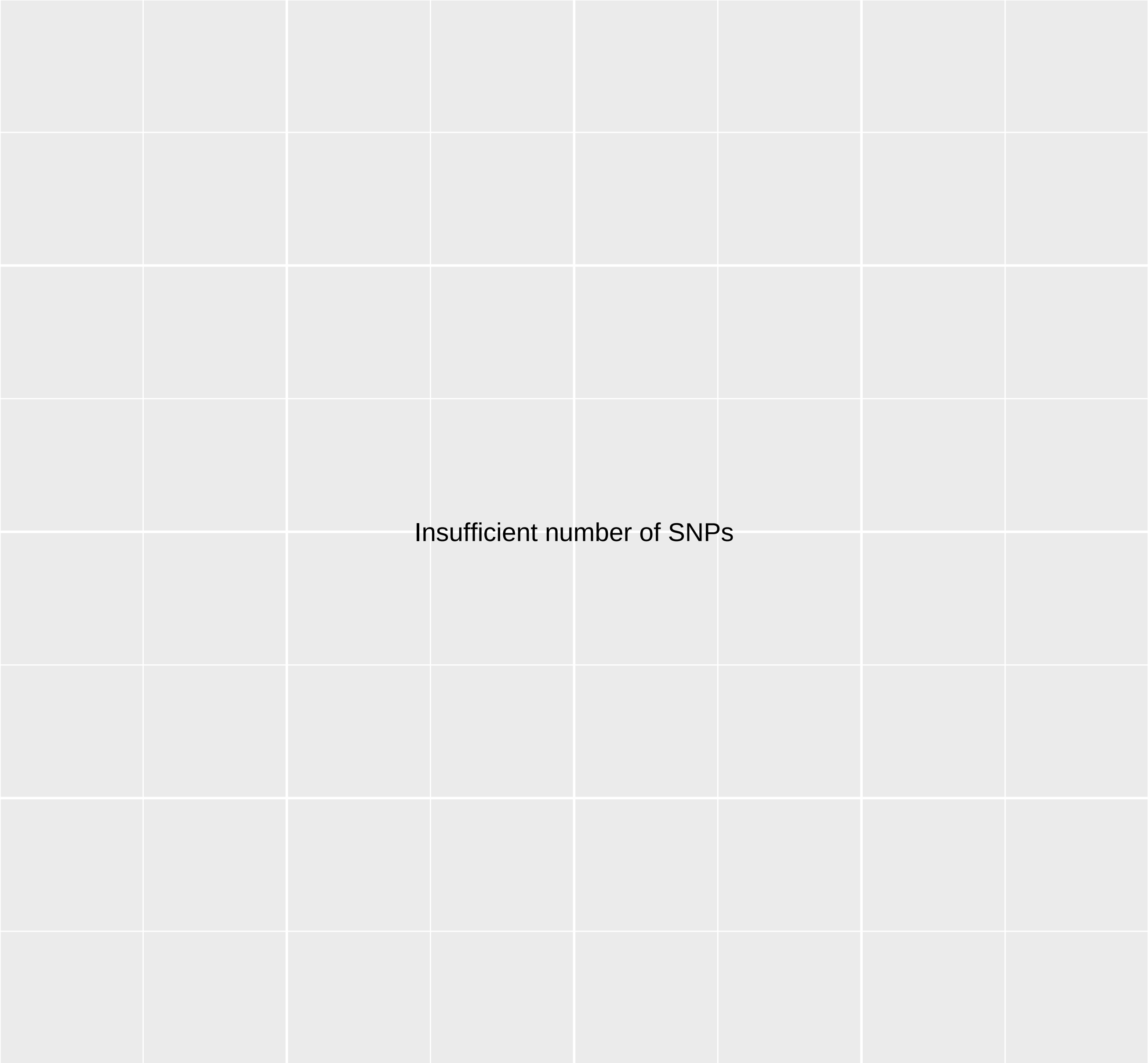
Insufficient number of SNPs

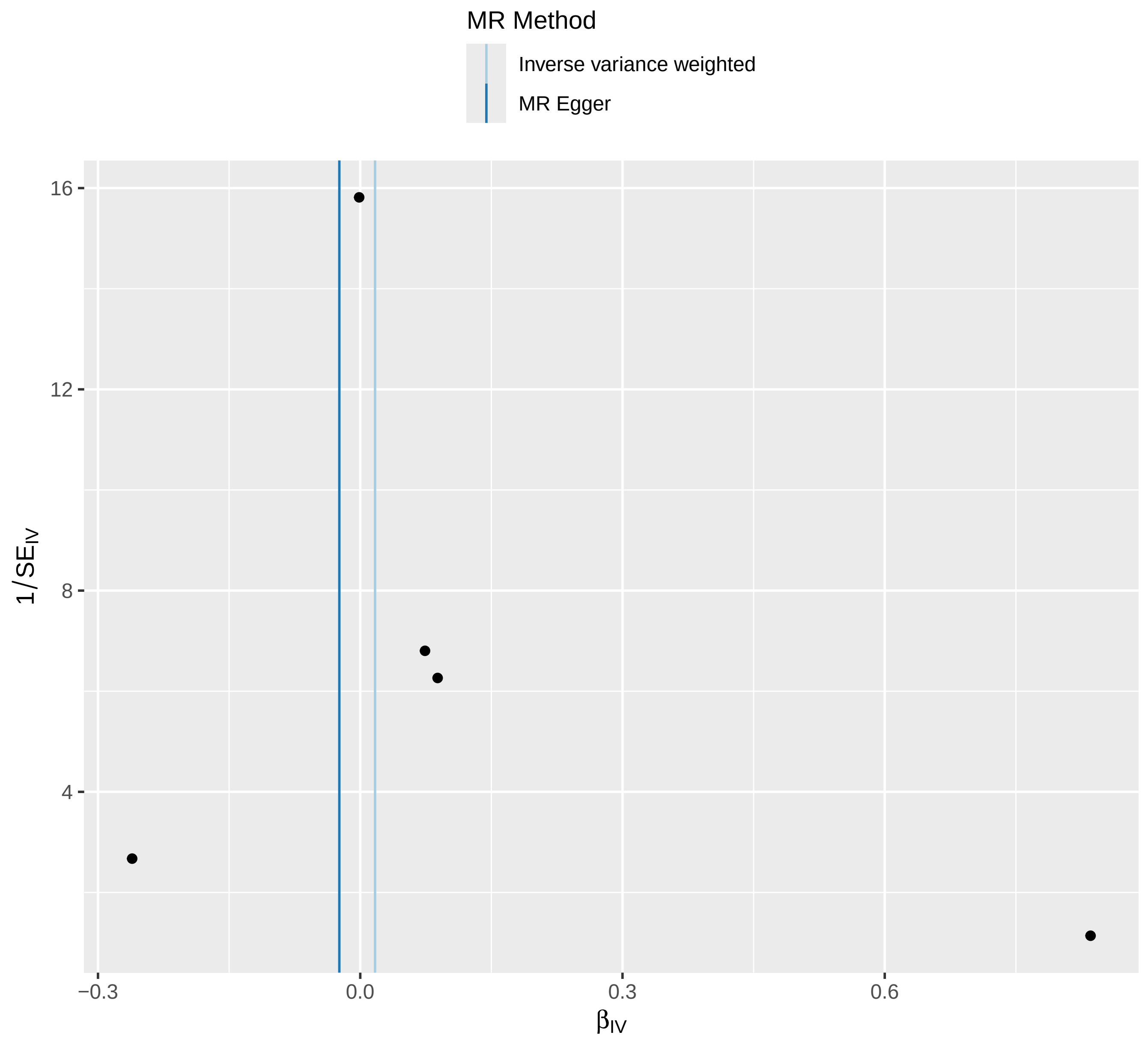

MR Method
Inverse variance weighted
MR Egger
16
12
8
4
1/SE$_{IV}$
−0.3
0.0
0.3
0.6
β$_{IV}$

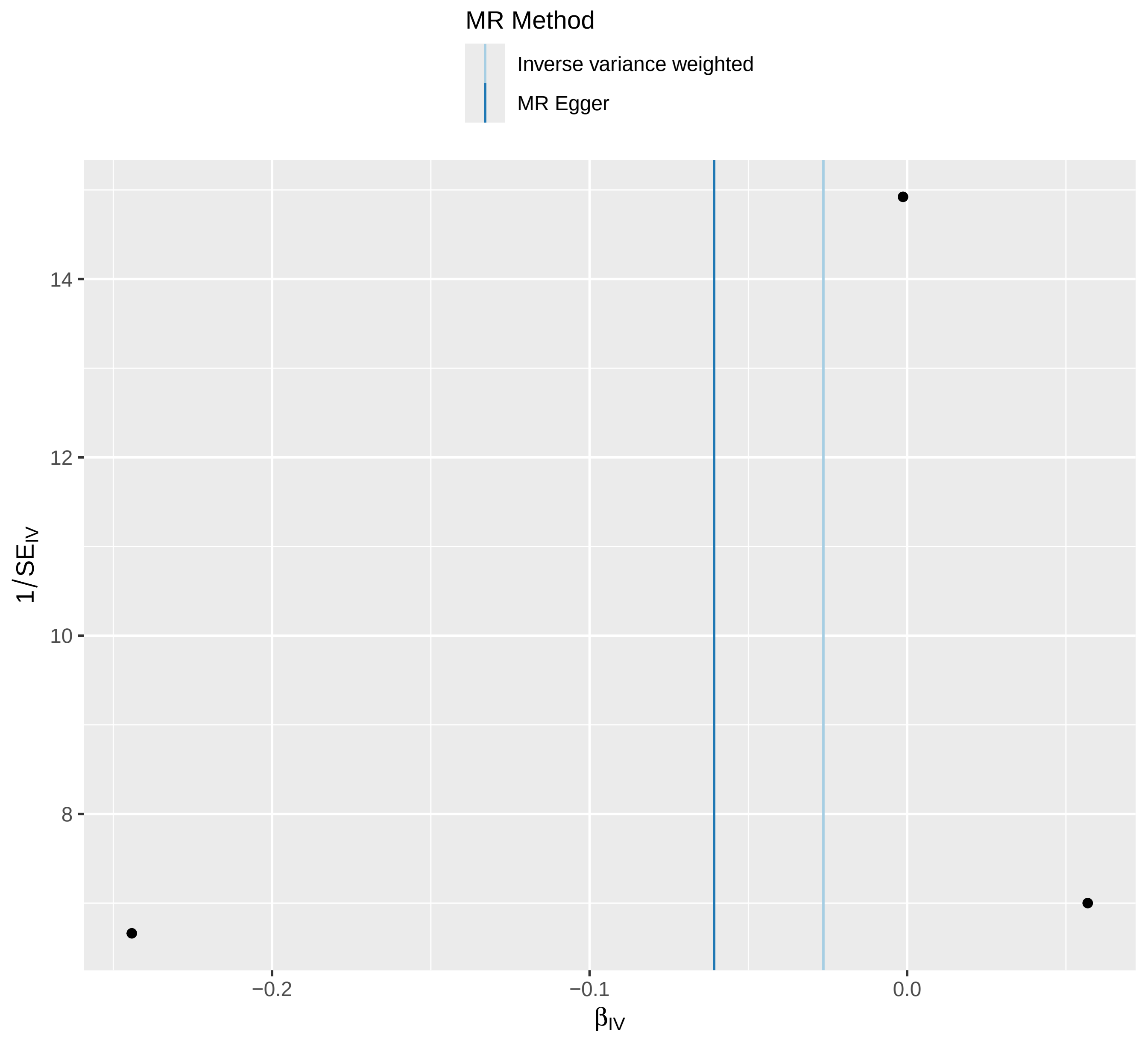

MR Method
Inverse variance weighted
MR Egger
14
12
10
8
1/SE_IV
−0.2
−0.1
0.0
β_IV

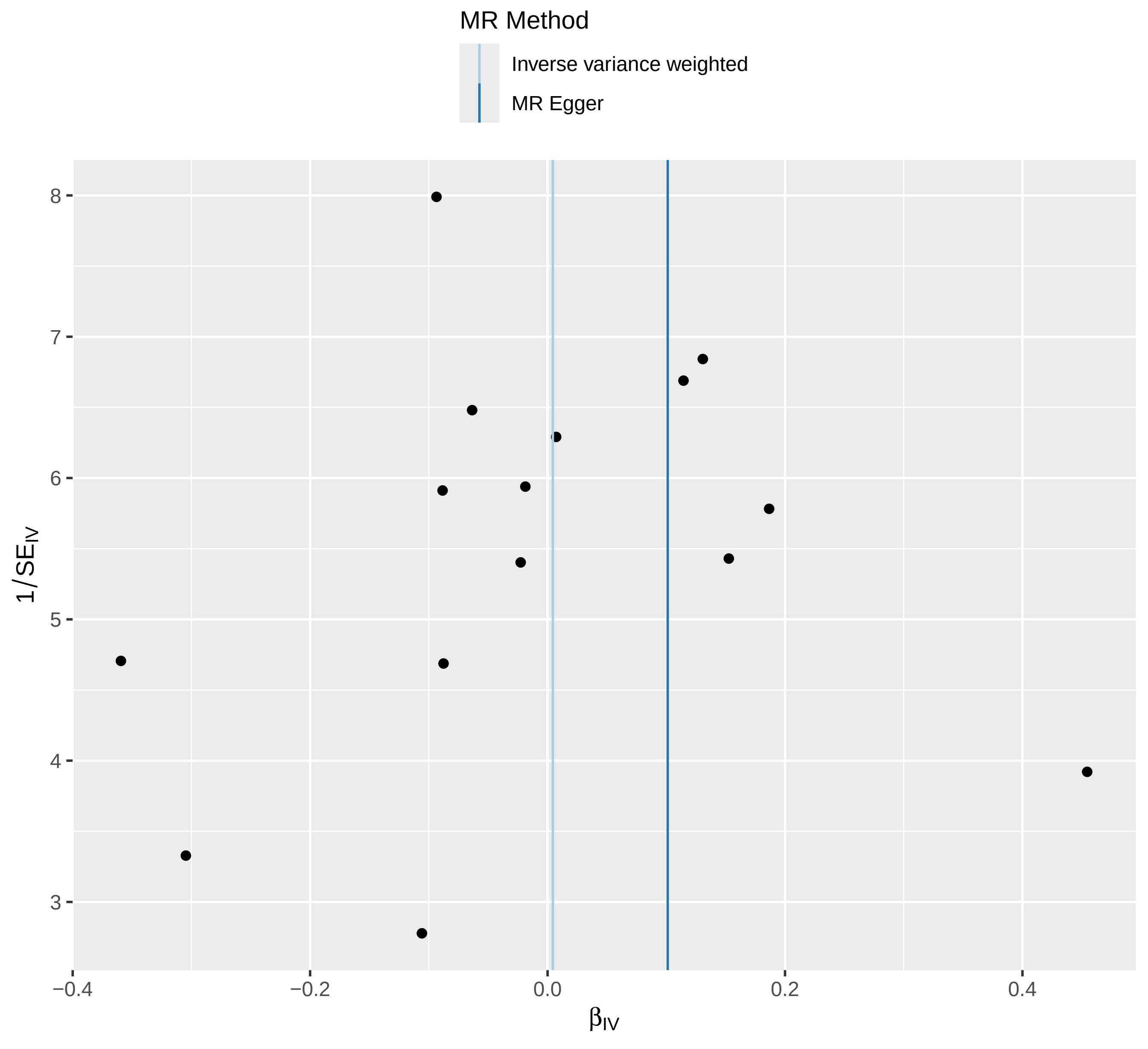
MR Method
Inverse variance weighted
MR Egger
1/$SE_{IV}$
$\beta_{IV}$
8
7
6
5
4
3
−0.4
−0.2
0.0
0.2
0.4

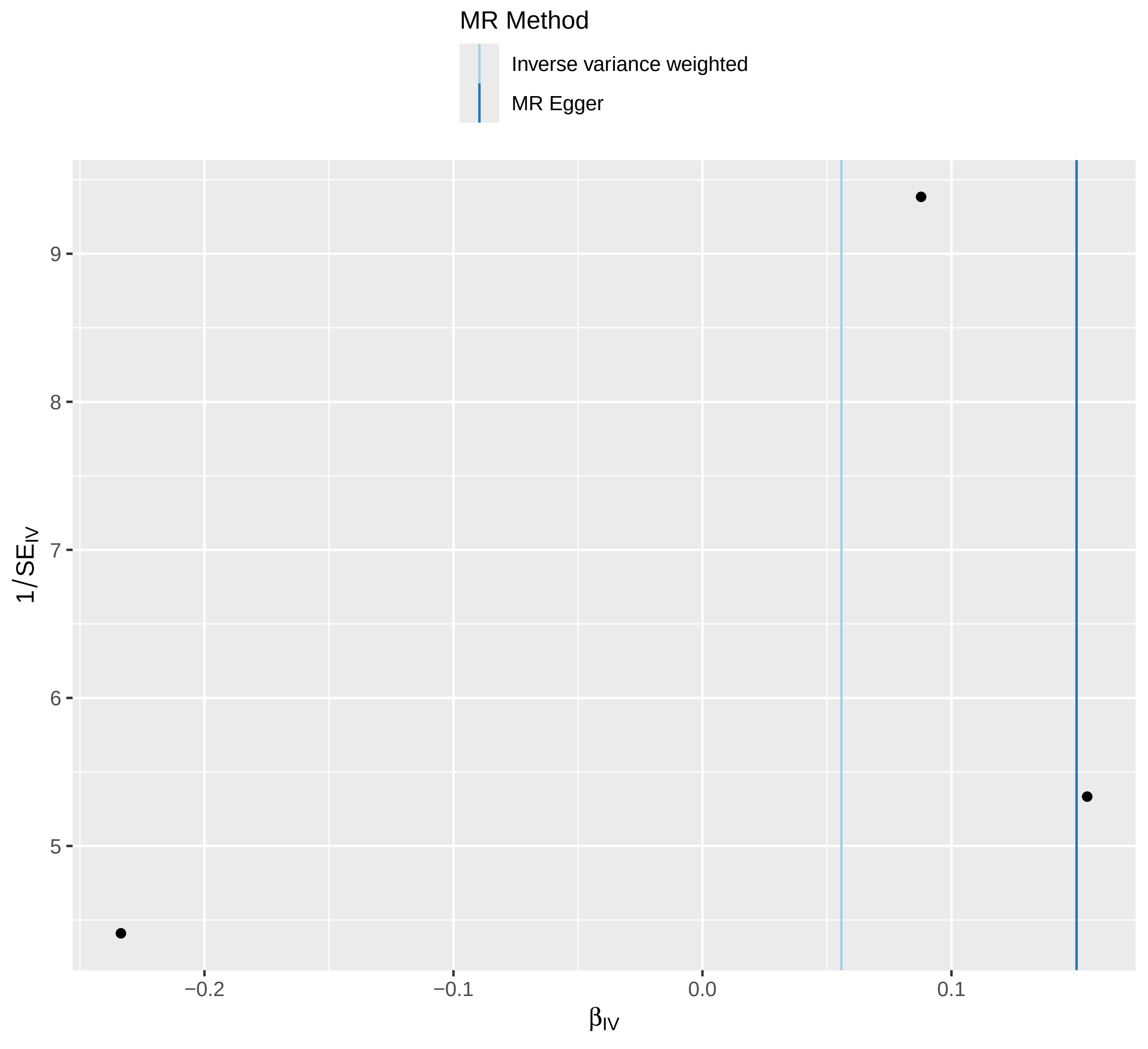

MR Method
Inverse variance weighted
MR Egger
1/SE$_{IV}$
$\beta_{IV}$
9
8
7
6
5
−0.2
−0.1
0.0
0.1

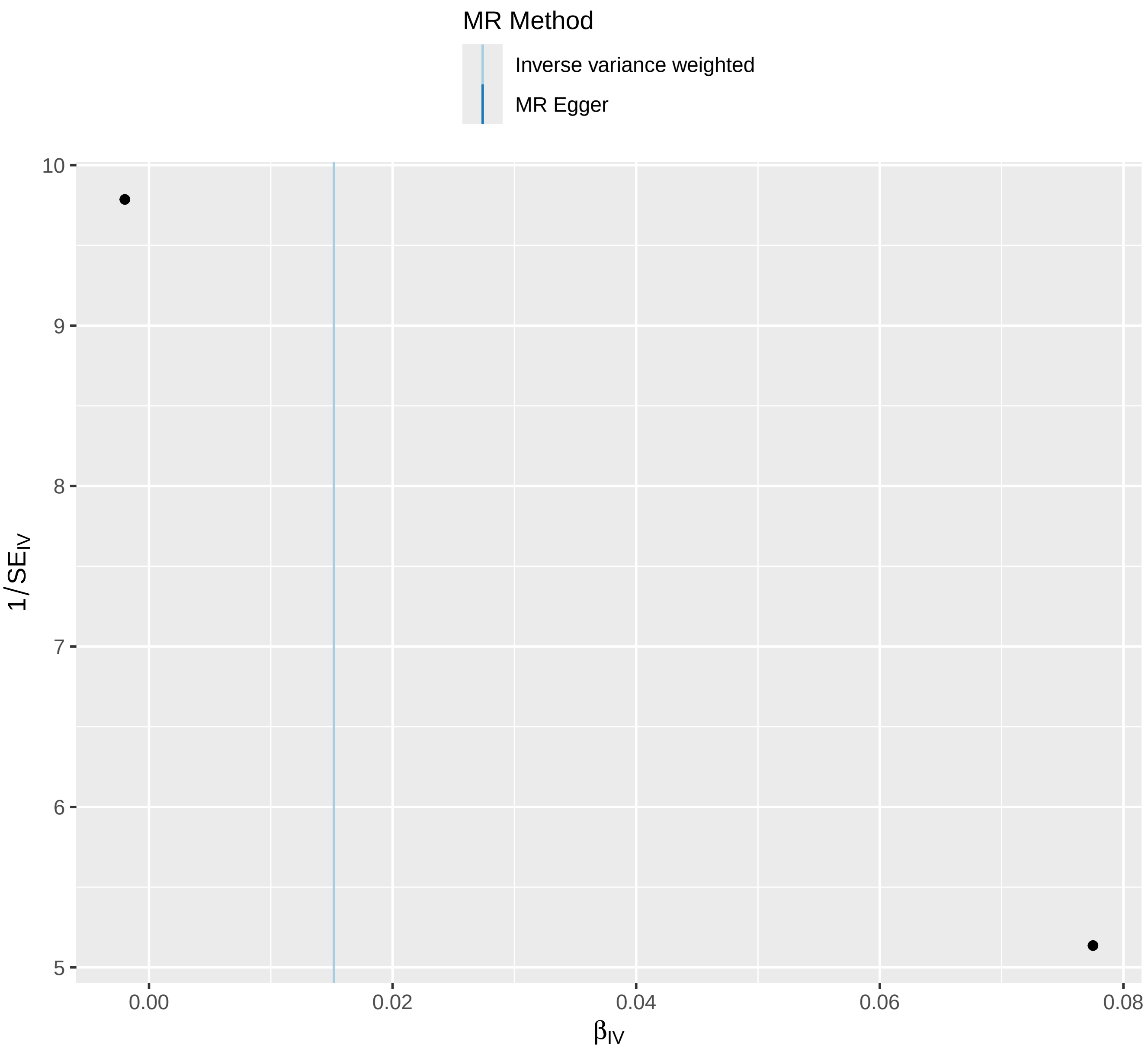

MR Method
Inverse variance weighted
MR Egger
10
9
8
7
6
5
1/SE$_{IV}$
0.00
0.02
0.04
0.06
0.08
β$_{IV}$

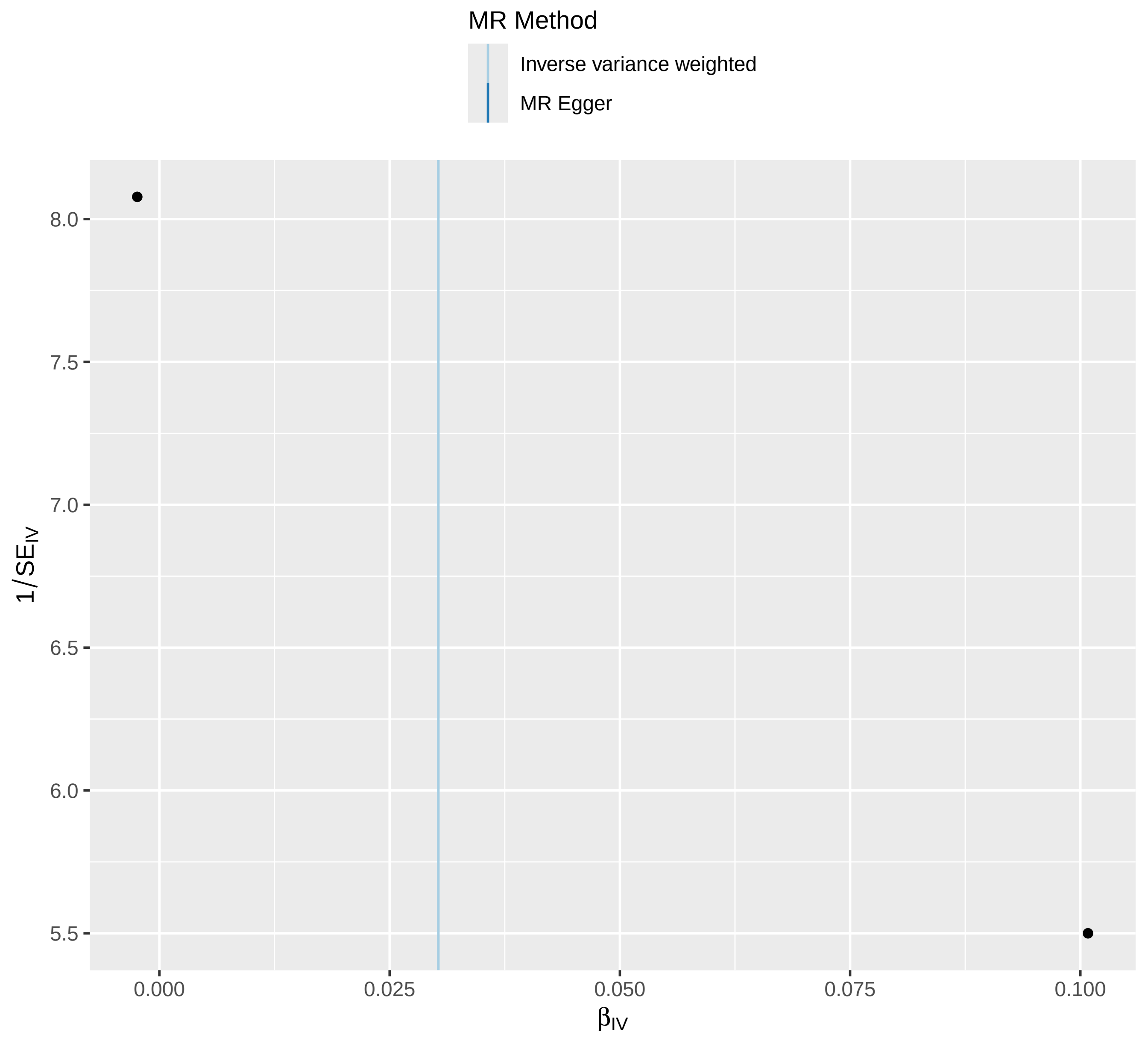

MR Method
Inverse variance weighted
MR Egger
8.0
7.5
7.0
6.5
6.0
5.5
1/SE_IV
0.000
0.025
0.050
0.075
0.100
β_IV

Insufficient number of SNPs

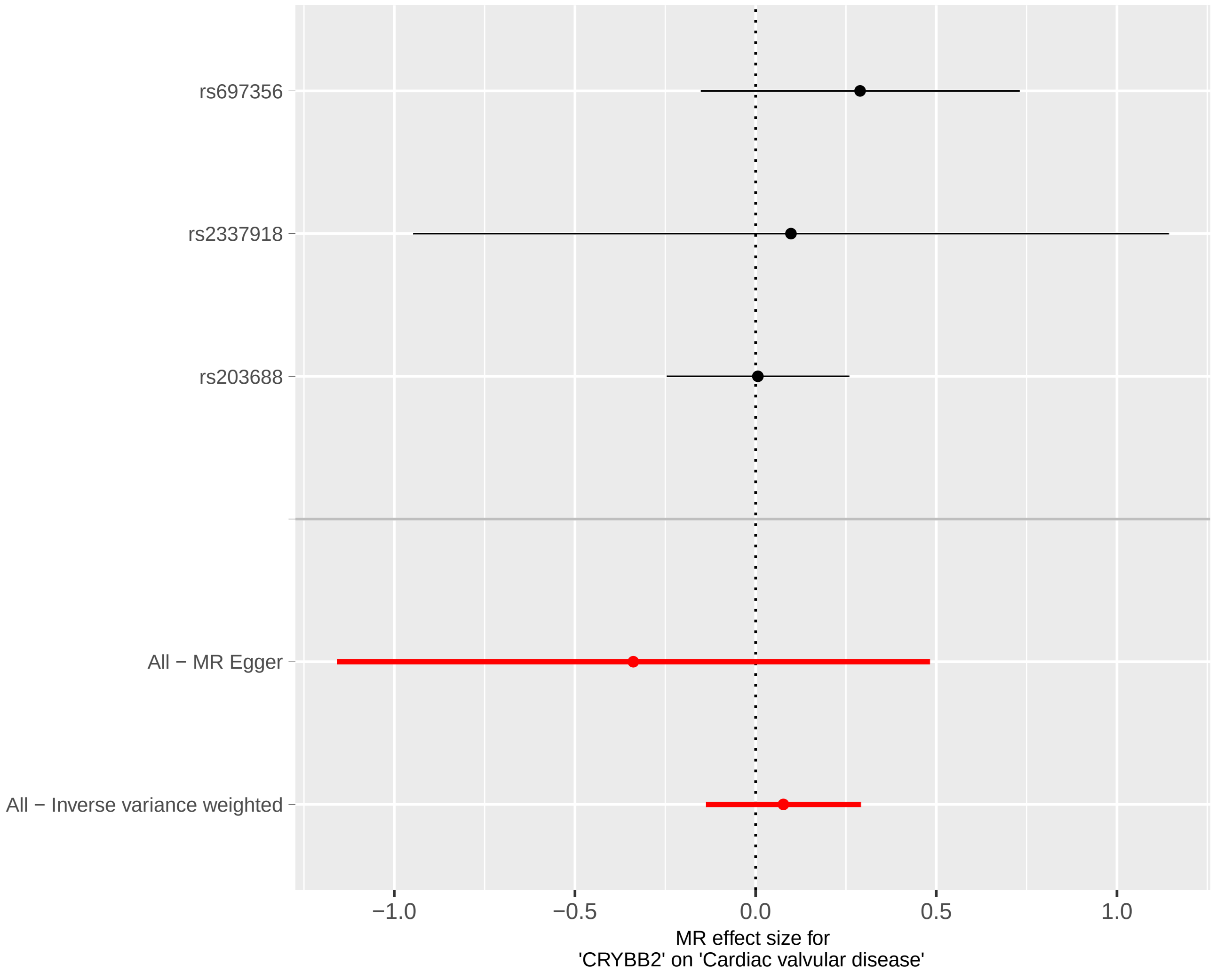
rs697356
rs2337918
rs203688
All − MR Egger
All − Inverse variance weighted
−1.0
−0.5
0.0
0.5
1.0
MR effect size for
'CRYBB2' on 'Cardiac valvular disease'

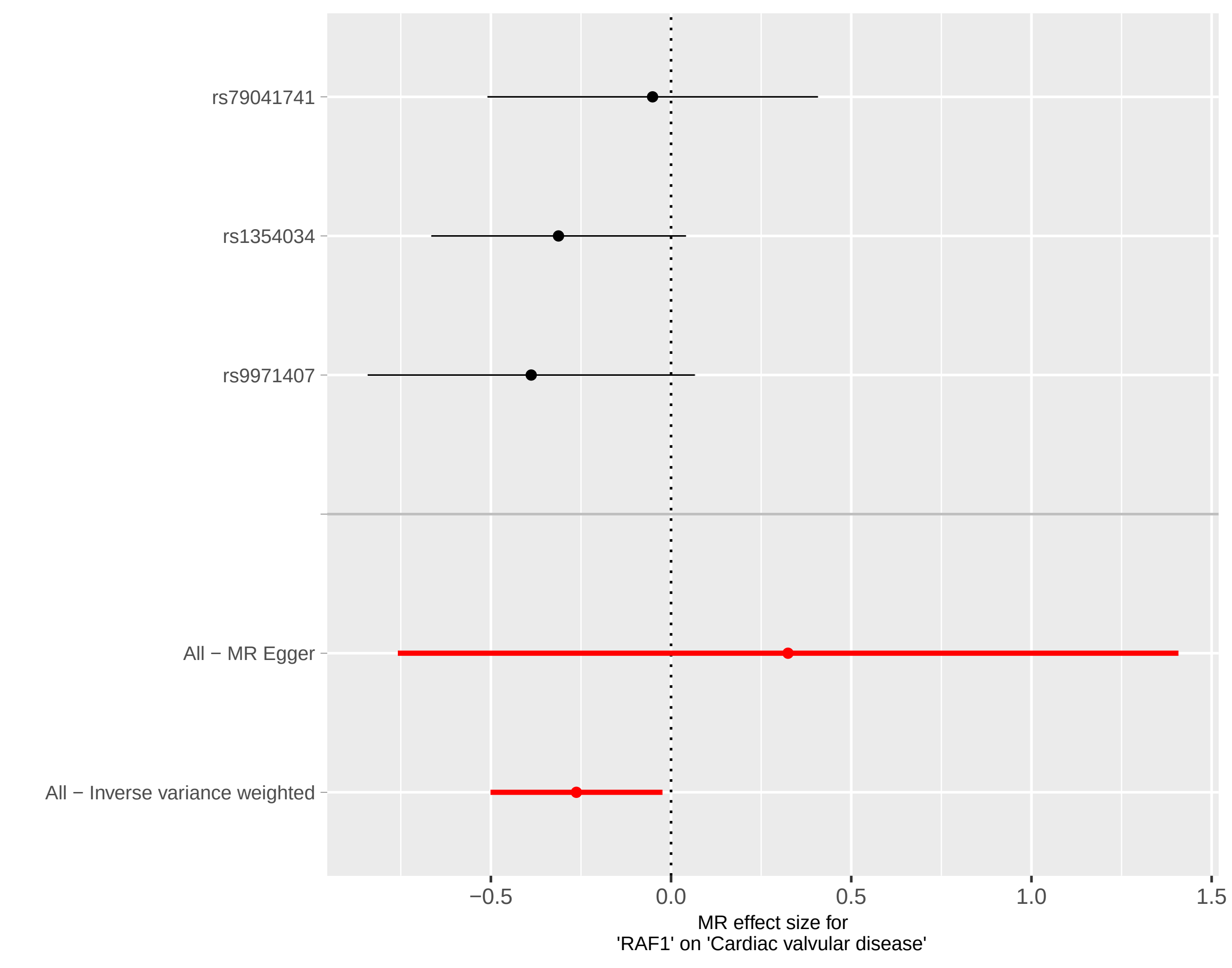

rs79041741
rs1354034
rs9971407
All − MR Egger
All − Inverse variance weighted
−0.5
0.0
0.5
1.0
1.5
MR effect size for
'RAF1' on 'Cardiac valvular disease'

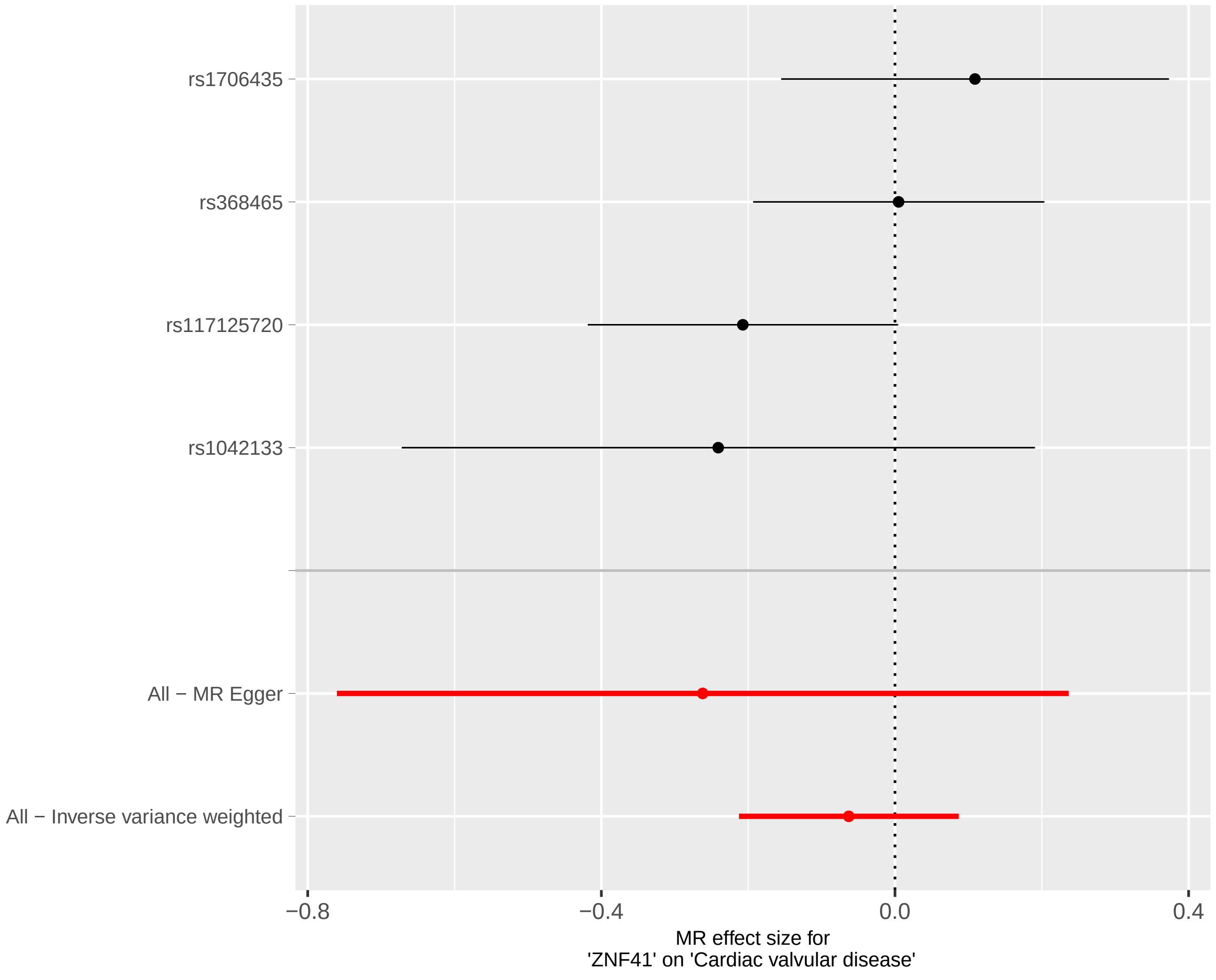

rs1706435
rs368465
rs117125720
rs1042133
All − MR Egger
All − Inverse variance weighted
−0.8
−0.4
0.0
0.4
MR effect size for
'ZNF41' on 'Cardiac valvular disease'

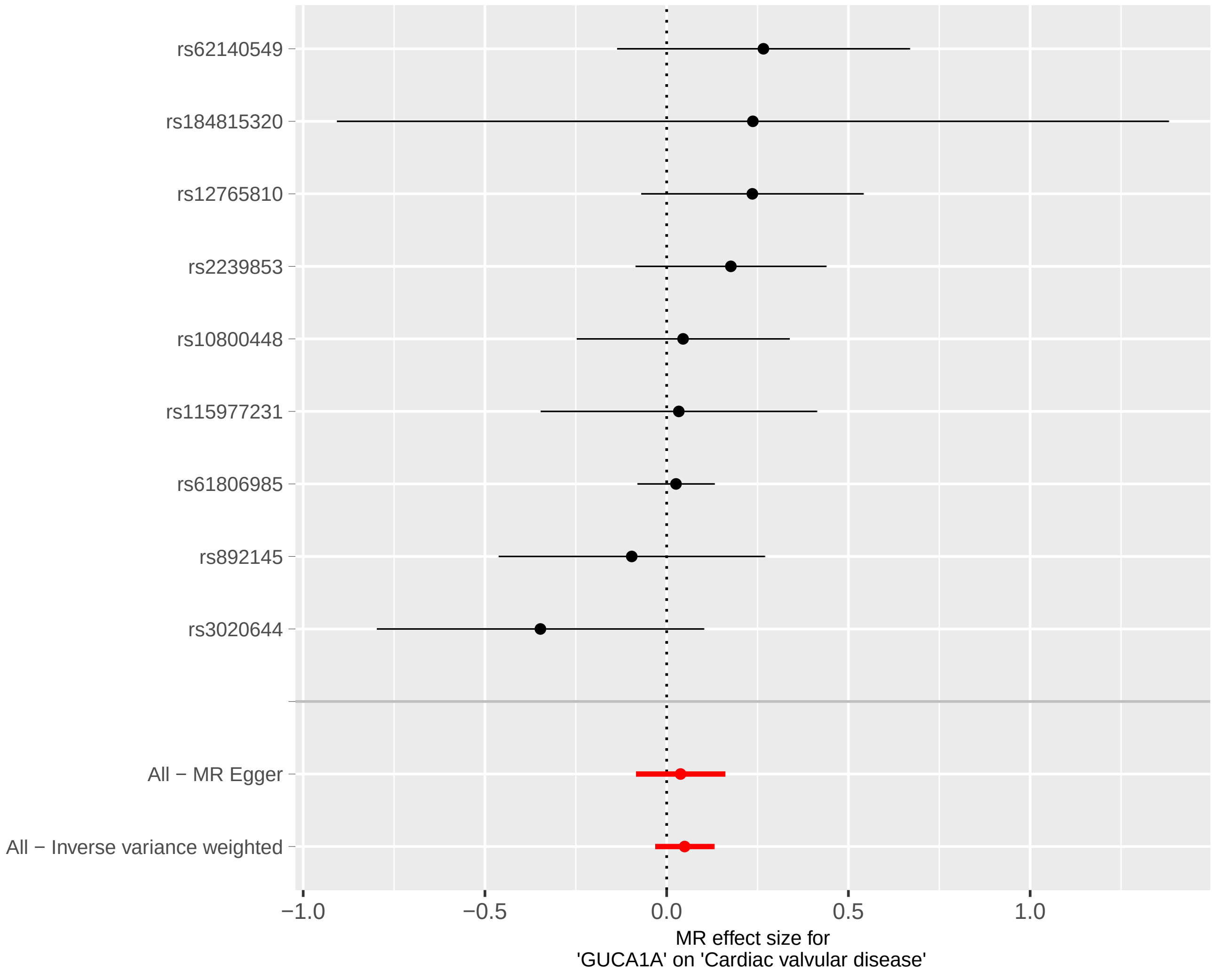
rs62140549
rs184815320
rs12765810
rs2239853
rs10800448
rs115977231
rs61806985
rs892145
rs3020644
All − MR Egger
All − Inverse variance weighted
−1.0
−0.5
0.0
0.5
1.0
MR effect size for
'GUCA1A' on 'Cardiac valvular disease'

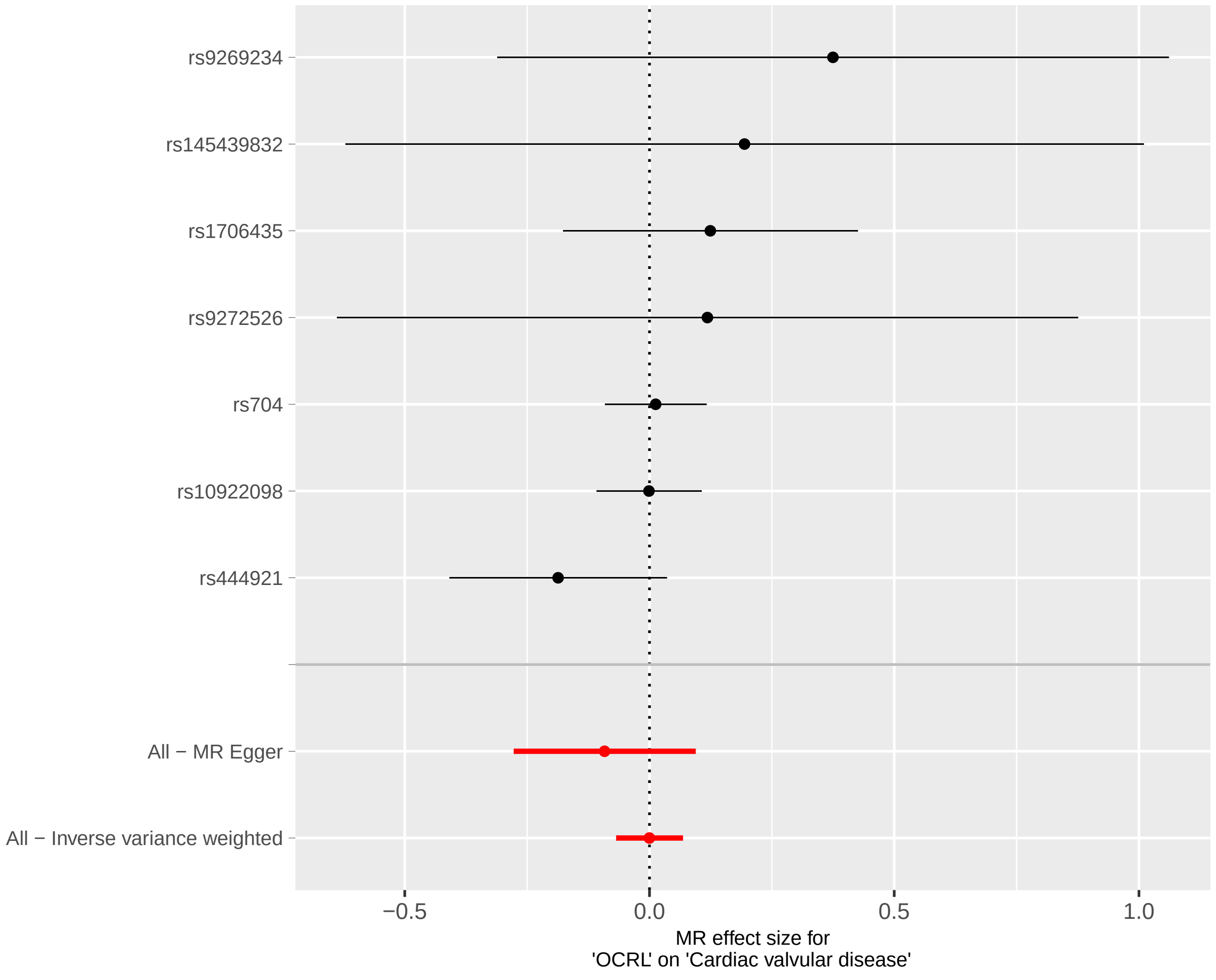

rs9269234
rs145439832
rs1706435
rs9272526
rs704
rs10922098
rs444921
All − MR Egger
All − Inverse variance weighted
−0.5
0.0
0.5
1.0
MR effect size for
'OCRL' on 'Cardiac valvular disease'

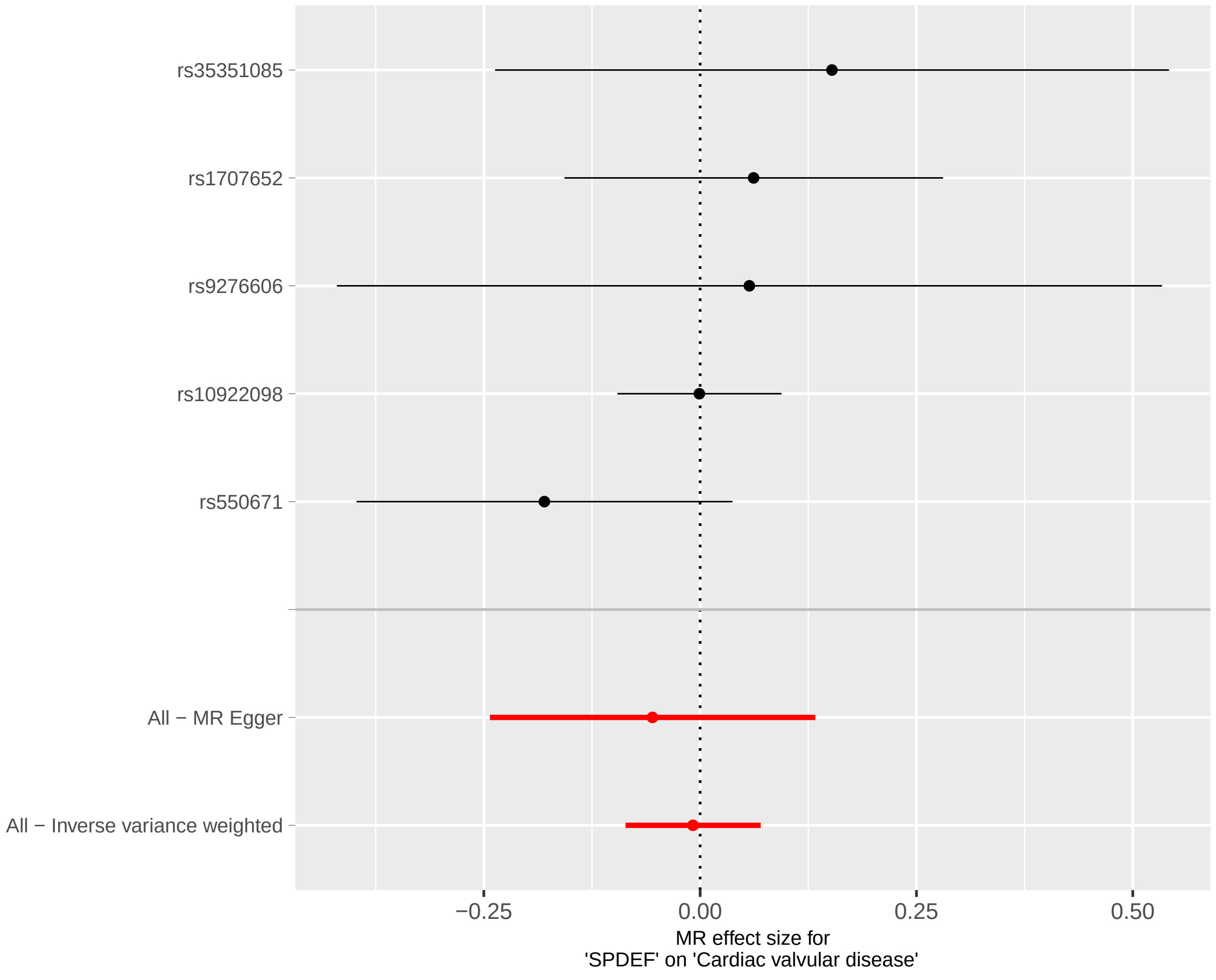
rs35351085
rs1707652
rs9276606
rs10922098
rs550671
All − MR Egger
All − Inverse variance weighted
−0.25
0.00
0.25
0.50
MR effect size for
'SPDEF' on 'Cardiac valvular disease'

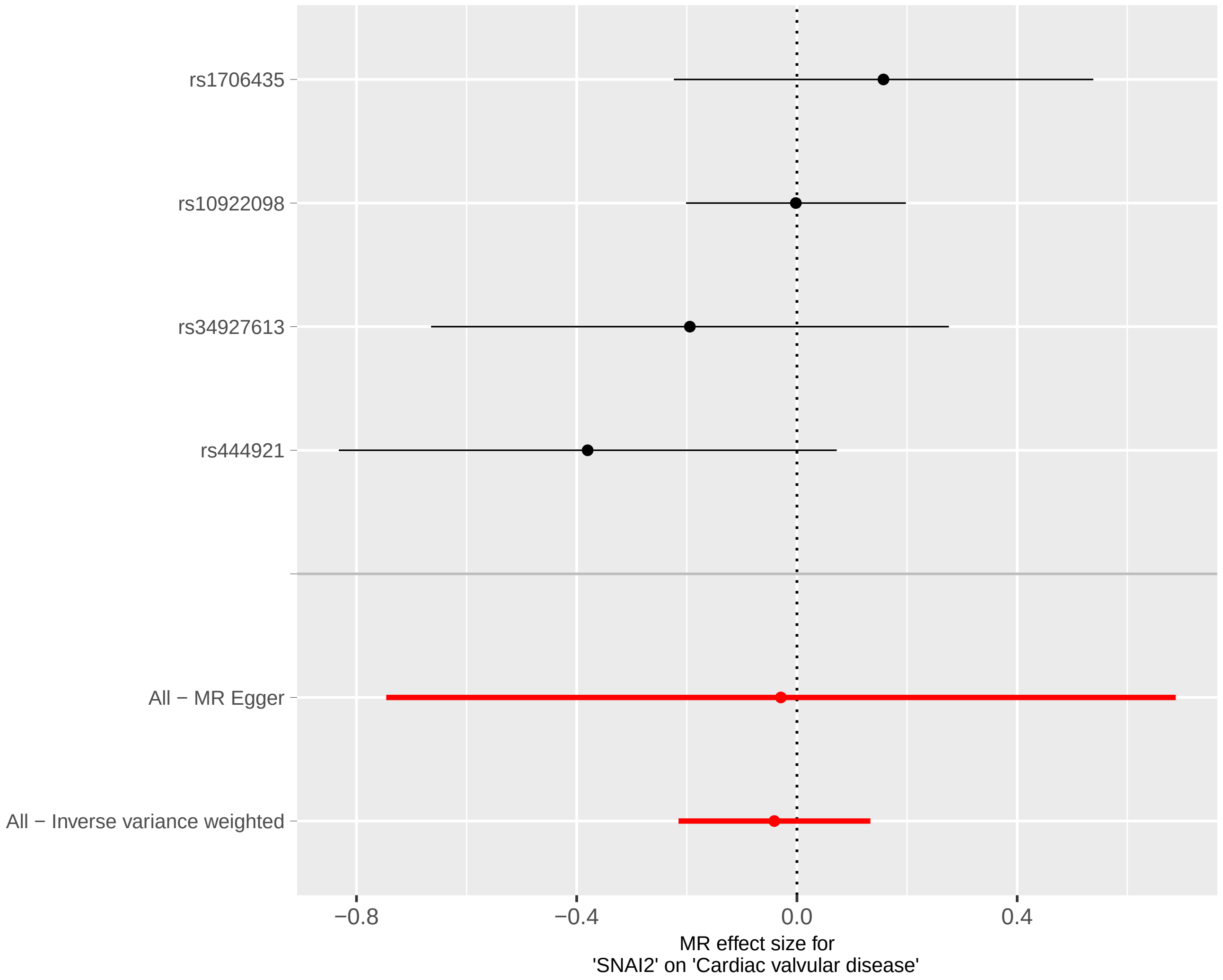
rs1706435
rs10922098
rs34927613
rs444921
All − MR Egger
All − Inverse variance weighted
−0.8
−0.4
0.0
0.4
MR effect size for
'SNAI2' on 'Cardiac valvular disease'

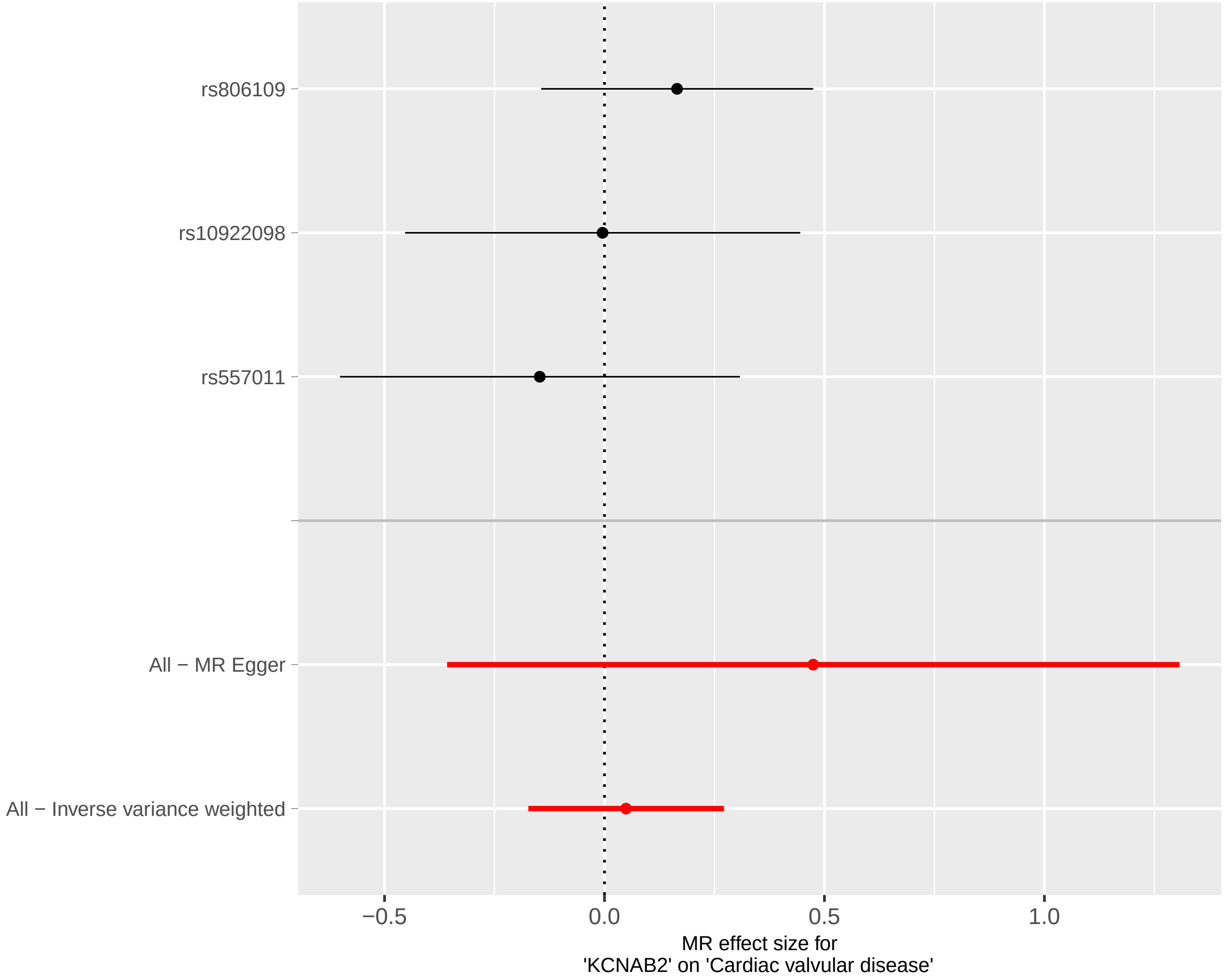
rs806109
rs10922098
rs557011
All − MR Egger
All − Inverse variance weighted
−0.5
0.0
0.5
1.0
MR effect size for
'KCNAB2' on 'Cardiac valvular disease'

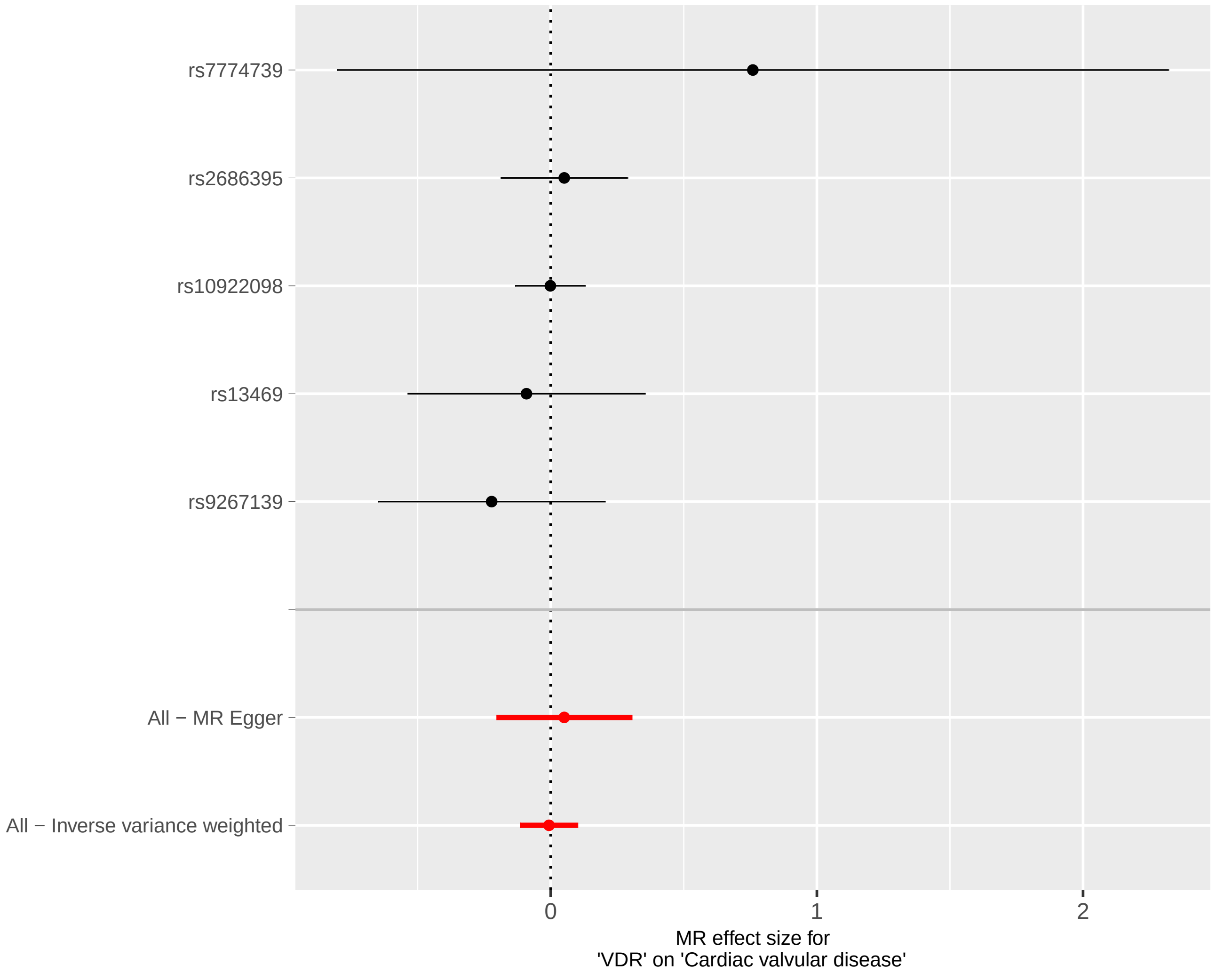
rs7774739
rs2686395
rs10922098
rs13469
rs9267139
All − MR Egger
All − Inverse variance weighted
0
1
2
MR effect size for
'VDR' on 'Cardiac valvular disease'

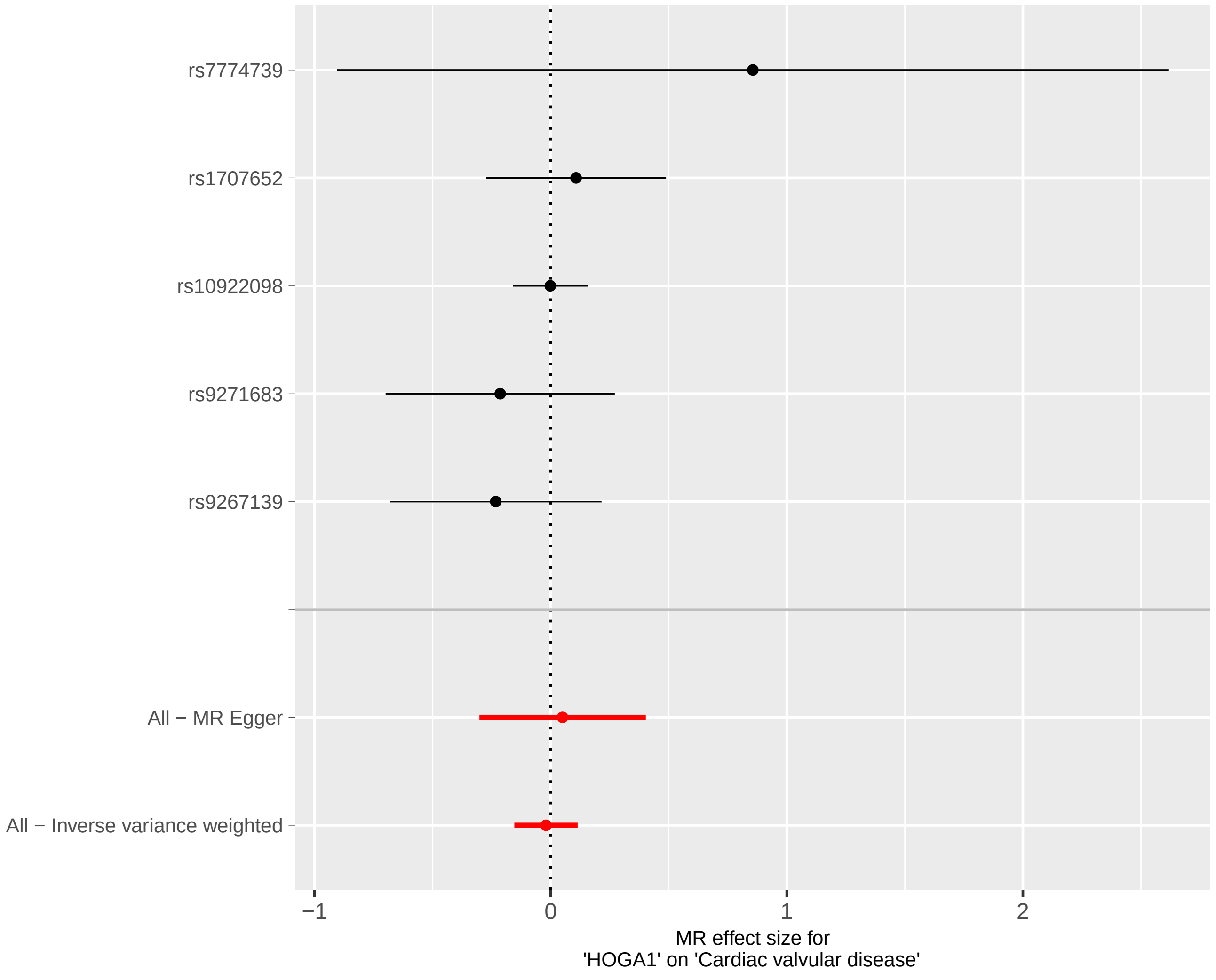
rs7774739
rs1707652
rs10922098
rs9271683
rs9267139
All − MR Egger
All − Inverse variance weighted
−1
0
1
2
MR effect size for
'HOGA1' on 'Cardiac valvular disease'

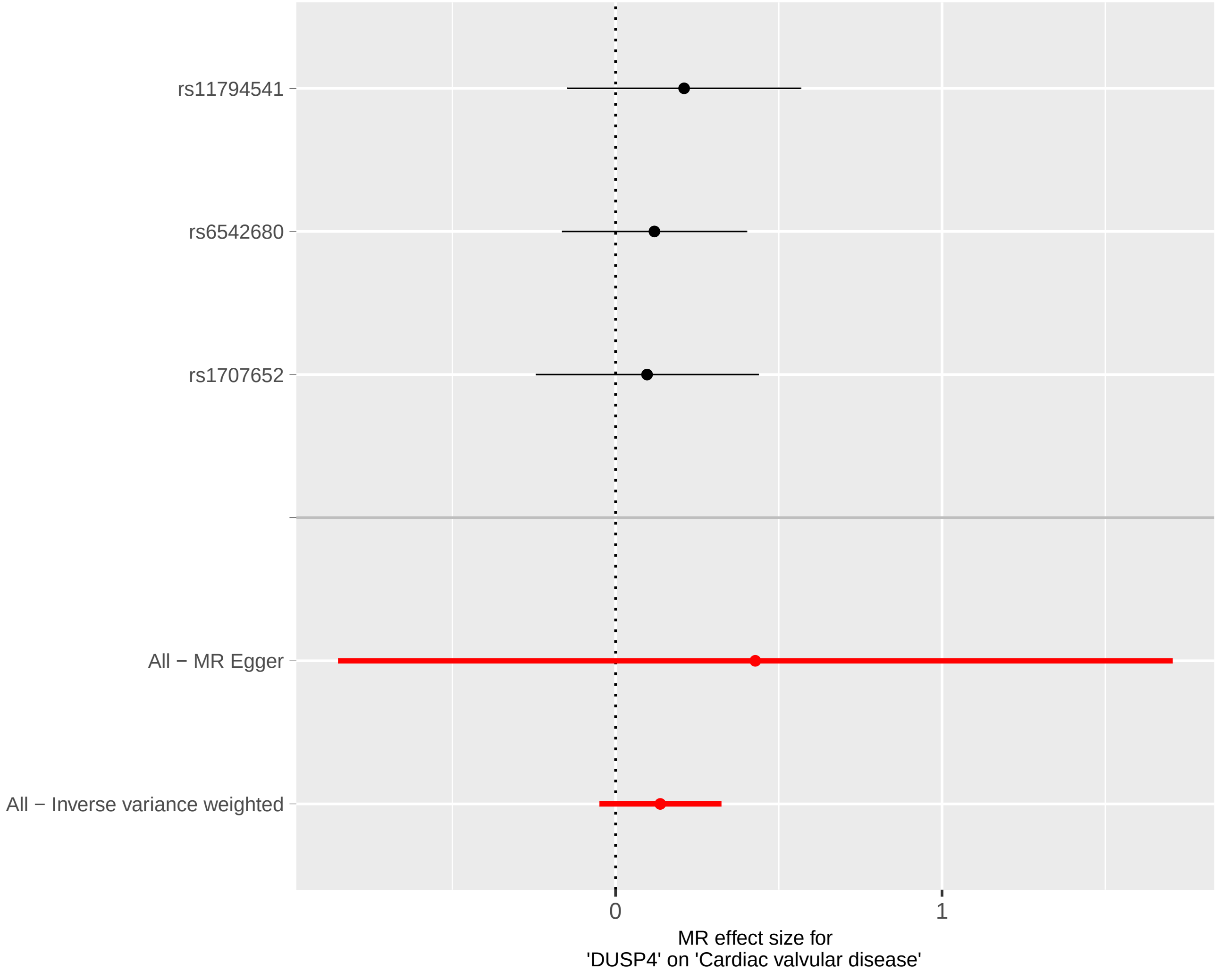
rs11794541
rs6542680
rs1707652
All − MR Egger
All − Inverse variance weighted
0
1
MR effect size for
'DUSP4' on 'Cardiac valvular disease'

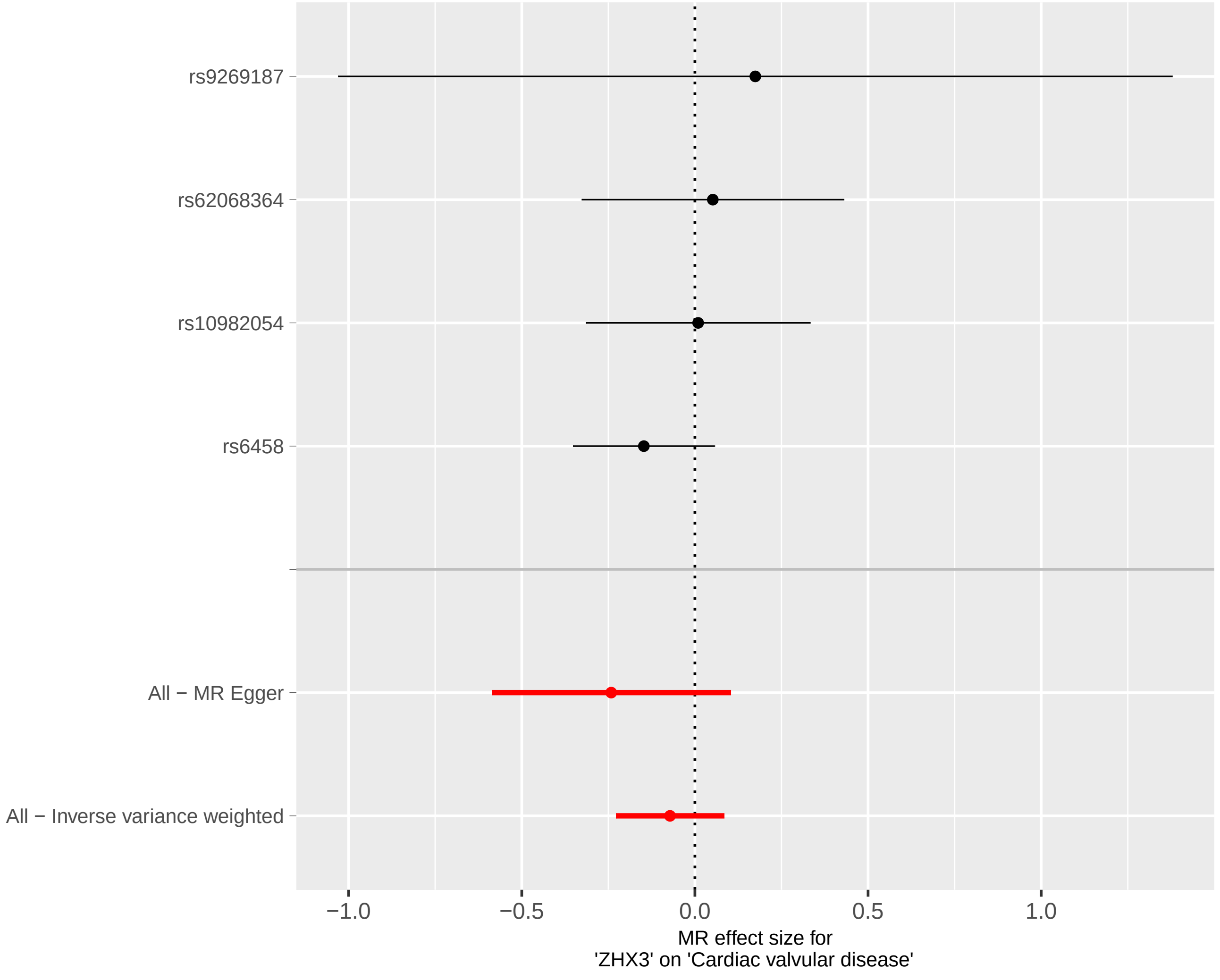
rs9269187
rs62068364
rs10982054
rs6458
All − MR Egger
All − Inverse variance weighted
−1.0
−0.5
0.0
0.5
1.0
MR effect size for
'ZHX3' on 'Cardiac valvular disease'

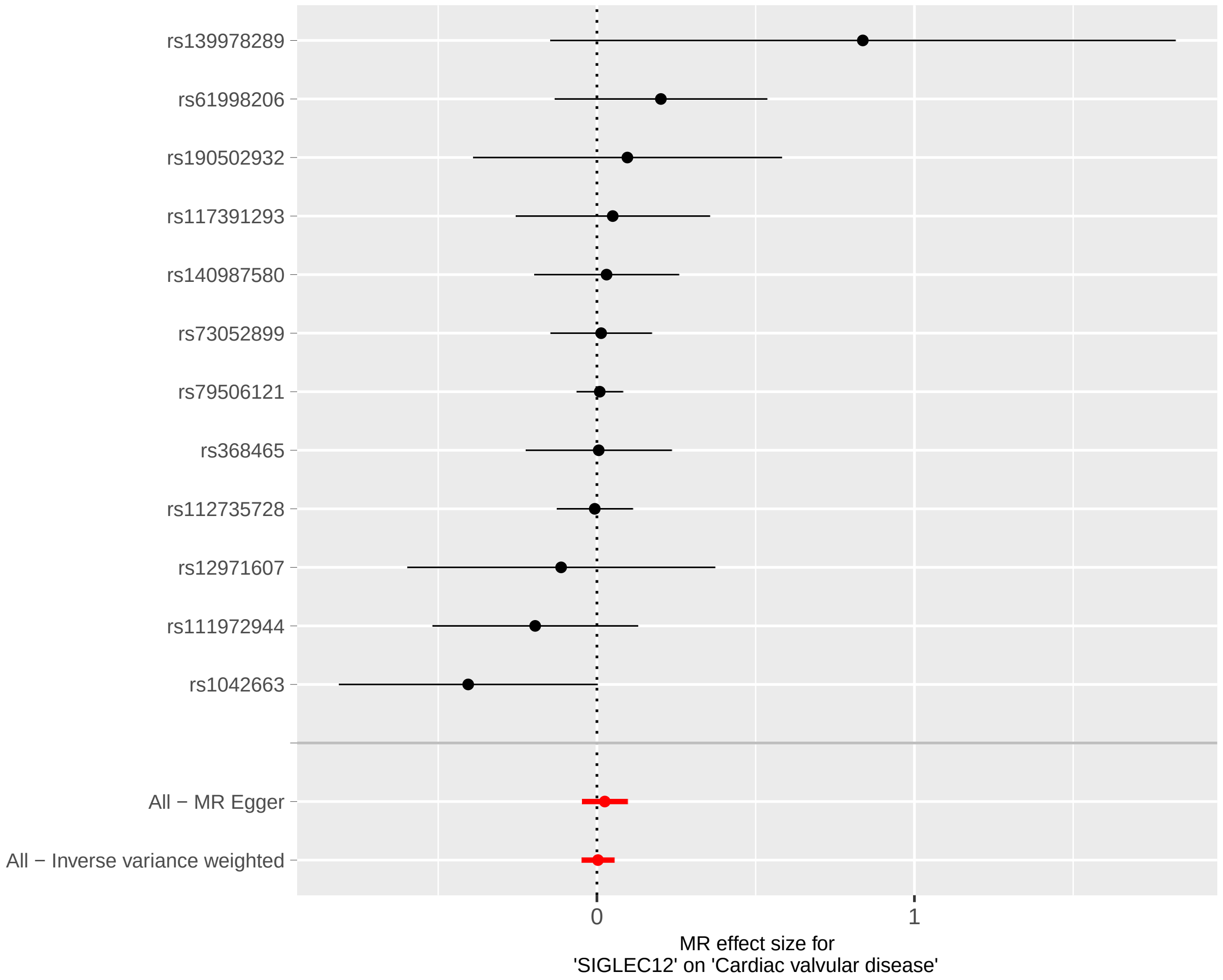

rs139978289
rs61998206
rs190502932
rs117391293
rs140987580
rs73052899
rs79506121
rs368465
rs112735728
rs12971607
rs111972944
rs1042663
All − MR Egger
All − Inverse variance weighted
0
1
MR effect size for
'SIGLEC12' on 'Cardiac valvular disease'

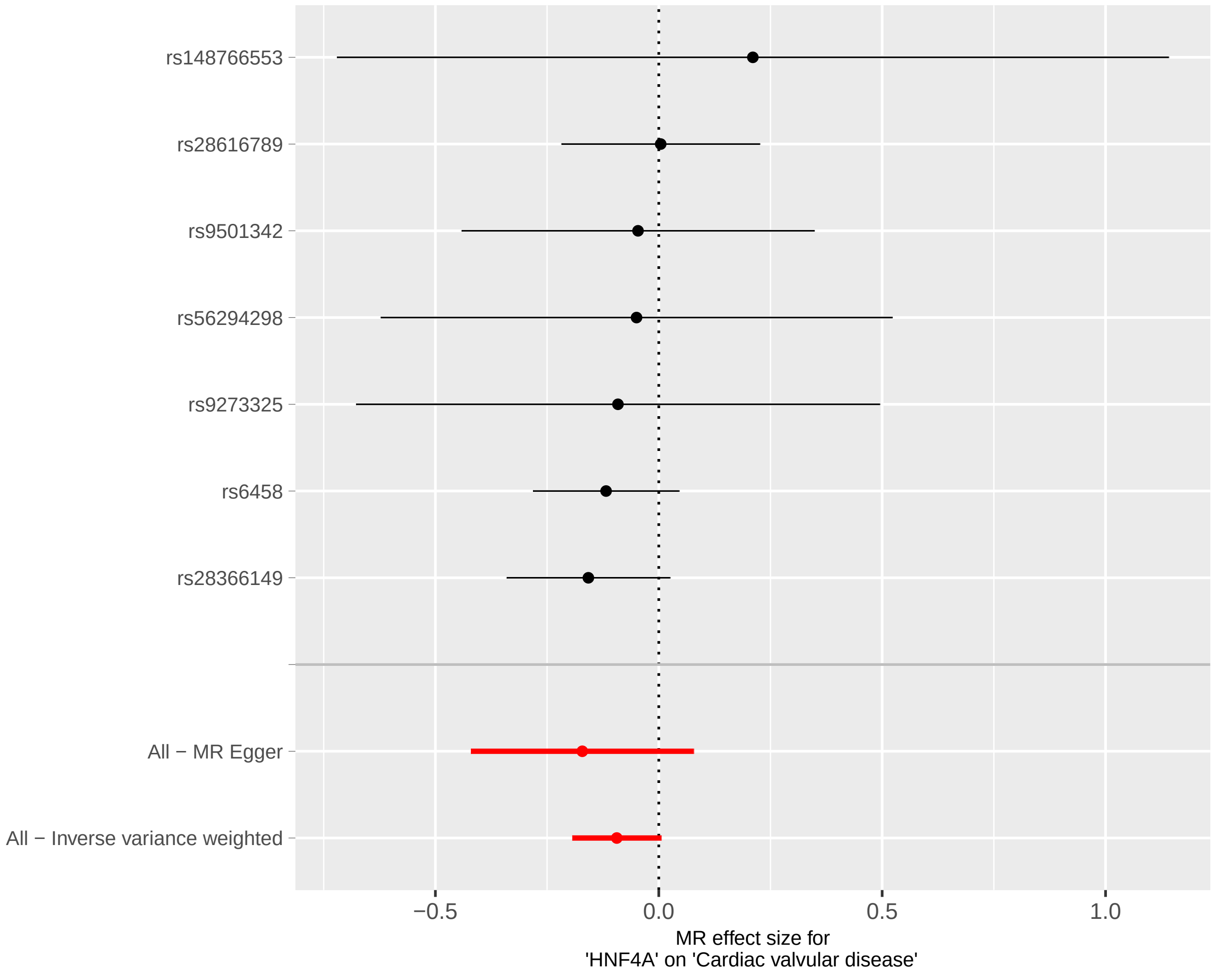

rs148766553
rs28616789
rs9501342
rs56294298
rs9273325
rs6458
rs28366149
All − MR Egger
All − Inverse variance weighted
−0.5
0.0
0.5
1.0
MR effect size for
'HNF4A' on 'Cardiac valvular disease'

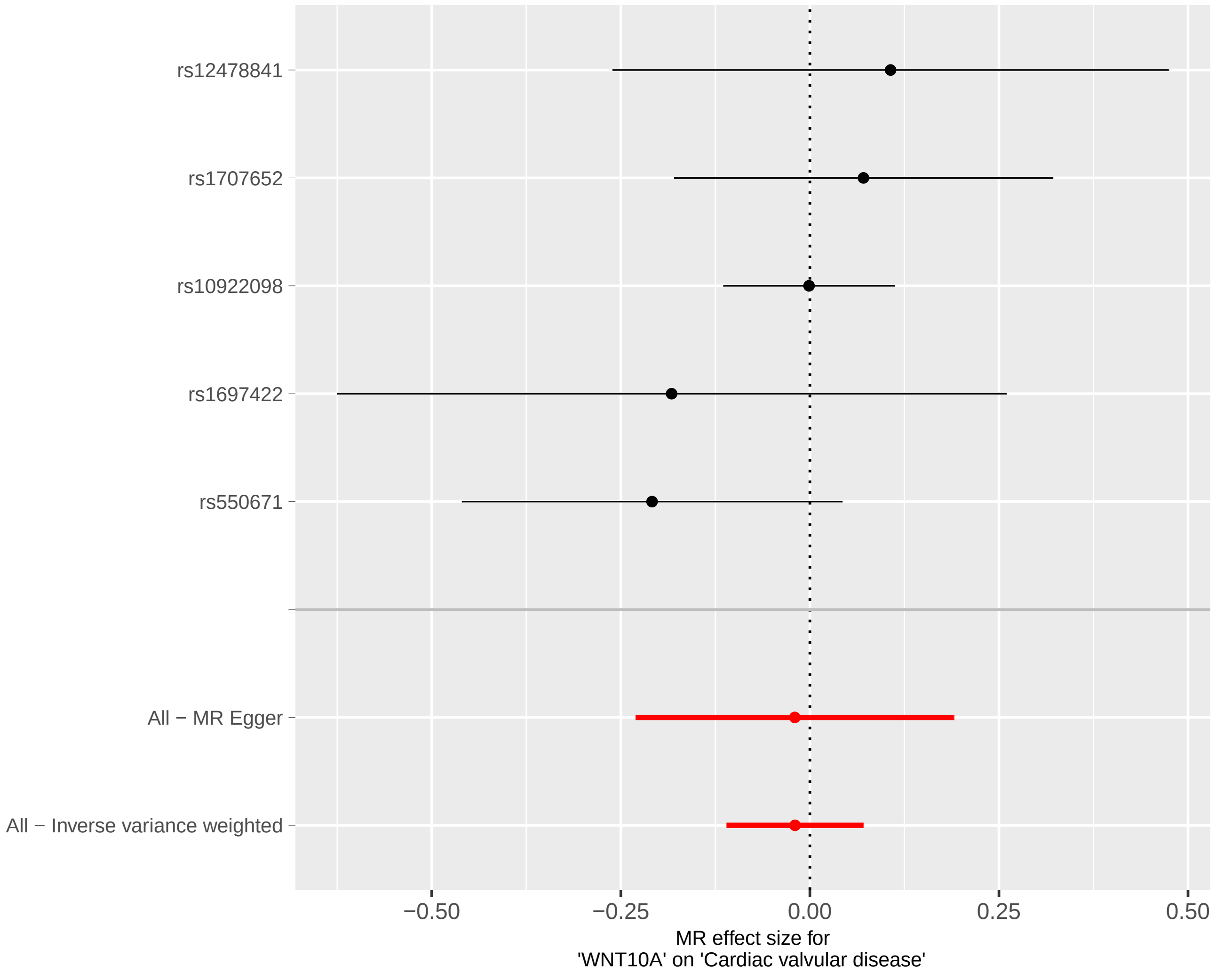

rs12478841
rs1707652
rs10922098
rs1697422
rs550671
All − MR Egger
All − Inverse variance weighted
−0.50
−0.25
0.00
0.25
0.50
MR effect size for
'WNT10A' on 'Cardiac valvular disease'

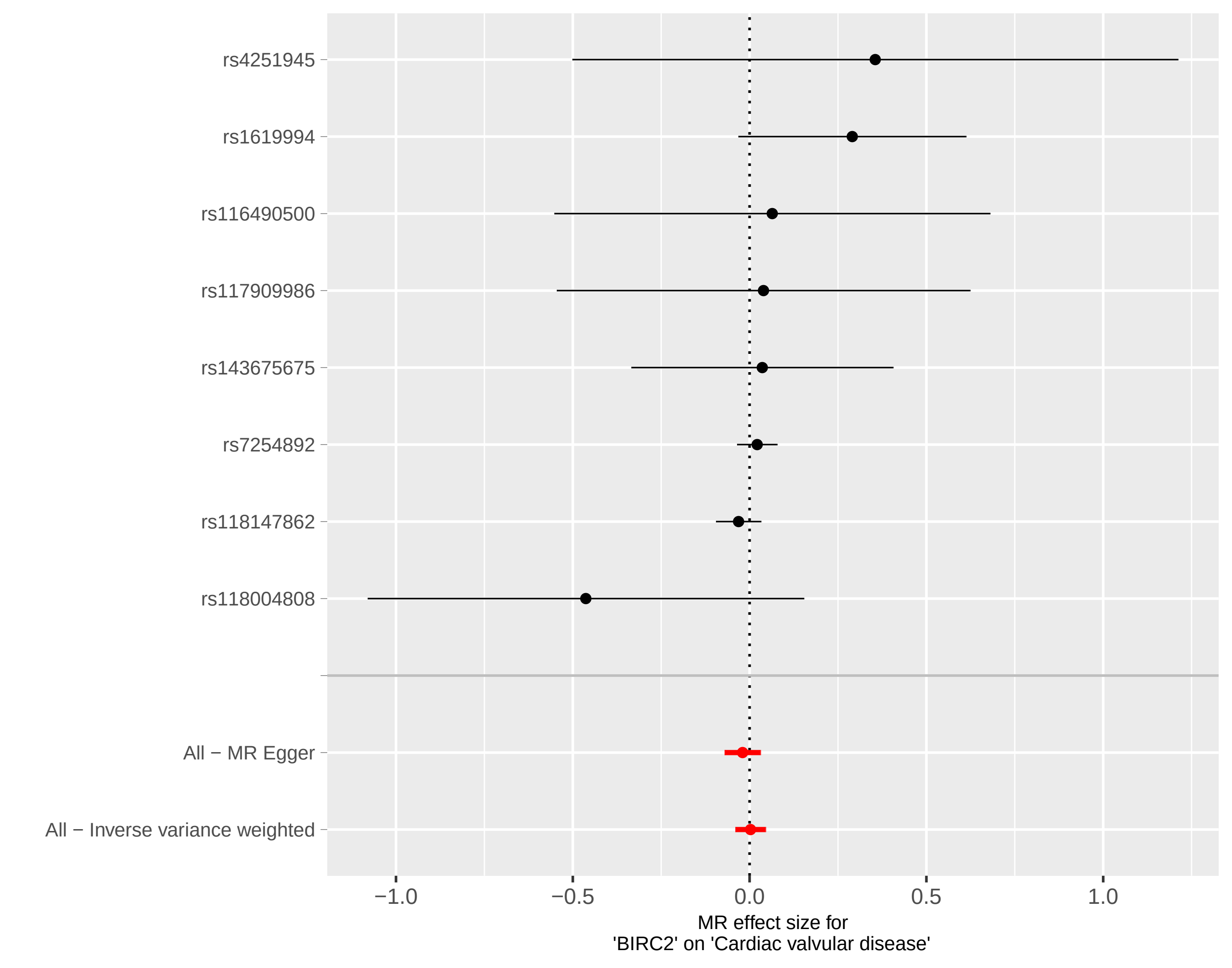
rs4251945
rs1619994
rs116490500
rs117909986
rs143675675
rs7254892
rs118147862
rs118004808
All − MR Egger
All − Inverse variance weighted
−1.0
−0.5
0.0
0.5
1.0
MR effect size for
'BIRC2' on 'Cardiac valvular disease'

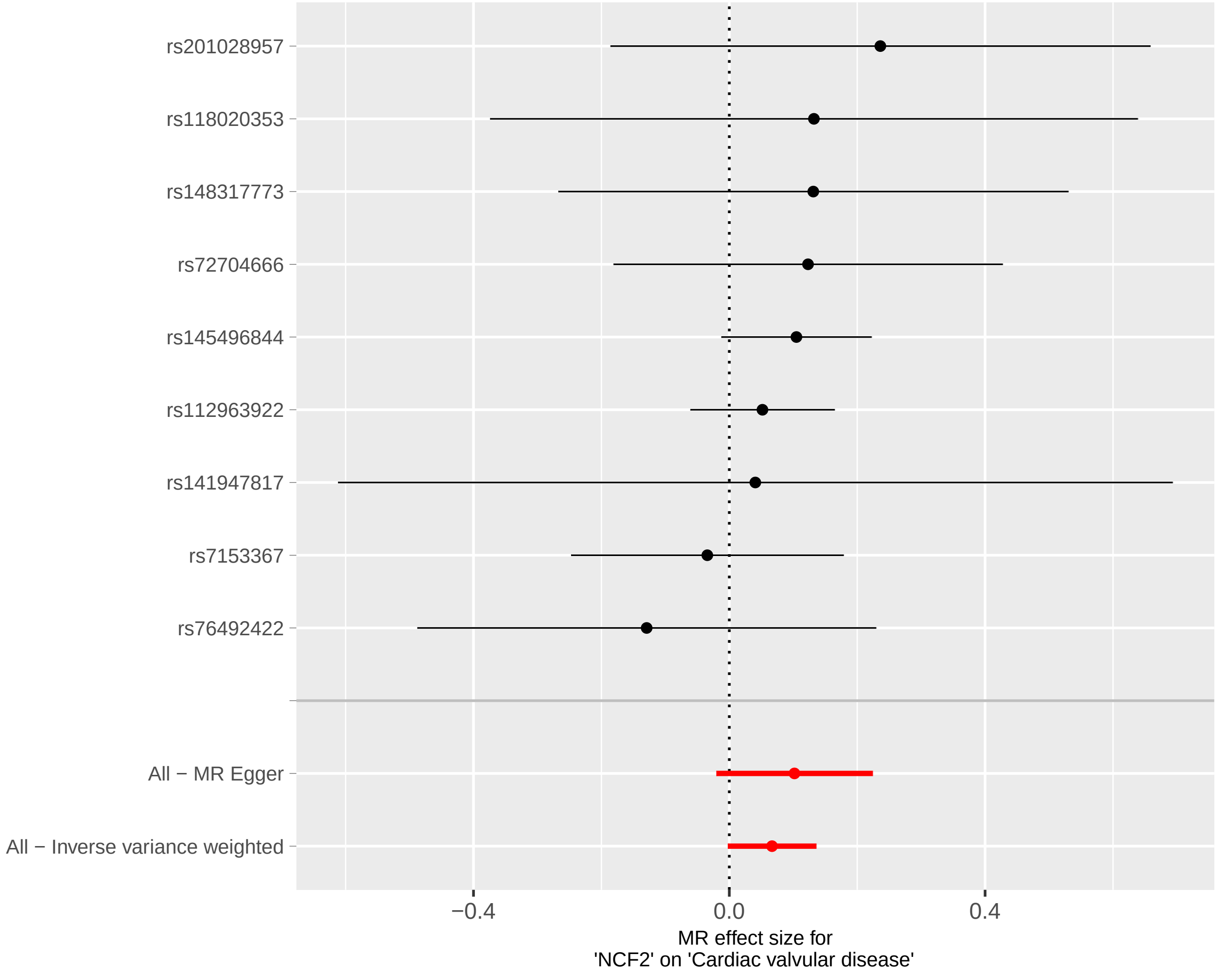
rs201028957
rs118020353
rs148317773
rs72704666
rs145496844
rs112963922
rs141947817
rs7153367
rs76492422
All − MR Egger
All − Inverse variance weighted
−0.4
0.0
0.4
MR effect size for
'NCF2' on 'Cardiac valvular disease'

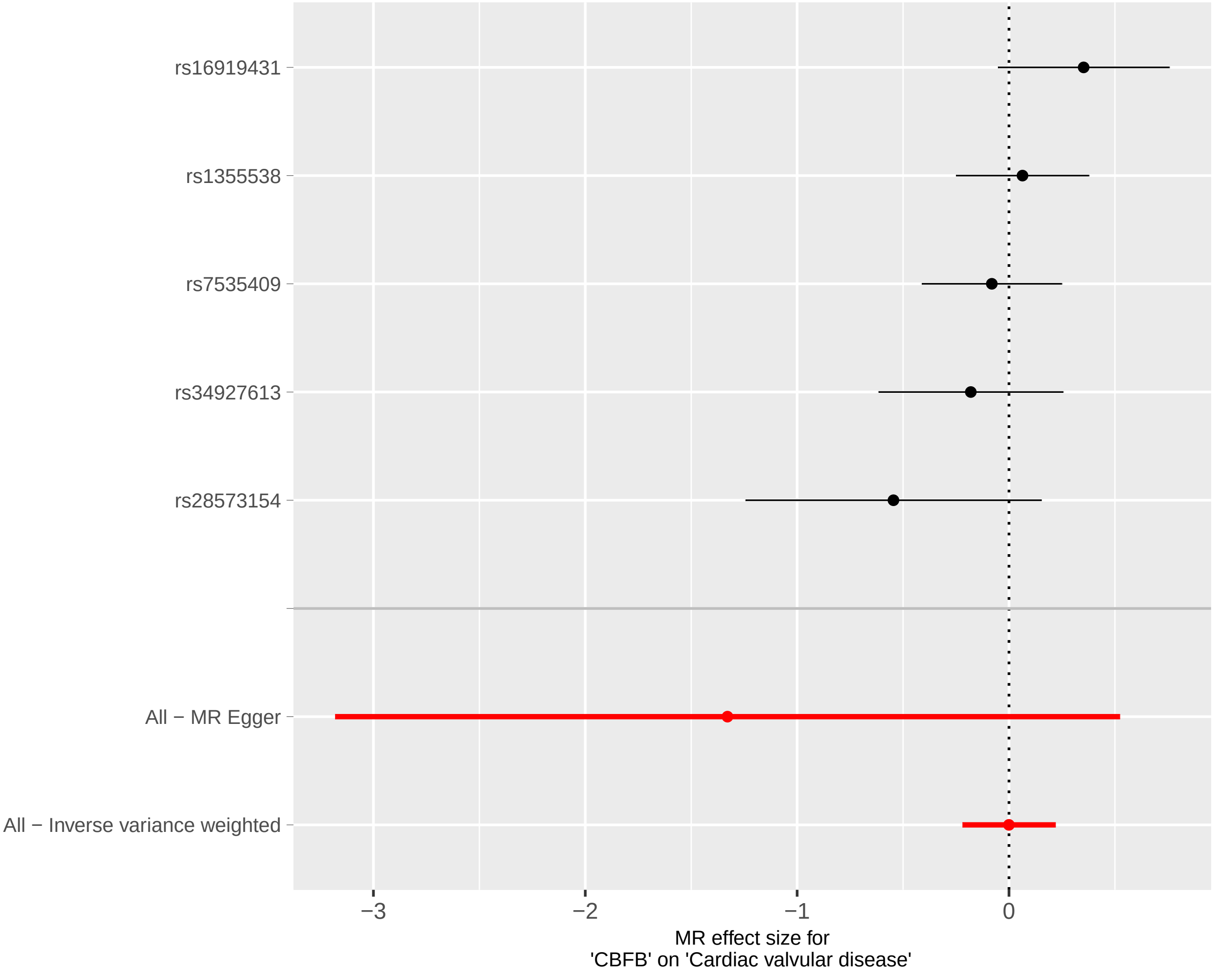
rs16919431
rs1355538
rs7535409
rs34927613
rs28573154
All − MR Egger
All − Inverse variance weighted
−3
−2
−1
0
MR effect size for
'CBFB' on 'Cardiac valvular disease'

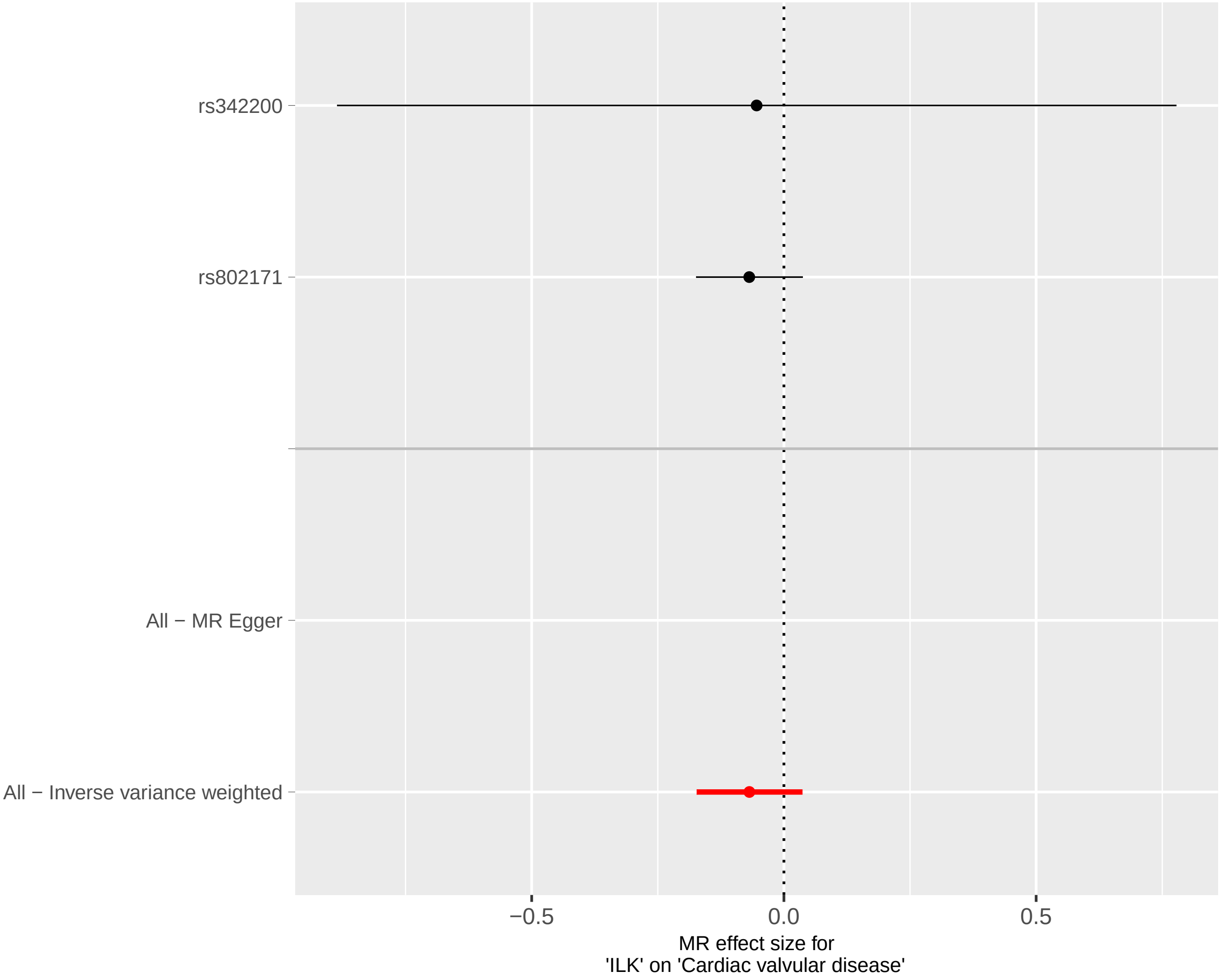

rs342200
rs802171
All − MR Egger
All − Inverse variance weighted
−0.5
0.0
0.5
MR effect size for
'ILK' on 'Cardiac valvular disease'

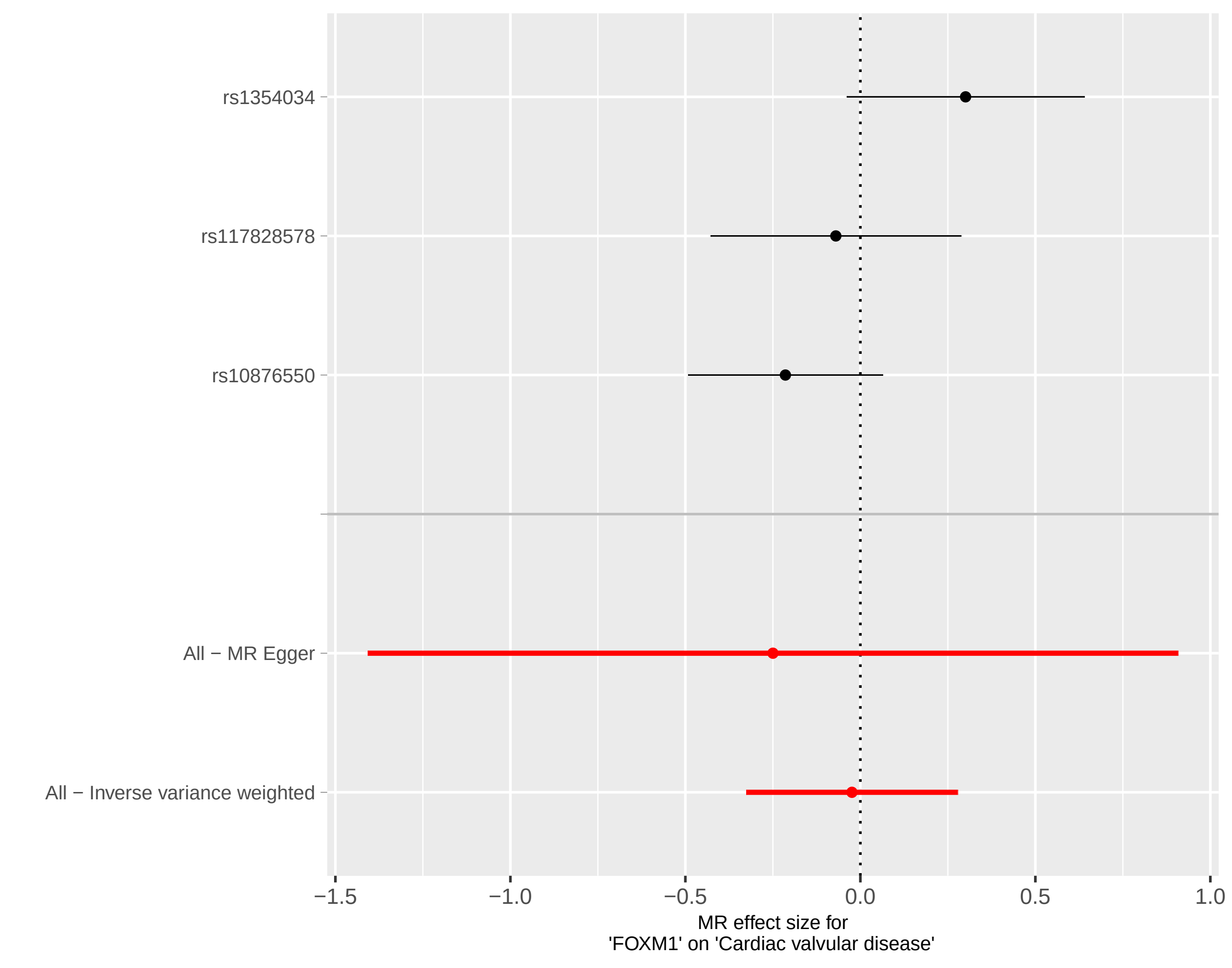
rs1354034
rs117828578
rs10876550
All − MR Egger
All − Inverse variance weighted
−1.5
−1.0
−0.5
0.0
0.5
1.0
MR effect size for
'FOXM1' on 'Cardiac valvular disease'

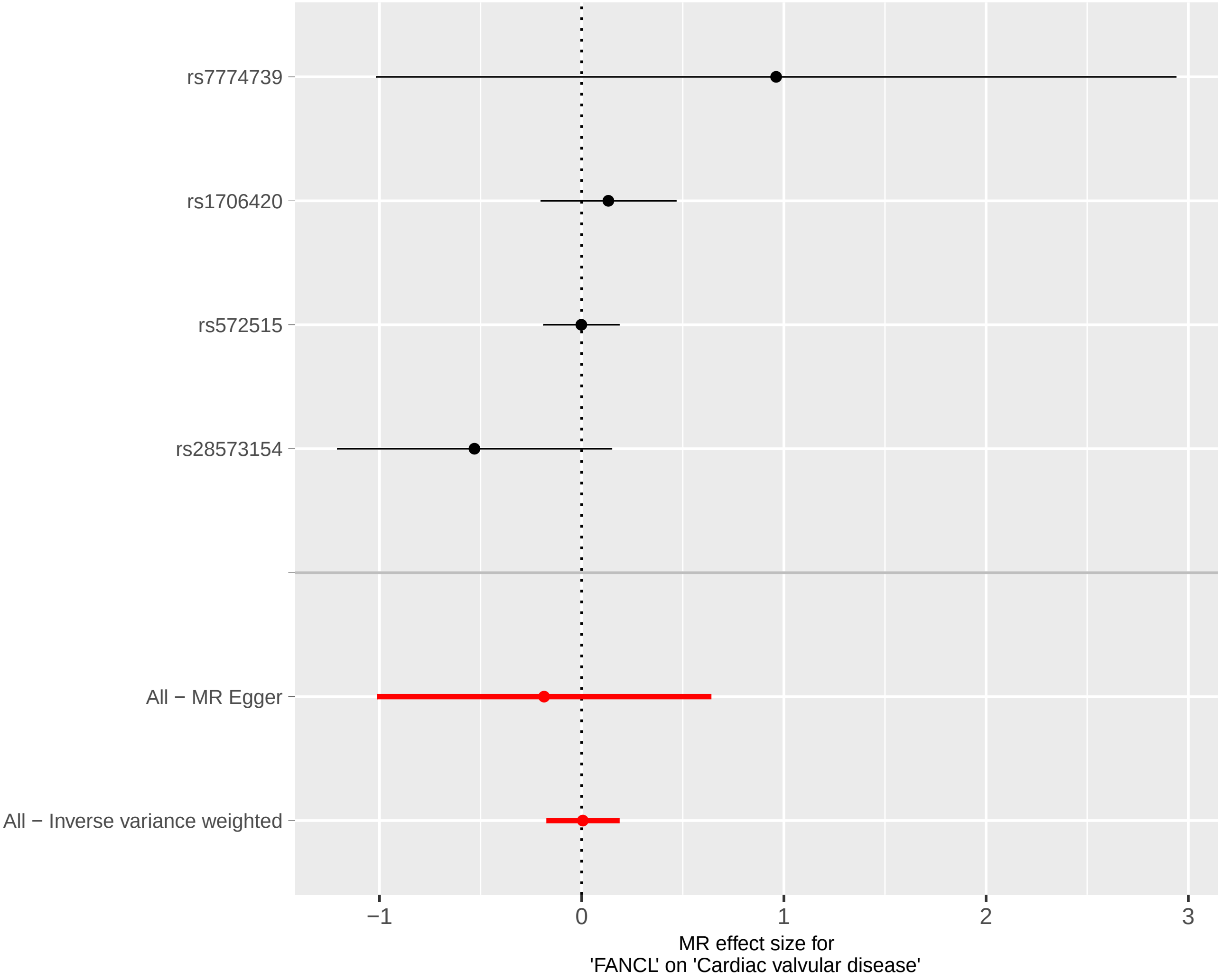
rs7774739
rs1706420
rs572515
rs28573154
All − MR Egger
All − Inverse variance weighted
−1
0
1
2
3
MR effect size for
'FANCL' on 'Cardiac valvular disease'

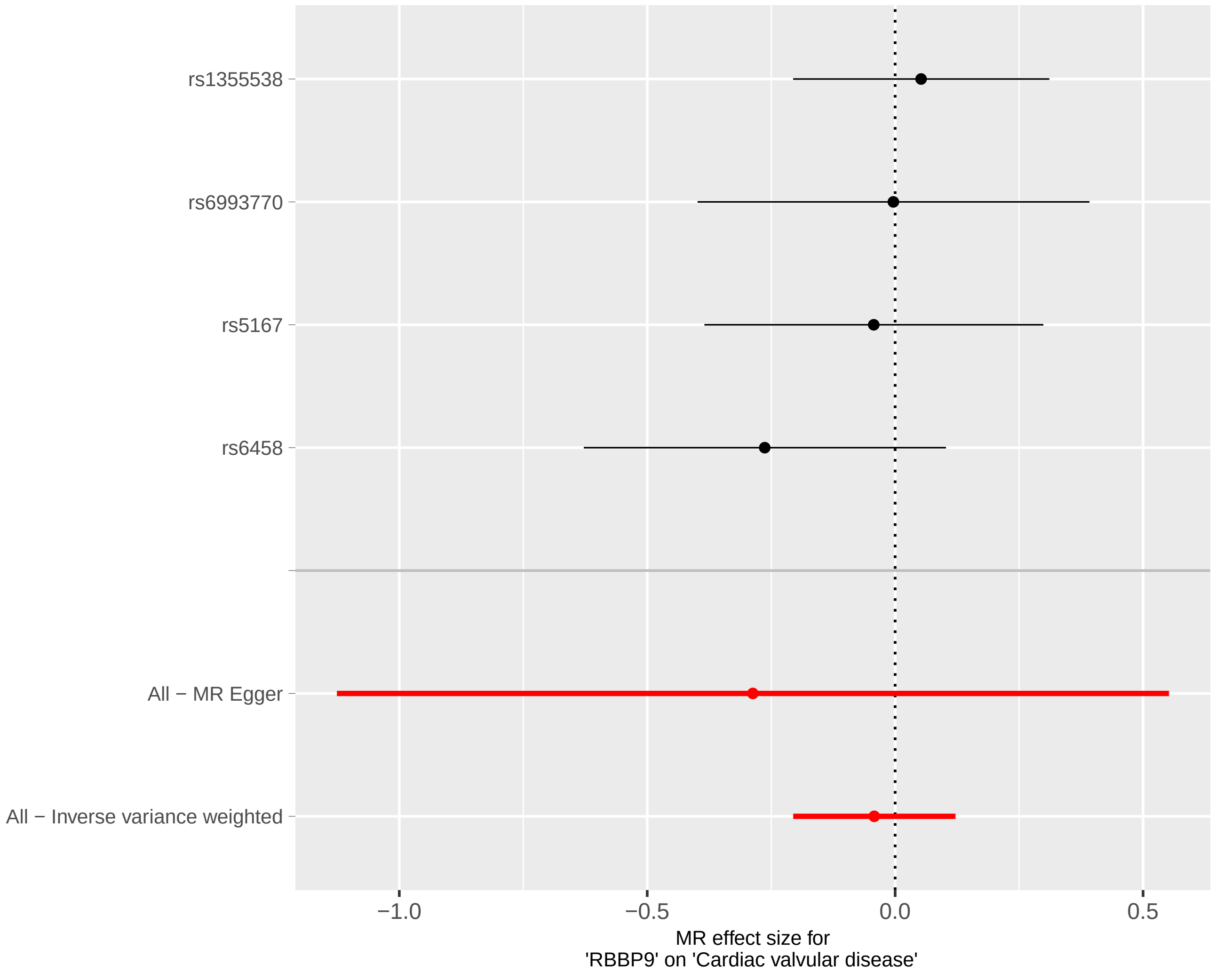
rs1355538
rs6993770
rs5167
rs6458
All − MR Egger
All − Inverse variance weighted
−1.0
−0.5
0.0
0.5
MR effect size for
'RBBP9' on 'Cardiac valvular disease'

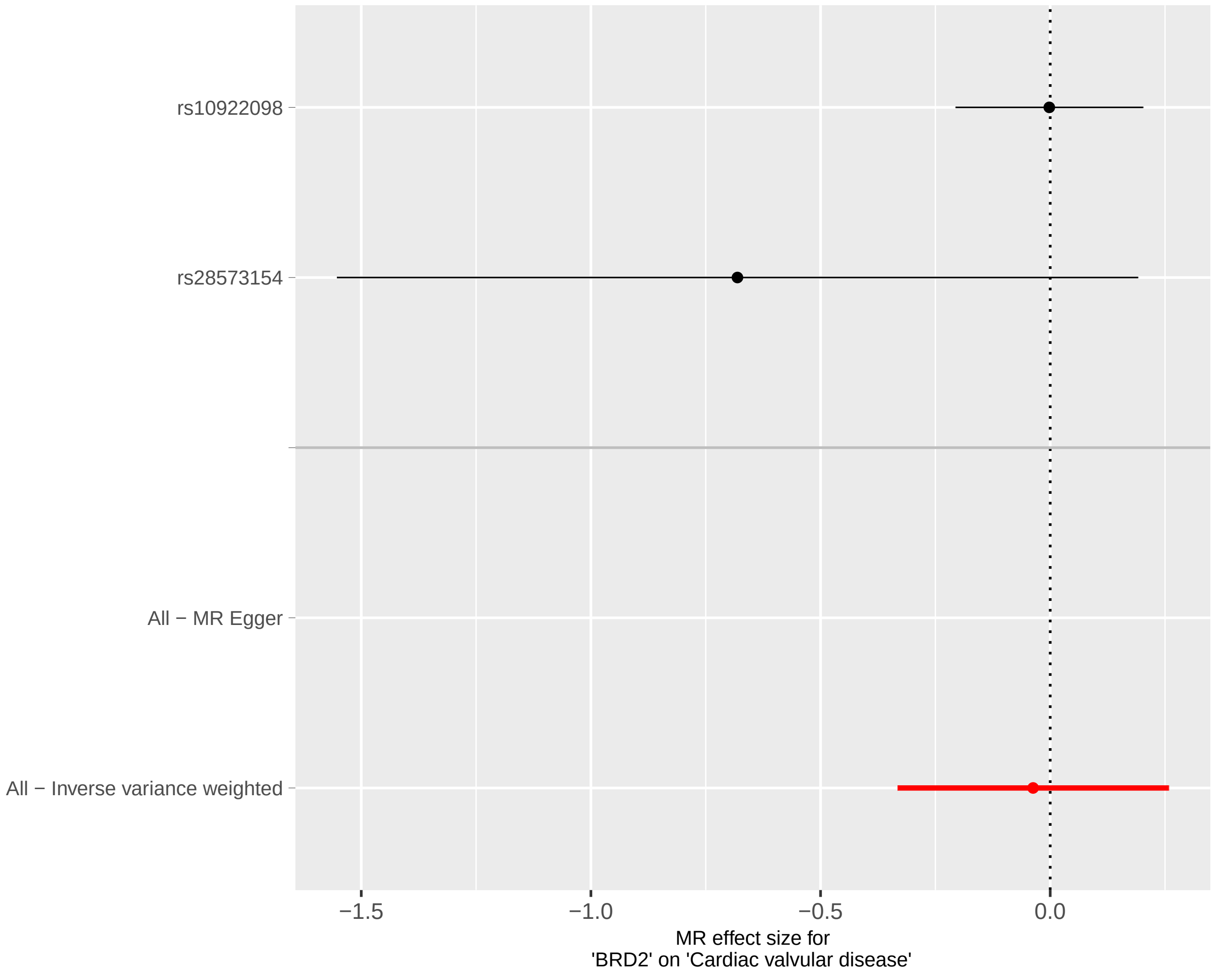

rs10922098
rs28573154
All − MR Egger
All − Inverse variance weighted
−1.5
−1.0
−0.5
0.0
MR effect size for
'BRD2' on 'Cardiac valvular disease'

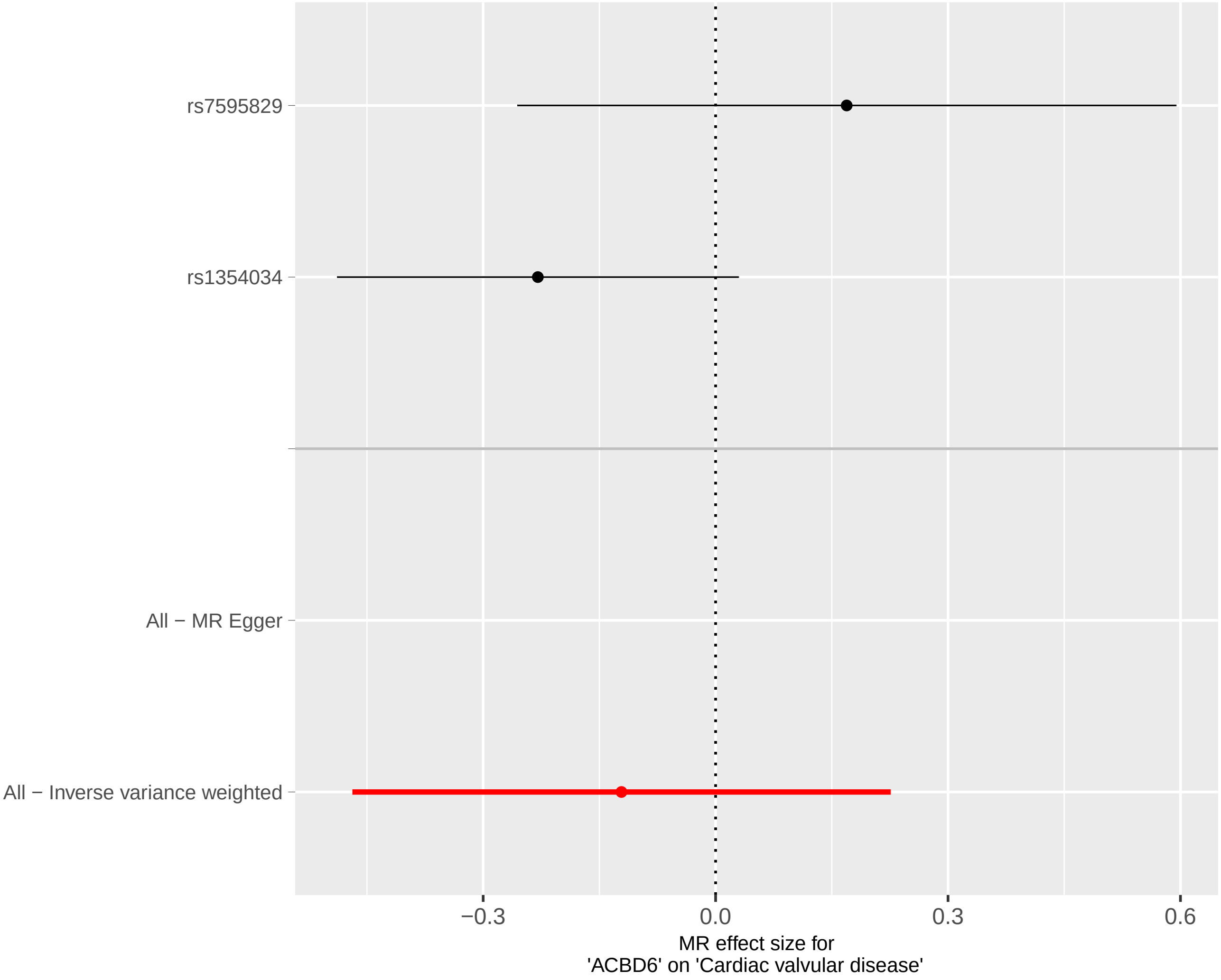
rs7595829
rs1354034
All − MR Egger
All − Inverse variance weighted
−0.3
0.0
0.3
0.6
MR effect size for
'ACBD6' on 'Cardiac valvular disease'

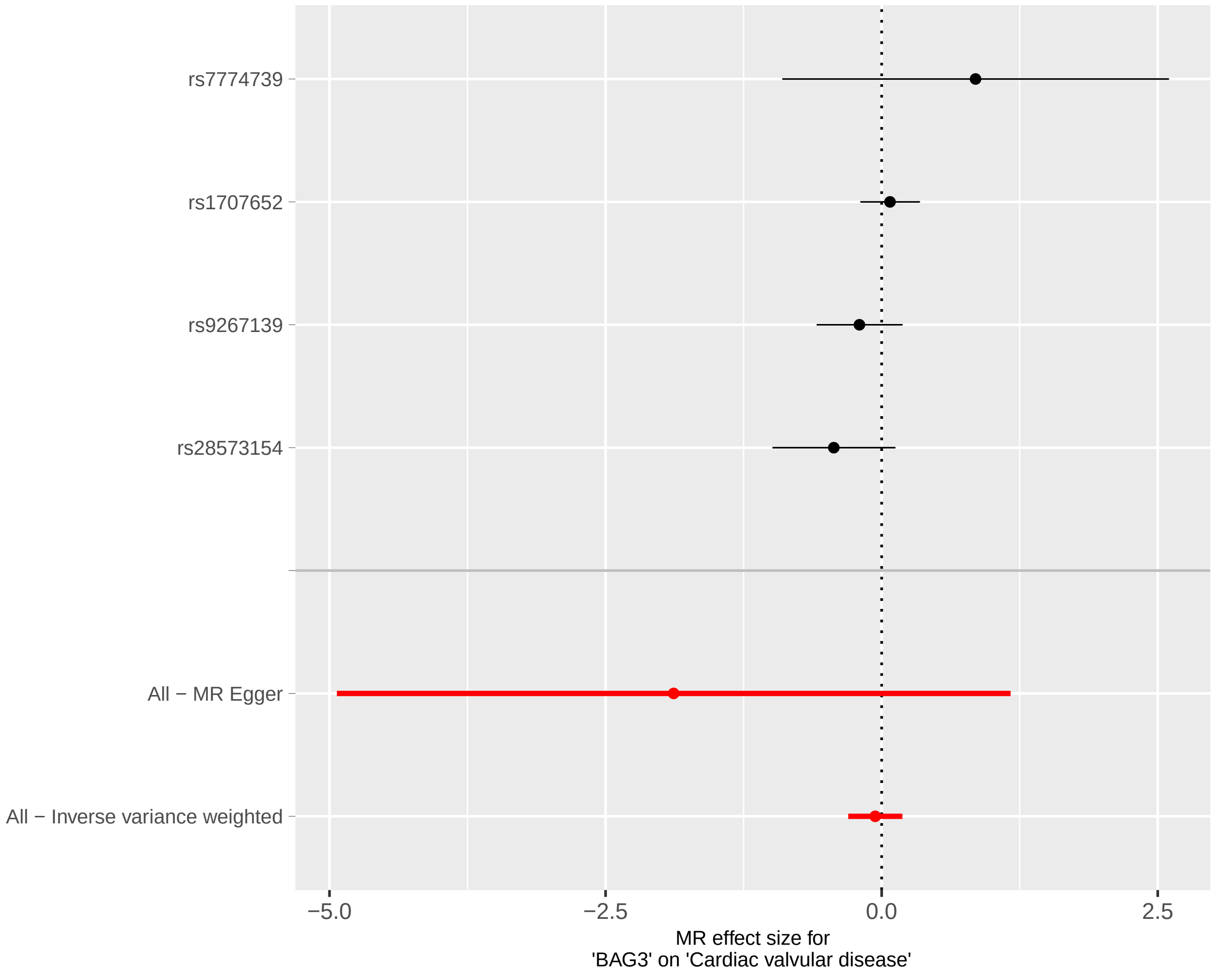
rs7774739
rs1707652
rs9267139
rs28573154
All − MR Egger
All − Inverse variance weighted
−5.0
−2.5
0.0
2.5
MR effect size for
'BAG3' on 'Cardiac valvular disease'

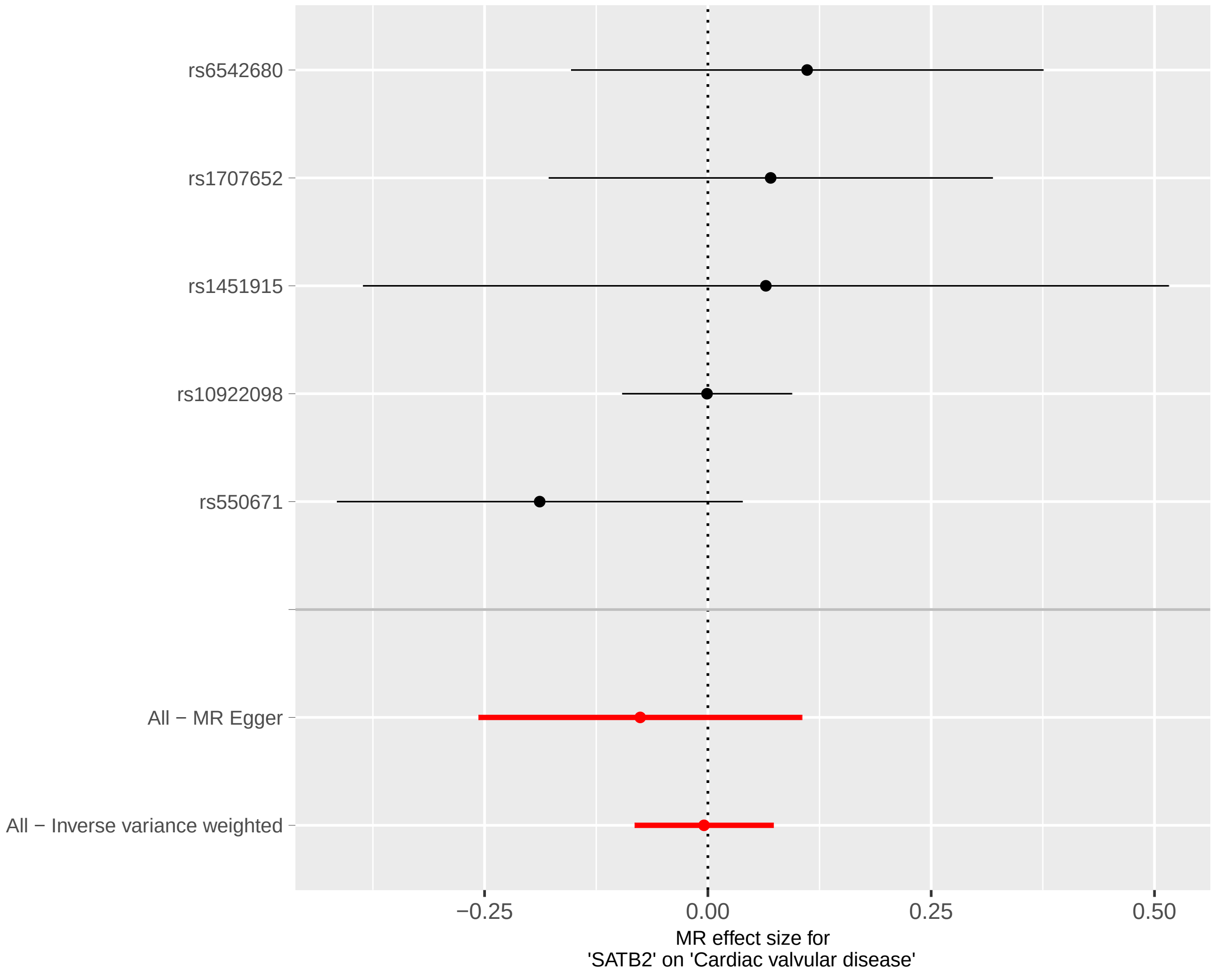
rs6542680
rs1707652
rs1451915
rs10922098
rs550671
All − MR Egger
All − Inverse variance weighted
−0.25
0.00
0.25
0.50
MR effect size for
'SATB2' on 'Cardiac valvular disease'

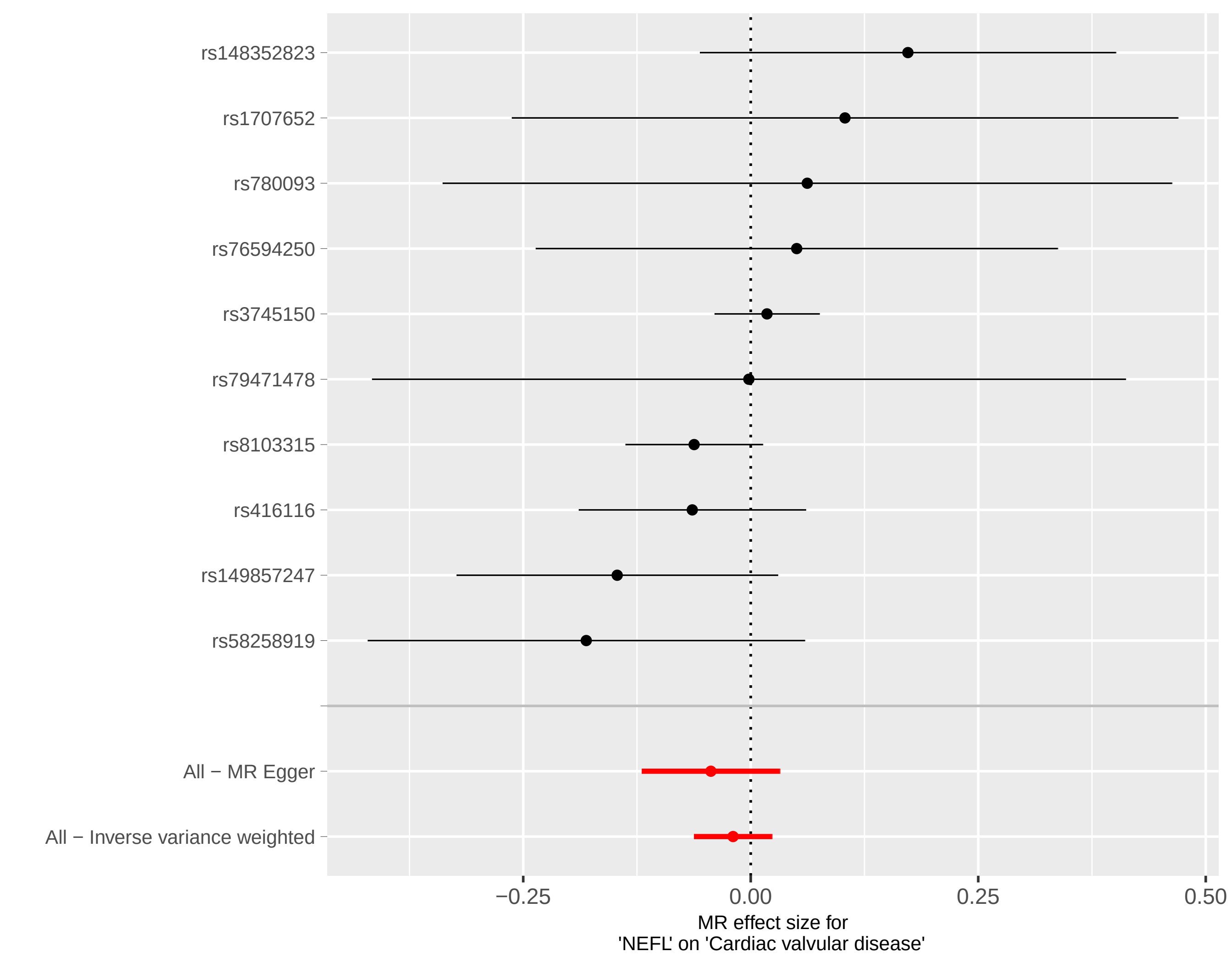
rs148352823
rs1707652
rs780093
rs76594250
rs3745150
rs79471478
rs8103315
rs416116
rs149857247
rs58258919
All − MR Egger
All − Inverse variance weighted
−0.25
0.00
0.25
0.50
MR effect size for
'NEFL' on 'Cardiac valvular disease'

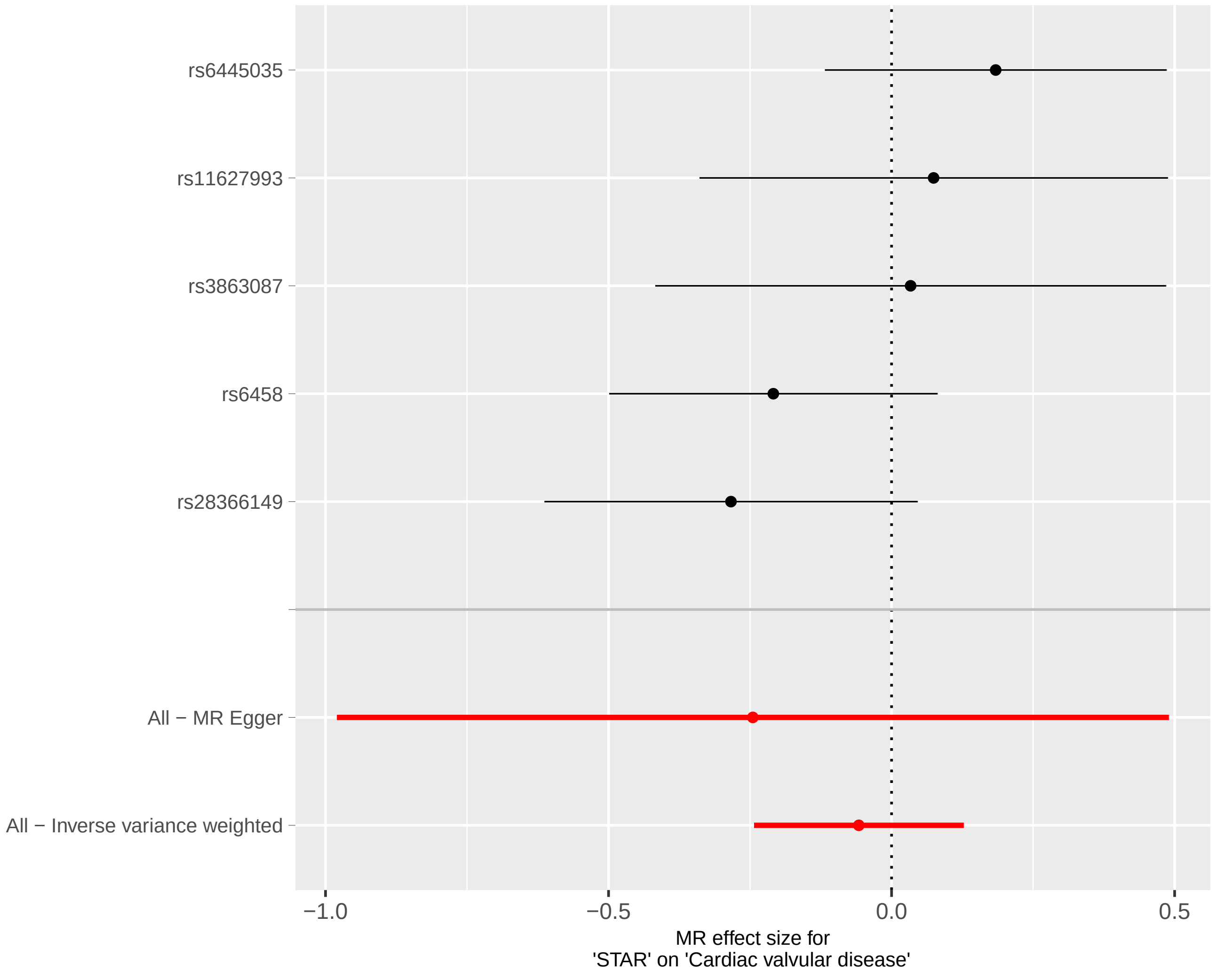
rs6445035
rs11627993
rs3863087
rs6458
rs28366149
All − MR Egger
All − Inverse variance weighted
−1.0
−0.5
0.0
0.5
MR effect size for
'STAR' on 'Cardiac valvular disease'

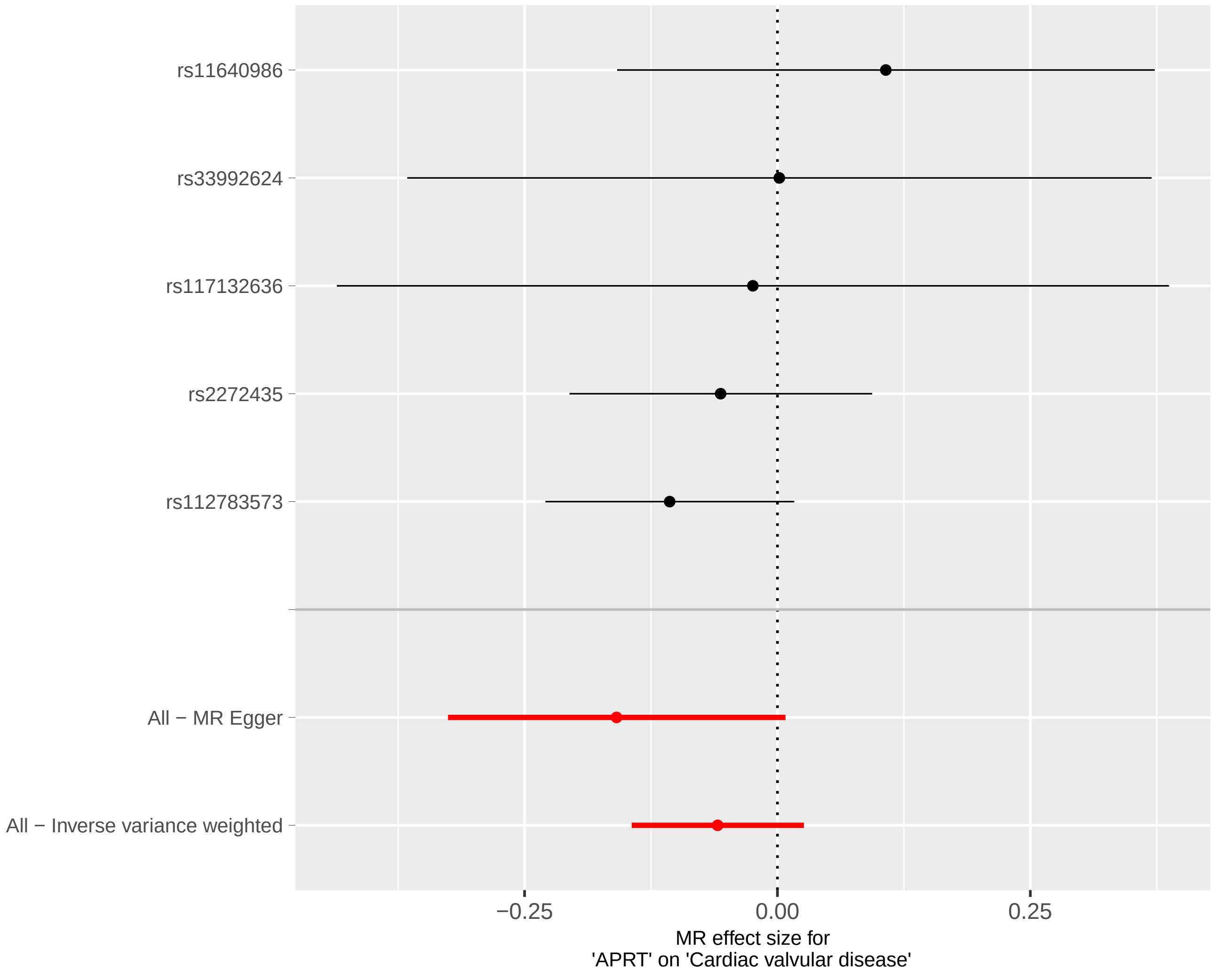

rs11640986
rs33992624
rs117132636
rs2272435
rs112783573
All − MR Egger
All − Inverse variance weighted
−0.25
0.00
0.25
MR effect size for
'APRT' on 'Cardiac valvular disease'

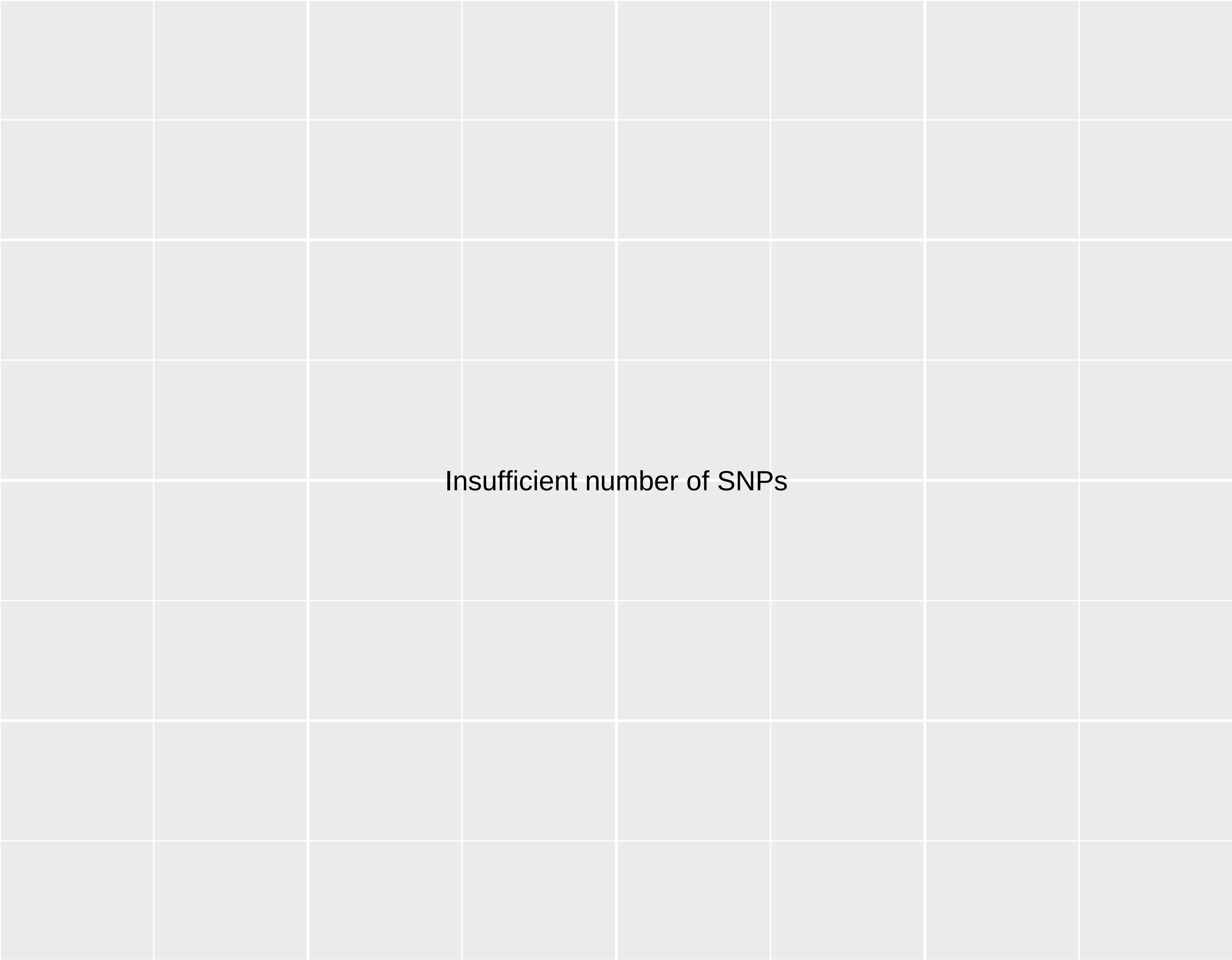
Insufficient number of SNPs

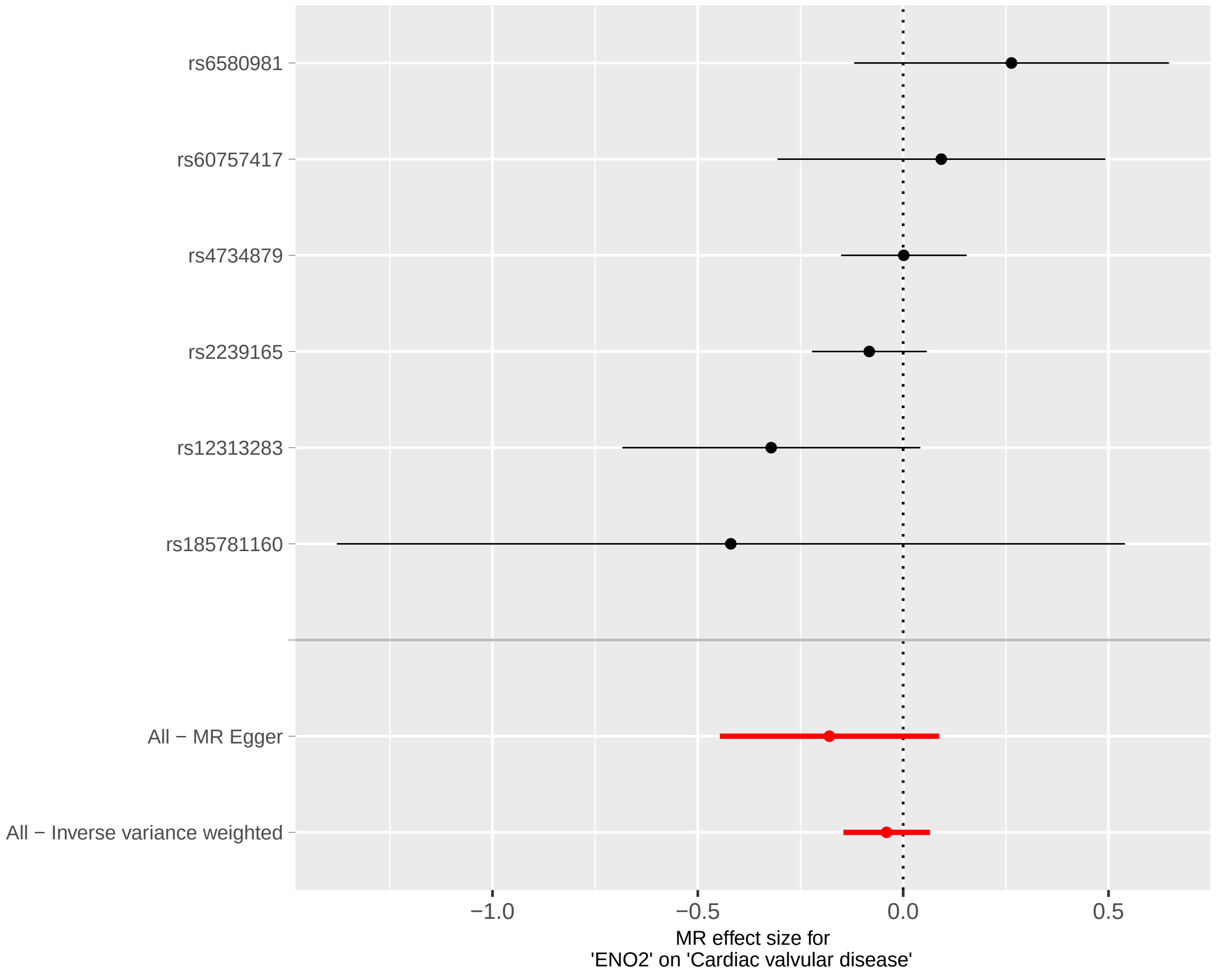
rs6580981
rs60757417
rs4734879
rs2239165
rs12313283
rs185781160
All − MR Egger
All − Inverse variance weighted
−1.0
−0.5
0.0
0.5
MR effect size for
'ENO2' on 'Cardiac valvular disease'

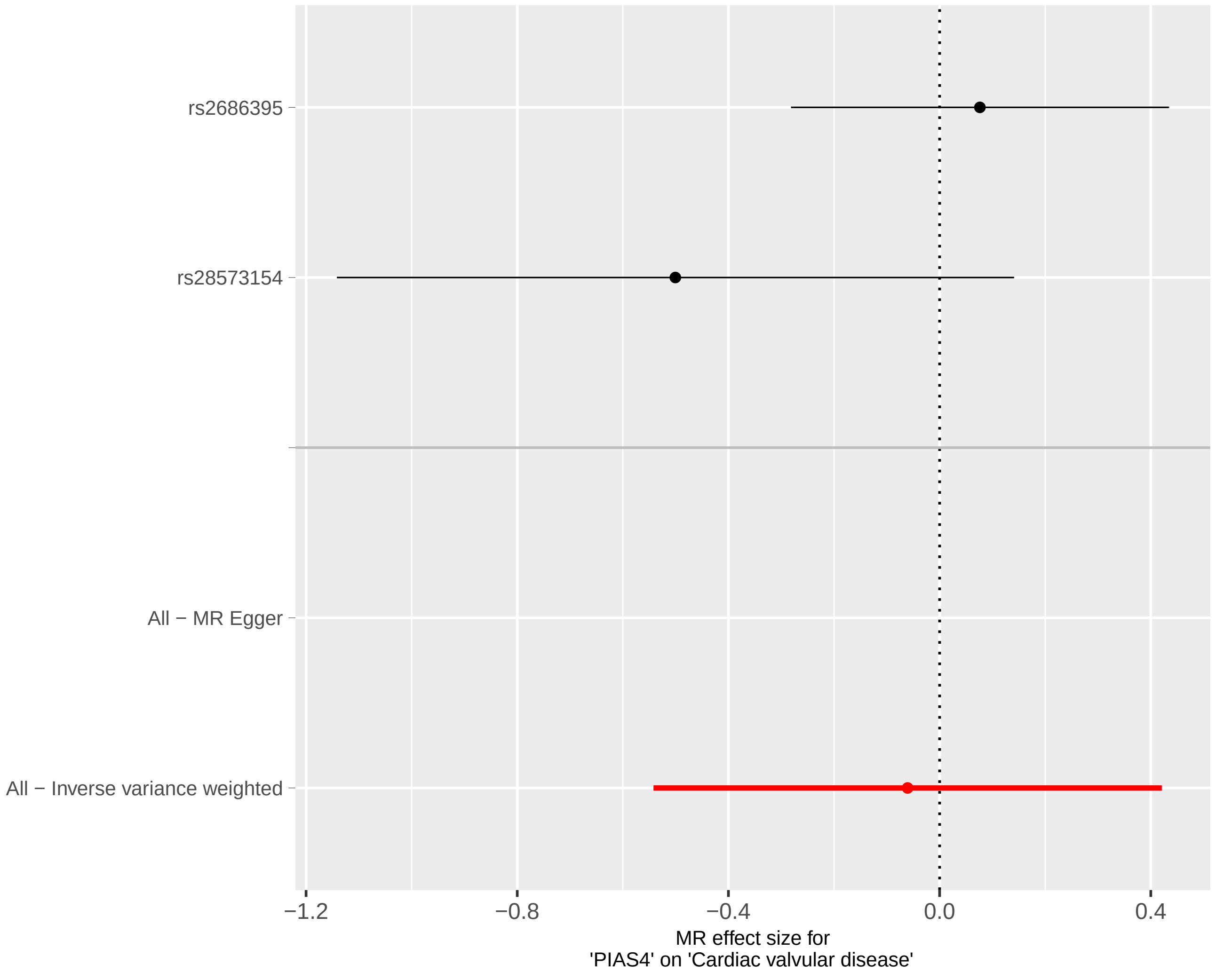
rs2686395
rs28573154
All − MR Egger
All − Inverse variance weighted
−1.2
−0.8
−0.4
0.0
0.4
MR effect size for
'PIAS4' on 'Cardiac valvular disease'

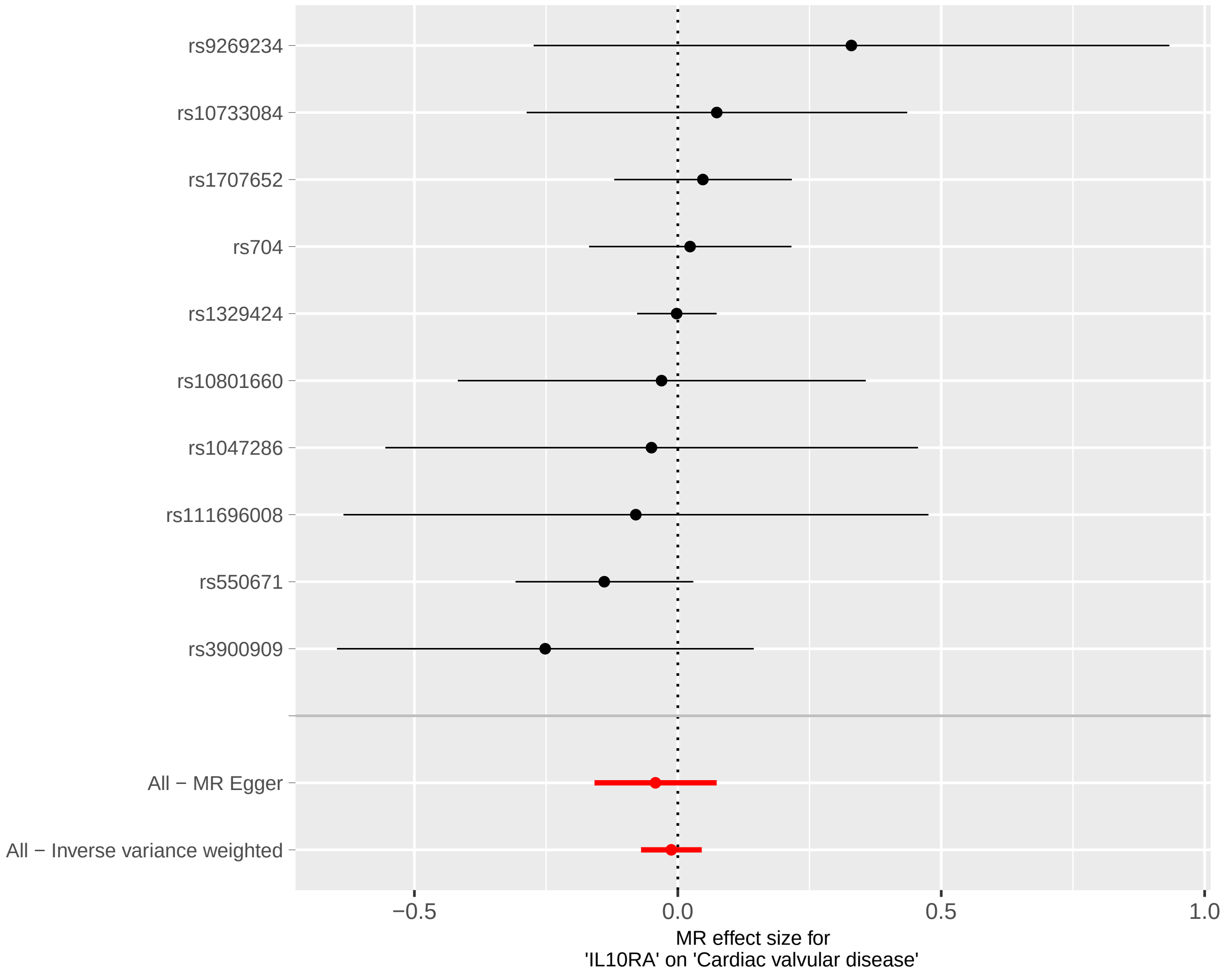
rs9269234
rs10733084
rs1707652
rs704
rs1329424
rs10801660
rs1047286
rs111696008
rs550671
rs3900909
All − MR Egger
All − Inverse variance weighted
−0.5
0.0
0.5
1.0
MR effect size for
'IL10RA' on 'Cardiac valvular disease'

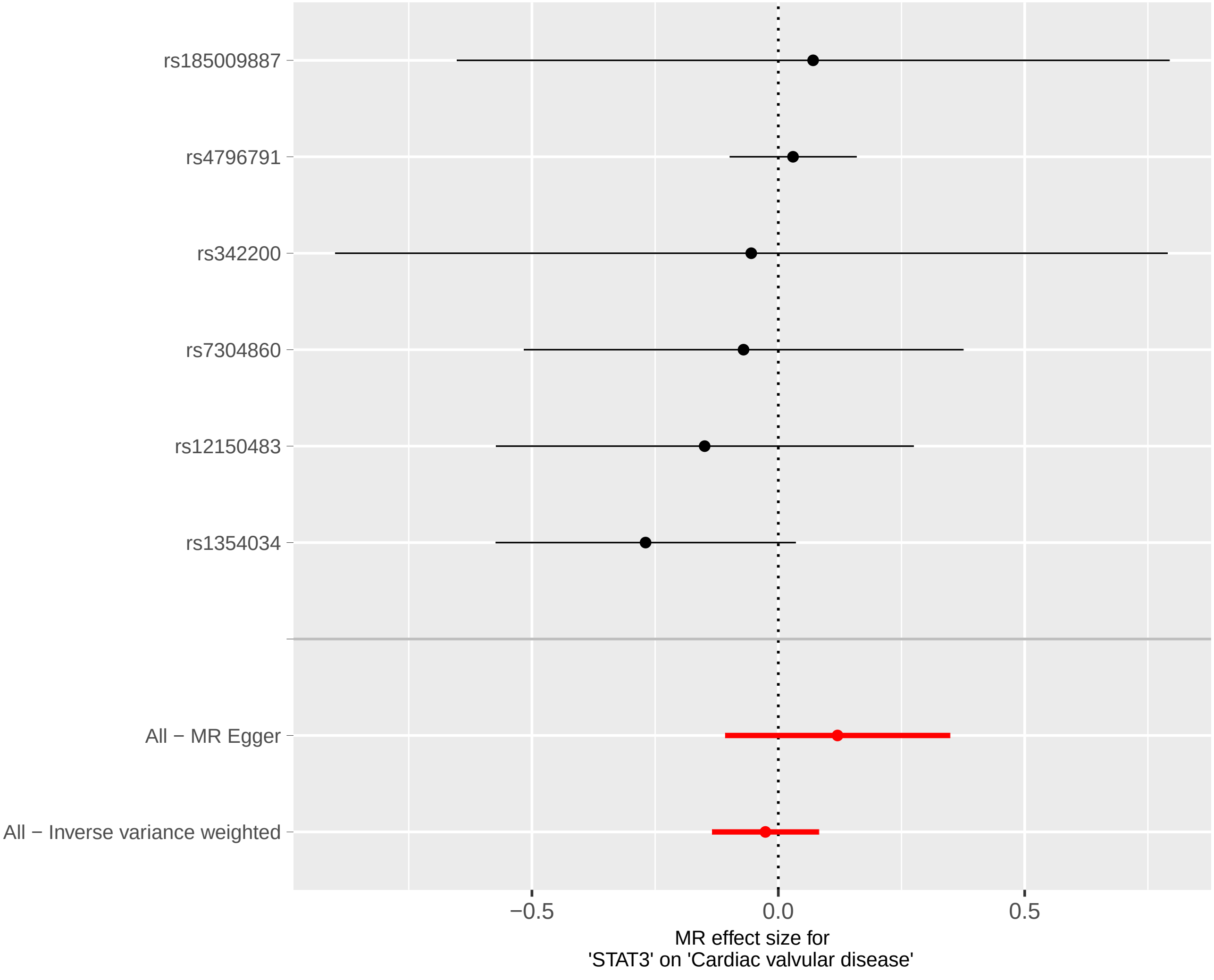
rs185009887
rs4796791
rs342200
rs7304860
rs12150483
rs1354034
All − MR Egger
All − Inverse variance weighted
−0.5
0.0
0.5
MR effect size for
'STAT3' on 'Cardiac valvular disease'

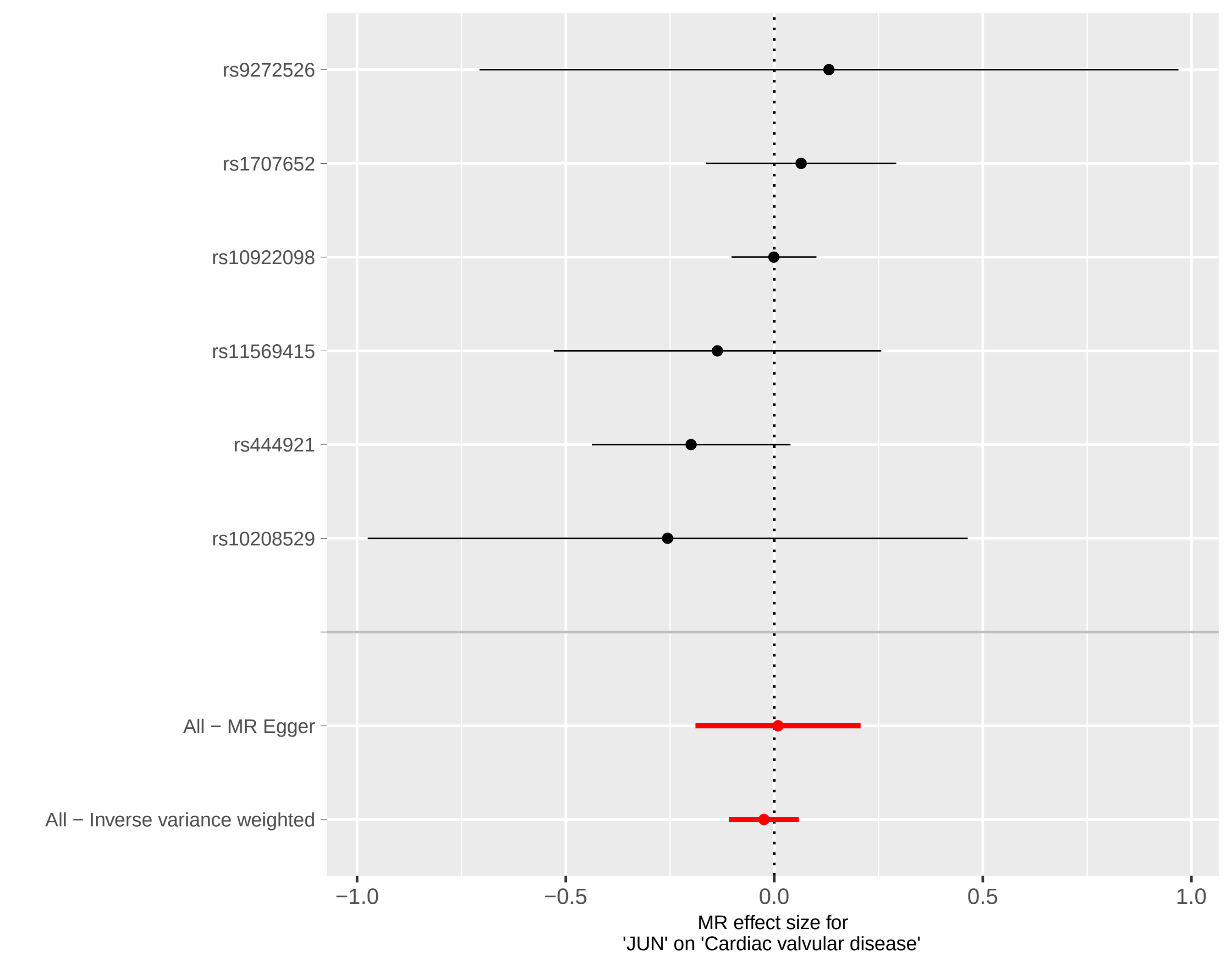

rs9272526
rs1707652
rs10922098
rs11569415
rs444921
rs10208529
All − MR Egger
All − Inverse variance weighted
−1.0
−0.5
0.0
0.5
1.0
MR effect size for
'JUN' on 'Cardiac valvular disease'

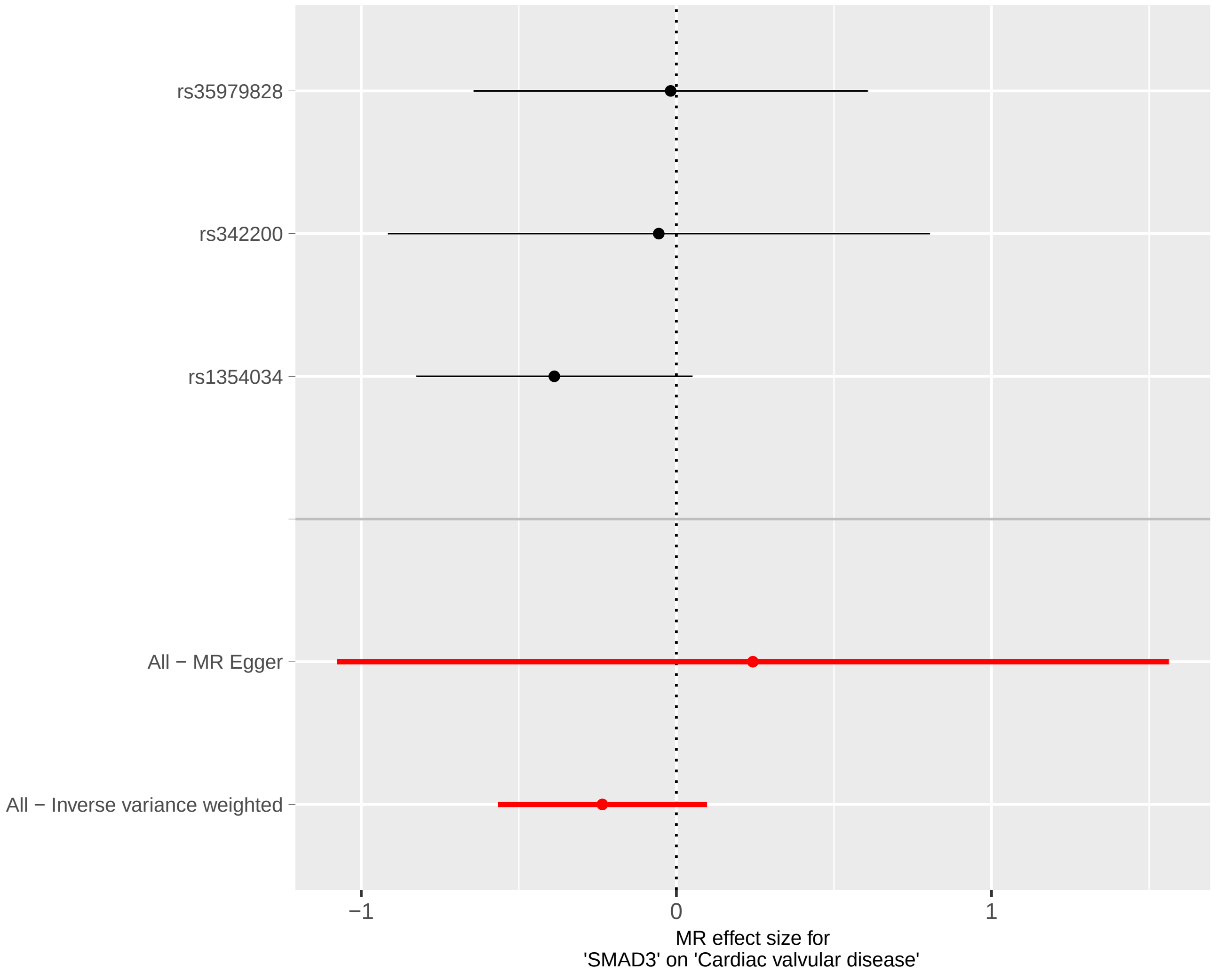
rs35979828
rs342200
rs1354034
All − MR Egger
All − Inverse variance weighted
−1
0
1
MR effect size for
'SMAD3' on 'Cardiac valvular disease'

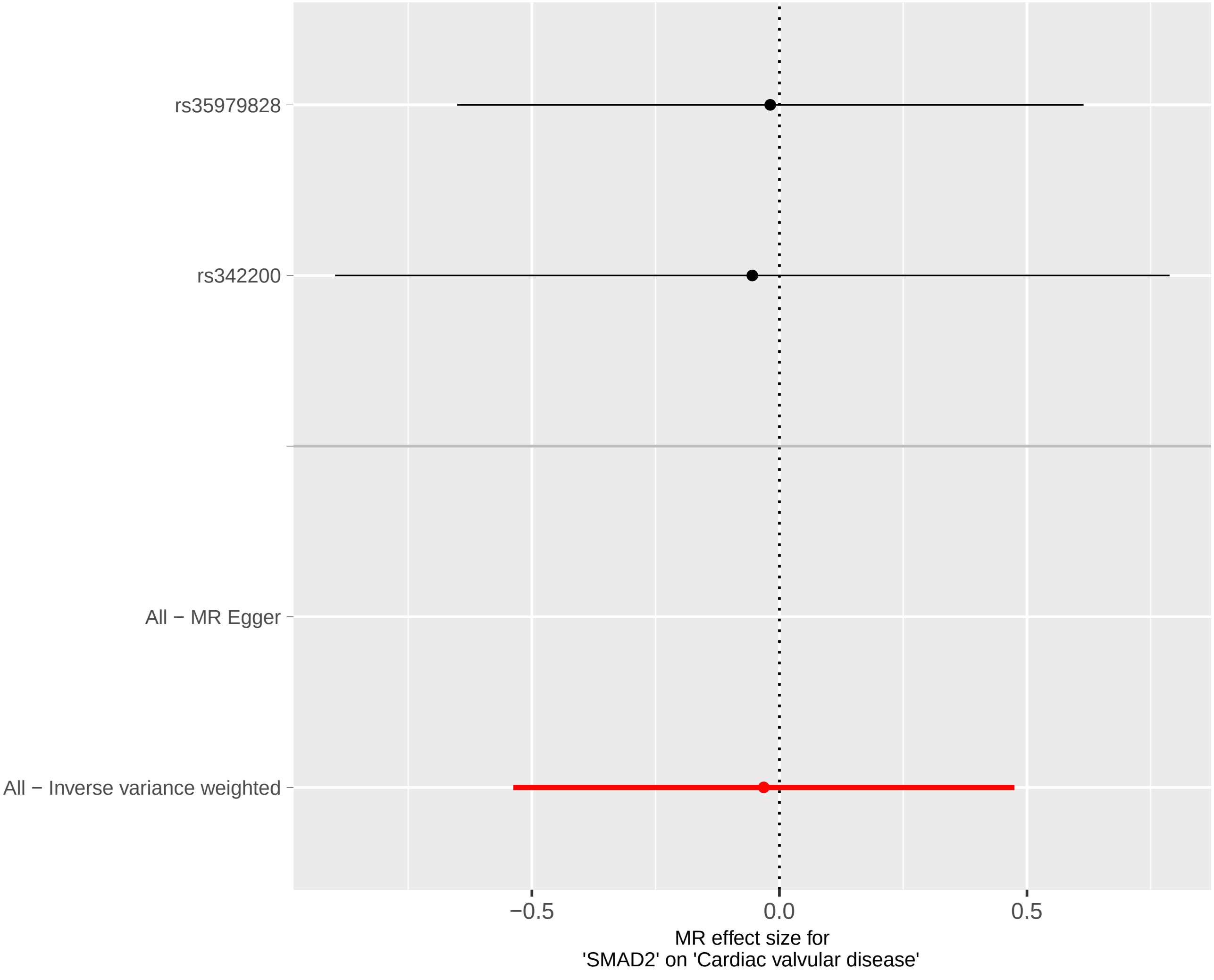

rs35979828
rs342200
All − MR Egger
All − Inverse variance weighted
−0.5
0.0
0.5
MR effect size for
'SMAD2' on 'Cardiac valvular disease'

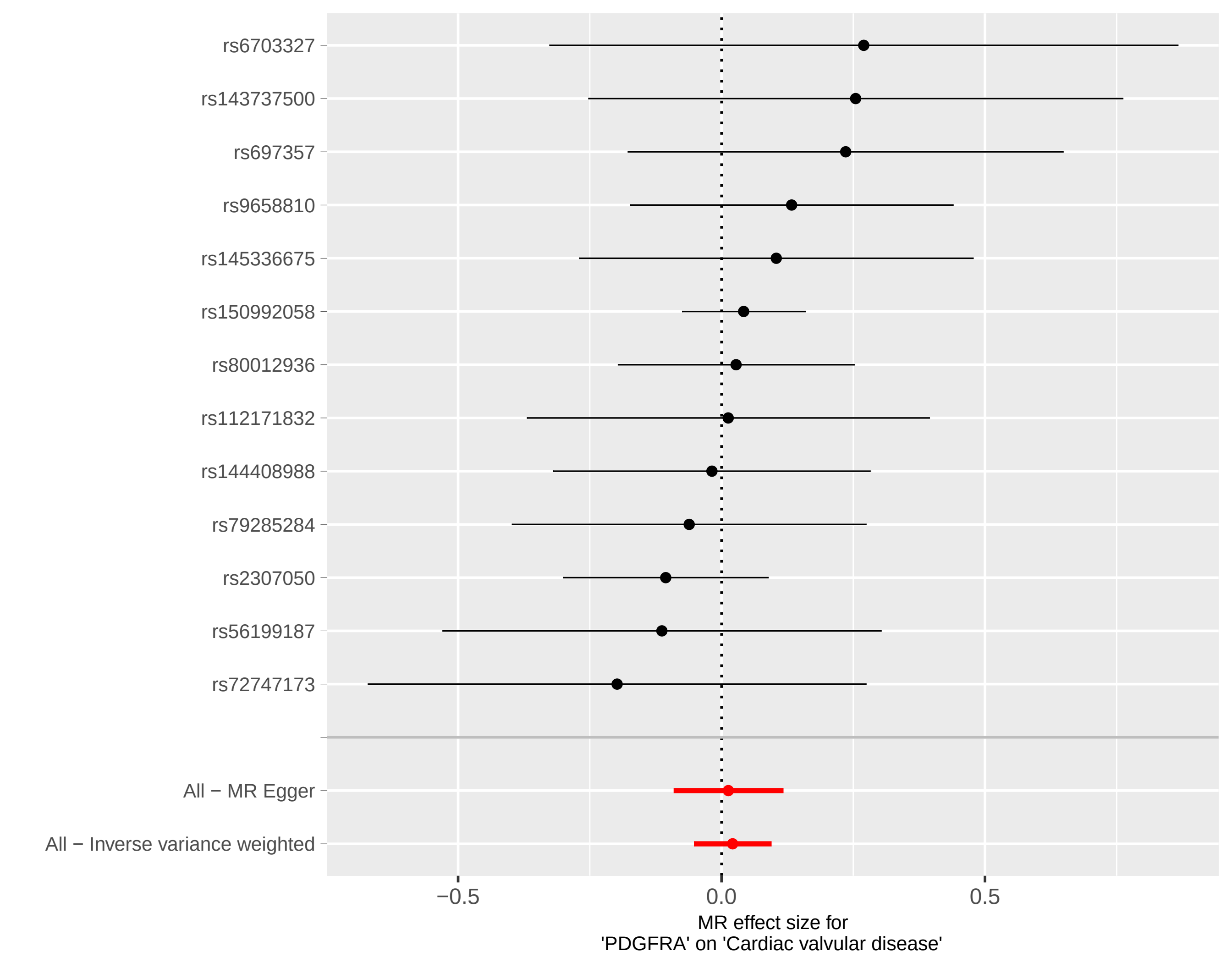
rs6703327
rs143737500
rs697357
rs9658810
rs145336675
rs150992058
rs80012936
rs112171832
rs144408988
rs79285284
rs2307050
rs56199187
rs72747173
All − MR Egger
All − Inverse variance weighted
−0.5
0.0
0.5
MR effect size for
'PDGFRA' on 'Cardiac valvular disease'

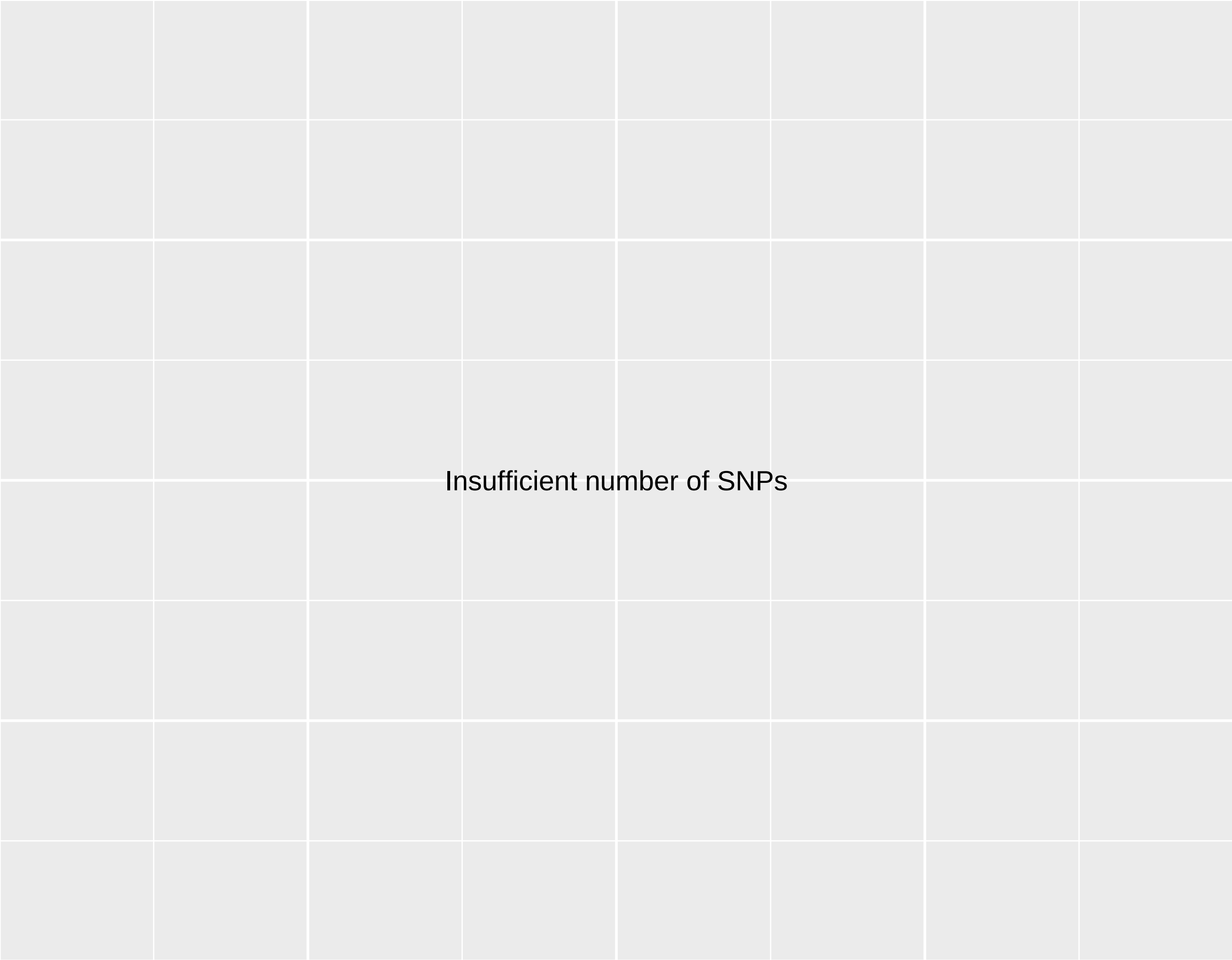
Insufficient number of SNPs

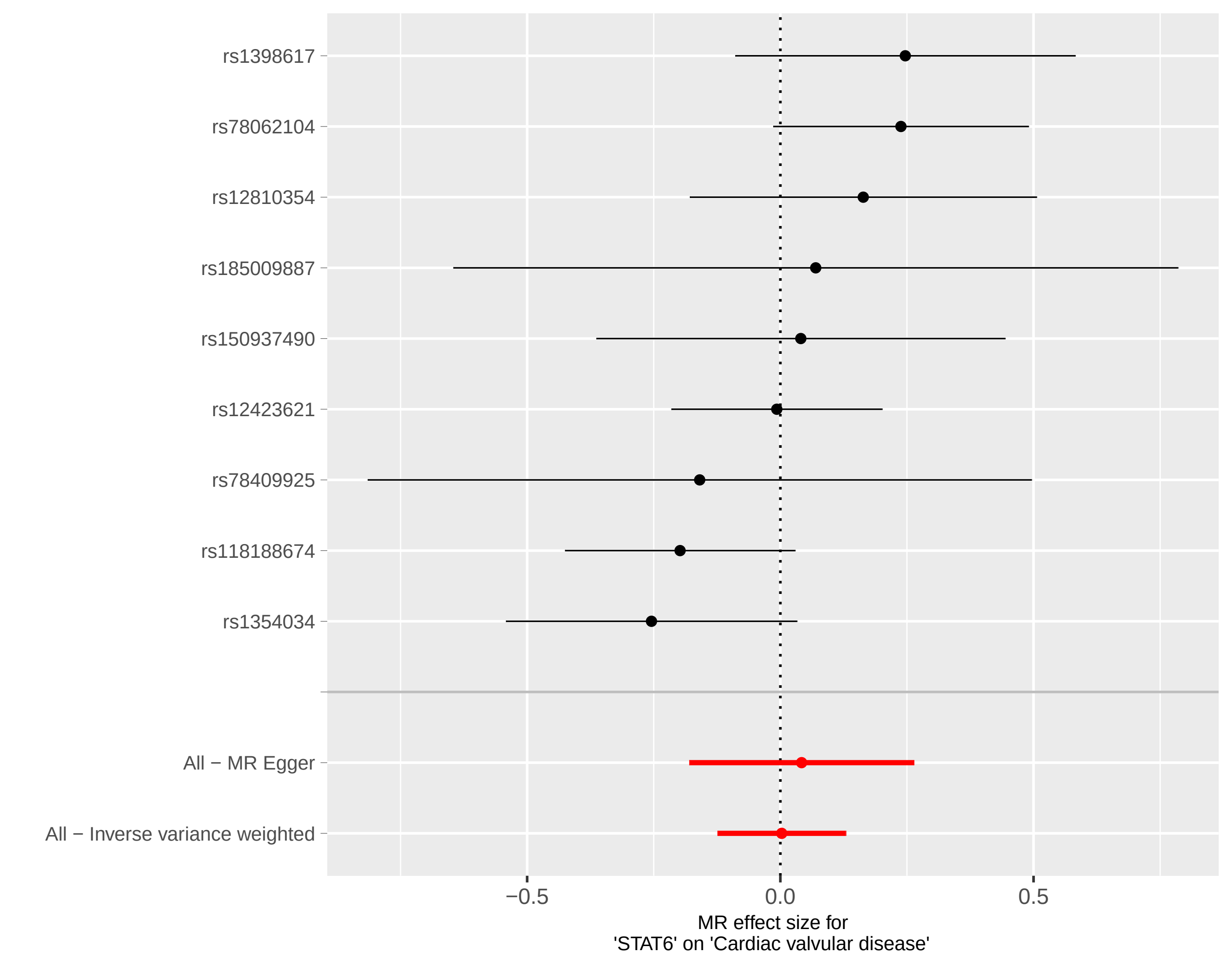
rs1398617
rs78062104
rs12810354
rs185009887
rs150937490
rs12423621
rs78409925
rs118188674
rs1354034
All − MR Egger
All − Inverse variance weighted
−0.5
0.0
0.5
MR effect size for
'STAT6' on 'Cardiac valvular disease'

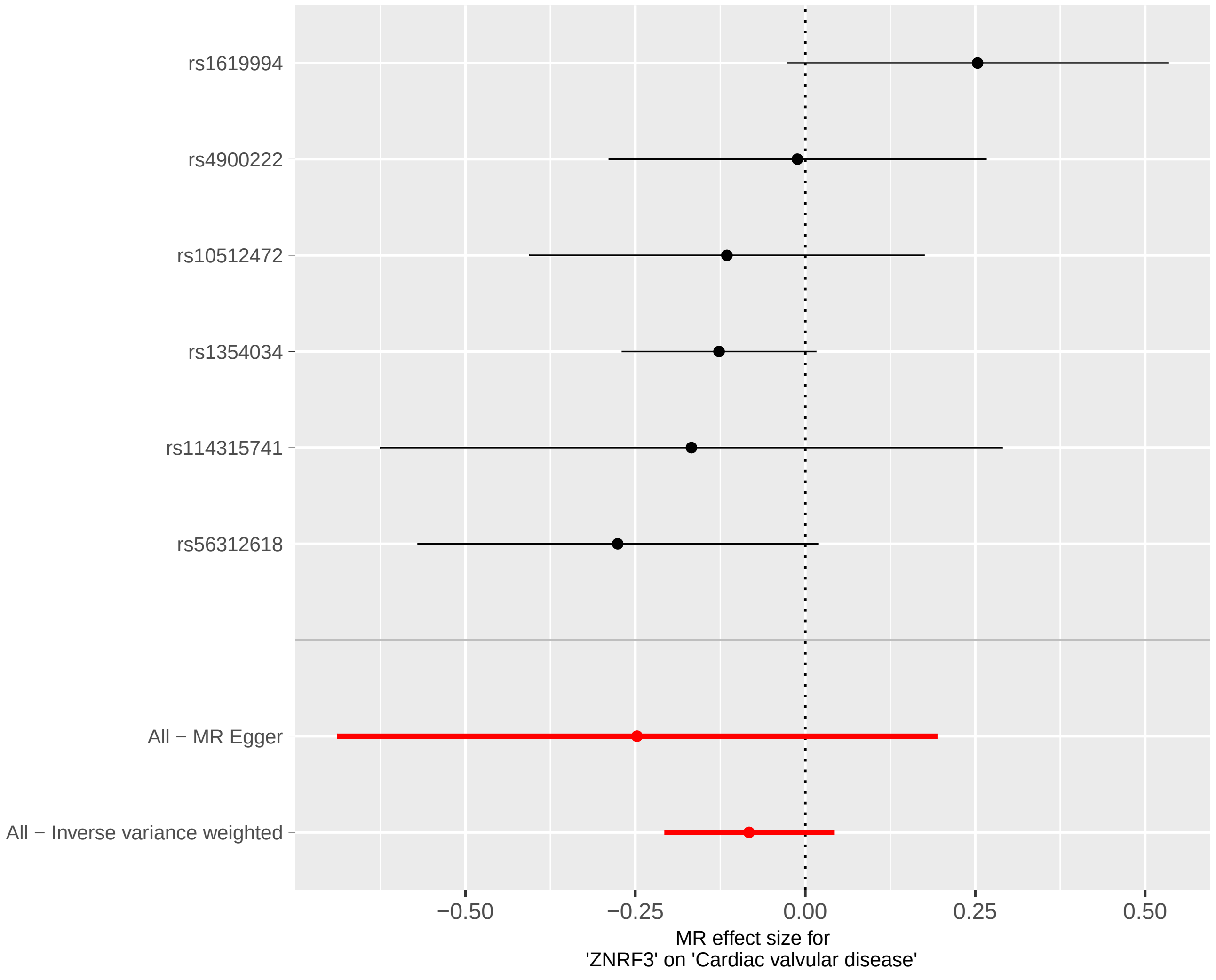
rs1619994
rs4900222
rs10512472
rs1354034
rs114315741
rs56312618
All − MR Egger
All − Inverse variance weighted
−0.50
−0.25
0.00
0.25
0.50
MR effect size for
'ZNRF3' on 'Cardiac valvular disease'

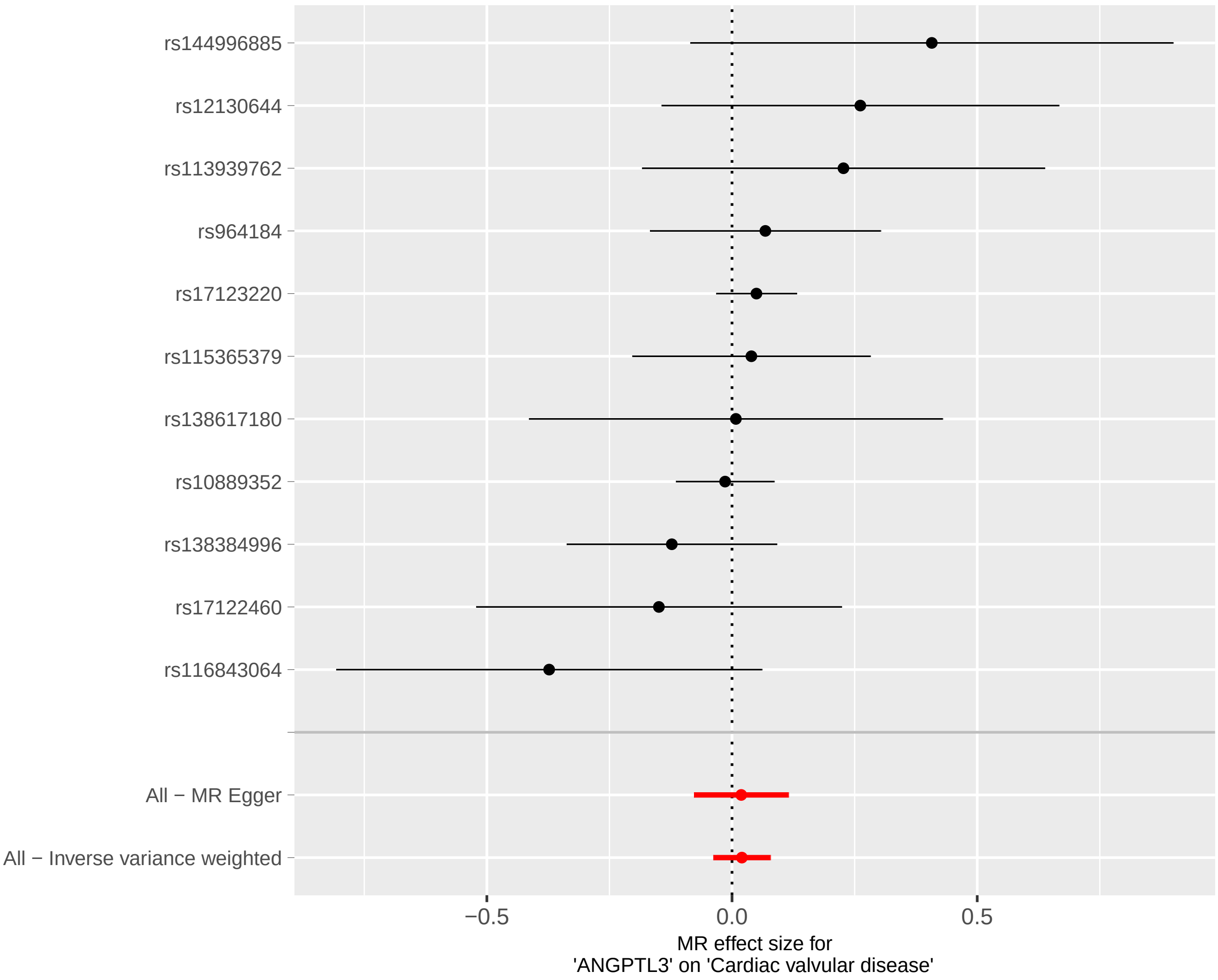
rs144996885
rs12130644
rs113939762
rs964184
rs17123220
rs115365379
rs138617180
rs10889352
rs138384996
rs17122460
rs116843064
All − MR Egger
All − Inverse variance weighted
−0.5
0.0
0.5
MR effect size for
'ANGPTL3' on 'Cardiac valvular disease'

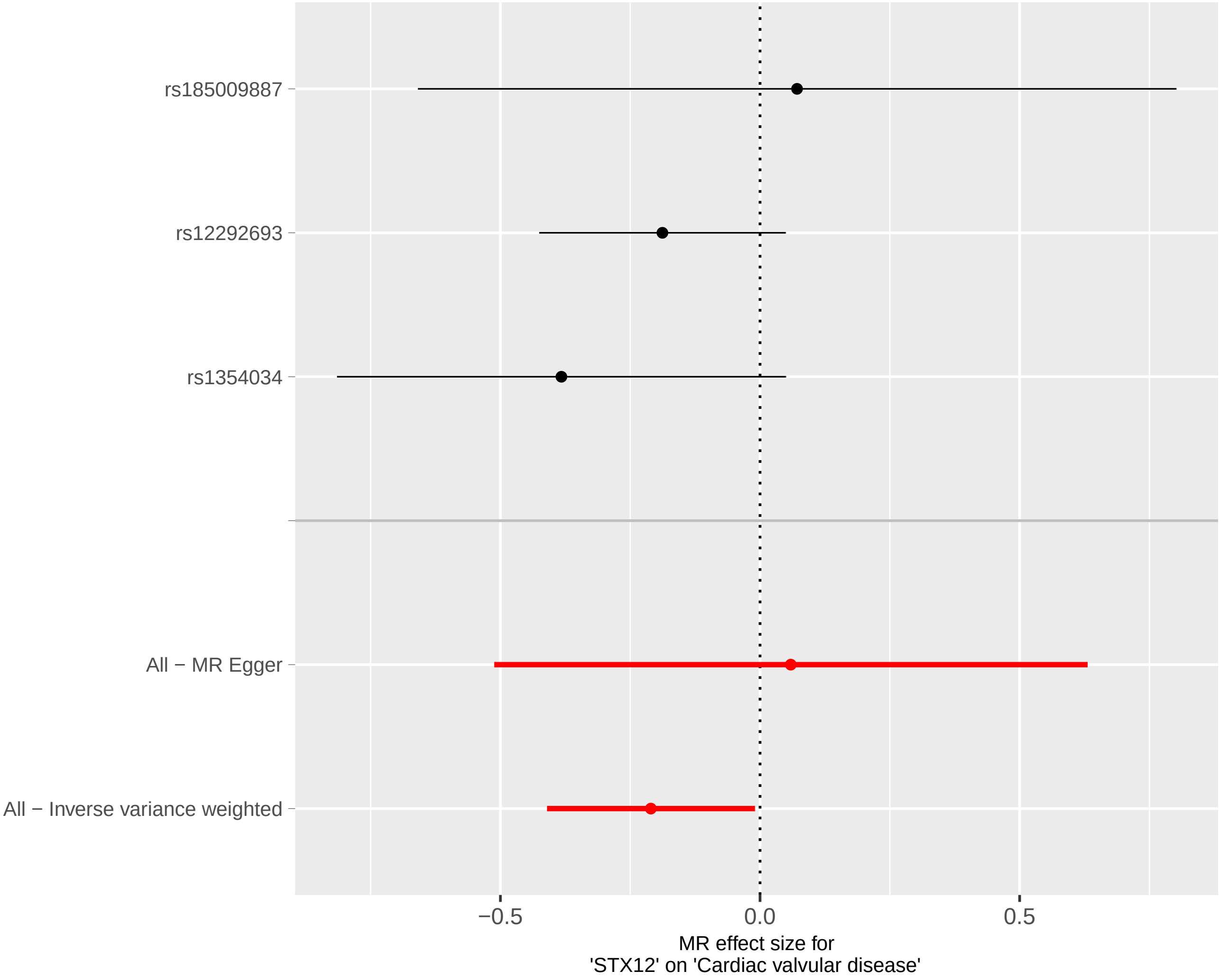

rs185009887
rs12292693
rs1354034
All − MR Egger
All − Inverse variance weighted
−0.5
0.0
0.5
MR effect size for
'STX12' on 'Cardiac valvular disease'

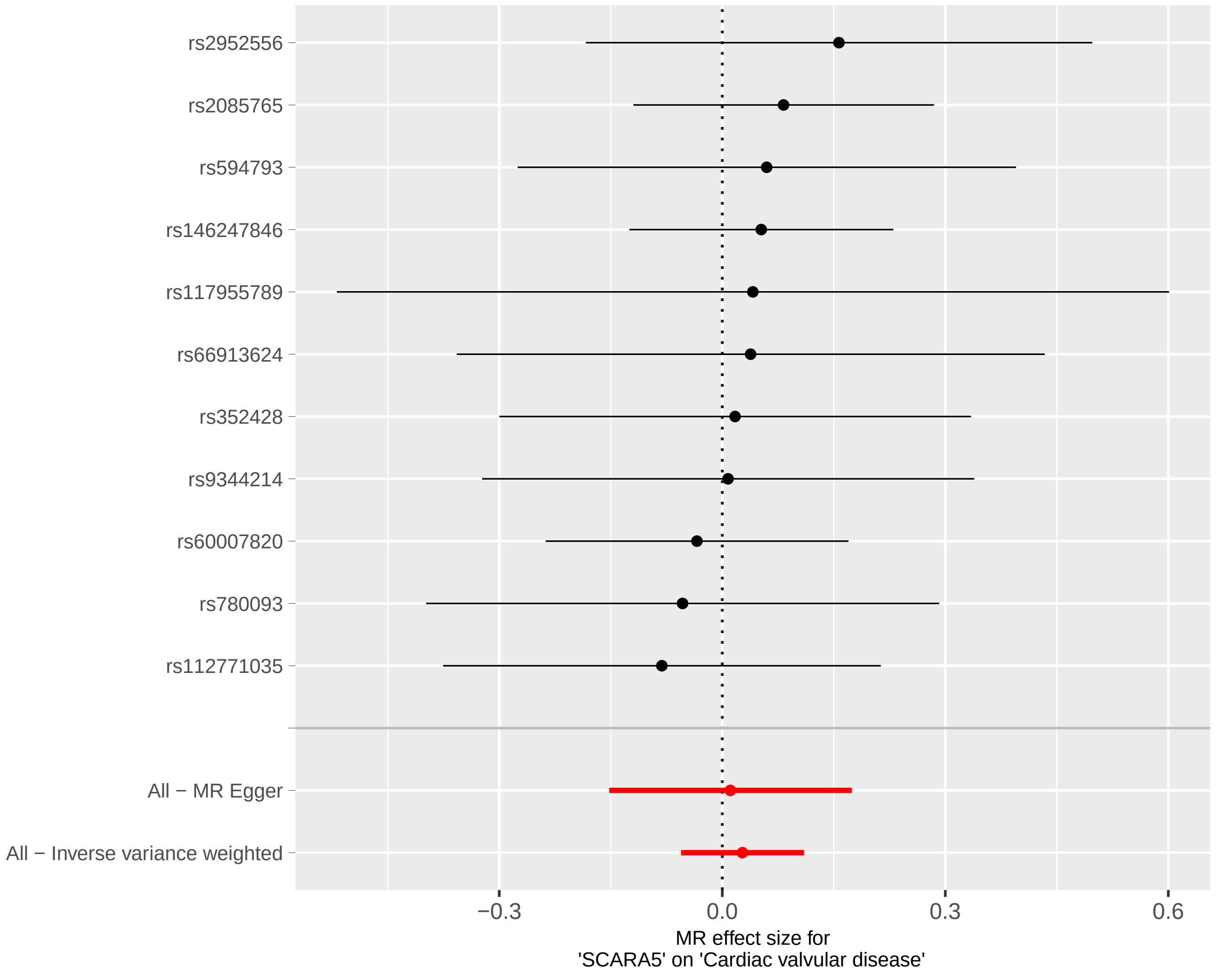
rs2952556
rs2085765
rs594793
rs146247846
rs117955789
rs66913624
rs352428
rs9344214
rs60007820
rs780093
rs112771035
All − MR Egger
All − Inverse variance weighted
−0.3
0.0
0.3
0.6
MR effect size for
'SCARA5' on 'Cardiac valvular disease'

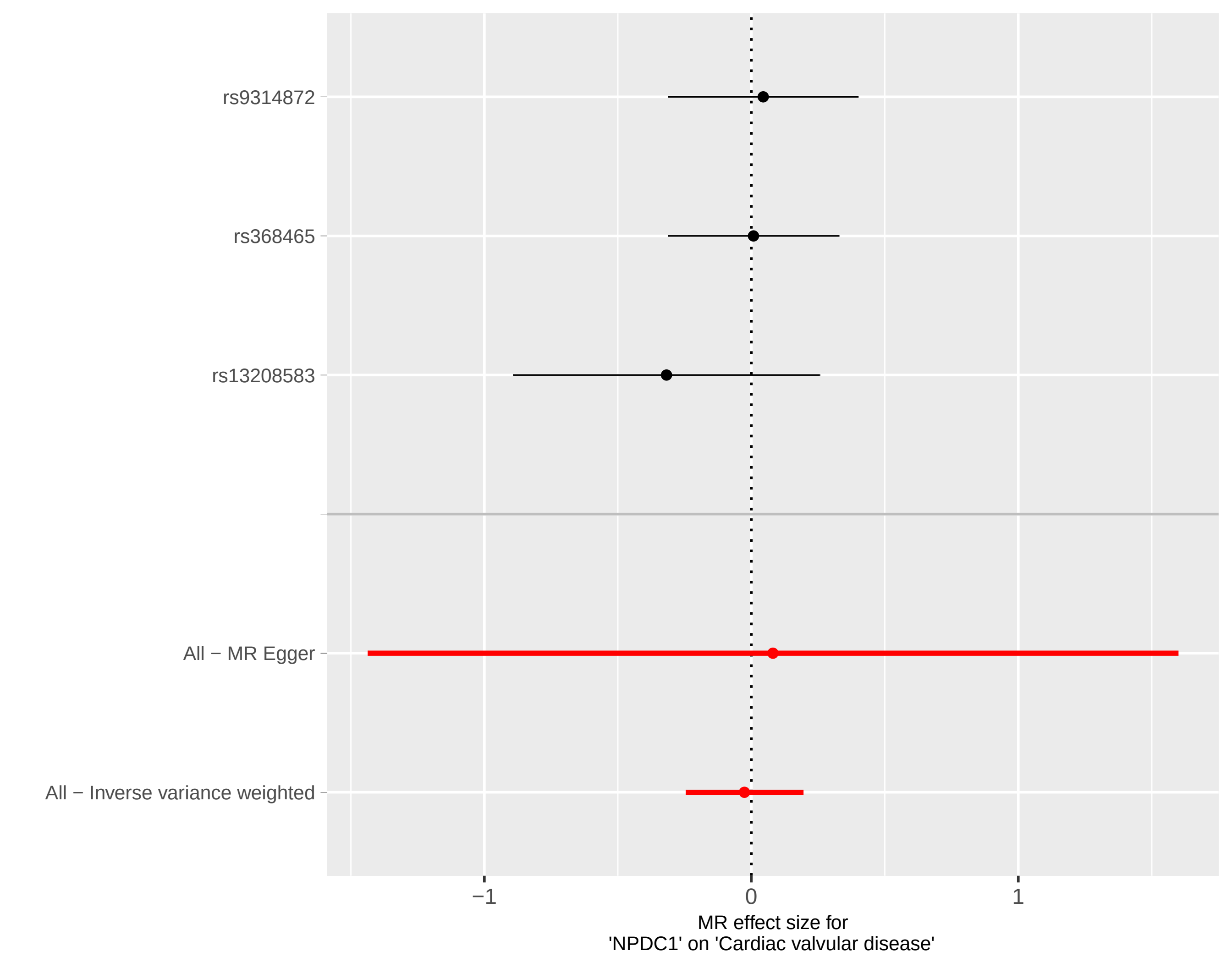
rs9314872
rs368465
rs13208583
All − MR Egger
All − Inverse variance weighted
−1
0
1
MR effect size for
'NPDC1' on 'Cardiac valvular disease'

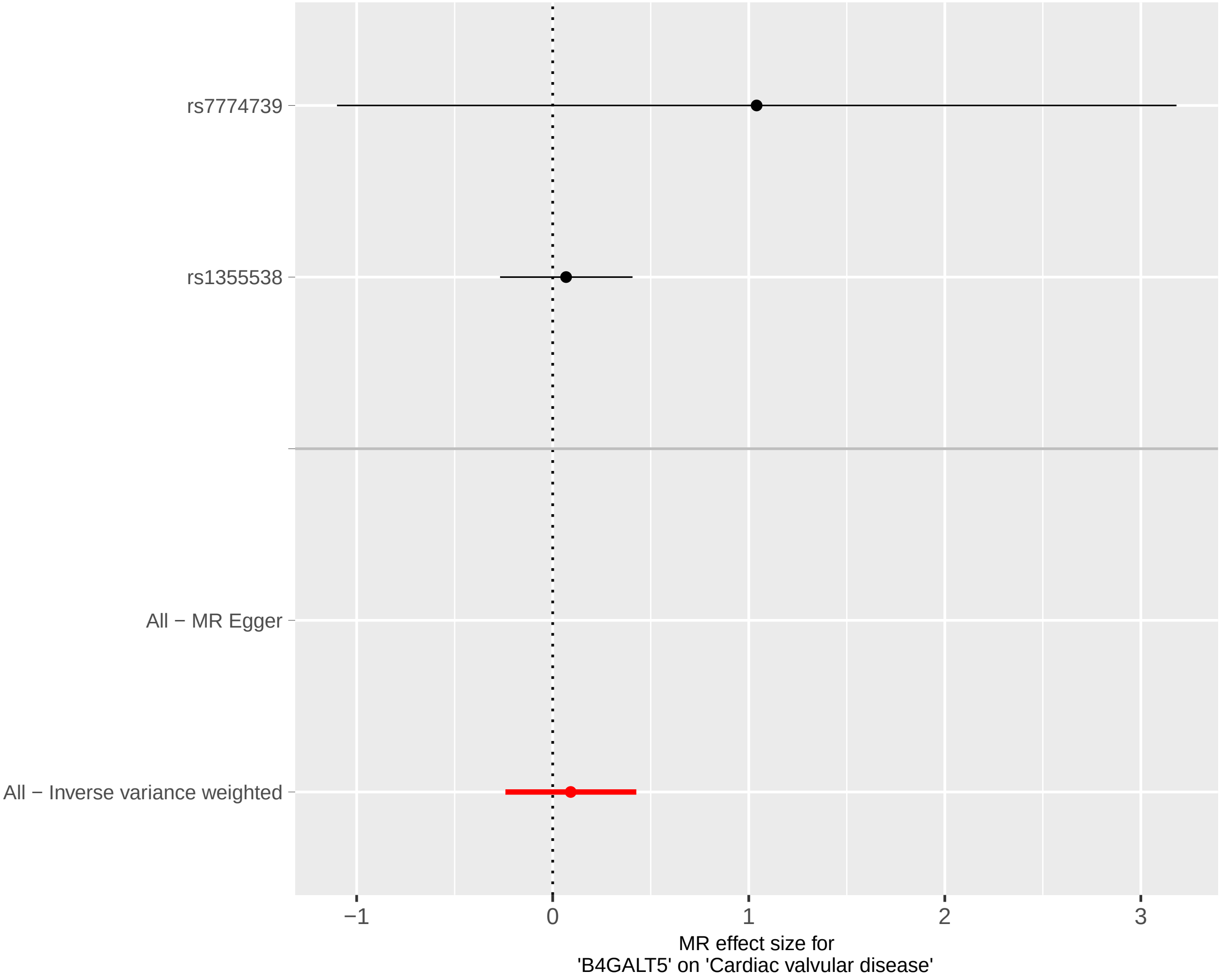
rs7774739
rs1355538
All − MR Egger
All − Inverse variance weighted
−1
0
1
2
3
MR effect size for
'B4GALT5' on 'Cardiac valvular disease'

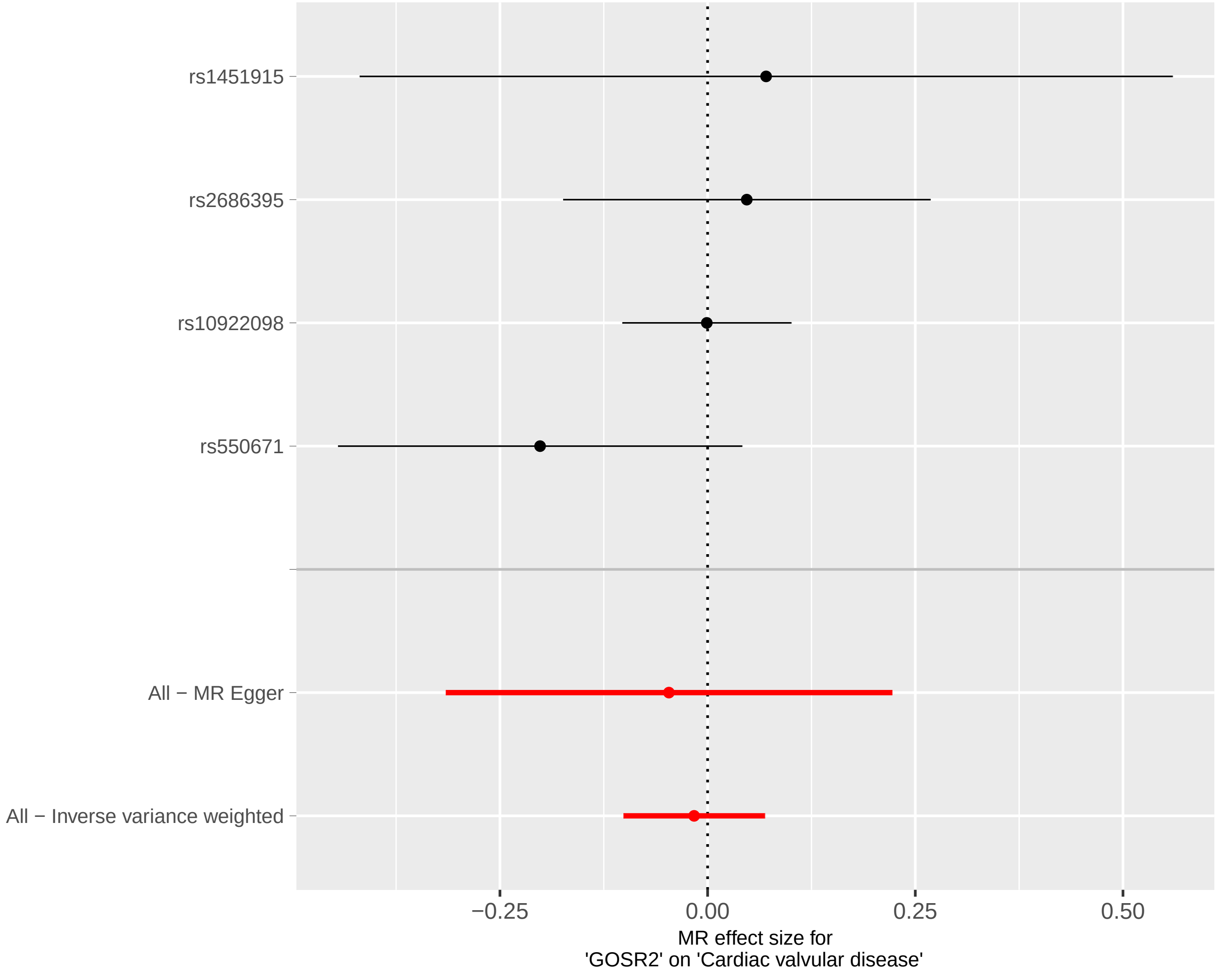

rs1451915
rs2686395
rs10922098
rs550671
All − MR Egger
All − Inverse variance weighted
−0.25
0.00
0.25
0.50
MR effect size for
'GOSR2' on 'Cardiac valvular disease'

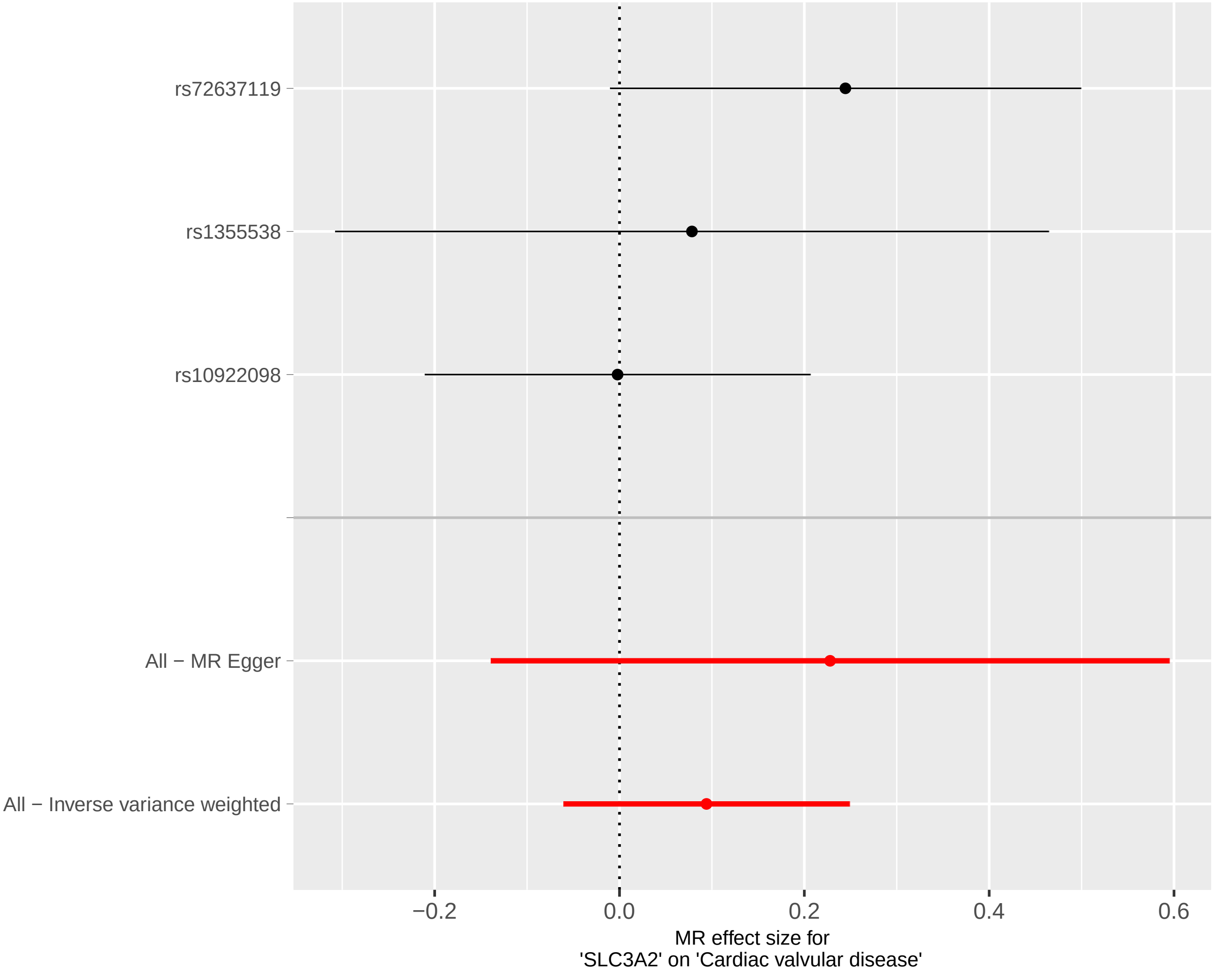

rs72637119
rs1355538
rs10922098
All − MR Egger
All − Inverse variance weighted
−0.2
0.0
0.2
0.4
0.6
MR effect size for
'SLC3A2' on 'Cardiac valvular disease'

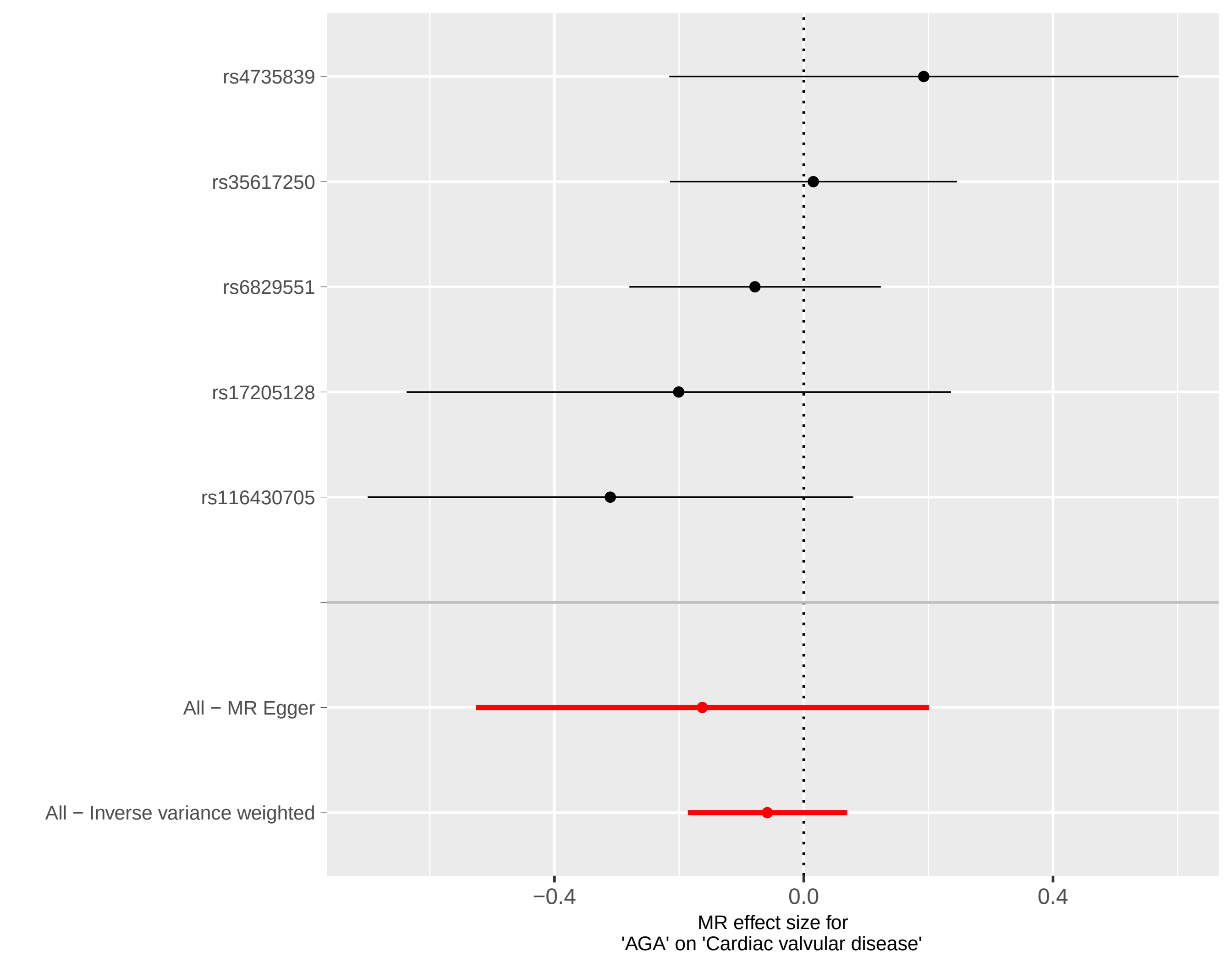

rs4735839
rs35617250
rs6829551
rs17205128
rs116430705
All − MR Egger
All − Inverse variance weighted
−0.4
0.0
0.4
MR effect size for
'AGA' on 'Cardiac valvular disease'

Insufficient number of SNPs

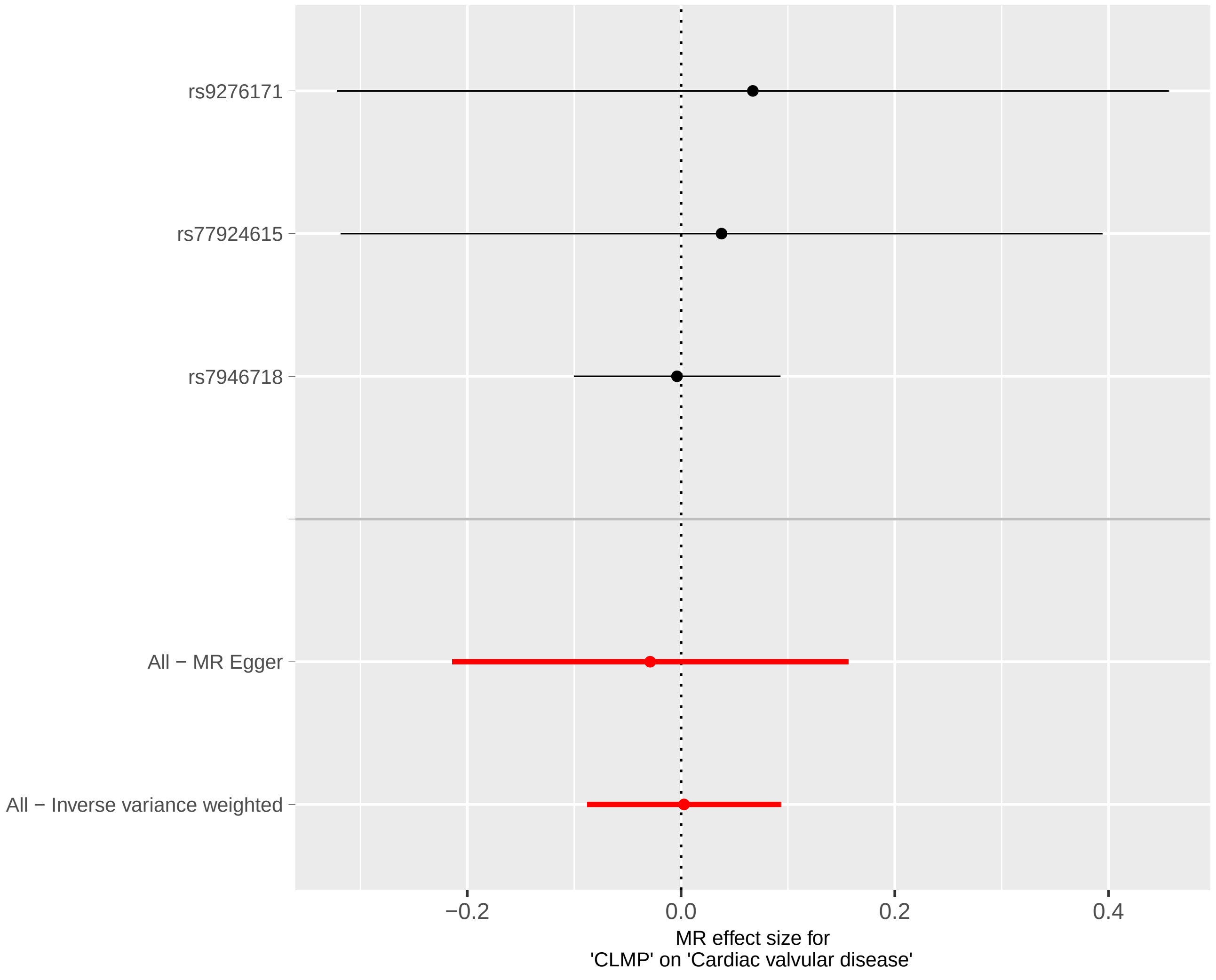
rs9276171
rs77924615
rs7946718
All − MR Egger
All − Inverse variance weighted
−0.2
0.0
0.2
0.4
MR effect size for
'CLMP' on 'Cardiac valvular disease'

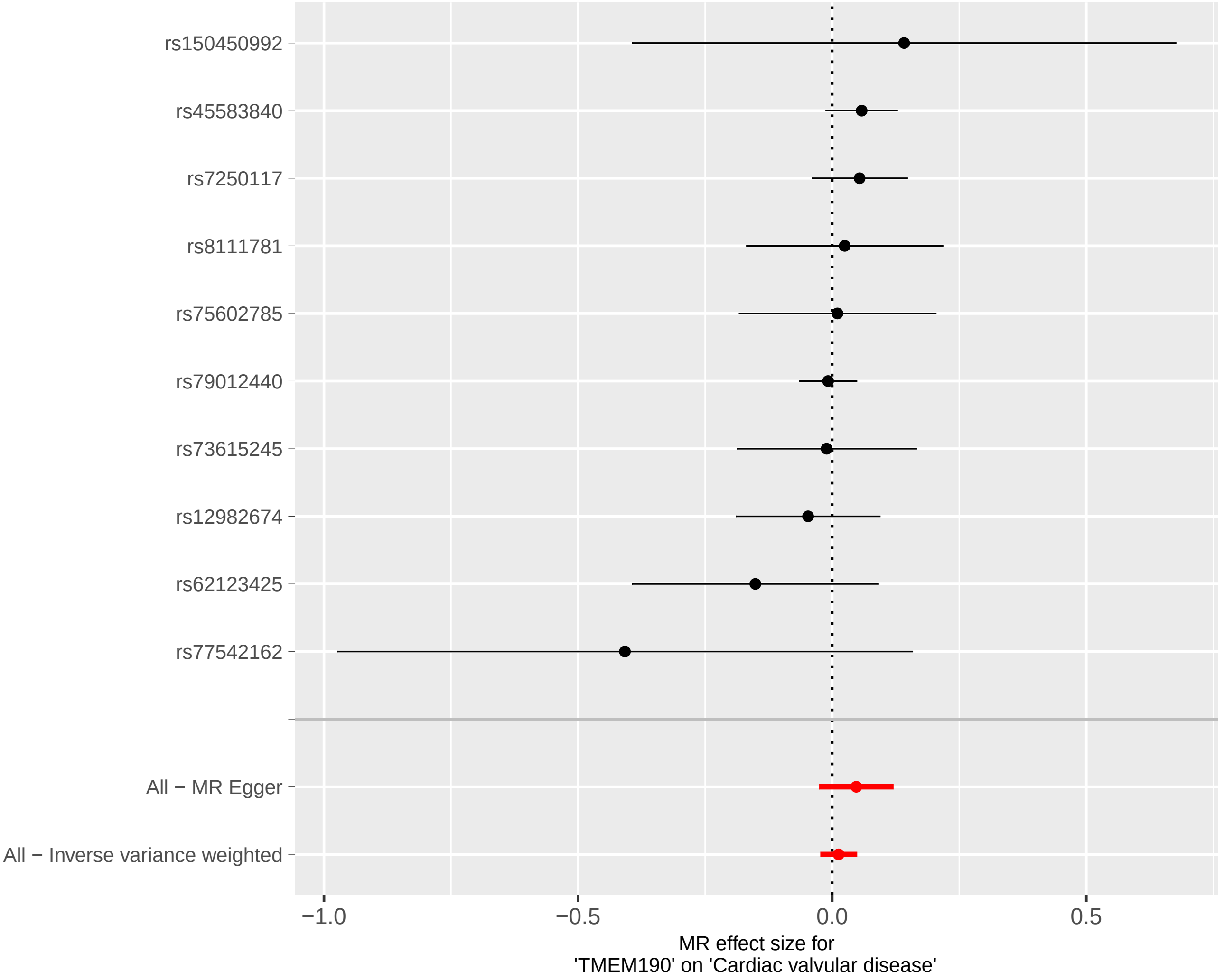
rs150450992
rs45583840
rs7250117
rs8111781
rs75602785
rs79012440
rs73615245
rs12982674
rs62123425
rs77542162
All − MR Egger
All − Inverse variance weighted
−1.0
−0.5
0.0
0.5
MR effect size for
'TMEM190' on 'Cardiac valvular disease'

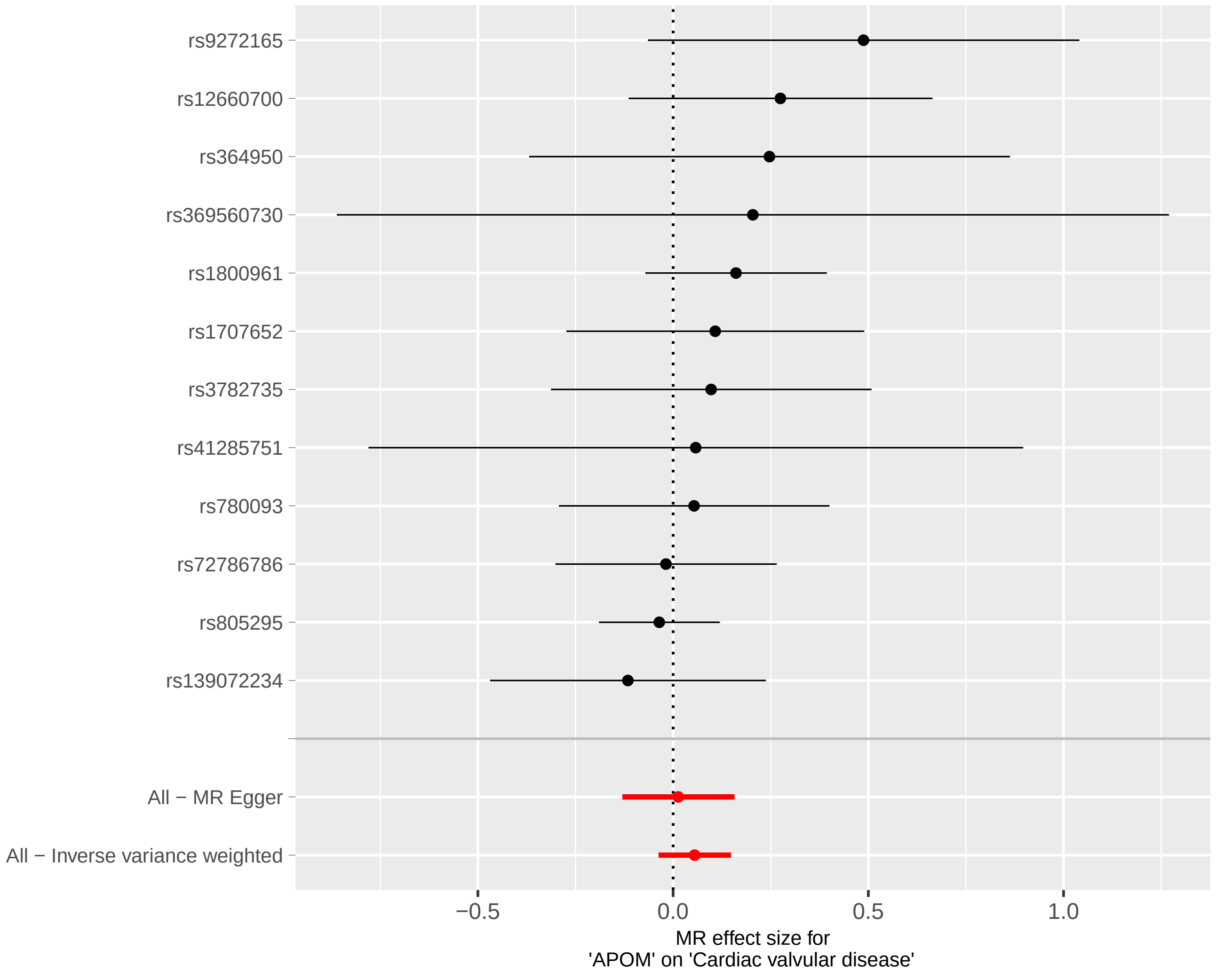
rs9272165
rs12660700
rs364950
rs369560730
rs1800961
rs1707652
rs3782735
rs41285751
rs780093
rs72786786
rs805295
rs139072234
All − MR Egger
All − Inverse variance weighted
−0.5
0.0
0.5
1.0
MR effect size for
'APOM' on 'Cardiac valvular disease'

Insufficient number of SNPs

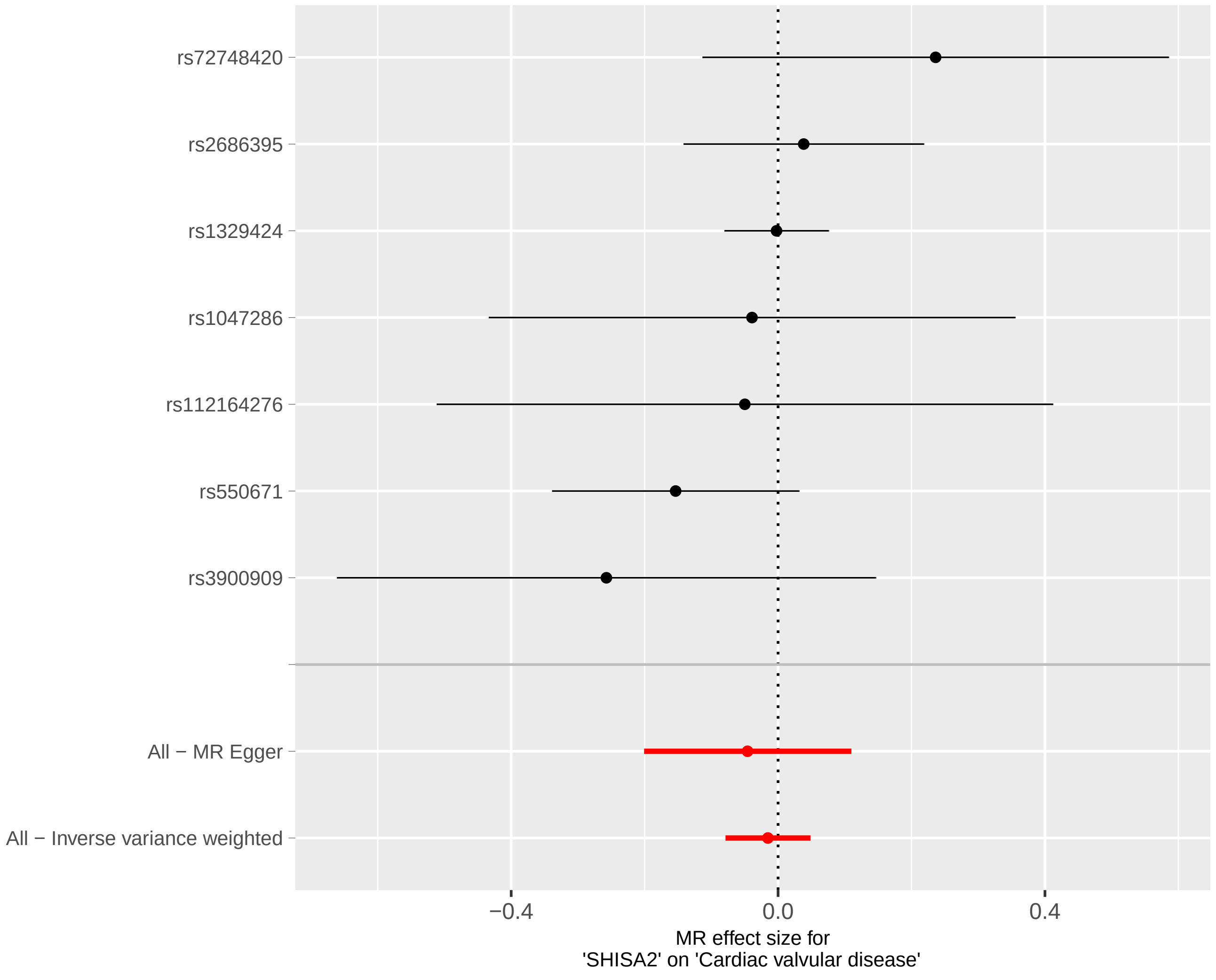
rs72748420
rs2686395
rs1329424
rs1047286
rs112164276
rs550671
rs3900909
All − MR Egger
All − Inverse variance weighted
−0.4
0.0
0.4
MR effect size for
'SHISA2' on 'Cardiac valvular disease'

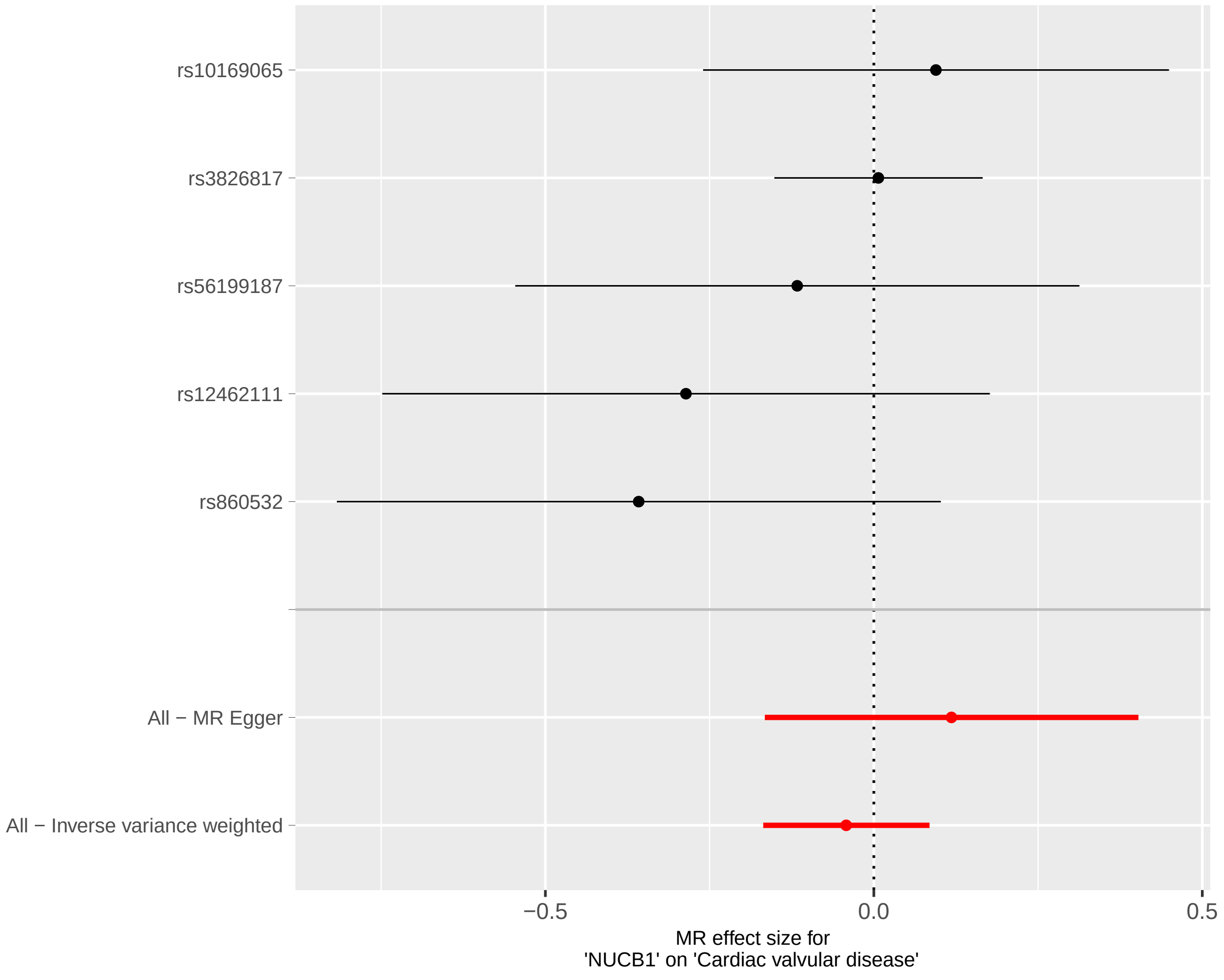
rs10169065
rs3826817
rs56199187
rs12462111
rs860532
All − MR Egger
All − Inverse variance weighted
−0.5
0.0
0.5
MR effect size for
'NUCB1' on 'Cardiac valvular disease'

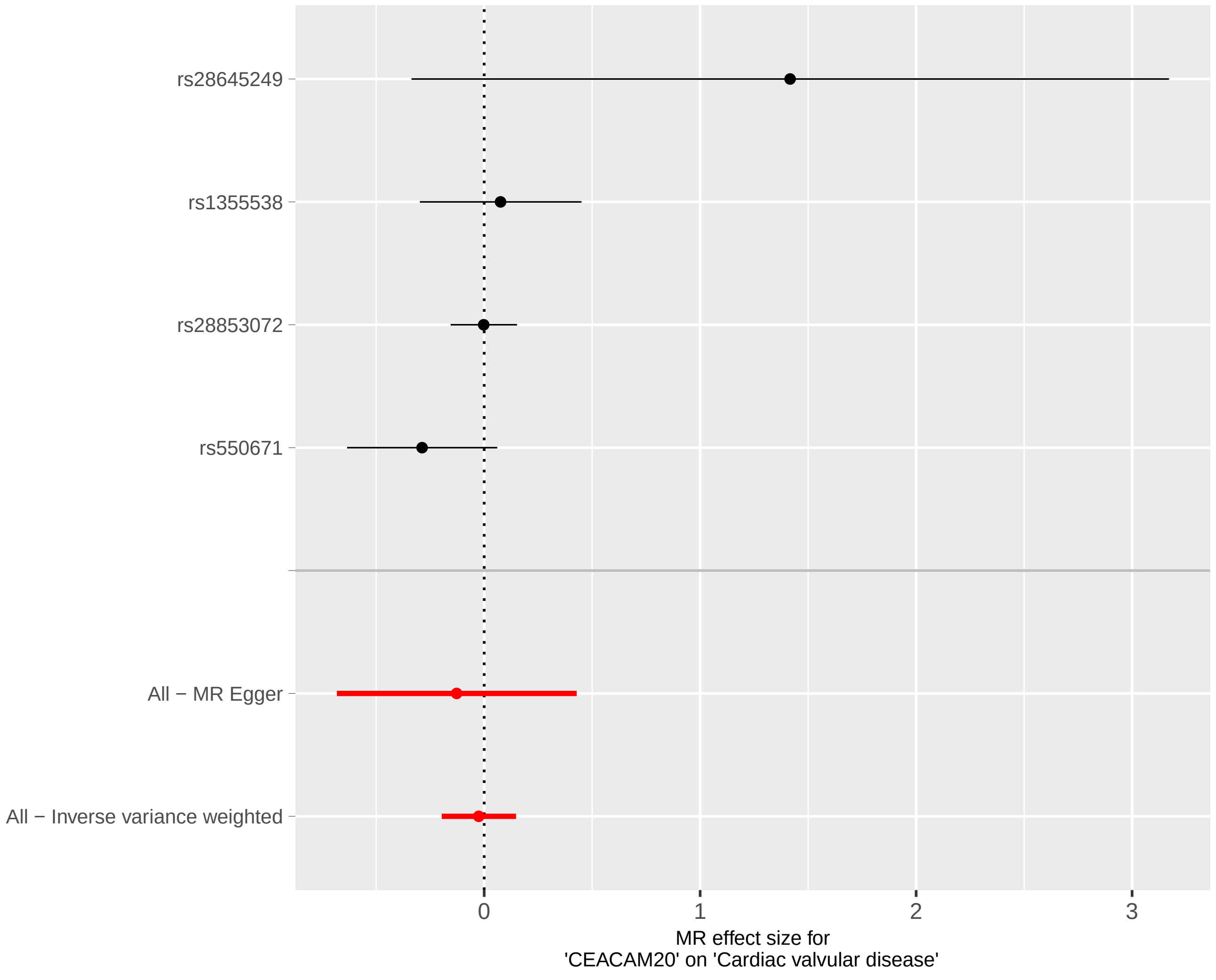
rs28645249
rs1355538
rs28853072
rs550671
All − MR Egger
All − Inverse variance weighted
0
1
2
3
MR effect size for
'CEACAM20' on 'Cardiac valvular disease'

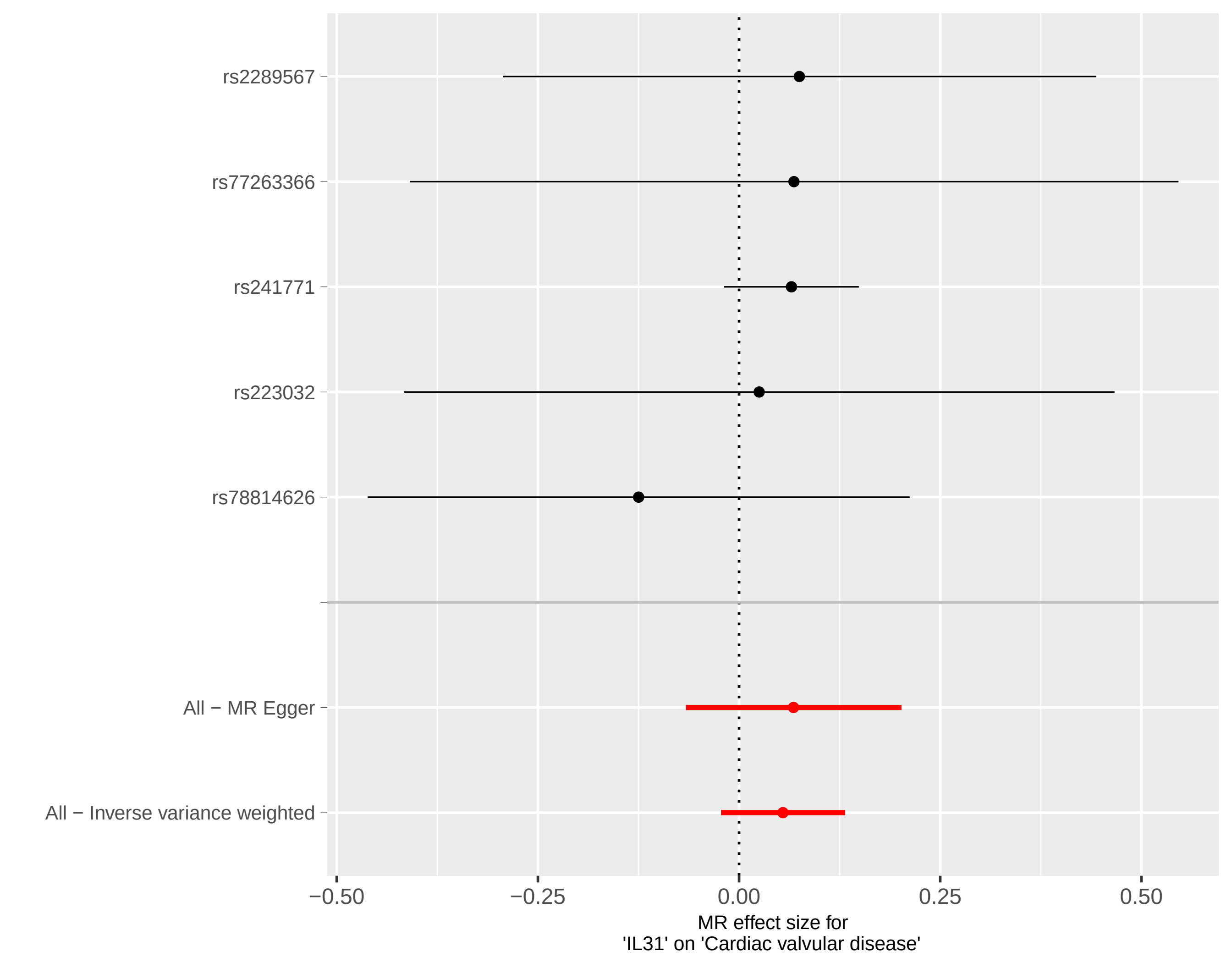
rs2289567
rs77263366
rs241771
rs223032
rs78814626
All − MR Egger
All − Inverse variance weighted
−0.50
−0.25
0.00
0.25
0.50
MR effect size for
'IL31' on 'Cardiac valvular disease'

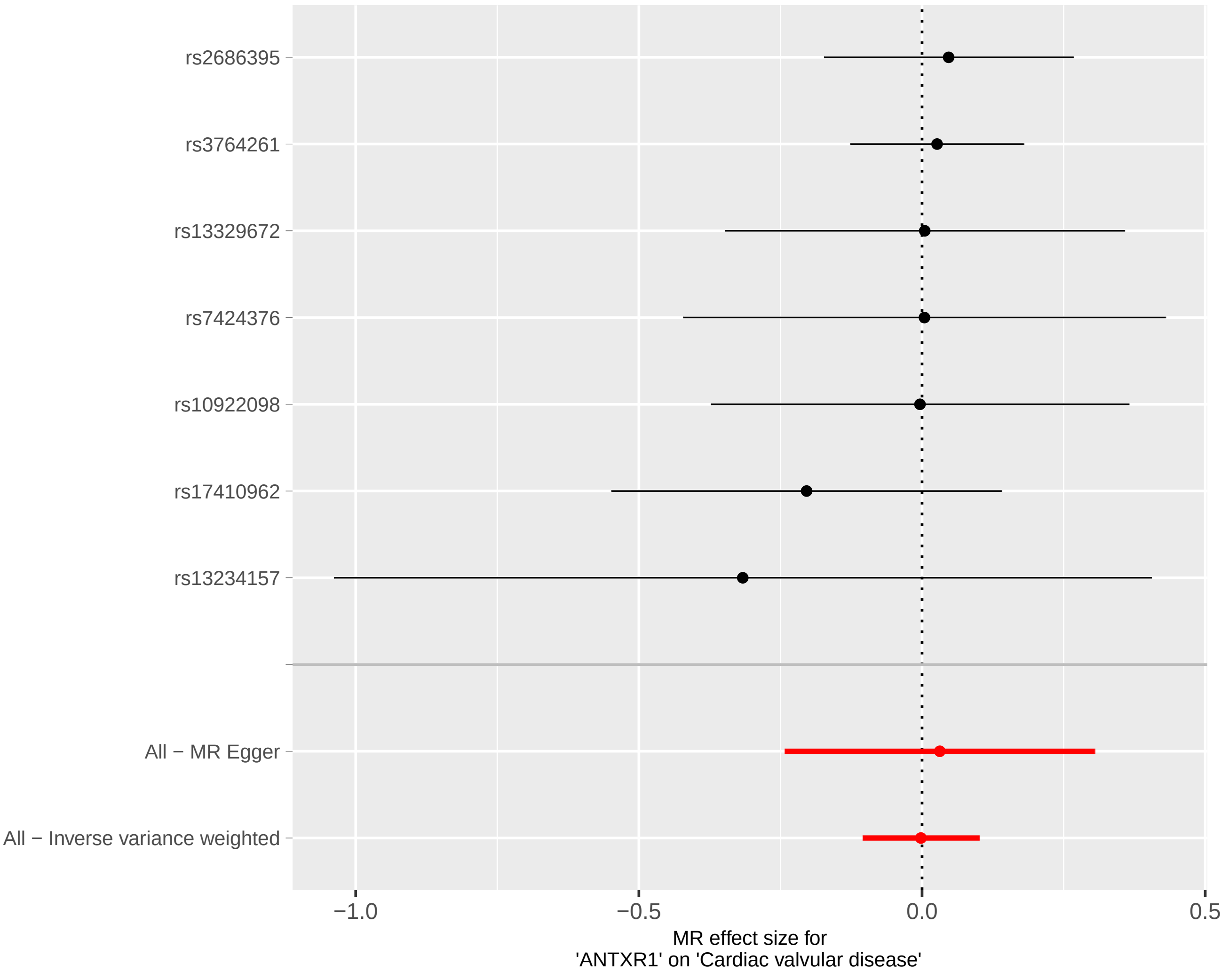
rs2686395
rs3764261
rs13329672
rs7424376
rs10922098
rs17410962
rs13234157
All − MR Egger
All − Inverse variance weighted
−1.0
−0.5
0.0
0.5
MR effect size for
'ANTXR1' on 'Cardiac valvular disease'

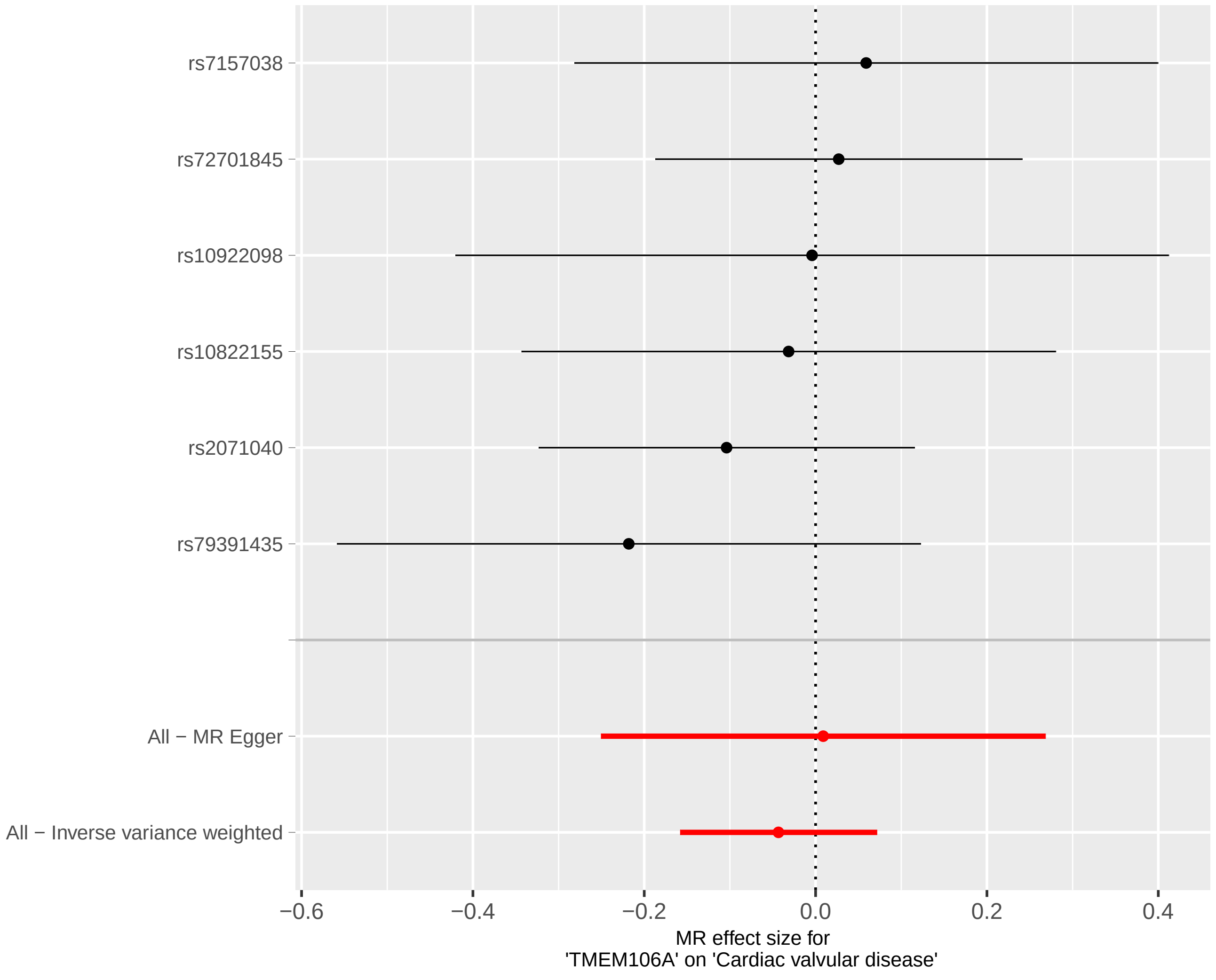
rs7157038
rs72701845
rs10922098
rs10822155
rs2071040
rs79391435
All − MR Egger
All − Inverse variance weighted
−0.6
−0.4
−0.2
0.0
0.2
0.4
MR effect size for
'TMEM106A' on 'Cardiac valvular disease'

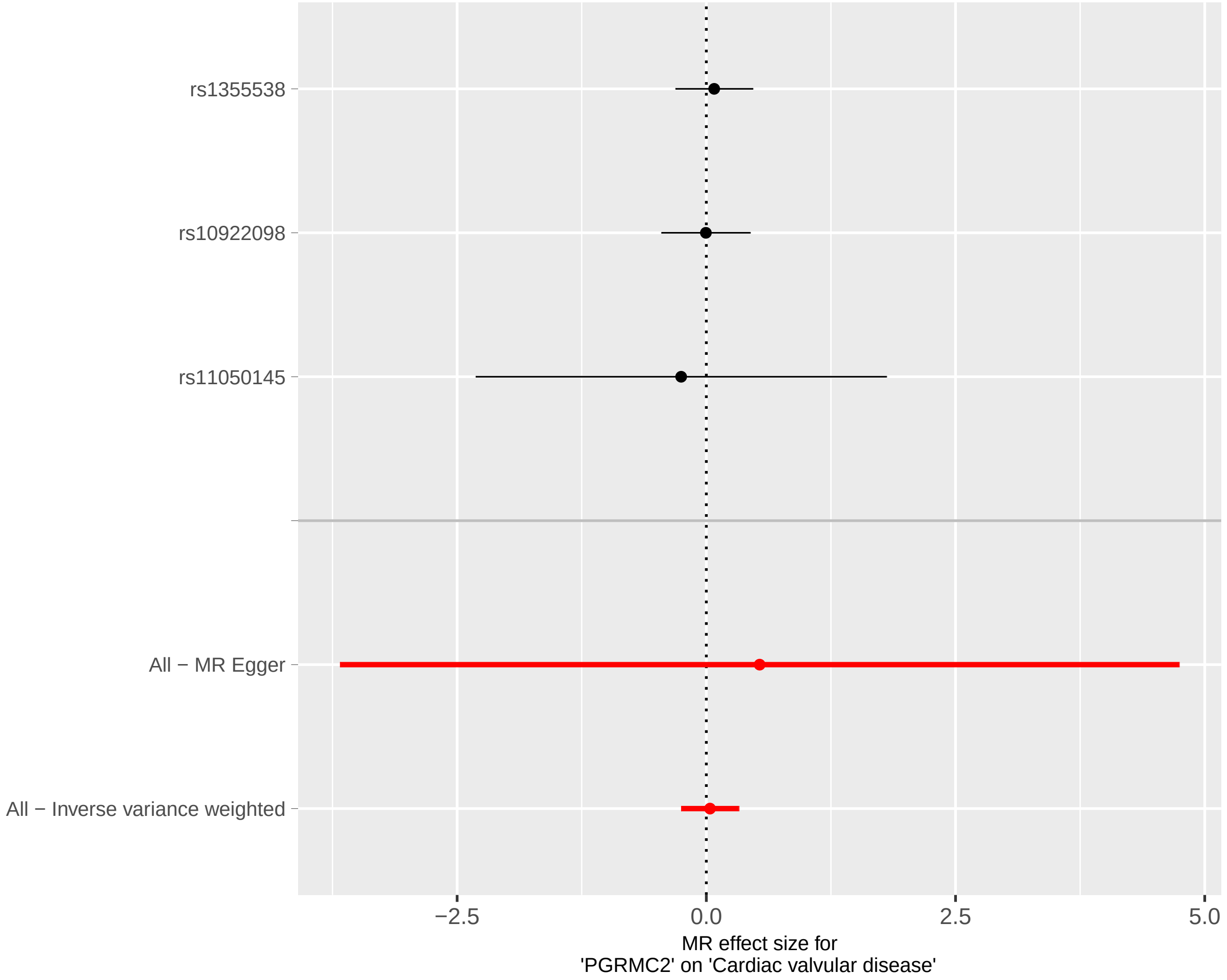
rs1355538
rs10922098
rs11050145
All − MR Egger
All − Inverse variance weighted
−2.5
0.0
2.5
5.0
MR effect size for
'PGRMC2' on 'Cardiac valvular disease'

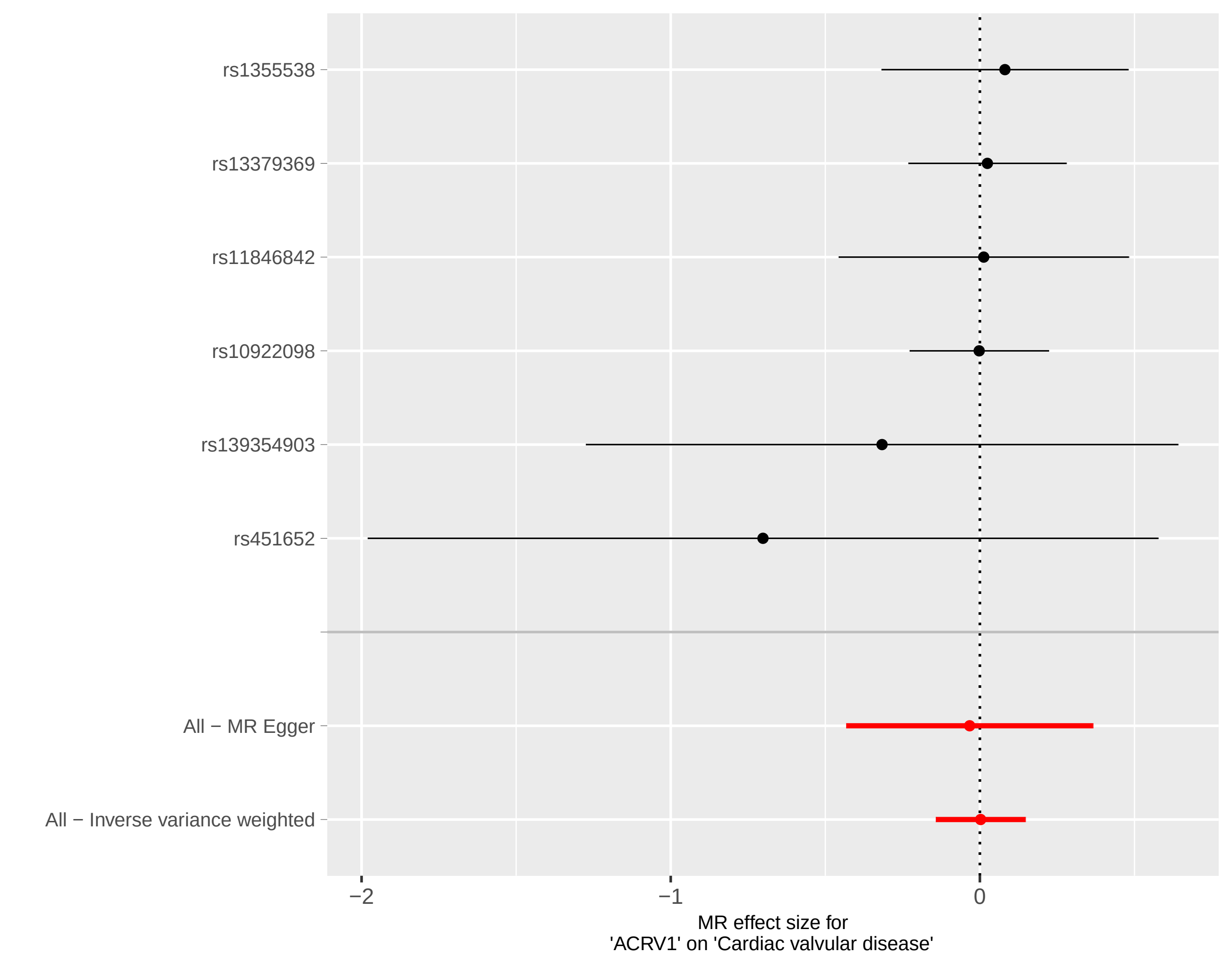
rs1355538
rs13379369
rs11846842
rs10922098
rs139354903
rs451652
All − MR Egger
All − Inverse variance weighted
−2
−1
0
MR effect size for
'ACRV1' on 'Cardiac valvular disease'

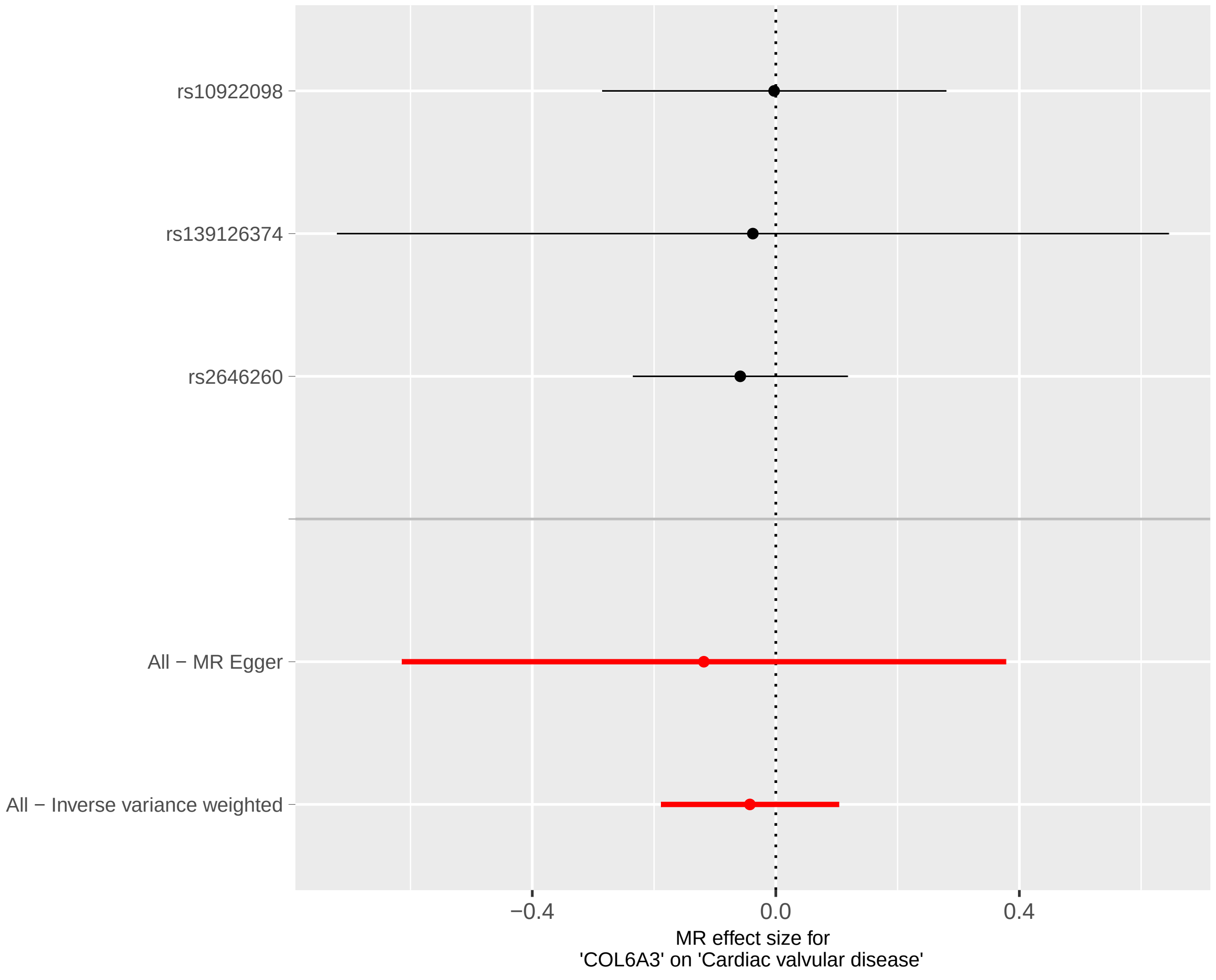
rs10922098
rs139126374
rs2646260
All − MR Egger
All − Inverse variance weighted
−0.4
0.0
0.4
MR effect size for
'COL6A3' on 'Cardiac valvular disease'

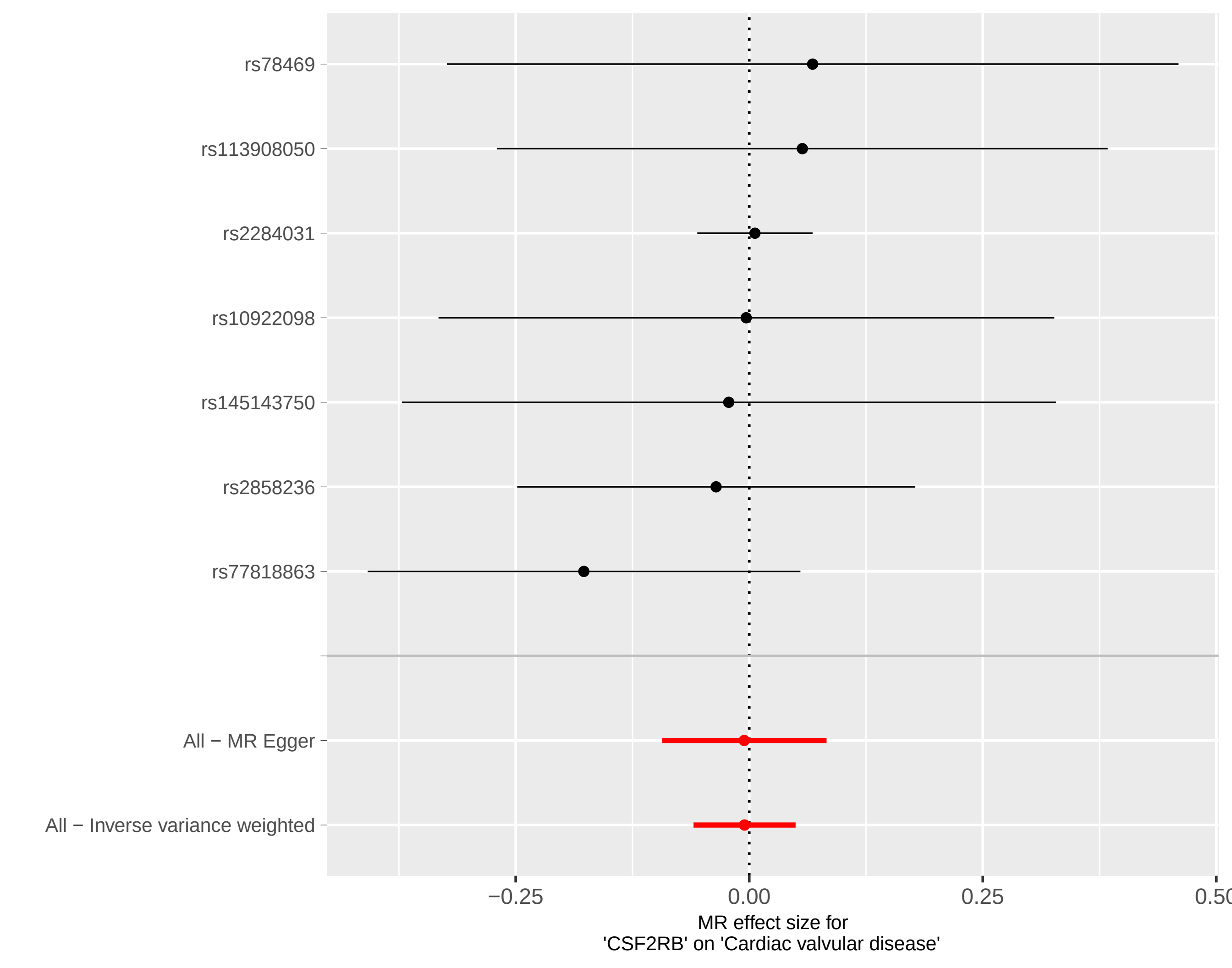
rs78469
rs113908050
rs2284031
rs10922098
rs145143750
rs2858236
rs77818863
All − MR Egger
All − Inverse variance weighted
−0.25
0.00
0.25
MR effect size for
'CSF2RB' on 'Cardiac valvular disease'

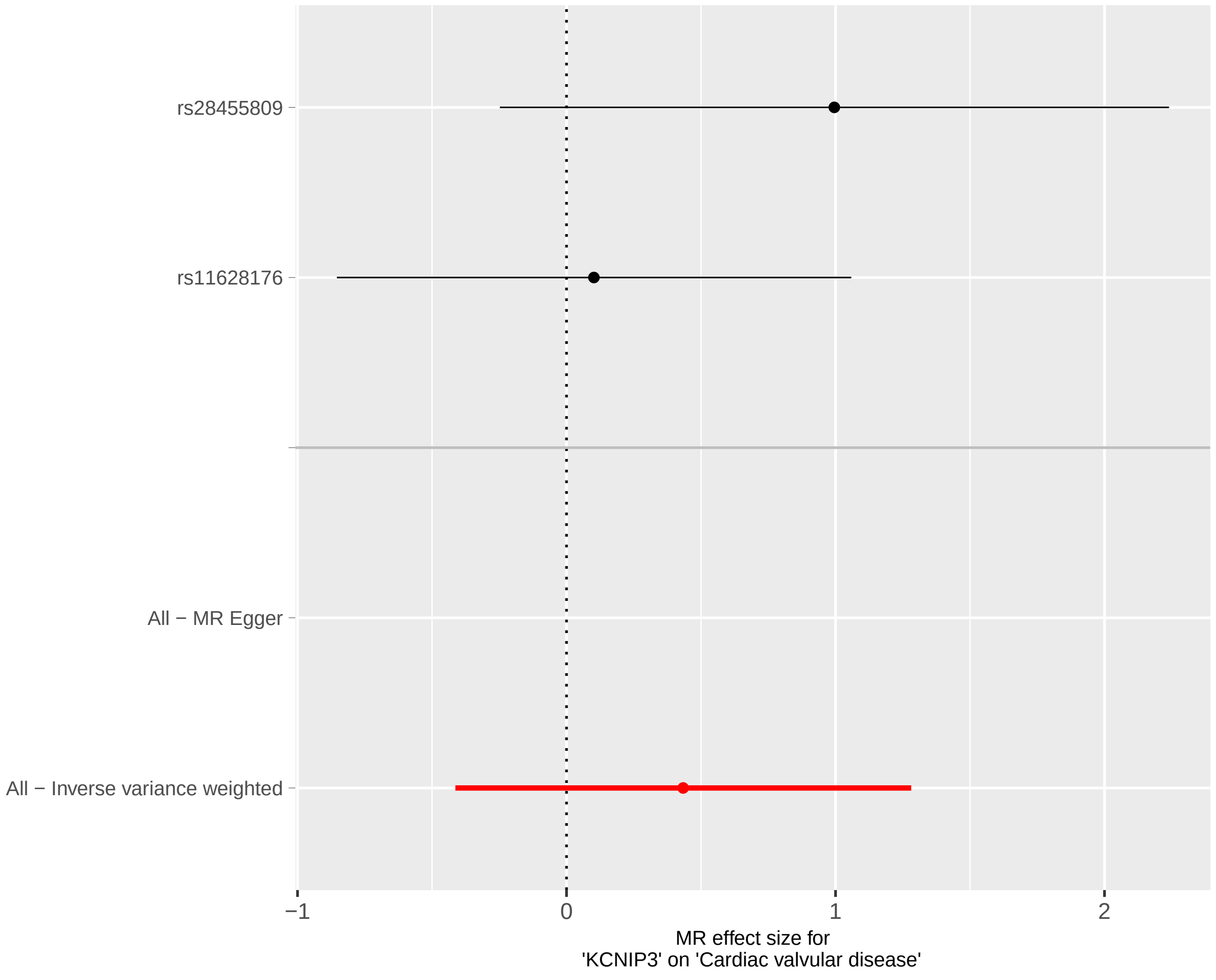
rs28455809
rs11628176
All − MR Egger
All − Inverse variance weighted
−1
0
1
2
MR effect size for
'KCNIP3' on 'Cardiac valvular disease'

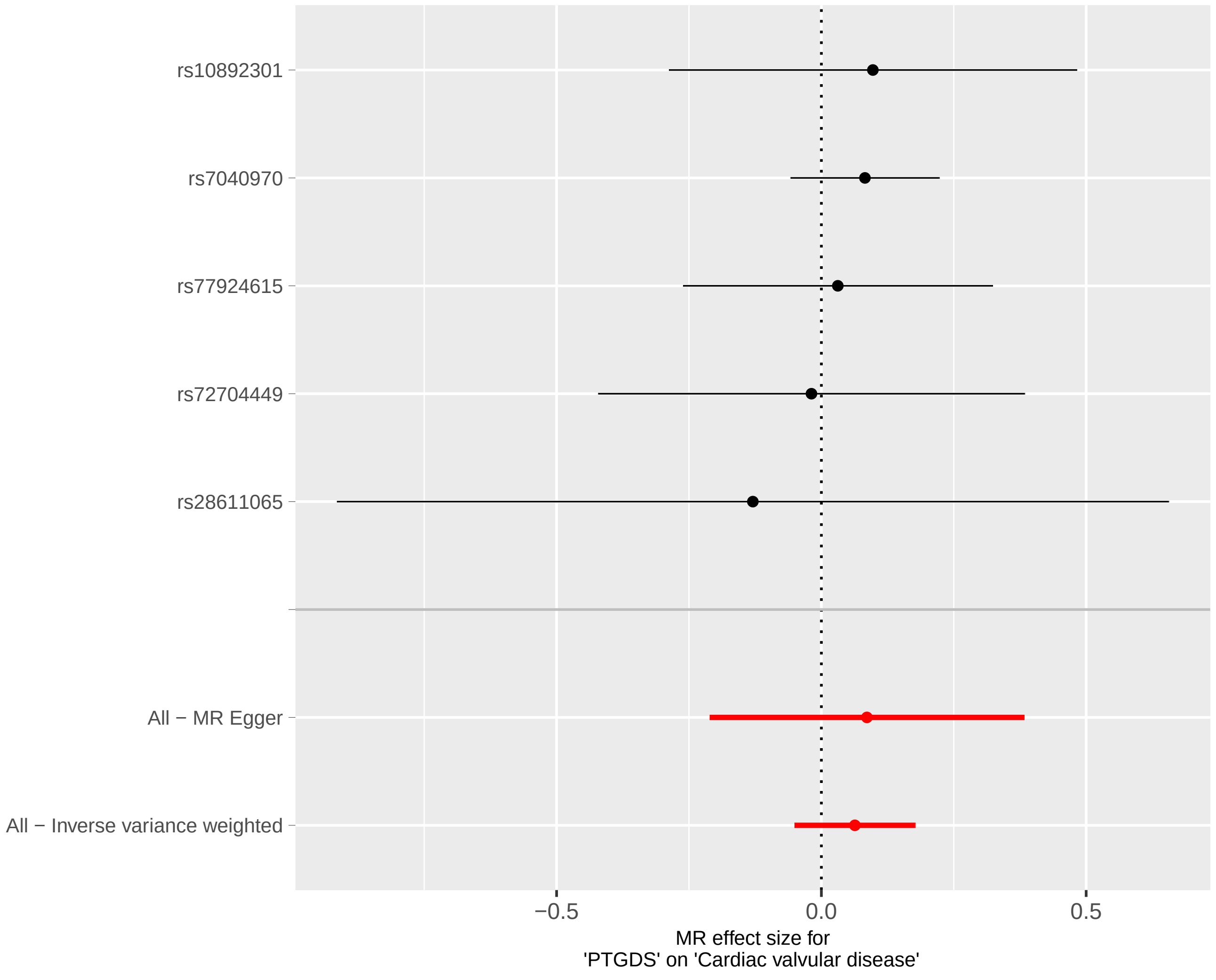
rs10892301
rs7040970
rs77924615
rs72704449
rs28611065
All − MR Egger
All − Inverse variance weighted
−0.5
0.0
0.5
MR effect size for
'PTGDS' on 'Cardiac valvular disease'

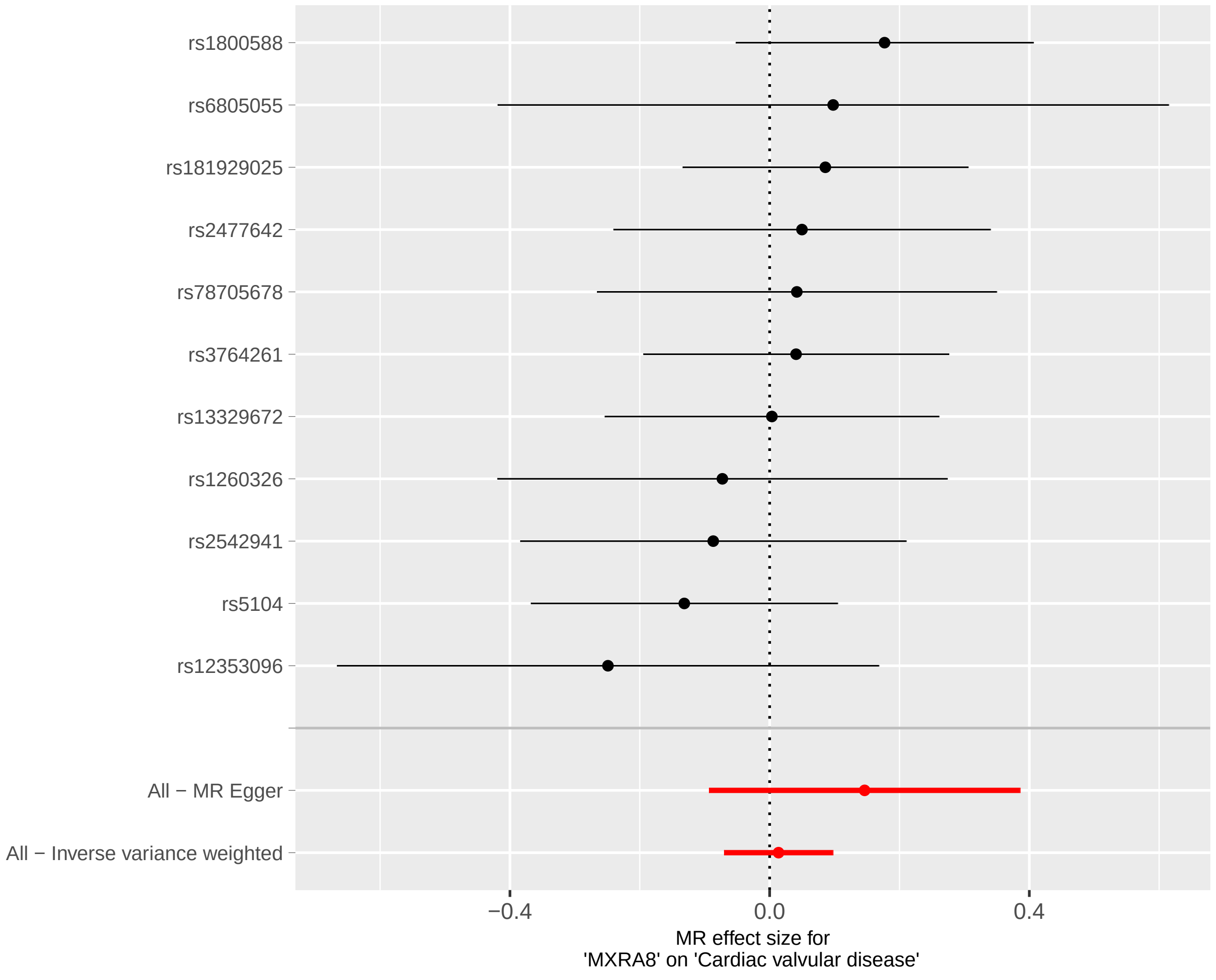
rs1800588
rs6805055
rs181929025
rs2477642
rs78705678
rs3764261
rs13329672
rs1260326
rs2542941
rs5104
rs12353096
All − MR Egger
All − Inverse variance weighted
−0.4
0.0
0.4
MR effect size for
'MXRA8' on 'Cardiac valvular disease'

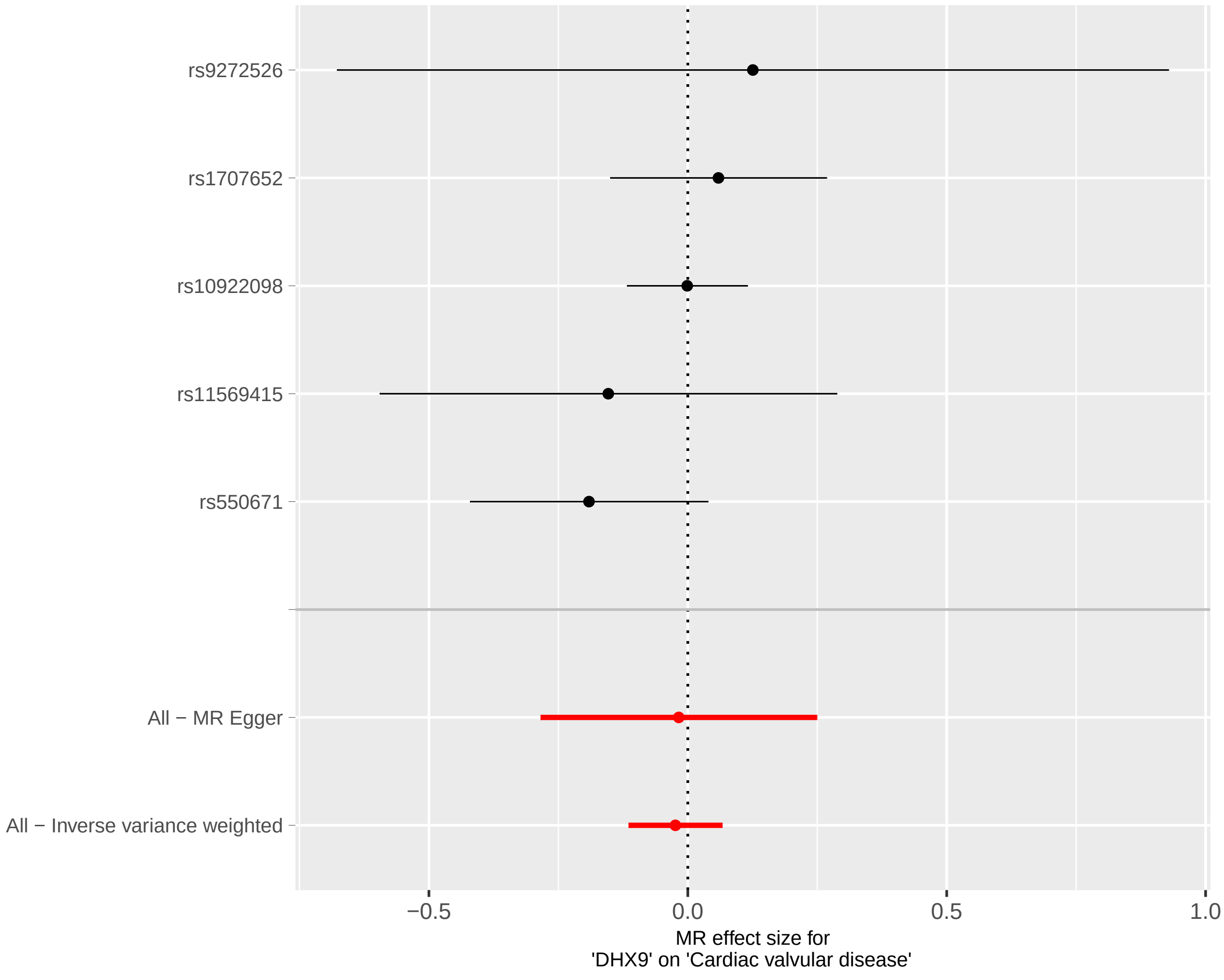
rs9272526
rs1707652
rs10922098
rs11569415
rs550671
All − MR Egger
All − Inverse variance weighted
−0.5
0.0
0.5
1.0
MR effect size for
'DHX9' on 'Cardiac valvular disease'

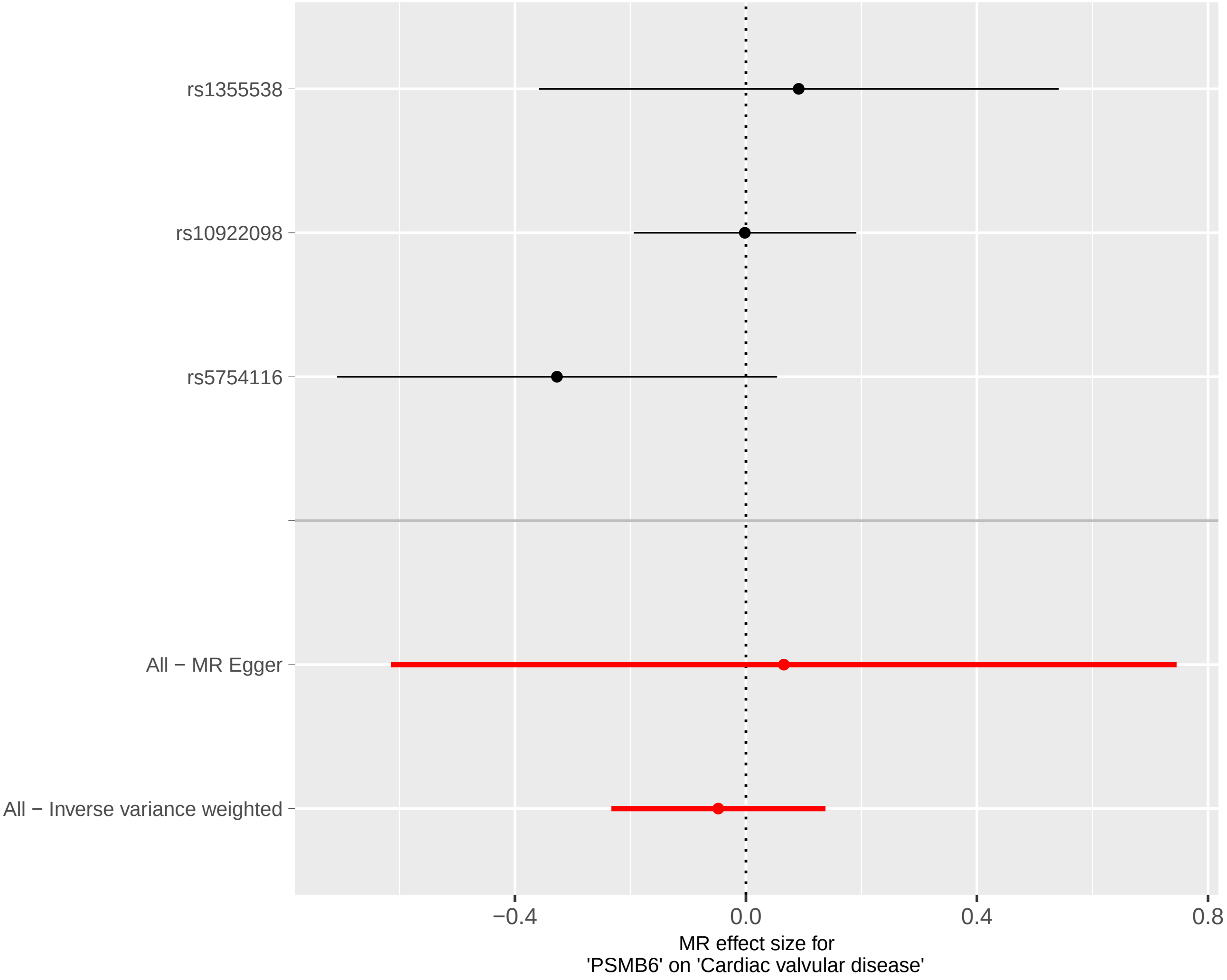
rs1355538
rs10922098
rs5754116
All − MR Egger
All − Inverse variance weighted
−0.4
0.0
0.4
0.8
MR effect size for
'PSMB6' on 'Cardiac valvular disease'

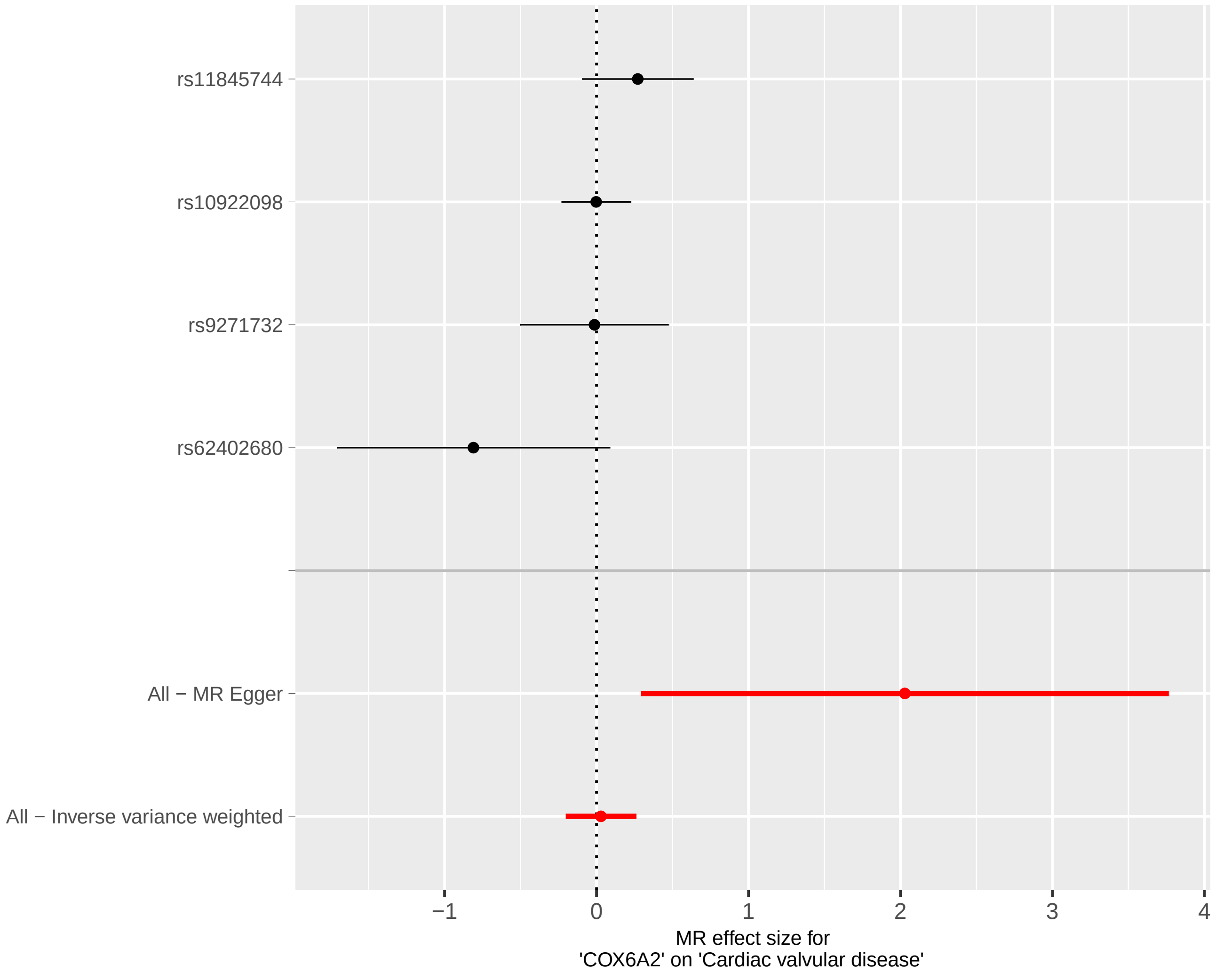
rs11845744
rs10922098
rs9271732
rs62402680
All − MR Egger
All − Inverse variance weighted
−1
0
1
2
3
4
MR effect size for
'COX6A2' on 'Cardiac valvular disease'

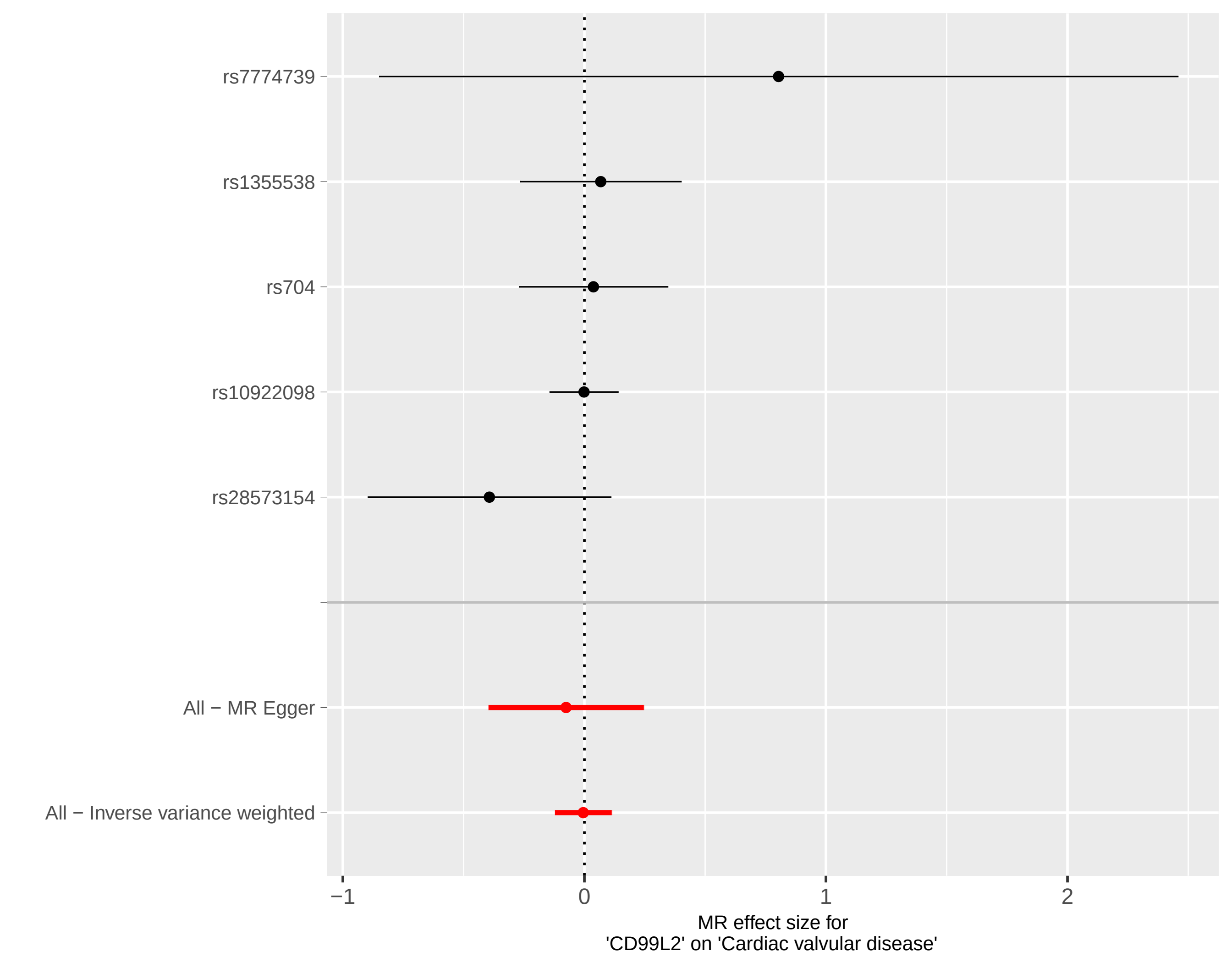

rs7774739
rs1355538
rs704
rs10922098
rs28573154
All − MR Egger
All − Inverse variance weighted
−1
0
1
2
MR effect size for
'CD99L2' on 'Cardiac valvular disease'

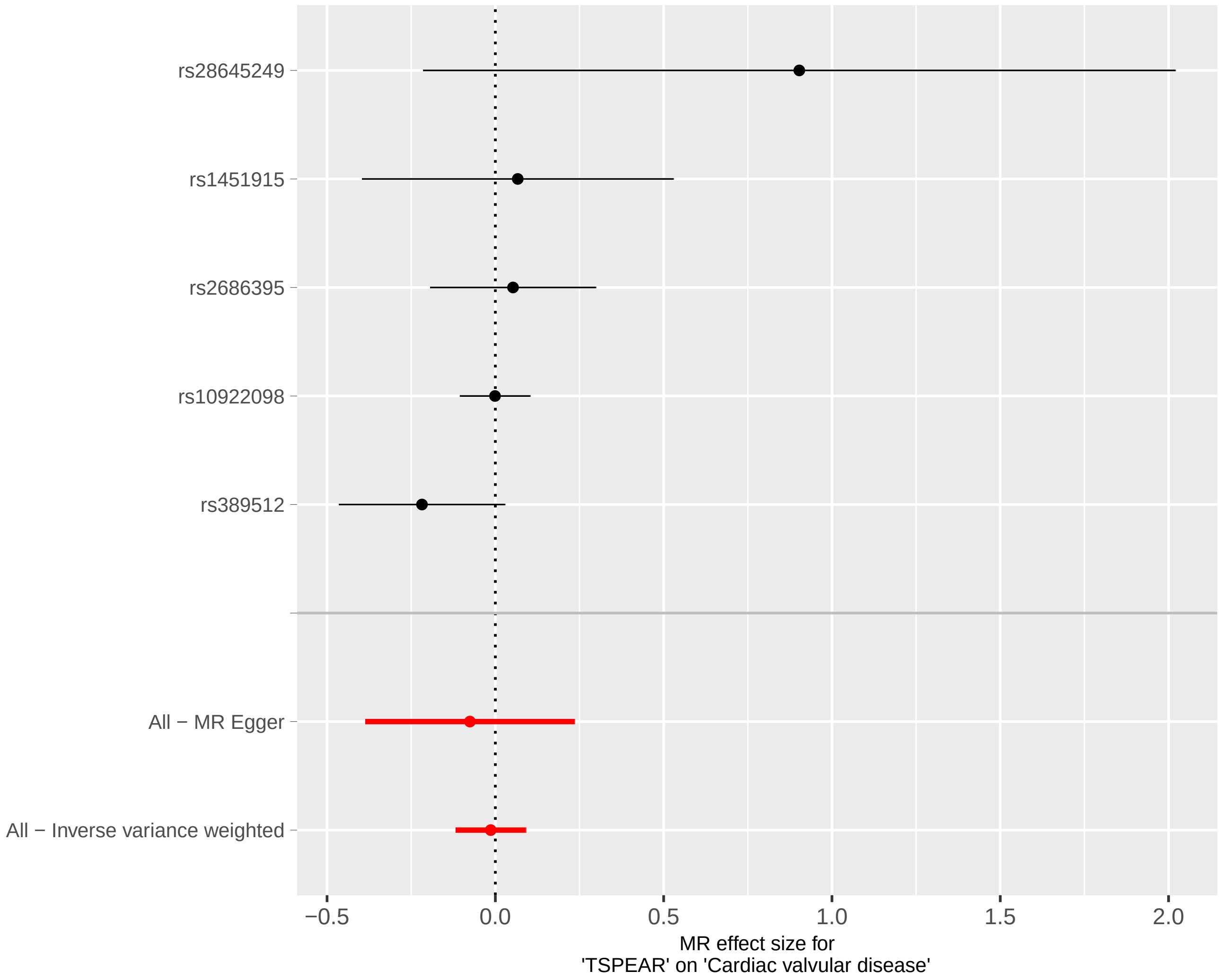
rs28645249
rs1451915
rs2686395
rs10922098
rs389512
All − MR Egger
All − Inverse variance weighted
−0.5
0.0
0.5
1.0
1.5
2.0
MR effect size for
'TSPEAR' on 'Cardiac valvular disease'

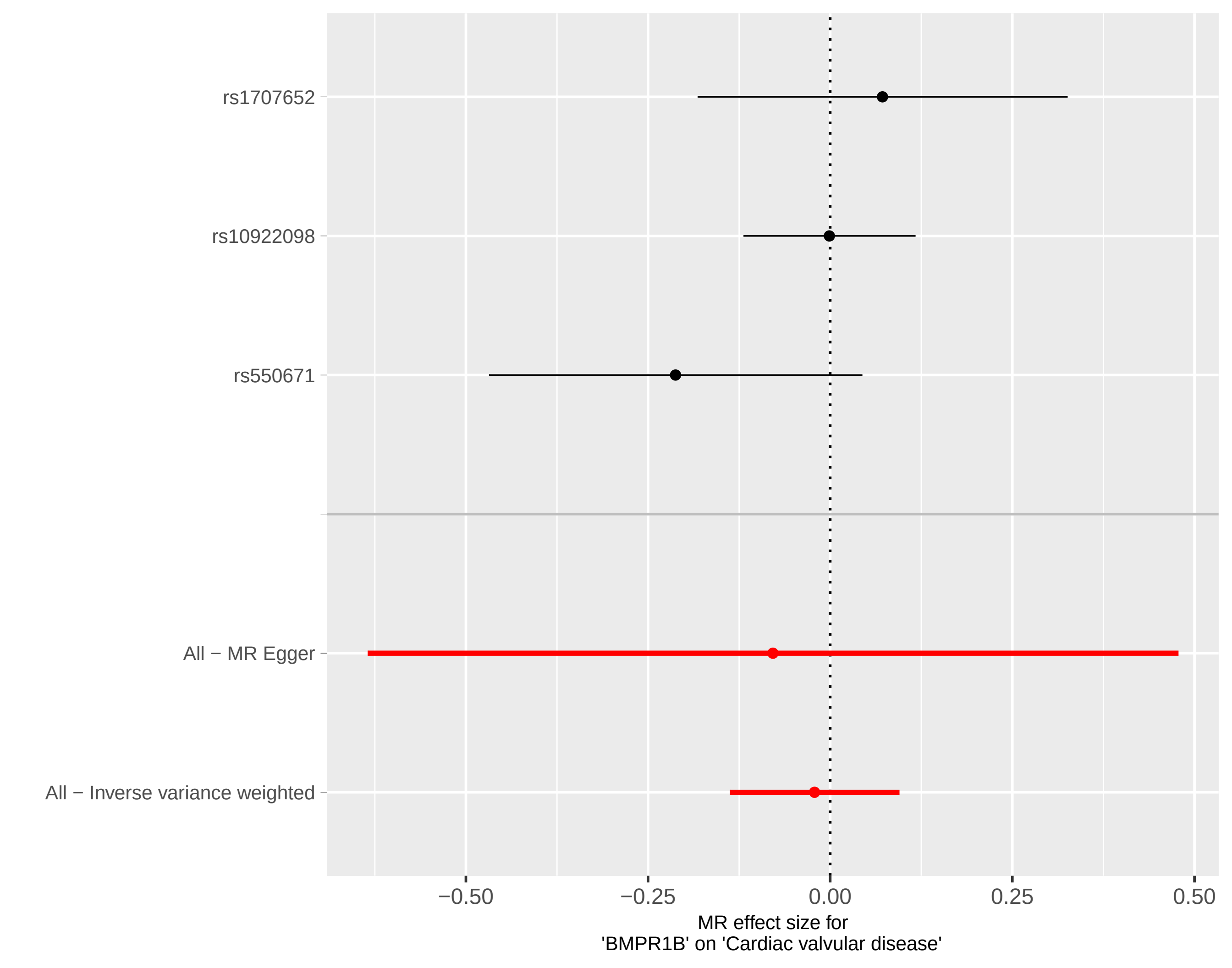
rs1707652
rs10922098
rs550671
All − MR Egger
All − Inverse variance weighted
−0.50
−0.25
0.00
0.25
0.50
MR effect size for
'BMPR1B' on 'Cardiac valvular disease'

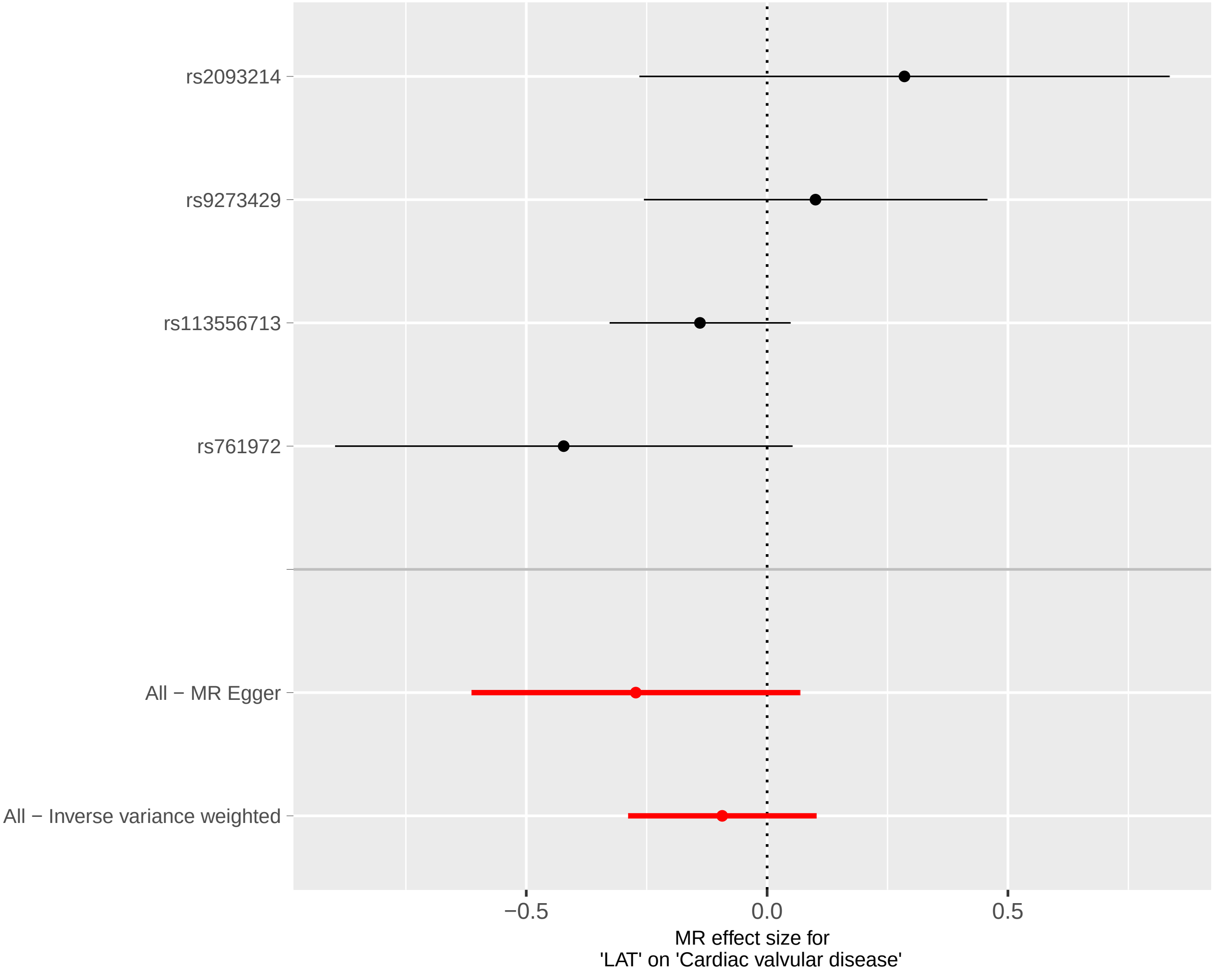

rs2093214
rs9273429
rs113556713
rs761972
All − MR Egger
All − Inverse variance weighted
−0.5
0.0
0.5
MR effect size for
'LAT' on 'Cardiac valvular disease'

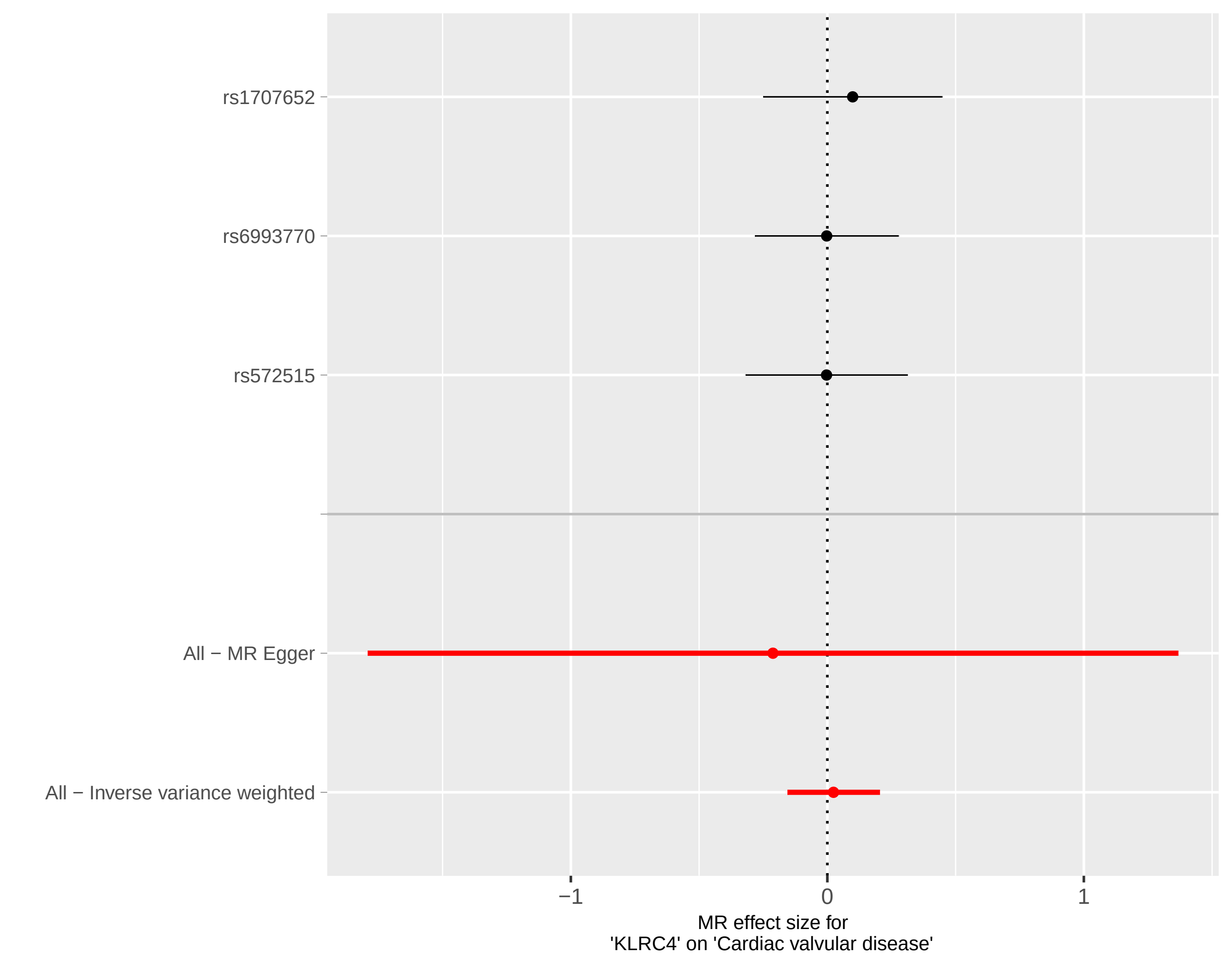
rs1707652
rs6993770
rs572515
All − MR Egger
All − Inverse variance weighted
−1
0
1
MR effect size for
'KLRC4' on 'Cardiac valvular disease'

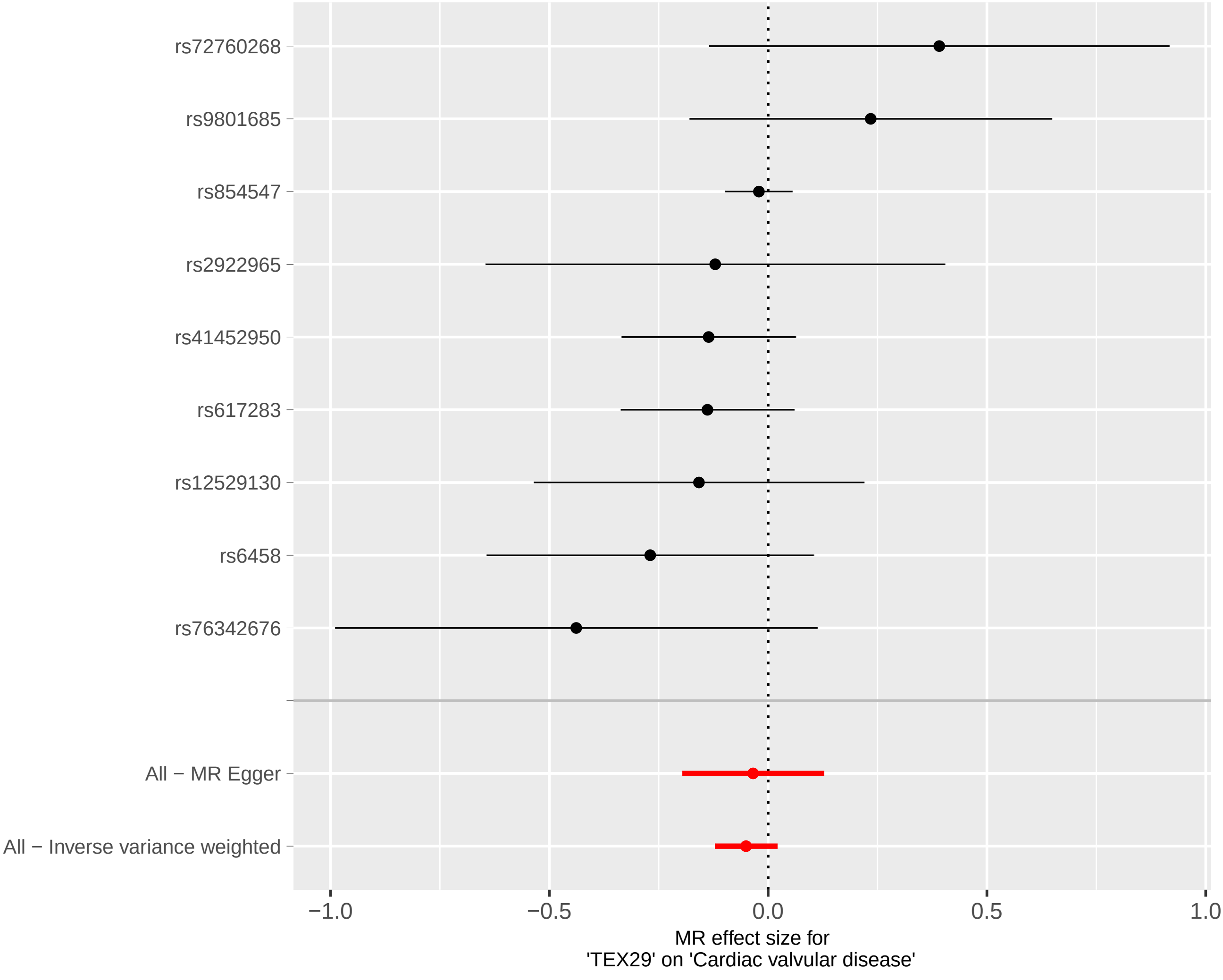
rs72760268
rs9801685
rs854547
rs2922965
rs41452950
rs617283
rs12529130
rs6458
rs76342676
All − MR Egger
All − Inverse variance weighted
−1.0
−0.5
0.0
0.5
1.0
MR effect size for
'TEX29' on 'Cardiac valvular disease'

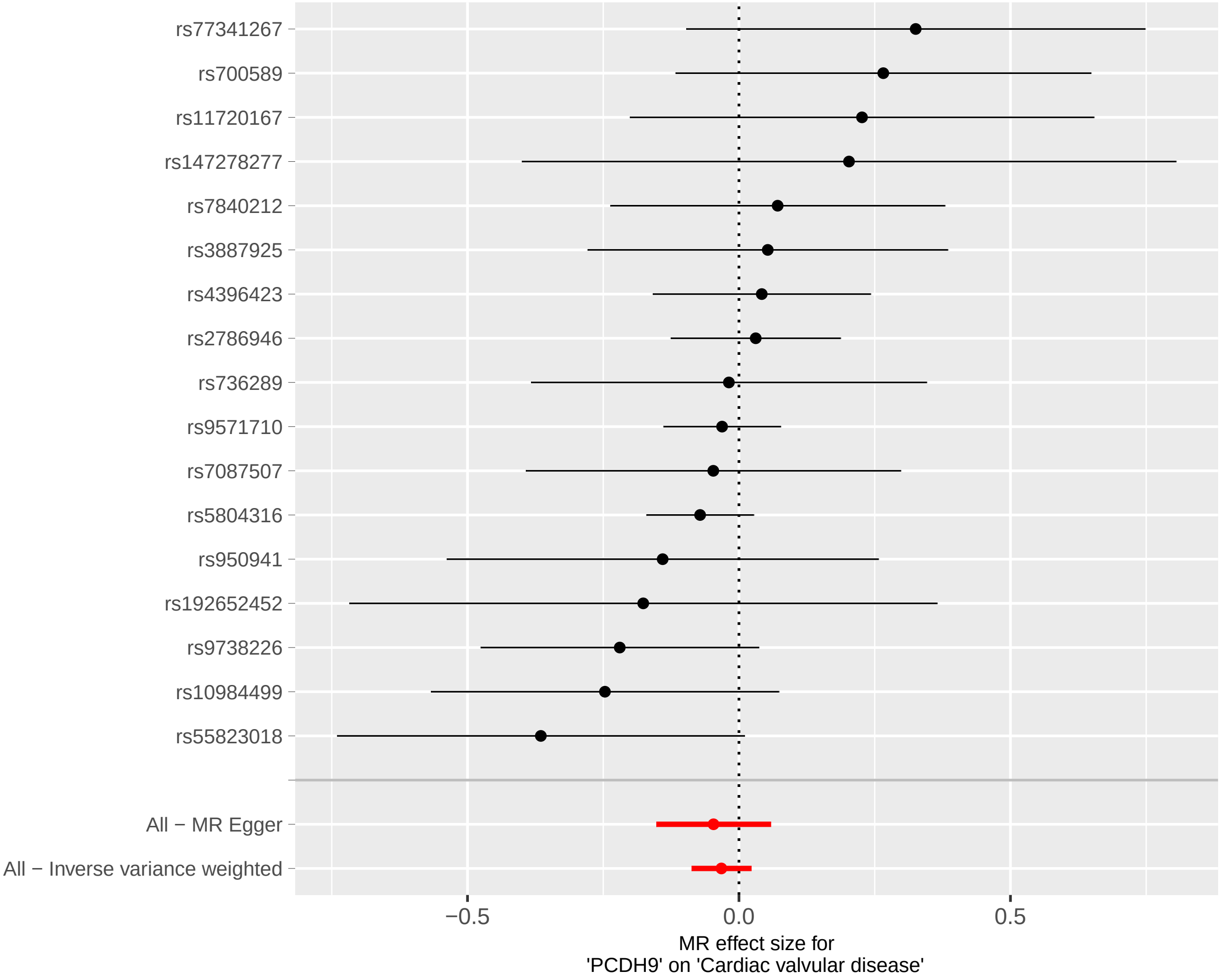
rs77341267
rs700589
rs11720167
rs147278277
rs7840212
rs3887925
rs4396423
rs2786946
rs736289
rs9571710
rs7087507
rs5804316
rs950941
rs192652452
rs9738226
rs10984499
rs55823018
All − MR Egger
All − Inverse variance weighted
−0.5
0.0
0.5
MR effect size for
'PCDH9' on 'Cardiac valvular disease'

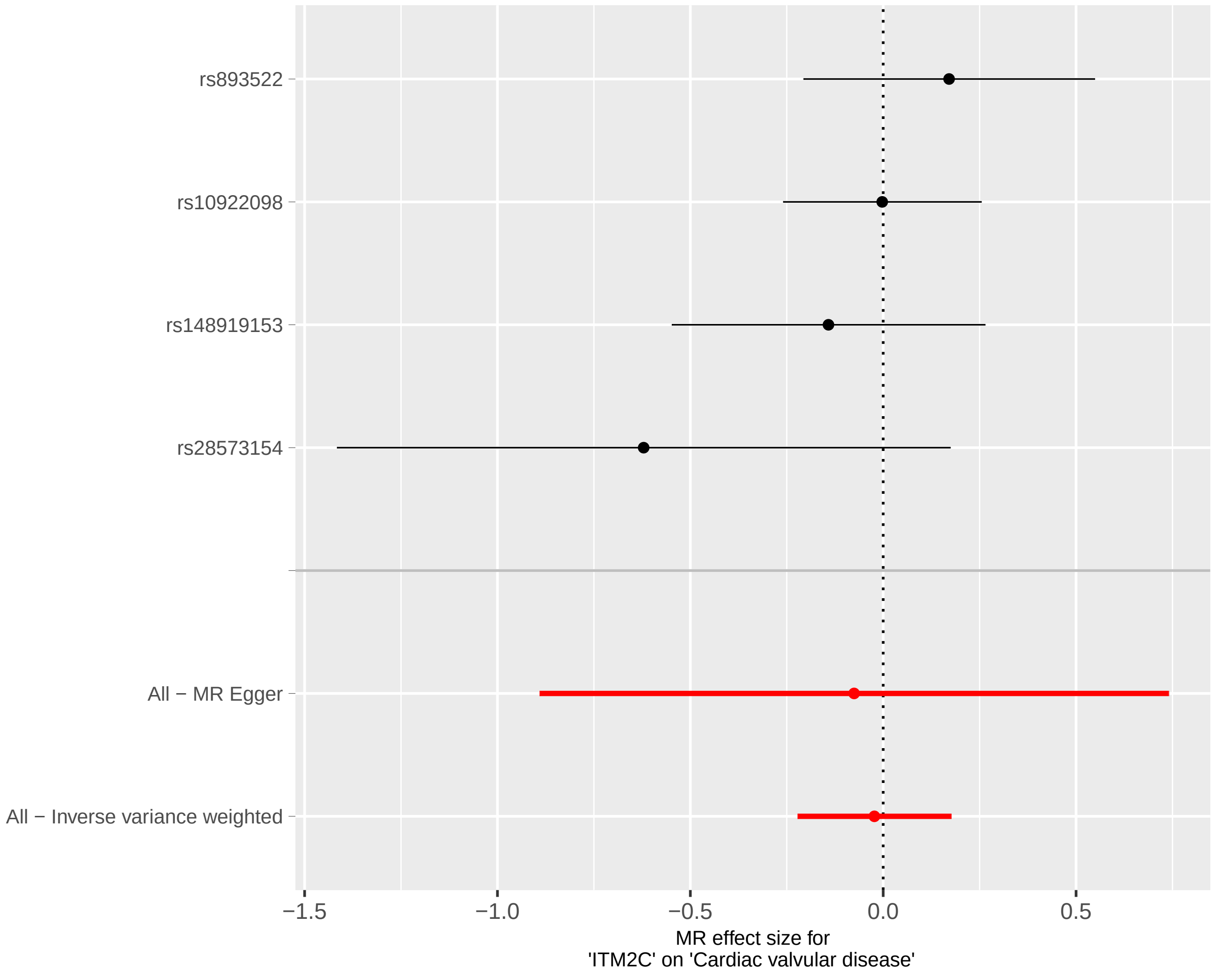
rs893522
rs10922098
rs148919153
rs28573154
All − MR Egger
All − Inverse variance weighted
−1.5
−1.0
−0.5
0.0
0.5
MR effect size for
'ITM2C' on 'Cardiac valvular disease'

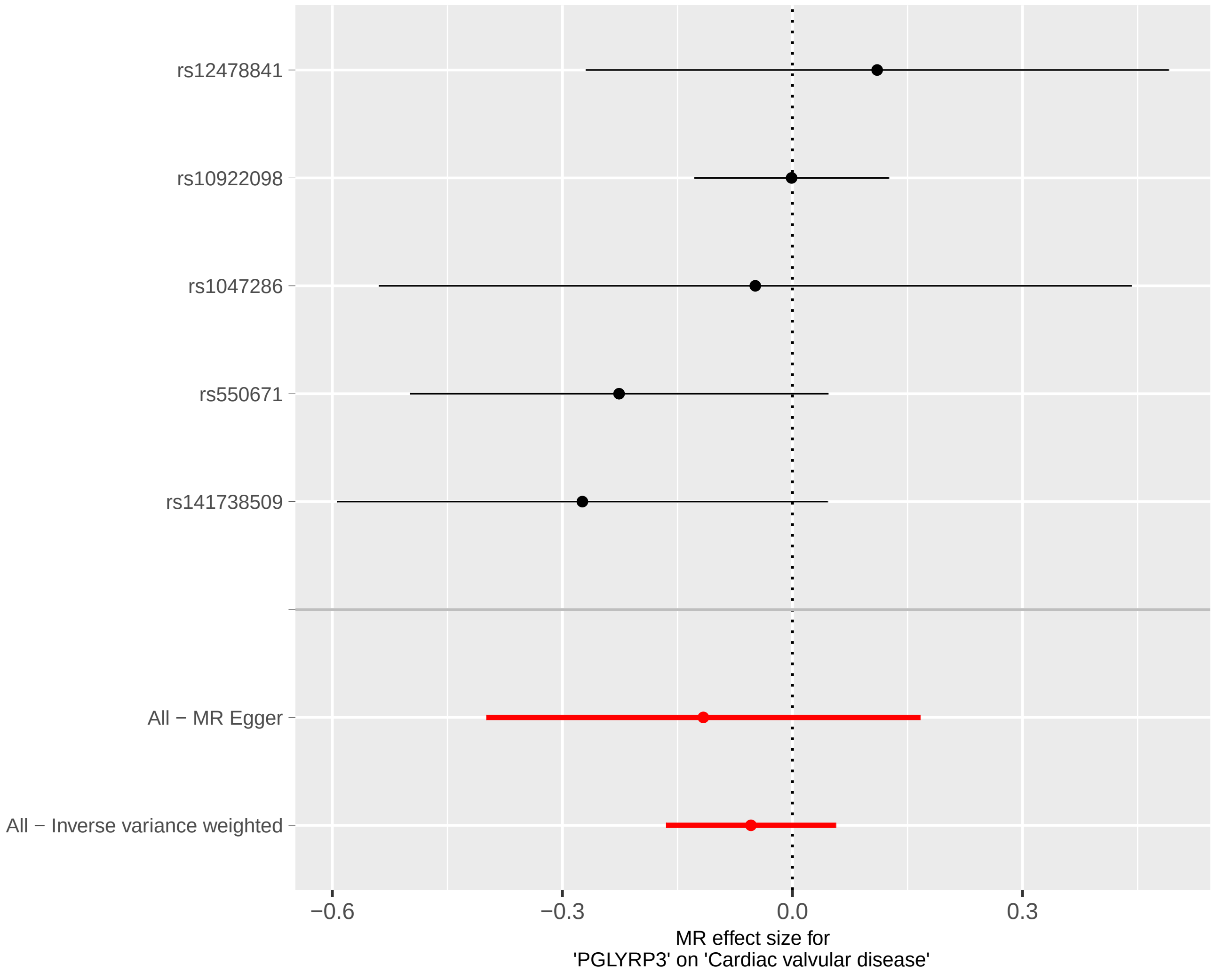
rs12478841
rs10922098
rs1047286
rs550671
rs141738509
All − MR Egger
All − Inverse variance weighted
−0.6
−0.3
0.0
0.3
MR effect size for
'PGLYRP3' on 'Cardiac valvular disease'

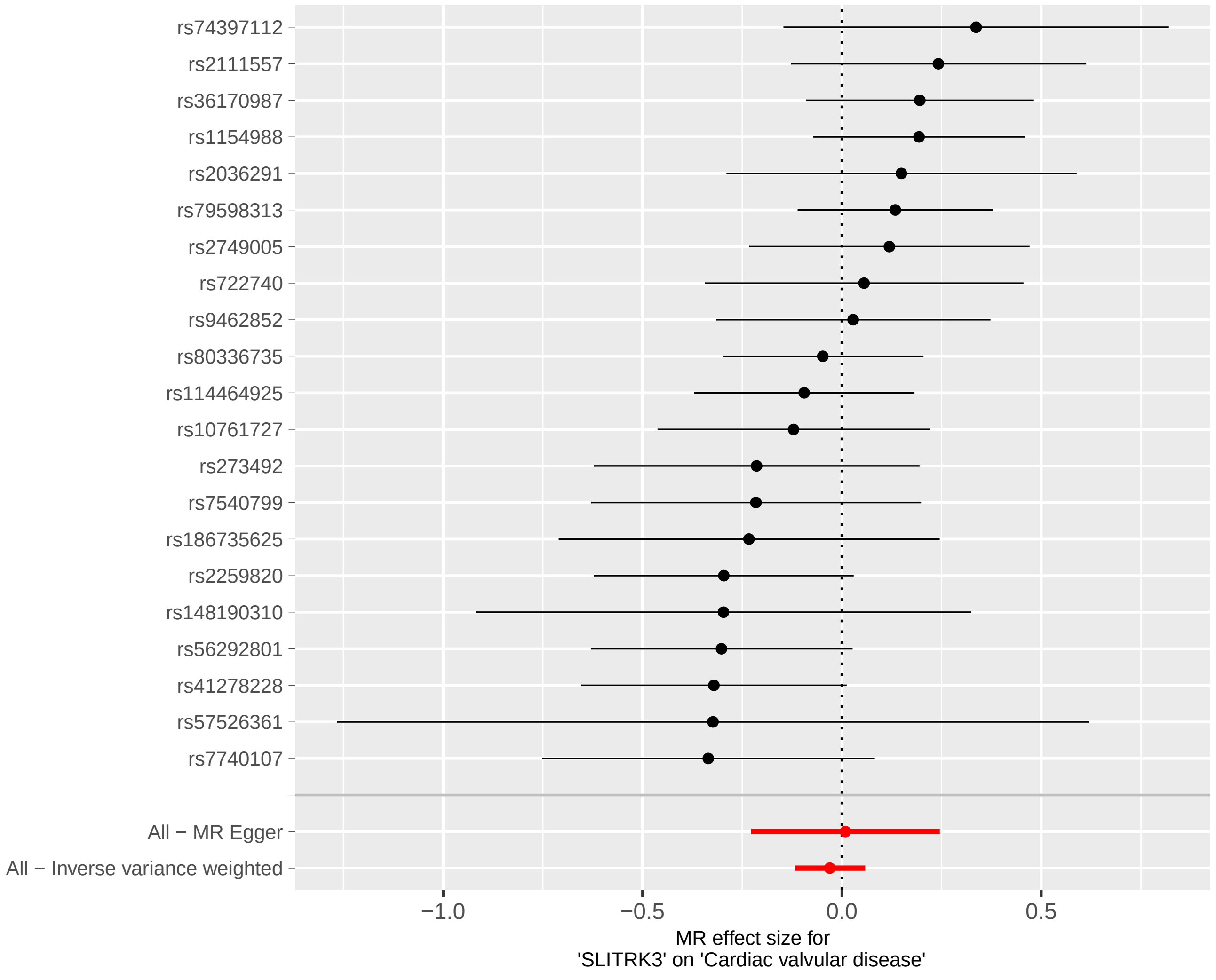
rs74397112
rs2111557
rs36170987
rs1154988
rs2036291
rs79598313
rs2749005
rs722740
rs9462852
rs80336735
rs114464925
rs10761727
rs273492
rs7540799
rs186735625
rs2259820
rs148190310
rs56292801
rs41278228
rs57526361
rs7740107
All − MR Egger
All − Inverse variance weighted
−1.0
−0.5
0.0
0.5
MR effect size for
'SLITRK3' on 'Cardiac valvular disease'

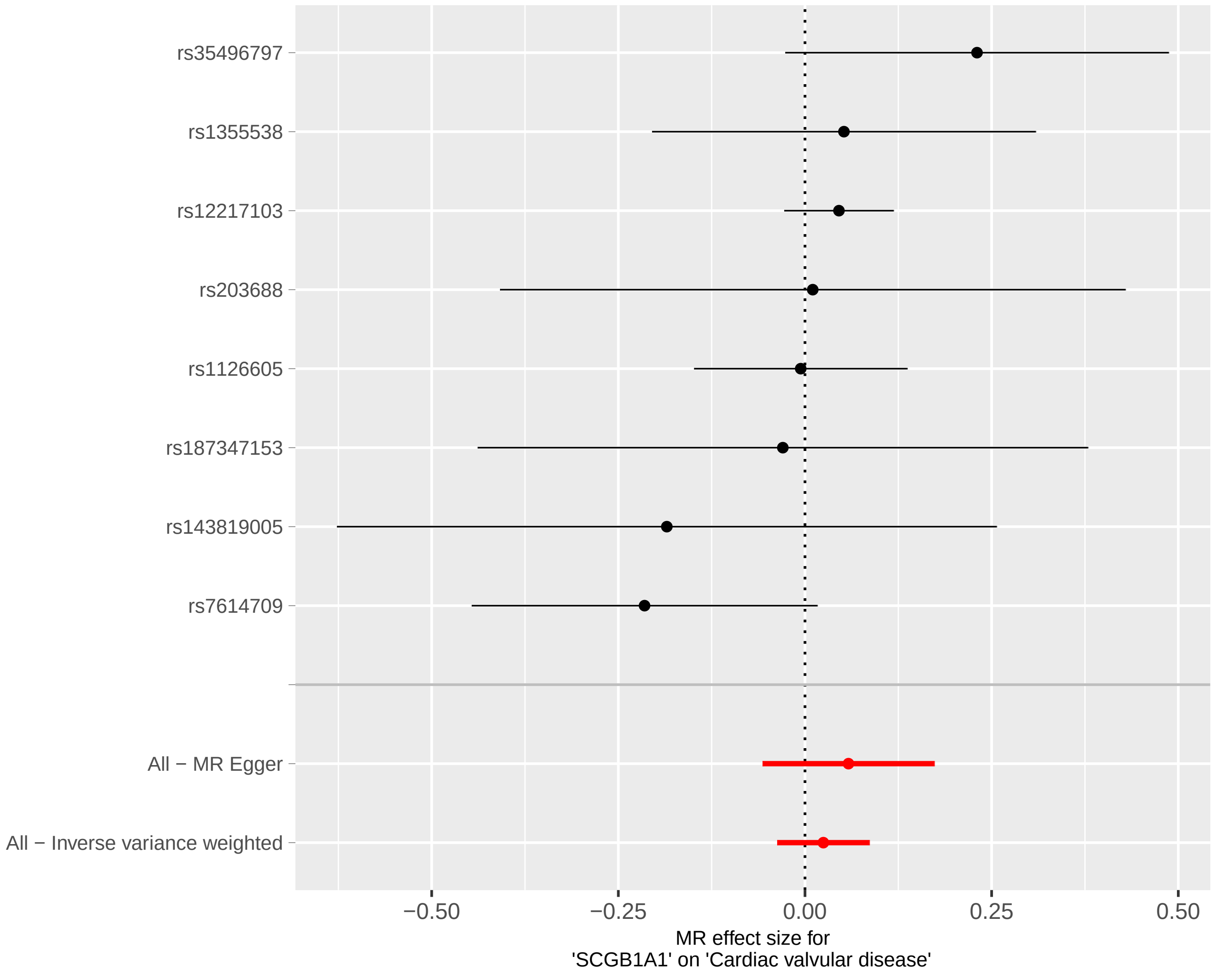
rs35496797
rs1355538
rs12217103
rs203688
rs1126605
rs187347153
rs143819005
rs7614709
All − MR Egger
All − Inverse variance weighted
−0.50
−0.25
0.00
0.25
0.50
MR effect size for
'SCGB1A1' on 'Cardiac valvular disease'

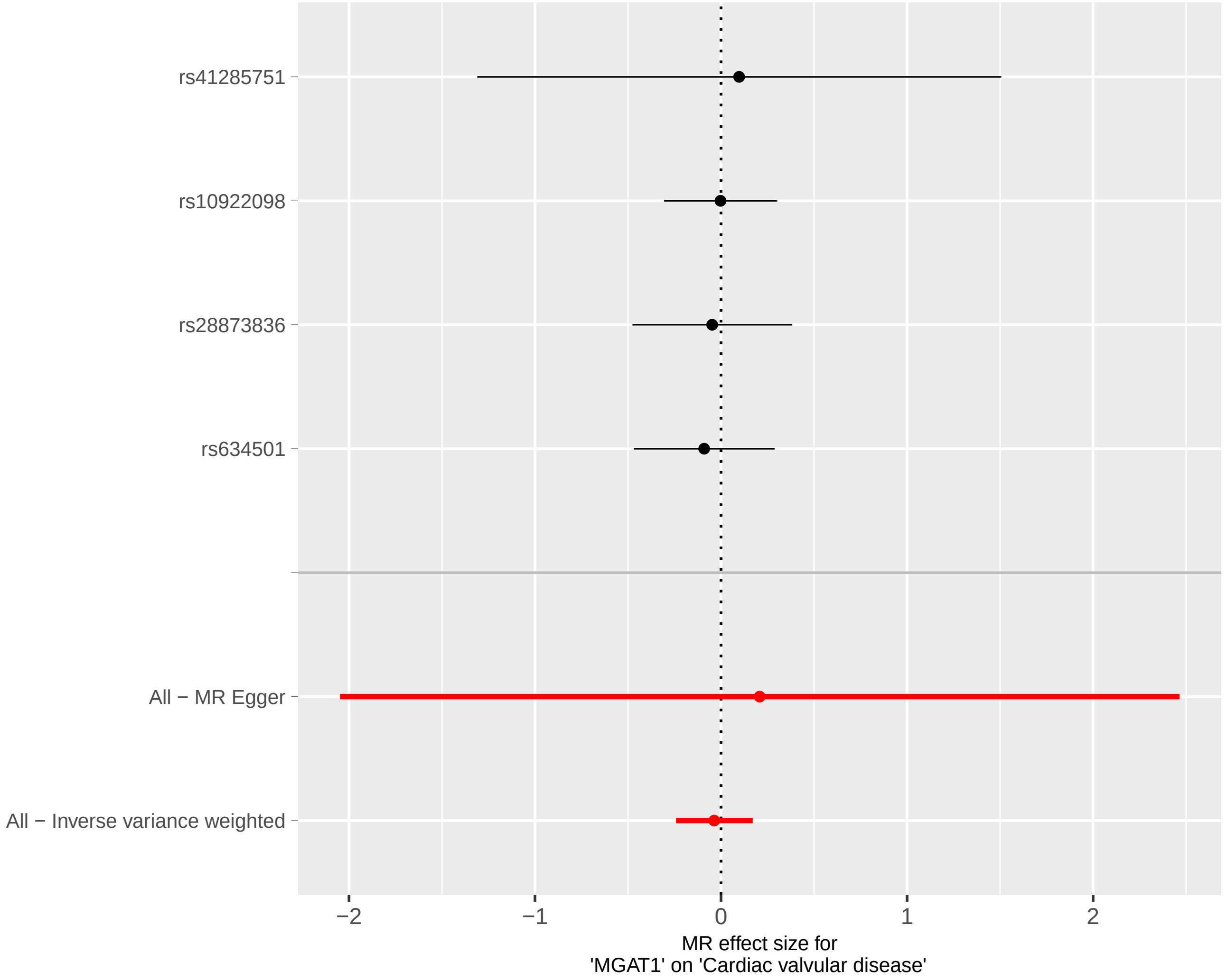
rs41285751
rs10922098
rs28873836
rs634501
All − MR Egger
All − Inverse variance weighted
−2
−1
0
1
2
MR effect size for
'MGAT1' on 'Cardiac valvular disease'

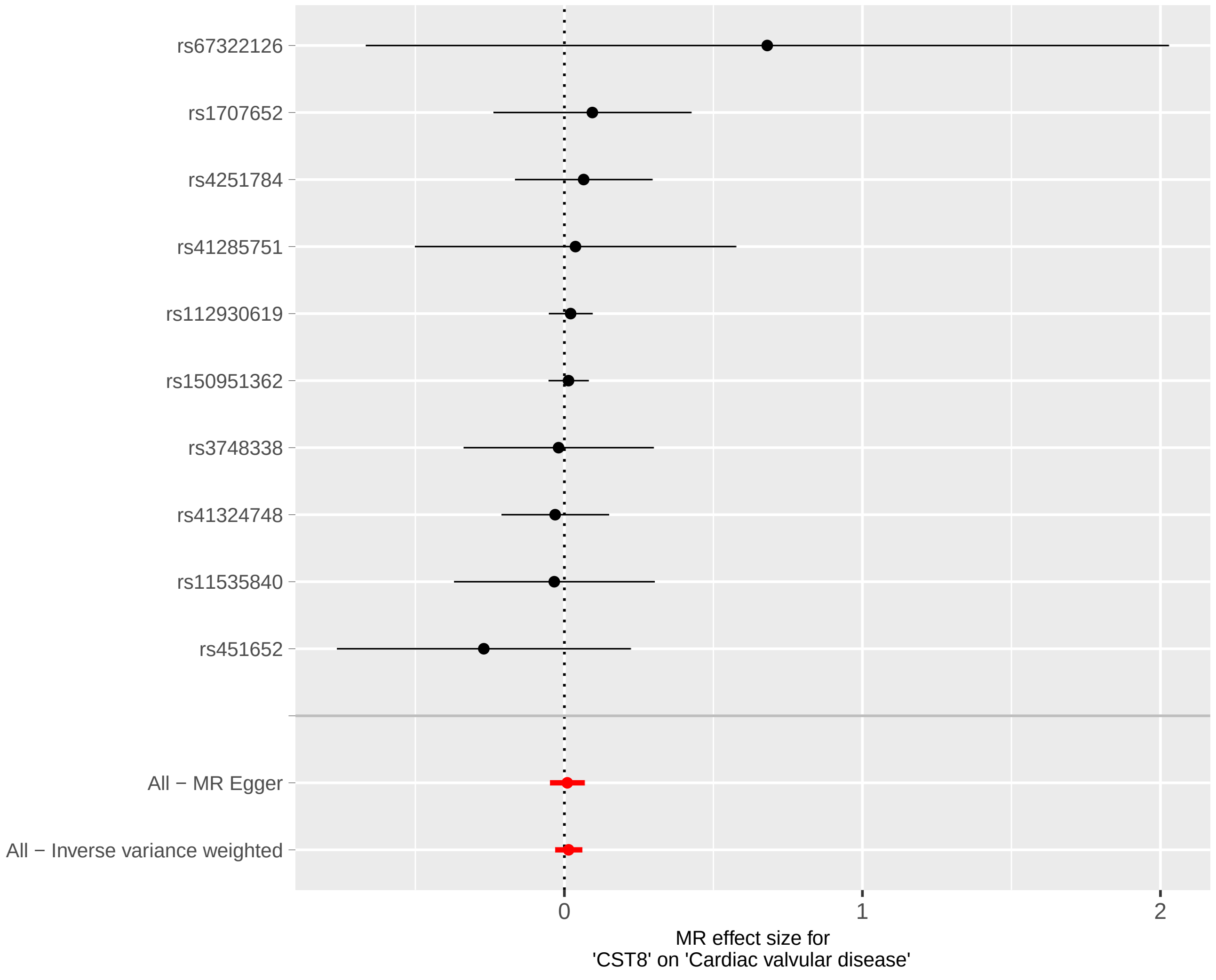
rs67322126
rs1707652
rs4251784
rs41285751
rs112930619
rs150951362
rs3748338
rs41324748
rs11535840
rs451652
All − MR Egger
All − Inverse variance weighted
0
1
2
MR effect size for
'CST8' on 'Cardiac valvular disease'

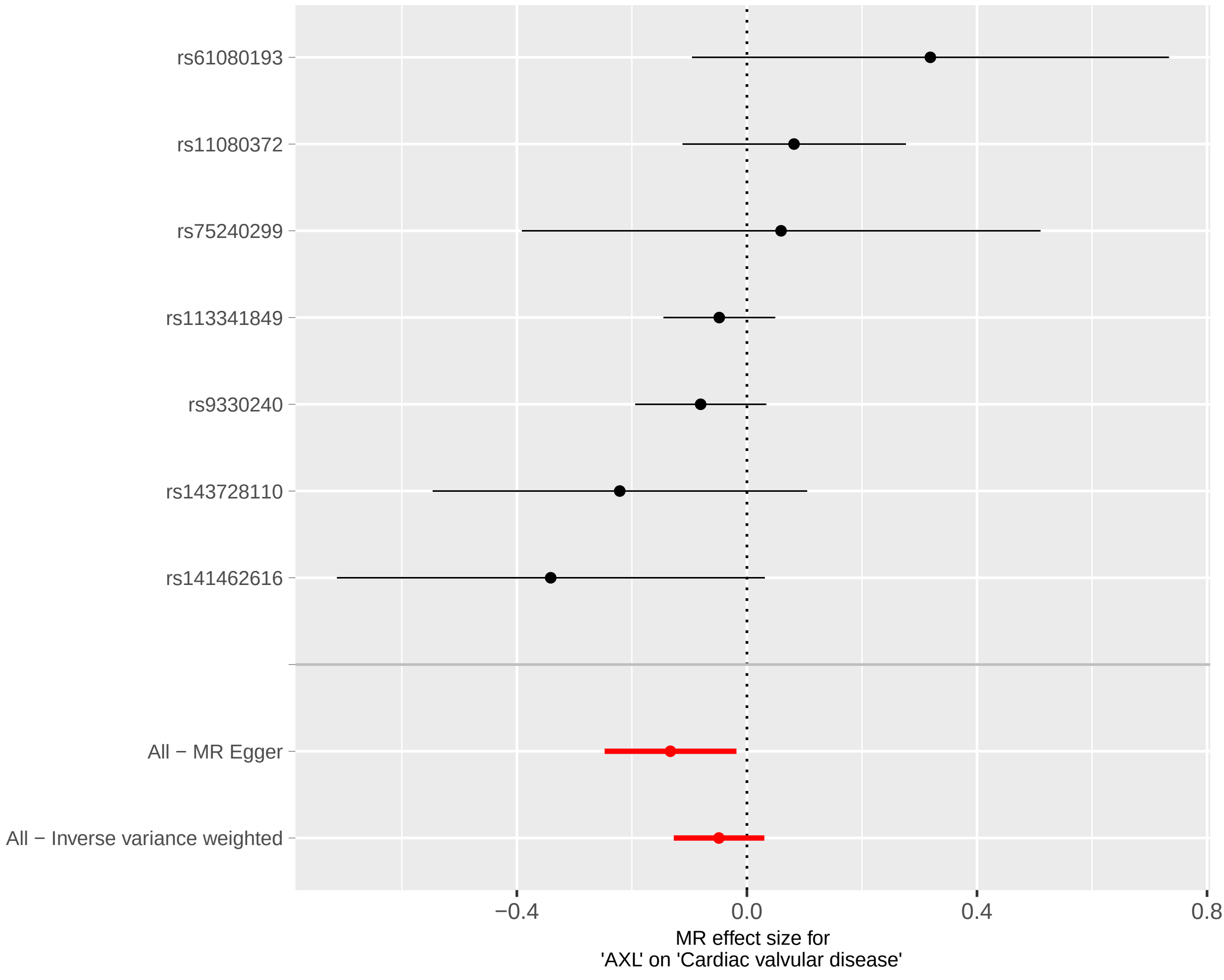
rs61080193
rs11080372
rs75240299
rs113341849
rs9330240
rs143728110
rs141462616
All − MR Egger
All − Inverse variance weighted
−0.4
0.0
0.4
0.8
MR effect size for
'AXL' on 'Cardiac valvular disease'

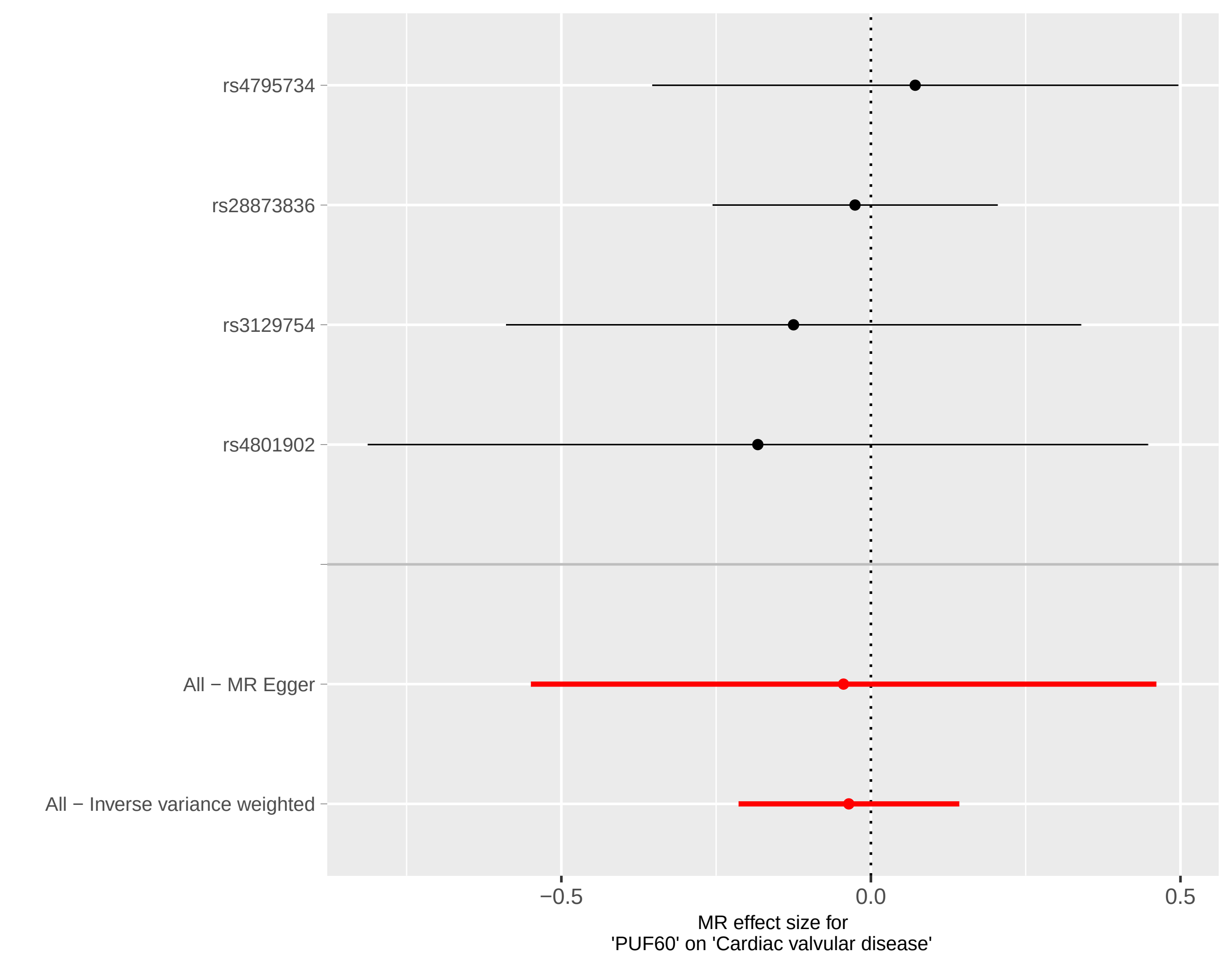
rs4795734
rs28873836
rs3129754
rs4801902
All − MR Egger
All − Inverse variance weighted
−0.5
0.0
0.5
MR effect size for
'PUF60' on 'Cardiac valvular disease'

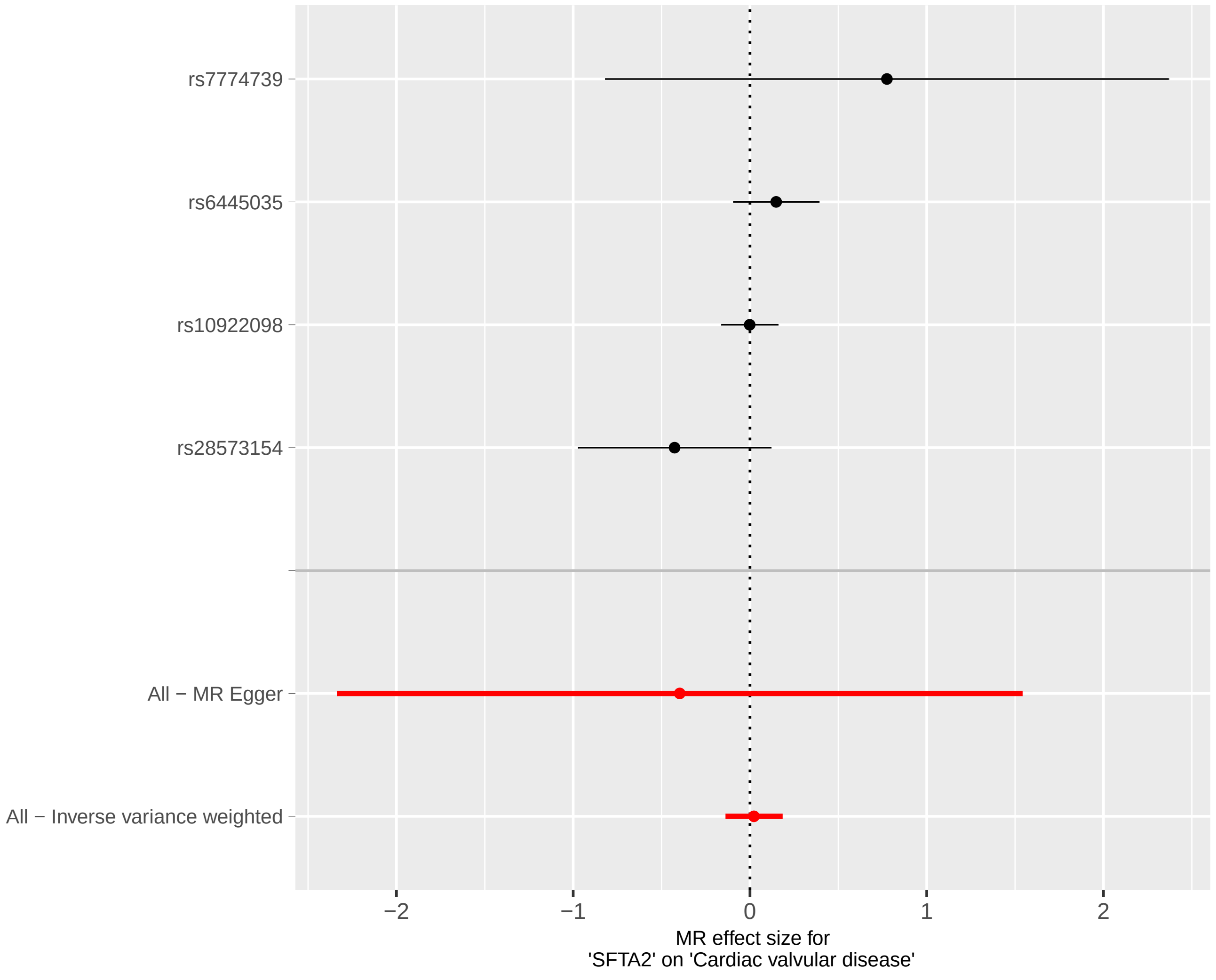

rs7774739
rs6445035
rs10922098
rs28573154
All − MR Egger
All − Inverse variance weighted
−2
−1
0
1
2
MR effect size for
'SFTA2' on 'Cardiac valvular disease'

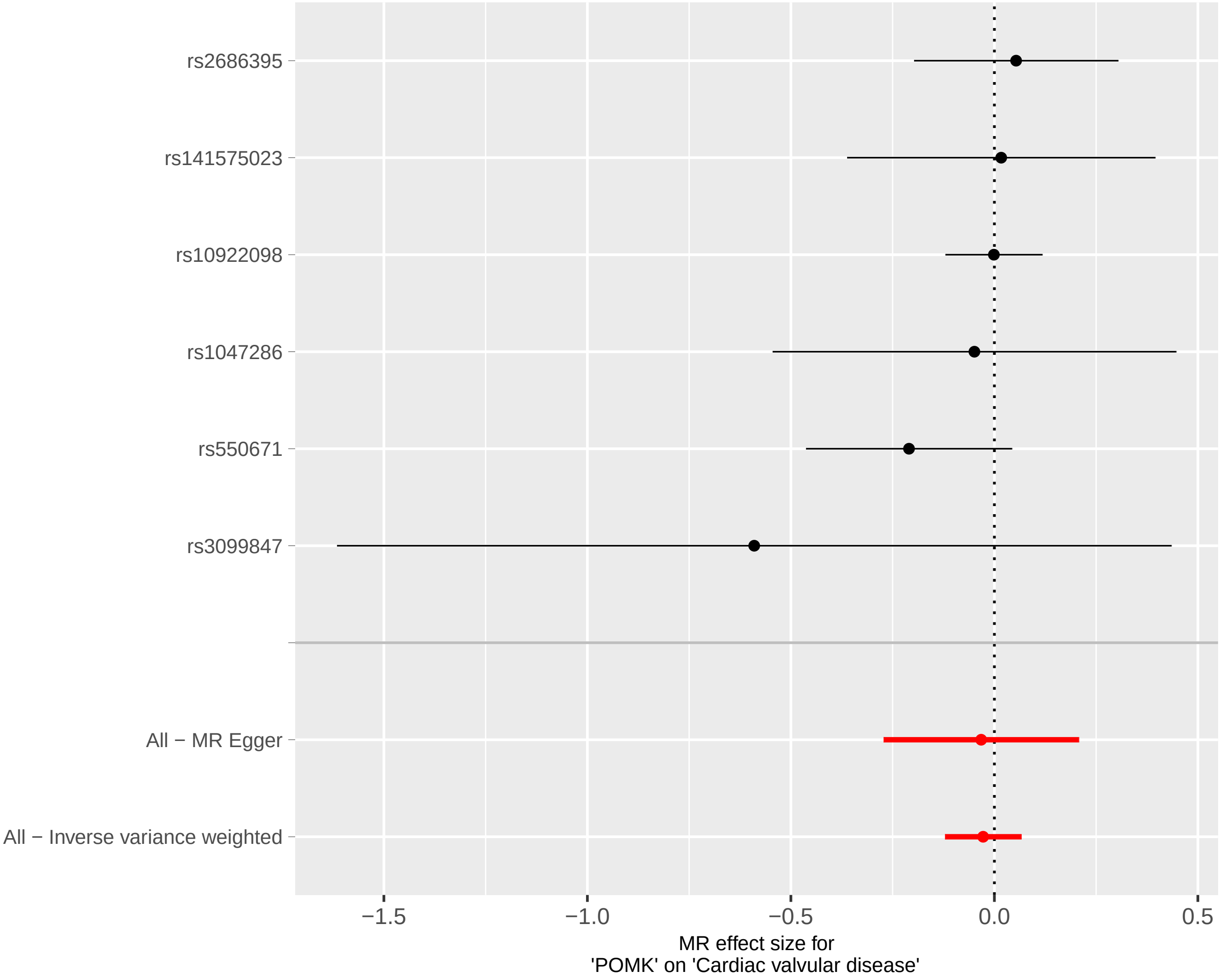
rs2686395
rs141575023
rs10922098
rs1047286
rs550671
rs3099847
All − MR Egger
All − Inverse variance weighted
−1.5
−1.0
−0.5
0.0
0.5
MR effect size for
'POMK' on 'Cardiac valvular disease'

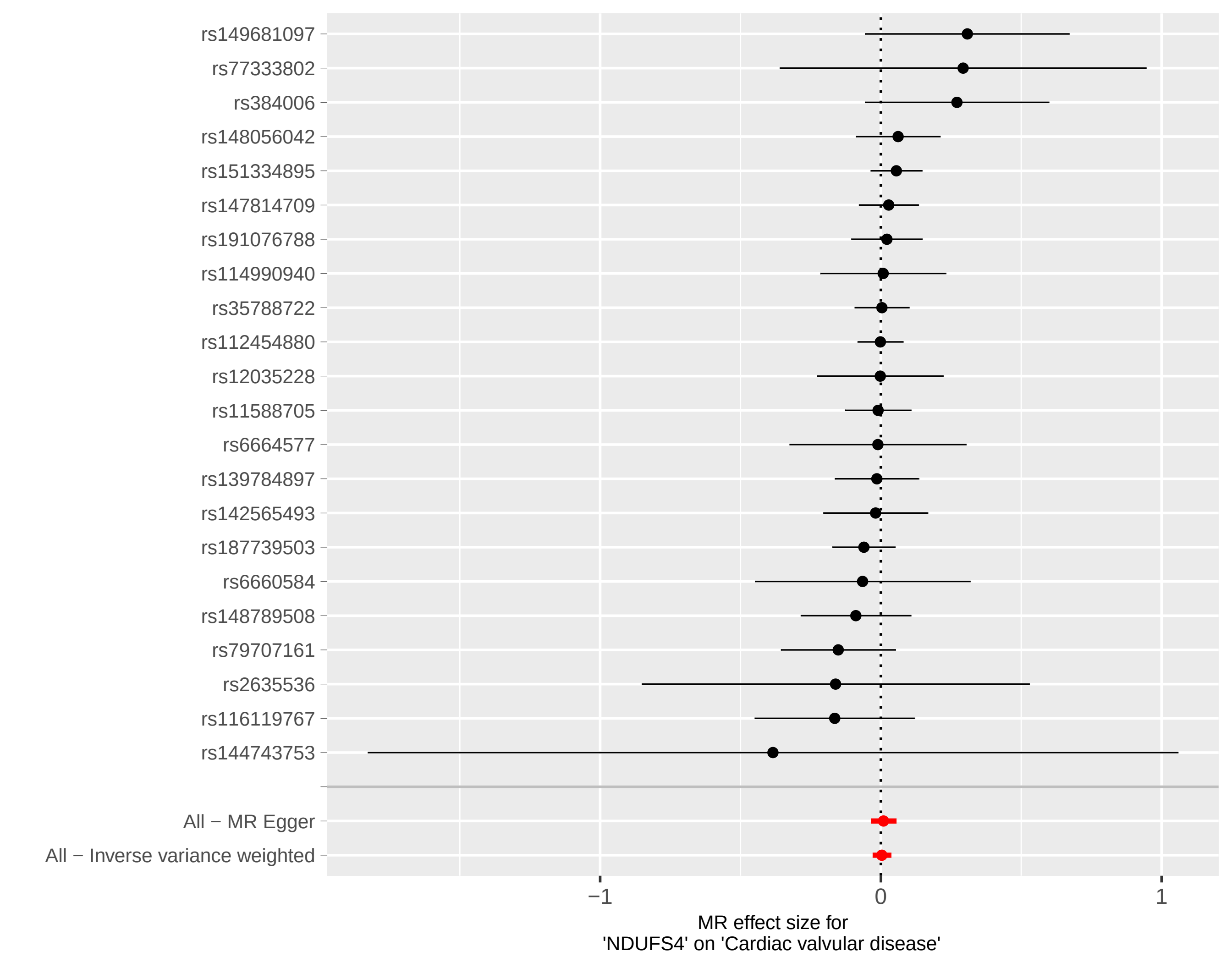
rs149681097
rs77333802
rs384006
rs148056042
rs151334895
rs147814709
rs191076788
rs114990940
rs35788722
rs112454880
rs12035228
rs11588705
rs6664577
rs139784897
rs142565493
rs187739503
rs6660584
rs148789508
rs79707161
rs2635536
rs116119767
rs144743753
All − MR Egger
All − Inverse variance weighted
−1
0
1
MR effect size for
'NDUFS4' on 'Cardiac valvular disease'

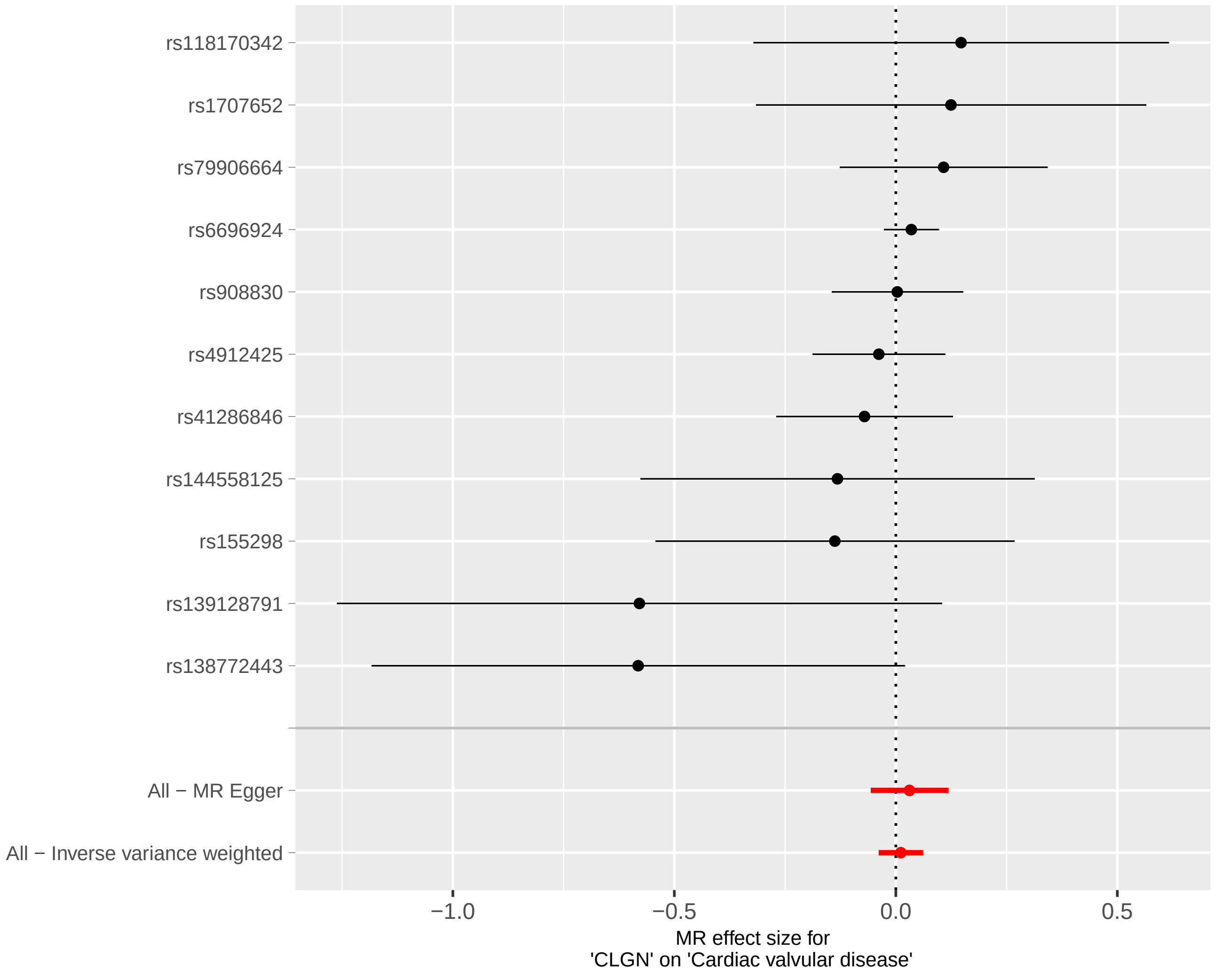

rs118170342
rs1707652
rs79906664
rs6696924
rs908830
rs4912425
rs41286846
rs144558125
rs155298
rs139128791
rs138772443
All − MR Egger
All − Inverse variance weighted
−1.0
−0.5
0.0
0.5
MR effect size for
'CLGN' on 'Cardiac valvular disease'

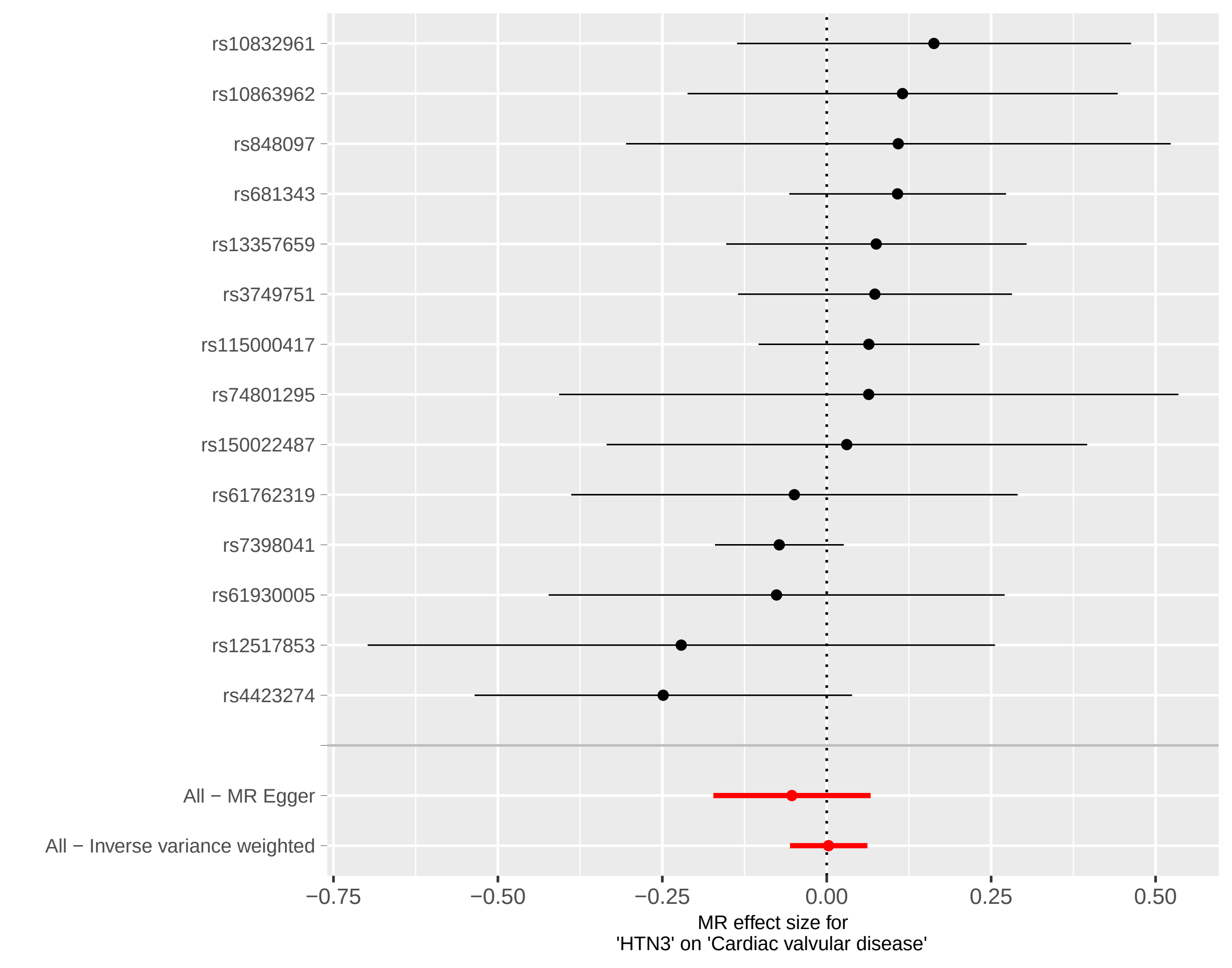
rs10832961
rs10863962
rs848097
rs681343
rs13357659
rs3749751
rs115000417
rs74801295
rs150022487
rs61762319
rs7398041
rs61930005
rs12517853
rs4423274
All − MR Egger
All − Inverse variance weighted
−0.75
−0.50
−0.25
0.00
0.25
0.50
MR effect size for
'HTN3' on 'Cardiac valvular disease'

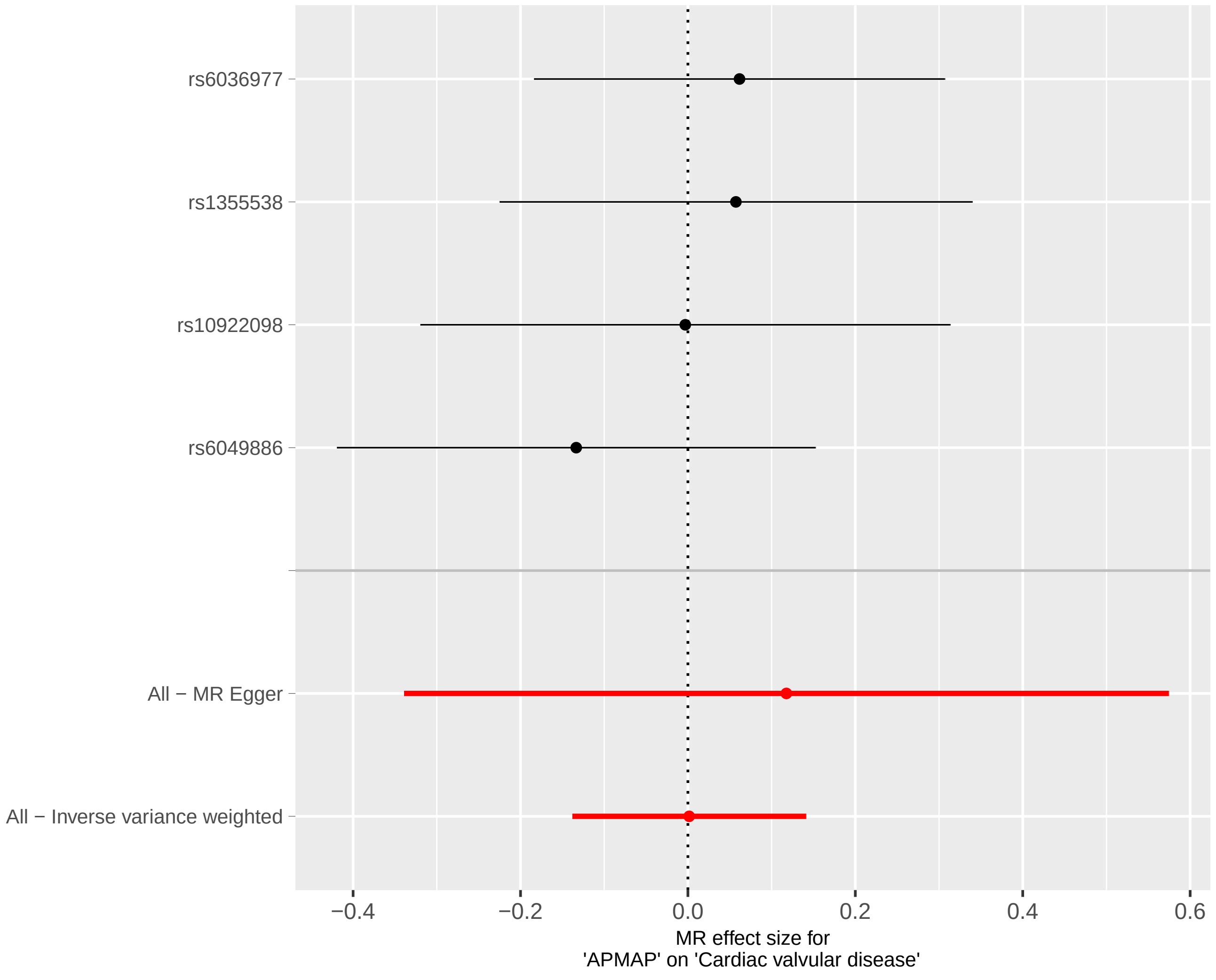
rs6036977
rs1355538
rs10922098
rs6049886
All − MR Egger
All − Inverse variance weighted
−0.4
−0.2
0.0
0.2
0.4
0.6
MR effect size for
'APMAP' on 'Cardiac valvular disease'

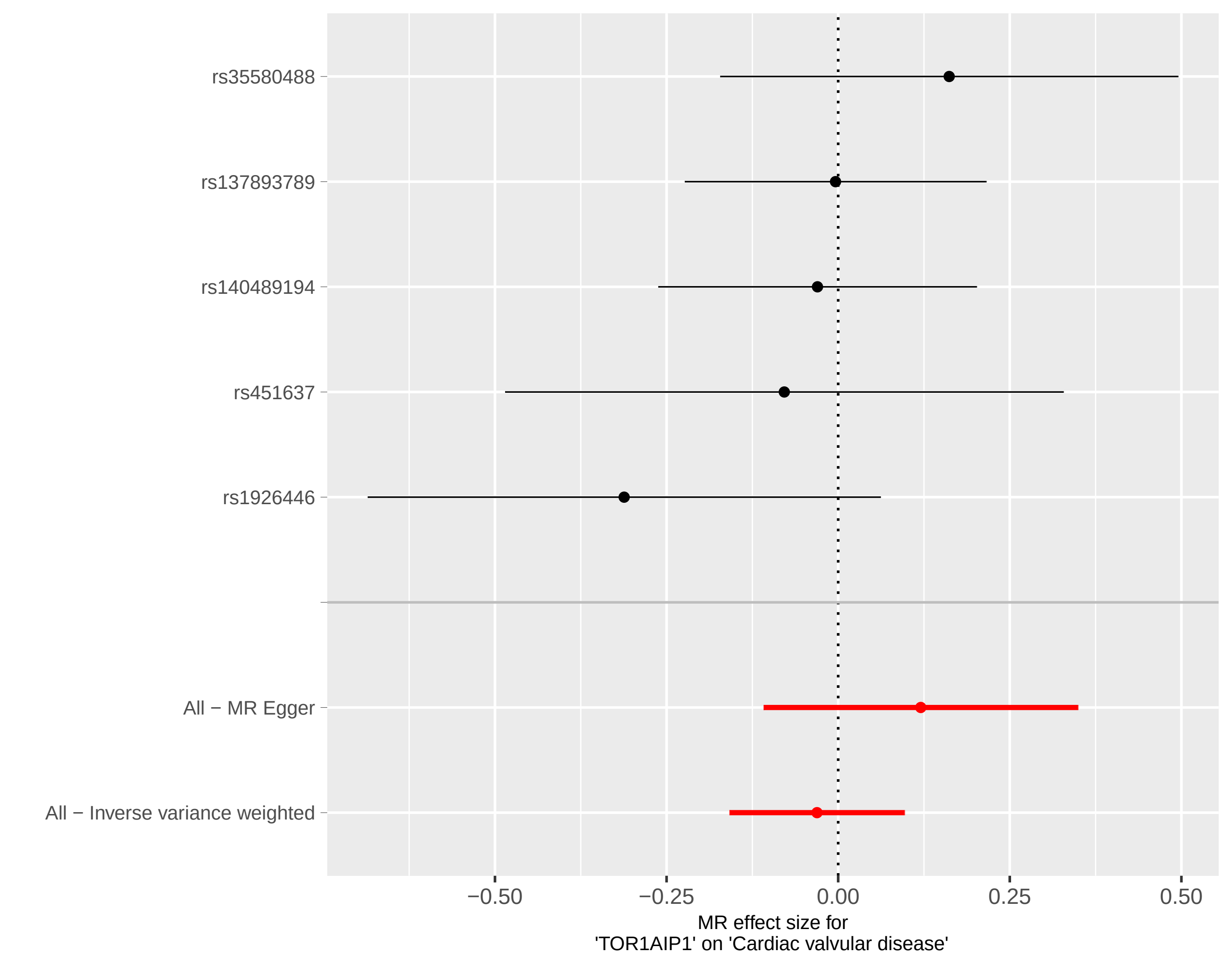
rs35580488
rs137893789
rs140489194
rs451637
rs1926446
All − MR Egger
All − Inverse variance weighted
−0.50
−0.25
0.00
0.25
0.50
MR effect size for
'TOR1AIP1' on 'Cardiac valvular disease'

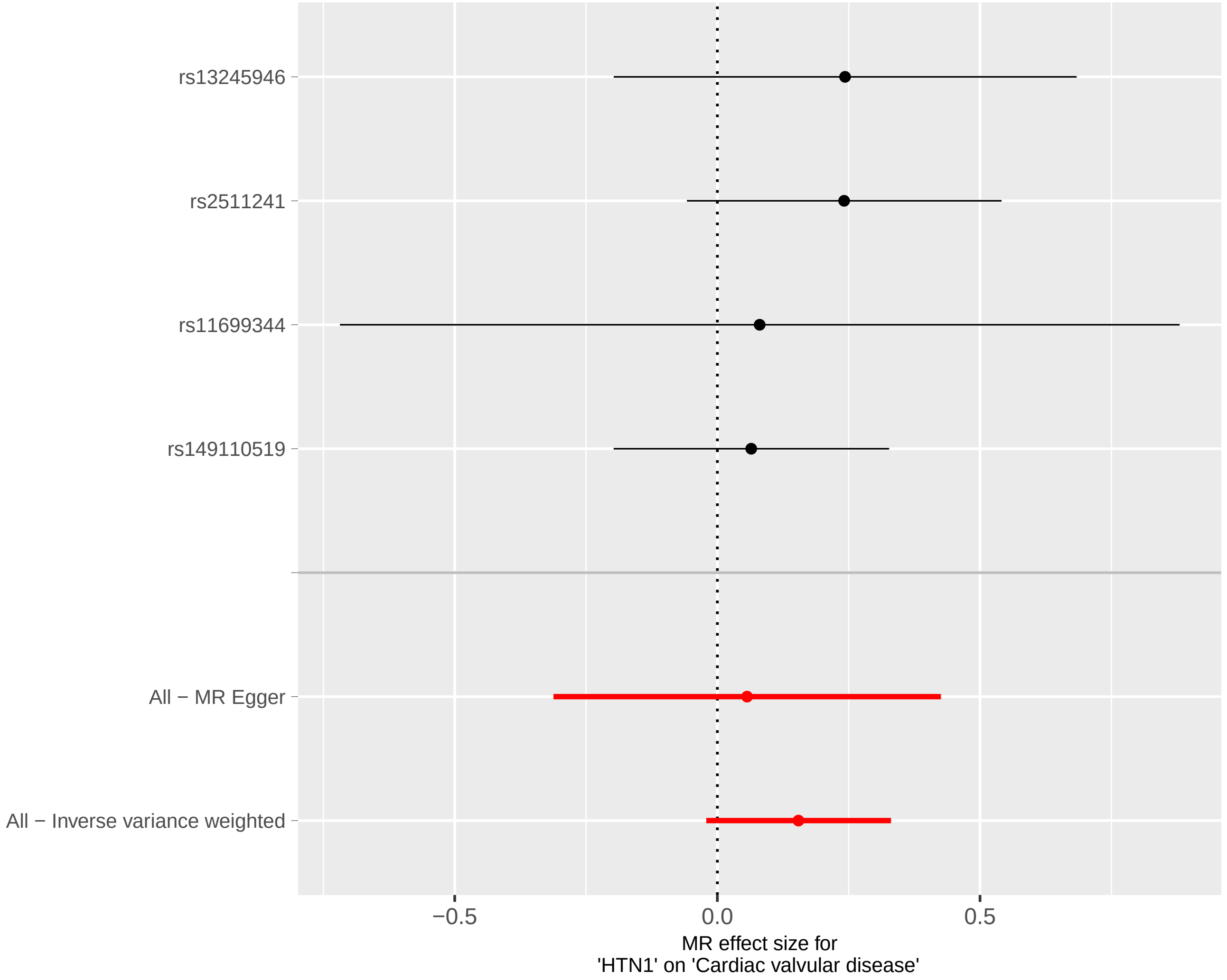

rs13245946
rs2511241
rs11699344
rs149110519
All − MR Egger
All − Inverse variance weighted
−0.5
0.0
0.5
MR effect size for
'HTN1' on 'Cardiac valvular disease'

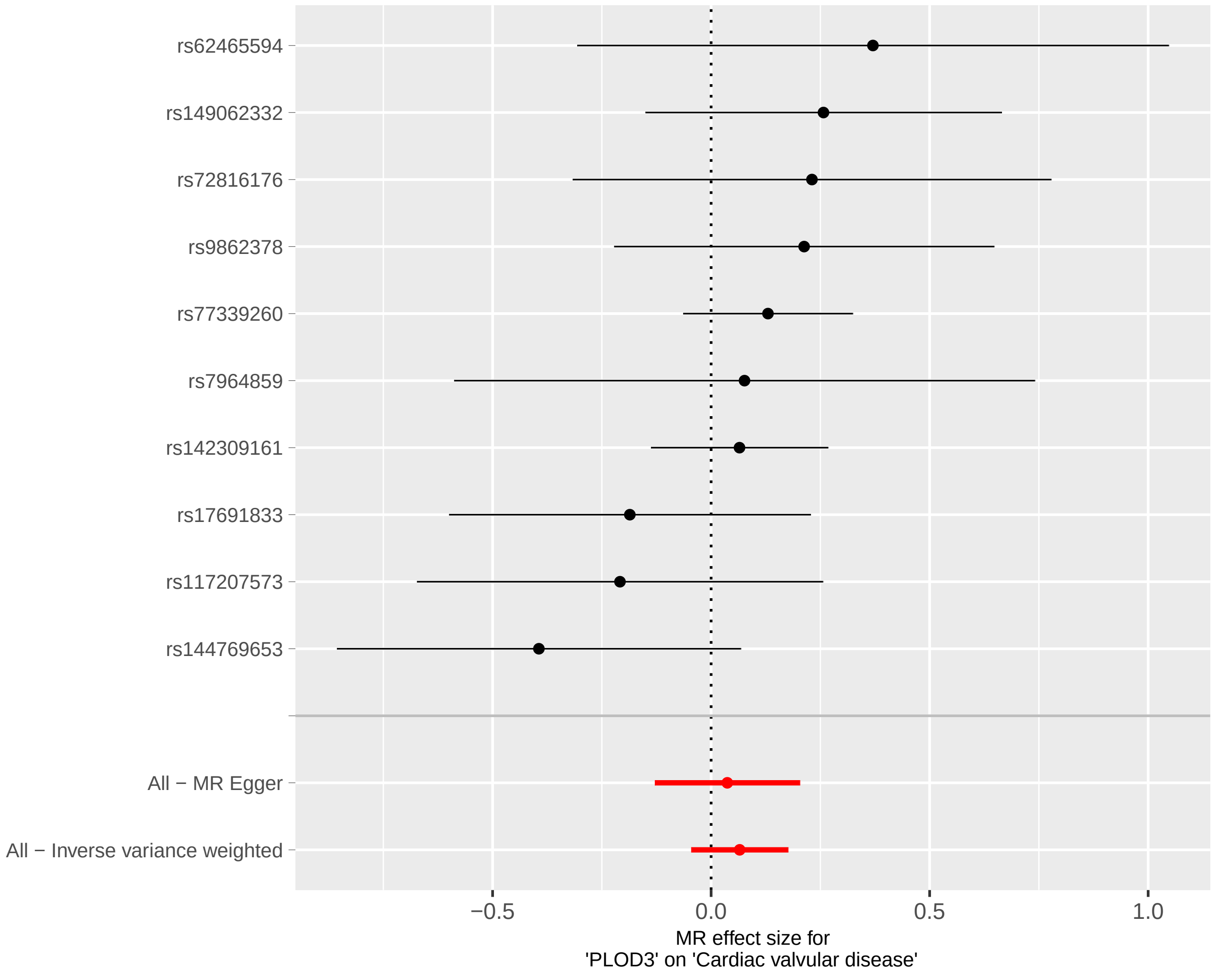
rs62465594
rs149062332
rs72816176
rs9862378
rs77339260
rs7964859
rs142309161
rs17691833
rs117207573
rs144769653
All − MR Egger
All − Inverse variance weighted
−0.5
0.0
0.5
1.0
MR effect size for
'PLOD3' on 'Cardiac valvular disease'

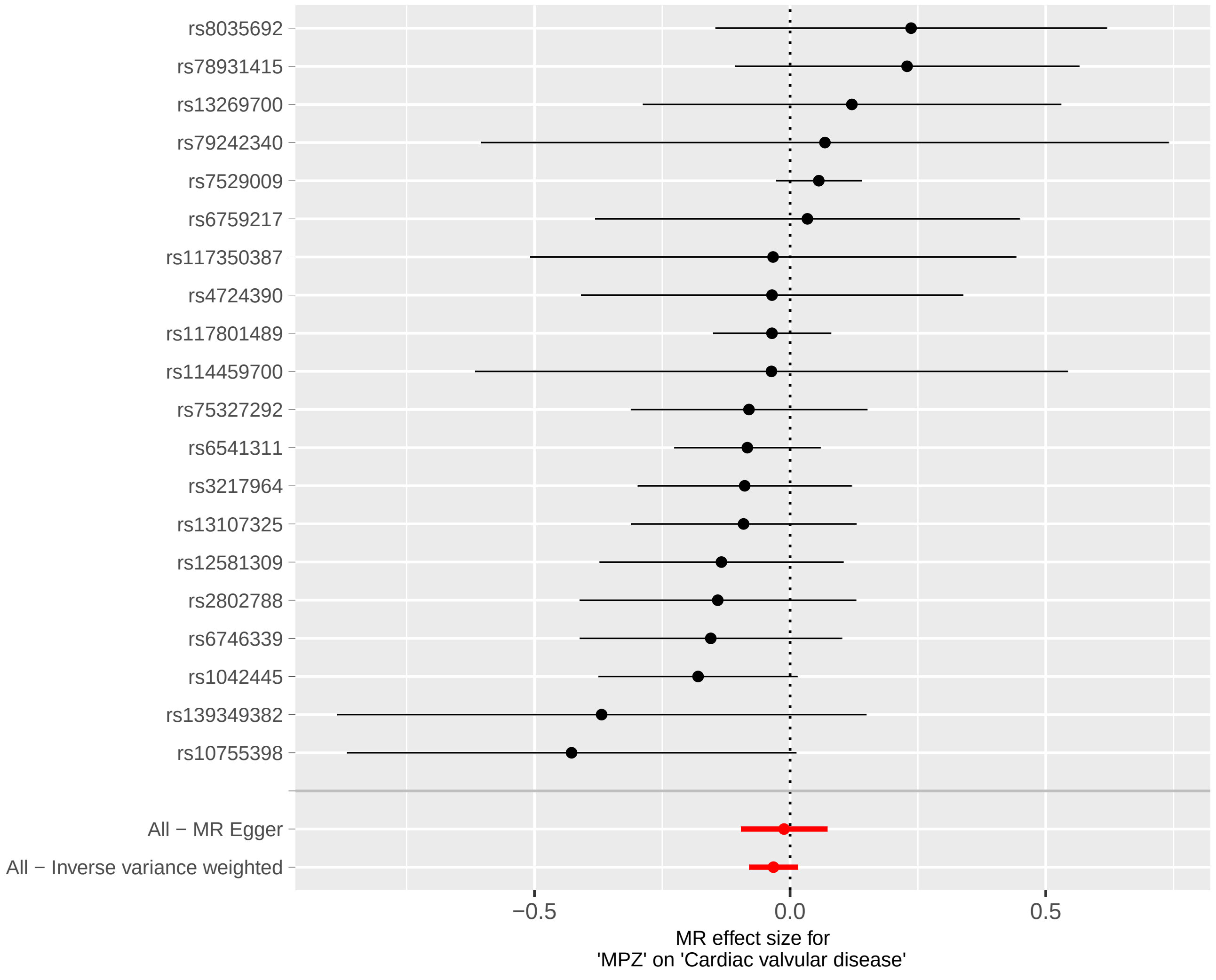
rs8035692
rs78931415
rs13269700
rs79242340
rs7529009
rs6759217
rs117350387
rs4724390
rs117801489
rs114459700
rs75327292
rs6541311
rs3217964
rs13107325
rs12581309
rs2802788
rs6746339
rs1042445
rs139349382
rs10755398
All − MR Egger
All − Inverse variance weighted
−0.5
0.0
0.5
MR effect size for
'MPZ' on 'Cardiac valvular disease'

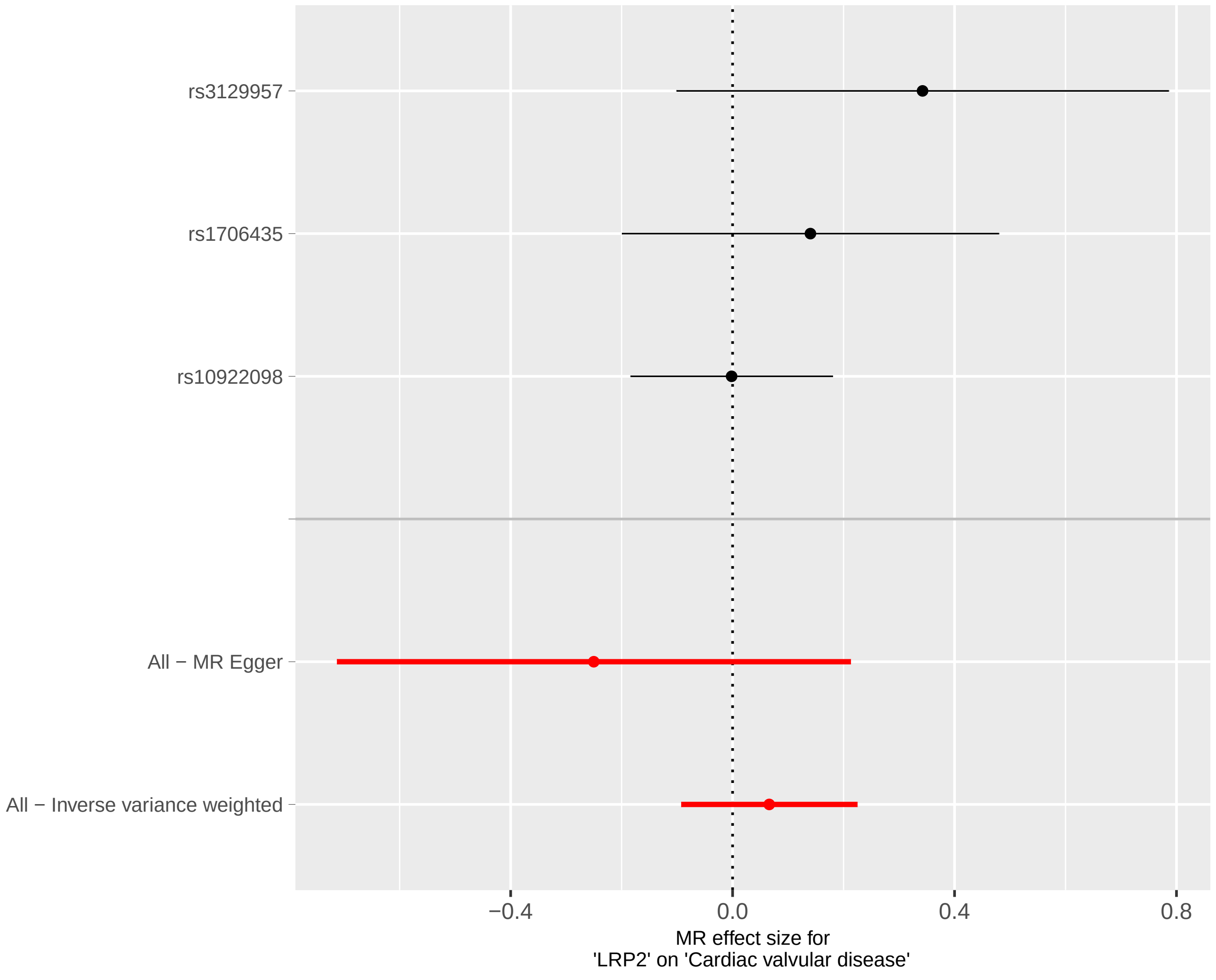
rs3129957
rs1706435
rs10922098
All − MR Egger
All − Inverse variance weighted
−0.4
0.0
0.4
0.8
MR effect size for
'LRP2' on 'Cardiac valvular disease'

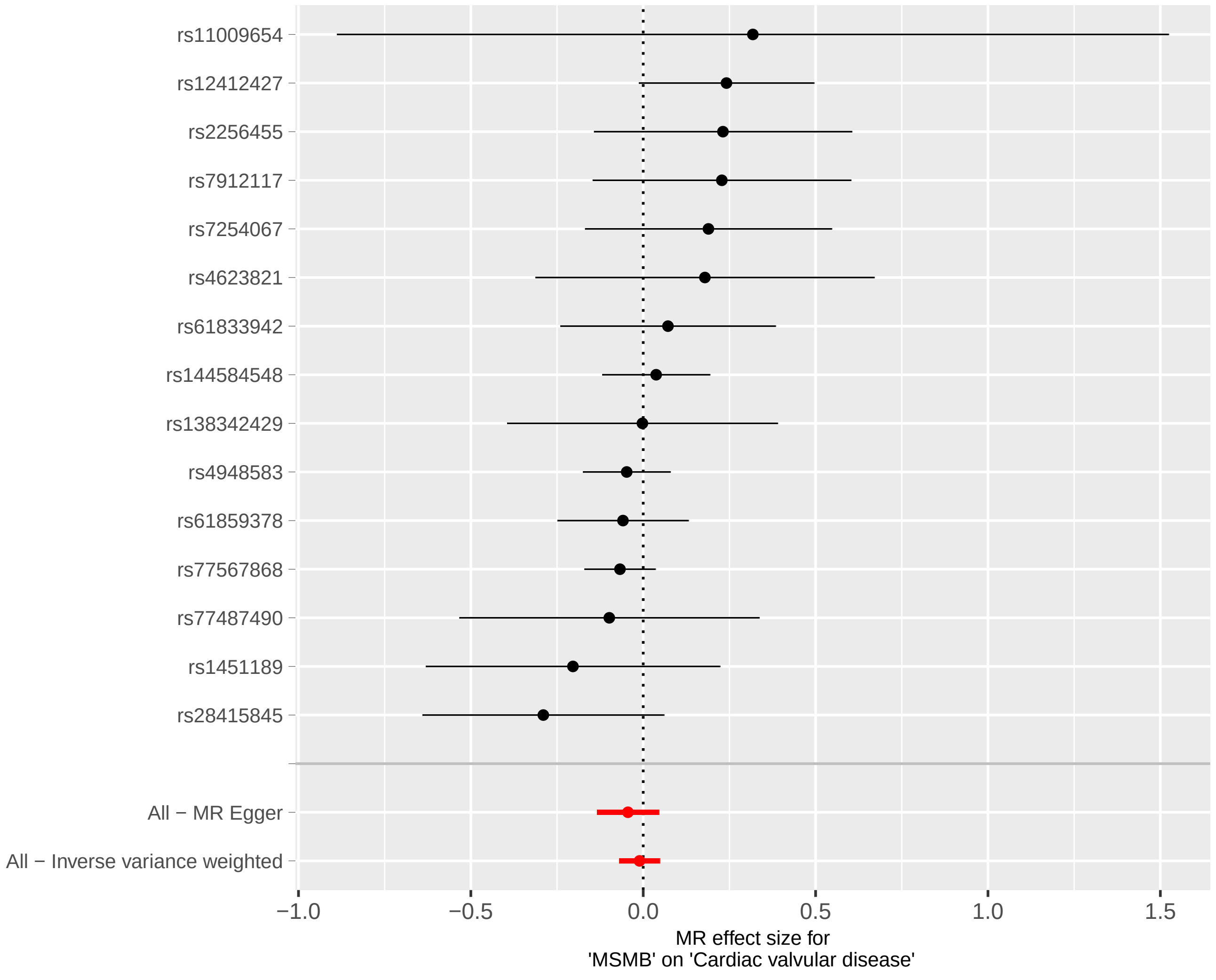

rs11009654
rs12412427
rs2256455
rs7912117
rs7254067
rs4623821
rs61833942
rs144584548
rs138342429
rs4948583
rs61859378
rs77567868
rs77487490
rs1451189
rs28415845
All − MR Egger
All − Inverse variance weighted
−1.0
−0.5
0.0
0.5
1.0
1.5
MR effect size for
'MSMB' on 'Cardiac valvular disease'

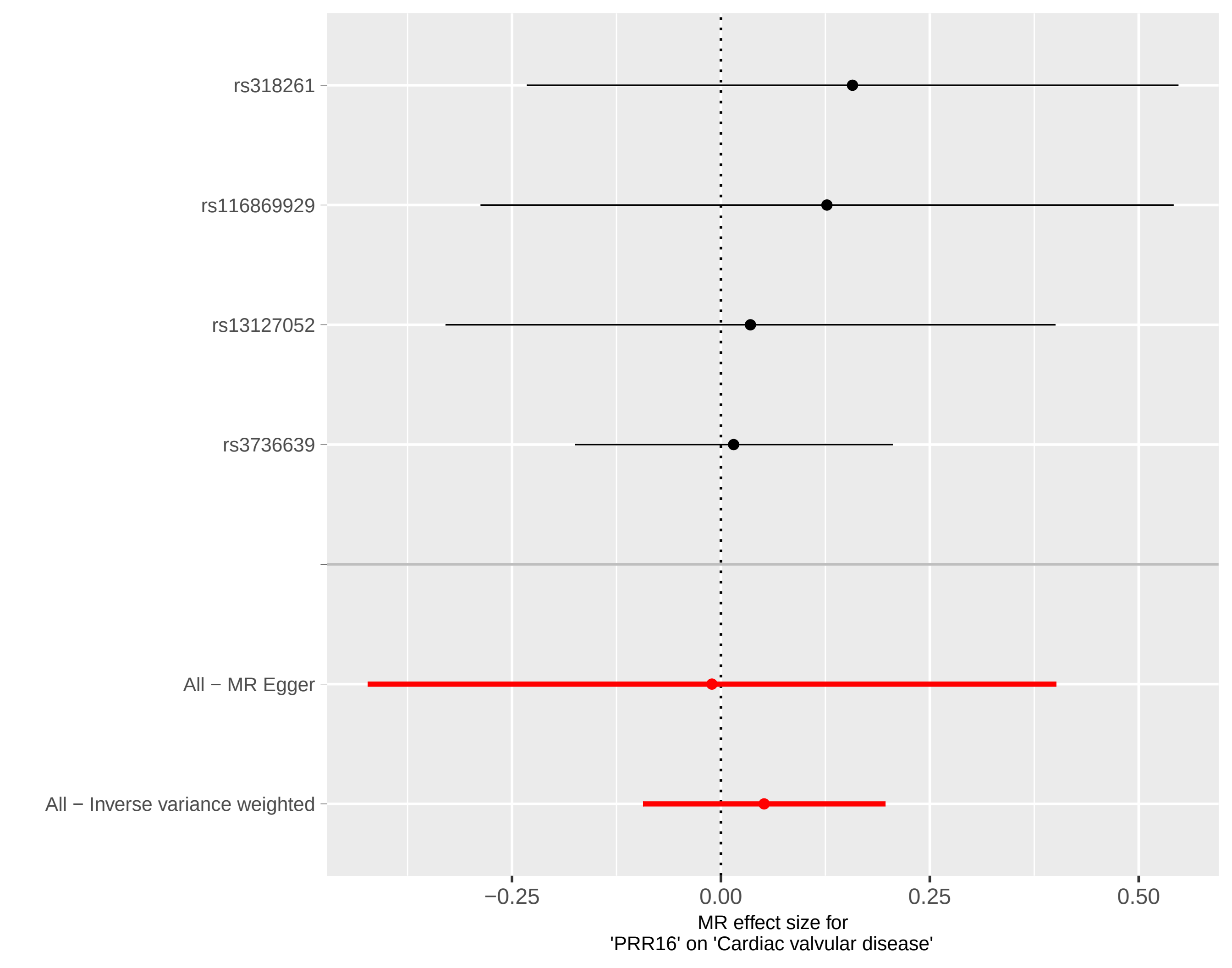
rs318261
rs116869929
rs13127052
rs3736639
All − MR Egger
All − Inverse variance weighted
−0.25
0.00
0.25
0.50
MR effect size for
'PRR16' on 'Cardiac valvular disease'

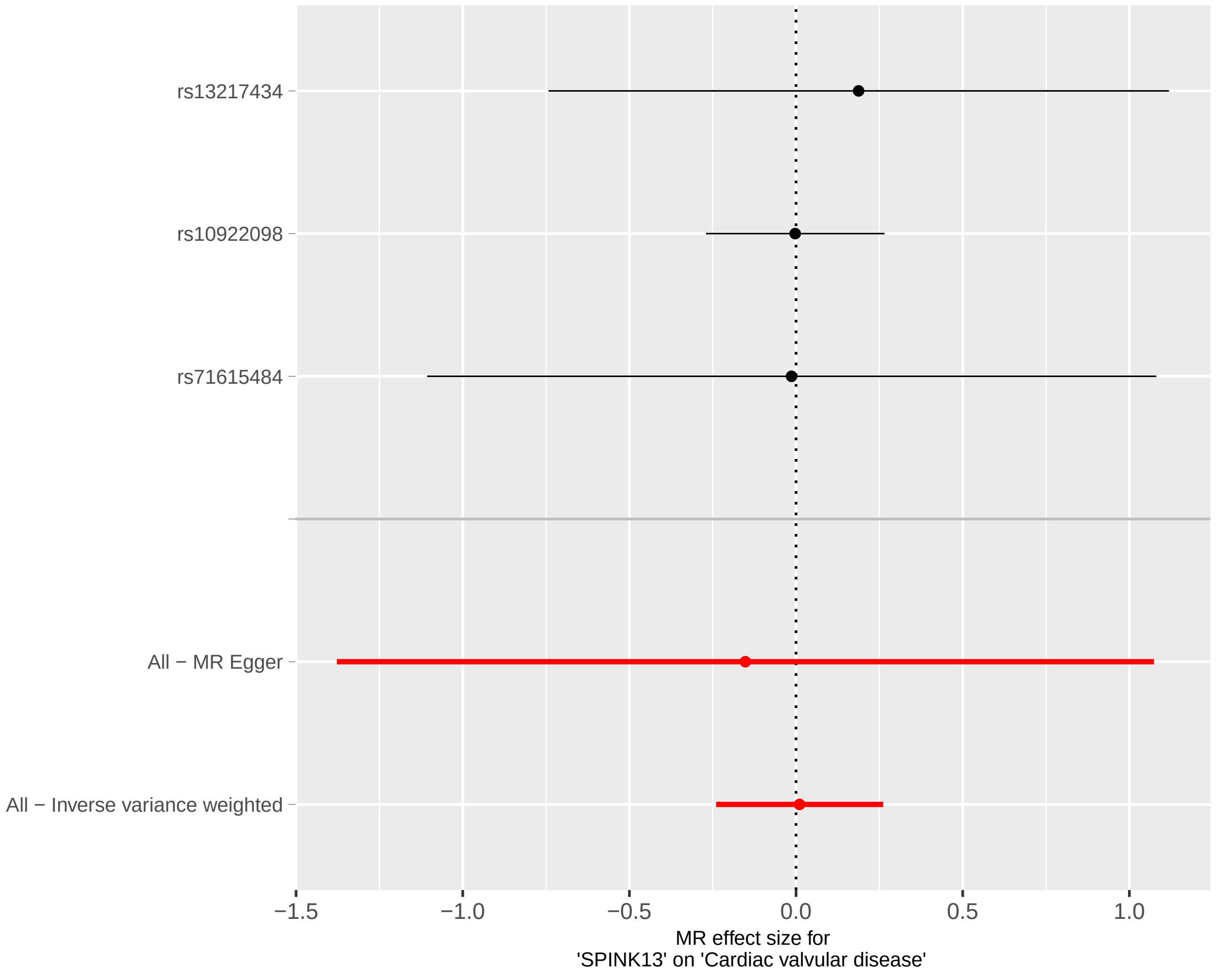

rs13217434
rs10922098
rs71615484
All − MR Egger
All − Inverse variance weighted
−1.5
−1.0
−0.5
0.0
0.5
1.0
MR effect size for
'SPINK13' on 'Cardiac valvular disease'

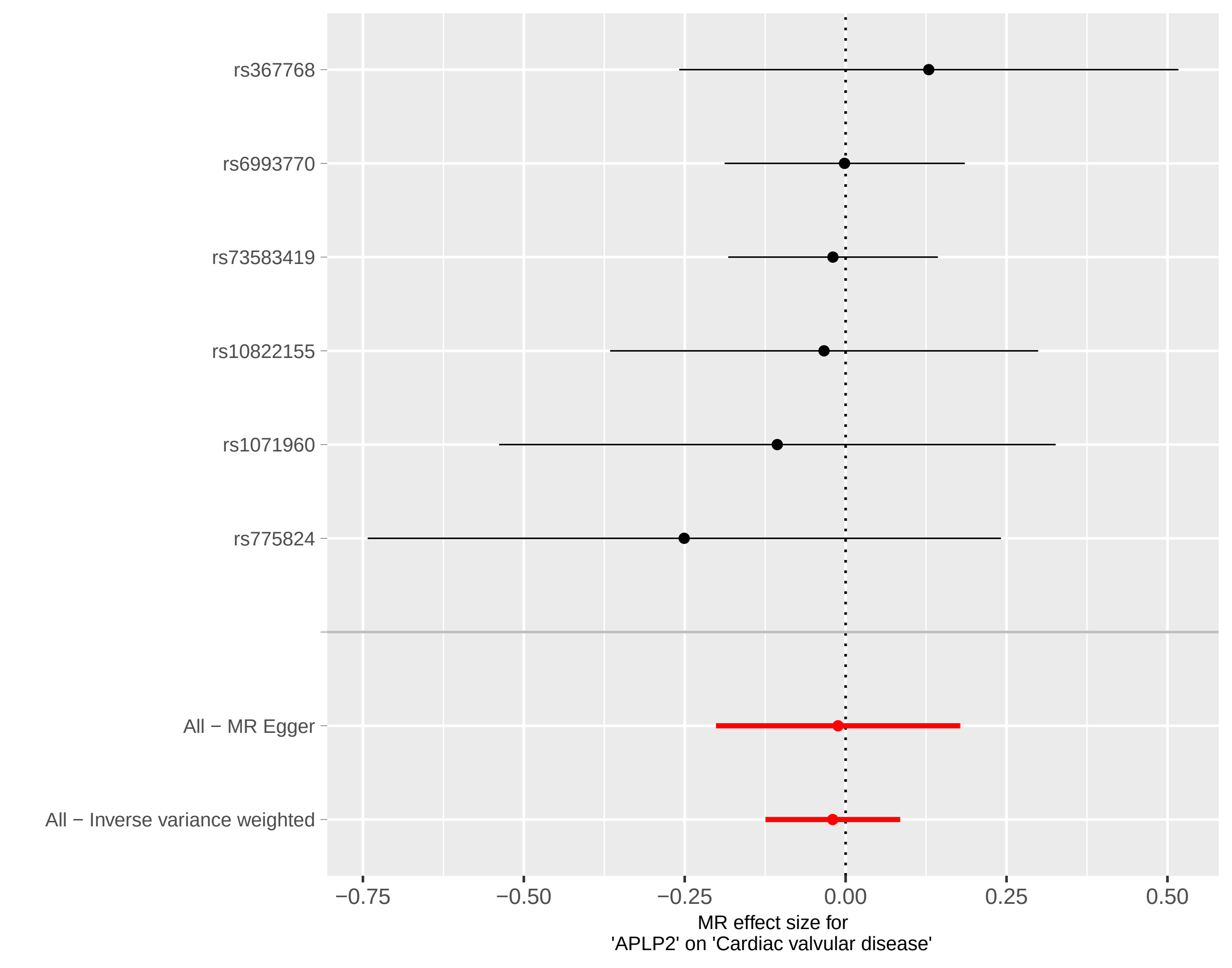
rs367768
rs6993770
rs73583419
rs10822155
rs1071960
rs775824
All − MR Egger
All − Inverse variance weighted
−0.75
−0.50
−0.25
0.00
0.25
0.50
MR effect size for
'APLP2' on 'Cardiac valvular disease'

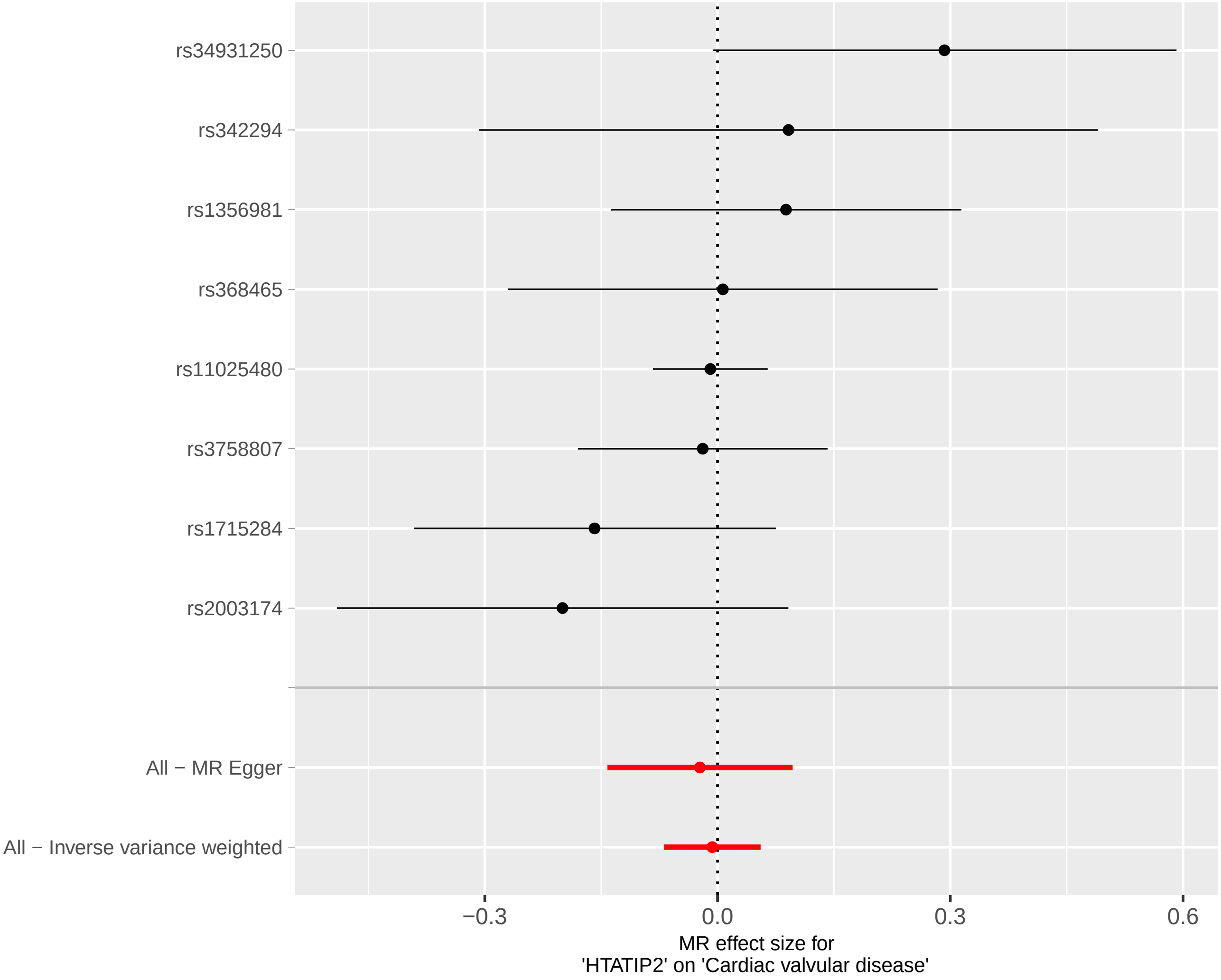
rs34931250
rs342294
rs1356981
rs368465
rs11025480
rs3758807
rs1715284
rs2003174
All − MR Egger
All − Inverse variance weighted
−0.3
0.0
0.3
0.6
MR effect size for
'HTATIP2' on 'Cardiac valvular disease'

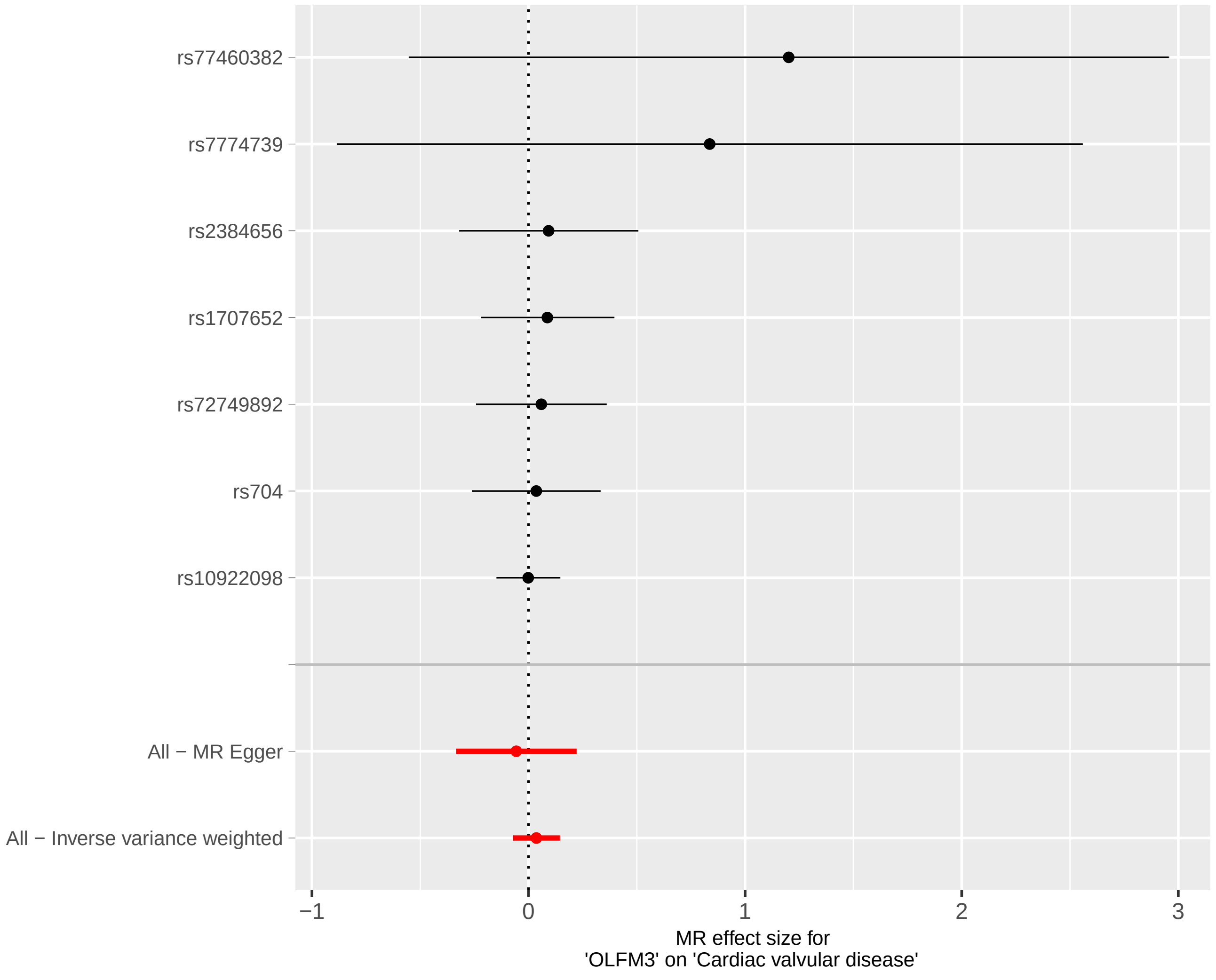
rs77460382
rs7774739
rs2384656
rs1707652
rs72749892
rs704
rs10922098
All − MR Egger
All − Inverse variance weighted
−1
0
1
2
3
MR effect size for
'OLFM3' on 'Cardiac valvular disease'

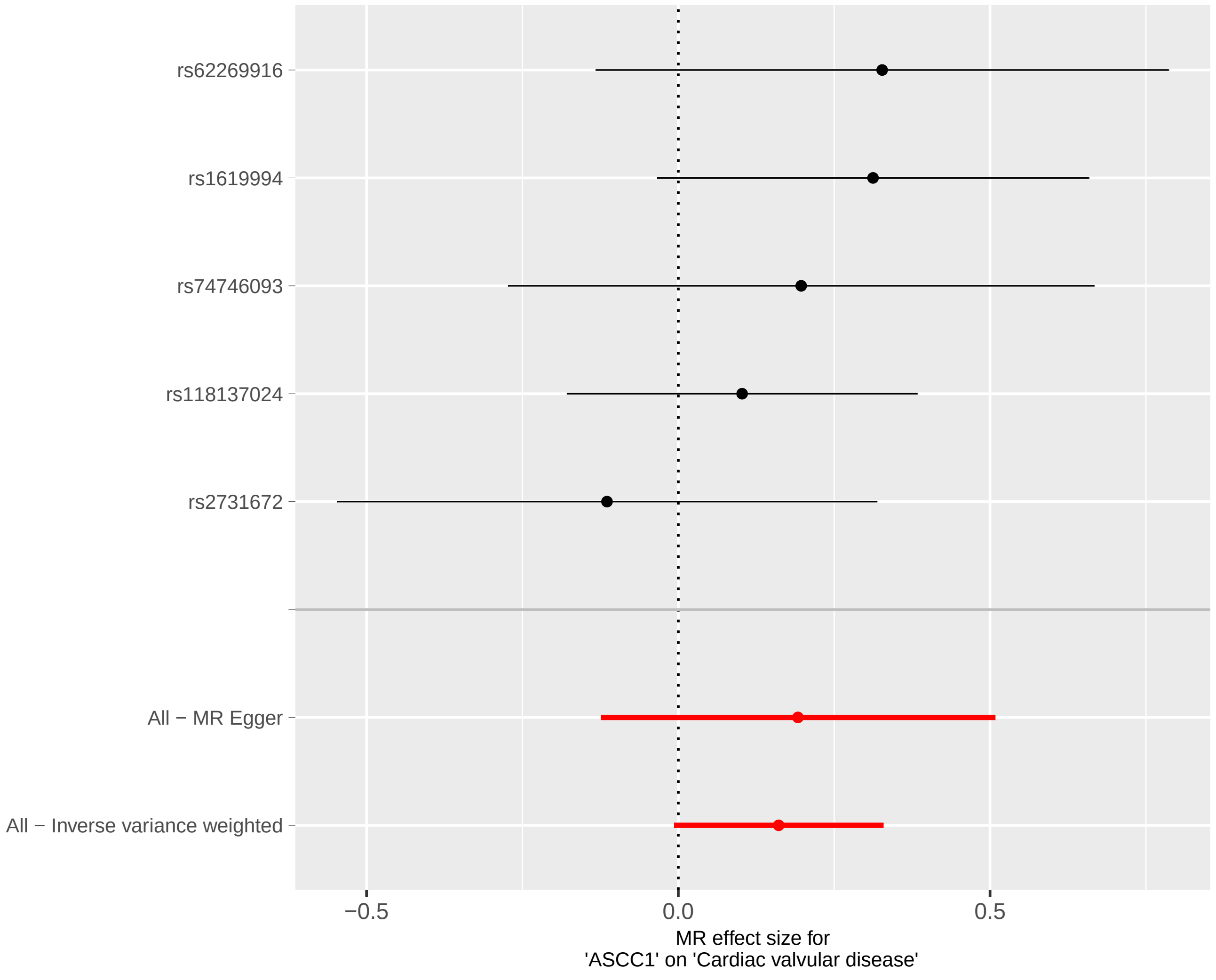
rs62269916
rs1619994
rs74746093
rs118137024
rs2731672
All − MR Egger
All − Inverse variance weighted
−0.5
0.0
0.5
MR effect size for
'ASCC1' on 'Cardiac valvular disease'

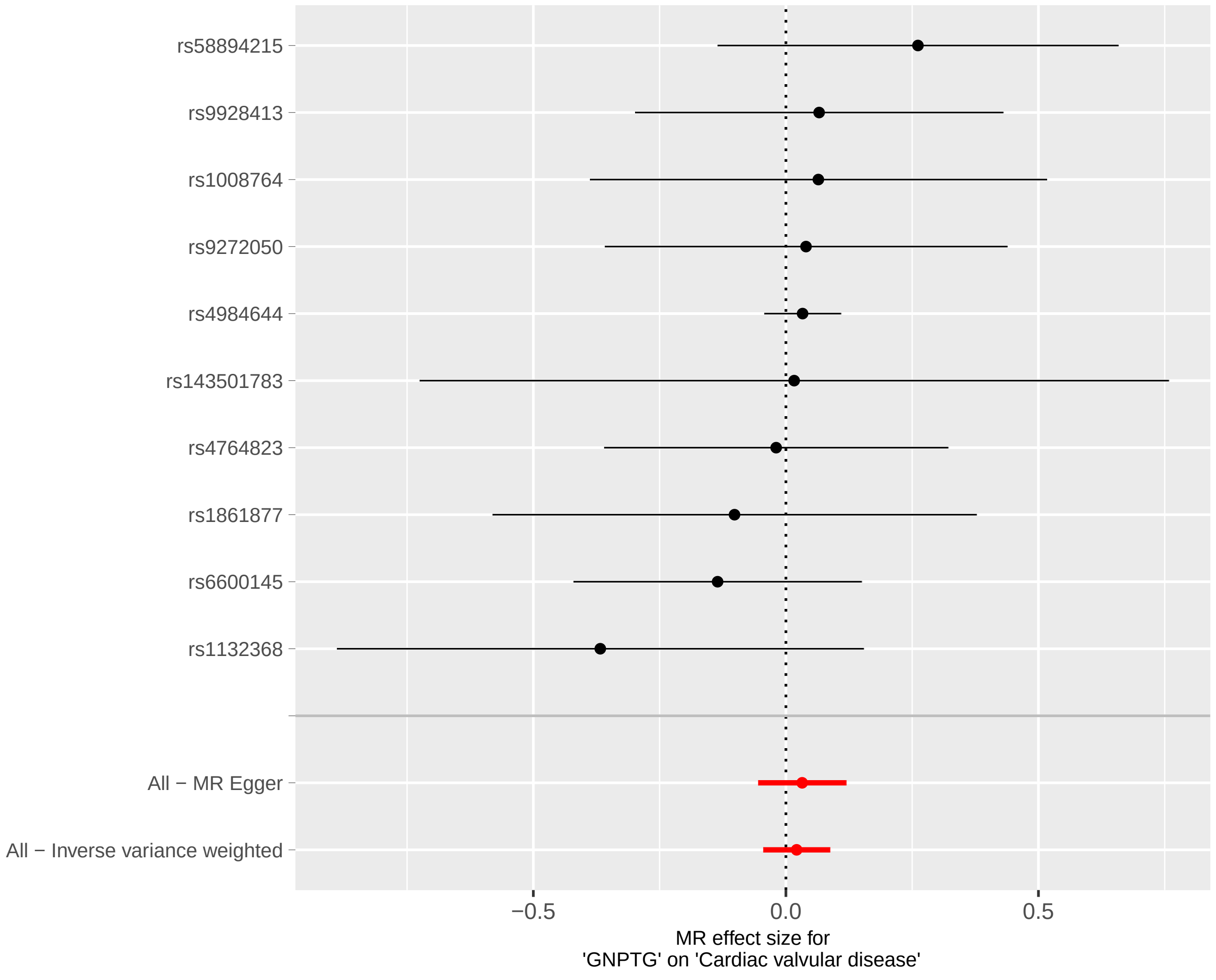
rs58894215
rs9928413
rs1008764
rs9272050
rs4984644
rs143501783
rs4764823
rs1861877
rs6600145
rs1132368
All − MR Egger
All − Inverse variance weighted
−0.5
0.0
0.5
MR effect size for
'GNPTG' on 'Cardiac valvular disease'

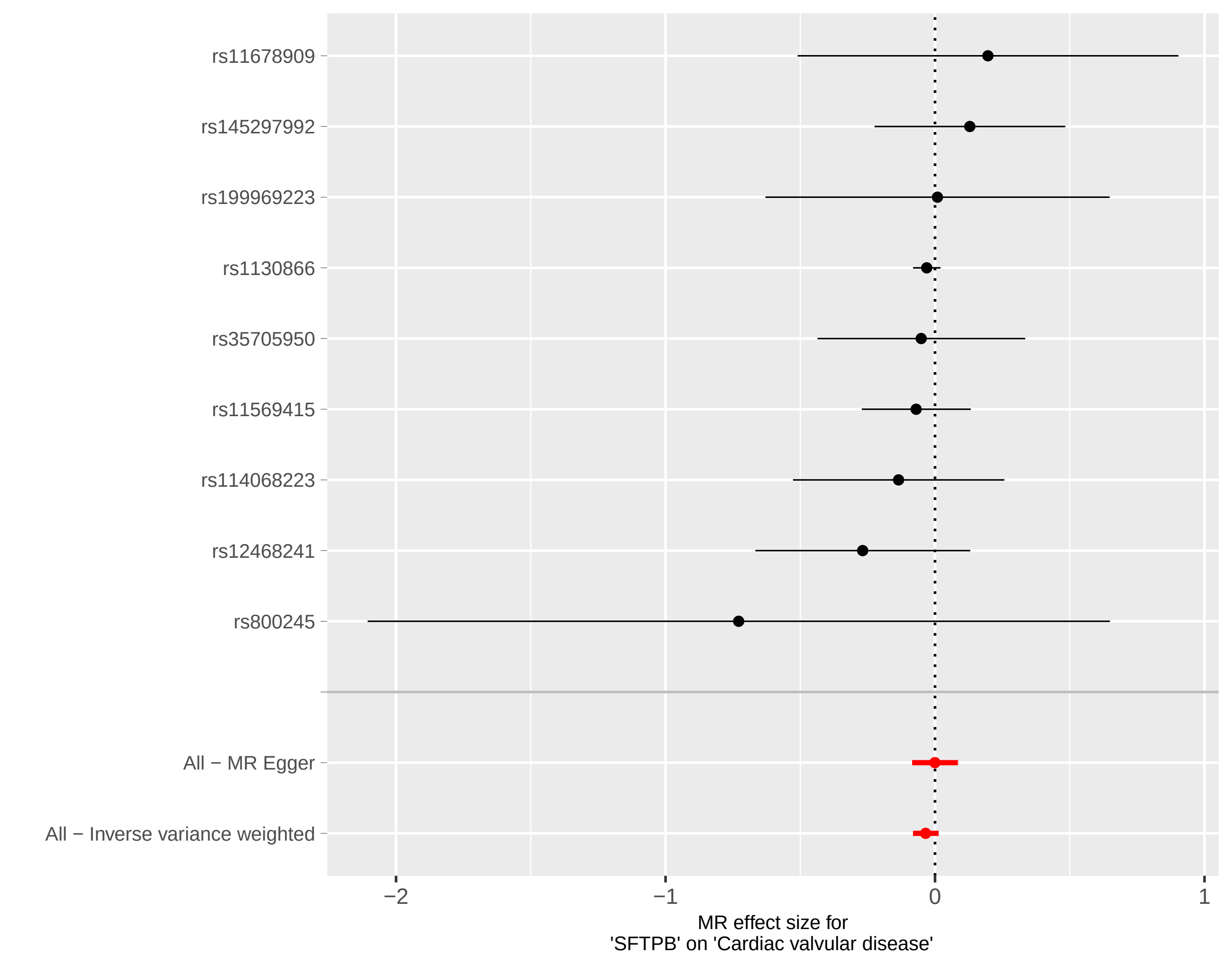
rs11678909
rs145297992
rs199969223
rs1130866
rs35705950
rs11569415
rs114068223
rs12468241
rs800245
All − MR Egger
All − Inverse variance weighted
−2
−1
0
1
MR effect size for
'SFTPB' on 'Cardiac valvular disease'

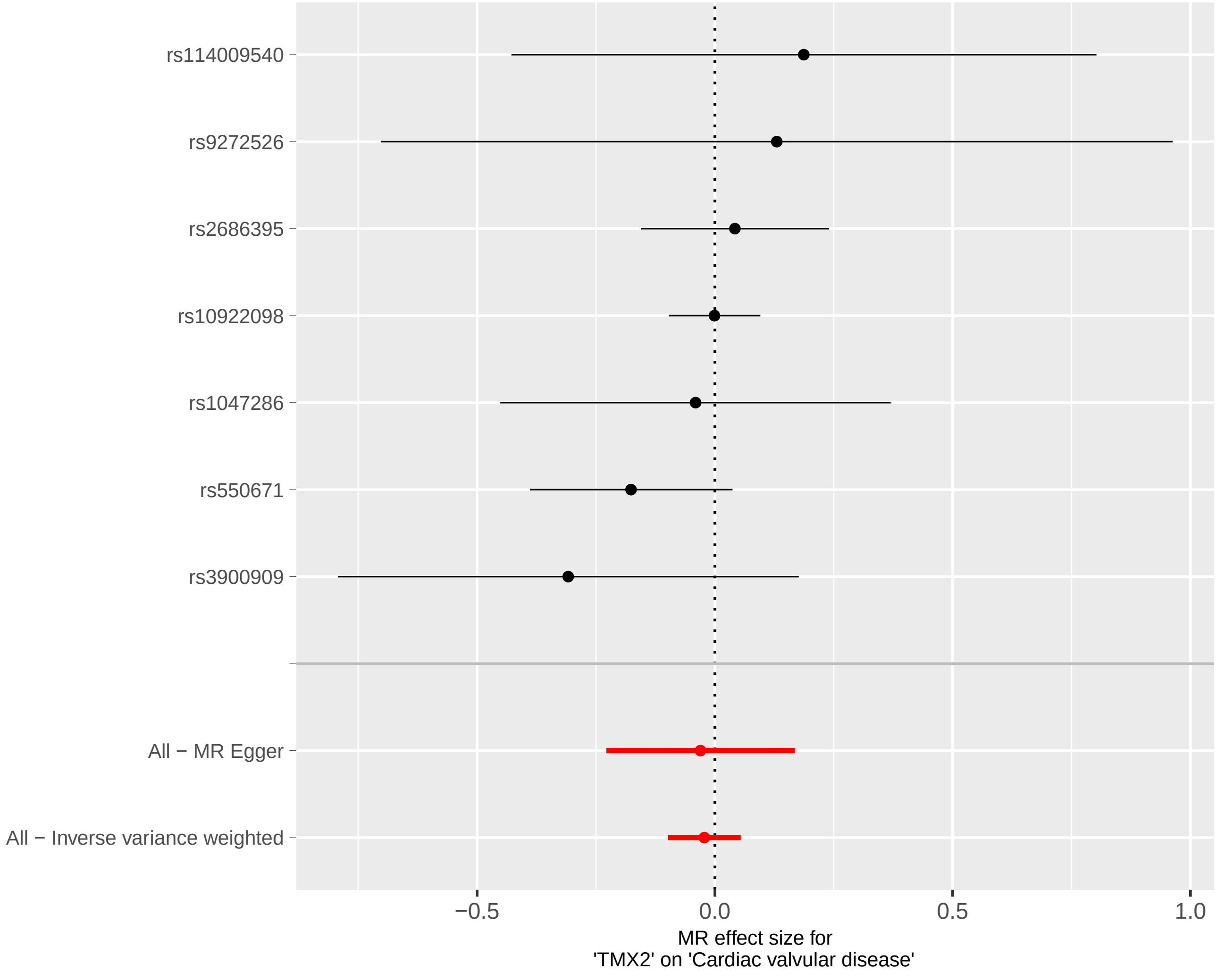

rs114009540
rs9272526
rs2686395
rs10922098
rs1047286
rs550671
rs3900909
All − MR Egger
All − Inverse variance weighted
−0.5
0.0
0.5
1.0
MR effect size for
'TMX2' on 'Cardiac valvular disease'

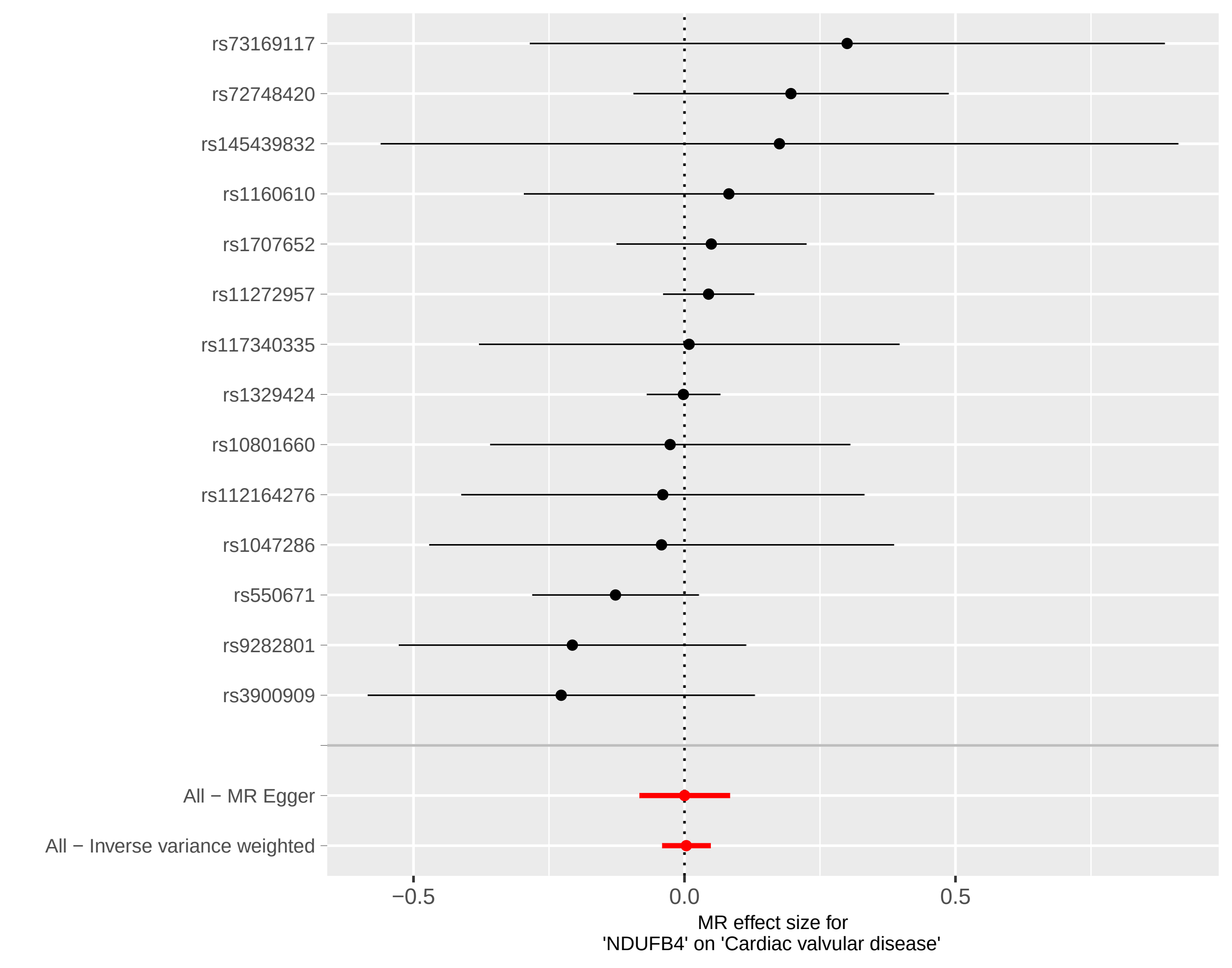
rs73169117
rs72748420
rs145439832
rs1160610
rs1707652
rs11272957
rs117340335
rs1329424
rs10801660
rs112164276
rs1047286
rs550671
rs9282801
rs3900909
All − MR Egger
All − Inverse variance weighted
−0.5
0.0
0.5
MR effect size for
'NDUFB4' on 'Cardiac valvular disease'

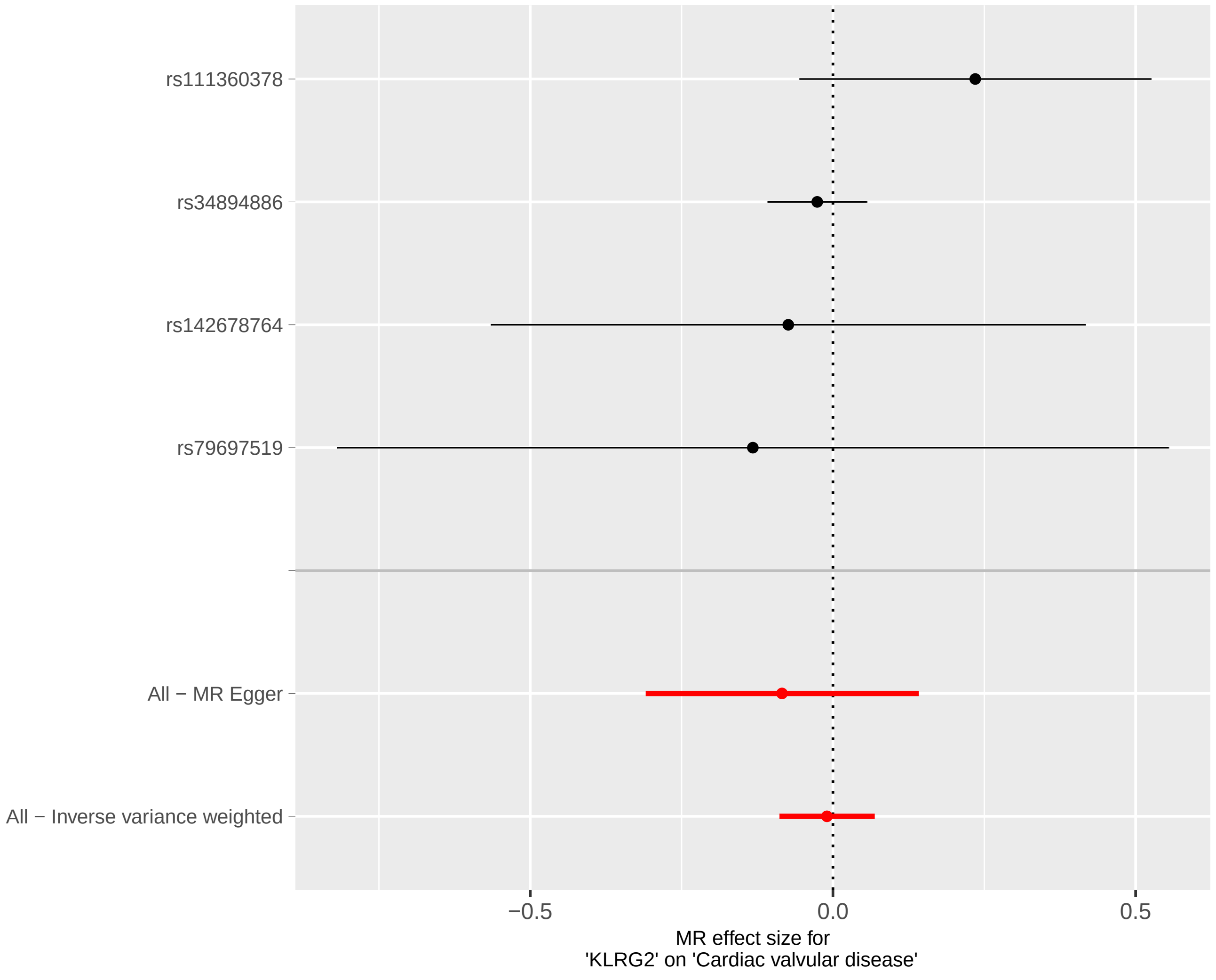
rs111360378
rs34894886
rs142678764
rs79697519
All − MR Egger
All − Inverse variance weighted
−0.5
0.0
0.5
MR effect size for
'KLRG2' on 'Cardiac valvular disease'

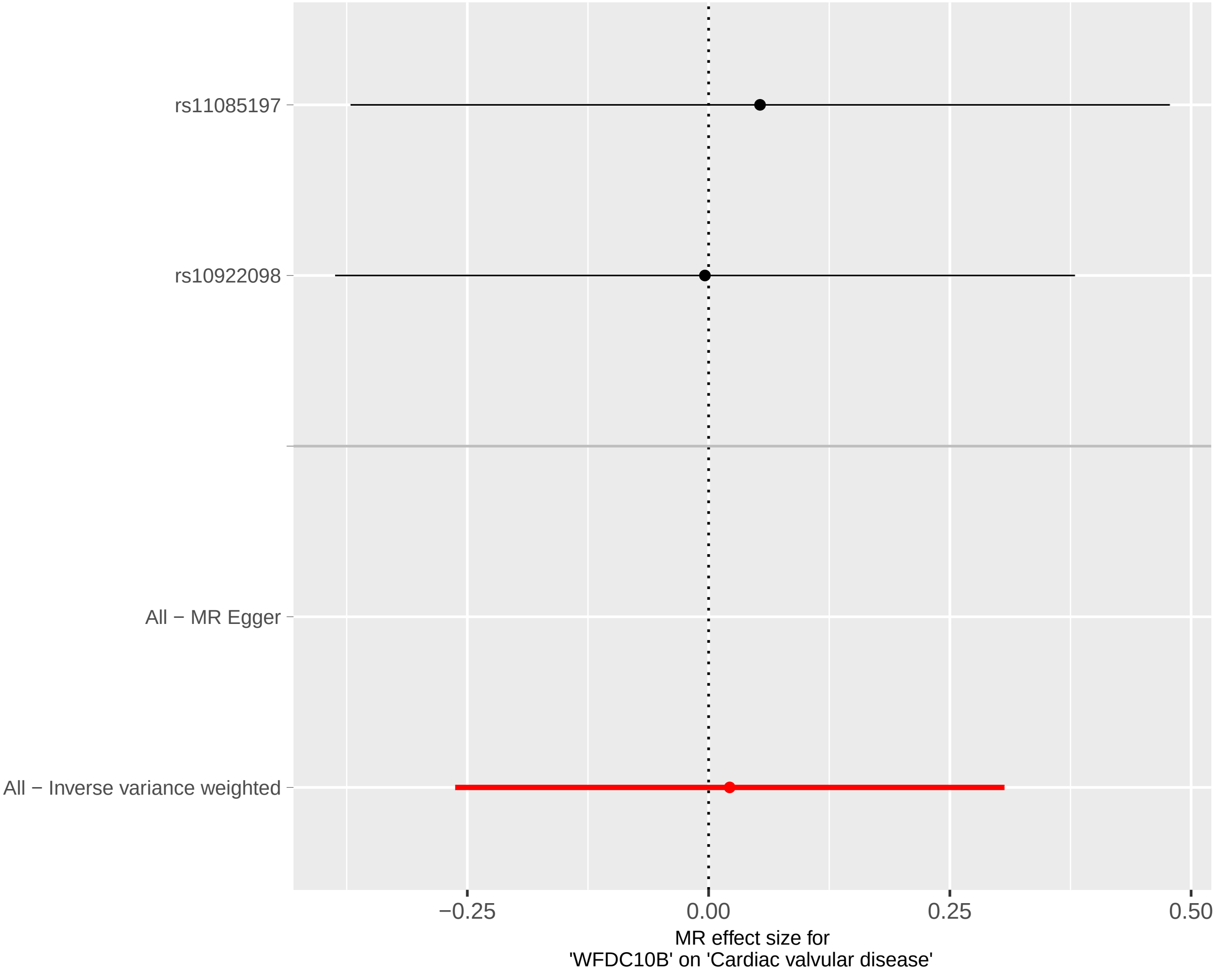
rs11085197
rs10922098
All − MR Egger
All − Inverse variance weighted
−0.25
0.00
0.25
0.50
MR effect size for
'WFDC10B' on 'Cardiac valvular disease'

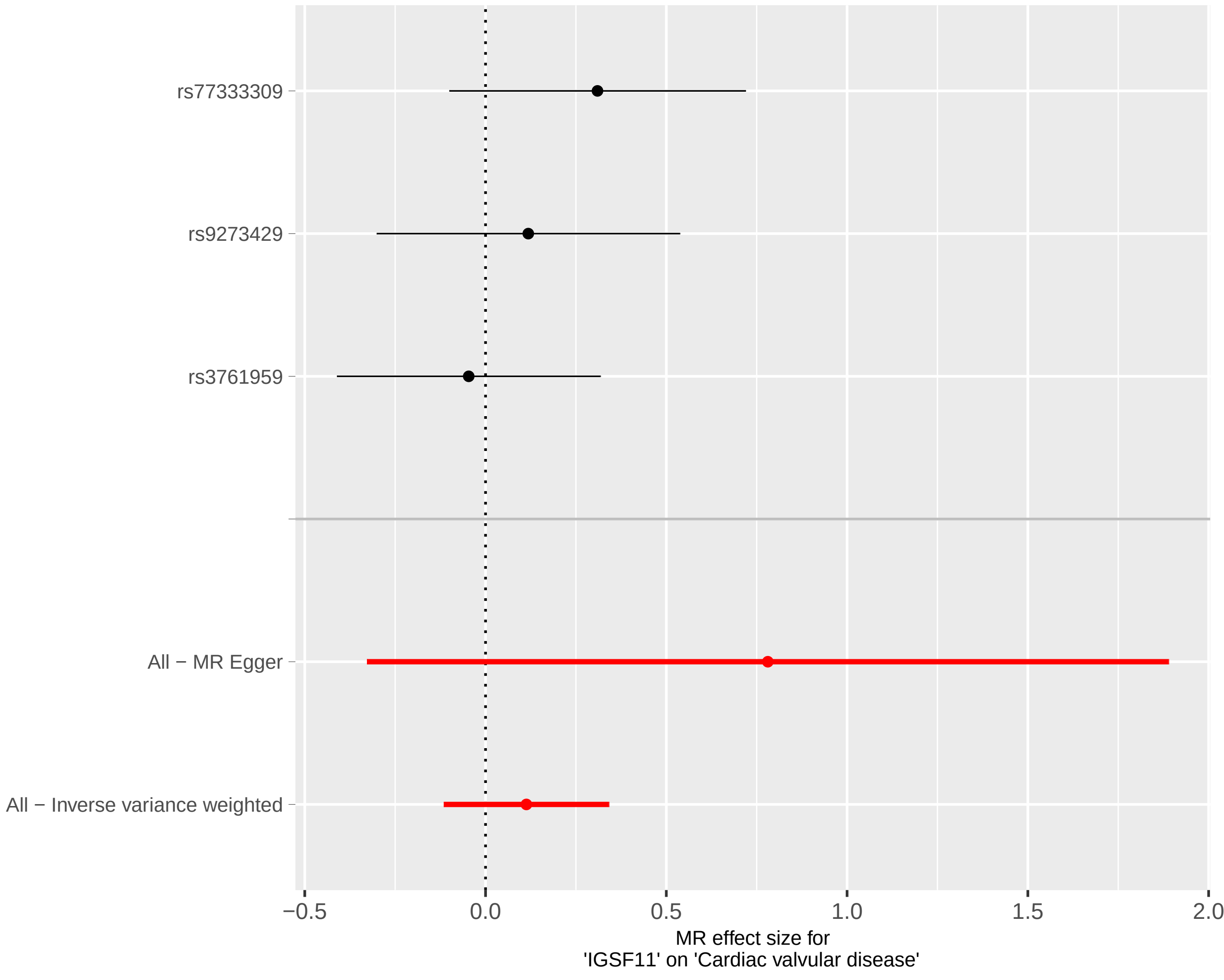
rs77333309
rs9273429
rs3761959
All − MR Egger
All − Inverse variance weighted
−0.5
0.0
0.5
1.0
1.5
2.0
MR effect size for
'IGSF11' on 'Cardiac valvular disease'

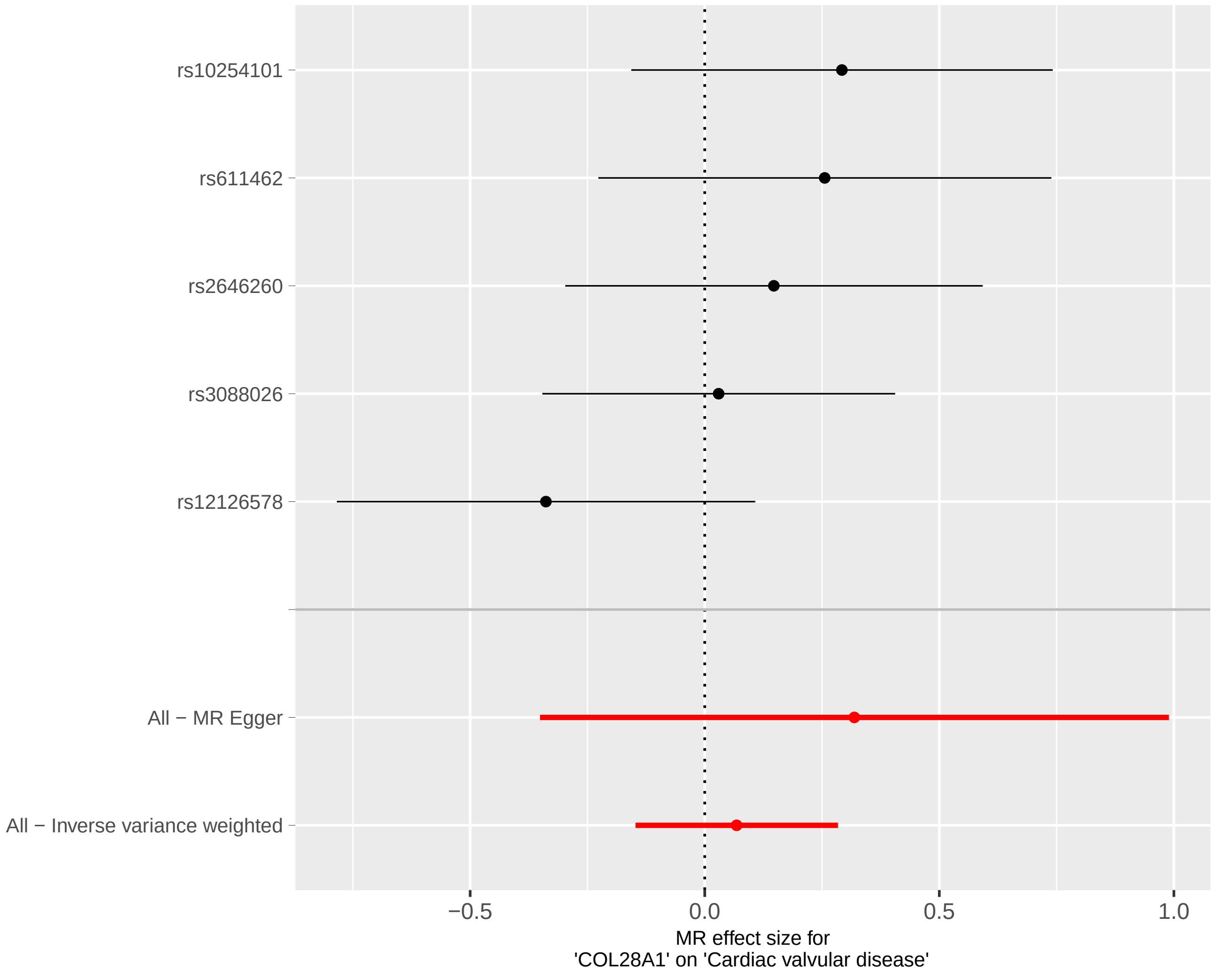
rs10254101
rs611462
rs2646260
rs3088026
rs12126578
All − MR Egger
All − Inverse variance weighted
−0.5
0.0
0.5
1.0
MR effect size for
'COL28A1' on 'Cardiac valvular disease'

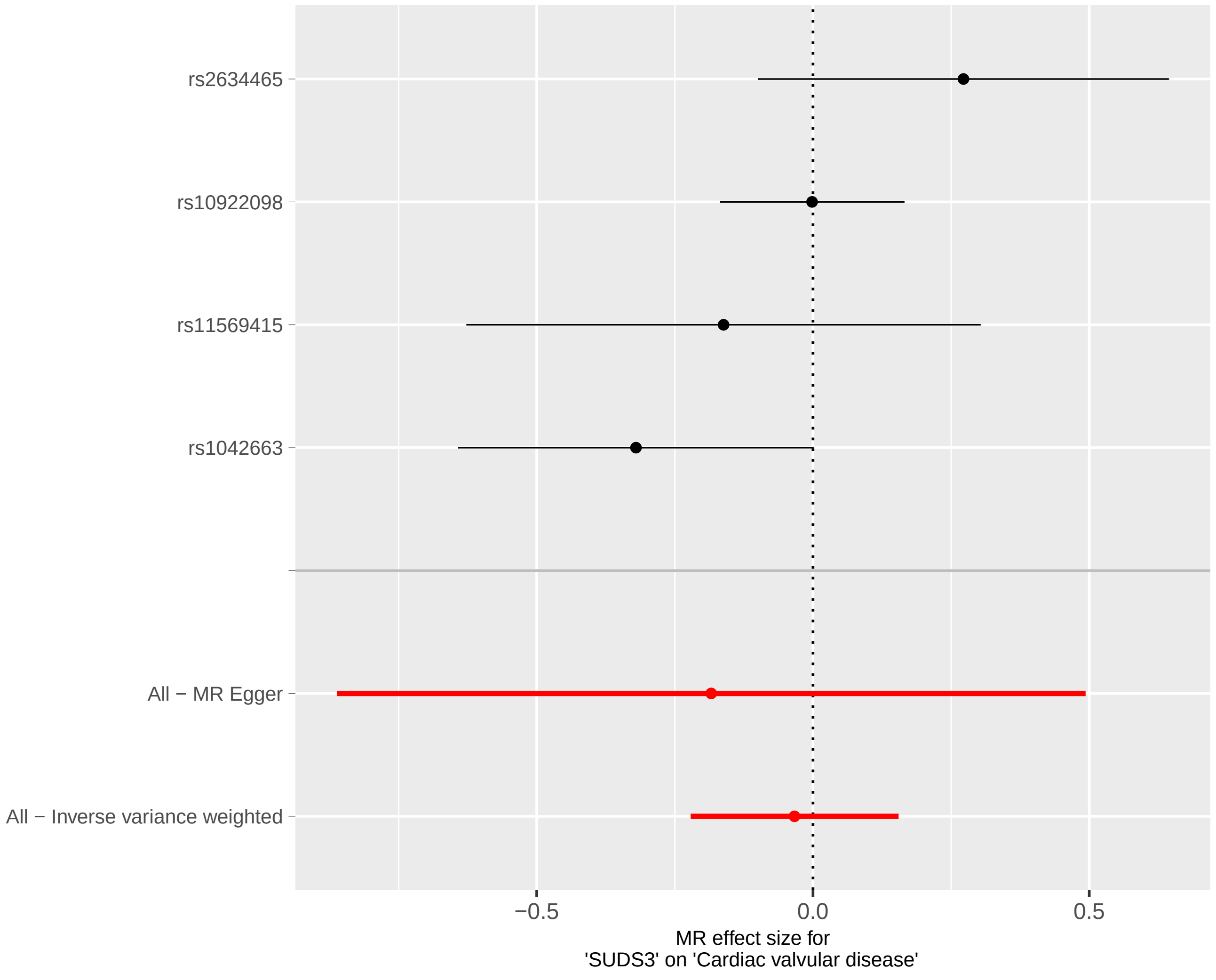
rs2634465
rs10922098
rs11569415
rs1042663
All − MR Egger
All − Inverse variance weighted
−0.5
0.0
0.5
MR effect size for
'SUDS3' on 'Cardiac valvular disease'

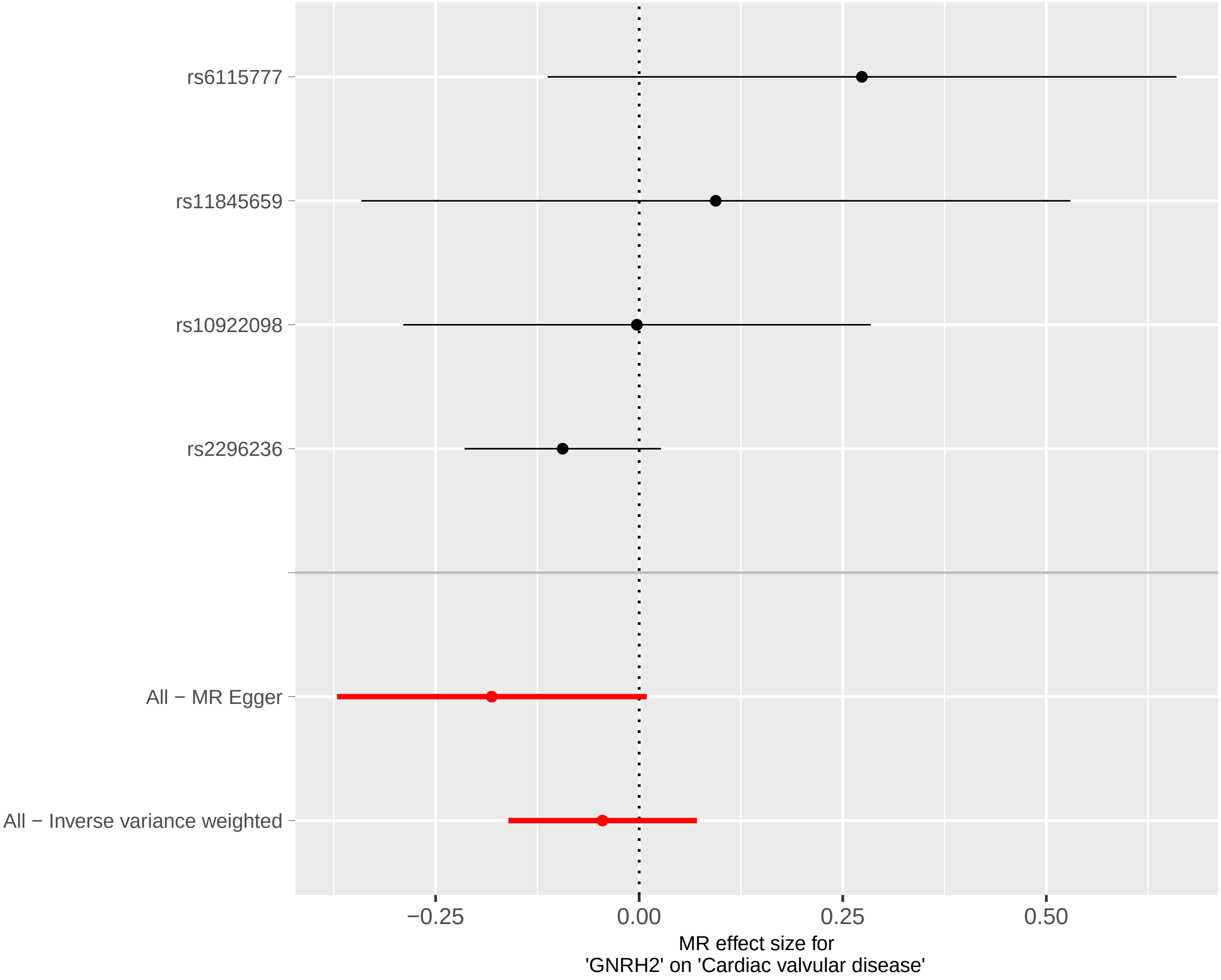

rs6115777
rs11845659
rs10922098
rs2296236
All − MR Egger
All − Inverse variance weighted
−0.25
0.00
0.25
0.50
MR effect size for
'GNRH2' on 'Cardiac valvular disease'

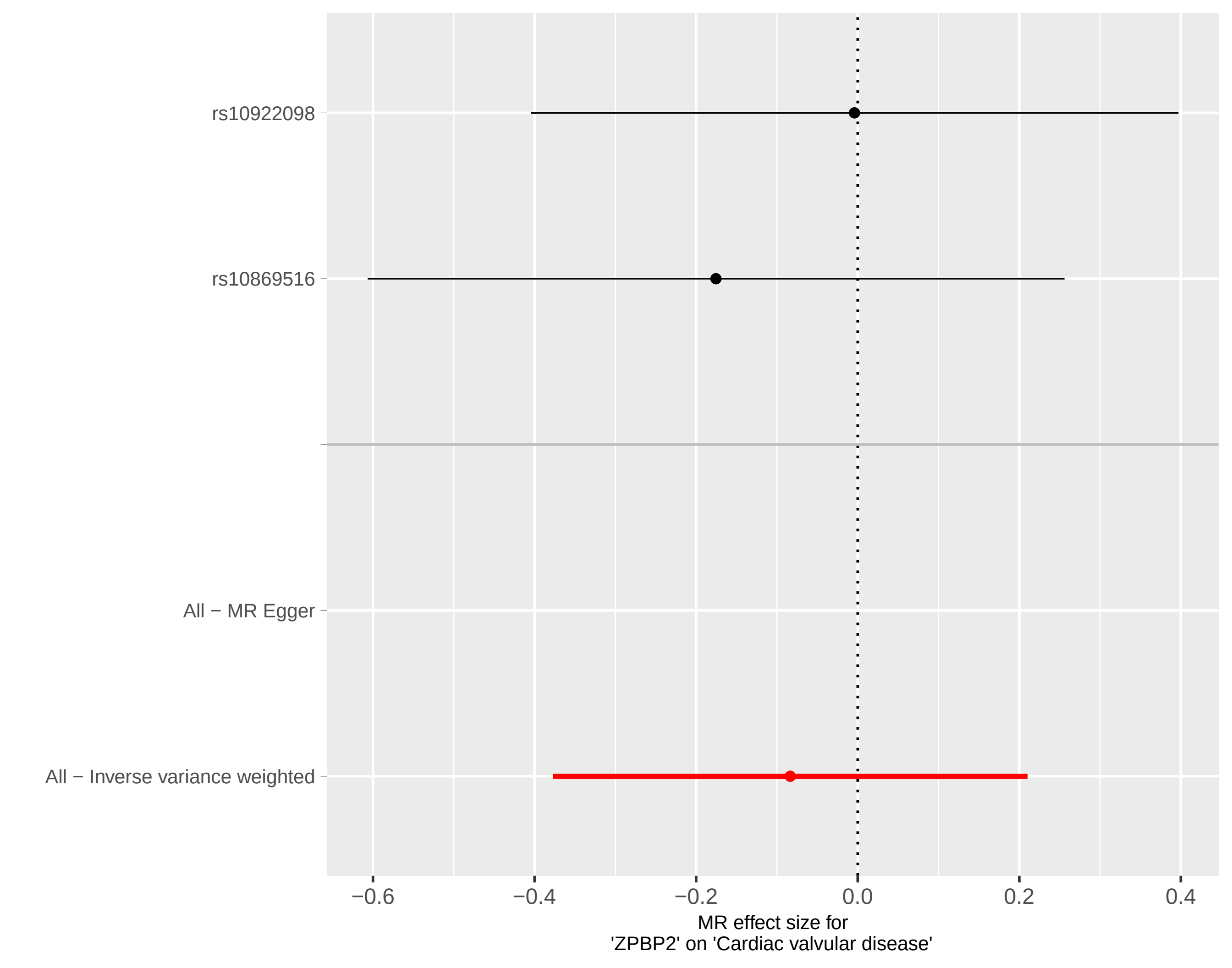
rs10922098
rs10869516
All − MR Egger
All − Inverse variance weighted
−0.6
−0.4
−0.2
0.0
0.2
0.4
MR effect size for
'ZPBP2' on 'Cardiac valvular disease'

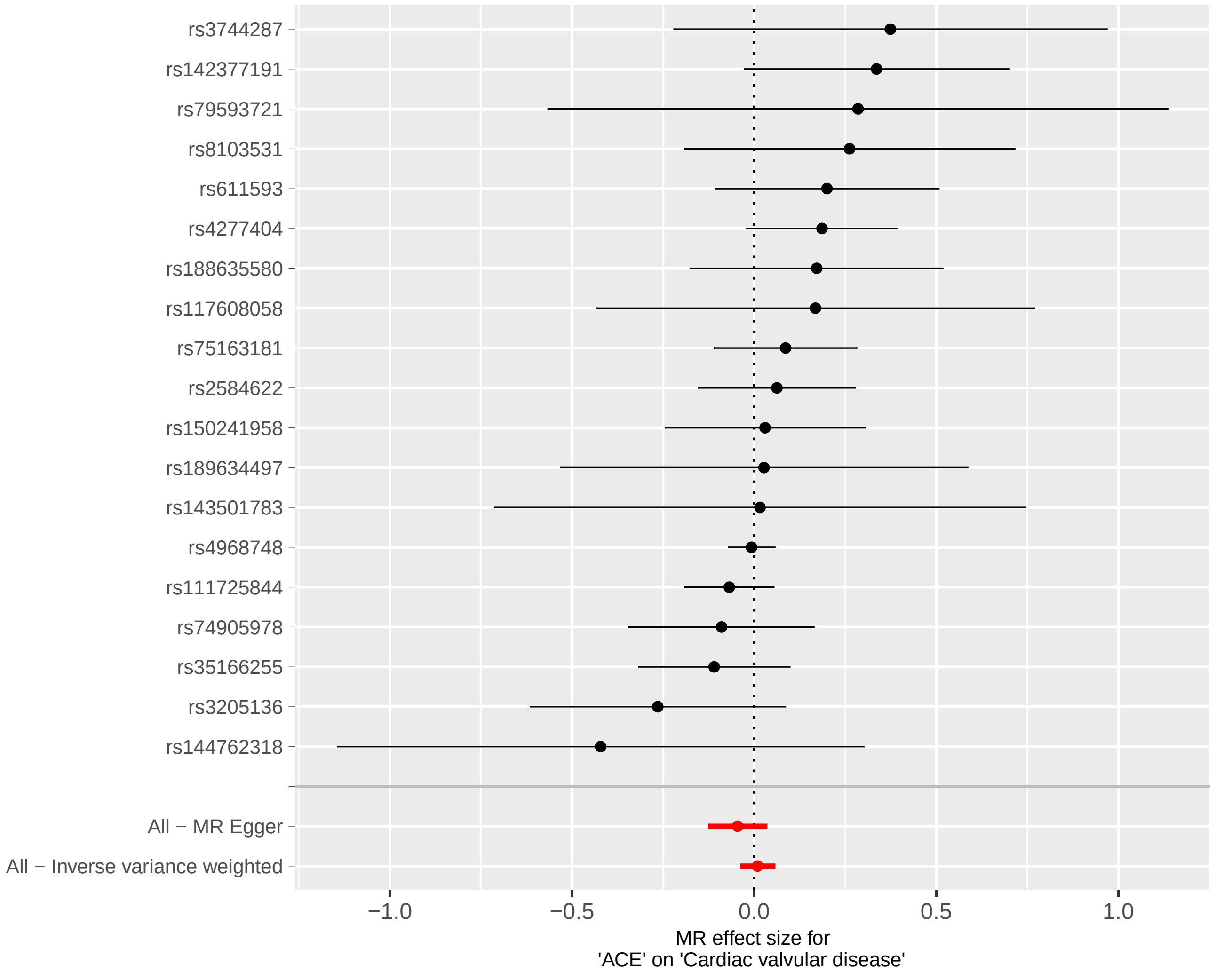

rs3744287
rs142377191
rs79593721
rs8103531
rs611593
rs4277404
rs188635580
rs117608058
rs75163181
rs2584622
rs150241958
rs189634497
rs143501783
rs4968748
rs111725844
rs74905978
rs35166255
rs3205136
rs144762318
All − MR Egger
All − Inverse variance weighted
−1.0
−0.5
0.0
0.5
1.0
MR effect size for
'ACE' on 'Cardiac valvular disease'

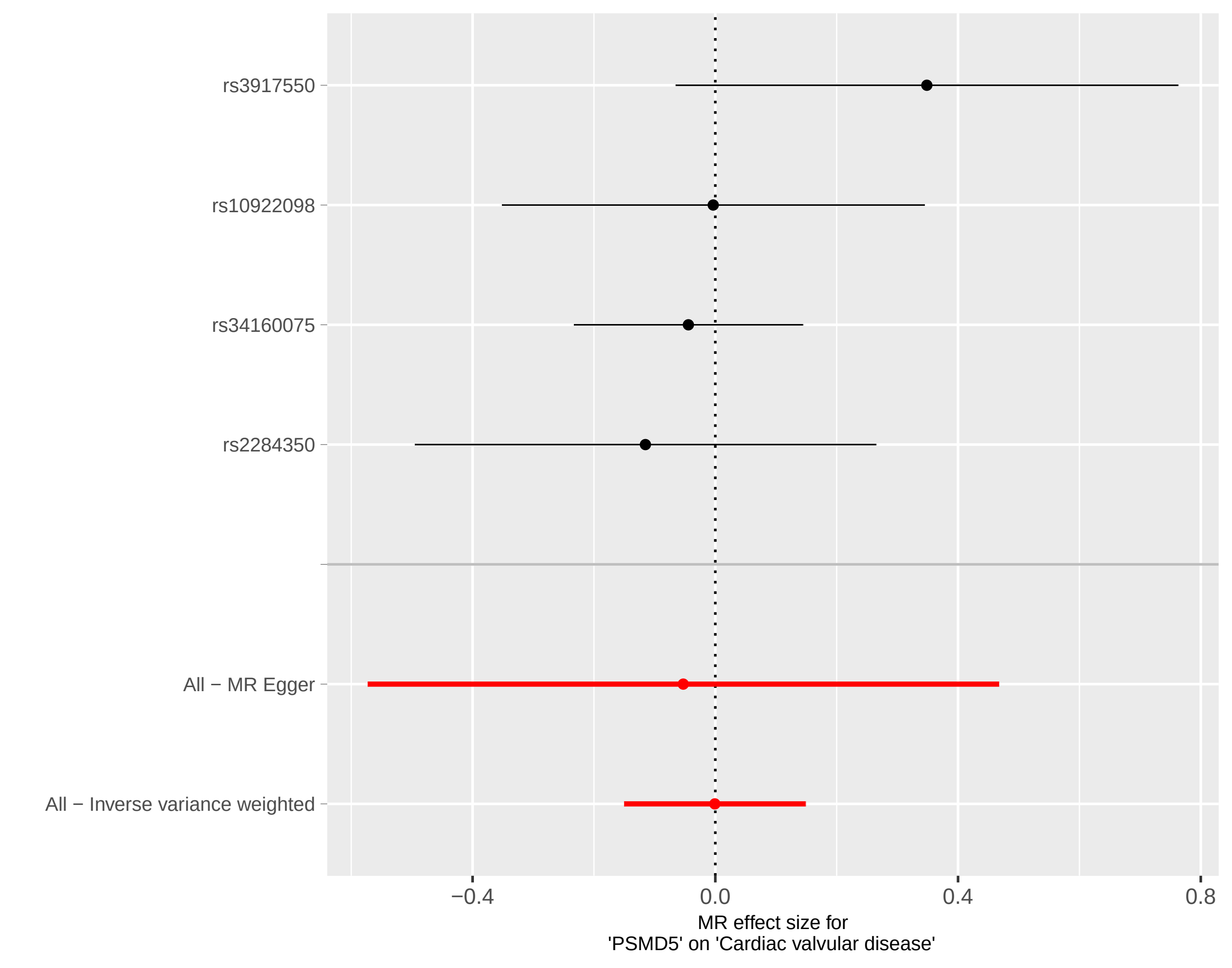
rs3917550
rs10922098
rs34160075
rs2284350
All − MR Egger
All − Inverse variance weighted
−0.4
0.0
0.4
0.8
MR effect size for
'PSMD5' on 'Cardiac valvular disease'

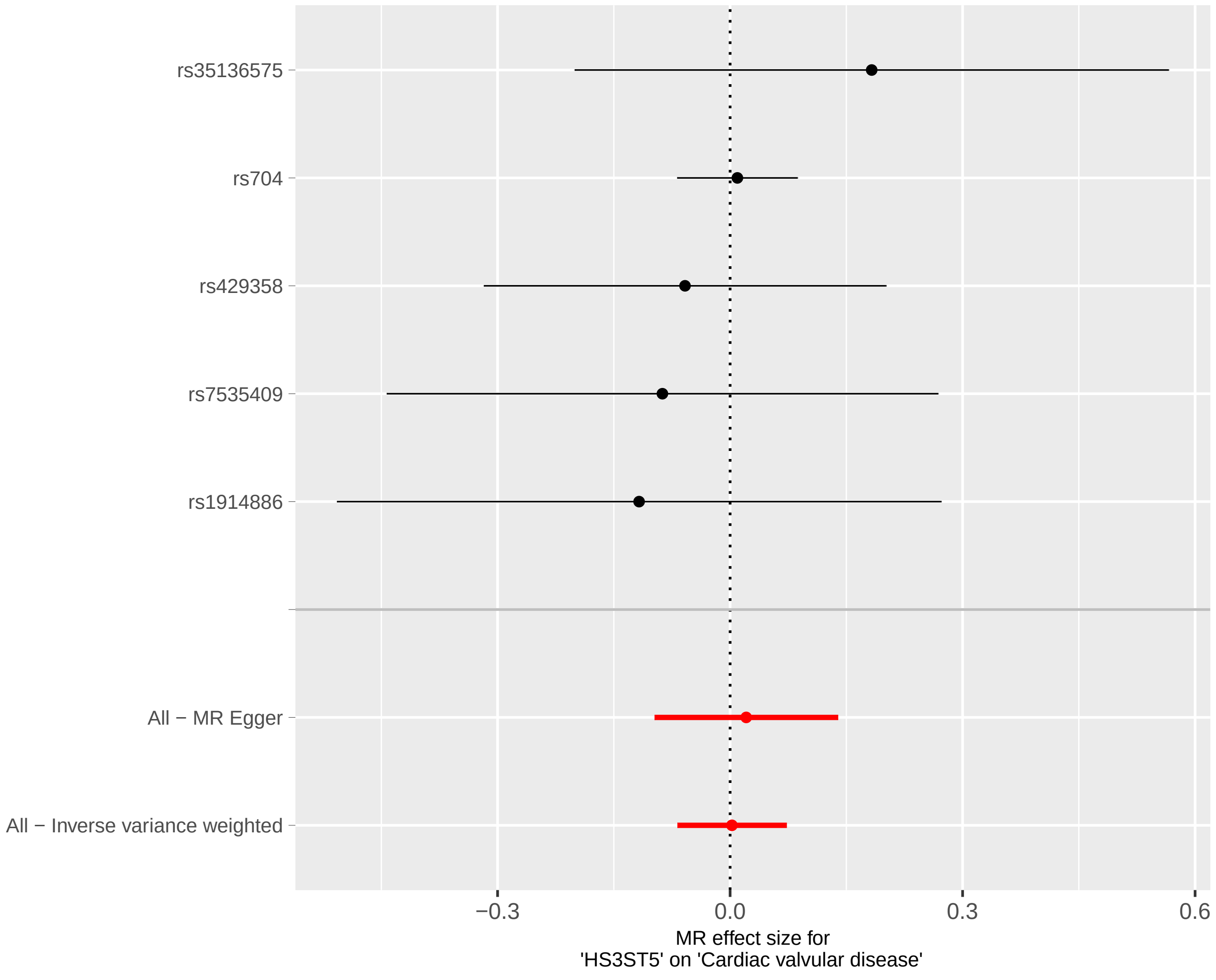
rs35136575
rs704
rs429358
rs7535409
rs1914886
All − MR Egger
All − Inverse variance weighted
−0.3
0.0
0.3
0.6
MR effect size for
'HS3ST5' on 'Cardiac valvular disease'

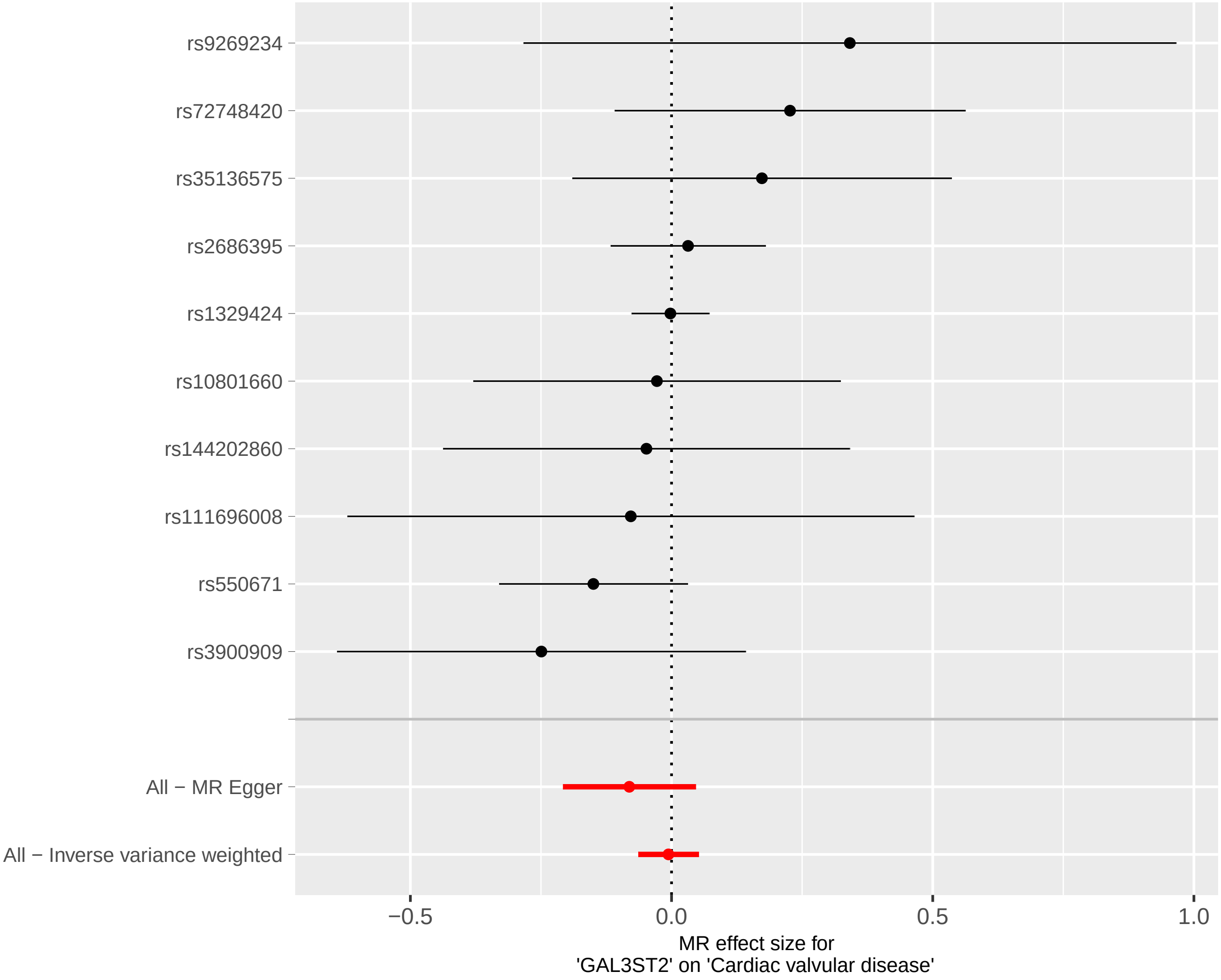
rs9269234
rs72748420
rs35136575
rs2686395
rs1329424
rs10801660
rs144202860
rs111696008
rs550671
rs3900909
All − MR Egger
All − Inverse variance weighted
−0.5
0.0
0.5
1.0
MR effect size for
'GAL3ST2' on 'Cardiac valvular disease'

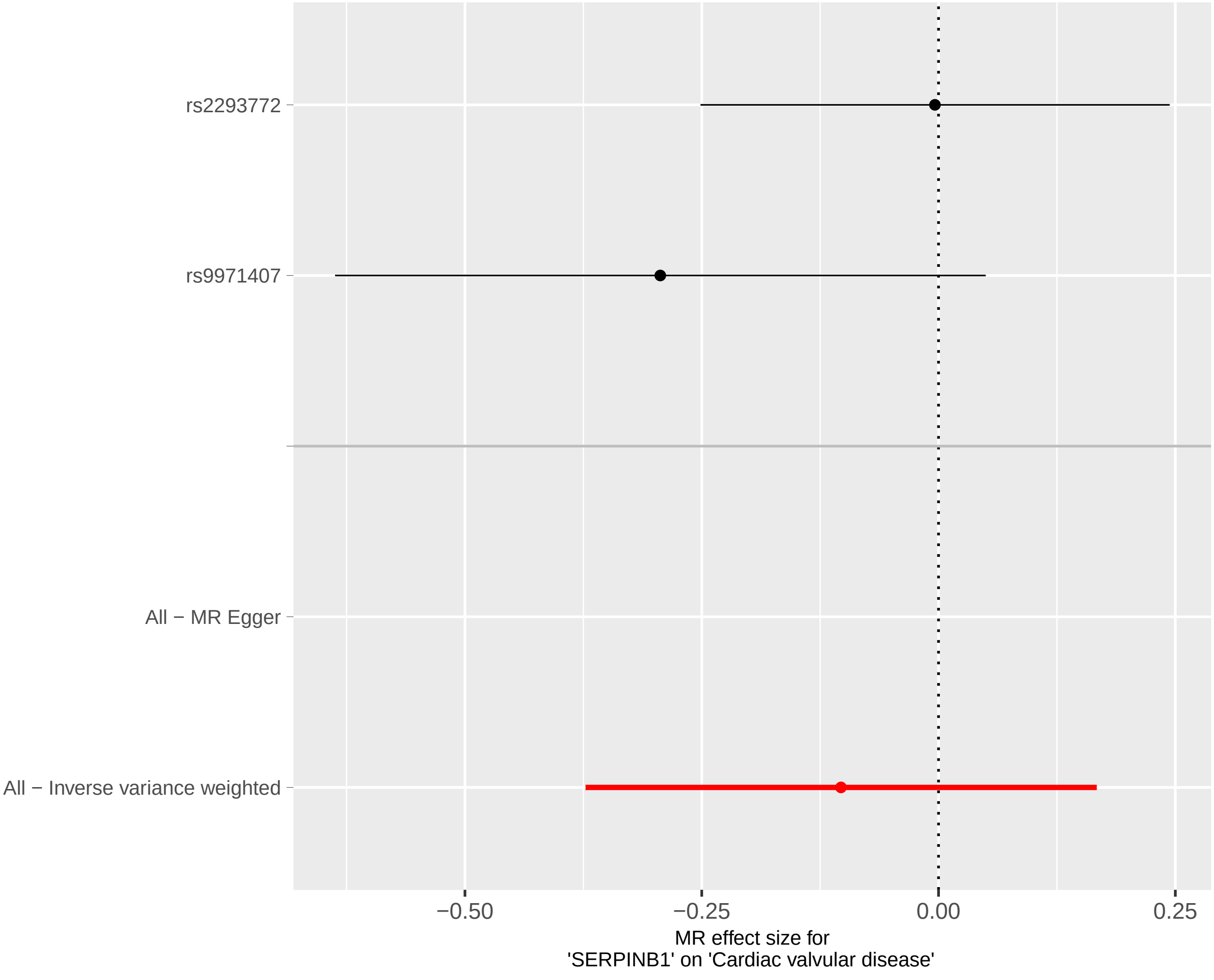
rs2293772
rs9971407
All − MR Egger
All − Inverse variance weighted
−0.50
−0.25
0.00
0.25
MR effect size for
'SERPINB1' on 'Cardiac valvular disease'

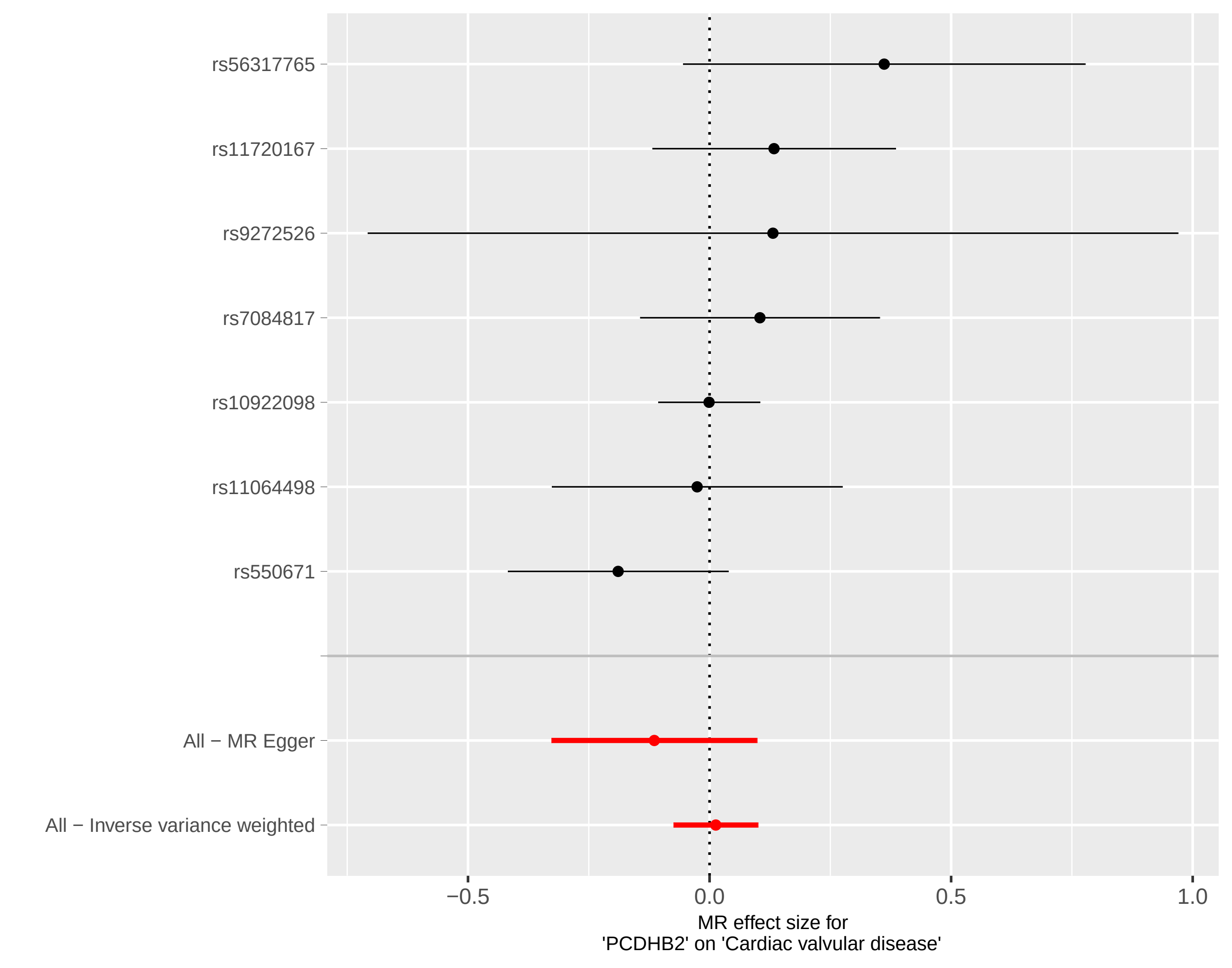
rs56317765
rs11720167
rs9272526
rs7084817
rs10922098
rs11064498
rs550671
All − MR Egger
All − Inverse variance weighted
−0.5
0.0
0.5
1.0
MR effect size for
'PCDHB2' on 'Cardiac valvular disease'

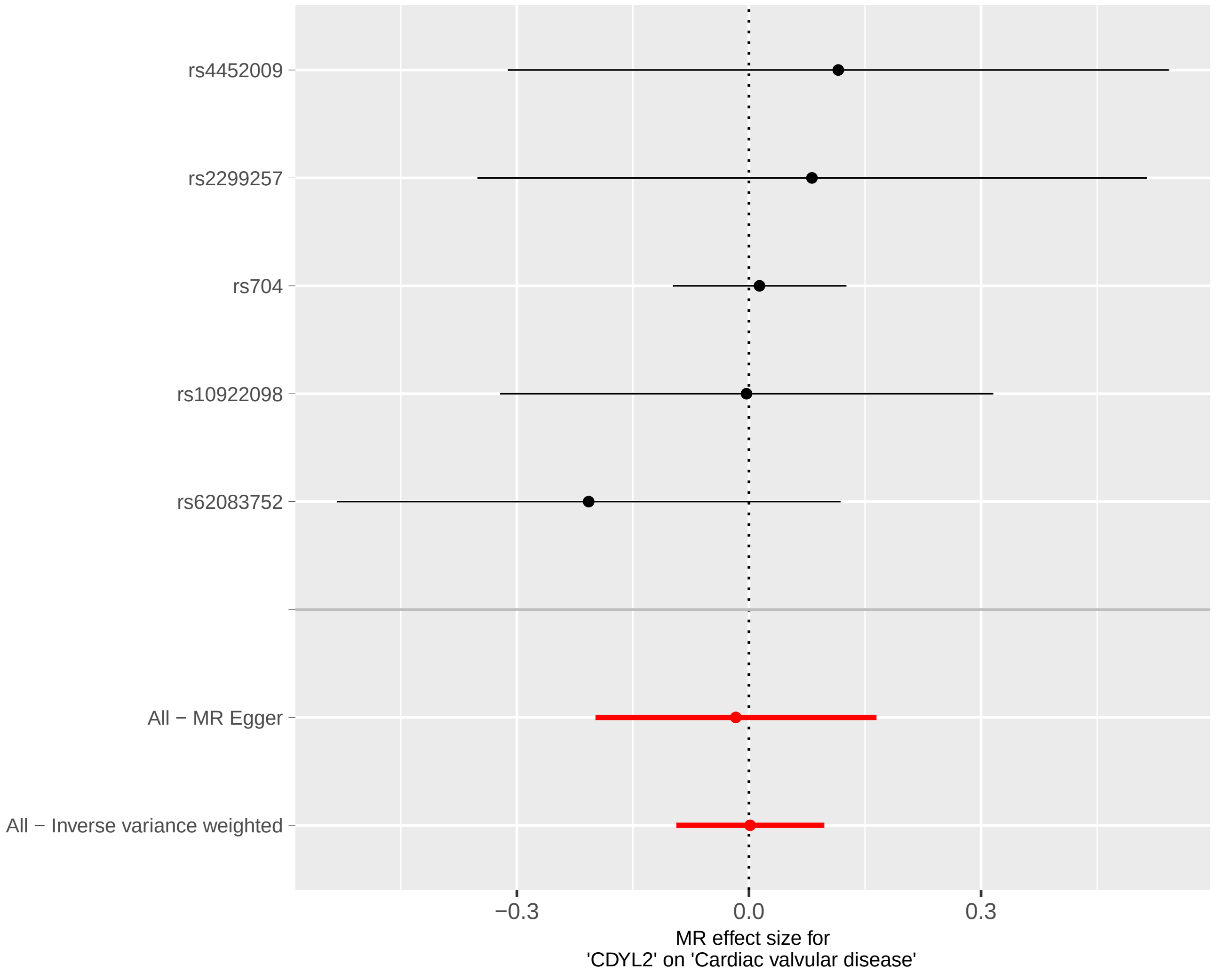
rs4452009
rs2299257
rs704
rs10922098
rs62083752
All − MR Egger
All − Inverse variance weighted
−0.3
0.0
0.3
MR effect size for
'CDYL2' on 'Cardiac valvular disease'

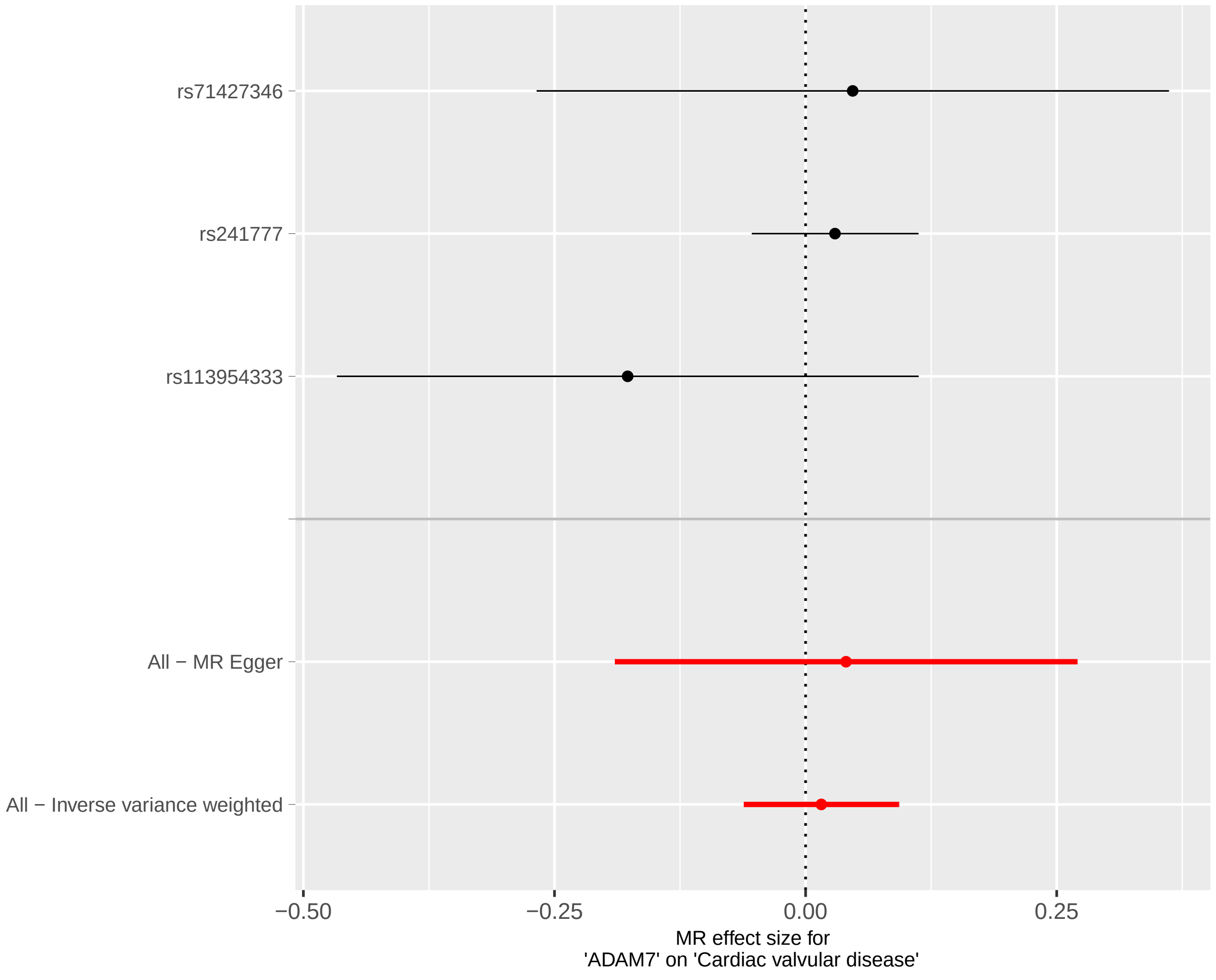
rs71427346
rs241777
rs113954333
All − MR Egger
All − Inverse variance weighted
−0.50
−0.25
0.00
0.25
MR effect size for
'ADAM7' on 'Cardiac valvular disease'

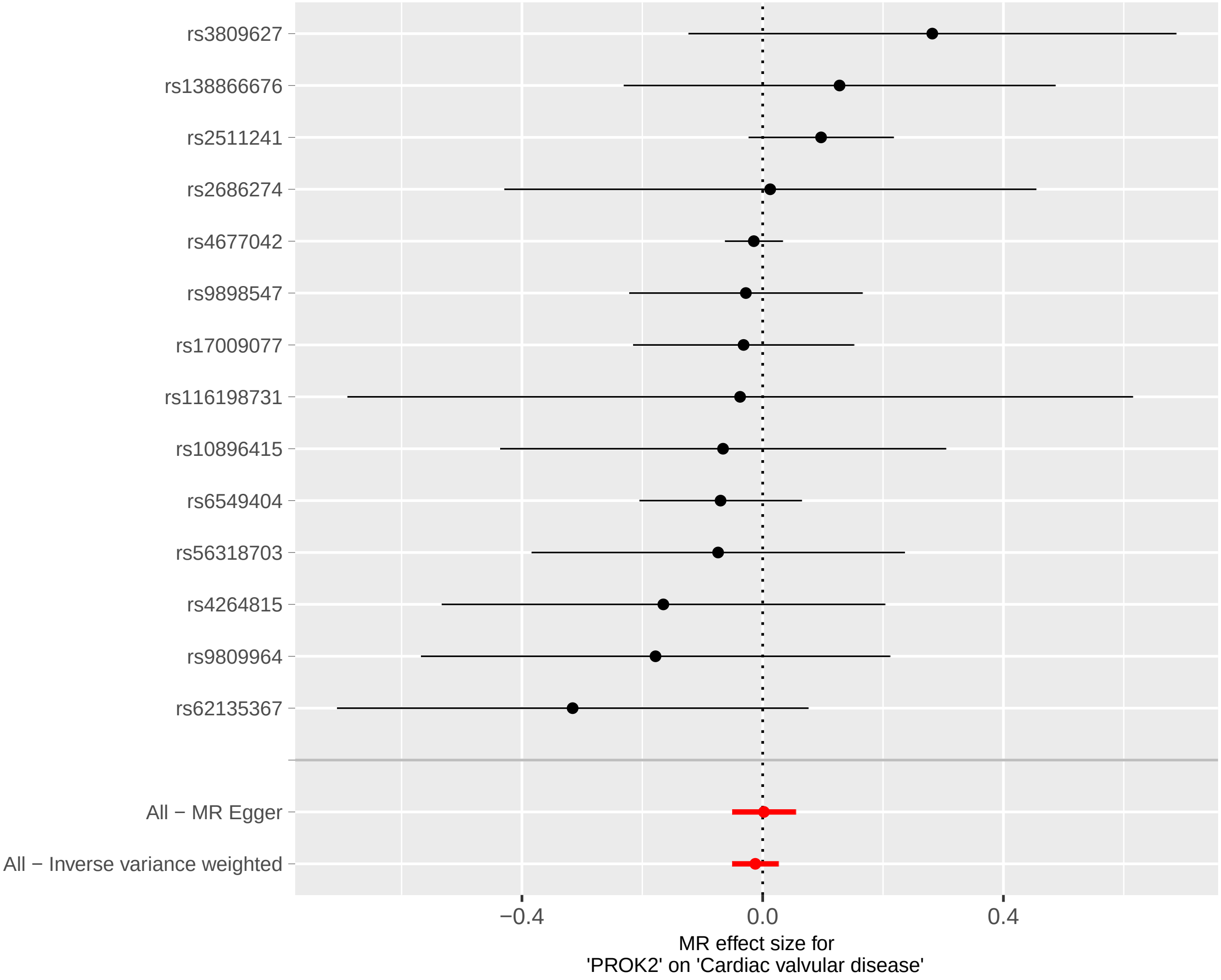

rs3809627
rs138866676
rs2511241
rs2686274
rs4677042
rs9898547
rs17009077
rs116198731
rs10896415
rs6549404
rs56318703
rs4264815
rs9809964
rs62135367
All − MR Egger
All − Inverse variance weighted
−0.4
0.0
0.4
MR effect size for
'PROK2' on 'Cardiac valvular disease'

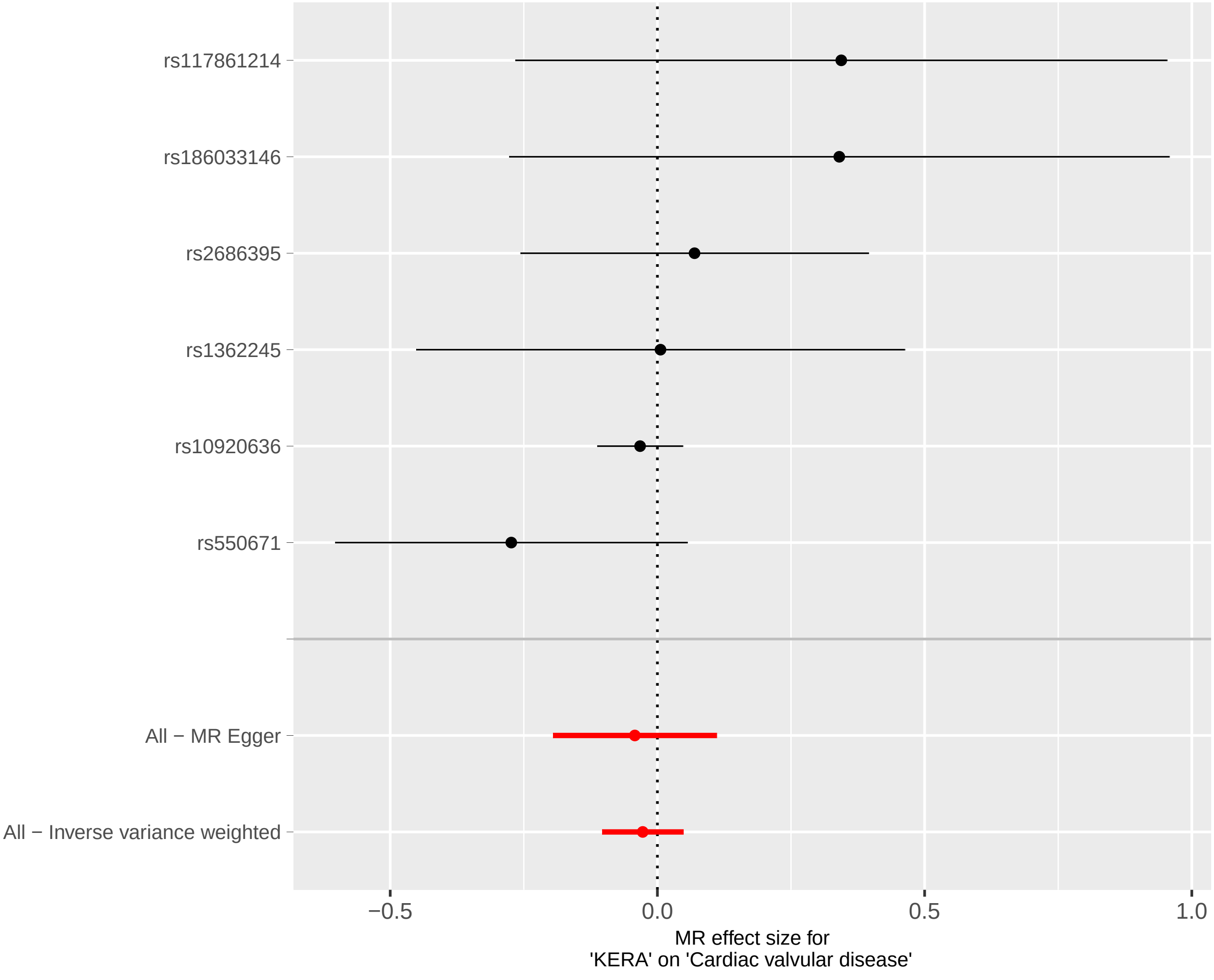
rs117861214
rs186033146
rs2686395
rs1362245
rs10920636
rs550671
All − MR Egger
All − Inverse variance weighted
−0.5
0.0
0.5
1.0
MR effect size for
'KERA' on 'Cardiac valvular disease'

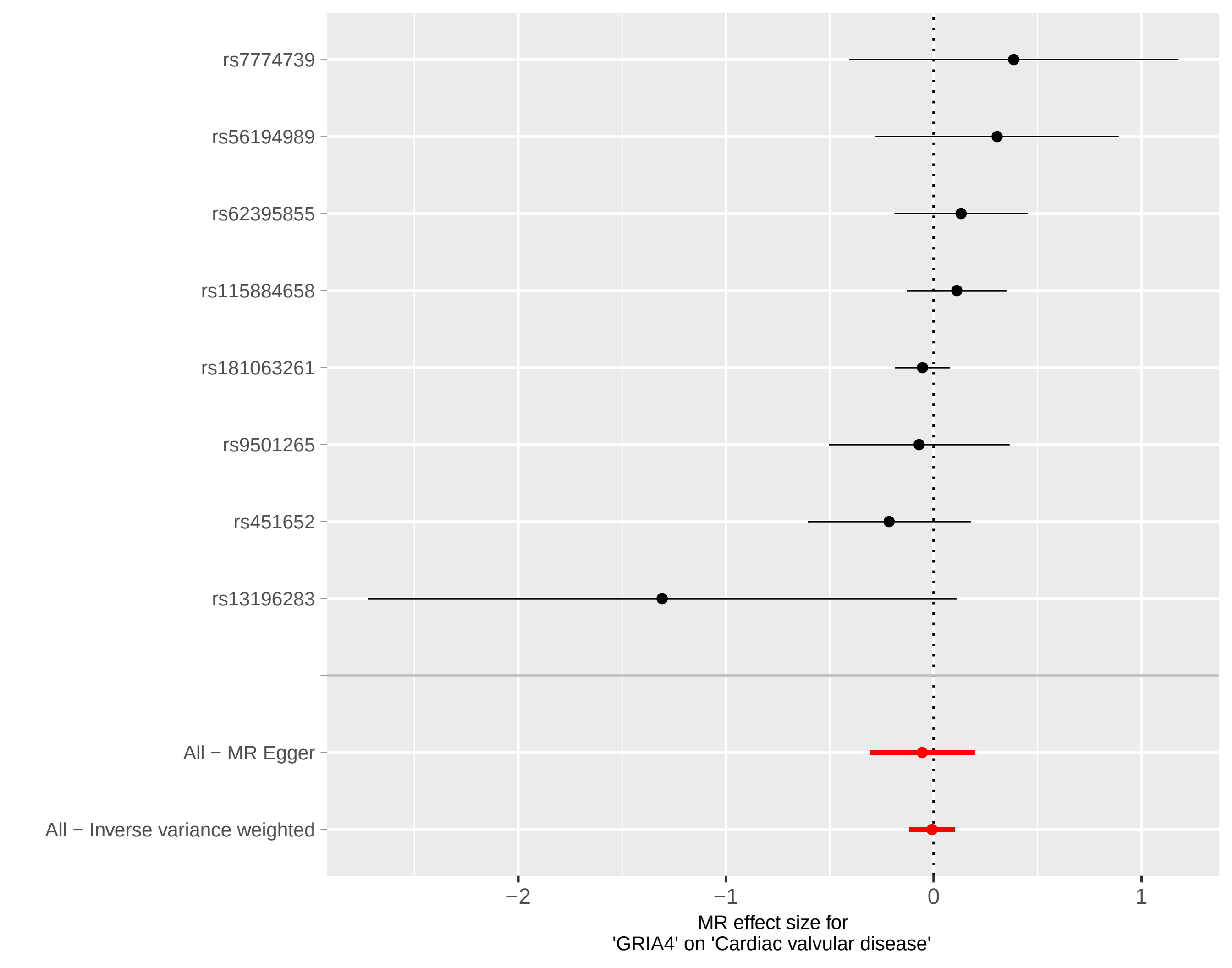
rs7774739
rs56194989
rs62395855
rs115884658
rs181063261
rs9501265
rs451652
rs13196283
All − MR Egger
All − Inverse variance weighted
−2
−1
0
1
MR effect size for
'GRIA4' on 'Cardiac valvular disease'

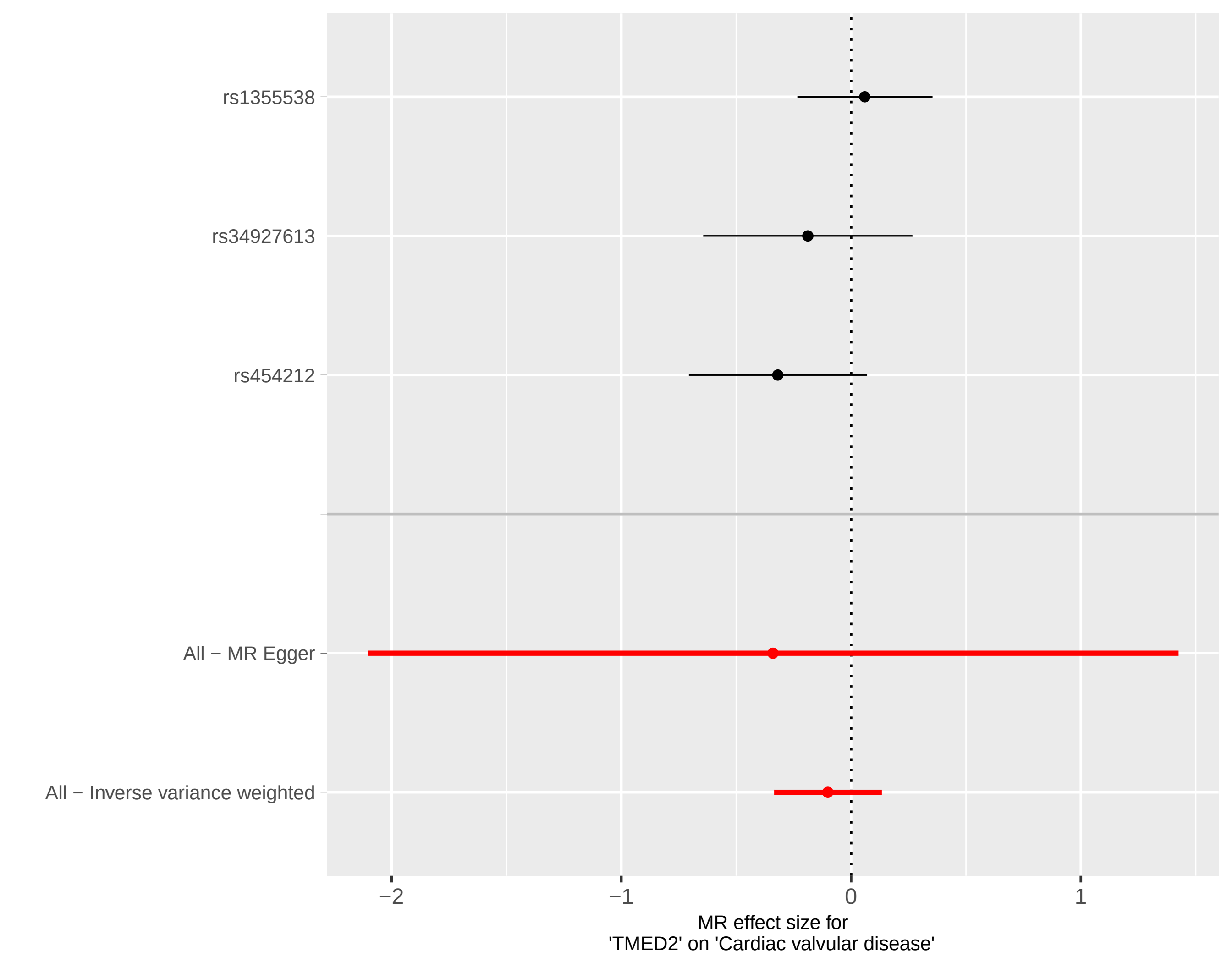
rs1355538
rs34927613
rs454212
All − MR Egger
All − Inverse variance weighted
−2
−1
0
1
MR effect size for
'TMED2' on 'Cardiac valvular disease'

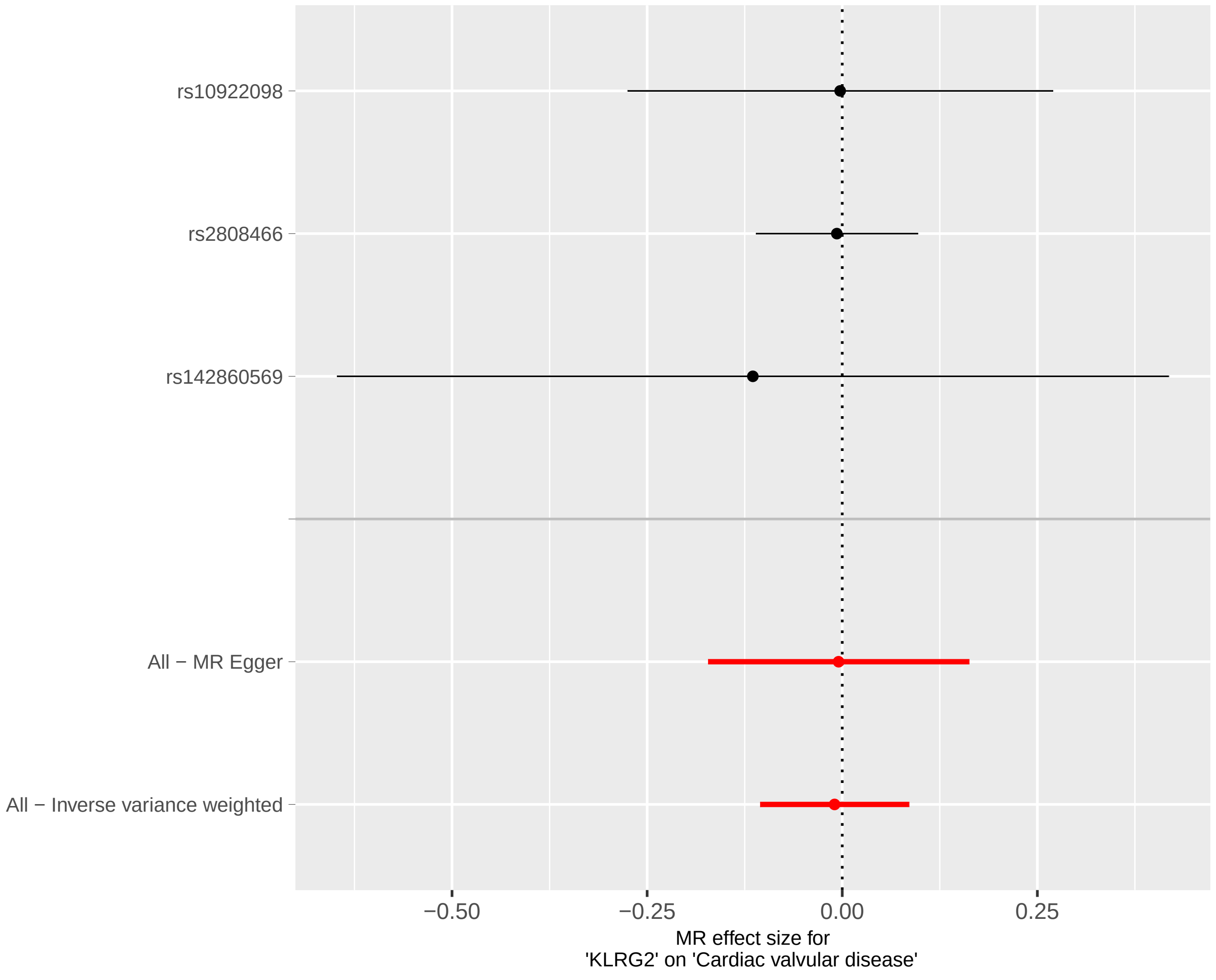
rs10922098
rs2808466
rs142860569
All − MR Egger
All − Inverse variance weighted
−0.50
−0.25
0.00
0.25
MR effect size for
'KLRG2' on 'Cardiac valvular disease'

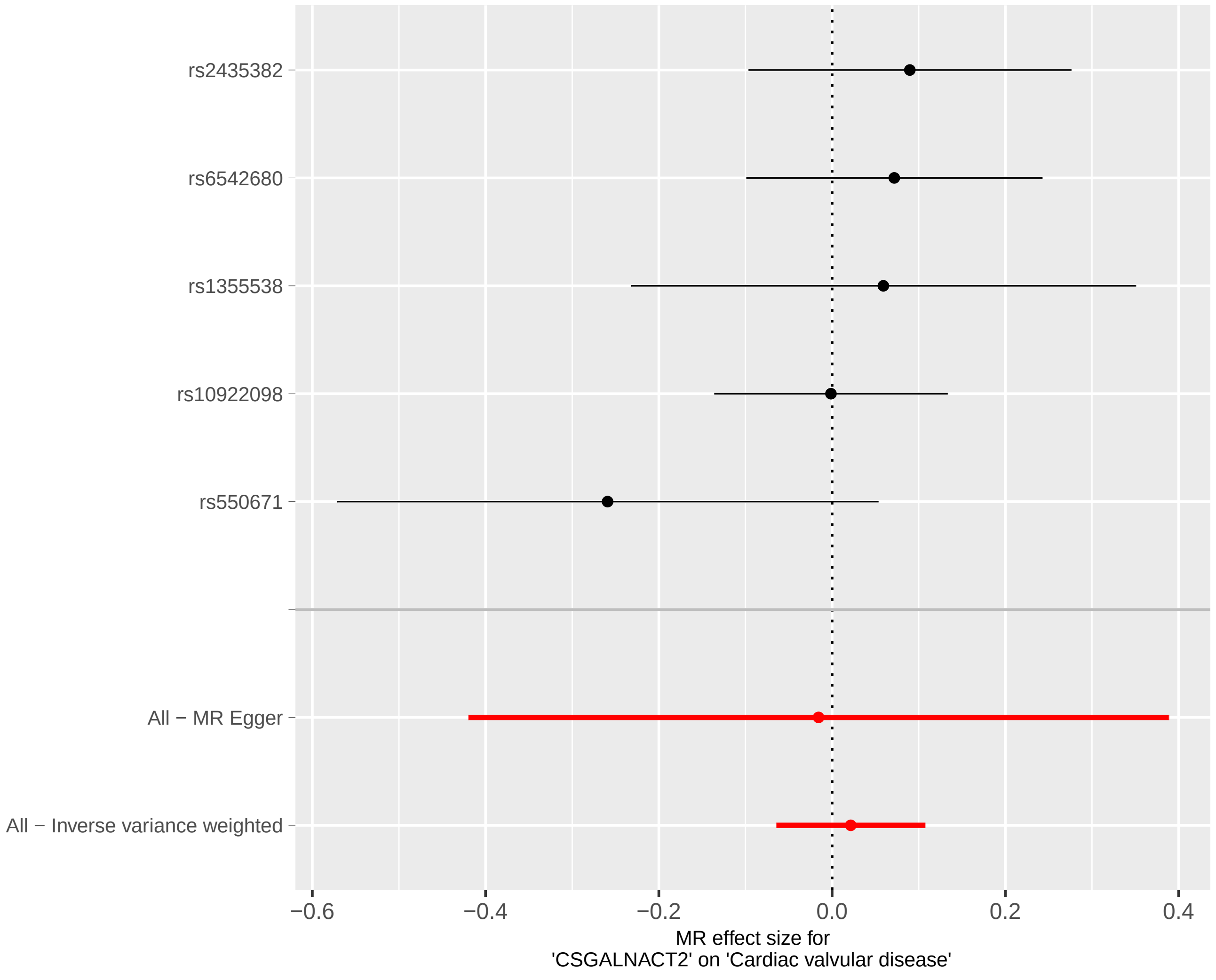
rs2435382
rs6542680
rs1355538
rs10922098
rs550671
All − MR Egger
All − Inverse variance weighted
−0.6
−0.4
−0.2
0.0
0.2
0.4
MR effect size for
'CSGALNACT2' on 'Cardiac valvular disease'

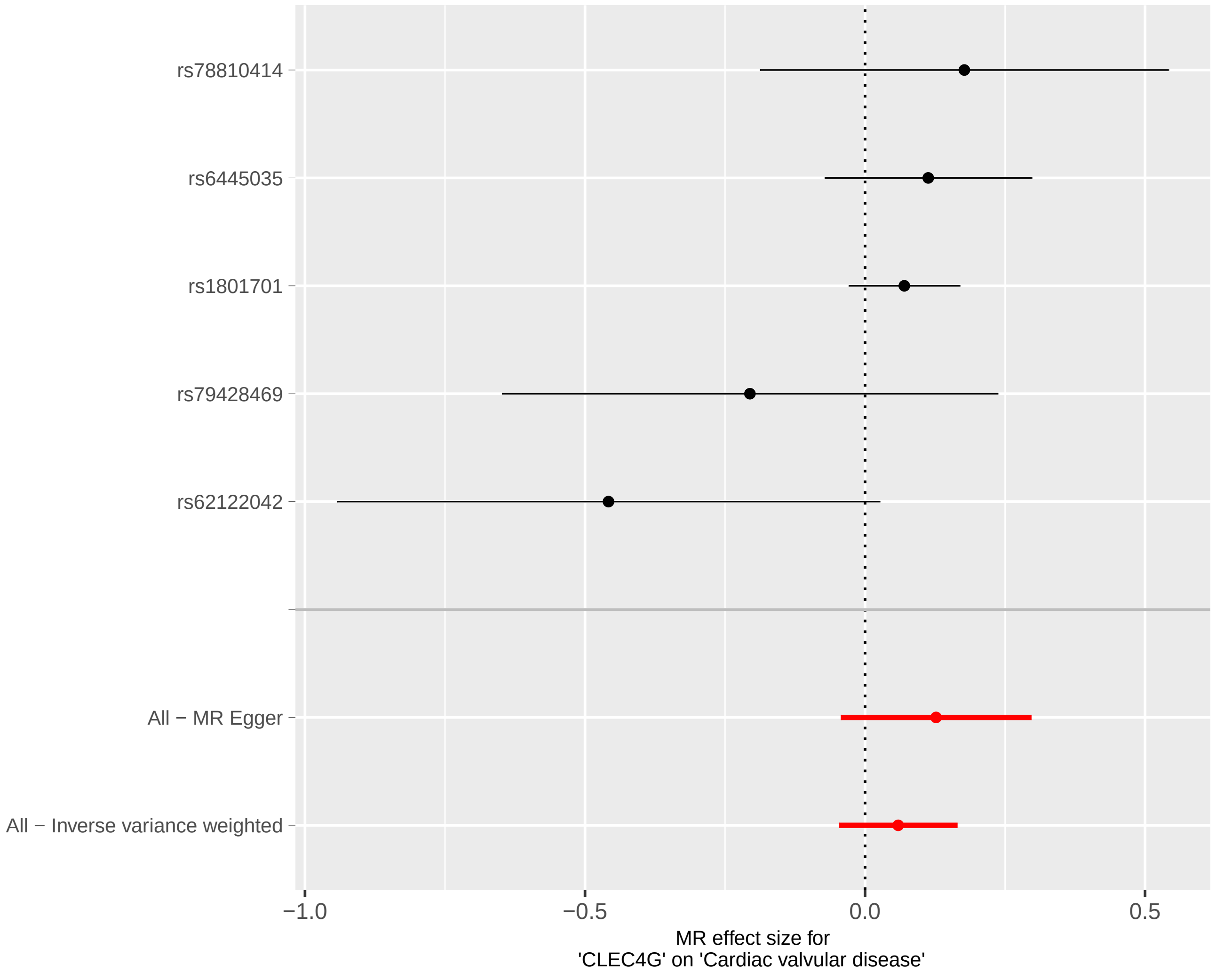
rs78810414
rs6445035
rs1801701
rs79428469
rs62122042
All − MR Egger
All − Inverse variance weighted
−1.0
−0.5
0.0
0.5
MR effect size for
'CLEC4G' on 'Cardiac valvular disease'

Insufficient number of SNPs

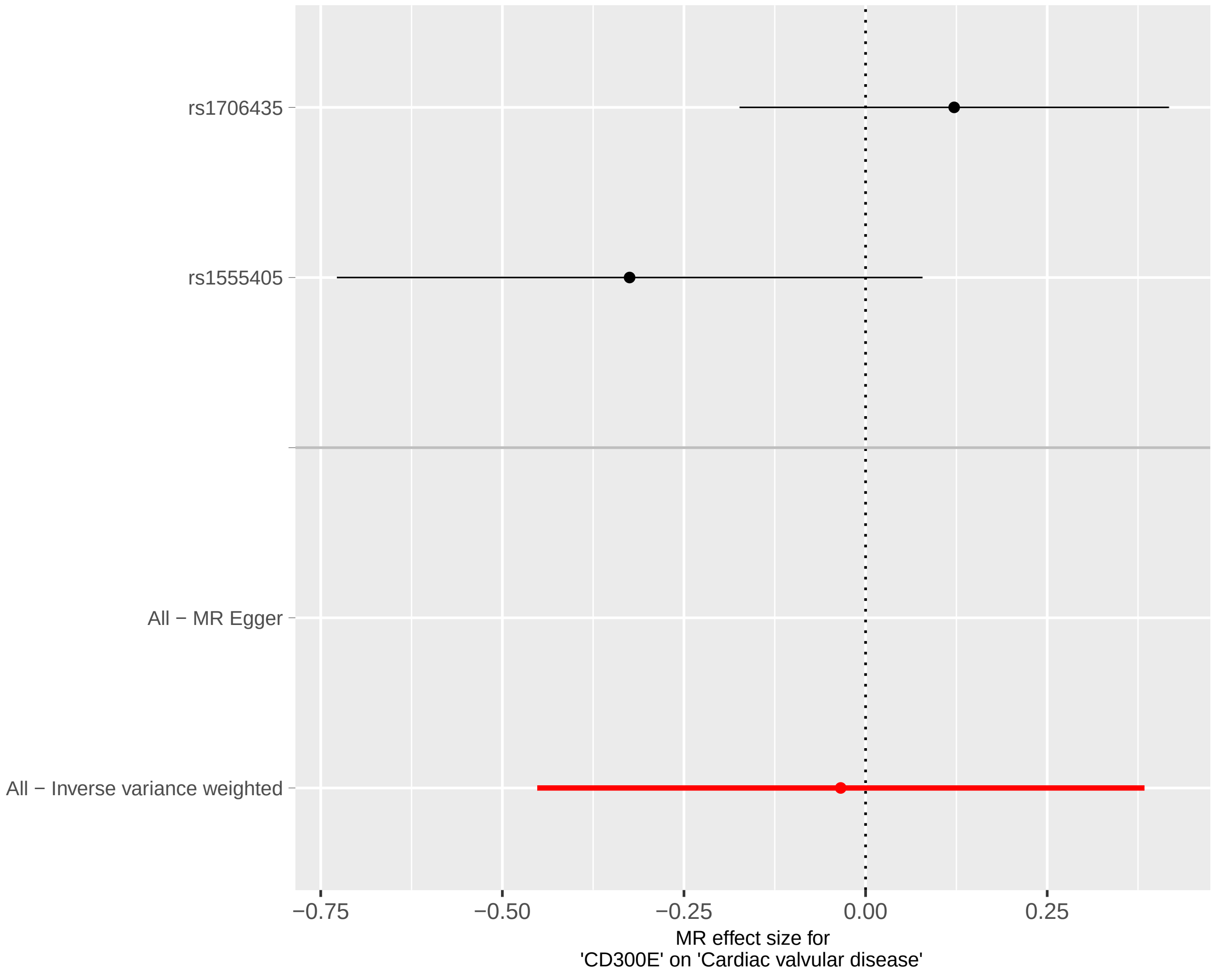
rs1706435
rs1555405
All − MR Egger
All − Inverse variance weighted
−0.75
−0.50
−0.25
0.00
0.25
MR effect size for
'CD300E' on 'Cardiac valvular disease'

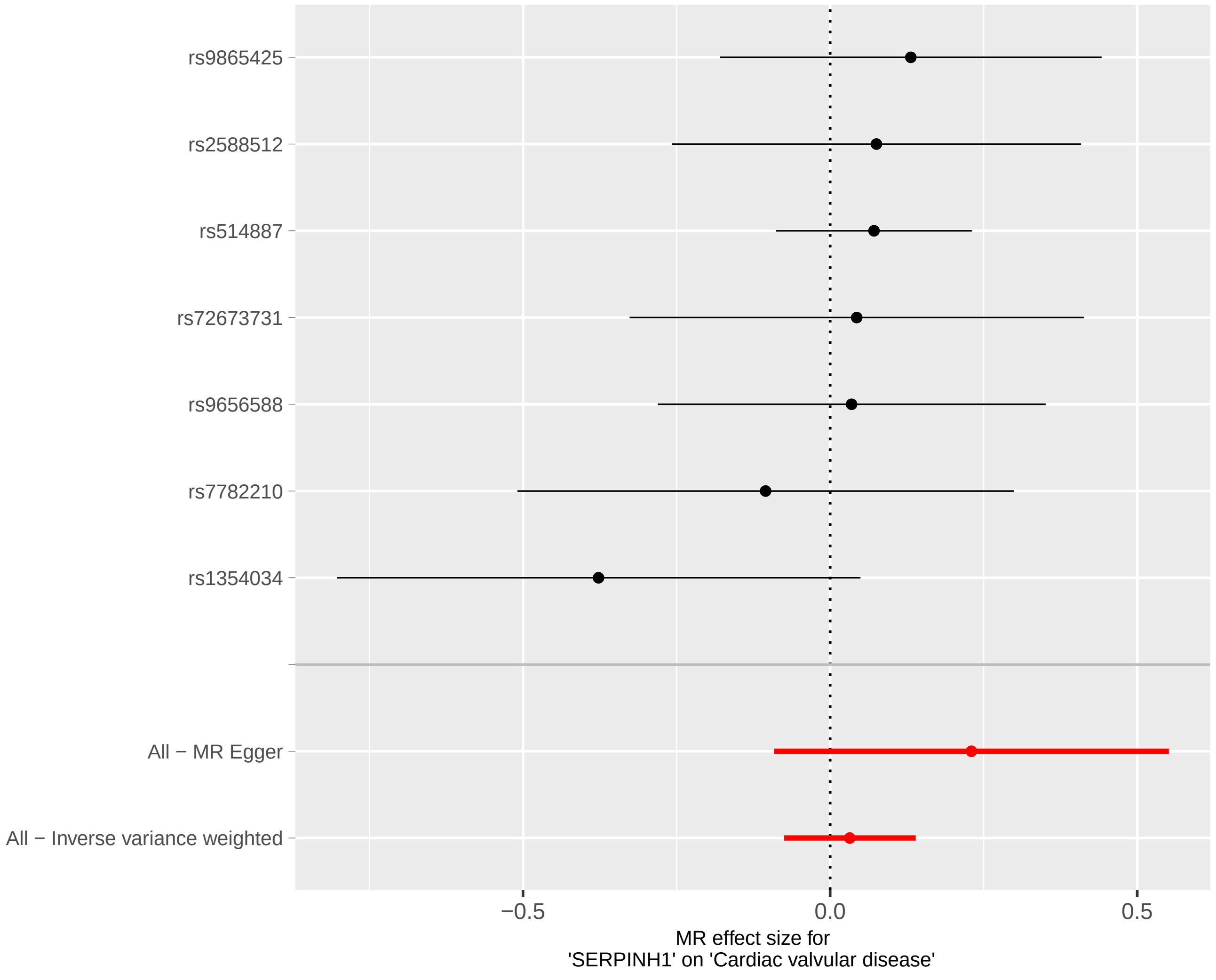
rs9865425
rs2588512
rs514887
rs72673731
rs9656588
rs7782210
rs1354034
All − MR Egger
All − Inverse variance weighted
−0.5
0.0
0.5
MR effect size for
'SERPINH1' on 'Cardiac valvular disease'

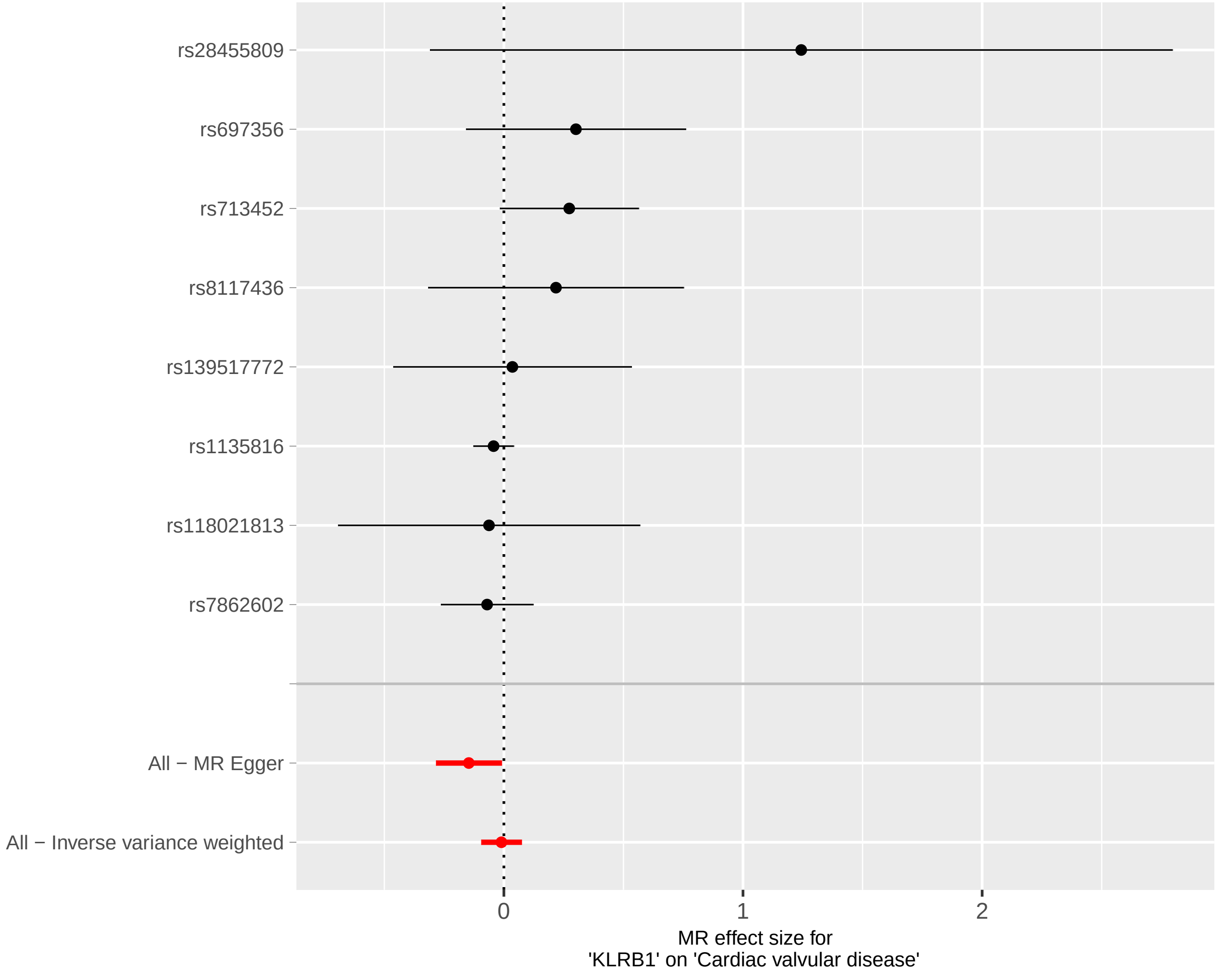
rs28455809
rs697356
rs713452
rs8117436
rs139517772
rs1135816
rs118021813
rs7862602
All − MR Egger
All − Inverse variance weighted
0
1
2
MR effect size for
'KLRB1' on 'Cardiac valvular disease'

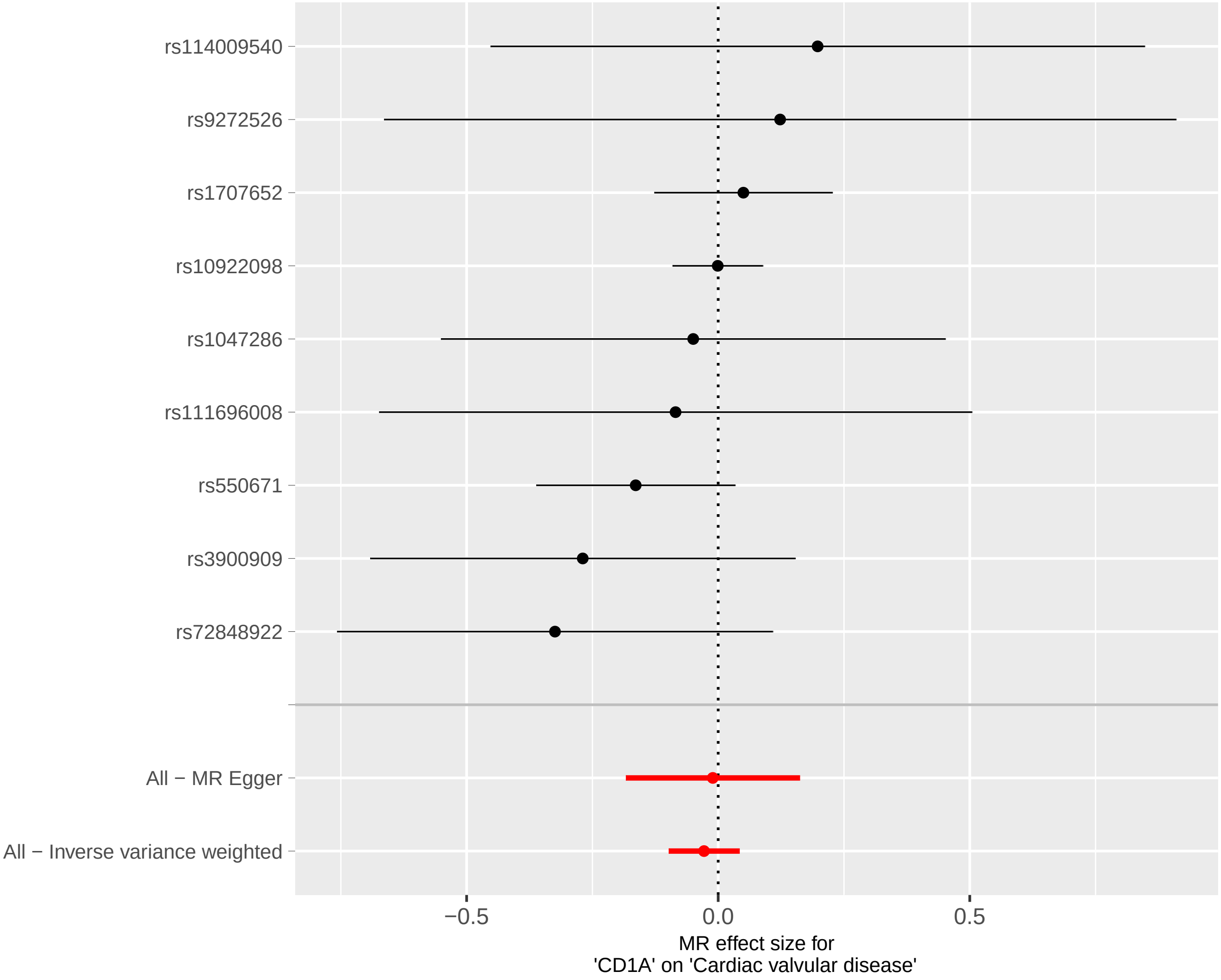
rs114009540
rs9272526
rs1707652
rs10922098
rs1047286
rs111696008
rs550671
rs3900909
rs72848922
All − MR Egger
All − Inverse variance weighted
−0.5
0.0
0.5
MR effect size for
'CD1A' on 'Cardiac valvular disease'

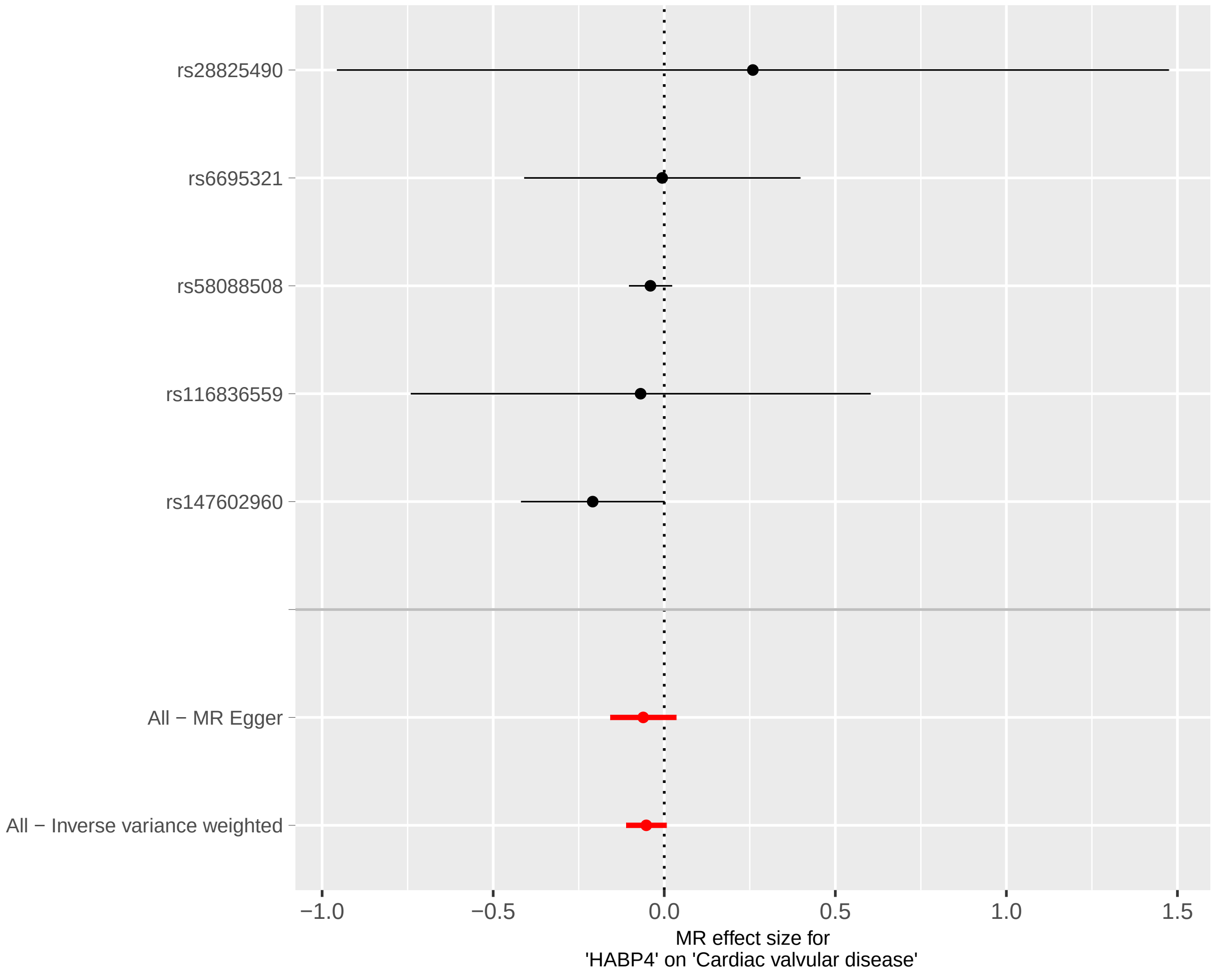
rs28825490
rs6695321
rs58088508
rs116836559
rs147602960
All − MR Egger
All − Inverse variance weighted
−1.0
−0.5
0.0
0.5
1.0
1.5
MR effect size for
'HABP4' on 'Cardiac valvular disease'

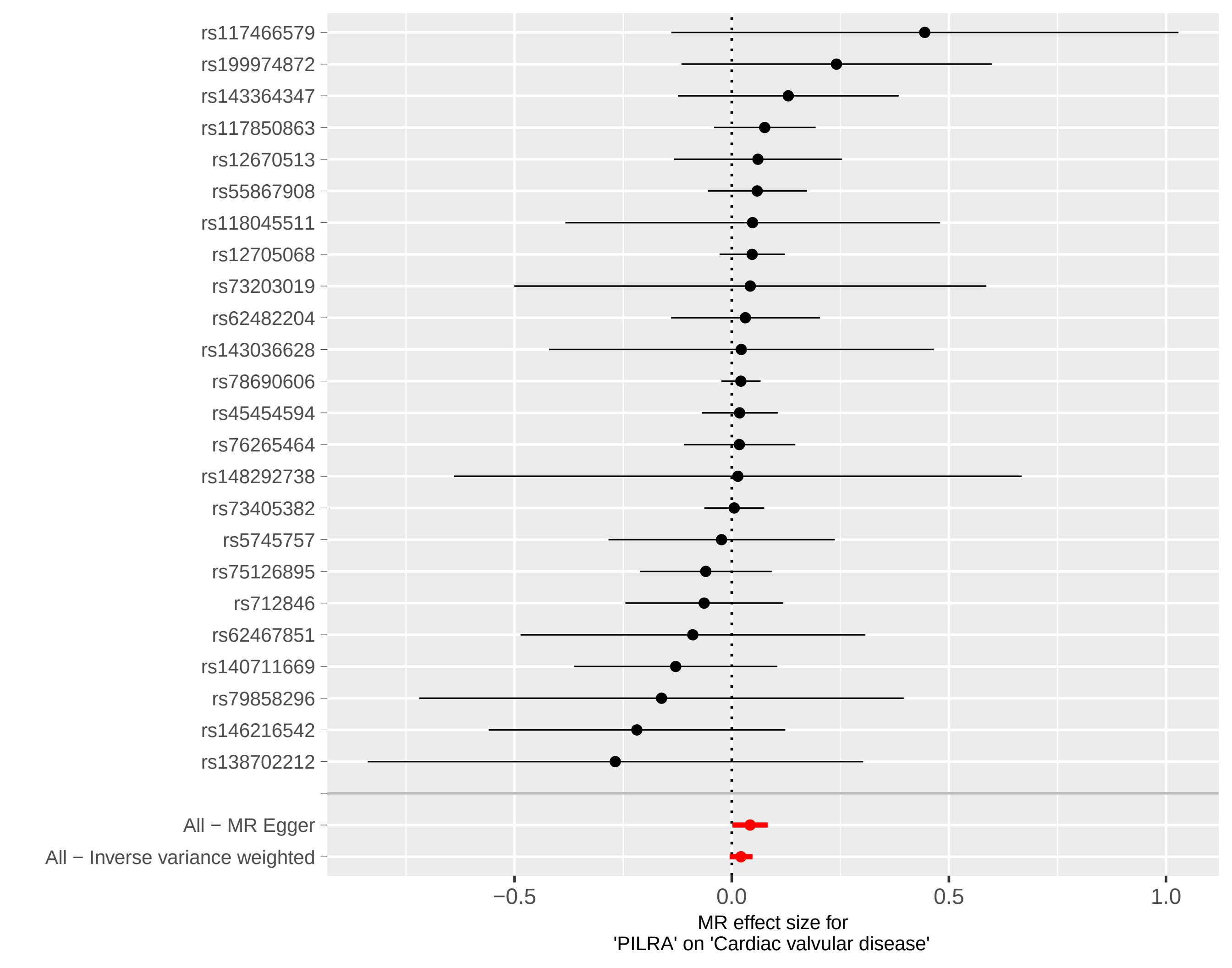
rs117466579
rs199974872
rs143364347
rs117850863
rs12670513
rs55867908
rs118045511
rs12705068
rs73203019
rs62482204
rs143036628
rs78690606
rs45454594
rs76265464
rs148292738
rs73405382
rs5745757
rs75126895
rs712846
rs62467851
rs140711669
rs79858296
rs146216542
rs138702212
All − MR Egger
All − Inverse variance weighted
−0.5
0.0
0.5
1.0
MR effect size for
'PILRA' on 'Cardiac valvular disease'

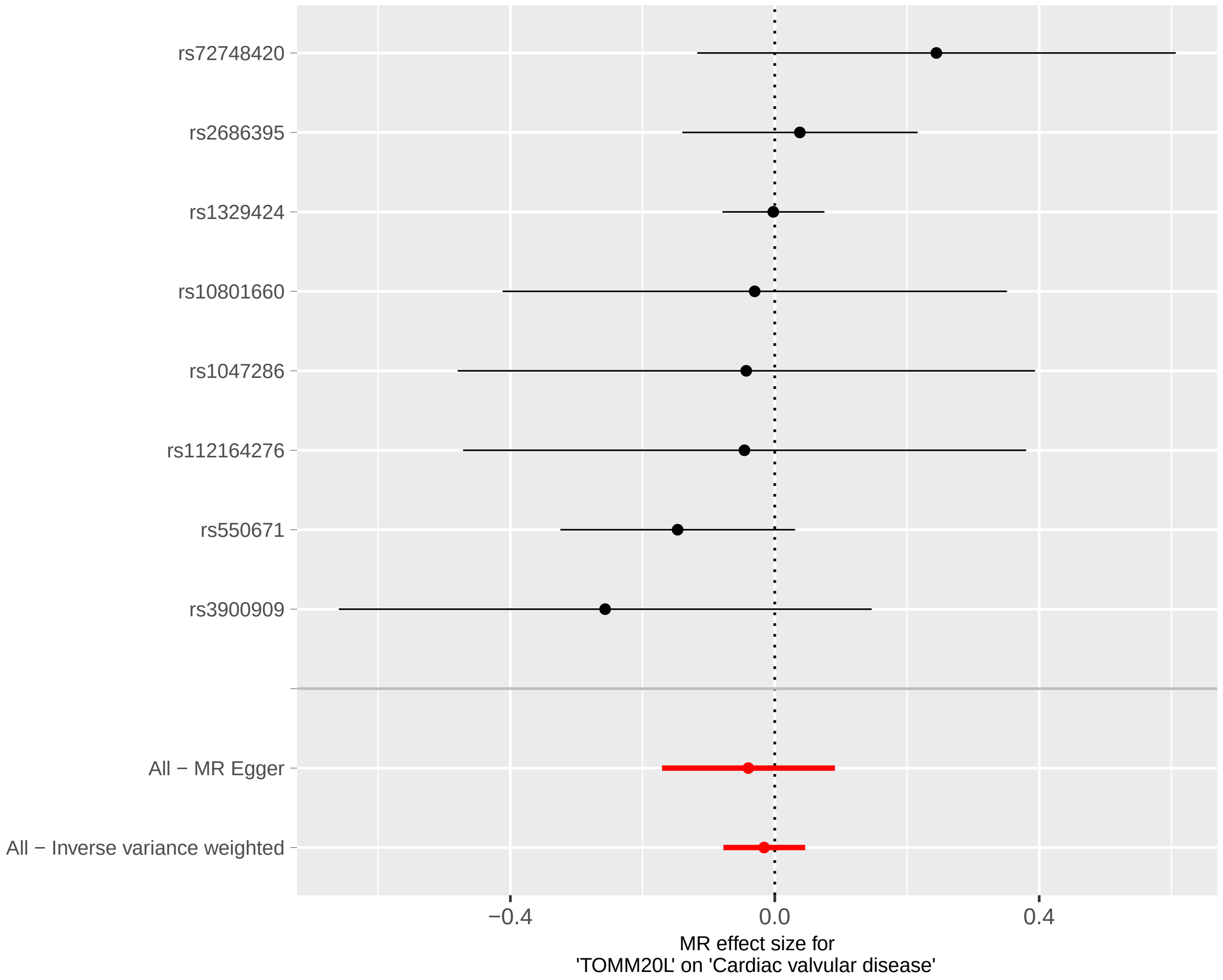

rs72748420
rs2686395
rs1329424
rs10801660
rs1047286
rs112164276
rs550671
rs3900909
All − MR Egger
All − Inverse variance weighted
−0.4
0.0
0.4
MR effect size for
'TOMM20L' on 'Cardiac valvular disease'

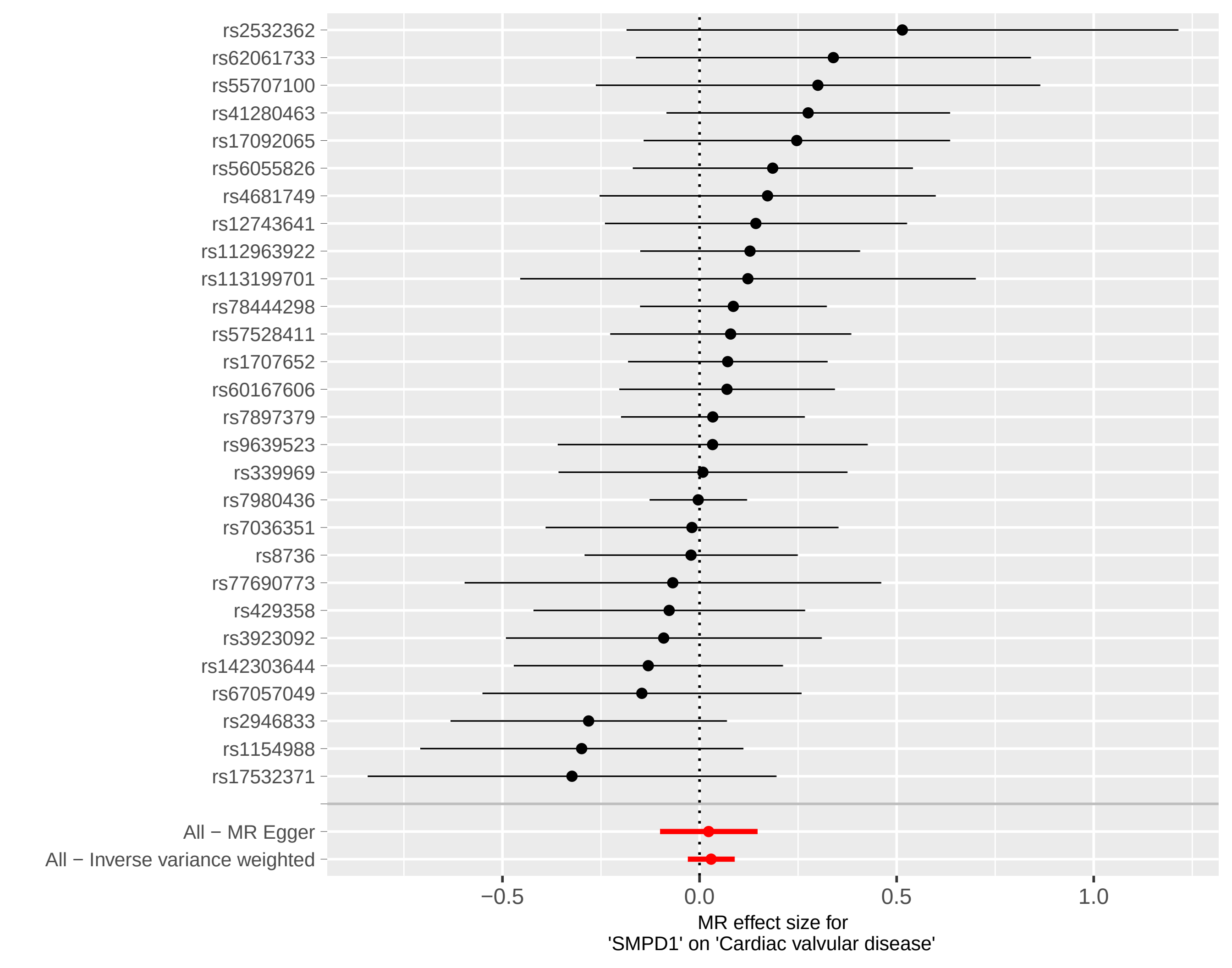

rs2532362
rs62061733
rs55707100
rs41280463
rs17092065
rs56055826
rs4681749
rs12743641
rs112963922
rs113199701
rs78444298
rs57528411
rs1707652
rs60167606
rs7897379
rs9639523
rs339969
rs7980436
rs7036351
rs8736
rs77690773
rs429358
rs3923092
rs142303644
rs67057049
rs2946833
rs1154988
rs17532371
All − MR Egger
All − Inverse variance weighted
−0.5
0.0
0.5
1.0
MR effect size for
'SMPD1' on 'Cardiac valvular disease'

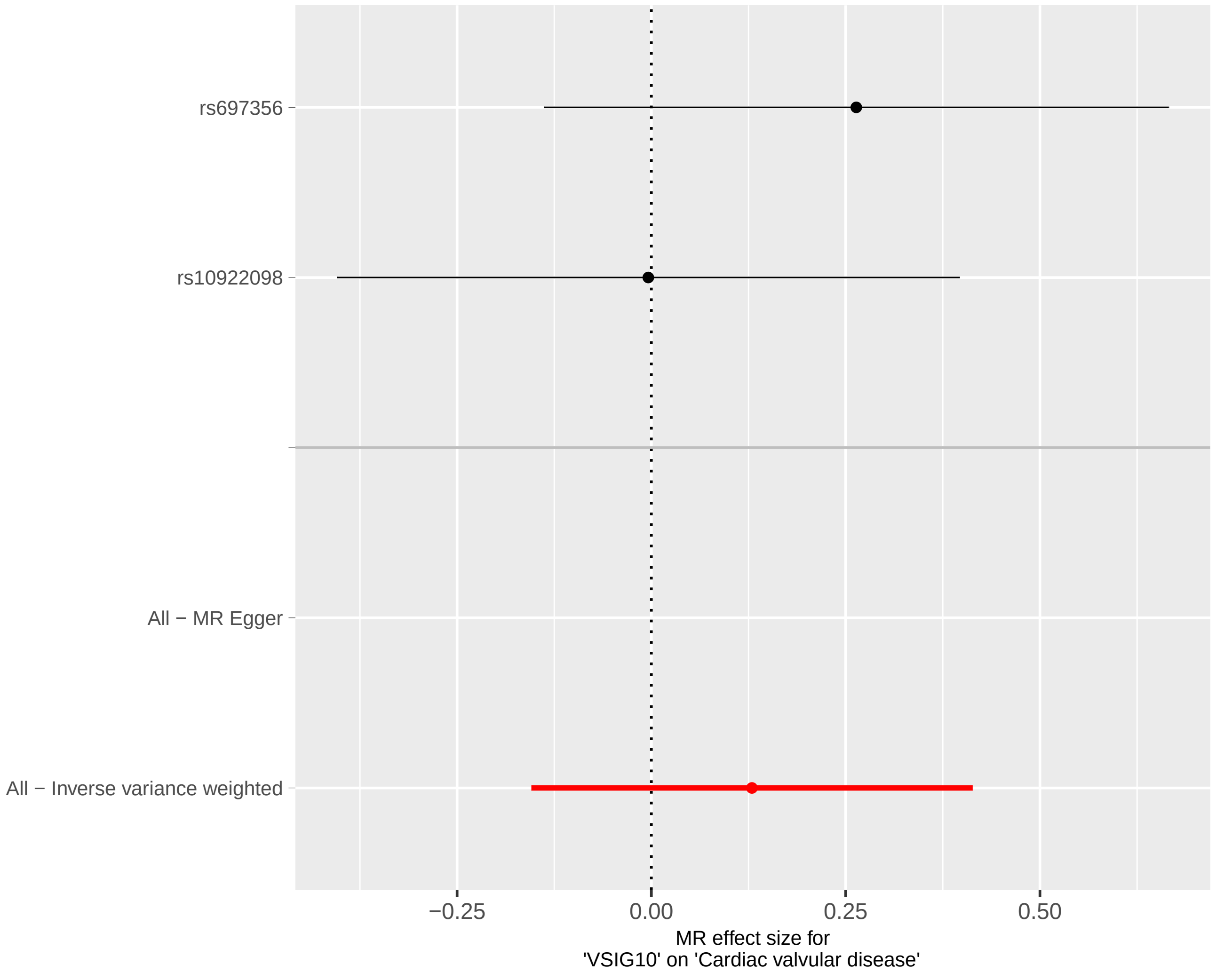

rs697356
rs10922098
All − MR Egger
All − Inverse variance weighted
−0.25
0.00
0.25
0.50
MR effect size for
'VSIG10' on 'Cardiac valvular disease'

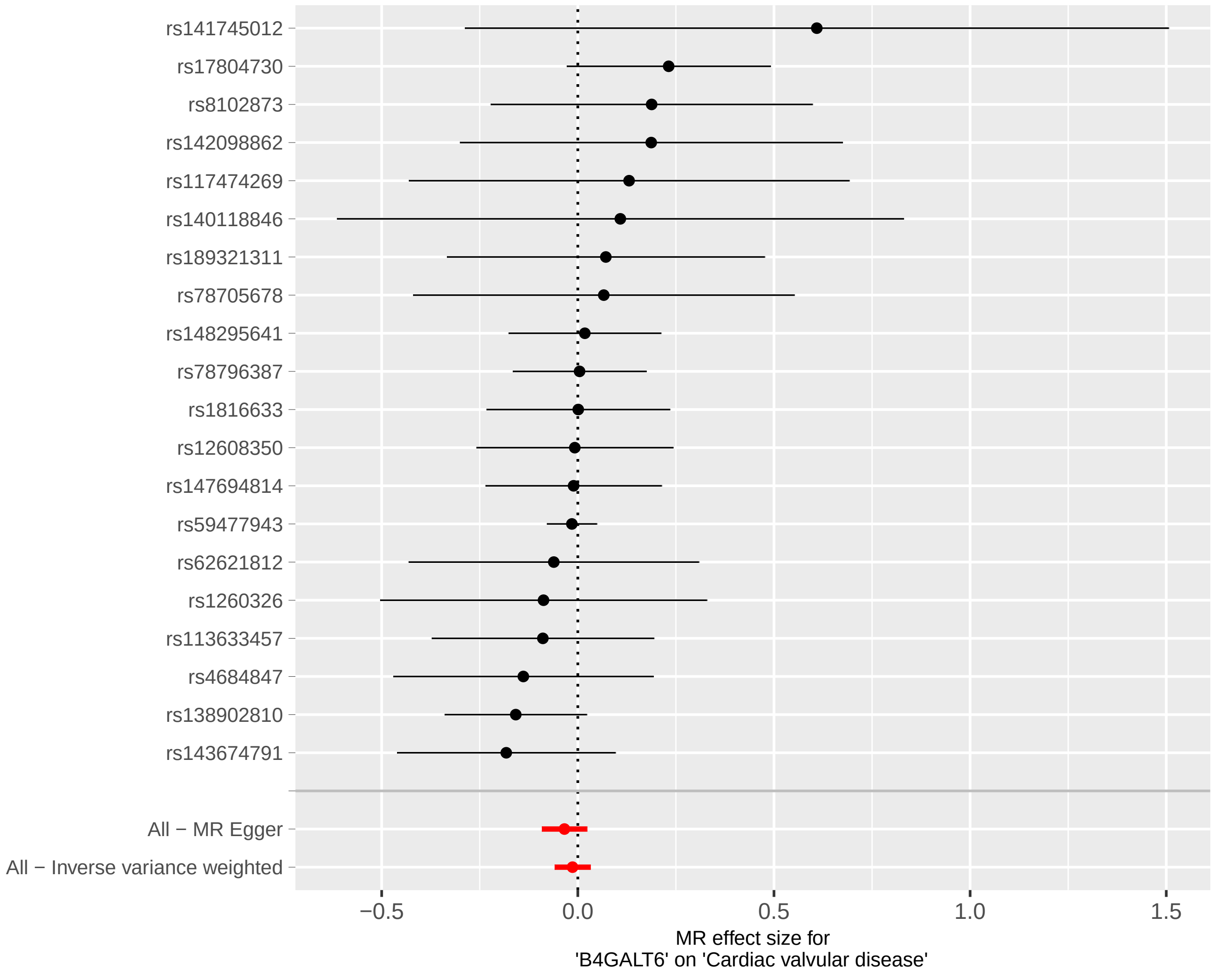
rs141745012
rs17804730
rs8102873
rs142098862
rs117474269
rs140118846
rs189321311
rs78705678
rs148295641
rs78796387
rs1816633
rs12608350
rs147694814
rs59477943
rs62621812
rs1260326
rs113633457
rs4684847
rs138902810
rs143674791
All − MR Egger
All − Inverse variance weighted
−0.5
0.0
0.5
1.0
1.5
MR effect size for
'B4GALT6' on 'Cardiac valvular disease'

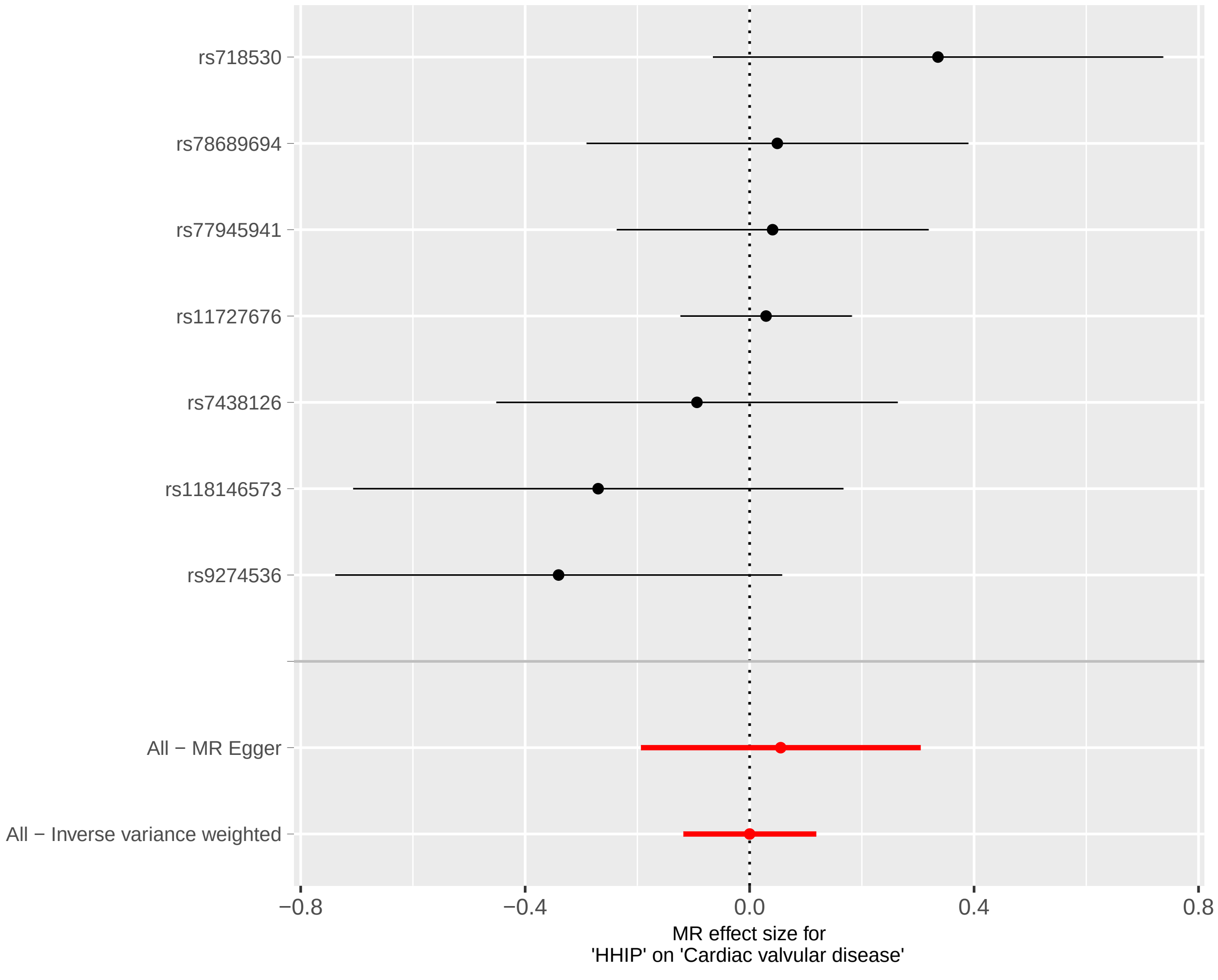
rs718530
rs78689694
rs77945941
rs11727676
rs7438126
rs118146573
rs9274536
All − MR Egger
All − Inverse variance weighted
−0.8
−0.4
0.0
0.4
0.8
MR effect size for
'HHIP' on 'Cardiac valvular disease'

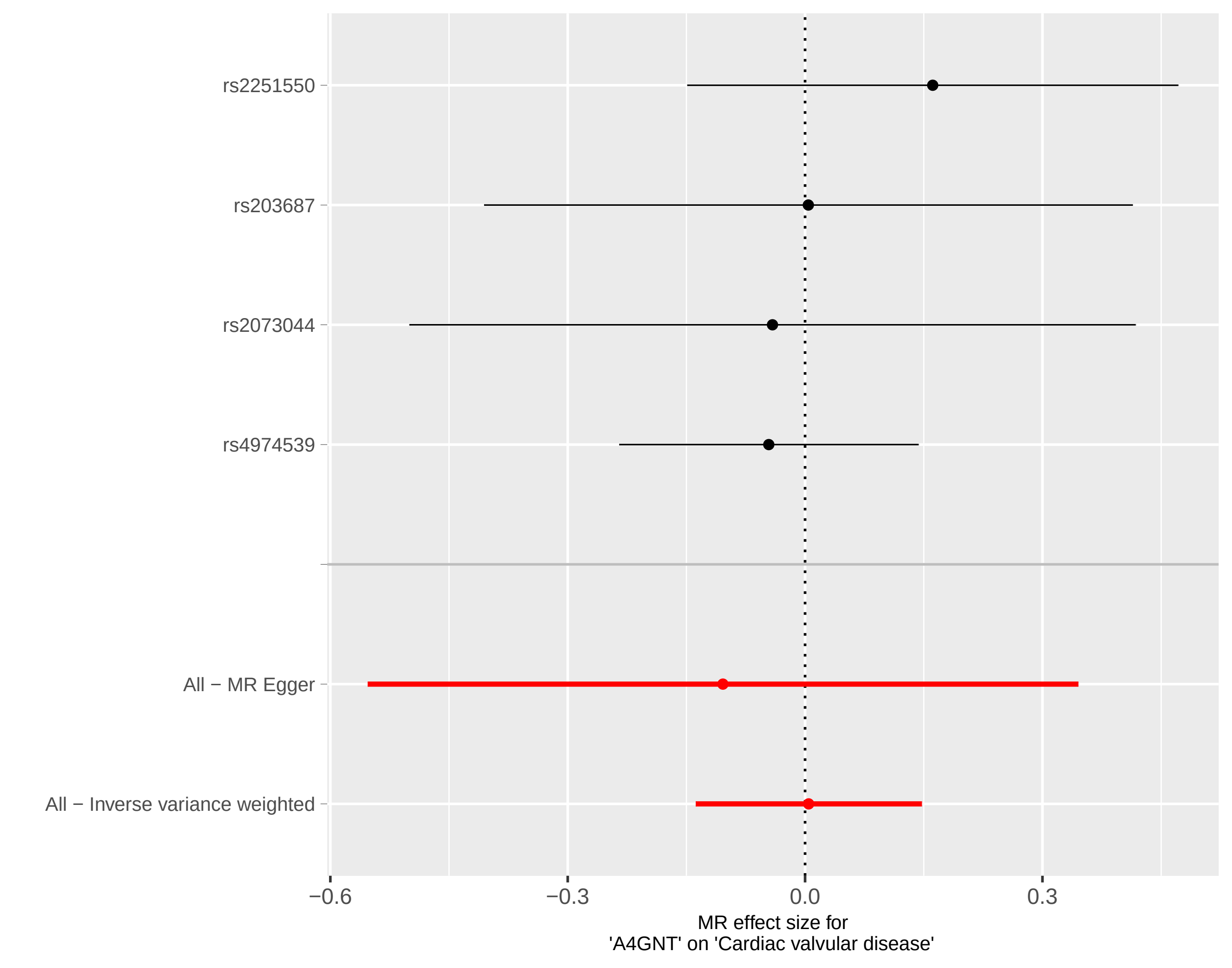

rs2251550
rs203687
rs2073044
rs4974539
All − MR Egger
All − Inverse variance weighted
−0.6
−0.3
0.0
0.3
MR effect size for
'A4GNT' on 'Cardiac valvular disease'

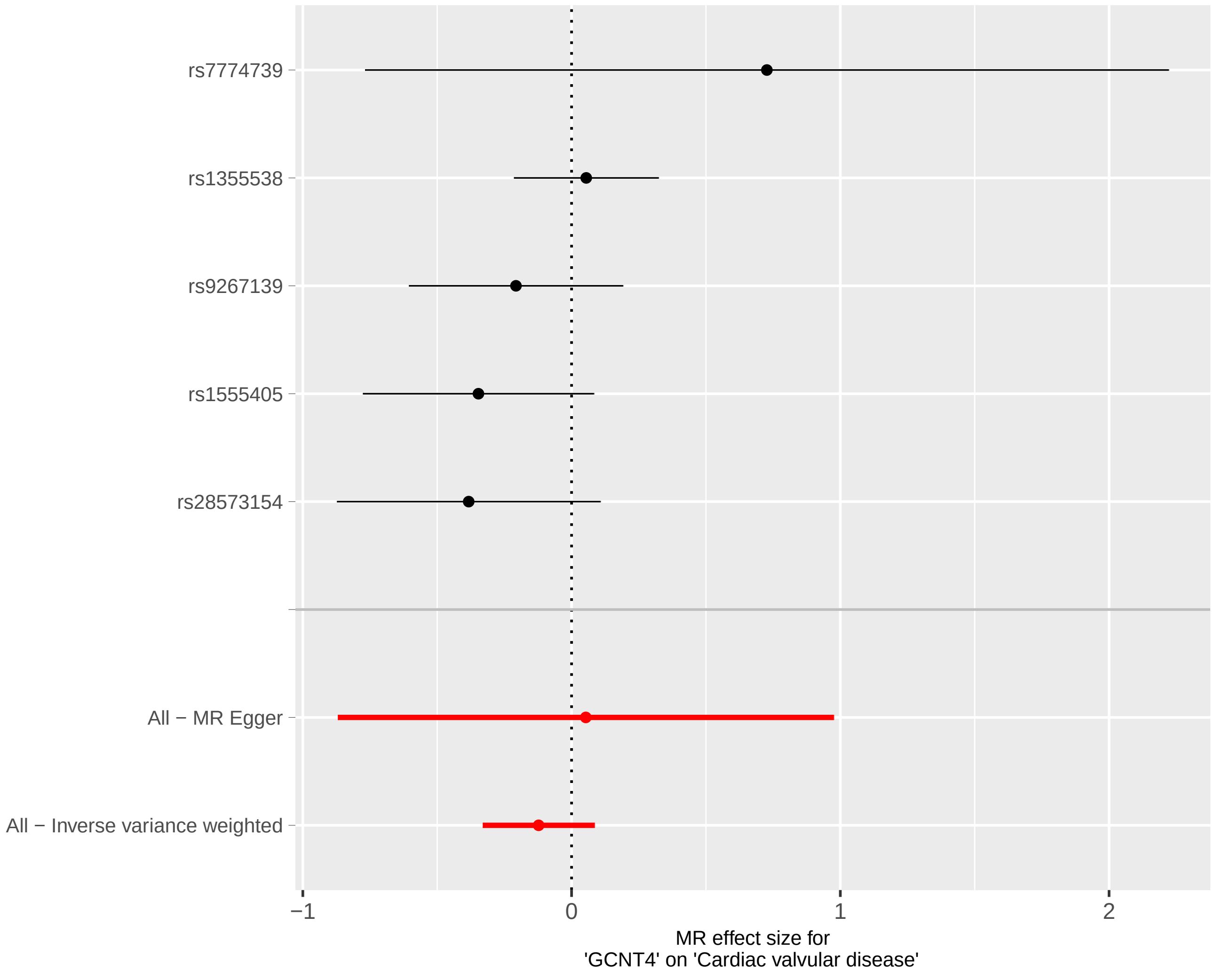
rs7774739
rs1355538
rs9267139
rs1555405
rs28573154
All − MR Egger
All − Inverse variance weighted
−1
0
1
2
MR effect size for
'GCNT4' on 'Cardiac valvular disease'

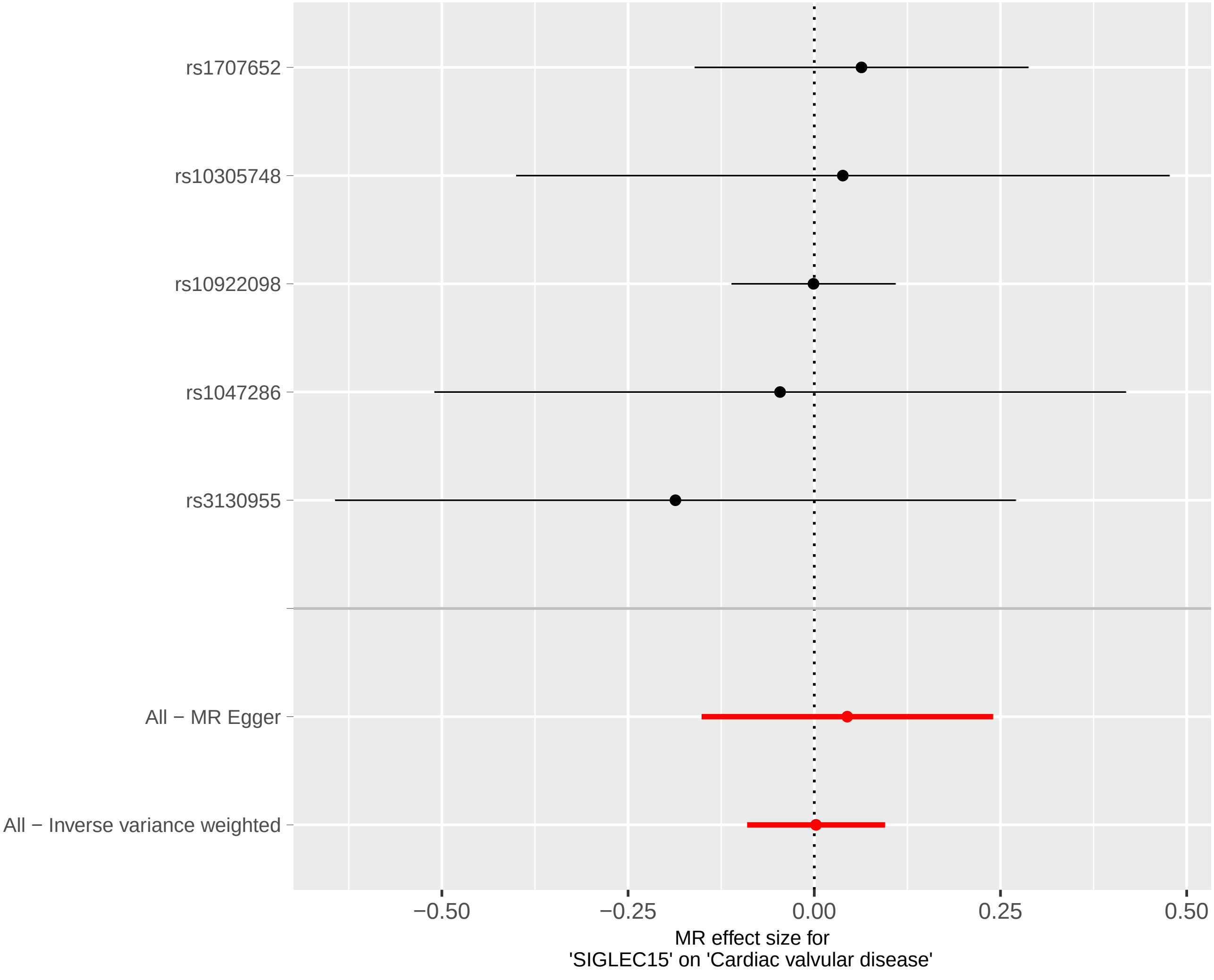
rs1707652
rs10305748
rs10922098
rs1047286
rs3130955
All − MR Egger
All − Inverse variance weighted
−0.50
−0.25
0.00
0.25
0.50
MR effect size for
'SIGLEC15' on 'Cardiac valvular disease'

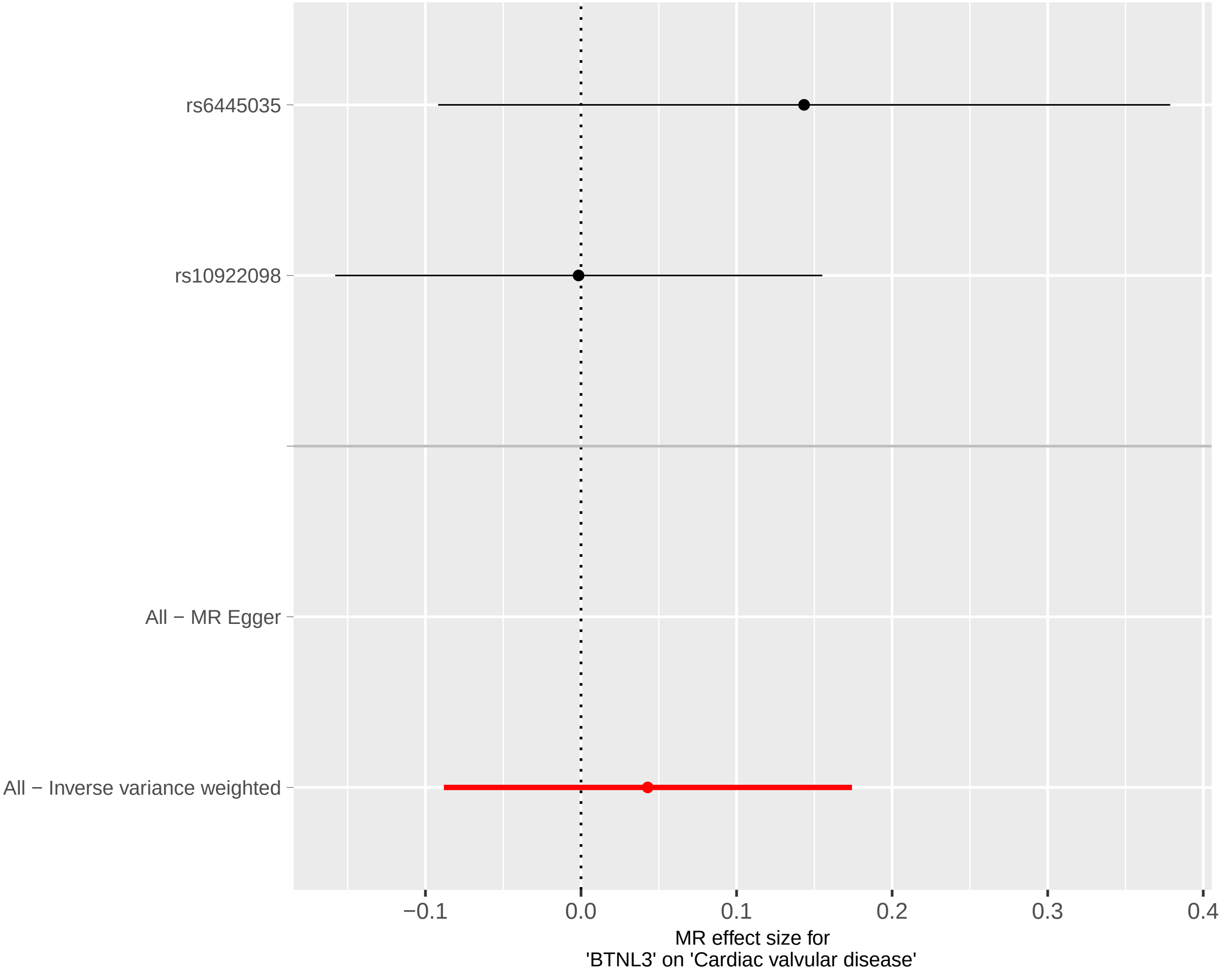
rs6445035
rs10922098
All − MR Egger
All − Inverse variance weighted
−0.1
0.0
0.1
0.2
0.3
0.4
MR effect size for
'BTNL3' on 'Cardiac valvular disease'

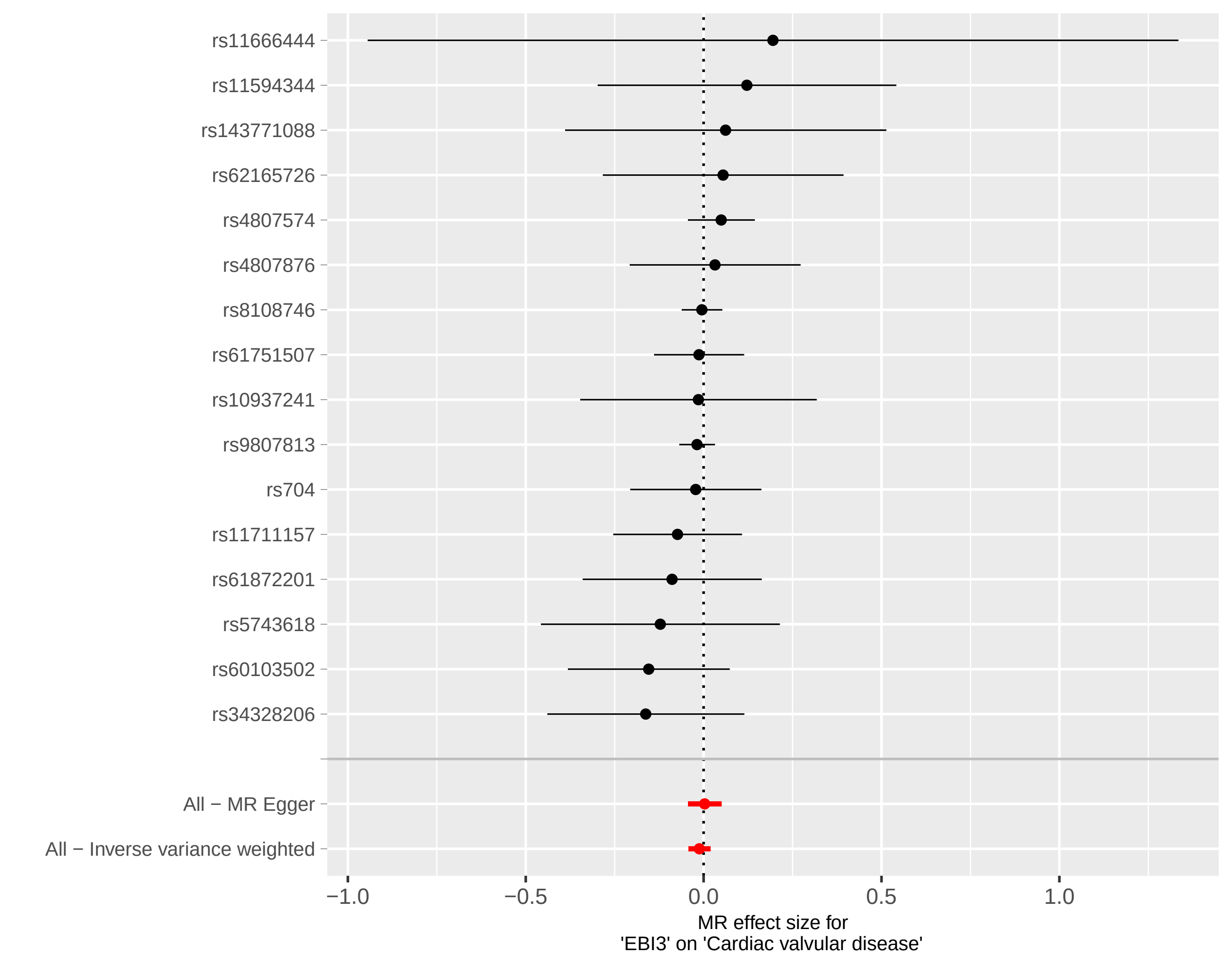

rs11666444
rs11594344
rs143771088
rs62165726
rs4807574
rs4807876
rs8108746
rs61751507
rs10937241
rs9807813
rs704
rs11711157
rs61872201
rs5743618
rs60103502
rs34328206
All − MR Egger
All − Inverse variance weighted
−1.0
−0.5
0.0
0.5
1.0
MR effect size for
'EBI3' on 'Cardiac valvular disease'

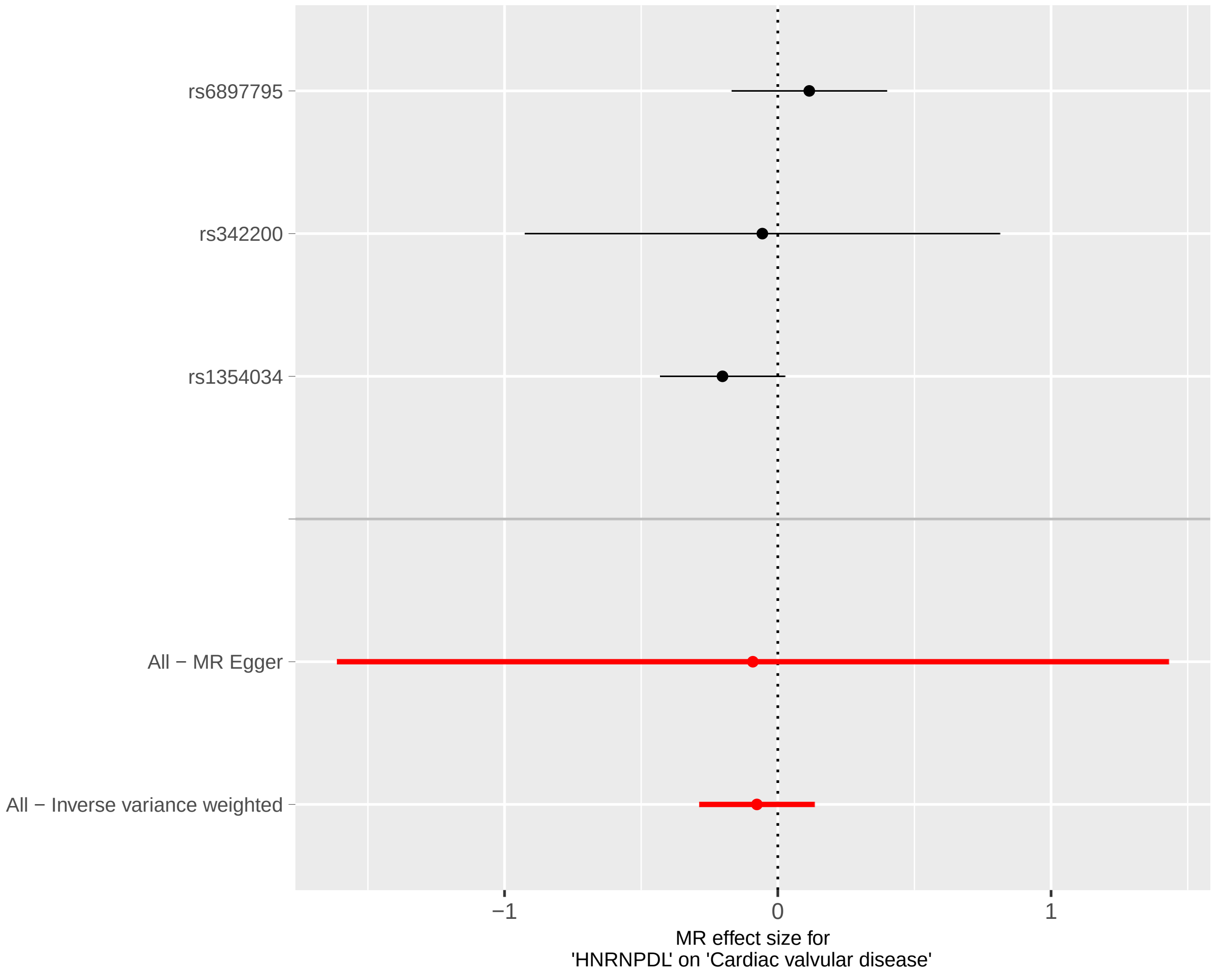
rs6897795
rs342200
rs1354034
All − MR Egger
All − Inverse variance weighted
−1
0
1
MR effect size for
'HNRNPDL' on 'Cardiac valvular disease'

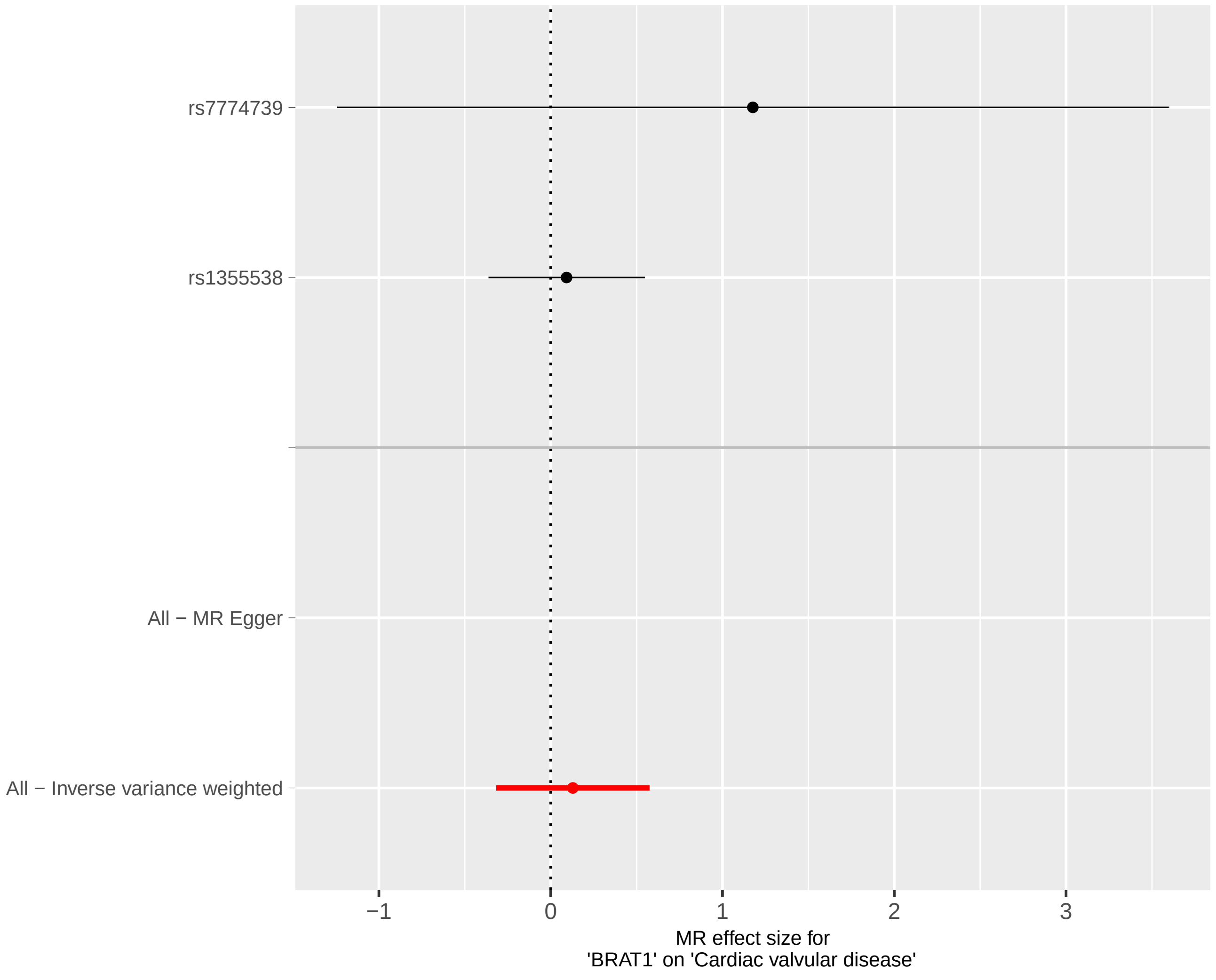
rs7774739
rs1355538
All − MR Egger
All − Inverse variance weighted
−1
0
1
2
3
MR effect size for
'BRAT1' on 'Cardiac valvular disease'

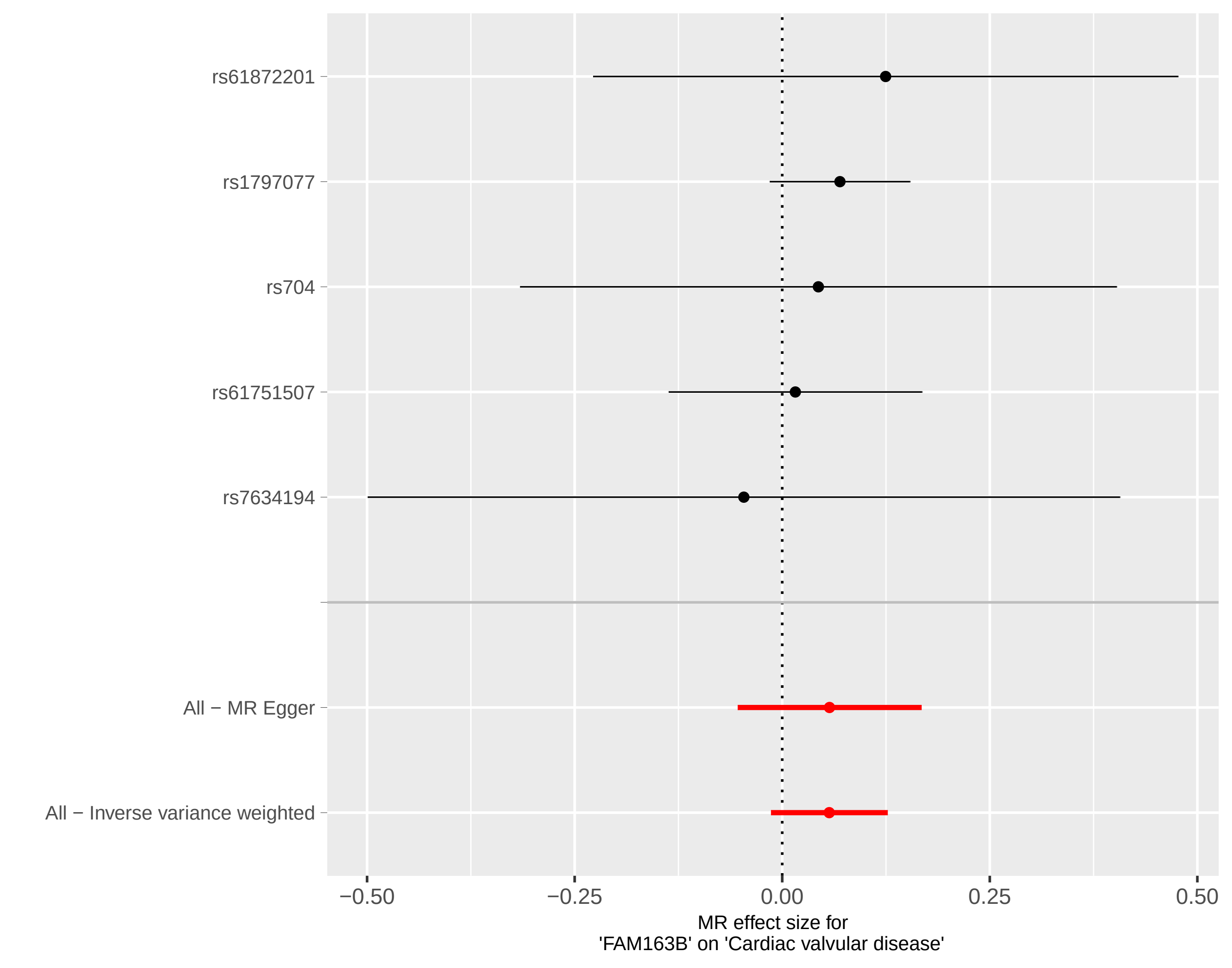

rs61872201
rs1797077
rs704
rs61751507
rs7634194
All − MR Egger
All − Inverse variance weighted
−0.50
−0.25
0.00
0.25
0.50
MR effect size for
'FAM163B' on 'Cardiac valvular disease'

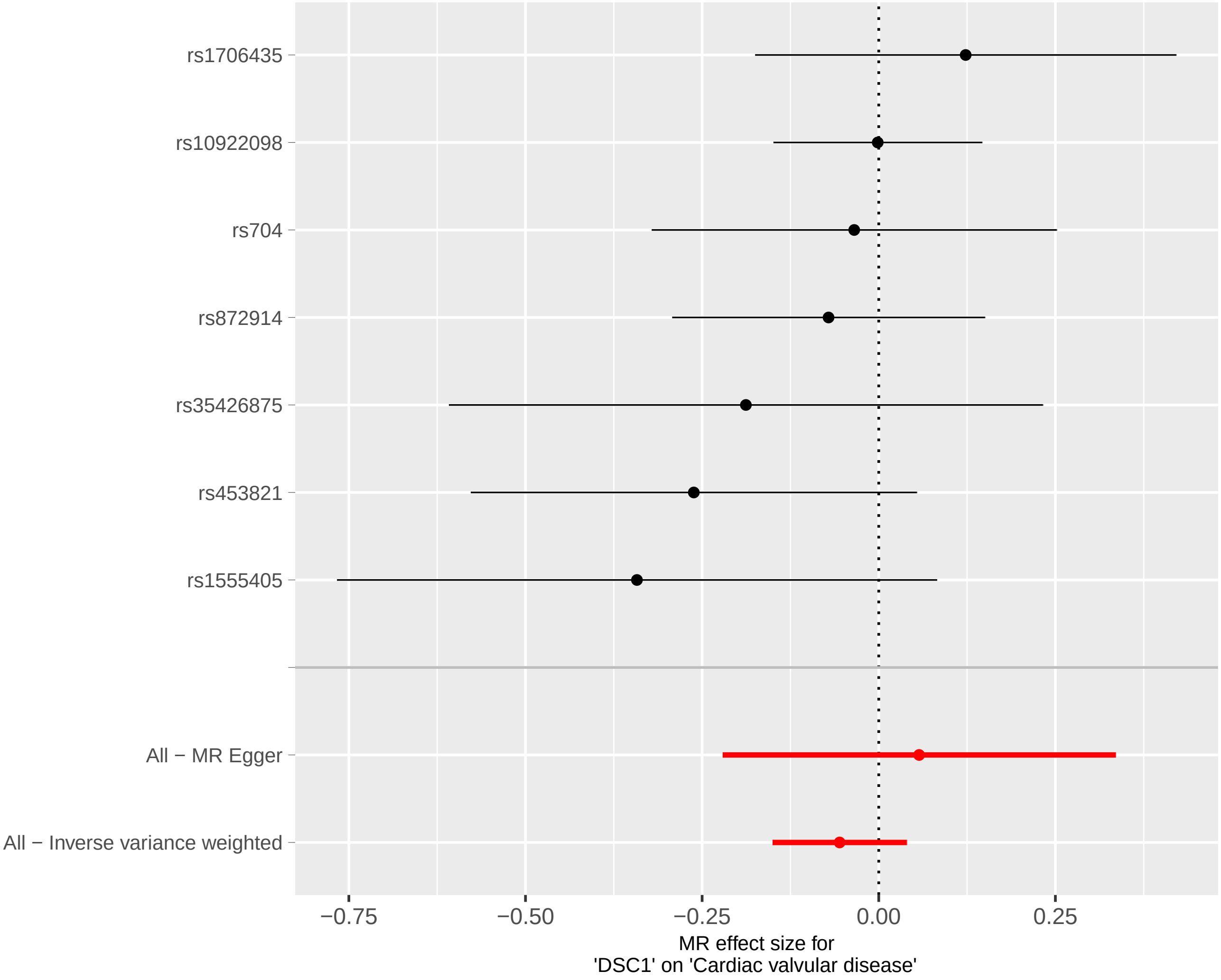
rs1706435
rs10922098
rs704
rs872914
rs35426875
rs453821
rs1555405
All − MR Egger
All − Inverse variance weighted
−0.75
−0.50
−0.25
0.00
0.25
MR effect size for
'DSC1' on 'Cardiac valvular disease'

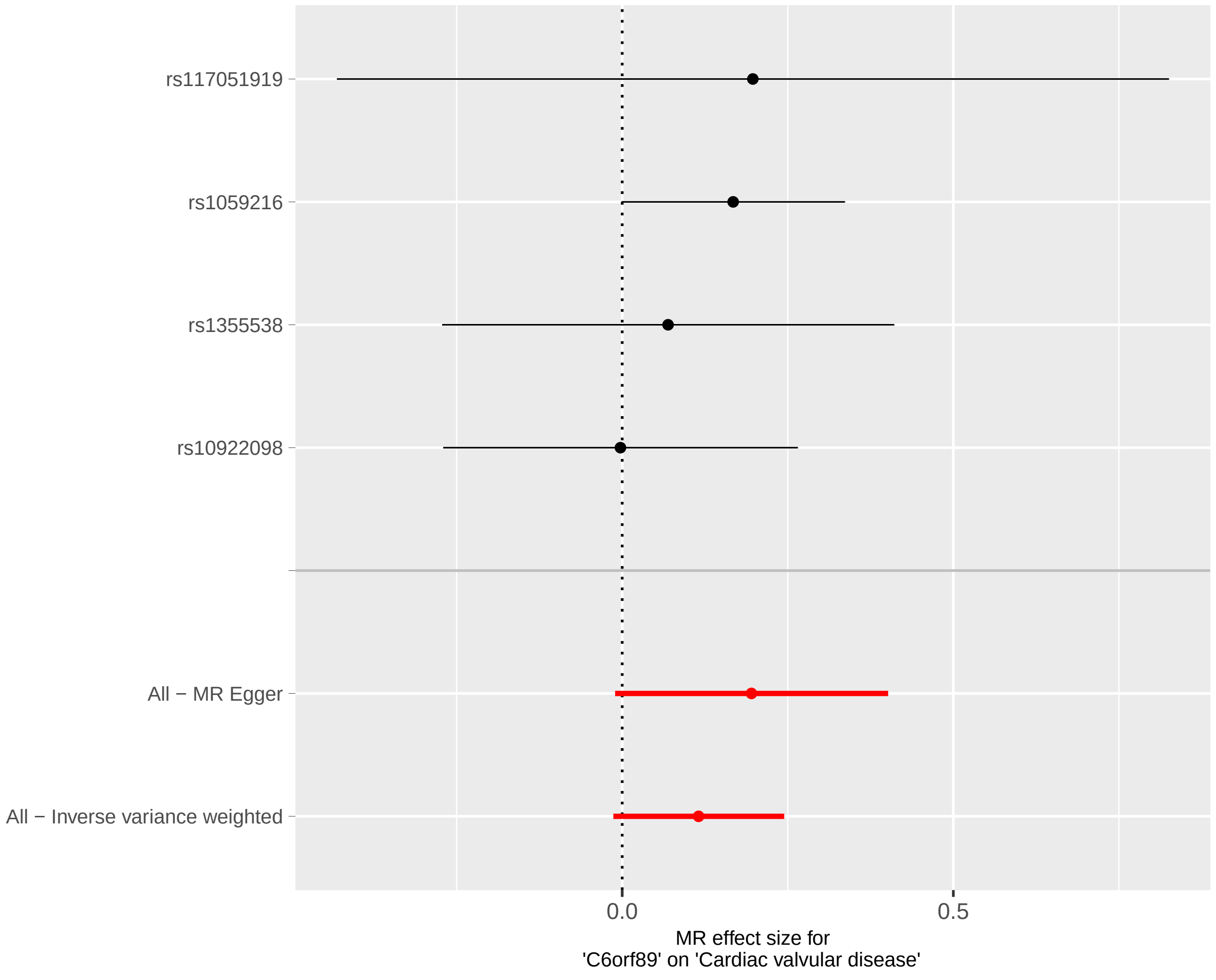
rs117051919
rs1059216
rs1355538
rs10922098
All − MR Egger
All − Inverse variance weighted
0.0
0.5
MR effect size for
'C6orf89' on 'Cardiac valvular disease'

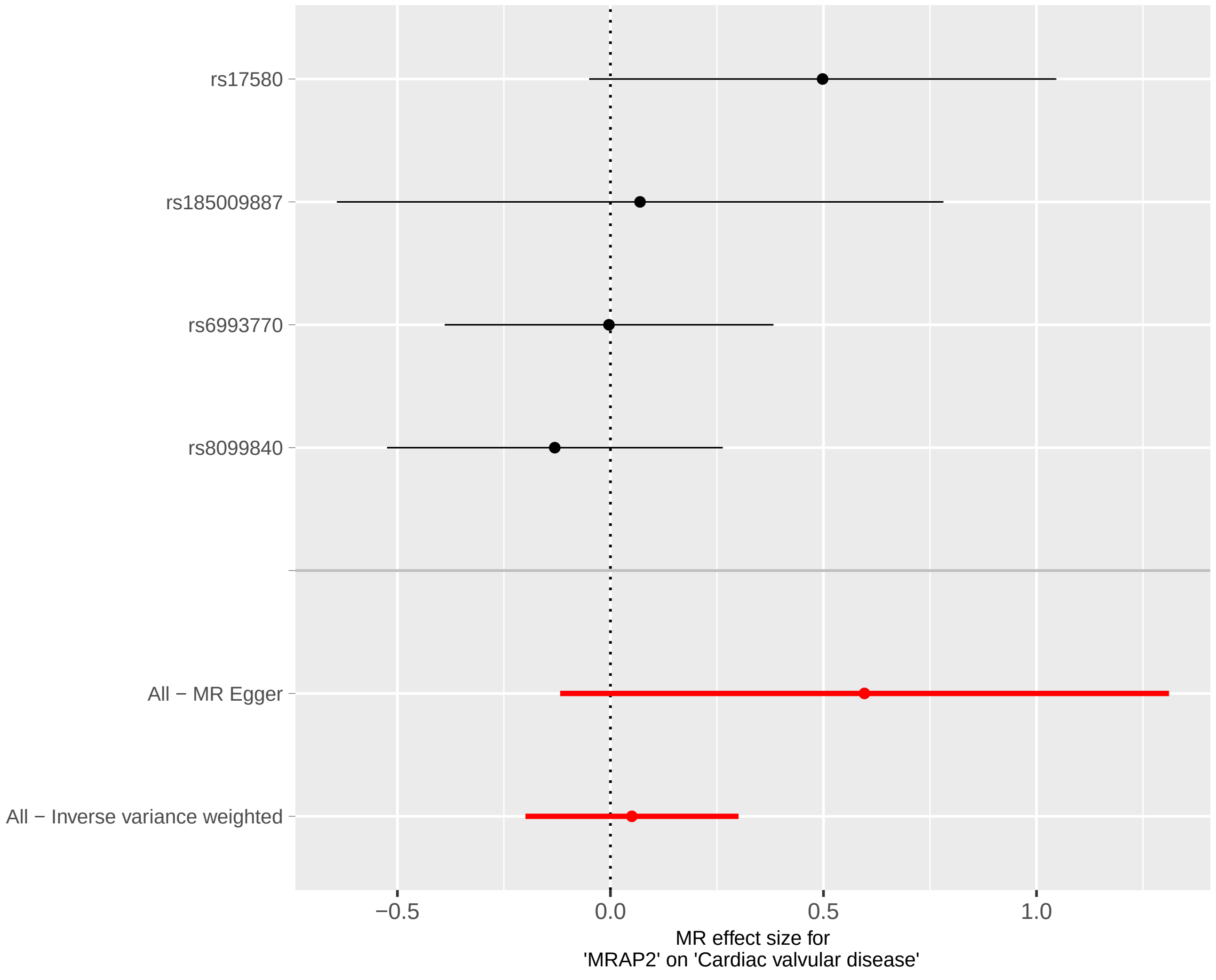
rs17580
rs185009887
rs6993770
rs8099840
All − MR Egger
All − Inverse variance weighted
−0.5
0.0
0.5
1.0
MR effect size for
'MRAP2' on 'Cardiac valvular disease'

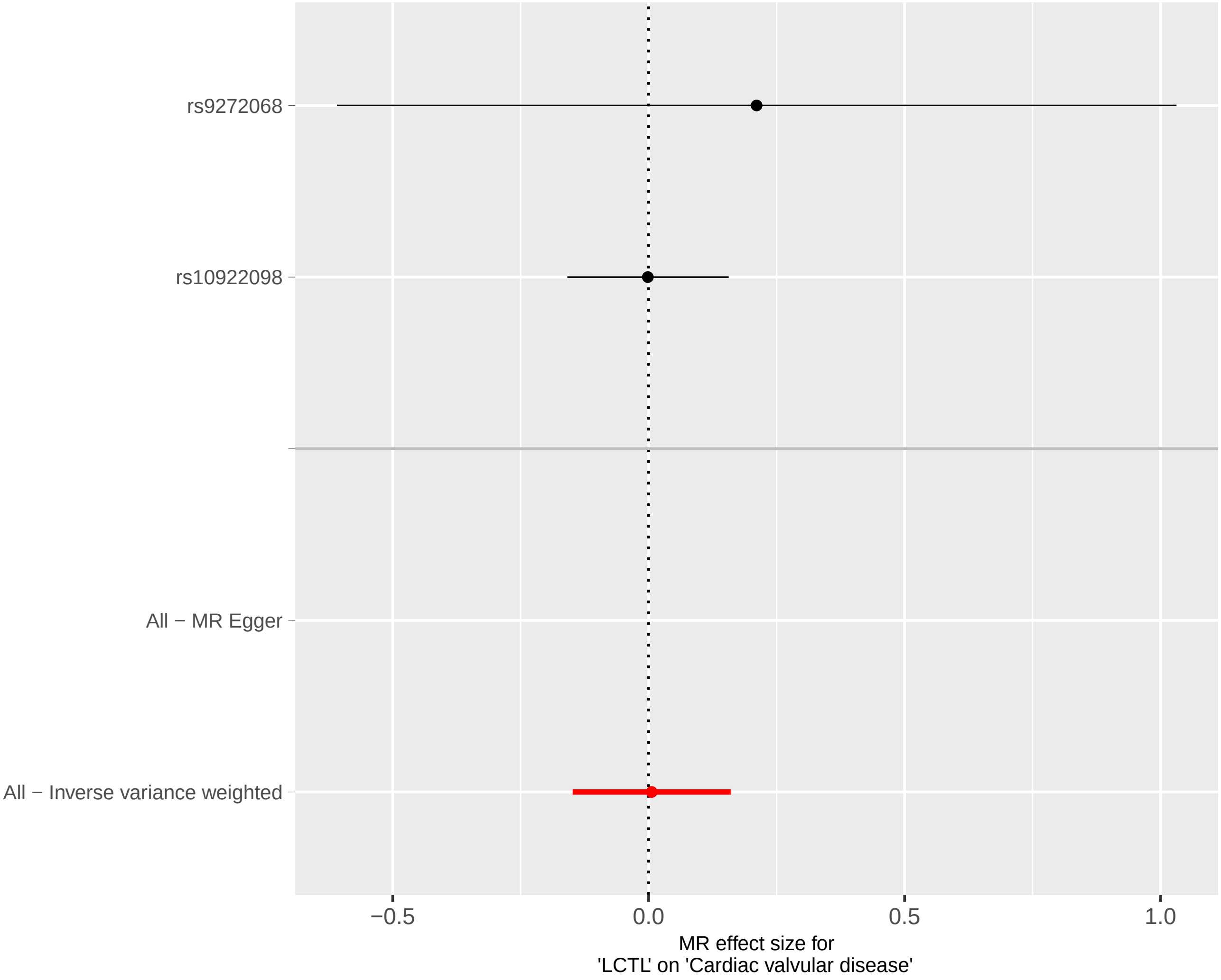

rs9272068
rs10922098
All − MR Egger
All − Inverse variance weighted
−0.5
0.0
0.5
1.0
MR effect size for
'LCTL' on 'Cardiac valvular disease'

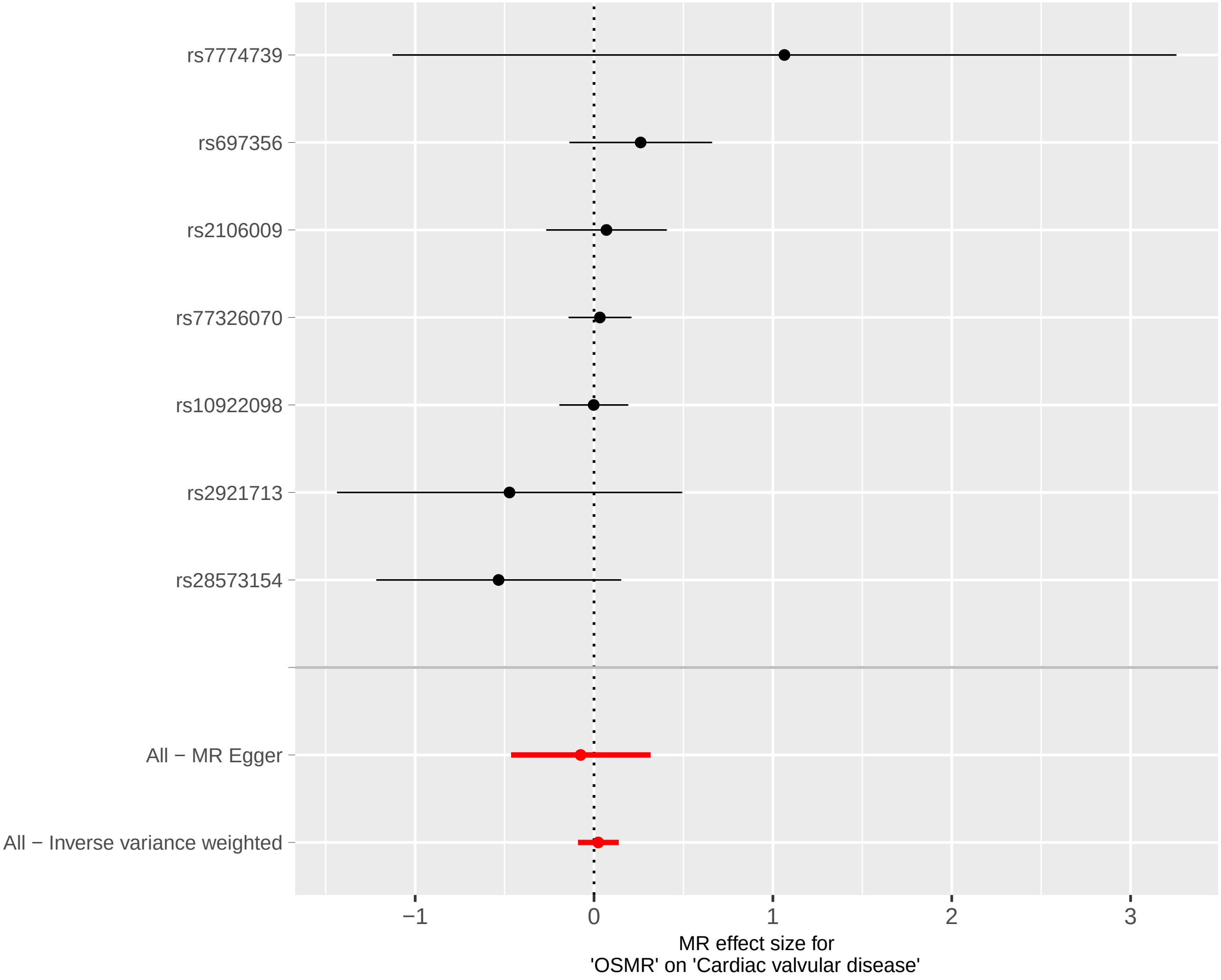
rs7774739
rs697356
rs2106009
rs77326070
rs10922098
rs2921713
rs28573154
All − MR Egger
All − Inverse variance weighted
−1
0
1
2
3
MR effect size for
'OSMR' on 'Cardiac valvular disease'

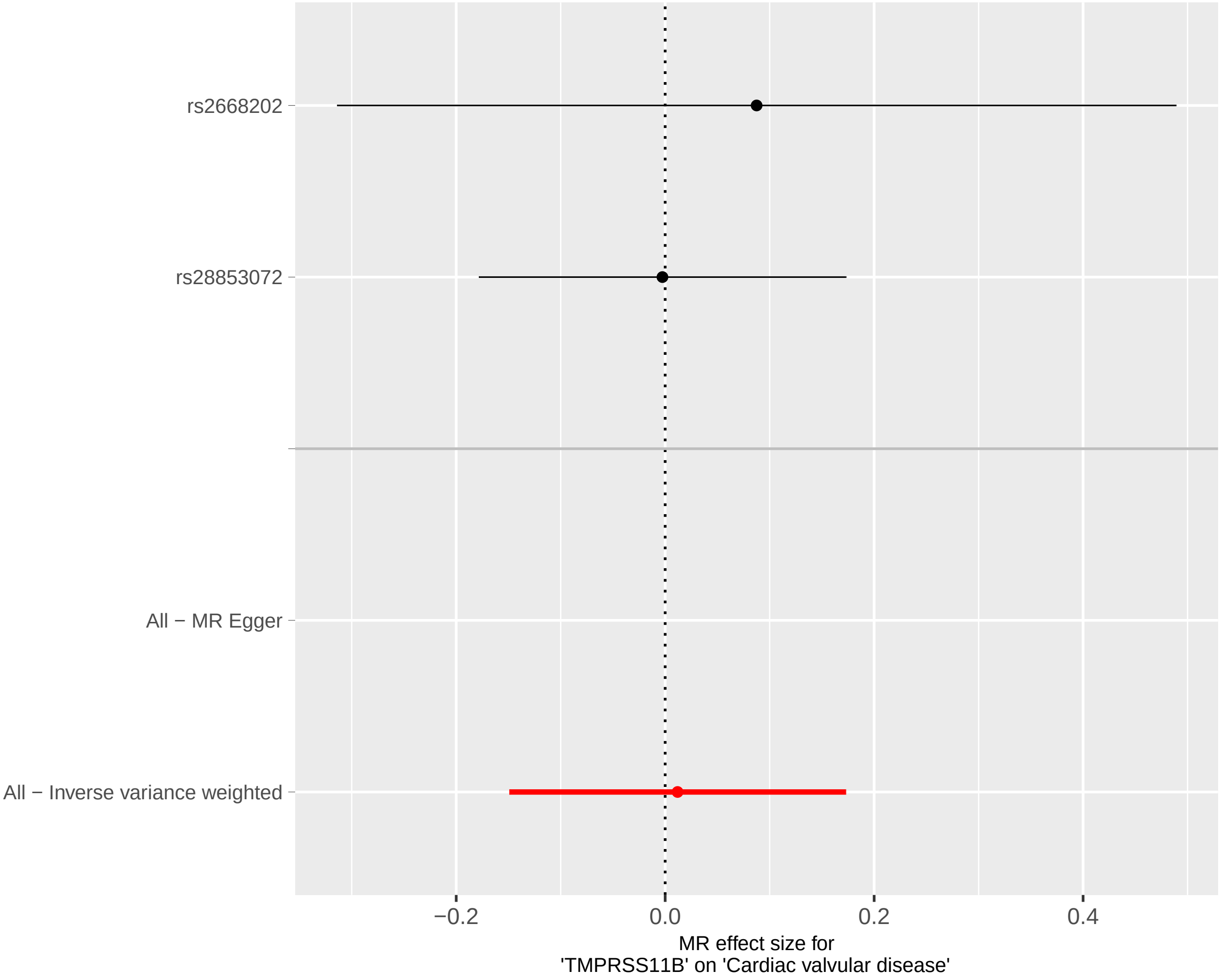
rs2668202
rs28853072
All − MR Egger
All − Inverse variance weighted
−0.2
0.0
0.2
0.4
MR effect size for
'TMPRSS11B' on 'Cardiac valvular disease'

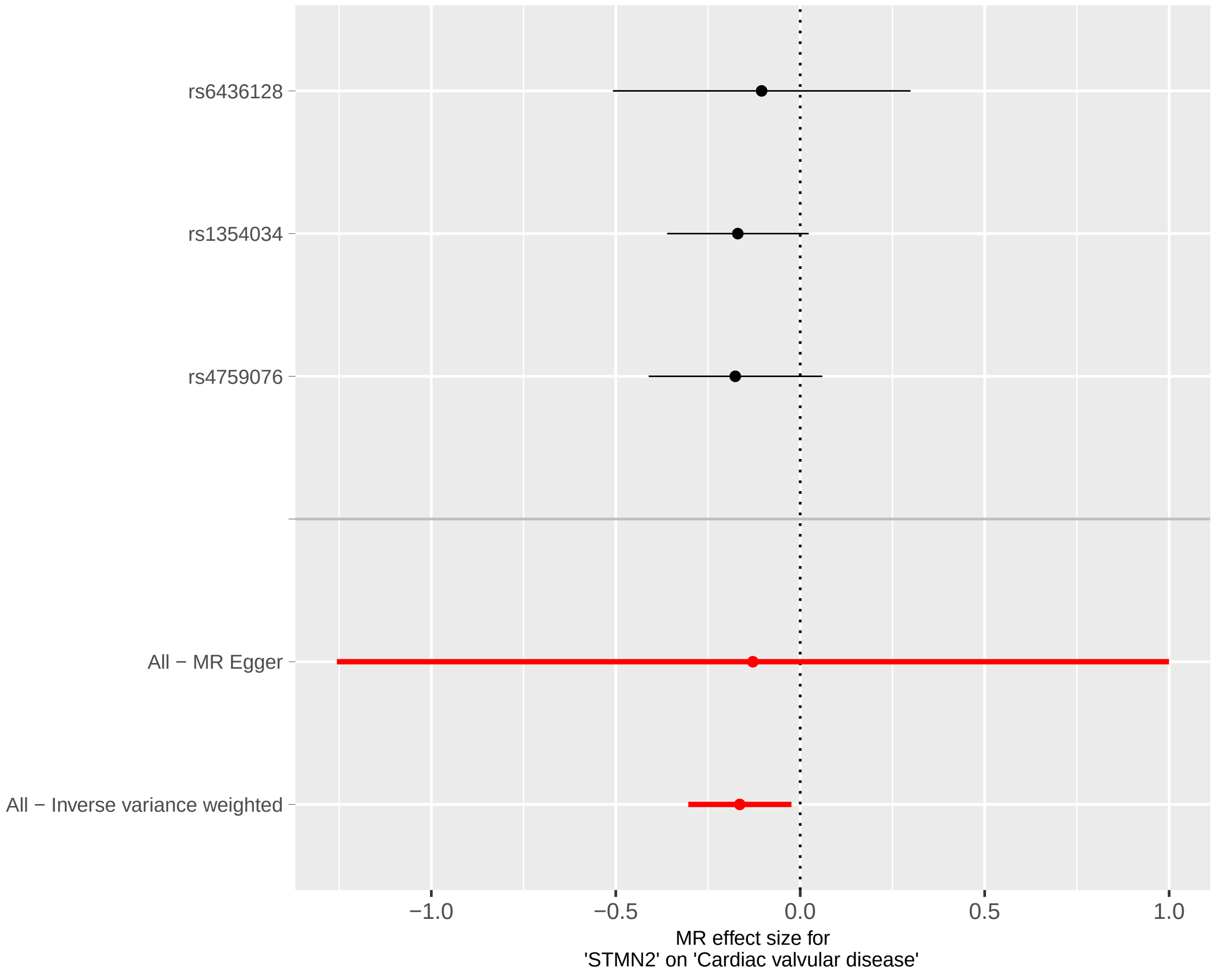
rs6436128
rs1354034
rs4759076
All − MR Egger
All − Inverse variance weighted
−1.0
−0.5
0.0
0.5
1.0
MR effect size for
'STMN2' on 'Cardiac valvular disease'

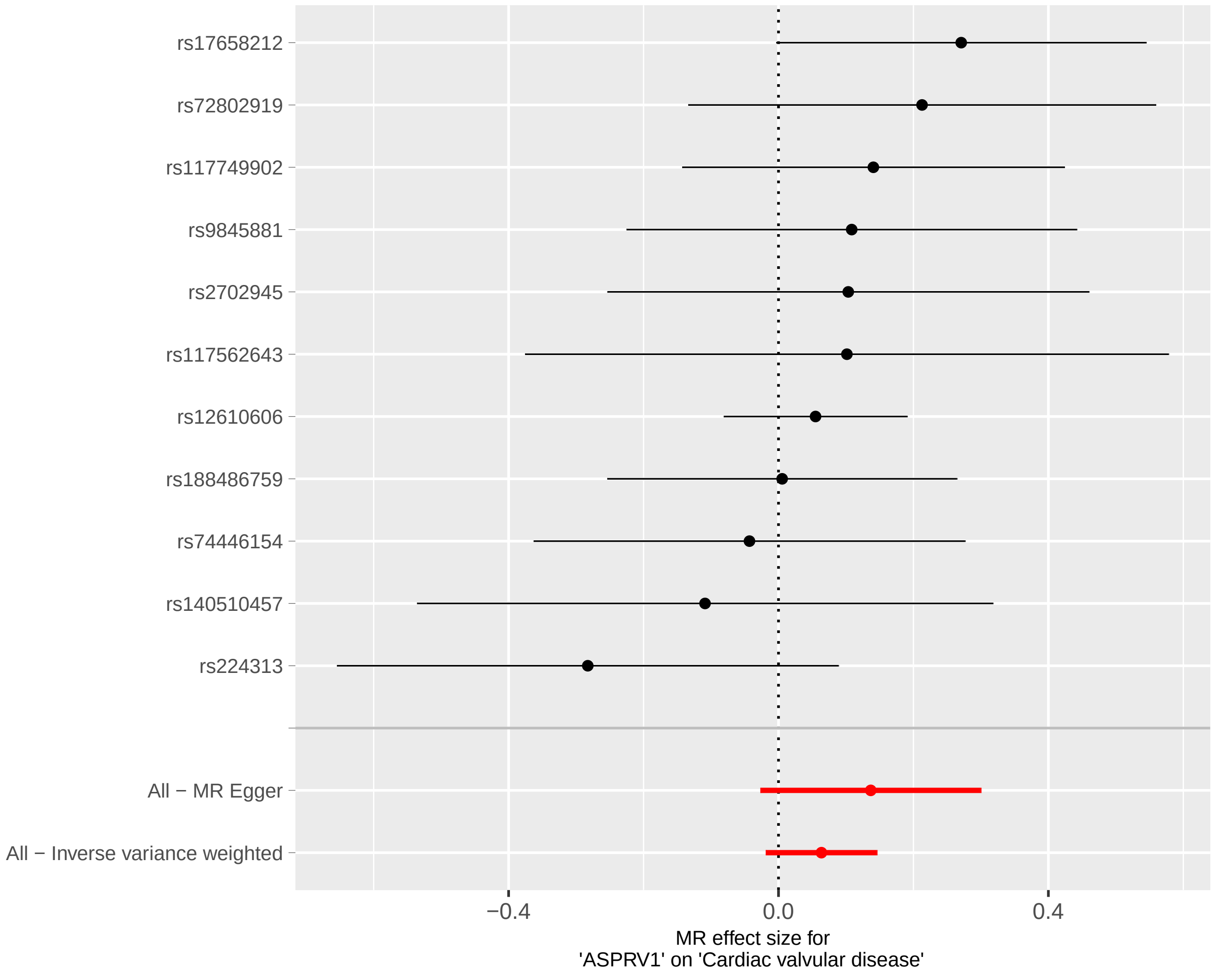

rs17658212
rs72802919
rs117749902
rs9845881
rs2702945
rs117562643
rs12610606
rs188486759
rs74446154
rs140510457
rs224313
All − MR Egger
All − Inverse variance weighted
−0.4
0.0
0.4
MR effect size for
'ASPRV1' on 'Cardiac valvular disease'

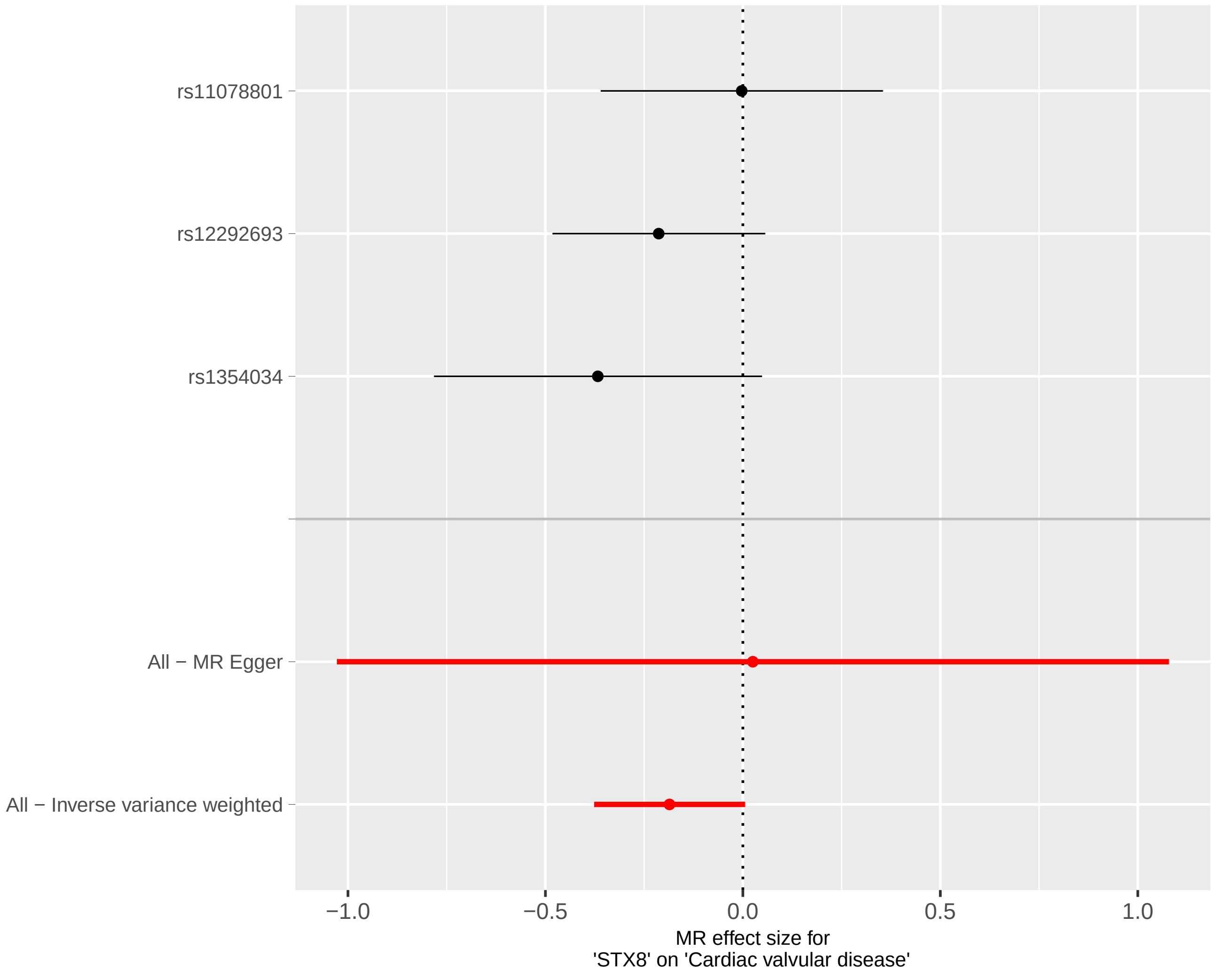
rs11078801
rs12292693
rs1354034
All − MR Egger
All − Inverse variance weighted
−1.0
−0.5
0.0
0.5
1.0
MR effect size for
'STX8' on 'Cardiac valvular disease'

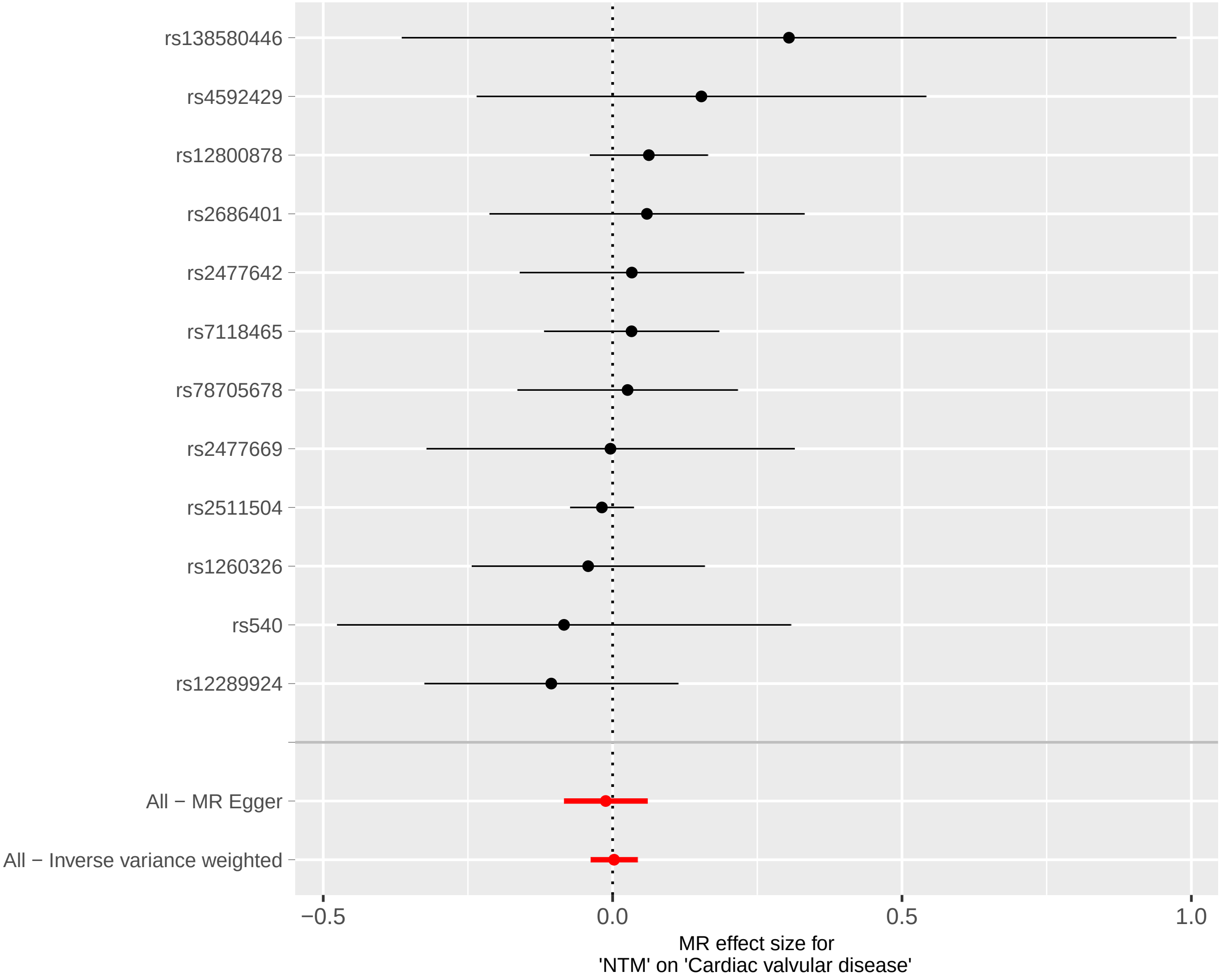

rs138580446
rs4592429
rs12800878
rs2686401
rs2477642
rs7118465
rs78705678
rs2477669
rs2511504
rs1260326
rs540
rs12289924
All − MR Egger
All − Inverse variance weighted
−0.5
0.0
0.5
1.0
MR effect size for
'NTM' on 'Cardiac valvular disease'

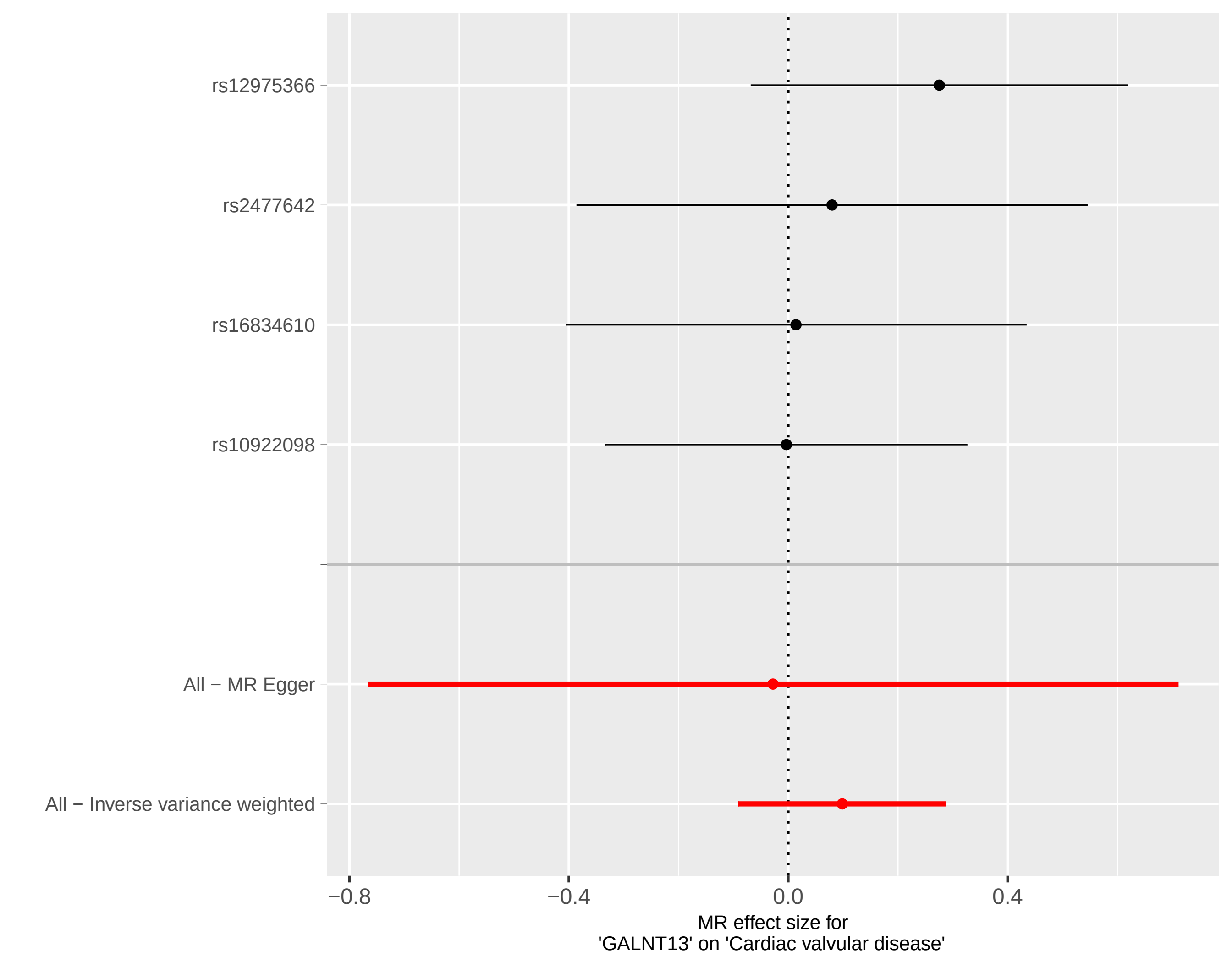
rs12975366
rs2477642
rs16834610
rs10922098
All − MR Egger
All − Inverse variance weighted
−0.8
−0.4
0.0
0.4
MR effect size for
'GALNT13' on 'Cardiac valvular disease'

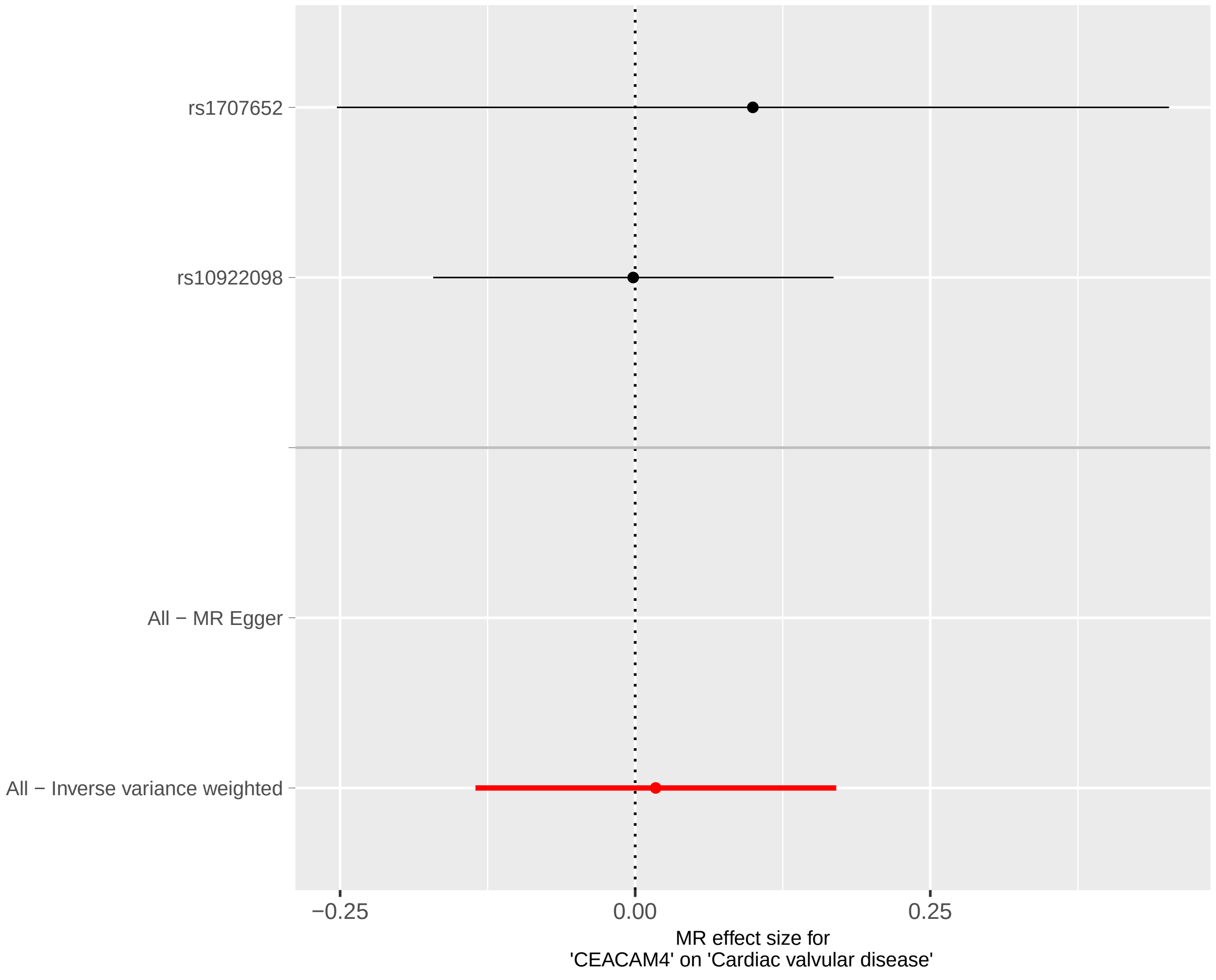
rs1707652
rs10922098
All − MR Egger
All − Inverse variance weighted
−0.25
0.00
0.25
MR effect size for
'CEACAM4' on 'Cardiac valvular disease'

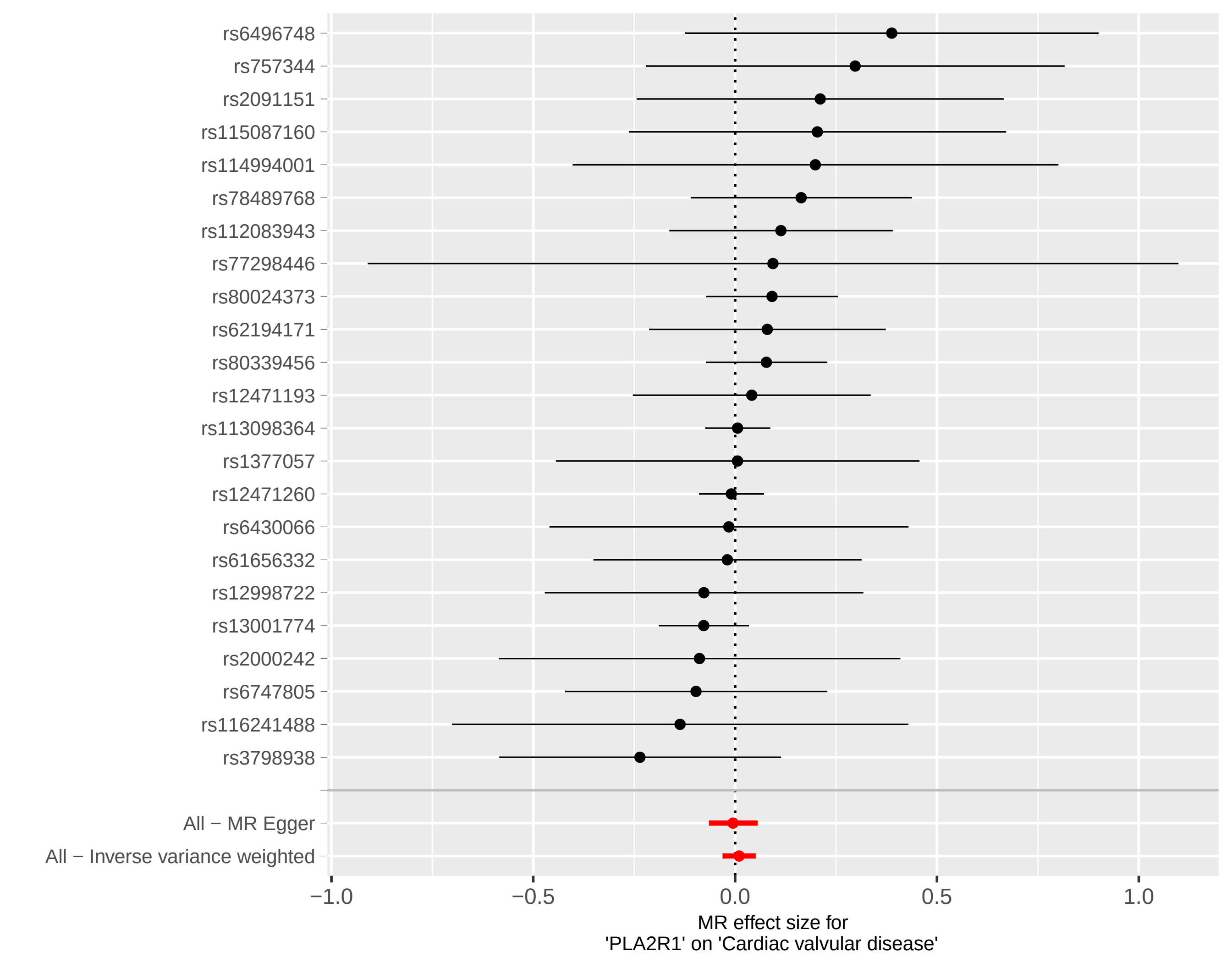
rs6496748
rs757344
rs2091151
rs115087160
rs114994001
rs78489768
rs112083943
rs77298446
rs80024373
rs62194171
rs80339456
rs12471193
rs113098364
rs1377057
rs12471260
rs6430066
rs61656332
rs12998722
rs13001774
rs2000242
rs6747805
rs116241488
rs3798938
All − MR Egger
All − Inverse variance weighted
−1.0
−0.5
0.0
0.5
1.0
MR effect size for
'PLA2R1' on 'Cardiac valvular disease'

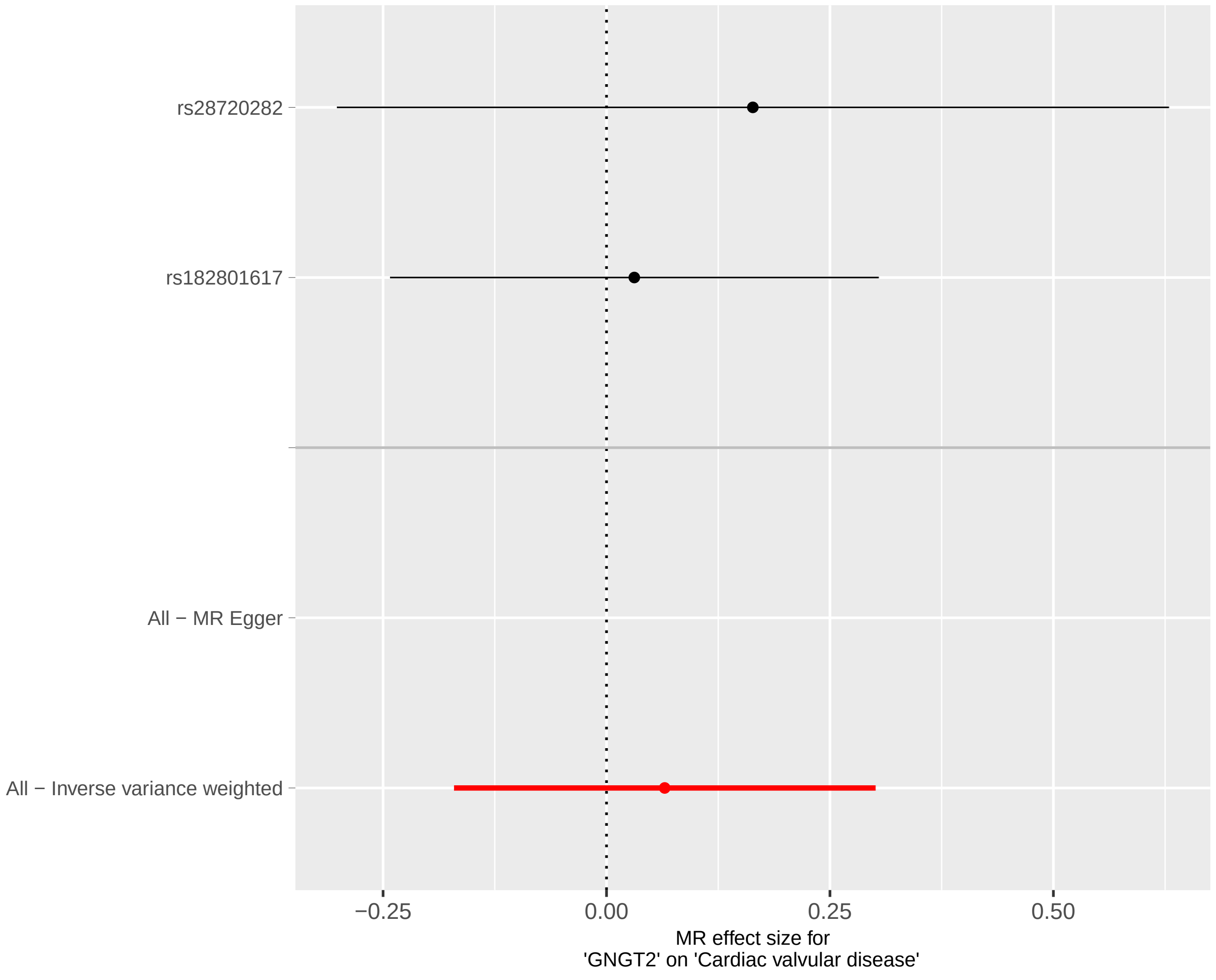
rs28720282
rs182801617
All − MR Egger
All − Inverse variance weighted
−0.25
0.00
0.25
0.50
MR effect size for
'GNGT2' on 'Cardiac valvular disease'

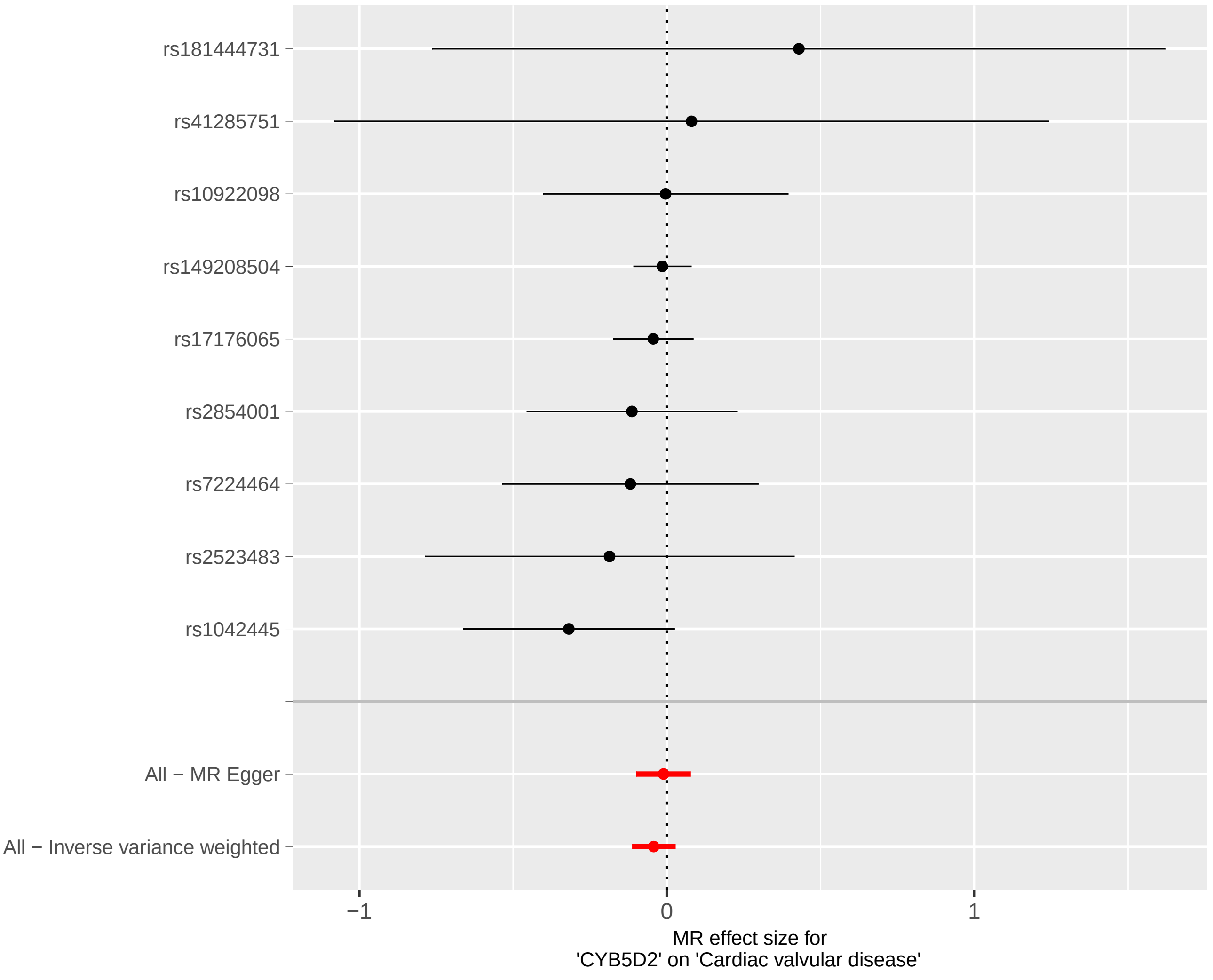

rs181444731
rs41285751
rs10922098
rs149208504
rs17176065
rs2854001
rs7224464
rs2523483
rs1042445
All − MR Egger
All − Inverse variance weighted
−1
0
1
MR effect size for
'CYB5D2' on 'Cardiac valvular disease'

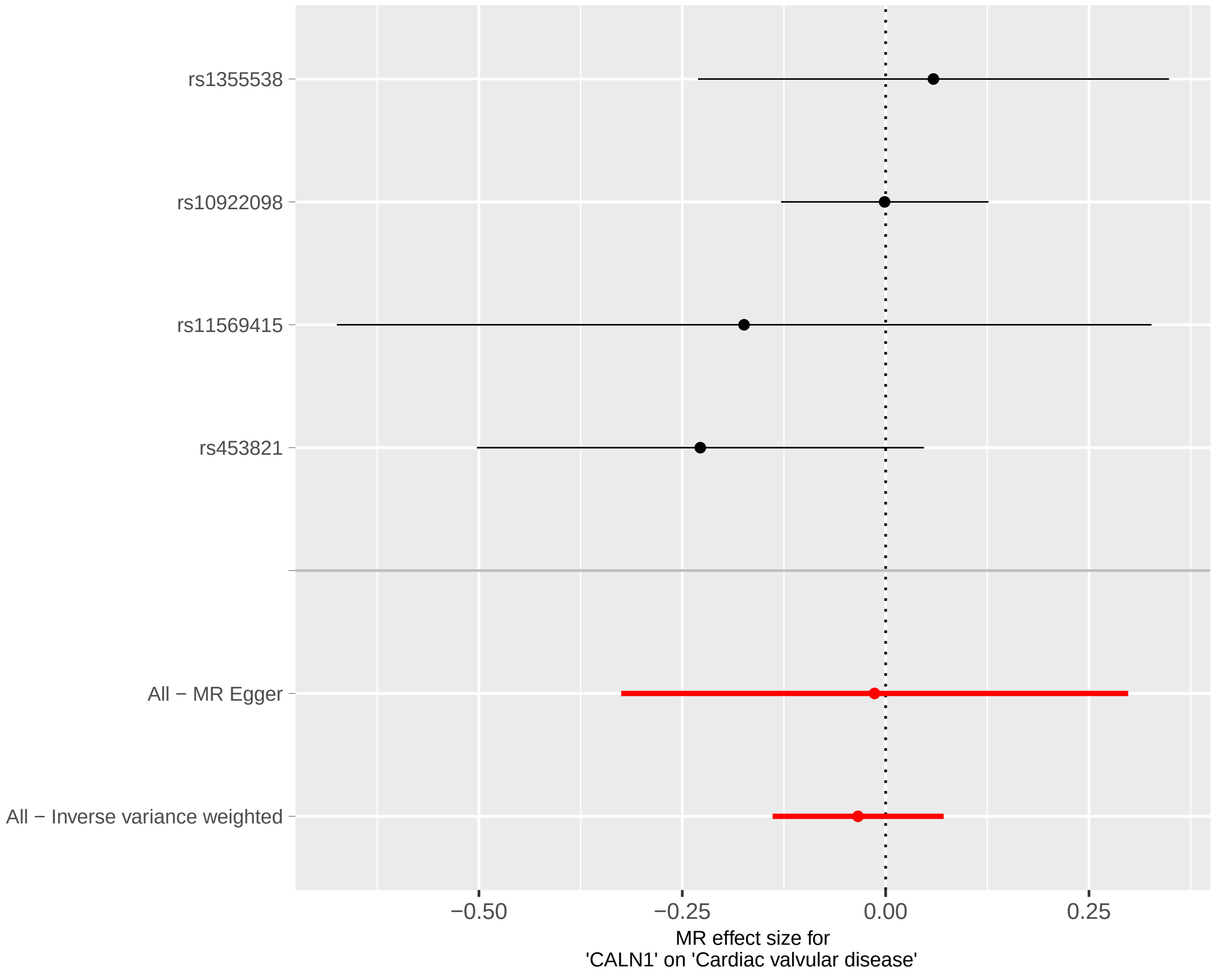
rs1355538
rs10922098
rs11569415
rs453821
All − MR Egger
All − Inverse variance weighted
−0.50
−0.25
0.00
0.25
MR effect size for
'CALN1' on 'Cardiac valvular disease'

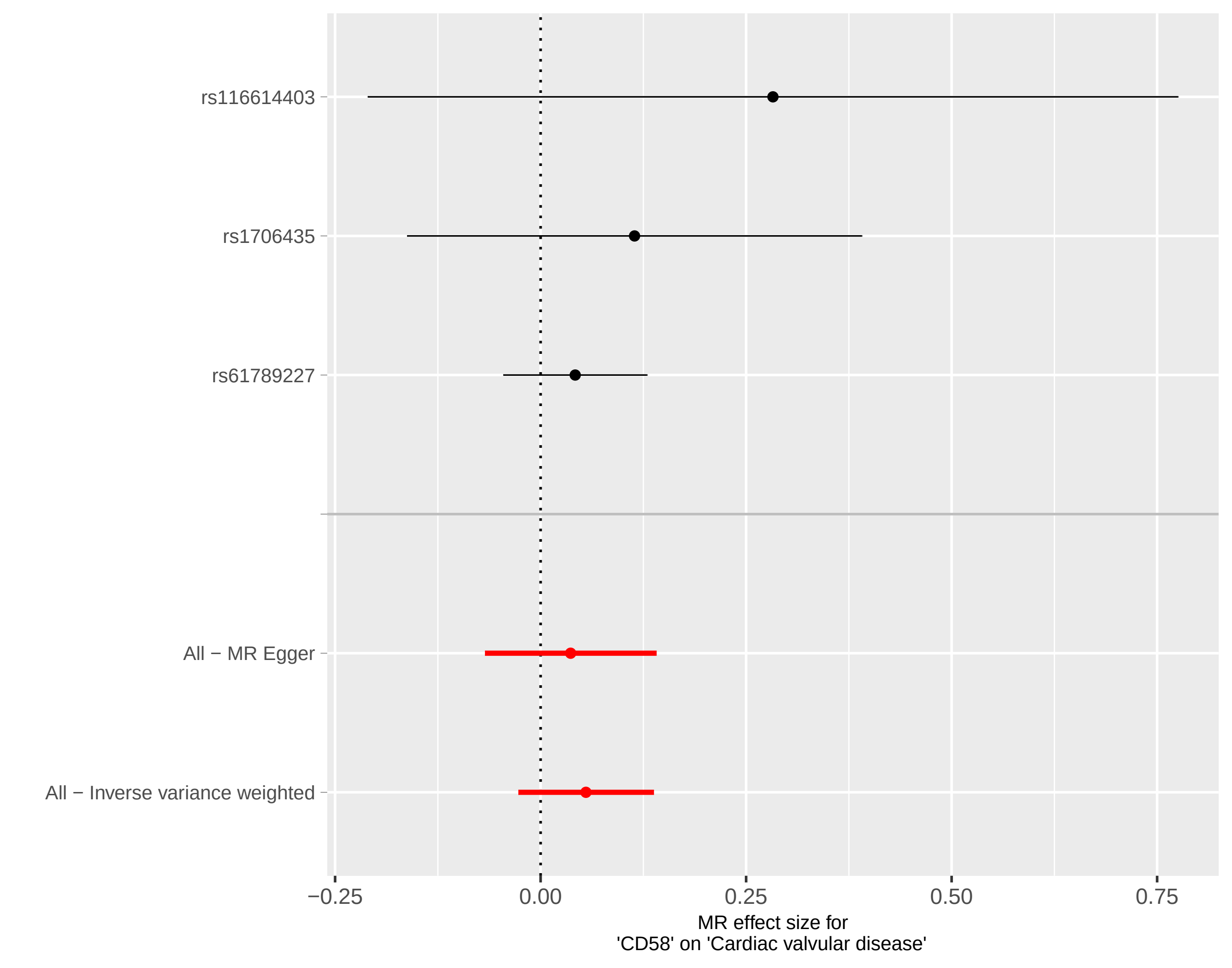
rs116614403
rs1706435
rs61789227
All − MR Egger
All − Inverse variance weighted
−0.25
0.00
0.25
0.50
0.75
MR effect size for
'CD58' on 'Cardiac valvular disease'

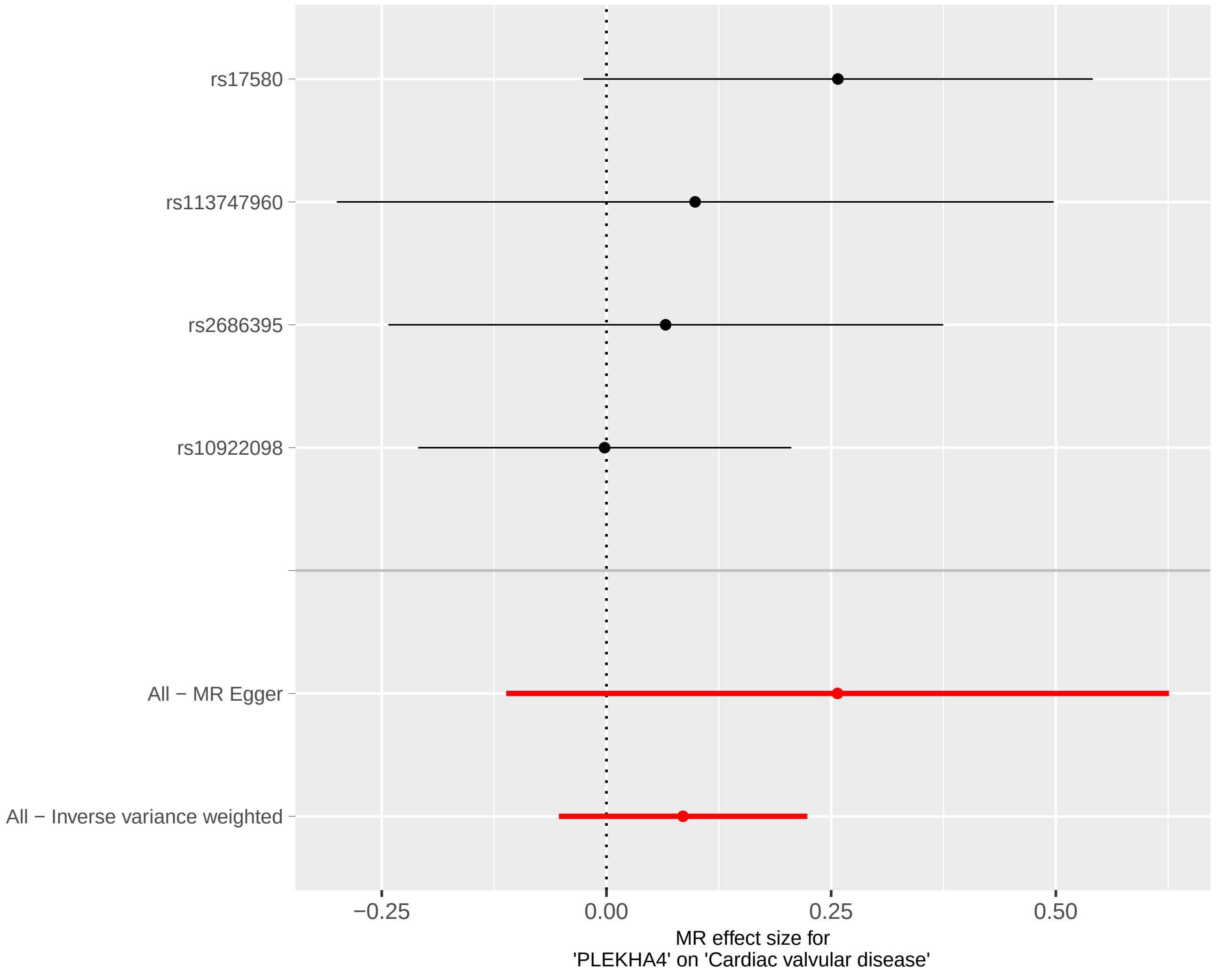
rs17580
rs113747960
rs2686395
rs10922098
All − MR Egger
All − Inverse variance weighted
−0.25
0.00
0.25
0.50
MR effect size for
'PLEKHA4' on 'Cardiac valvular disease'

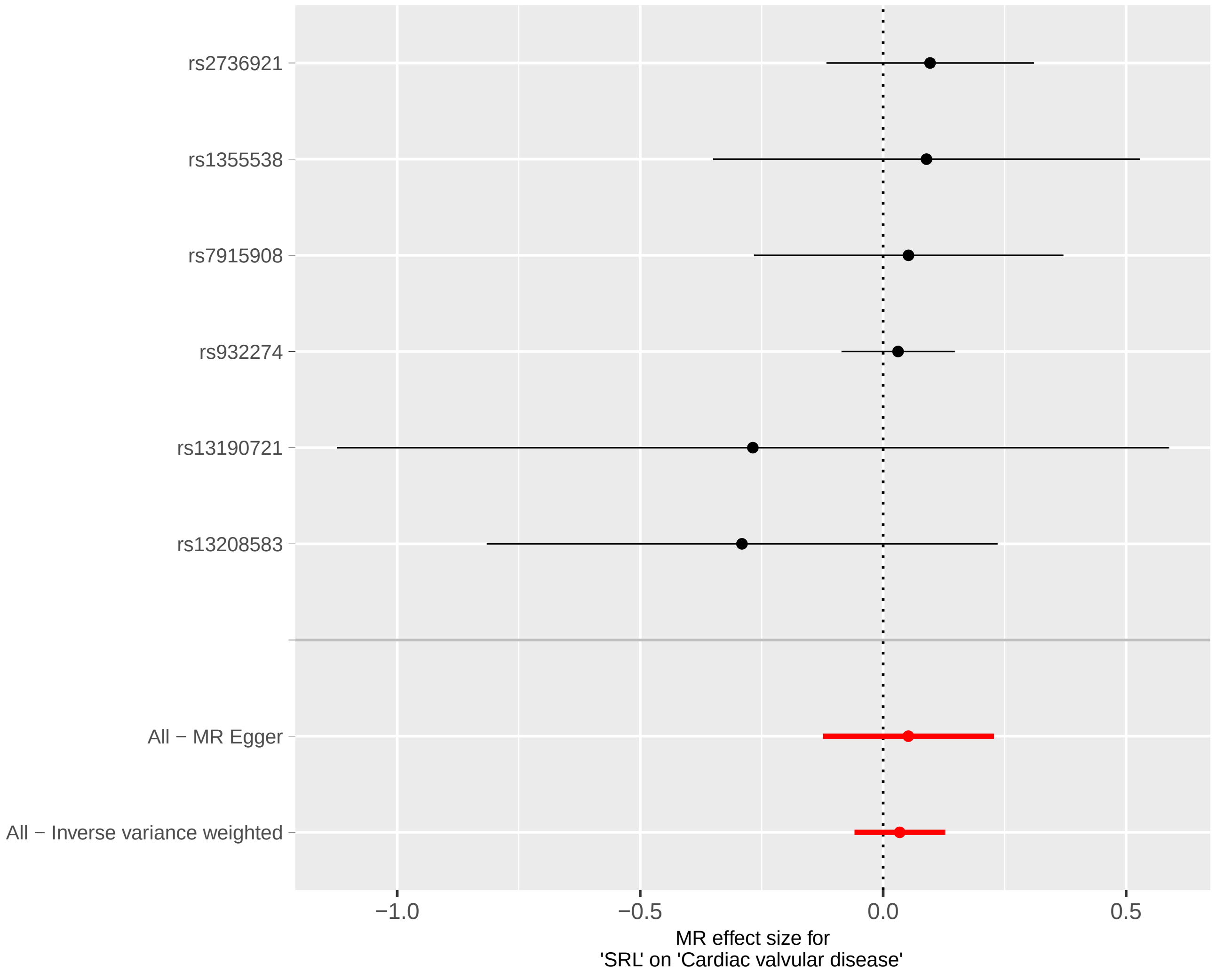
rs2736921
rs1355538
rs7915908
rs932274
rs13190721
rs13208583
All − MR Egger
All − Inverse variance weighted
−1.0
−0.5
0.0
0.5
MR effect size for
'SRL' on 'Cardiac valvular disease'

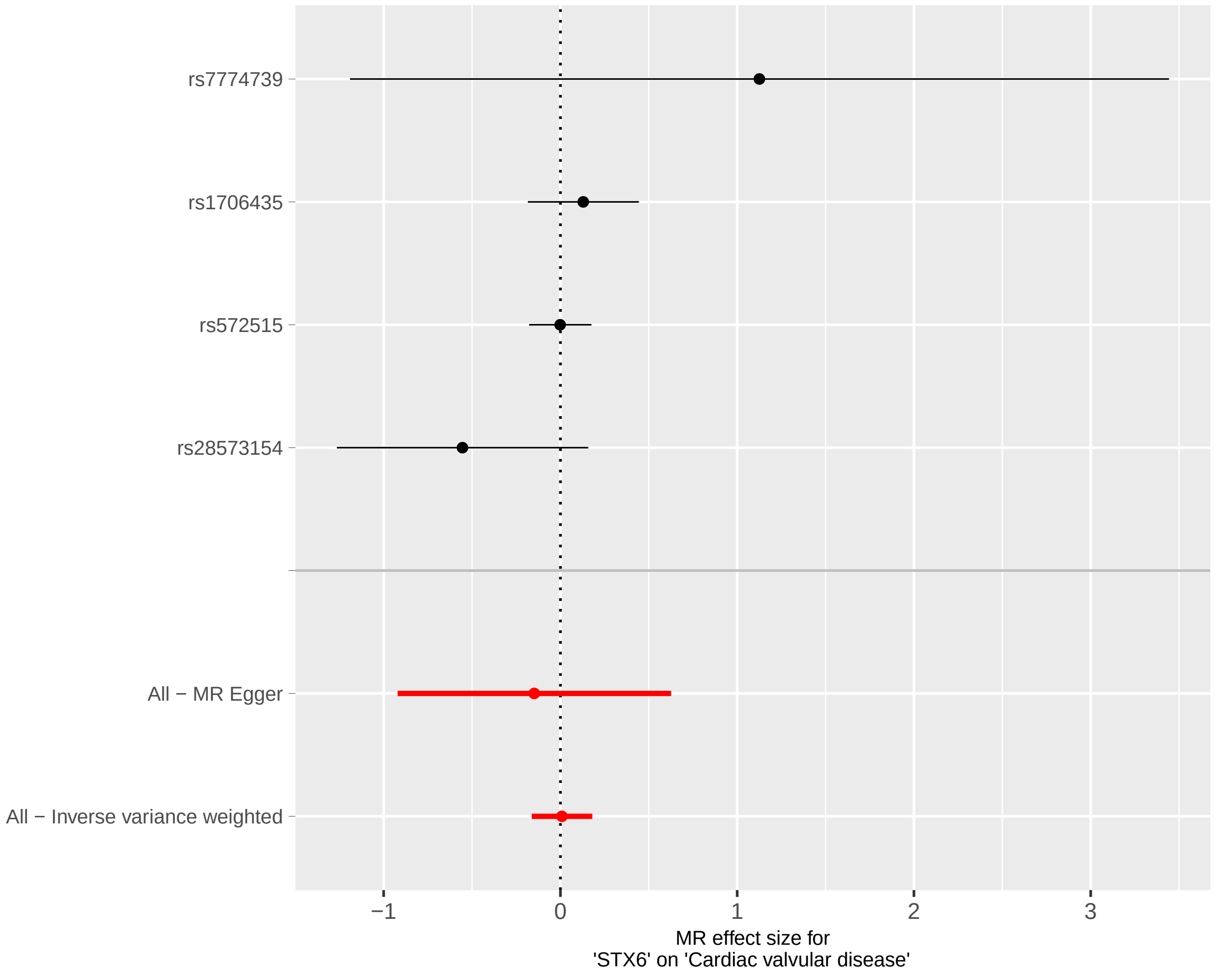

rs7774739
rs1706435
rs572515
rs28573154
All − MR Egger
All − Inverse variance weighted
−1
0
1
2
3
MR effect size for
'STX6' on 'Cardiac valvular disease'

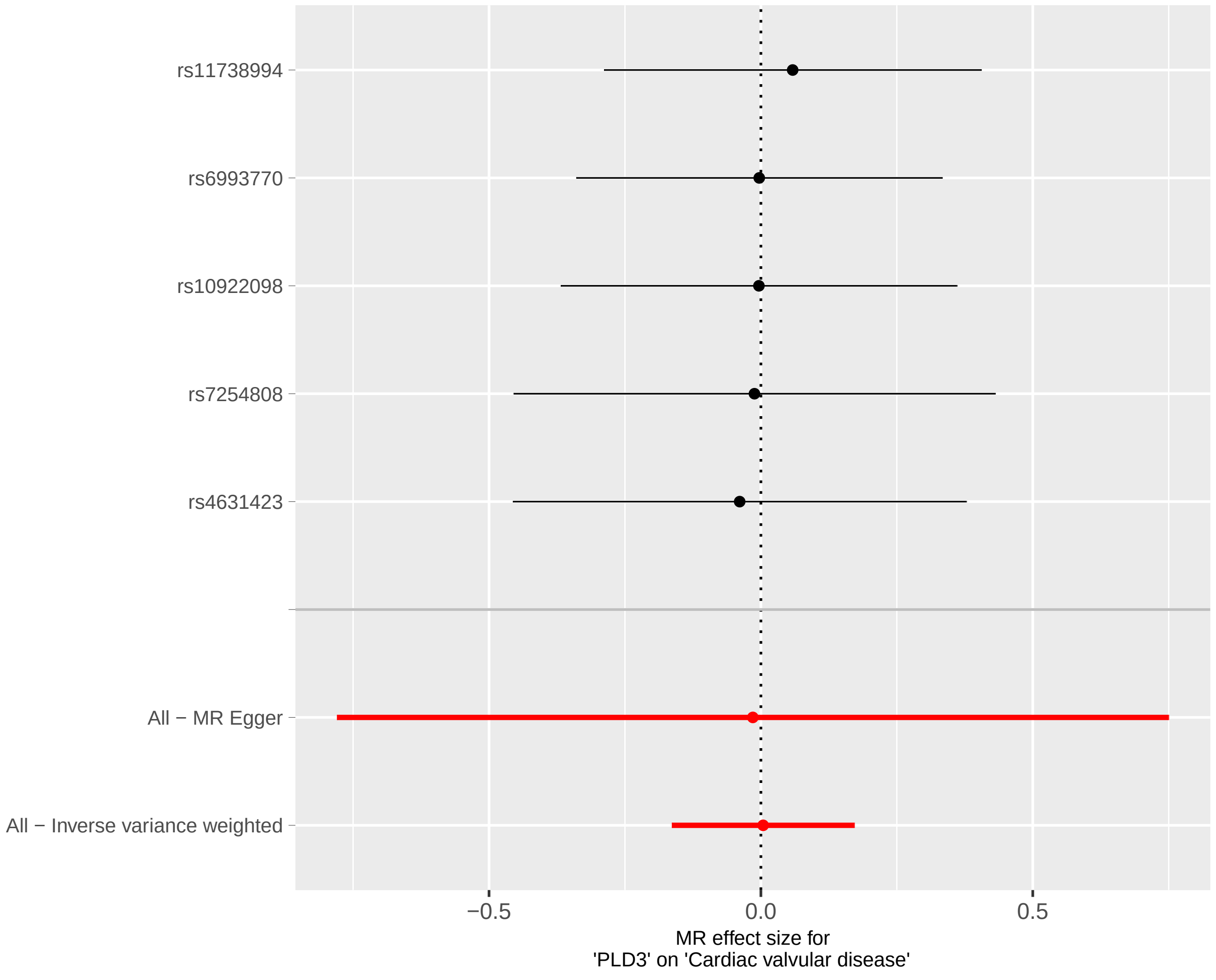

rs11738994
rs6993770
rs10922098
rs7254808
rs4631423
All − MR Egger
All − Inverse variance weighted
−0.5
0.0
0.5
MR effect size for
'PLD3' on 'Cardiac valvular disease'

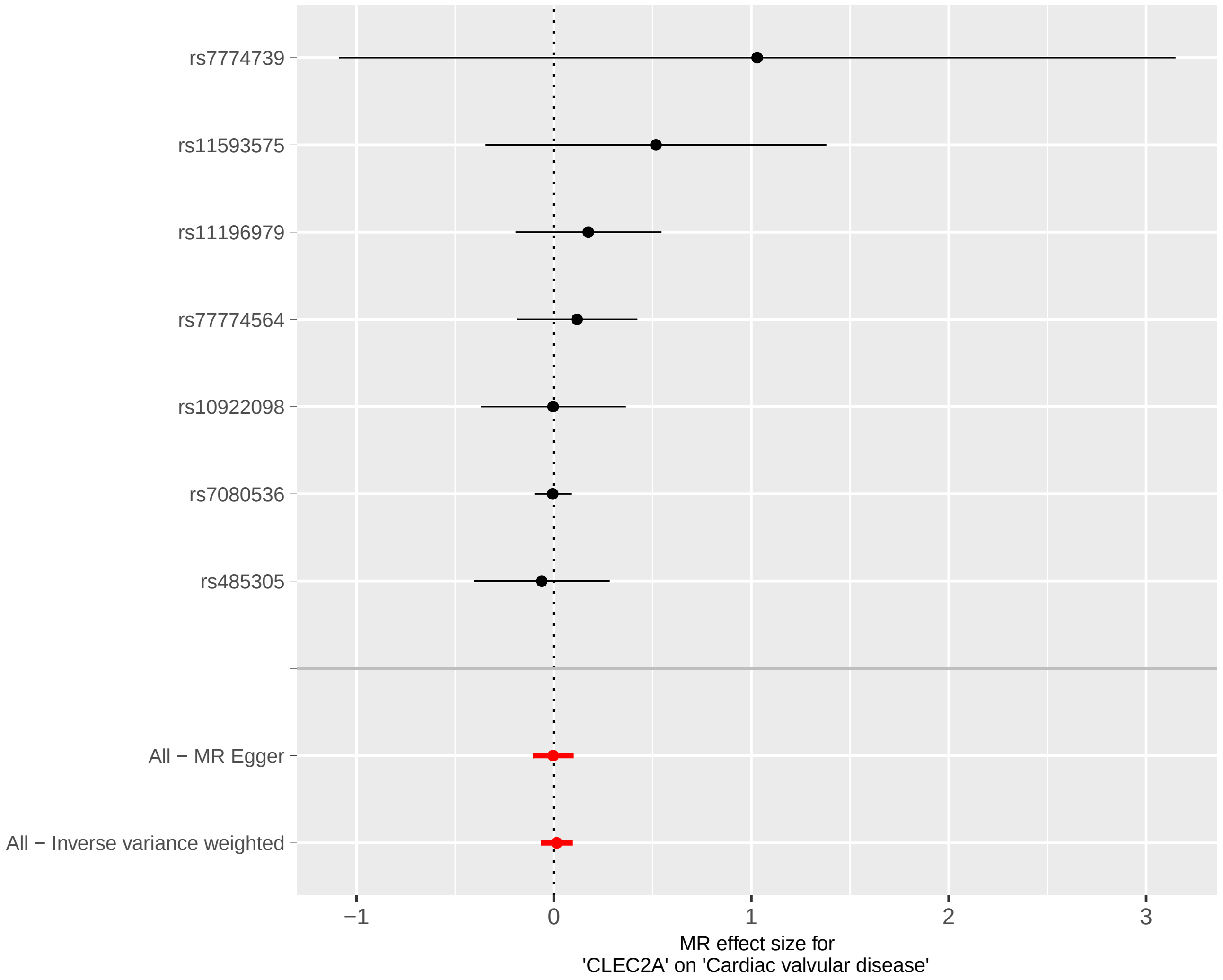

rs7774739
rs11593575
rs11196979
rs77774564
rs10922098
rs7080536
rs485305
All − MR Egger
All − Inverse variance weighted
−1
0
1
2
3
MR effect size for
'CLEC2A' on 'Cardiac valvular disease'

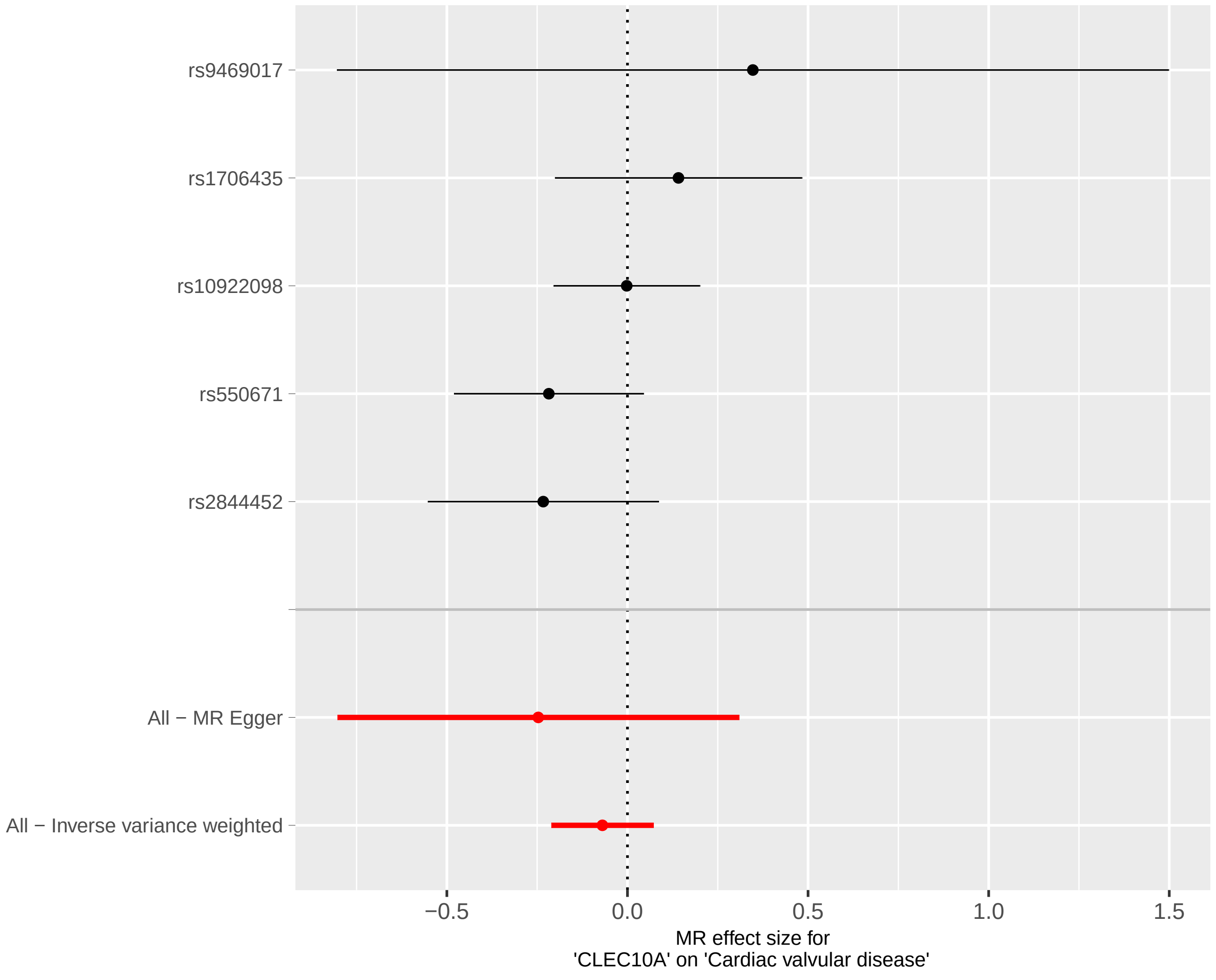
rs9469017
rs1706435
rs10922098
rs550671
rs2844452
All − MR Egger
All − Inverse variance weighted
−0.5
0.0
0.5
1.0
1.5
MR effect size for
'CLEC10A' on 'Cardiac valvular disease'

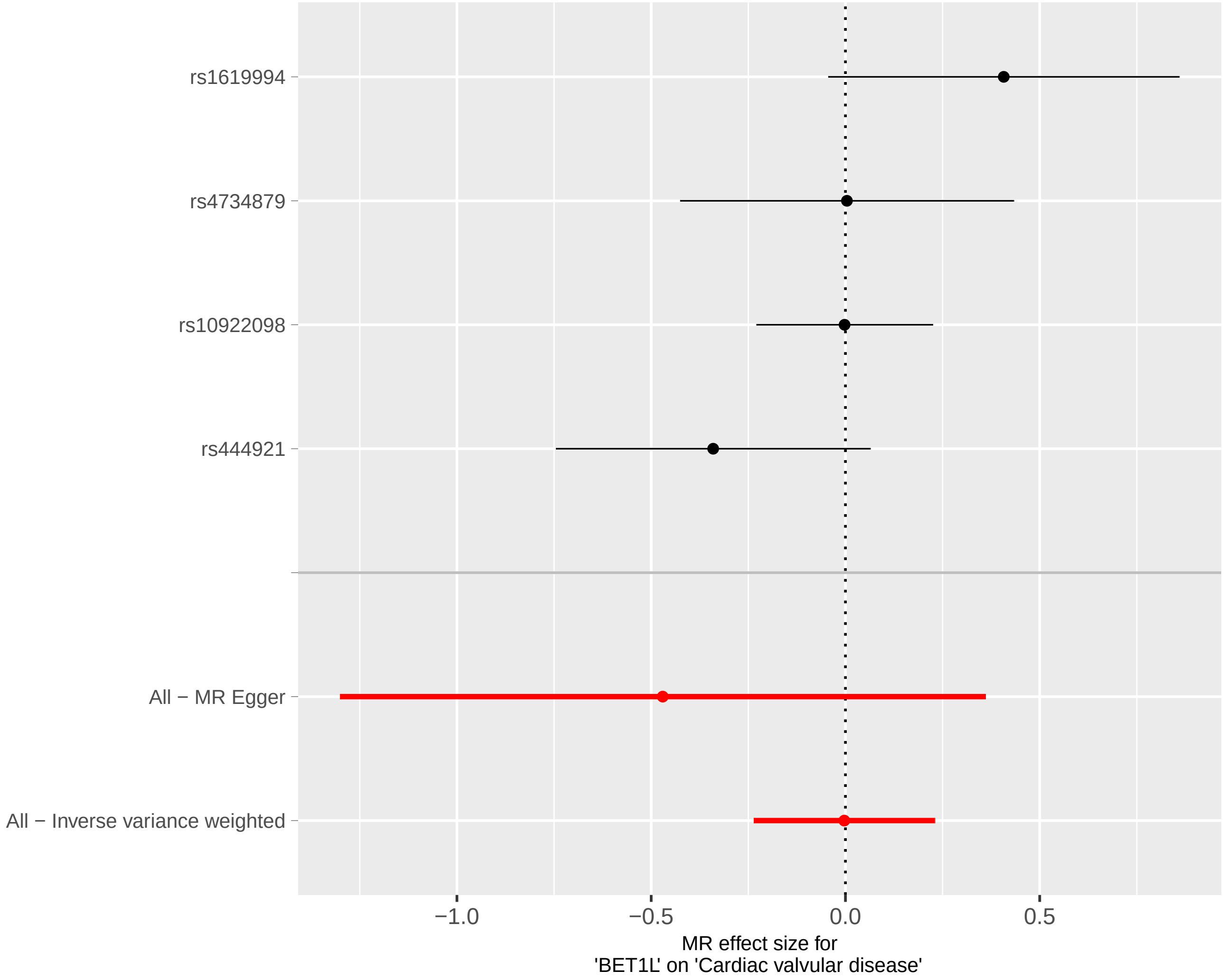

rs1619994
rs4734879
rs10922098
rs444921
All − MR Egger
All − Inverse variance weighted
−1.0
−0.5
0.0
0.5
MR effect size for
'BET1L' on 'Cardiac valvular disease'

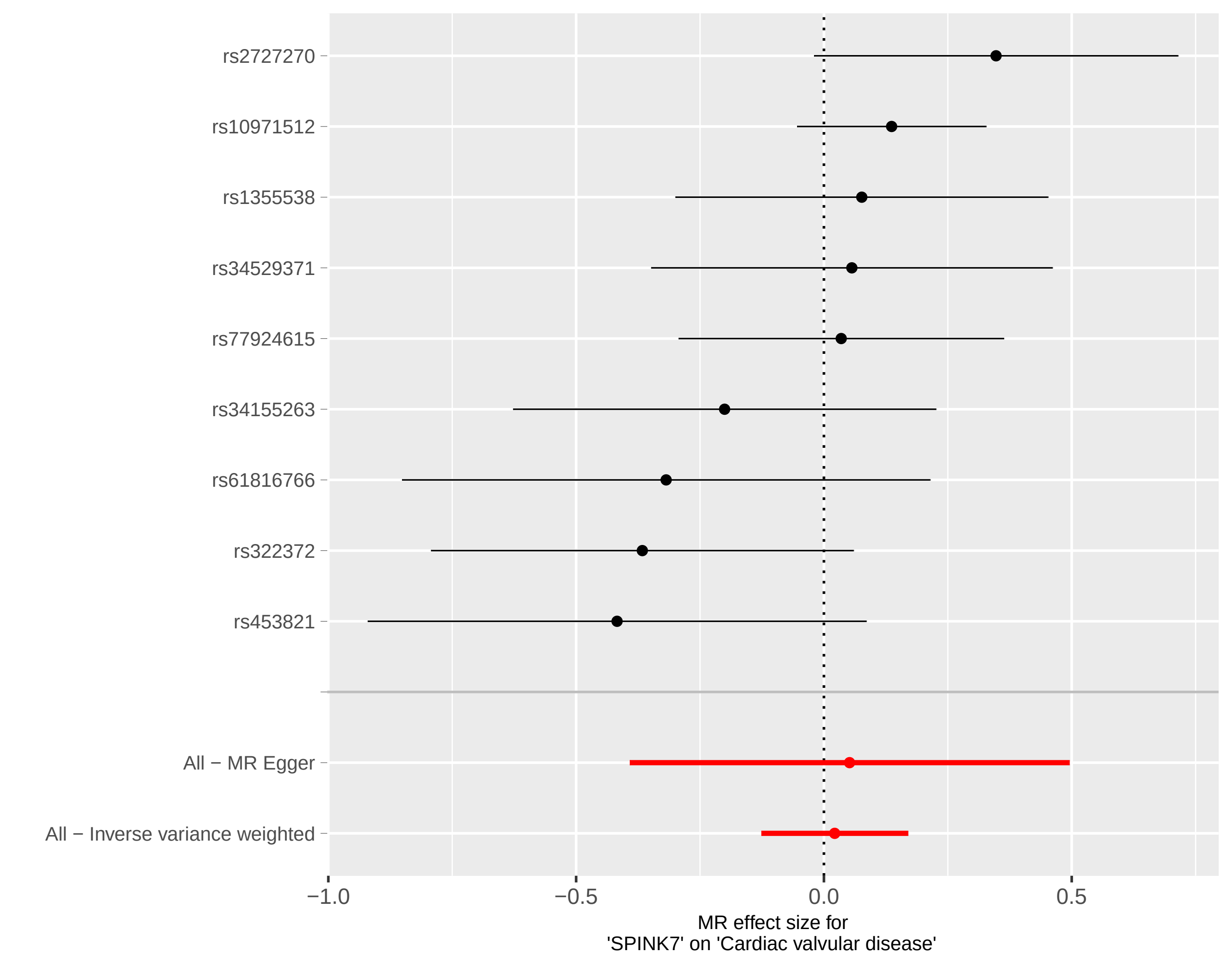
rs2727270
rs10971512
rs1355538
rs34529371
rs77924615
rs34155263
rs61816766
rs322372
rs453821
All − MR Egger
All − Inverse variance weighted
−1.0
−0.5
0.0
0.5
MR effect size for
'SPINK7' on 'Cardiac valvular disease'

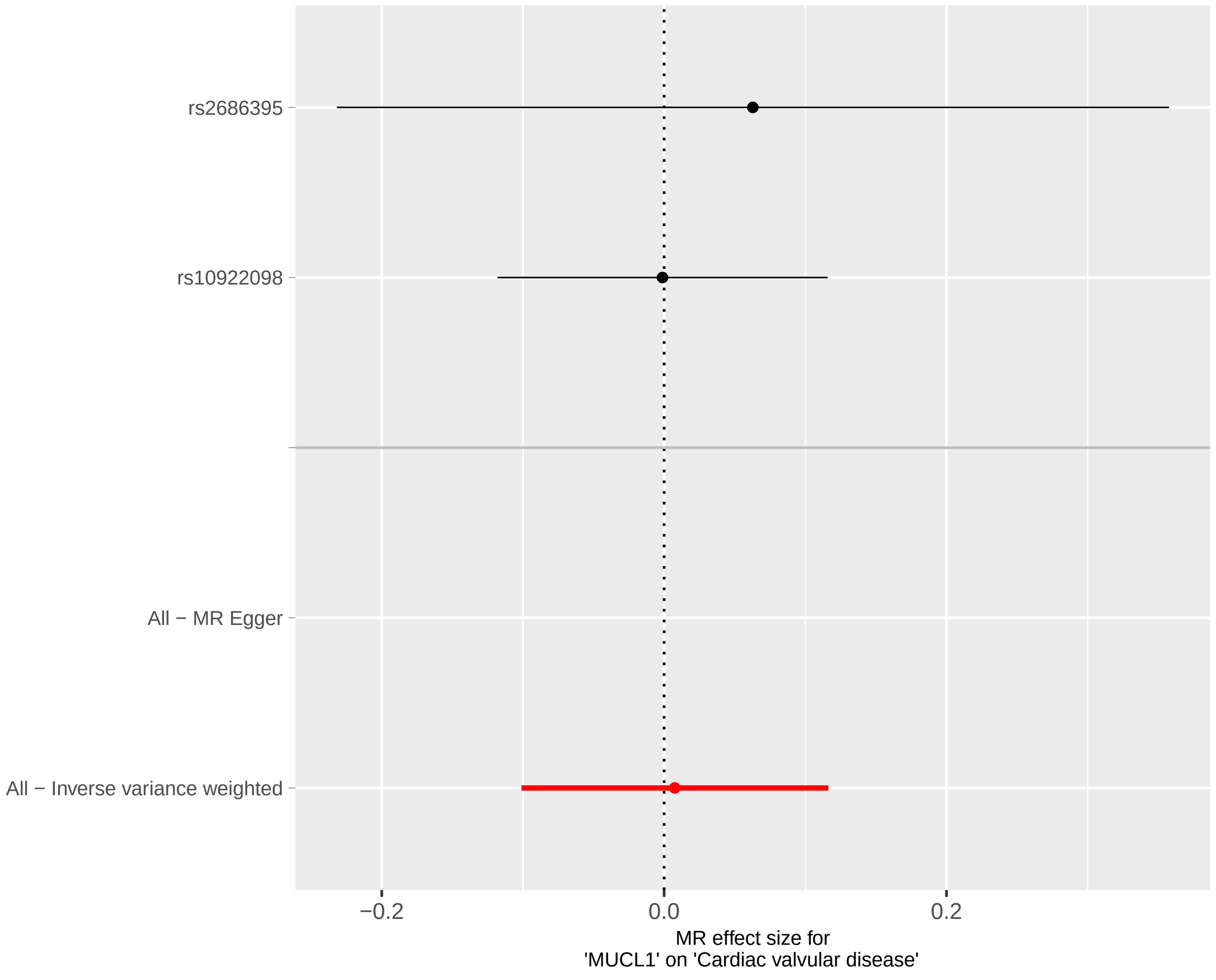
rs2686395
rs10922098
All − MR Egger
All − Inverse variance weighted
−0.2
0.0
0.2
MR effect size for
'MUCL1' on 'Cardiac valvular disease'

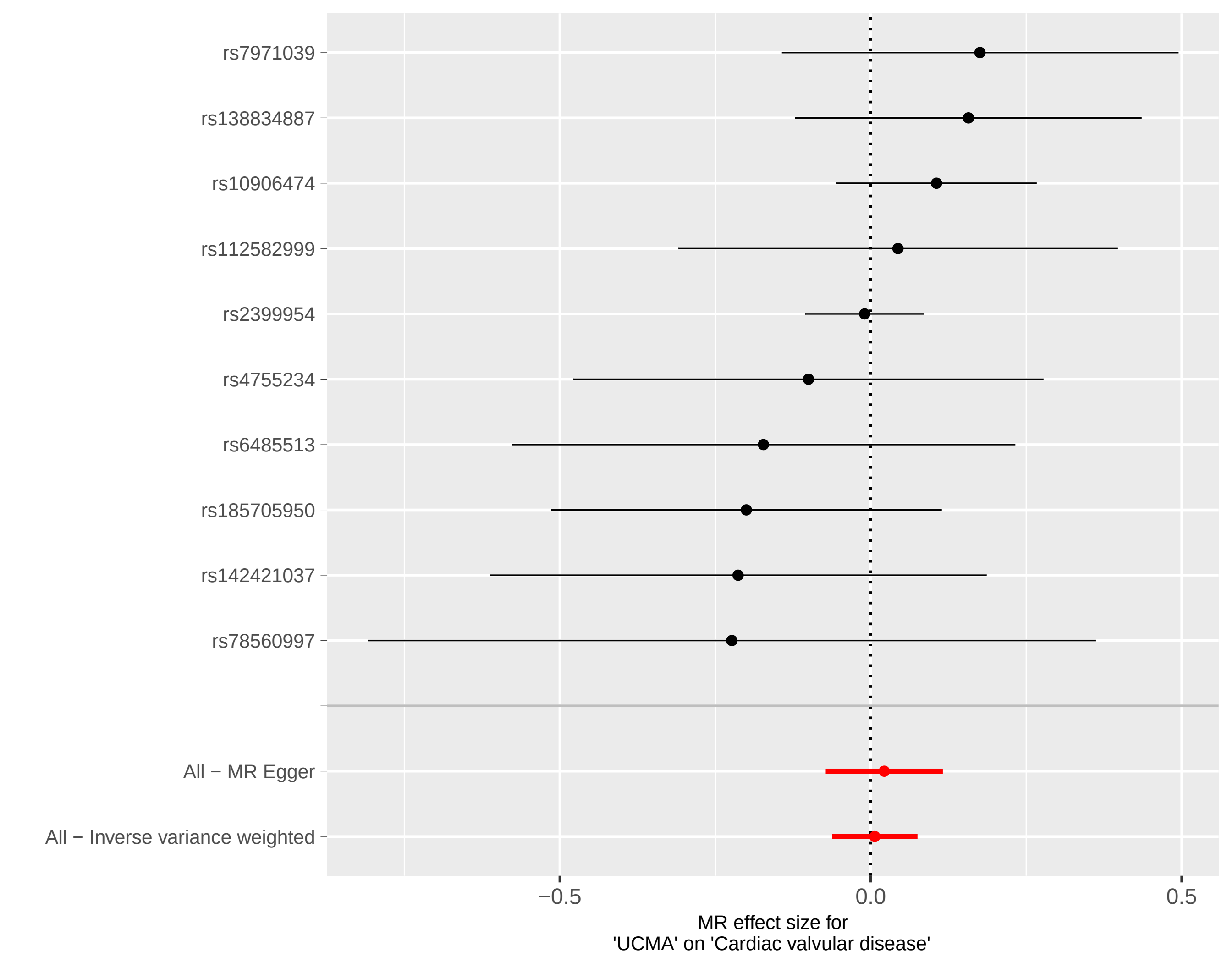
rs7971039
rs138834887
rs10906474
rs112582999
rs2399954
rs4755234
rs6485513
rs185705950
rs142421037
rs78560997
All − MR Egger
All − Inverse variance weighted
−0.5
0.0
0.5
MR effect size for
'UCMA' on 'Cardiac valvular disease'

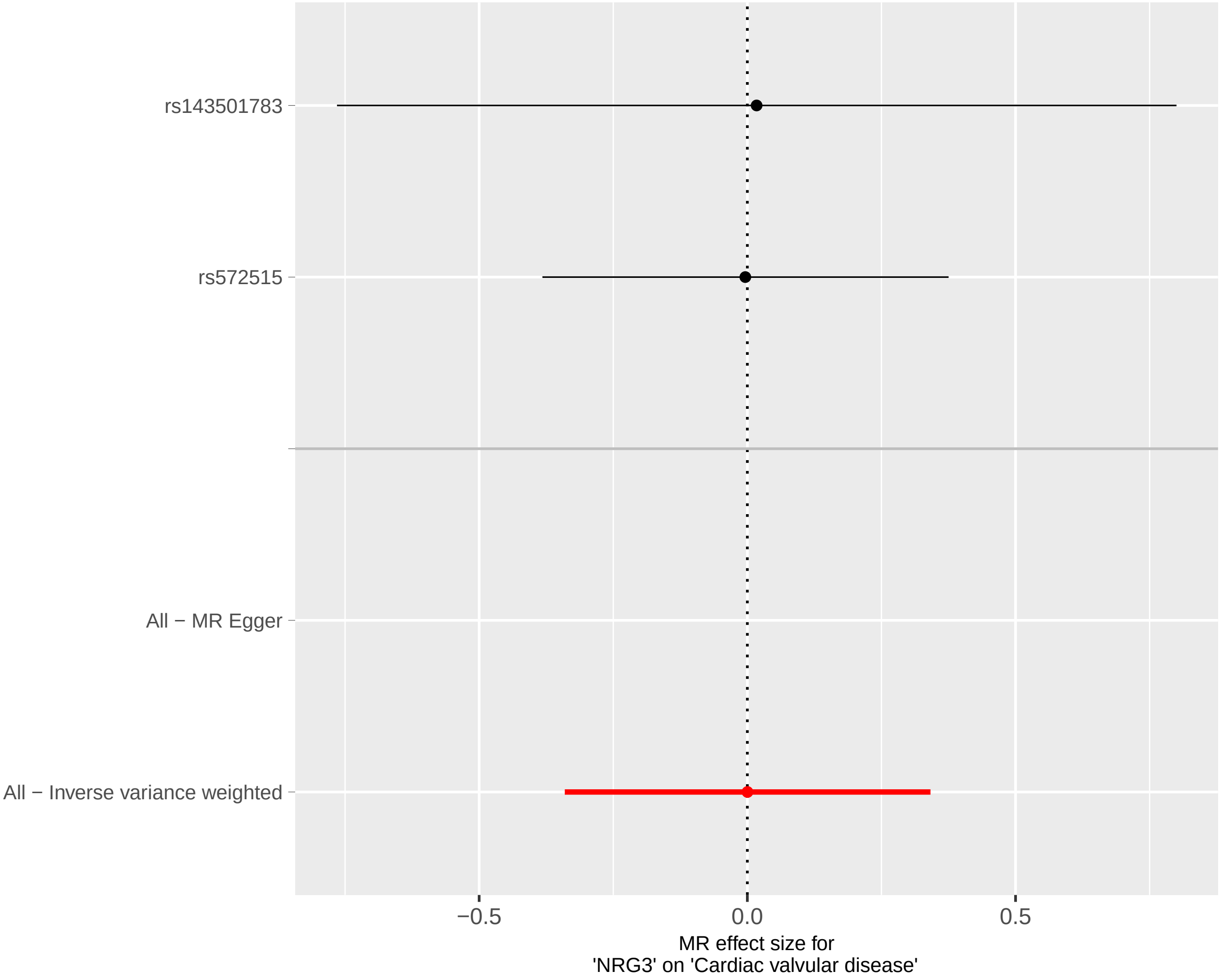
rs143501783
rs572515
All − MR Egger
All − Inverse variance weighted
−0.5
0.0
0.5
MR effect size for
'NRG3' on 'Cardiac valvular disease'

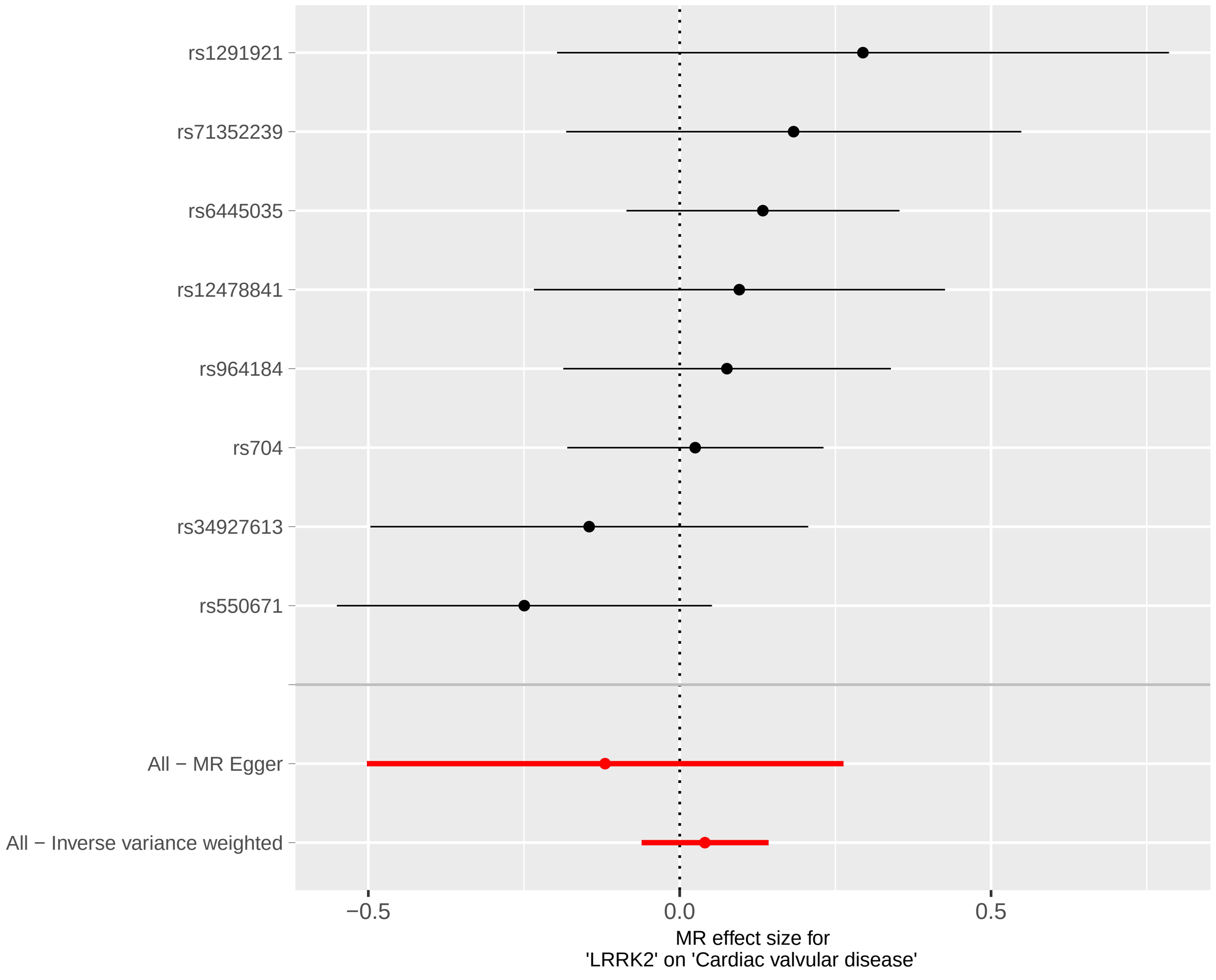
rs1291921
rs71352239
rs6445035
rs12478841
rs964184
rs704
rs34927613
rs550671
All − MR Egger
All − Inverse variance weighted
−0.5
0.0
0.5
MR effect size for
'LRRK2' on 'Cardiac valvular disease'

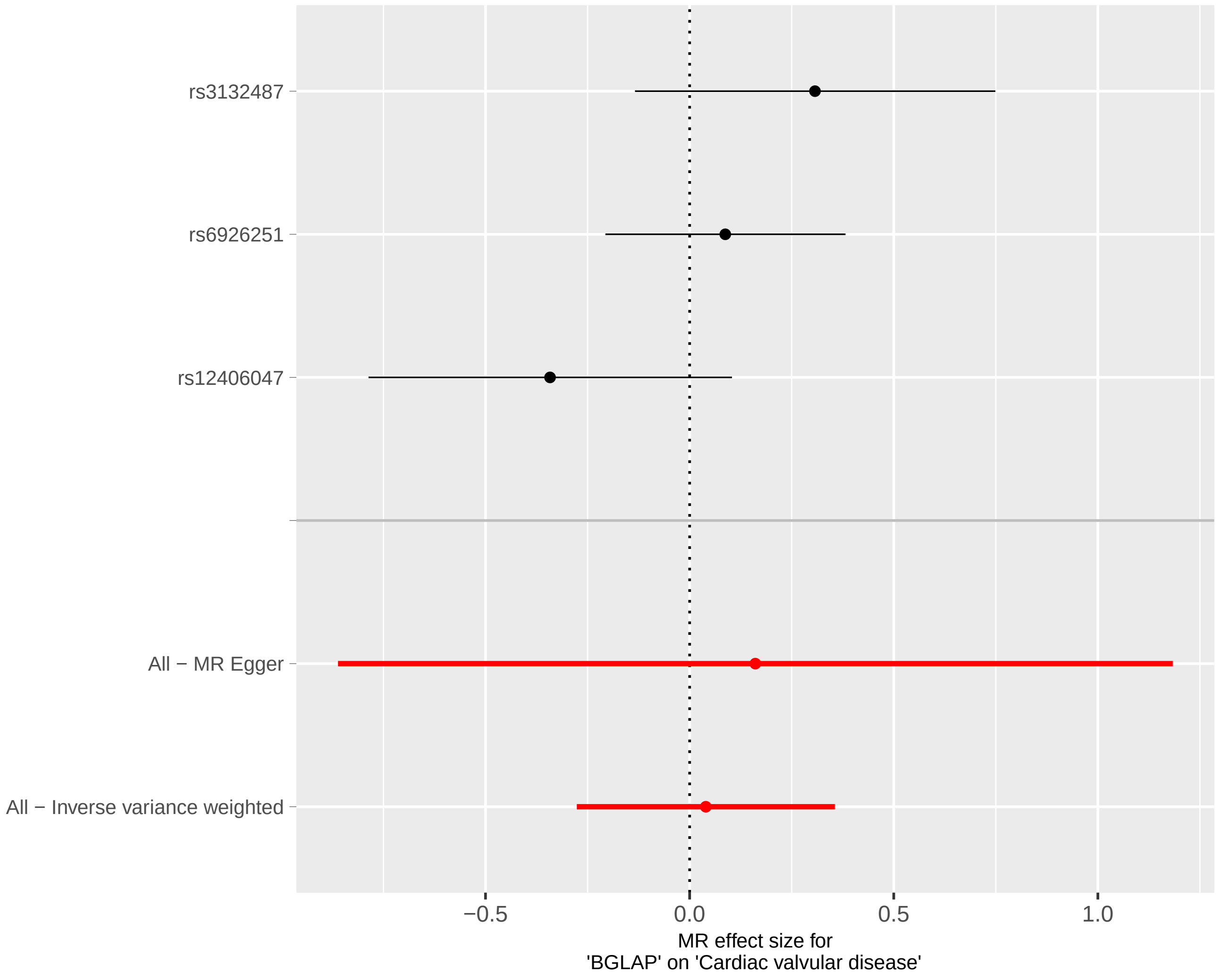
rs3132487
rs6926251
rs12406047
All − MR Egger
All − Inverse variance weighted
−0.5
0.0
0.5
1.0
MR effect size for
'BGLAP' on 'Cardiac valvular disease'

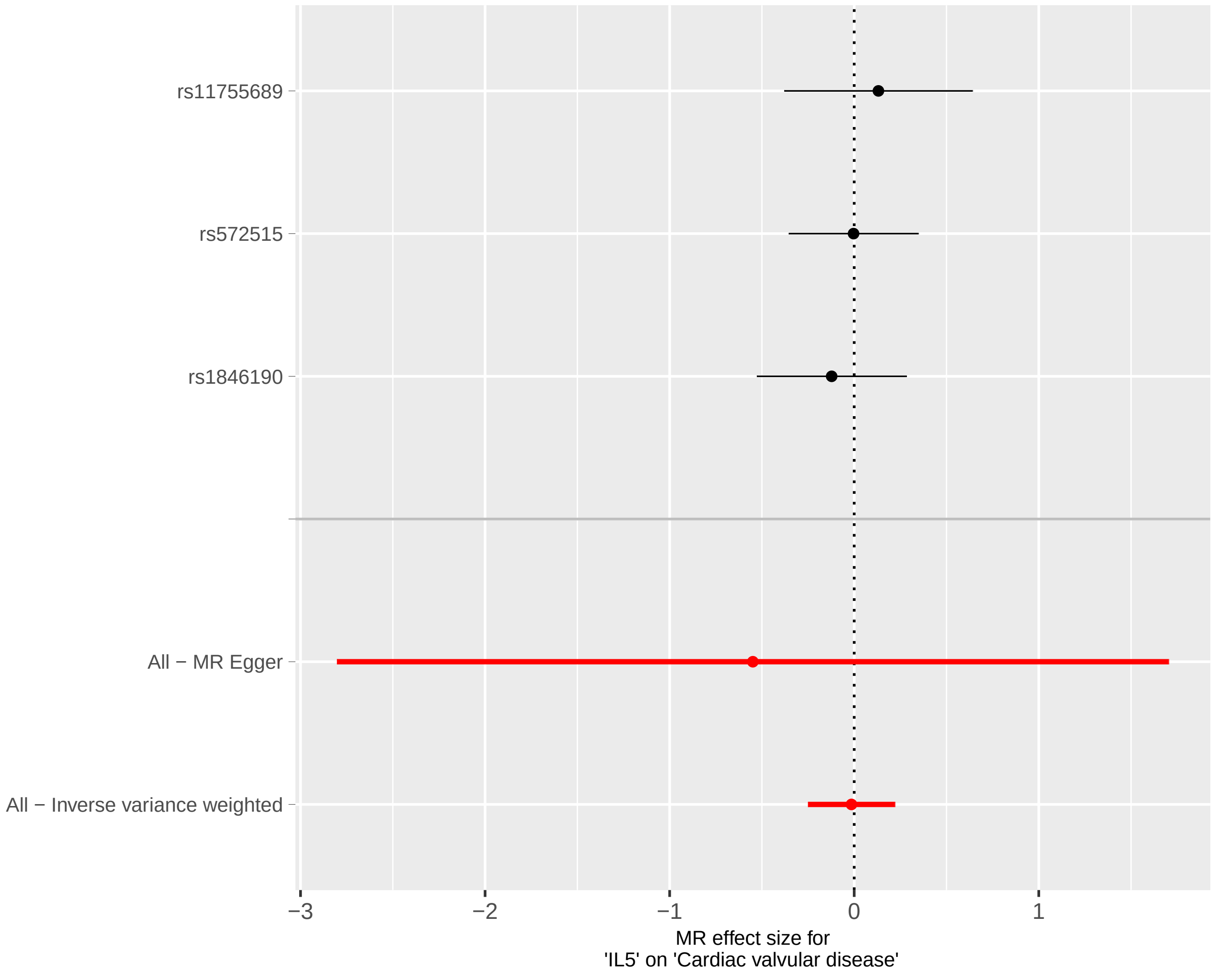
rs11755689
rs572515
rs1846190
All − MR Egger
All − Inverse variance weighted
−3
−2
−1
0
1
MR effect size for
'IL5' on 'Cardiac valvular disease'

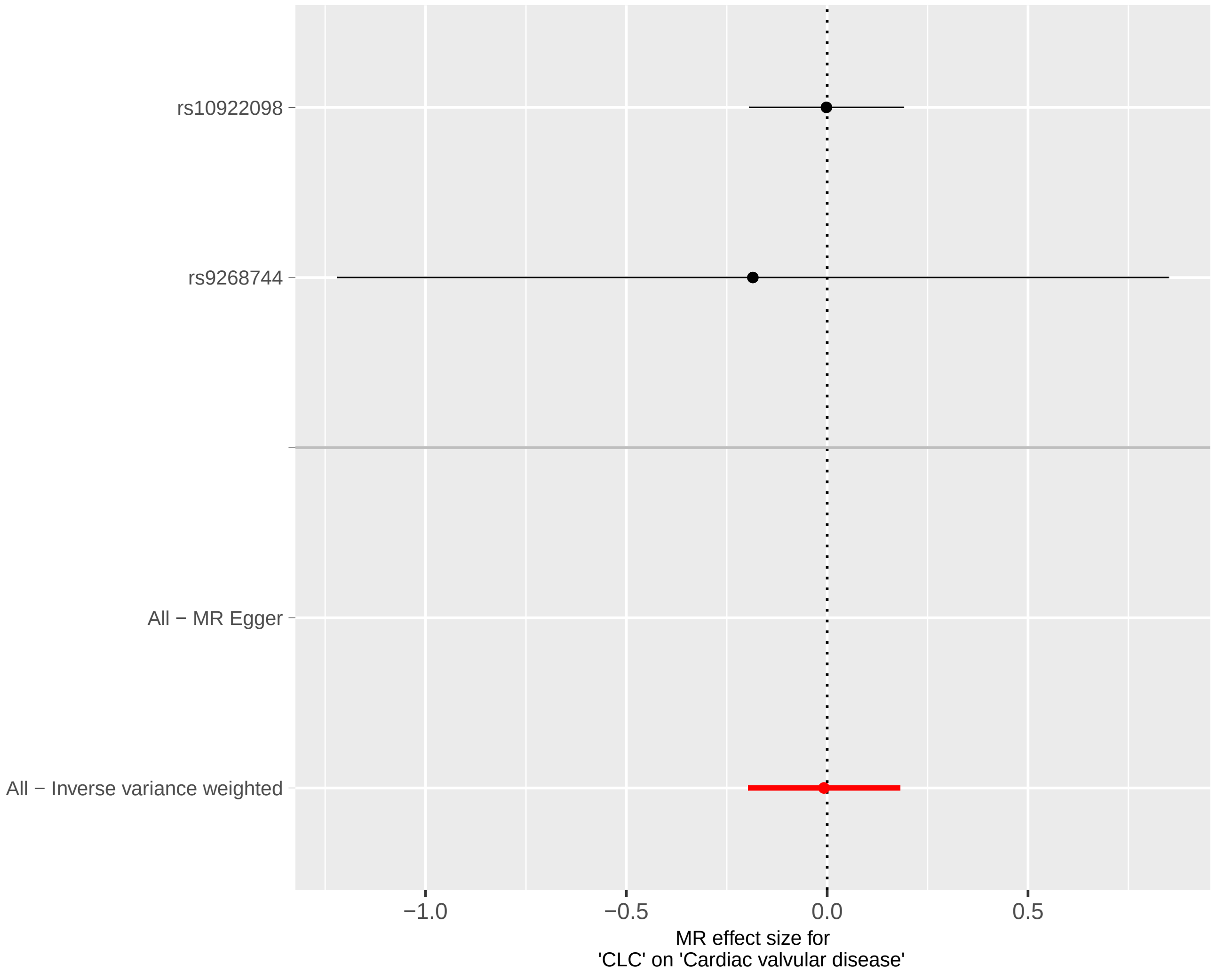
rs10922098
rs9268744
All − MR Egger
All − Inverse variance weighted
−1.0
−0.5
0.0
0.5
MR effect size for
'CLC' on 'Cardiac valvular disease'

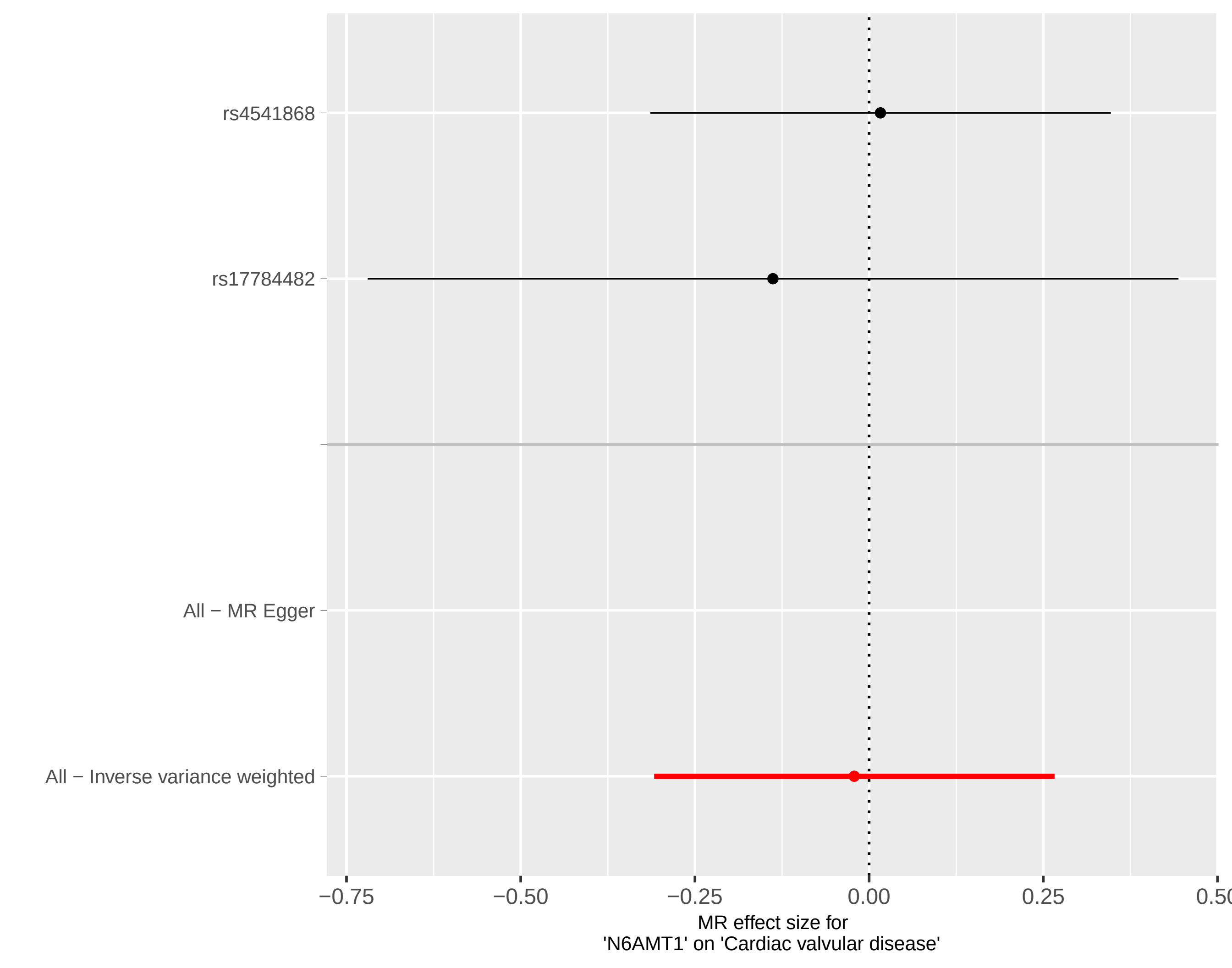

rs4541868
rs17784482
All − MR Egger
All − Inverse variance weighted
−0.75
−0.50
−0.25
0.00
0.25
0.50
MR effect size for
'N6AMT1' on 'Cardiac valvular disease'

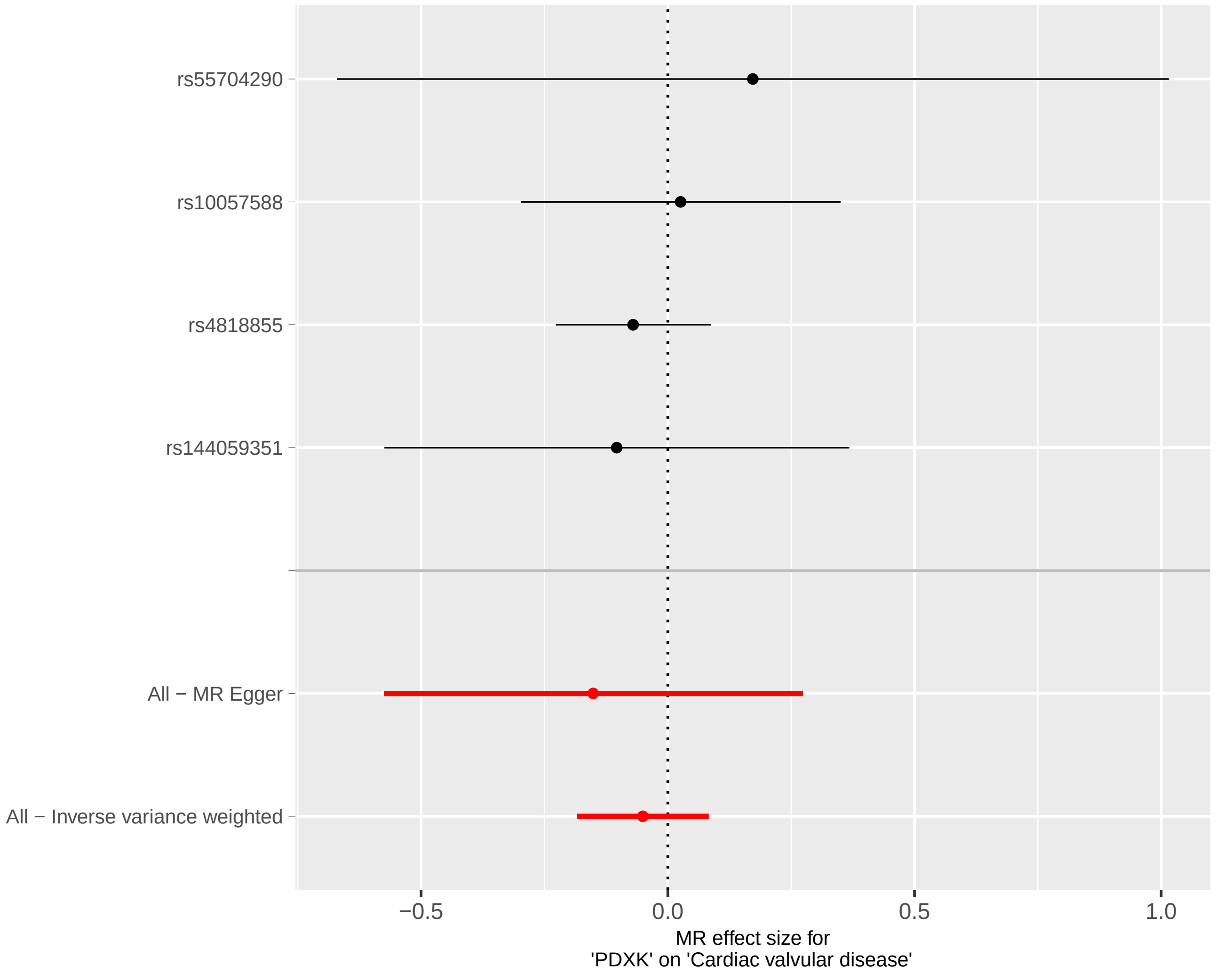
rs55704290
rs10057588
rs4818855
rs144059351
All − MR Egger
All − Inverse variance weighted
−0.5
0.0
0.5
1.0
MR effect size for
'PDXK' on 'Cardiac valvular disease'

Insufficient number of SNPs

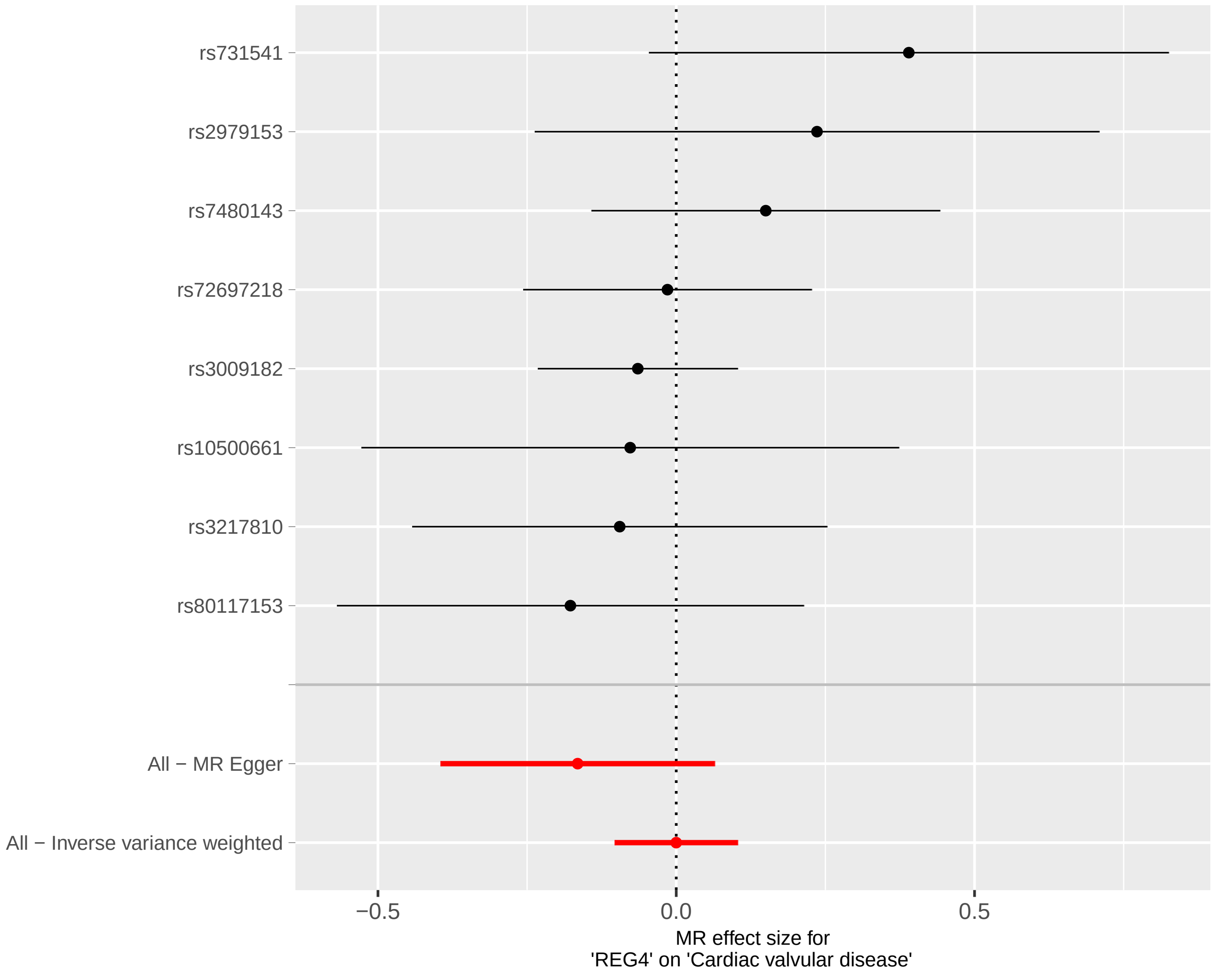
rs731541
rs2979153
rs7480143
rs72697218
rs3009182
rs10500661
rs3217810
rs80117153
All − MR Egger
All − Inverse variance weighted
−0.5
0.0
0.5
MR effect size for
'REG4' on 'Cardiac valvular disease'

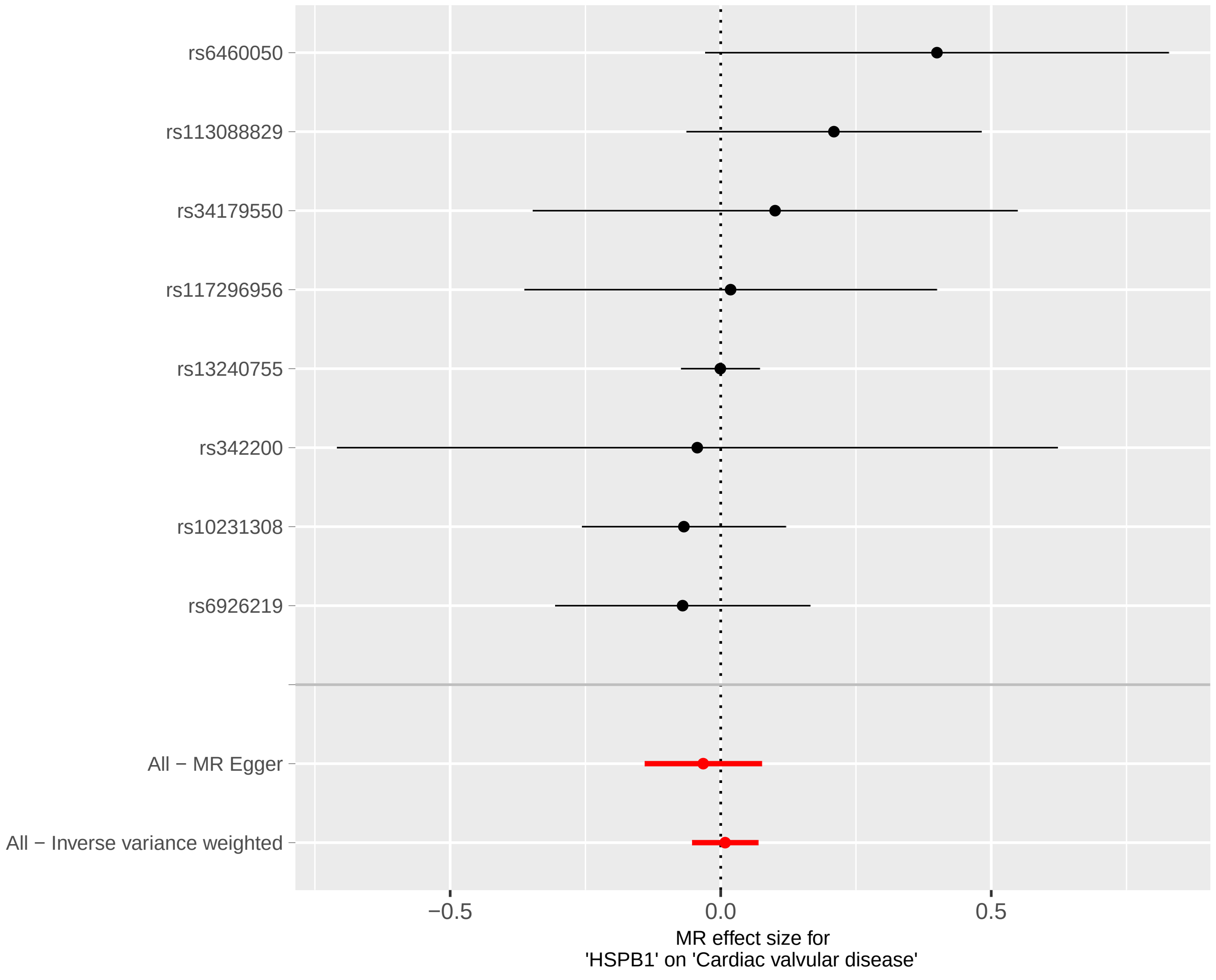
rs6460050
rs113088829
rs34179550
rs117296956
rs13240755
rs342200
rs10231308
rs6926219
All − MR Egger
All − Inverse variance weighted
−0.5
0.0
0.5
MR effect size for
'HSPB1' on 'Cardiac valvular disease'

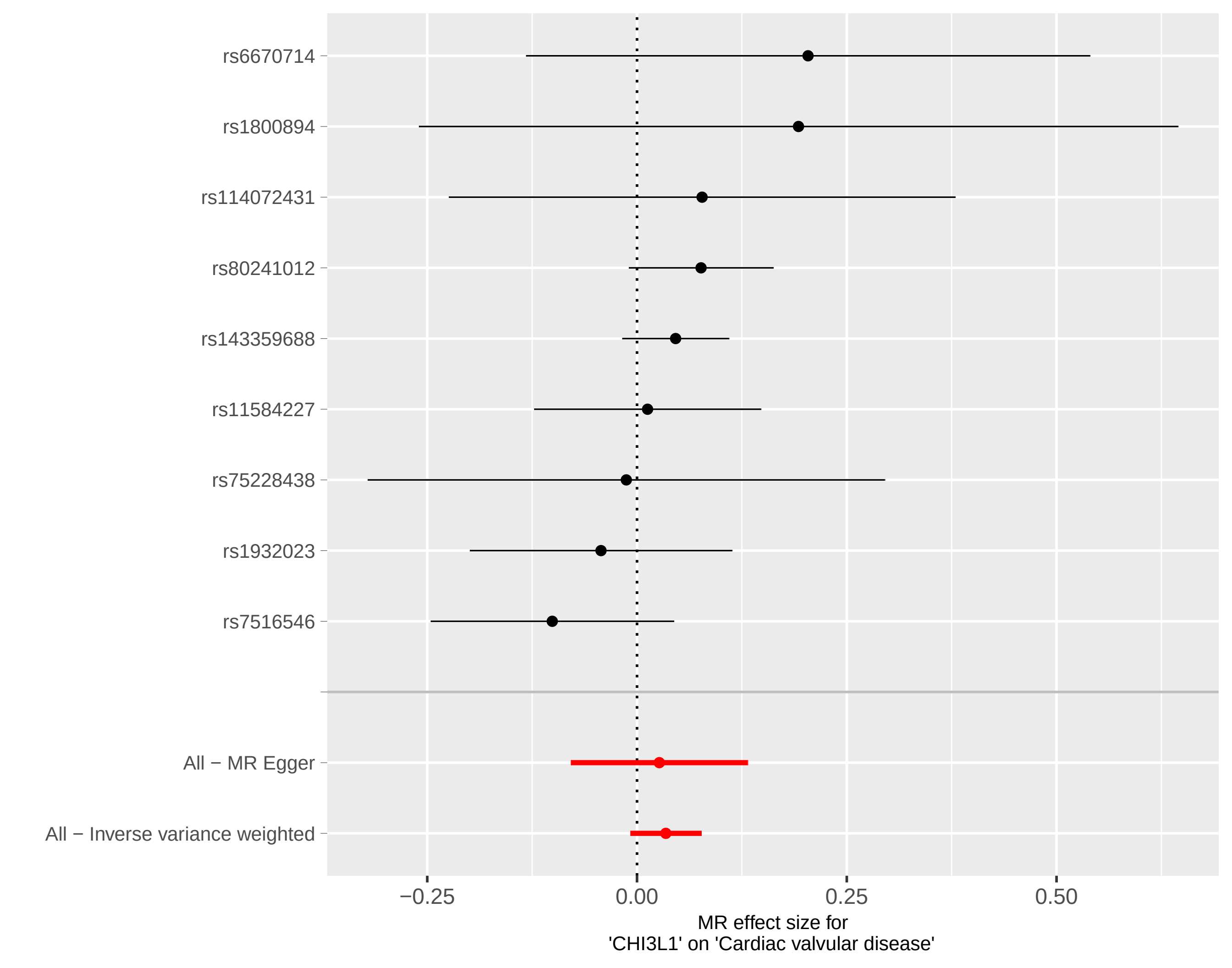
rs6670714
rs1800894
rs114072431
rs80241012
rs143359688
rs11584227
rs75228438
rs1932023
rs7516546
All − MR Egger
All − Inverse variance weighted
−0.25
0.00
0.25
0.50
MR effect size for
'CHI3L1' on 'Cardiac valvular disease'

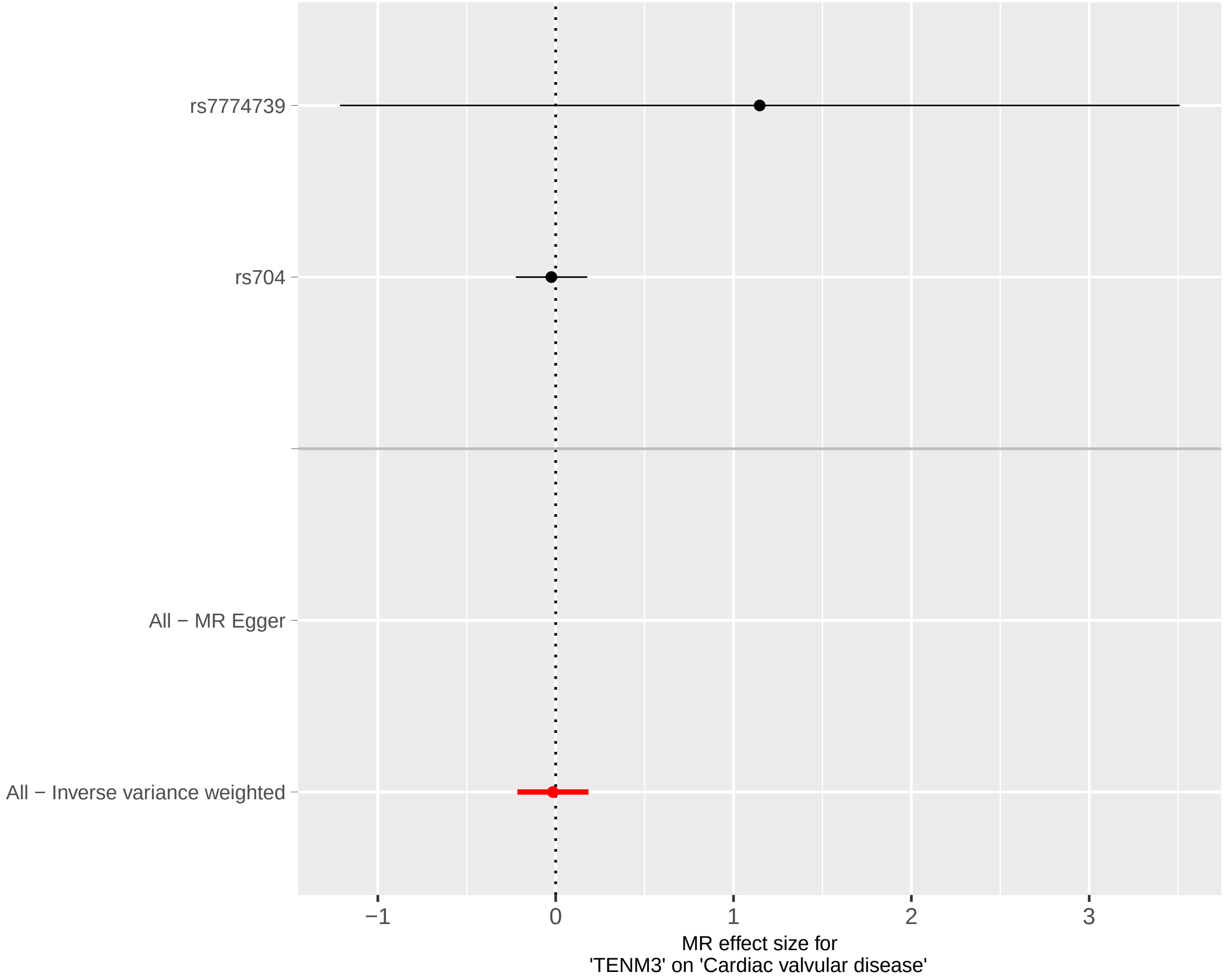
rs7774739
rs704
All − MR Egger
All − Inverse variance weighted
−1
0
1
2
3
MR effect size for
'TENM3' on 'Cardiac valvular disease'

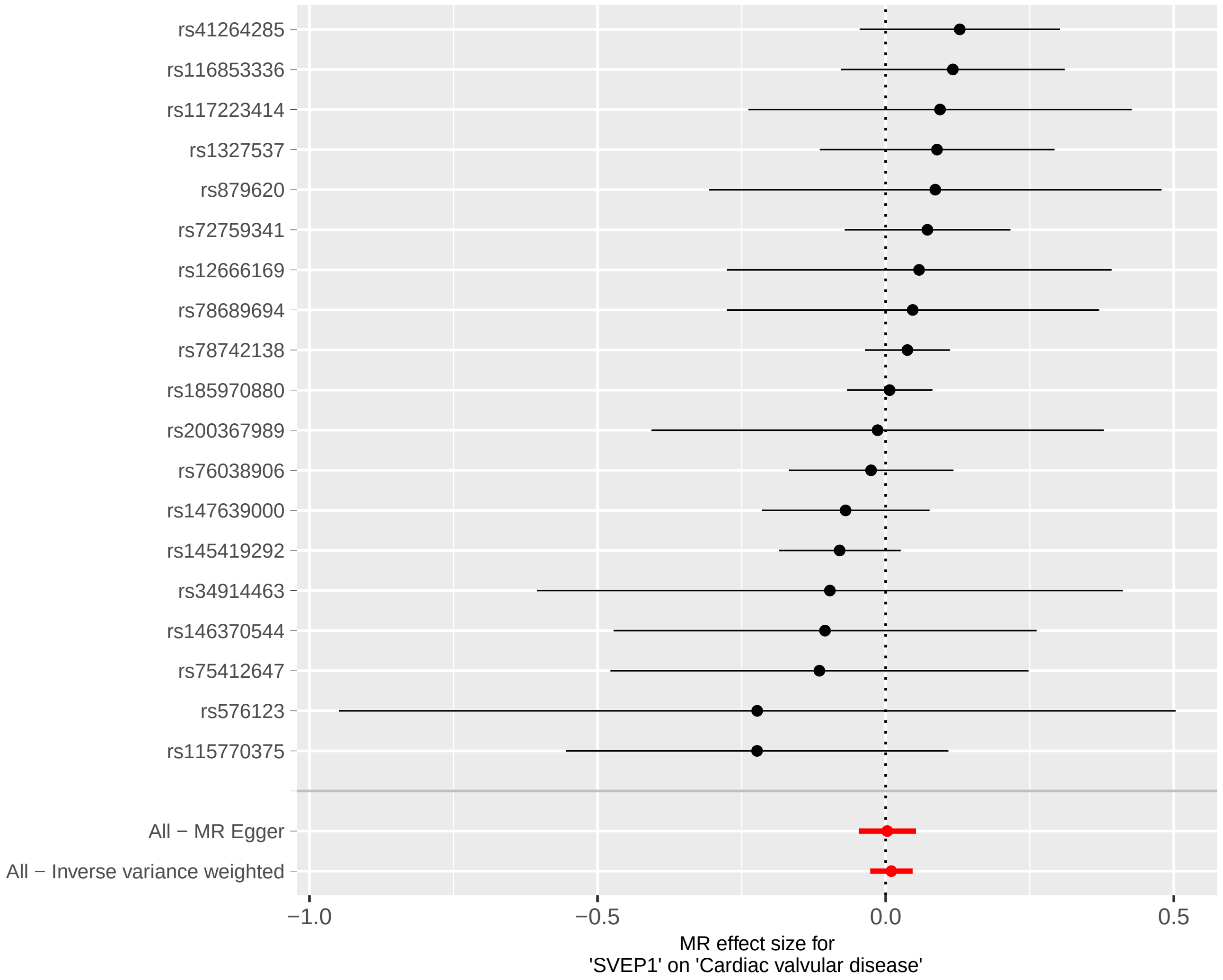

rs41264285
rs116853336
rs117223414
rs1327537
rs879620
rs72759341
rs12666169
rs78689694
rs78742138
rs185970880
rs200367989
rs76038906
rs147639000
rs145419292
rs34914463
rs146370544
rs75412647
rs576123
rs115770375
All − MR Egger
All − Inverse variance weighted
−1.0
−0.5
0.0
0.5
MR effect size for
'SVEP1' on 'Cardiac valvular disease'

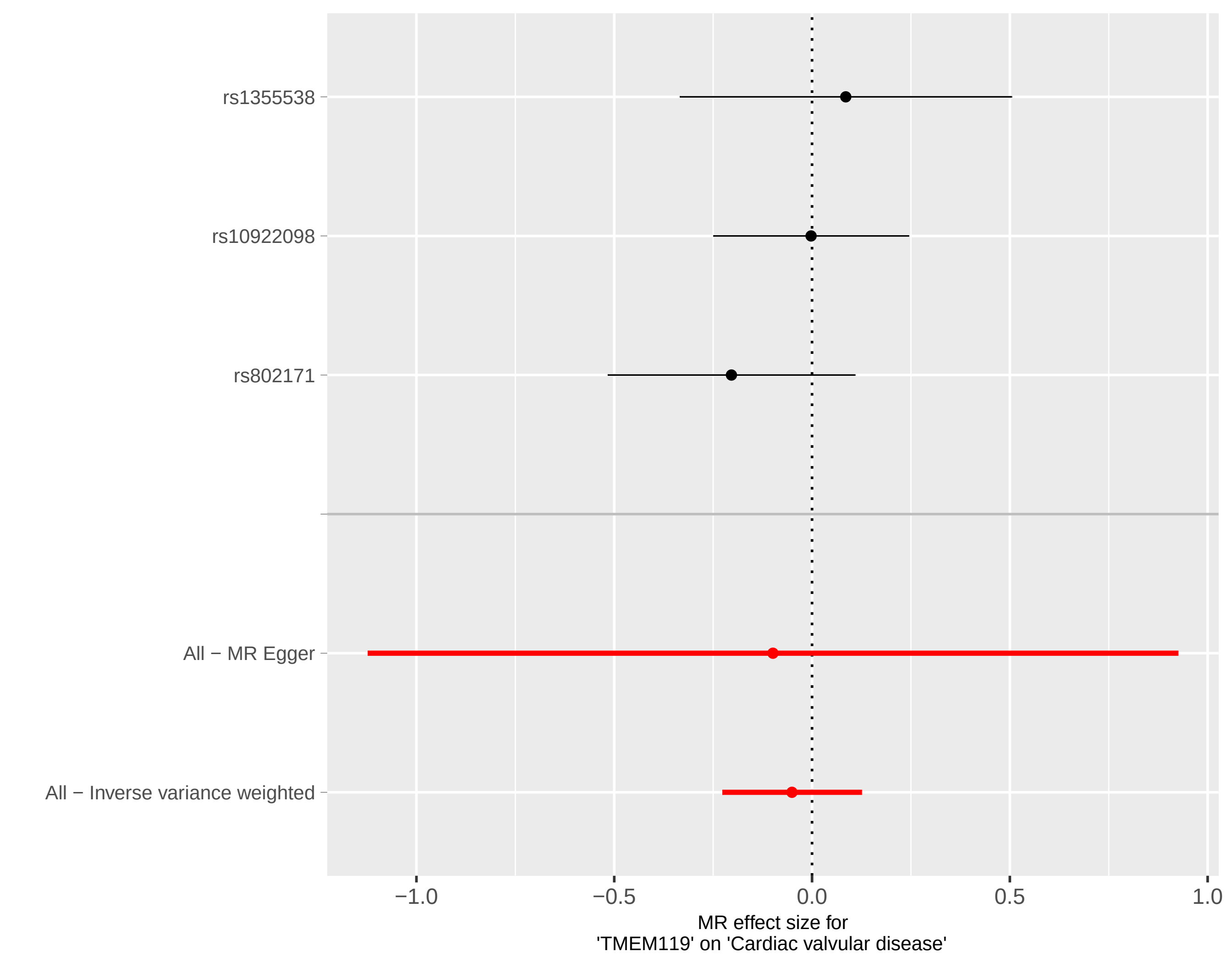

rs1355538
rs10922098
rs802171
All − MR Egger
All − Inverse variance weighted
−1.0
−0.5
0.0
0.5
1.0
MR effect size for
'TMEM119' on 'Cardiac valvular disease'

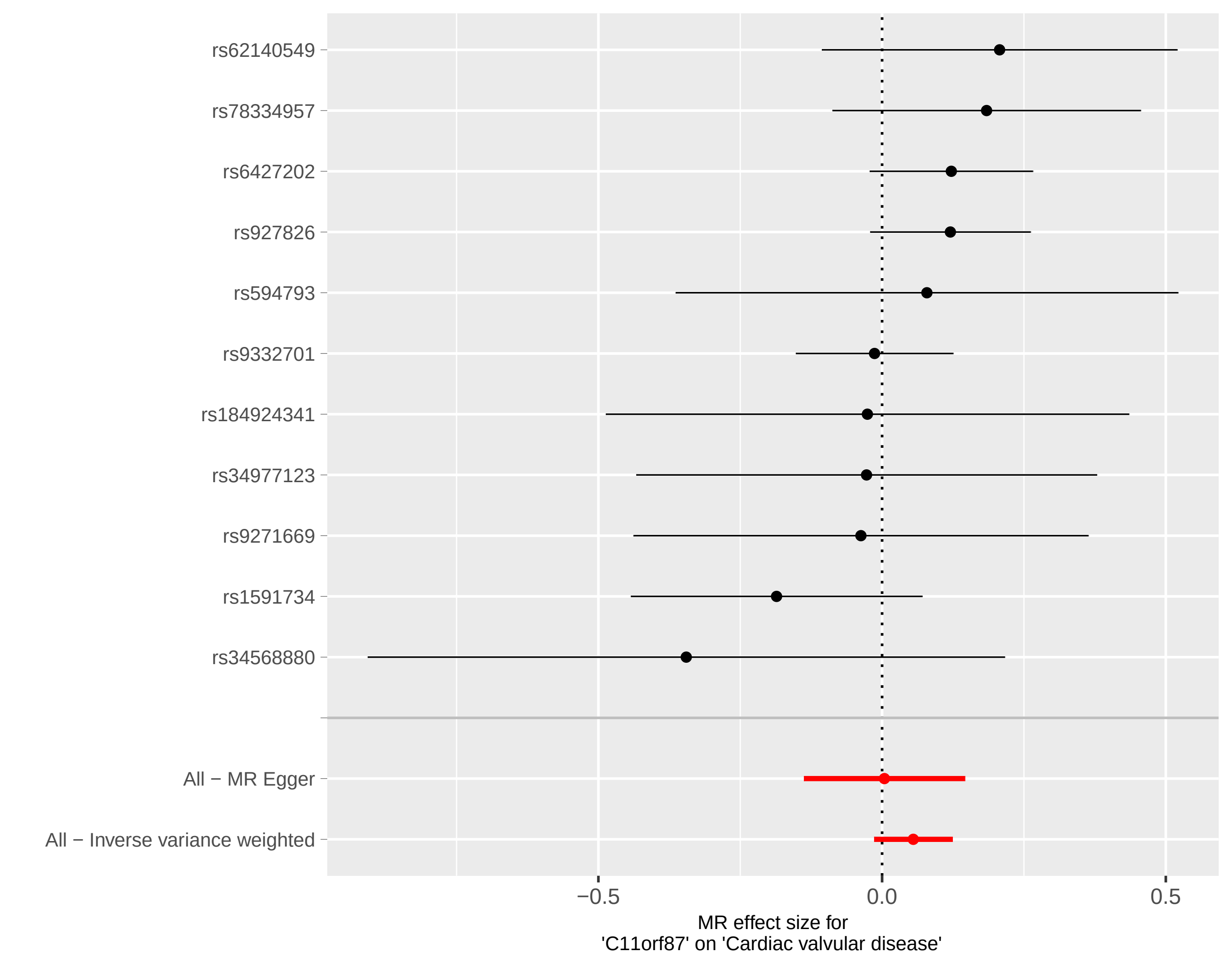

rs62140549
rs78334957
rs6427202
rs927826
rs594793
rs9332701
rs184924341
rs34977123
rs9271669
rs1591734
rs34568880
All − MR Egger
All − Inverse variance weighted
−0.5
0.0
0.5
MR effect size for
'C11orf87' on 'Cardiac valvular disease'

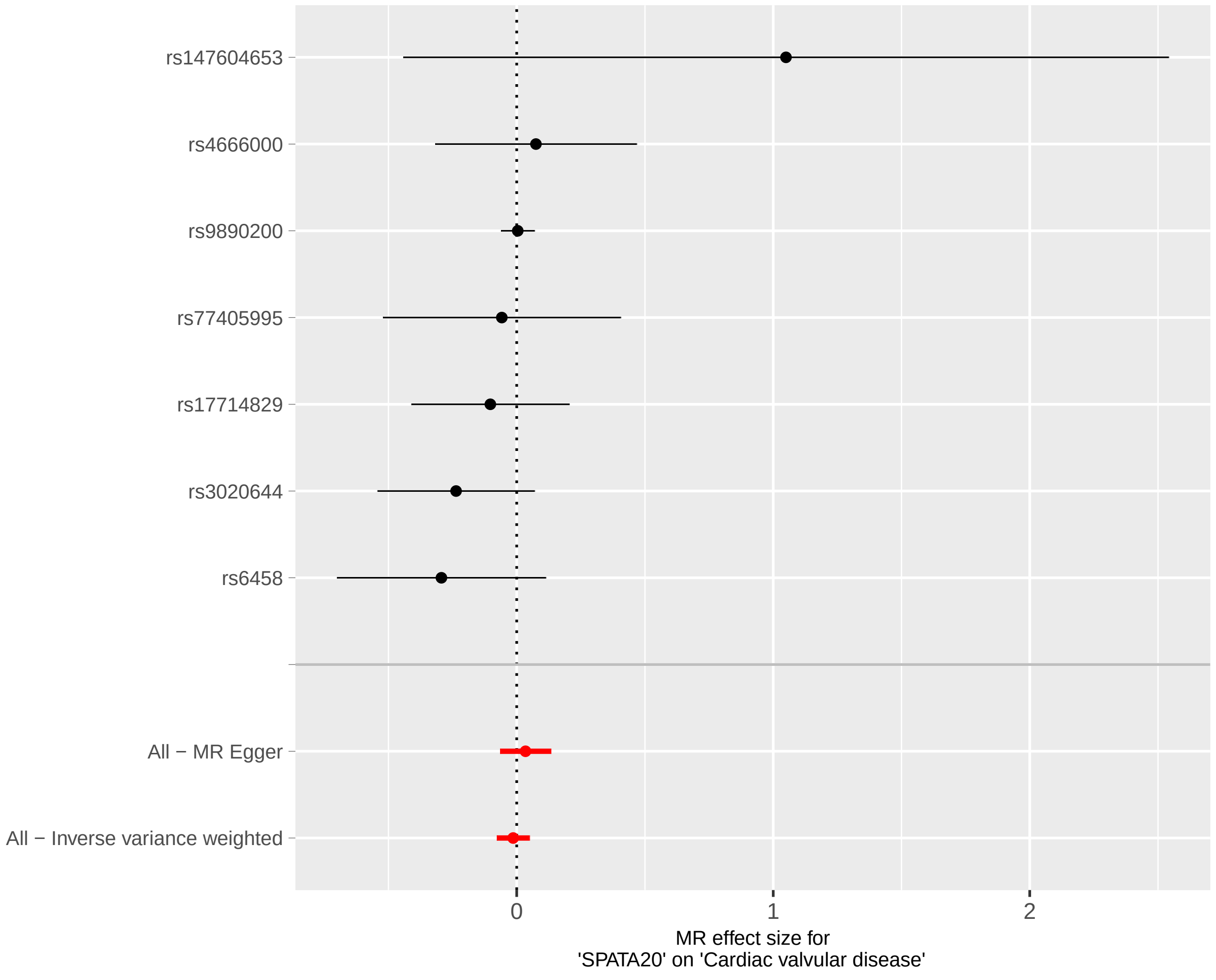
rs147604653
rs4666000
rs9890200
rs77405995
rs17714829
rs3020644
rs6458
All − MR Egger
All − Inverse variance weighted
0
1
2
MR effect size for
'SPATA20' on 'Cardiac valvular disease'

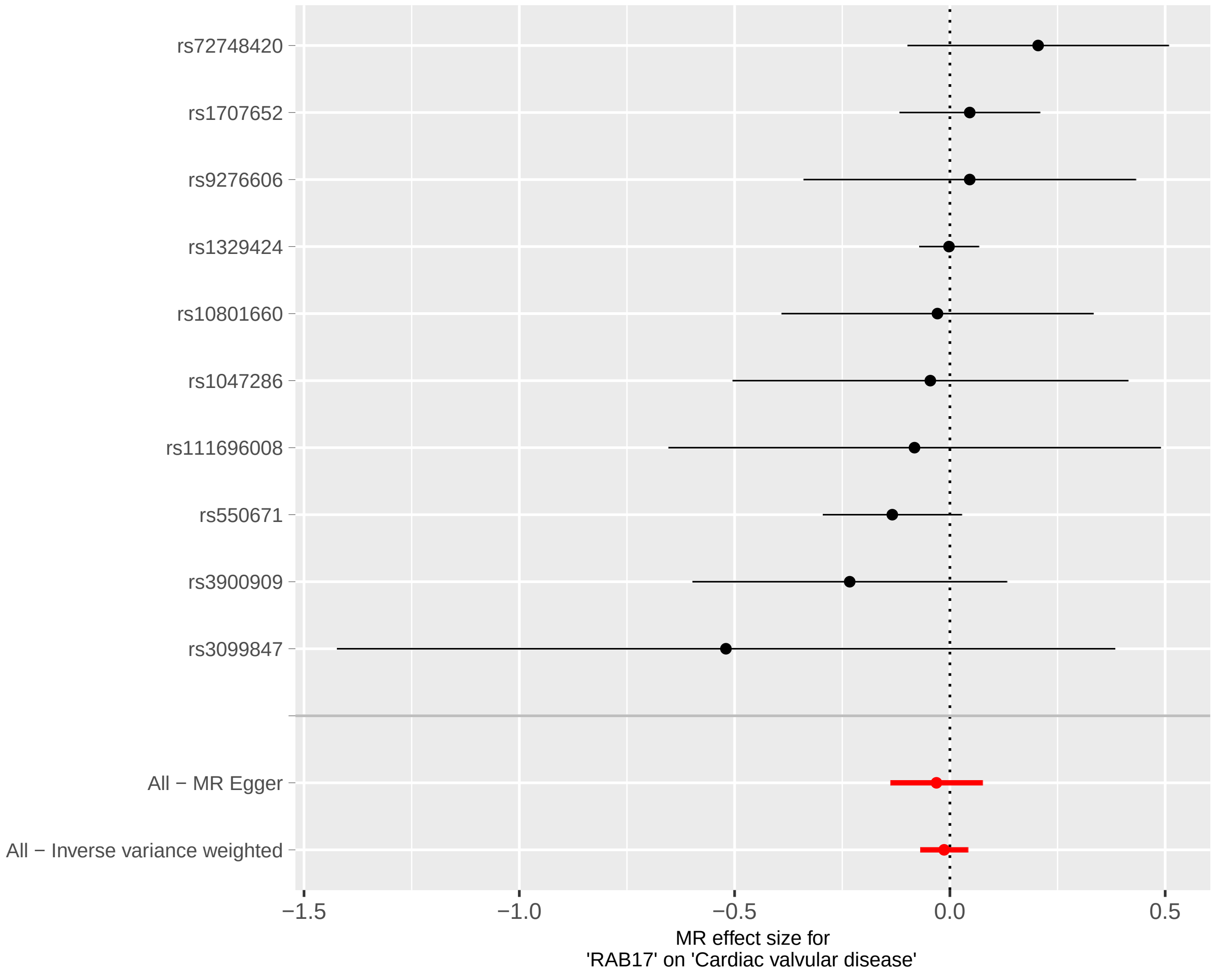
rs72748420
rs1707652
rs9276606
rs1329424
rs10801660
rs1047286
rs111696008
rs550671
rs3900909
rs3099847
All − MR Egger
All − Inverse variance weighted
−1.5
−1.0
−0.5
0.0
0.5
MR effect size for
'RAB17' on 'Cardiac valvular disease'

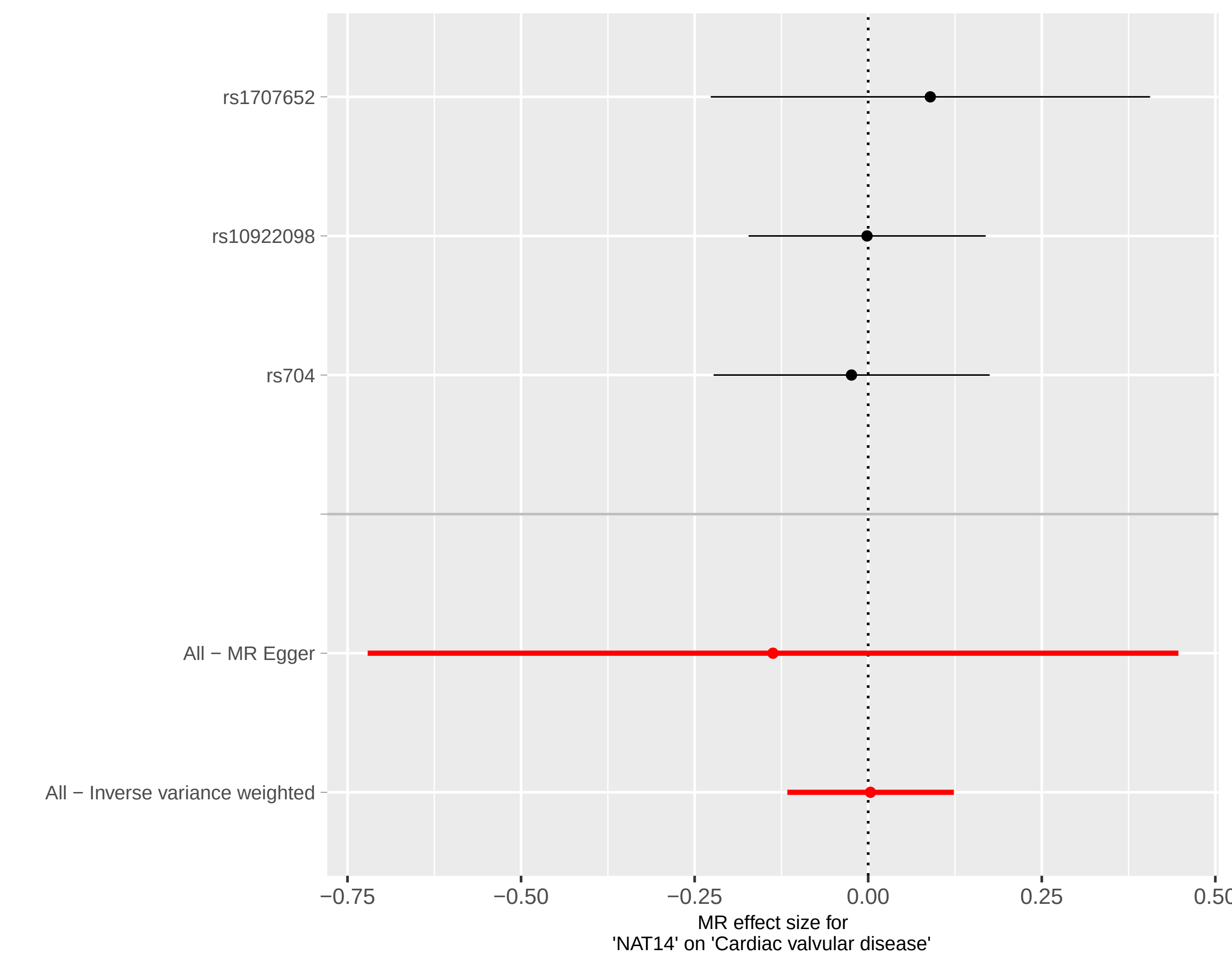
rs1707652
rs10922098
rs704
All − MR Egger
All − Inverse variance weighted
−0.75
−0.50
−0.25
0.00
0.25
0.50
MR effect size for
'NAT14' on 'Cardiac valvular disease'

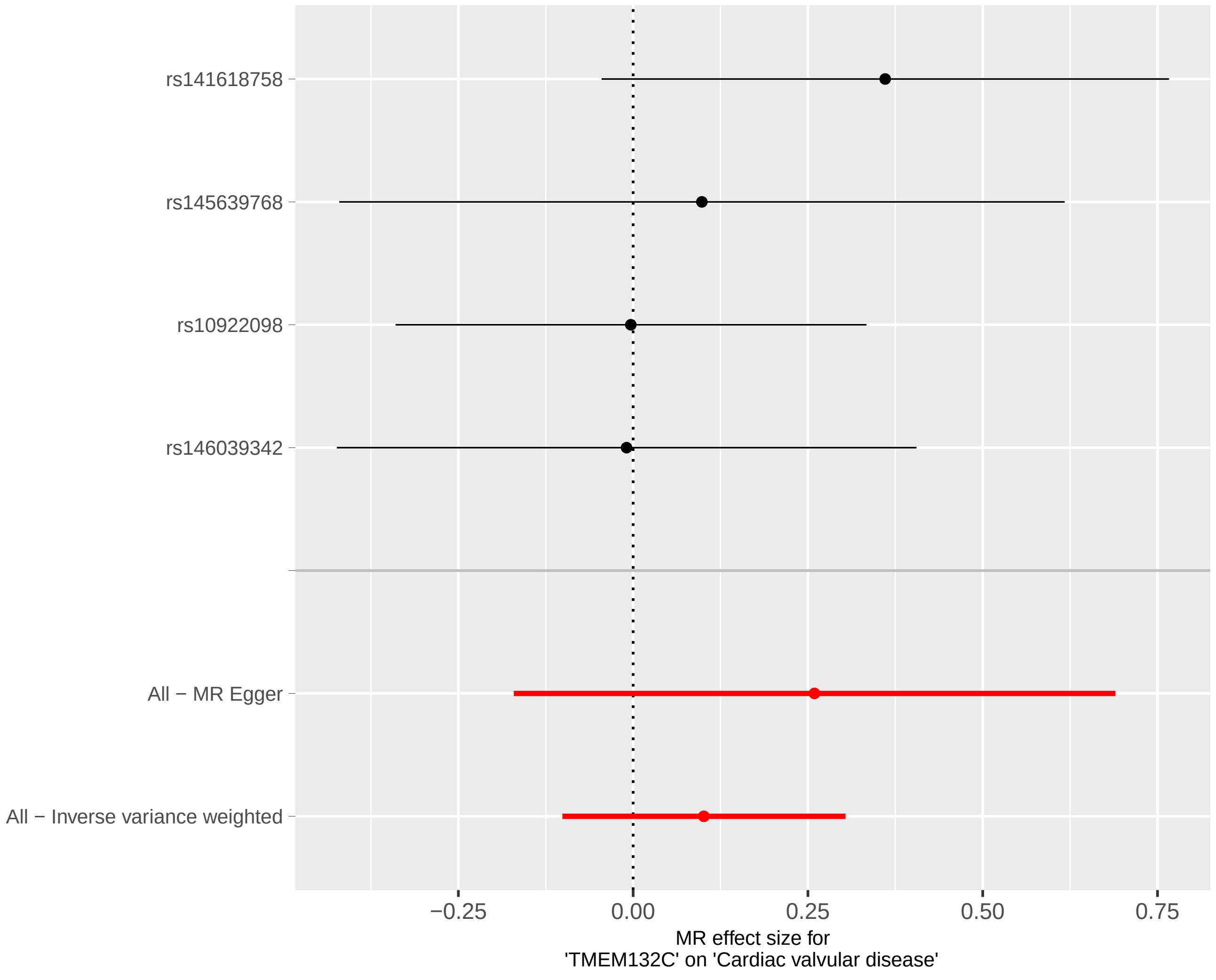
rs141618758
rs145639768
rs10922098
rs146039342
All − MR Egger
All − Inverse variance weighted
−0.25
0.00
0.25
0.50
0.75
MR effect size for
'TMEM132C' on 'Cardiac valvular disease'

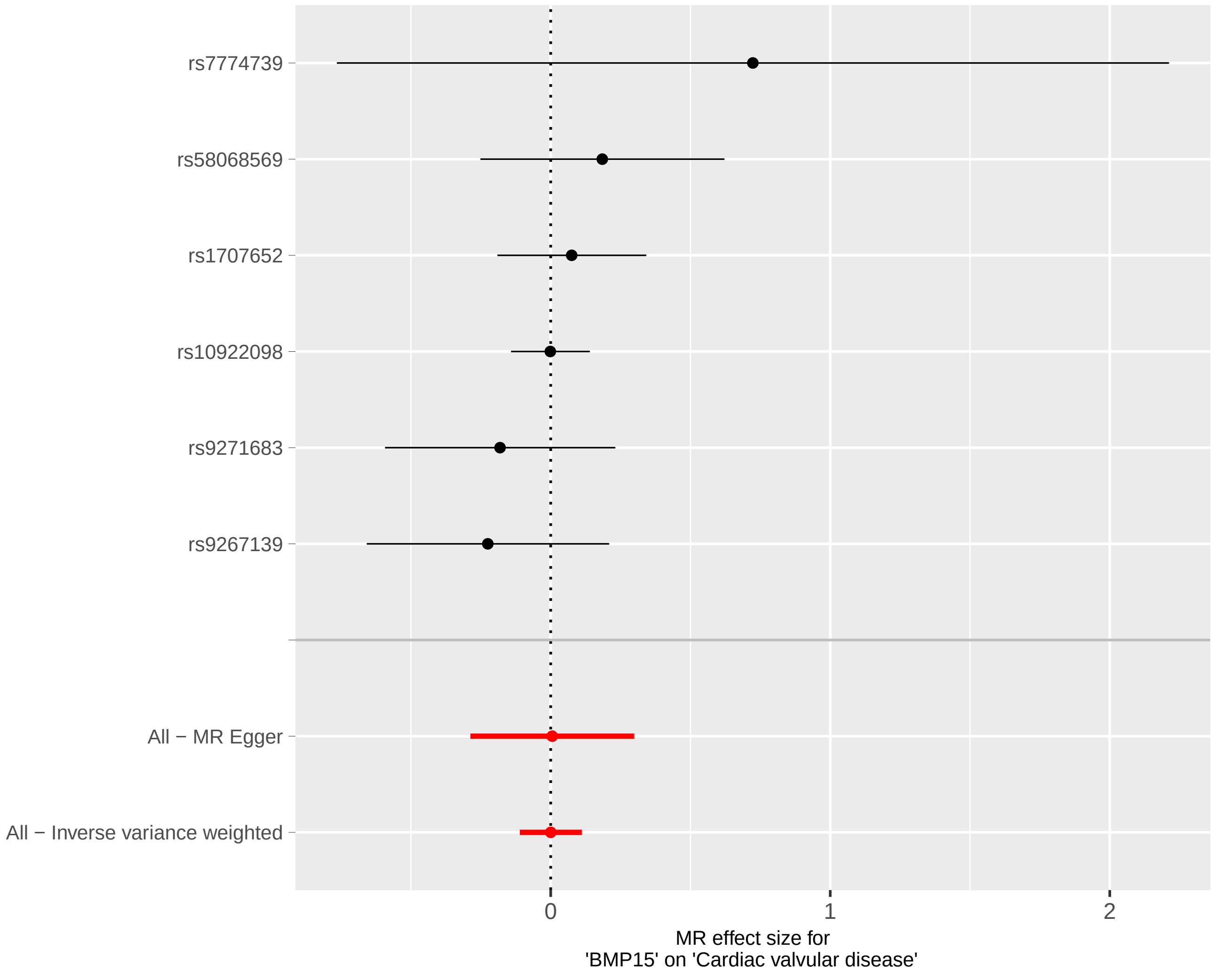
rs7774739
rs58068569
rs1707652
rs10922098
rs9271683
rs9267139
All − MR Egger
All − Inverse variance weighted
0
1
2
MR effect size for
'BMP15' on 'Cardiac valvular disease'

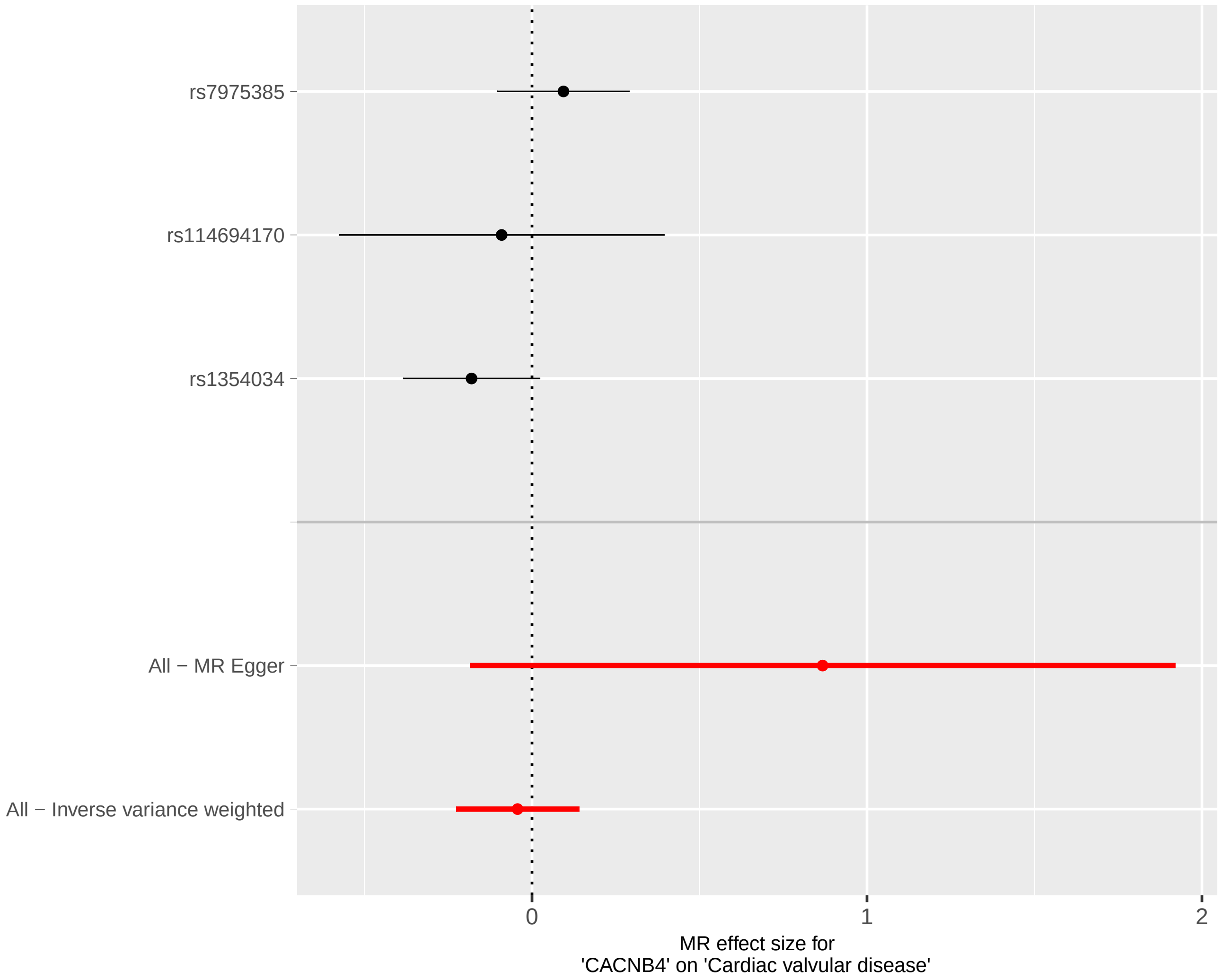

rs7975385
rs114694170
rs1354034
All − MR Egger
All − Inverse variance weighted
0
1
2
MR effect size for
'CACNB4' on 'Cardiac valvular disease'

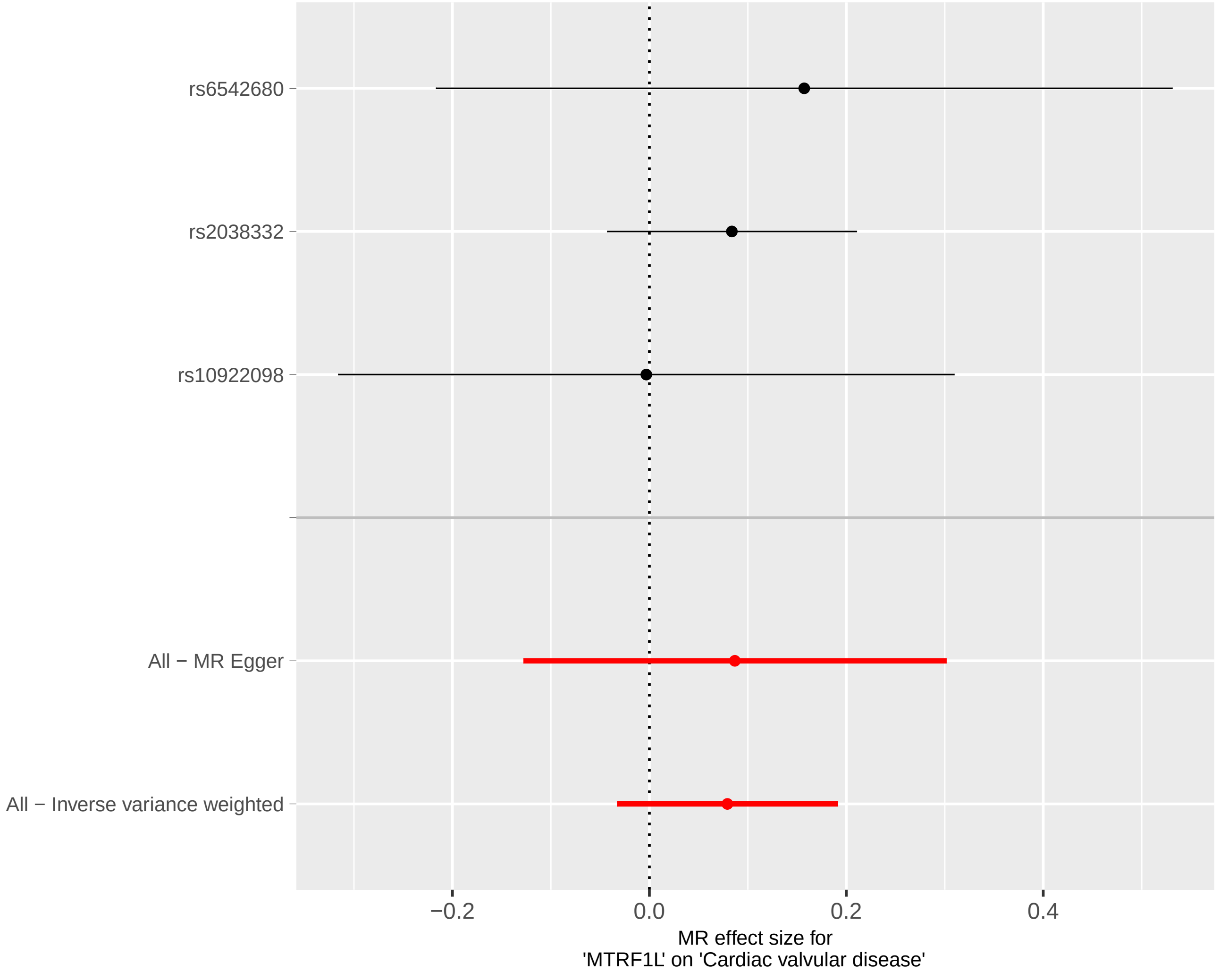
rs6542680
rs2038332
rs10922098
All − MR Egger
All − Inverse variance weighted
−0.2
0.0
0.2
0.4
MR effect size for
'MTRF1L' on 'Cardiac valvular disease'

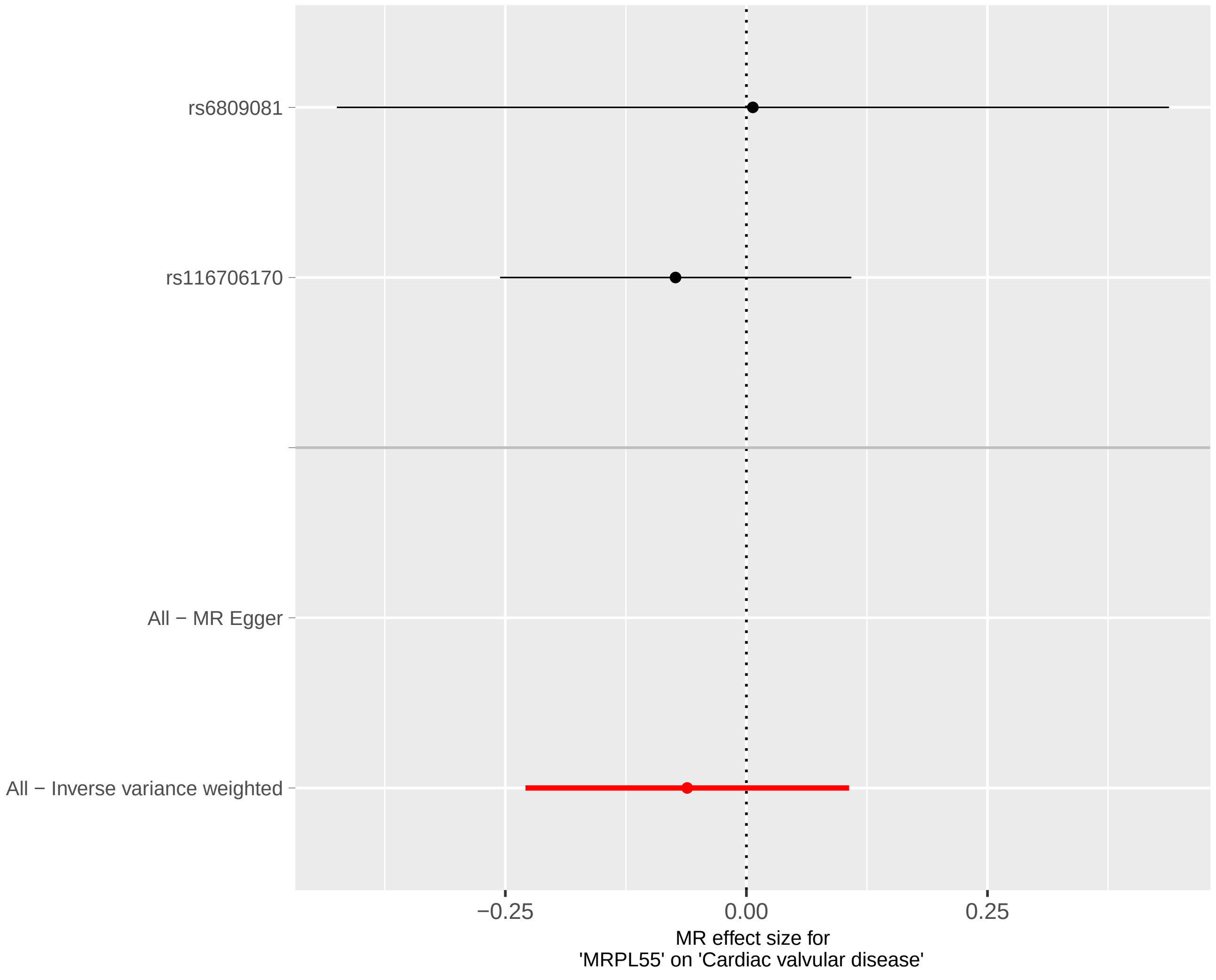

rs6809081
rs116706170
All − MR Egger
All − Inverse variance weighted
−0.25
0.00
0.25
MR effect size for
'MRPL55' on 'Cardiac valvular disease'

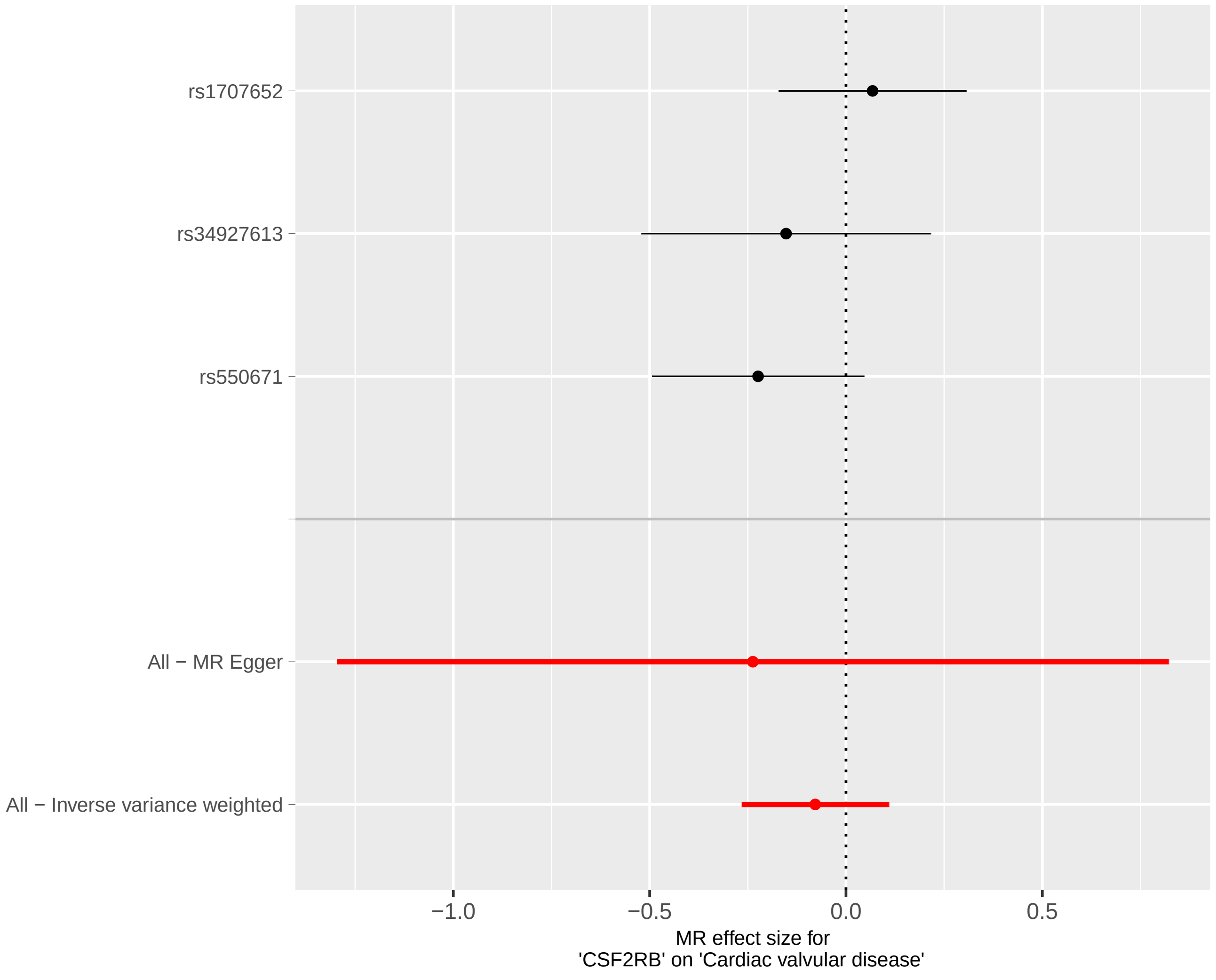
rs1707652
rs34927613
rs550671
All − MR Egger
All − Inverse variance weighted
−1.0
−0.5
0.0
0.5
MR effect size for
'CSF2RB' on 'Cardiac valvular disease'

Insufficient number of SNPs

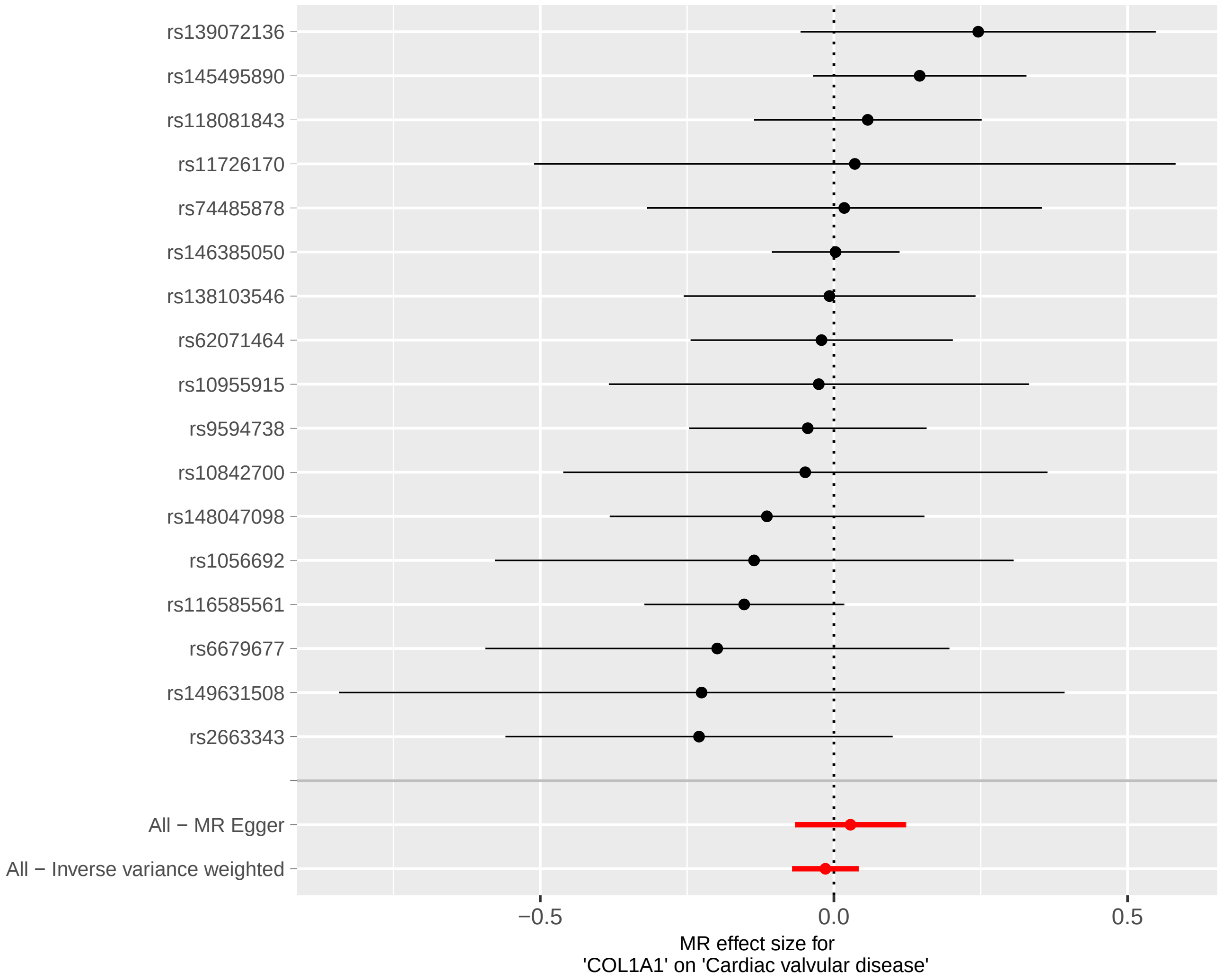
rs139072136
rs145495890
rs118081843
rs11726170
rs74485878
rs146385050
rs138103546
rs62071464
rs10955915
rs9594738
rs10842700
rs148047098
rs1056692
rs116585561
rs6679677
rs149631508
rs2663343
All − MR Egger
All − Inverse variance weighted
−0.5
0.0
0.5
MR effect size for
'COL1A1' on 'Cardiac valvular disease'

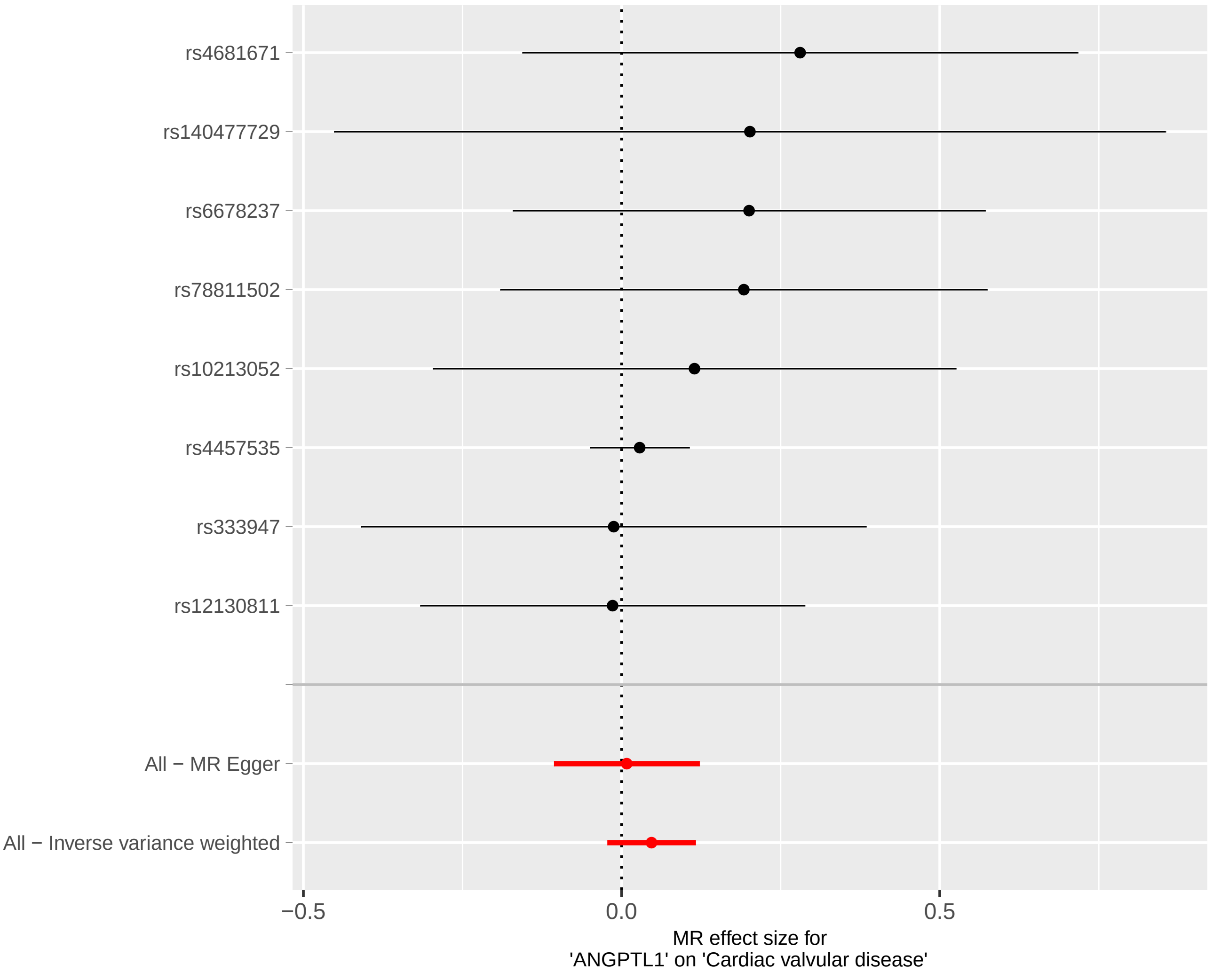
rs4681671
rs140477729
rs6678237
rs78811502
rs10213052
rs4457535
rs333947
rs12130811
All − MR Egger
All − Inverse variance weighted
−0.5
0.0
0.5
MR effect size for
'ANGPTL1' on 'Cardiac valvular disease'

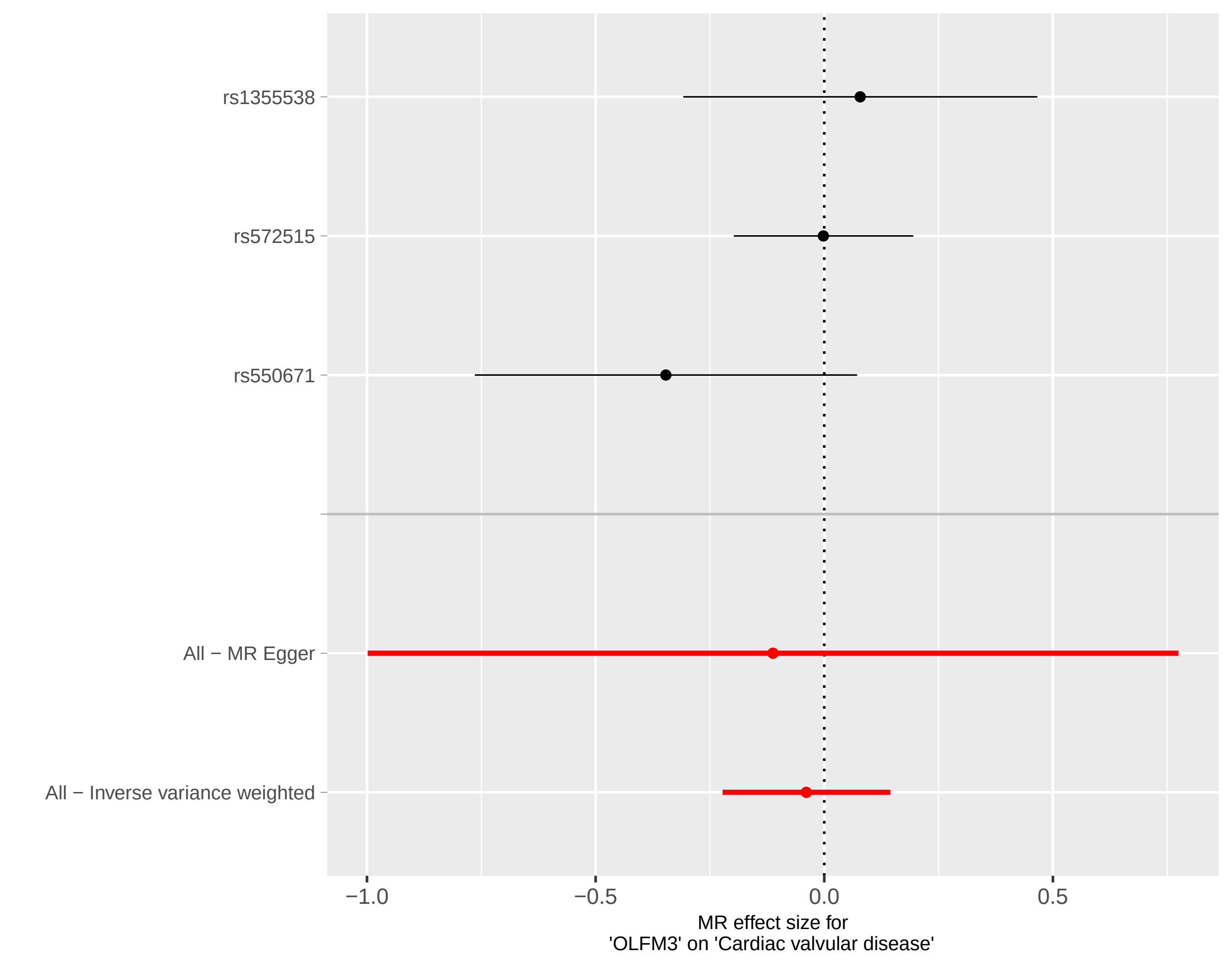
rs1355538
rs572515
rs550671
All − MR Egger
All − Inverse variance weighted
−1.0
−0.5
0.0
0.5
MR effect size for
'OLFM3' on 'Cardiac valvular disease'

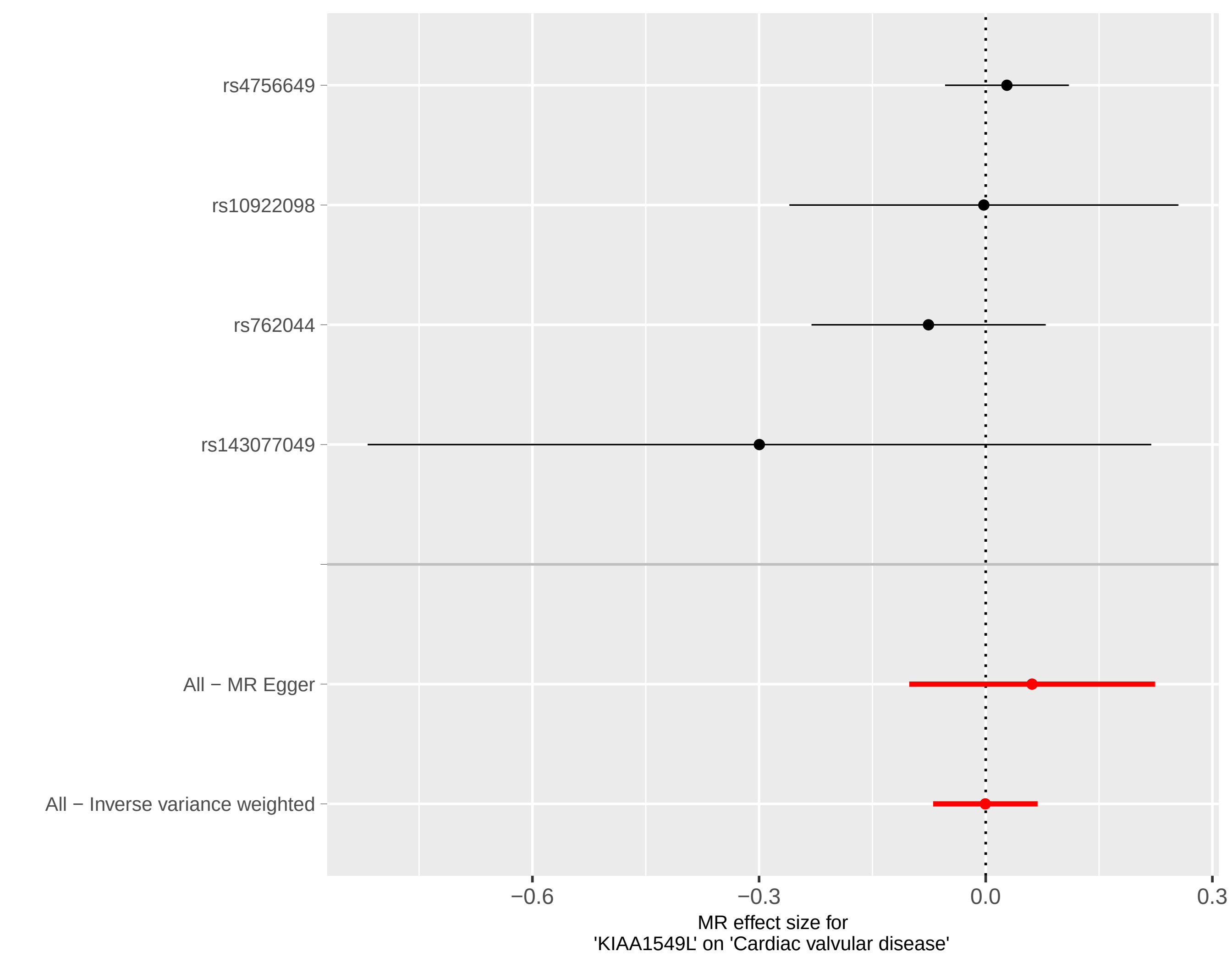
rs4756649
rs10922098
rs762044
rs143077049
All − MR Egger
All − Inverse variance weighted
−0.6
−0.3
0.0
0.3
MR effect size for
'KIAA1549L' on 'Cardiac valvular disease'

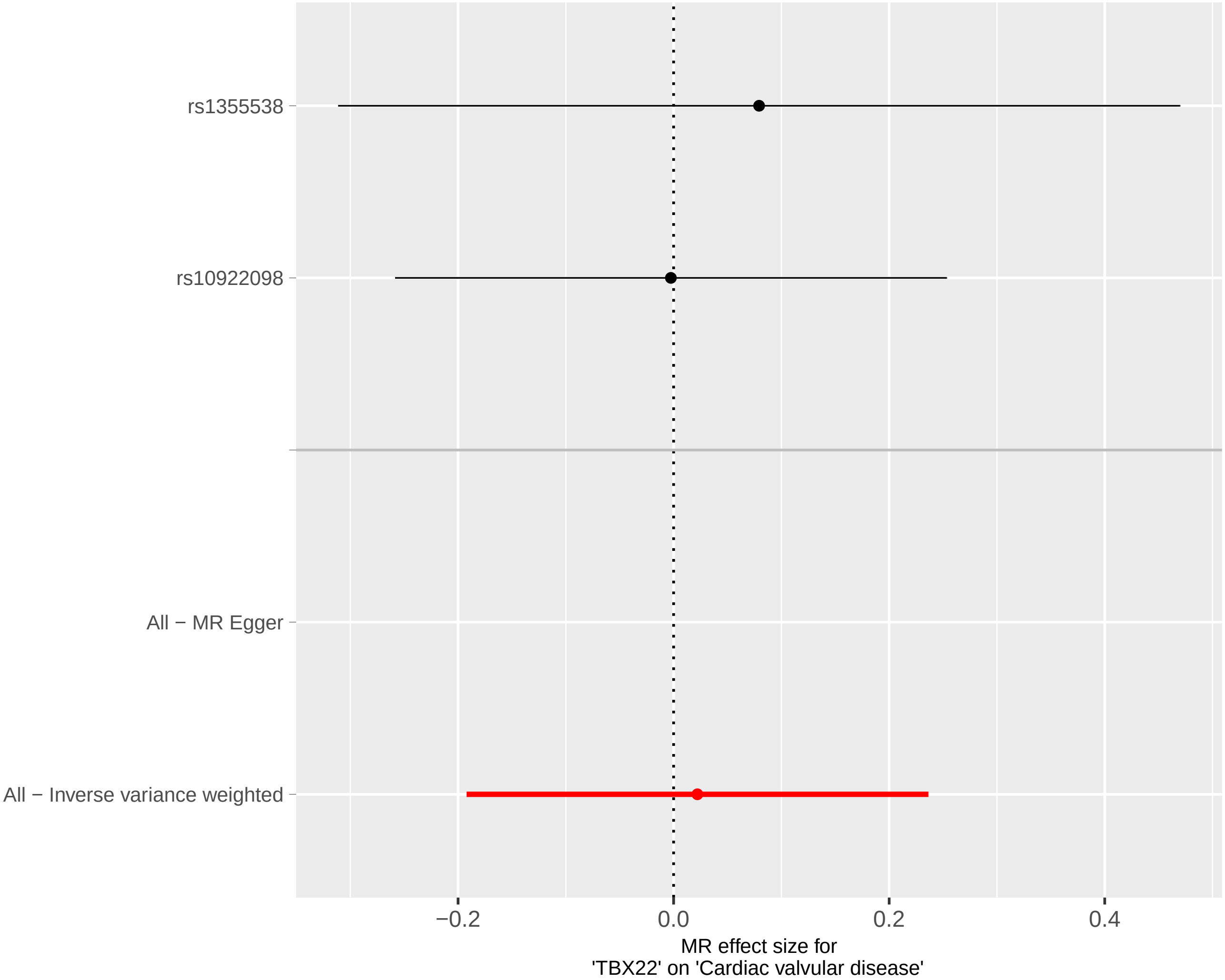
rs1355538
rs10922098
All − MR Egger
All − Inverse variance weighted
−0.2
0.0
0.2
0.4
MR effect size for
'TBX22' on 'Cardiac valvular disease'

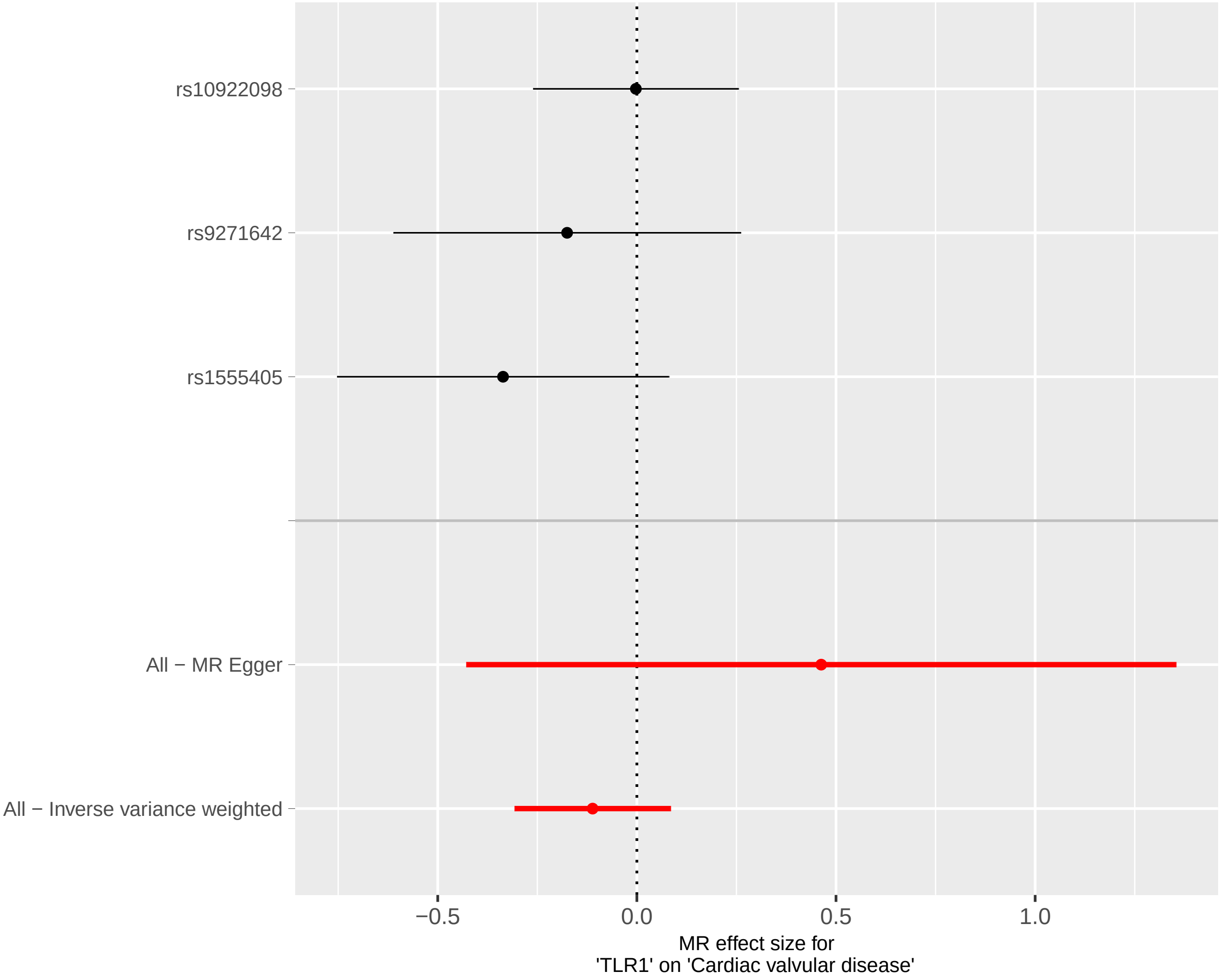

rs10922098
rs9271642
rs1555405
All − MR Egger
All − Inverse variance weighted
−0.5
0.0
0.5
1.0
MR effect size for
'TLR1' on 'Cardiac valvular disease'

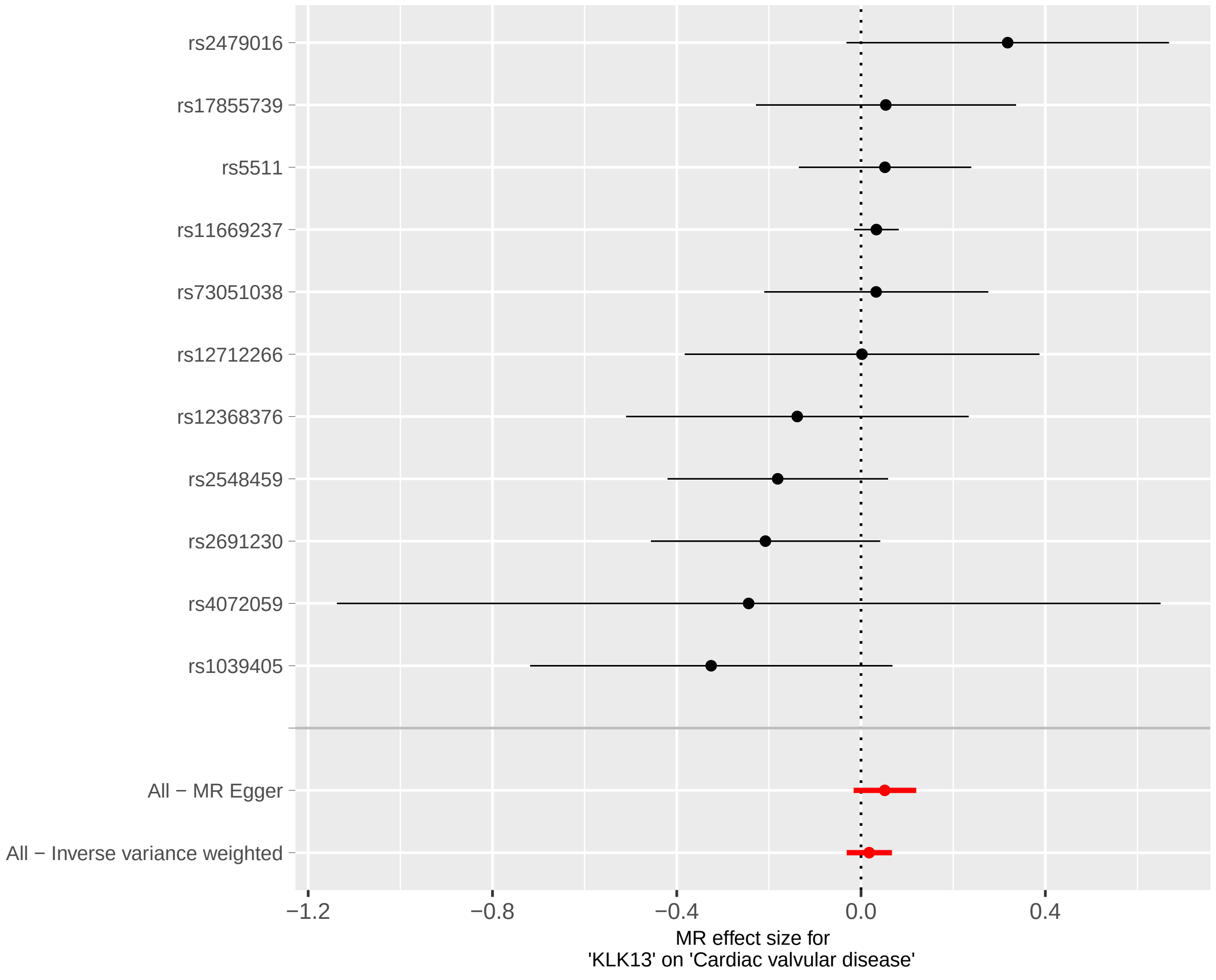
rs2479016
rs17855739
rs5511
rs11669237
rs73051038
rs12712266
rs12368376
rs2548459
rs2691230
rs4072059
rs1039405
All − MR Egger
All − Inverse variance weighted
−1.2
−0.8
−0.4
0.0
0.4
MR effect size for
'KLK13' on 'Cardiac valvular disease'

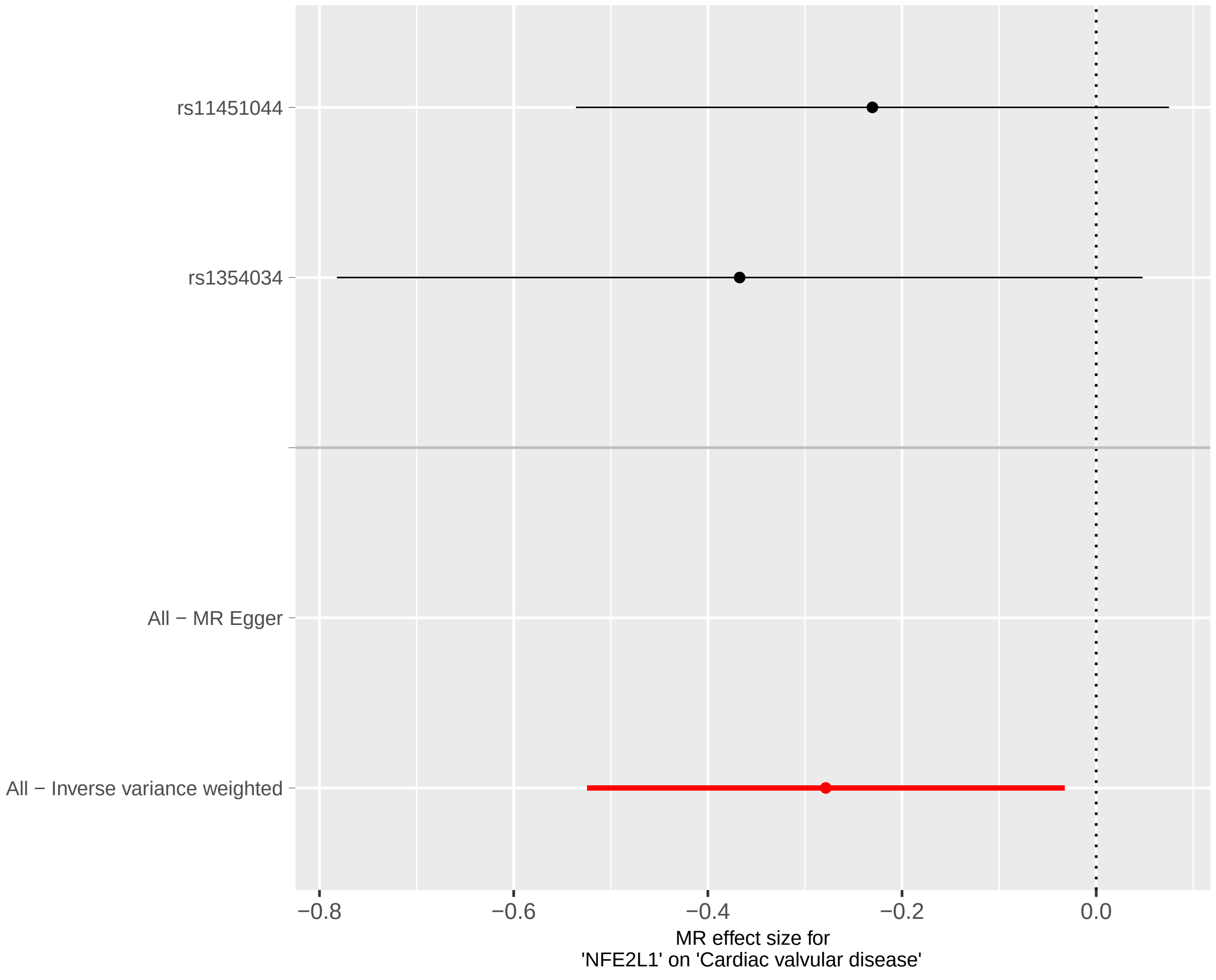
rs11451044
rs1354034
All − MR Egger
All − Inverse variance weighted
−0.8
−0.6
−0.4
−0.2
0.0
MR effect size for
'NFE2L1' on 'Cardiac valvular disease'

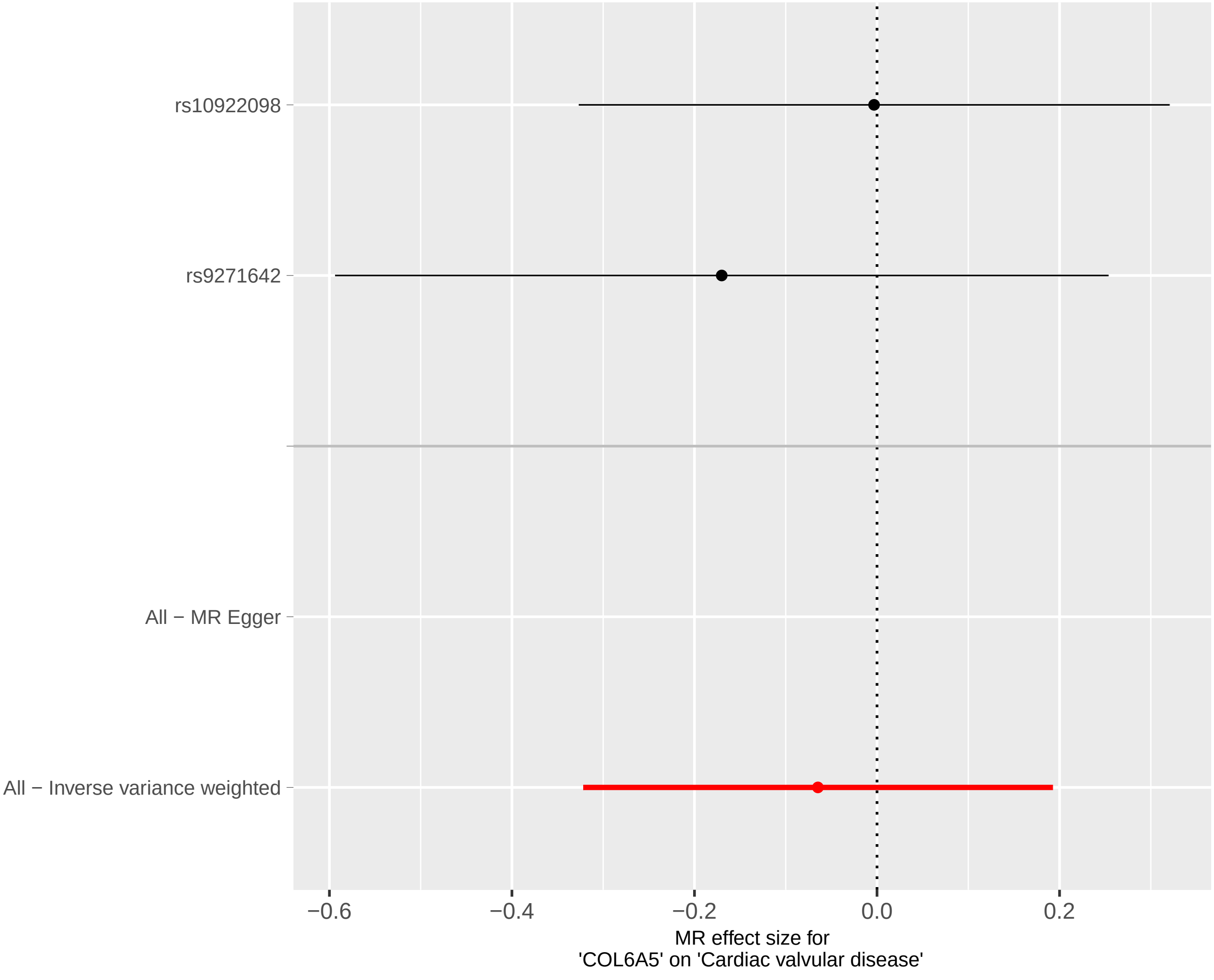
rs10922098
rs9271642
All − MR Egger
All − Inverse variance weighted
−0.6
−0.4
−0.2
0.0
0.2
MR effect size for
'COL6A5' on 'Cardiac valvular disease'

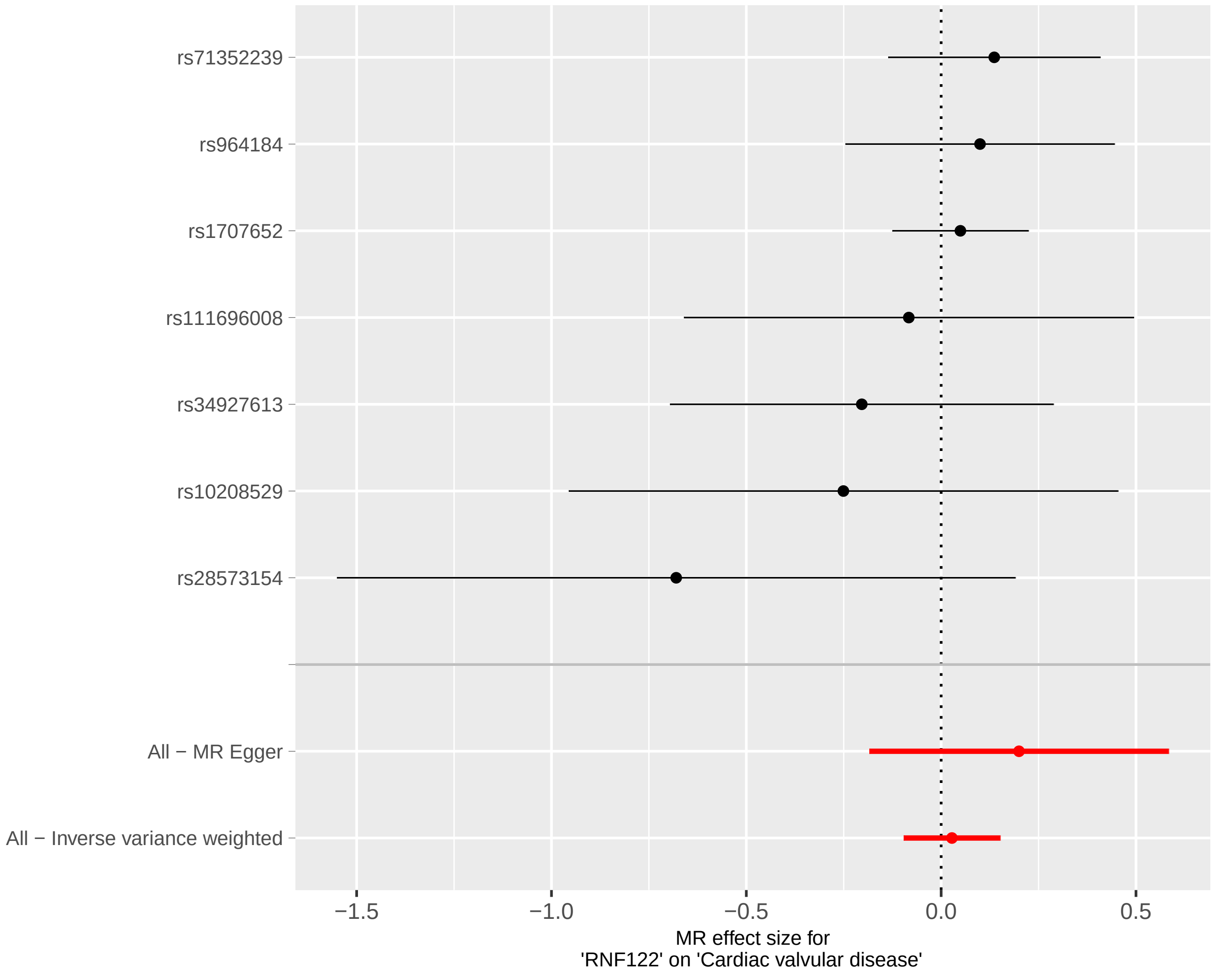

rs71352239
rs964184
rs1707652
rs111696008
rs34927613
rs10208529
rs28573154
All − MR Egger
All − Inverse variance weighted
−1.5
−1.0
−0.5
0.0
0.5
MR effect size for
'RNF122' on 'Cardiac valvular disease'

Insufficient number of SNPs

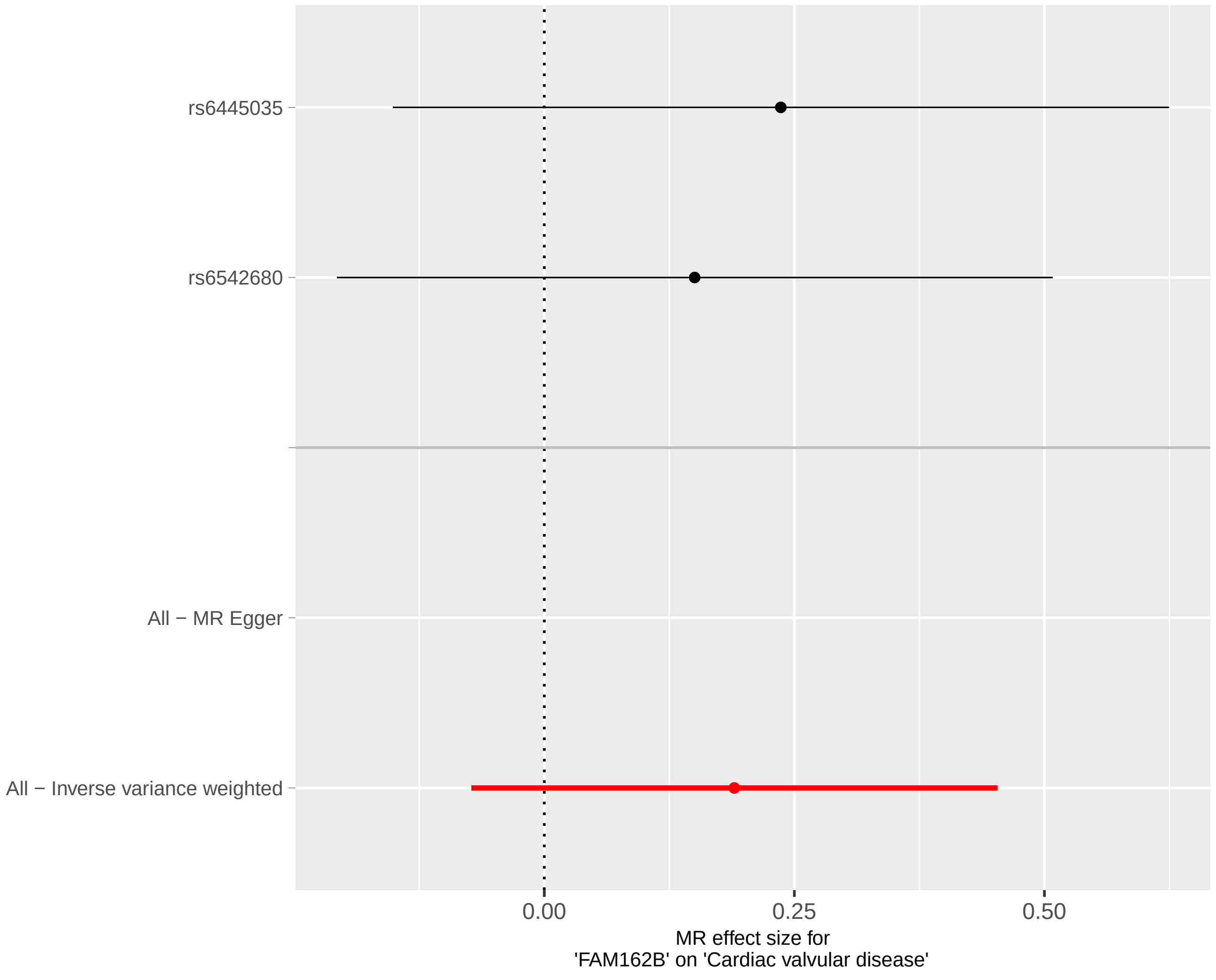
rs6445035
rs6542680
All − MR Egger
All − Inverse variance weighted
0.00
0.25
0.50
MR effect size for
'FAM162B' on 'Cardiac valvular disease'

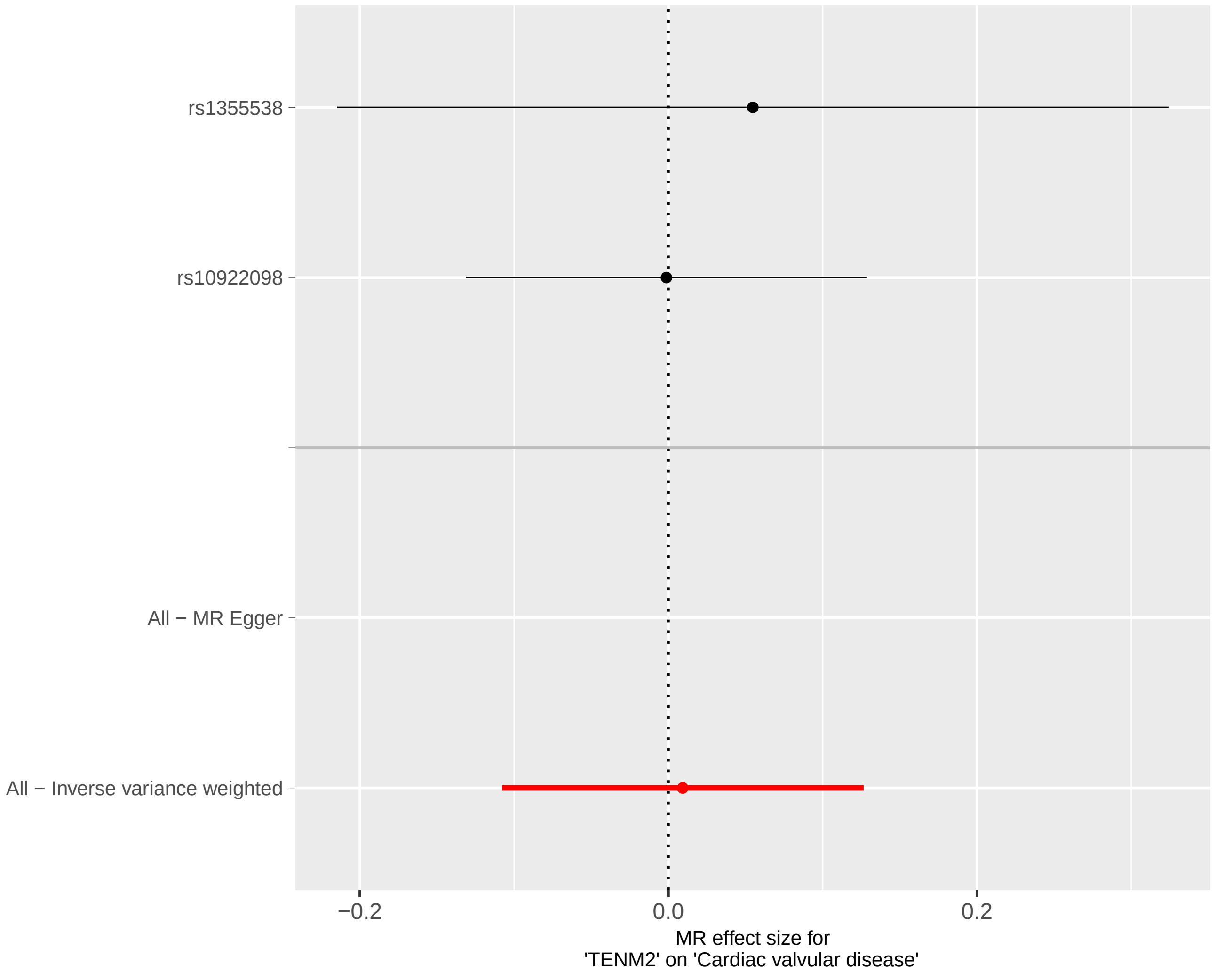
rs1355538
rs10922098
All − MR Egger
All − Inverse variance weighted
−0.2
0.0
0.2
MR effect size for
'TENM2' on 'Cardiac valvular disease'

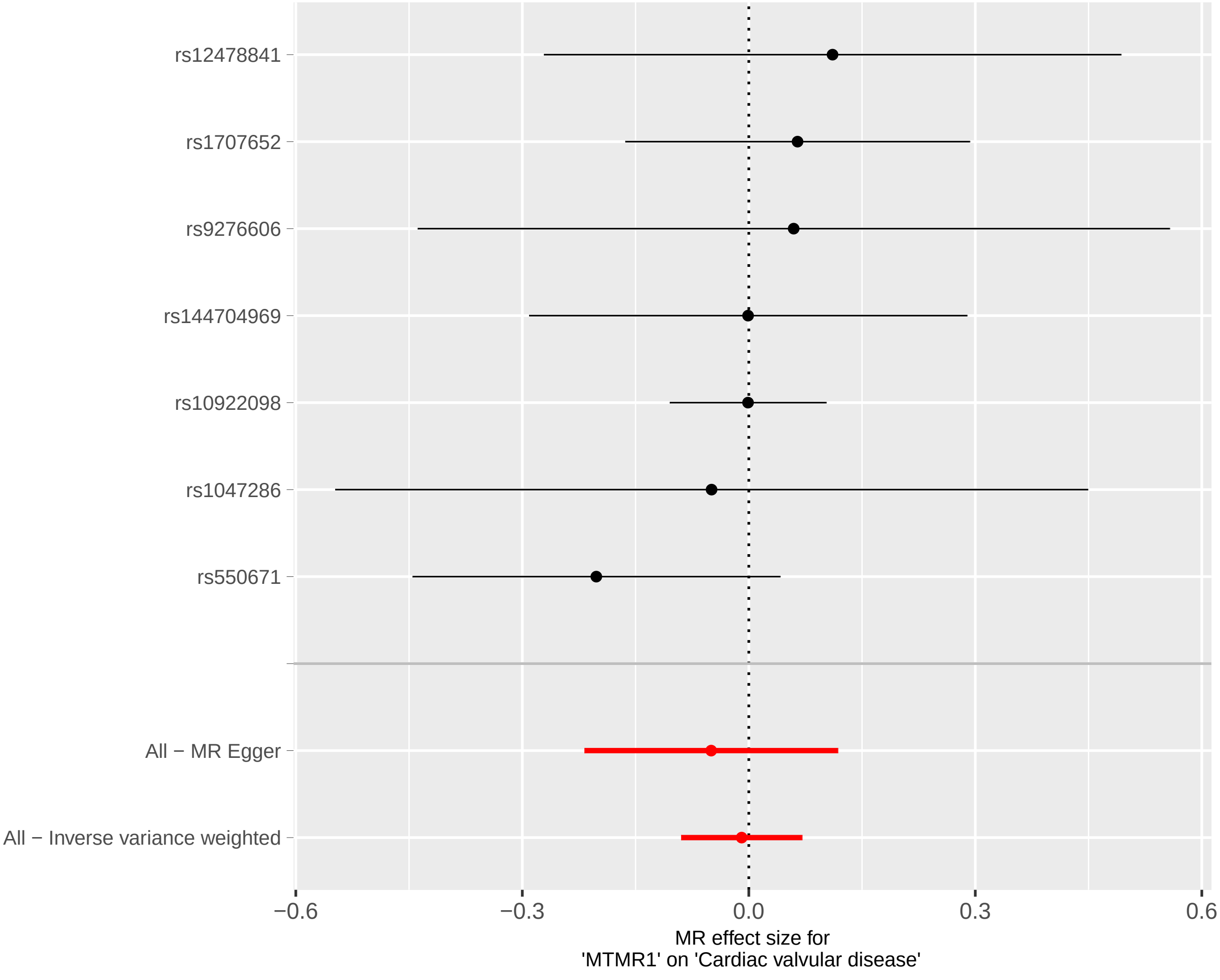
rs12478841
rs1707652
rs9276606
rs144704969
rs10922098
rs1047286
rs550671
All − MR Egger
All − Inverse variance weighted
−0.6
−0.3
0.0
0.3
0.6
MR effect size for
'MTMR1' on 'Cardiac valvular disease'

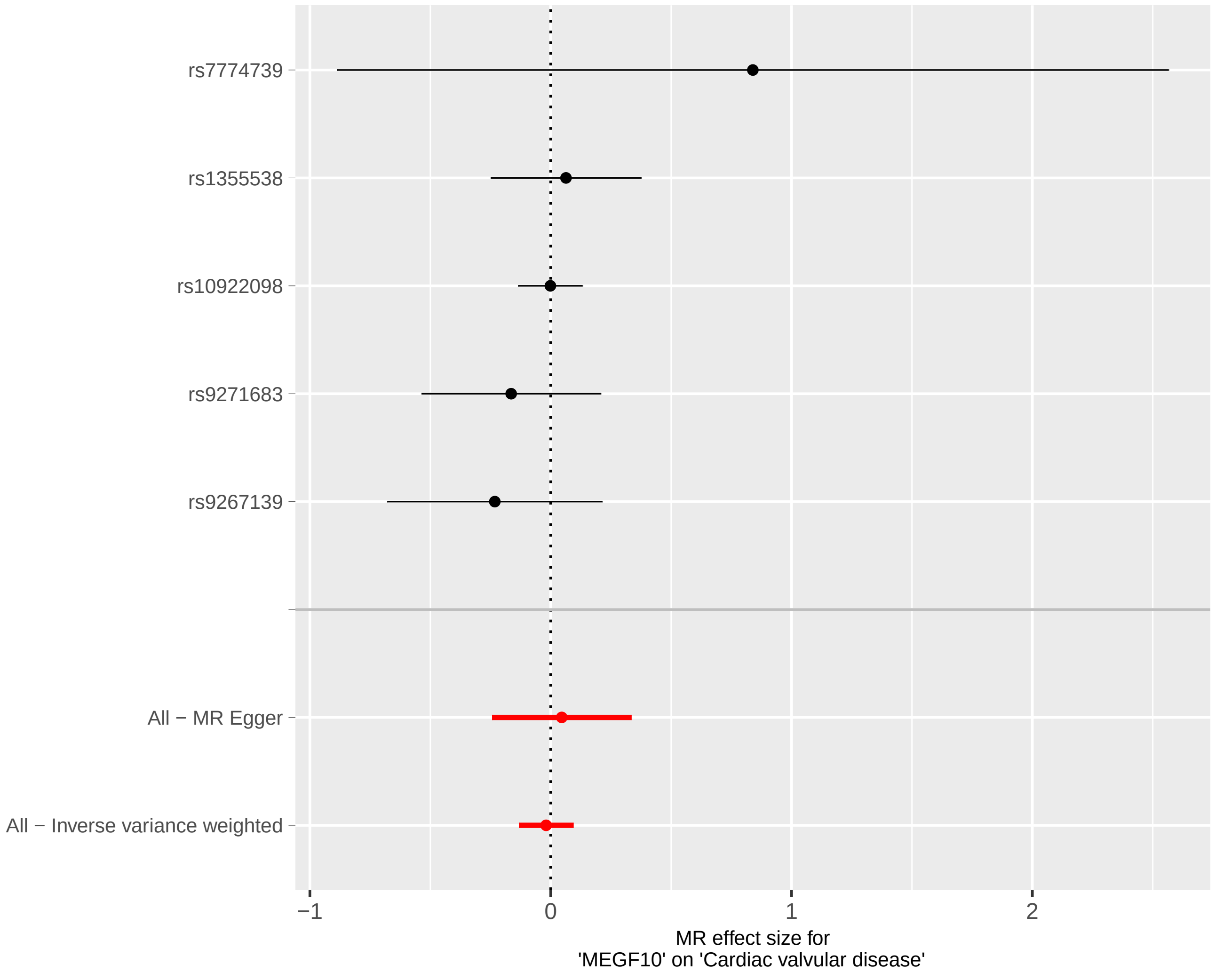
rs7774739
rs1355538
rs10922098
rs9271683
rs9267139
All − MR Egger
All − Inverse variance weighted
−1
0
1
2
MR effect size for
'MEGF10' on 'Cardiac valvular disease'

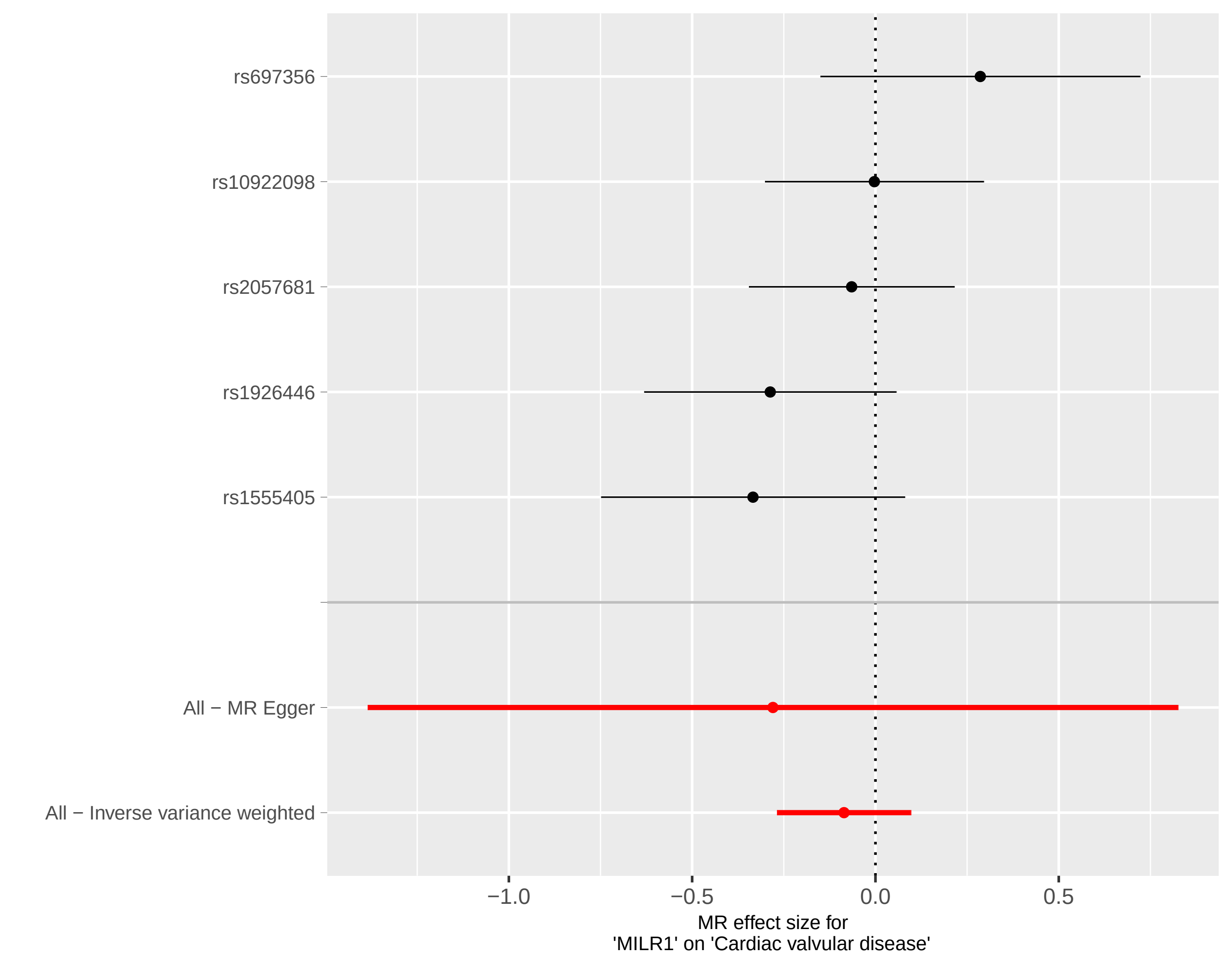
rs697356
rs10922098
rs2057681
rs1926446
rs1555405
All − MR Egger
All − Inverse variance weighted
−1.0
−0.5
0.0
0.5
MR effect size for
'MILR1' on 'Cardiac valvular disease'

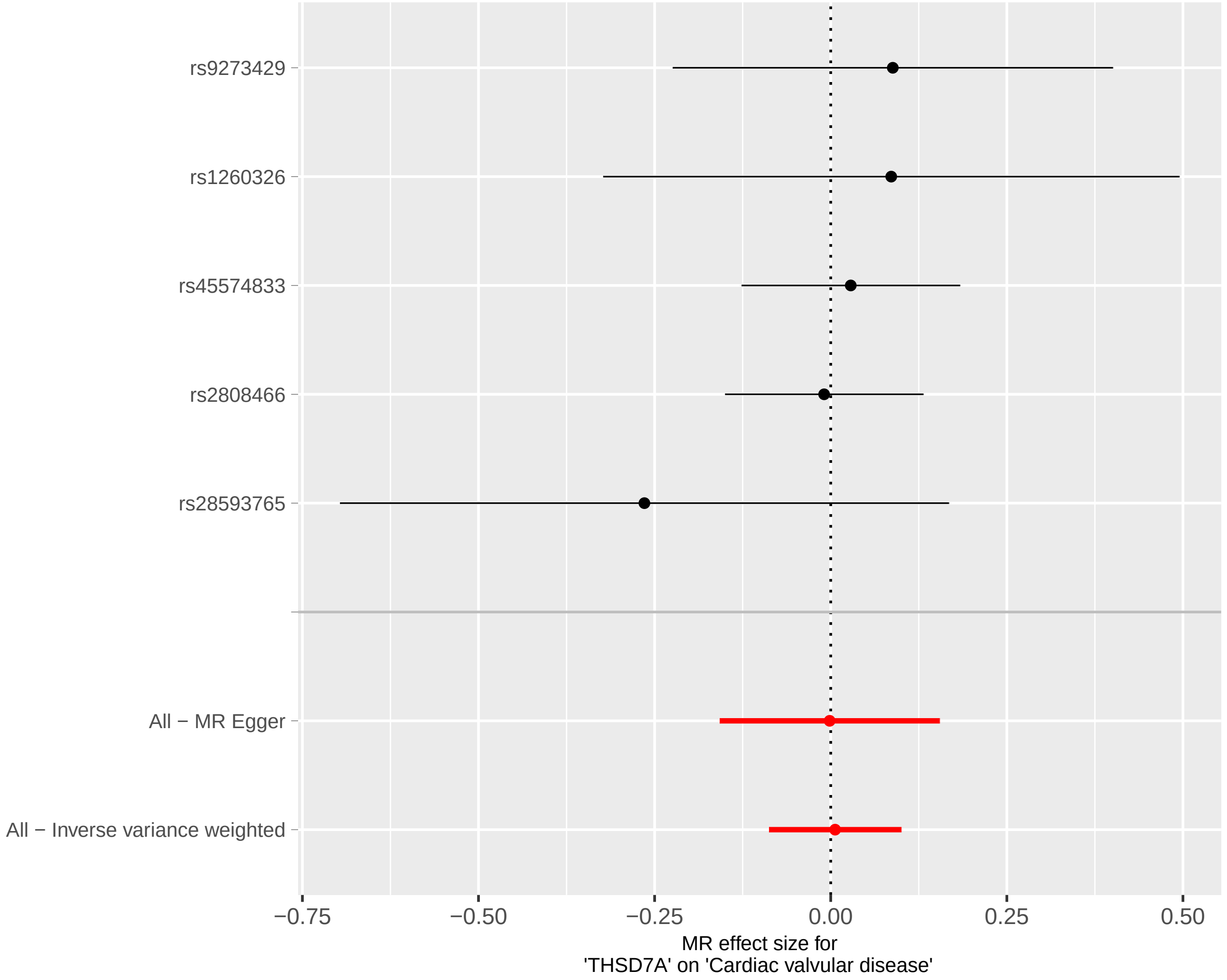
rs9273429
rs1260326
rs45574833
rs2808466
rs28593765
All − MR Egger
All − Inverse variance weighted
−0.75
−0.50
−0.25
0.00
0.25
0.50
MR effect size for
'THSD7A' on 'Cardiac valvular disease'

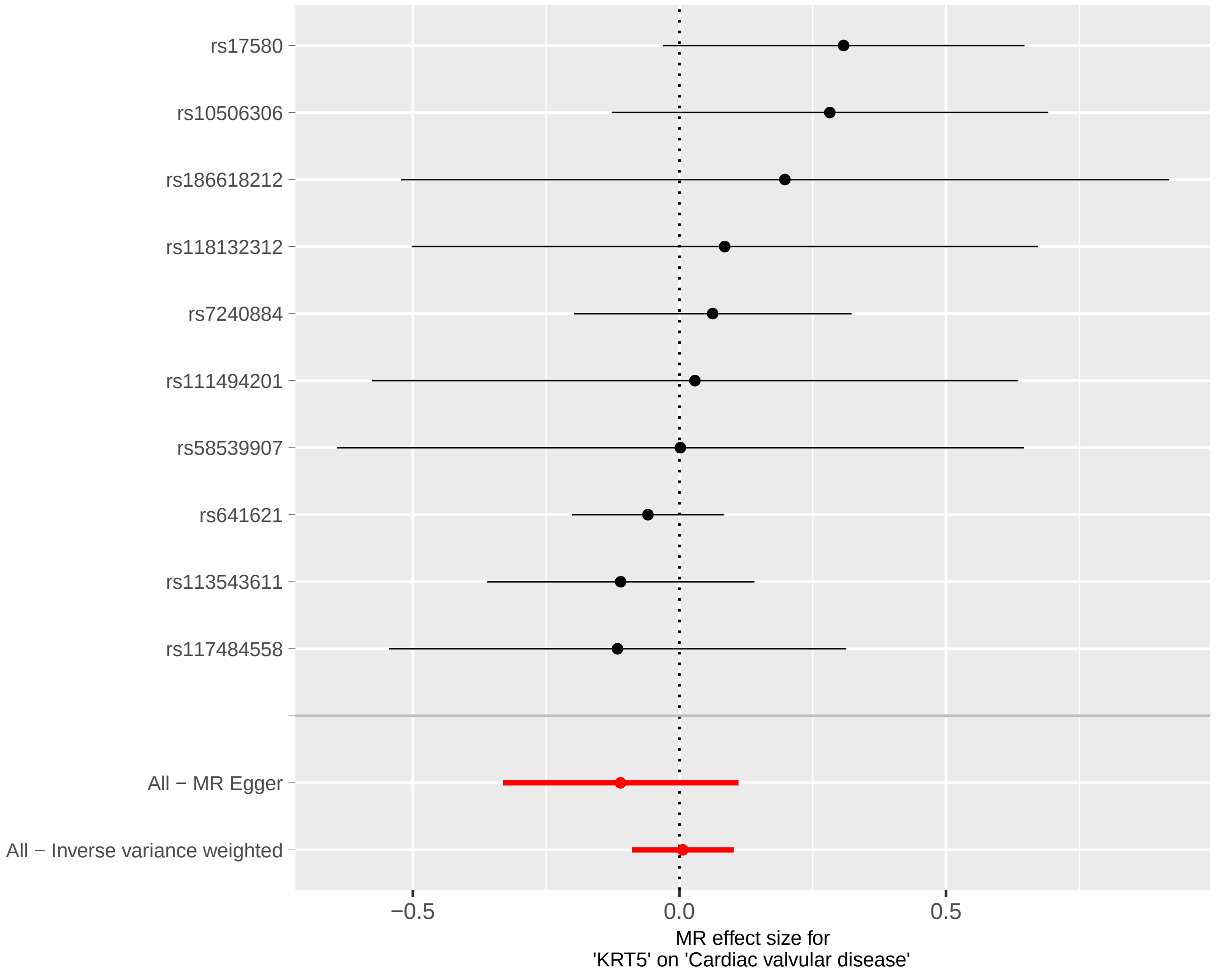
rs17580
rs10506306
rs186618212
rs118132312
rs7240884
rs111494201
rs58539907
rs641621
rs113543611
rs117484558
All − MR Egger
All − Inverse variance weighted
−0.5
0.0
0.5
MR effect size for
'KRT5' on 'Cardiac valvular disease'

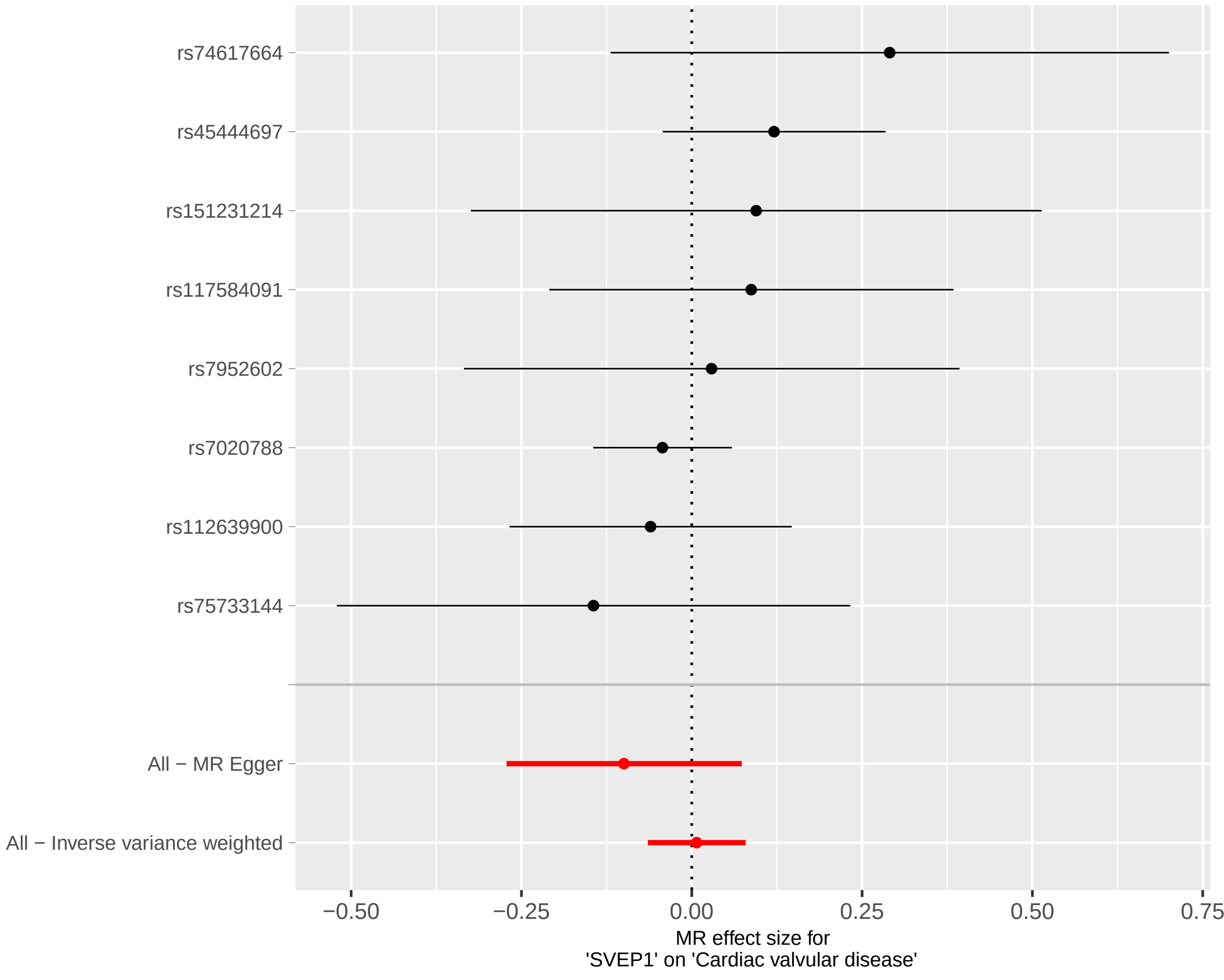

rs74617664
rs45444697
rs151231214
rs117584091
rs7952602
rs7020788
rs112639900
rs75733144
All − MR Egger
All − Inverse variance weighted
−0.50
−0.25
0.00
0.25
0.50
0.75
MR effect size for
'SVEP1' on 'Cardiac valvular disease'

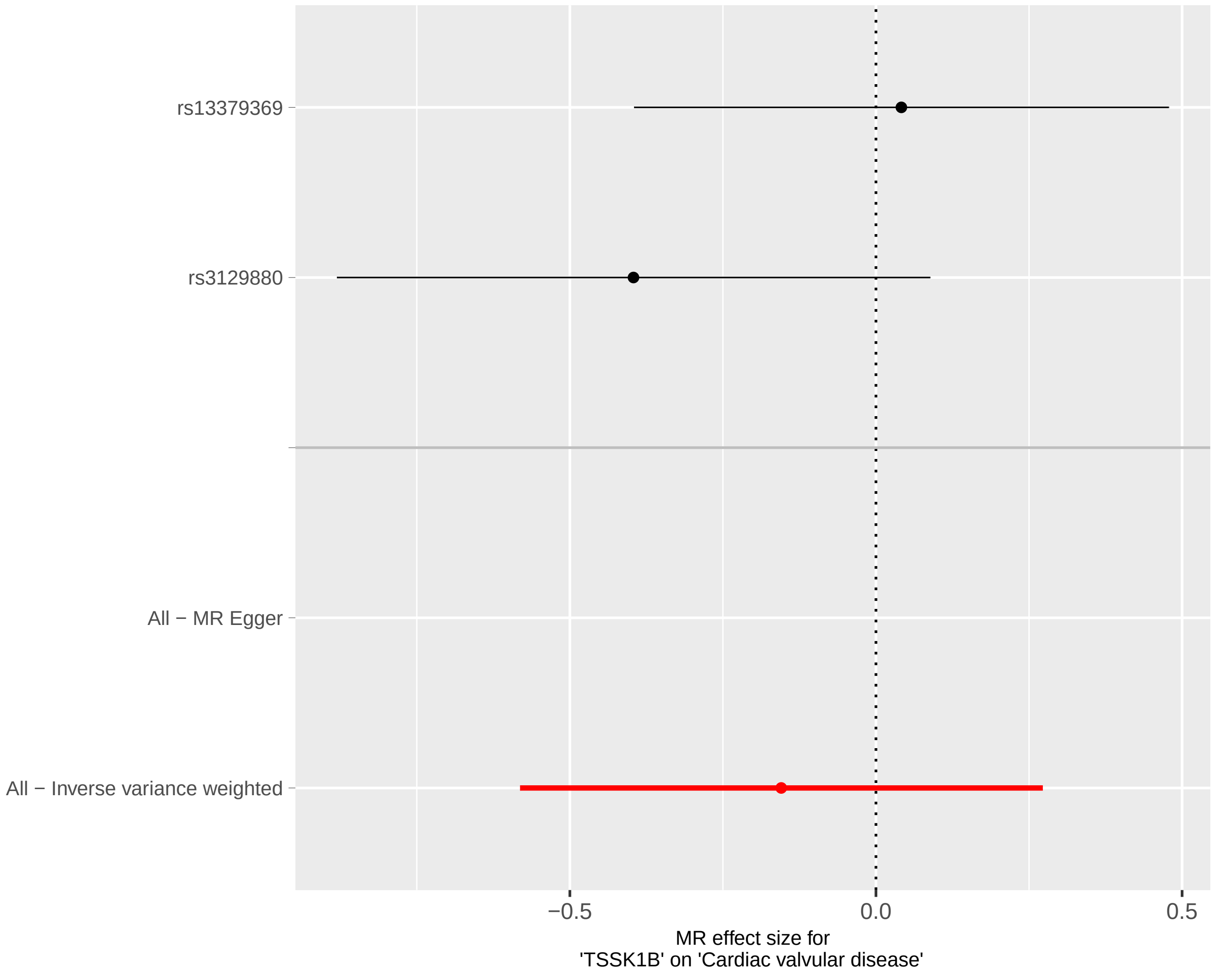

rs13379369
rs3129880
All − MR Egger
All − Inverse variance weighted
−0.5
0.0
0.5
MR effect size for
'TSSK1B' on 'Cardiac valvular disease'

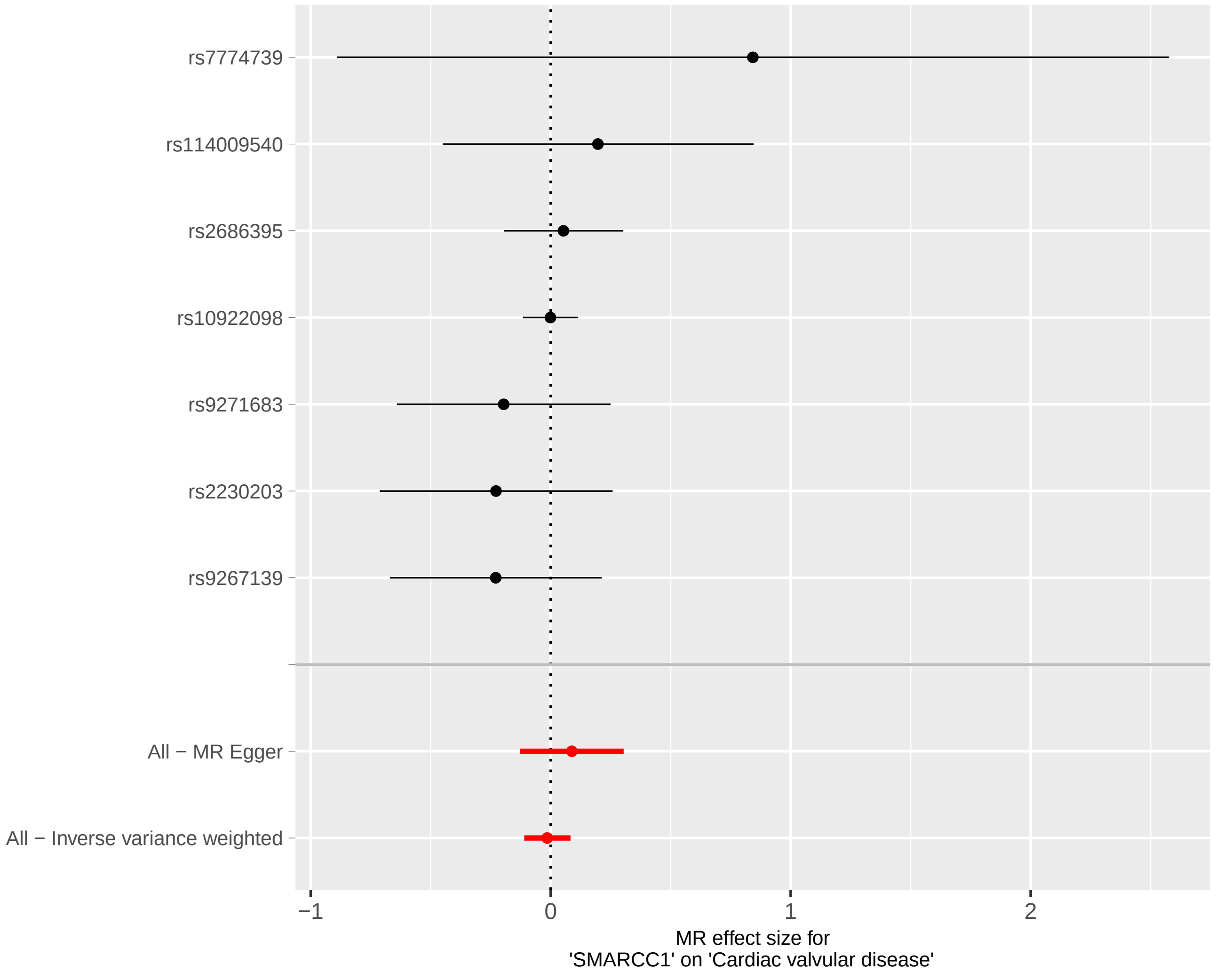

rs7774739
rs114009540
rs2686395
rs10922098
rs9271683
rs2230203
rs9267139
All − MR Egger
All − Inverse variance weighted
−1
0
1
2
MR effect size for
'SMARCC1' on 'Cardiac valvular disease'

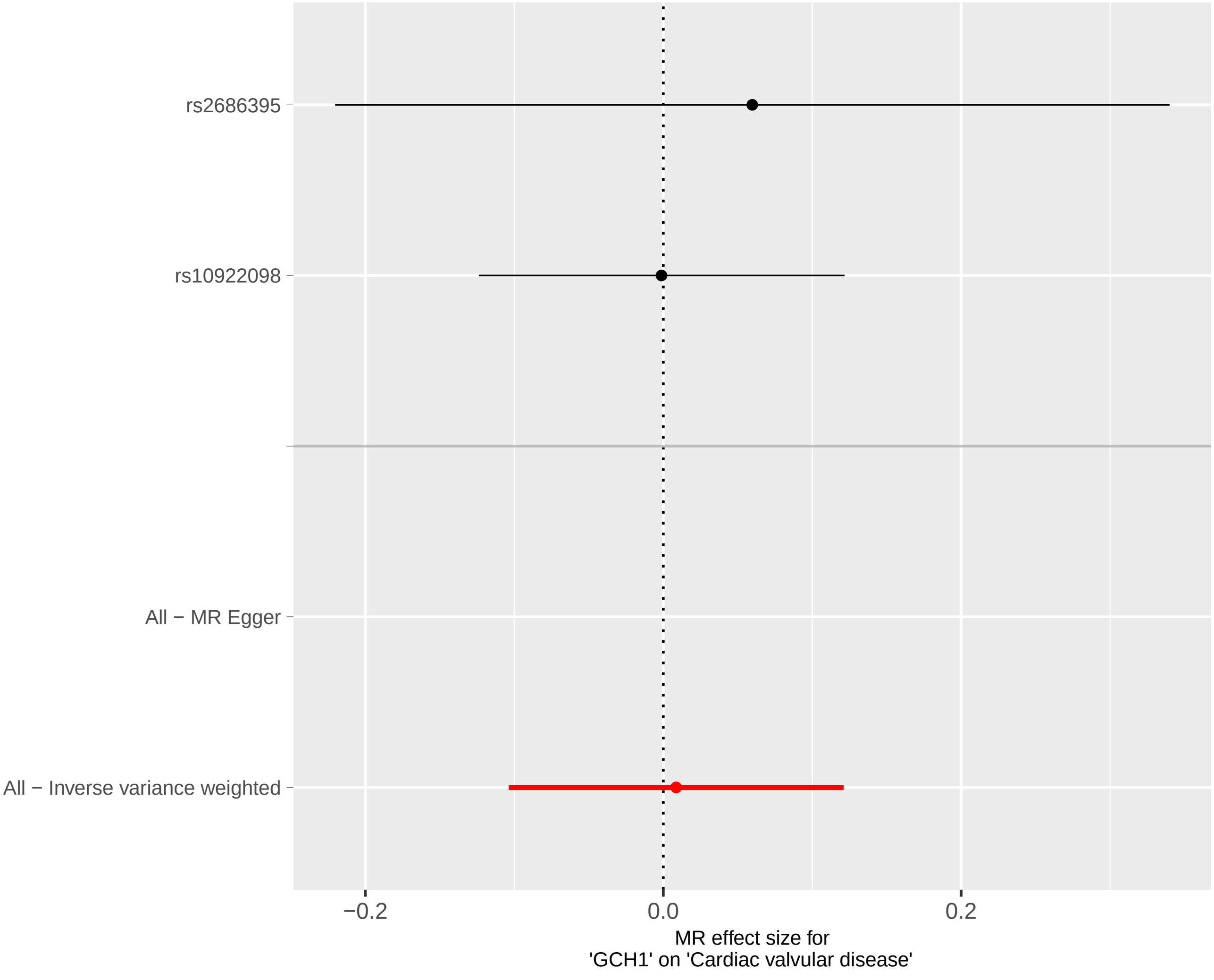
rs2686395
rs10922098
All − MR Egger
All − Inverse variance weighted
−0.2
0.0
0.2
MR effect size for
'GCH1' on 'Cardiac valvular disease'

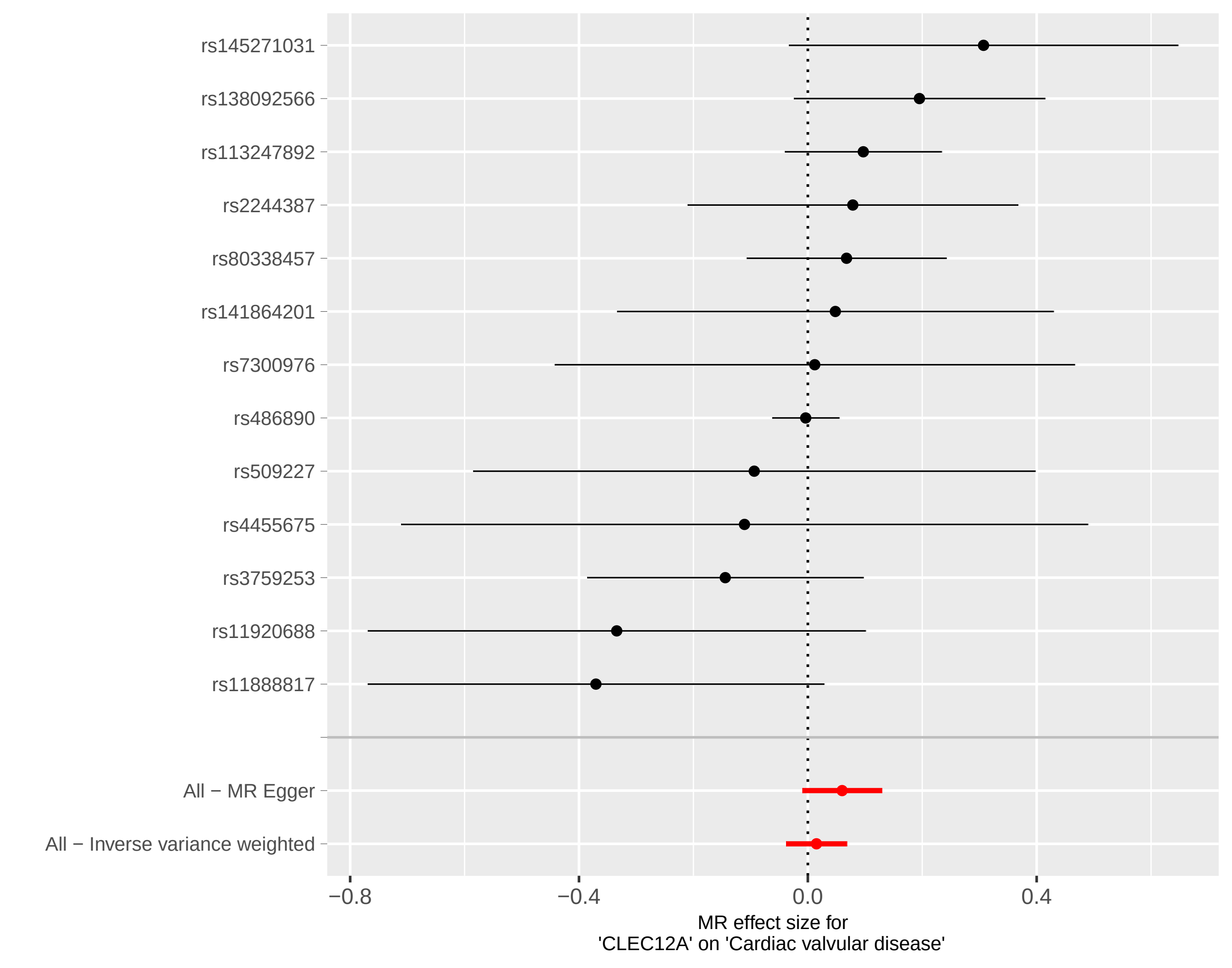
rs145271031
rs138092566
rs113247892
rs2244387
rs80338457
rs141864201
rs7300976
rs486890
rs509227
rs4455675
rs3759253
rs11920688
rs11888817
All − MR Egger
All − Inverse variance weighted
−0.8
−0.4
0.0
0.4
MR effect size for
'CLEC12A' on 'Cardiac valvular disease'

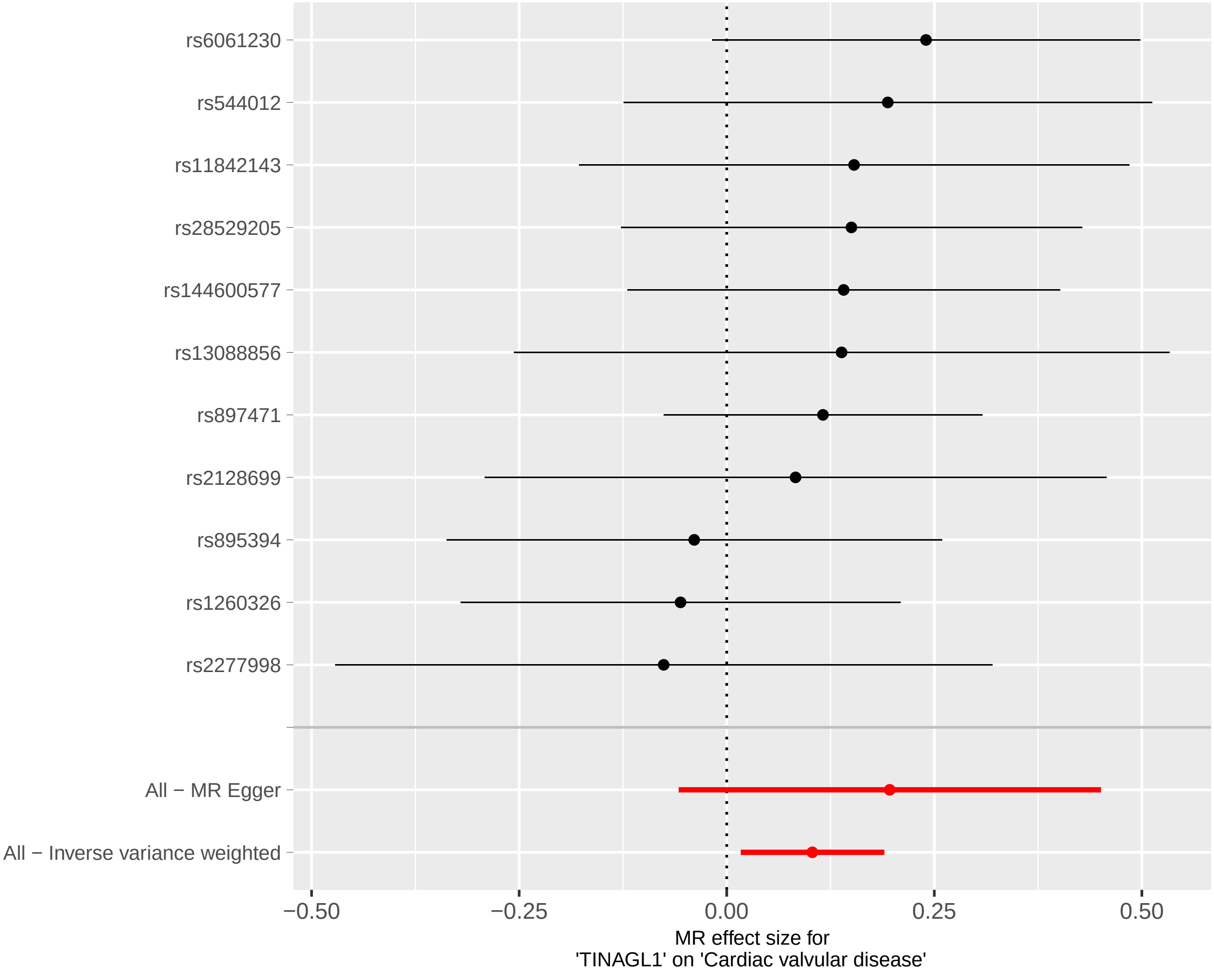
rs6061230
rs544012
rs11842143
rs28529205
rs144600577
rs13088856
rs897471
rs2128699
rs895394
rs1260326
rs2277998
All − MR Egger
All − Inverse variance weighted
−0.50
−0.25
0.00
0.25
0.50
MR effect size for
'TINAGL1' on 'Cardiac valvular disease'

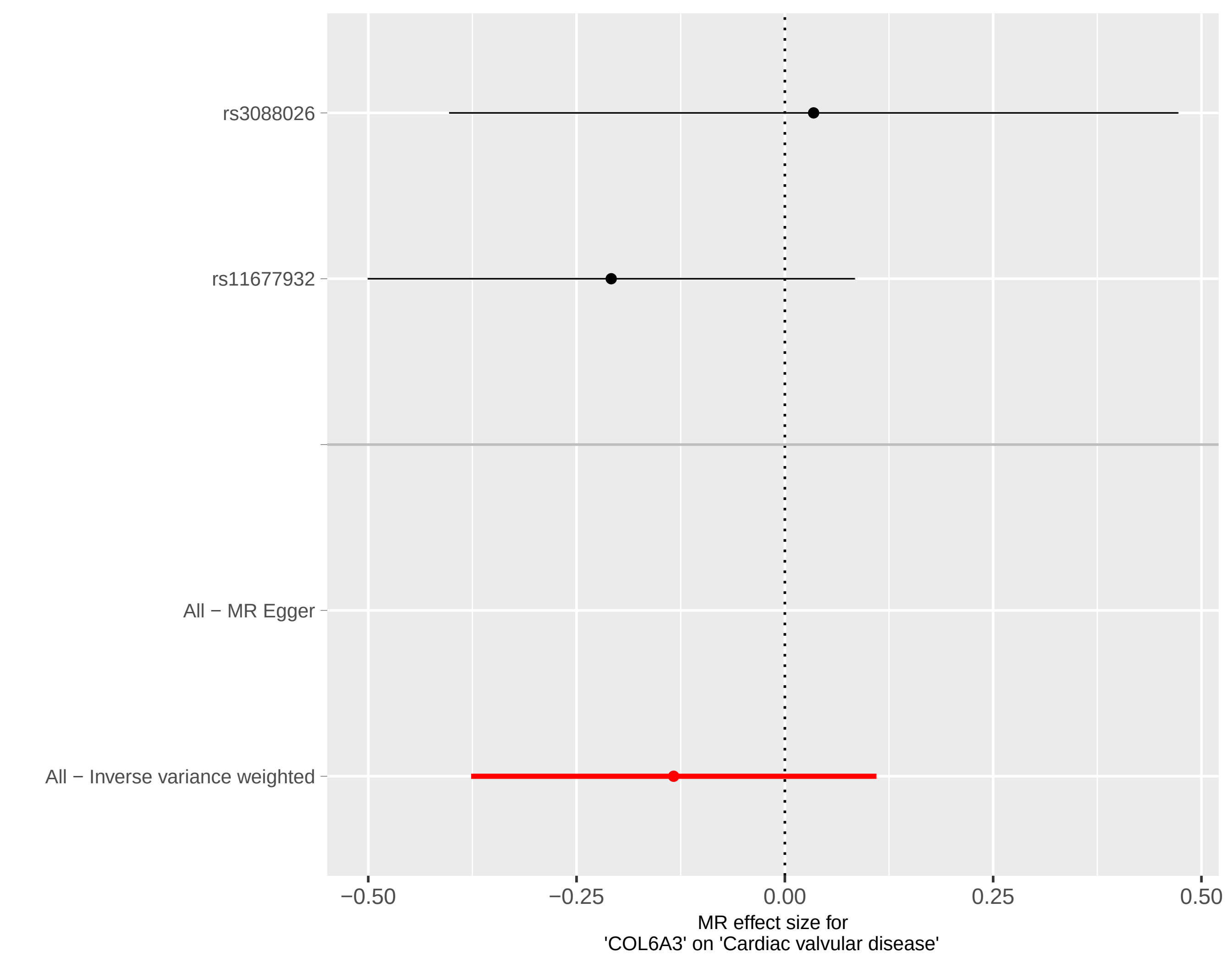
rs3088026
rs11677932
All − MR Egger
All − Inverse variance weighted
−0.50
−0.25
0.00
0.25
0.50
MR effect size for
'COL6A3' on 'Cardiac valvular disease'

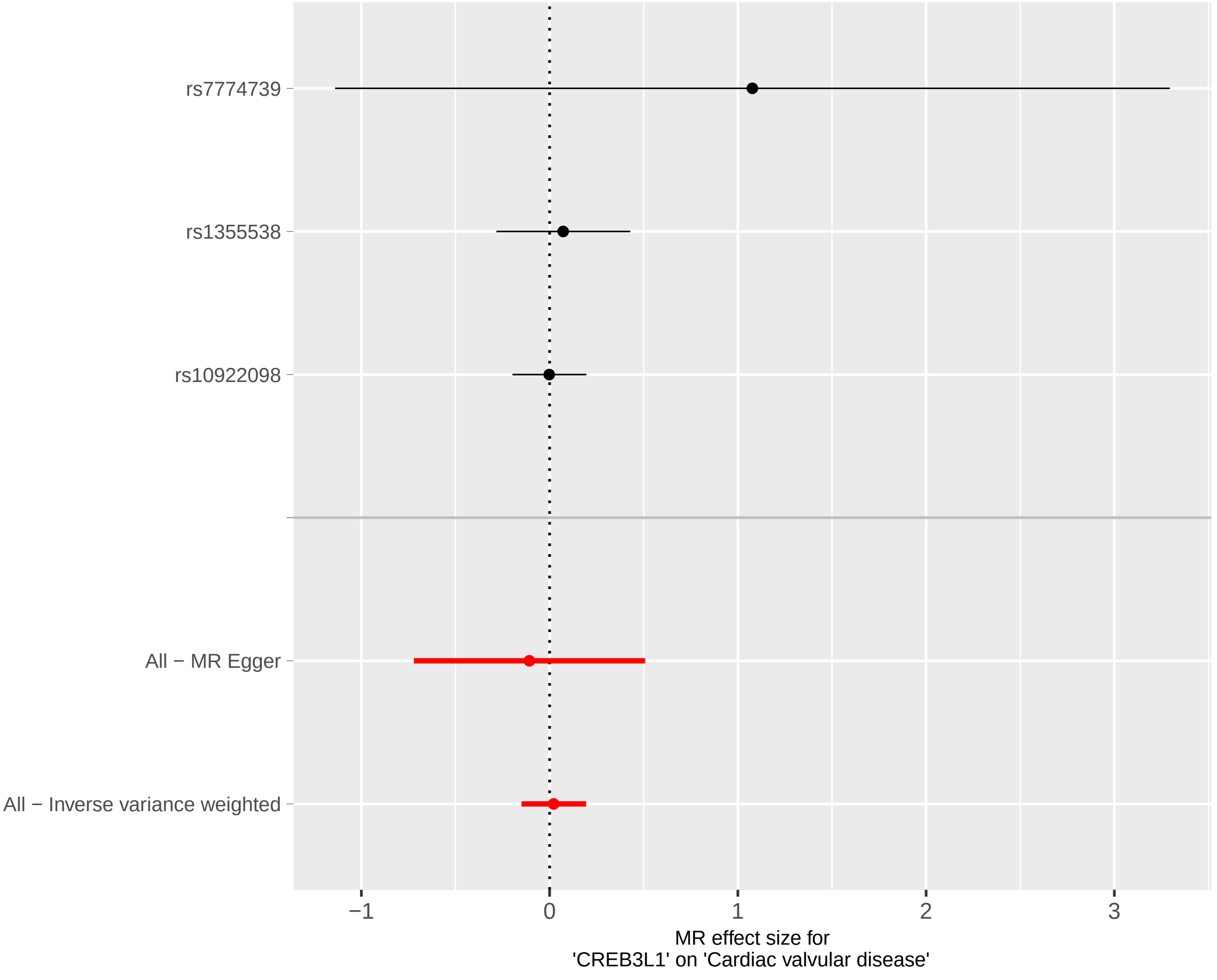

rs7774739
rs1355538
rs10922098
All − MR Egger
All − Inverse variance weighted
−1
0
1
2
3
MR effect size for
'CREB3L1' on 'Cardiac valvular disease'

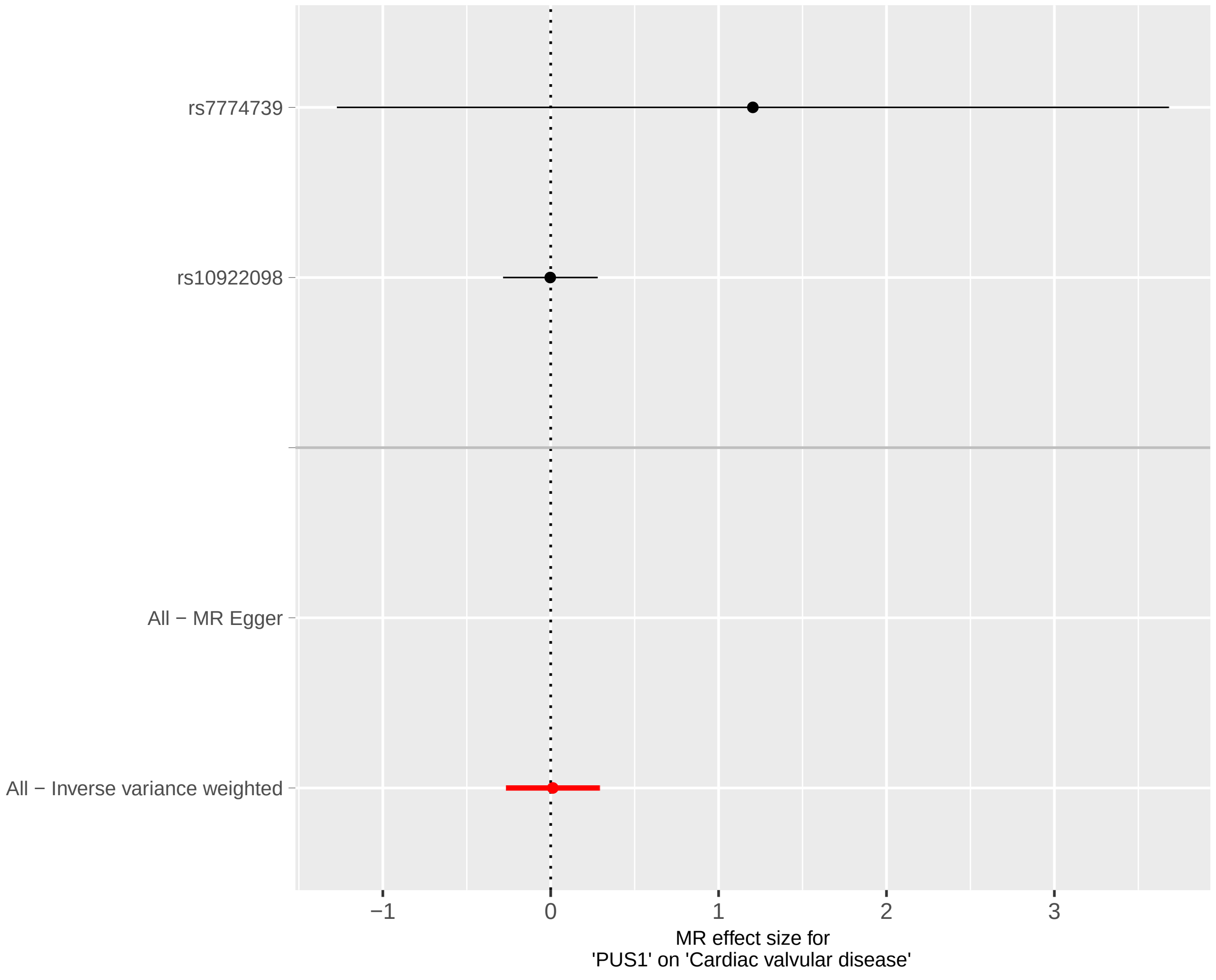
rs7774739
rs10922098
All − MR Egger
All − Inverse variance weighted
−1
0
1
2
3
MR effect size for
'PUS1' on 'Cardiac valvular disease'

Insufficient number of SNPs

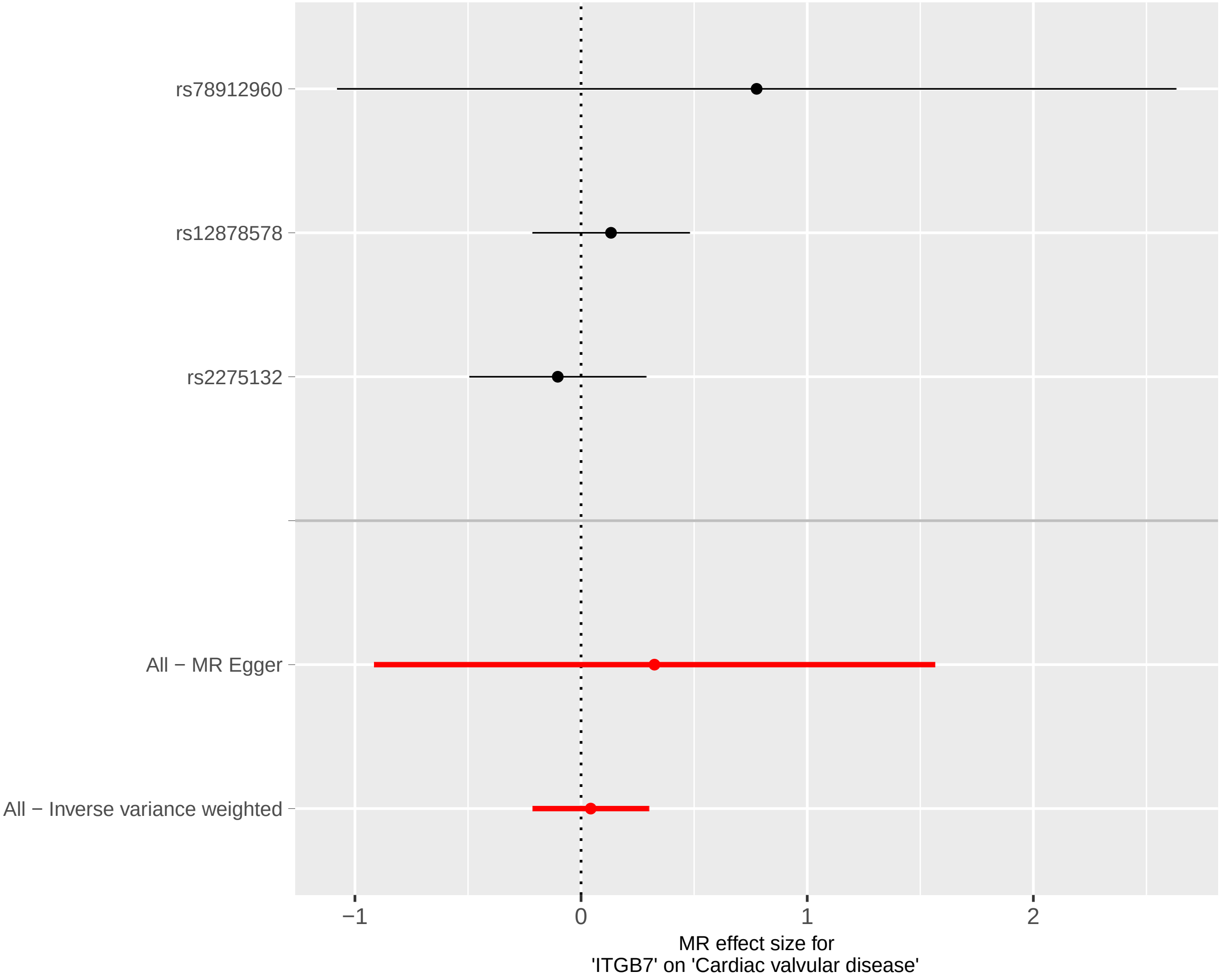
rs78912960
rs12878578
rs2275132
All − MR Egger
All − Inverse variance weighted
−1
0
1
2
MR effect size for
'ITGB7' on 'Cardiac valvular disease'

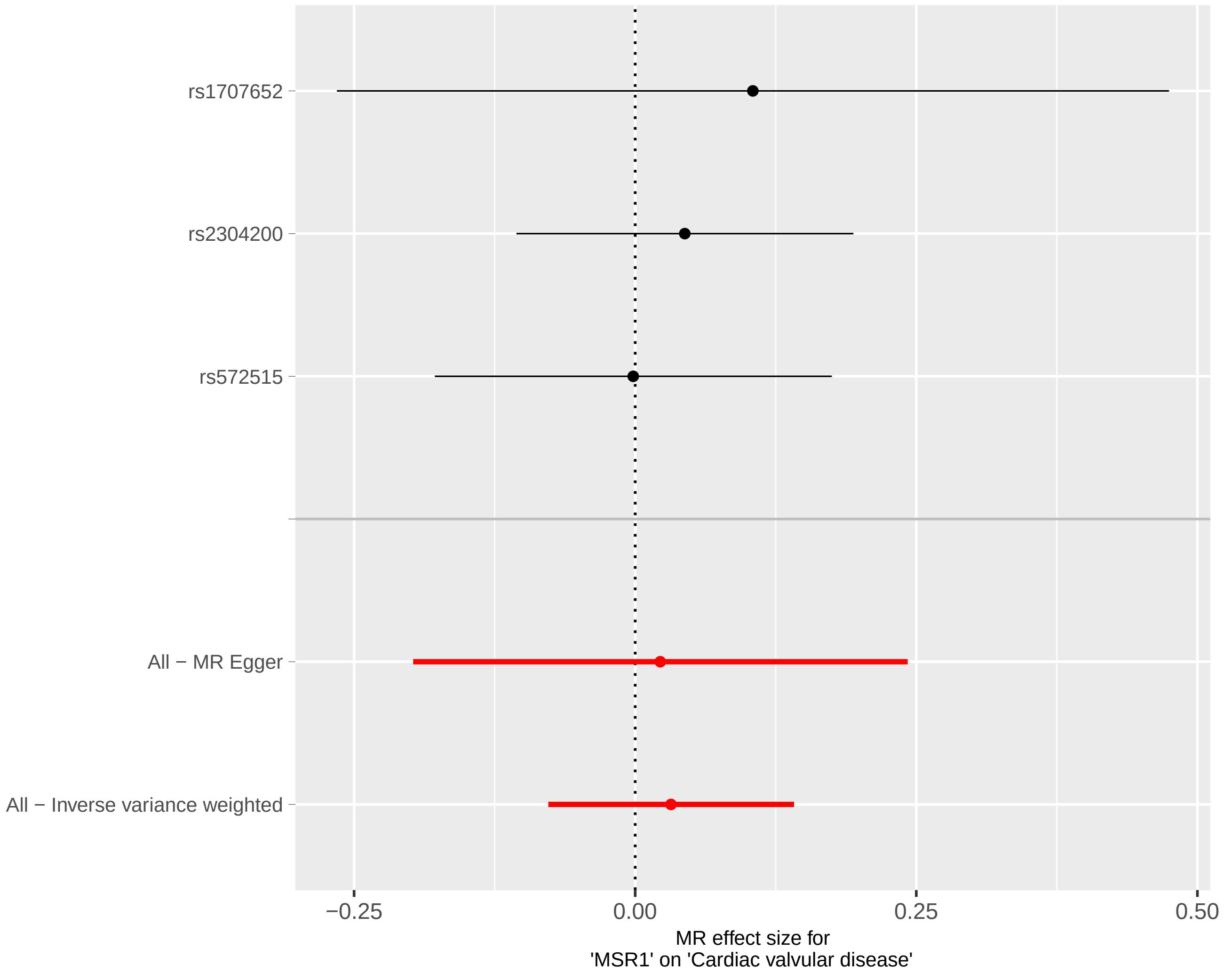

rs1707652
rs2304200
rs572515
All − MR Egger
All − Inverse variance weighted
−0.25
0.00
0.25
0.50
MR effect size for
'MSR1' on 'Cardiac valvular disease'

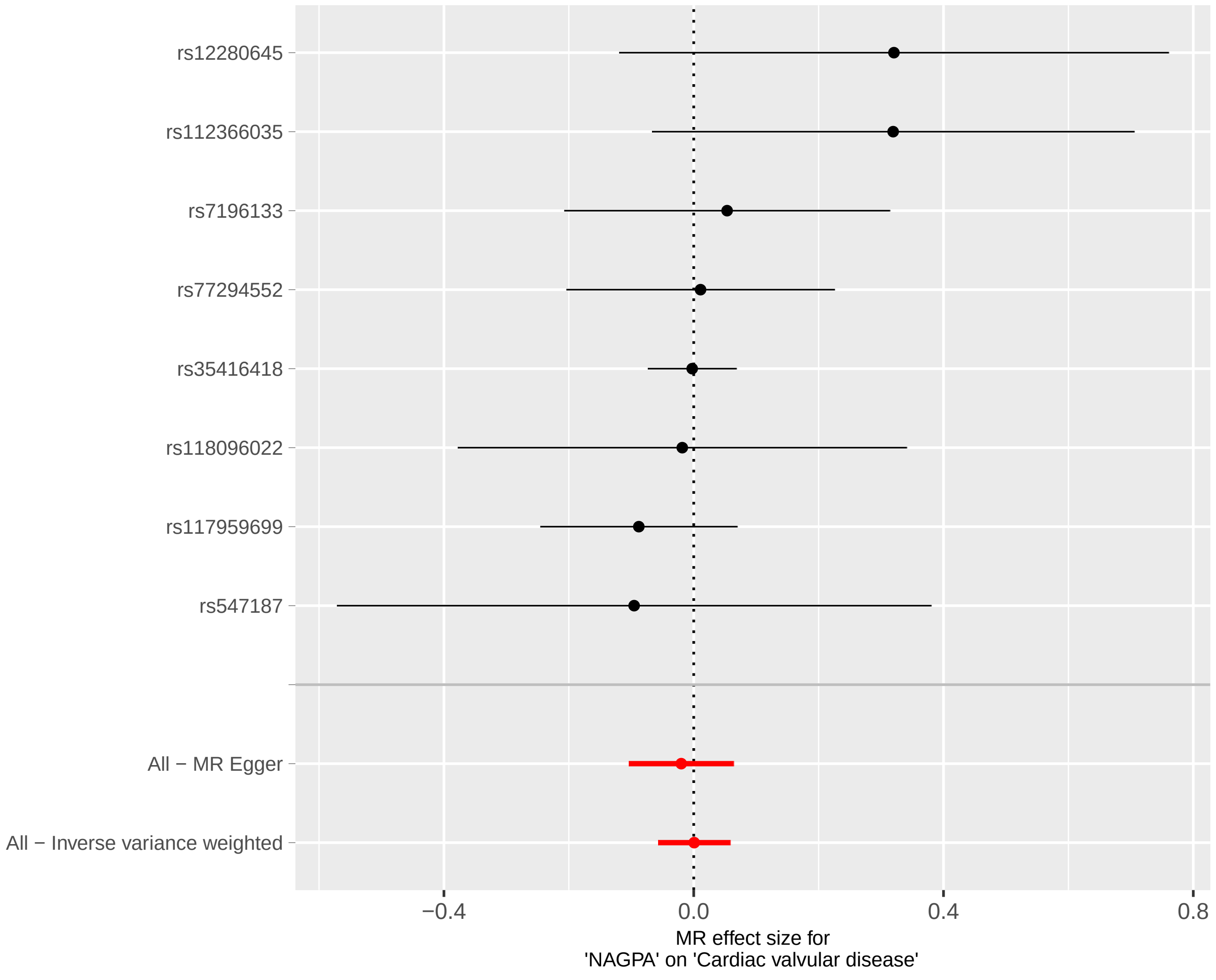
rs12280645
rs112366035
rs7196133
rs77294552
rs35416418
rs118096022
rs117959699
rs547187
All − MR Egger
All − Inverse variance weighted
−0.4
0.0
0.4
0.8
MR effect size for
'NAGPA' on 'Cardiac valvular disease'

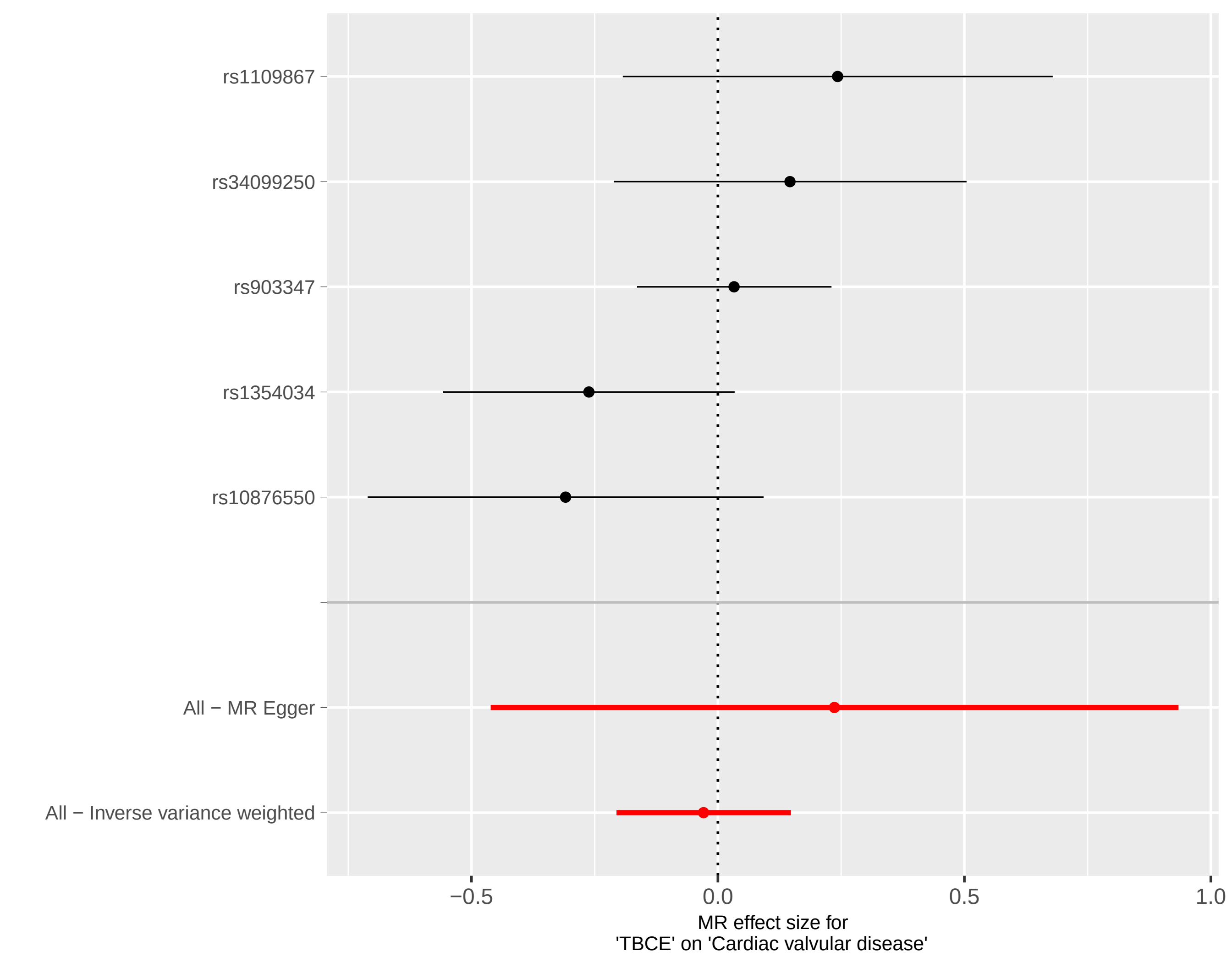
rs1109867
rs34099250
rs903347
rs1354034
rs10876550
All − MR Egger
All − Inverse variance weighted
−0.5
0.0
0.5
1.0
MR effect size for
'TBCE' on 'Cardiac valvular disease'

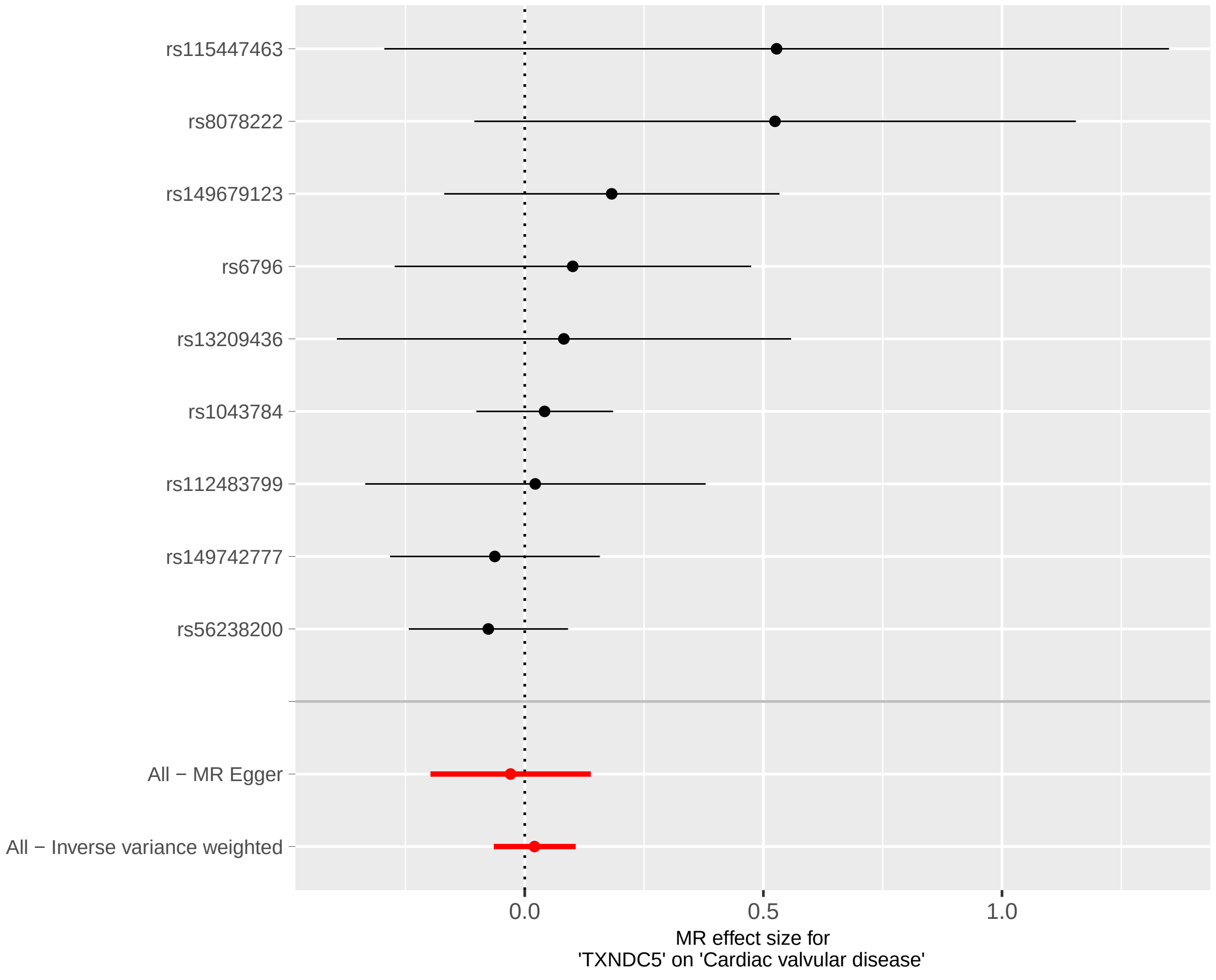
rs115447463
rs8078222
rs149679123
rs6796
rs13209436
rs1043784
rs112483799
rs149742777
rs56238200
All − MR Egger
All − Inverse variance weighted
0.0
0.5
1.0
MR effect size for
'TXNDC5' on 'Cardiac valvular disease'

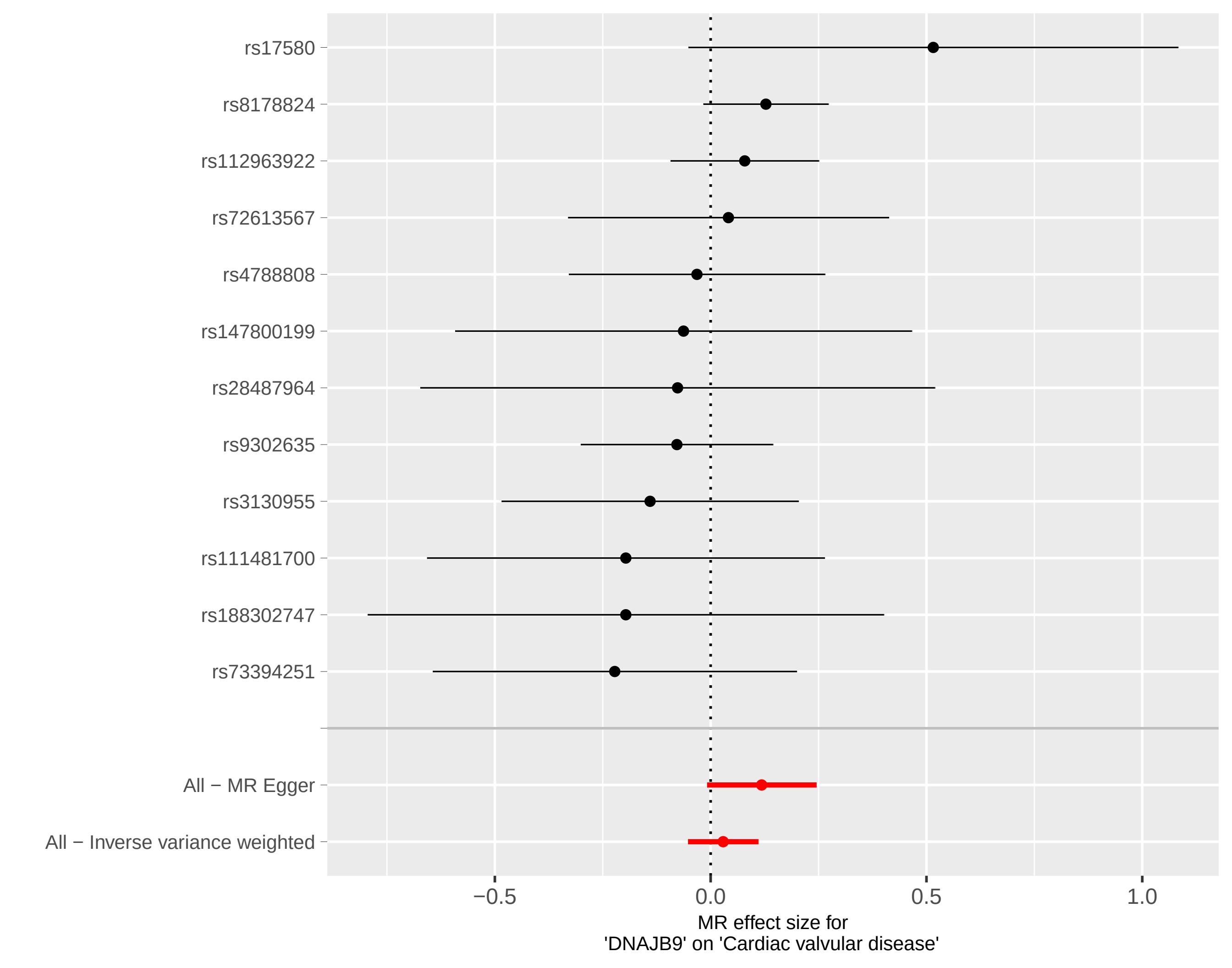
rs17580
rs8178824
rs112963922
rs72613567
rs4788808
rs147800199
rs28487964
rs9302635
rs3130955
rs111481700
rs188302747
rs73394251
All − MR Egger
All − Inverse variance weighted
−0.5
0.0
0.5
1.0
MR effect size for
'DNAJB9' on 'Cardiac valvular disease'

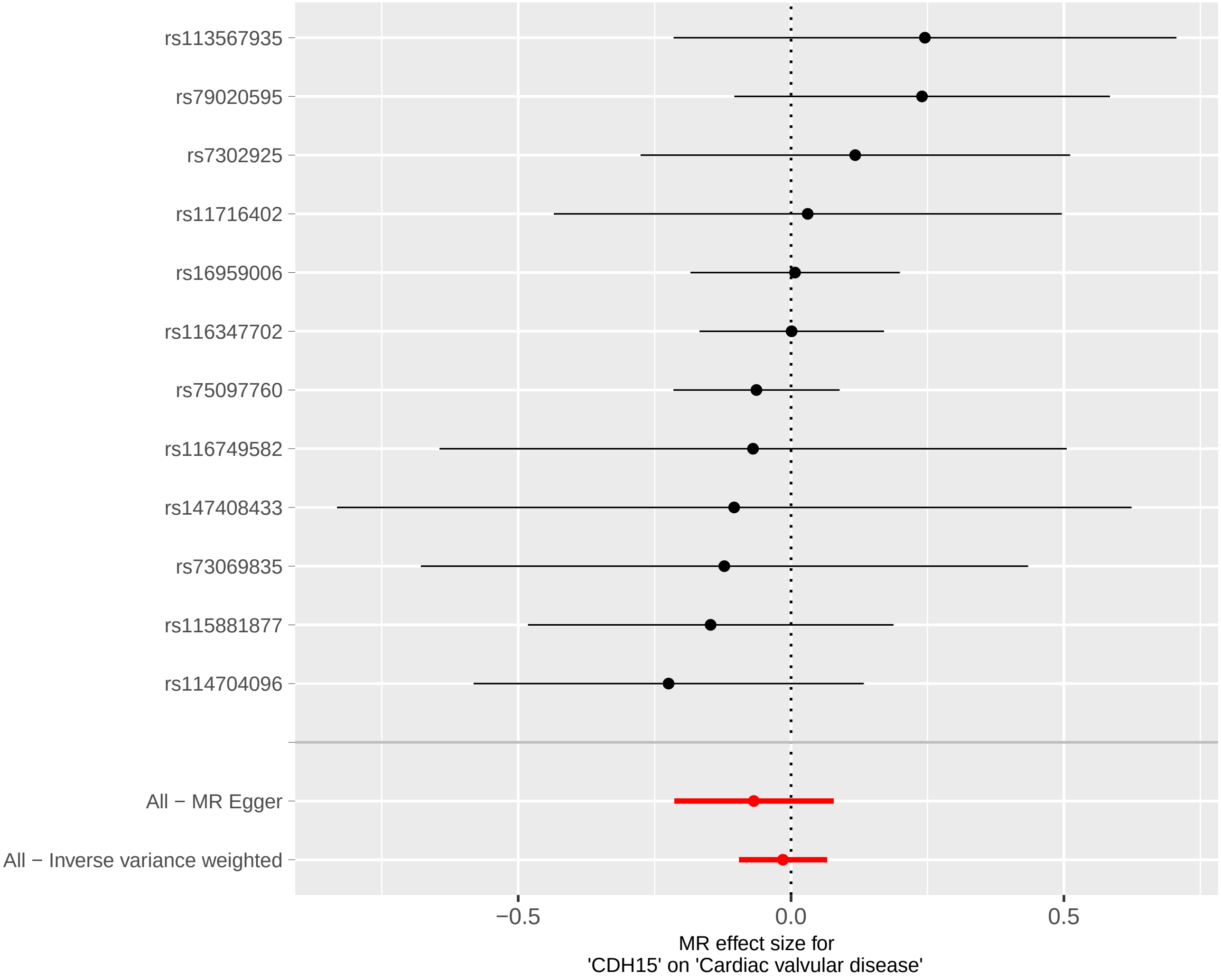

rs113567935
rs79020595
rs7302925
rs11716402
rs16959006
rs116347702
rs75097760
rs116749582
rs147408433
rs73069835
rs115881877
rs114704096
All − MR Egger
All − Inverse variance weighted
−0.5
0.0
0.5
MR effect size for
'CDH15' on 'Cardiac valvular disease'

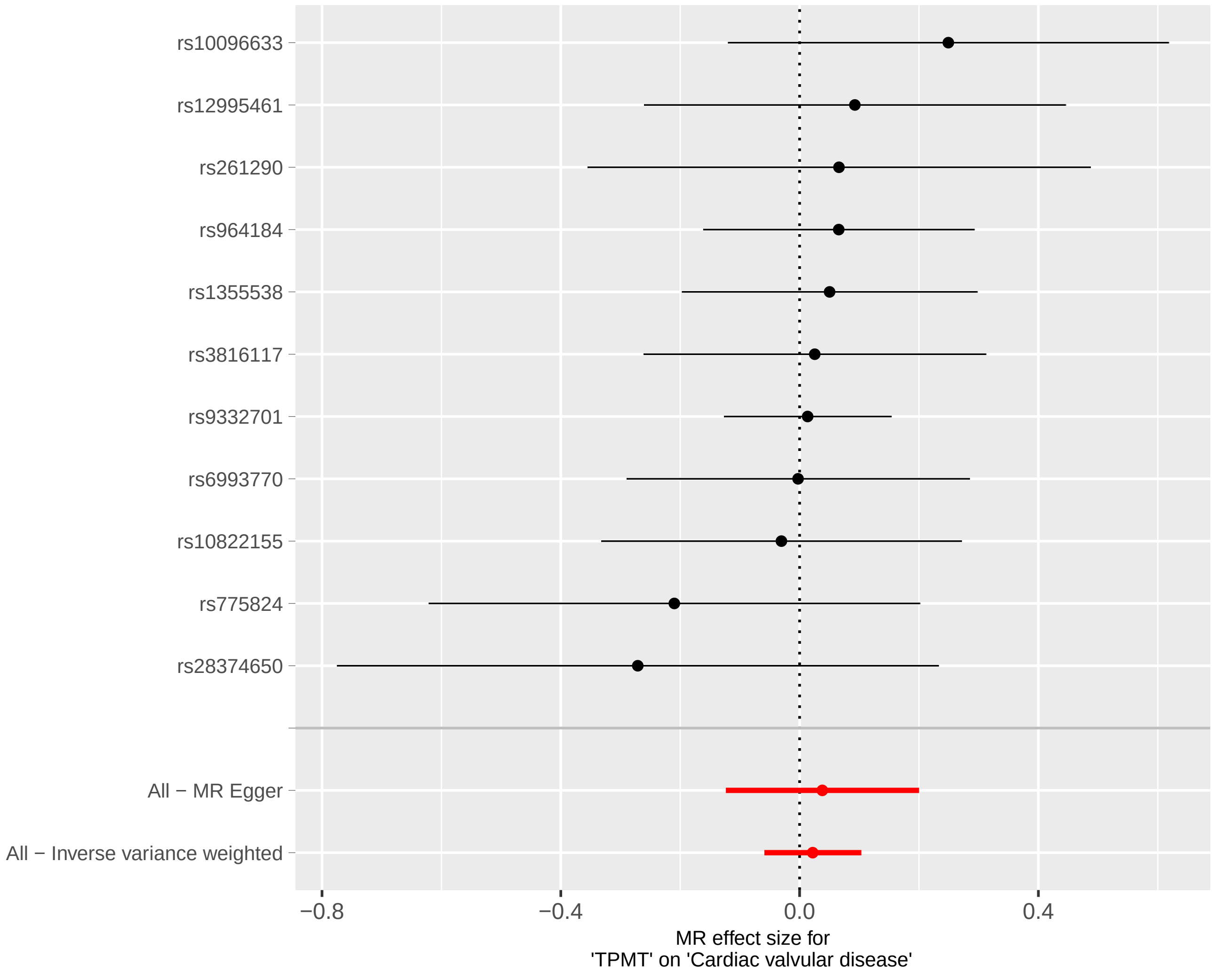
rs10096633
rs12995461
rs261290
rs964184
rs1355538
rs3816117
rs9332701
rs6993770
rs10822155
rs775824
rs28374650
All − MR Egger
All − Inverse variance weighted
−0.8
−0.4
0.0
0.4
MR effect size for
'TPMT' on 'Cardiac valvular disease'

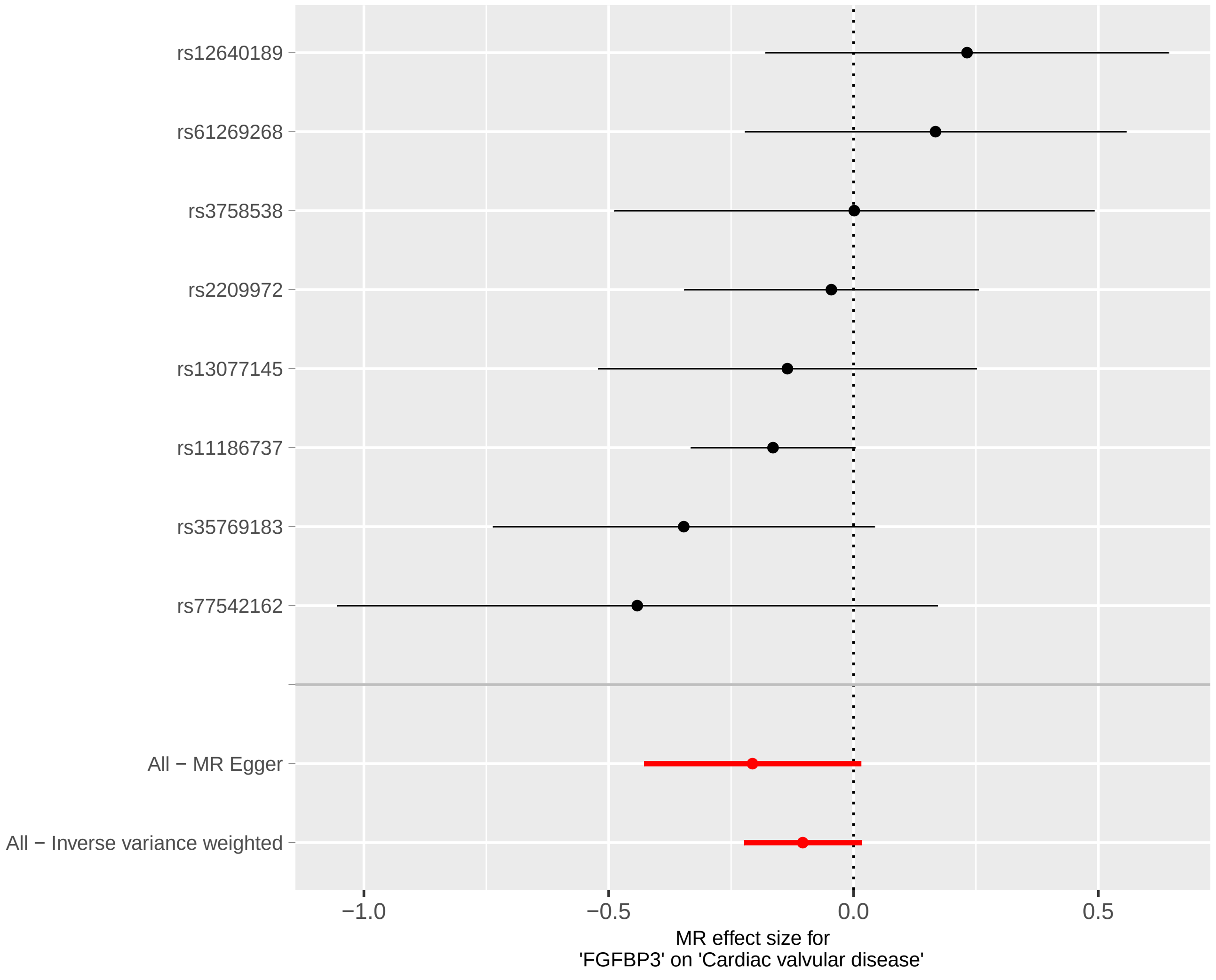
rs12640189
rs61269268
rs3758538
rs2209972
rs13077145
rs11186737
rs35769183
rs77542162
All − MR Egger
All − Inverse variance weighted
−1.0
−0.5
0.0
0.5
MR effect size for
'FGFBP3' on 'Cardiac valvular disease'

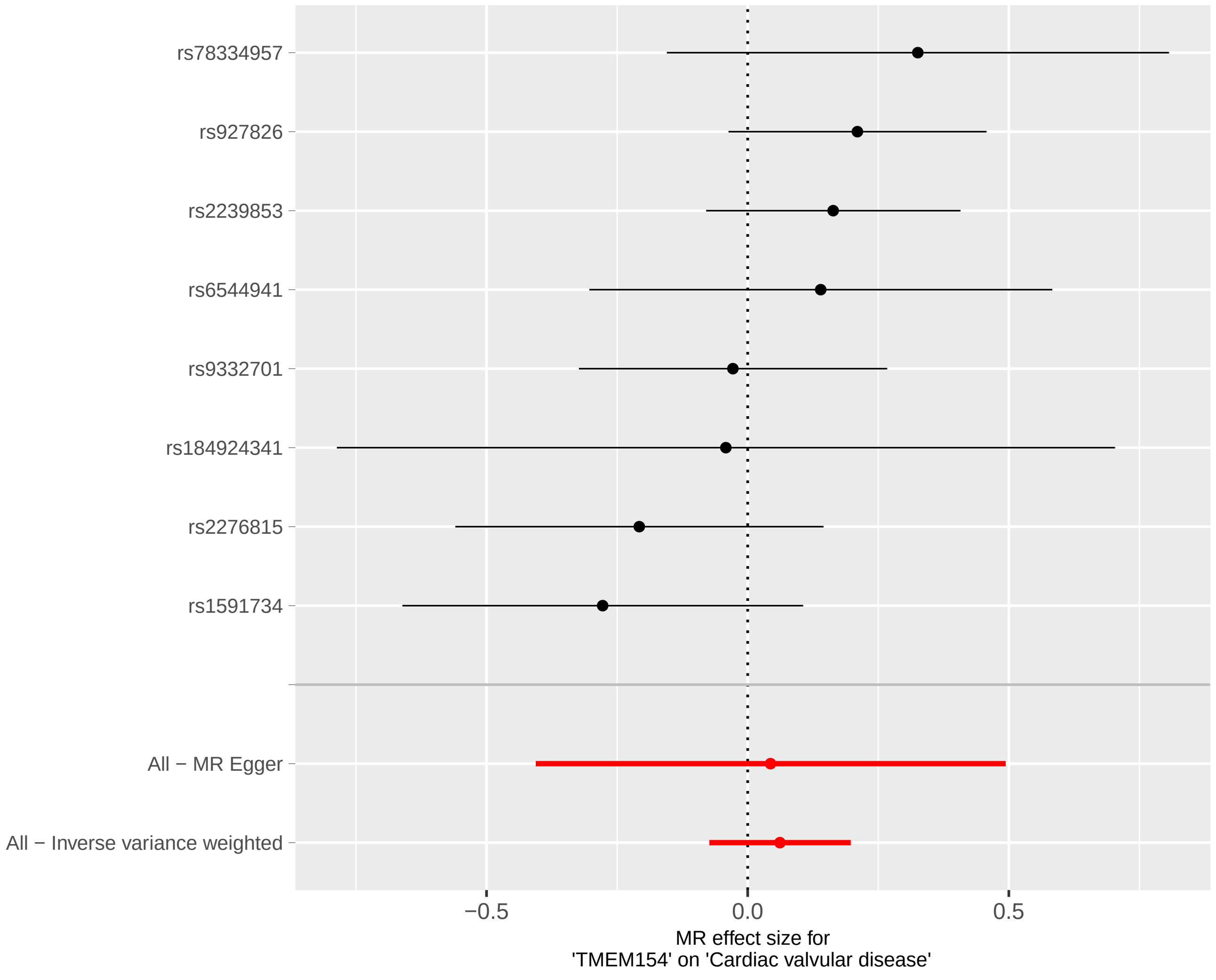
rs78334957
rs927826
rs2239853
rs6544941
rs9332701
rs184924341
rs2276815
rs1591734
All − MR Egger
All − Inverse variance weighted
−0.5
0.0
0.5
MR effect size for
'TMEM154' on 'Cardiac valvular disease'

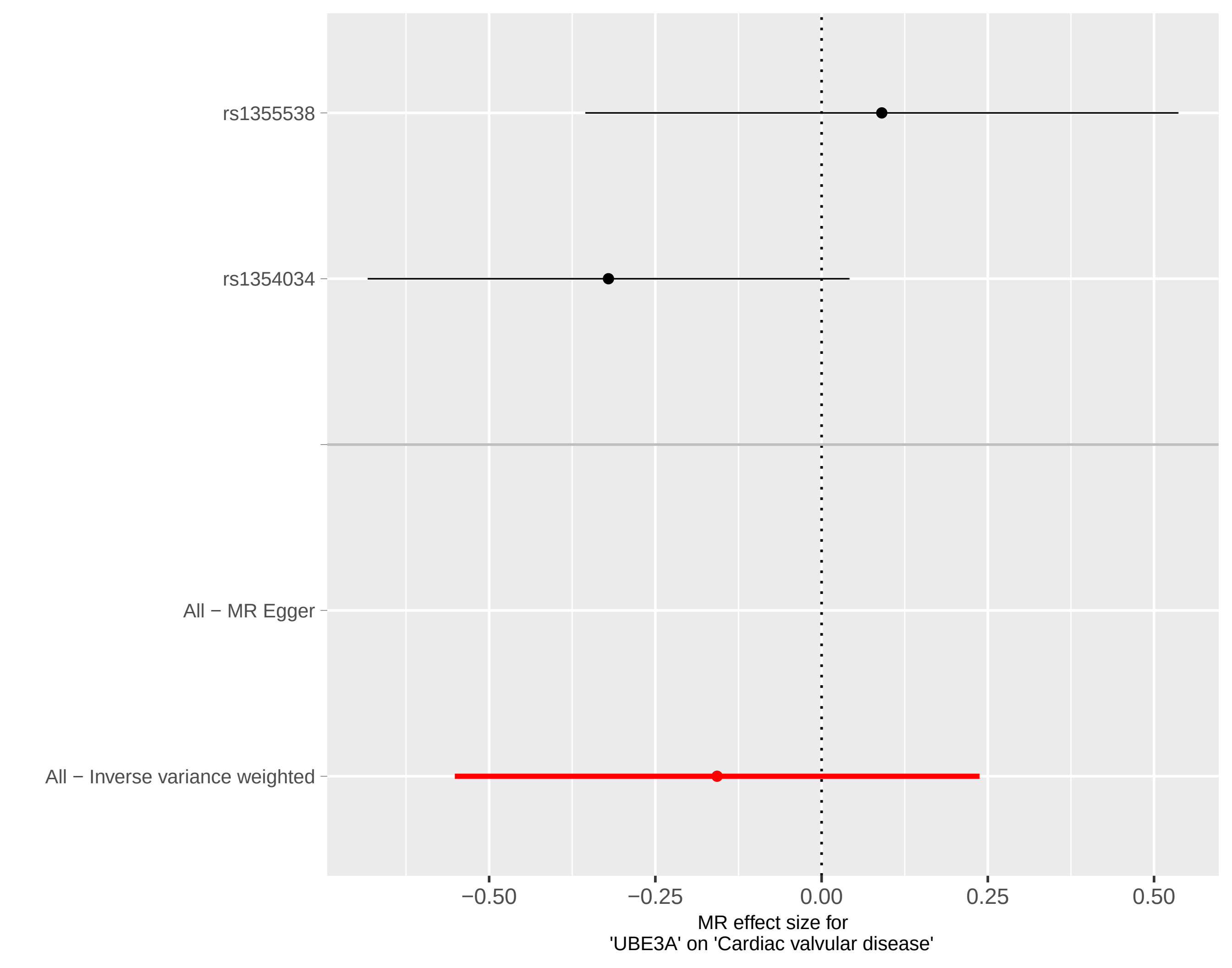
rs1355538
rs1354034
All − MR Egger
All − Inverse variance weighted
−0.50
−0.25
0.00
0.25
0.50
MR effect size for
'UBE3A' on 'Cardiac valvular disease'

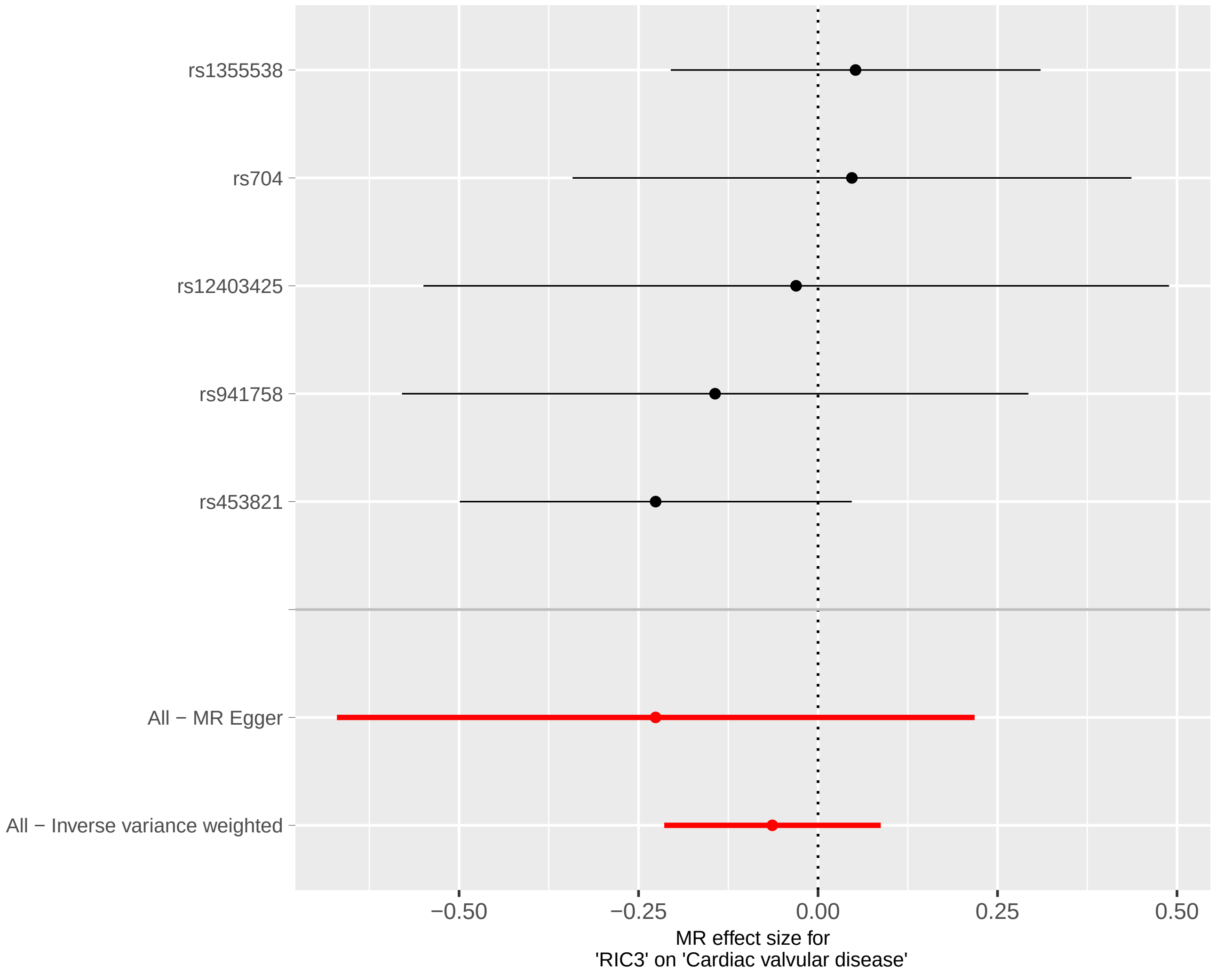

rs1355538
rs704
rs12403425
rs941758
rs453821
All − MR Egger
All − Inverse variance weighted
−0.50
−0.25
0.00
0.25
0.50
MR effect size for
'RIC3' on 'Cardiac valvular disease'

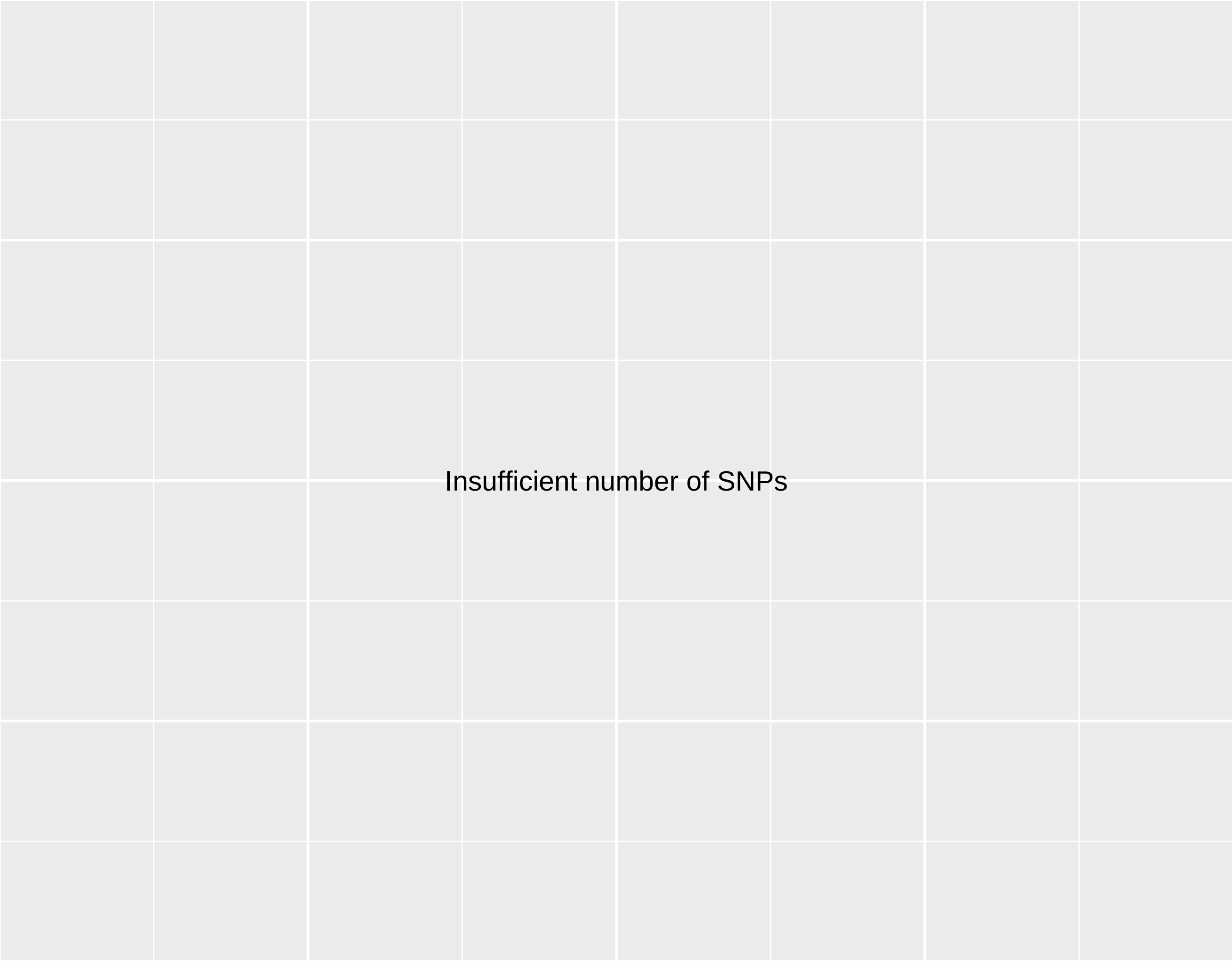
Insufficient number of SNPs

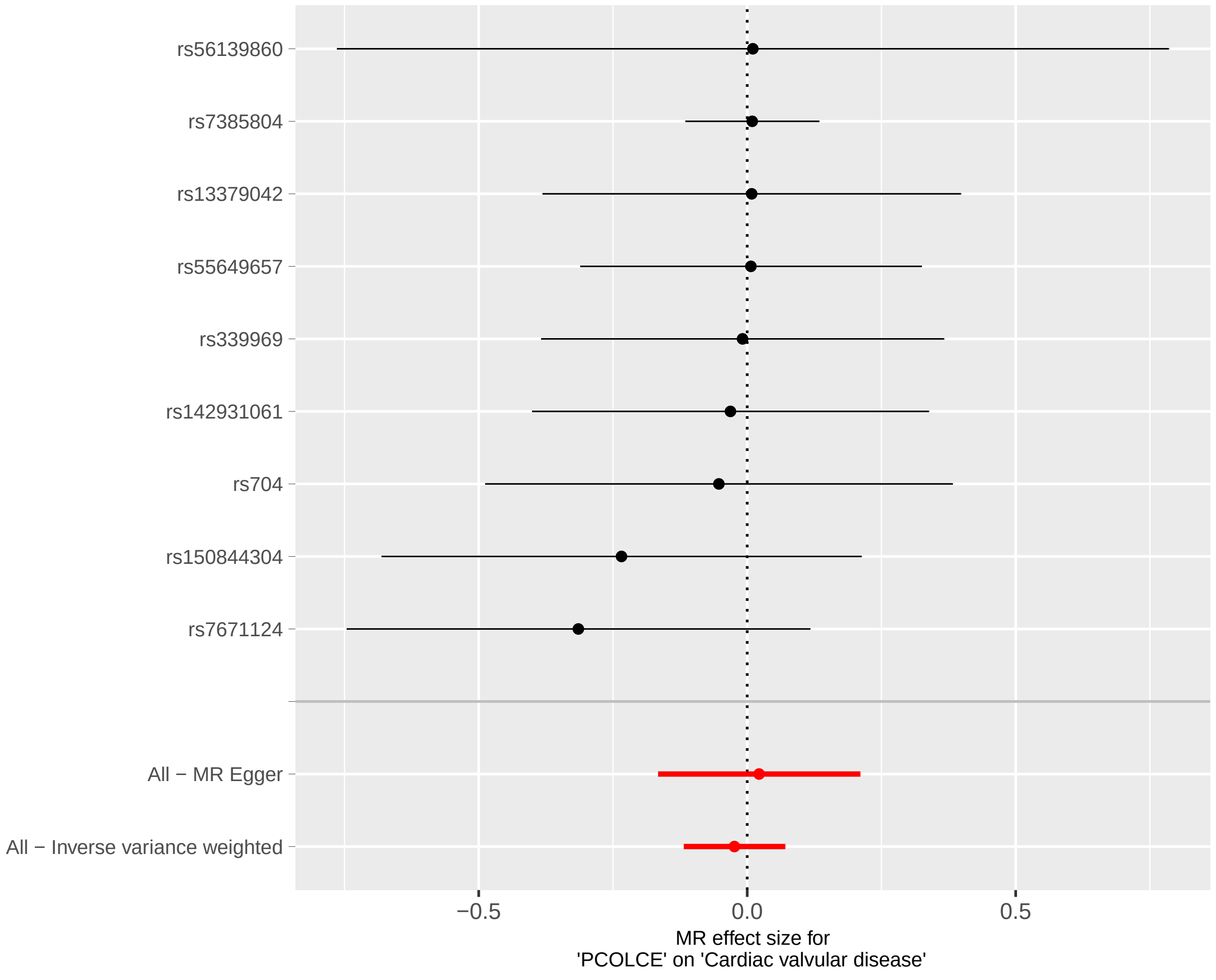
rs56139860
rs7385804
rs13379042
rs55649657
rs339969
rs142931061
rs704
rs150844304
rs7671124
All − MR Egger
All − Inverse variance weighted
−0.5
0.0
0.5
MR effect size for
'PCOLCE' on 'Cardiac valvular disease'

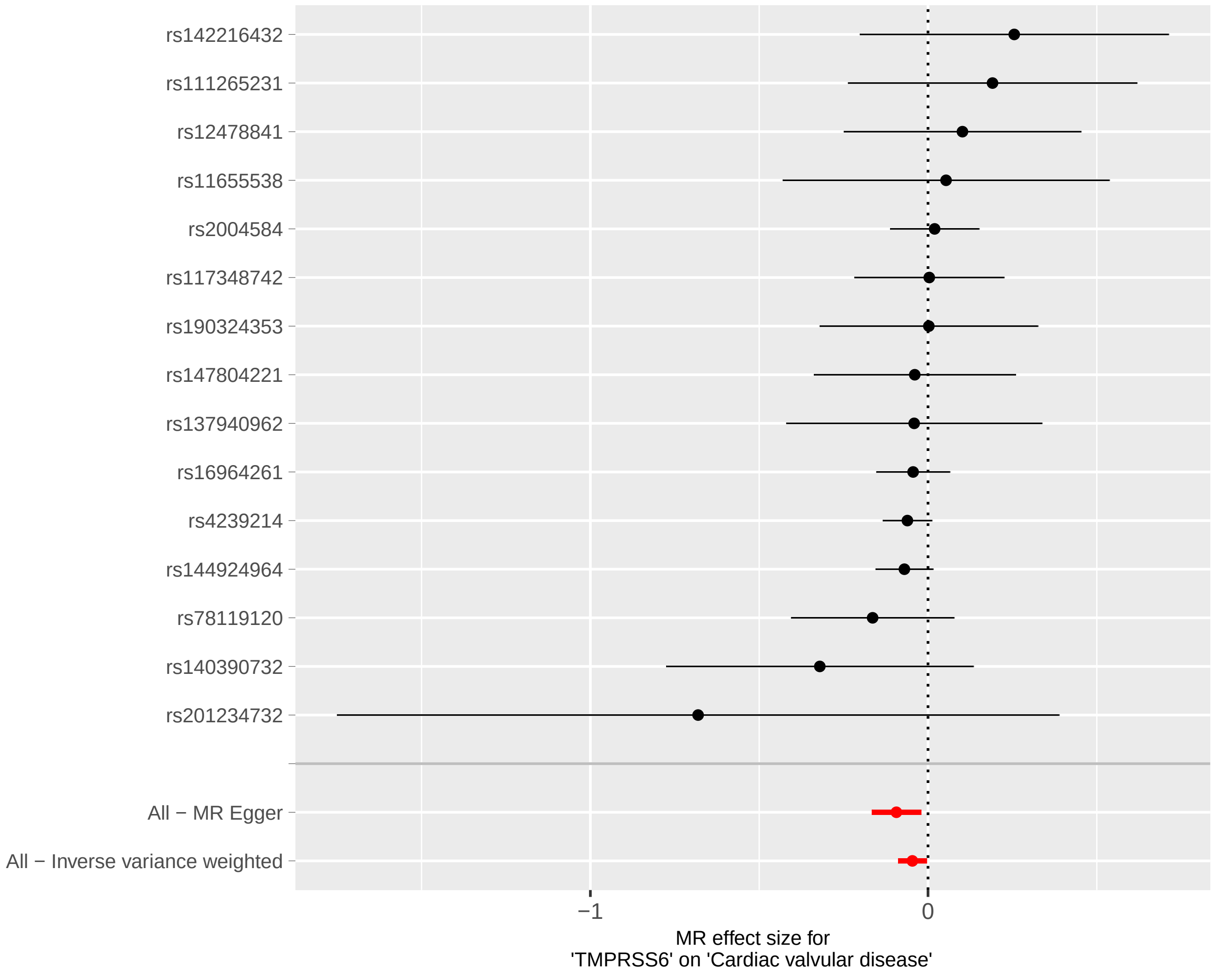
rs142216432
rs111265231
rs12478841
rs11655538
rs2004584
rs117348742
rs190324353
rs147804221
rs137940962
rs16964261
rs4239214
rs144924964
rs78119120
rs140390732
rs201234732
All − MR Egger
All − Inverse variance weighted
−1
0
MR effect size for
'TMPRSS6' on 'Cardiac valvular disease'

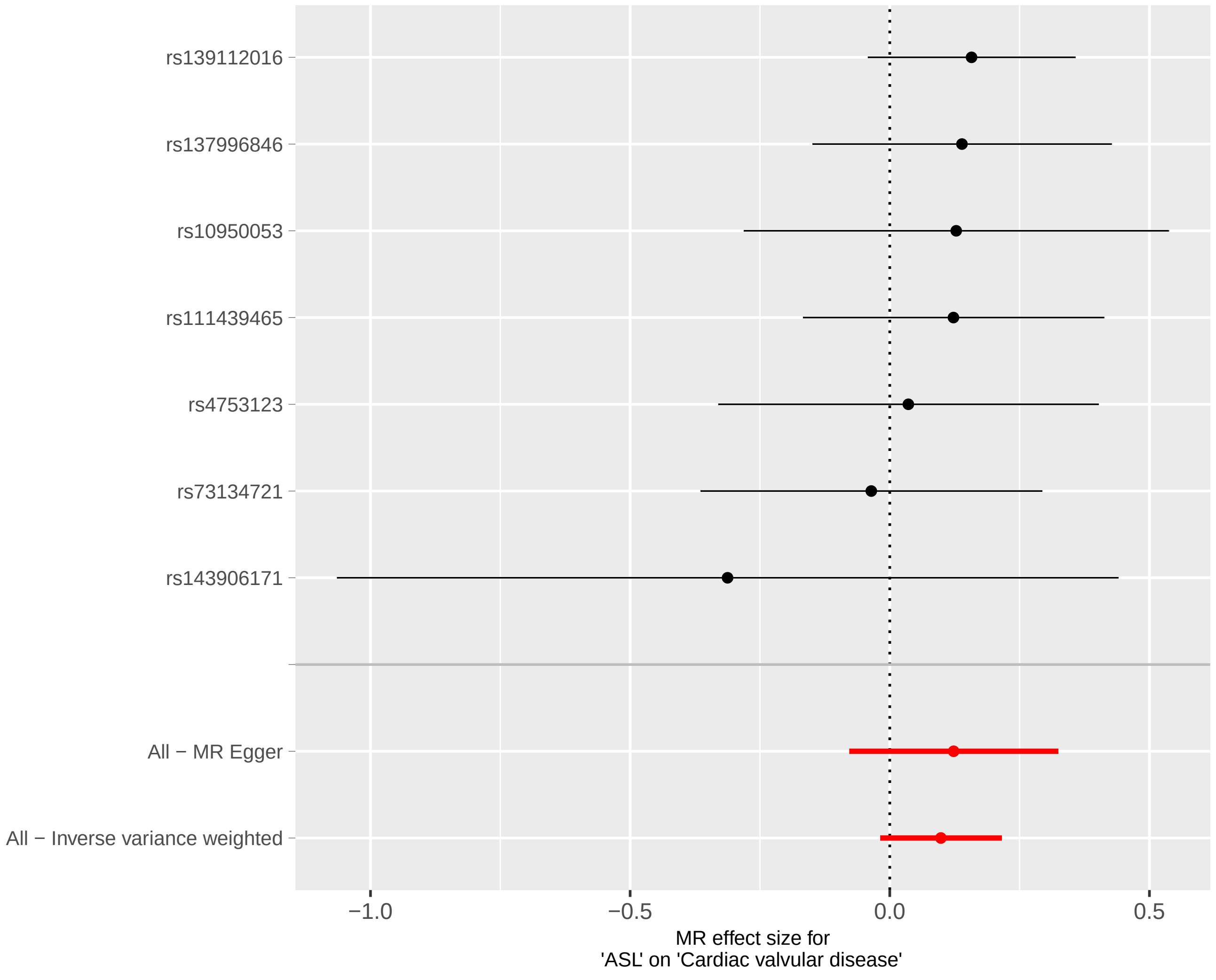
rs139112016
rs137996846
rs10950053
rs111439465
rs4753123
rs73134721
rs143906171
All − MR Egger
All − Inverse variance weighted
−1.0
−0.5
0.0
0.5
MR effect size for
'ASL' on 'Cardiac valvular disease'

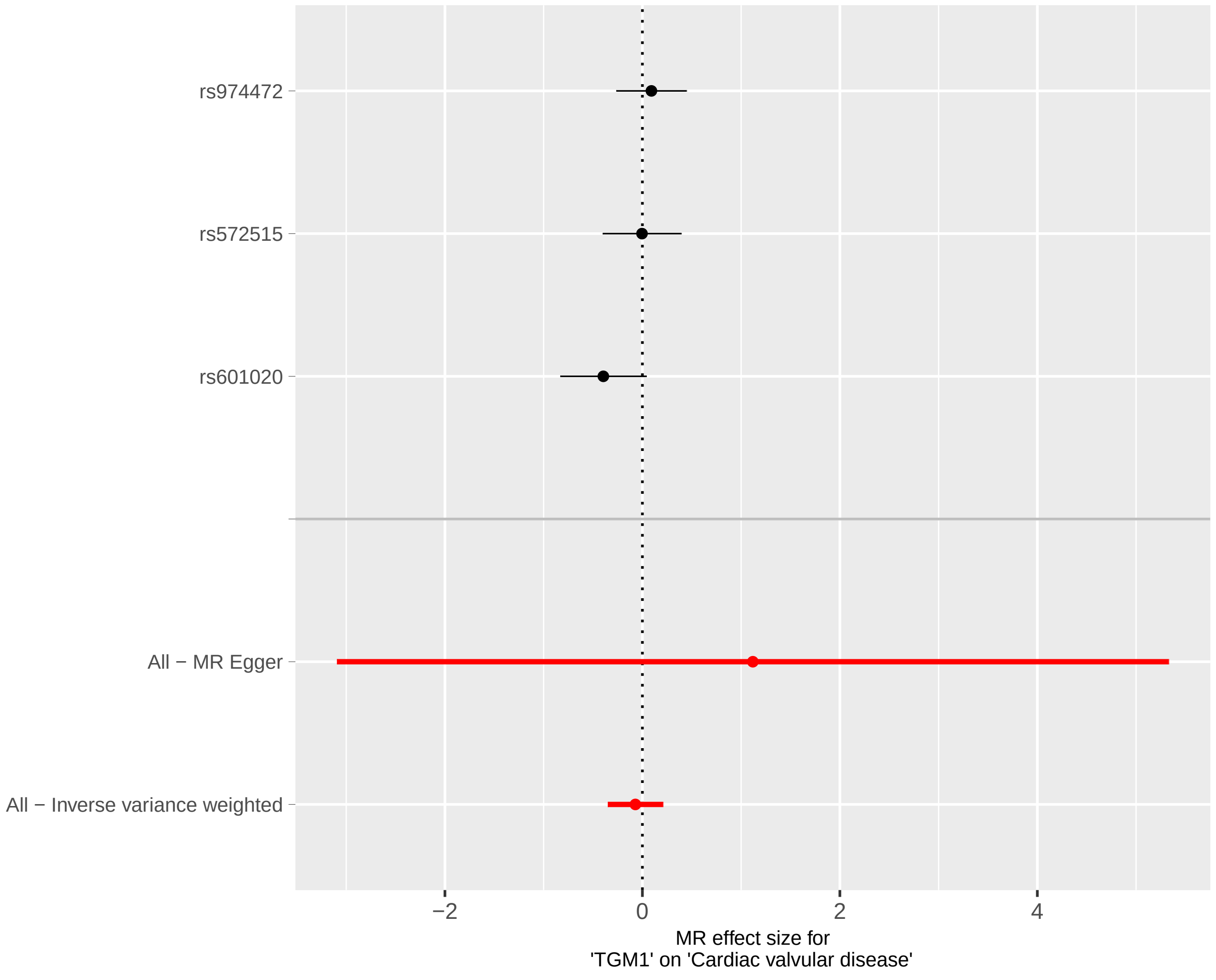
rs974472
rs572515
rs601020
All − MR Egger
All − Inverse variance weighted
−2
0
2
4
MR effect size for
'TGM1' on 'Cardiac valvular disease'

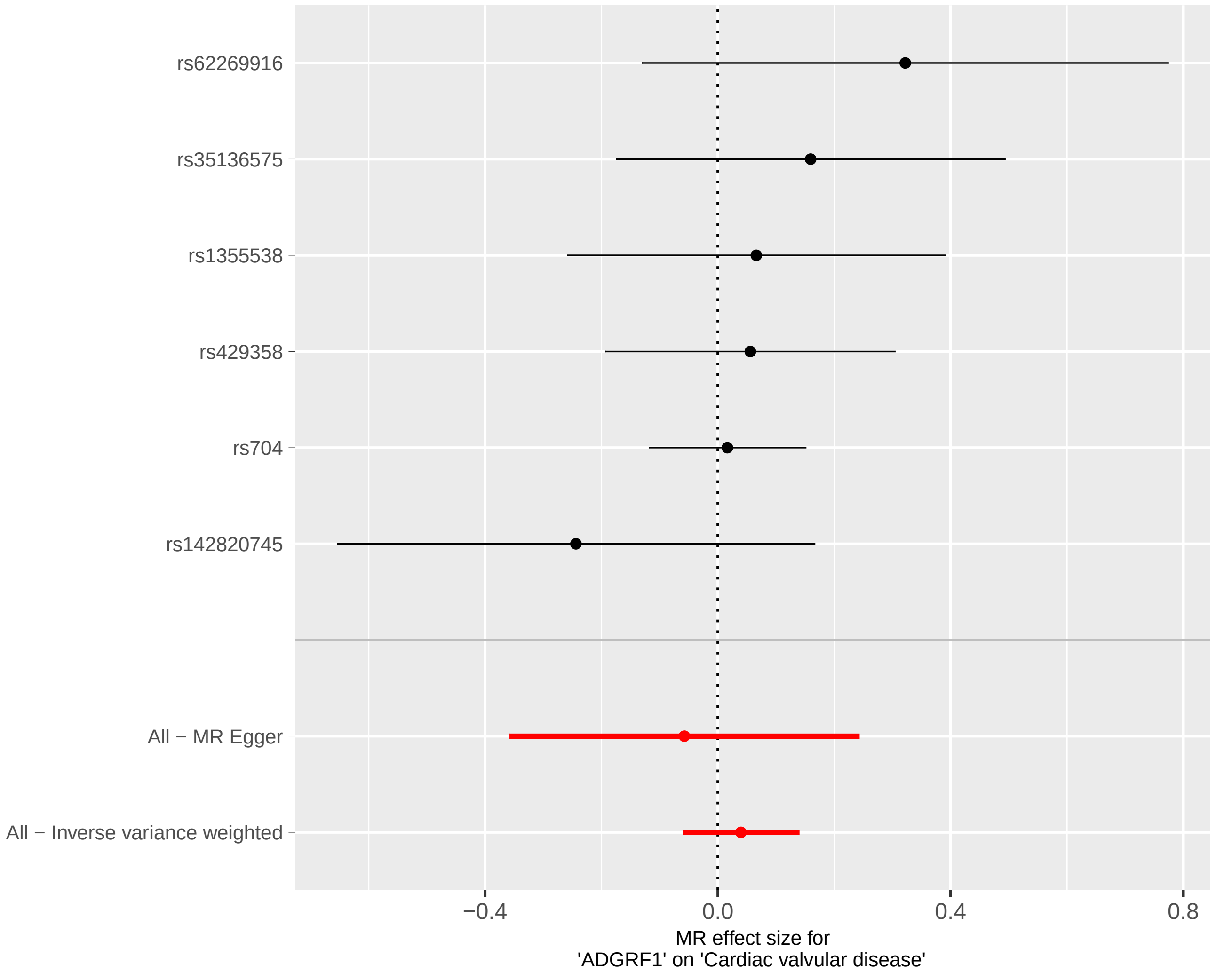
rs62269916
rs35136575
rs1355538
rs429358
rs704
rs142820745
All − MR Egger
All − Inverse variance weighted
−0.4
0.0
0.4
0.8
MR effect size for
'ADGRF1' on 'Cardiac valvular disease'

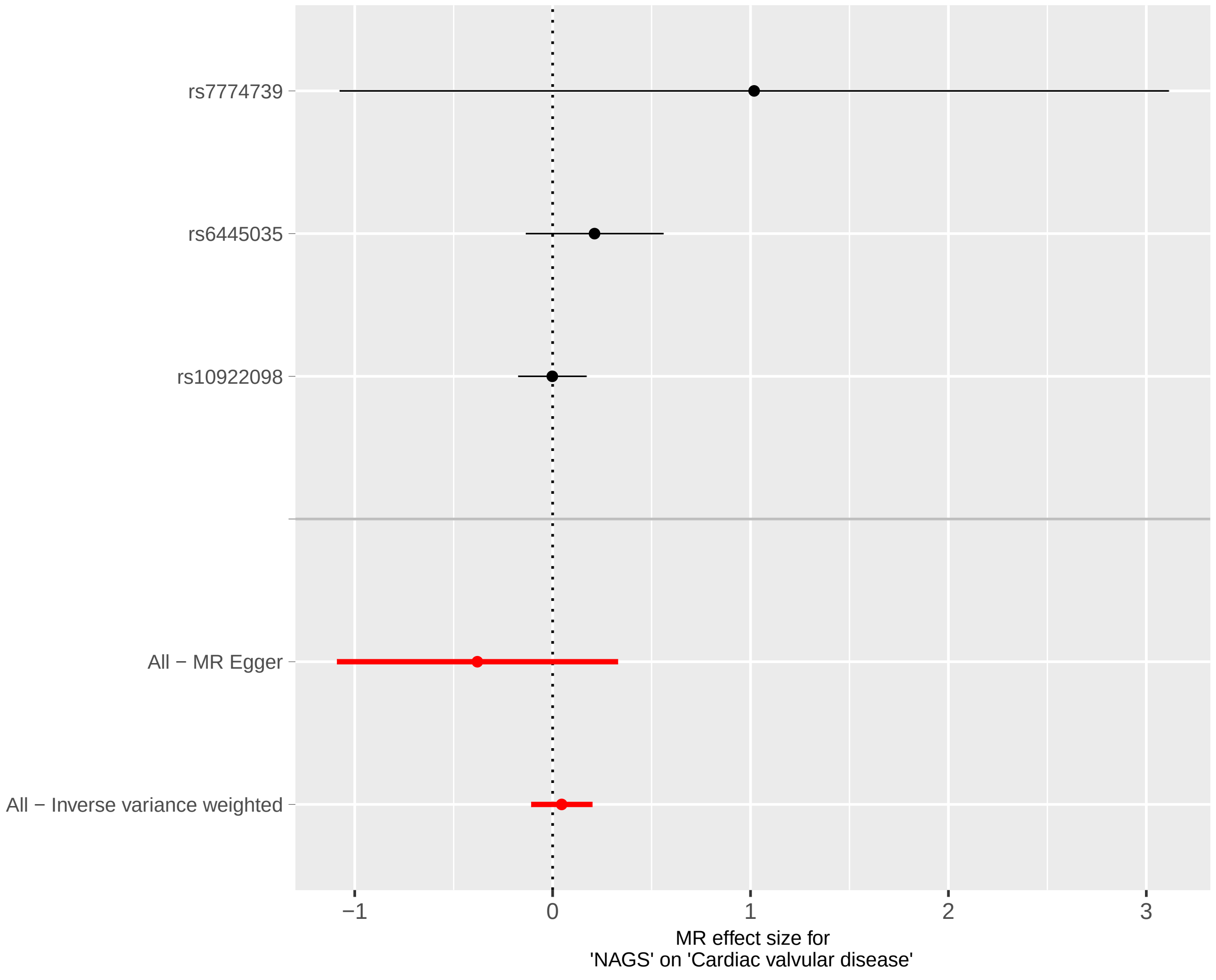

rs7774739
rs6445035
rs10922098
All − MR Egger
All − Inverse variance weighted
−1
0
1
2
3
MR effect size for
'NAGS' on 'Cardiac valvular disease'

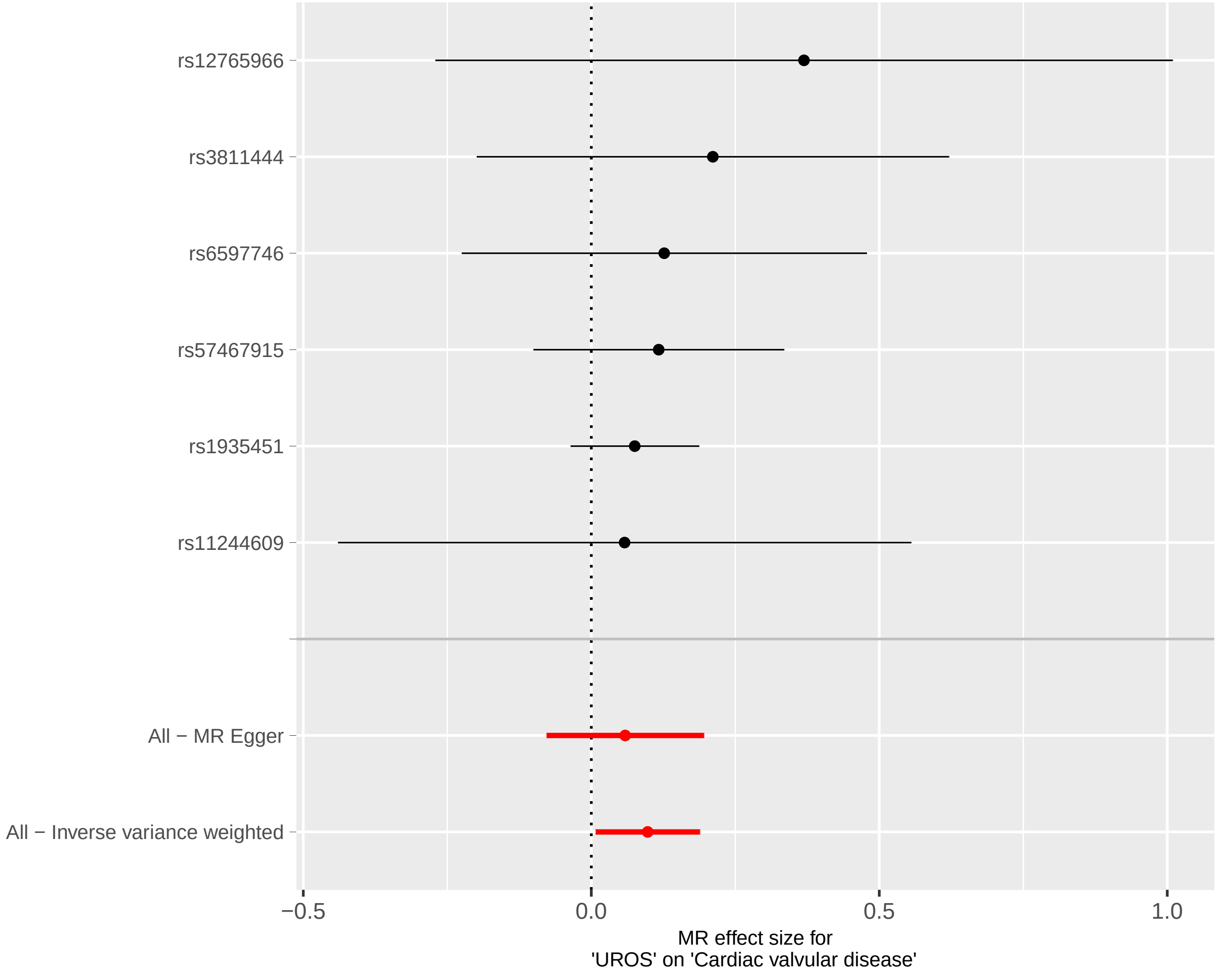
rs12765966
rs3811444
rs6597746
rs57467915
rs1935451
rs11244609
All − MR Egger
All − Inverse variance weighted
−0.5
0.0
0.5
1.0
MR effect size for
'UROS' on 'Cardiac valvular disease'

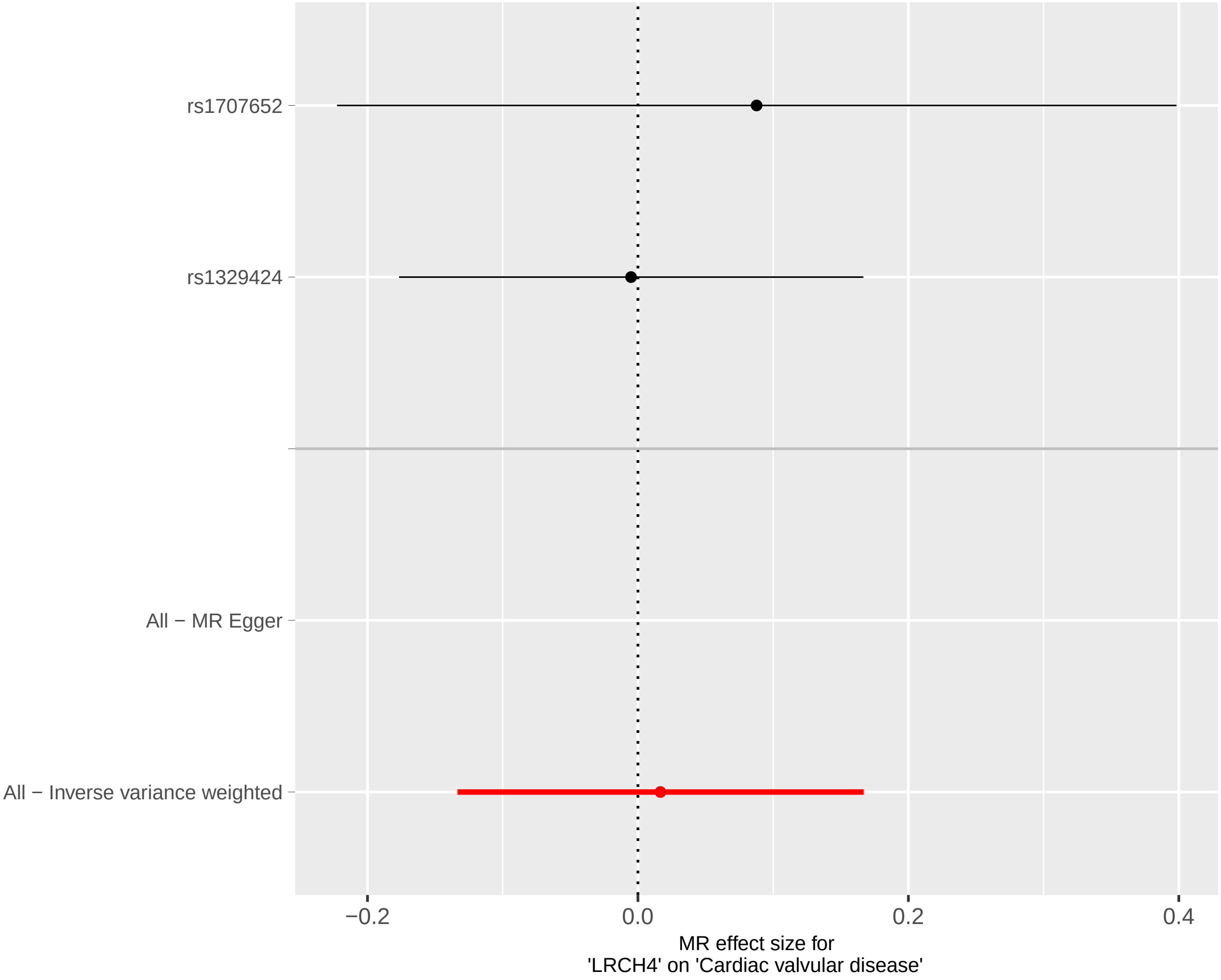
rs1707652
rs1329424
All − MR Egger
All − Inverse variance weighted
−0.2
0.0
0.2
0.4
MR effect size for
'LRCH4' on 'Cardiac valvular disease'

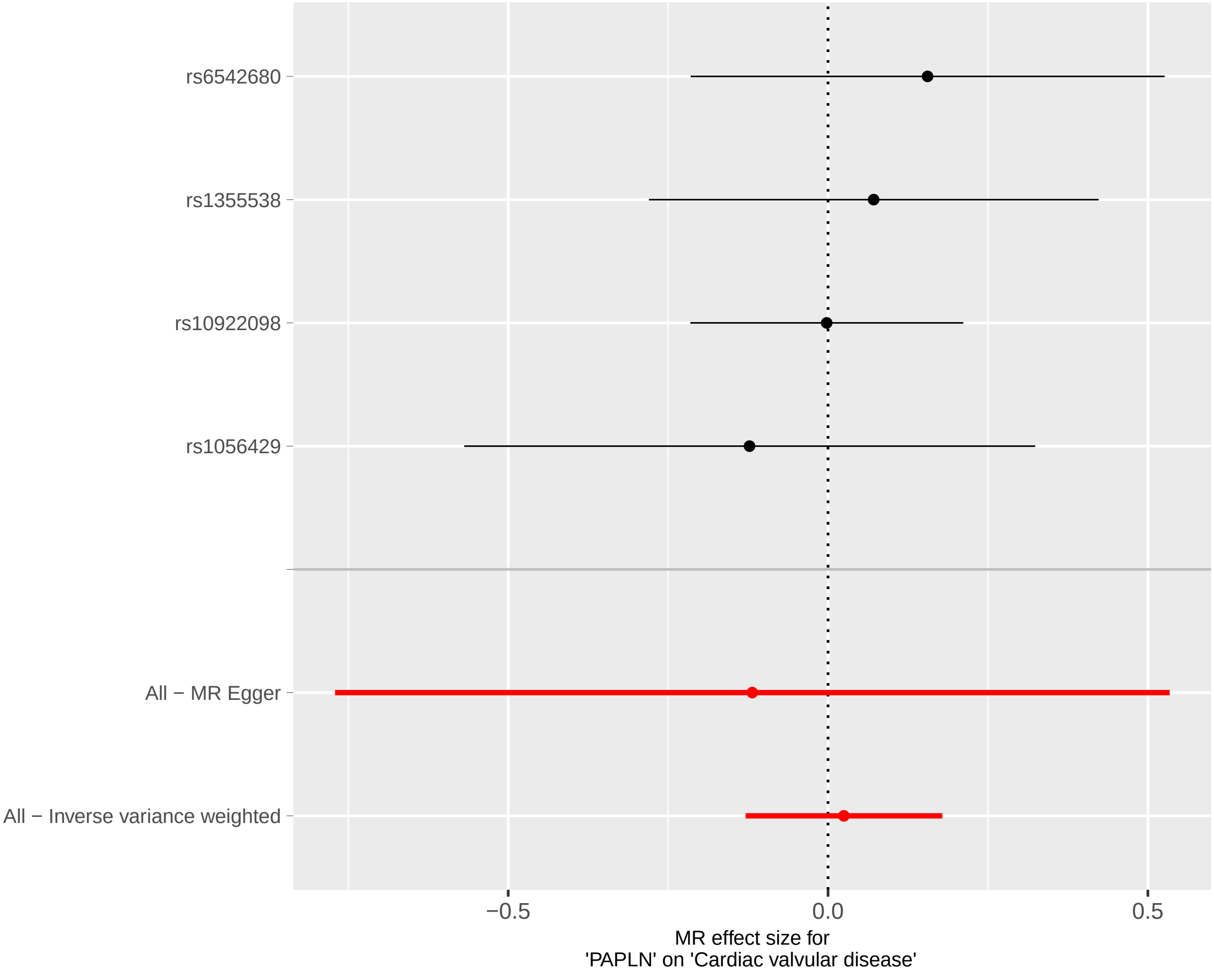

rs6542680
rs1355538
rs10922098
rs1056429
All − MR Egger
All − Inverse variance weighted
−0.5
0.0
0.5
MR effect size for
'PAPLN' on 'Cardiac valvular disease'

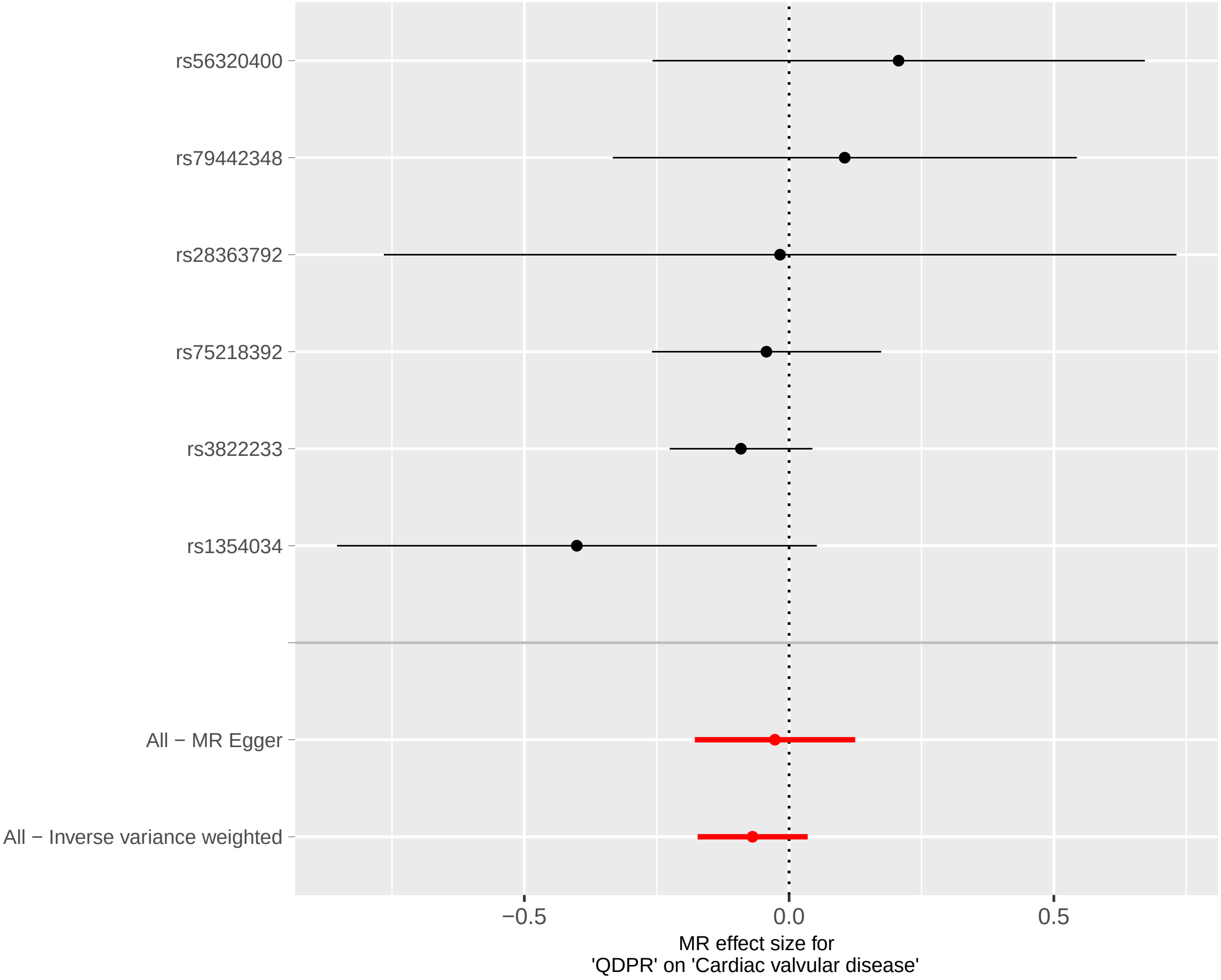
rs56320400
rs79442348
rs28363792
rs75218392
rs3822233
rs1354034
All − MR Egger
All − Inverse variance weighted
−0.5
0.0
0.5
MR effect size for
'QDPR' on 'Cardiac valvular disease'

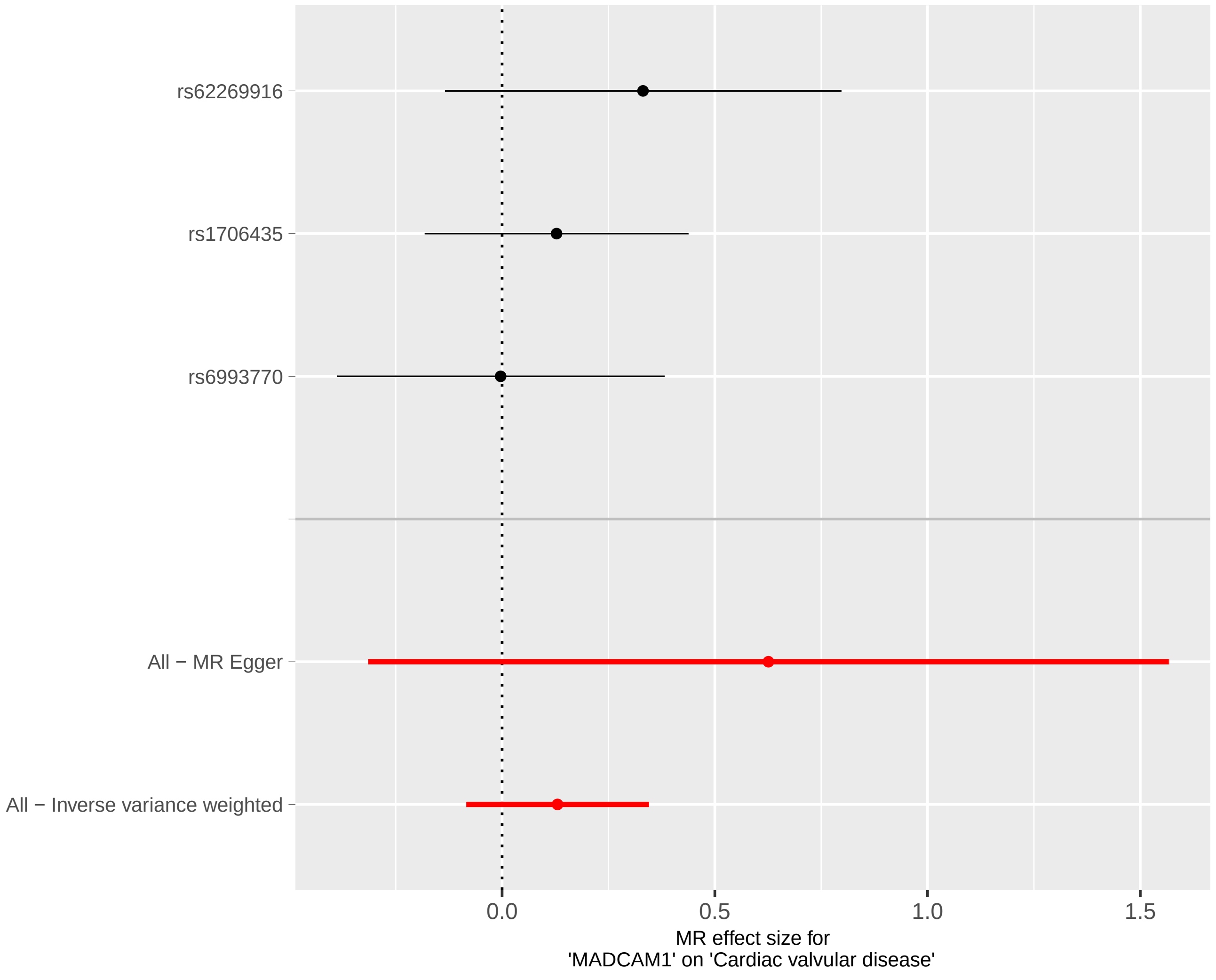

rs62269916
rs1706435
rs6993770
All − MR Egger
All − Inverse variance weighted
0.0
0.5
1.0
1.5
MR effect size for
'MADCAM1' on 'Cardiac valvular disease'

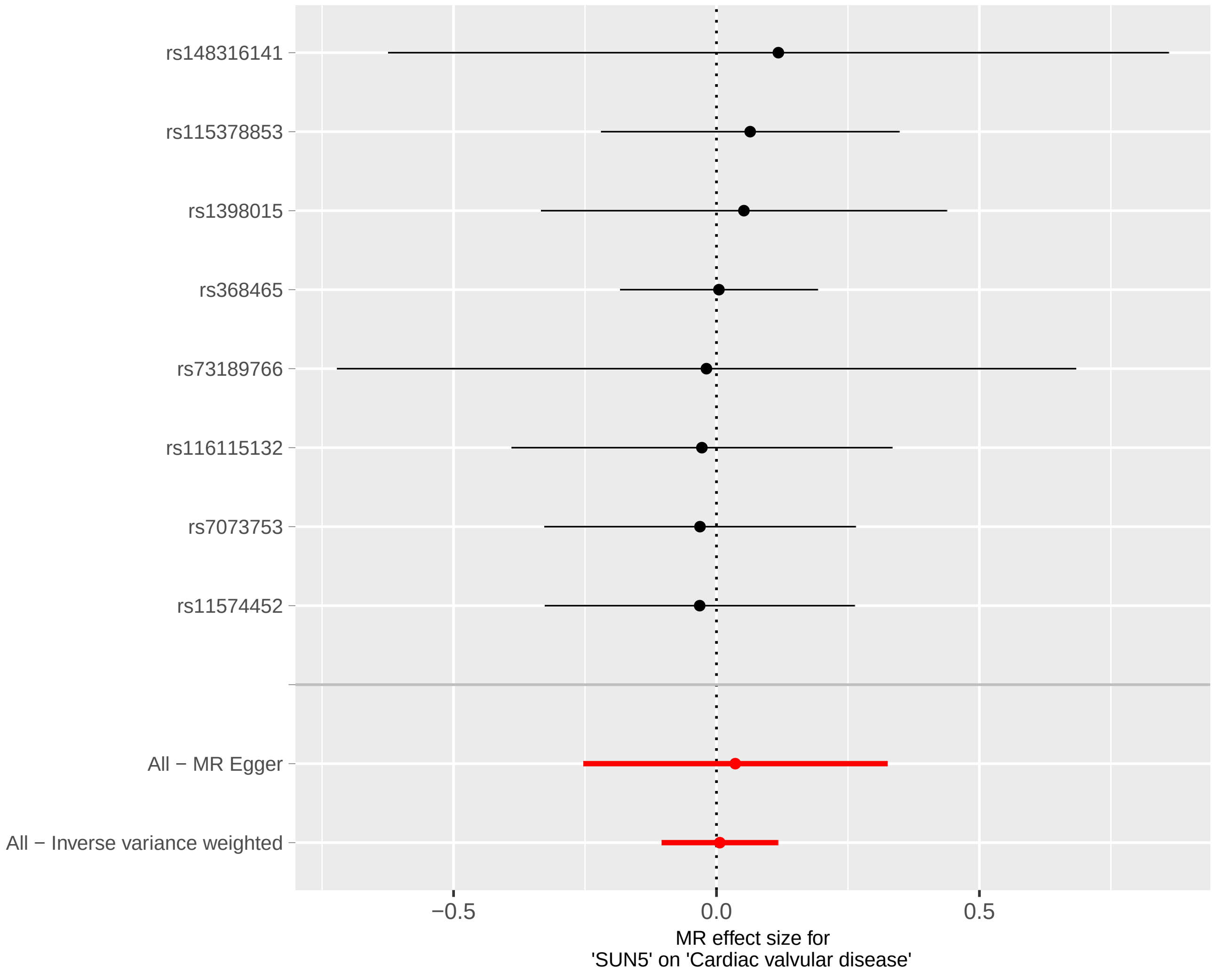
rs148316141
rs115378853
rs1398015
rs368465
rs73189766
rs116115132
rs7073753
rs11574452
All − MR Egger
All − Inverse variance weighted
−0.5
0.0
0.5
MR effect size for
'SUN5' on 'Cardiac valvular disease'

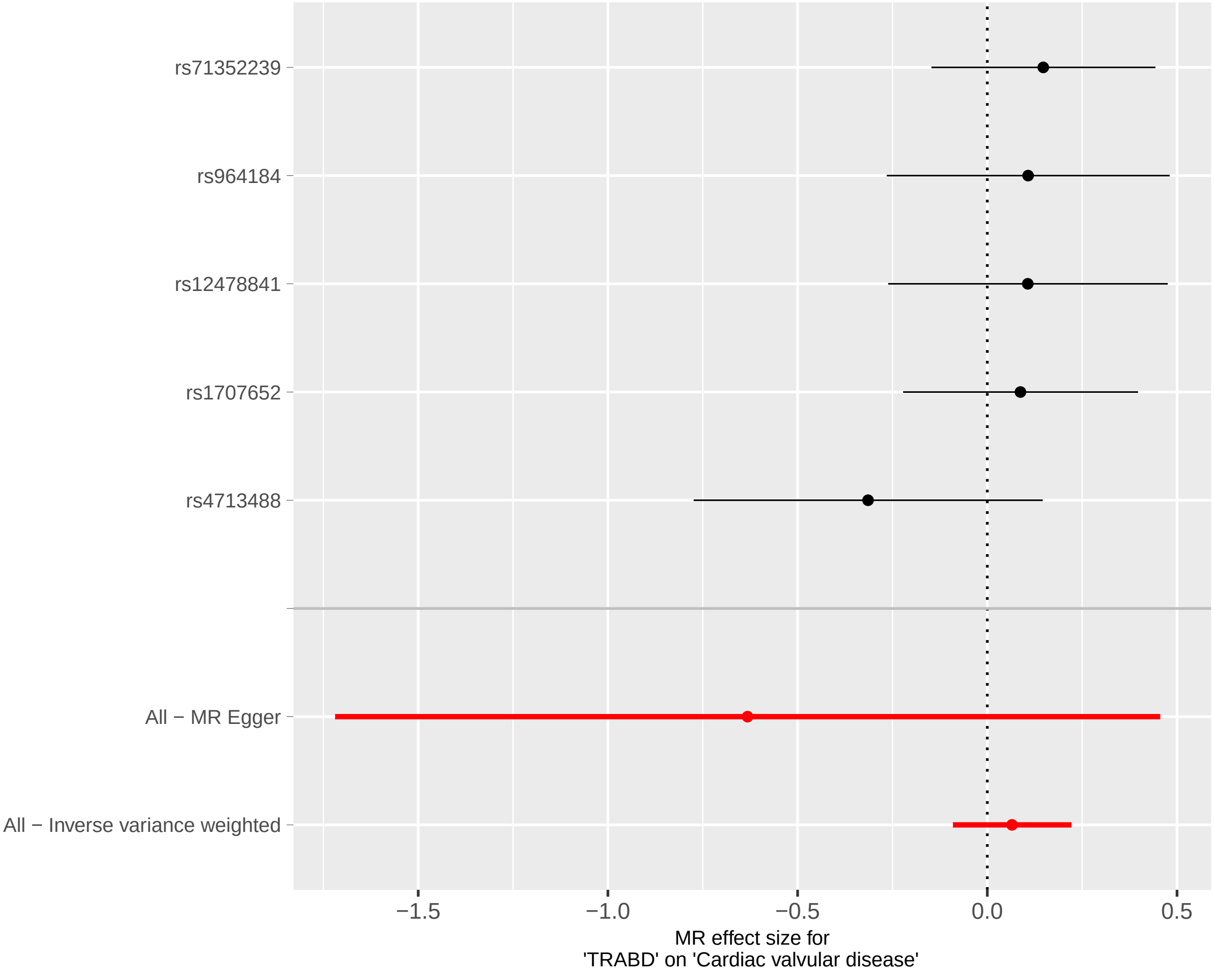

rs71352239
rs964184
rs12478841
rs1707652
rs4713488
All − MR Egger
All − Inverse variance weighted
−1.5
−1.0
−0.5
0.0
0.5
MR effect size for
'TRABD' on 'Cardiac valvular disease'

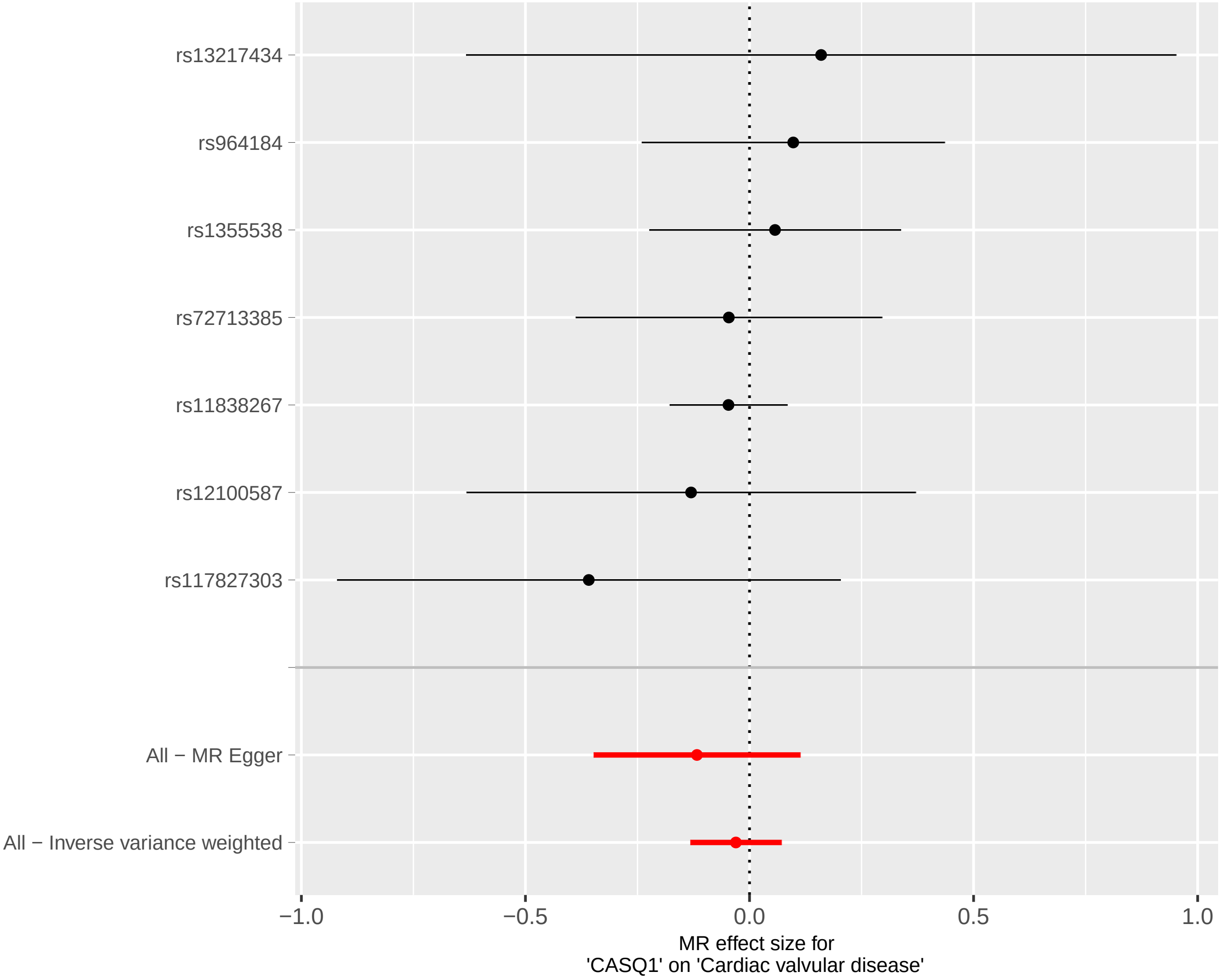

rs13217434
rs964184
rs1355538
rs72713385
rs11838267
rs12100587
rs117827303
All − MR Egger
All − Inverse variance weighted
−1.0
−0.5
0.0
0.5
1.0
MR effect size for
'CASQ1' on 'Cardiac valvular disease'

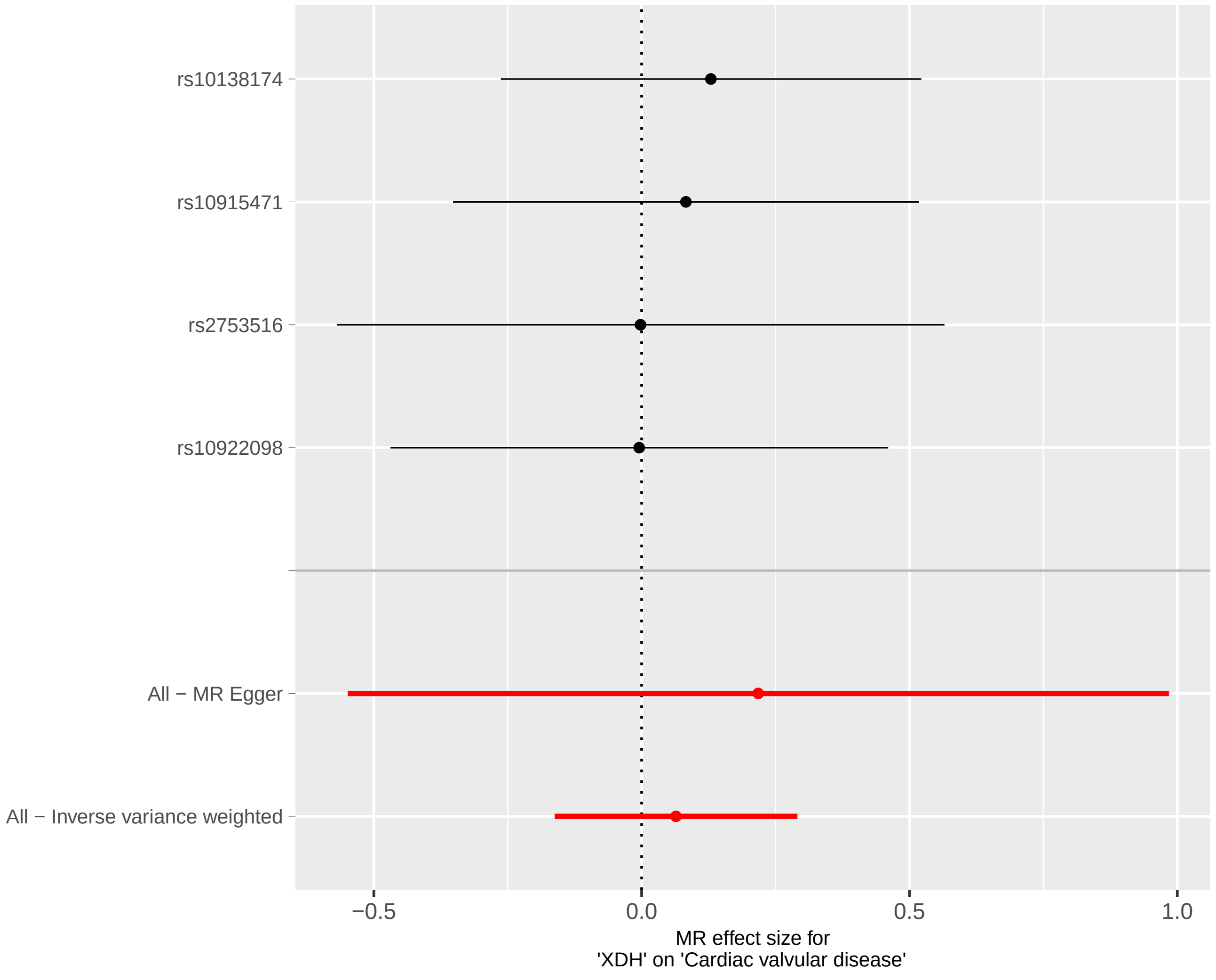

rs10138174
rs10915471
rs2753516
rs10922098
All − MR Egger
All − Inverse variance weighted
−0.5
0.0
0.5
1.0
MR effect size for
'XDH' on 'Cardiac valvular disease'

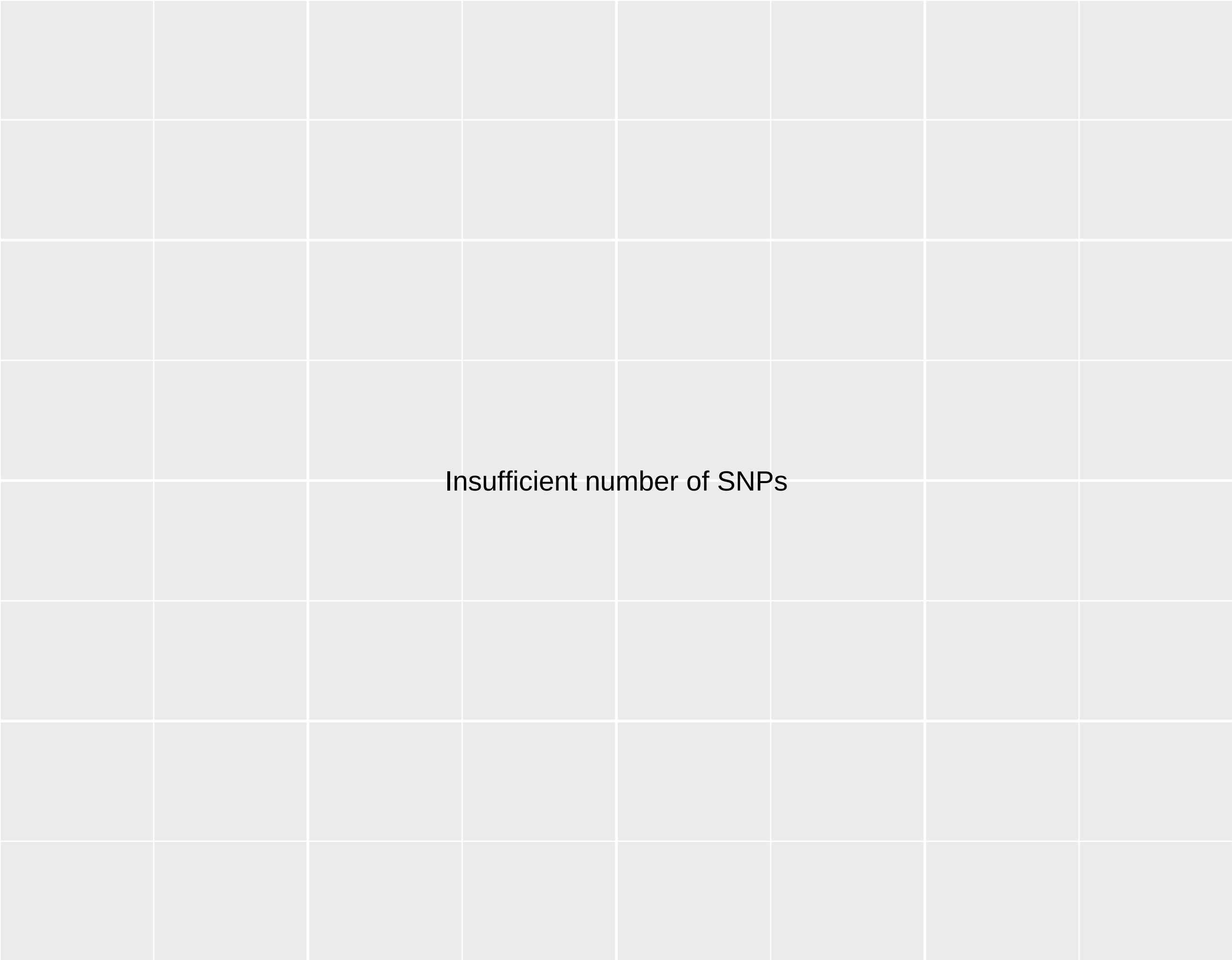
Insufficient number of SNPs

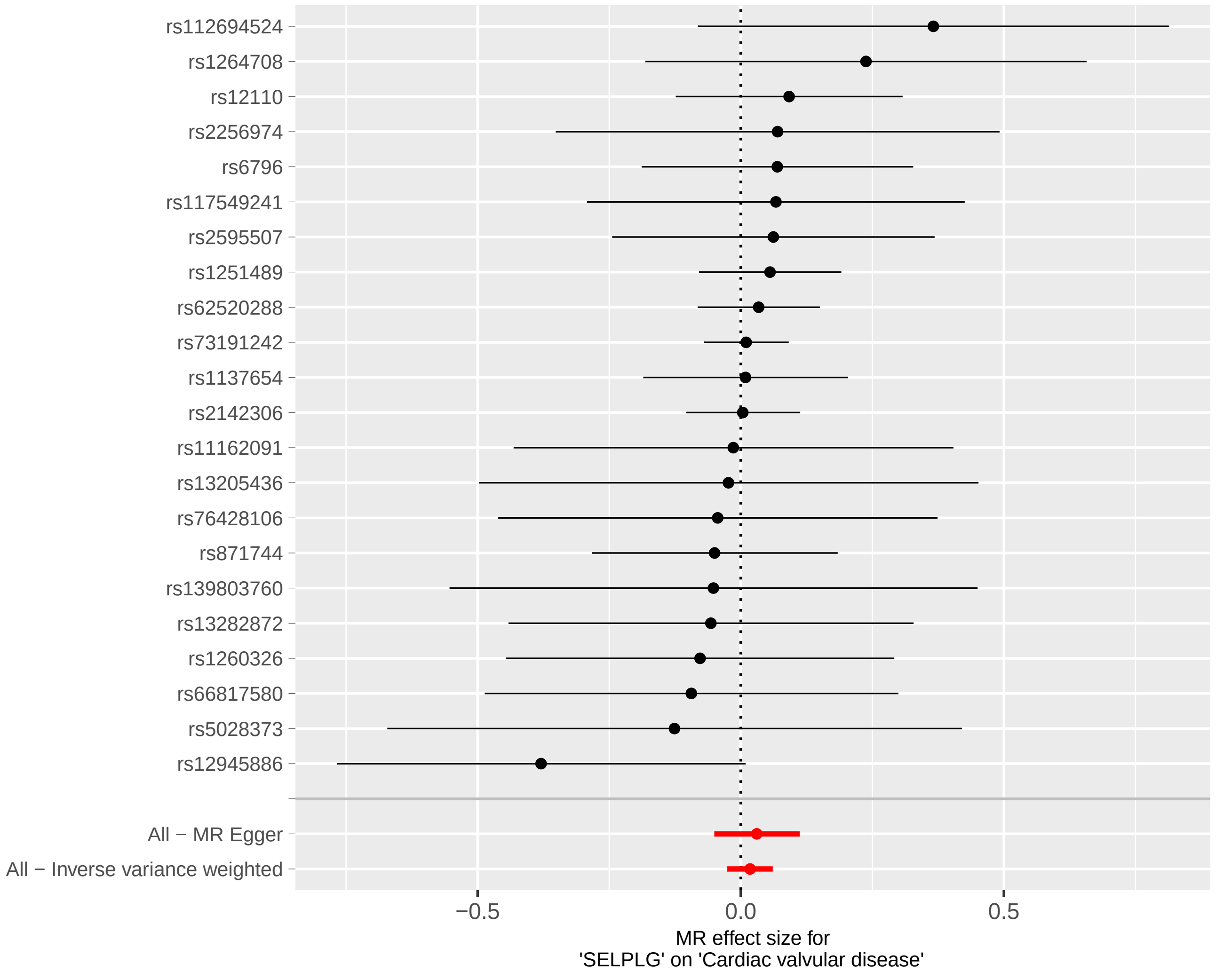
rs112694524
rs1264708
rs12110
rs2256974
rs6796
rs117549241
rs2595507
rs1251489
rs62520288
rs73191242
rs1137654
rs2142306
rs11162091
rs13205436
rs76428106
rs871744
rs139803760
rs13282872
rs1260326
rs66817580
rs5028373
rs12945886
All − MR Egger
All − Inverse variance weighted
−0.5
0.0
0.5
MR effect size for
'SELPLG' on 'Cardiac valvular disease'

Insufficient number of SNPs

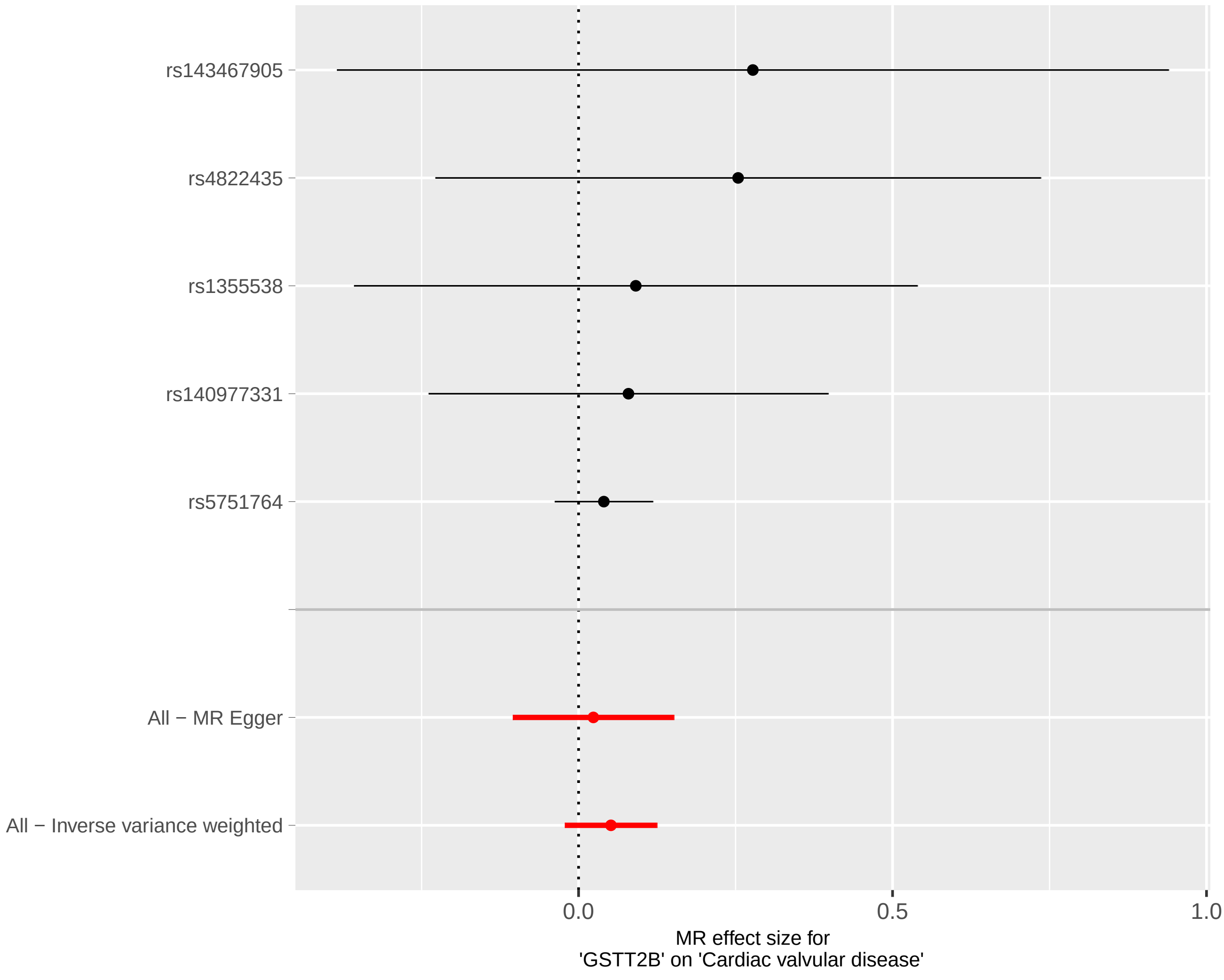

rs143467905
rs4822435
rs1355538
rs140977331
rs5751764
All − MR Egger
All − Inverse variance weighted
0.0
0.5
1.0
MR effect size for
'GSTT2B' on 'Cardiac valvular disease'

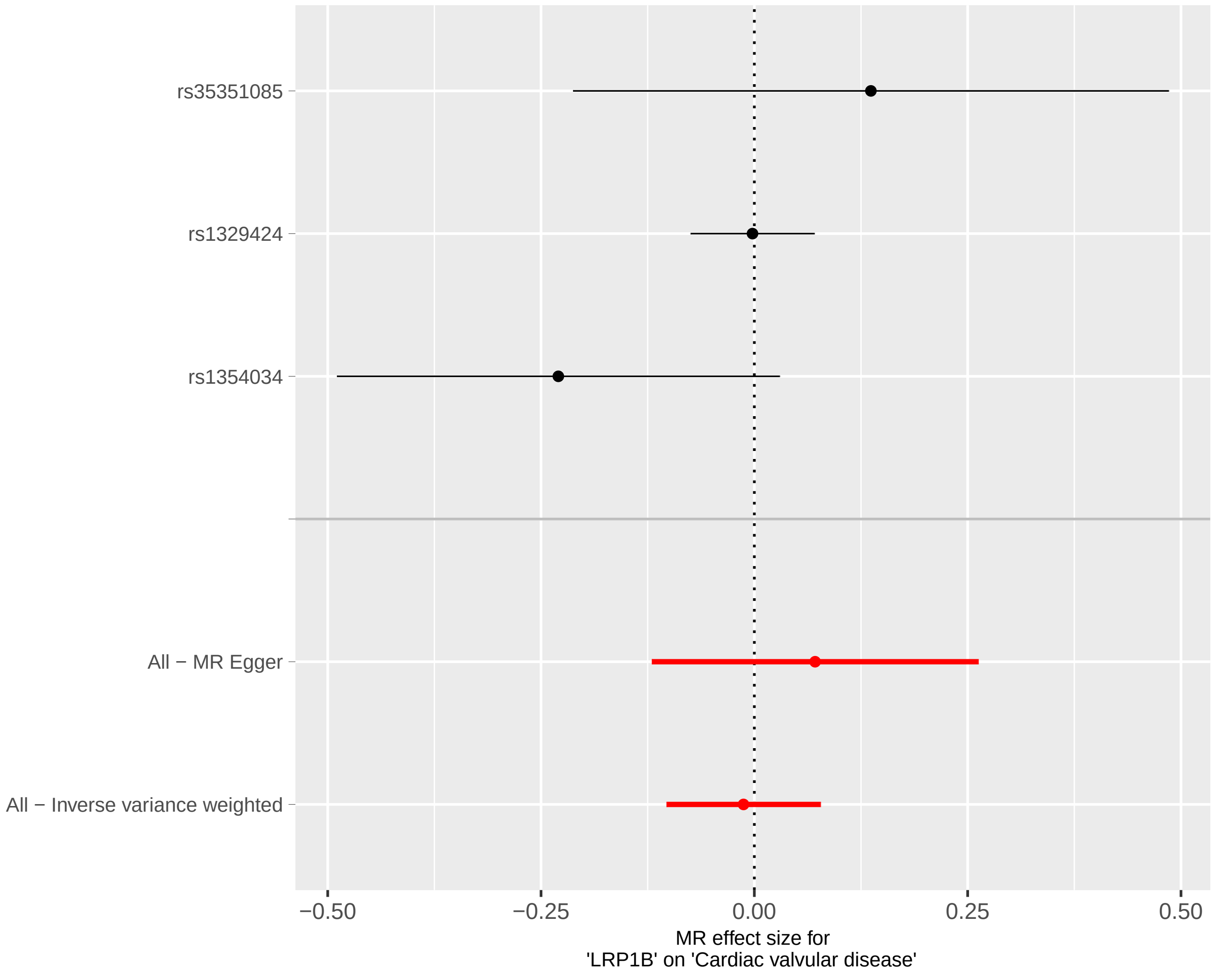
rs35351085
rs1329424
rs1354034
All − MR Egger
All − Inverse variance weighted
−0.50
−0.25
0.00
0.25
0.50
MR effect size for
'LRP1B' on 'Cardiac valvular disease'

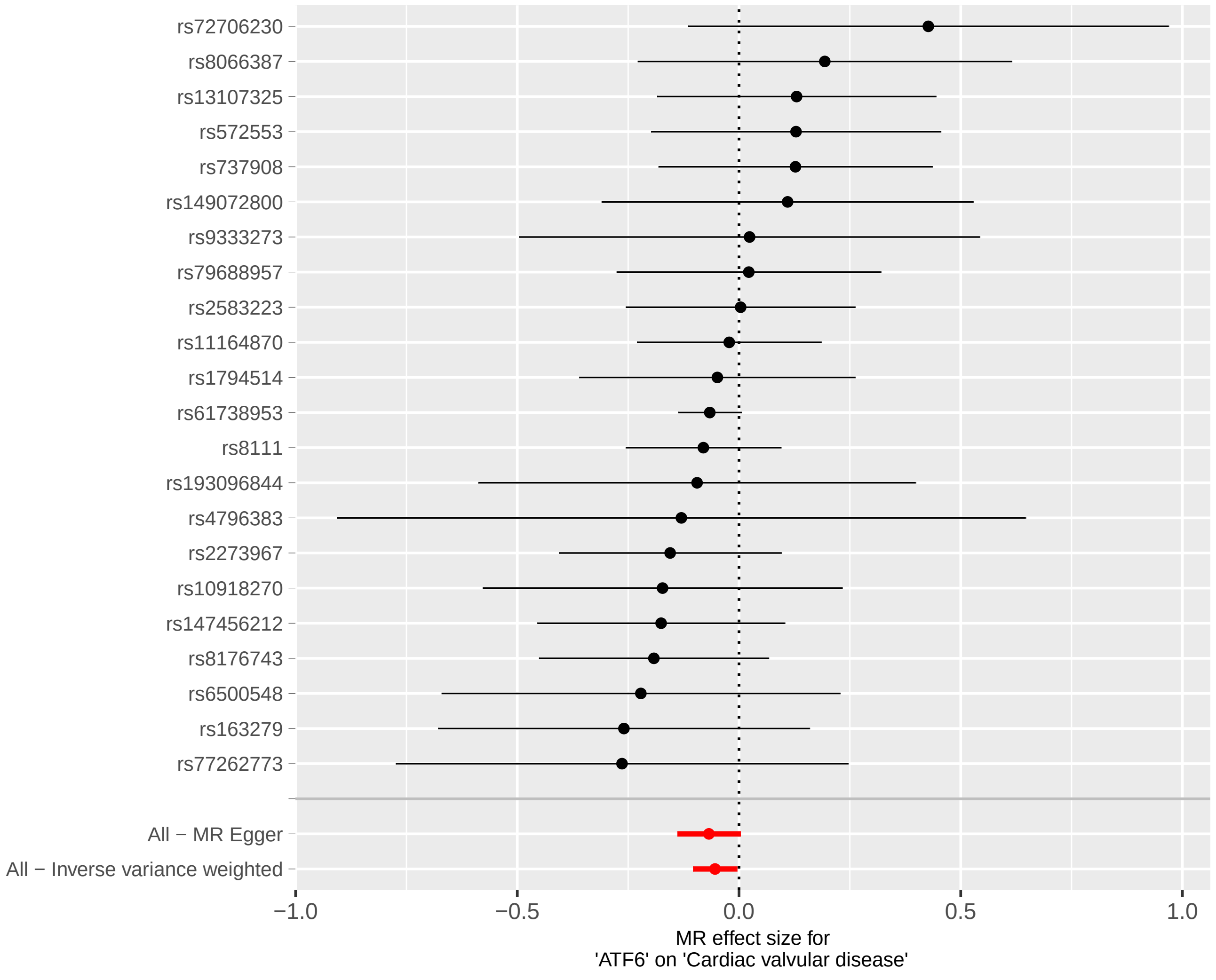
rs72706230
rs8066387
rs13107325
rs572553
rs737908
rs149072800
rs9333273
rs79688957
rs2583223
rs11164870
rs1794514
rs61738953
rs8111
rs193096844
rs4796383
rs2273967
rs10918270
rs147456212
rs8176743
rs6500548
rs163279
rs77262773
All − MR Egger
All − Inverse variance weighted
−1.0
−0.5
0.0
0.5
1.0
MR effect size for
'ATF6' on 'Cardiac valvular disease'

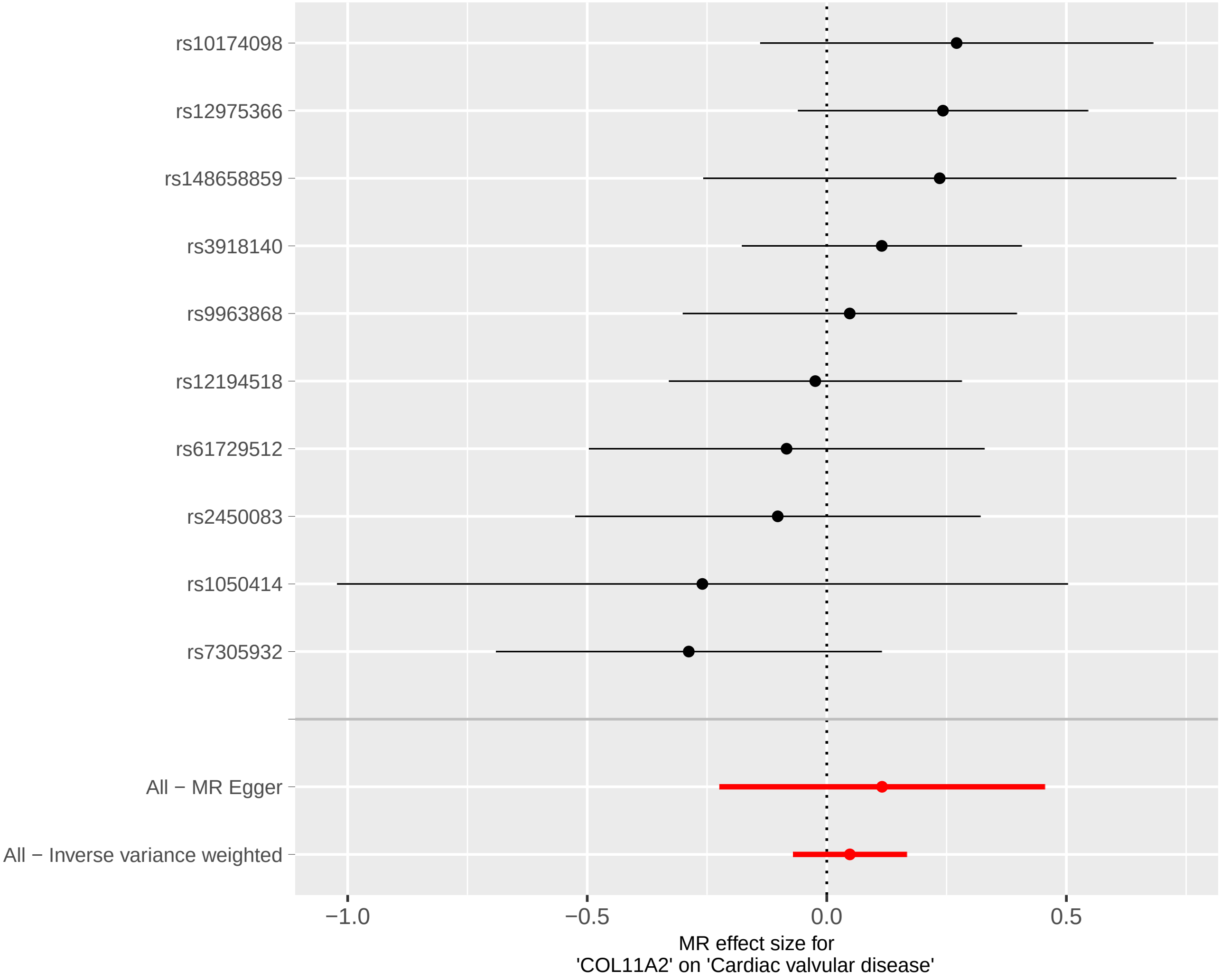
rs10174098
rs12975366
rs148658859
rs3918140
rs9963868
rs12194518
rs61729512
rs2450083
rs1050414
rs7305932
All − MR Egger
All − Inverse variance weighted
−1.0
−0.5
0.0
0.5
MR effect size for
'COL11A2' on 'Cardiac valvular disease'

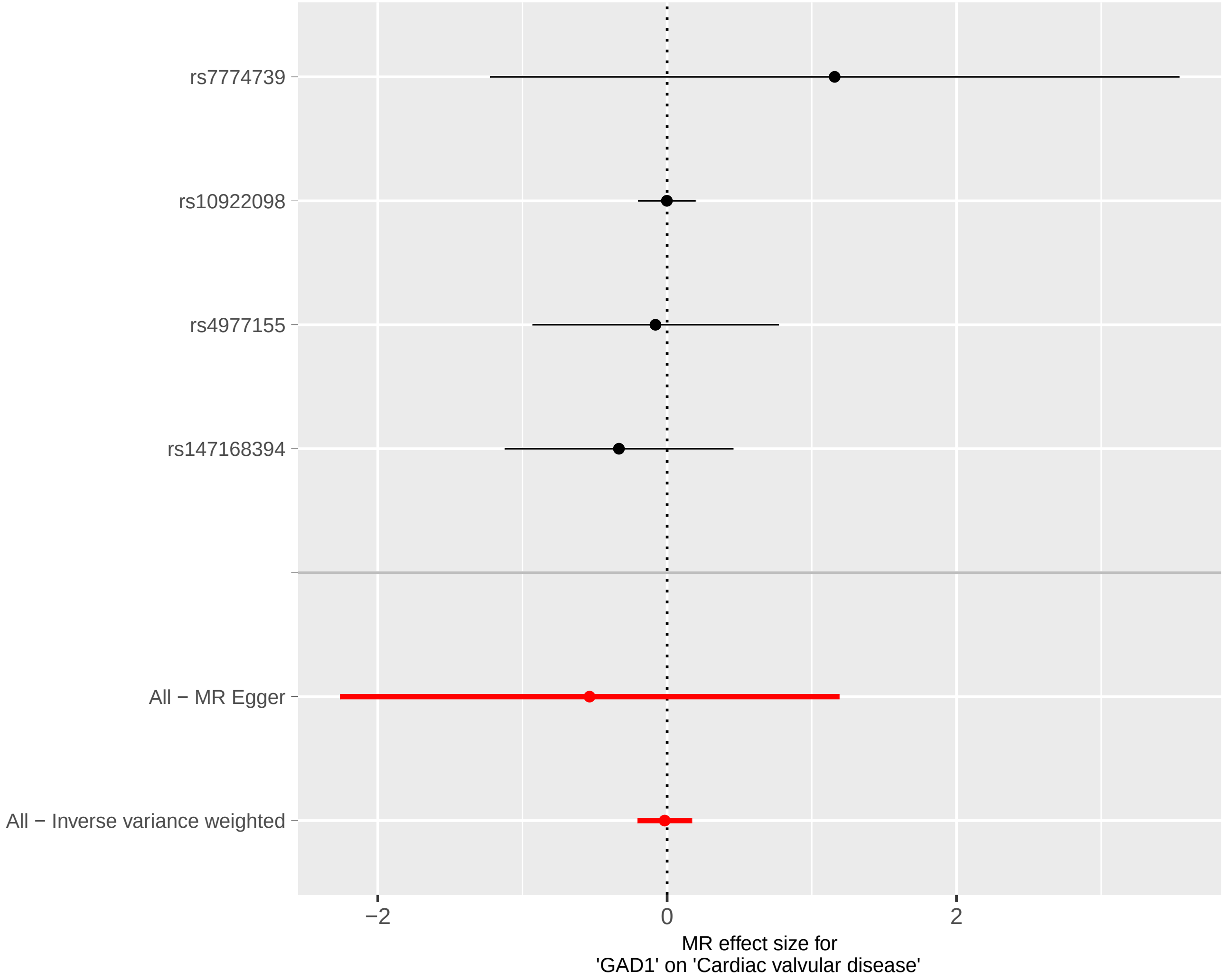
rs7774739
rs10922098
rs4977155
rs147168394
All − MR Egger
All − Inverse variance weighted
−2
0
2
MR effect size for
'GAD1' on 'Cardiac valvular disease'

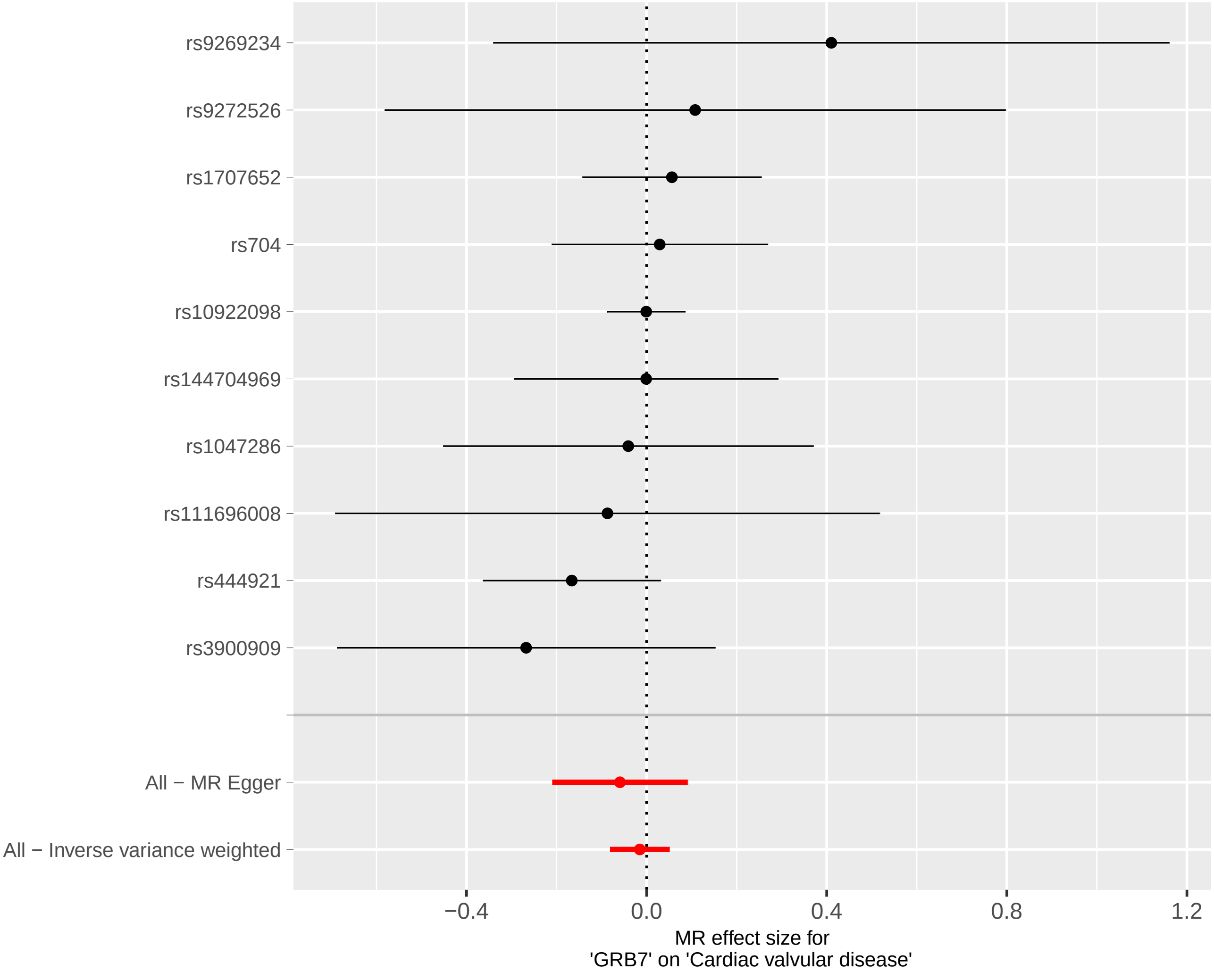
rs9269234
rs9272526
rs1707652
rs704
rs10922098
rs144704969
rs1047286
rs111696008
rs444921
rs3900909
All − MR Egger
All − Inverse variance weighted
−0.4
0.0
0.4
0.8
1.2
MR effect size for
'GRB7' on 'Cardiac valvular disease'

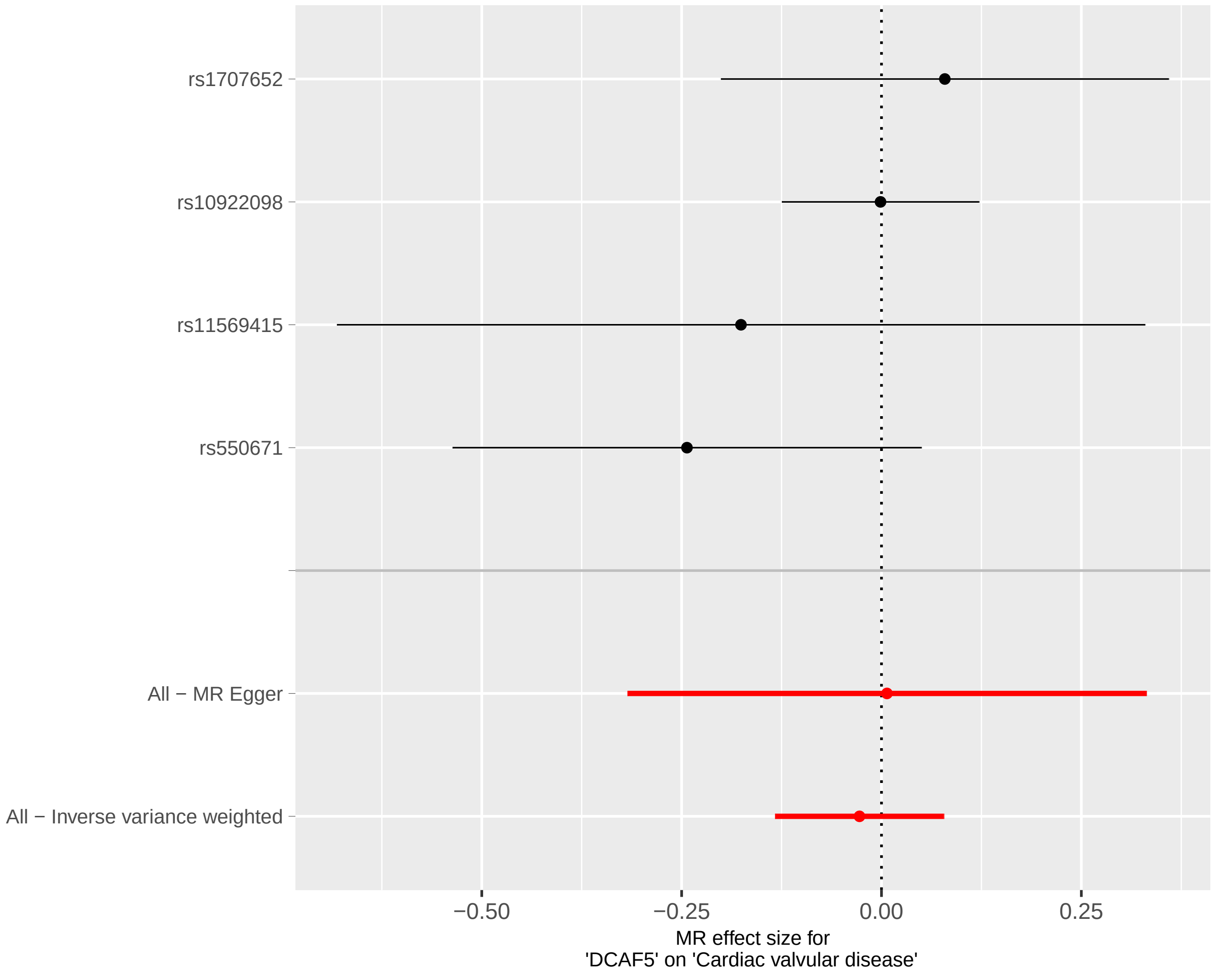
rs1707652
rs10922098
rs11569415
rs550671
All − MR Egger
All − Inverse variance weighted
−0.50
−0.25
0.00
0.25
MR effect size for
'DCAF5' on 'Cardiac valvular disease'

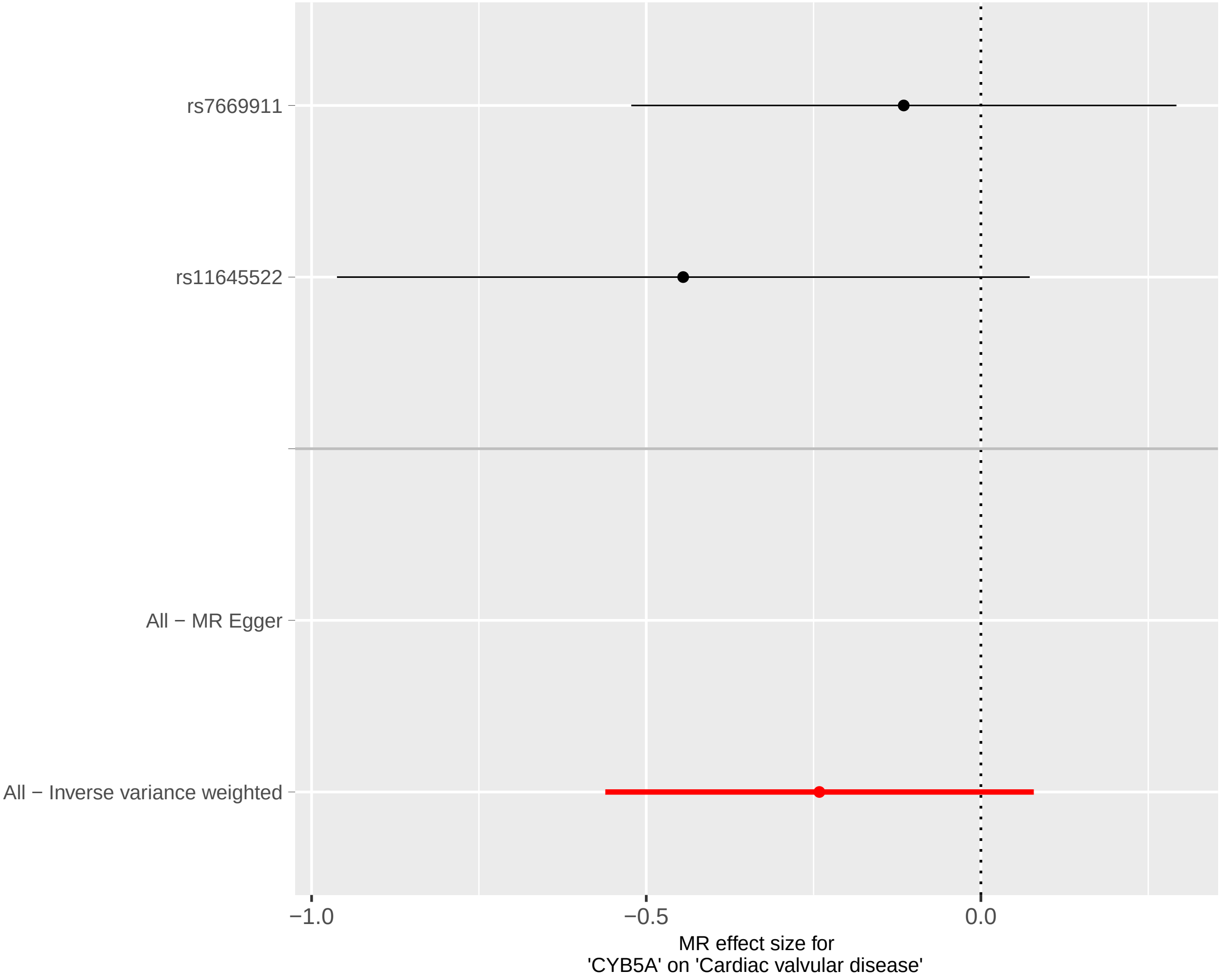
rs7669911
rs11645522
All − MR Egger
All − Inverse variance weighted
−1.0
−0.5
0.0
MR effect size for
'CYB5A' on 'Cardiac valvular disease'

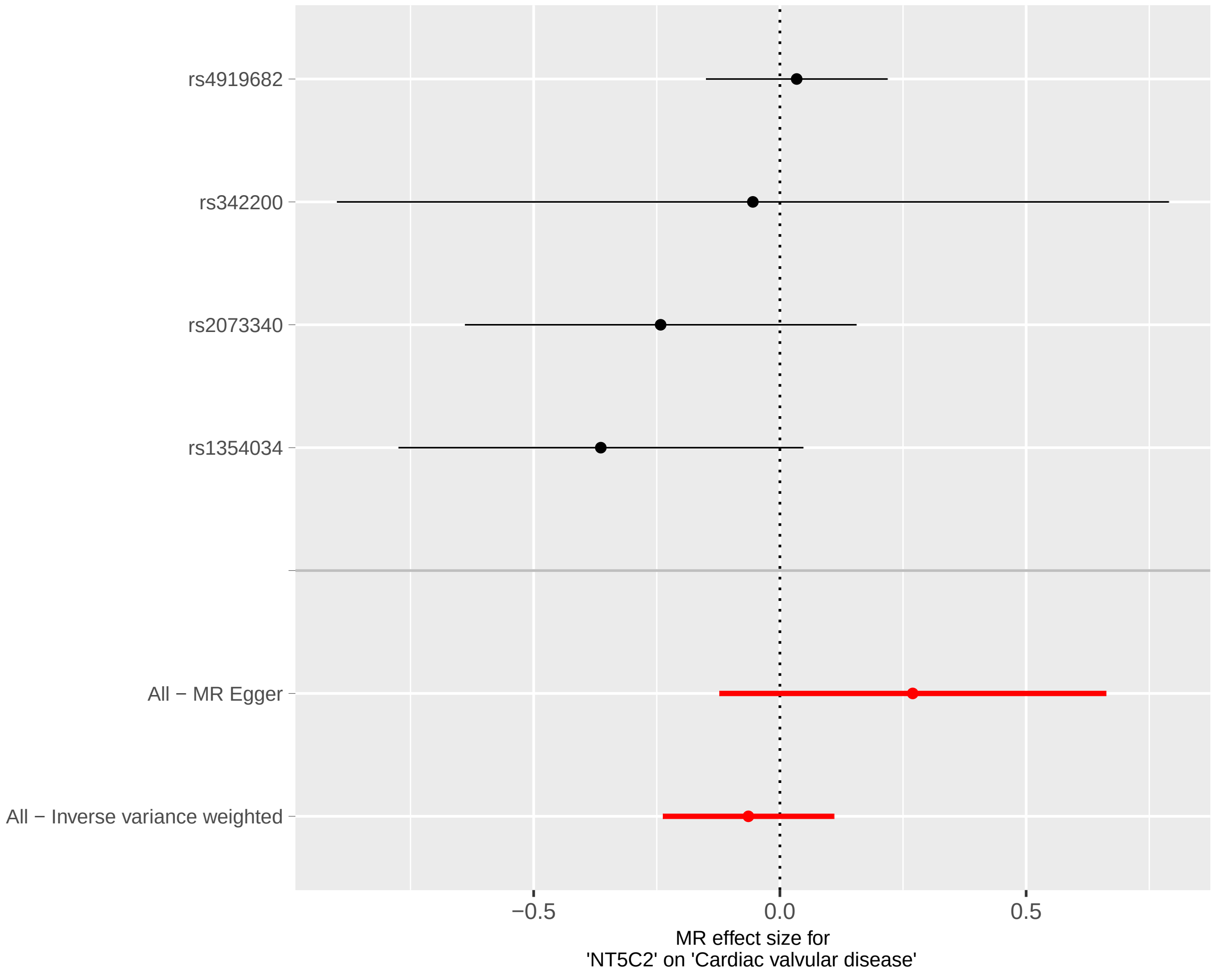

rs4919682
rs342200
rs2073340
rs1354034
All − MR Egger
All − Inverse variance weighted
−0.5
0.0
0.5
MR effect size for
'NT5C2' on 'Cardiac valvular disease'

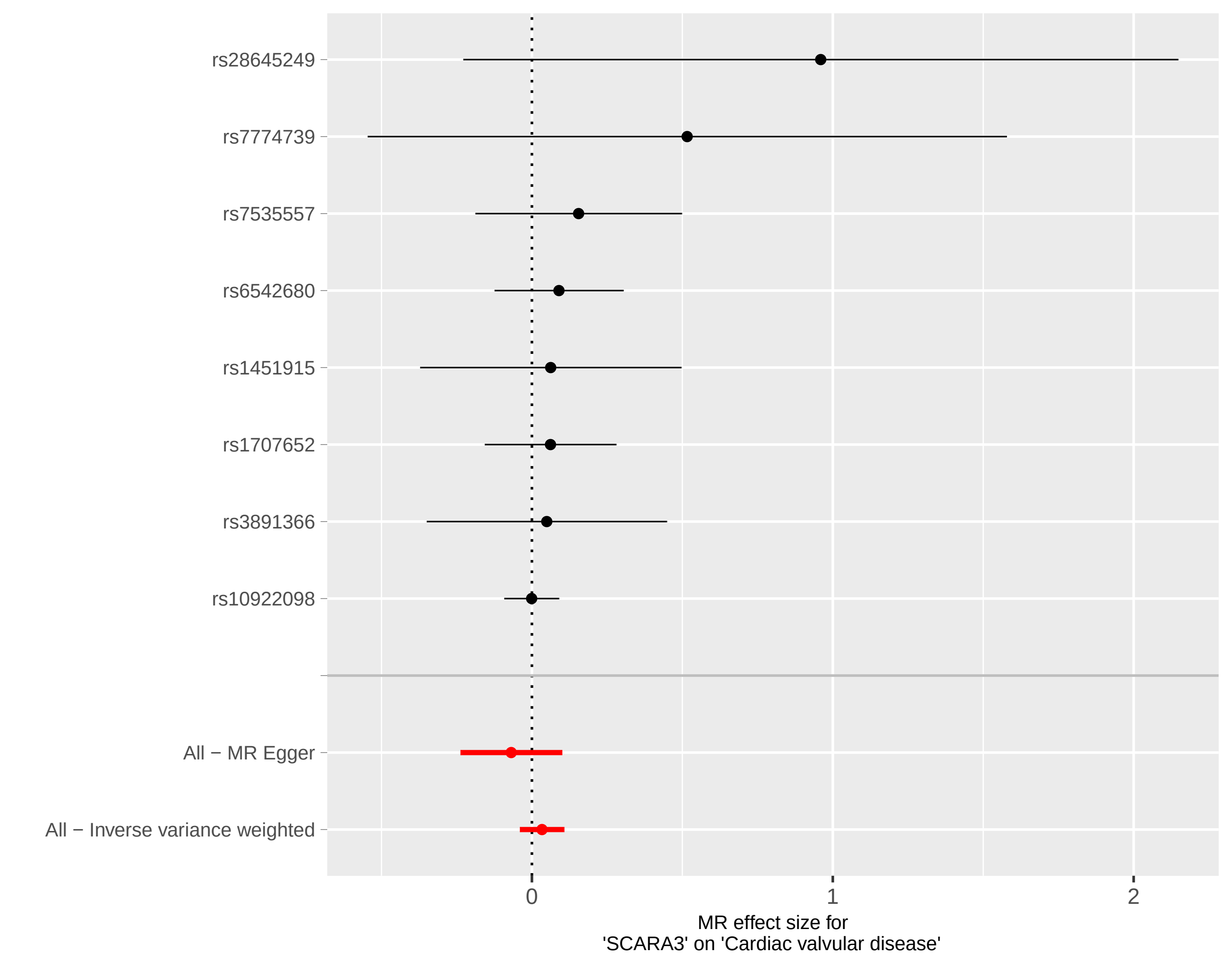
rs28645249
rs7774739
rs7535557
rs6542680
rs1451915
rs1707652
rs3891366
rs10922098
All − MR Egger
All − Inverse variance weighted
0
1
2
MR effect size for
'SCARA3' on 'Cardiac valvular disease'

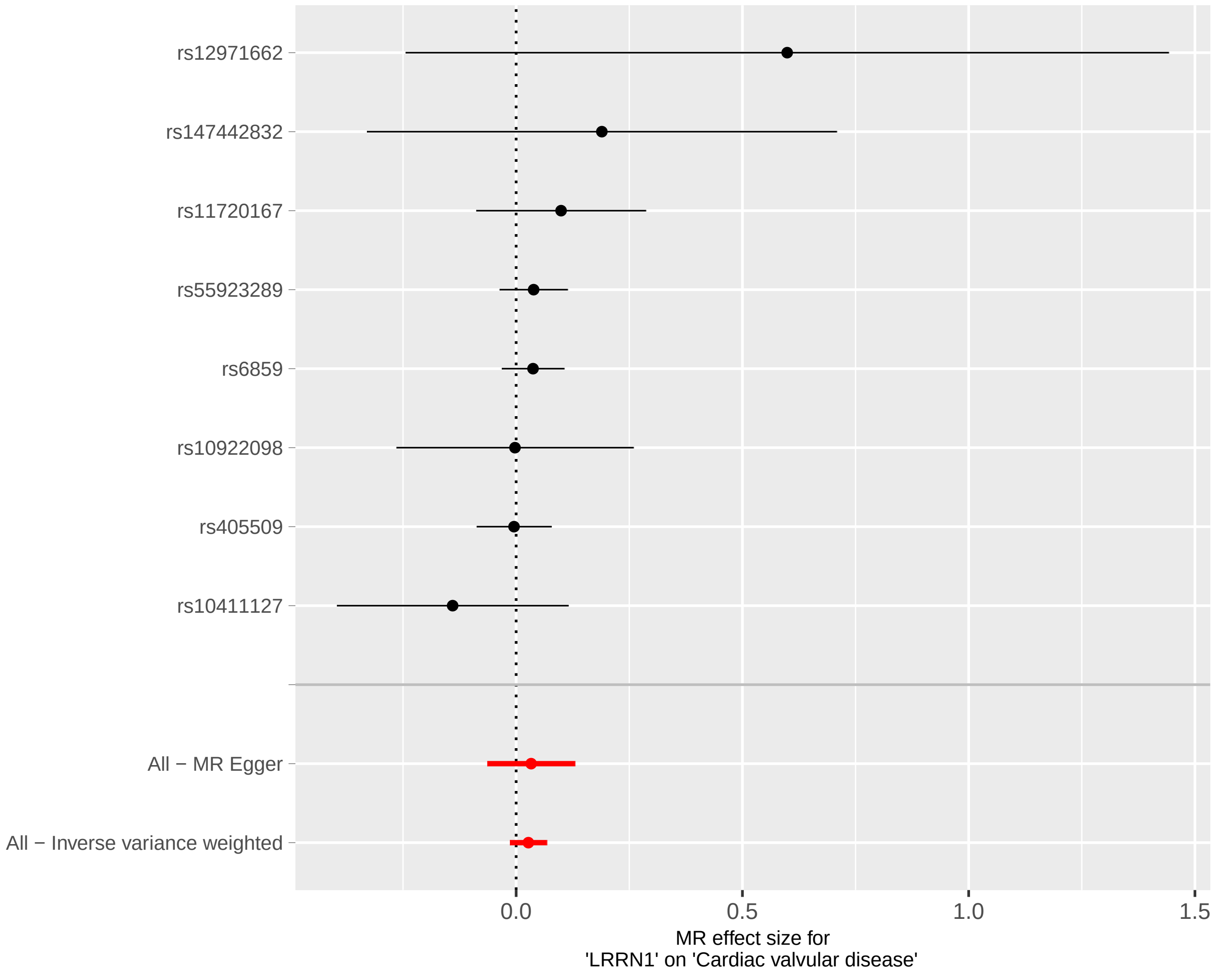
rs12971662
rs147442832
rs11720167
rs55923289
rs6859
rs10922098
rs405509
rs10411127
All − MR Egger
All − Inverse variance weighted
0.0
0.5
1.0
1.5
MR effect size for
'LRRN1' on 'Cardiac valvular disease'

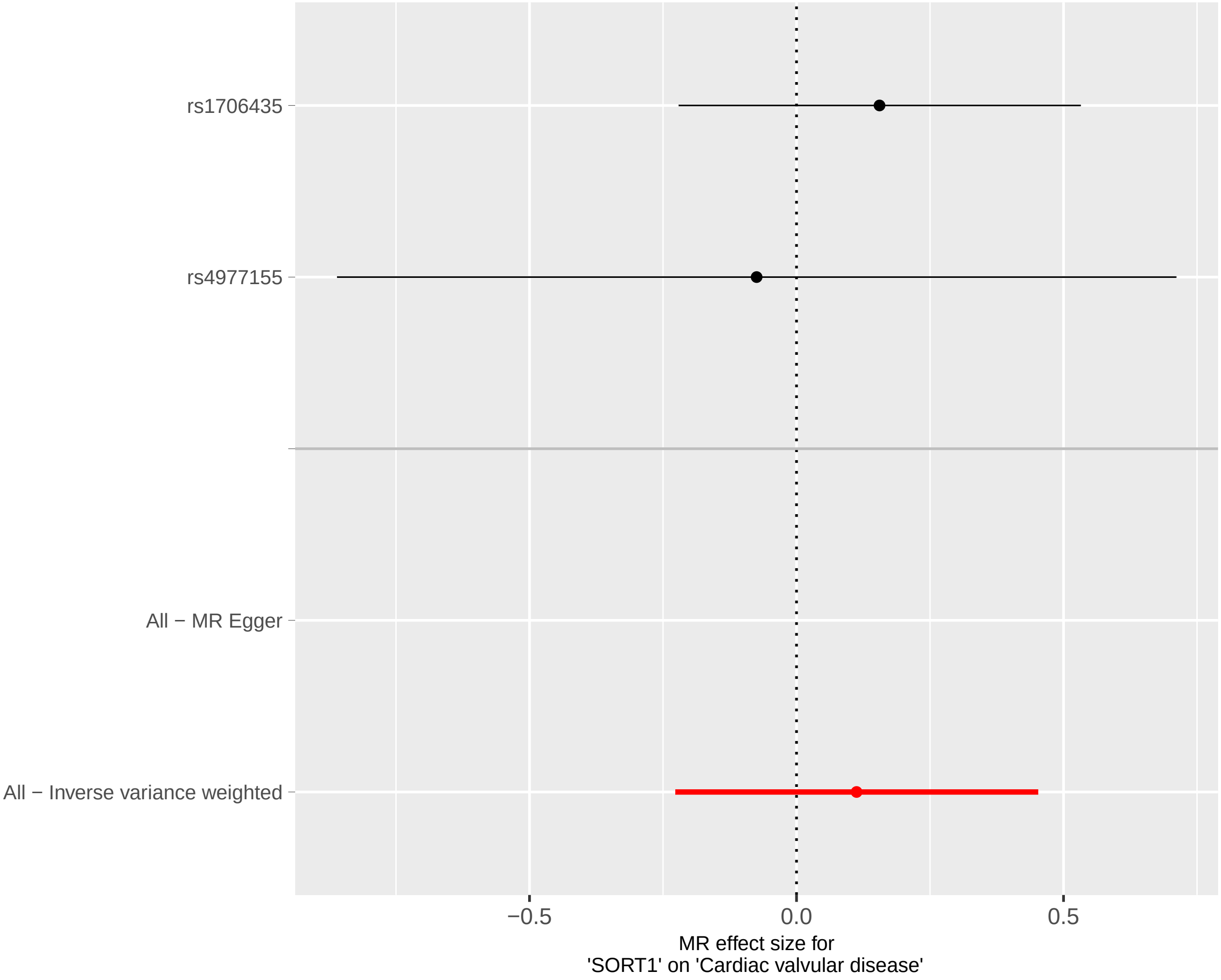
rs1706435
rs4977155
All − MR Egger
All − Inverse variance weighted
−0.5
0.0
0.5
MR effect size for
'SORT1' on 'Cardiac valvular disease'

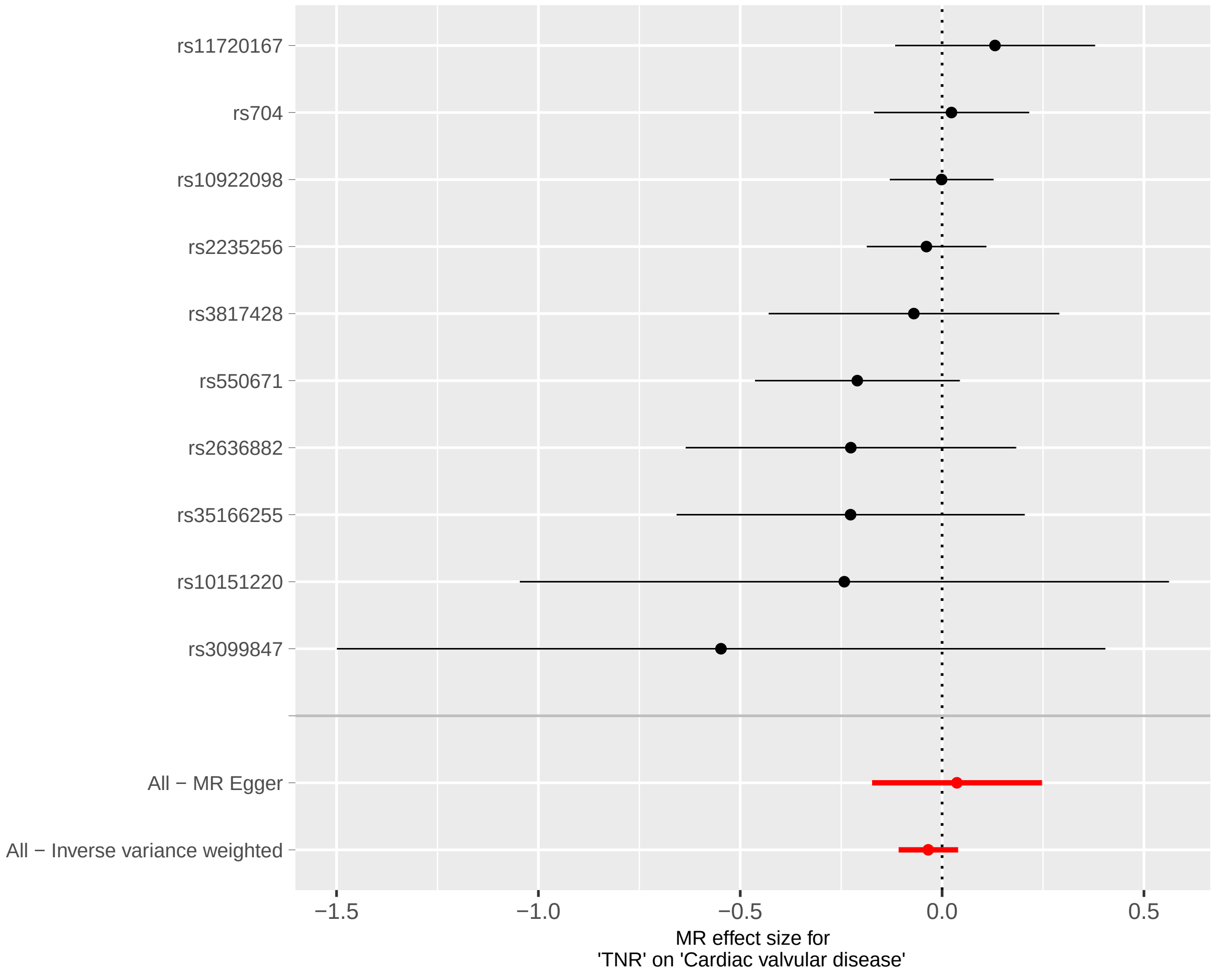
rs11720167
rs704
rs10922098
rs2235256
rs3817428
rs550671
rs2636882
rs35166255
rs10151220
rs3099847
All − MR Egger
All − Inverse variance weighted
−1.5
−1.0
−0.5
0.0
0.5
MR effect size for
'TNR' on 'Cardiac valvular disease'

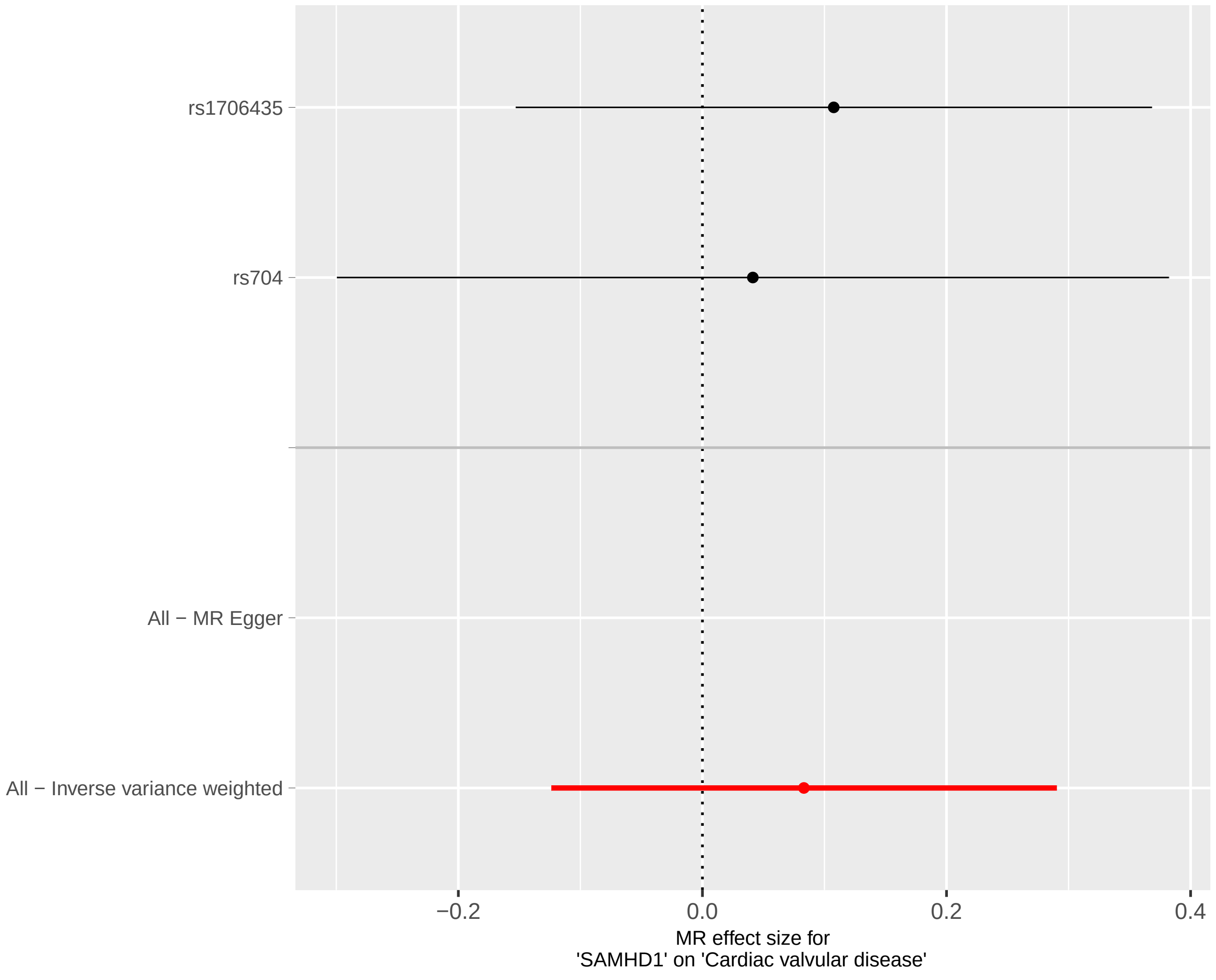
rs1706435
rs704
All − MR Egger
All − Inverse variance weighted
−0.2
0.0
0.2
0.4
MR effect size for
'SAMHD1' on 'Cardiac valvular disease'

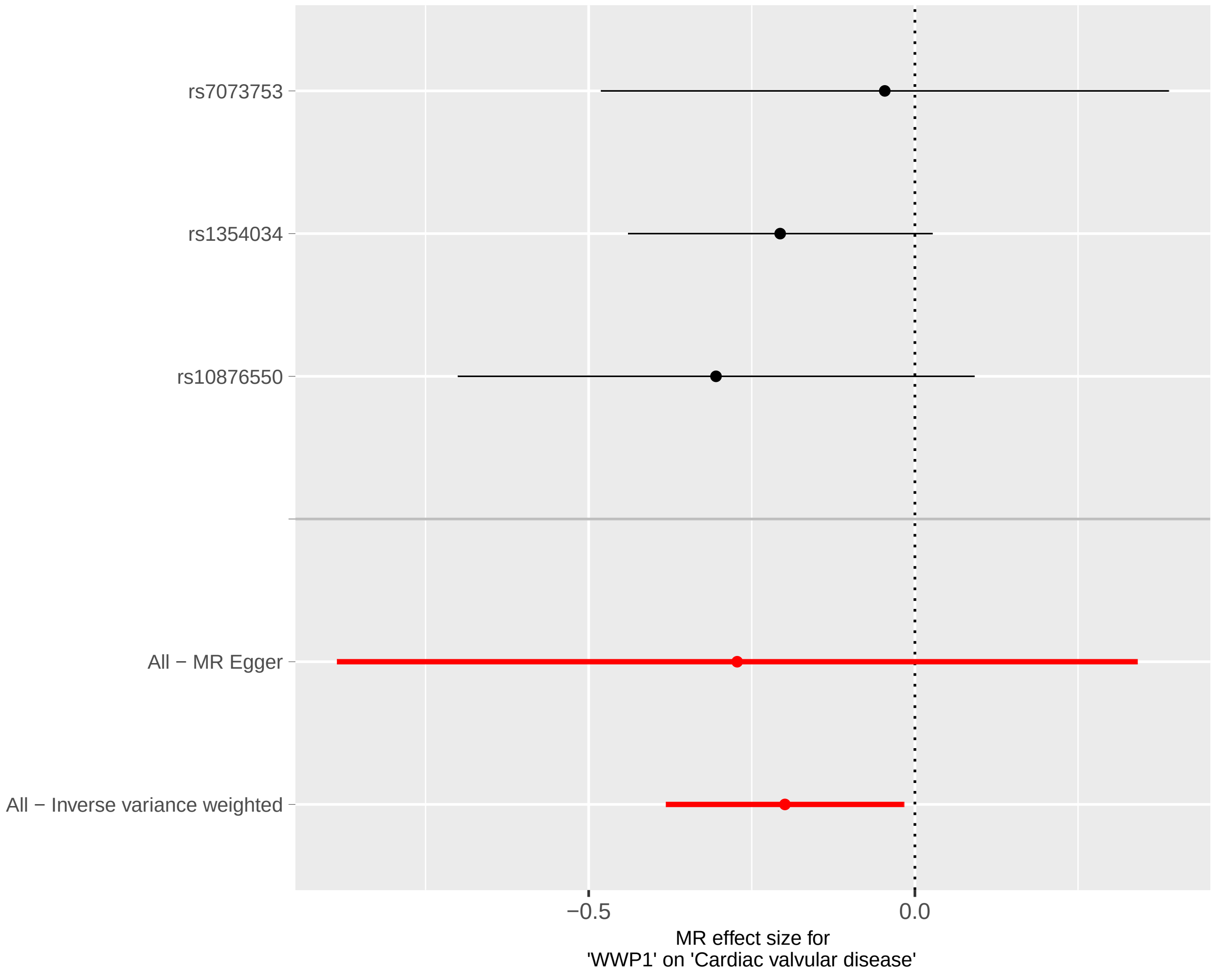
rs7073753
rs1354034
rs10876550
All − MR Egger
All − Inverse variance weighted
−0.5
0.0
MR effect size for
'WWP1' on 'Cardiac valvular disease'

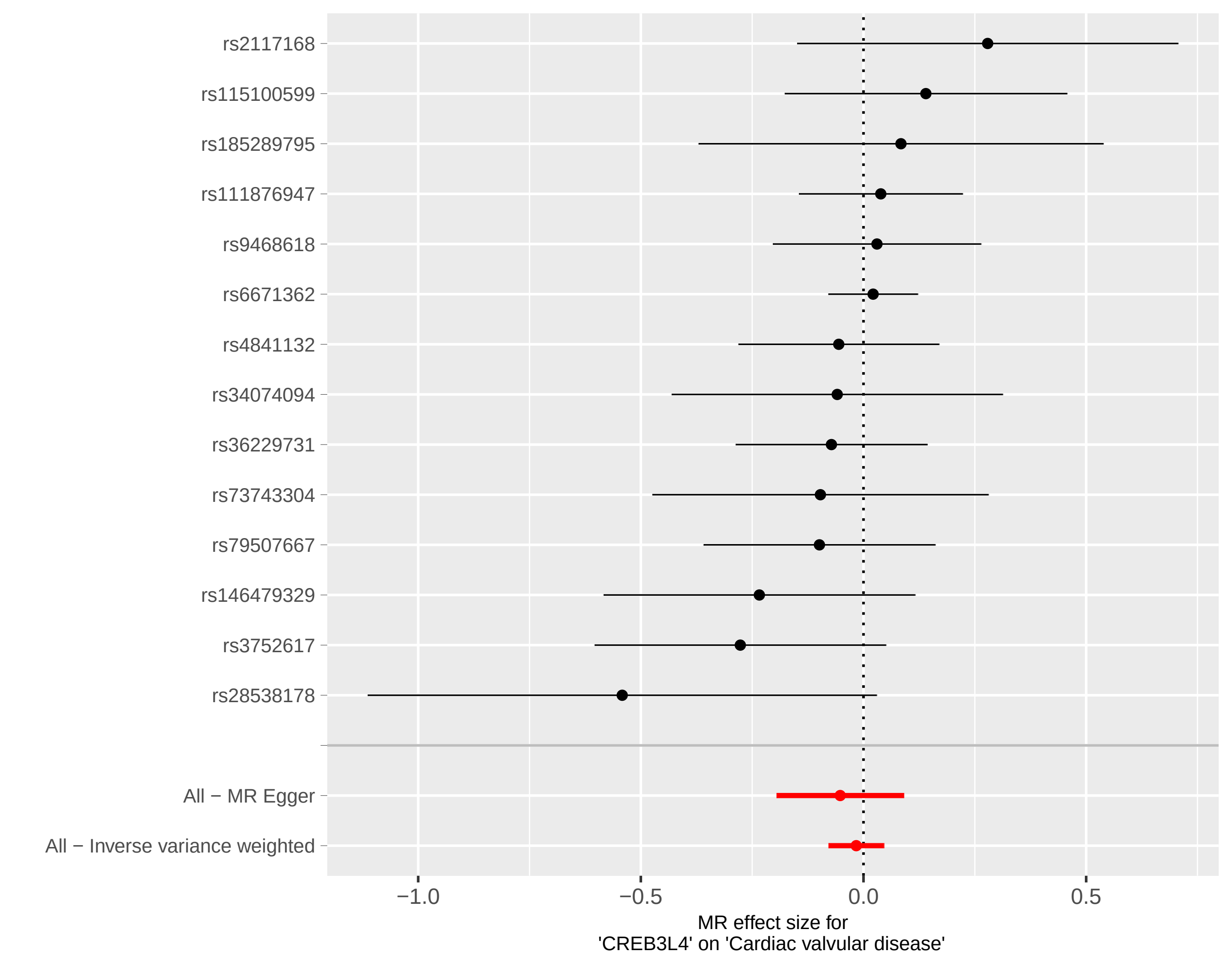
rs2117168
rs115100599
rs185289795
rs111876947
rs9468618
rs6671362
rs4841132
rs34074094
rs36229731
rs73743304
rs79507667
rs146479329
rs3752617
rs28538178
All − MR Egger
All − Inverse variance weighted
−1.0
−0.5
0.0
0.5
MR effect size for
'CREB3L4' on 'Cardiac valvular disease'

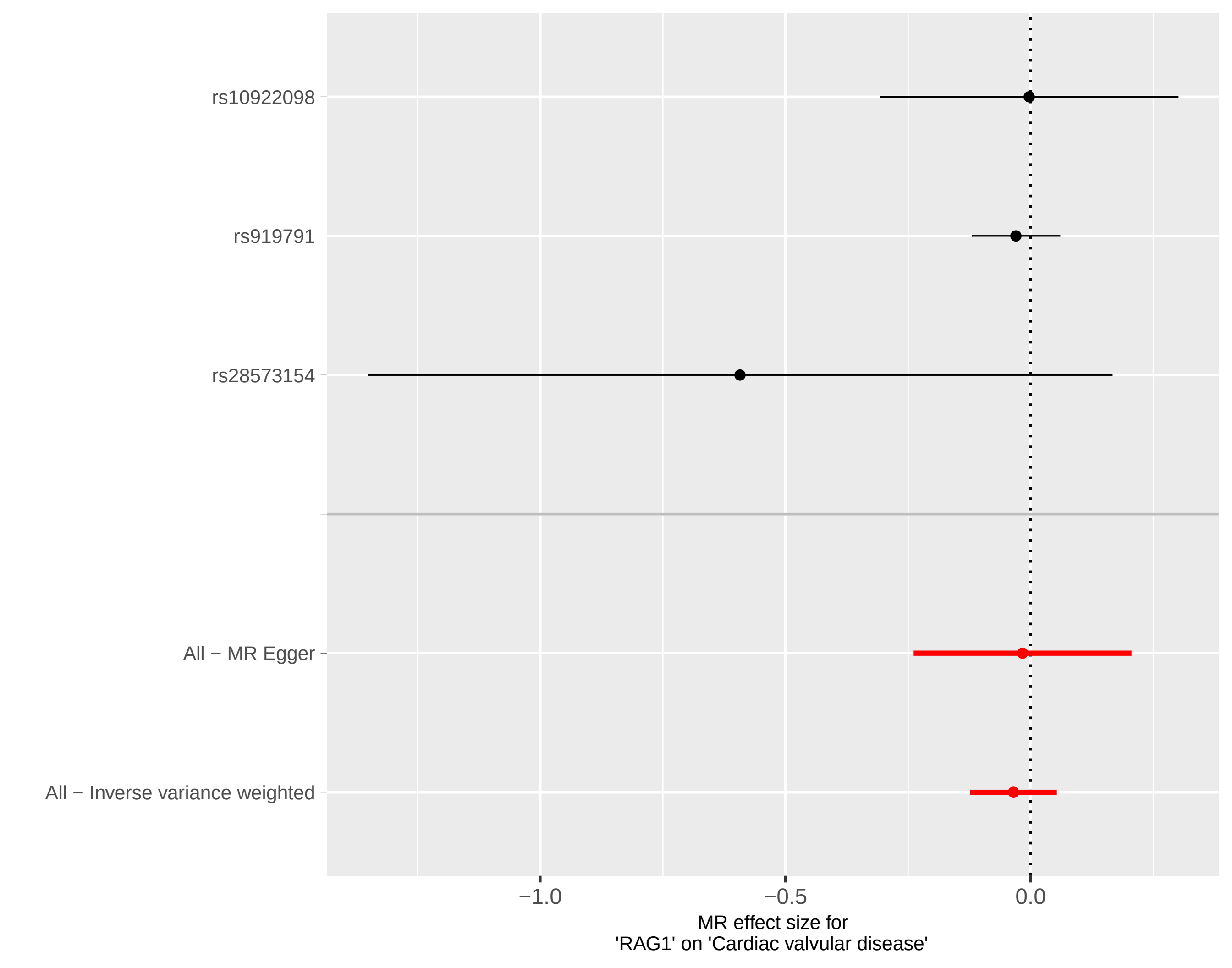
rs10922098
rs919791
rs28573154
All − MR Egger
All − Inverse variance weighted
−1.0
−0.5
0.0
MR effect size for
'RAG1' on 'Cardiac valvular disease'

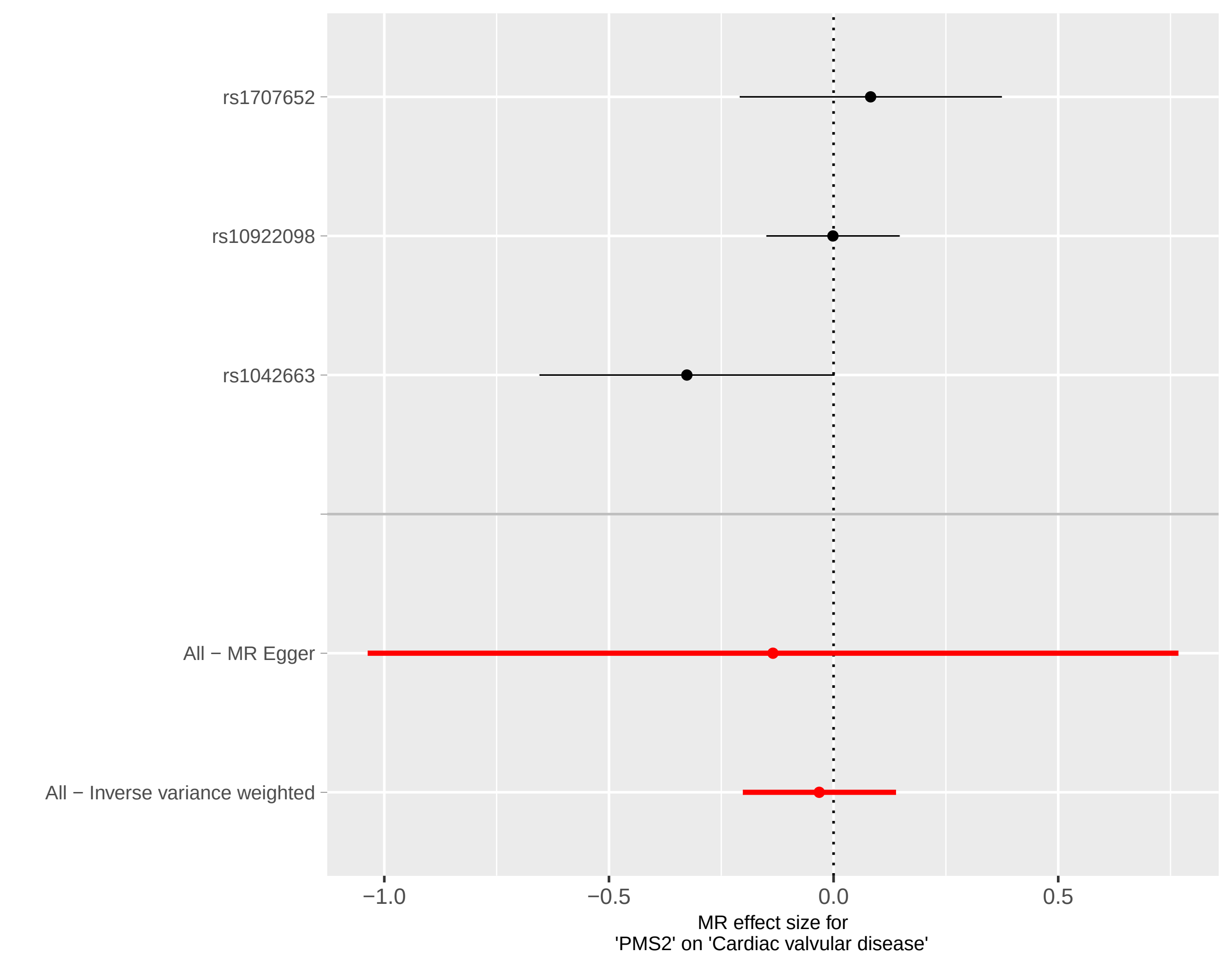
rs1707652
rs10922098
rs1042663
All − MR Egger
All − Inverse variance weighted
−1.0
−0.5
0.0
0.5
MR effect size for
'PMS2' on 'Cardiac valvular disease'

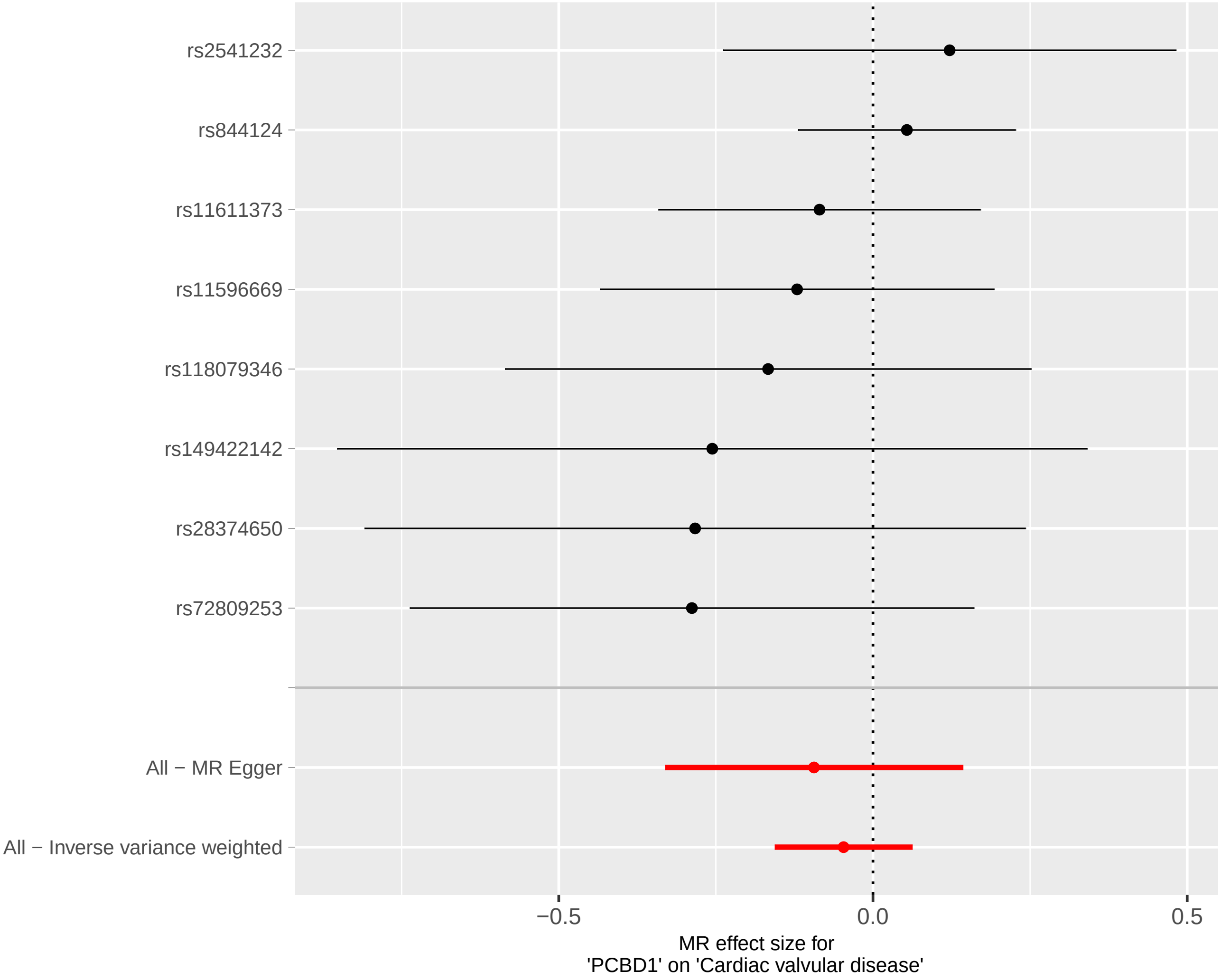

rs2541232
rs844124
rs11611373
rs11596669
rs118079346
rs149422142
rs28374650
rs72809253
All − MR Egger
All − Inverse variance weighted
−0.5
0.0
0.5
MR effect size for
'PCBD1' on 'Cardiac valvular disease'

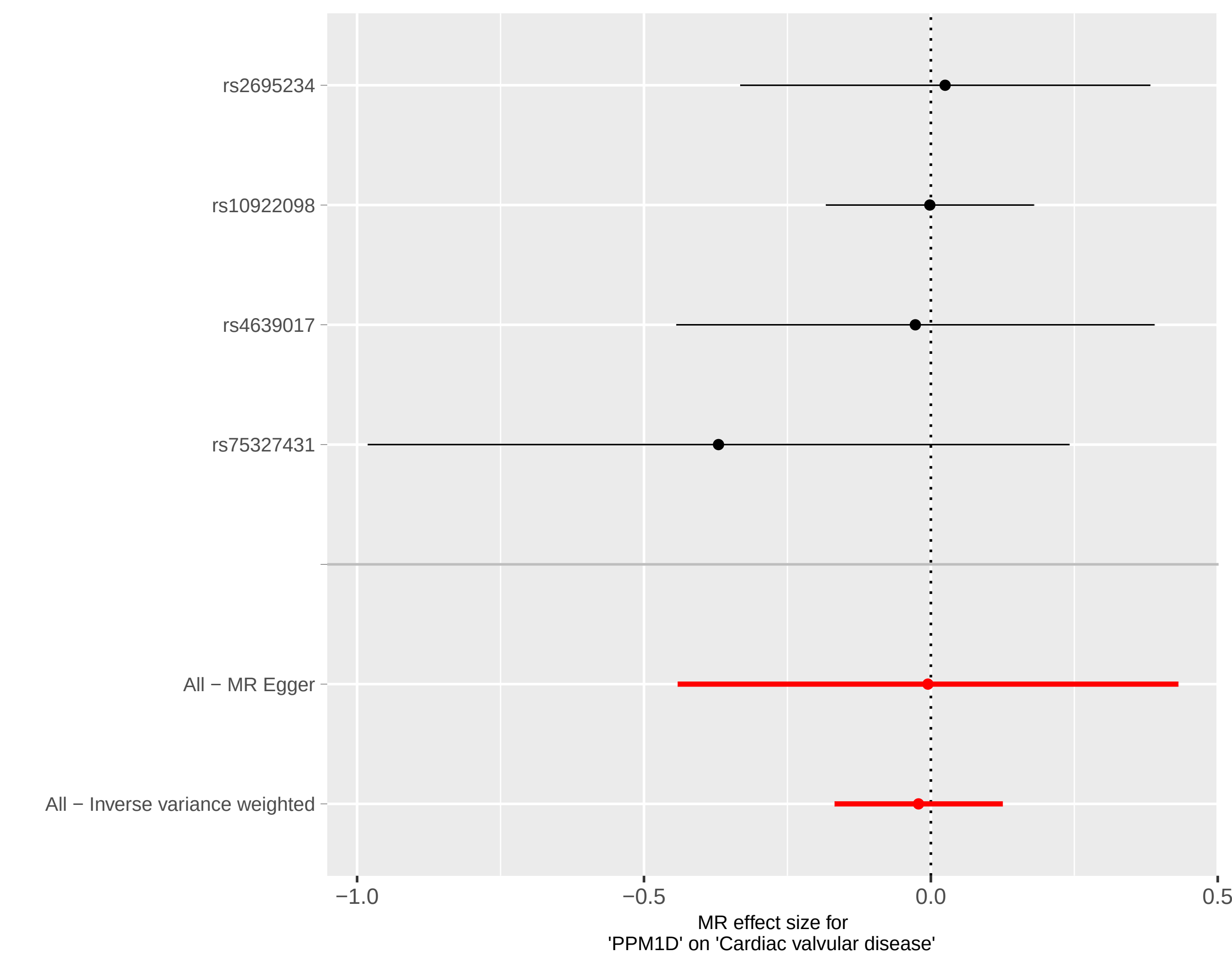

rs2695234
rs10922098
rs4639017
rs75327431
All − MR Egger
All − Inverse variance weighted
−1.0
−0.5
0.0
0.5
MR effect size for
'PPM1D' on 'Cardiac valvular disease'

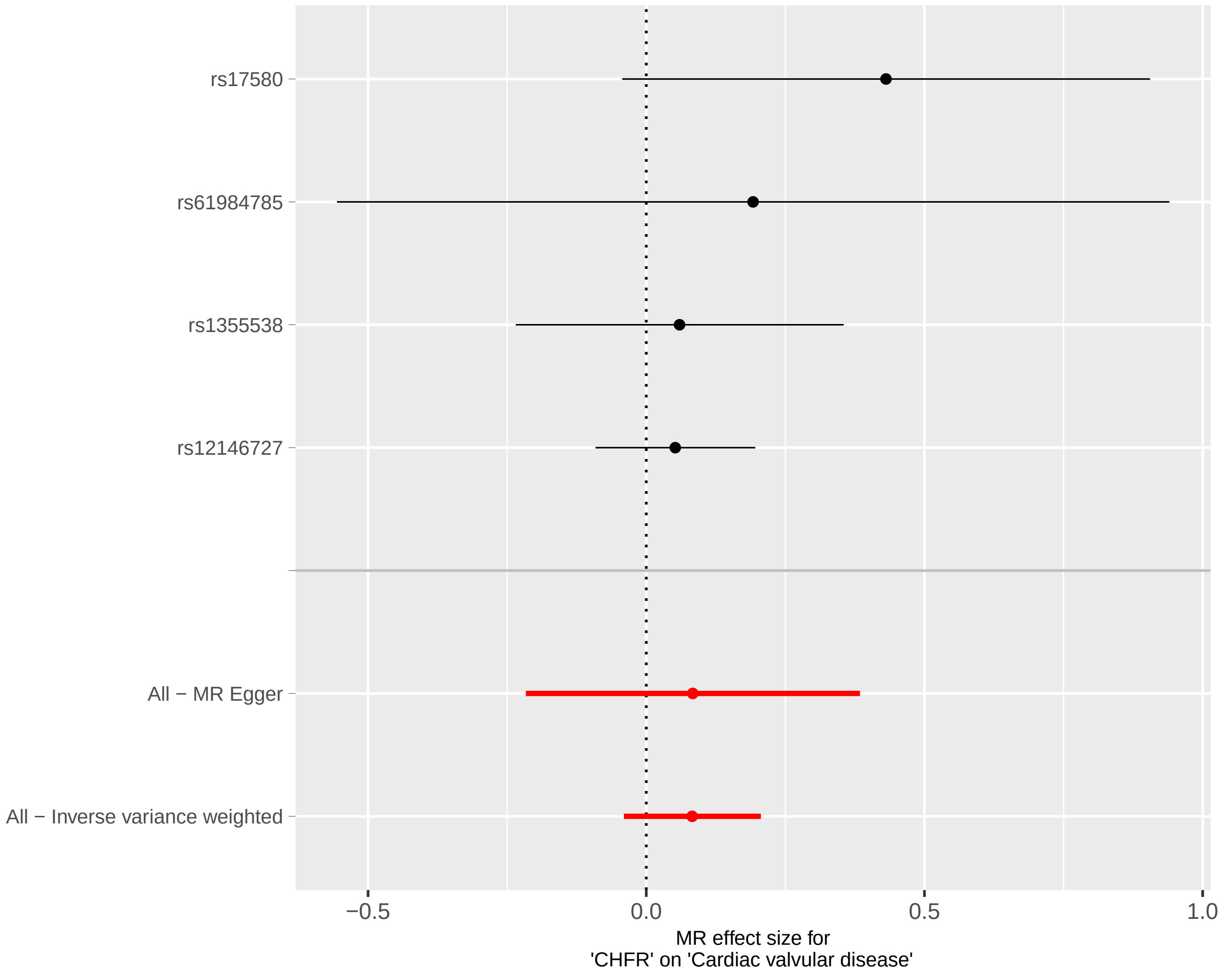

rs17580
rs61984785
rs1355538
rs12146727
All − MR Egger
All − Inverse variance weighted
−0.5
0.0
0.5
1.0
MR effect size for
'CHFR' on 'Cardiac valvular disease'

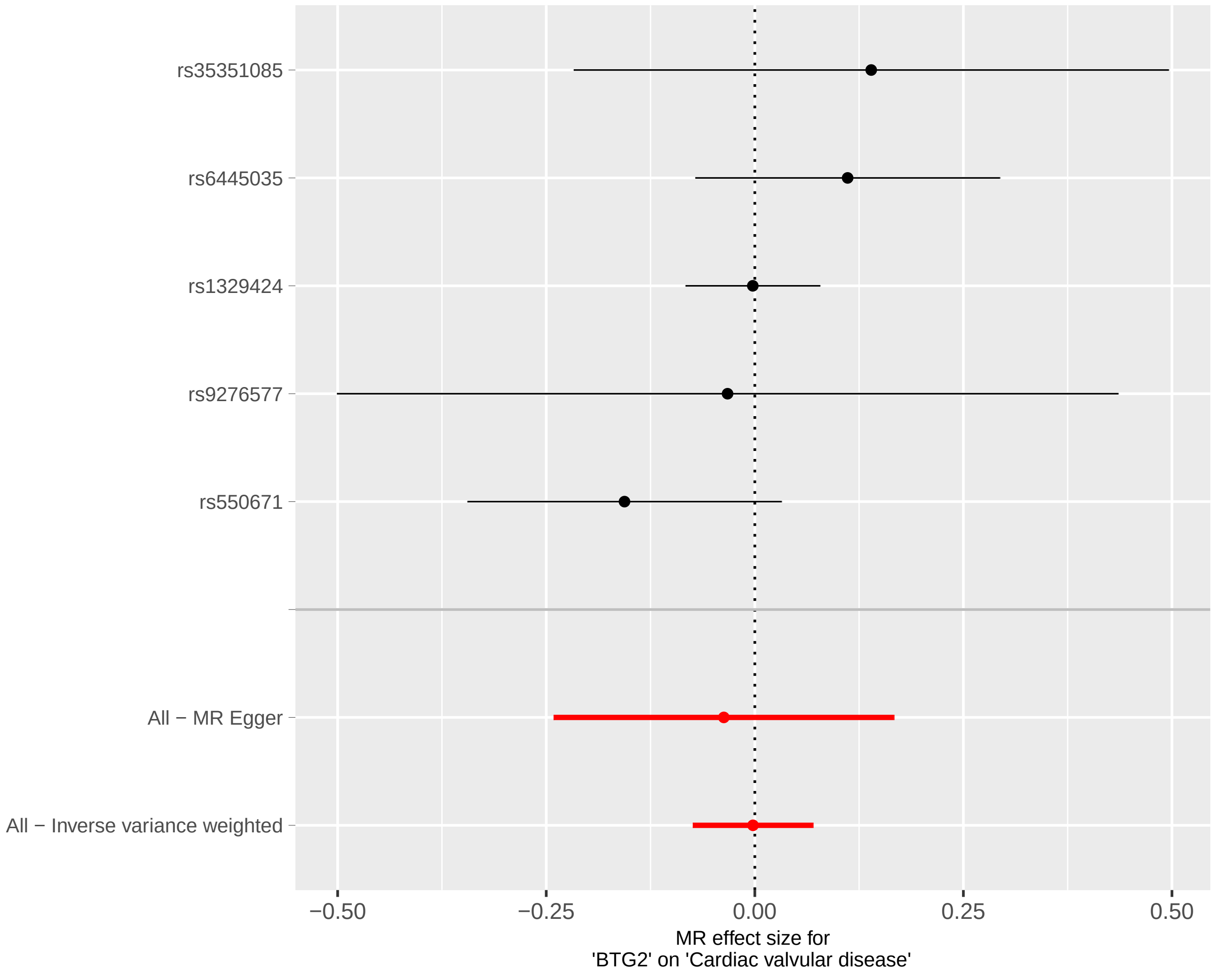
rs35351085
rs6445035
rs1329424
rs9276577
rs550671
All − MR Egger
All − Inverse variance weighted
−0.50
−0.25
0.00
0.25
0.50
MR effect size for
'BTG2' on 'Cardiac valvular disease'

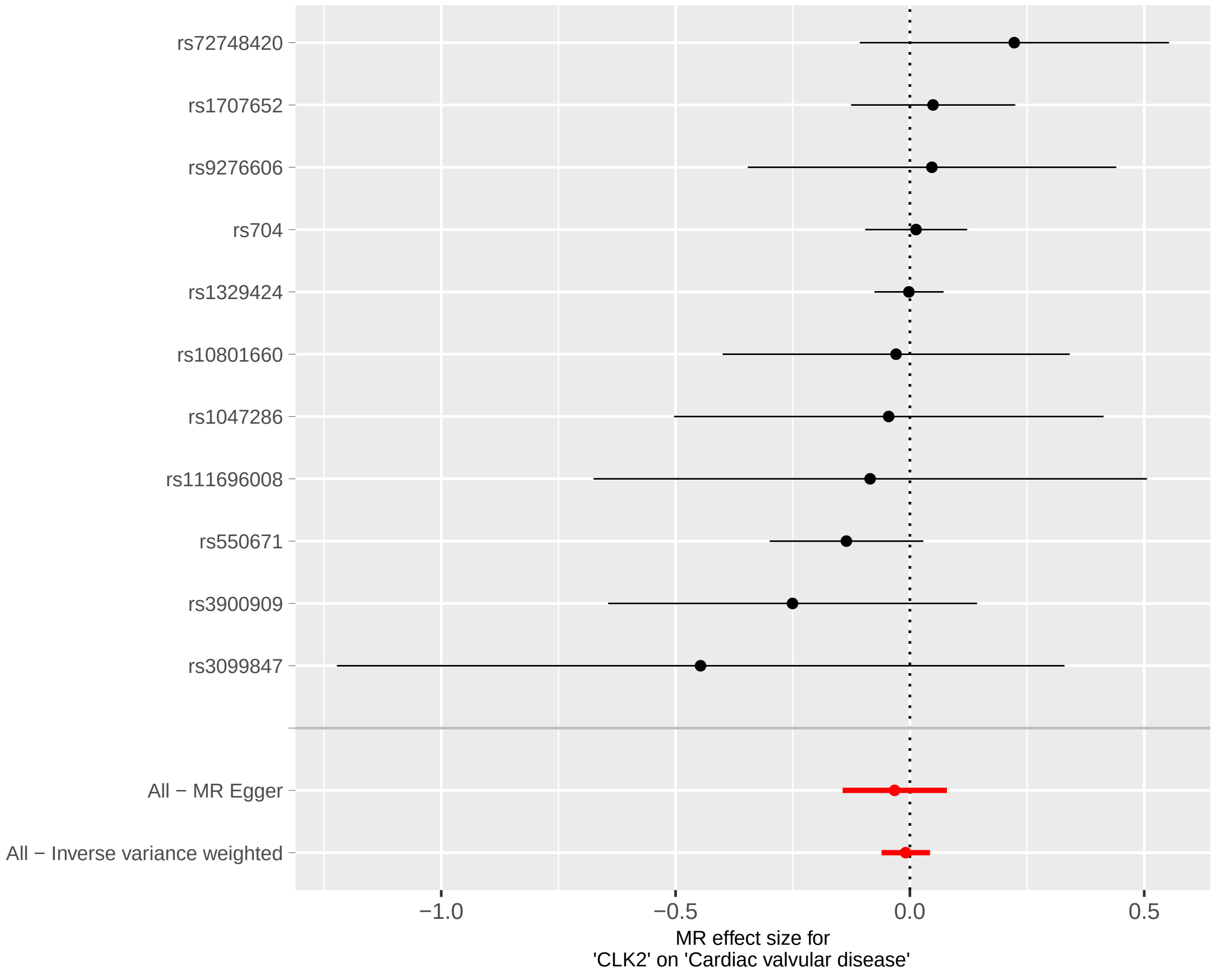
rs72748420
rs1707652
rs9276606
rs704
rs1329424
rs10801660
rs1047286
rs111696008
rs550671
rs3900909
rs3099847
All − MR Egger
All − Inverse variance weighted
−1.0
−0.5
0.0
0.5
MR effect size for
'CLK2' on 'Cardiac valvular disease'

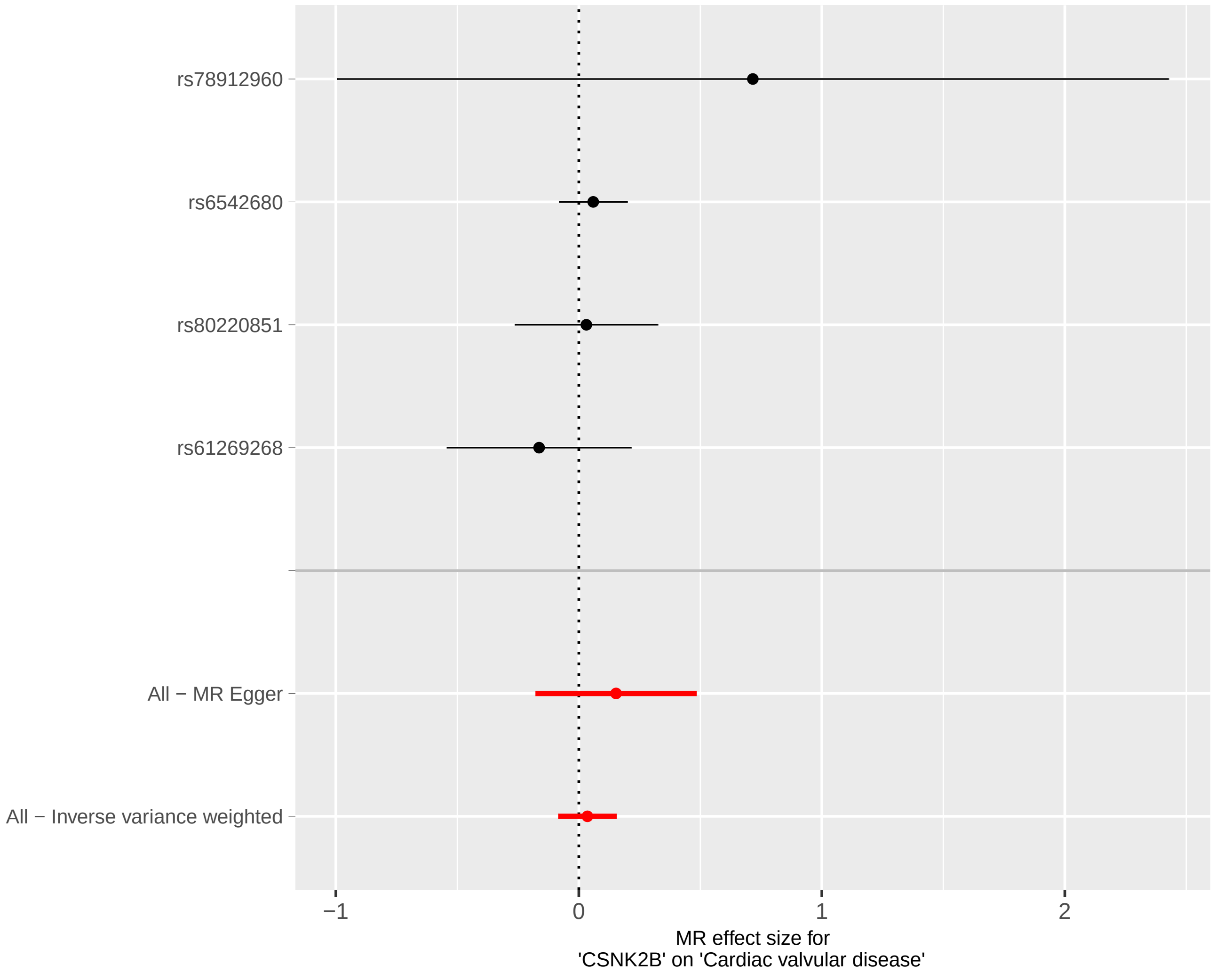

rs78912960
rs6542680
rs80220851
rs61269268
All − MR Egger
All − Inverse variance weighted
−1
0
1
2
MR effect size for
'CSNK2B' on 'Cardiac valvular disease'

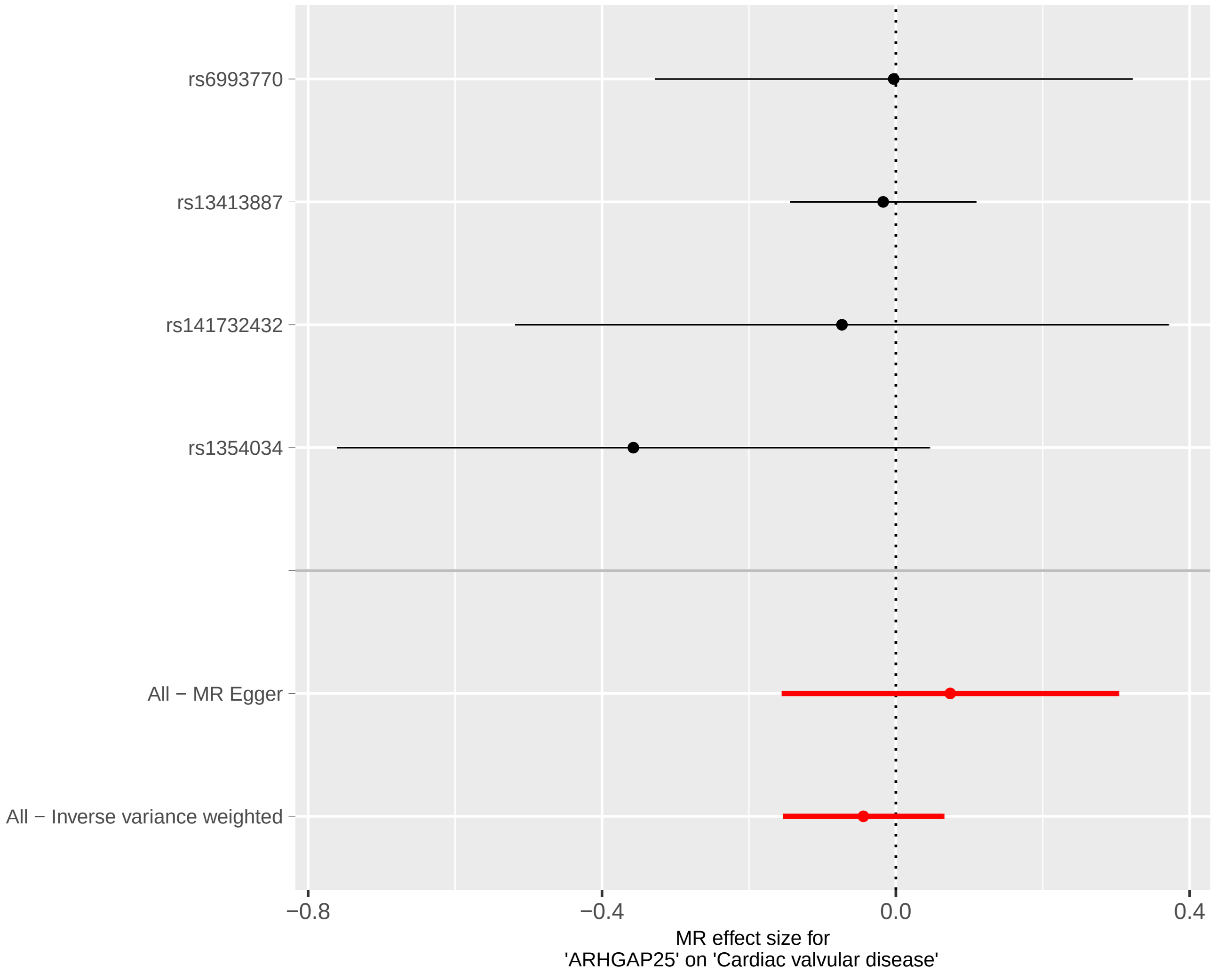
rs6993770
rs13413887
rs141732432
rs1354034
All − MR Egger
All − Inverse variance weighted
−0.8
−0.4
0.0
0.4
MR effect size for
'ARHGAP25' on 'Cardiac valvular disease'

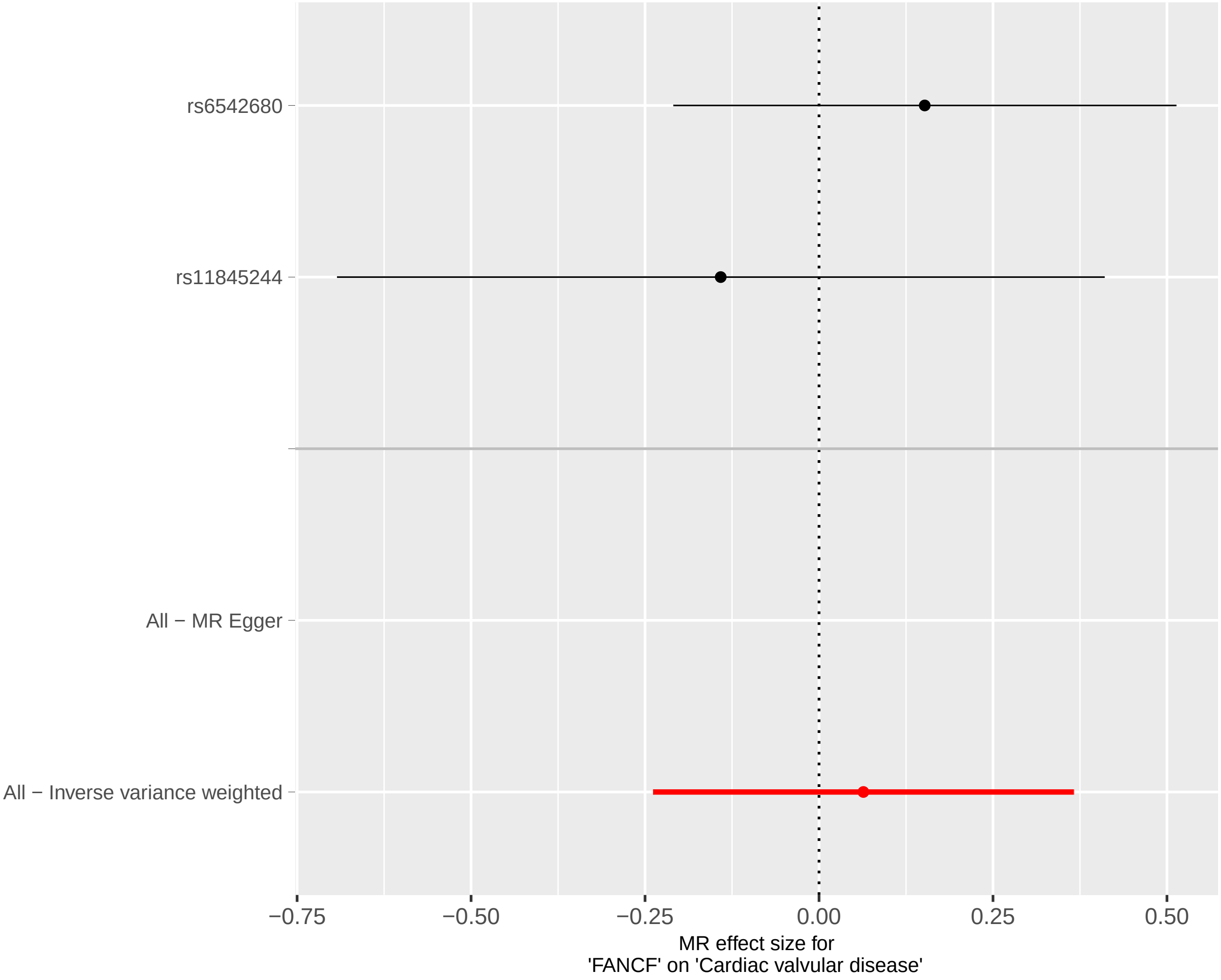
rs6542680
rs11845244
All − MR Egger
All − Inverse variance weighted
−0.75
−0.50
−0.25
0.00
0.25
0.50
MR effect size for
'FANCF' on 'Cardiac valvular disease'

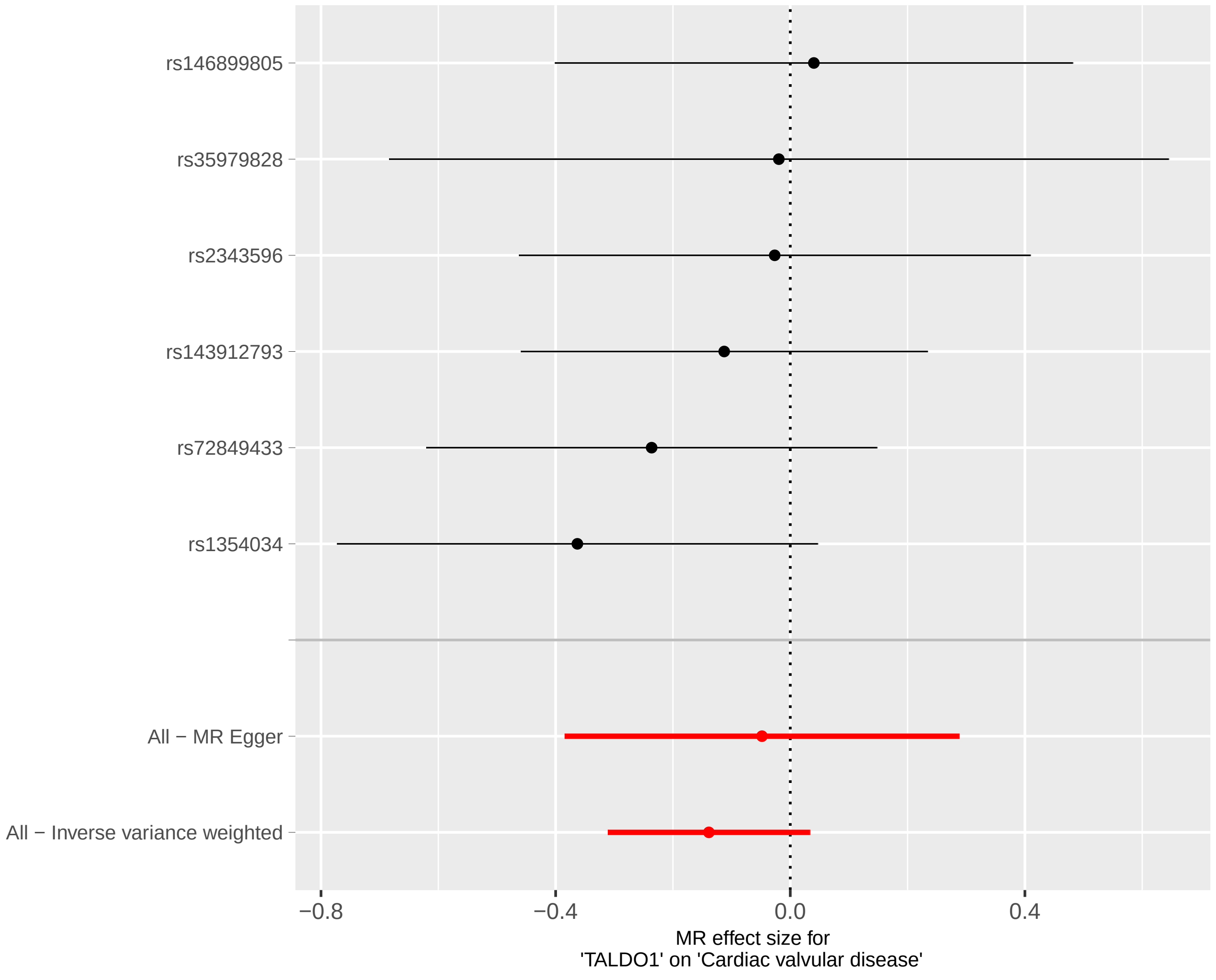

rs146899805
rs35979828
rs2343596
rs143912793
rs72849433
rs1354034
All − MR Egger
All − Inverse variance weighted
−0.8
−0.4
0.0
0.4
MR effect size for
'TALDO1' on 'Cardiac valvular disease'

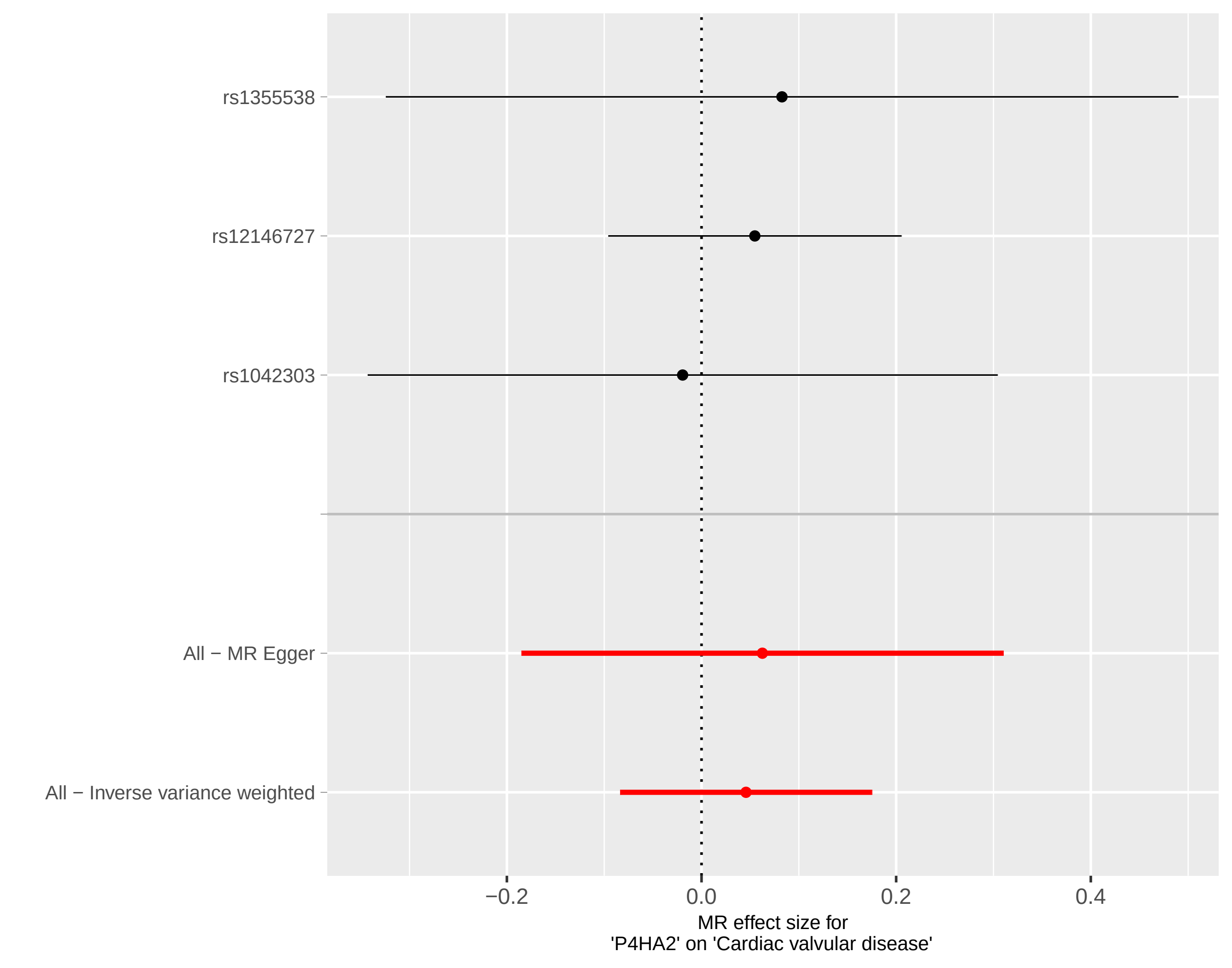
rs1355538
rs12146727
rs1042303
All − MR Egger
All − Inverse variance weighted
−0.2
0.0
0.2
0.4
MR effect size for
'P4HA2' on 'Cardiac valvular disease'

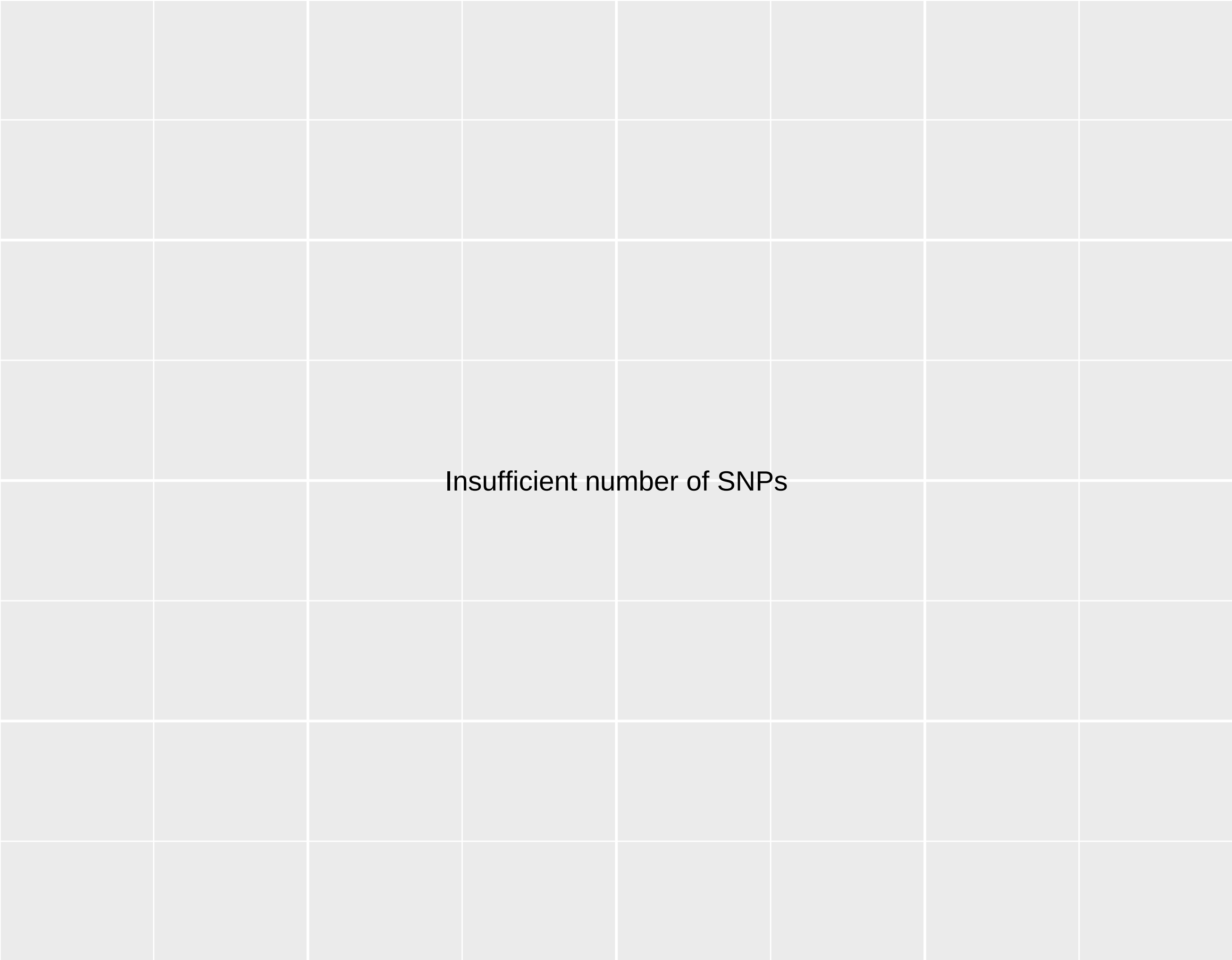
Insufficient number of SNPs

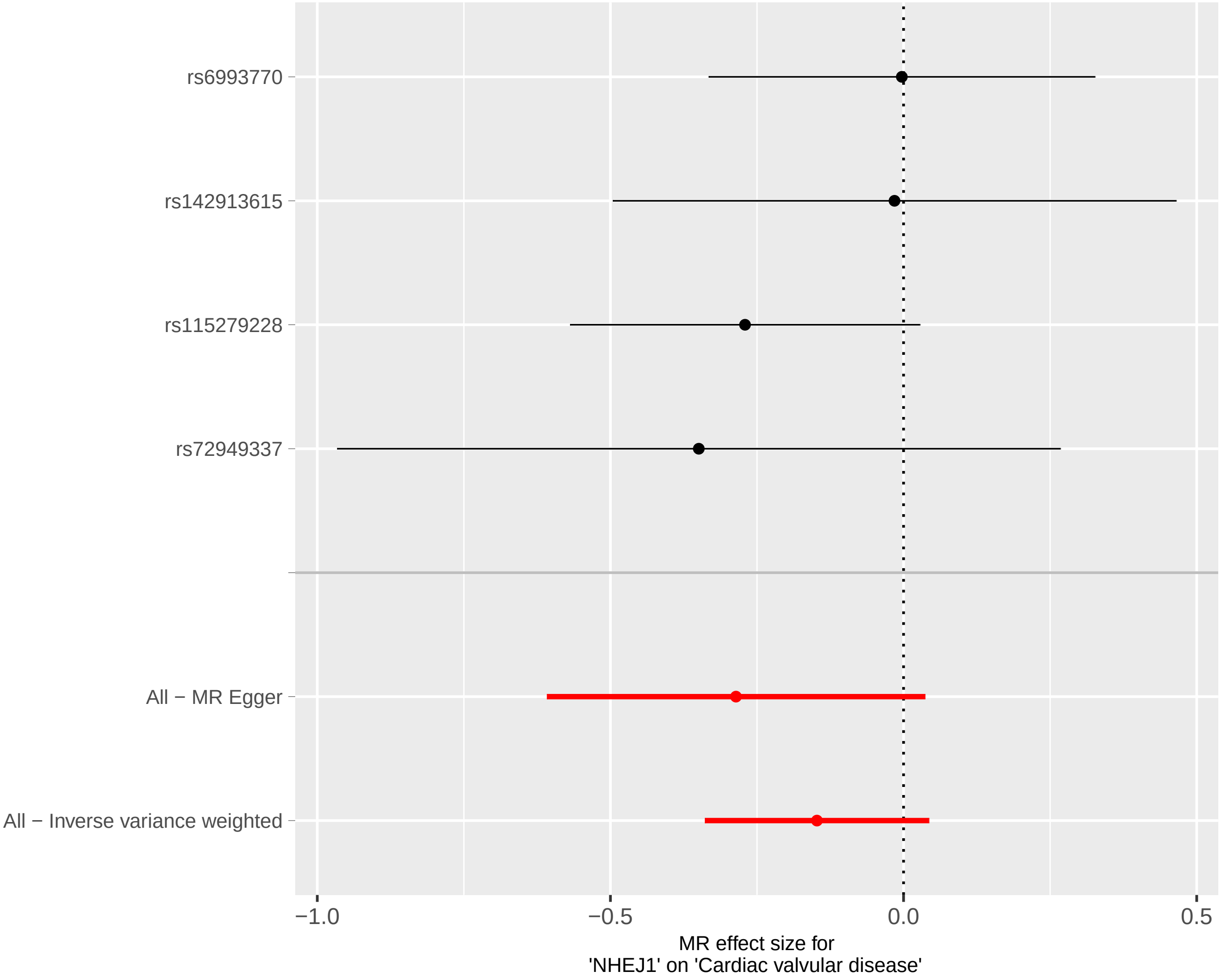
rs6993770
rs142913615
rs115279228
rs72949337
All − MR Egger
All − Inverse variance weighted
−1.0
−0.5
0.0
0.5
MR effect size for
'NHEJ1' on 'Cardiac valvular disease'

Insufficient number of SNPs

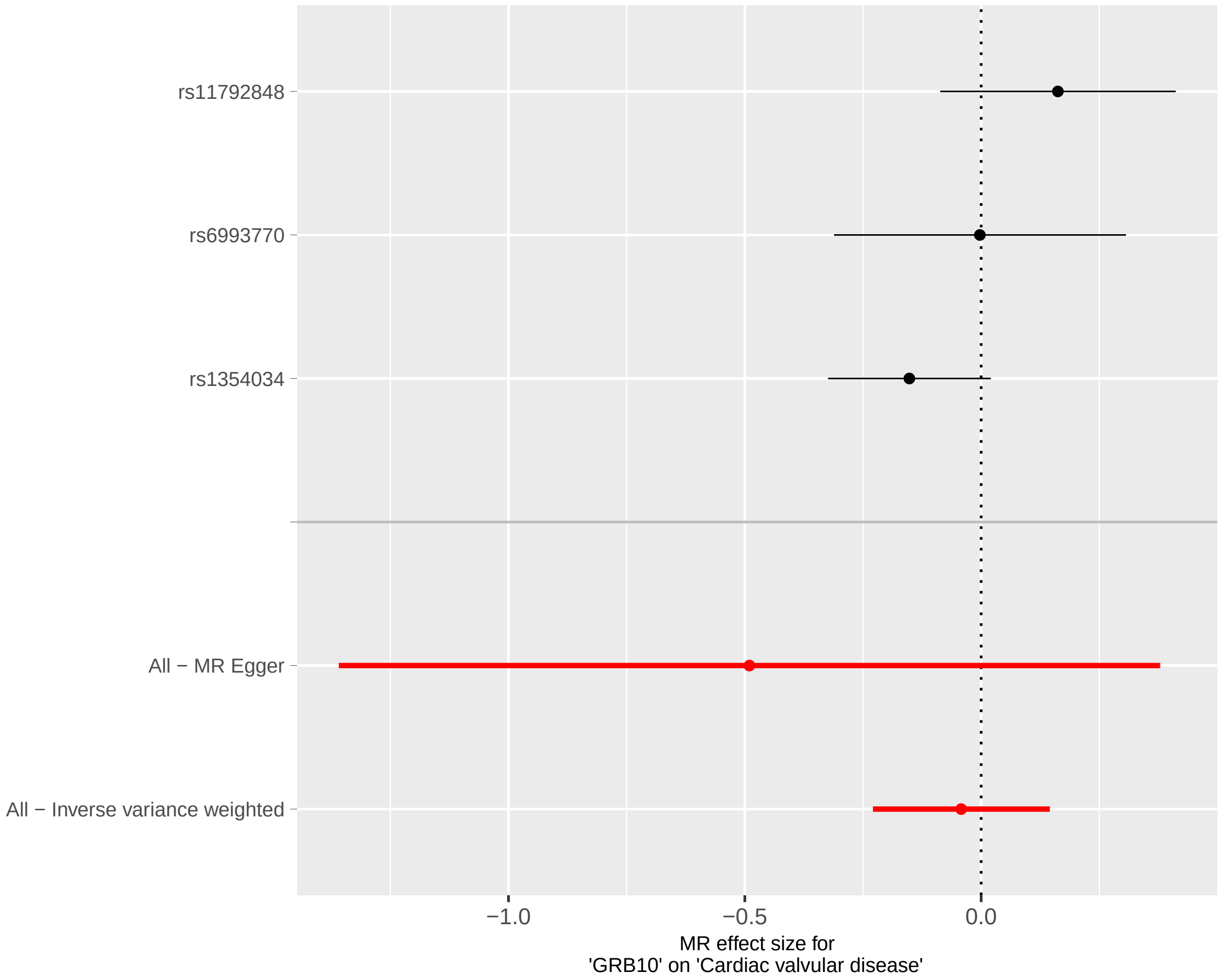
rs11792848
rs6993770
rs1354034
All − MR Egger
All − Inverse variance weighted
−1.0
−0.5
0.0
MR effect size for
'GRB10' on 'Cardiac valvular disease'

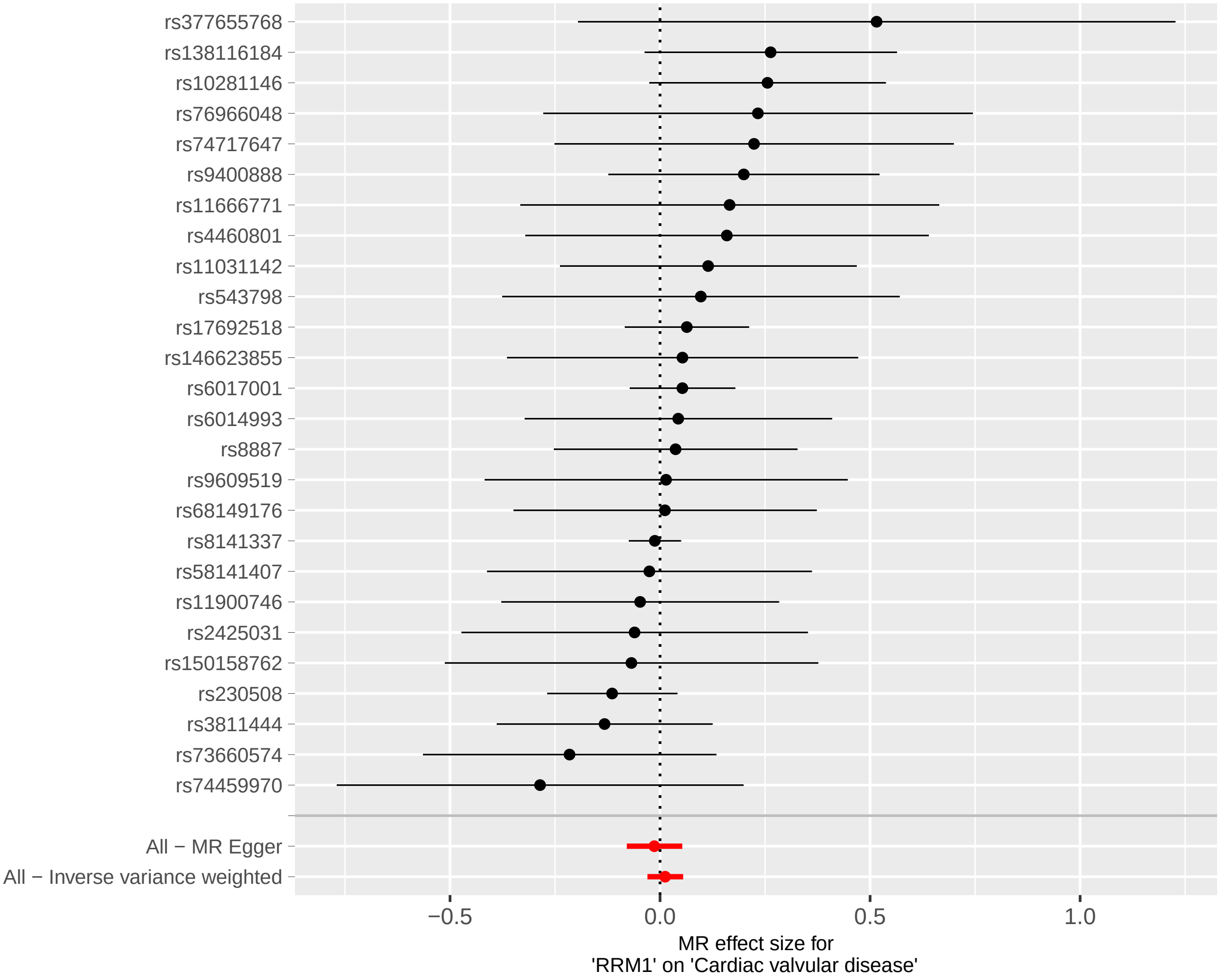

rs377655768
rs138116184
rs10281146
rs76966048
rs74717647
rs9400888
rs11666771
rs4460801
rs11031142
rs543798
rs17692518
rs146623855
rs6017001
rs6014993
rs8887
rs9609519
rs68149176
rs8141337
rs58141407
rs11900746
rs2425031
rs150158762
rs230508
rs3811444
rs73660574
rs74459970
All − MR Egger
All − Inverse variance weighted
−0.5
0.0
0.5
1.0
MR effect size for
'RRM1' on 'Cardiac valvular disease'

Insufficient number of SNPs

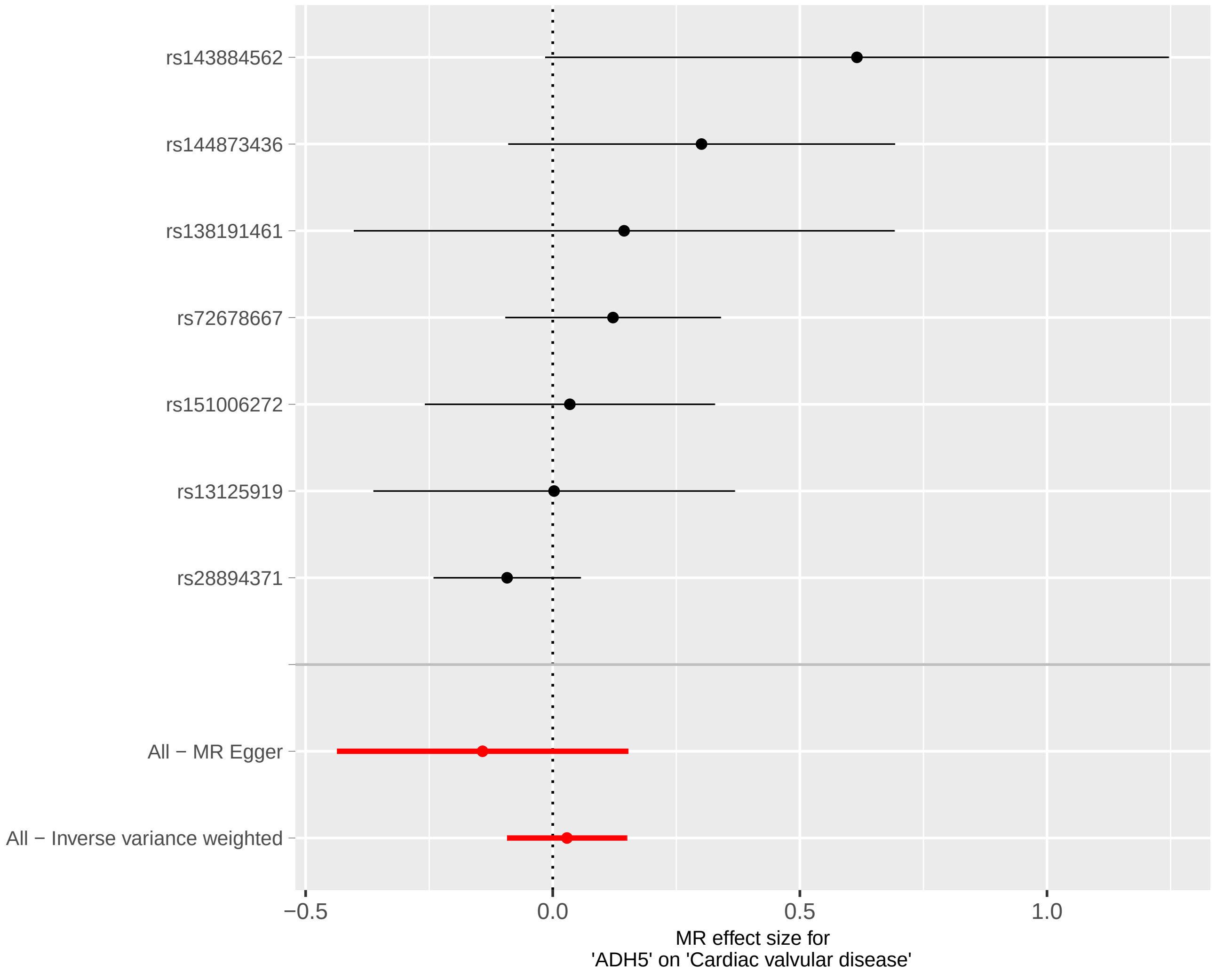
rs143884562
rs144873436
rs138191461
rs72678667
rs151006272
rs13125919
rs28894371
All − MR Egger
All − Inverse variance weighted
−0.5
0.0
0.5
1.0
MR effect size for
'ADH5' on 'Cardiac valvular disease'

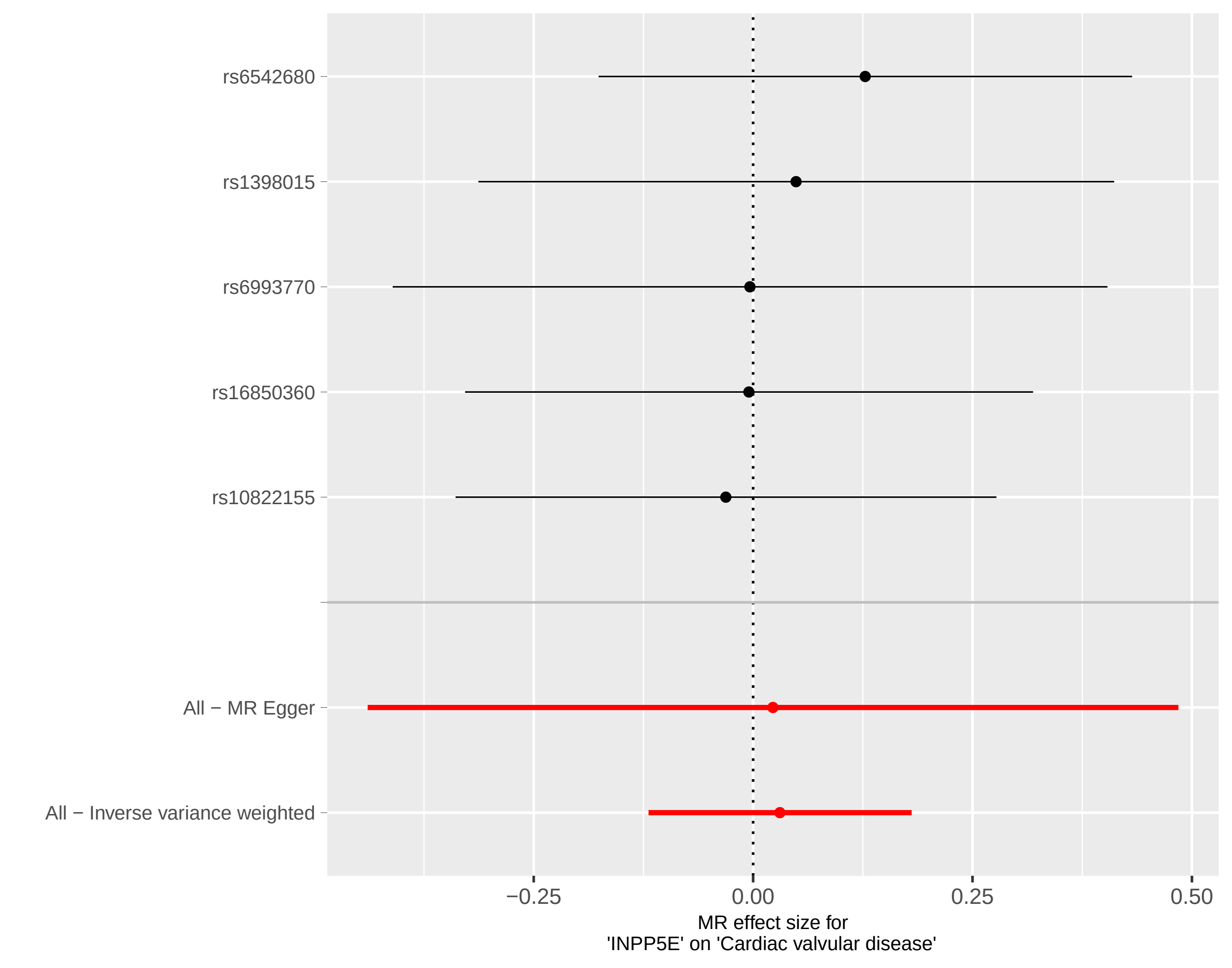
rs6542680
rs1398015
rs6993770
rs16850360
rs10822155
All − MR Egger
All − Inverse variance weighted
−0.25
0.00
0.25
0.50
MR effect size for
'INPP5E' on 'Cardiac valvular disease'

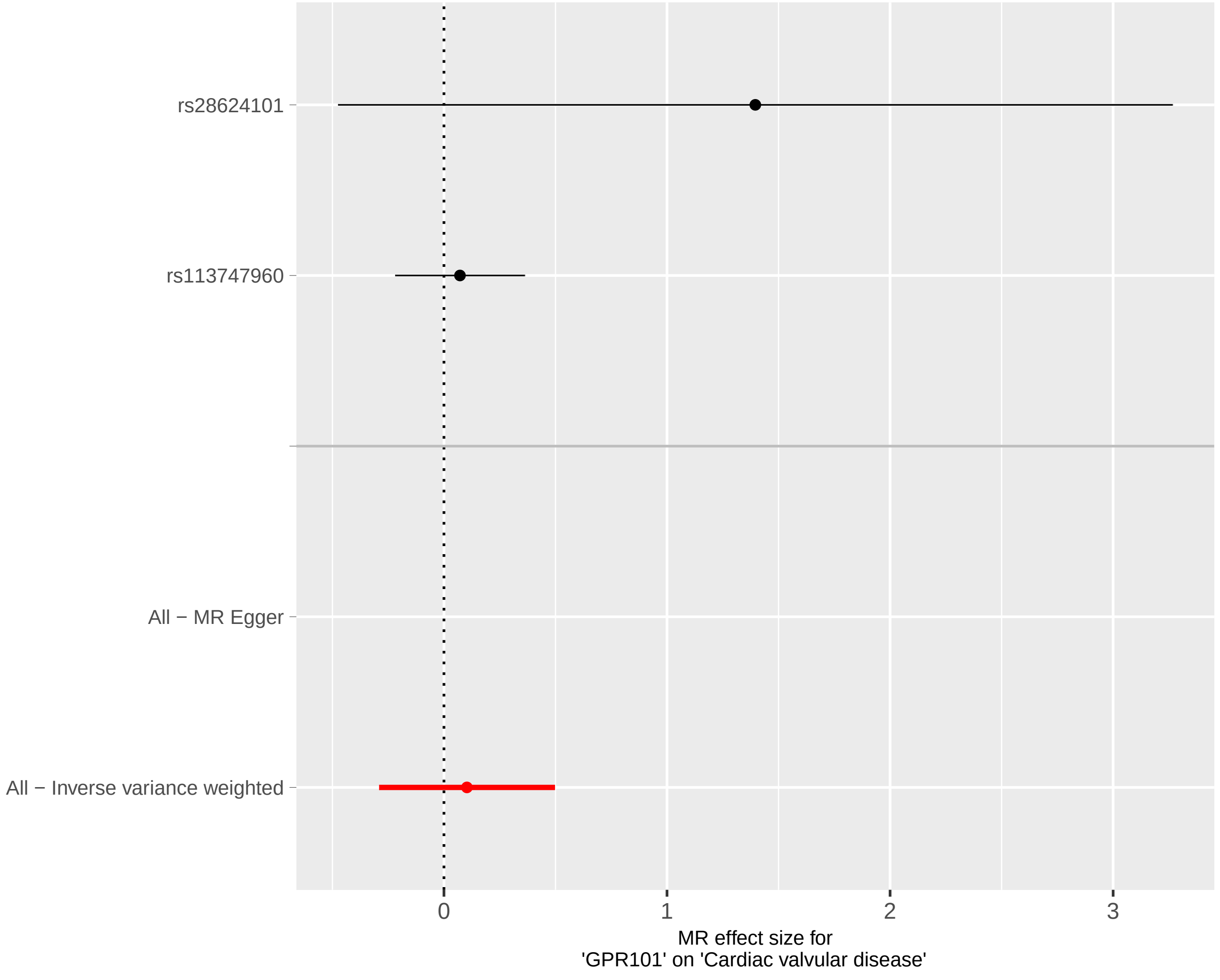
rs28624101
rs113747960
All − MR Egger
All − Inverse variance weighted
0
1
2
3
MR effect size for
'GPR101' on 'Cardiac valvular disease'

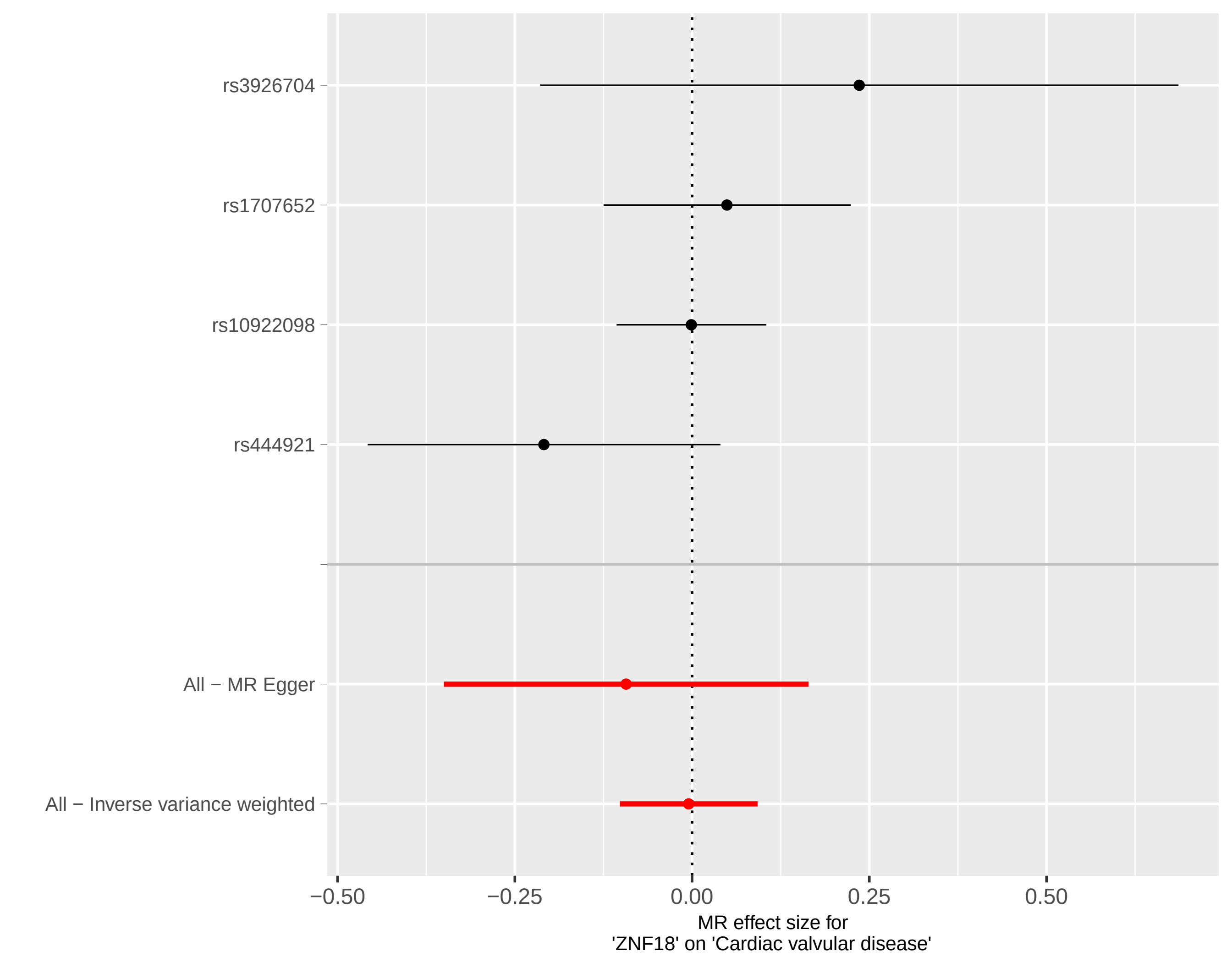
rs3926704
rs1707652
rs10922098
rs444921
All − MR Egger
All − Inverse variance weighted
−0.50
−0.25
0.00
0.25
0.50
MR effect size for
'ZNF18' on 'Cardiac valvular disease'

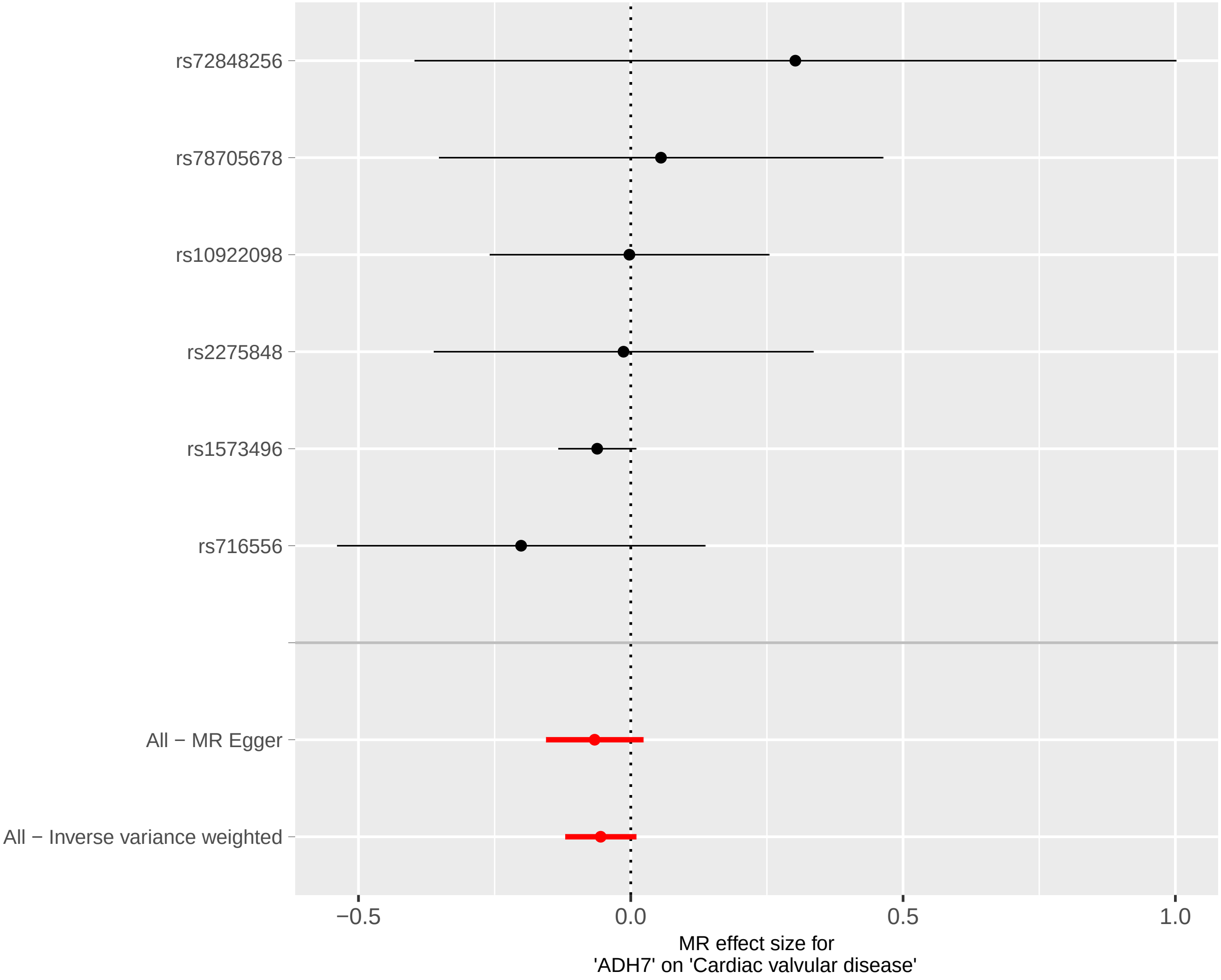

rs72848256
rs78705678
rs10922098
rs2275848
rs1573496
rs716556
All − MR Egger
All − Inverse variance weighted
−0.5
0.0
0.5
1.0
MR effect size for
'ADH7' on 'Cardiac valvular disease'

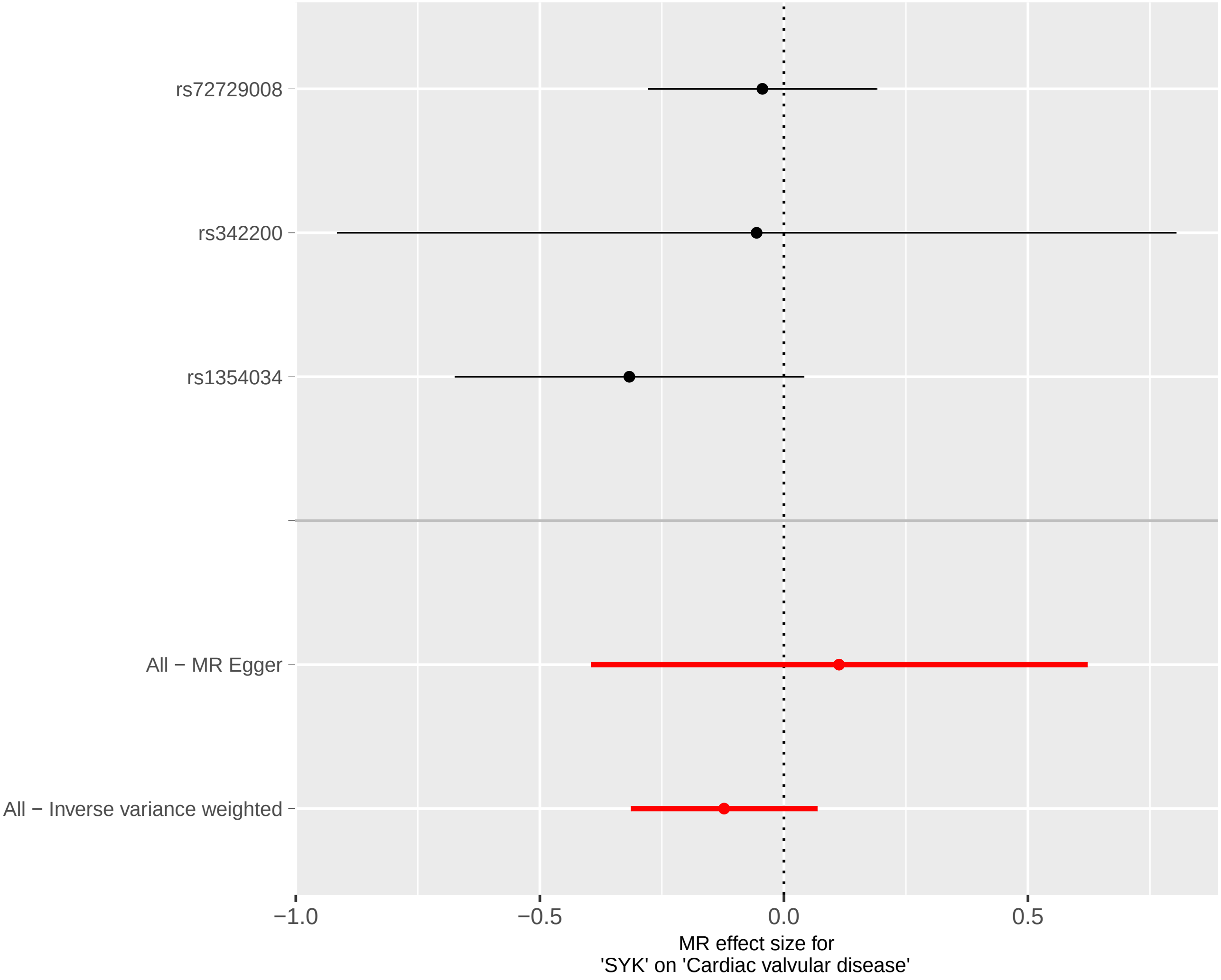
rs72729008
rs342200
rs1354034
All − MR Egger
All − Inverse variance weighted
−1.0
−0.5
0.0
0.5
MR effect size for
'SYK' on 'Cardiac valvular disease'

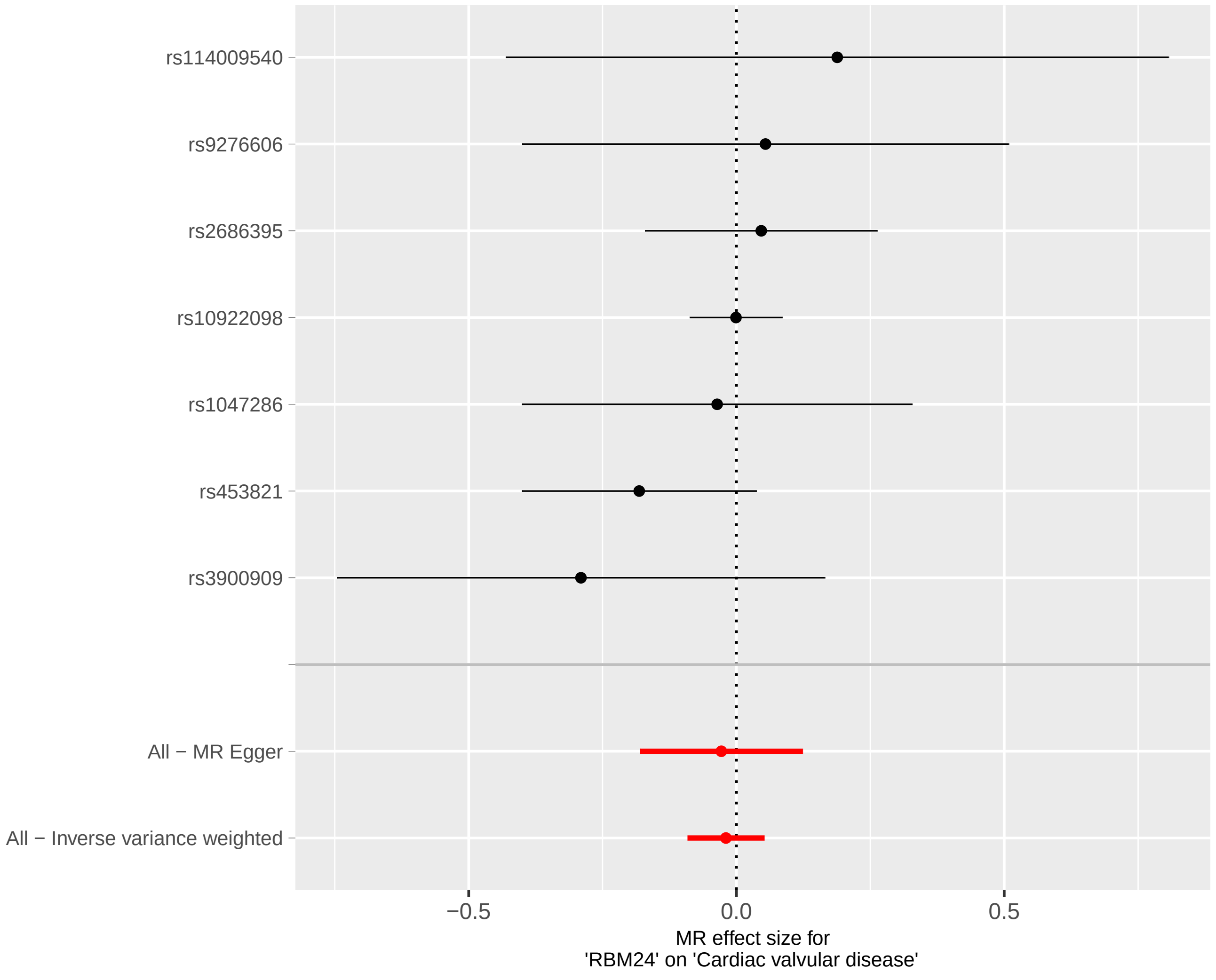
rs114009540
rs9276606
rs2686395
rs10922098
rs1047286
rs453821
rs3900909
All − MR Egger
All − Inverse variance weighted
−0.5
0.0
0.5
MR effect size for
'RBM24' on 'Cardiac valvular disease'

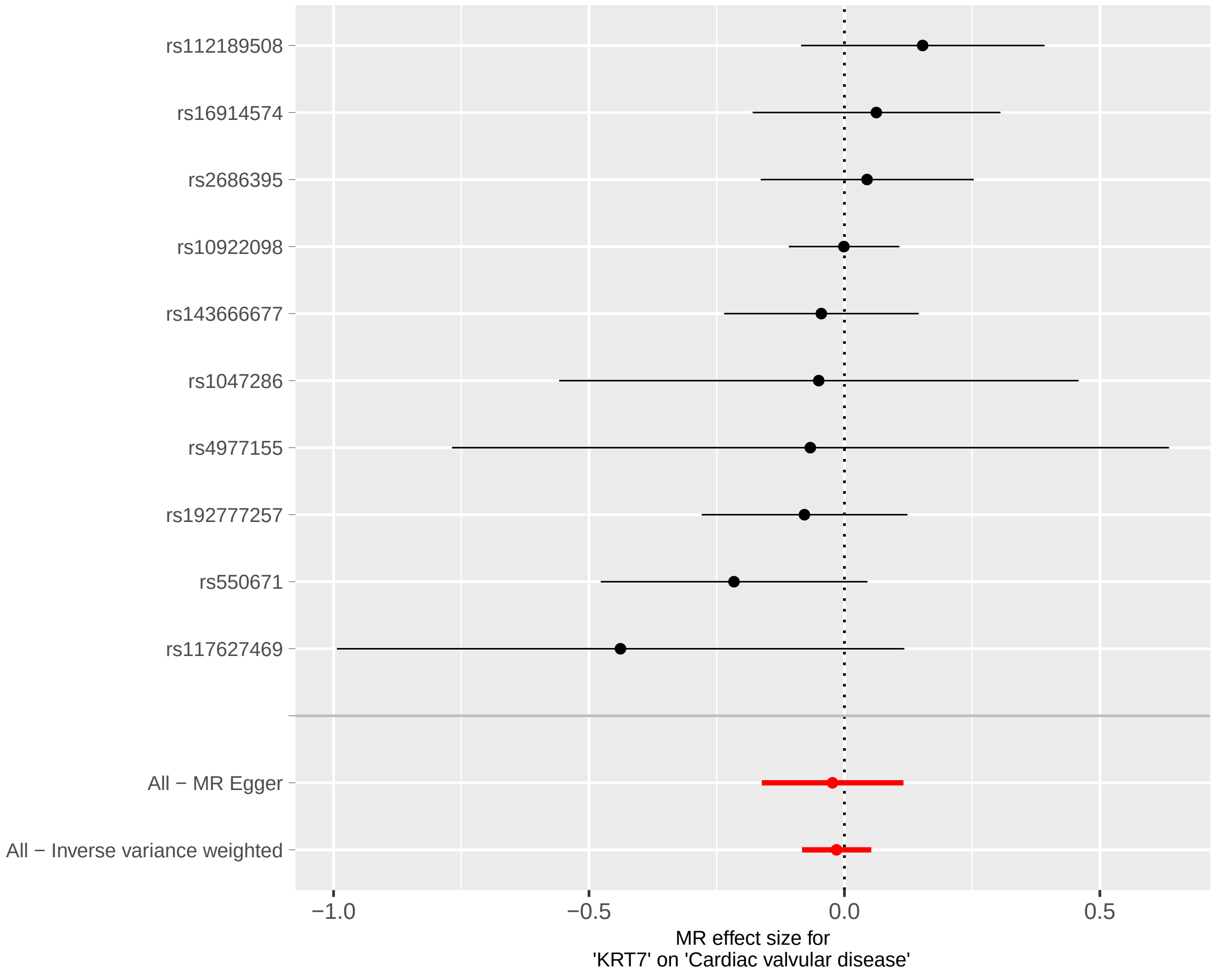
rs112189508
rs16914574
rs2686395
rs10922098
rs143666677
rs1047286
rs4977155
rs192777257
rs550671
rs117627469
All − MR Egger
All − Inverse variance weighted
−1.0
−0.5
0.0
0.5
MR effect size for
'KRT7' on 'Cardiac valvular disease'

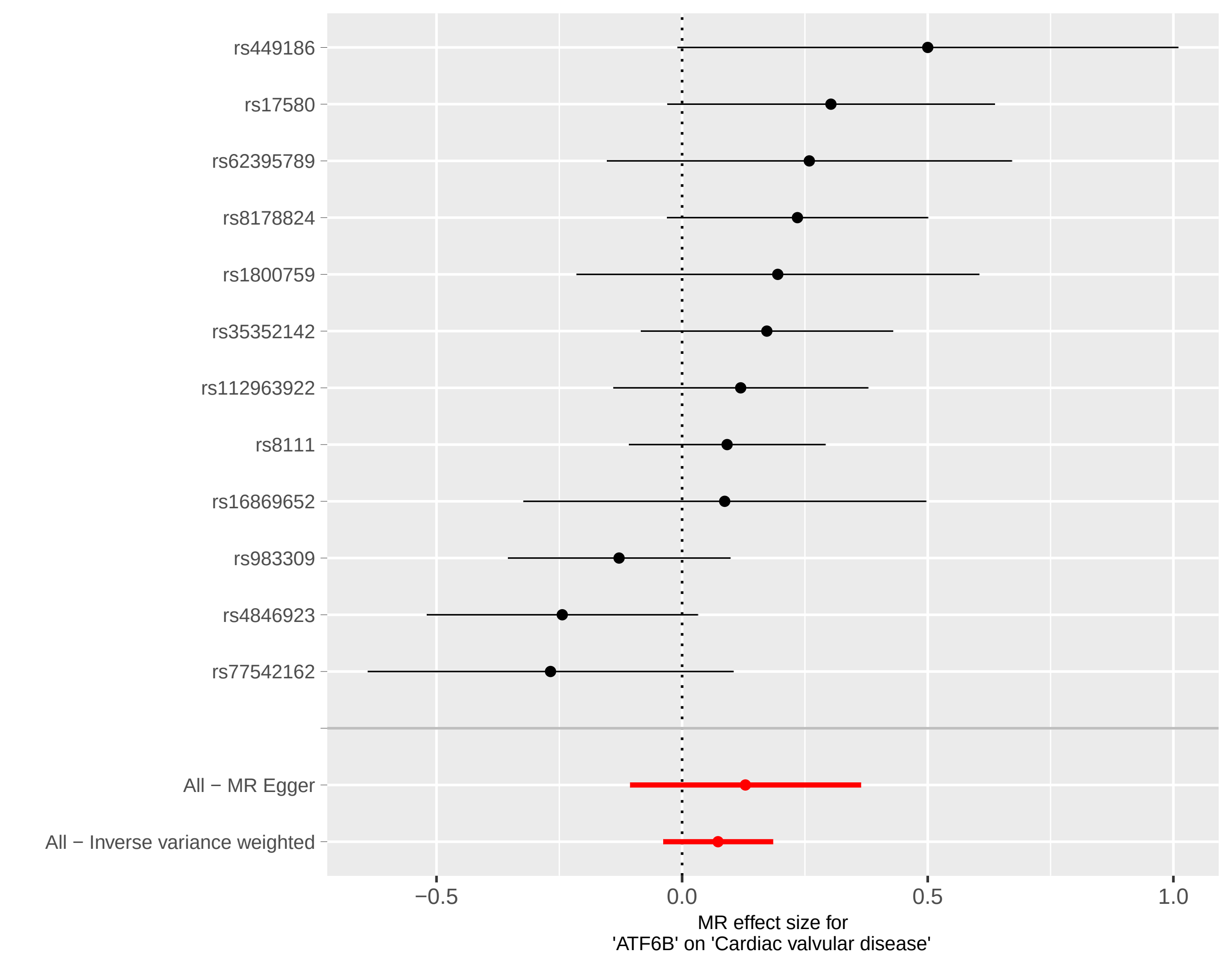
rs449186
rs17580
rs62395789
rs8178824
rs1800759
rs35352142
rs112963922
rs8111
rs16869652
rs983309
rs4846923
rs77542162
All − MR Egger
All − Inverse variance weighted
−0.5
0.0
0.5
1.0
MR effect size for
'ATF6B' on 'Cardiac valvular disease'

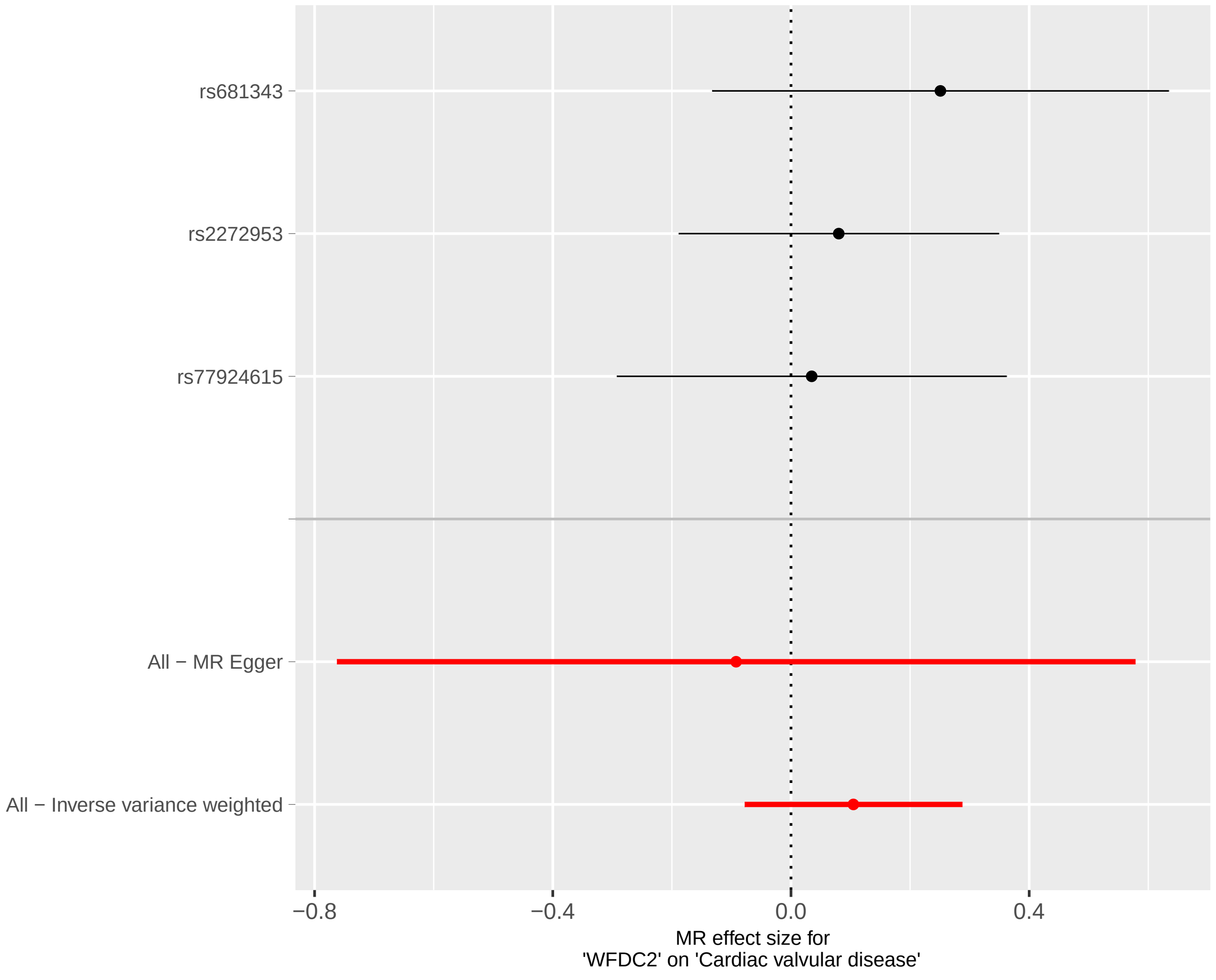
rs681343
rs2272953
rs77924615
All − MR Egger
All − Inverse variance weighted
−0.8
−0.4
0.0
0.4
MR effect size for
'WFDC2' on 'Cardiac valvular disease'

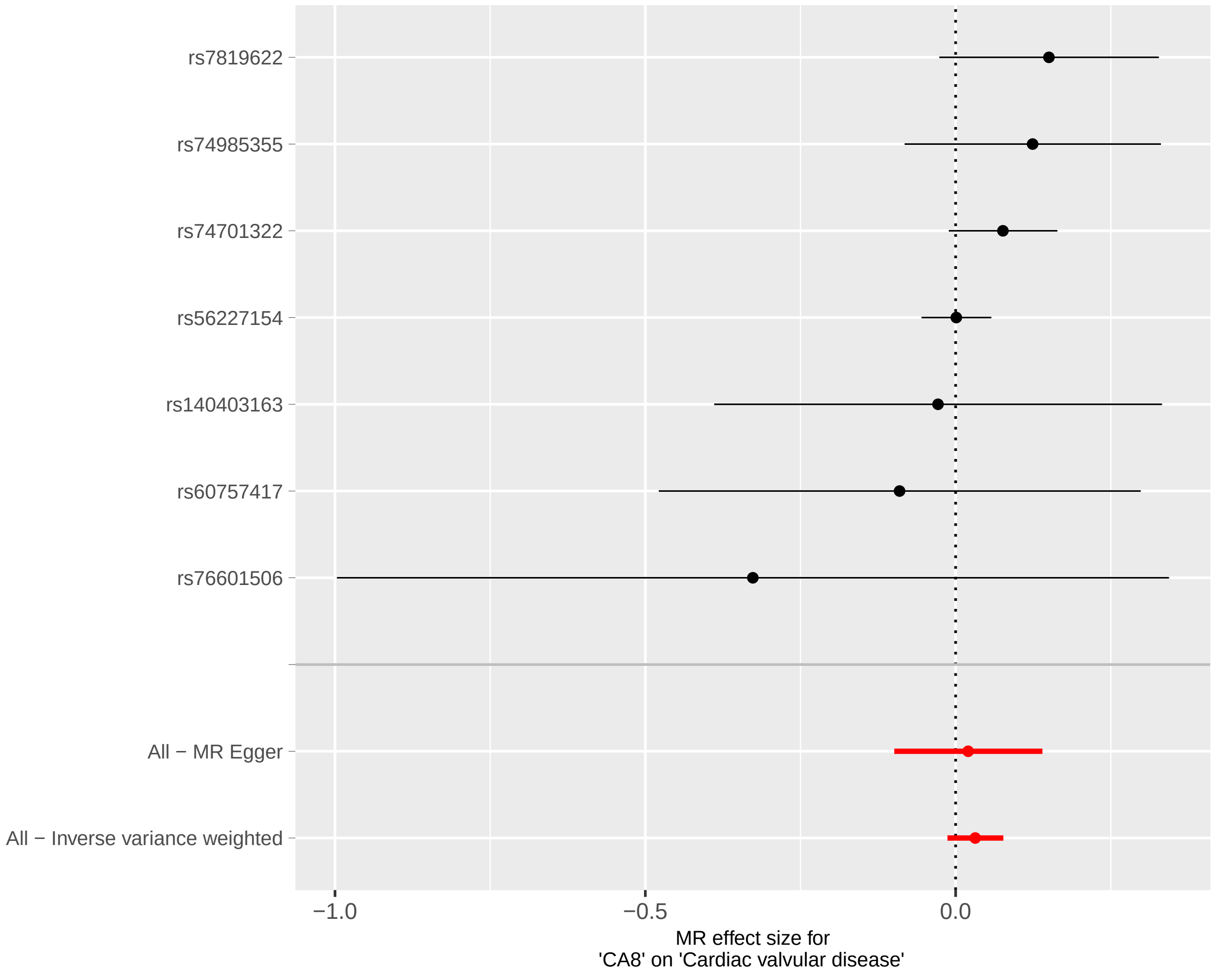
rs7819622
rs74985355
rs74701322
rs56227154
rs140403163
rs60757417
rs76601506
All − MR Egger
All − Inverse variance weighted
−1.0
−0.5
0.0
MR effect size for
'CA8' on 'Cardiac valvular disease'

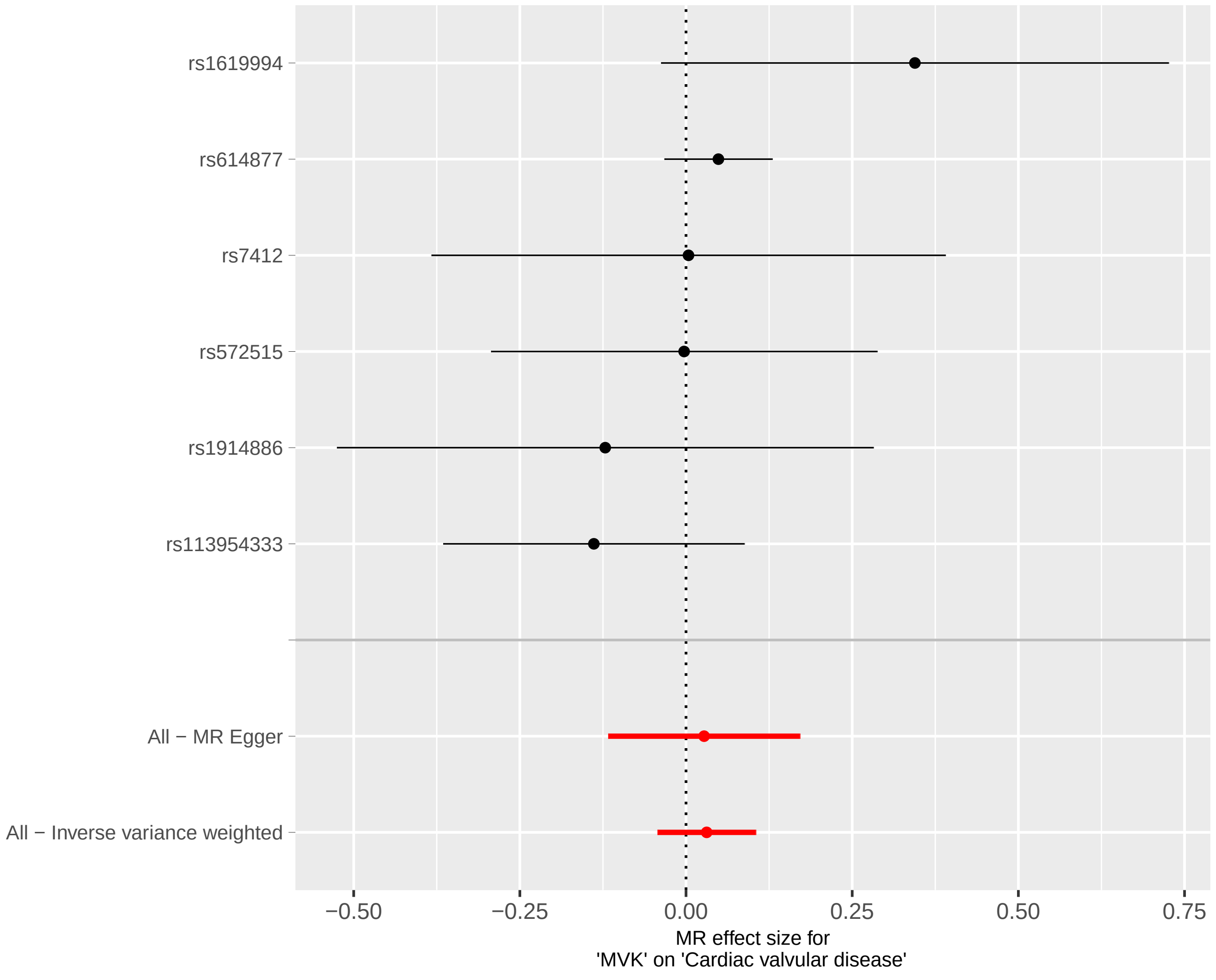
rs1619994
rs614877
rs7412
rs572515
rs1914886
rs113954333
All − MR Egger
All − Inverse variance weighted
−0.50
−0.25
0.00
0.25
0.50
0.75
MR effect size for
'MVK' on 'Cardiac valvular disease'

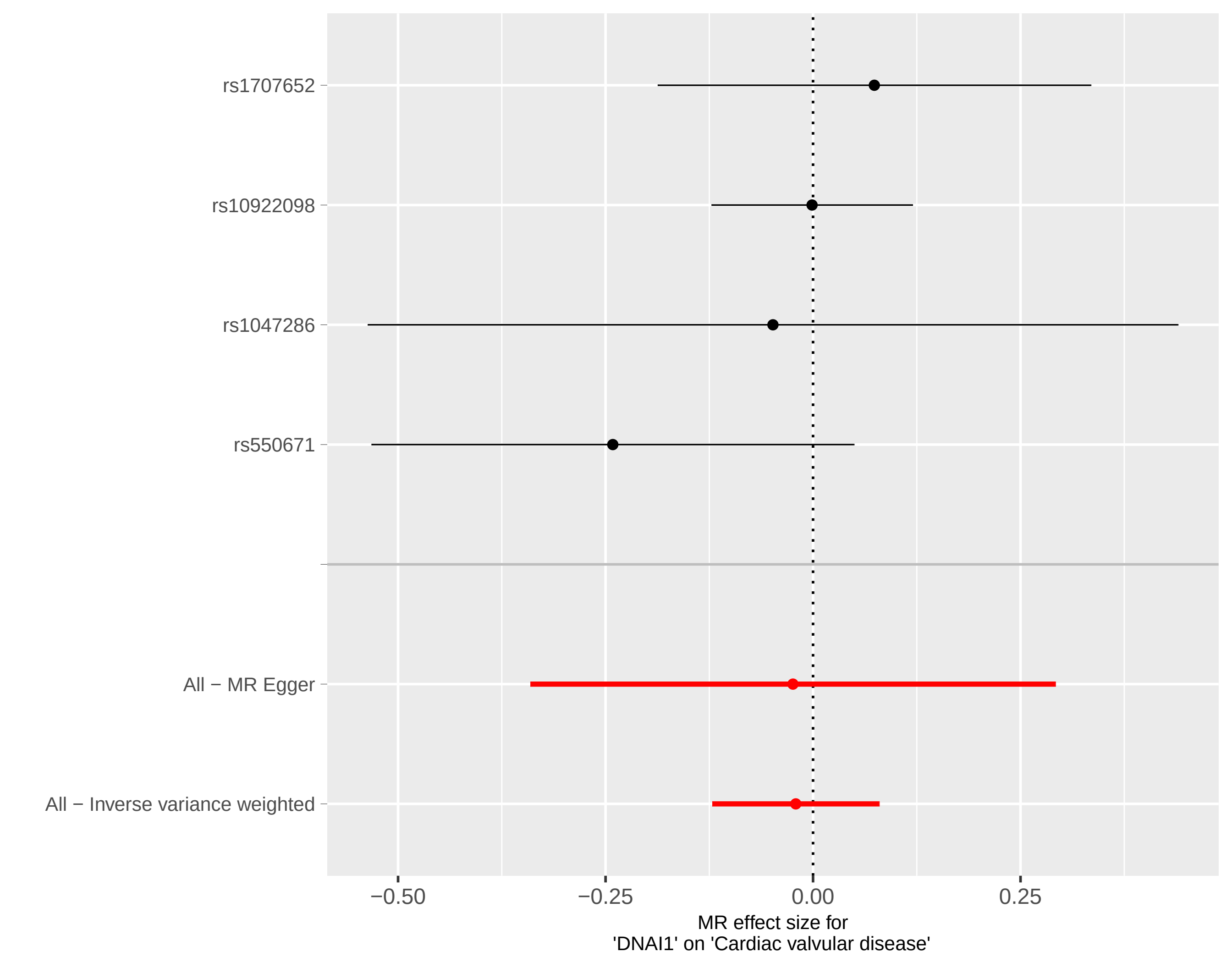

rs1707652
rs10922098
rs1047286
rs550671
All − MR Egger
All − Inverse variance weighted
−0.50
−0.25
0.00
0.25
MR effect size for
'DNAI1' on 'Cardiac valvular disease'

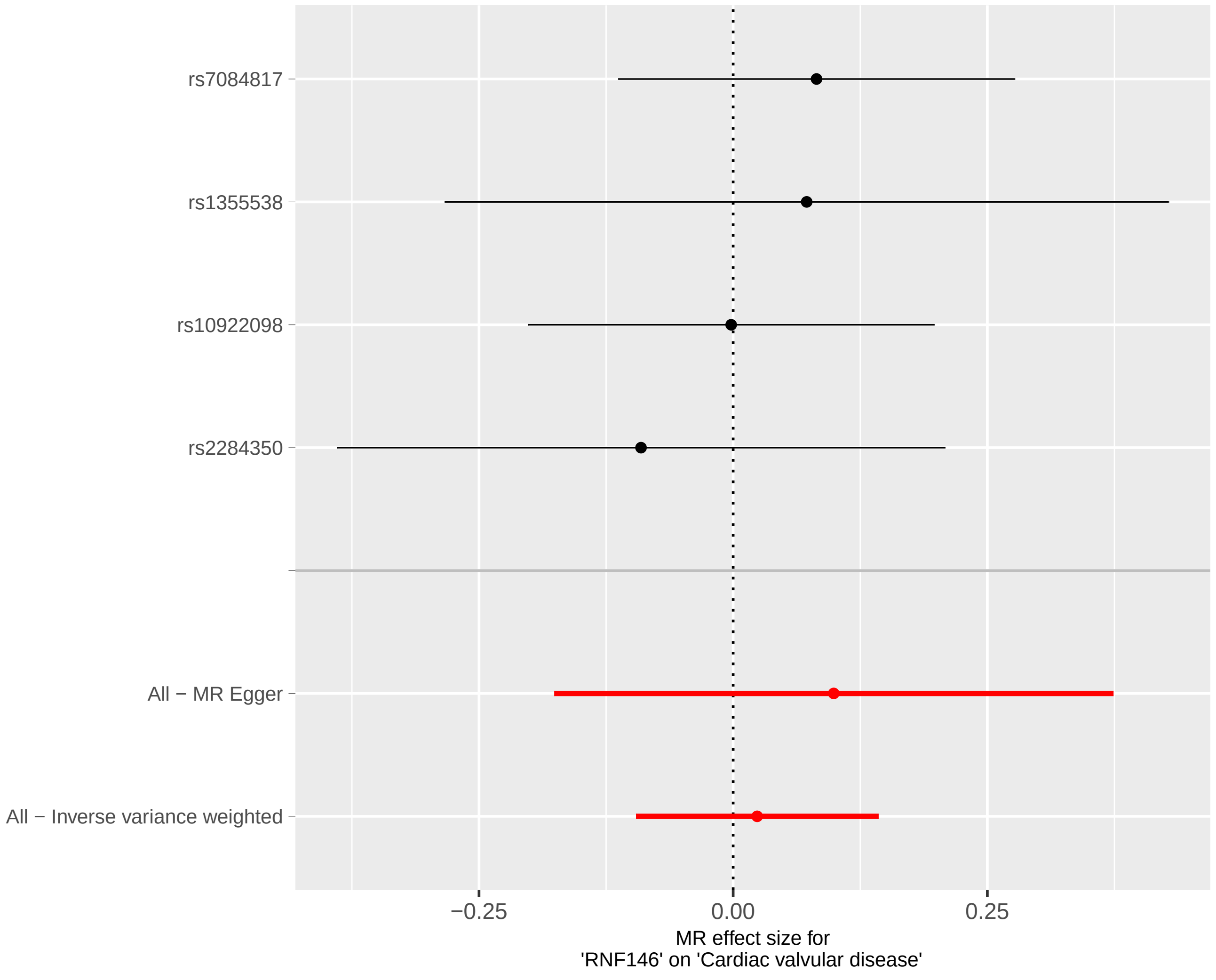

rs7084817
rs1355538
rs10922098
rs2284350
All − MR Egger
All − Inverse variance weighted
−0.25
0.00
0.25
MR effect size for
'RNF146' on 'Cardiac valvular disease'

Insufficient number of SNPs

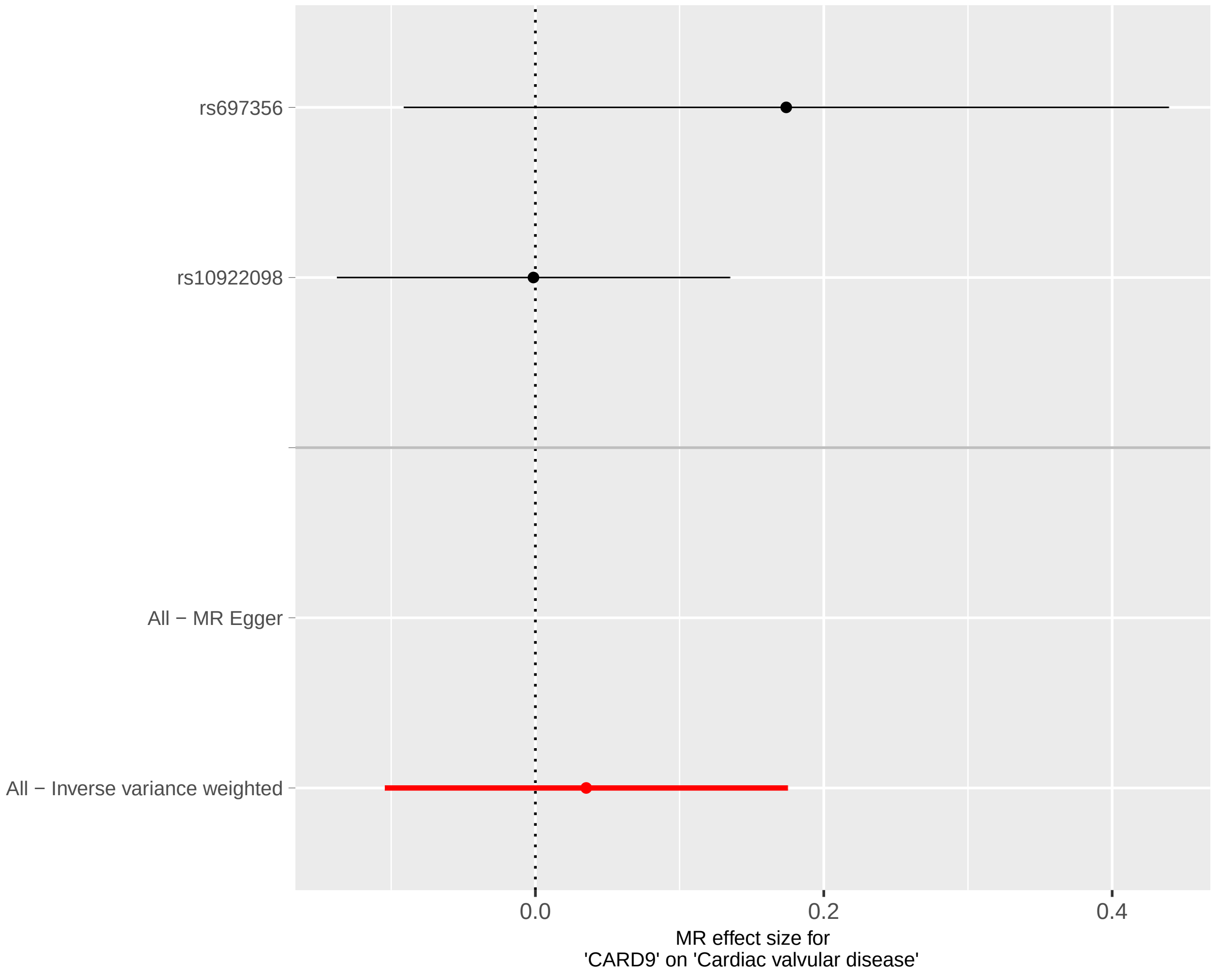

rs697356
rs10922098
All − MR Egger
All − Inverse variance weighted
0.0
0.2
0.4
MR effect size for
'CARD9' on 'Cardiac valvular disease'

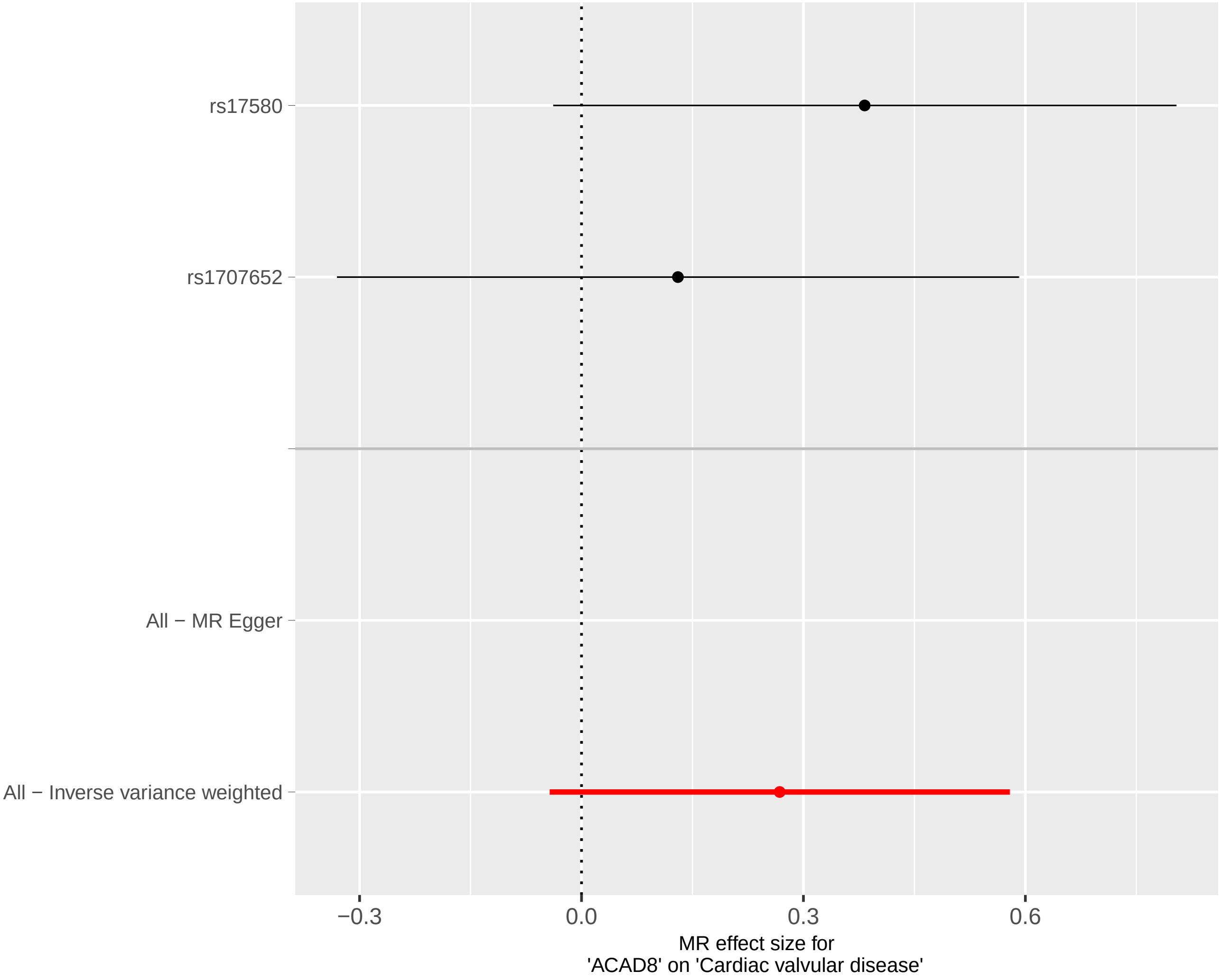

rs17580
rs1707652
All − MR Egger
All − Inverse variance weighted
−0.3
0.0
0.3
0.6
MR effect size for
'ACAD8' on 'Cardiac valvular disease'

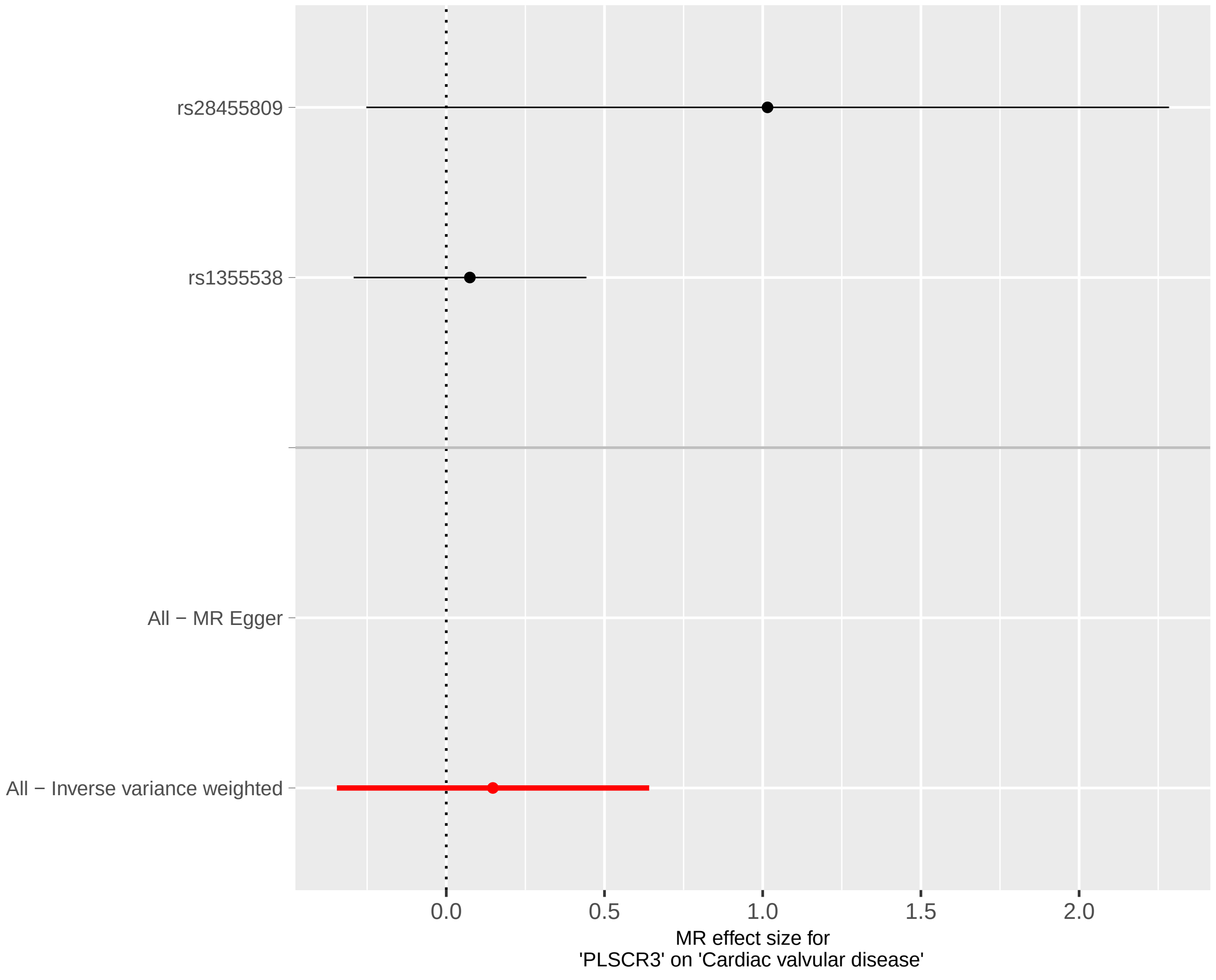
rs28455809
rs1355538
All − MR Egger
All − Inverse variance weighted
0.0
0.5
1.0
1.5
2.0
MR effect size for
'PLSCR3' on 'Cardiac valvular disease'

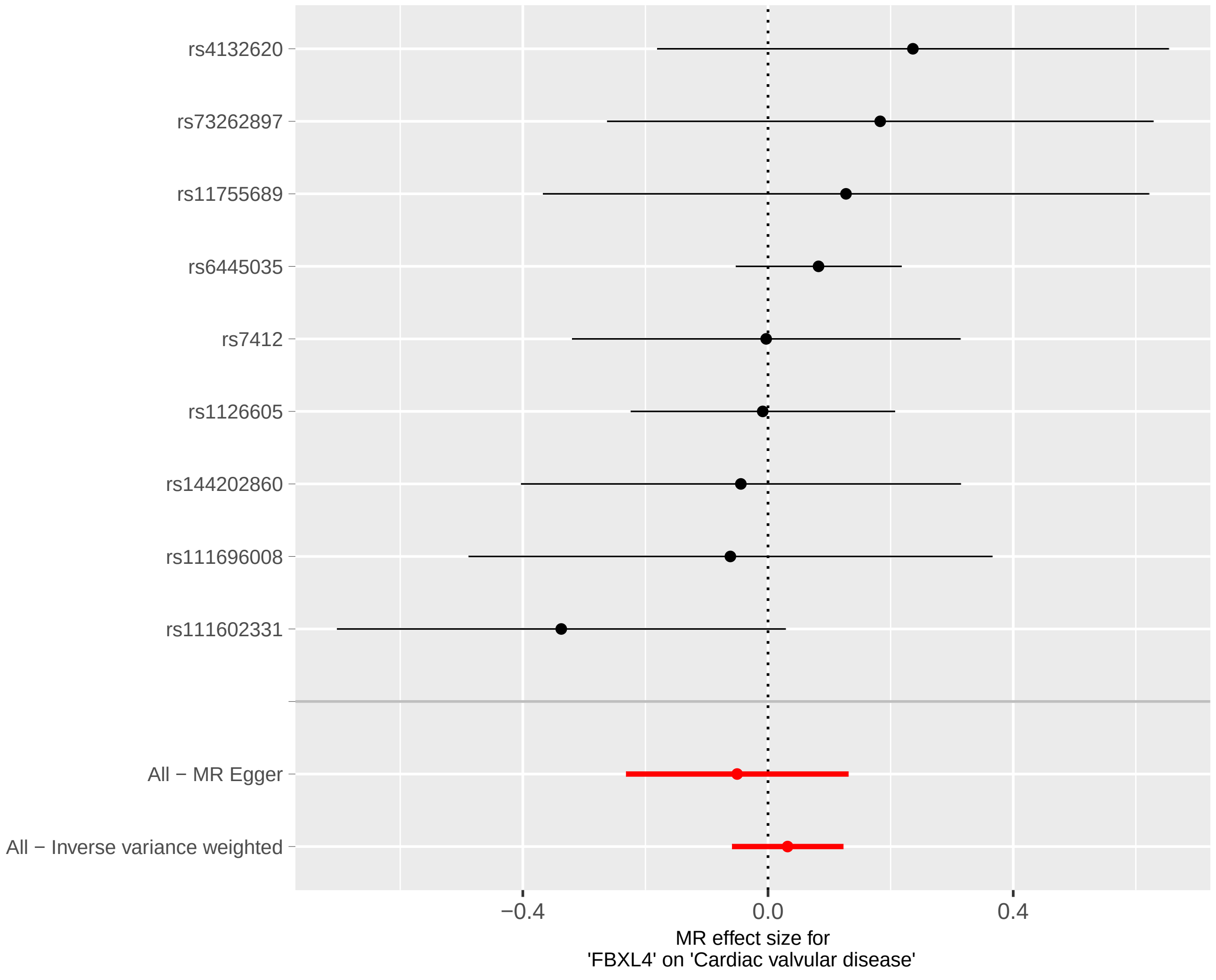
rs4132620
rs73262897
rs11755689
rs6445035
rs7412
rs1126605
rs144202860
rs111696008
rs111602331
All − MR Egger
All − Inverse variance weighted
−0.4
0.0
0.4
MR effect size for
'FBXL4' on 'Cardiac valvular disease'

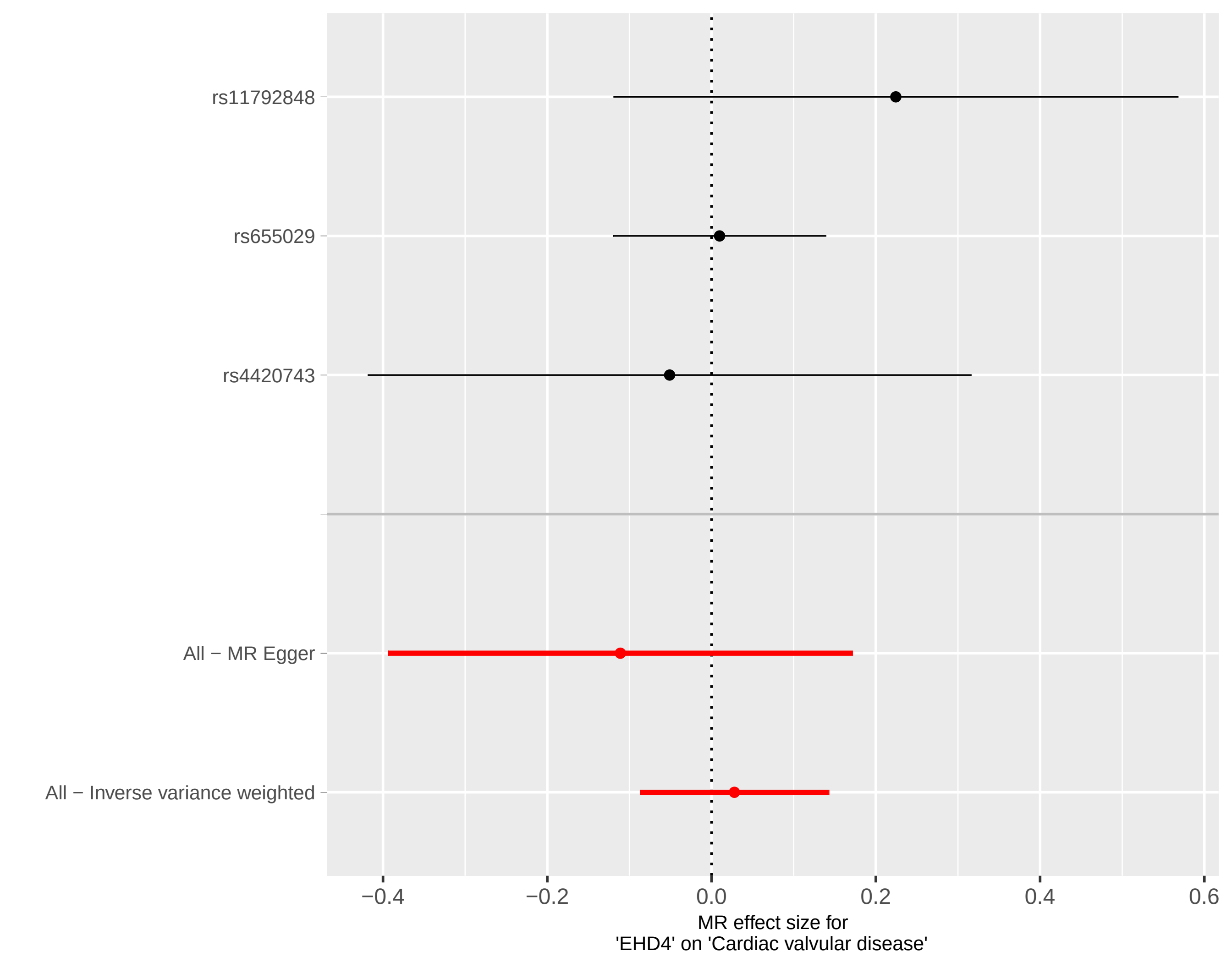

rs11792848
rs655029
rs4420743
All − MR Egger
All − Inverse variance weighted
−0.4
−0.2
0.0
0.2
0.4
0.6
MR effect size for
'EHD4' on 'Cardiac valvular disease'

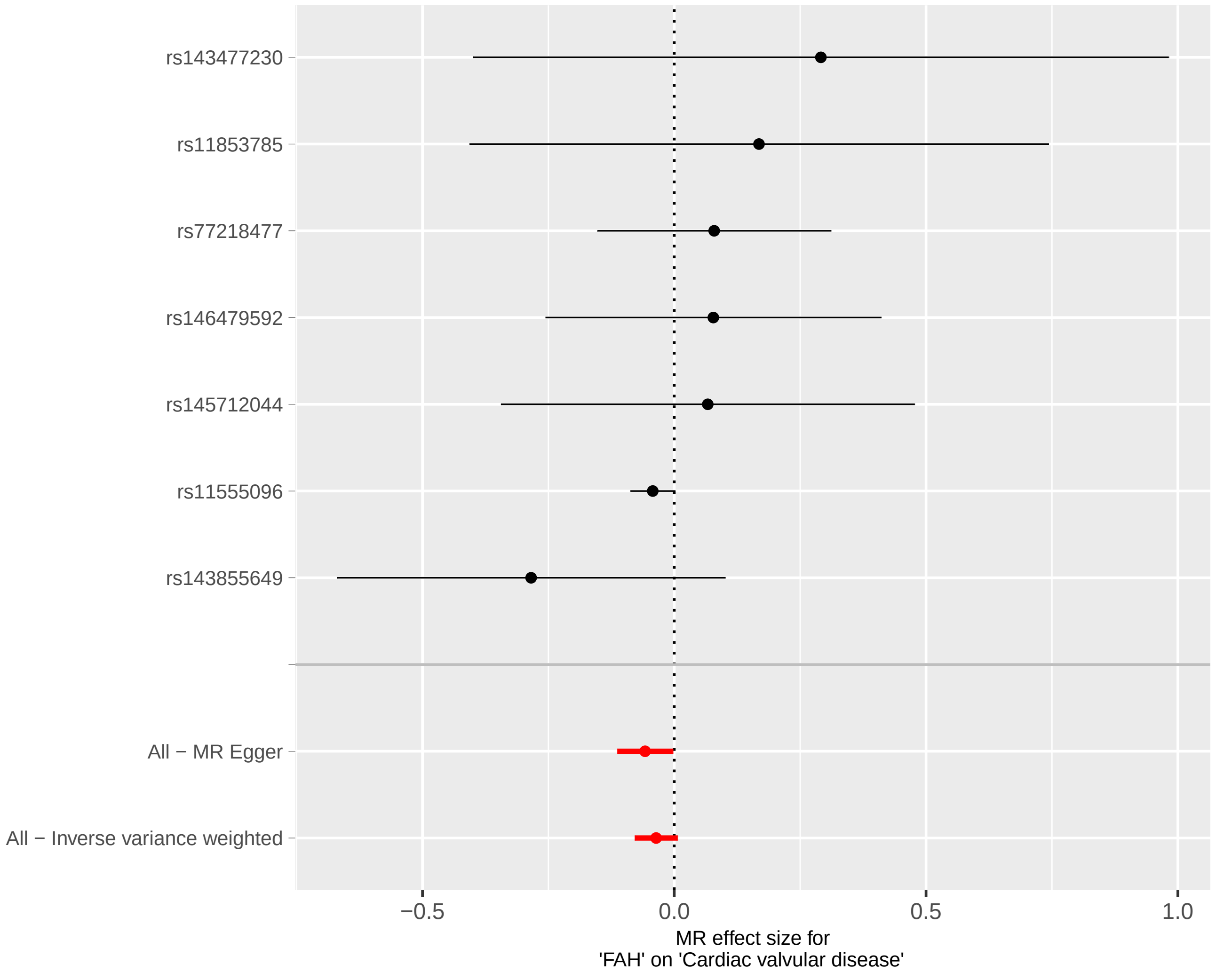

rs143477230
rs11853785
rs77218477
rs146479592
rs145712044
rs11555096
rs143855649
All − MR Egger
All − Inverse variance weighted
−0.5
0.0
0.5
1.0
MR effect size for
'FAH' on 'Cardiac valvular disease'

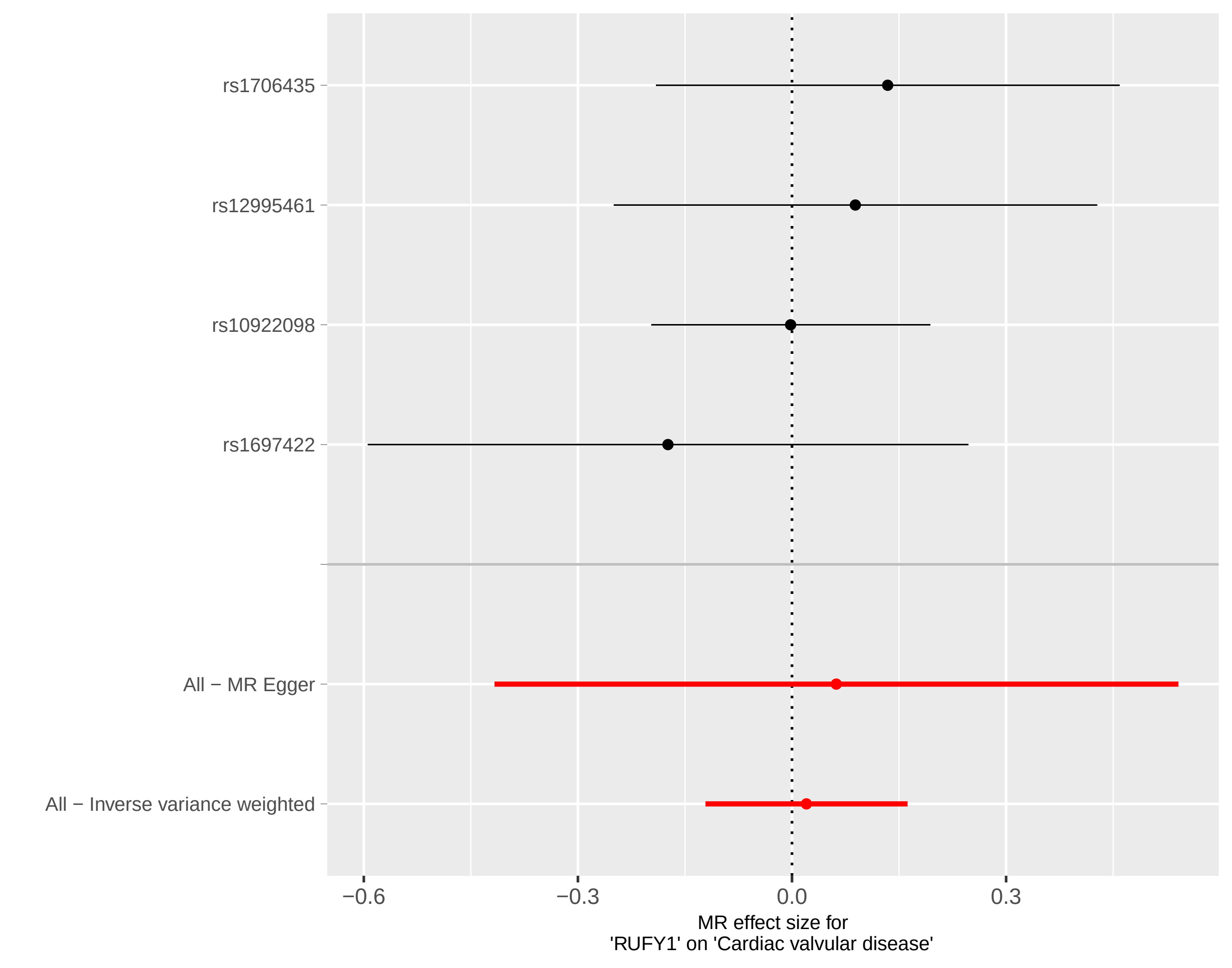
rs1706435
rs12995461
rs10922098
rs1697422
All − MR Egger
All − Inverse variance weighted
−0.6
−0.3
0.0
0.3
MR effect size for
'RUFY1' on 'Cardiac valvular disease'

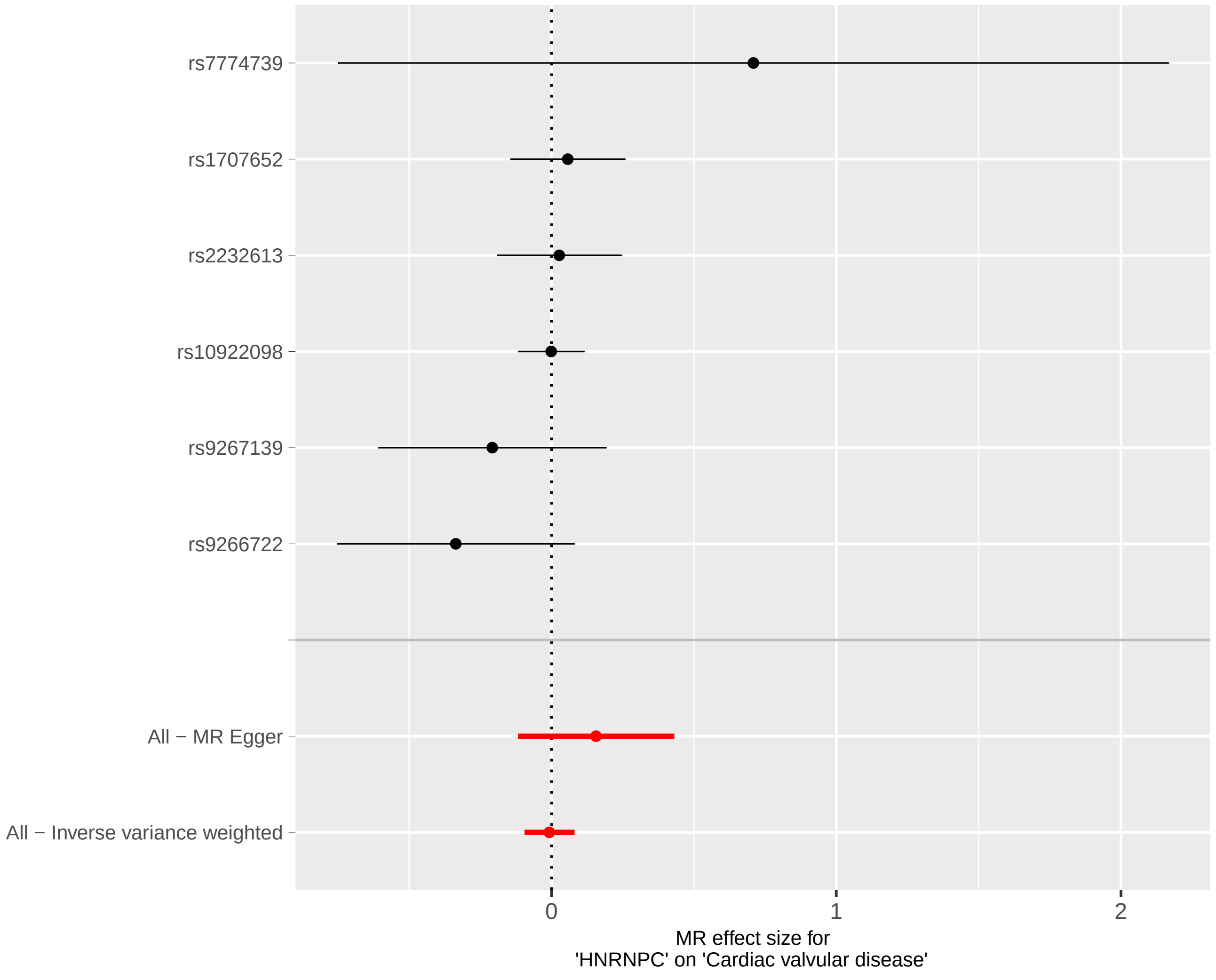
rs7774739
rs1707652
rs2232613
rs10922098
rs9267139
rs9266722
All − MR Egger
All − Inverse variance weighted
0
1
2
MR effect size for
'HNRNPC' on 'Cardiac valvular disease'

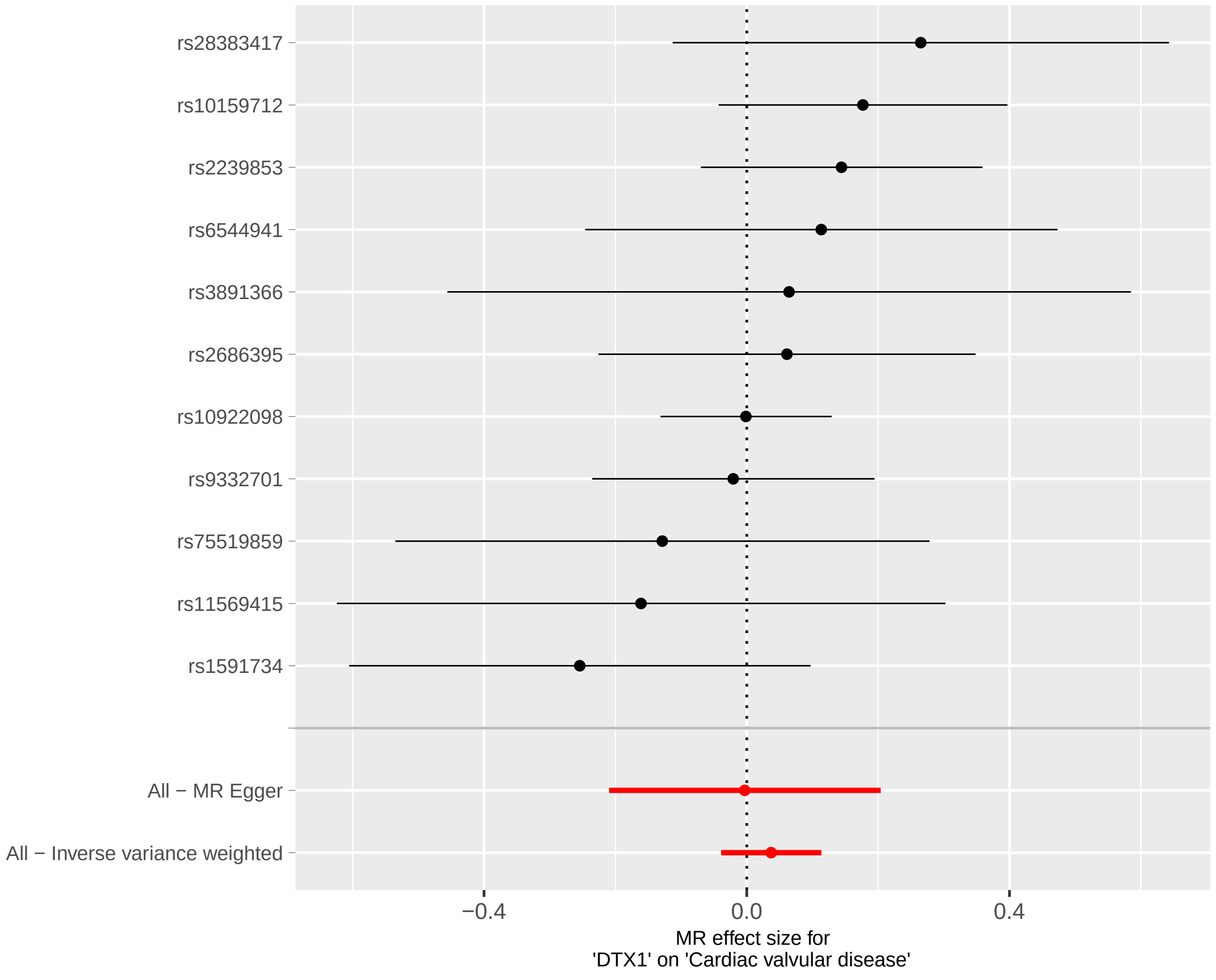

rs28383417
rs10159712
rs2239853
rs6544941
rs3891366
rs2686395
rs10922098
rs9332701
rs75519859
rs11569415
rs1591734
All − MR Egger
All − Inverse variance weighted
−0.4
0.0
0.4
MR effect size for
'DTX1' on 'Cardiac valvular disease'

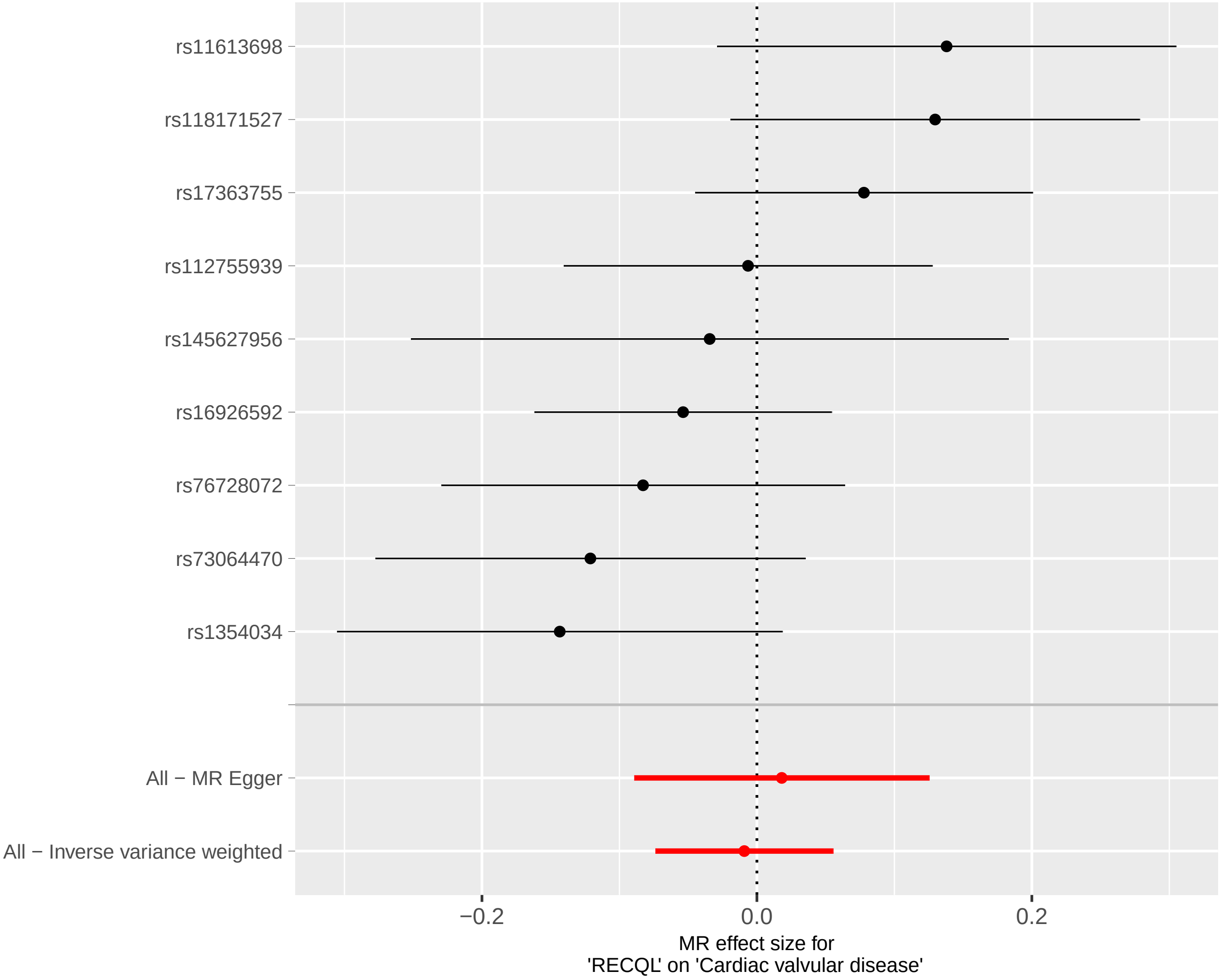
rs11613698
rs118171527
rs17363755
rs112755939
rs145627956
rs16926592
rs76728072
rs73064470
rs1354034
All − MR Egger
All − Inverse variance weighted
−0.2
0.0
0.2
MR effect size for
'RECQL' on 'Cardiac valvular disease'

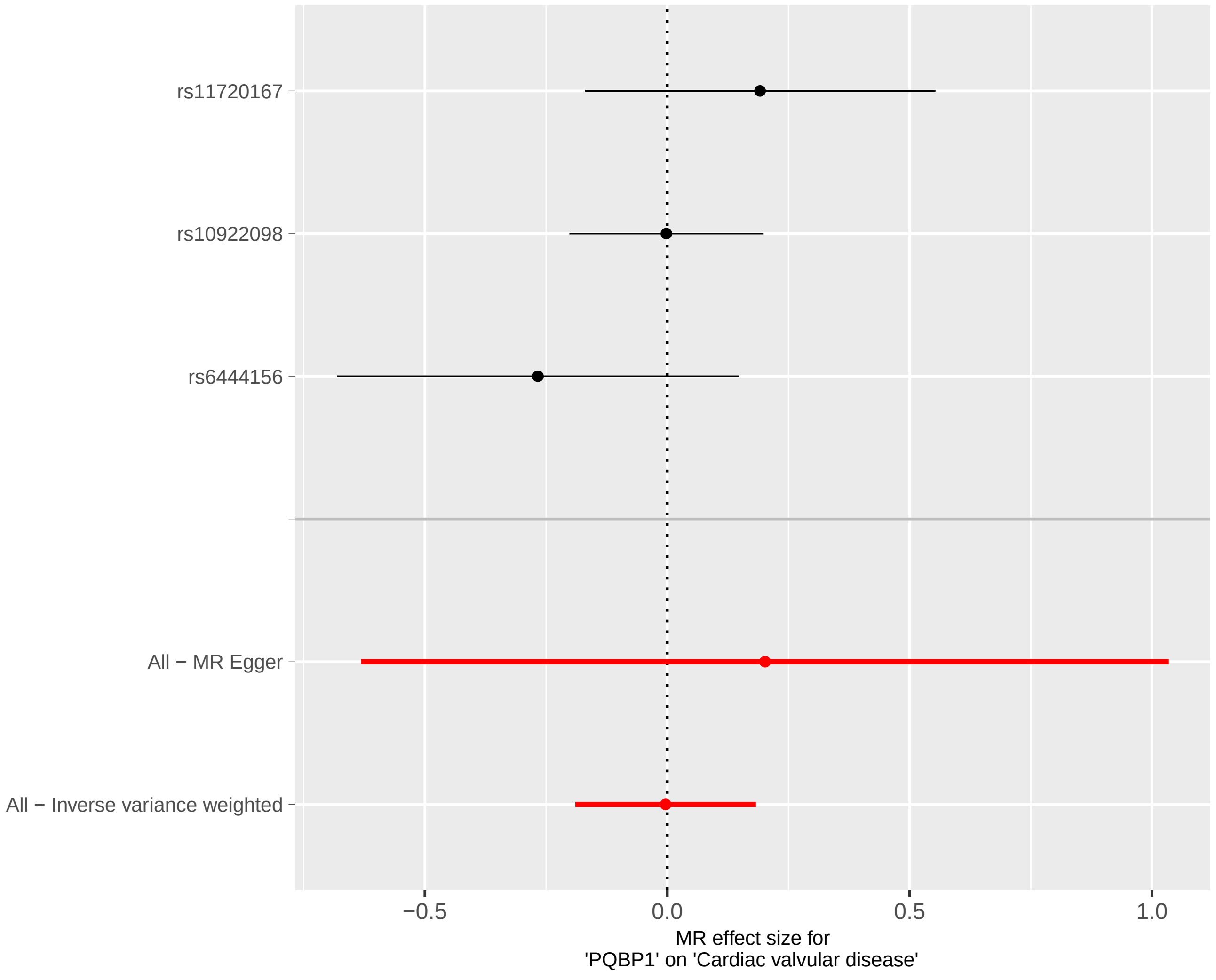

rs11720167
rs10922098
rs6444156
All − MR Egger
All − Inverse variance weighted
−0.5
0.0
0.5
1.0
MR effect size for
'PQBP1' on 'Cardiac valvular disease'

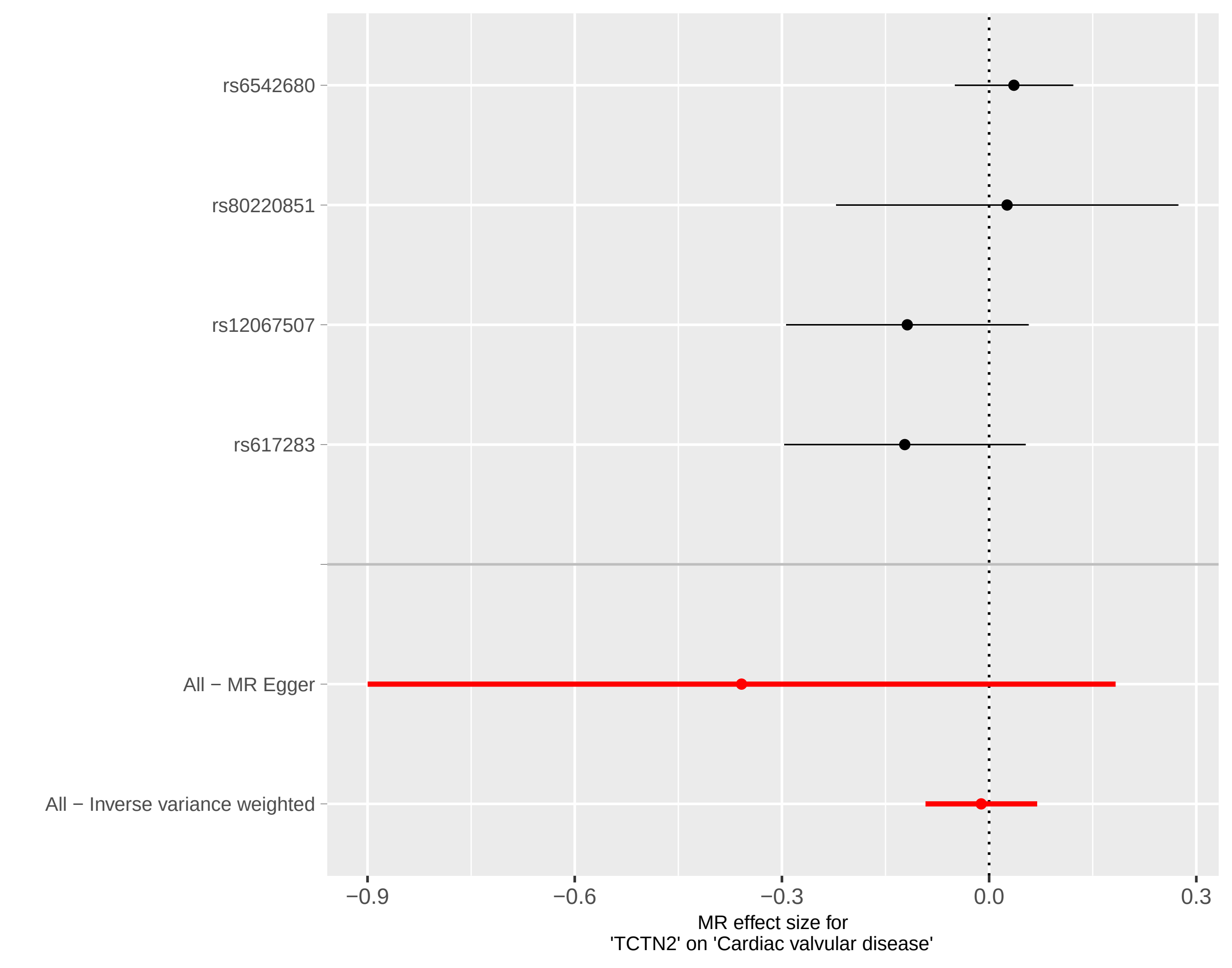

rs6542680
rs80220851
rs12067507
rs617283
All − MR Egger
All − Inverse variance weighted
−0.9
−0.6
−0.3
0.0
0.3
MR effect size for
'TCTN2' on 'Cardiac valvular disease'

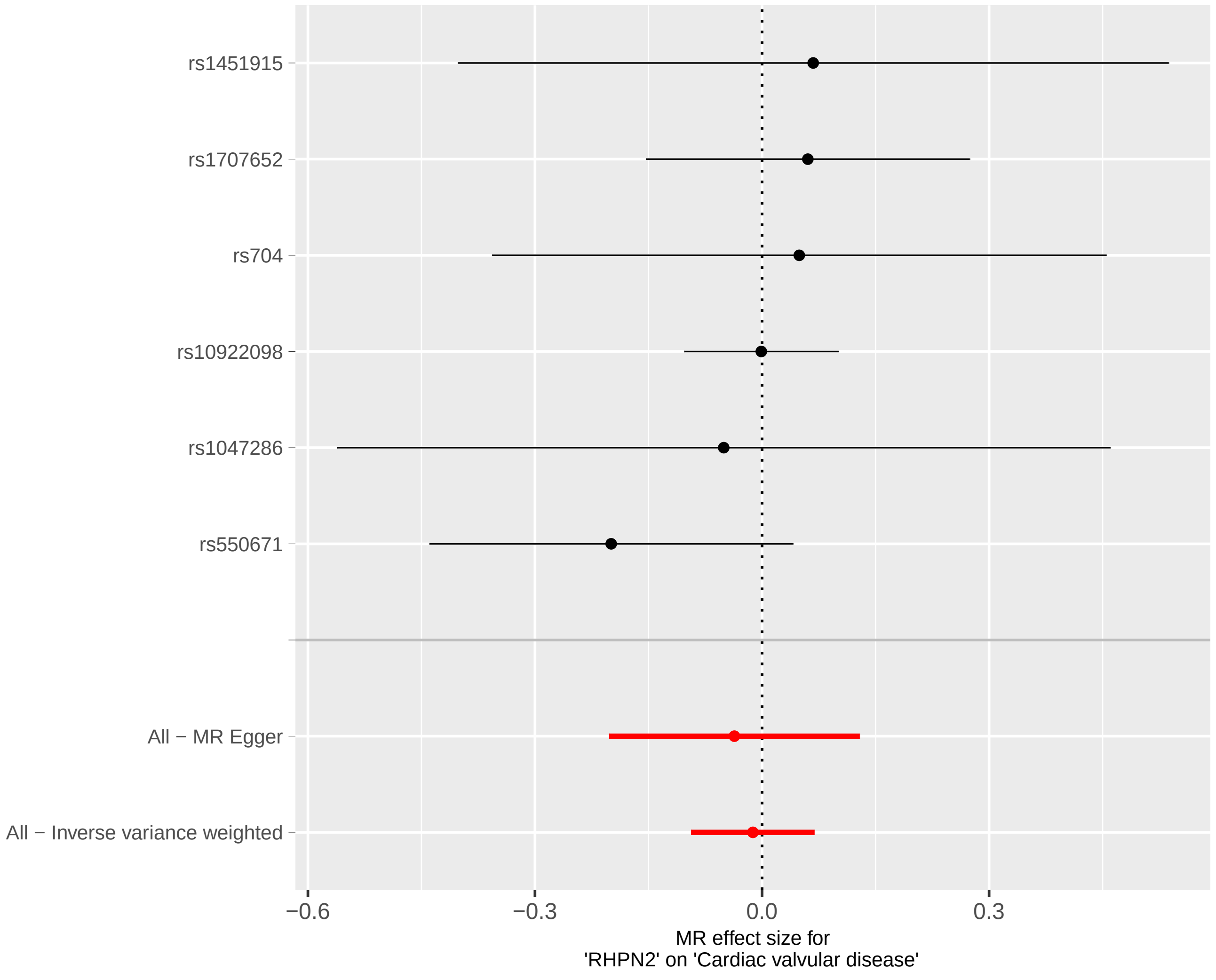

rs1451915
rs1707652
rs704
rs10922098
rs1047286
rs550671
All − MR Egger
All − Inverse variance weighted
−0.6
−0.3
0.0
0.3
MR effect size for
'RHPN2' on 'Cardiac valvular disease'

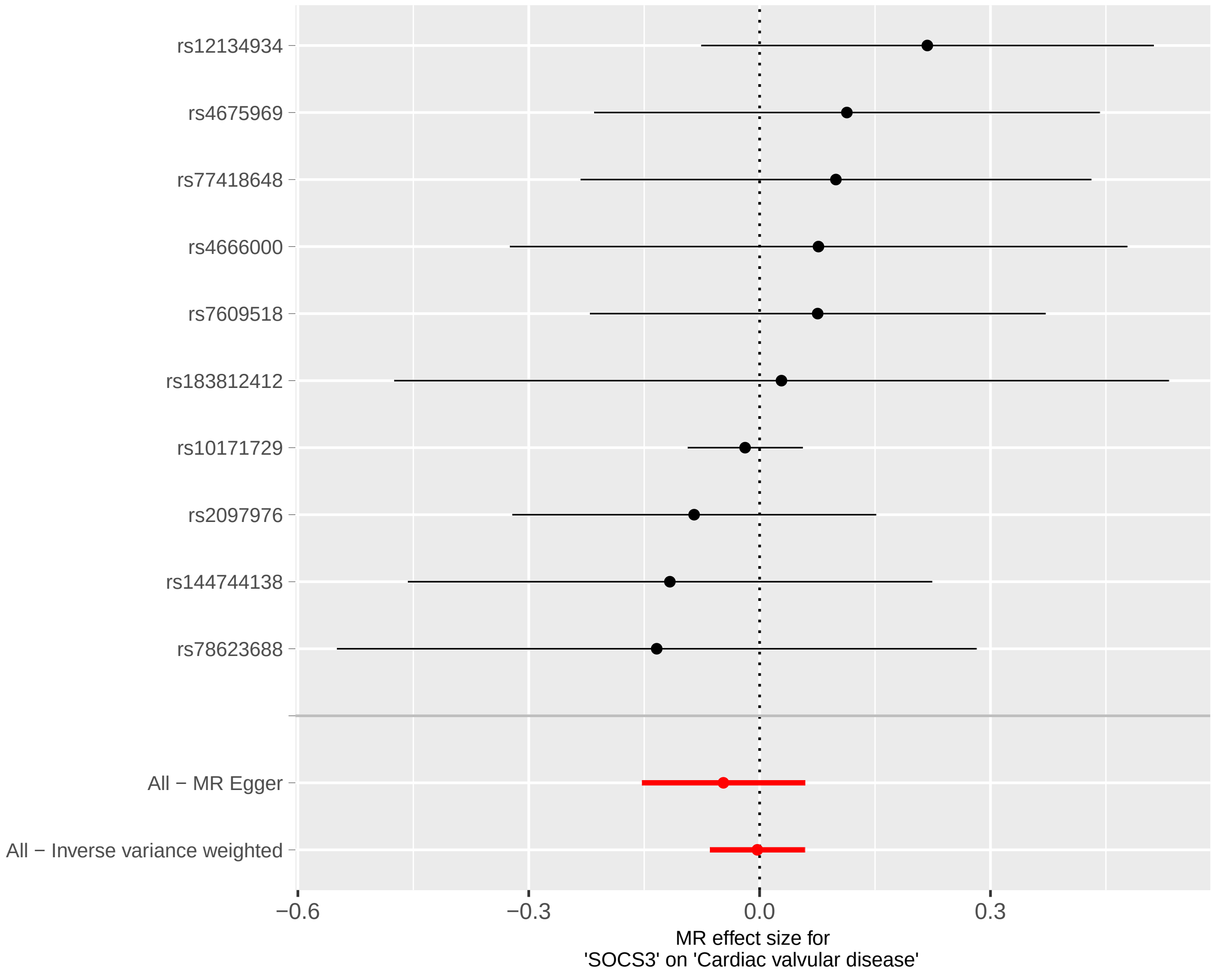

rs12134934
rs4675969
rs77418648
rs4666000
rs7609518
rs183812412
rs10171729
rs2097976
rs144744138
rs78623688
All − MR Egger
All − Inverse variance weighted
−0.6
−0.3
0.0
0.3
MR effect size for
'SOCS3' on 'Cardiac valvular disease'

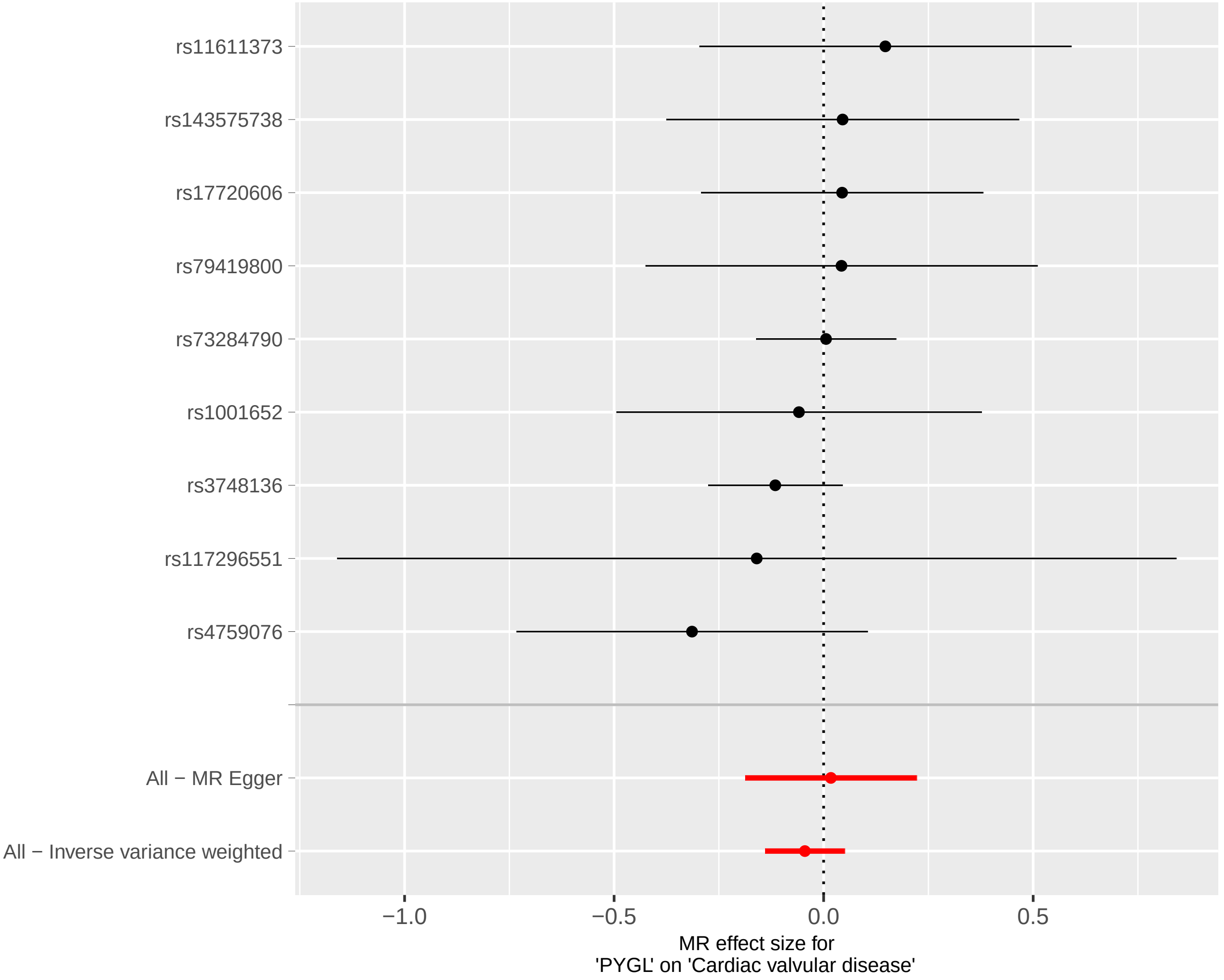
rs11611373
rs143575738
rs17720606
rs79419800
rs73284790
rs1001652
rs3748136
rs117296551
rs4759076
All − MR Egger
All − Inverse variance weighted
−1.0
−0.5
0.0
0.5
MR effect size for
'PYGL' on 'Cardiac valvular disease'

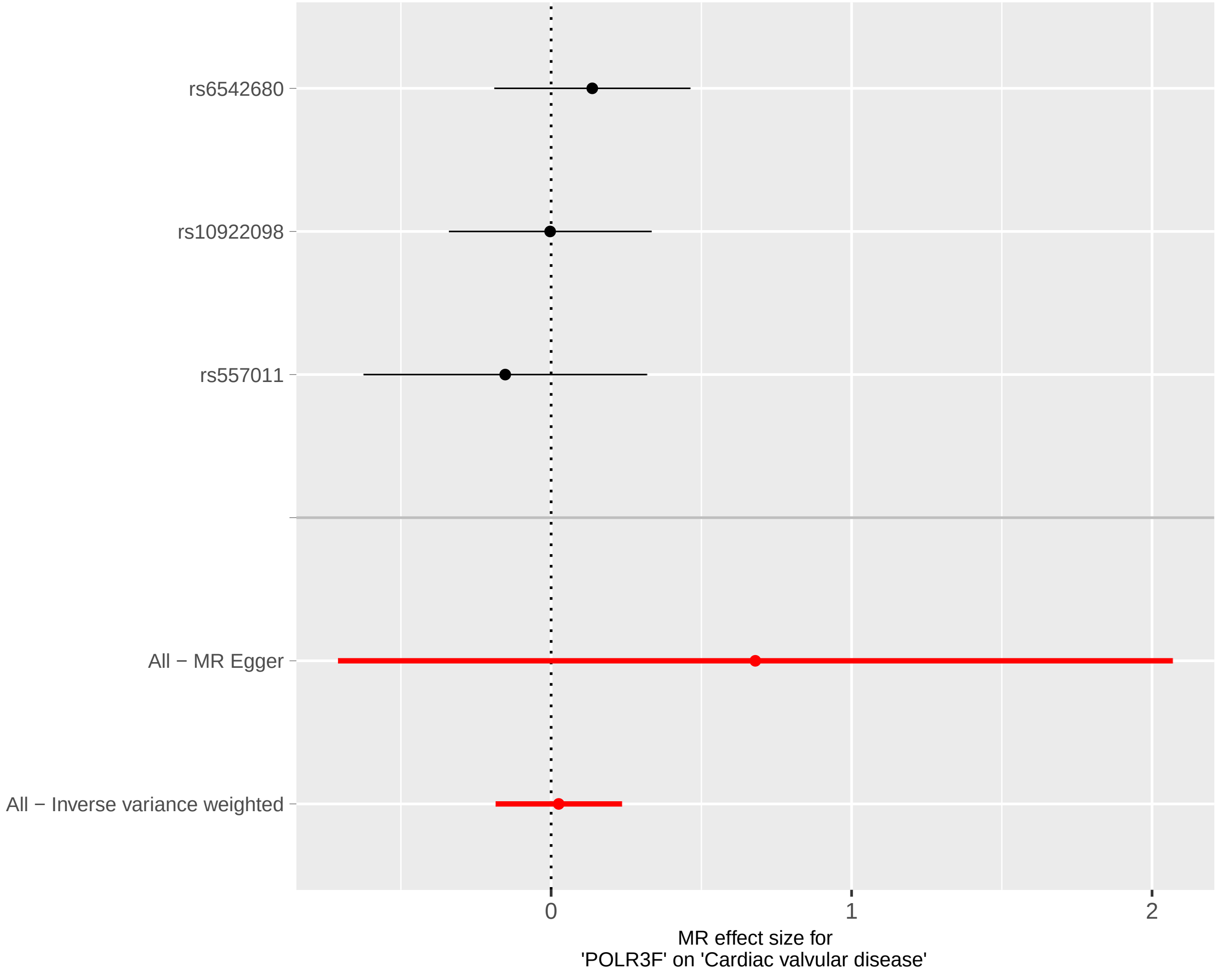
rs6542680
rs10922098
rs557011
All − MR Egger
All − Inverse variance weighted
0
1
2
MR effect size for
'POLR3F' on 'Cardiac valvular disease'

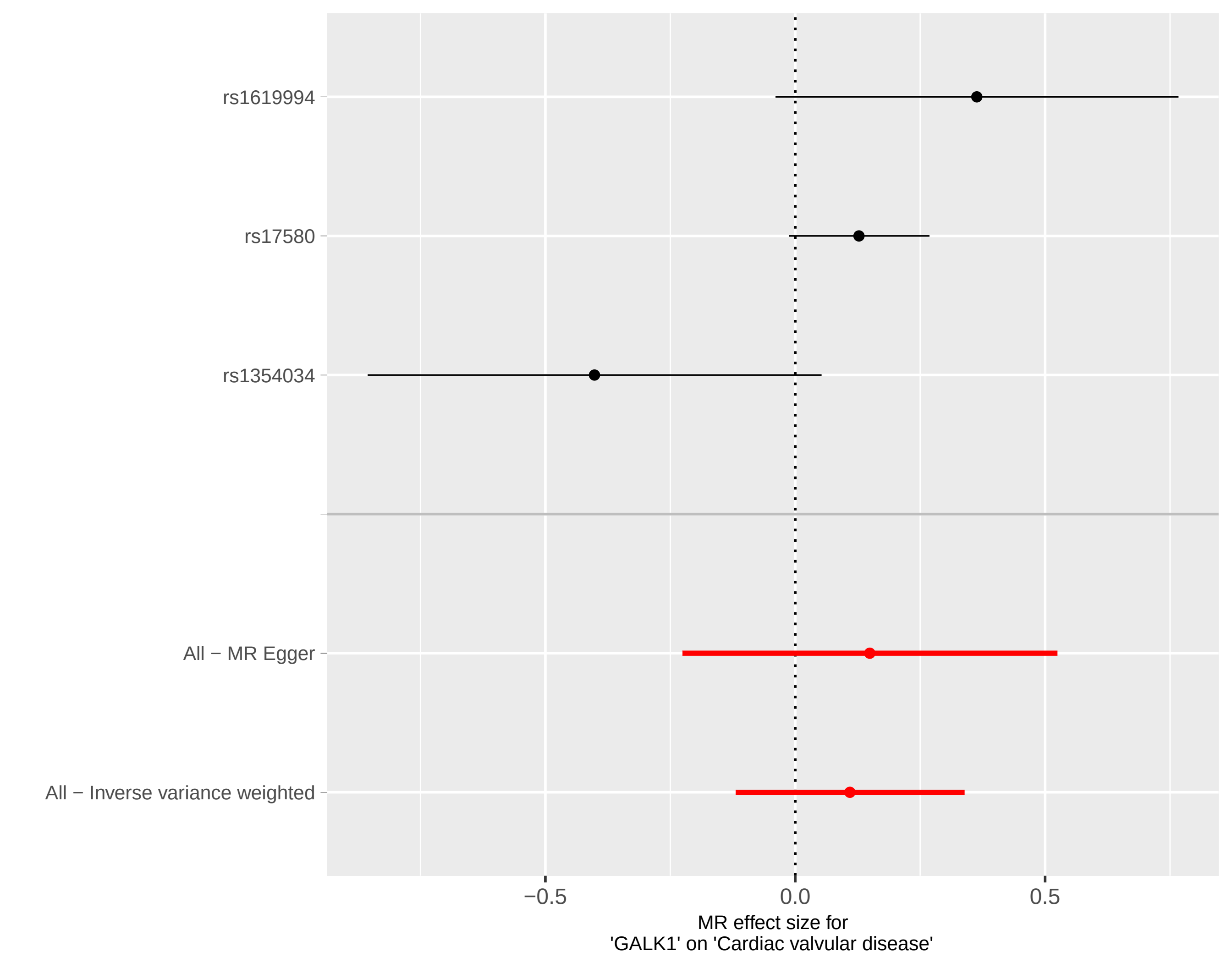
rs1619994
rs17580
rs1354034
All − MR Egger
All − Inverse variance weighted
−0.5
0.0
0.5
MR effect size for
'GALK1' on 'Cardiac valvular disease'

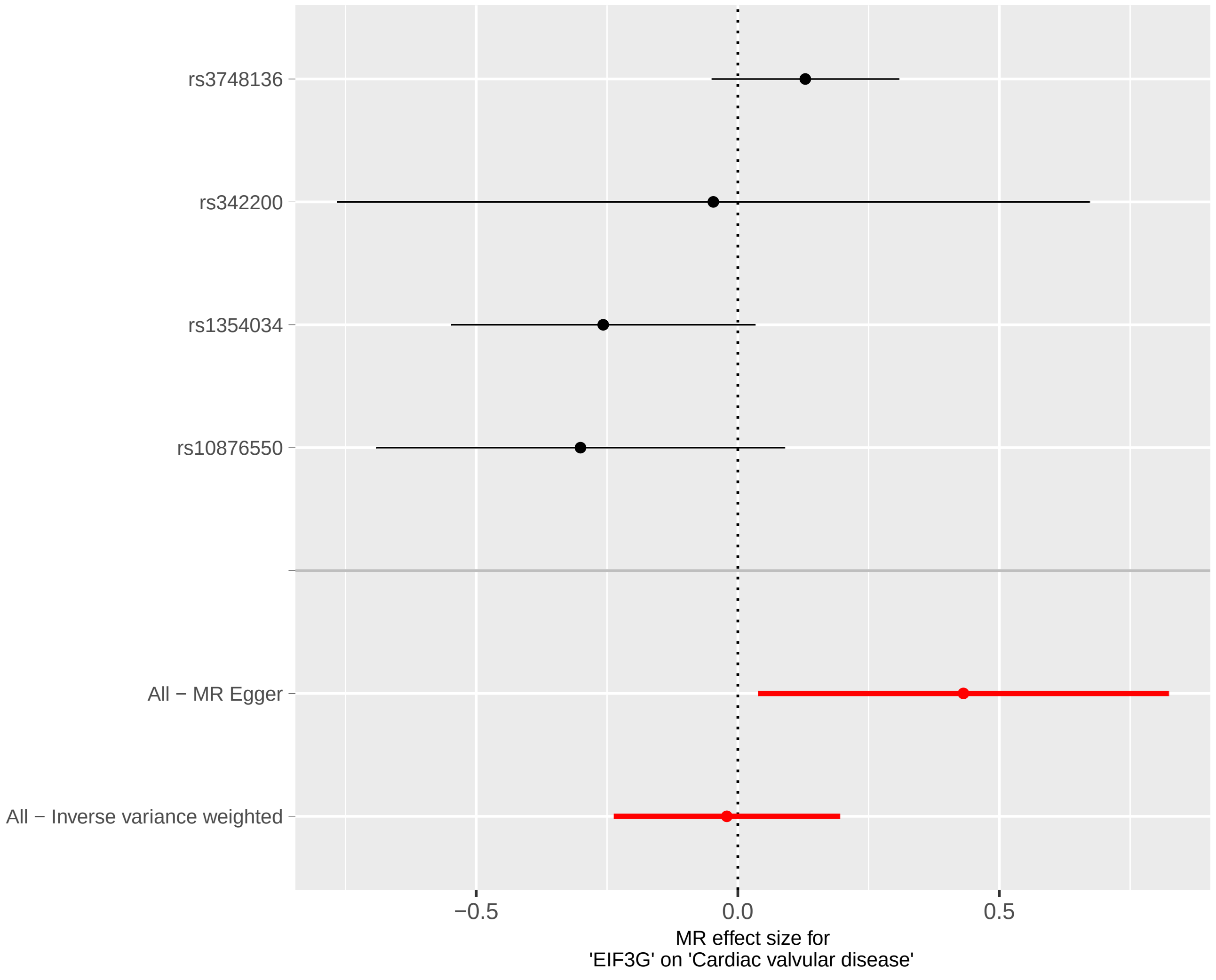
rs3748136
rs342200
rs1354034
rs10876550
All − MR Egger
All − Inverse variance weighted
−0.5
0.0
0.5
MR effect size for
'EIF3G' on 'Cardiac valvular disease'

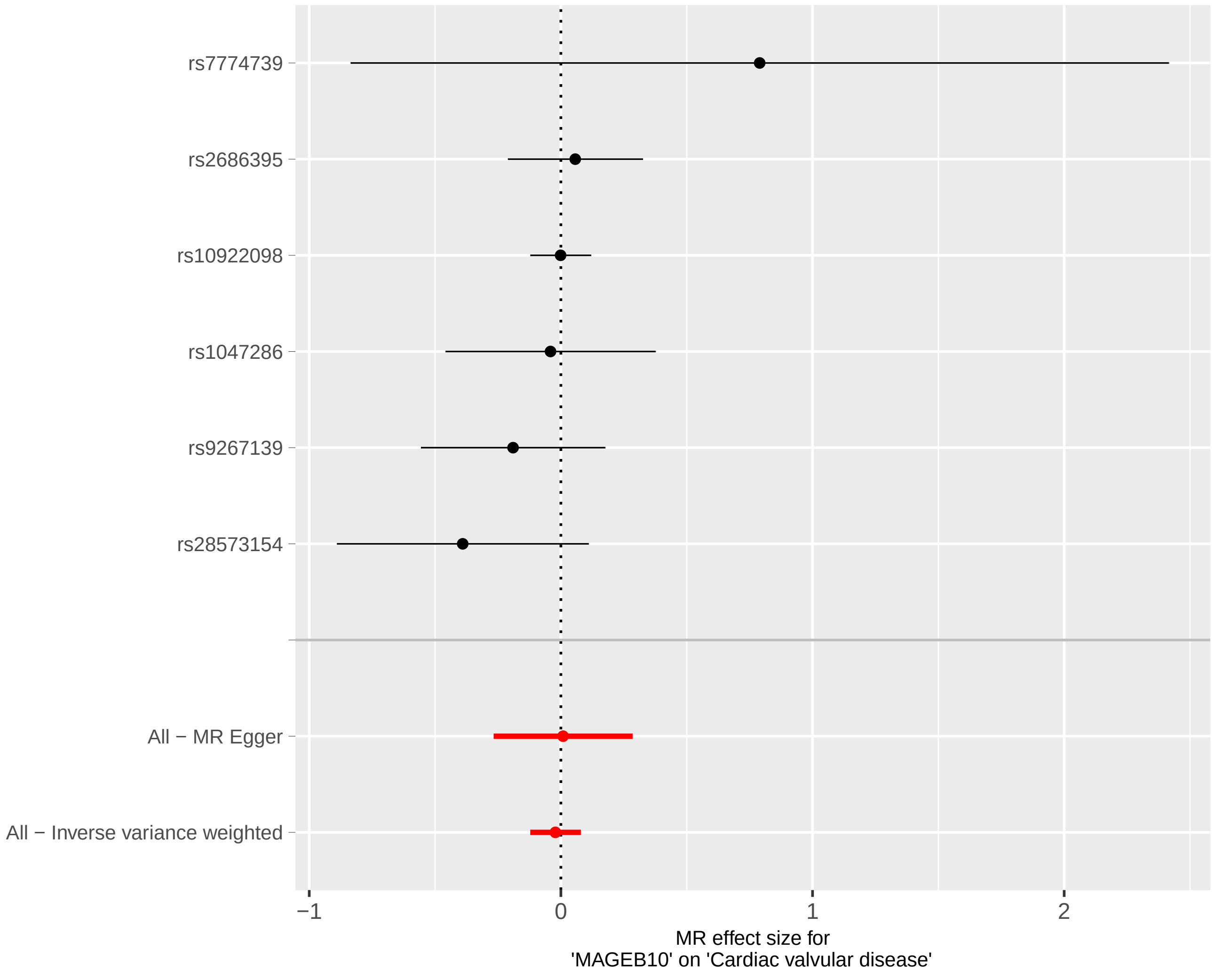
rs7774739
rs2686395
rs10922098
rs1047286
rs9267139
rs28573154
All − MR Egger
All − Inverse variance weighted
−1
0
1
2
MR effect size for
'MAGEB10' on 'Cardiac valvular disease'

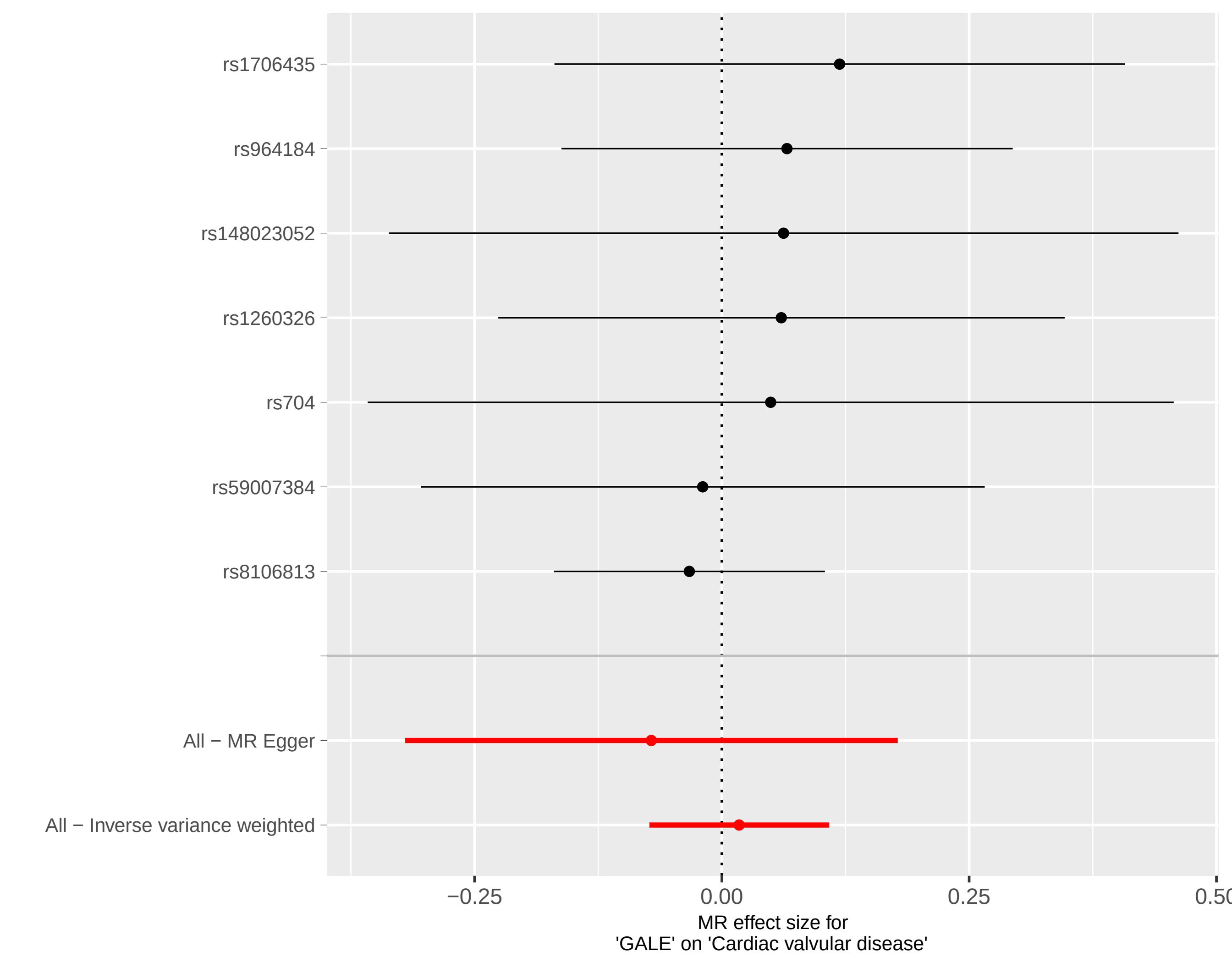

rs1706435
rs964184
rs148023052
rs1260326
rs704
rs59007384
rs8106813
All − MR Egger
All − Inverse variance weighted
−0.25
0.00
0.25
MR effect size for
'GALE' on 'Cardiac valvular disease'

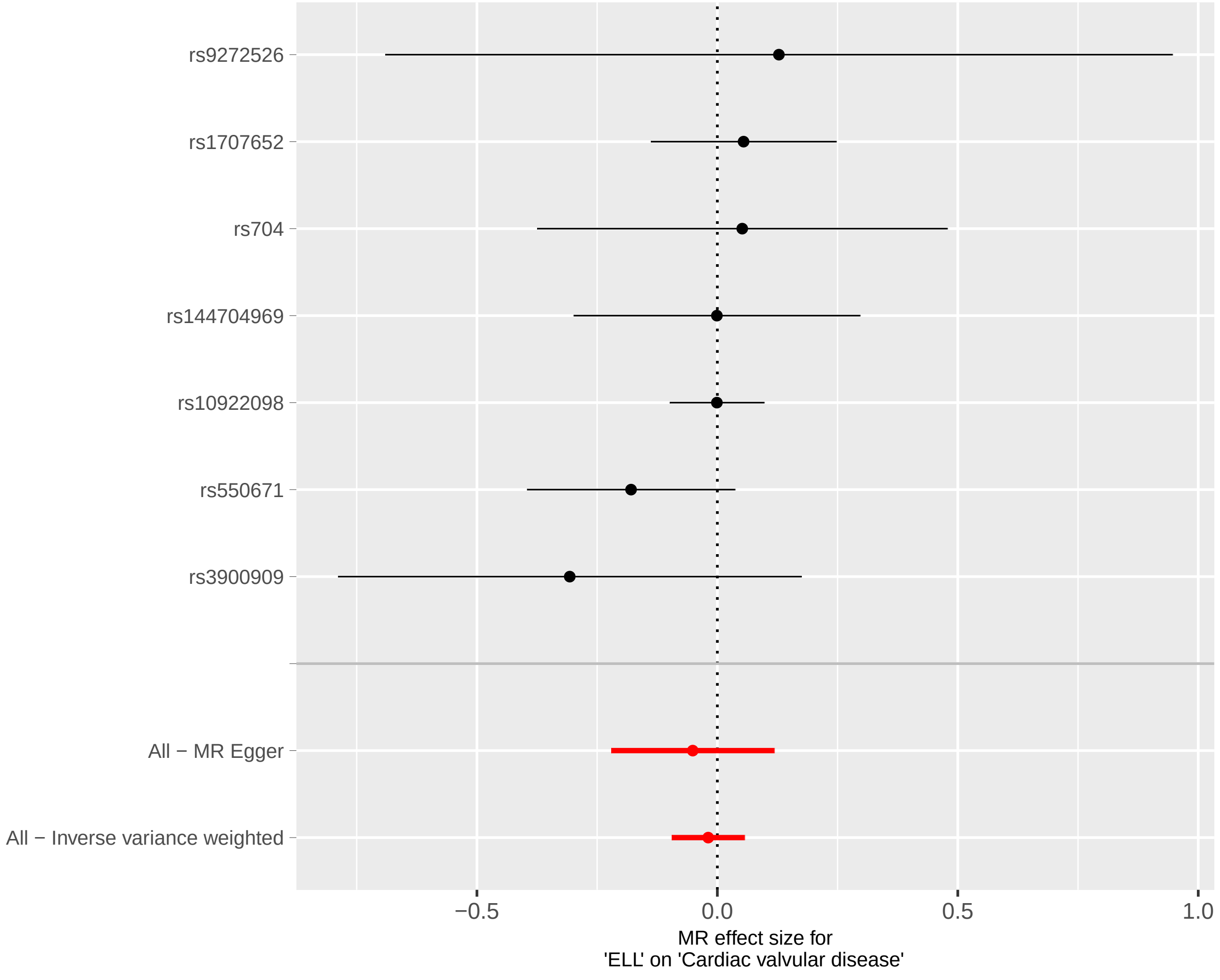

rs9272526
rs1707652
rs704
rs144704969
rs10922098
rs550671
rs3900909
All − MR Egger
All − Inverse variance weighted
−0.5
0.0
0.5
1.0
MR effect size for
'ELL' on 'Cardiac valvular disease'

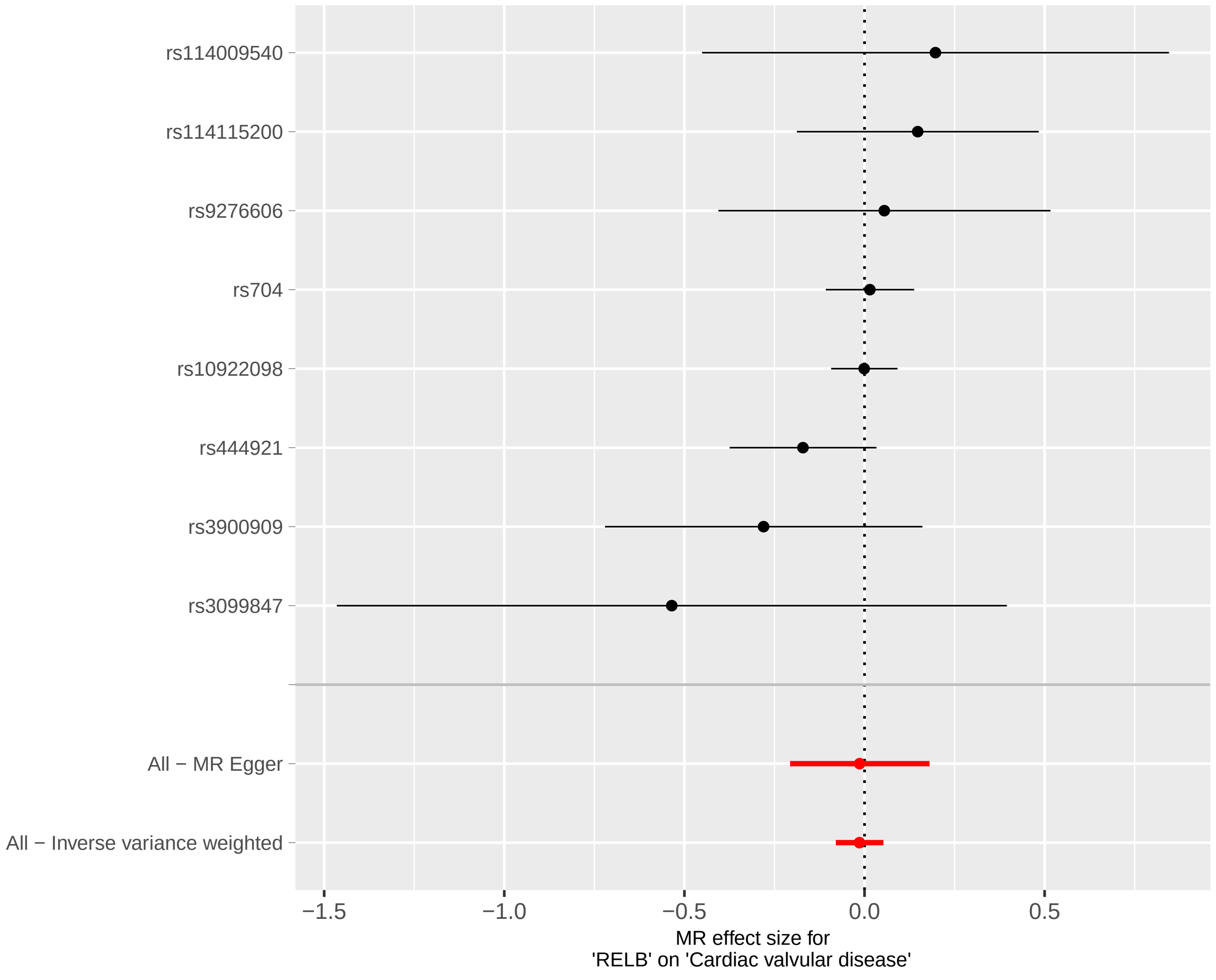
rs114009540
rs114115200
rs9276606
rs704
rs10922098
rs444921
rs3900909
rs3099847
All − MR Egger
All − Inverse variance weighted
−1.5
−1.0
−0.5
0.0
0.5
MR effect size for
'RELB' on 'Cardiac valvular disease'

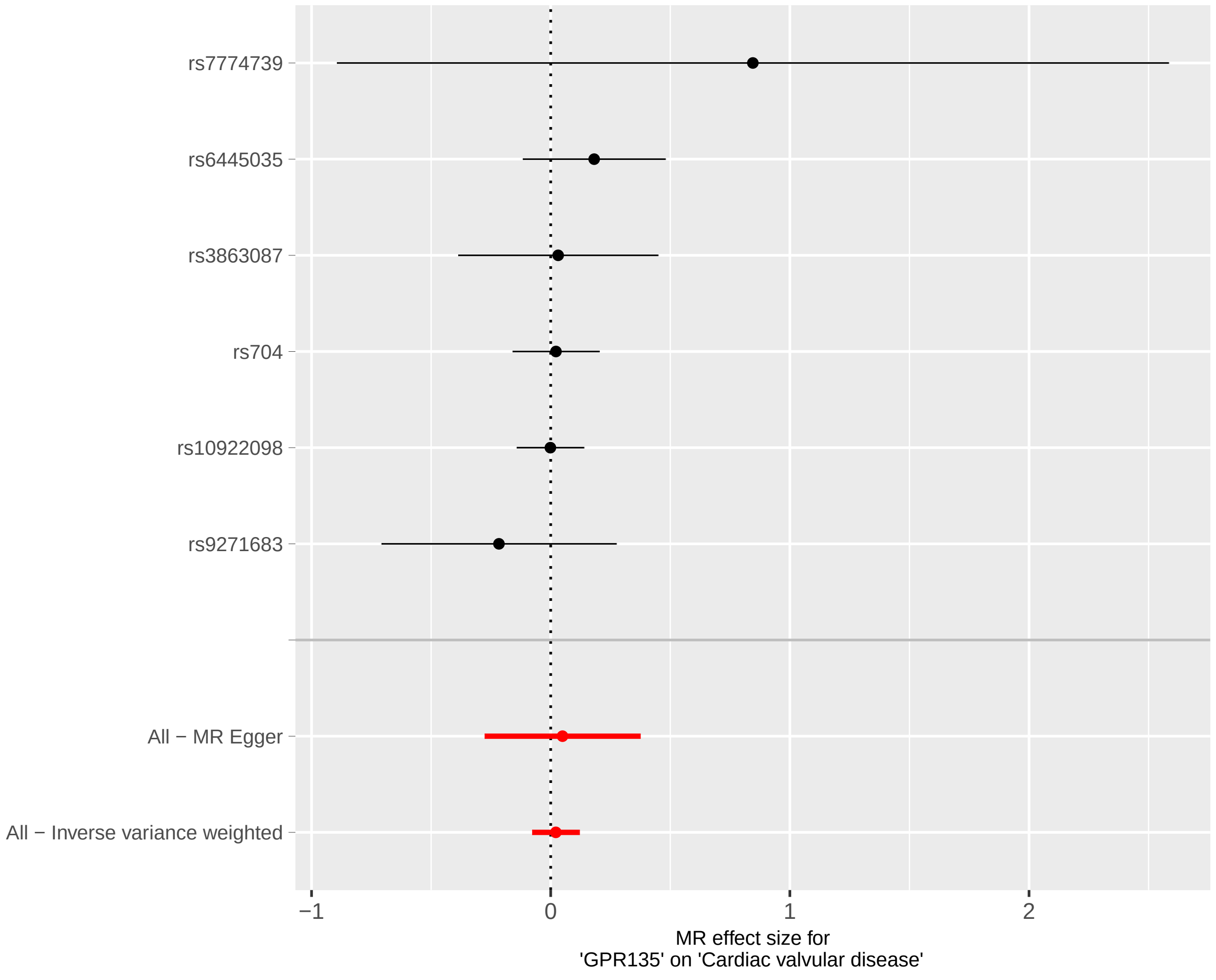
rs7774739
rs6445035
rs3863087
rs704
rs10922098
rs9271683
All − MR Egger
All − Inverse variance weighted
−1
0
1
2
MR effect size for
'GPR135' on 'Cardiac valvular disease'

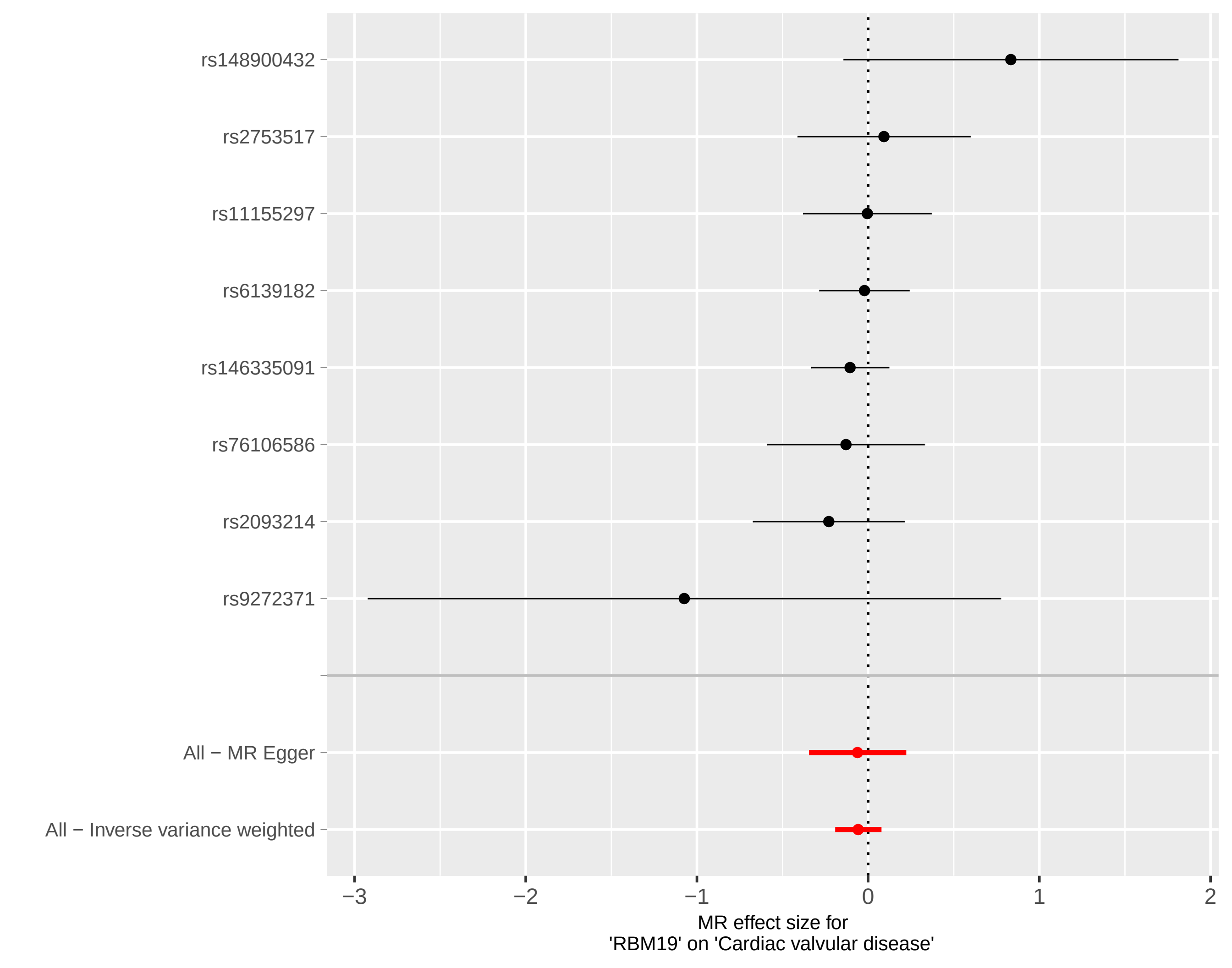
rs148900432
rs2753517
rs11155297
rs6139182
rs146335091
rs76106586
rs2093214
rs9272371
All − MR Egger
All − Inverse variance weighted
−3
−2
−1
0
1
2
MR effect size for
'RBM19' on 'Cardiac valvular disease'

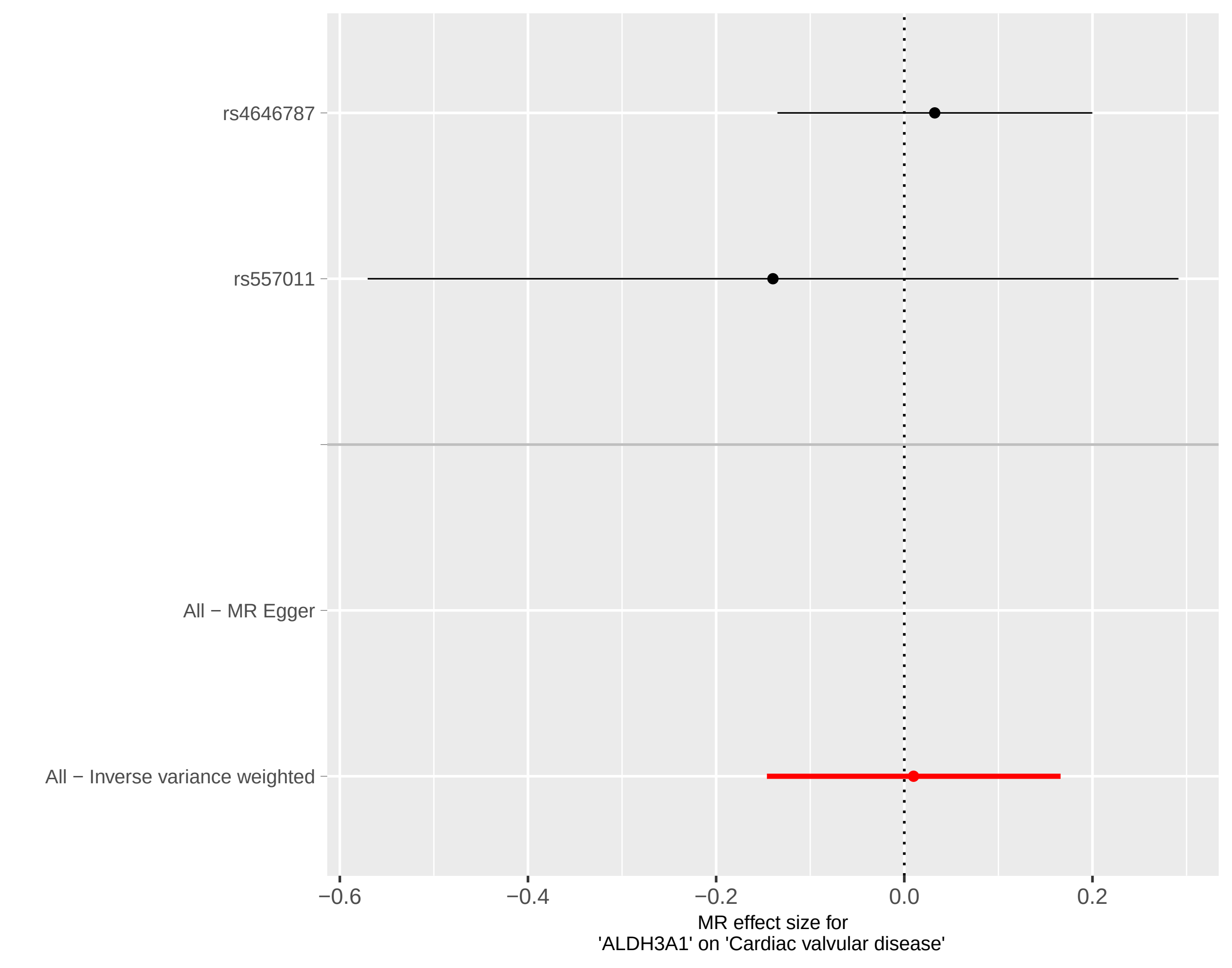

rs4646787
rs557011
All − MR Egger
All − Inverse variance weighted
−0.6
−0.4
−0.2
0.0
0.2
MR effect size for
'ALDH3A1' on 'Cardiac valvular disease'

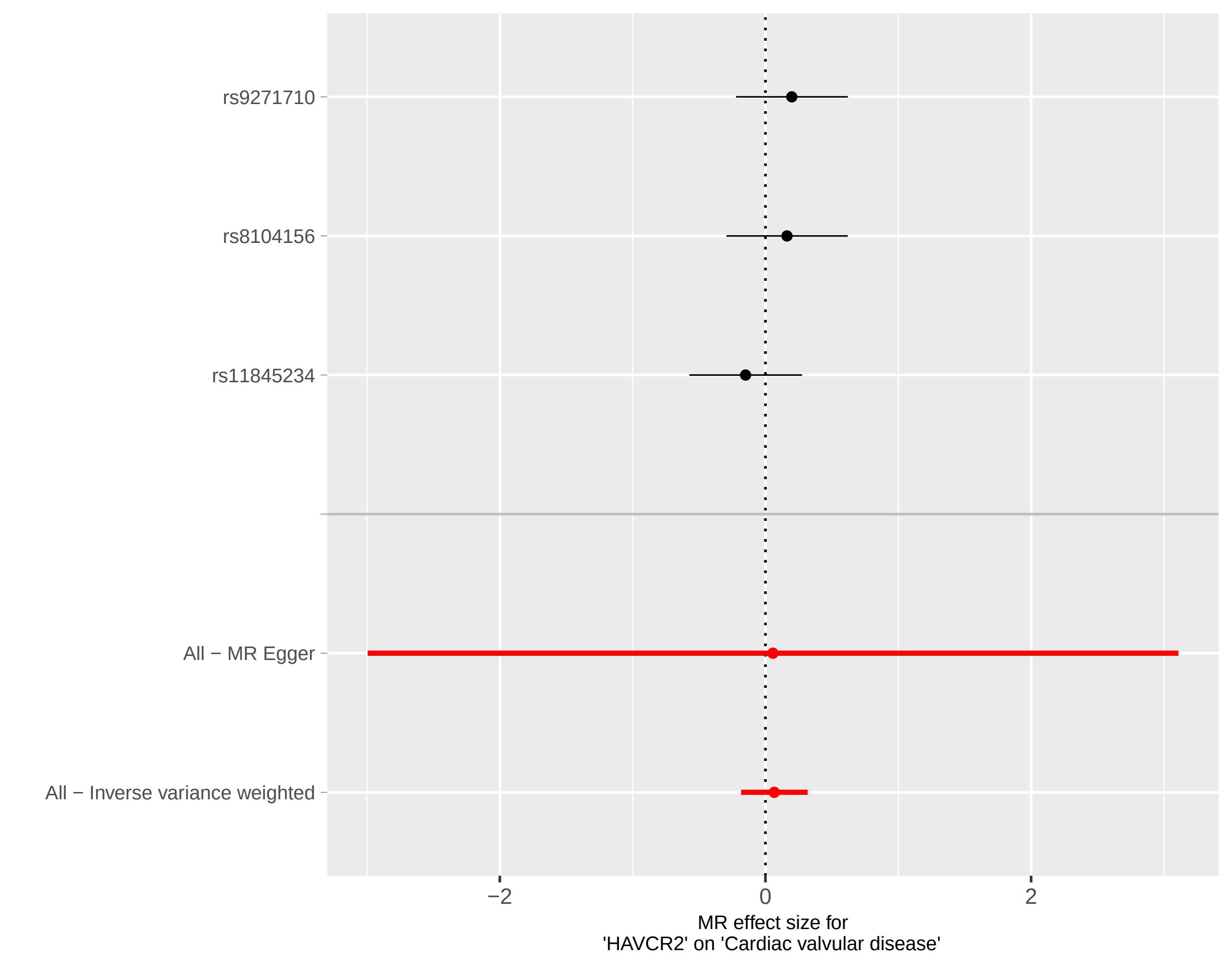
rs9271710
rs8104156
rs11845234
All − MR Egger
All − Inverse variance weighted
−2
0
2
MR effect size for
'HAVCR2' on 'Cardiac valvular disease'

Insufficient number of SNPs

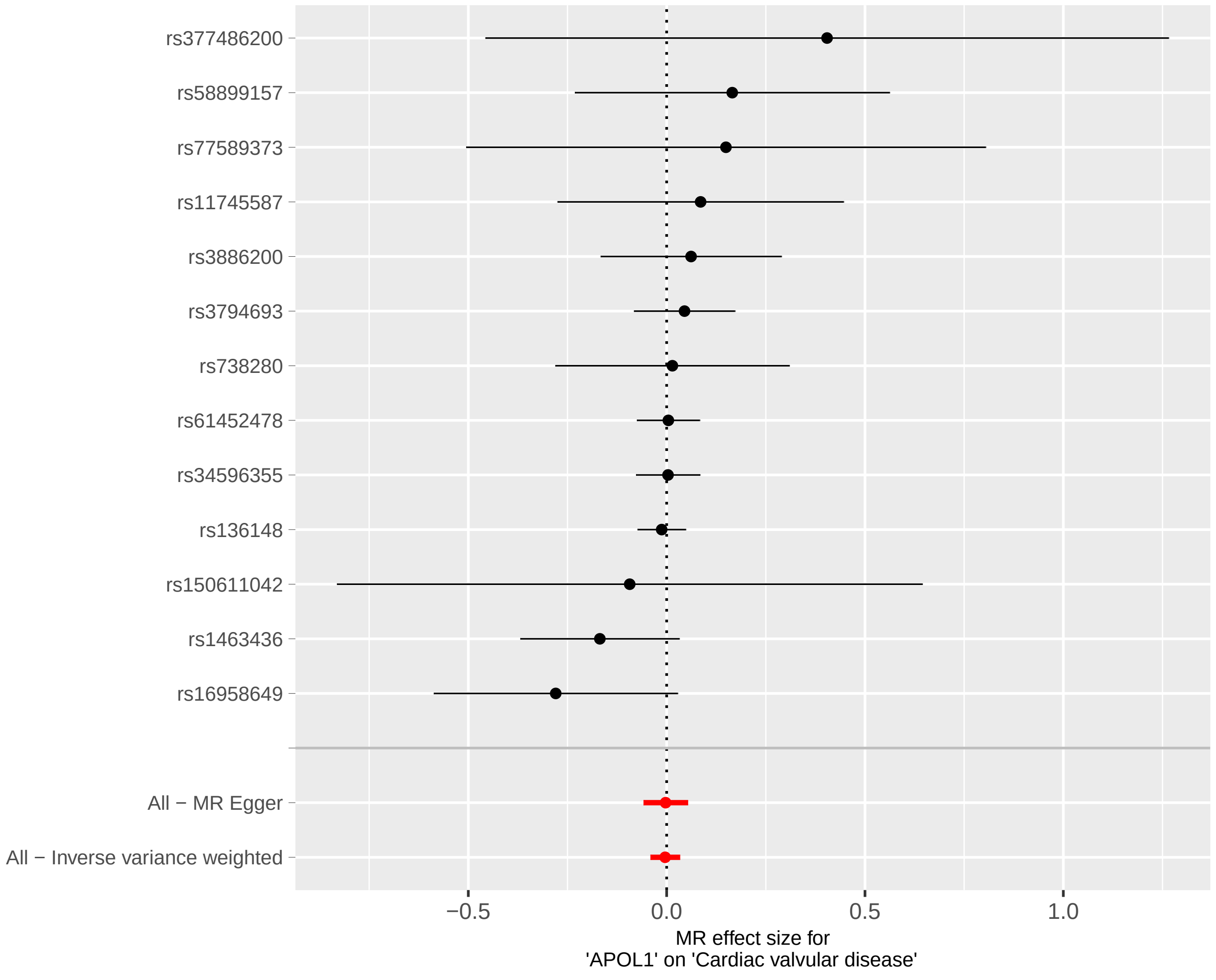
rs377486200
rs58899157
rs77589373
rs11745587
rs3886200
rs3794693
rs738280
rs61452478
rs34596355
rs136148
rs150611042
rs1463436
rs16958649
All − MR Egger
All − Inverse variance weighted
−0.5
0.0
0.5
1.0
MR effect size for
'APOL1' on 'Cardiac valvular disease'

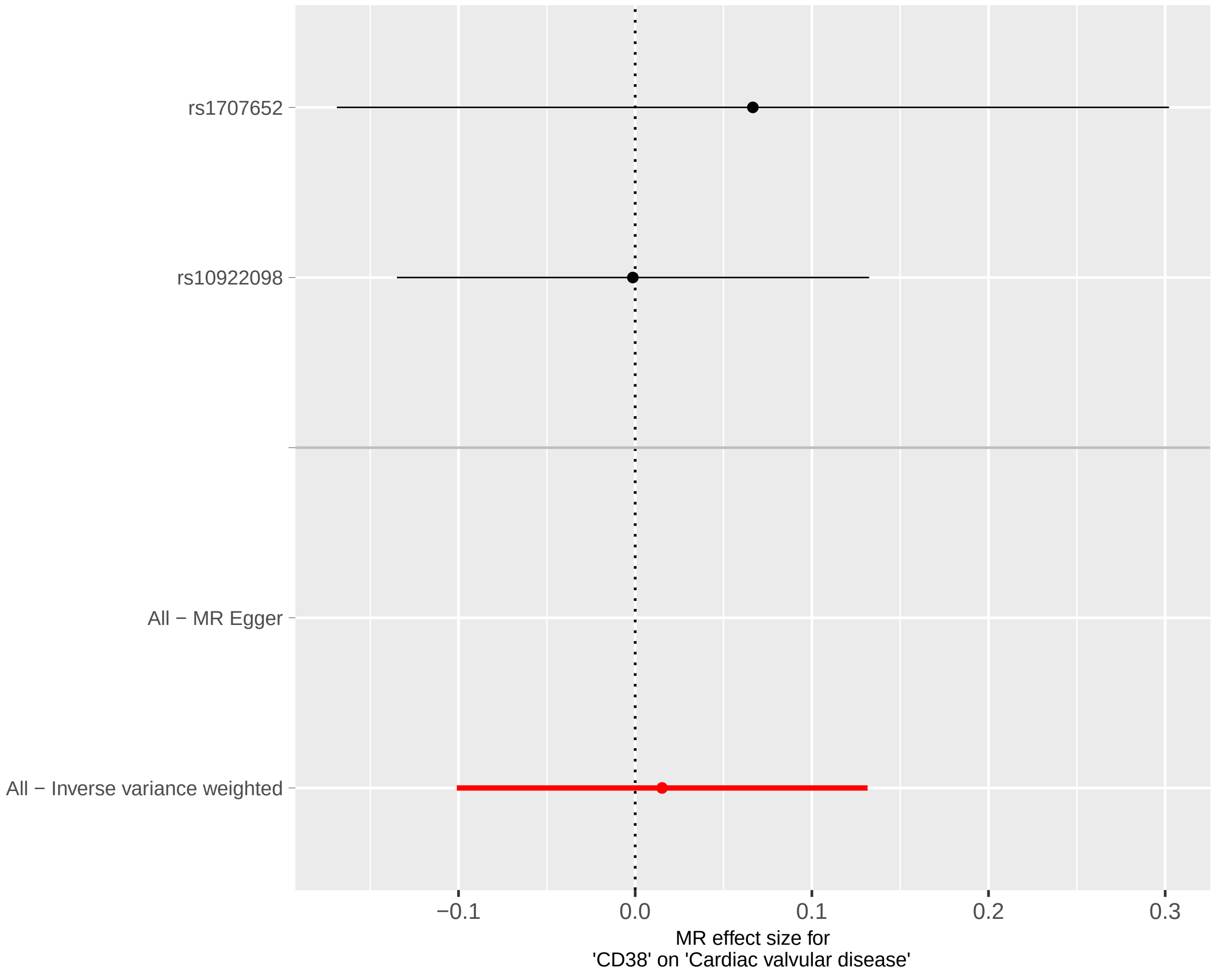
rs1707652
rs10922098
All − MR Egger
All − Inverse variance weighted
−0.1
0.0
0.1
0.2
0.3
MR effect size for
'CD38' on 'Cardiac valvular disease'

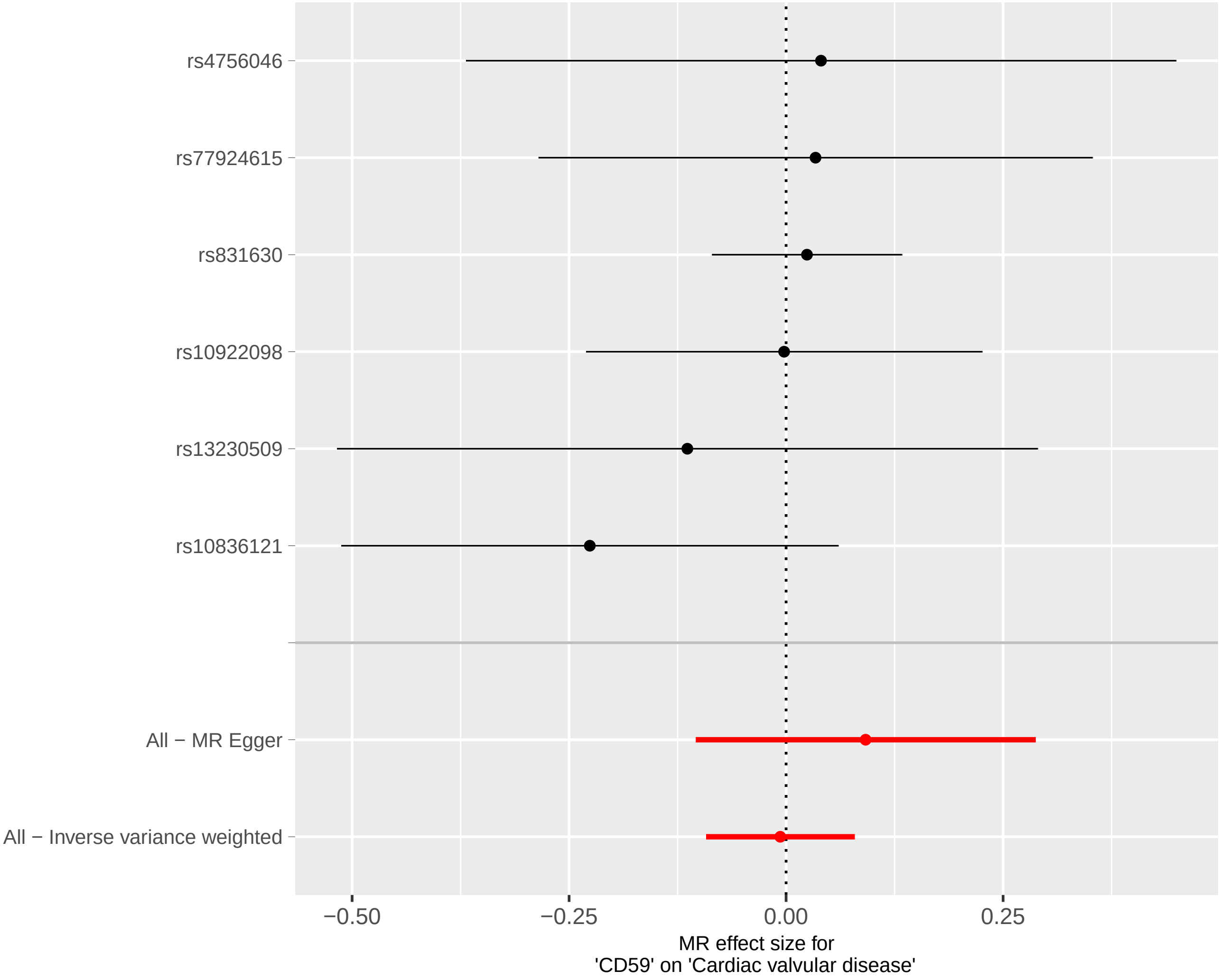
rs4756046
rs77924615
rs831630
rs10922098
rs13230509
rs10836121
All − MR Egger
All − Inverse variance weighted
−0.50
−0.25
0.00
0.25
MR effect size for
'CD59' on 'Cardiac valvular disease'

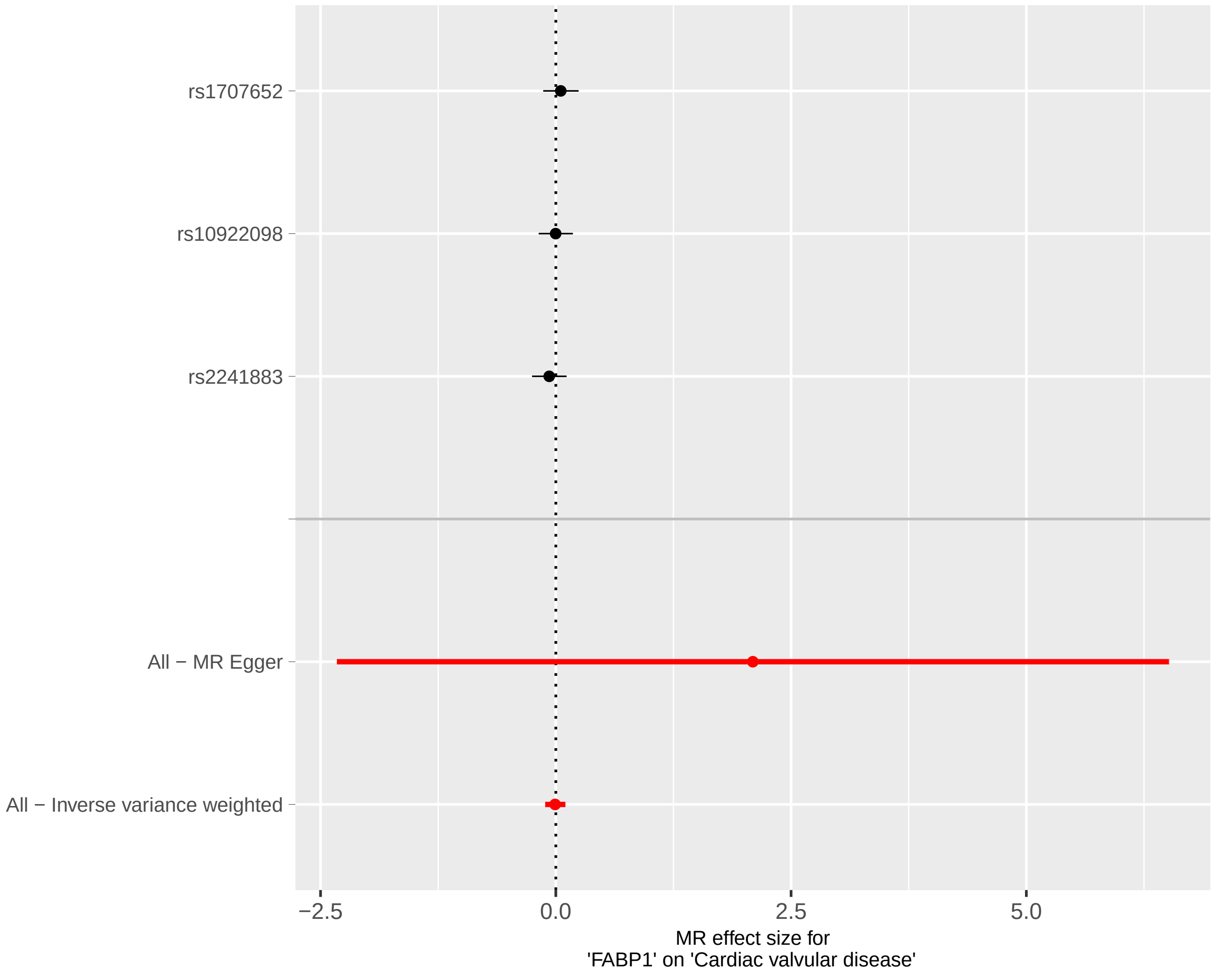
rs1707652
rs10922098
rs2241883
All − MR Egger
All − Inverse variance weighted
−2.5
0.0
2.5
5.0
MR effect size for
'FABP1' on 'Cardiac valvular disease'

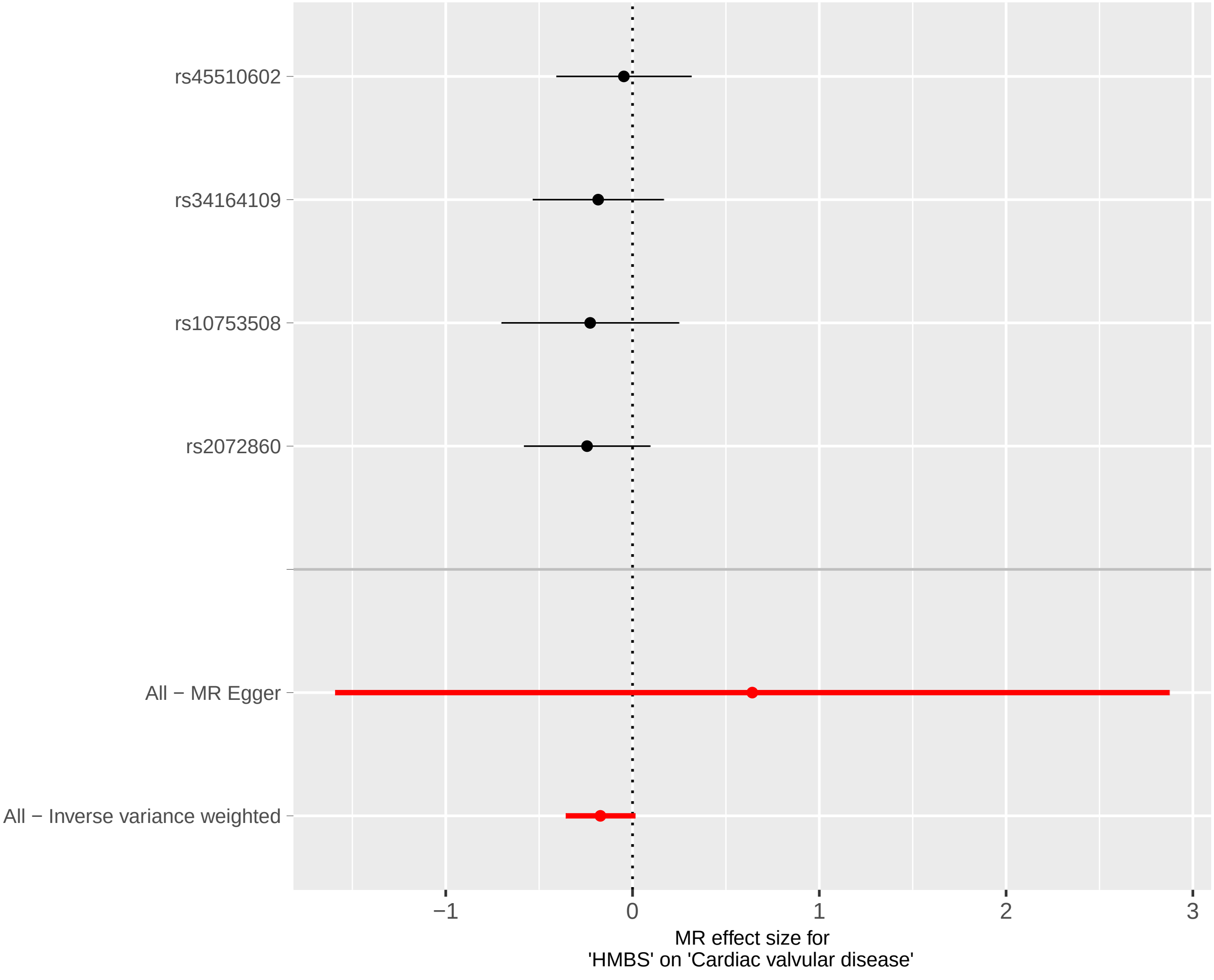

rs45510602
rs34164109
rs10753508
rs2072860
All − MR Egger
All − Inverse variance weighted
−1
0
1
2
3
MR effect size for
'HMBS' on 'Cardiac valvular disease'

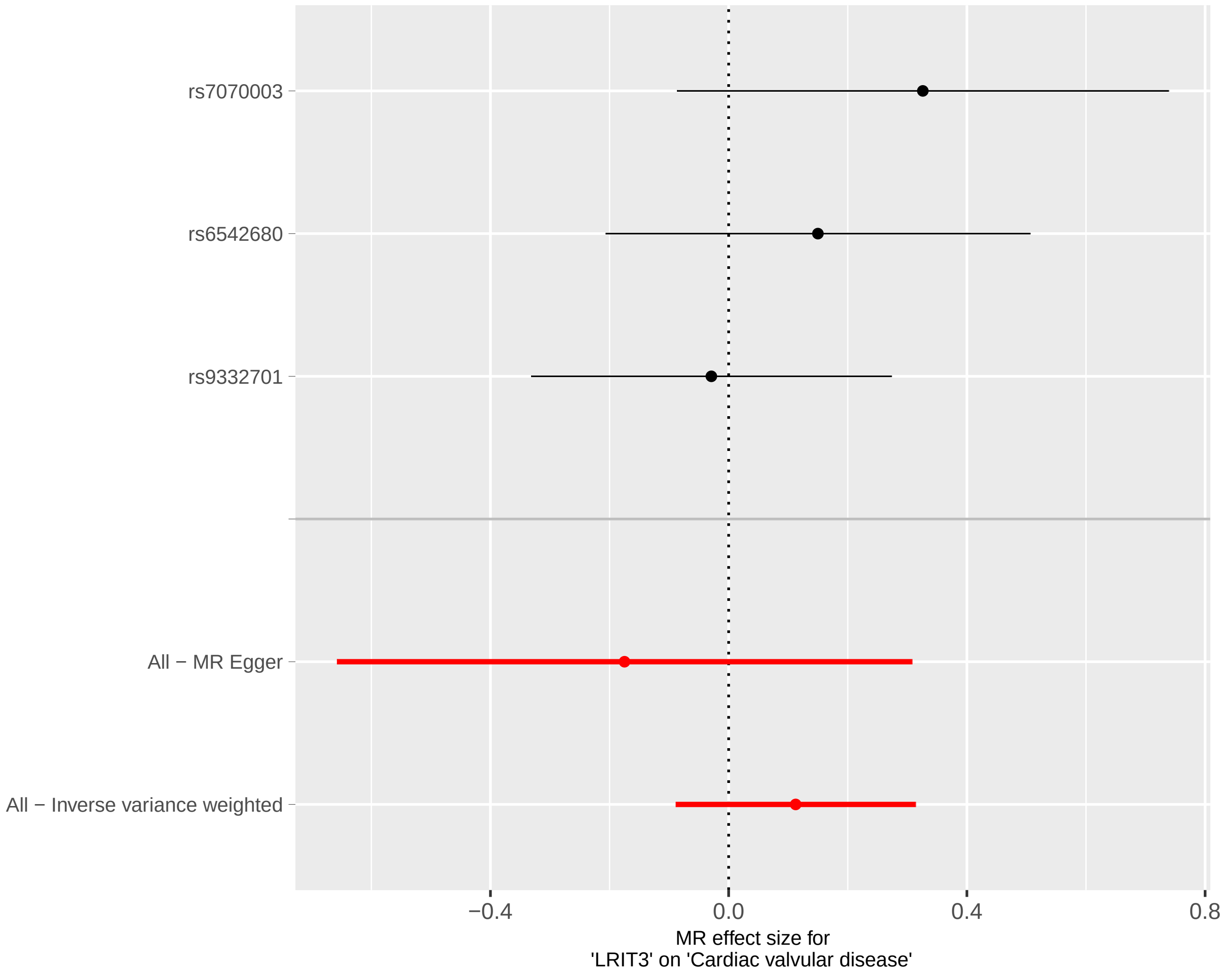
rs7070003
rs6542680
rs9332701
All − MR Egger
All − Inverse variance weighted
−0.4
0.0
0.4
0.8
MR effect size for
'LRIT3' on 'Cardiac valvular disease'

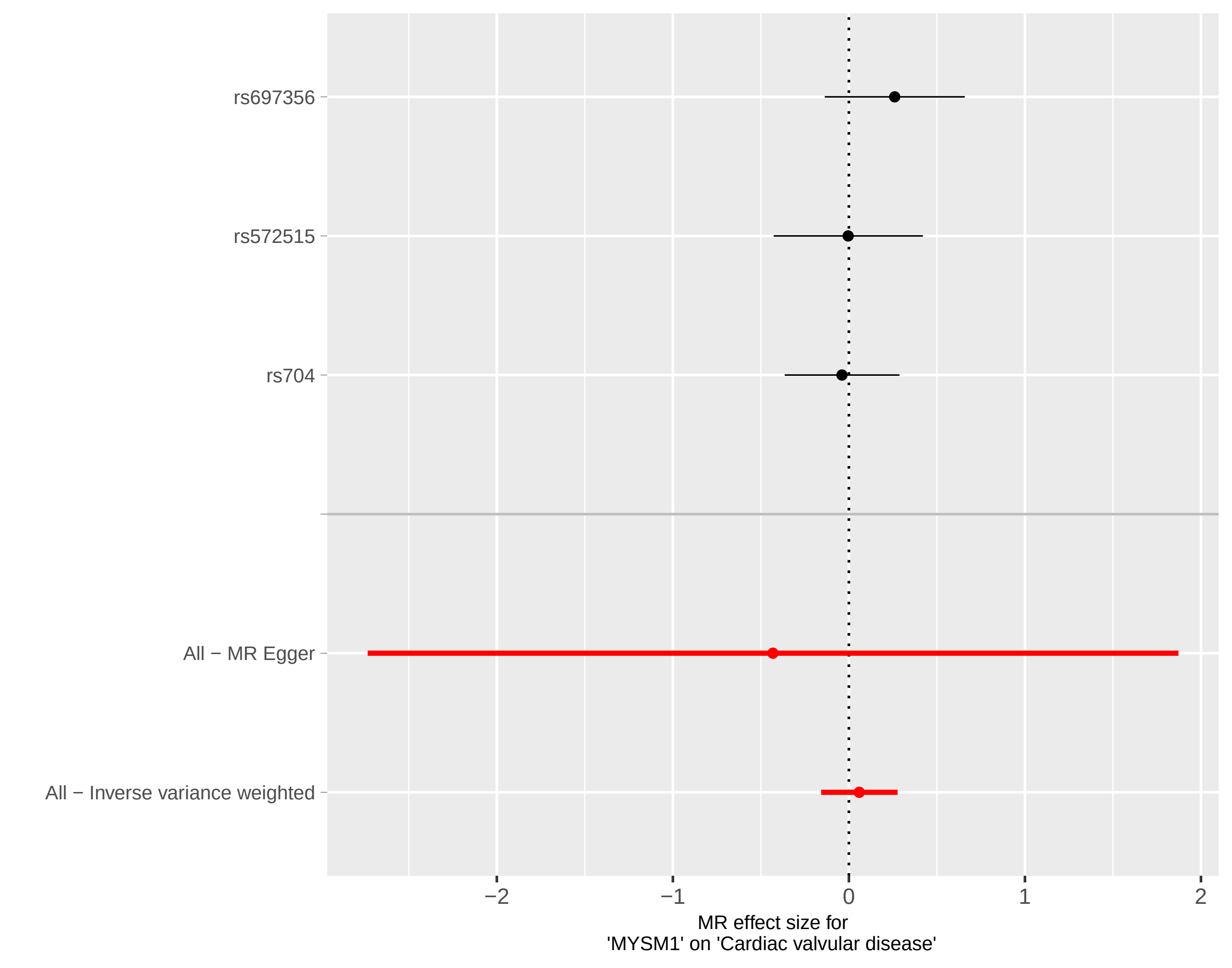

rs697356
rs572515
rs704
All − MR Egger
All − Inverse variance weighted
−2
−1
0
1
2
MR effect size for
'MYSM1' on 'Cardiac valvular disease'

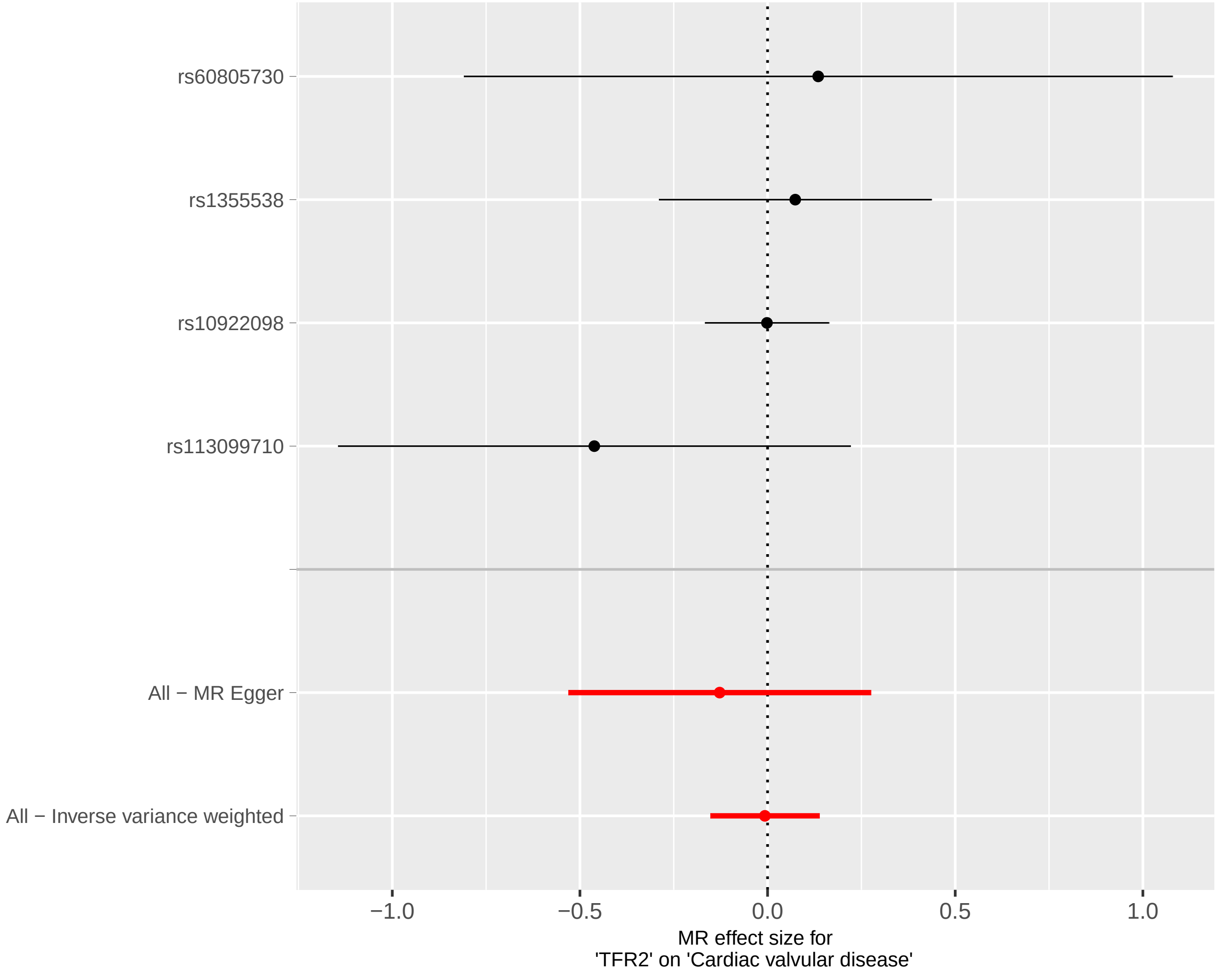
rs60805730
rs1355538
rs10922098
rs113099710
All − MR Egger
All − Inverse variance weighted
−1.0
−0.5
0.0
0.5
1.0
MR effect size for
'TFR2' on 'Cardiac valvular disease'

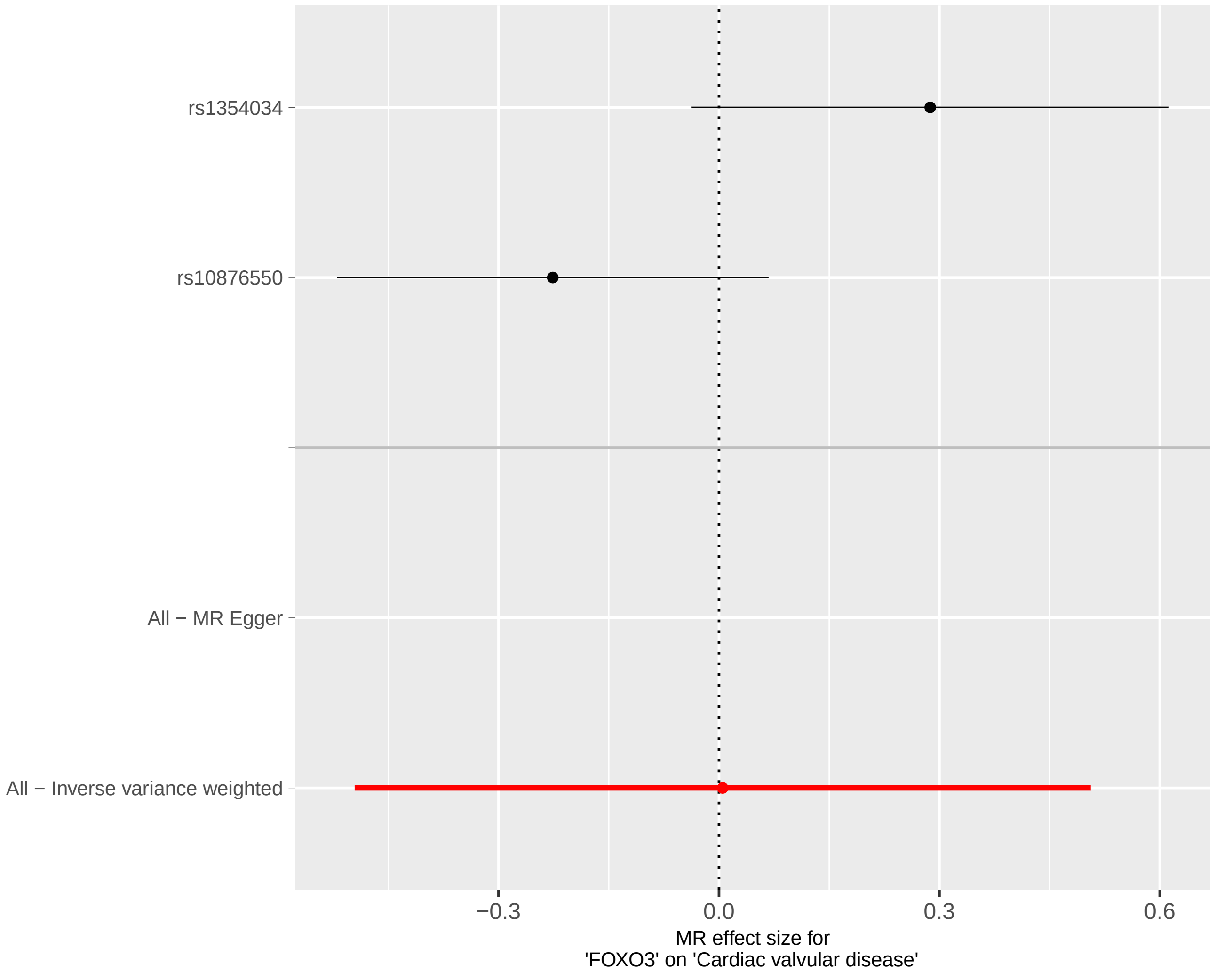
rs1354034
rs10876550
All − MR Egger
All − Inverse variance weighted
−0.3
0.0
0.3
0.6
MR effect size for
'FOXO3' on 'Cardiac valvular disease'

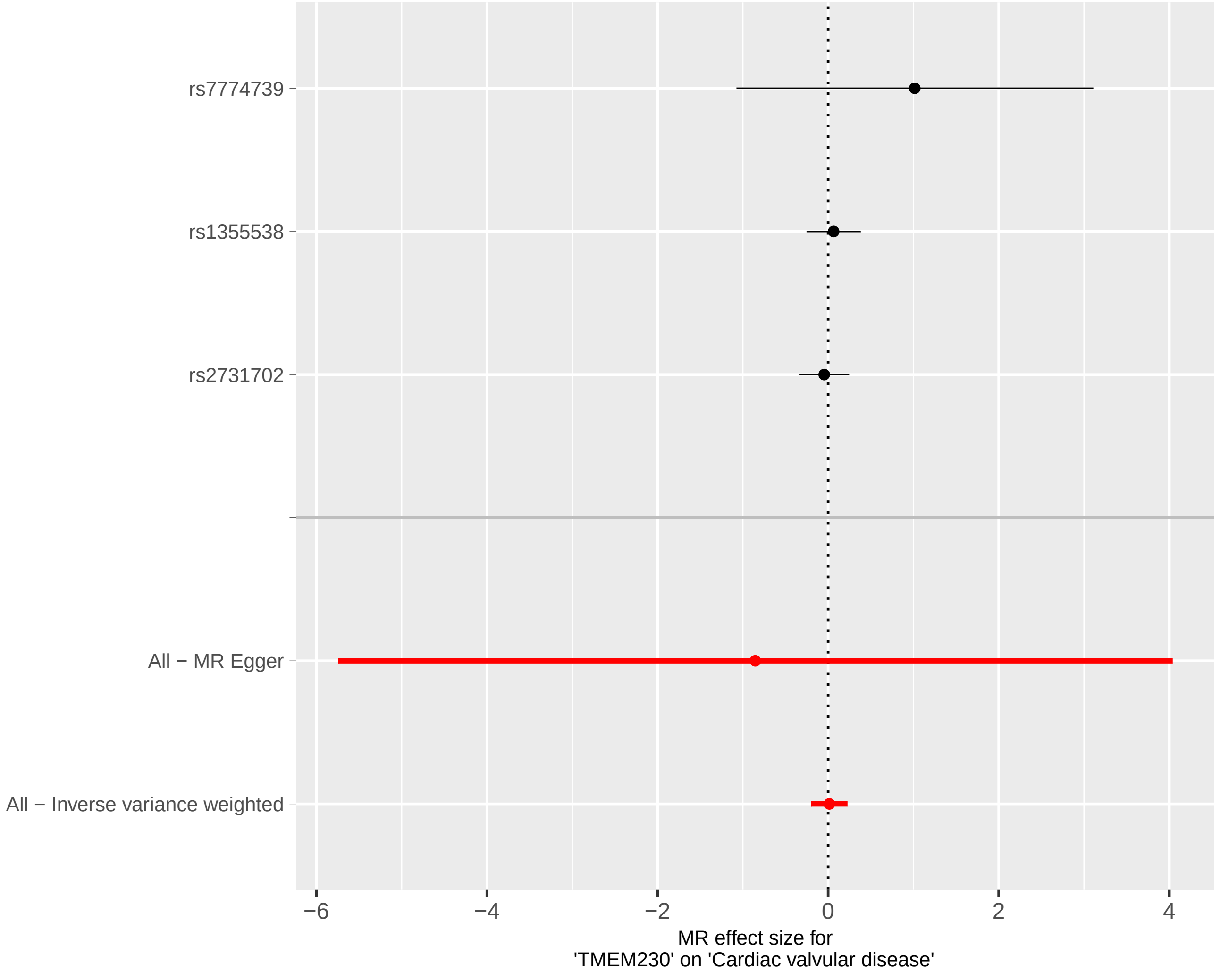

rs7774739
rs1355538
rs2731702
All − MR Egger
All − Inverse variance weighted
−6
−4
−2
0
2
4
MR effect size for
'TMEM230' on 'Cardiac valvular disease'

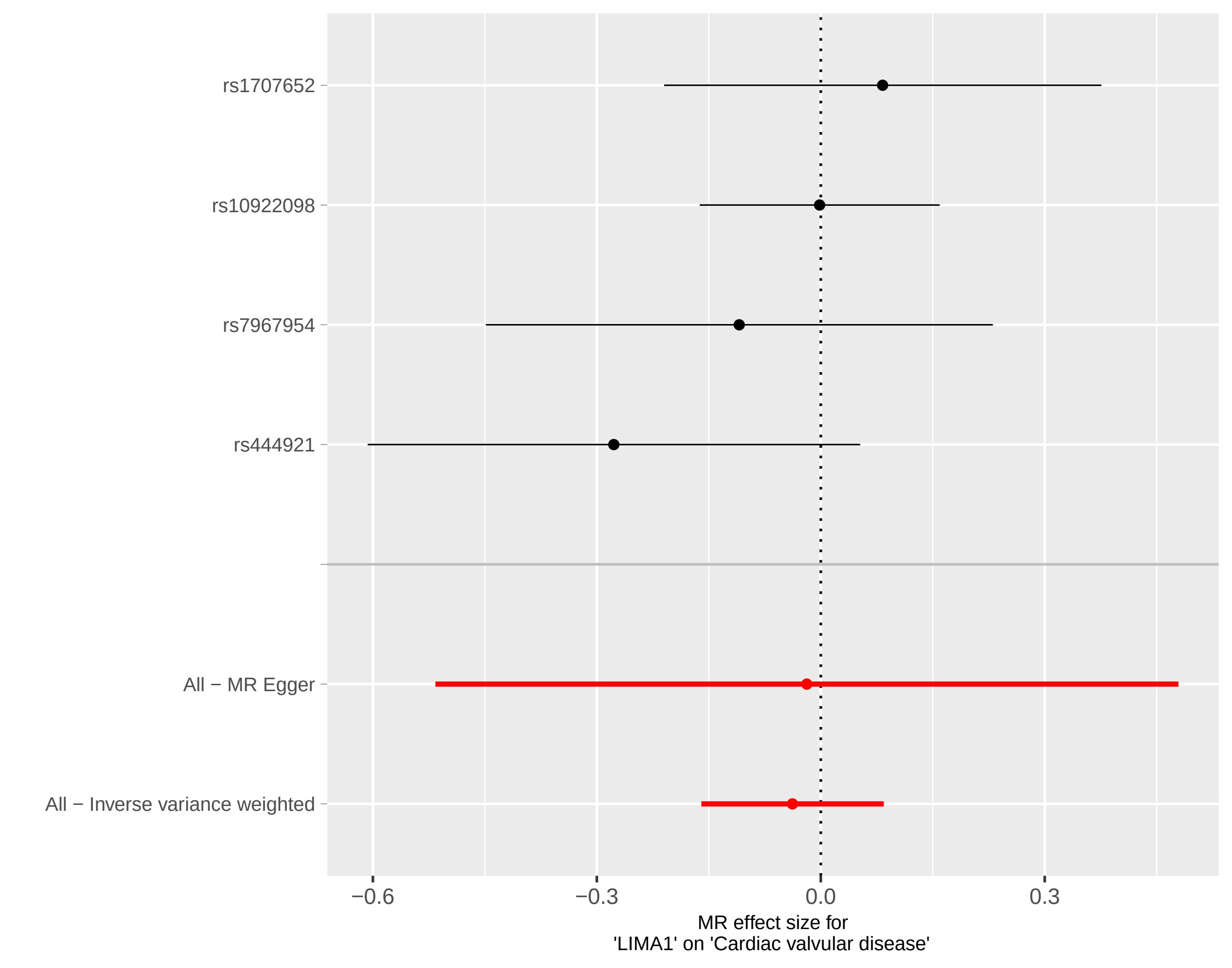
rs1707652
rs10922098
rs7967954
rs444921
All − MR Egger
All − Inverse variance weighted
−0.6
−0.3
0.0
0.3
MR effect size for
'LIMA1' on 'Cardiac valvular disease'

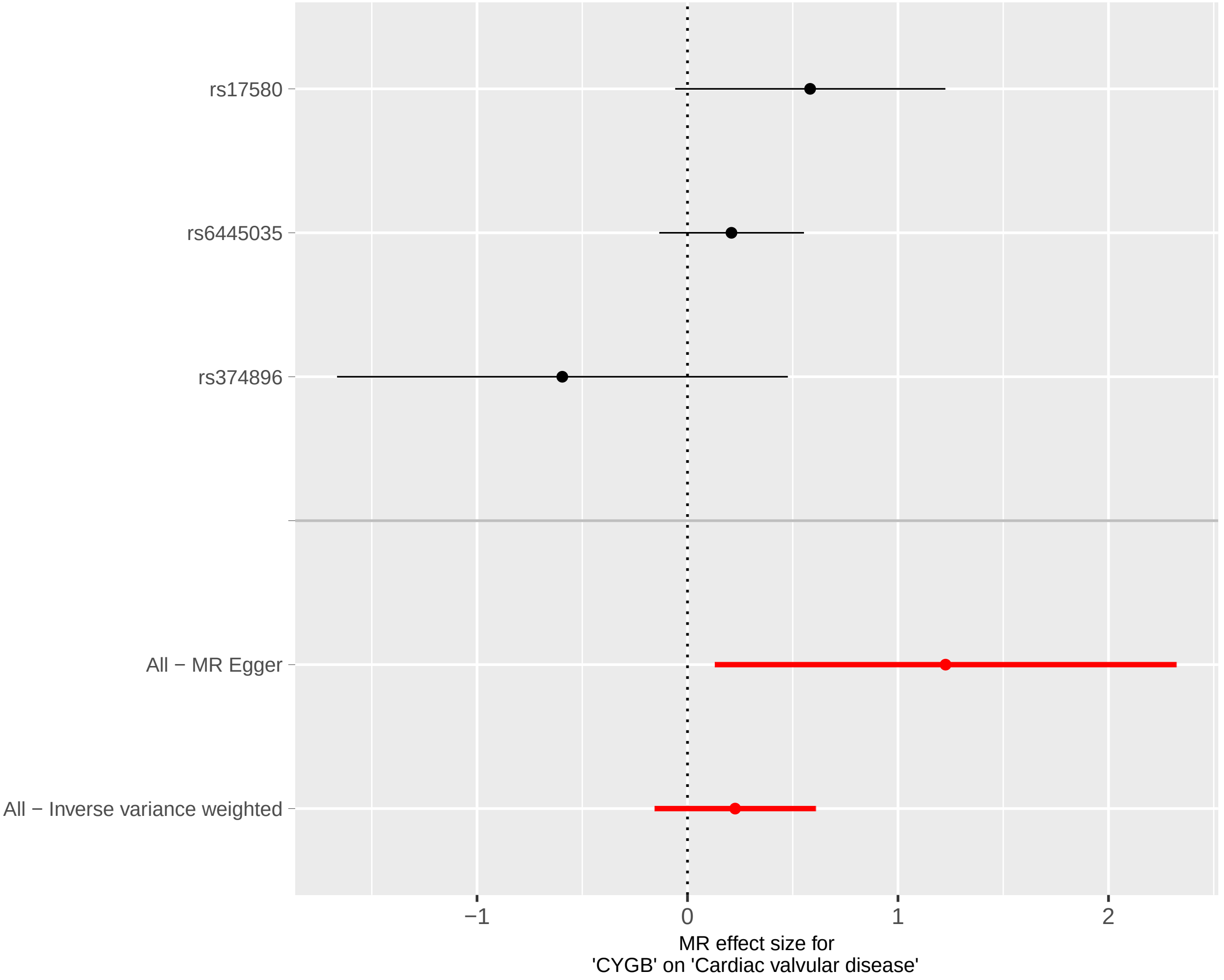
rs17580
rs6445035
rs374896
All − MR Egger
All − Inverse variance weighted
−1
0
1
2
MR effect size for
'CYGB' on 'Cardiac valvular disease'

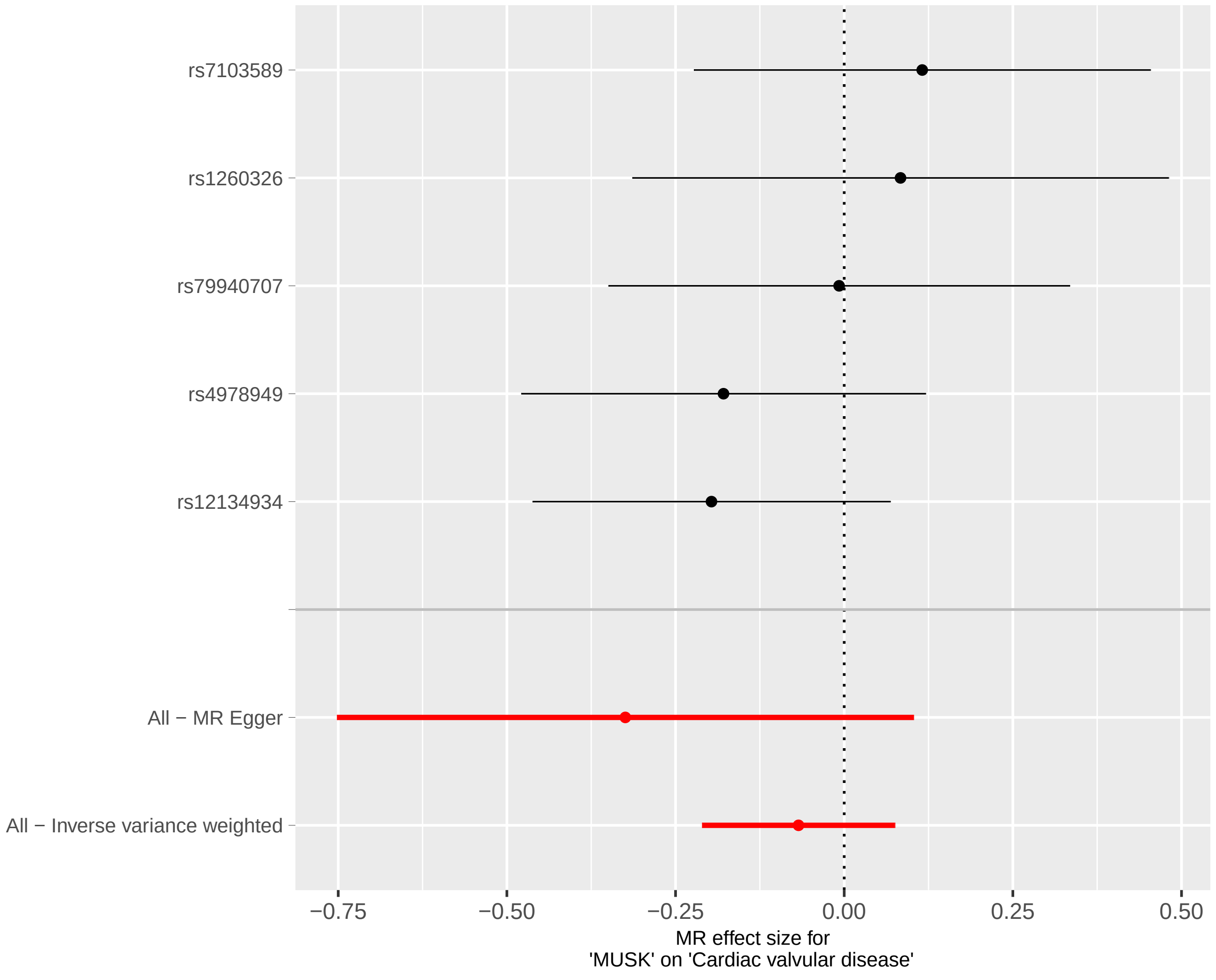
rs7103589
rs1260326
rs79940707
rs4978949
rs12134934
All − MR Egger
All − Inverse variance weighted
−0.75
−0.50
−0.25
0.00
0.25
0.50
MR effect size for
'MUSK' on 'Cardiac valvular disease'

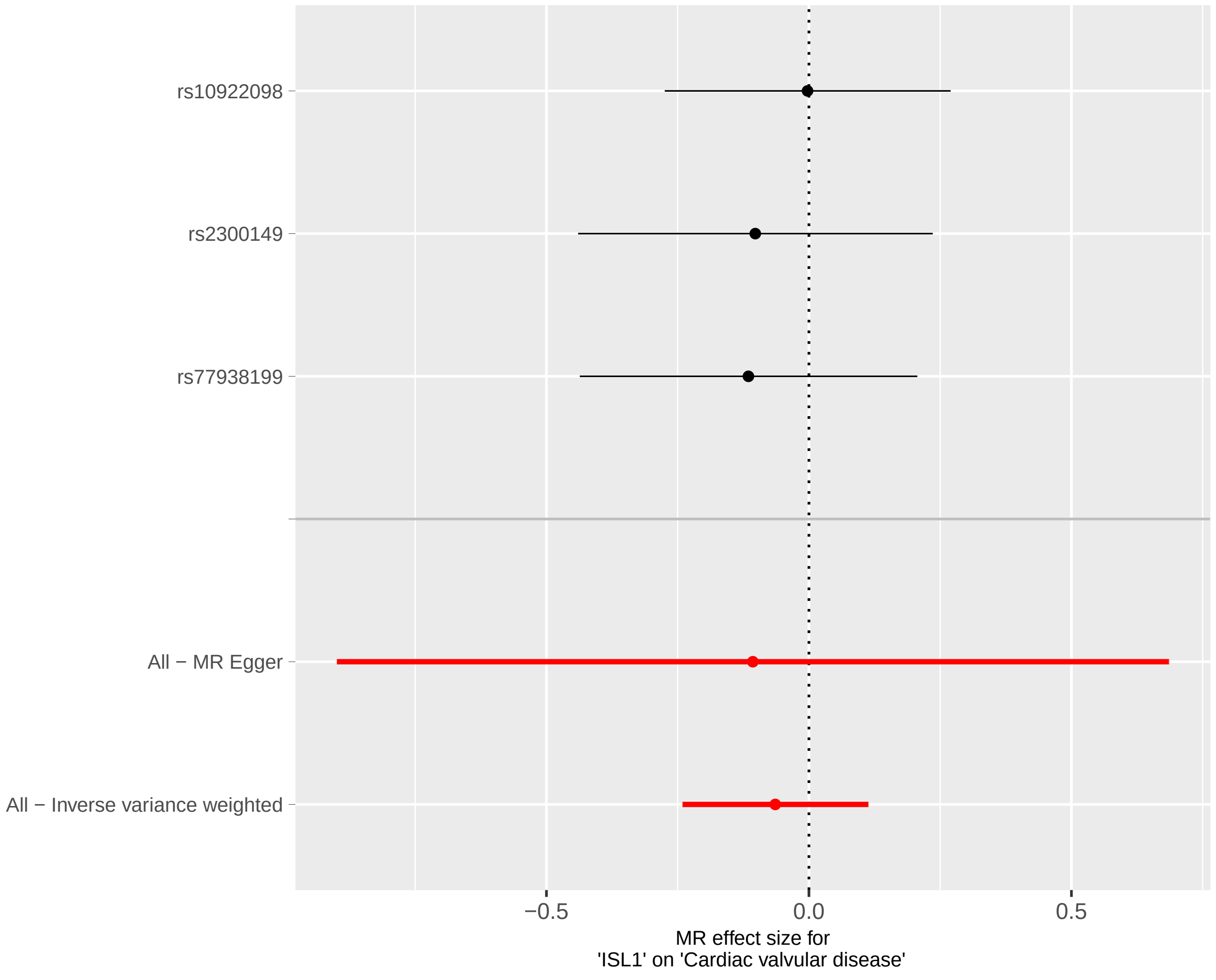
rs10922098
rs2300149
rs77938199
All − MR Egger
All − Inverse variance weighted
−0.5
0.0
0.5
MR effect size for
'ISL1' on 'Cardiac valvular disease'

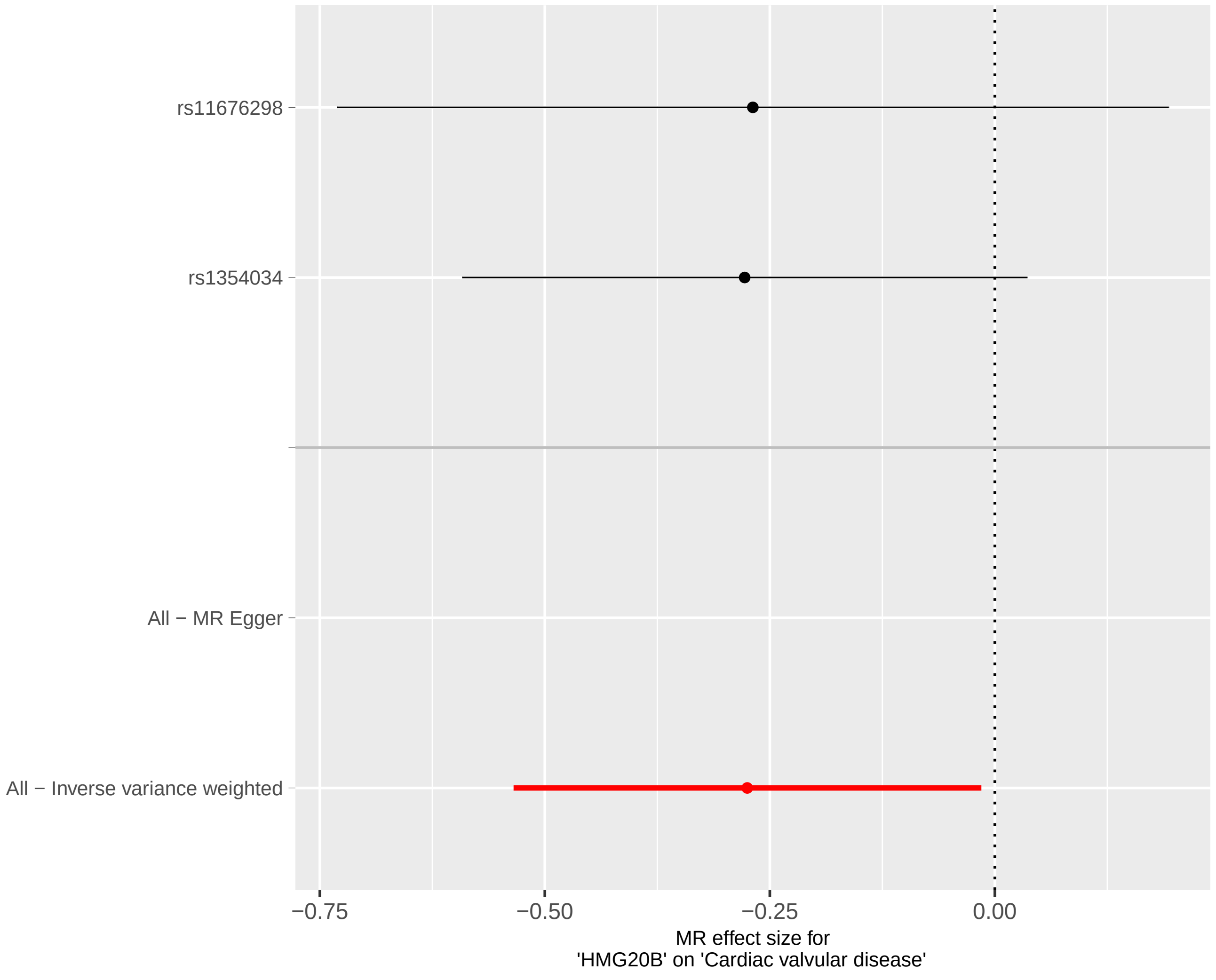
rs11676298
rs1354034
All − MR Egger
All − Inverse variance weighted
−0.75
−0.50
−0.25
0.00
MR effect size for
'HMG20B' on 'Cardiac valvular disease'

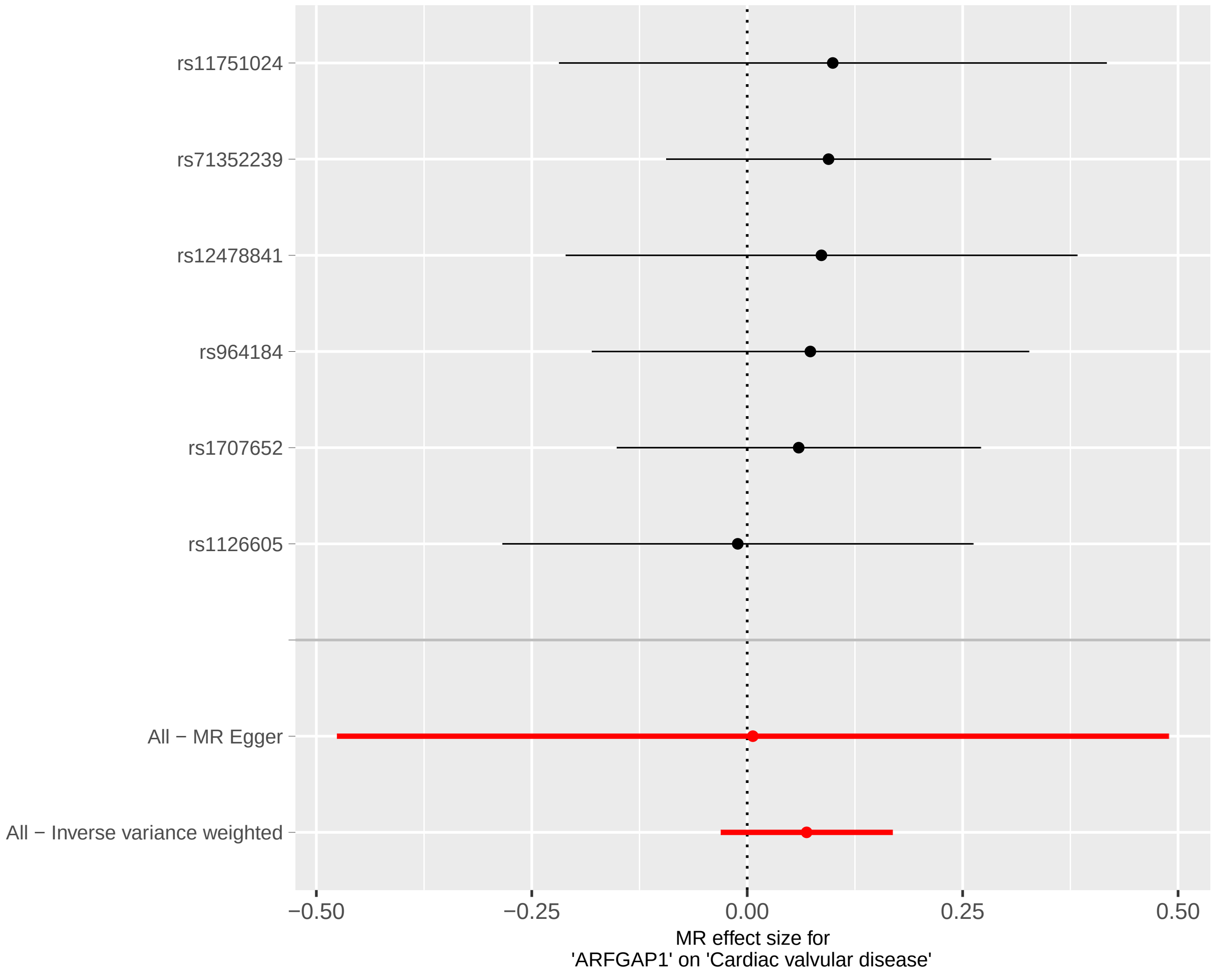
rs11751024
rs71352239
rs12478841
rs964184
rs1707652
rs1126605
All − MR Egger
All − Inverse variance weighted
−0.50
−0.25
0.00
0.25
0.50
MR effect size for
'ARFGAP1' on 'Cardiac valvular disease'

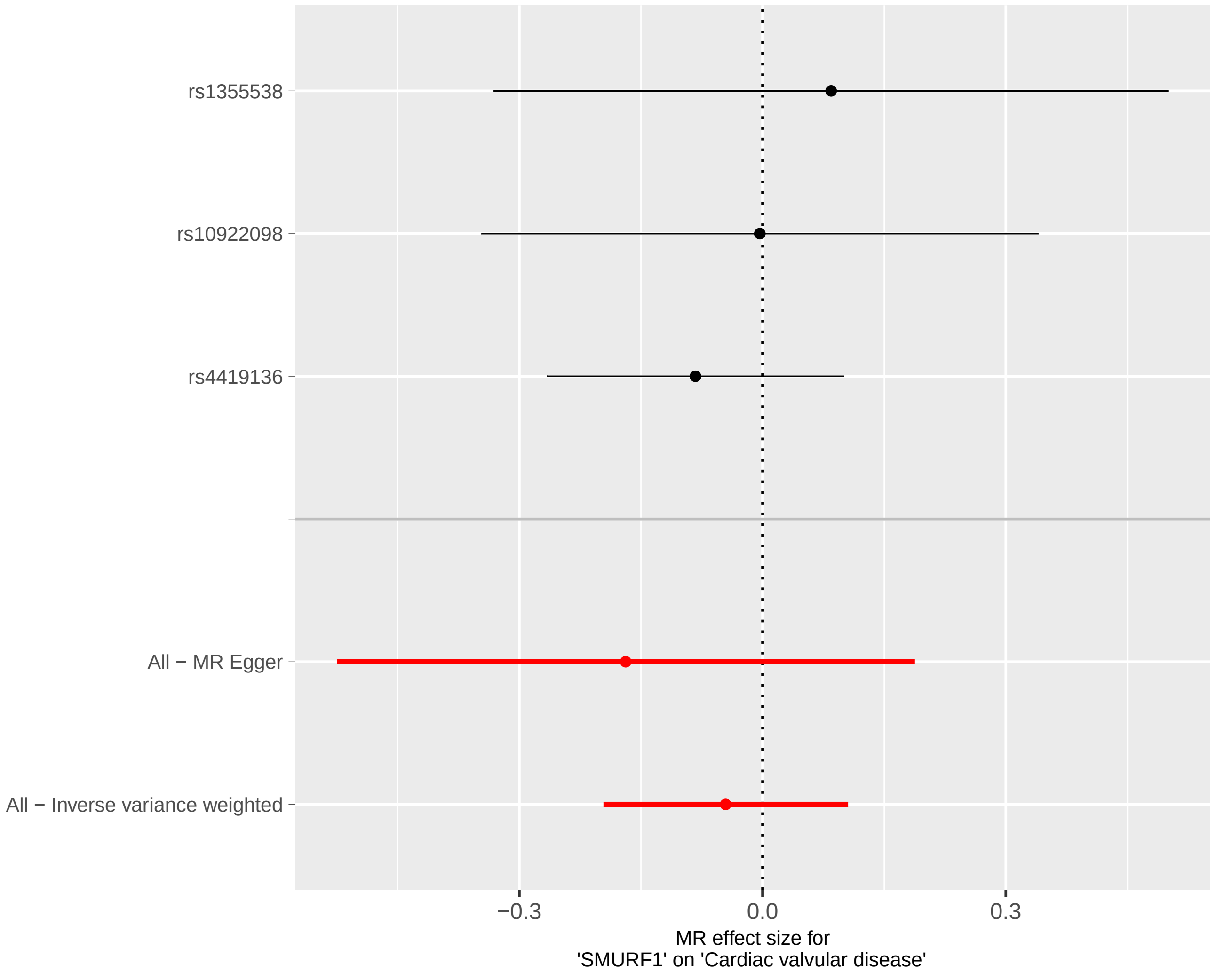

rs1355538
rs10922098
rs4419136
All − MR Egger
All − Inverse variance weighted
−0.3
0.0
0.3
MR effect size for
'SMURF1' on 'Cardiac valvular disease'

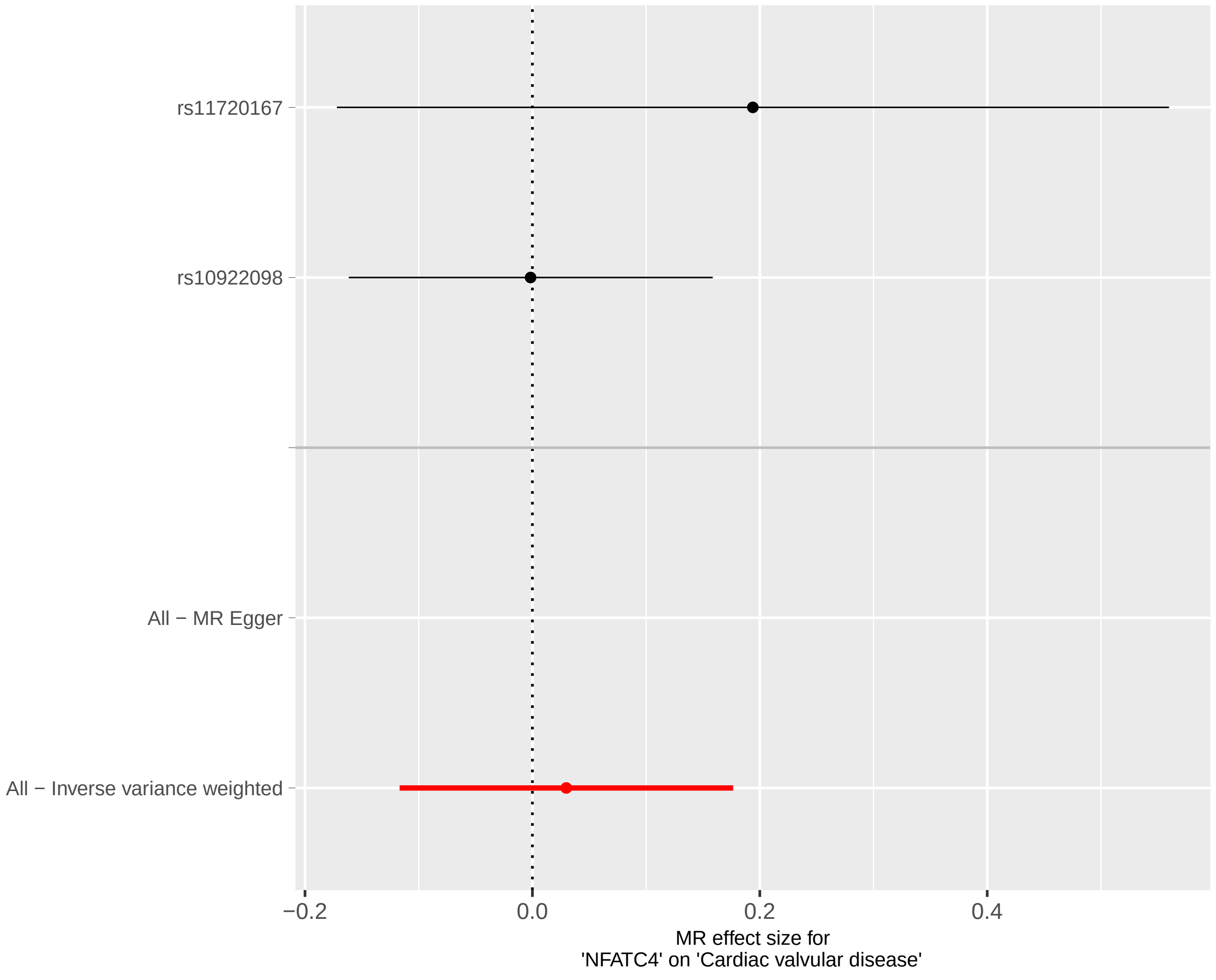

rs11720167
rs10922098
All − MR Egger
All − Inverse variance weighted
−0.2
0.0
0.2
0.4
MR effect size for
'NFATC4' on 'Cardiac valvular disease'

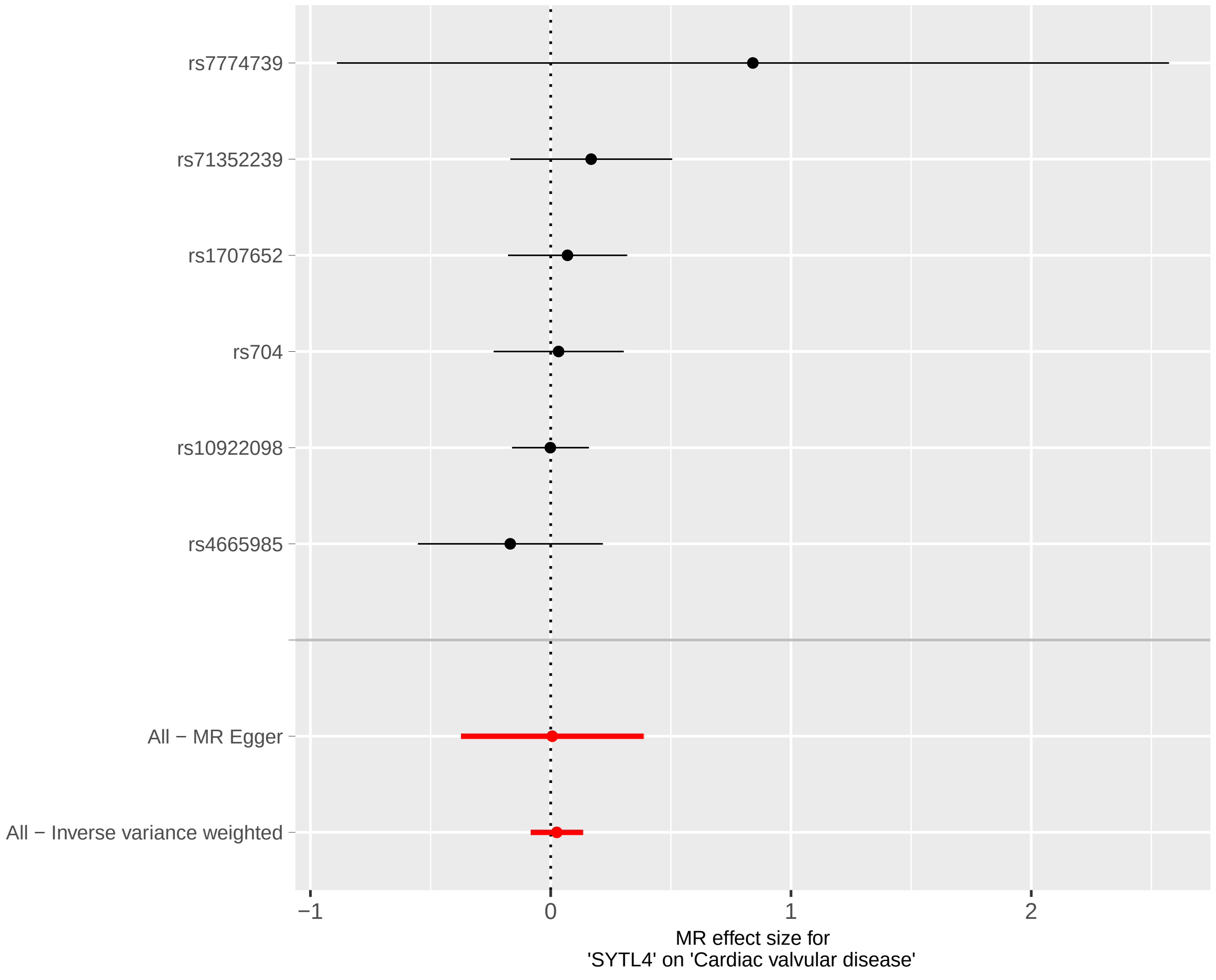
rs7774739
rs71352239
rs1707652
rs704
rs10922098
rs4665985
All − MR Egger
All − Inverse variance weighted
−1
0
1
2
MR effect size for
'SYTL4' on 'Cardiac valvular disease'

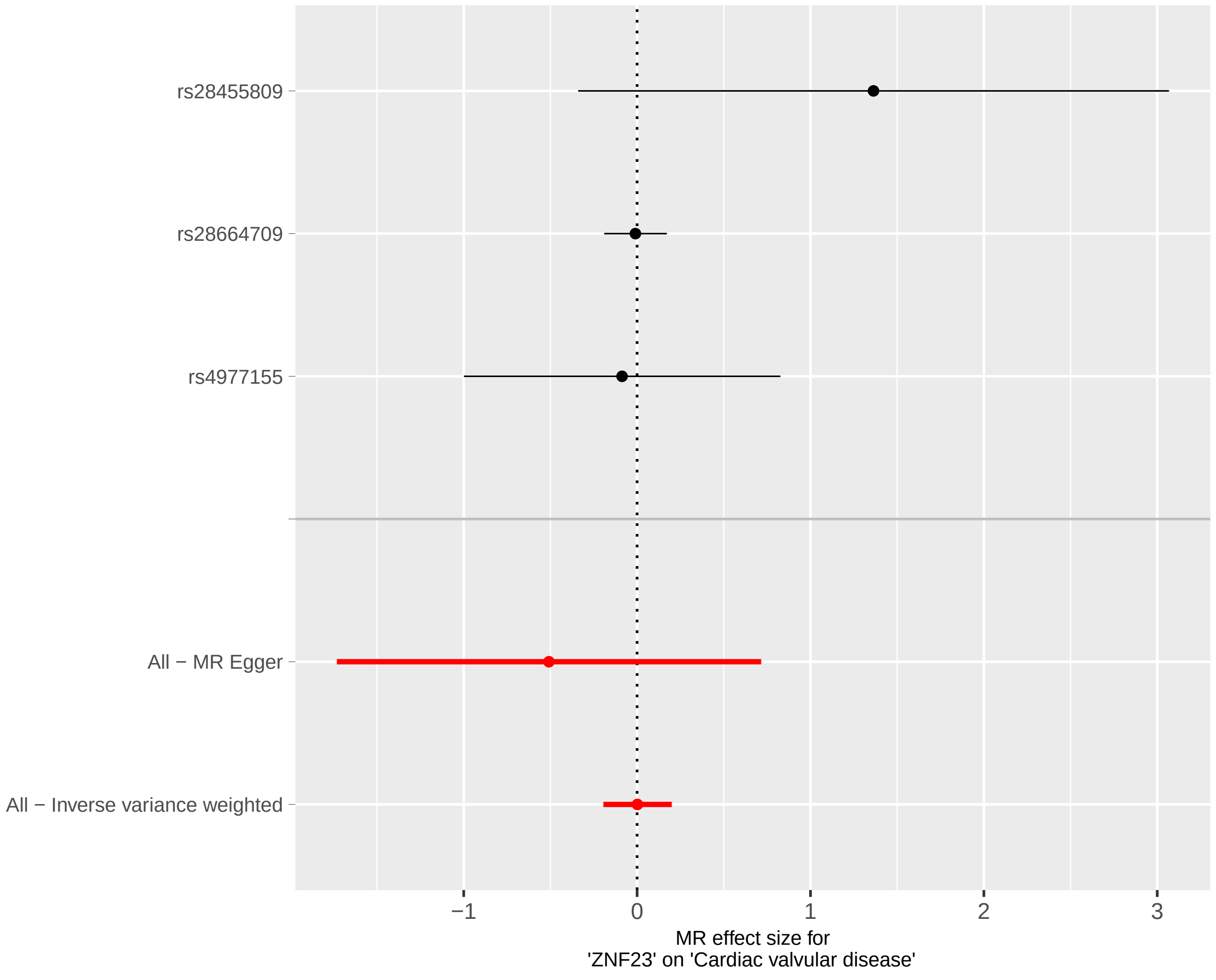
rs28455809
rs28664709
rs4977155
All − MR Egger
All − Inverse variance weighted
−1
0
1
2
3
MR effect size for
'ZNF23' on 'Cardiac valvular disease'

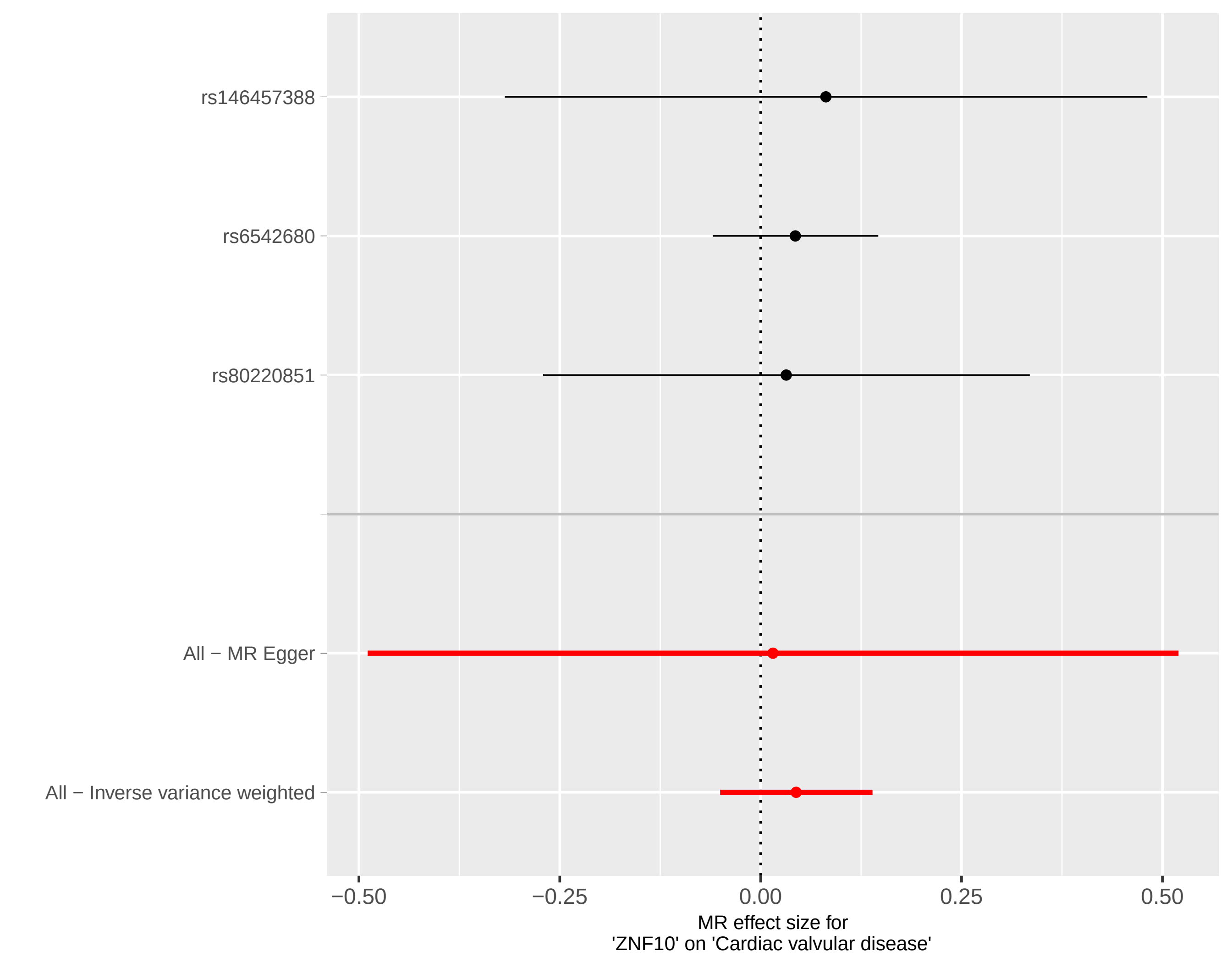
rs146457388
rs6542680
rs80220851
All − MR Egger
All − Inverse variance weighted
−0.50
−0.25
0.00
0.25
0.50
MR effect size for
'ZNF10' on 'Cardiac valvular disease'

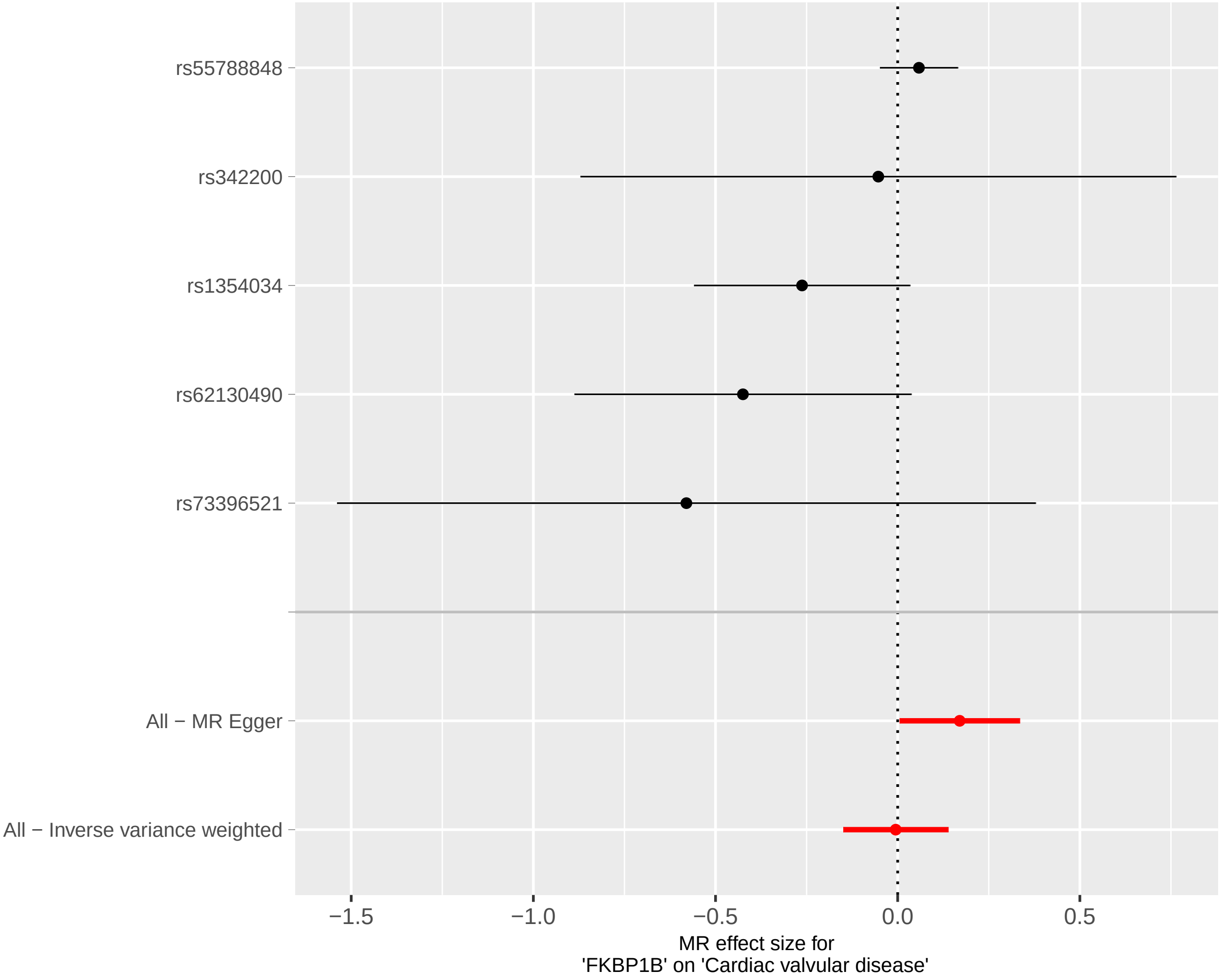
rs55788848
rs342200
rs1354034
rs62130490
rs73396521
All − MR Egger
All − Inverse variance weighted
−1.5
−1.0
−0.5
0.0
0.5
MR effect size for
'FKBP1B' on 'Cardiac valvular disease'

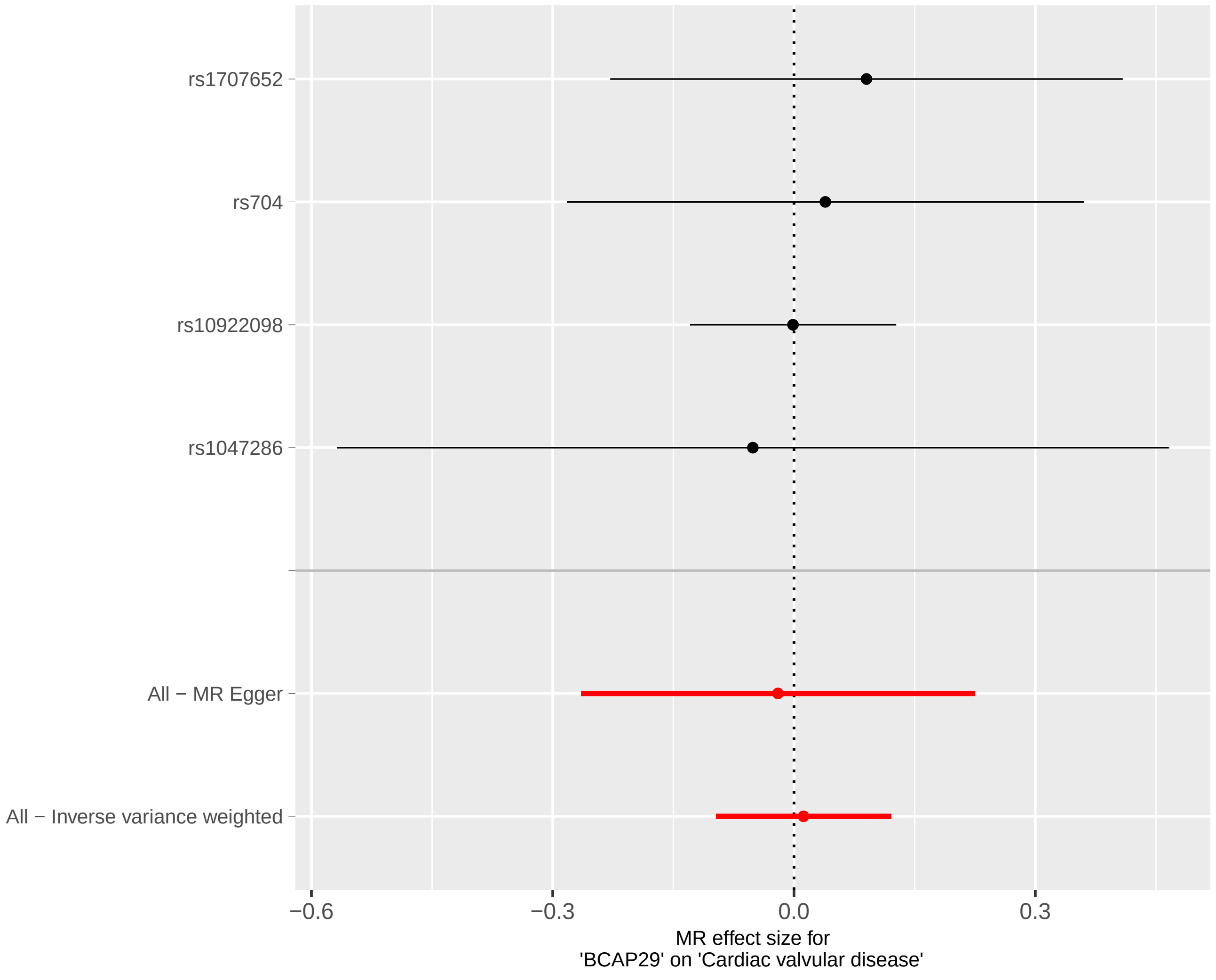
rs1707652
rs704
rs10922098
rs1047286
All − MR Egger
All − Inverse variance weighted
−0.6
−0.3
0.0
0.3
MR effect size for
'BCAP29' on 'Cardiac valvular disease'

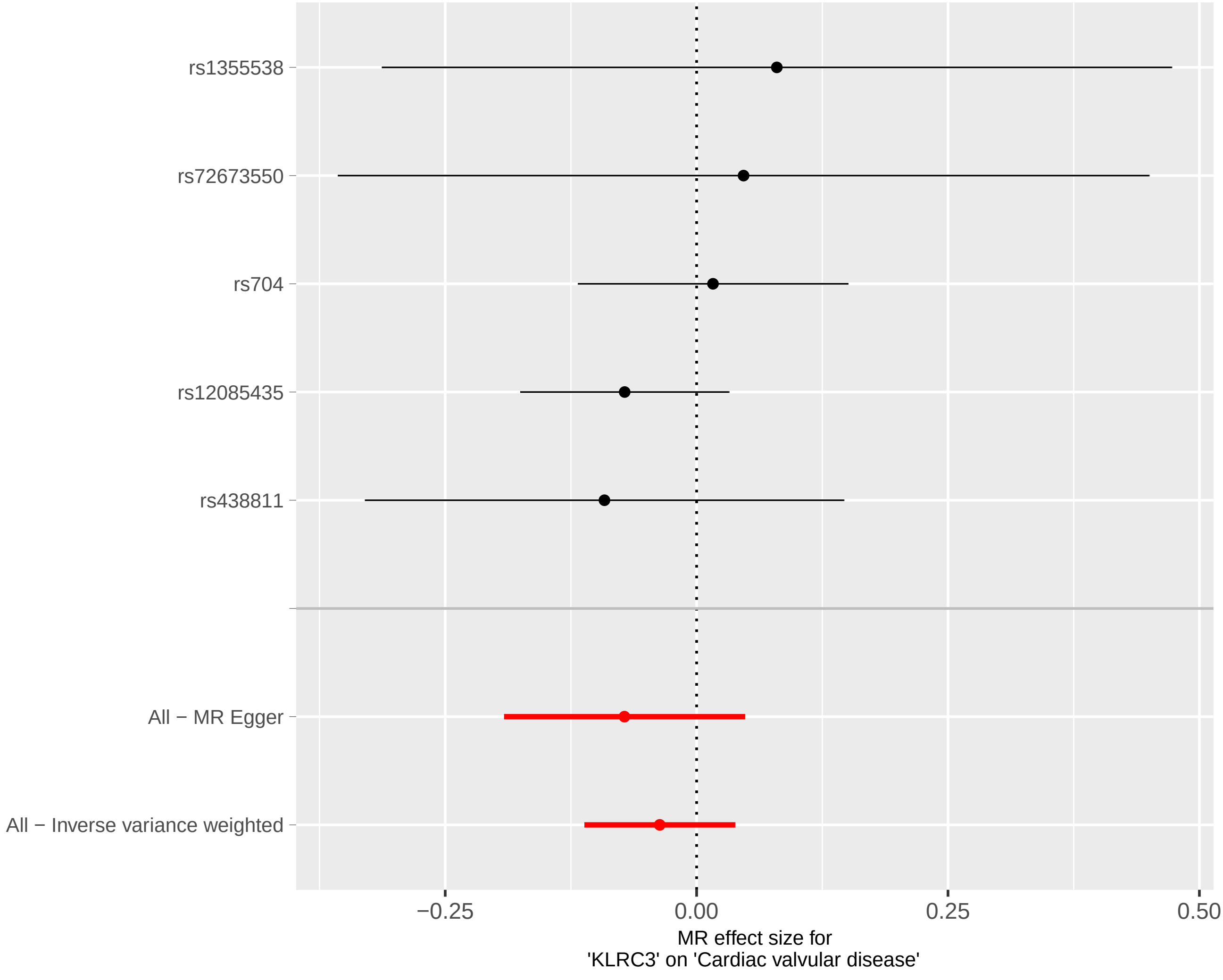

rs1355538
rs72673550
rs704
rs12085435
rs438811
All − MR Egger
All − Inverse variance weighted
−0.25
0.00
0.25
0.50
MR effect size for
'KLRC3' on 'Cardiac valvular disease'

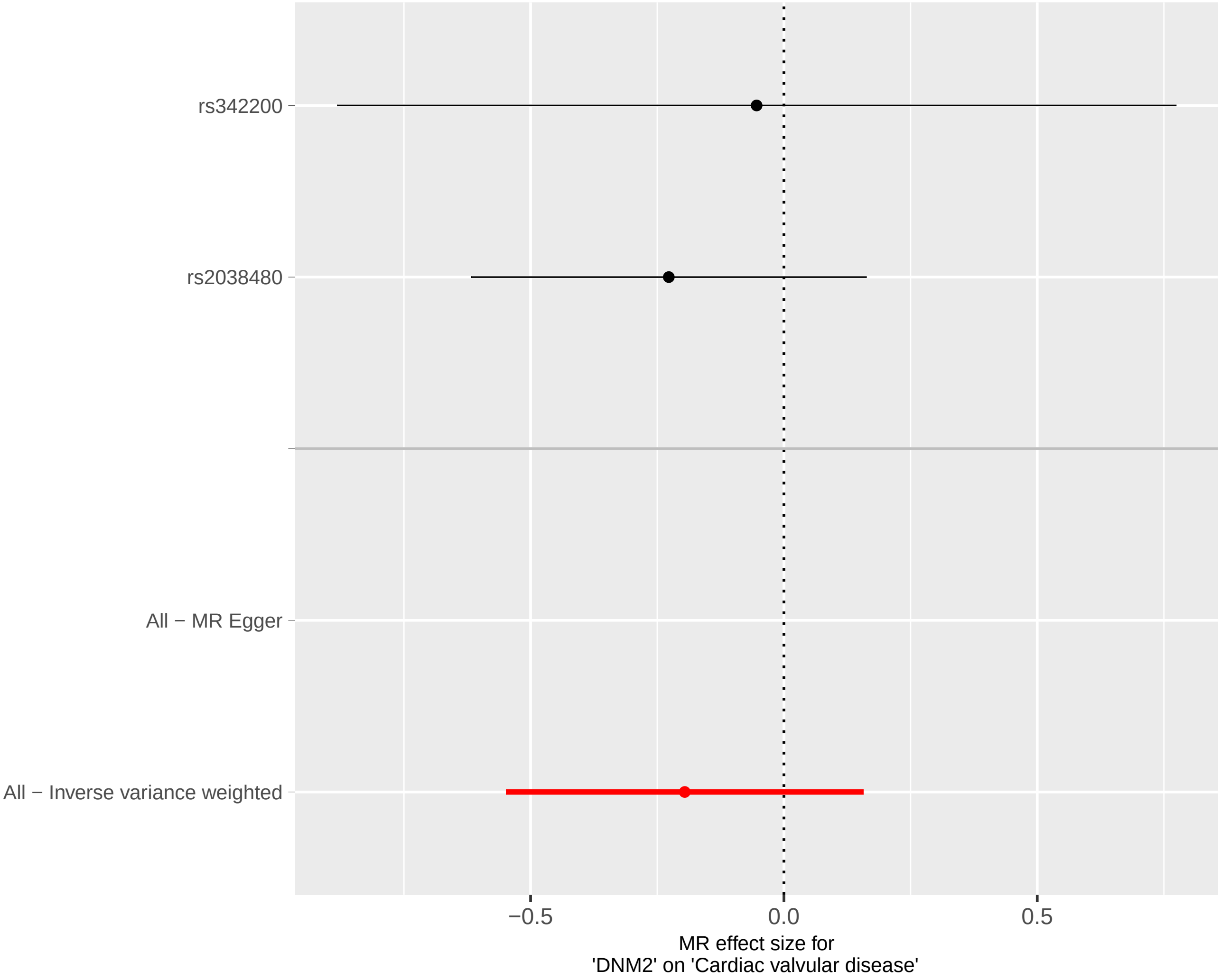
rs342200
rs2038480
All − MR Egger
All − Inverse variance weighted
−0.5
0.0
0.5
MR effect size for
'DNM2' on 'Cardiac valvular disease'

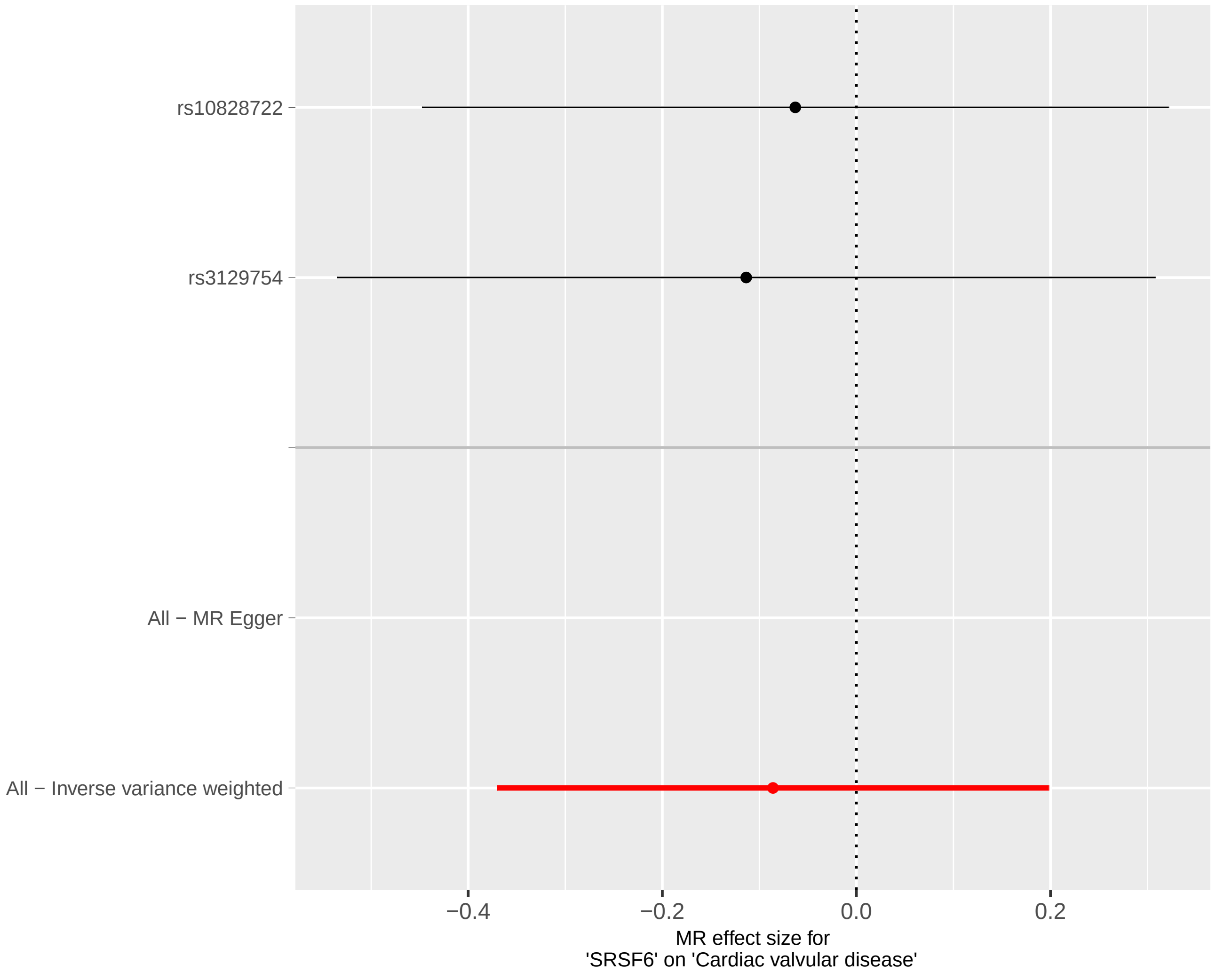

rs10828722
rs3129754
All − MR Egger
All − Inverse variance weighted
−0.4
−0.2
0.0
0.2
MR effect size for
'SRSF6' on 'Cardiac valvular disease'

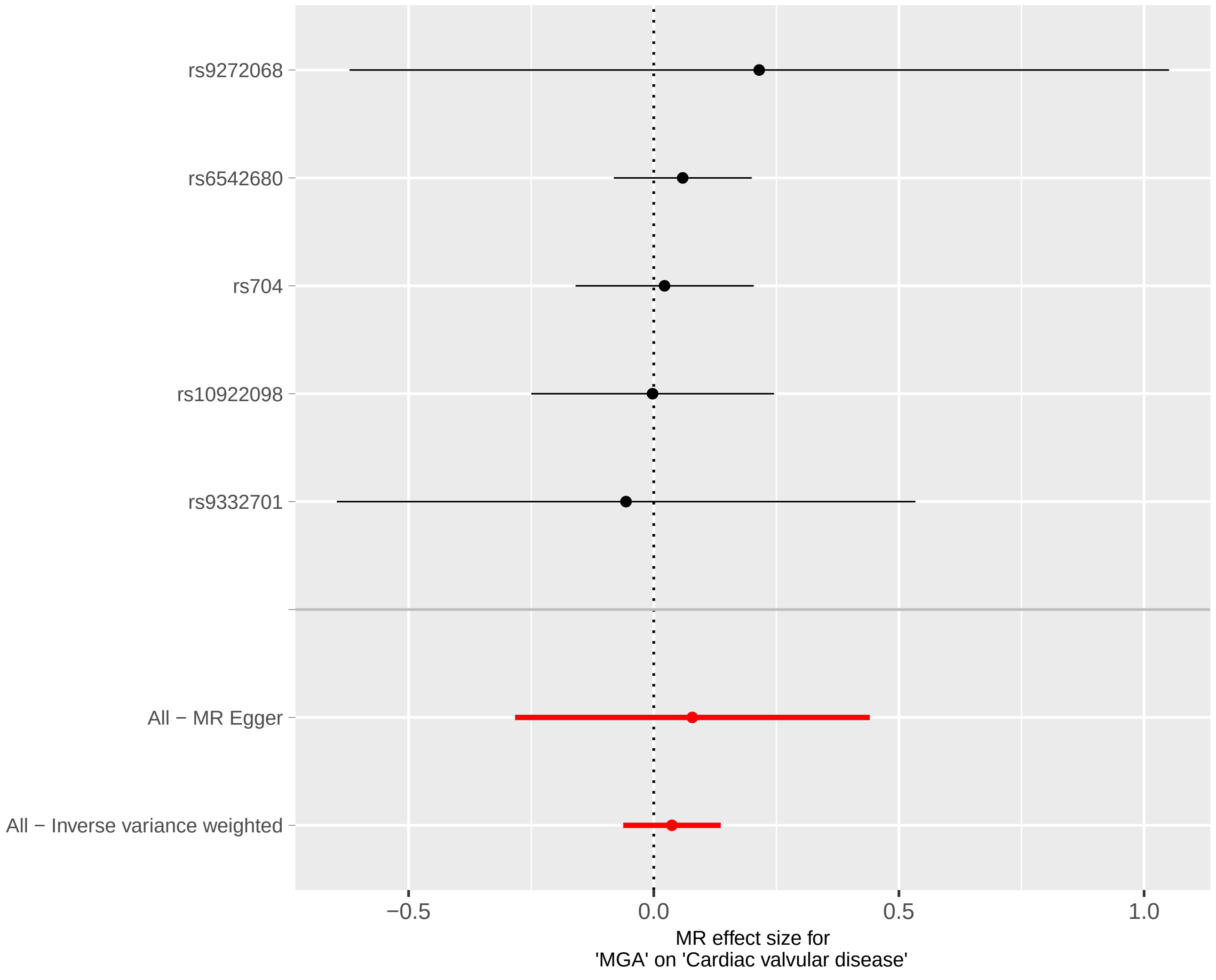
rs9272068
rs6542680
rs704
rs10922098
rs9332701
All − MR Egger
All − Inverse variance weighted
−0.5
0.0
0.5
1.0
MR effect size for
'MGA' on 'Cardiac valvular disease'

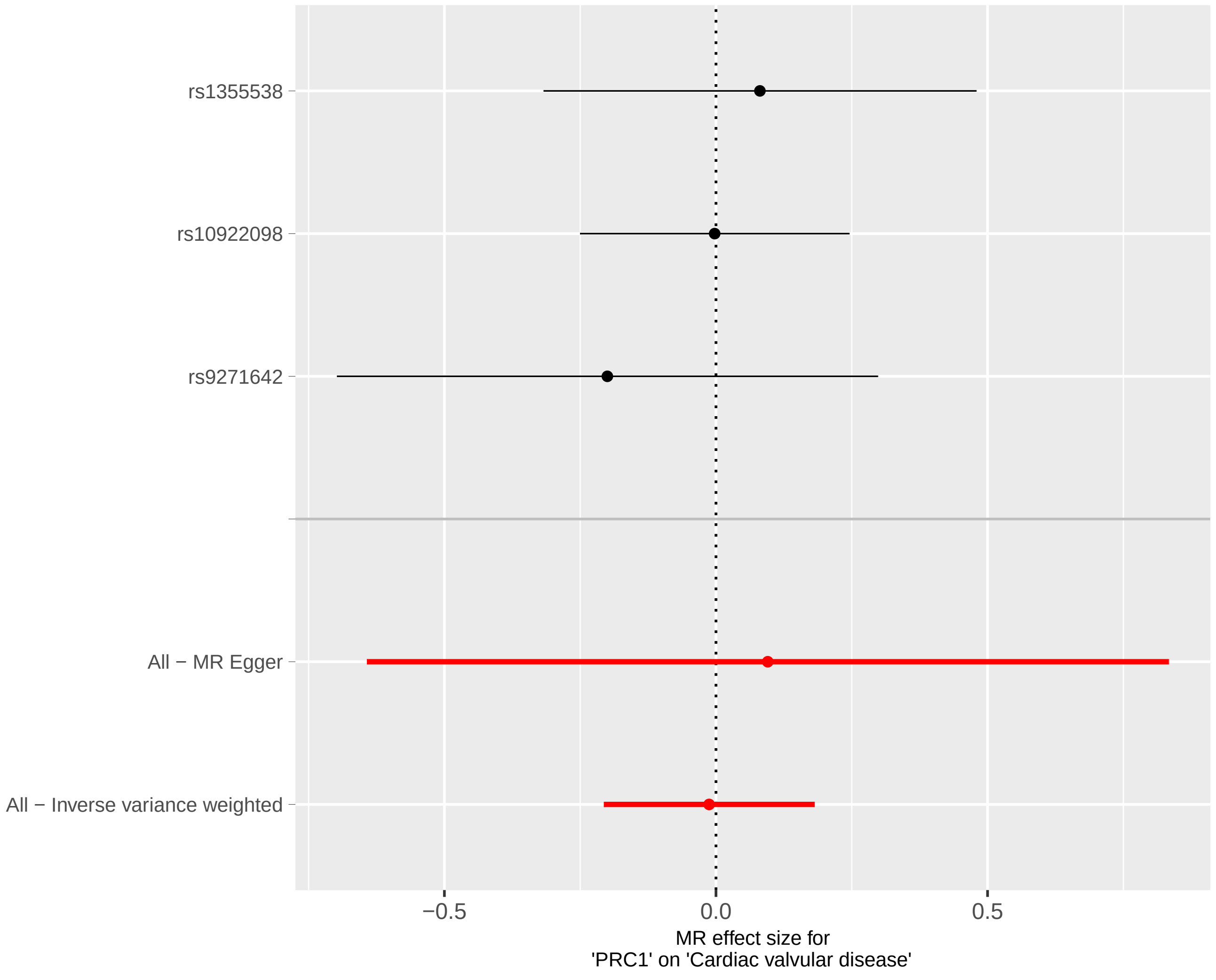

rs1355538
rs10922098
rs9271642
All − MR Egger
All − Inverse variance weighted
−0.5
0.0
0.5
MR effect size for
'PRC1' on 'Cardiac valvular disease'

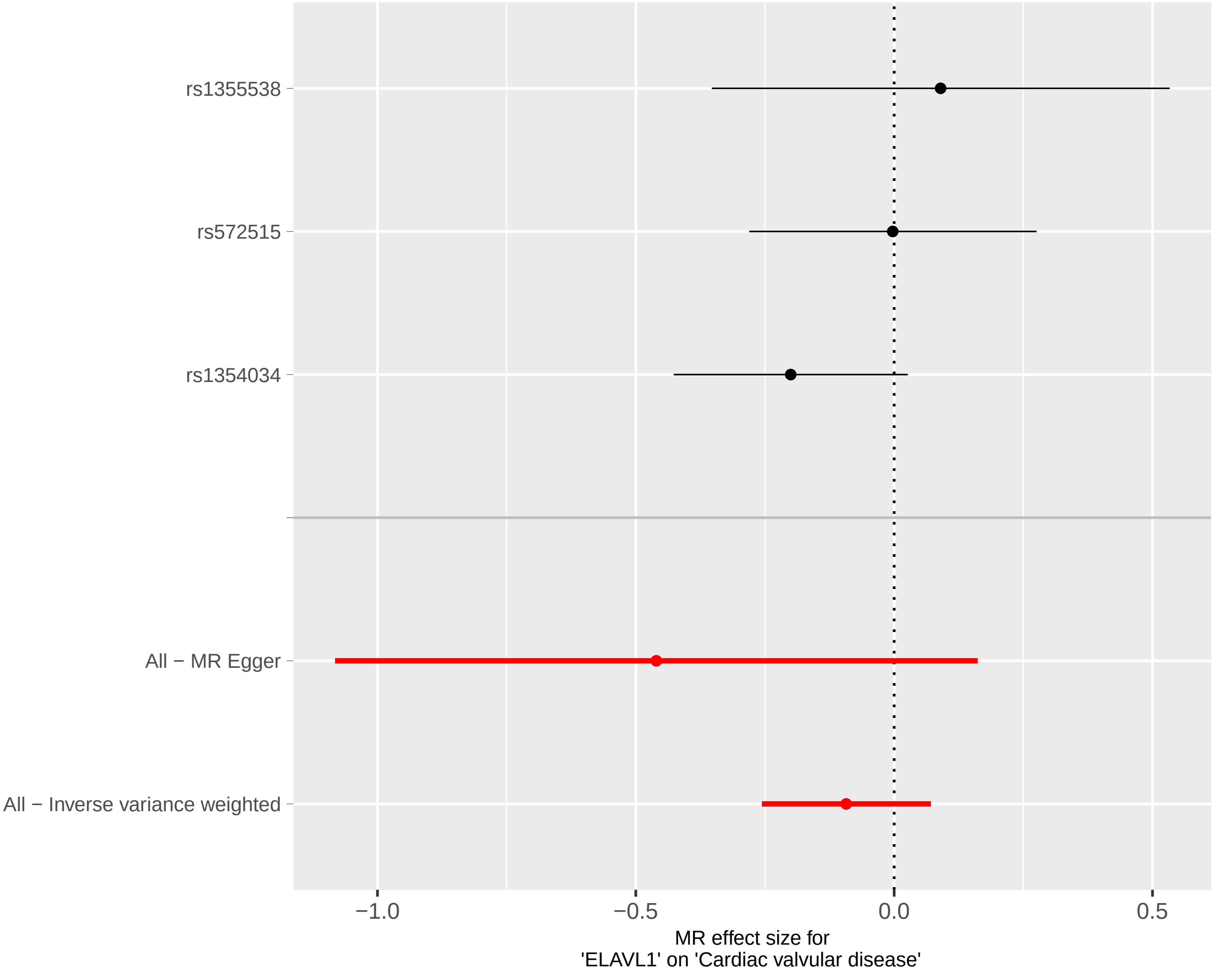

rs1355538
rs572515
rs1354034
All − MR Egger
All − Inverse variance weighted
−1.0
−0.5
0.0
0.5
MR effect size for
'ELAVL1' on 'Cardiac valvular disease'

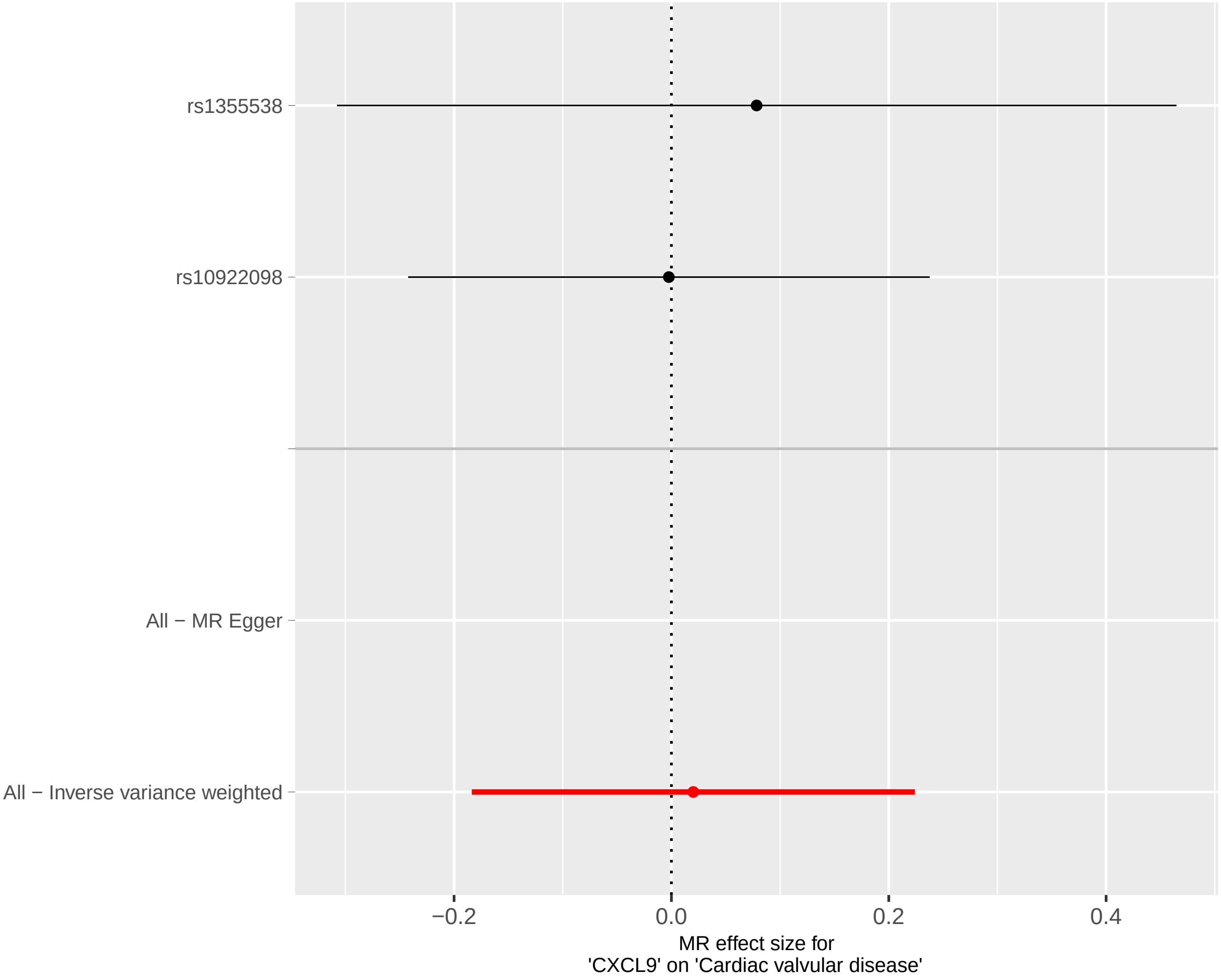

rs1355538
rs10922098
All − MR Egger
All − Inverse variance weighted
−0.2
0.0
0.2
0.4
MR effect size for
'CXCL9' on 'Cardiac valvular disease'

Insufficient number of SNPs

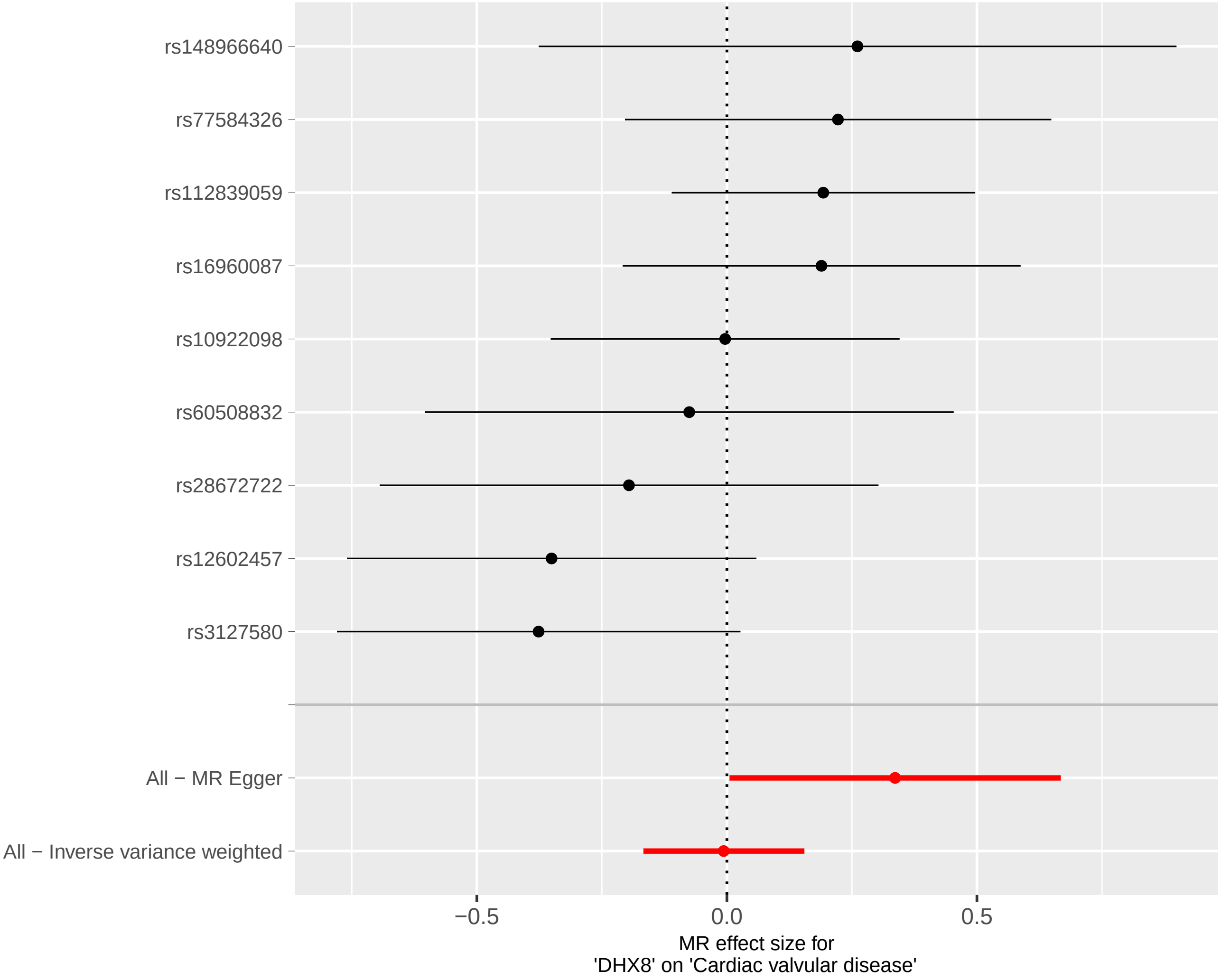
rs148966640
rs77584326
rs112839059
rs16960087
rs10922098
rs60508832
rs28672722
rs12602457
rs3127580
All − MR Egger
All − Inverse variance weighted
−0.5
0.0
0.5
MR effect size for
'DHX8' on 'Cardiac valvular disease'

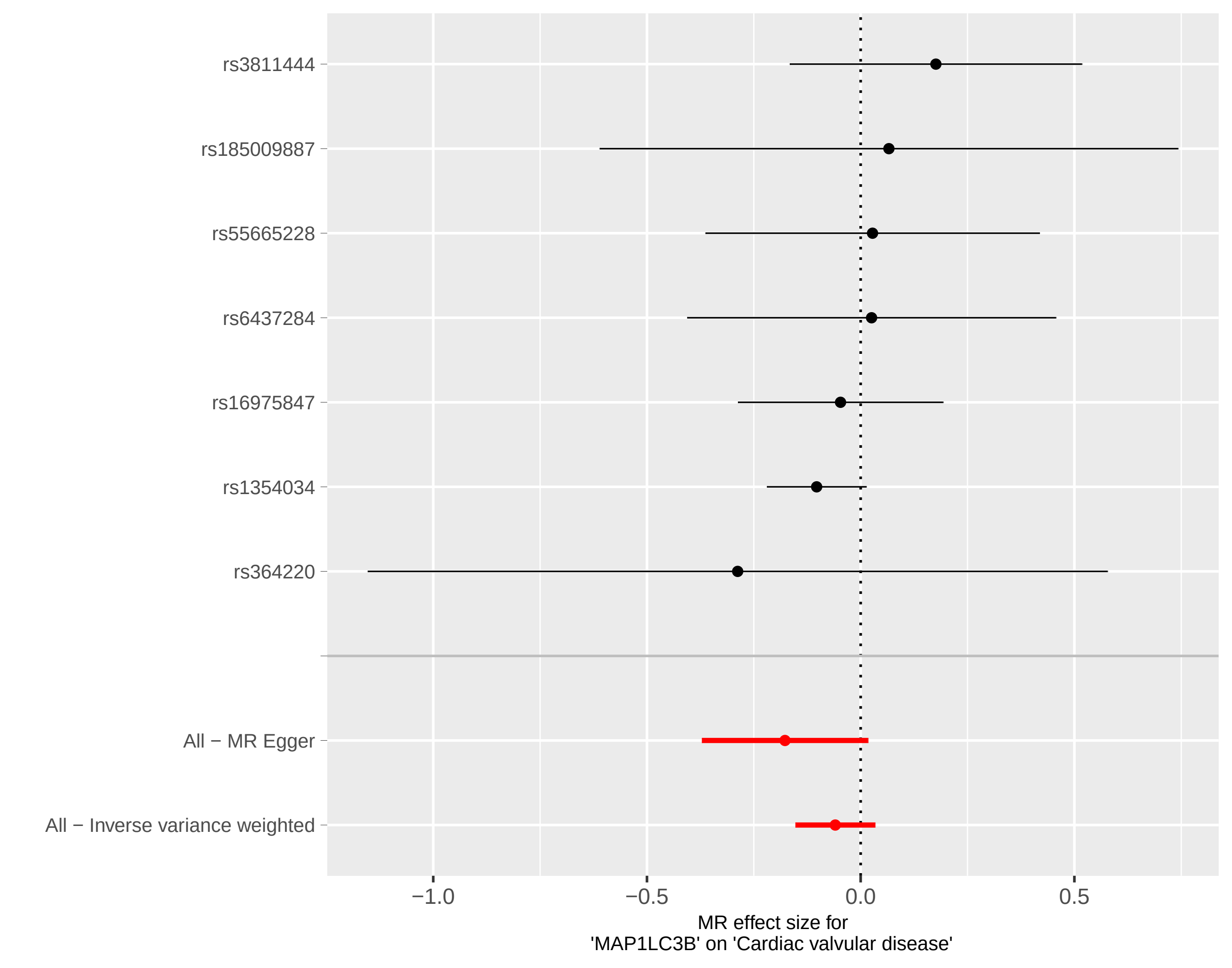
rs3811444
rs185009887
rs55665228
rs6437284
rs16975847
rs1354034
rs364220
All − MR Egger
All − Inverse variance weighted
−1.0
−0.5
0.0
0.5
MR effect size for
'MAP1LC3B' on 'Cardiac valvular disease'

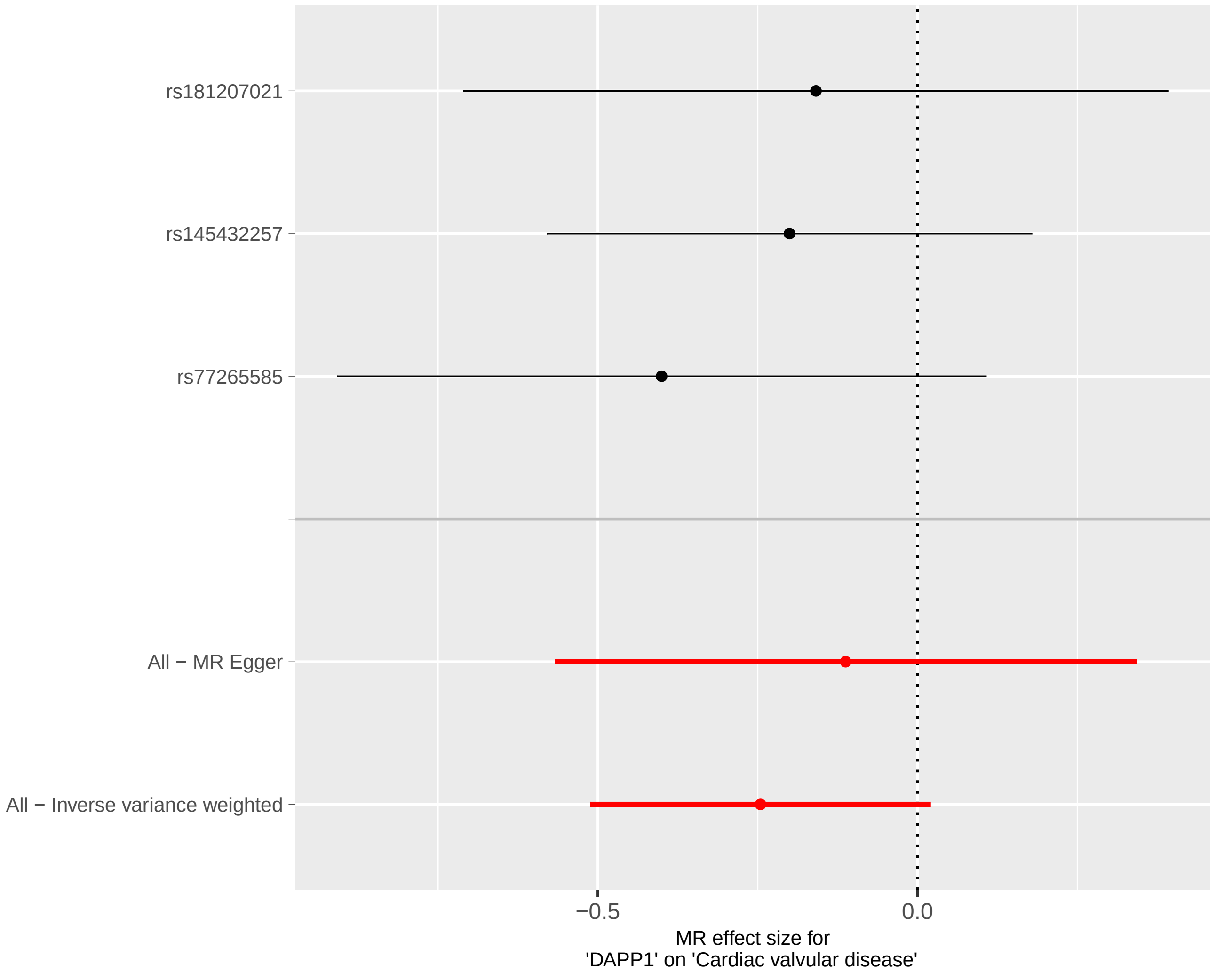
rs181207021
rs145432257
rs77265585
All − MR Egger
All − Inverse variance weighted
−0.5
0.0
MR effect size for
'DAPP1' on 'Cardiac valvular disease'

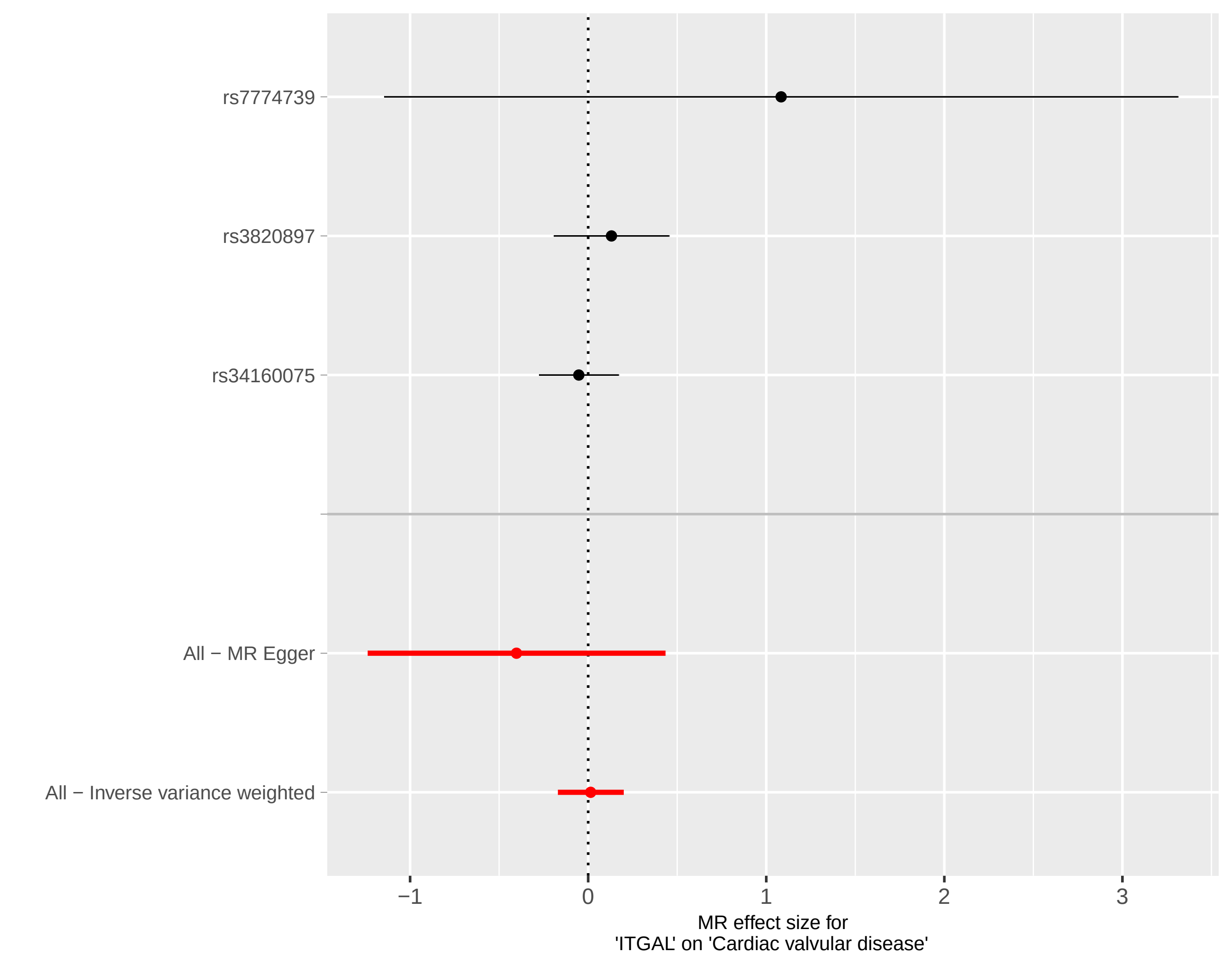
rs7774739
rs3820897
rs34160075
All − MR Egger
All − Inverse variance weighted
−1
0
1
2
3
MR effect size for
'ITGAL' on 'Cardiac valvular disease'

Insufficient number of SNPs

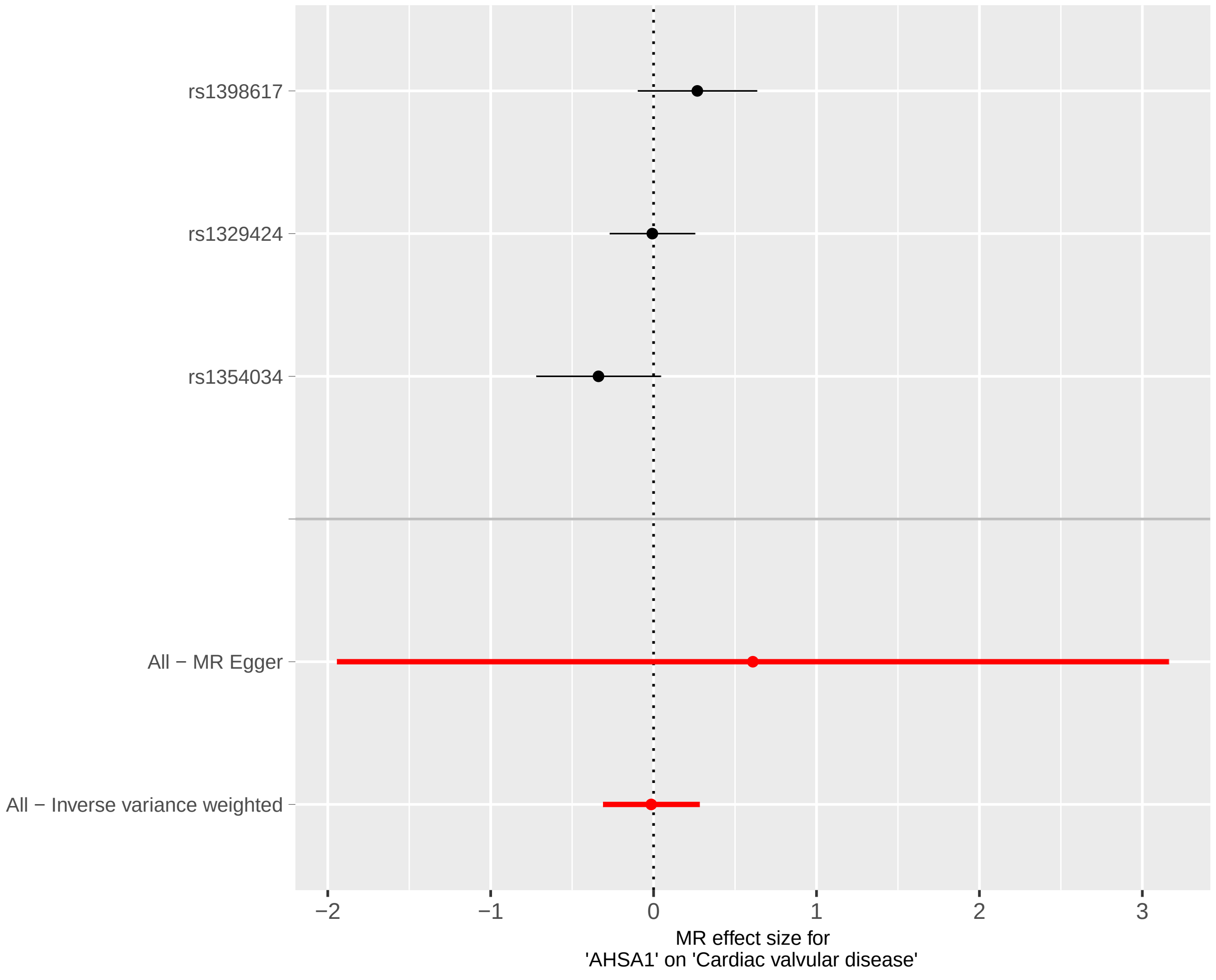
rs1398617
rs1329424
rs1354034
All − MR Egger
All − Inverse variance weighted
−2
−1
0
1
2
3
MR effect size for
'AHSA1' on 'Cardiac valvular disease'

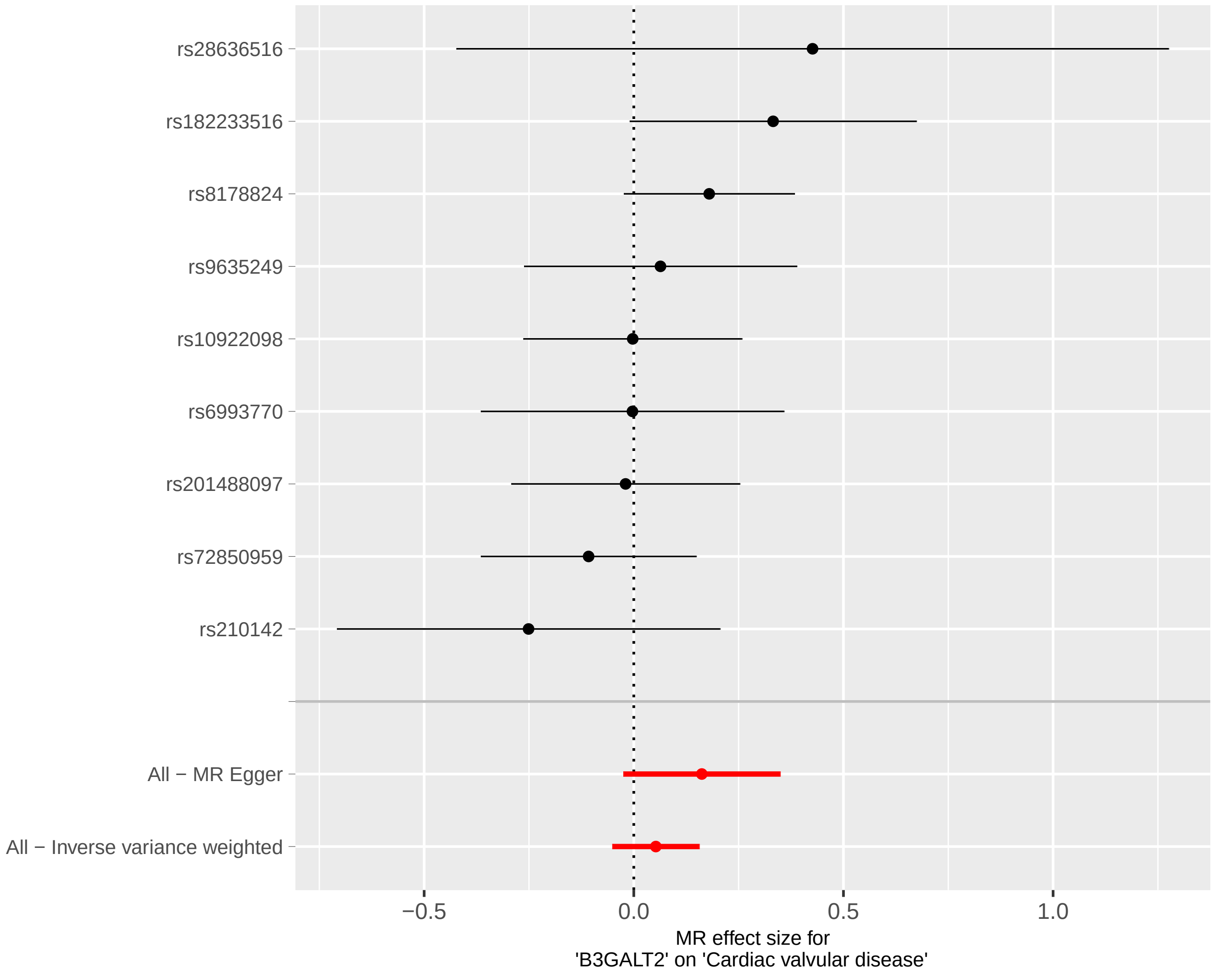
rs28636516
rs182233516
rs8178824
rs9635249
rs10922098
rs6993770
rs201488097
rs72850959
rs210142
All − MR Egger
All − Inverse variance weighted
−0.5
0.0
0.5
1.0
MR effect size for
'B3GALT2' on 'Cardiac valvular disease'

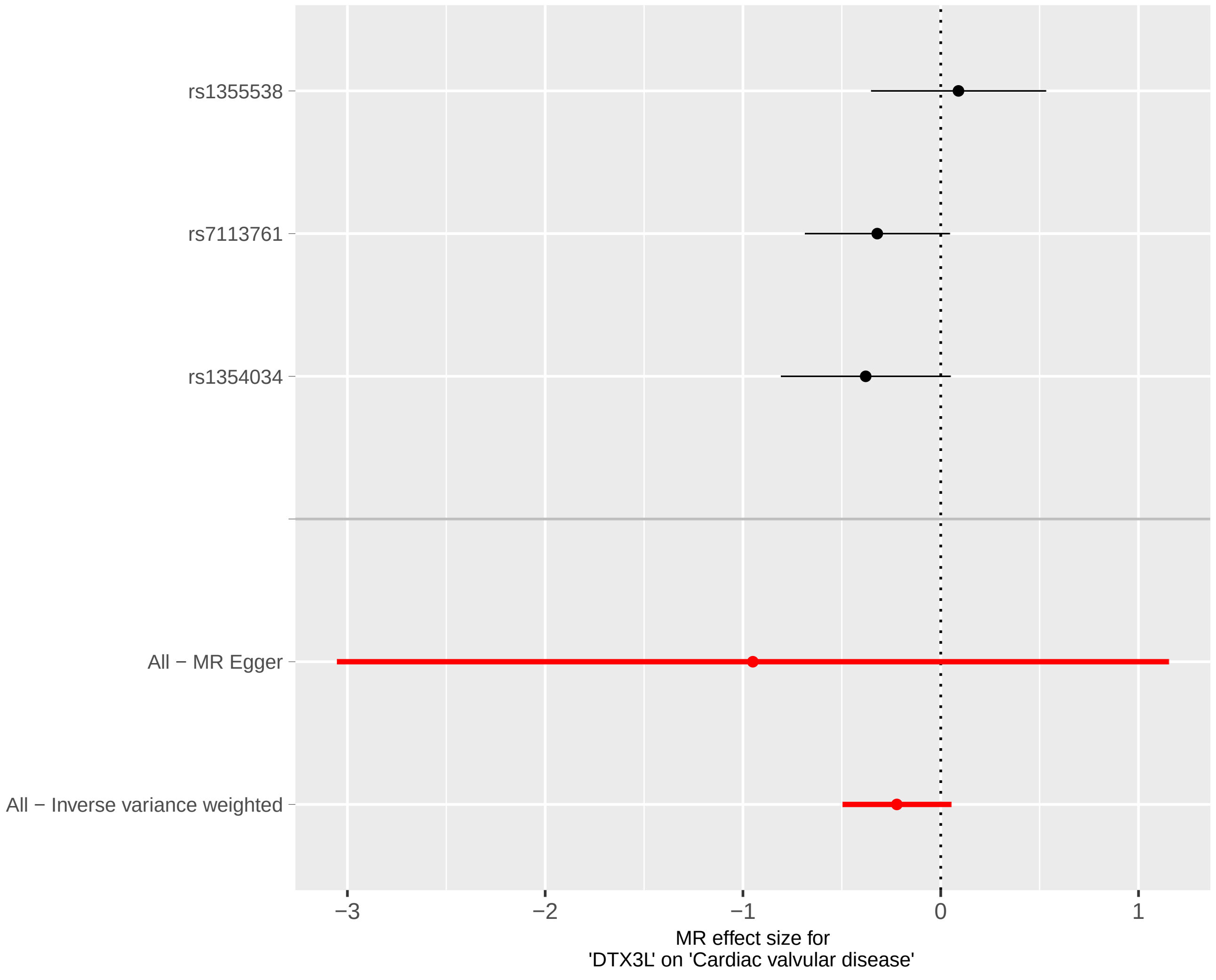
rs1355538
rs7113761
rs1354034
All − MR Egger
All − Inverse variance weighted
−3
−2
−1
0
1
MR effect size for
'DTX3L' on 'Cardiac valvular disease'

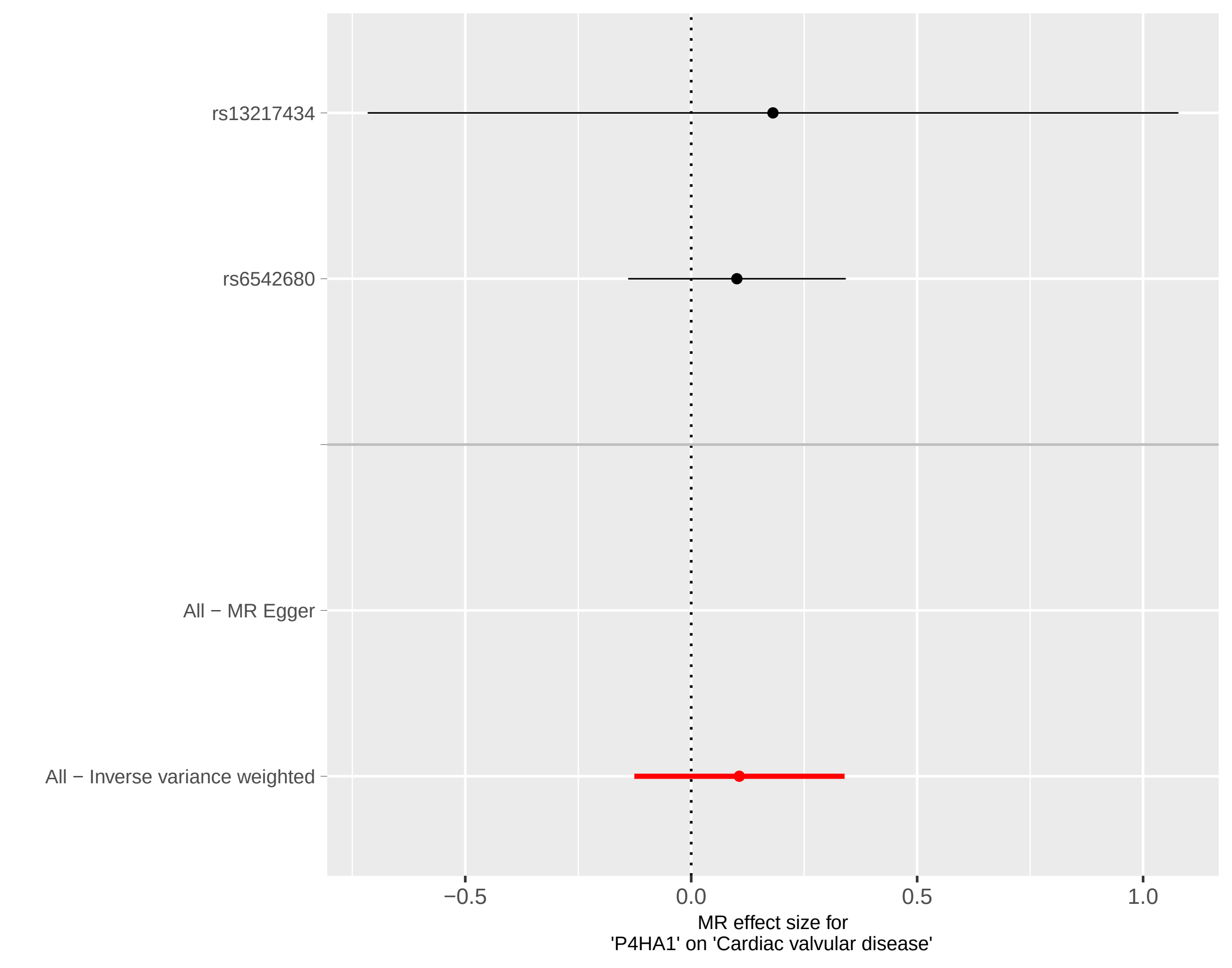
rs13217434
rs6542680
All − MR Egger
All − Inverse variance weighted
−0.5
0.0
0.5
1.0
MR effect size for
'P4HA1' on 'Cardiac valvular disease'

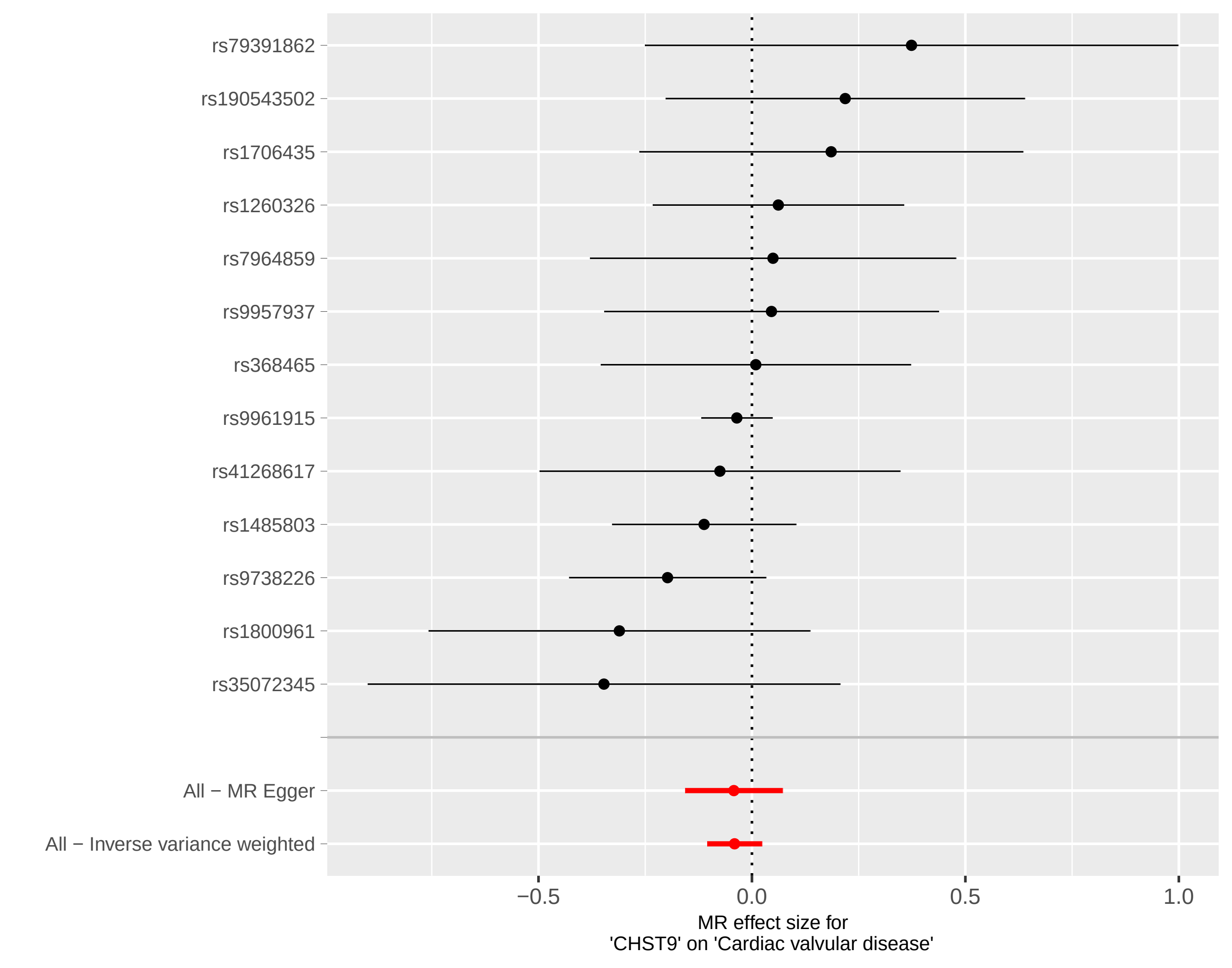
rs79391862
rs190543502
rs1706435
rs1260326
rs7964859
rs9957937
rs368465
rs9961915
rs41268617
rs1485803
rs9738226
rs1800961
rs35072345
All − MR Egger
All − Inverse variance weighted
−0.5
0.0
0.5
1.0
MR effect size for
'CHST9' on 'Cardiac valvular disease'

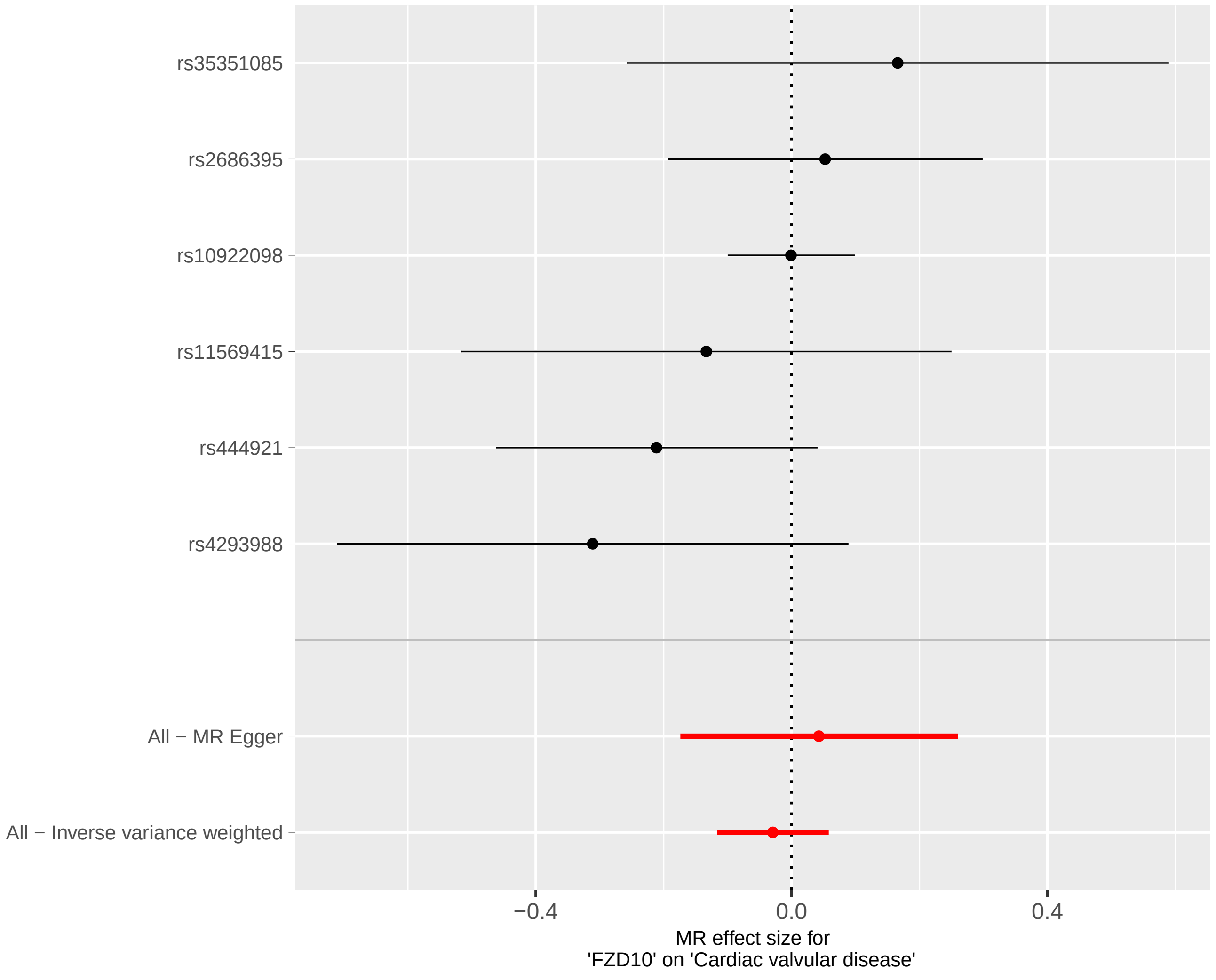

rs35351085
rs2686395
rs10922098
rs11569415
rs444921
rs4293988
All − MR Egger
All − Inverse variance weighted
−0.4
0.0
0.4
MR effect size for
'FZD10' on 'Cardiac valvular disease'

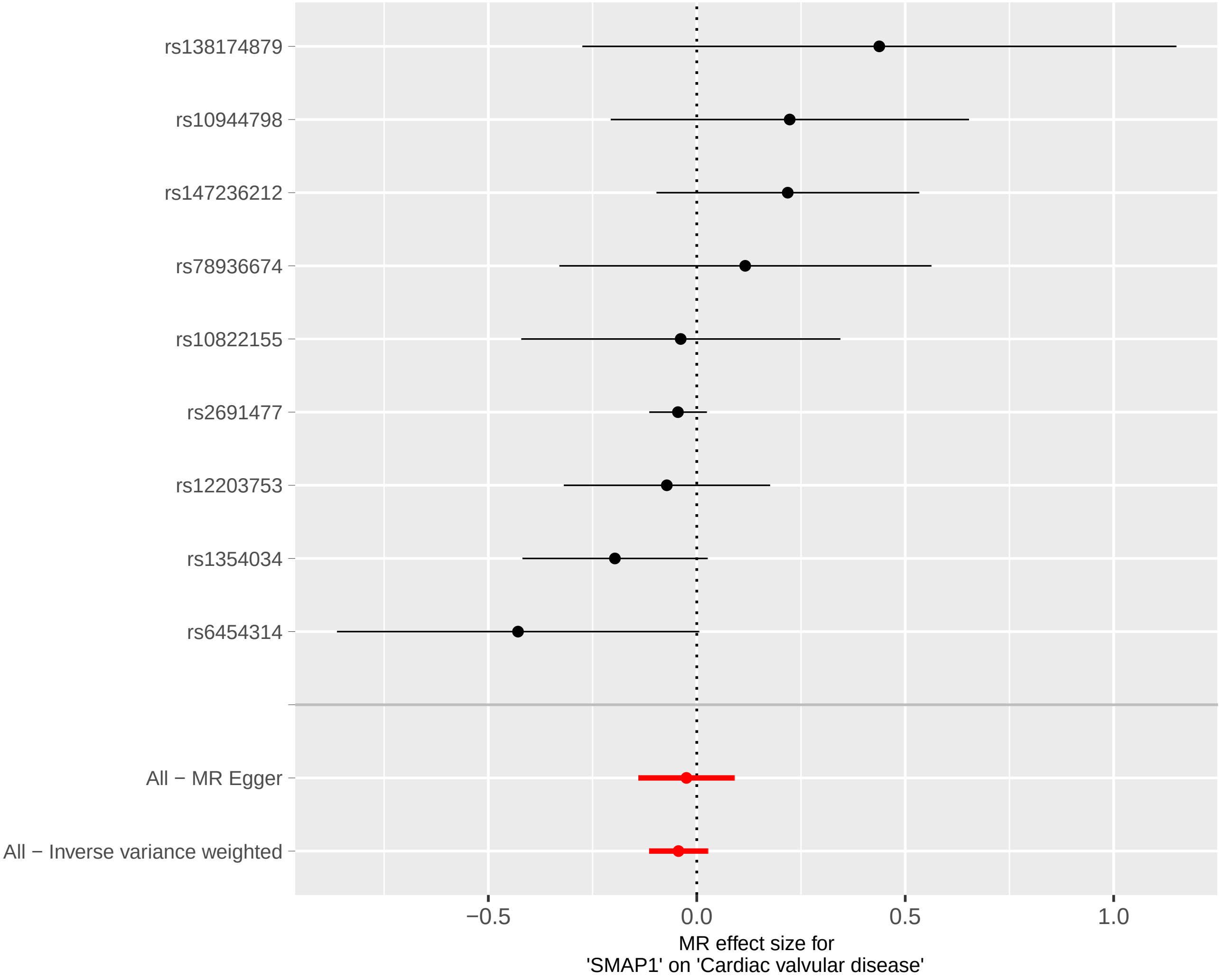
rs138174879
rs10944798
rs147236212
rs78936674
rs10822155
rs2691477
rs12203753
rs1354034
rs6454314
All − MR Egger
All − Inverse variance weighted
−0.5
0.0
0.5
1.0
MR effect size for
'SMAP1' on 'Cardiac valvular disease'

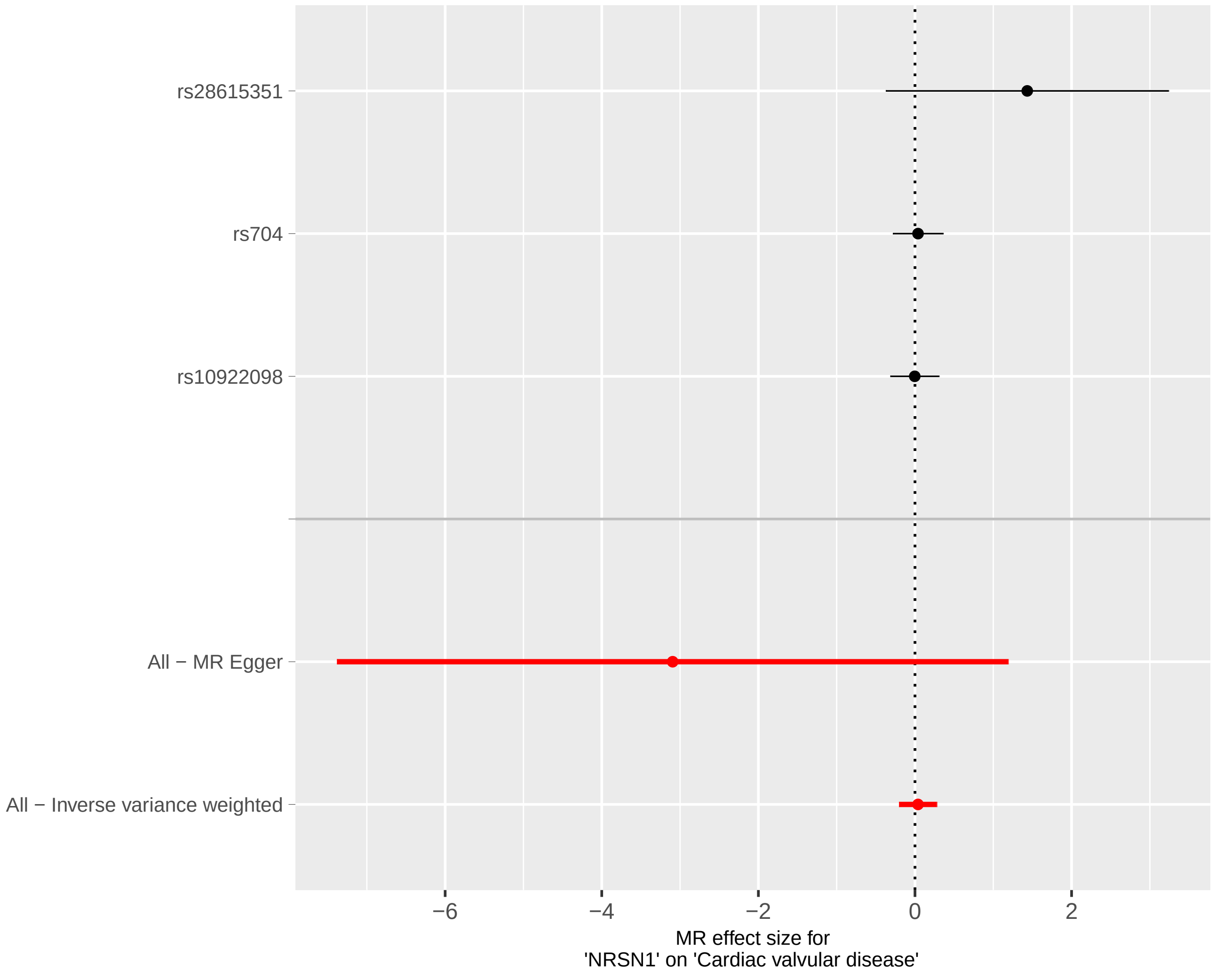

rs28615351
rs704
rs10922098
All − MR Egger
All − Inverse variance weighted
−6
−4
−2
0
2
MR effect size for
'NRSN1' on 'Cardiac valvular disease'

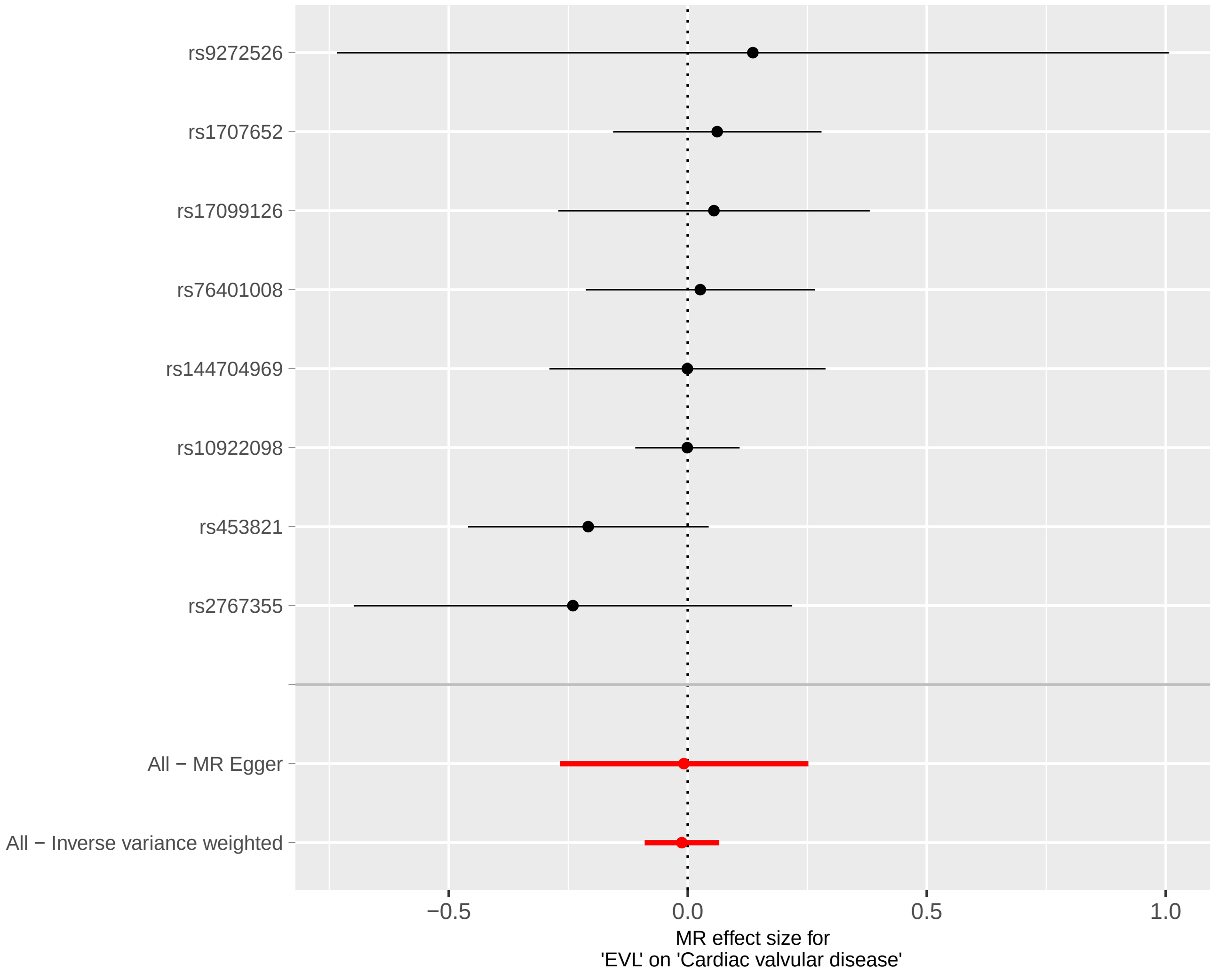

rs9272526
rs1707652
rs17099126
rs76401008
rs144704969
rs10922098
rs453821
rs2767355
All − MR Egger
All − Inverse variance weighted
−0.5
0.0
0.5
1.0
MR effect size for
'EVL' on 'Cardiac valvular disease'

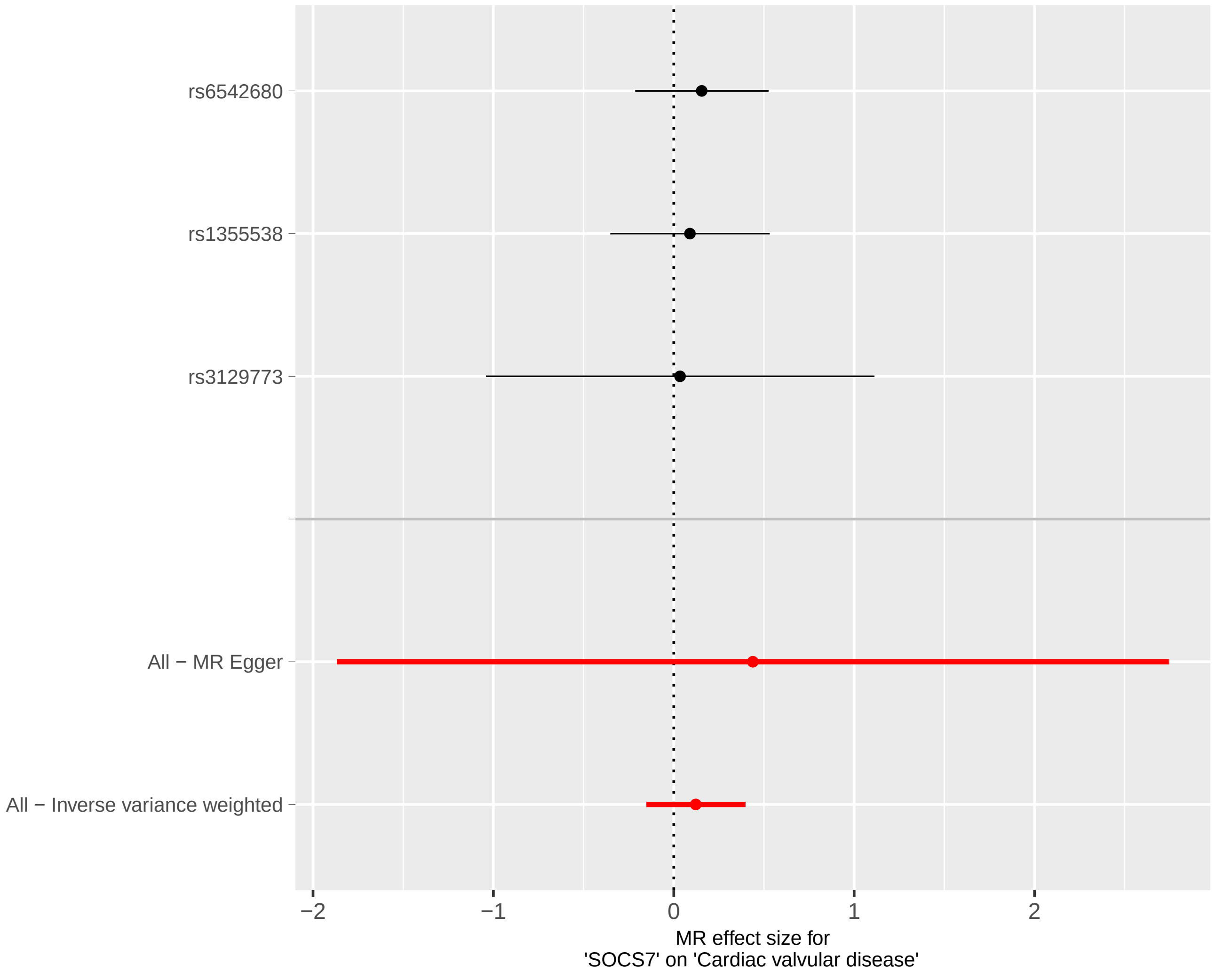

rs6542680
rs1355538
rs3129773
All − MR Egger
All − Inverse variance weighted
−2
−1
0
1
2
MR effect size for
'SOCS7' on 'Cardiac valvular disease'

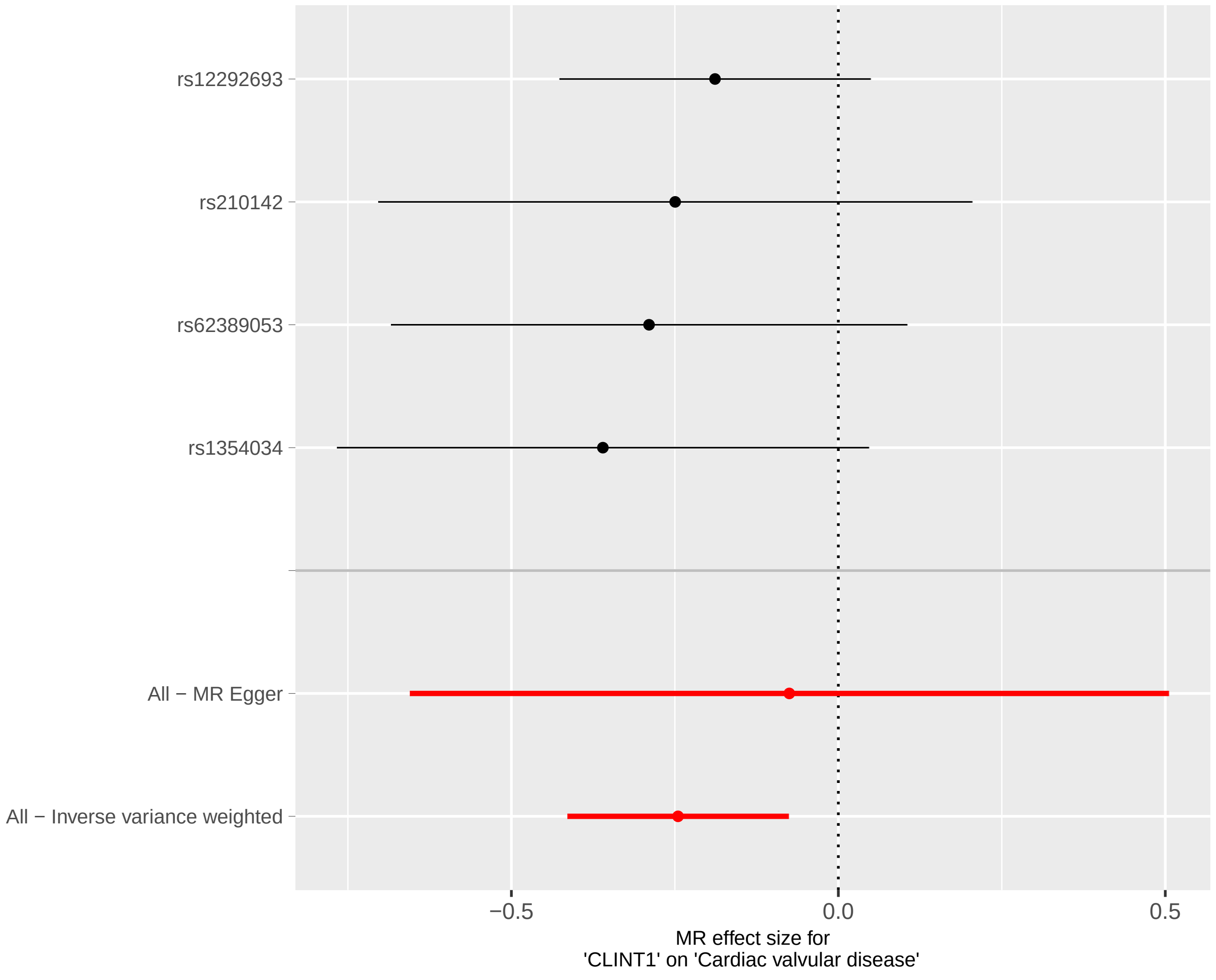

rs12292693
rs210142
rs62389053
rs1354034
All − MR Egger
All − Inverse variance weighted
−0.5
0.0
0.5
MR effect size for
'CLINT1' on 'Cardiac valvular disease'

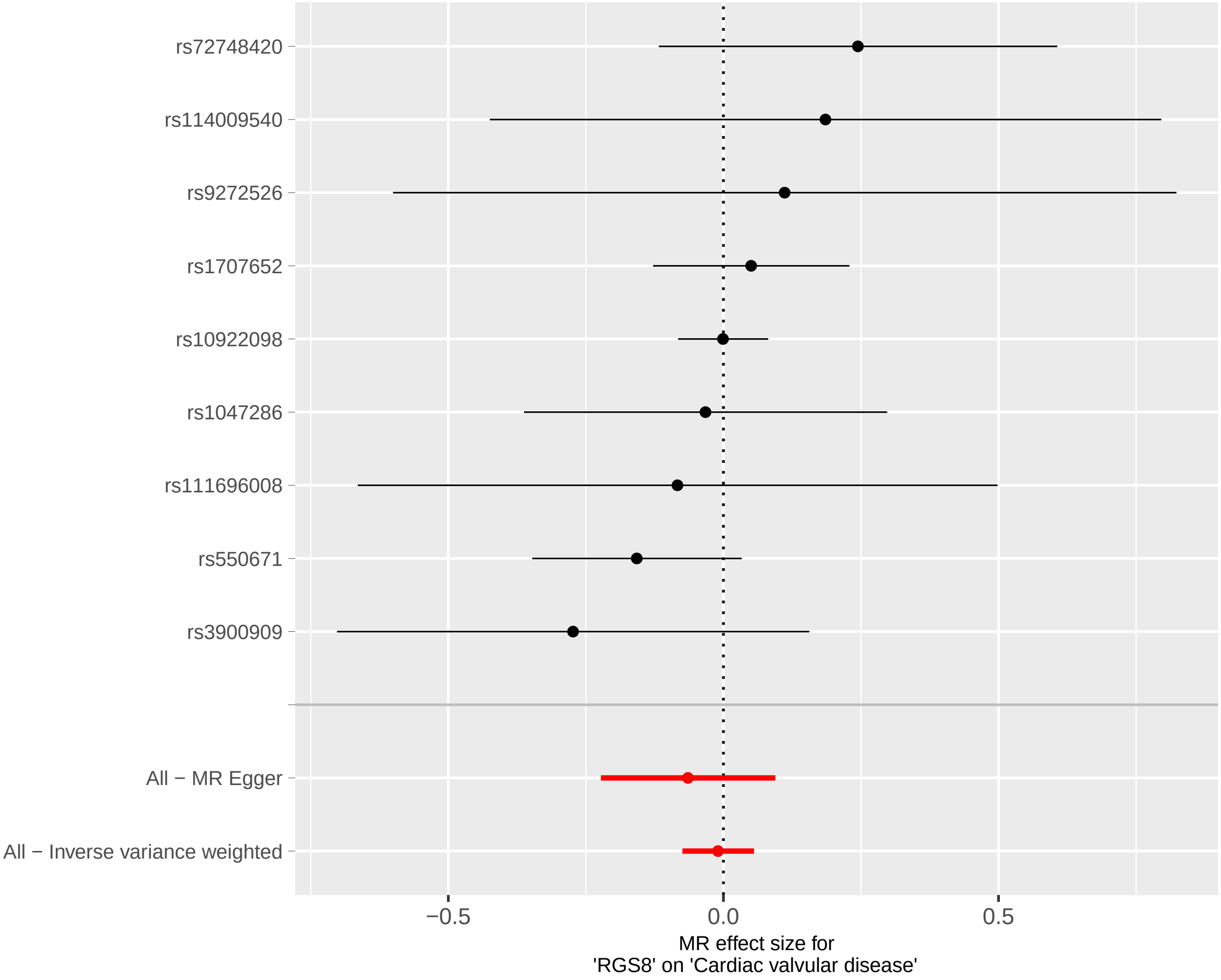
rs72748420
rs114009540
rs9272526
rs1707652
rs10922098
rs1047286
rs111696008
rs550671
rs3900909
All − MR Egger
All − Inverse variance weighted
−0.5
0.0
0.5
MR effect size for
'RGS8' on 'Cardiac valvular disease'

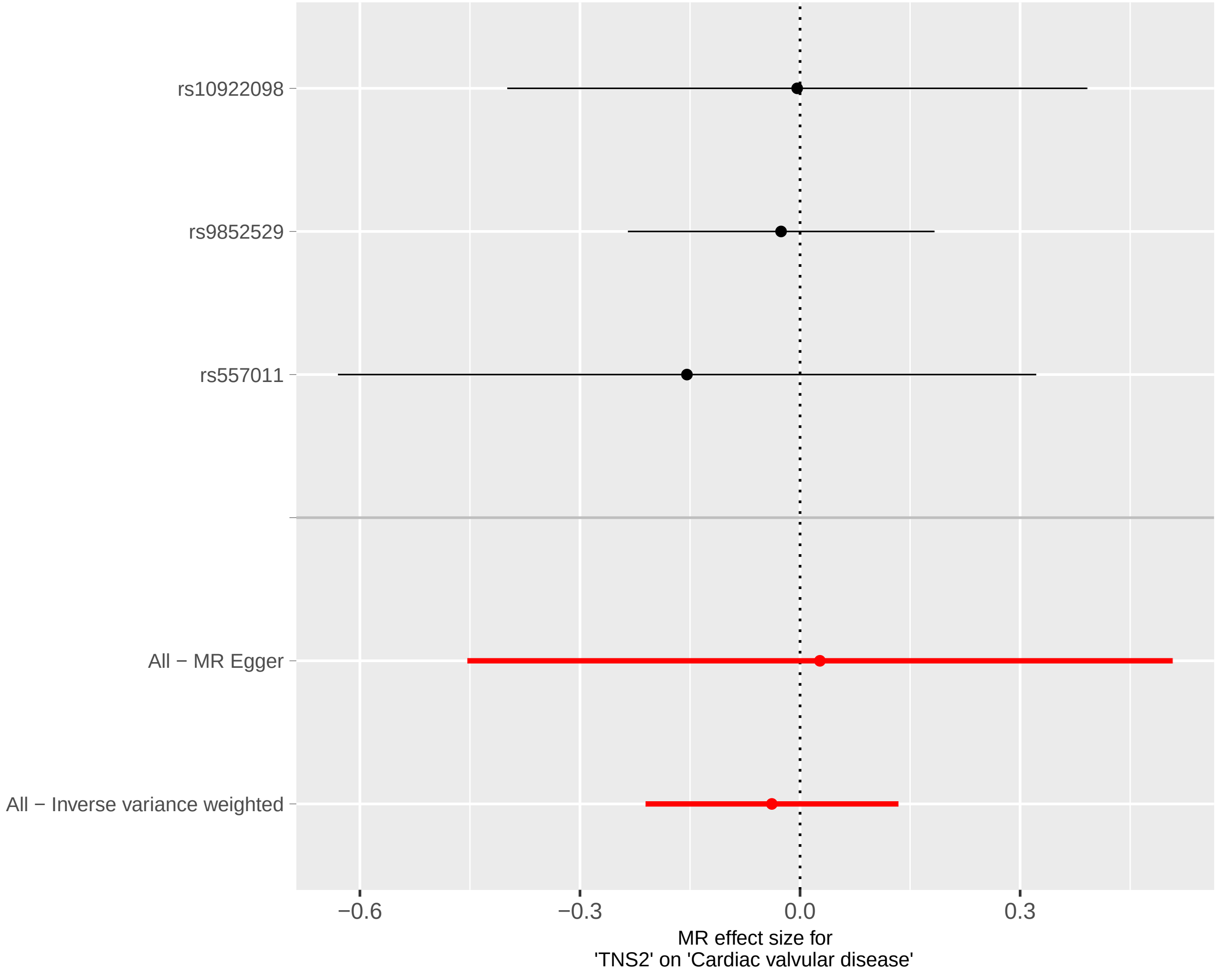
rs10922098
rs9852529
rs557011
All − MR Egger
All − Inverse variance weighted
−0.6
−0.3
0.0
0.3
MR effect size for
'TNS2' on 'Cardiac valvular disease'

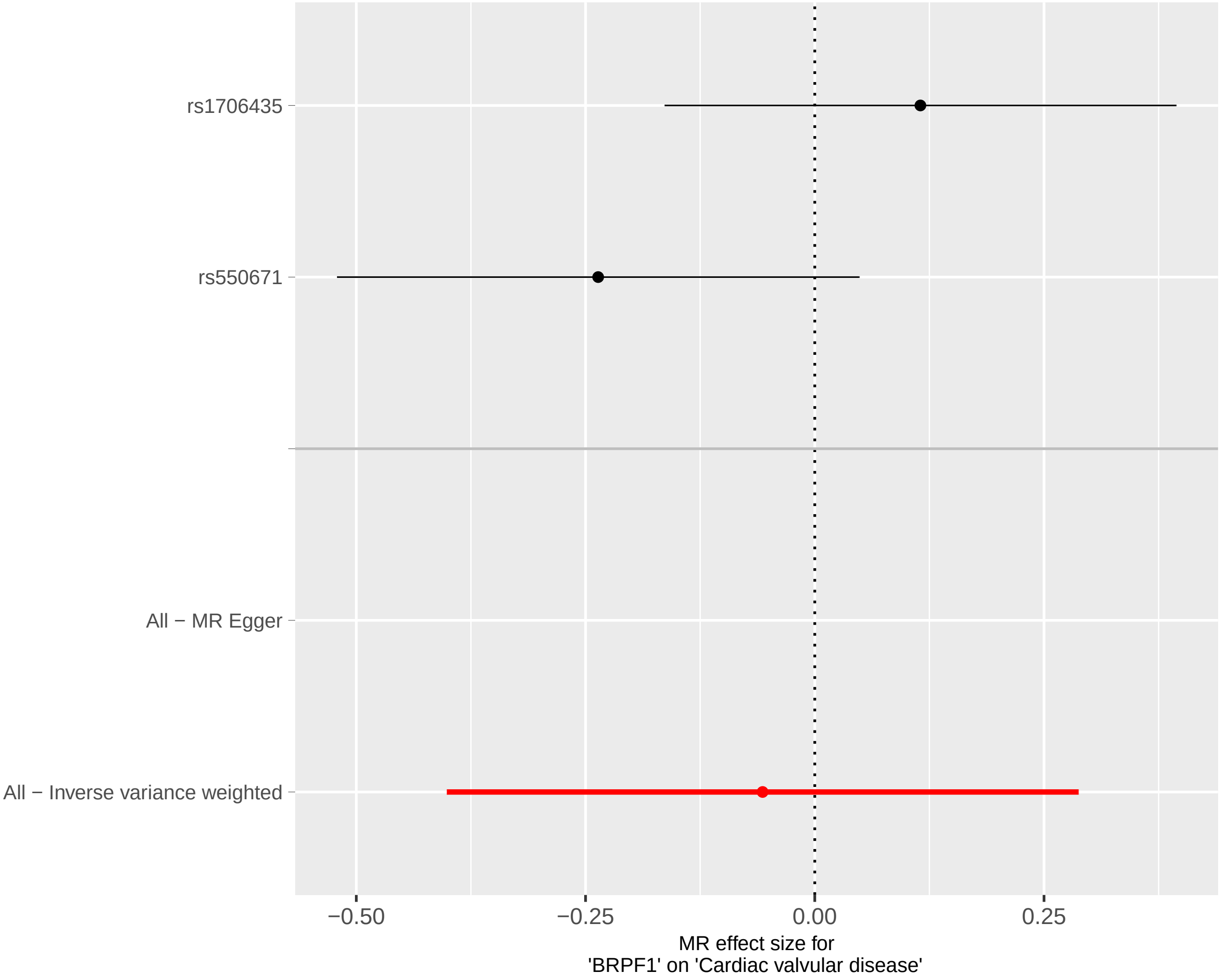
rs1706435
rs550671
All − MR Egger
All − Inverse variance weighted
−0.50
−0.25
0.00
0.25
MR effect size for
'BRPF1' on 'Cardiac valvular disease'

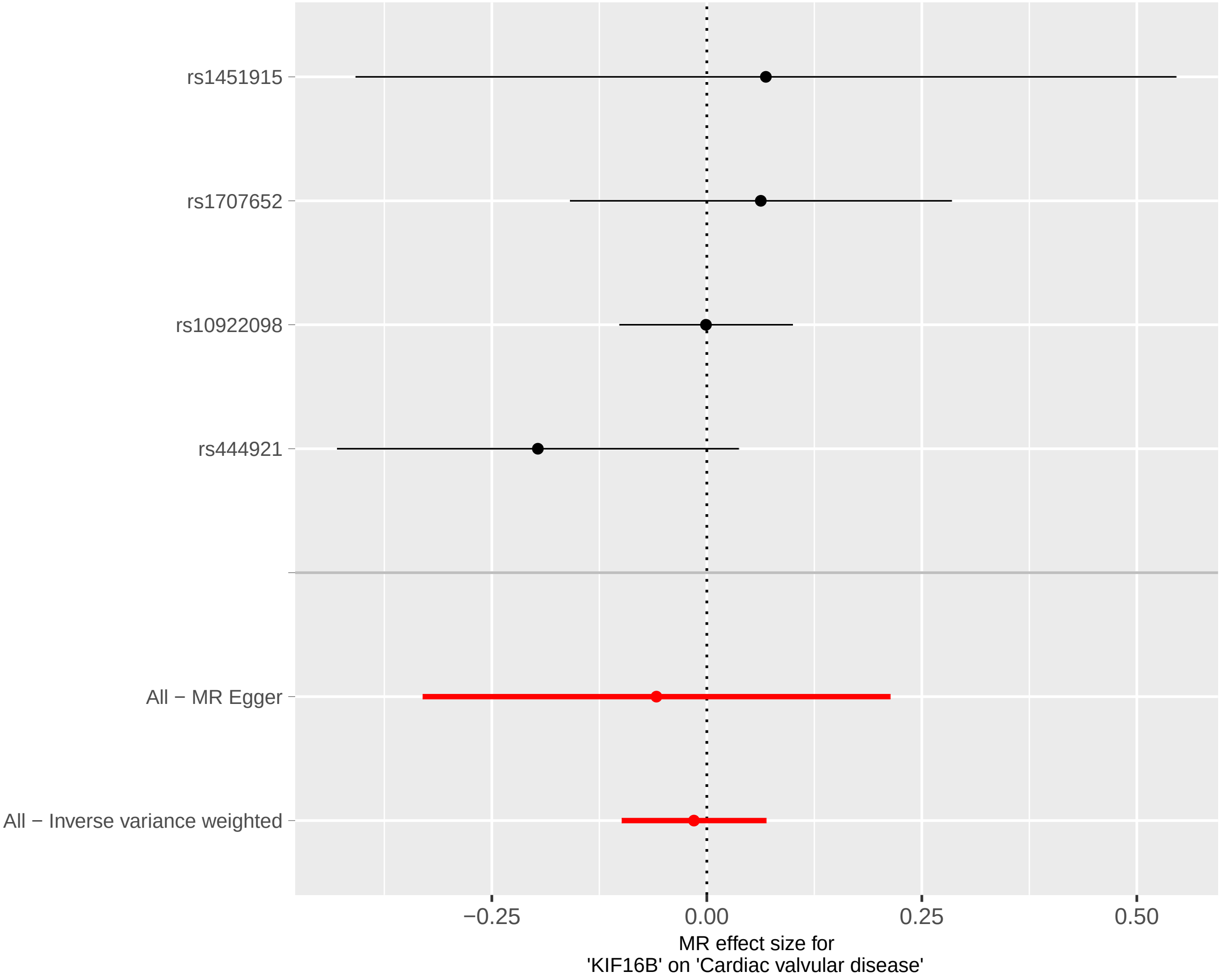
rs1451915
rs1707652
rs10922098
rs444921
All − MR Egger
All − Inverse variance weighted
−0.25
0.00
0.25
0.50
MR effect size for
'KIF16B' on 'Cardiac valvular disease'

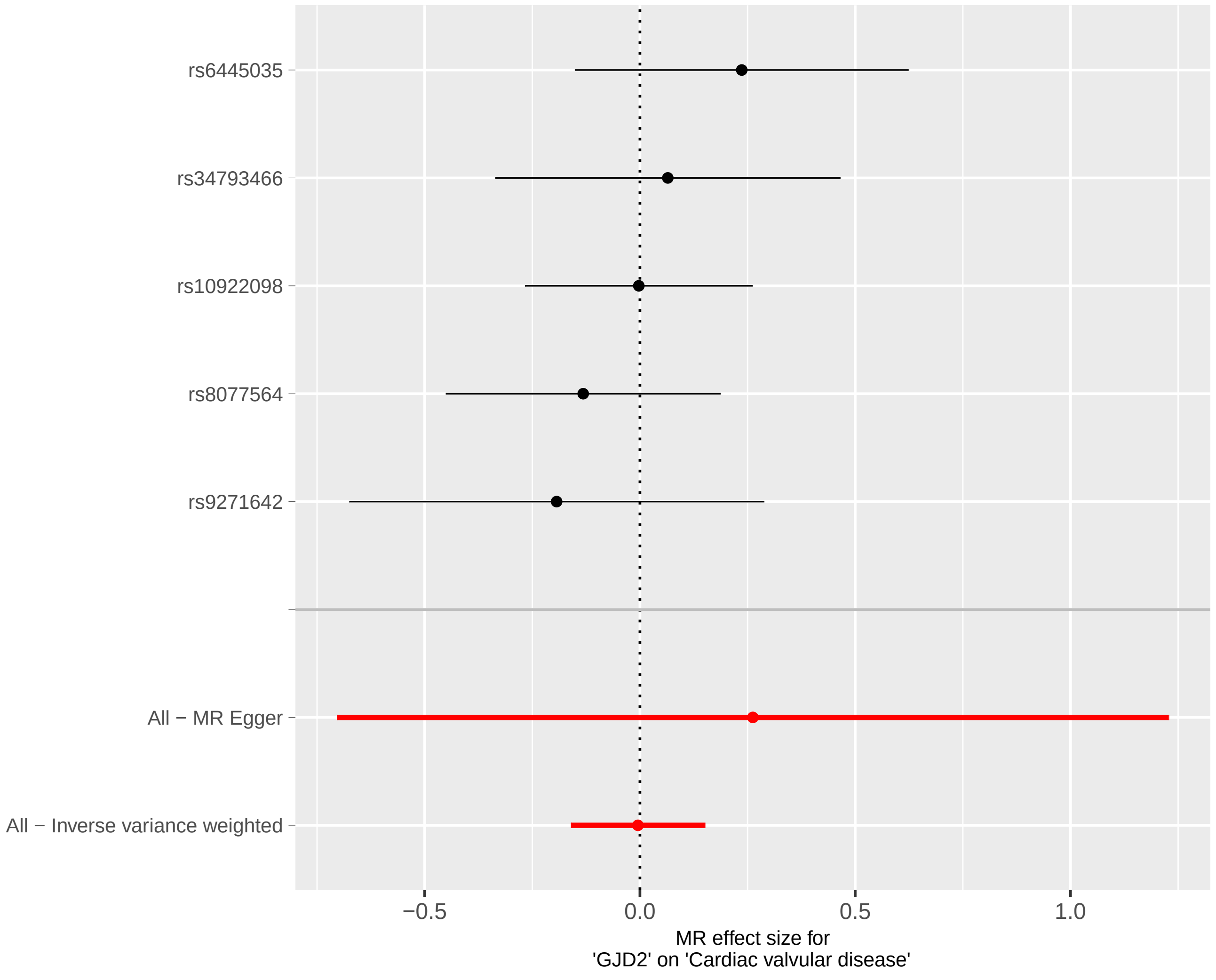
rs6445035
rs34793466
rs10922098
rs8077564
rs9271642
All − MR Egger
All − Inverse variance weighted
−0.5
0.0
0.5
1.0
MR effect size for
'GJD2' on 'Cardiac valvular disease'

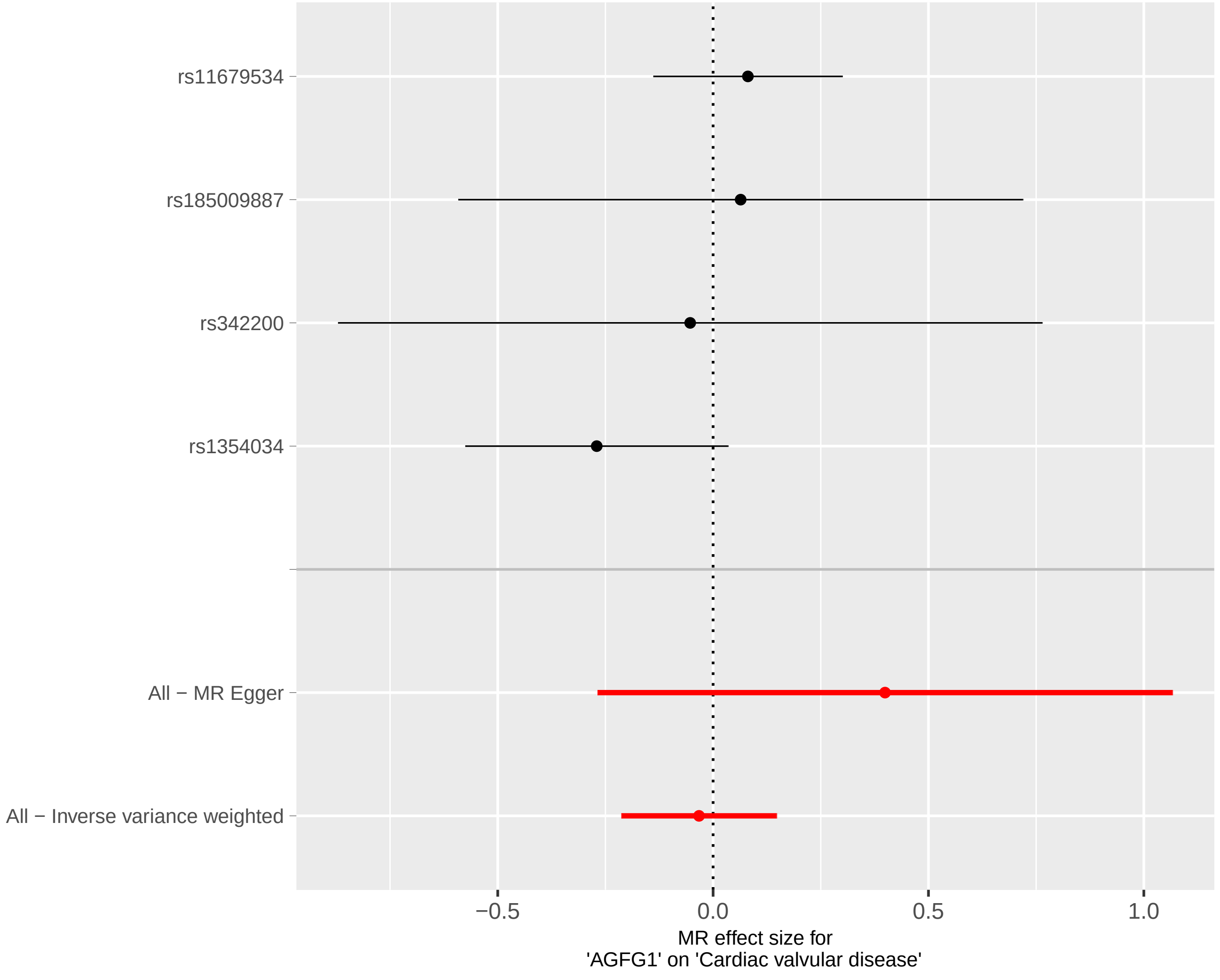
rs11679534
rs185009887
rs342200
rs1354034
All − MR Egger
All − Inverse variance weighted
−0.5
0.0
0.5
1.0
MR effect size for
'AGFG1' on 'Cardiac valvular disease'

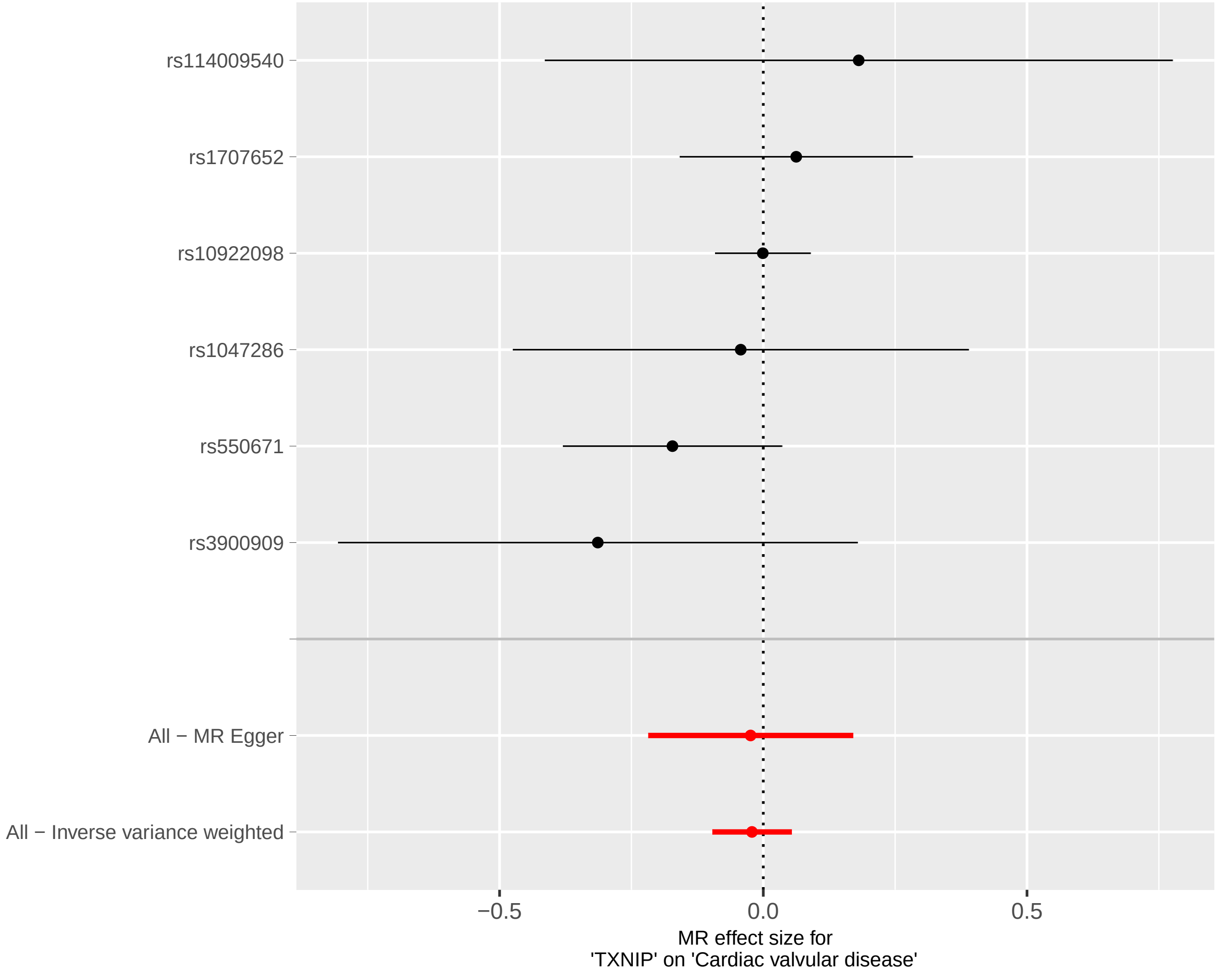
rs114009540
rs1707652
rs10922098
rs1047286
rs550671
rs3900909
All − MR Egger
All − Inverse variance weighted
−0.5
0.0
0.5
MR effect size for
'TXNIP' on 'Cardiac valvular disease'

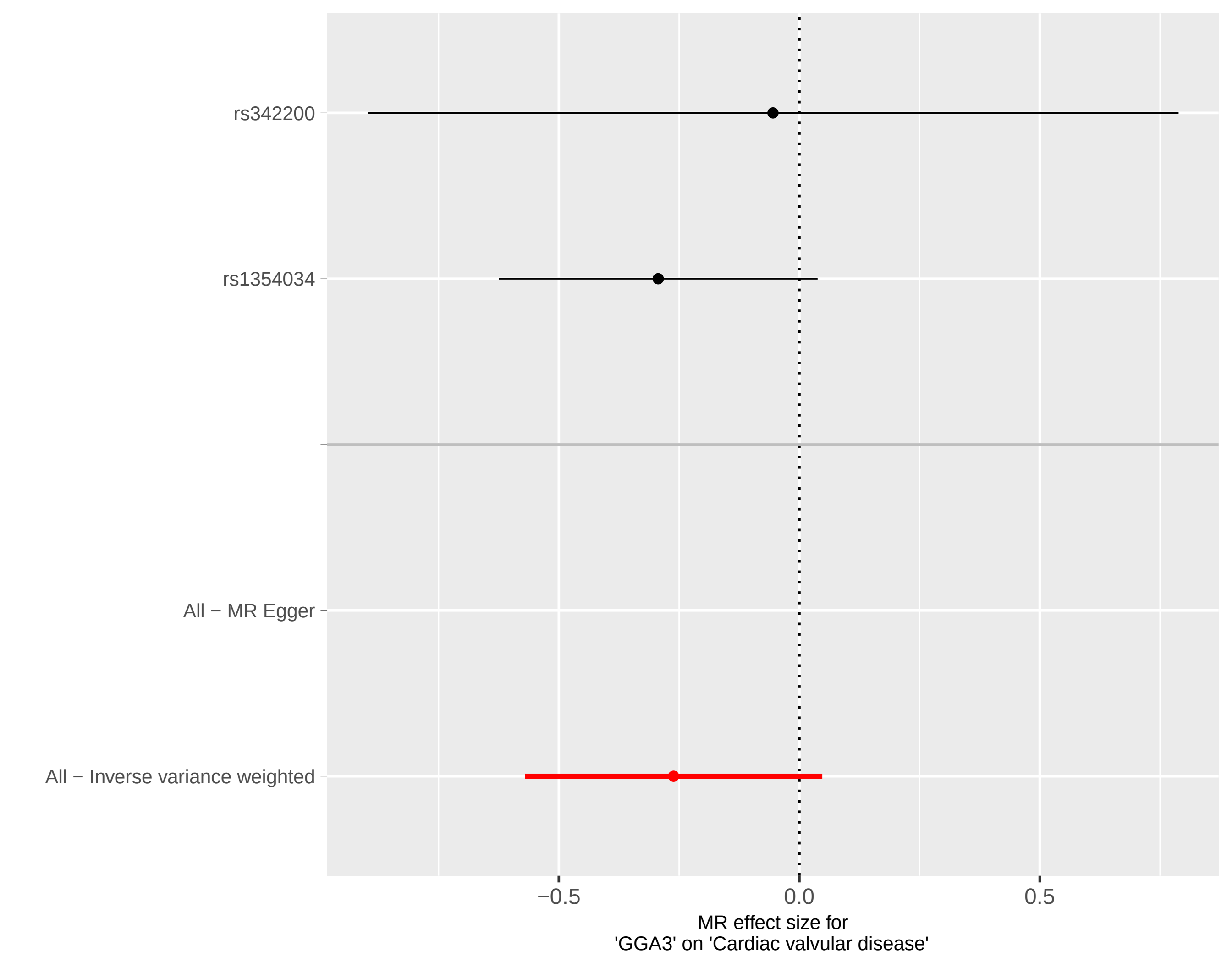

rs342200
rs1354034
All − MR Egger
All − Inverse variance weighted
−0.5
0.0
0.5
MR effect size for
'GGA3' on 'Cardiac valvular disease'

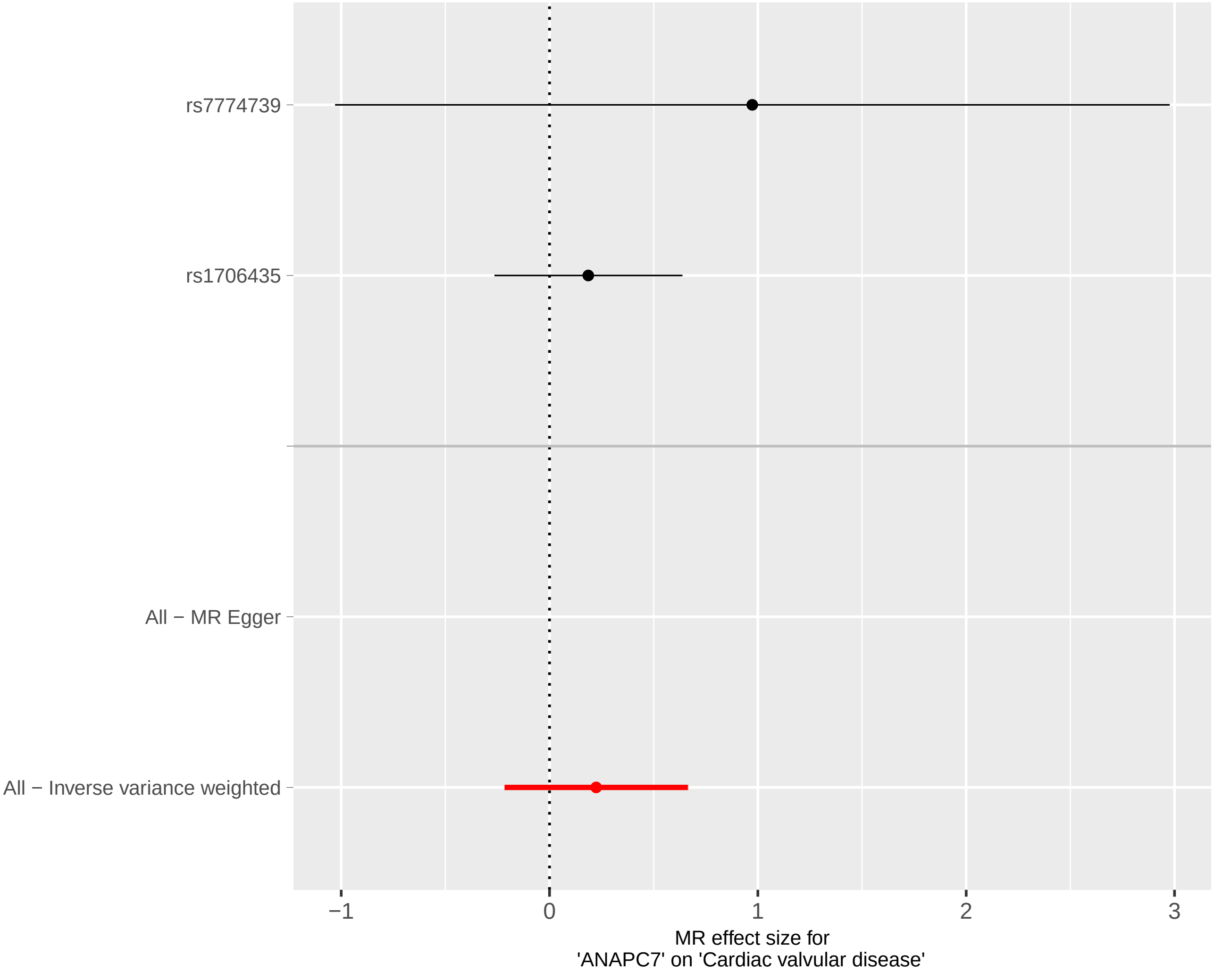

rs7774739
rs1706435
All − MR Egger
All − Inverse variance weighted
−1
0
1
2
3
MR effect size for
'ANAPC7' on 'Cardiac valvular disease'

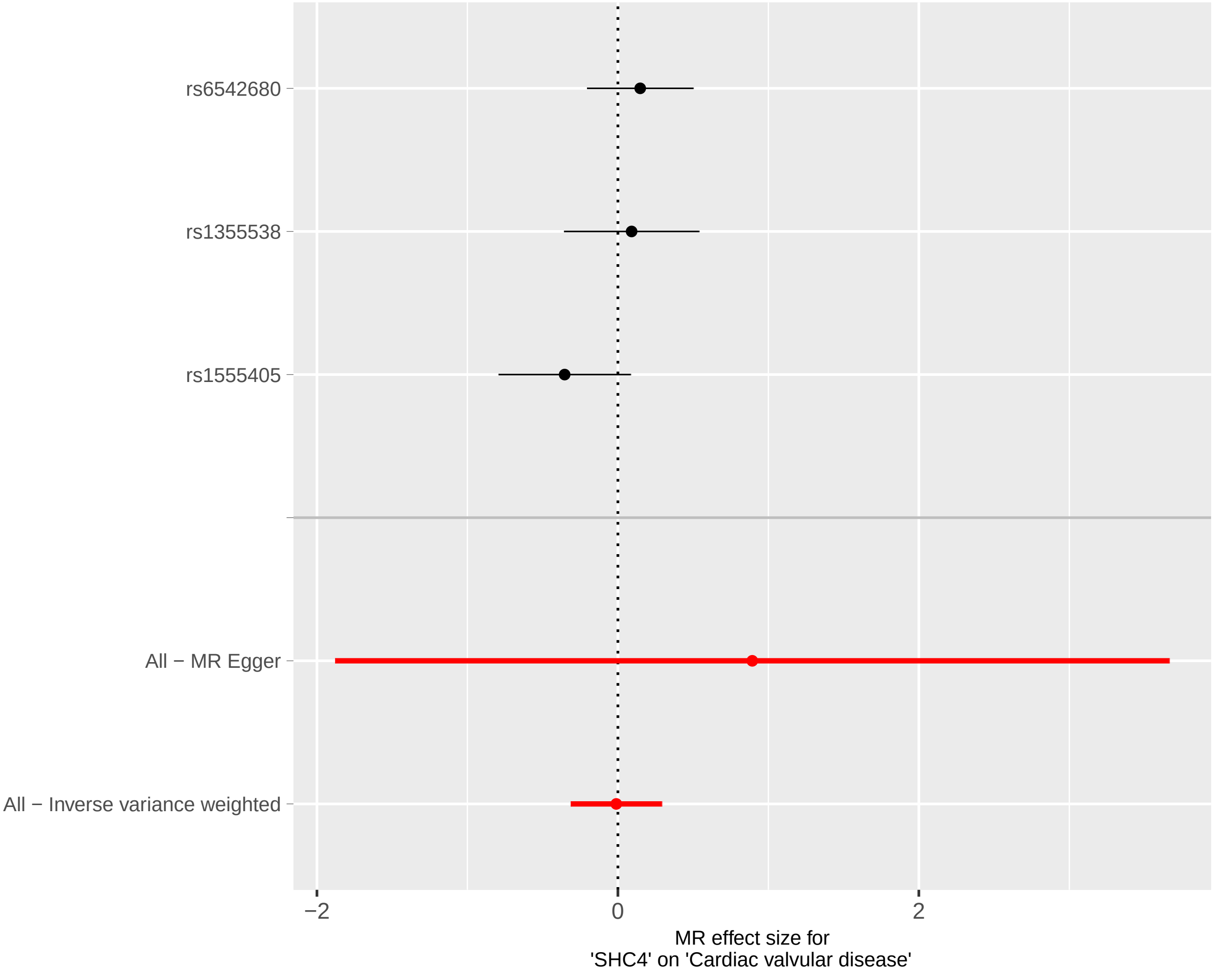
rs6542680
rs1355538
rs1555405
All − MR Egger
All − Inverse variance weighted
−2
0
2
MR effect size for
'SHC4' on 'Cardiac valvular disease'

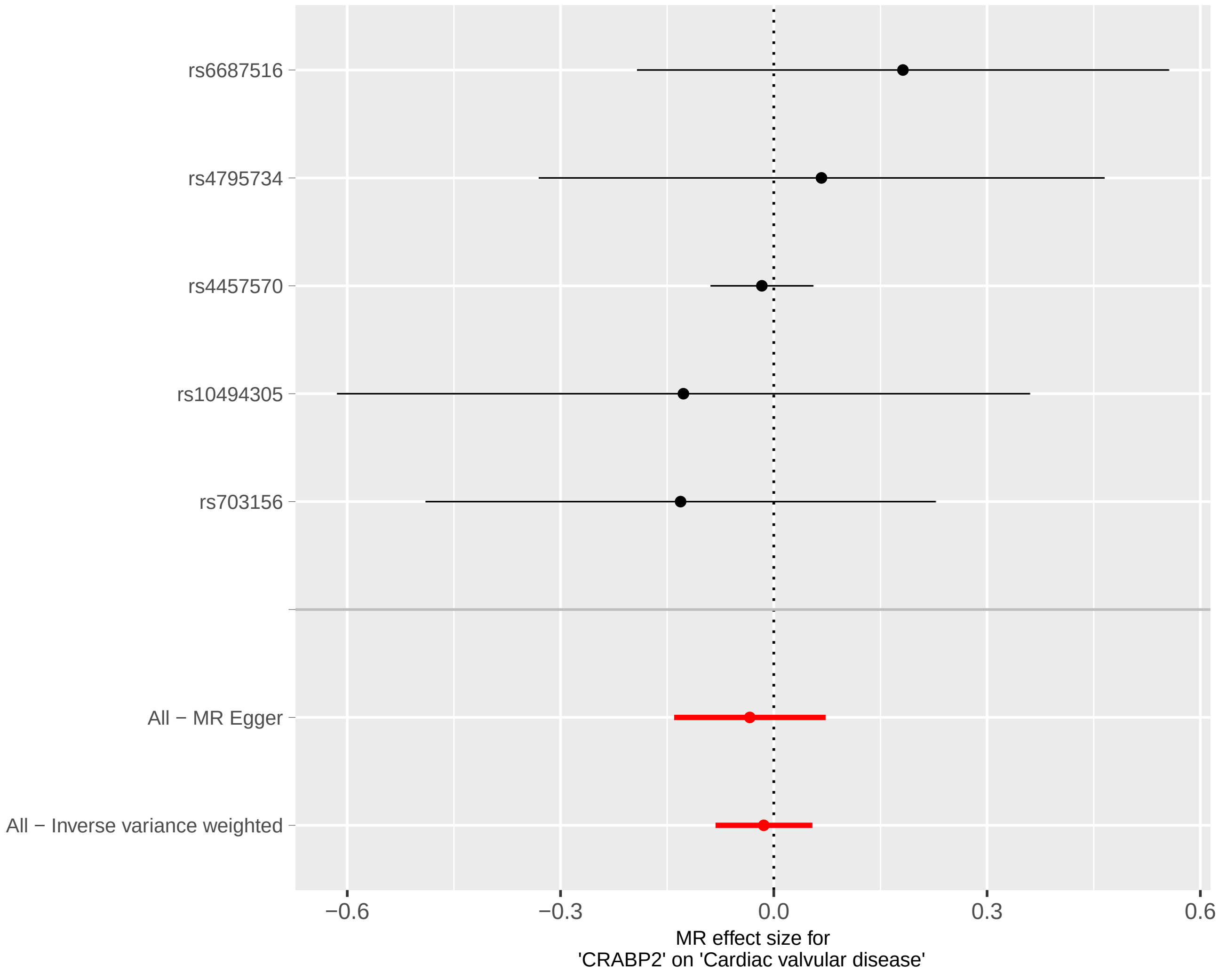
rs6687516
rs4795734
rs4457570
rs10494305
rs703156
All − MR Egger
All − Inverse variance weighted
−0.6
−0.3
0.0
0.3
0.6
MR effect size for
'CRABP2' on 'Cardiac valvular disease'

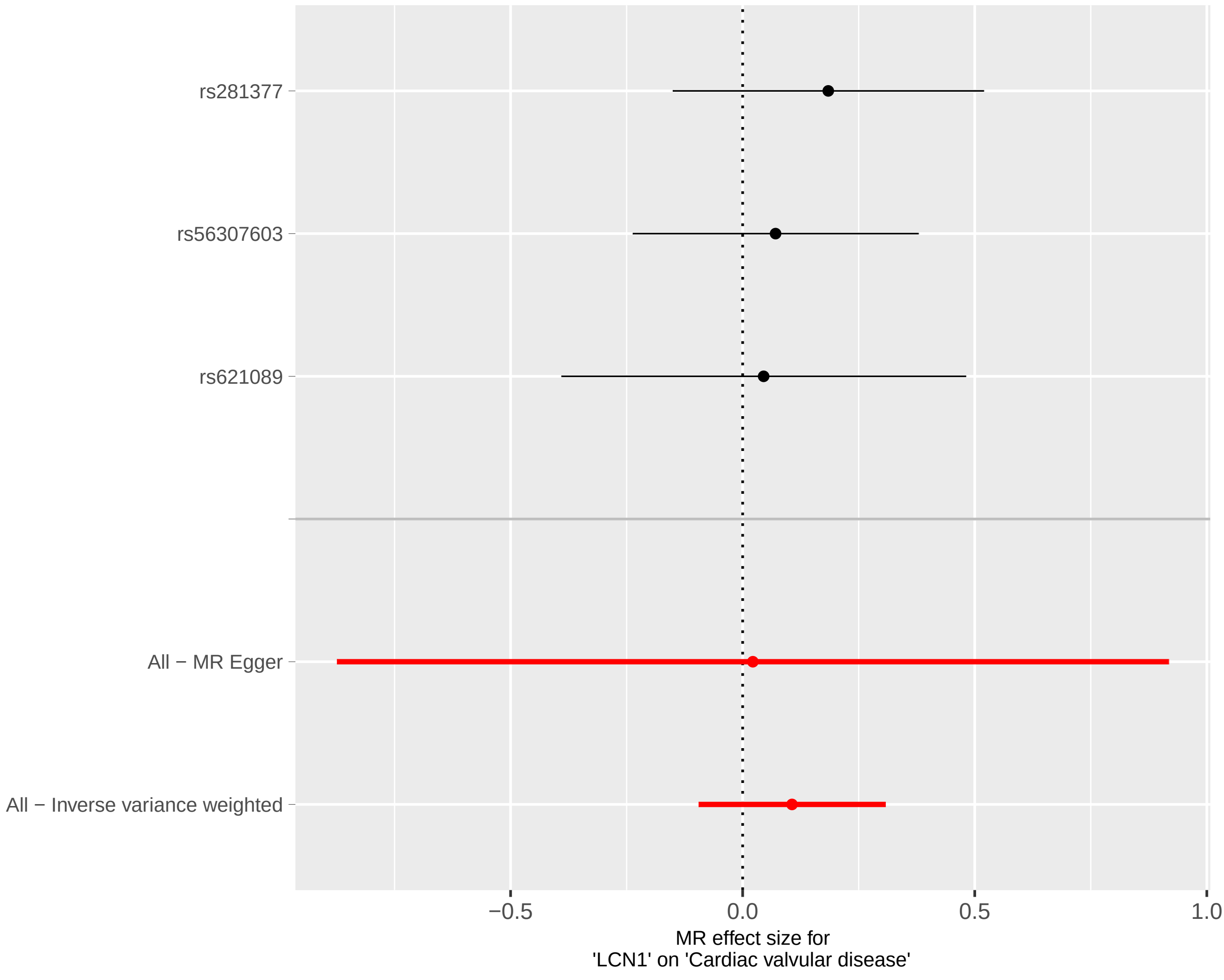
rs281377
rs56307603
rs621089
All − MR Egger
All − Inverse variance weighted
−0.5
0.0
0.5
1.0
MR effect size for
'LCN1' on 'Cardiac valvular disease'

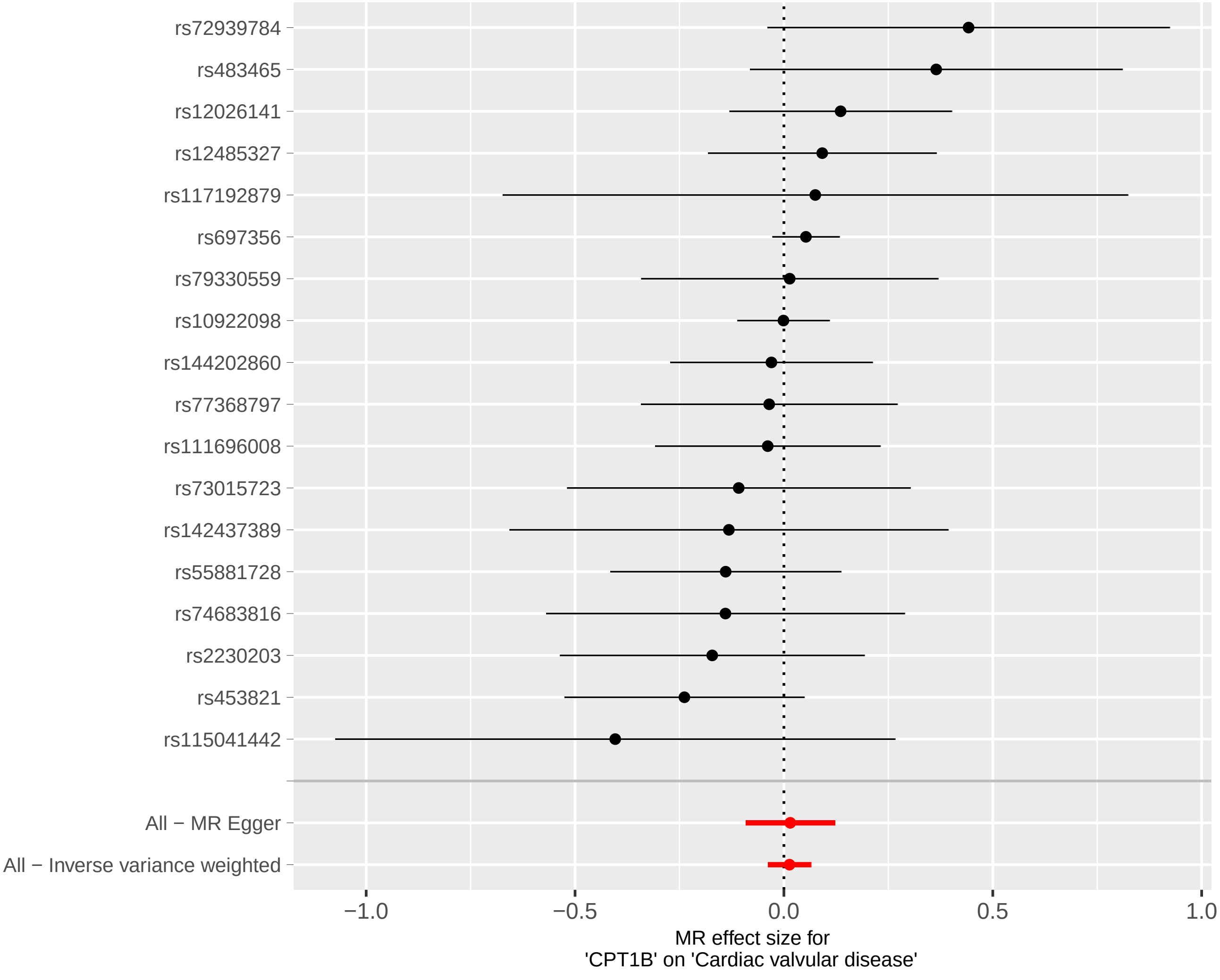

rs72939784
rs483465
rs12026141
rs12485327
rs117192879
rs697356
rs79330559
rs10922098
rs144202860
rs77368797
rs111696008
rs73015723
rs142437389
rs55881728
rs74683816
rs2230203
rs453821
rs115041442
All − MR Egger
All − Inverse variance weighted
−1.0
−0.5
0.0
0.5
1.0
MR effect size for
'CPT1B' on 'Cardiac valvular disease'

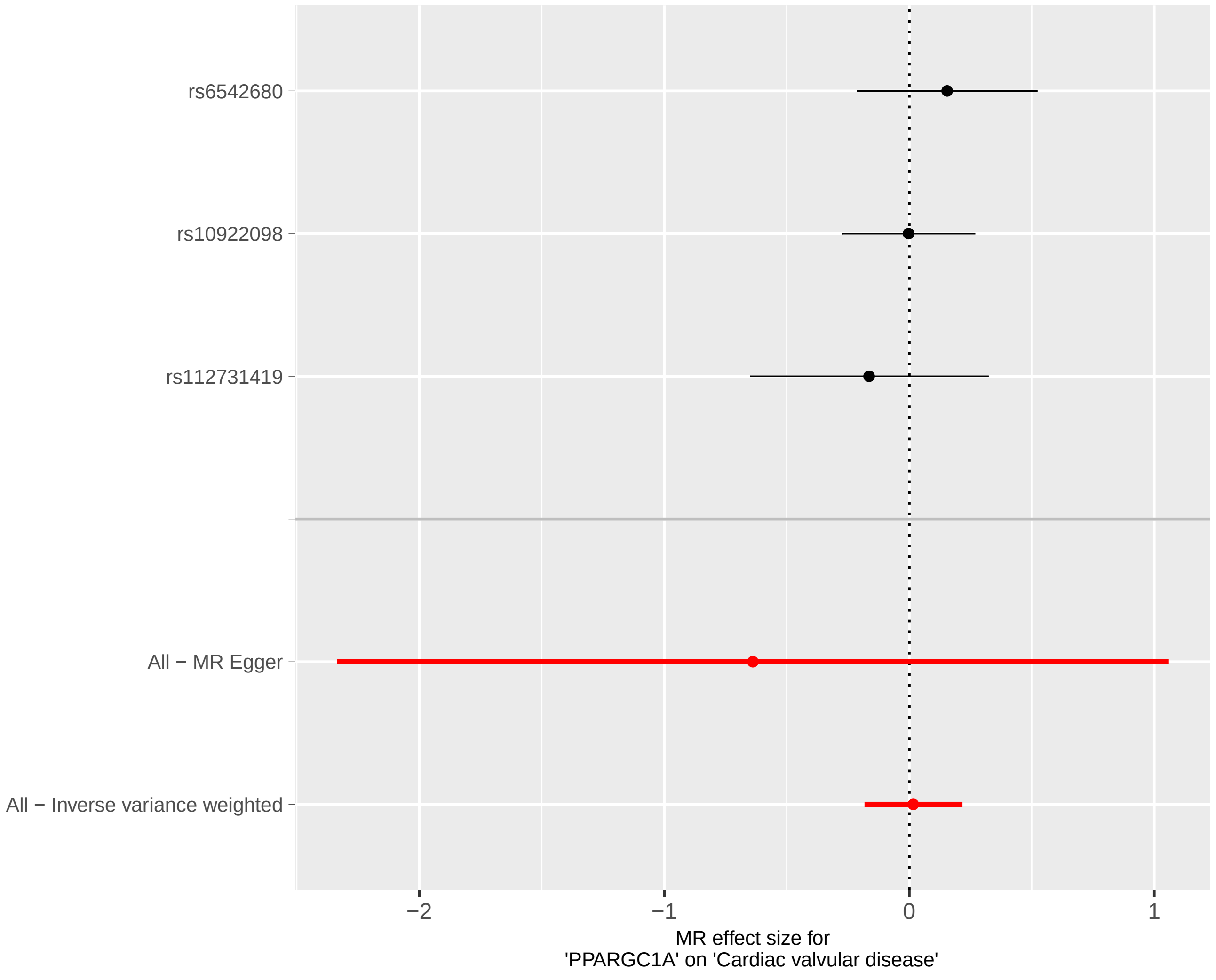

rs6542680
rs10922098
rs112731419
All − MR Egger
All − Inverse variance weighted
−2
−1
0
1
MR effect size for
'PPARGC1A' on 'Cardiac valvular disease'

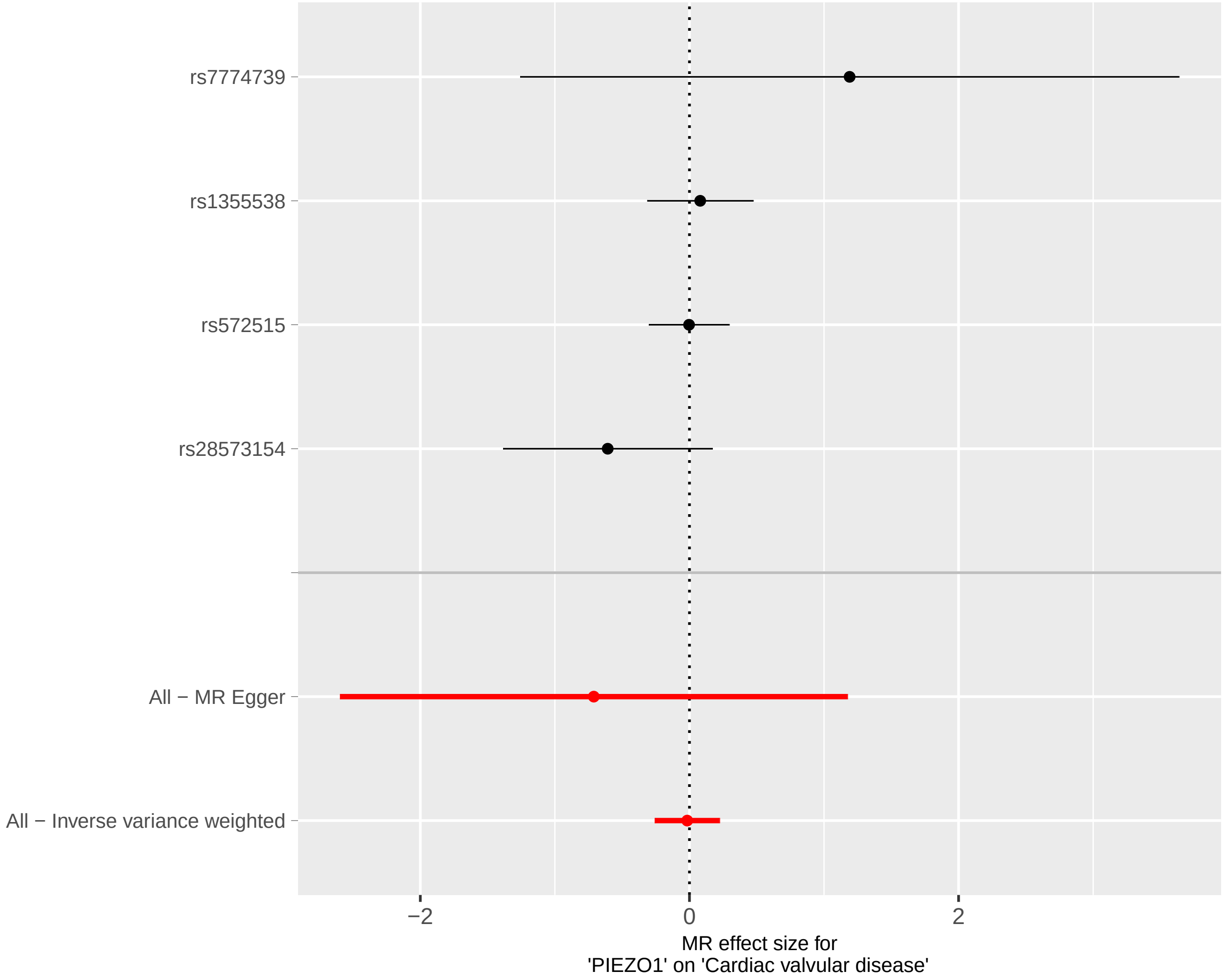

rs7774739
rs1355538
rs572515
rs28573154
All − MR Egger
All − Inverse variance weighted
−2
0
2
MR effect size for
'PIEZO1' on 'Cardiac valvular disease'

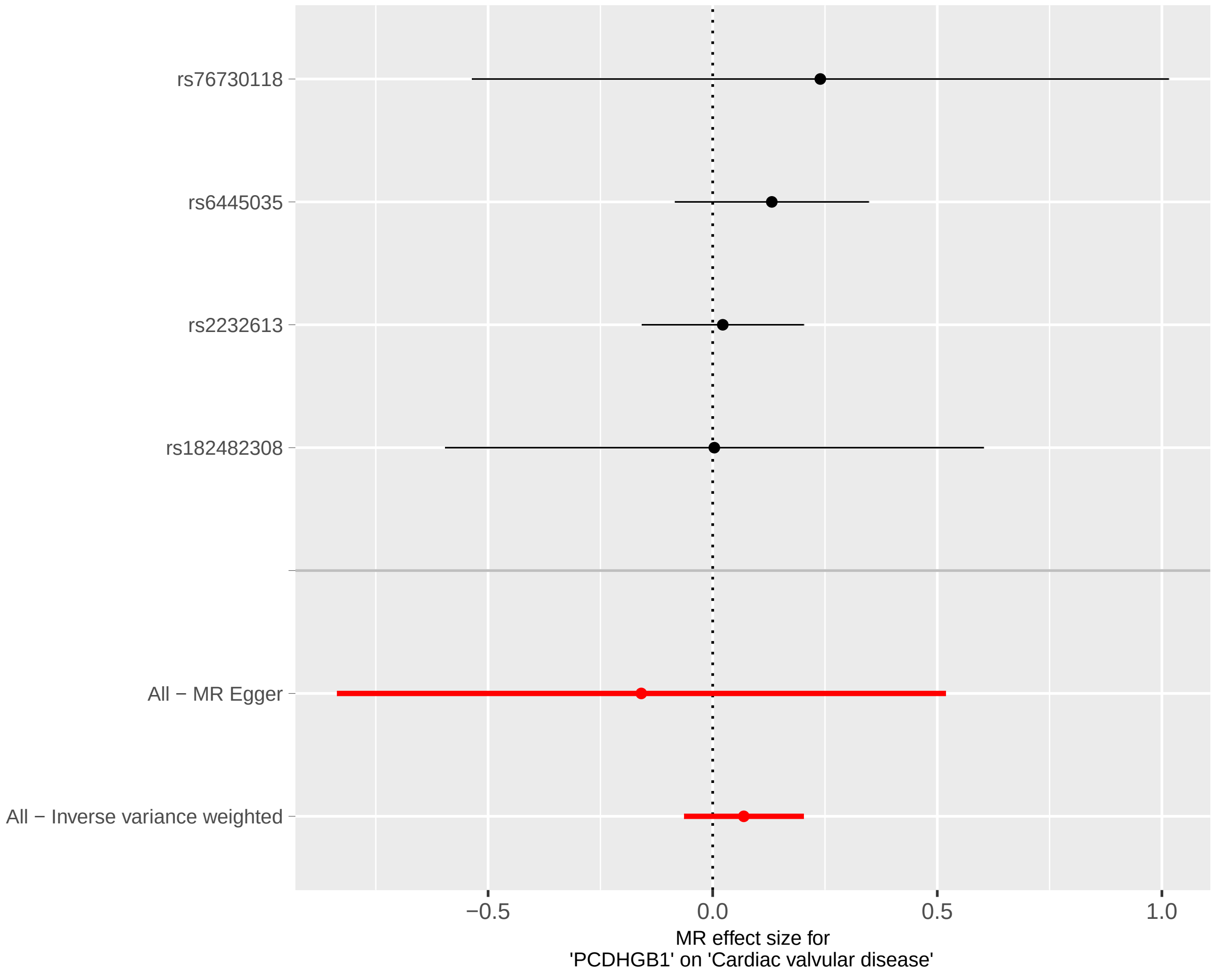
rs76730118
rs6445035
rs2232613
rs182482308
All − MR Egger
All − Inverse variance weighted
−0.5
0.0
0.5
1.0
MR effect size for
'PCDHGB1' on 'Cardiac valvular disease'

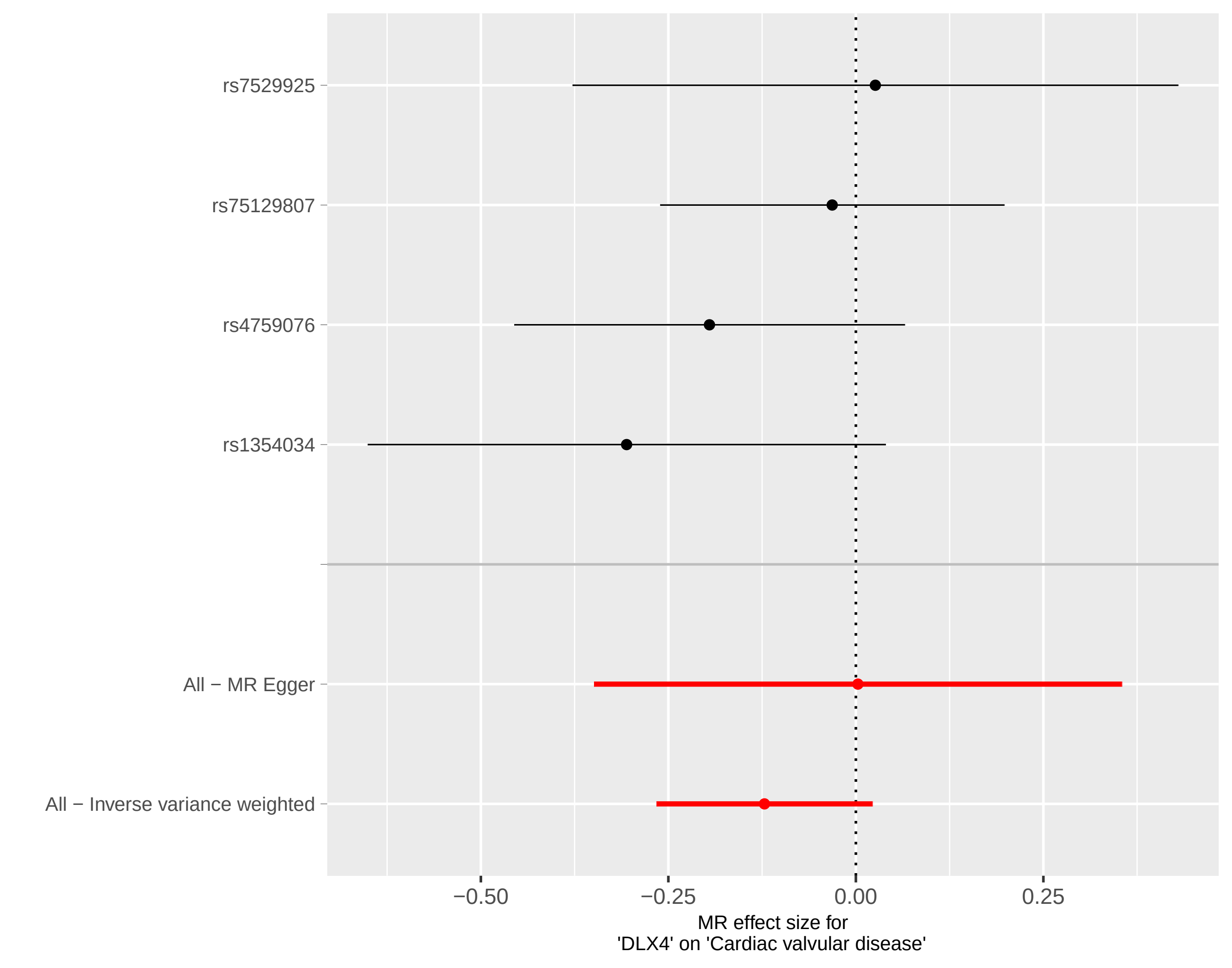

rs7529925
rs75129807
rs4759076
rs1354034
All − MR Egger
All − Inverse variance weighted
−0.50
−0.25
0.00
0.25
MR effect size for
'DLX4' on 'Cardiac valvular disease'

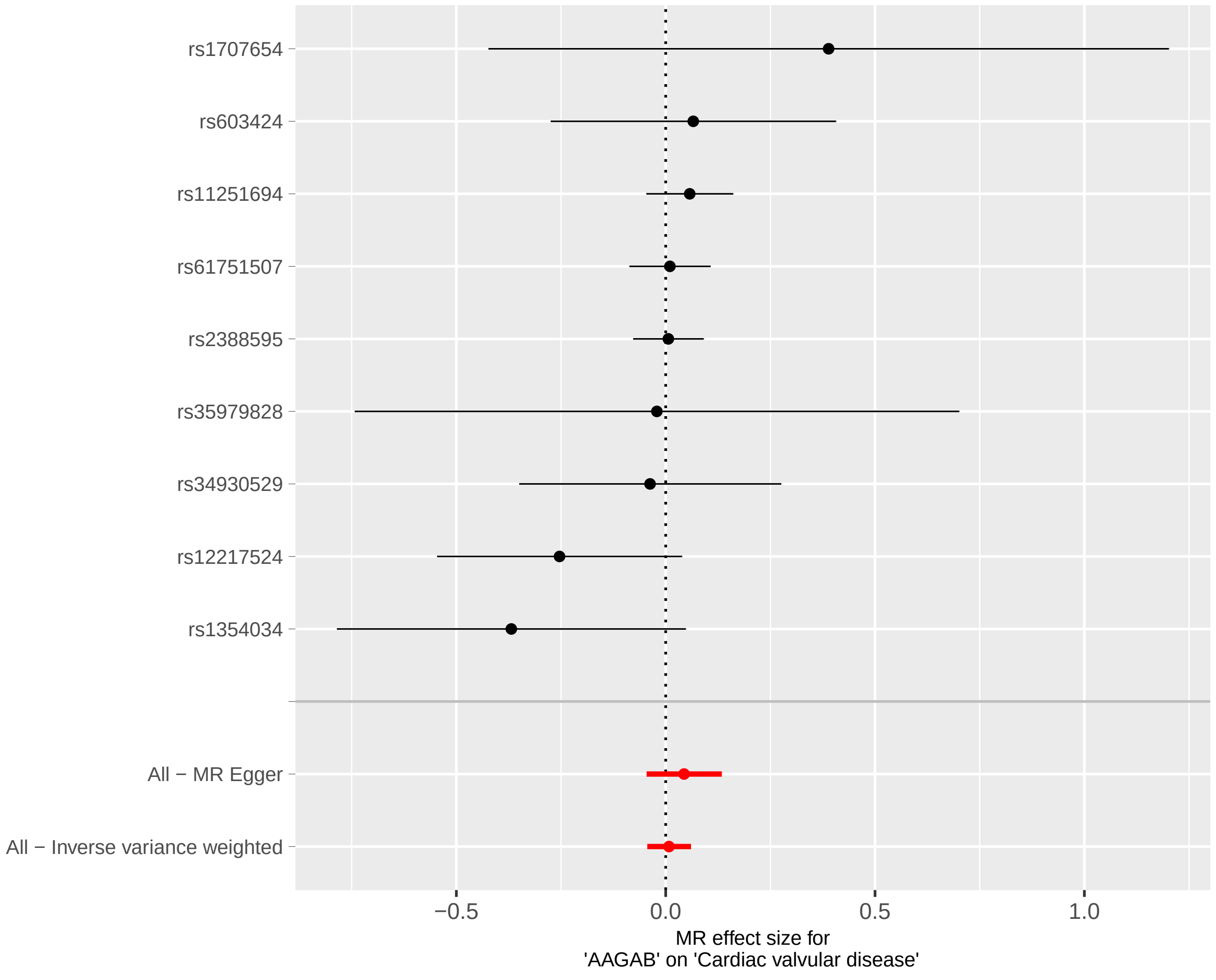
rs1707654
rs603424
rs11251694
rs61751507
rs2388595
rs35979828
rs34930529
rs12217524
rs1354034
All − MR Egger
All − Inverse variance weighted
−0.5
0.0
0.5
1.0
MR effect size for
'AAGAB' on 'Cardiac valvular disease'

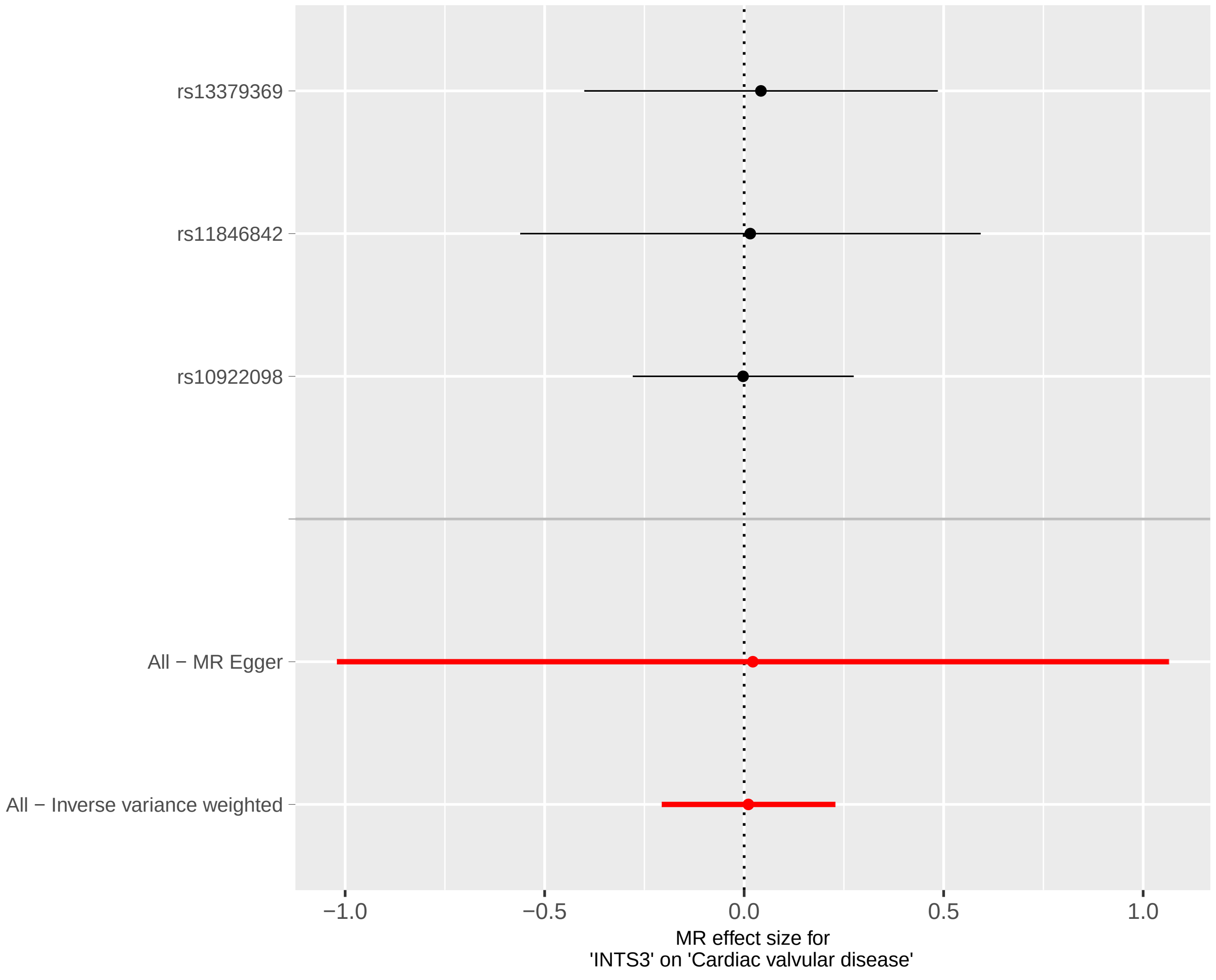
rs13379369
rs11846842
rs10922098
All − MR Egger
All − Inverse variance weighted
−1.0
−0.5
0.0
0.5
1.0
MR effect size for
'INTS3' on 'Cardiac valvular disease'

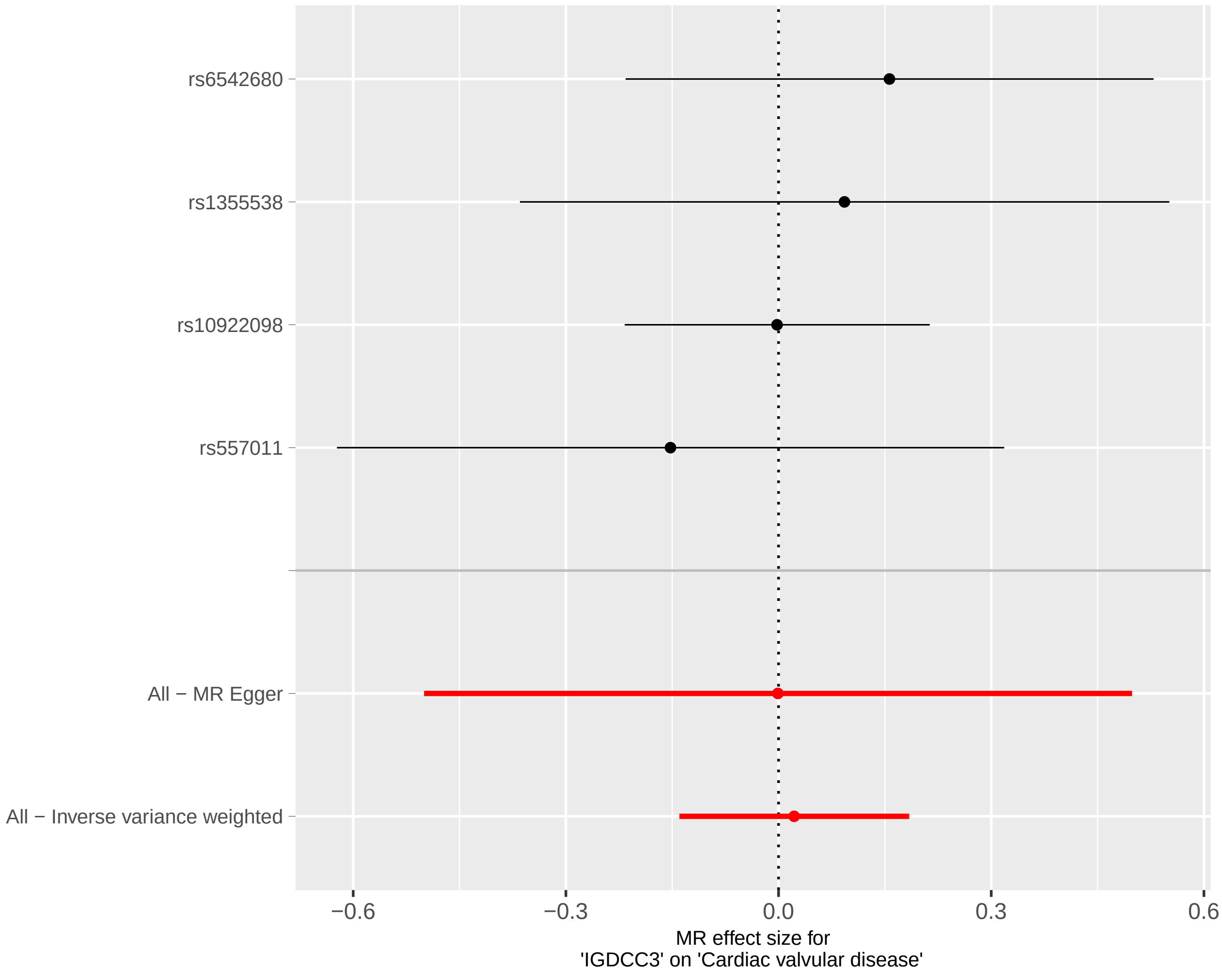
rs6542680
rs1355538
rs10922098
rs557011
All − MR Egger
All − Inverse variance weighted
−0.6
−0.3
0.0
0.3
0.6
MR effect size for
'IGDCC3' on 'Cardiac valvular disease'

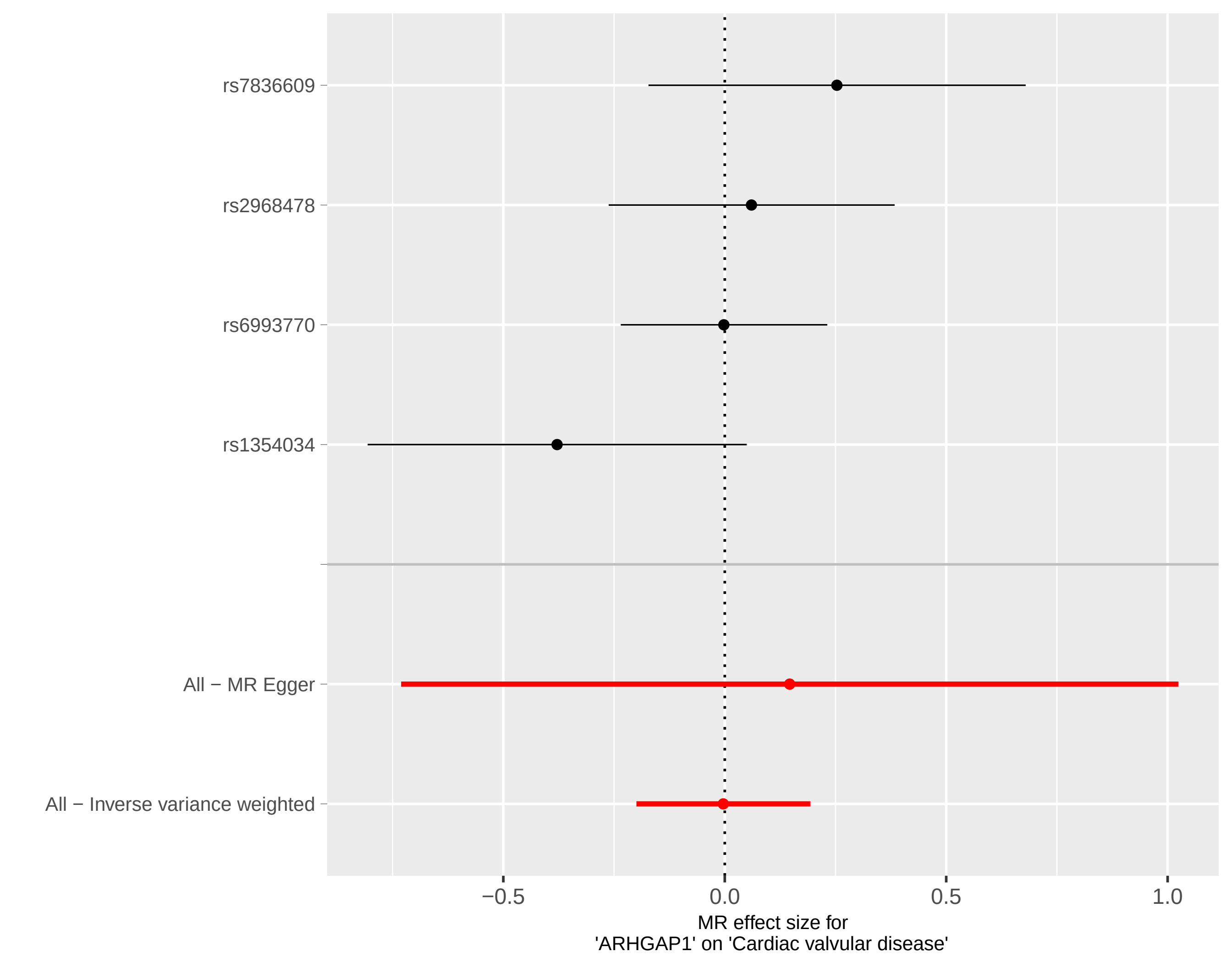
rs7836609
rs2968478
rs6993770
rs1354034
All − MR Egger
All − Inverse variance weighted
−0.5
0.0
0.5
1.0
MR effect size for
'ARHGAP1' on 'Cardiac valvular disease'

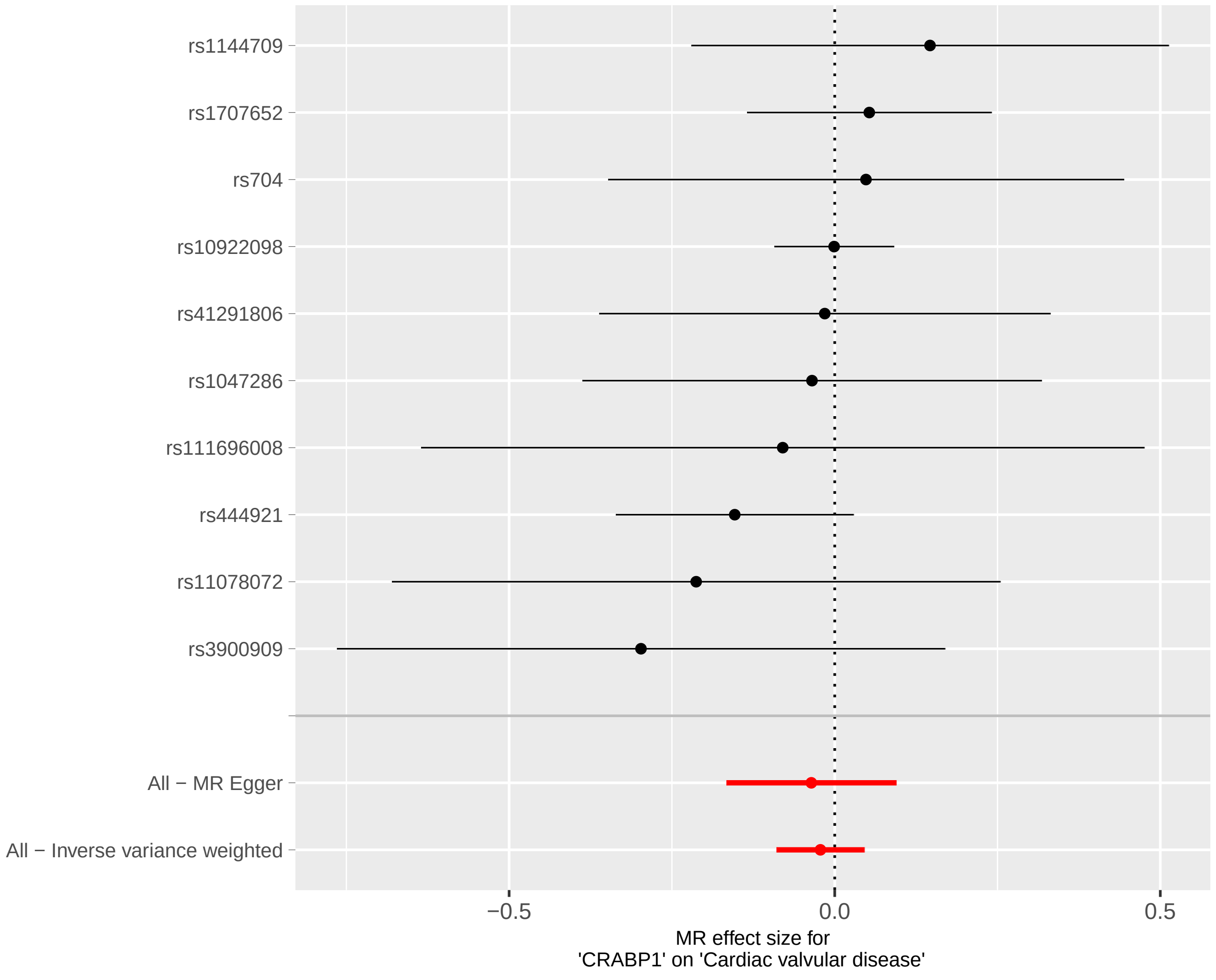
rs1144709
rs1707652
rs704
rs10922098
rs41291806
rs1047286
rs111696008
rs444921
rs11078072
rs3900909
All − MR Egger
All − Inverse variance weighted
−0.5
0.0
0.5
MR effect size for
'CRABP1' on 'Cardiac valvular disease'

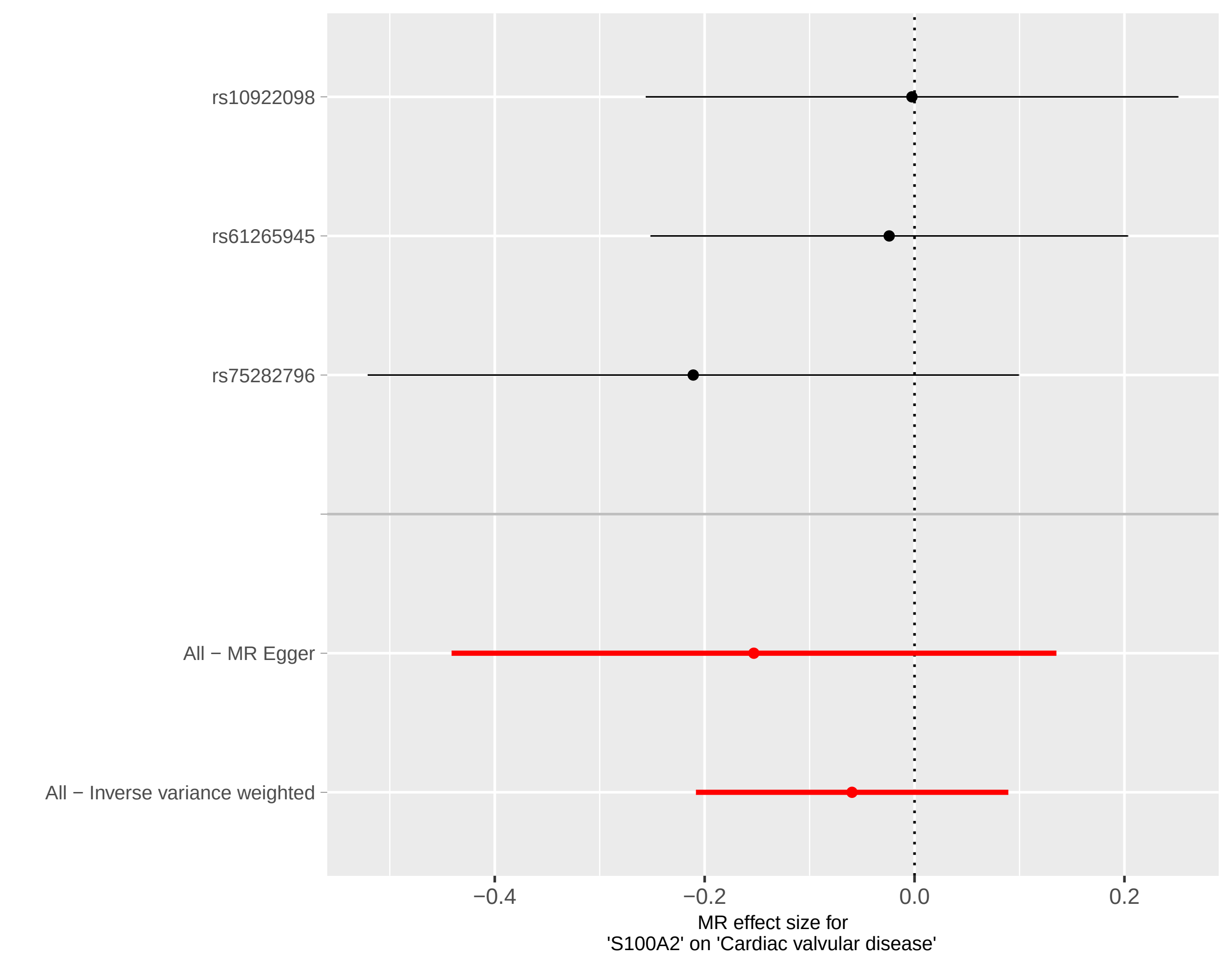
rs10922098
rs61265945
rs75282796
All − MR Egger
All − Inverse variance weighted
−0.4
−0.2
0.0
0.2
MR effect size for
'S100A2' on 'Cardiac valvular disease'

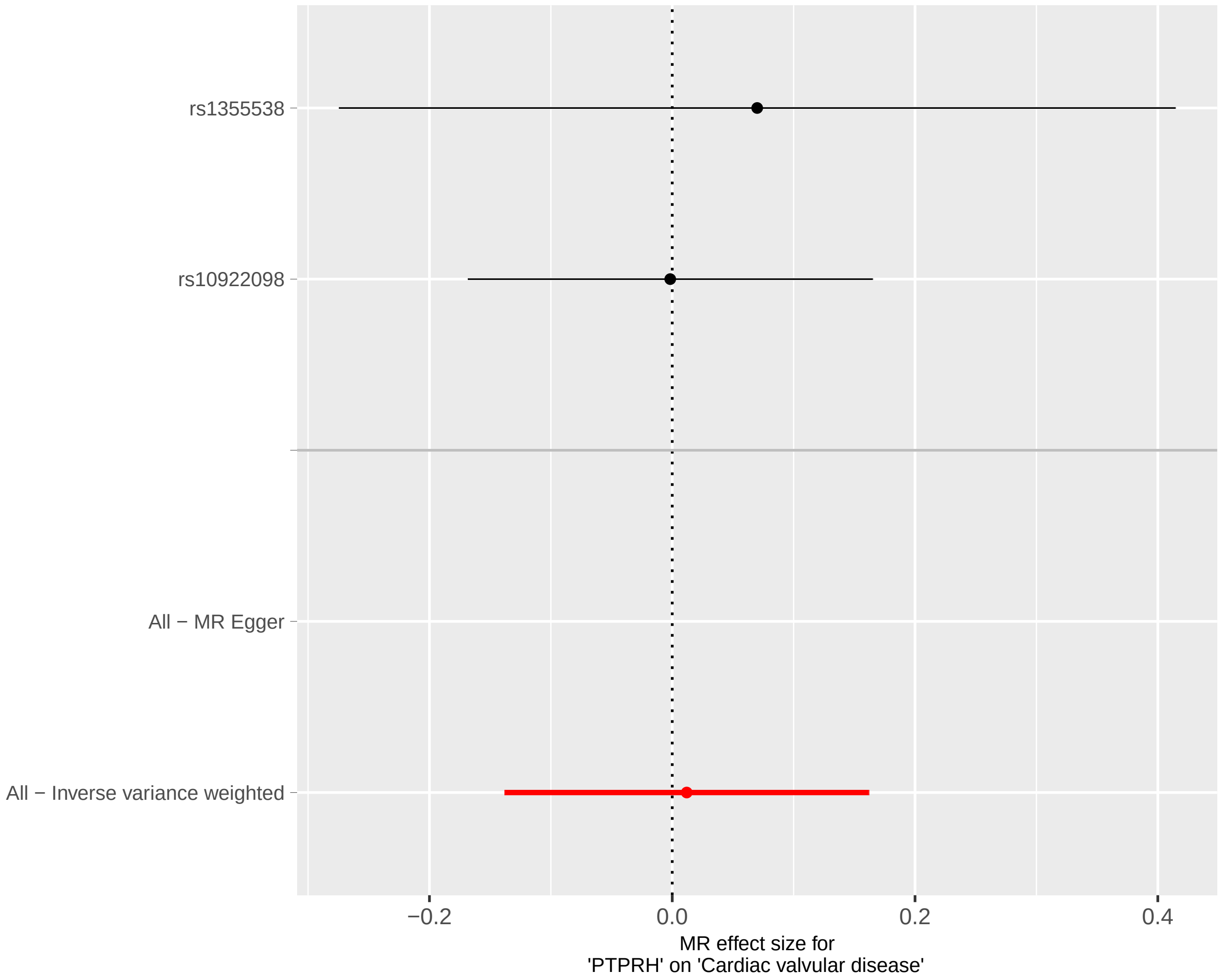
rs1355538
rs10922098
All − MR Egger
All − Inverse variance weighted
−0.2
0.0
0.2
0.4
MR effect size for
'PTPRH' on 'Cardiac valvular disease'

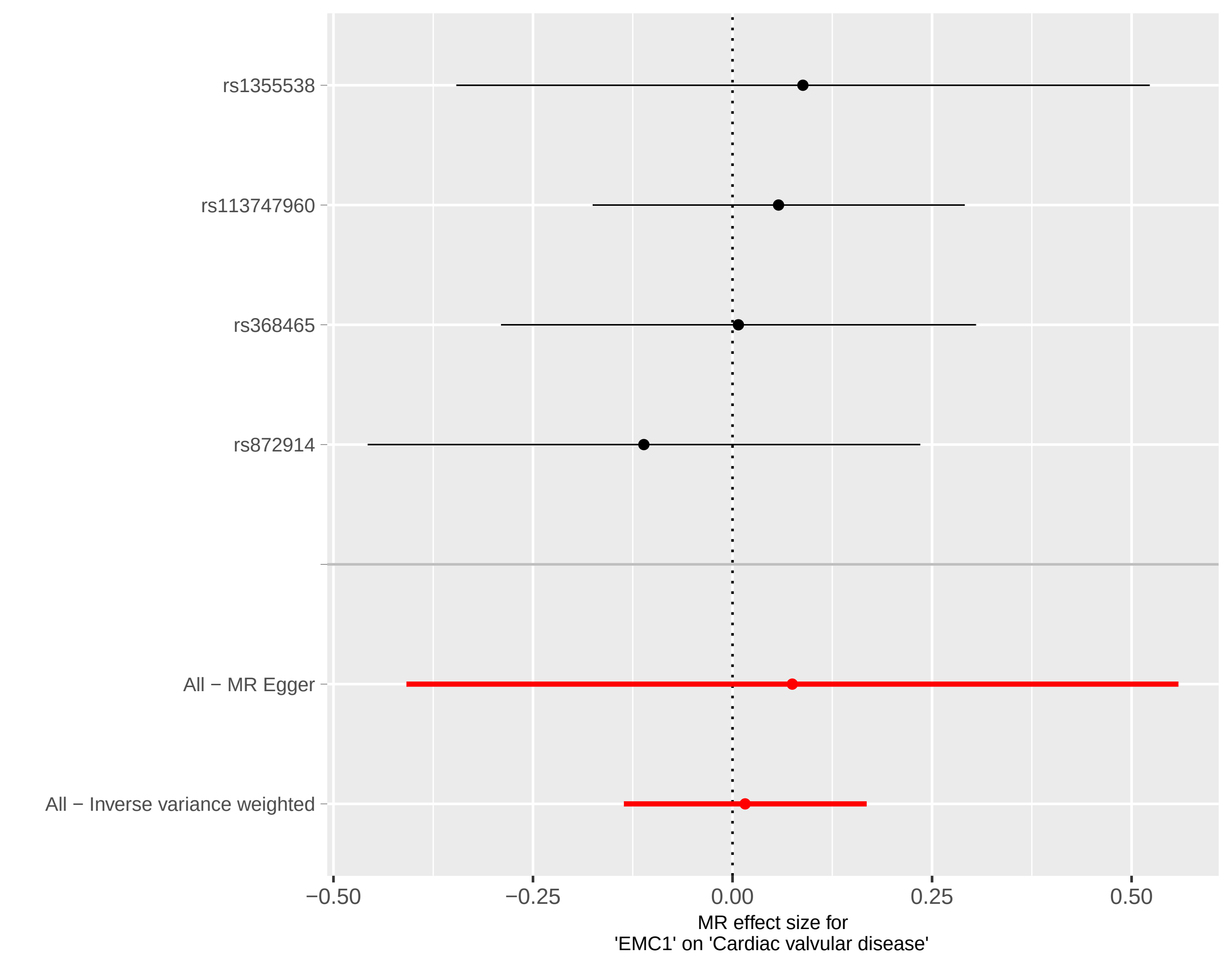

rs1355538
rs113747960
rs368465
rs872914
All − MR Egger
All − Inverse variance weighted
−0.50
−0.25
0.00
0.25
0.50
MR effect size for
'EMC1' on 'Cardiac valvular disease'

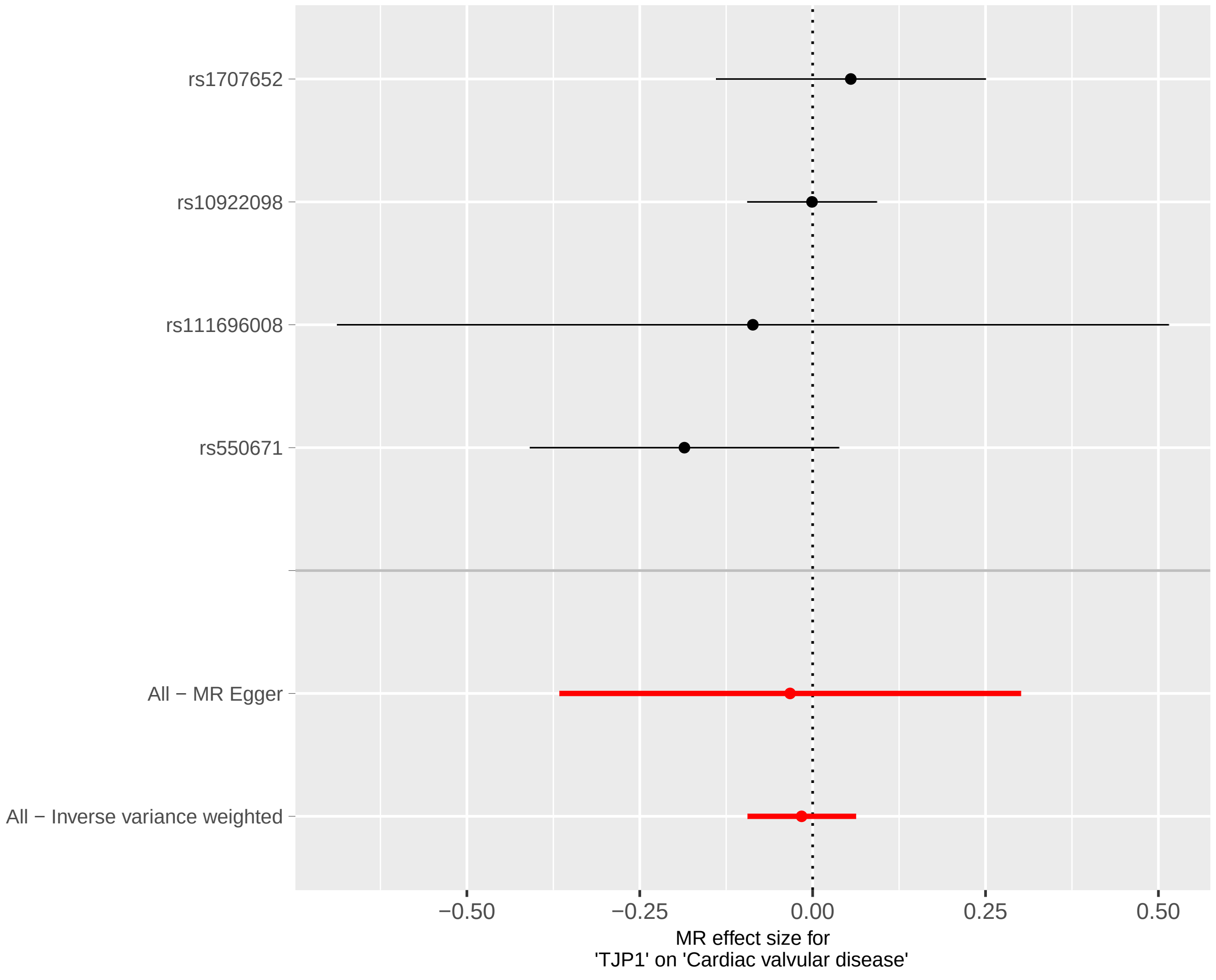
rs1707652
rs10922098
rs111696008
rs550671
All − MR Egger
All − Inverse variance weighted
−0.50
−0.25
0.00
0.25
0.50
MR effect size for
'TJP1' on 'Cardiac valvular disease'

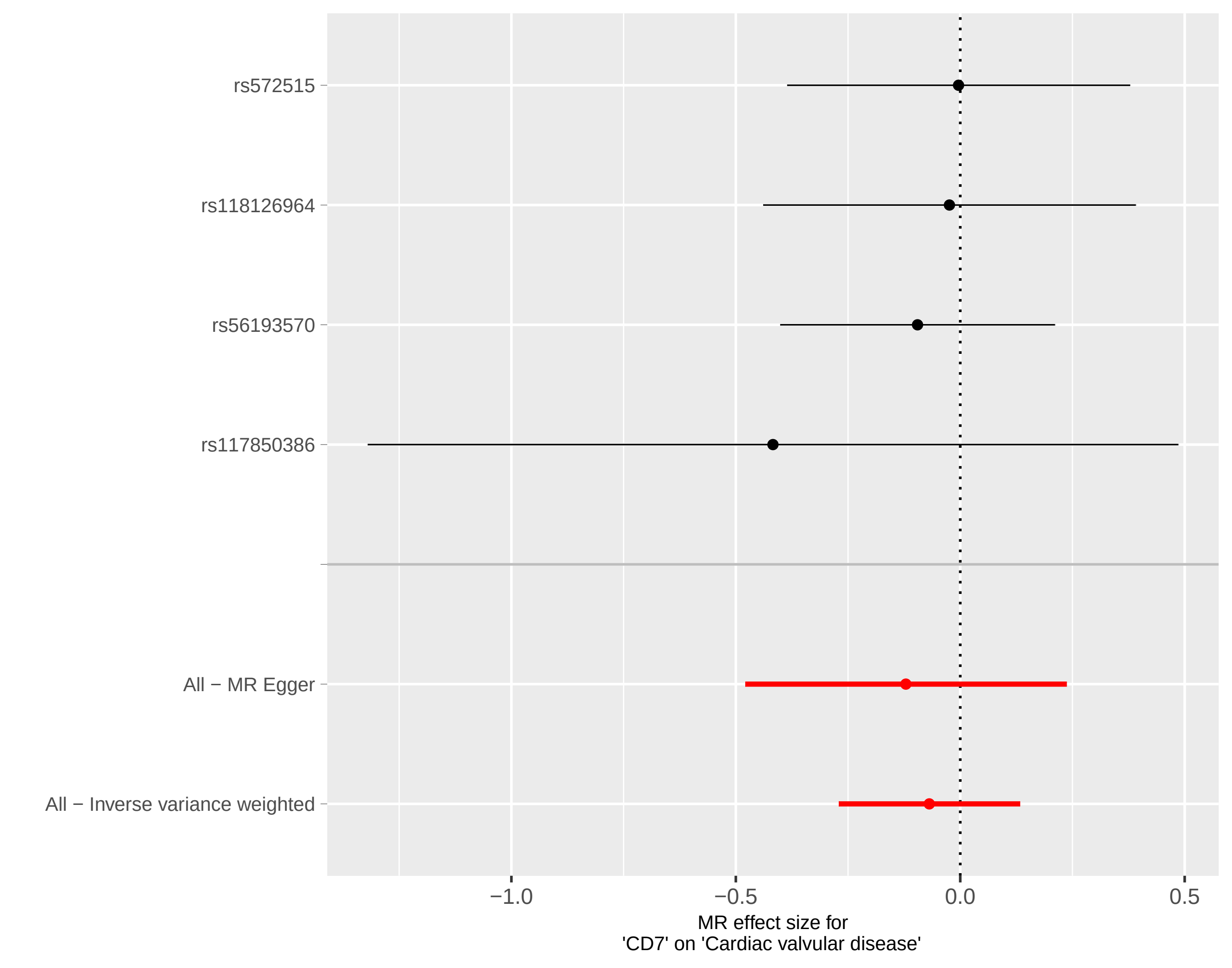
rs572515
rs118126964
rs56193570
rs117850386
All − MR Egger
All − Inverse variance weighted
−1.0
−0.5
0.0
0.5
MR effect size for
'CD7' on 'Cardiac valvular disease'

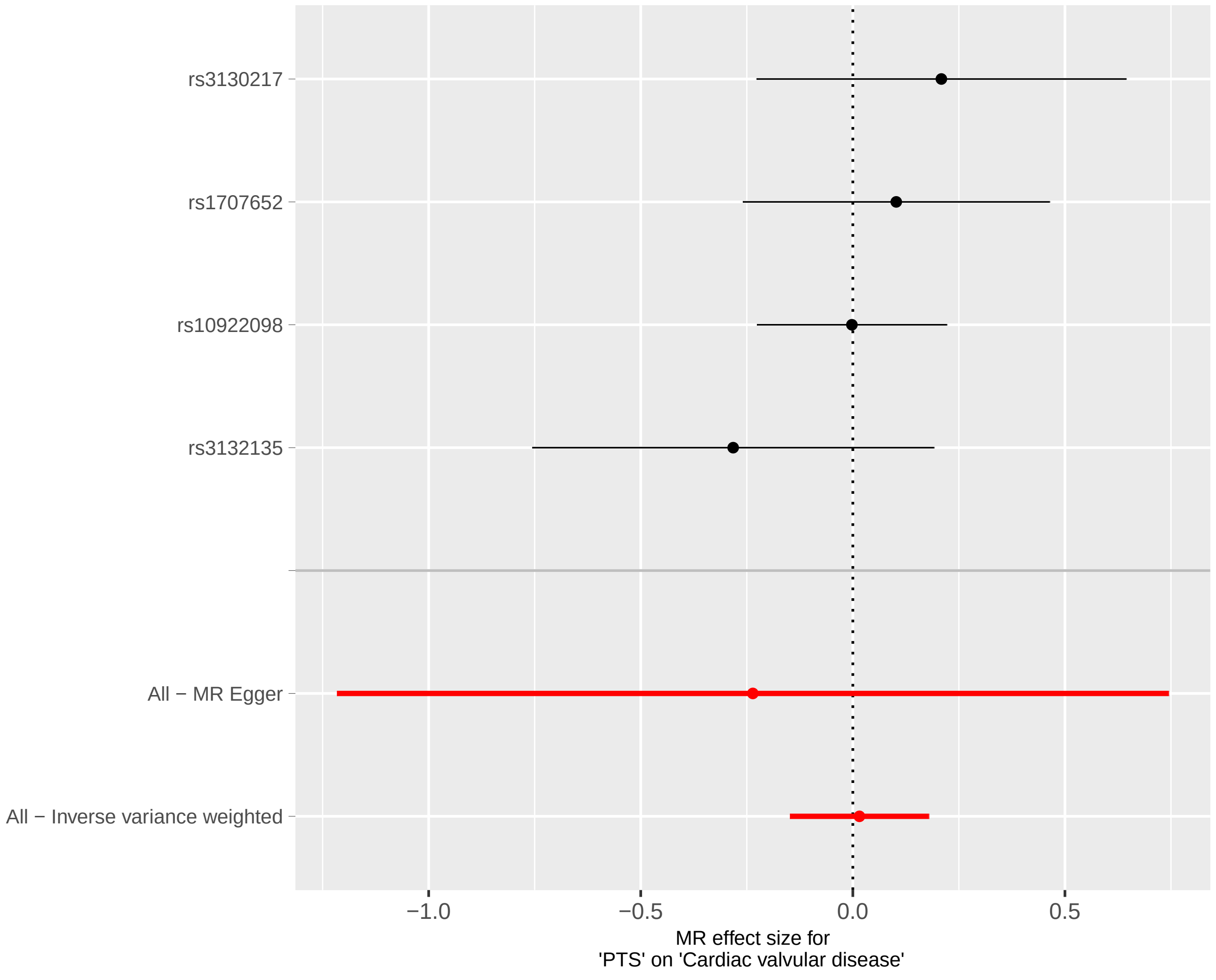
rs3130217
rs1707652
rs10922098
rs3132135
All − MR Egger
All − Inverse variance weighted
−1.0
−0.5
0.0
0.5
MR effect size for
'PTS' on 'Cardiac valvular disease'

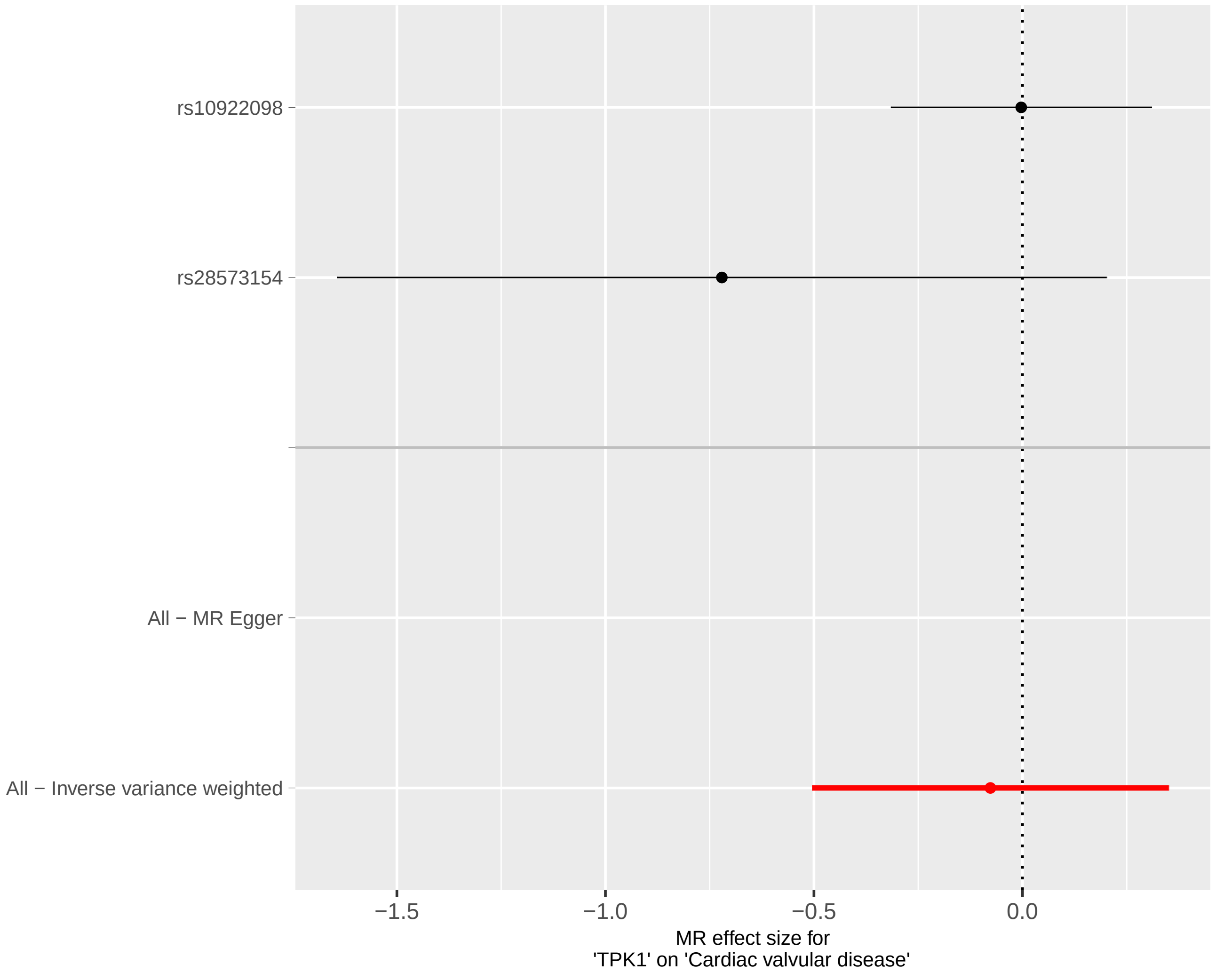
rs10922098
rs28573154
All − MR Egger
All − Inverse variance weighted
−1.5
−1.0
−0.5
0.0
MR effect size for
'TPK1' on 'Cardiac valvular disease'

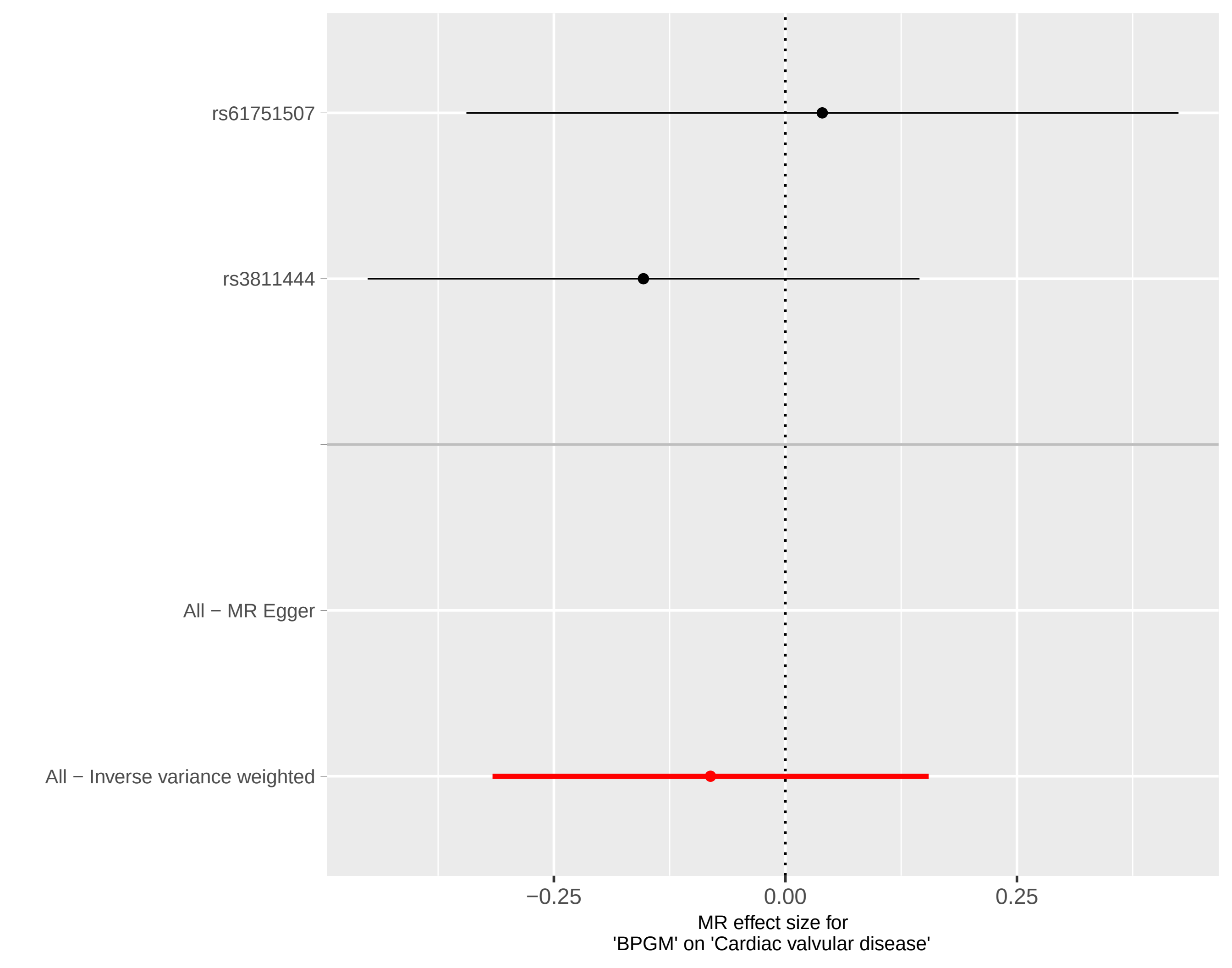
rs61751507
rs3811444
All − MR Egger
All − Inverse variance weighted
−0.25
0.00
0.25
MR effect size for
'BPGM' on 'Cardiac valvular disease'

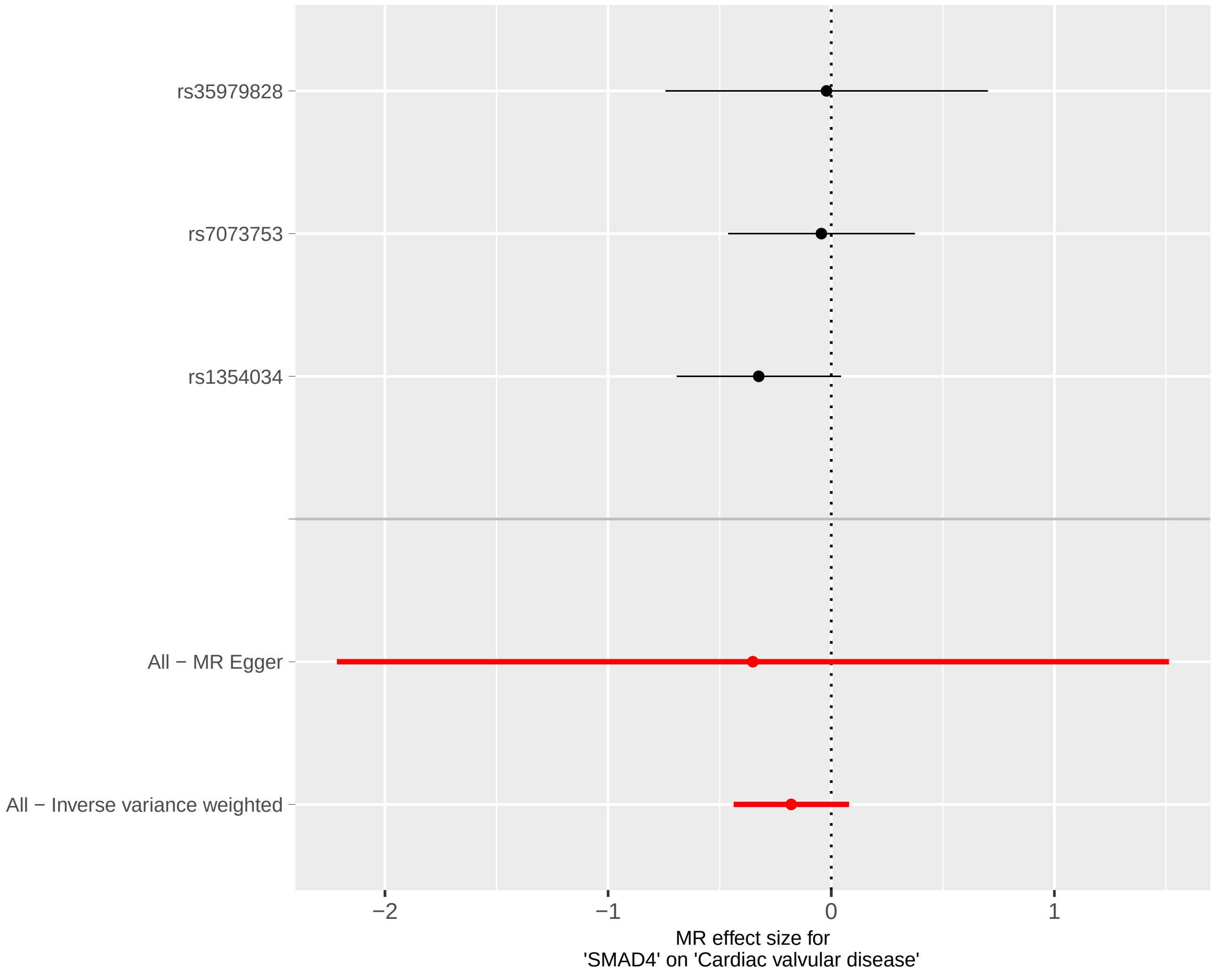

rs35979828
rs7073753
rs1354034
All − MR Egger
All − Inverse variance weighted
−2
−1
0
1
MR effect size for
'SMAD4' on 'Cardiac valvular disease'

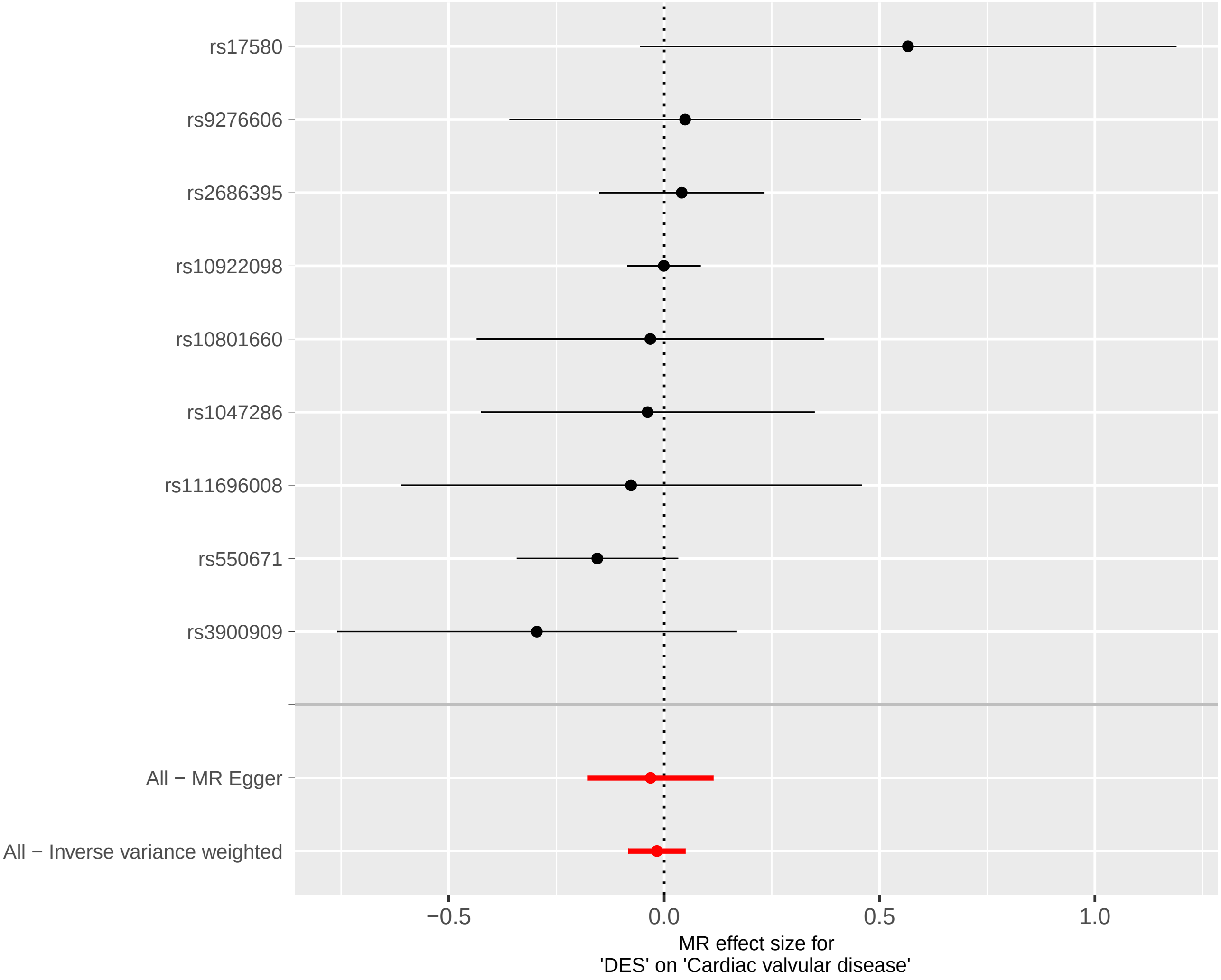
rs17580
rs9276606
rs2686395
rs10922098
rs10801660
rs1047286
rs111696008
rs550671
rs3900909
All − MR Egger
All − Inverse variance weighted
−0.5
0.0
0.5
1.0
MR effect size for
'DES' on 'Cardiac valvular disease'

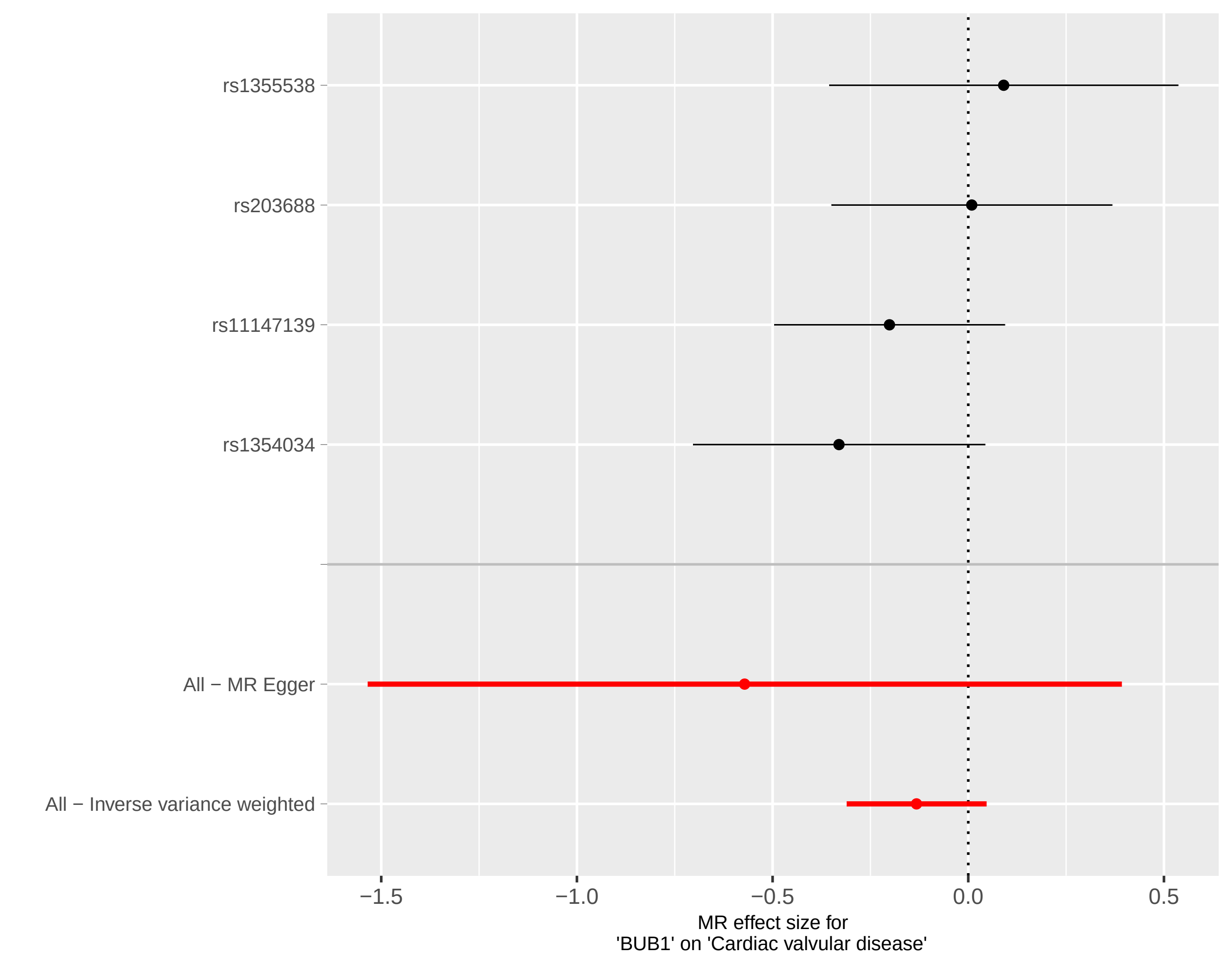
rs1355538
rs203688
rs11147139
rs1354034
All − MR Egger
All − Inverse variance weighted
−1.5
−1.0
−0.5
0.0
0.5
MR effect size for
'BUB1' on 'Cardiac valvular disease'

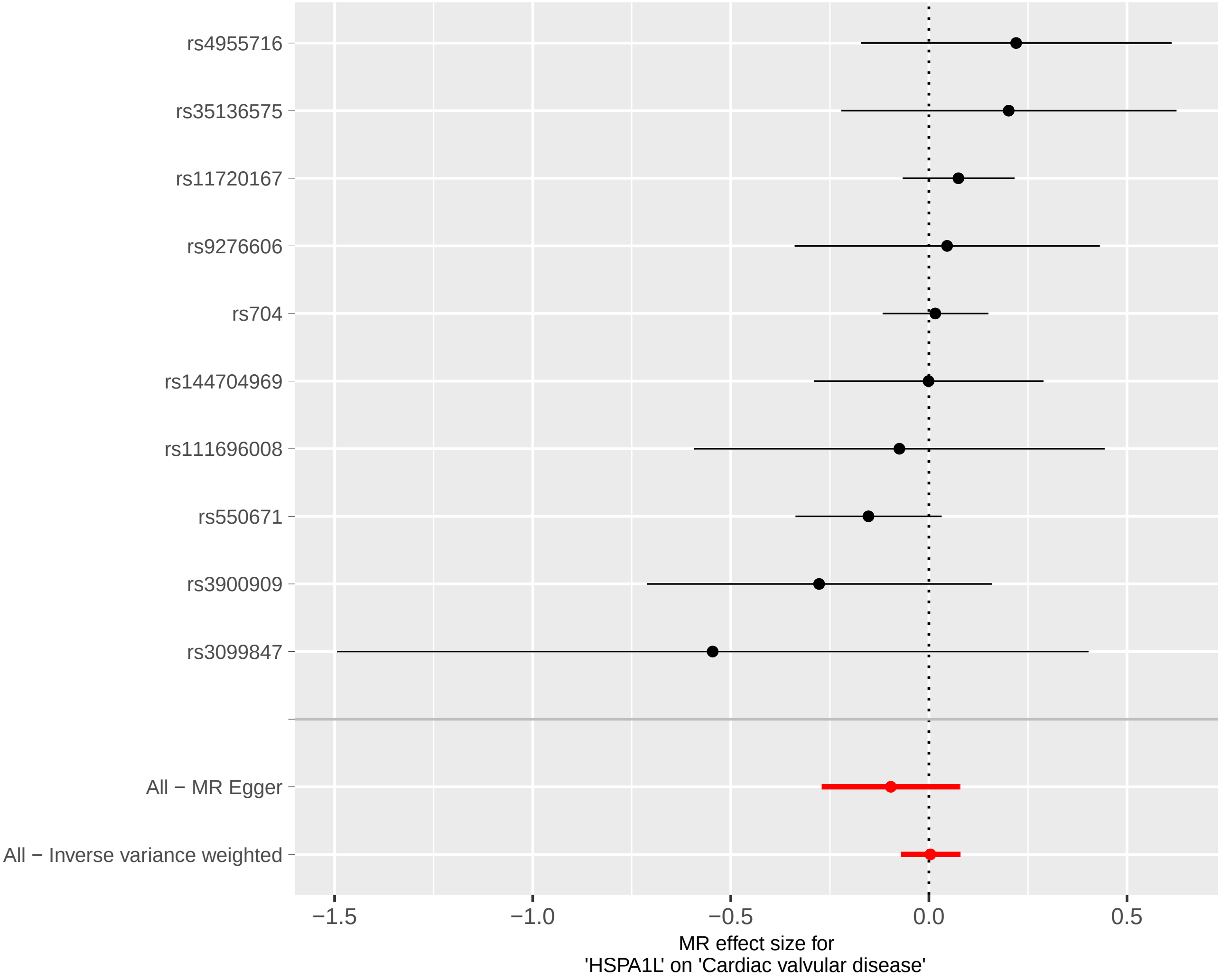
rs4955716
rs35136575
rs11720167
rs9276606
rs704
rs144704969
rs111696008
rs550671
rs3900909
rs3099847
All − MR Egger
All − Inverse variance weighted
−1.5
−1.0
−0.5
0.0
0.5
MR effect size for
'HSPA1L' on 'Cardiac valvular disease'

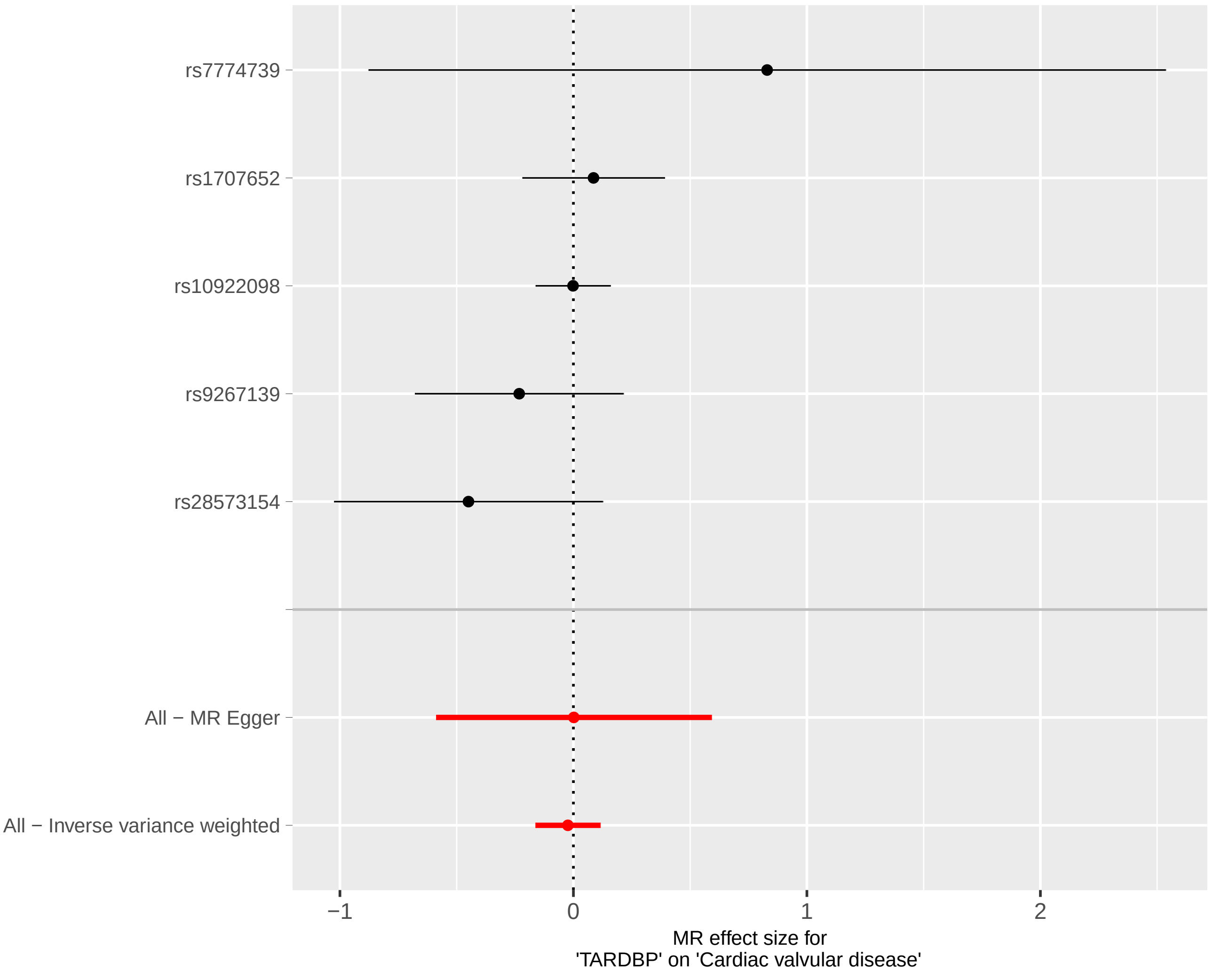

rs7774739
rs1707652
rs10922098
rs9267139
rs28573154
All − MR Egger
All − Inverse variance weighted
−1
0
1
2
MR effect size for
'TARDBP' on 'Cardiac valvular disease'

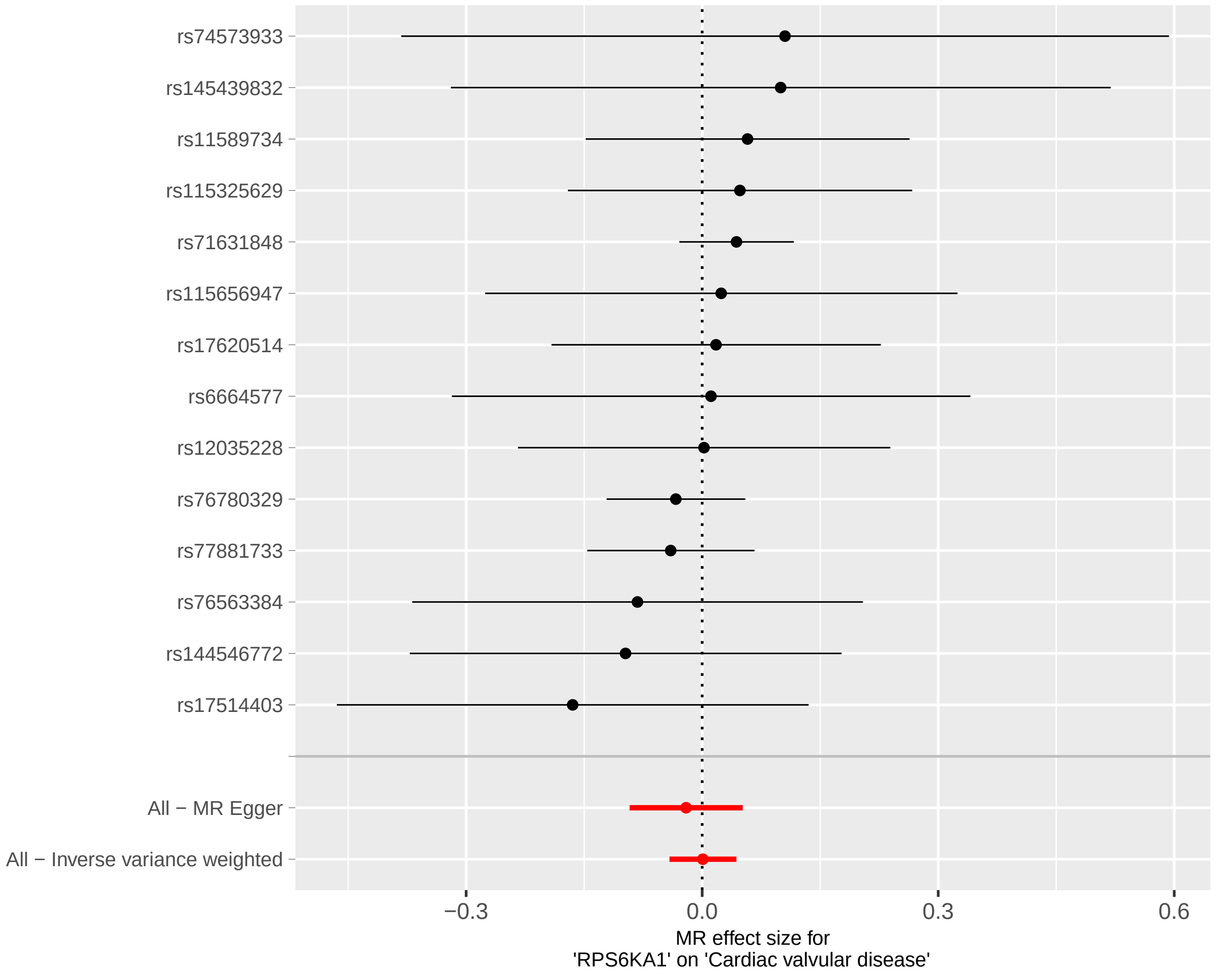
rs74573933
rs145439832
rs11589734
rs115325629
rs71631848
rs115656947
rs17620514
rs6664577
rs12035228
rs76780329
rs77881733
rs76563384
rs144546772
rs17514403
All − MR Egger
All − Inverse variance weighted
−0.3
0.0
0.3
0.6
MR effect size for
'RPS6KA1' on 'Cardiac valvular disease'

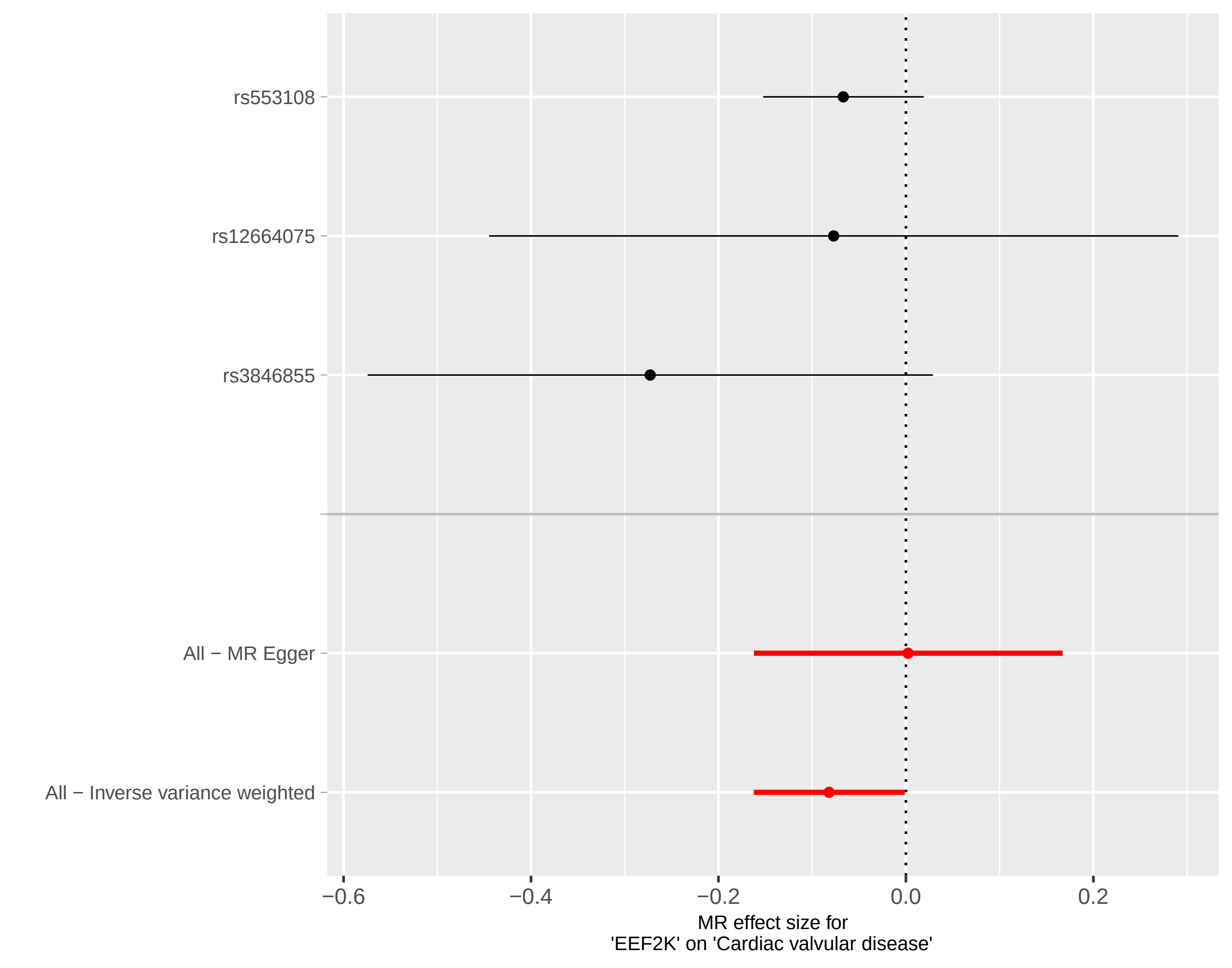
rs553108
rs12664075
rs3846855
All − MR Egger
All − Inverse variance weighted
−0.6
−0.4
−0.2
0.0
0.2
MR effect size for
'EEF2K' on 'Cardiac valvular disease'

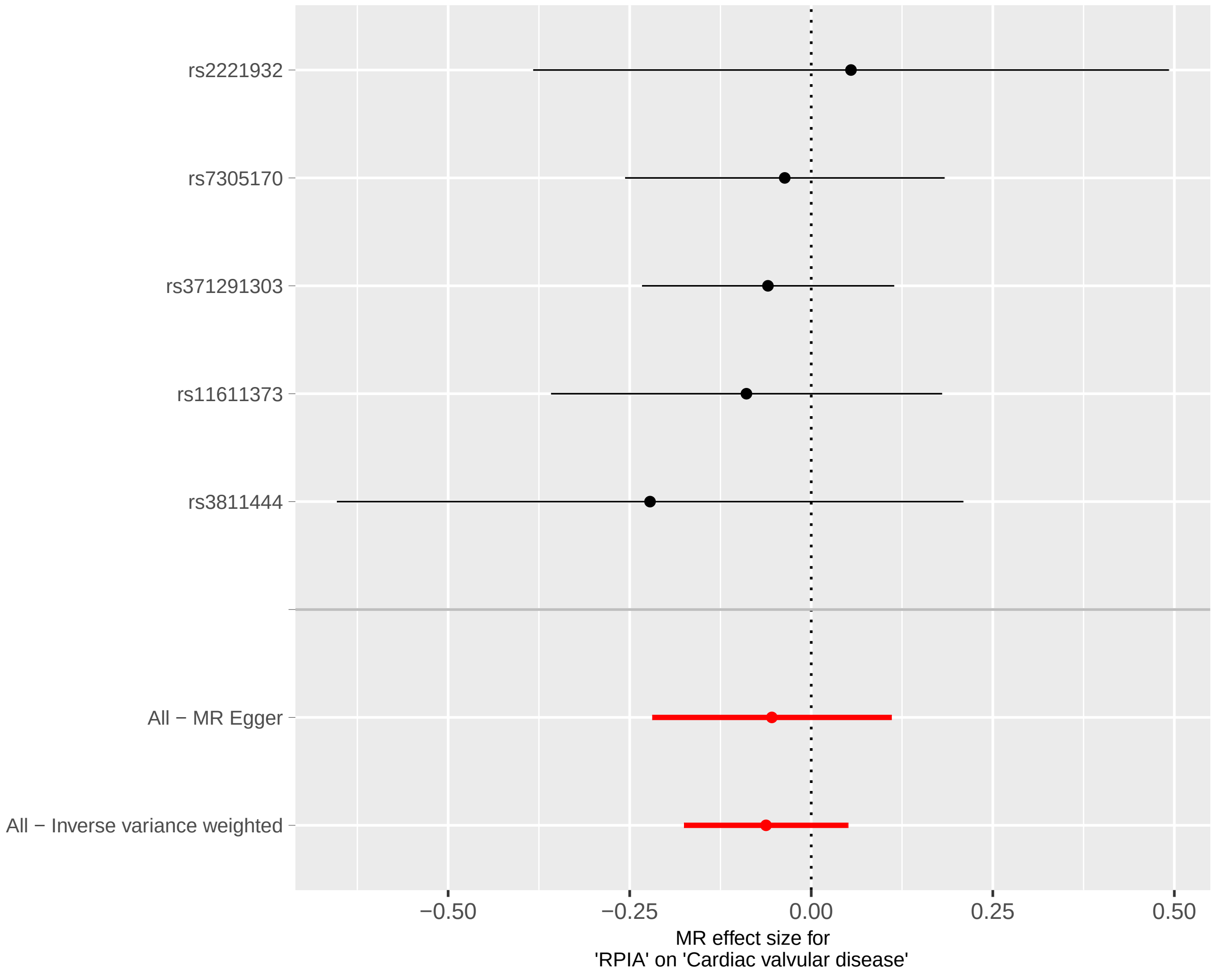
rs2221932
rs7305170
rs371291303
rs11611373
rs3811444
All − MR Egger
All − Inverse variance weighted
−0.50
−0.25
0.00
0.25
0.50
MR effect size for
'RPIA' on 'Cardiac valvular disease'

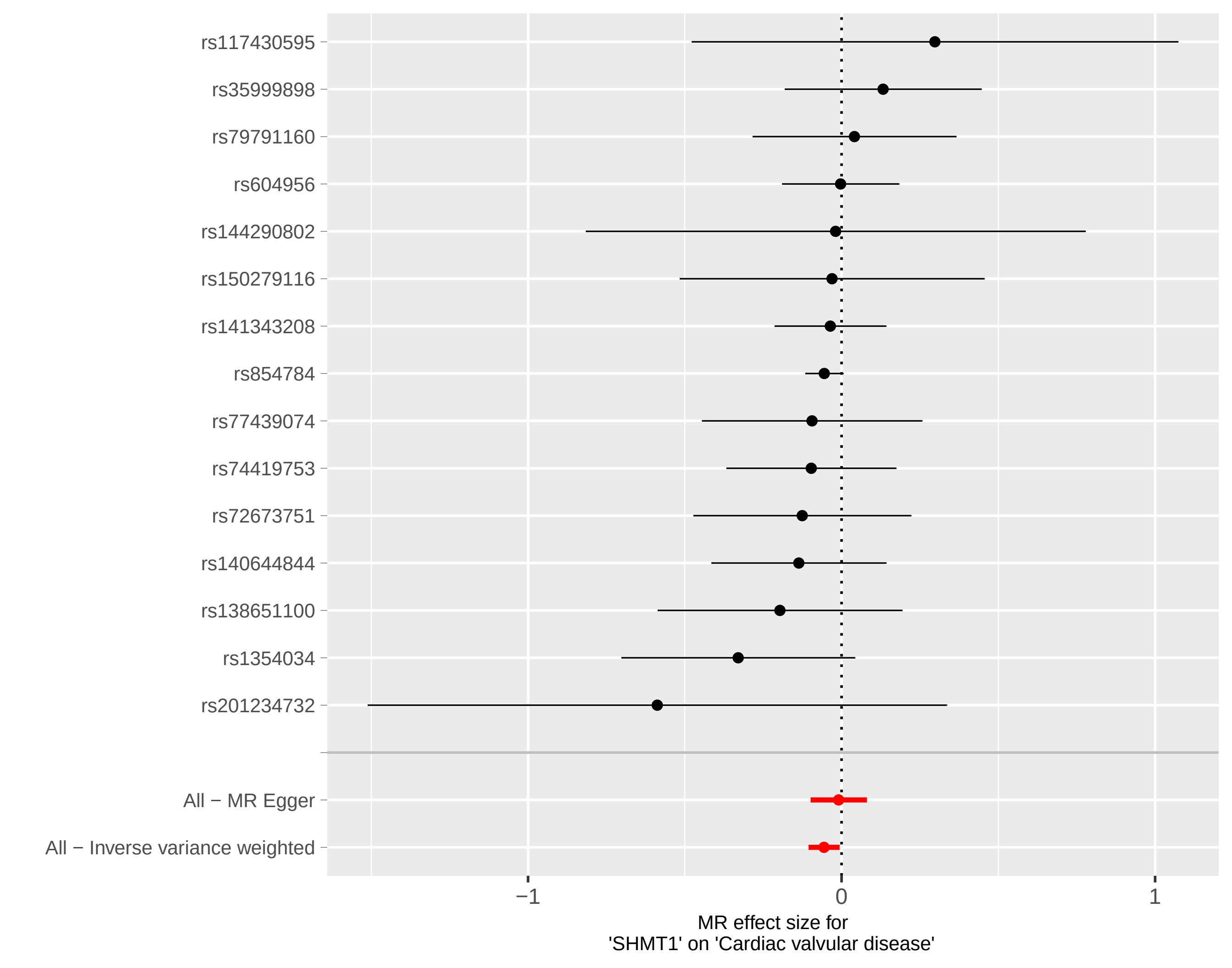
rs117430595
rs35999898
rs79791160
rs604956
rs144290802
rs150279116
rs141343208
rs854784
rs77439074
rs74419753
rs72673751
rs140644844
rs138651100
rs1354034
rs201234732
All − MR Egger
All − Inverse variance weighted
−1
0
1
MR effect size for
'SHMT1' on 'Cardiac valvular disease'

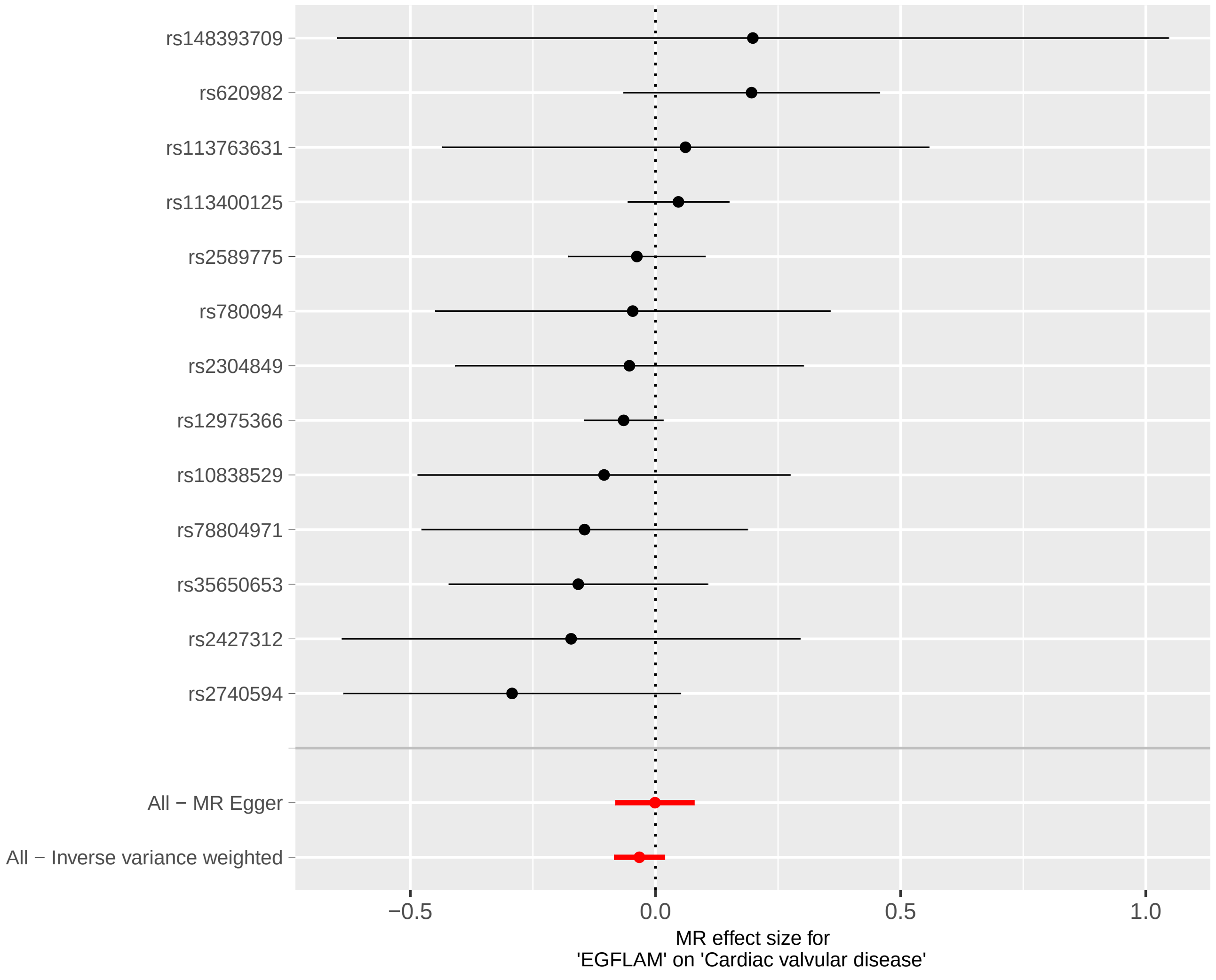

rs148393709
rs620982
rs113763631
rs113400125
rs2589775
rs780094
rs2304849
rs12975366
rs10838529
rs78804971
rs35650653
rs2427312
rs2740594
All − MR Egger
All − Inverse variance weighted
−0.5
0.0
0.5
1.0
MR effect size for
'EGFLAM' on 'Cardiac valvular disease'

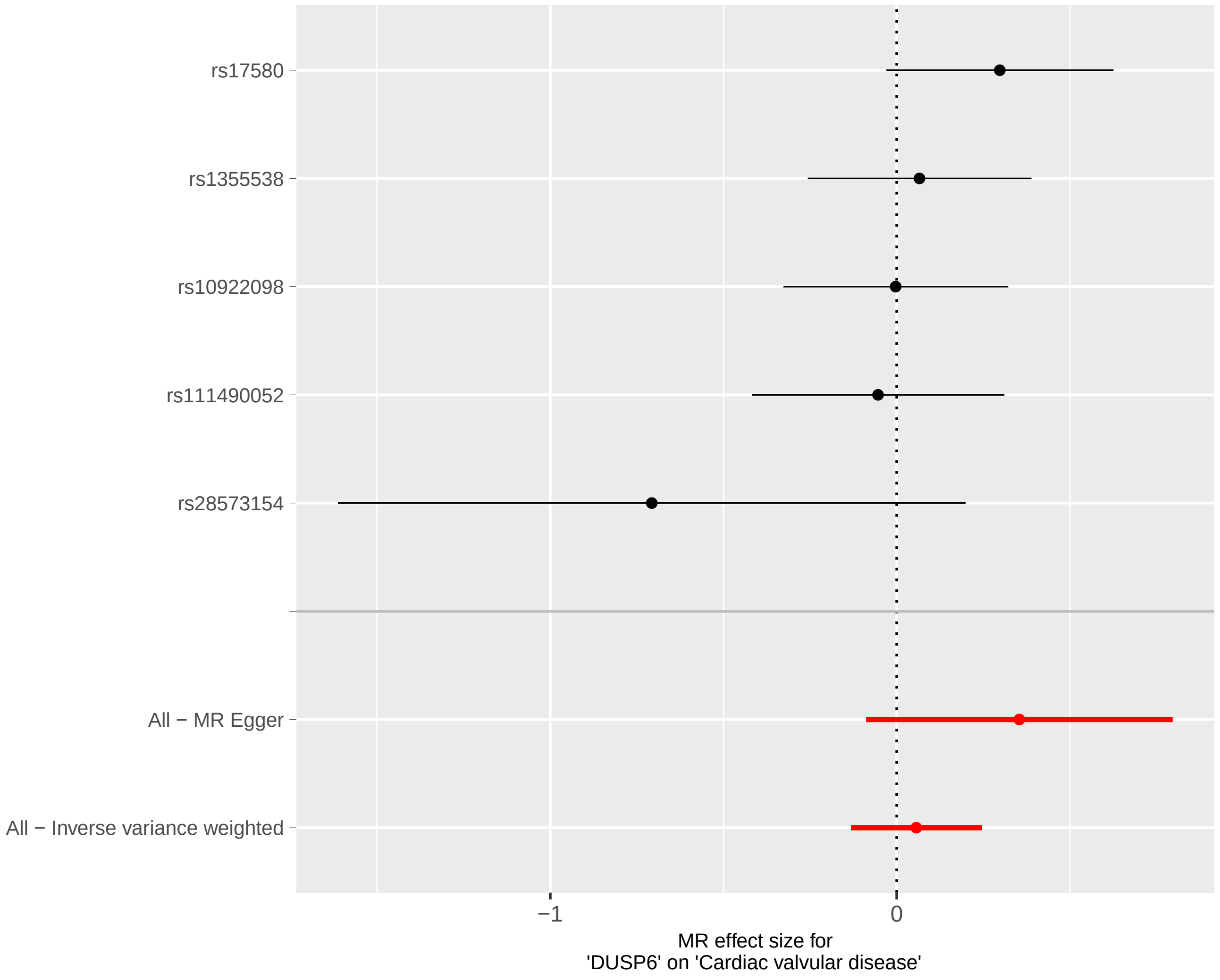
rs17580
rs1355538
rs10922098
rs111490052
rs28573154
All − MR Egger
All − Inverse variance weighted
−1
0
MR effect size for
'DUSP6' on 'Cardiac valvular disease'

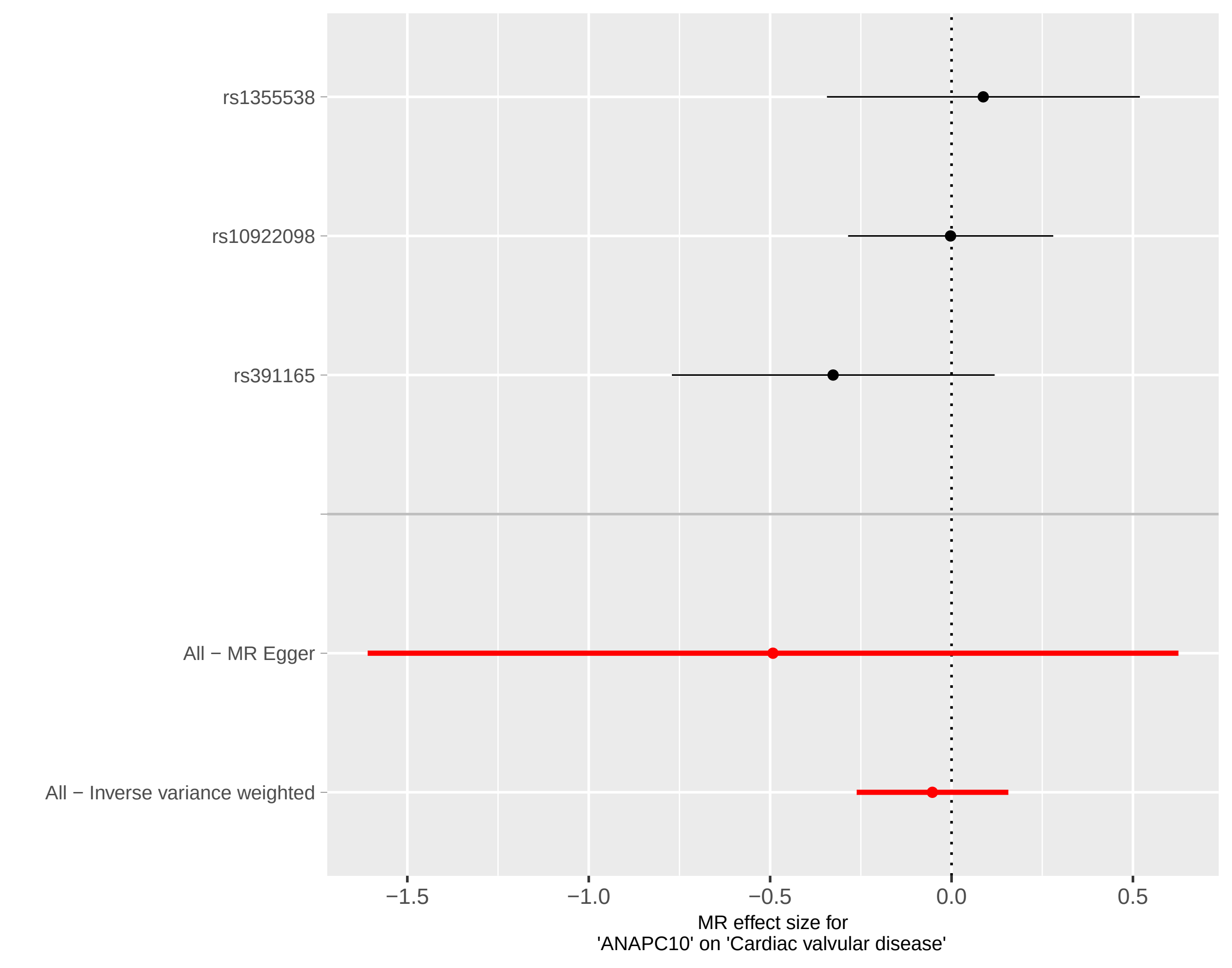
rs1355538
rs10922098
rs391165
All − MR Egger
All − Inverse variance weighted
−1.5
−1.0
−0.5
0.0
0.5
MR effect size for
'ANAPC10' on 'Cardiac valvular disease'

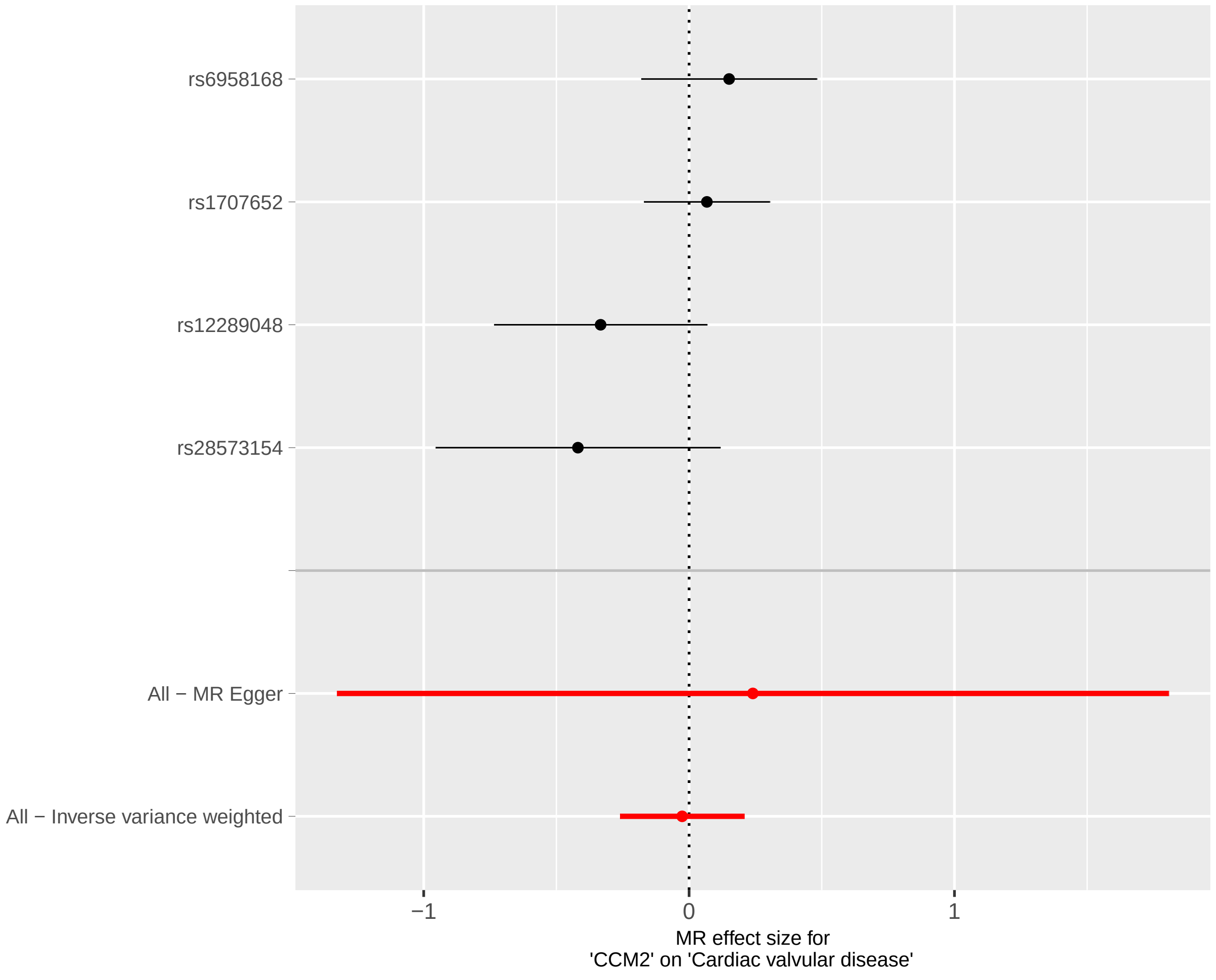
rs6958168
rs1707652
rs12289048
rs28573154
All − MR Egger
All − Inverse variance weighted
−1
0
1
MR effect size for
'CCM2' on 'Cardiac valvular disease'

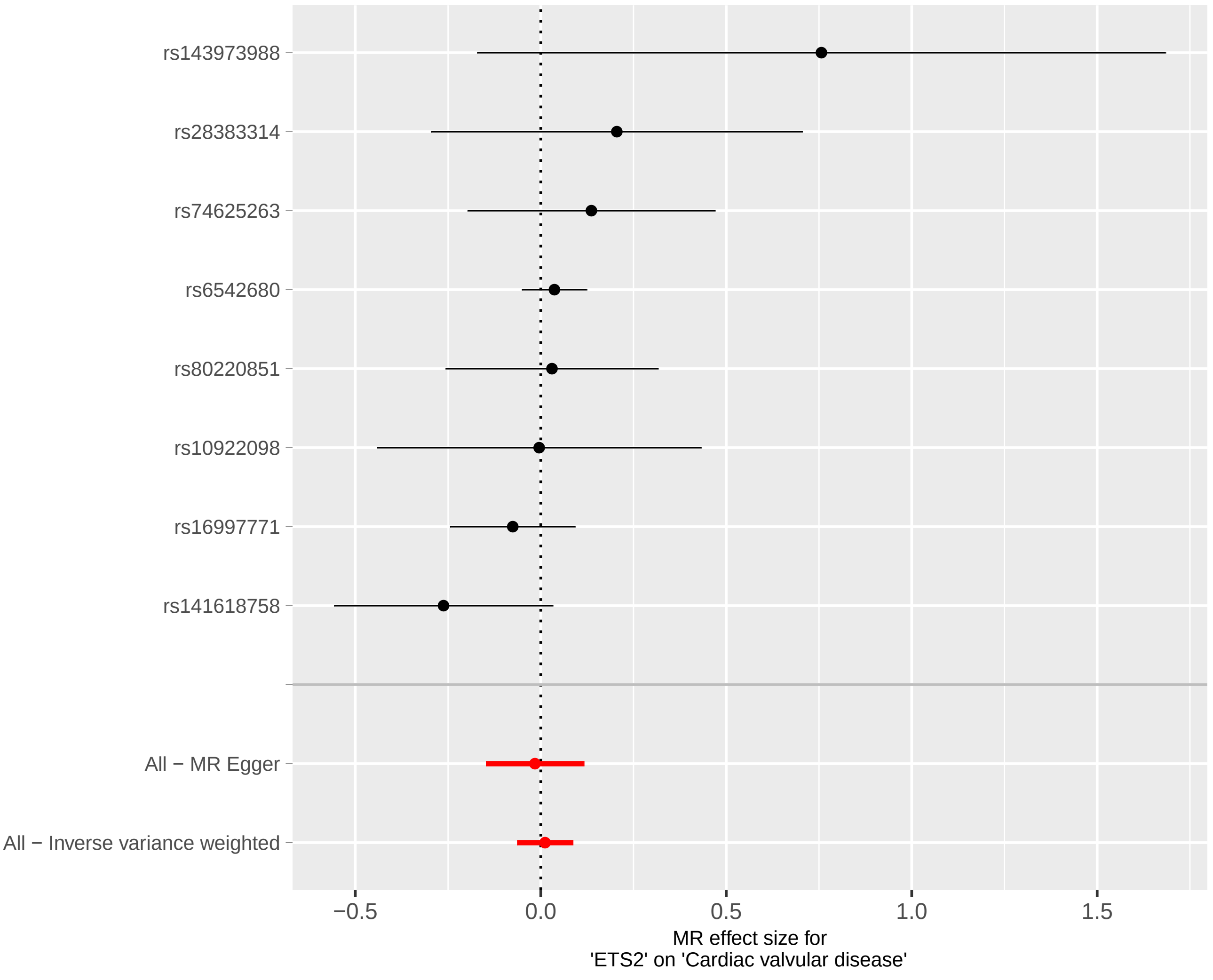

rs143973988
rs28383314
rs74625263
rs6542680
rs80220851
rs10922098
rs16997771
rs141618758
All − MR Egger
All − Inverse variance weighted
−0.5
0.0
0.5
1.0
1.5
MR effect size for
'ETS2' on 'Cardiac valvular disease'

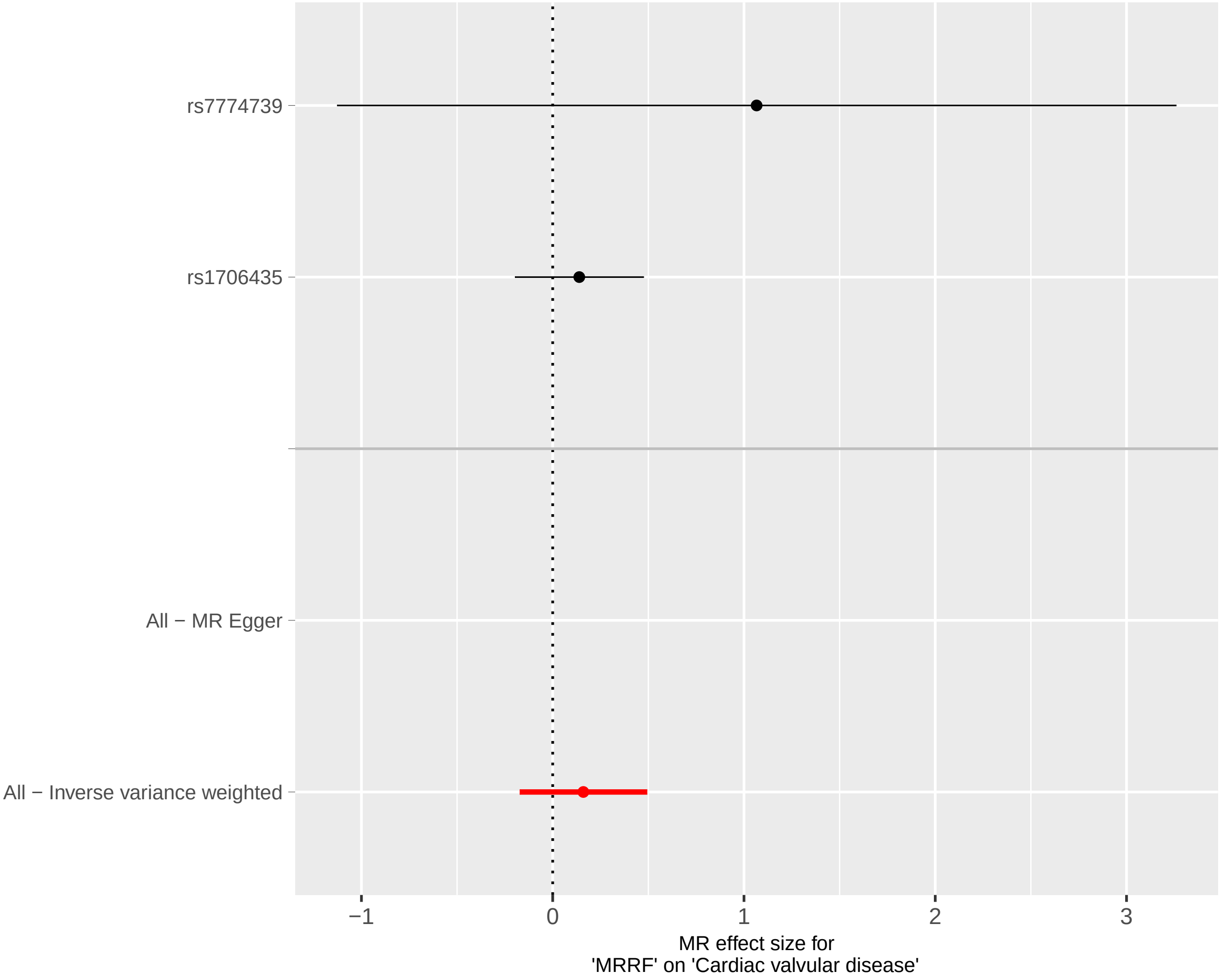
rs7774739
rs1706435
All − MR Egger
All − Inverse variance weighted
−1
0
1
2
3
MR effect size for
'MRRF' on 'Cardiac valvular disease'

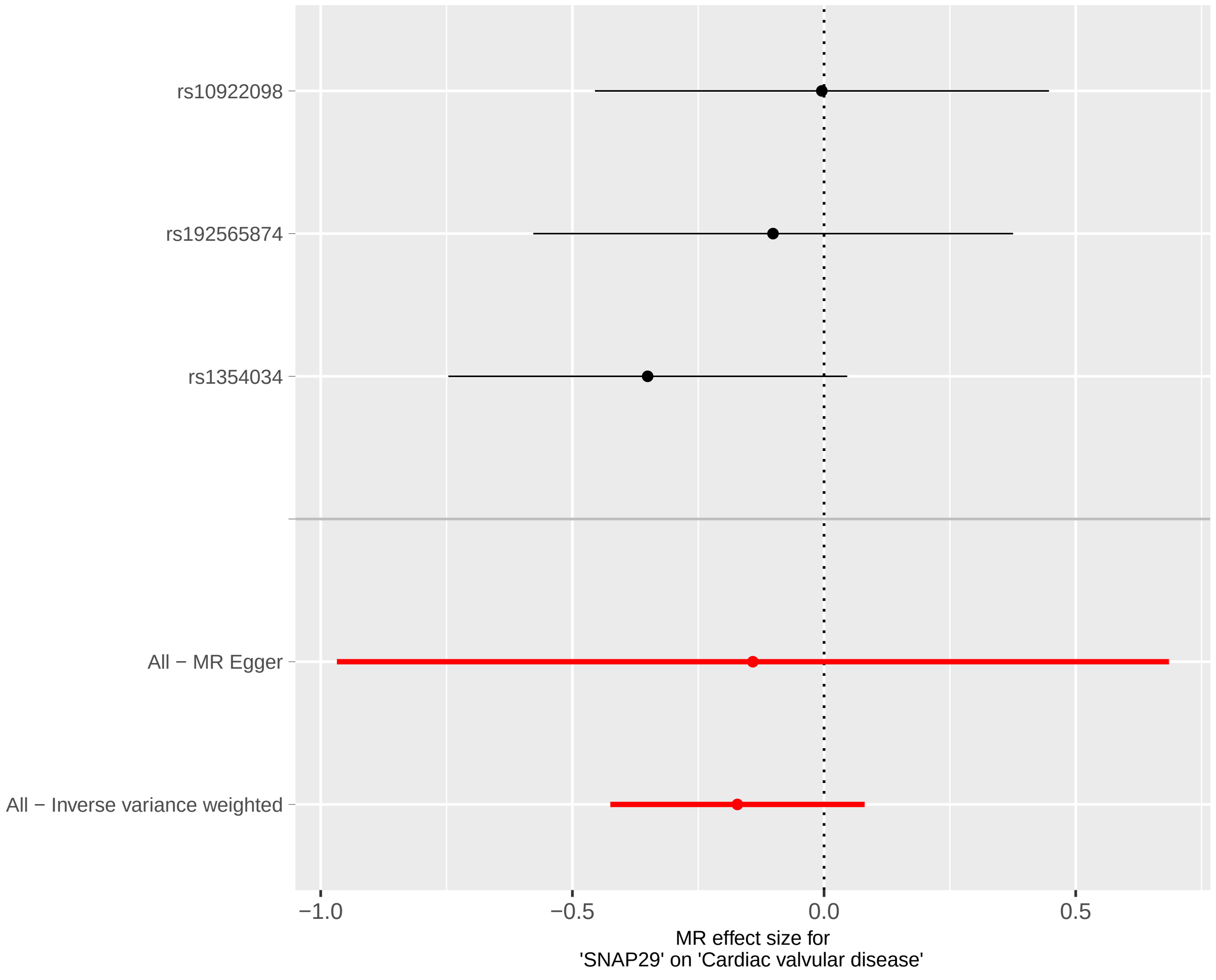
rs10922098
rs192565874
rs1354034
All − MR Egger
All − Inverse variance weighted
−1.0
−0.5
0.0
0.5
MR effect size for
'SNAP29' on 'Cardiac valvular disease'

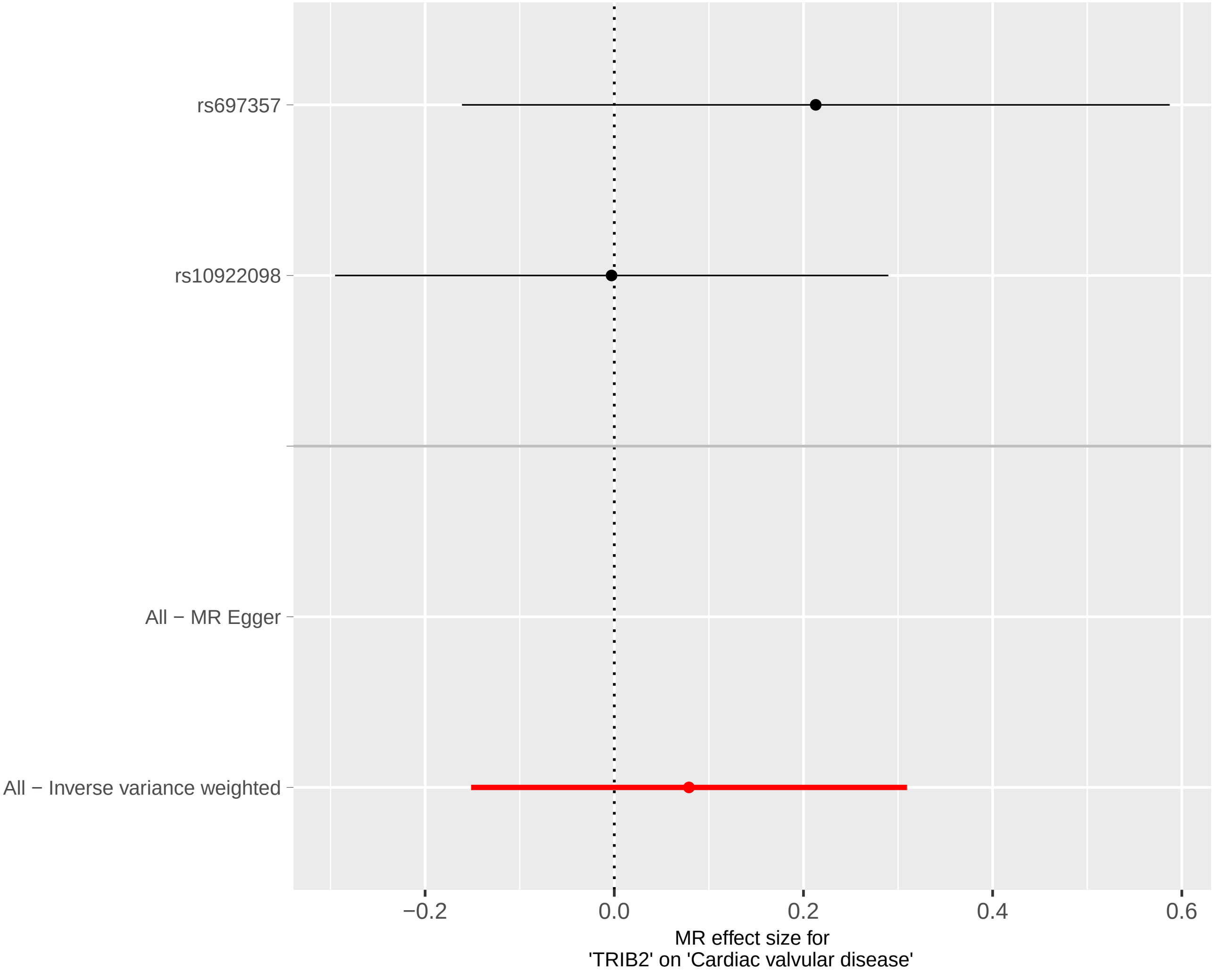
rs697357
rs10922098
All − MR Egger
All − Inverse variance weighted
−0.2
0.0
0.2
0.4
0.6
MR effect size for
'TRIB2' on 'Cardiac valvular disease'

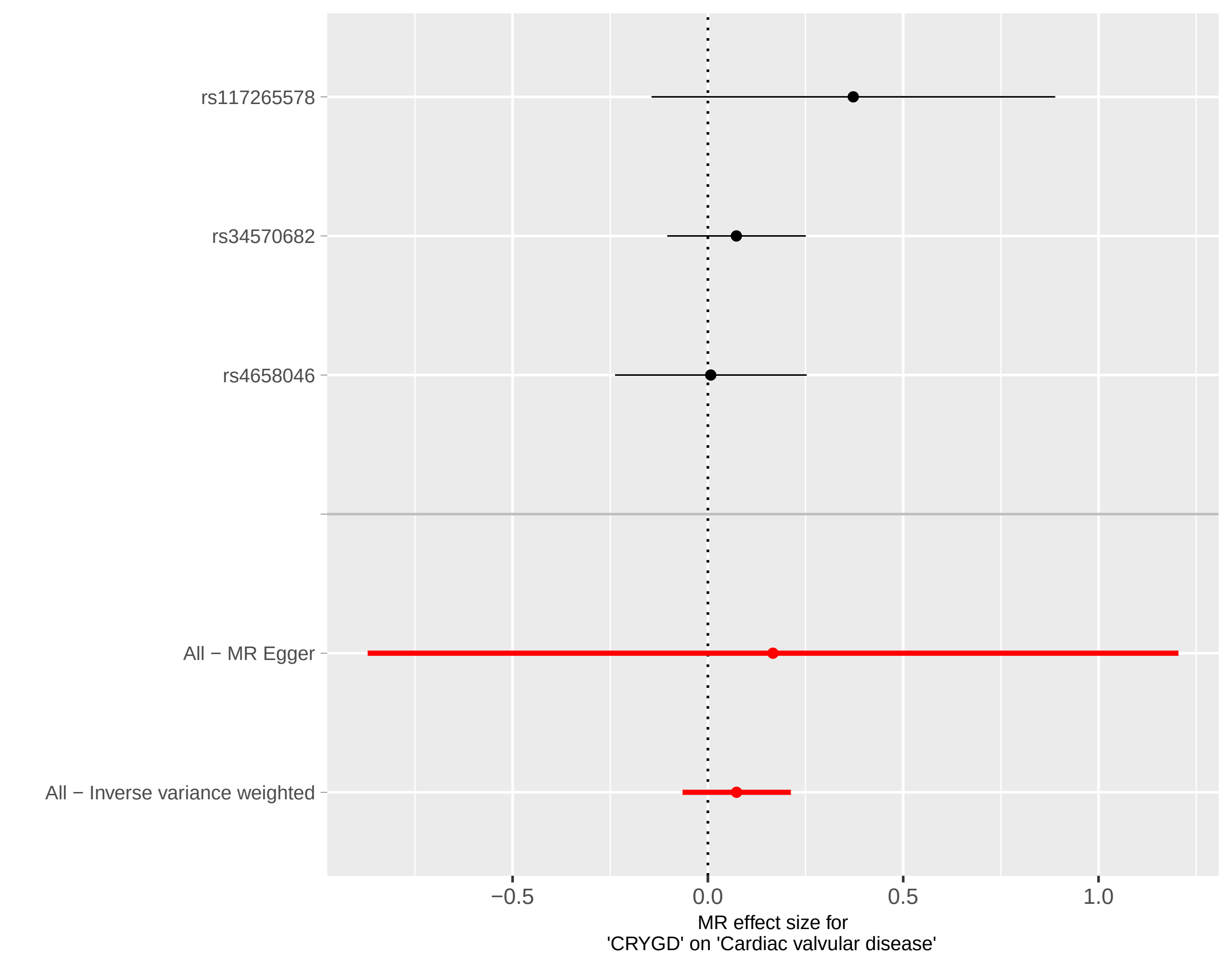
rs117265578
rs34570682
rs4658046
All − MR Egger
All − Inverse variance weighted
−0.5
0.0
0.5
1.0
MR effect size for
'CRYGD' on 'Cardiac valvular disease'

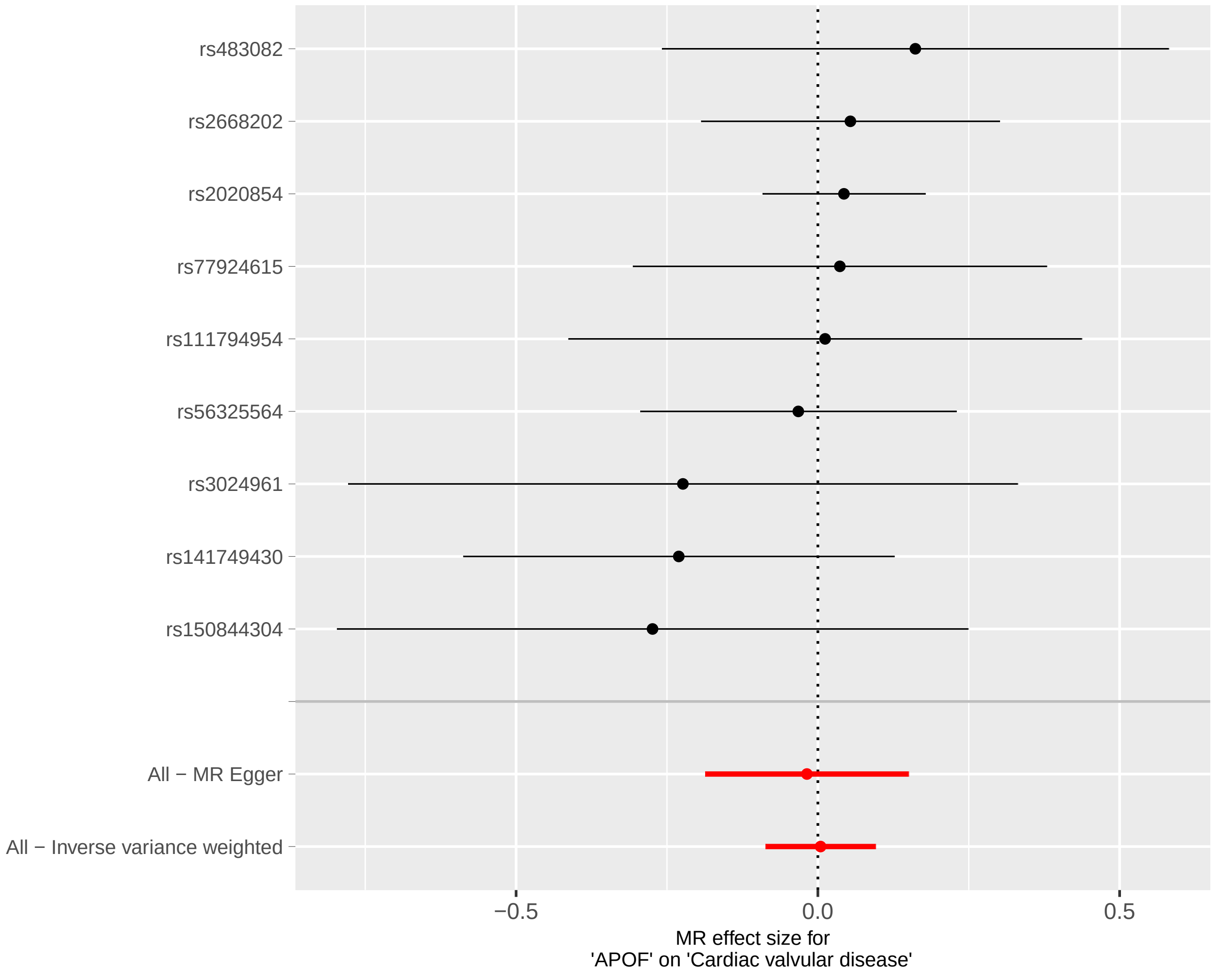

rs483082
rs2668202
rs2020854
rs77924615
rs111794954
rs56325564
rs3024961
rs141749430
rs150844304
All − MR Egger
All − Inverse variance weighted
−0.5
0.0
0.5
MR effect size for
'APOF' on 'Cardiac valvular disease'

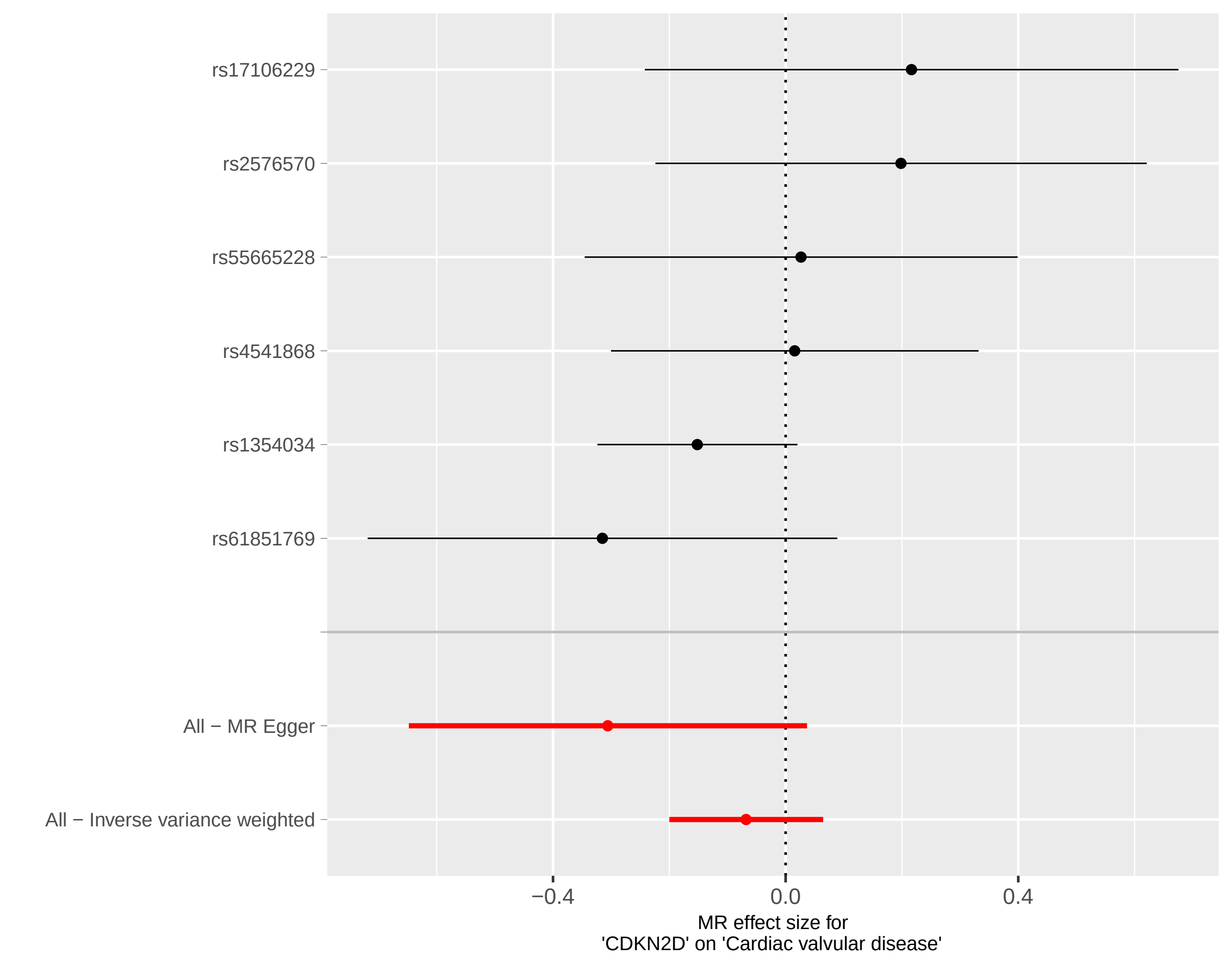

rs17106229
rs2576570
rs55665228
rs4541868
rs1354034
rs61851769
All − MR Egger
All − Inverse variance weighted
−0.4
0.0
0.4
MR effect size for
'CDKN2D' on 'Cardiac valvular disease'

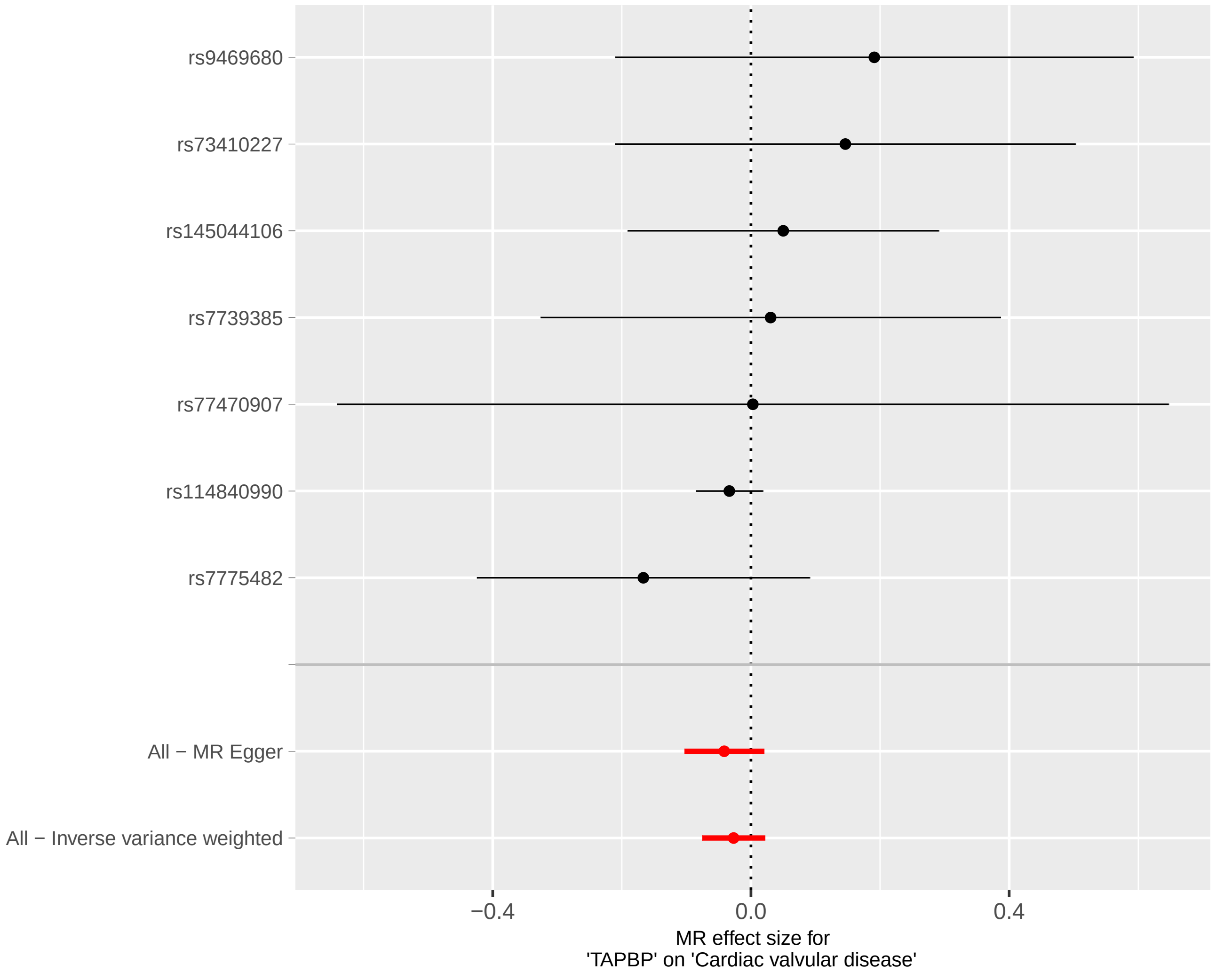
rs9469680
rs73410227
rs145044106
rs7739385
rs77470907
rs114840990
rs7775482
All − MR Egger
All − Inverse variance weighted
−0.4
0.0
0.4
MR effect size for
'TAPBP' on 'Cardiac valvular disease'

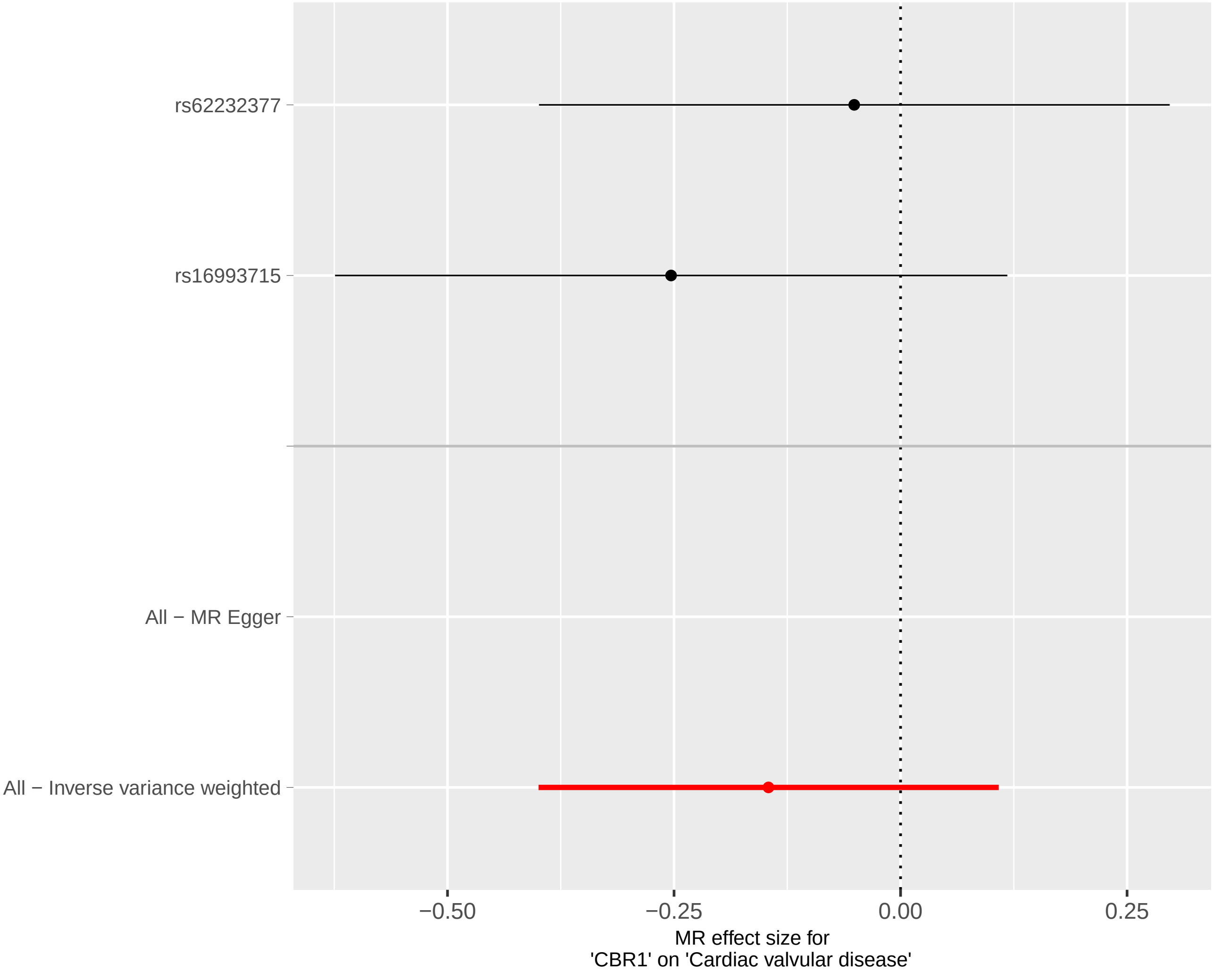
rs62232377
rs16993715
All − MR Egger
All − Inverse variance weighted
−0.50
−0.25
0.00
0.25
MR effect size for
'CBR1' on 'Cardiac valvular disease'

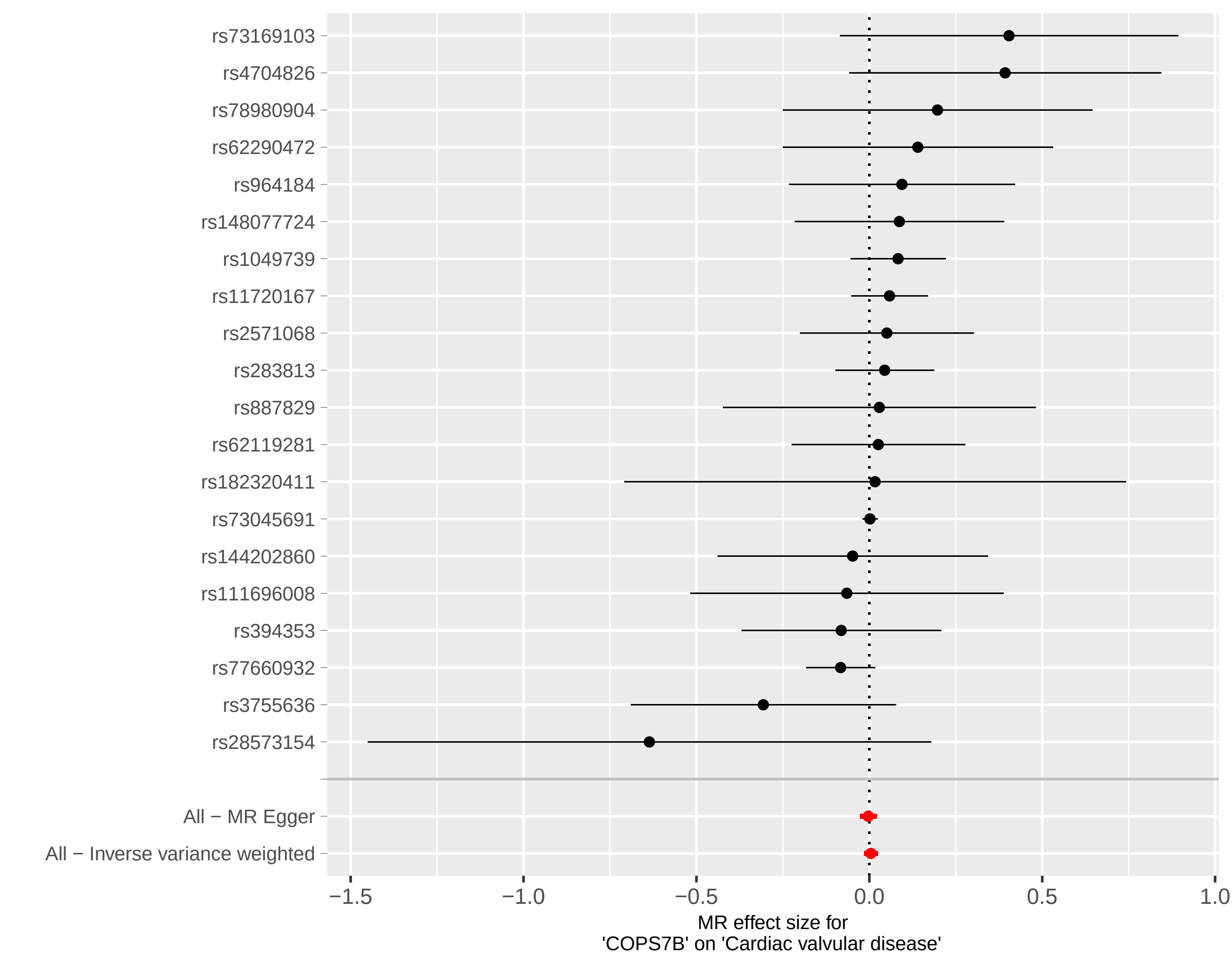

rs73169103
rs4704826
rs78980904
rs62290472
rs964184
rs148077724
rs1049739
rs11720167
rs2571068
rs283813
rs887829
rs62119281
rs182320411
rs73045691
rs144202860
rs111696008
rs394353
rs77660932
rs3755636
rs28573154
All − MR Egger
All − Inverse variance weighted
−1.5
−1.0
−0.5
0.0
0.5
1.0
MR effect size for
'COPS7B' on 'Cardiac valvular disease'

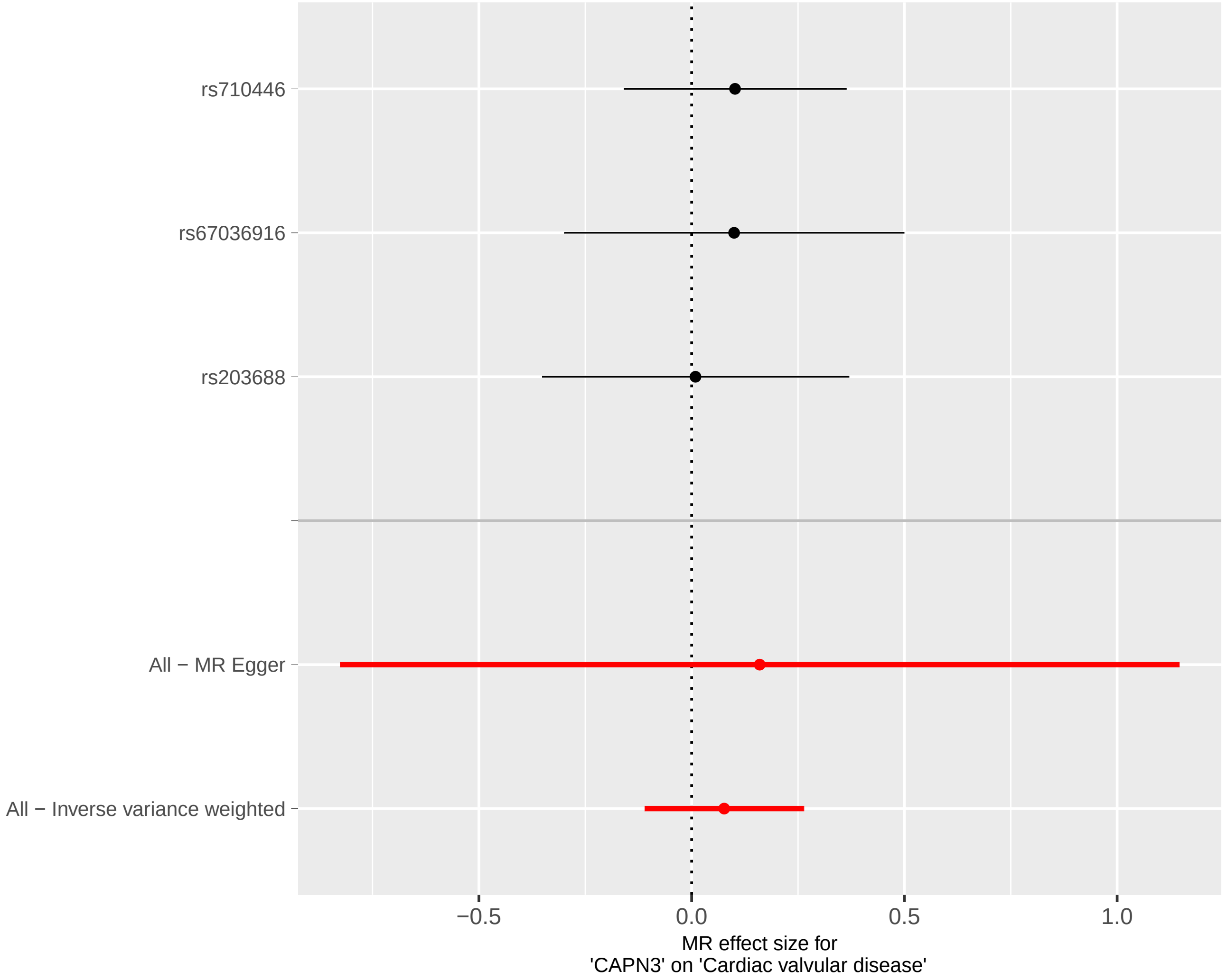

rs710446
rs67036916
rs203688
All − MR Egger
All − Inverse variance weighted
−0.5
0.0
0.5
1.0
MR effect size for
'CAPN3' on 'Cardiac valvular disease'

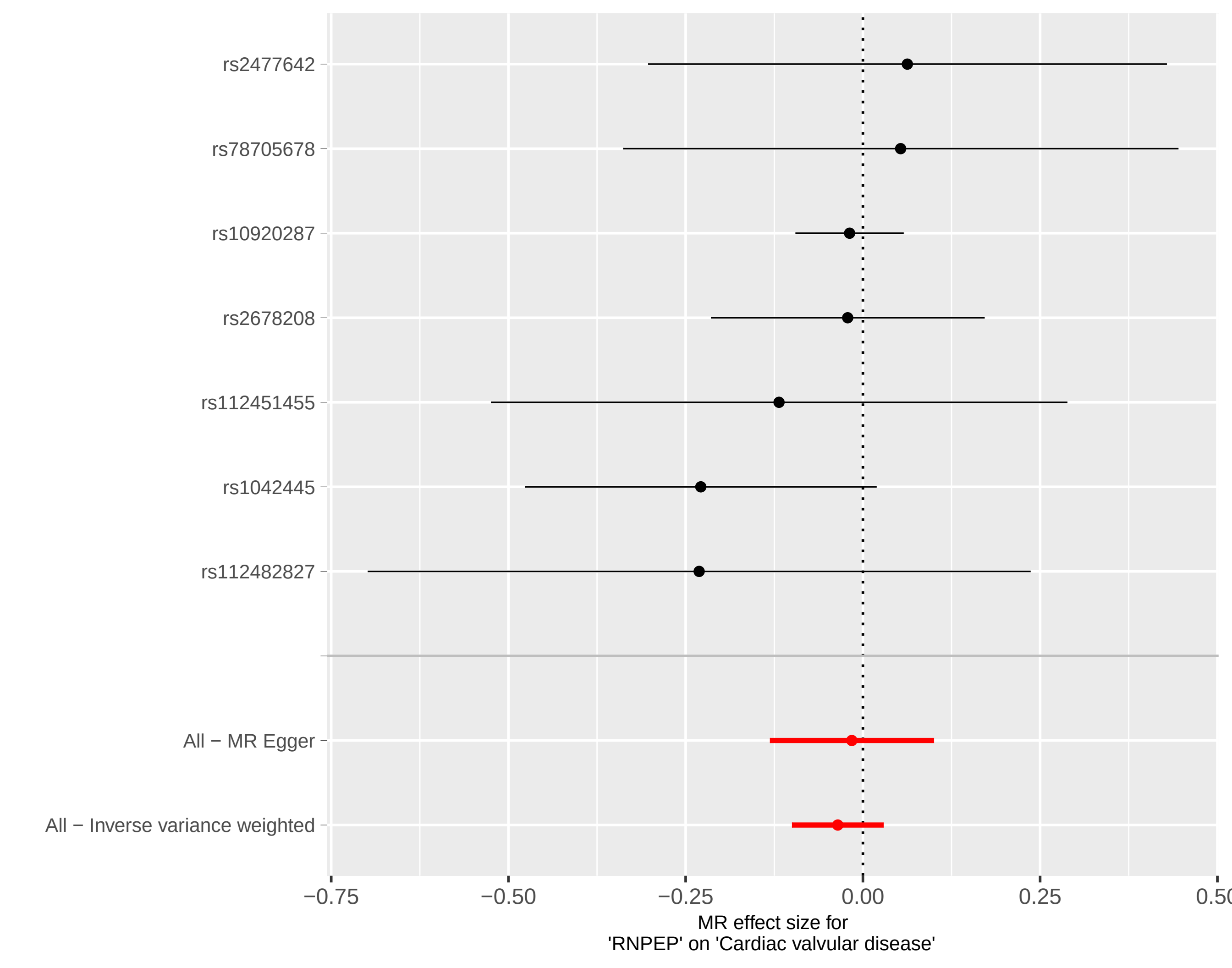
rs2477642
rs78705678
rs10920287
rs2678208
rs112451455
rs1042445
rs112482827
All − MR Egger
All − Inverse variance weighted
−0.75
−0.50
−0.25
0.00
0.25
MR effect size for
'RNPEP' on 'Cardiac valvular disease'

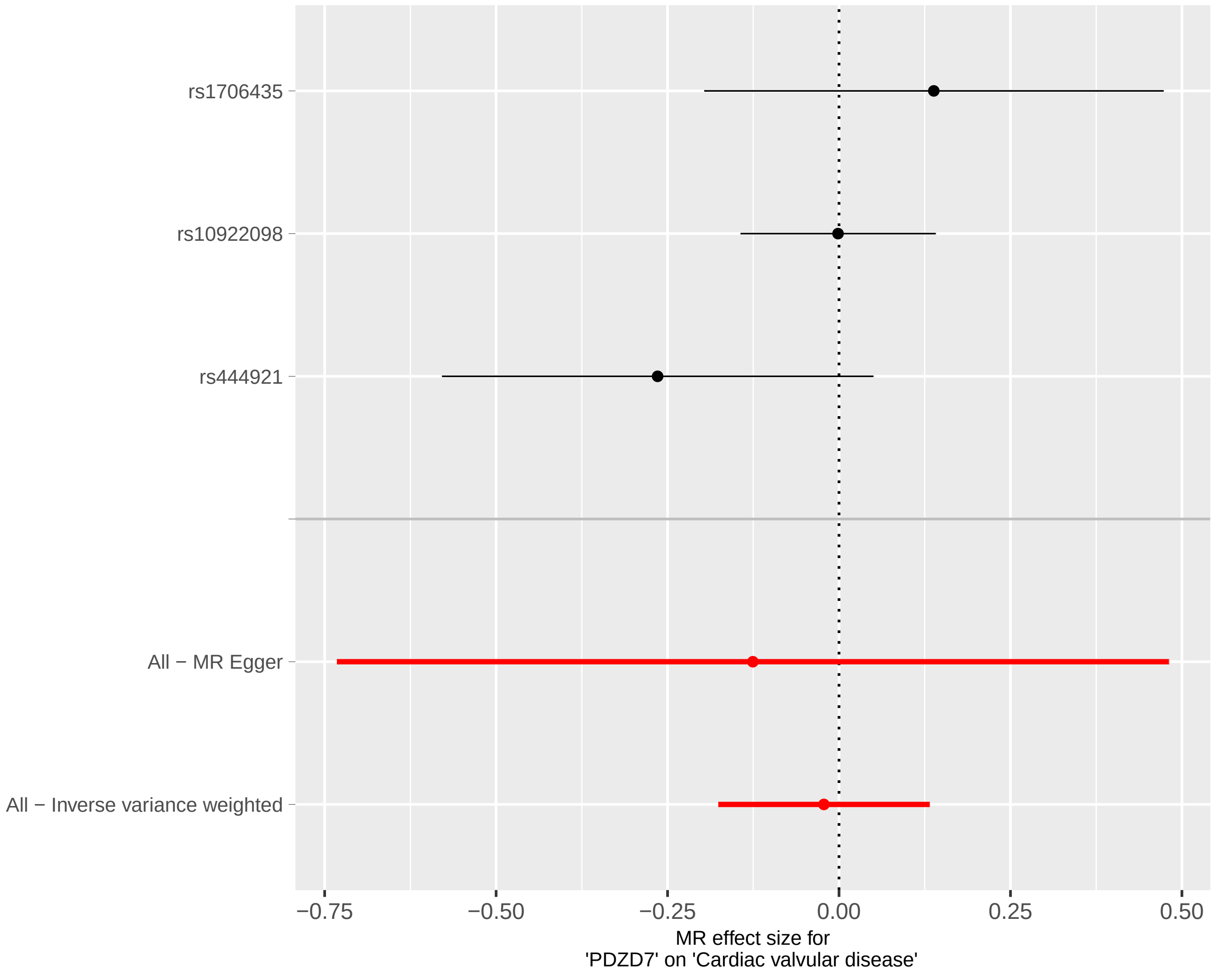
rs1706435
rs10922098
rs444921
All − MR Egger
All − Inverse variance weighted
−0.75
−0.50
−0.25
0.00
0.25
0.50
MR effect size for
'PDZD7' on 'Cardiac valvular disease'

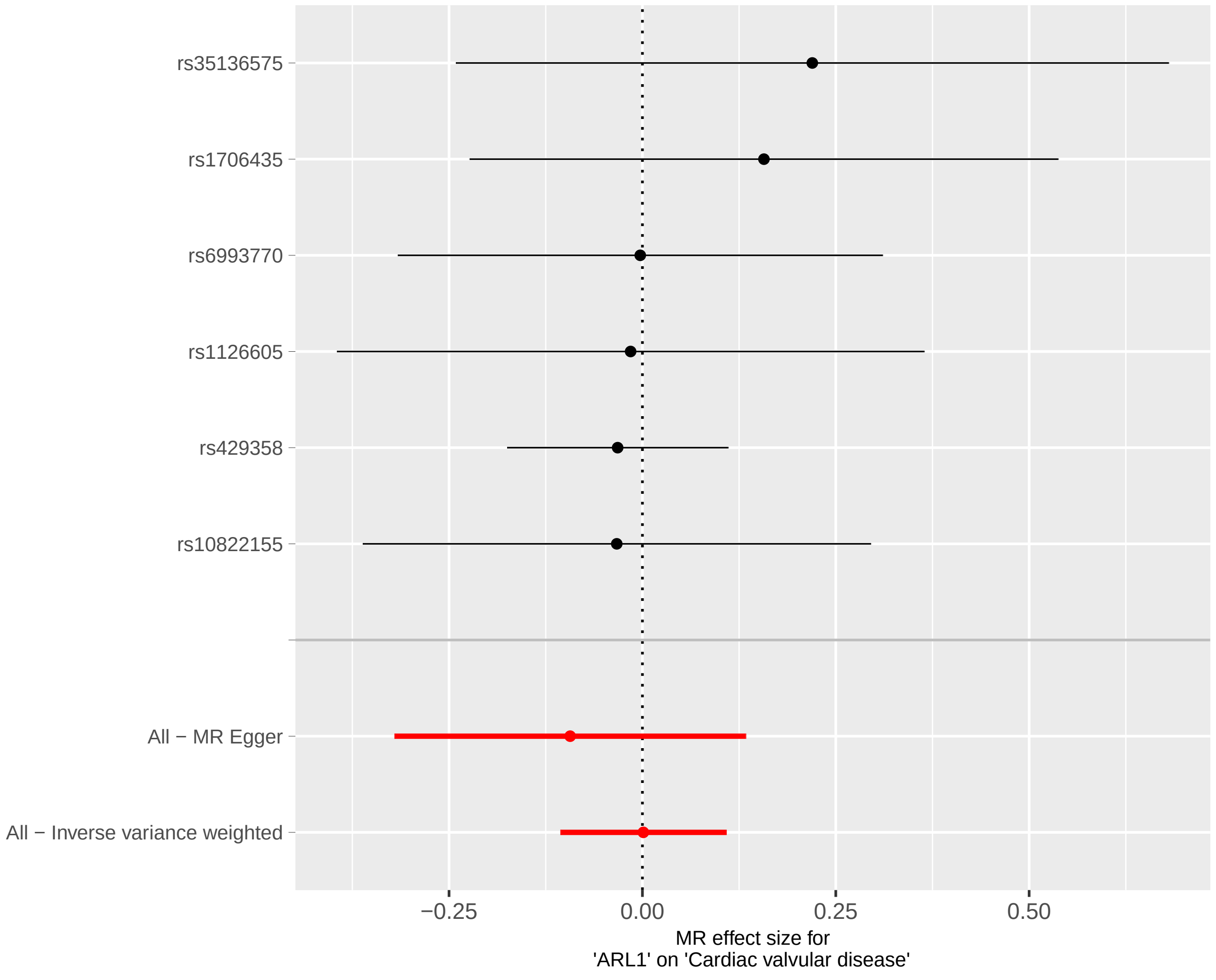

rs35136575
rs1706435
rs6993770
rs1126605
rs429358
rs10822155
All − MR Egger
All − Inverse variance weighted
−0.25
0.00
0.25
0.50
MR effect size for
'ARL1' on 'Cardiac valvular disease'

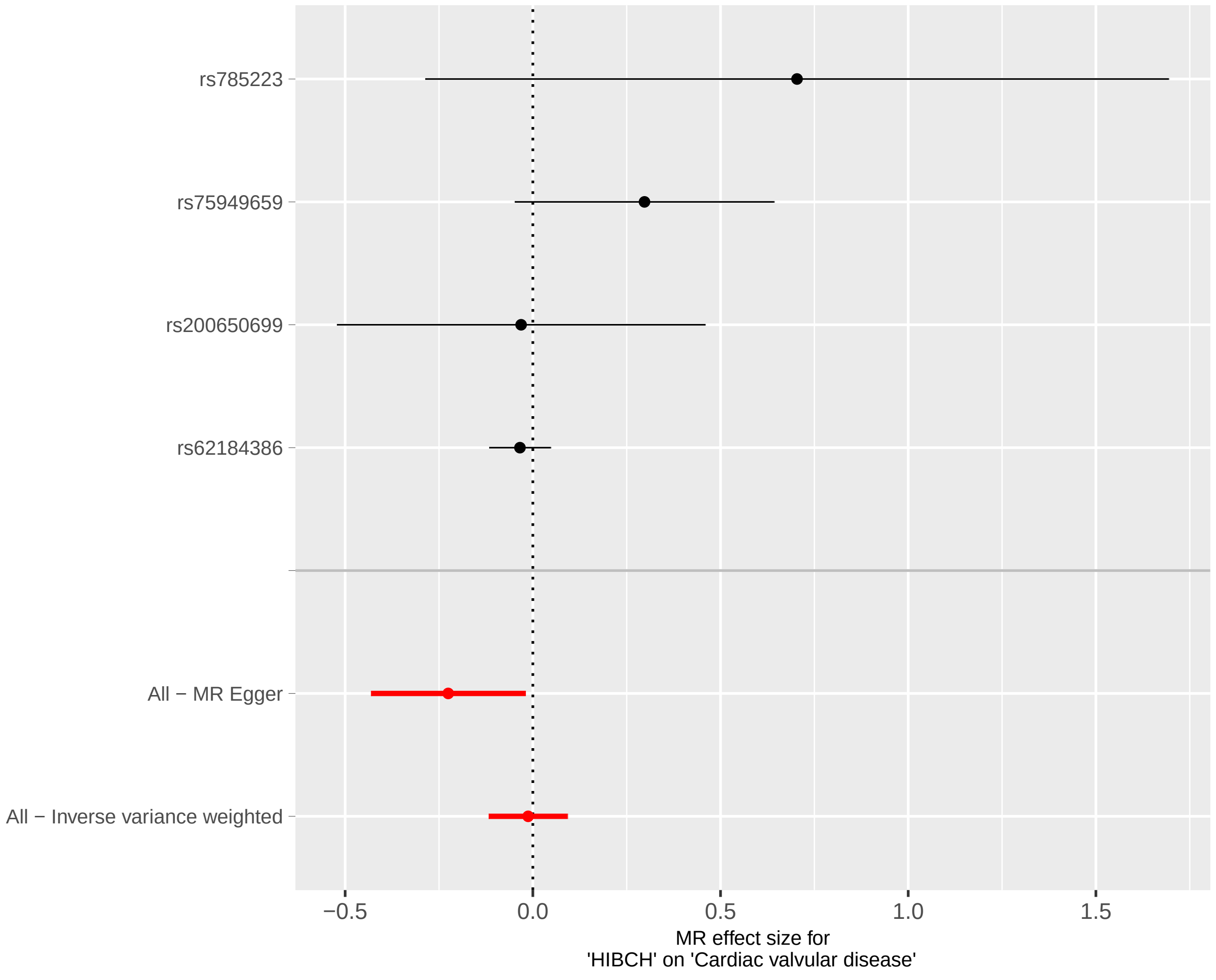
rs785223
rs75949659
rs200650699
rs62184386
All − MR Egger
All − Inverse variance weighted
−0.5
0.0
0.5
1.0
1.5
MR effect size for
'HIBCH' on 'Cardiac valvular disease'

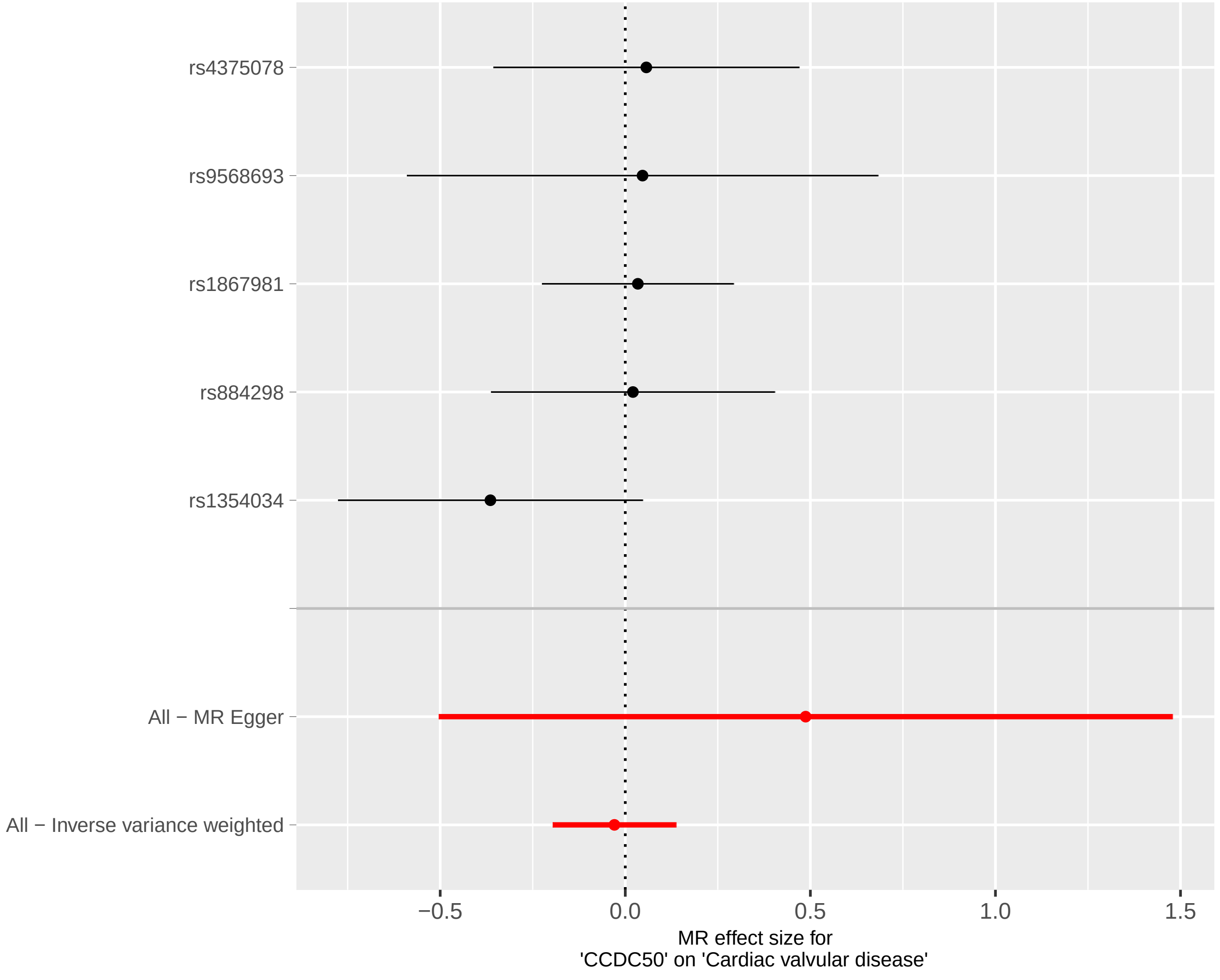

rs4375078
rs9568693
rs1867981
rs884298
rs1354034
All − MR Egger
All − Inverse variance weighted
−0.5
0.0
0.5
1.0
1.5
MR effect size for
'CCDC50' on 'Cardiac valvular disease'

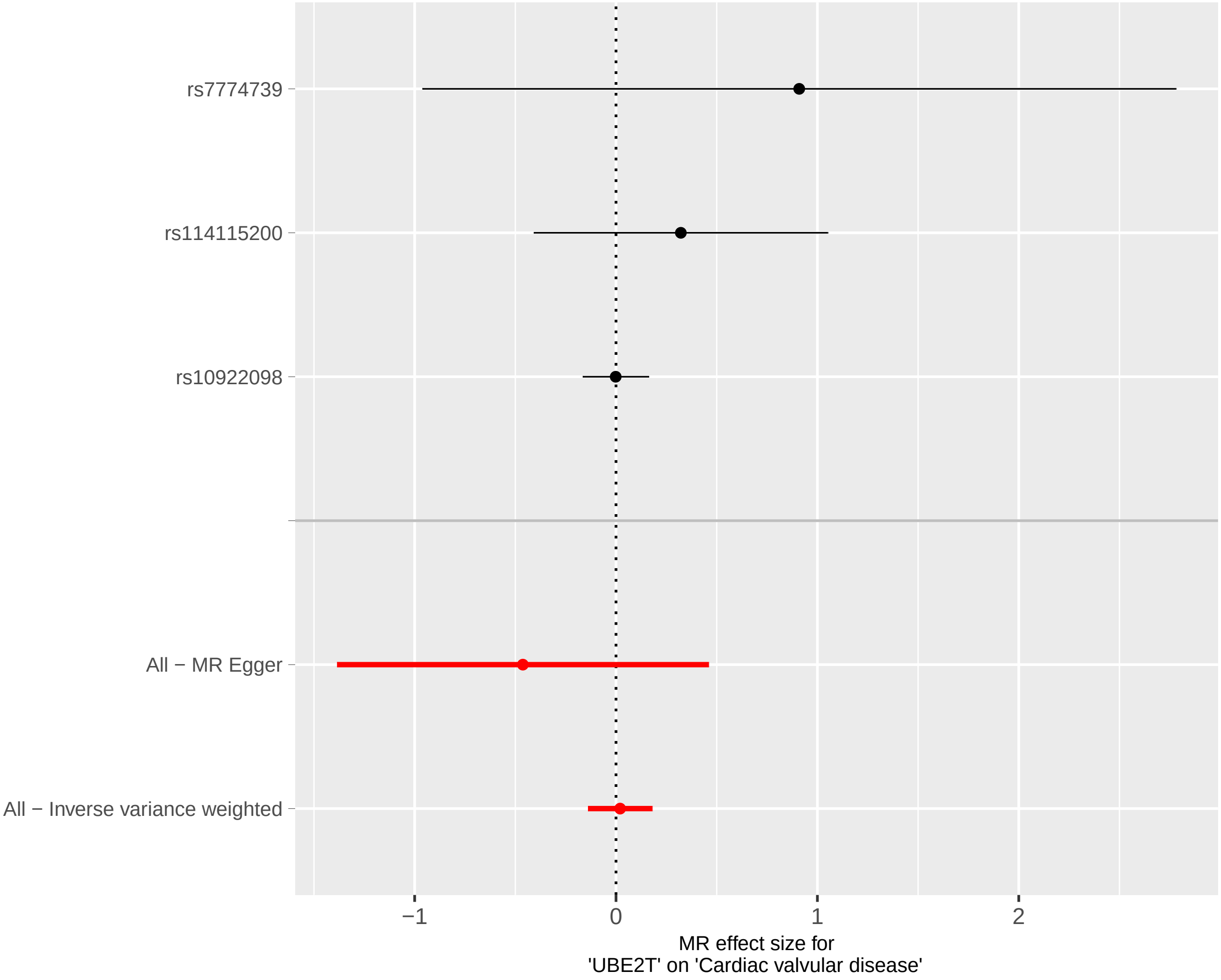
rs7774739
rs114115200
rs10922098
All − MR Egger
All − Inverse variance weighted
−1
0
1
2
MR effect size for
'UBE2T' on 'Cardiac valvular disease'

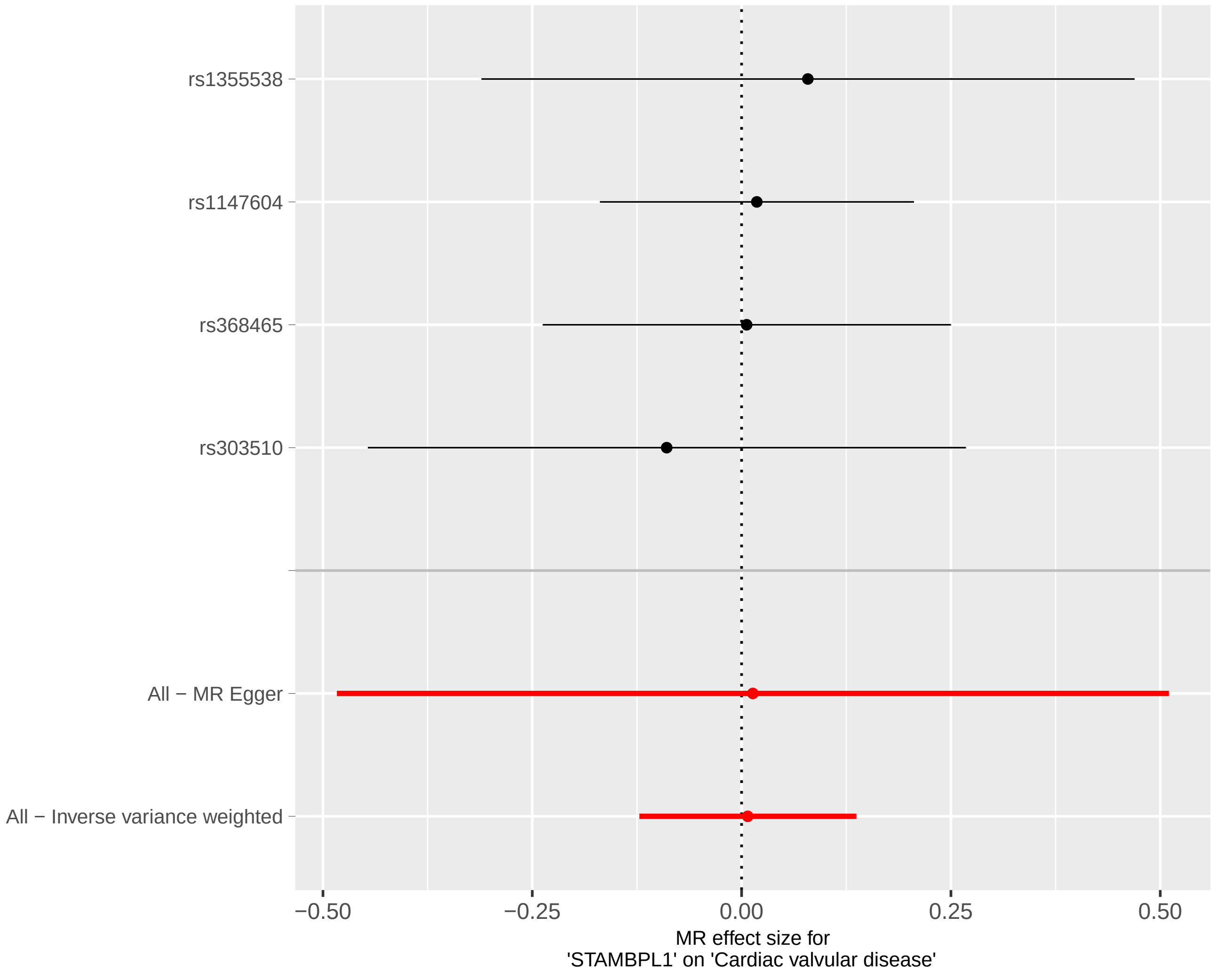
rs1355538
rs1147604
rs368465
rs303510
All − MR Egger
All − Inverse variance weighted
−0.50
−0.25
0.00
0.25
0.50
MR effect size for
'STAMBPL1' on 'Cardiac valvular disease'

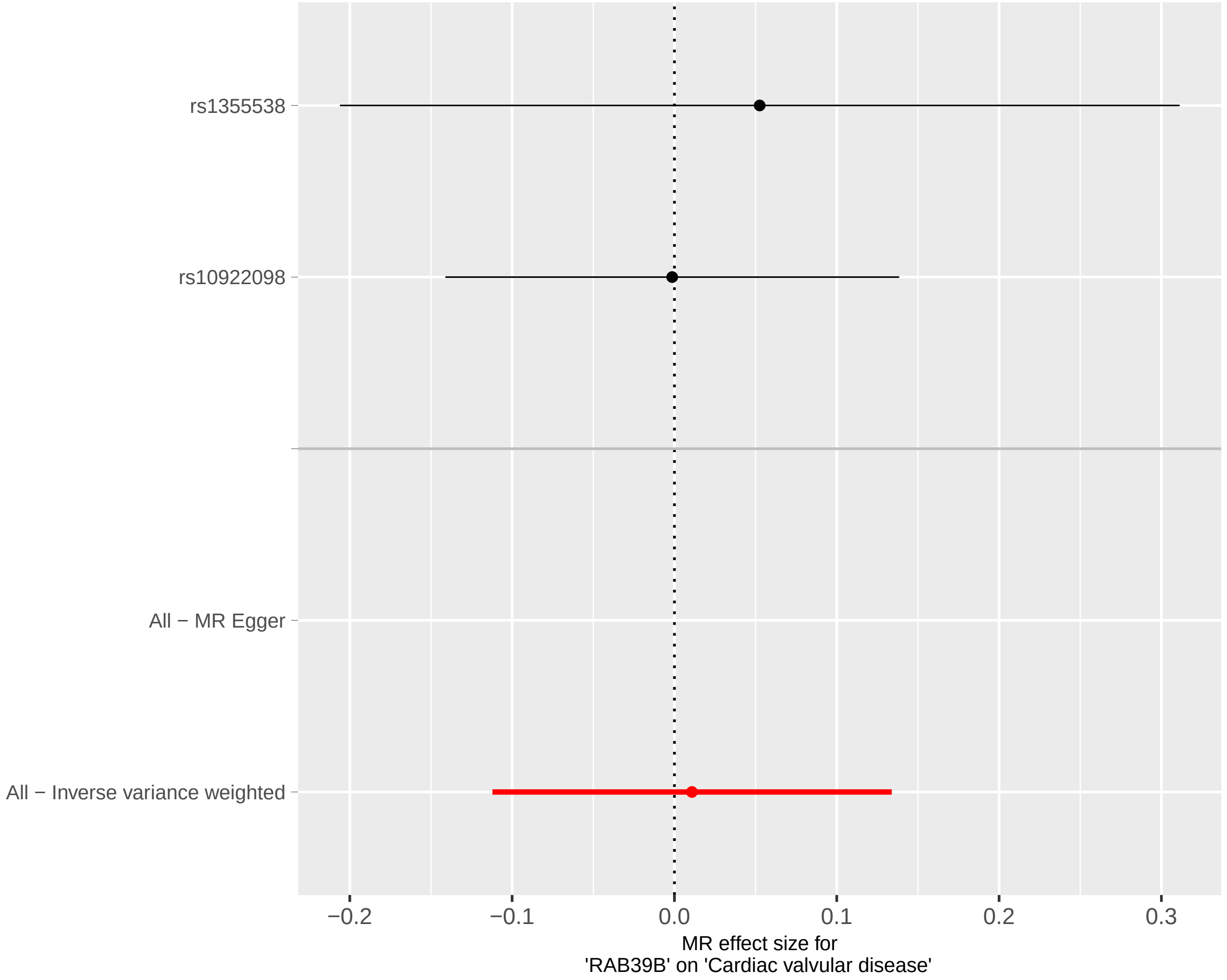
rs1355538
rs10922098
All − MR Egger
All − Inverse variance weighted
−0.2
−0.1
0.0
0.1
0.2
0.3
MR effect size for
'RAB39B' on 'Cardiac valvular disease'

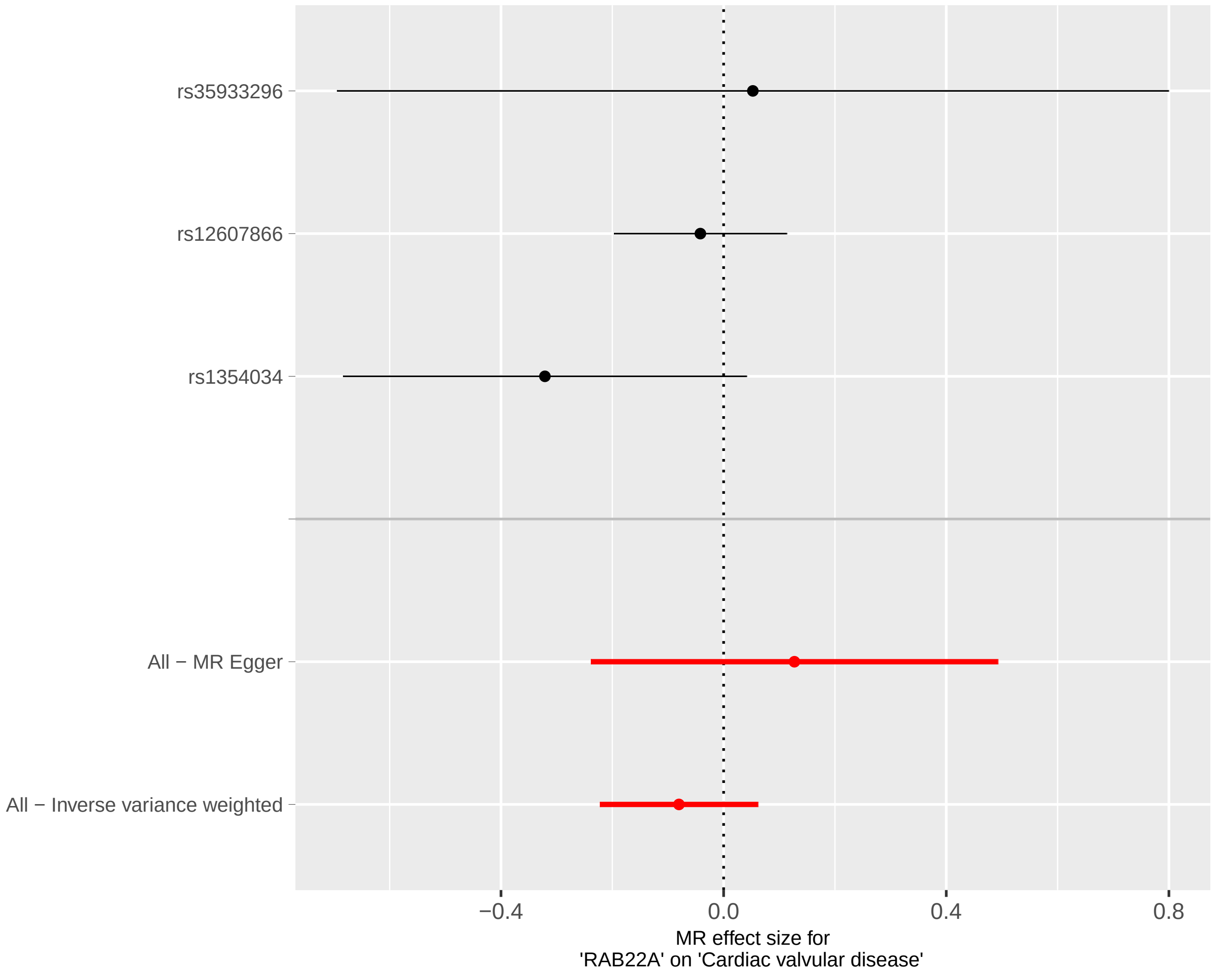
rs35933296
rs12607866
rs1354034
All − MR Egger
All − Inverse variance weighted
−0.4
0.0
0.4
0.8
MR effect size for
'RAB22A' on 'Cardiac valvular disease'

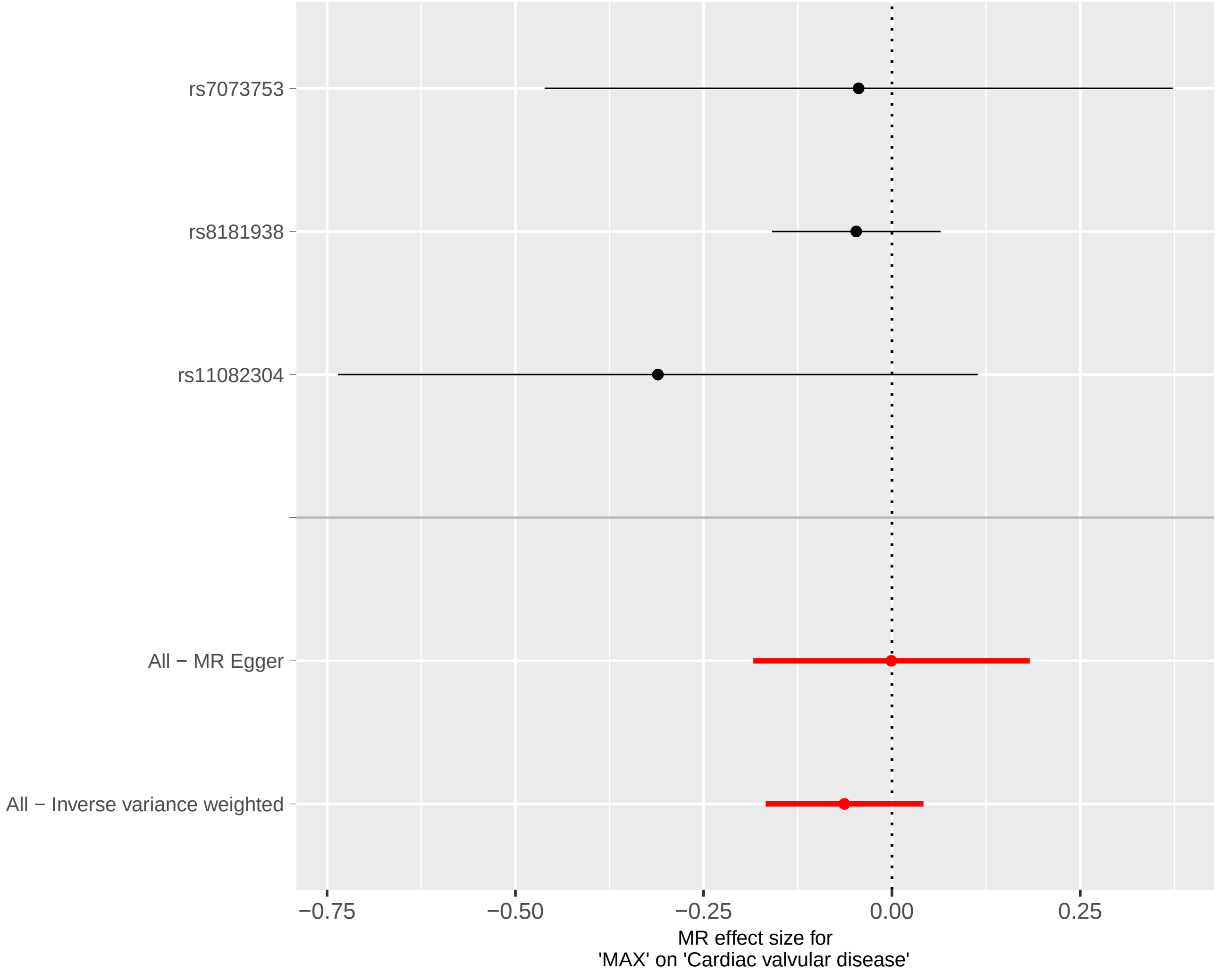

rs7073753
rs8181938
rs11082304
All − MR Egger
All − Inverse variance weighted
−0.75
−0.50
−0.25
0.00
0.25
MR effect size for
'MAX' on 'Cardiac valvular disease'

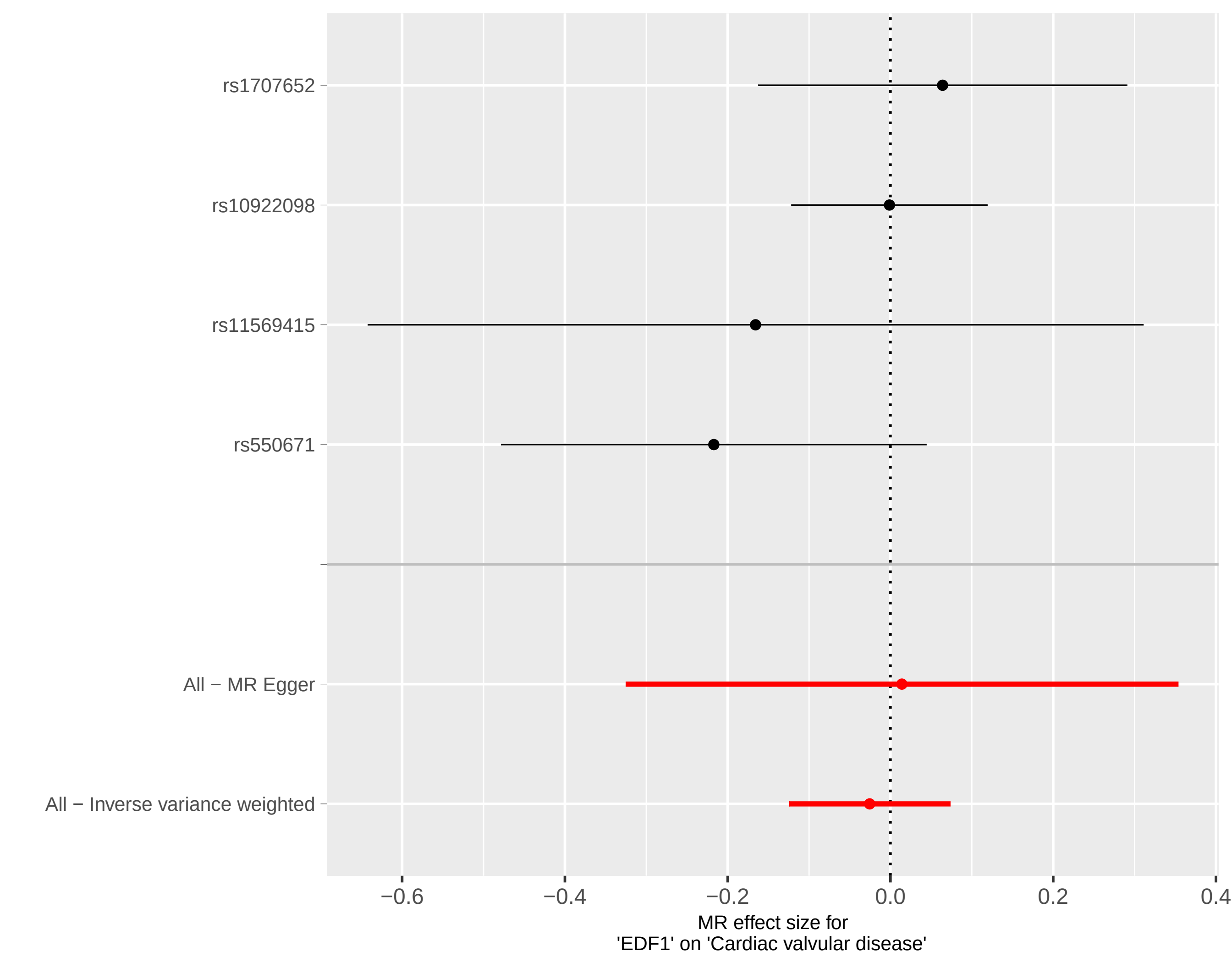

rs1707652
rs10922098
rs11569415
rs550671
All − MR Egger
All − Inverse variance weighted
−0.6
−0.4
−0.2
0.0
0.2
0.4
MR effect size for
'EDF1' on 'Cardiac valvular disease'

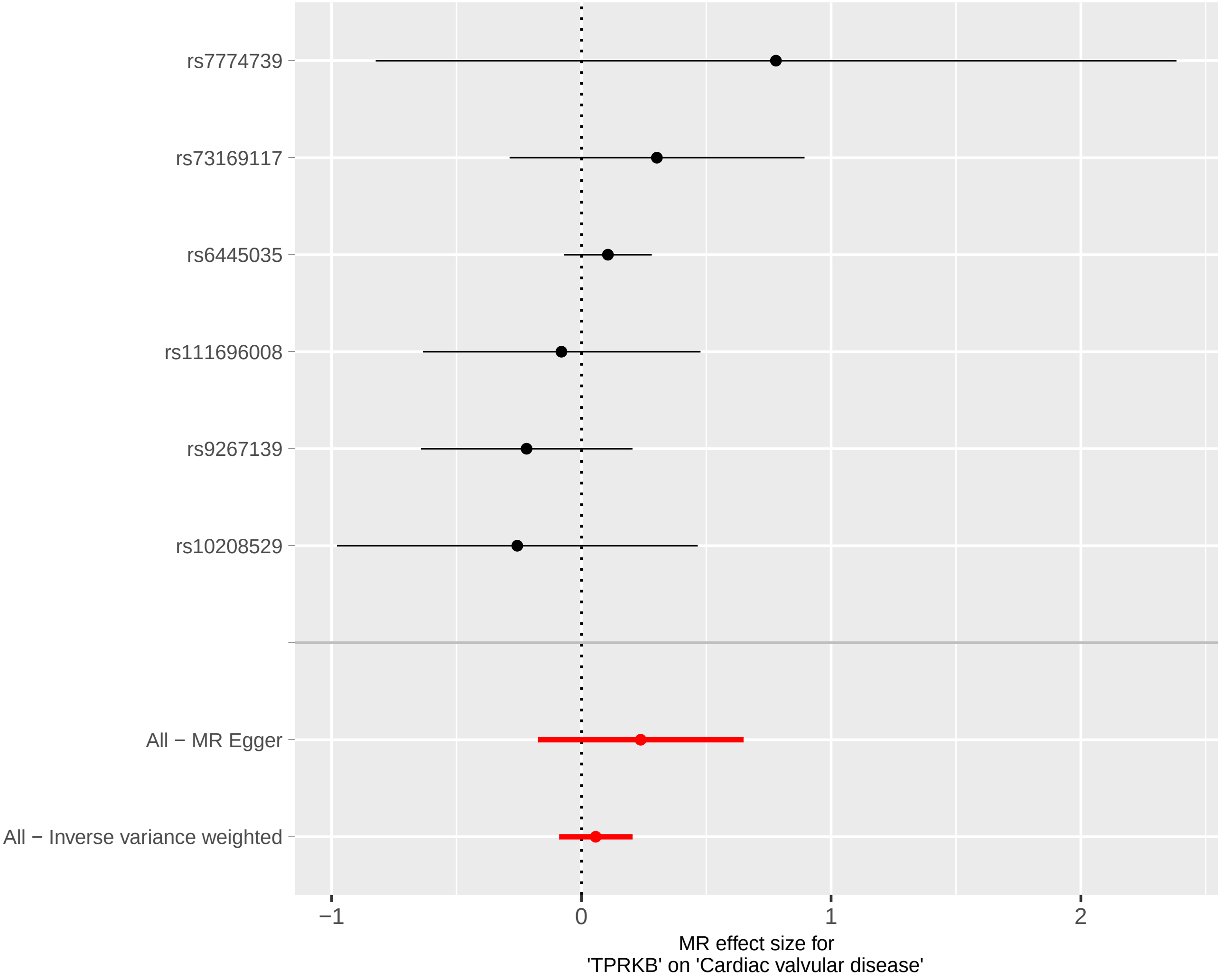
rs7774739
rs73169117
rs6445035
rs111696008
rs9267139
rs10208529
All − MR Egger
All − Inverse variance weighted
−1
0
1
2
MR effect size for
'TPRKB' on 'Cardiac valvular disease'

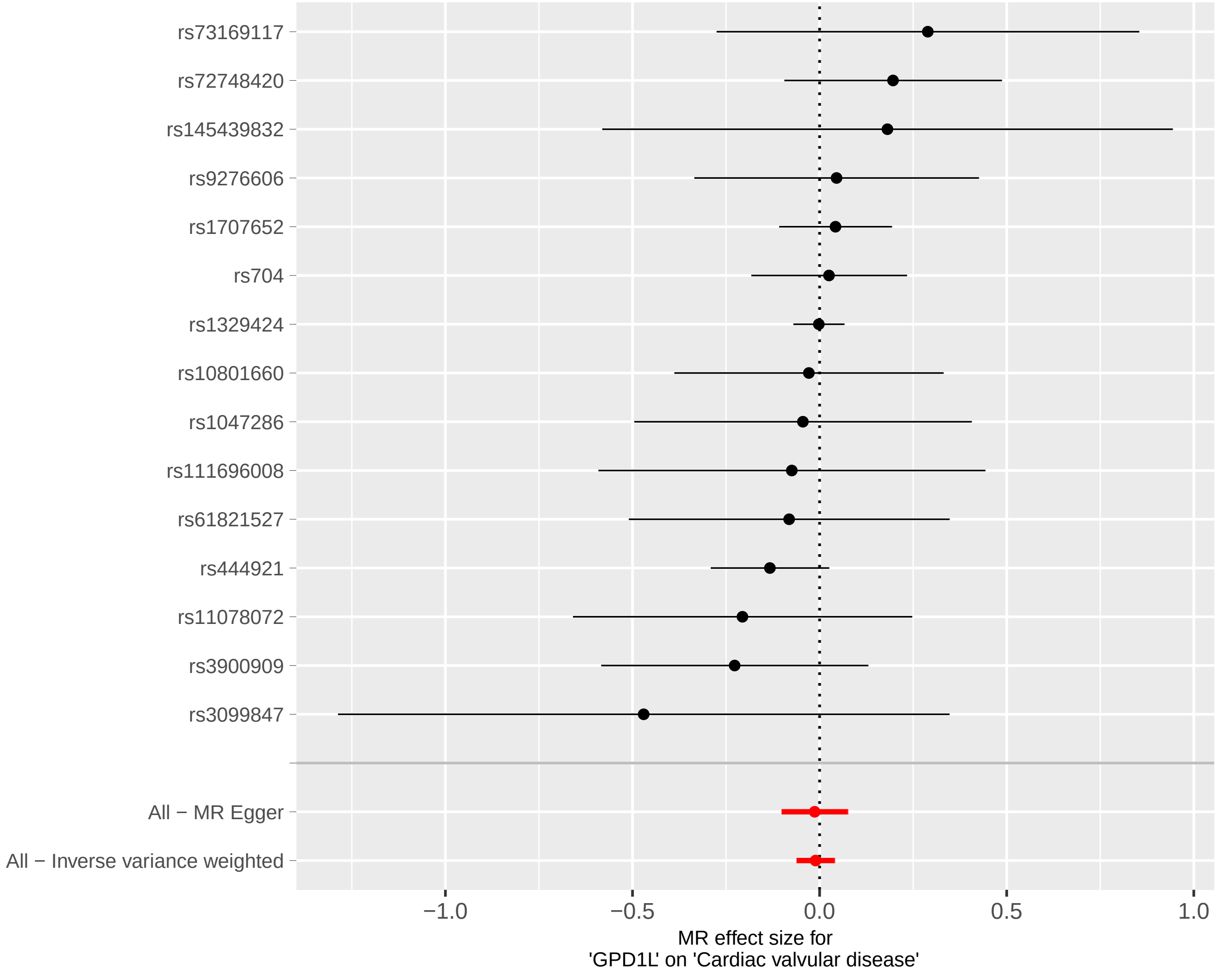
rs73169117
rs72748420
rs145439832
rs9276606
rs1707652
rs704
rs1329424
rs10801660
rs1047286
rs111696008
rs61821527
rs444921
rs11078072
rs3900909
rs3099847
All − MR Egger
All − Inverse variance weighted
−1.0
−0.5
0.0
0.5
1.0
MR effect size for
'GPD1L' on 'Cardiac valvular disease'

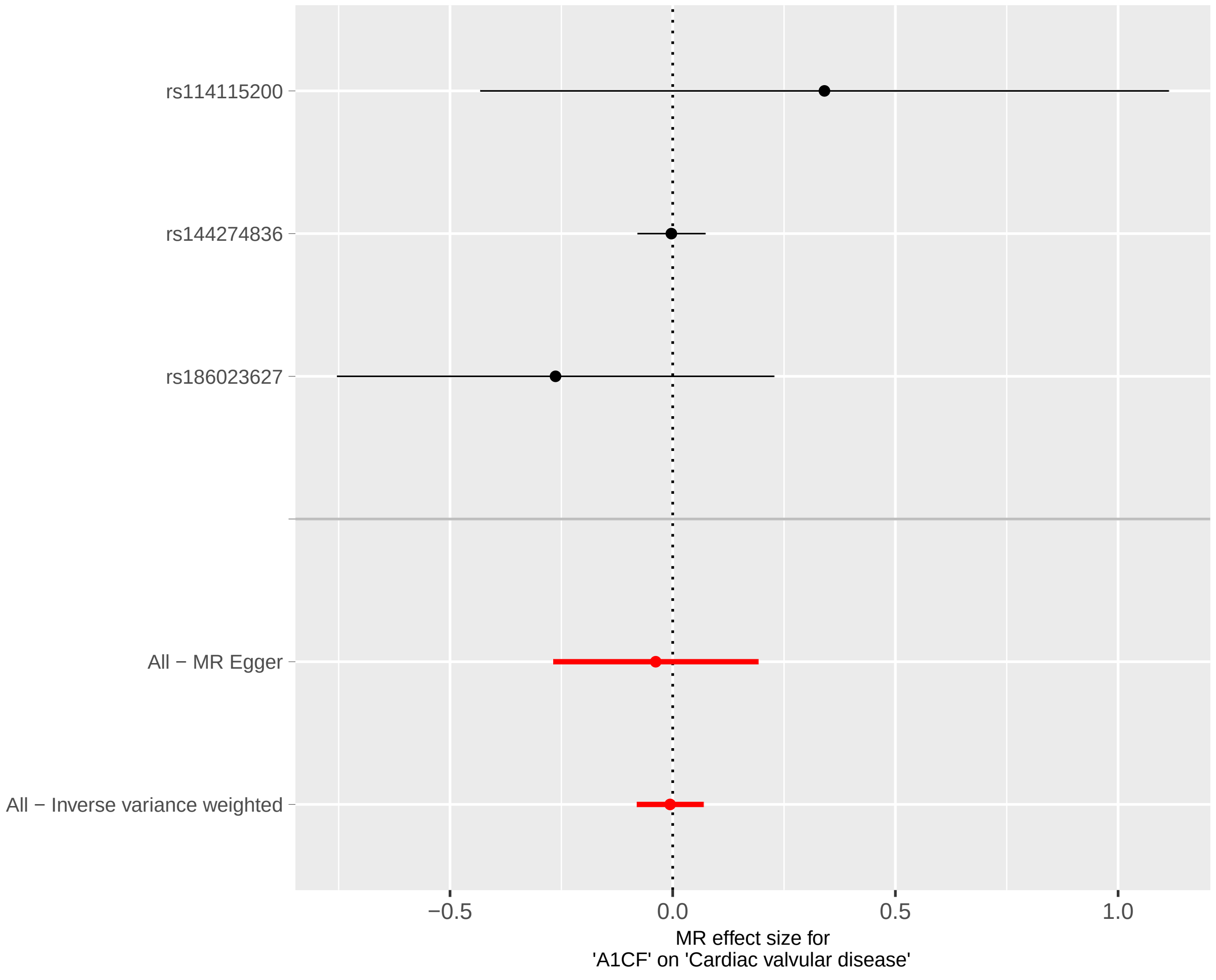

rs114115200
rs144274836
rs186023627
All − MR Egger
All − Inverse variance weighted
−0.5
0.0
0.5
1.0
MR effect size for
'A1CF' on 'Cardiac valvular disease'

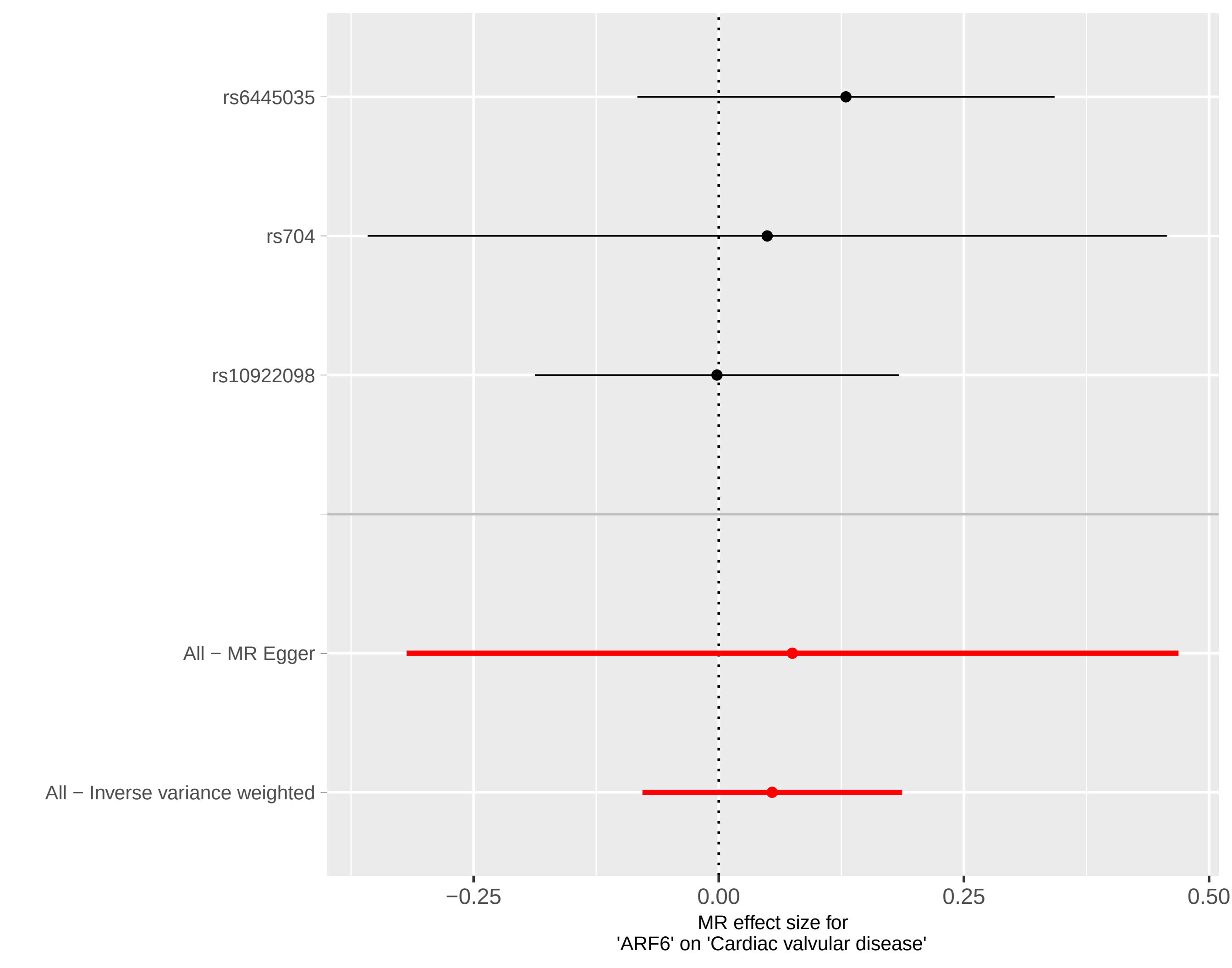
rs6445035
rs704
rs10922098
All − MR Egger
All − Inverse variance weighted
−0.25
0.00
0.25
0.50
MR effect size for
'ARF6' on 'Cardiac valvular disease'

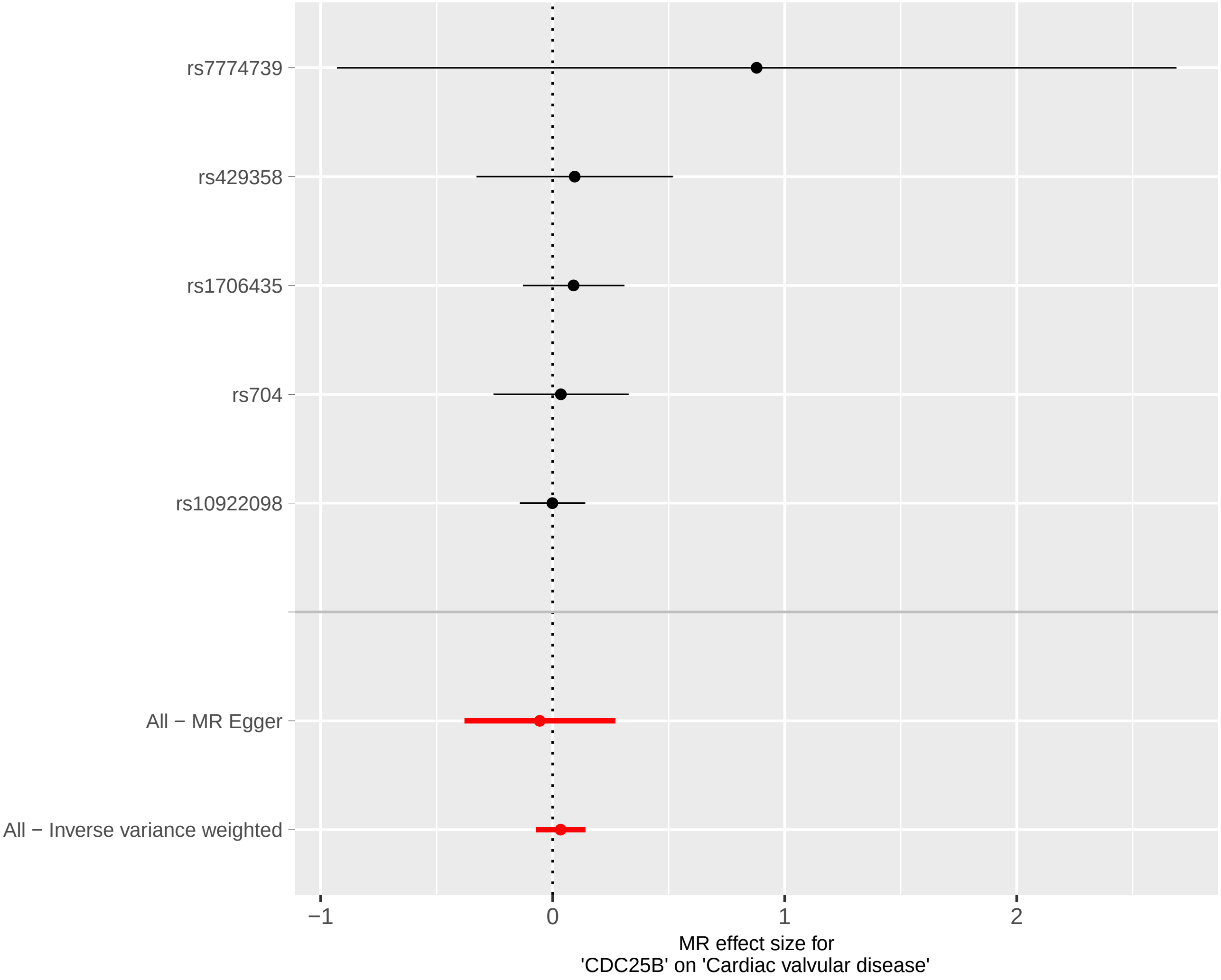
rs7774739
rs429358
rs1706435
rs704
rs10922098
All − MR Egger
All − Inverse variance weighted
−1
0
1
2
MR effect size for
'CDC25B' on 'Cardiac valvular disease'

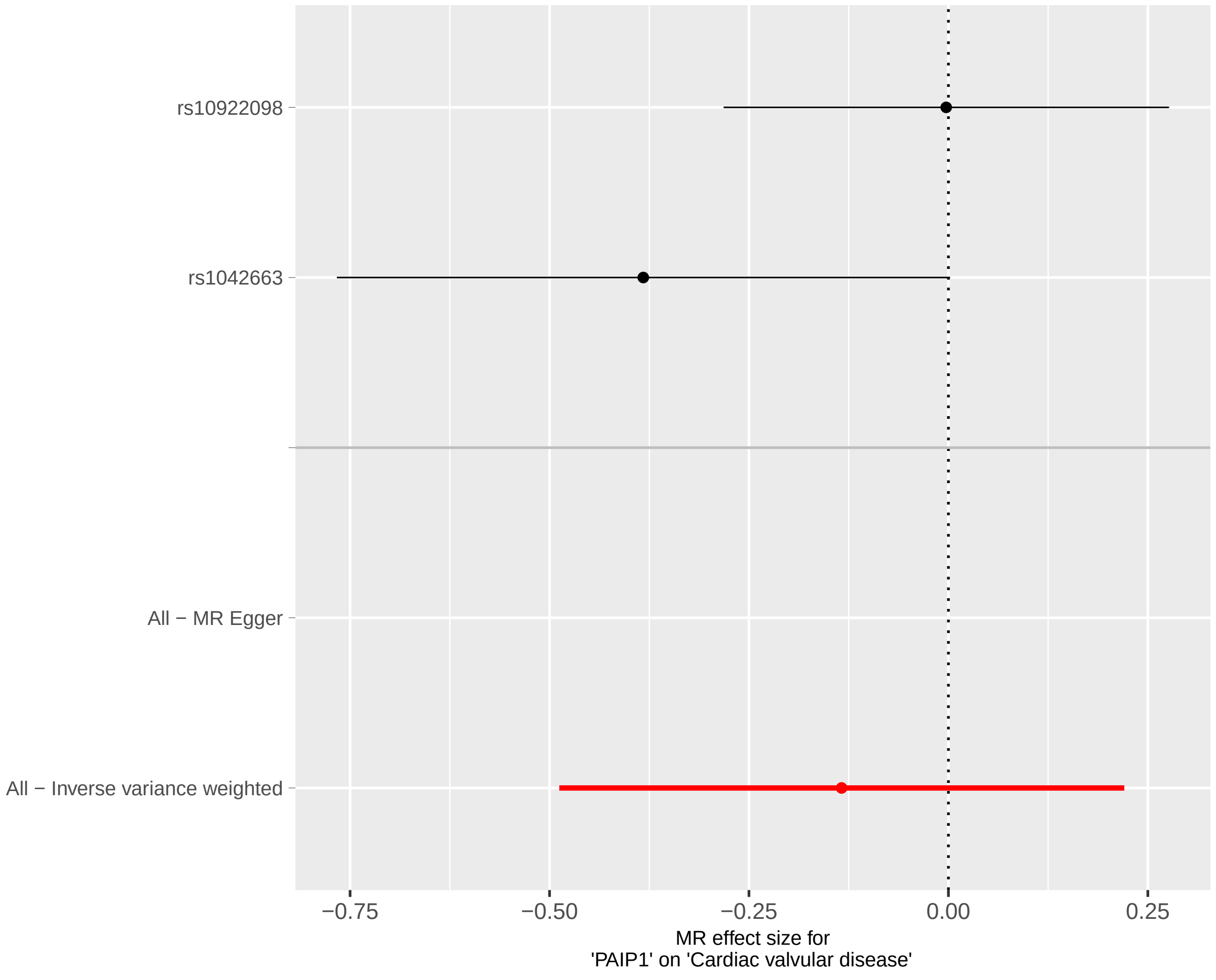
rs10922098
rs1042663
All − MR Egger
All − Inverse variance weighted
−0.75
−0.50
−0.25
0.00
0.25
MR effect size for
'PAIP1' on 'Cardiac valvular disease'

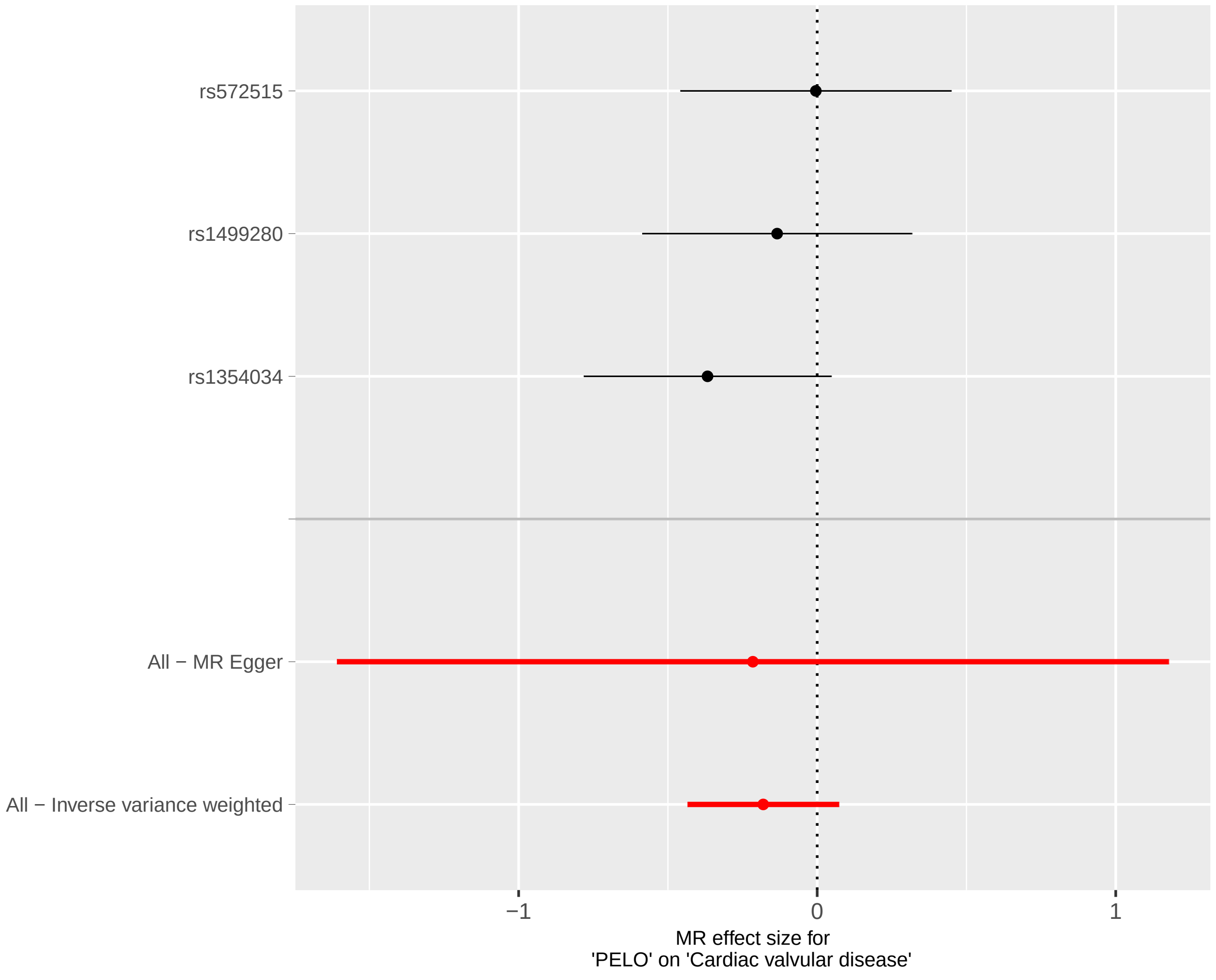

rs572515
rs1499280
rs1354034
All − MR Egger
All − Inverse variance weighted
−1
0
1
MR effect size for
'PELO' on 'Cardiac valvular disease'

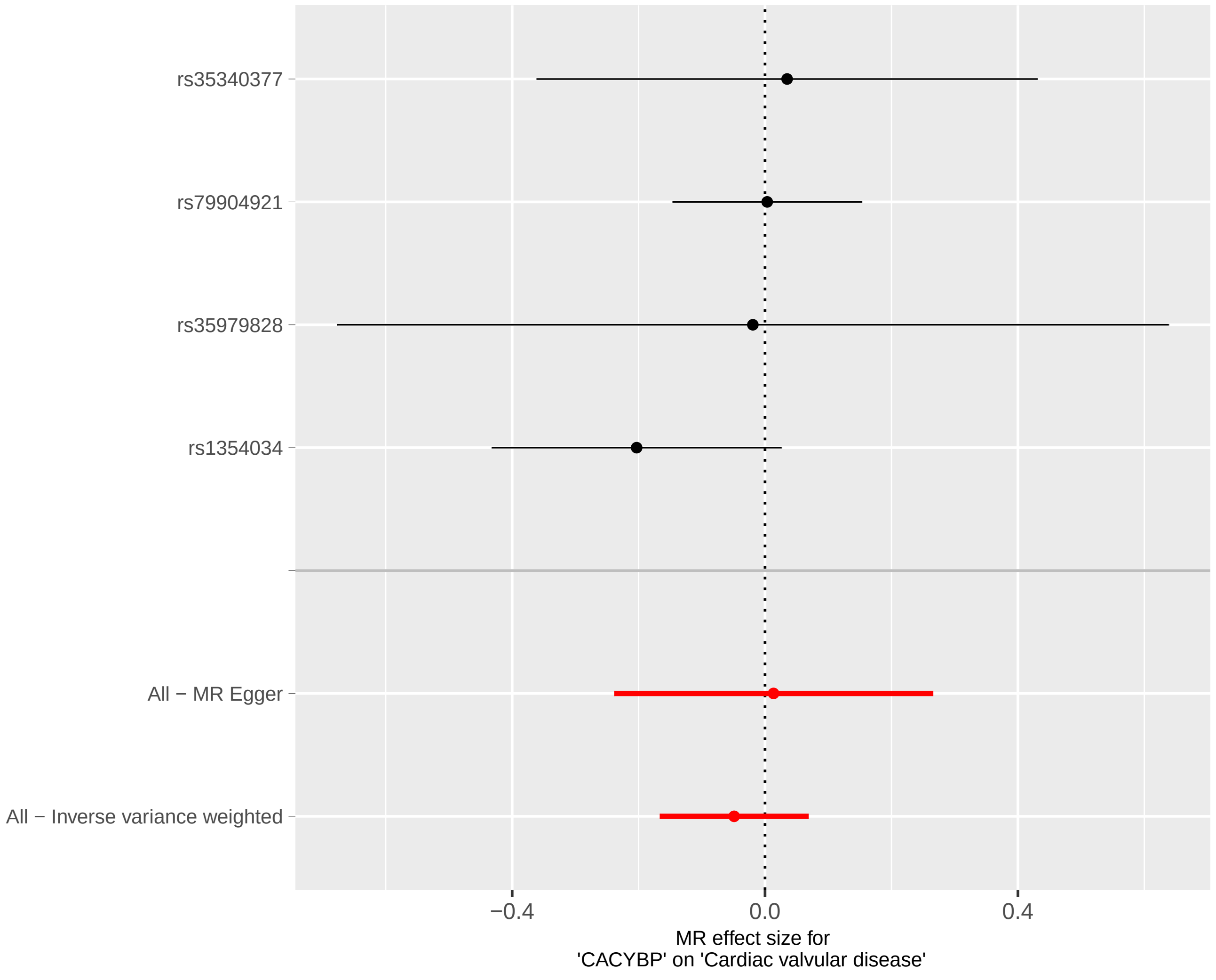
rs35340377
rs79904921
rs35979828
rs1354034
All − MR Egger
All − Inverse variance weighted
−0.4
0.0
0.4
MR effect size for
'CACYBP' on 'Cardiac valvular disease'

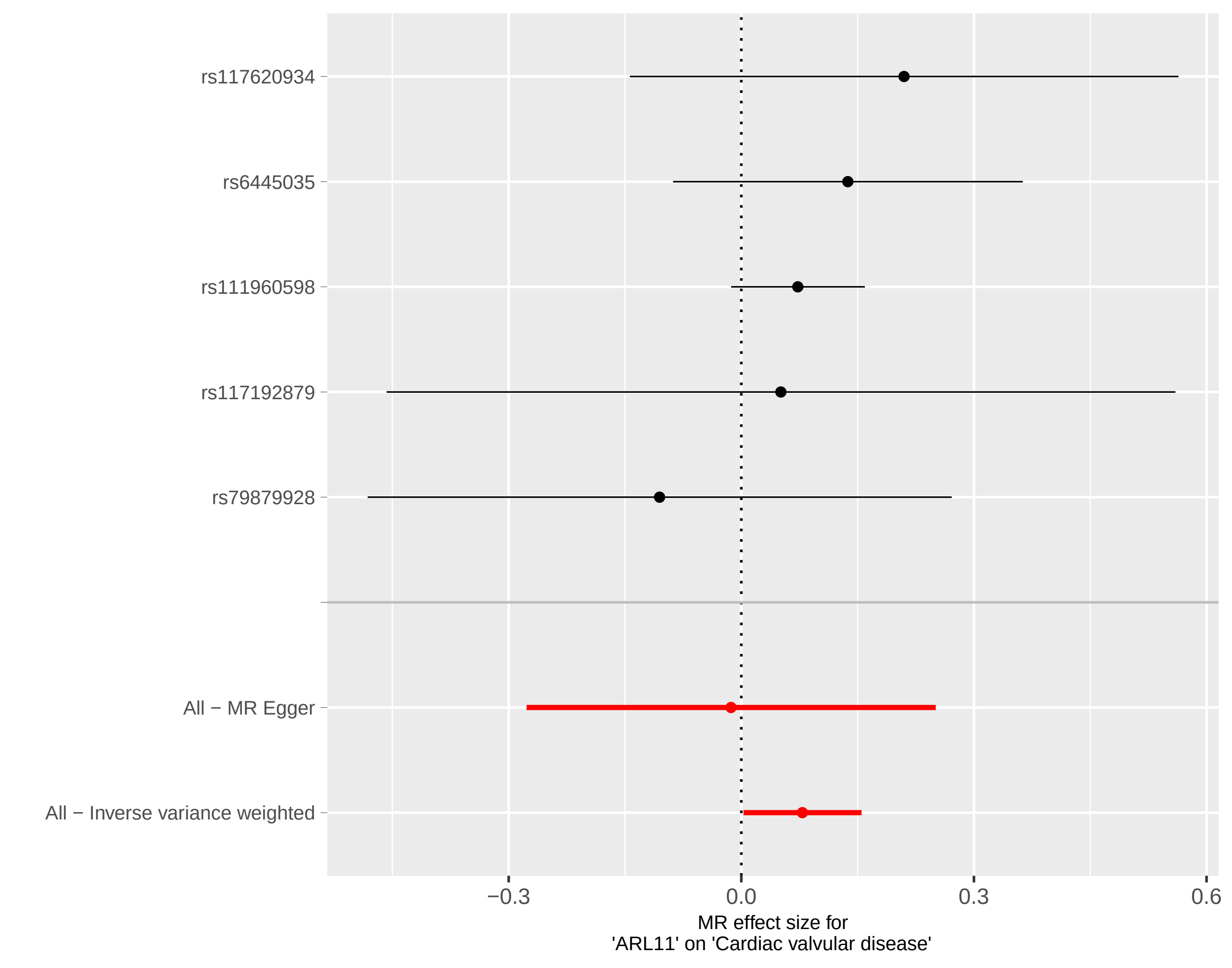
rs117620934
rs6445035
rs111960598
rs117192879
rs79879928
All − MR Egger
All − Inverse variance weighted
−0.3
0.0
0.3
0.6
MR effect size for
'ARL11' on 'Cardiac valvular disease'

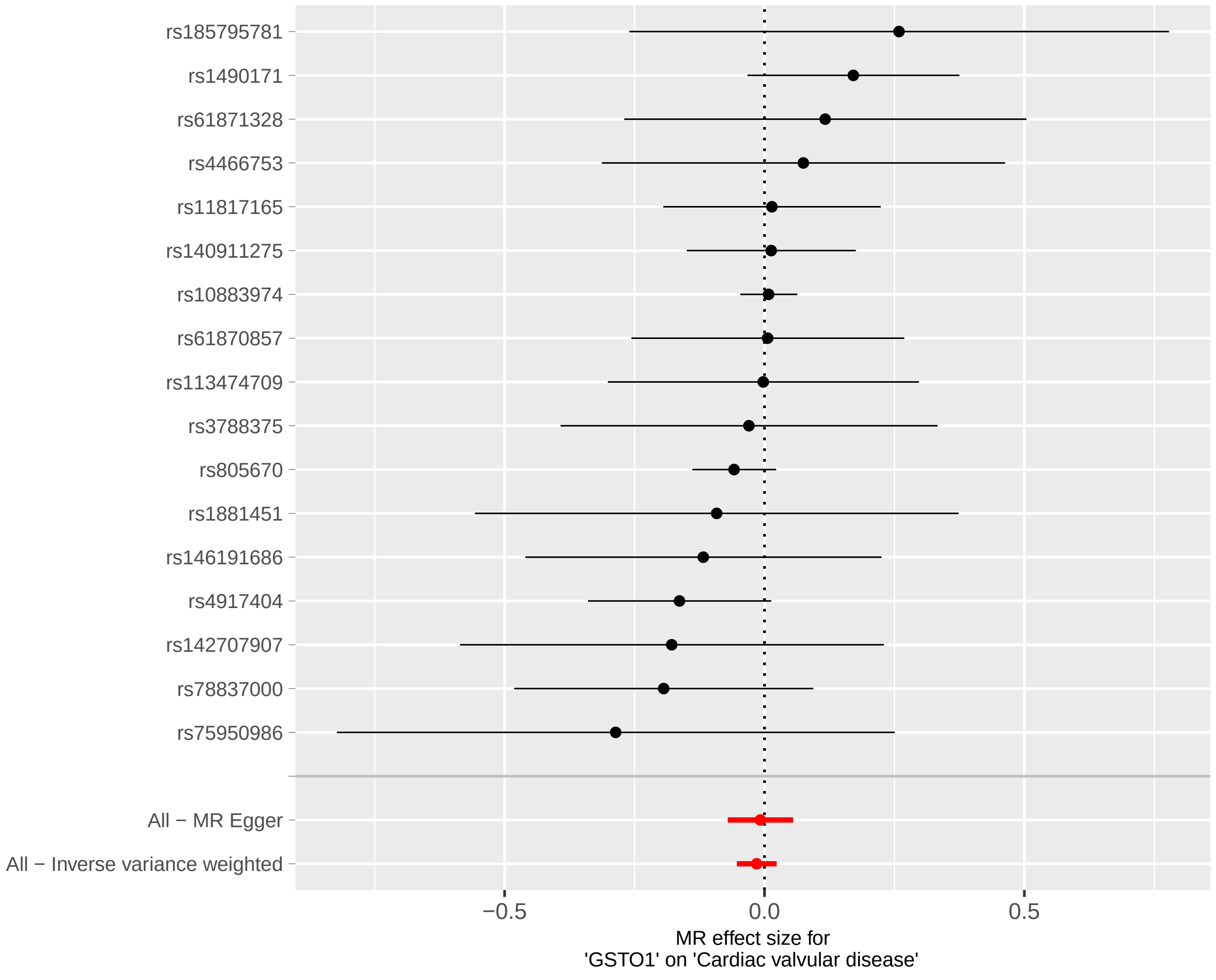
rs185795781
rs1490171
rs61871328
rs4466753
rs11817165
rs140911275
rs10883974
rs61870857
rs113474709
rs3788375
rs805670
rs1881451
rs146191686
rs4917404
rs142707907
rs78837000
rs75950986
All − MR Egger
All − Inverse variance weighted
−0.5
0.0
0.5
MR effect size for
'GSTO1' on 'Cardiac valvular disease'

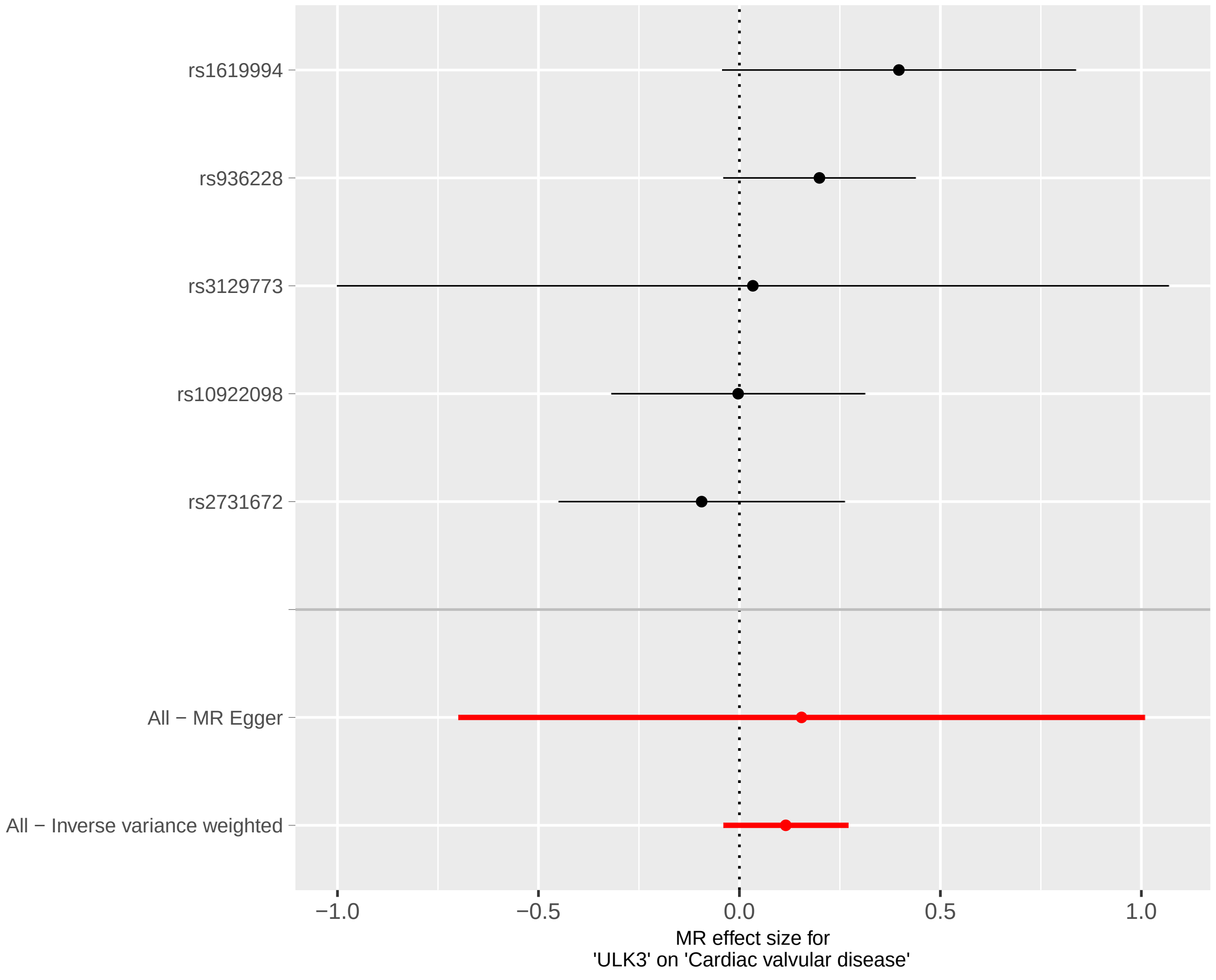

rs1619994
rs936228
rs3129773
rs10922098
rs2731672
All − MR Egger
All − Inverse variance weighted
−1.0
−0.5
0.0
0.5
1.0
MR effect size for
'ULK3' on 'Cardiac valvular disease'

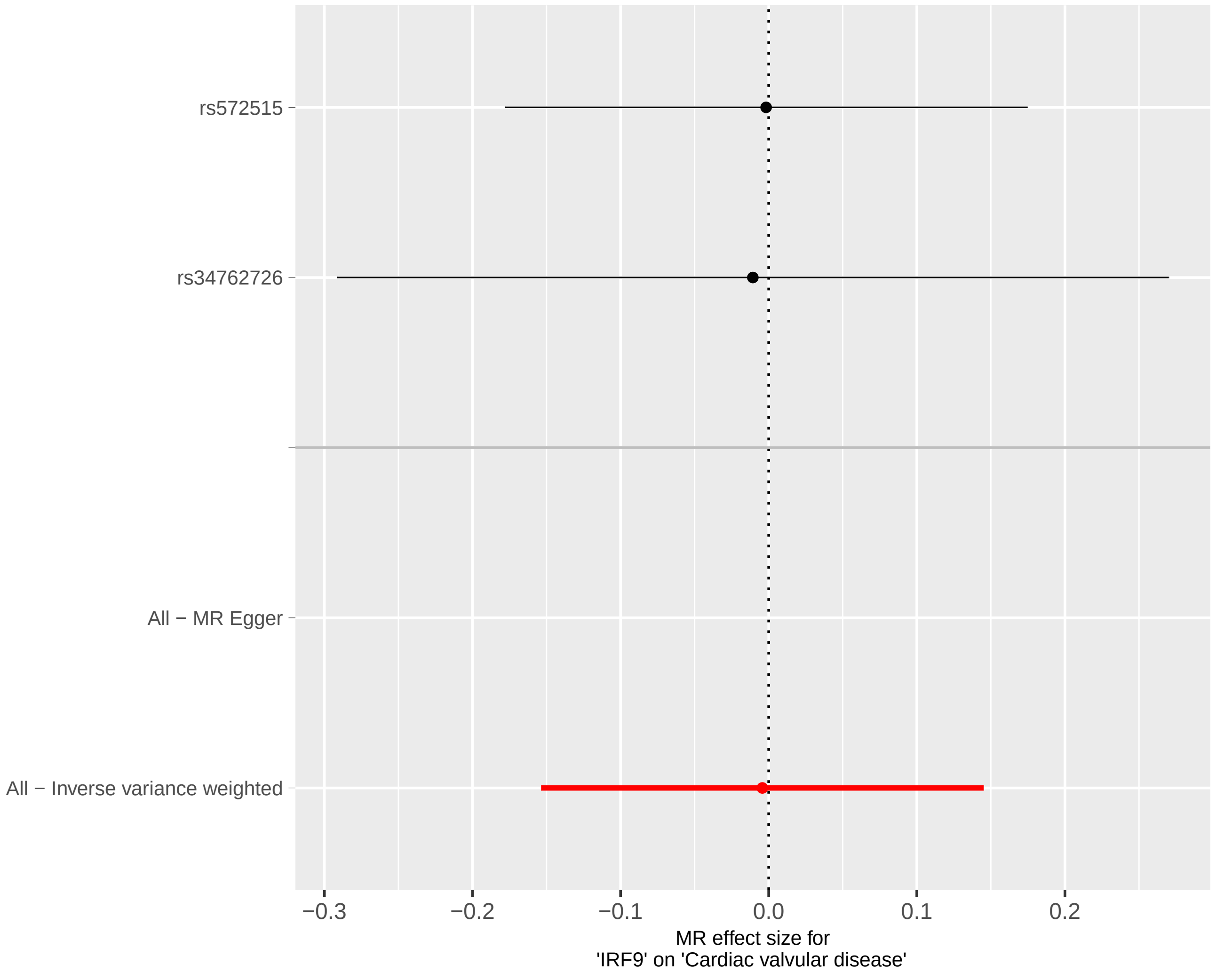
rs572515
rs34762726
All − MR Egger
All − Inverse variance weighted
−0.3
−0.2
−0.1
0.0
0.1
0.2
MR effect size for
'IRF9' on 'Cardiac valvular disease'

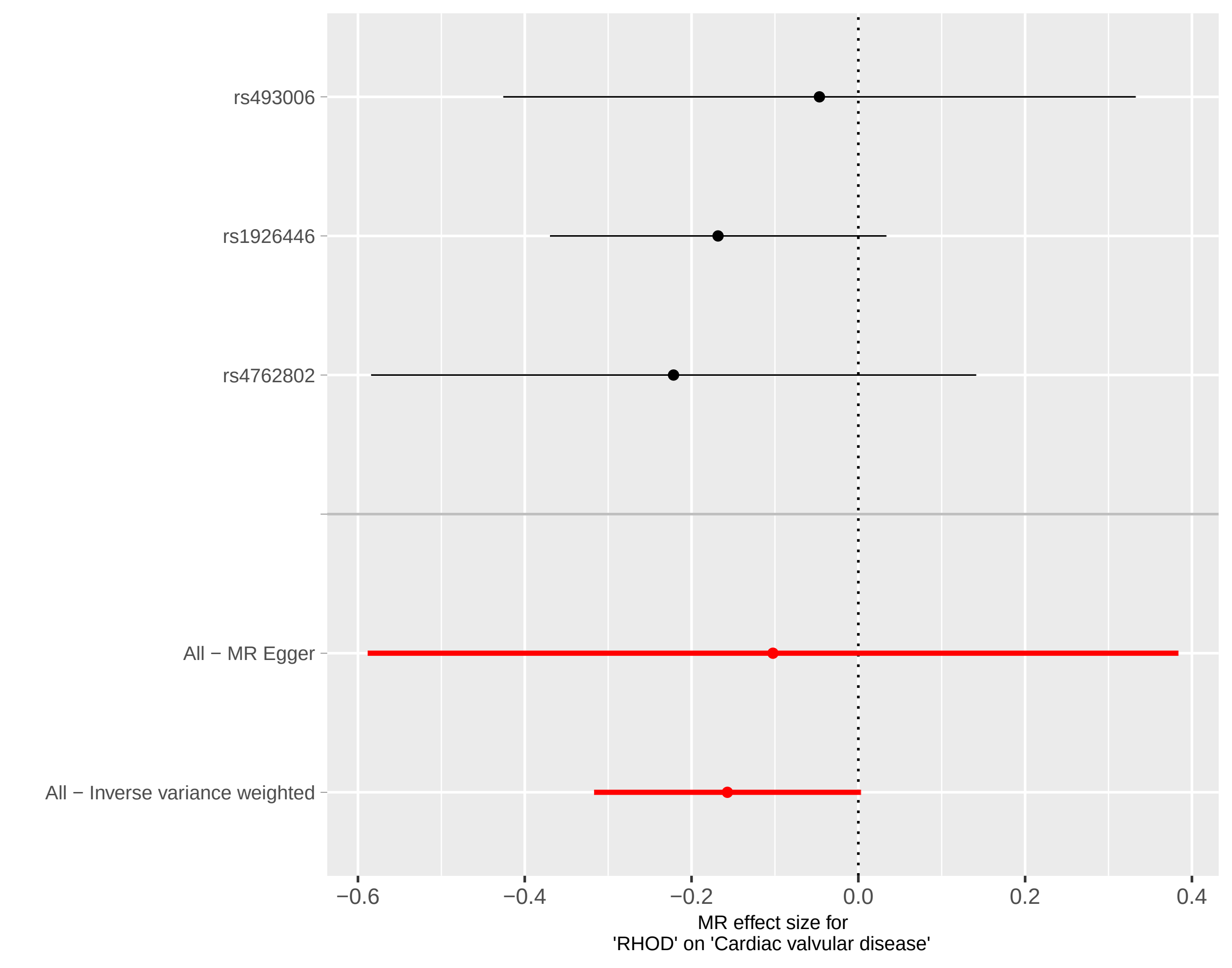
rs493006
rs1926446
rs4762802
All − MR Egger
All − Inverse variance weighted
−0.6
−0.4
−0.2
0.0
0.2
0.4
MR effect size for
'RHOD' on 'Cardiac valvular disease'

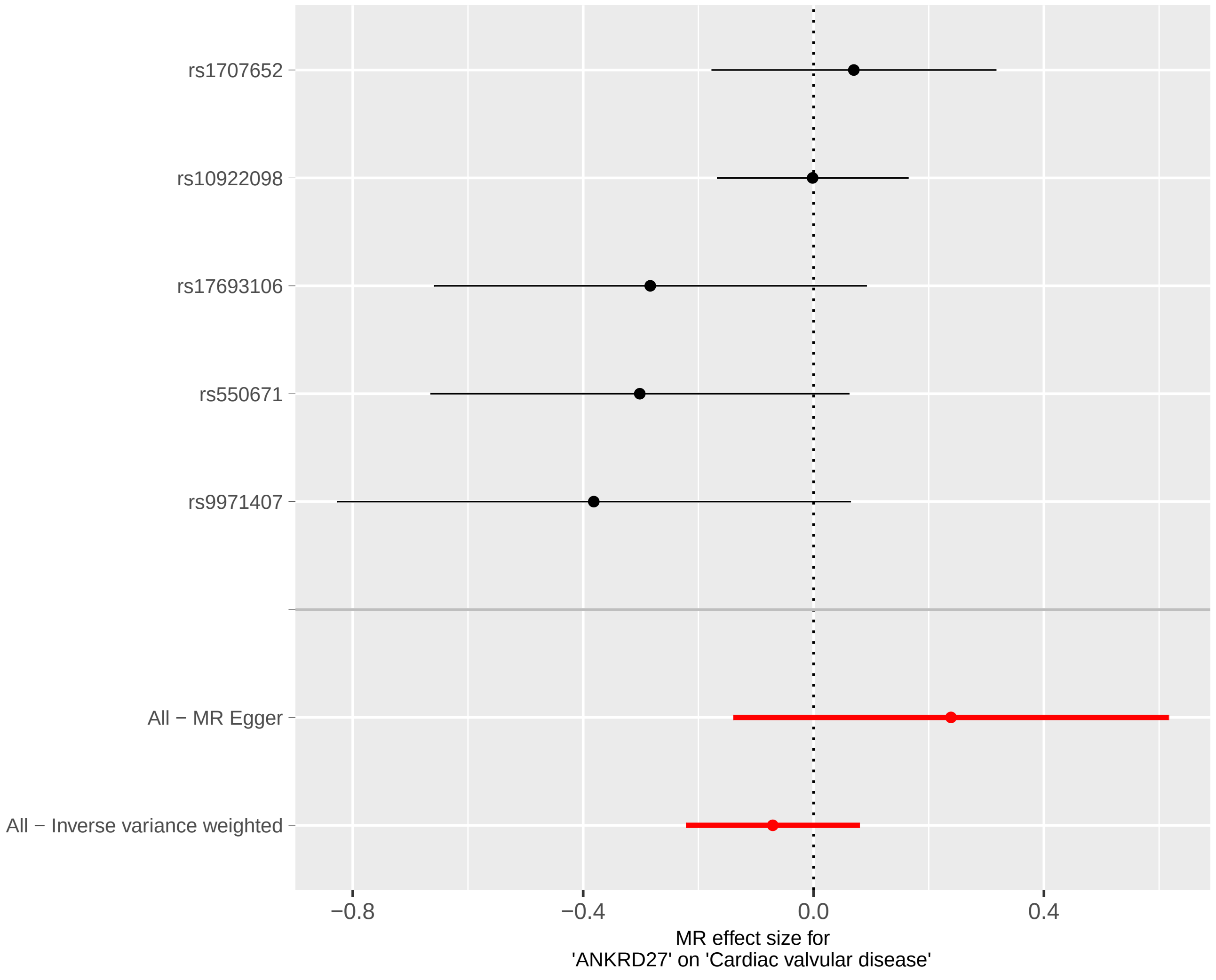
rs1707652
rs10922098
rs17693106
rs550671
rs9971407
All − MR Egger
All − Inverse variance weighted
−0.8
−0.4
0.0
0.4
MR effect size for
'ANKRD27' on 'Cardiac valvular disease'

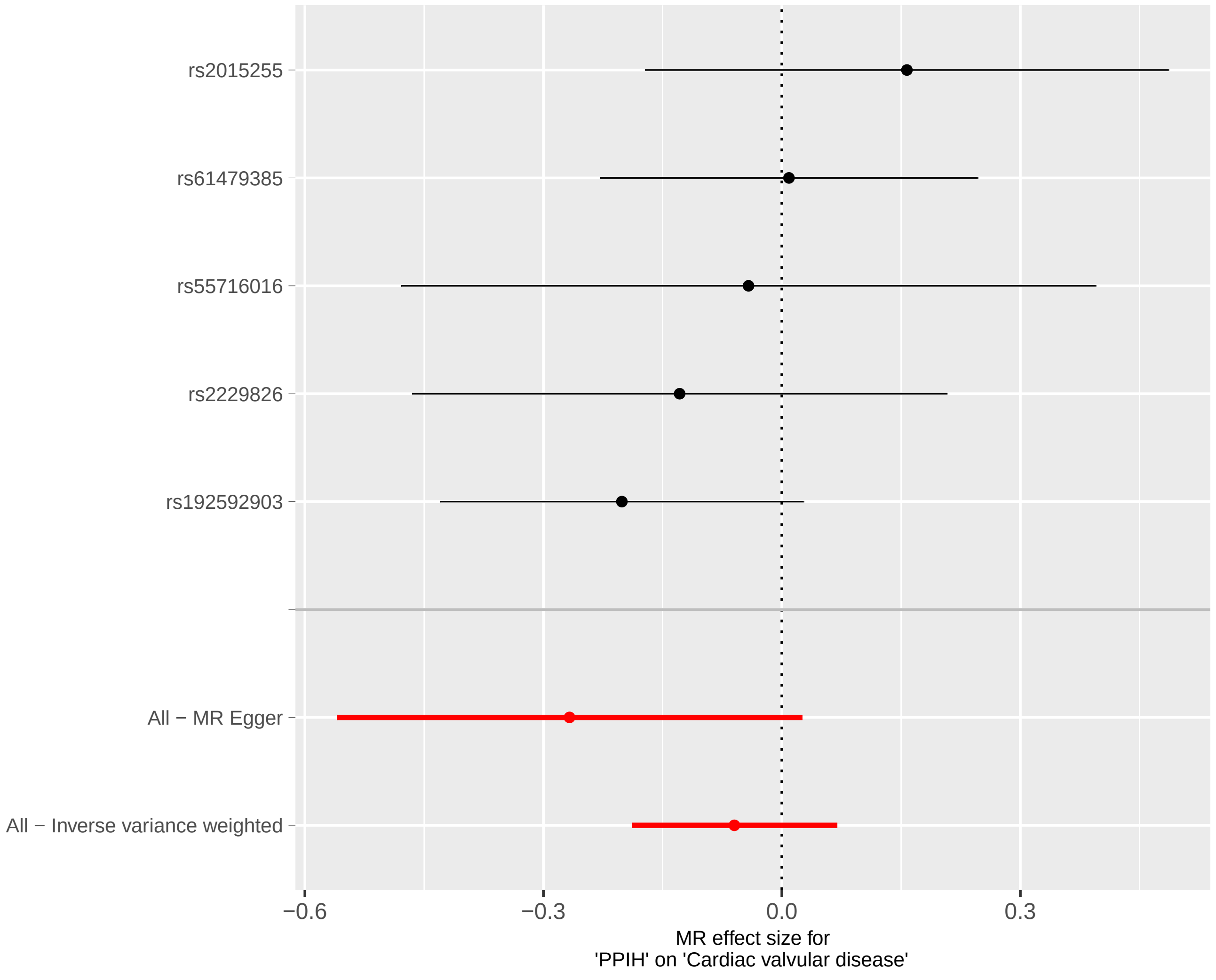
rs2015255
rs61479385
rs55716016
rs2229826
rs192592903
All − MR Egger
All − Inverse variance weighted
−0.6
−0.3
0.0
0.3
MR effect size for
'PPIH' on 'Cardiac valvular disease'

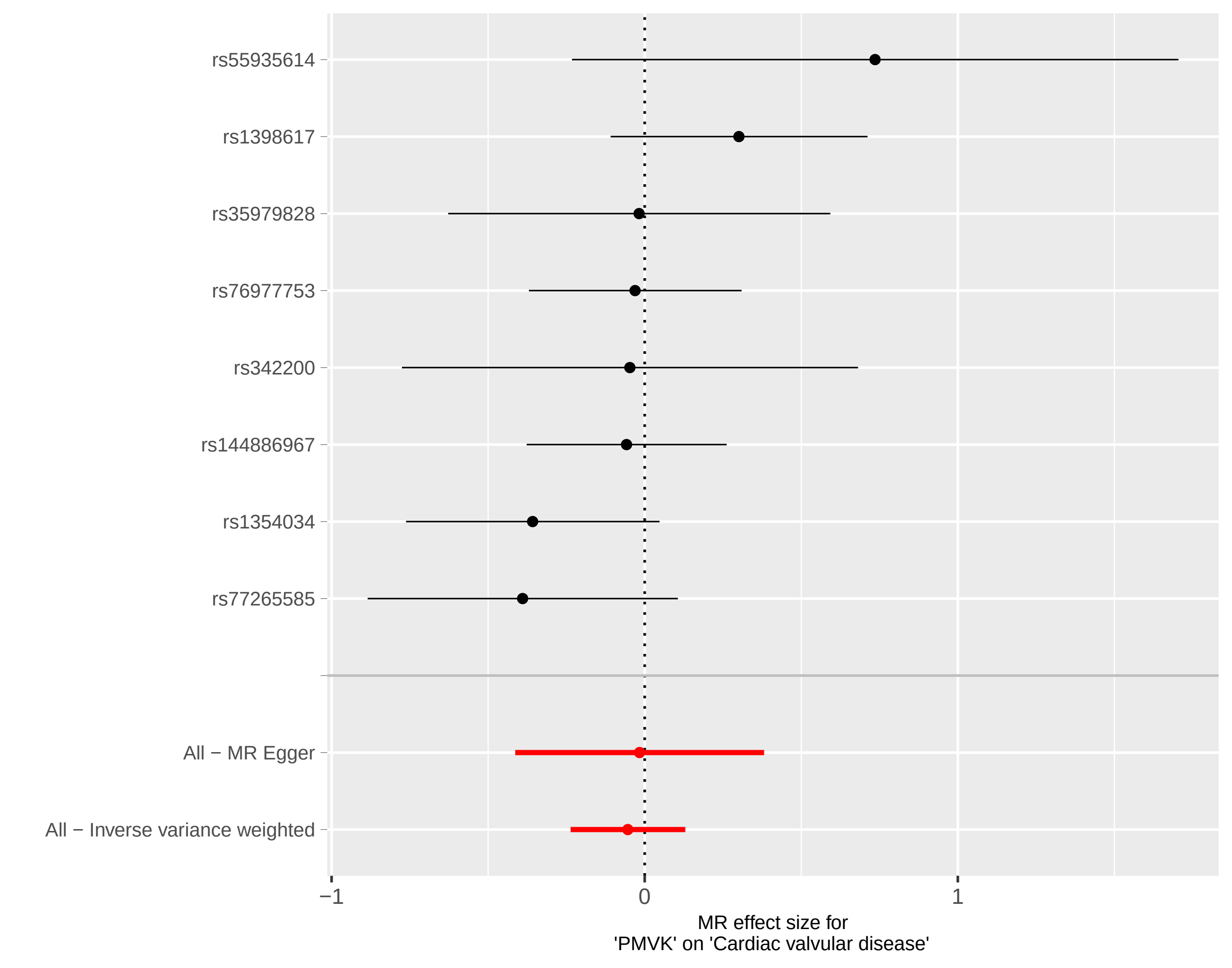
rs55935614
rs1398617
rs35979828
rs76977753
rs342200
rs144886967
rs1354034
rs77265585
All − MR Egger
All − Inverse variance weighted
−1
0
1
MR effect size for
'PMVK' on 'Cardiac valvular disease'

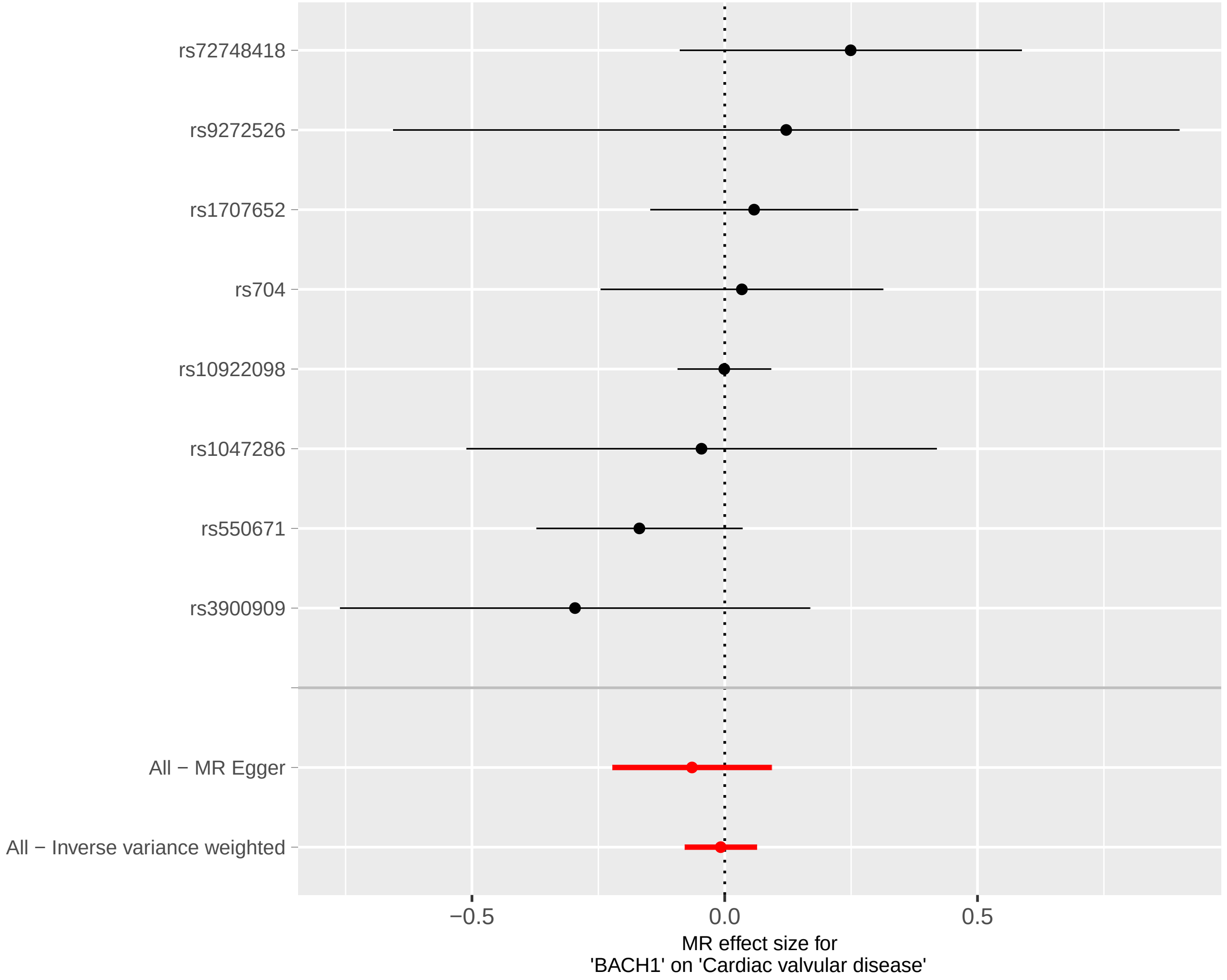

rs72748418
rs9272526
rs1707652
rs704
rs10922098
rs1047286
rs550671
rs3900909
All − MR Egger
All − Inverse variance weighted
−0.5
0.0
0.5
MR effect size for
'BACH1' on 'Cardiac valvular disease'

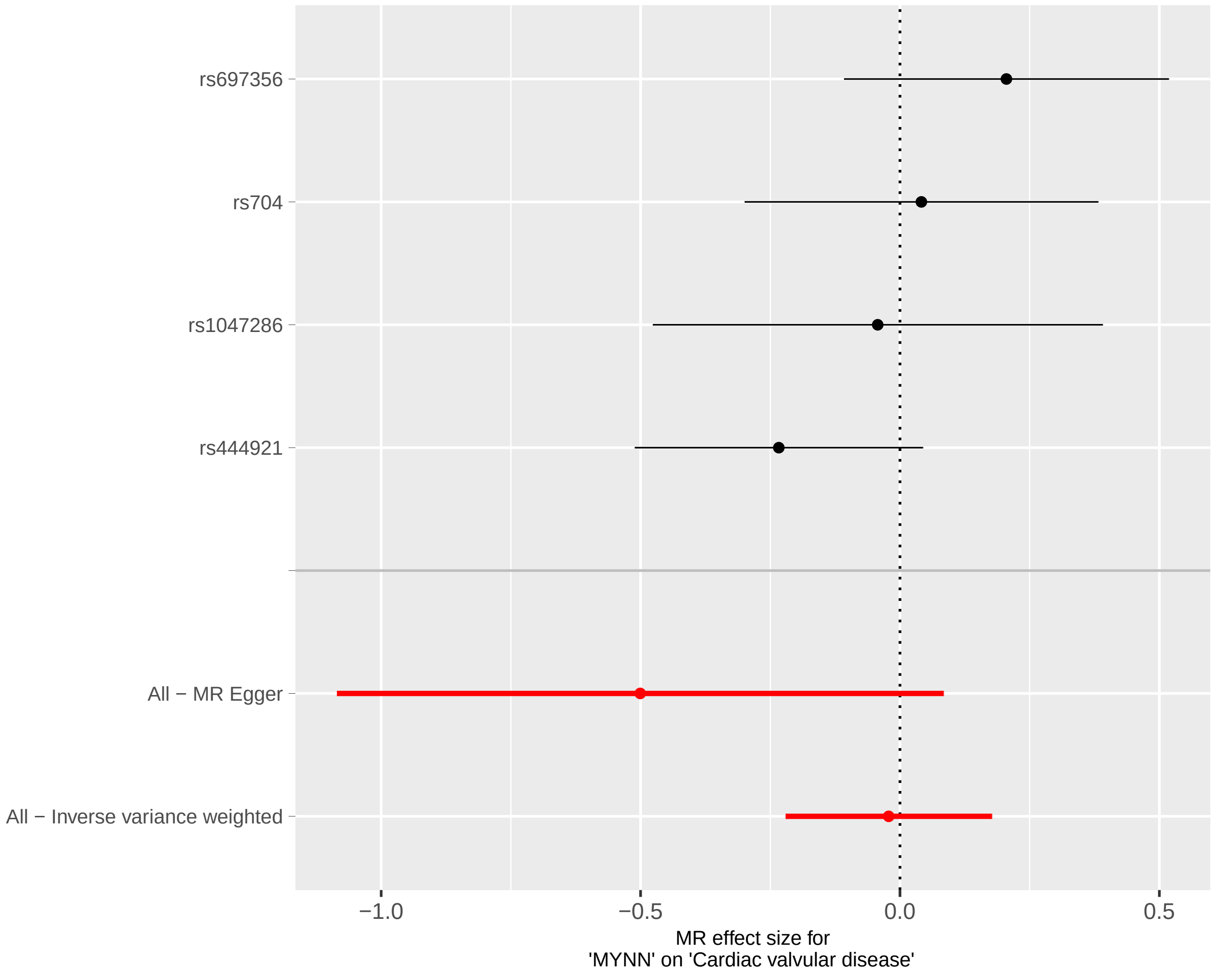
rs697356
rs704
rs1047286
rs444921
All − MR Egger
All − Inverse variance weighted
−1.0
−0.5
0.0
0.5
MR effect size for
'MYNN' on 'Cardiac valvular disease'

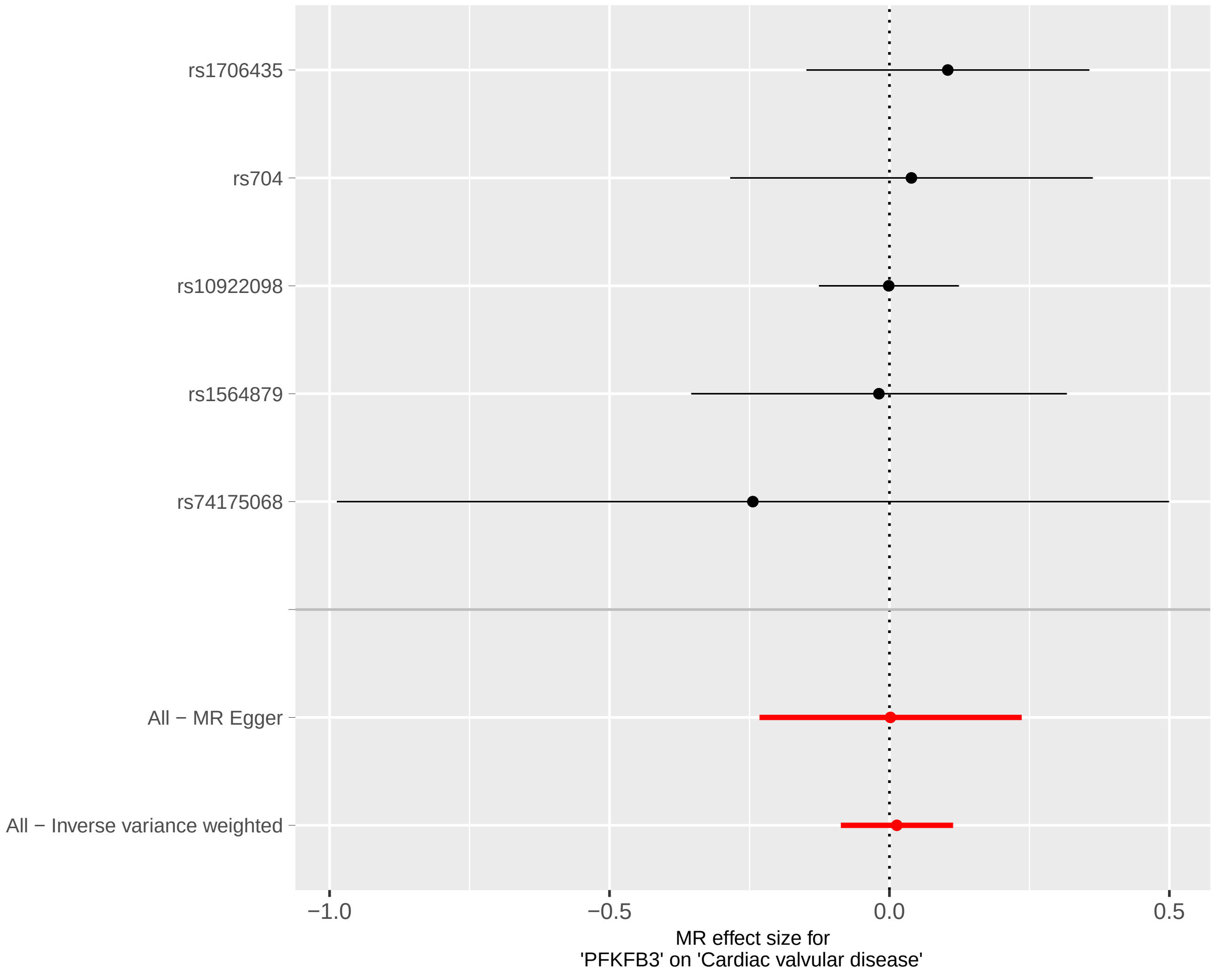

rs1706435
rs704
rs10922098
rs1564879
rs74175068
All − MR Egger
All − Inverse variance weighted
−1.0
−0.5
0.0
0.5
MR effect size for
'PFKFB3' on 'Cardiac valvular disease'

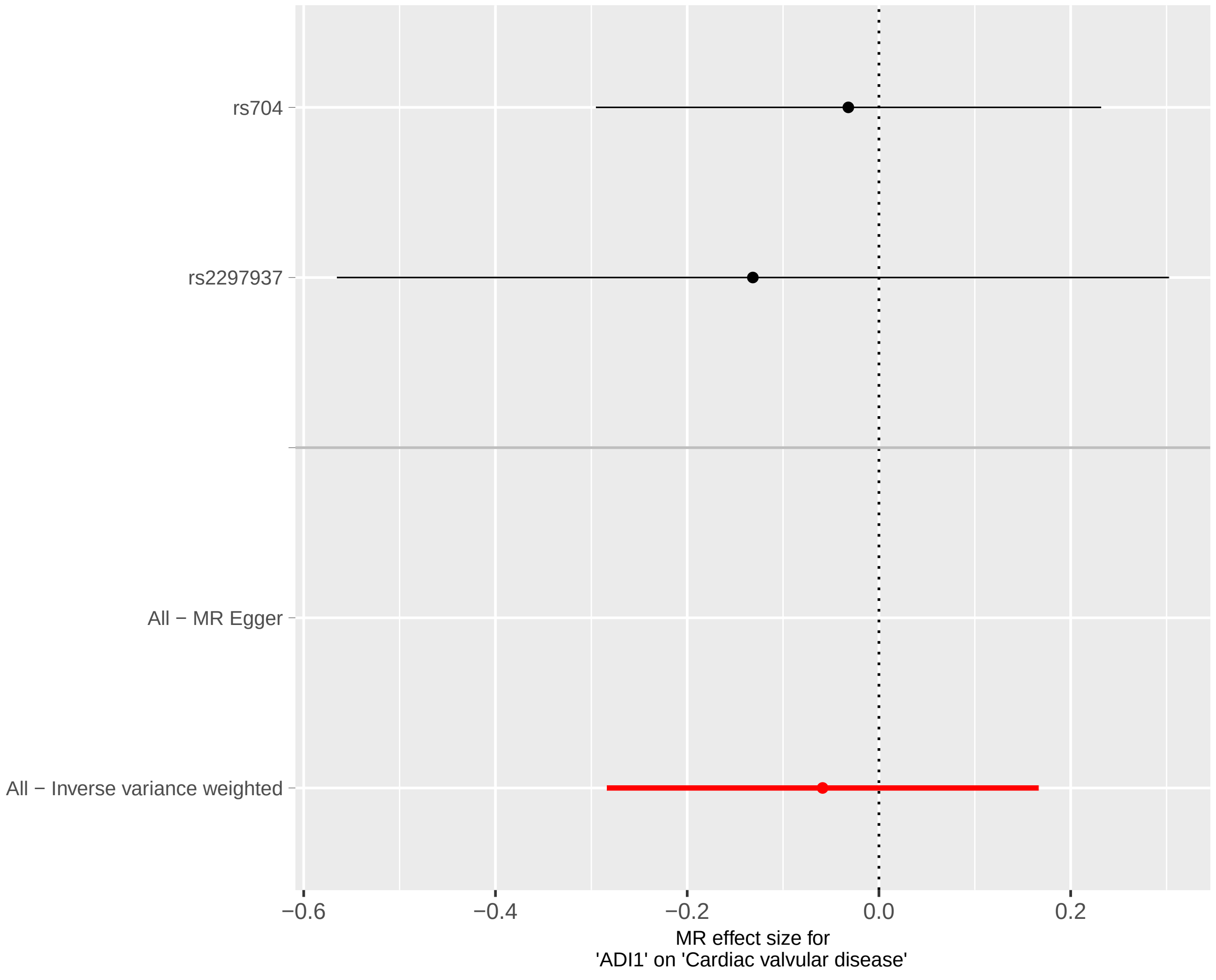
rs704
rs2297937
All − MR Egger
All − Inverse variance weighted
−0.6
−0.4
−0.2
0.0
0.2
MR effect size for
'ADI1' on 'Cardiac valvular disease'

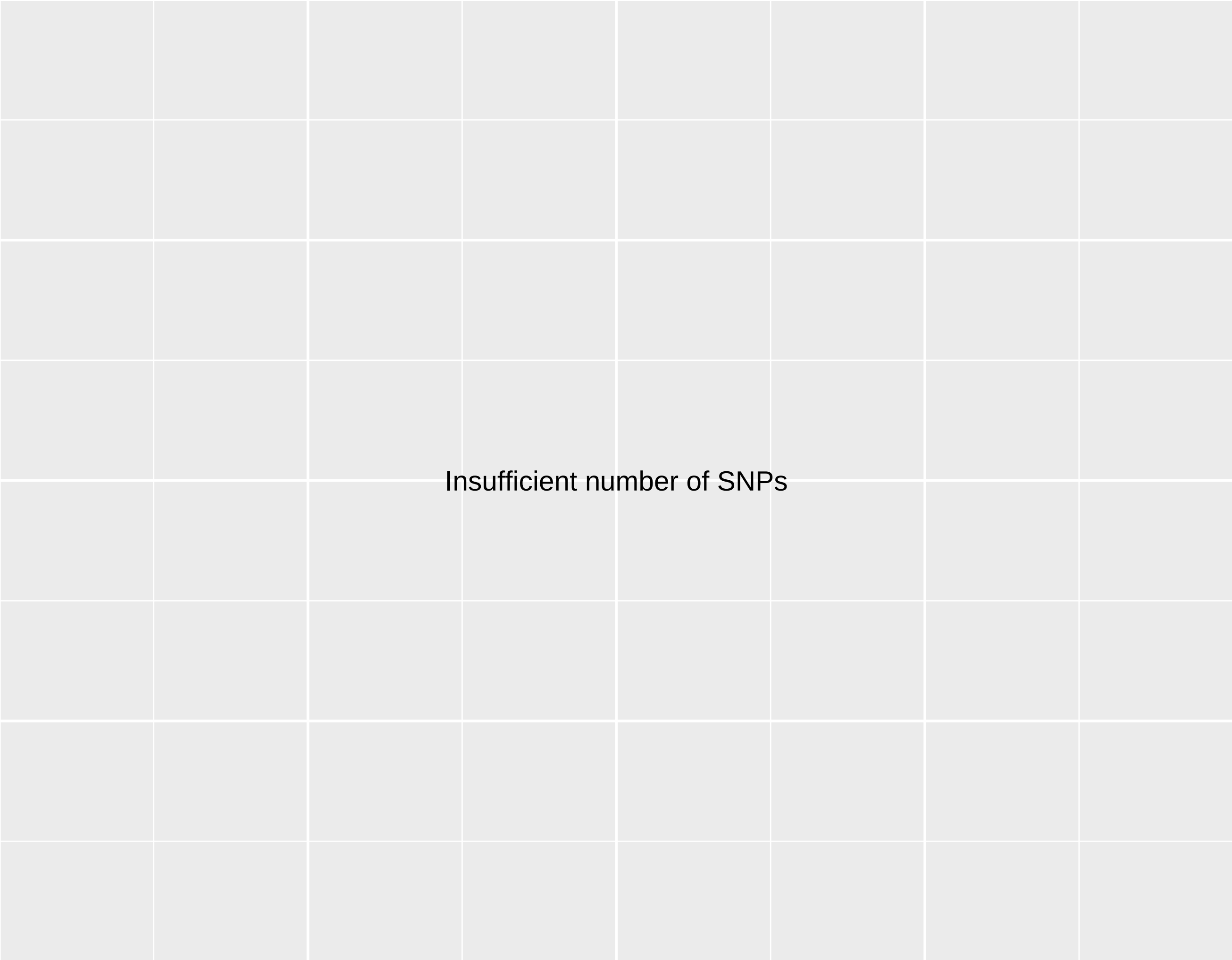
Insufficient number of SNPs

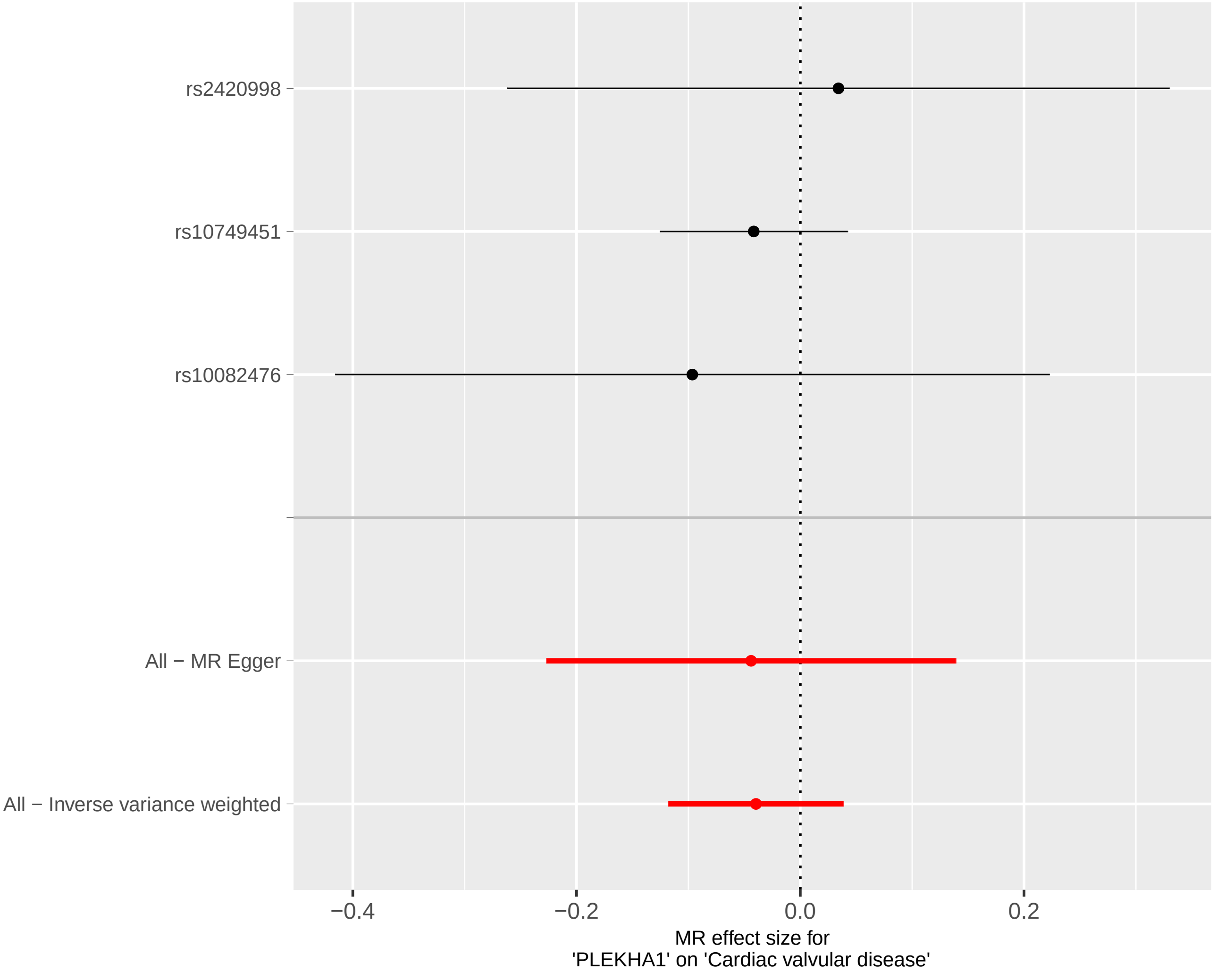
rs2420998
rs10749451
rs10082476
All − MR Egger
All − Inverse variance weighted
−0.4
−0.2
0.0
0.2
MR effect size for
'PLEKHA1' on 'Cardiac valvular disease'

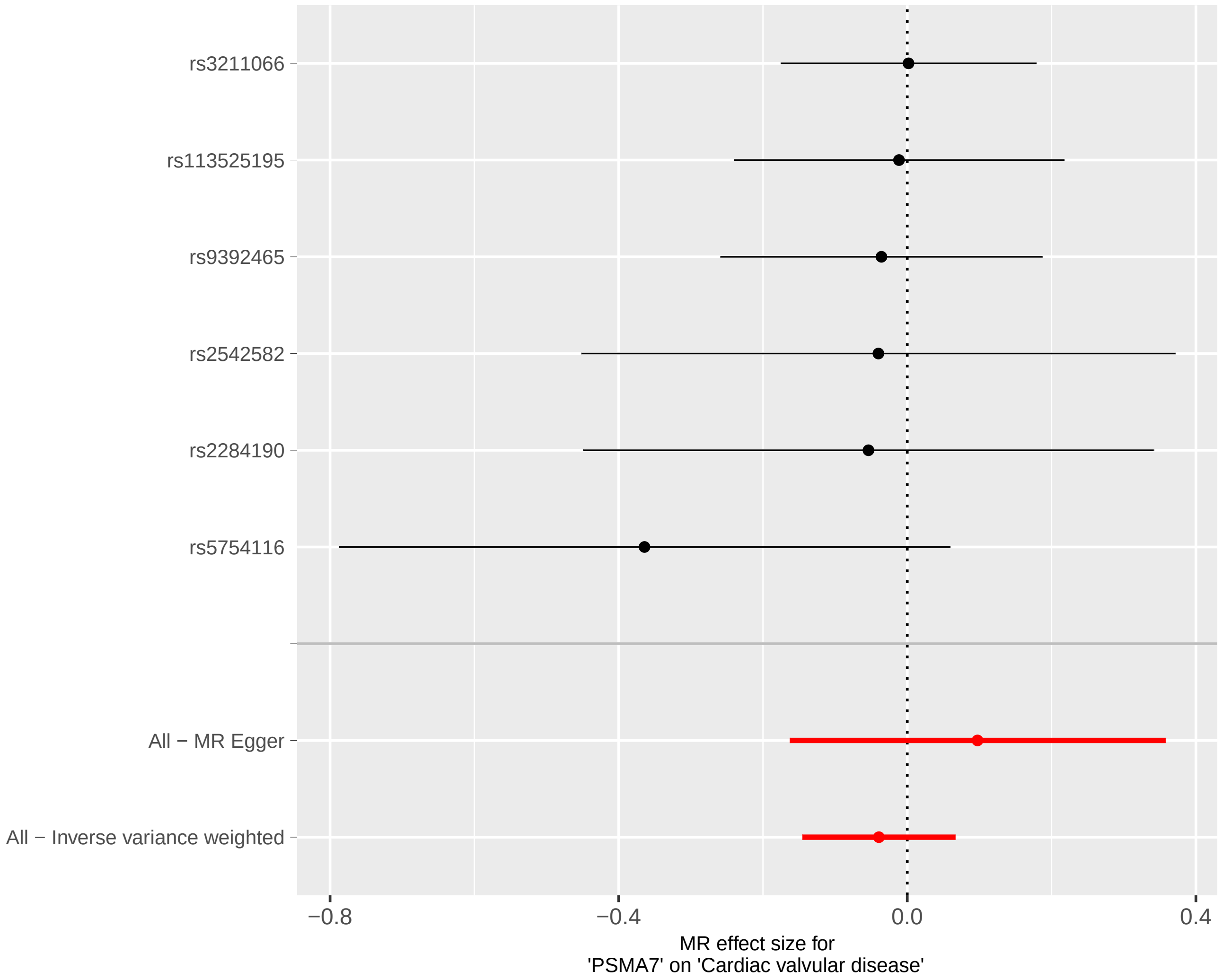
rs3211066
rs113525195
rs9392465
rs2542582
rs2284190
rs5754116
All − MR Egger
All − Inverse variance weighted
−0.8
−0.4
0.0
0.4
MR effect size for
'PSMA7' on 'Cardiac valvular disease'

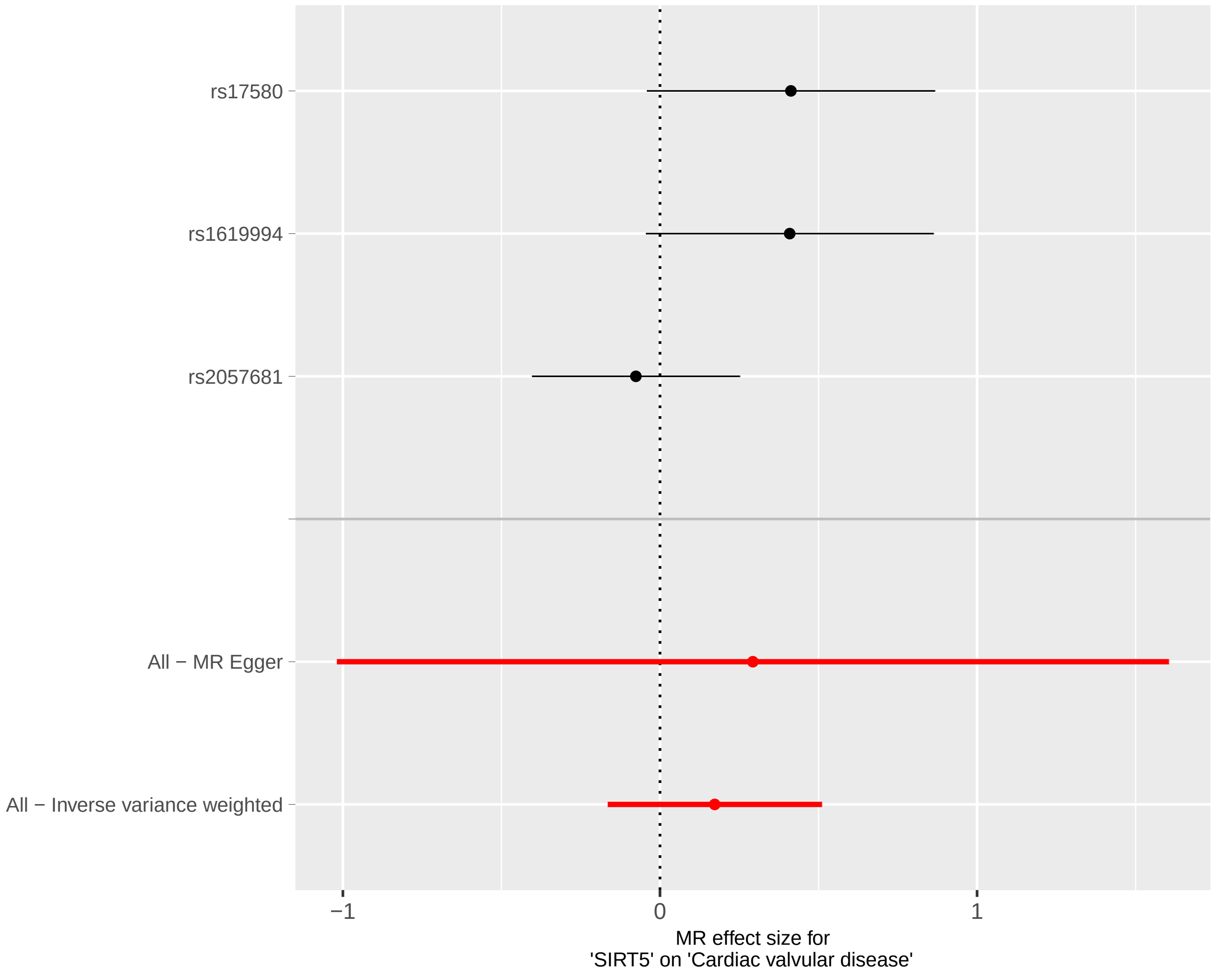

rs17580
rs1619994
rs2057681
All − MR Egger
All − Inverse variance weighted
−1
0
1
MR effect size for
'SIRT5' on 'Cardiac valvular disease'

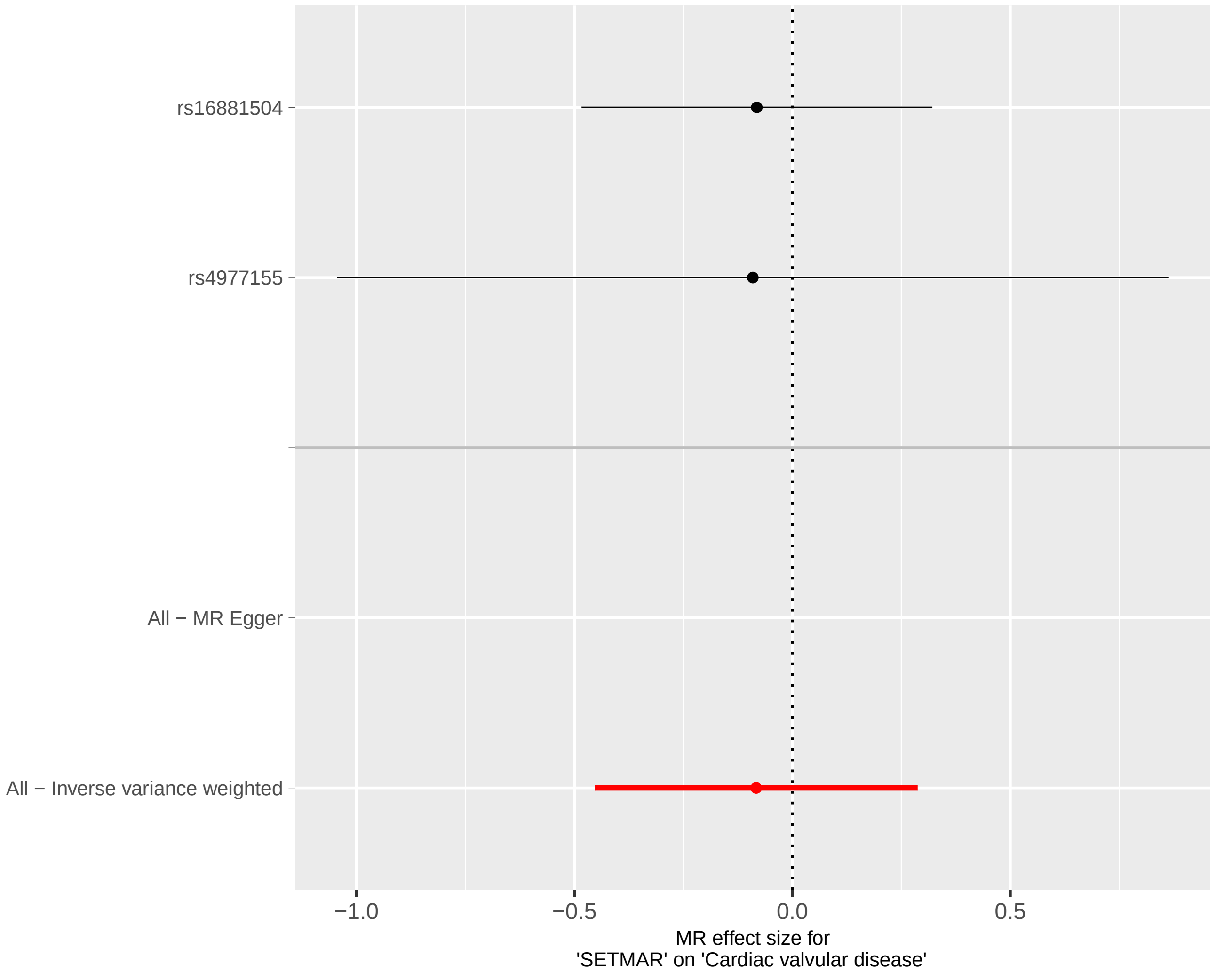

rs16881504
rs4977155
All − MR Egger
All − Inverse variance weighted
−1.0
−0.5
0.0
0.5
MR effect size for
'SETMAR' on 'Cardiac valvular disease'

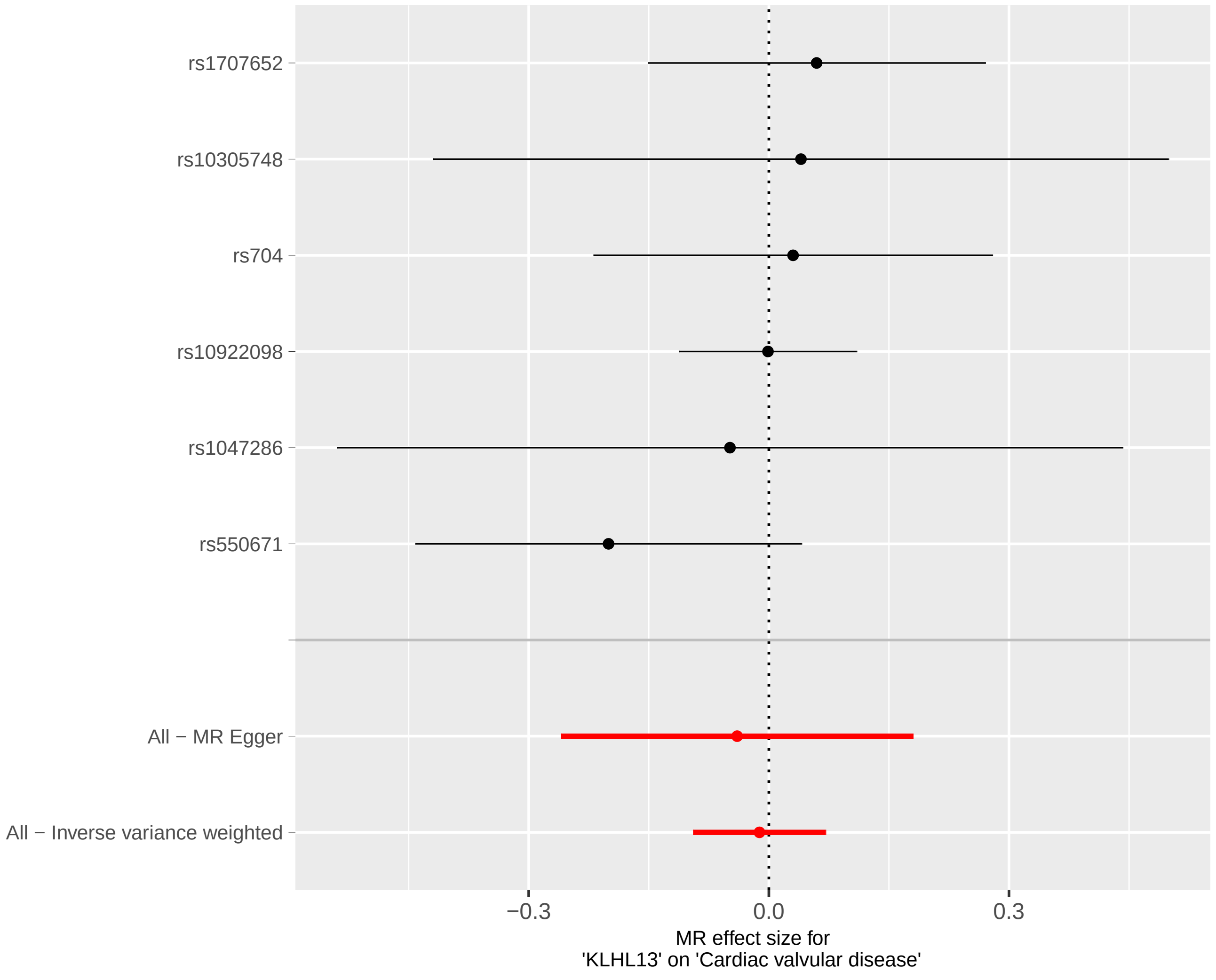

rs1707652
rs10305748
rs704
rs10922098
rs1047286
rs550671
All − MR Egger
All − Inverse variance weighted
−0.3
0.0
0.3
MR effect size for
'KLHL13' on 'Cardiac valvular disease'

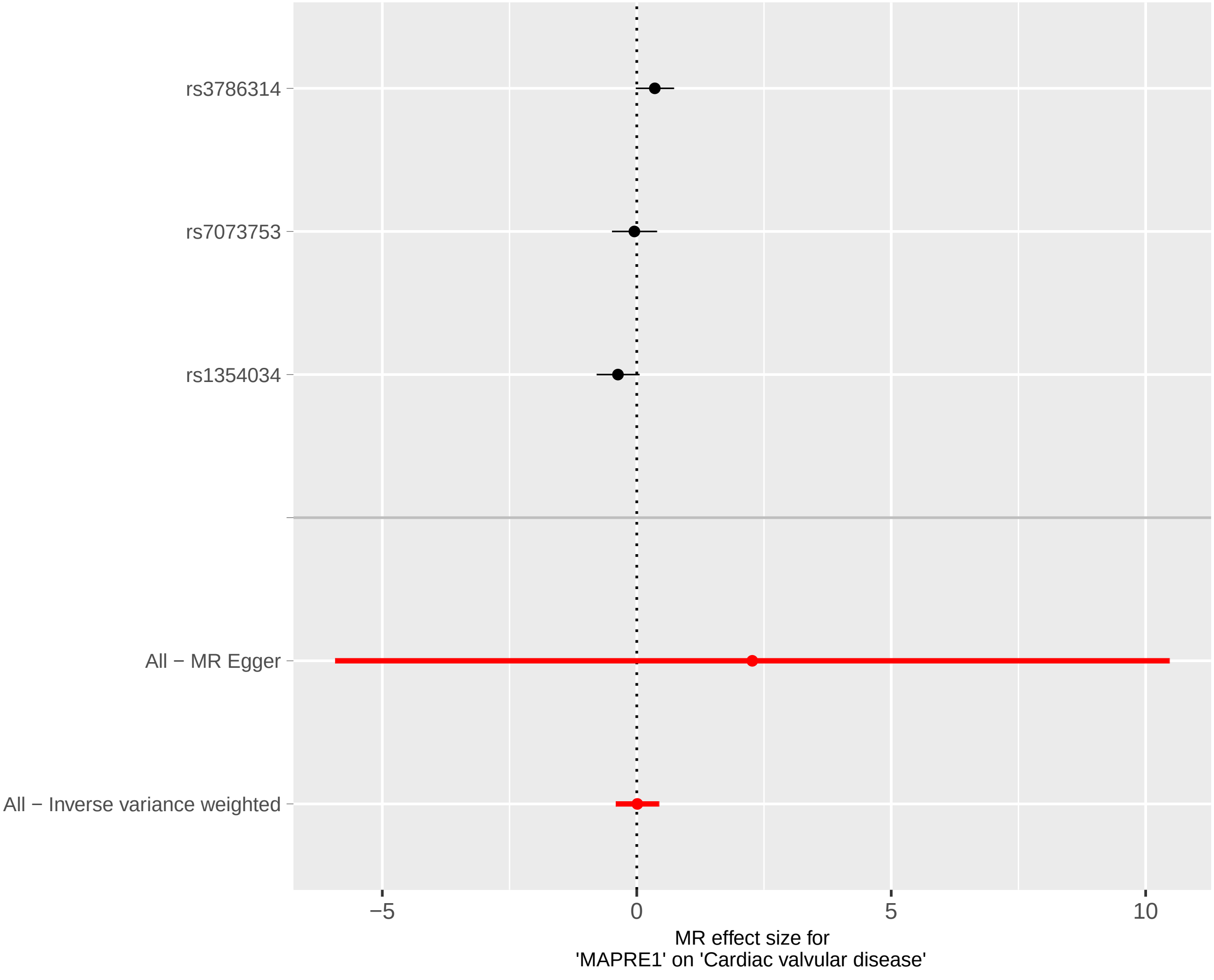

rs3786314
rs7073753
rs1354034
All − MR Egger
All − Inverse variance weighted
−5
0
5
10
MR effect size for
'MAPRE1' on 'Cardiac valvular disease'

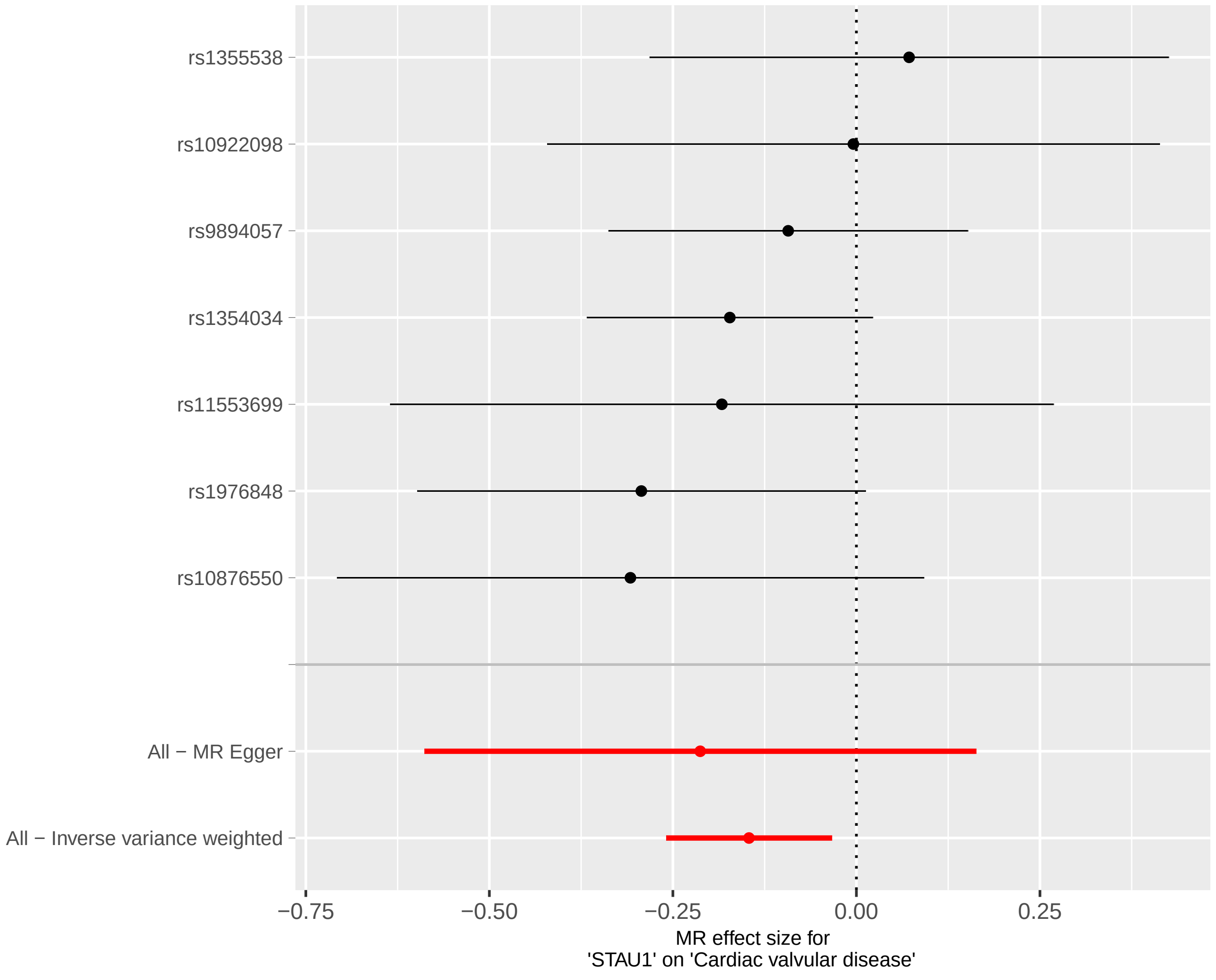
rs1355538
rs10922098
rs9894057
rs1354034
rs11553699
rs1976848
rs10876550
All − MR Egger
All − Inverse variance weighted
−0.75
−0.50
−0.25
0.00
0.25
MR effect size for
'STAU1' on 'Cardiac valvular disease'

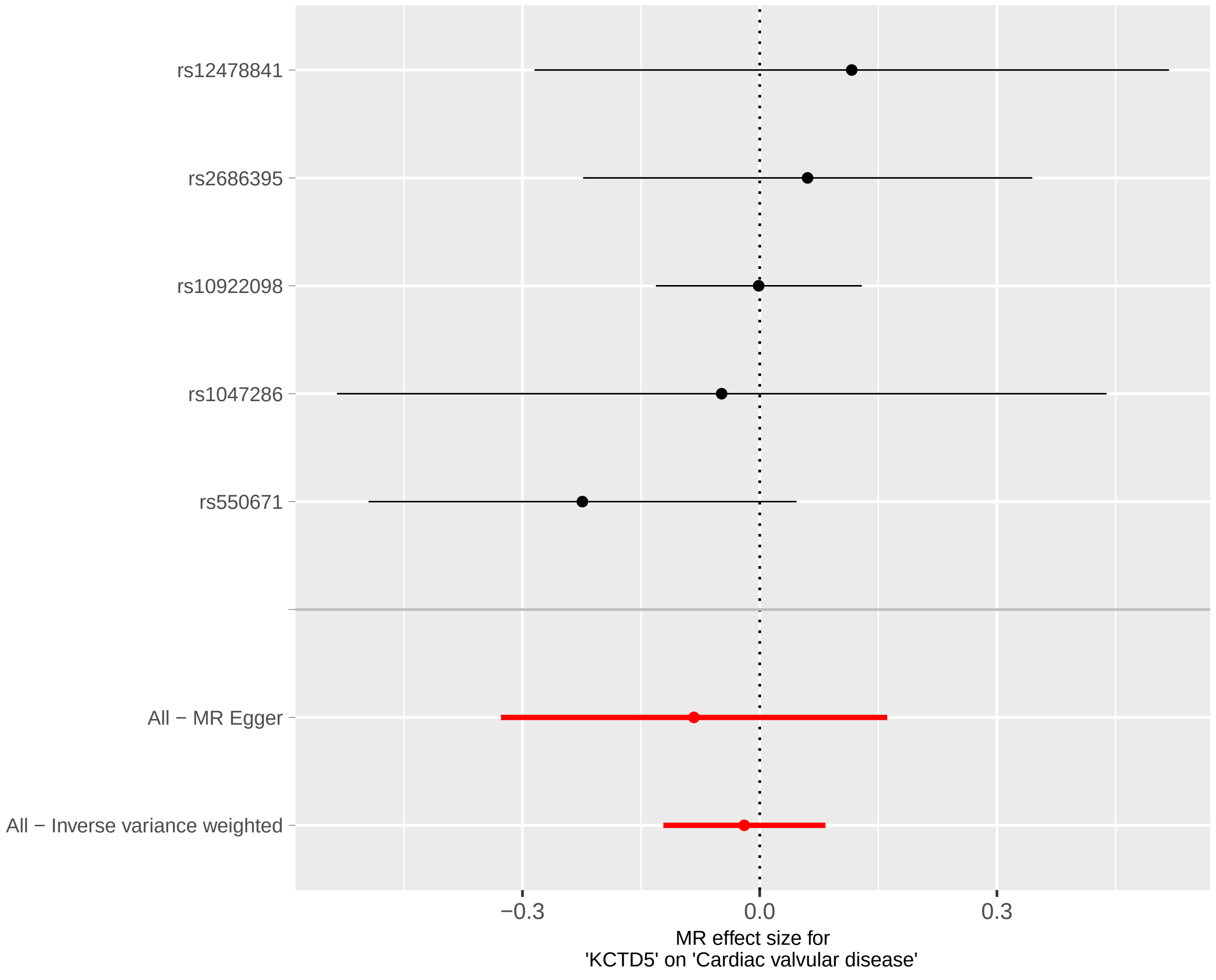
rs12478841
rs2686395
rs10922098
rs1047286
rs550671
All − MR Egger
All − Inverse variance weighted
−0.3
0.0
0.3
MR effect size for
'KCTD5' on 'Cardiac valvular disease'

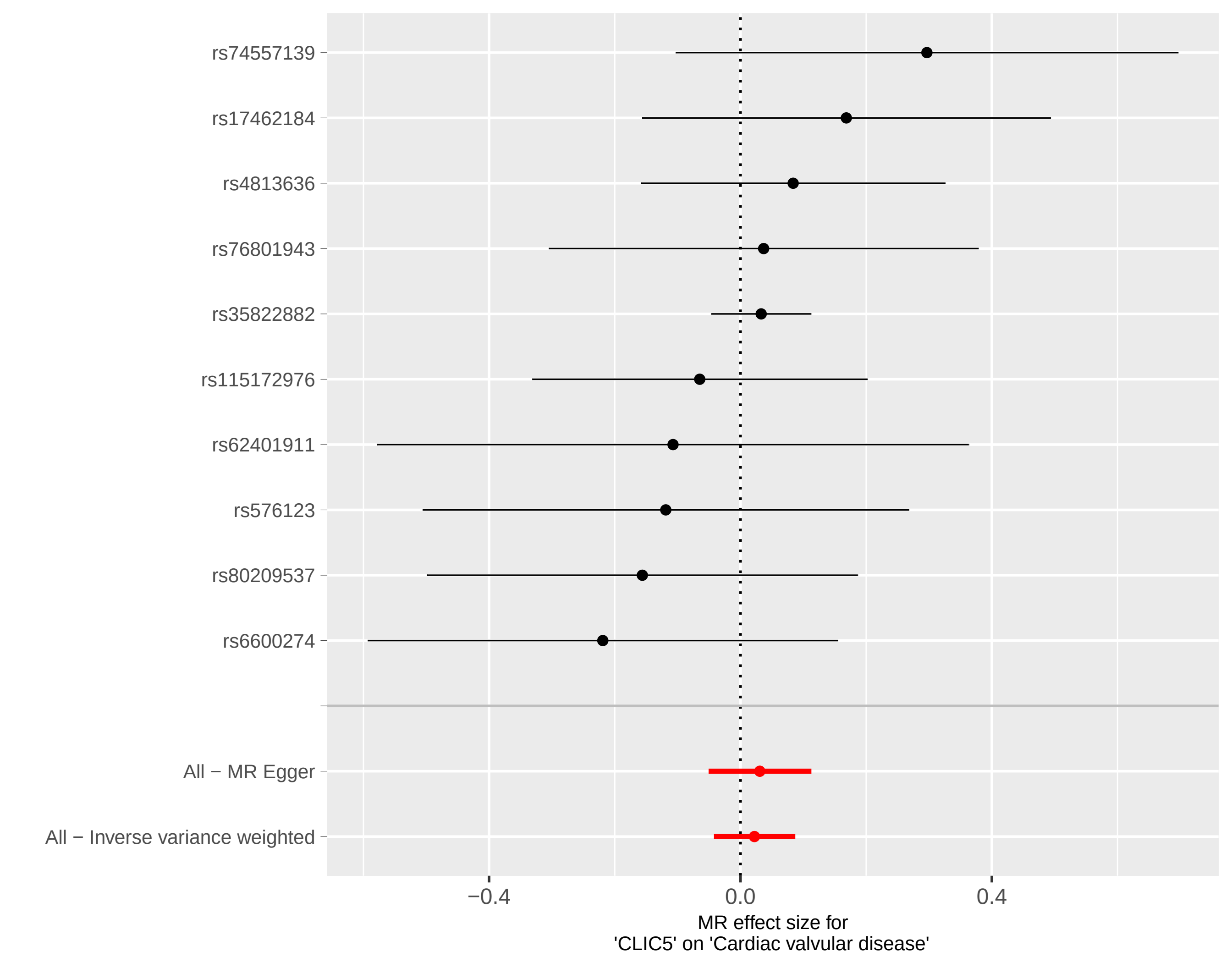

rs74557139
rs17462184
rs4813636
rs76801943
rs35822882
rs115172976
rs62401911
rs576123
rs80209537
rs6600274
All − MR Egger
All − Inverse variance weighted
−0.4
0.0
0.4
MR effect size for
'CLIC5' on 'Cardiac valvular disease'

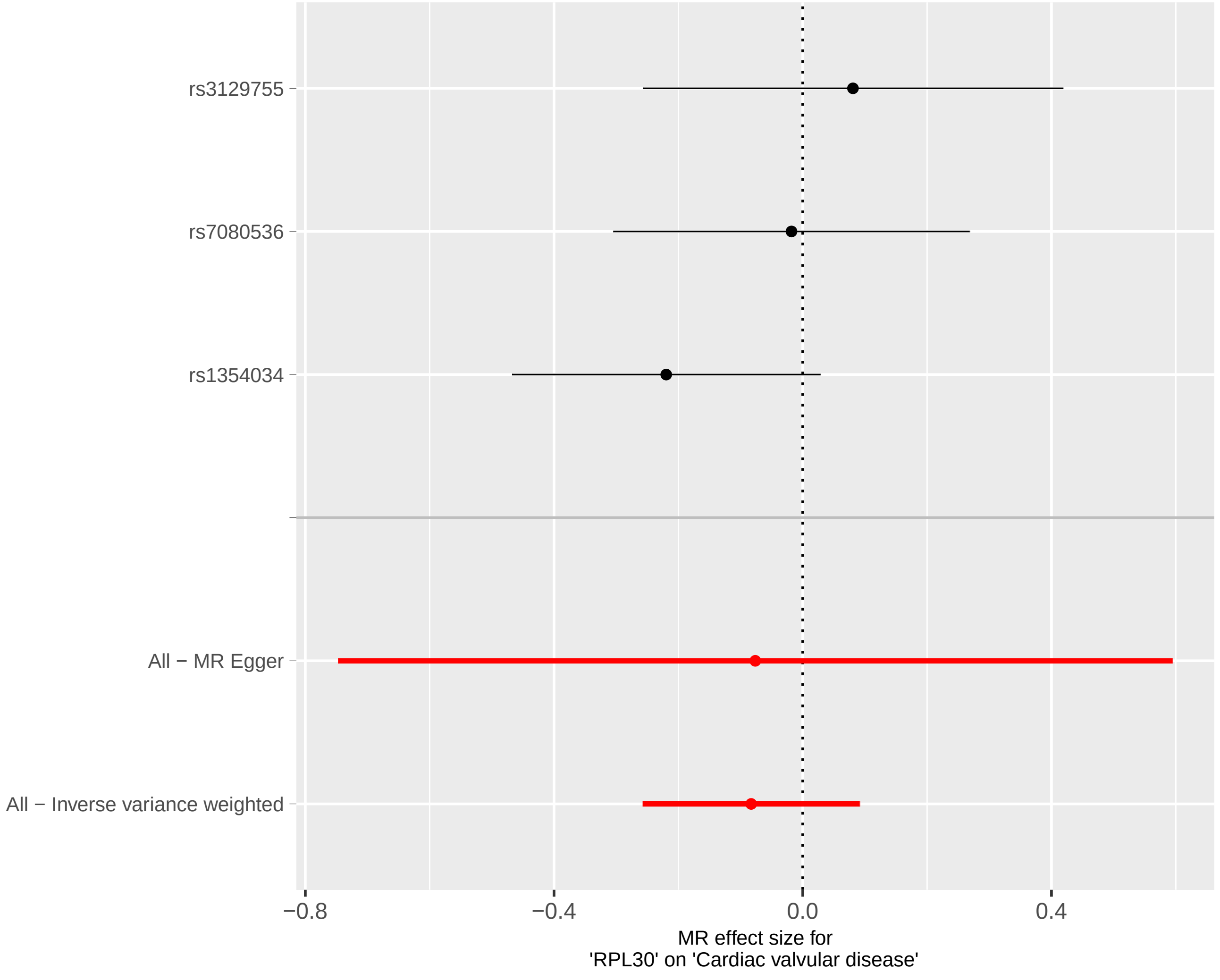
rs3129755
rs7080536
rs1354034
All − MR Egger
All − Inverse variance weighted
−0.8
−0.4
0.0
0.4
MR effect size for
'RPL30' on 'Cardiac valvular disease'

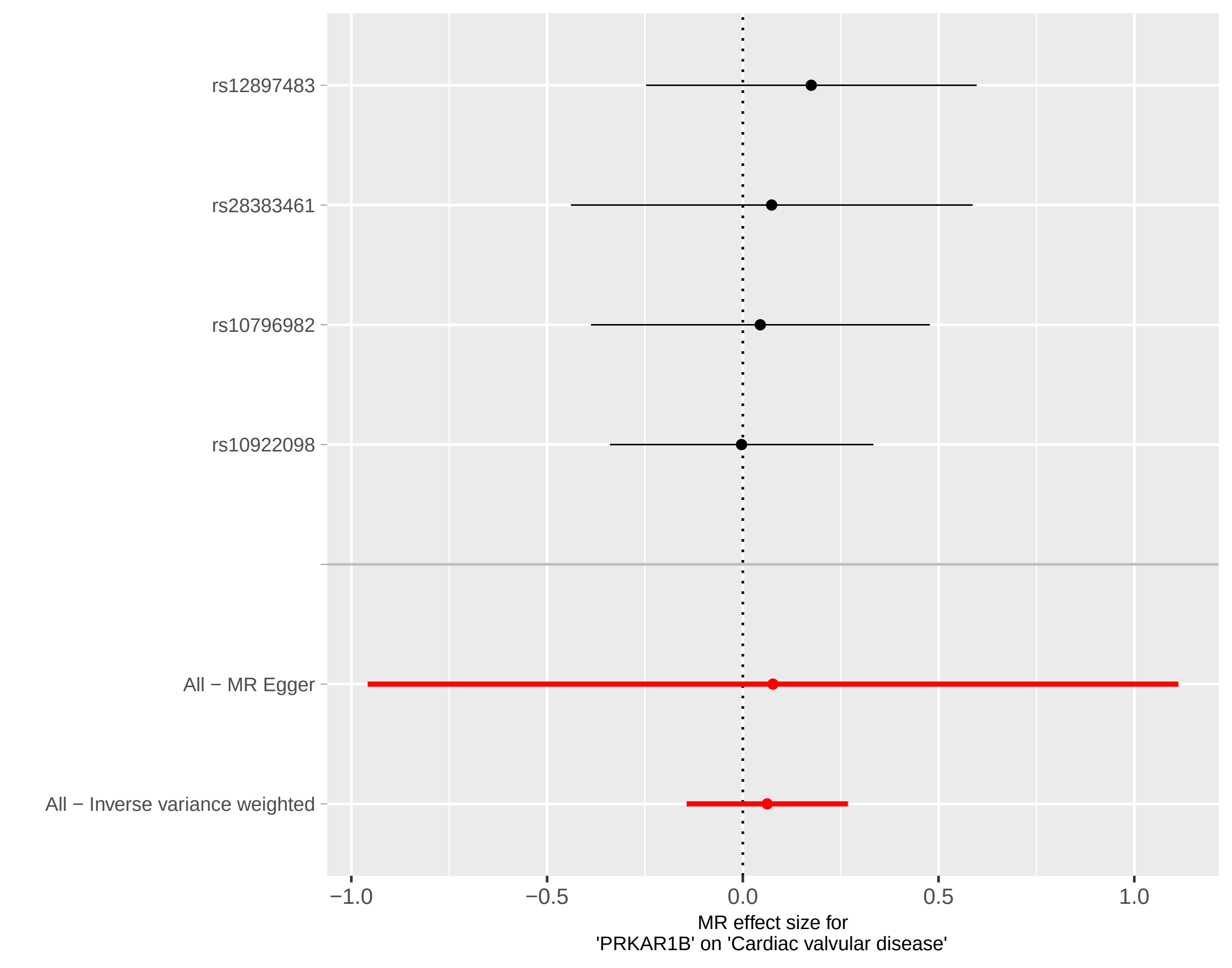
rs12897483
rs28383461
rs10796982
rs10922098
All − MR Egger
All − Inverse variance weighted
−1.0
−0.5
0.0
0.5
1.0
MR effect size for
'PRKAR1B' on 'Cardiac valvular disease'

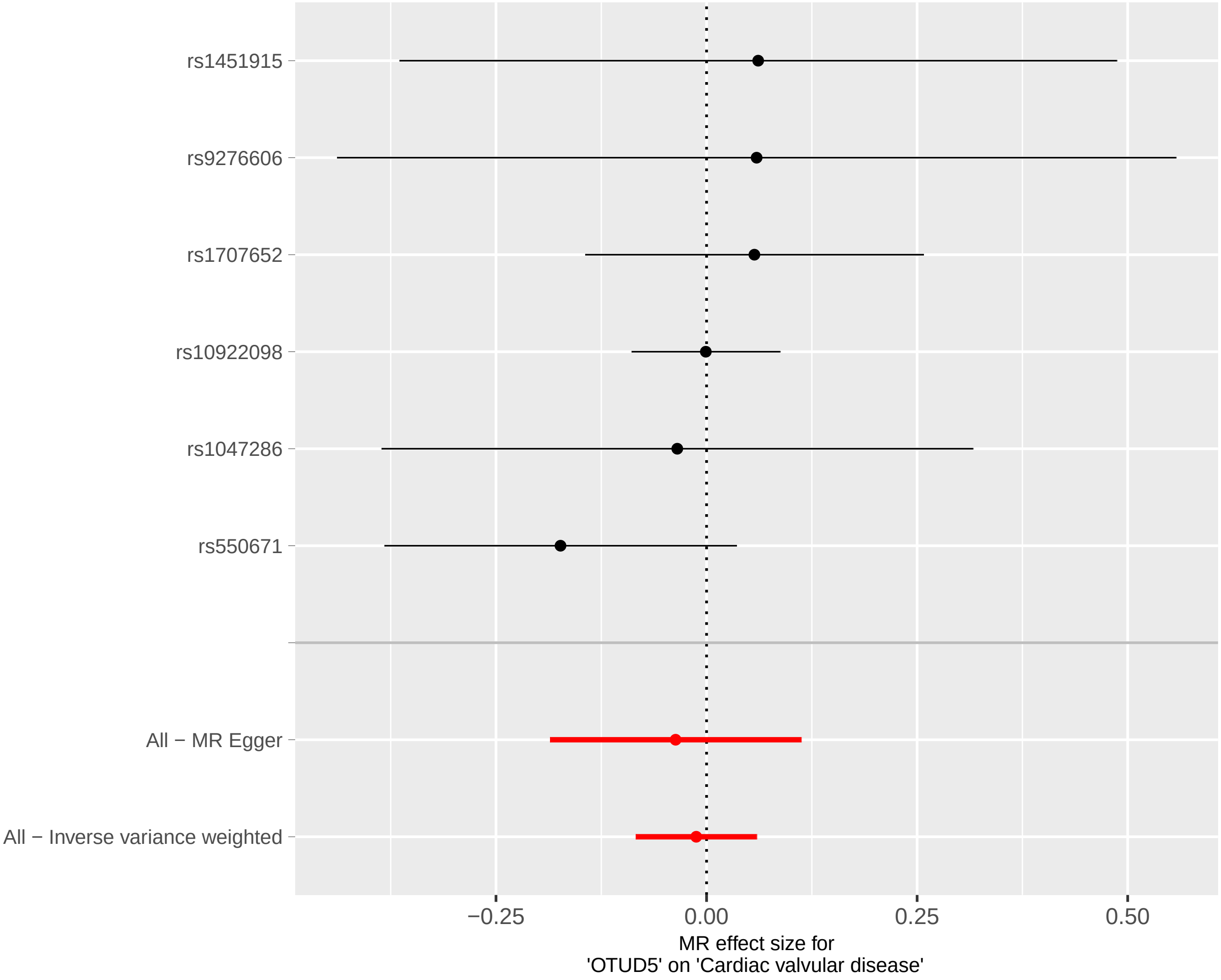
rs1451915
rs9276606
rs1707652
rs10922098
rs1047286
rs550671
All − MR Egger
All − Inverse variance weighted
−0.25
0.00
0.25
0.50
MR effect size for
'OTUD5' on 'Cardiac valvular disease'

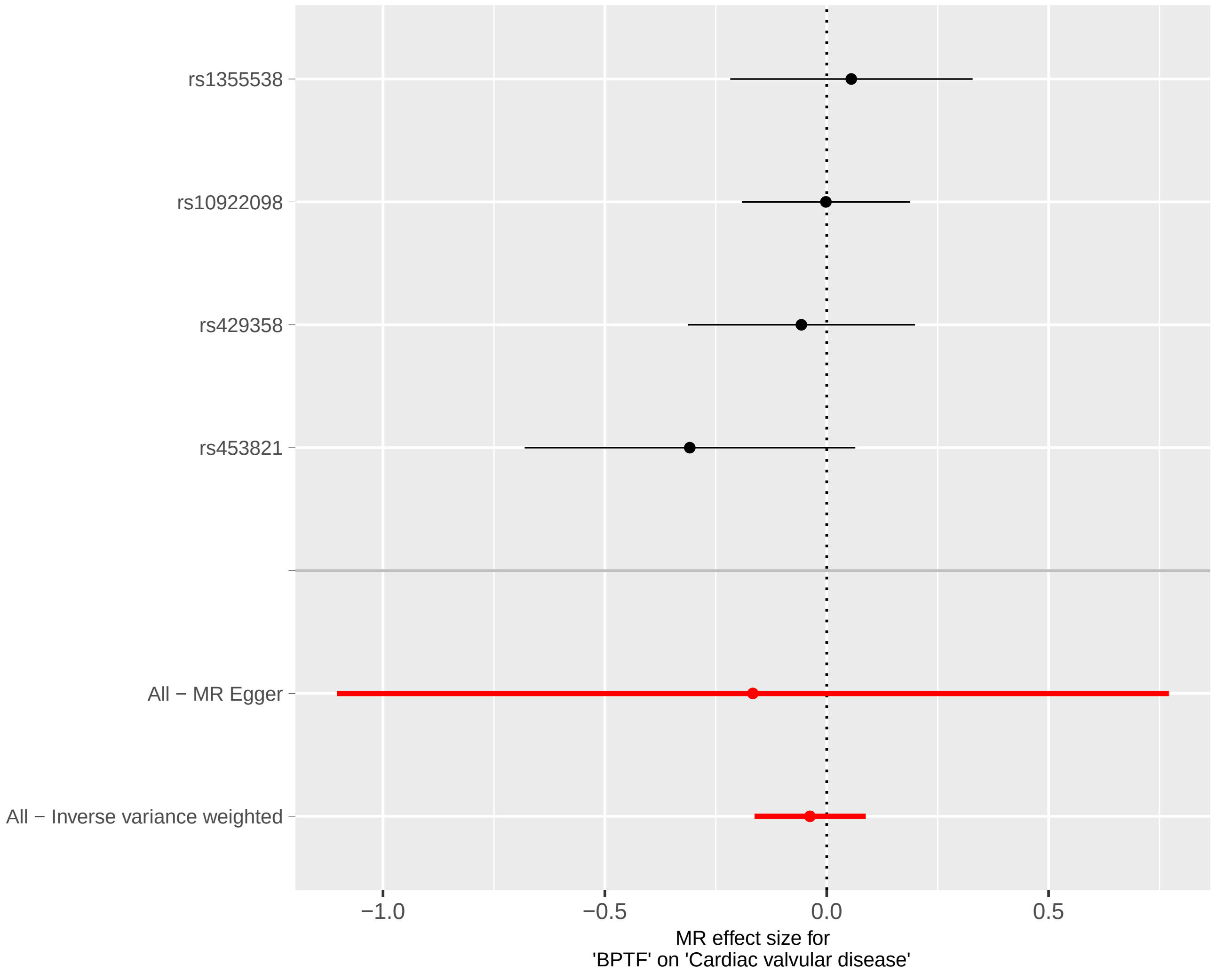

rs1355538
rs10922098
rs429358
rs453821
All − MR Egger
All − Inverse variance weighted
−1.0
−0.5
0.0
0.5
MR effect size for
'BPTF' on 'Cardiac valvular disease'

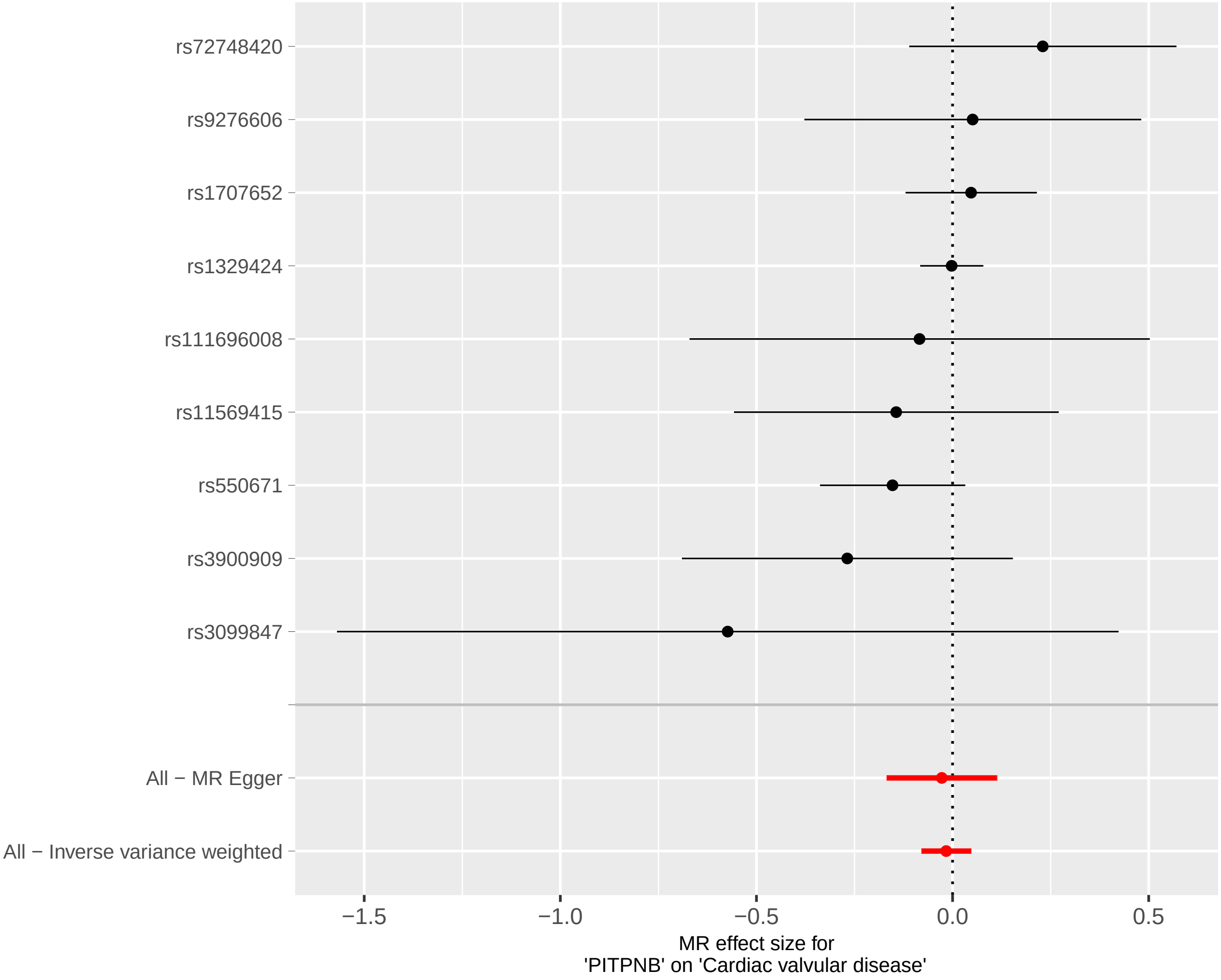

rs72748420
rs9276606
rs1707652
rs1329424
rs111696008
rs11569415
rs550671
rs3900909
rs3099847
All − MR Egger
All − Inverse variance weighted
−1.5
−1.0
−0.5
0.0
0.5
MR effect size for
'PITPNB' on 'Cardiac valvular disease'

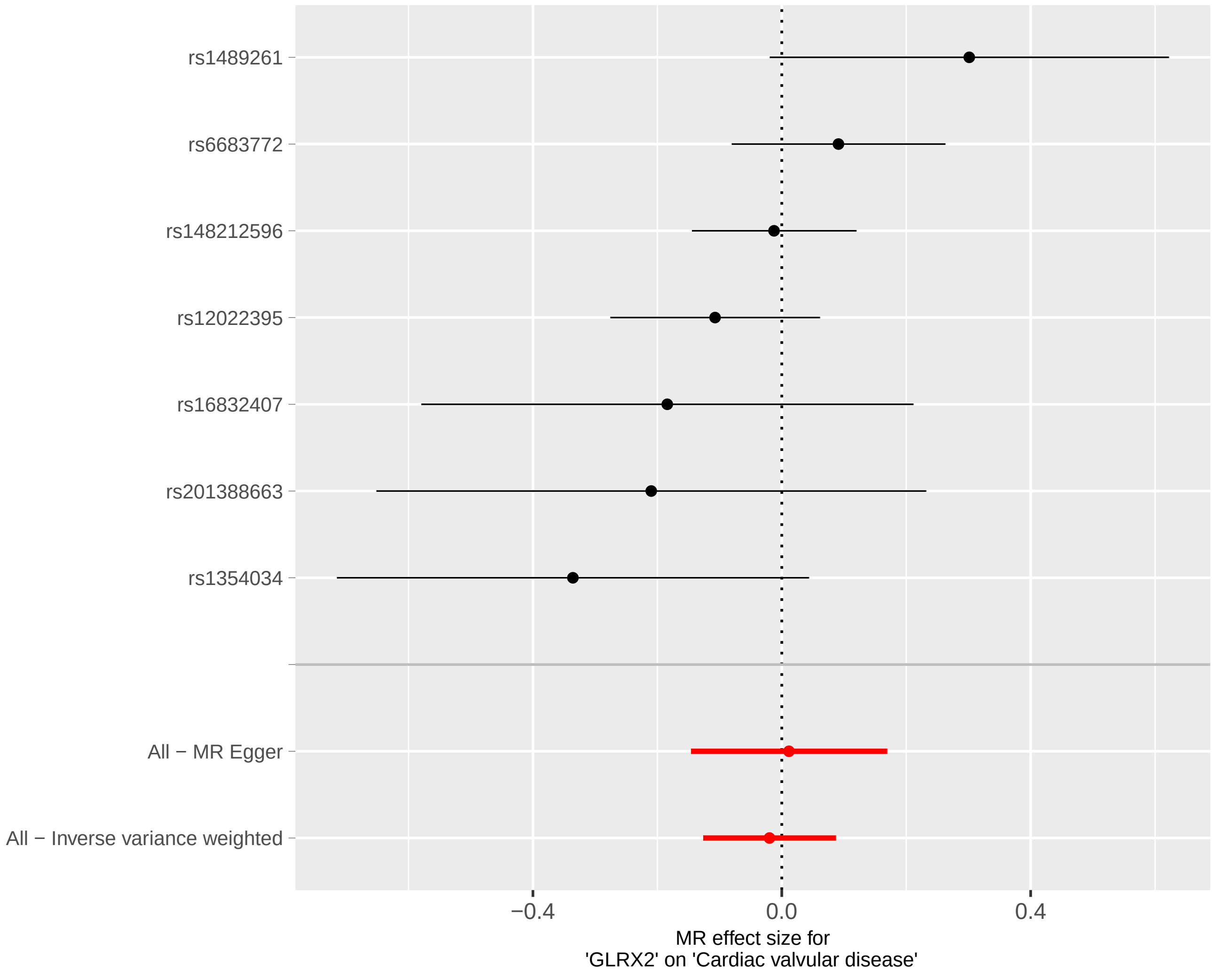
rs1489261
rs6683772
rs148212596
rs12022395
rs16832407
rs201388663
rs1354034
All − MR Egger
All − Inverse variance weighted
−0.4
0.0
0.4
MR effect size for
'GLRX2' on 'Cardiac valvular disease'

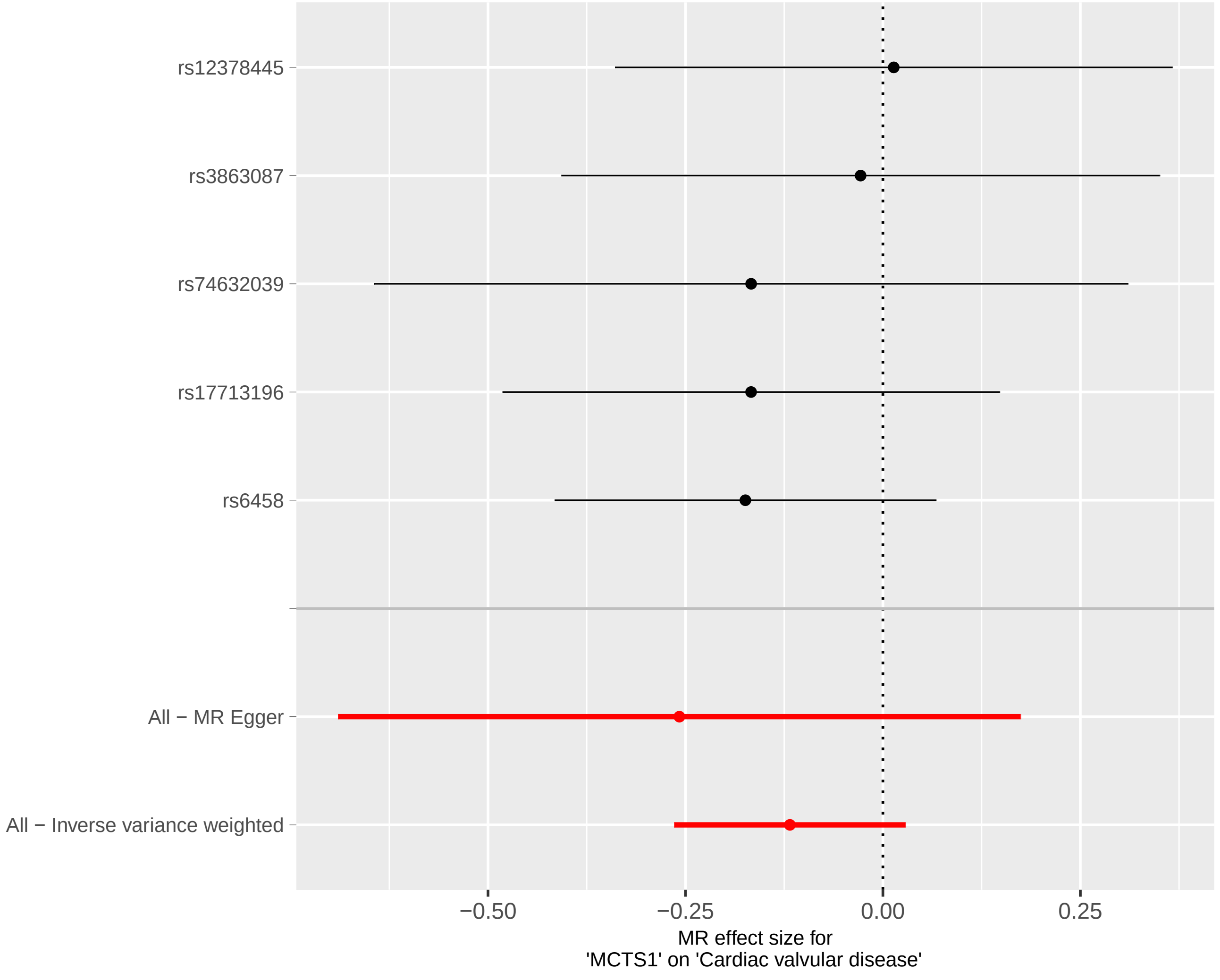
rs12378445
rs3863087
rs74632039
rs17713196
rs6458
All − MR Egger
All − Inverse variance weighted
−0.50
−0.25
0.00
0.25
MR effect size for
'MCTS1' on 'Cardiac valvular disease'

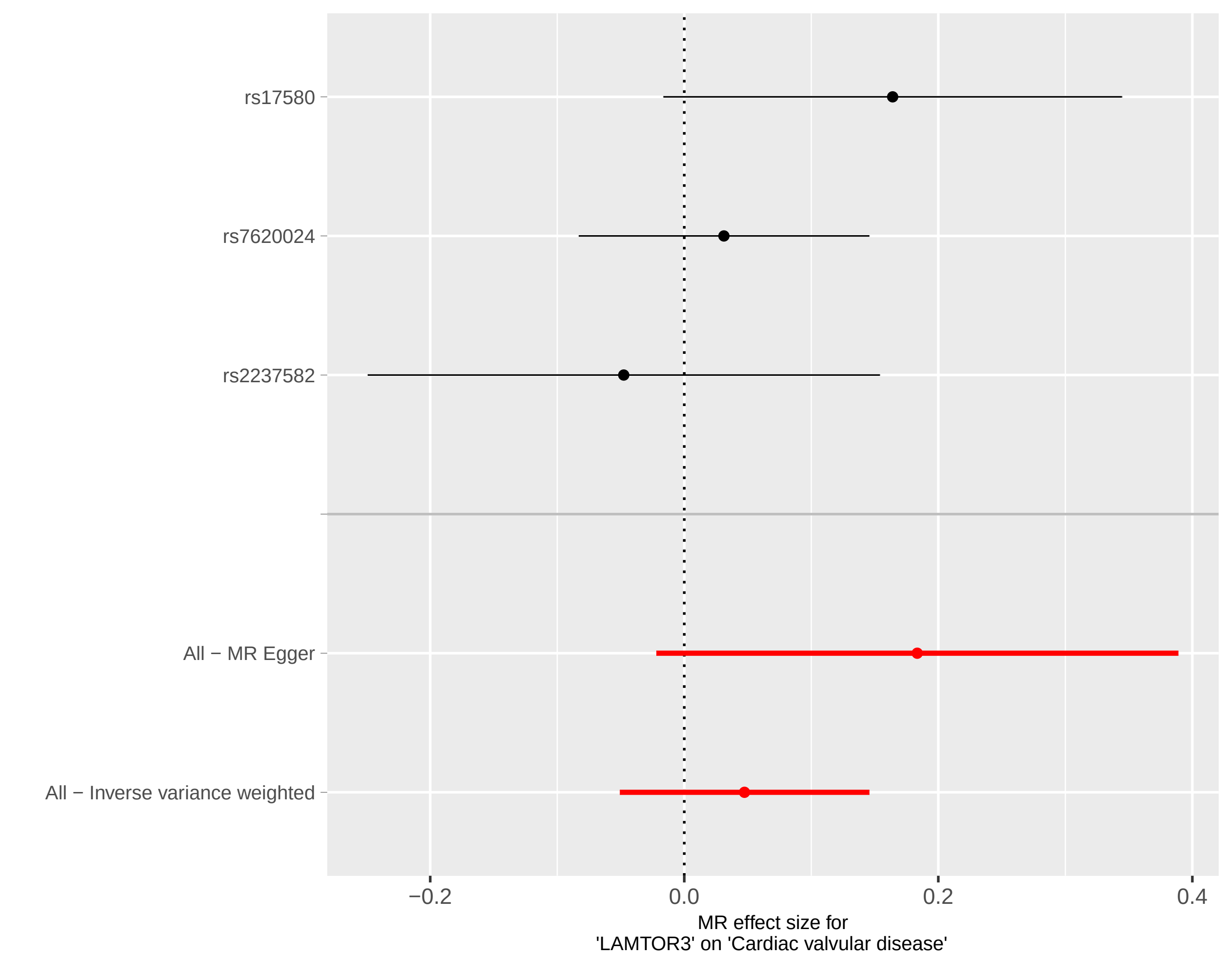
rs17580
rs7620024
rs2237582
All − MR Egger
All − Inverse variance weighted
−0.2
0.0
0.2
0.4
MR effect size for
'LAMTOR3' on 'Cardiac valvular disease'

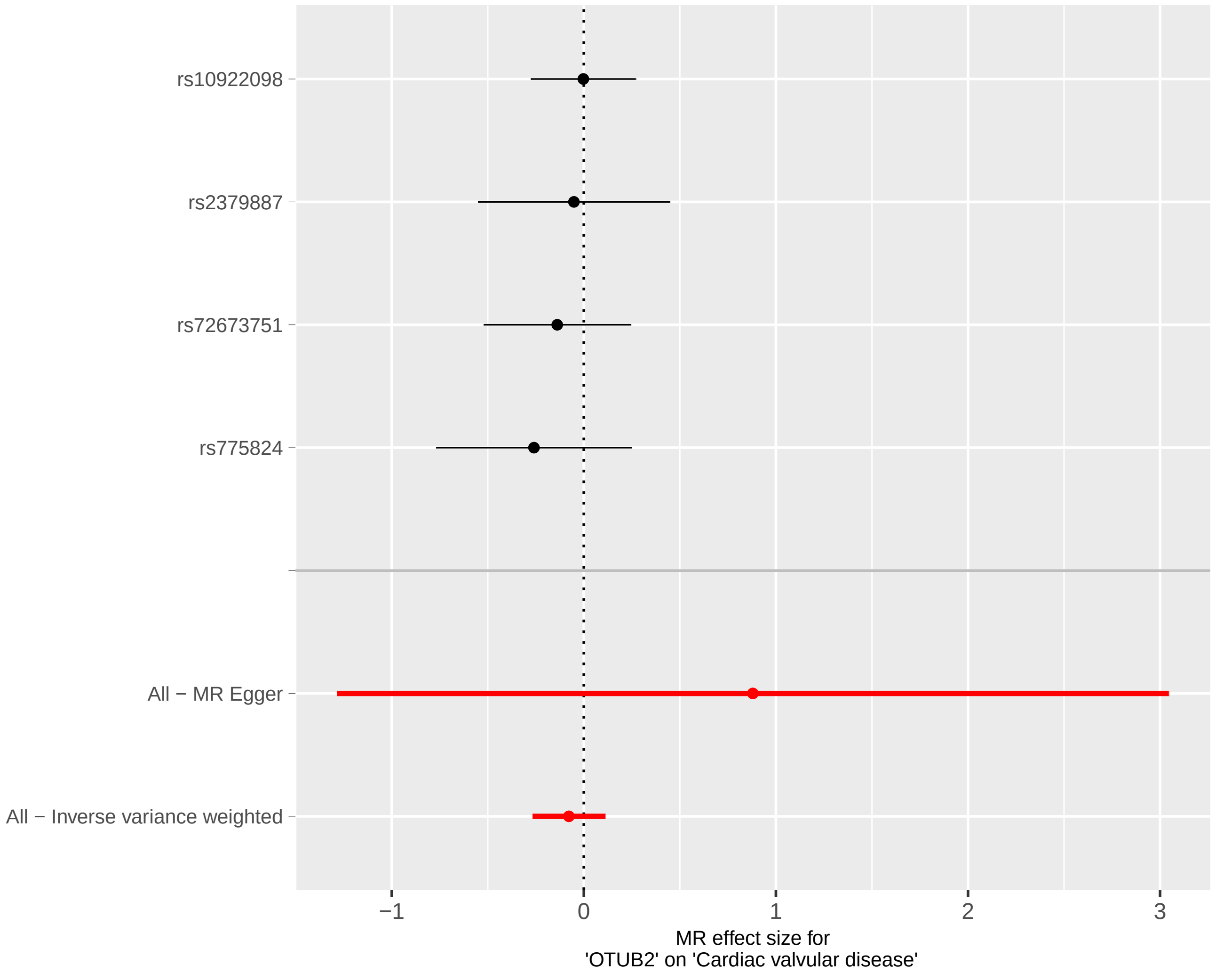

rs10922098
rs2379887
rs72673751
rs775824
All − MR Egger
All − Inverse variance weighted
−1
0
1
2
3
MR effect size for
'OTUB2' on 'Cardiac valvular disease'

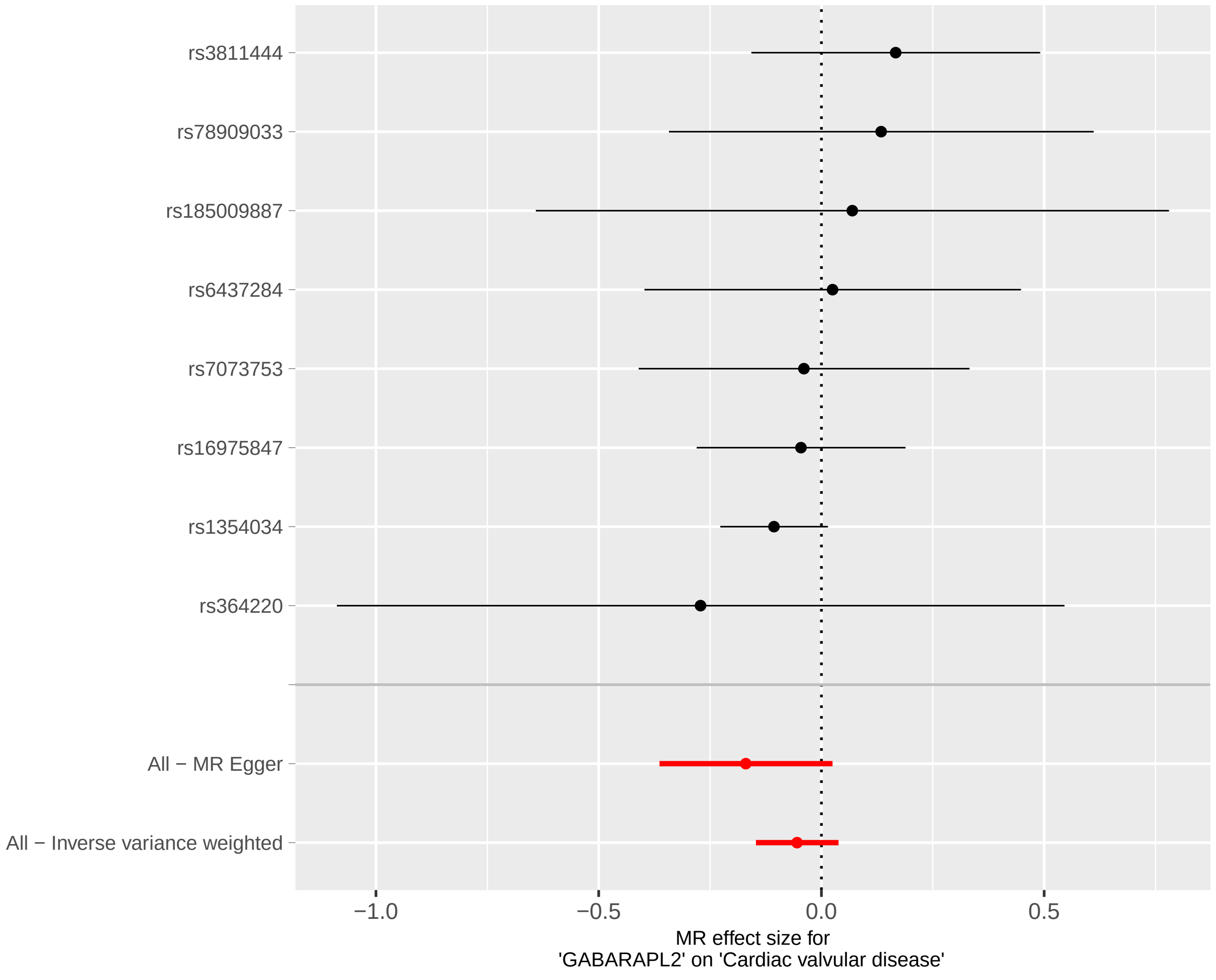
rs3811444
rs78909033
rs185009887
rs6437284
rs7073753
rs16975847
rs1354034
rs364220
All − MR Egger
All − Inverse variance weighted
−1.0
−0.5
0.0
0.5
MR effect size for
'GABARAPL2' on 'Cardiac valvular disease'

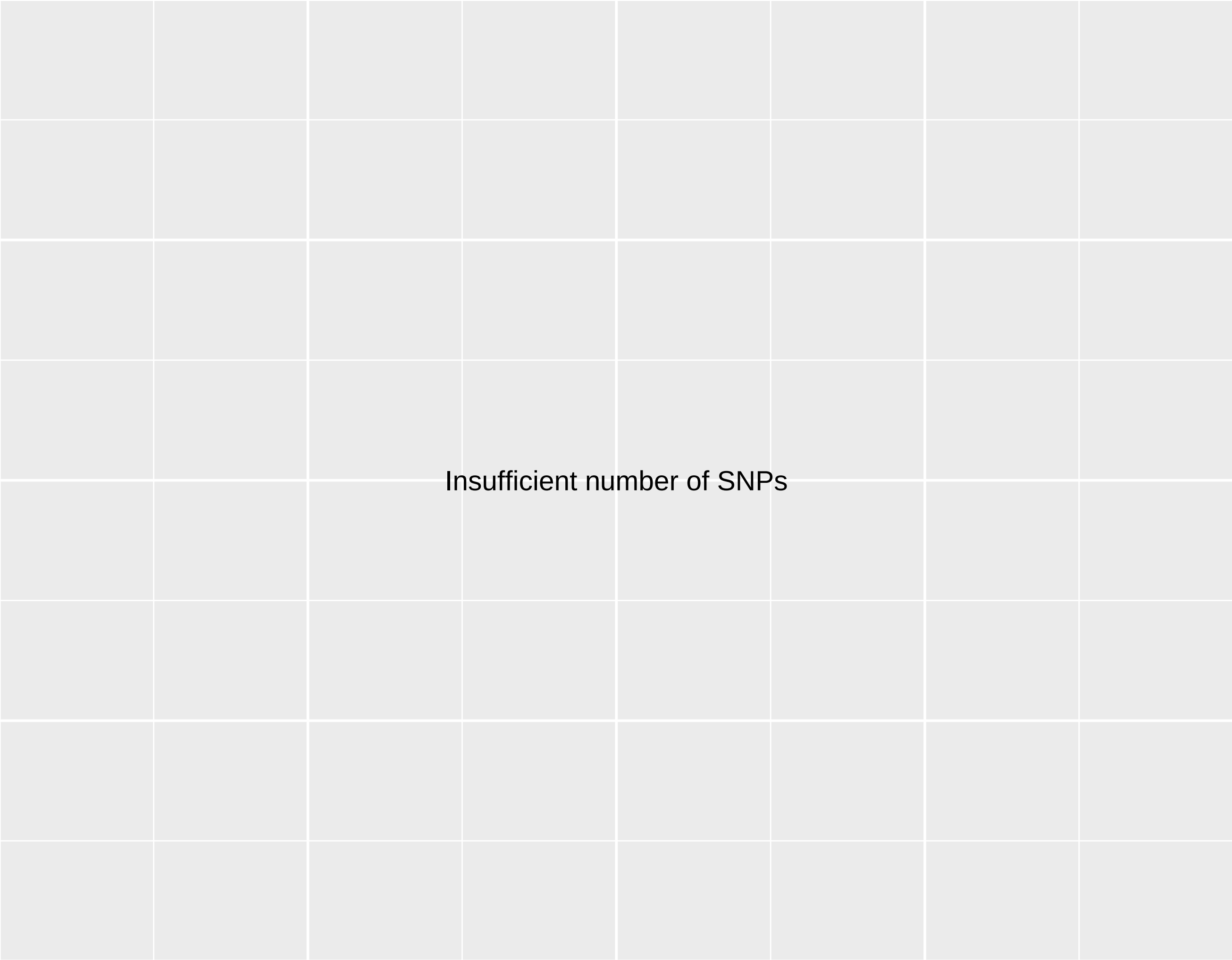
Insufficient number of SNPs

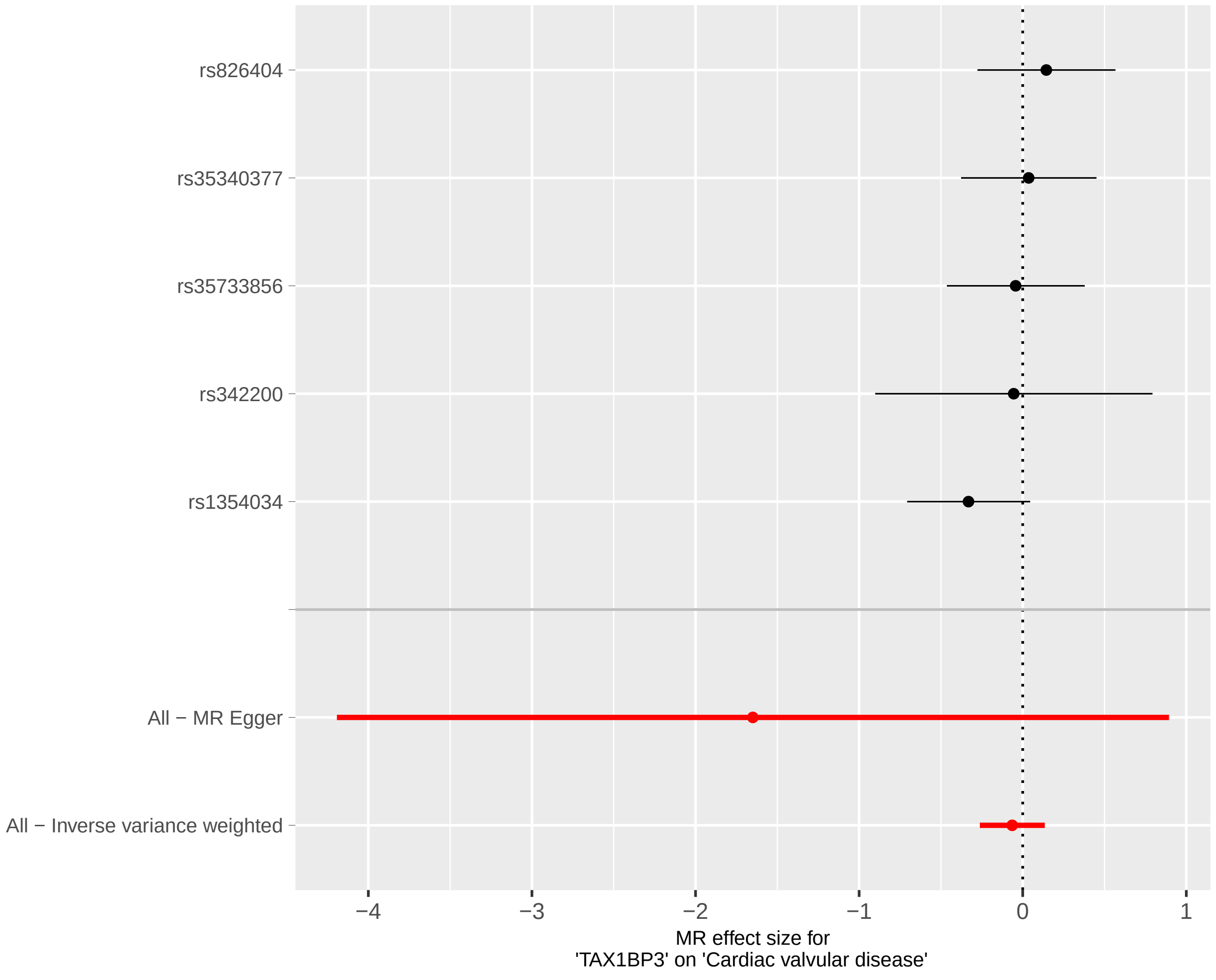
rs826404
rs35340377
rs35733856
rs342200
rs1354034
All − MR Egger
All − Inverse variance weighted
−4
−3
−2
−1
0
1
MR effect size for
'TAX1BP3' on 'Cardiac valvular disease'

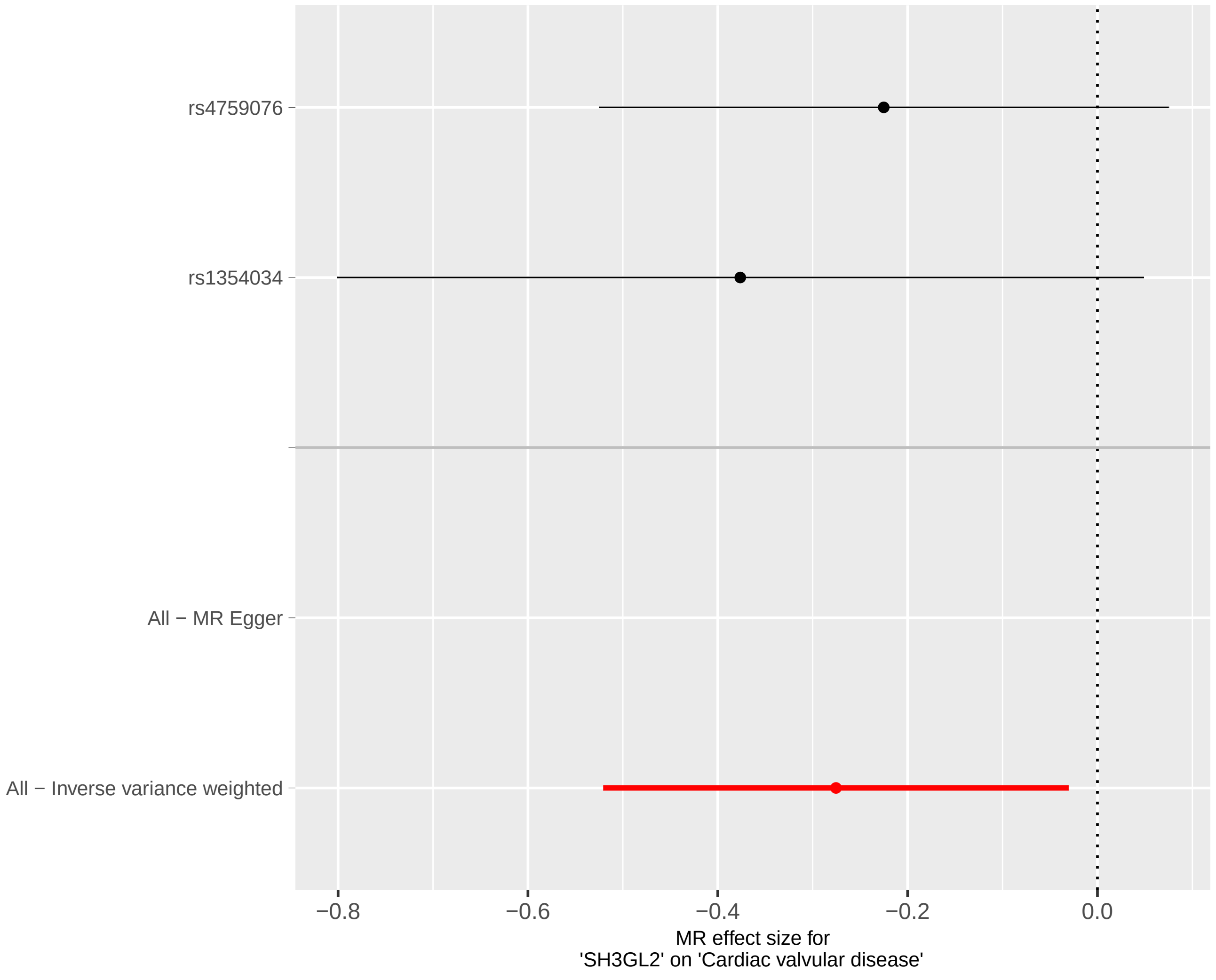
rs4759076
rs1354034
All − MR Egger
All − Inverse variance weighted
−0.8
−0.6
−0.4
−0.2
0.0
MR effect size for
'SH3GL2' on 'Cardiac valvular disease'

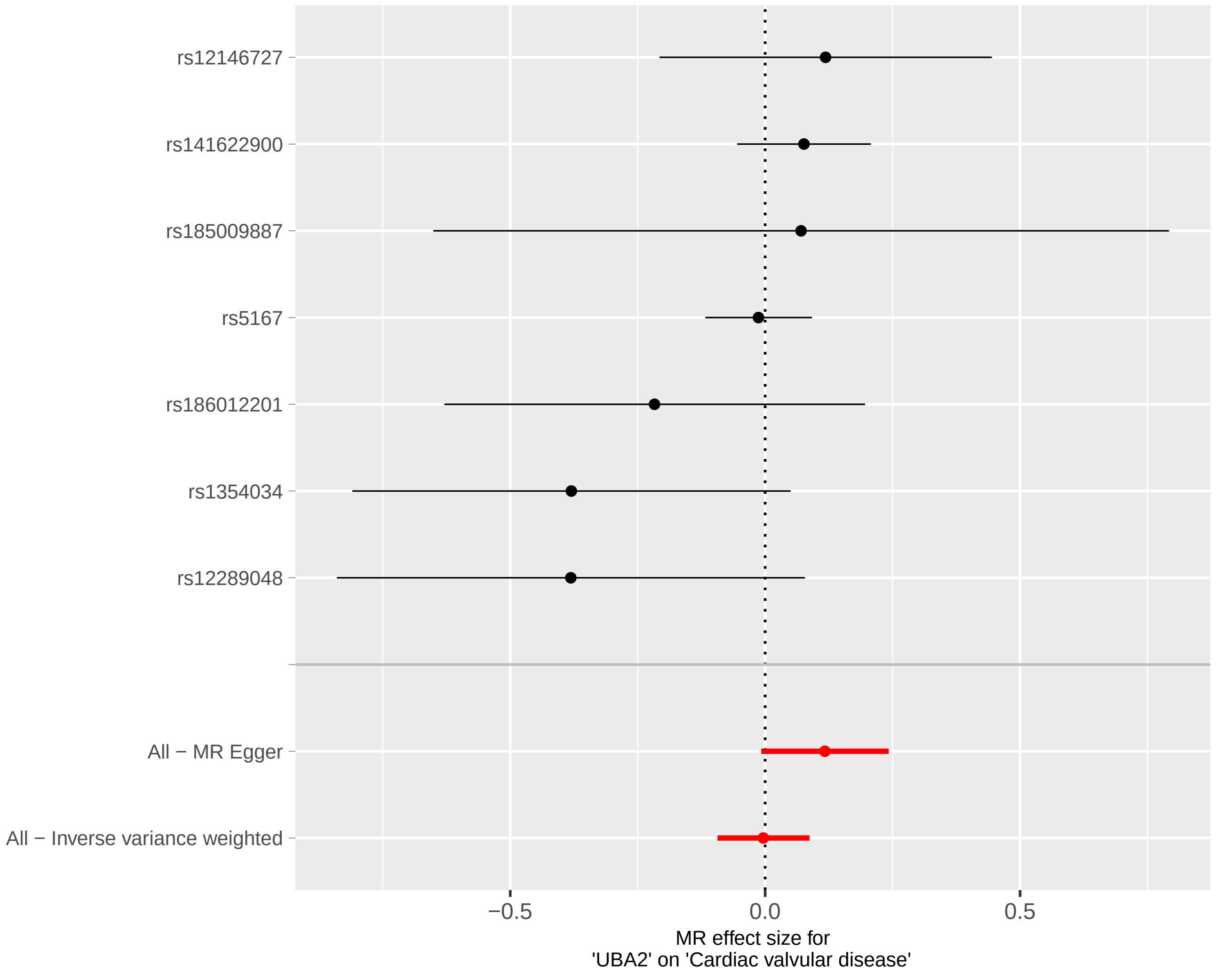

rs12146727
rs141622900
rs185009887
rs5167
rs186012201
rs1354034
rs12289048
All − MR Egger
All − Inverse variance weighted
−0.5
0.0
0.5
MR effect size for
'UBA2' on 'Cardiac valvular disease'

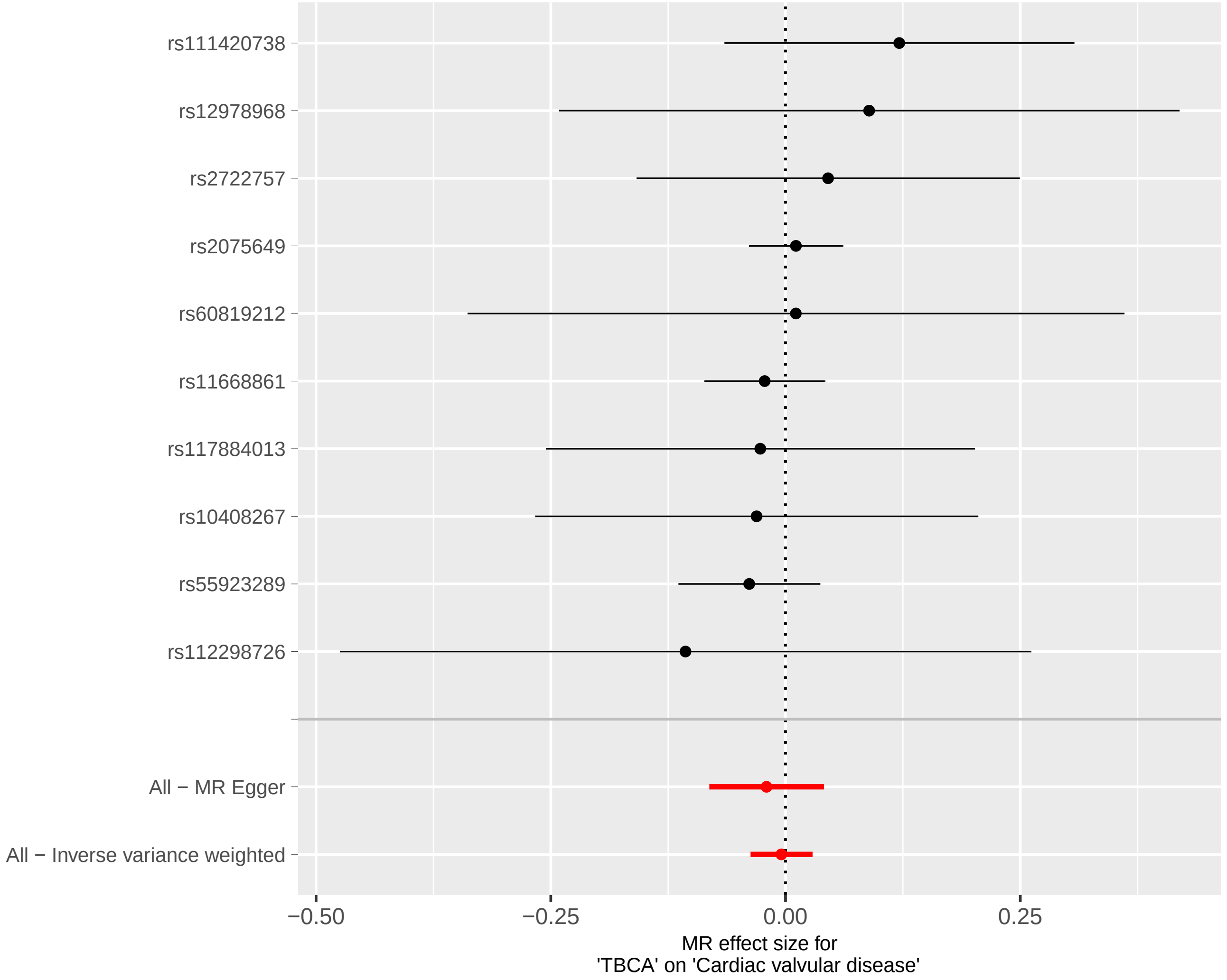

rs111420738
rs12978968
rs2722757
rs2075649
rs60819212
rs11668861
rs117884013
rs10408267
rs55923289
rs112298726
All − MR Egger
All − Inverse variance weighted
−0.50
−0.25
0.00
0.25
MR effect size for
'TBCA' on 'Cardiac valvular disease'

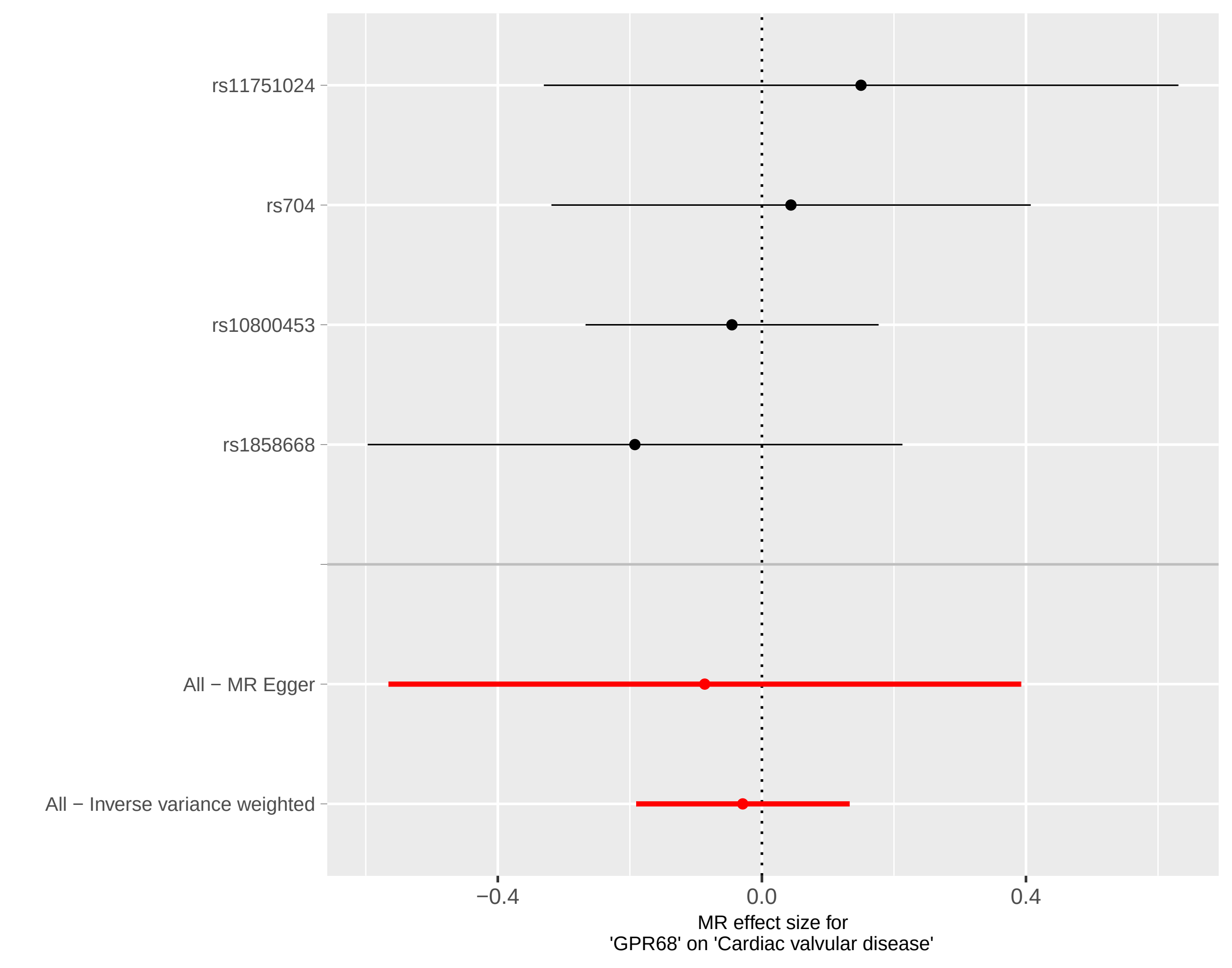
rs11751024
rs704
rs10800453
rs1858668
All − MR Egger
All − Inverse variance weighted
−0.4
0.0
0.4
MR effect size for
'GPR68' on 'Cardiac valvular disease'

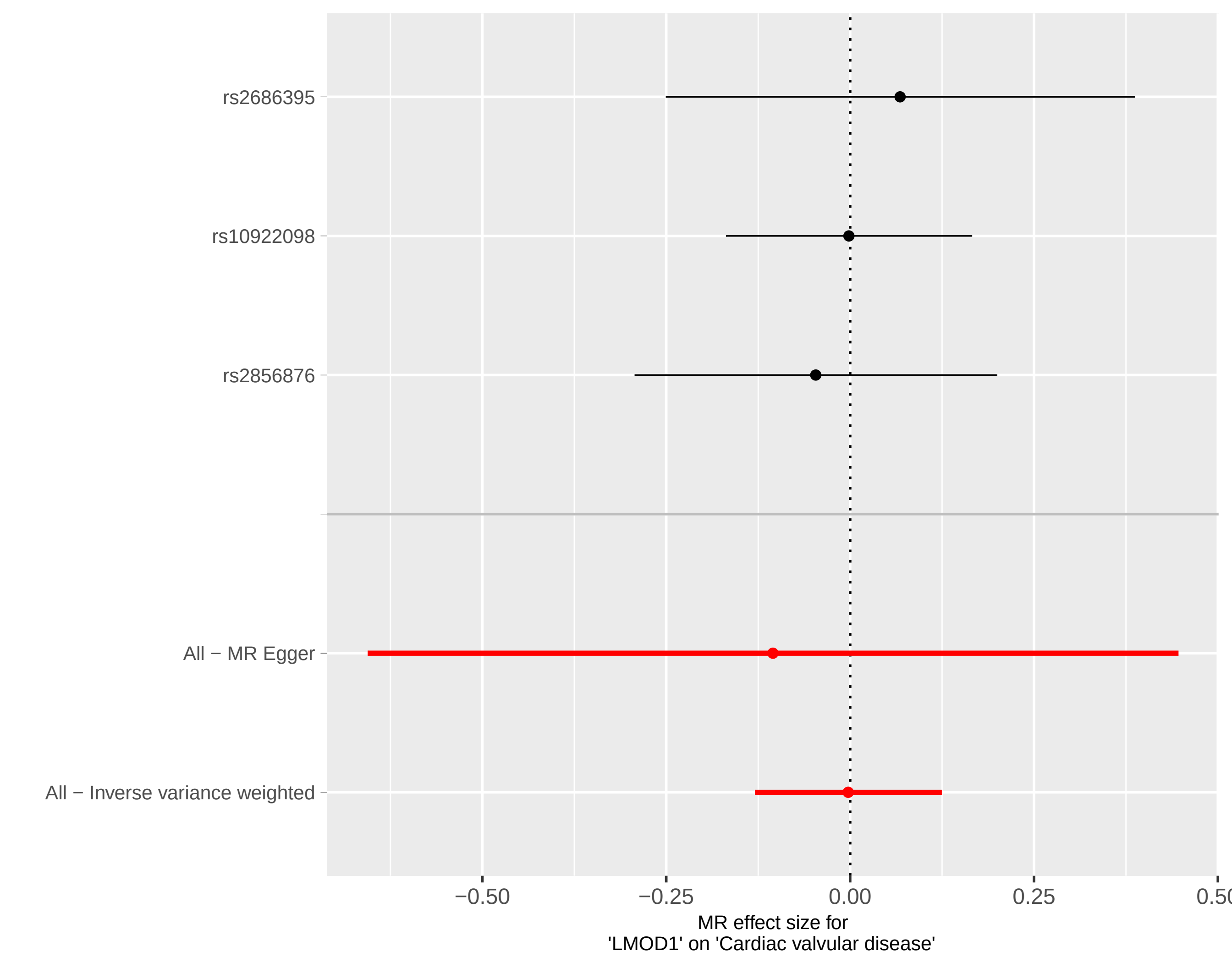
rs2686395
rs10922098
rs2856876
All − MR Egger
All − Inverse variance weighted
−0.50
−0.25
0.00
0.25
MR effect size for
'LMOD1' on 'Cardiac valvular disease'

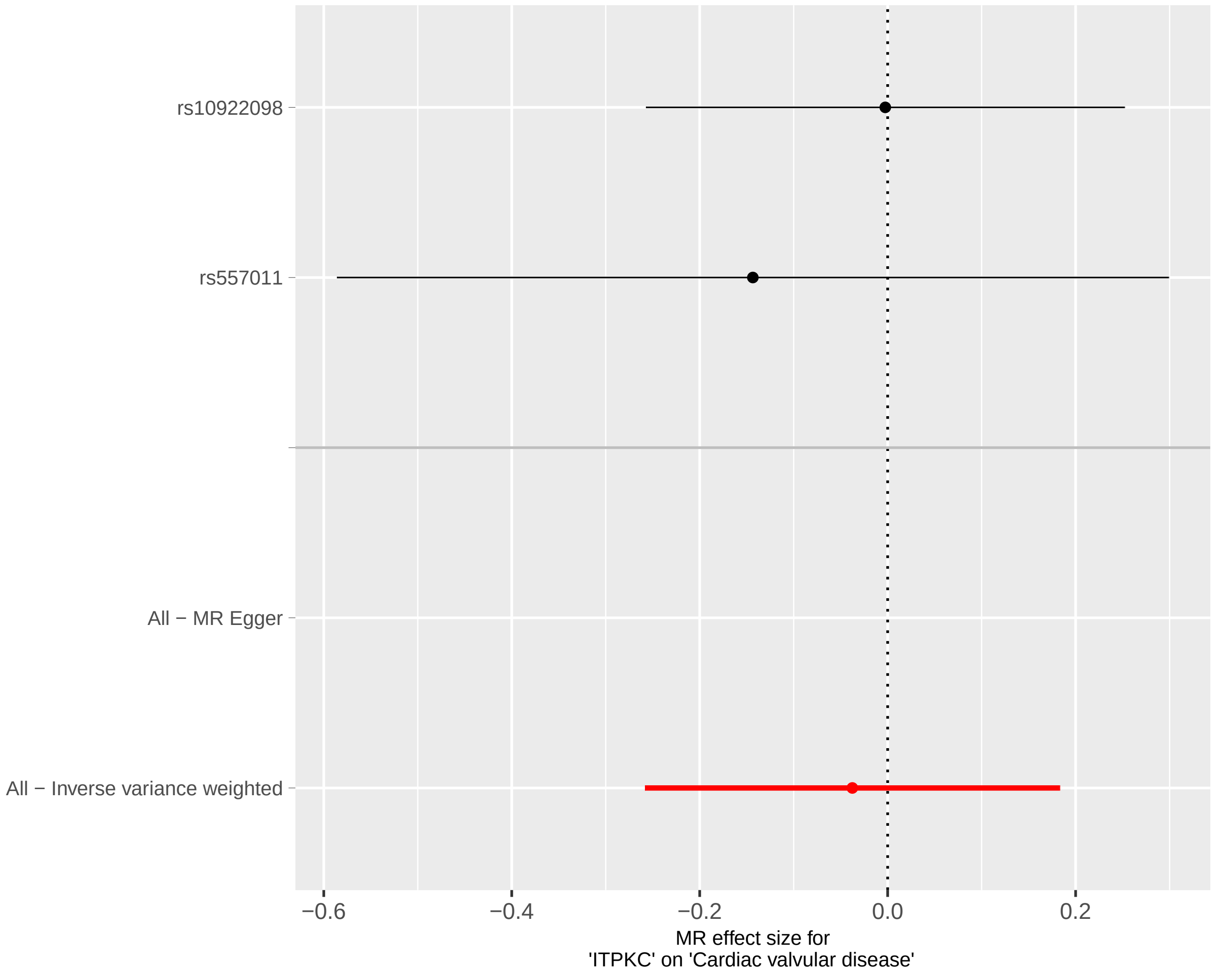
rs10922098
rs557011
All − MR Egger
All − Inverse variance weighted
−0.6
−0.4
−0.2
0.0
0.2
MR effect size for
'ITPKC' on 'Cardiac valvular disease'

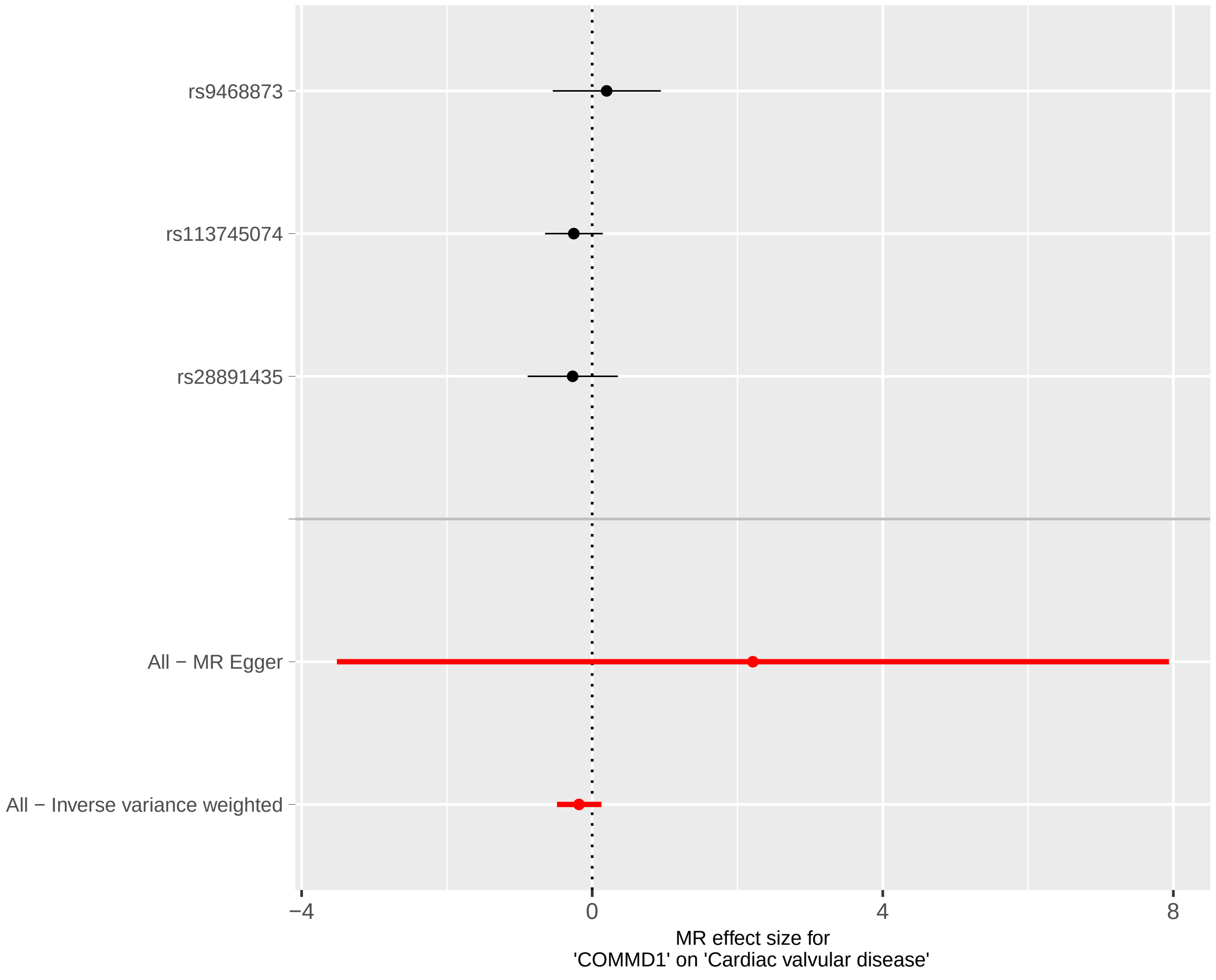
rs9468873
rs113745074
rs28891435
All − MR Egger
All − Inverse variance weighted
−4
0
4
8
MR effect size for
'COMMD1' on 'Cardiac valvular disease'

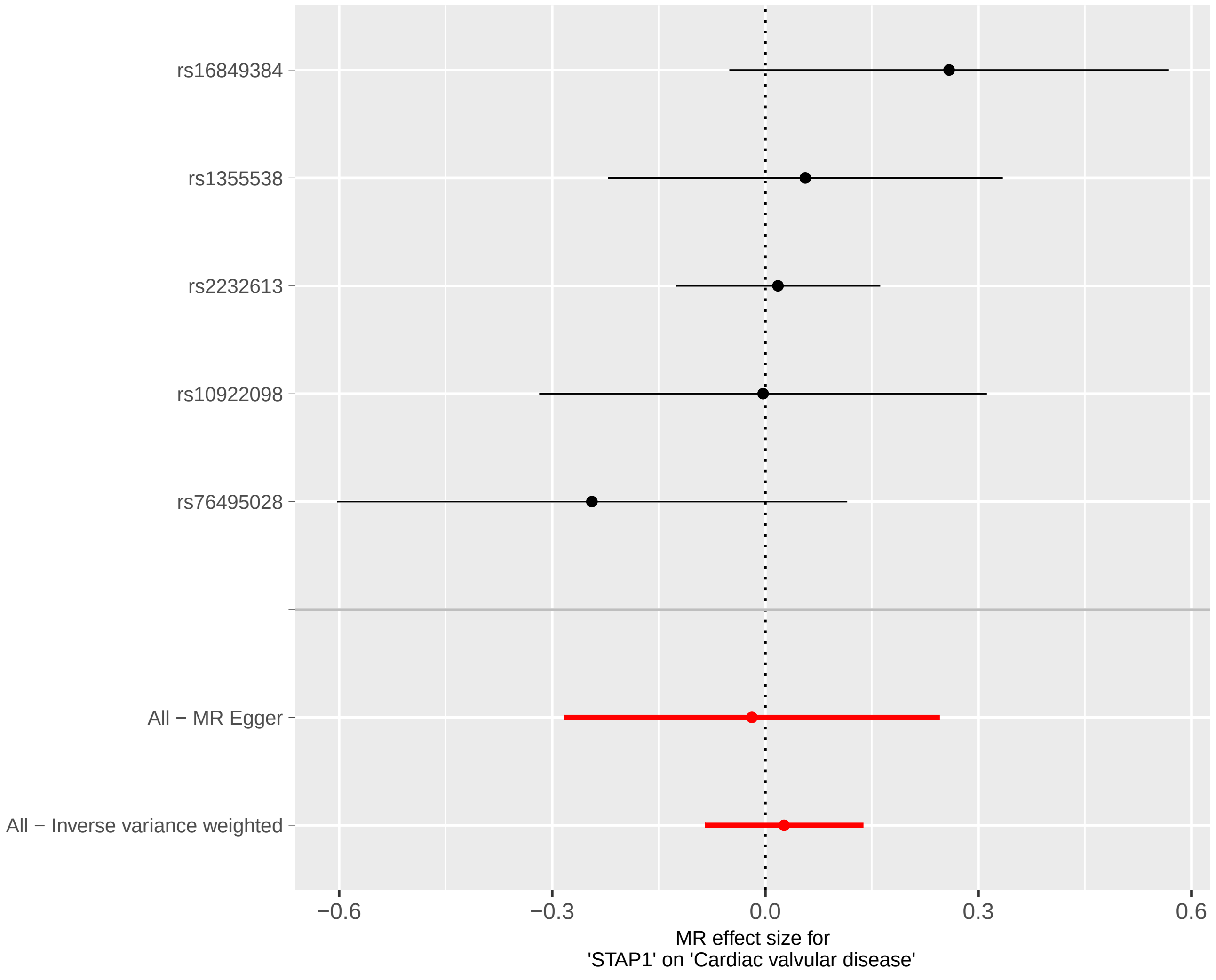
rs16849384
rs1355538
rs2232613
rs10922098
rs76495028
All − MR Egger
All − Inverse variance weighted
−0.6
−0.3
0.0
0.3
0.6
MR effect size for
'STAP1' on 'Cardiac valvular disease'

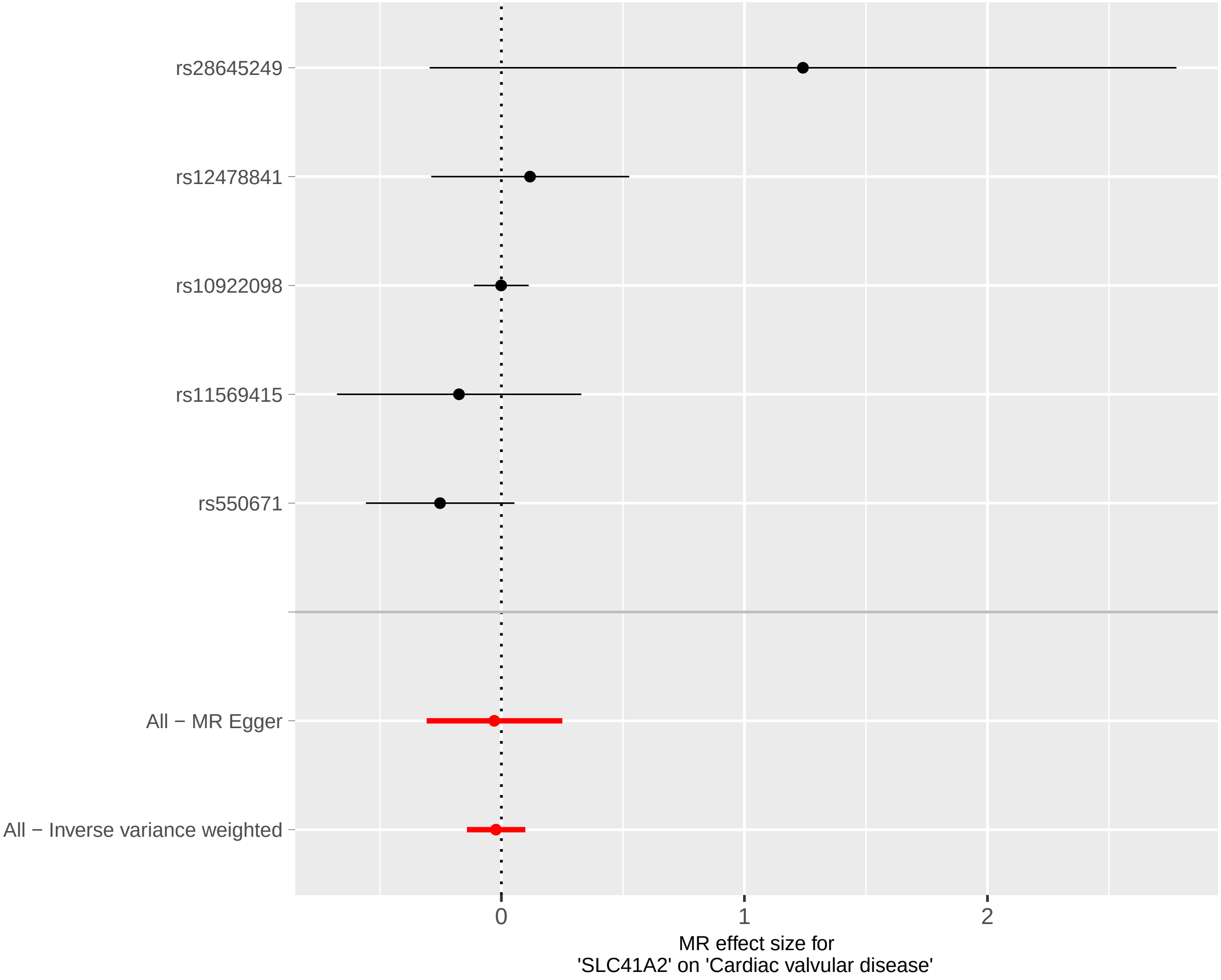
rs28645249
rs12478841
rs10922098
rs11569415
rs550671
All − MR Egger
All − Inverse variance weighted
0
1
2
MR effect size for
'SLC41A2' on 'Cardiac valvular disease'

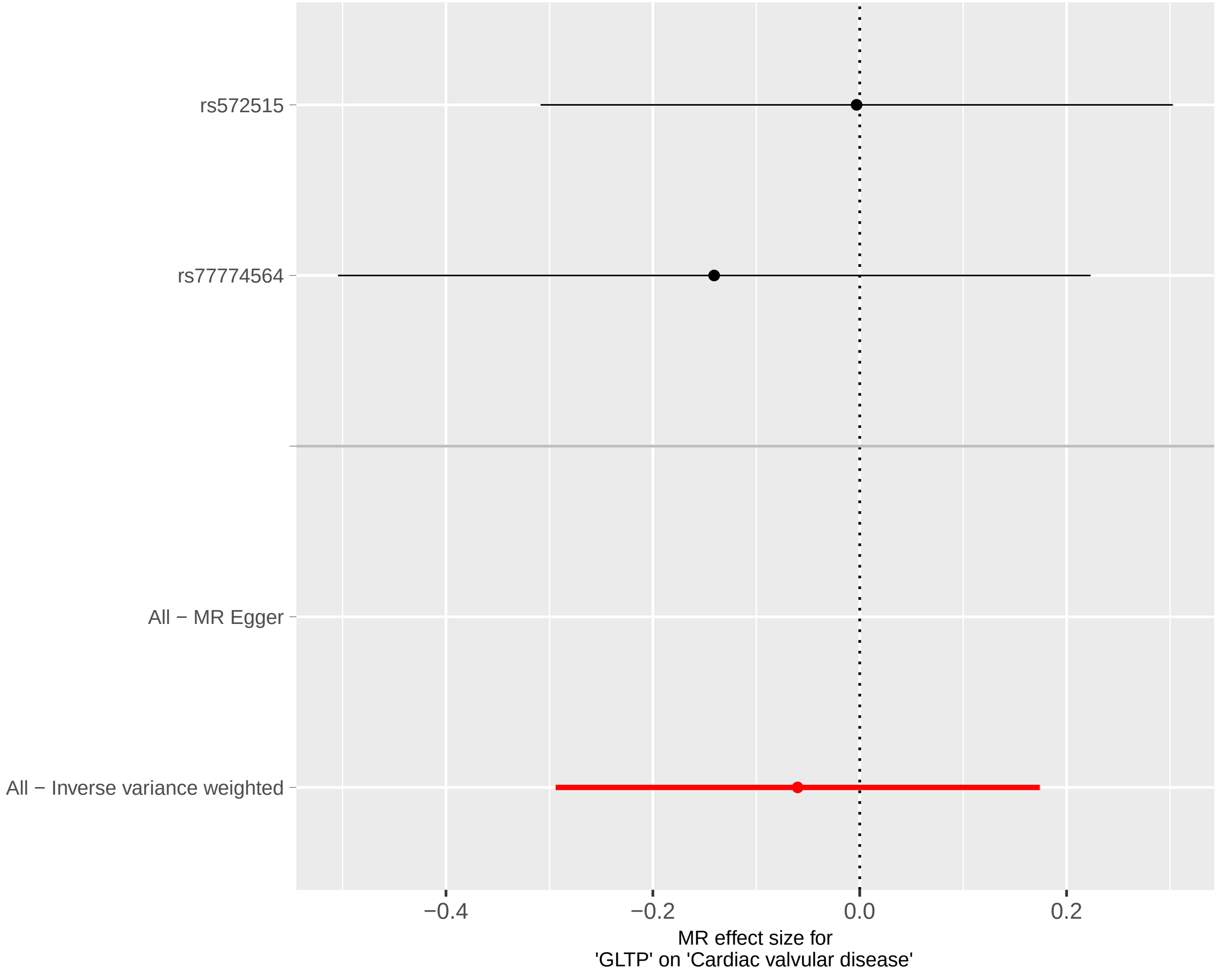

rs572515
rs77774564
All − MR Egger
All − Inverse variance weighted
−0.4
−0.2
0.0
0.2
MR effect size for
'GLTP' on 'Cardiac valvular disease'

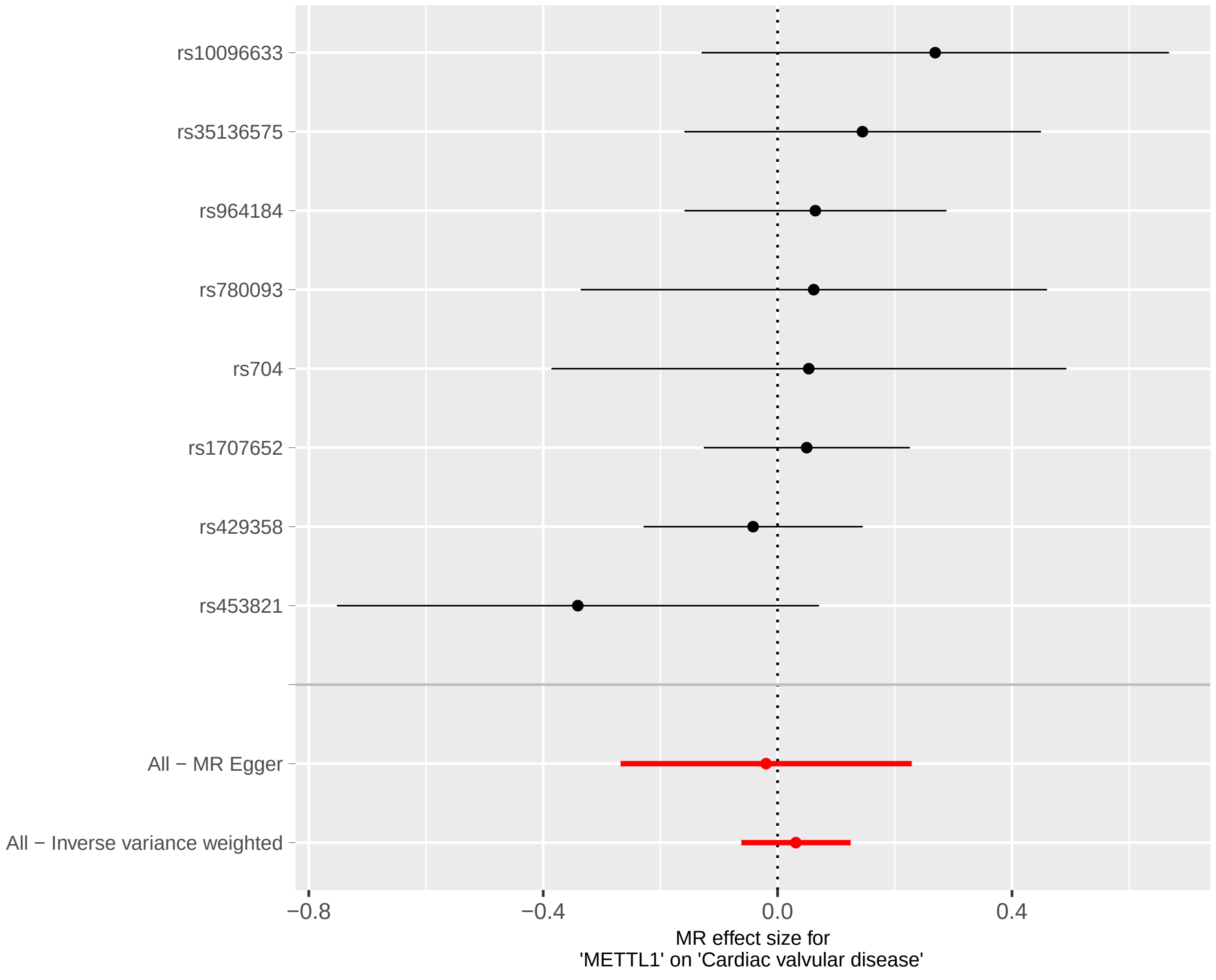

rs10096633
rs35136575
rs964184
rs780093
rs704
rs1707652
rs429358
rs453821
All − MR Egger
All − Inverse variance weighted
−0.8
−0.4
0.0
0.4
MR effect size for
'METTL1' on 'Cardiac valvular disease'

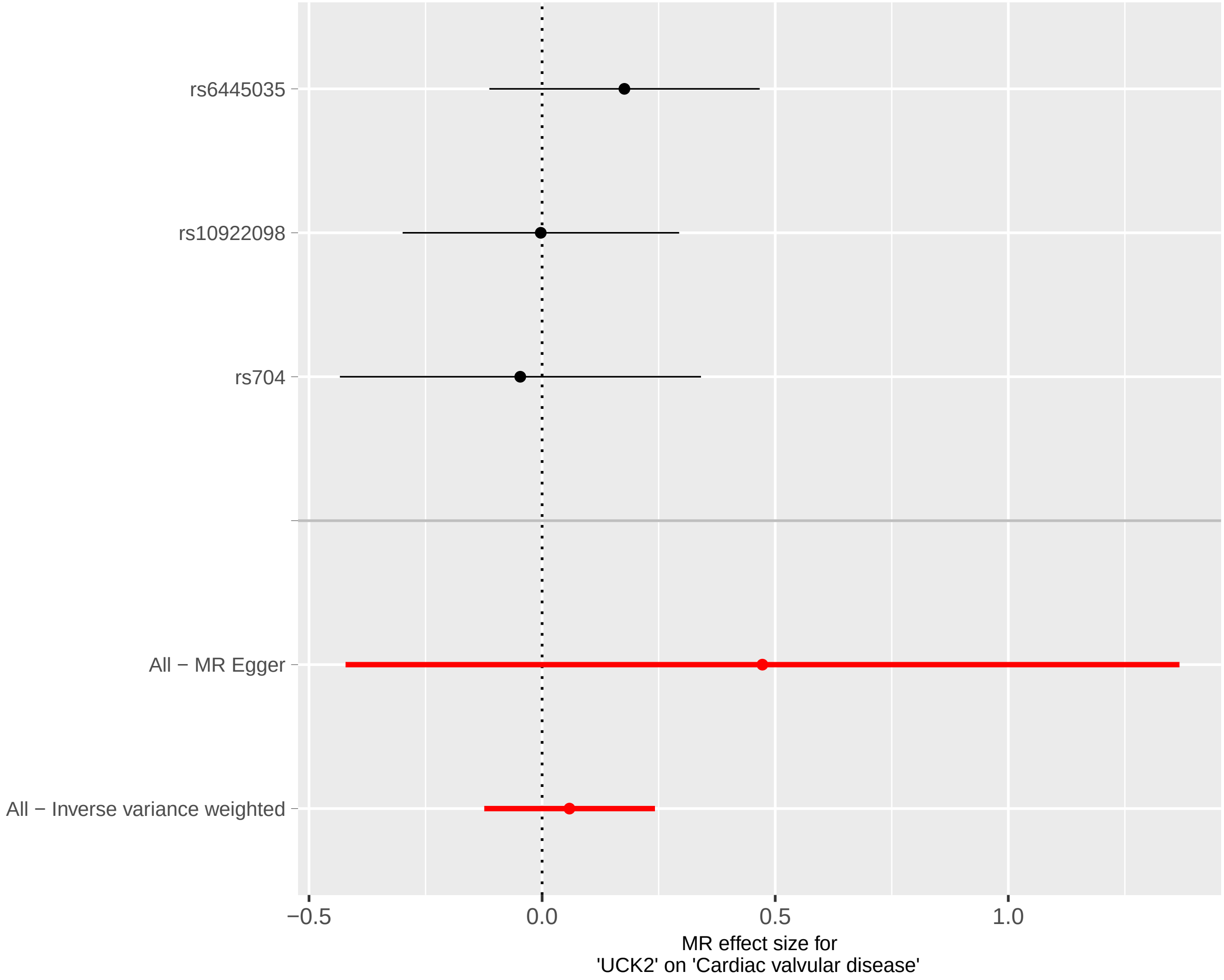

rs6445035
rs10922098
rs704
All − MR Egger
All − Inverse variance weighted
−0.5
0.0
0.5
1.0
MR effect size for
'UCK2' on 'Cardiac valvular disease'

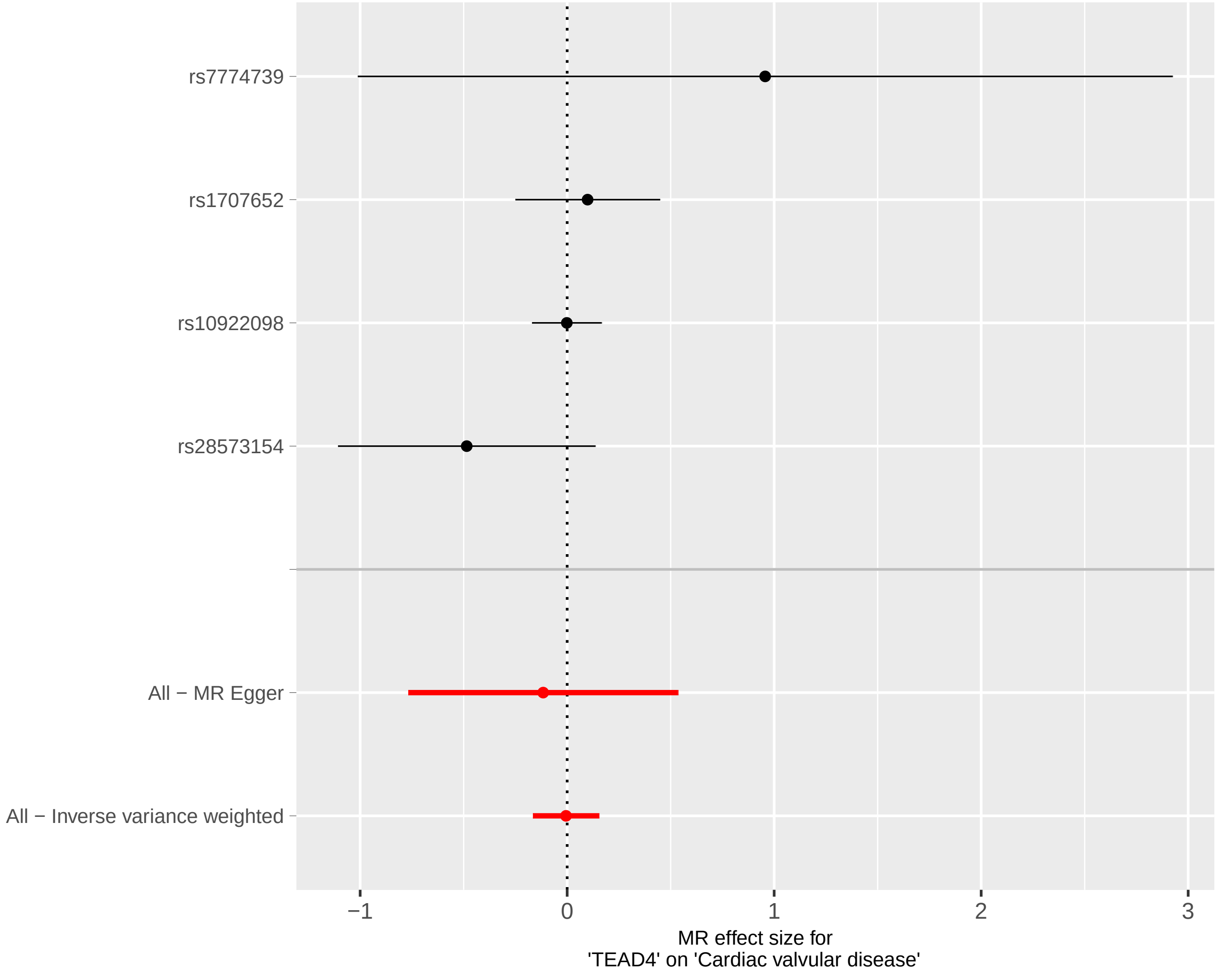
rs7774739
rs1707652
rs10922098
rs28573154
All − MR Egger
All − Inverse variance weighted
−1
0
1
2
3
MR effect size for
'TEAD4' on 'Cardiac valvular disease'

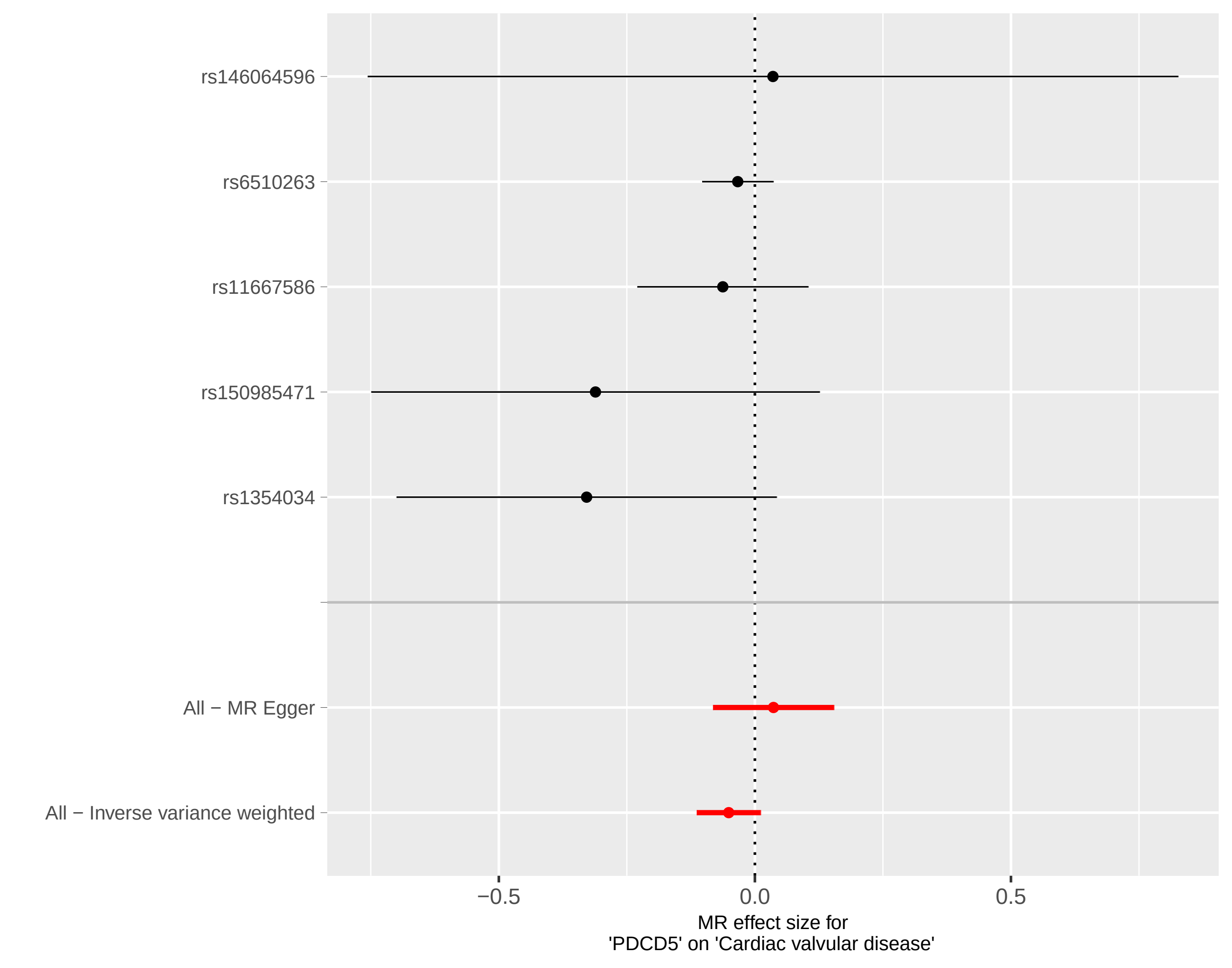

rs146064596
rs6510263
rs11667586
rs150985471
rs1354034
All − MR Egger
All − Inverse variance weighted
−0.5
0.0
0.5
MR effect size for
'PDCD5' on 'Cardiac valvular disease'

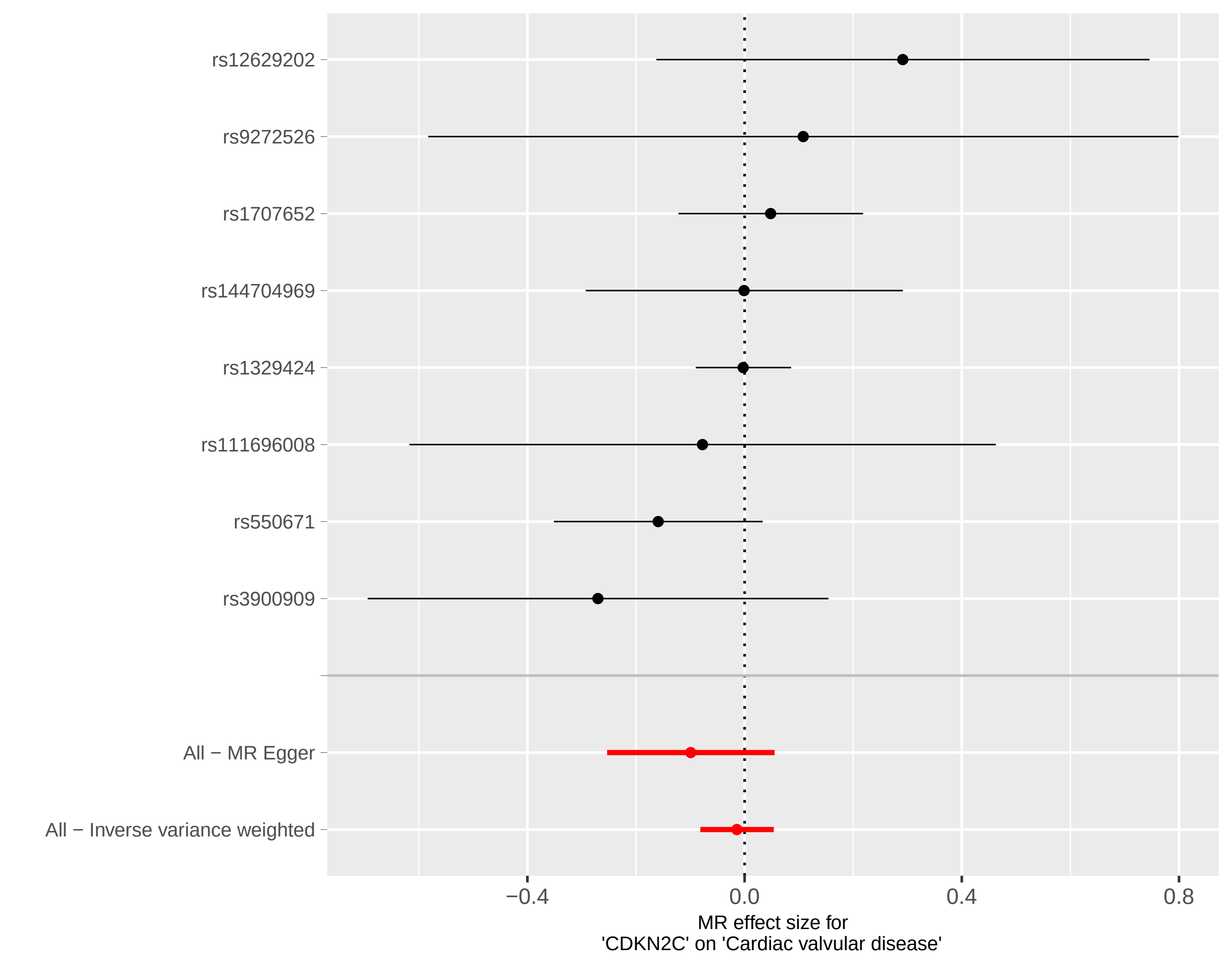
rs12629202
rs9272526
rs1707652
rs144704969
rs1329424
rs111696008
rs550671
rs3900909
All − MR Egger
All − Inverse variance weighted
−0.4
0.0
0.4
0.8
MR effect size for
'CDKN2C' on 'Cardiac valvular disease'

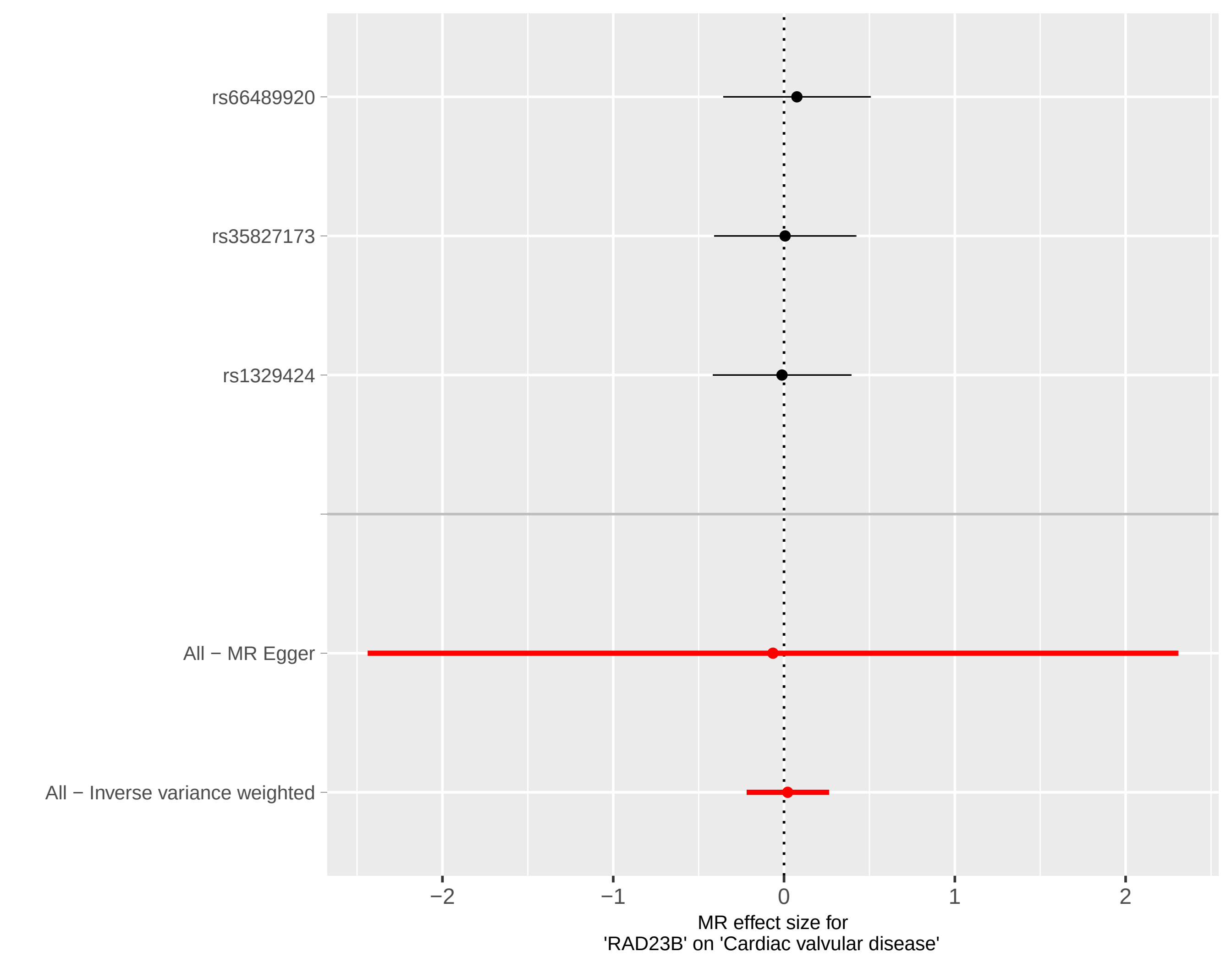
rs66489920
rs35827173
rs1329424
All − MR Egger
All − Inverse variance weighted
−2
−1
0
1
2
MR effect size for
'RAD23B' on 'Cardiac valvular disease'

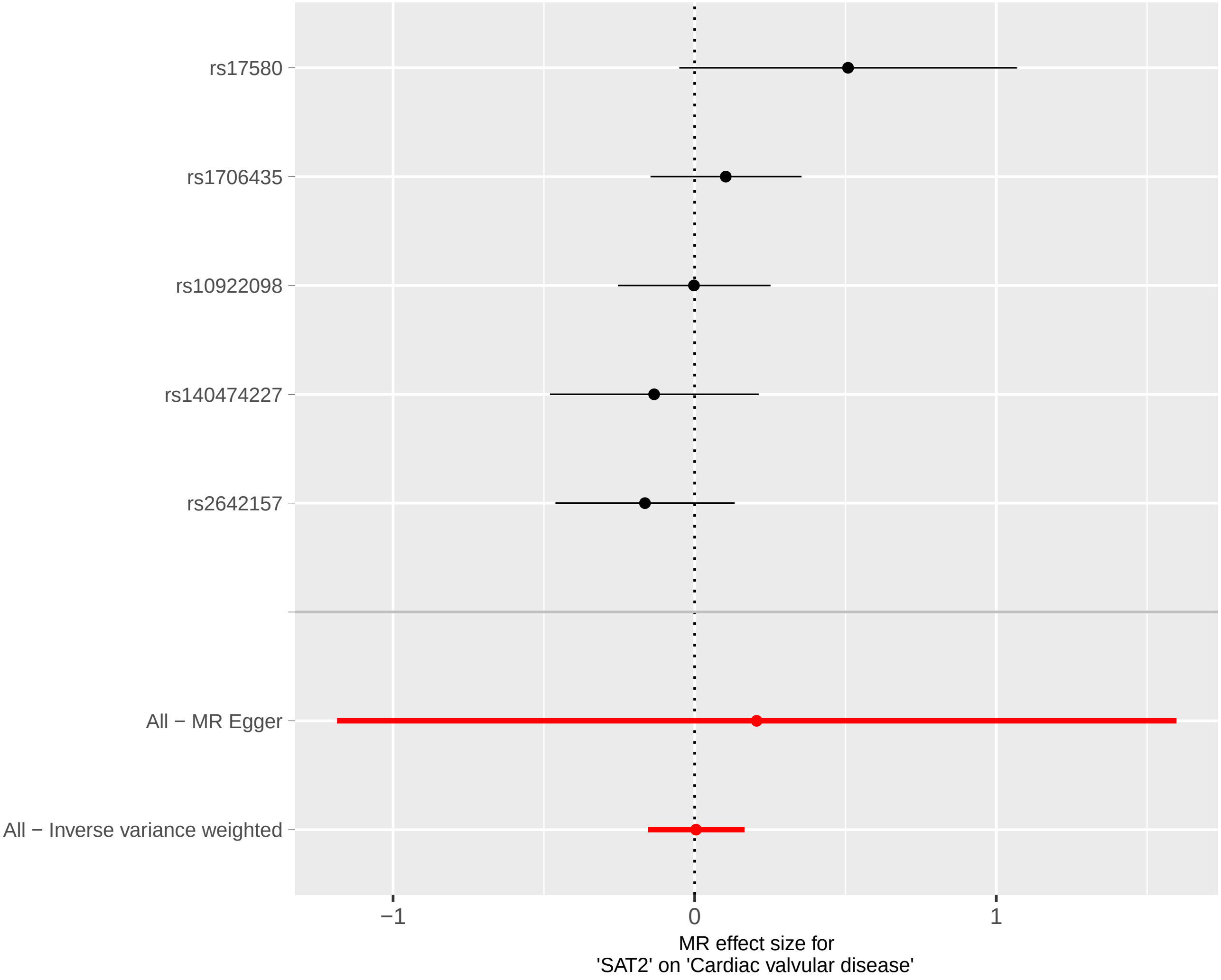
rs17580
rs1706435
rs10922098
rs140474227
rs2642157
All − MR Egger
All − Inverse variance weighted
−1
0
1
MR effect size for
'SAT2' on 'Cardiac valvular disease'

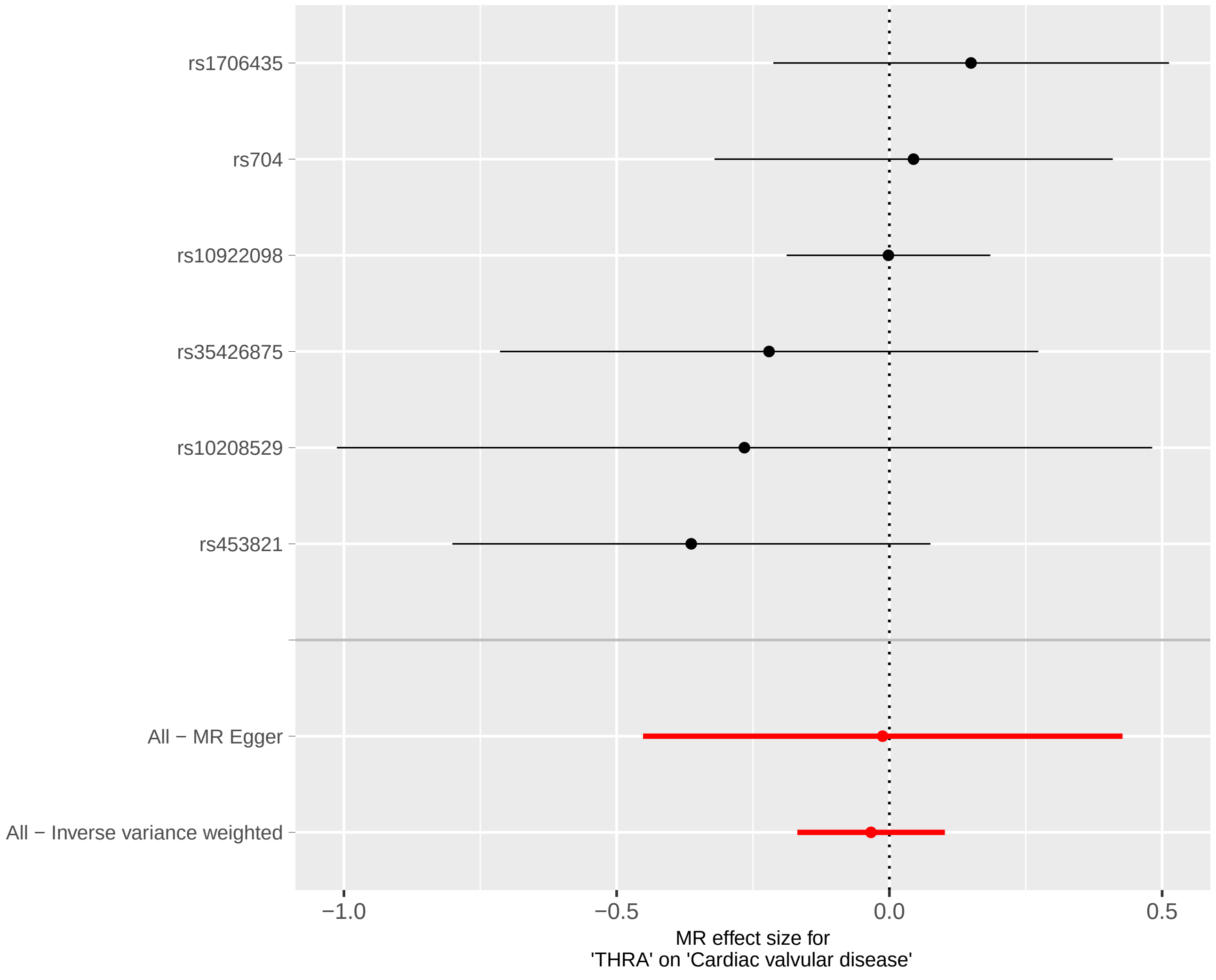
rs1706435
rs704
rs10922098
rs35426875
rs10208529
rs453821
All − MR Egger
All − Inverse variance weighted
−1.0
−0.5
0.0
0.5
MR effect size for
'THRA' on 'Cardiac valvular disease'

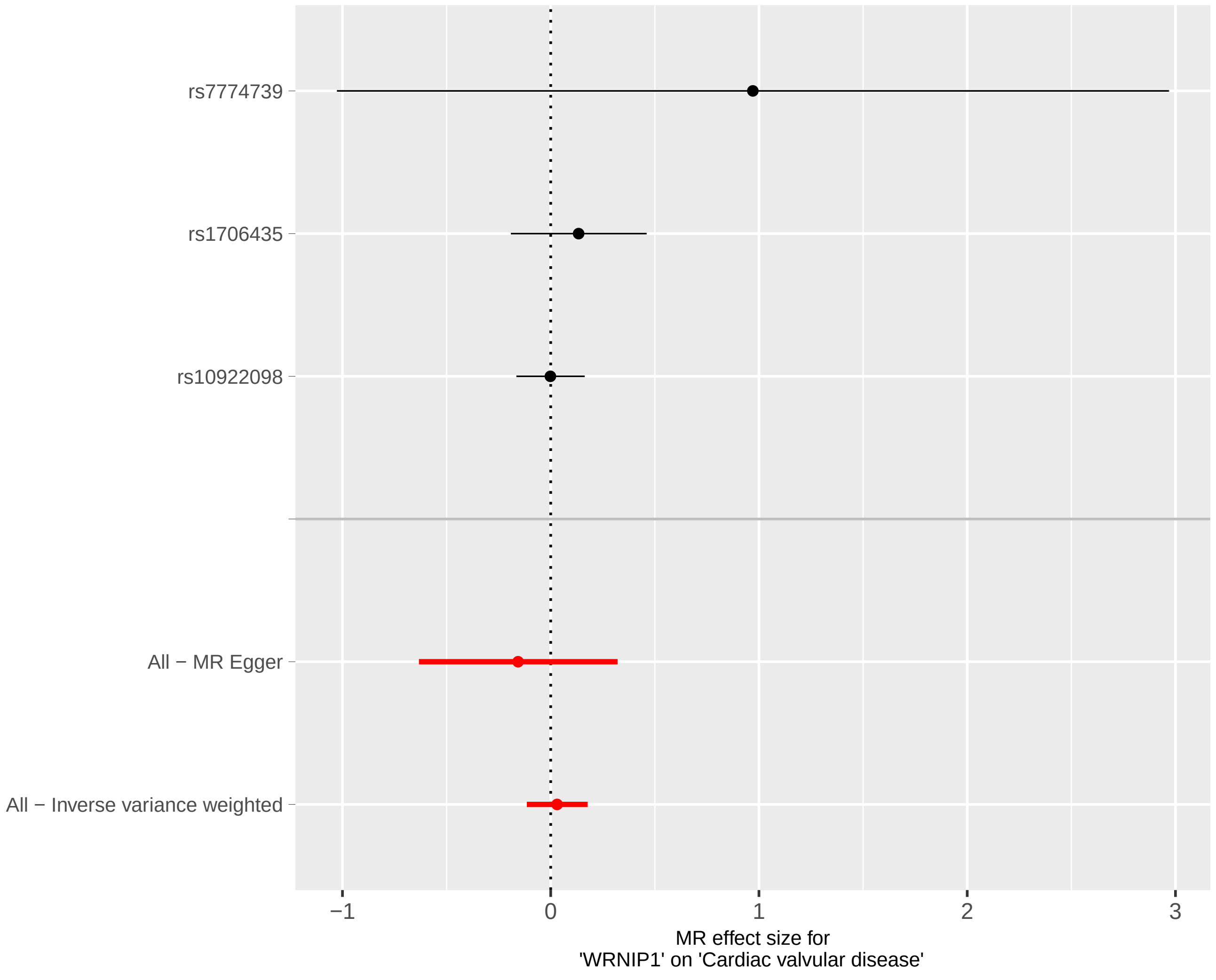
rs7774739
rs1706435
rs10922098
All − MR Egger
All − Inverse variance weighted
−1
0
1
2
3
MR effect size for
'WRNIP1' on 'Cardiac valvular disease'

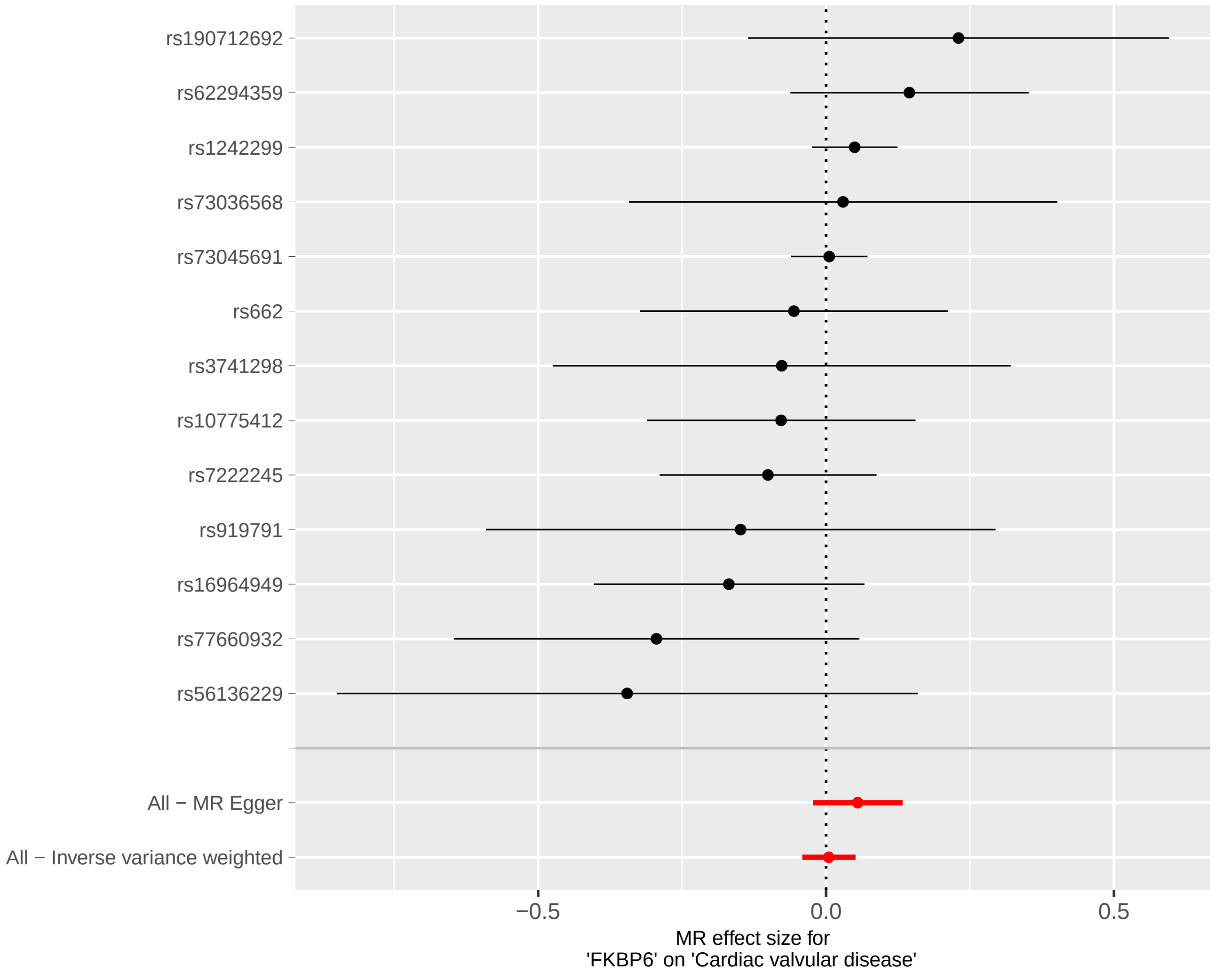
rs190712692
rs62294359
rs1242299
rs73036568
rs73045691
rs662
rs3741298
rs10775412
rs7222245
rs919791
rs16964949
rs77660932
rs56136229
All − MR Egger
All − Inverse variance weighted
−0.5
0.0
0.5
MR effect size for
'FKBP6' on 'Cardiac valvular disease'

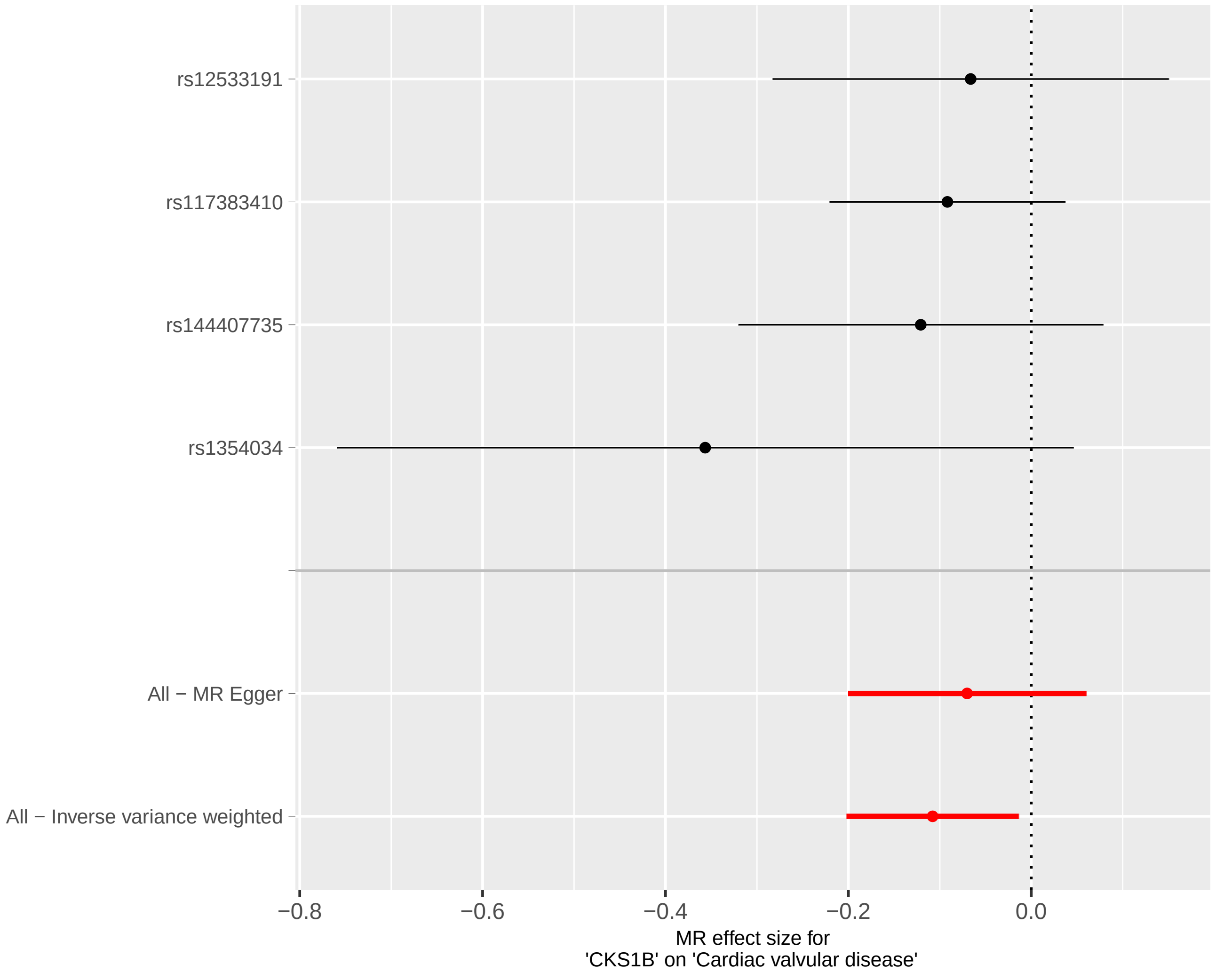

rs12533191
rs117383410
rs144407735
rs1354034
All − MR Egger
All − Inverse variance weighted
−0.8
−0.6
−0.4
−0.2
0.0
MR effect size for
'CKS1B' on 'Cardiac valvular disease'

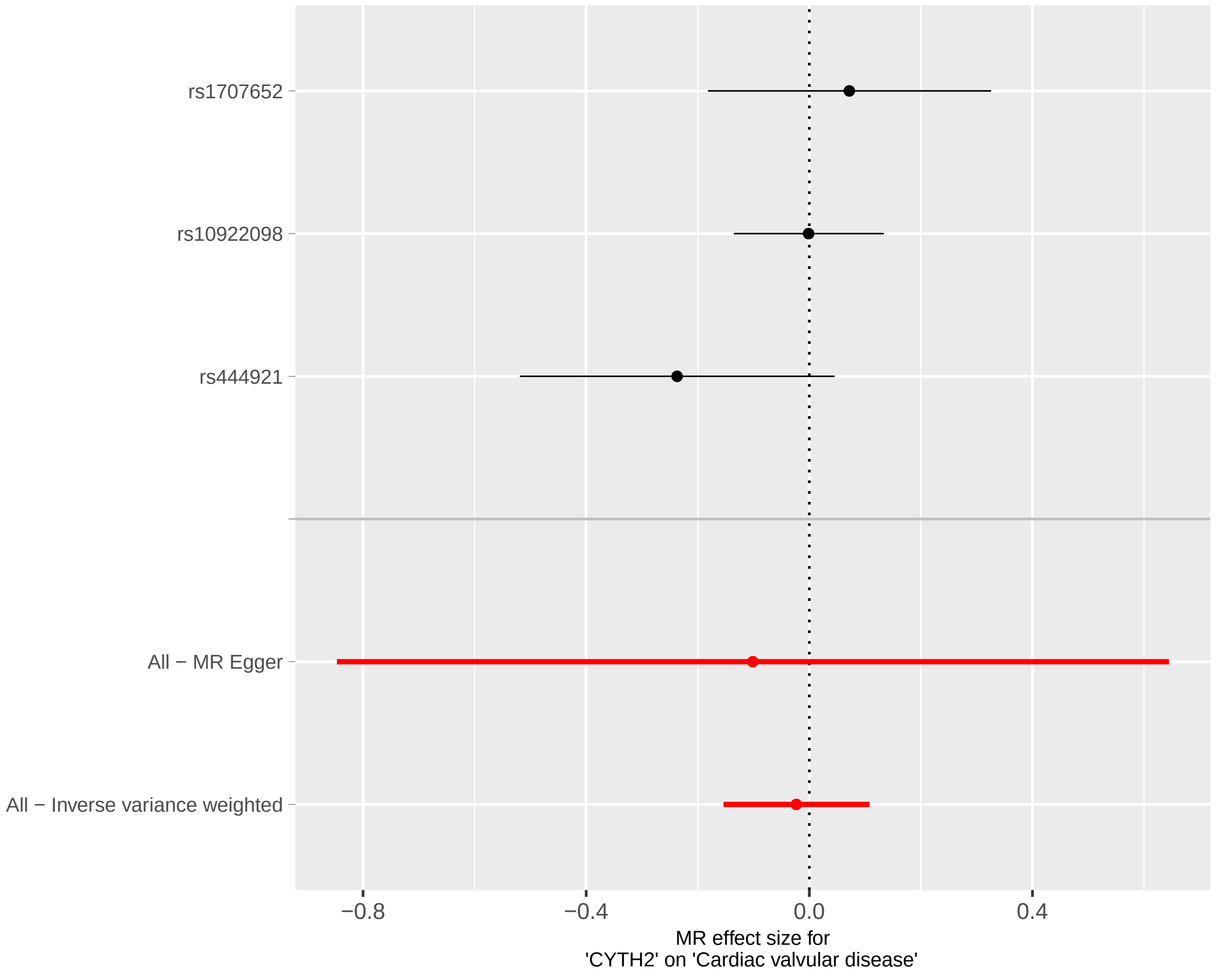
rs1707652
rs10922098
rs444921
All − MR Egger
All − Inverse variance weighted
−0.8
−0.4
0.0
0.4
MR effect size for
'CYTH2' on 'Cardiac valvular disease'

Insufficient number of SNPs

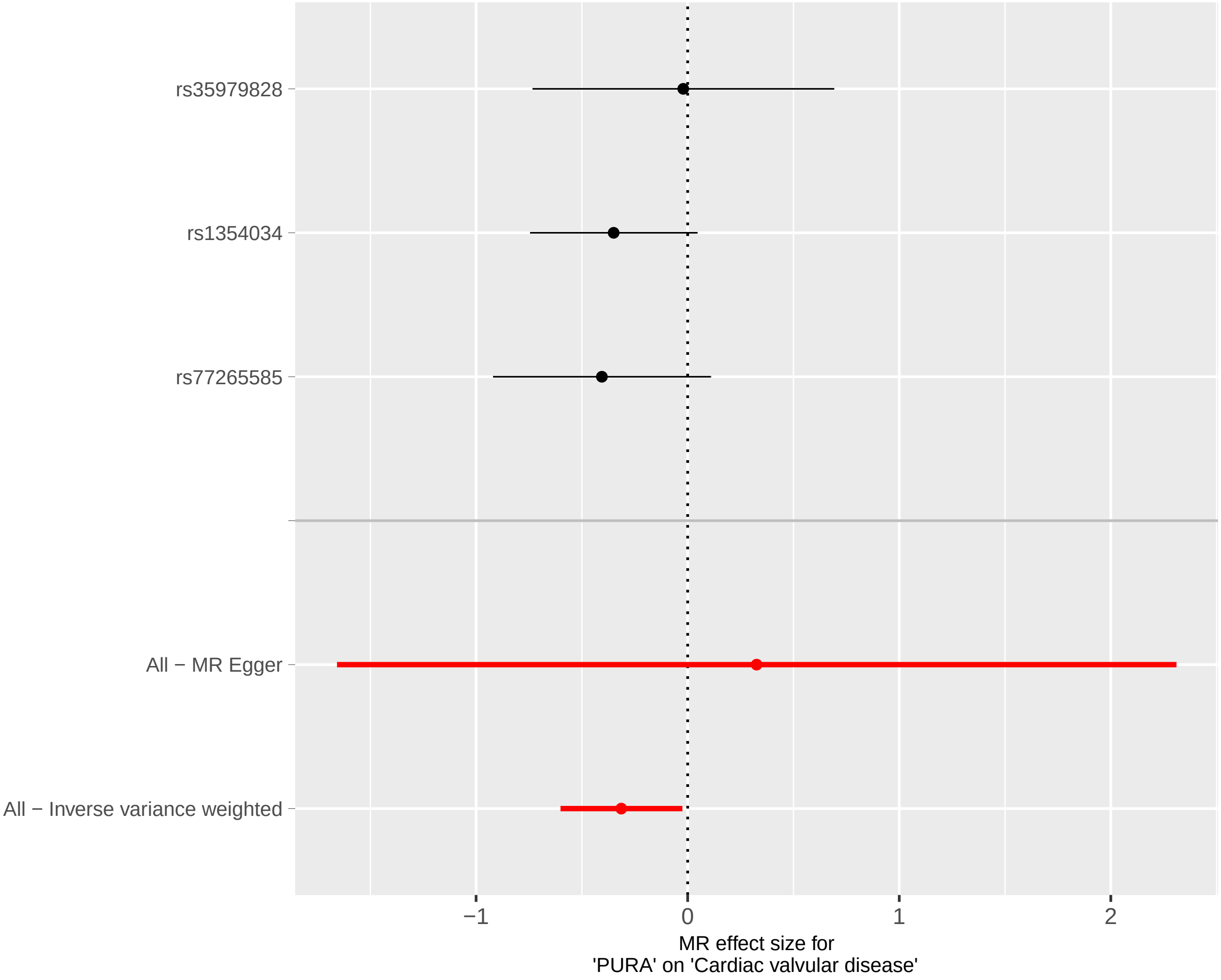
rs35979828
rs1354034
rs77265585
All − MR Egger
All − Inverse variance weighted
−1
0
1
2
MR effect size for
'PURA' on 'Cardiac valvular disease'

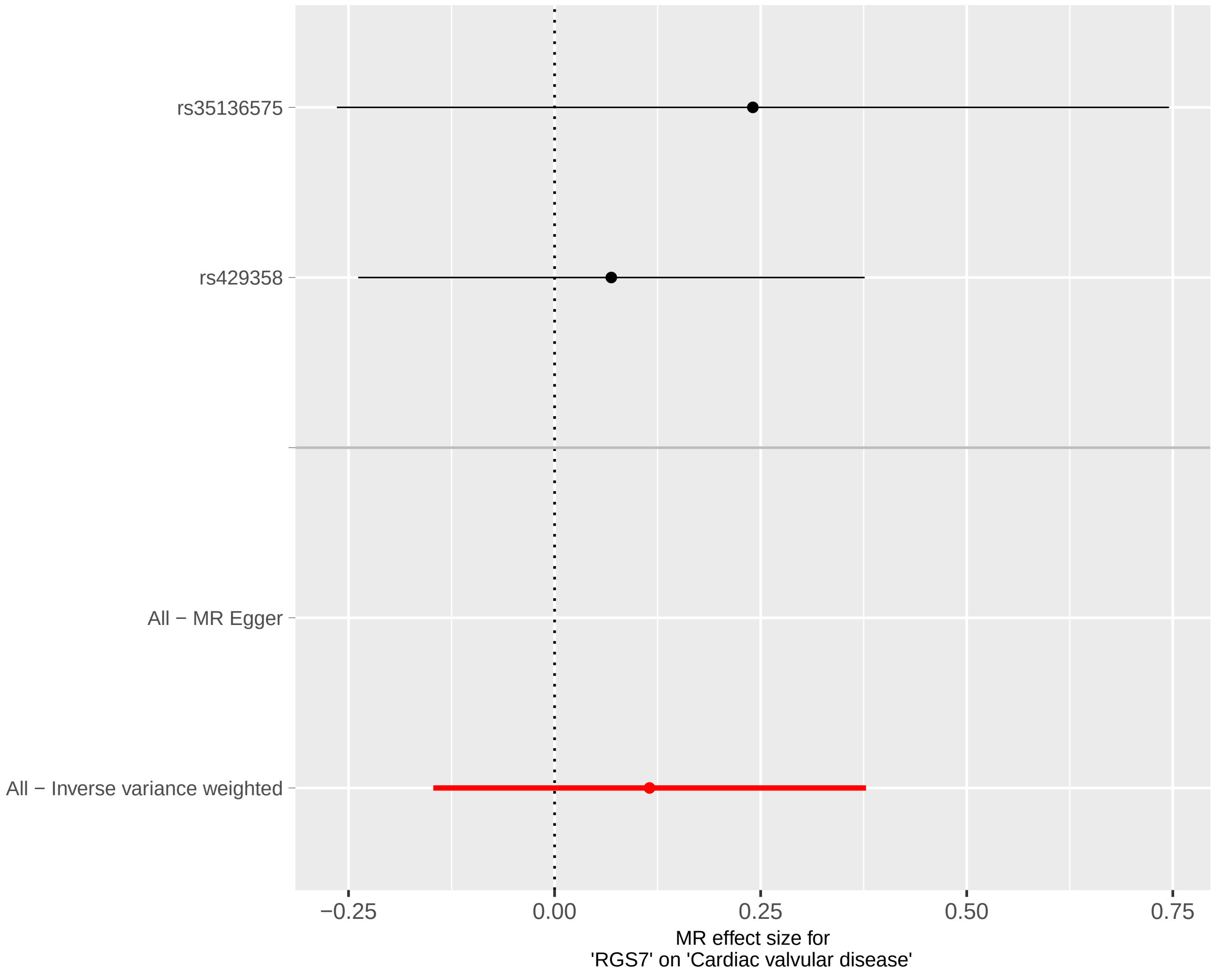

rs35136575
rs429358
All − MR Egger
All − Inverse variance weighted
−0.25
0.00
0.25
0.50
0.75
MR effect size for
'RGS7' on 'Cardiac valvular disease'

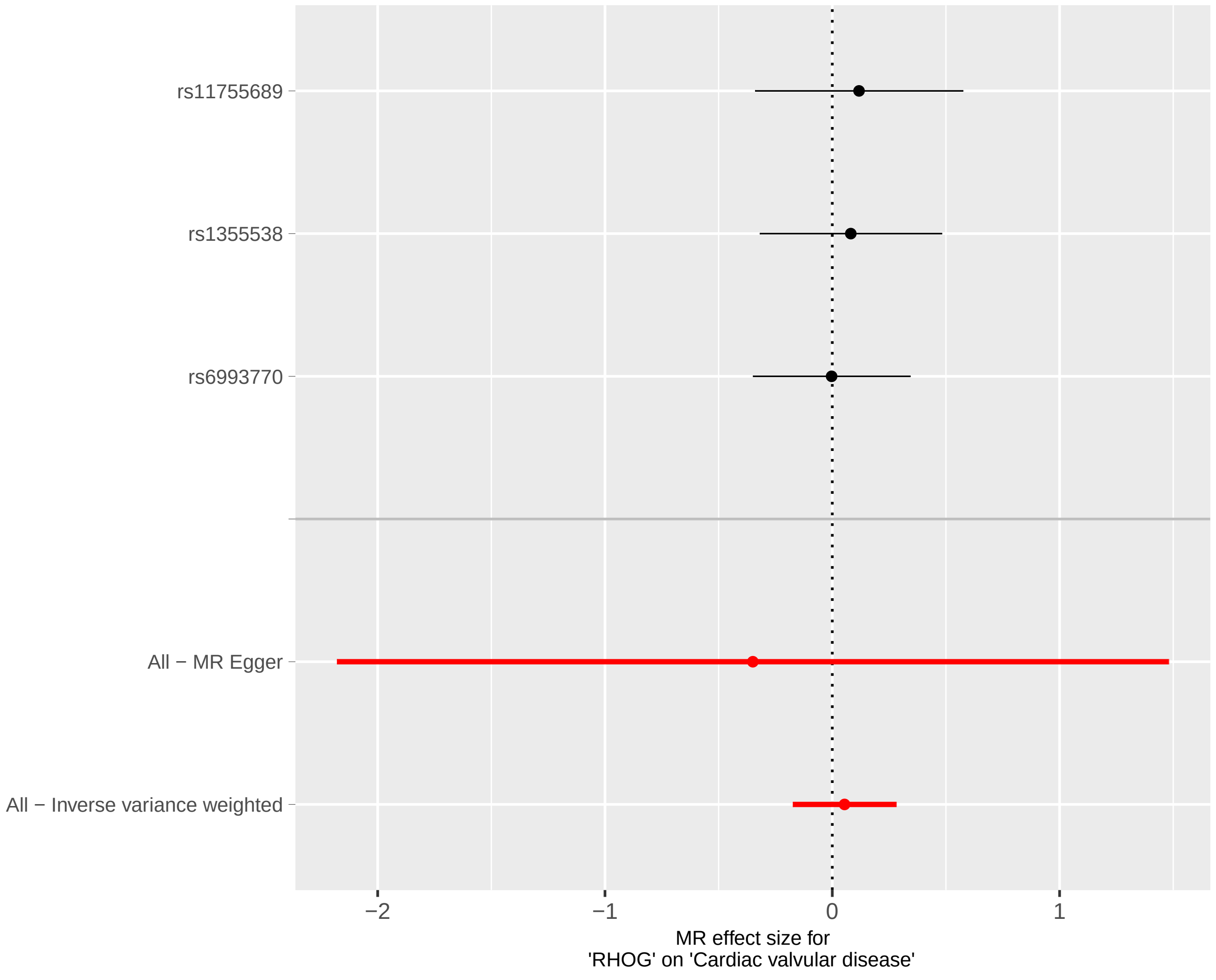
rs11755689
rs1355538
rs6993770
All − MR Egger
All − Inverse variance weighted
−2
−1
0
1
MR effect size for
'RHOG' on 'Cardiac valvular disease'

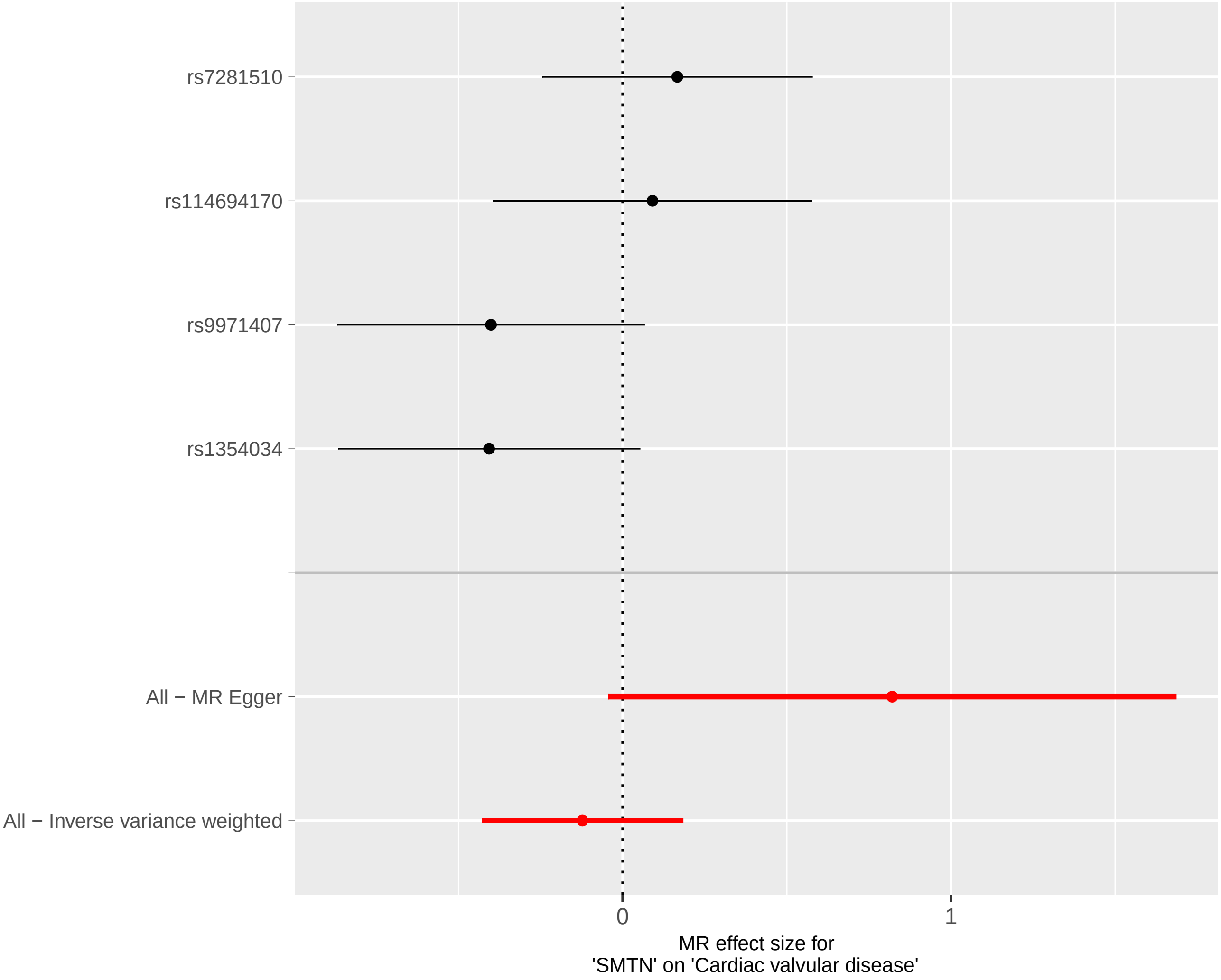
rs7281510
rs114694170
rs9971407
rs1354034
All − MR Egger
All − Inverse variance weighted
0
1
MR effect size for
'SMTN' on 'Cardiac valvular disease'

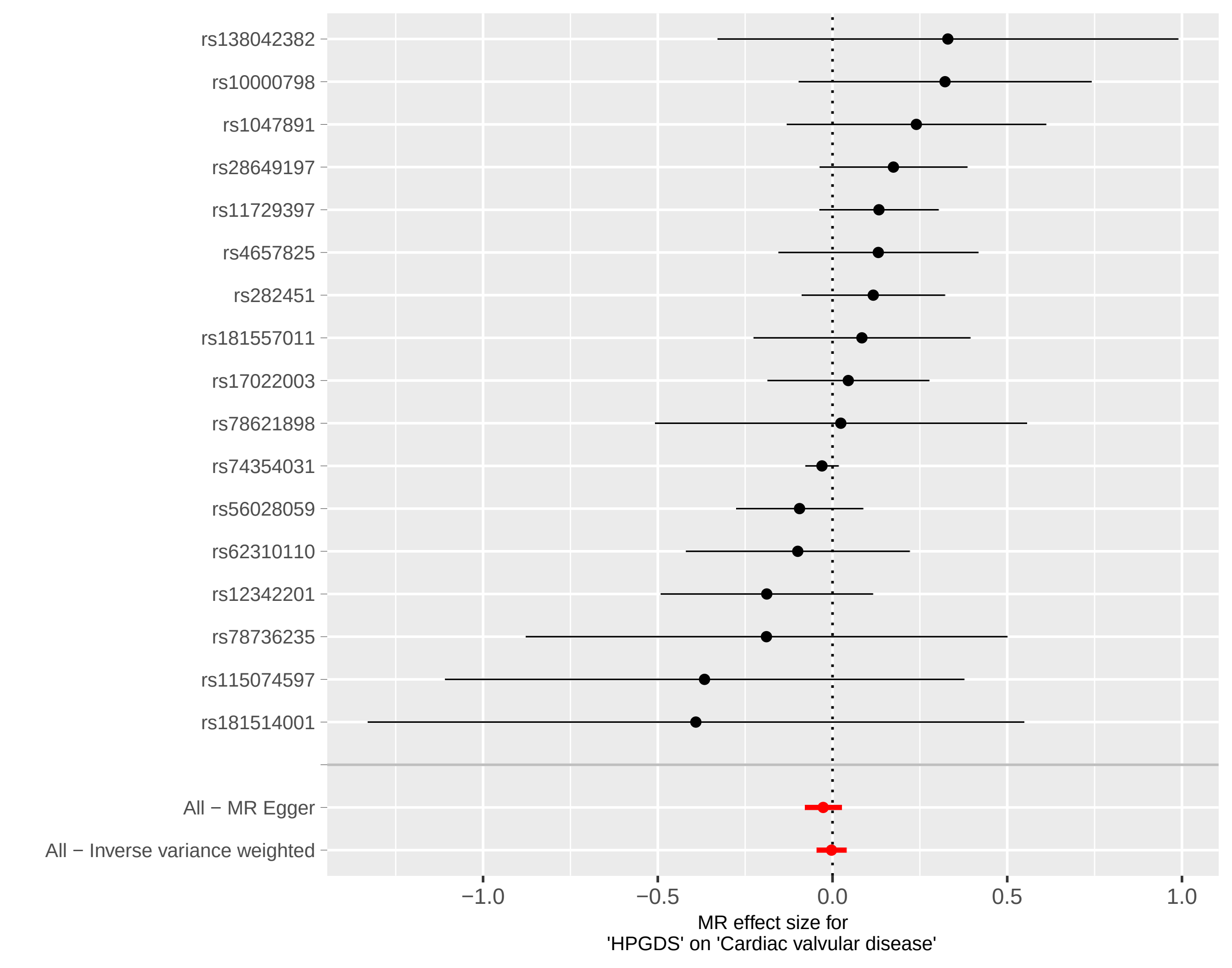
rs138042382
rs10000798
rs1047891
rs28649197
rs11729397
rs4657825
rs282451
rs181557011
rs17022003
rs78621898
rs74354031
rs56028059
rs62310110
rs12342201
rs78736235
rs115074597
rs181514001
All − MR Egger
All − Inverse variance weighted
−1.0
−0.5
0.0
0.5
1.0
MR effect size for
'HPGDS' on 'Cardiac valvular disease'

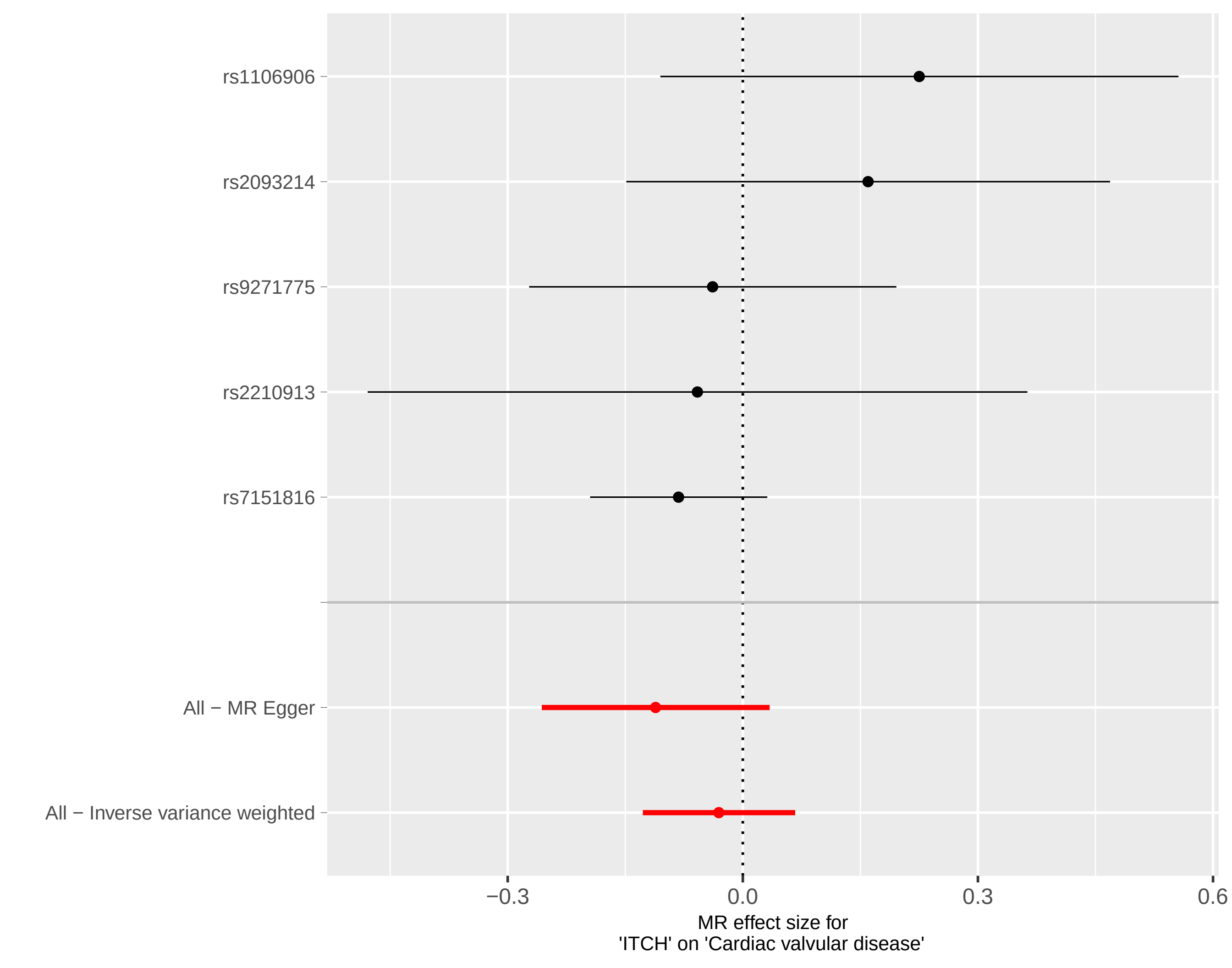
rs1106906
rs2093214
rs9271775
rs2210913
rs7151816
All − MR Egger
All − Inverse variance weighted
−0.3
0.0
0.3
0.6
MR effect size for
'ITCH' on 'Cardiac valvular disease'

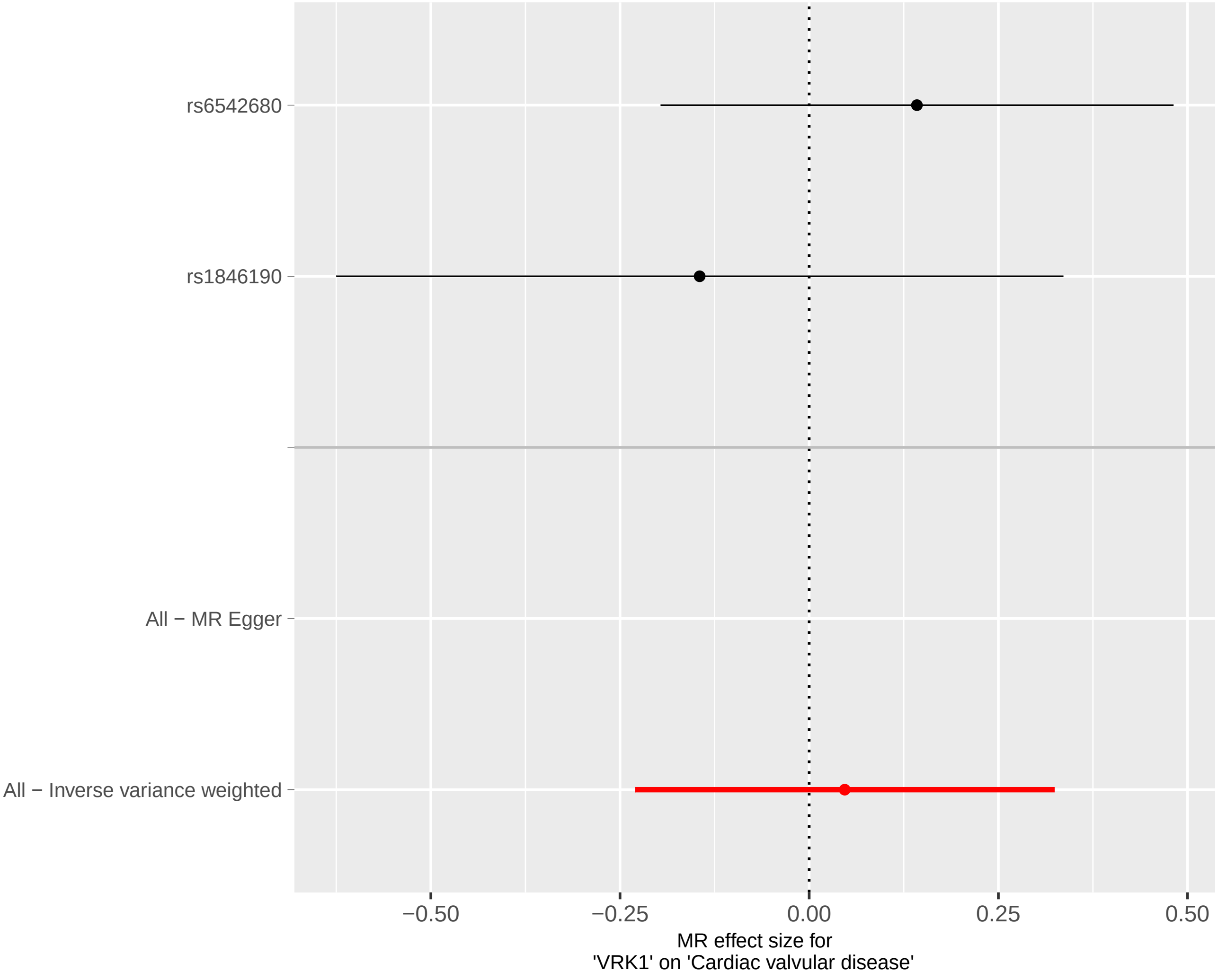
rs6542680
rs1846190
All − MR Egger
All − Inverse variance weighted
−0.50
−0.25
0.00
0.25
0.50
MR effect size for
'VRK1' on 'Cardiac valvular disease'

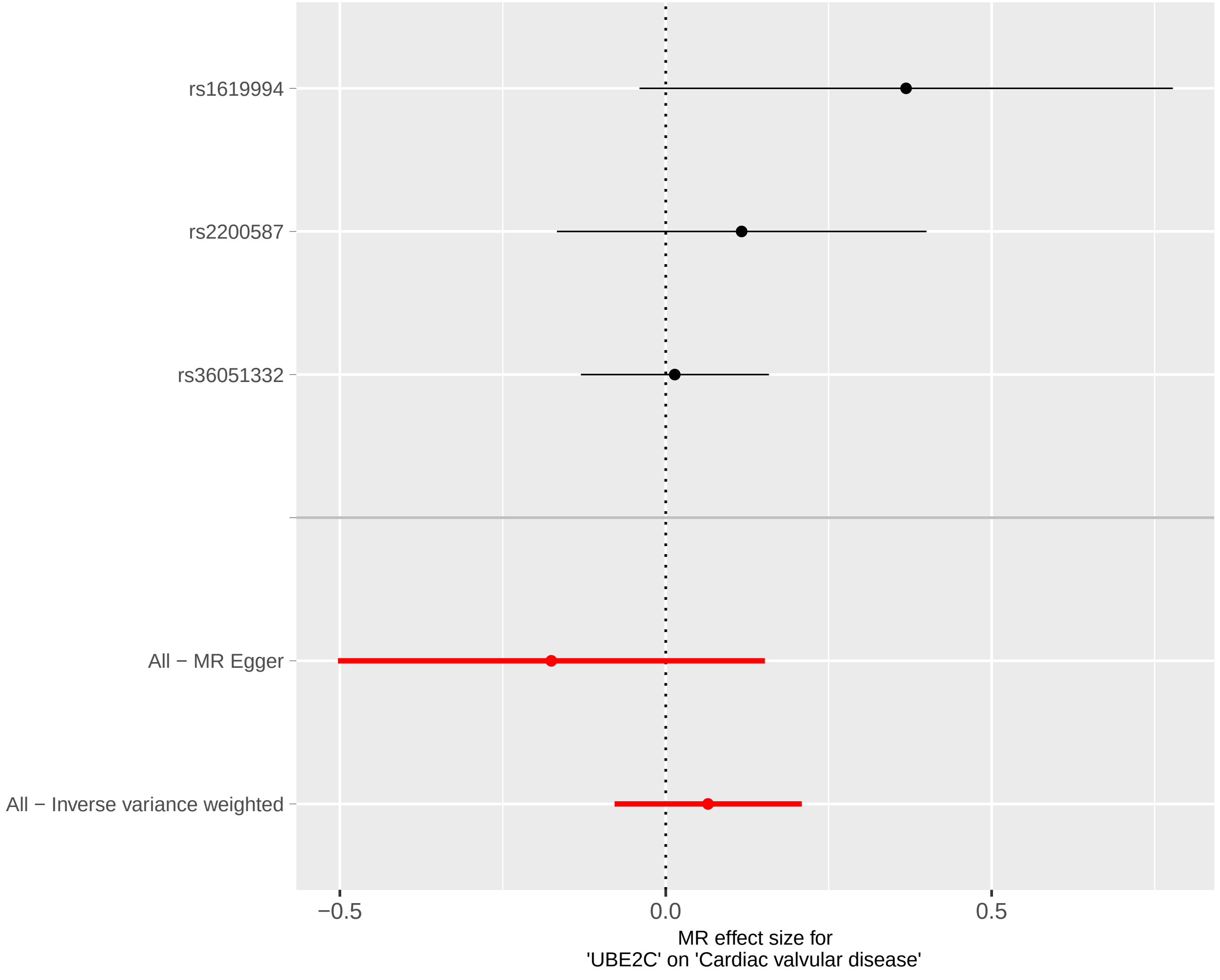
rs1619994
rs2200587
rs36051332
All − MR Egger
All − Inverse variance weighted
−0.5
0.0
0.5
MR effect size for
'UBE2C' on 'Cardiac valvular disease'

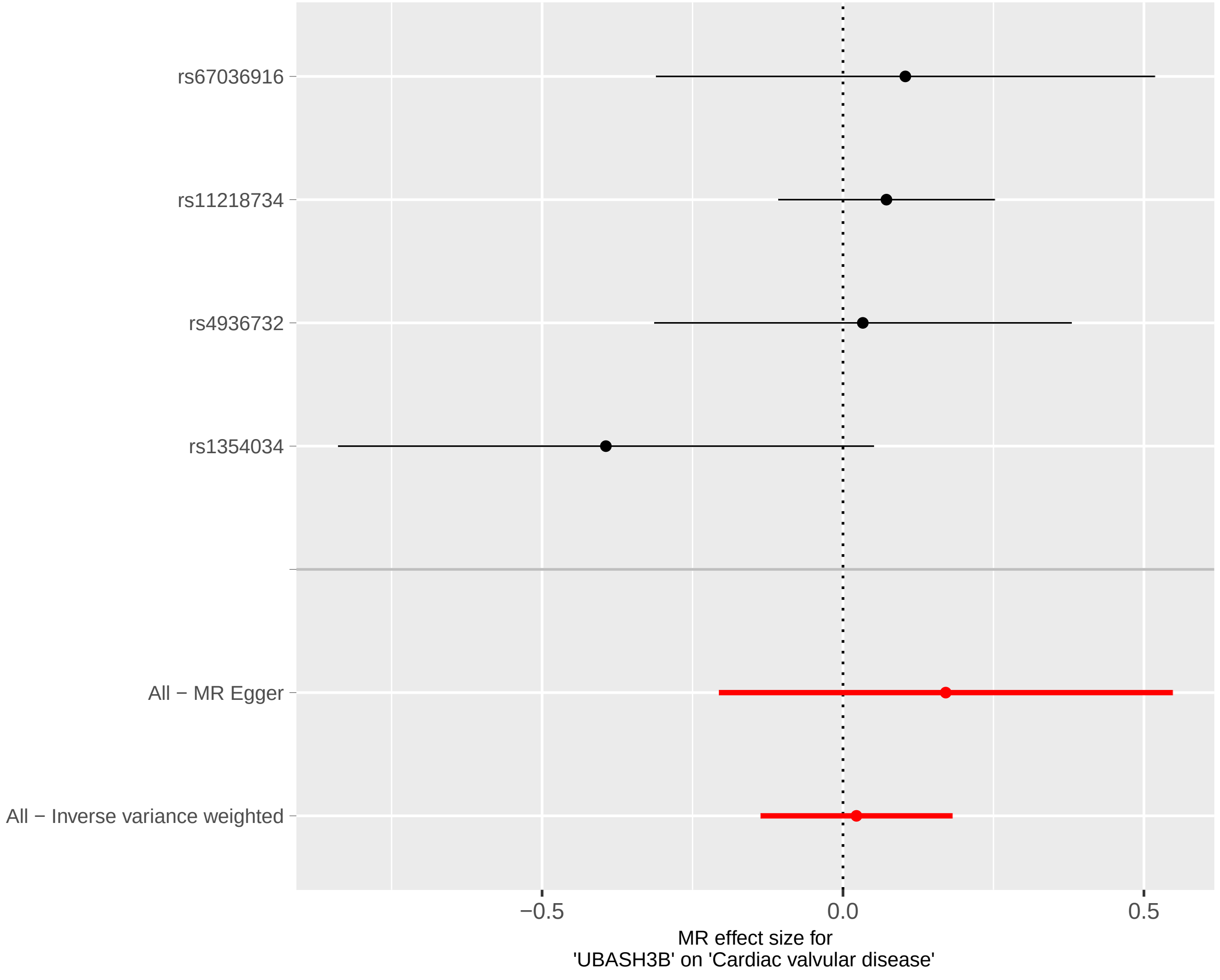
rs67036916
rs11218734
rs4936732
rs1354034
All − MR Egger
All − Inverse variance weighted
−0.5
0.0
0.5
MR effect size for
'UBASH3B' on 'Cardiac valvular disease'

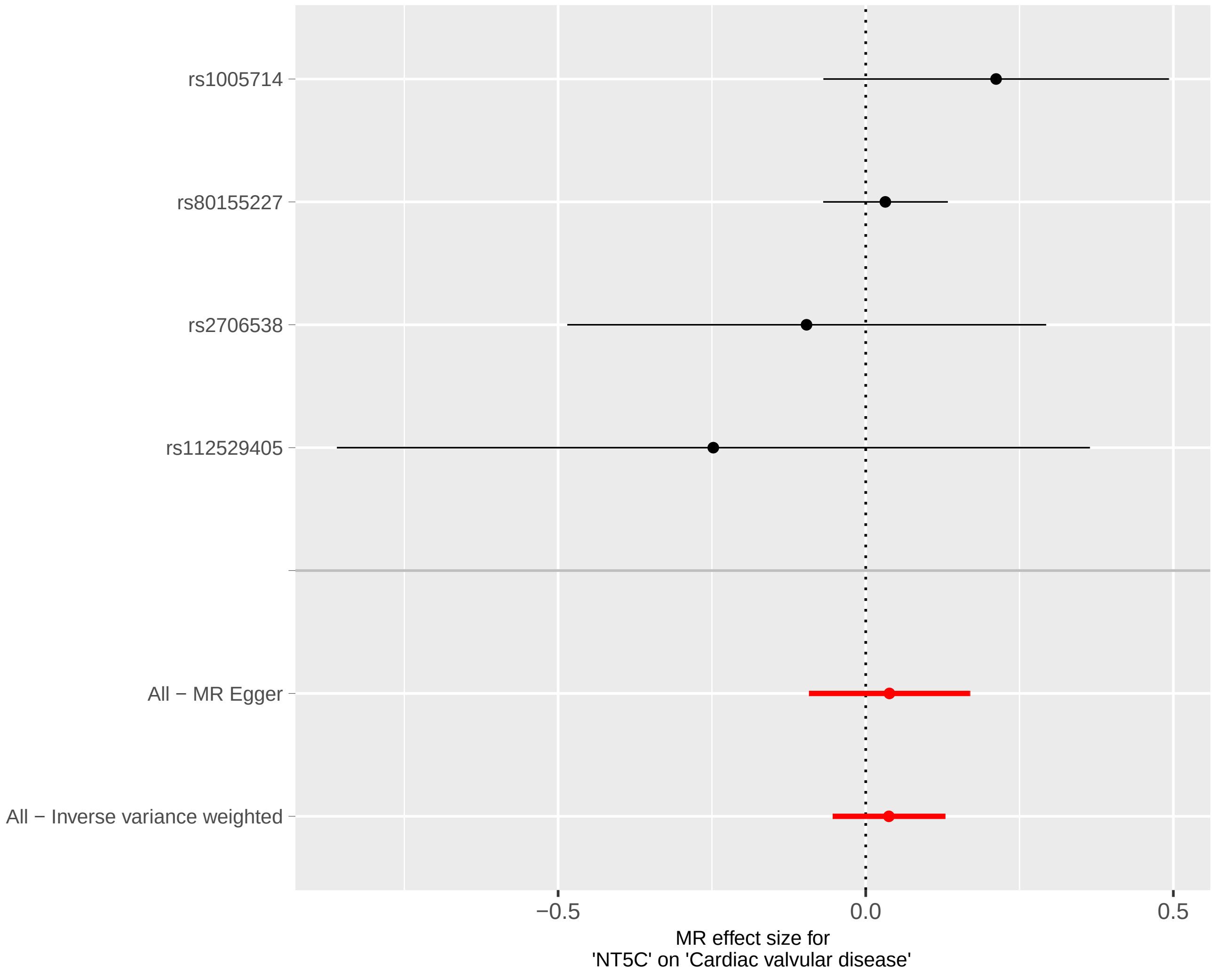

rs1005714
rs80155227
rs2706538
rs112529405
All − MR Egger
All − Inverse variance weighted
−0.5
0.0
0.5
MR effect size for
'NT5C' on 'Cardiac valvular disease'

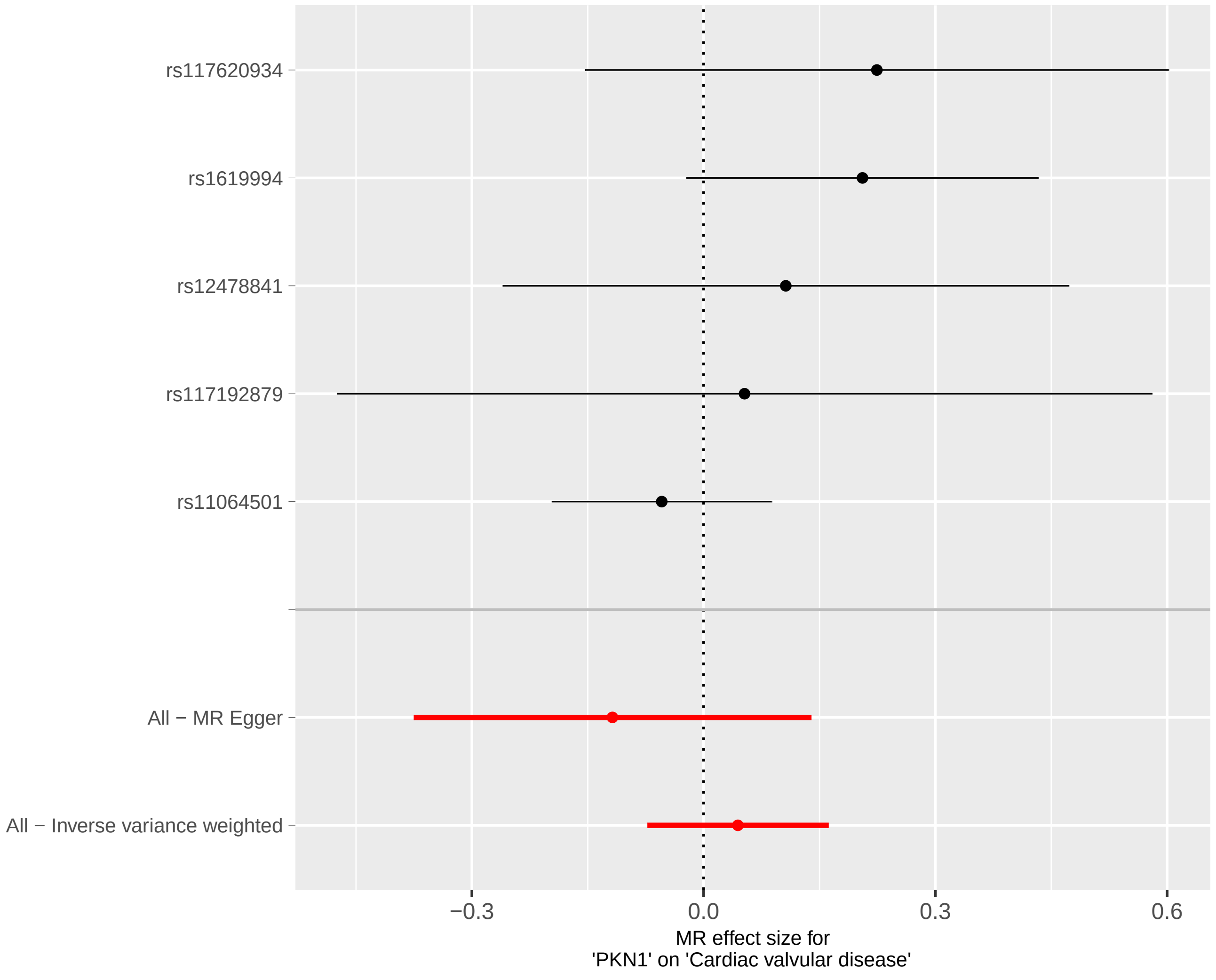
rs117620934
rs1619994
rs12478841
rs117192879
rs11064501
All − MR Egger
All − Inverse variance weighted
−0.3
0.0
0.3
0.6
MR effect size for
'PKN1' on 'Cardiac valvular disease'

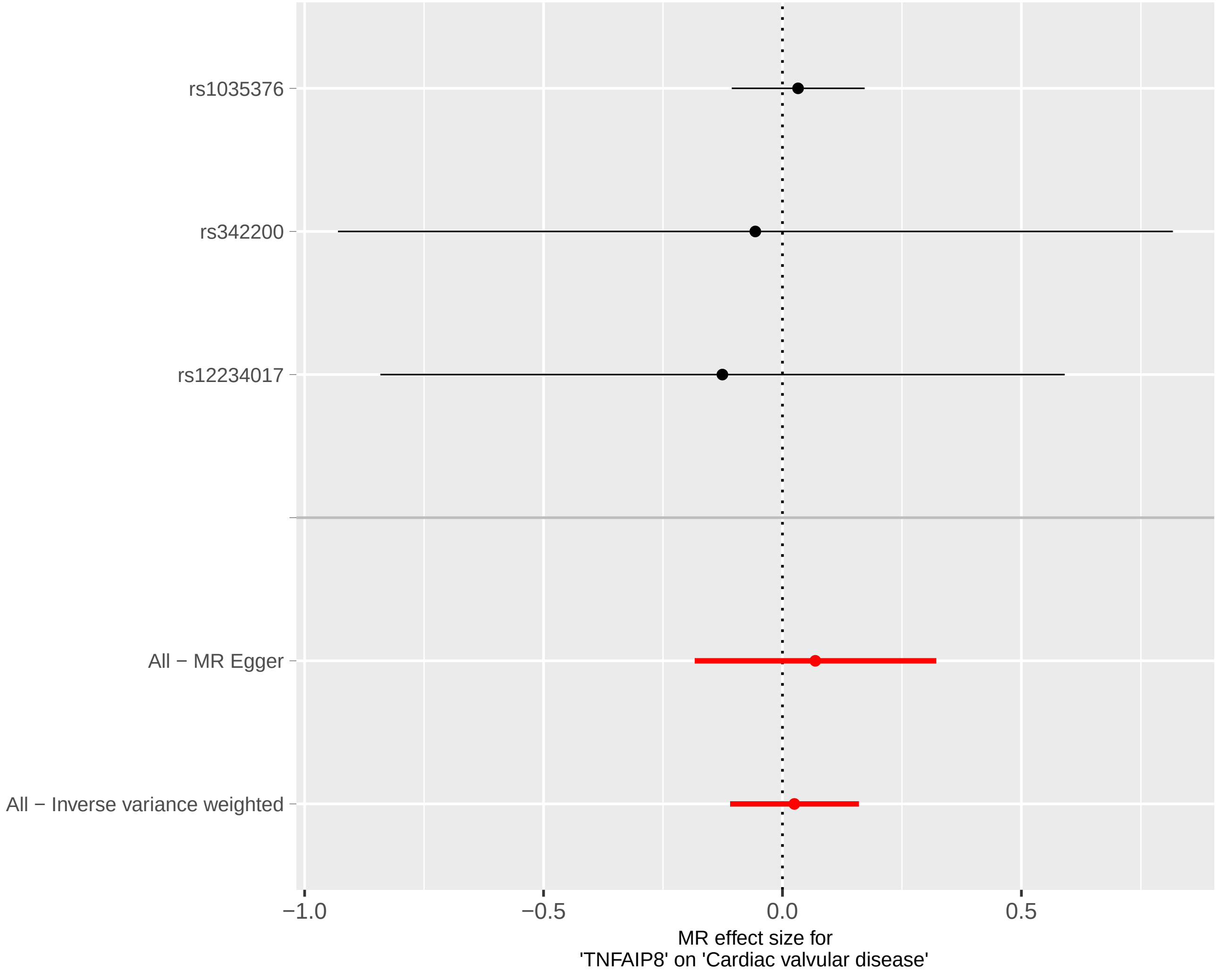
rs1035376
rs342200
rs12234017
All − MR Egger
All − Inverse variance weighted
−1.0
−0.5
0.0
0.5
MR effect size for
'TNFAIP8' on 'Cardiac valvular disease'

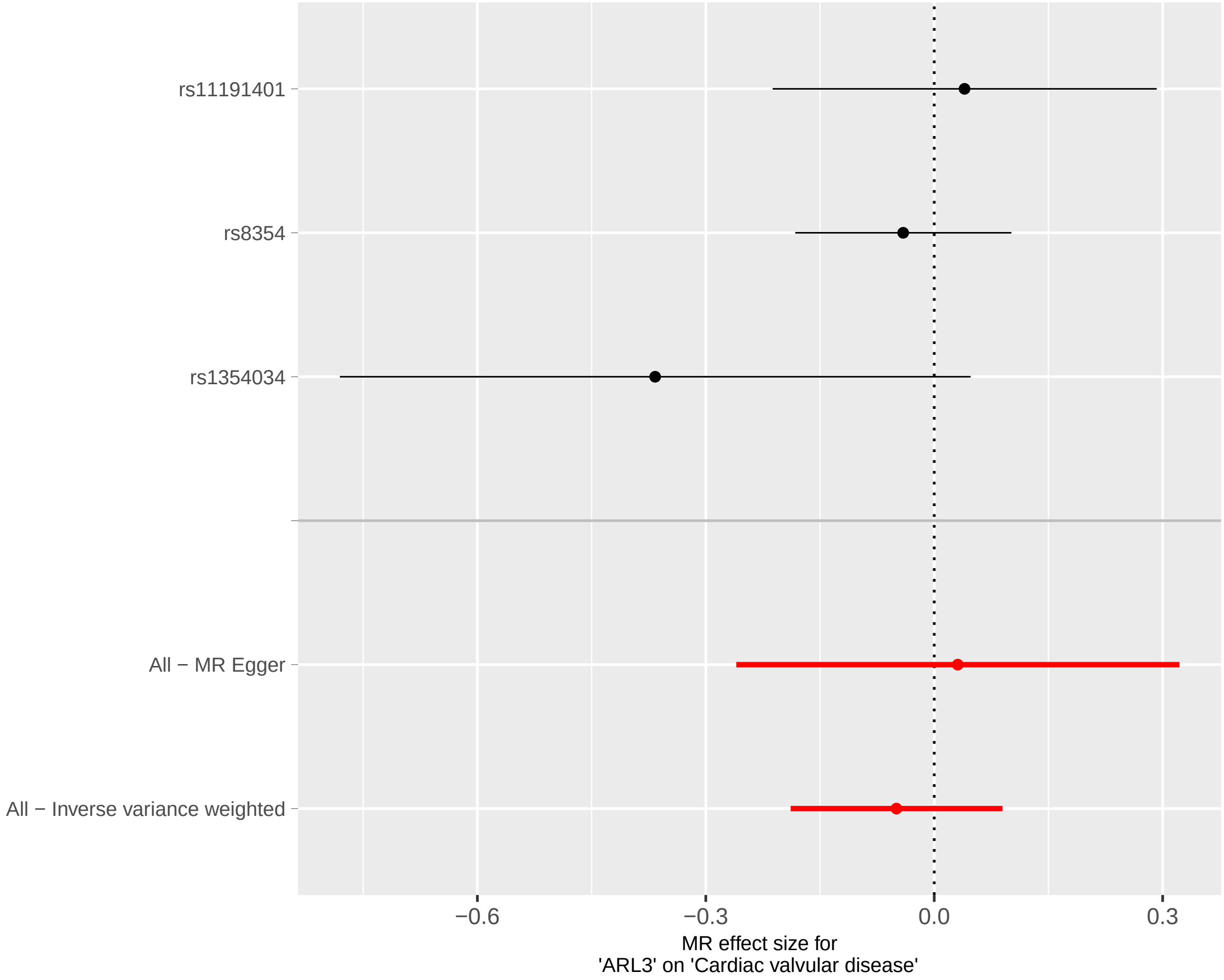

rs11191401
rs8354
rs1354034
All − MR Egger
All − Inverse variance weighted
−0.6
−0.3
0.0
0.3
MR effect size for
'ARL3' on 'Cardiac valvular disease'

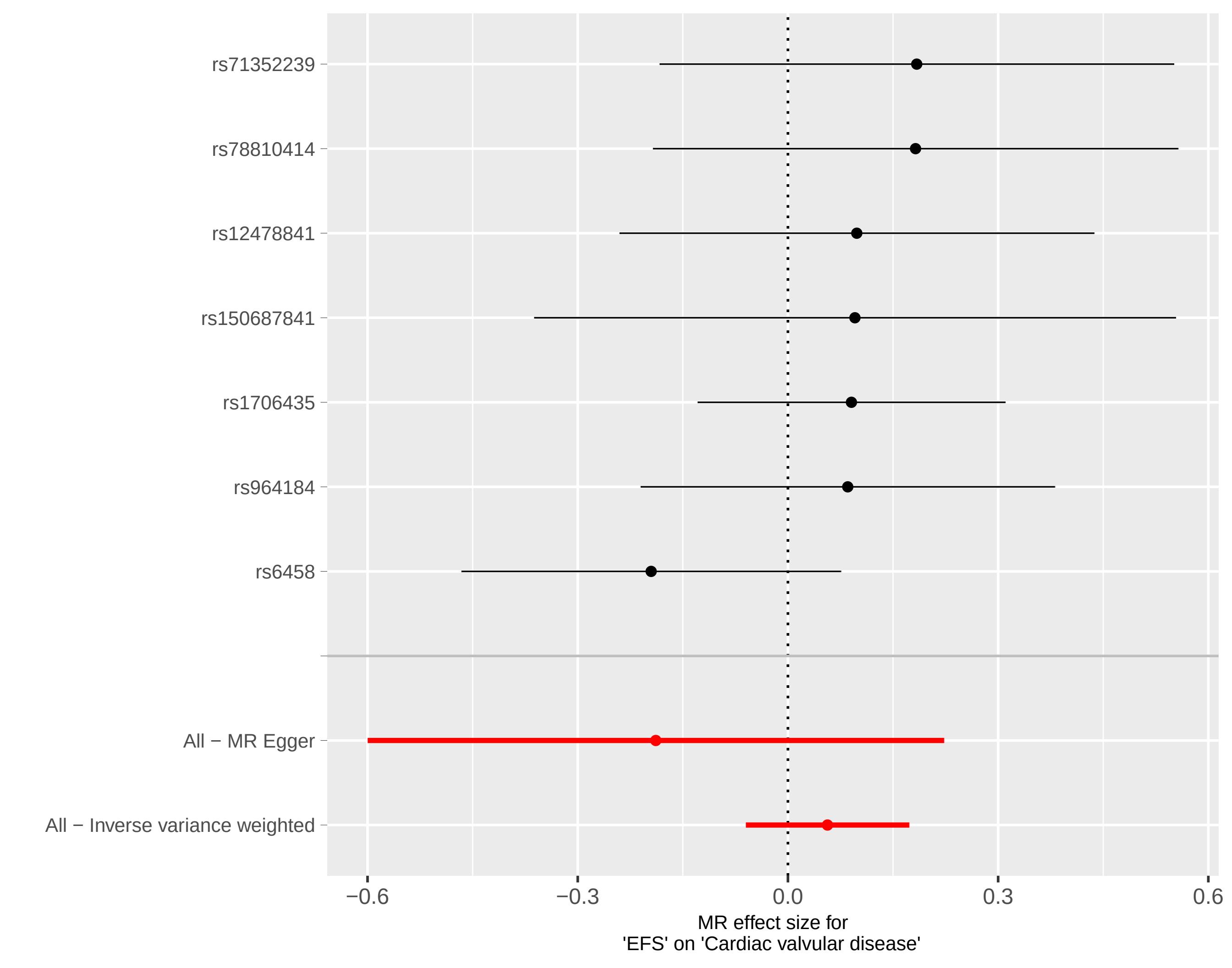
rs71352239
rs78810414
rs12478841
rs150687841
rs1706435
rs964184
rs6458
All − MR Egger
All − Inverse variance weighted
−0.6
−0.3
0.0
0.3
0.6
MR effect size for
'EFS' on 'Cardiac valvular disease'

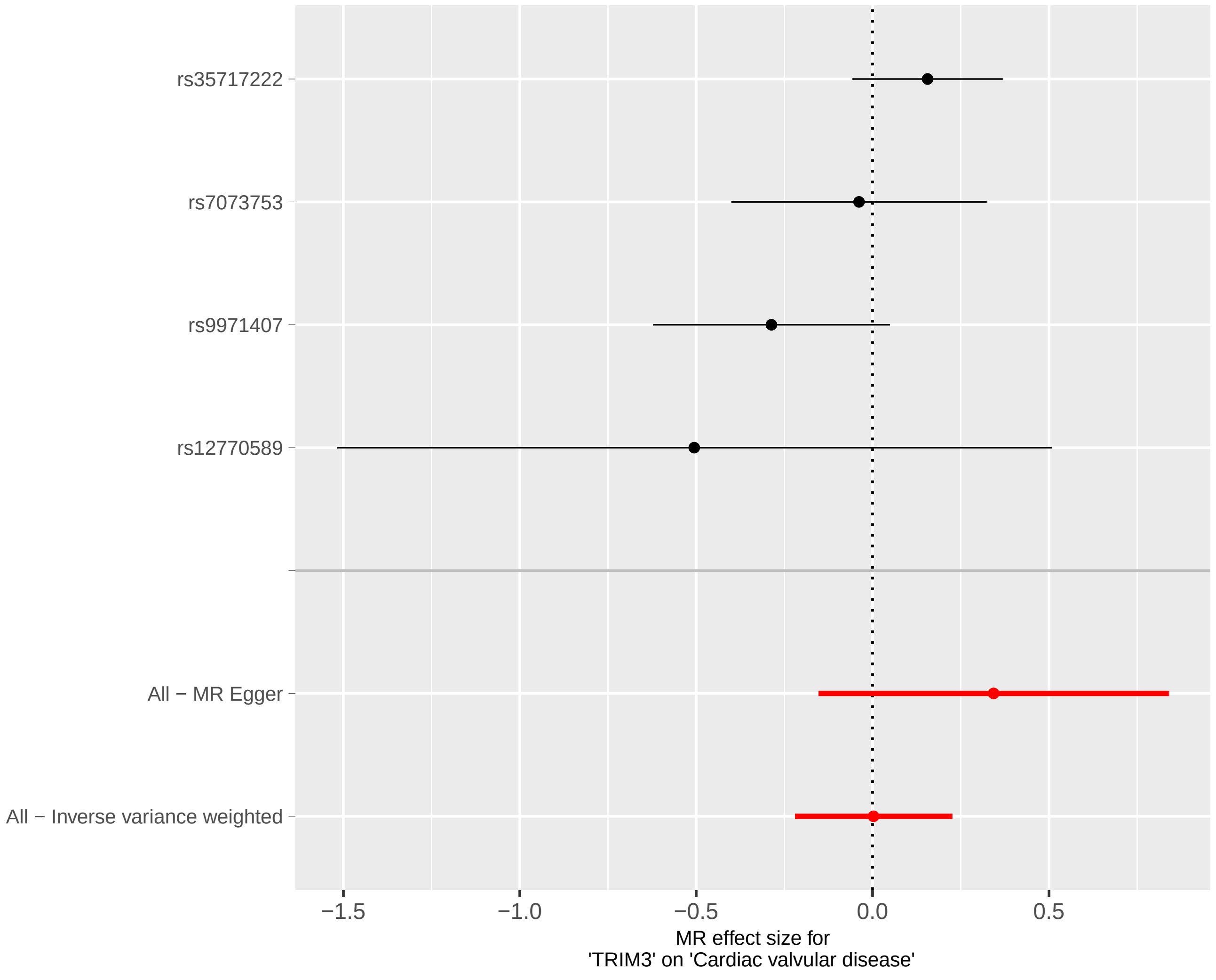
rs35717222
rs7073753
rs9971407
rs12770589
All − MR Egger
All − Inverse variance weighted
−1.5
−1.0
−0.5
0.0
0.5
MR effect size for
'TRIM3' on 'Cardiac valvular disease'

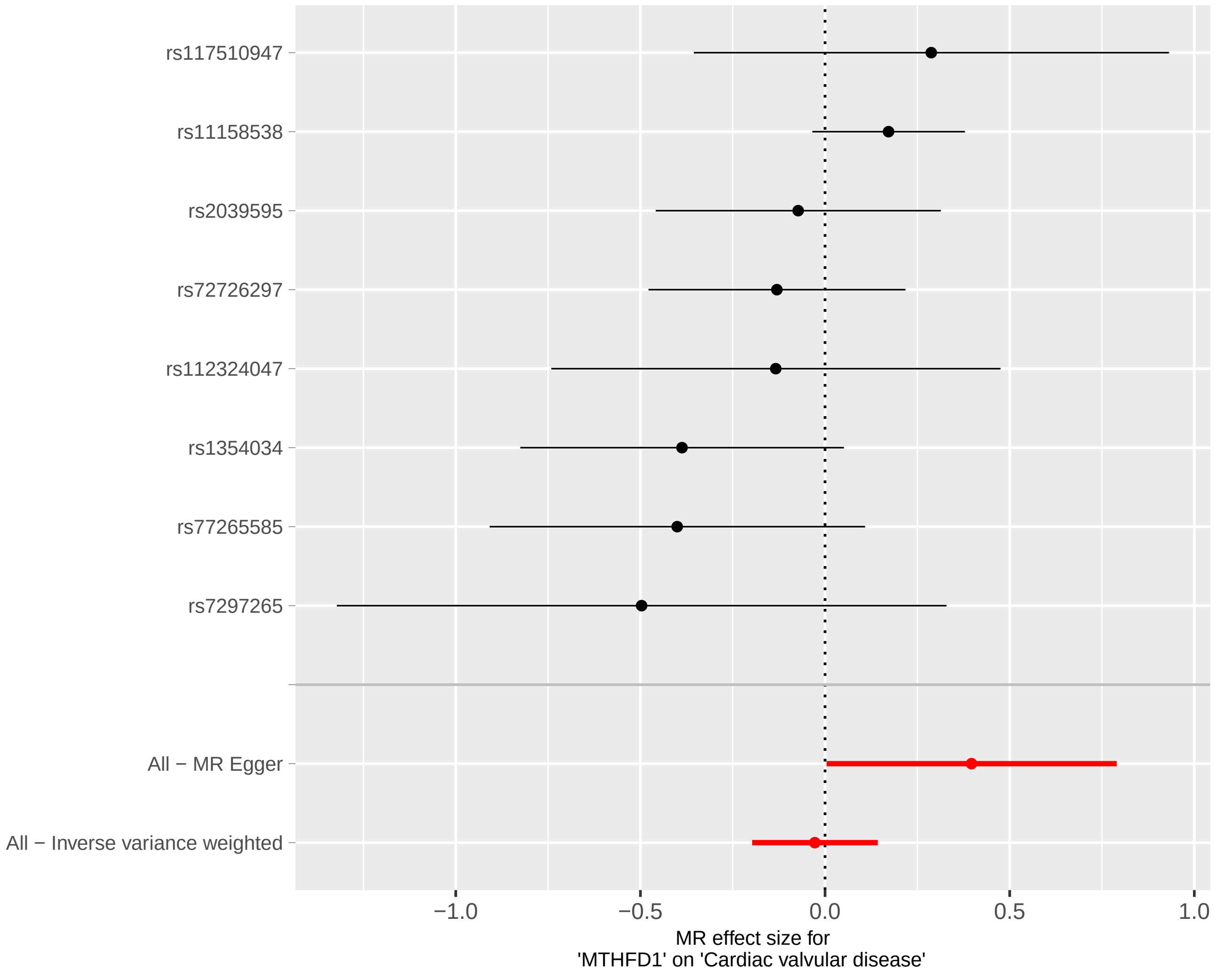
rs117510947
rs11158538
rs2039595
rs72726297
rs112324047
rs1354034
rs77265585
rs7297265
All − MR Egger
All − Inverse variance weighted
−1.0
−0.5
0.0
0.5
1.0
MR effect size for
'MTHFD1' on 'Cardiac valvular disease'

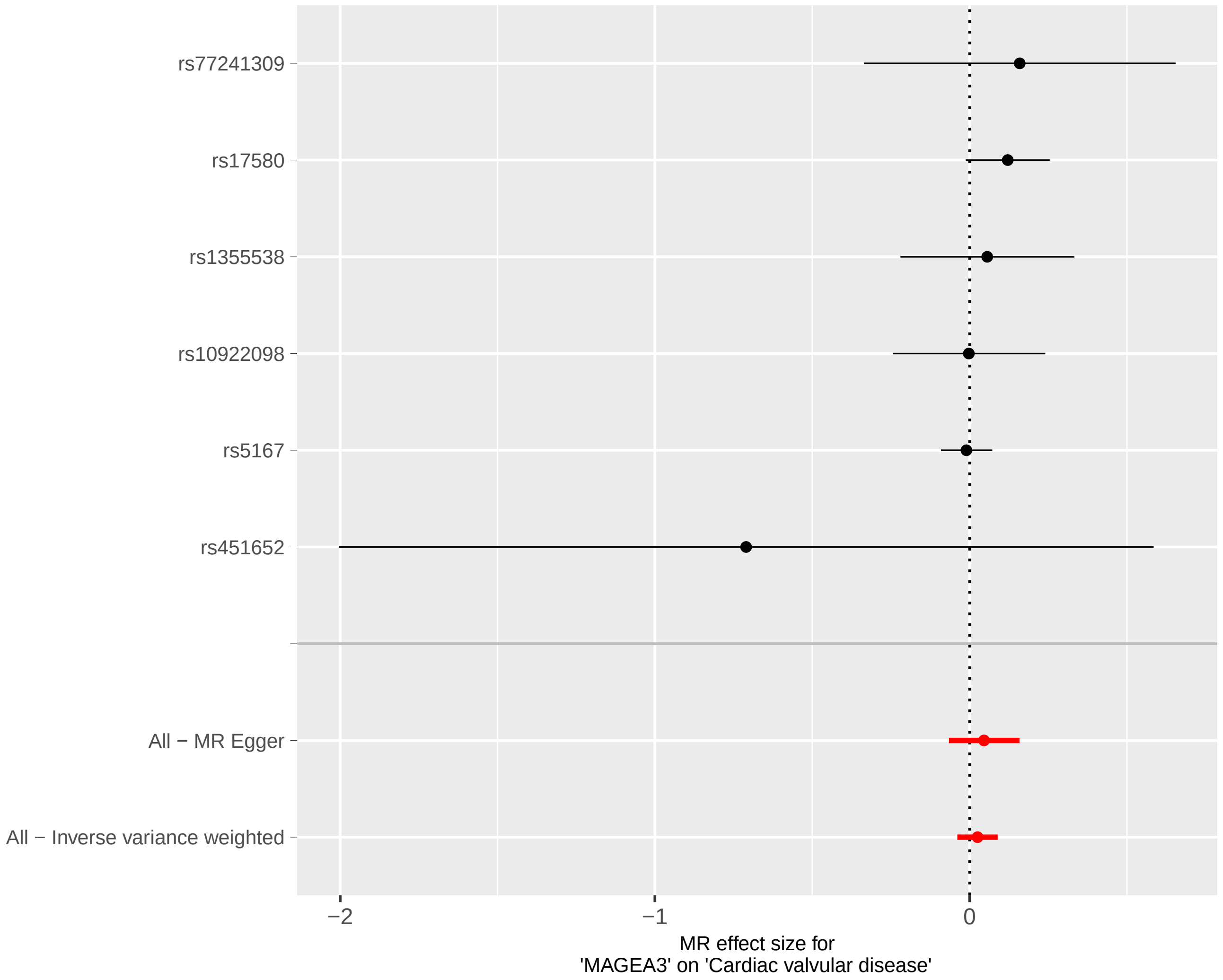
rs77241309
rs17580
rs1355538
rs10922098
rs5167
rs451652
All − MR Egger
All − Inverse variance weighted
−2
−1
0
MR effect size for
'MAGEA3' on 'Cardiac valvular disease'

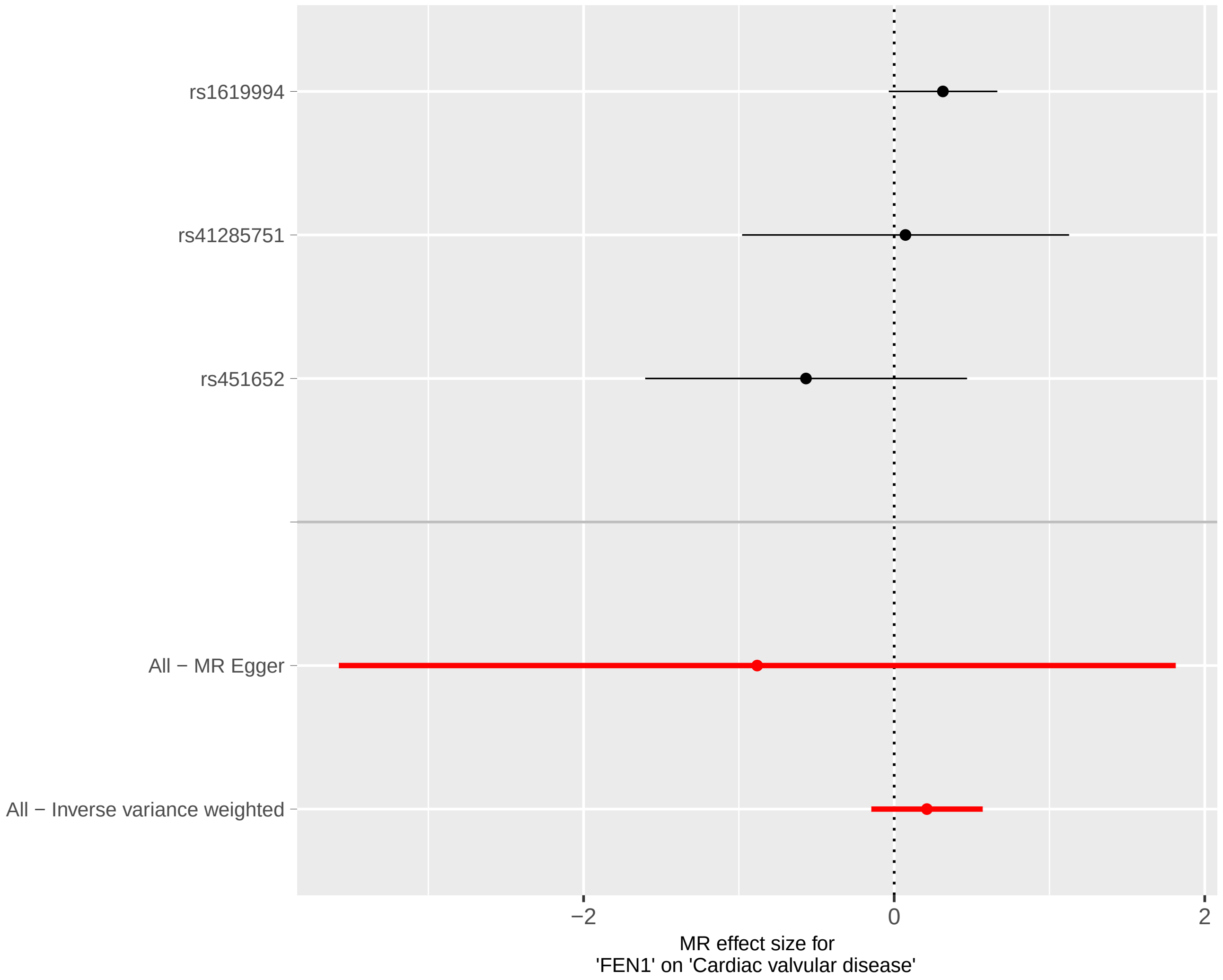
rs1619994
rs41285751
rs451652
All − MR Egger
All − Inverse variance weighted
−2
0
2
MR effect size for
'FEN1' on 'Cardiac valvular disease'

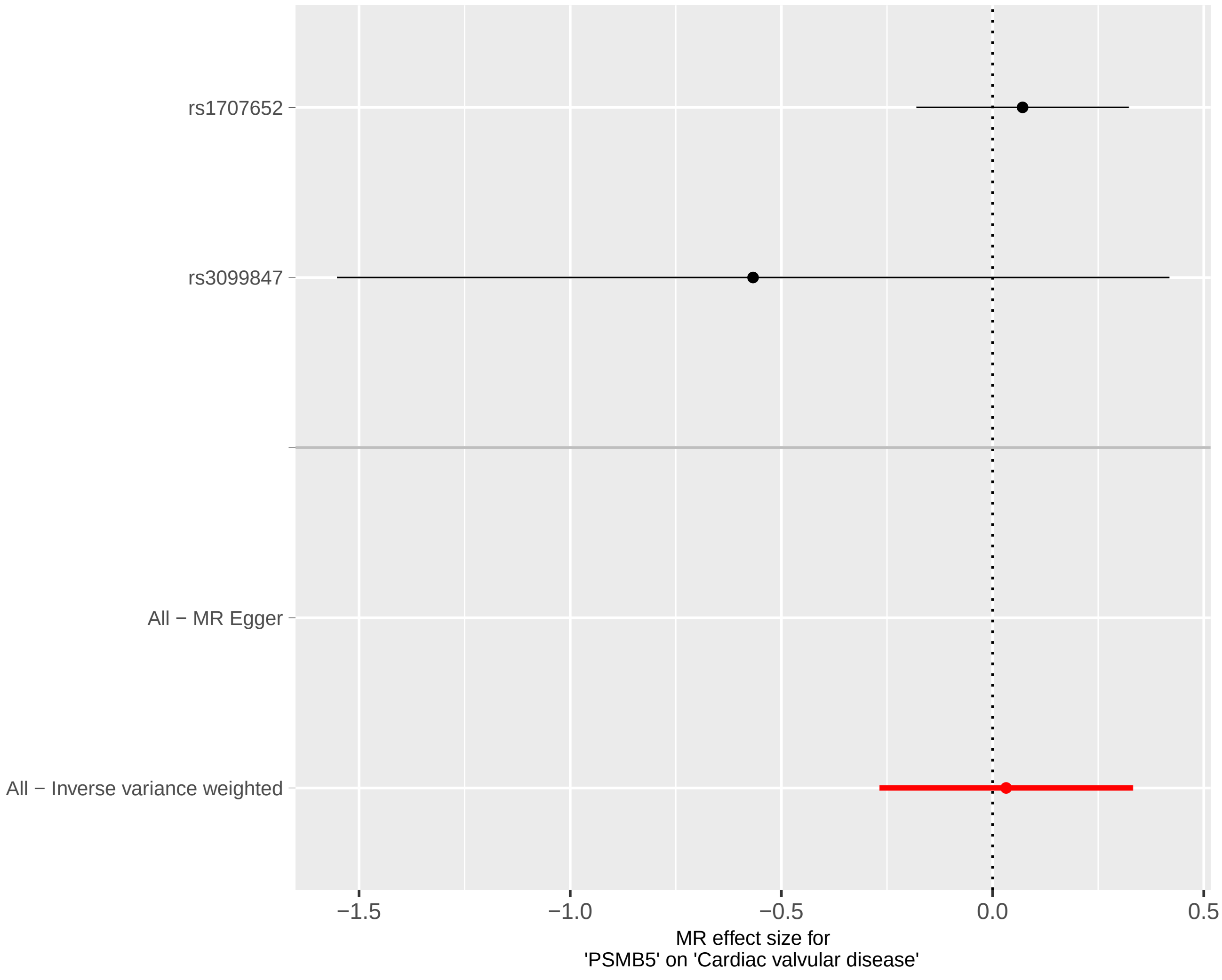

rs1707652
rs3099847
All − MR Egger
All − Inverse variance weighted
−1.5
−1.0
−0.5
0.0
0.5
MR effect size for
'PSMB5' on 'Cardiac valvular disease'

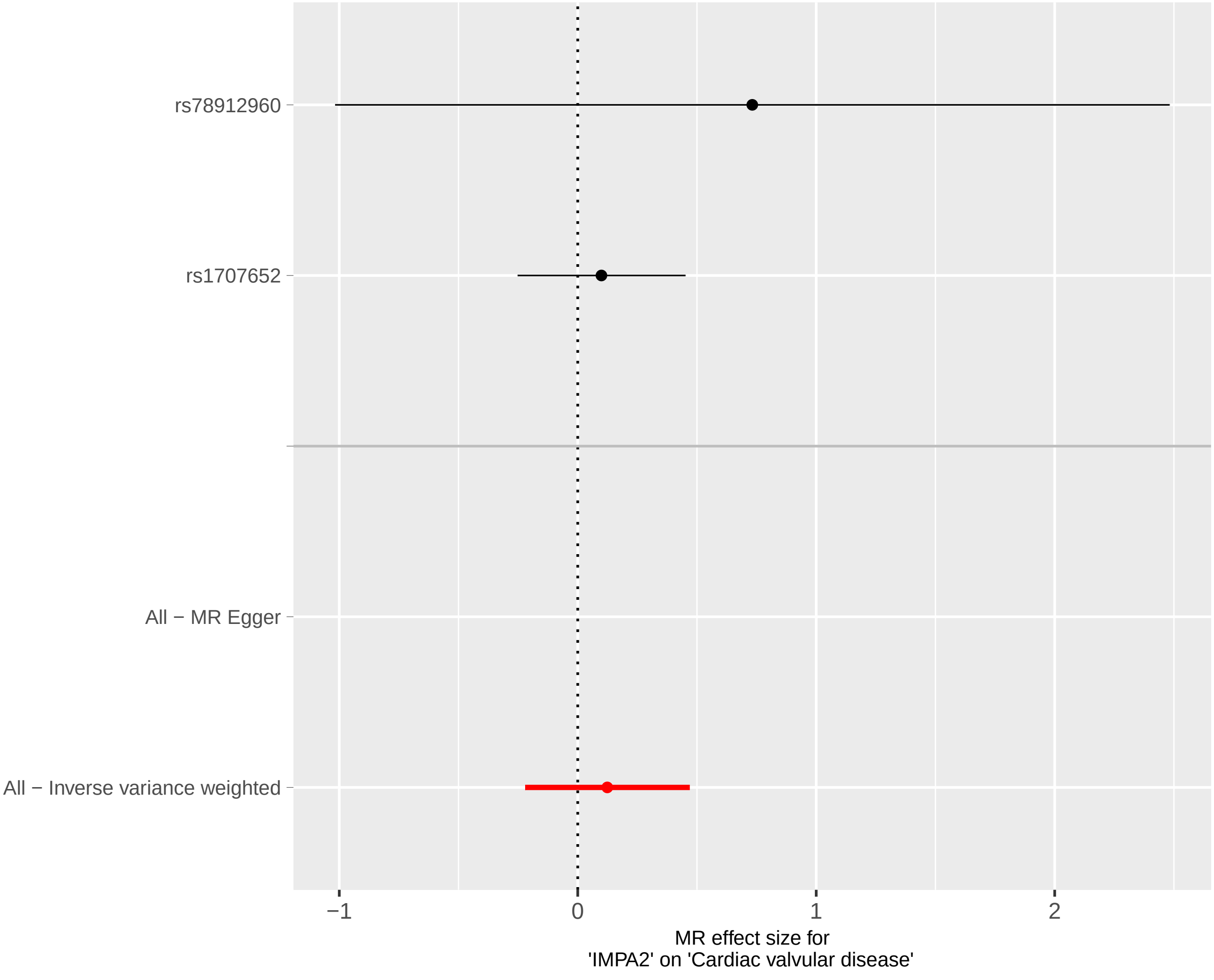
rs78912960
rs1707652
All − MR Egger
All − Inverse variance weighted
−1
0
1
2
MR effect size for
'IMPA2' on 'Cardiac valvular disease'

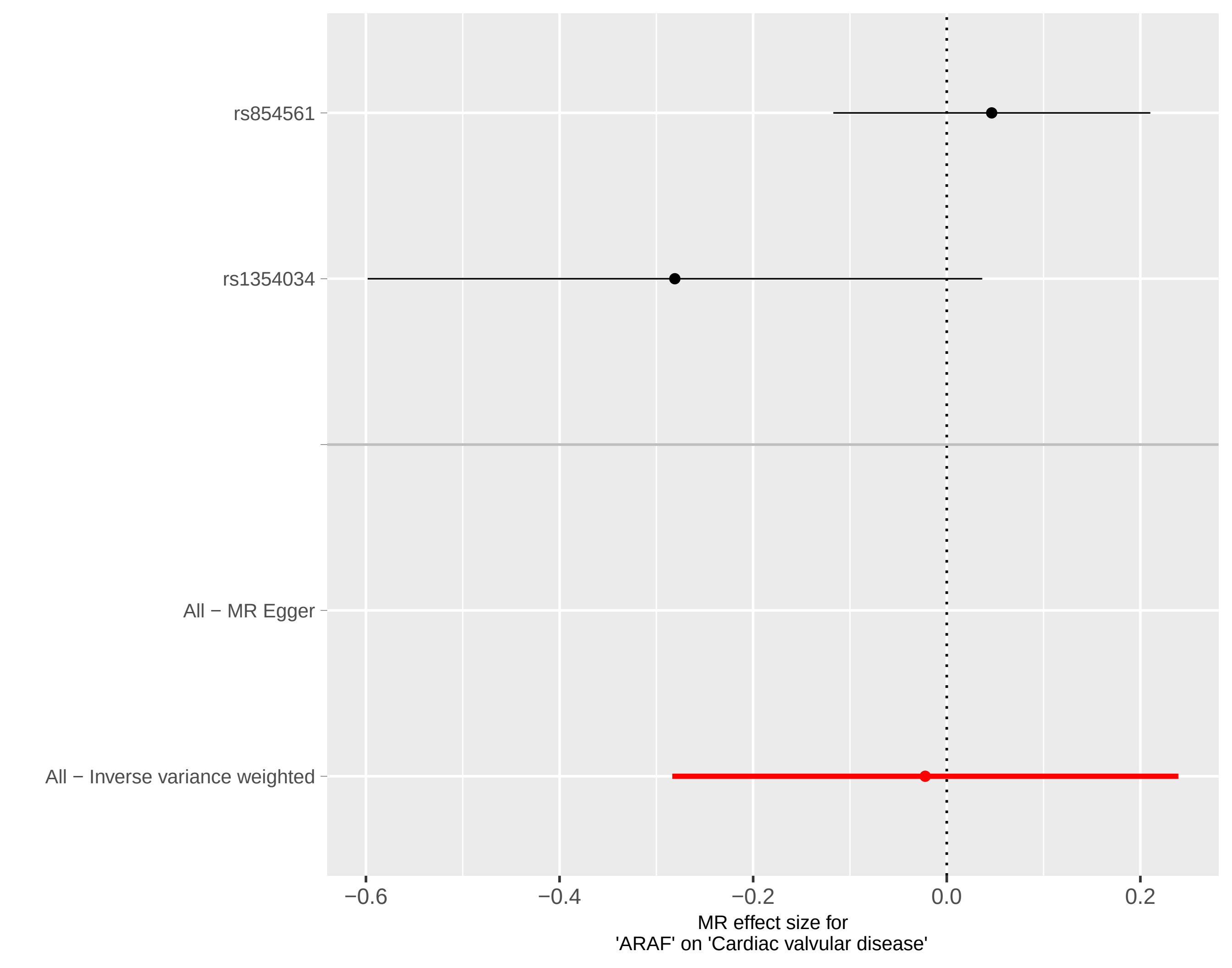

rs854561
rs1354034
All − MR Egger
All − Inverse variance weighted
−0.6
−0.4
−0.2
0.0
0.2
MR effect size for
'ARAF' on 'Cardiac valvular disease'

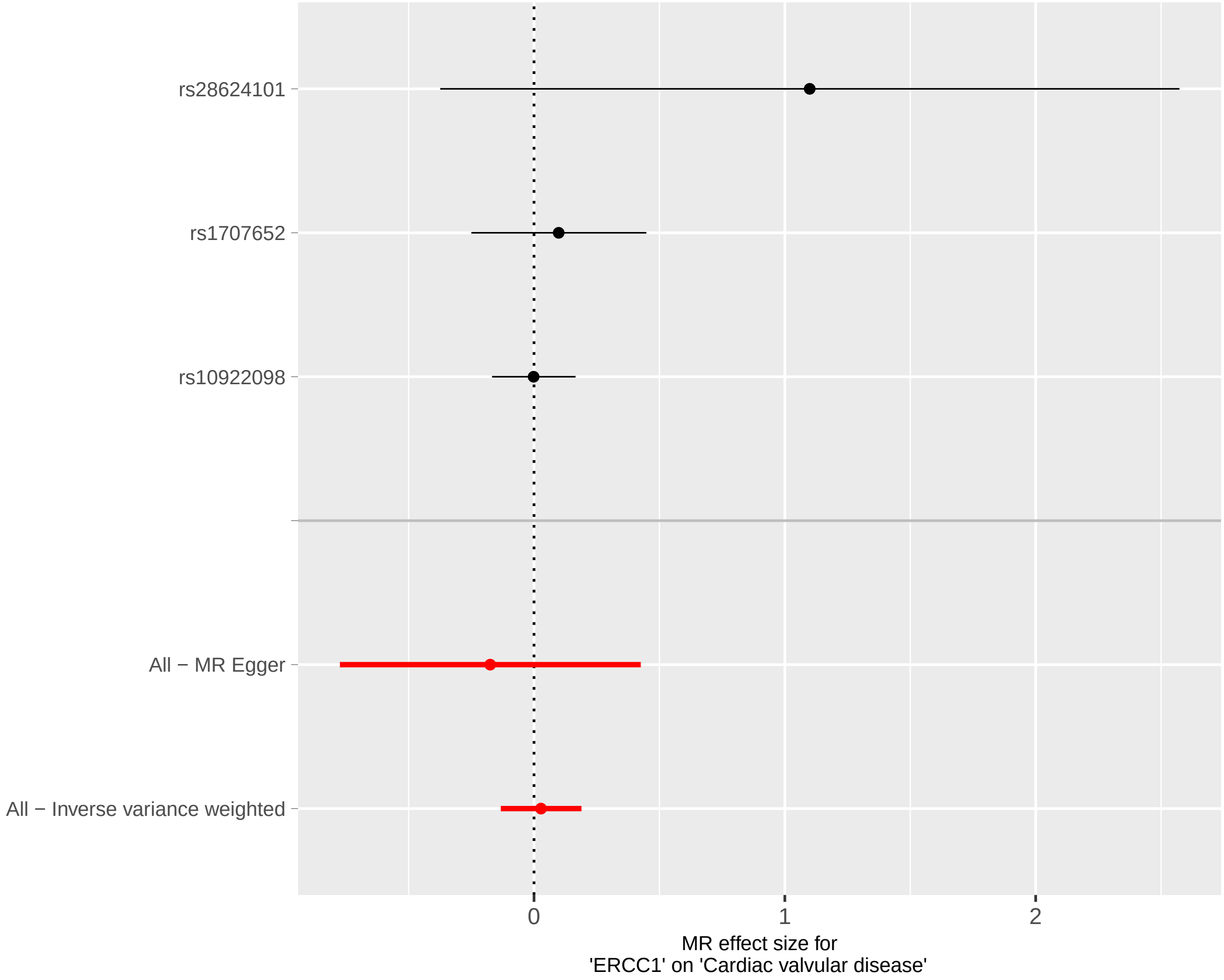
rs28624101
rs1707652
rs10922098
All − MR Egger
All − Inverse variance weighted
0
1
2
MR effect size for
'ERCC1' on 'Cardiac valvular disease'

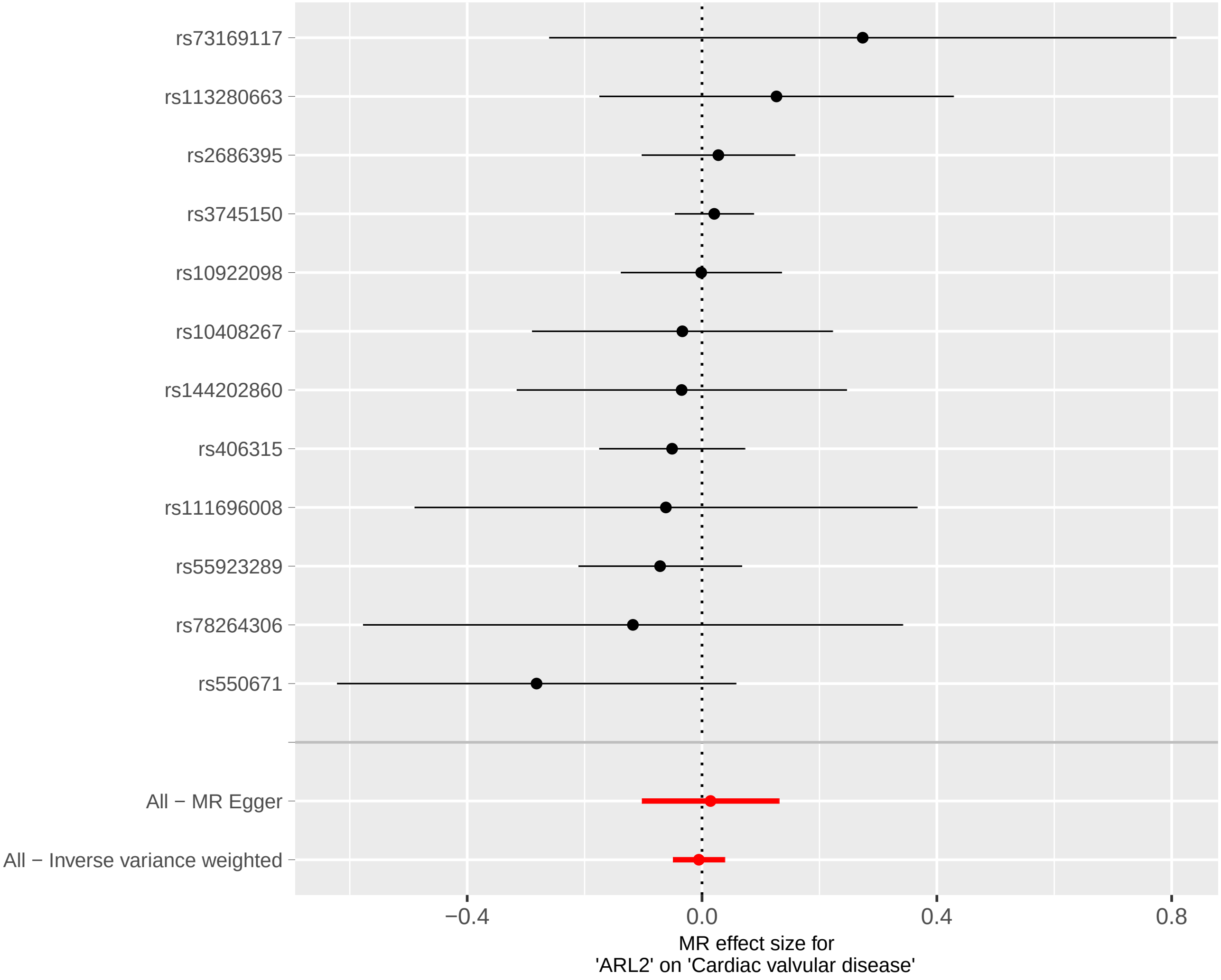
rs73169117
rs113280663
rs2686395
rs3745150
rs10922098
rs10408267
rs144202860
rs406315
rs111696008
rs55923289
rs78264306
rs550671
All − MR Egger
All − Inverse variance weighted
−0.4
0.0
0.4
0.8
MR effect size for
'ARL2' on 'Cardiac valvular disease'

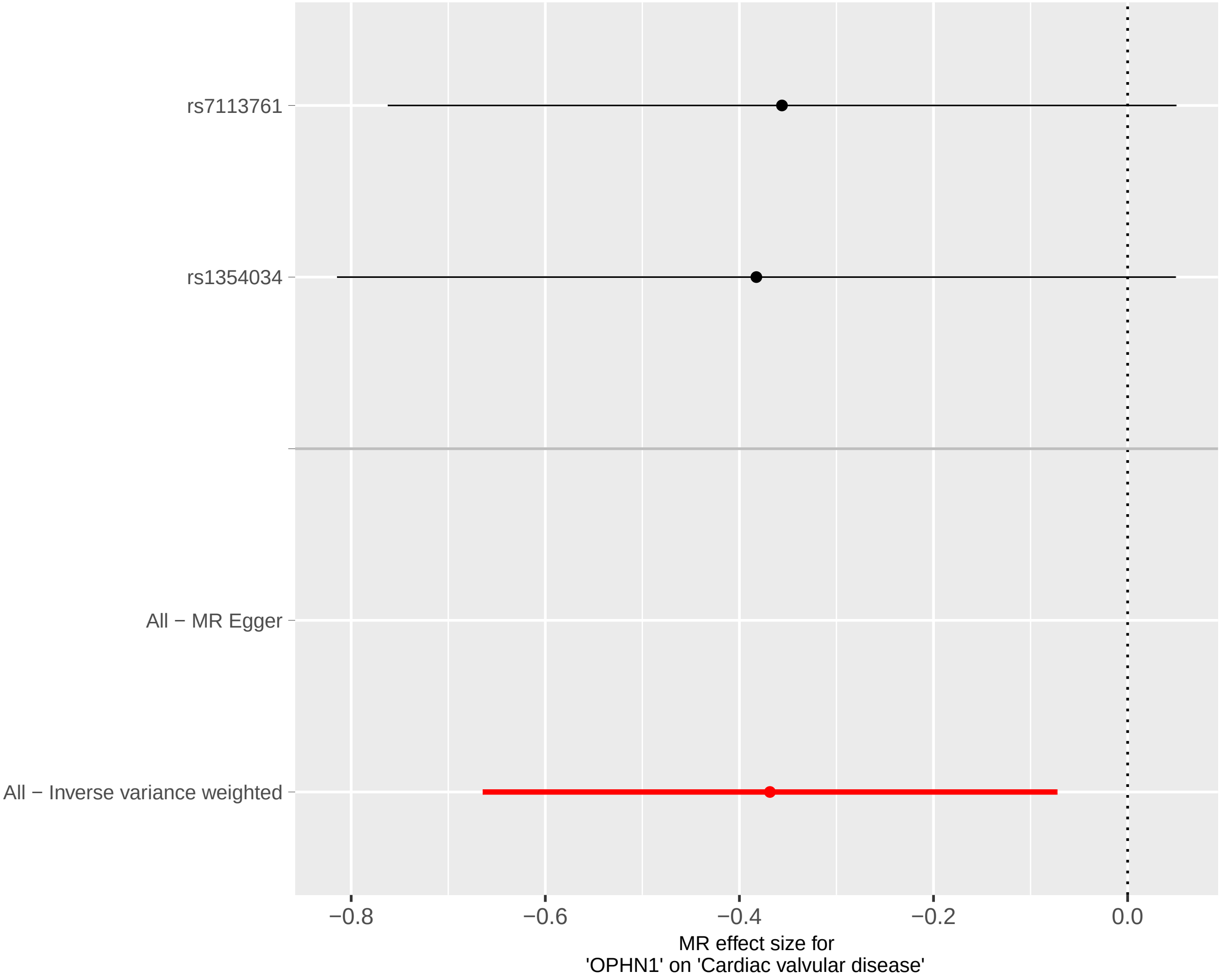
rs7113761
rs1354034
All − MR Egger
All − Inverse variance weighted
−0.8
−0.6
−0.4
−0.2
0.0
MR effect size for
'OPHN1' on 'Cardiac valvular disease'

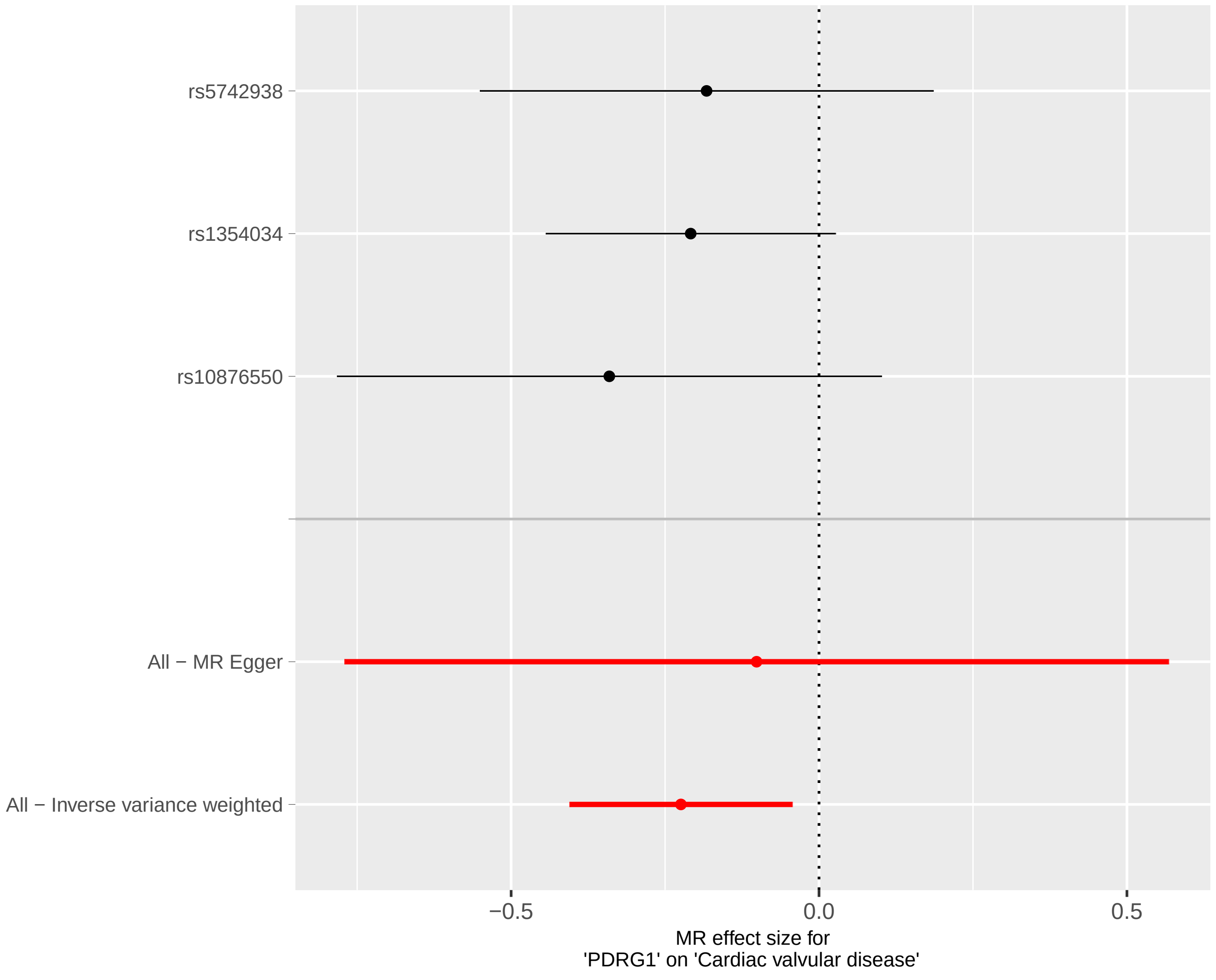
rs5742938
rs1354034
rs10876550
All − MR Egger
All − Inverse variance weighted
−0.5
0.0
0.5
MR effect size for
'PDRG1' on 'Cardiac valvular disease'

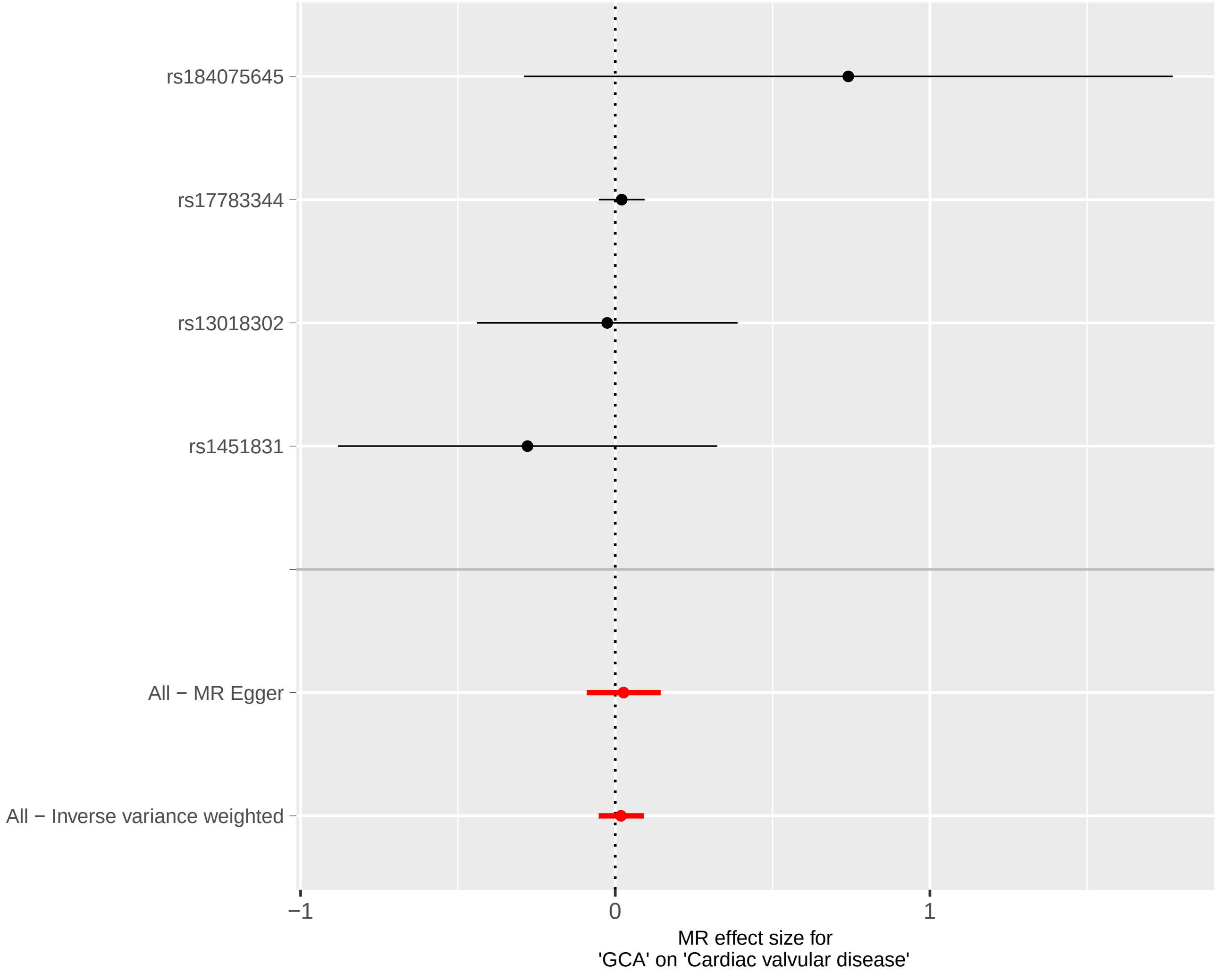
rs184075645
rs17783344
rs13018302
rs1451831
All − MR Egger
All − Inverse variance weighted
−1
0
1
MR effect size for
'GCA' on 'Cardiac valvular disease'

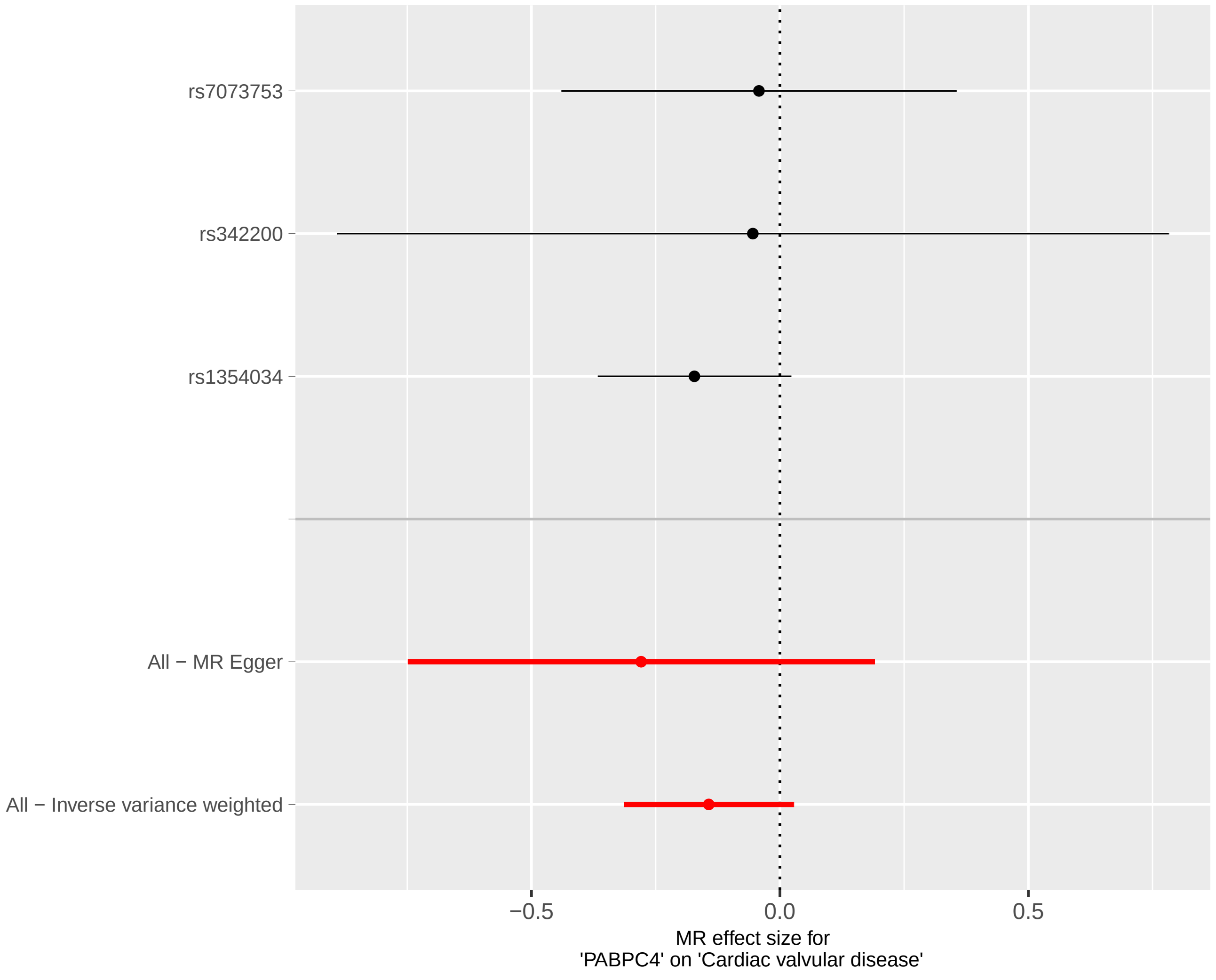
rs7073753
rs342200
rs1354034
All − MR Egger
All − Inverse variance weighted
−0.5
0.0
0.5
MR effect size for
'PABPC4' on 'Cardiac valvular disease'

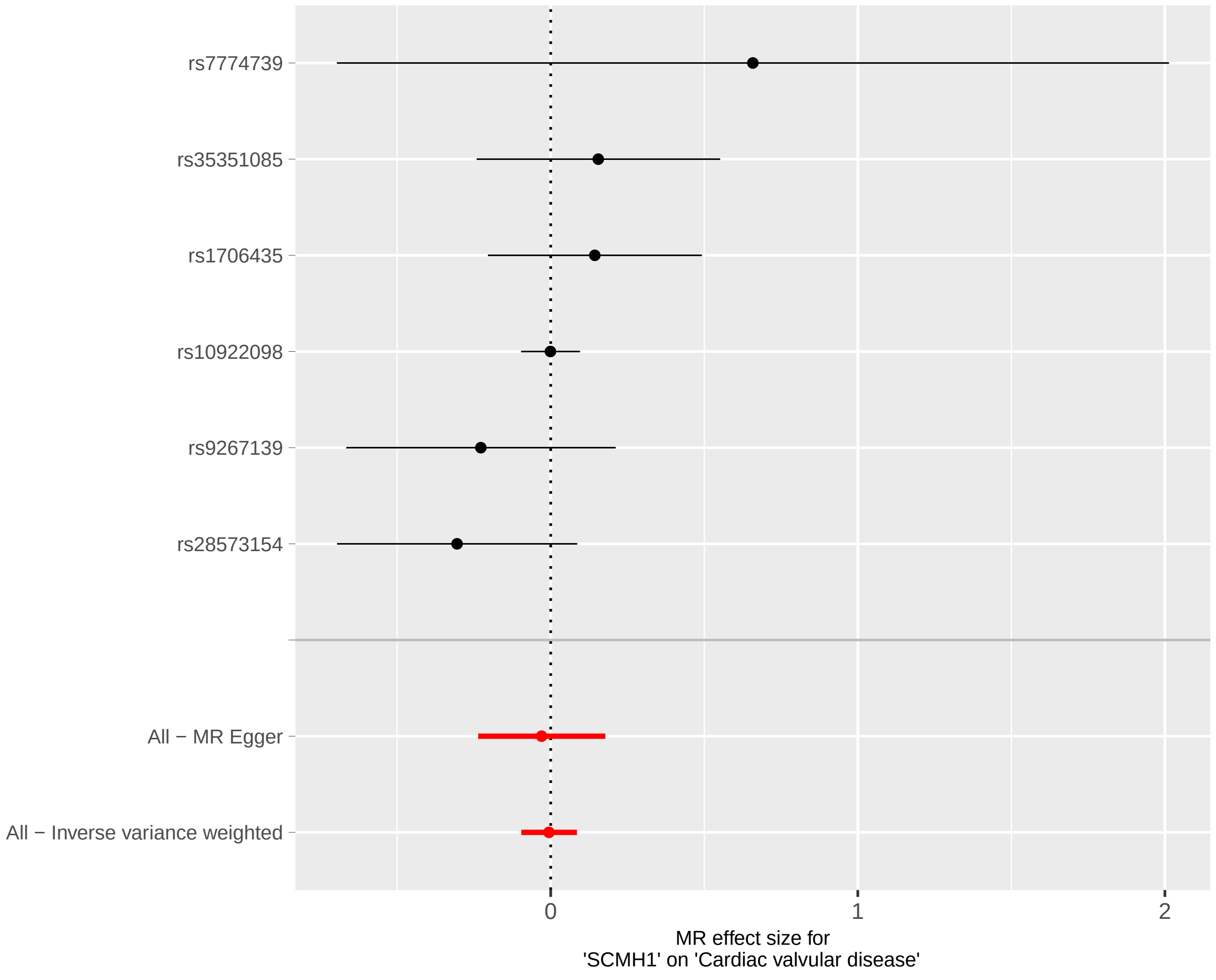
rs7774739
rs35351085
rs1706435
rs10922098
rs9267139
rs28573154
All − MR Egger
All − Inverse variance weighted
0
1
2
MR effect size for
'SCMH1' on 'Cardiac valvular disease'

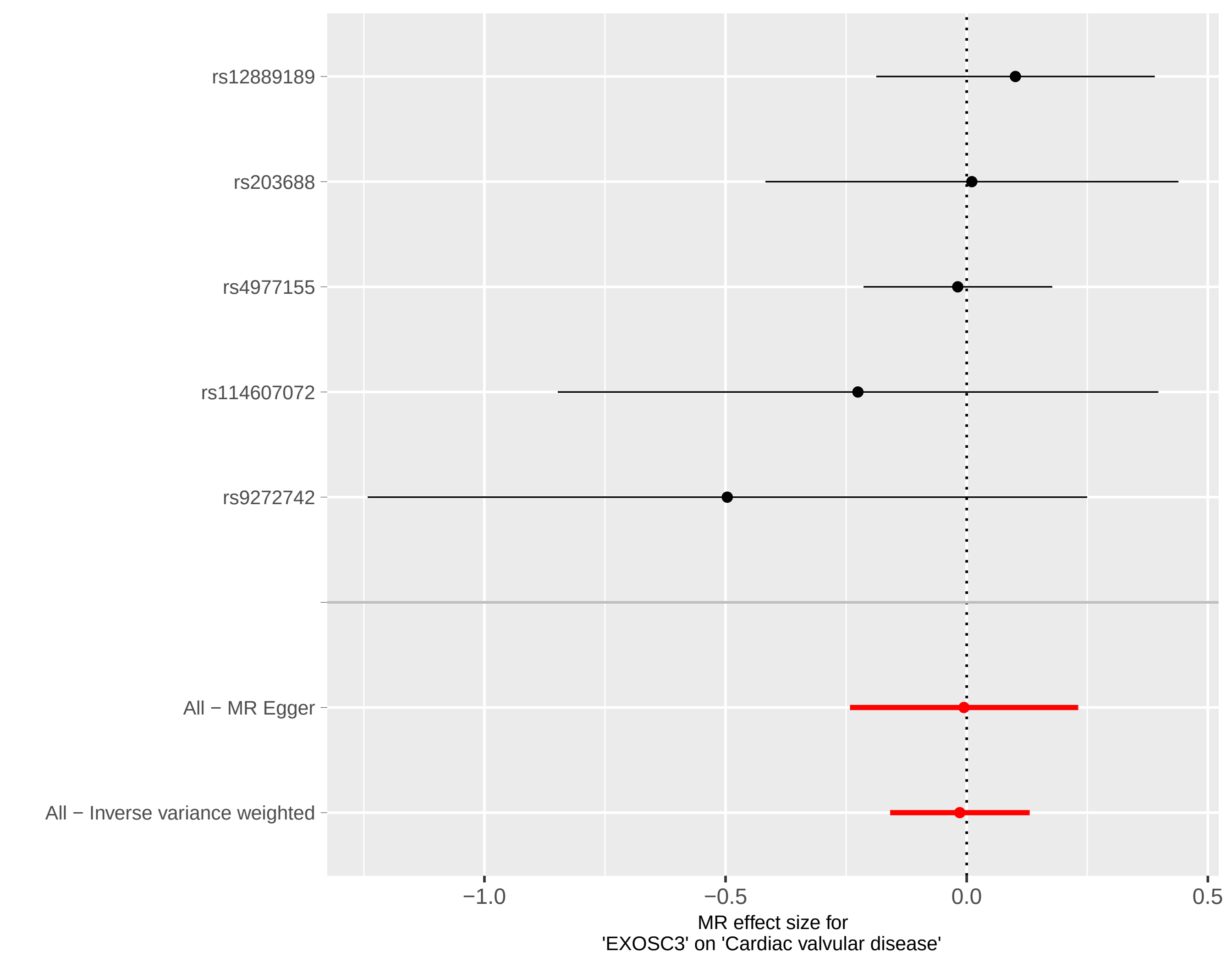
rs12889189
rs203688
rs4977155
rs114607072
rs9272742
All − MR Egger
All − Inverse variance weighted
−1.0
−0.5
0.0
0.5
MR effect size for
'EXOSC3' on 'Cardiac valvular disease'

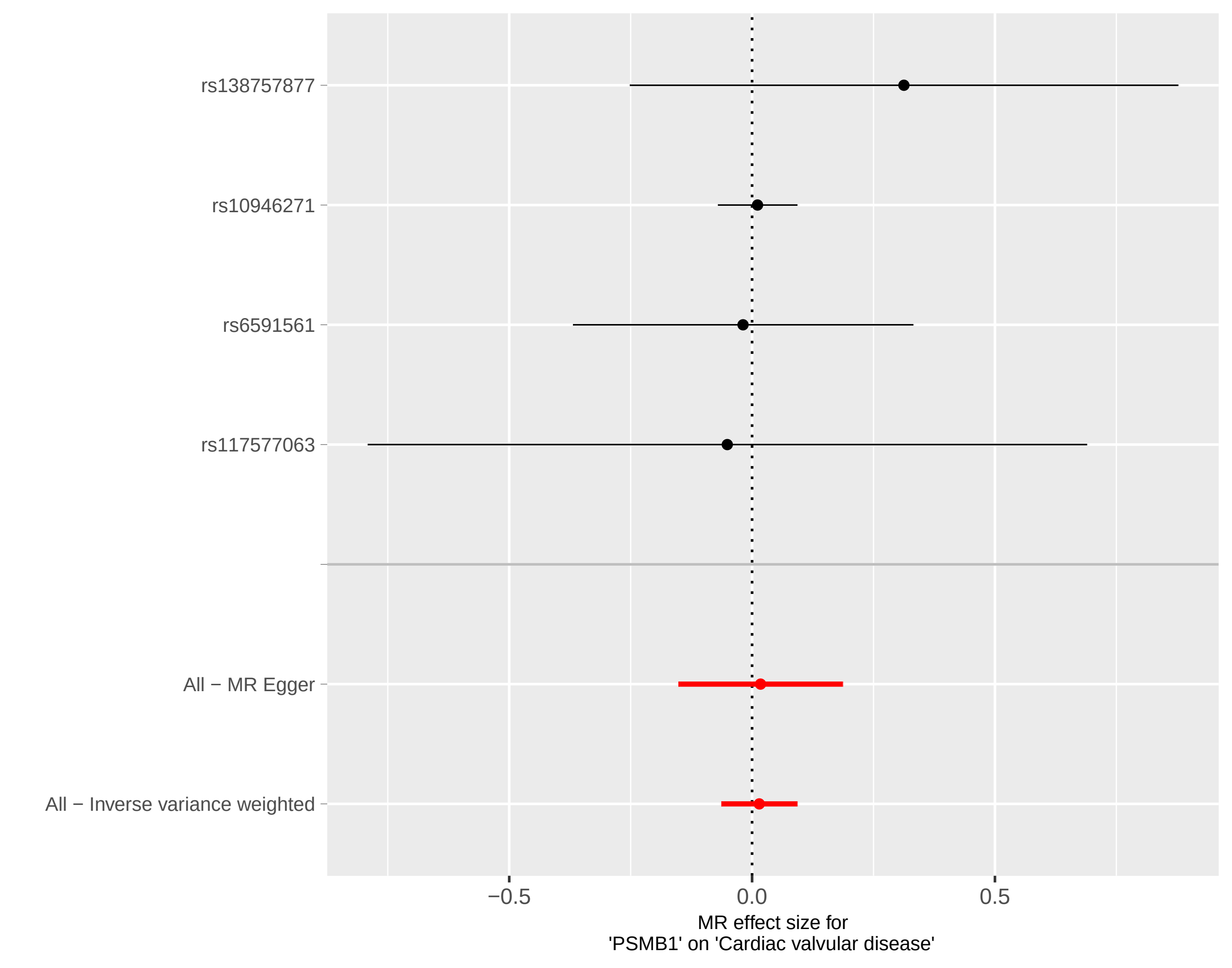
rs138757877
rs10946271
rs6591561
rs117577063
All − MR Egger
All − Inverse variance weighted
−0.5
0.0
0.5
MR effect size for
'PSMB1' on 'Cardiac valvular disease'

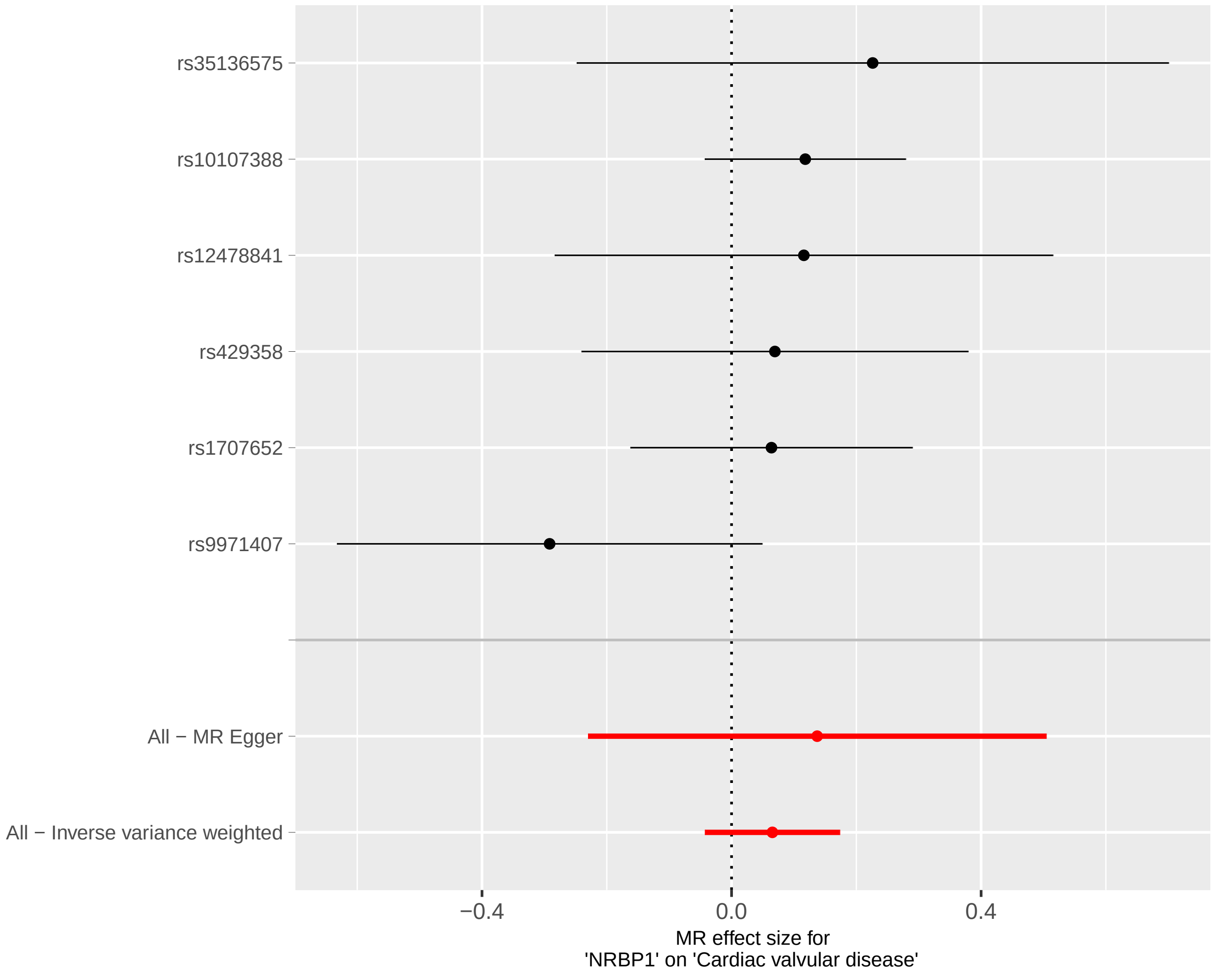
rs35136575
rs10107388
rs12478841
rs429358
rs1707652
rs9971407
All − MR Egger
All − Inverse variance weighted
−0.4
0.0
0.4
MR effect size for
'NRBP1' on 'Cardiac valvular disease'

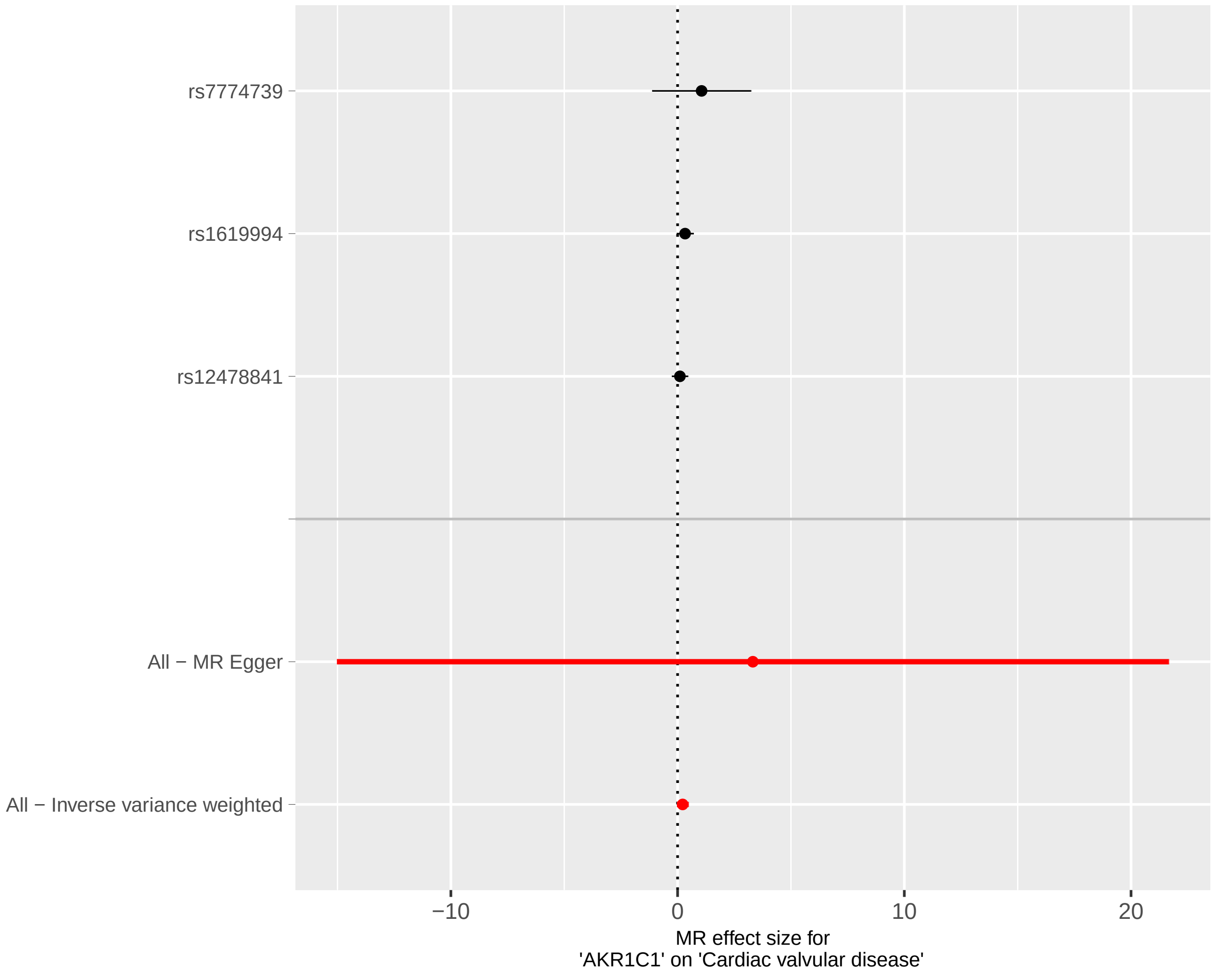
rs7774739
rs1619994
rs12478841
All − MR Egger
All − Inverse variance weighted
−10
0
10
20
MR effect size for
'AKR1C1' on 'Cardiac valvular disease'

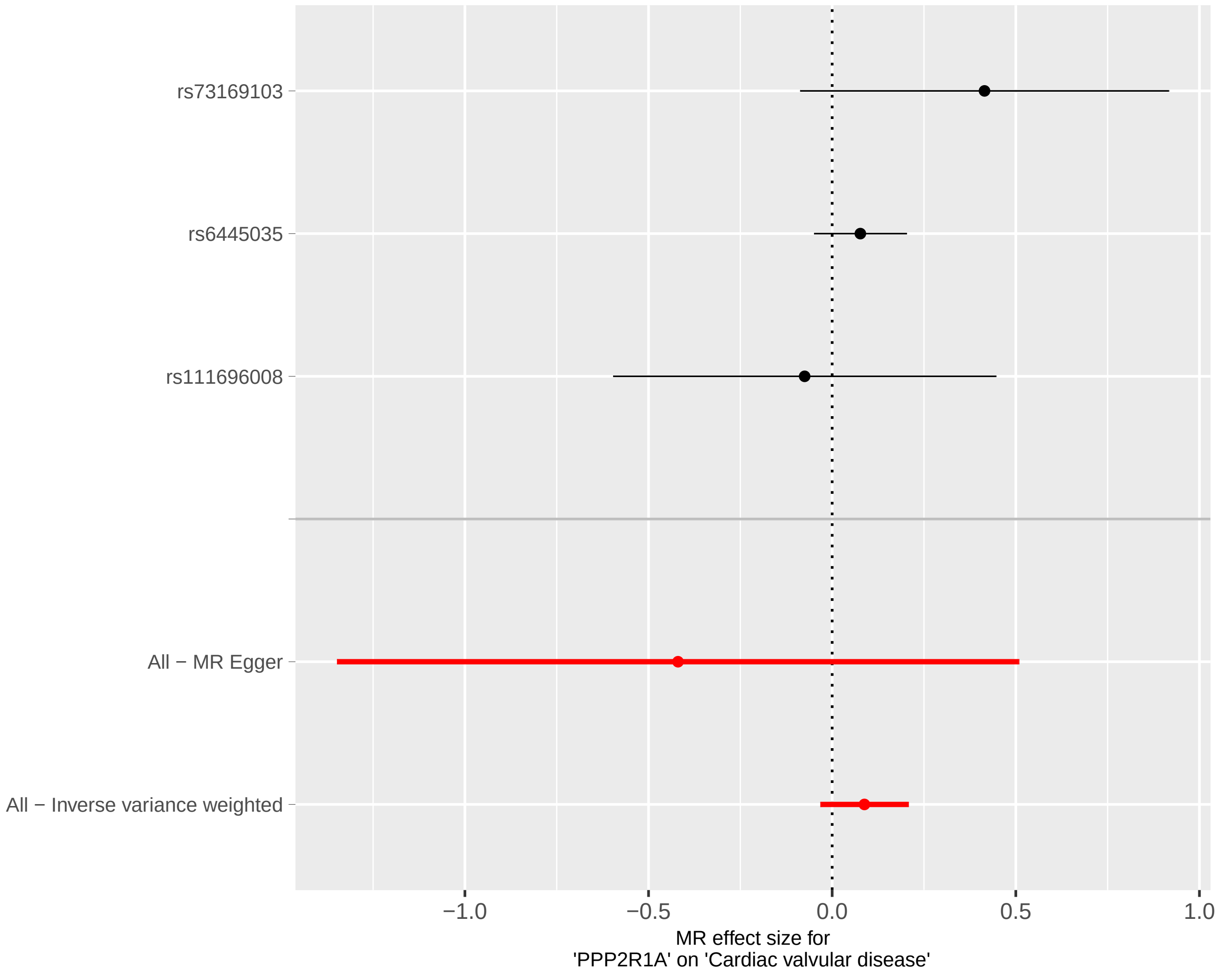
rs73169103
rs6445035
rs111696008
All − MR Egger
All − Inverse variance weighted
−1.0
−0.5
0.0
0.5
1.0
MR effect size for
'PPP2R1A' on 'Cardiac valvular disease'

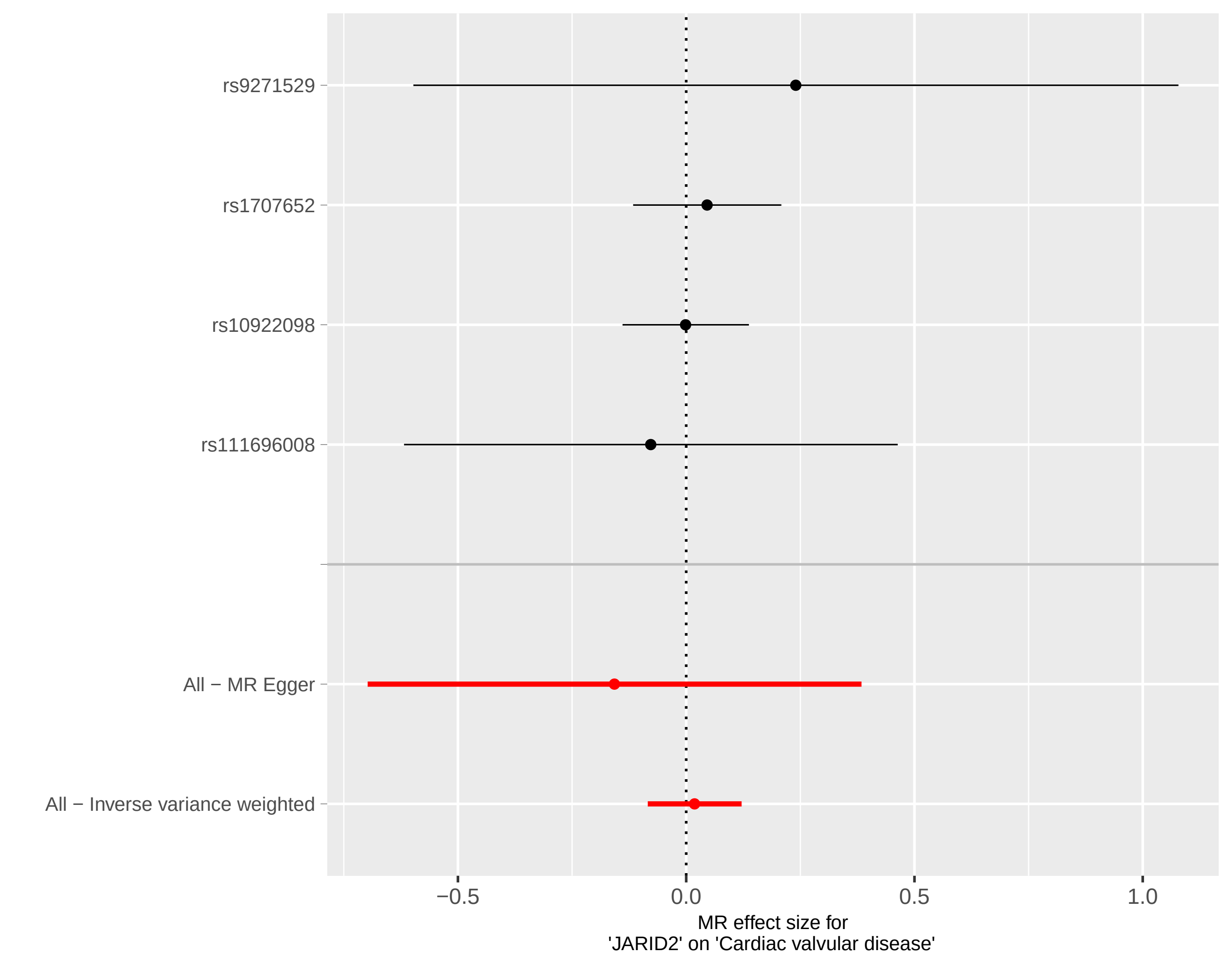
rs9271529
rs1707652
rs10922098
rs111696008
All − MR Egger
All − Inverse variance weighted
−0.5
0.0
0.5
1.0
MR effect size for
'JARID2' on 'Cardiac valvular disease'

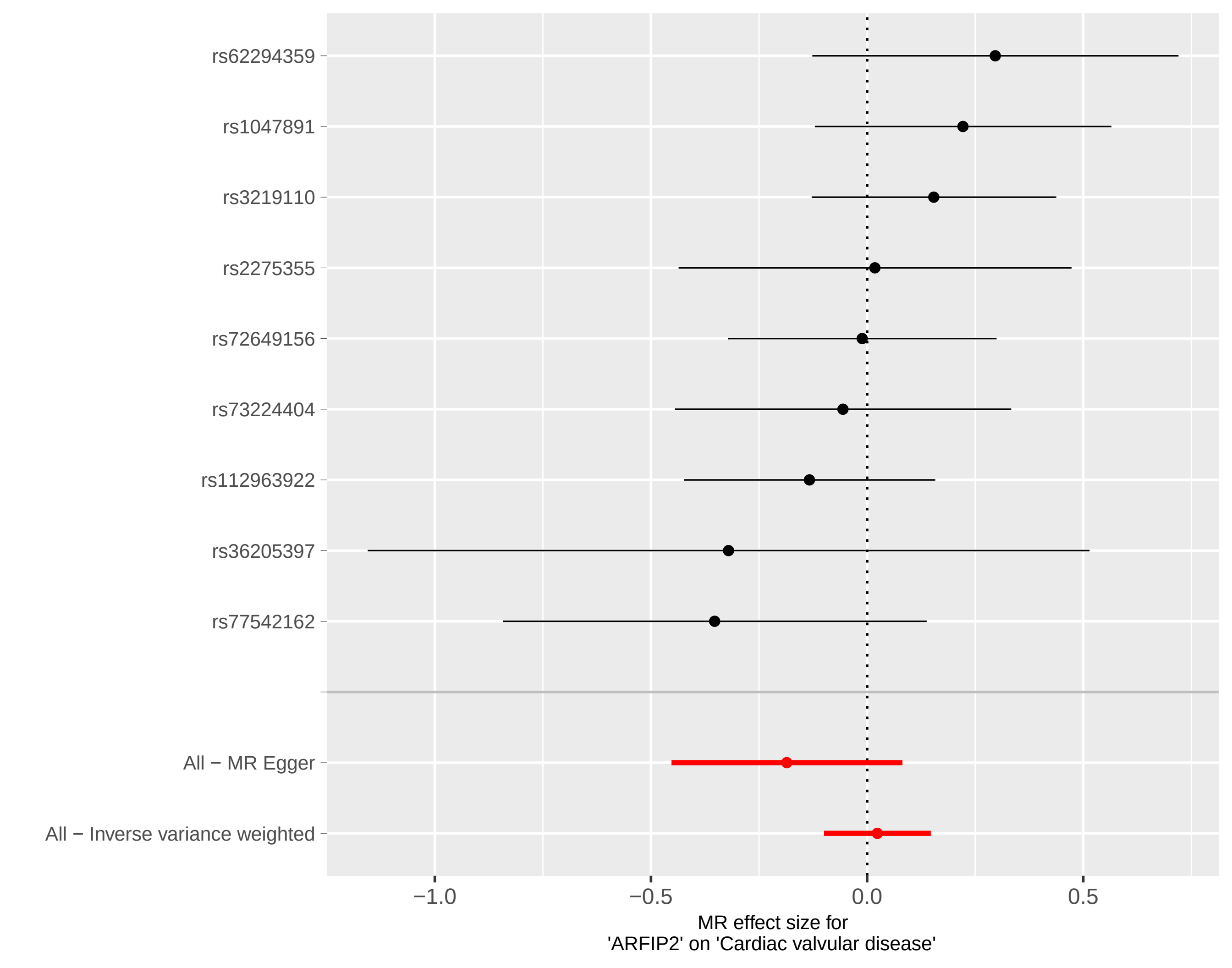
rs62294359
rs1047891
rs3219110
rs2275355
rs72649156
rs73224404
rs112963922
rs36205397
rs77542162
All − MR Egger
All − Inverse variance weighted
−1.0
−0.5
0.0
0.5
MR effect size for
'ARFIP2' on 'Cardiac valvular disease'

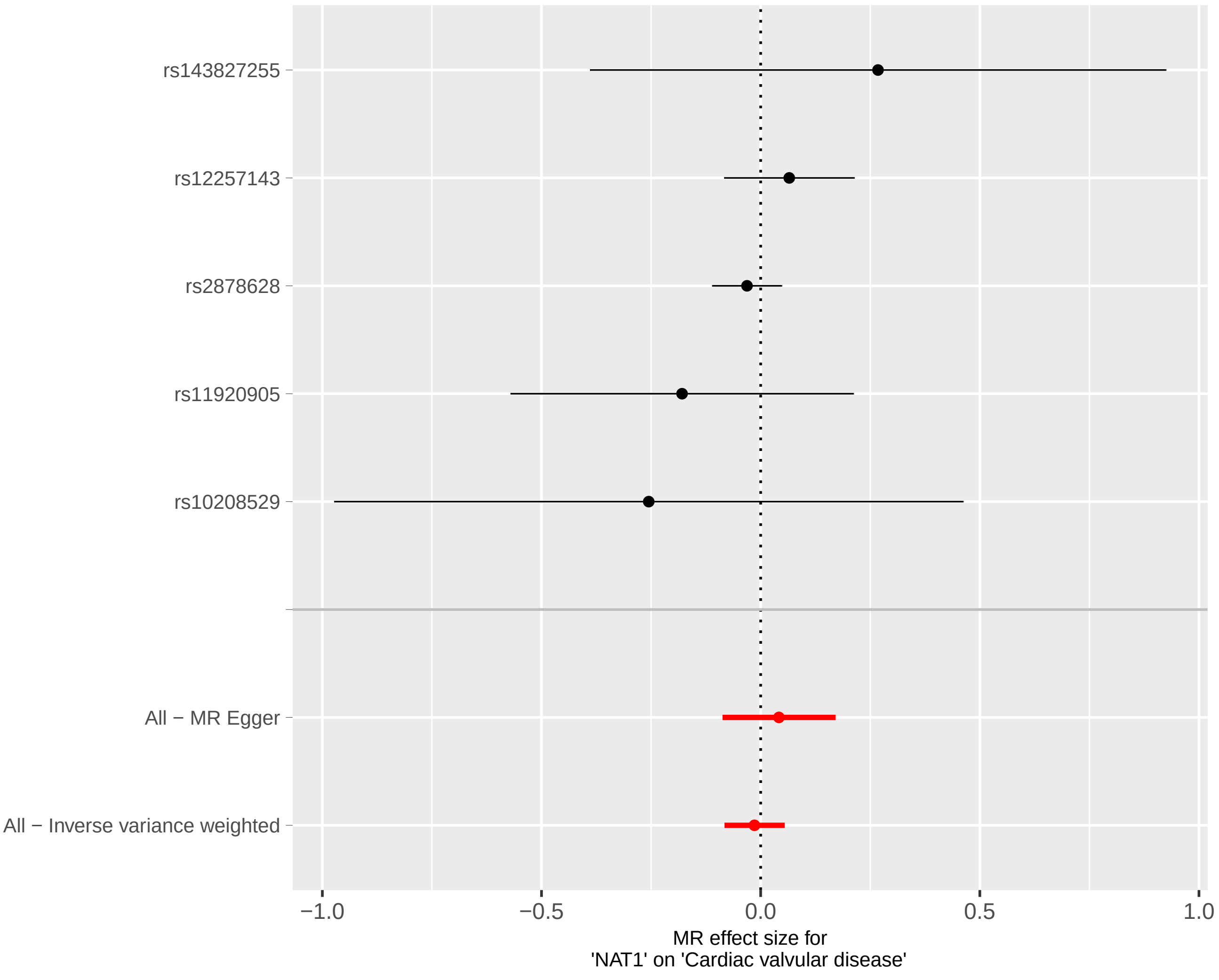
rs143827255
rs12257143
rs2878628
rs11920905
rs10208529
All − MR Egger
All − Inverse variance weighted
−1.0
−0.5
0.0
0.5
1.0
MR effect size for
'NAT1' on 'Cardiac valvular disease'

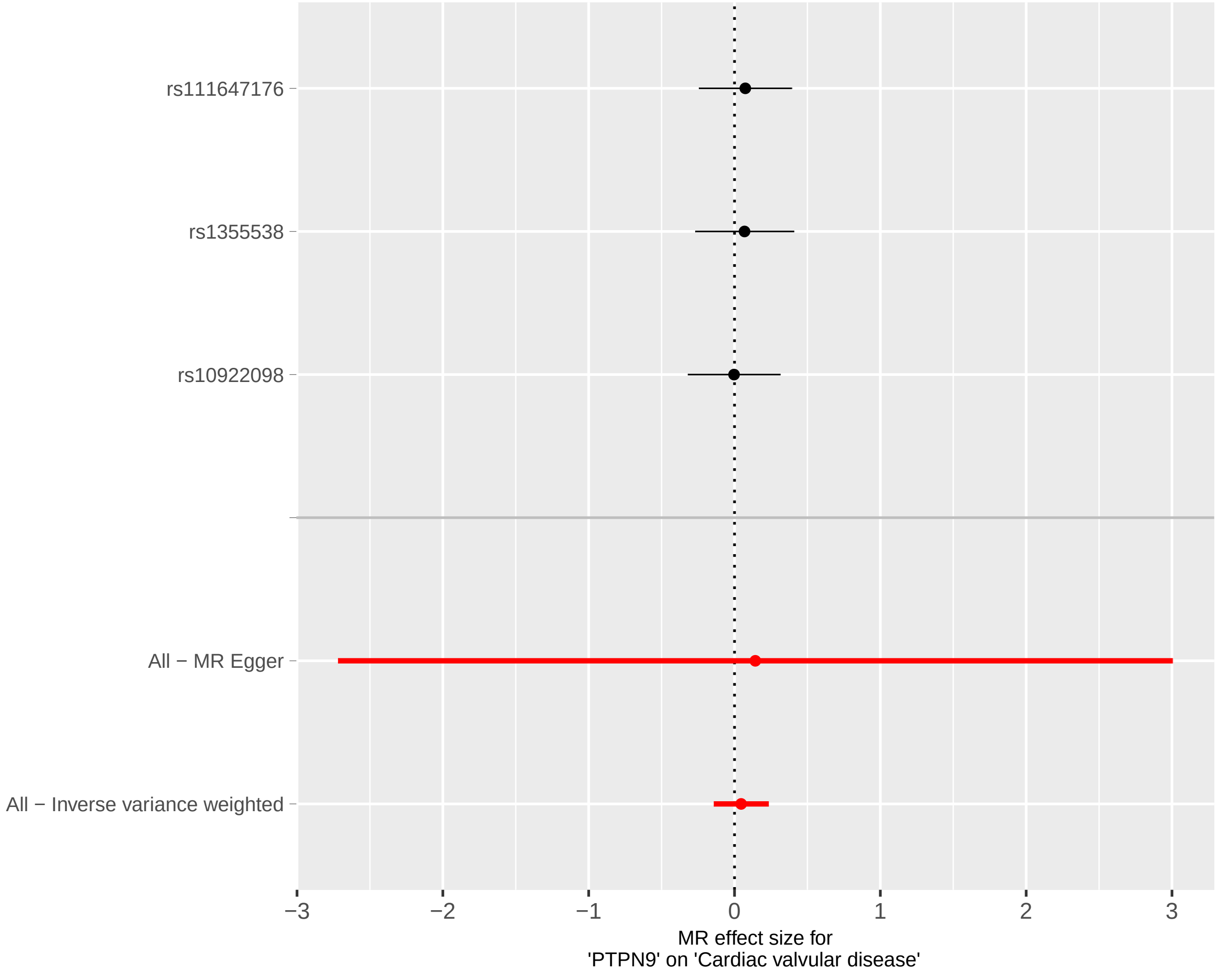
rs111647176
rs1355538
rs10922098
All − MR Egger
All − Inverse variance weighted
−3
−2
−1
0
1
2
3
MR effect size for
'PTPN9' on 'Cardiac valvular disease'

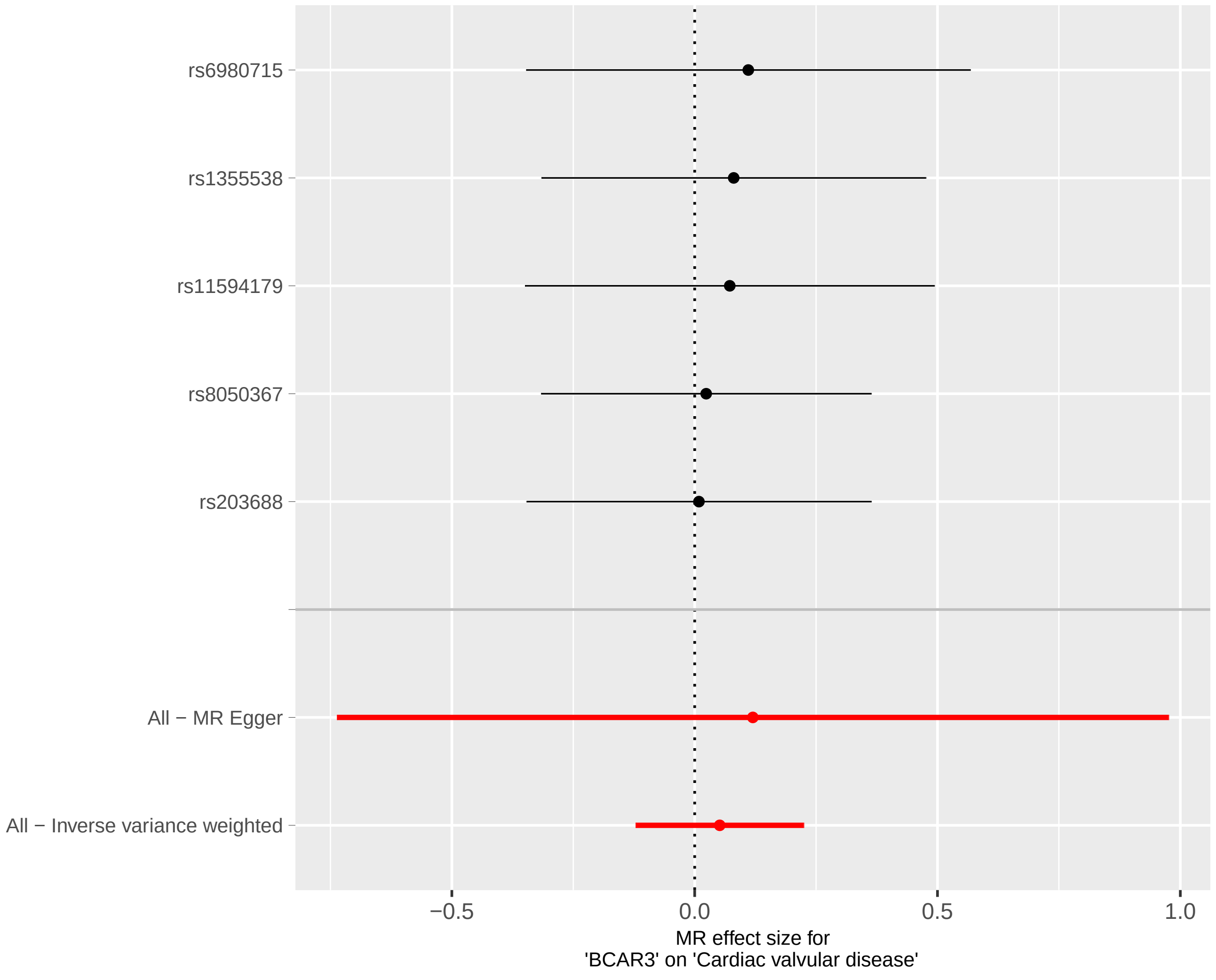

rs6980715
rs1355538
rs11594179
rs8050367
rs203688
All − MR Egger
All − Inverse variance weighted
−0.5
0.0
0.5
1.0
MR effect size for
'BCAR3' on 'Cardiac valvular disease'

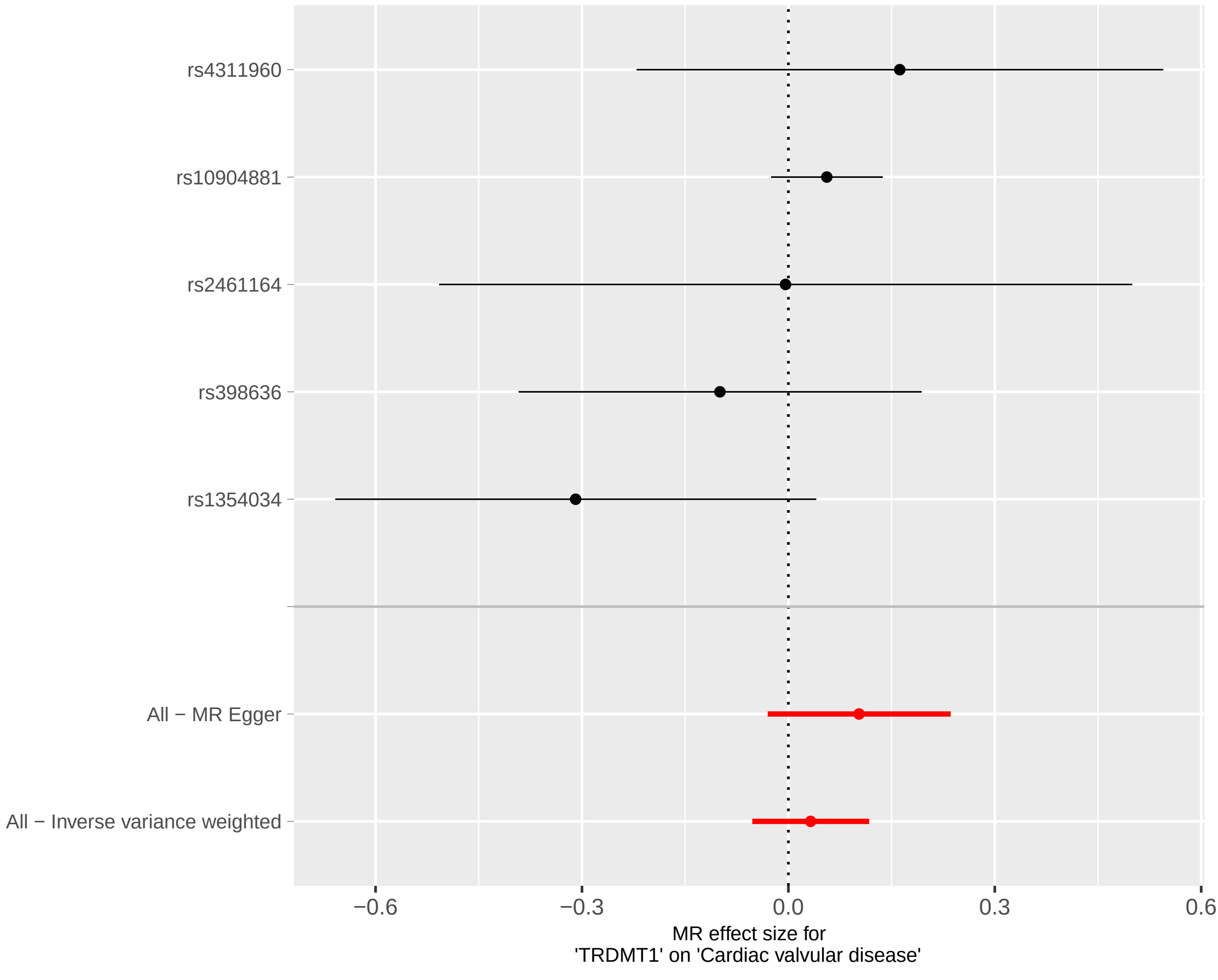
rs4311960
rs10904881
rs2461164
rs398636
rs1354034
All − MR Egger
All − Inverse variance weighted
−0.6
−0.3
0.0
0.3
0.6
MR effect size for
'TRDMT1' on 'Cardiac valvular disease'

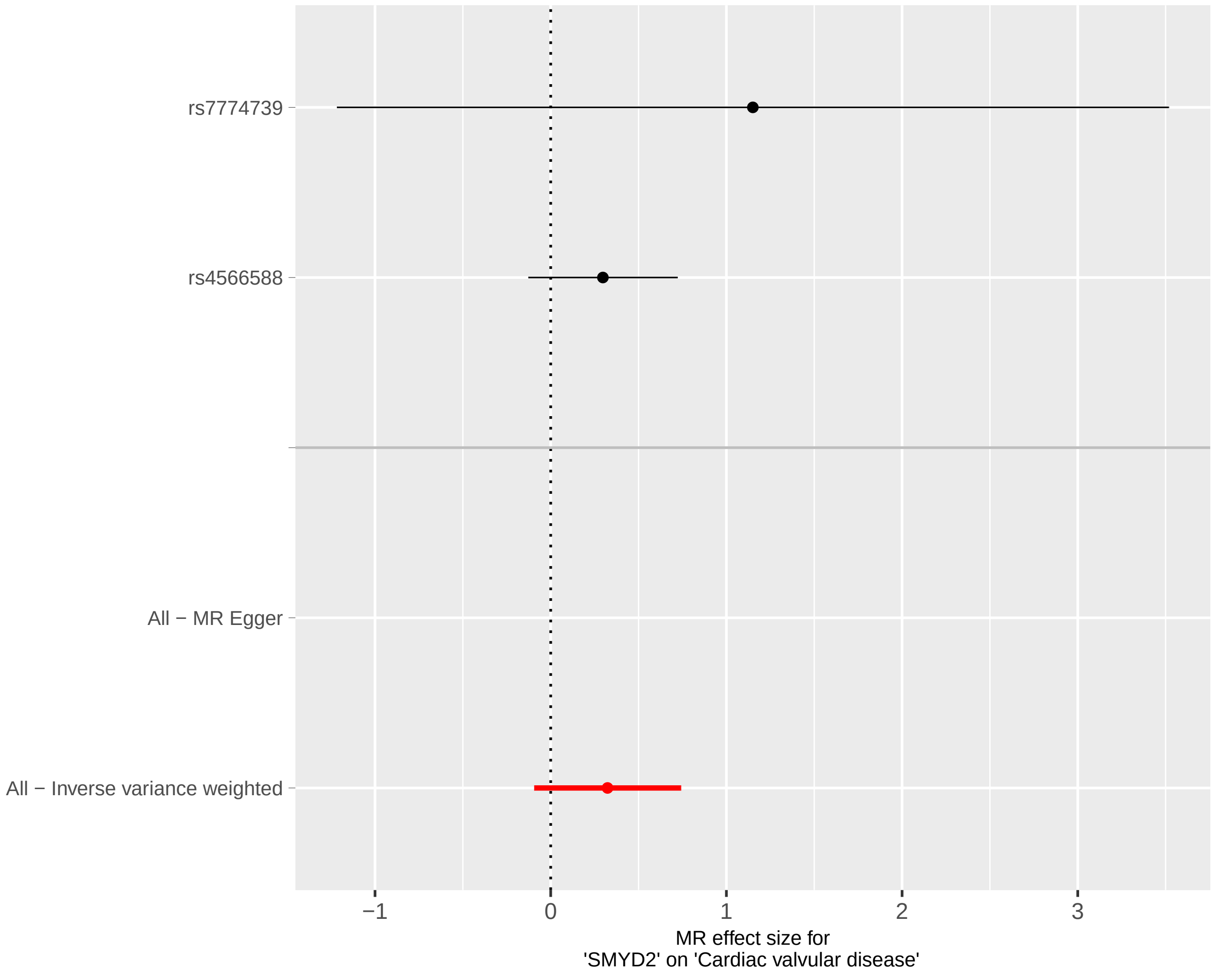
rs7774739
rs4566588
All − MR Egger
All − Inverse variance weighted
−1
0
1
2
3
MR effect size for
'SMYD2' on 'Cardiac valvular disease'

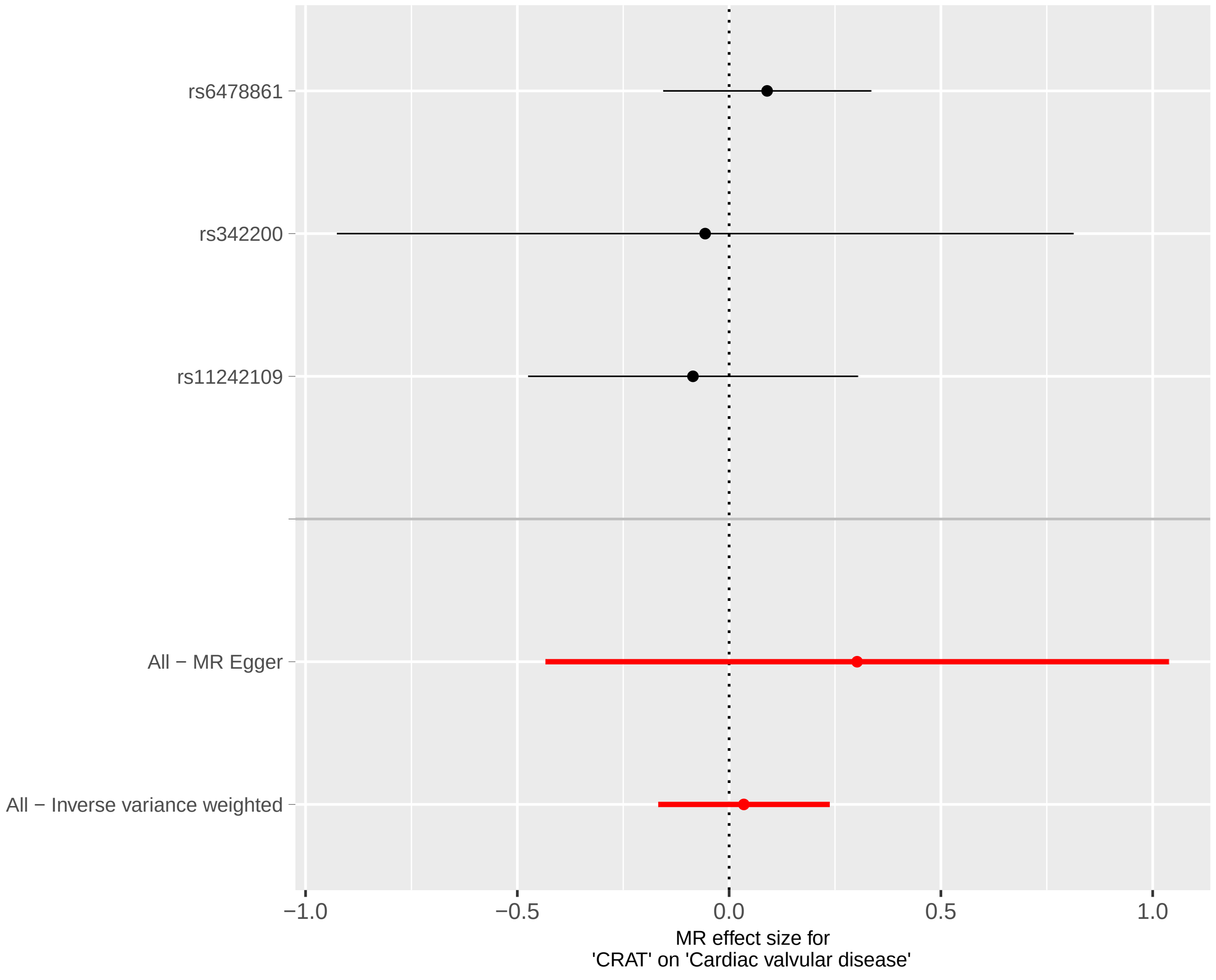
rs6478861
rs342200
rs11242109
All − MR Egger
All − Inverse variance weighted
−1.0
−0.5
0.0
0.5
1.0
MR effect size for
'CRAT' on 'Cardiac valvular disease'

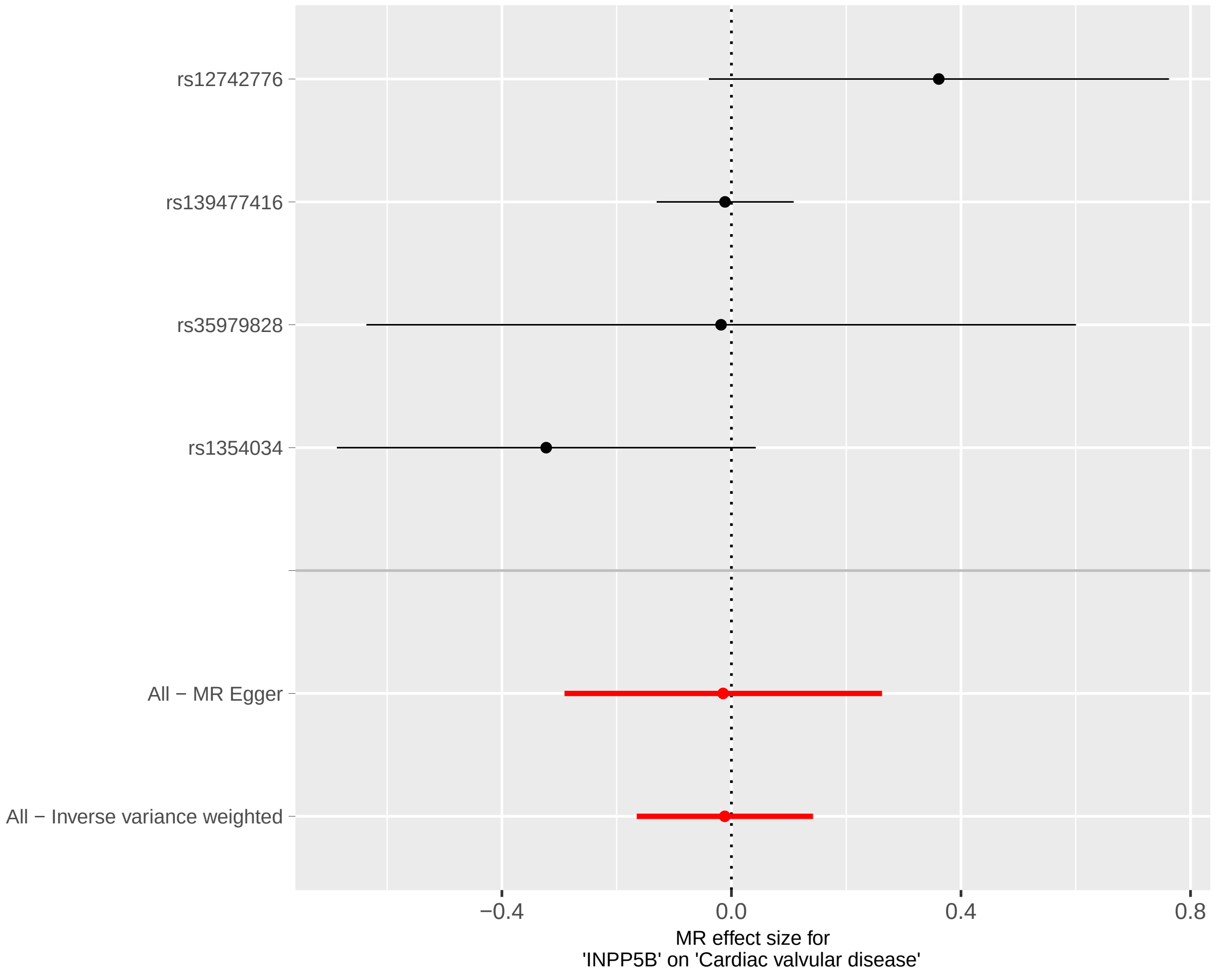
rs12742776
rs139477416
rs35979828
rs1354034
All − MR Egger
All − Inverse variance weighted
−0.4
0.0
0.4
0.8
MR effect size for
'INPP5B' on 'Cardiac valvular disease'

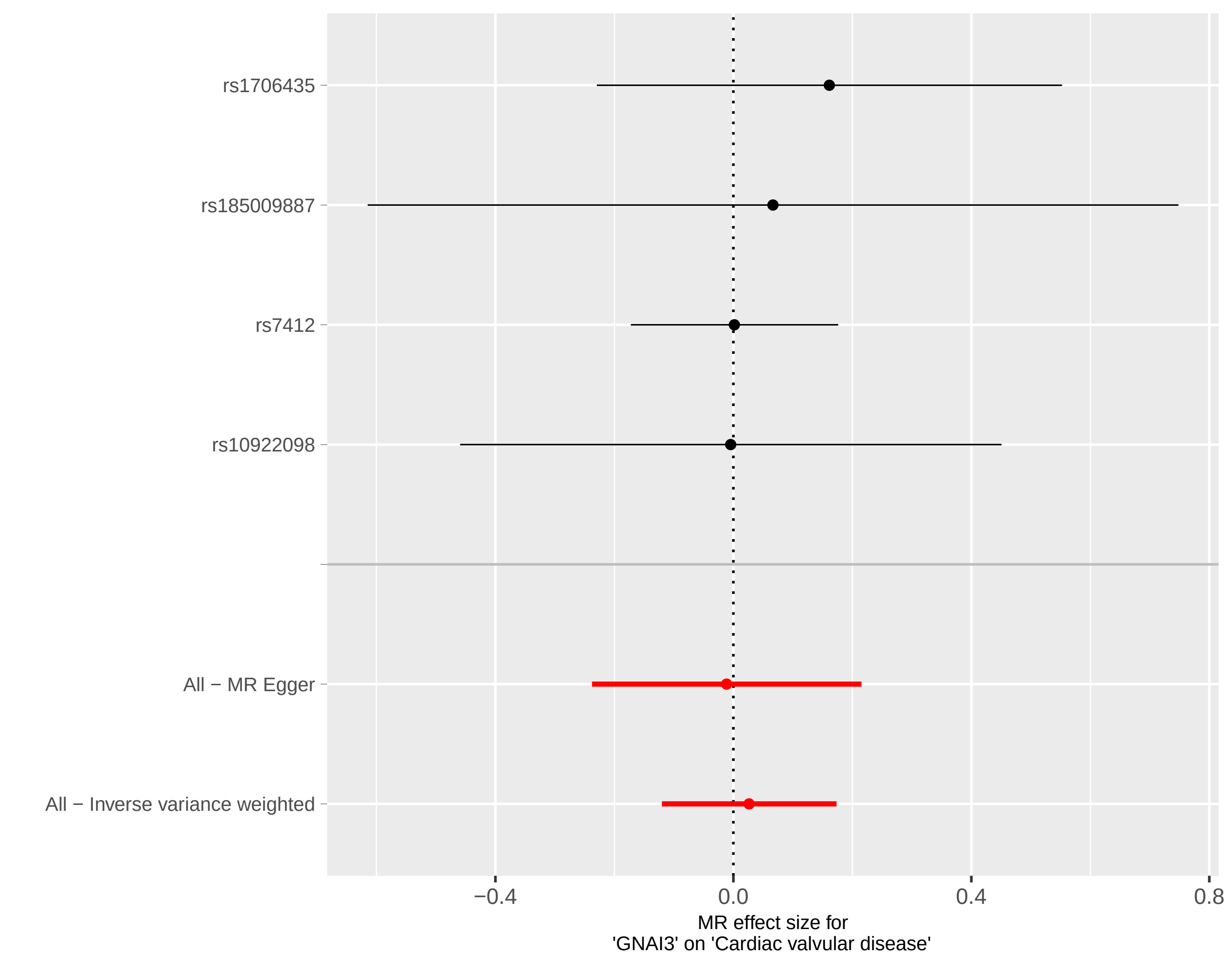
rs1706435
rs185009887
rs7412
rs10922098
All − MR Egger
All − Inverse variance weighted
−0.4
0.0
0.4
0.8
MR effect size for
'GNAI3' on 'Cardiac valvular disease'

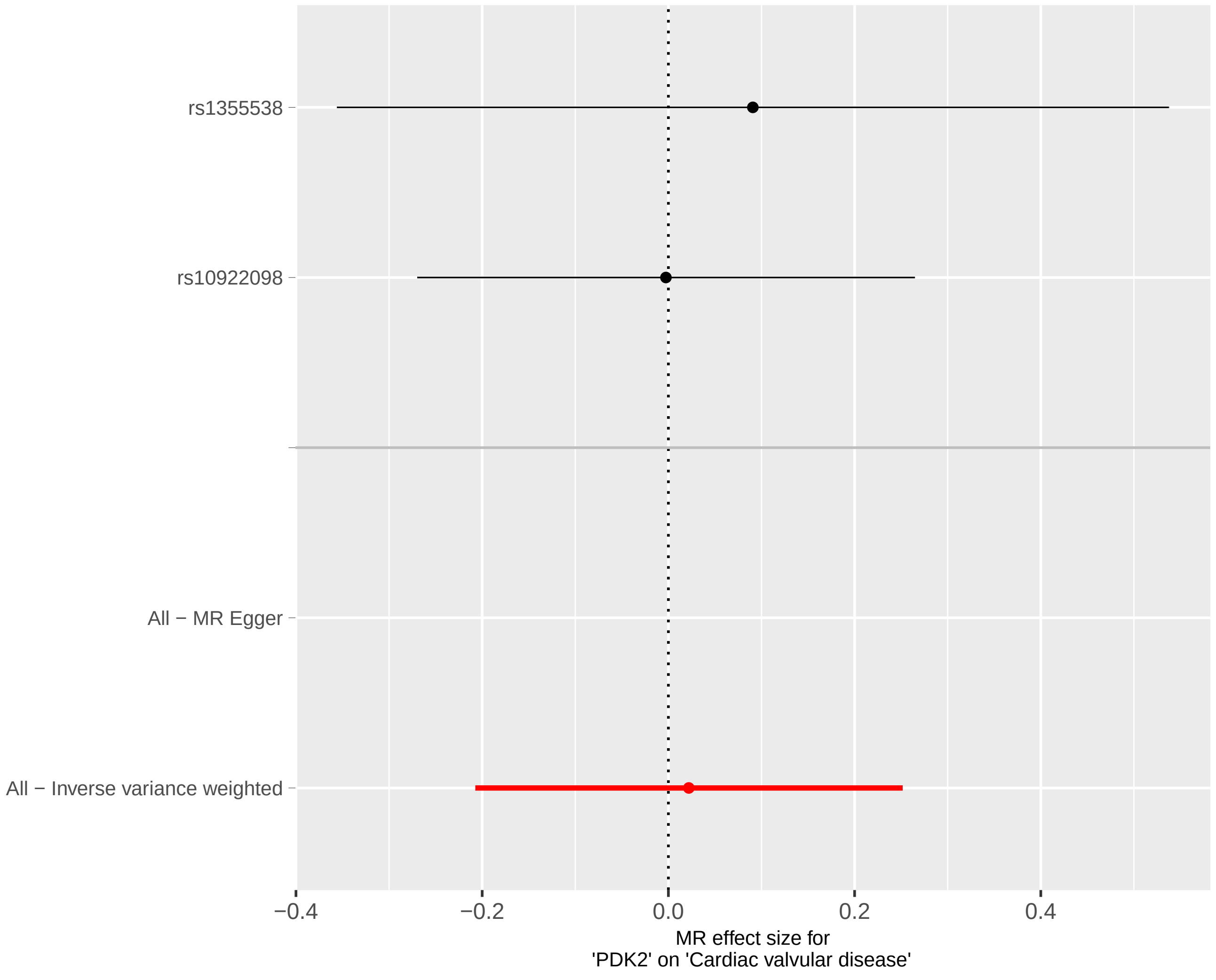
rs1355538
rs10922098
All − MR Egger
All − Inverse variance weighted
−0.4
−0.2
0.0
0.2
0.4
MR effect size for
'PDK2' on 'Cardiac valvular disease'

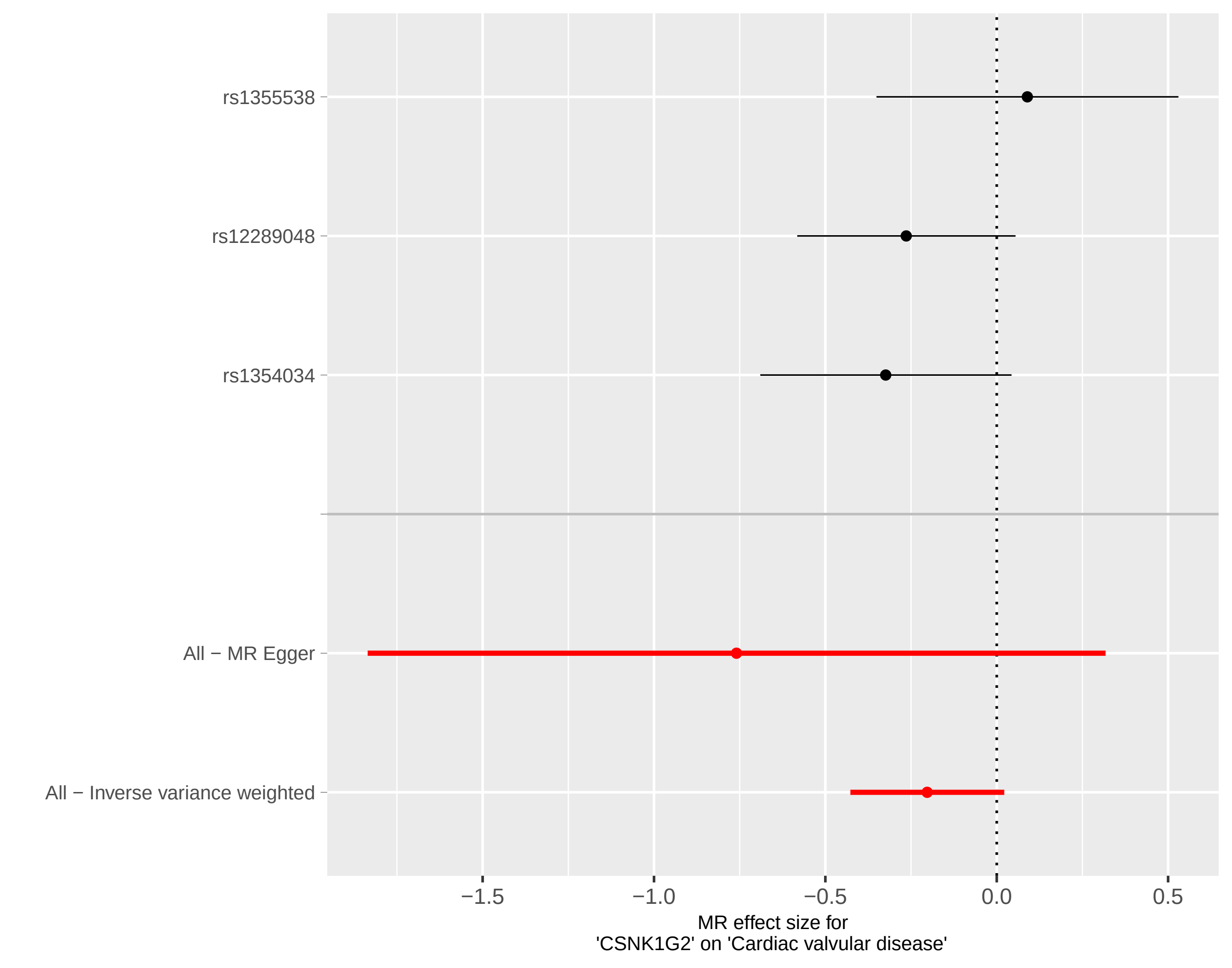
rs1355538
rs12289048
rs1354034
All − MR Egger
All − Inverse variance weighted
−1.5
−1.0
−0.5
0.0
0.5
MR effect size for
'CSNK1G2' on 'Cardiac valvular disease'

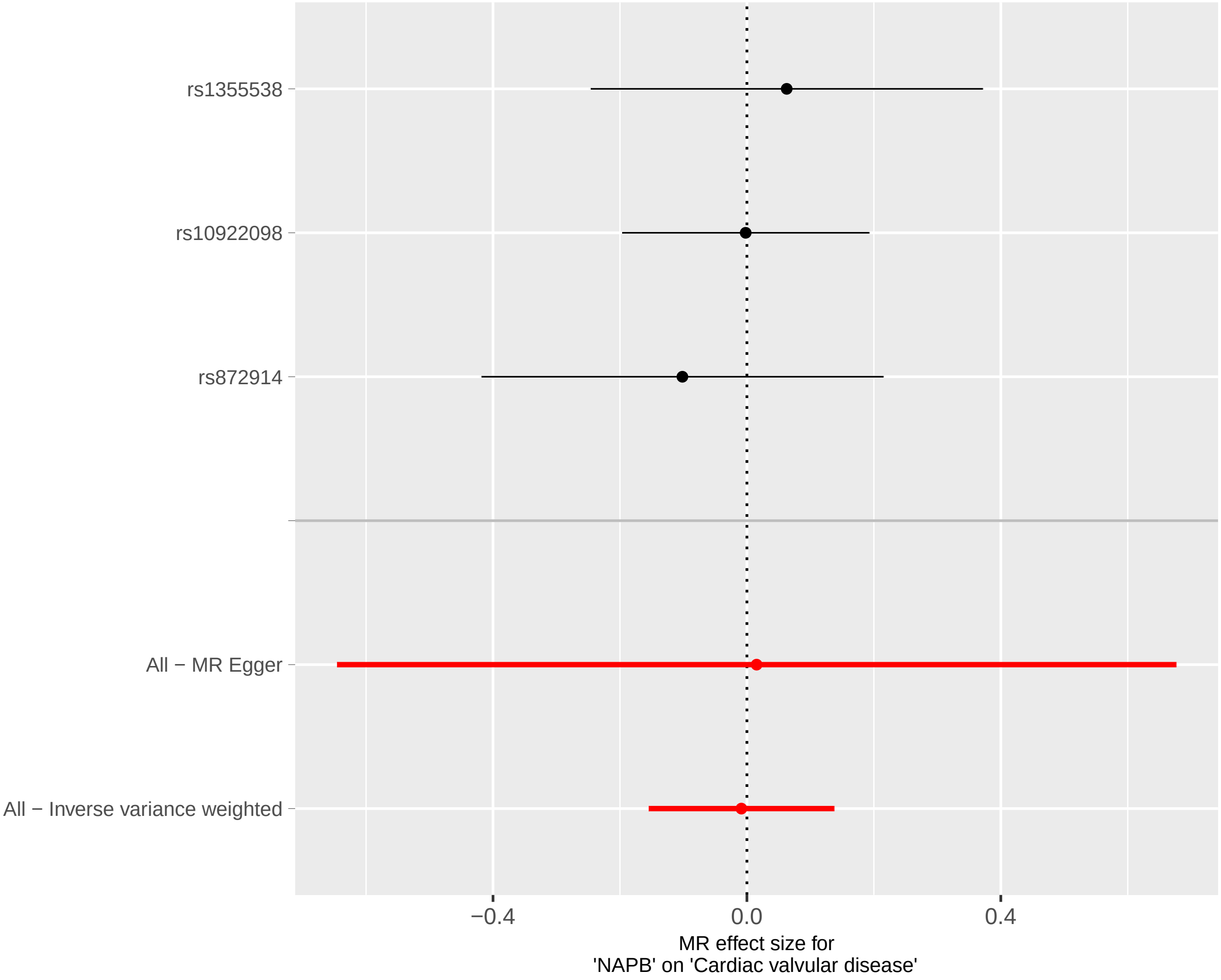
rs1355538
rs10922098
rs872914
All − MR Egger
All − Inverse variance weighted
−0.4
0.0
0.4
MR effect size for
'NAPB' on 'Cardiac valvular disease'

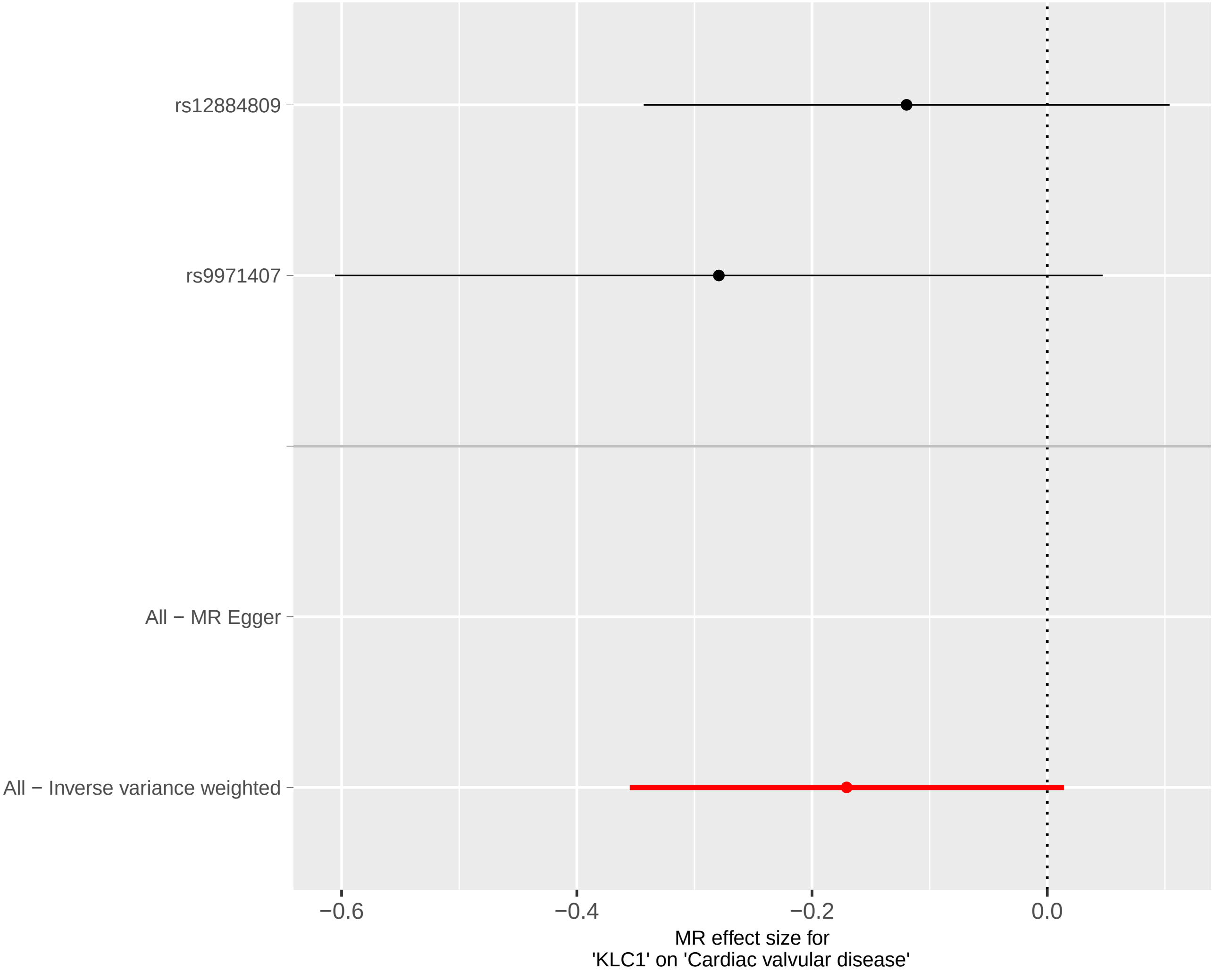
rs12884809
rs9971407
All − MR Egger
All − Inverse variance weighted
−0.6
−0.4
−0.2
0.0
MR effect size for
'KLC1' on 'Cardiac valvular disease'

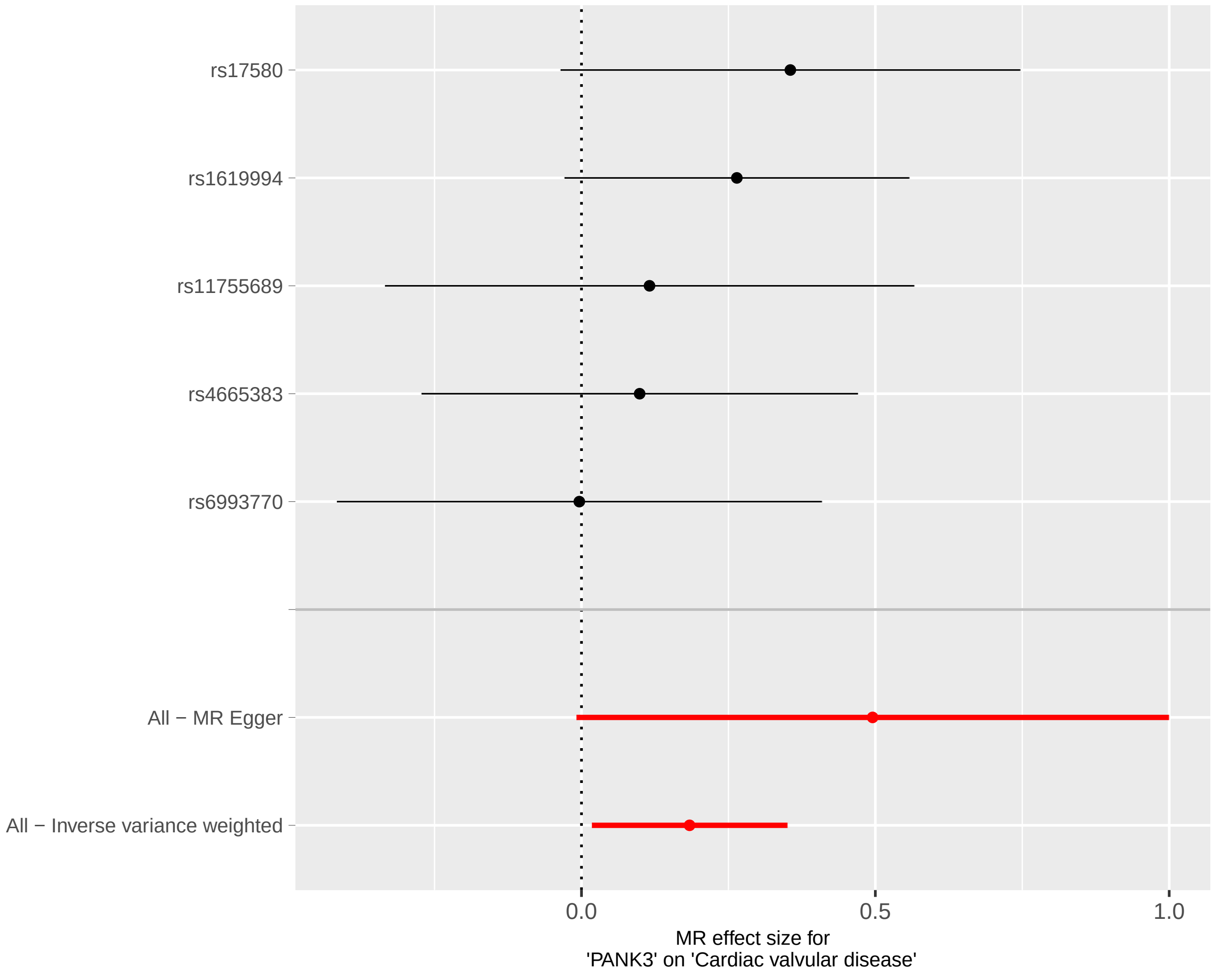
rs17580
rs1619994
rs11755689
rs4665383
rs6993770
All − MR Egger
All − Inverse variance weighted
0.0
0.5
1.0
MR effect size for
'PANK3' on 'Cardiac valvular disease'

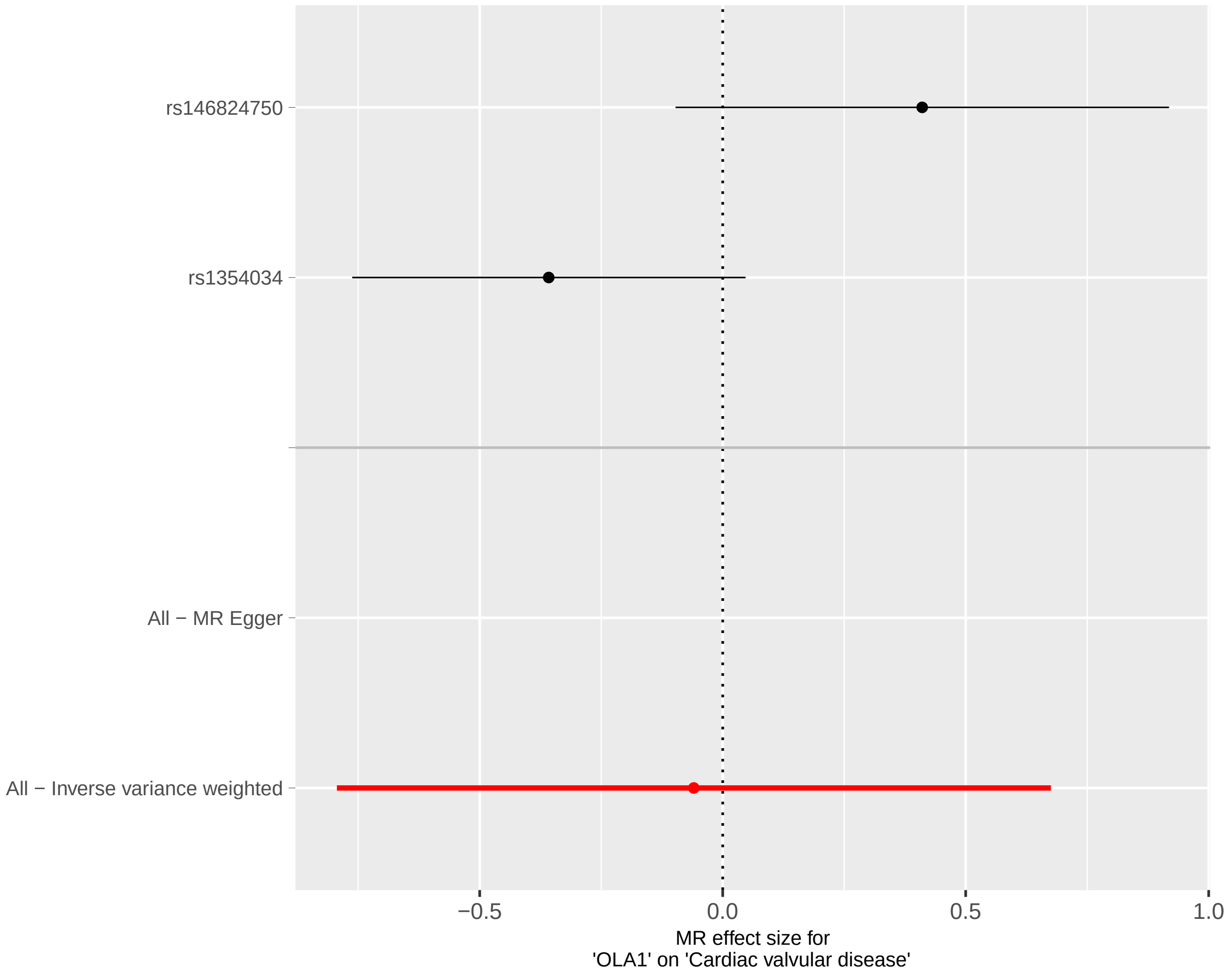

rs146824750
rs1354034
All − MR Egger
All − Inverse variance weighted
−0.5
0.0
0.5
1.0
MR effect size for
'OLA1' on 'Cardiac valvular disease'

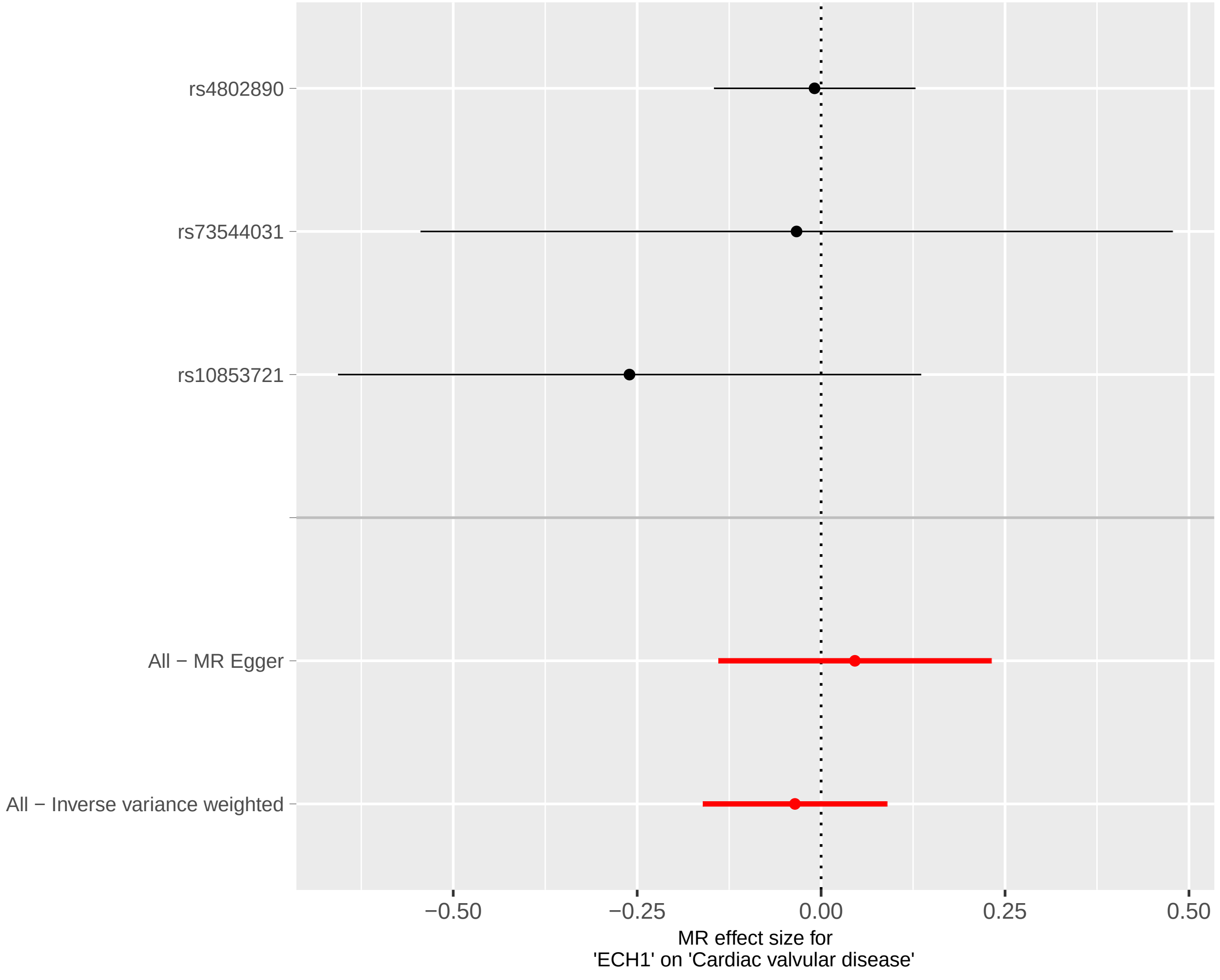
rs4802890
rs73544031
rs10853721
All − MR Egger
All − Inverse variance weighted
−0.50
−0.25
0.00
0.25
0.50
MR effect size for
'ECH1' on 'Cardiac valvular disease'

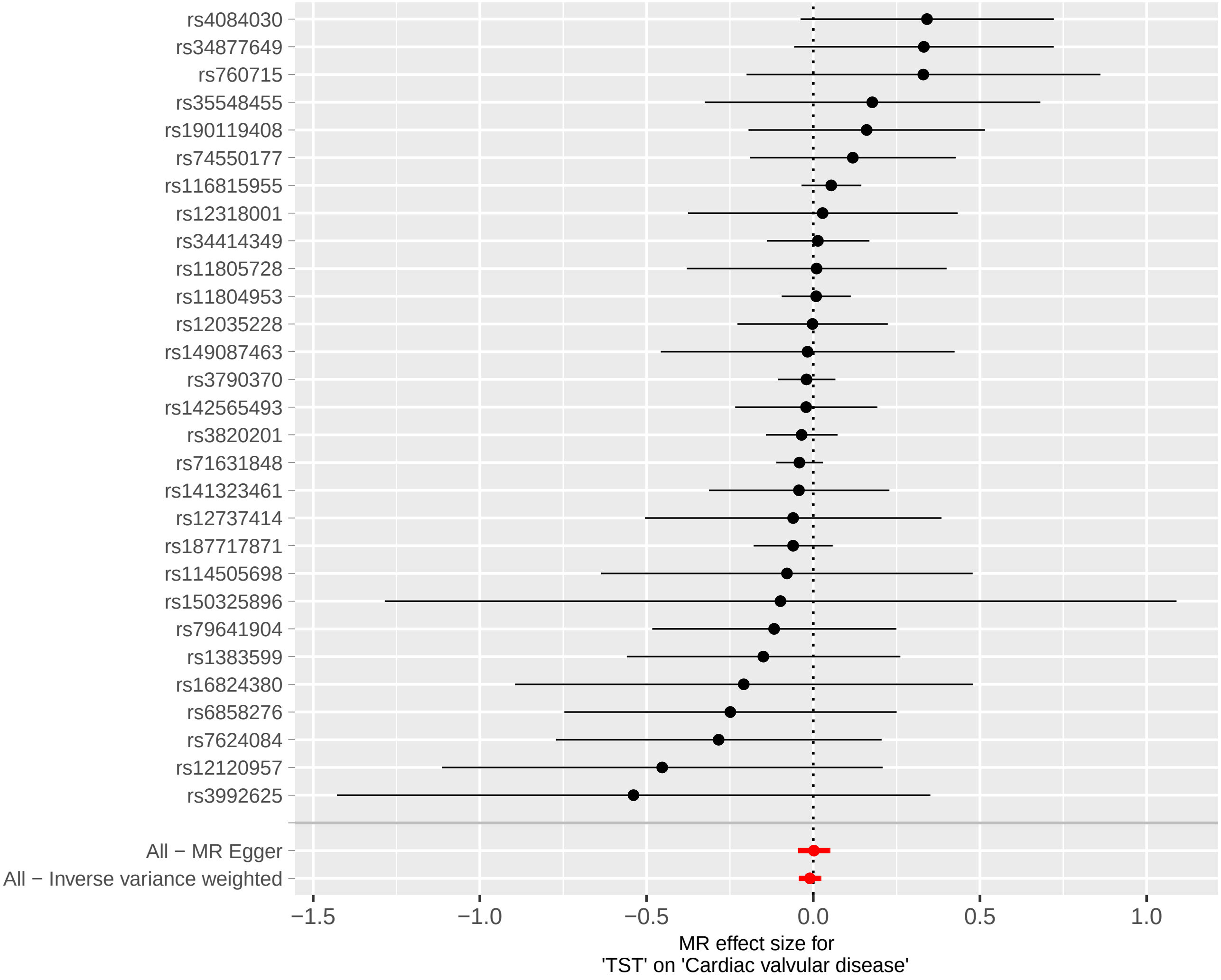

rs4084030
rs34877649
rs760715
rs35548455
rs190119408
rs74550177
rs116815955
rs12318001
rs34414349
rs11805728
rs11804953
rs12035228
rs149087463
rs3790370
rs142565493
rs3820201
rs71631848
rs141323461
rs12737414
rs187717871
rs114505698
rs150325896
rs79641904
rs1383599
rs16824380
rs6858276
rs7624084
rs12120957
rs3992625
All − MR Egger
All − Inverse variance weighted
−1.5
−1.0
−0.5
0.0
0.5
1.0
MR effect size for
'TST' on 'Cardiac valvular disease'

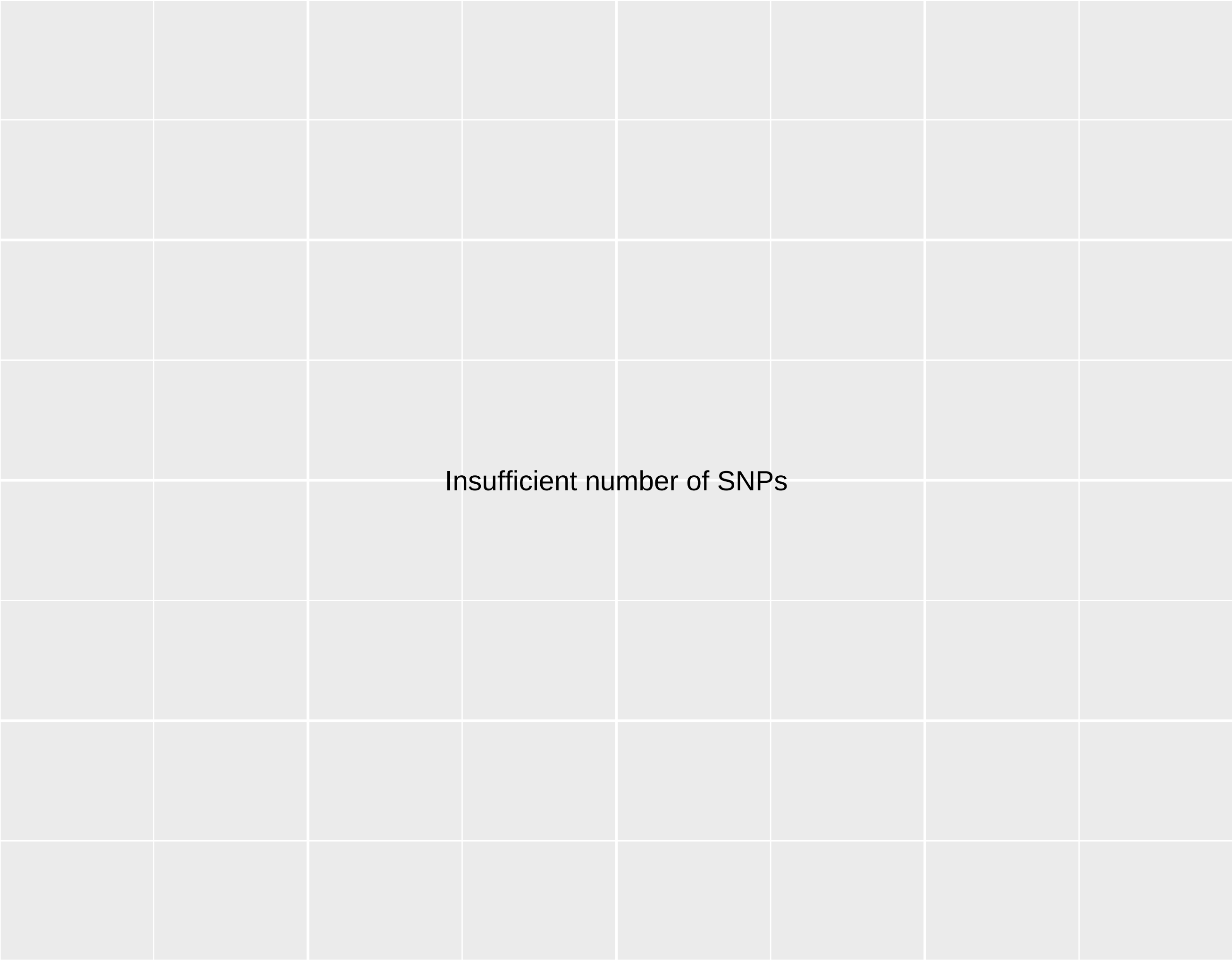
Insufficient number of SNPs

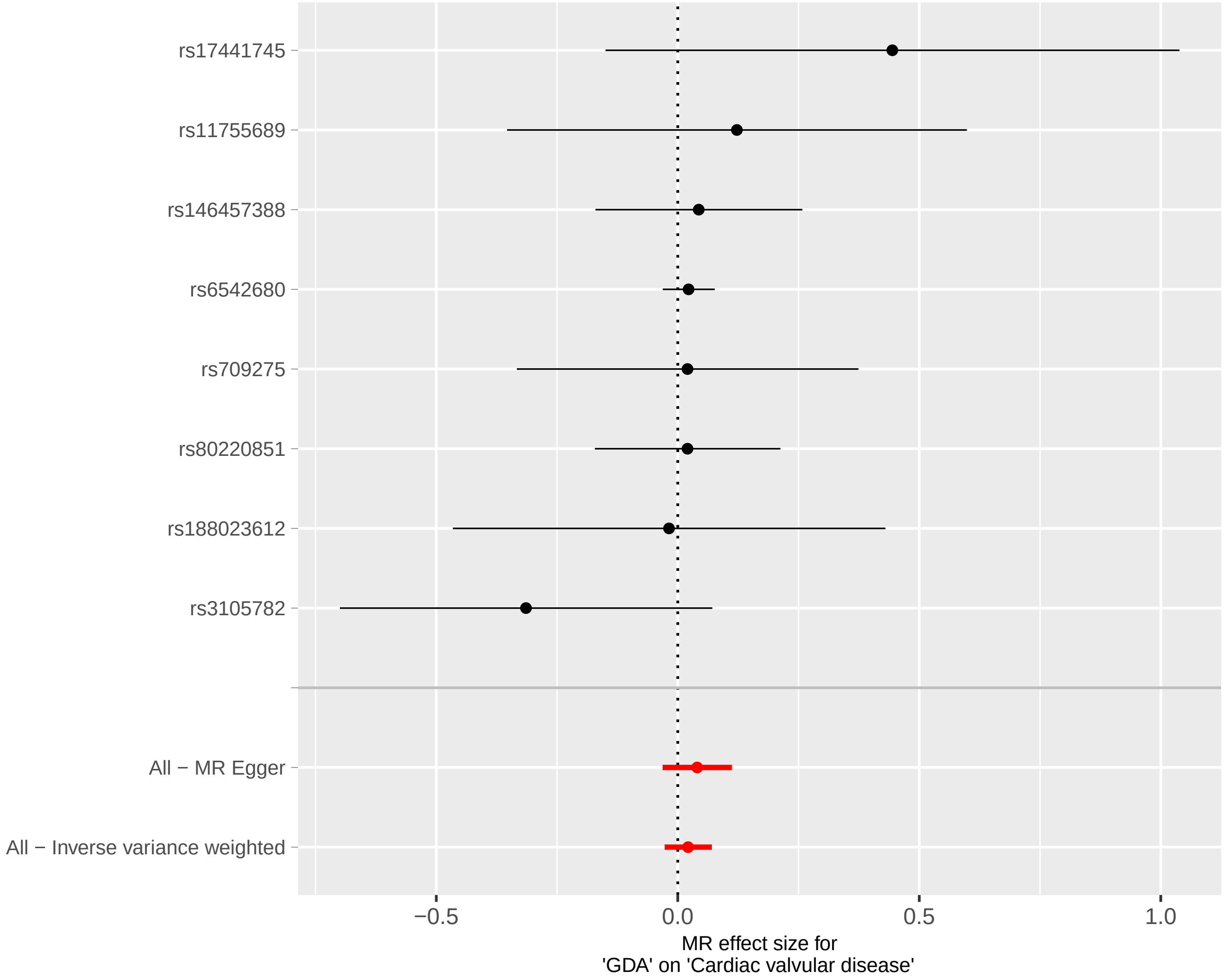
rs17441745
rs11755689
rs146457388
rs6542680
rs709275
rs80220851
rs188023612
rs3105782
All − MR Egger
All − Inverse variance weighted
−0.5
0.0
0.5
1.0
MR effect size for
'GDA' on 'Cardiac valvular disease'

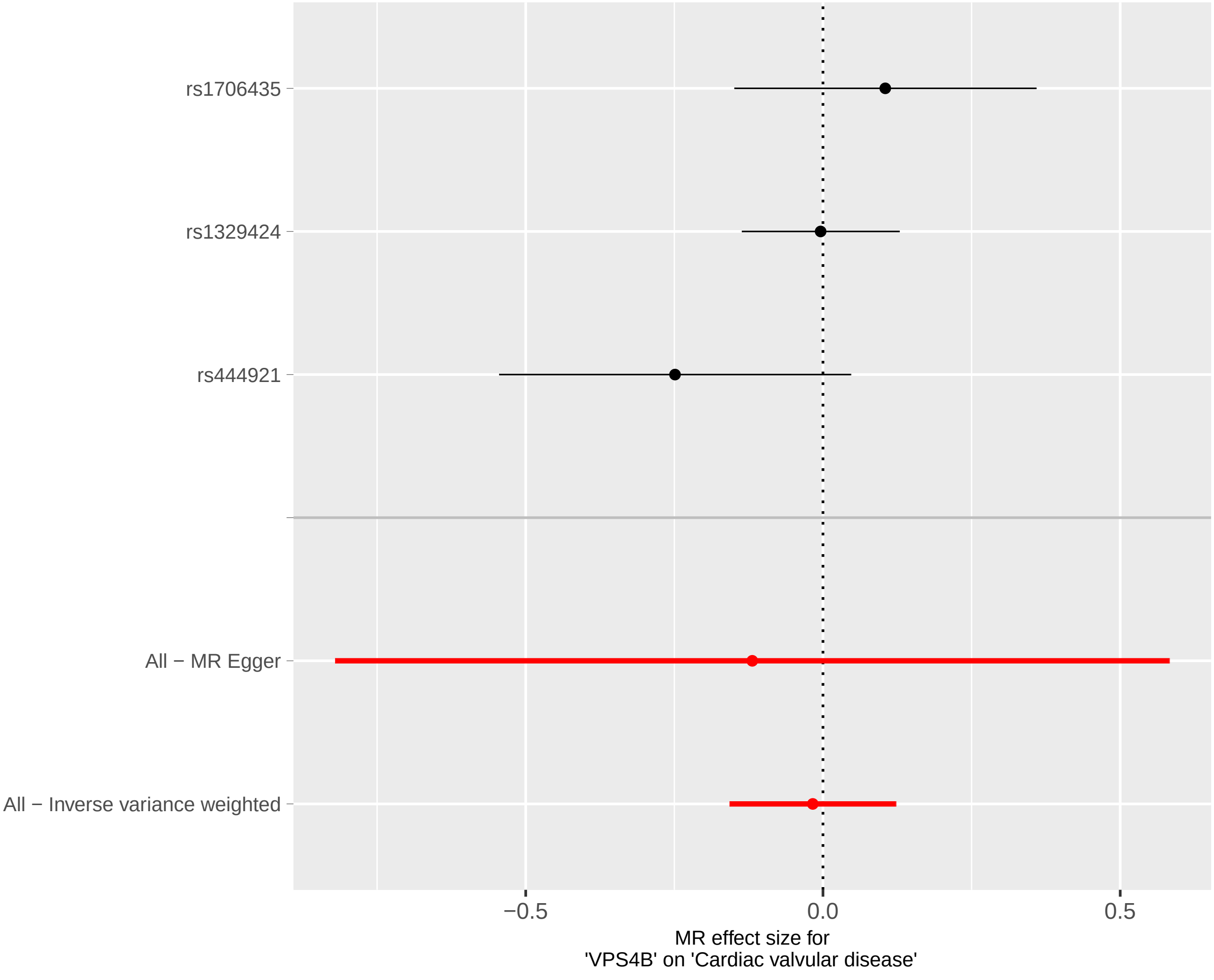
rs1706435
rs1329424
rs444921
All − MR Egger
All − Inverse variance weighted
−0.5
0.0
0.5
MR effect size for
'VPS4B' on 'Cardiac valvular disease'

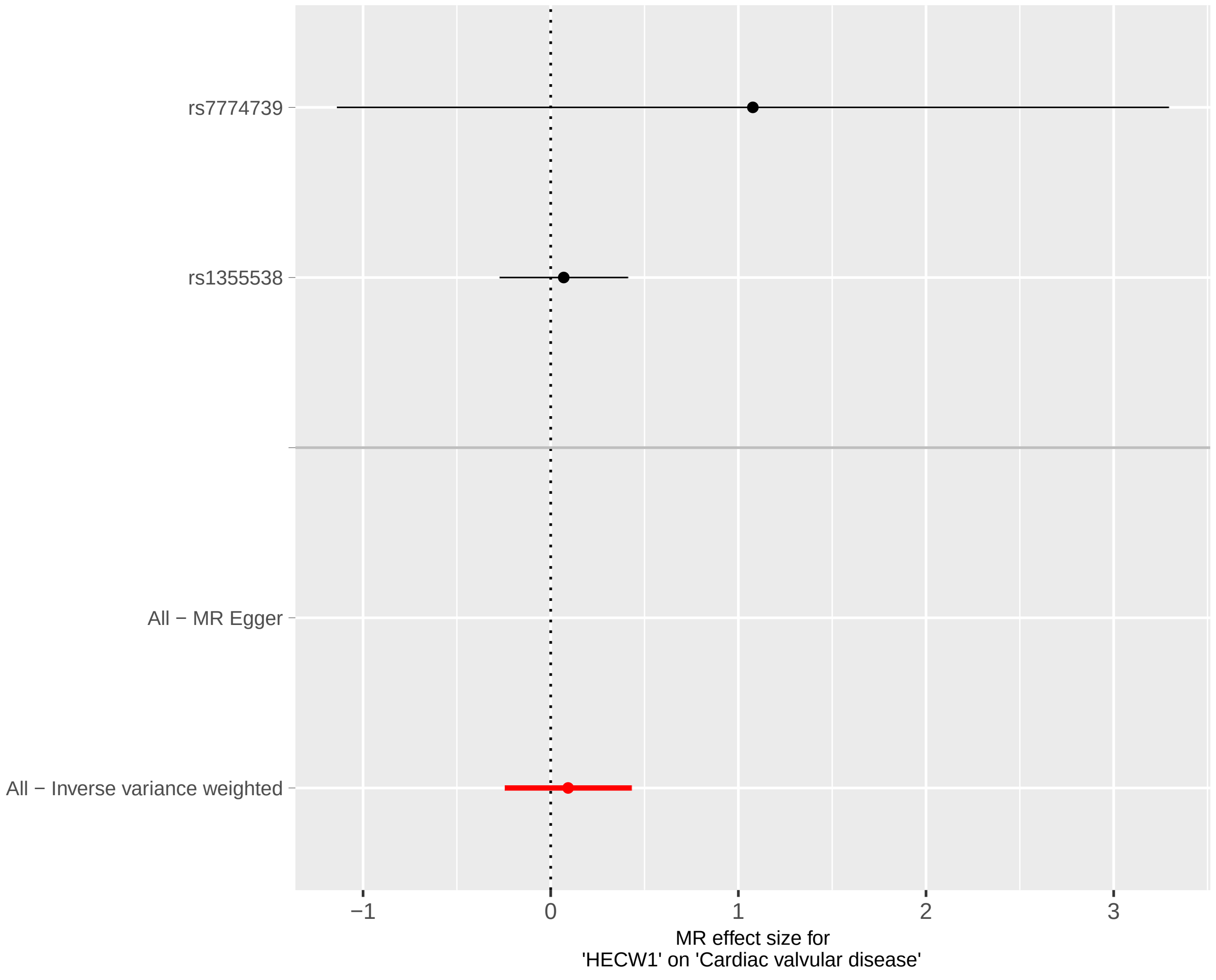
rs7774739
rs1355538
All − MR Egger
All − Inverse variance weighted
−1
0
1
2
3
MR effect size for
'HECW1' on 'Cardiac valvular disease'

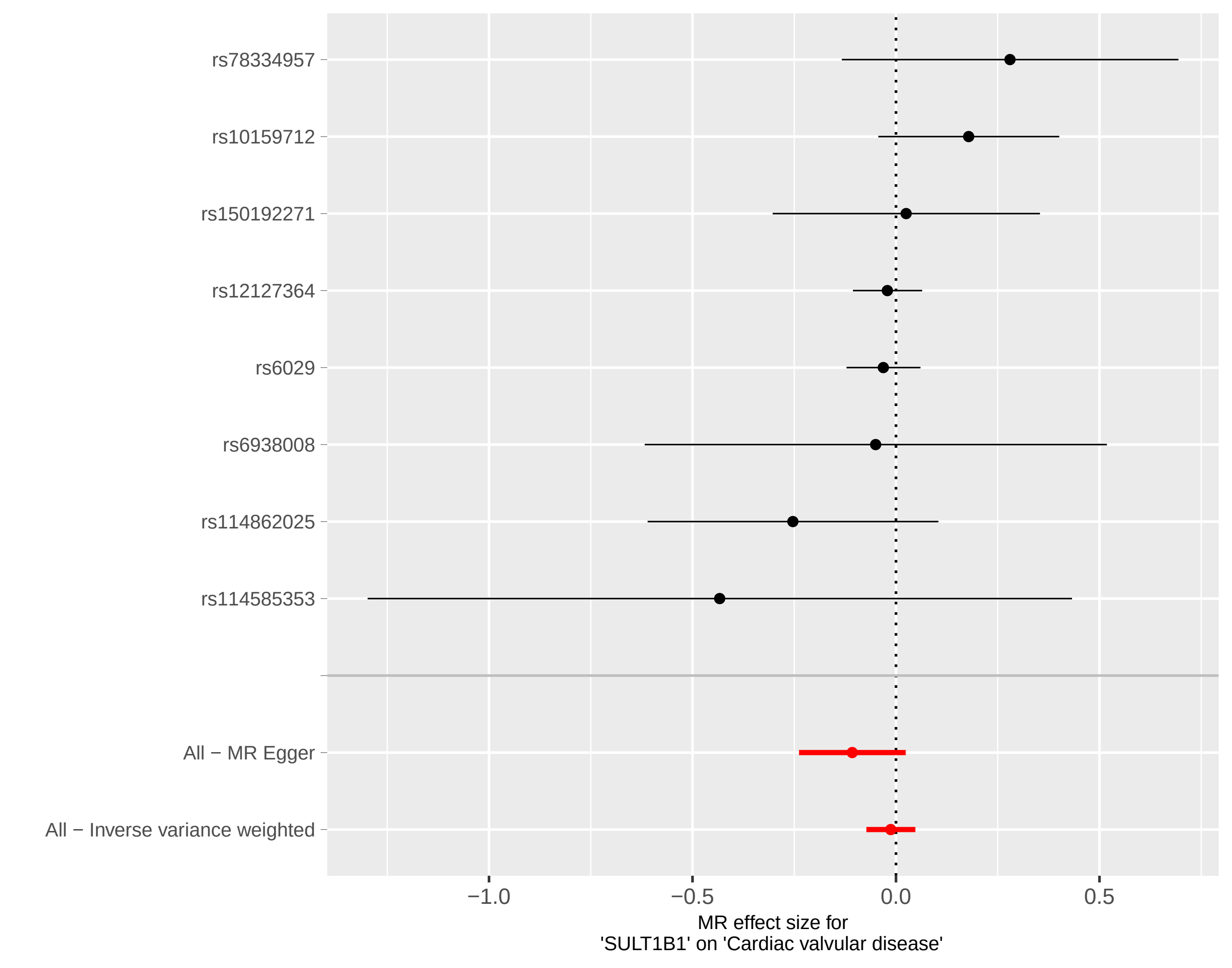

rs78334957
rs10159712
rs150192271
rs12127364
rs6029
rs6938008
rs114862025
rs114585353
All − MR Egger
All − Inverse variance weighted
−1.0
−0.5
0.0
0.5
MR effect size for
'SULT1B1' on 'Cardiac valvular disease'

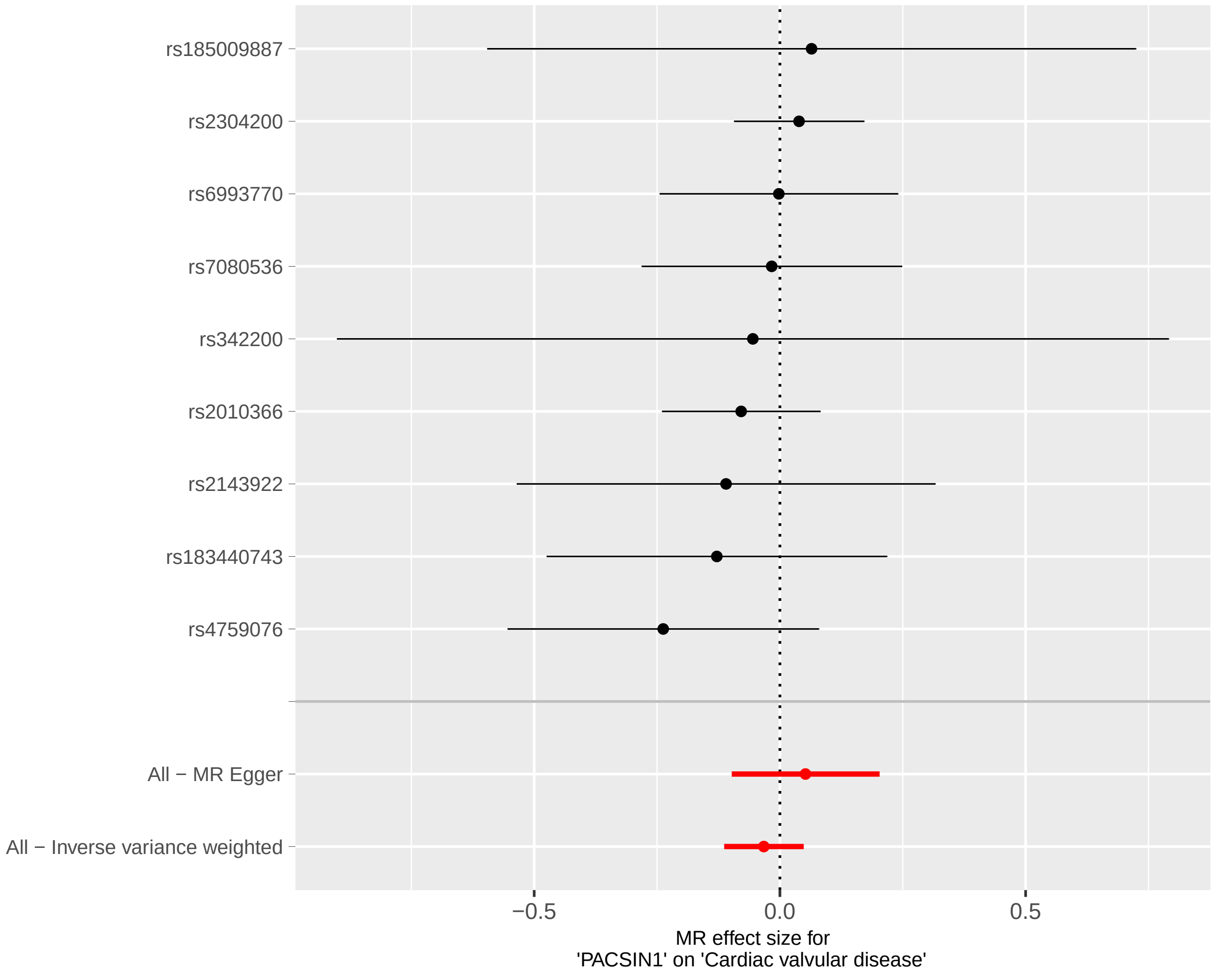
rs185009887
rs2304200
rs6993770
rs7080536
rs342200
rs2010366
rs2143922
rs183440743
rs4759076
All − MR Egger
All − Inverse variance weighted
−0.5
0.0
0.5
MR effect size for
'PACSIN1' on 'Cardiac valvular disease'

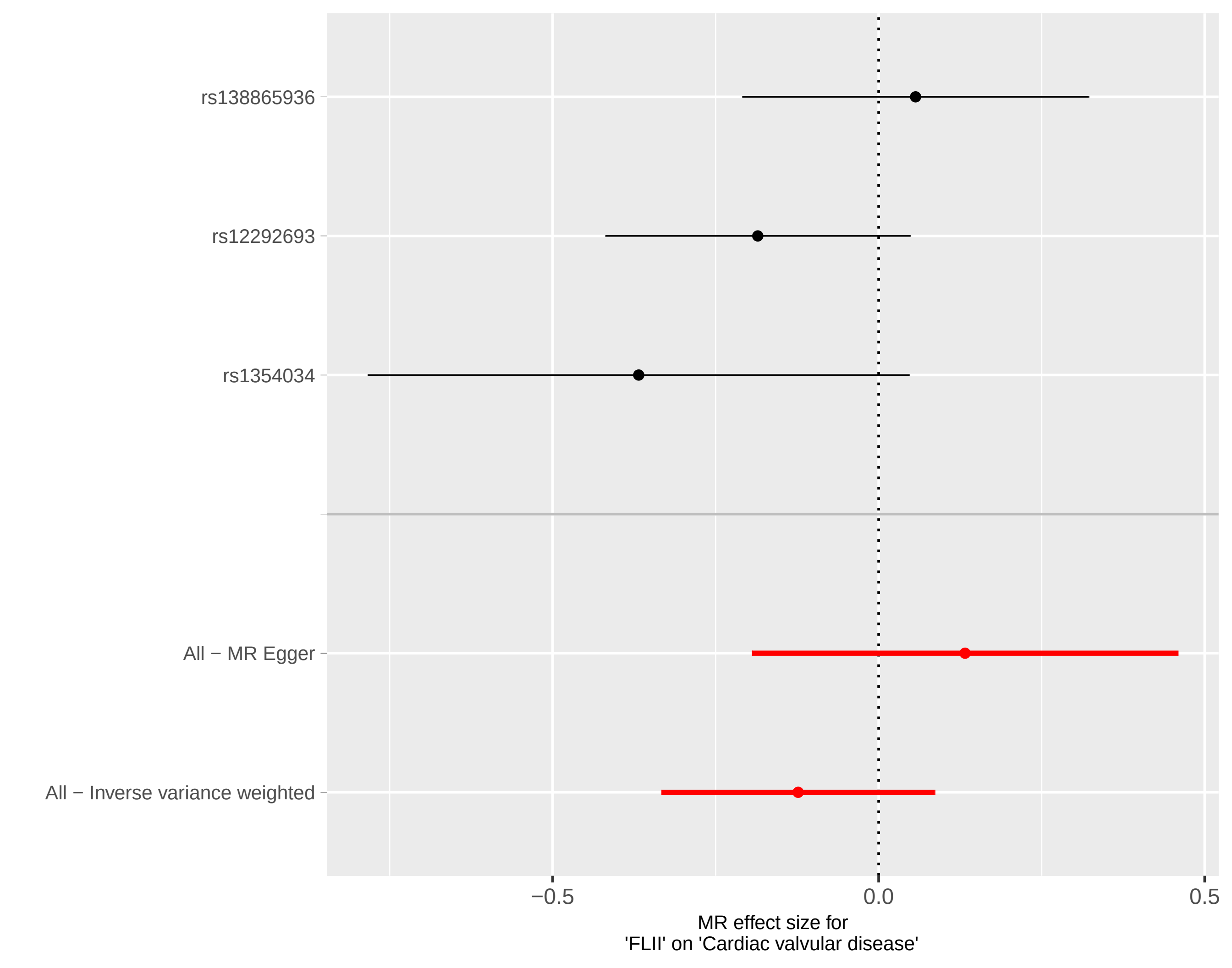
rs138865936
rs12292693
rs1354034
All − MR Egger
All − Inverse variance weighted
−0.5
0.0
0.5
MR effect size for
'FLII' on 'Cardiac valvular disease'

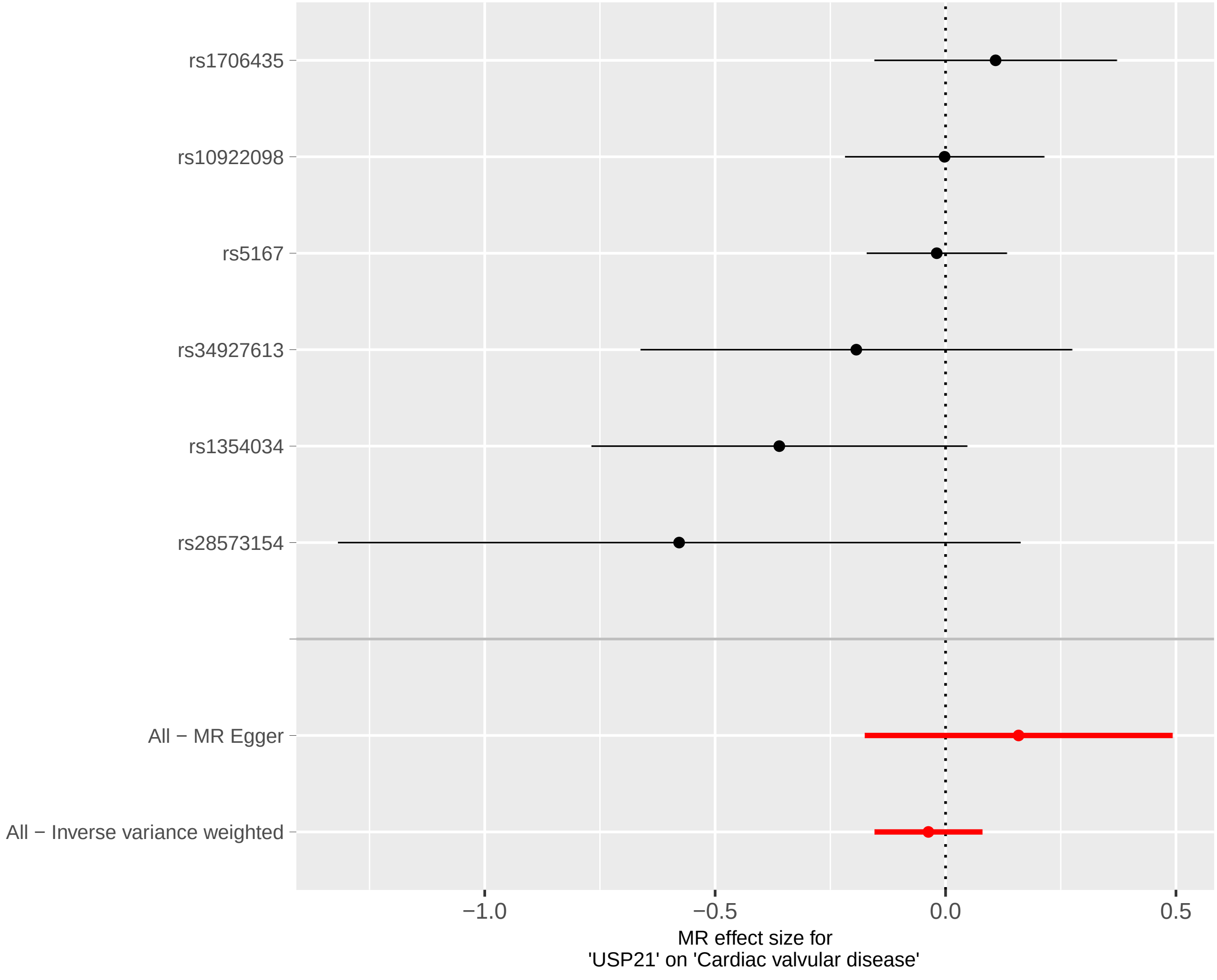
rs1706435
rs10922098
rs5167
rs34927613
rs1354034
rs28573154
All − MR Egger
All − Inverse variance weighted
−1.0
−0.5
0.0
0.5
MR effect size for
'USP21' on 'Cardiac valvular disease'

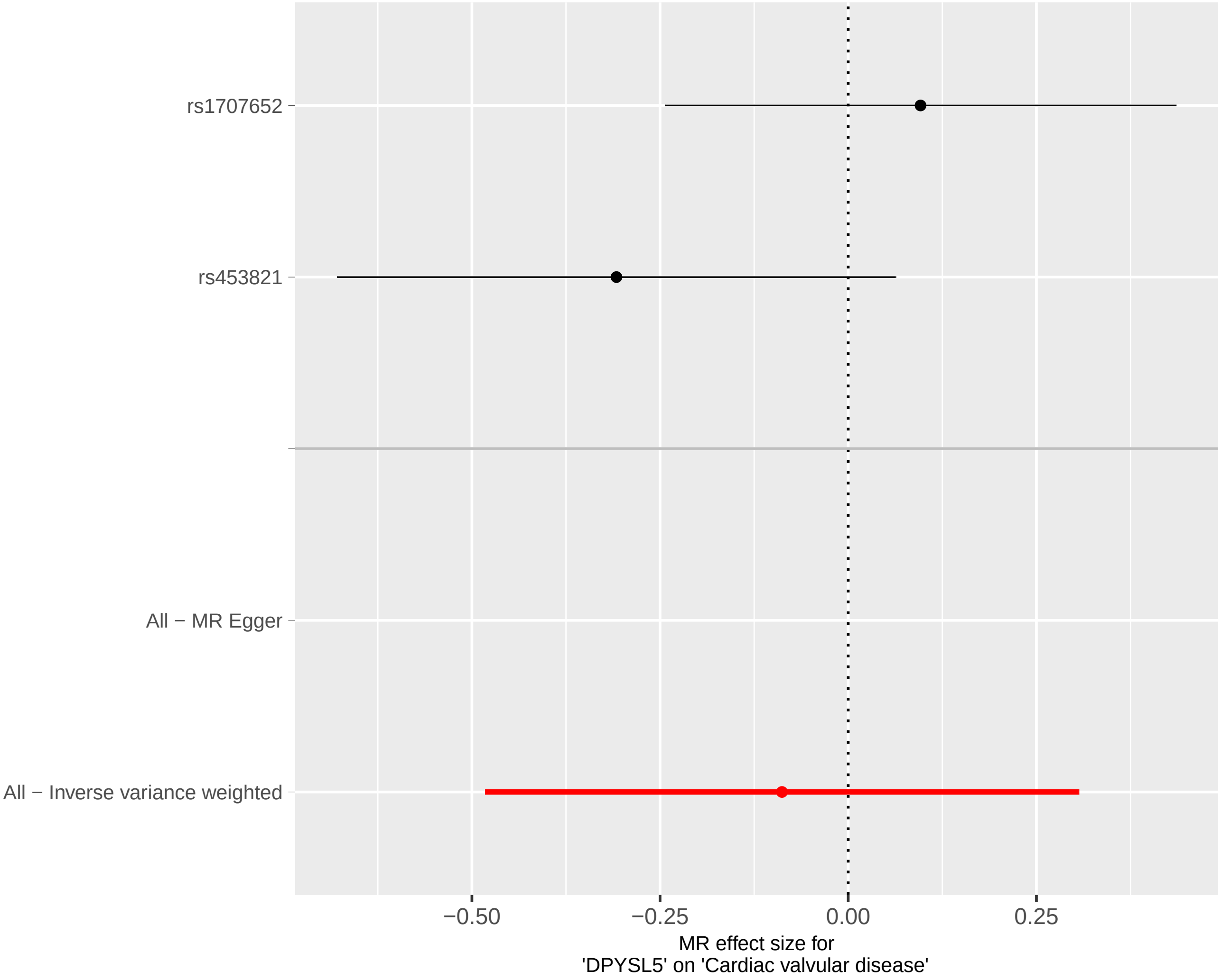
rs1707652
rs453821
All − MR Egger
All − Inverse variance weighted
−0.50
−0.25
0.00
0.25
MR effect size for
'DPYSL5' on 'Cardiac valvular disease'

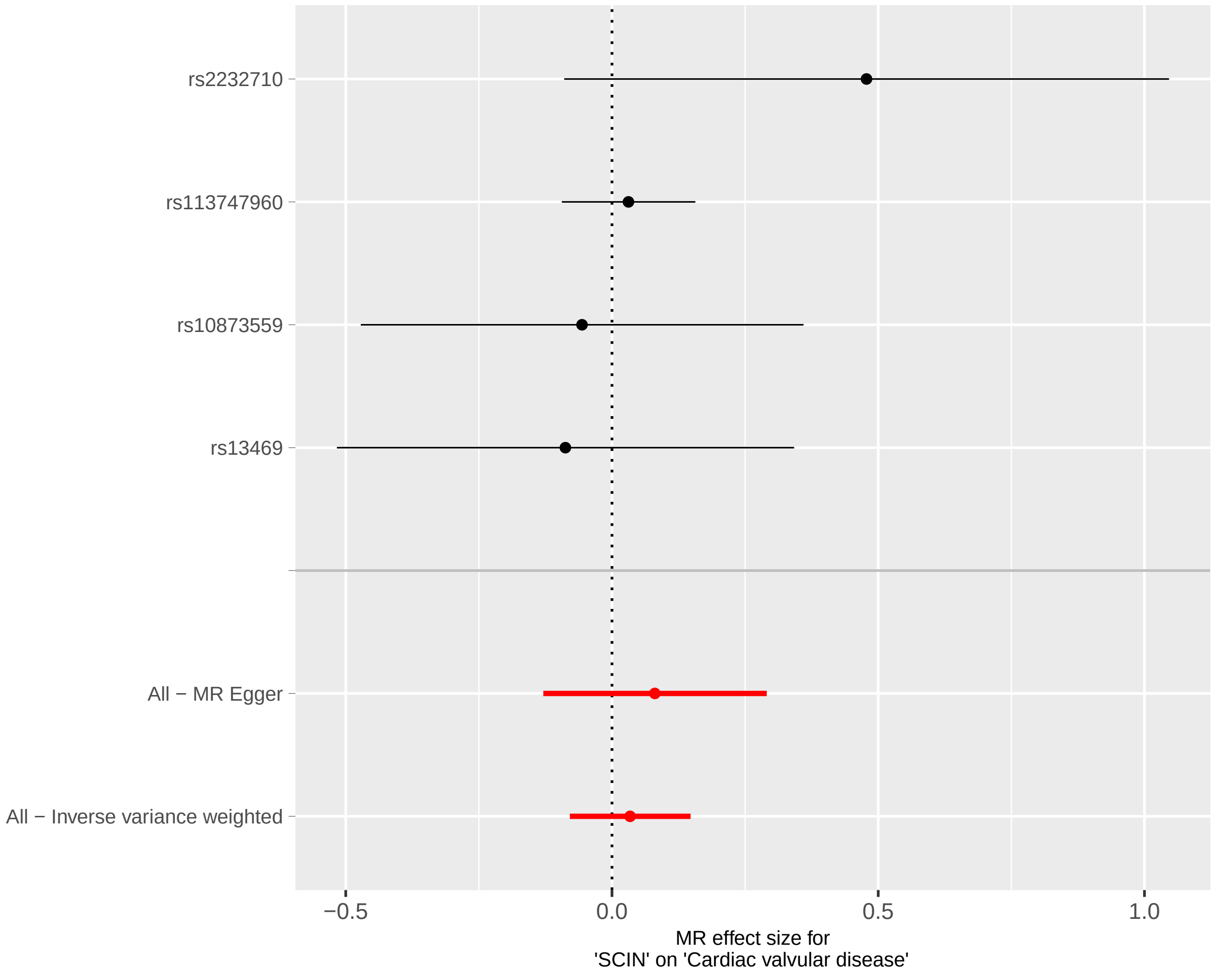
rs2232710
rs113747960
rs10873559
rs13469
All − MR Egger
All − Inverse variance weighted
−0.5
0.0
0.5
1.0
MR effect size for
'SCIN' on 'Cardiac valvular disease'

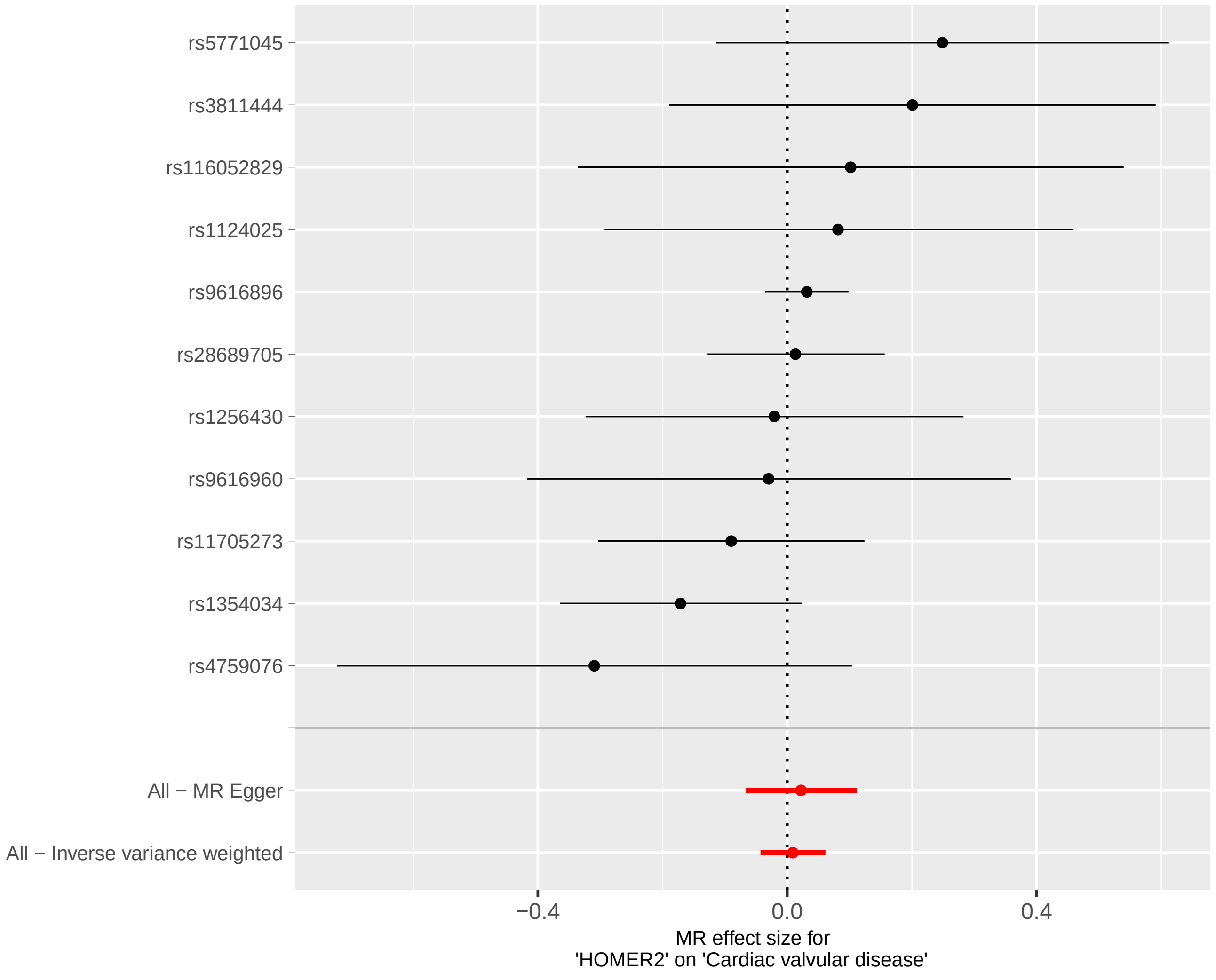
rs5771045
rs3811444
rs116052829
rs1124025
rs9616896
rs28689705
rs1256430
rs9616960
rs11705273
rs1354034
rs4759076
All − MR Egger
All − Inverse variance weighted
−0.4
0.0
0.4
MR effect size for
'HOMER2' on 'Cardiac valvular disease'

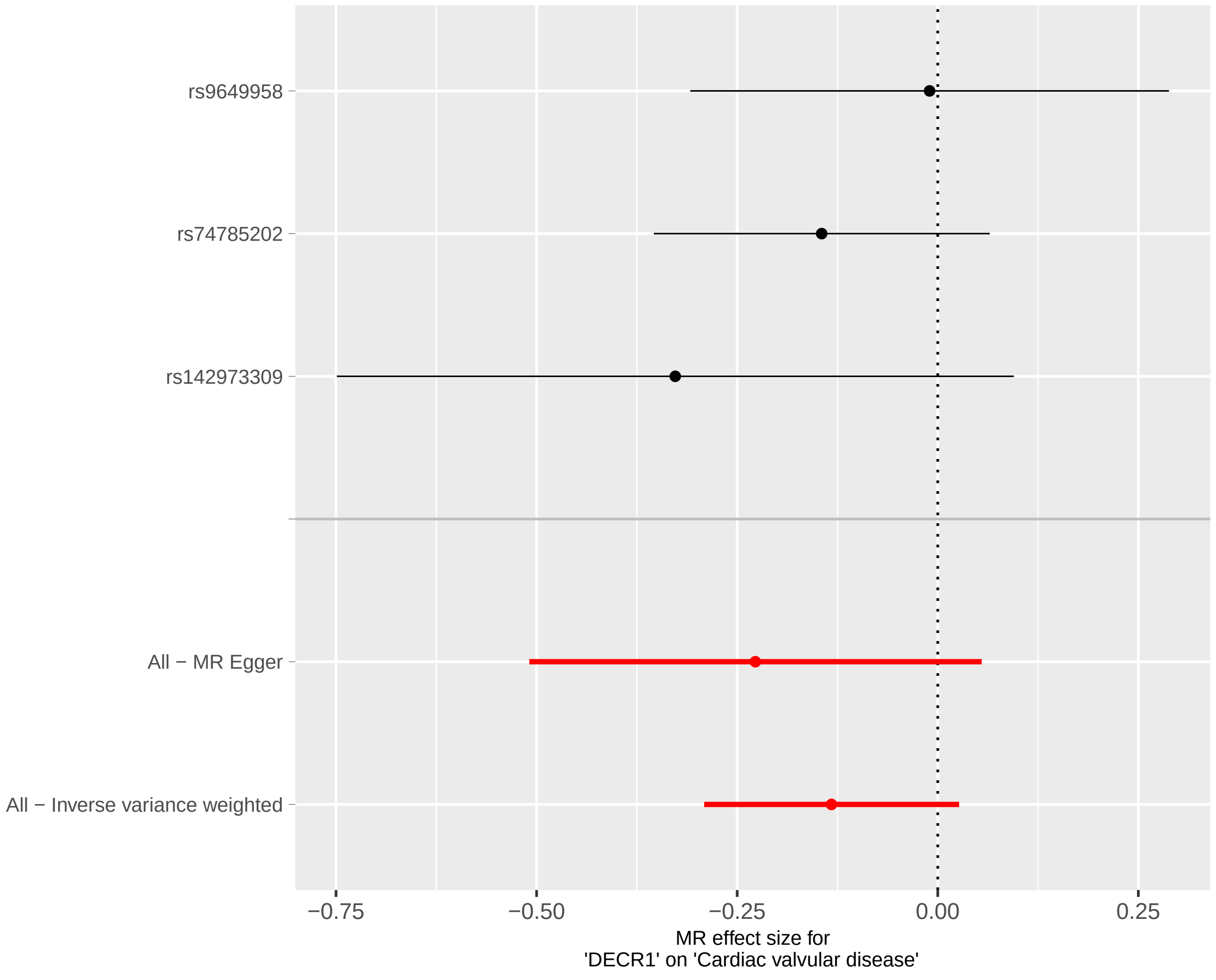

rs9649958
rs74785202
rs142973309
All − MR Egger
All − Inverse variance weighted
−0.75
−0.50
−0.25
0.00
0.25
MR effect size for
'DECR1' on 'Cardiac valvular disease'

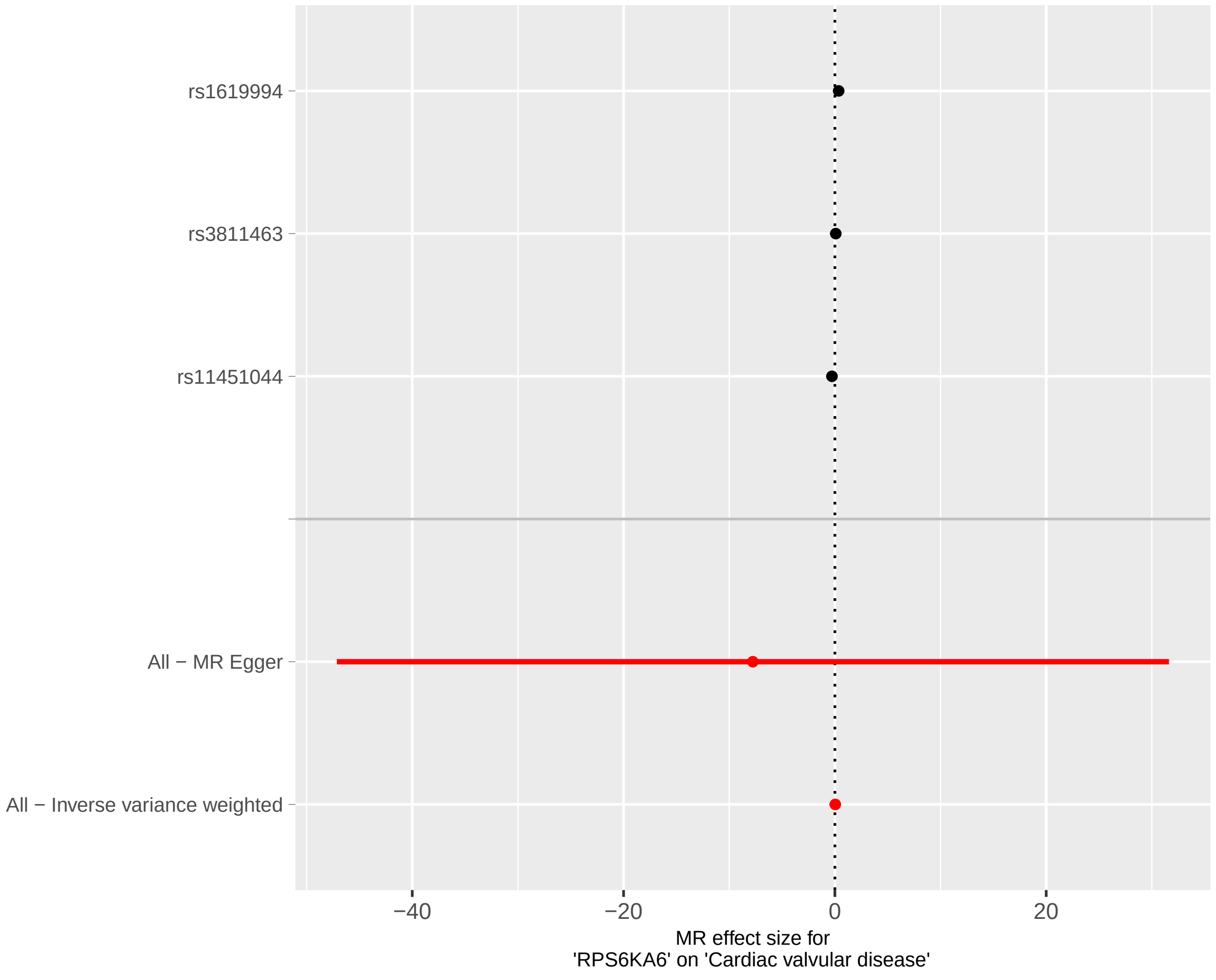

rs1619994
rs3811463
rs11451044
All − MR Egger
All − Inverse variance weighted
−40
−20
0
20
MR effect size for
'RPS6KA6' on 'Cardiac valvular disease'

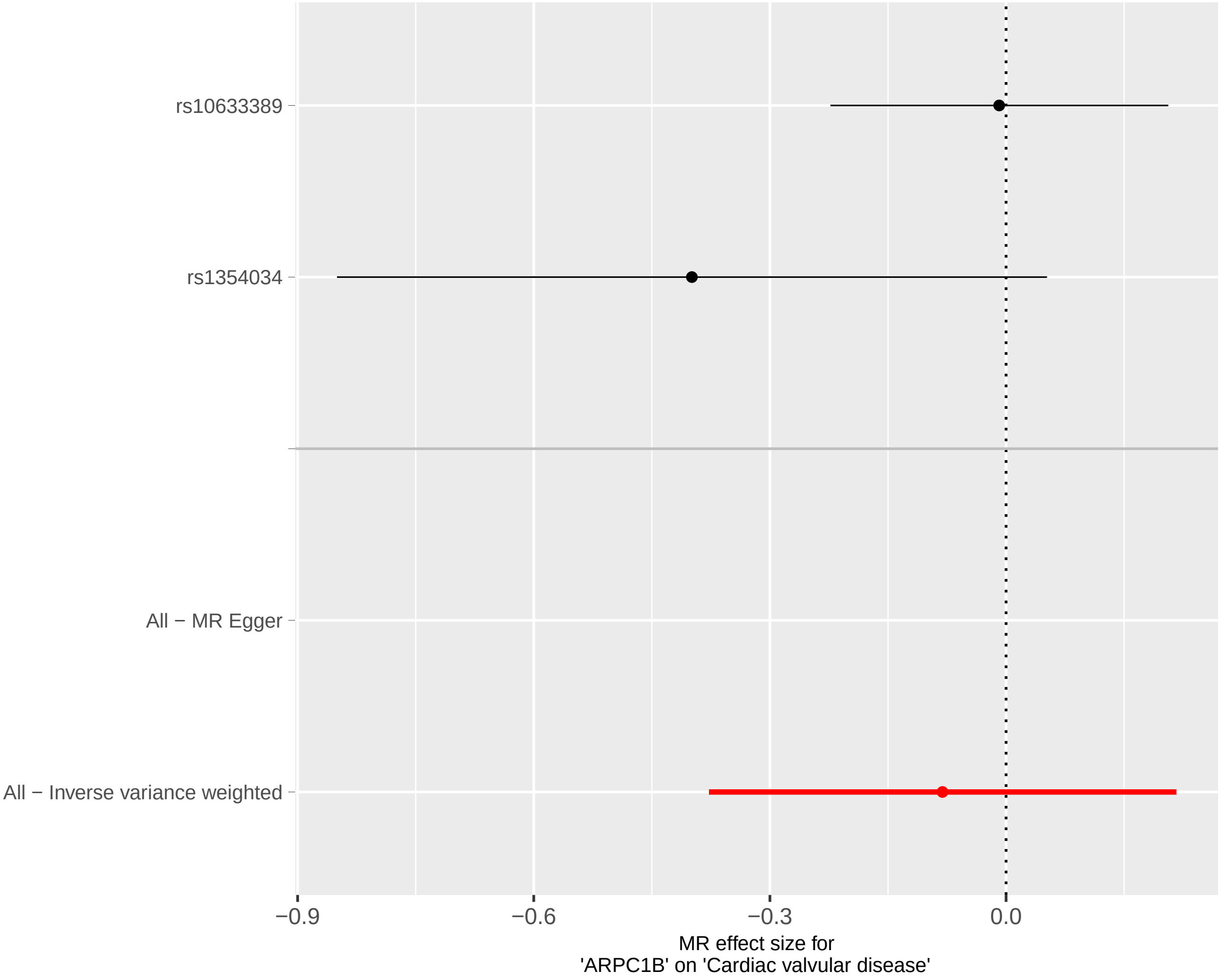
rs10633389
rs1354034
All − MR Egger
All − Inverse variance weighted
−0.9
−0.6
−0.3
0.0
MR effect size for
'ARPC1B' on 'Cardiac valvular disease'

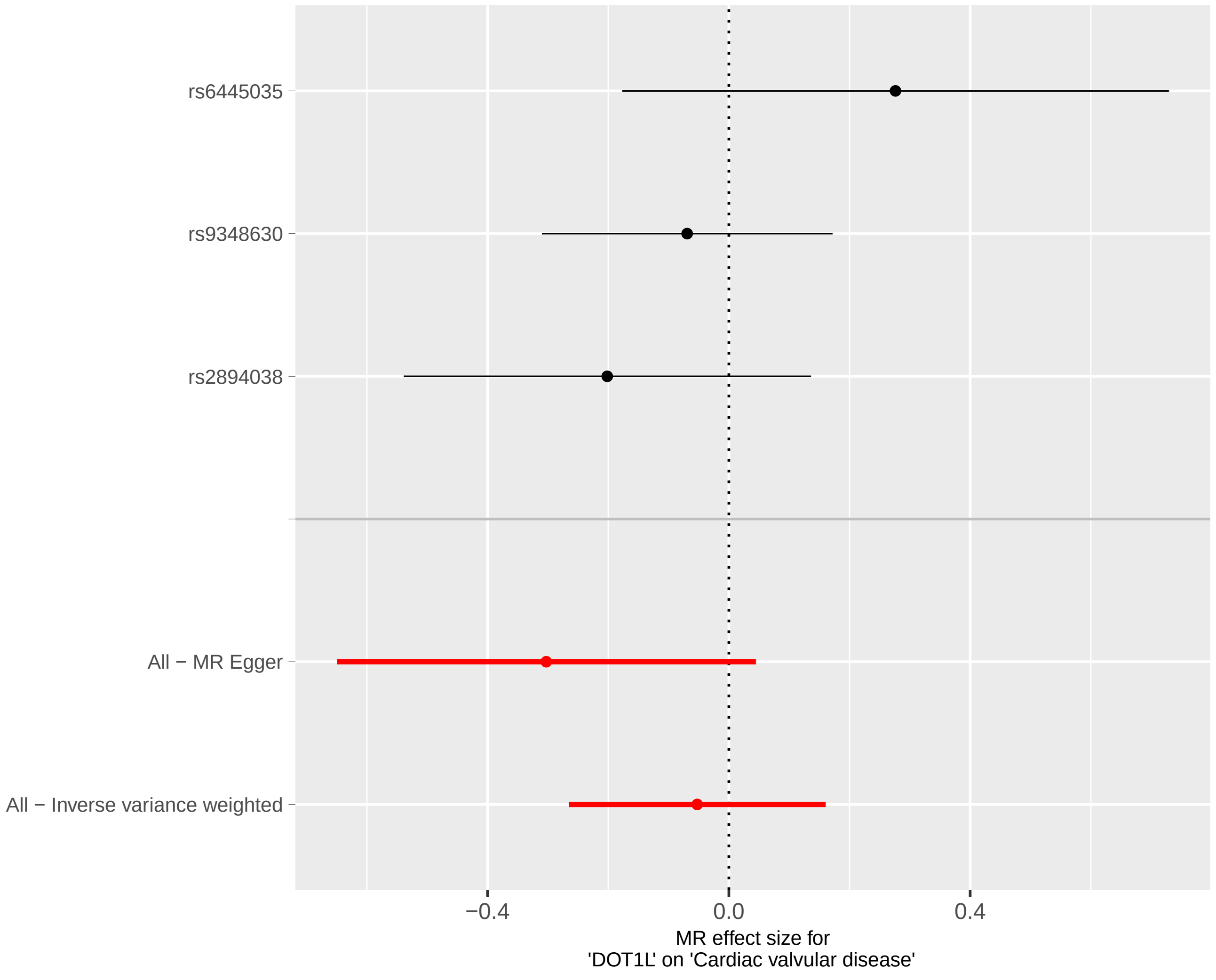
rs6445035
rs9348630
rs2894038
All − MR Egger
All − Inverse variance weighted
−0.4
0.0
0.4
MR effect size for
'DOT1L' on 'Cardiac valvular disease'

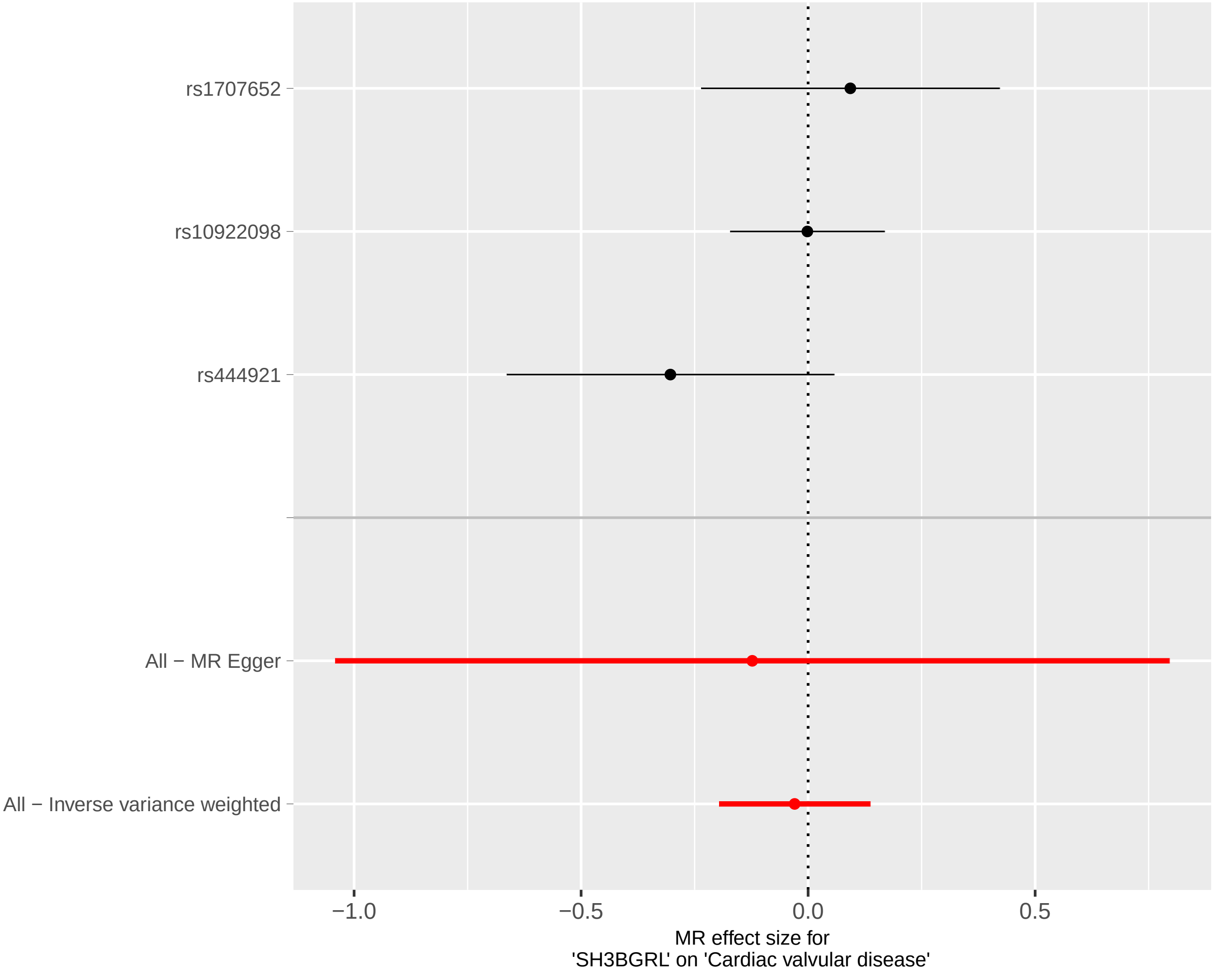
rs1707652
rs10922098
rs444921
All − MR Egger
All − Inverse variance weighted
−1.0
−0.5
0.0
0.5
MR effect size for
'SH3BGRL' on 'Cardiac valvular disease'

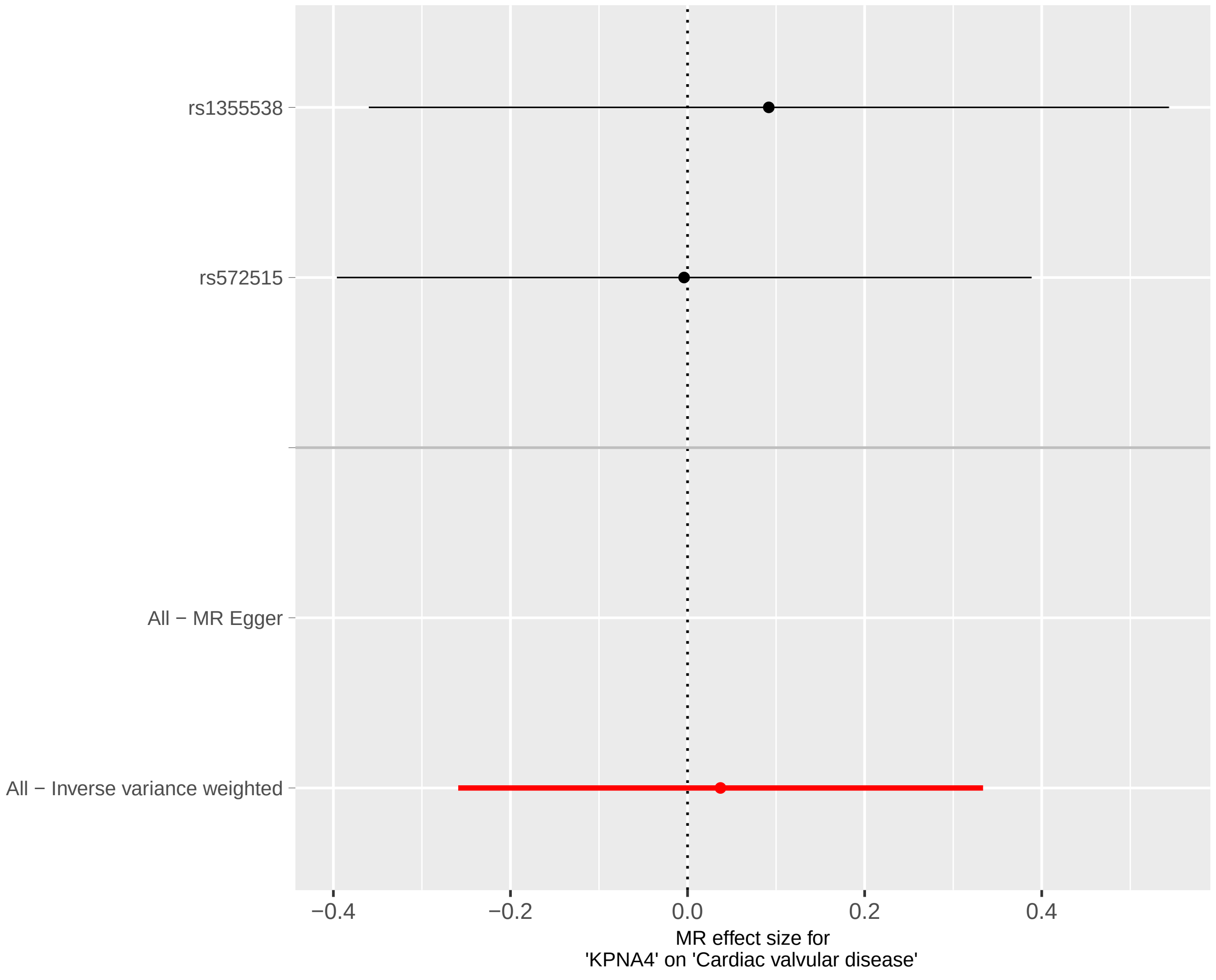

rs1355538
rs572515
All − MR Egger
All − Inverse variance weighted
−0.4
−0.2
0.0
0.2
0.4
MR effect size for
'KPNA4' on 'Cardiac valvular disease'

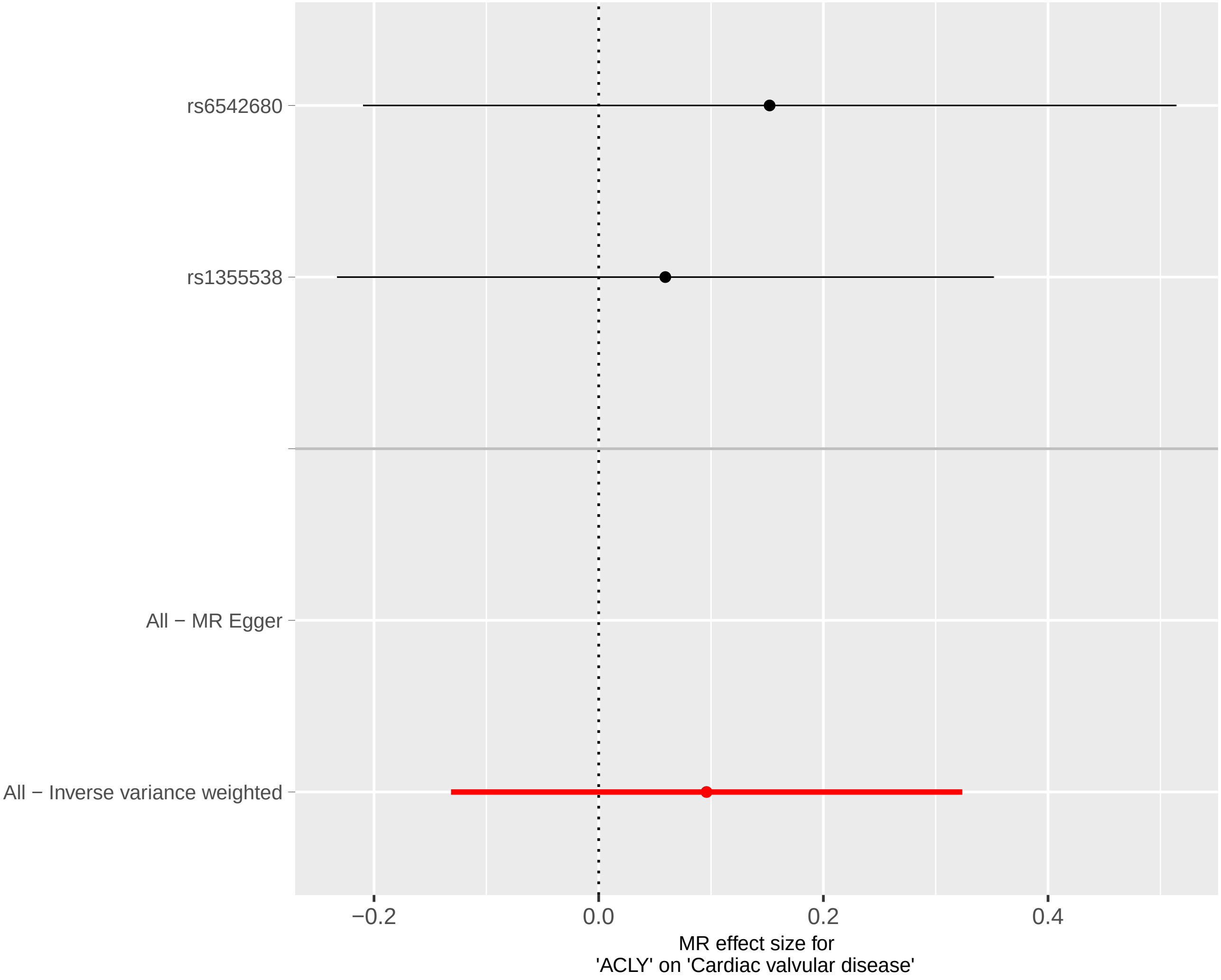

rs6542680
rs1355538
All − MR Egger
All − Inverse variance weighted
−0.2
0.0
0.2
0.4
MR effect size for
'ACLY' on 'Cardiac valvular disease'

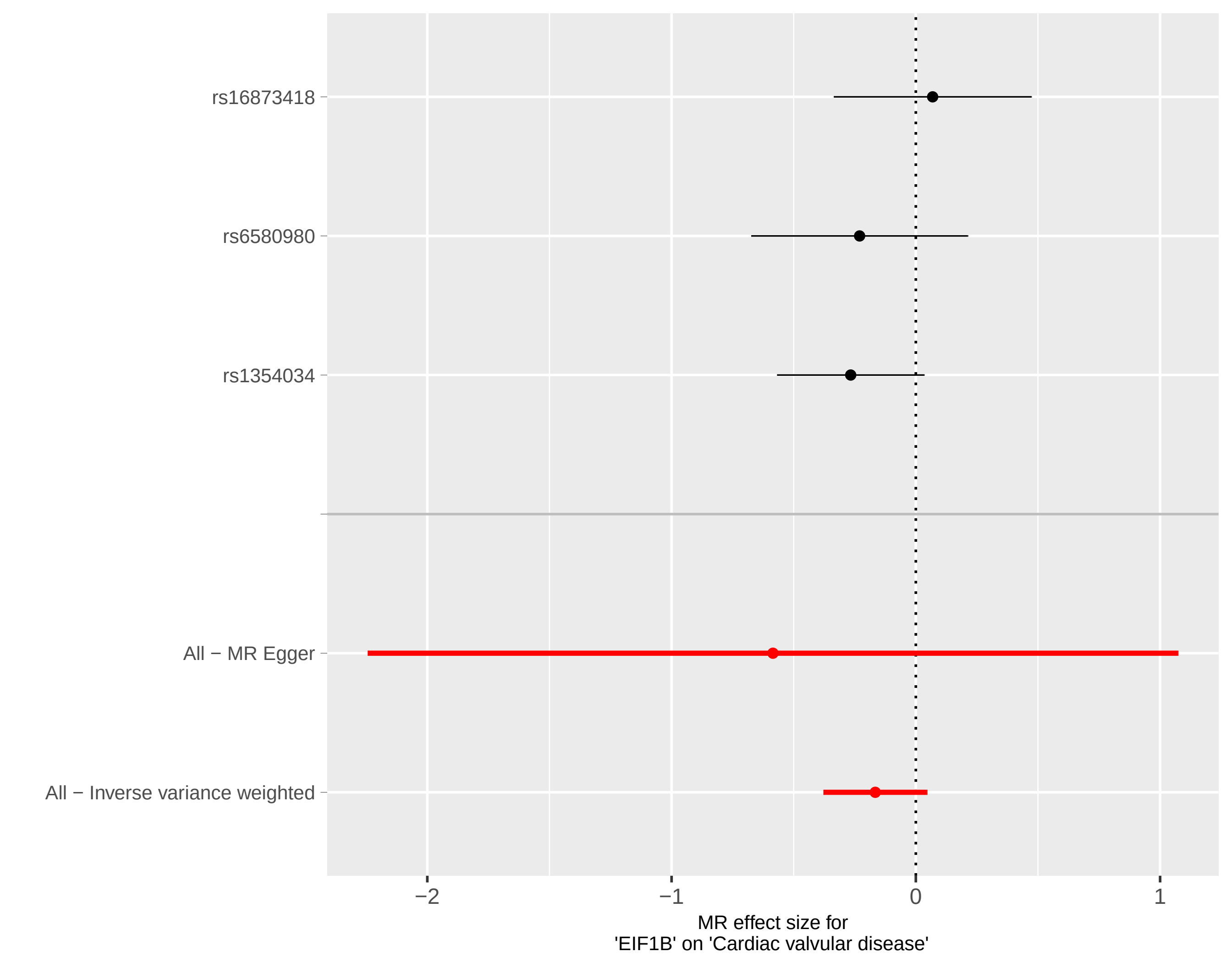
rs16873418
rs6580980
rs1354034
All − MR Egger
All − Inverse variance weighted
−2
−1
0
1
MR effect size for
'EIF1B' on 'Cardiac valvular disease'

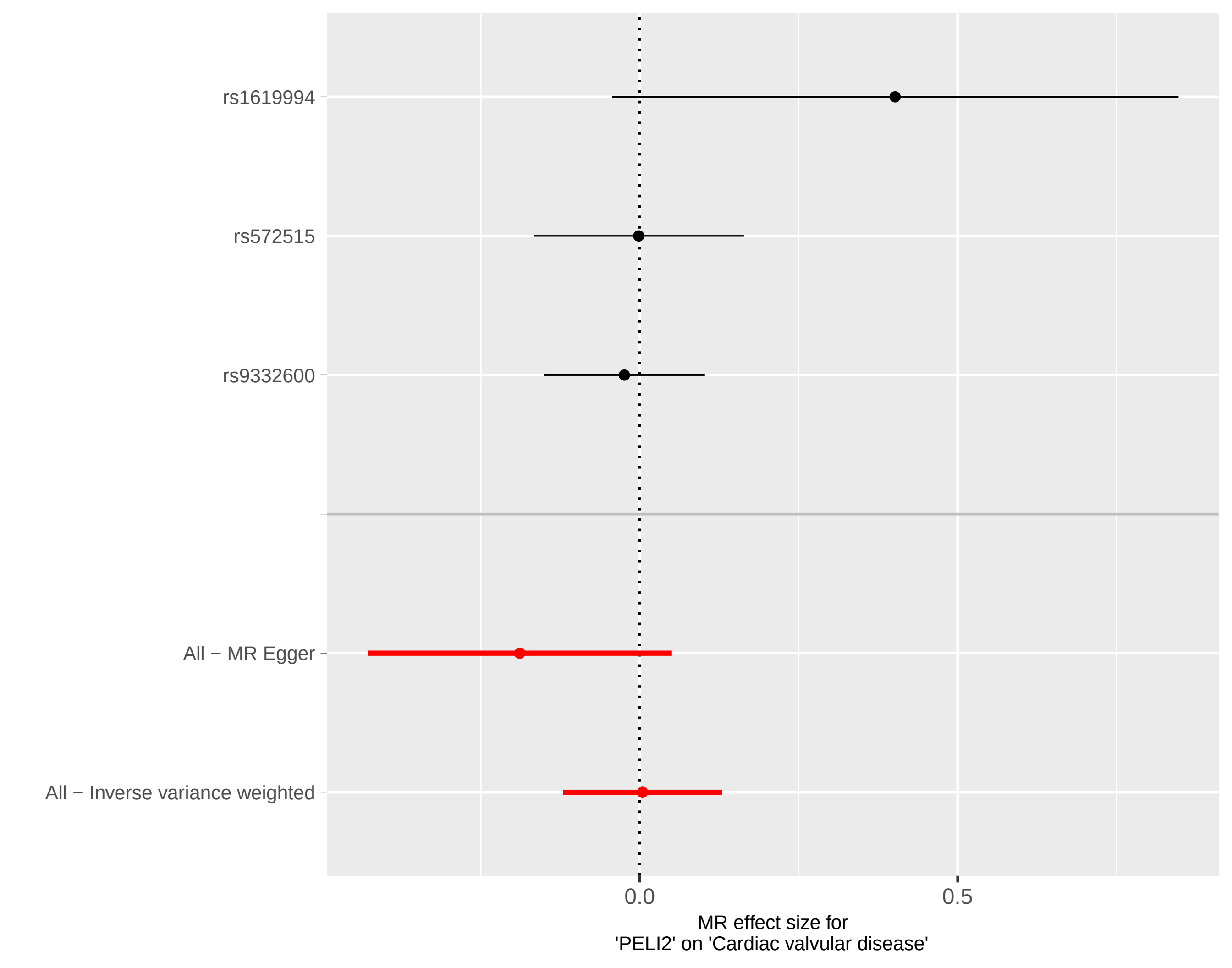
rs1619994
rs572515
rs9332600
All − MR Egger
All − Inverse variance weighted
0.0
0.5
MR effect size for
'PELI2' on 'Cardiac valvular disease'

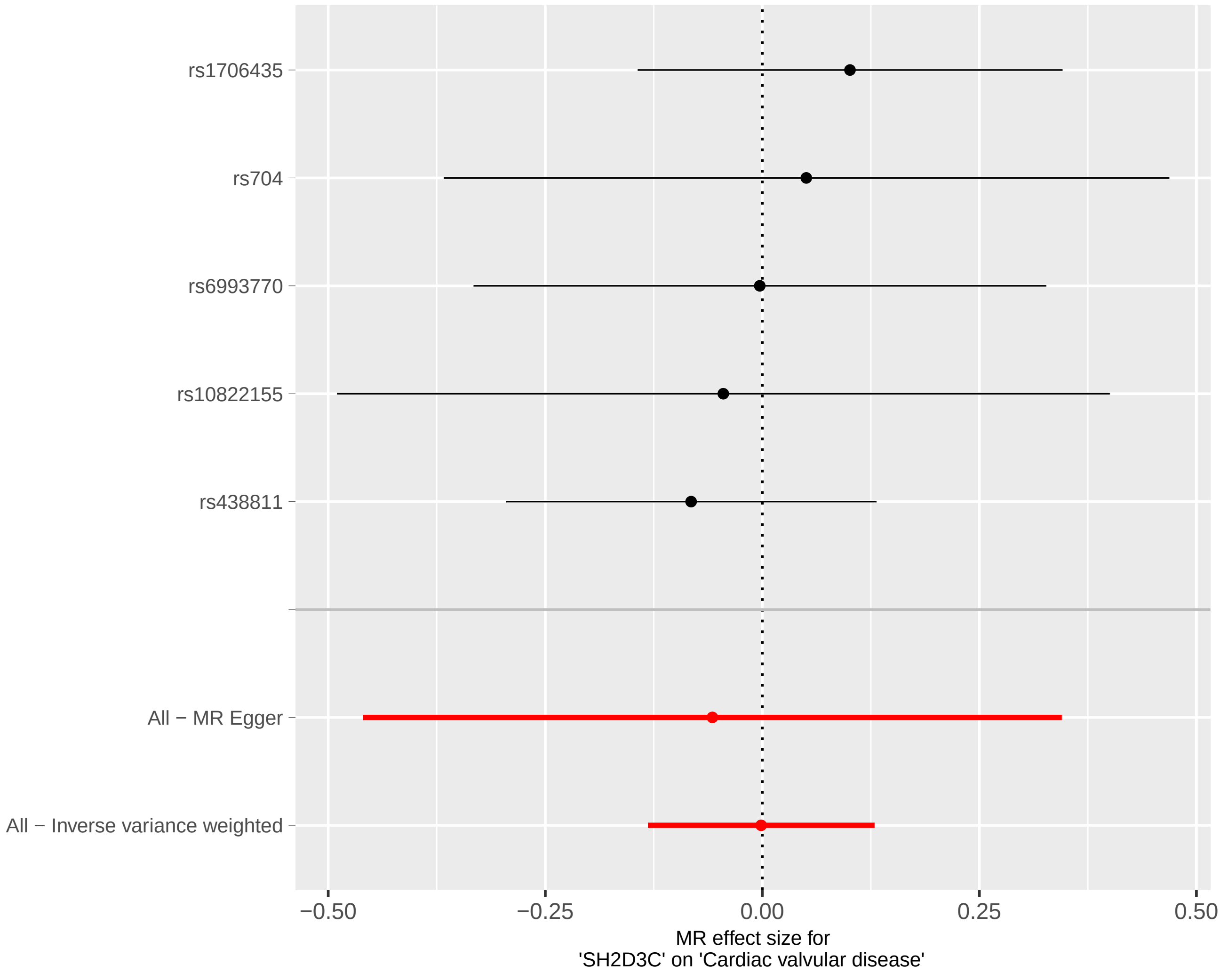
rs1706435
rs704
rs6993770
rs10822155
rs438811
All − MR Egger
All − Inverse variance weighted
−0.50
−0.25
0.00
0.25
0.50
MR effect size for
'SH2D3C' on 'Cardiac valvular disease'

Insufficient number of SNPs

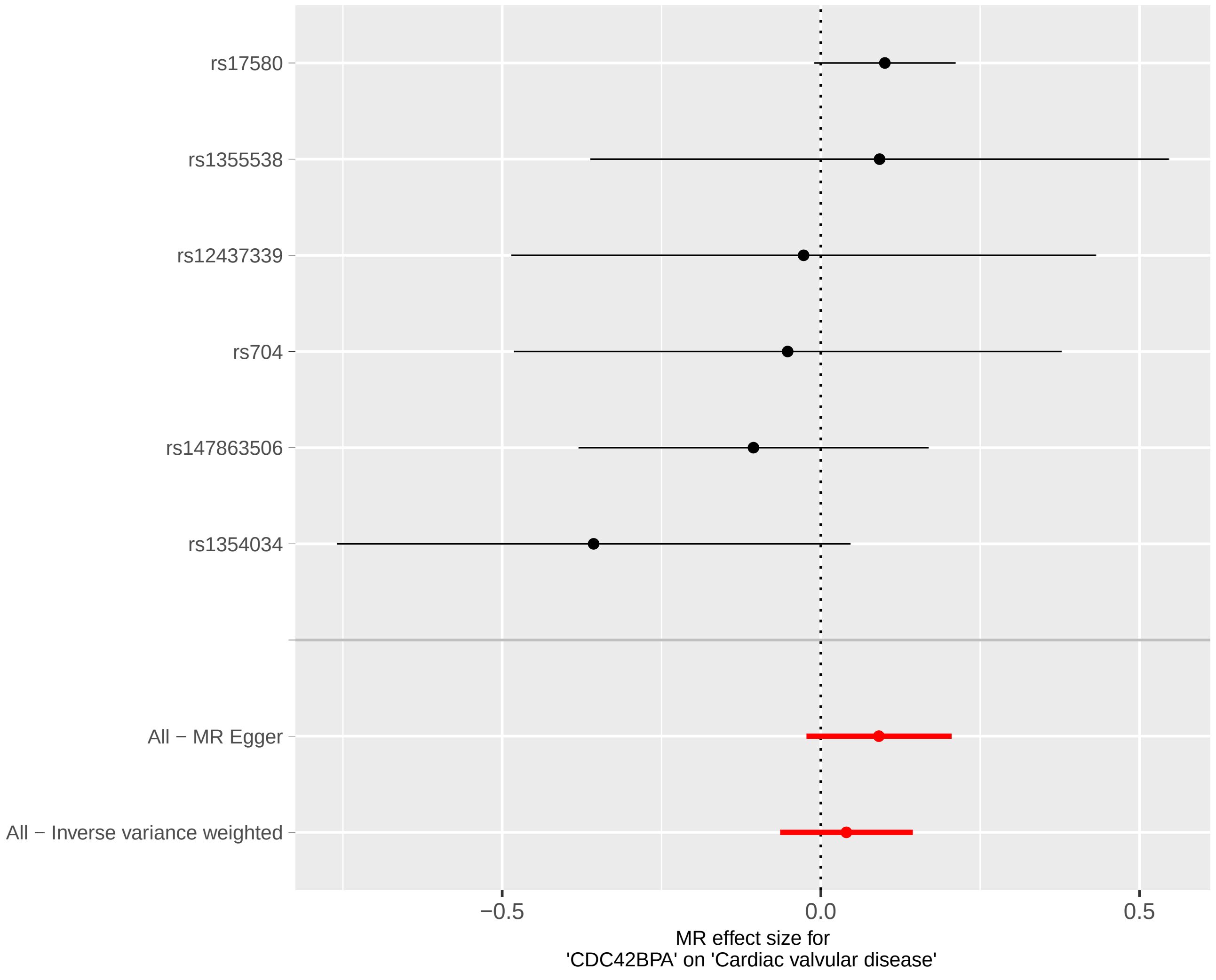
rs17580
rs1355538
rs12437339
rs704
rs147863506
rs1354034
All − MR Egger
All − Inverse variance weighted
−0.5
0.0
0.5
MR effect size for
'CDC42BPA' on 'Cardiac valvular disease'

Insufficient number of SNPs

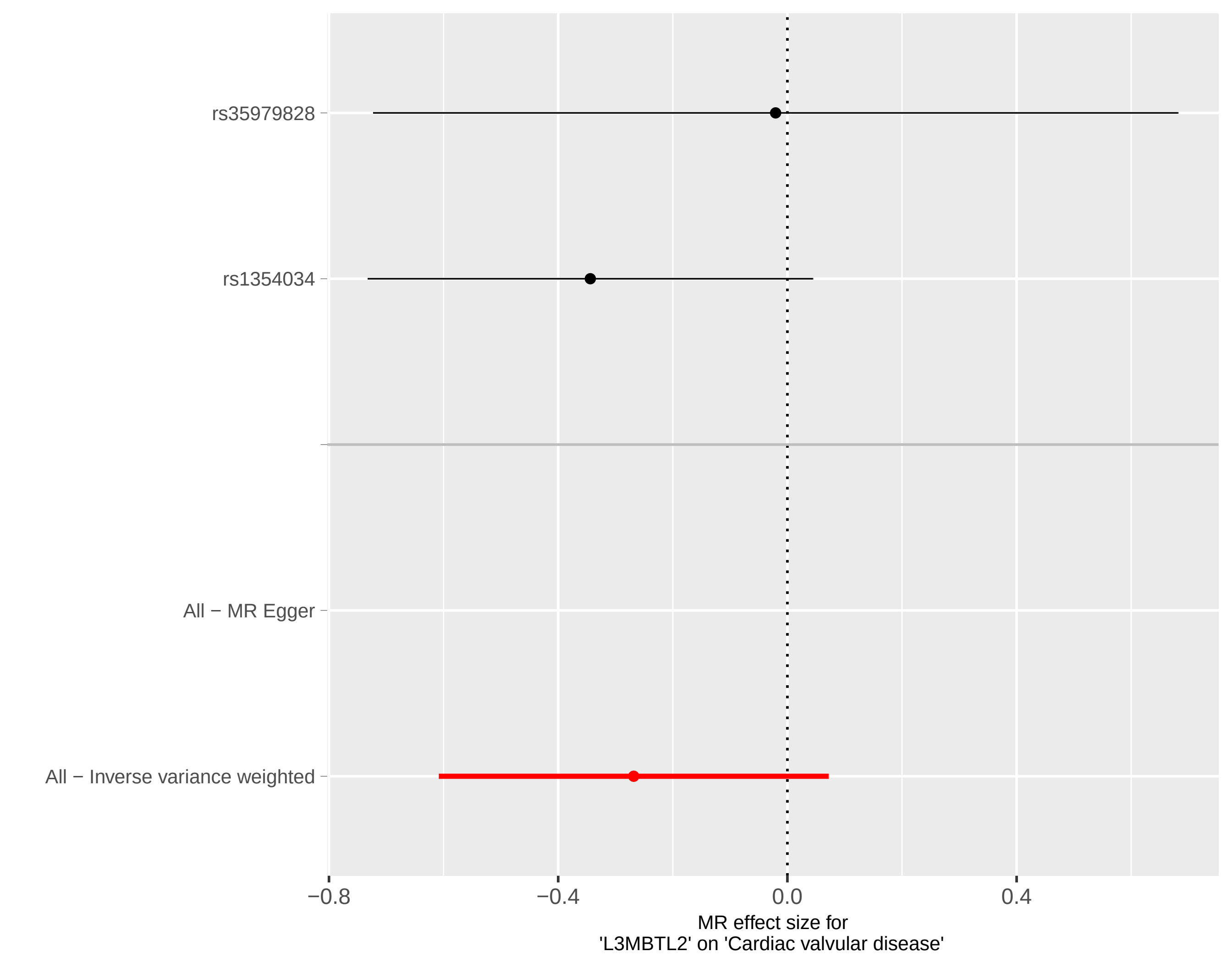
rs35979828
rs1354034
All − MR Egger
All − Inverse variance weighted
−0.8
−0.4
0.0
0.4
MR effect size for
'L3MBTL2' on 'Cardiac valvular disease'

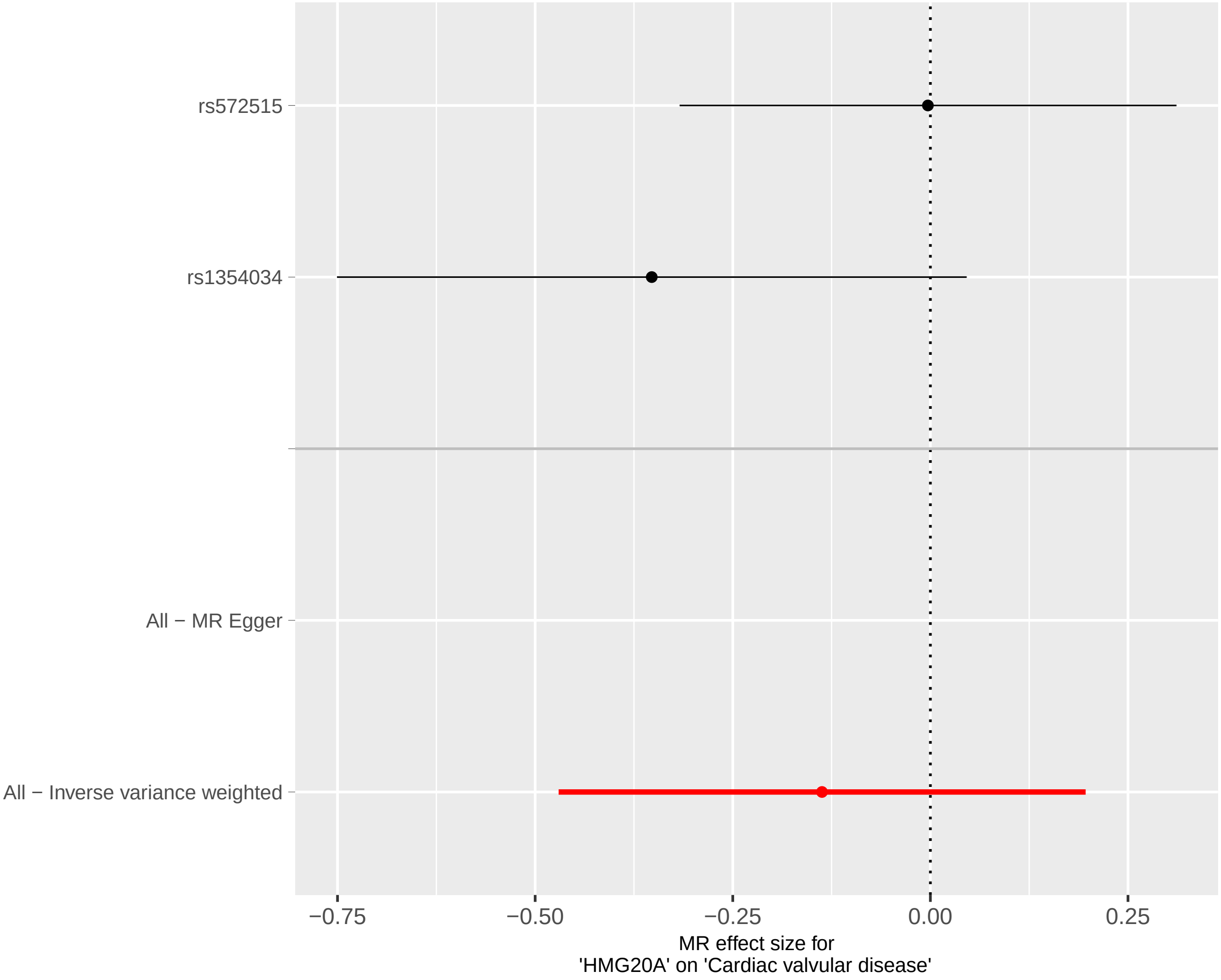

rs572515
rs1354034
All − MR Egger
All − Inverse variance weighted
−0.75
−0.50
−0.25
0.00
0.25
MR effect size for
'HMG20A' on 'Cardiac valvular disease'

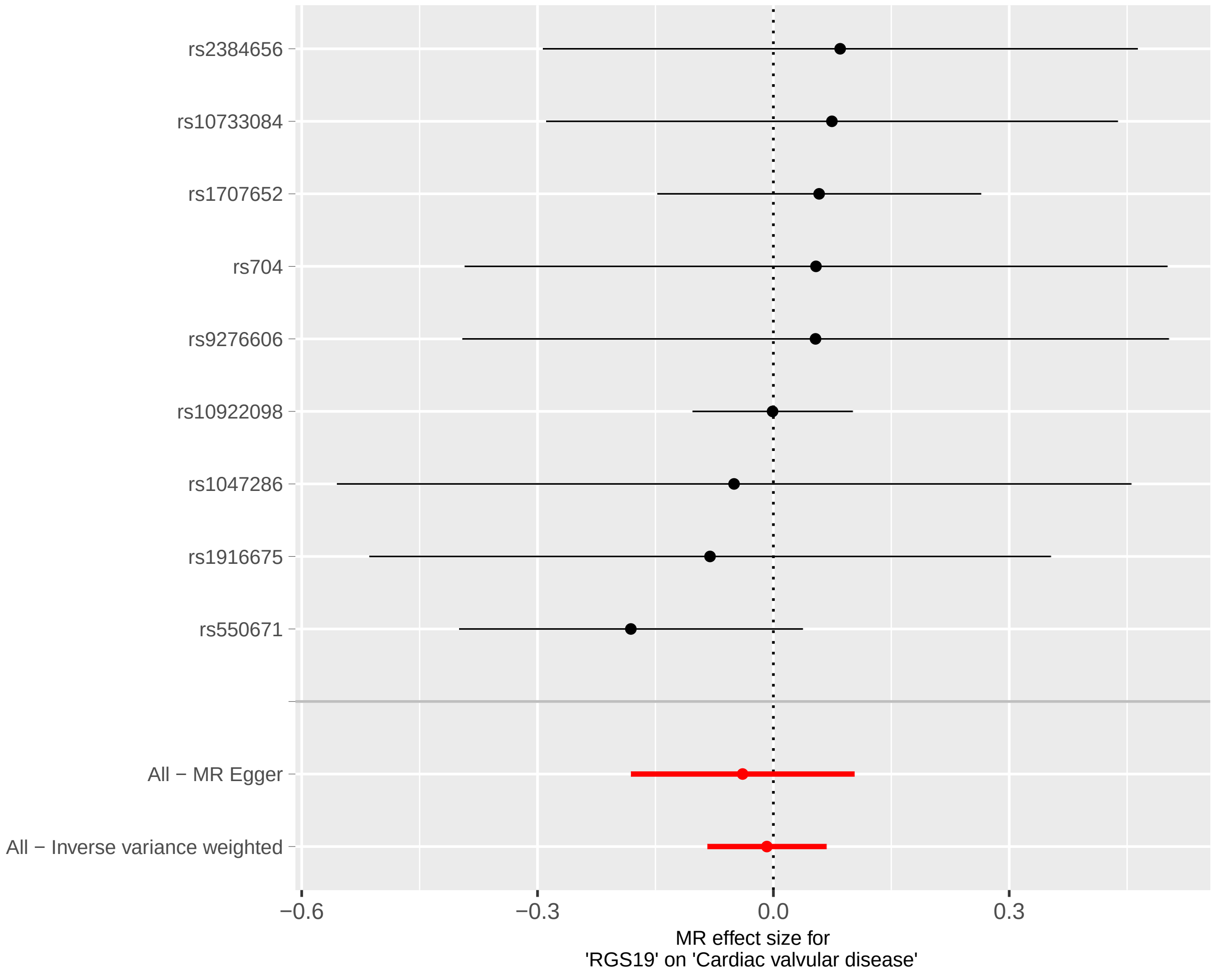
rs2384656
rs10733084
rs1707652
rs704
rs9276606
rs10922098
rs1047286
rs1916675
rs550671
All − MR Egger
All − Inverse variance weighted
−0.6
−0.3
0.0
0.3
MR effect size for
'RGS19' on 'Cardiac valvular disease'

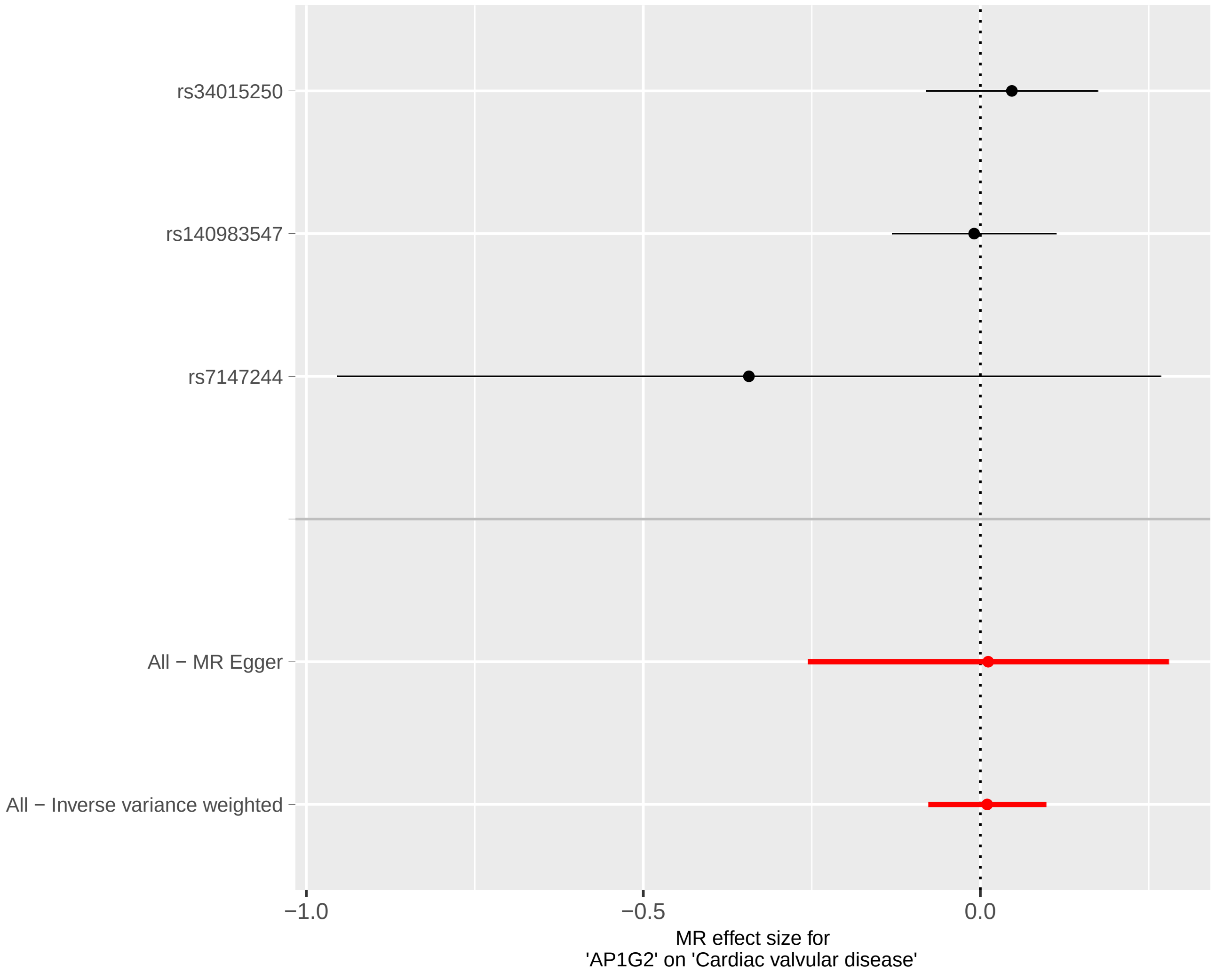

rs34015250
rs140983547
rs7147244
All − MR Egger
All − Inverse variance weighted
−1.0
−0.5
0.0
MR effect size for
'AP1G2' on 'Cardiac valvular disease'

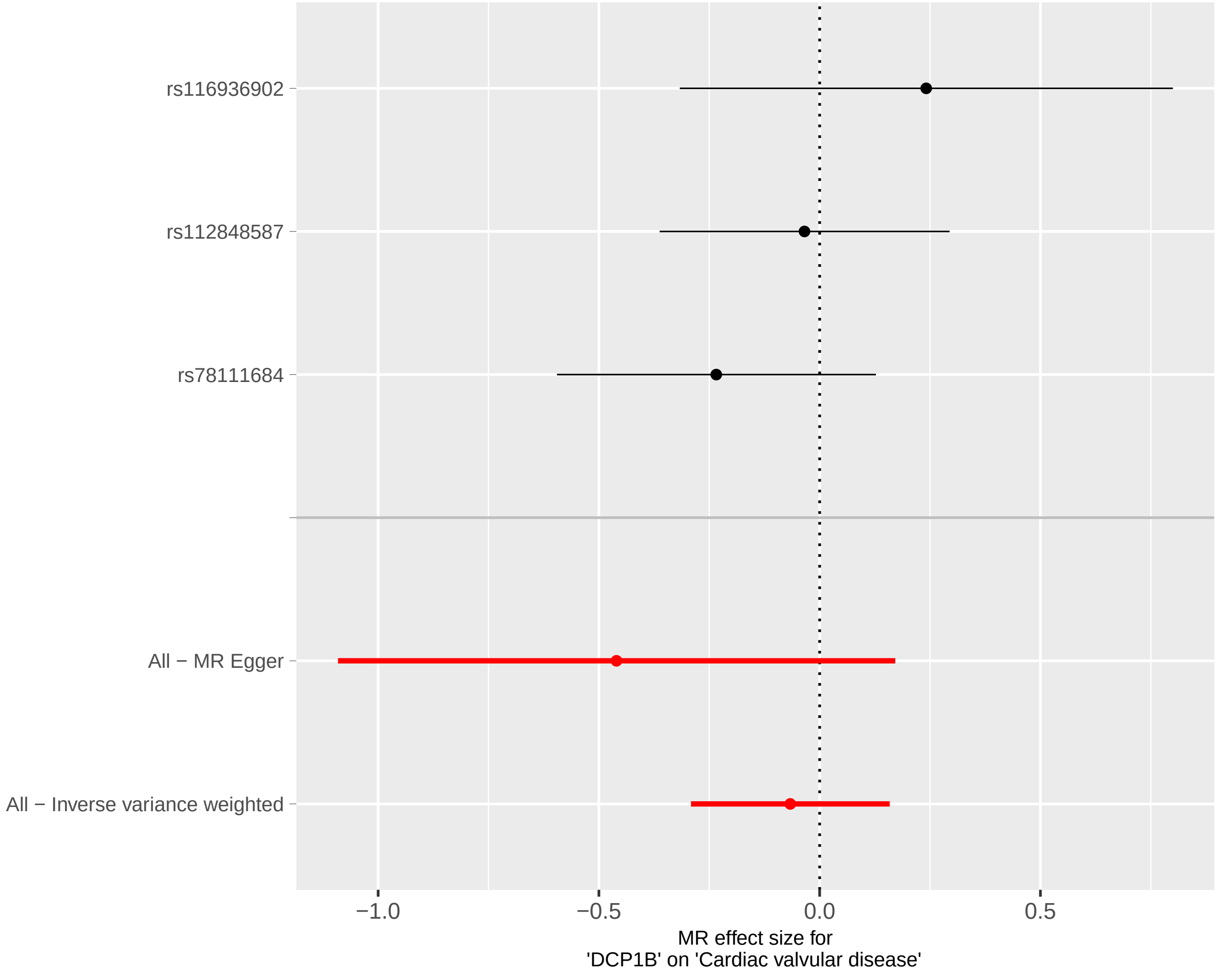
rs116936902
rs112848587
rs78111684
All − MR Egger
All − Inverse variance weighted
−1.0
−0.5
0.0
0.5
MR effect size for
'DCP1B' on 'Cardiac valvular disease'

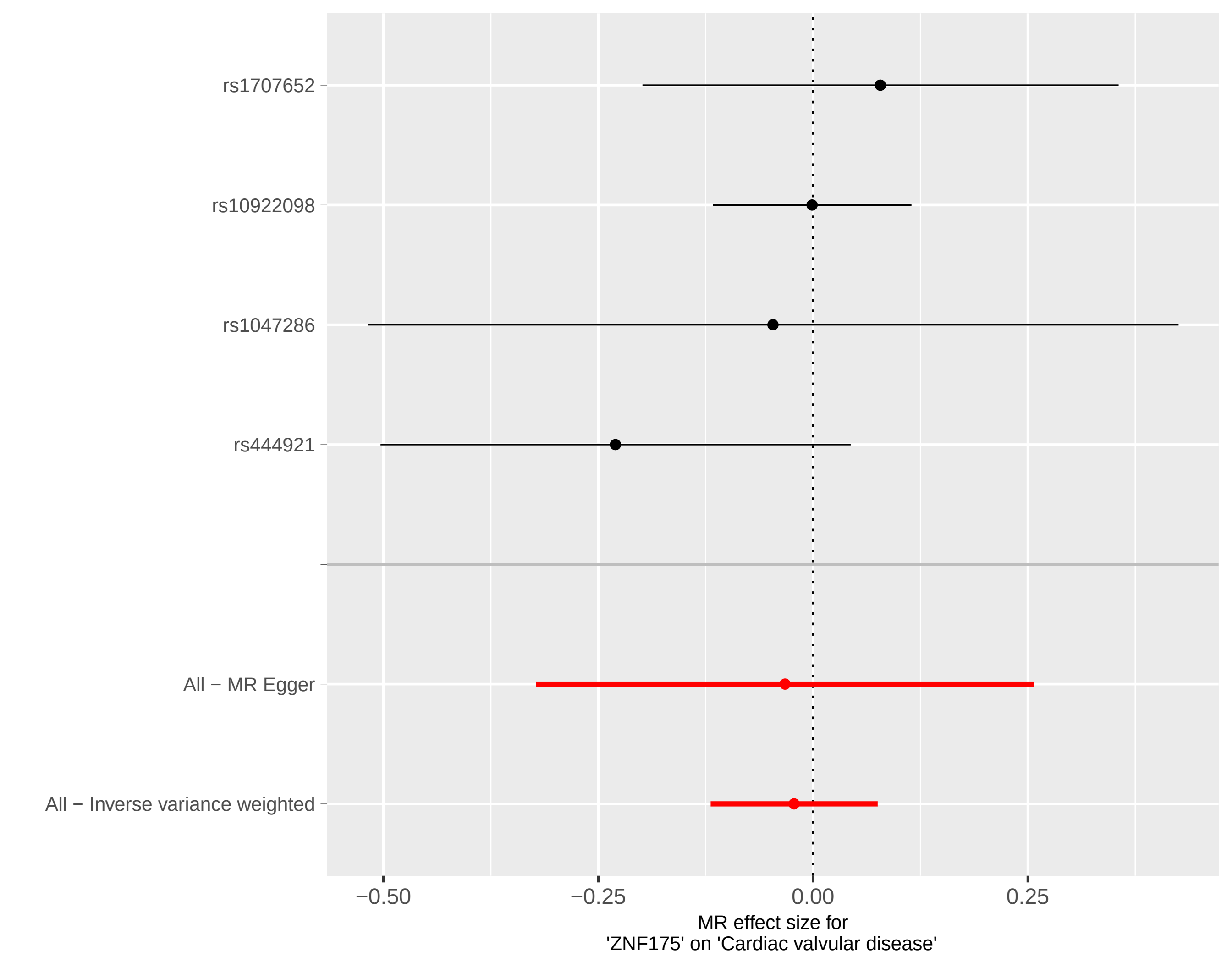
rs1707652
rs10922098
rs1047286
rs444921
All − MR Egger
All − Inverse variance weighted
−0.50
−0.25
0.00
0.25
MR effect size for
'ZNF175' on 'Cardiac valvular disease'

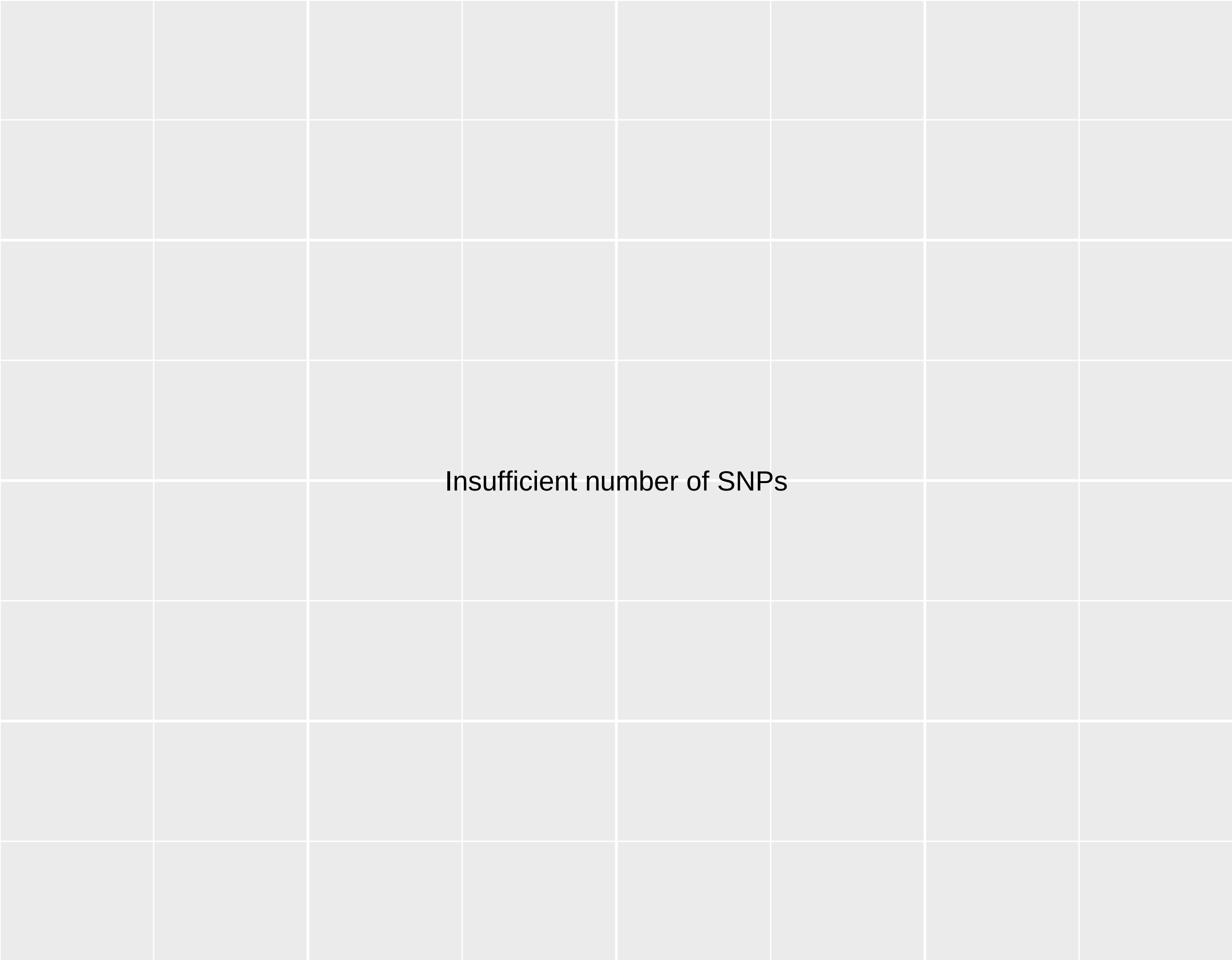
Insufficient number of SNPs

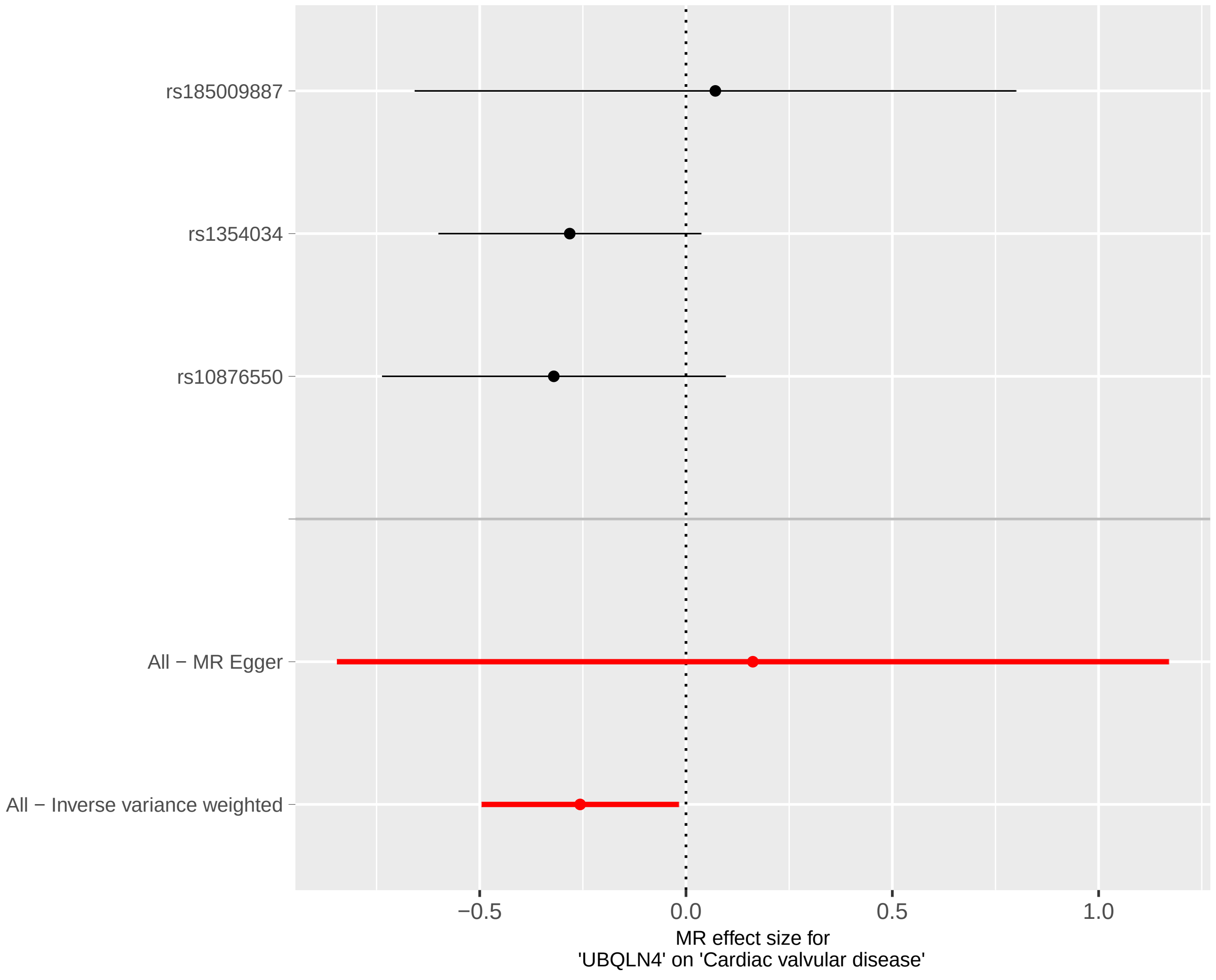
rs185009887
rs1354034
rs10876550
All − MR Egger
All − Inverse variance weighted
−0.5
0.0
0.5
1.0
MR effect size for
'UBQLN4' on 'Cardiac valvular disease'

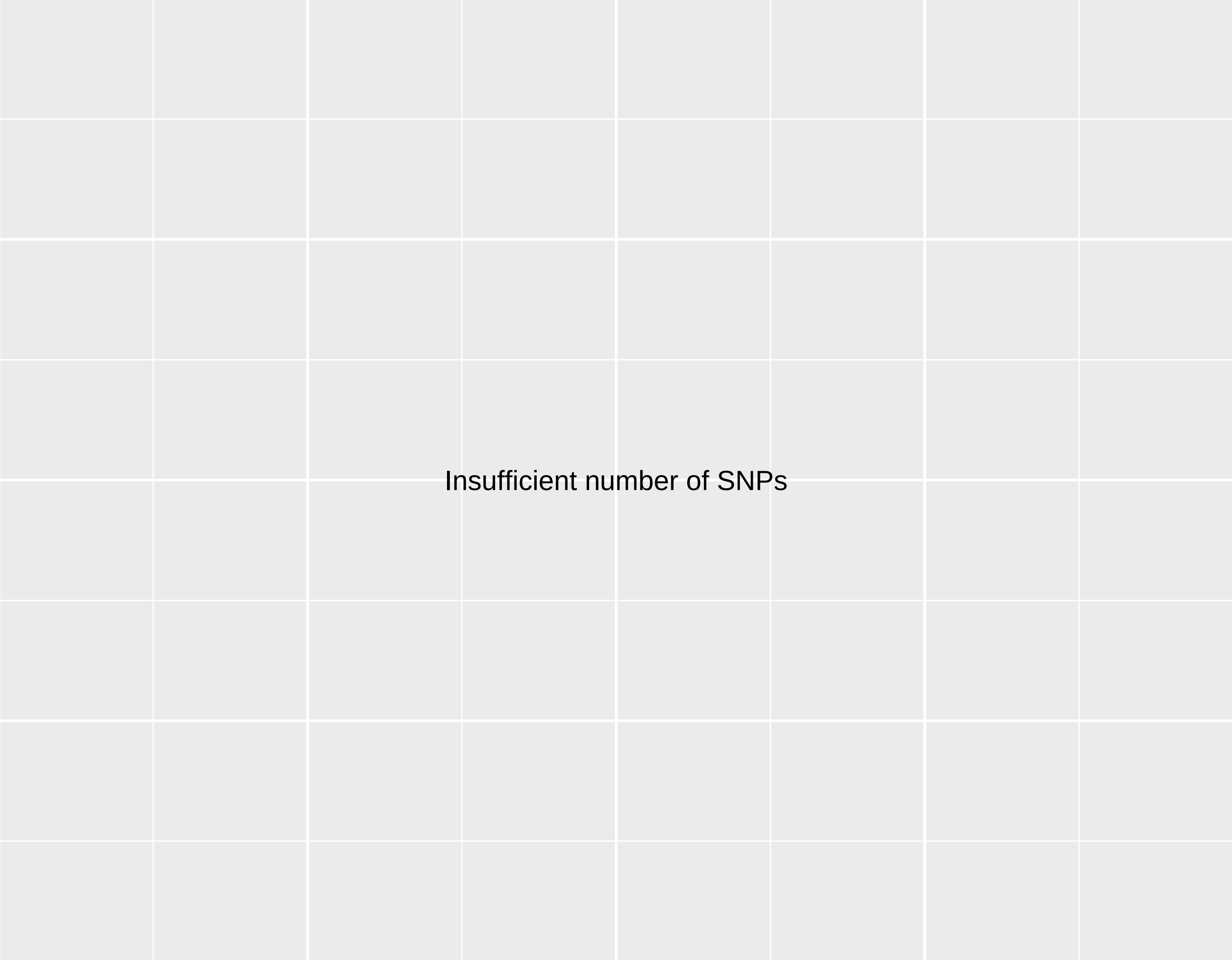
Insufficient number of SNPs

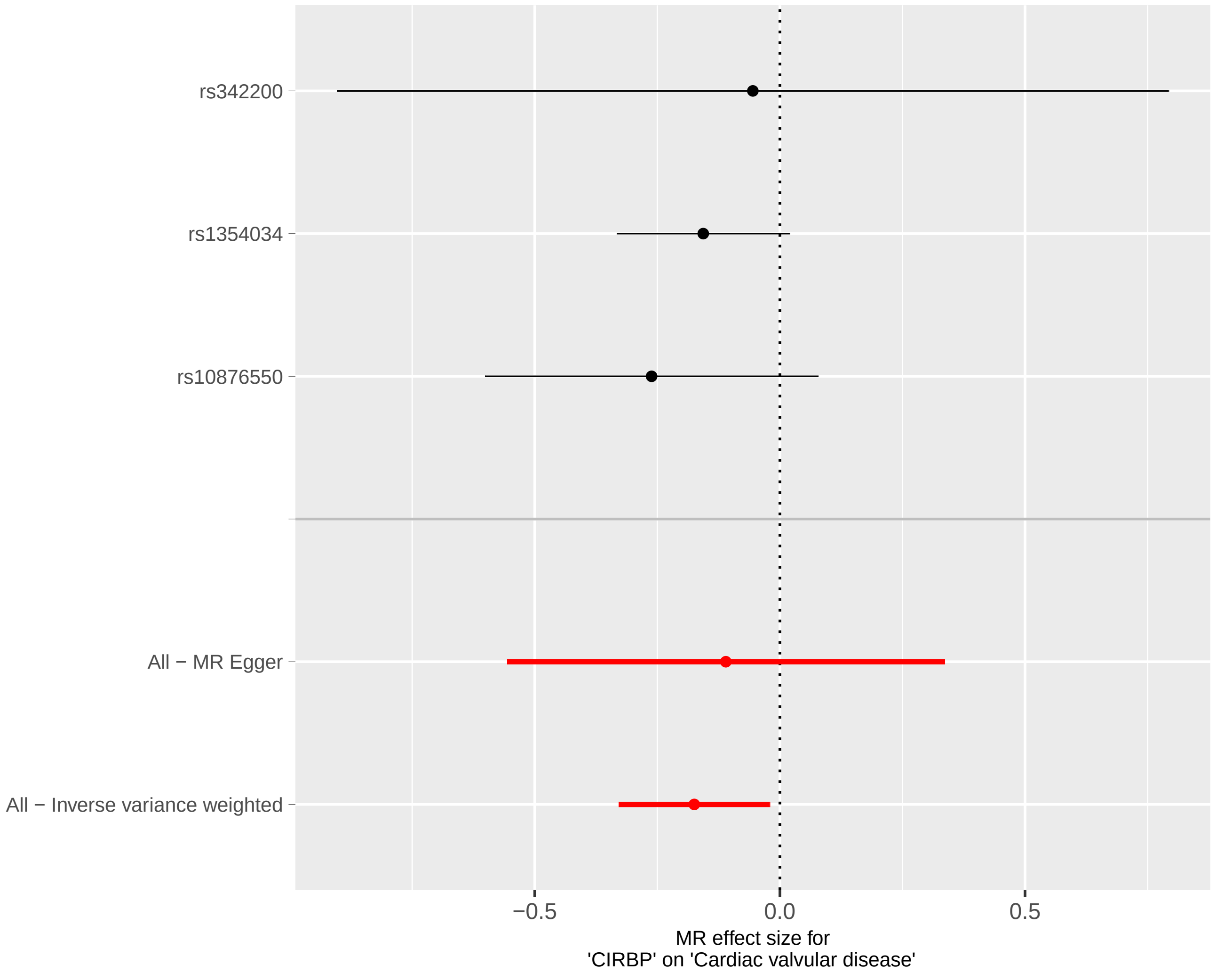
rs342200
rs1354034
rs10876550
All − MR Egger
All − Inverse variance weighted
−0.5
0.0
0.5
MR effect size for
'CIRBP' on 'Cardiac valvular disease'

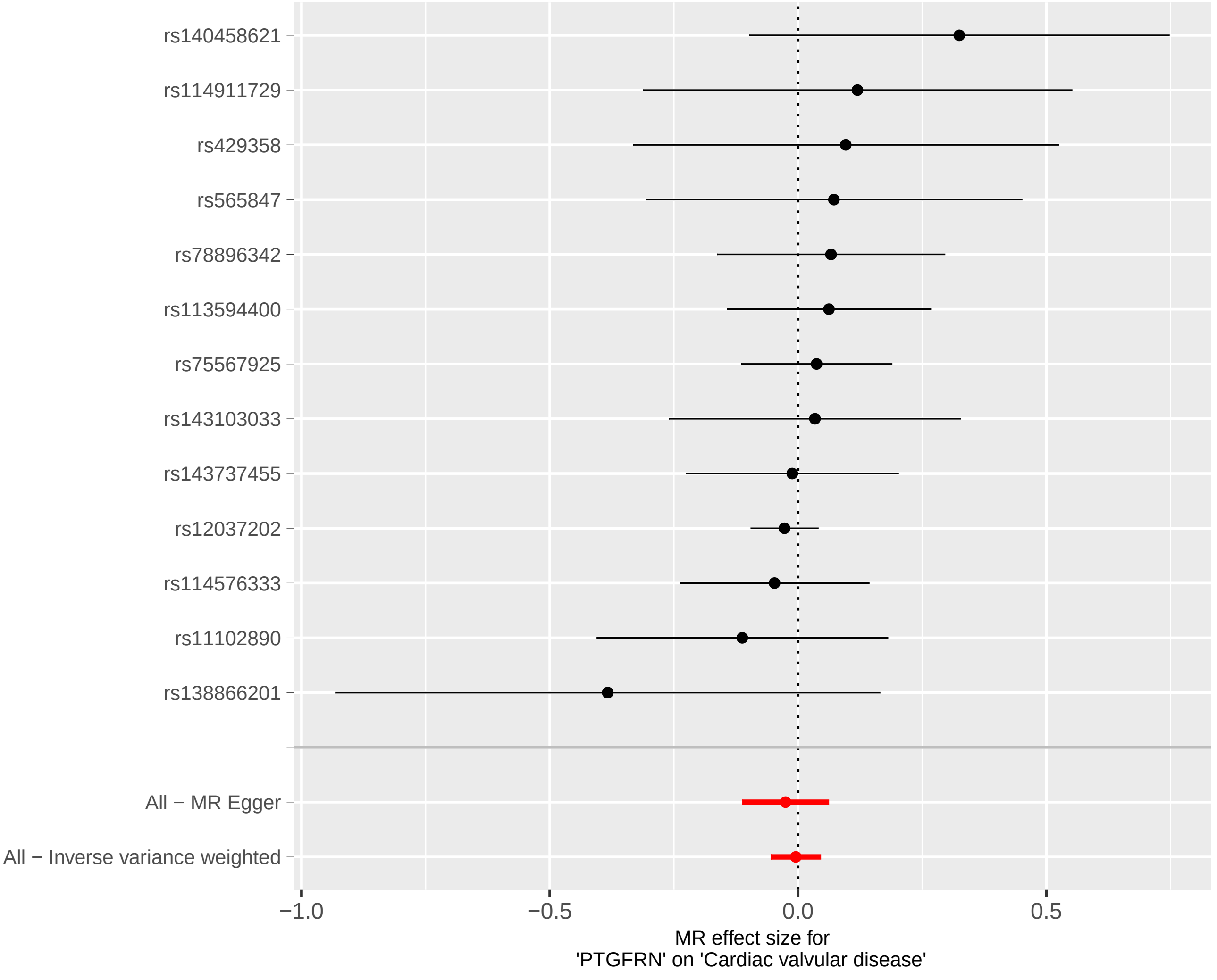
rs140458621
rs114911729
rs429358
rs565847
rs78896342
rs113594400
rs75567925
rs143103033
rs143737455
rs12037202
rs114576333
rs11102890
rs138866201
All − MR Egger
All − Inverse variance weighted
−1.0
−0.5
0.0
0.5
MR effect size for
'PTGFRN' on 'Cardiac valvular disease'

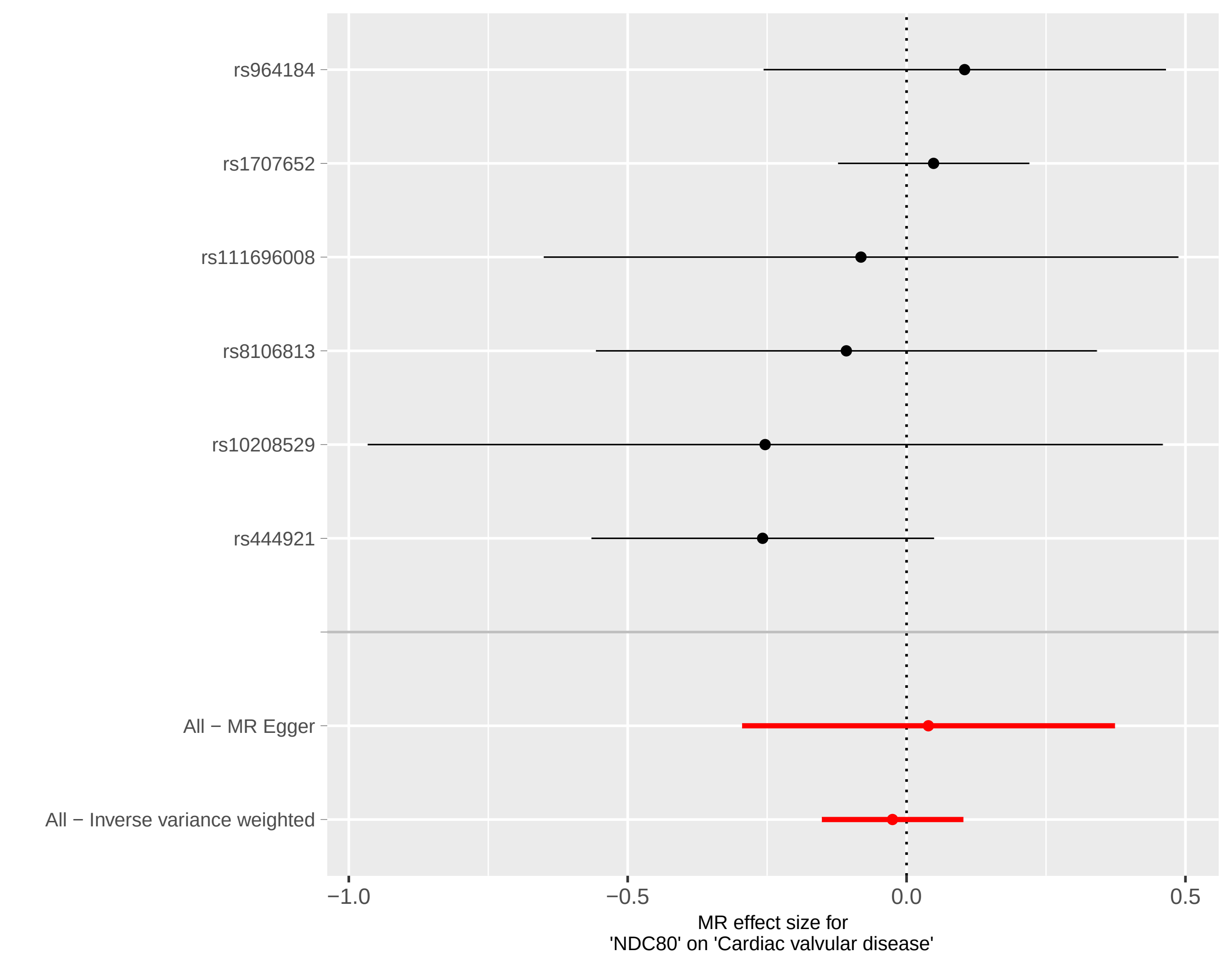
rs964184
rs1707652
rs111696008
rs8106813
rs10208529
rs444921
All − MR Egger
All − Inverse variance weighted
−1.0
−0.5
0.0
0.5
MR effect size for
'NDC80' on 'Cardiac valvular disease'

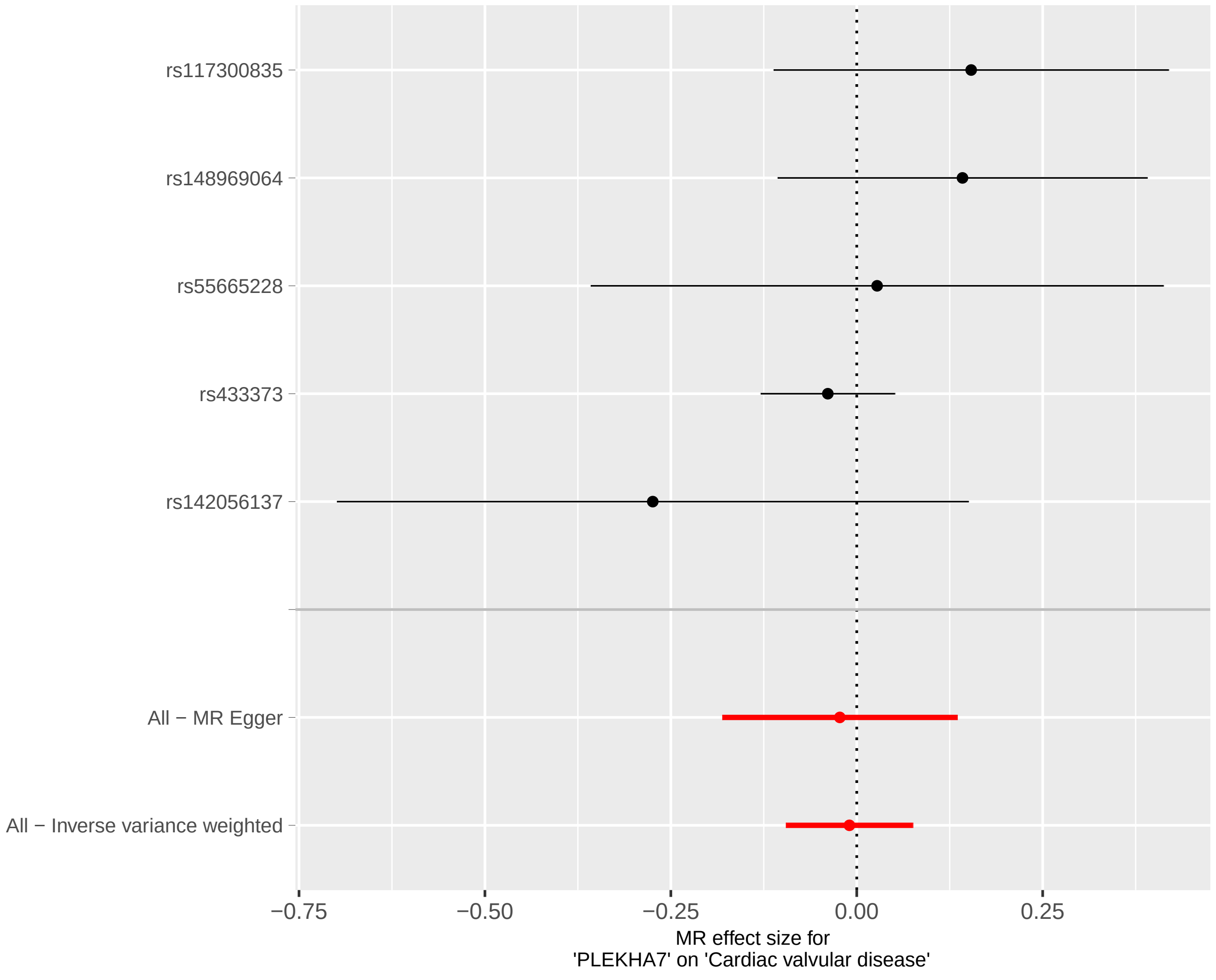

rs117300835
rs148969064
rs55665228
rs433373
rs142056137
All − MR Egger
All − Inverse variance weighted
−0.75
−0.50
−0.25
0.00
0.25
MR effect size for
'PLEKHA7' on 'Cardiac valvular disease'

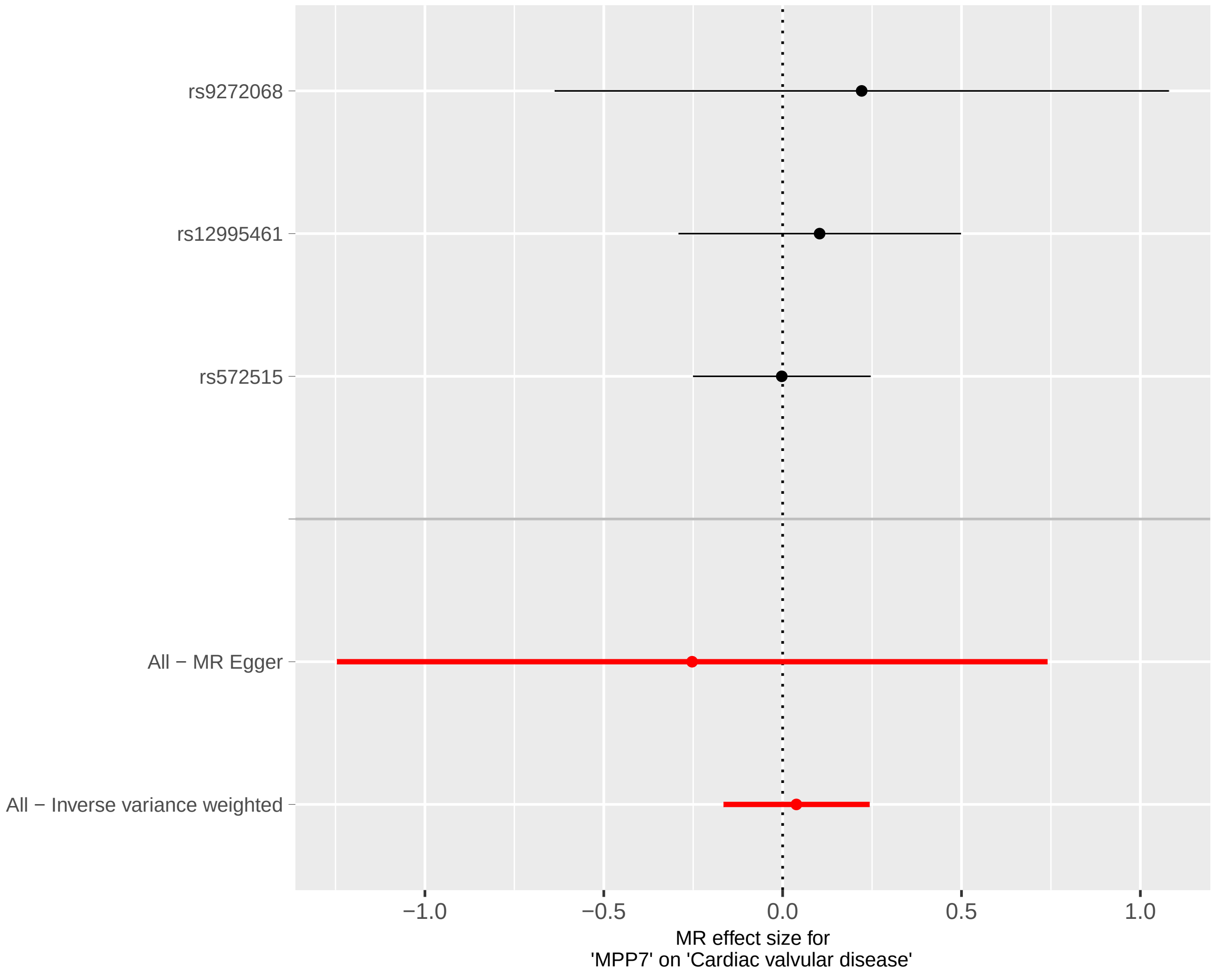
rs9272068
rs12995461
rs572515
All − MR Egger
All − Inverse variance weighted
−1.0
−0.5
0.0
0.5
1.0
MR effect size for
'MPP7' on 'Cardiac valvular disease'

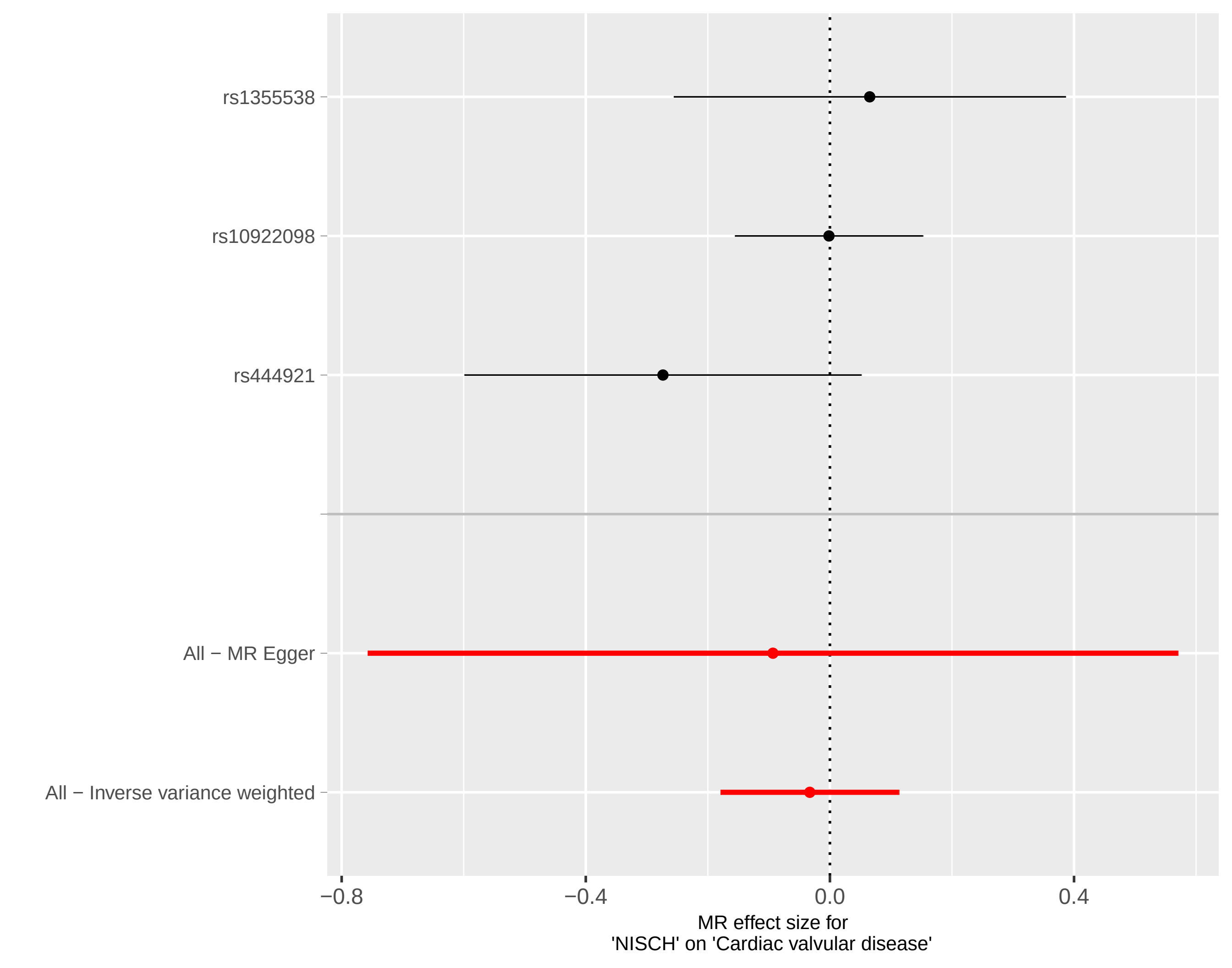

rs1355538
rs10922098
rs444921
All − MR Egger
All − Inverse variance weighted
−0.8
−0.4
0.0
0.4
MR effect size for
'NISCH' on 'Cardiac valvular disease'

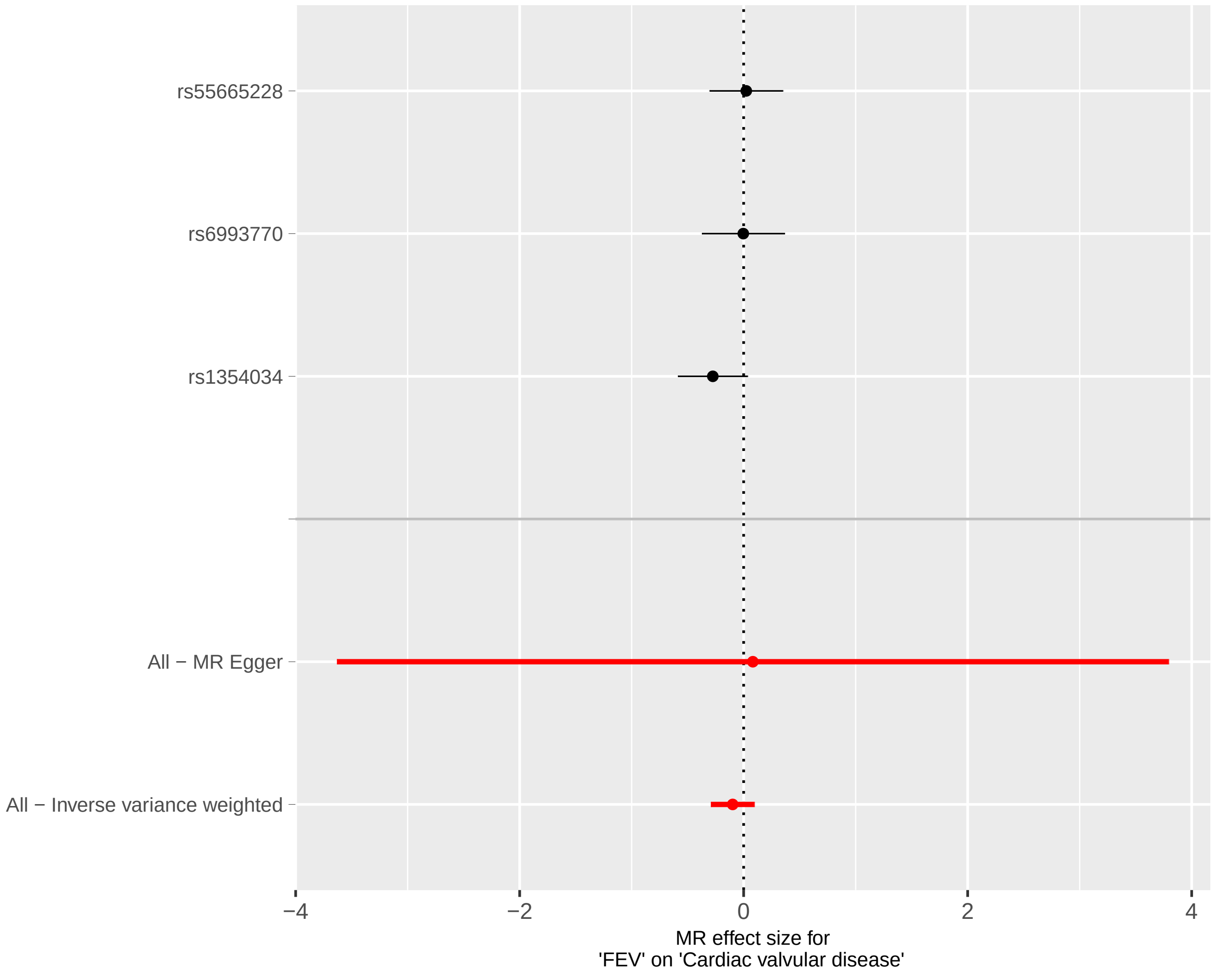

rs55665228
rs6993770
rs1354034
All − MR Egger
All − Inverse variance weighted
−4
−2
0
2
4
MR effect size for
'FEV' on 'Cardiac valvular disease'

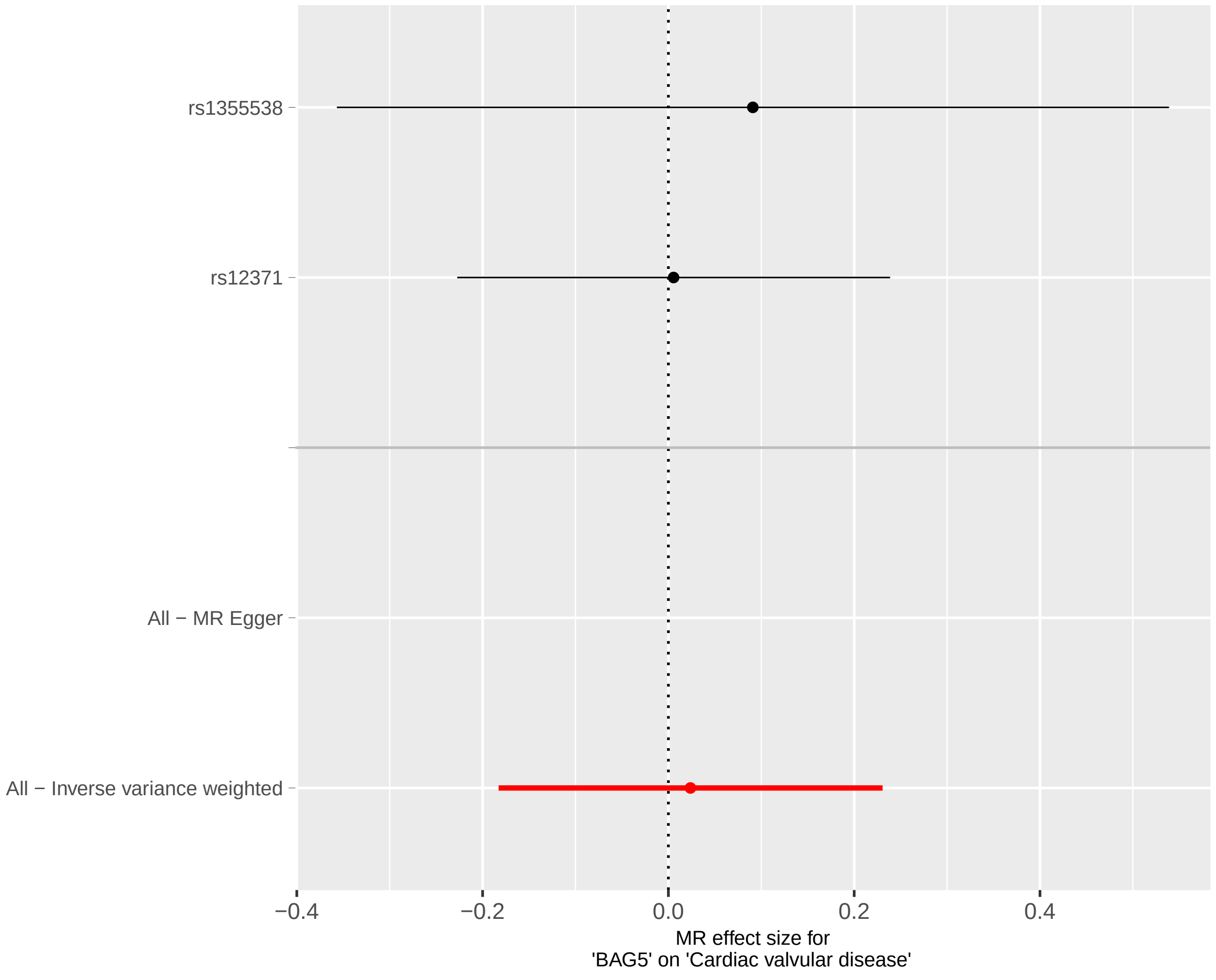
rs1355538
rs12371
All − MR Egger
All − Inverse variance weighted
−0.4
−0.2
0.0
0.2
0.4
MR effect size for
'BAG5' on 'Cardiac valvular disease'

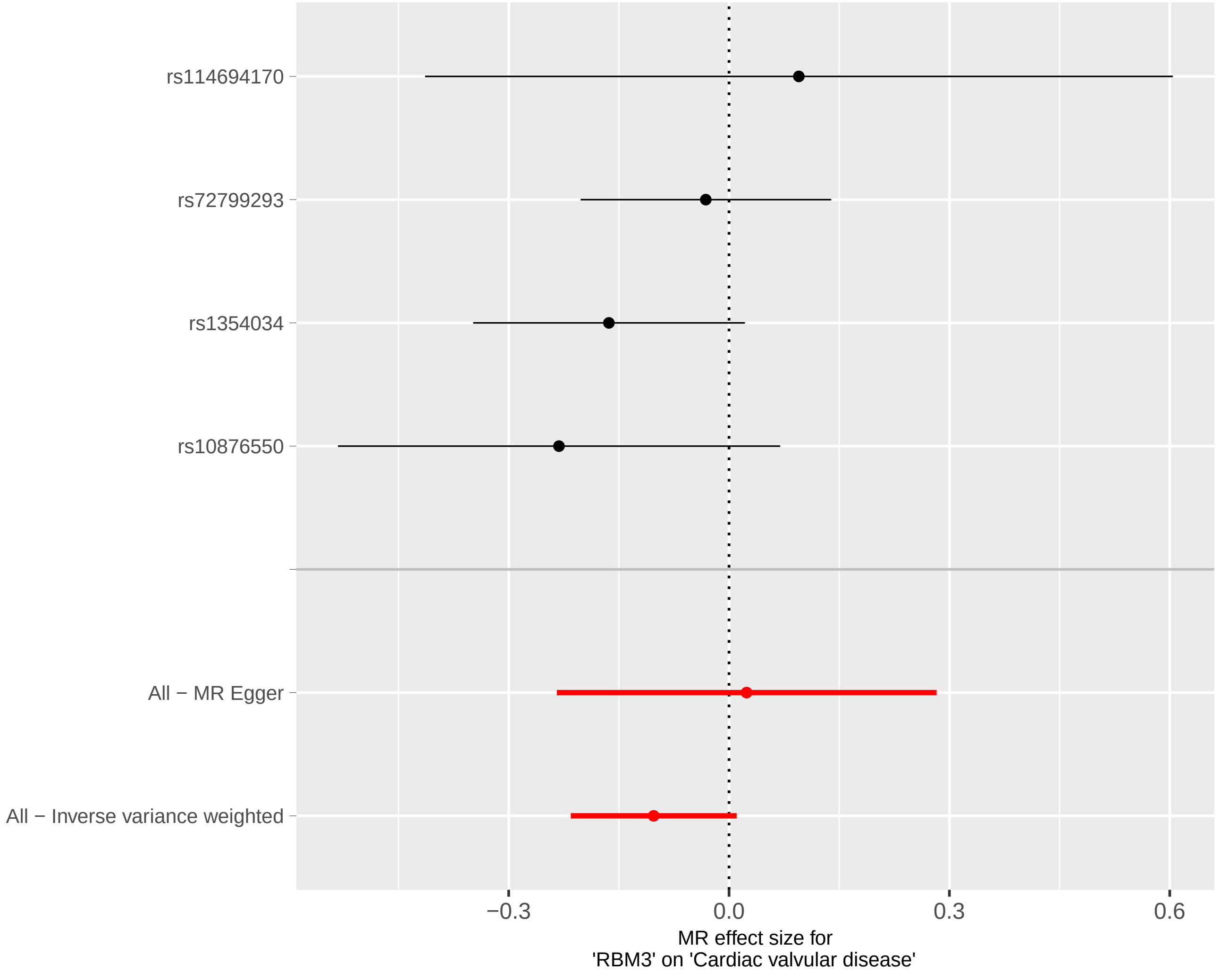
rs114694170
rs72799293
rs1354034
rs10876550
All − MR Egger
All − Inverse variance weighted
−0.3
0.0
0.3
0.6
MR effect size for
'RBM3' on 'Cardiac valvular disease'

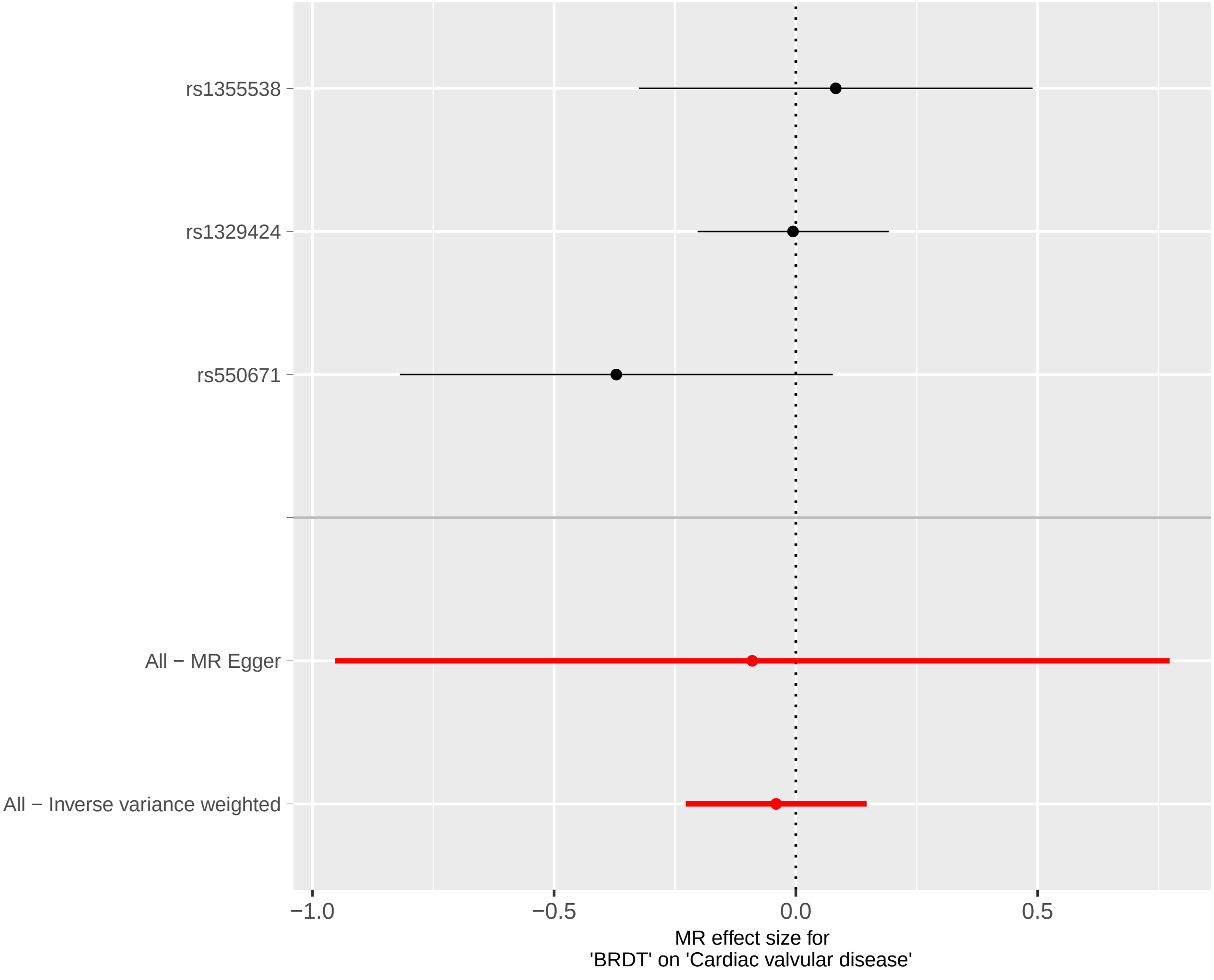
rs1355538
rs1329424
rs550671
All − MR Egger
All − Inverse variance weighted
−1.0
−0.5
0.0
0.5
MR effect size for
'BRDT' on 'Cardiac valvular disease'

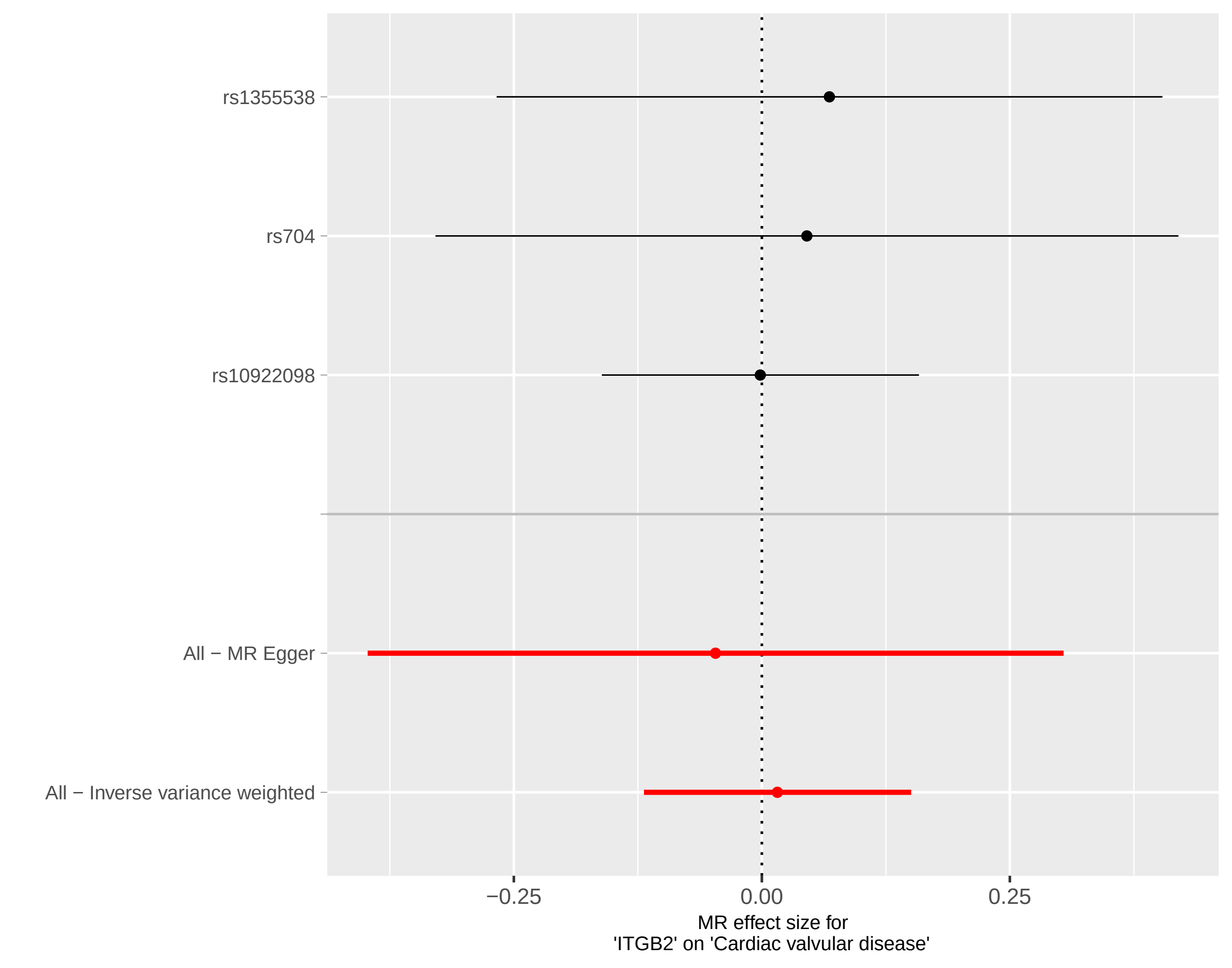

rs1355538
rs704
rs10922098
All − MR Egger
All − Inverse variance weighted
−0.25
0.00
0.25
MR effect size for
'ITGB2' on 'Cardiac valvular disease'

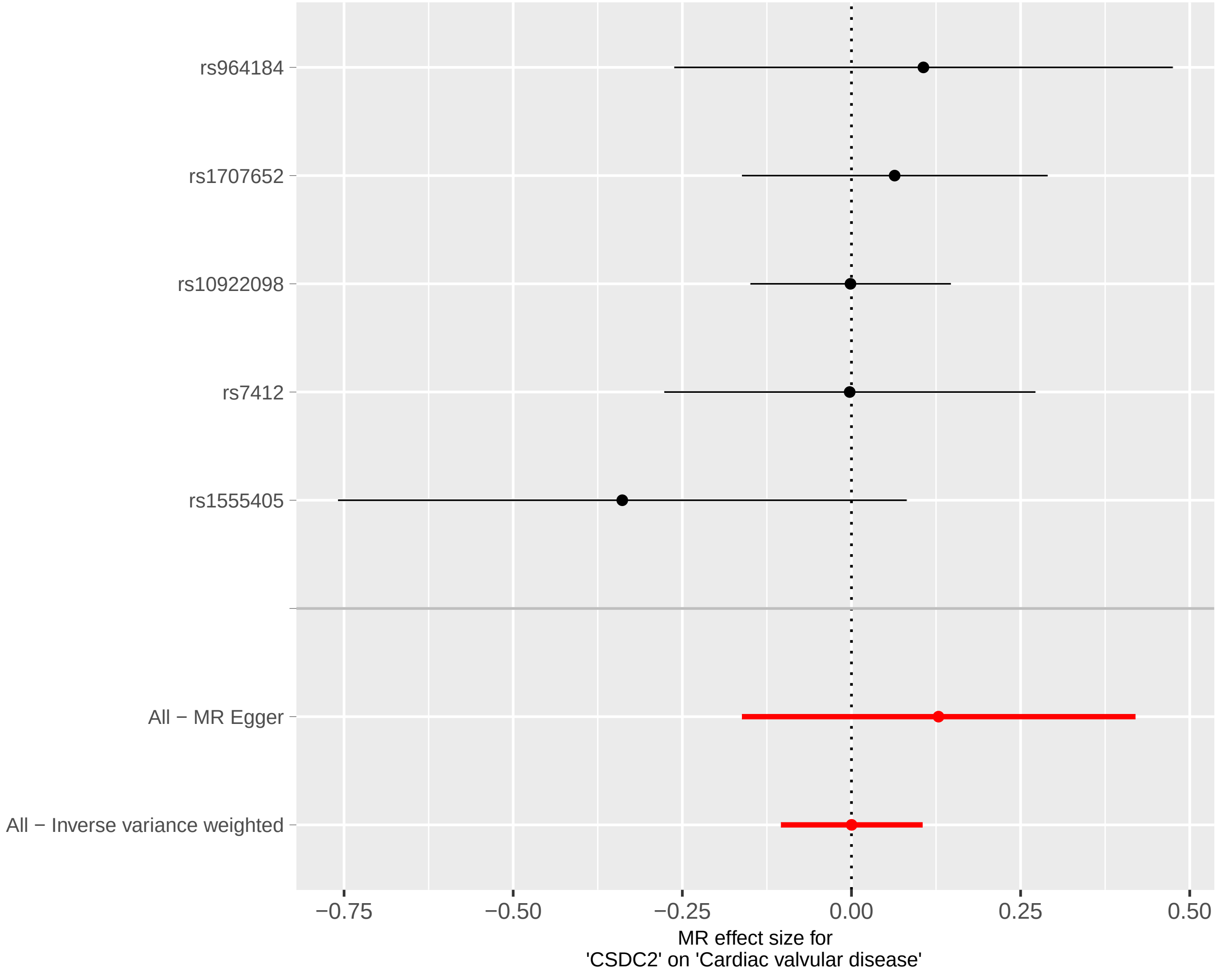
rs964184
rs1707652
rs10922098
rs7412
rs1555405
All − MR Egger
All − Inverse variance weighted
−0.75
−0.50
−0.25
0.00
0.25
0.50
MR effect size for
'CSDC2' on 'Cardiac valvular disease'

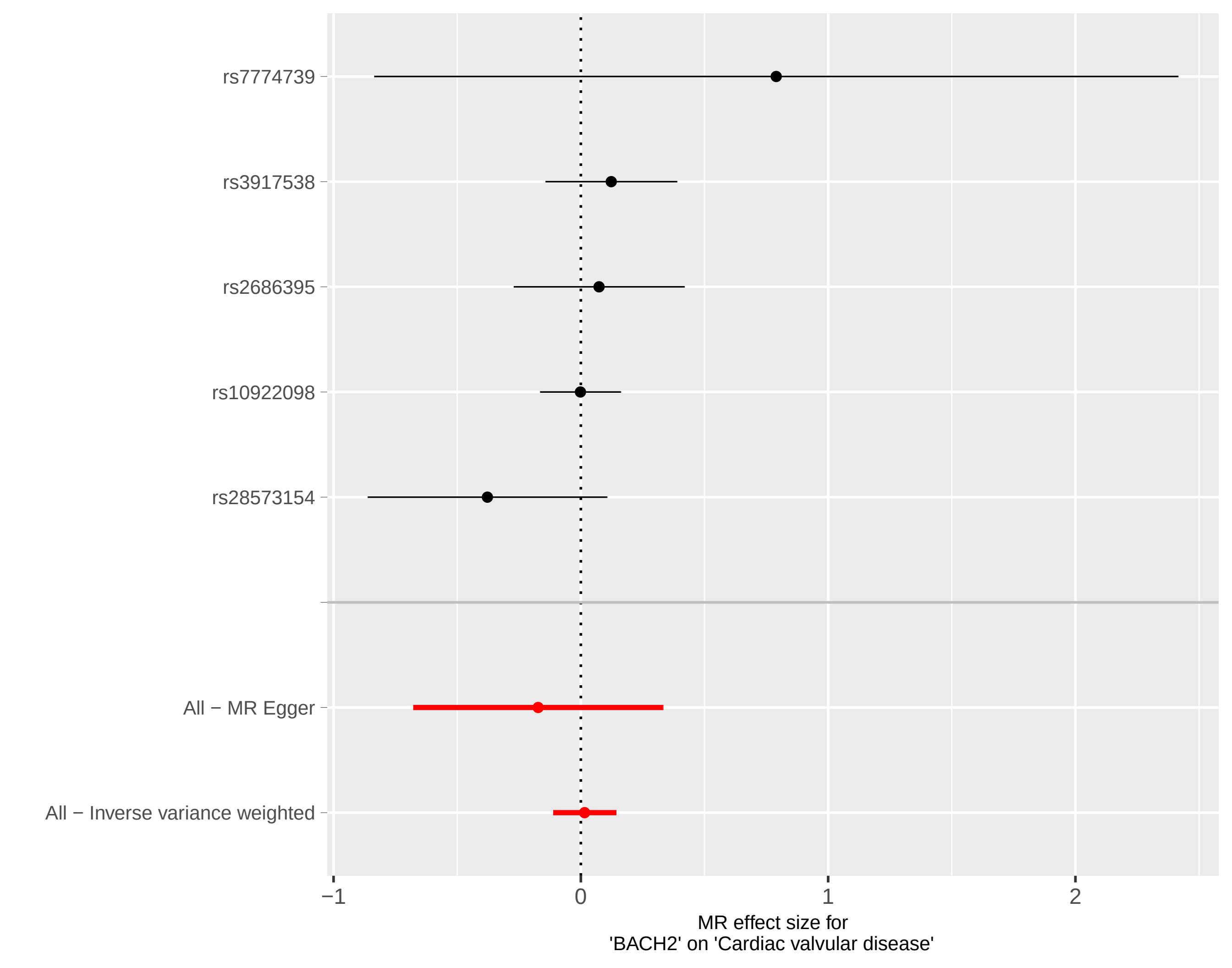
rs7774739
rs3917538
rs2686395
rs10922098
rs28573154
All − MR Egger
All − Inverse variance weighted
−1
0
1
2
MR effect size for
'BACH2' on 'Cardiac valvular disease'

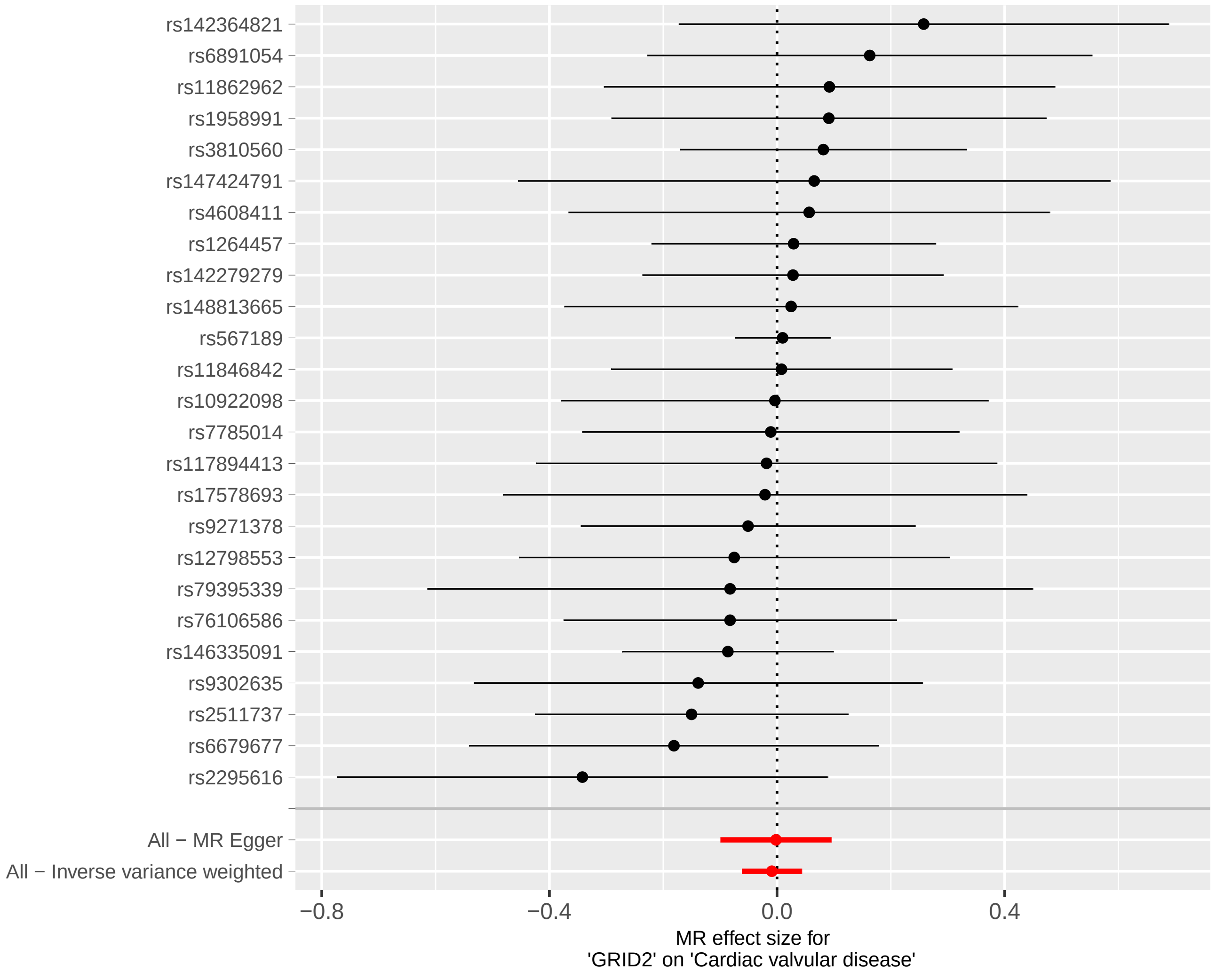
rs142364821
rs6891054
rs11862962
rs1958991
rs3810560
rs147424791
rs4608411
rs1264457
rs142279279
rs148813665
rs567189
rs11846842
rs10922098
rs7785014
rs117894413
rs17578693
rs9271378
rs12798553
rs79395339
rs76106586
rs146335091
rs9302635
rs2511737
rs6679677
rs2295616
All − MR Egger
All − Inverse variance weighted
−0.8
−0.4
0.0
0.4
MR effect size for
'GRID2' on 'Cardiac valvular disease'

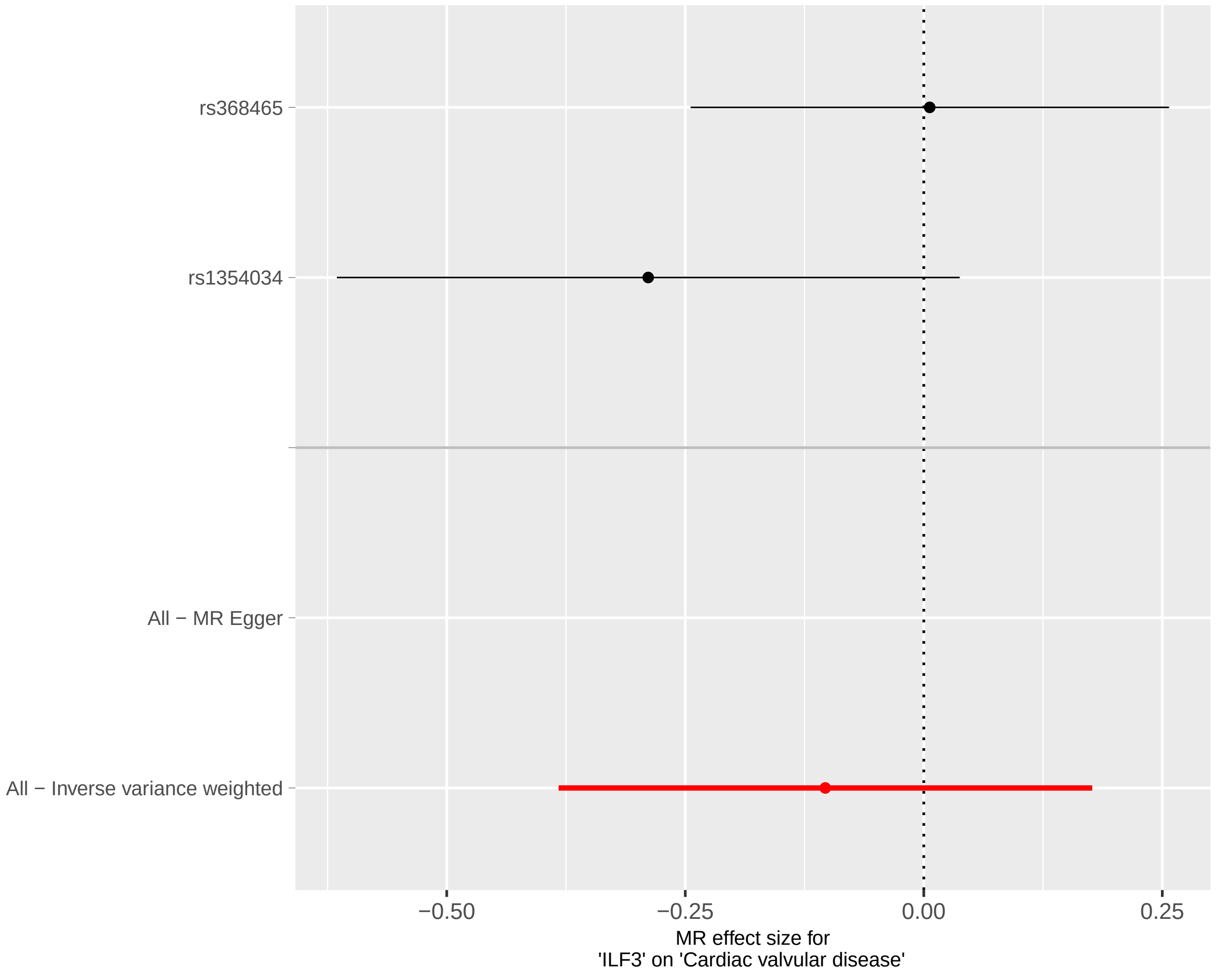

rs368465
rs1354034
All − MR Egger
All − Inverse variance weighted
−0.50
−0.25
0.00
0.25
MR effect size for
'ILF3' on 'Cardiac valvular disease'

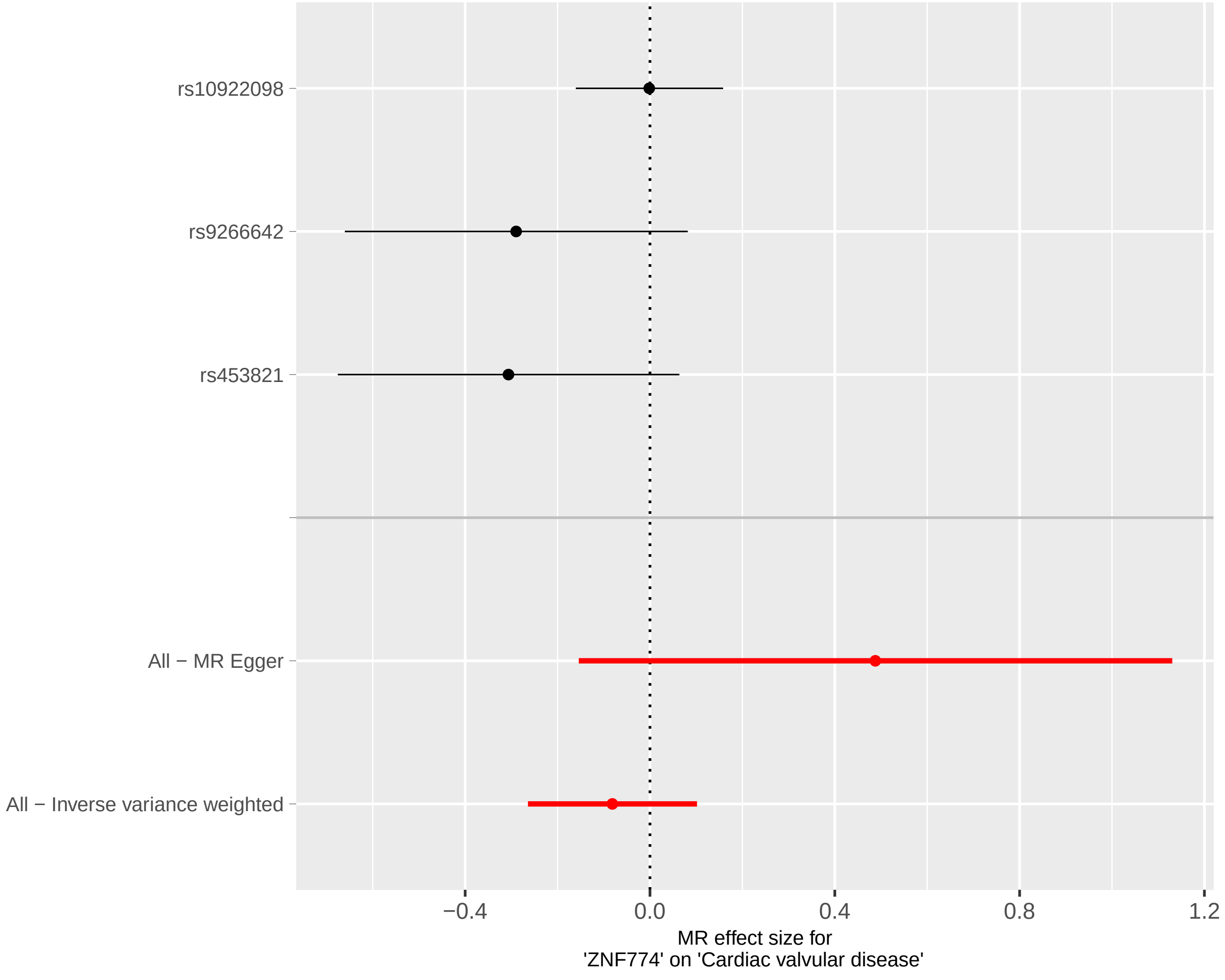
rs10922098
rs9266642
rs453821
All − MR Egger
All − Inverse variance weighted
−0.4
0.0
0.4
0.8
1.2
MR effect size for
'ZNF774' on 'Cardiac valvular disease'

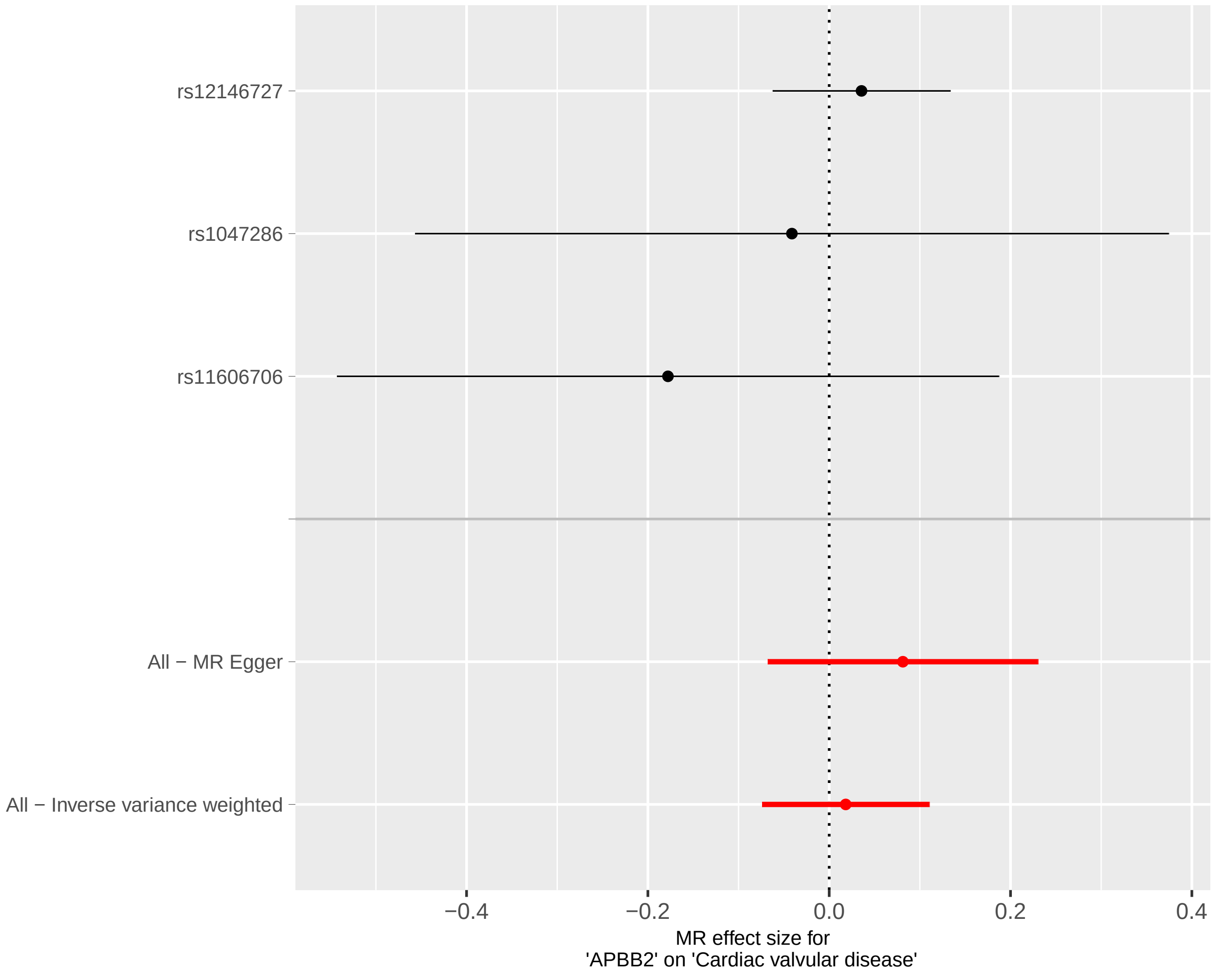
rs12146727
rs1047286
rs11606706
All − MR Egger
All − Inverse variance weighted
−0.4
−0.2
0.0
0.2
0.4
MR effect size for
'APBB2' on 'Cardiac valvular disease'

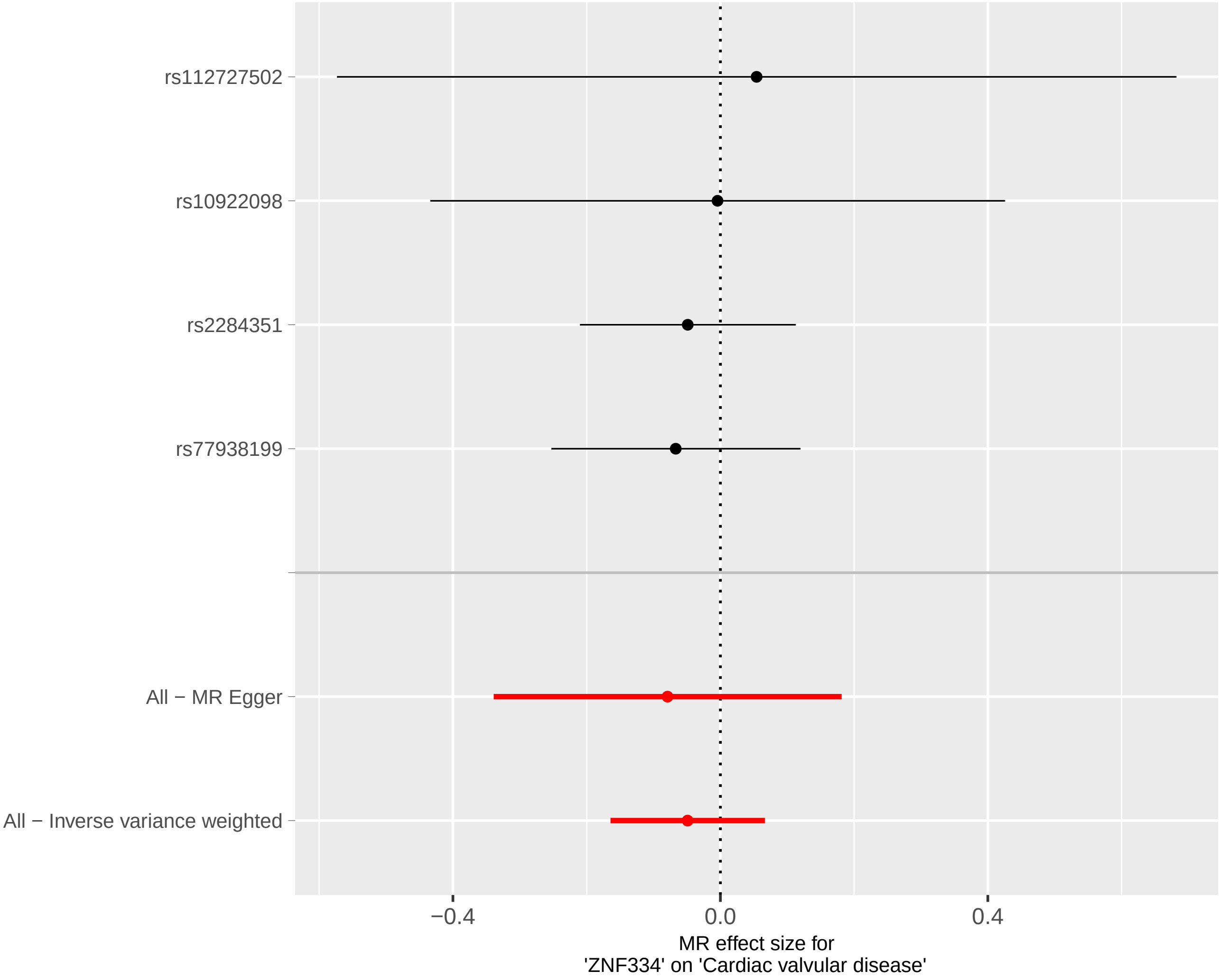
rs112727502
rs10922098
rs2284351
rs77938199
All − MR Egger
All − Inverse variance weighted
−0.4
0.0
0.4
MR effect size for
'ZNF334' on 'Cardiac valvular disease'

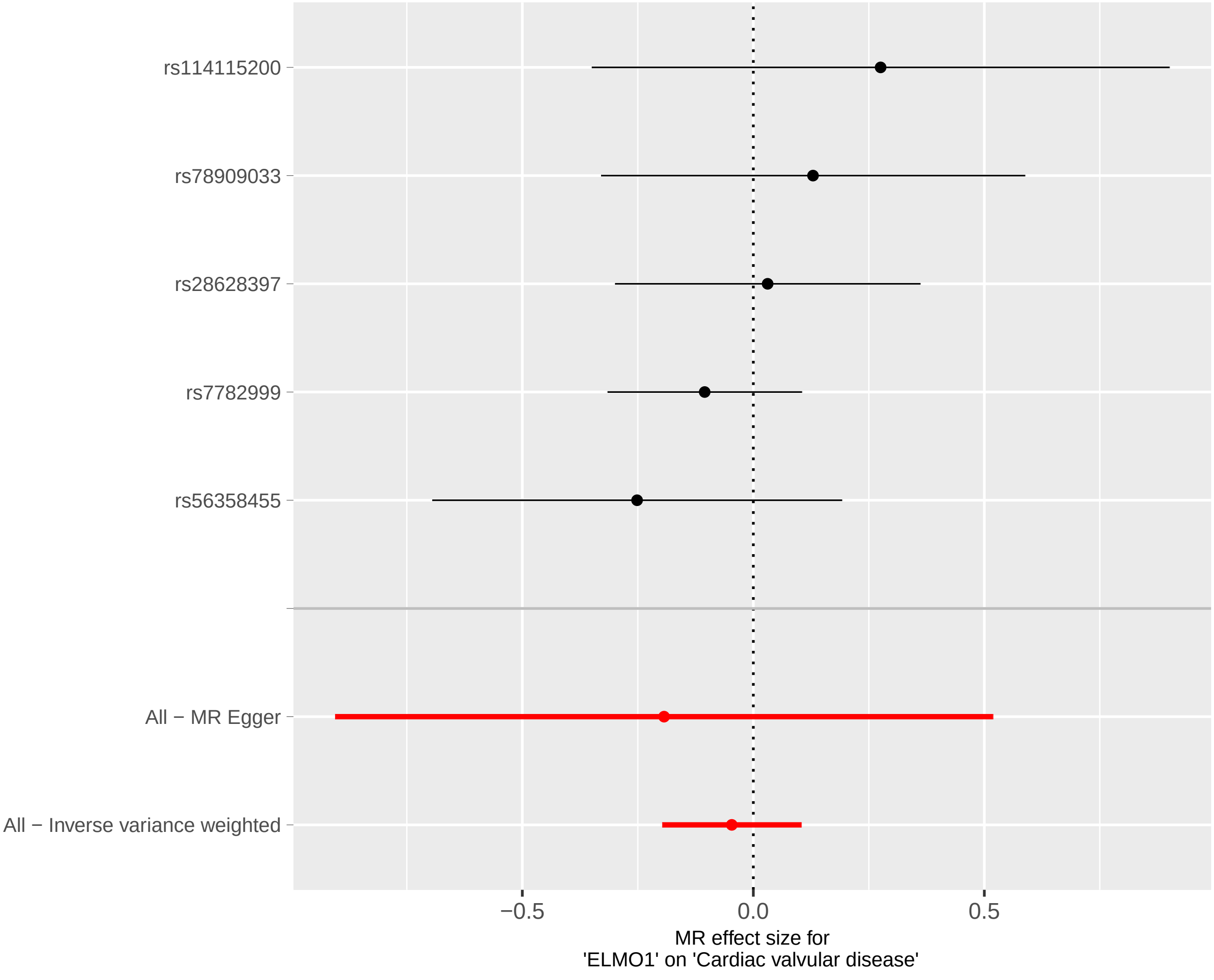
rs114115200
rs78909033
rs28628397
rs7782999
rs56358455
All − MR Egger
All − Inverse variance weighted
−0.5
0.0
0.5
MR effect size for
'ELMO1' on 'Cardiac valvular disease'

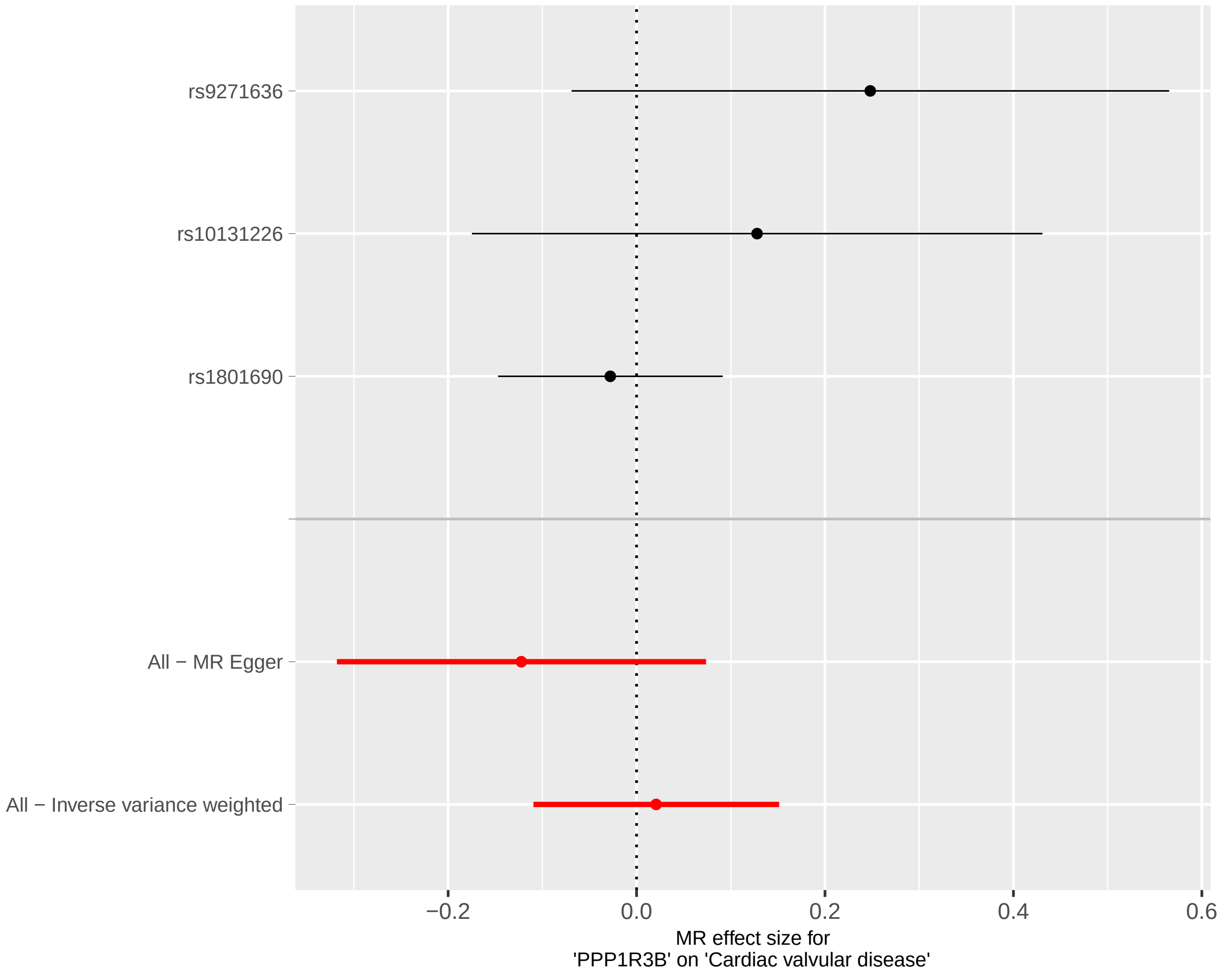

rs9271636
rs10131226
rs1801690
All − MR Egger
All − Inverse variance weighted
−0.2
0.0
0.2
0.4
0.6
MR effect size for
'PPP1R3B' on 'Cardiac valvular disease'

Insufficient number of SNPs

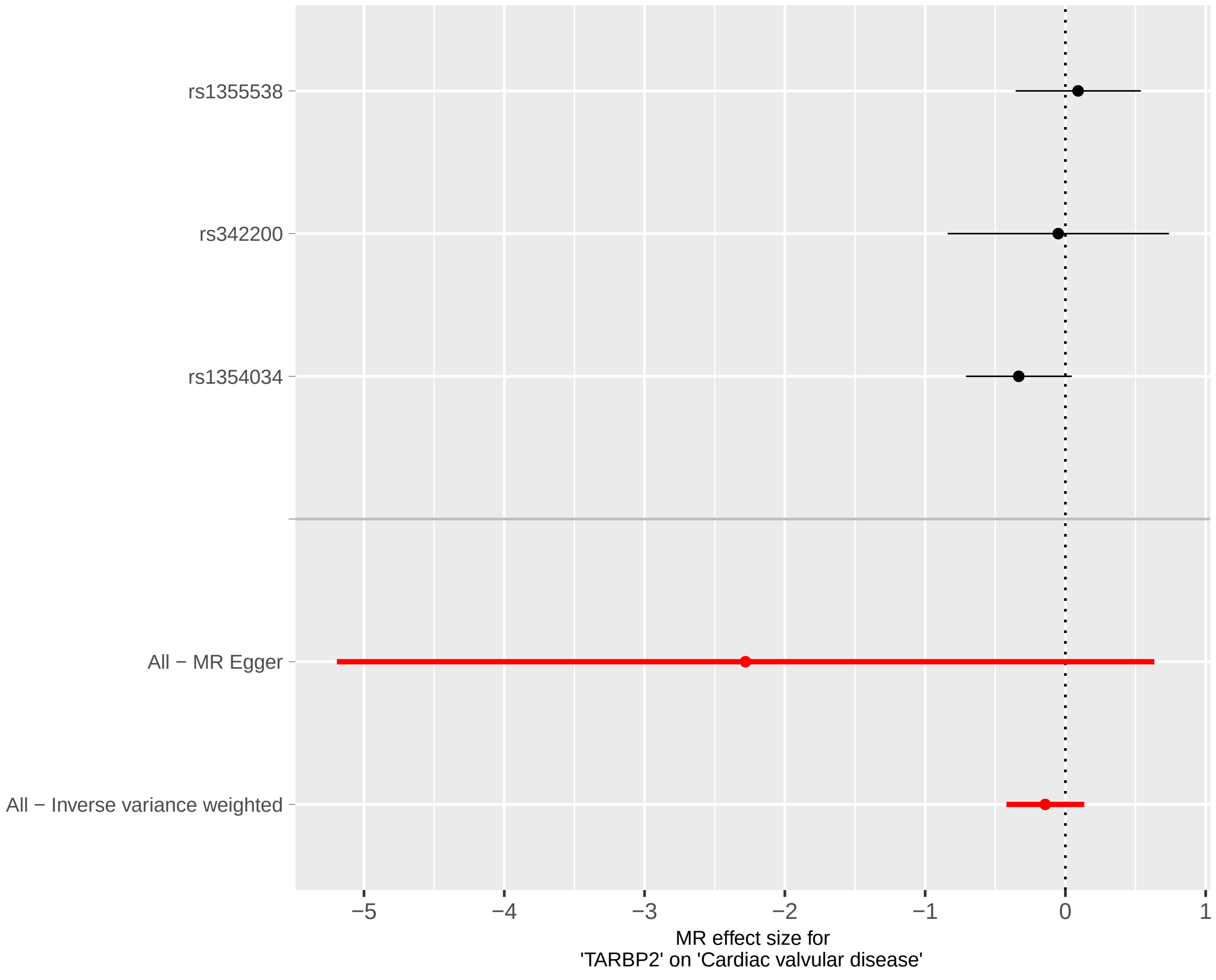
rs1355538
rs342200
rs1354034
All − MR Egger
All − Inverse variance weighted
−5
−4
−3
−2
−1
0
1
MR effect size for
'TARBP2' on 'Cardiac valvular disease'

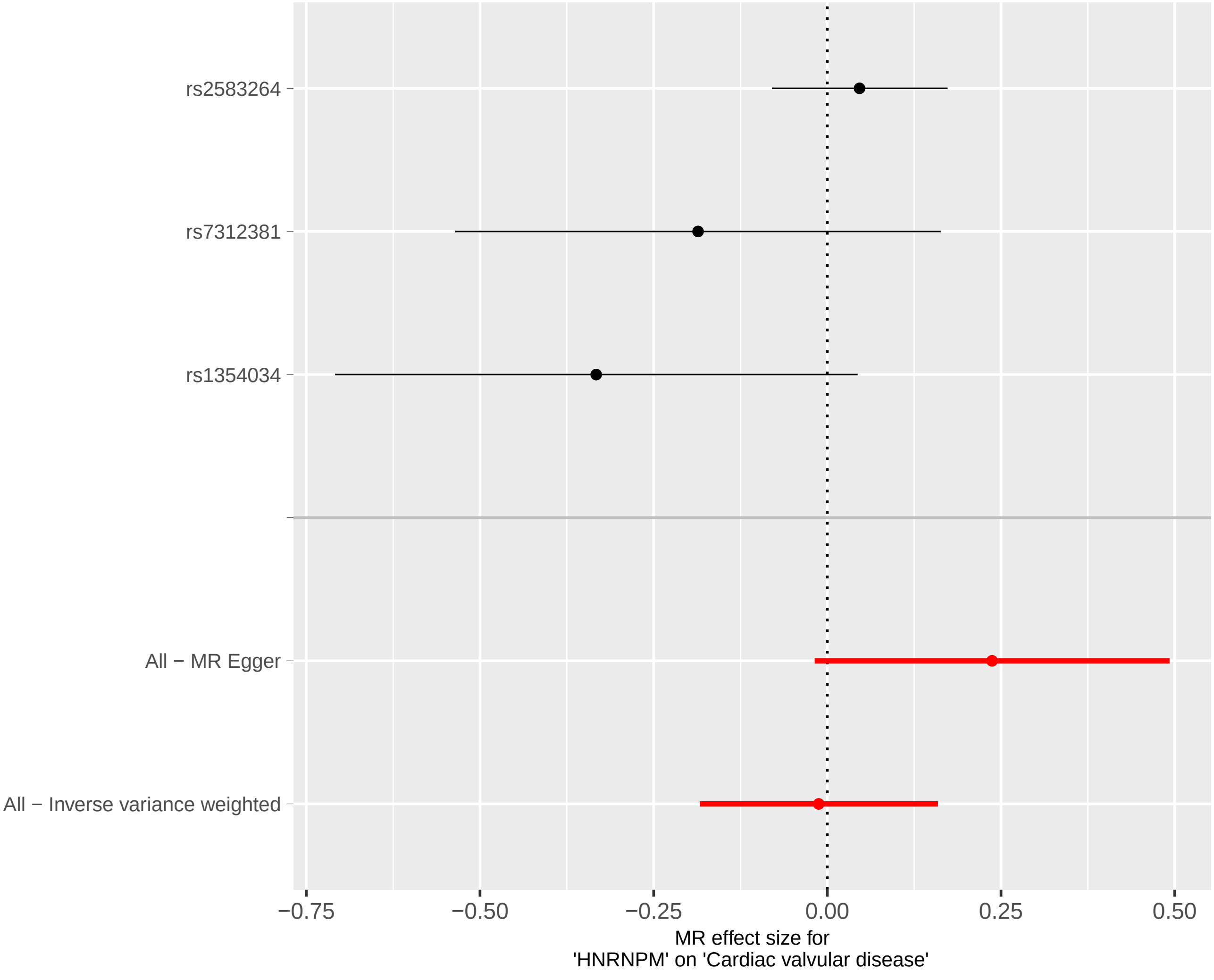
rs2583264
rs7312381
rs1354034
All − MR Egger
All − Inverse variance weighted
−0.75
−0.50
−0.25
0.00
0.25
0.50
MR effect size for
'HNRNPM' on 'Cardiac valvular disease'

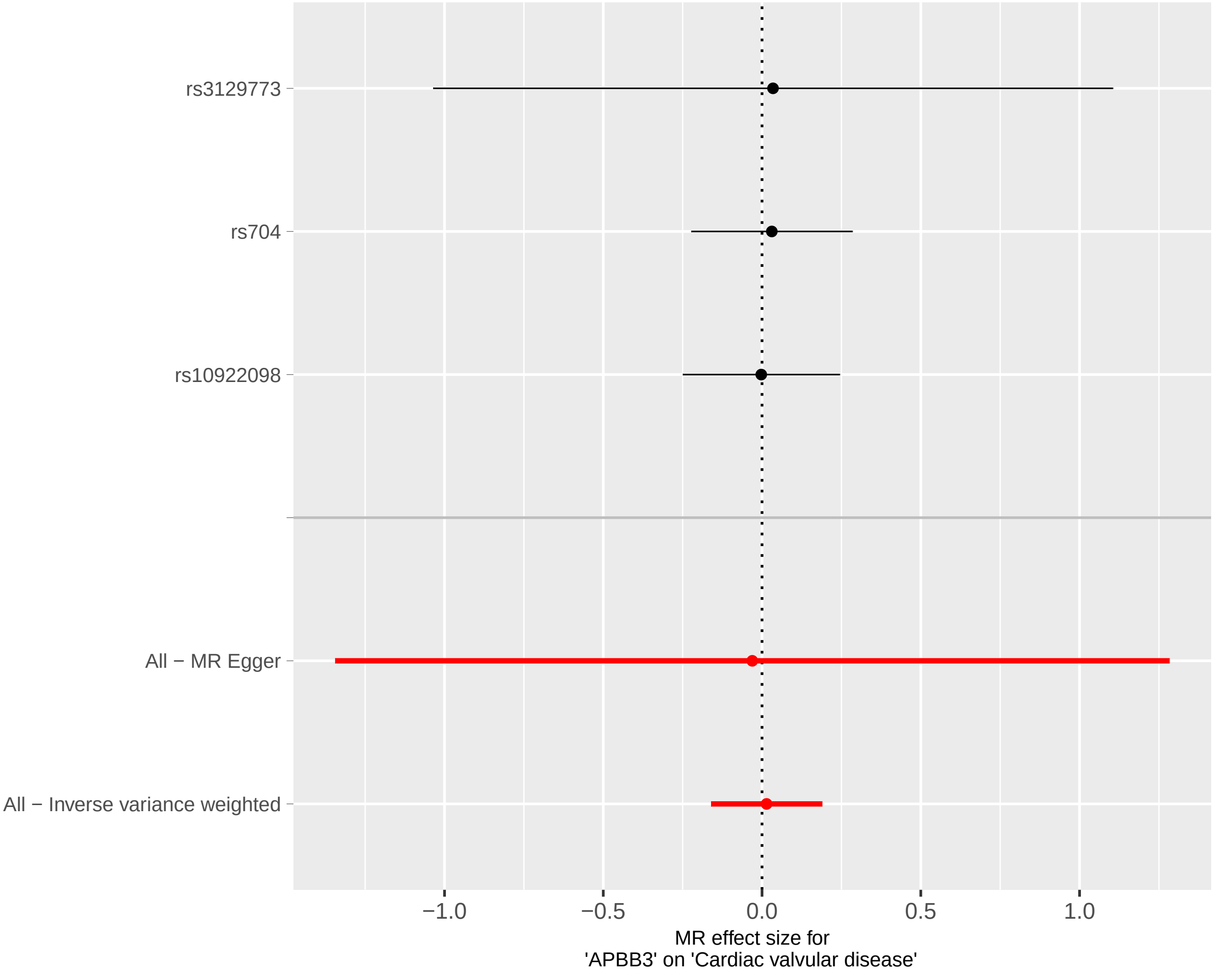
rs3129773
rs704
rs10922098
All − MR Egger
All − Inverse variance weighted
−1.0
−0.5
0.0
0.5
1.0
MR effect size for
'APBB3' on 'Cardiac valvular disease'

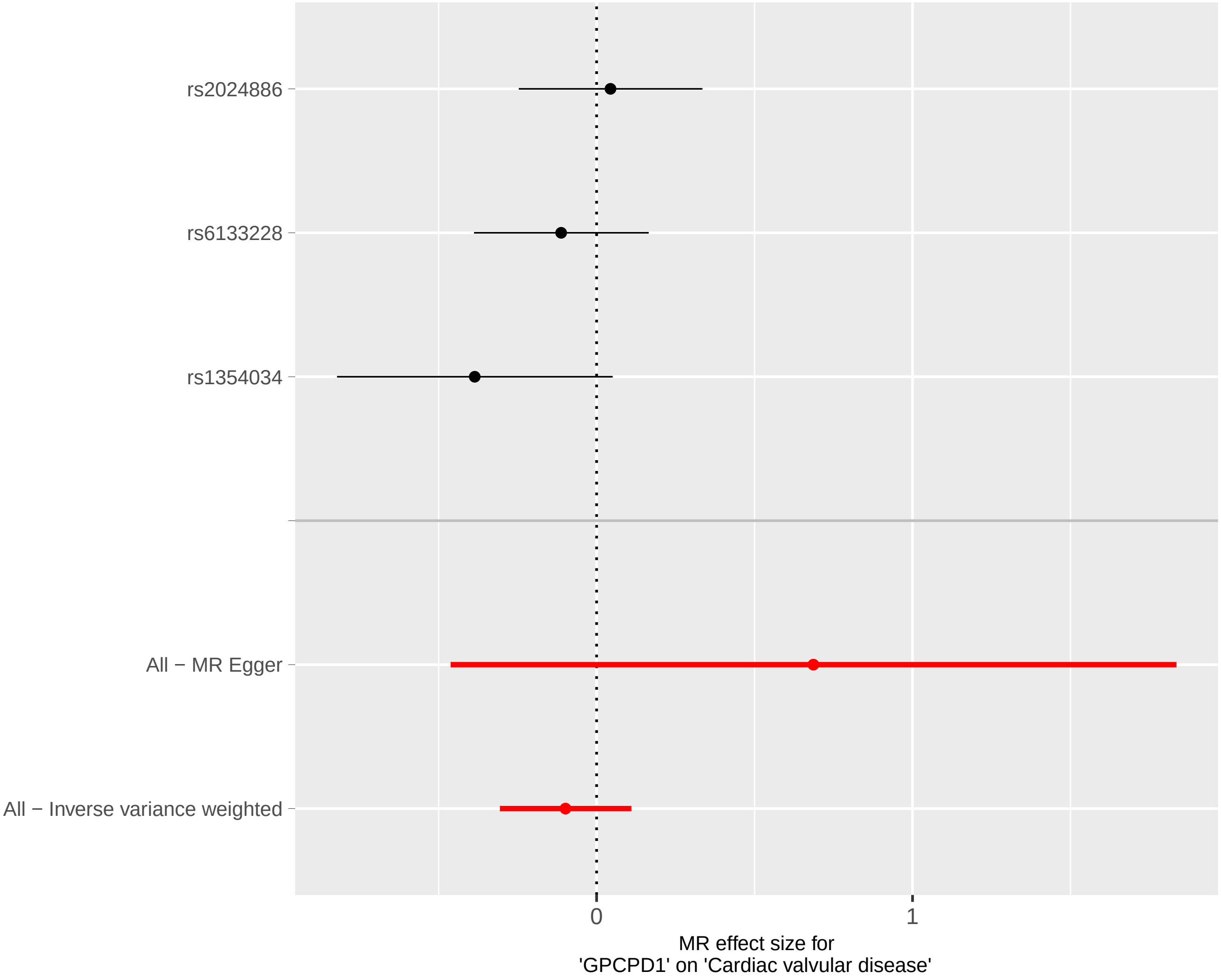
rs2024886
rs6133228
rs1354034
All − MR Egger
All − Inverse variance weighted
0
1
MR effect size for
'GPCPD1' on 'Cardiac valvular disease'

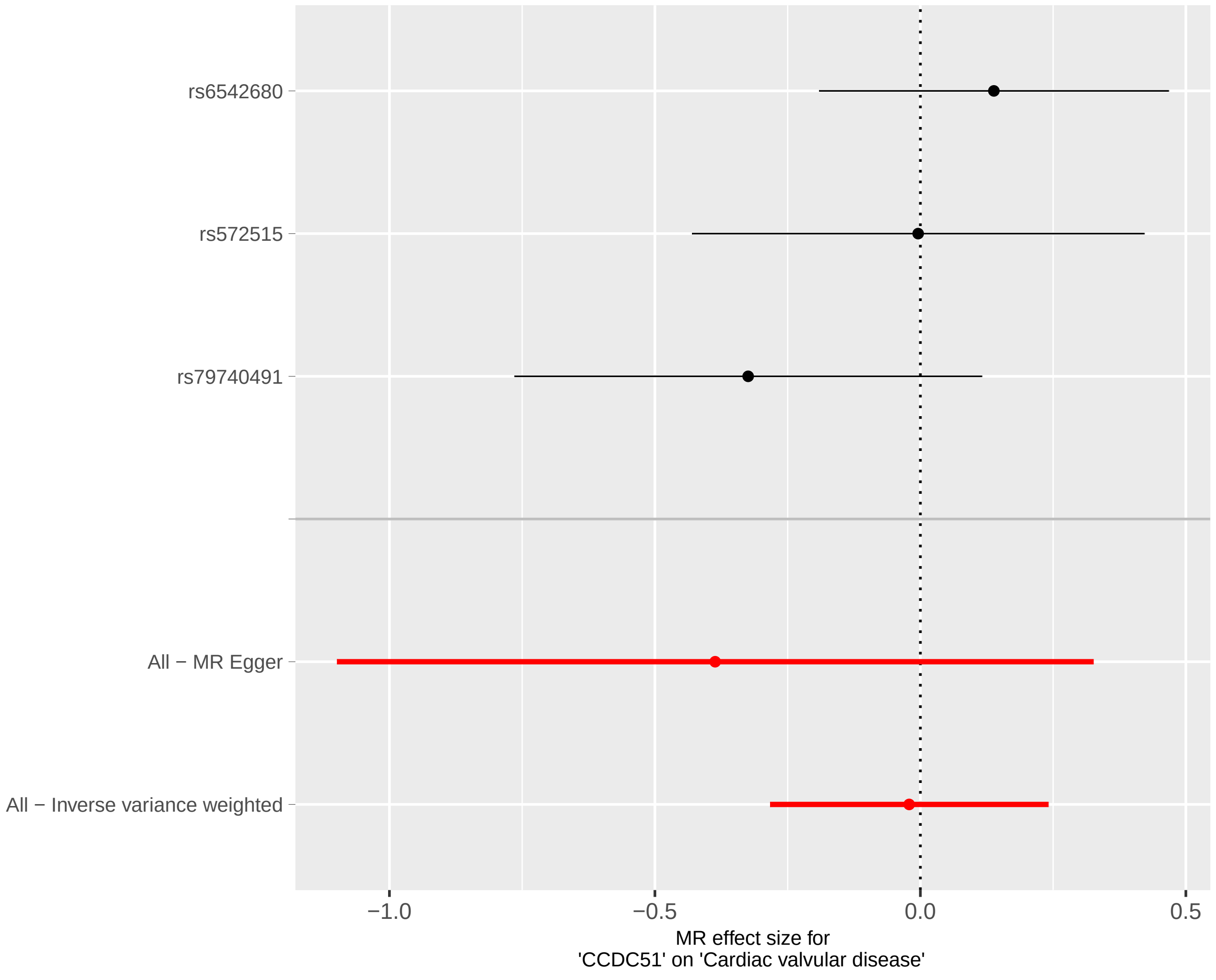
rs6542680
rs572515
rs79740491
All − MR Egger
All − Inverse variance weighted
−1.0
−0.5
0.0
0.5
MR effect size for
'CCDC51' on 'Cardiac valvular disease'

Insufficient number of SNPs

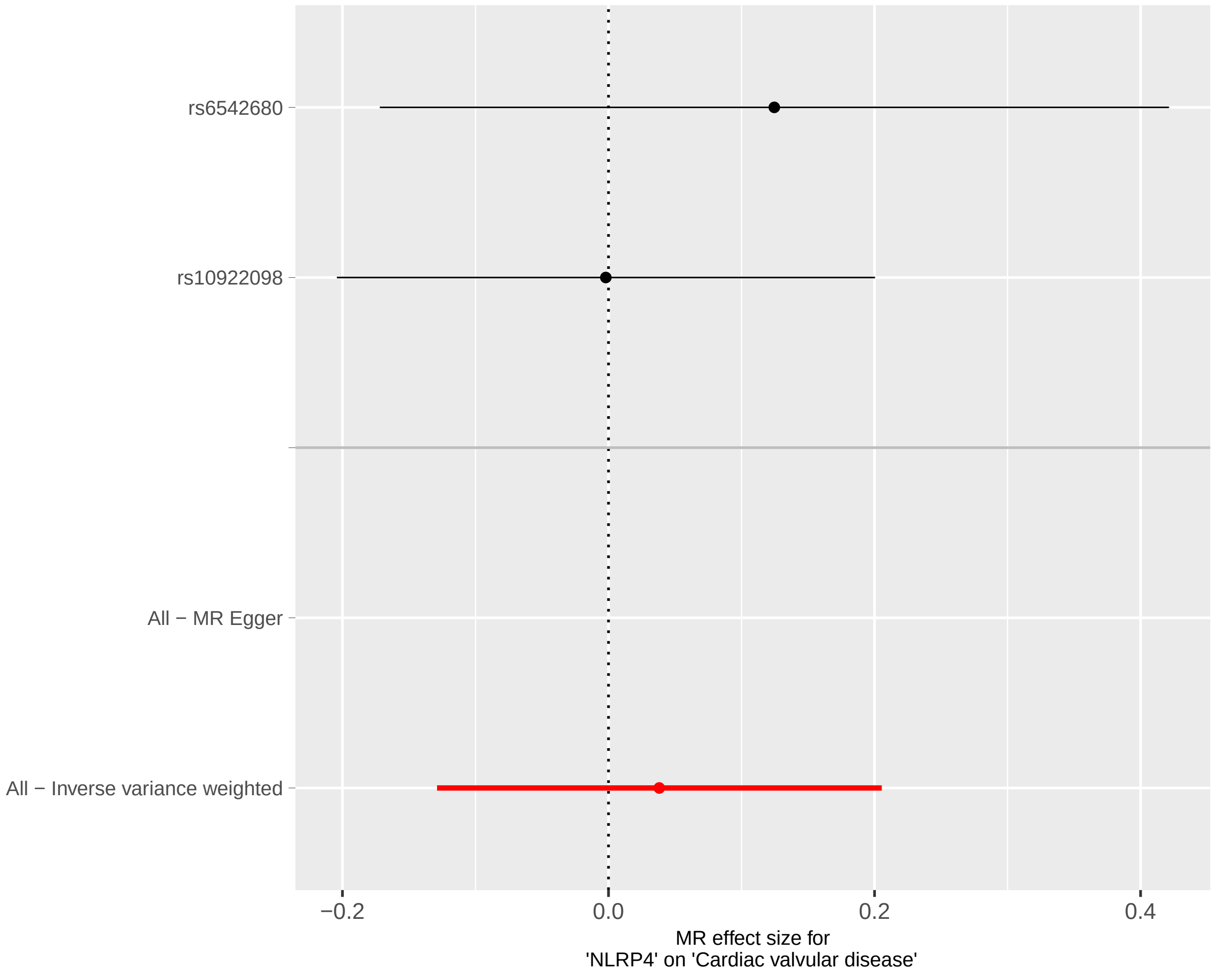
rs6542680
rs10922098
All − MR Egger
All − Inverse variance weighted
−0.2
0.0
0.2
0.4
MR effect size for
'NLRP4' on 'Cardiac valvular disease'

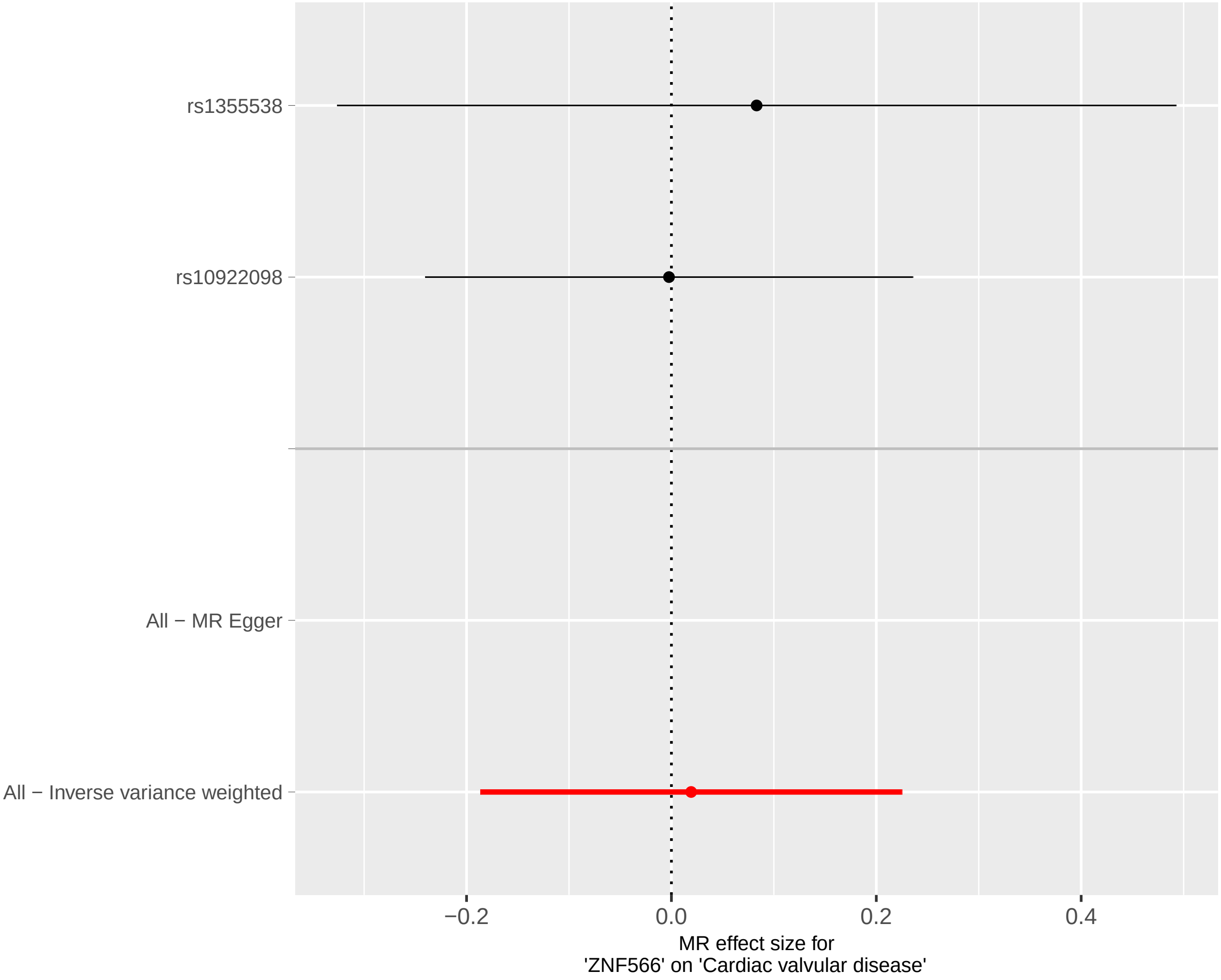

rs1355538
rs10922098
All − MR Egger
All − Inverse variance weighted
−0.2
0.0
0.2
0.4
MR effect size for
'ZNF566' on 'Cardiac valvular disease'

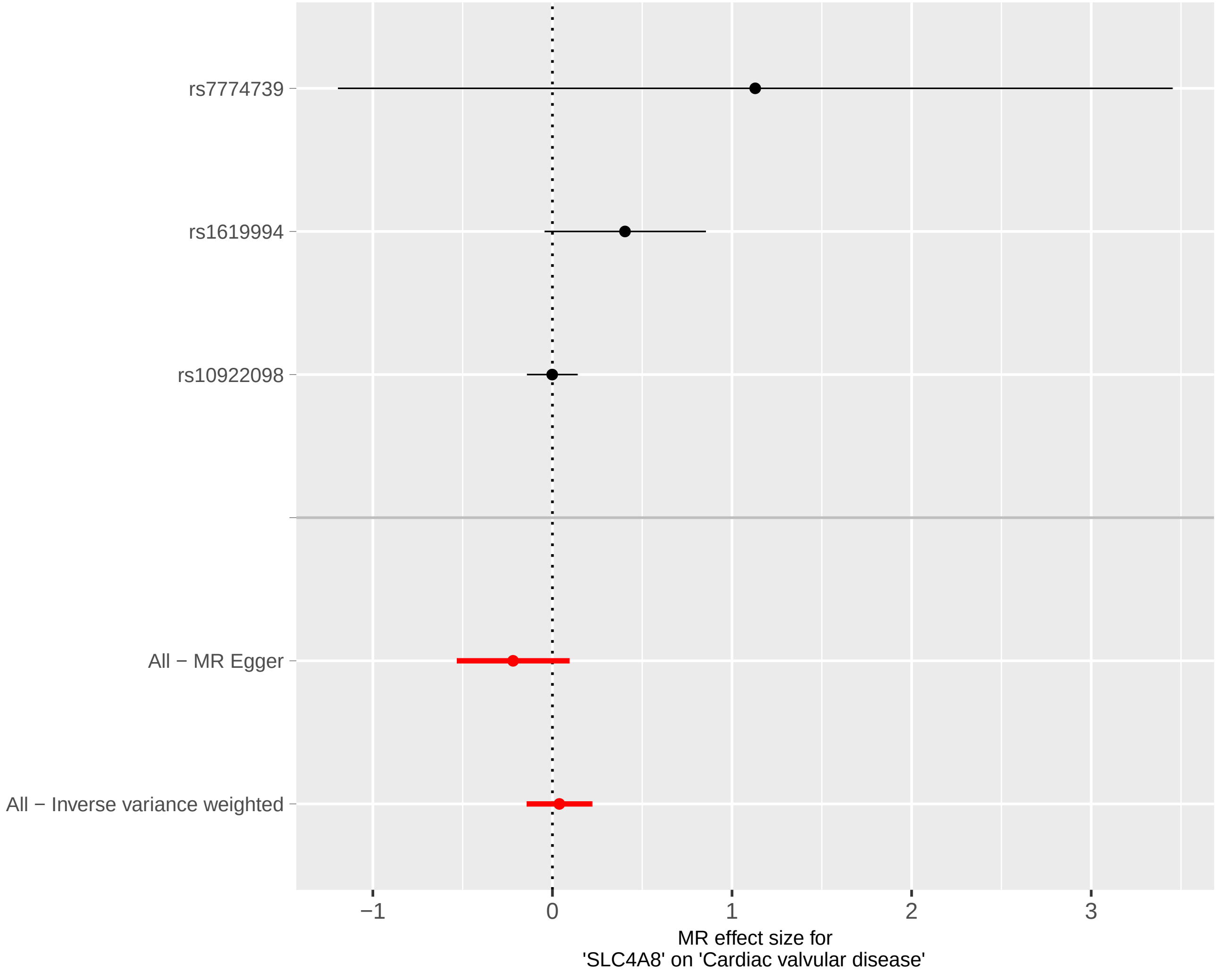

rs7774739
rs1619994
rs10922098
All − MR Egger
All − Inverse variance weighted
−1
0
1
2
3
MR effect size for
'SLC4A8' on 'Cardiac valvular disease'

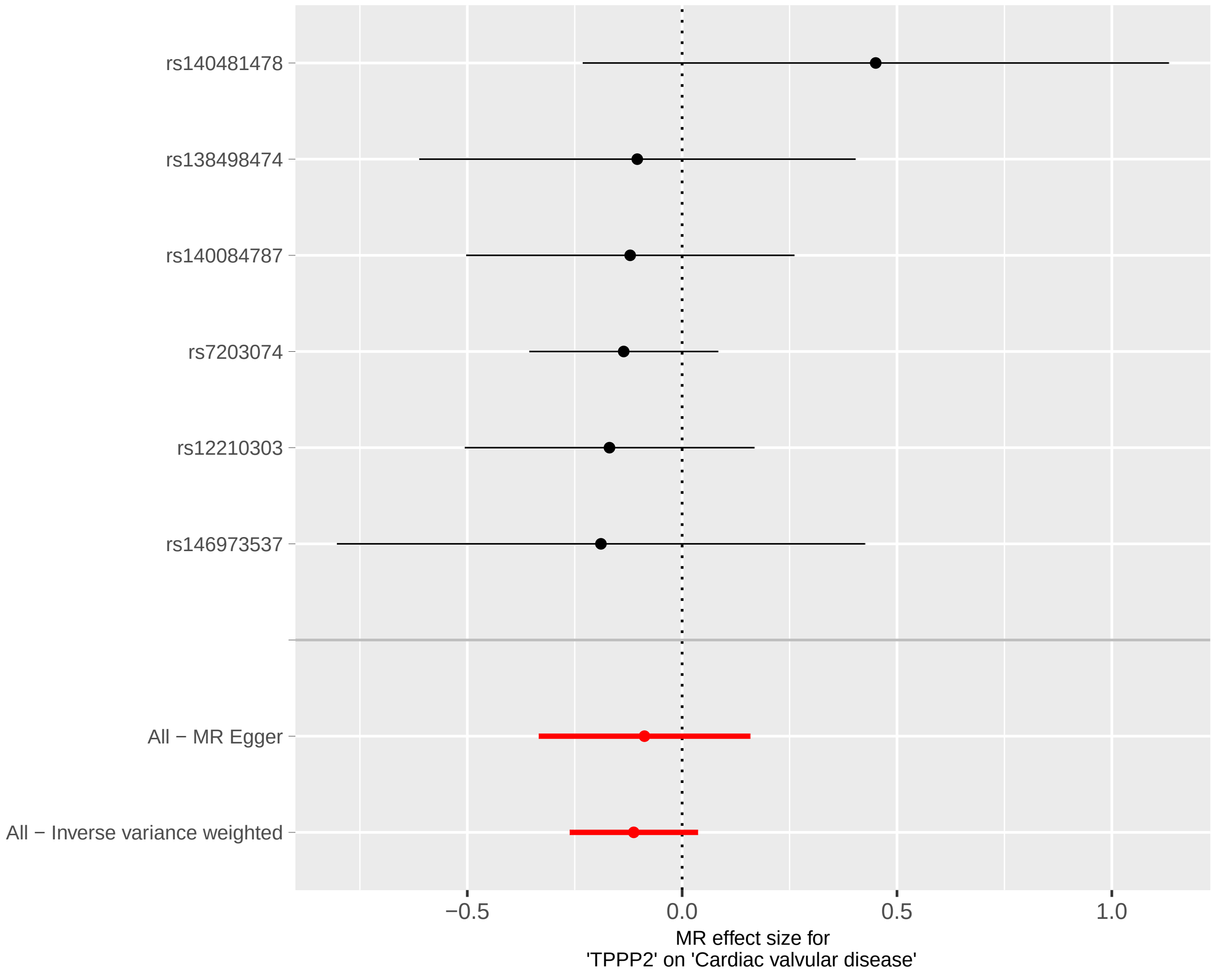
rs140481478
rs138498474
rs140084787
rs7203074
rs12210303
rs146973537
All − MR Egger
All − Inverse variance weighted
−0.5
0.0
0.5
1.0
MR effect size for
'TPPP2' on 'Cardiac valvular disease'

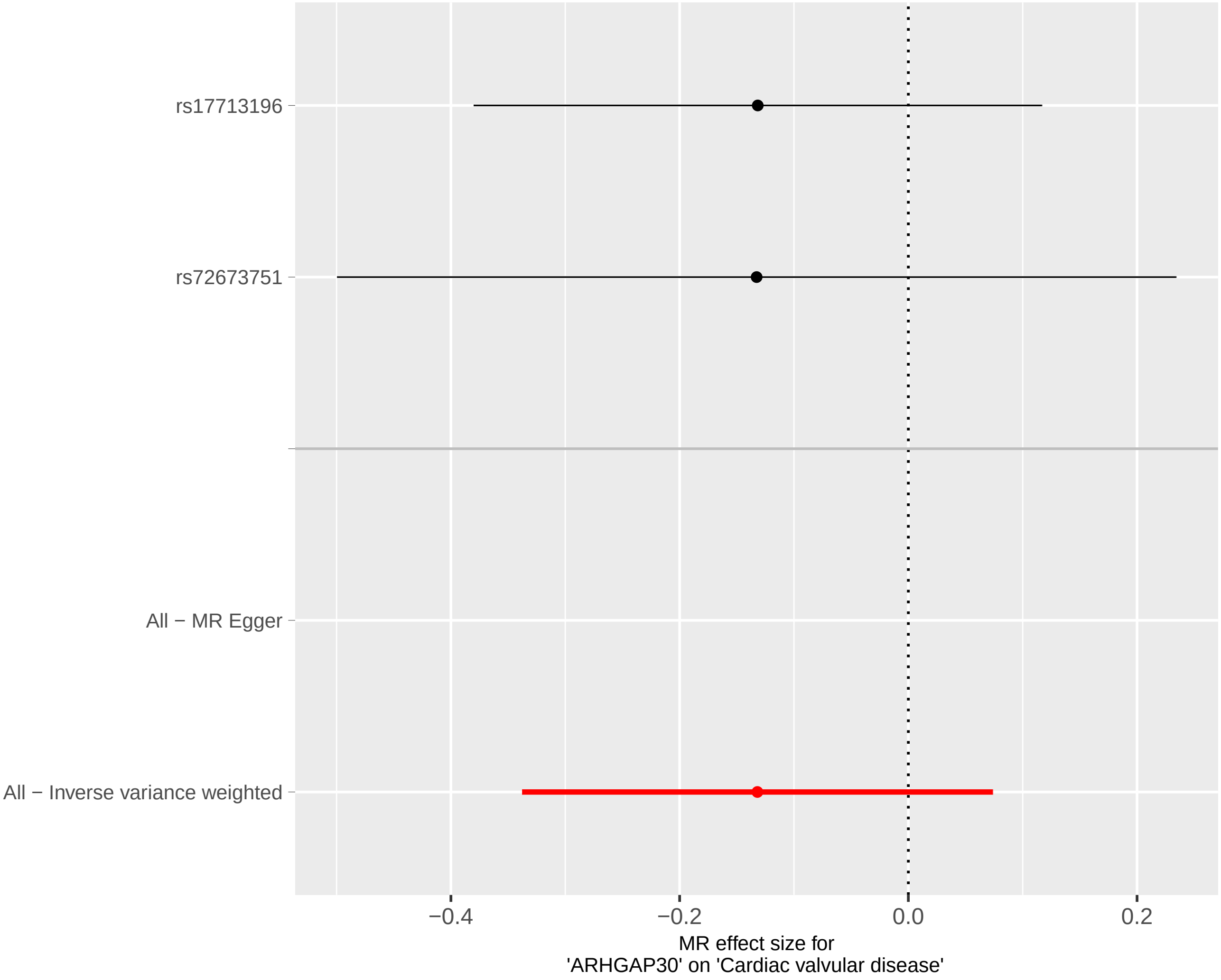

rs17713196
rs72673751
All − MR Egger
All − Inverse variance weighted
−0.4
−0.2
0.0
0.2
MR effect size for
'ARHGAP30' on 'Cardiac valvular disease'

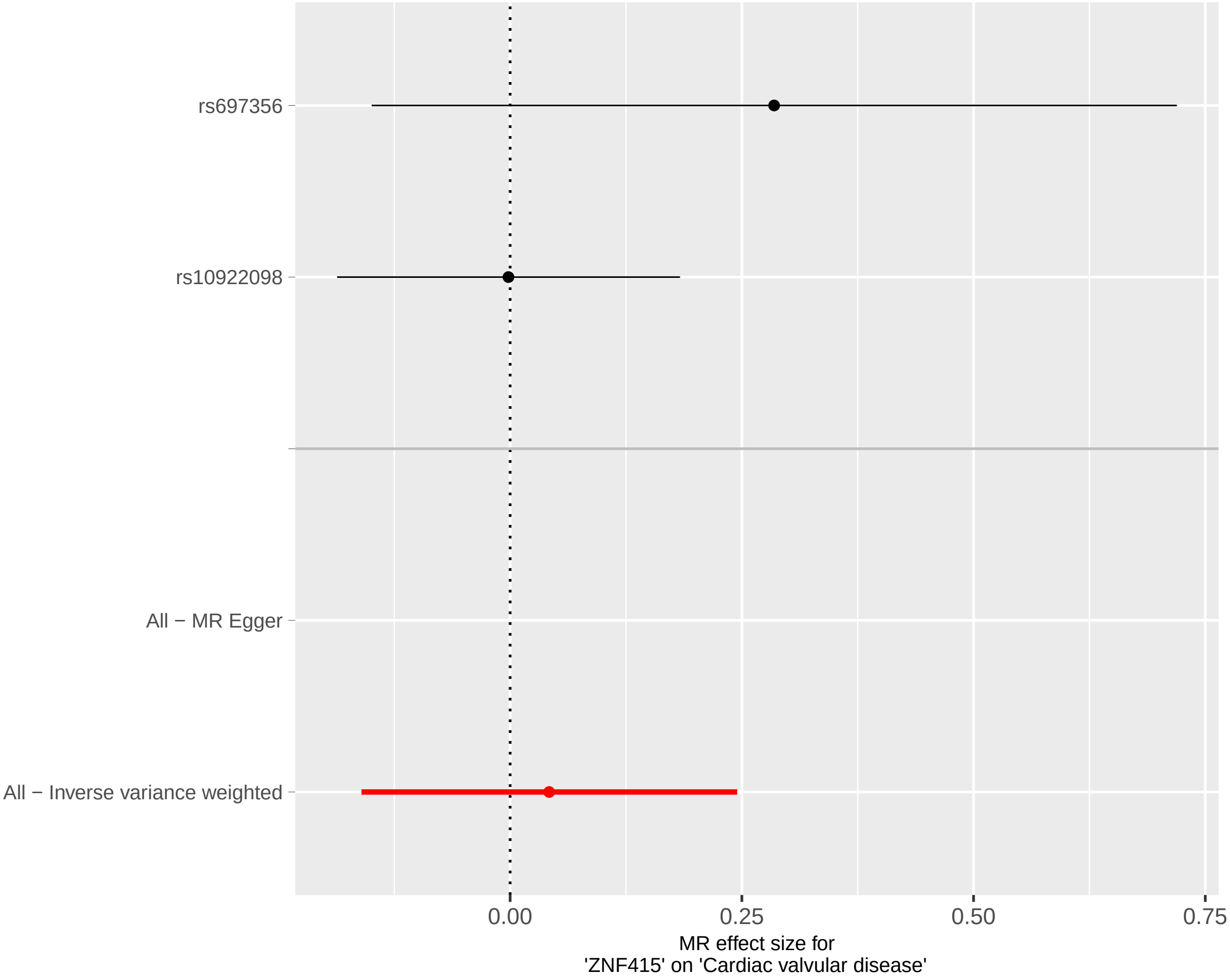
rs697356
rs10922098
All − MR Egger
All − Inverse variance weighted
0.00
0.25
0.50
0.75
MR effect size for
'ZNF415' on 'Cardiac valvular disease'

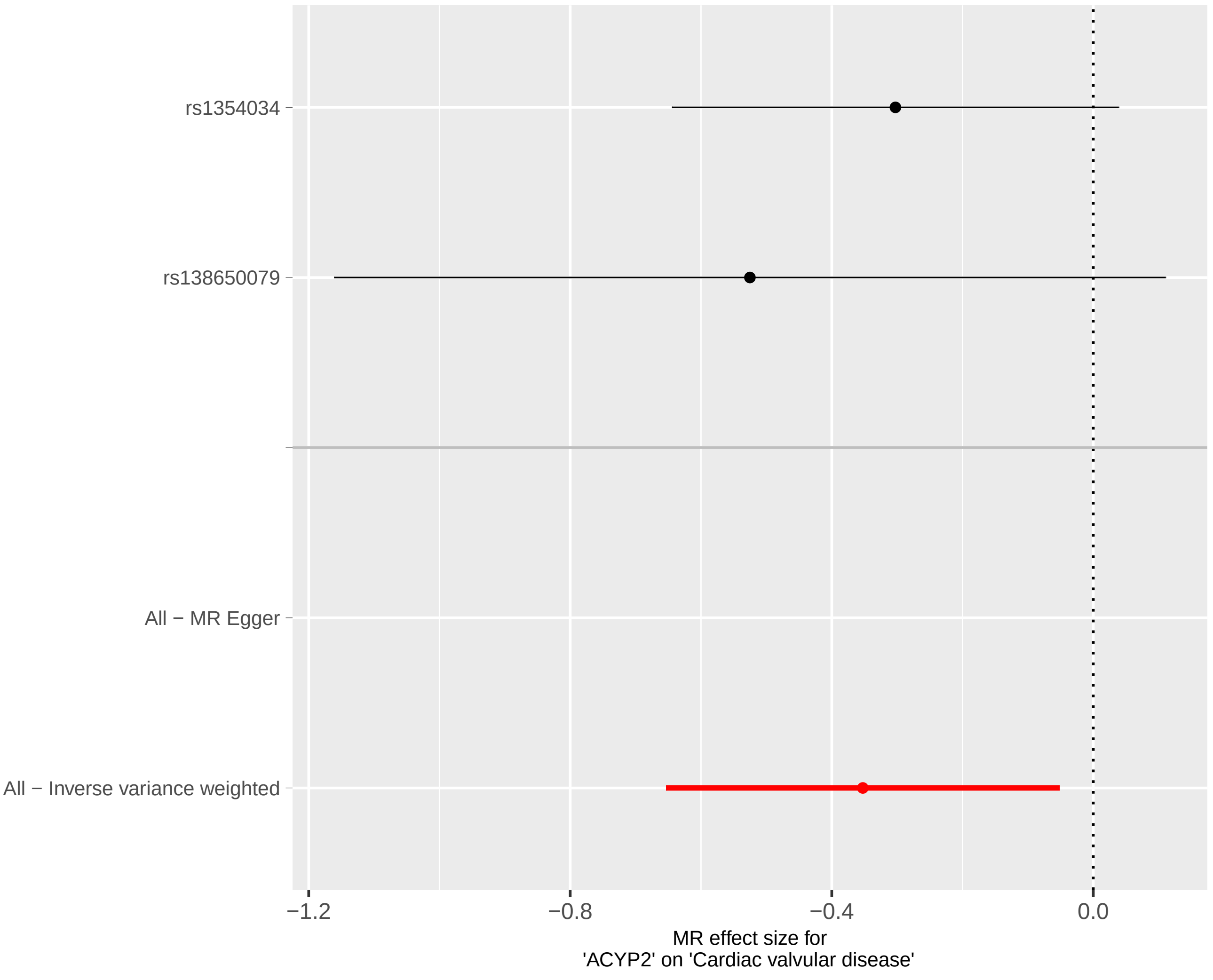
rs1354034
rs138650079
All − MR Egger
All − Inverse variance weighted
−1.2
−0.8
−0.4
0.0
MR effect size for
'ACYP2' on 'Cardiac valvular disease'

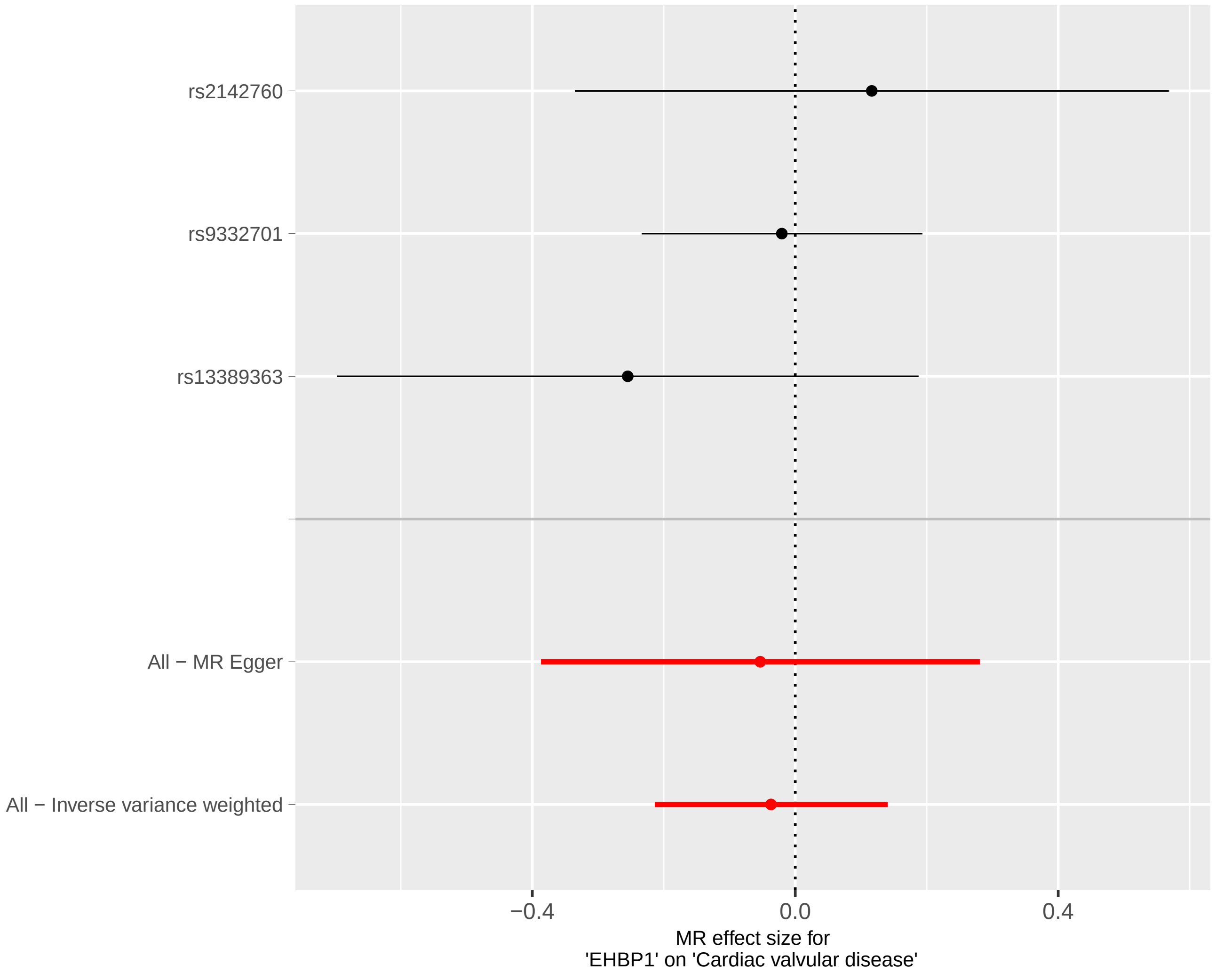

rs2142760
rs9332701
rs13389363
All − MR Egger
All − Inverse variance weighted
−0.4
0.0
0.4
MR effect size for
'EHBP1' on 'Cardiac valvular disease'

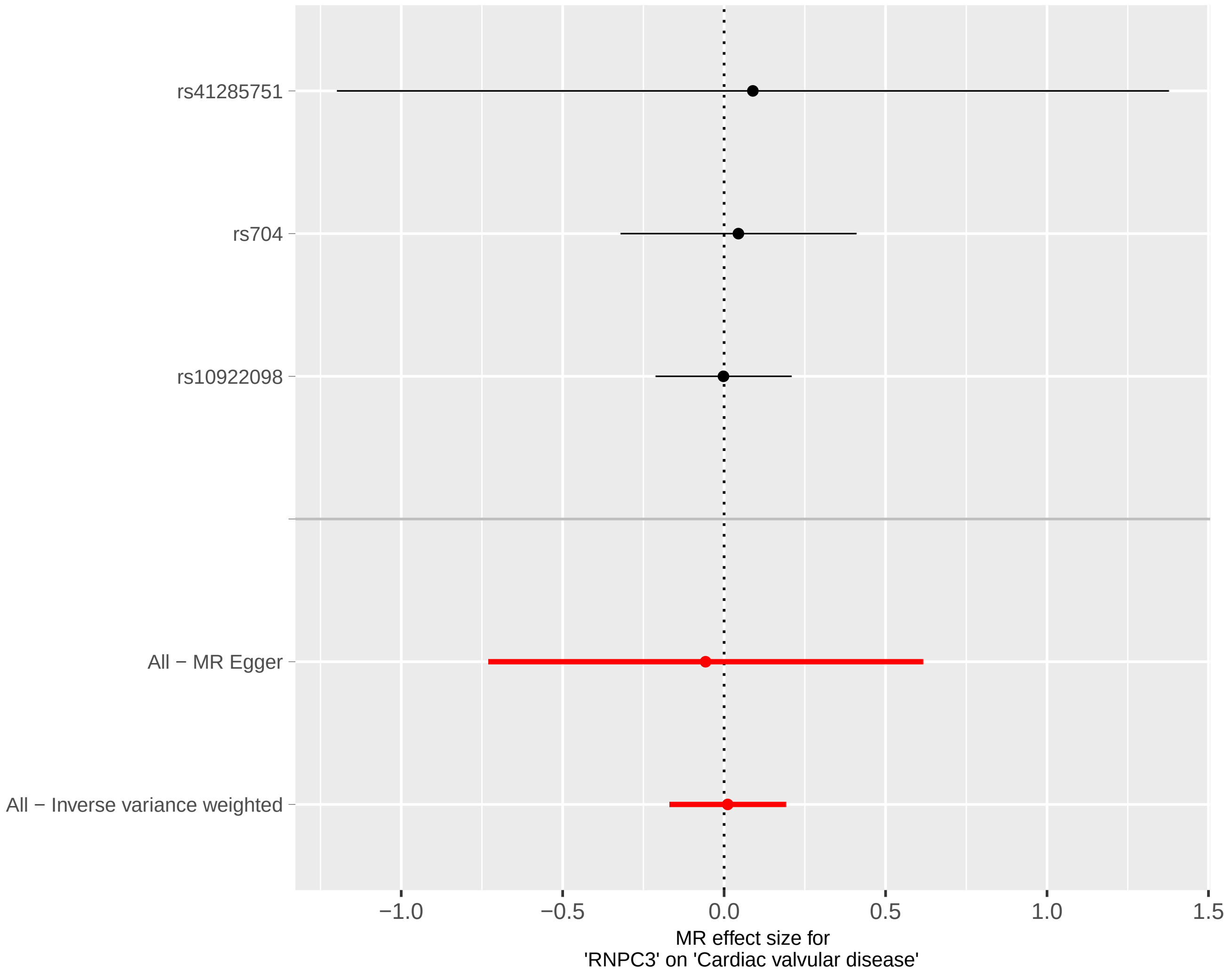
rs41285751
rs704
rs10922098
All − MR Egger
All − Inverse variance weighted
−1.0
−0.5
0.0
0.5
1.0
1.5
MR effect size for
'RNPC3' on 'Cardiac valvular disease'

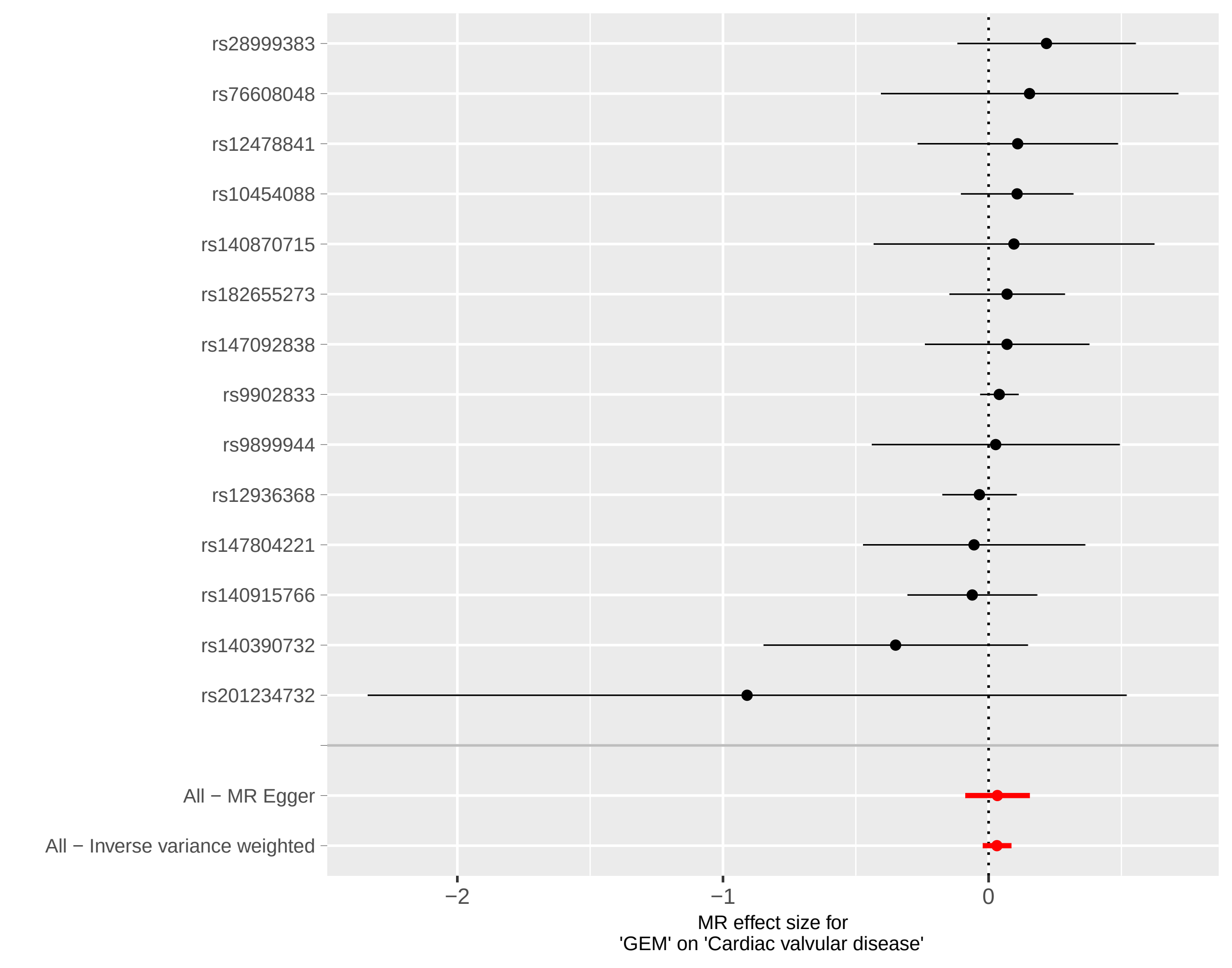
rs28999383
rs76608048
rs12478841
rs10454088
rs140870715
rs182655273
rs147092838
rs9902833
rs9899944
rs12936368
rs147804221
rs140915766
rs140390732
rs201234732
All − MR Egger
All − Inverse variance weighted
−2
−1
0
MR effect size for
'GEM' on 'Cardiac valvular disease'

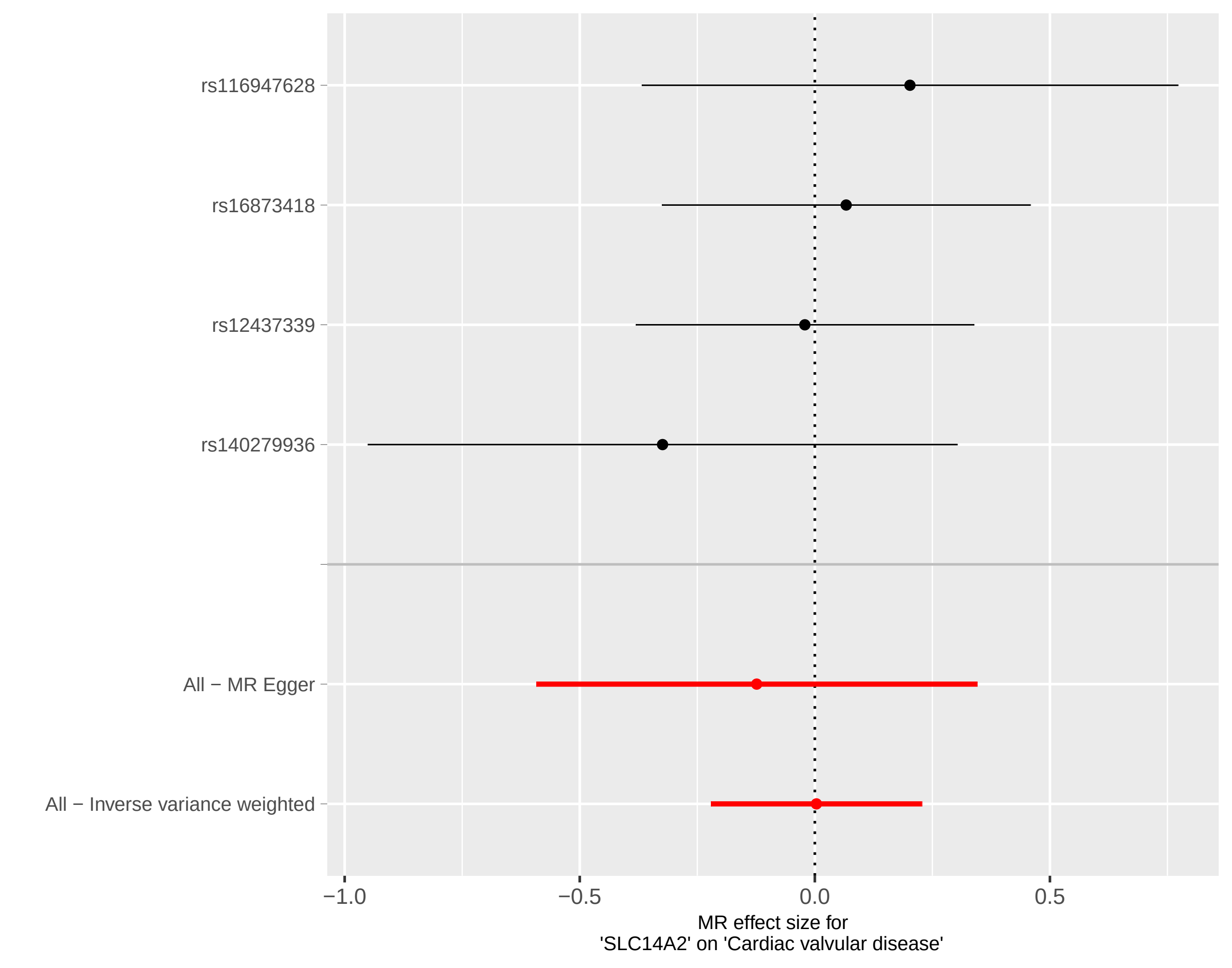
rs116947628
rs16873418
rs12437339
rs140279936
All − MR Egger
All − Inverse variance weighted
−1.0
−0.5
0.0
0.5
MR effect size for
'SLC14A2' on 'Cardiac valvular disease'

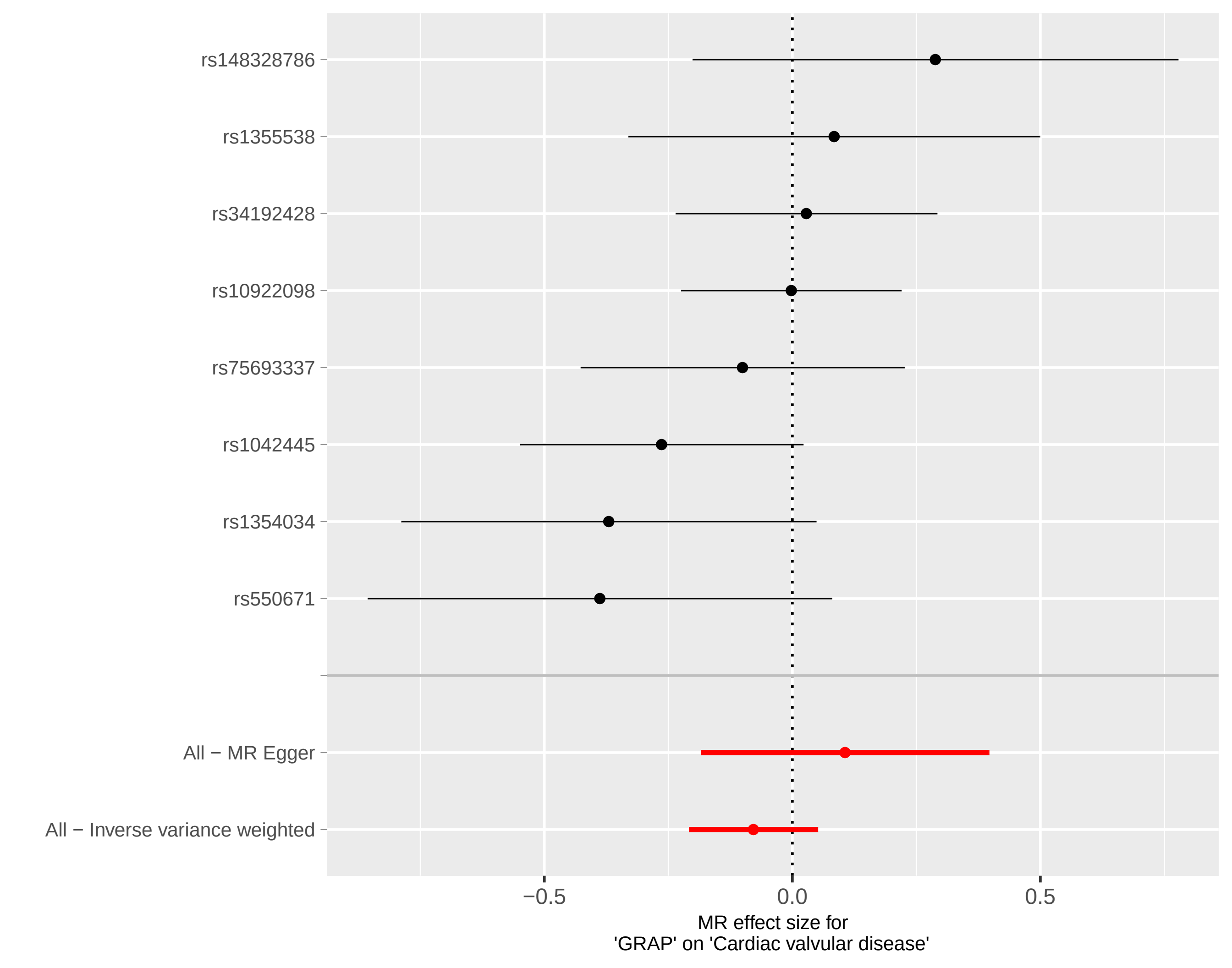
rs148328786
rs1355538
rs34192428
rs10922098
rs75693337
rs1042445
rs1354034
rs550671
All − MR Egger
All − Inverse variance weighted
−0.5
0.0
0.5
MR effect size for
'GRAP' on 'Cardiac valvular disease'

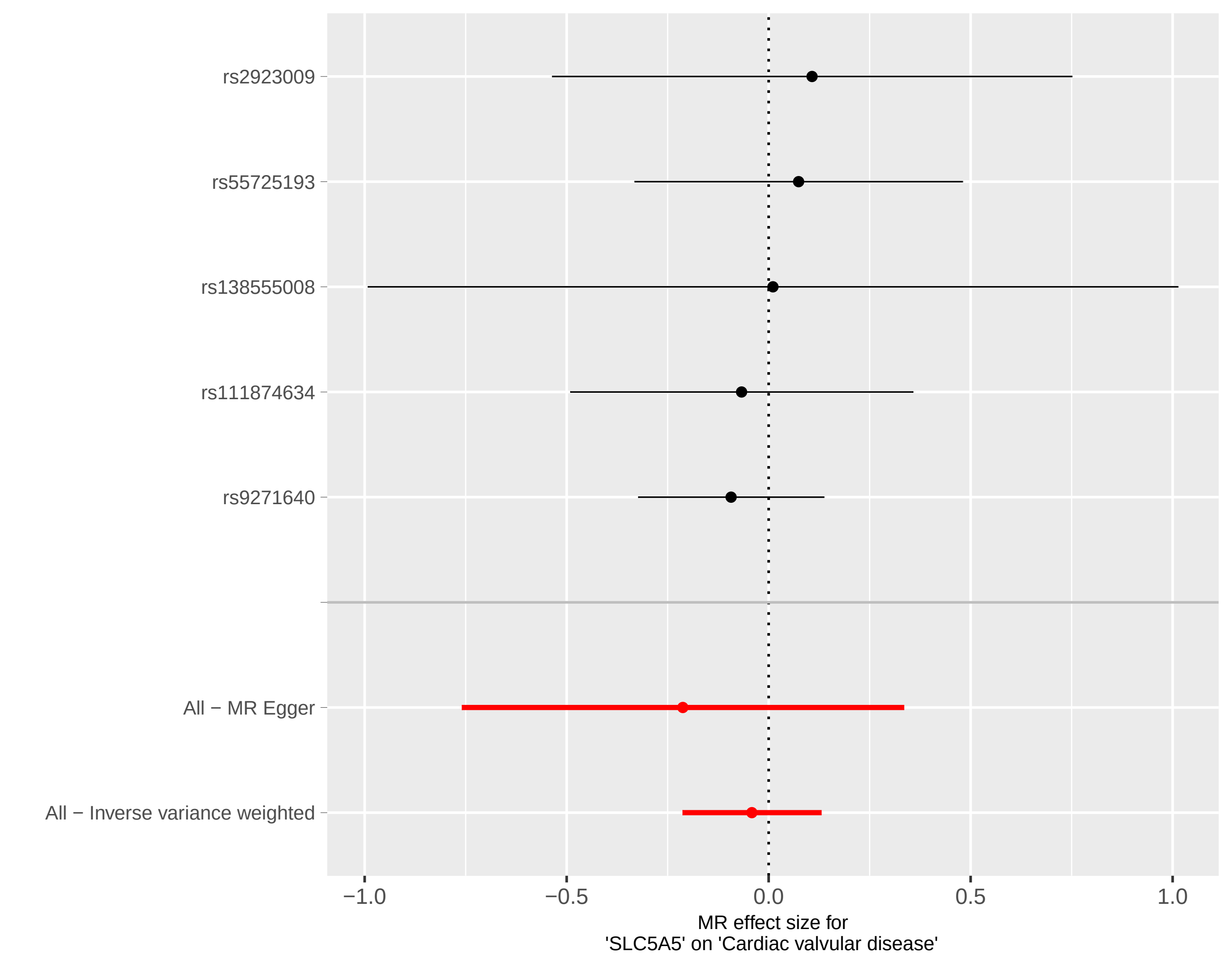
rs2923009
rs55725193
rs138555008
rs111874634
rs9271640
All − MR Egger
All − Inverse variance weighted
−1.0
−0.5
0.0
0.5
1.0
MR effect size for
'SLC5A5' on 'Cardiac valvular disease'

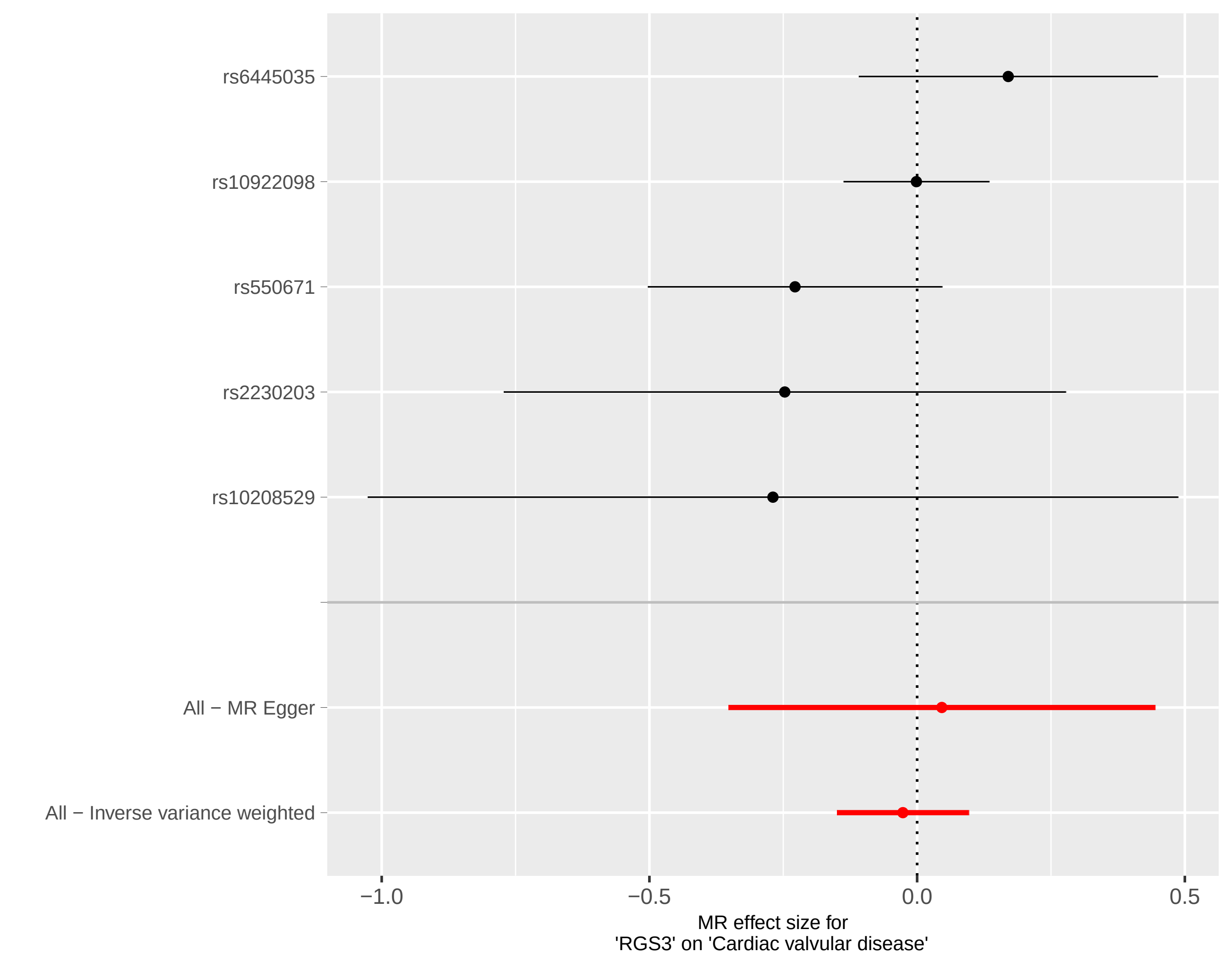
rs6445035
rs10922098
rs550671
rs2230203
rs10208529
All − MR Egger
All − Inverse variance weighted
−1.0
−0.5
0.0
0.5
MR effect size for
'RGS3' on 'Cardiac valvular disease'

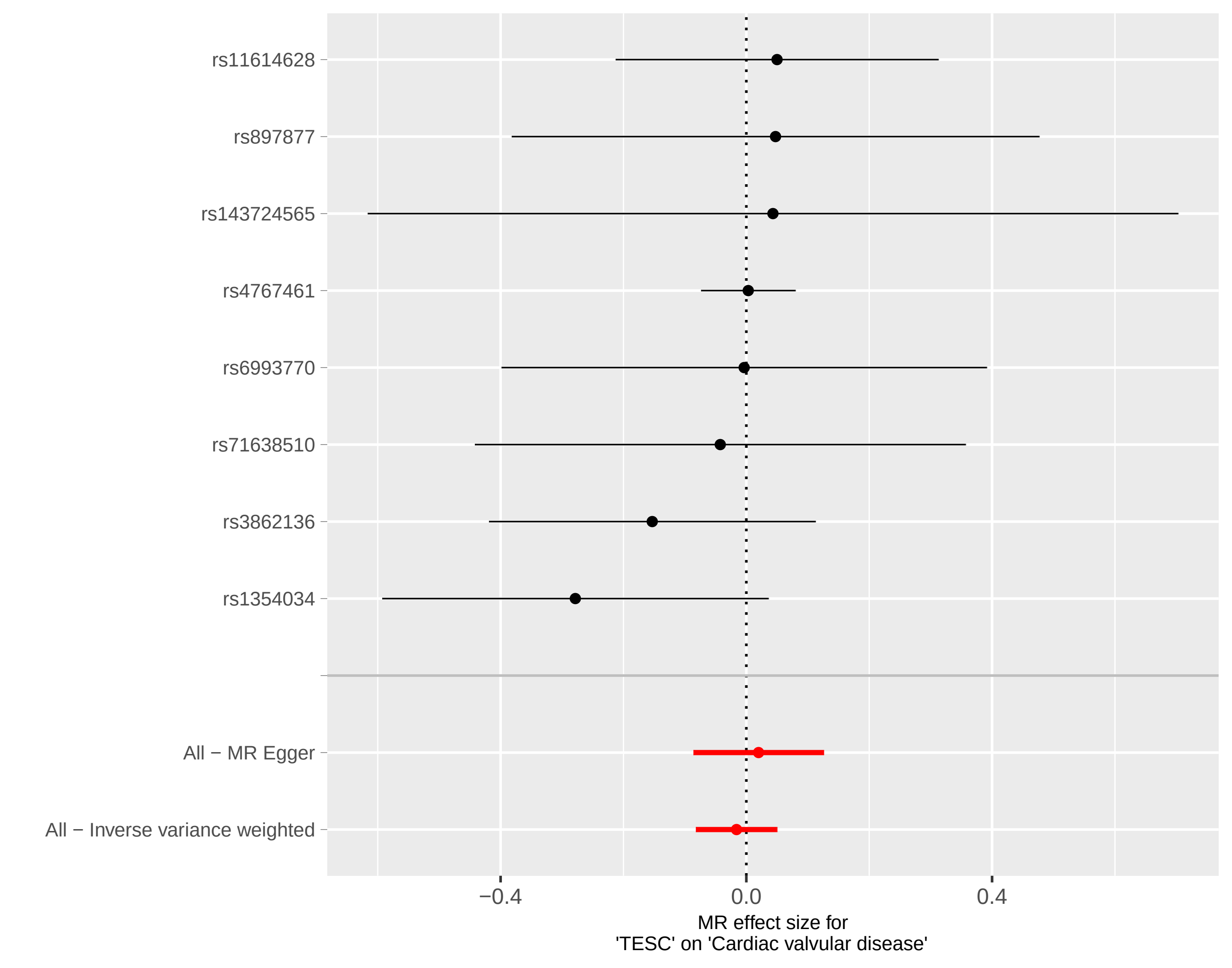

rs11614628
rs897877
rs143724565
rs4767461
rs6993770
rs71638510
rs3862136
rs1354034
All − MR Egger
All − Inverse variance weighted
−0.4
0.0
0.4
MR effect size for
'TESC' on 'Cardiac valvular disease'

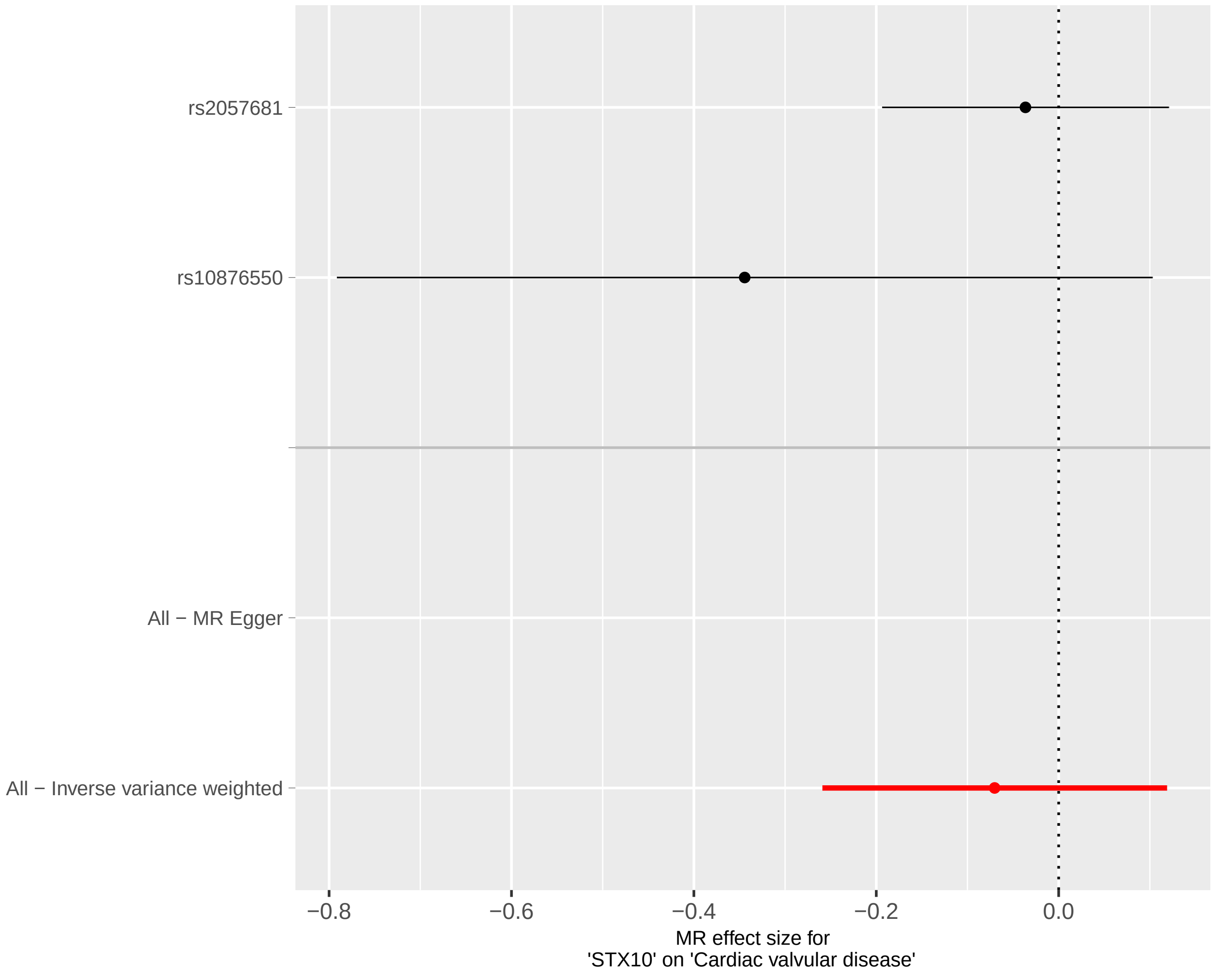
rs2057681
rs10876550
All − MR Egger
All − Inverse variance weighted
−0.8
−0.6
−0.4
−0.2
0.0
MR effect size for
'STX10' on 'Cardiac valvular disease'

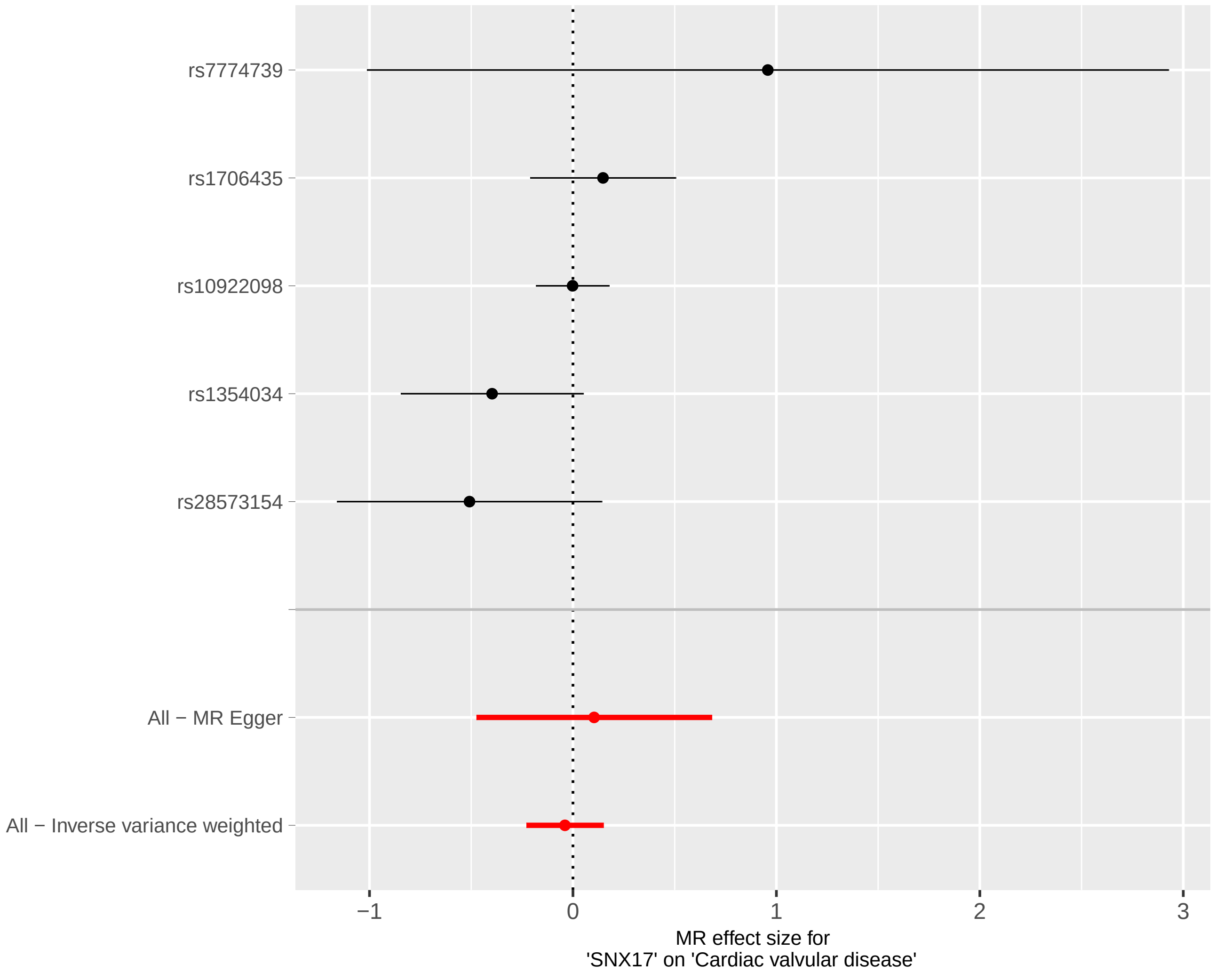

rs7774739
rs1706435
rs10922098
rs1354034
rs28573154
All − MR Egger
All − Inverse variance weighted
−1
0
1
2
3
MR effect size for
'SNX17' on 'Cardiac valvular disease'

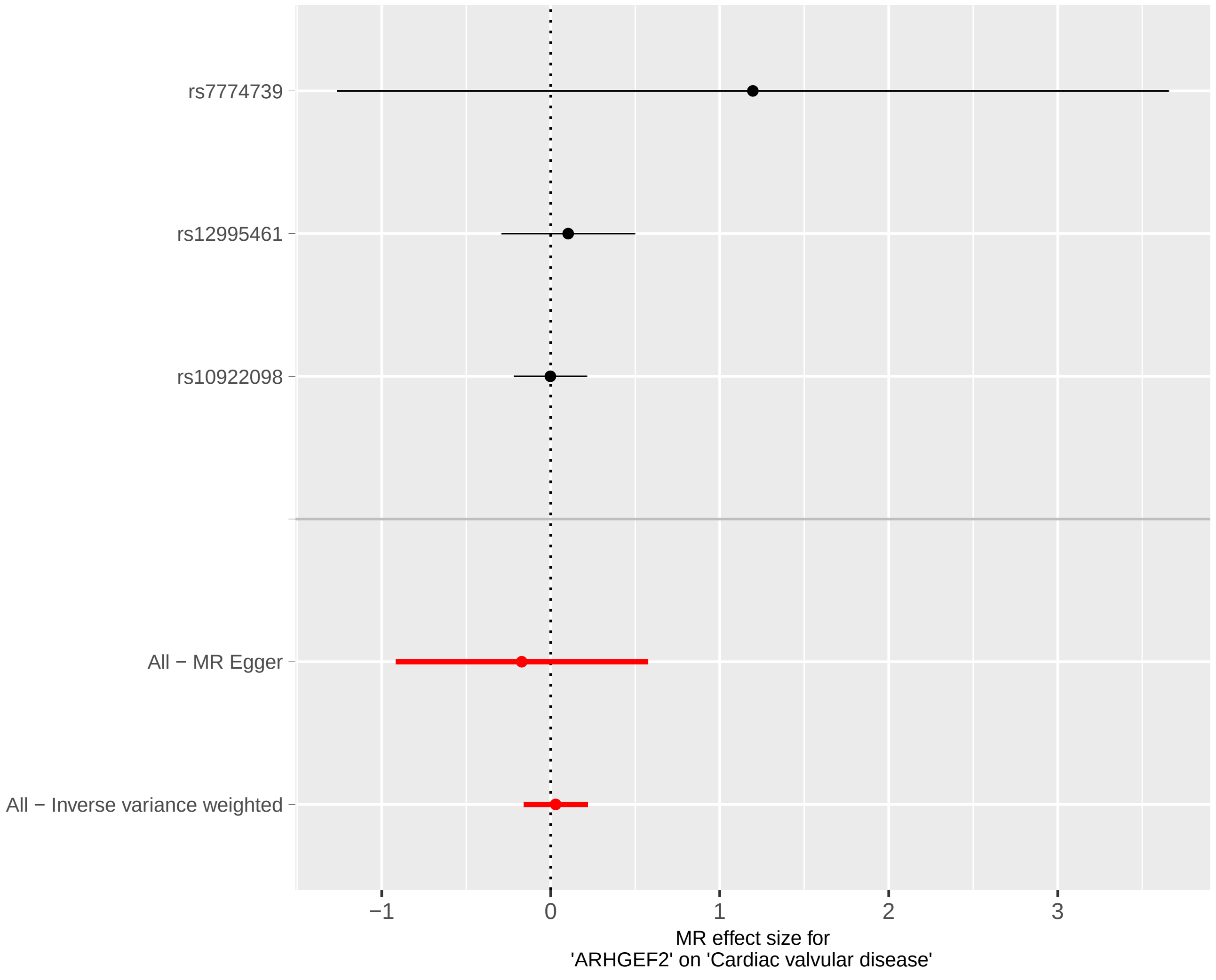
rs7774739
rs12995461
rs10922098
All − MR Egger
All − Inverse variance weighted
−1
0
1
2
3
MR effect size for
'ARHGEF2' on 'Cardiac valvular disease'

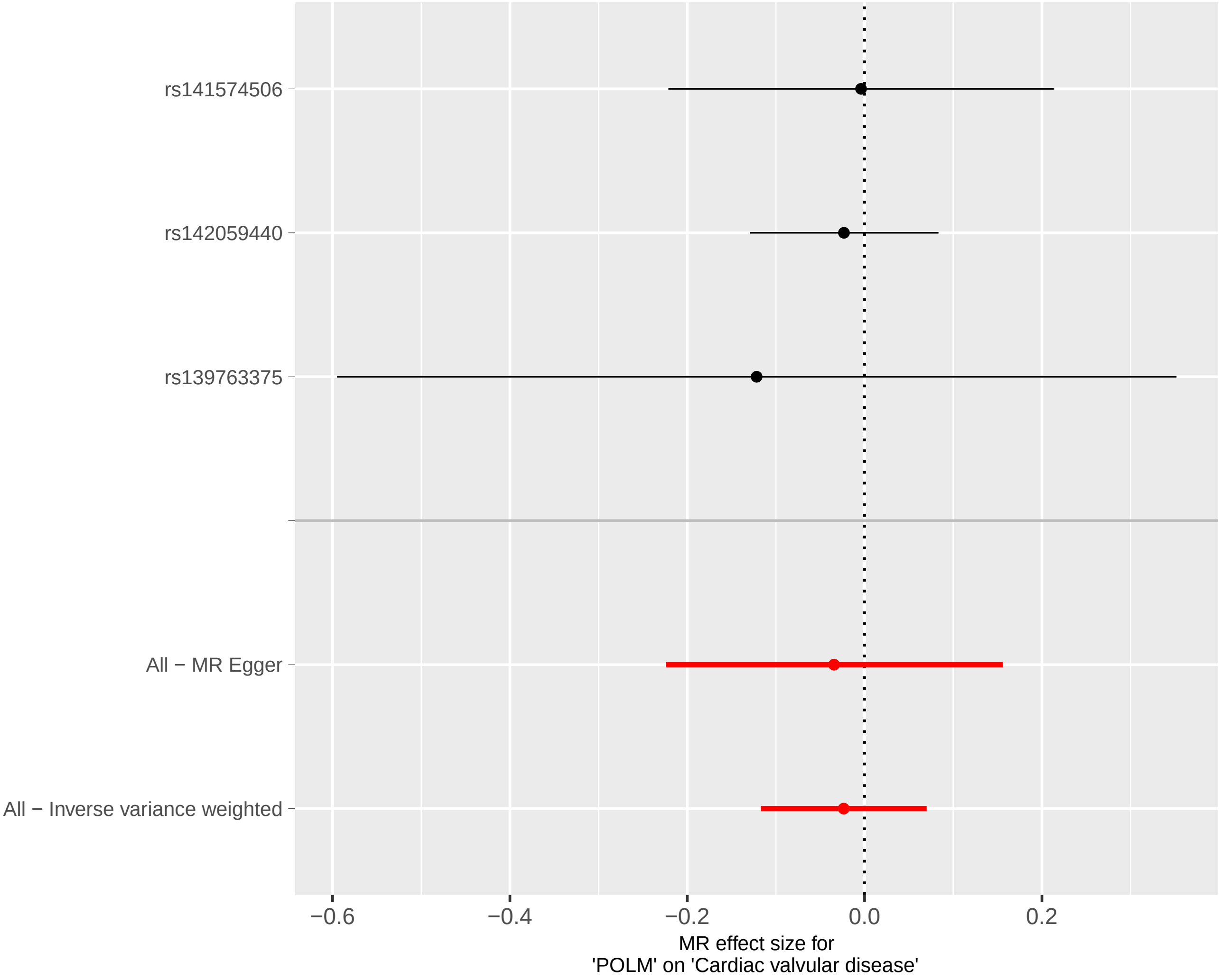
rs141574506
rs142059440
rs139763375
All − MR Egger
All − Inverse variance weighted
−0.6
−0.4
−0.2
0.0
0.2
MR effect size for
'POLM' on 'Cardiac valvular disease'

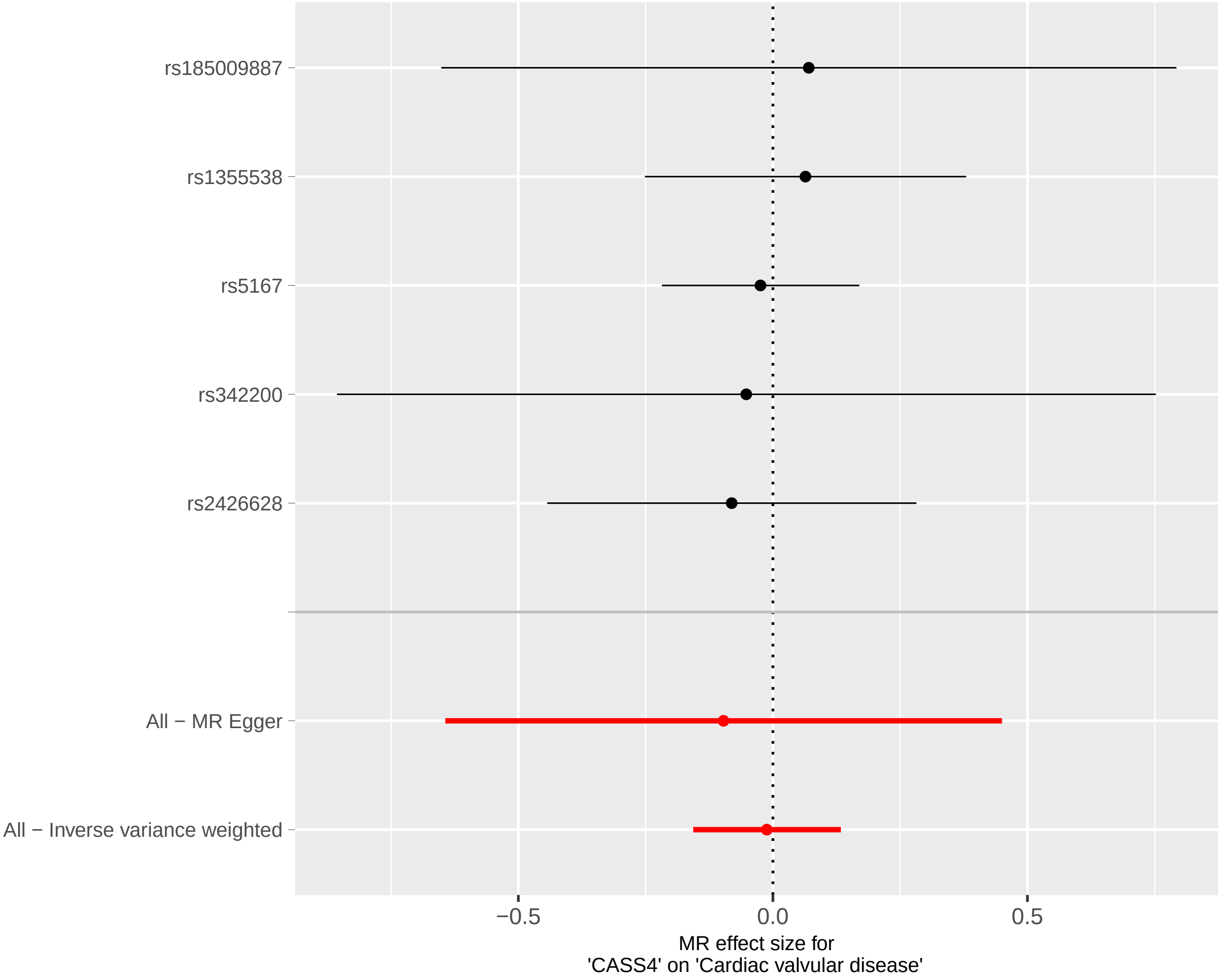
rs185009887
rs1355538
rs5167
rs342200
rs2426628
All − MR Egger
All − Inverse variance weighted
−0.5
0.0
0.5
MR effect size for
'CASS4' on 'Cardiac valvular disease'

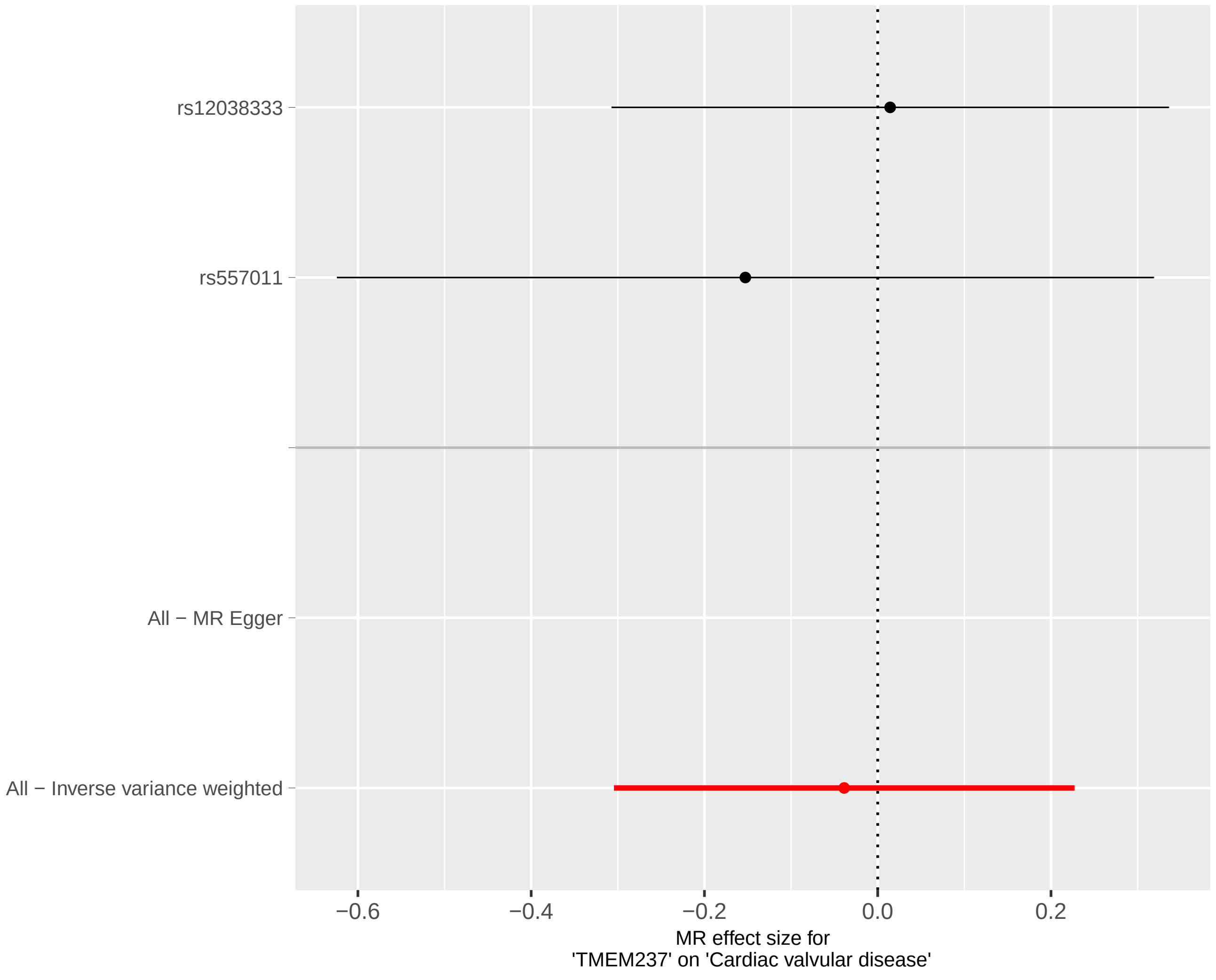
rs12038333
rs557011
All − MR Egger
All − Inverse variance weighted
−0.6
−0.4
−0.2
0.0
0.2
MR effect size for
'TMEM237' on 'Cardiac valvular disease'

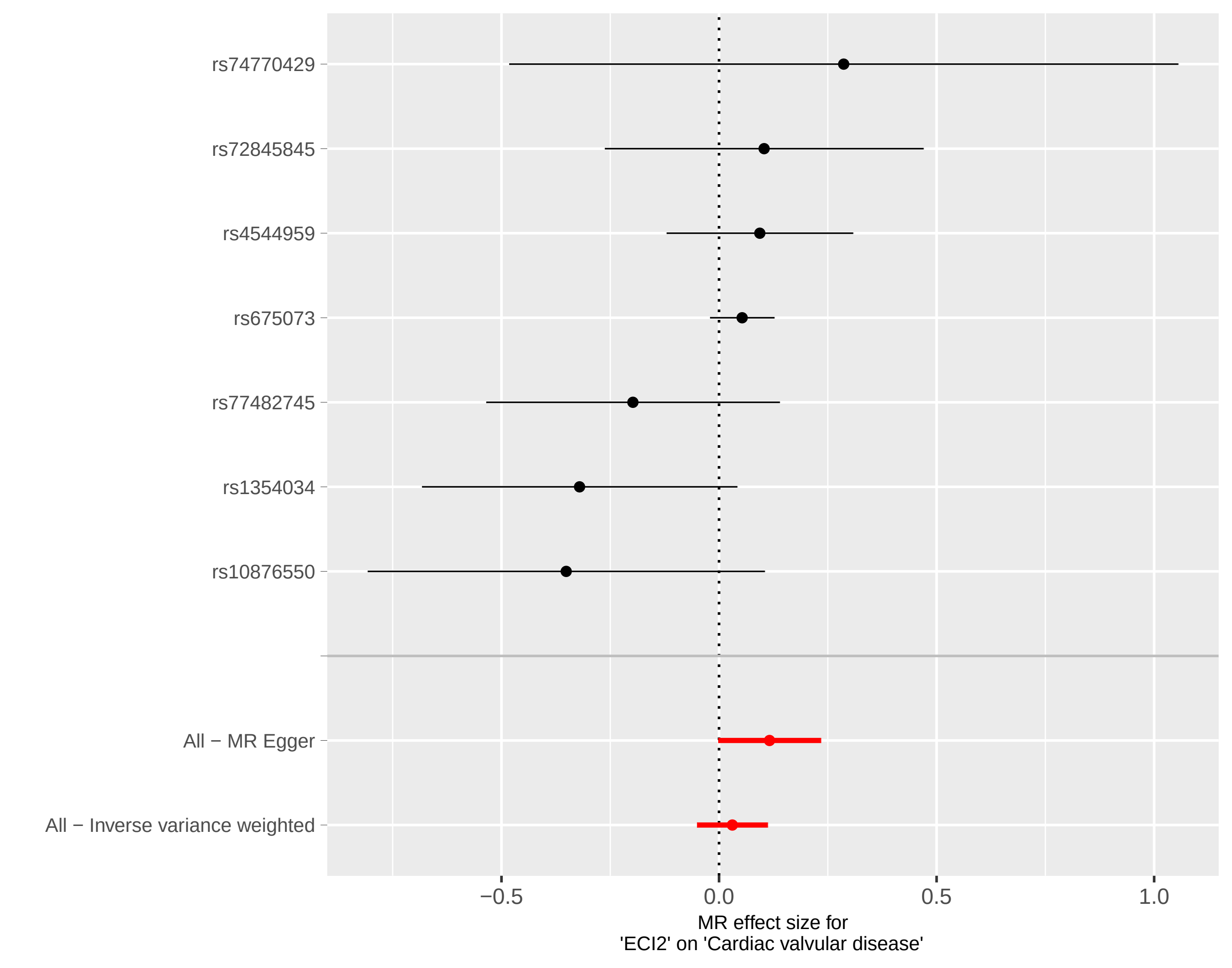
rs74770429
rs72845845
rs4544959
rs675073
rs77482745
rs1354034
rs10876550
All − MR Egger
All − Inverse variance weighted
−0.5
0.0
0.5
1.0
MR effect size for
'ECI2' on 'Cardiac valvular disease'

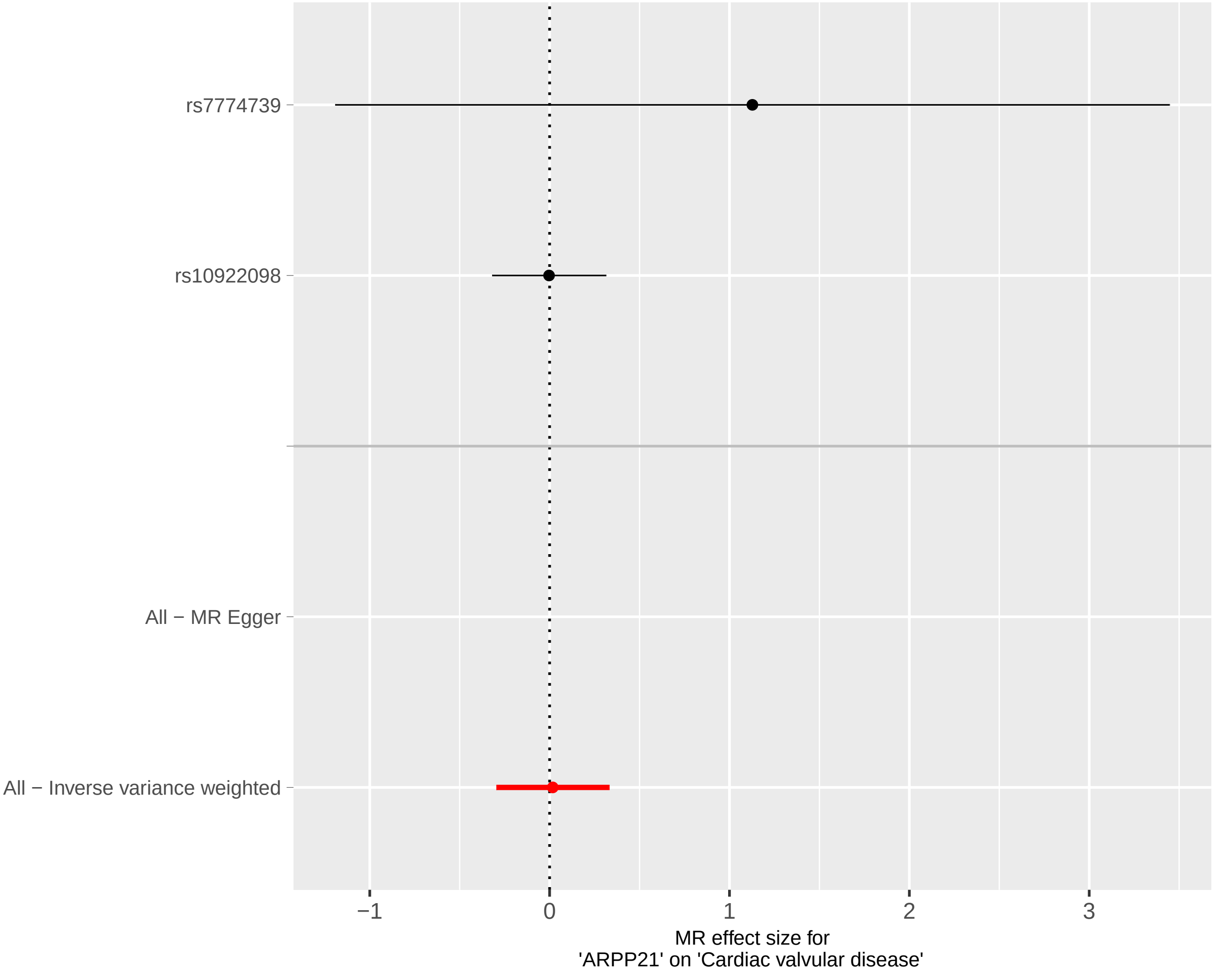

rs7774739
rs10922098
All − MR Egger
All − Inverse variance weighted
−1
0
1
2
3
MR effect size for
'ARPP21' on 'Cardiac valvular disease'

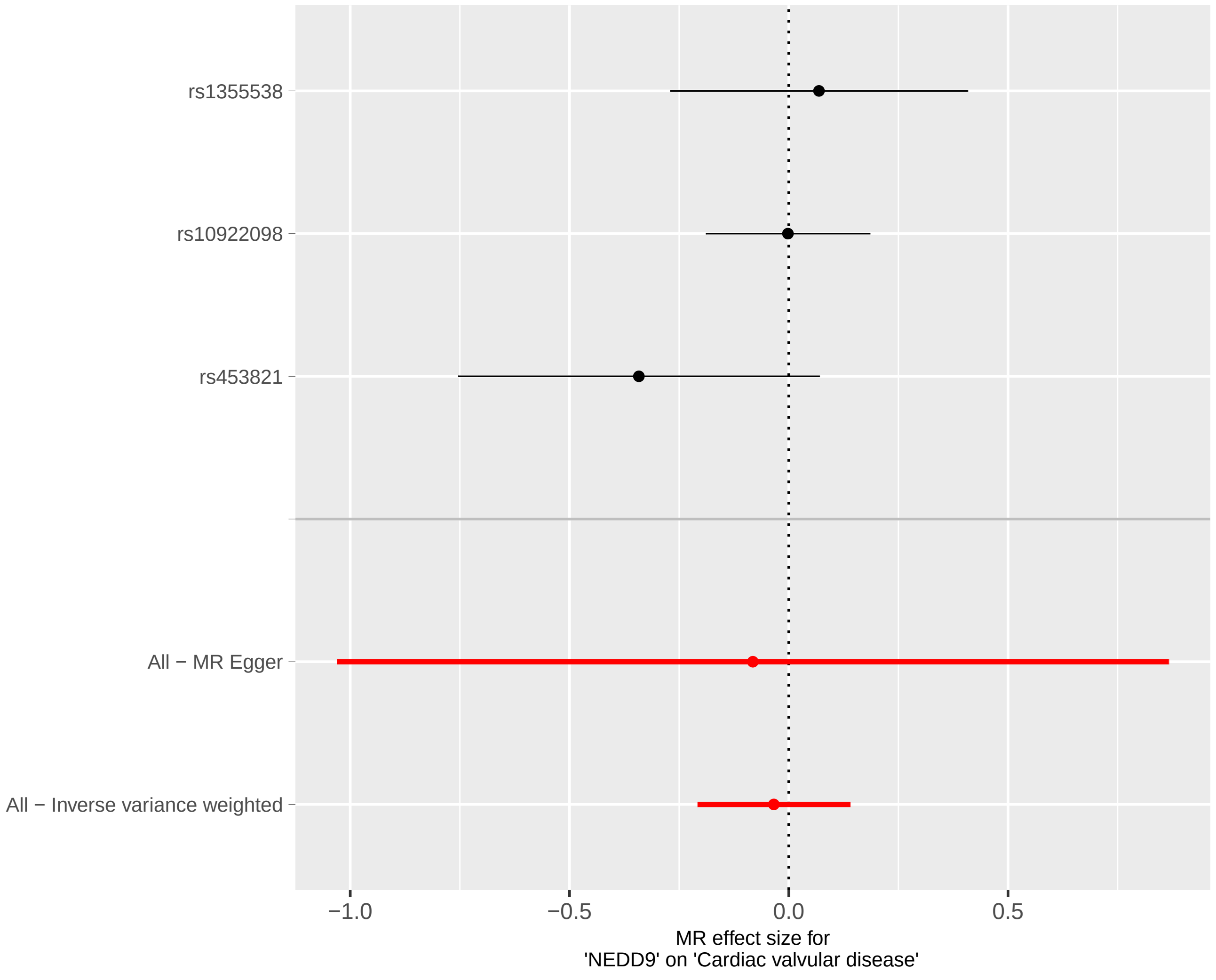
rs1355538
rs10922098
rs453821
All − MR Egger
All − Inverse variance weighted
−1.0
−0.5
0.0
0.5
MR effect size for
'NEDD9' on 'Cardiac valvular disease'

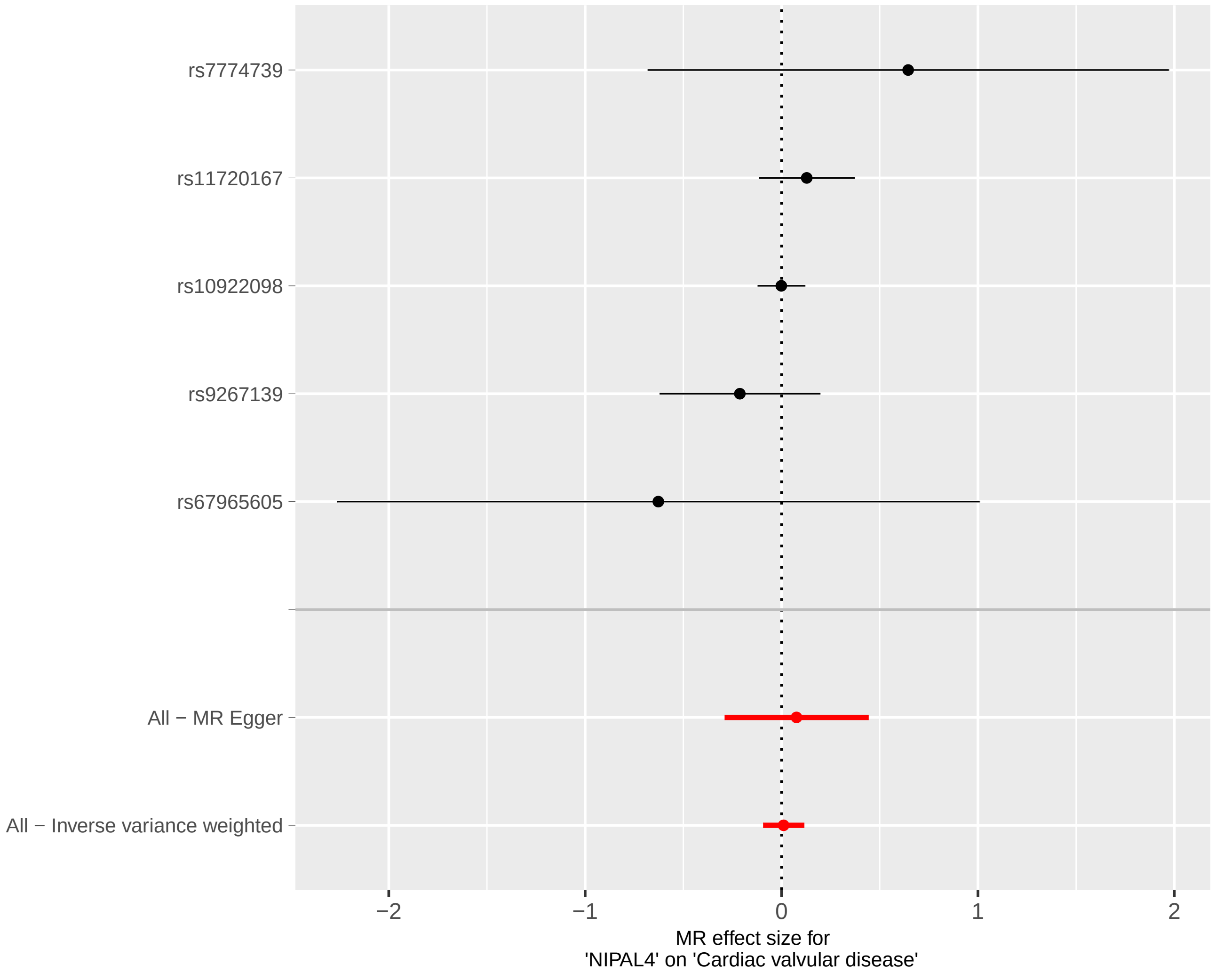
rs7774739
rs11720167
rs10922098
rs9267139
rs67965605
All − MR Egger
All − Inverse variance weighted
−2
−1
0
1
2
MR effect size for
'NIPAL4' on 'Cardiac valvular disease'

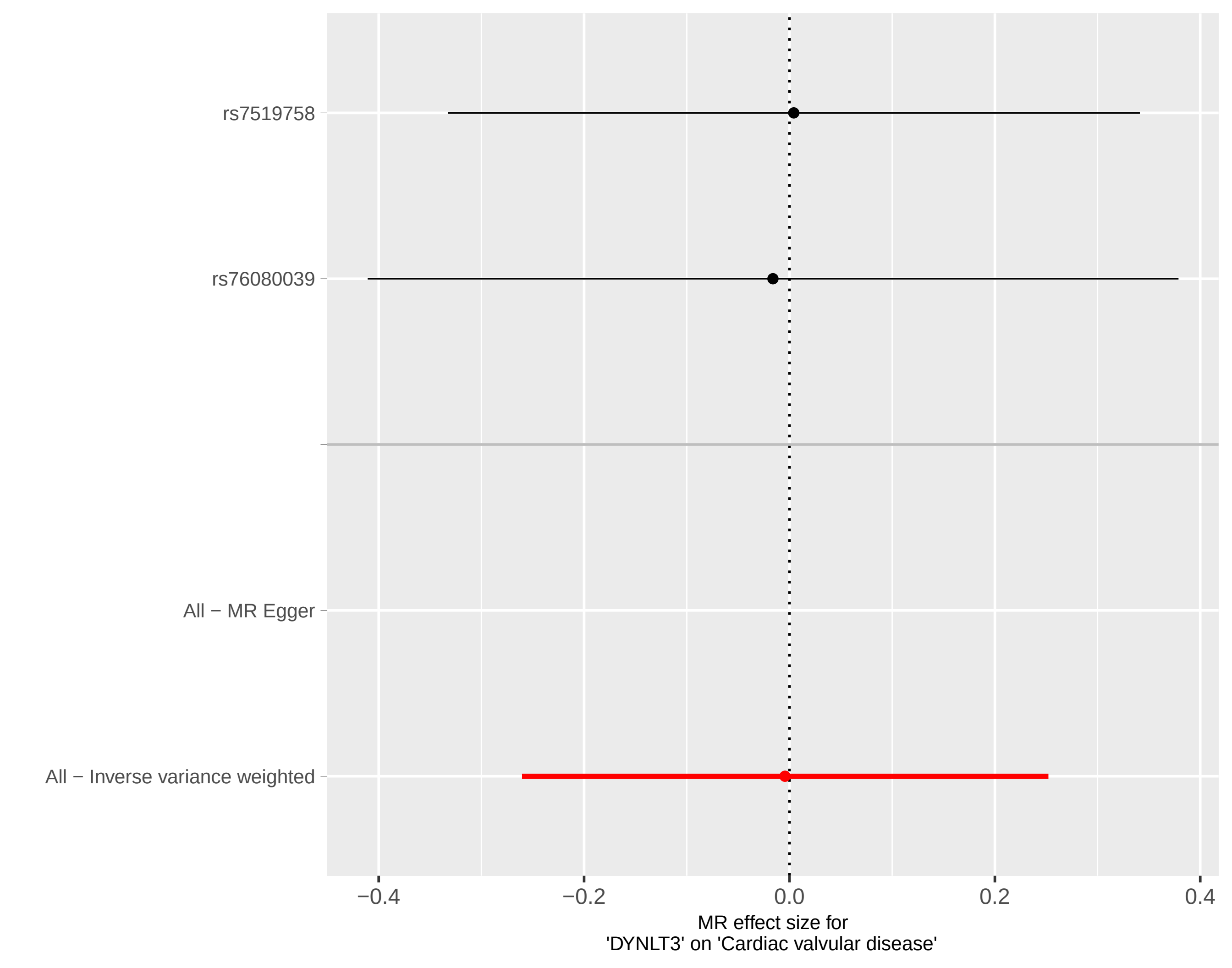
rs7519758
rs76080039
All − MR Egger
All − Inverse variance weighted
−0.4
−0.2
0.0
0.2
0.4
MR effect size for
'DYNLT3' on 'Cardiac valvular disease'

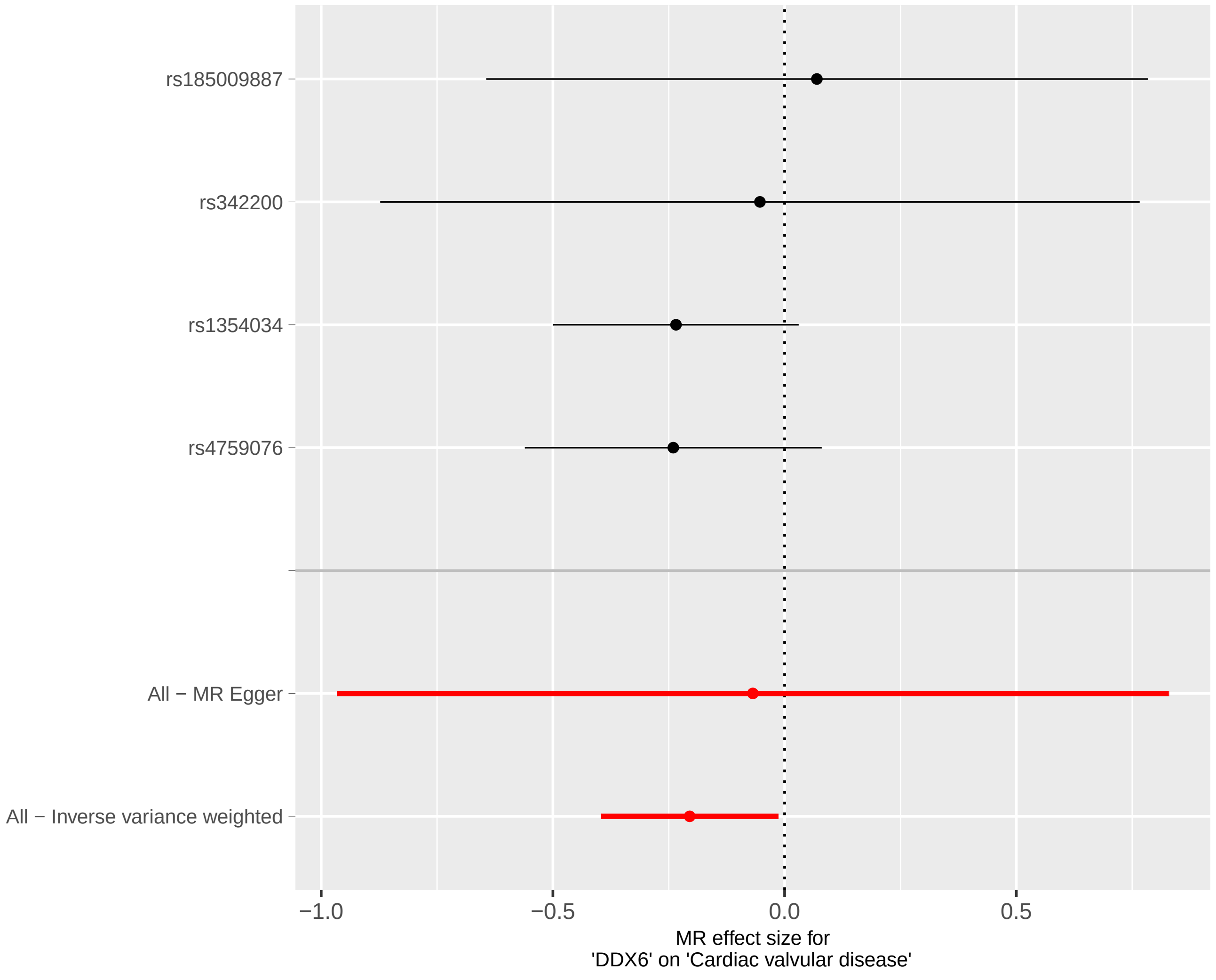
rs185009887
rs342200
rs1354034
rs4759076
All − MR Egger
All − Inverse variance weighted
−1.0
−0.5
0.0
0.5
MR effect size for
'DDX6' on 'Cardiac valvular disease'

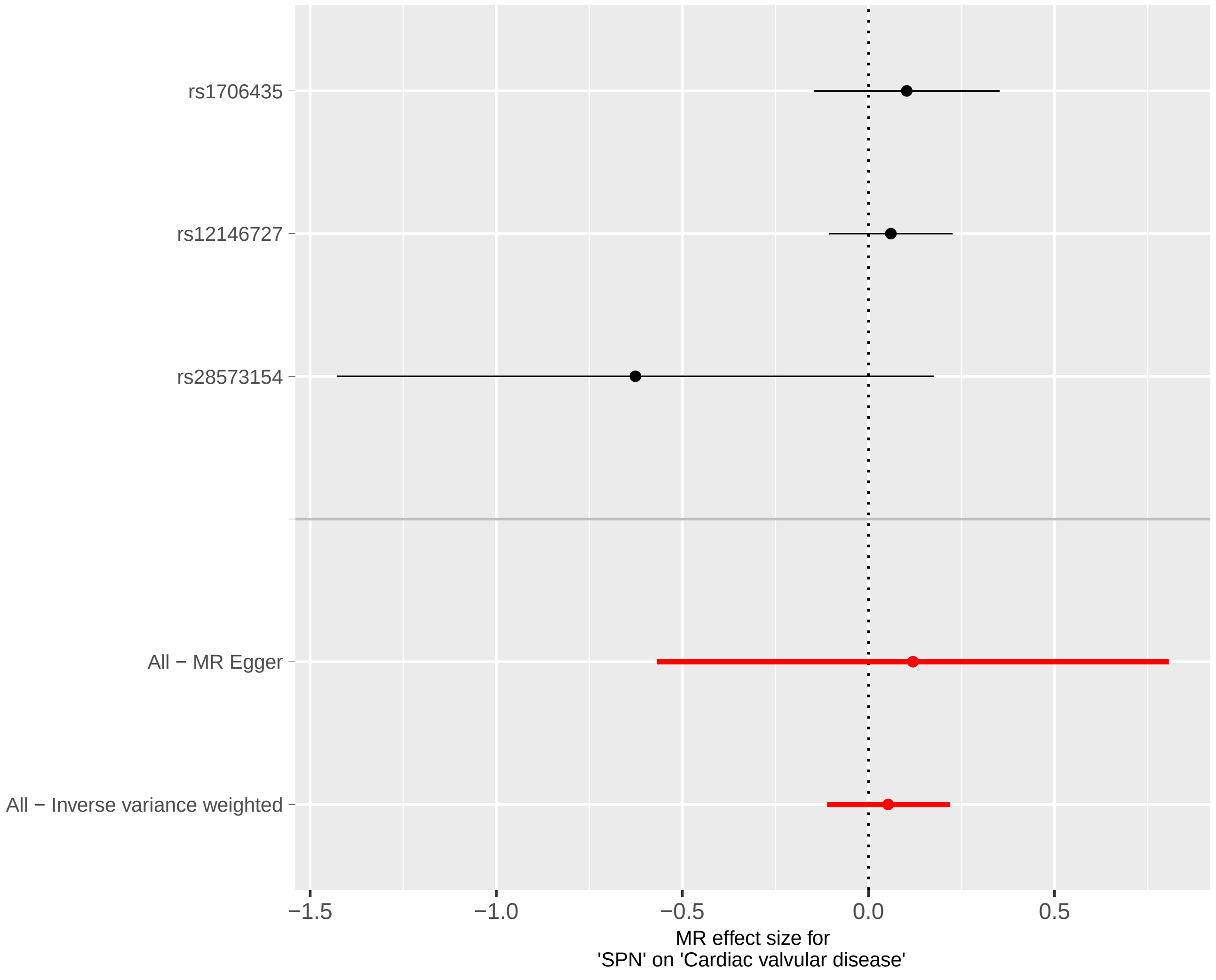

rs1706435
rs12146727
rs28573154
All − MR Egger
All − Inverse variance weighted
−1.5
−1.0
−0.5
0.0
0.5
MR effect size for
'SPN' on 'Cardiac valvular disease'

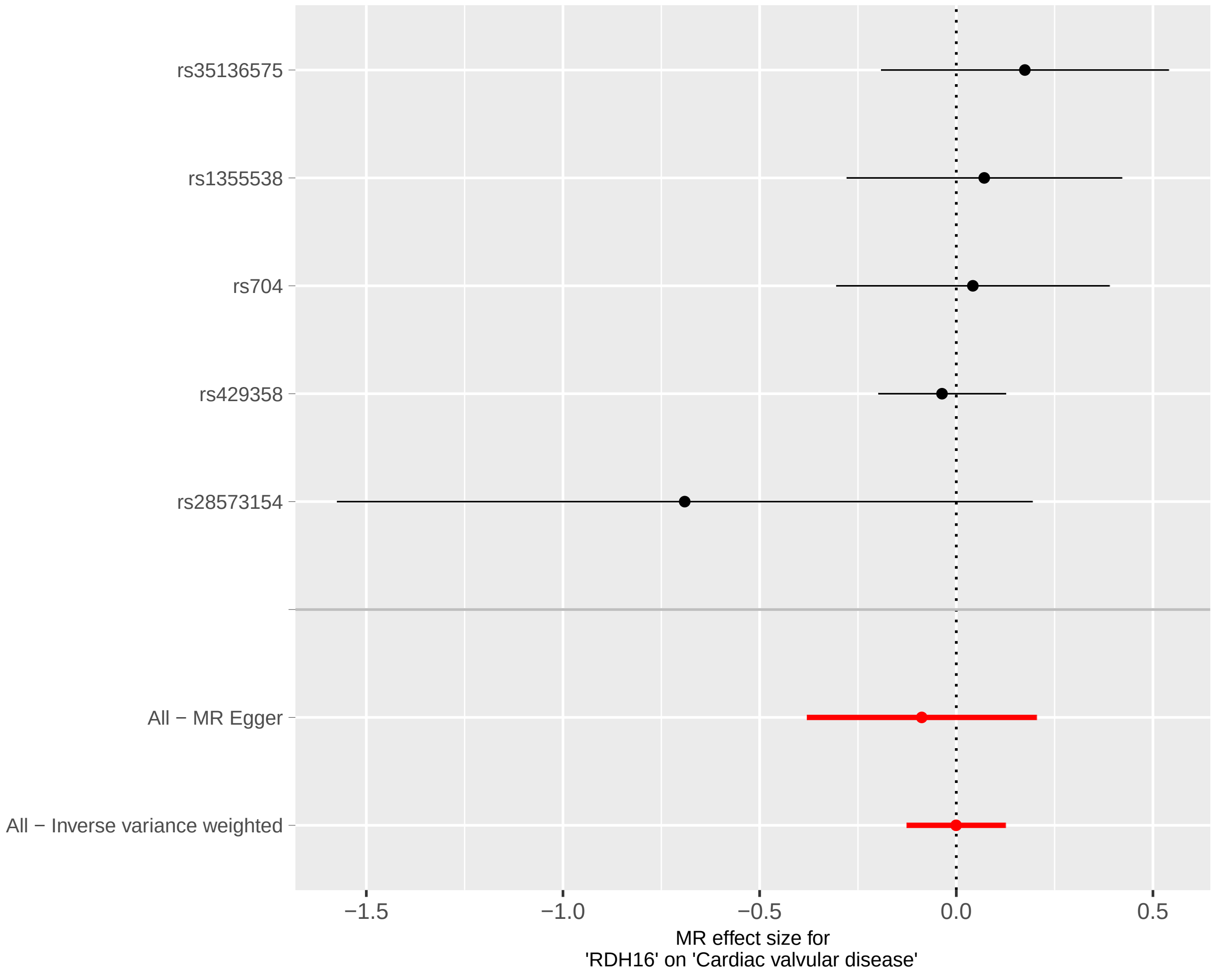
rs35136575
rs1355538
rs704
rs429358
rs28573154
All − MR Egger
All − Inverse variance weighted
−1.5
−1.0
−0.5
0.0
0.5
MR effect size for
'RDH16' on 'Cardiac valvular disease'

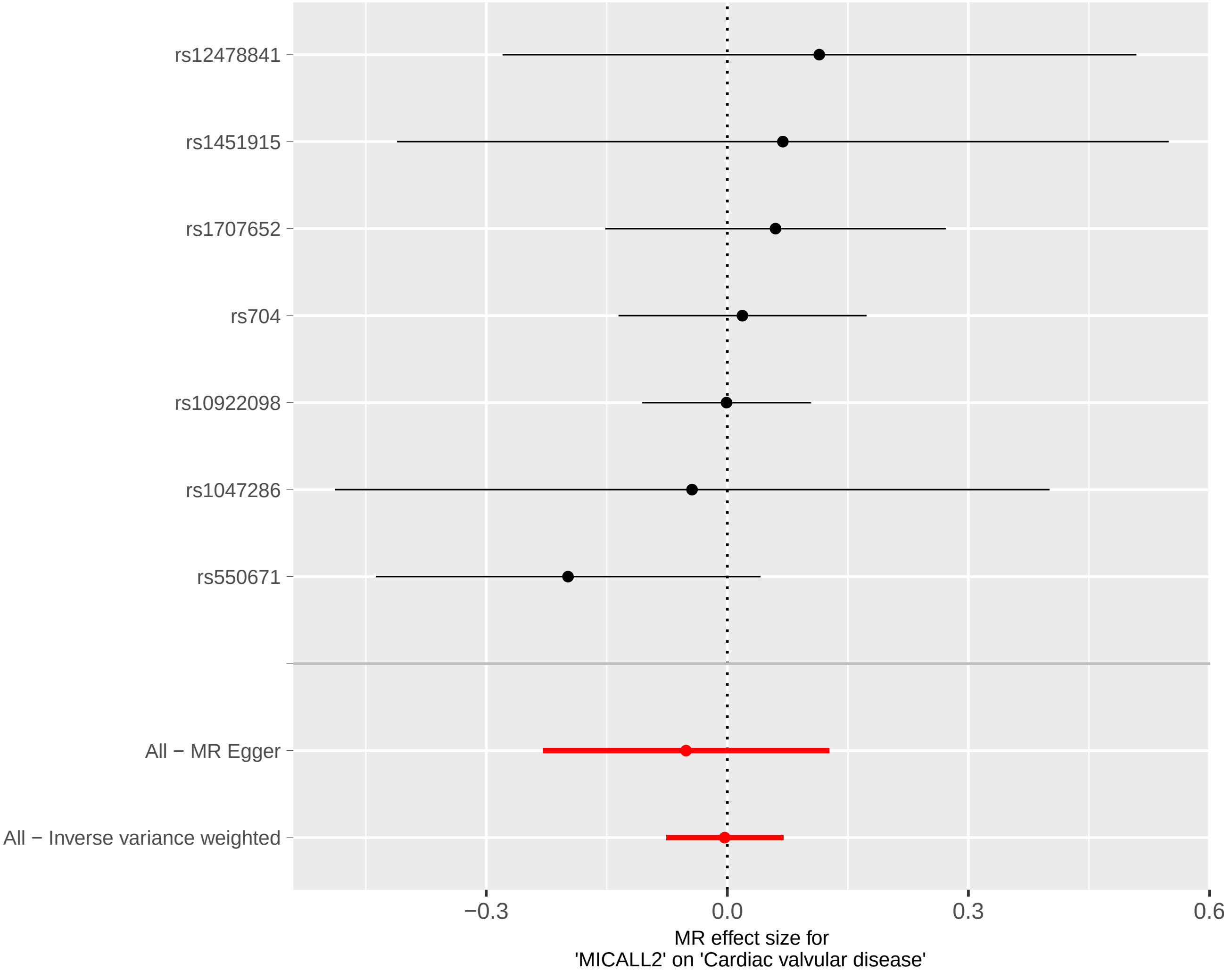

rs12478841
rs1451915
rs1707652
rs704
rs10922098
rs1047286
rs550671
All − MR Egger
All − Inverse variance weighted
−0.3
0.0
0.3
0.6
MR effect size for
'MICALL2' on 'Cardiac valvular disease'

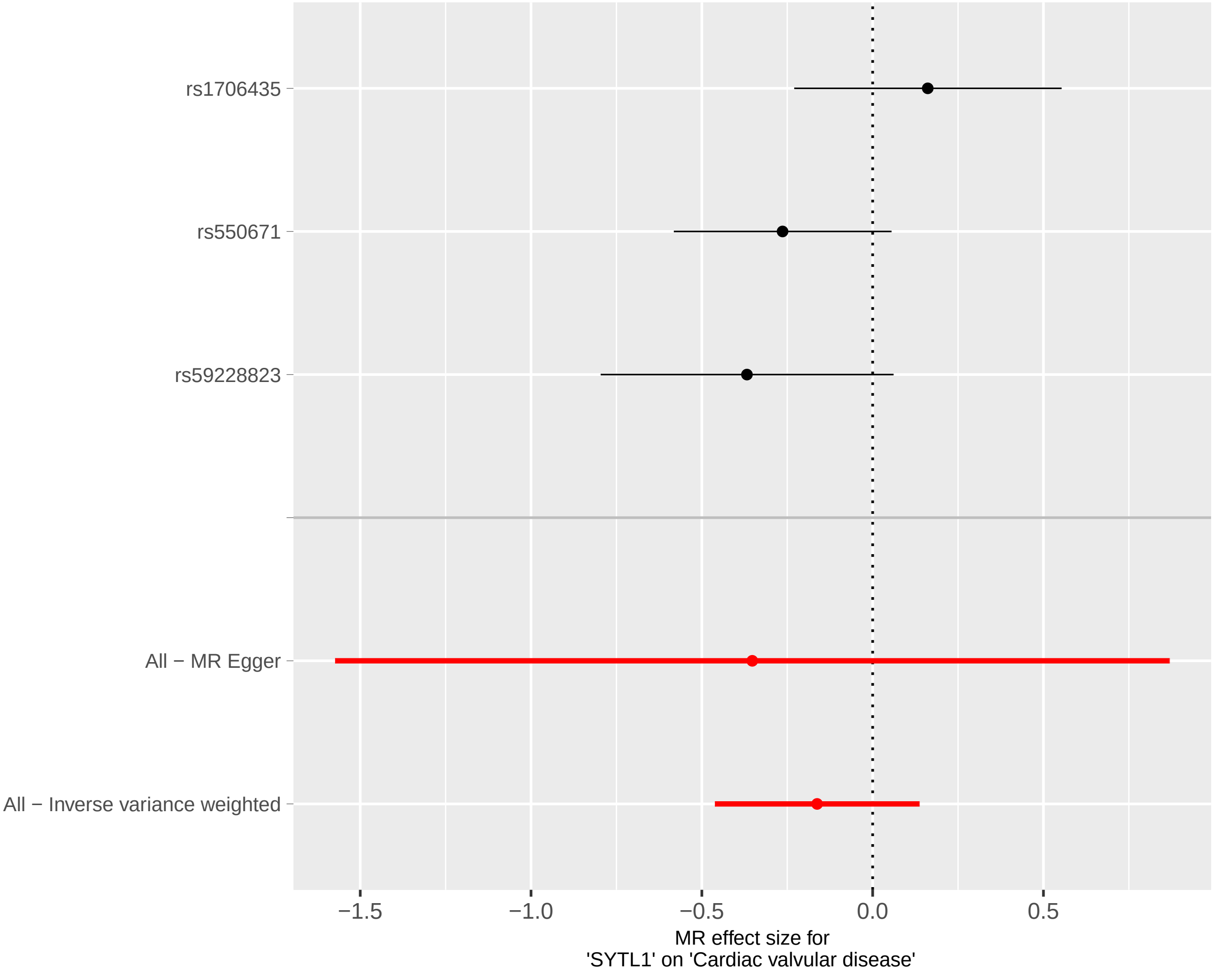
rs1706435
rs550671
rs59228823
All − MR Egger
All − Inverse variance weighted
−1.5
−1.0
−0.5
0.0
0.5
MR effect size for
'SYTL1' on 'Cardiac valvular disease'

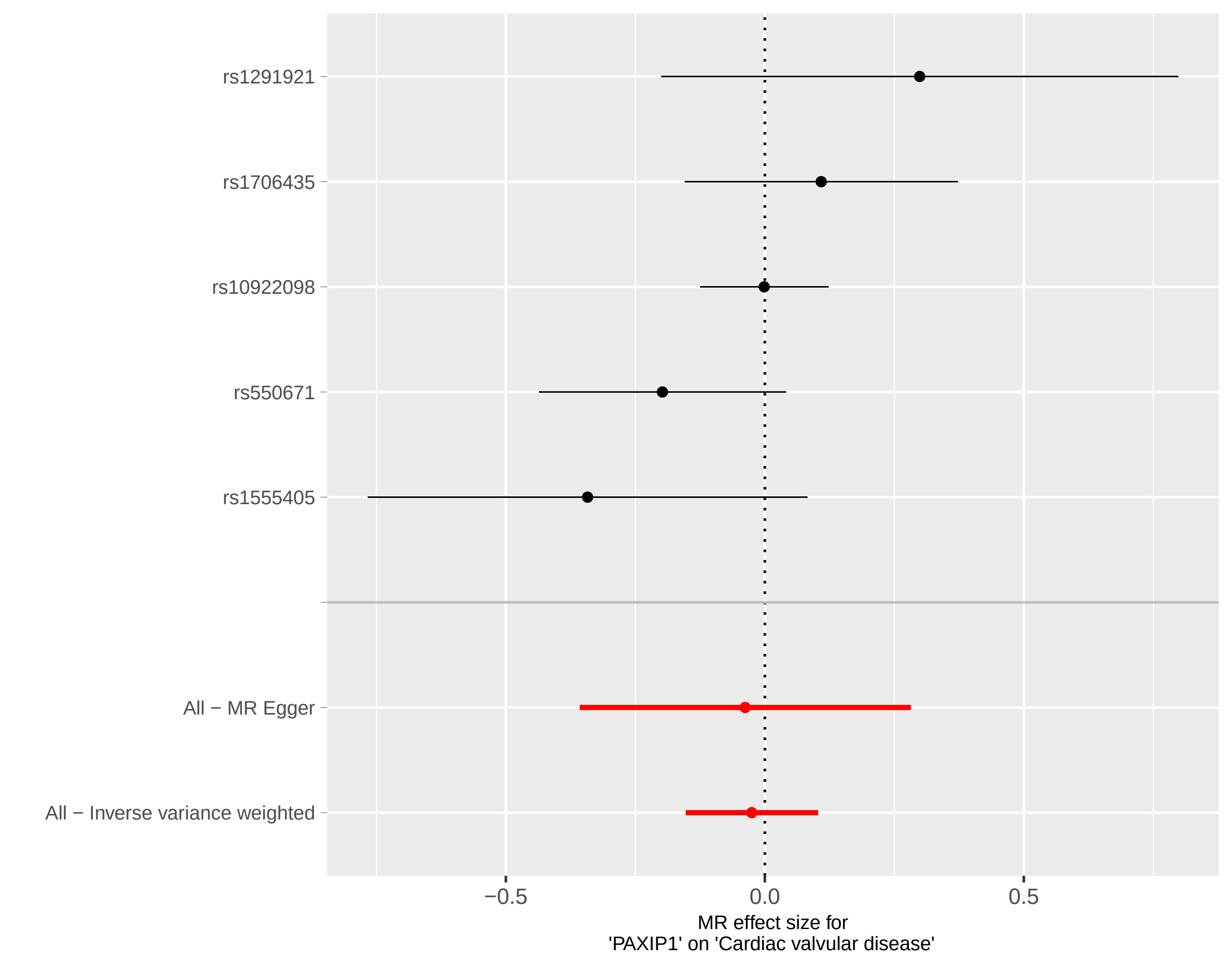
rs1291921
rs1706435
rs10922098
rs550671
rs1555405
All − MR Egger
All − Inverse variance weighted
−0.5
0.0
0.5
MR effect size for
'PAXIP1' on 'Cardiac valvular disease'

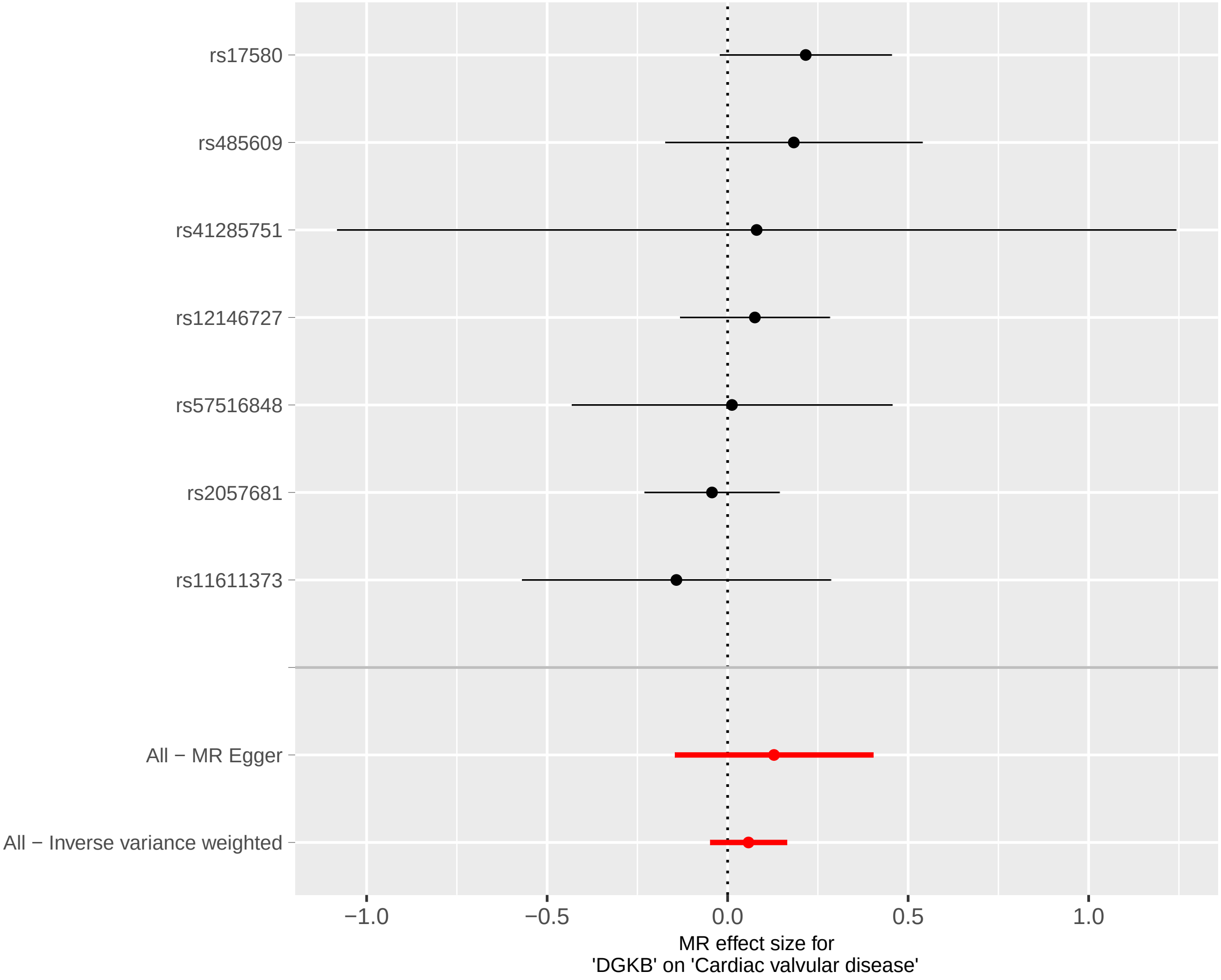
rs17580
rs485609
rs41285751
rs12146727
rs57516848
rs2057681
rs11611373
All − MR Egger
All − Inverse variance weighted
−1.0
−0.5
0.0
0.5
1.0
MR effect size for
'DGKB' on 'Cardiac valvular disease'

Insufficient number of SNPs

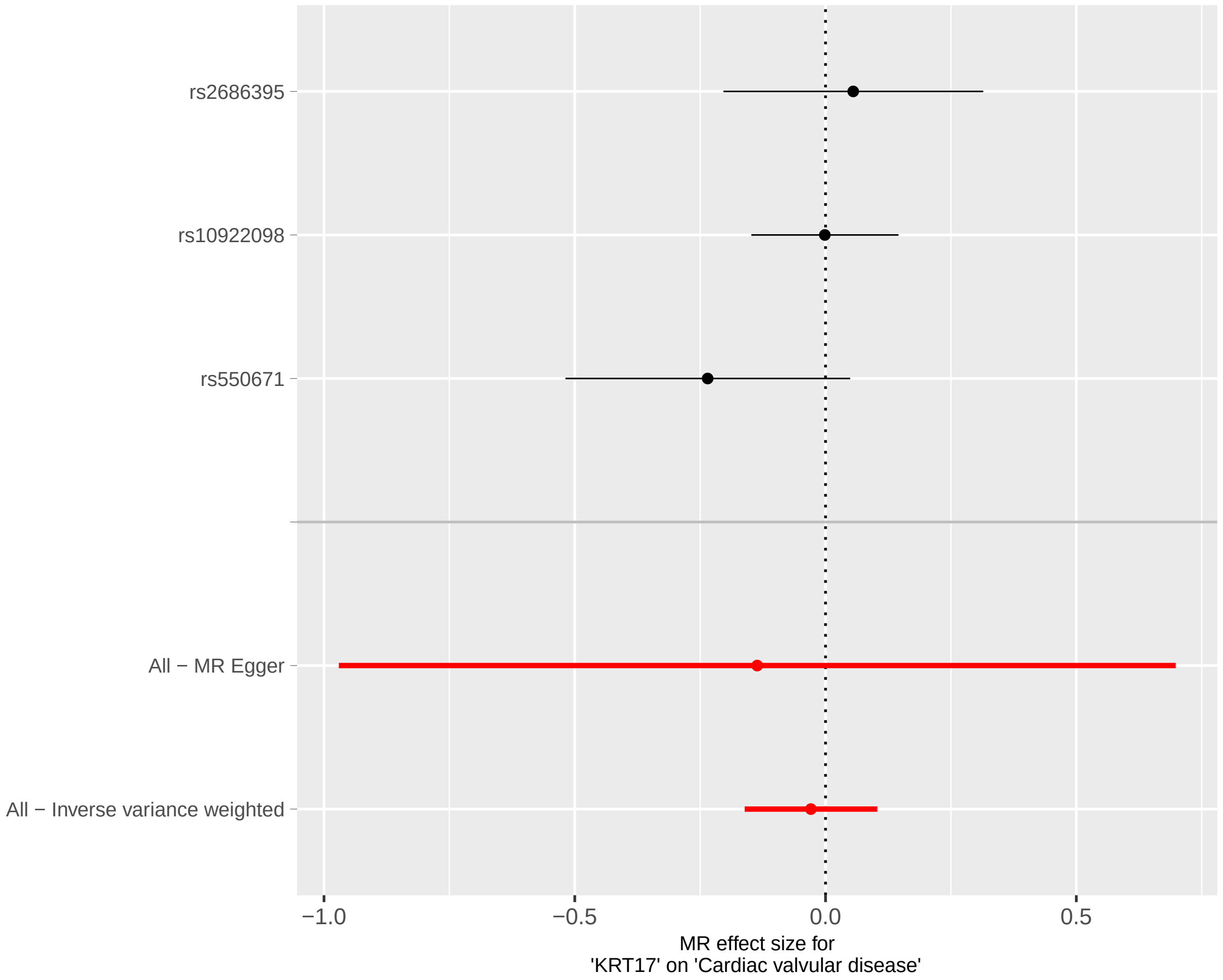
rs2686395
rs10922098
rs550671
All − MR Egger
All − Inverse variance weighted
−1.0
−0.5
0.0
0.5
MR effect size for
'KRT17' on 'Cardiac valvular disease'

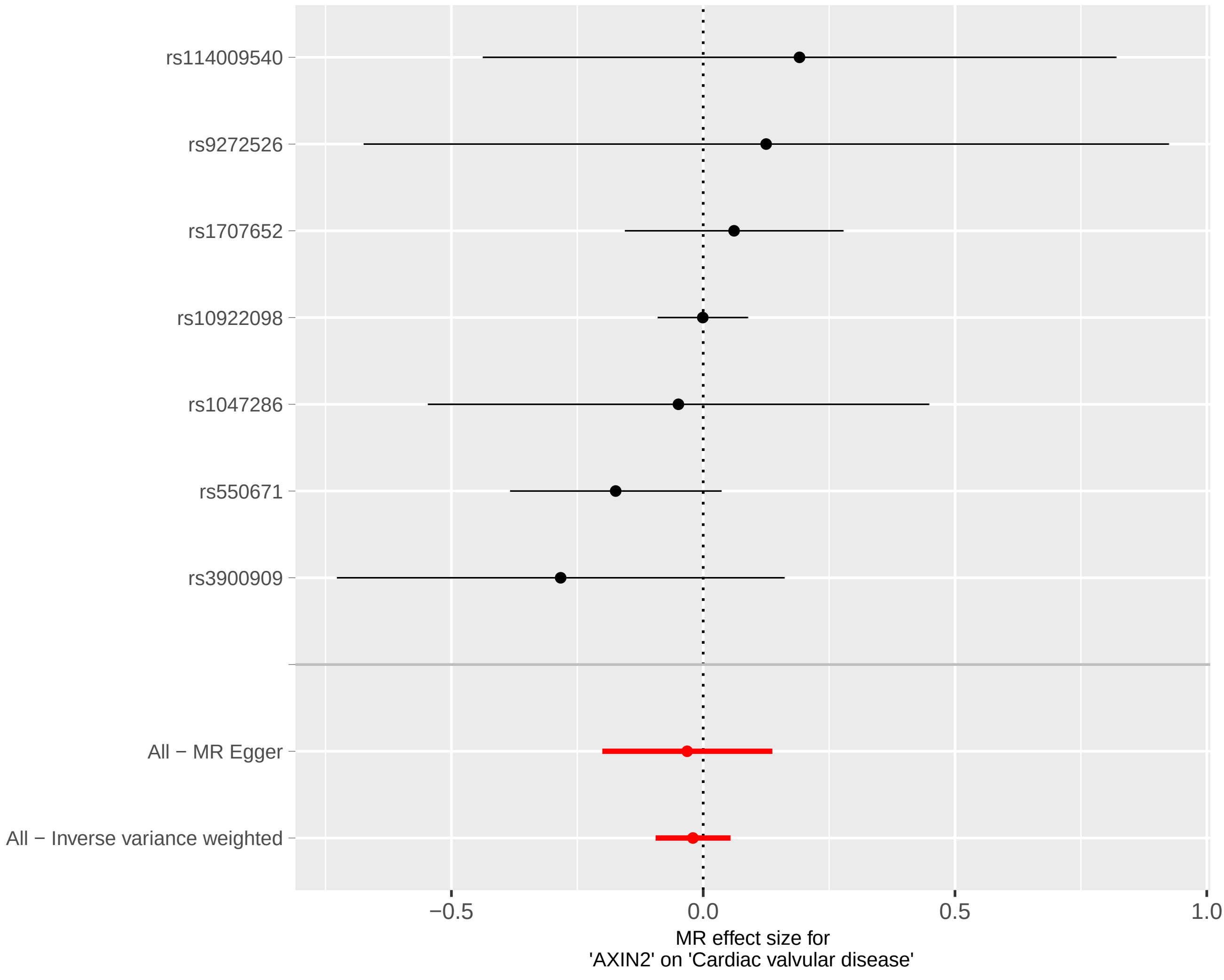
rs114009540
rs9272526
rs1707652
rs10922098
rs1047286
rs550671
rs3900909
All − MR Egger
All − Inverse variance weighted
−0.5
0.0
0.5
1.0
MR effect size for
'AXIN2' on 'Cardiac valvular disease'

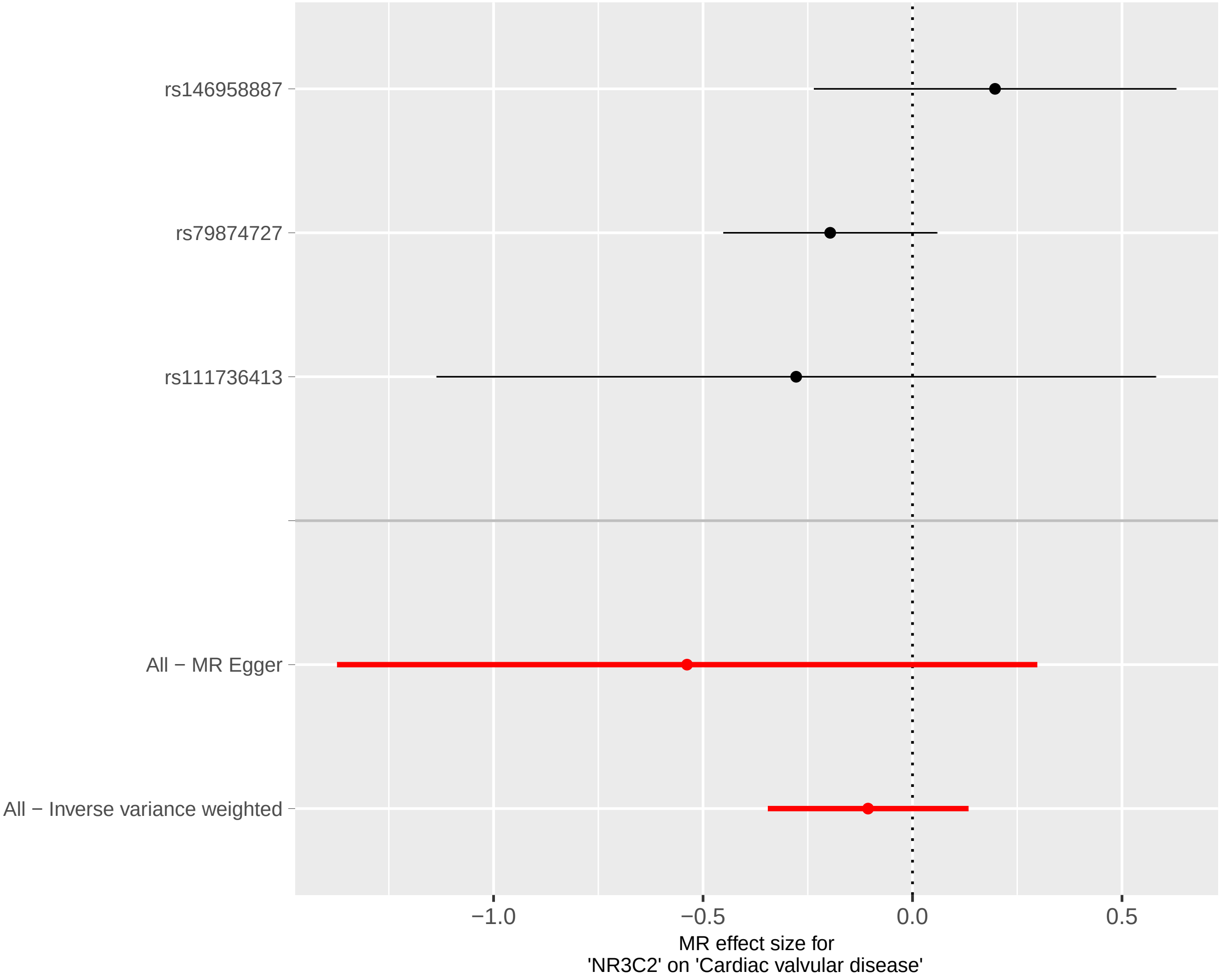
rs146958887
rs79874727
rs111736413
All − MR Egger
All − Inverse variance weighted
−1.0
−0.5
0.0
0.5
MR effect size for
'NR3C2' on 'Cardiac valvular disease'

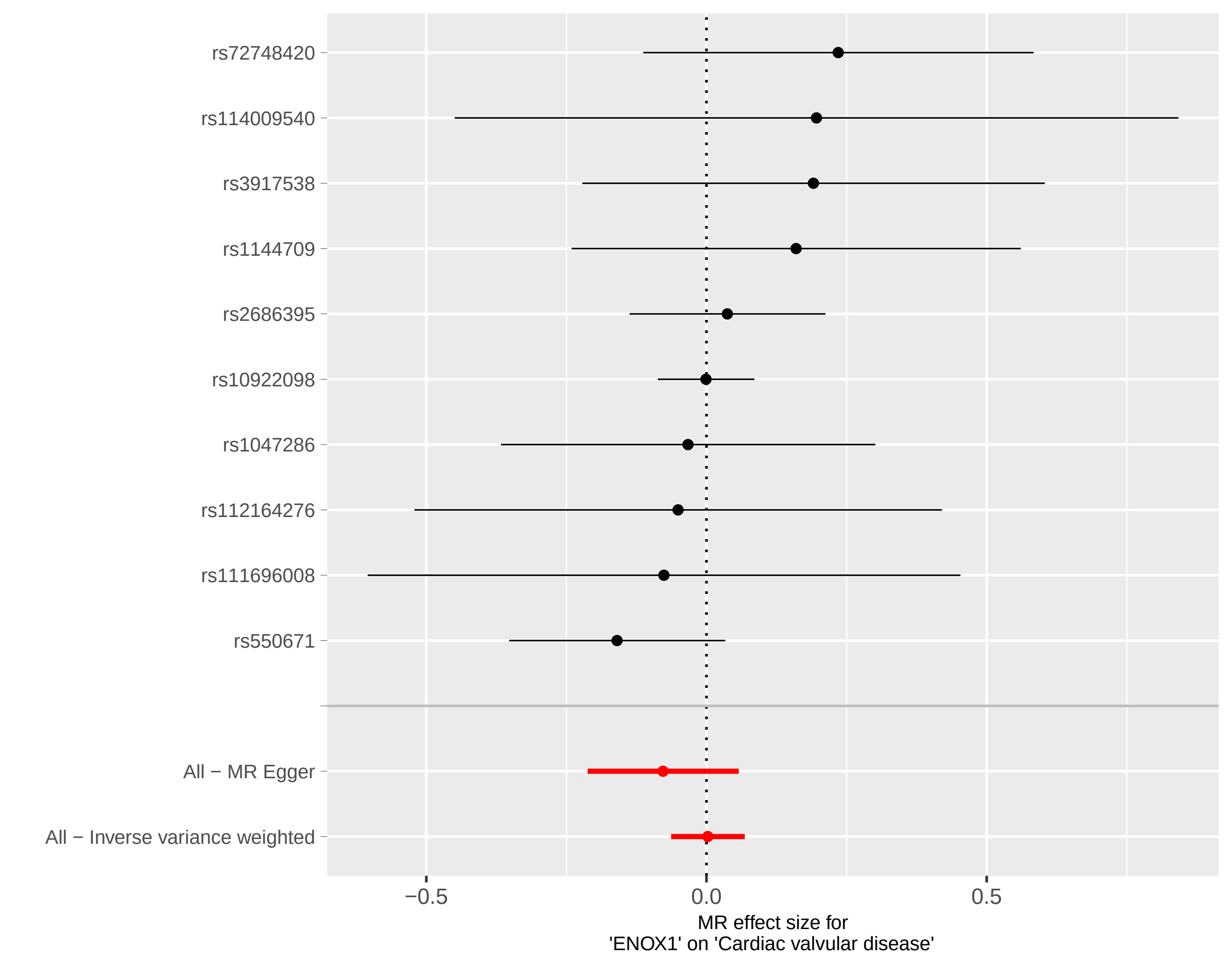

rs72748420
rs114009540
rs3917538
rs1144709
rs2686395
rs10922098
rs1047286
rs112164276
rs111696008
rs550671
All − MR Egger
All − Inverse variance weighted
−0.5
0.0
0.5
MR effect size for
'ENOX1' on 'Cardiac valvular disease'

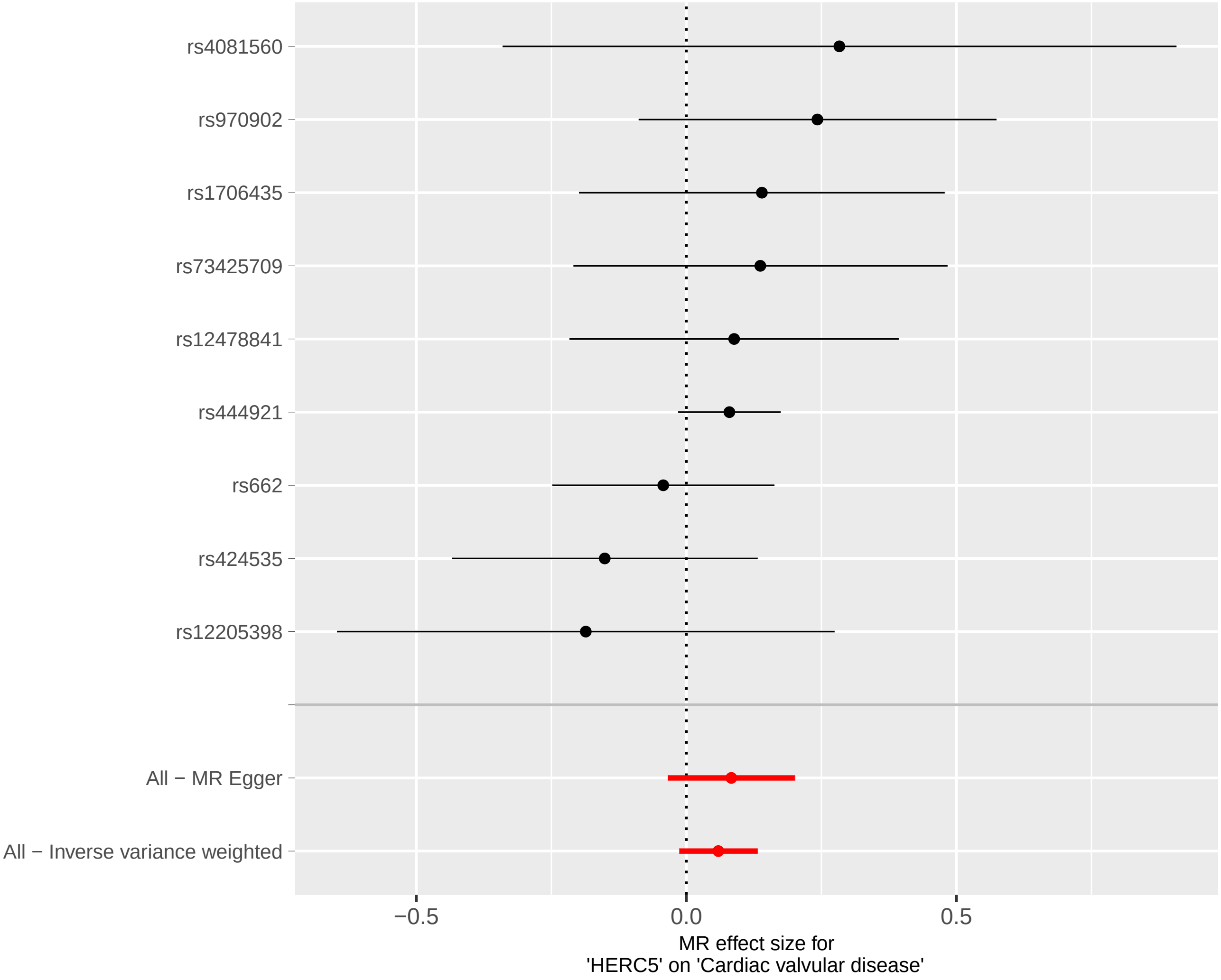
rs4081560
rs970902
rs1706435
rs73425709
rs12478841
rs444921
rs662
rs424535
rs12205398
All − MR Egger
All − Inverse variance weighted
−0.5
0.0
0.5
MR effect size for
'HERC5' on 'Cardiac valvular disease'

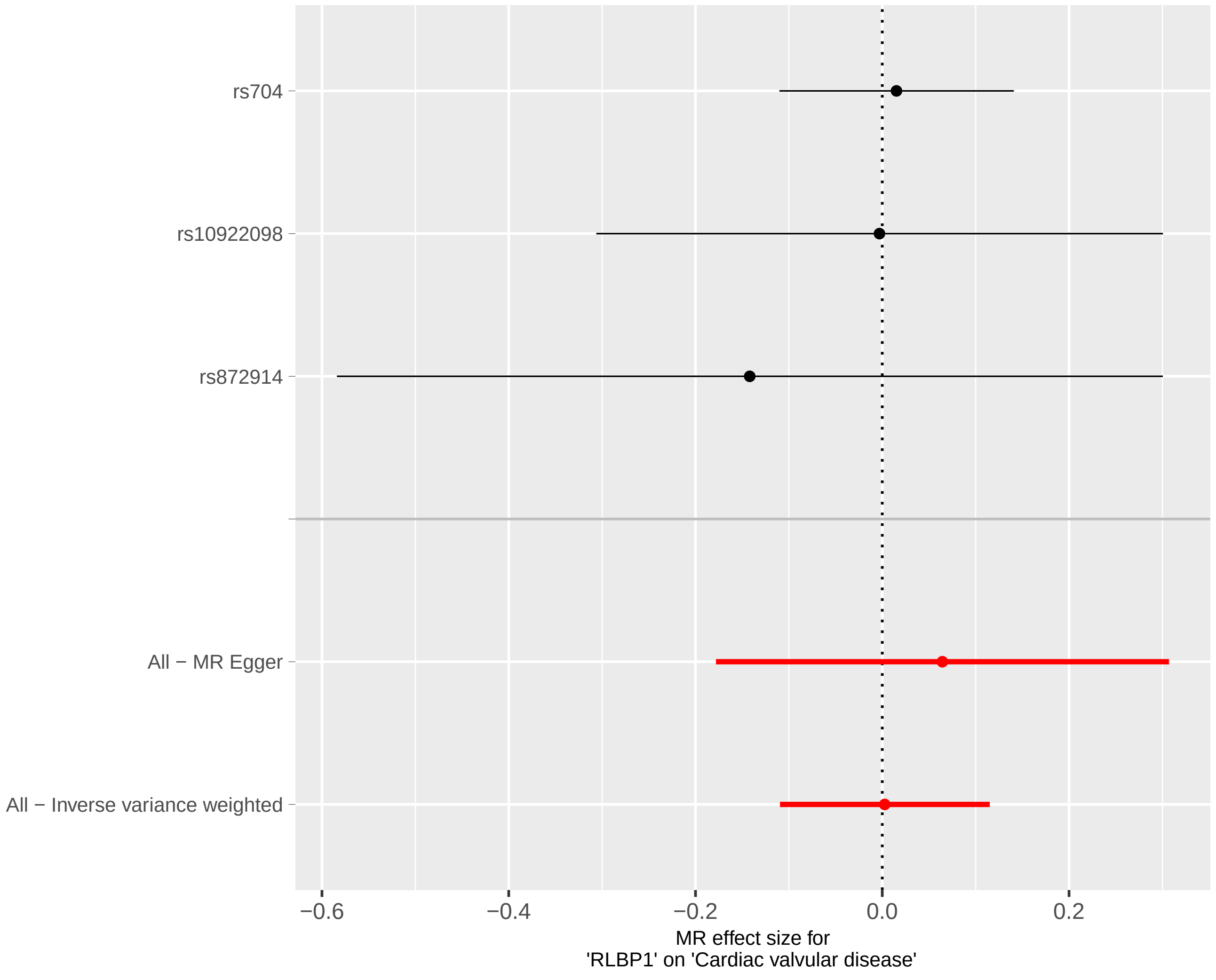
rs704
rs10922098
rs872914
All − MR Egger
All − Inverse variance weighted
−0.6
−0.4
−0.2
0.0
0.2
MR effect size for
'RLBP1' on 'Cardiac valvular disease'

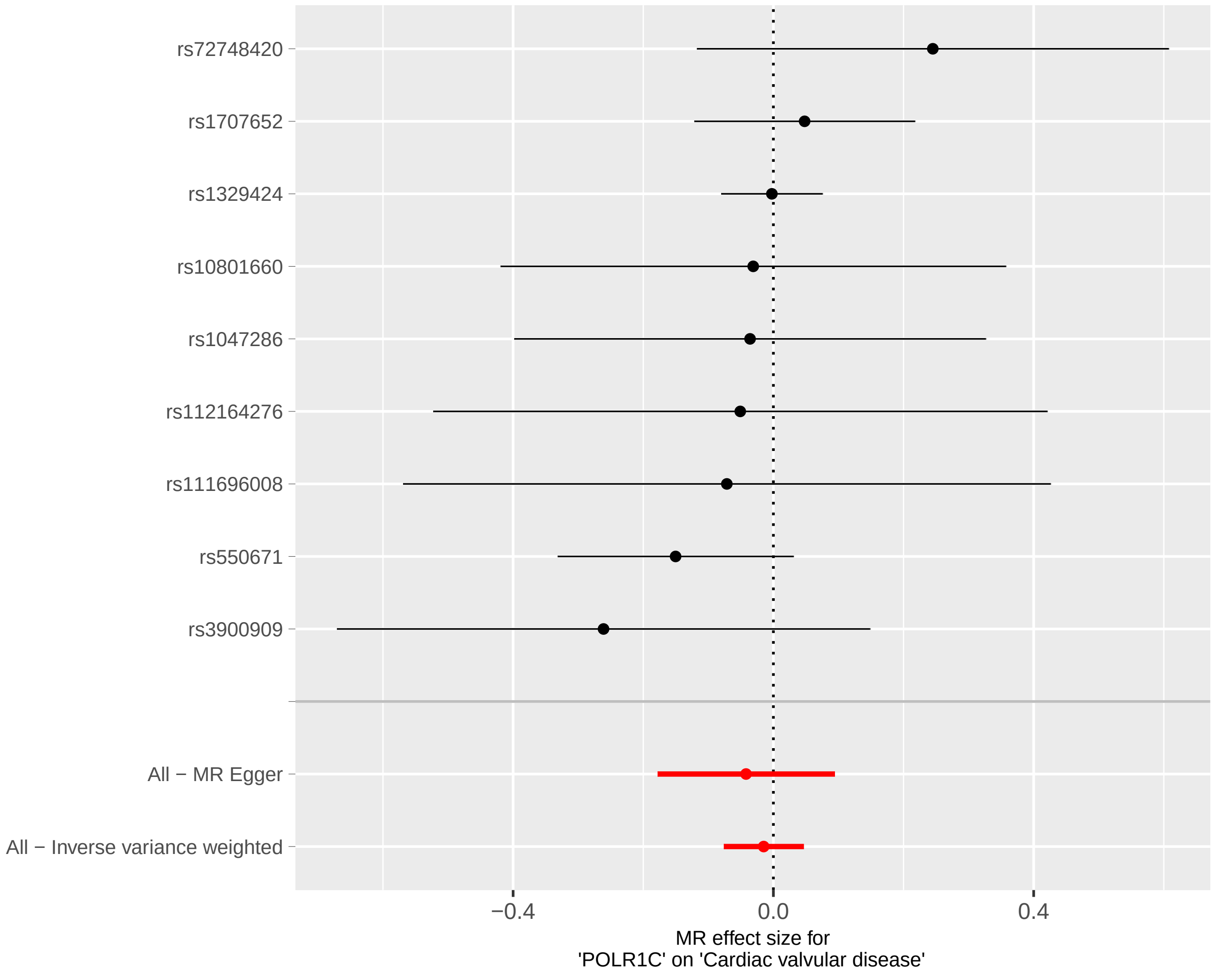
rs72748420
rs1707652
rs1329424
rs10801660
rs1047286
rs112164276
rs111696008
rs550671
rs3900909
All − MR Egger
All − Inverse variance weighted
−0.4
0.0
0.4
MR effect size for
'POLR1C' on 'Cardiac valvular disease'

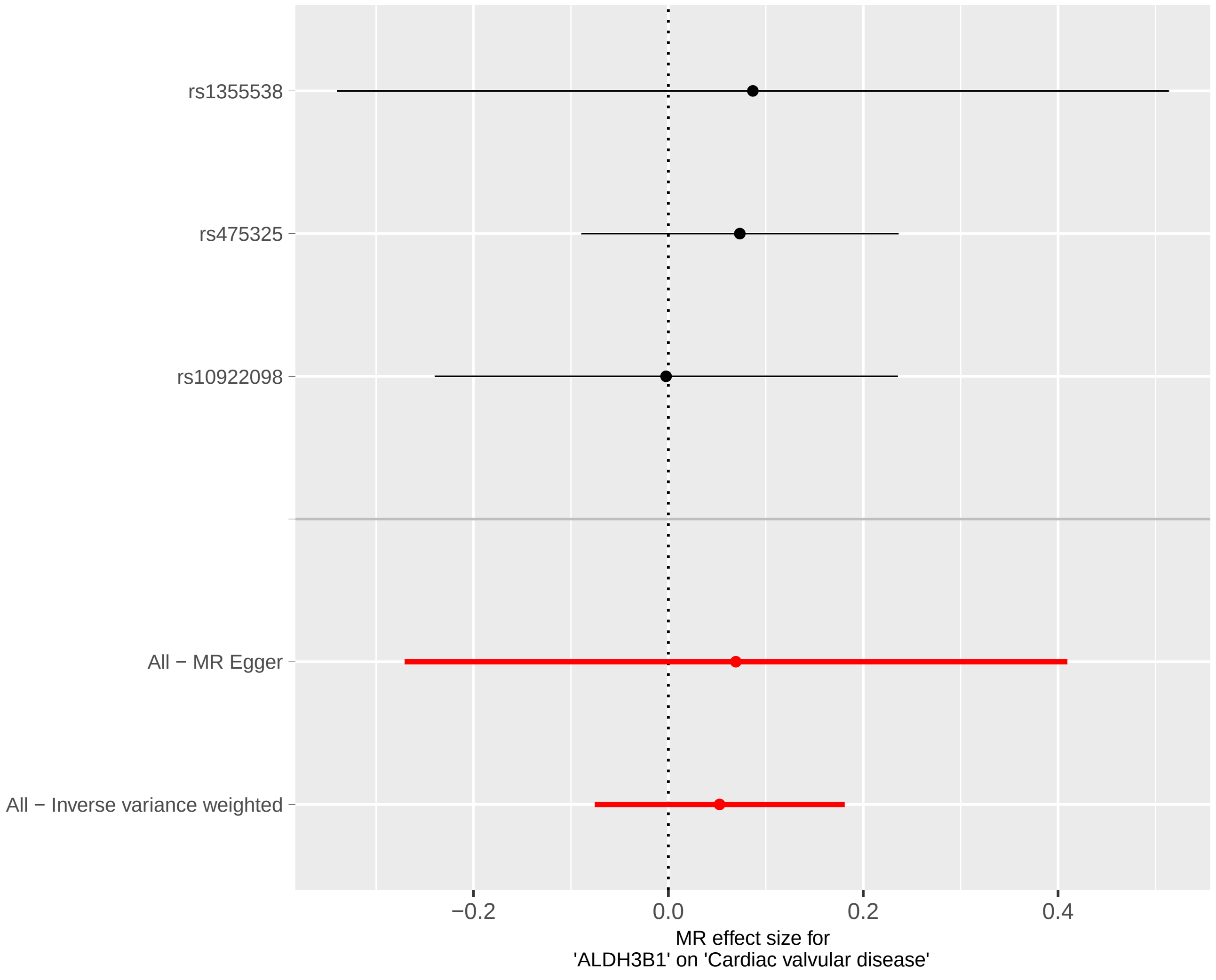
rs1355538
rs475325
rs10922098
All − MR Egger
All − Inverse variance weighted
−0.2
0.0
0.2
0.4
MR effect size for
'ALDH3B1' on 'Cardiac valvular disease'

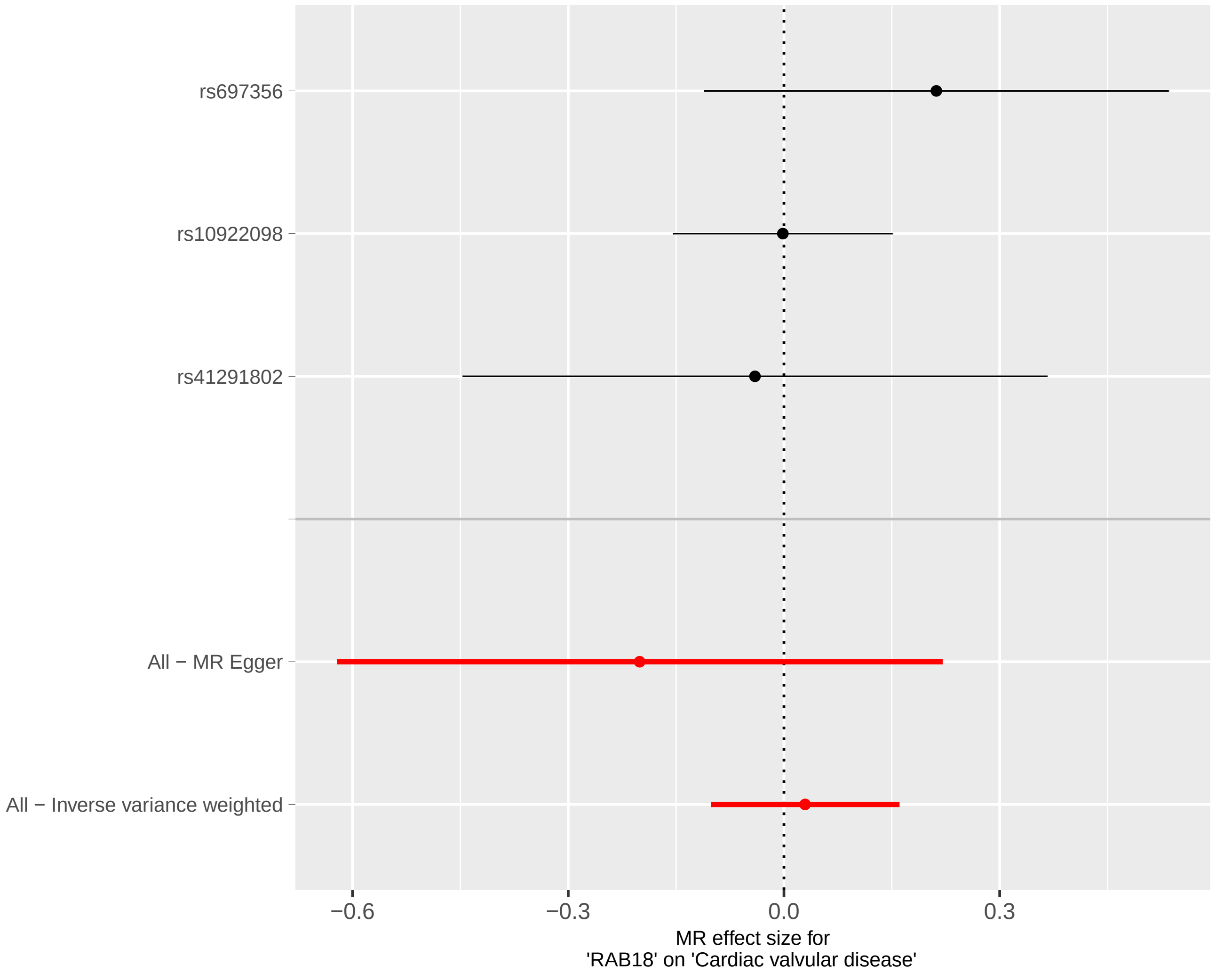
rs697356
rs10922098
rs41291802
All − MR Egger
All − Inverse variance weighted
−0.6
−0.3
0.0
0.3
MR effect size for
'RAB18' on 'Cardiac valvular disease'

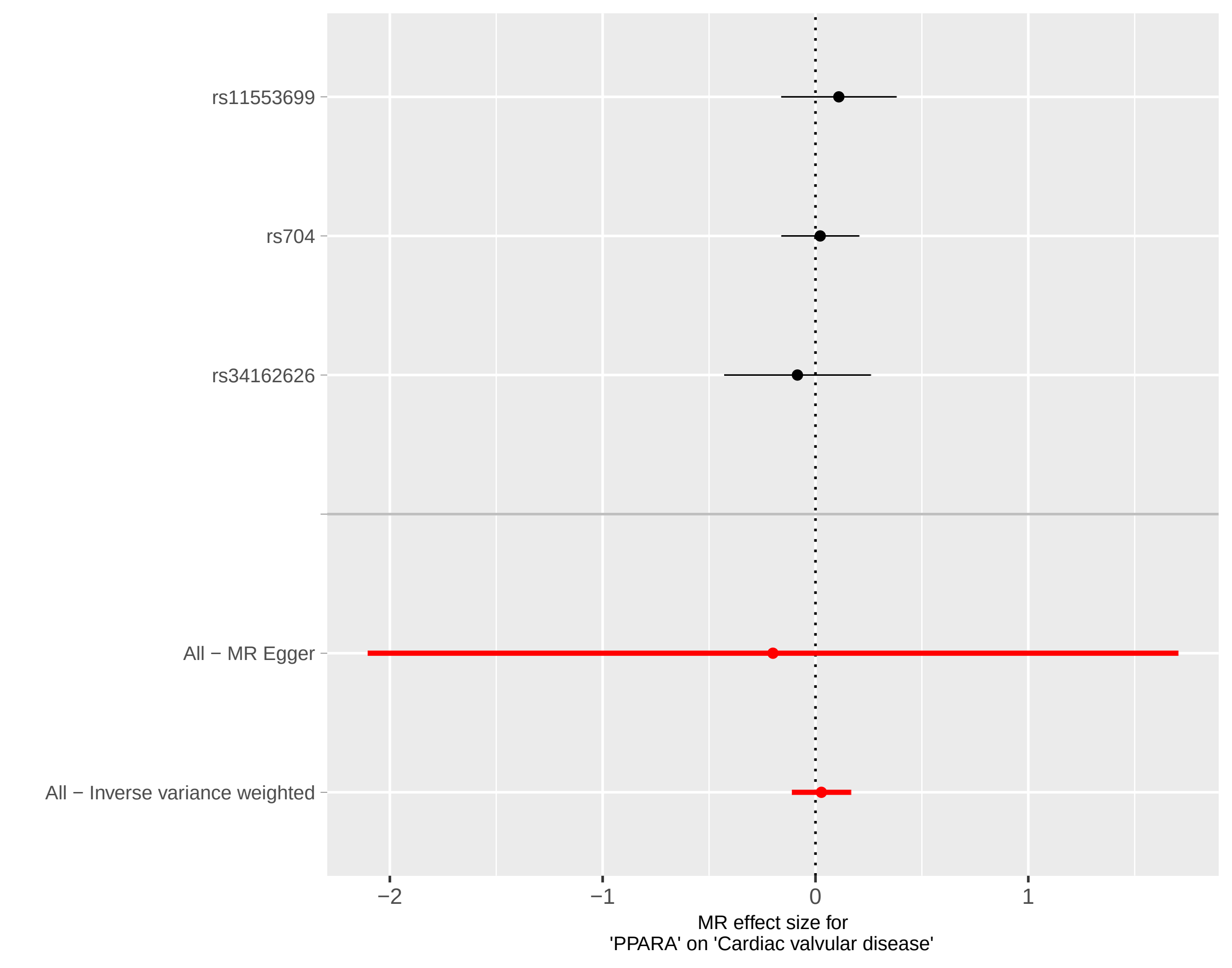

rs11553699
rs704
rs34162626
All − MR Egger
All − Inverse variance weighted
−2
−1
0
1
MR effect size for
'PPARA' on 'Cardiac valvular disease'

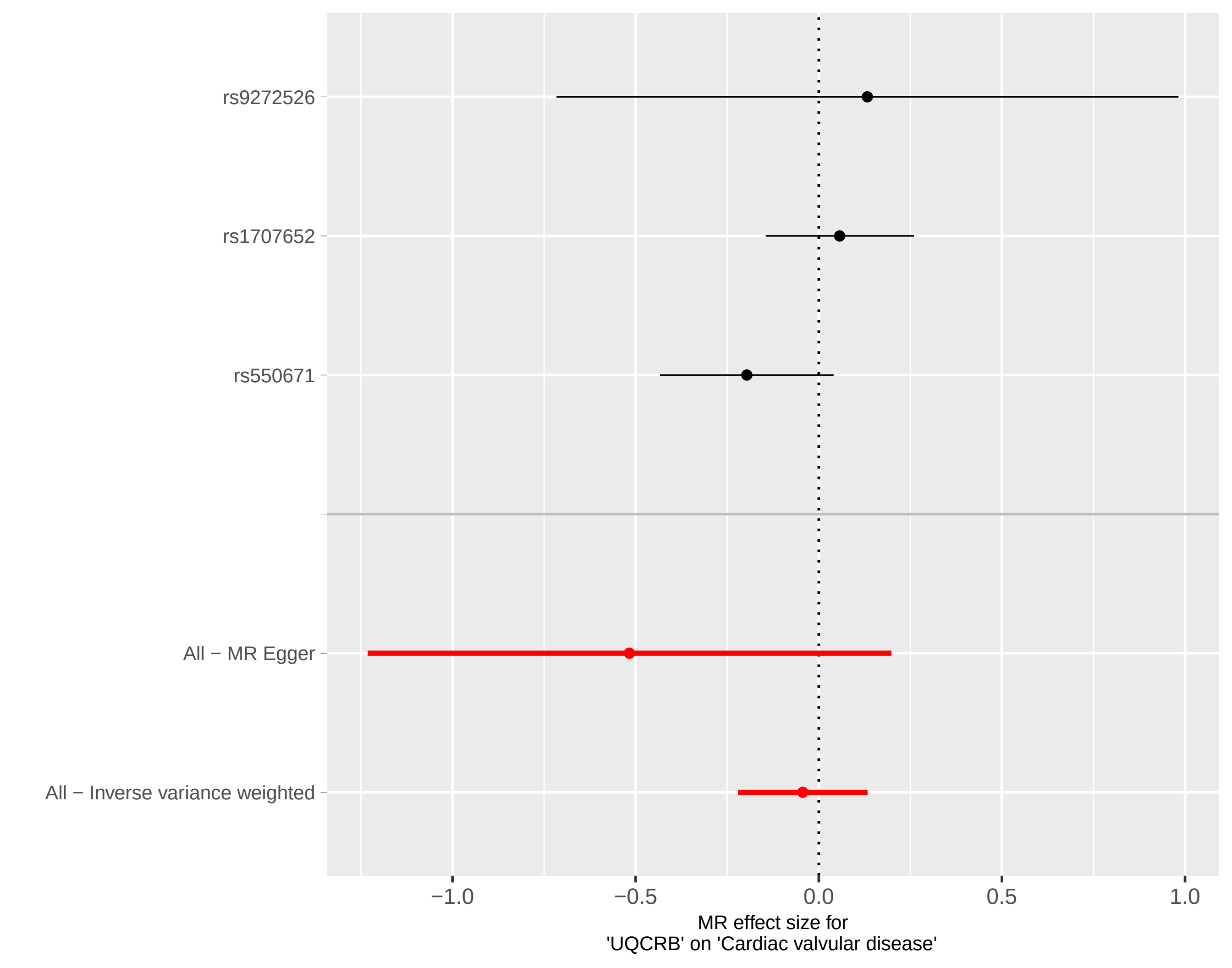

rs9272526
rs1707652
rs550671
All − MR Egger
All − Inverse variance weighted
−1.0
−0.5
0.0
0.5
1.0
MR effect size for
'UQCRB' on 'Cardiac valvular disease'

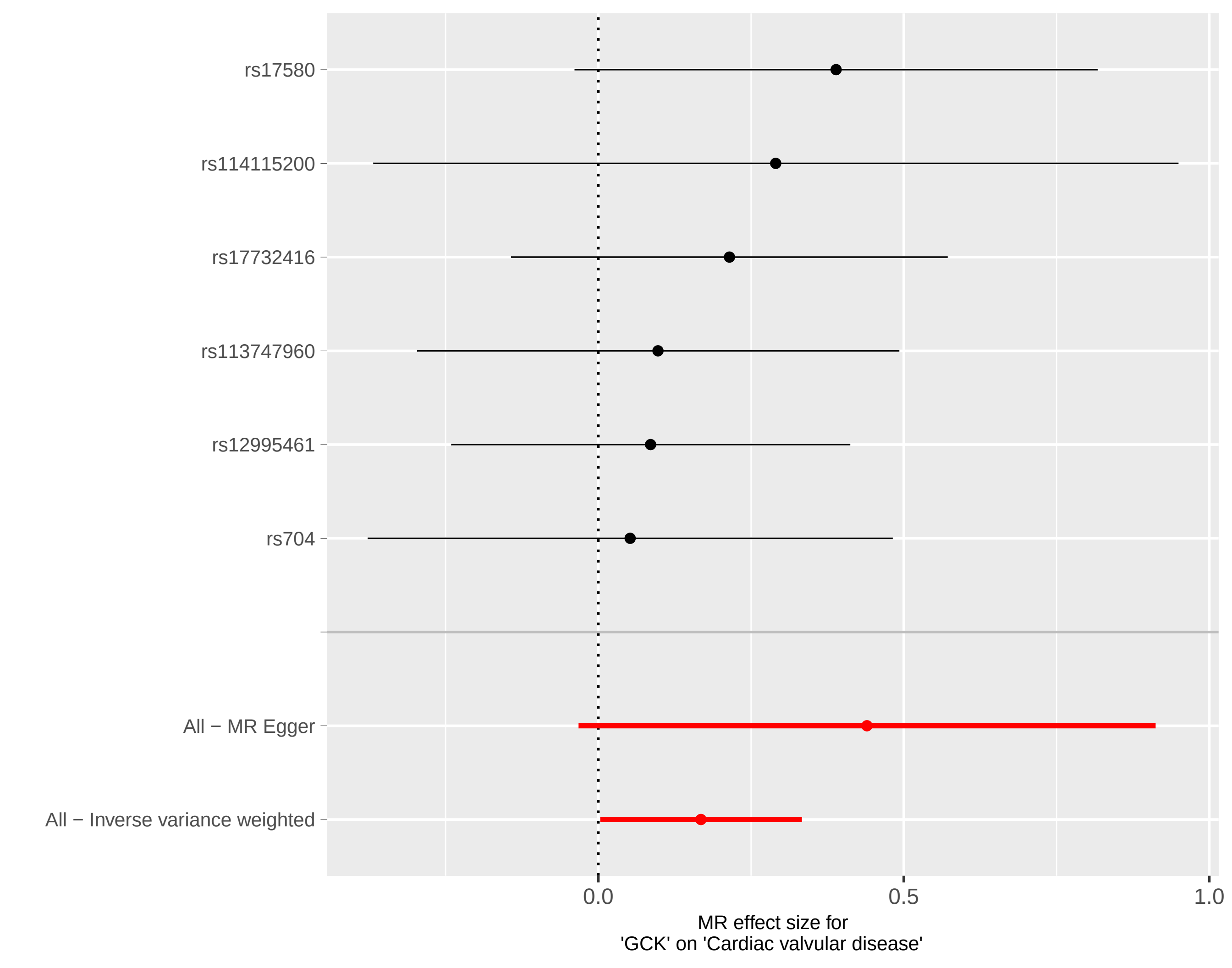

rs17580
rs114115200
rs17732416
rs113747960
rs12995461
rs704
All − MR Egger
All − Inverse variance weighted
0.0
0.5
1.0
MR effect size for
'GCK' on 'Cardiac valvular disease'

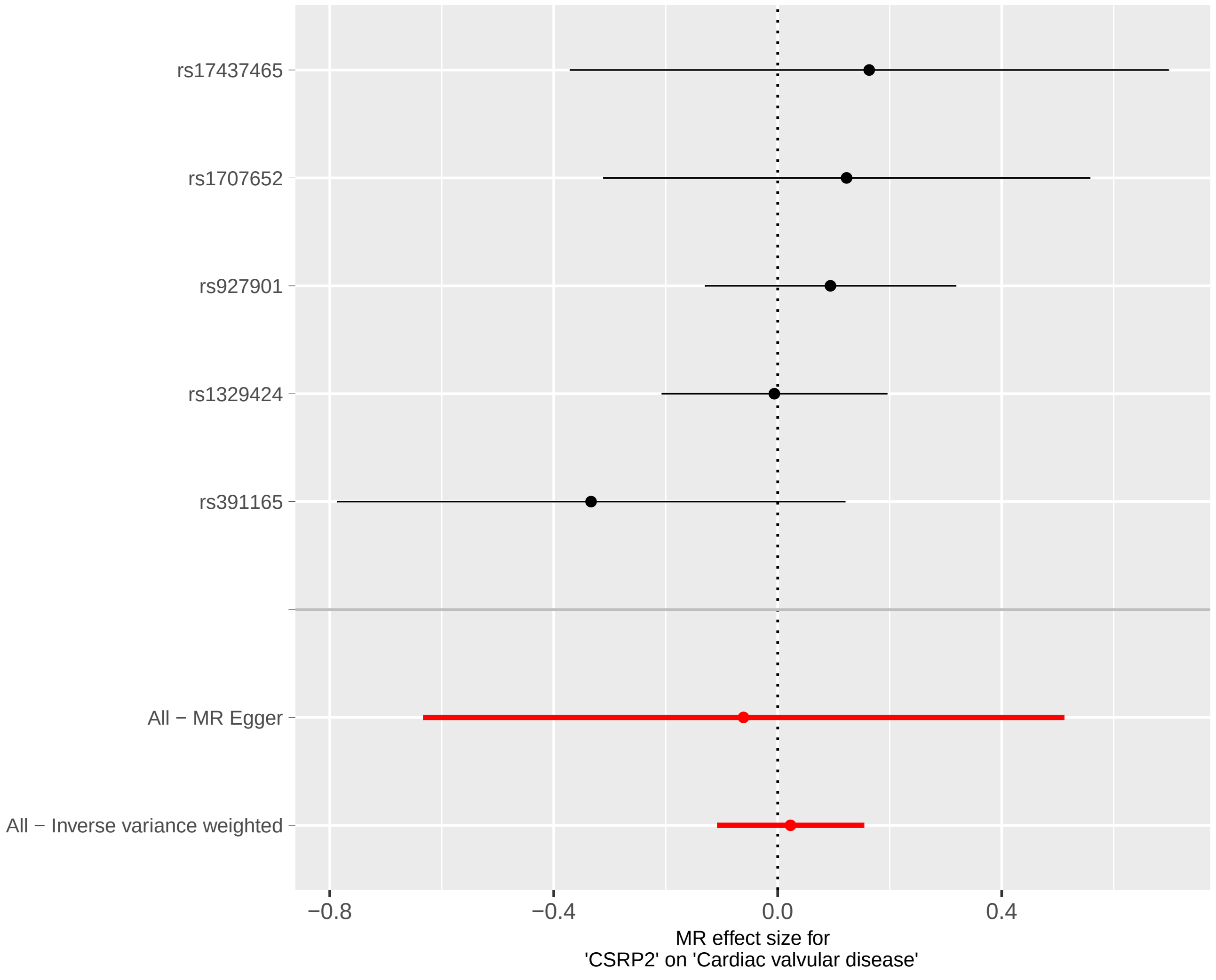

rs17437465
rs1707652
rs927901
rs1329424
rs391165
All − MR Egger
All − Inverse variance weighted
−0.8
−0.4
0.0
0.4
MR effect size for
'CSRP2' on 'Cardiac valvular disease'

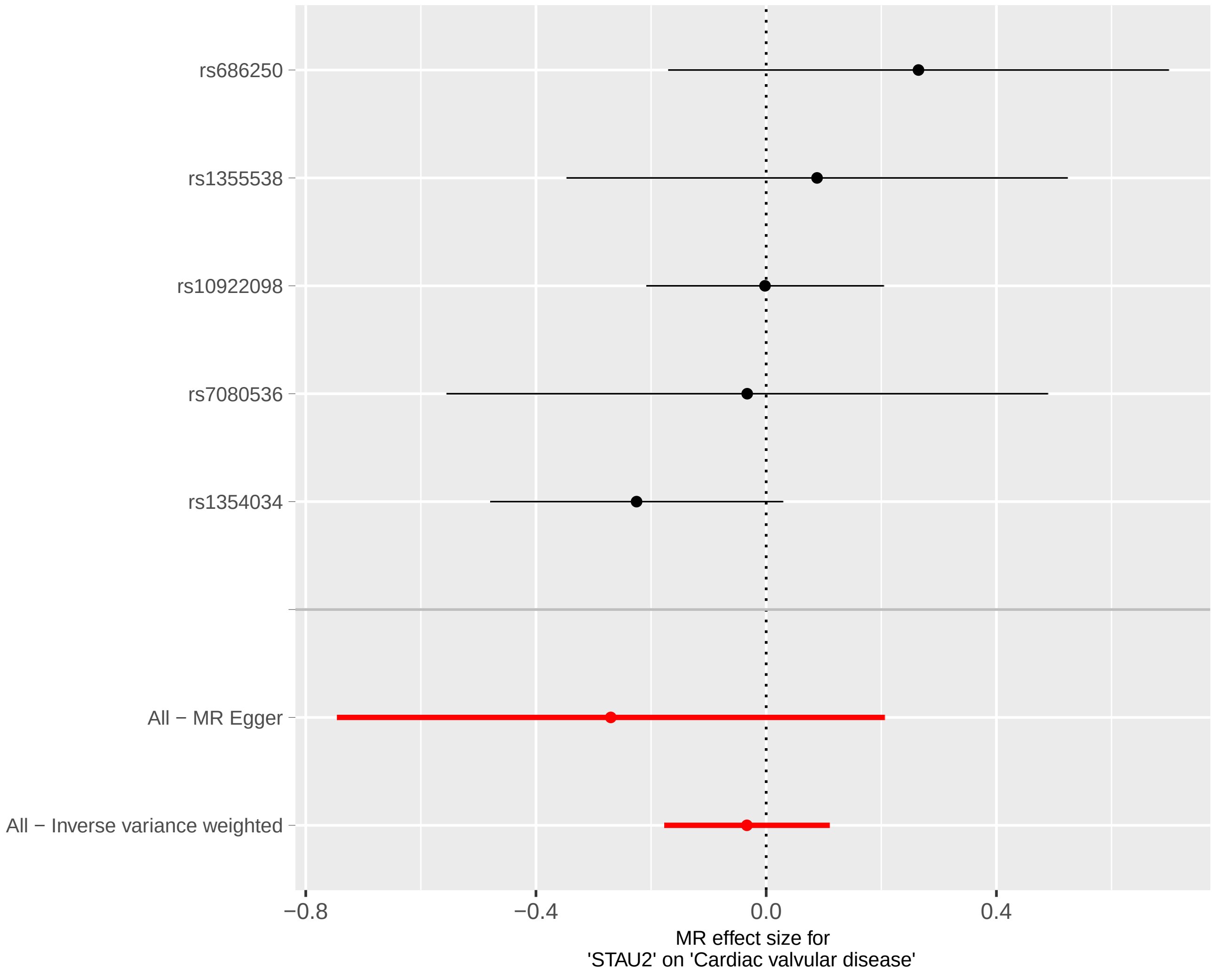
rs686250
rs1355538
rs10922098
rs7080536
rs1354034
All − MR Egger
All − Inverse variance weighted
−0.8
−0.4
0.0
0.4
MR effect size for
'STAU2' on 'Cardiac valvular disease'

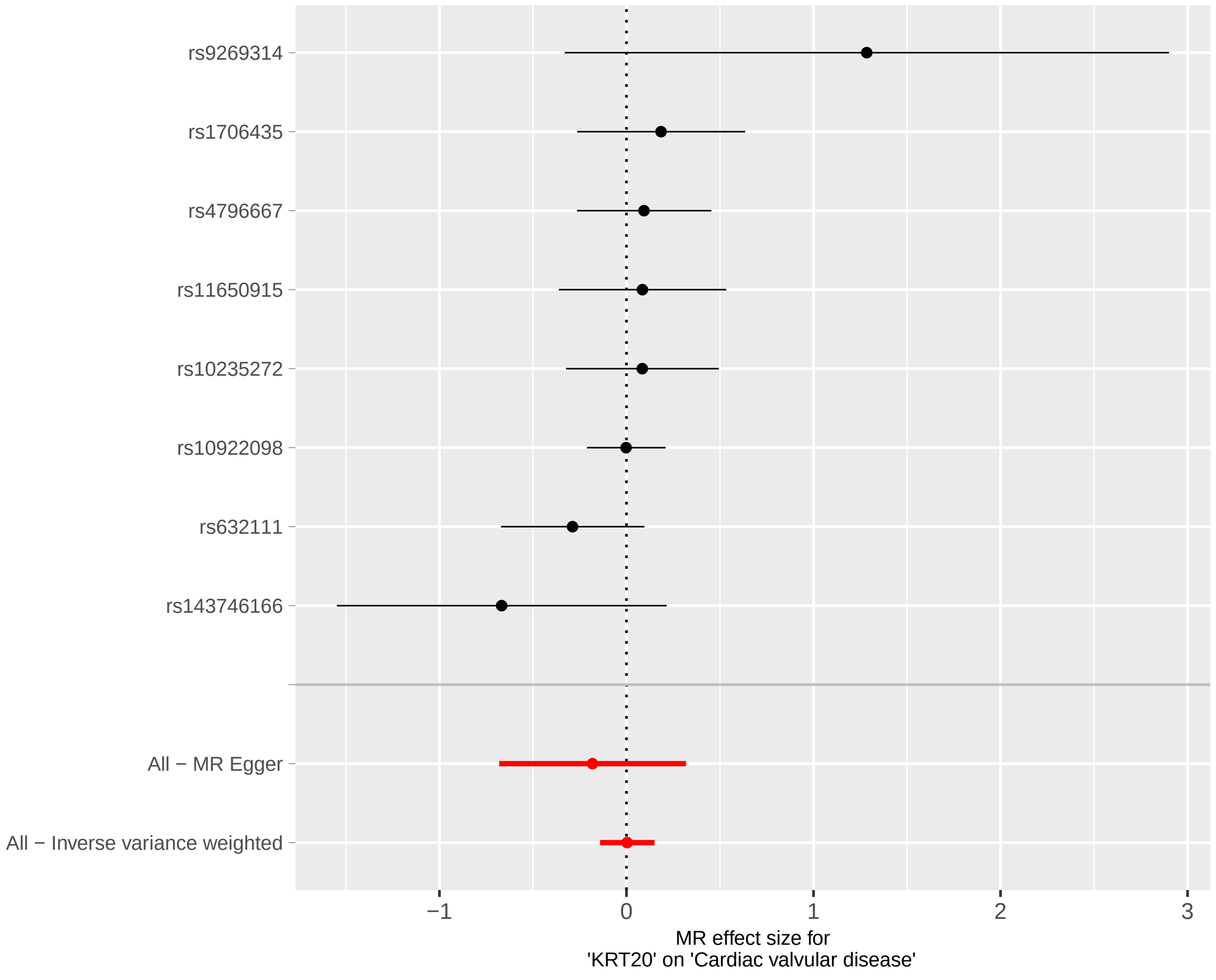
rs9269314
rs1706435
rs4796667
rs11650915
rs10235272
rs10922098
rs632111
rs143746166
All − MR Egger
All − Inverse variance weighted
−1
0
1
2
3
MR effect size for
'KRT20' on 'Cardiac valvular disease'

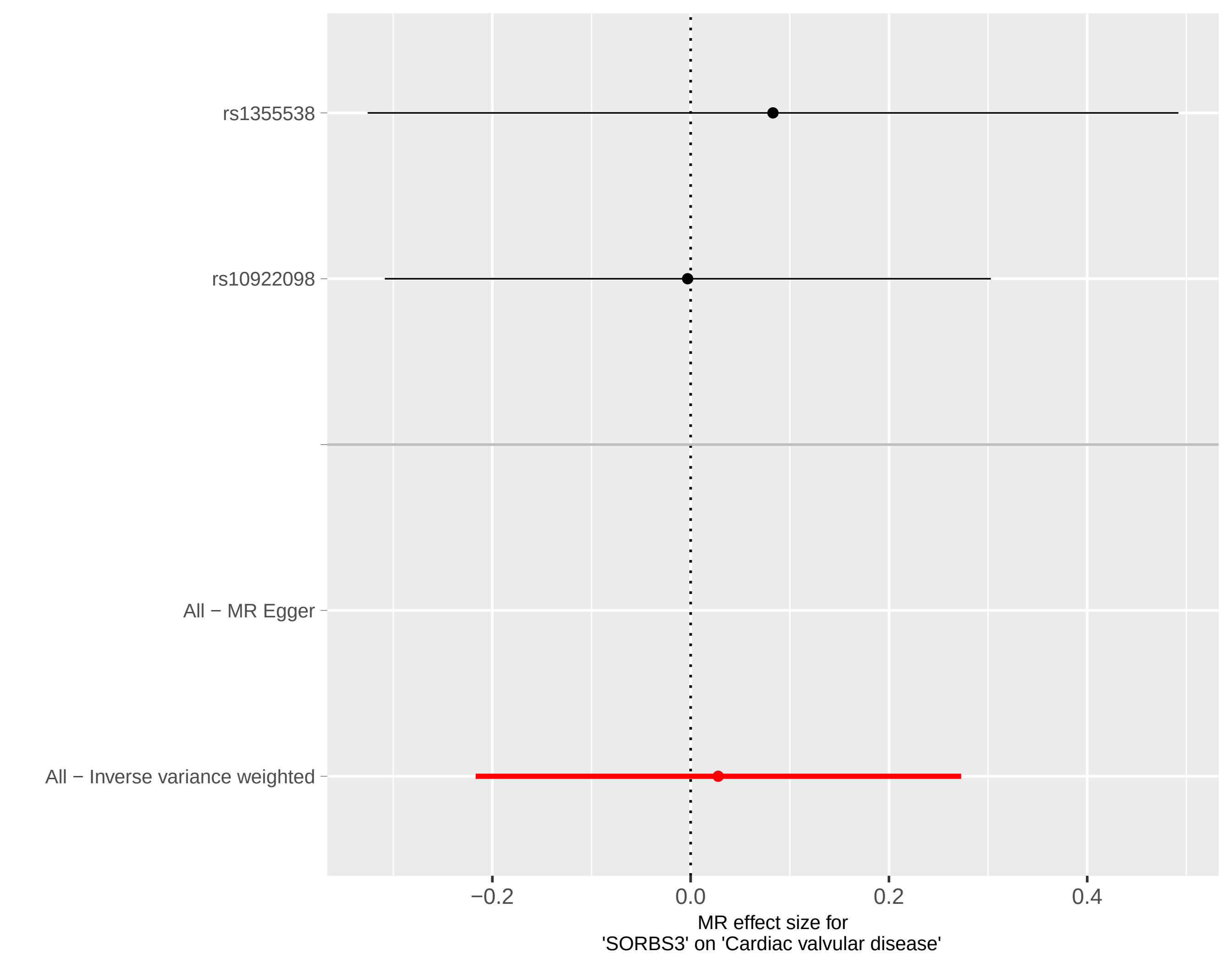
rs1355538
rs10922098
All − MR Egger
All − Inverse variance weighted
−0.2
0.0
0.2
0.4
MR effect size for
'SORBS3' on 'Cardiac valvular disease'

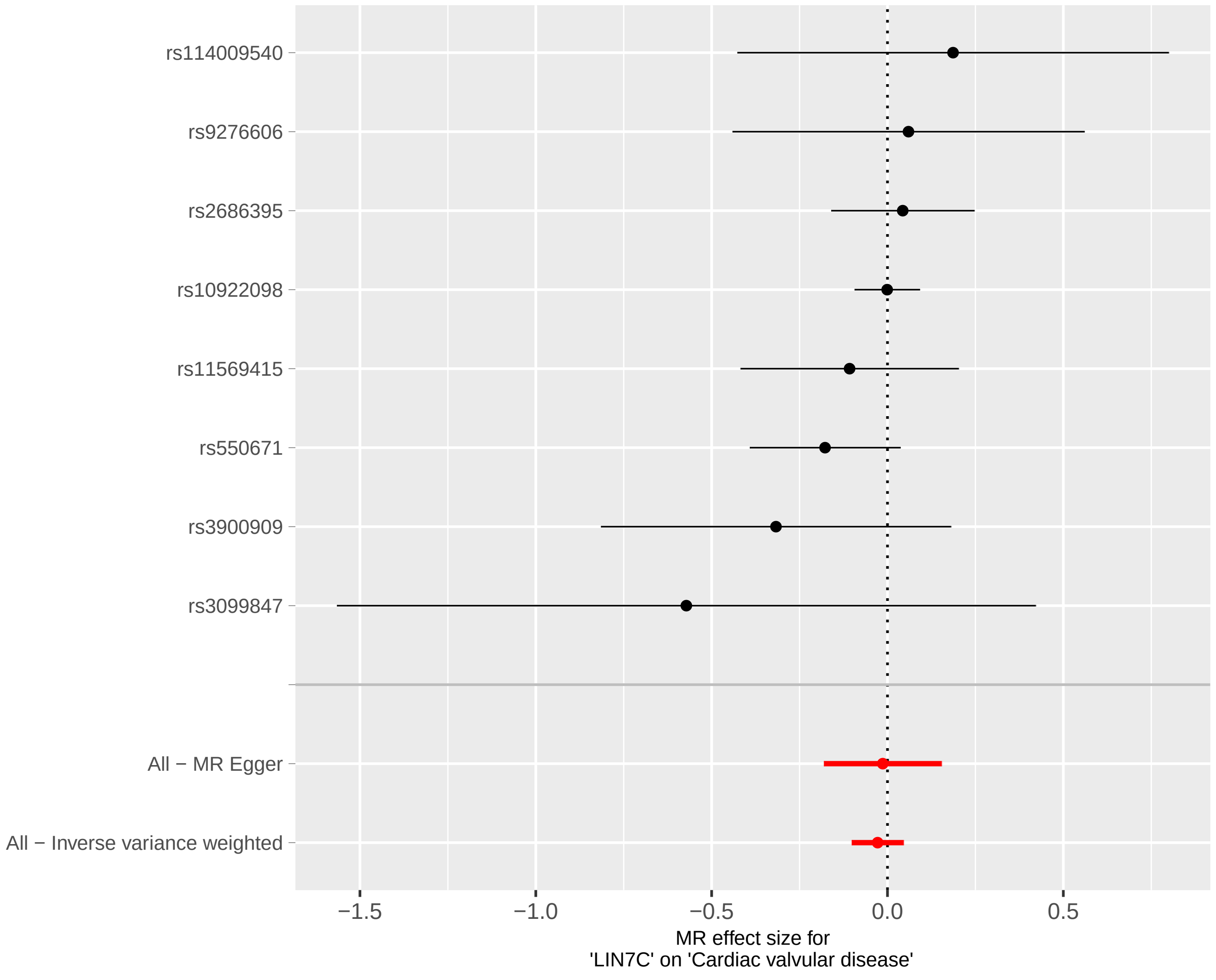
rs114009540
rs9276606
rs2686395
rs10922098
rs11569415
rs550671
rs3900909
rs3099847
All − MR Egger
All − Inverse variance weighted
−1.5
−1.0
−0.5
0.0
0.5
MR effect size for
'LIN7C' on 'Cardiac valvular disease'

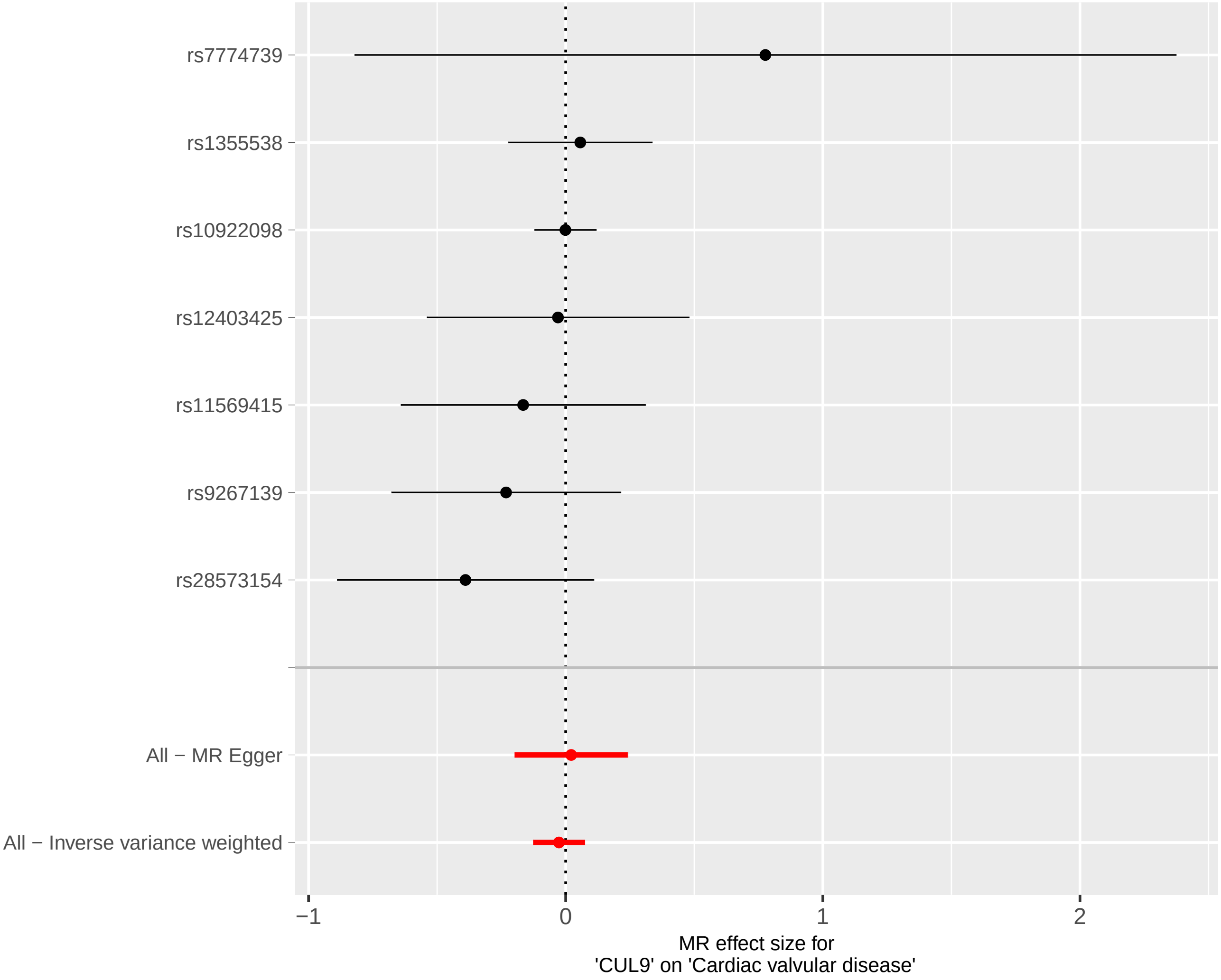

rs7774739
rs1355538
rs10922098
rs12403425
rs11569415
rs9267139
rs28573154
All − MR Egger
All − Inverse variance weighted
−1
0
1
2
MR effect size for
'CUL9' on 'Cardiac valvular disease'

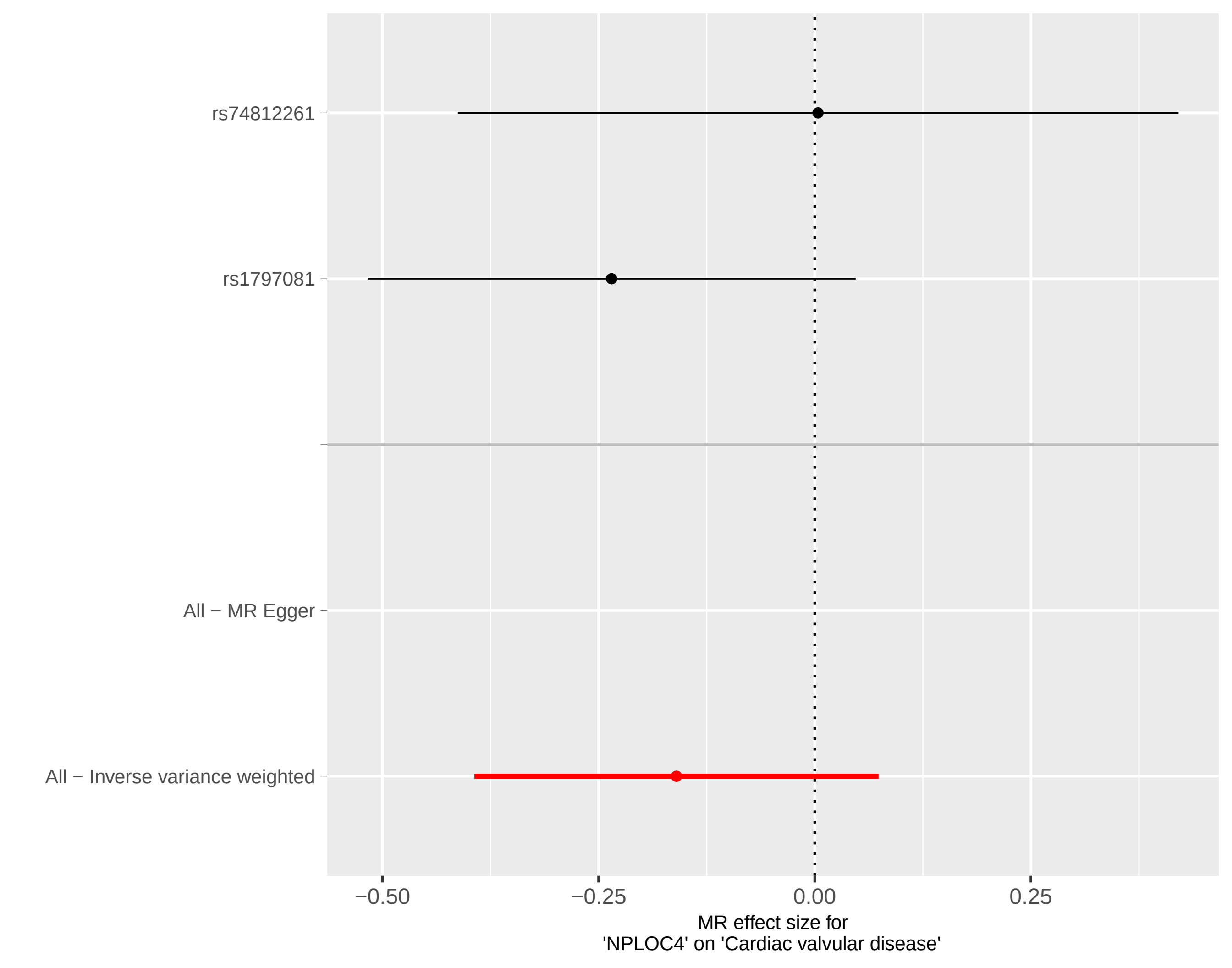
rs74812261
rs1797081
All − MR Egger
All − Inverse variance weighted
−0.50
−0.25
0.00
0.25
MR effect size for
'NPLOC4' on 'Cardiac valvular disease'

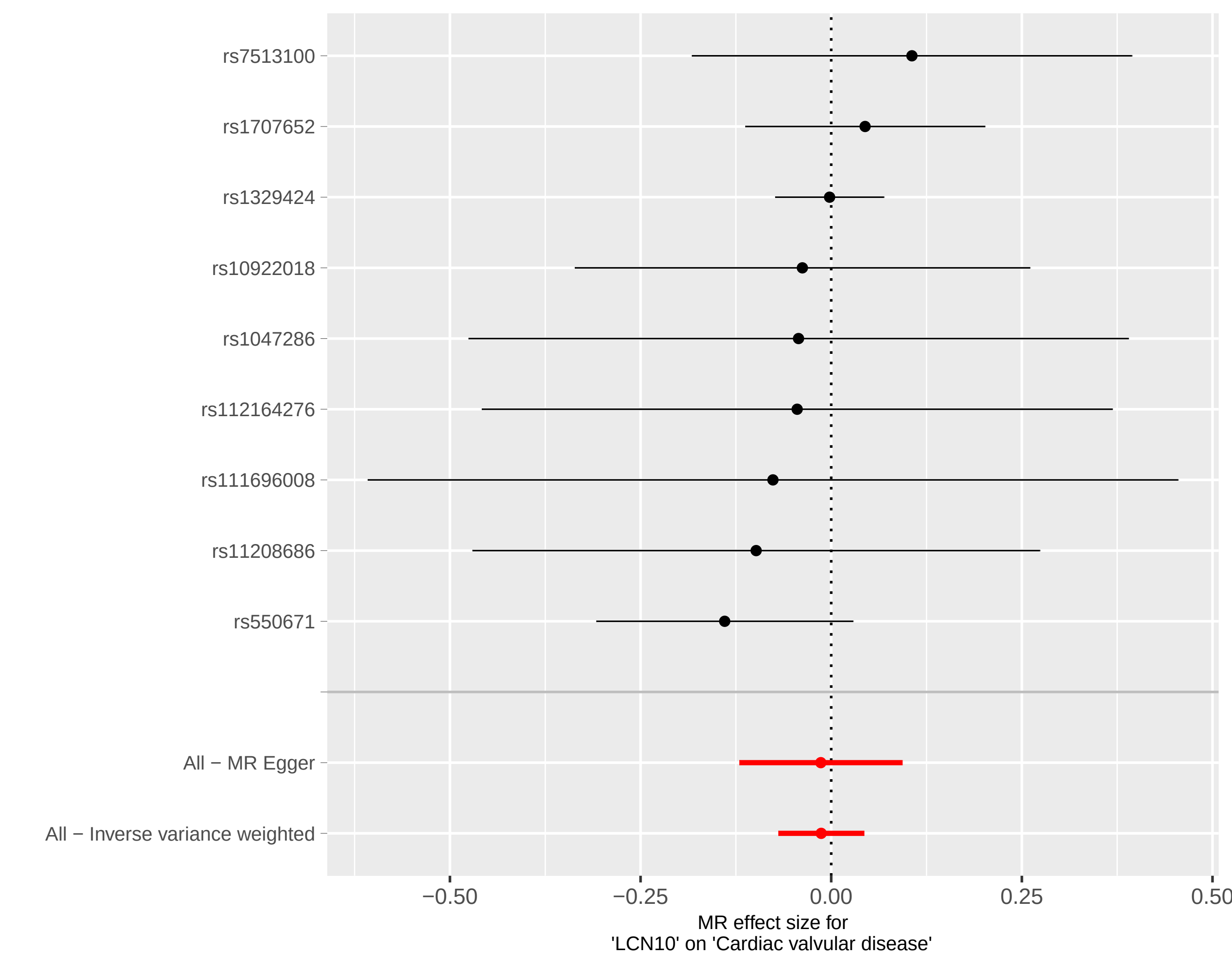

rs7513100
rs1707652
rs1329424
rs10922018
rs1047286
rs112164276
rs111696008
rs11208686
rs550671
All − MR Egger
All − Inverse variance weighted
−0.50
−0.25
0.00
0.25
0.50
MR effect size for
'LCN10' on 'Cardiac valvular disease'

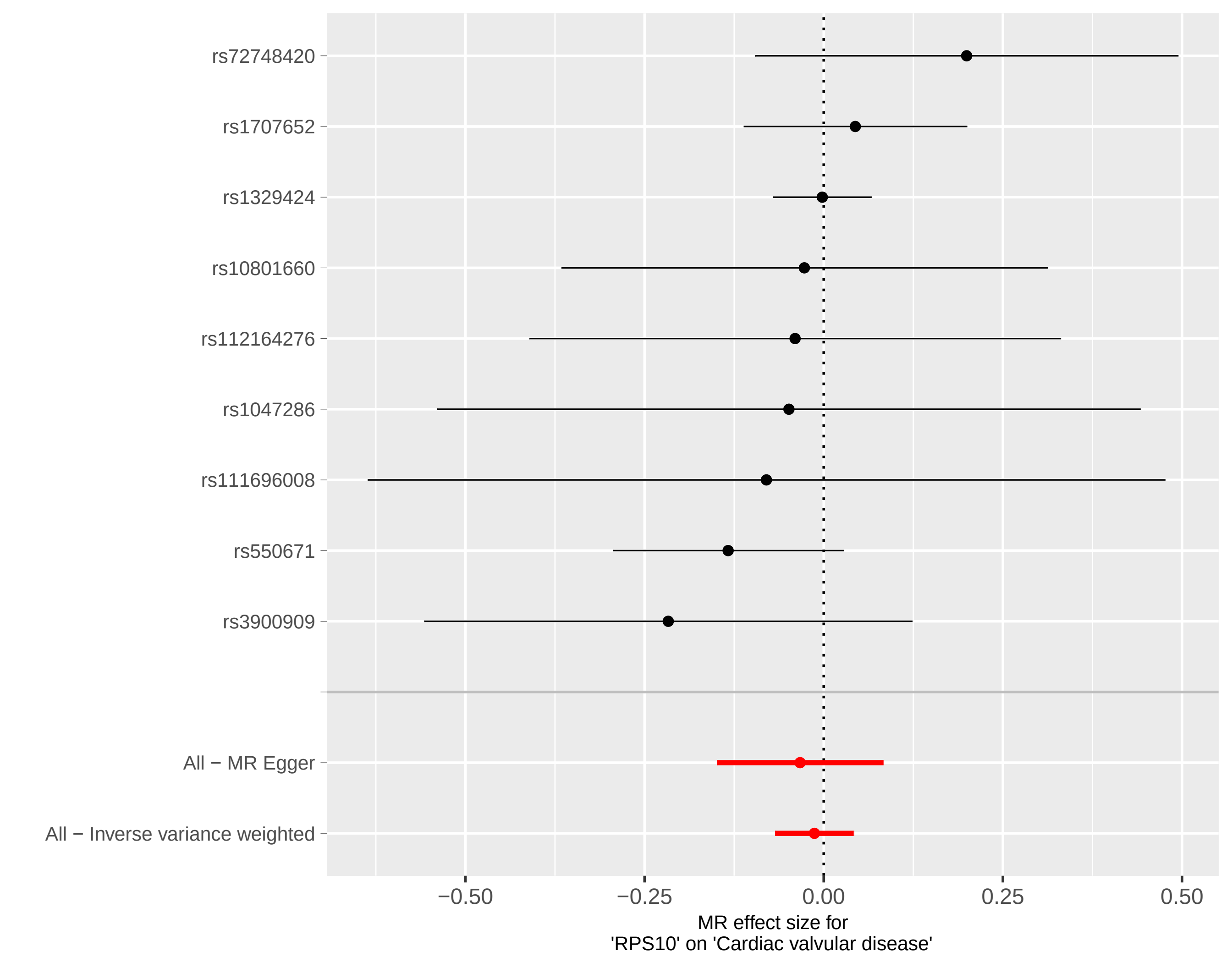

rs72748420
rs1707652
rs1329424
rs10801660
rs112164276
rs1047286
rs111696008
rs550671
rs3900909
All − MR Egger
All − Inverse variance weighted
−0.50
−0.25
0.00
0.25
0.50
MR effect size for
'RPS10' on 'Cardiac valvular disease'

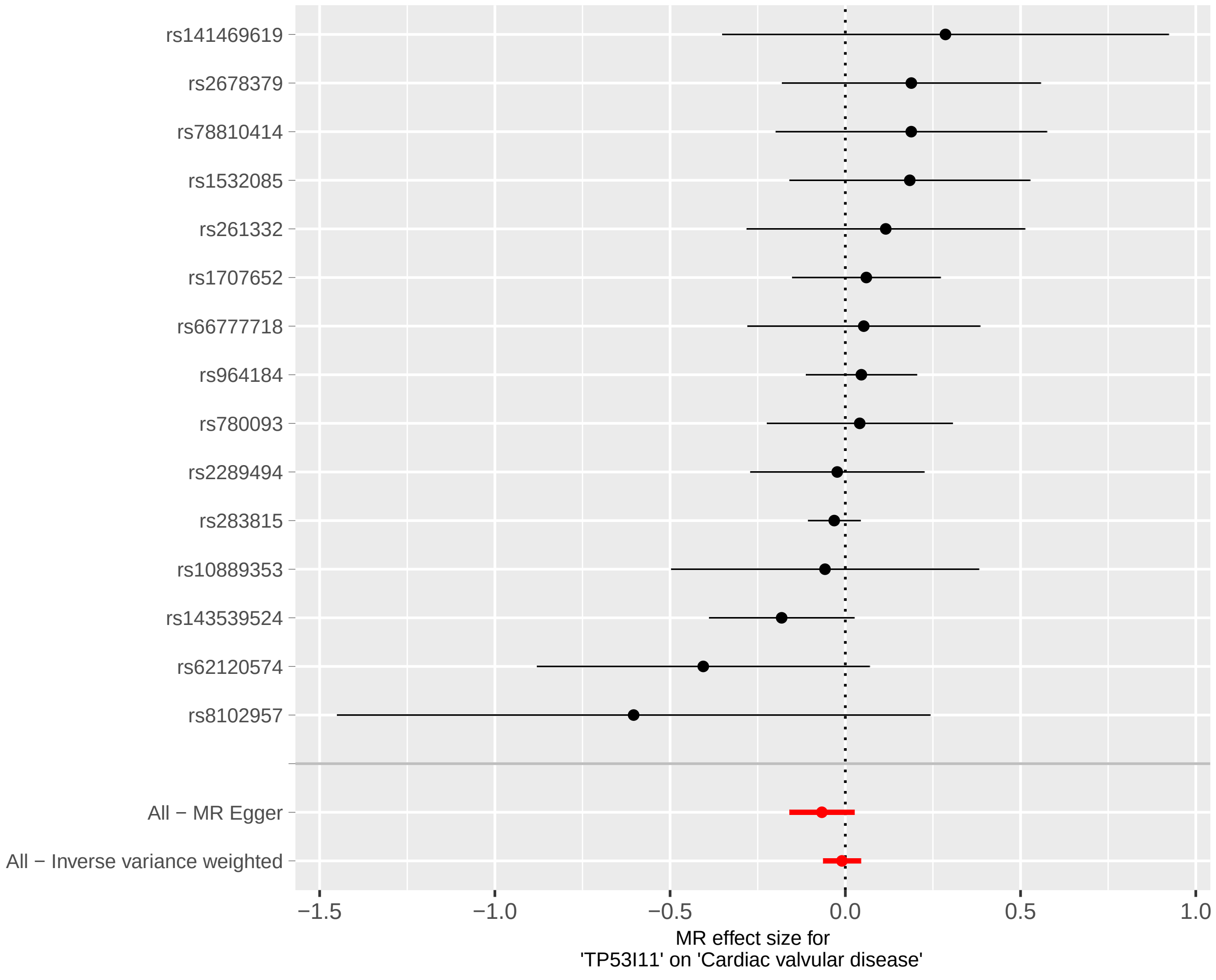

rs141469619
rs2678379
rs78810414
rs1532085
rs261332
rs1707652
rs66777718
rs964184
rs780093
rs2289494
rs283815
rs10889353
rs143539524
rs62120574
rs8102957
All − MR Egger
All − Inverse variance weighted
−1.5
−1.0
−0.5
0.0
0.5
1.0
MR effect size for
'TP53I11' on 'Cardiac valvular disease'

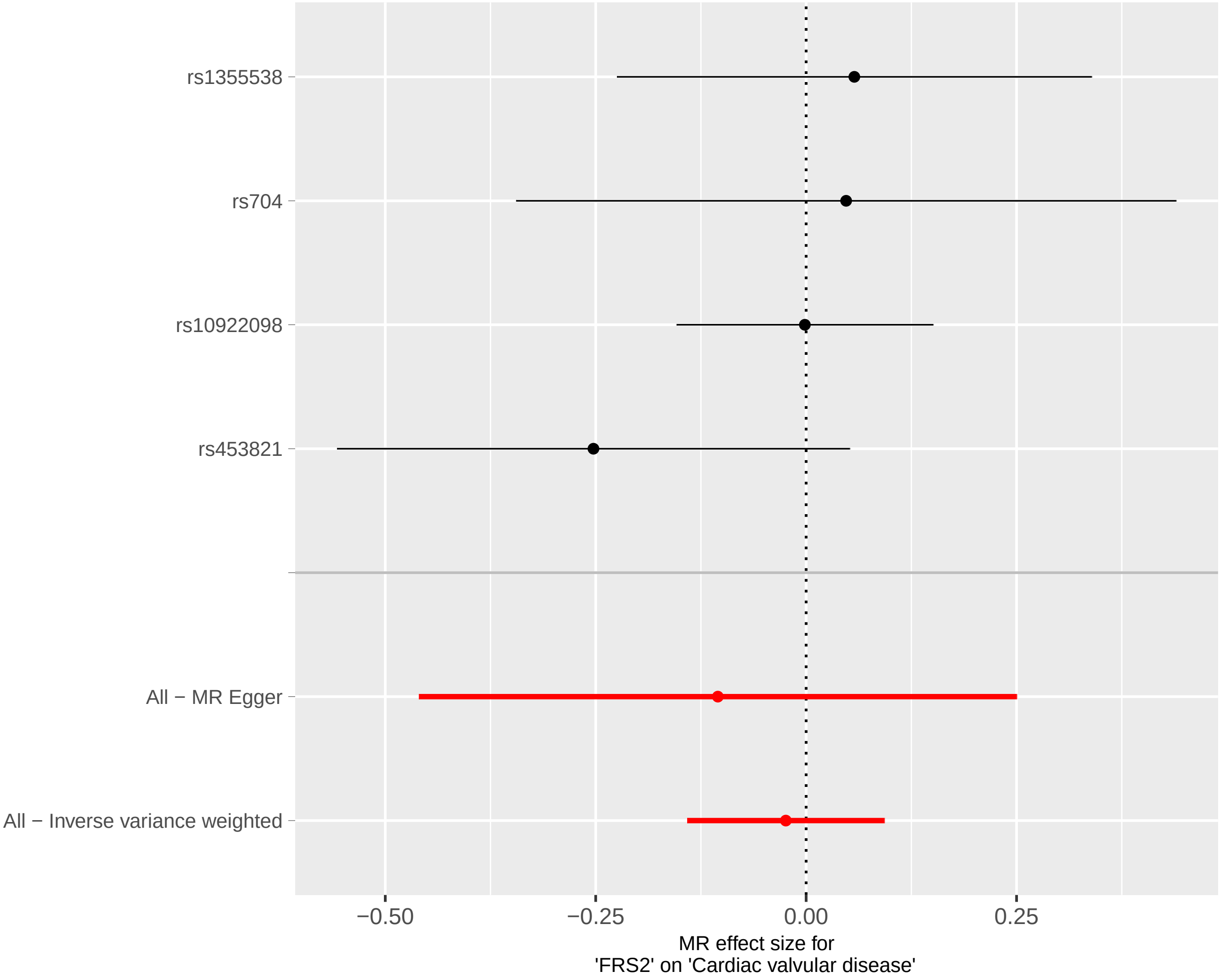

rs1355538
rs704
rs10922098
rs453821
All − MR Egger
All − Inverse variance weighted
−0.50
−0.25
0.00
0.25
MR effect size for
'FRS2' on 'Cardiac valvular disease'

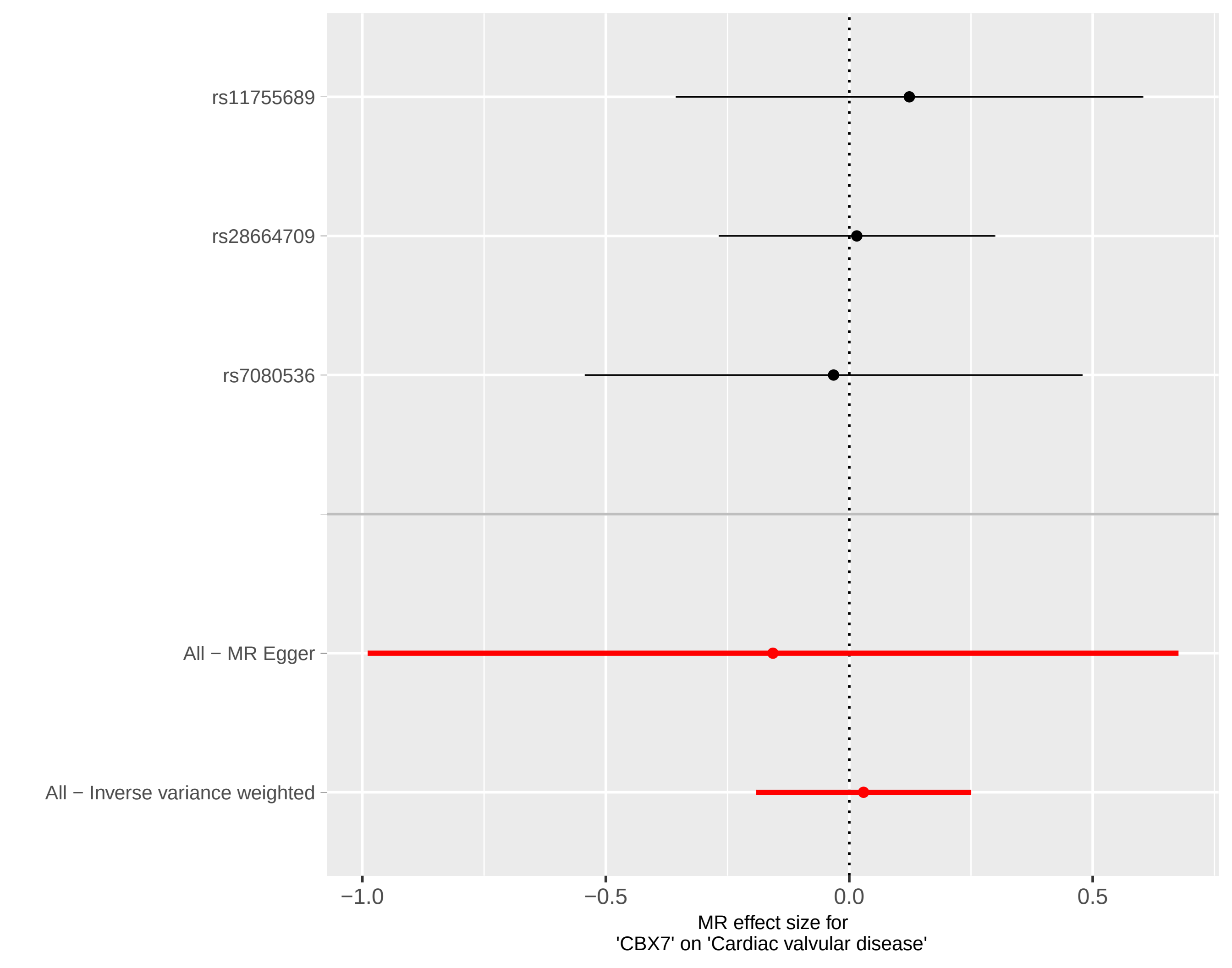
rs11755689
rs28664709
rs7080536
All − MR Egger
All − Inverse variance weighted
−1.0
−0.5
0.0
0.5
MR effect size for
'CBX7' on 'Cardiac valvular disease'

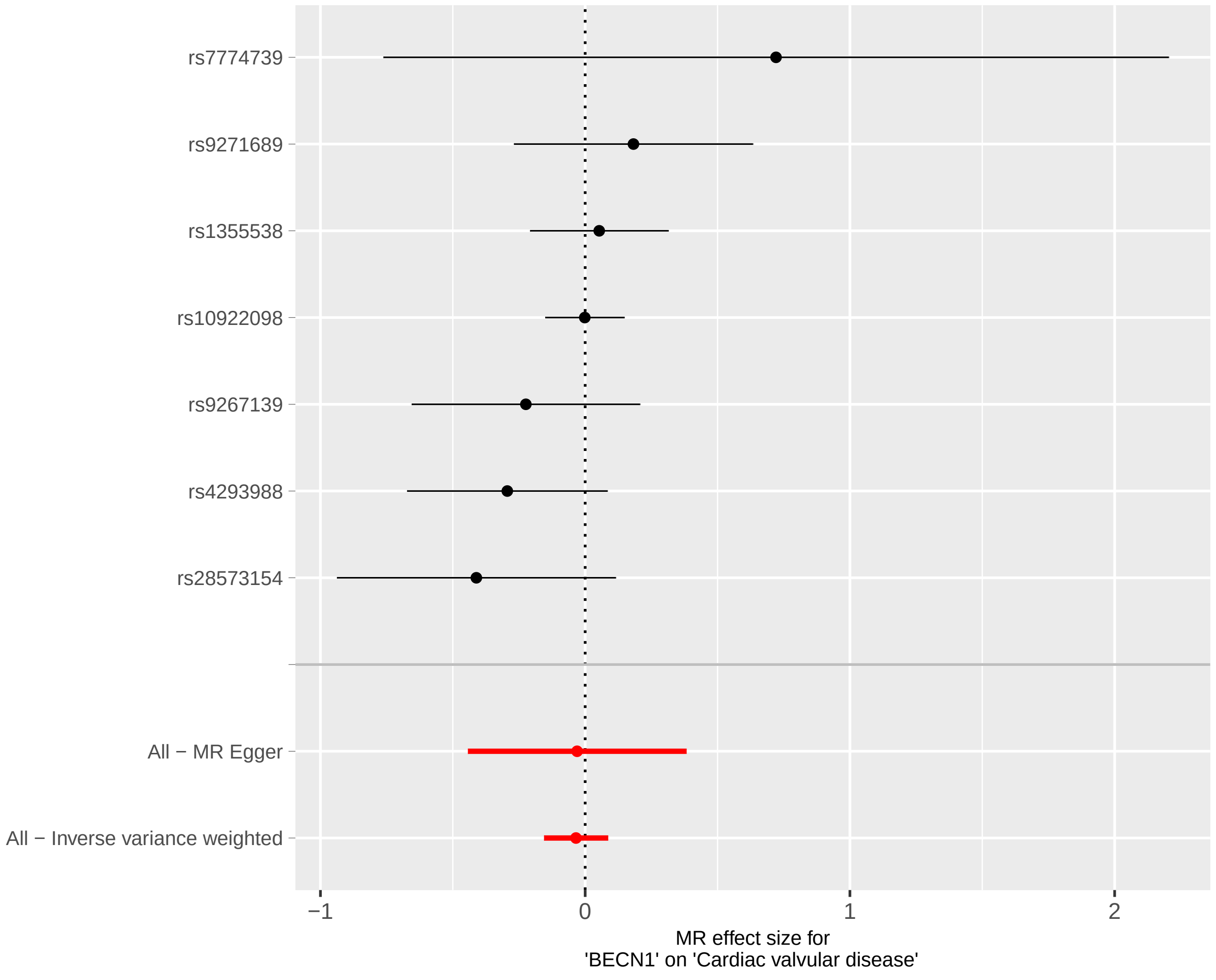
rs7774739
rs9271689
rs1355538
rs10922098
rs9267139
rs4293988
rs28573154
All − MR Egger
All − Inverse variance weighted
−1
0
1
2
MR effect size for
'BECN1' on 'Cardiac valvular disease'

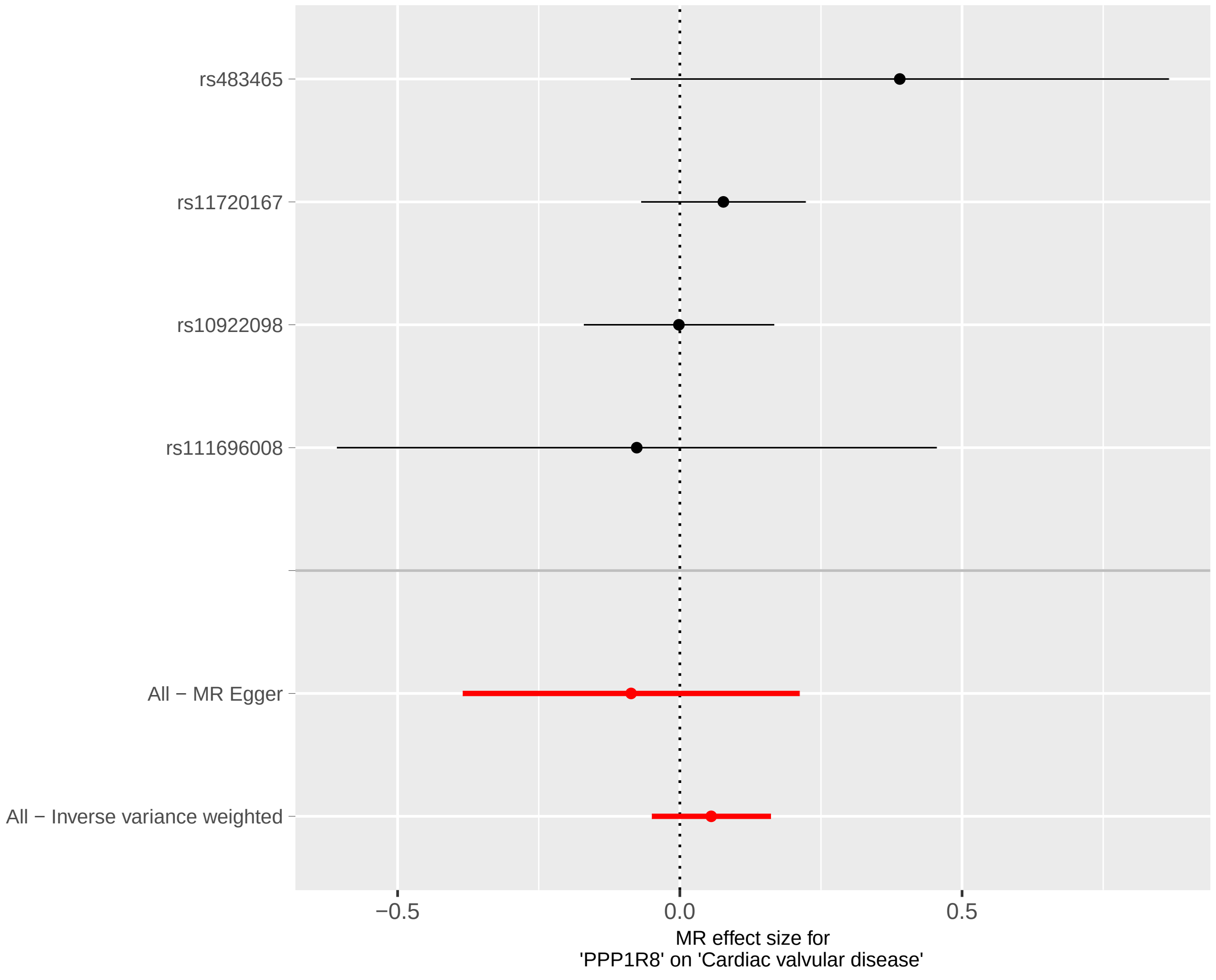
rs483465
rs11720167
rs10922098
rs111696008
All − MR Egger
All − Inverse variance weighted
−0.5
0.0
0.5
MR effect size for
'PPP1R8' on 'Cardiac valvular disease'

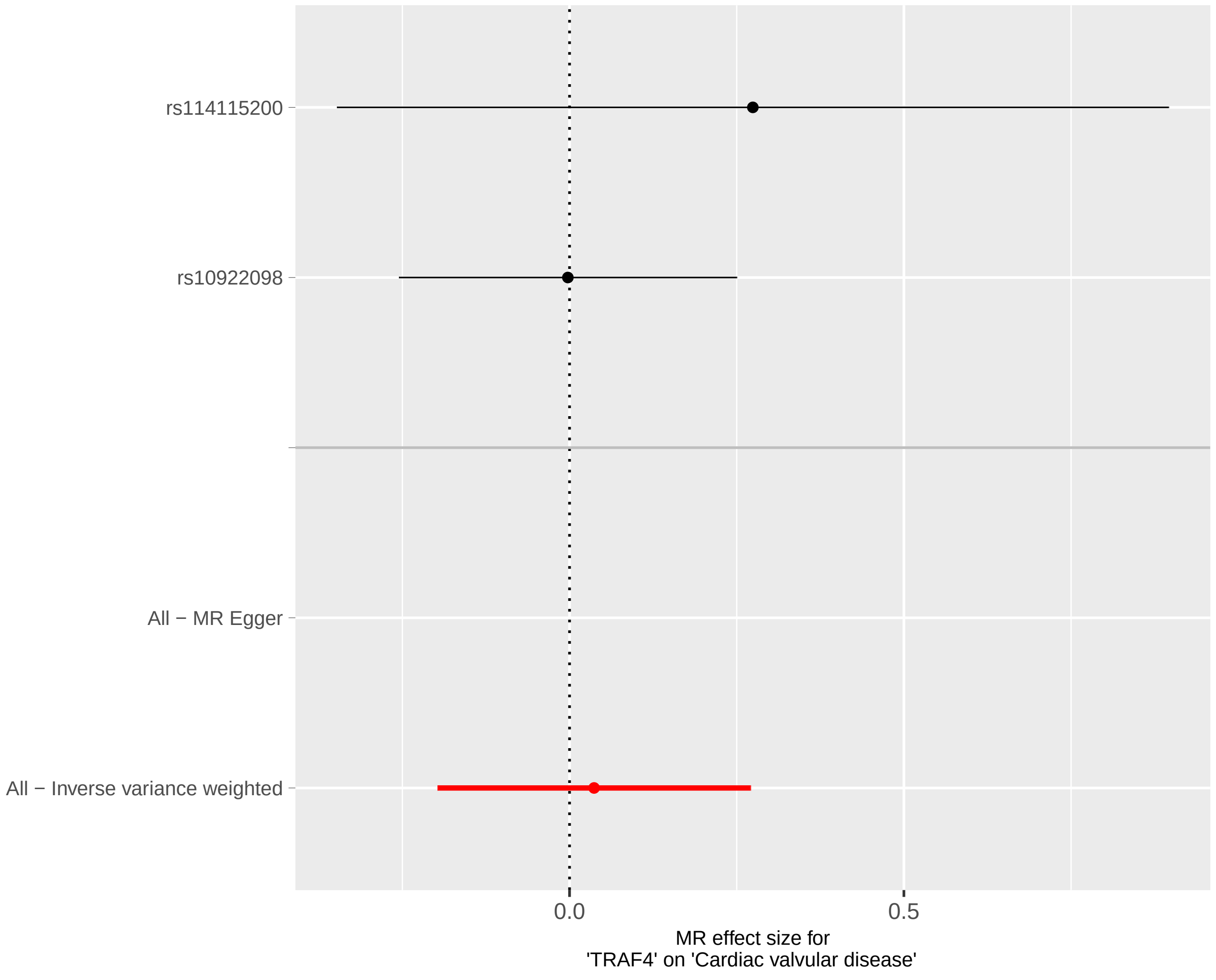

rs114115200
rs10922098
All − MR Egger
All − Inverse variance weighted
0.0
0.5
MR effect size for
'TRAF4' on 'Cardiac valvular disease'

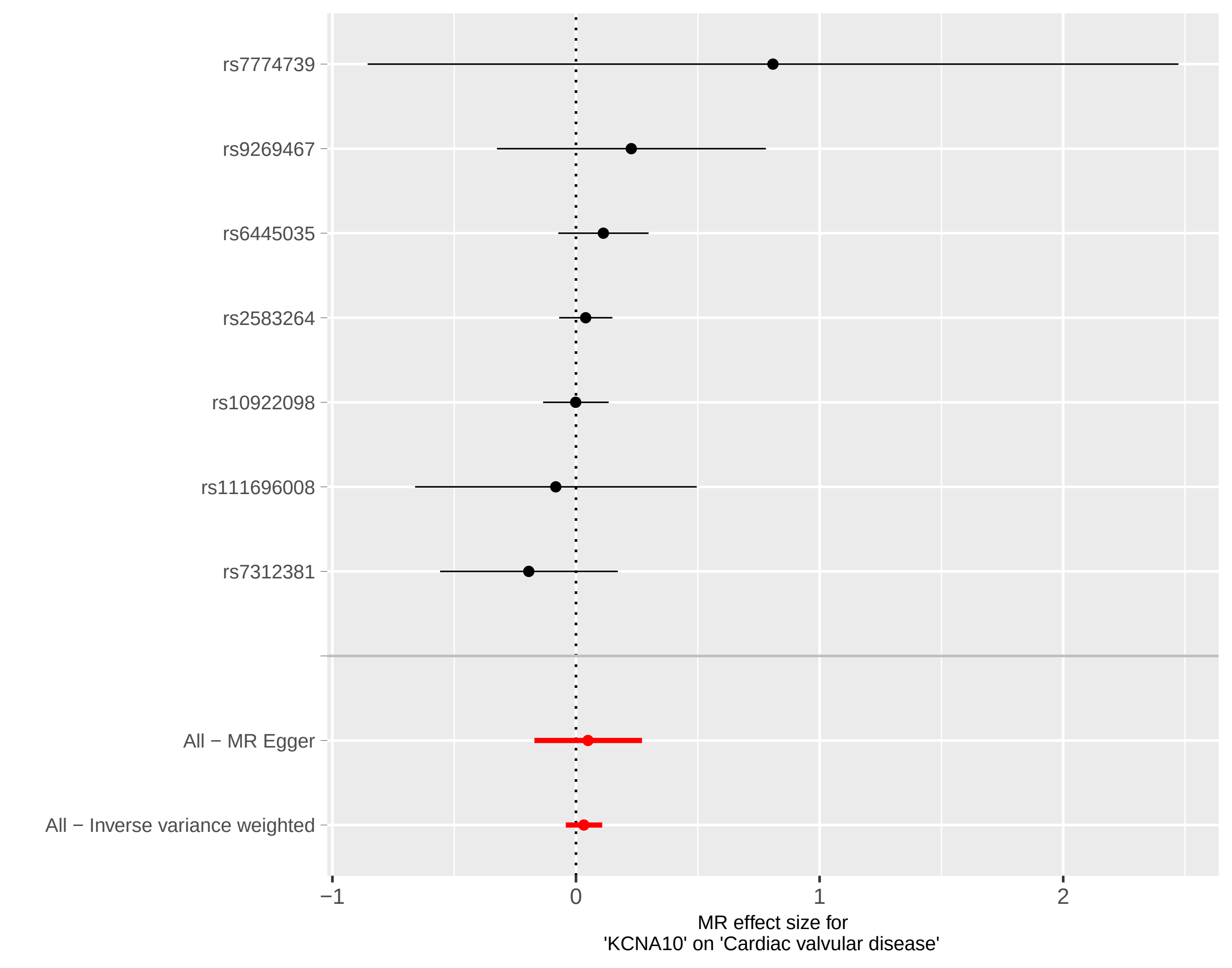

rs7774739
rs9269467
rs6445035
rs2583264
rs10922098
rs111696008
rs7312381
All − MR Egger
All − Inverse variance weighted
−1
0
1
2
MR effect size for
'KCNA10' on 'Cardiac valvular disease'

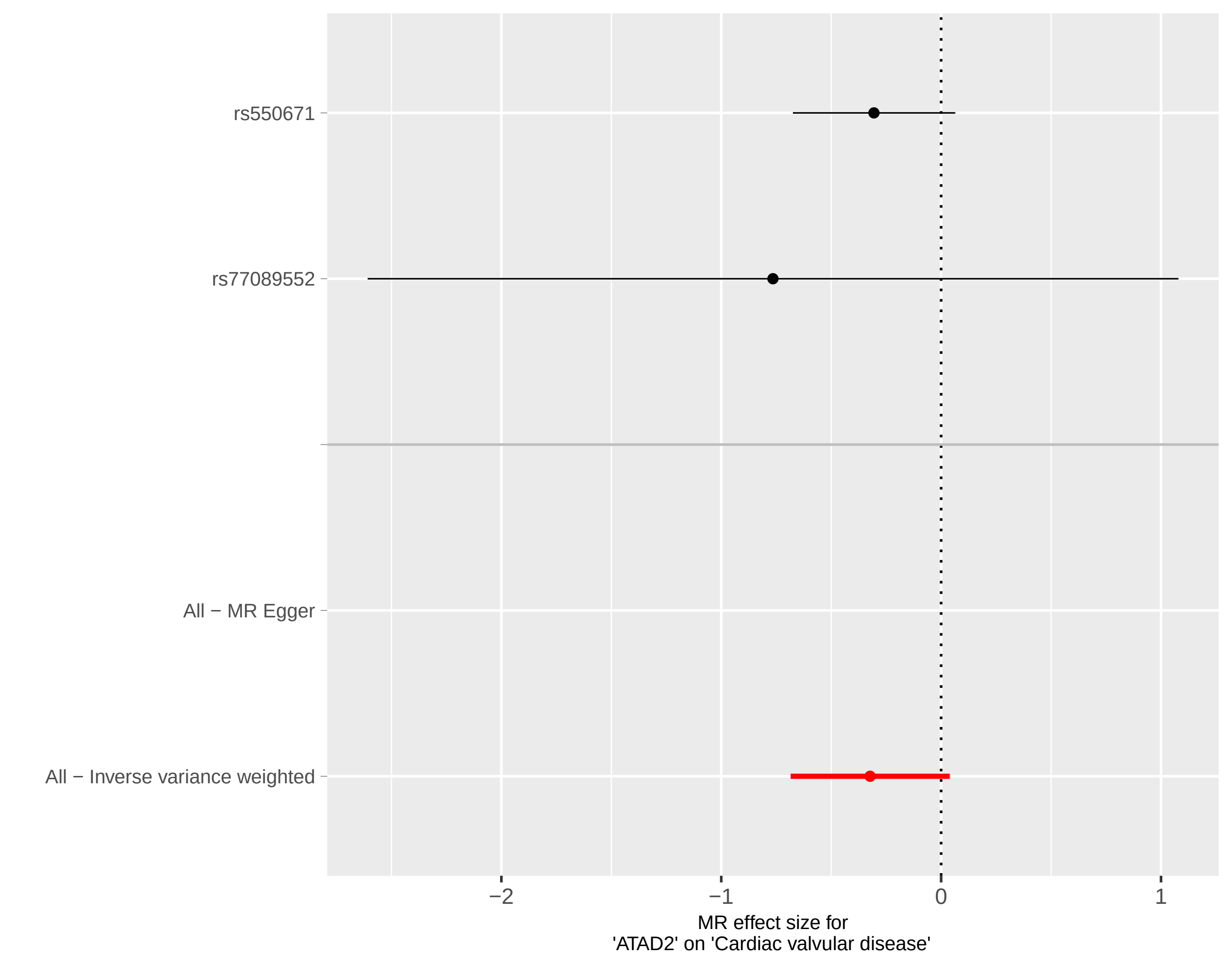

rs550671
rs77089552
All − MR Egger
All − Inverse variance weighted
−2
−1
0
1
MR effect size for
'ATAD2' on 'Cardiac valvular disease'

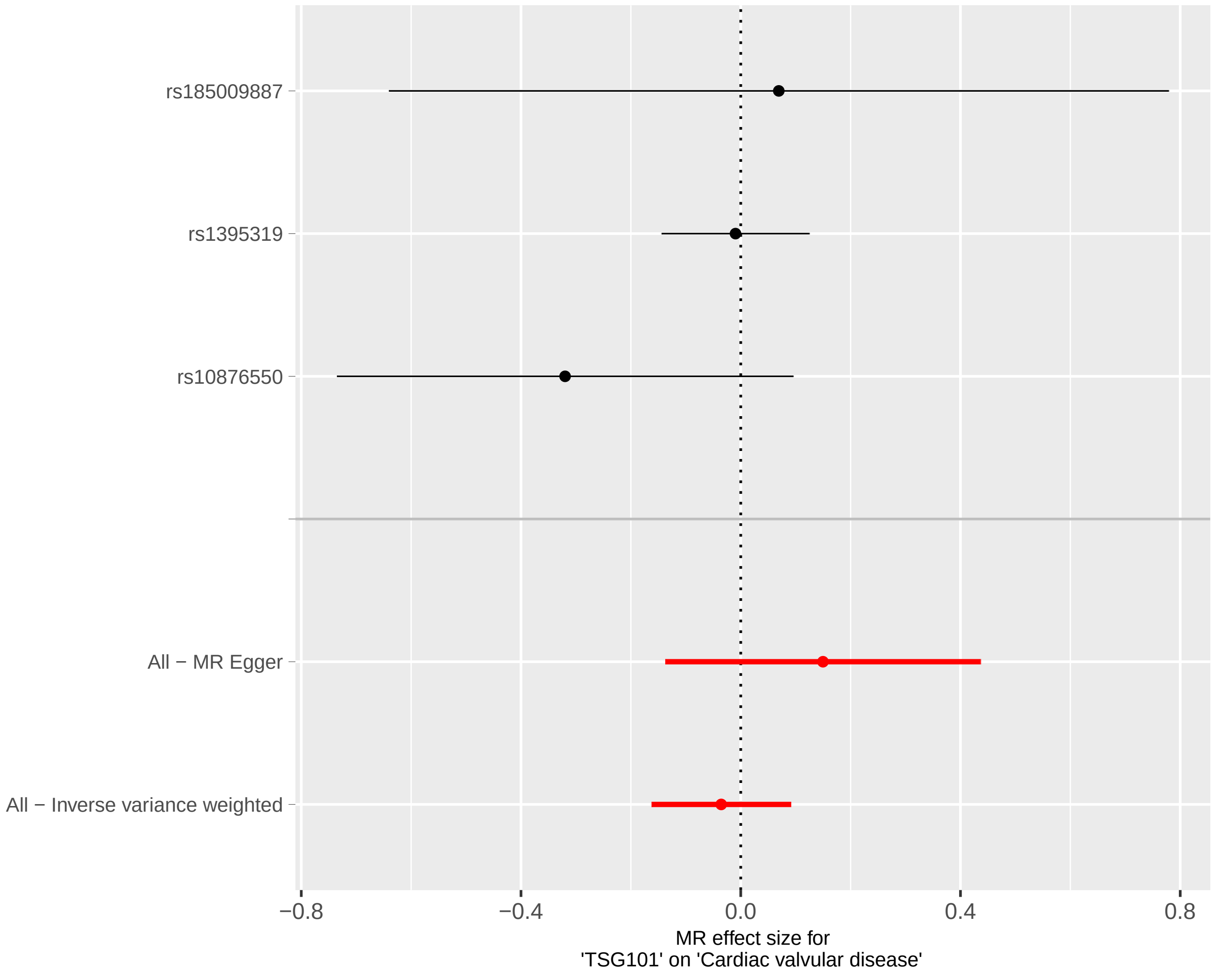
rs185009887
rs1395319
rs10876550
All − MR Egger
All − Inverse variance weighted
−0.8
−0.4
0.0
0.4
0.8
MR effect size for
'TSG101' on 'Cardiac valvular disease'

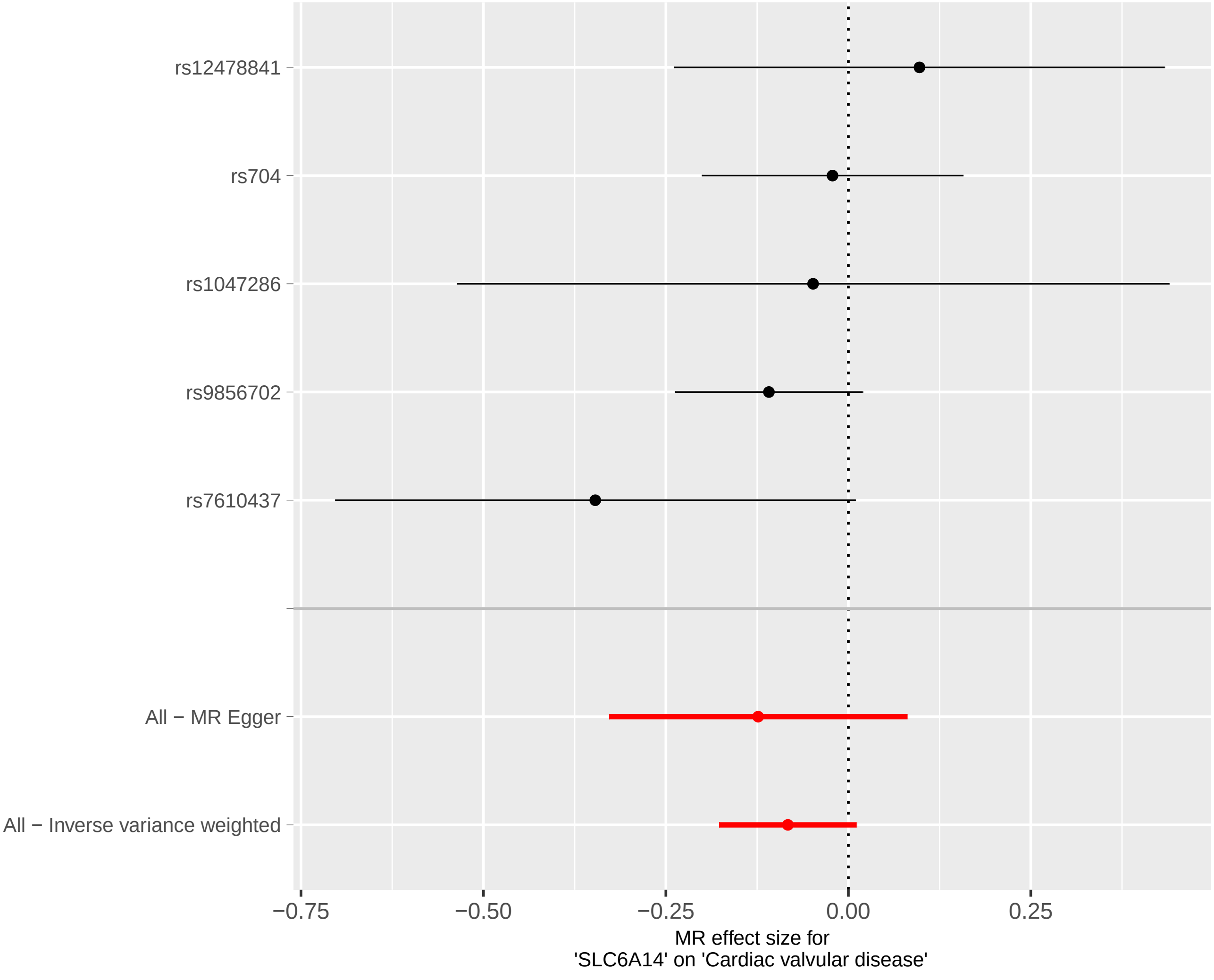
rs12478841
rs704
rs1047286
rs9856702
rs7610437
All − MR Egger
All − Inverse variance weighted
−0.75
−0.50
−0.25
0.00
0.25
MR effect size for
'SLC6A14' on 'Cardiac valvular disease'

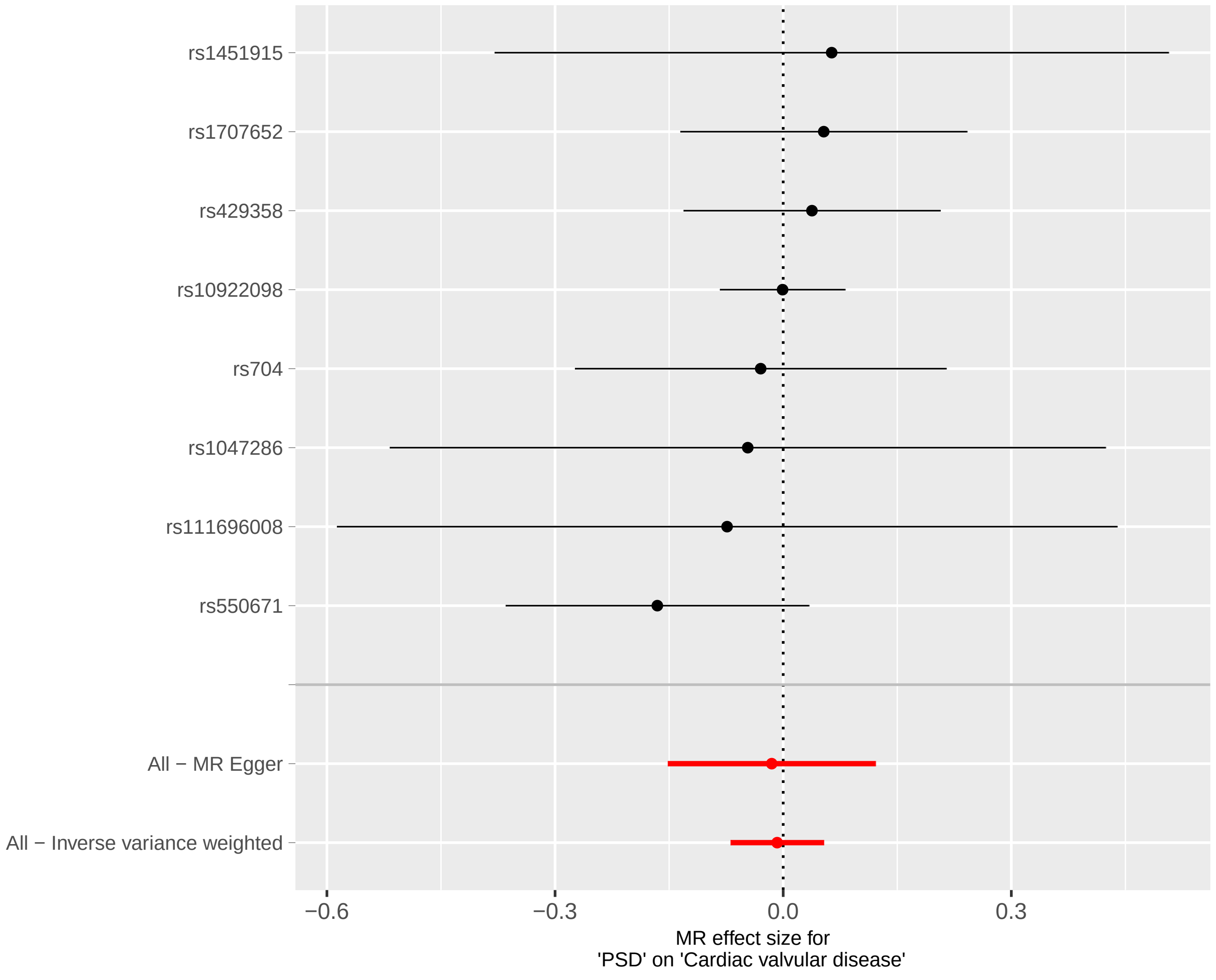
rs1451915
rs1707652
rs429358
rs10922098
rs704
rs1047286
rs111696008
rs550671
All − MR Egger
All − Inverse variance weighted
−0.6
−0.3
0.0
0.3
MR effect size for
'PSD' on 'Cardiac valvular disease'

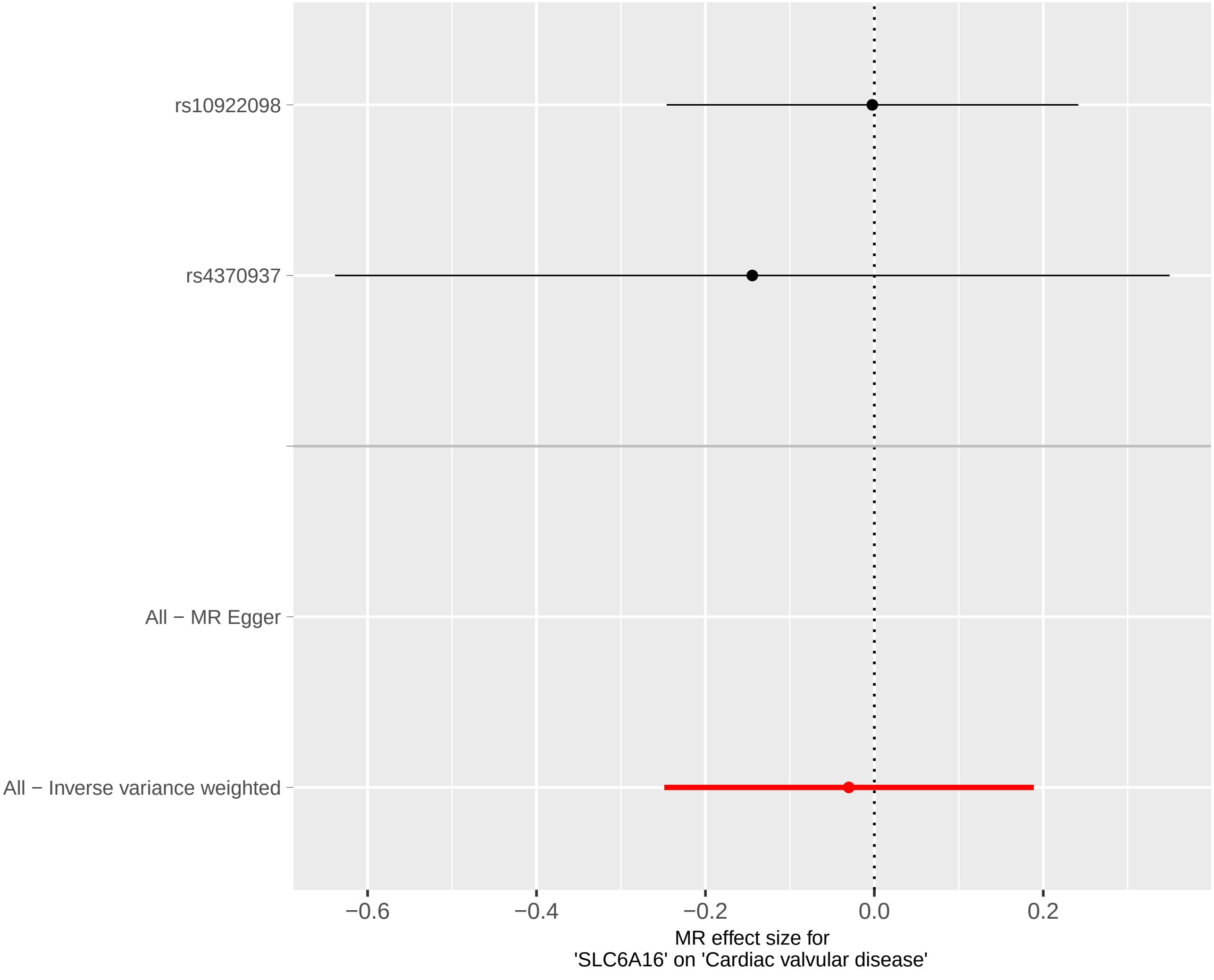
rs10922098
rs4370937
All − MR Egger
All − Inverse variance weighted
−0.6
−0.4
−0.2
0.0
0.2
MR effect size for
'SLC6A16' on 'Cardiac valvular disease'

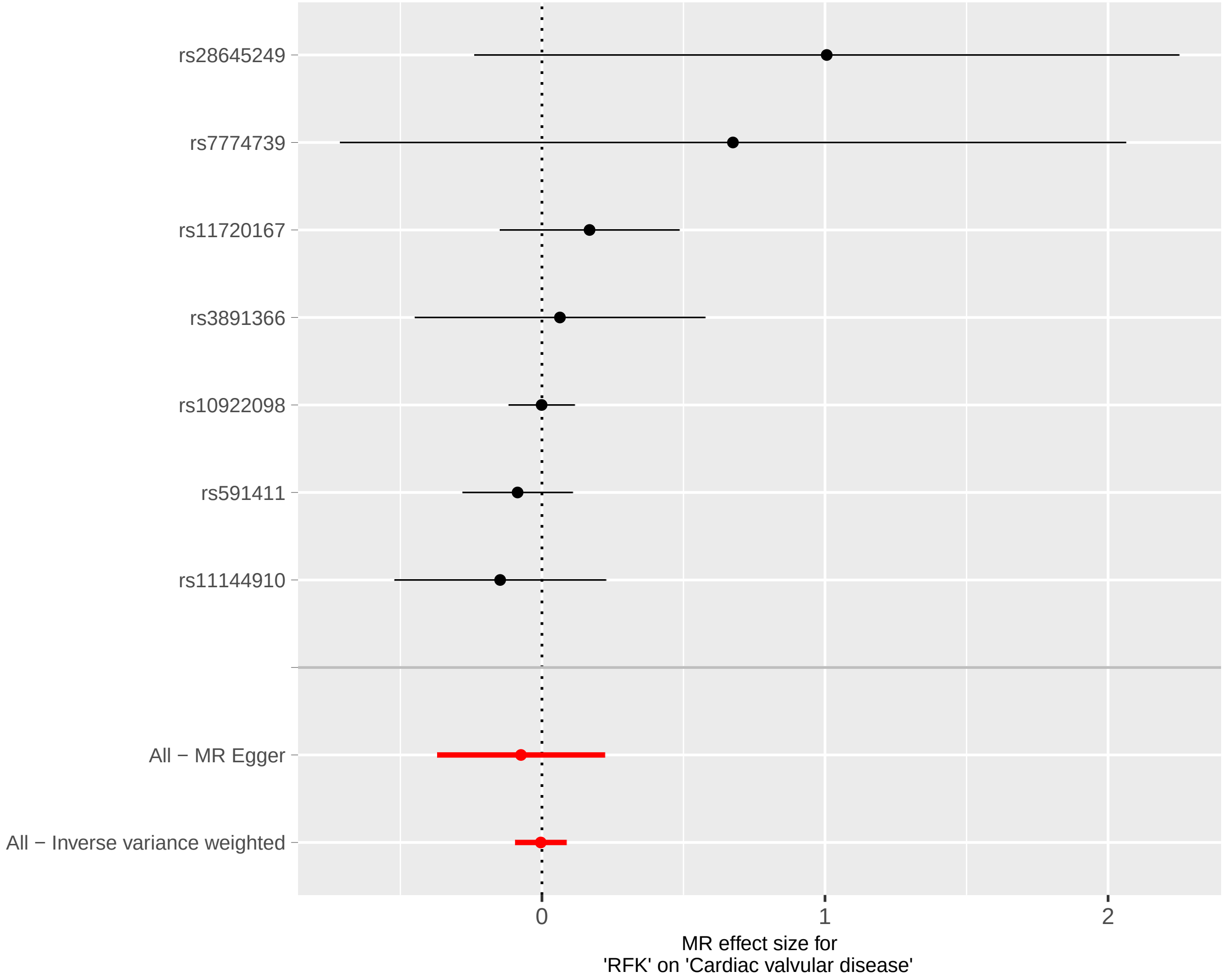
rs28645249
rs7774739
rs11720167
rs3891366
rs10922098
rs591411
rs11144910
All − MR Egger
All − Inverse variance weighted
0
1
2
MR effect size for
'RFK' on 'Cardiac valvular disease'

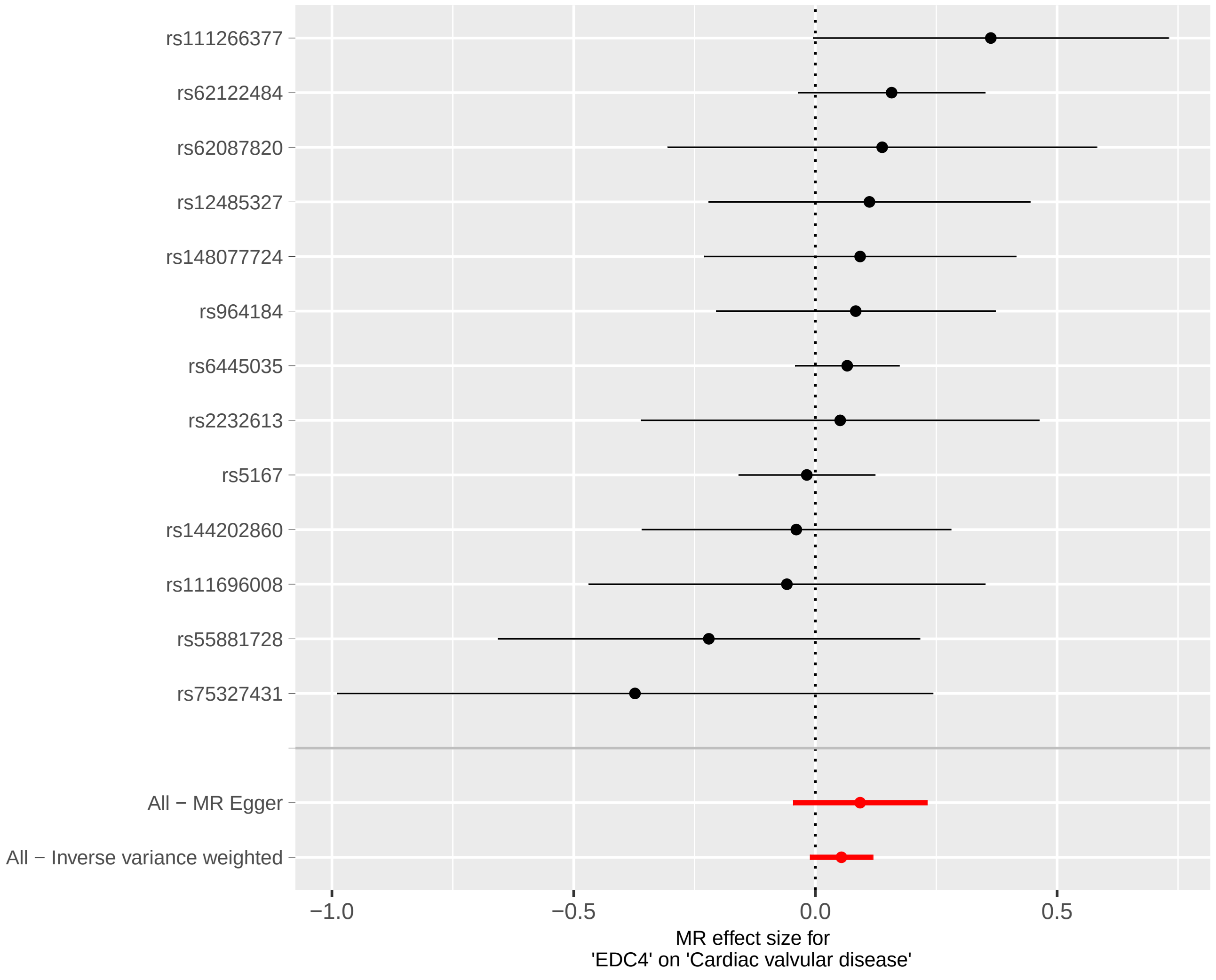
rs111266377
rs62122484
rs62087820
rs12485327
rs148077724
rs964184
rs6445035
rs2232613
rs5167
rs144202860
rs111696008
rs55881728
rs75327431
All − MR Egger
All − Inverse variance weighted
−1.0
−0.5
0.0
0.5
MR effect size for
'EDC4' on 'Cardiac valvular disease'

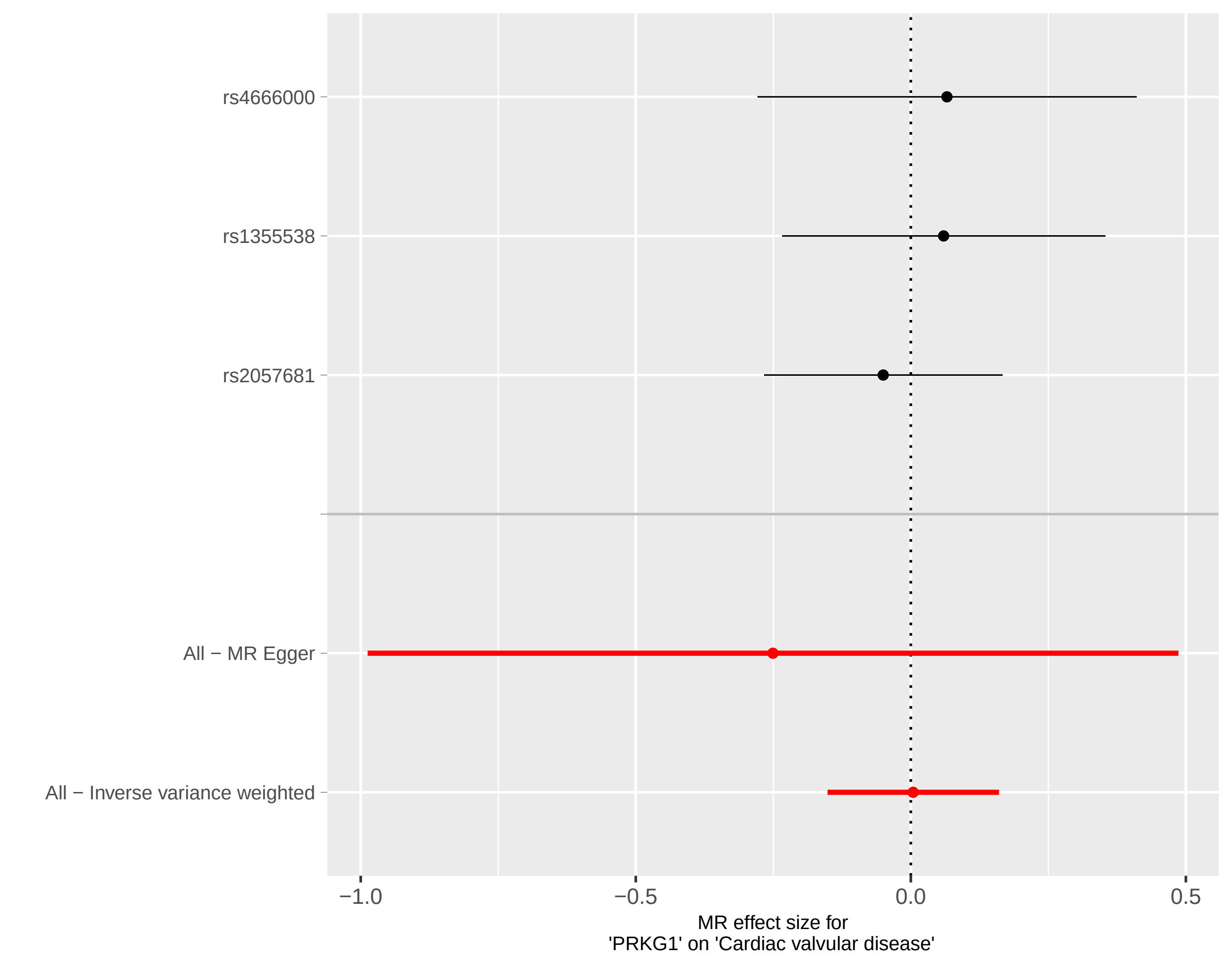
rs4666000
rs1355538
rs2057681
All − MR Egger
All − Inverse variance weighted
−1.0
−0.5
0.0
0.5
MR effect size for
'PRKG1' on 'Cardiac valvular disease'

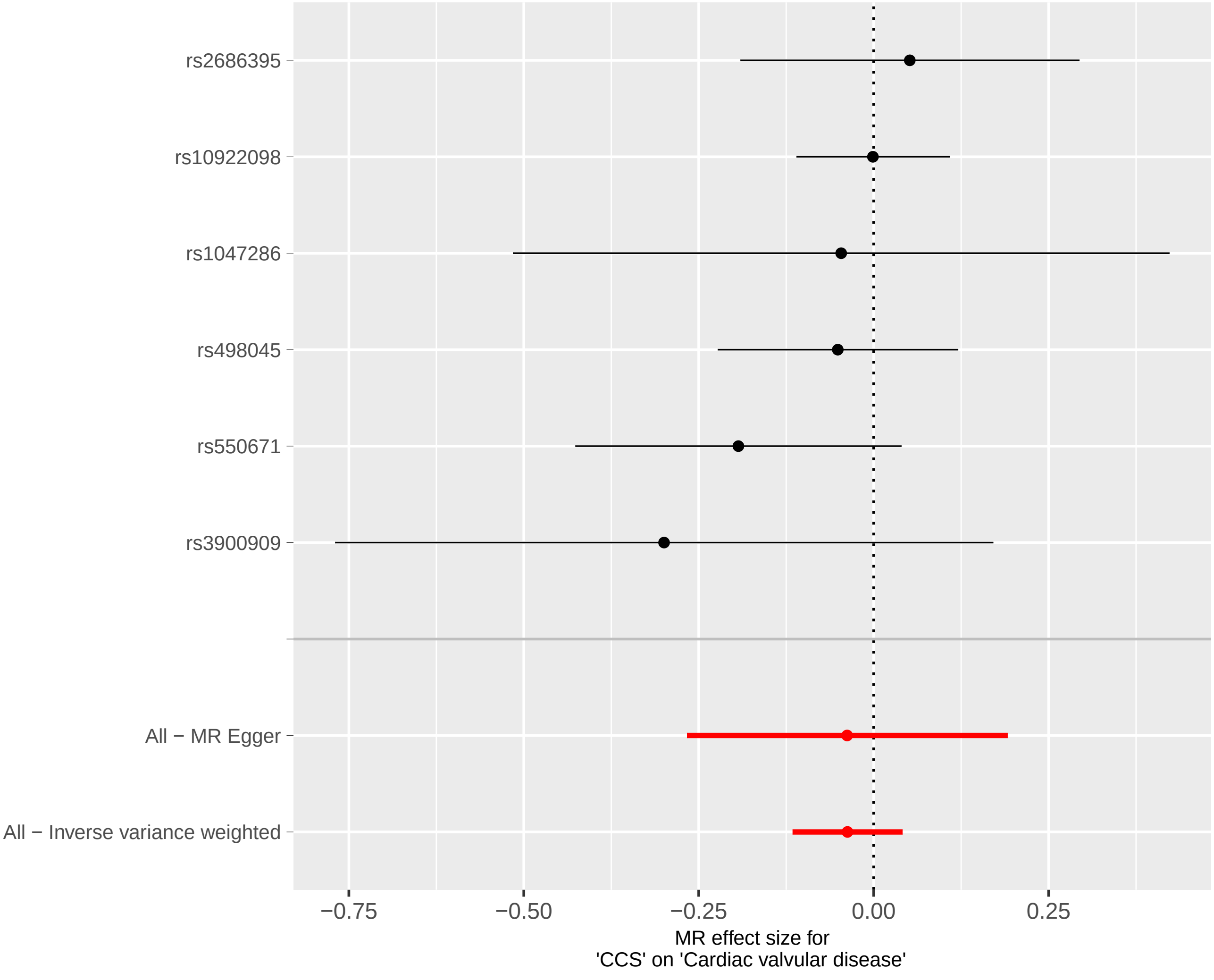

rs2686395
rs10922098
rs1047286
rs498045
rs550671
rs3900909
All − MR Egger
All − Inverse variance weighted
−0.75
−0.50
−0.25
0.00
0.25
MR effect size for
'CCS' on 'Cardiac valvular disease'

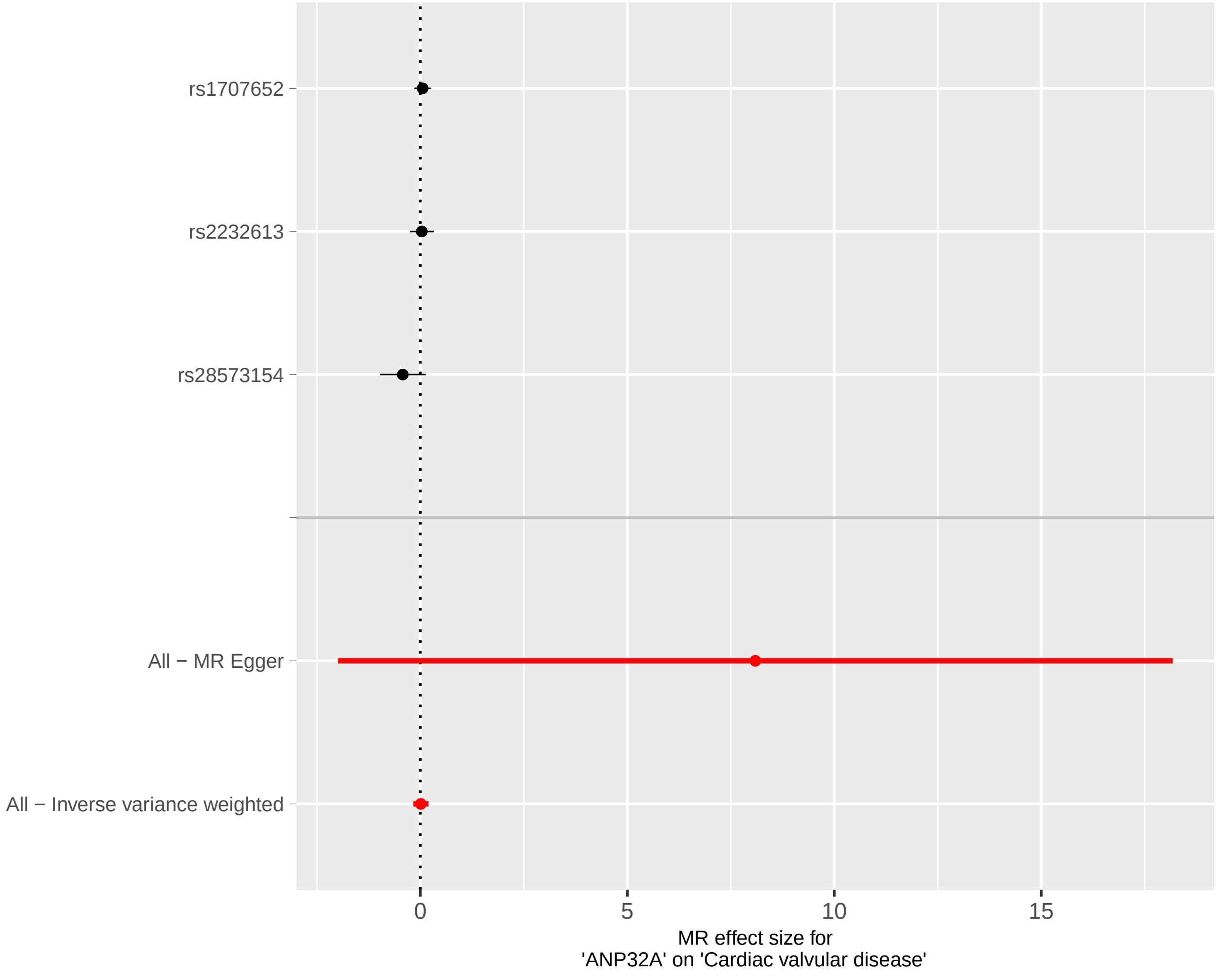
rs1707652
rs2232613
rs28573154
All − MR Egger
All − Inverse variance weighted
0
5
10
15
MR effect size for
'ANP32A' on 'Cardiac valvular disease'

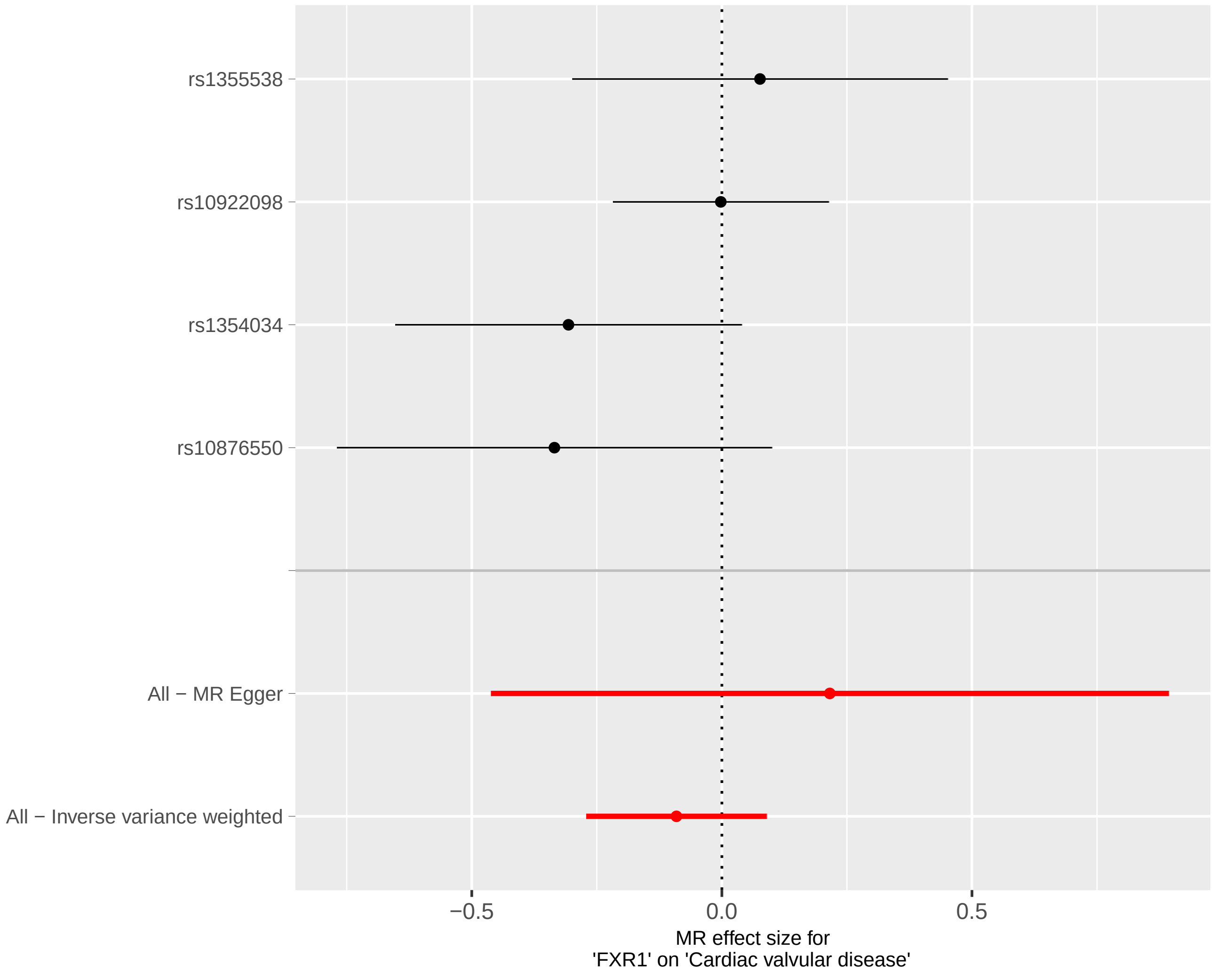

rs1355538
rs10922098
rs1354034
rs10876550
All − MR Egger
All − Inverse variance weighted
−0.5
0.0
0.5
MR effect size for
'FXR1' on 'Cardiac valvular disease'

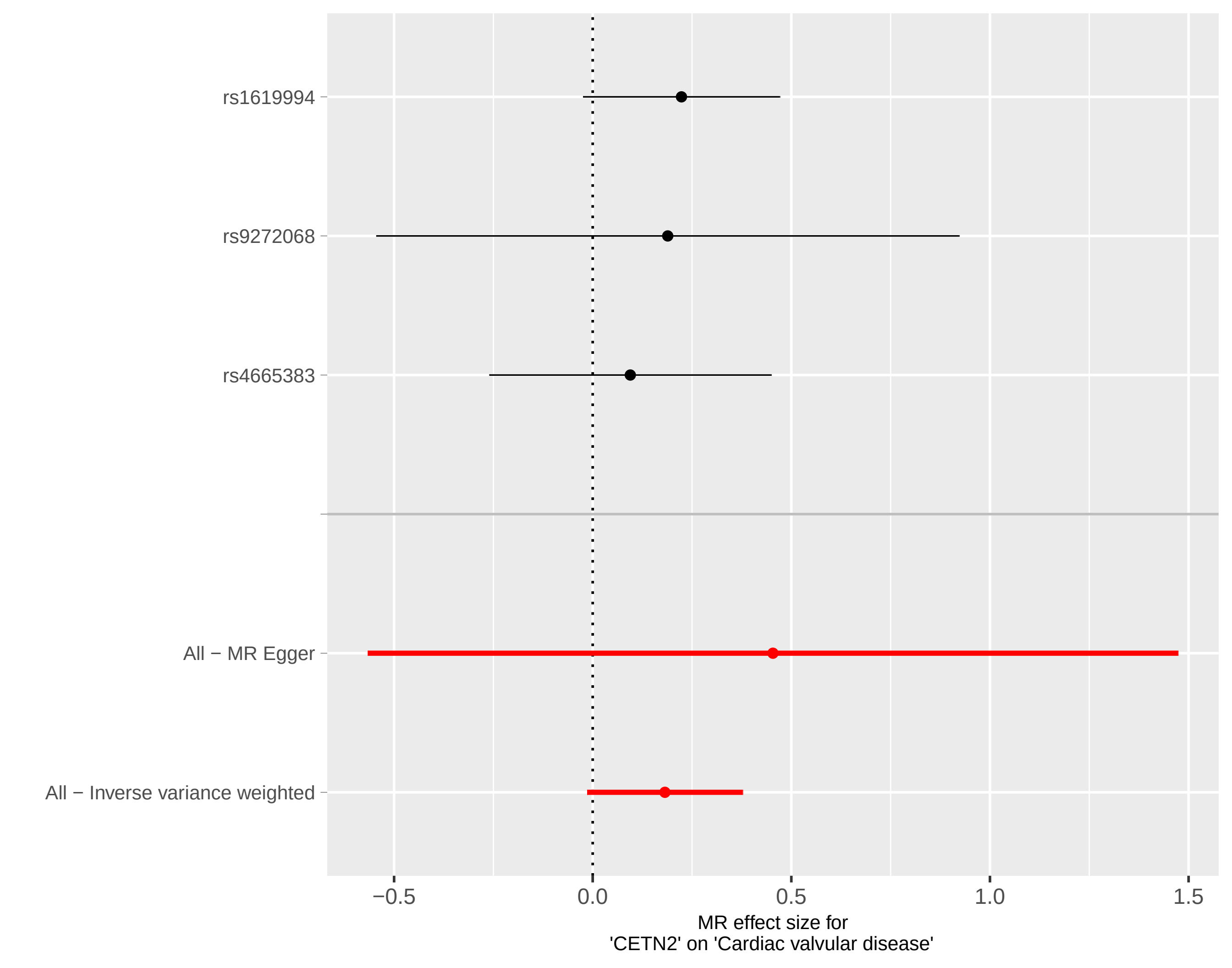
rs1619994
rs9272068
rs4665383
All − MR Egger
All − Inverse variance weighted
−0.5
0.0
0.5
1.0
1.5
MR effect size for
'CETN2' on 'Cardiac valvular disease'

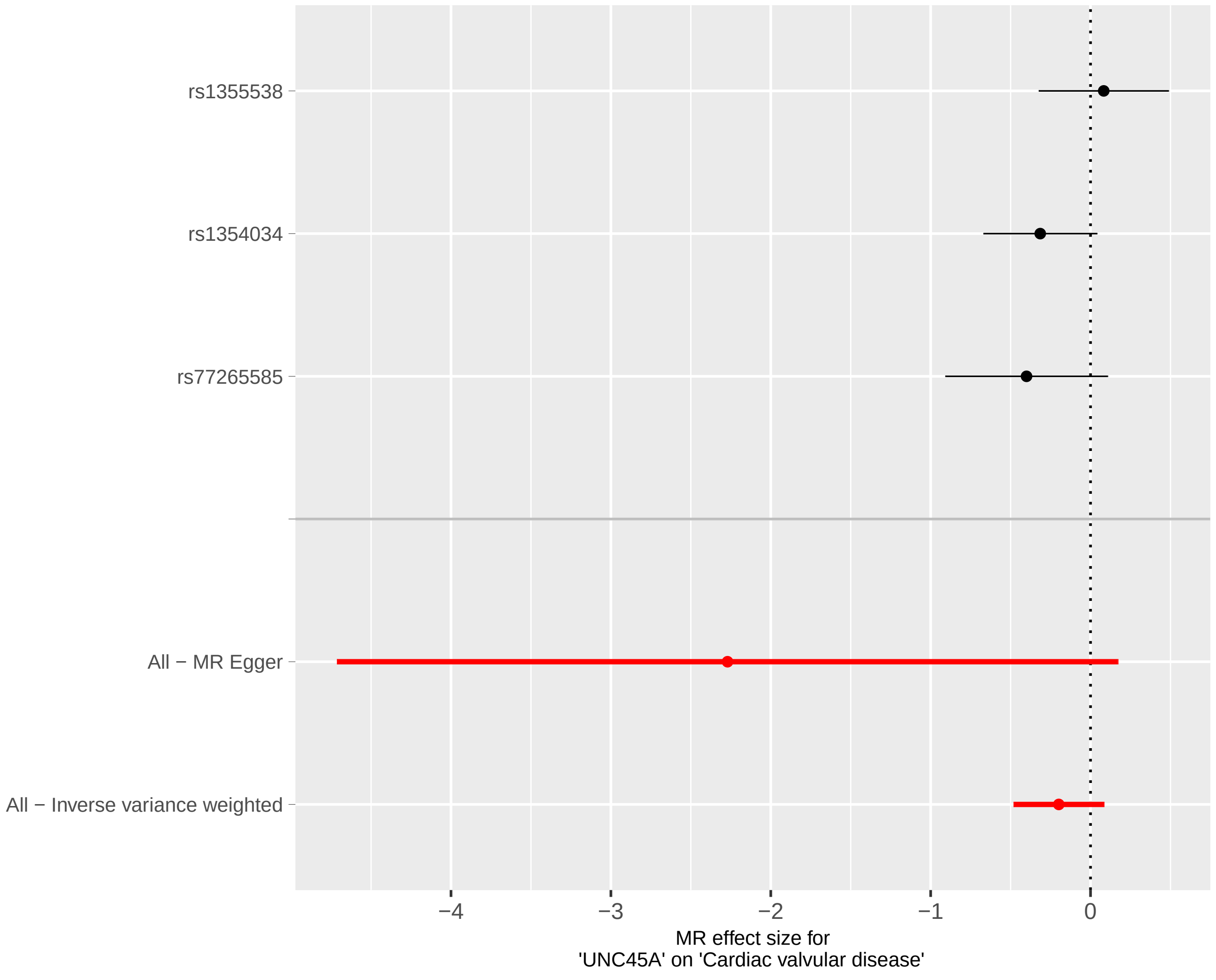

rs1355538
rs1354034
rs77265585
All − MR Egger
All − Inverse variance weighted
−4
−3
−2
−1
0
MR effect size for
'UNC45A' on 'Cardiac valvular disease'

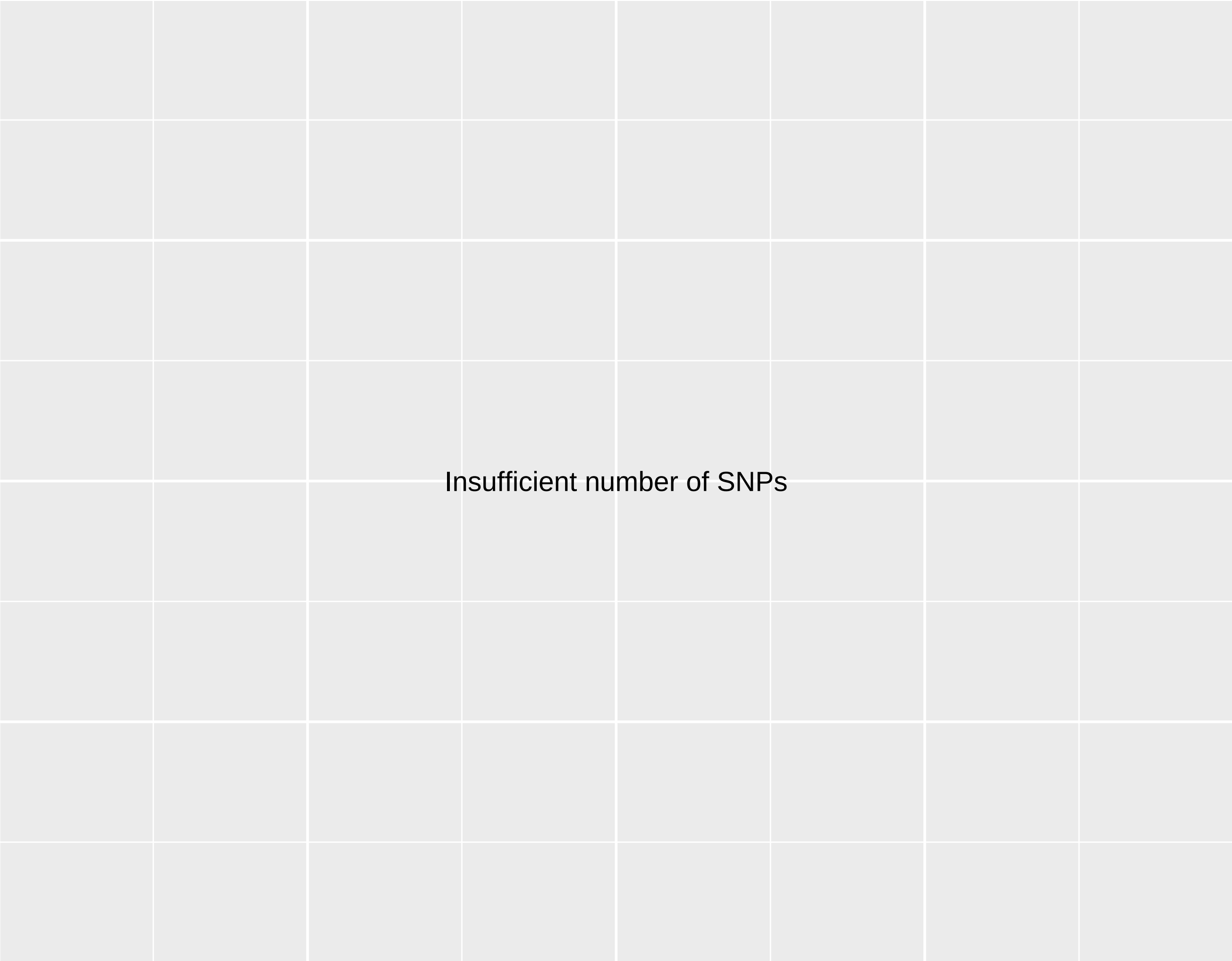
Insufficient number of SNPs

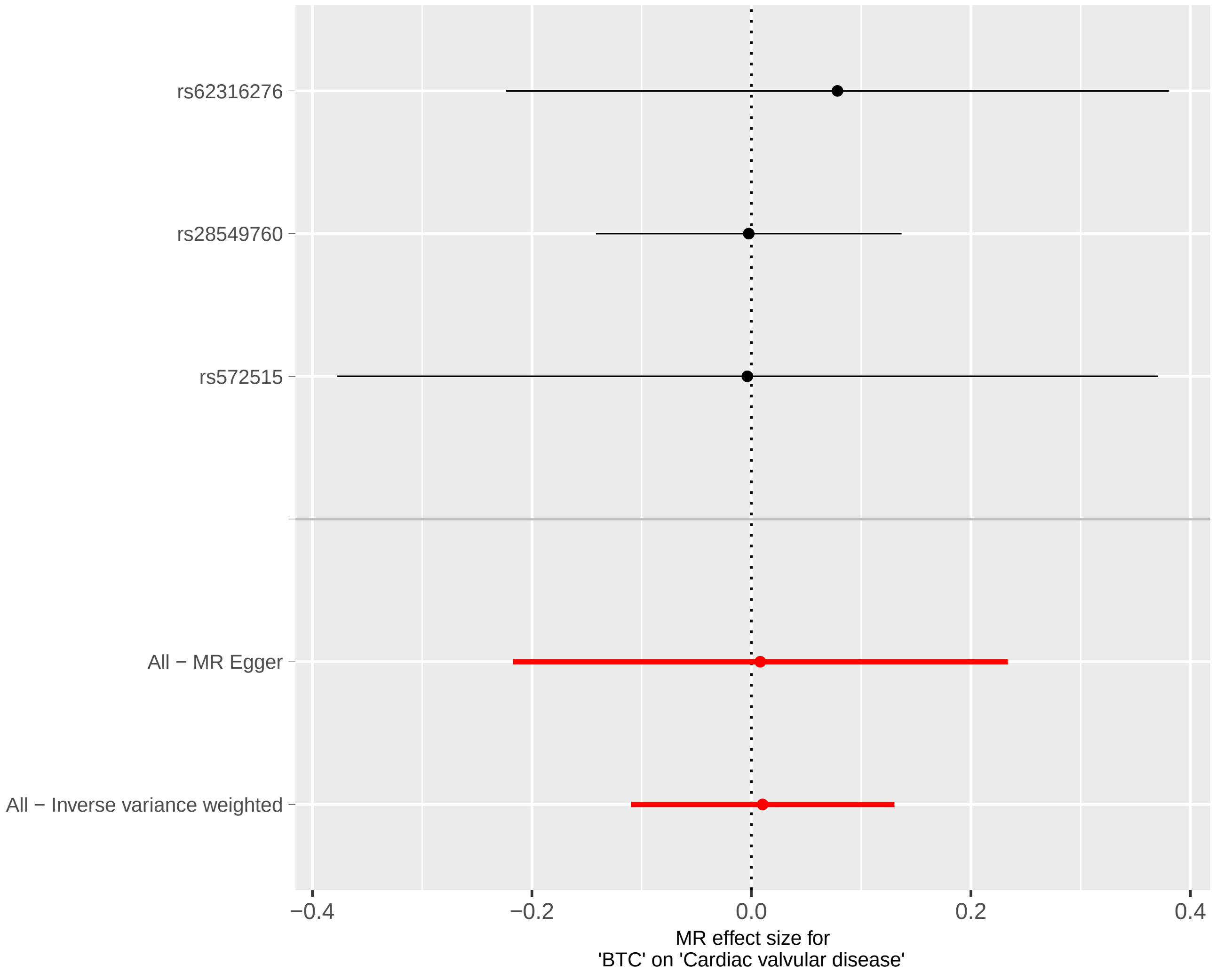
rs62316276
rs28549760
rs572515
All − MR Egger
All − Inverse variance weighted
−0.4
−0.2
0.0
0.2
0.4
MR effect size for
'BTC' on 'Cardiac valvular disease'

Insufficient number of SNPs

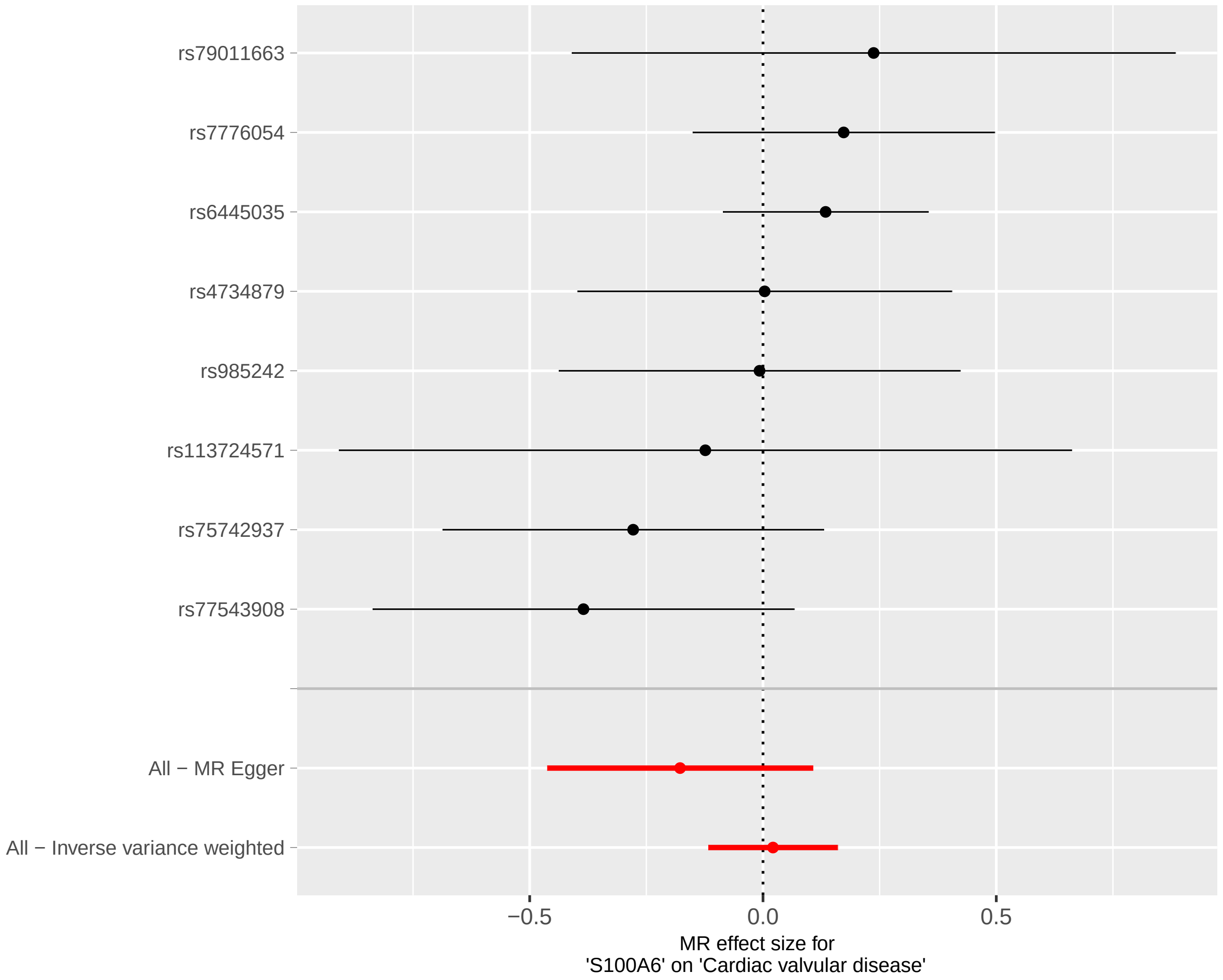
rs79011663
rs7776054
rs6445035
rs4734879
rs985242
rs113724571
rs75742937
rs77543908
All − MR Egger
All − Inverse variance weighted
−0.5
0.0
0.5
MR effect size for
'S100A6' on 'Cardiac valvular disease'

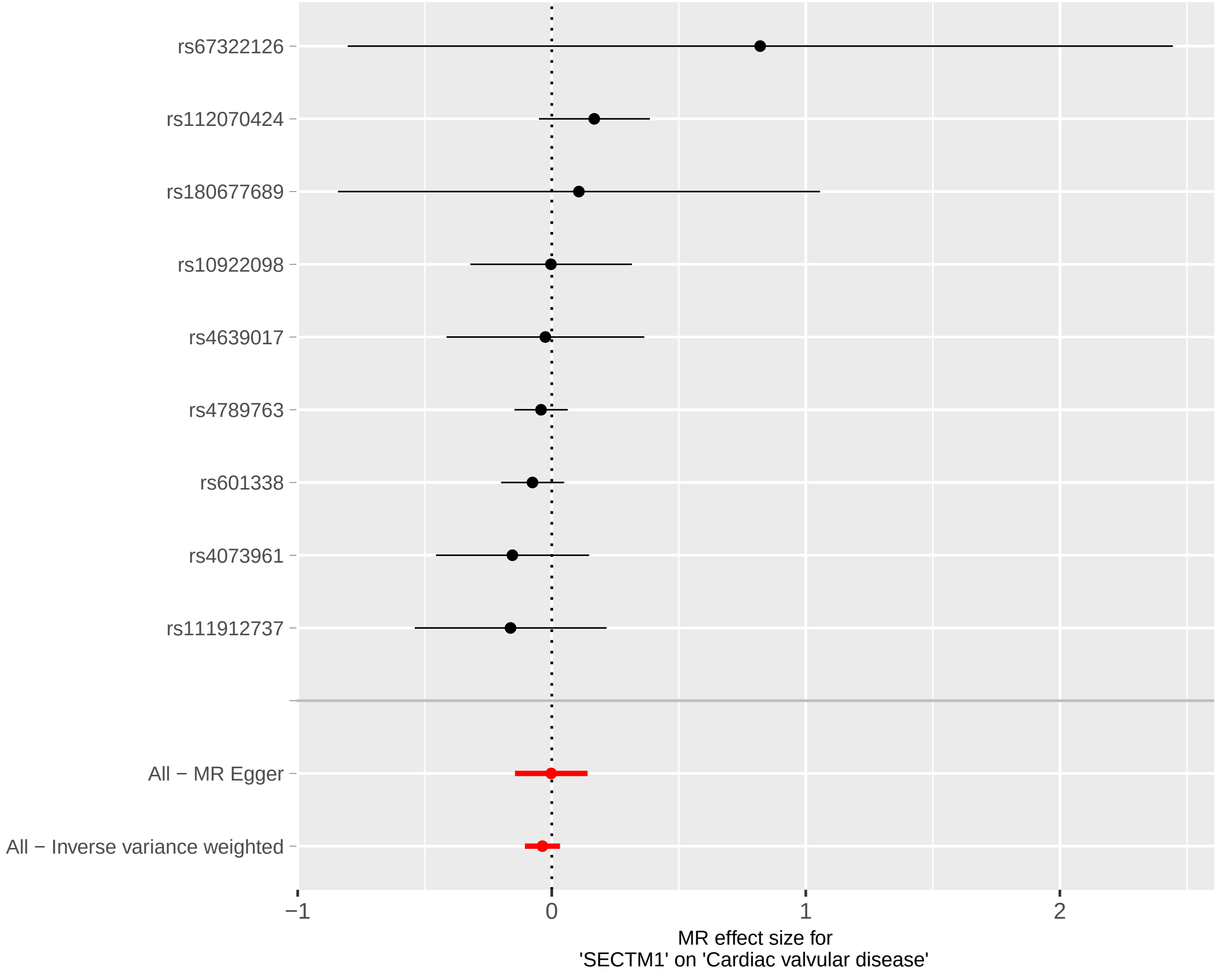
rs67322126
rs112070424
rs180677689
rs10922098
rs4639017
rs4789763
rs601338
rs4073961
rs111912737
All − MR Egger
All − Inverse variance weighted
−1
0
1
2
MR effect size for
'SECTM1' on 'Cardiac valvular disease'

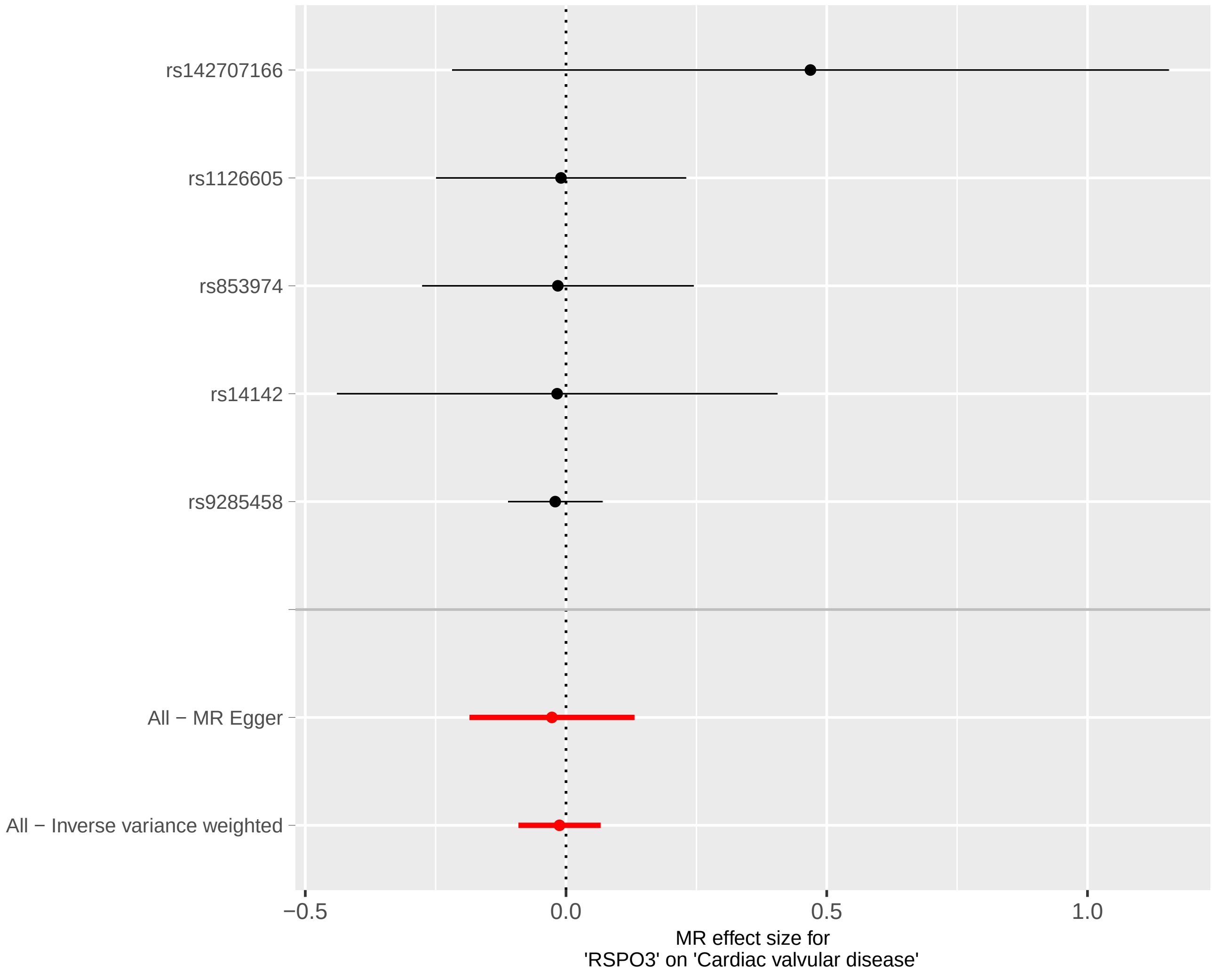
rs142707166
rs1126605
rs853974
rs14142
rs9285458
All − MR Egger
All − Inverse variance weighted
−0.5
0.0
0.5
1.0
MR effect size for
'RSPO3' on 'Cardiac valvular disease'

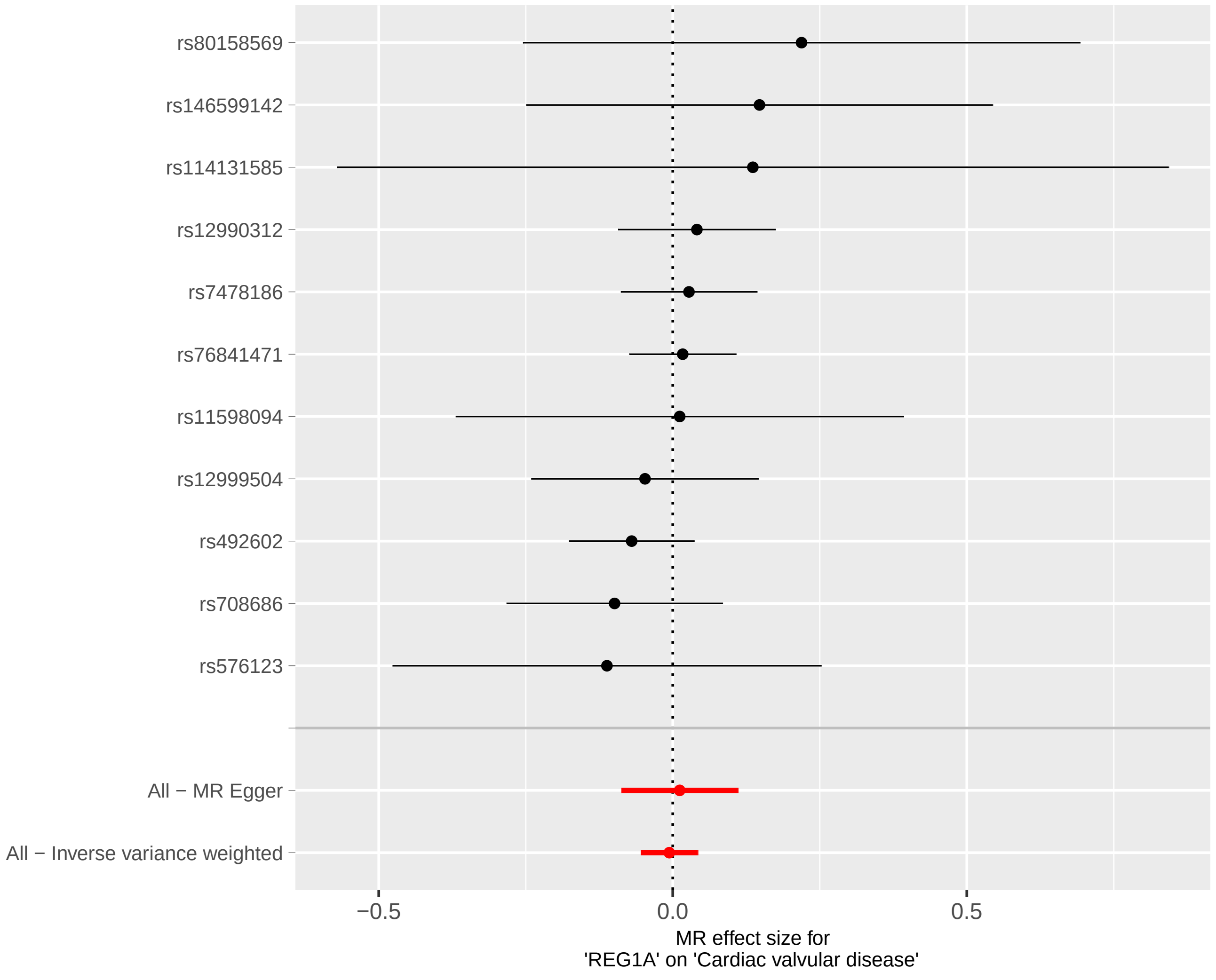
rs80158569
rs146599142
rs114131585
rs12990312
rs7478186
rs76841471
rs11598094
rs12999504
rs492602
rs708686
rs576123
All − MR Egger
All − Inverse variance weighted
−0.5
0.0
0.5
MR effect size for
'REG1A' on 'Cardiac valvular disease'

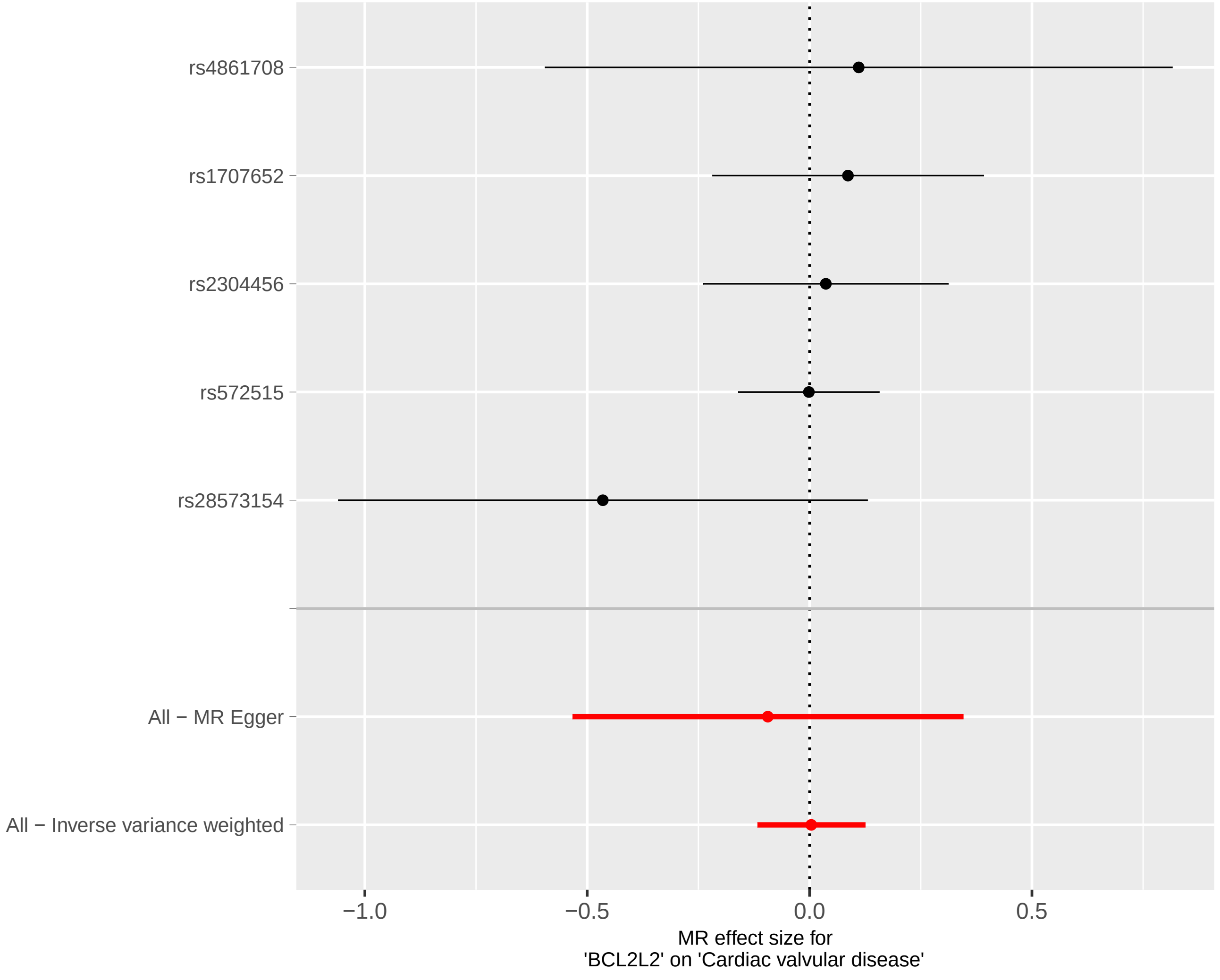
rs4861708
rs1707652
rs2304456
rs572515
rs28573154
All − MR Egger
All − Inverse variance weighted
−1.0
−0.5
0.0
0.5
MR effect size for
'BCL2L2' on 'Cardiac valvular disease'

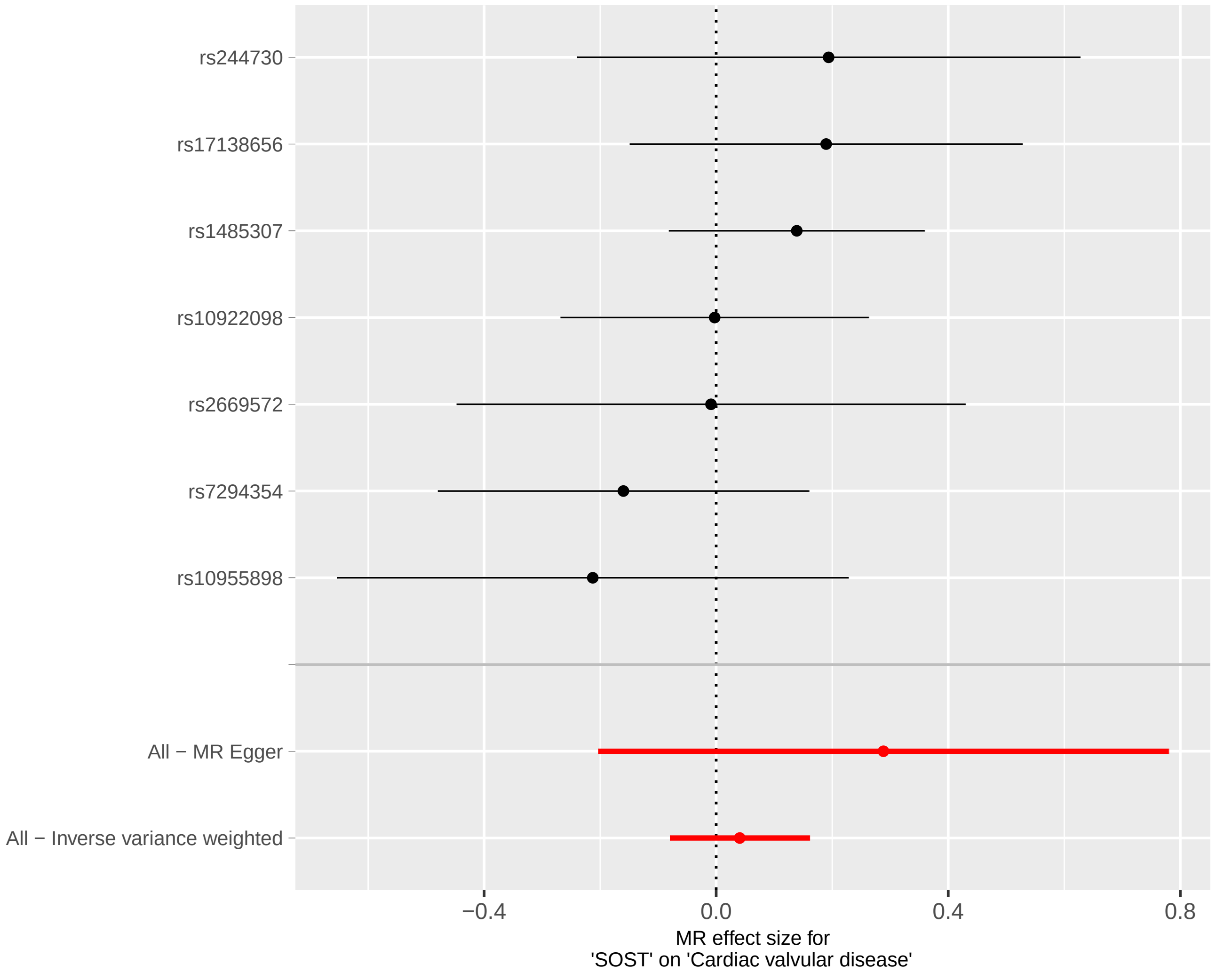
rs244730
rs17138656
rs1485307
rs10922098
rs2669572
rs7294354
rs10955898
All − MR Egger
All − Inverse variance weighted
−0.4
0.0
0.4
0.8
MR effect size for
'SOST' on 'Cardiac valvular disease'

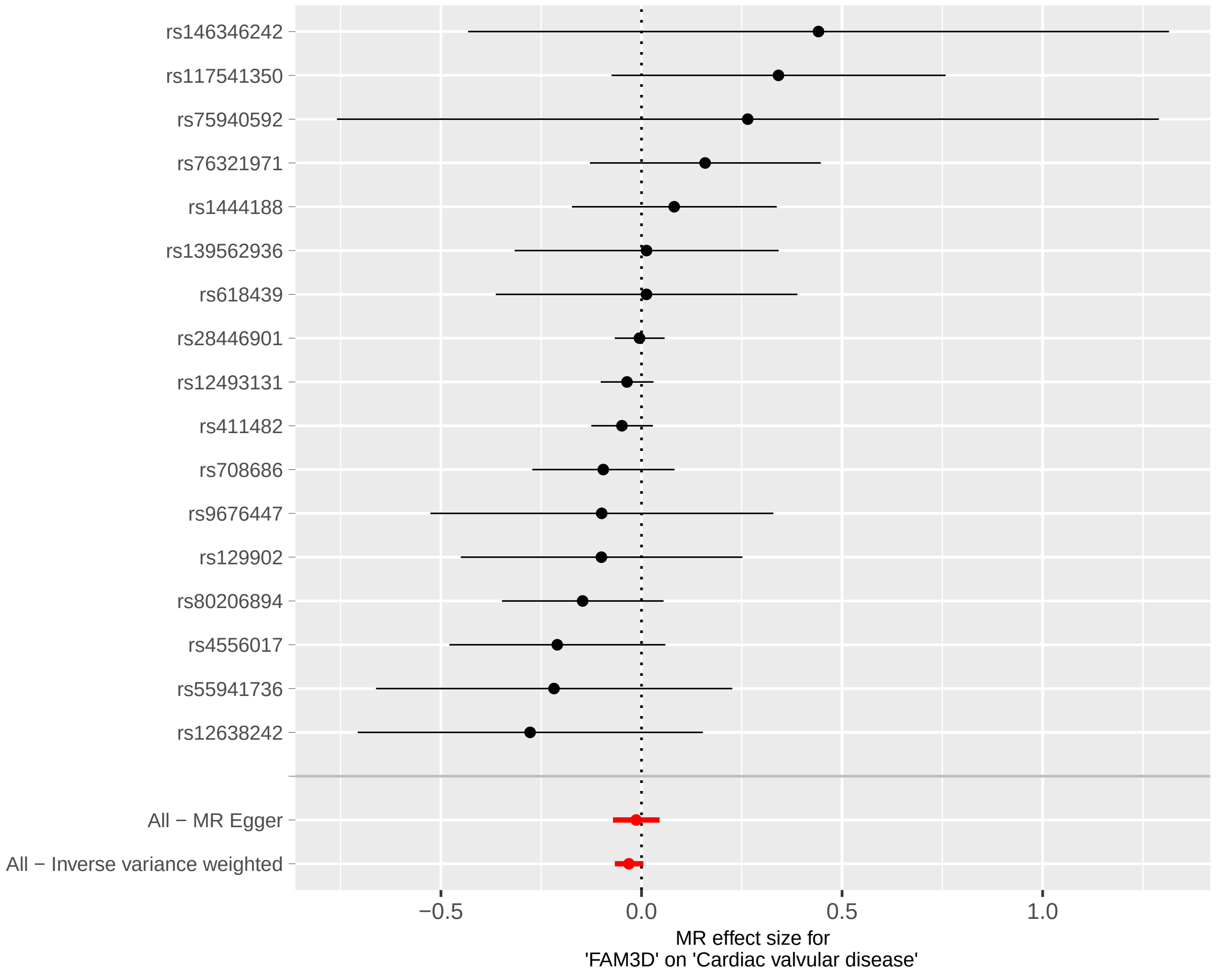
rs146346242
rs117541350
rs75940592
rs76321971
rs1444188
rs139562936
rs618439
rs28446901
rs12493131
rs411482
rs708686
rs9676447
rs129902
rs80206894
rs4556017
rs55941736
rs12638242
All − MR Egger
All − Inverse variance weighted
−0.5
0.0
0.5
1.0
MR effect size for
'FAM3D' on 'Cardiac valvular disease'

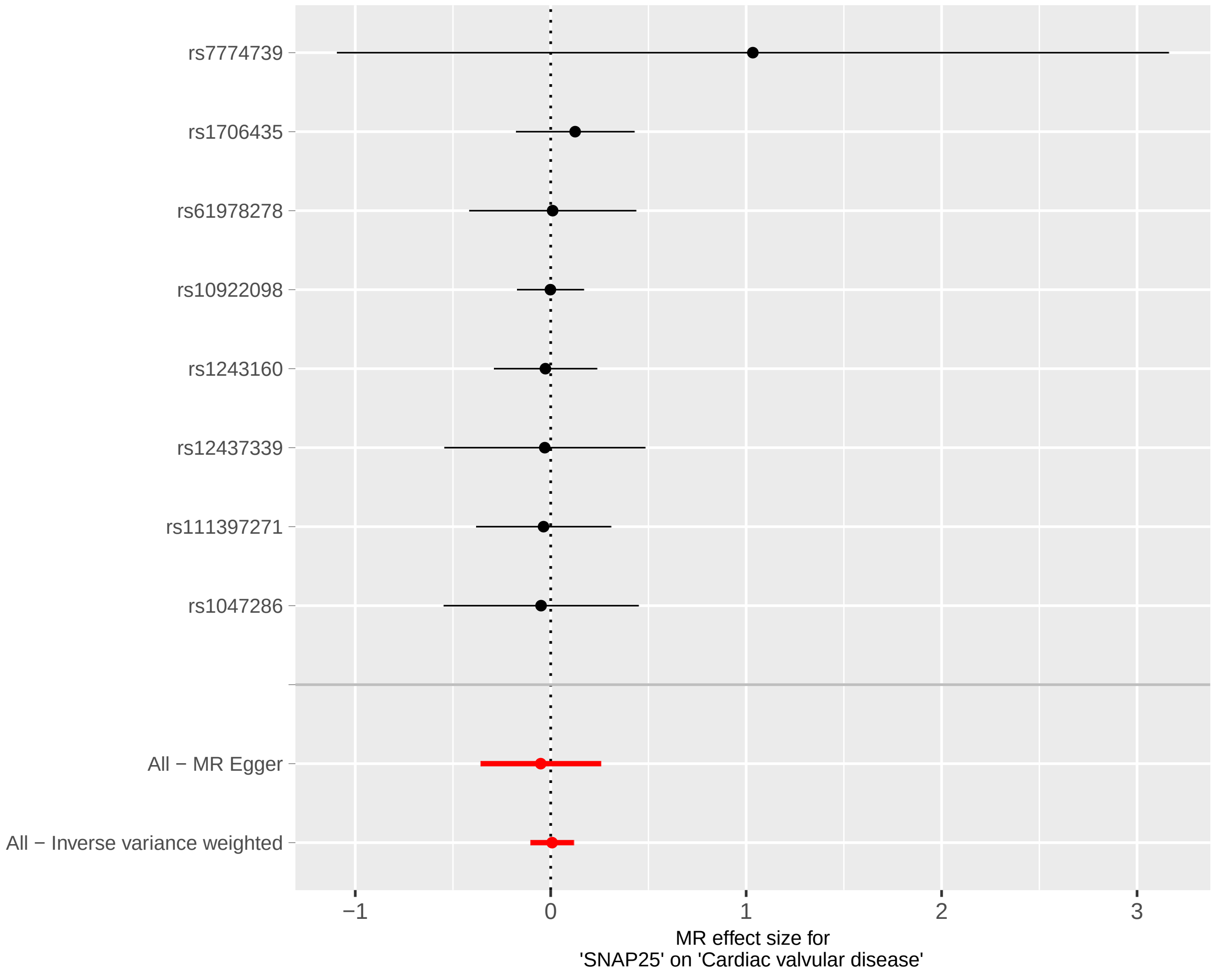
rs7774739
rs1706435
rs61978278
rs10922098
rs1243160
rs12437339
rs111397271
rs1047286
All − MR Egger
All − Inverse variance weighted
−1
0
1
2
3
MR effect size for
'SNAP25' on 'Cardiac valvular disease'

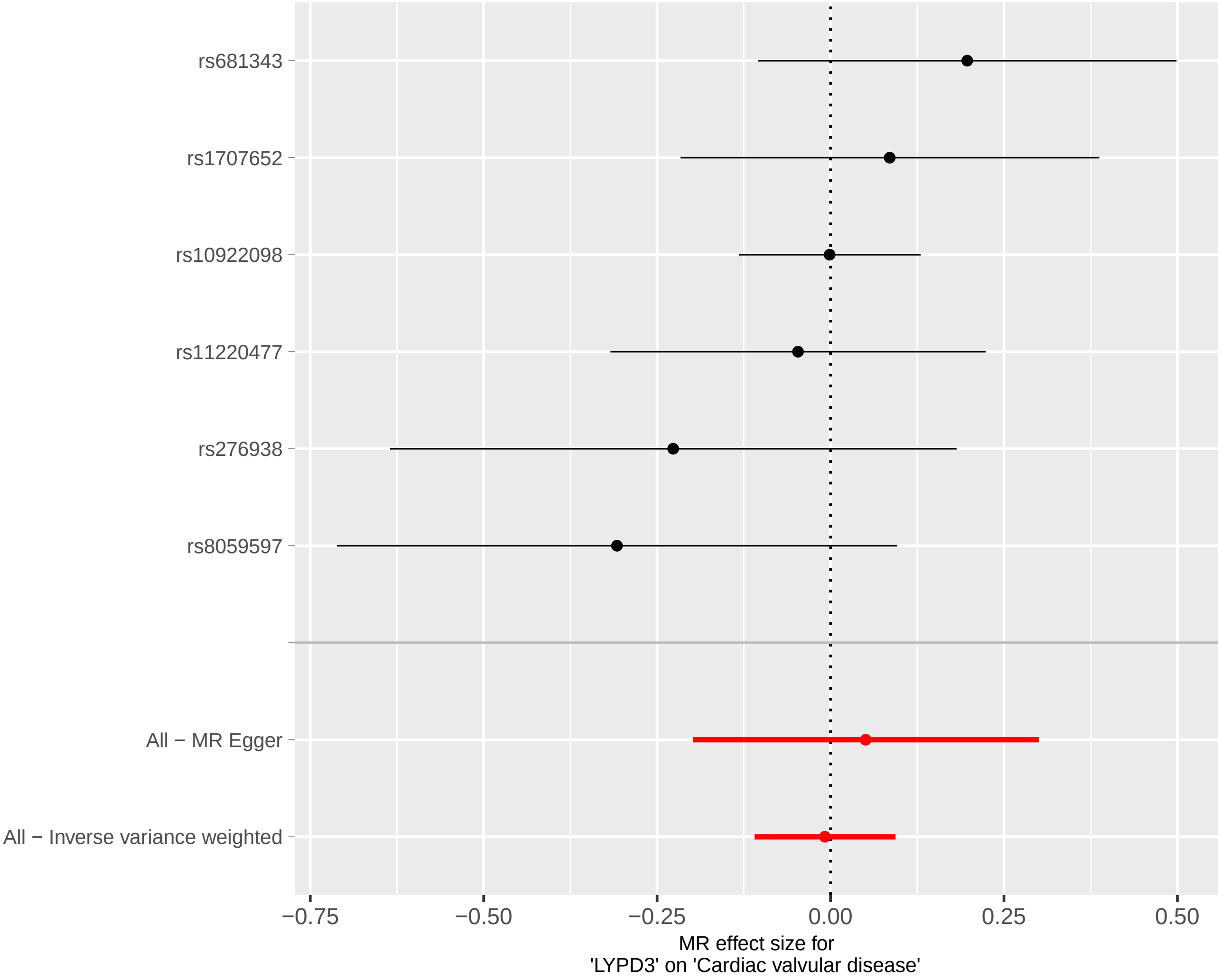

rs681343
rs1707652
rs10922098
rs11220477
rs276938
rs8059597
All − MR Egger
All − Inverse variance weighted
−0.75
−0.50
−0.25
0.00
0.25
0.50
MR effect size for
'LYPD3' on 'Cardiac valvular disease'

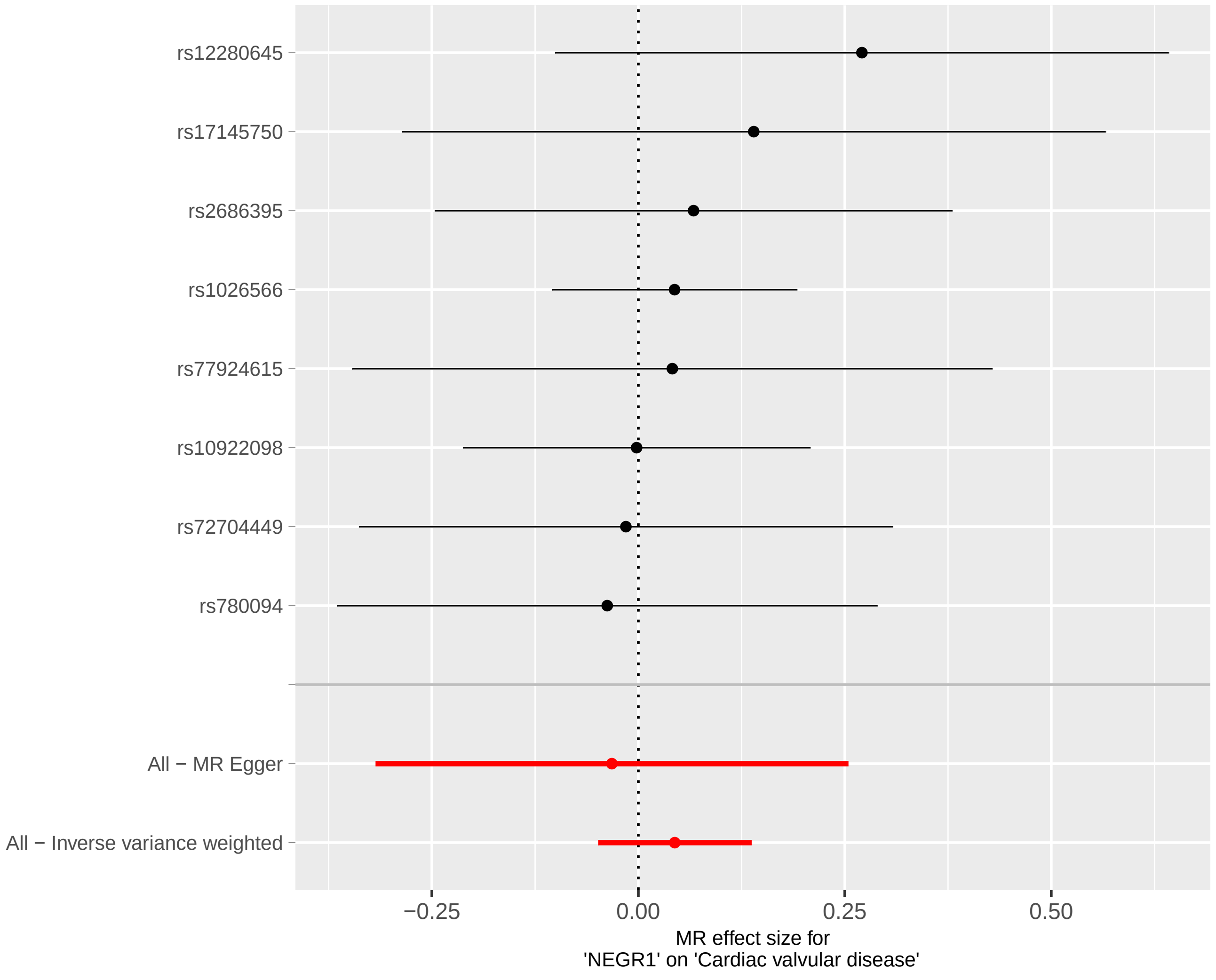
rs12280645
rs17145750
rs2686395
rs1026566
rs77924615
rs10922098
rs72704449
rs780094
All − MR Egger
All − Inverse variance weighted
−0.25
0.00
0.25
0.50
MR effect size for
'NEGR1' on 'Cardiac valvular disease'

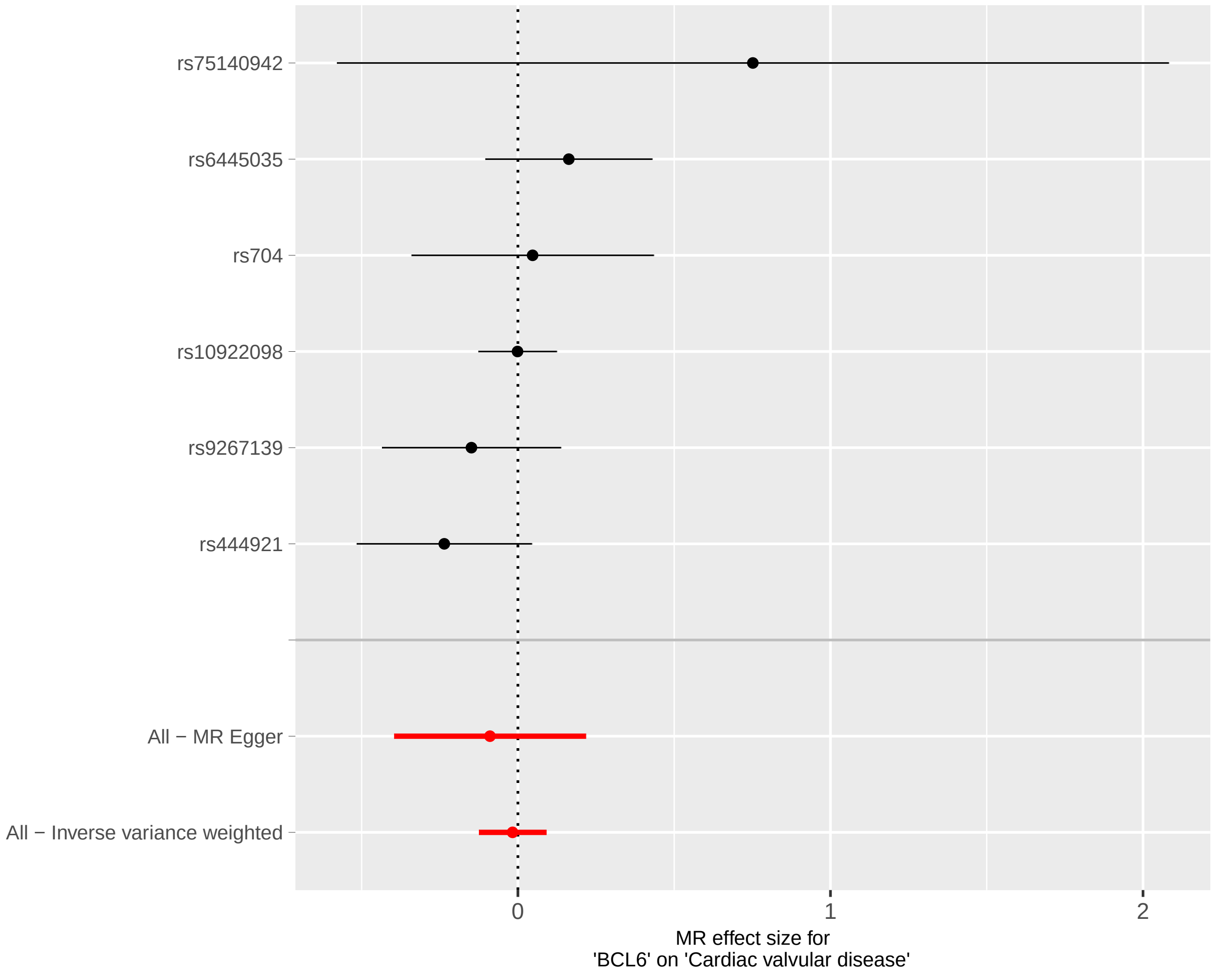
rs75140942
rs6445035
rs704
rs10922098
rs9267139
rs444921
All − MR Egger
All − Inverse variance weighted
0
1
2
MR effect size for
'BCL6' on 'Cardiac valvular disease'

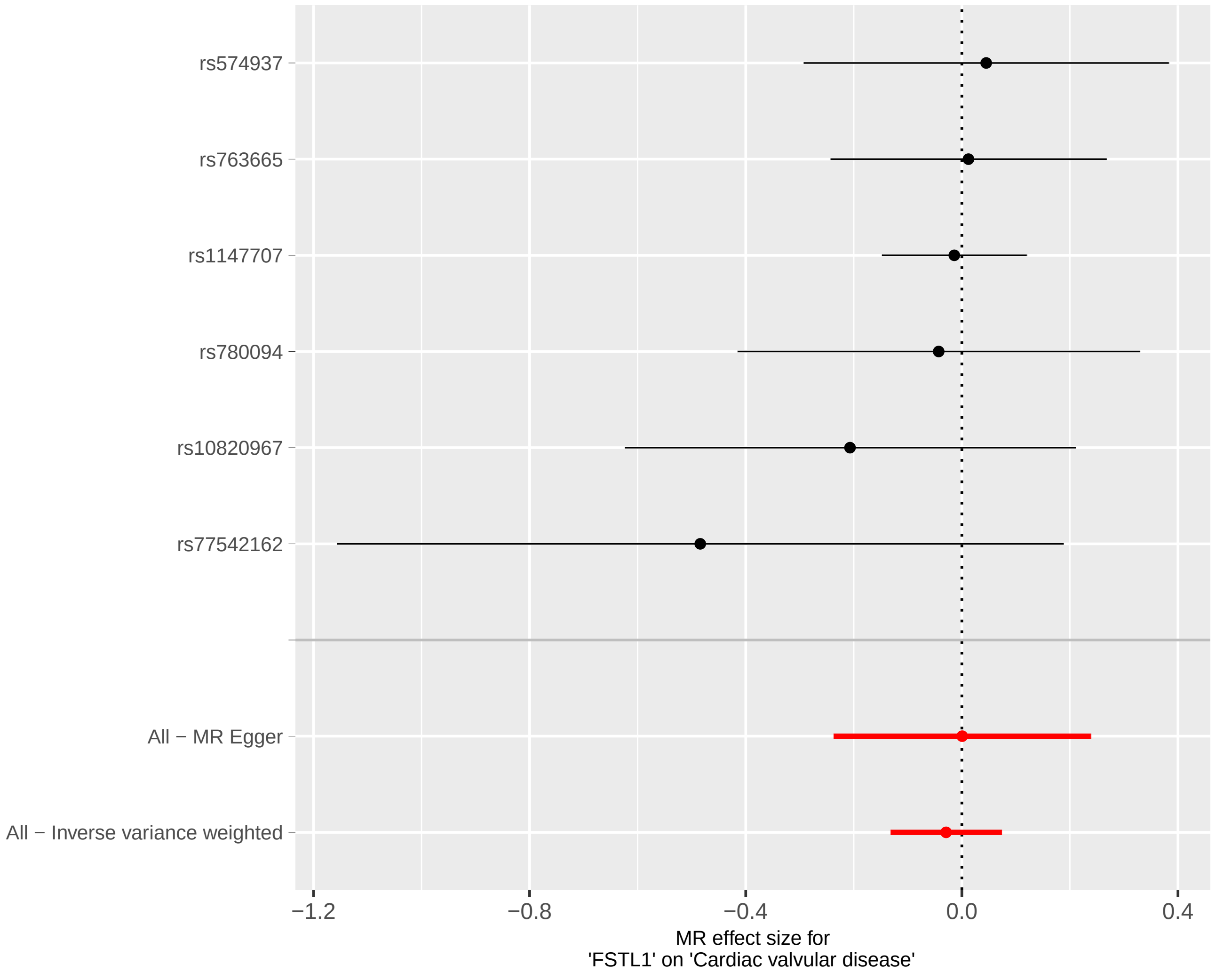
rs574937
rs763665
rs1147707
rs780094
rs10820967
rs77542162
All − MR Egger
All − Inverse variance weighted
−1.2
−0.8
−0.4
0.0
0.4
MR effect size for
'FSTL1' on 'Cardiac valvular disease'

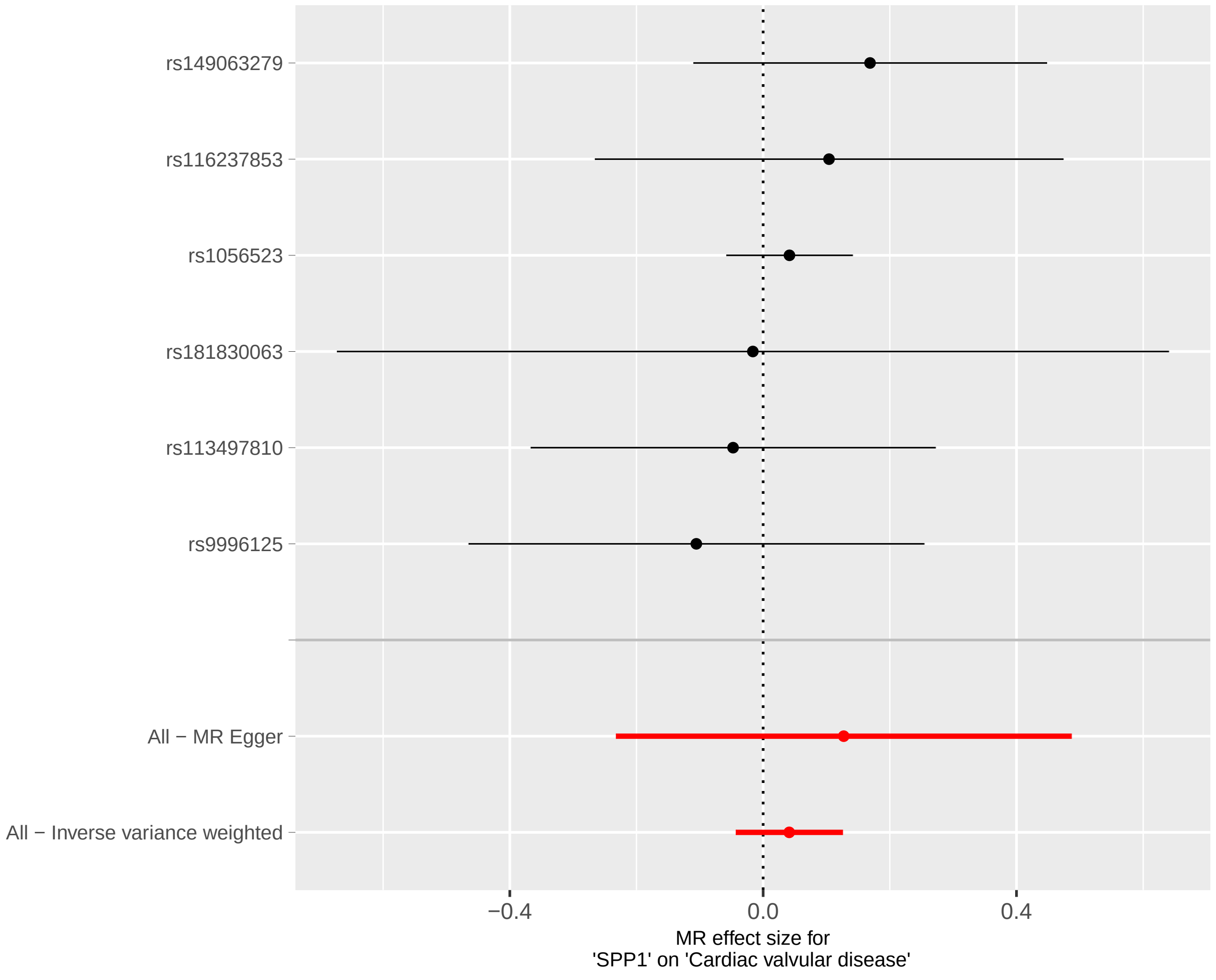

rs149063279
rs116237853
rs1056523
rs181830063
rs113497810
rs9996125
All − MR Egger
All − Inverse variance weighted
−0.4
0.0
0.4
MR effect size for
'SPP1' on 'Cardiac valvular disease'

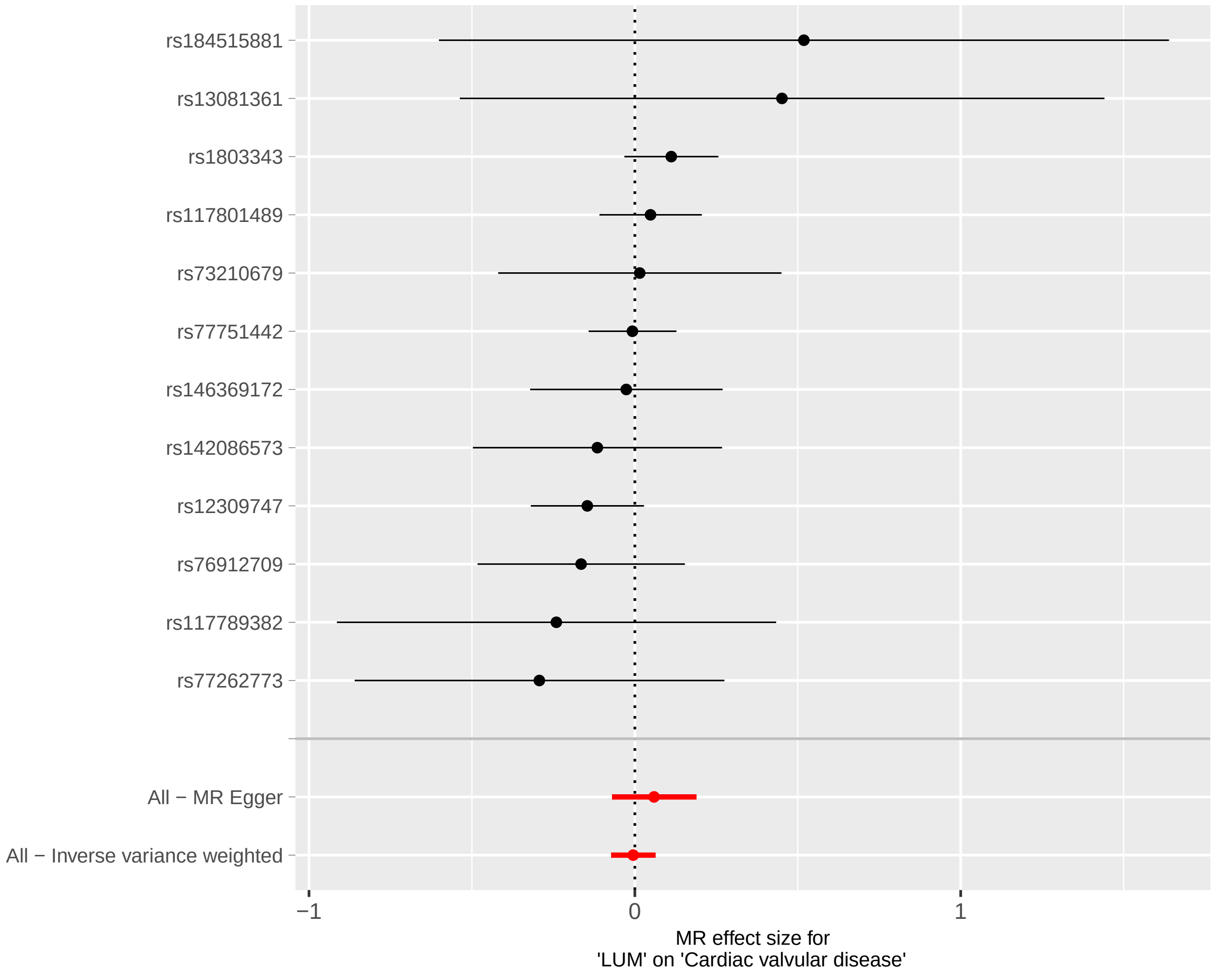
rs184515881
rs13081361
rs1803343
rs117801489
rs73210679
rs77751442
rs146369172
rs142086573
rs12309747
rs76912709
rs117789382
rs77262773
All − MR Egger
All − Inverse variance weighted
−1
0
1
MR effect size for
'LUM' on 'Cardiac valvular disease'

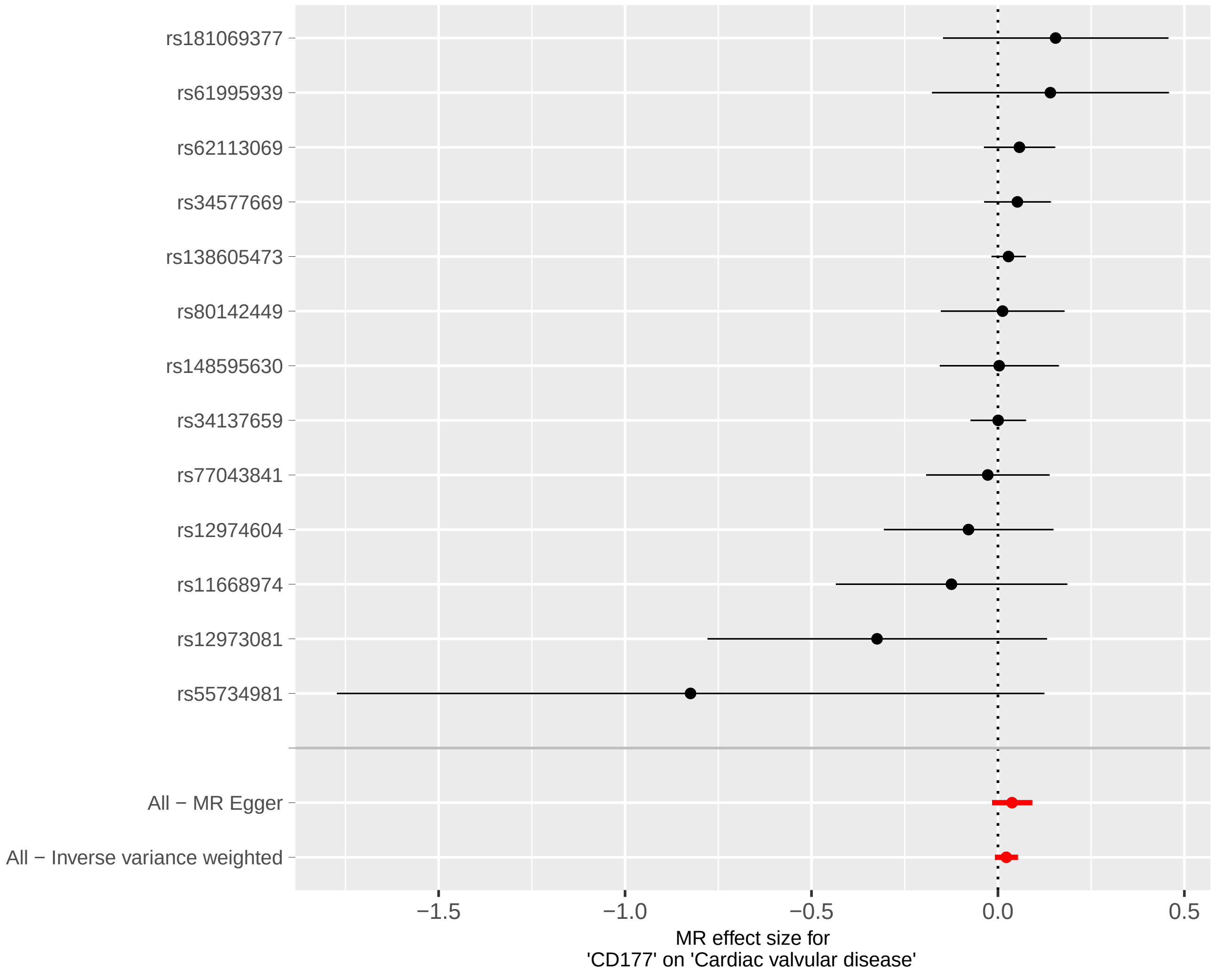

rs181069377
rs61995939
rs62113069
rs34577669
rs138605473
rs80142449
rs148595630
rs34137659
rs77043841
rs12974604
rs11668974
rs12973081
rs55734981
All − MR Egger
All − Inverse variance weighted
−1.5
−1.0
−0.5
0.0
0.5
MR effect size for
'CD177' on 'Cardiac valvular disease'

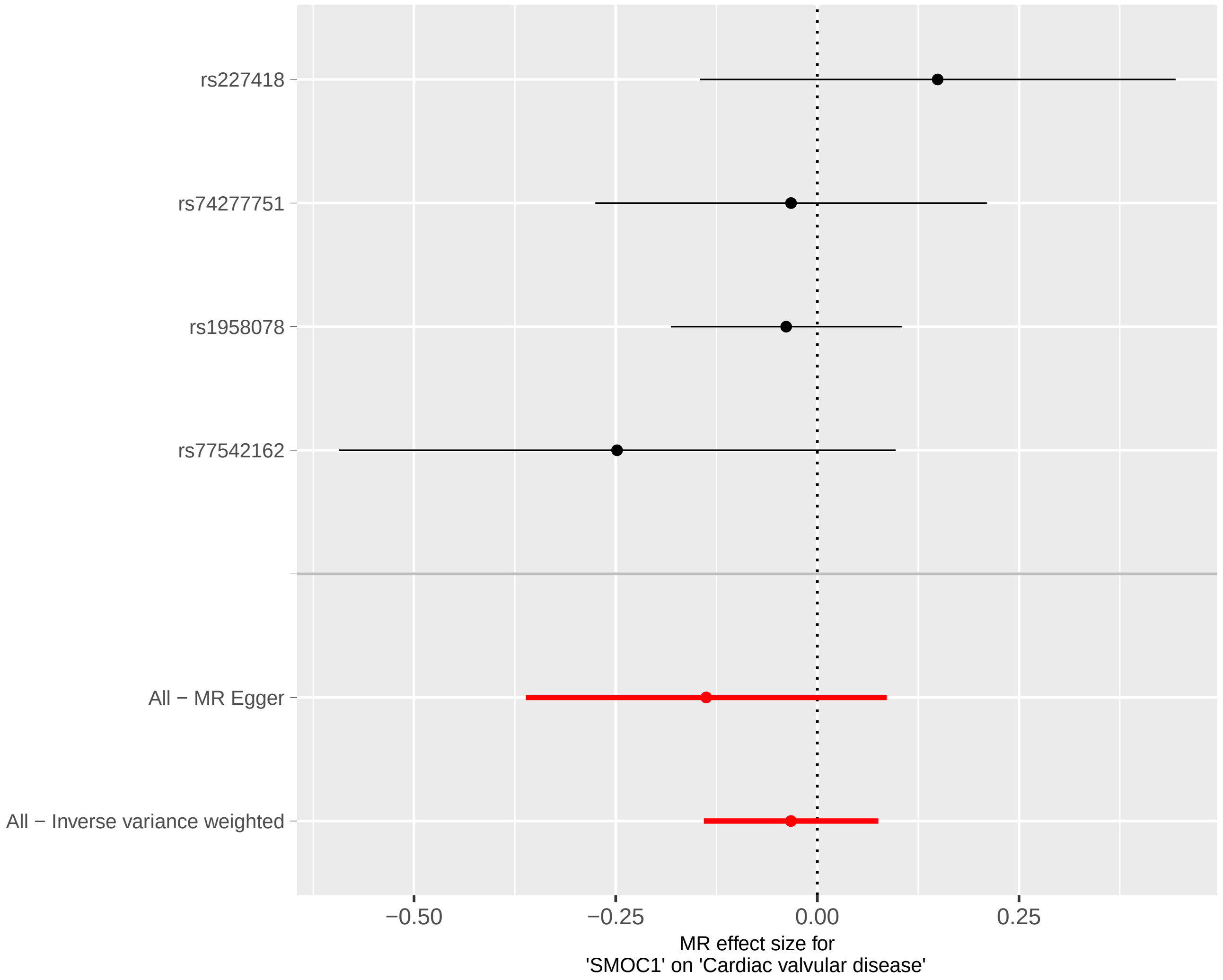
rs227418
rs74277751
rs1958078
rs77542162
All − MR Egger
All − Inverse variance weighted
−0.50
−0.25
0.00
0.25
MR effect size for
'SMOC1' on 'Cardiac valvular disease'

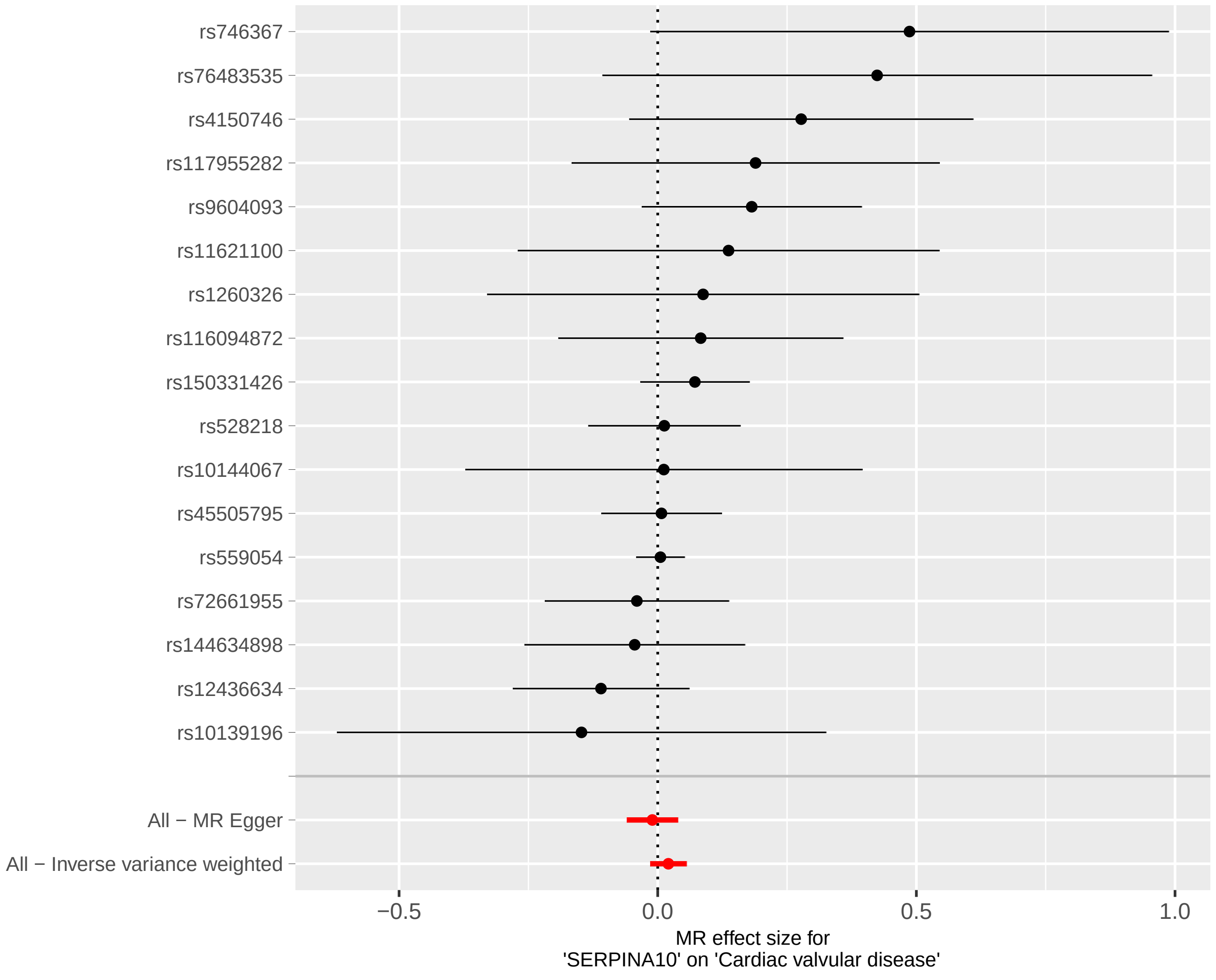
rs746367
rs76483535
rs4150746
rs117955282
rs9604093
rs11621100
rs1260326
rs116094872
rs150331426
rs528218
rs10144067
rs45505795
rs559054
rs72661955
rs144634898
rs12436634
rs10139196
All − MR Egger
All − Inverse variance weighted
−0.5
0.0
0.5
1.0
MR effect size for
'SERPINA10' on 'Cardiac valvular disease'

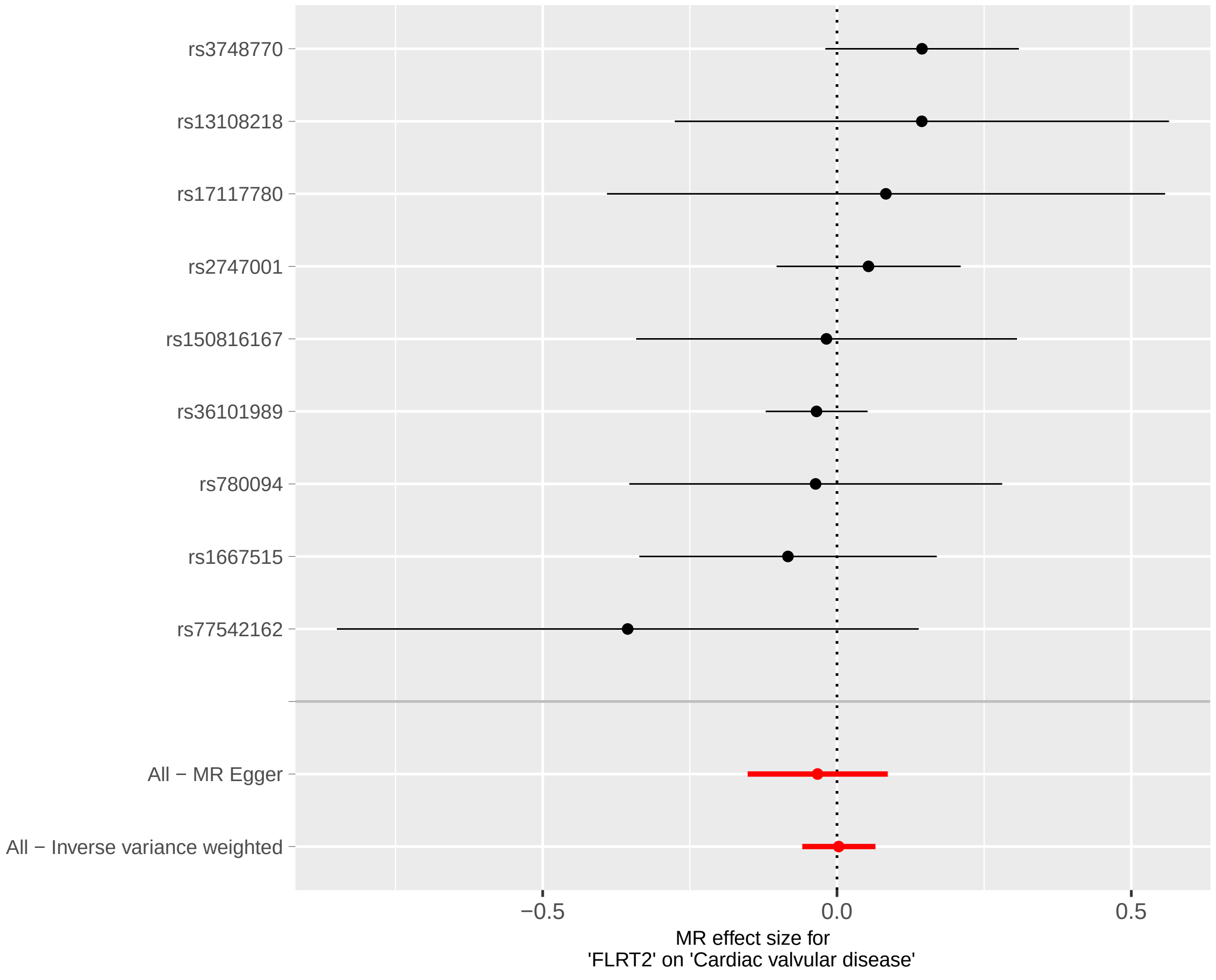
rs3748770
rs13108218
rs17117780
rs2747001
rs150816167
rs36101989
rs780094
rs1667515
rs77542162
All − MR Egger
All − Inverse variance weighted
−0.5
0.0
0.5
MR effect size for
'FLRT2' on 'Cardiac valvular disease'

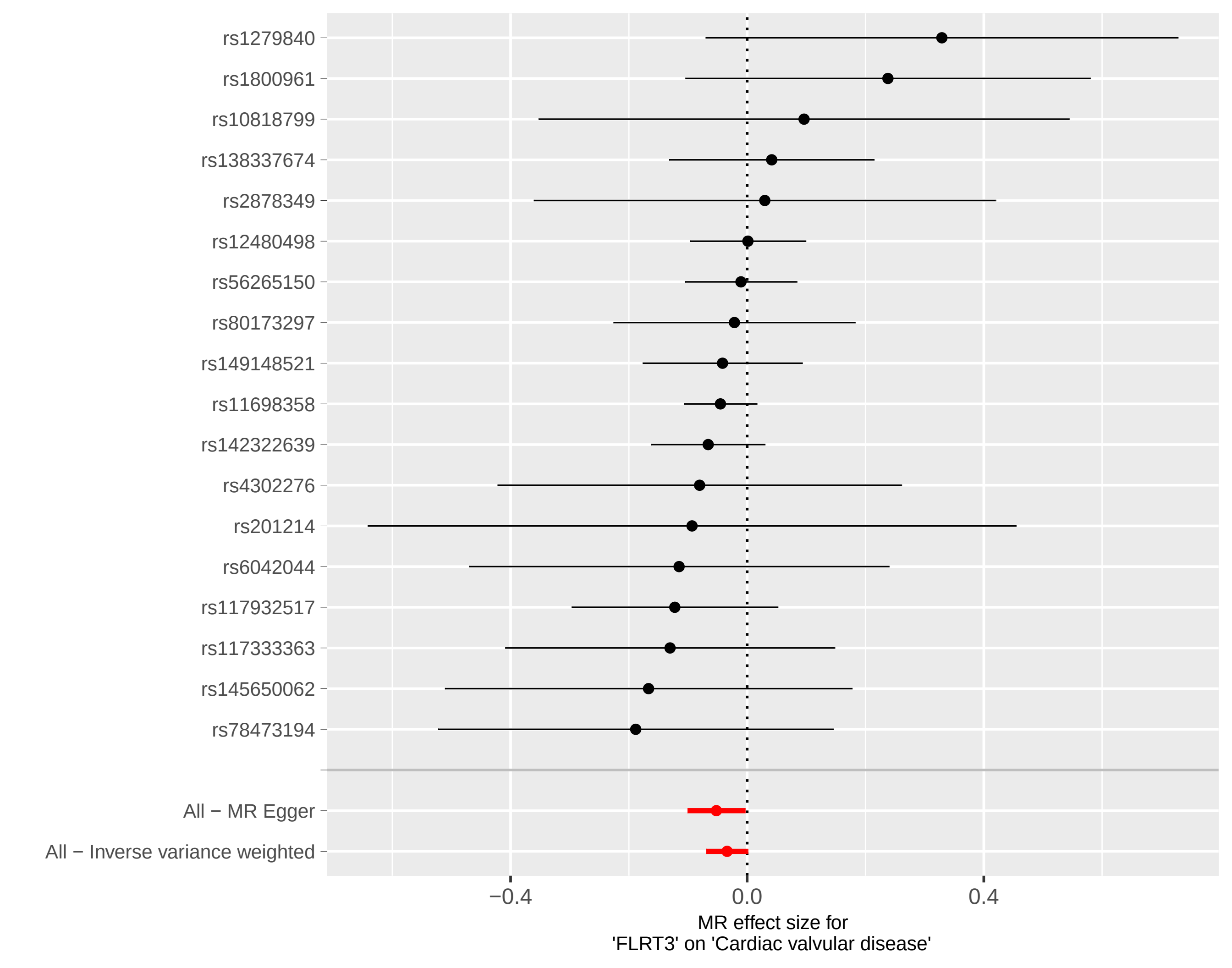
rs1279840
rs1800961
rs10818799
rs138337674
rs2878349
rs12480498
rs56265150
rs80173297
rs149148521
rs11698358
rs142322639
rs4302276
rs201214
rs6042044
rs117932517
rs117333363
rs145650062
rs78473194
All − MR Egger
All − Inverse variance weighted
−0.4
0.0
0.4
MR effect size for
'FLRT3' on 'Cardiac valvular disease'

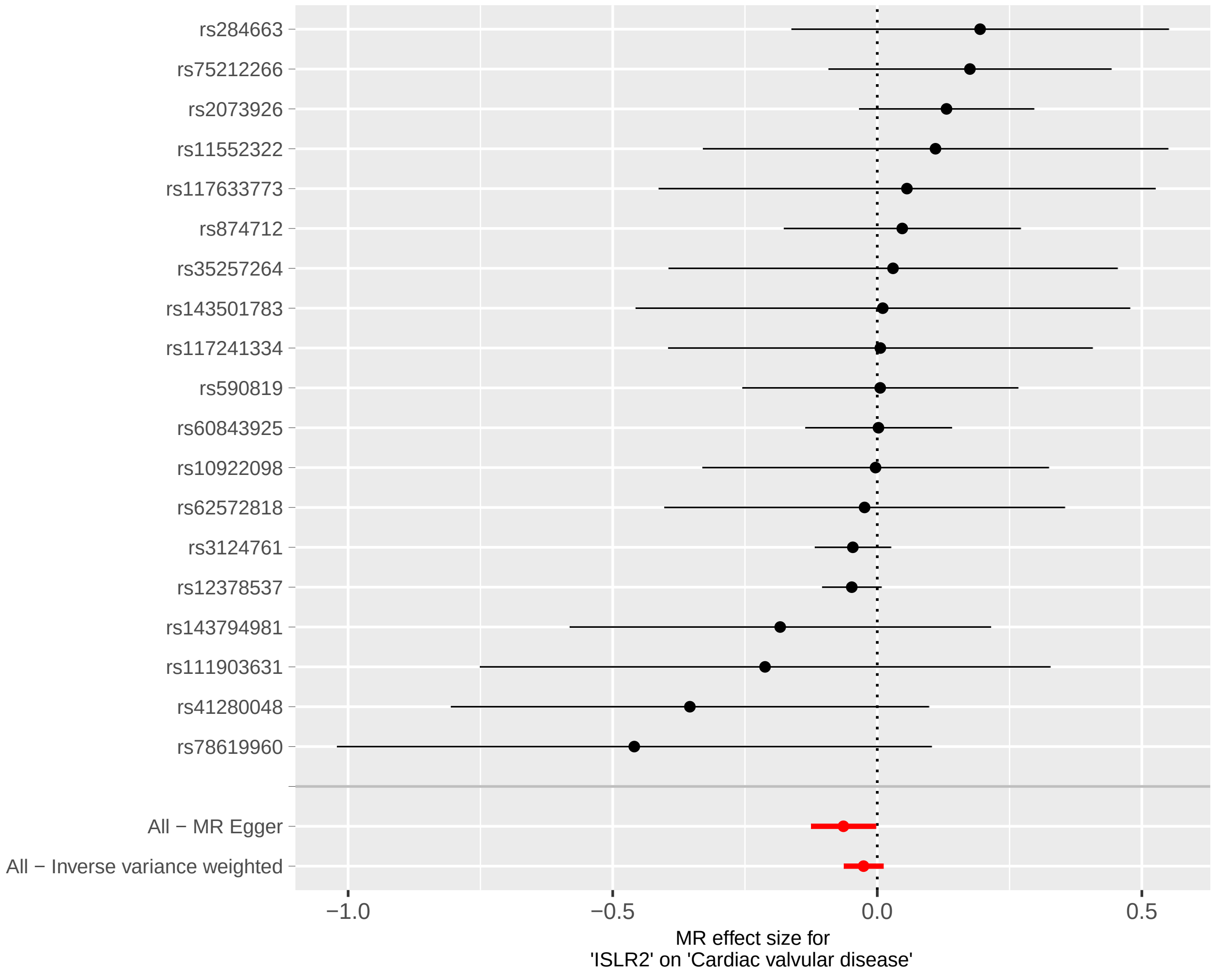
rs284663
rs75212266
rs2073926
rs11552322
rs117633773
rs874712
rs35257264
rs143501783
rs117241334
rs590819
rs60843925
rs10922098
rs62572818
rs3124761
rs12378537
rs143794981
rs111903631
rs41280048
rs78619960
All − MR Egger
All − Inverse variance weighted
−1.0
−0.5
0.0
0.5
MR effect size for
'ISLR2' on 'Cardiac valvular disease'

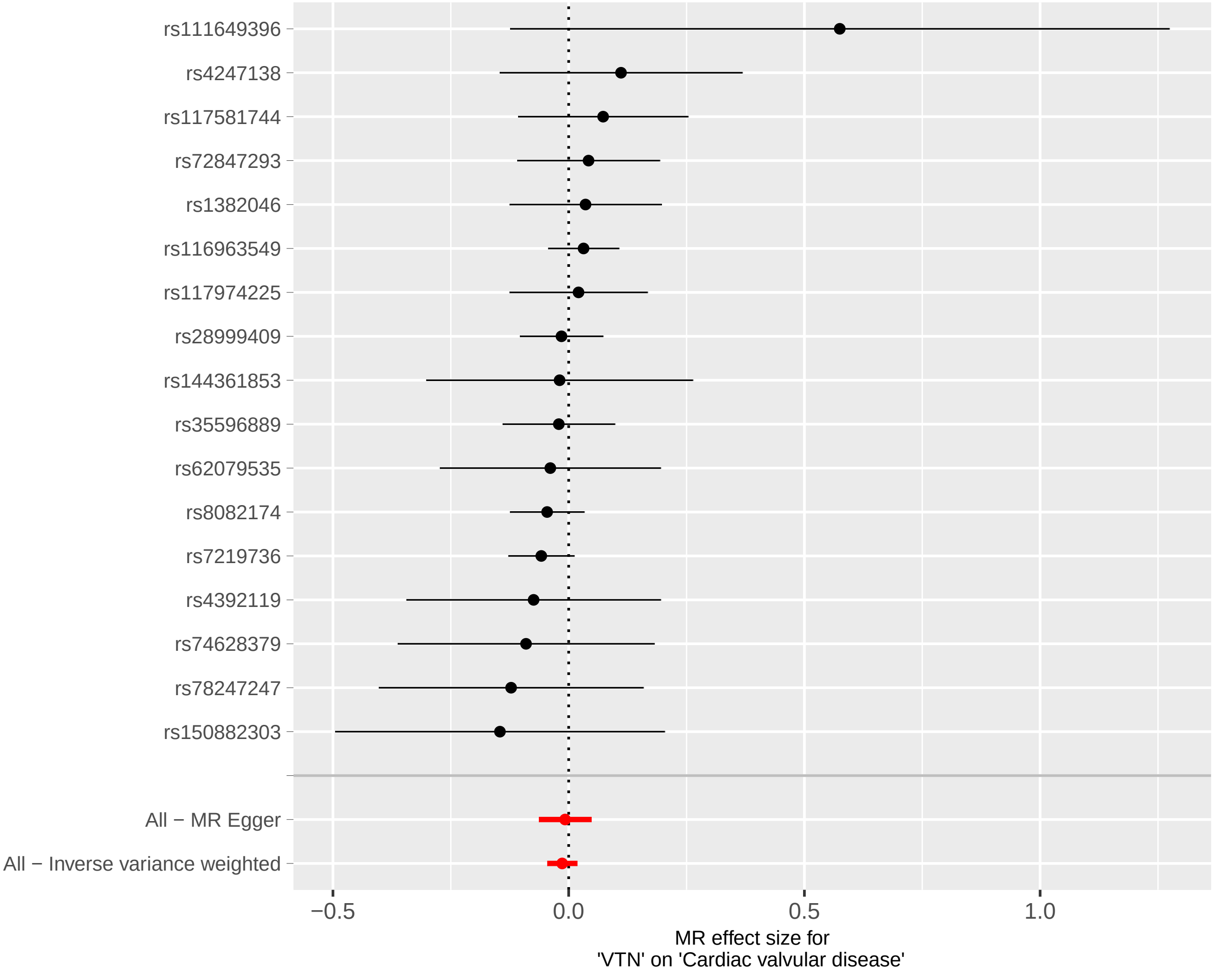
rs111649396
rs4247138
rs117581744
rs72847293
rs1382046
rs116963549
rs117974225
rs28999409
rs144361853
rs35596889
rs62079535
rs8082174
rs7219736
rs4392119
rs74628379
rs78247247
rs150882303
All − MR Egger
All − Inverse variance weighted
−0.5
0.0
0.5
1.0
MR effect size for
'VTN' on 'Cardiac valvular disease'

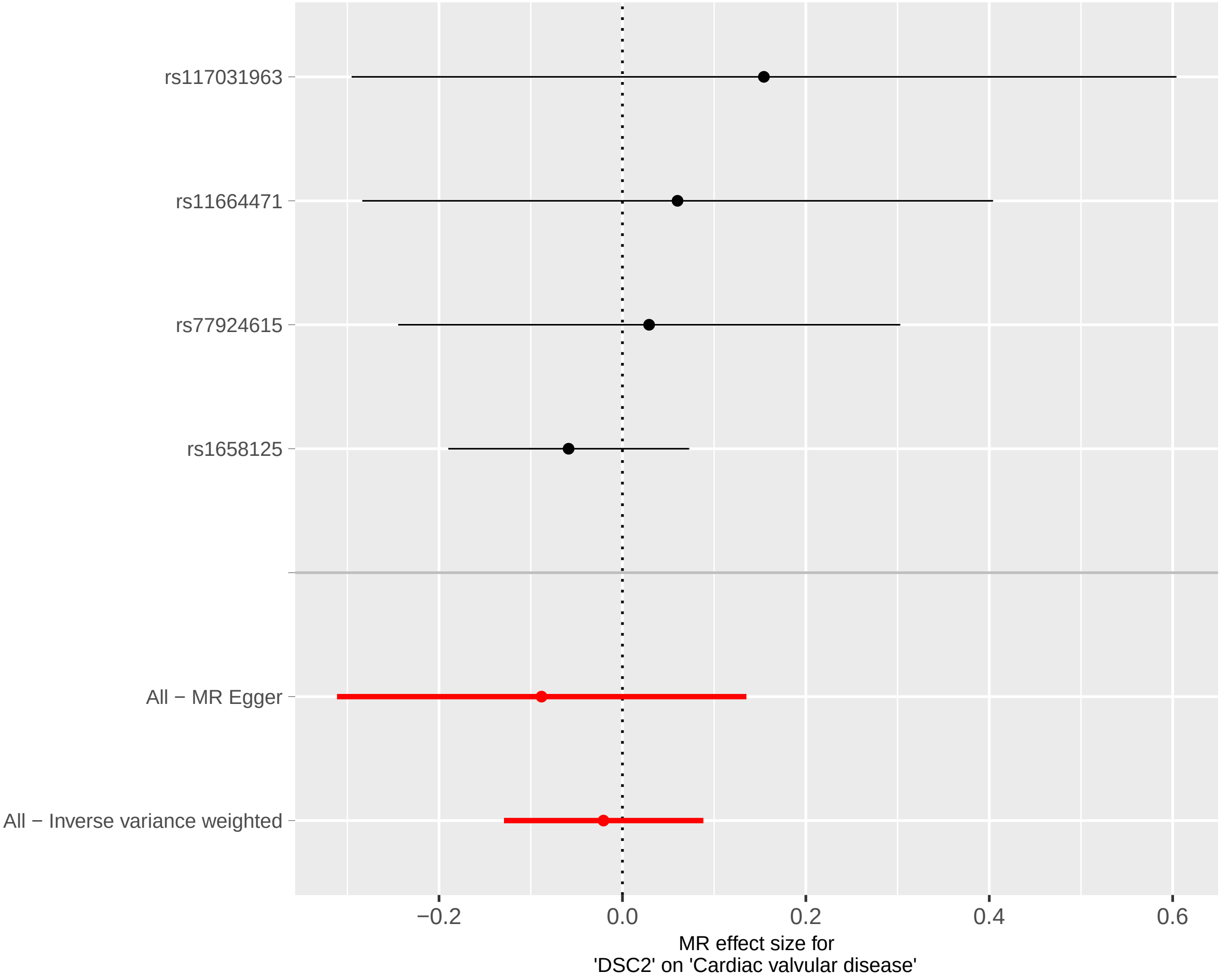
rs117031963
rs11664471
rs77924615
rs1658125
All − MR Egger
All − Inverse variance weighted
−0.2
0.0
0.2
0.4
0.6
MR effect size for
'DSC2' on 'Cardiac valvular disease'

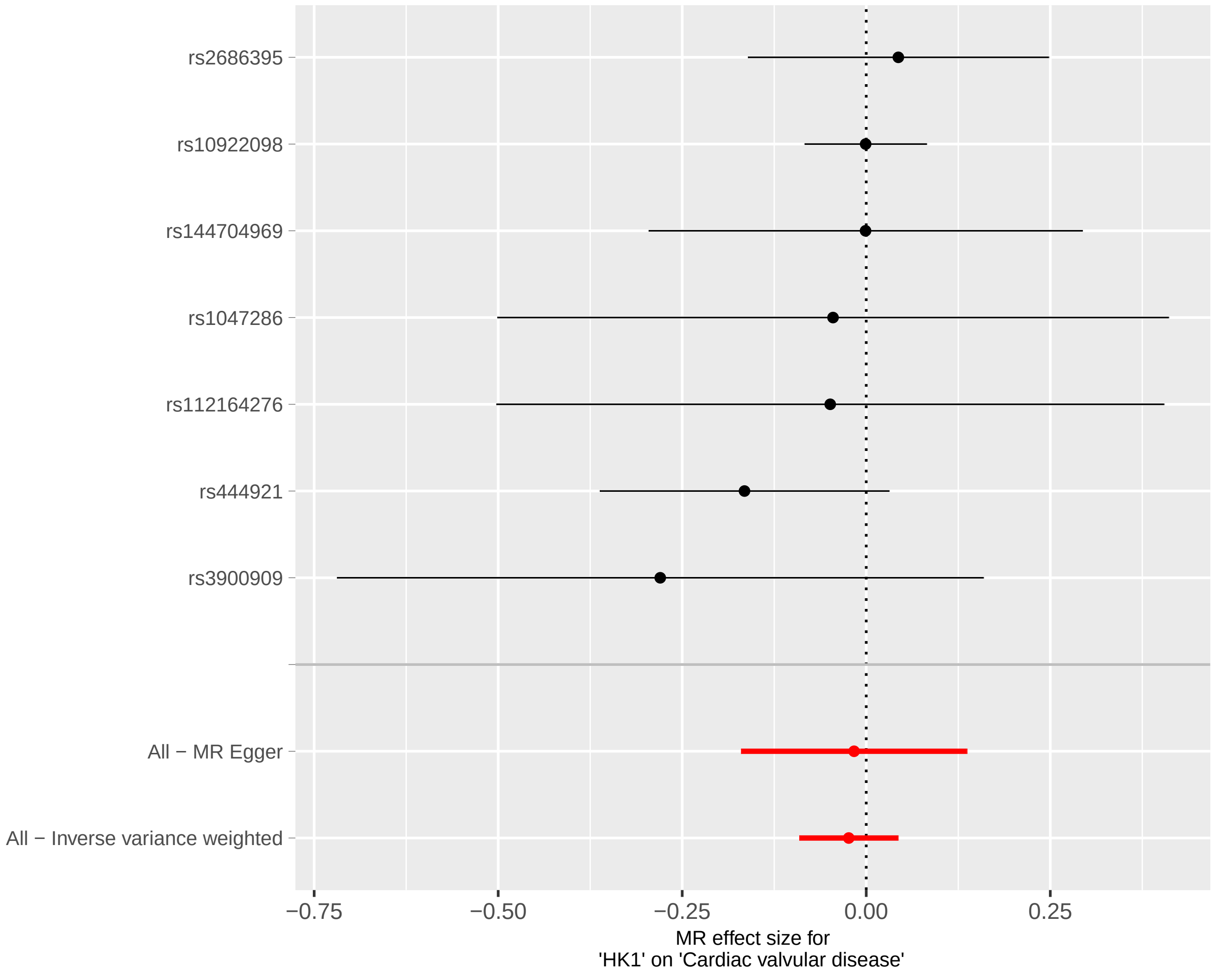
rs2686395
rs10922098
rs144704969
rs1047286
rs112164276
rs444921
rs3900909
All − MR Egger
All − Inverse variance weighted
−0.75
−0.50
−0.25
0.00
0.25
MR effect size for
'HK1' on 'Cardiac valvular disease'

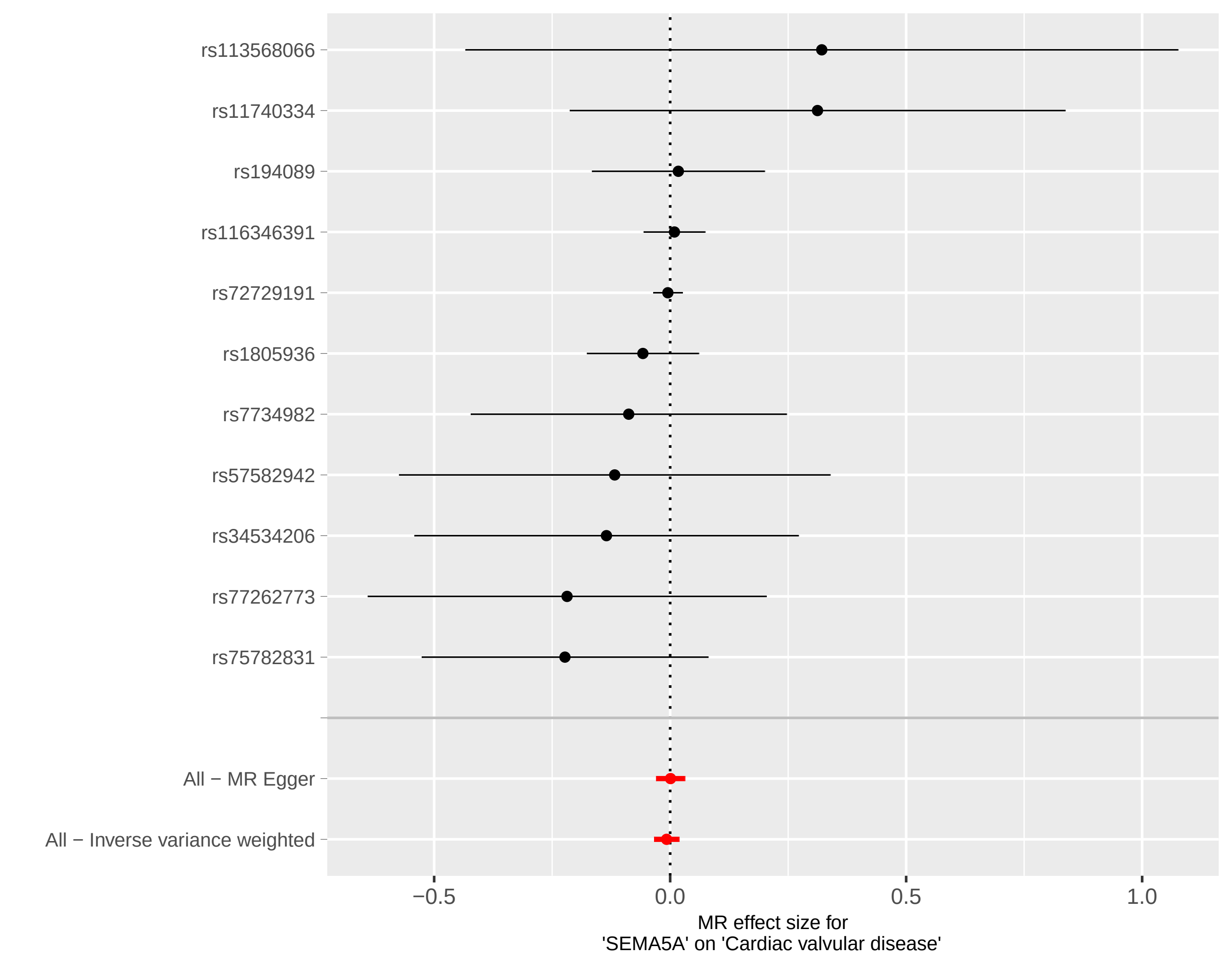

rs113568066
rs11740334
rs194089
rs116346391
rs72729191
rs1805936
rs7734982
rs57582942
rs34534206
rs77262773
rs75782831
All − MR Egger
All − Inverse variance weighted
−0.5
0.0
0.5
1.0
MR effect size for
'SEMA5A' on 'Cardiac valvular disease'

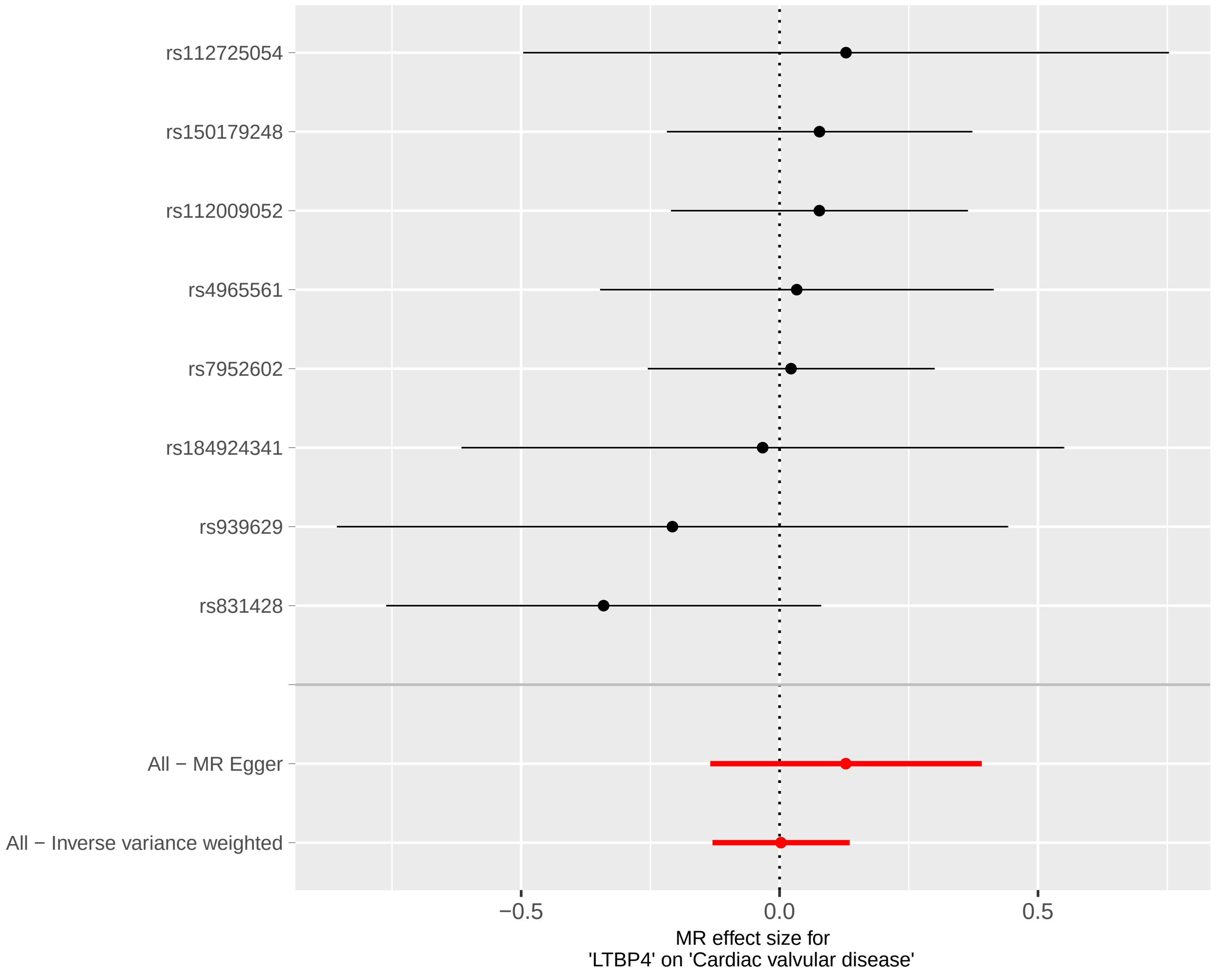
rs112725054
rs150179248
rs112009052
rs4965561
rs7952602
rs184924341
rs939629
rs831428
All − MR Egger
All − Inverse variance weighted
−0.5
0.0
0.5
MR effect size for
'LTBP4' on 'Cardiac valvular disease'

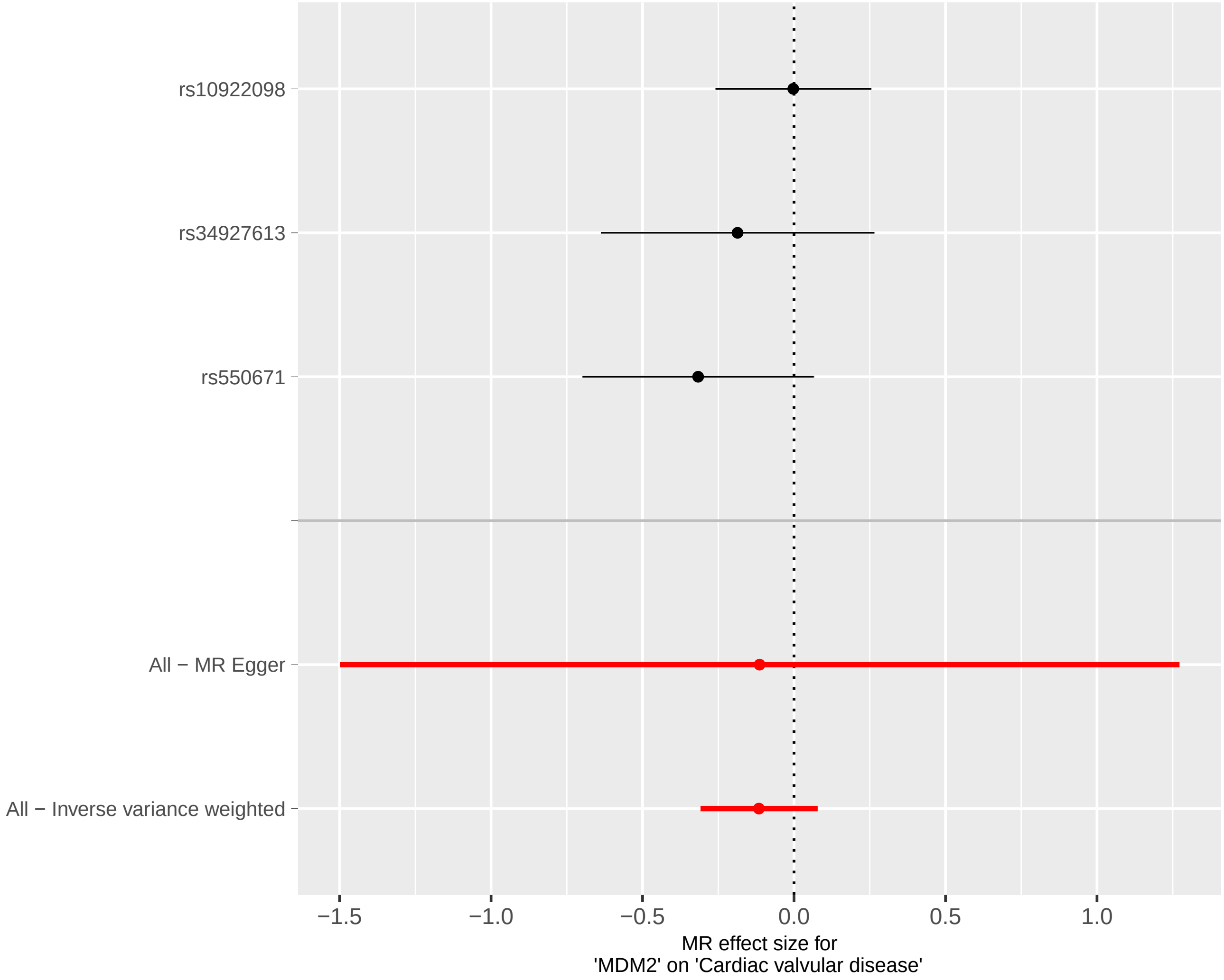
rs10922098
rs34927613
rs550671
All − MR Egger
All − Inverse variance weighted
−1.5
−1.0
−0.5
0.0
0.5
1.0
MR effect size for
'MDM2' on 'Cardiac valvular disease'

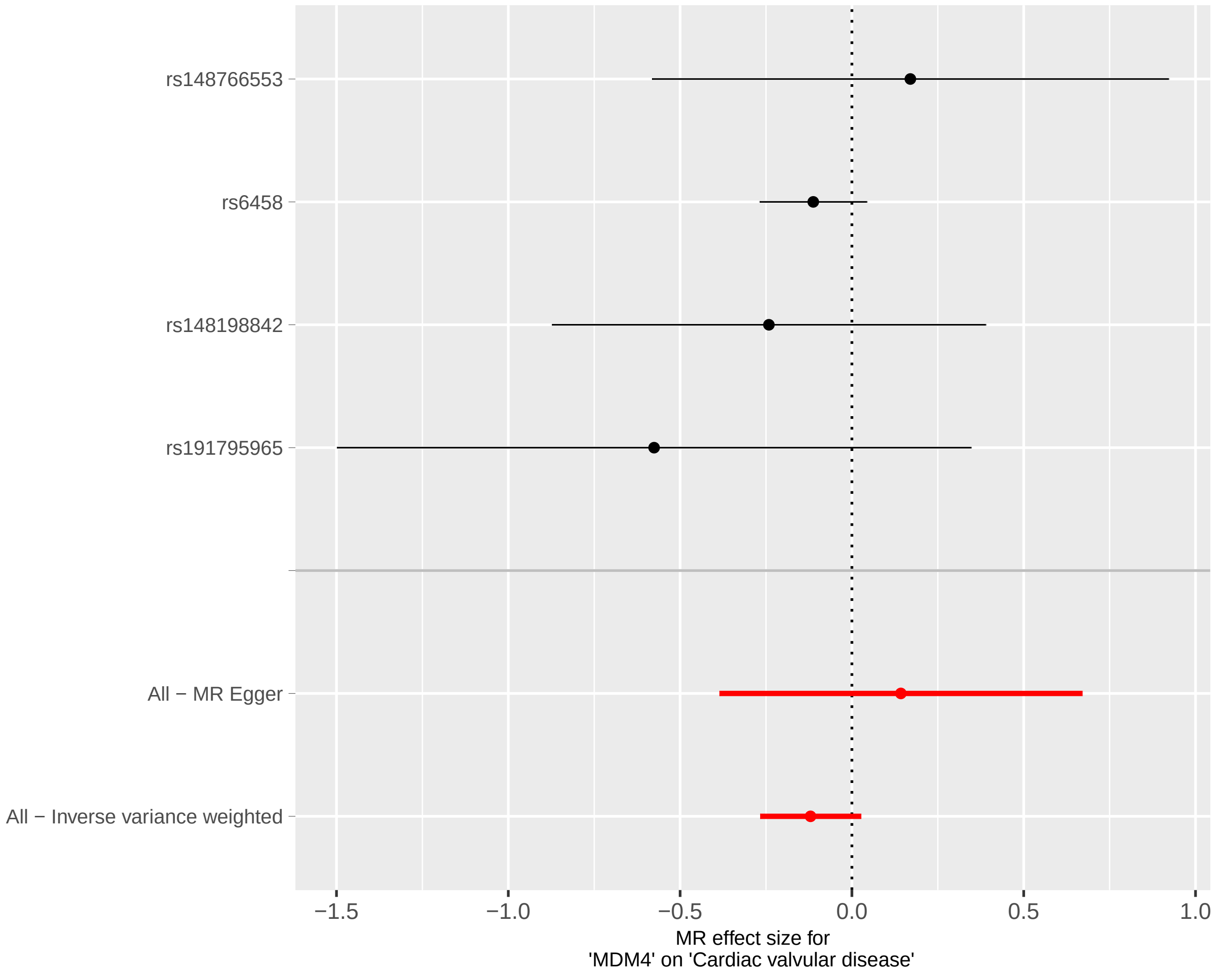
rs148766553
rs6458
rs148198842
rs191795965
All − MR Egger
All − Inverse variance weighted
−1.5
−1.0
−0.5
0.0
0.5
1.0
MR effect size for
'MDM4' on 'Cardiac valvular disease'

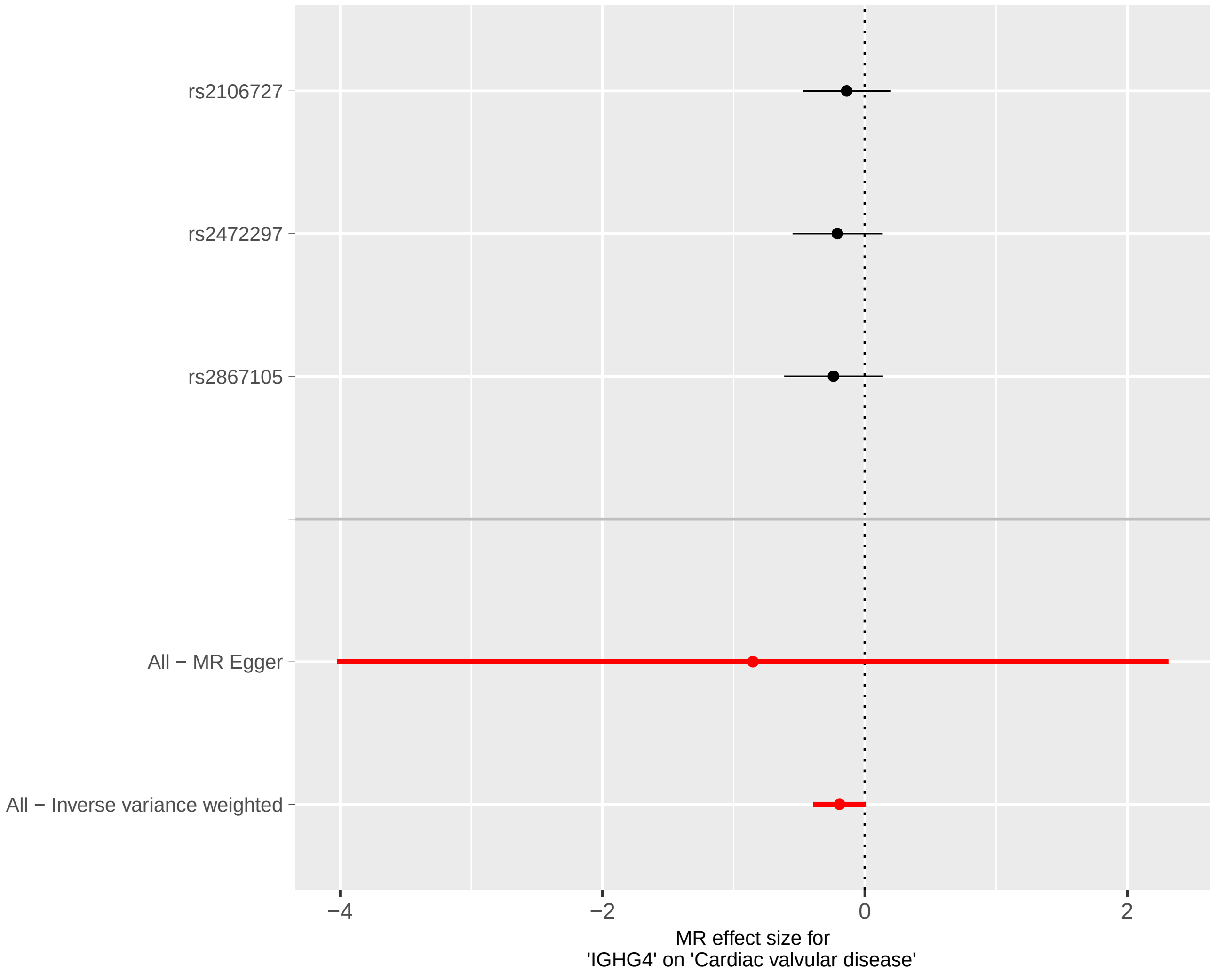
rs2106727
rs2472297
rs2867105
All − MR Egger
All − Inverse variance weighted
−4
−2
0
2
MR effect size for
'IGHG4' on 'Cardiac valvular disease'

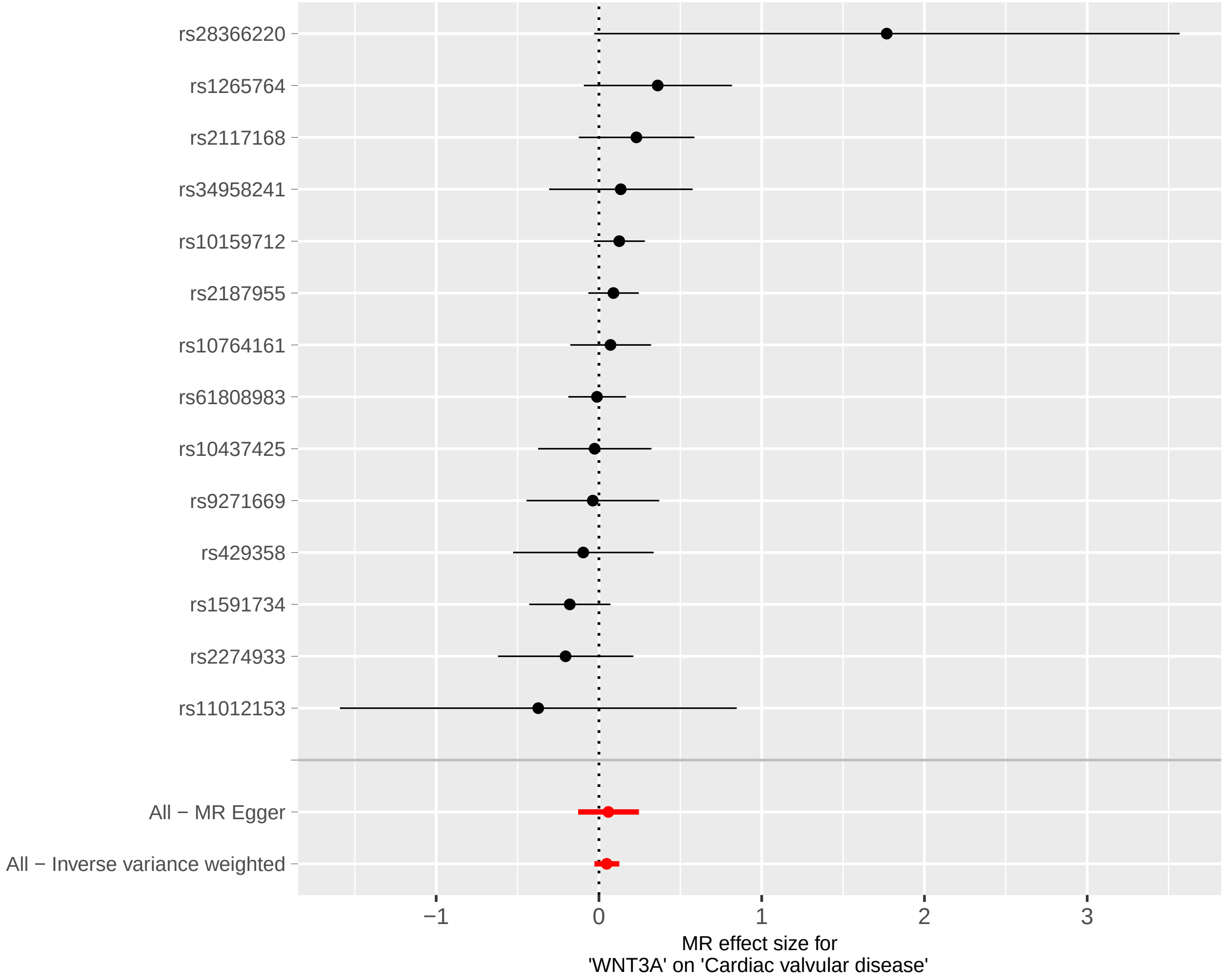

rs28366220
rs1265764
rs2117168
rs34958241
rs10159712
rs2187955
rs10764161
rs61808983
rs10437425
rs9271669
rs429358
rs1591734
rs2274933
rs11012153
All − MR Egger
All − Inverse variance weighted
−1
0
1
2
3
MR effect size for
'WNT3A' on 'Cardiac valvular disease'

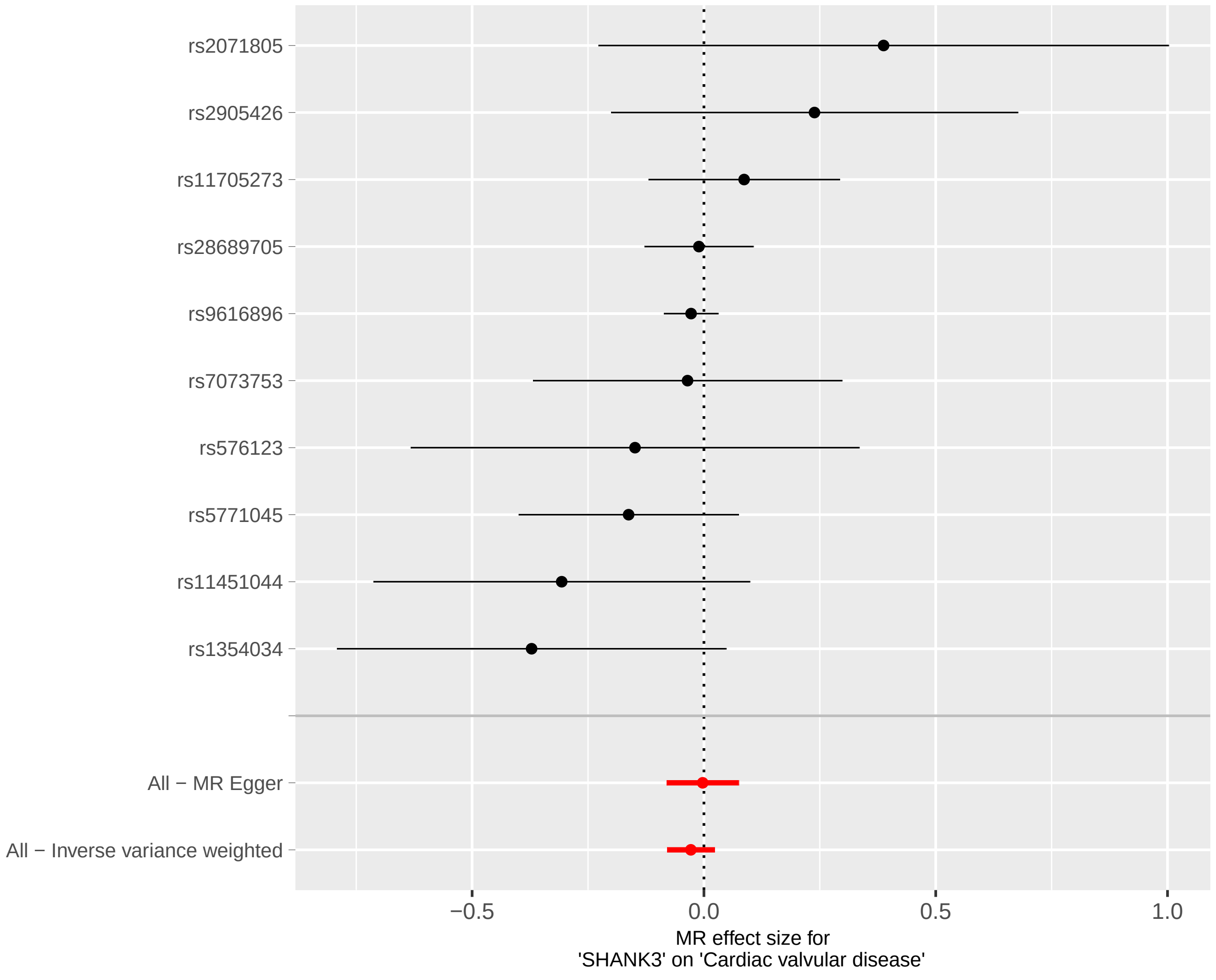

rs2071805
rs2905426
rs11705273
rs28689705
rs9616896
rs7073753
rs576123
rs5771045
rs11451044
rs1354034
All − MR Egger
All − Inverse variance weighted
−0.5
0.0
0.5
1.0
MR effect size for
'SHANK3' on 'Cardiac valvular disease'

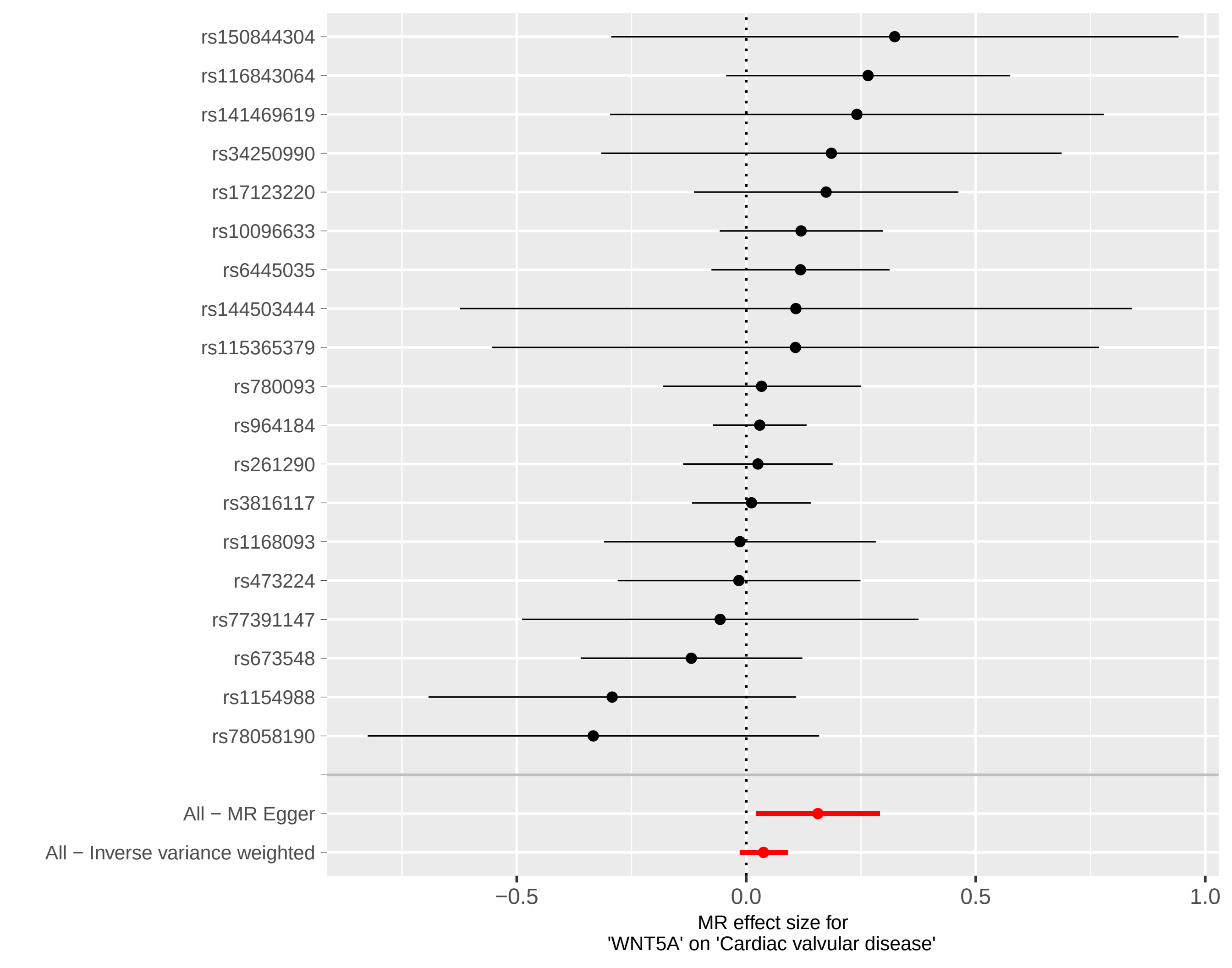

rs150844304
rs116843064
rs141469619
rs34250990
rs17123220
rs10096633
rs6445035
rs144503444
rs115365379
rs780093
rs964184
rs261290
rs3816117
rs1168093
rs473224
rs77391147
rs673548
rs1154988
rs78058190
All − MR Egger
All − Inverse variance weighted
−0.5
0.0
0.5
1.0
MR effect size for
'WNT5A' on 'Cardiac valvular disease'

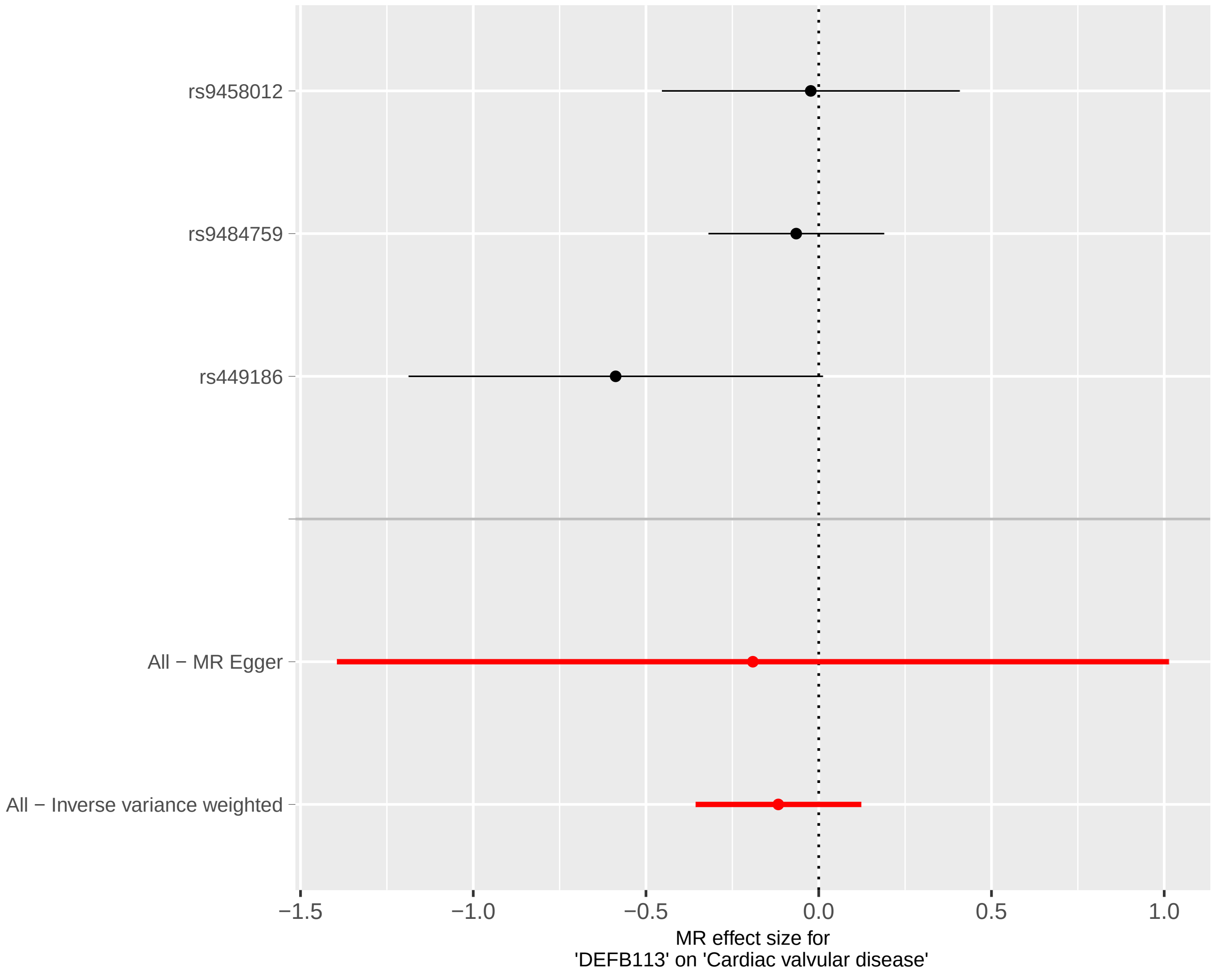
rs9458012
rs9484759
rs449186
All − MR Egger
All − Inverse variance weighted
−1.5
−1.0
−0.5
0.0
0.5
1.0
MR effect size for
'DEFB113' on 'Cardiac valvular disease'

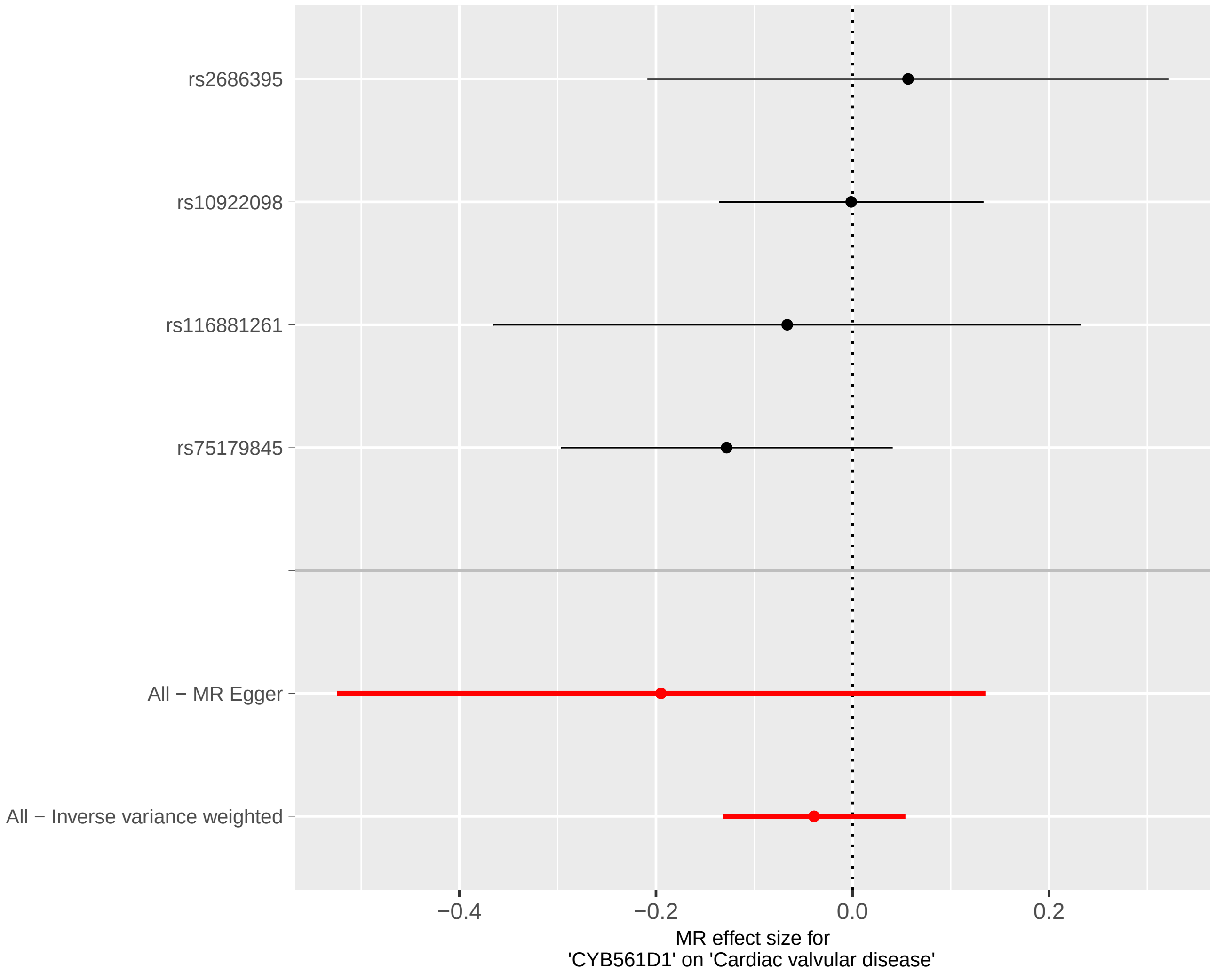

rs2686395
rs10922098
rs116881261
rs75179845
All − MR Egger
All − Inverse variance weighted
−0.4
−0.2
0.0
0.2
MR effect size for
'CYB561D1' on 'Cardiac valvular disease'

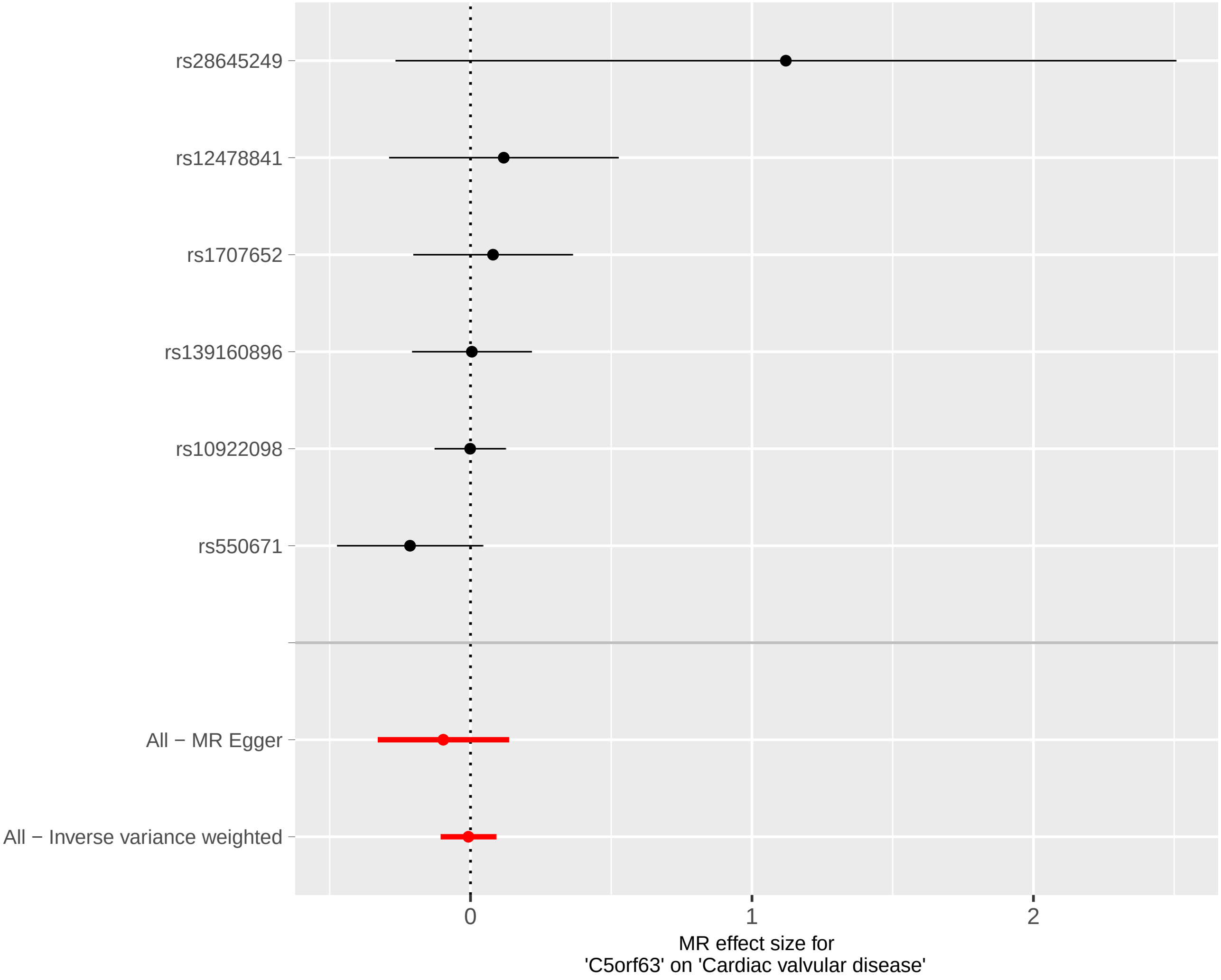

rs28645249
rs12478841
rs1707652
rs139160896
rs10922098
rs550671
All − MR Egger
All − Inverse variance weighted
0
1
2
MR effect size for
'C5orf63' on 'Cardiac valvular disease'

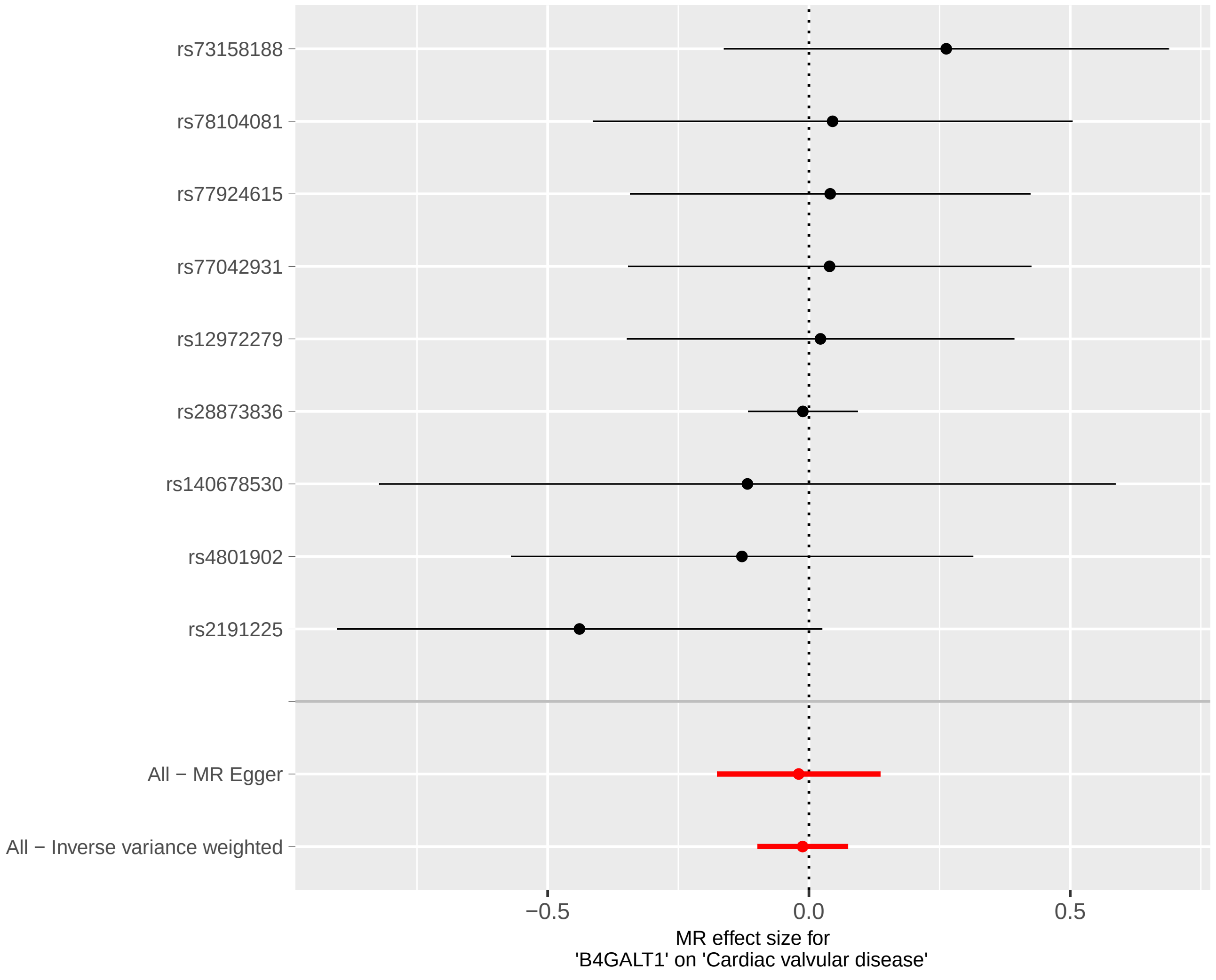
rs73158188
rs78104081
rs77924615
rs77042931
rs12972279
rs28873836
rs140678530
rs4801902
rs2191225
All − MR Egger
All − Inverse variance weighted
−0.5
0.0
0.5
MR effect size for
'B4GALT1' on 'Cardiac valvular disease'

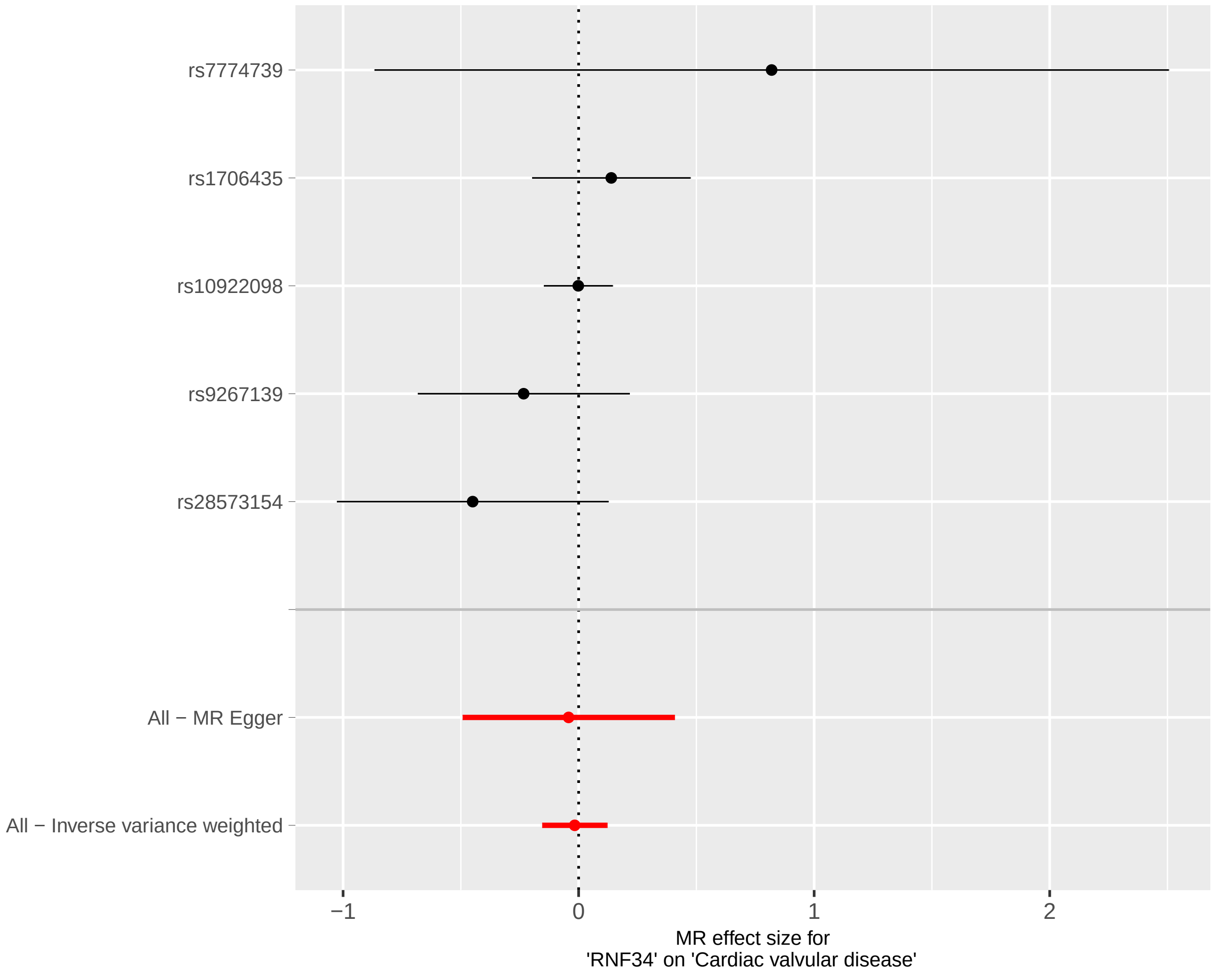

rs7774739
rs1706435
rs10922098
rs9267139
rs28573154
All − MR Egger
All − Inverse variance weighted
−1
0
1
2
MR effect size for
'RNF34' on 'Cardiac valvular disease'

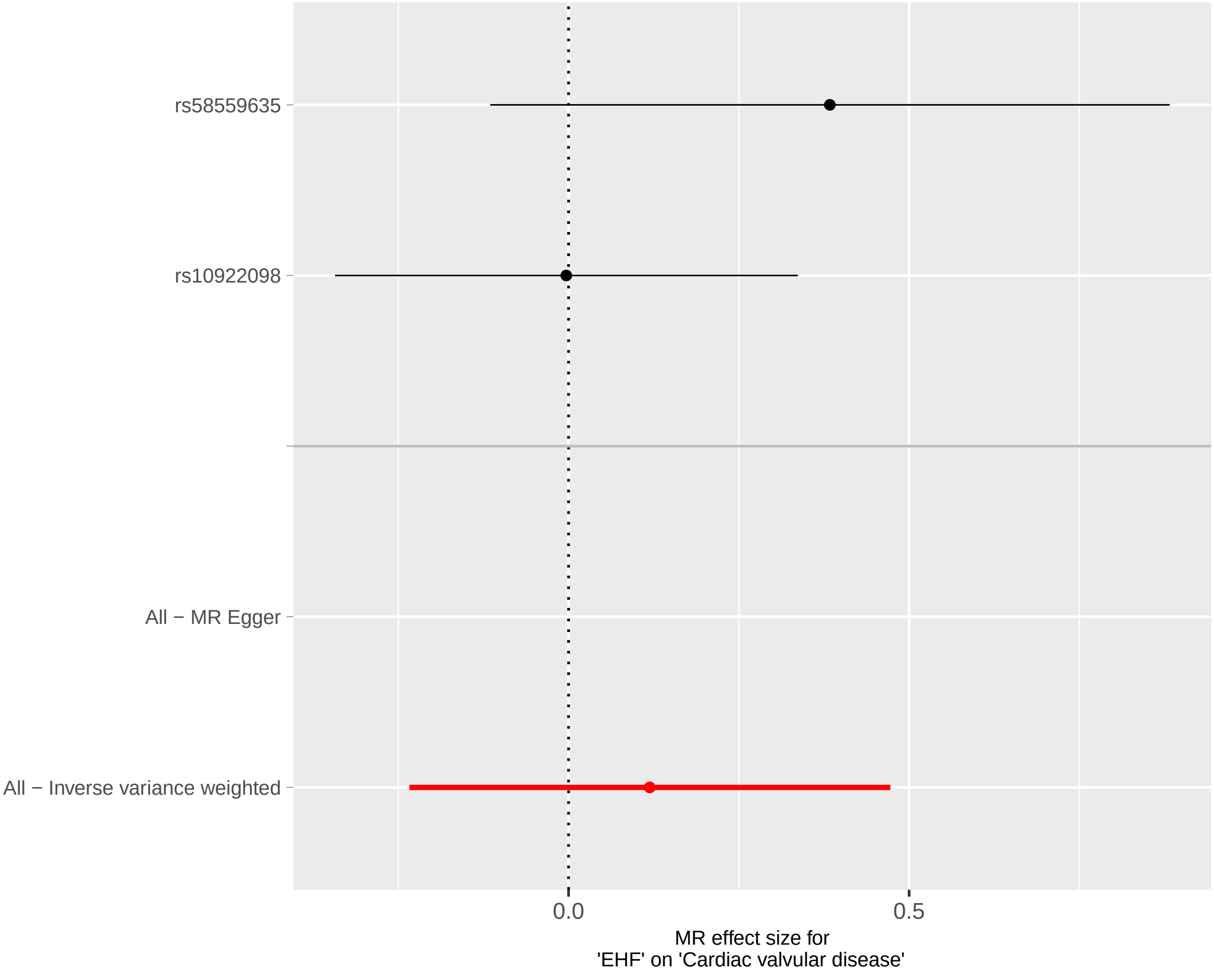
rs58559635
rs10922098
All − MR Egger
All − Inverse variance weighted
0.0
0.5
MR effect size for
'EHF' on 'Cardiac valvular disease'

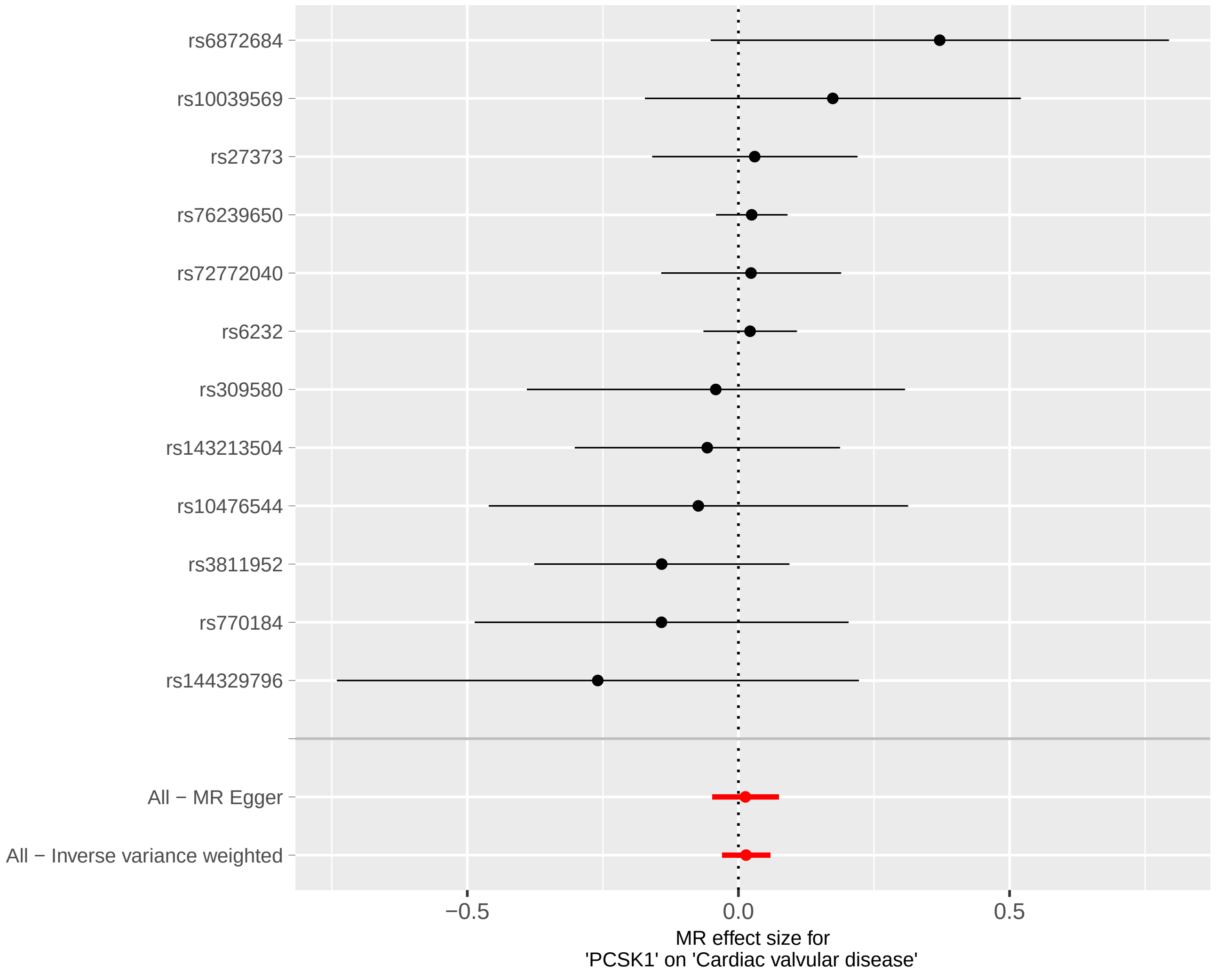
rs6872684
rs10039569
rs27373
rs76239650
rs72772040
rs6232
rs309580
rs143213504
rs10476544
rs3811952
rs770184
rs144329796
All − MR Egger
All − Inverse variance weighted
−0.5
0.0
0.5
MR effect size for
'PCSK1' on 'Cardiac valvular disease'

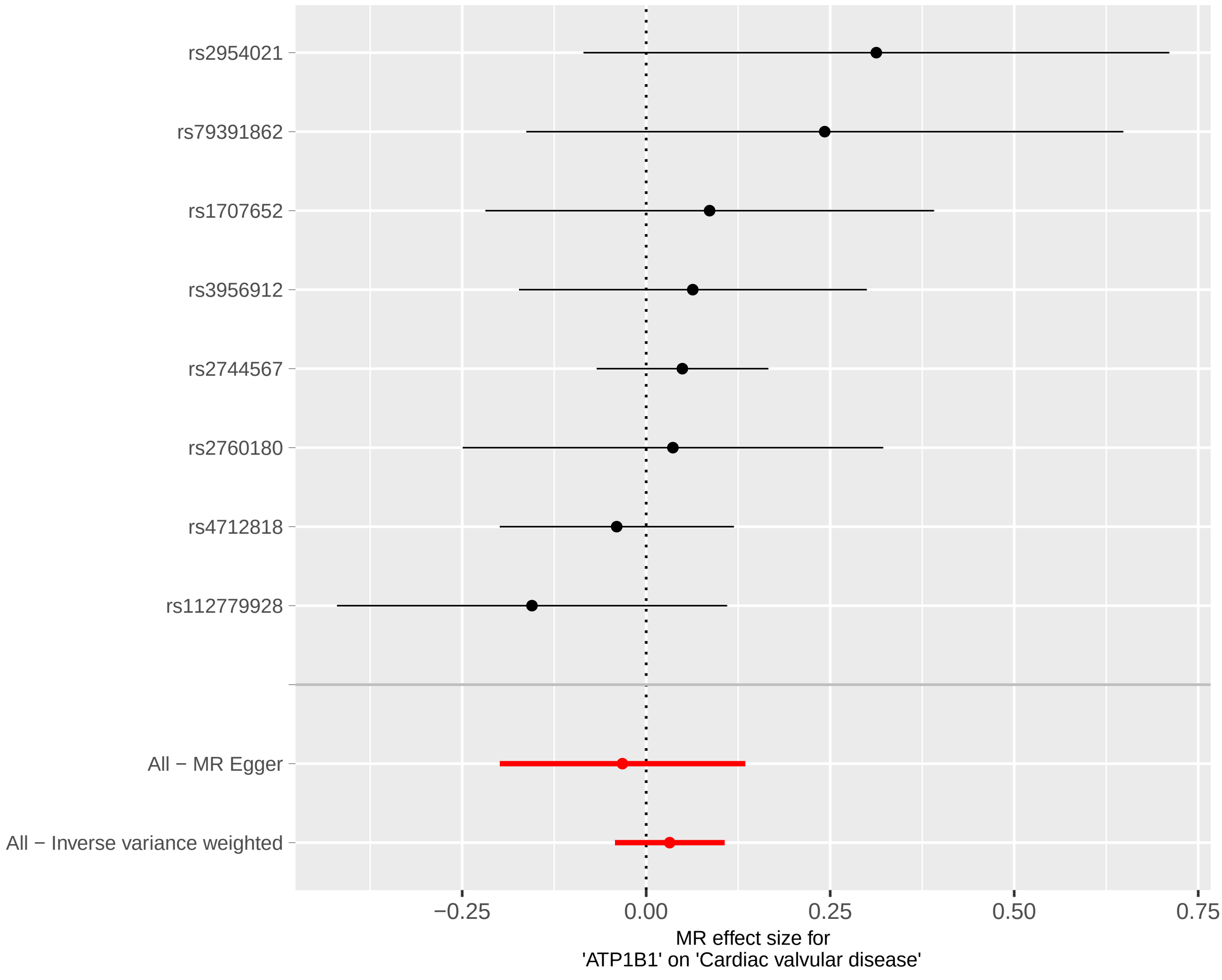

rs2954021
rs79391862
rs1707652
rs3956912
rs2744567
rs2760180
rs4712818
rs112779928
All − MR Egger
All − Inverse variance weighted
−0.25
0.00
0.25
0.50
0.75
MR effect size for
'ATP1B1' on 'Cardiac valvular disease'

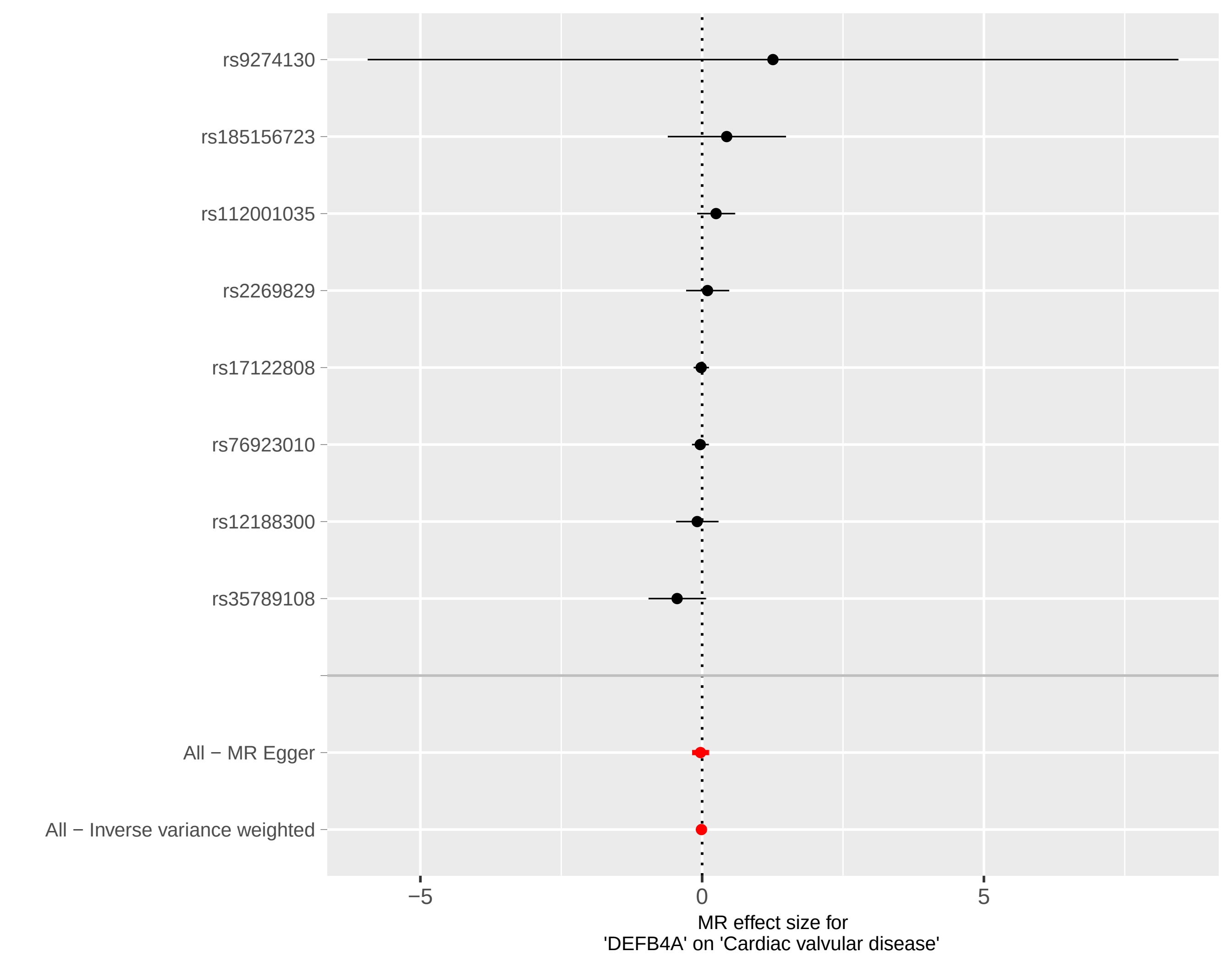
rs9274130
rs185156723
rs112001035
rs2269829
rs17122808
rs76923010
rs12188300
rs35789108
All − MR Egger
All − Inverse variance weighted
−5
0
5
MR effect size for
'DEFB4A' on 'Cardiac valvular disease'

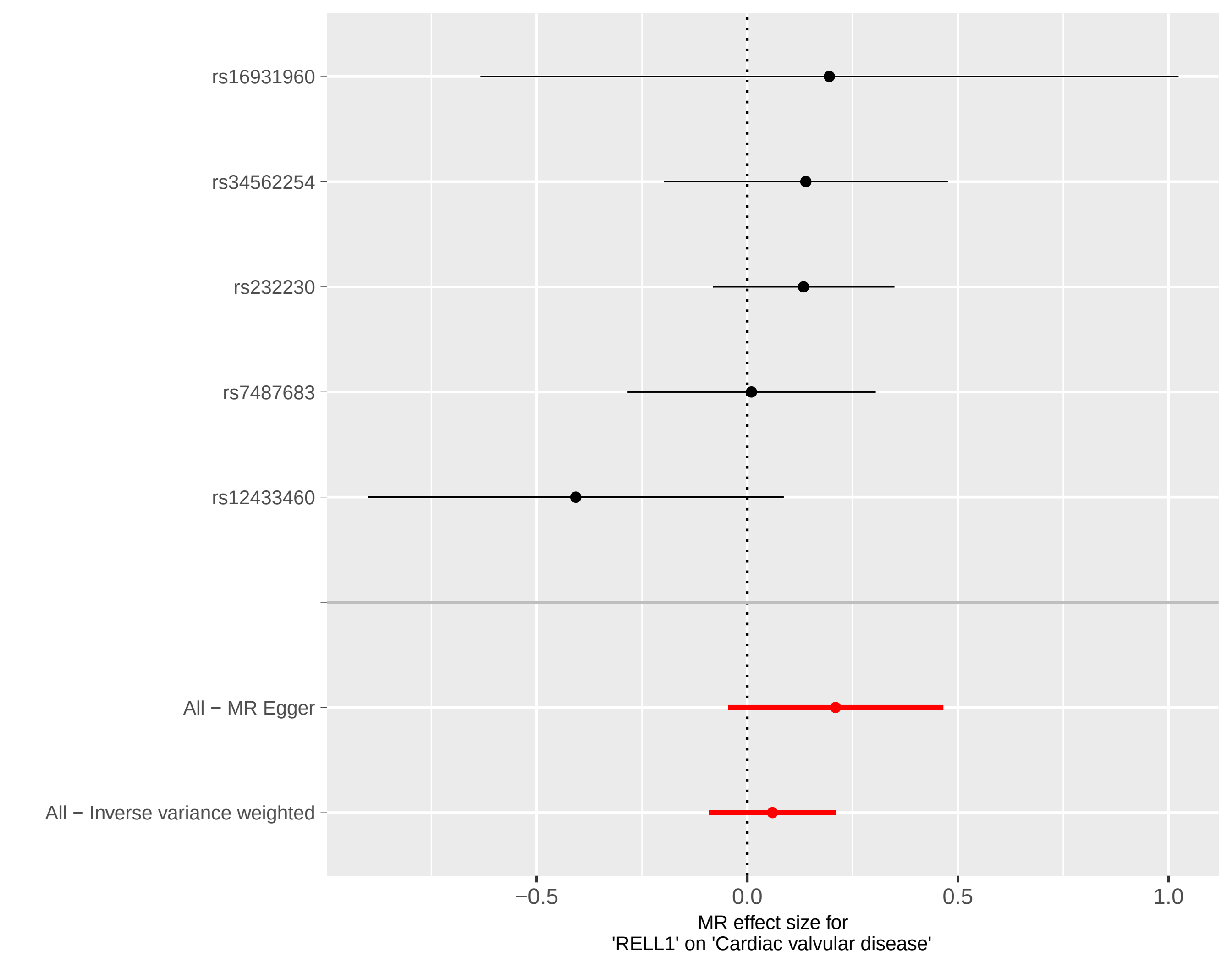
rs16931960
rs34562254
rs232230
rs7487683
rs12433460
All − MR Egger
All − Inverse variance weighted
−0.5
0.0
0.5
1.0
MR effect size for
'RELL1' on 'Cardiac valvular disease'

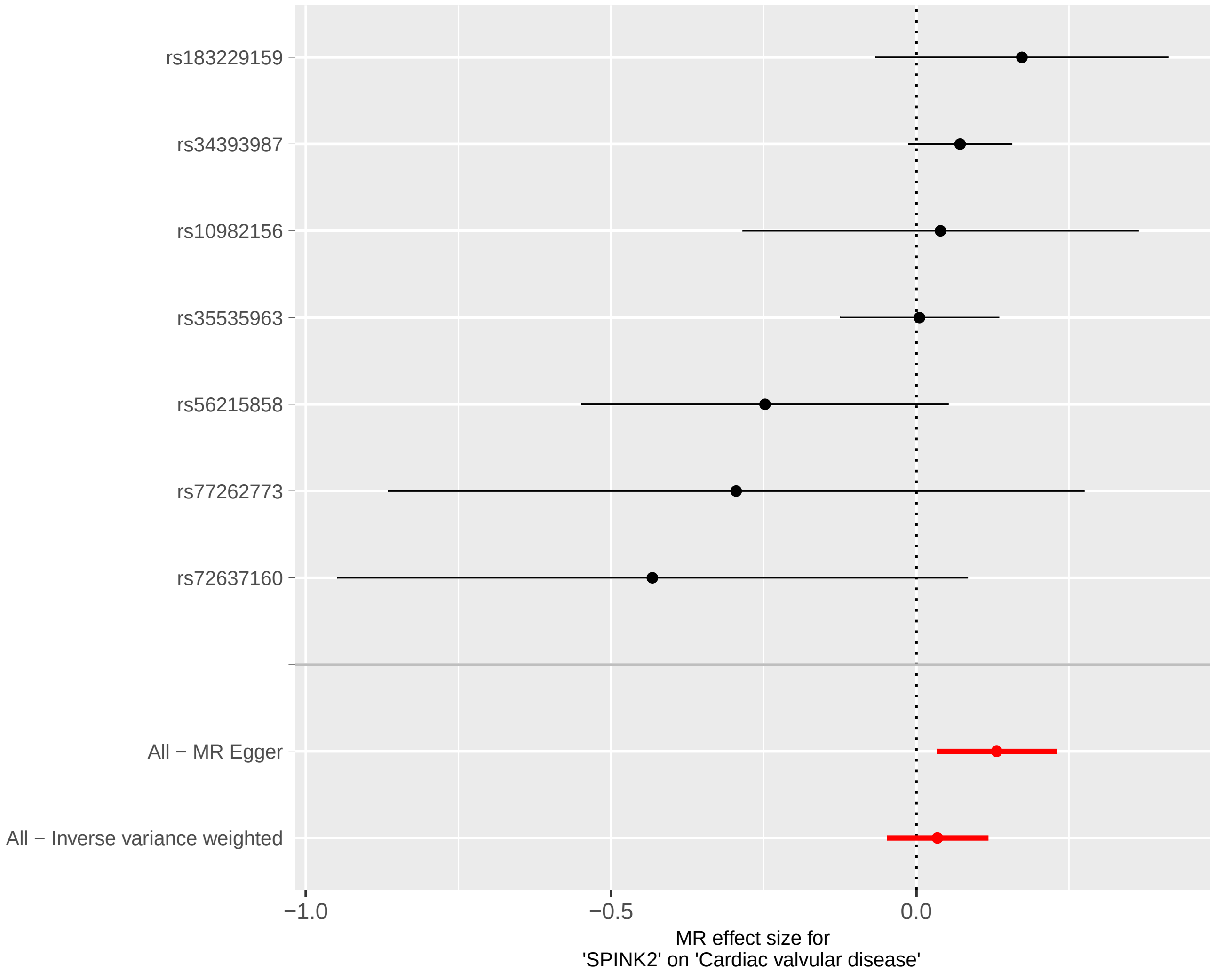
rs183229159
rs34393987
rs10982156
rs35535963
rs56215858
rs77262773
rs72637160
All − MR Egger
All − Inverse variance weighted
−1.0
−0.5
0.0
MR effect size for
'SPINK2' on 'Cardiac valvular disease'

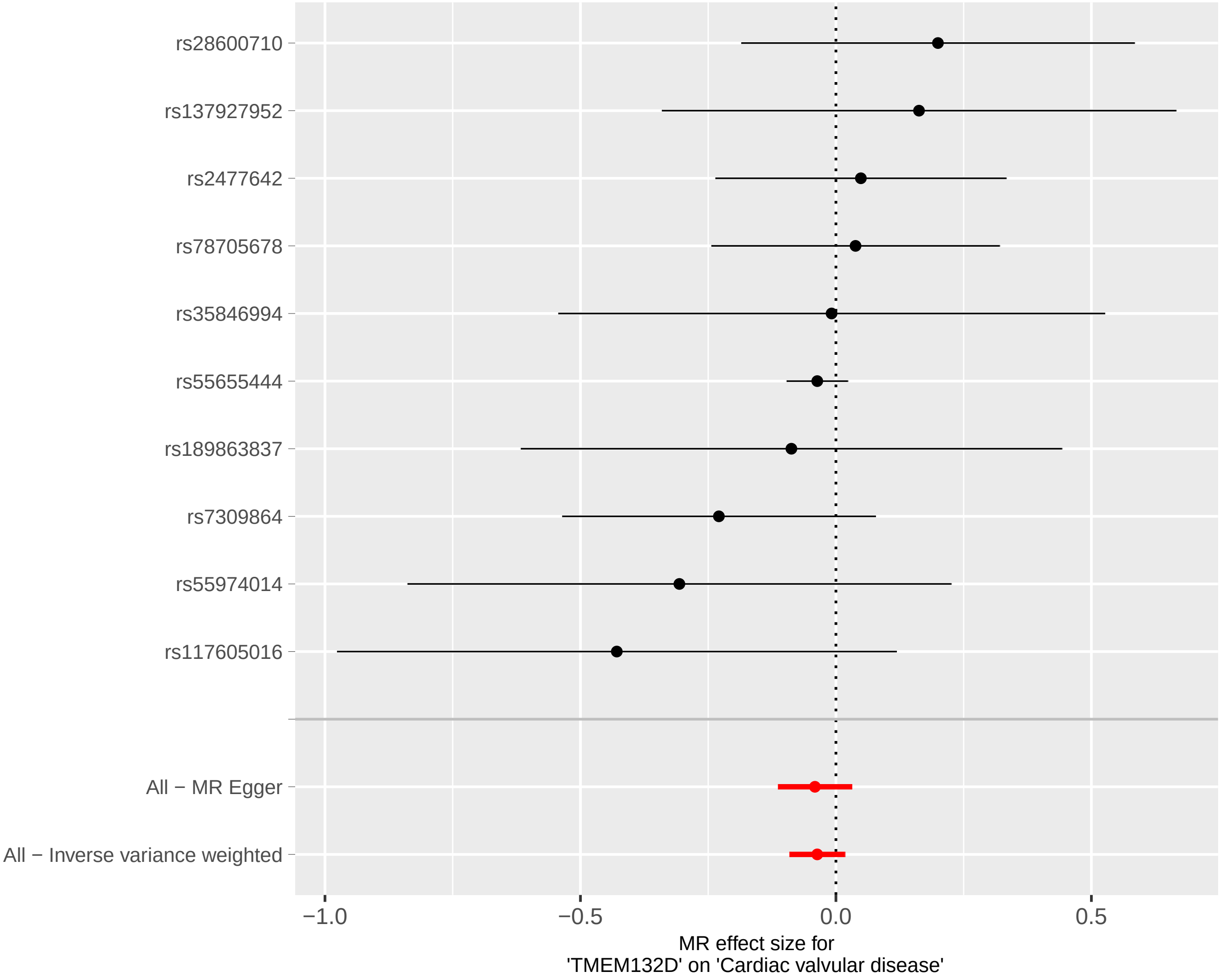
rs28600710
rs137927952
rs2477642
rs78705678
rs35846994
rs55655444
rs189863837
rs7309864
rs55974014
rs117605016
All − MR Egger
All − Inverse variance weighted
−1.0
−0.5
0.0
0.5
MR effect size for
'TMEM132D' on 'Cardiac valvular disease'

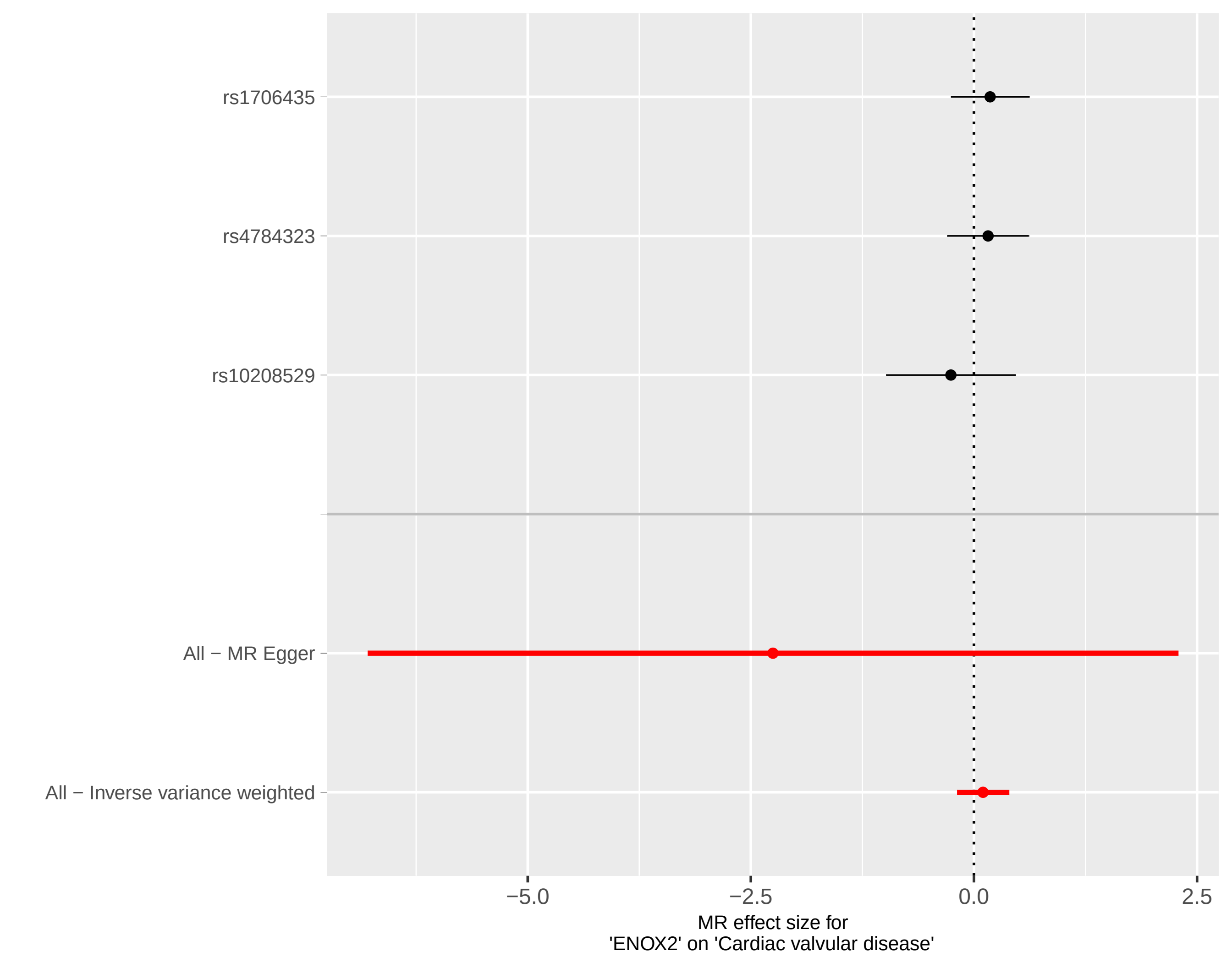
rs1706435
rs4784323
rs10208529
All − MR Egger
All − Inverse variance weighted
−5.0
−2.5
0.0
2.5
MR effect size for
'ENOX2' on 'Cardiac valvular disease'

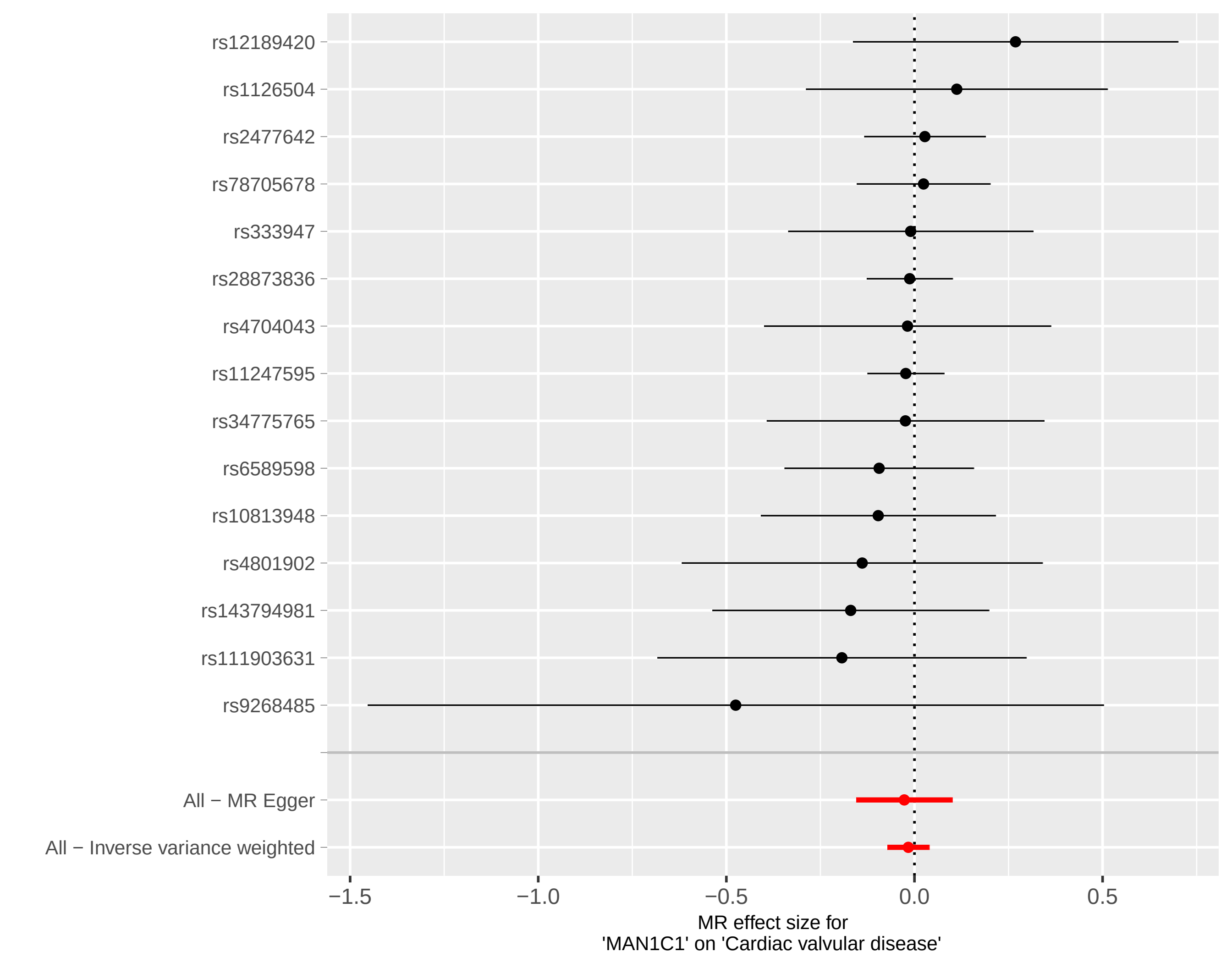
rs12189420
rs1126504
rs2477642
rs78705678
rs333947
rs28873836
rs4704043
rs11247595
rs34775765
rs6589598
rs10813948
rs4801902
rs143794981
rs111903631
rs9268485
All − MR Egger
All − Inverse variance weighted
−1.5
−1.0
−0.5
0.0
0.5
MR effect size for
'MAN1C1' on 'Cardiac valvular disease'

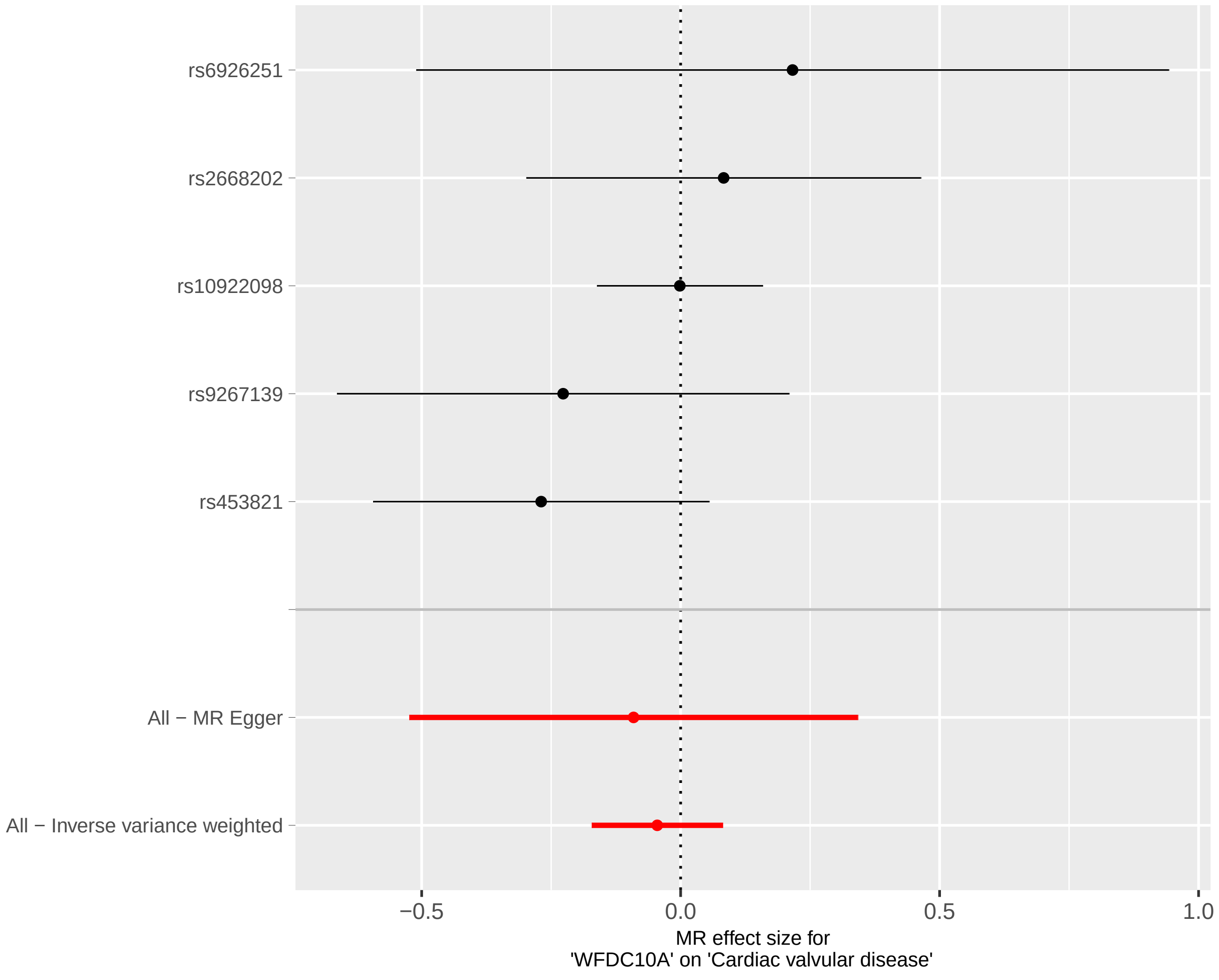
rs6926251
rs2668202
rs10922098
rs9267139
rs453821
All − MR Egger
All − Inverse variance weighted
−0.5
0.0
0.5
1.0
MR effect size for
'WFDC10A' on 'Cardiac valvular disease'

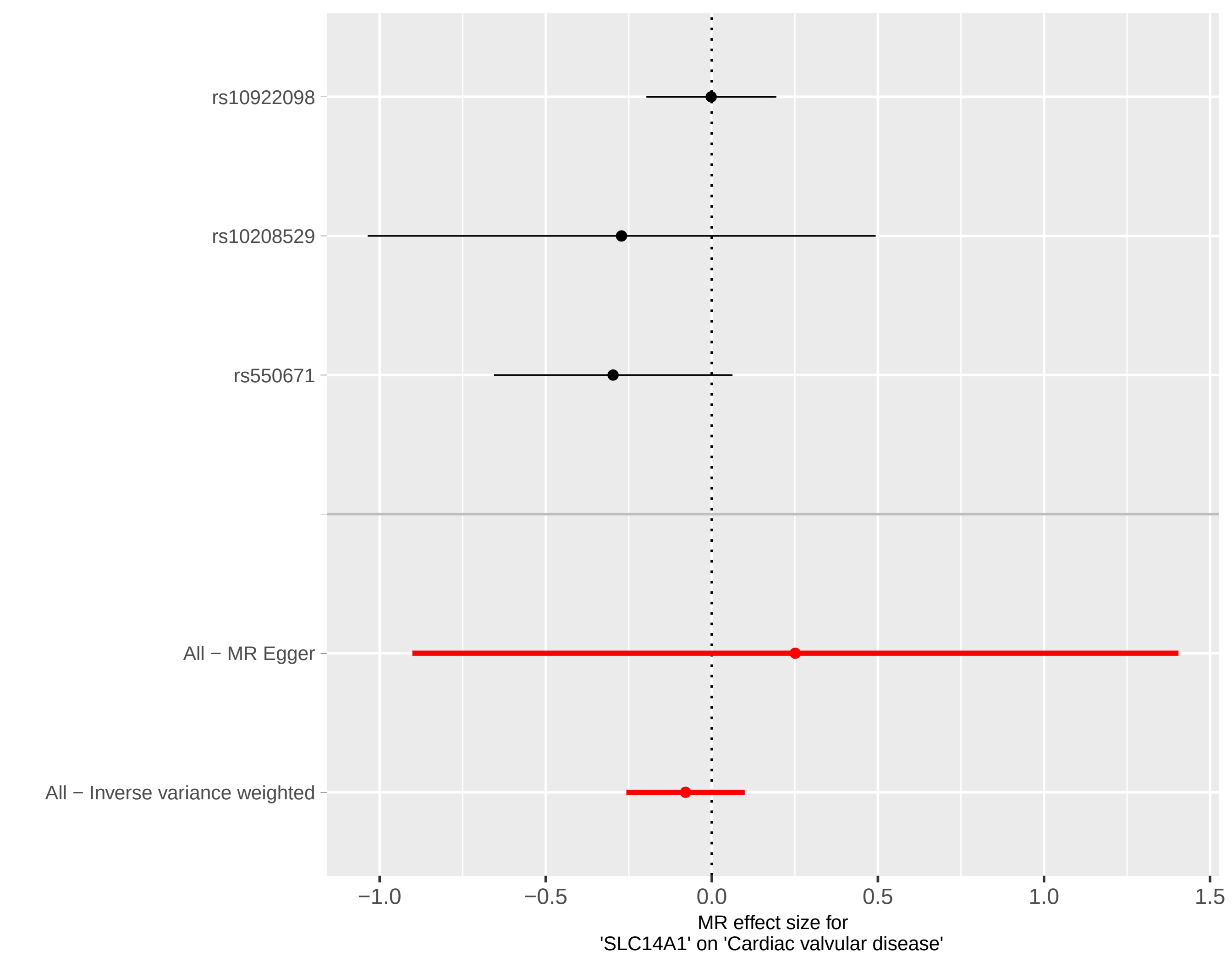

rs10922098
rs10208529
rs550671
All − MR Egger
All − Inverse variance weighted
−1.0
−0.5
0.0
0.5
1.0
1.5
MR effect size for
'SLC14A1' on 'Cardiac valvular disease'

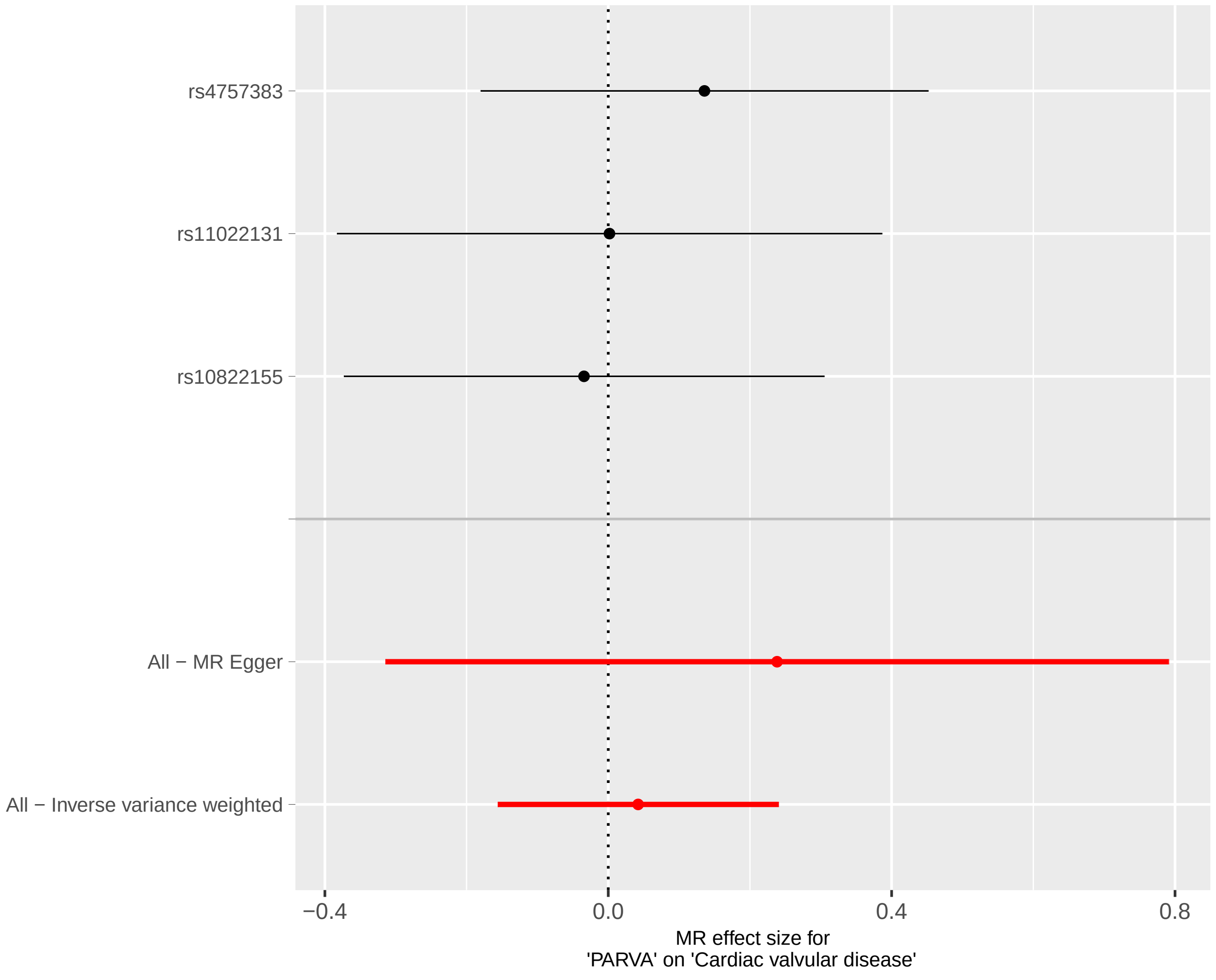
rs4757383
rs11022131
rs10822155
All − MR Egger
All − Inverse variance weighted
−0.4
0.0
0.4
0.8
MR effect size for
'PARVA' on 'Cardiac valvular disease'

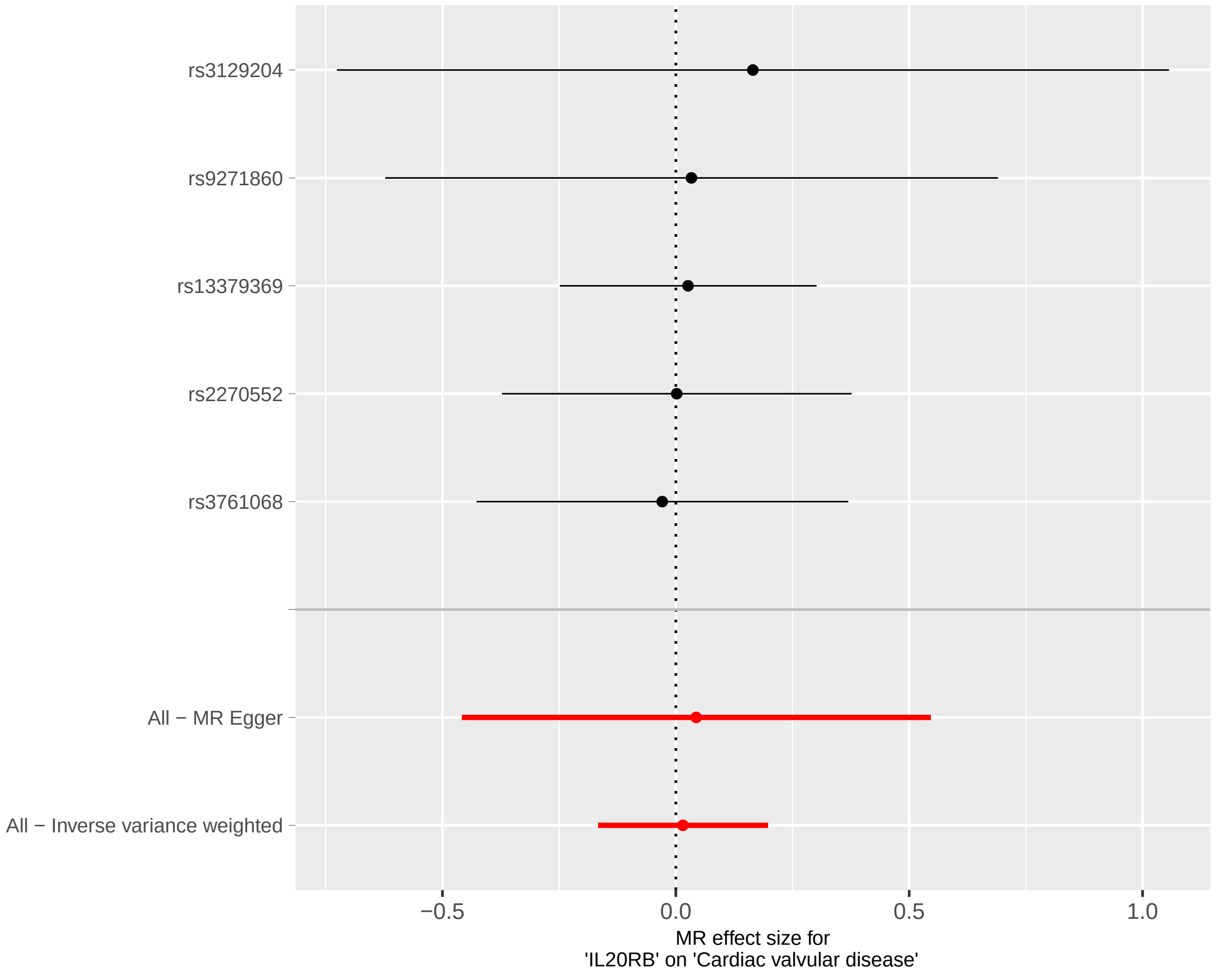

rs3129204
rs9271860
rs13379369
rs2270552
rs3761068
All − MR Egger
All − Inverse variance weighted
−0.5
0.0
0.5
1.0
MR effect size for
'IL20RB' on 'Cardiac valvular disease'

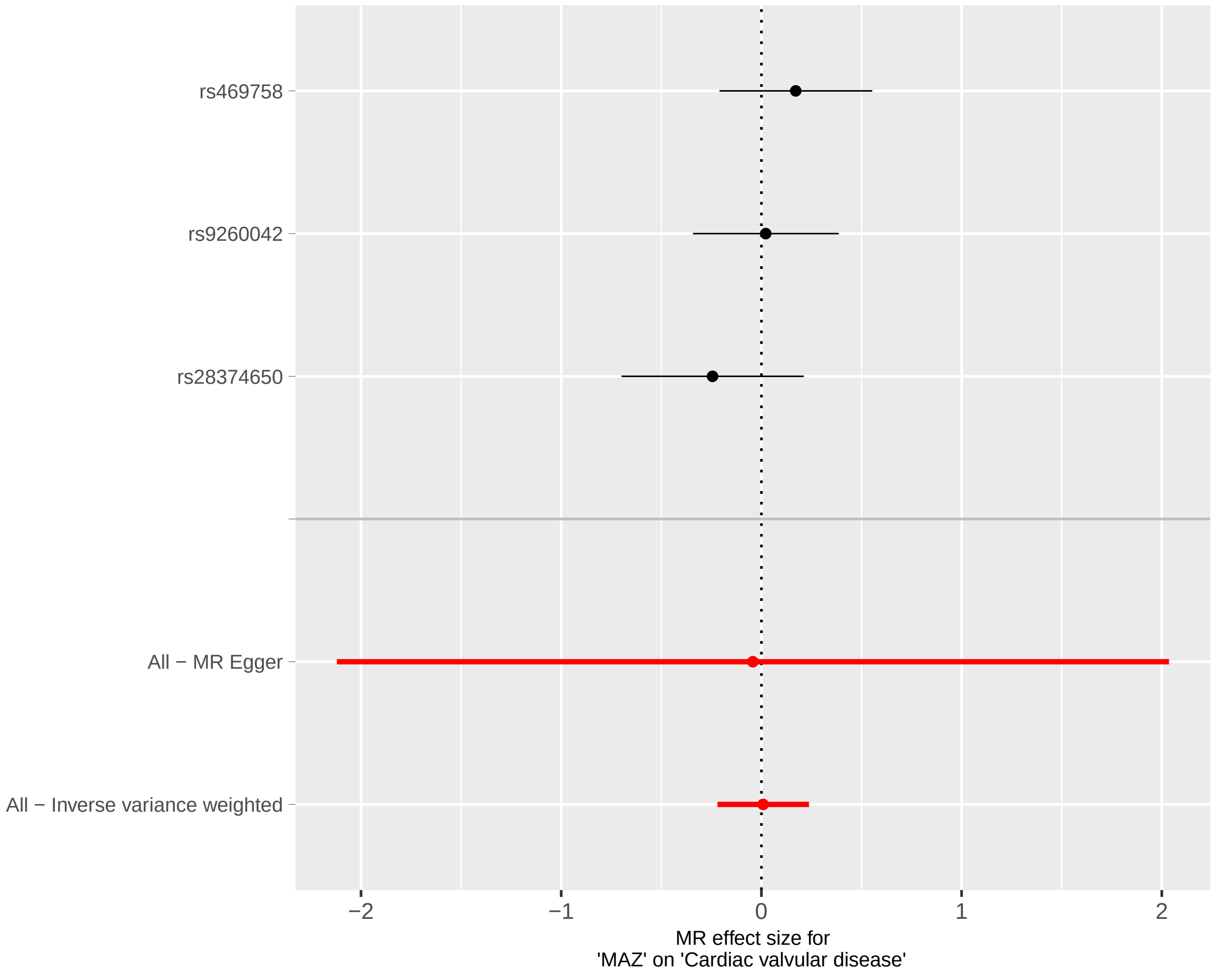

rs469758
rs9260042
rs28374650
All − MR Egger
All − Inverse variance weighted
−2
−1
0
1
2
MR effect size for
'MAZ' on 'Cardiac valvular disease'

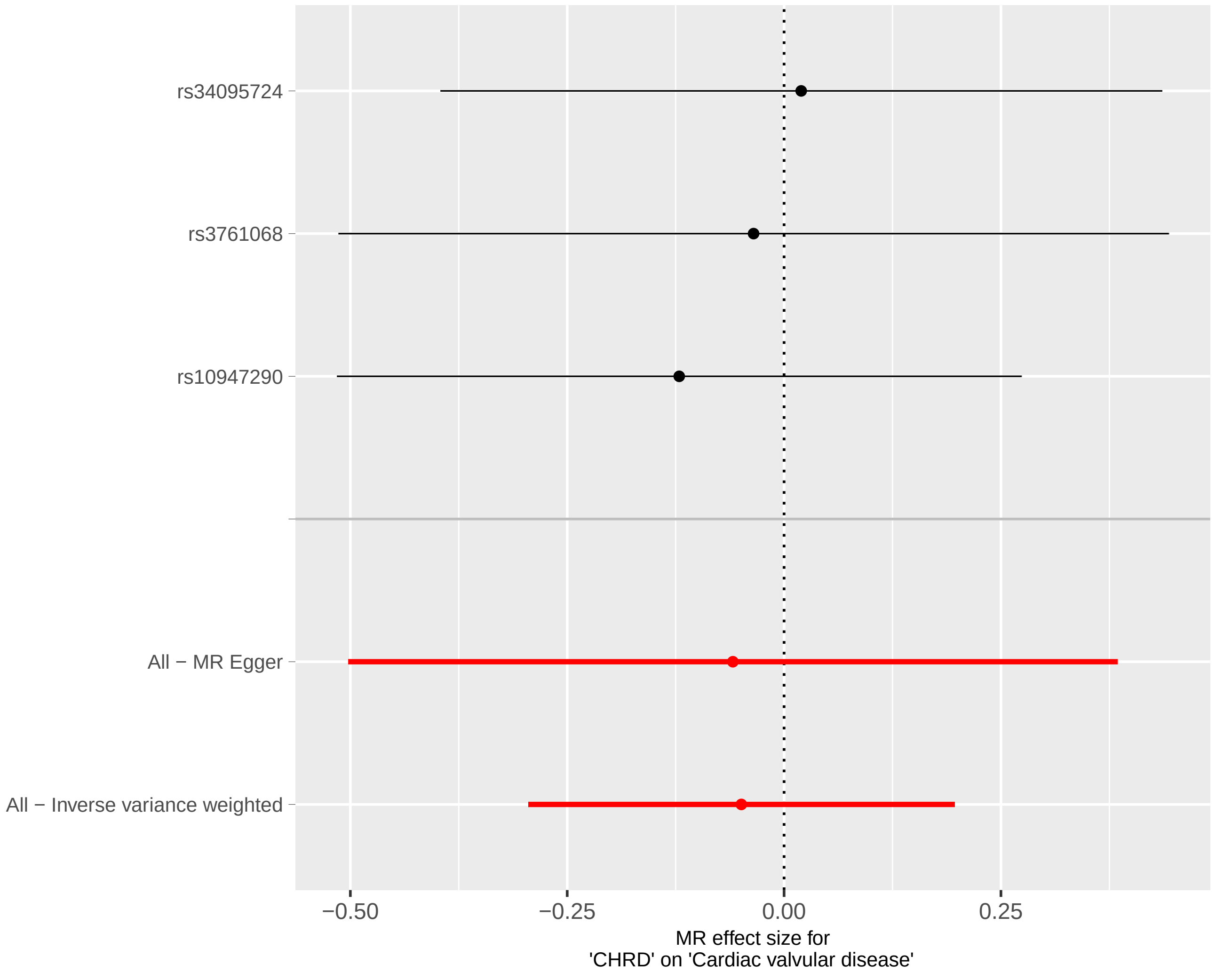

rs34095724
rs3761068
rs10947290
All − MR Egger
All − Inverse variance weighted
−0.50
−0.25
0.00
0.25
MR effect size for
'CHRD' on 'Cardiac valvular disease'

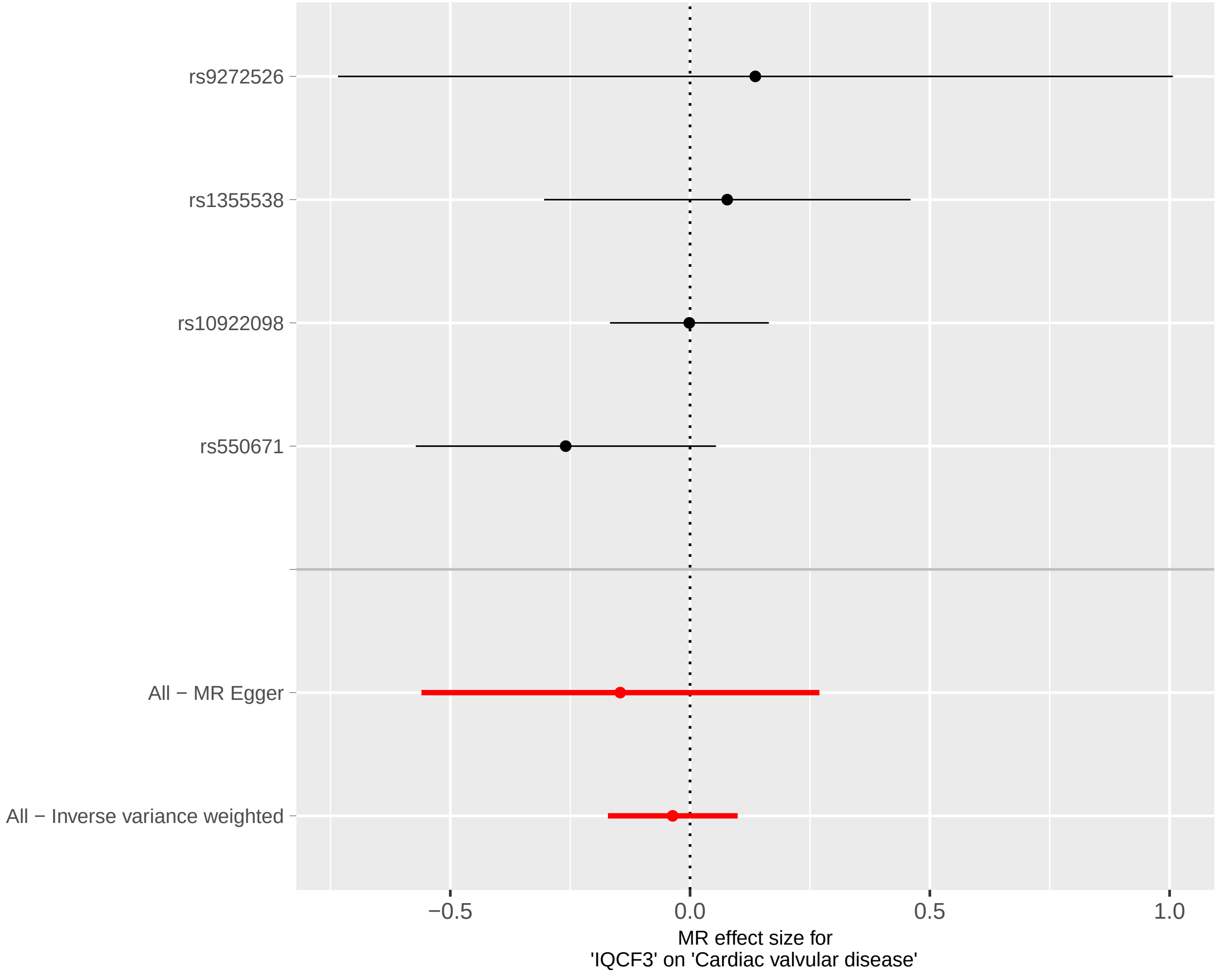

rs9272526
rs1355538
rs10922098
rs550671
All − MR Egger
All − Inverse variance weighted
−0.5
0.0
0.5
1.0
MR effect size for
'IQCF3' on 'Cardiac valvular disease'

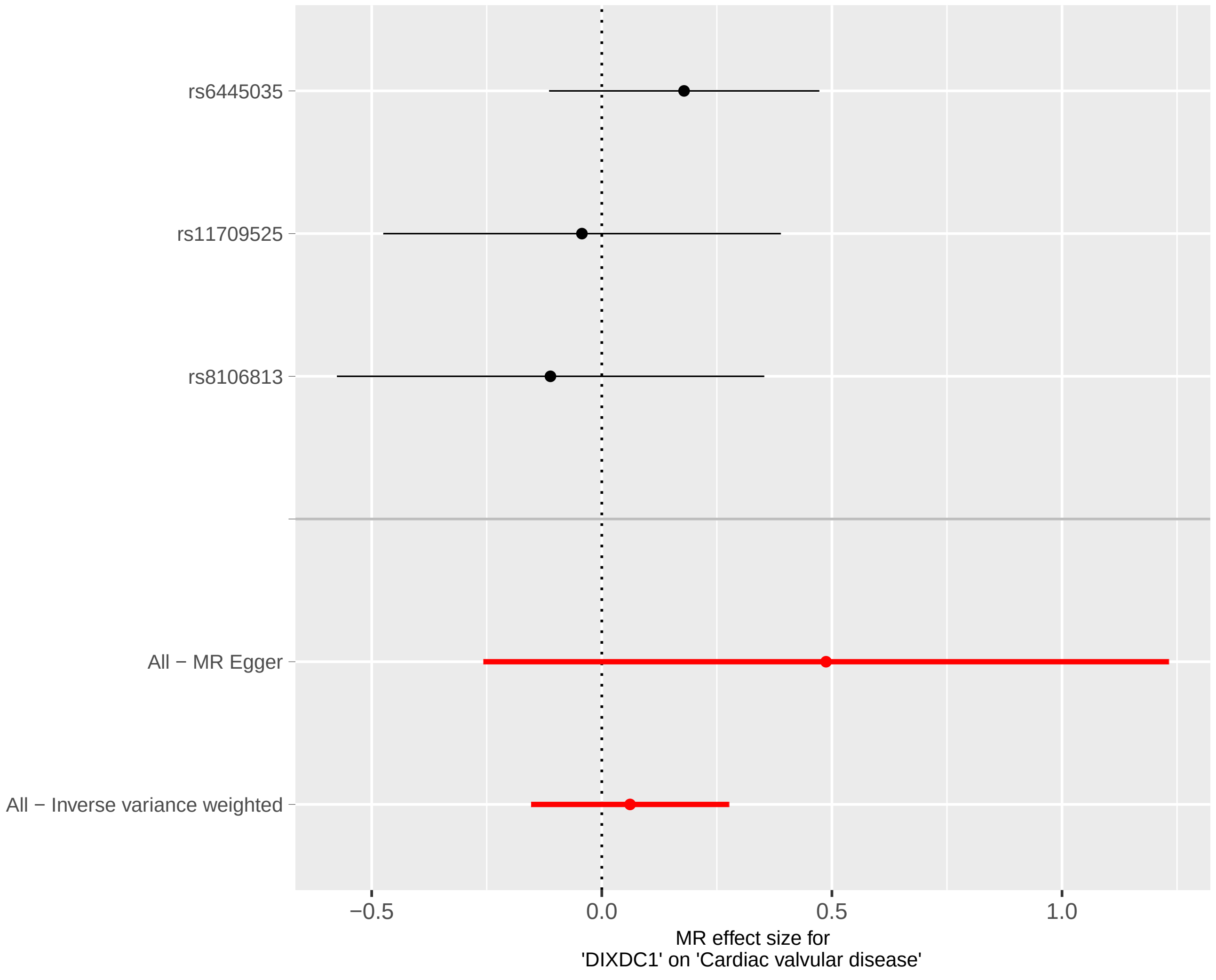
rs6445035
rs11709525
rs8106813
All − MR Egger
All − Inverse variance weighted
−0.5
0.0
0.5
1.0
MR effect size for
'DIXDC1' on 'Cardiac valvular disease'

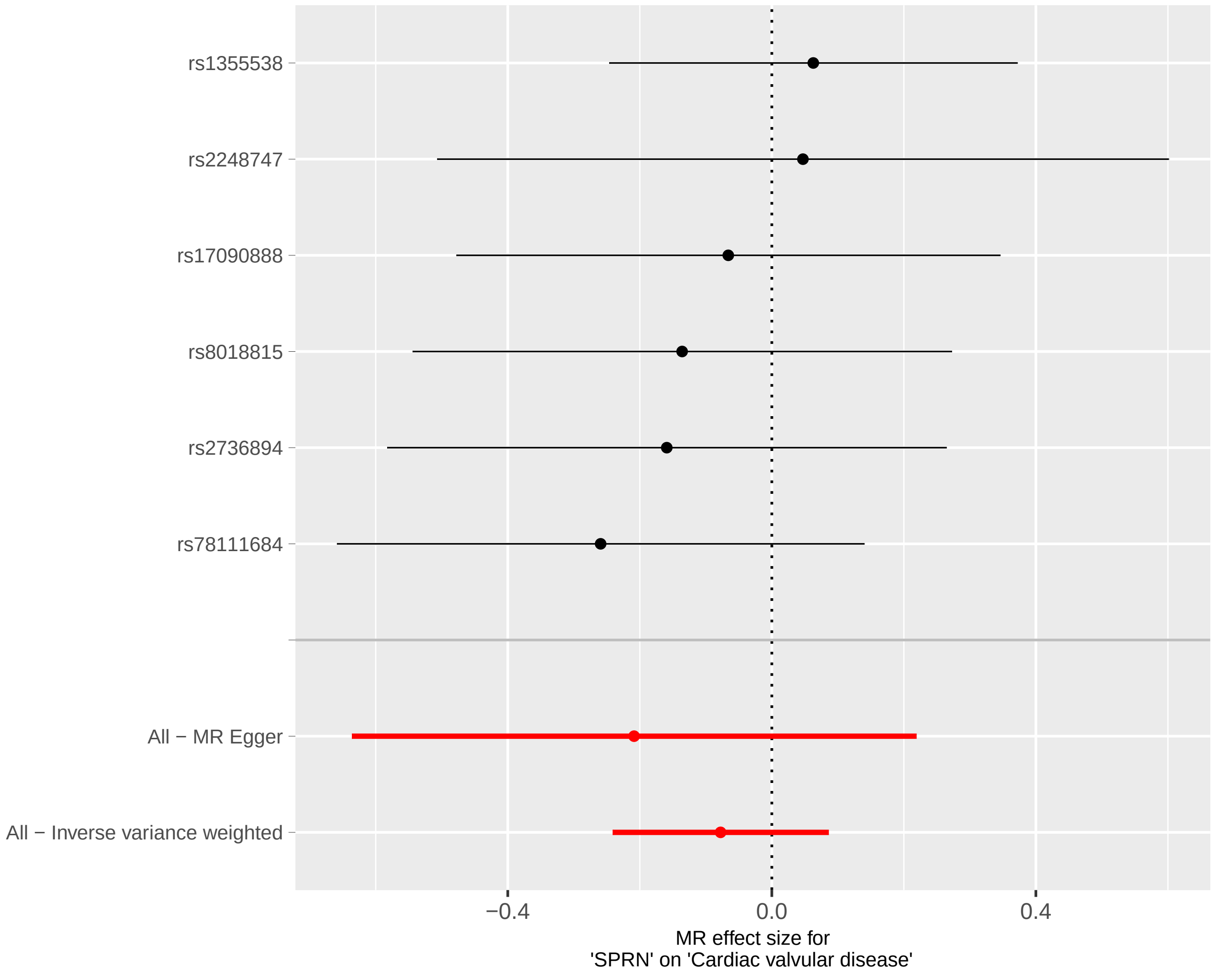
rs1355538
rs2248747
rs17090888
rs8018815
rs2736894
rs78111684
All − MR Egger
All − Inverse variance weighted
−0.4
0.0
0.4
MR effect size for
'SPRN' on 'Cardiac valvular disease'

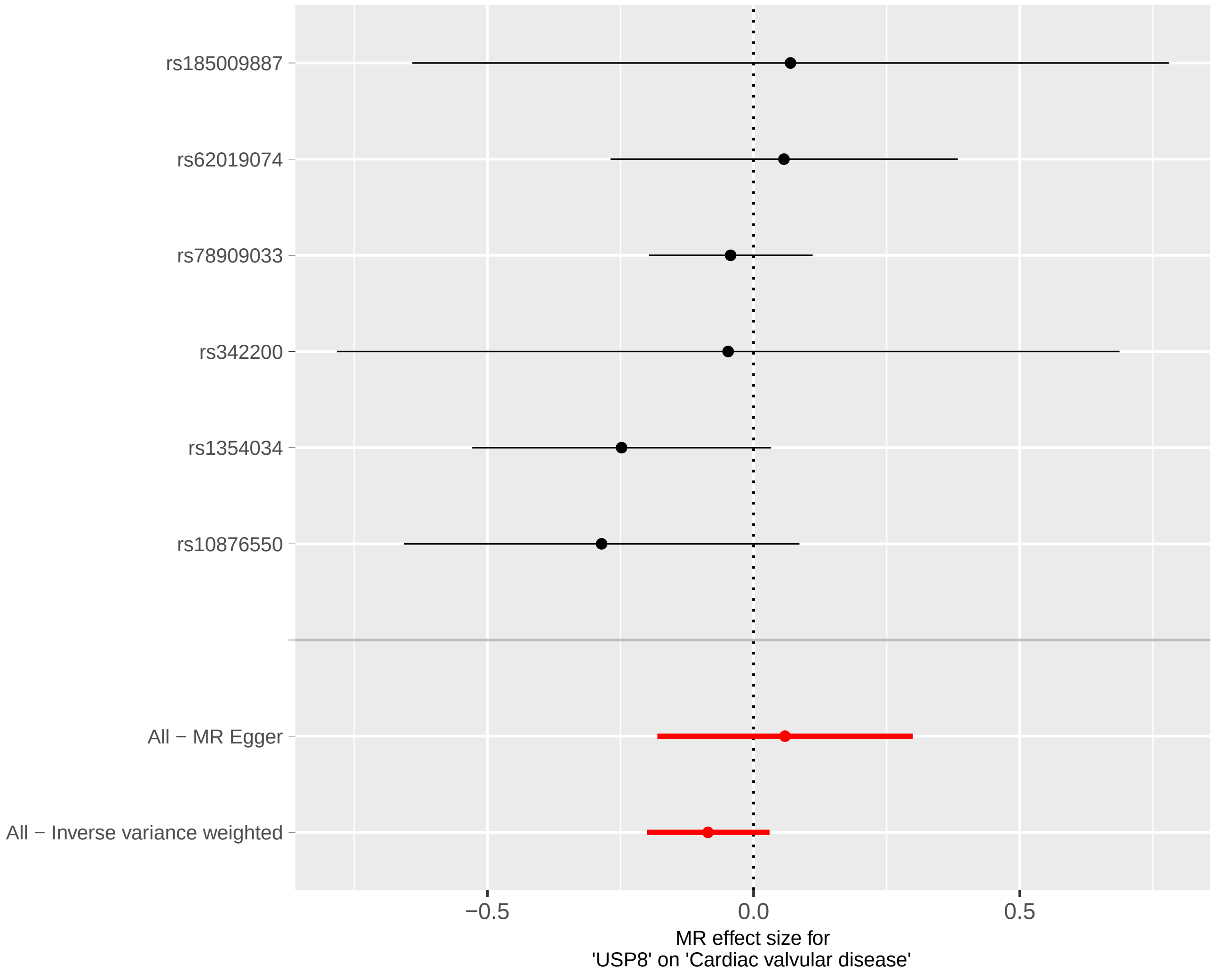
rs185009887
rs62019074
rs78909033
rs342200
rs1354034
rs10876550
All − MR Egger
All − Inverse variance weighted
−0.5
0.0
0.5
MR effect size for
'USP8' on 'Cardiac valvular disease'

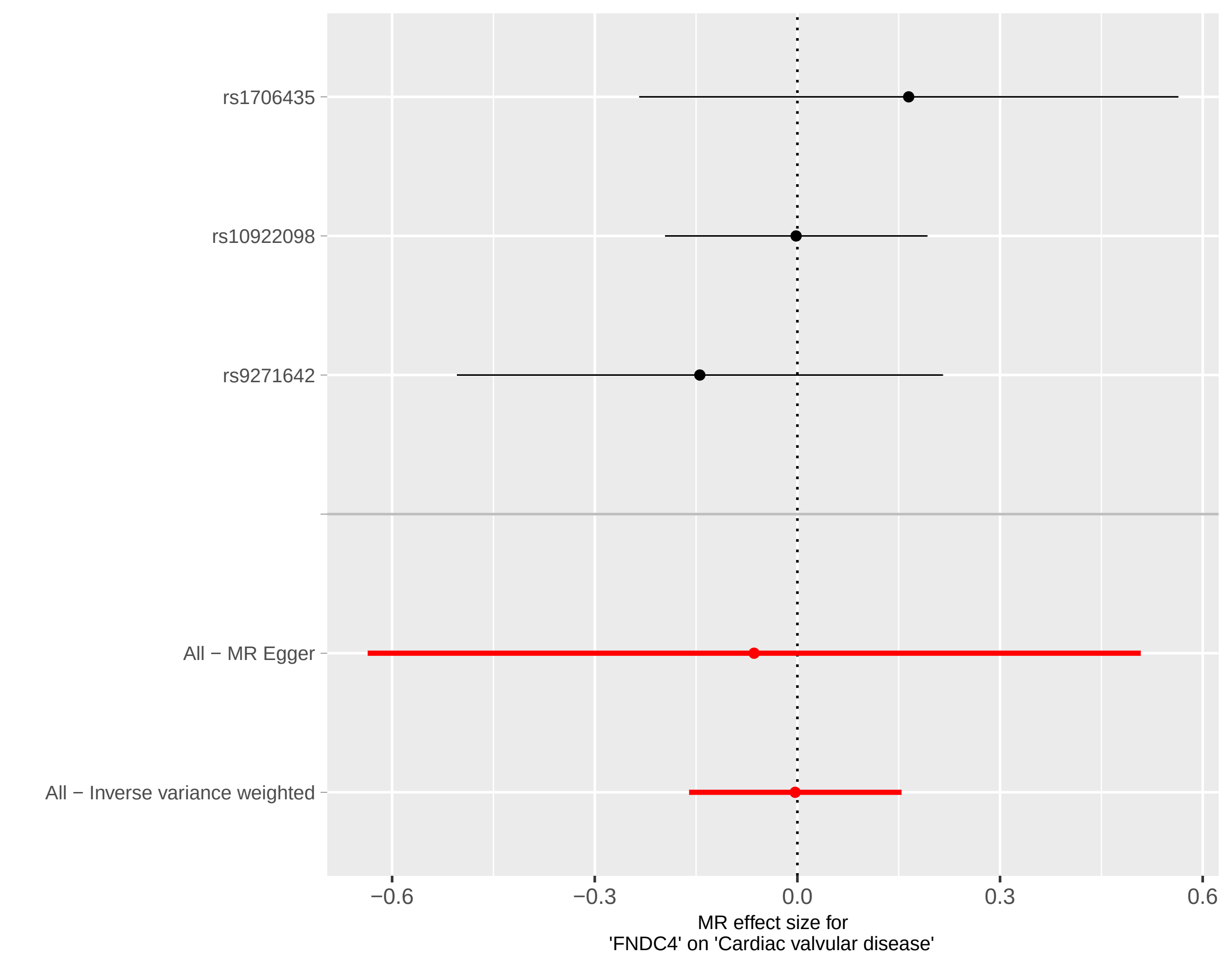

rs1706435
rs10922098
rs9271642
All − MR Egger
All − Inverse variance weighted
−0.6
−0.3
0.0
0.3
0.6
MR effect size for
'FNDC4' on 'Cardiac valvular disease'

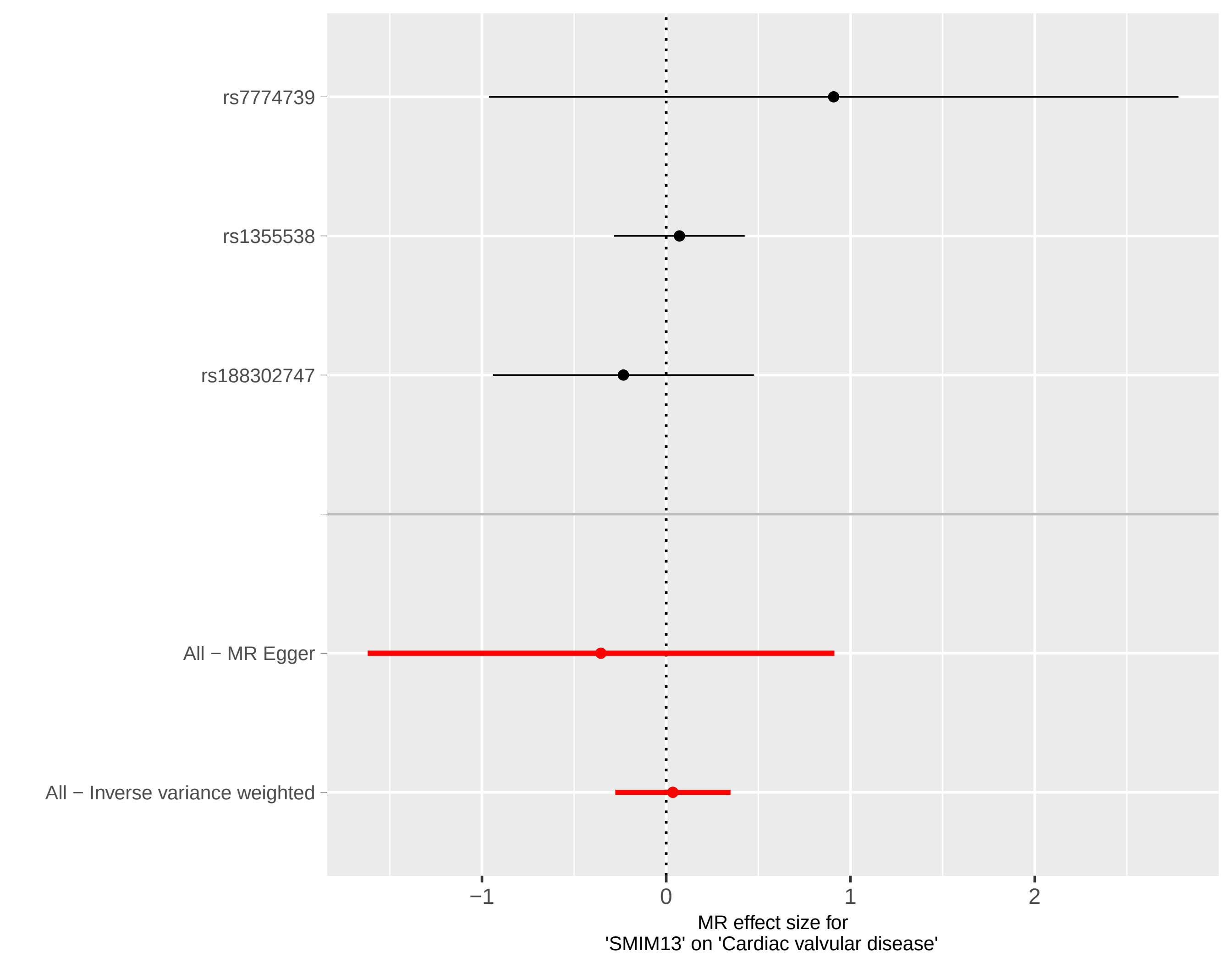
rs7774739
rs1355538
rs188302747
All − MR Egger
All − Inverse variance weighted
−1
0
1
2
MR effect size for
'SMIM13' on 'Cardiac valvular disease'

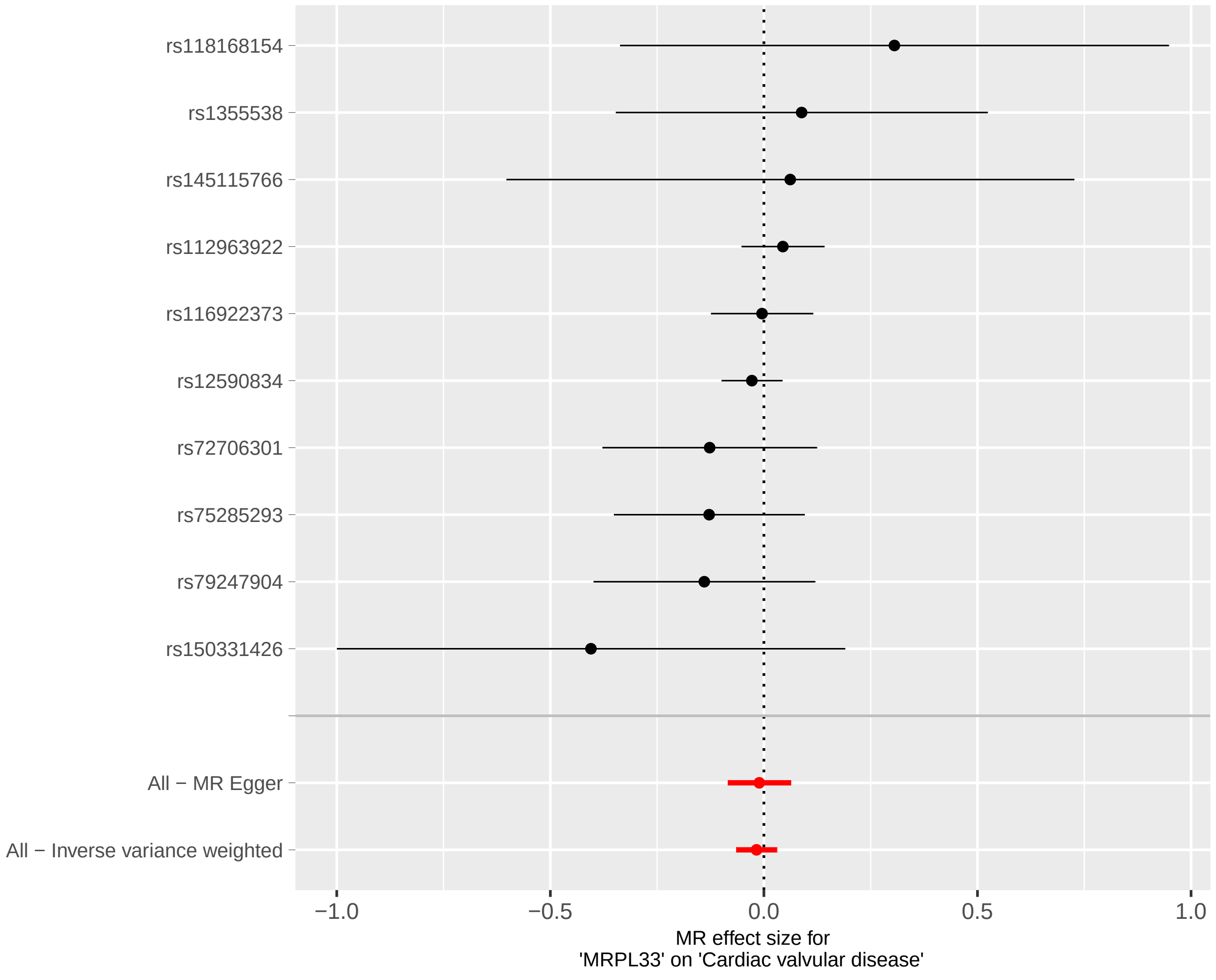

rs118168154
rs1355538
rs145115766
rs112963922
rs116922373
rs12590834
rs72706301
rs75285293
rs79247904
rs150331426
All − MR Egger
All − Inverse variance weighted
−1.0
−0.5
0.0
0.5
1.0
MR effect size for
'MRPL33' on 'Cardiac valvular disease'

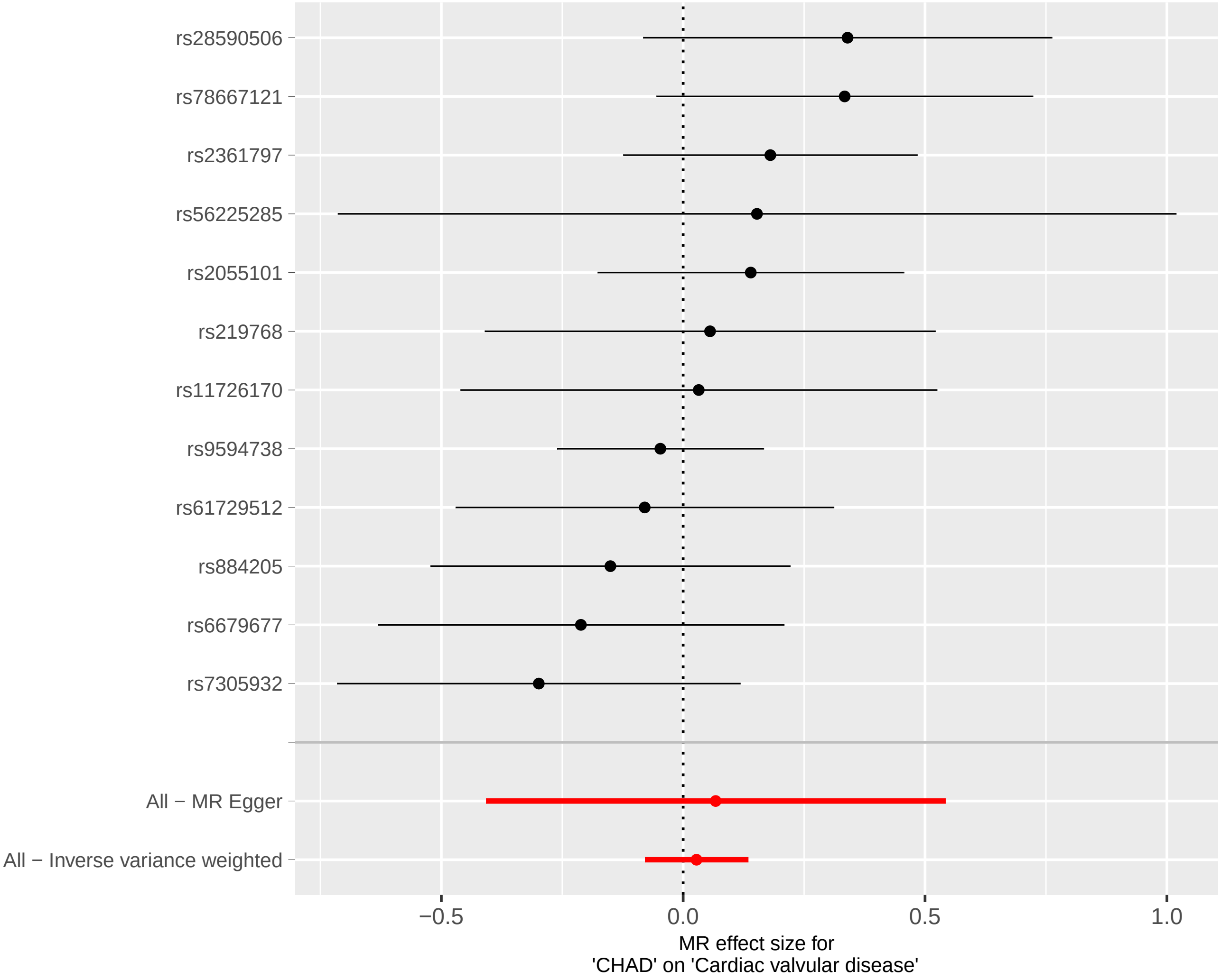
rs28590506
rs78667121
rs2361797
rs56225285
rs2055101
rs219768
rs11726170
rs9594738
rs61729512
rs884205
rs6679677
rs7305932
All − MR Egger
All − Inverse variance weighted
−0.5
0.0
0.5
1.0
MR effect size for
'CHAD' on 'Cardiac valvular disease'

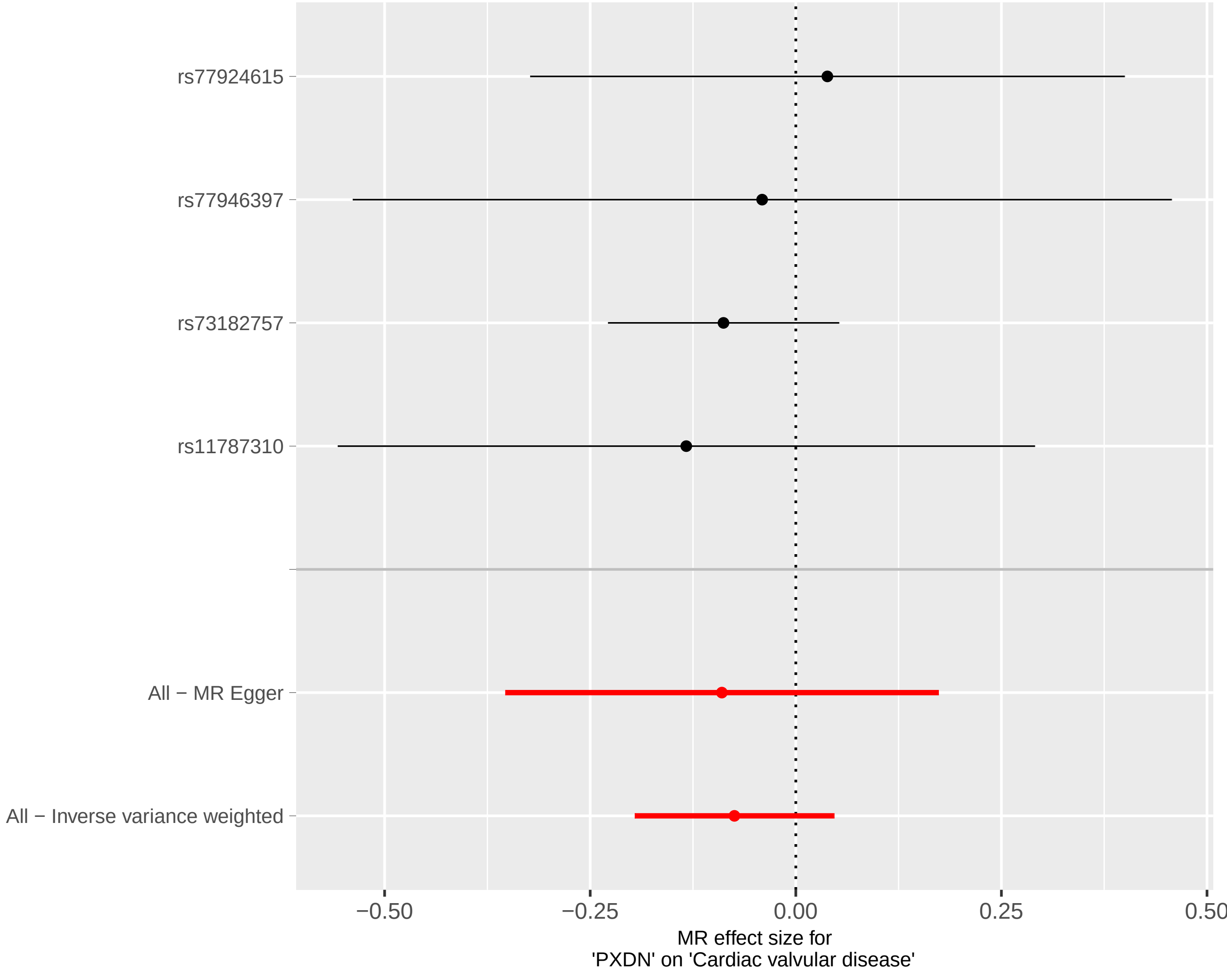

rs77924615
rs77946397
rs73182757
rs11787310
All − MR Egger
All − Inverse variance weighted
−0.50
−0.25
0.00
0.25
0.50
MR effect size for
'PXDN' on 'Cardiac valvular disease'

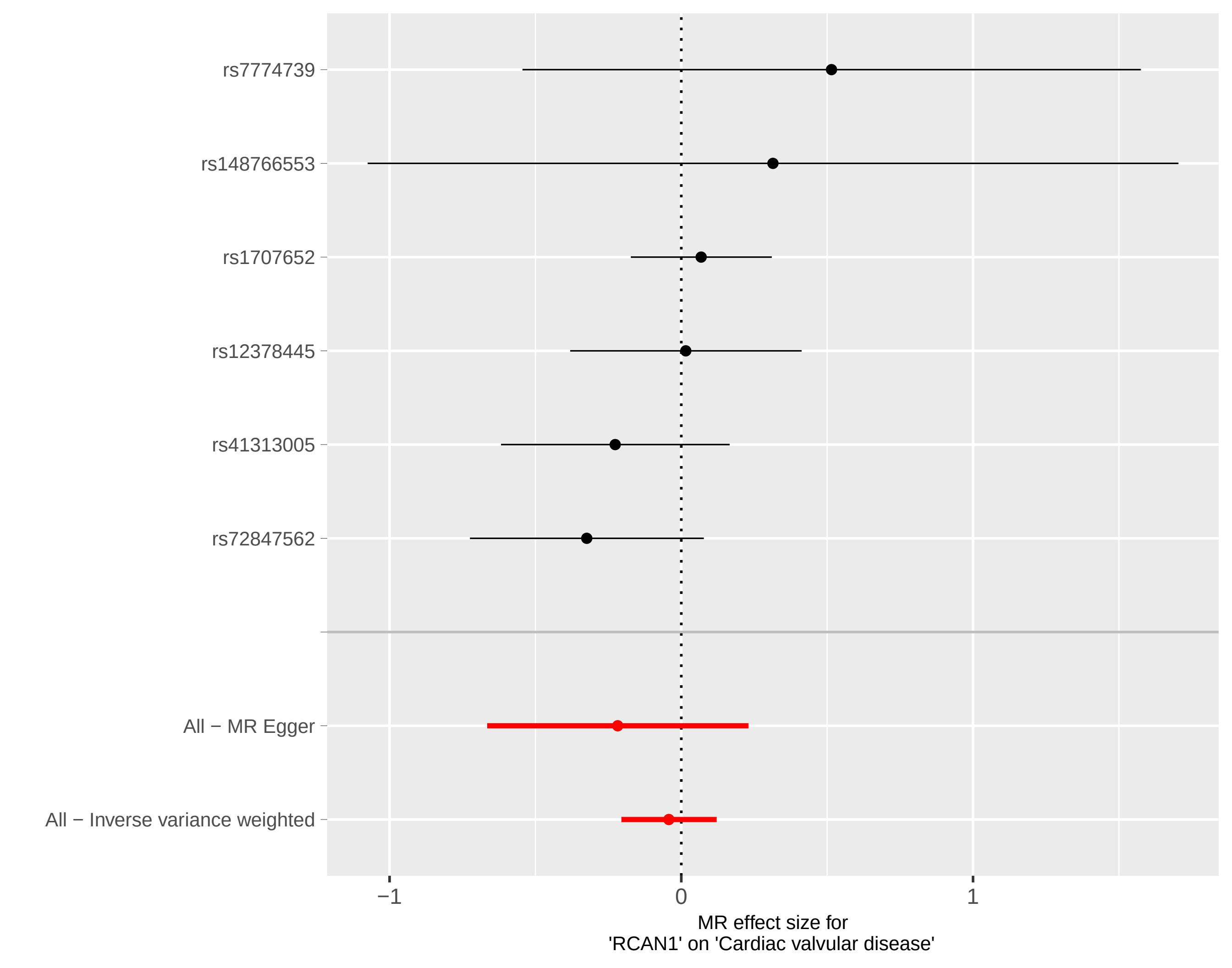
rs7774739
rs148766553
rs1707652
rs12378445
rs41313005
rs72847562
All − MR Egger
All − Inverse variance weighted
−1
0
1
MR effect size for
'RCAN1' on 'Cardiac valvular disease'

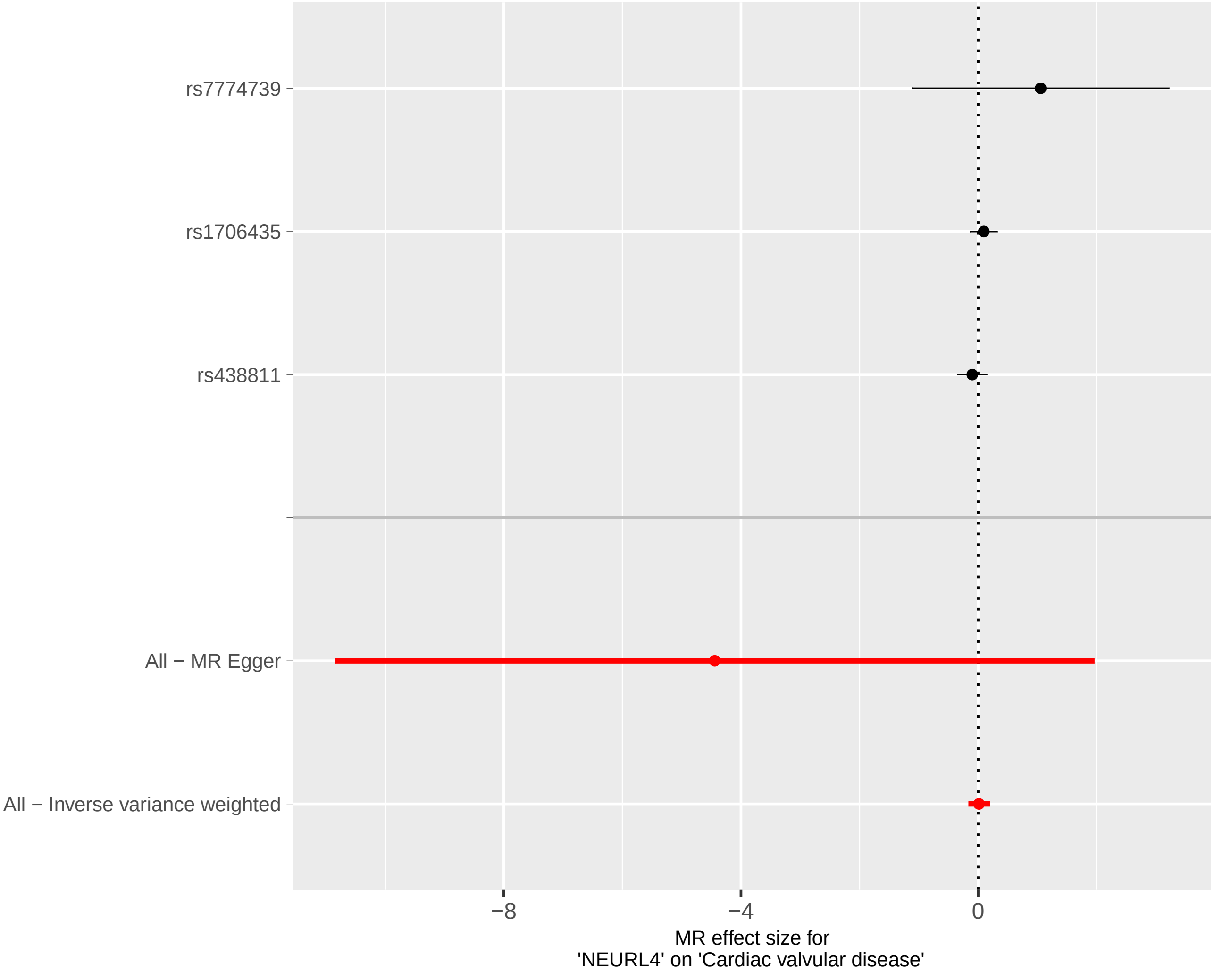
rs7774739
rs1706435
rs438811
All − MR Egger
All − Inverse variance weighted
−8
−4
0
MR effect size for
'NEURL4' on 'Cardiac valvular disease'

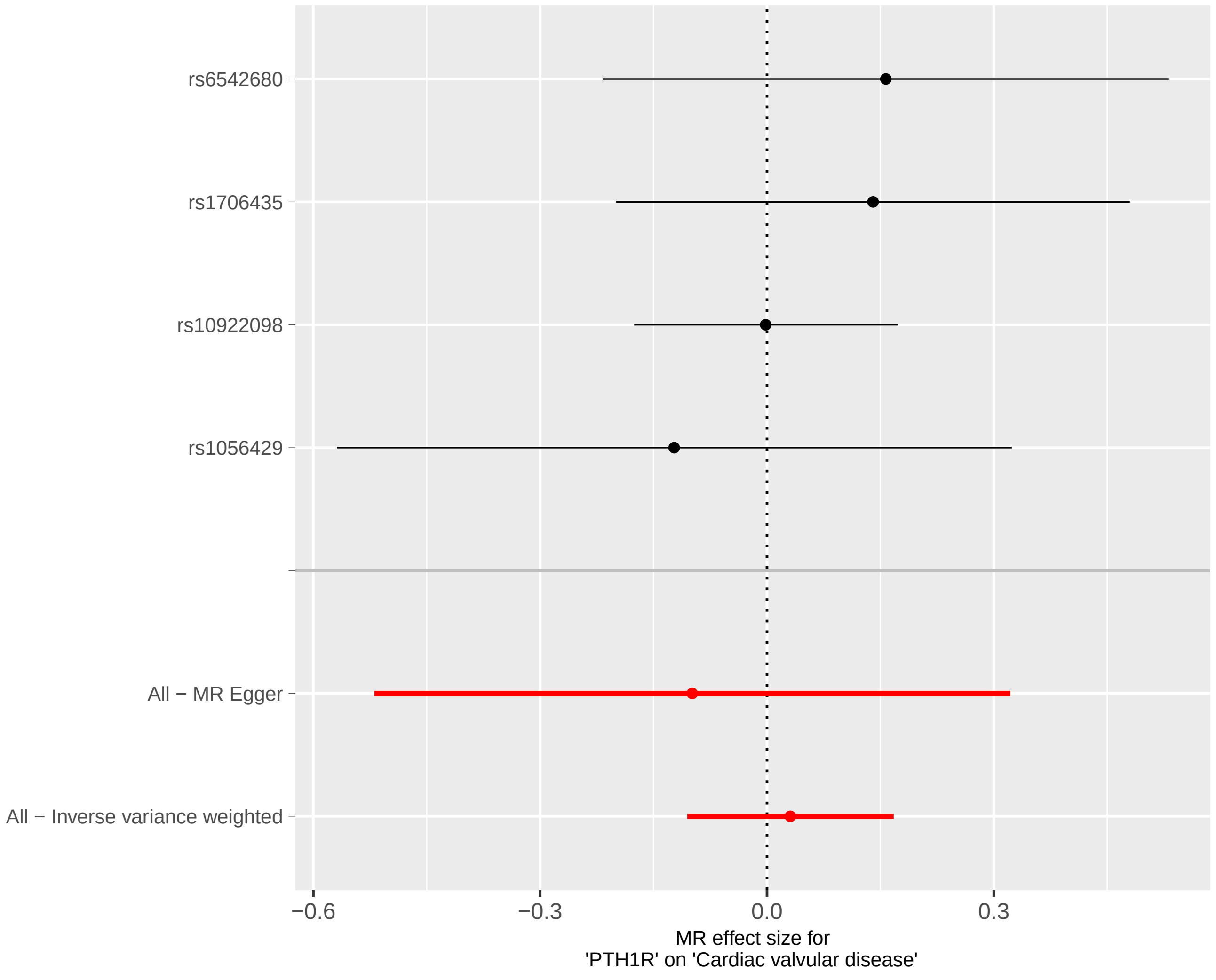
rs6542680
rs1706435
rs10922098
rs1056429
All − MR Egger
All − Inverse variance weighted
−0.6
−0.3
0.0
0.3
MR effect size for
'PTH1R' on 'Cardiac valvular disease'

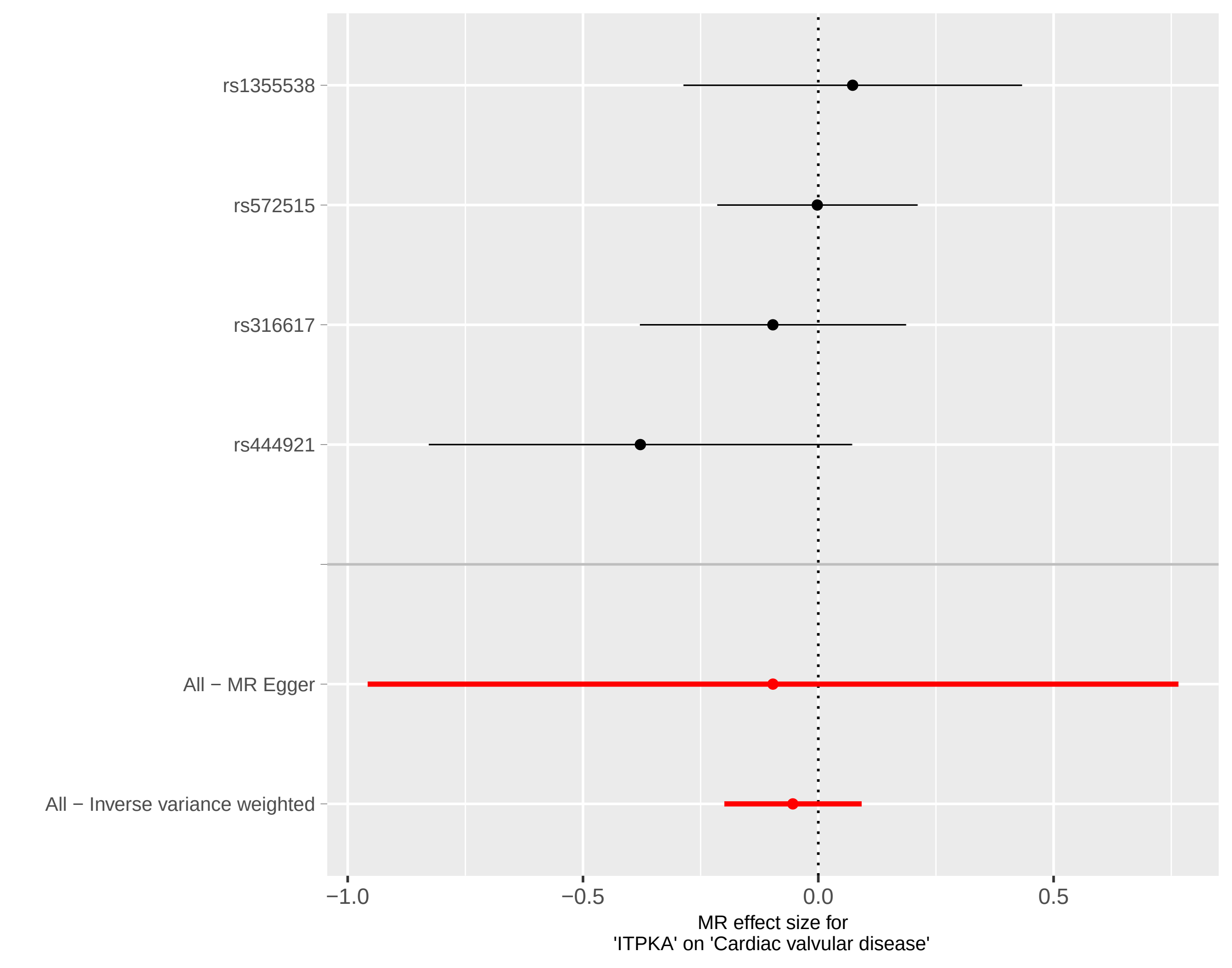

rs1355538
rs572515
rs316617
rs444921
All − MR Egger
All − Inverse variance weighted
−1.0
−0.5
0.0
0.5
MR effect size for
'ITPKA' on 'Cardiac valvular disease'

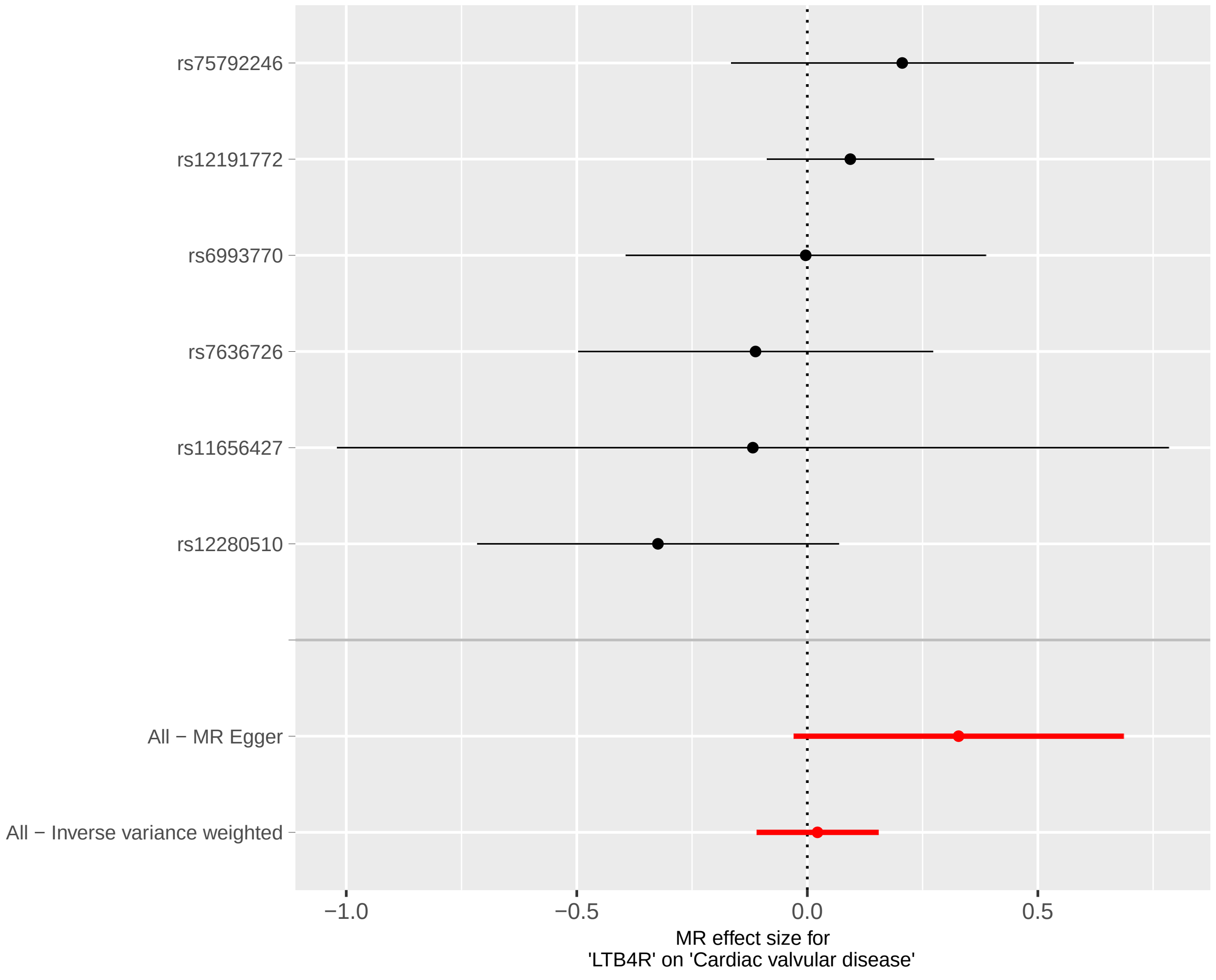

rs75792246
rs12191772
rs6993770
rs7636726
rs11656427
rs12280510
All − MR Egger
All − Inverse variance weighted
−1.0
−0.5
0.0
0.5
MR effect size for
'LTB4R' on 'Cardiac valvular disease'

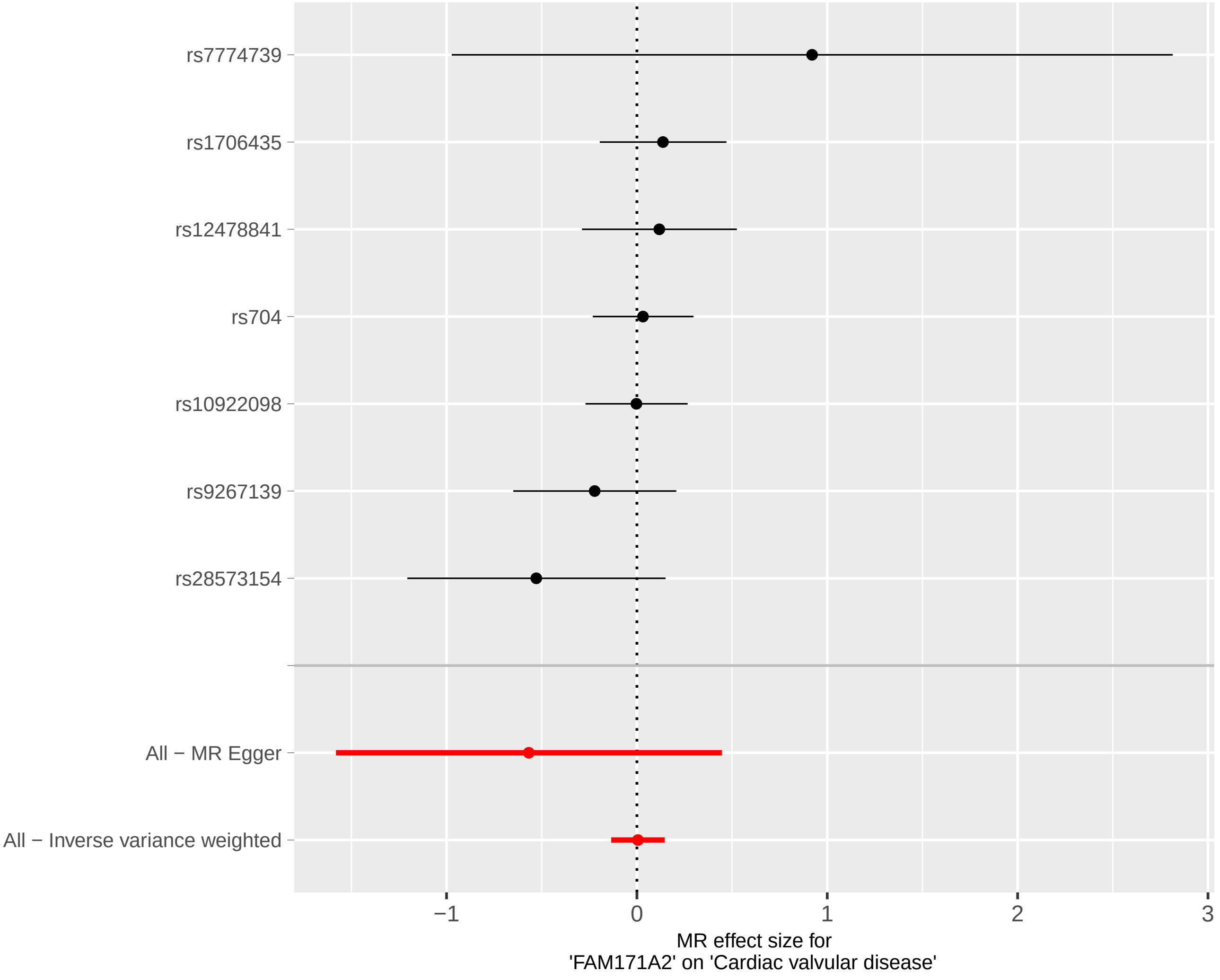

rs7774739
rs1706435
rs12478841
rs704
rs10922098
rs9267139
rs28573154
All − MR Egger
All − Inverse variance weighted
−1
0
1
2
3
MR effect size for
'FAM171A2' on 'Cardiac valvular disease'

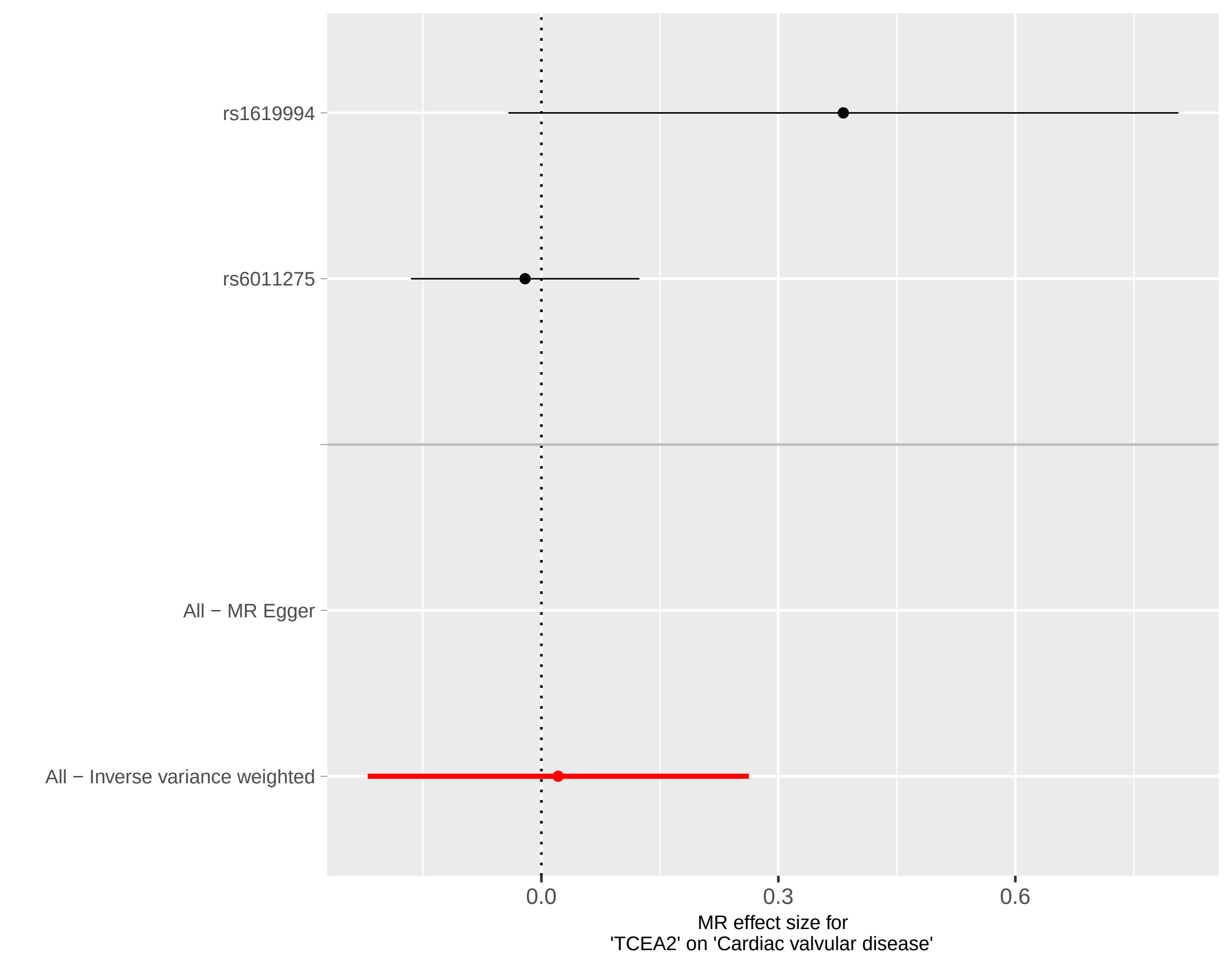
rs1619994
rs6011275
All − MR Egger
All − Inverse variance weighted
0.0
0.3
0.6
MR effect size for
'TCEA2' on 'Cardiac valvular disease'

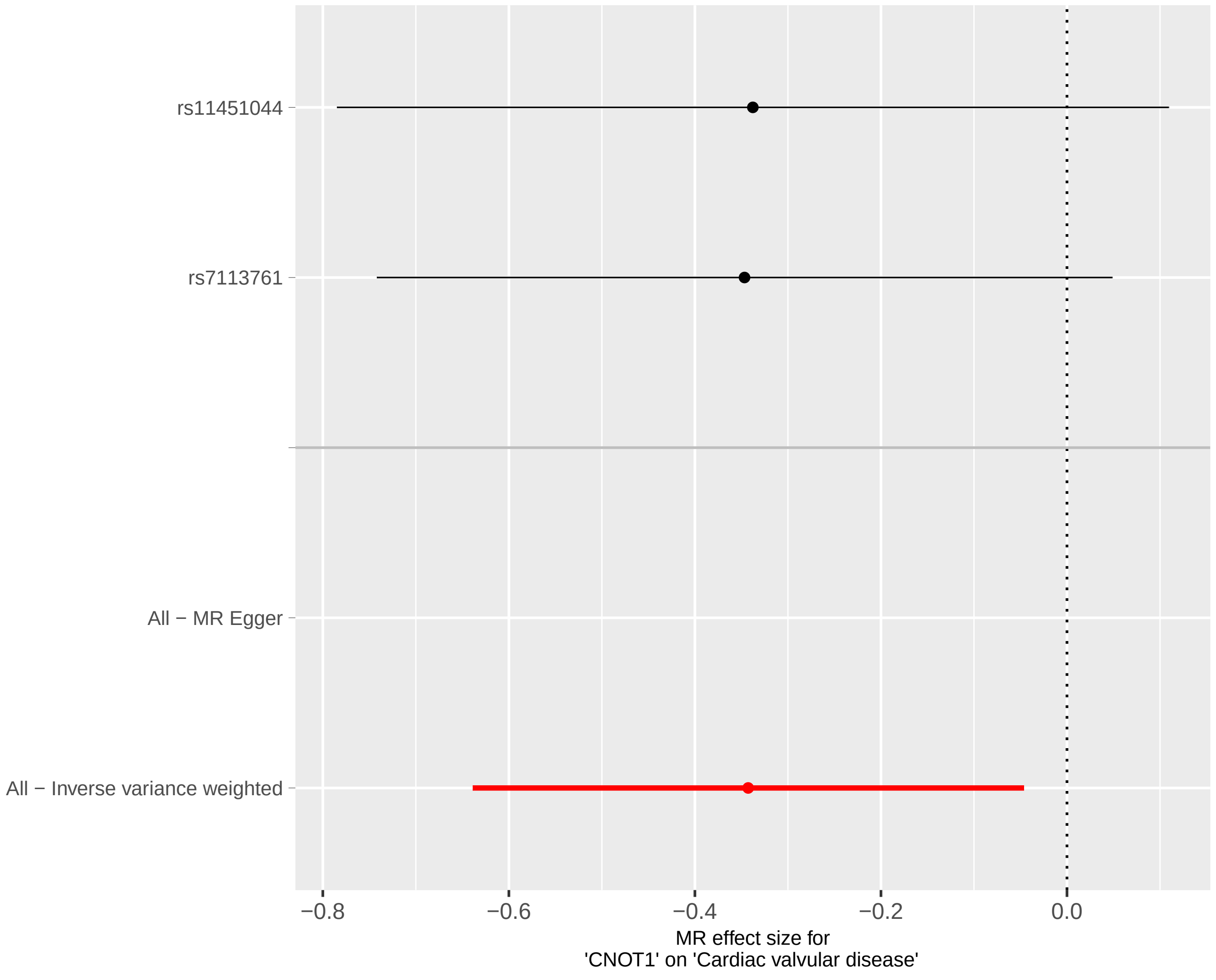

rs11451044
rs7113761
All − MR Egger
All − Inverse variance weighted
−0.8
−0.6
−0.4
−0.2
0.0
MR effect size for
'CNOT1' on 'Cardiac valvular disease'

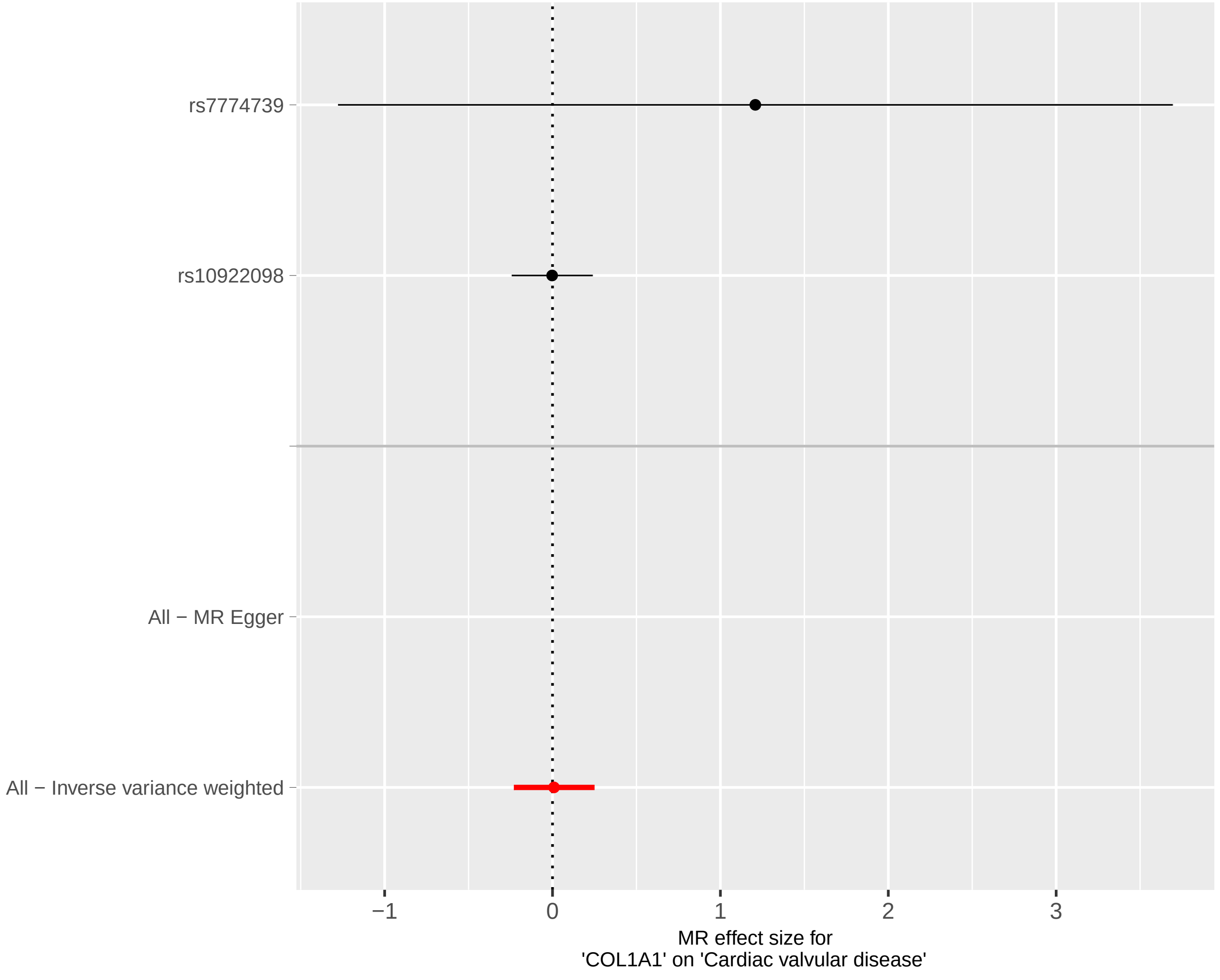
rs7774739
rs10922098
All − MR Egger
All − Inverse variance weighted
−1
0
1
2
3
MR effect size for
'COL1A1' on 'Cardiac valvular disease'

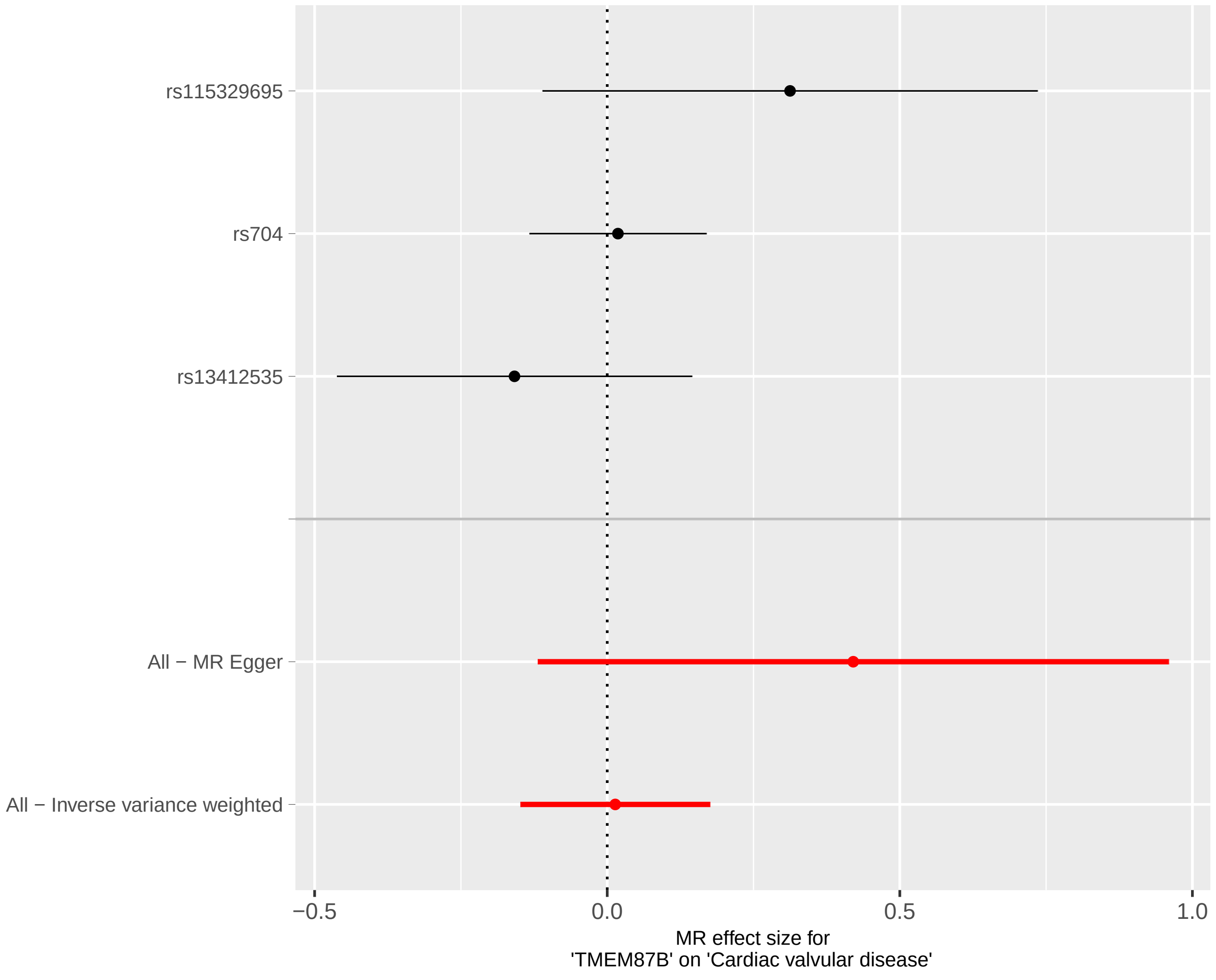
rs115329695
rs704
rs13412535
All − MR Egger
All − Inverse variance weighted
−0.5
0.0
0.5
1.0
MR effect size for
'TMEM87B' on 'Cardiac valvular disease'

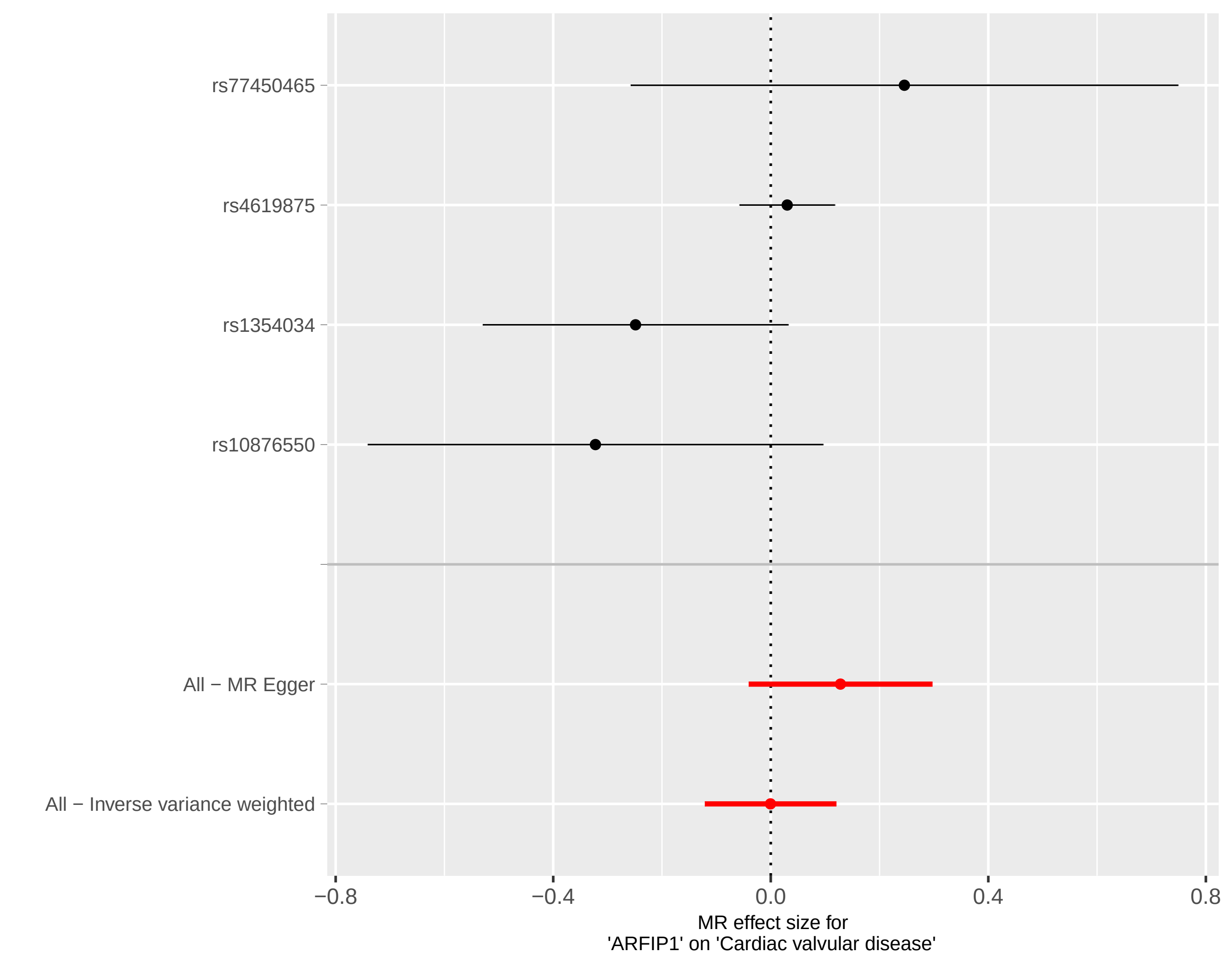

rs77450465
rs4619875
rs1354034
rs10876550
All − MR Egger
All − Inverse variance weighted
−0.8
−0.4
0.0
0.4
0.8
MR effect size for
'ARFIP1' on 'Cardiac valvular disease'

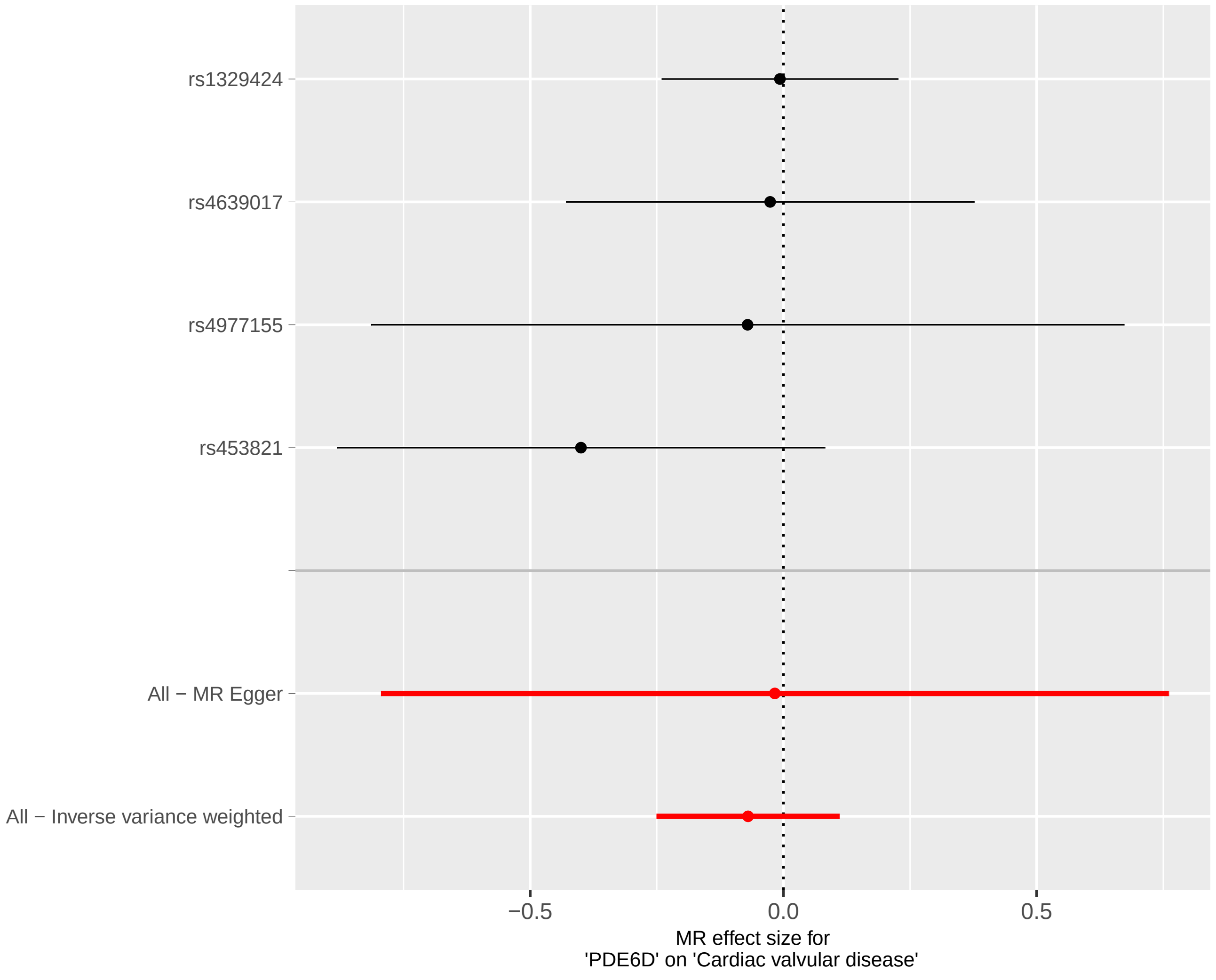
rs1329424
rs4639017
rs4977155
rs453821
All − MR Egger
All − Inverse variance weighted
−0.5
0.0
0.5
MR effect size for
'PDE6D' on 'Cardiac valvular disease'

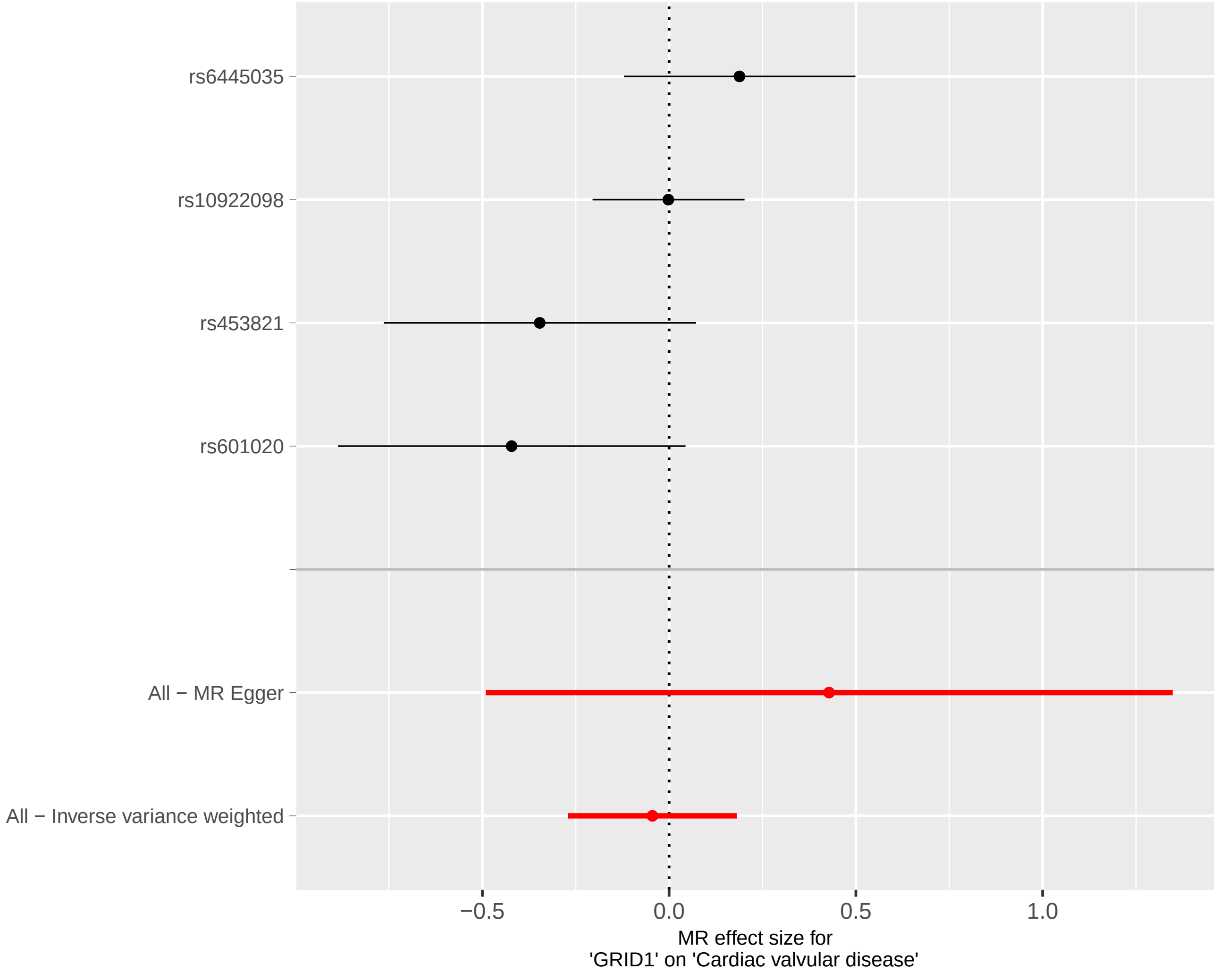
rs6445035
rs10922098
rs453821
rs601020
All − MR Egger
All − Inverse variance weighted
−0.5
0.0
0.5
1.0
MR effect size for
'GRID1' on 'Cardiac valvular disease'

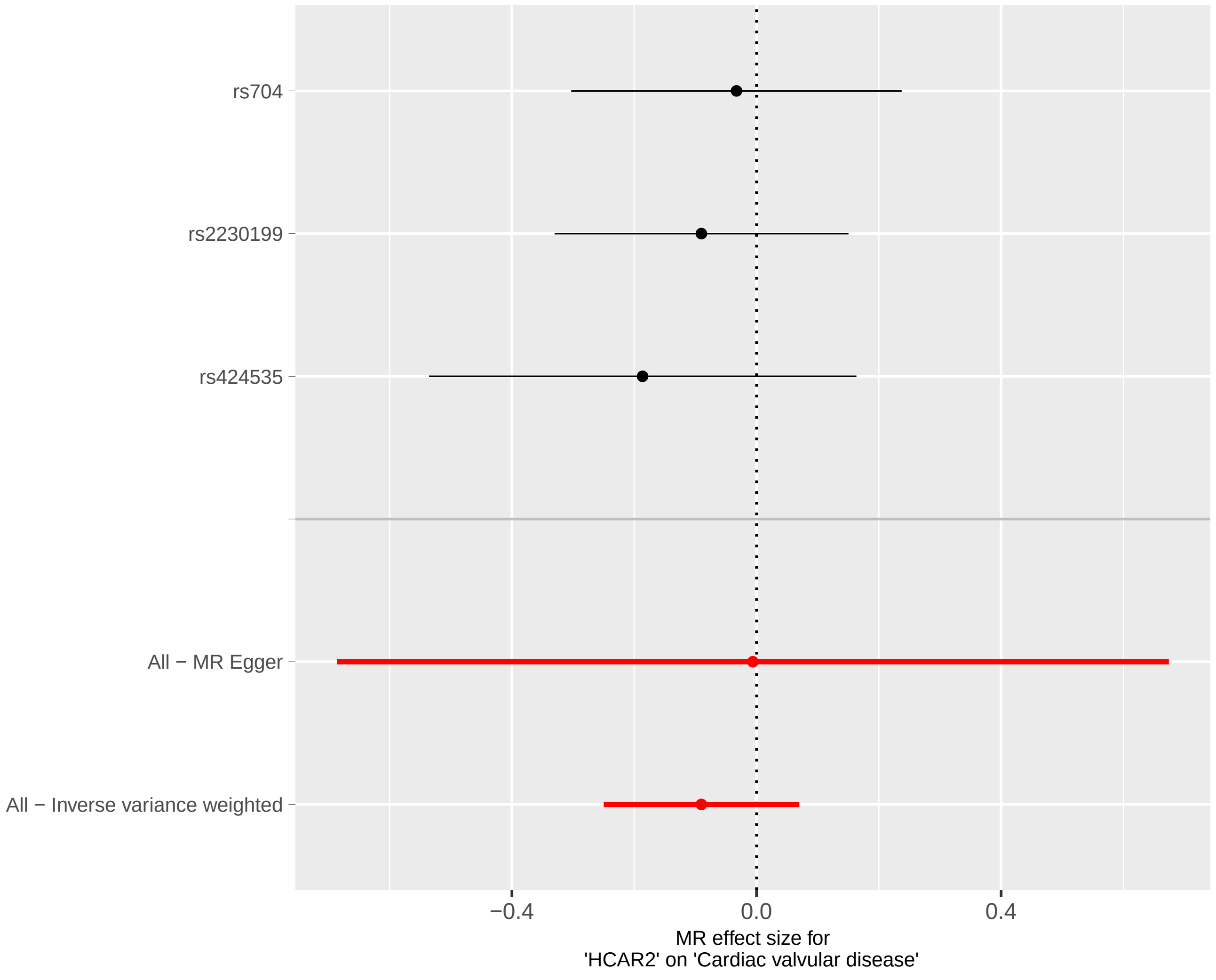
rs704
rs2230199
rs424535
All − MR Egger
All − Inverse variance weighted
−0.4
0.0
0.4
MR effect size for
'HCAR2' on 'Cardiac valvular disease'

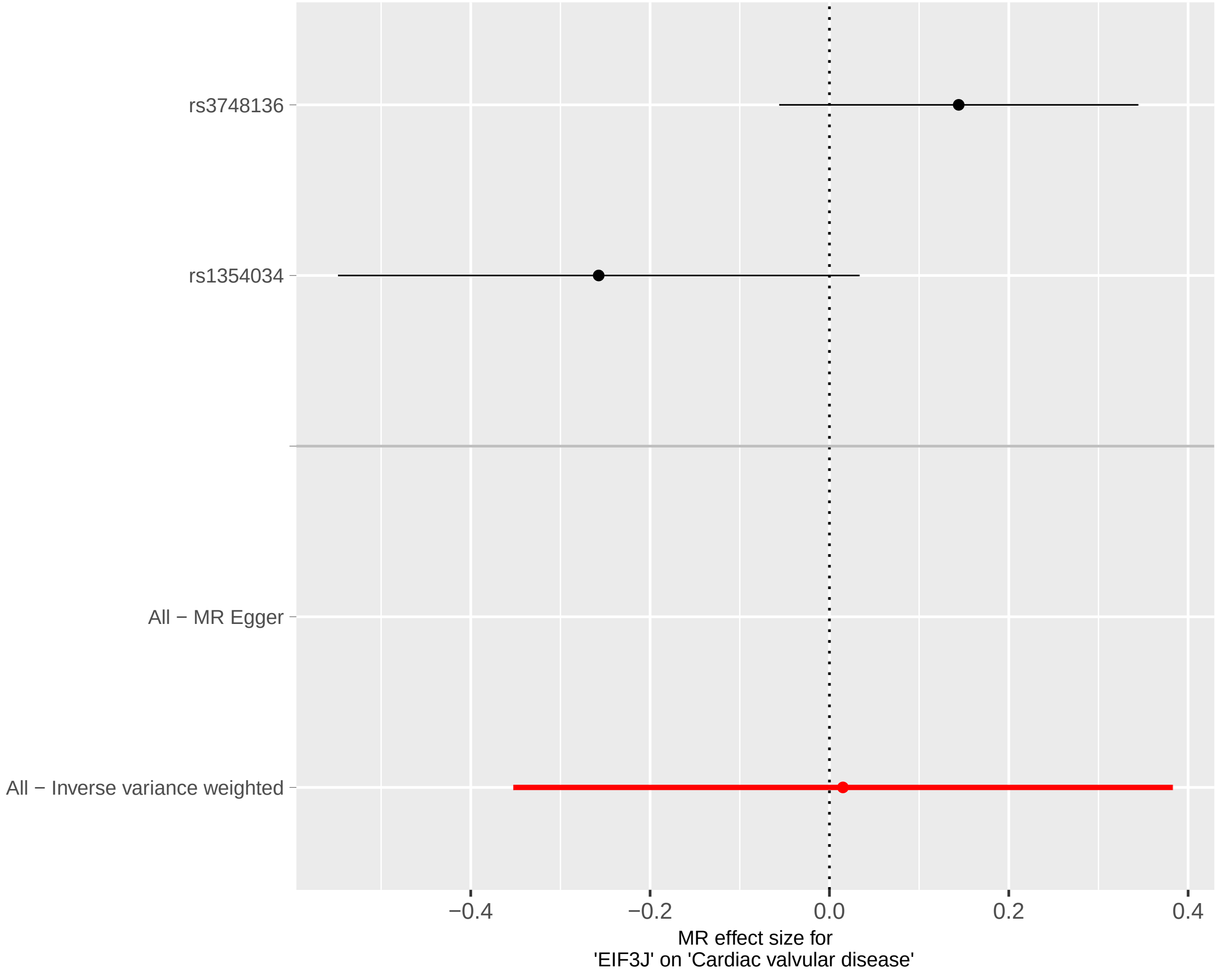
rs3748136
rs1354034
All − MR Egger
All − Inverse variance weighted
−0.4
−0.2
0.0
0.2
0.4
MR effect size for
'EIF3J' on 'Cardiac valvular disease'

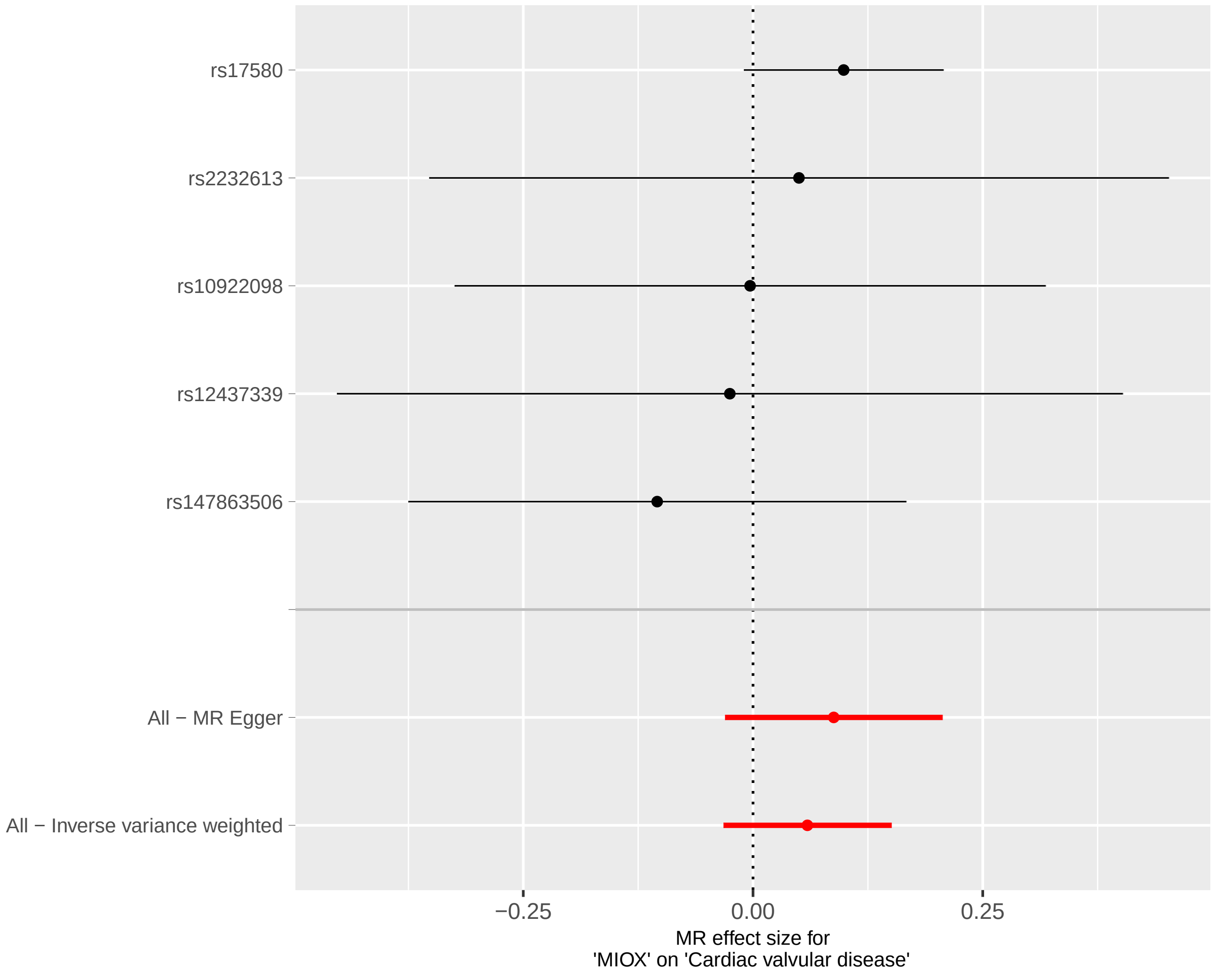
rs17580
rs2232613
rs10922098
rs12437339
rs147863506
All − MR Egger
All − Inverse variance weighted
−0.25
0.00
0.25
MR effect size for
'MIOX' on 'Cardiac valvular disease'

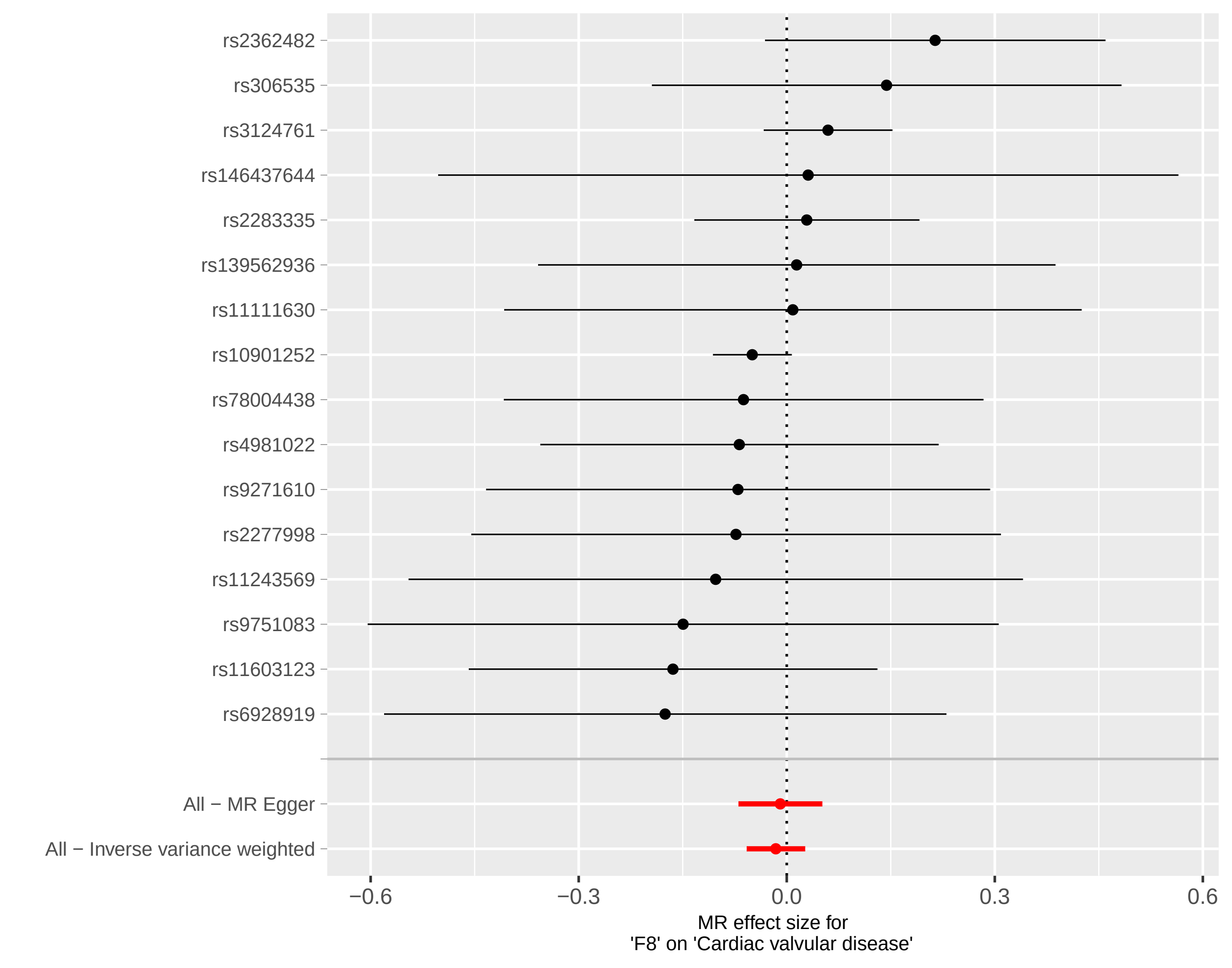

rs2362482
rs306535
rs3124761
rs146437644
rs2283335
rs139562936
rs11111630
rs10901252
rs78004438
rs4981022
rs9271610
rs2277998
rs11243569
rs9751083
rs11603123
rs6928919
All − MR Egger
All − Inverse variance weighted
−0.6
−0.3
0.0
0.3
0.6
MR effect size for
'F8' on 'Cardiac valvular disease'

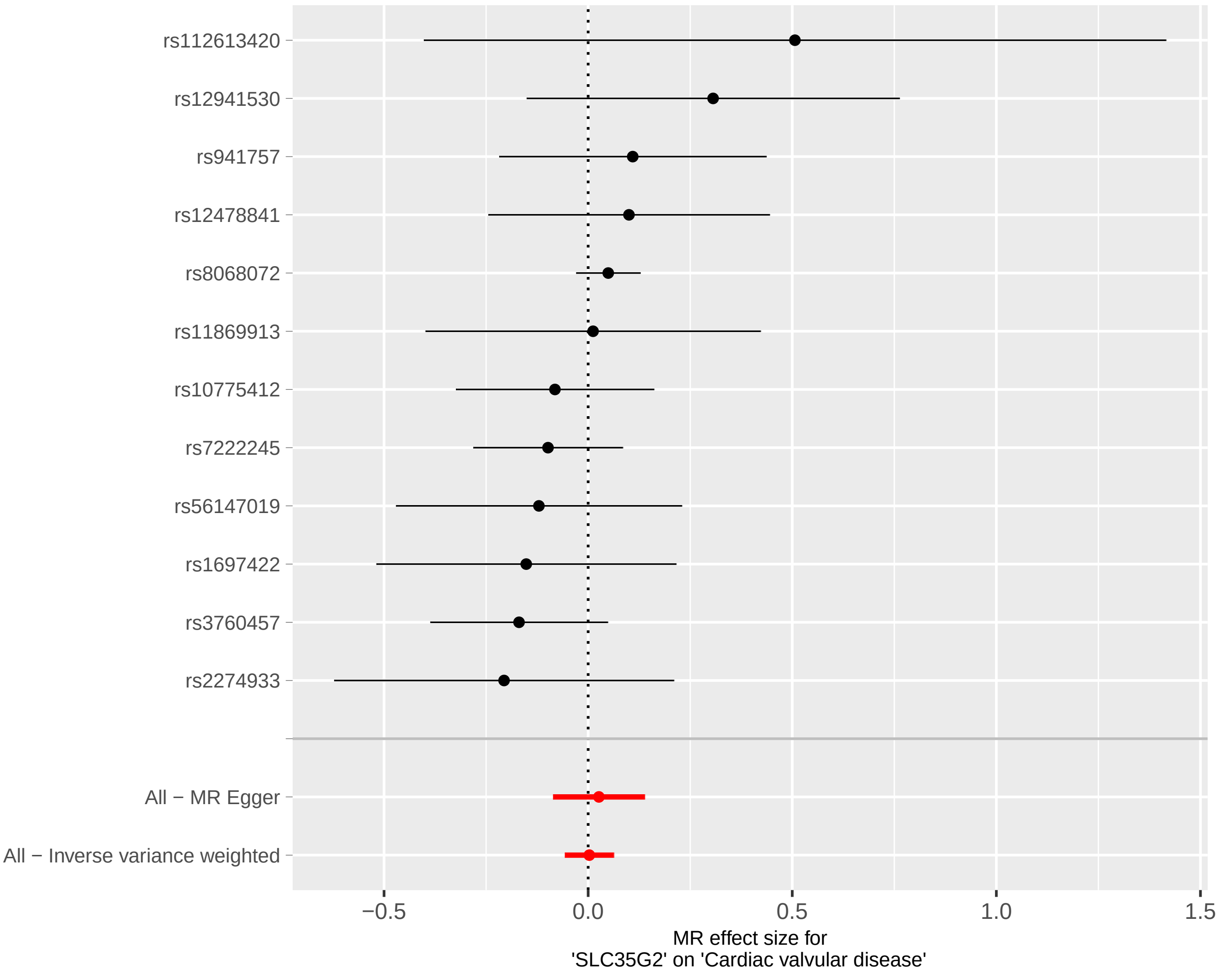
rs112613420
rs12941530
rs941757
rs12478841
rs8068072
rs11869913
rs10775412
rs7222245
rs56147019
rs1697422
rs3760457
rs2274933
All − MR Egger
All − Inverse variance weighted
−0.5
0.0
0.5
1.0
1.5
MR effect size for
'SLC35G2' on 'Cardiac valvular disease'

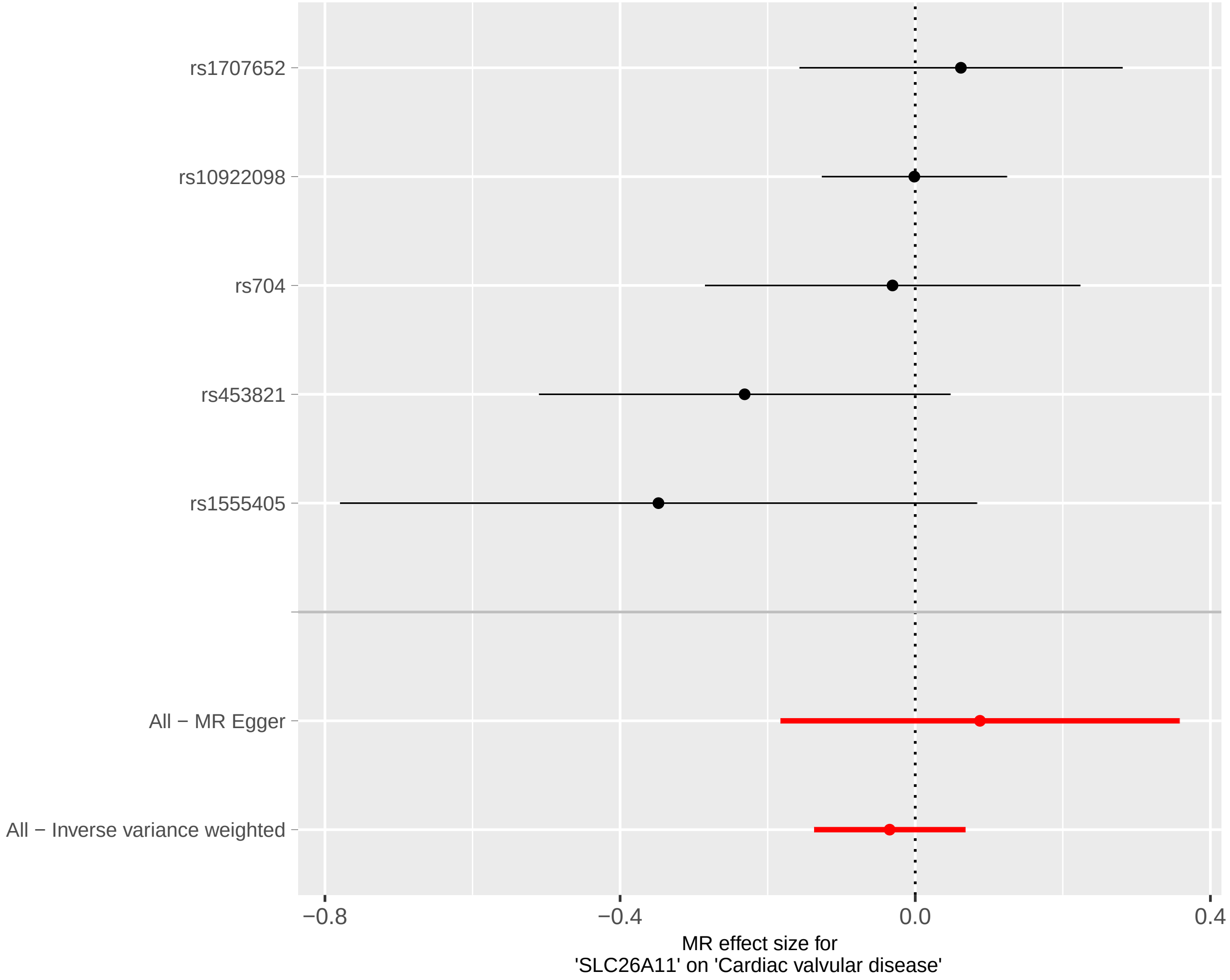
rs1707652
rs10922098
rs704
rs453821
rs1555405
All − MR Egger
All − Inverse variance weighted
−0.8
−0.4
0.0
0.4
MR effect size for
'SLC26A11' on 'Cardiac valvular disease'

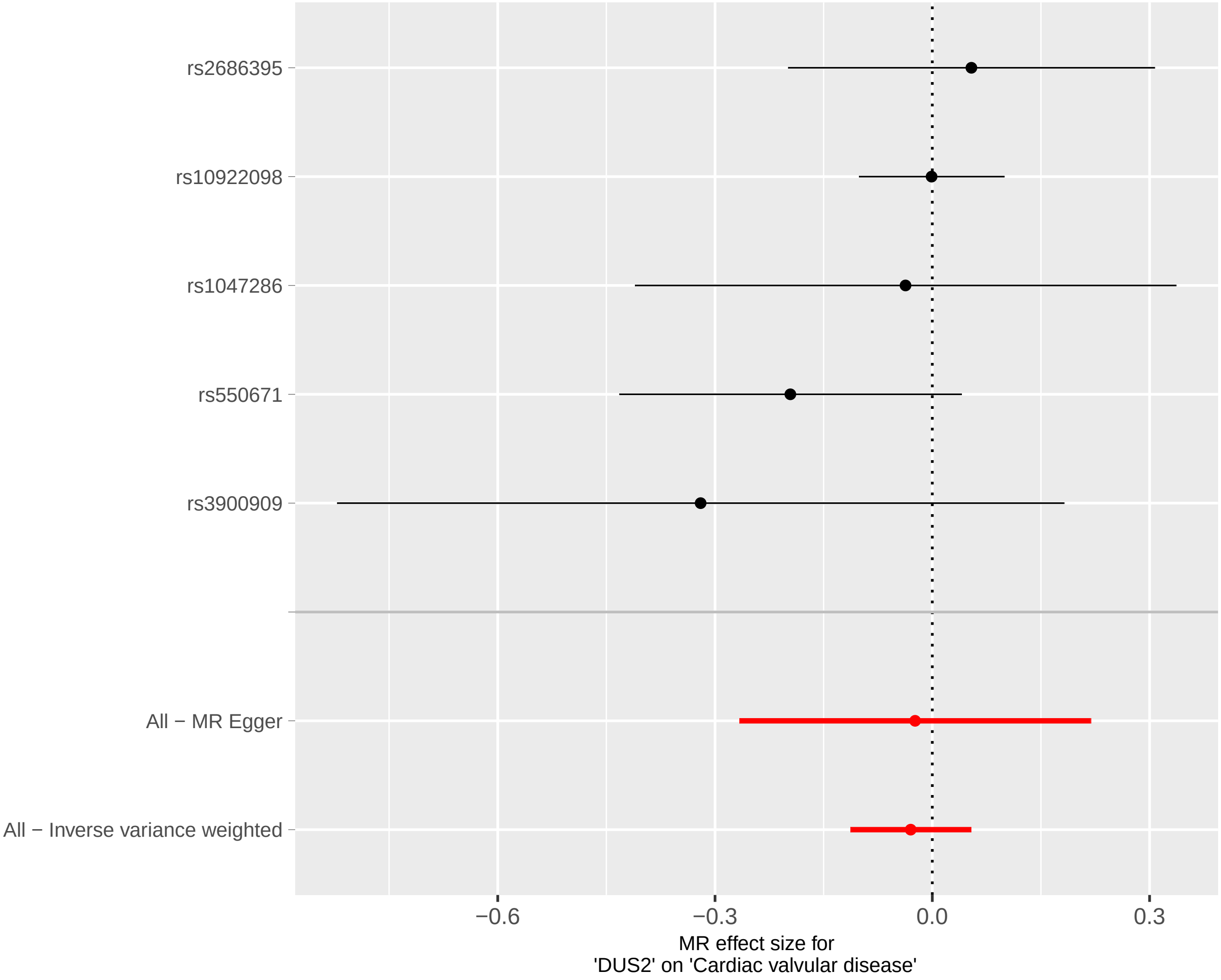

rs2686395
rs10922098
rs1047286
rs550671
rs3900909
All − MR Egger
All − Inverse variance weighted
−0.6
−0.3
0.0
0.3
MR effect size for
'DUS2' on 'Cardiac valvular disease'

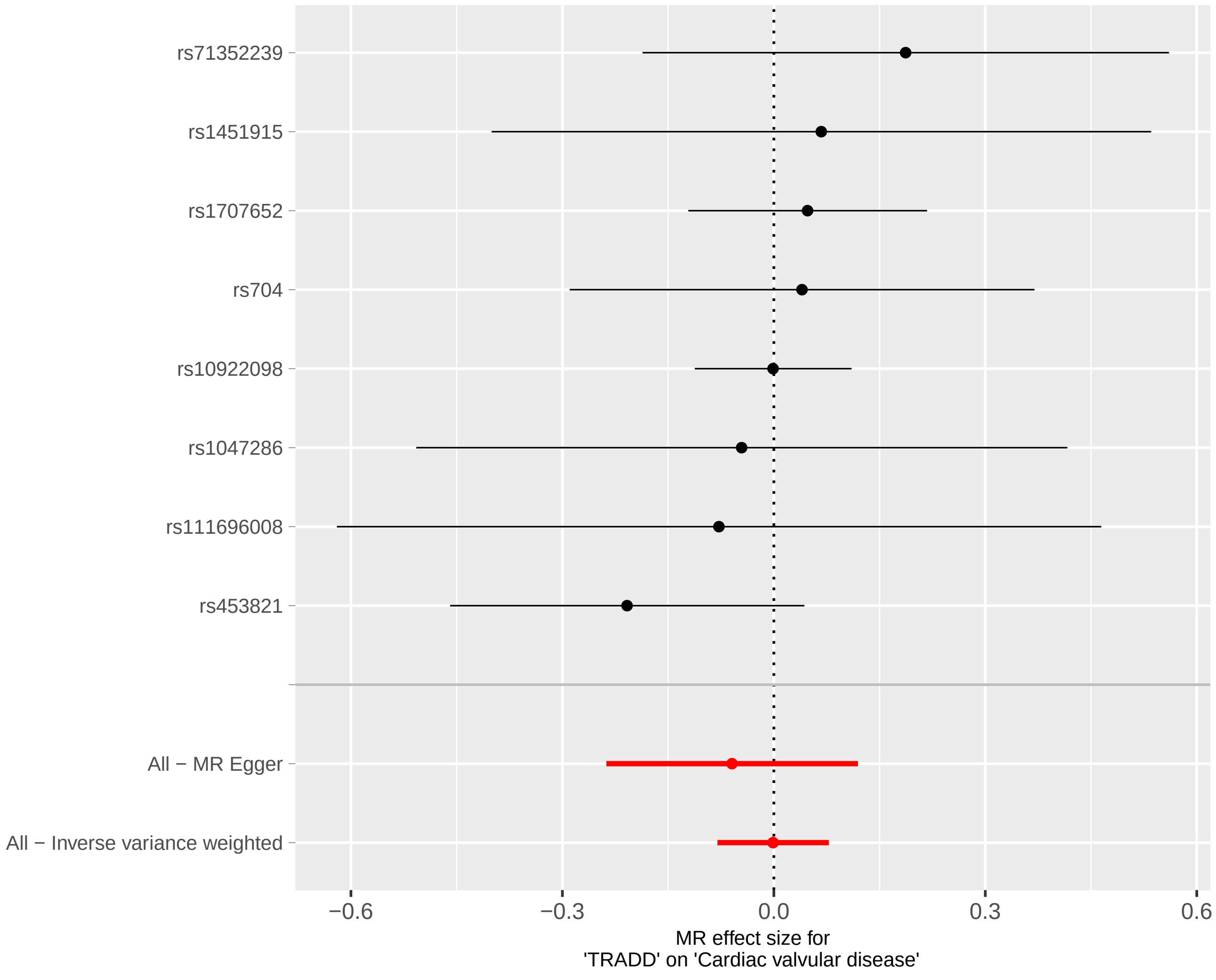
rs71352239
rs1451915
rs1707652
rs704
rs10922098
rs1047286
rs111696008
rs453821
All − MR Egger
All − Inverse variance weighted
−0.6
−0.3
0.0
0.3
0.6
MR effect size for
'TRADD' on 'Cardiac valvular disease'

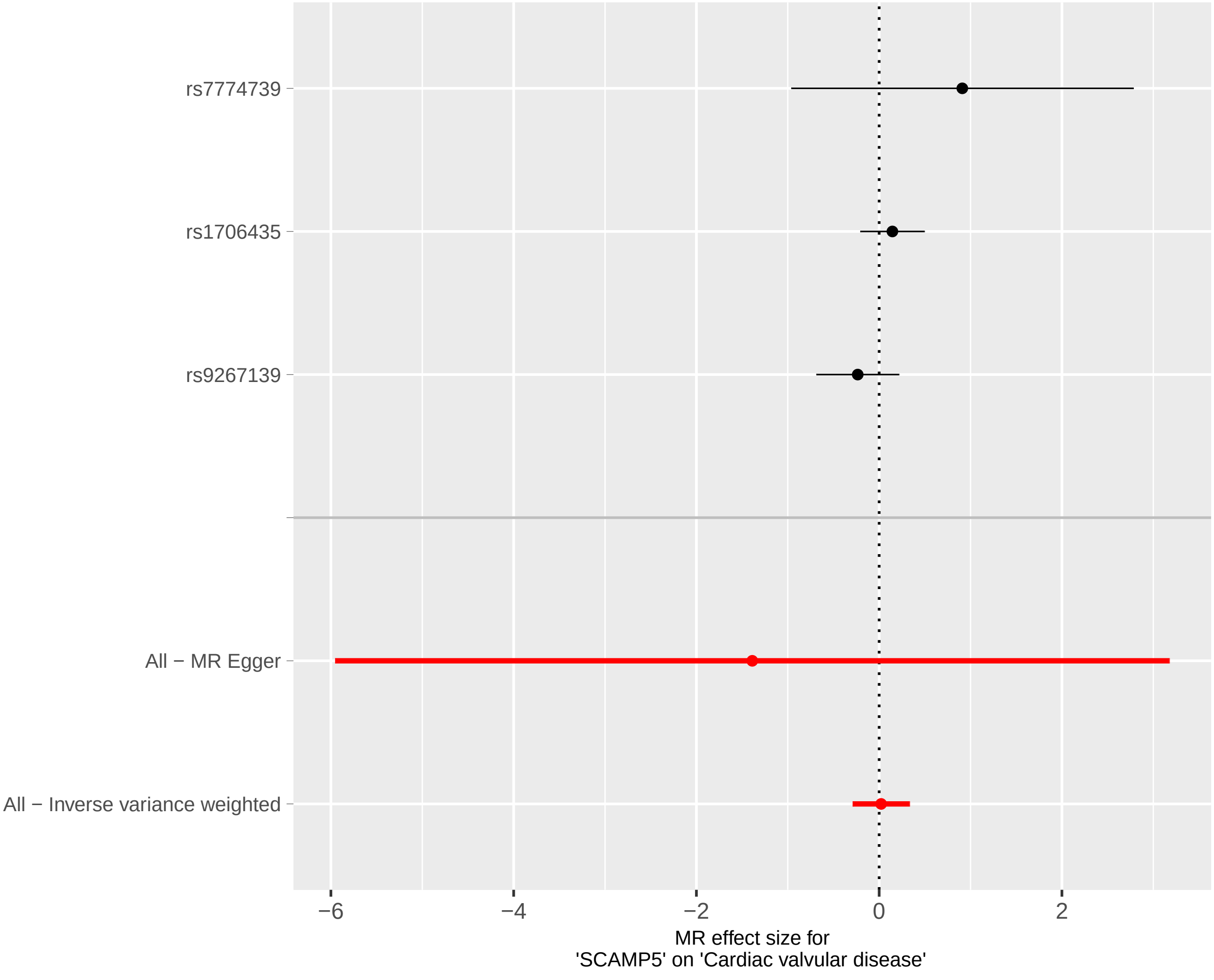

rs7774739
rs1706435
rs9267139
All − MR Egger
All − Inverse variance weighted
−6
−4
−2
0
2
MR effect size for
'SCAMP5' on 'Cardiac valvular disease'

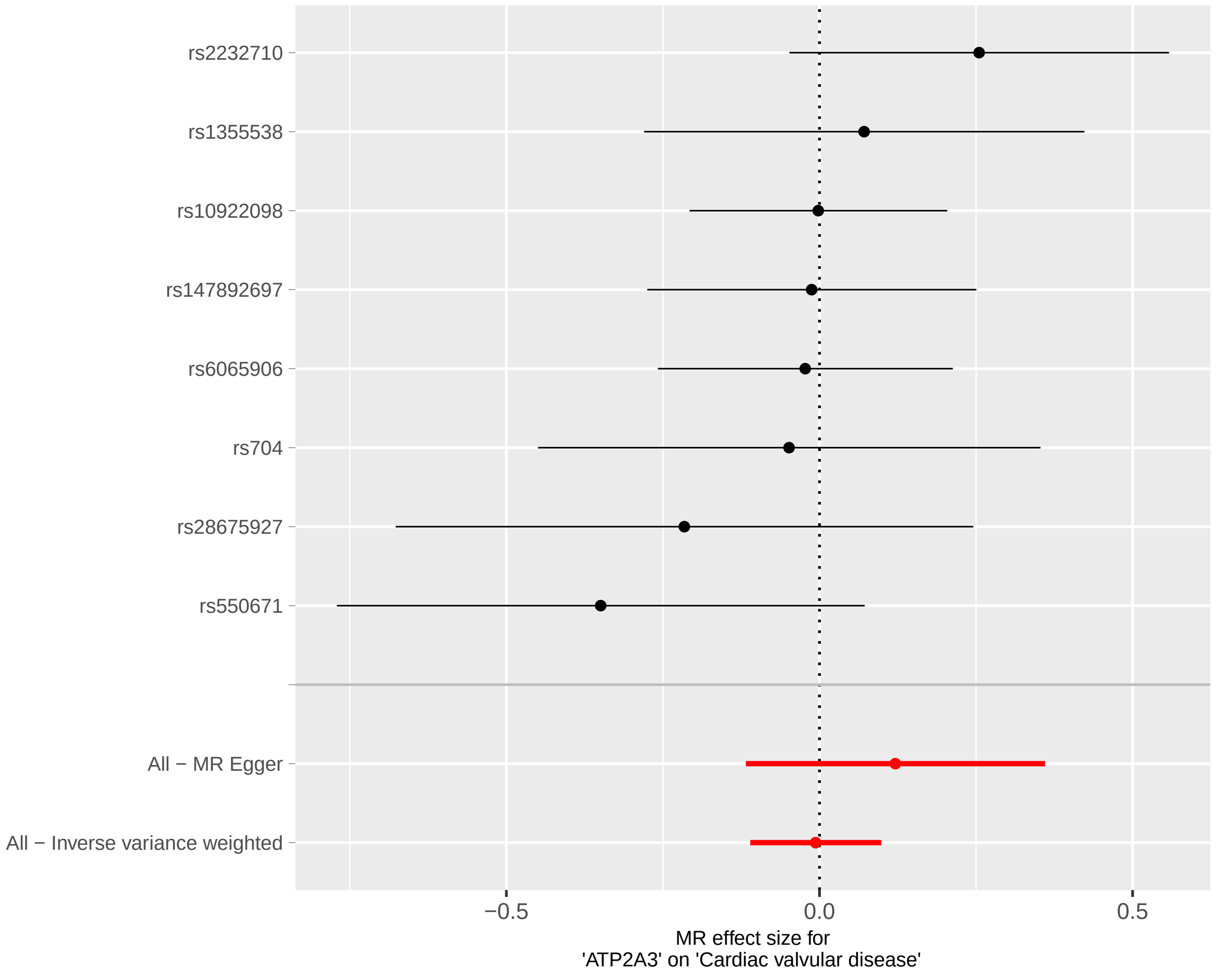
rs2232710
rs1355538
rs10922098
rs147892697
rs6065906
rs704
rs28675927
rs550671
All − MR Egger
All − Inverse variance weighted
−0.5
0.0
0.5
MR effect size for
'ATP2A3' on 'Cardiac valvular disease'

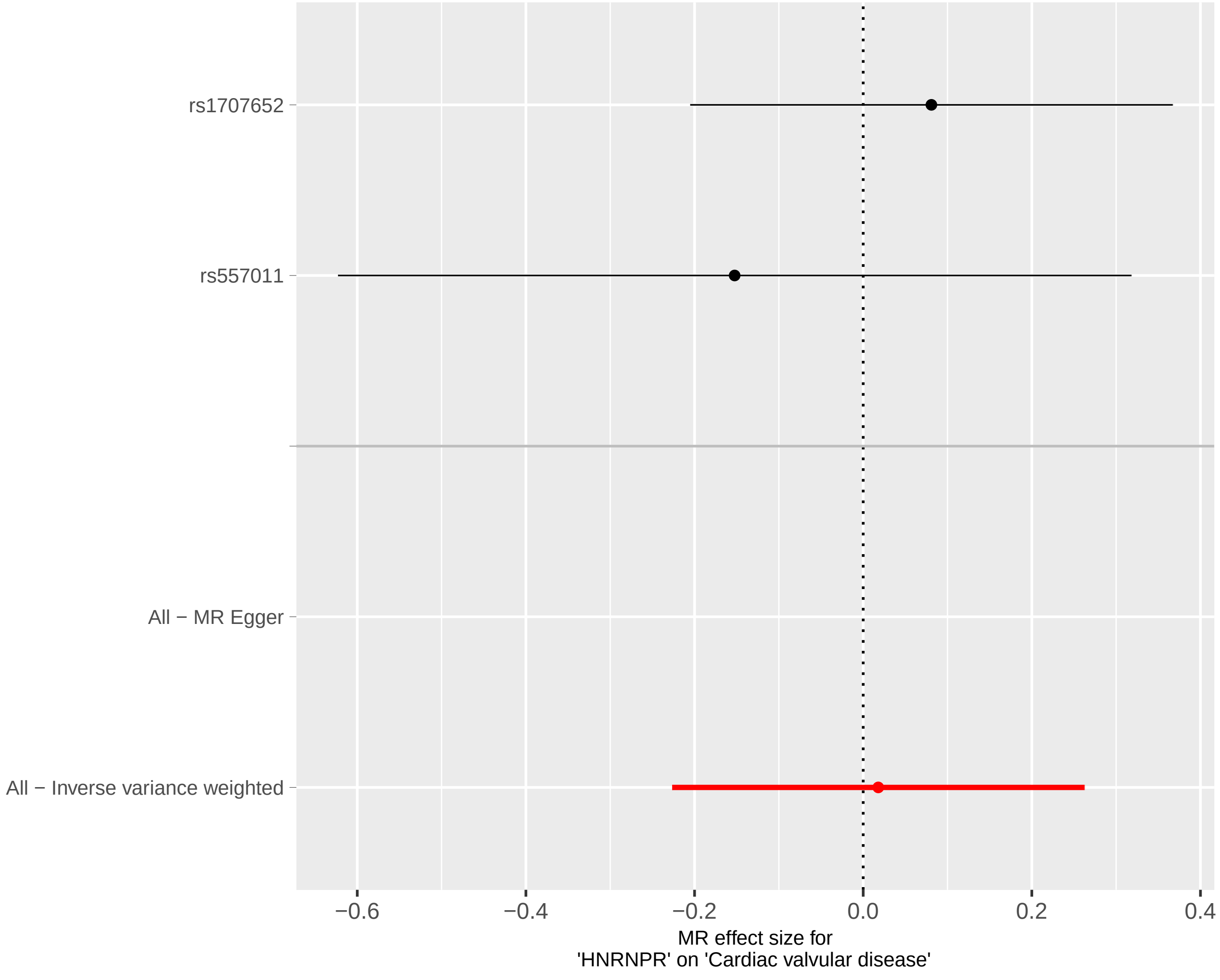
rs1707652
rs557011
All − MR Egger
All − Inverse variance weighted
−0.6
−0.4
−0.2
0.0
0.2
0.4
MR effect size for
'HNRNPR' on 'Cardiac valvular disease'

Insufficient number of SNPs

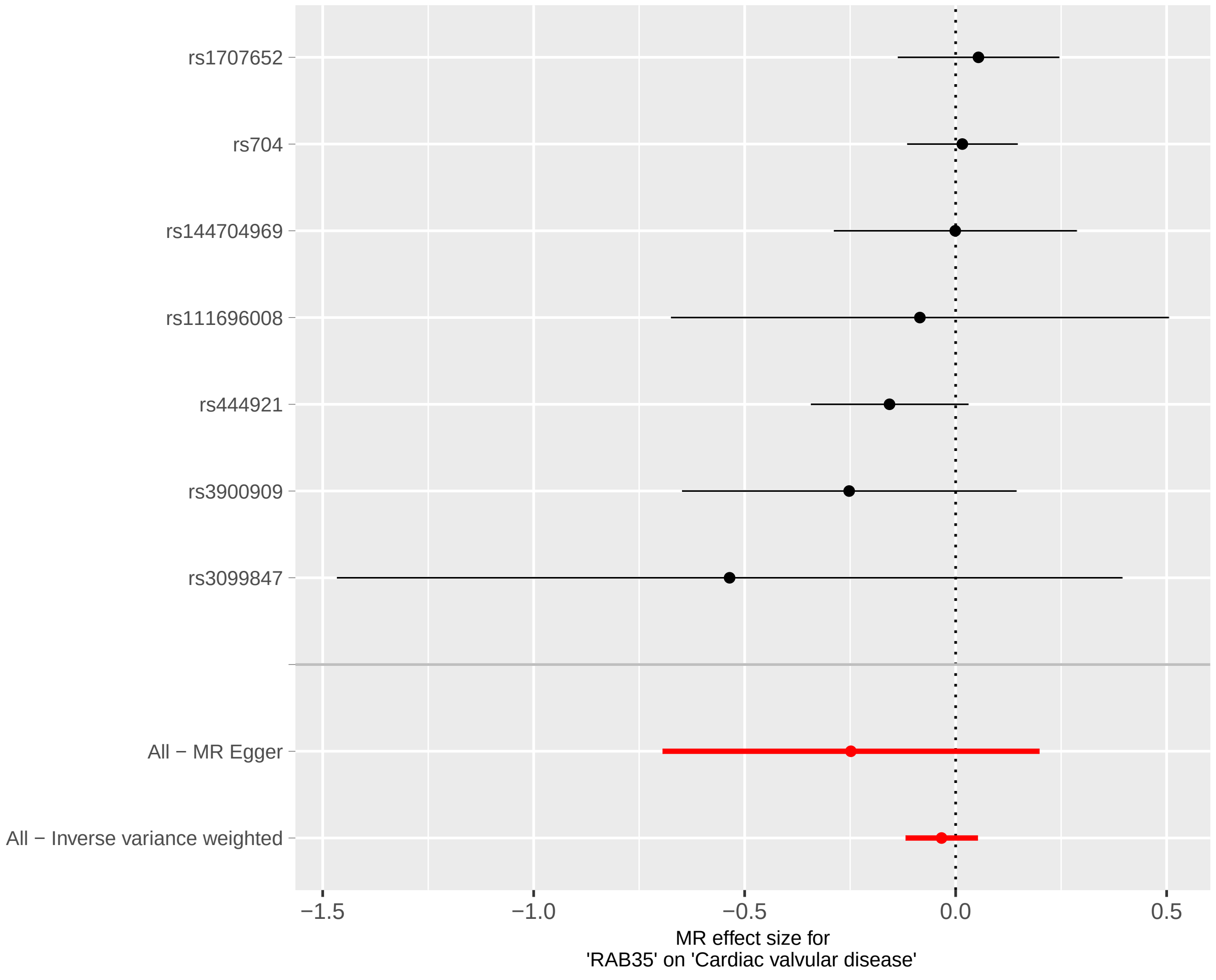

rs1707652
rs704
rs144704969
rs111696008
rs444921
rs3900909
rs3099847
All − MR Egger
All − Inverse variance weighted
−1.5
−1.0
−0.5
0.0
0.5
MR effect size for
'RAB35' on 'Cardiac valvular disease'

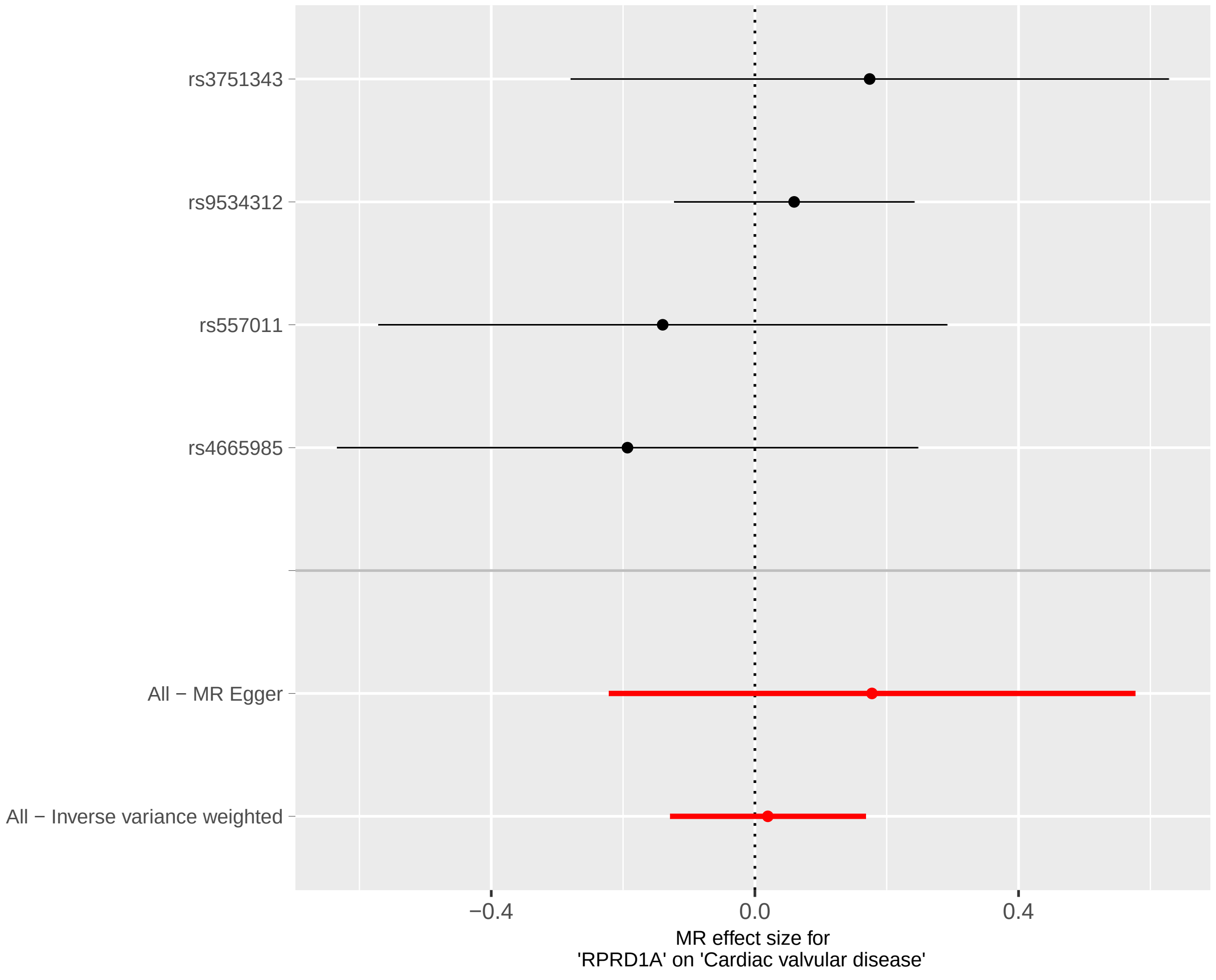
rs3751343
rs9534312
rs557011
rs4665985
All − MR Egger
All − Inverse variance weighted
−0.4
0.0
0.4
MR effect size for
'RPRD1A' on 'Cardiac valvular disease'

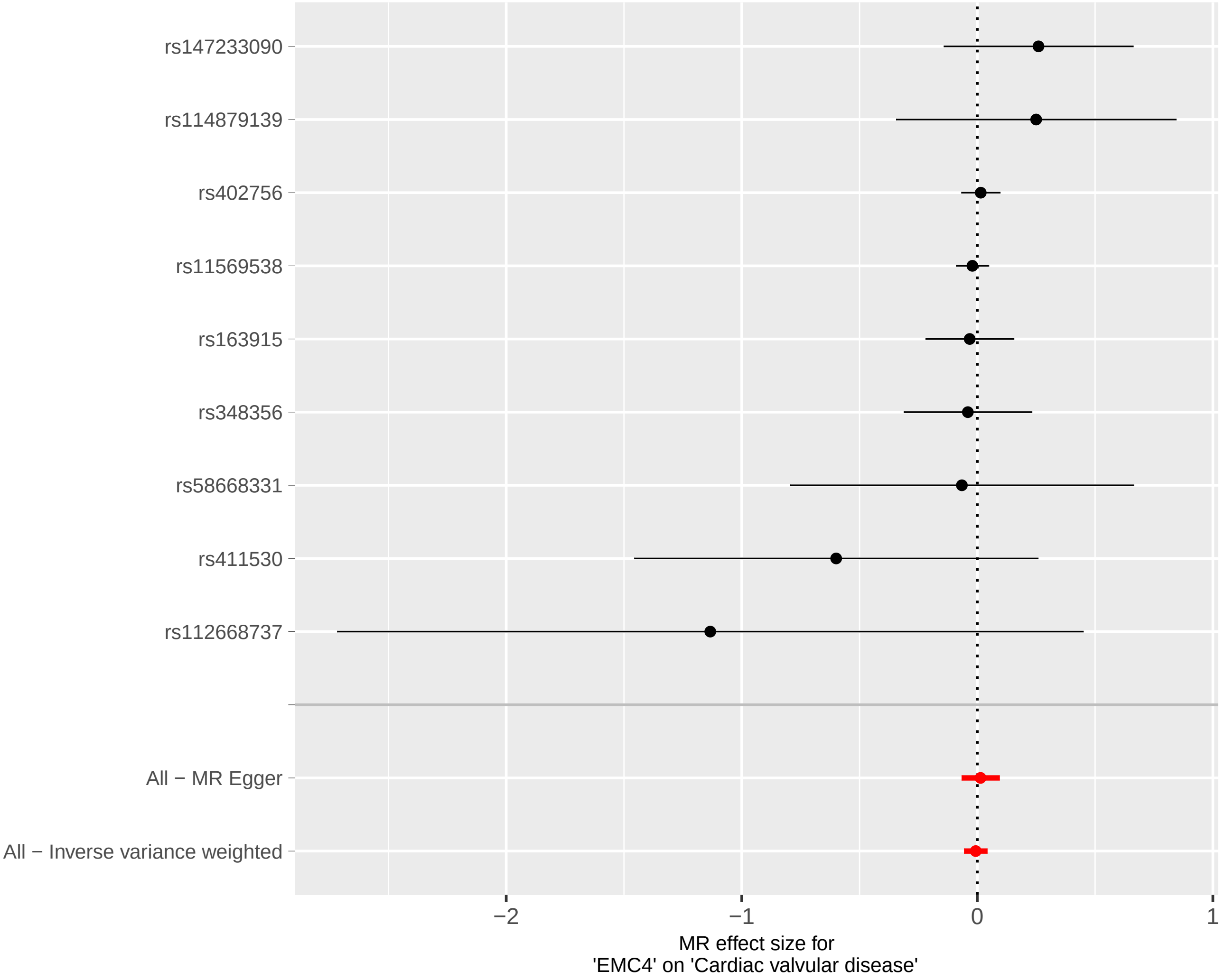
rs147233090
rs114879139
rs402756
rs11569538
rs163915
rs348356
rs58668331
rs411530
rs112668737
All − MR Egger
All − Inverse variance weighted
−2
−1
0
1
MR effect size for
'EMC4' on 'Cardiac valvular disease'

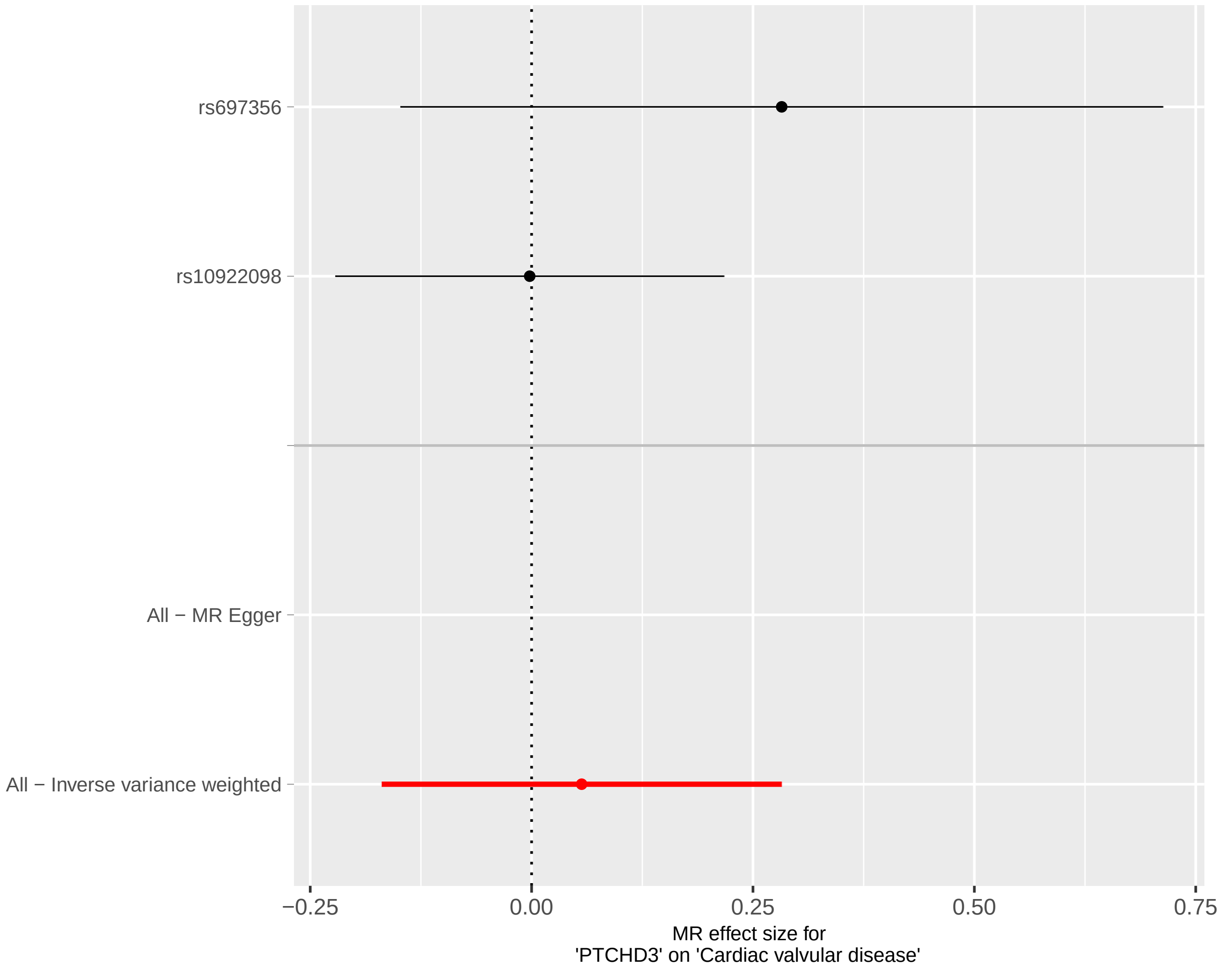
rs697356
rs10922098
All − MR Egger
All − Inverse variance weighted
−0.25
0.00
0.25
0.50
0.75
MR effect size for
'PTCHD3' on 'Cardiac valvular disease'

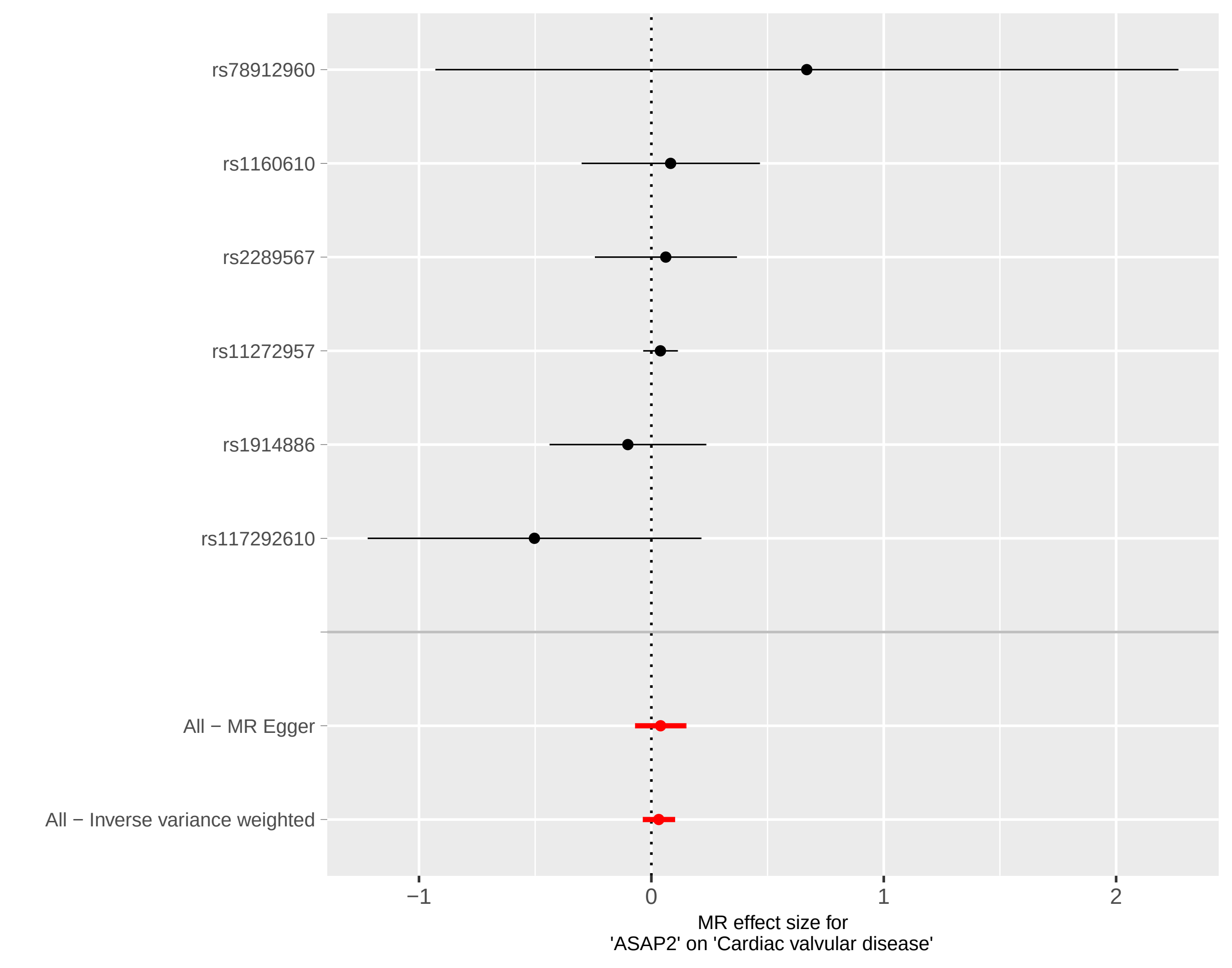
rs78912960
rs1160610
rs2289567
rs11272957
rs1914886
rs117292610
All − MR Egger
All − Inverse variance weighted
−1
0
1
2
MR effect size for
'ASAP2' on 'Cardiac valvular disease'

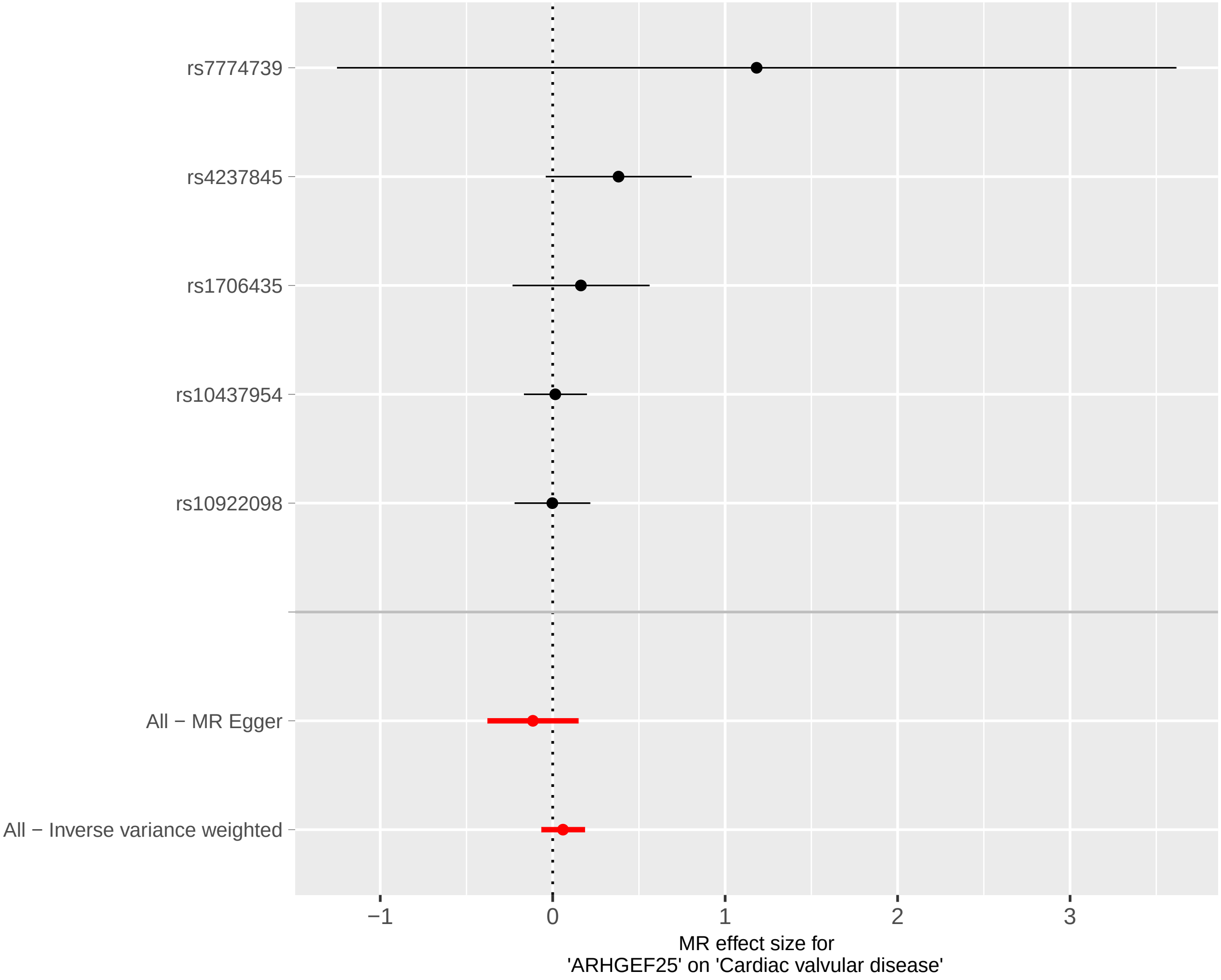
rs7774739
rs4237845
rs1706435
rs10437954
rs10922098
All − MR Egger
All − Inverse variance weighted
−1
0
1
2
3
MR effect size for
'ARHGEF25' on 'Cardiac valvular disease'

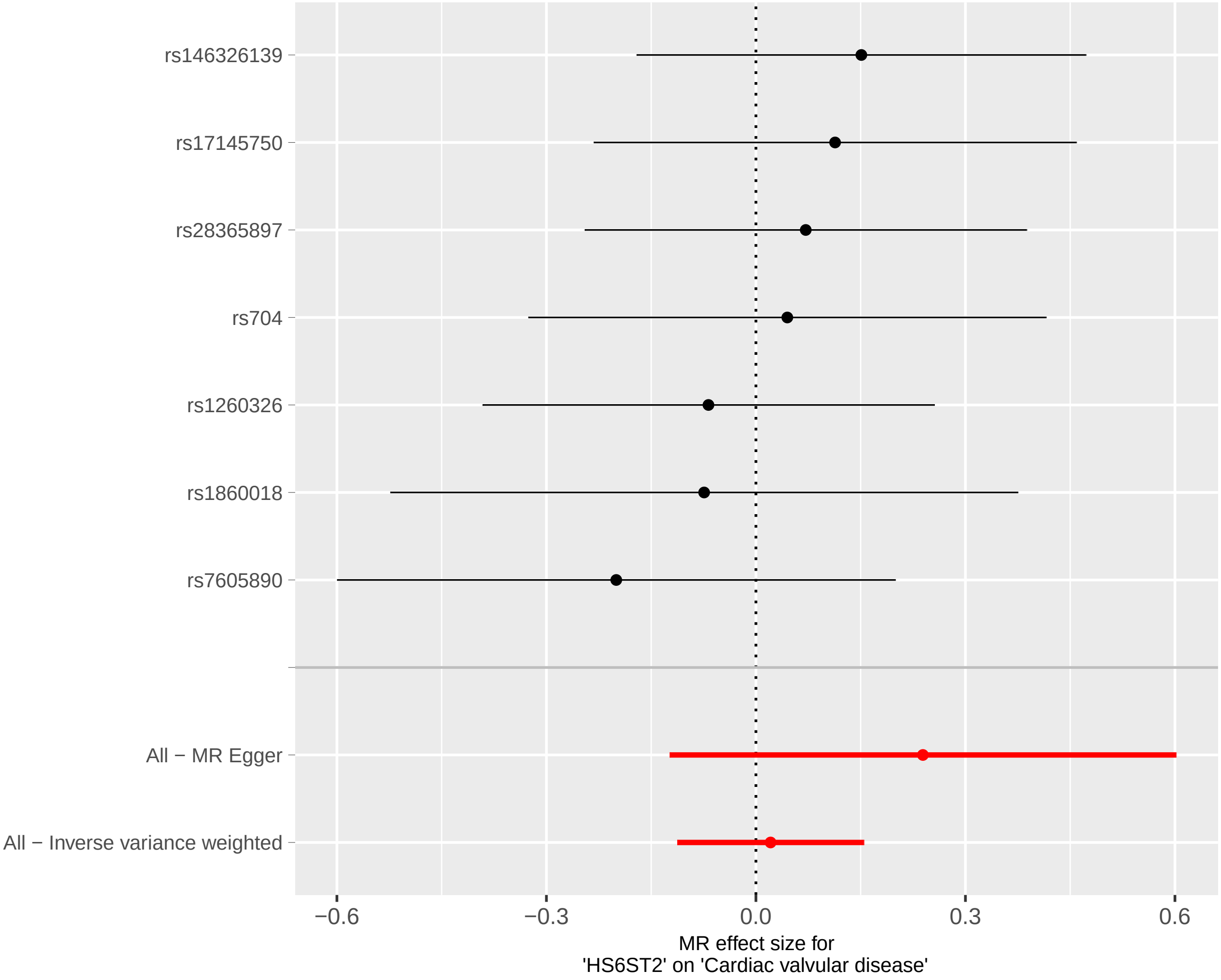

rs146326139
rs17145750
rs28365897
rs704
rs1260326
rs1860018
rs7605890
All − MR Egger
All − Inverse variance weighted
−0.6
−0.3
0.0
0.3
0.6
MR effect size for
'HS6ST2' on 'Cardiac valvular disease'

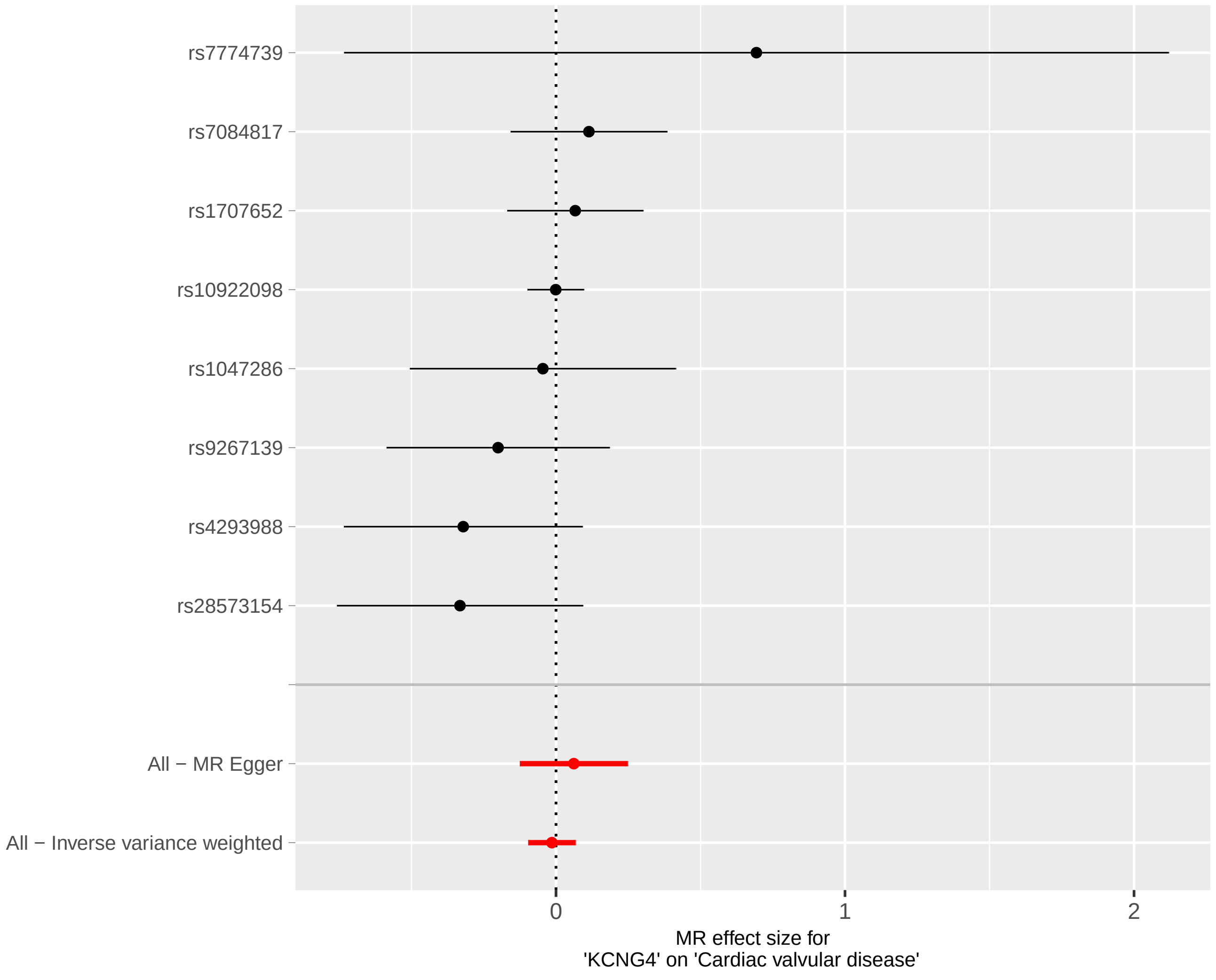

rs7774739
rs7084817
rs1707652
rs10922098
rs1047286
rs9267139
rs4293988
rs28573154
All − MR Egger
All − Inverse variance weighted
0
1
2
MR effect size for
'KCNG4' on 'Cardiac valvular disease'

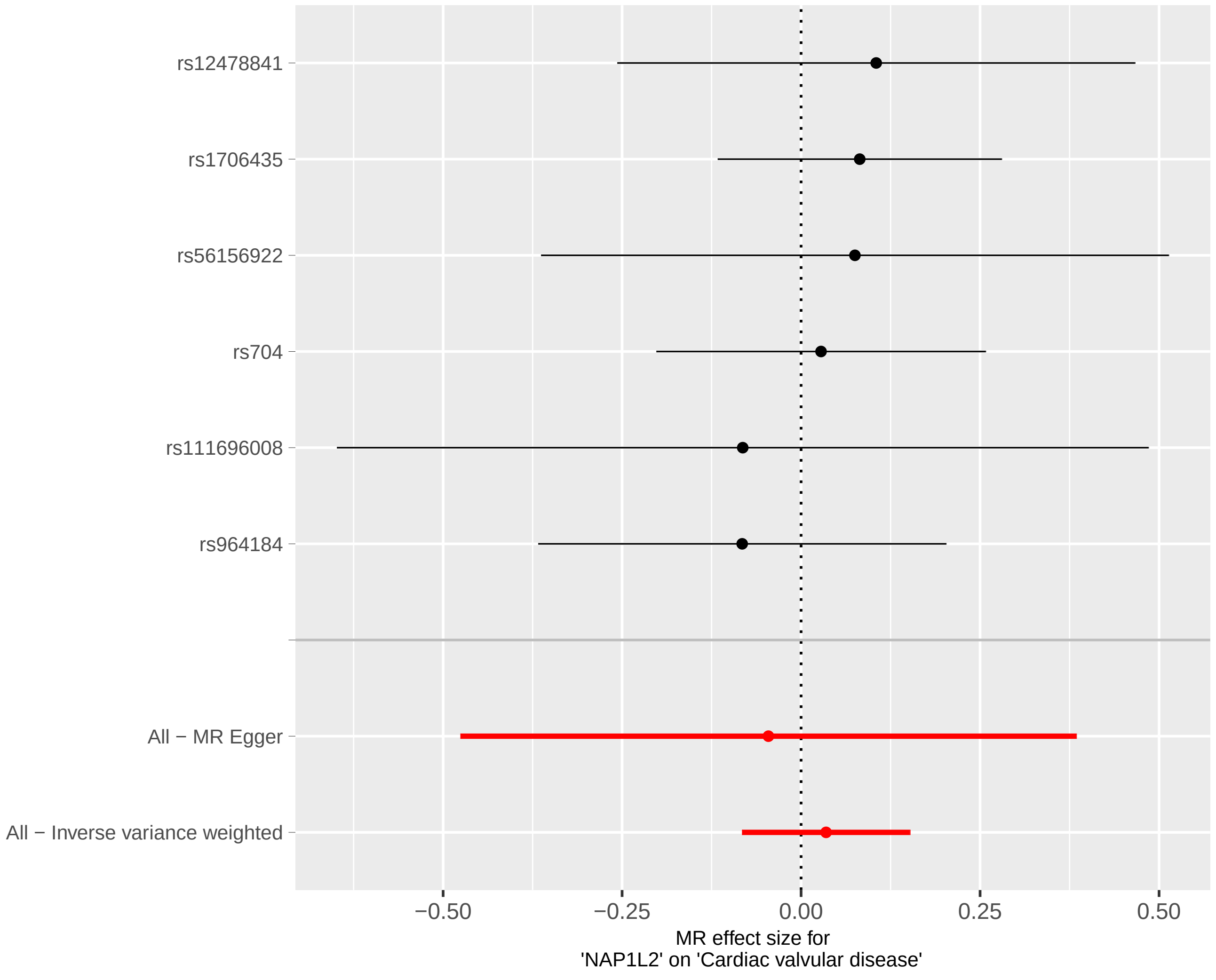

rs12478841
rs1706435
rs56156922
rs704
rs111696008
rs964184
All − MR Egger
All − Inverse variance weighted
−0.50
−0.25
0.00
0.25
0.50
MR effect size for
'NAP1L2' on 'Cardiac valvular disease'

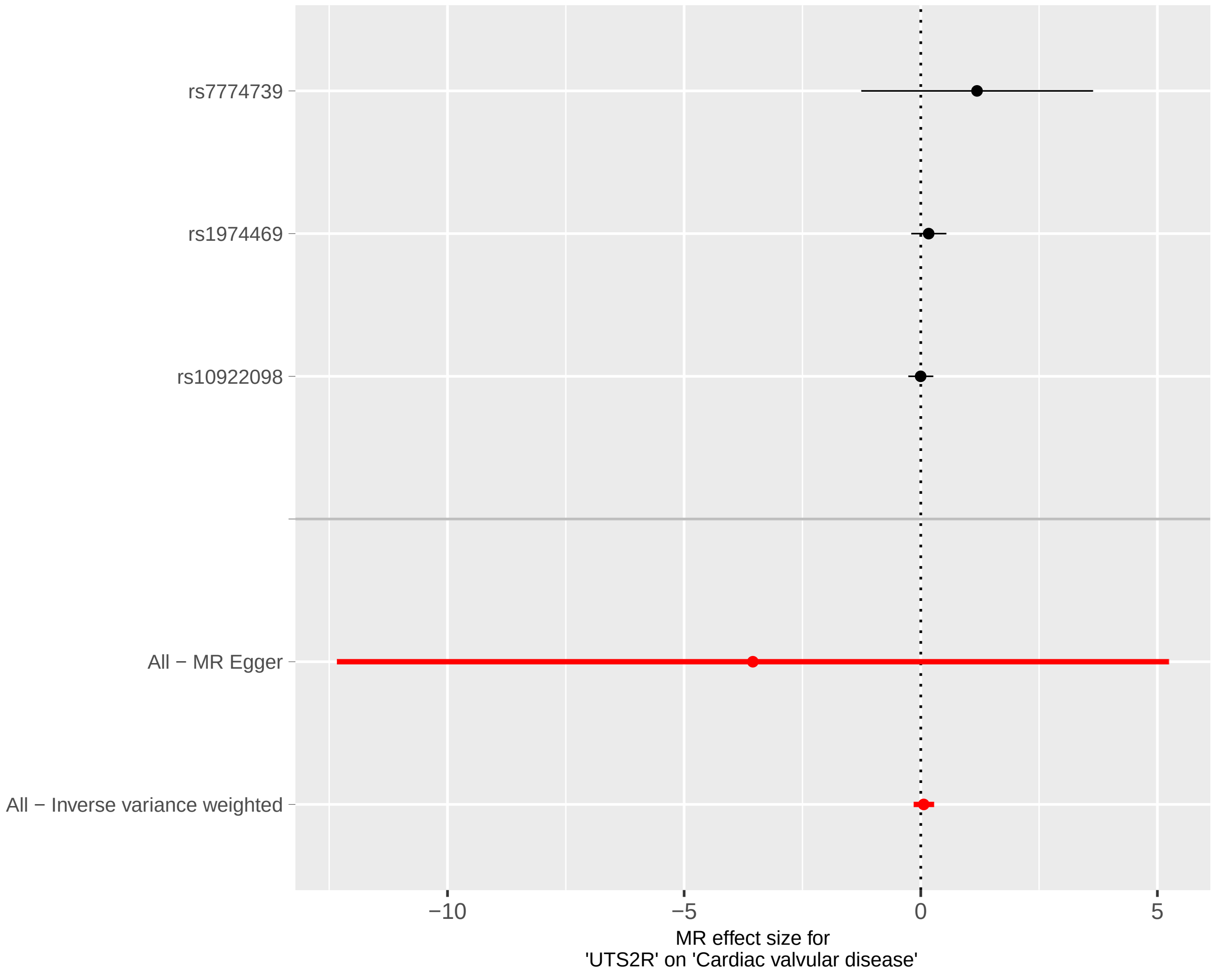

rs7774739
rs1974469
rs10922098
All − MR Egger
All − Inverse variance weighted
−10
−5
0
5
MR effect size for
'UTS2R' on 'Cardiac valvular disease'

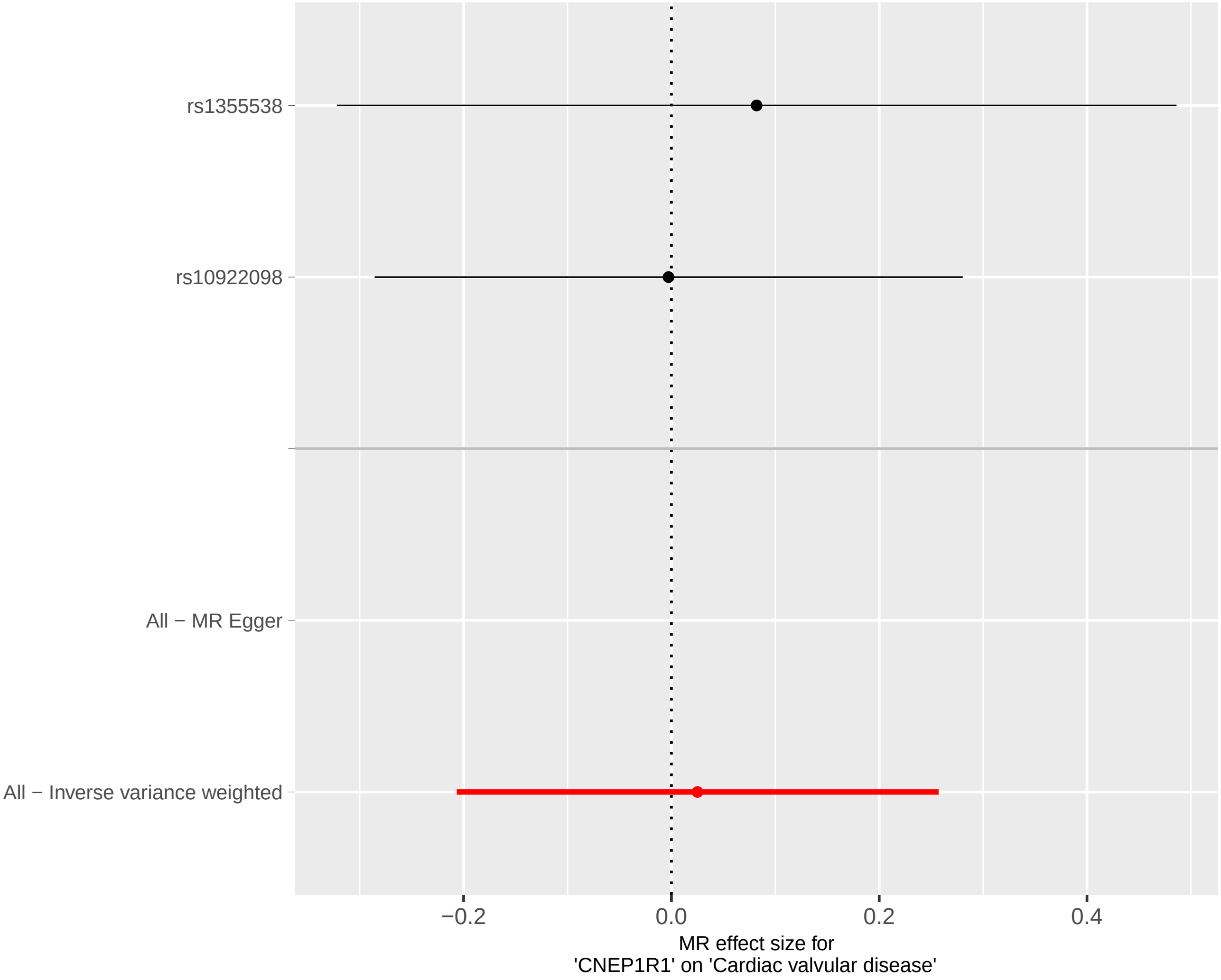
rs1355538
rs10922098
All − MR Egger
All − Inverse variance weighted
−0.2
0.0
0.2
0.4
MR effect size for
'CNEP1R1' on 'Cardiac valvular disease'

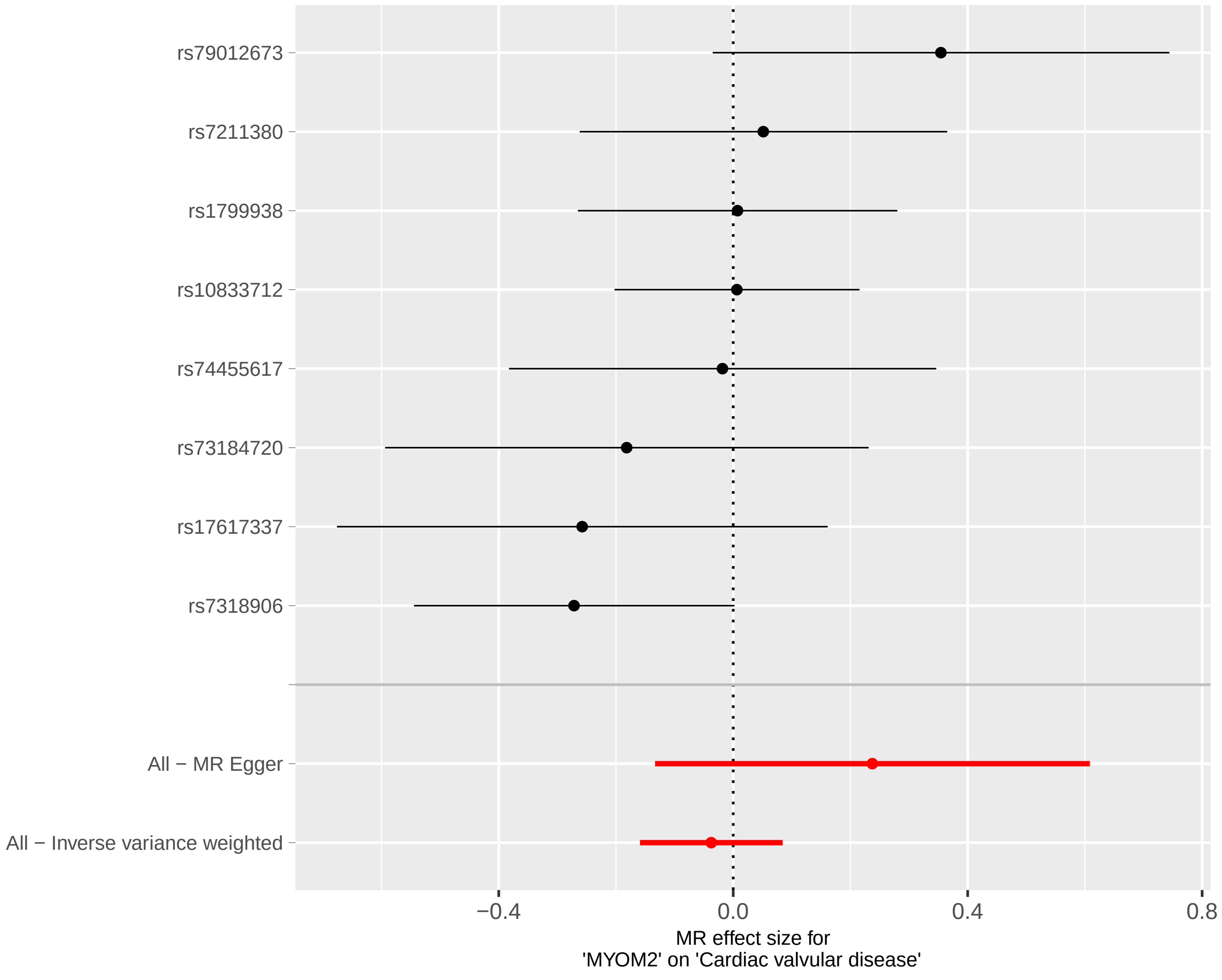
rs79012673
rs7211380
rs1799938
rs10833712
rs74455617
rs73184720
rs17617337
rs7318906
All − MR Egger
All − Inverse variance weighted
−0.4
0.0
0.4
0.8
MR effect size for
'MYOM2' on 'Cardiac valvular disease'

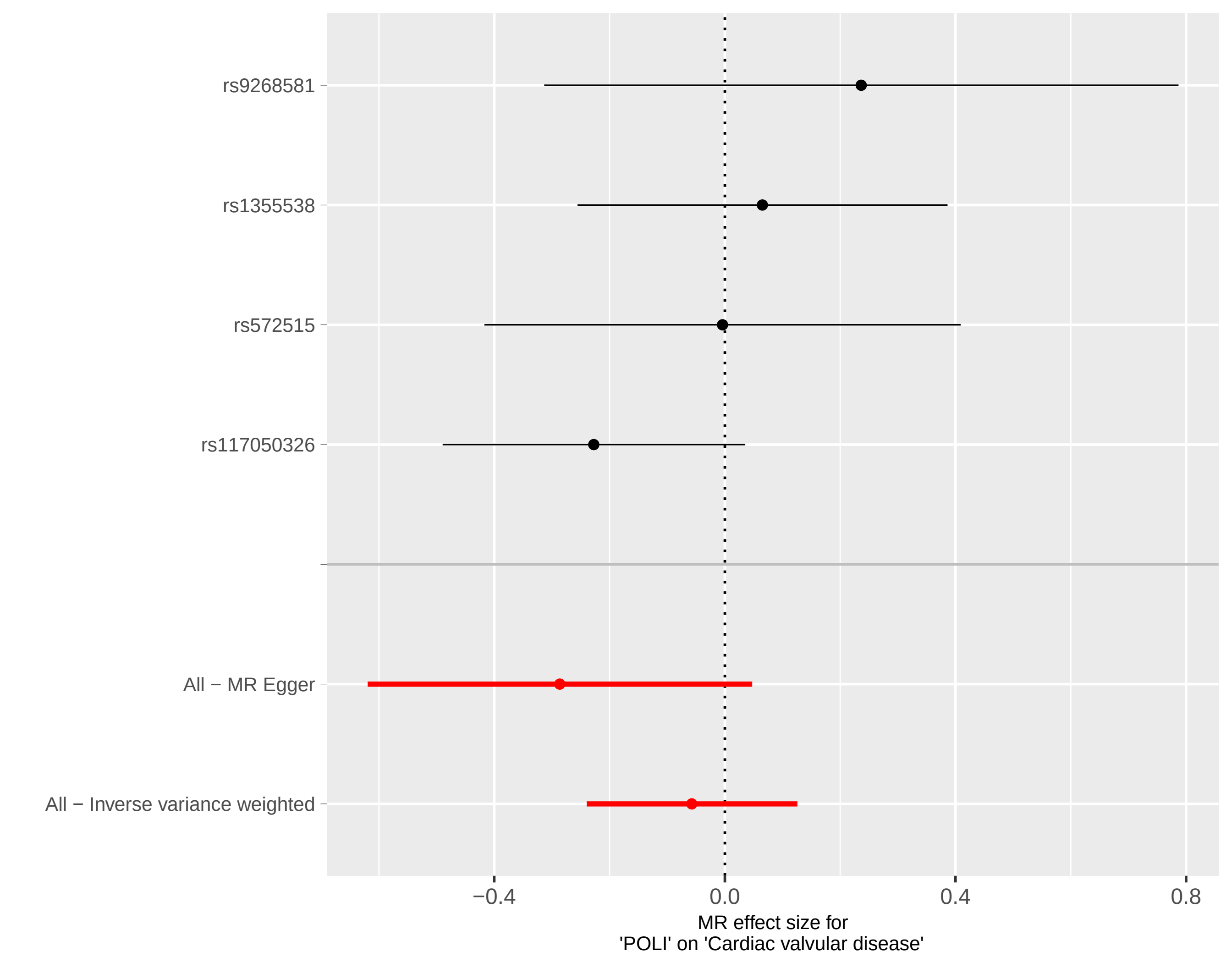
rs9268581
rs1355538
rs572515
rs117050326
All − MR Egger
All − Inverse variance weighted
−0.4
0.0
0.4
0.8
MR effect size for
'POLI' on 'Cardiac valvular disease'

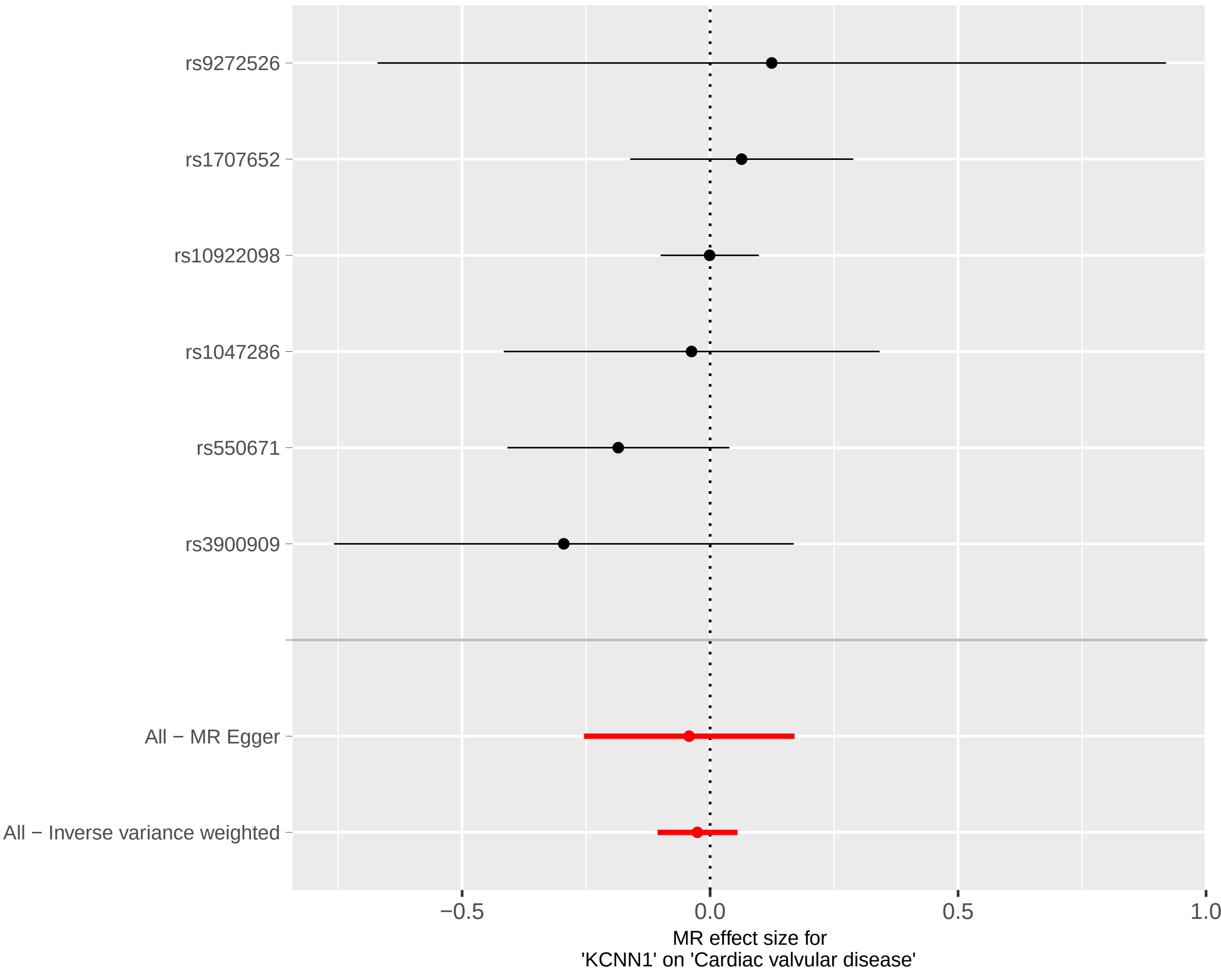
rs9272526
rs1707652
rs10922098
rs1047286
rs550671
rs3900909
All − MR Egger
All − Inverse variance weighted
−0.5
0.0
0.5
1.0
MR effect size for
'KCNN1' on 'Cardiac valvular disease'

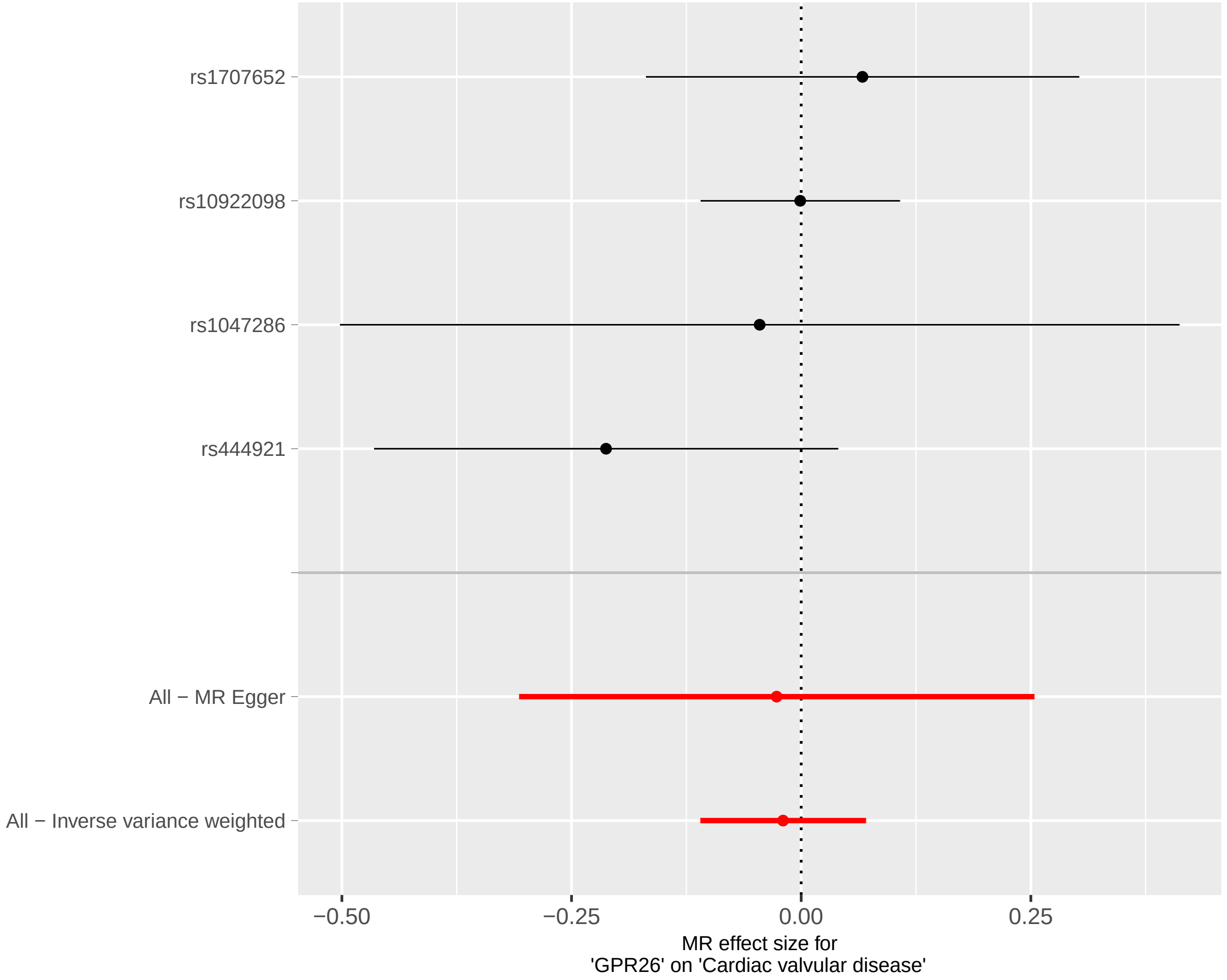
rs1707652
rs10922098
rs1047286
rs444921
All − MR Egger
All − Inverse variance weighted
−0.50
−0.25
0.00
0.25
MR effect size for
'GPR26' on 'Cardiac valvular disease'

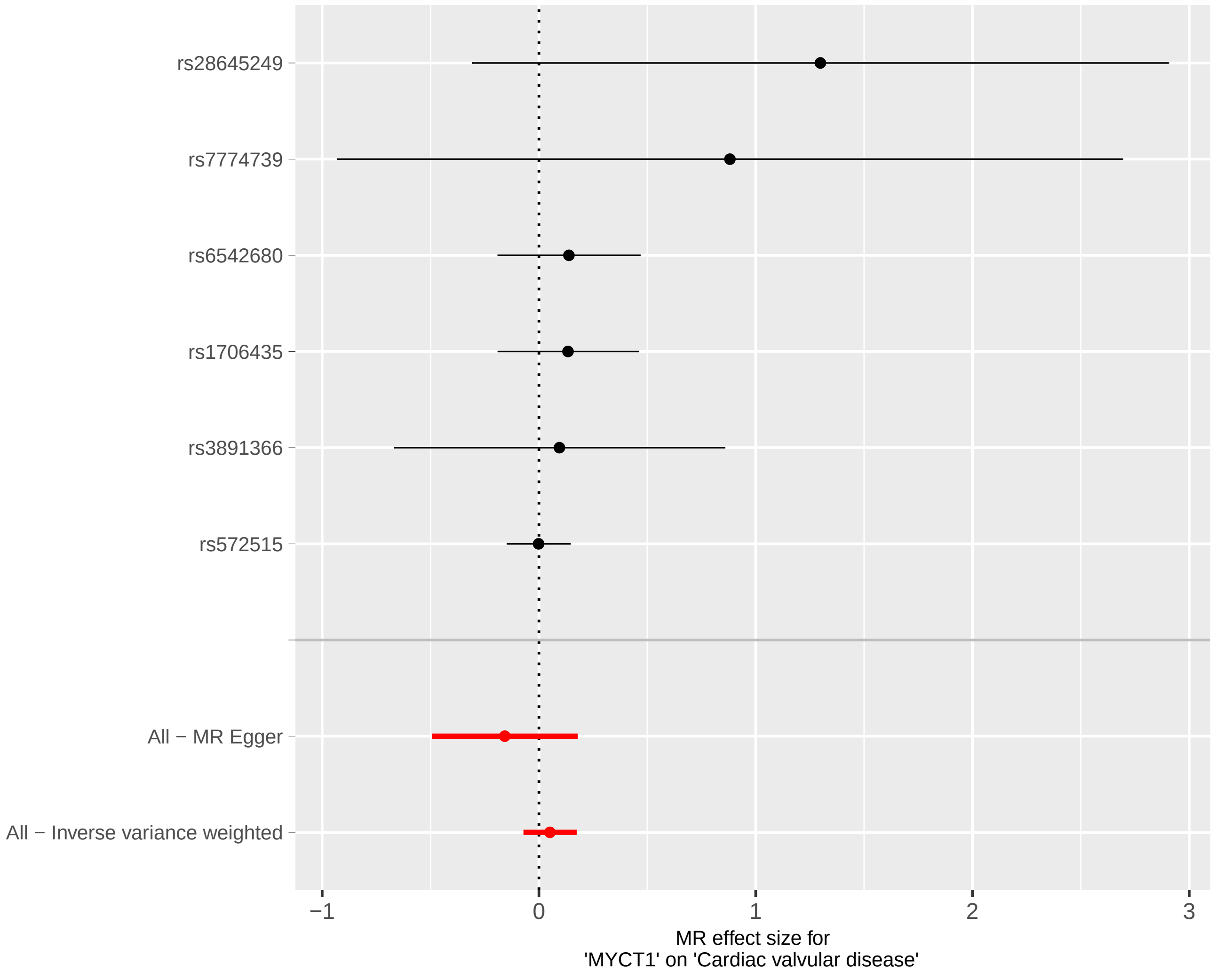
rs28645249
rs7774739
rs6542680
rs1706435
rs3891366
rs572515
All − MR Egger
All − Inverse variance weighted
−1
0
1
2
3
MR effect size for
'MYCT1' on 'Cardiac valvular disease'

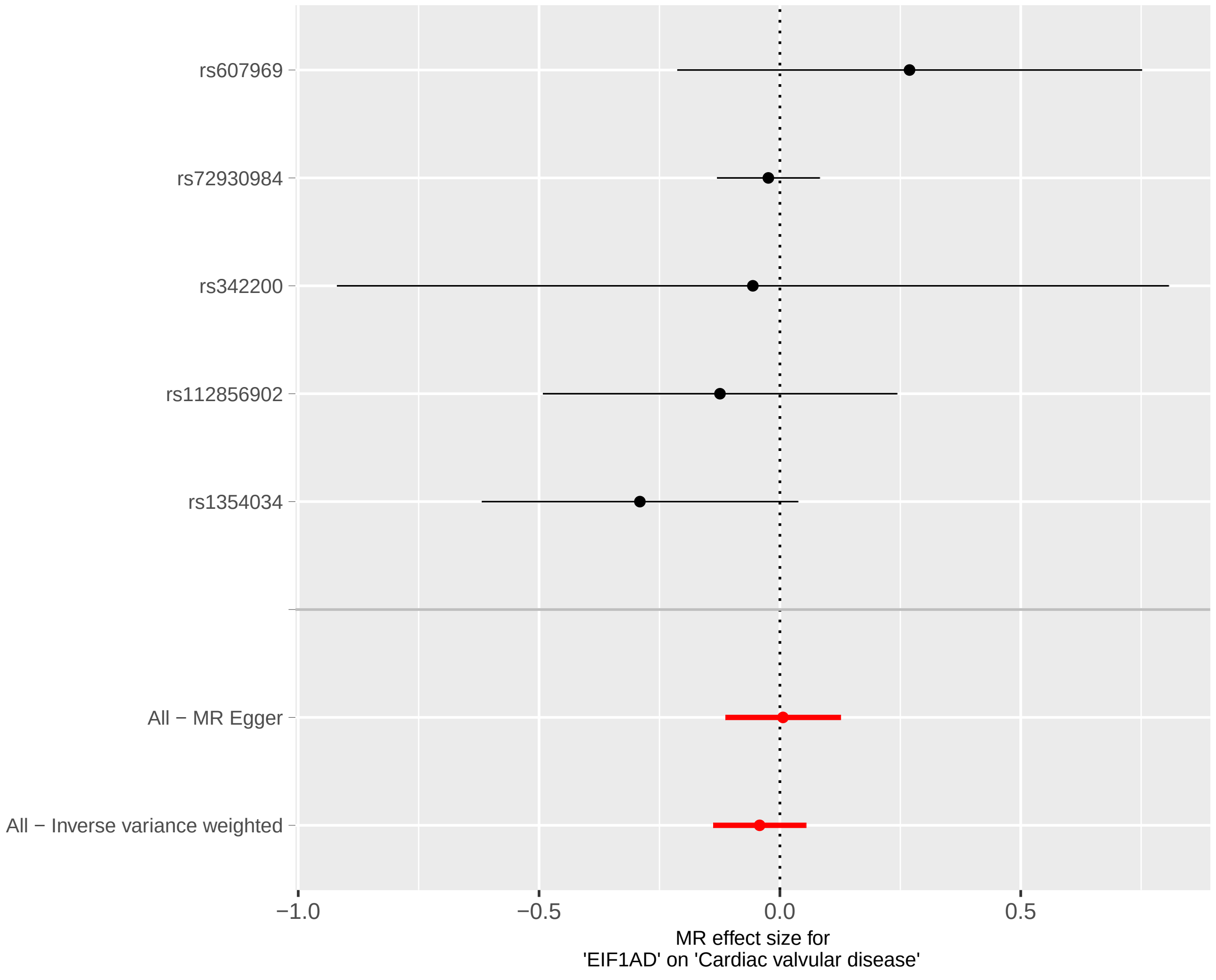
rs607969
rs72930984
rs342200
rs112856902
rs1354034
All − MR Egger
All − Inverse variance weighted
−1.0
−0.5
0.0
0.5
MR effect size for
'EIF1AD' on 'Cardiac valvular disease'

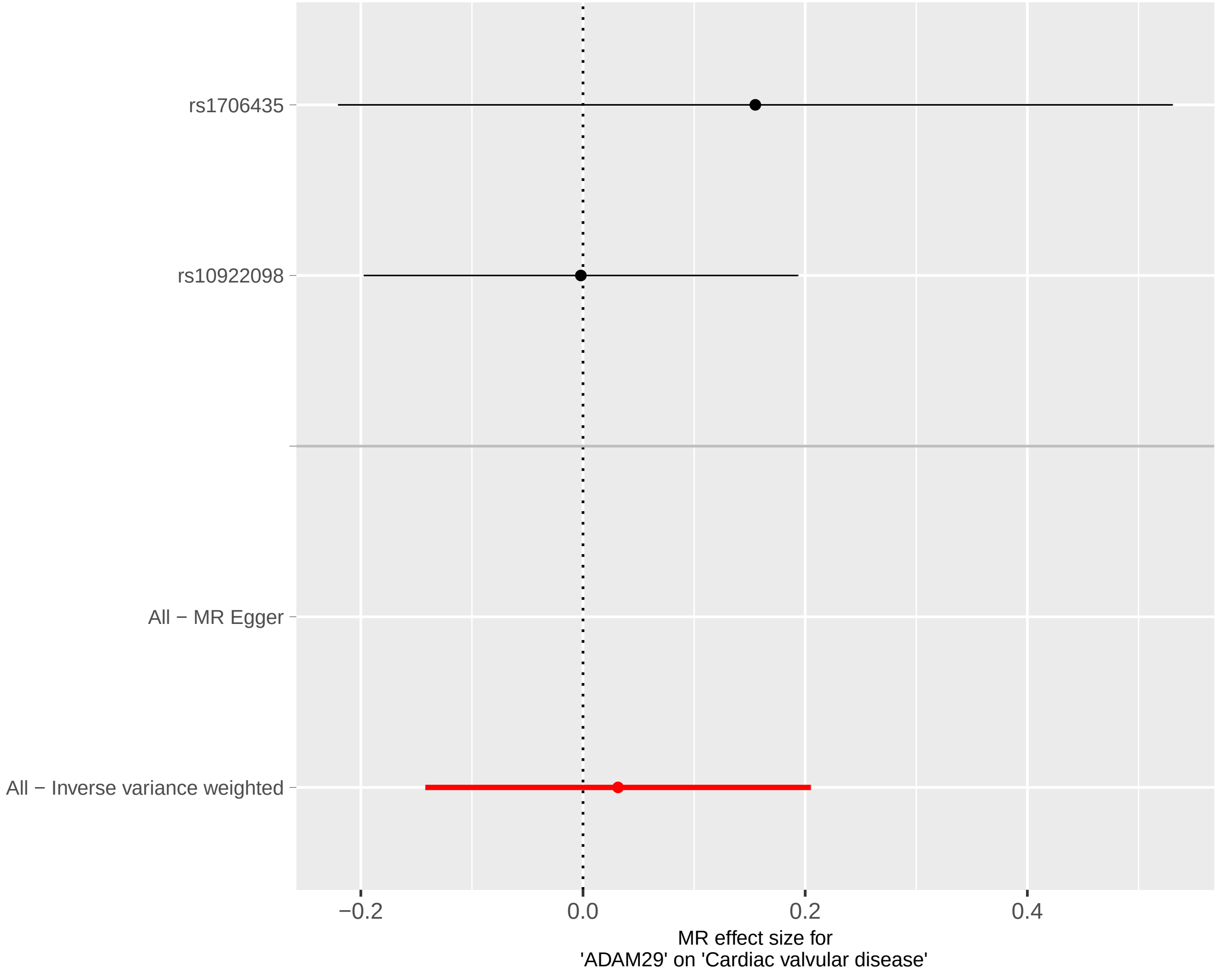
rs1706435
rs10922098
All − MR Egger
All − Inverse variance weighted
−0.2
0.0
0.2
0.4
MR effect size for
'ADAM29' on 'Cardiac valvular disease'

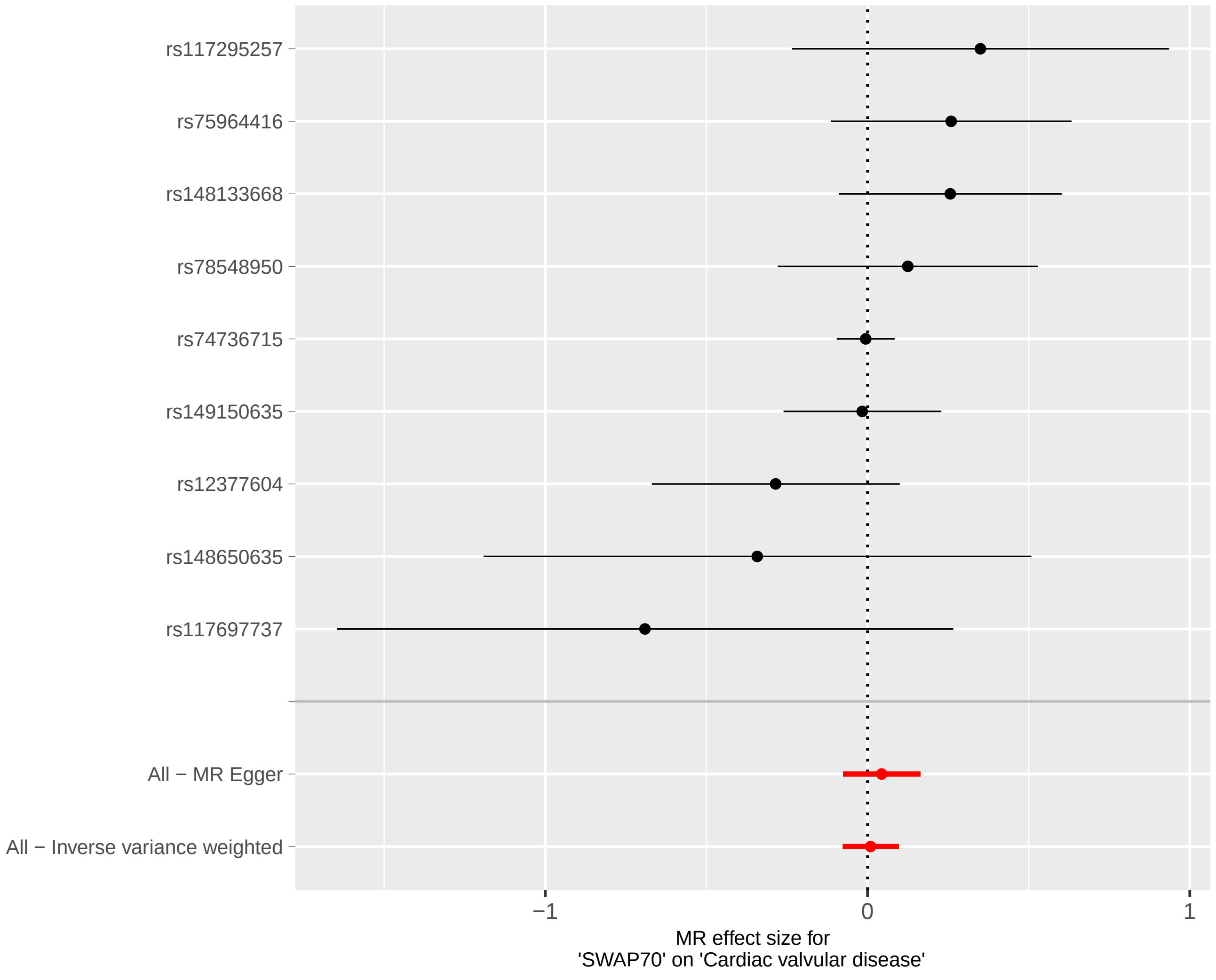
rs117295257
rs75964416
rs148133668
rs78548950
rs74736715
rs149150635
rs12377604
rs148650635
rs117697737
All − MR Egger
All − Inverse variance weighted
−1
0
1
MR effect size for
'SWAP70' on 'Cardiac valvular disease'

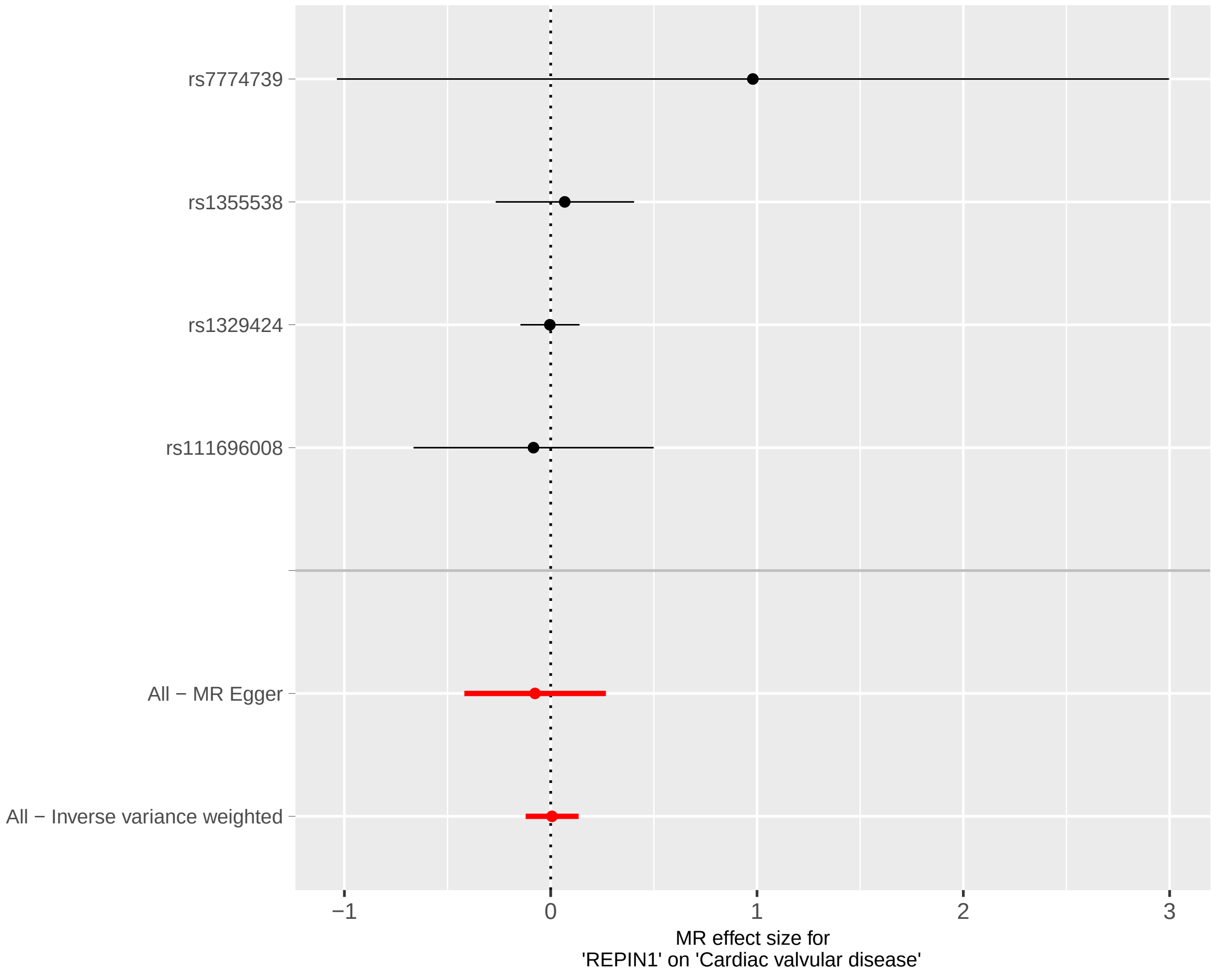
rs7774739
rs1355538
rs1329424
rs111696008
All − MR Egger
All − Inverse variance weighted
−1
0
1
2
3
MR effect size for
'REPIN1' on 'Cardiac valvular disease'

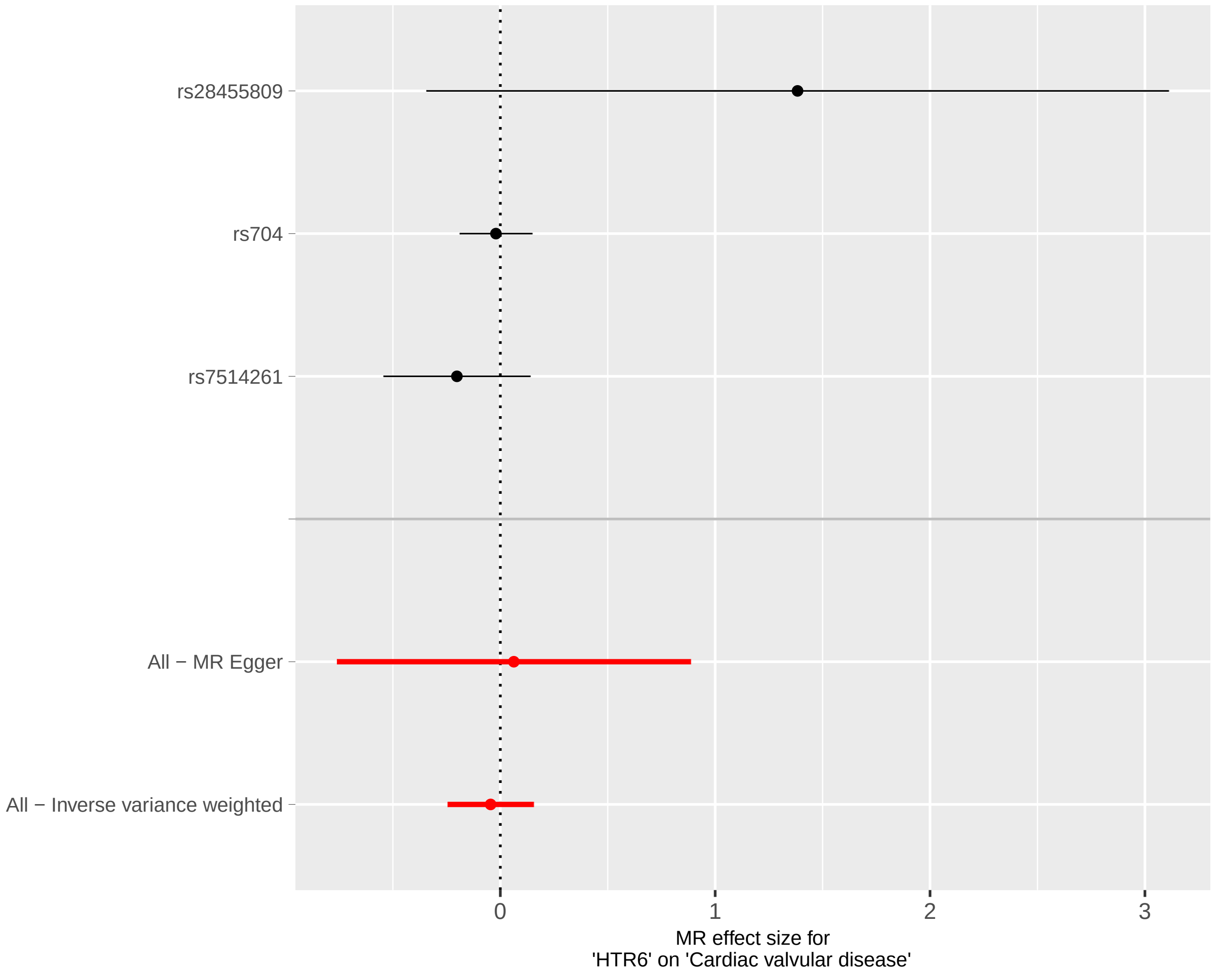
rs28455809
rs704
rs7514261
All − MR Egger
All − Inverse variance weighted
0
1
2
3
MR effect size for
'HTR6' on 'Cardiac valvular disease'

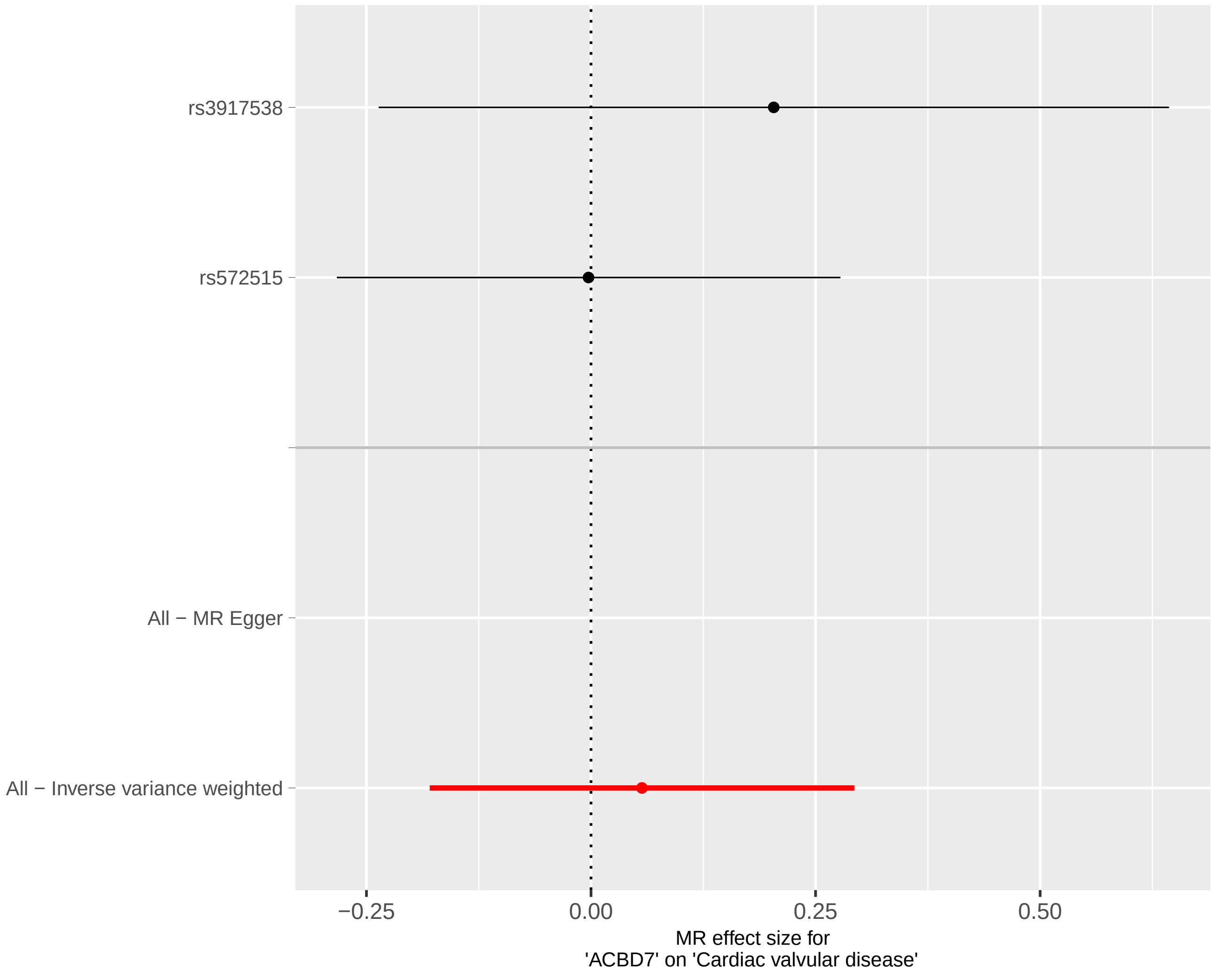
rs3917538
rs572515
All − MR Egger
All − Inverse variance weighted
−0.25
0.00
0.25
0.50
MR effect size for
'ACBD7' on 'Cardiac valvular disease'

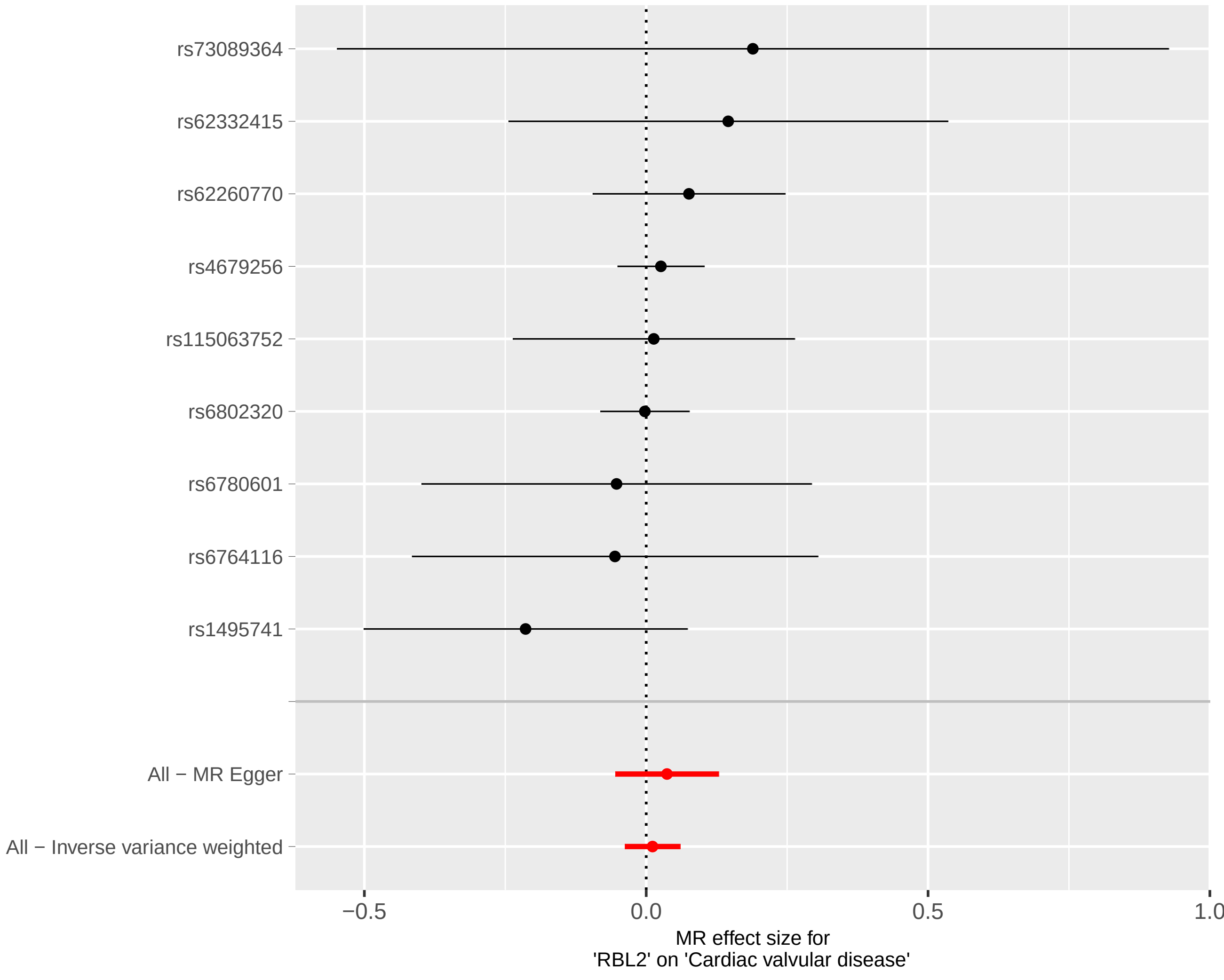
rs73089364
rs62332415
rs62260770
rs4679256
rs115063752
rs6802320
rs6780601
rs6764116
rs1495741
All − MR Egger
All − Inverse variance weighted
−0.5
0.0
0.5
1.0
MR effect size for
'RBL2' on 'Cardiac valvular disease'

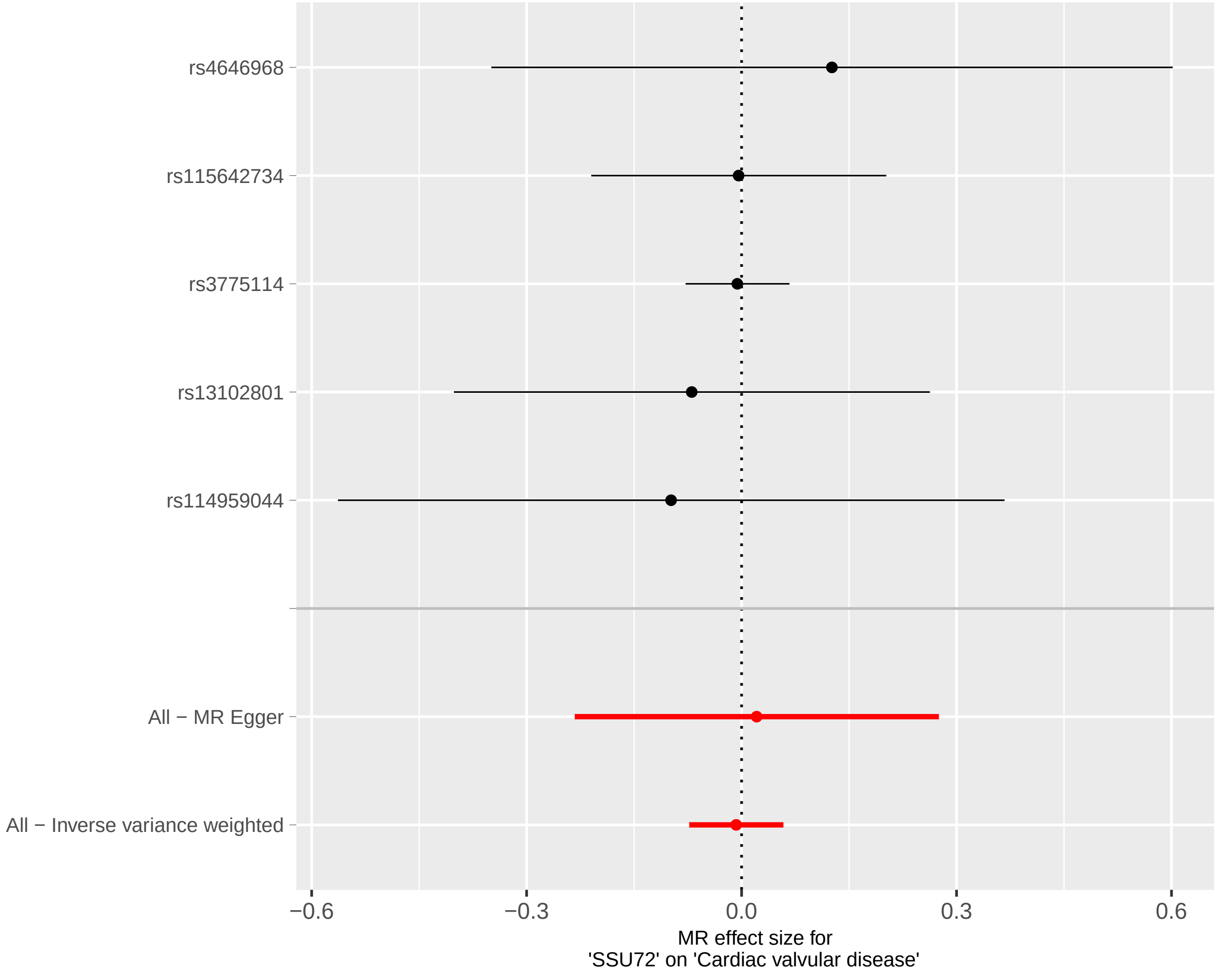

rs4646968
rs115642734
rs3775114
rs13102801
rs114959044
All − MR Egger
All − Inverse variance weighted
−0.6
−0.3
0.0
0.3
0.6
MR effect size for
'SSU72' on 'Cardiac valvular disease'

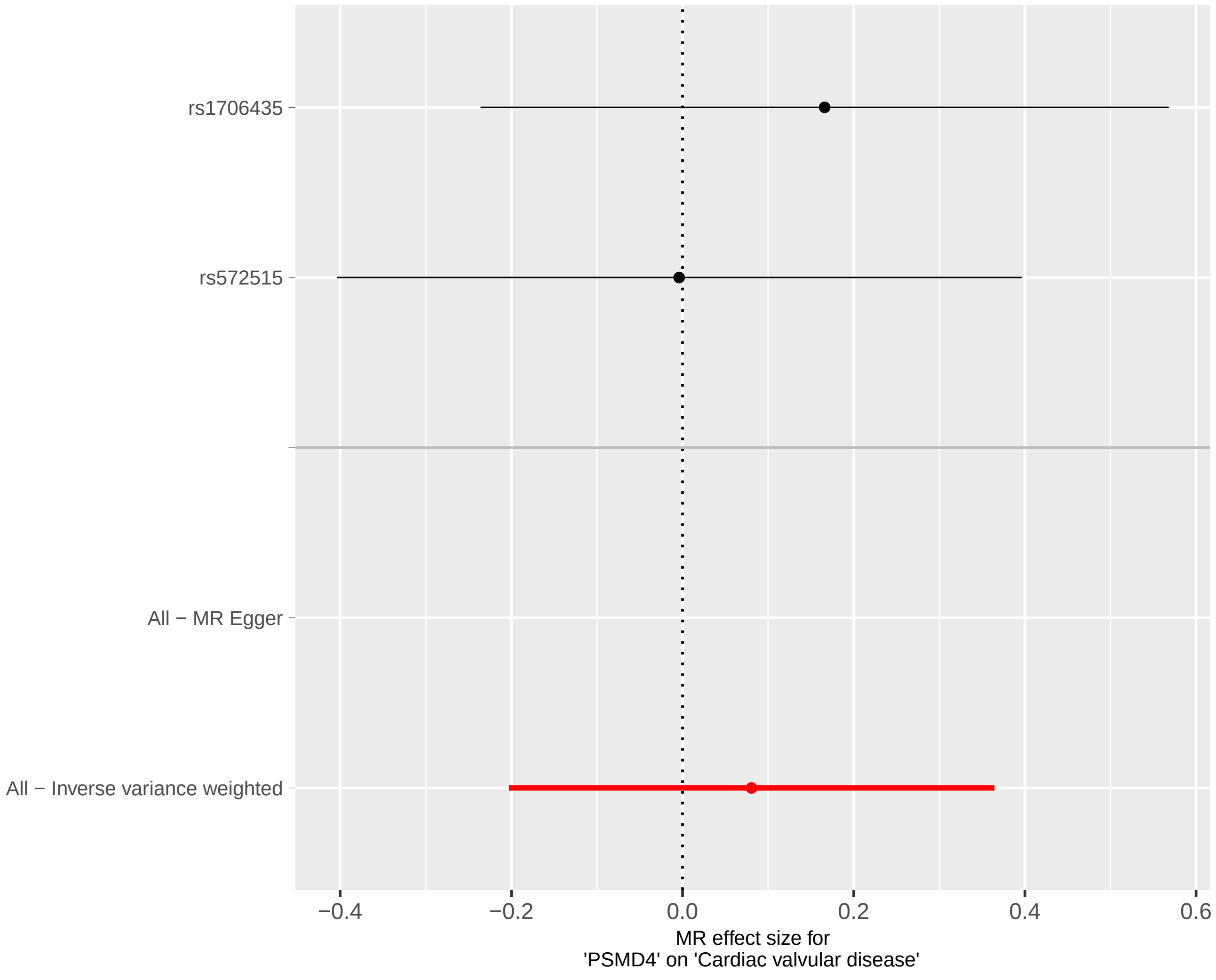
rs1706435
rs572515
All − MR Egger
All − Inverse variance weighted
−0.4
−0.2
0.0
0.2
0.4
0.6
MR effect size for
'PSMD4' on 'Cardiac valvular disease'

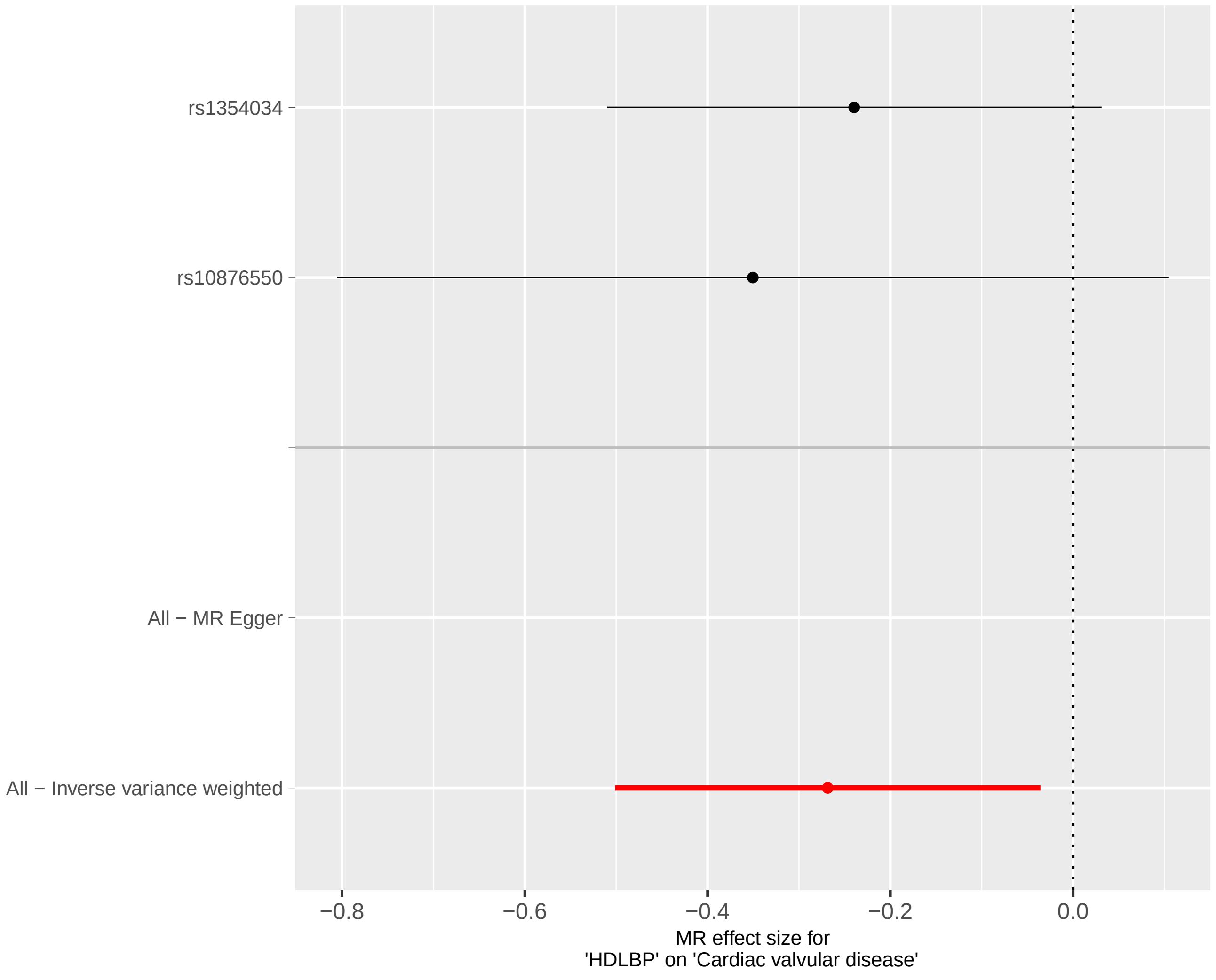
rs1354034
rs10876550
All − MR Egger
All − Inverse variance weighted
−0.8
−0.6
−0.4
−0.2
0.0
MR effect size for
'HDLBP' on 'Cardiac valvular disease'

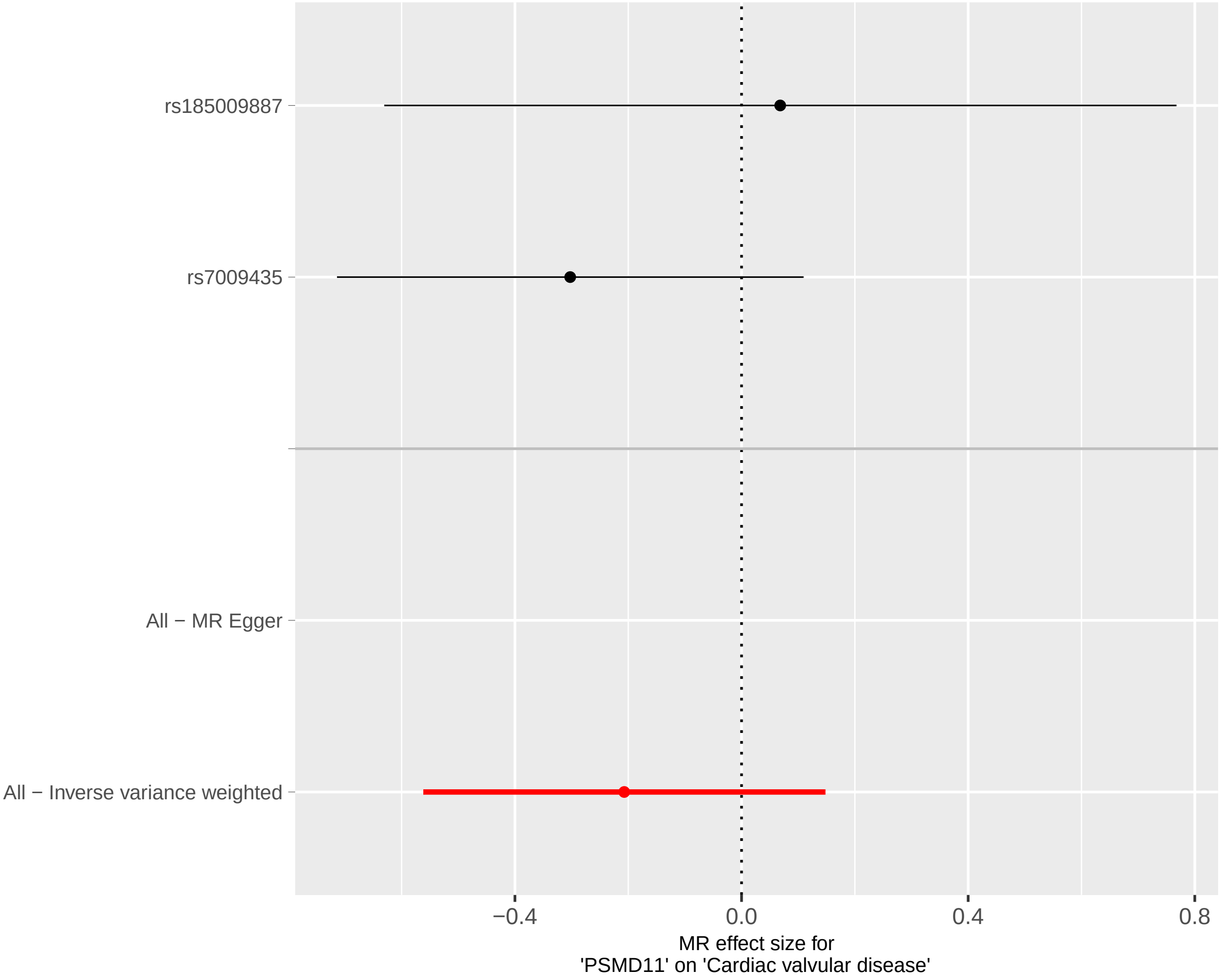

rs185009887
rs7009435
All − MR Egger
All − Inverse variance weighted
−0.4
0.0
0.4
0.8
MR effect size for
'PSMD11' on 'Cardiac valvular disease'

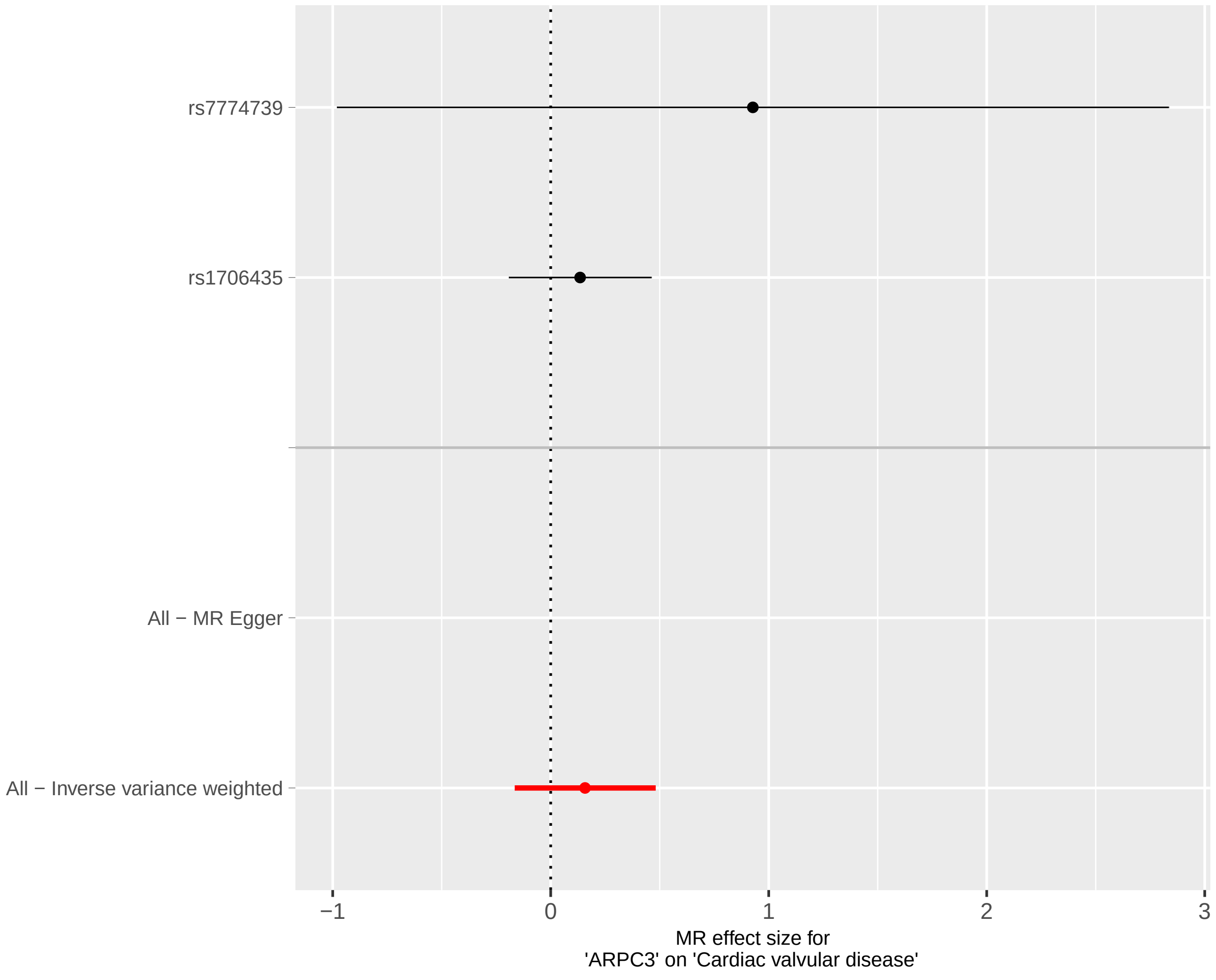
rs7774739
rs1706435
All − MR Egger
All − Inverse variance weighted
−1
0
1
2
3
MR effect size for
'ARPC3' on 'Cardiac valvular disease'

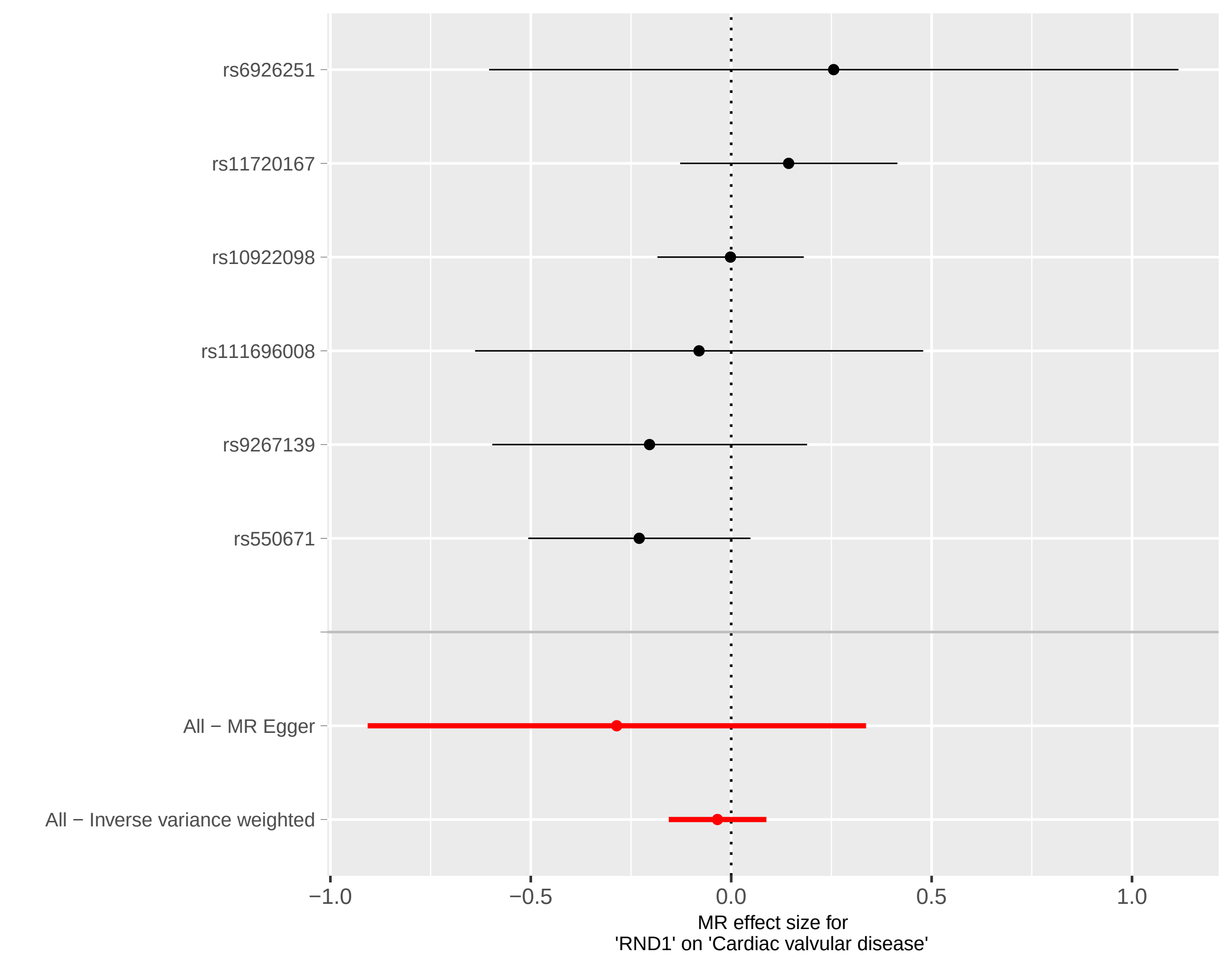
rs6926251
rs11720167
rs10922098
rs111696008
rs9267139
rs550671
All − MR Egger
All − Inverse variance weighted
−1.0
−0.5
0.0
0.5
1.0
MR effect size for
'RND1' on 'Cardiac valvular disease'

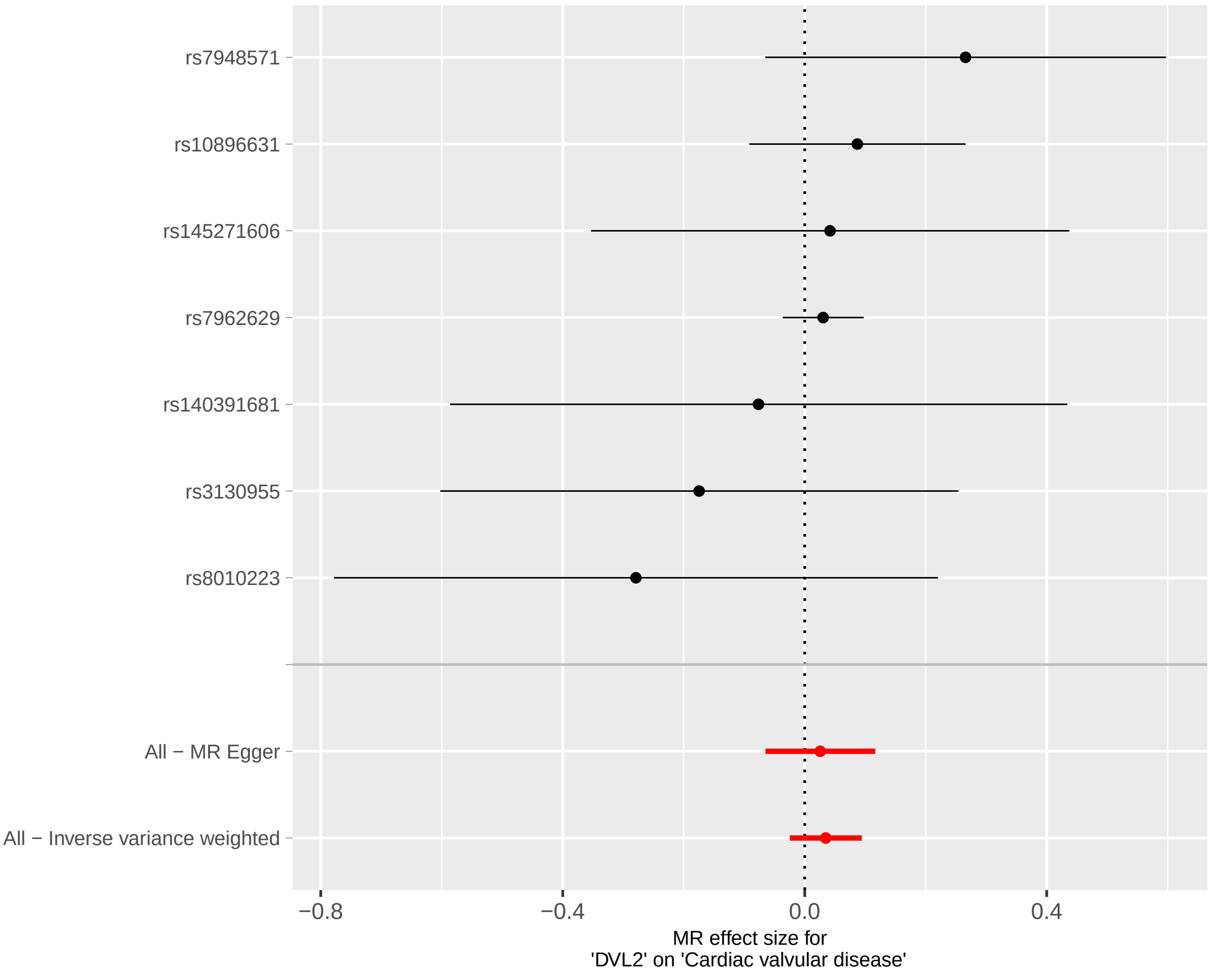
rs7948571
rs10896631
rs145271606
rs7962629
rs140391681
rs3130955
rs8010223
All − MR Egger
All − Inverse variance weighted
−0.8
−0.4
0.0
0.4
MR effect size for
'DVL2' on 'Cardiac valvular disease'

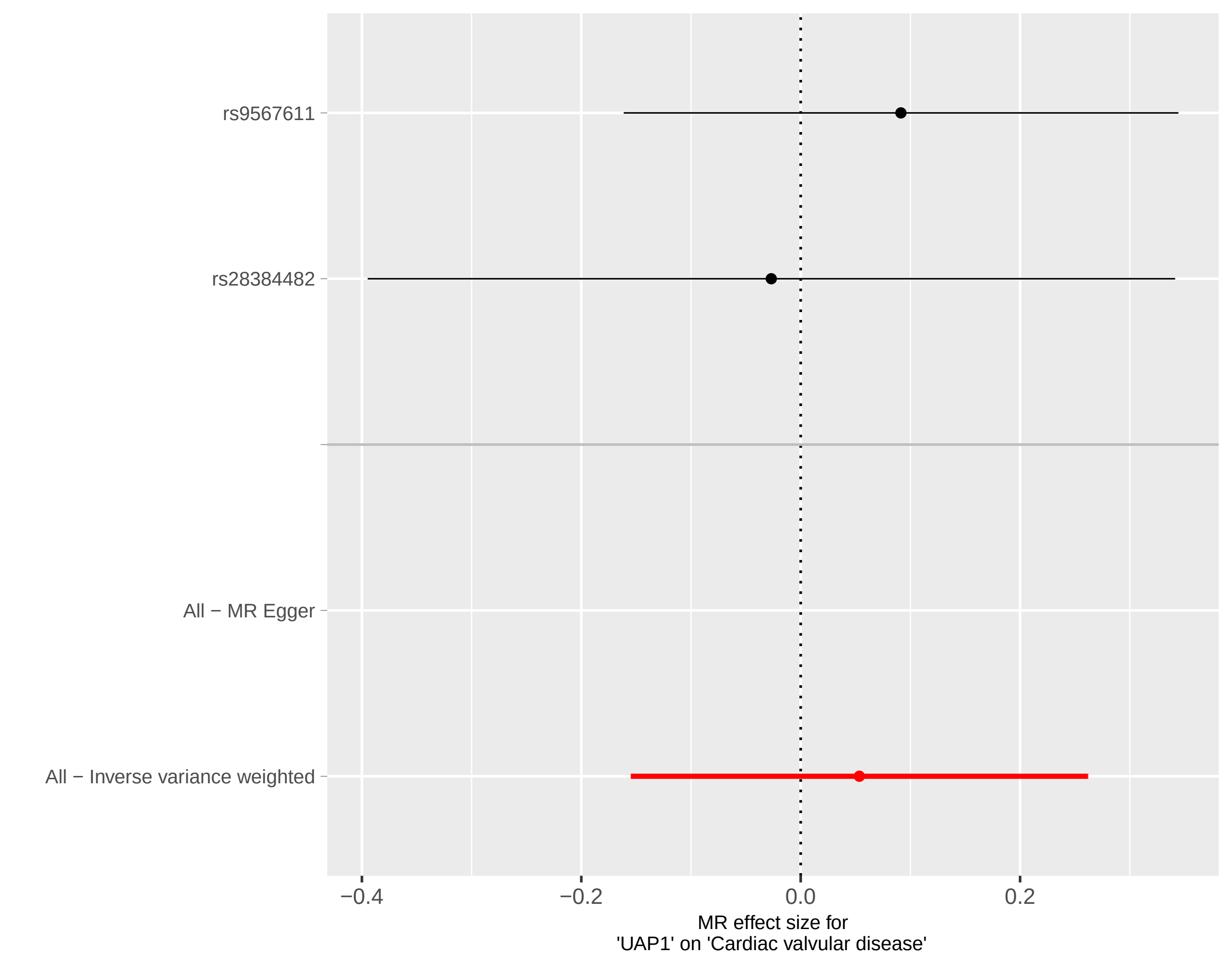
rs9567611
rs28384482
All − MR Egger
All − Inverse variance weighted
−0.4
−0.2
0.0
0.2
MR effect size for
'UAP1' on 'Cardiac valvular disease'

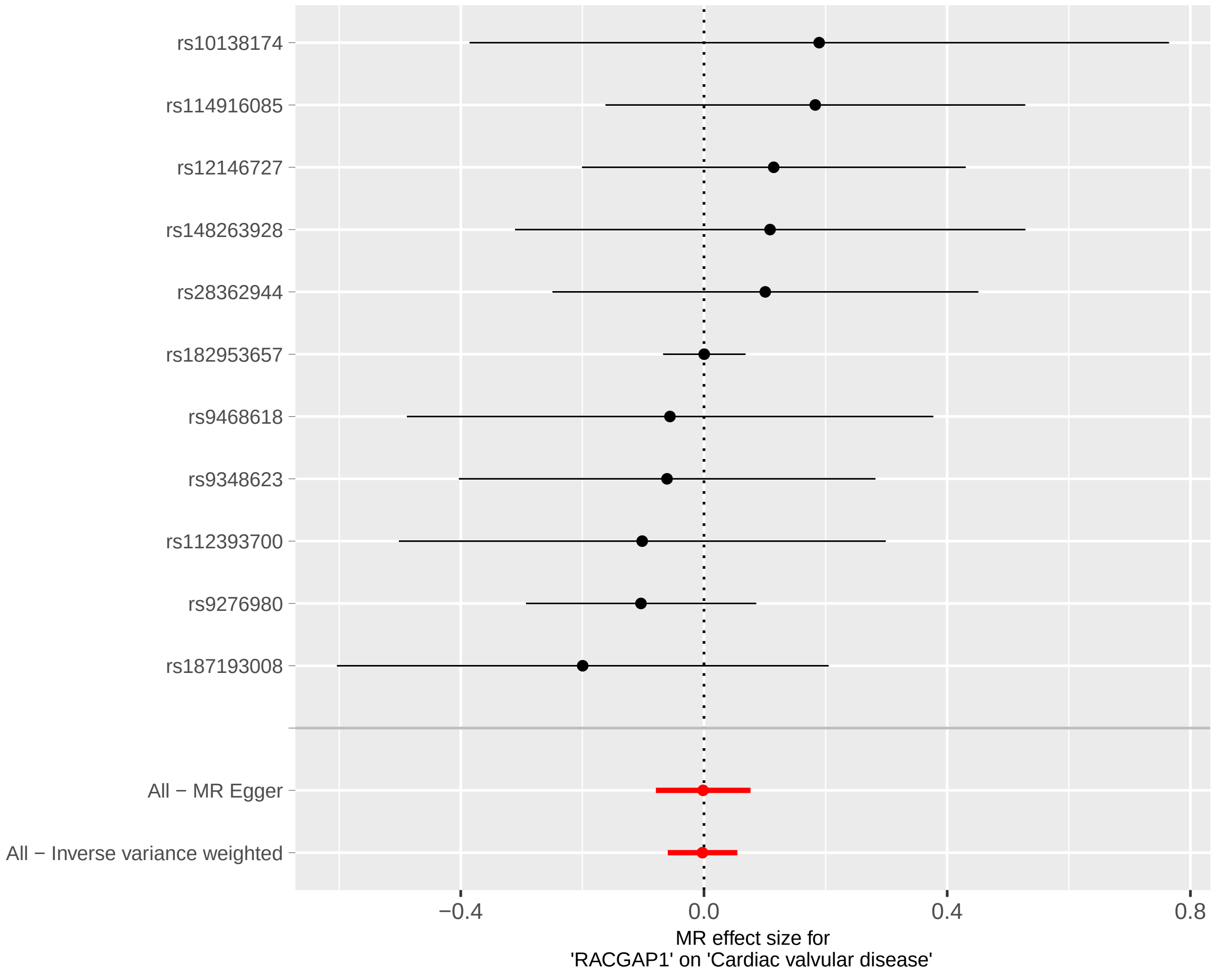
rs10138174
rs114916085
rs12146727
rs148263928
rs28362944
rs182953657
rs9468618
rs9348623
rs112393700
rs9276980
rs187193008
All − MR Egger
All − Inverse variance weighted
−0.4
0.0
0.4
0.8
MR effect size for
'RACGAP1' on 'Cardiac valvular disease'

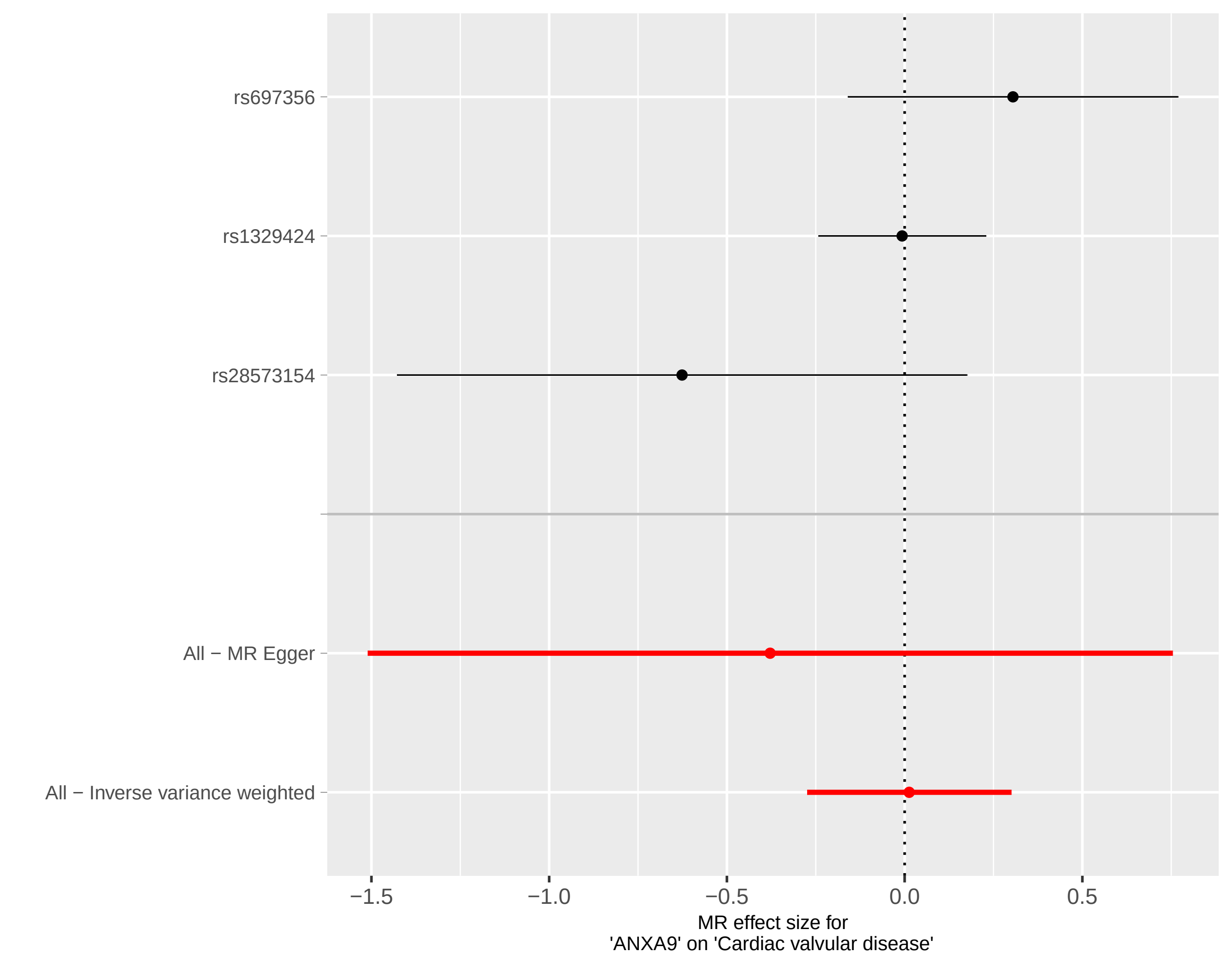
rs697356
rs1329424
rs28573154
All − MR Egger
All − Inverse variance weighted
−1.5
−1.0
−0.5
0.0
0.5
MR effect size for
'ANXA9' on 'Cardiac valvular disease'

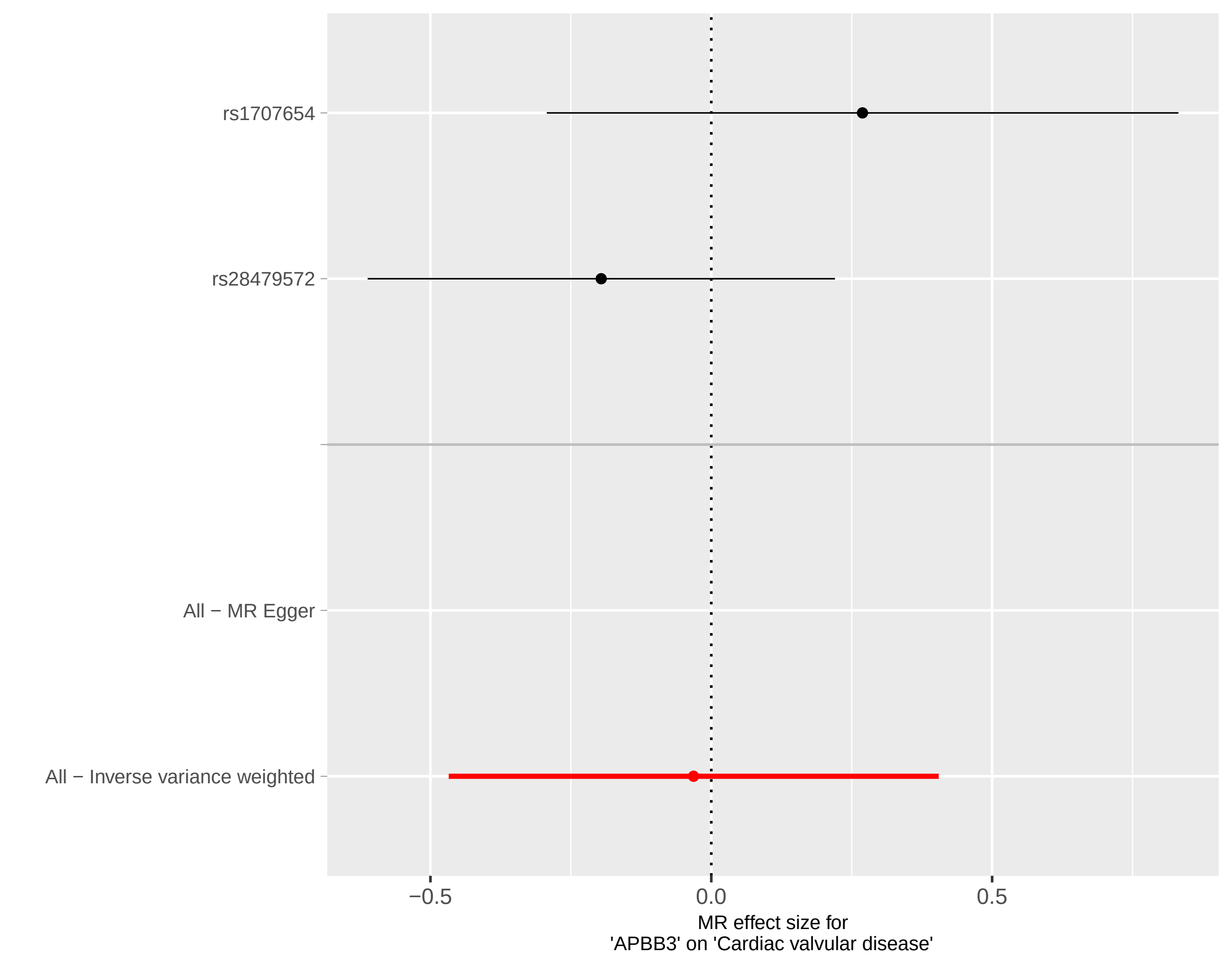
rs1707654
rs28479572
All − MR Egger
All − Inverse variance weighted
−0.5
0.0
0.5
MR effect size for
'APBB3' on 'Cardiac valvular disease'

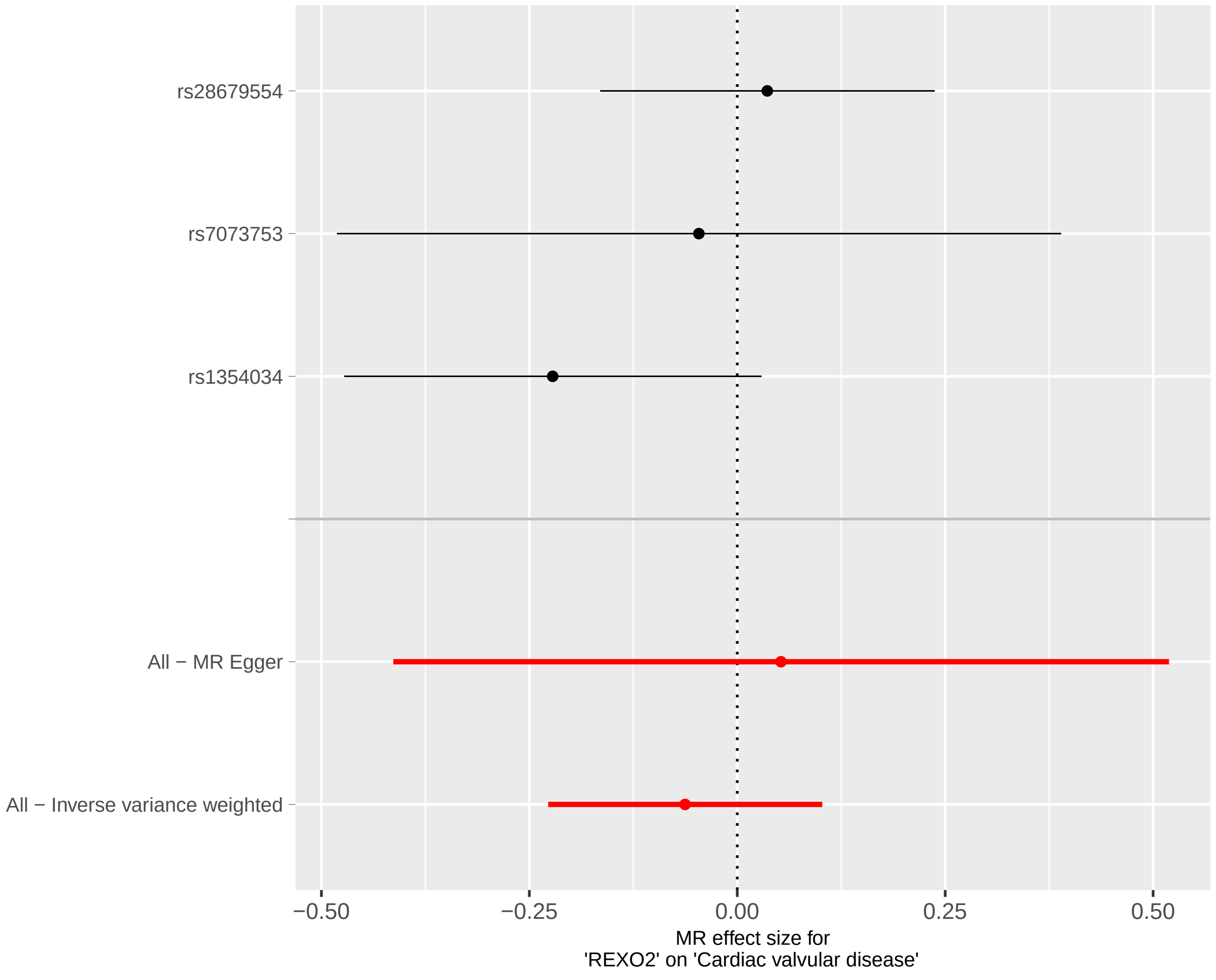
rs28679554
rs7073753
rs1354034
All − MR Egger
All − Inverse variance weighted
−0.50
−0.25
0.00
0.25
0.50
MR effect size for
'REXO2' on 'Cardiac valvular disease'

Insufficient number of SNPs

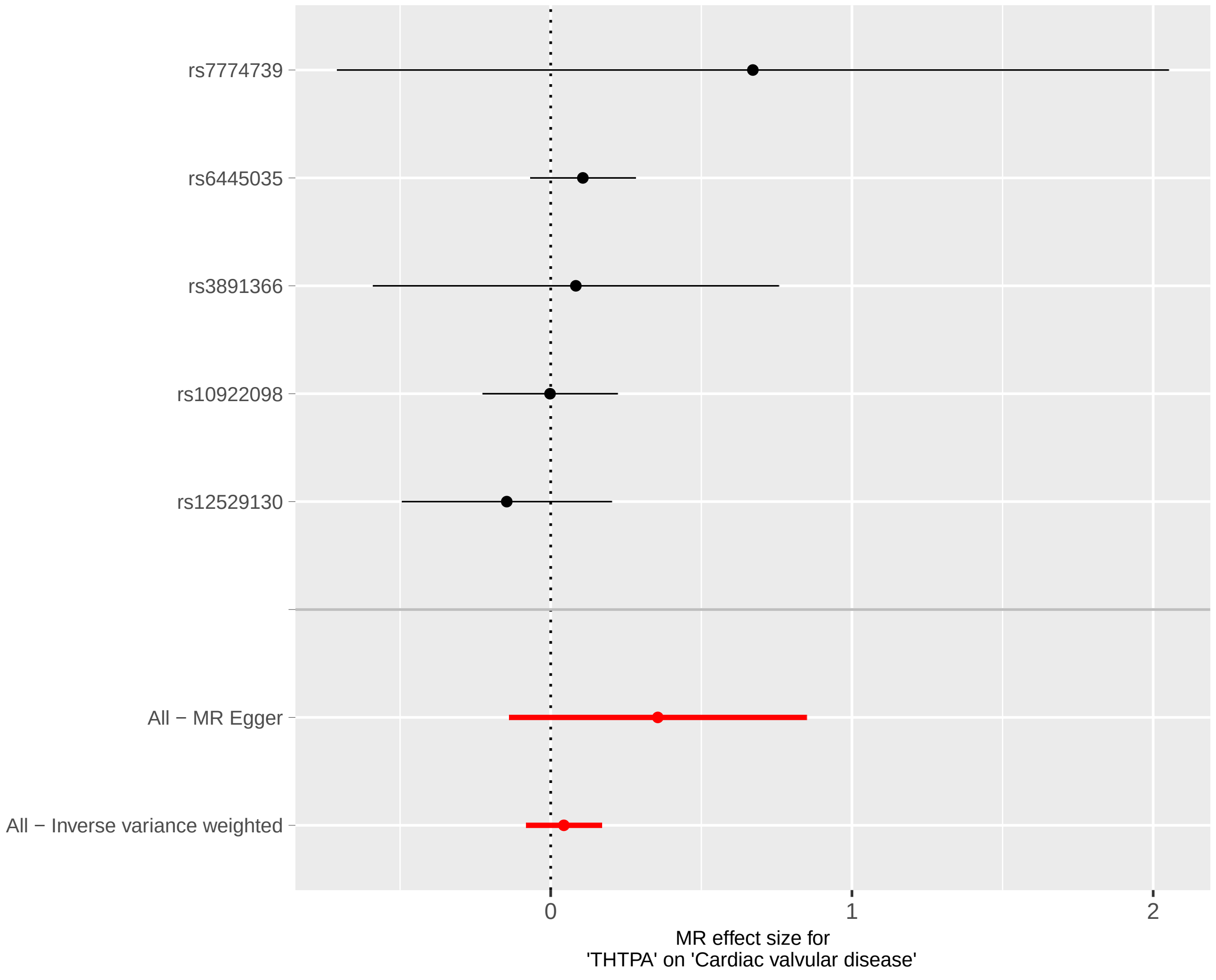
rs7774739
rs6445035
rs3891366
rs10922098
rs12529130
All − MR Egger
All − Inverse variance weighted
0
1
2
MR effect size for
'THTPA' on 'Cardiac valvular disease'

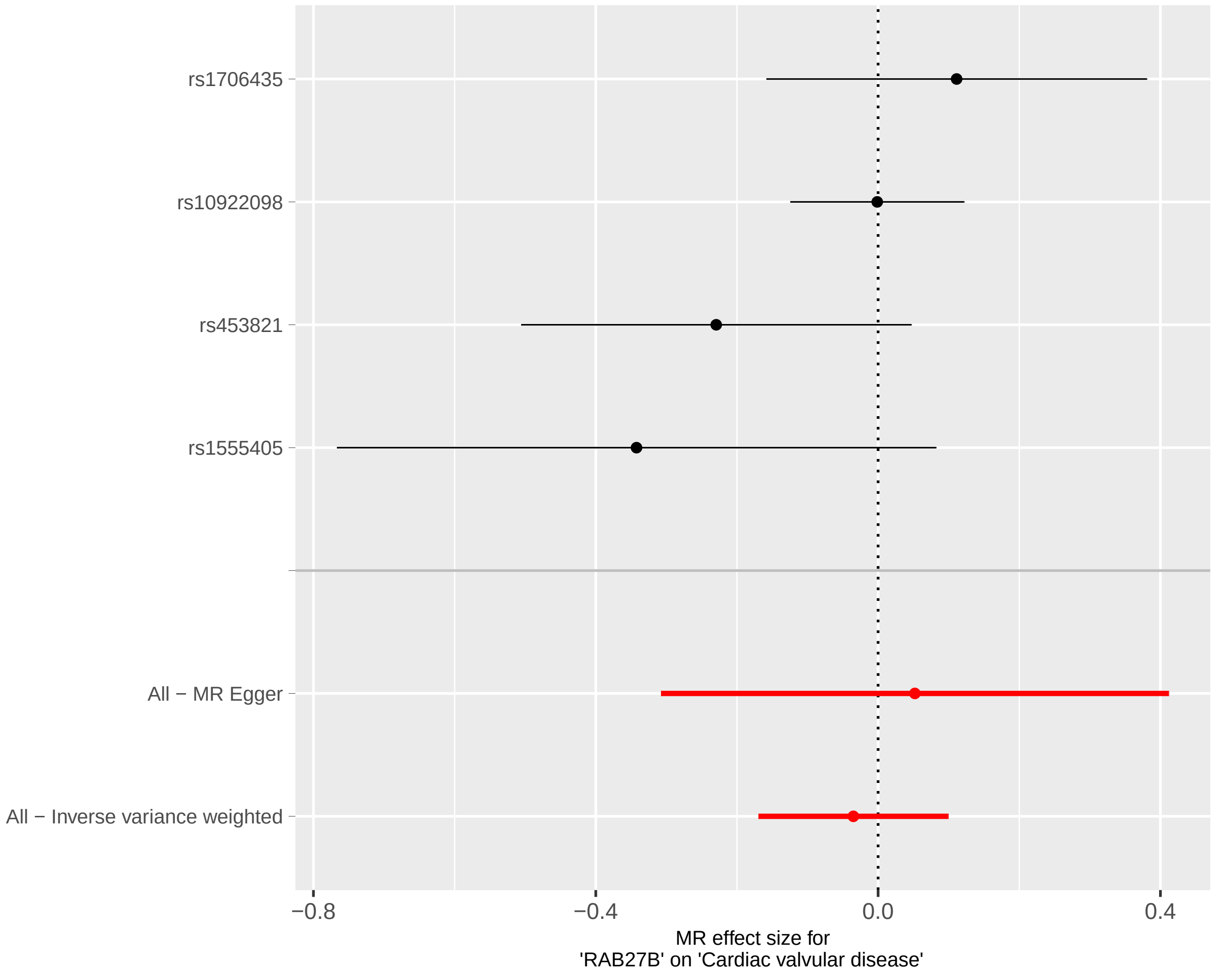
rs1706435
rs10922098
rs453821
rs1555405
All − MR Egger
All − Inverse variance weighted
−0.8
−0.4
0.0
0.4
MR effect size for
'RAB27B' on 'Cardiac valvular disease'

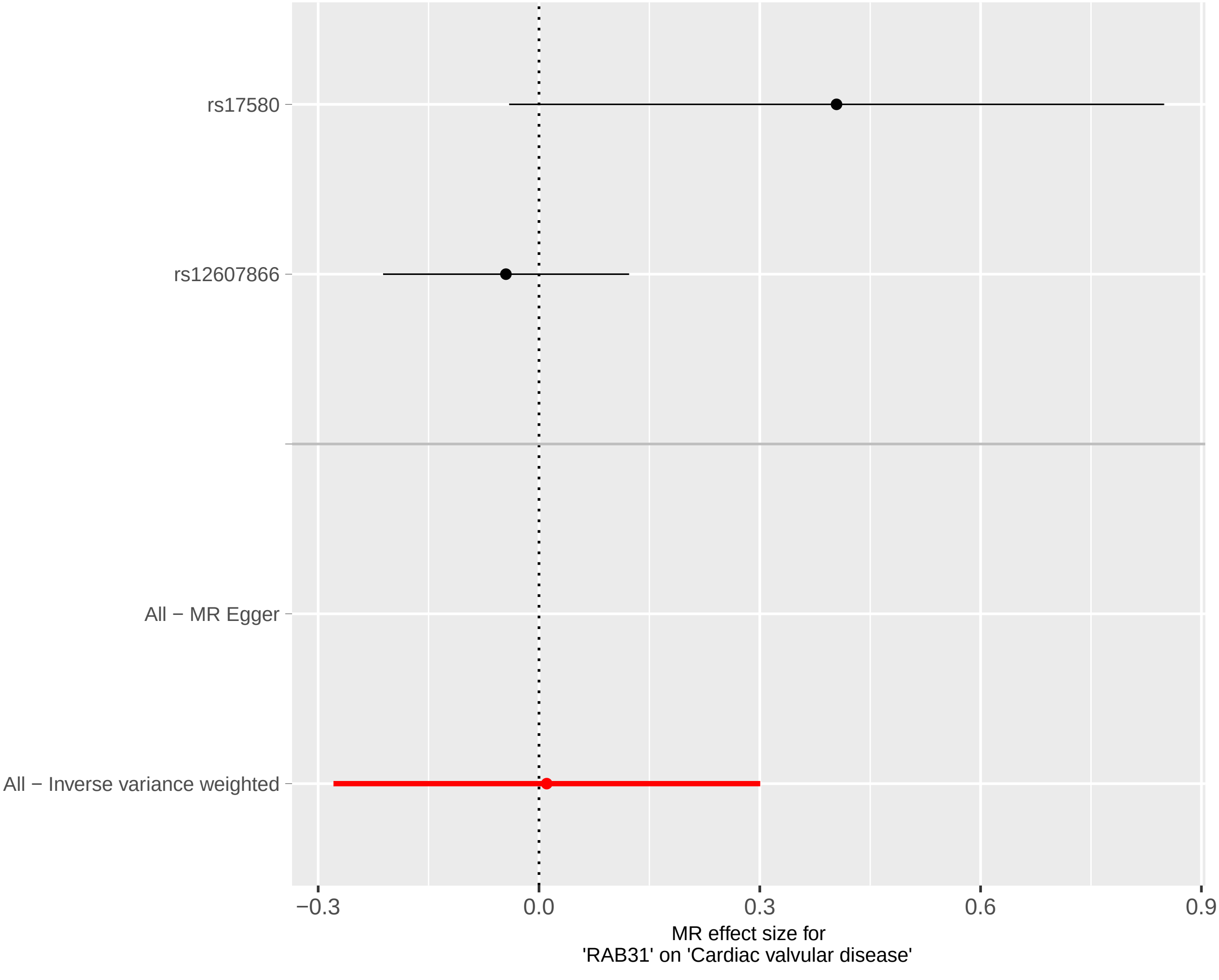
rs17580
rs12607866
All − MR Egger
All − Inverse variance weighted
−0.3
0.0
0.3
0.6
0.9
MR effect size for
'RAB31' on 'Cardiac valvular disease'

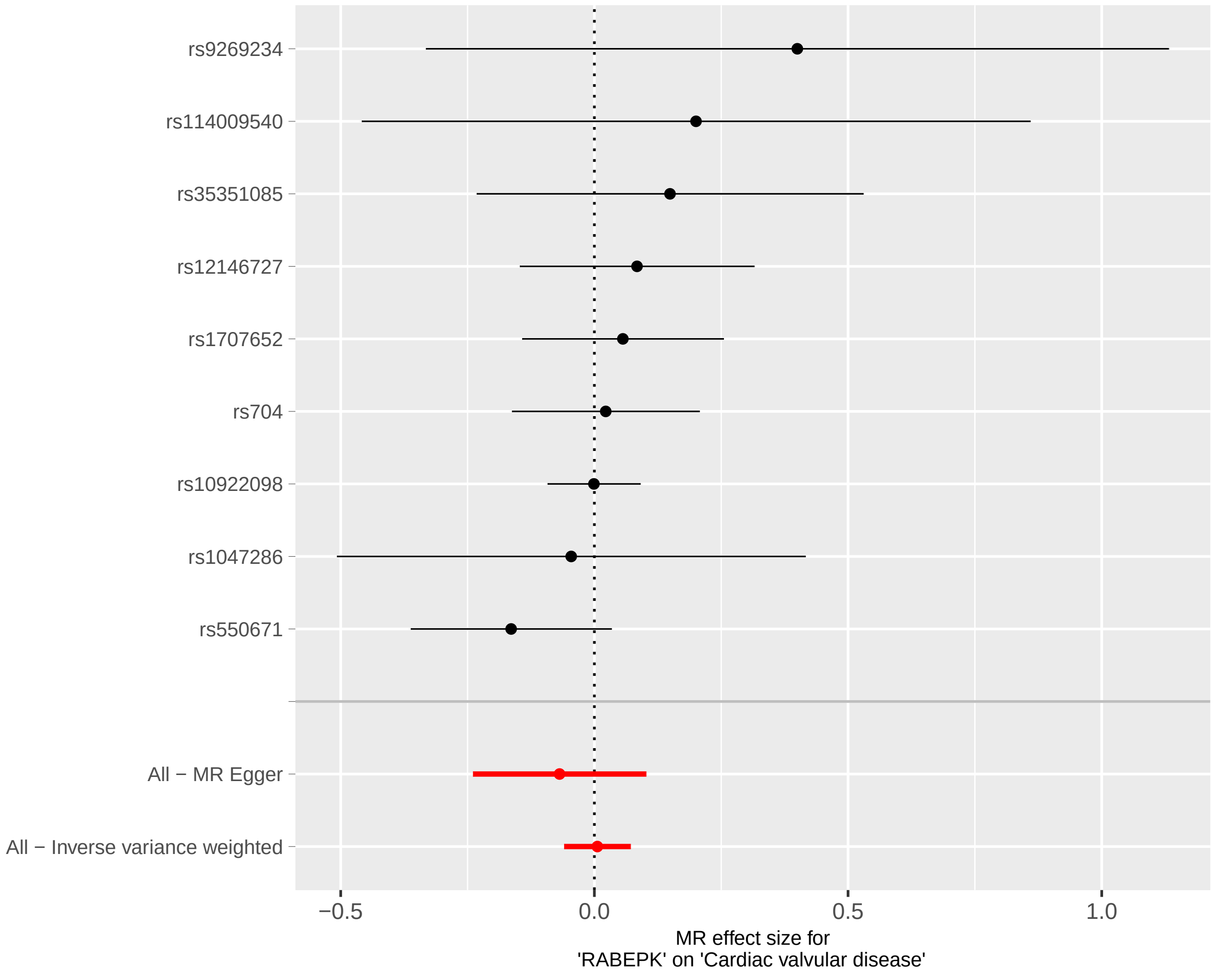
rs9269234
rs114009540
rs35351085
rs12146727
rs1707652
rs704
rs10922098
rs1047286
rs550671
All − MR Egger
All − Inverse variance weighted
−0.5
0.0
0.5
1.0
MR effect size for
'RABEPK' on 'Cardiac valvular disease'

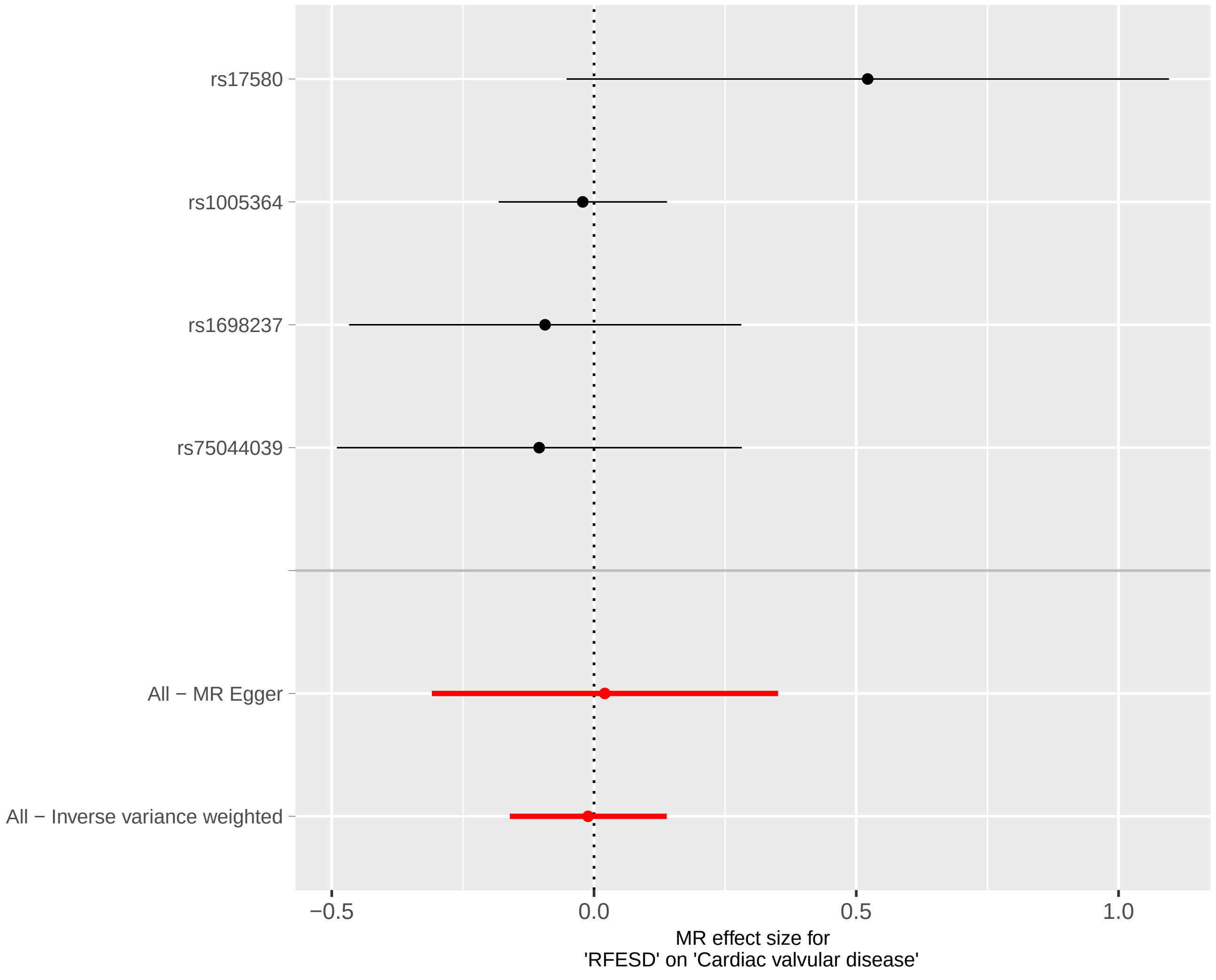
rs17580
rs1005364
rs1698237
rs75044039
All − MR Egger
All − Inverse variance weighted
−0.5
0.0
0.5
1.0
MR effect size for
'RFESD' on 'Cardiac valvular disease'

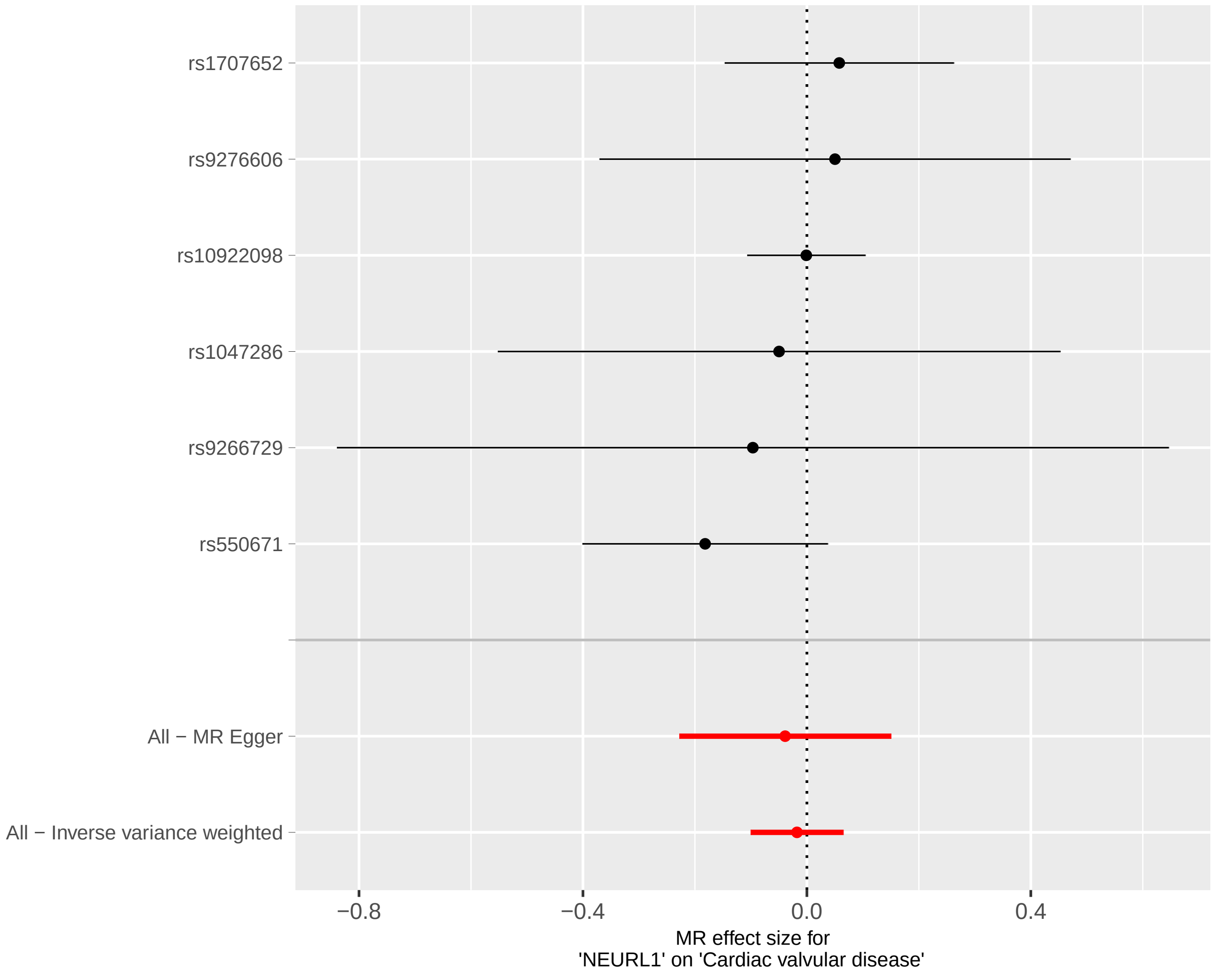
rs1707652
rs9276606
rs10922098
rs1047286
rs9266729
rs550671
All − MR Egger
All − Inverse variance weighted
−0.8
−0.4
0.0
0.4
MR effect size for
'NEURL1' on 'Cardiac valvular disease'

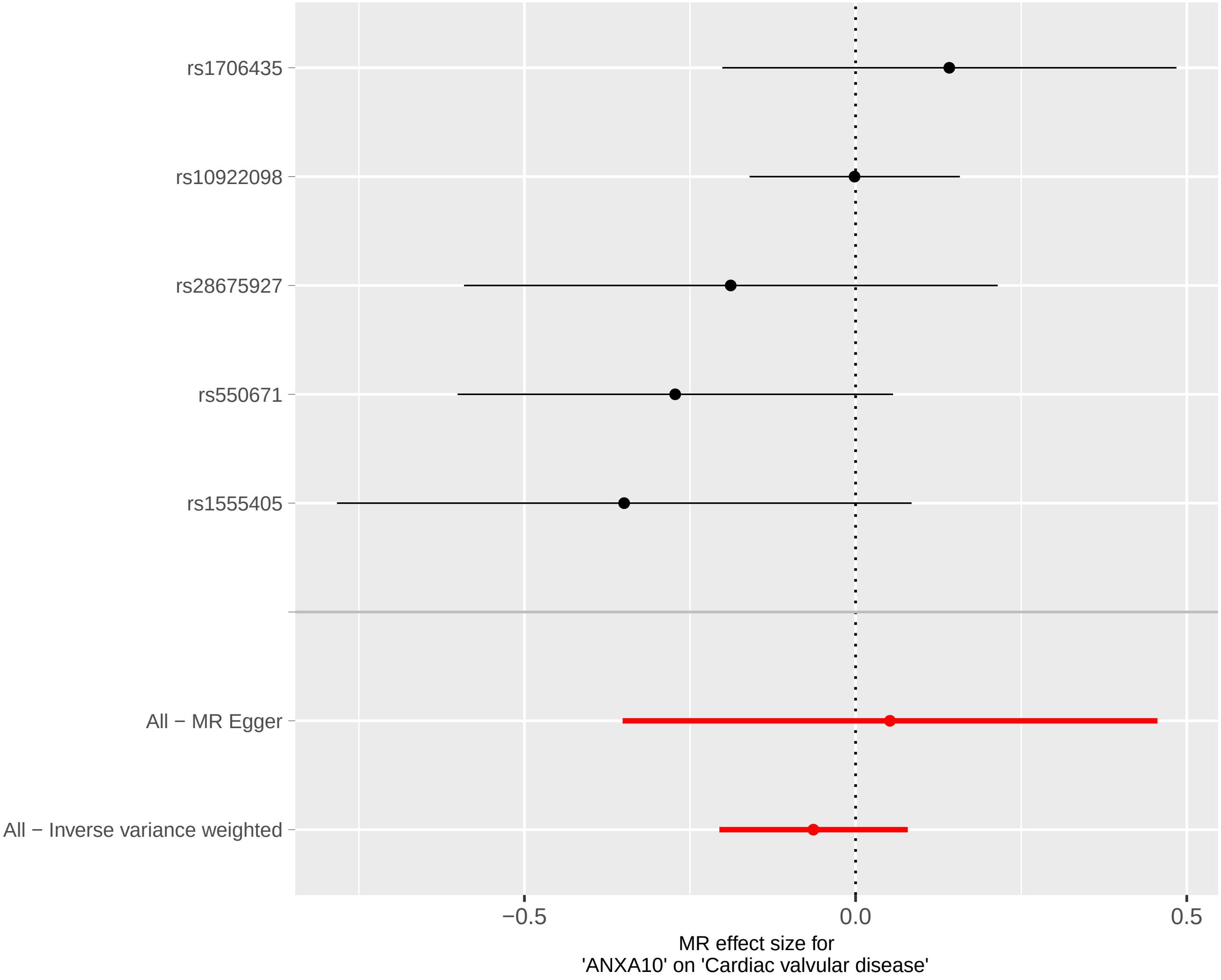

rs1706435
rs10922098
rs28675927
rs550671
rs1555405
All − MR Egger
All − Inverse variance weighted
−0.5
0.0
0.5
MR effect size for
'ANXA10' on 'Cardiac valvular disease'

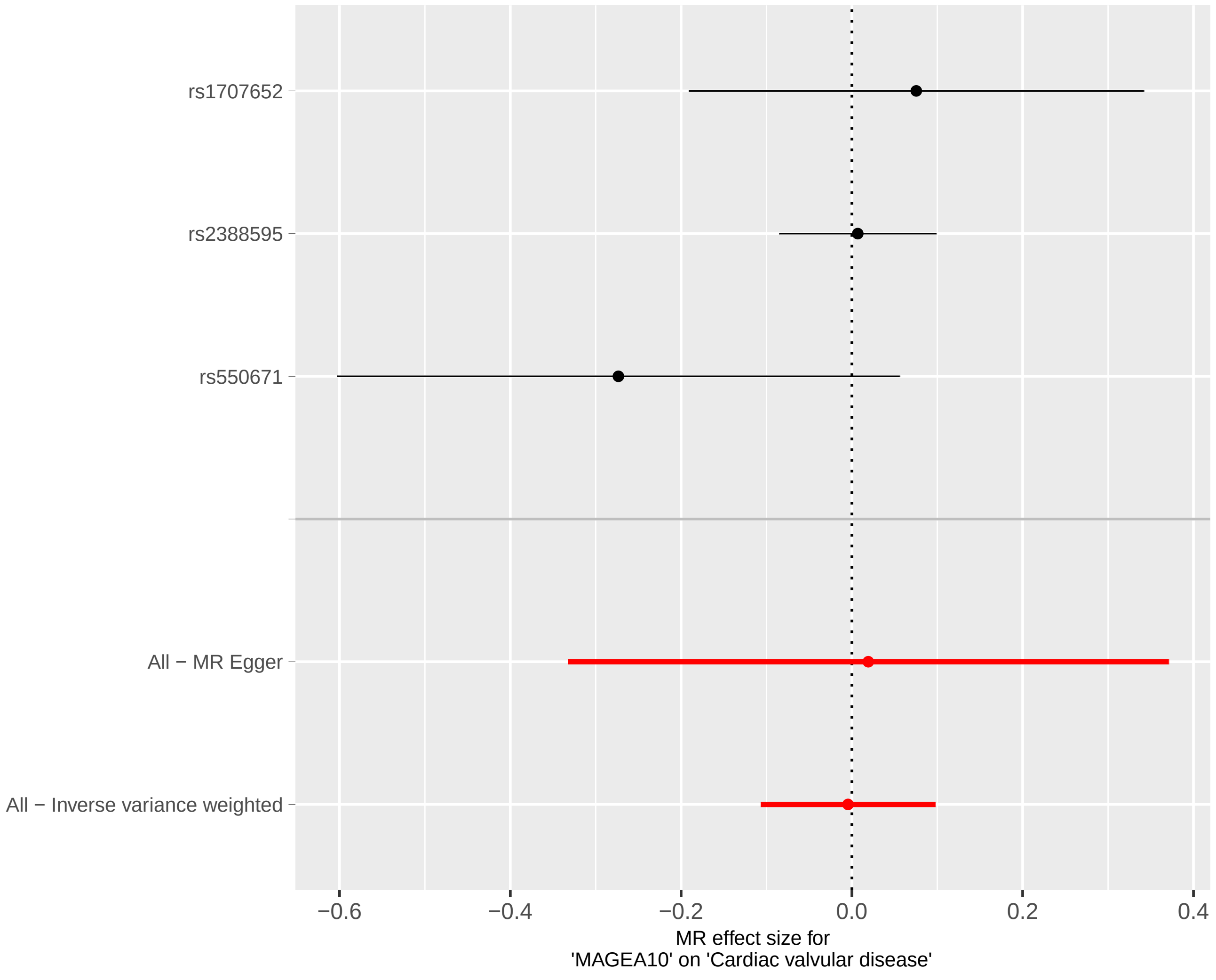
rs1707652
rs2388595
rs550671
All − MR Egger
All − Inverse variance weighted
−0.6
−0.4
−0.2
0.0
0.2
0.4
MR effect size for
'MAGEA10' on 'Cardiac valvular disease'

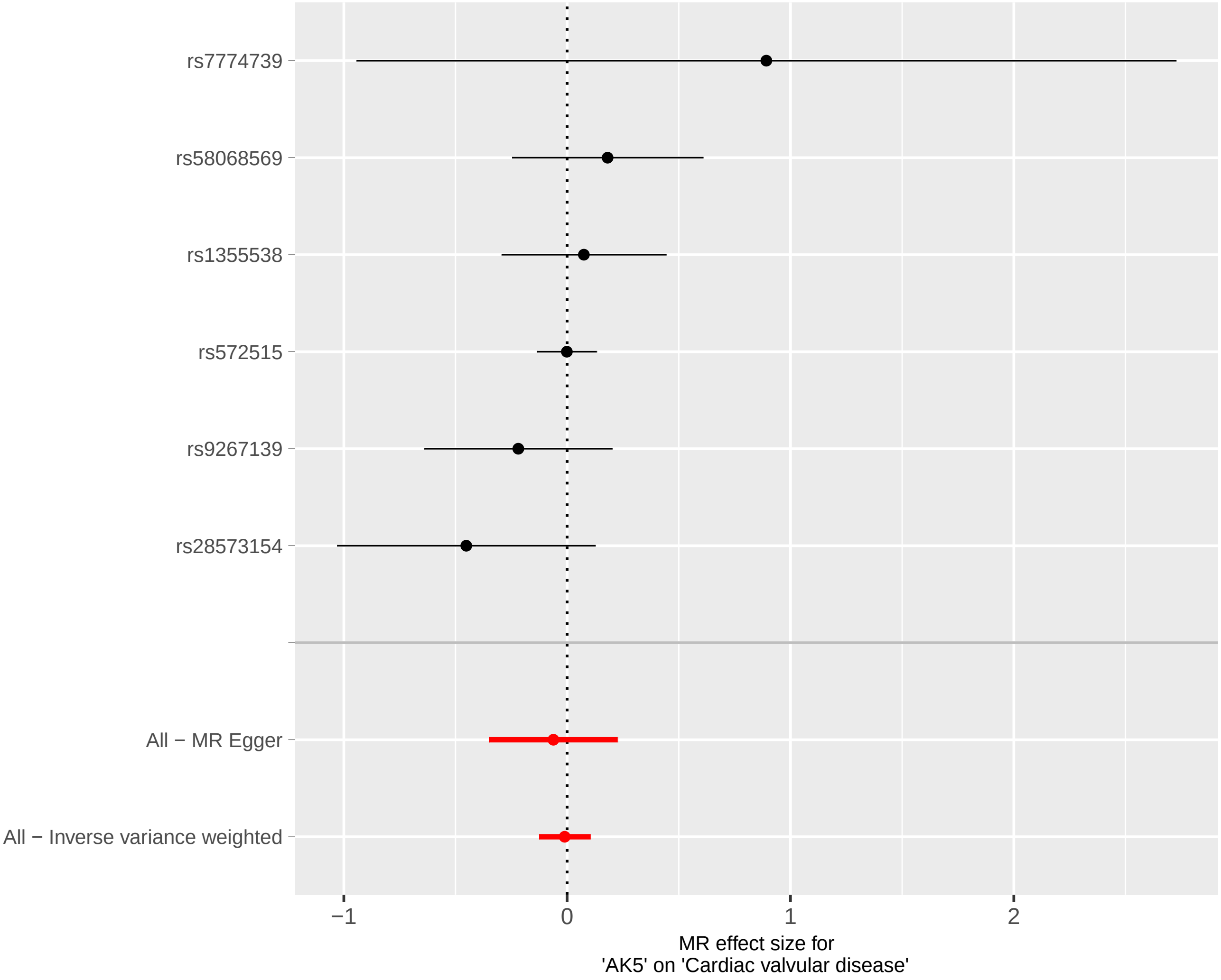

rs7774739
rs58068569
rs1355538
rs572515
rs9267139
rs28573154
All − MR Egger
All − Inverse variance weighted
−1
0
1
2
MR effect size for
'AK5' on 'Cardiac valvular disease'

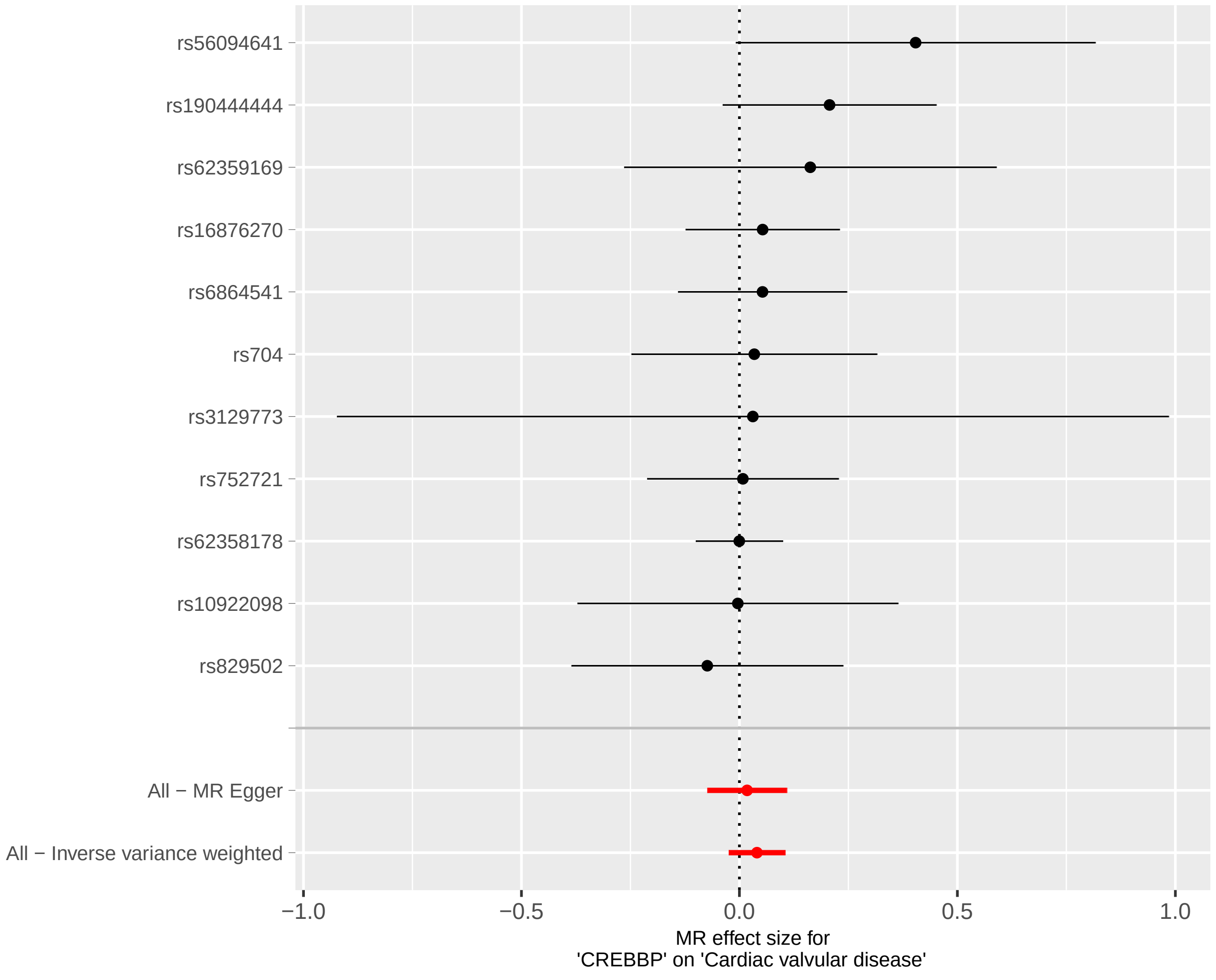
rs56094641
rs190444444
rs62359169
rs16876270
rs6864541
rs704
rs3129773
rs752721
rs62358178
rs10922098
rs829502
All − MR Egger
All − Inverse variance weighted
−1.0
−0.5
0.0
0.5
1.0
MR effect size for
'CREBBP' on 'Cardiac valvular disease'

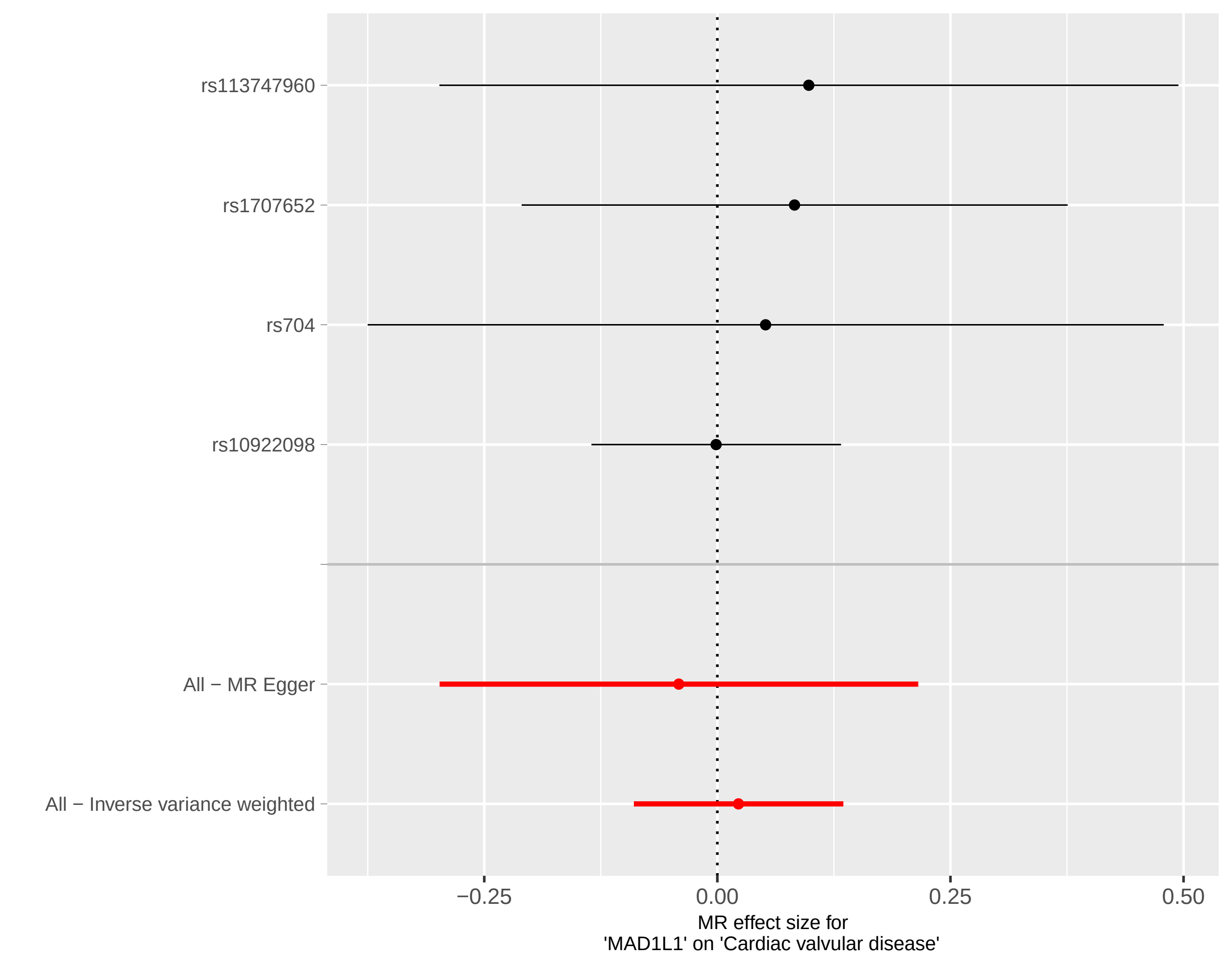
rs113747960
rs1707652
rs704
rs10922098
All − MR Egger
All − Inverse variance weighted
−0.25
0.00
0.25
0.50
MR effect size for
'MAD1L1' on 'Cardiac valvular disease'

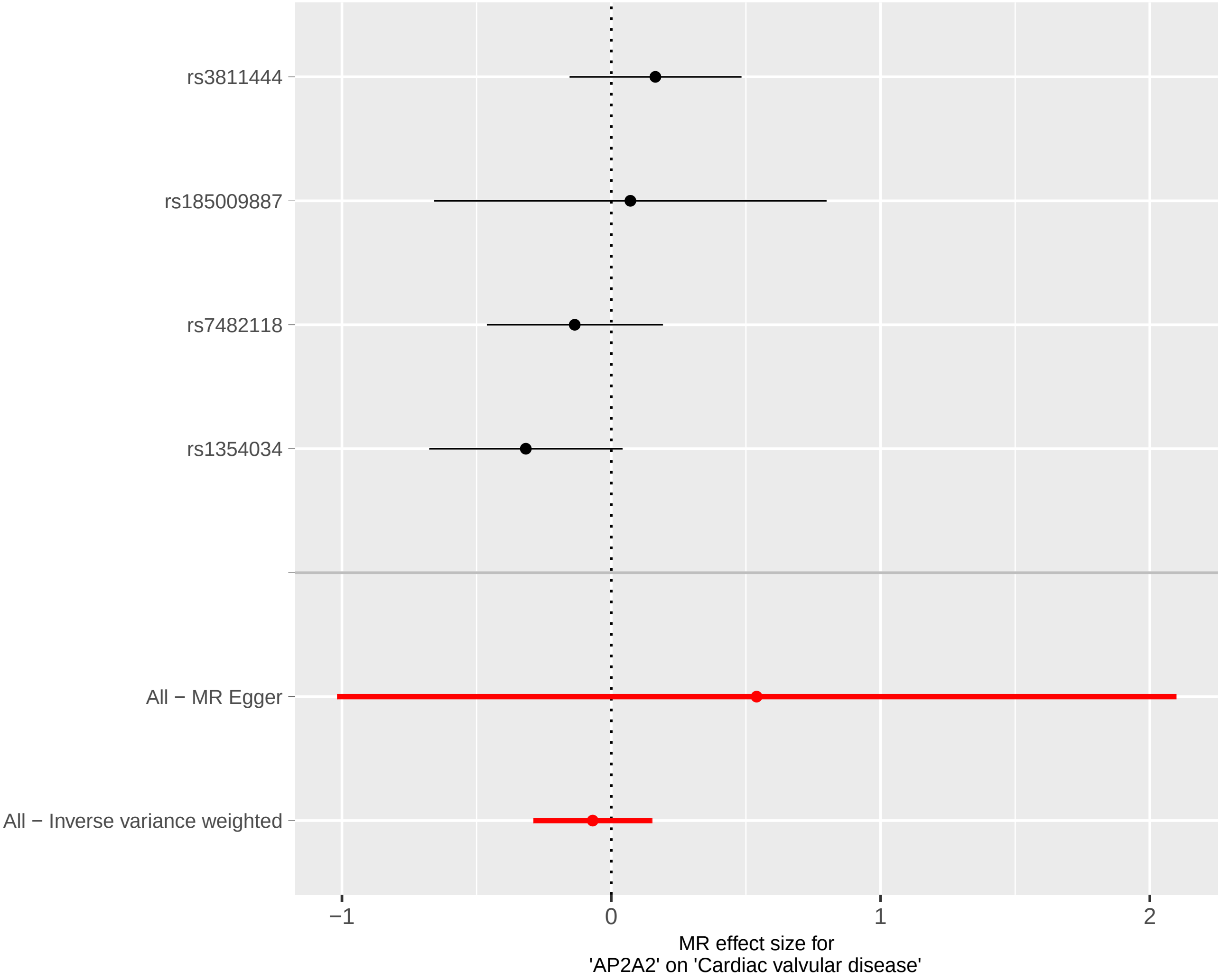
rs3811444
rs185009887
rs7482118
rs1354034
All − MR Egger
All − Inverse variance weighted
−1
0
1
2
MR effect size for
'AP2A2' on 'Cardiac valvular disease'

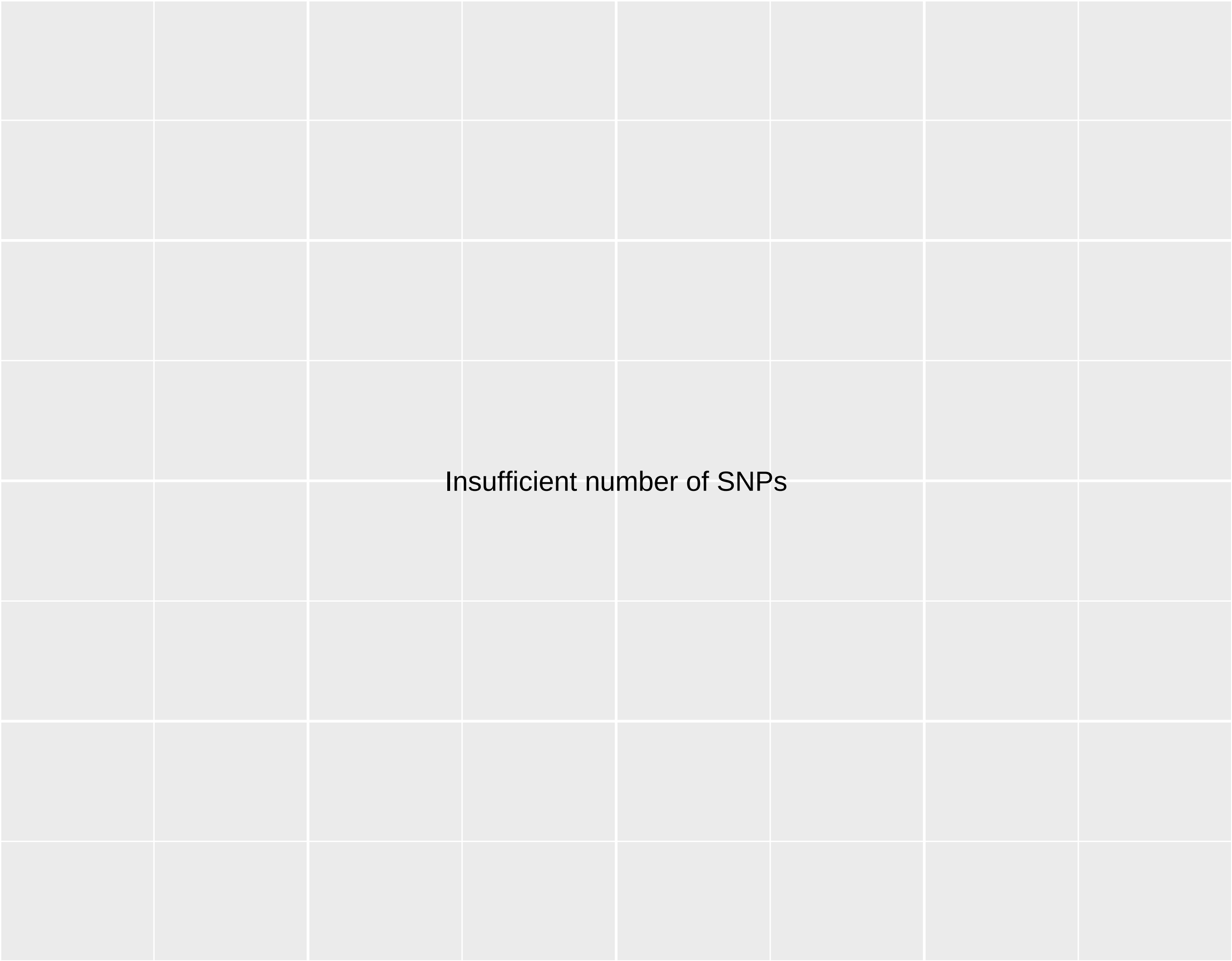
Insufficient number of SNPs

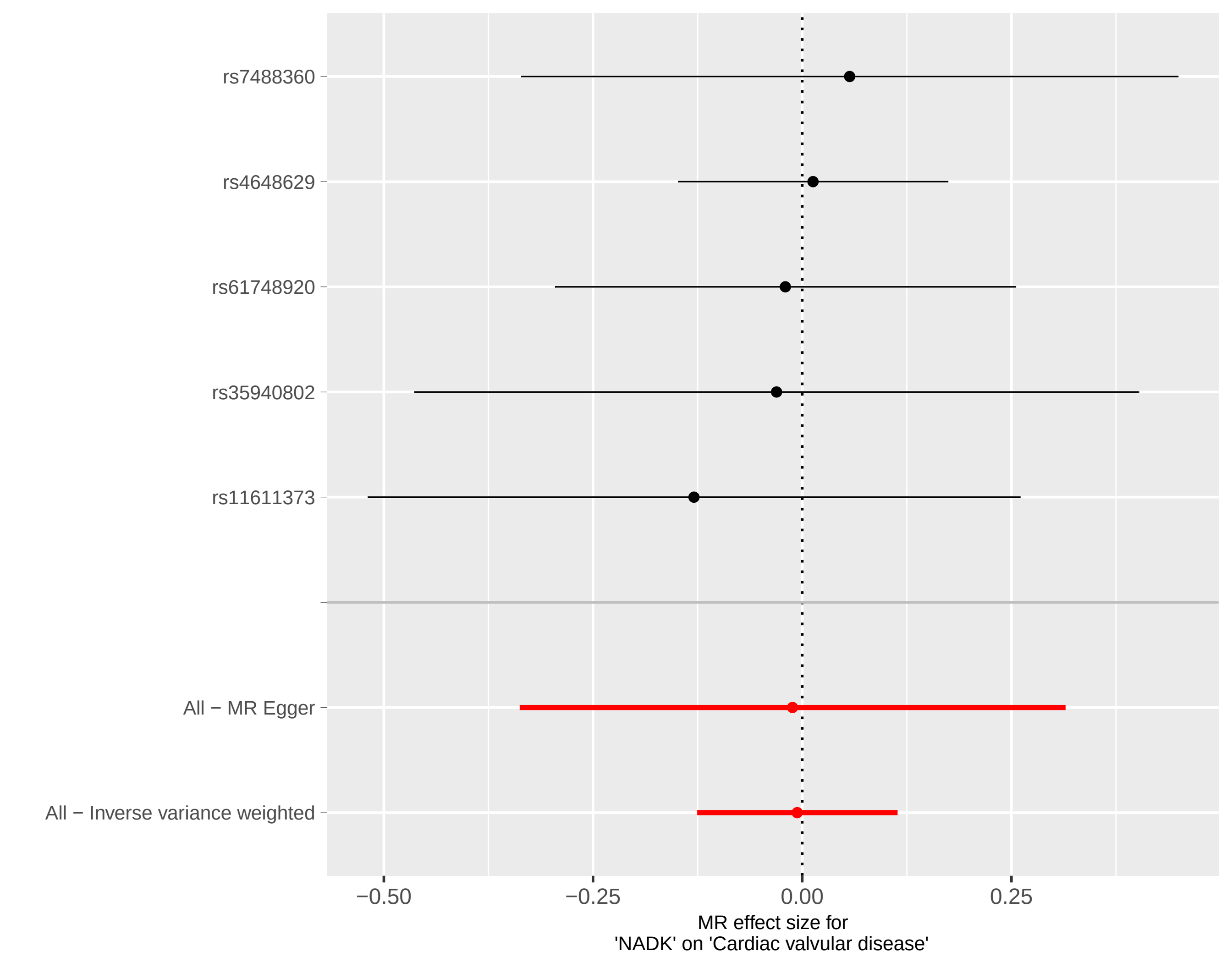

rs7488360
rs4648629
rs61748920
rs35940802
rs11611373
All − MR Egger
All − Inverse variance weighted
−0.50
−0.25
0.00
0.25
MR effect size for
'NADK' on 'Cardiac valvular disease'

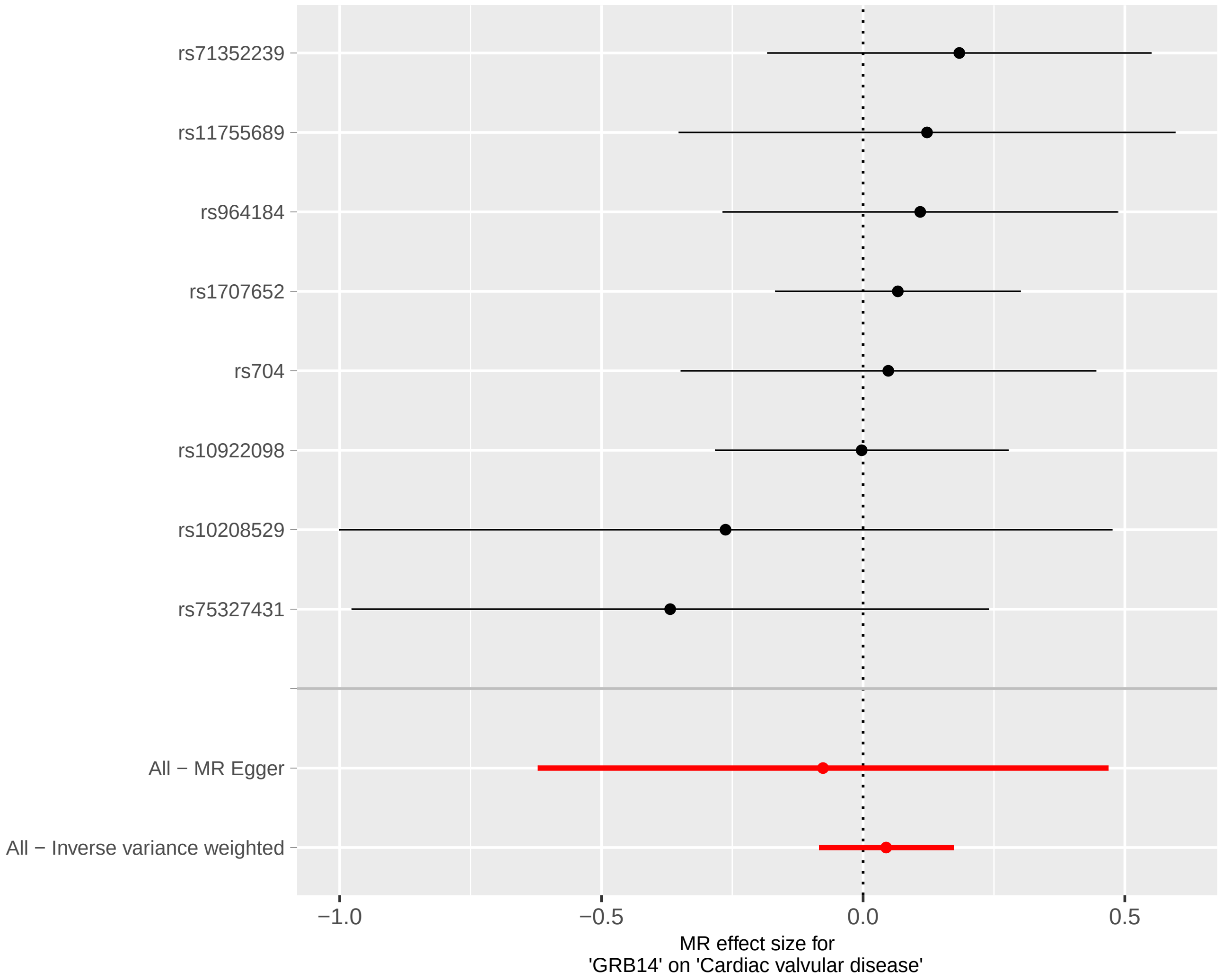

rs71352239
rs11755689
rs964184
rs1707652
rs704
rs10922098
rs10208529
rs75327431
All − MR Egger
All − Inverse variance weighted
−1.0
−0.5
0.0
0.5
MR effect size for
'GRB14' on 'Cardiac valvular disease'

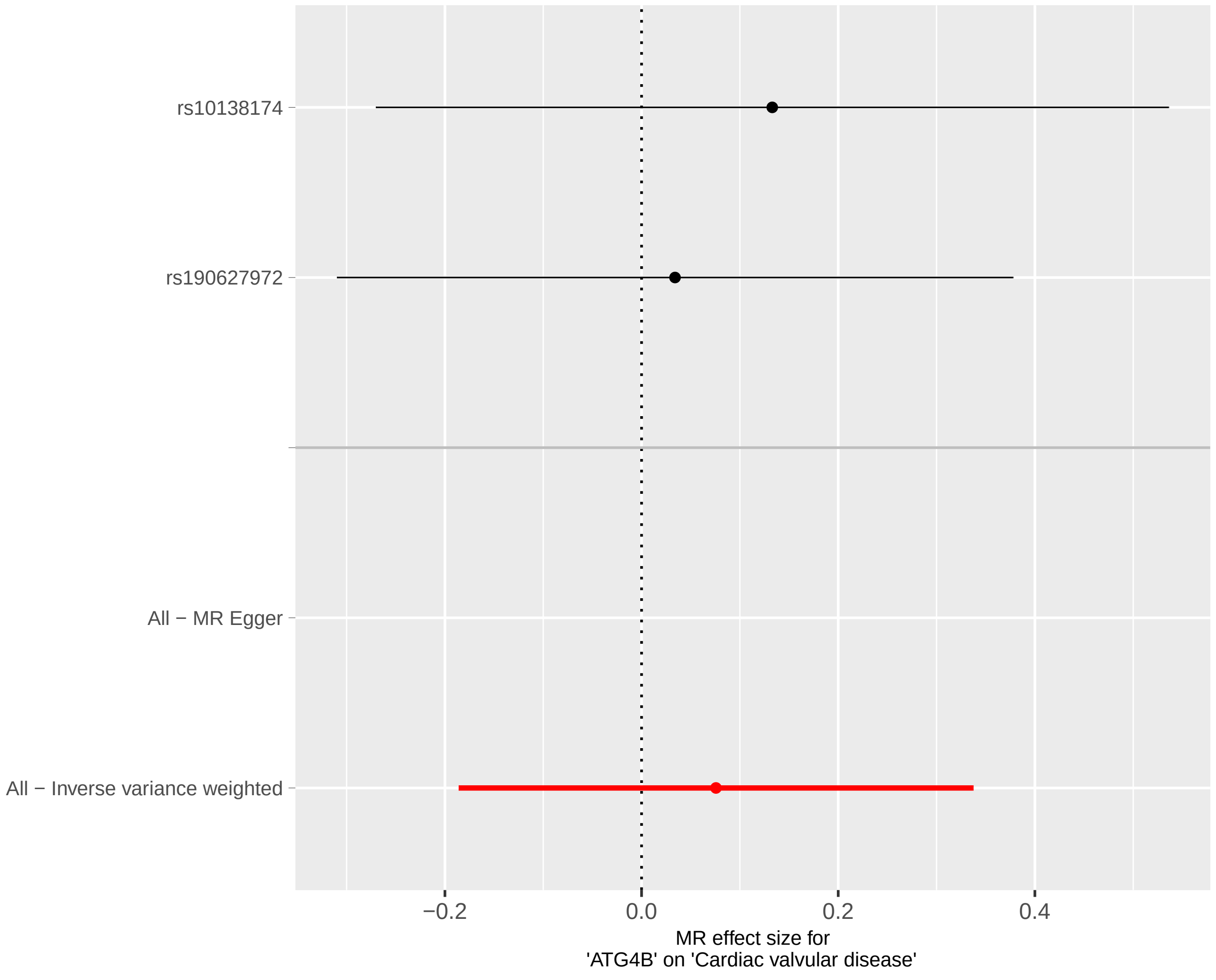
rs10138174
rs190627972
All − MR Egger
All − Inverse variance weighted
−0.2
0.0
0.2
0.4
MR effect size for
'ATG4B' on 'Cardiac valvular disease'

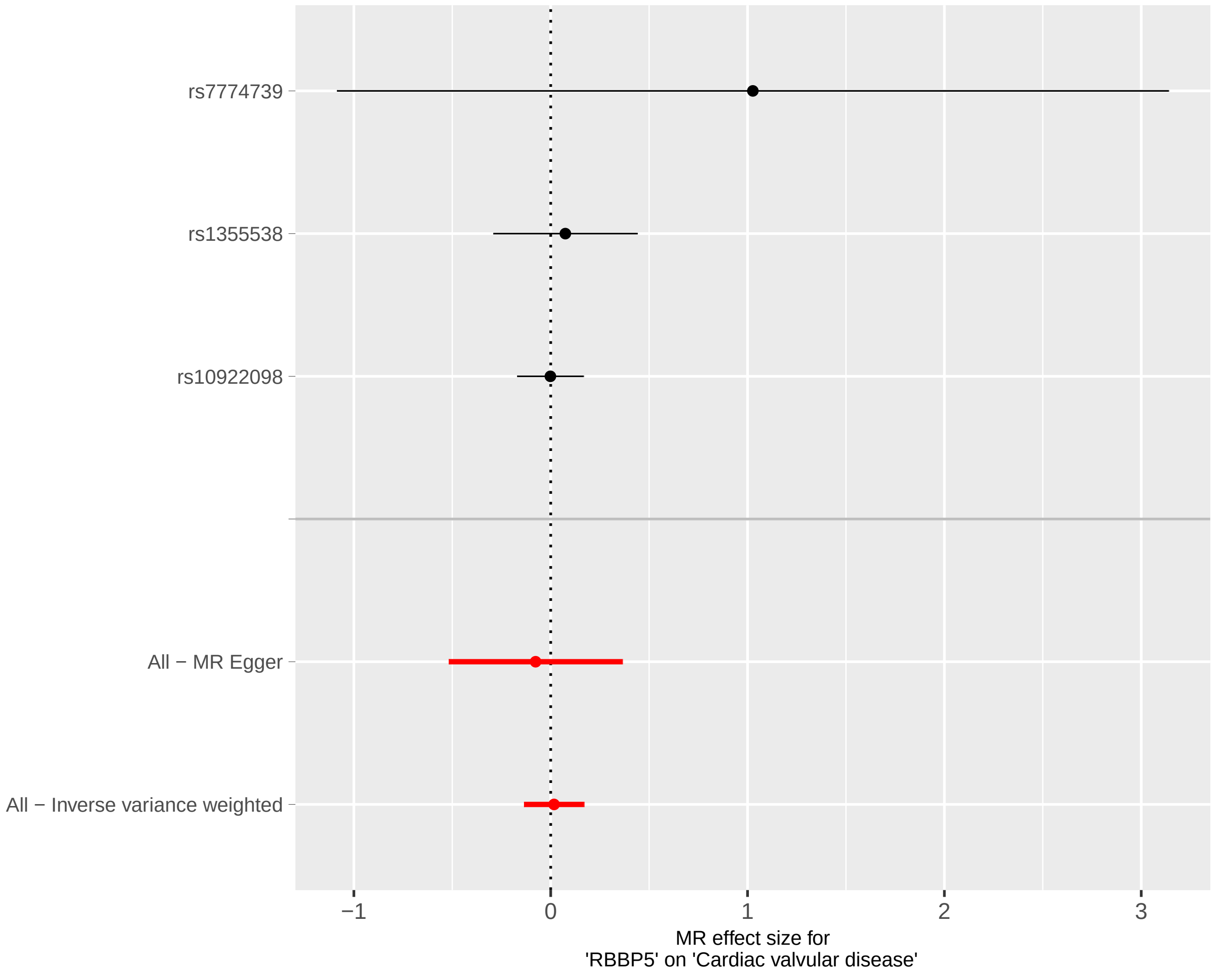
rs7774739
rs1355538
rs10922098
All − MR Egger
All − Inverse variance weighted
−1
0
1
2
3
MR effect size for
'RBBP5' on 'Cardiac valvular disease'

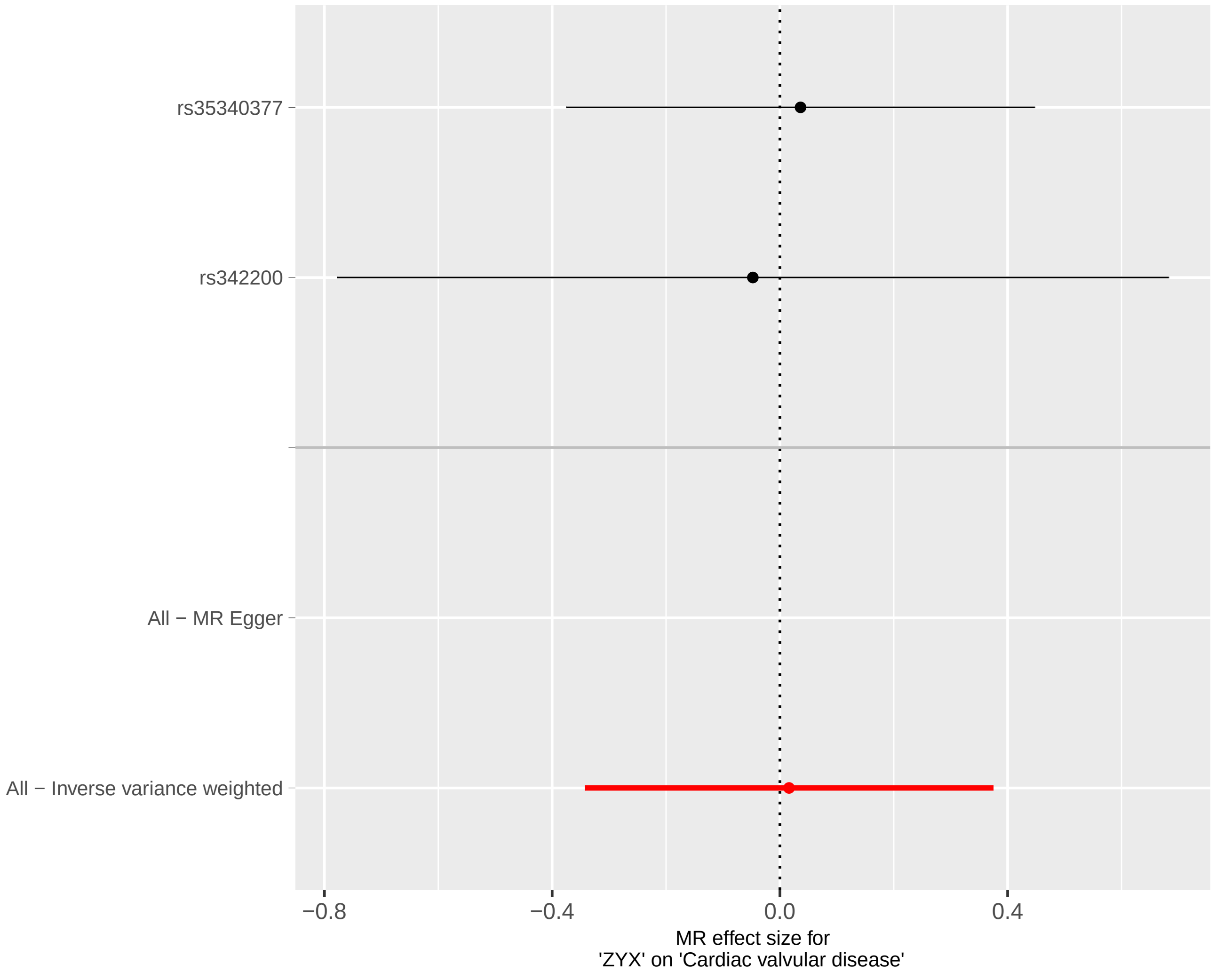
rs35340377
rs342200
All − MR Egger
All − Inverse variance weighted
−0.8
−0.4
0.0
0.4
MR effect size for
'ZYX' on 'Cardiac valvular disease'

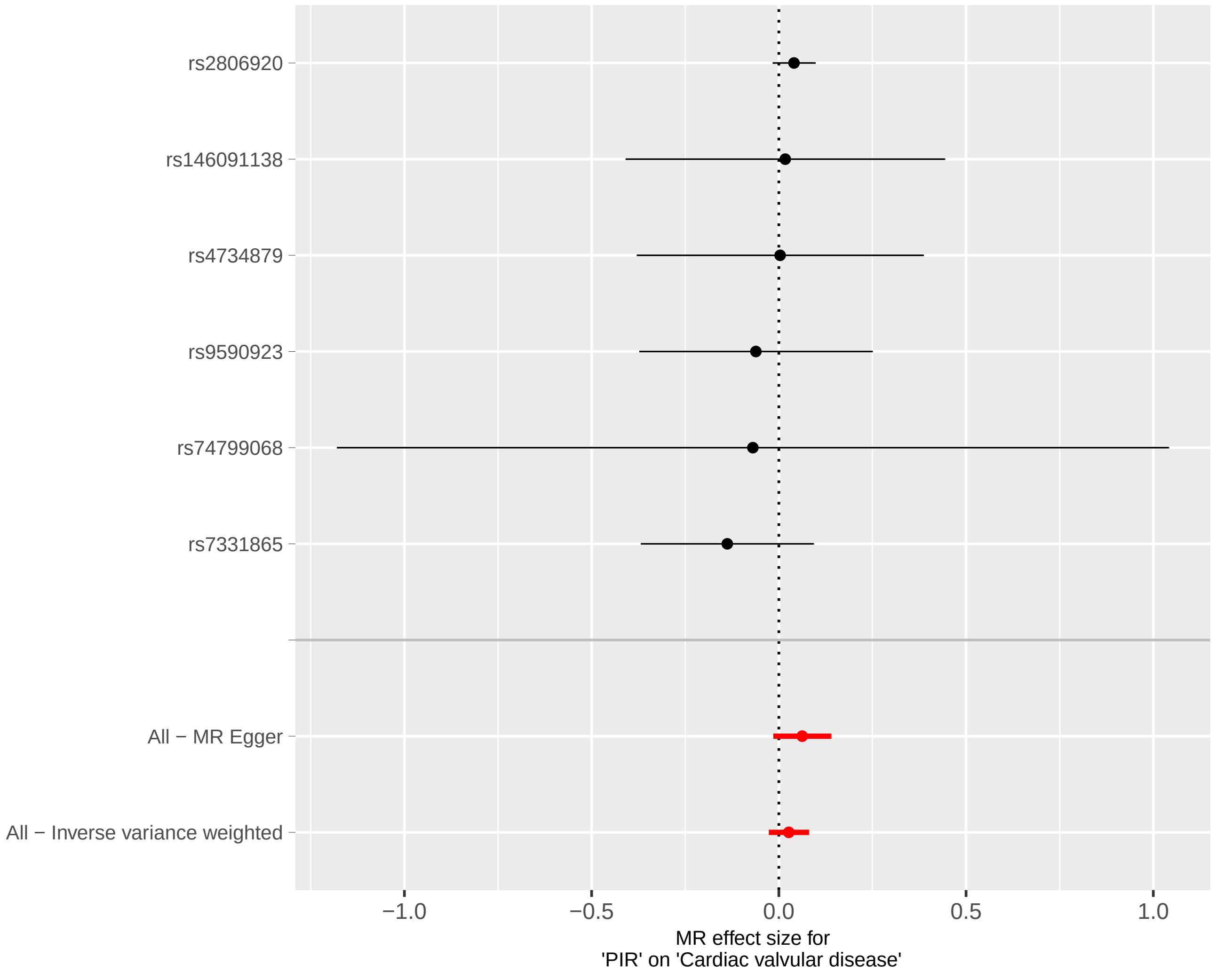
rs2806920
rs146091138
rs4734879
rs9590923
rs74799068
rs7331865
All − MR Egger
All − Inverse variance weighted
−1.0
−0.5
0.0
0.5
1.0
MR effect size for
'PIR' on 'Cardiac valvular disease'

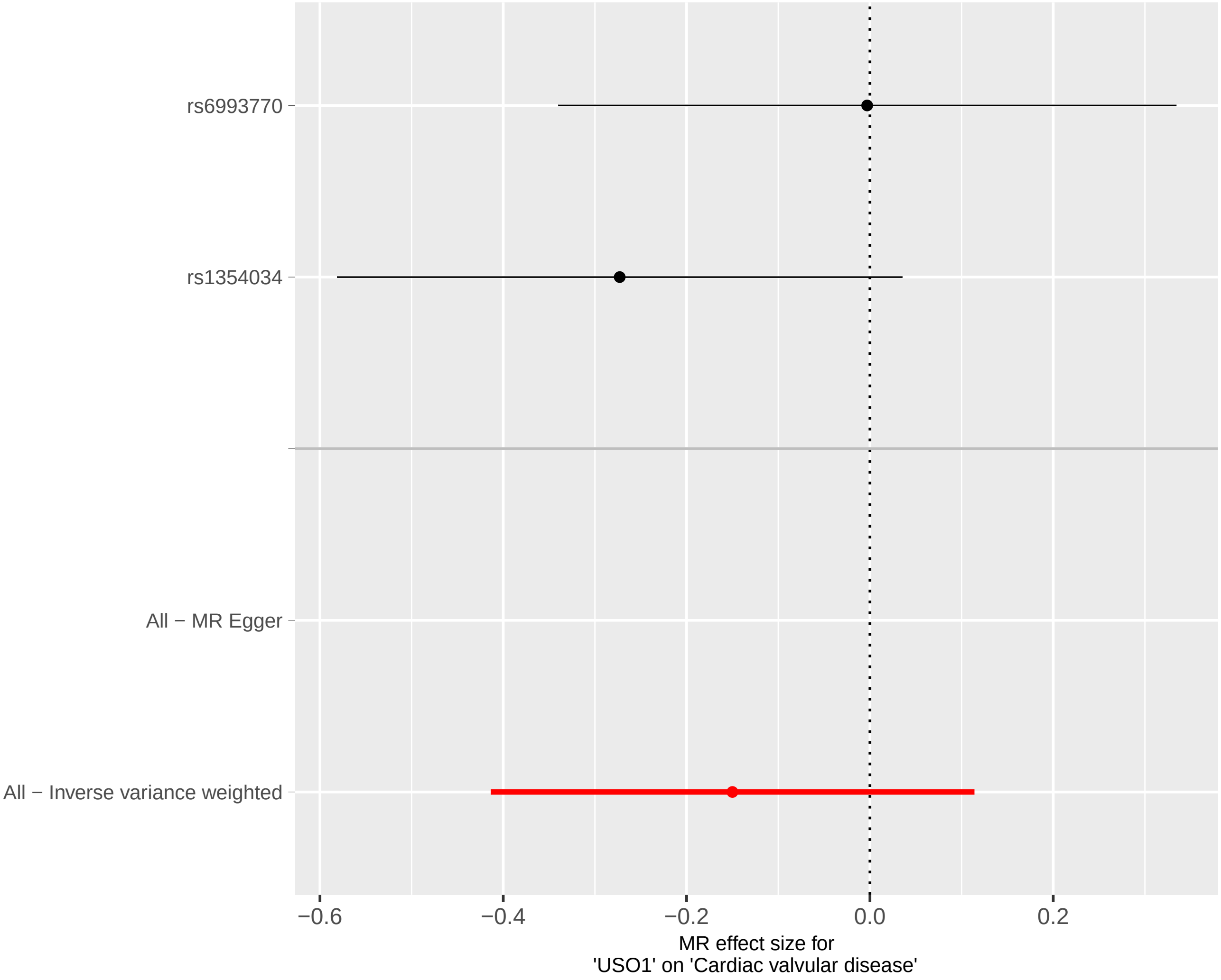
rs6993770
rs1354034
All − MR Egger
All − Inverse variance weighted
−0.6
−0.4
−0.2
0.0
0.2
MR effect size for
'USO1' on 'Cardiac valvular disease'

Insufficient number of SNPs

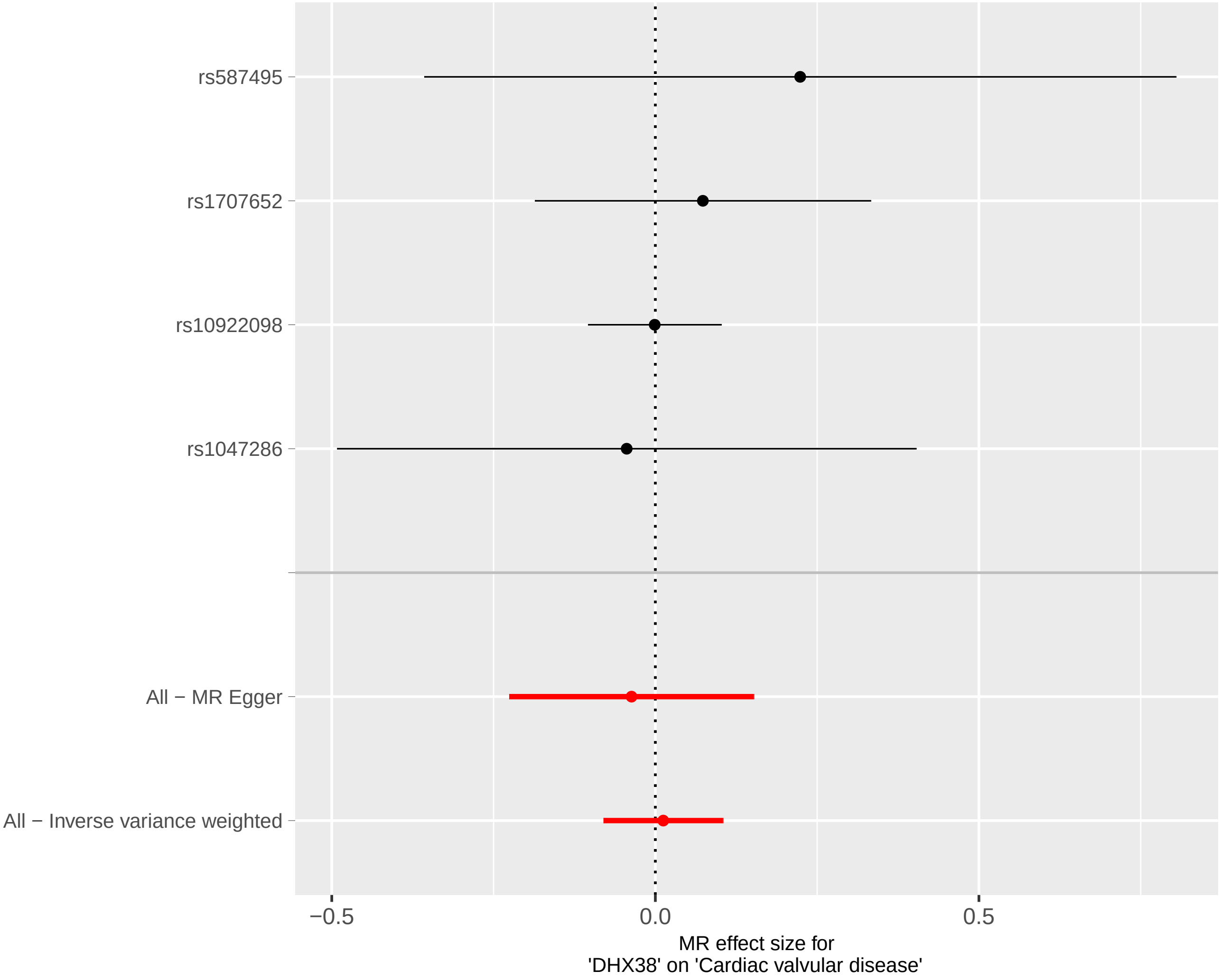
rs587495
rs1707652
rs10922098
rs1047286
All − MR Egger
All − Inverse variance weighted
−0.5
0.0
0.5
MR effect size for
'DHX38' on 'Cardiac valvular disease'

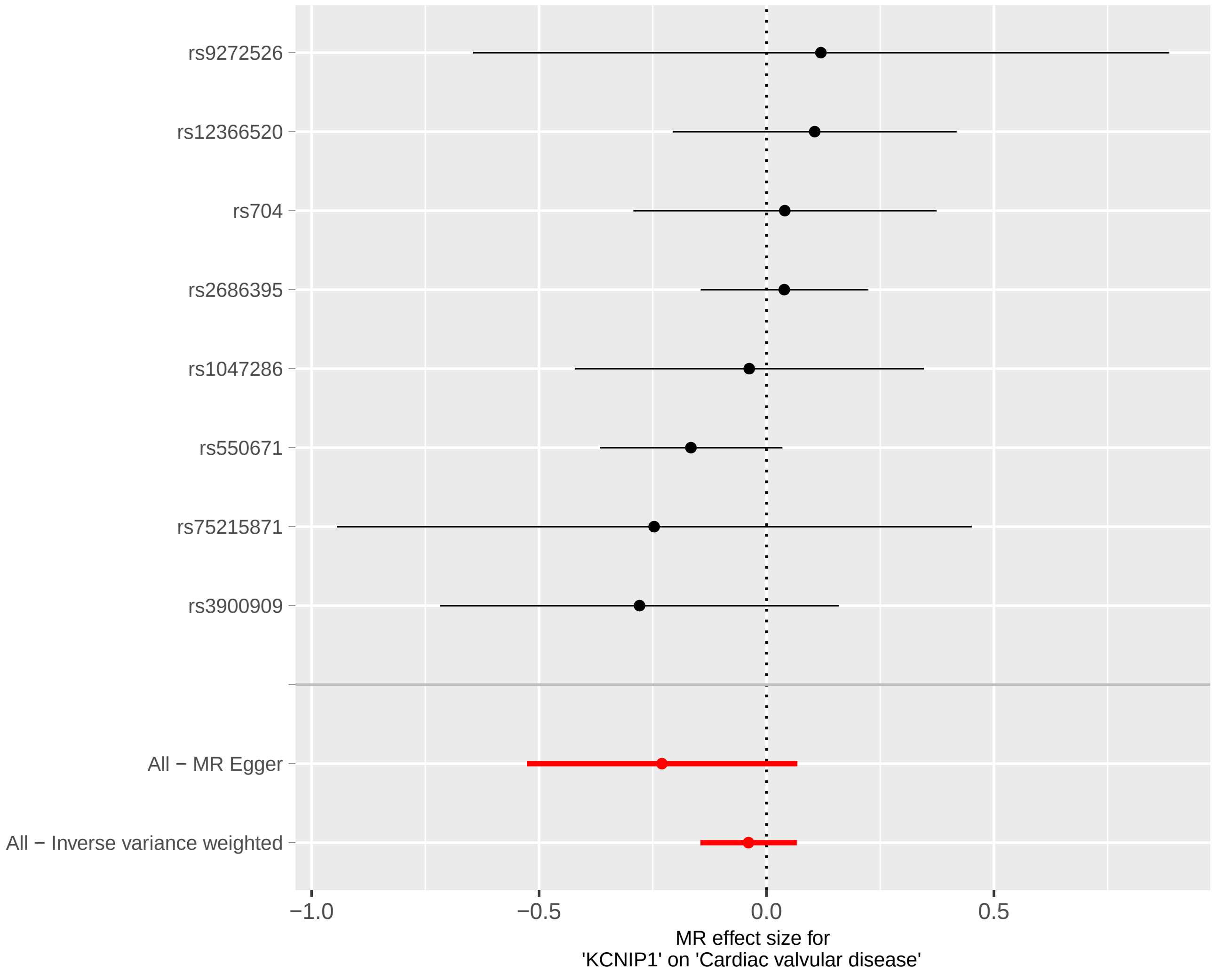
rs9272526
rs12366520
rs704
rs2686395
rs1047286
rs550671
rs75215871
rs3900909
All − MR Egger
All − Inverse variance weighted
−1.0
−0.5
0.0
0.5
MR effect size for
'KCNIP1' on 'Cardiac valvular disease'

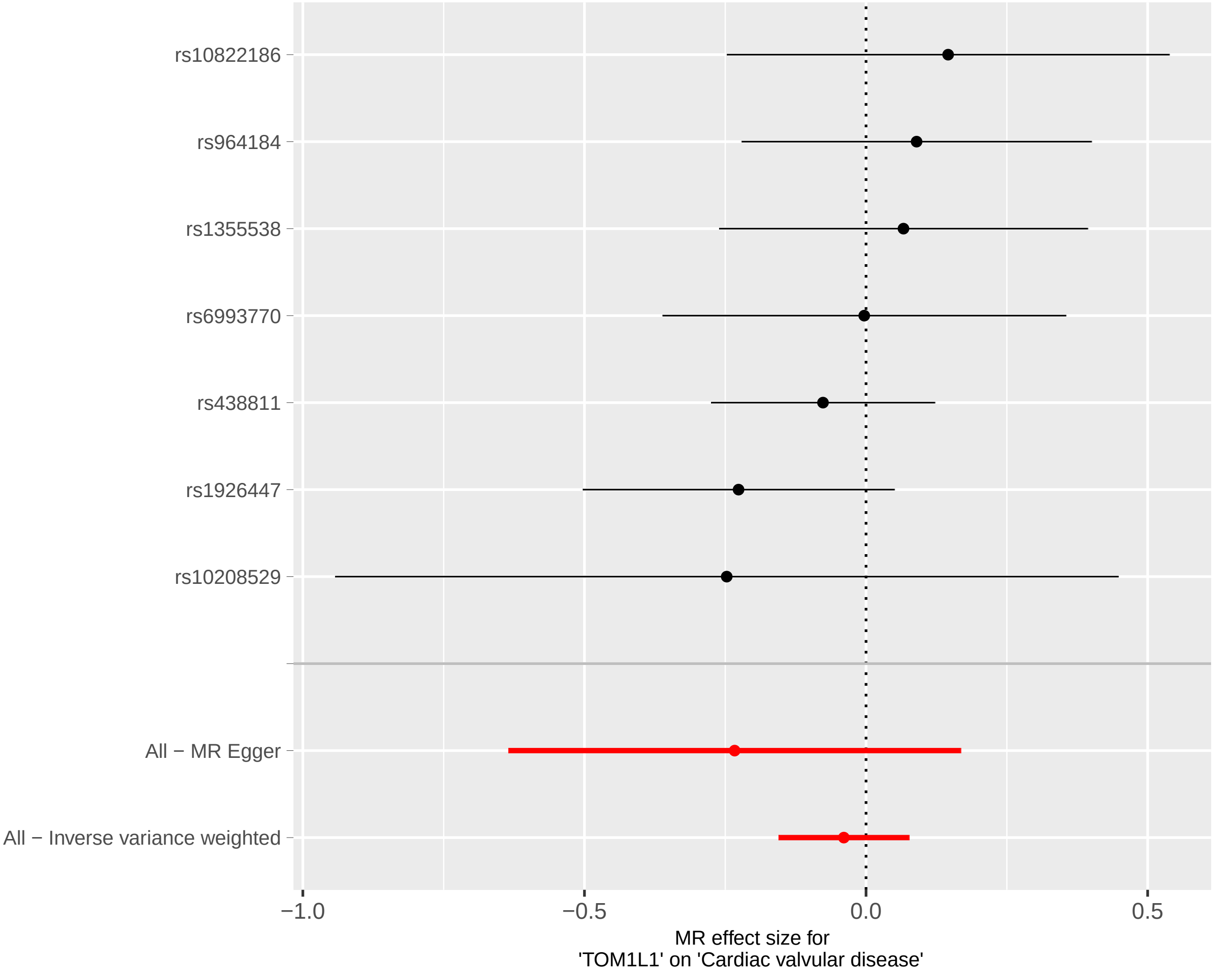
rs10822186
rs964184
rs1355538
rs6993770
rs438811
rs1926447
rs10208529
All − MR Egger
All − Inverse variance weighted
−1.0
−0.5
0.0
0.5
MR effect size for
'TOM1L1' on 'Cardiac valvular disease'

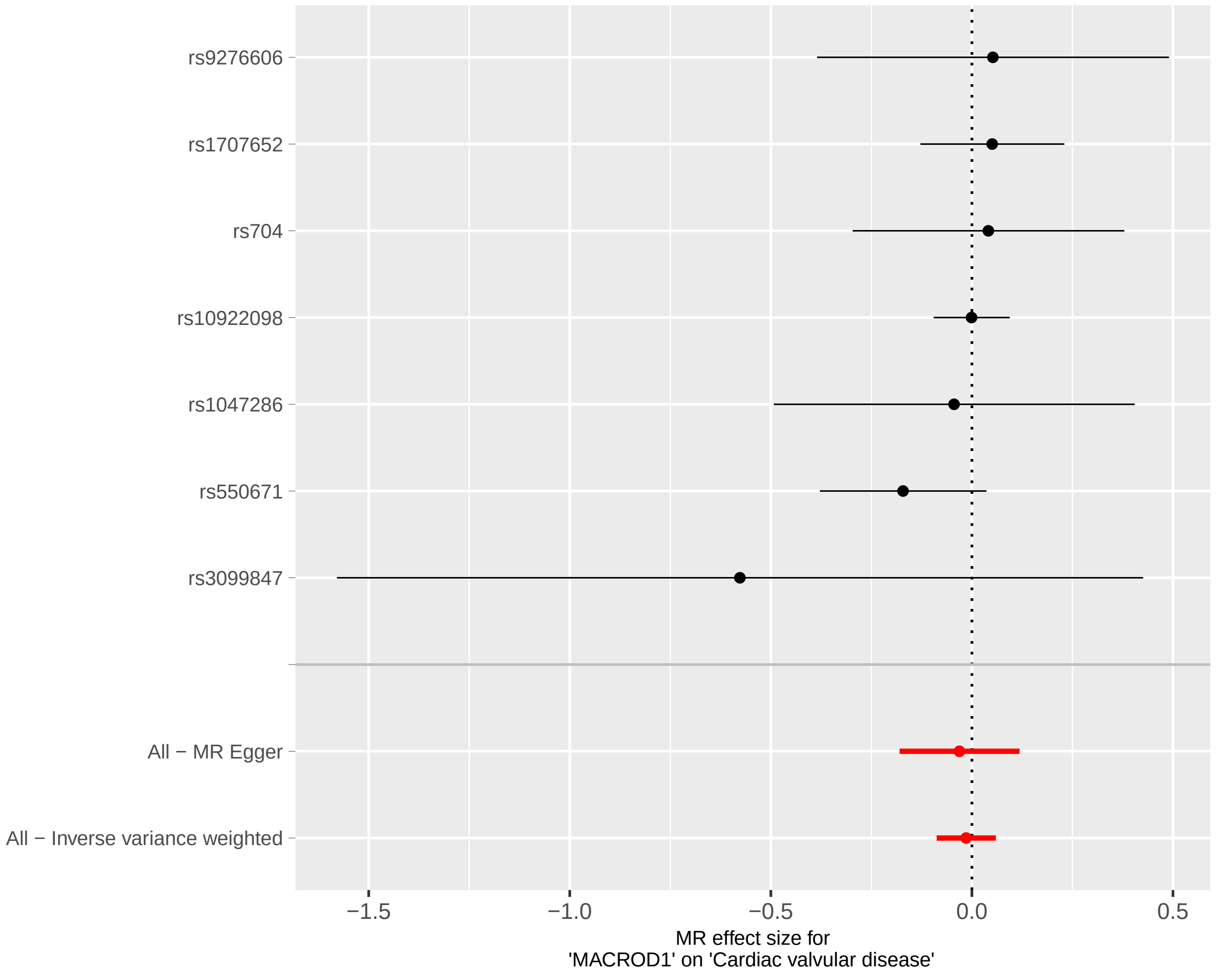
rs9276606
rs1707652
rs704
rs10922098
rs1047286
rs550671
rs3099847
All − MR Egger
All − Inverse variance weighted
−1.5
−1.0
−0.5
0.0
0.5
MR effect size for
'MACROD1' on 'Cardiac valvular disease'

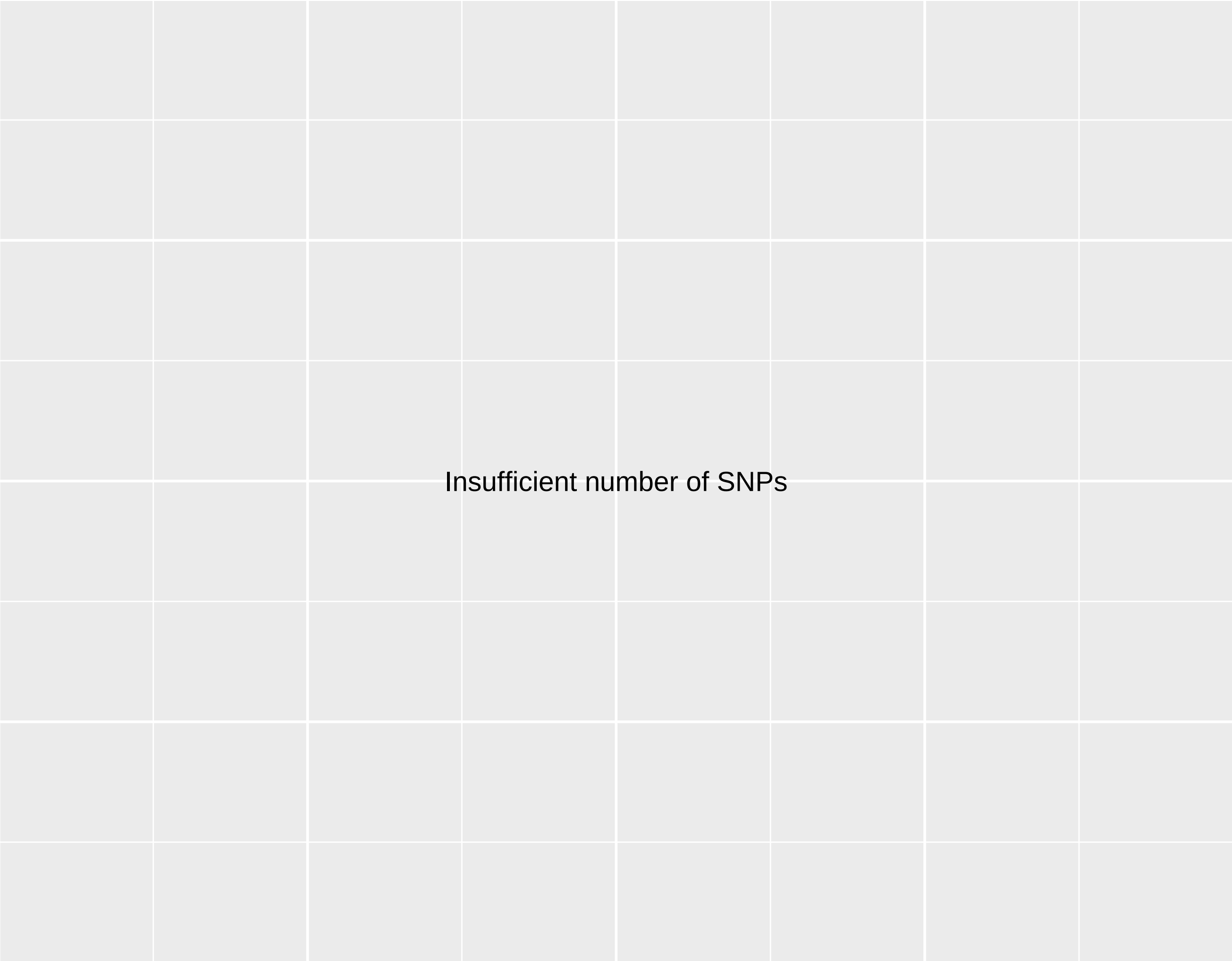
Insufficient number of SNPs

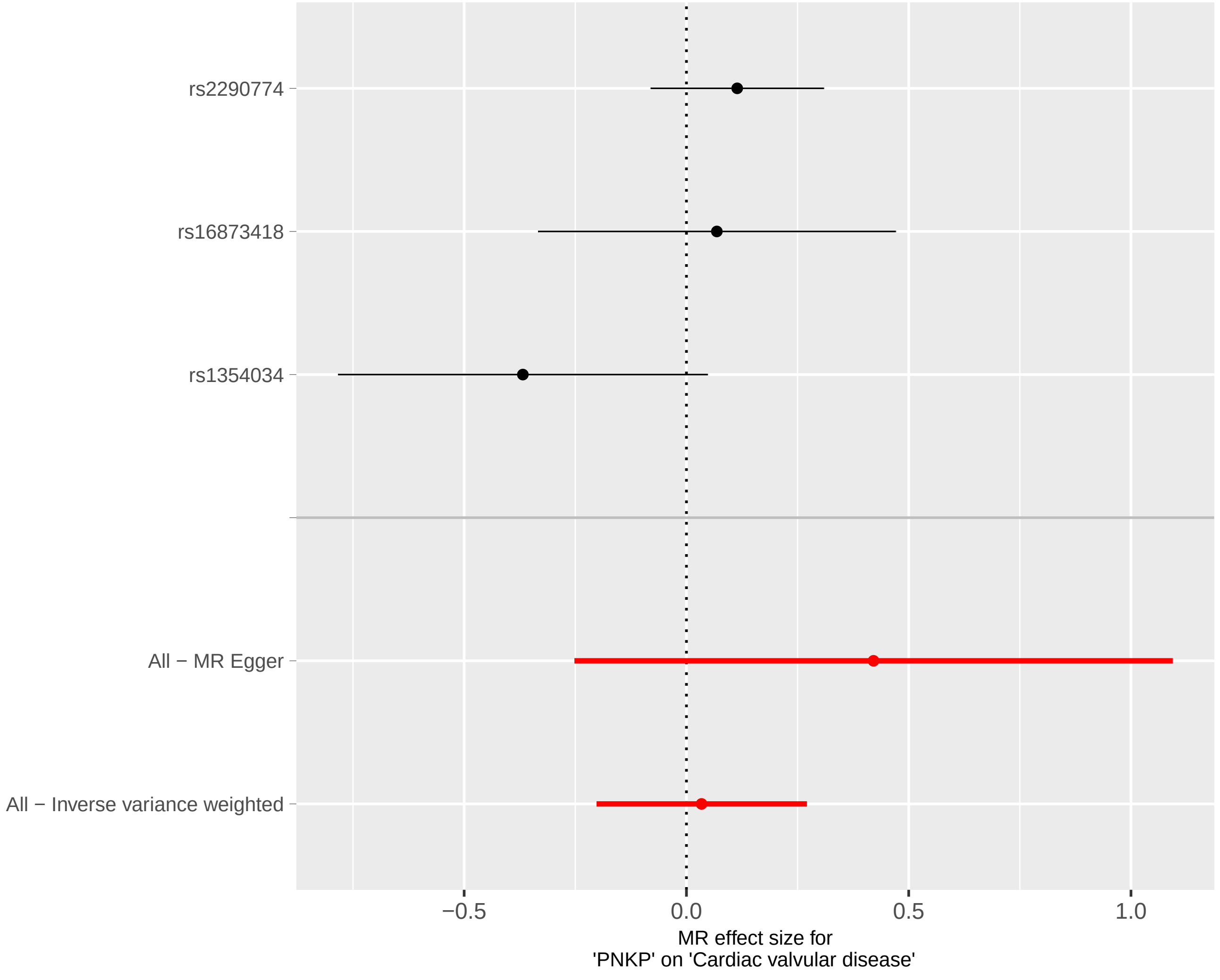
rs2290774
rs16873418
rs1354034
All − MR Egger
All − Inverse variance weighted
−0.5
0.0
0.5
1.0
MR effect size for
'PNKP' on 'Cardiac valvular disease'

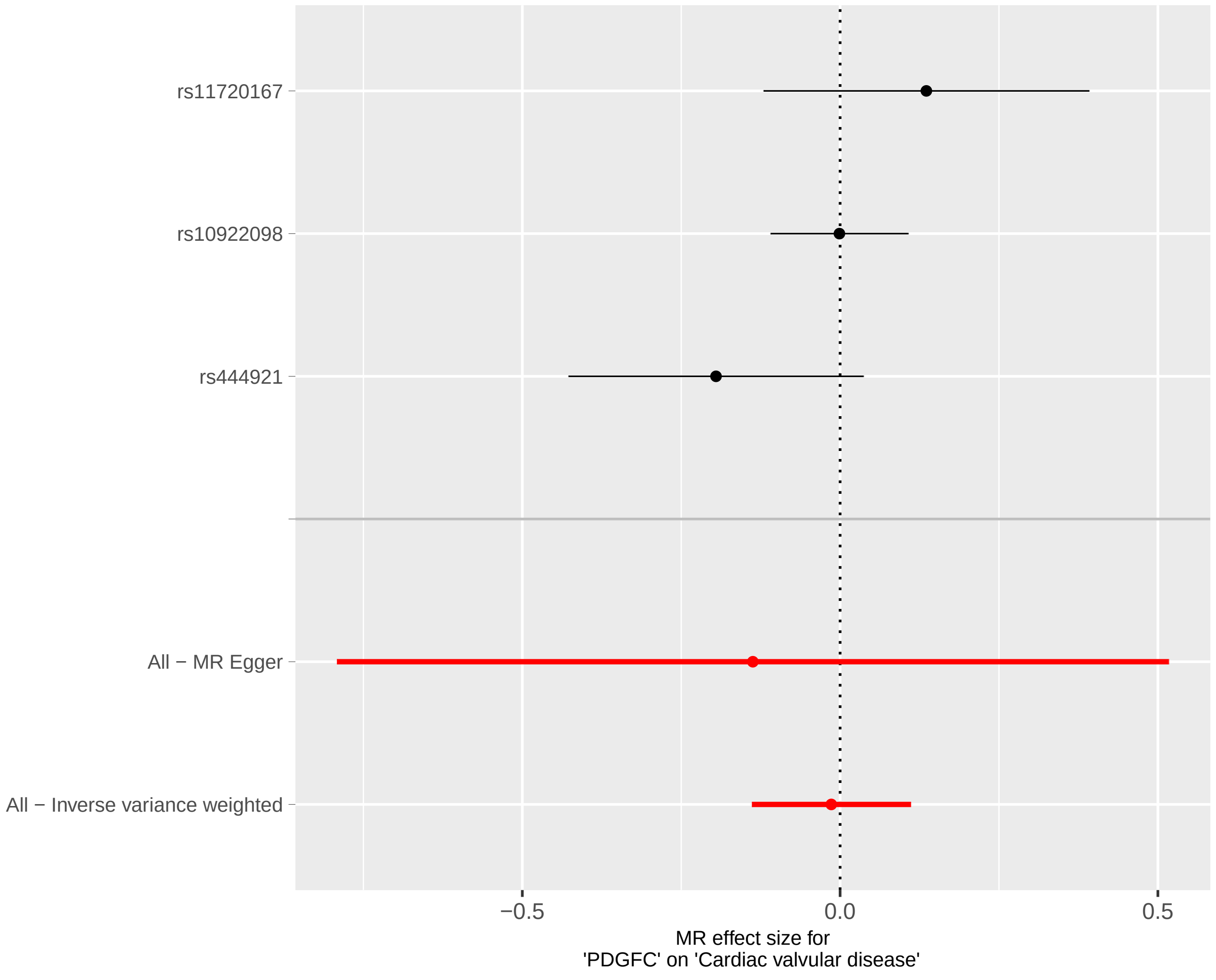
rs11720167
rs10922098
rs444921
All − MR Egger
All − Inverse variance weighted
−0.5
0.0
0.5
MR effect size for
'PDGFC' on 'Cardiac valvular disease'

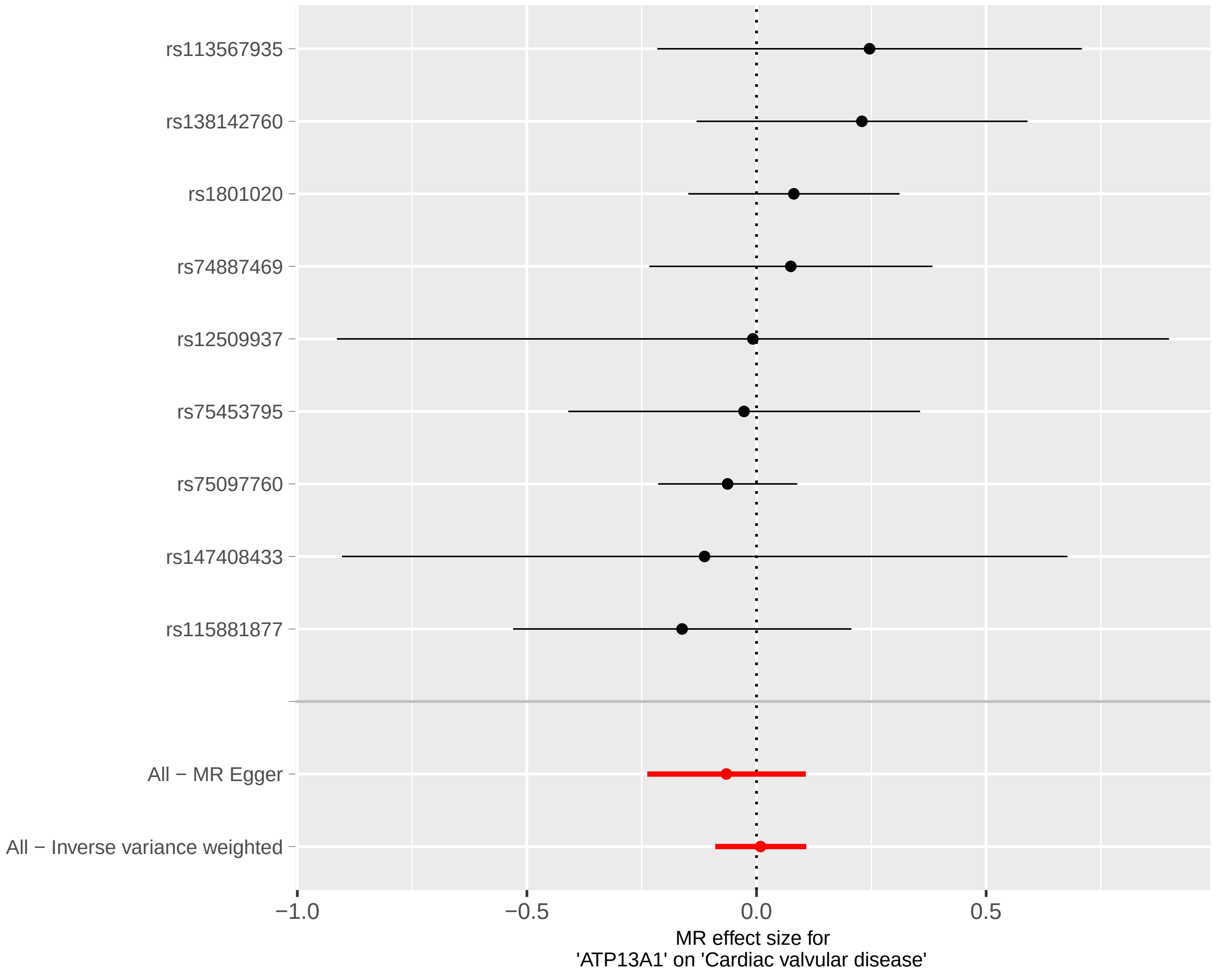
rs113567935
rs138142760
rs1801020
rs74887469
rs12509937
rs75453795
rs75097760
rs147408433
rs115881877
All − MR Egger
All − Inverse variance weighted
−1.0
−0.5
0.0
0.5
MR effect size for
'ATP13A1' on 'Cardiac valvular disease'

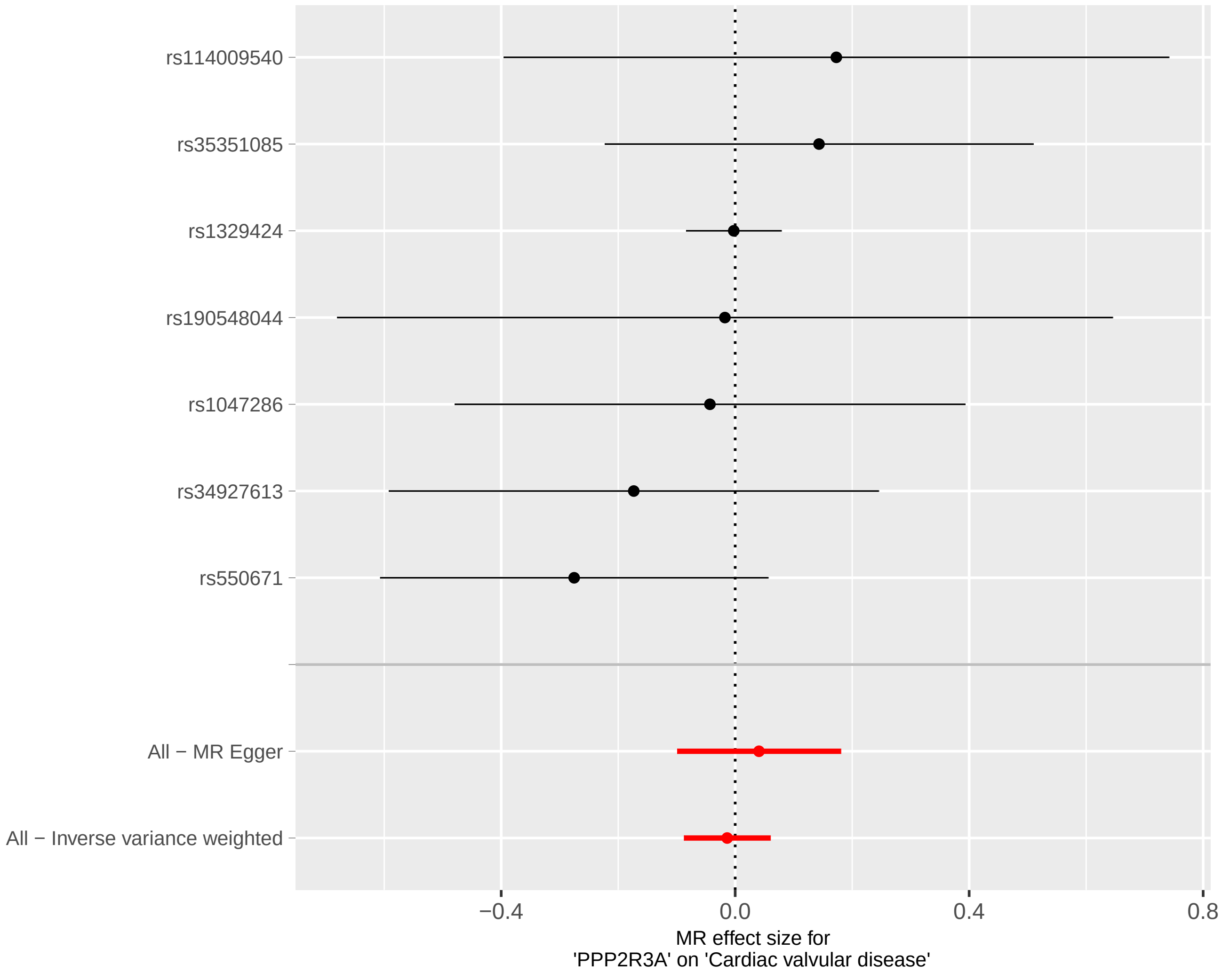
rs114009540
rs35351085
rs1329424
rs190548044
rs1047286
rs34927613
rs550671
All − MR Egger
All − Inverse variance weighted
−0.4
0.0
0.4
0.8
MR effect size for
'PPP2R3A' on 'Cardiac valvular disease'

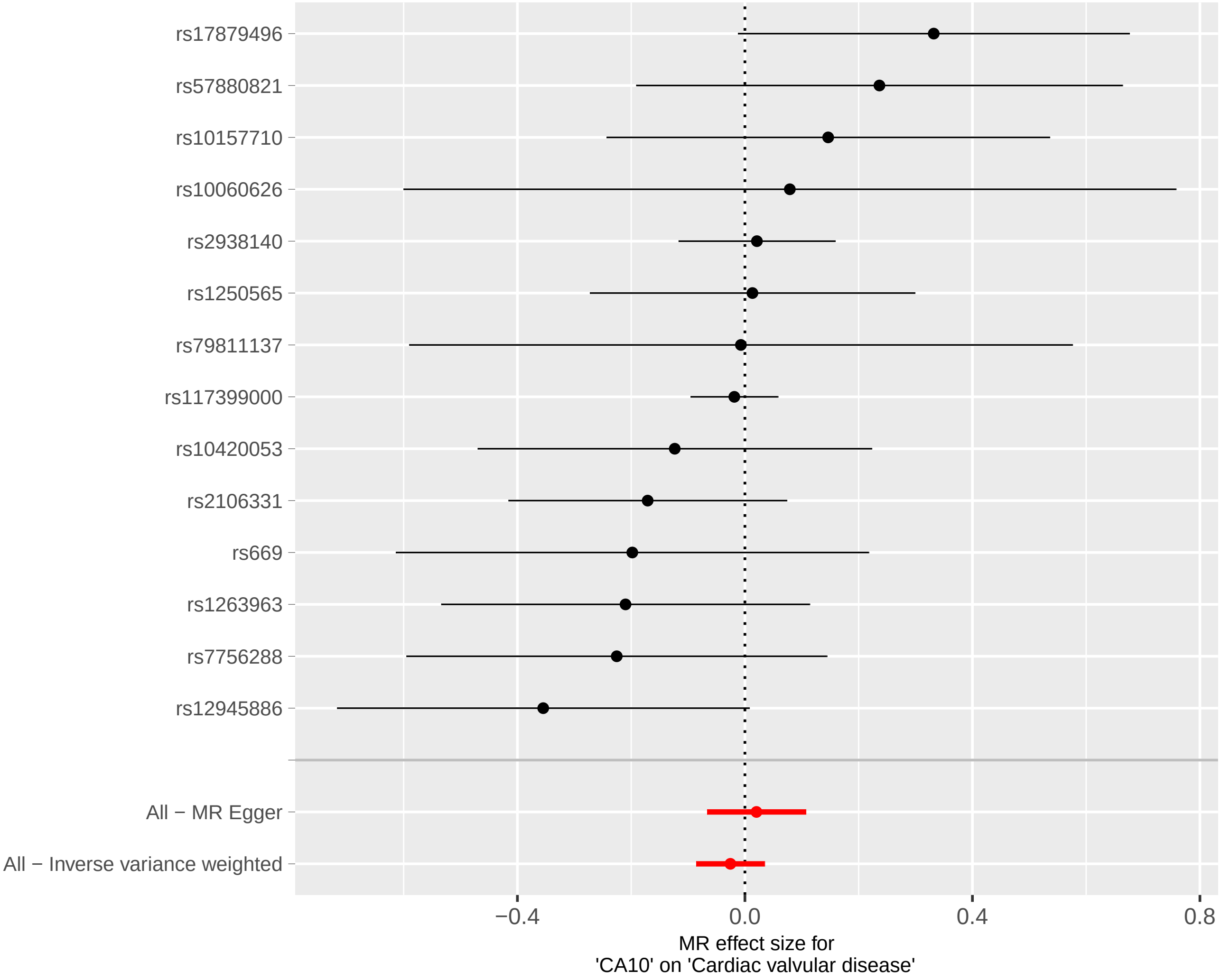

rs17879496
rs57880821
rs10157710
rs10060626
rs2938140
rs1250565
rs79811137
rs117399000
rs10420053
rs2106331
rs669
rs1263963
rs7756288
rs12945886
All − MR Egger
All − Inverse variance weighted
−0.4
0.0
0.4
0.8
MR effect size for
'CA10' on 'Cardiac valvular disease'

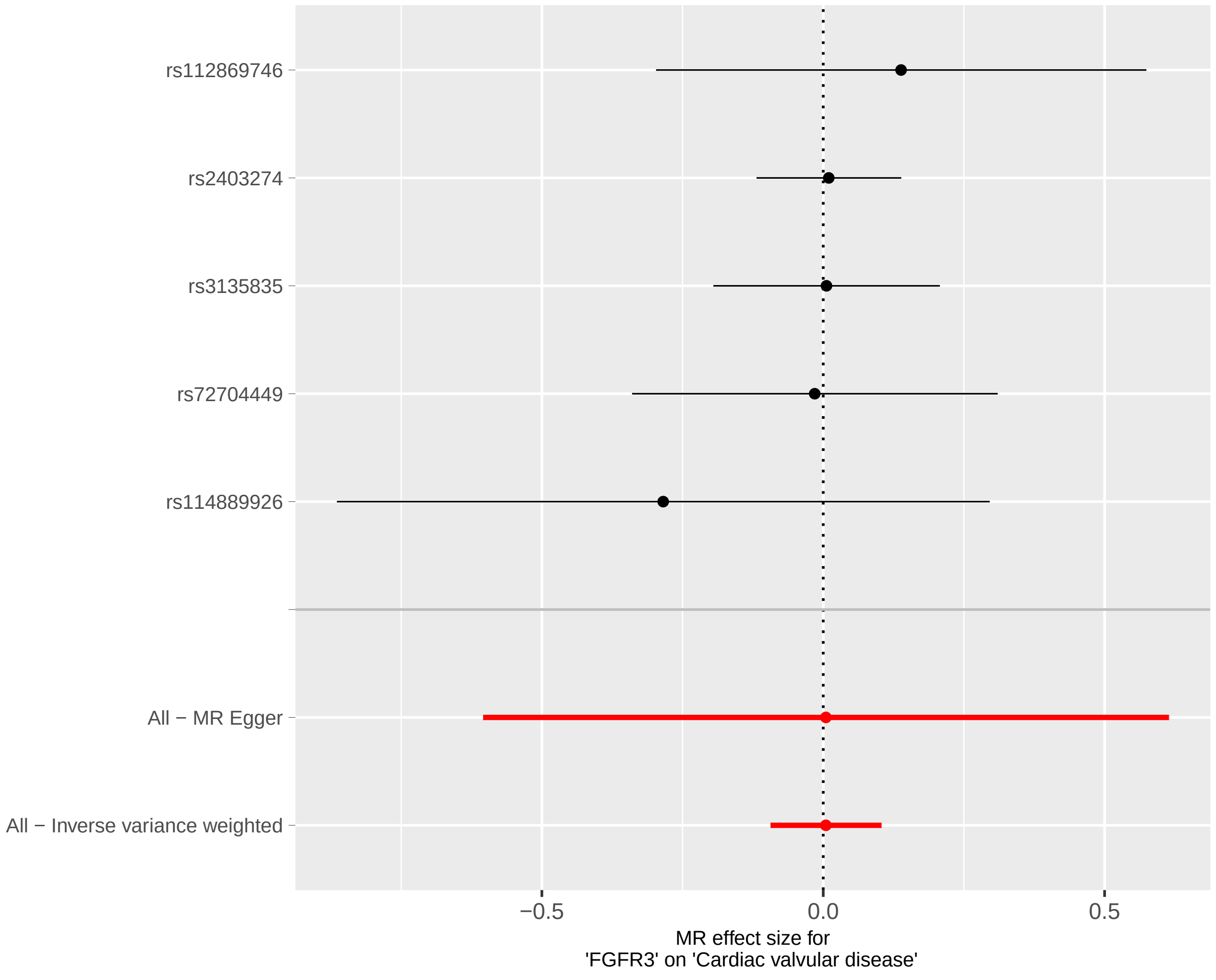

rs112869746
rs2403274
rs3135835
rs72704449
rs114889926
All − MR Egger
All − Inverse variance weighted
−0.5
0.0
0.5
MR effect size for
'FGFR3' on 'Cardiac valvular disease'

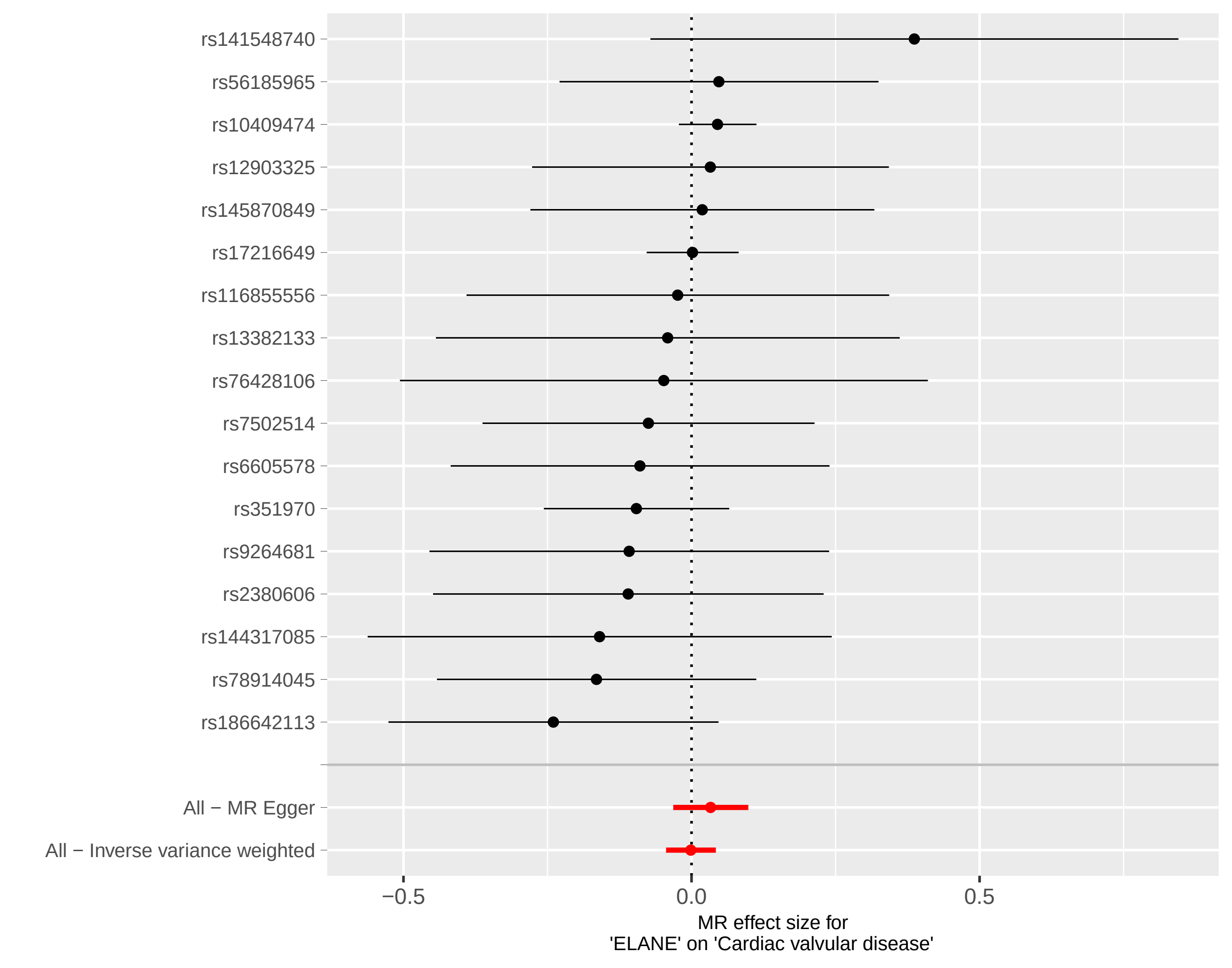
rs141548740
rs56185965
rs10409474
rs12903325
rs145870849
rs17216649
rs116855556
rs13382133
rs76428106
rs7502514
rs6605578
rs351970
rs9264681
rs2380606
rs144317085
rs78914045
rs186642113
All − MR Egger
All − Inverse variance weighted
−0.5
0.0
0.5
MR effect size for
'ELANE' on 'Cardiac valvular disease'

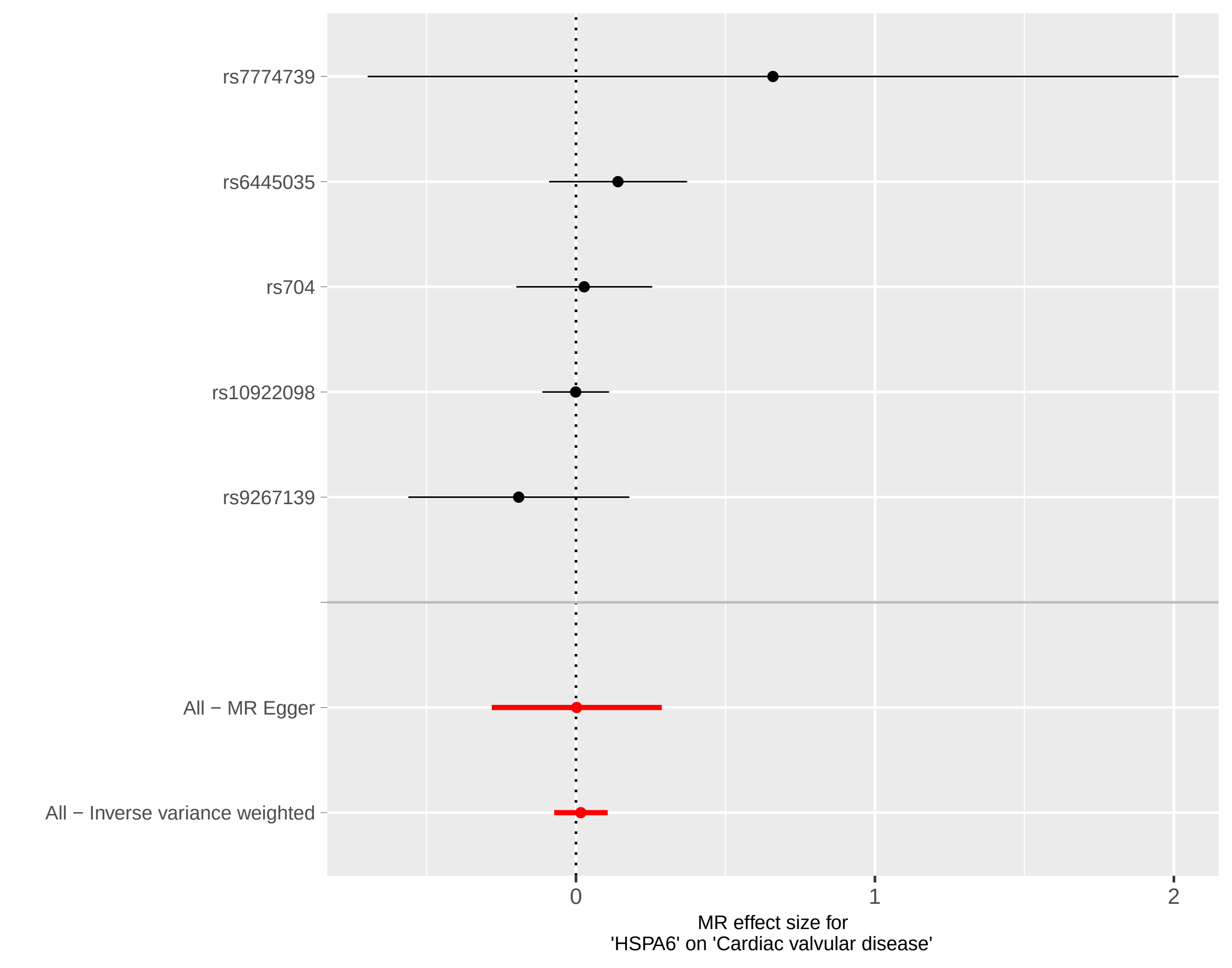
rs7774739
rs6445035
rs704
rs10922098
rs9267139
All − MR Egger
All − Inverse variance weighted
0
1
2
MR effect size for
'HSPA6' on 'Cardiac valvular disease'

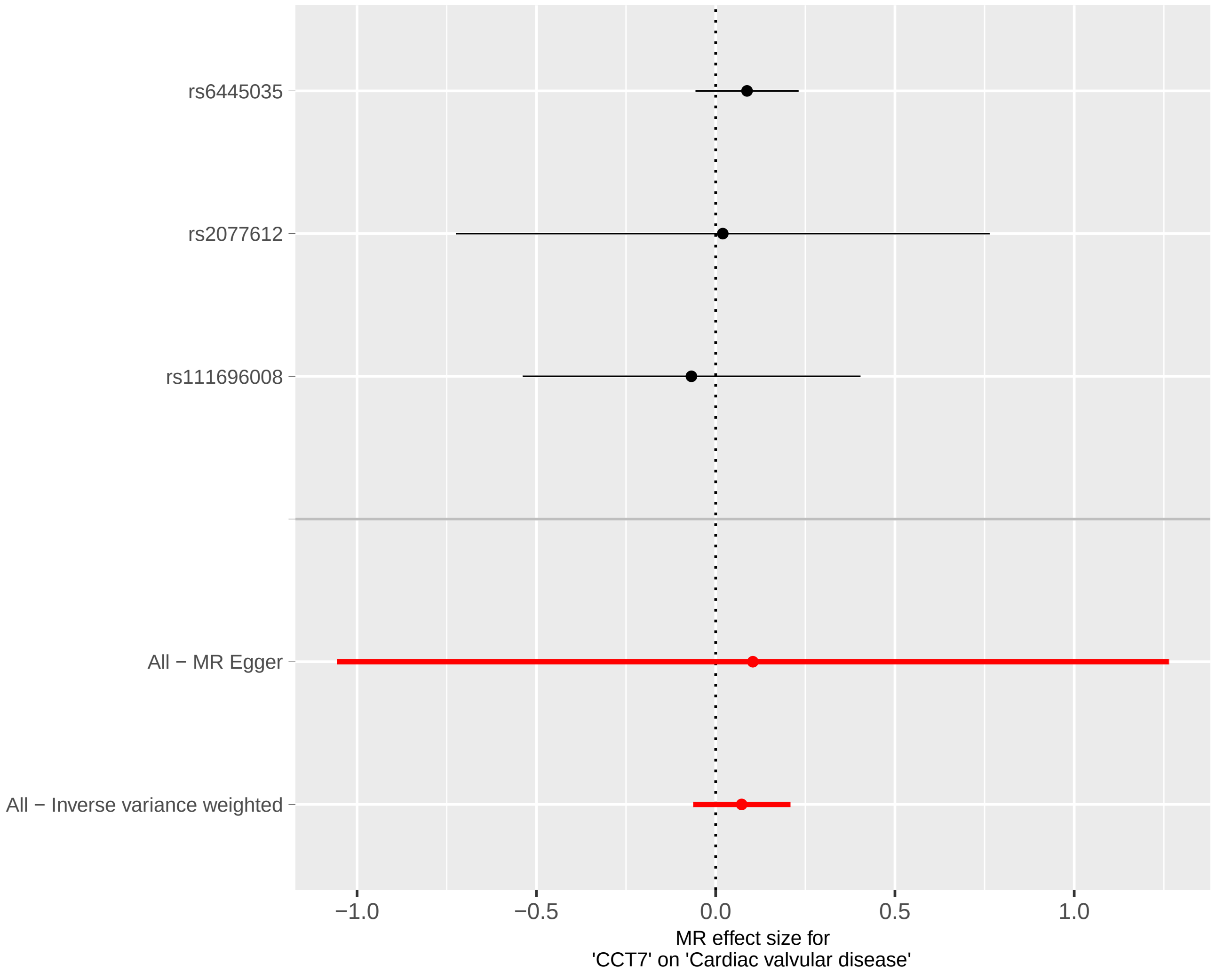
rs6445035
rs2077612
rs111696008
All − MR Egger
All − Inverse variance weighted
−1.0
−0.5
0.0
0.5
1.0
MR effect size for
'CCT7' on 'Cardiac valvular disease'

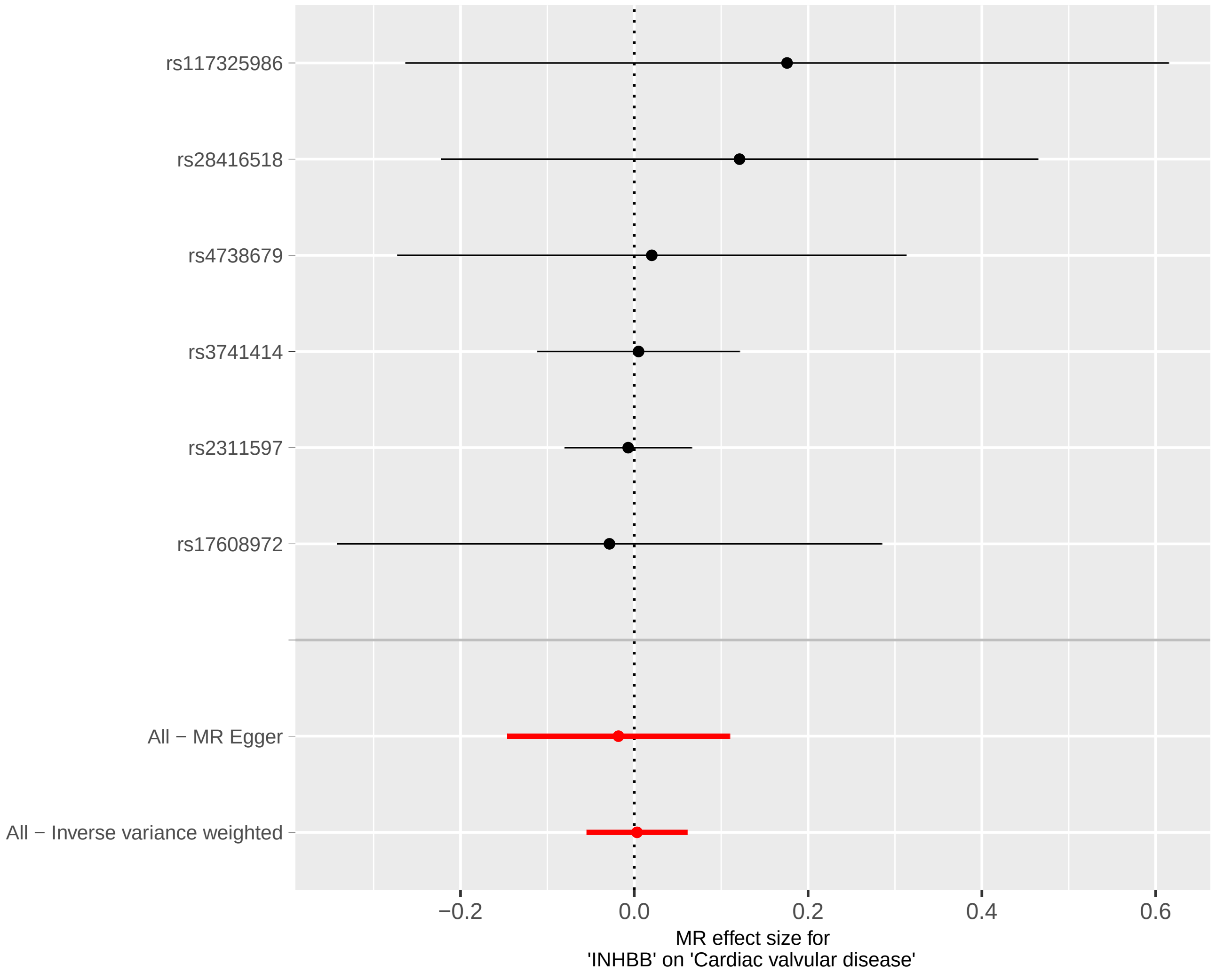
rs117325986
rs28416518
rs4738679
rs3741414
rs2311597
rs17608972
All − MR Egger
All − Inverse variance weighted
−0.2
0.0
0.2
0.4
0.6
MR effect size for
'INHBB' on 'Cardiac valvular disease'

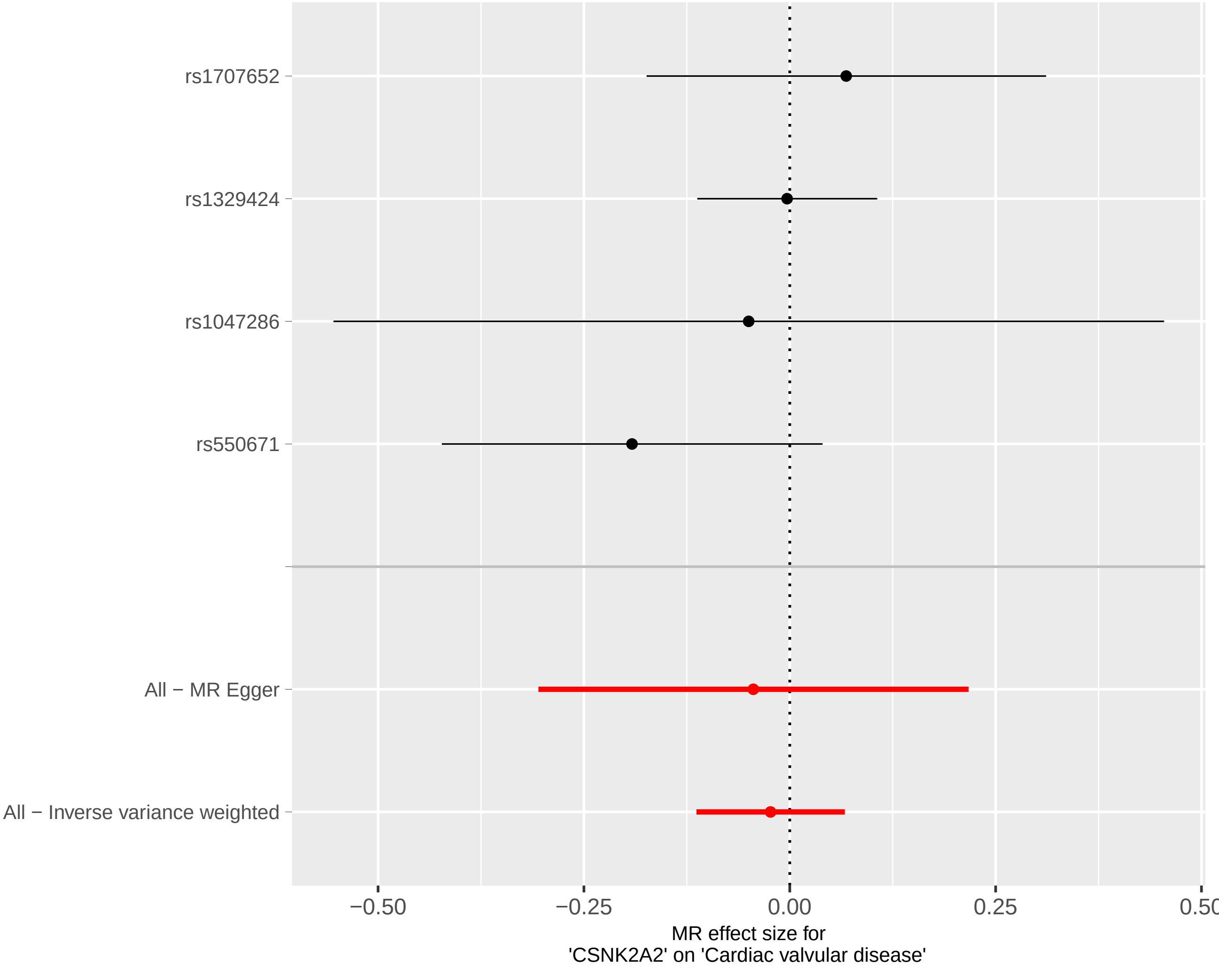
rs1707652
rs1329424
rs1047286
rs550671
All − MR Egger
All − Inverse variance weighted
−0.50
−0.25
0.00
0.25
0.50
MR effect size for
'CSNK2A2' on 'Cardiac valvular disease'

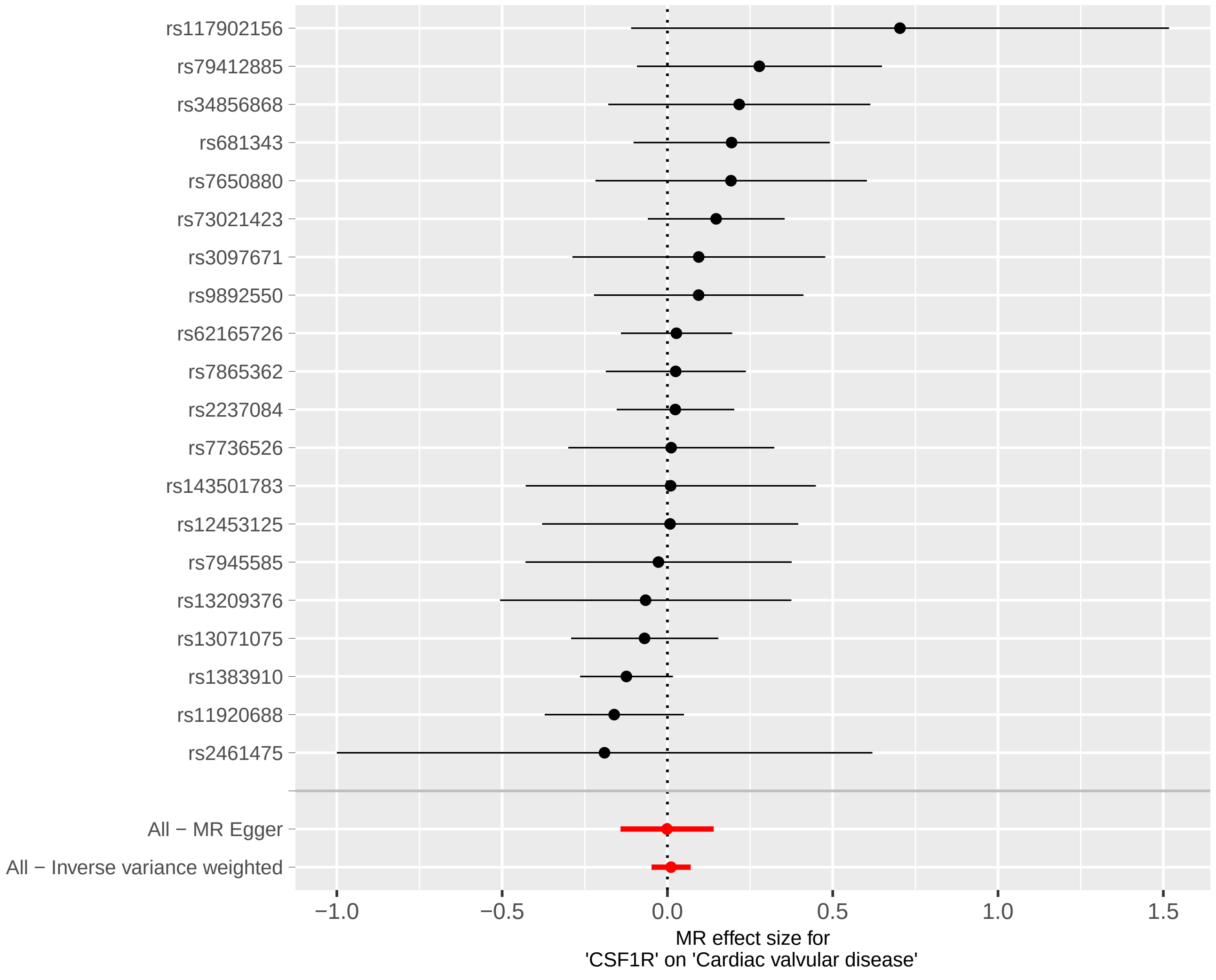
rs117902156
rs79412885
rs34856868
rs681343
rs7650880
rs73021423
rs3097671
rs9892550
rs62165726
rs7865362
rs2237084
rs7736526
rs143501783
rs12453125
rs7945585
rs13209376
rs13071075
rs1383910
rs11920688
rs2461475
All − MR Egger
All − Inverse variance weighted
−1.0
−0.5
0.0
0.5
1.0
1.5
MR effect size for
'CSF1R' on 'Cardiac valvular disease'

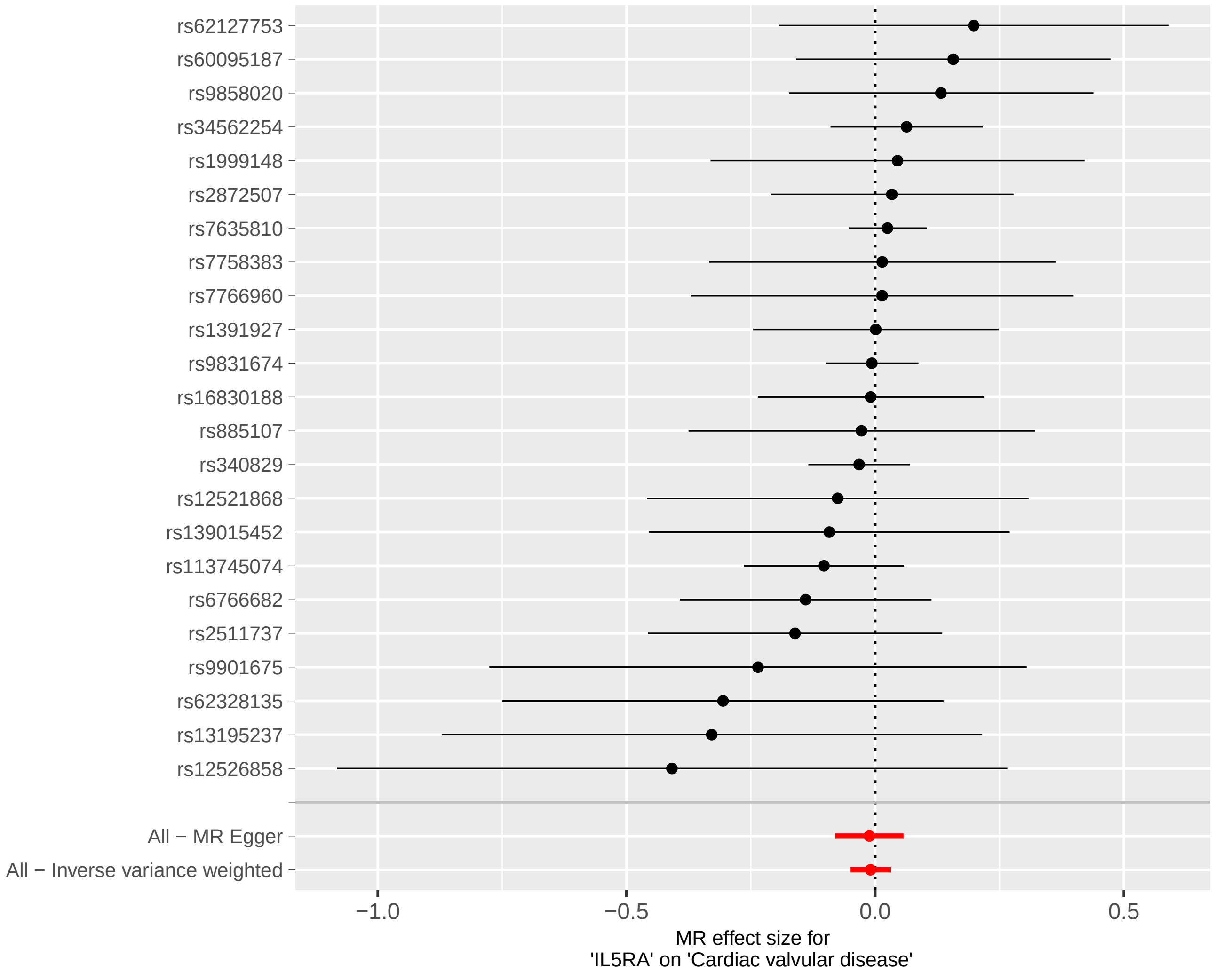

rs62127753
rs60095187
rs9858020
rs34562254
rs1999148
rs2872507
rs7635810
rs7758383
rs7766960
rs1391927
rs9831674
rs16830188
rs885107
rs340829
rs12521868
rs139015452
rs113745074
rs6766682
rs2511737
rs9901675
rs62328135
rs13195237
rs12526858
All − MR Egger
All − Inverse variance weighted
−1.0
−0.5
0.0
0.5
MR effect size for
'IL5RA' on 'Cardiac valvular disease'

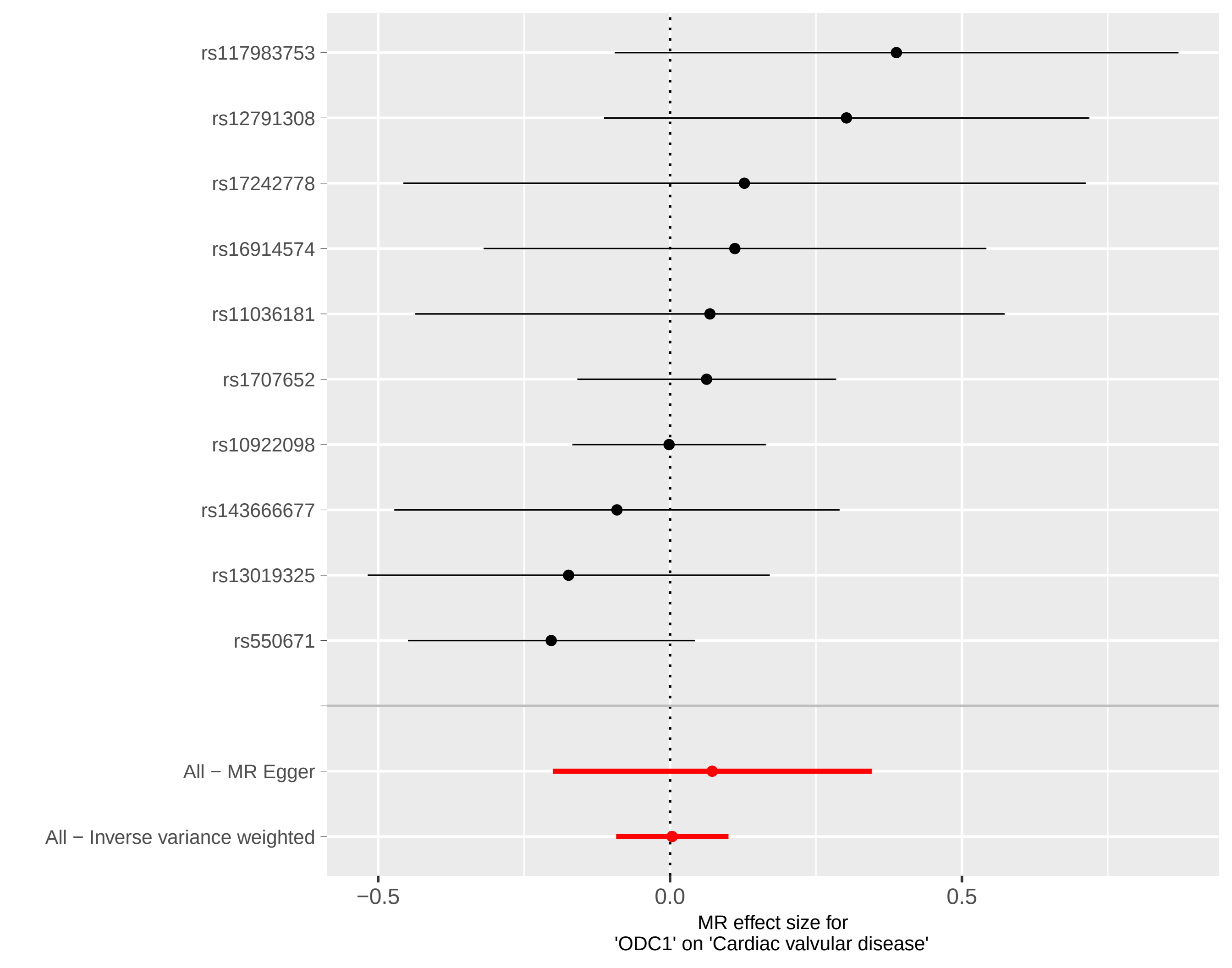
rs117983753
rs12791308
rs17242778
rs16914574
rs11036181
rs1707652
rs10922098
rs143666677
rs13019325
rs550671
All − MR Egger
All − Inverse variance weighted
−0.5
0.0
0.5
MR effect size for
'ODC1' on 'Cardiac valvular disease'

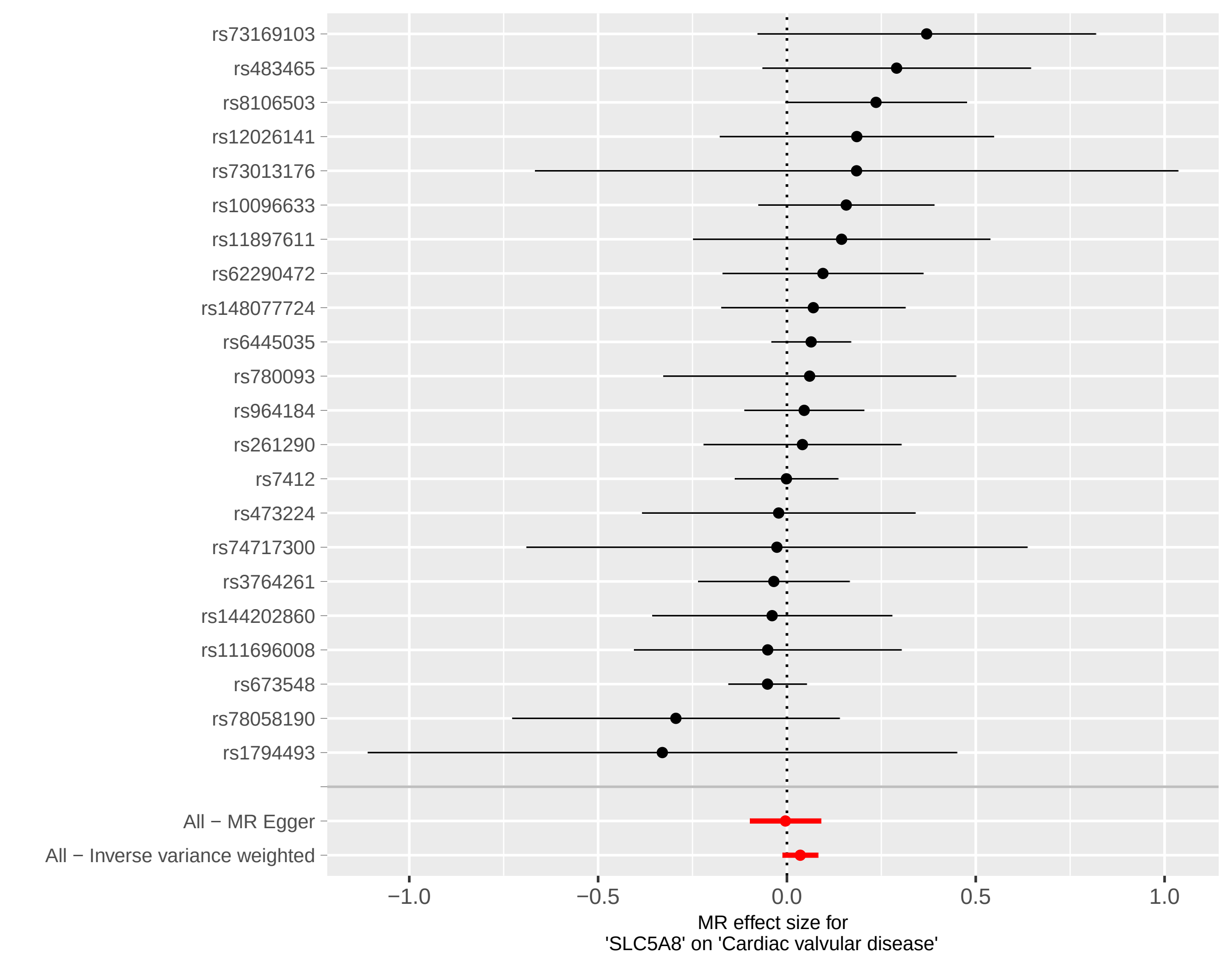

rs73169103
rs483465
rs8106503
rs12026141
rs73013176
rs10096633
rs11897611
rs62290472
rs148077724
rs6445035
rs780093
rs964184
rs261290
rs7412
rs473224
rs74717300
rs3764261
rs144202860
rs111696008
rs673548
rs78058190
rs1794493
All − MR Egger
All − Inverse variance weighted
−1.0
−0.5
0.0
0.5
1.0
MR effect size for
'SLC5A8' on 'Cardiac valvular disease'

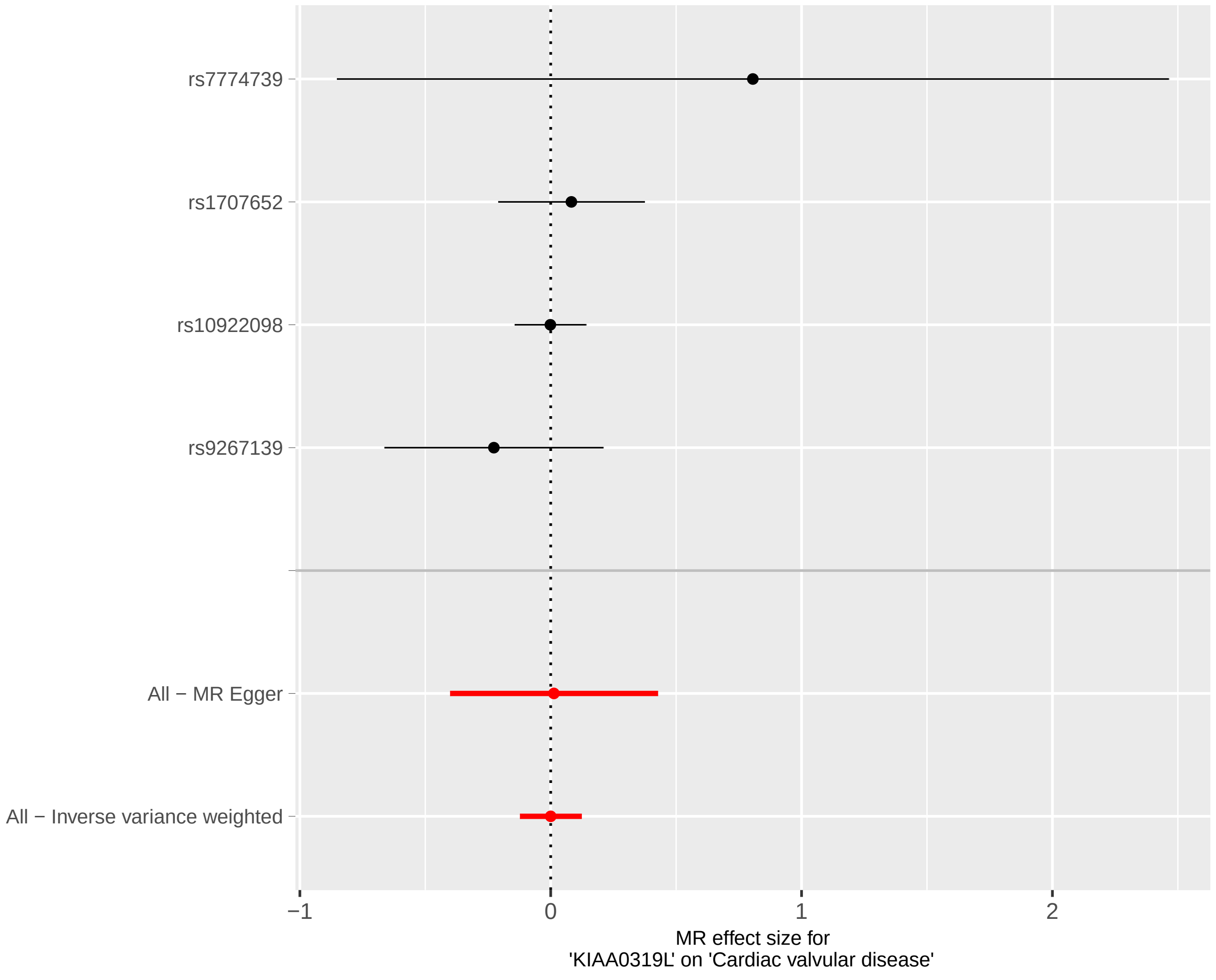
rs7774739
rs1707652
rs10922098
rs9267139
All − MR Egger
All − Inverse variance weighted
−1
0
1
2
MR effect size for
'KIAA0319L' on 'Cardiac valvular disease'

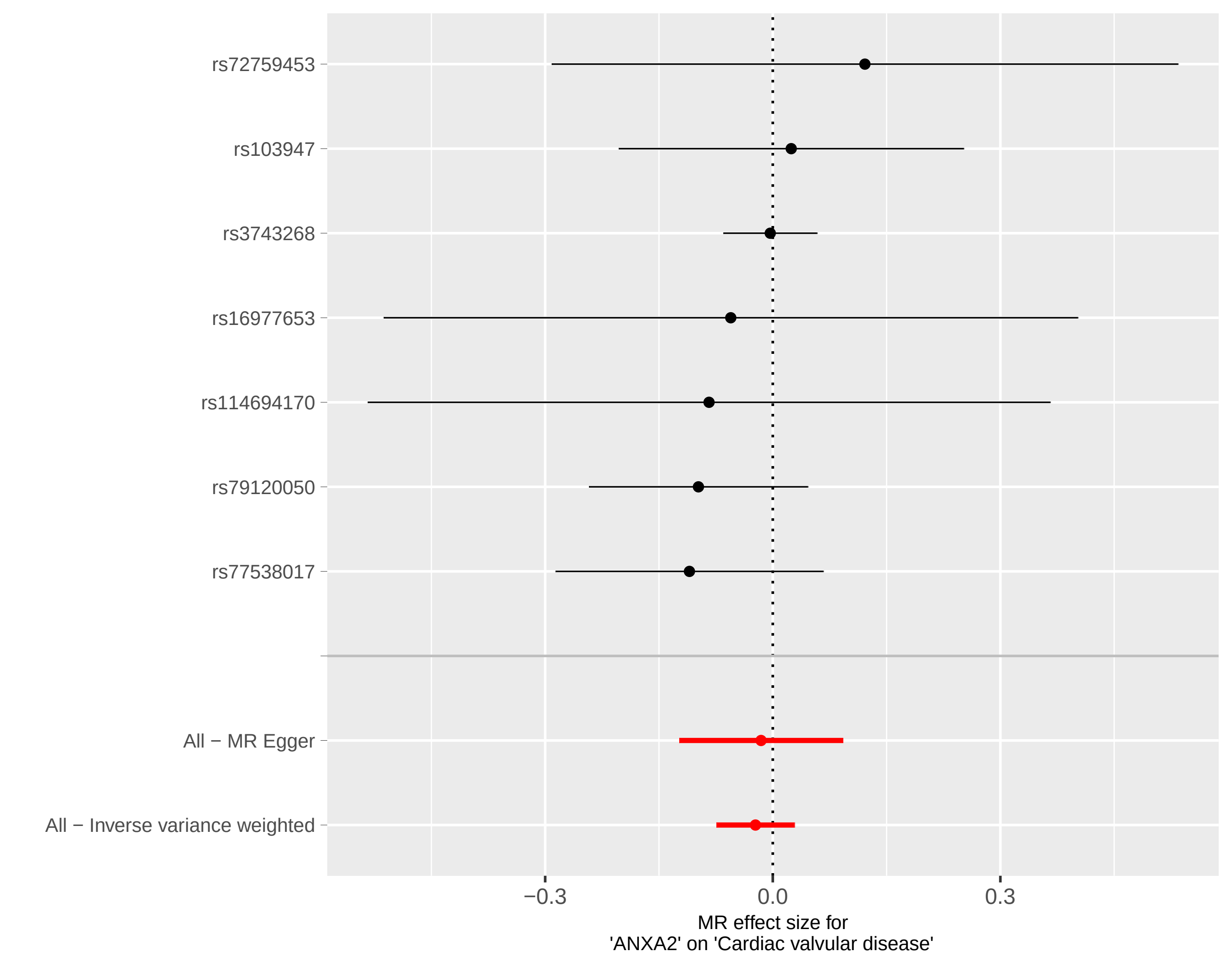
rs72759453
rs103947
rs3743268
rs16977653
rs114694170
rs79120050
rs77538017
All − MR Egger
All − Inverse variance weighted
−0.3
0.0
0.3
MR effect size for
'ANXA2' on 'Cardiac valvular disease'

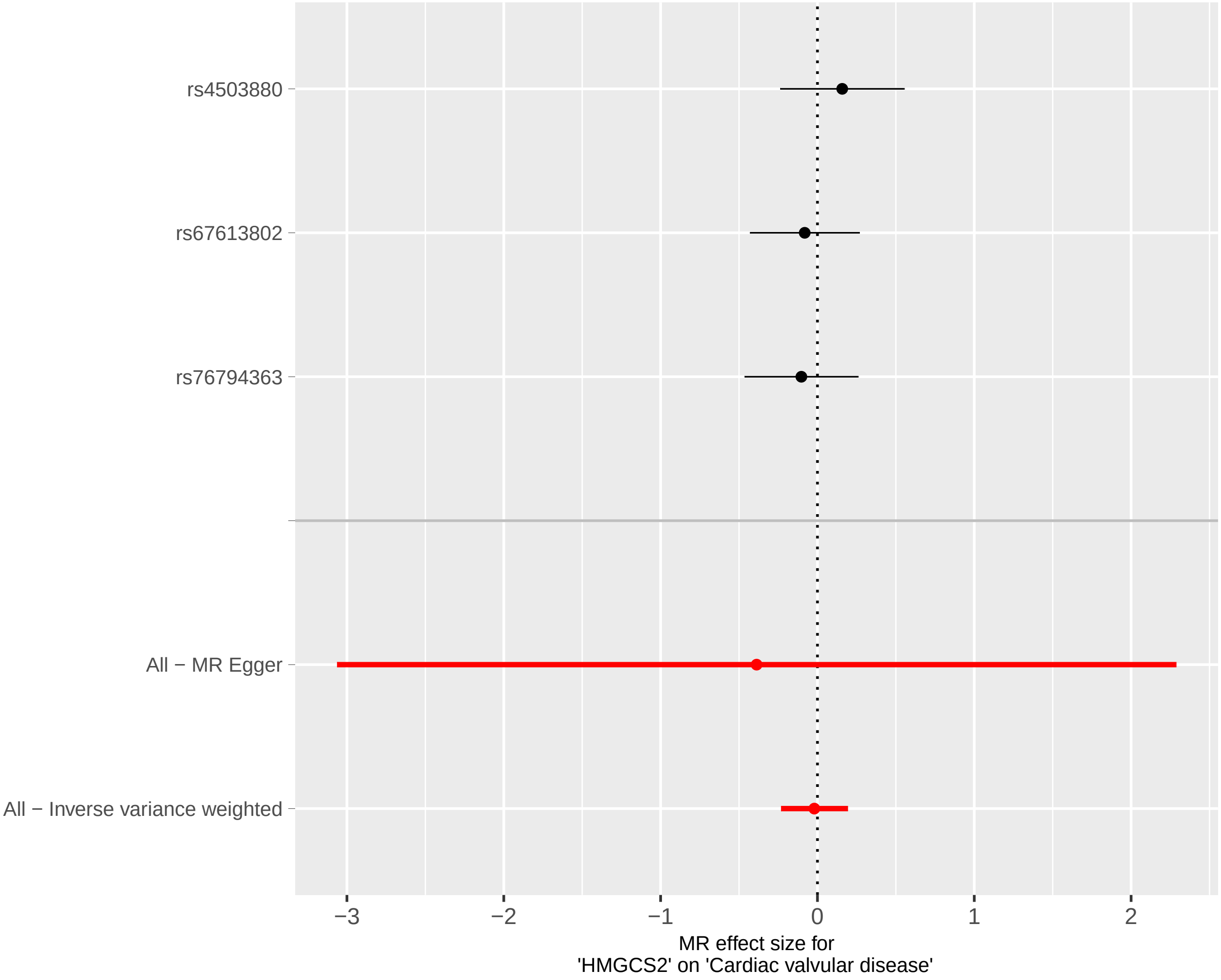
rs4503880
rs67613802
rs76794363
All − MR Egger
All − Inverse variance weighted
−3
−2
−1
0
1
2
MR effect size for
'HMGCS2' on 'Cardiac valvular disease'

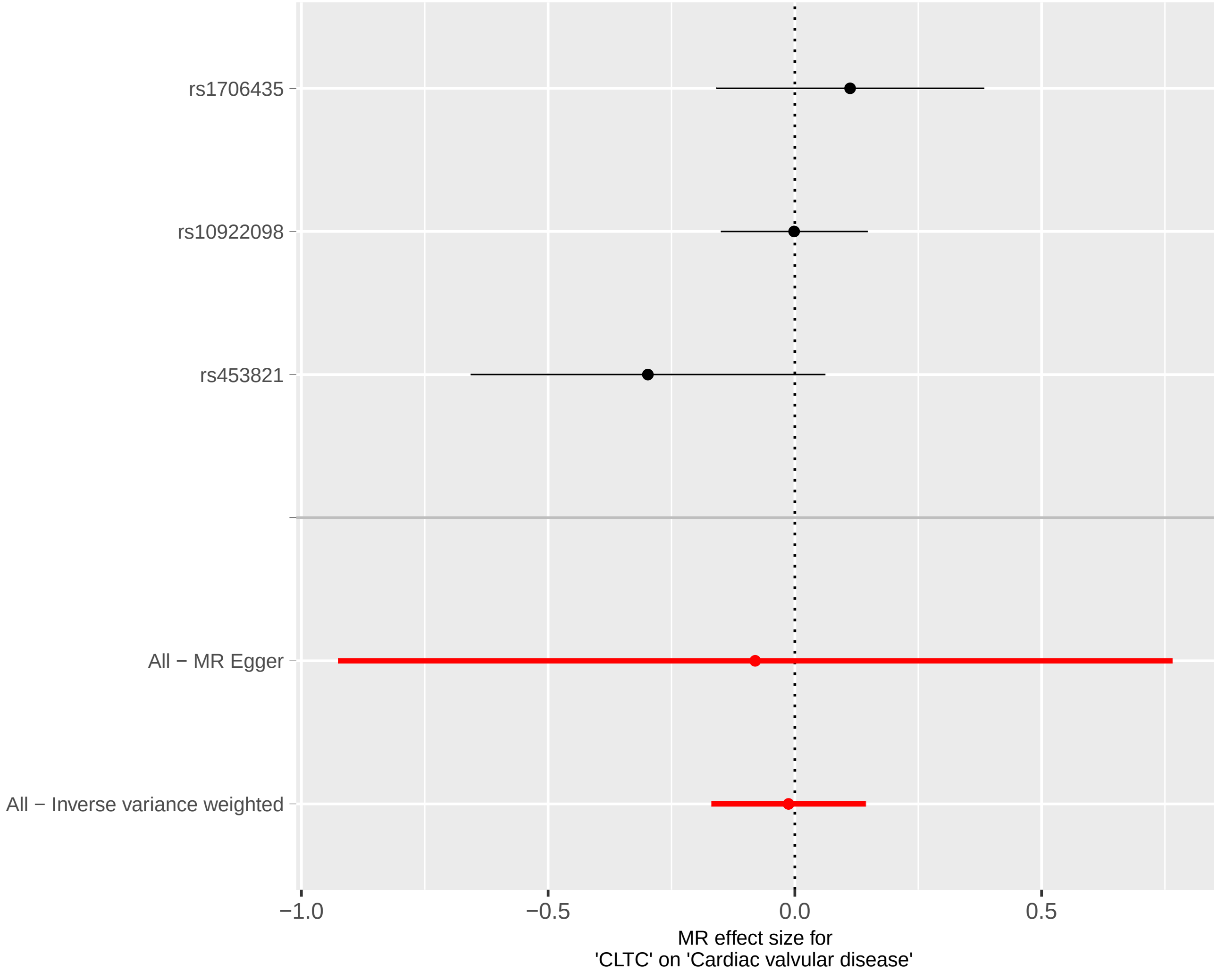

rs1706435
rs10922098
rs453821
All − MR Egger
All − Inverse variance weighted
−1.0
−0.5
0.0
0.5
MR effect size for
'CLTC' on 'Cardiac valvular disease'

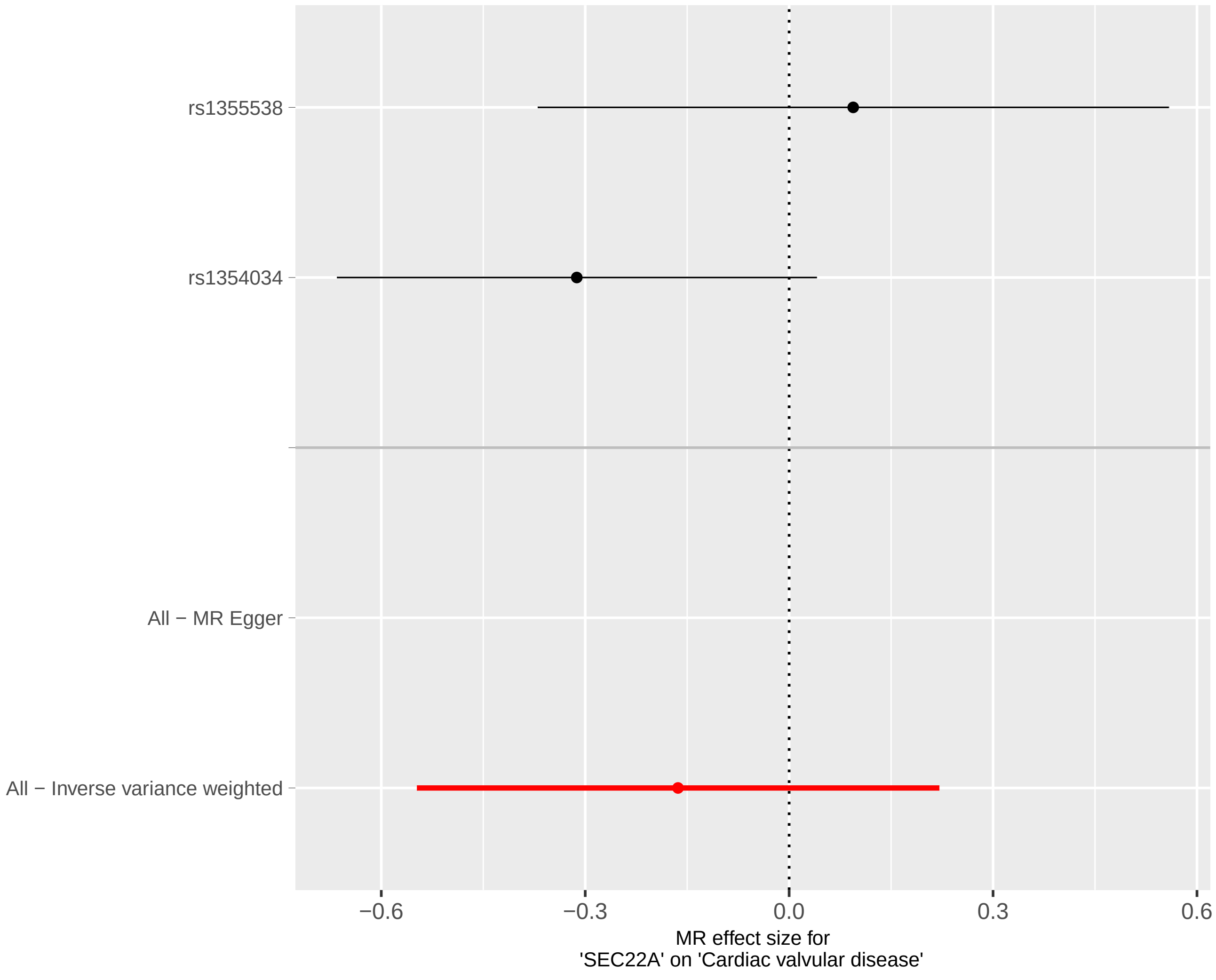
rs1355538
rs1354034
All − MR Egger
All − Inverse variance weighted
−0.6
−0.3
0.0
0.3
0.6
MR effect size for
'SEC22A' on 'Cardiac valvular disease'

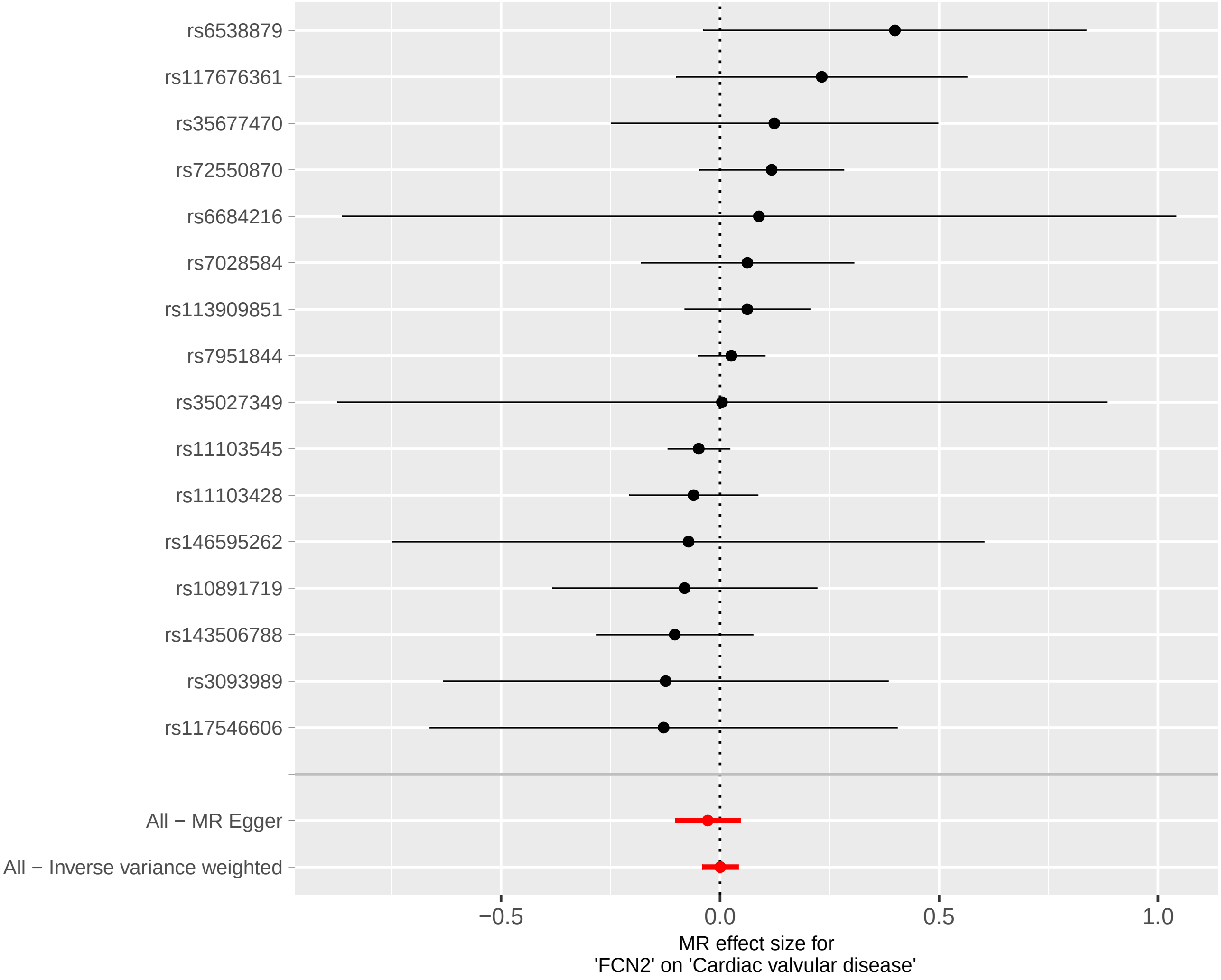
rs6538879
rs117676361
rs35677470
rs72550870
rs6684216
rs7028584
rs113909851
rs7951844
rs35027349
rs11103545
rs11103428
rs146595262
rs10891719
rs143506788
rs3093989
rs117546606
All − MR Egger
All − Inverse variance weighted
−0.5
0.0
0.5
1.0
MR effect size for
'FCN2' on 'Cardiac valvular disease'

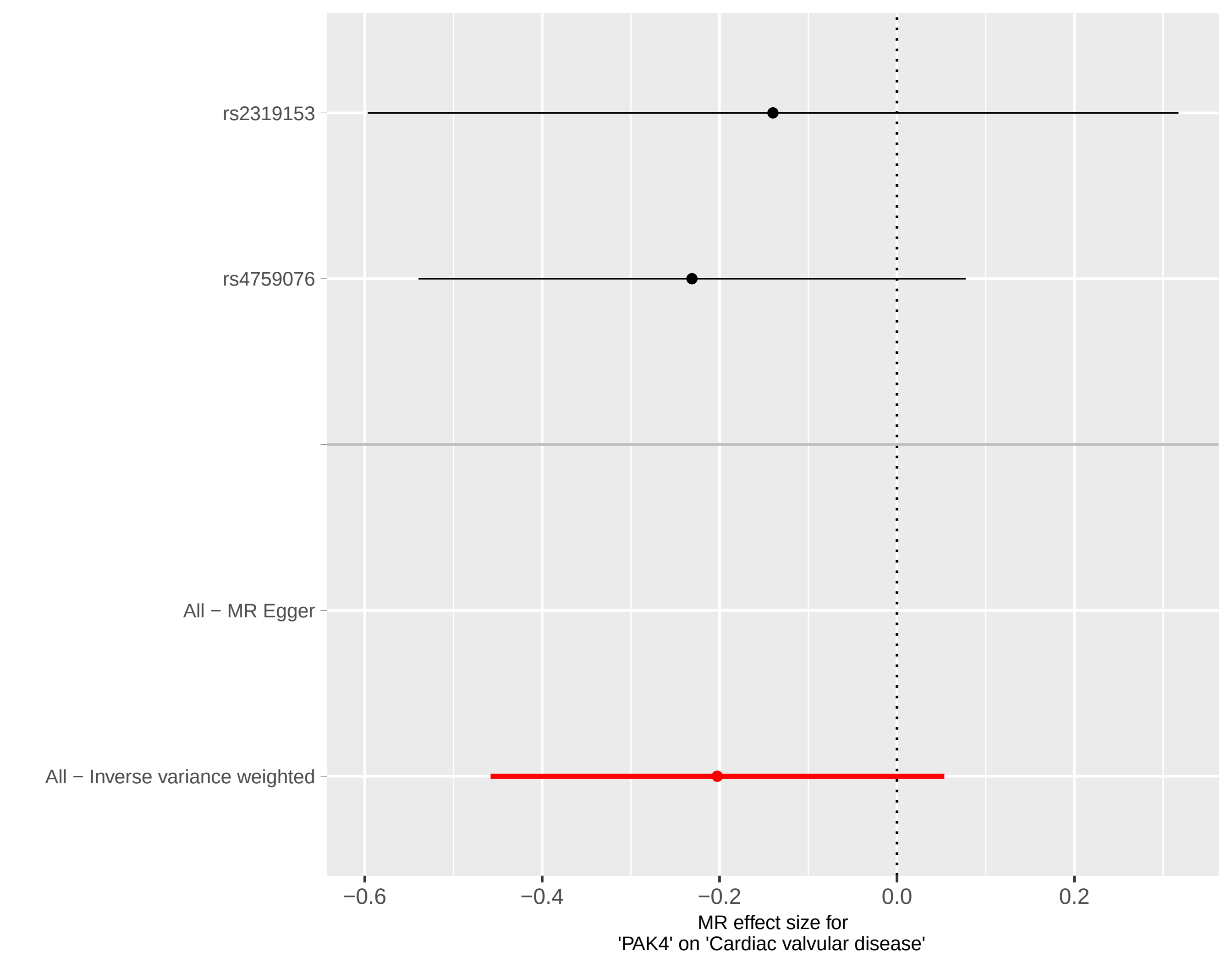

rs2319153
rs4759076
All − MR Egger
All − Inverse variance weighted
−0.6
−0.4
−0.2
0.0
0.2
MR effect size for
'PAK4' on 'Cardiac valvular disease'

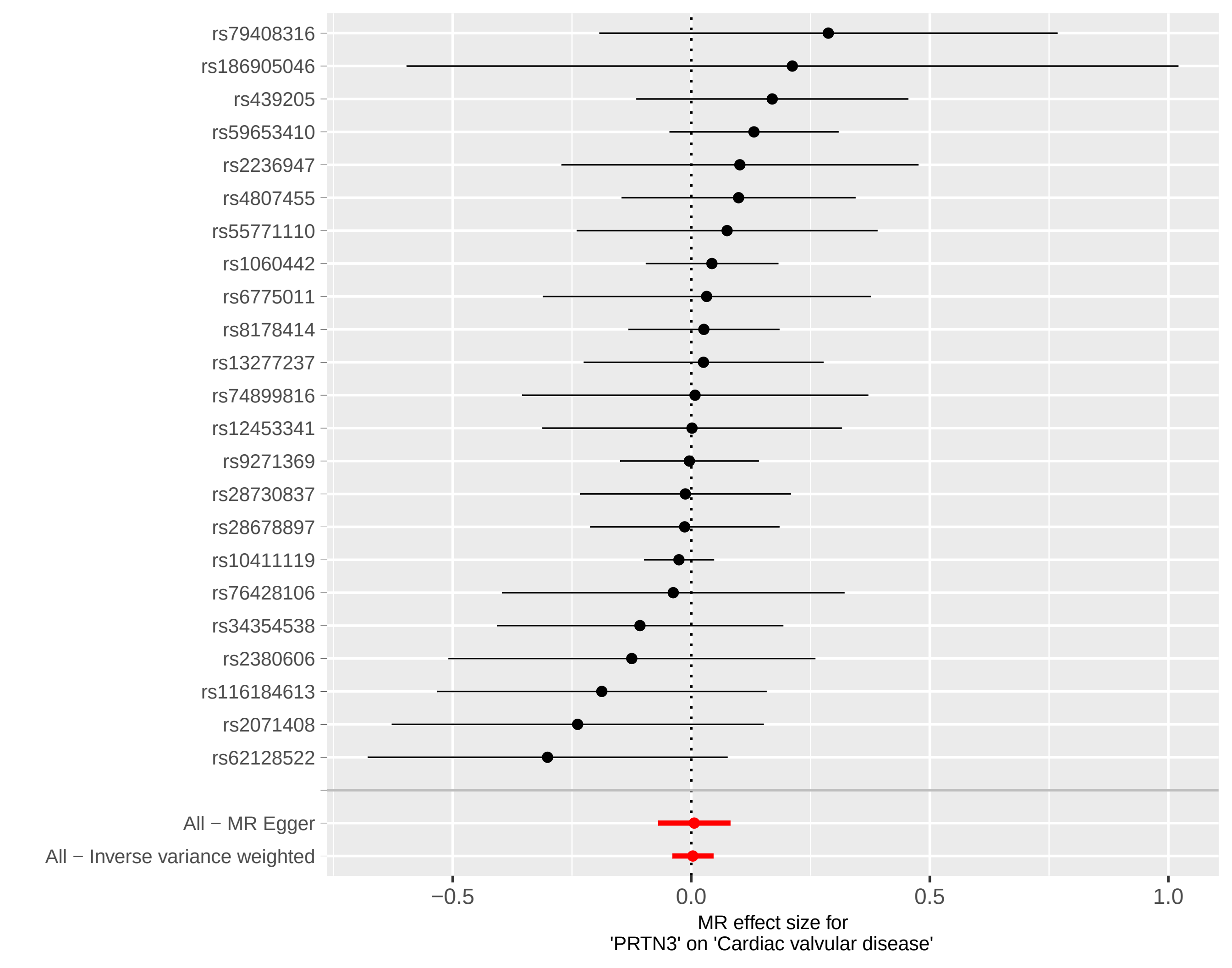
rs79408316
rs186905046
rs439205
rs59653410
rs2236947
rs4807455
rs55771110
rs1060442
rs6775011
rs8178414
rs13277237
rs74899816
rs12453341
rs9271369
rs28730837
rs28678897
rs10411119
rs76428106
rs34354538
rs2380606
rs116184613
rs2071408
rs62128522
All − MR Egger
All − Inverse variance weighted
−0.5
0.0
0.5
1.0
MR effect size for
'PRTN3' on 'Cardiac valvular disease'

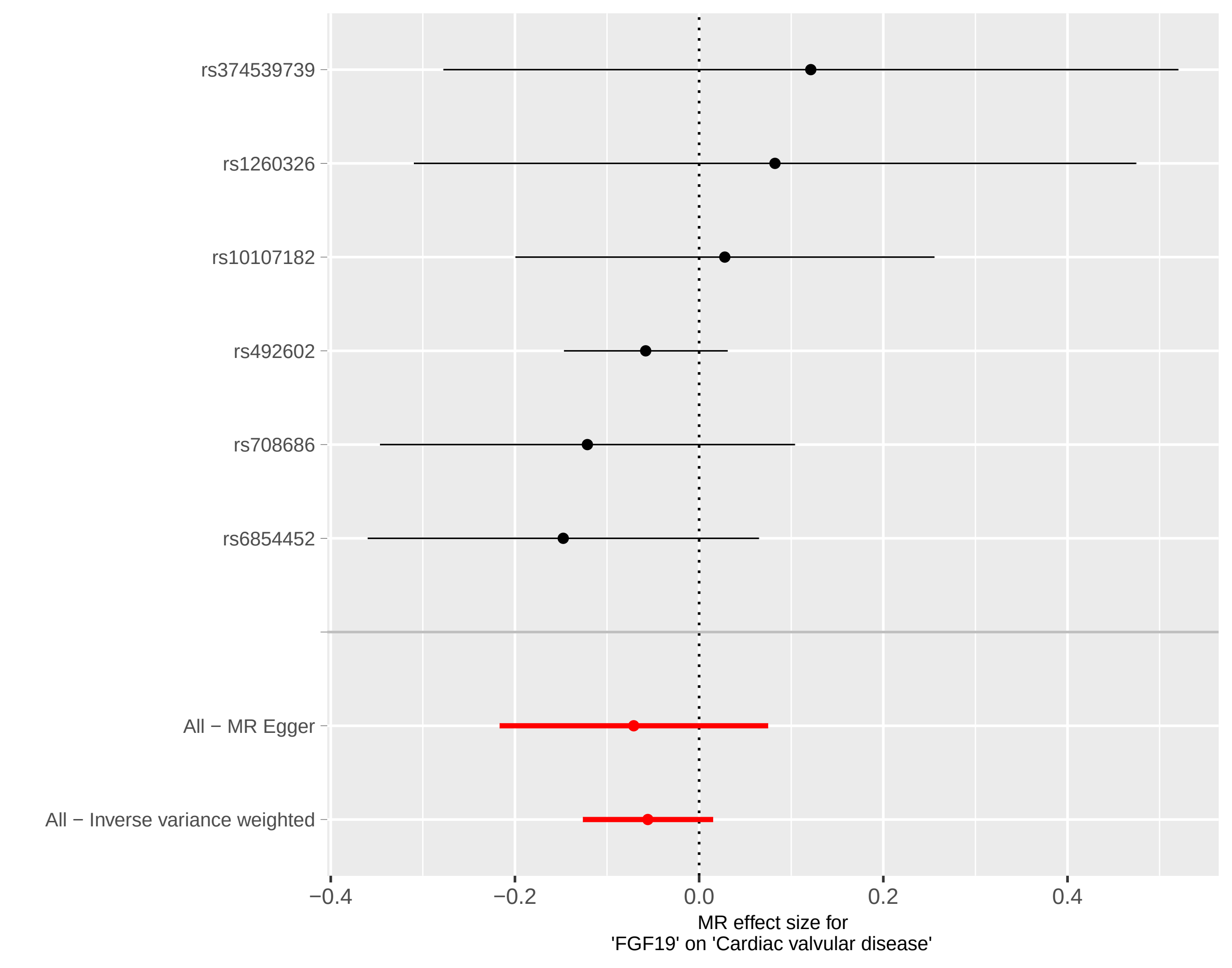

rs374539739
rs1260326
rs10107182
rs492602
rs708686
rs6854452
All − MR Egger
All − Inverse variance weighted
−0.4
−0.2
0.0
0.2
0.4
MR effect size for
'FGF19' on 'Cardiac valvular disease'

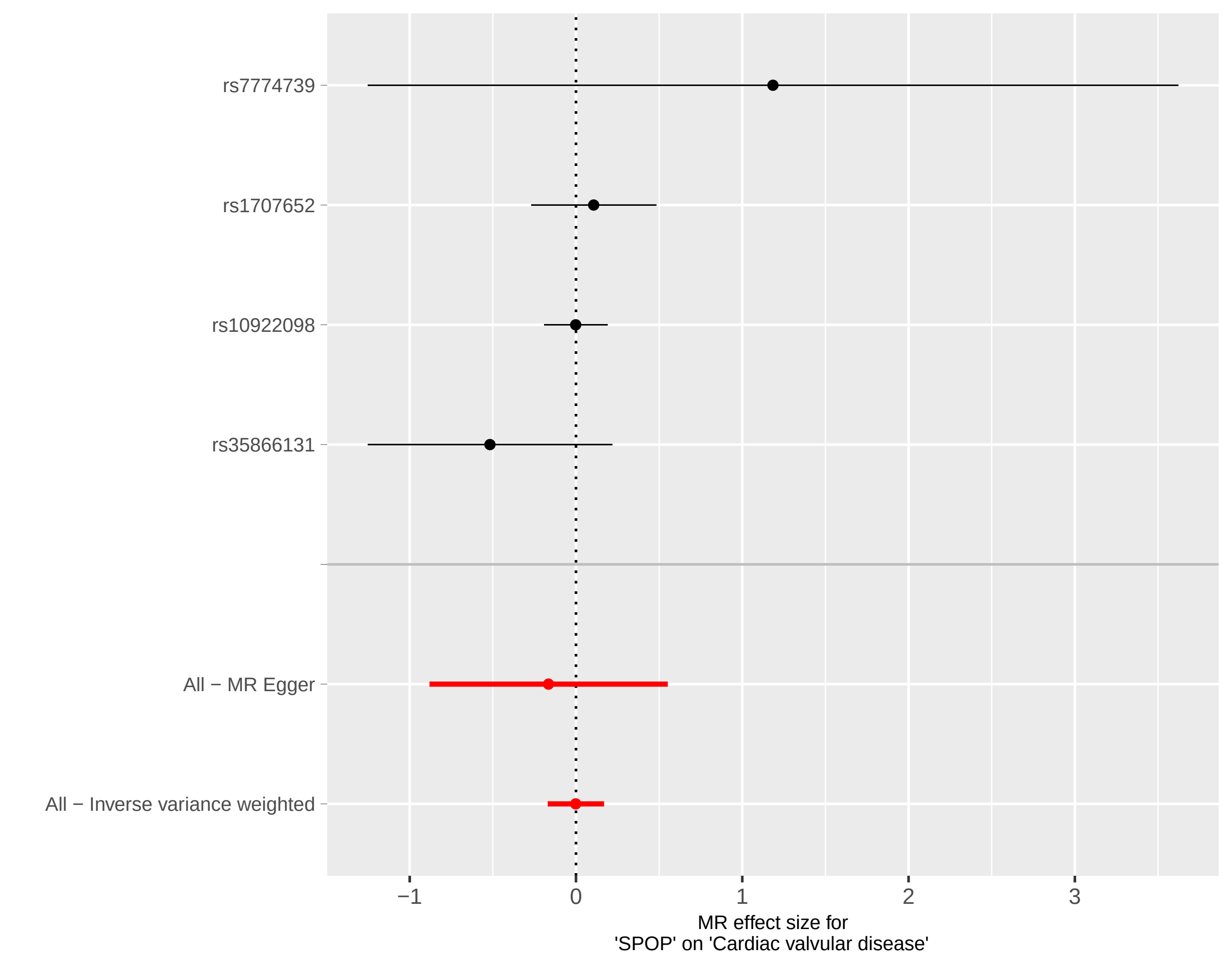

rs7774739
rs1707652
rs10922098
rs35866131
All − MR Egger
All − Inverse variance weighted
−1
0
1
2
3
MR effect size for
'SPOP' on 'Cardiac valvular disease'

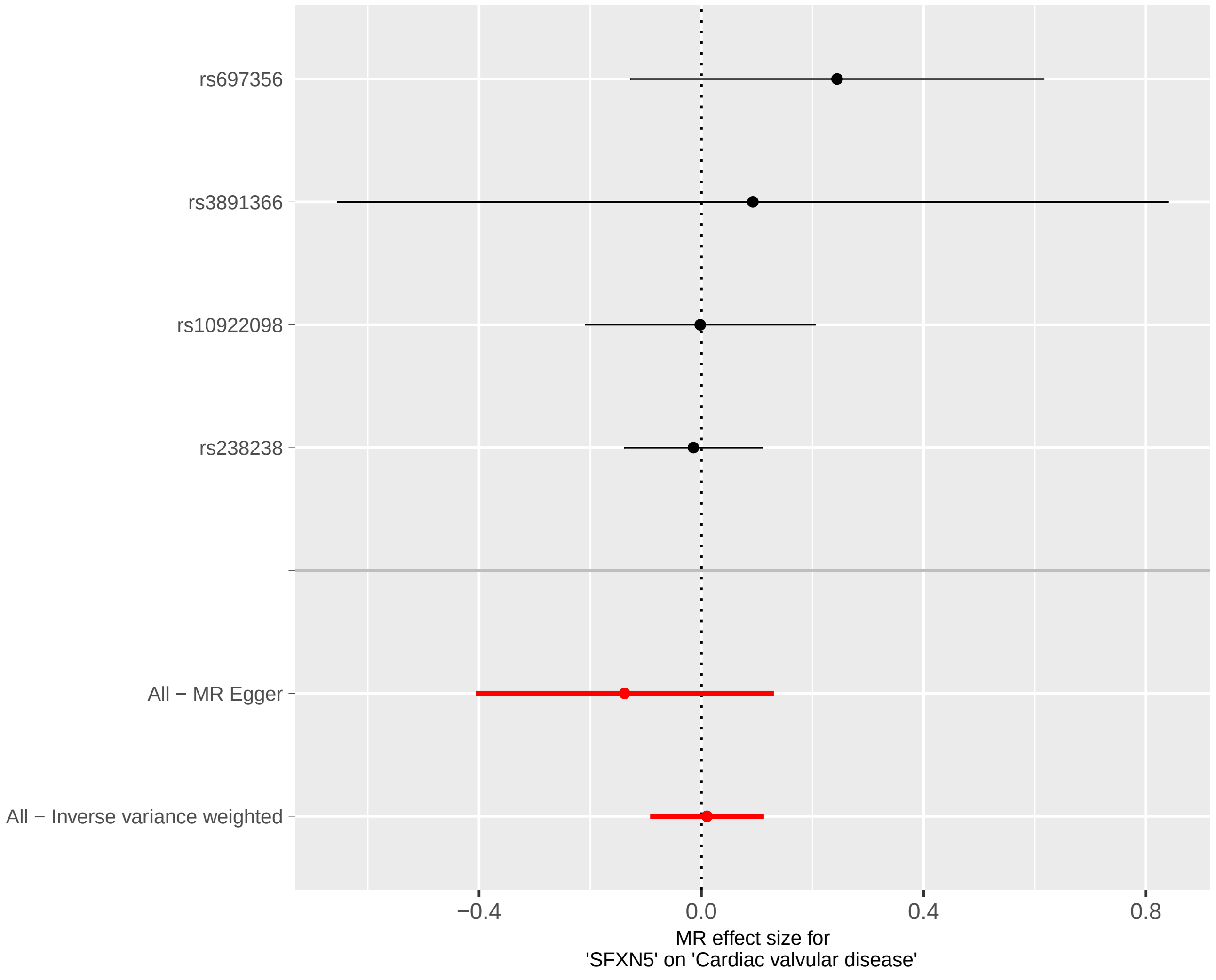
rs697356
rs3891366
rs10922098
rs238238
All − MR Egger
All − Inverse variance weighted
−0.4
0.0
0.4
0.8
MR effect size for
'SFXN5' on 'Cardiac valvular disease'

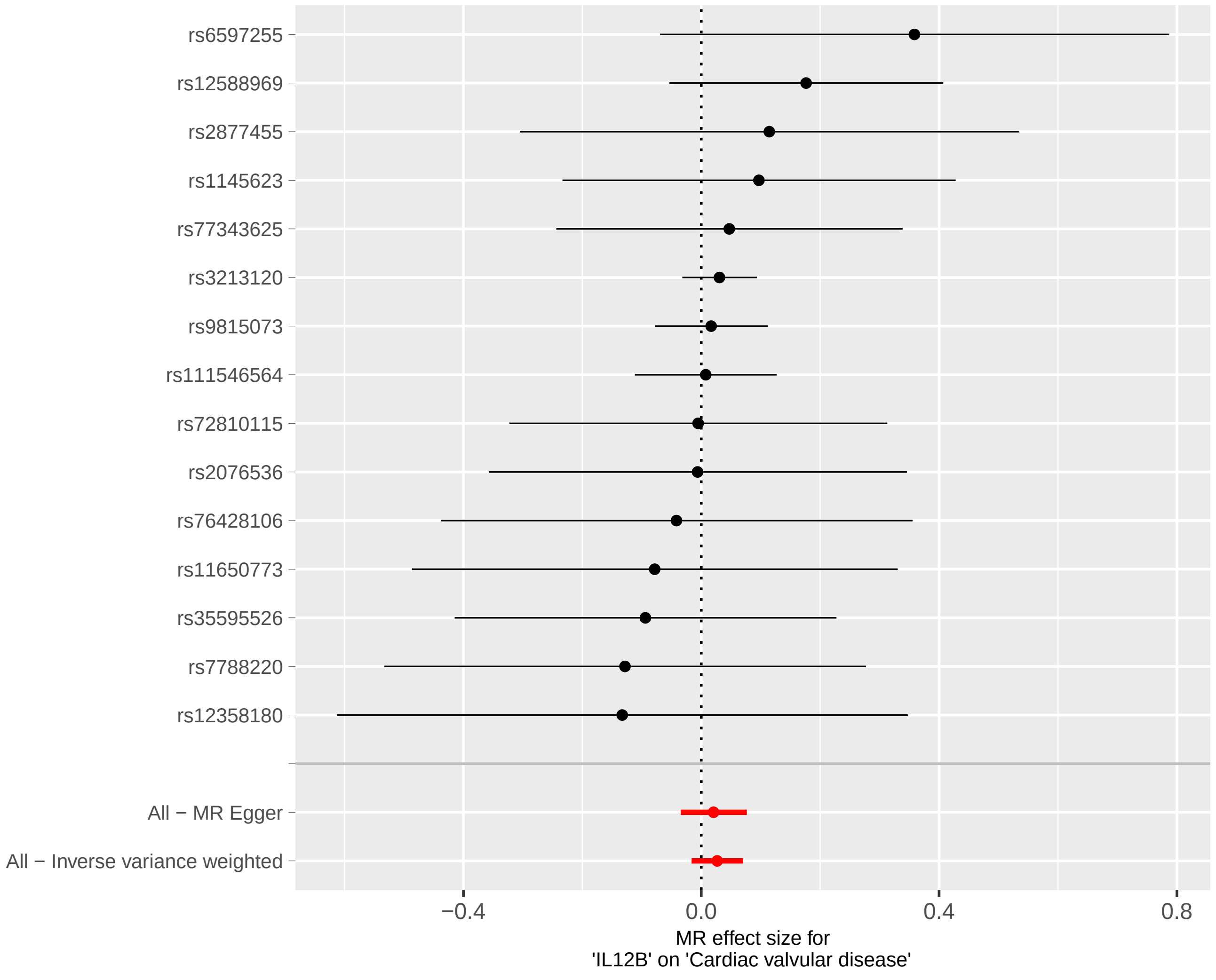
rs6597255
rs12588969
rs2877455
rs1145623
rs77343625
rs3213120
rs9815073
rs111546564
rs72810115
rs2076536
rs76428106
rs11650773
rs35595526
rs7788220
rs12358180
All − MR Egger
All − Inverse variance weighted
−0.4
0.0
0.4
0.8
MR effect size for
'IL12B' on 'Cardiac valvular disease'

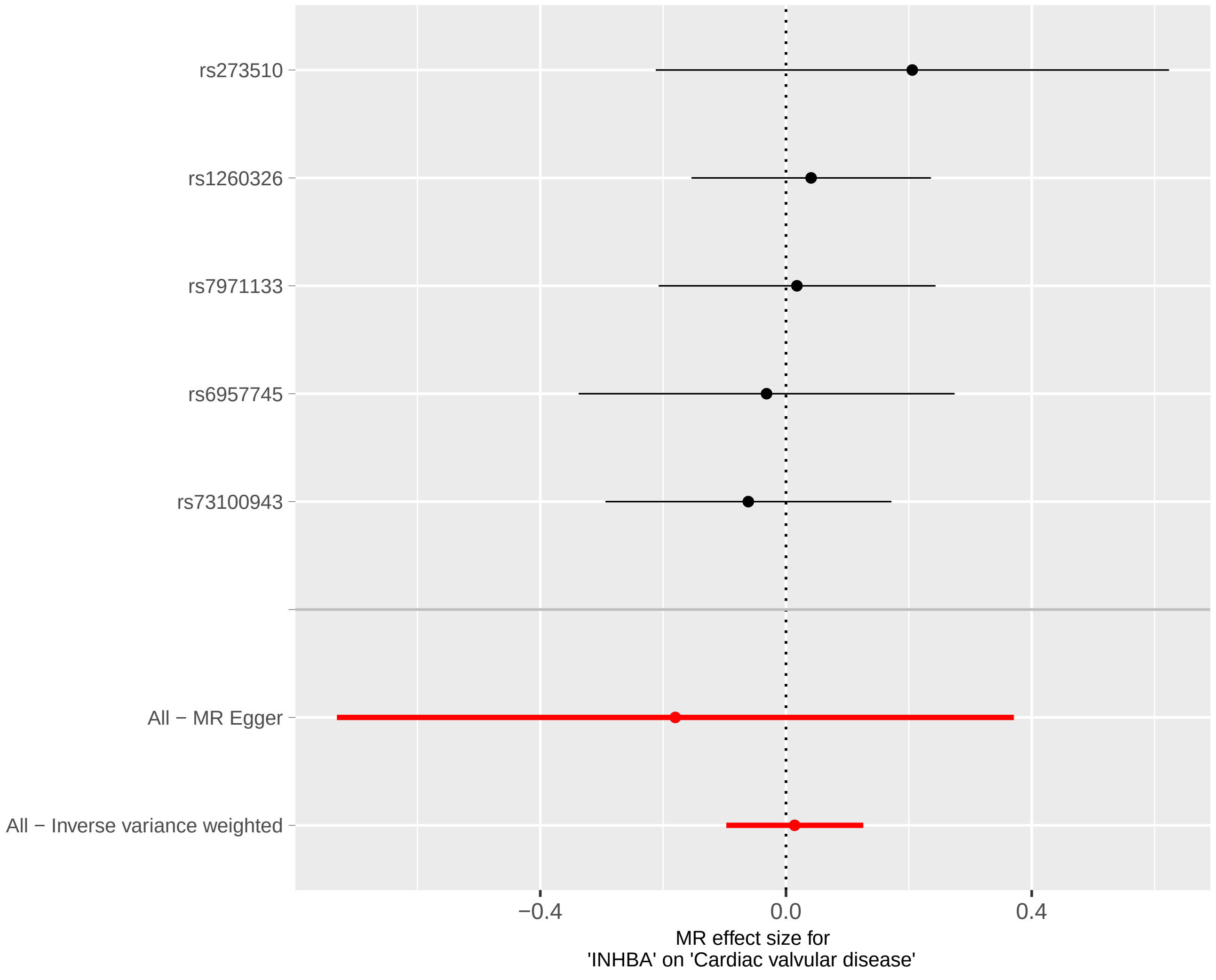
rs273510
rs1260326
rs7971133
rs6957745
rs73100943
All − MR Egger
All − Inverse variance weighted
−0.4
0.0
0.4
MR effect size for
'INHBA' on 'Cardiac valvular disease'

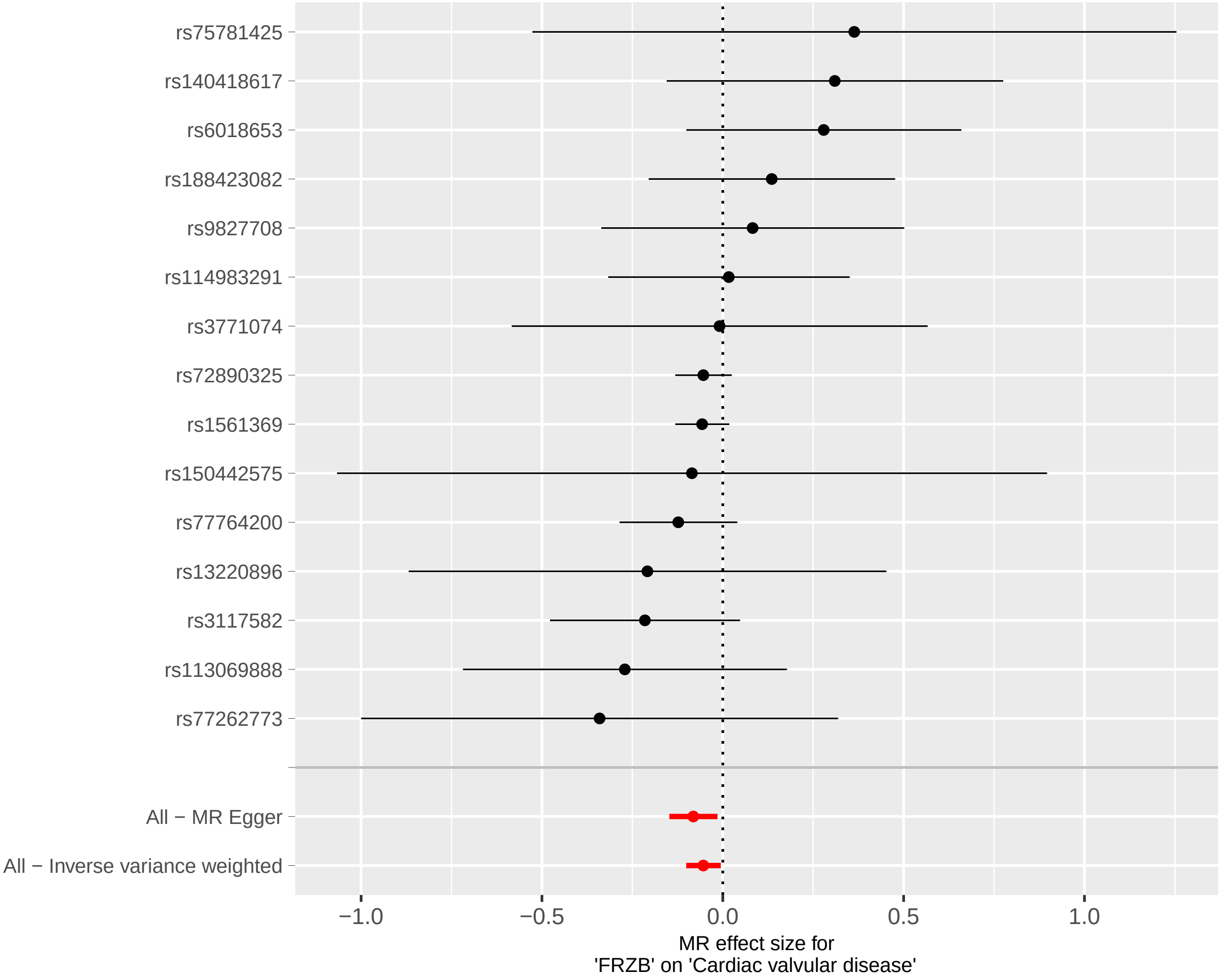
rs75781425
rs140418617
rs6018653
rs188423082
rs9827708
rs114983291
rs3771074
rs72890325
rs1561369
rs150442575
rs77764200
rs13220896
rs3117582
rs113069888
rs77262773
All − MR Egger
All − Inverse variance weighted
−1.0
−0.5
0.0
0.5
1.0
MR effect size for
'FRZB' on 'Cardiac valvular disease'

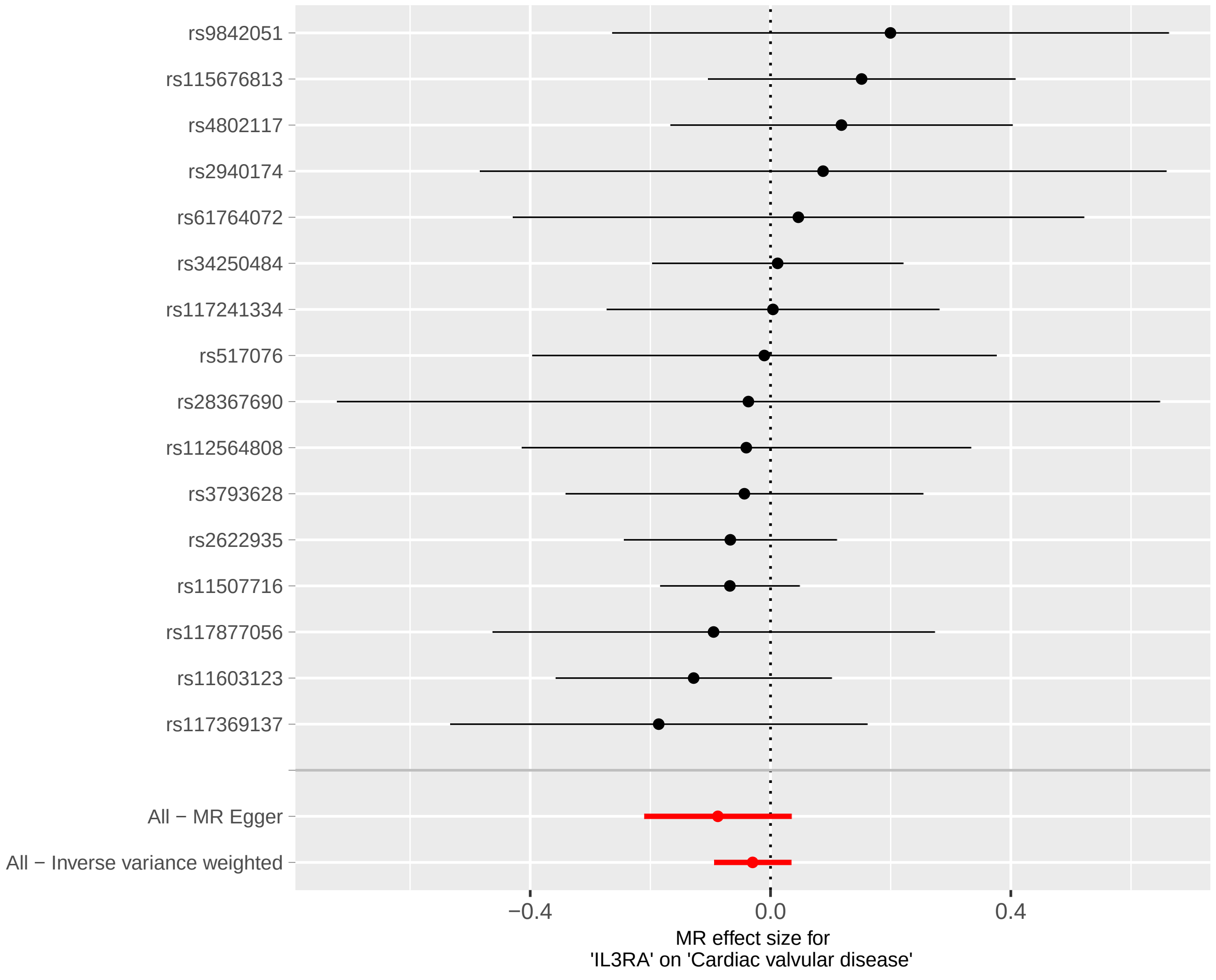

rs9842051
rs115676813
rs4802117
rs2940174
rs61764072
rs34250484
rs117241334
rs517076
rs28367690
rs112564808
rs3793628
rs2622935
rs11507716
rs117877056
rs11603123
rs117369137
All − MR Egger
All − Inverse variance weighted
−0.4
0.0
0.4
MR effect size for
'IL3RA' on 'Cardiac valvular disease'

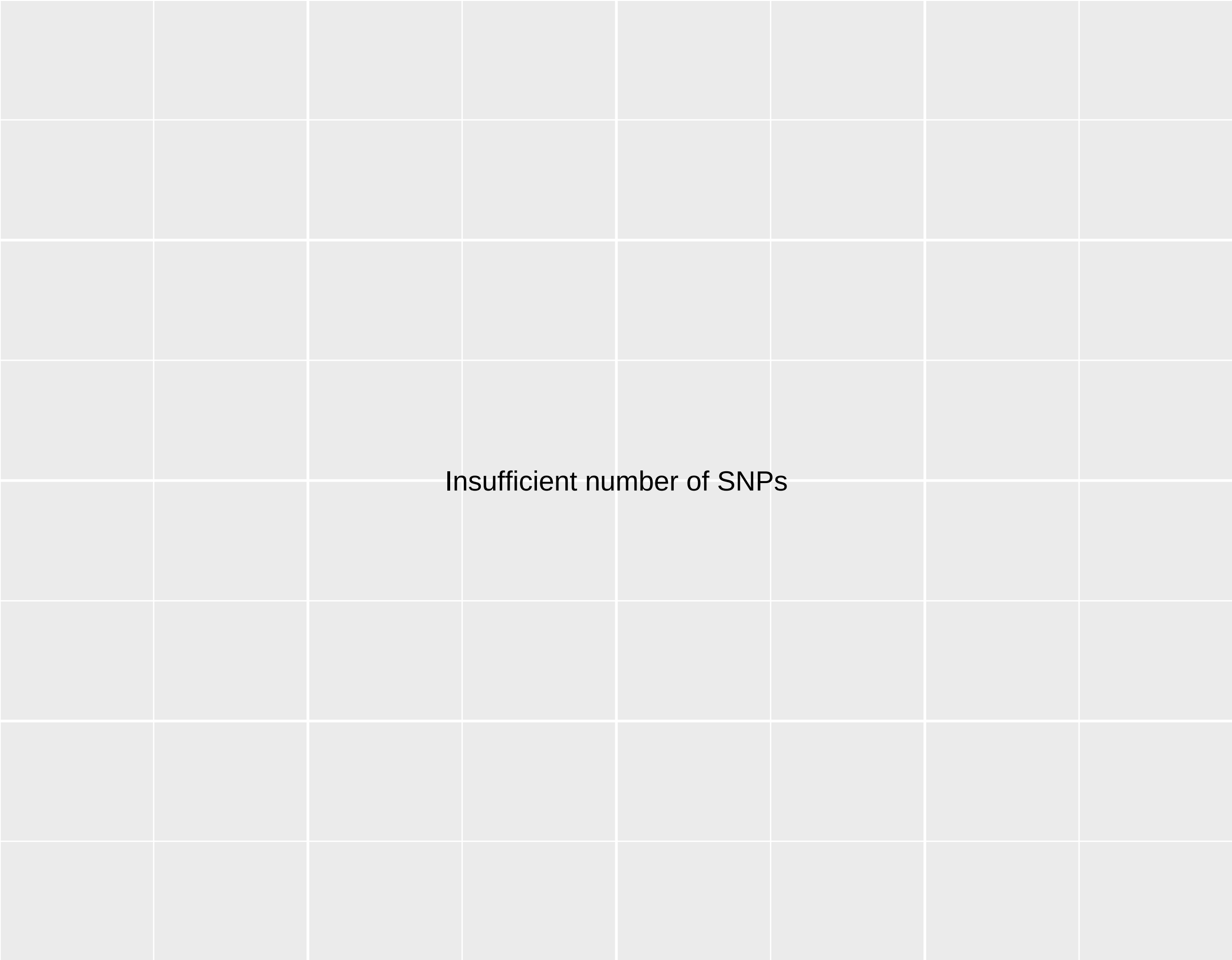
Insufficient number of SNPs

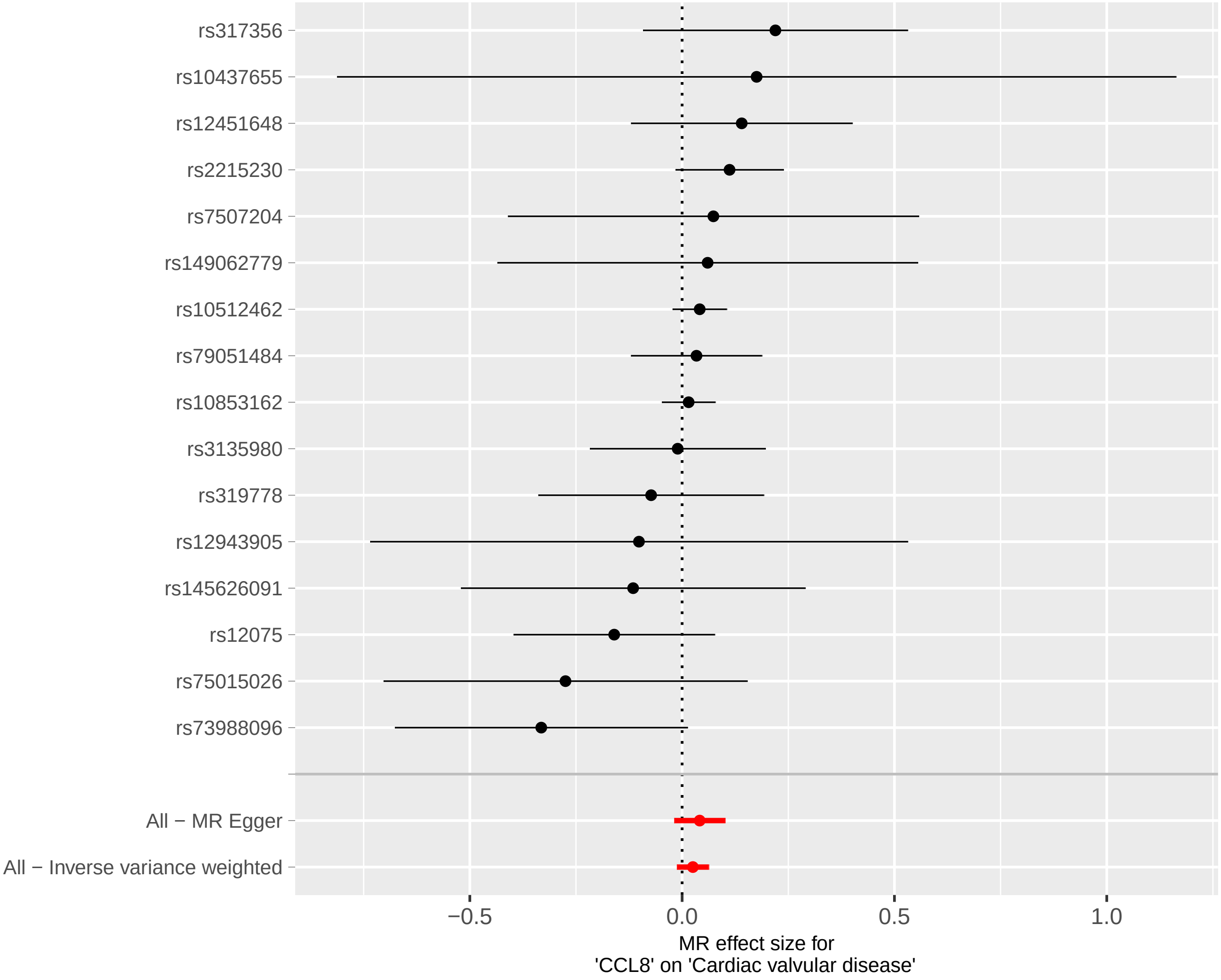
rs317356
rs10437655
rs12451648
rs2215230
rs7507204
rs149062779
rs10512462
rs79051484
rs10853162
rs3135980
rs319778
rs12943905
rs145626091
rs12075
rs75015026
rs73988096
All − MR Egger
All − Inverse variance weighted
−0.5
0.0
0.5
1.0
MR effect size for
'CCL8' on 'Cardiac valvular disease'

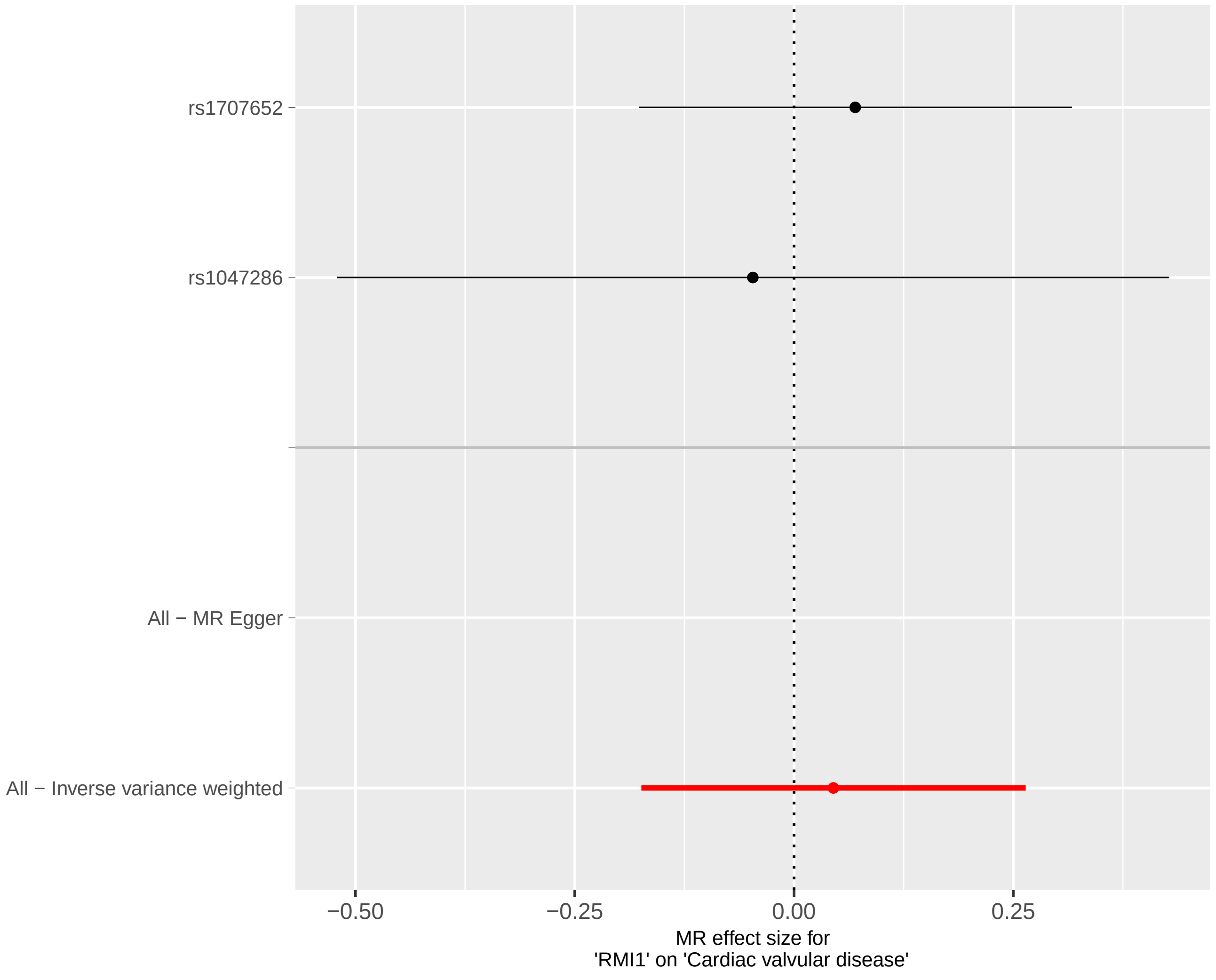
rs1707652
rs1047286
All − MR Egger
All − Inverse variance weighted
−0.50
−0.25
0.00
0.25
MR effect size for
'RMI1' on 'Cardiac valvular disease'

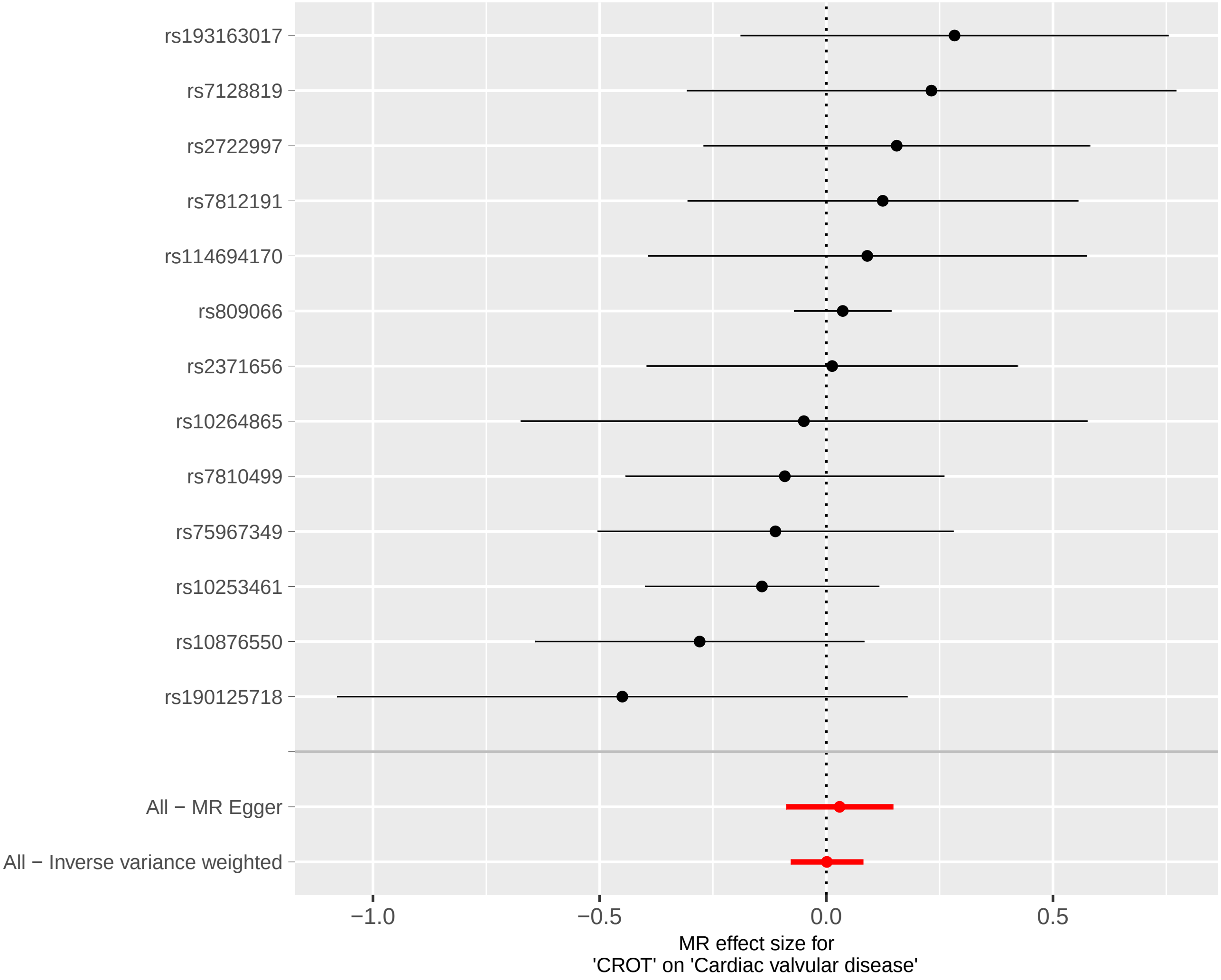
rs193163017
rs7128819
rs2722997
rs7812191
rs114694170
rs809066
rs2371656
rs10264865
rs7810499
rs75967349
rs10253461
rs10876550
rs190125718
All − MR Egger
All − Inverse variance weighted
−1.0
−0.5
0.0
0.5
MR effect size for
'CROT' on 'Cardiac valvular disease'

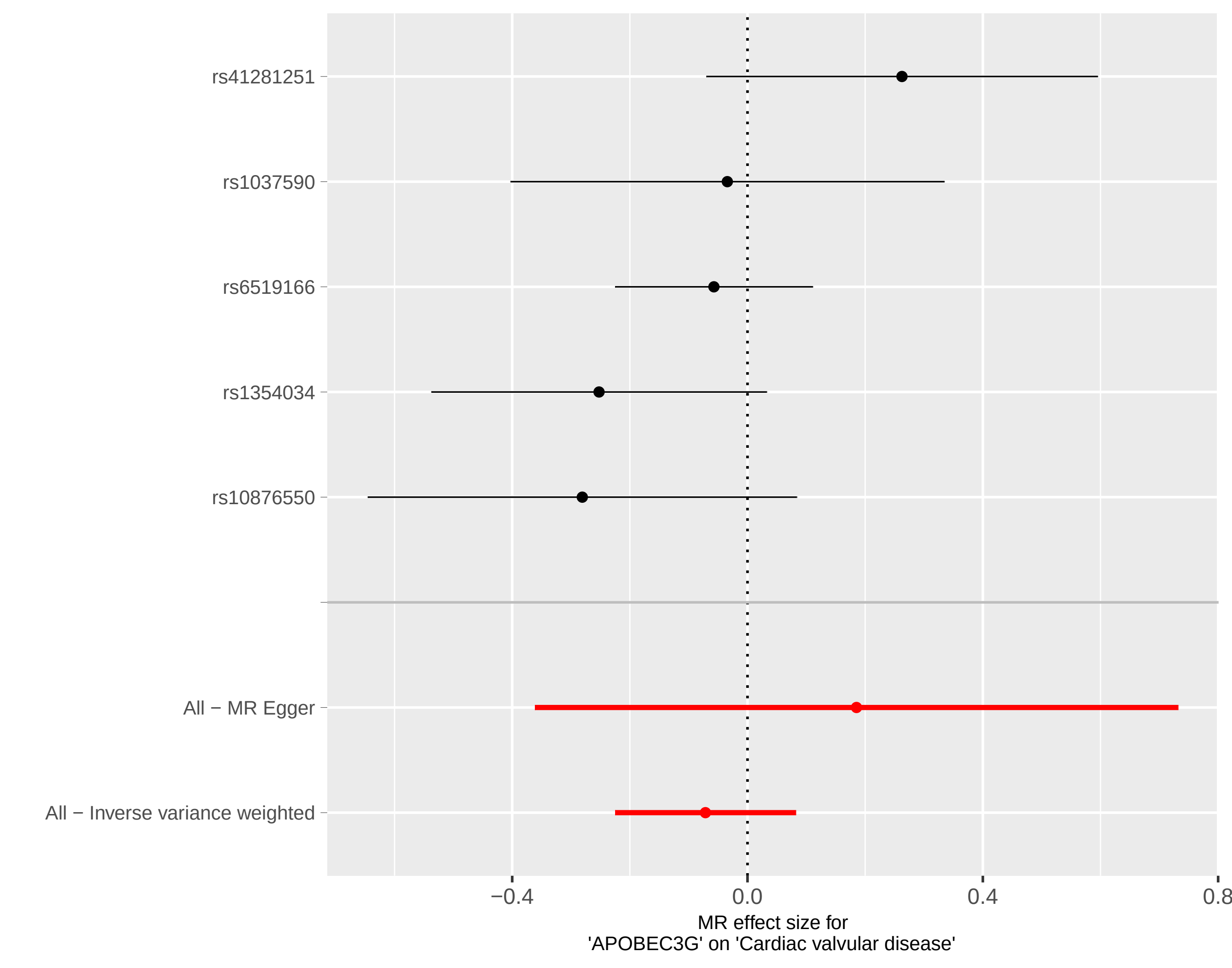

rs41281251
rs1037590
rs6519166
rs1354034
rs10876550
All − MR Egger
All − Inverse variance weighted
−0.4
0.0
0.4
0.8
MR effect size for
'APOBEC3G' on 'Cardiac valvular disease'

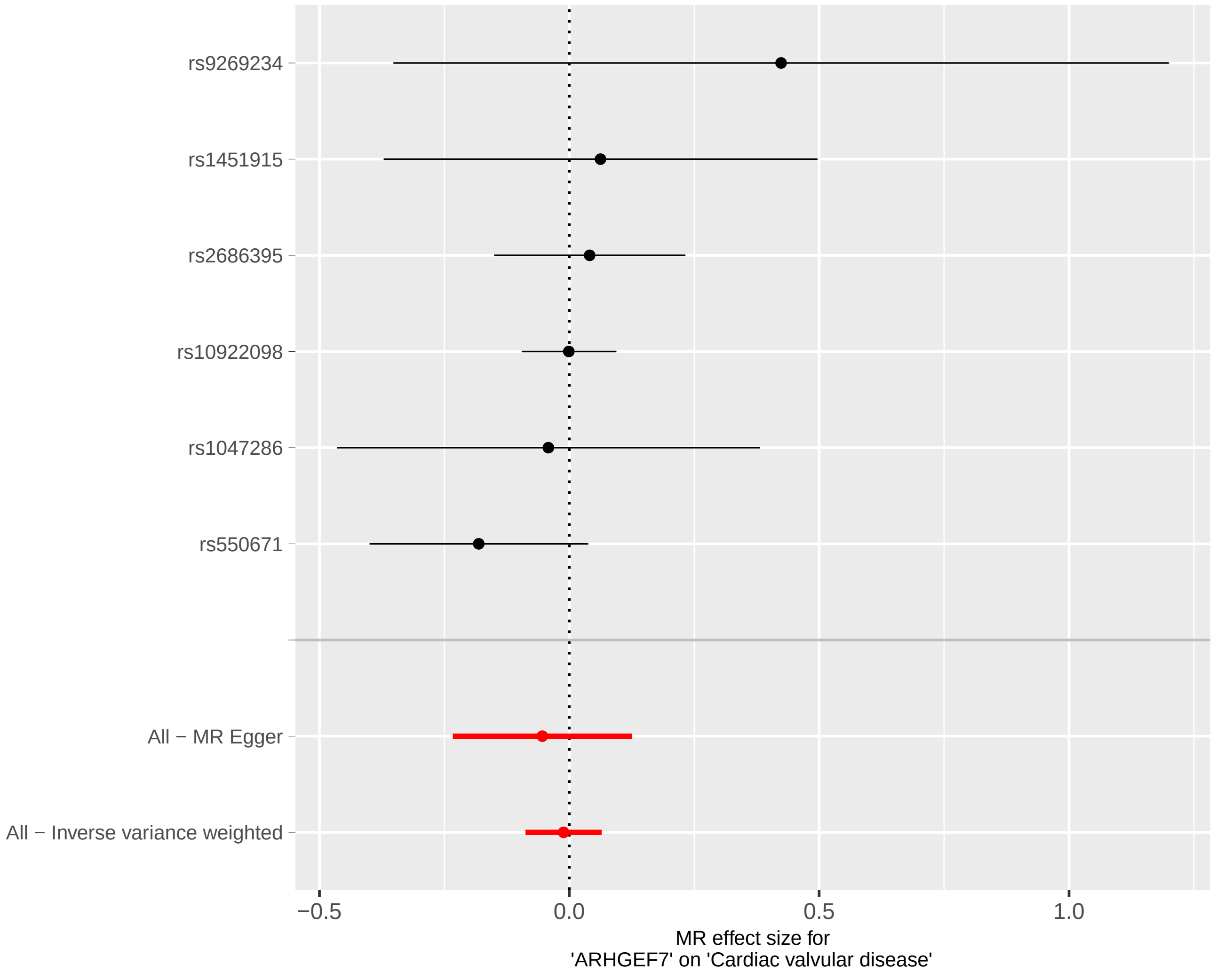
rs9269234
rs1451915
rs2686395
rs10922098
rs1047286
rs550671
All − MR Egger
All − Inverse variance weighted
−0.5
0.0
0.5
1.0
MR effect size for
'ARHGEF7' on 'Cardiac valvular disease'

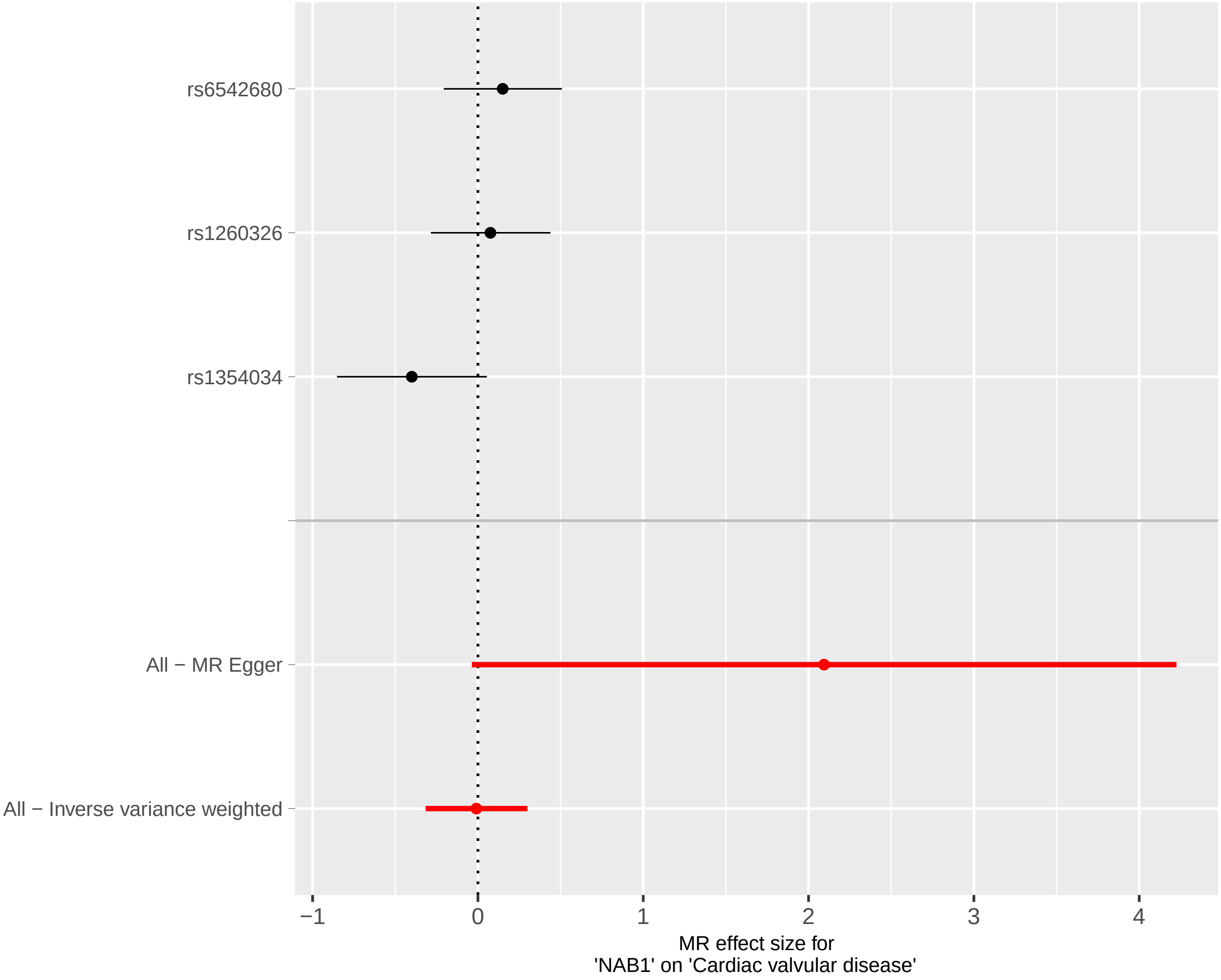
rs6542680
rs1260326
rs1354034
All − MR Egger
All − Inverse variance weighted
−1
0
1
2
3
4
MR effect size for
'NAB1' on 'Cardiac valvular disease'

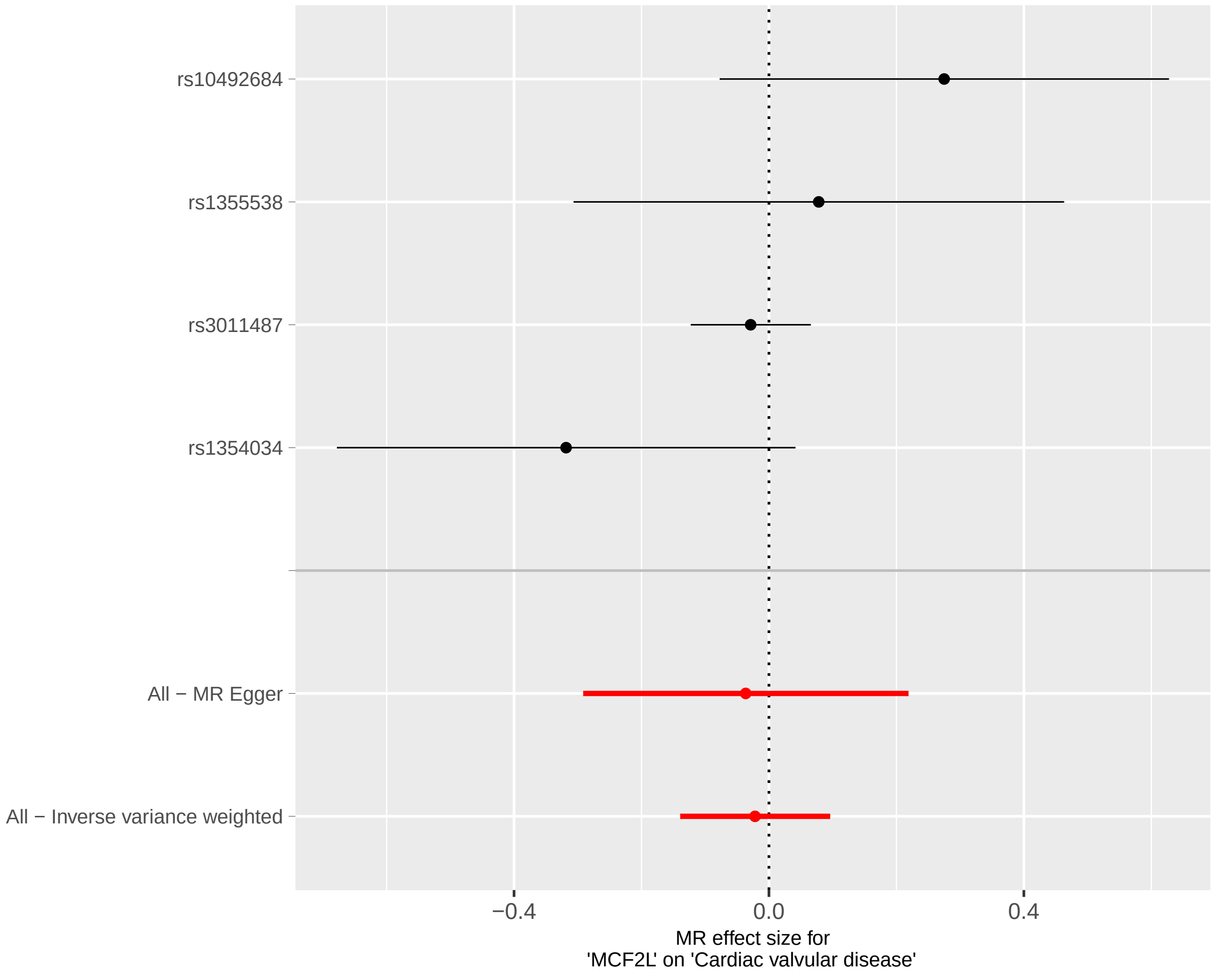
rs10492684
rs1355538
rs3011487
rs1354034
All − MR Egger
All − Inverse variance weighted
−0.4
0.0
0.4
MR effect size for
'MCF2L' on 'Cardiac valvular disease'

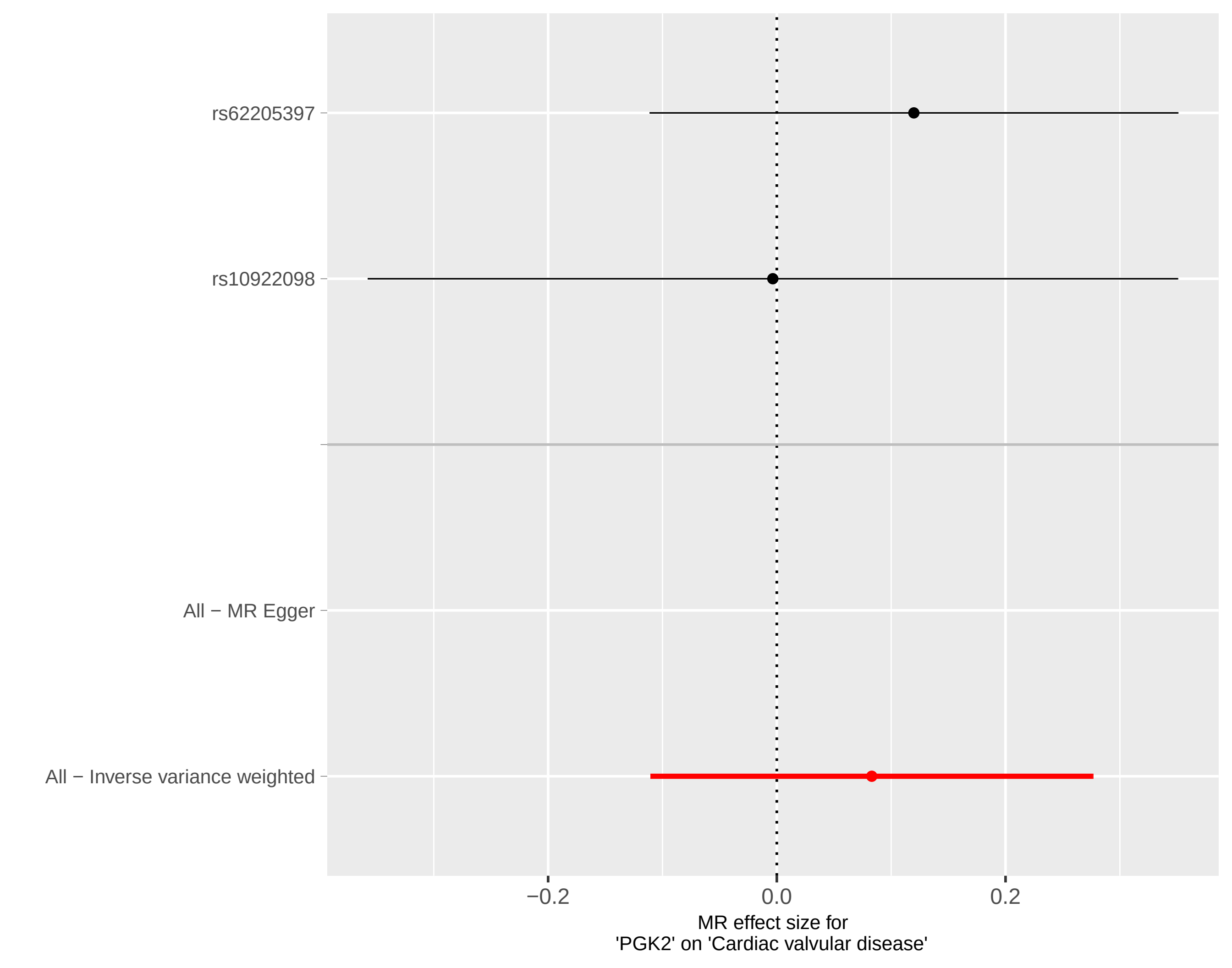
rs62205397
rs10922098
All − MR Egger
All − Inverse variance weighted
−0.2
0.0
0.2
MR effect size for
'PGK2' on 'Cardiac valvular disease'

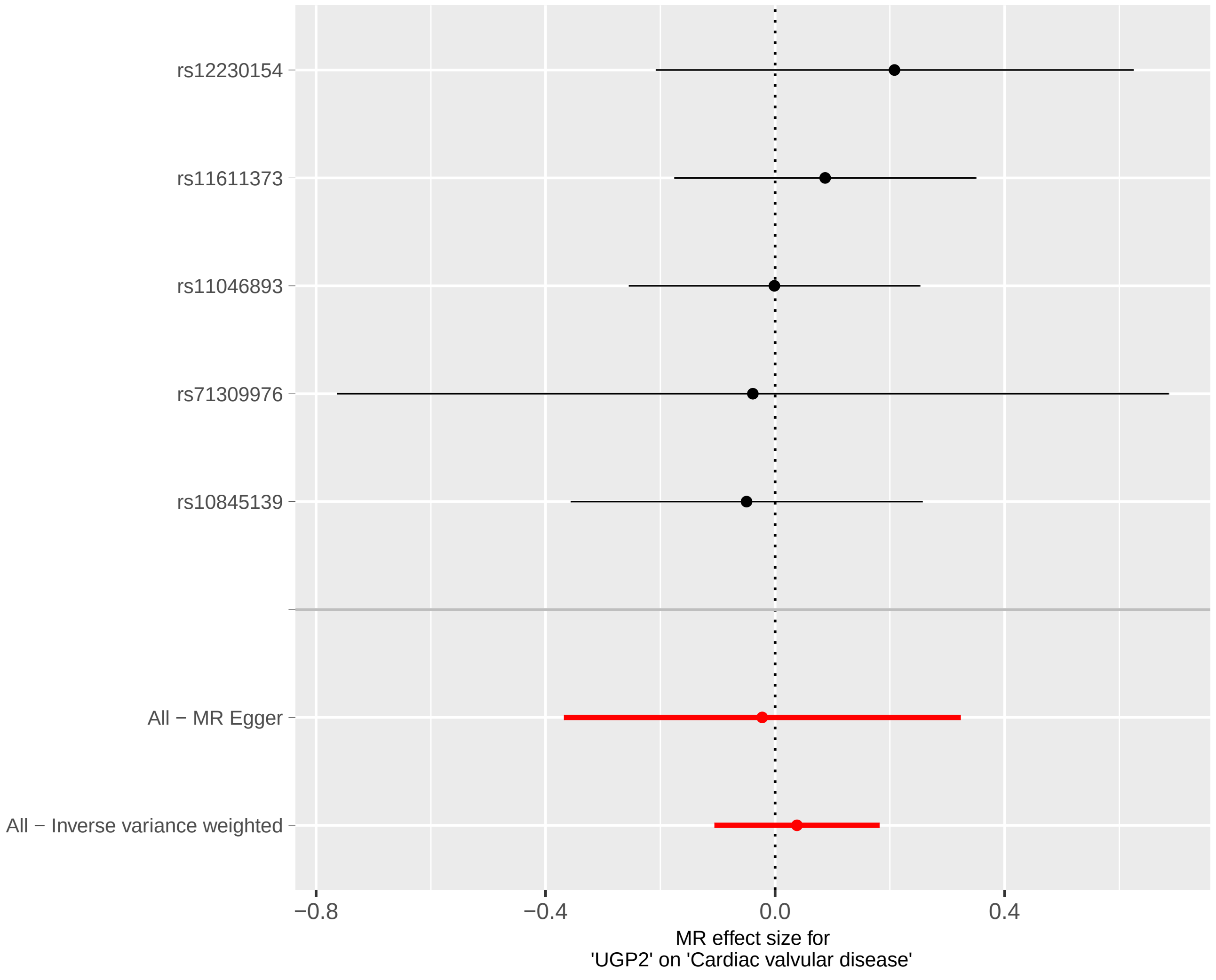
rs12230154
rs11611373
rs11046893
rs71309976
rs10845139
All − MR Egger
All − Inverse variance weighted
−0.8
−0.4
0.0
0.4
MR effect size for
'UGP2' on 'Cardiac valvular disease'

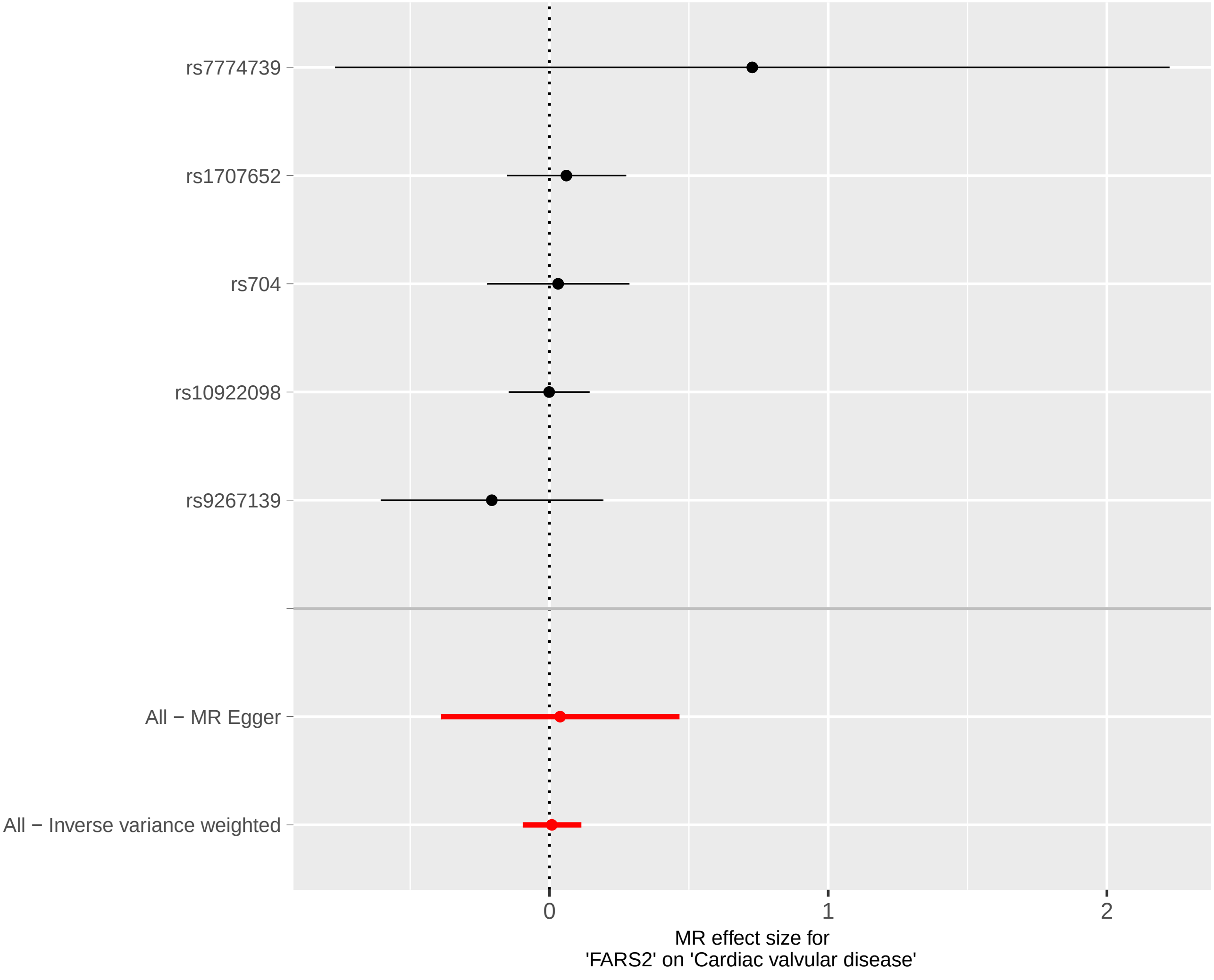
rs7774739
rs1707652
rs704
rs10922098
rs9267139
All − MR Egger
All − Inverse variance weighted
0
1
2
MR effect size for
'FARS2' on 'Cardiac valvular disease'

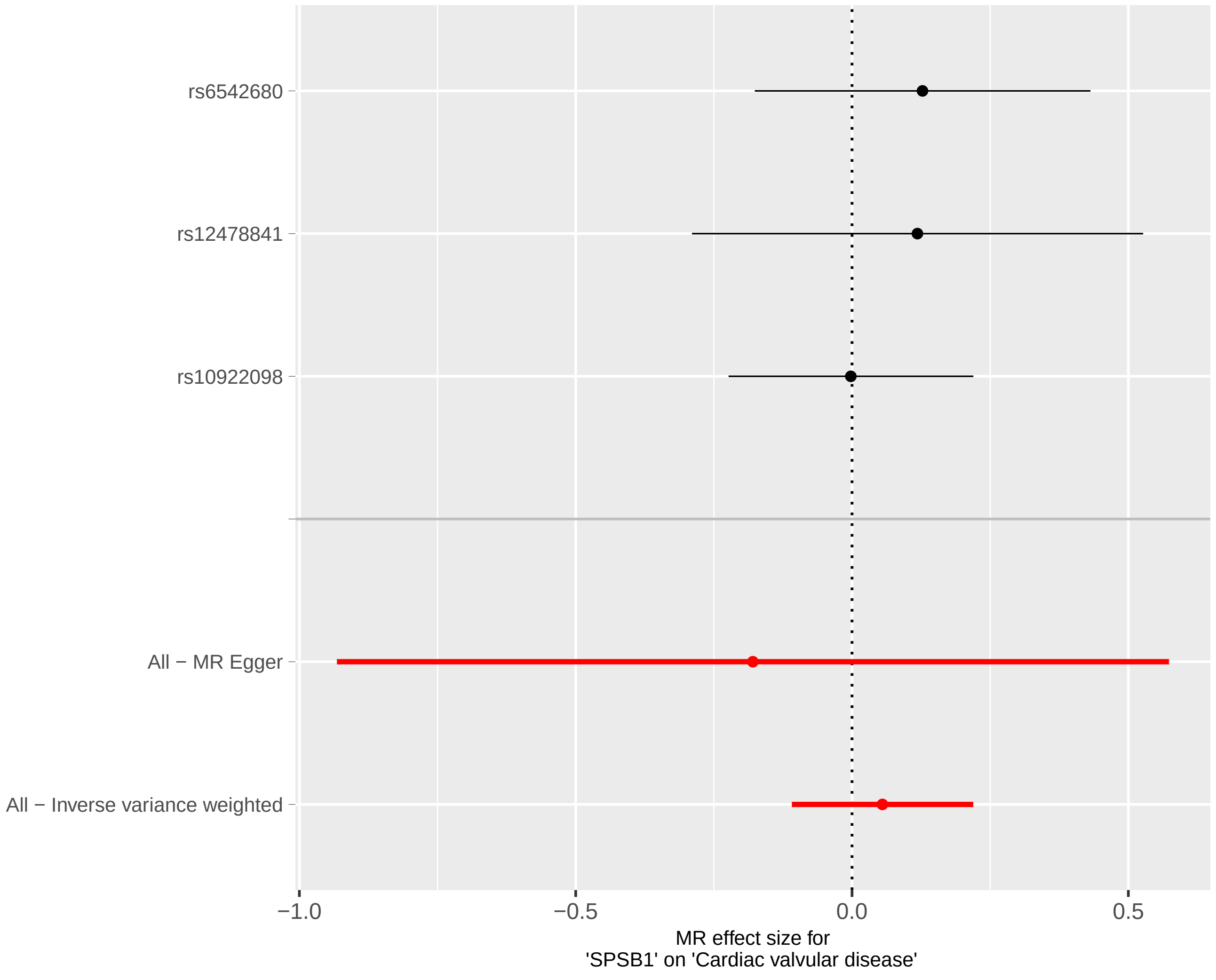
rs6542680
rs12478841
rs10922098
All − MR Egger
All − Inverse variance weighted
−1.0
−0.5
0.0
0.5
MR effect size for
'SPSB1' on 'Cardiac valvular disease'

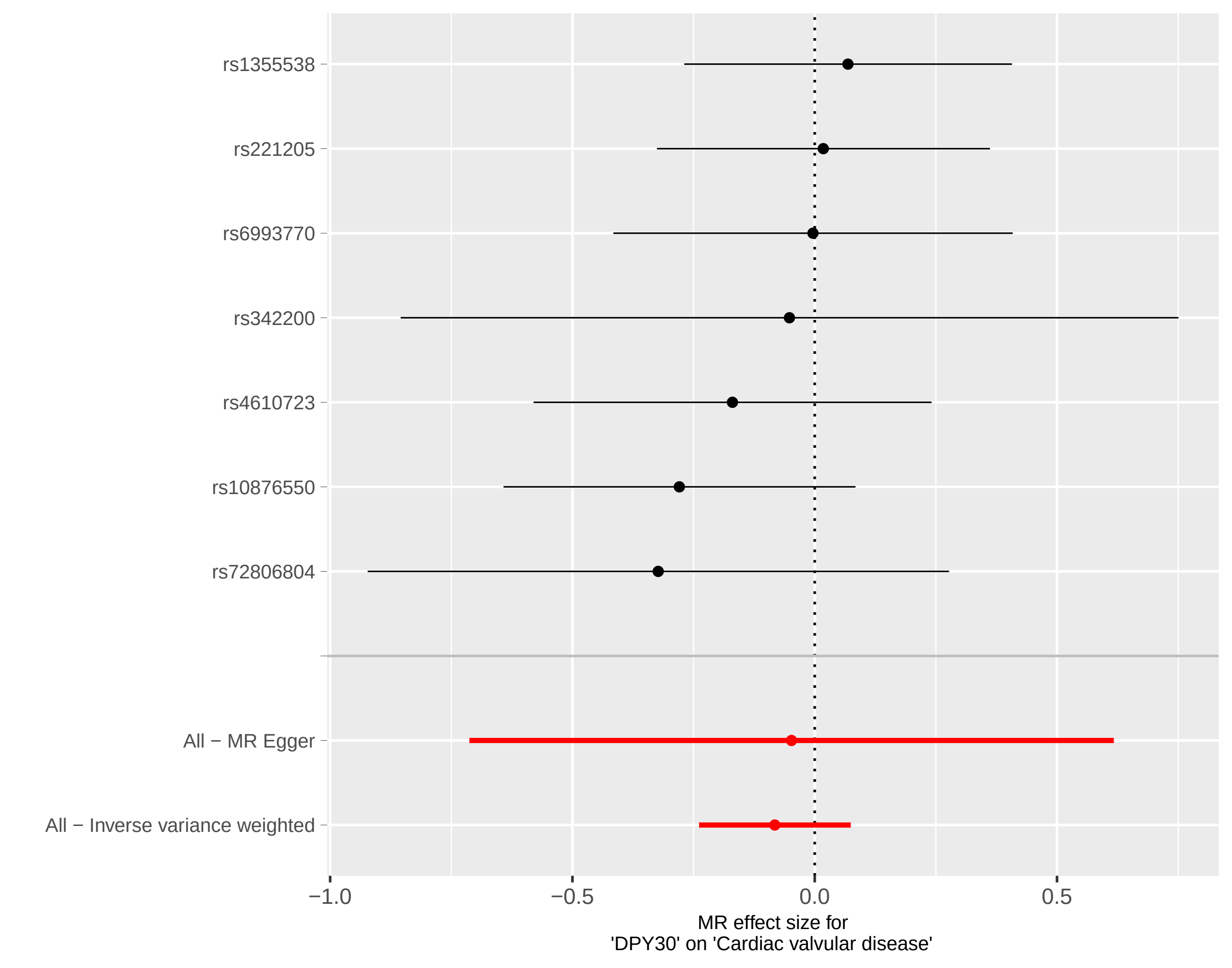

rs1355538
rs221205
rs6993770
rs342200
rs4610723
rs10876550
rs72806804
All − MR Egger
All − Inverse variance weighted
−1.0
−0.5
0.0
0.5
MR effect size for
'DPY30' on 'Cardiac valvular disease'

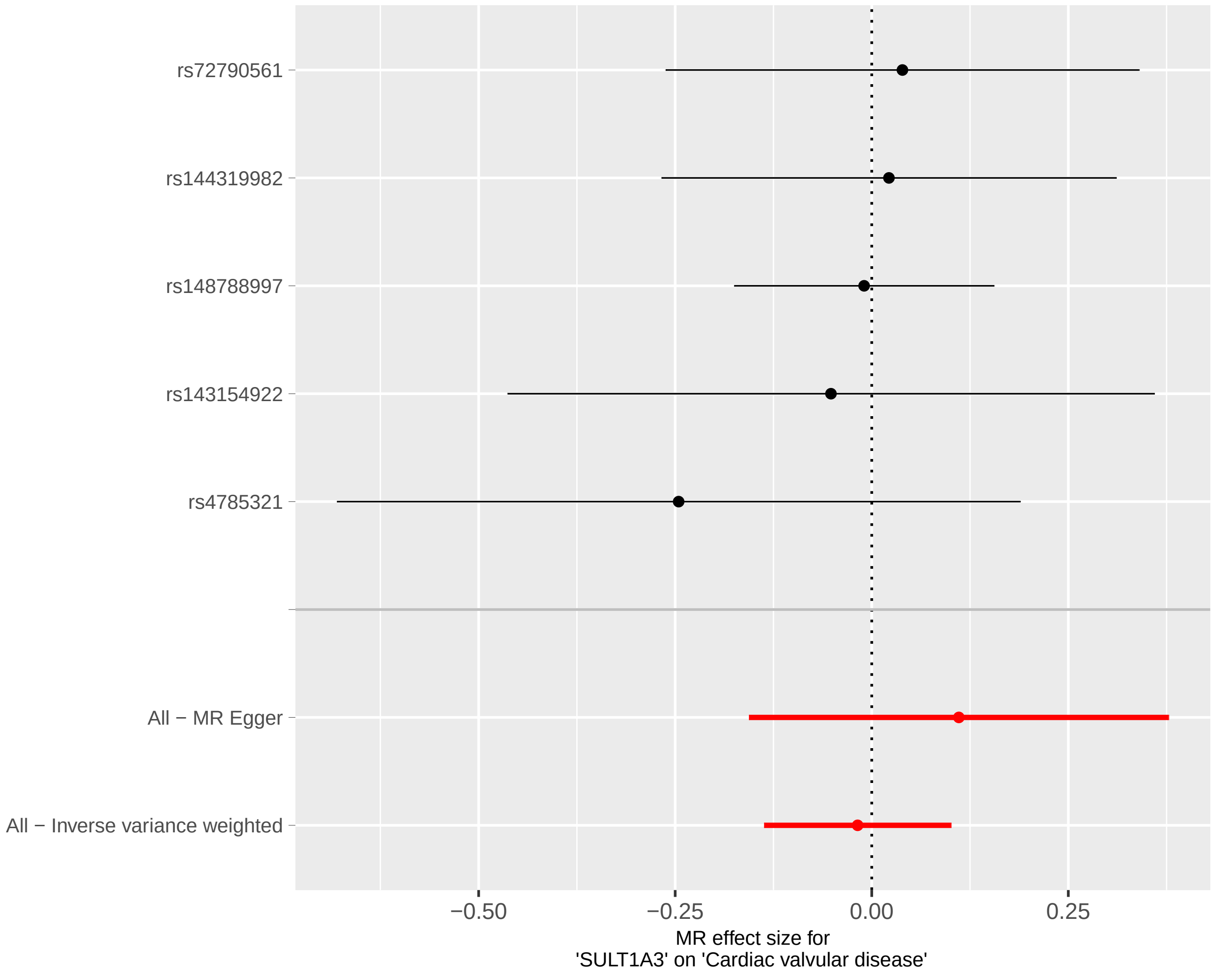
rs72790561
rs144319982
rs148788997
rs143154922
rs4785321
All − MR Egger
All − Inverse variance weighted
−0.50
−0.25
0.00
0.25
MR effect size for
'SULT1A3' on 'Cardiac valvular disease'

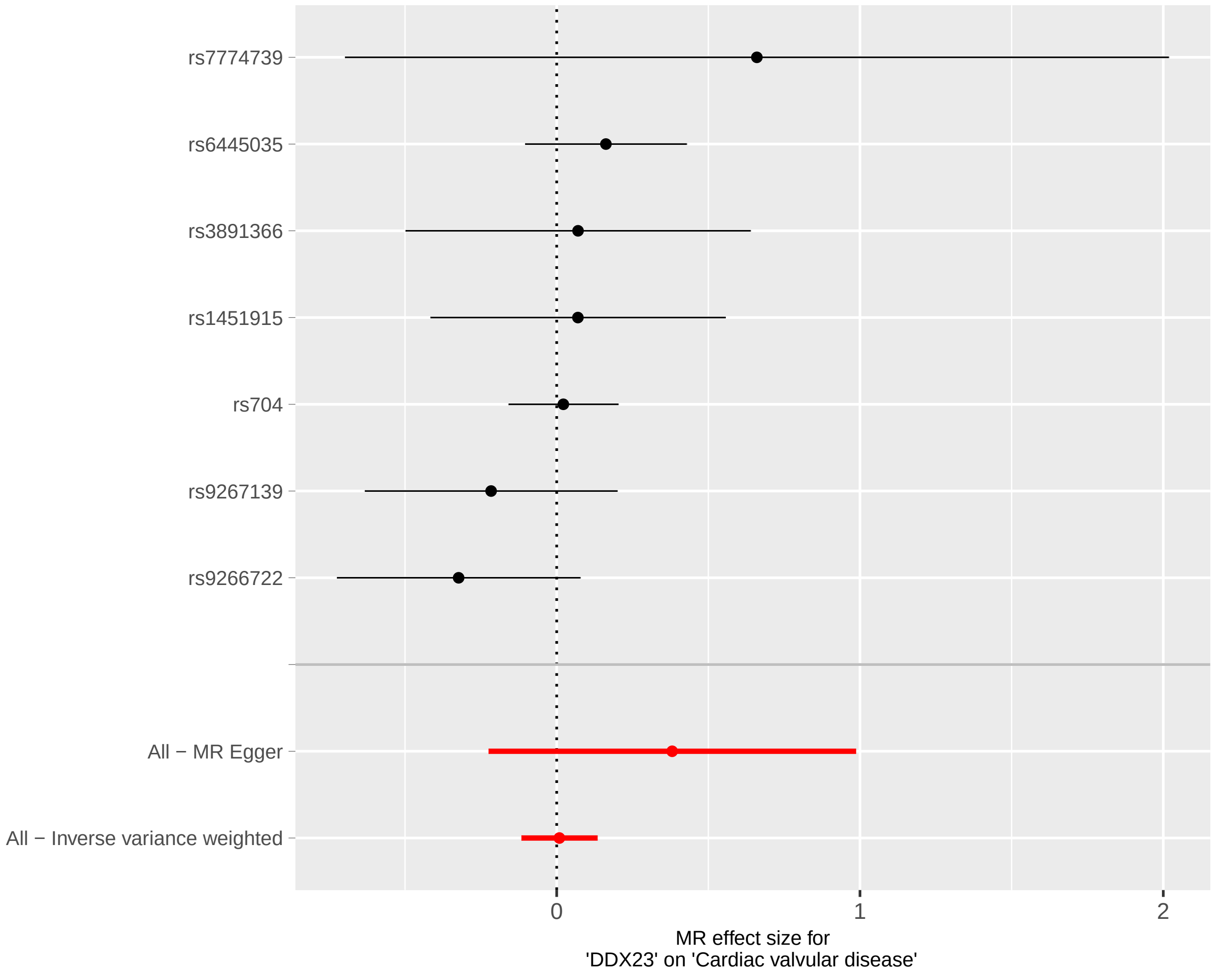
rs7774739
rs6445035
rs3891366
rs1451915
rs704
rs9267139
rs9266722
All − MR Egger
All − Inverse variance weighted
0
1
2
MR effect size for
'DDX23' on 'Cardiac valvular disease'

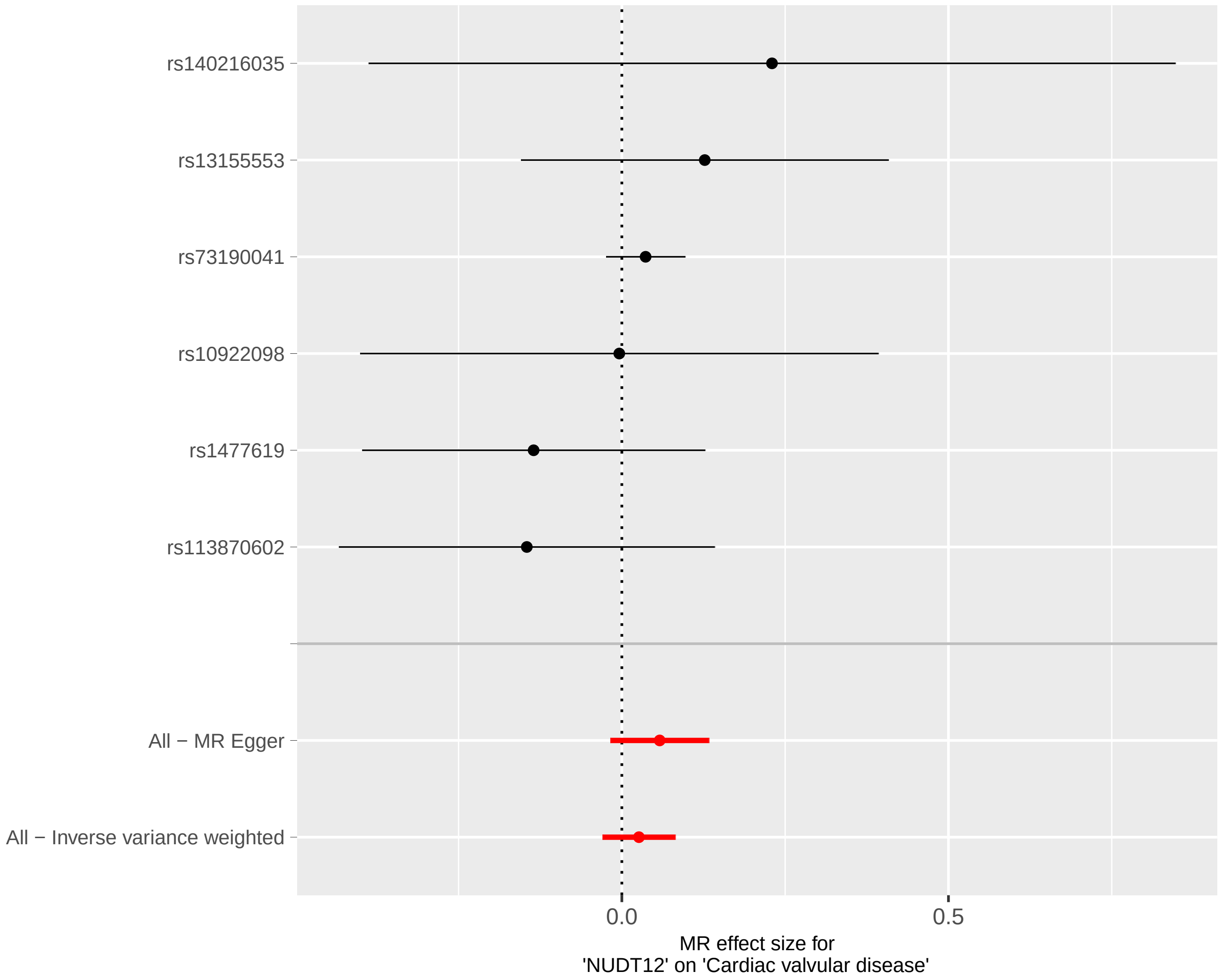
rs140216035
rs13155553
rs73190041
rs10922098
rs1477619
rs113870602
All − MR Egger
All − Inverse variance weighted
0.0
0.5
MR effect size for
'NUDT12' on 'Cardiac valvular disease'

Insufficient number of SNPs

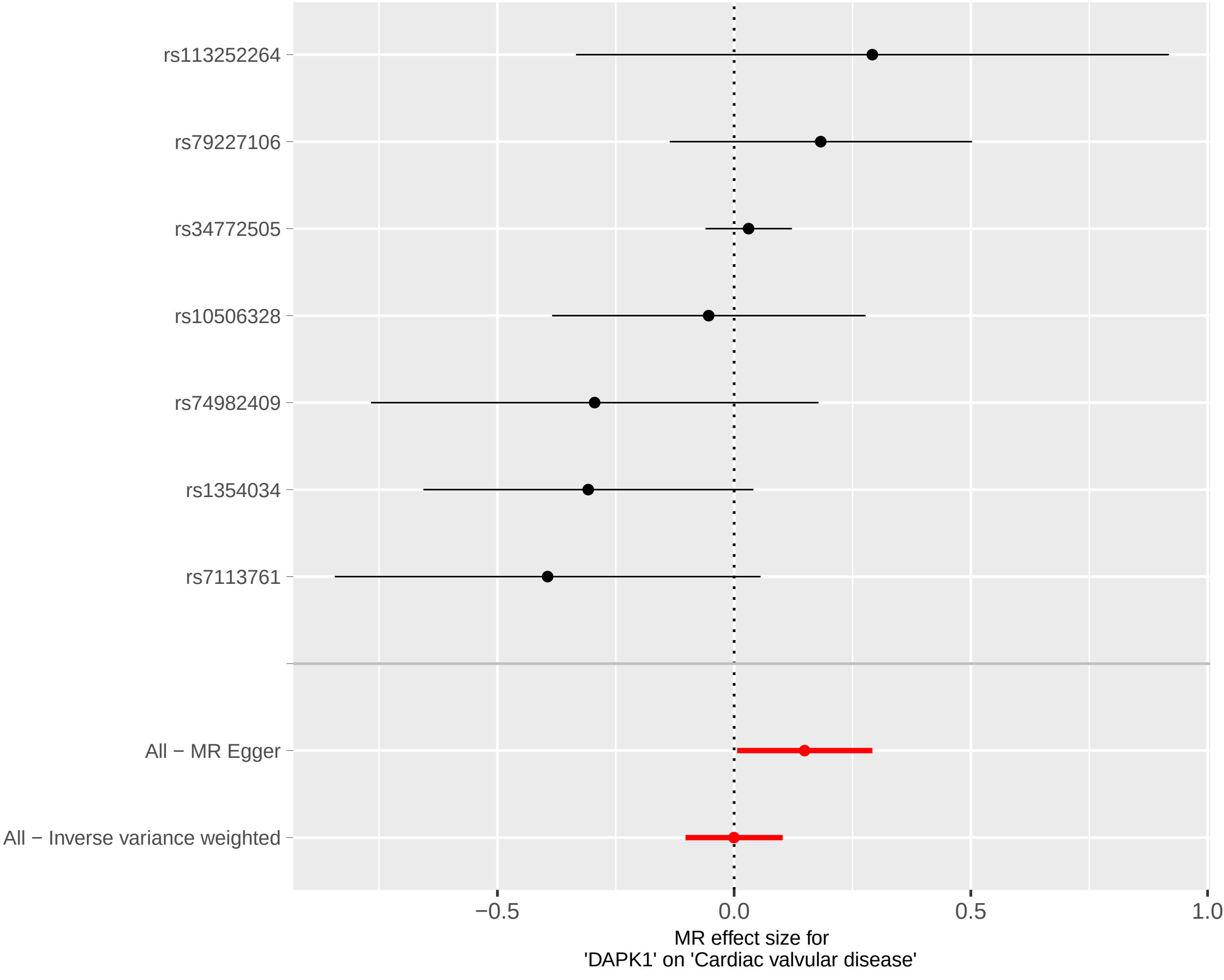
rs113252264
rs79227106
rs34772505
rs10506328
rs74982409
rs1354034
rs7113761
All − MR Egger
All − Inverse variance weighted
−0.5
0.0
0.5
1.0
MR effect size for
'DAPK1' on 'Cardiac valvular disease'

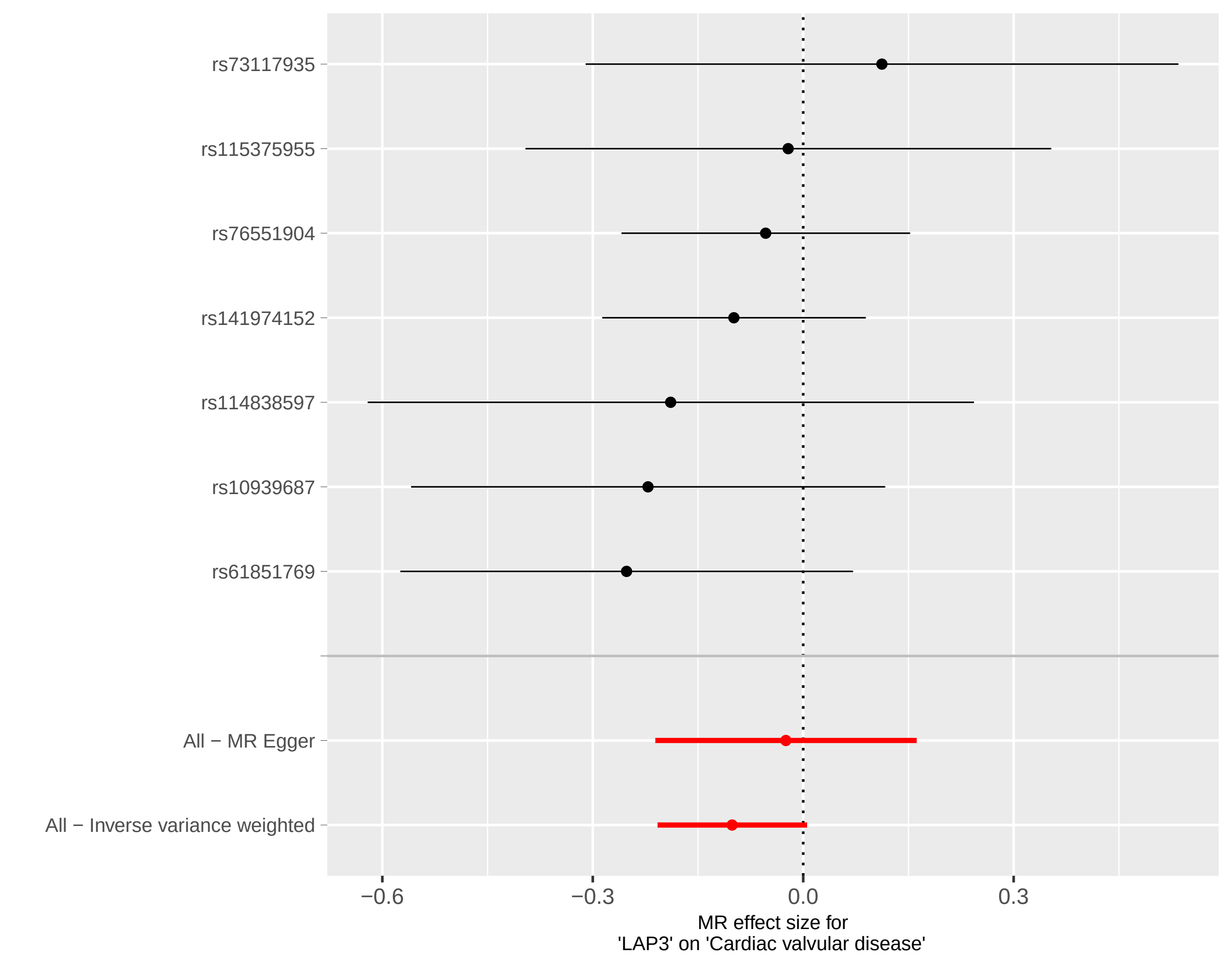
rs73117935
rs115375955
rs76551904
rs141974152
rs114838597
rs10939687
rs61851769
All − MR Egger
All − Inverse variance weighted
−0.6
−0.3
0.0
0.3
MR effect size for
'LAP3' on 'Cardiac valvular disease'

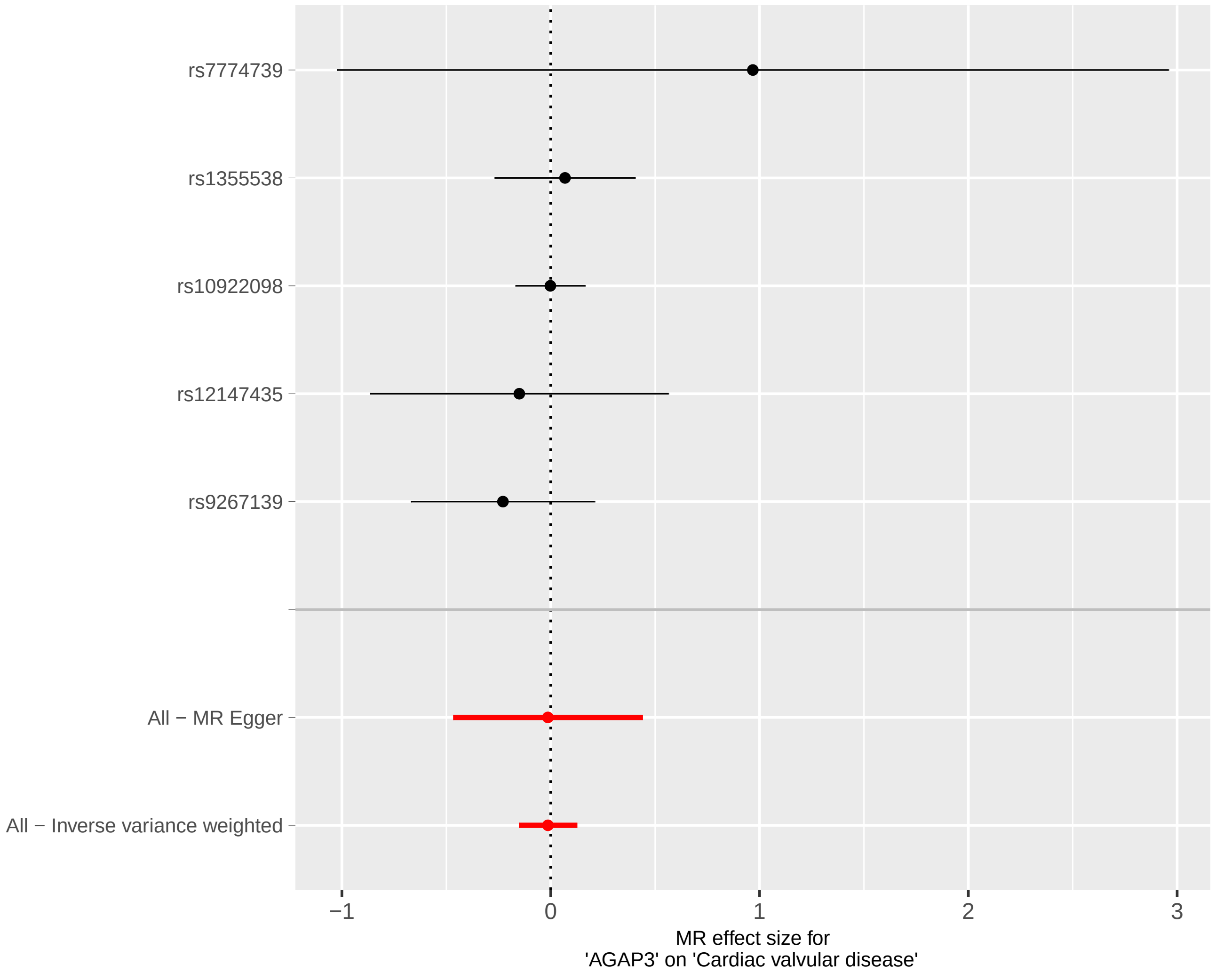
rs7774739
rs1355538
rs10922098
rs12147435
rs9267139
All − MR Egger
All − Inverse variance weighted
−1
0
1
2
3
MR effect size for
'AGAP3' on 'Cardiac valvular disease'

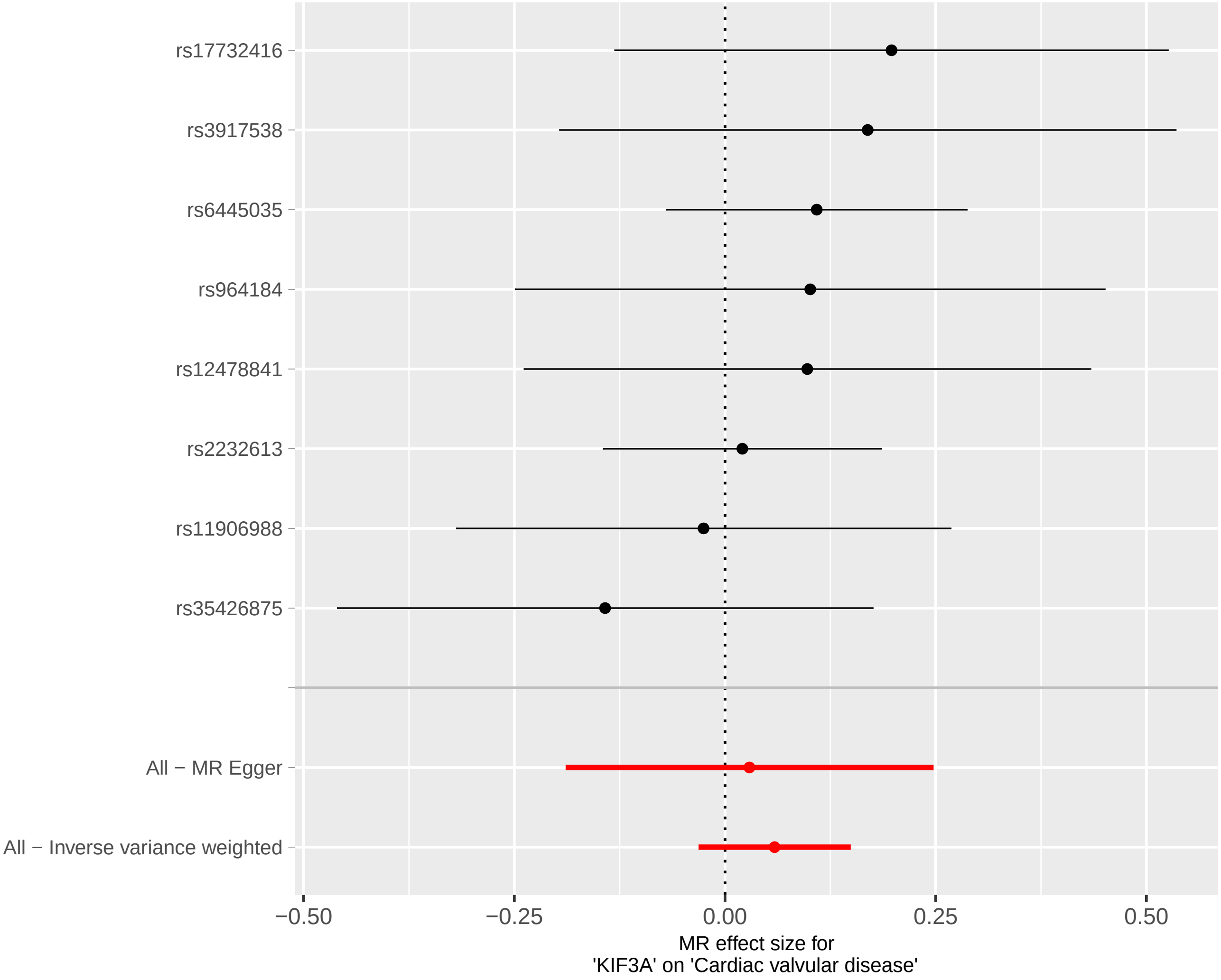
rs17732416
rs3917538
rs6445035
rs964184
rs12478841
rs2232613
rs11906988
rs35426875
All − MR Egger
All − Inverse variance weighted
−0.50
−0.25
0.00
0.25
0.50
MR effect size for
'KIF3A' on 'Cardiac valvular disease'

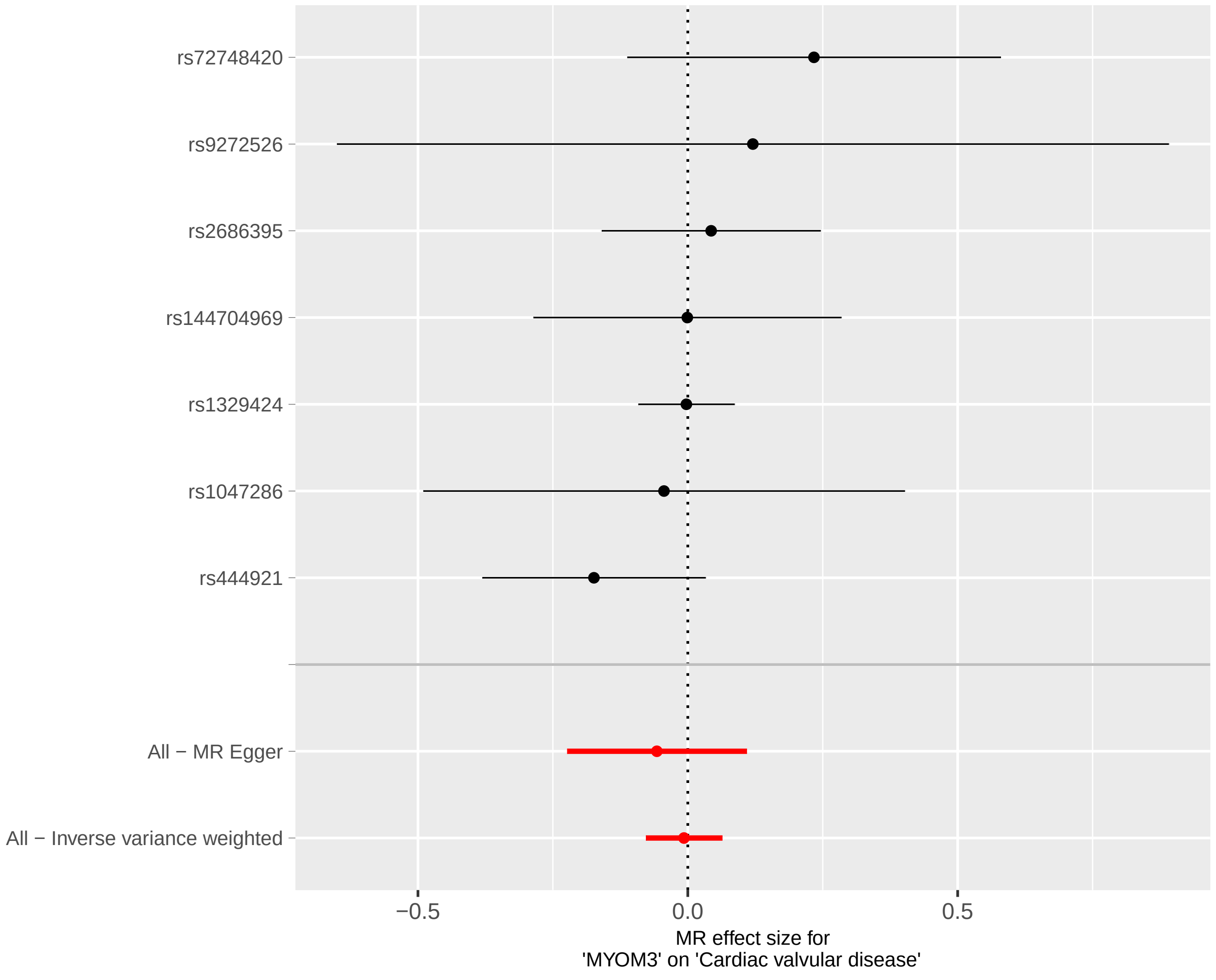
rs72748420
rs9272526
rs2686395
rs144704969
rs1329424
rs1047286
rs444921
All − MR Egger
All − Inverse variance weighted
−0.5
0.0
0.5
MR effect size for
'MYOM3' on 'Cardiac valvular disease'

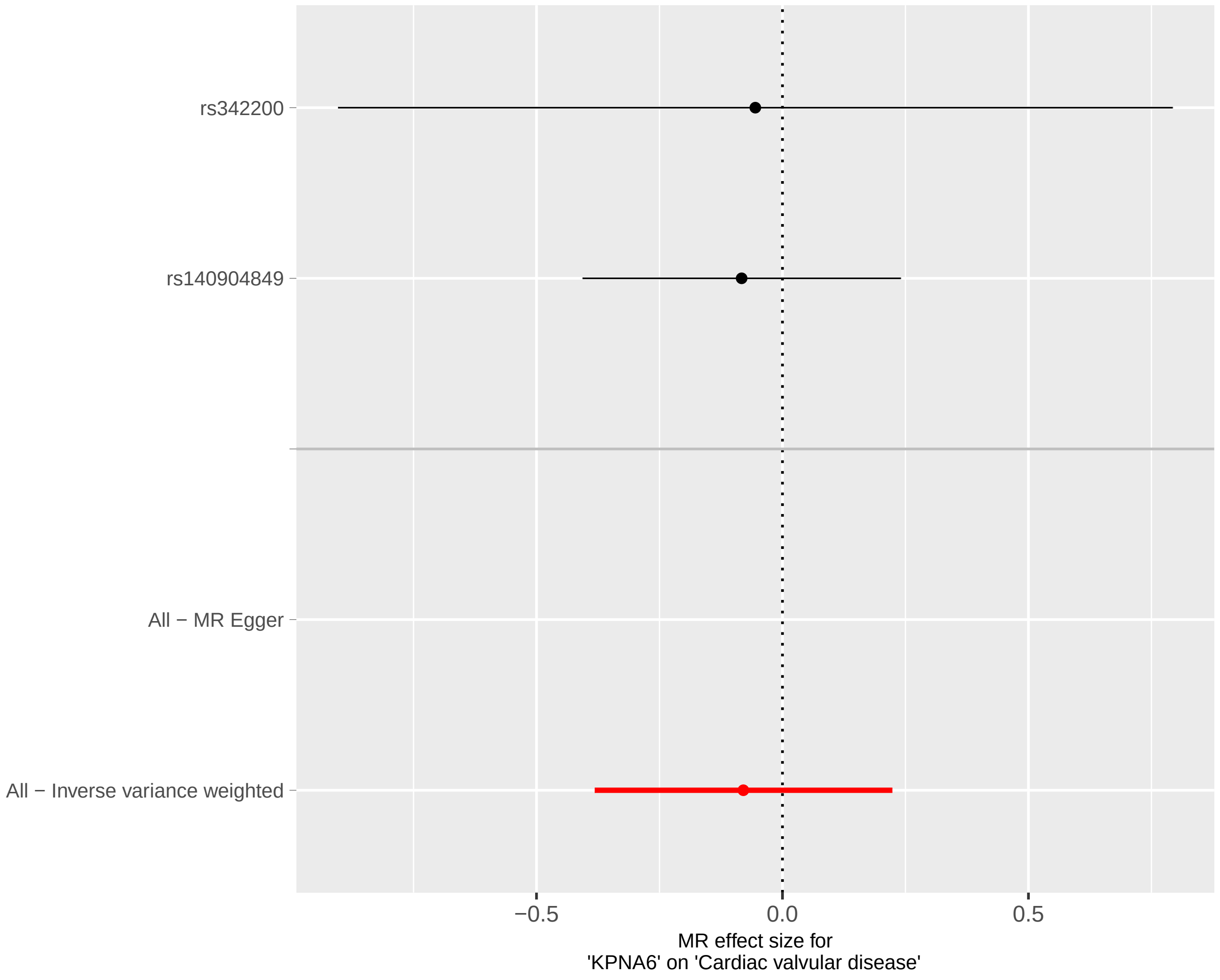

rs342200
rs140904849
All − MR Egger
All − Inverse variance weighted
−0.5
0.0
0.5
MR effect size for
'KPNA6' on 'Cardiac valvular disease'

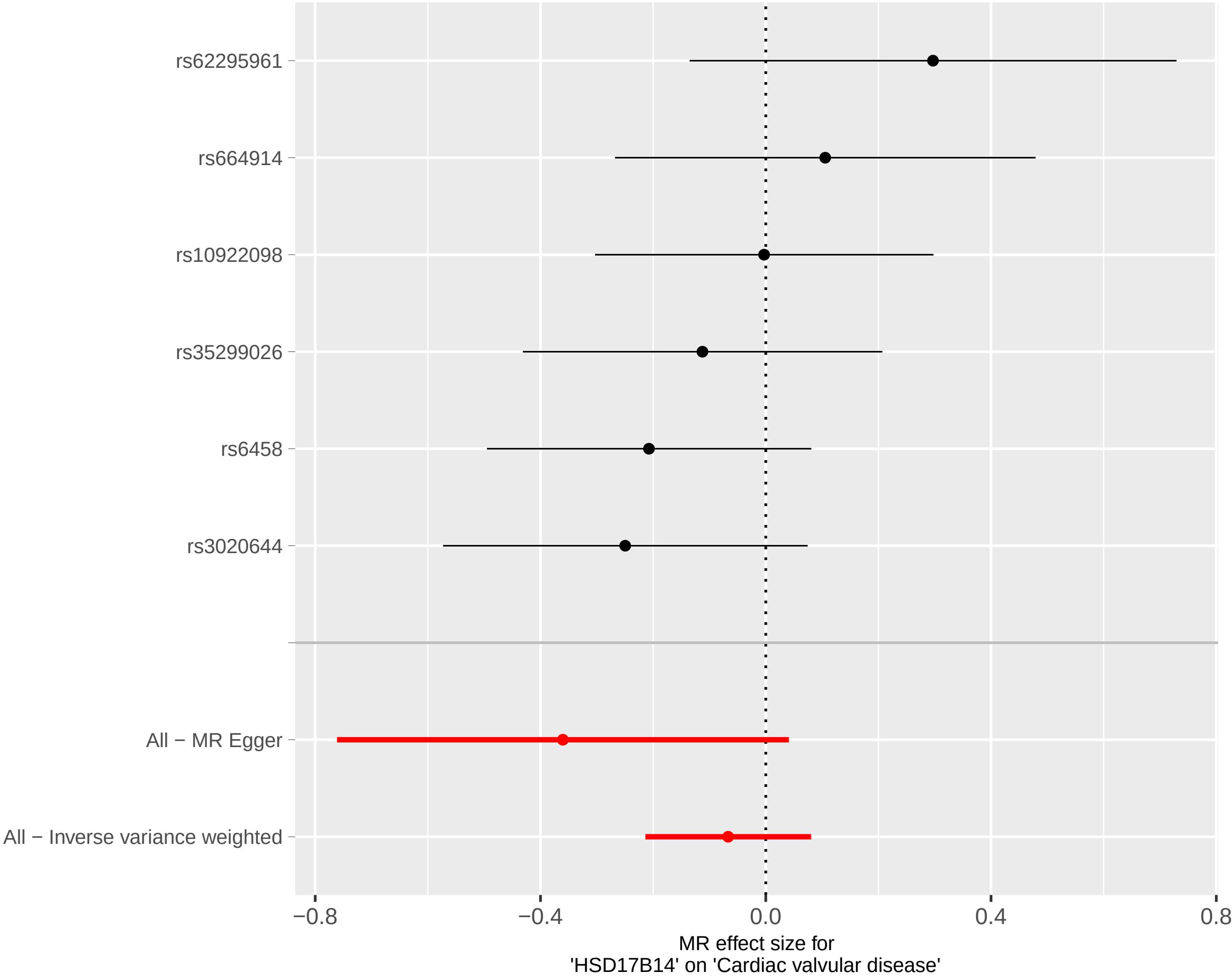

rs62295961
rs664914
rs10922098
rs35299026
rs6458
rs3020644
All − MR Egger
All − Inverse variance weighted
−0.8
−0.4
0.0
0.4
0.8
MR effect size for
'HSD17B14' on 'Cardiac valvular disease'

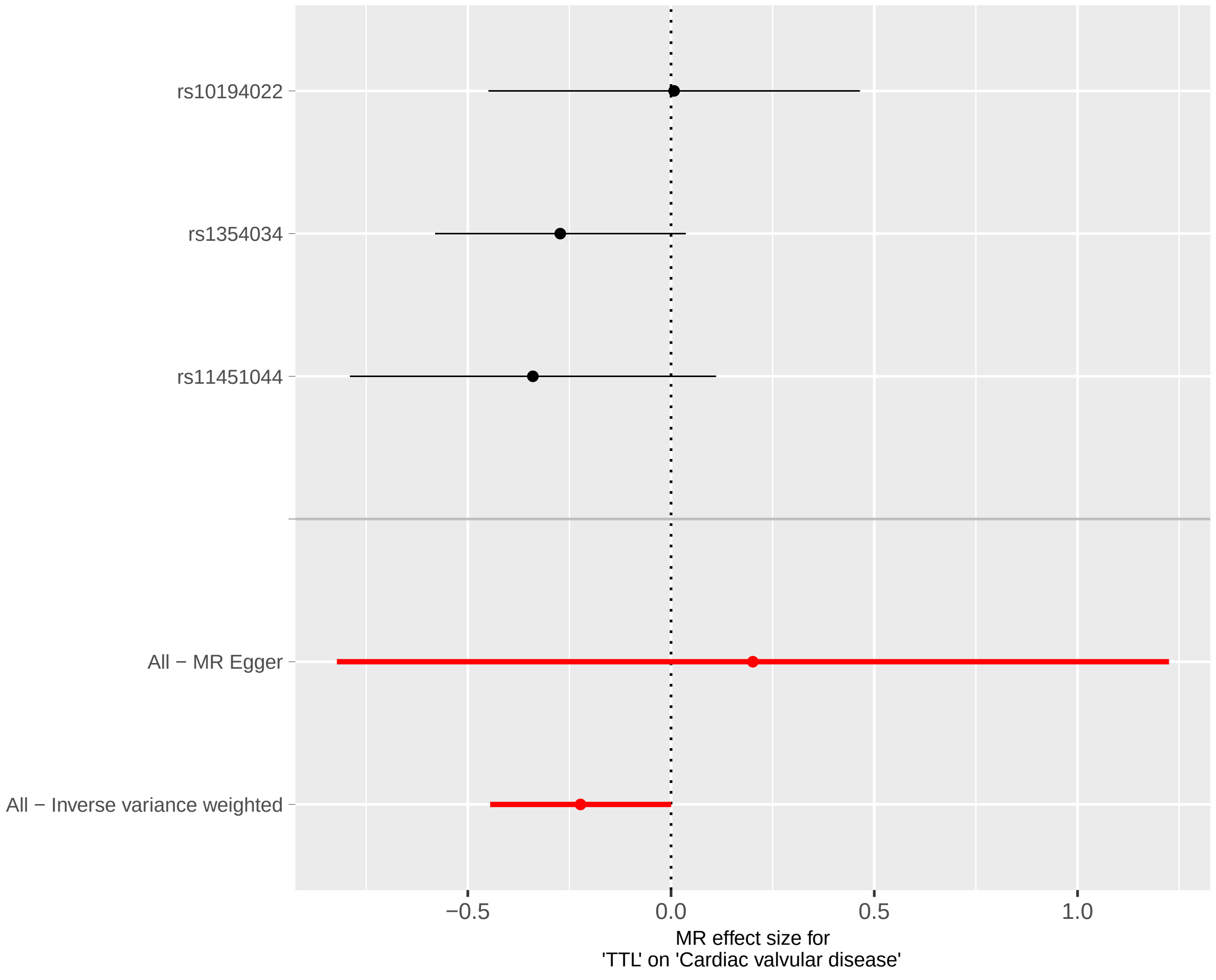
rs10194022
rs1354034
rs11451044
All − MR Egger
All − Inverse variance weighted
−0.5
0.0
0.5
1.0
MR effect size for
'TTL' on 'Cardiac valvular disease'

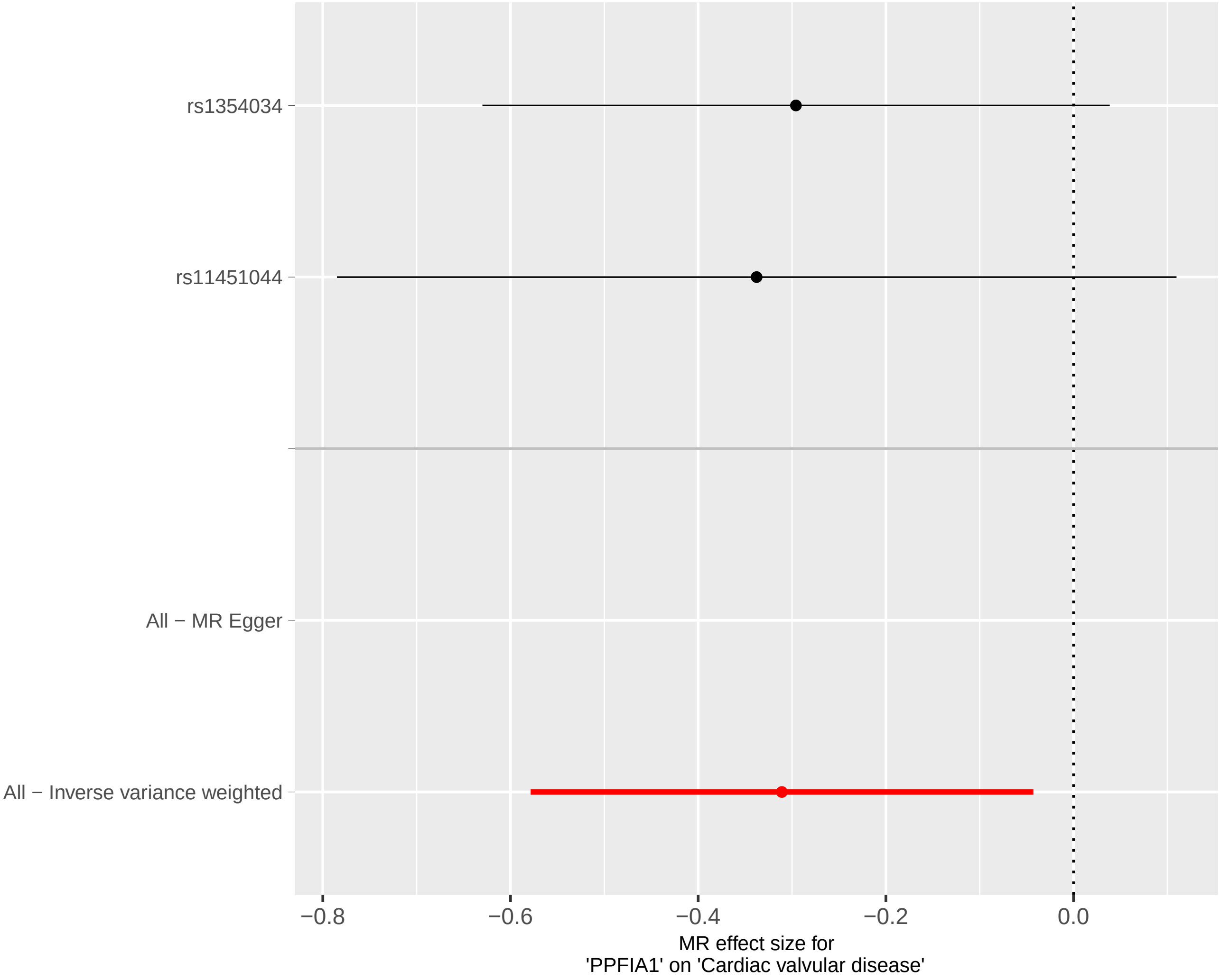
rs1354034
rs11451044
All − MR Egger
All − Inverse variance weighted
−0.8
−0.6
−0.4
−0.2
0.0
MR effect size for
'PPFIA1' on 'Cardiac valvular disease'

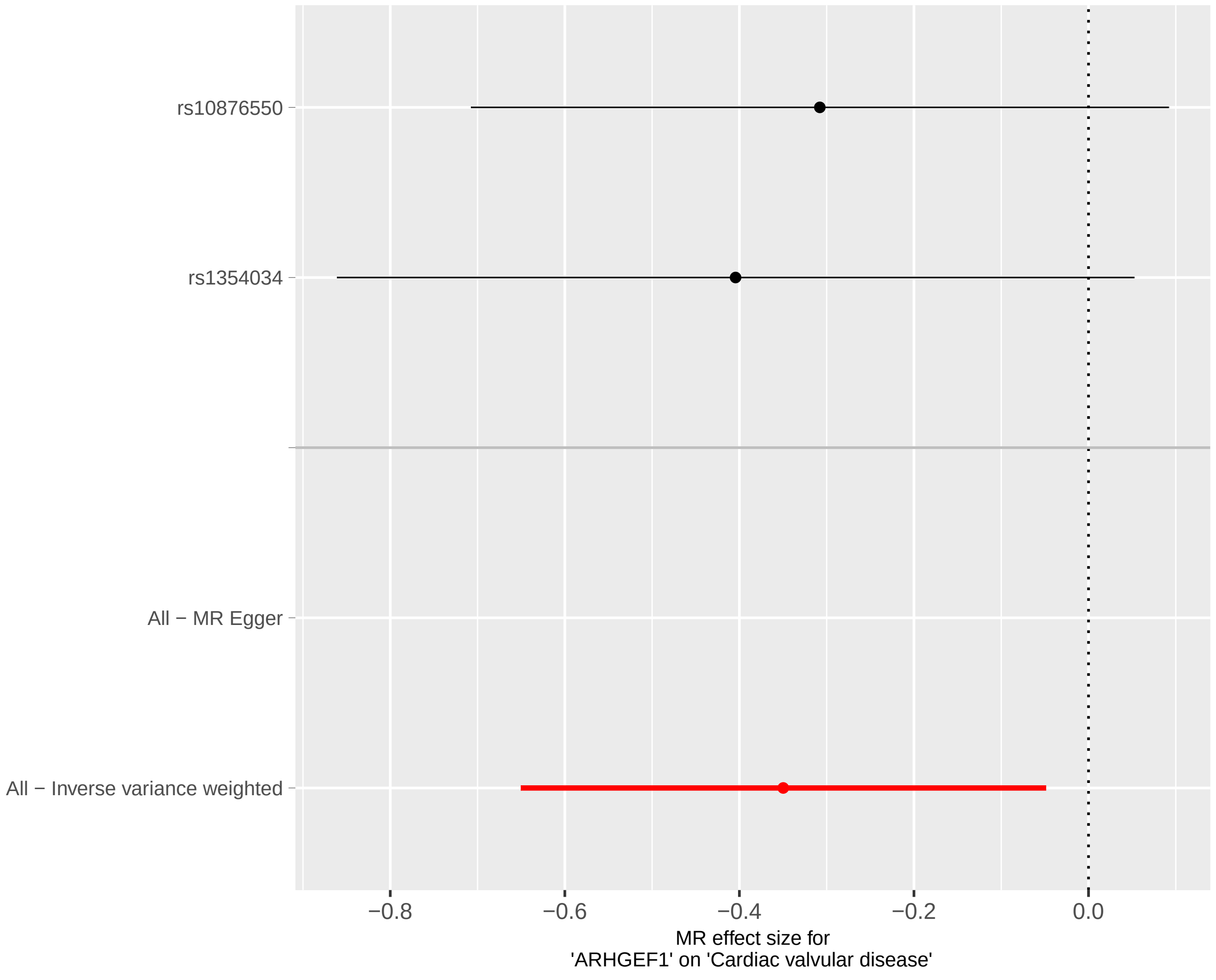
rs10876550
rs1354034
All − MR Egger
All − Inverse variance weighted
−0.8
−0.6
−0.4
−0.2
0.0
MR effect size for
'ARHGEF1' on 'Cardiac valvular disease'

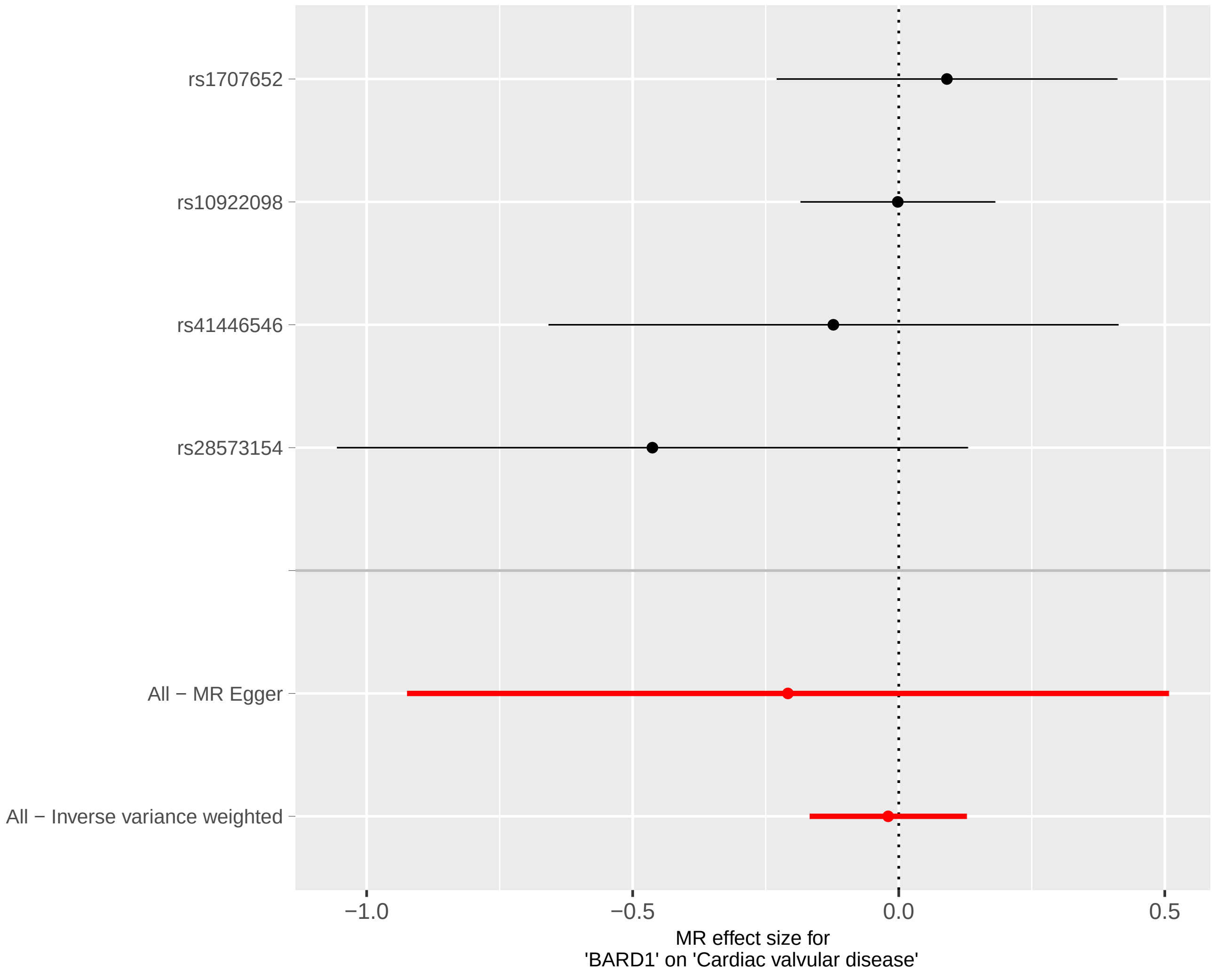
rs1707652
rs10922098
rs41446546
rs28573154
All − MR Egger
All − Inverse variance weighted
−1.0
−0.5
0.0
0.5
MR effect size for
'BARD1' on 'Cardiac valvular disease'

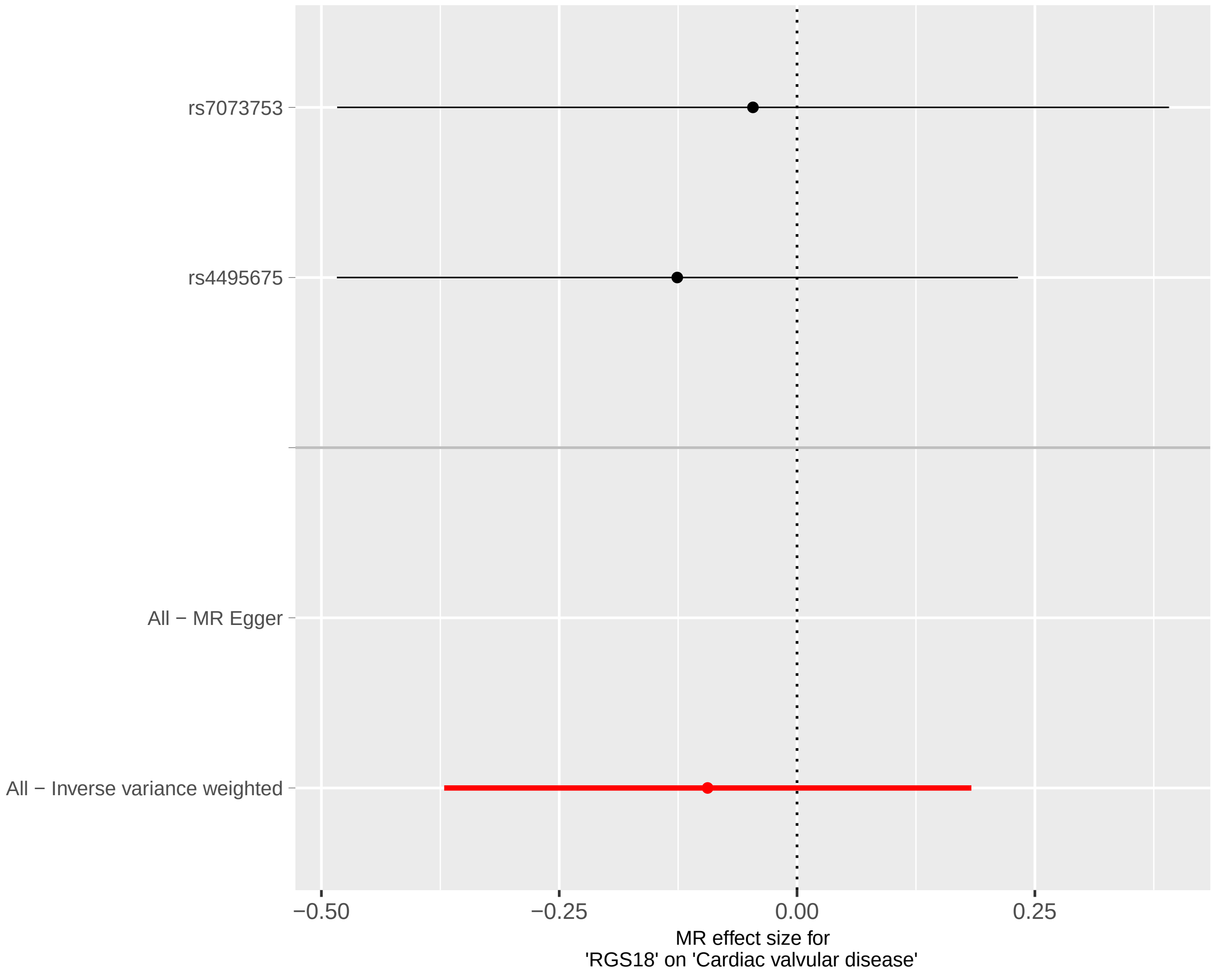
rs7073753
rs4495675
All − MR Egger
All − Inverse variance weighted
−0.50
−0.25
0.00
0.25
MR effect size for
'RGS18' on 'Cardiac valvular disease'

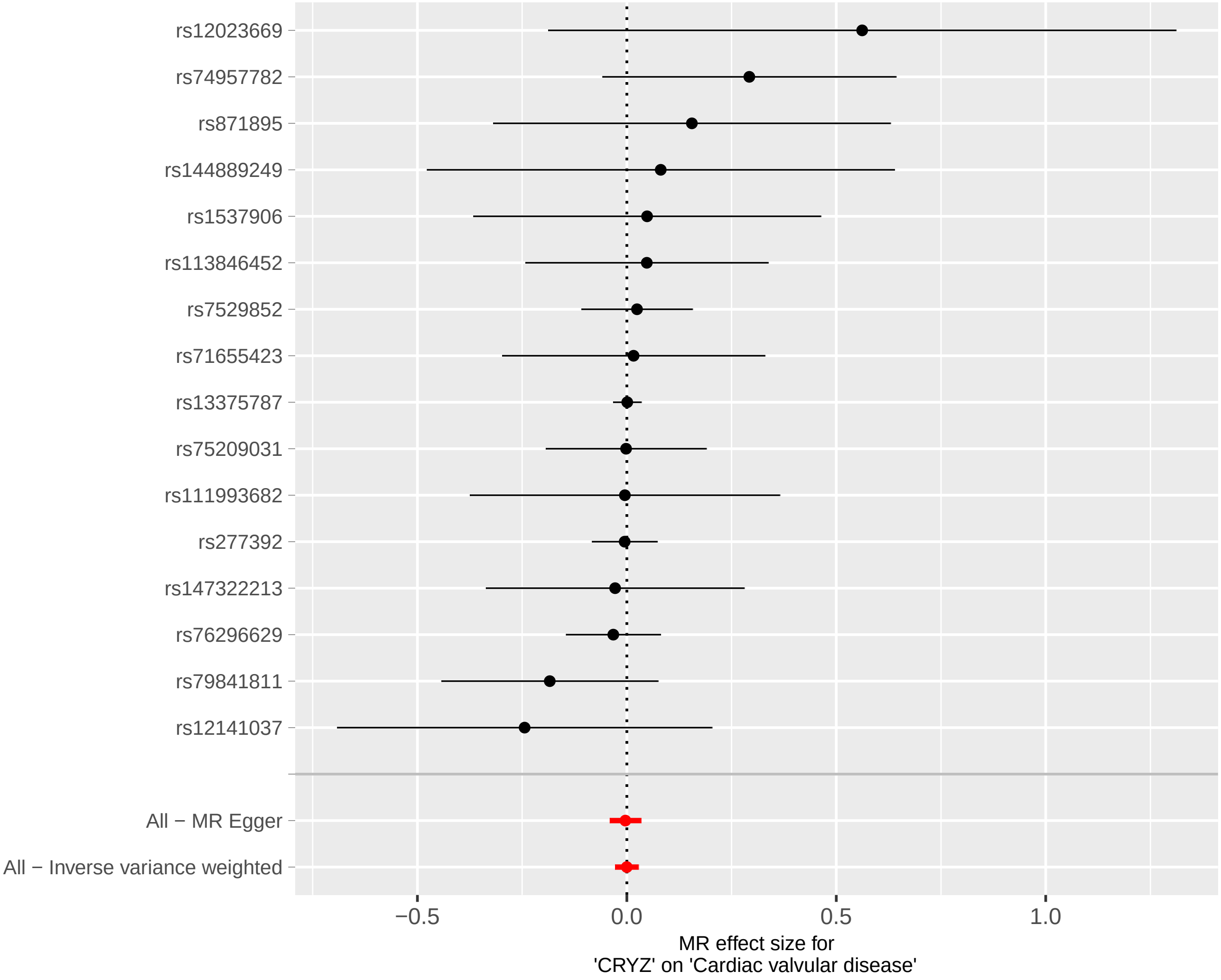
rs12023669
rs74957782
rs871895
rs144889249
rs1537906
rs113846452
rs7529852
rs71655423
rs13375787
rs75209031
rs111993682
rs277392
rs147322213
rs76296629
rs79841811
rs12141037
All − MR Egger
All − Inverse variance weighted
−0.5
0.0
0.5
1.0
MR effect size for
'CRYZ' on 'Cardiac valvular disease'

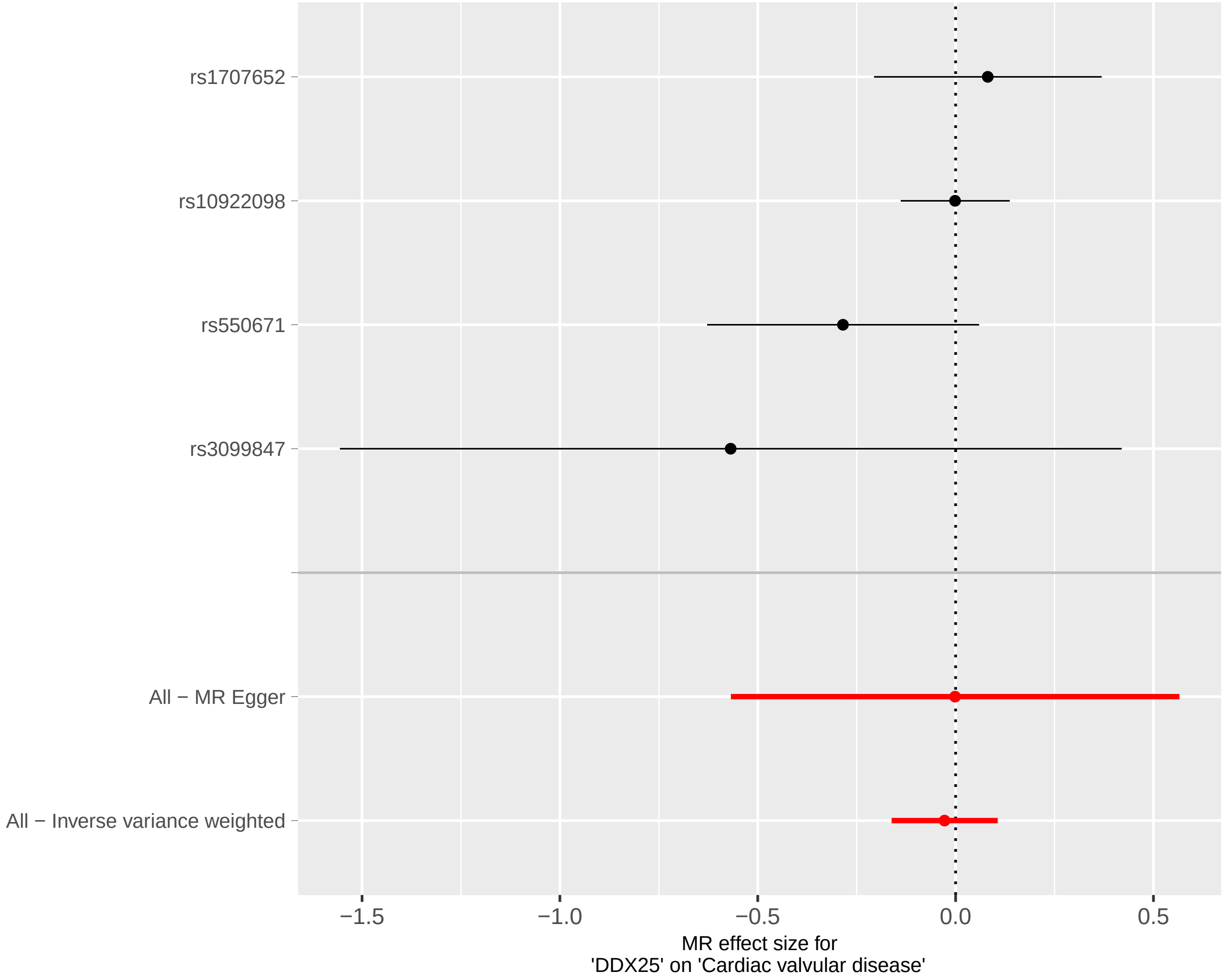
rs1707652
rs10922098
rs550671
rs3099847
All − MR Egger
All − Inverse variance weighted
−1.5
−1.0
−0.5
0.0
0.5
MR effect size for
'DDX25' on 'Cardiac valvular disease'

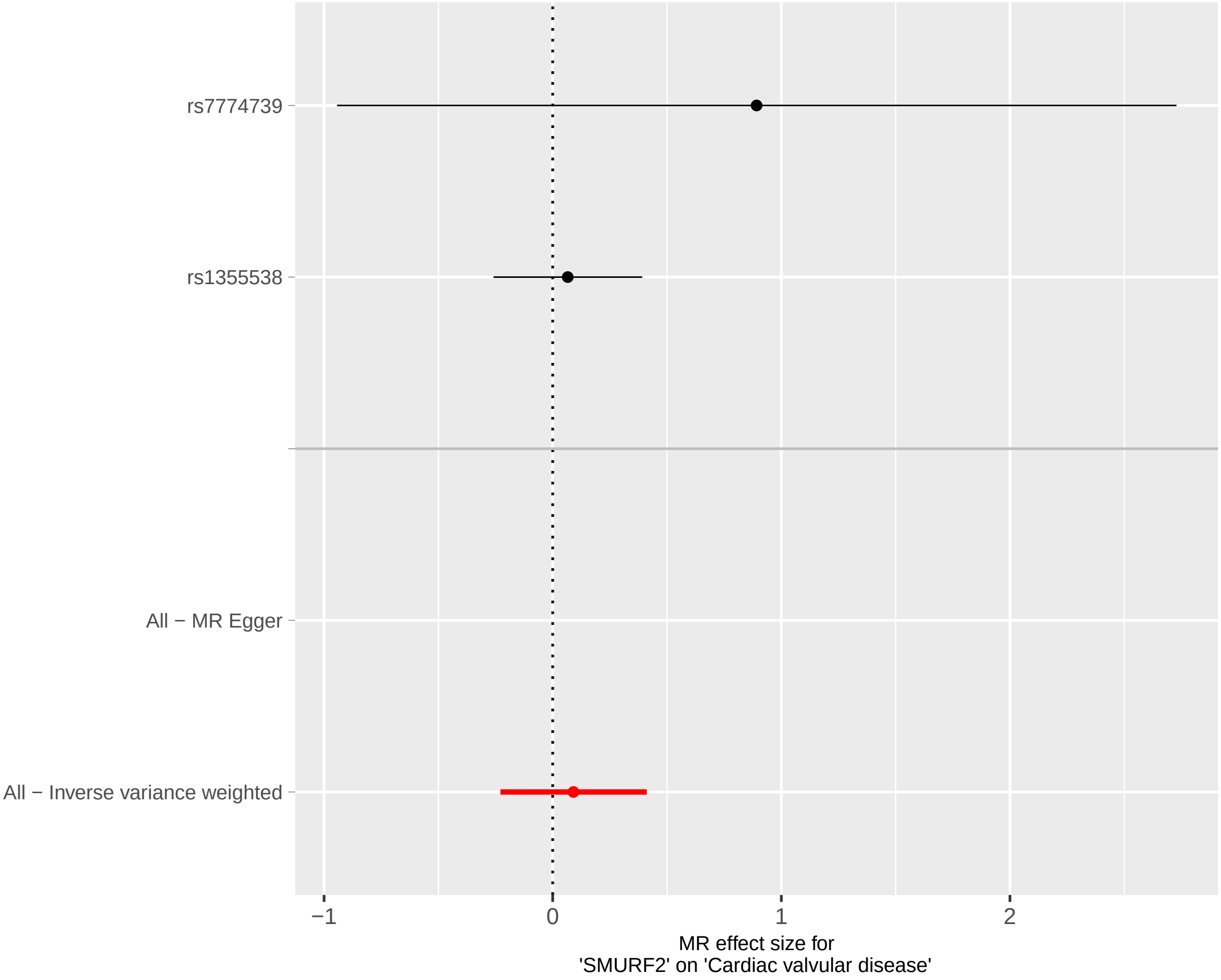
rs7774739
rs1355538
All − MR Egger
All − Inverse variance weighted
−1
0
1
2
MR effect size for
'SMURF2' on 'Cardiac valvular disease'

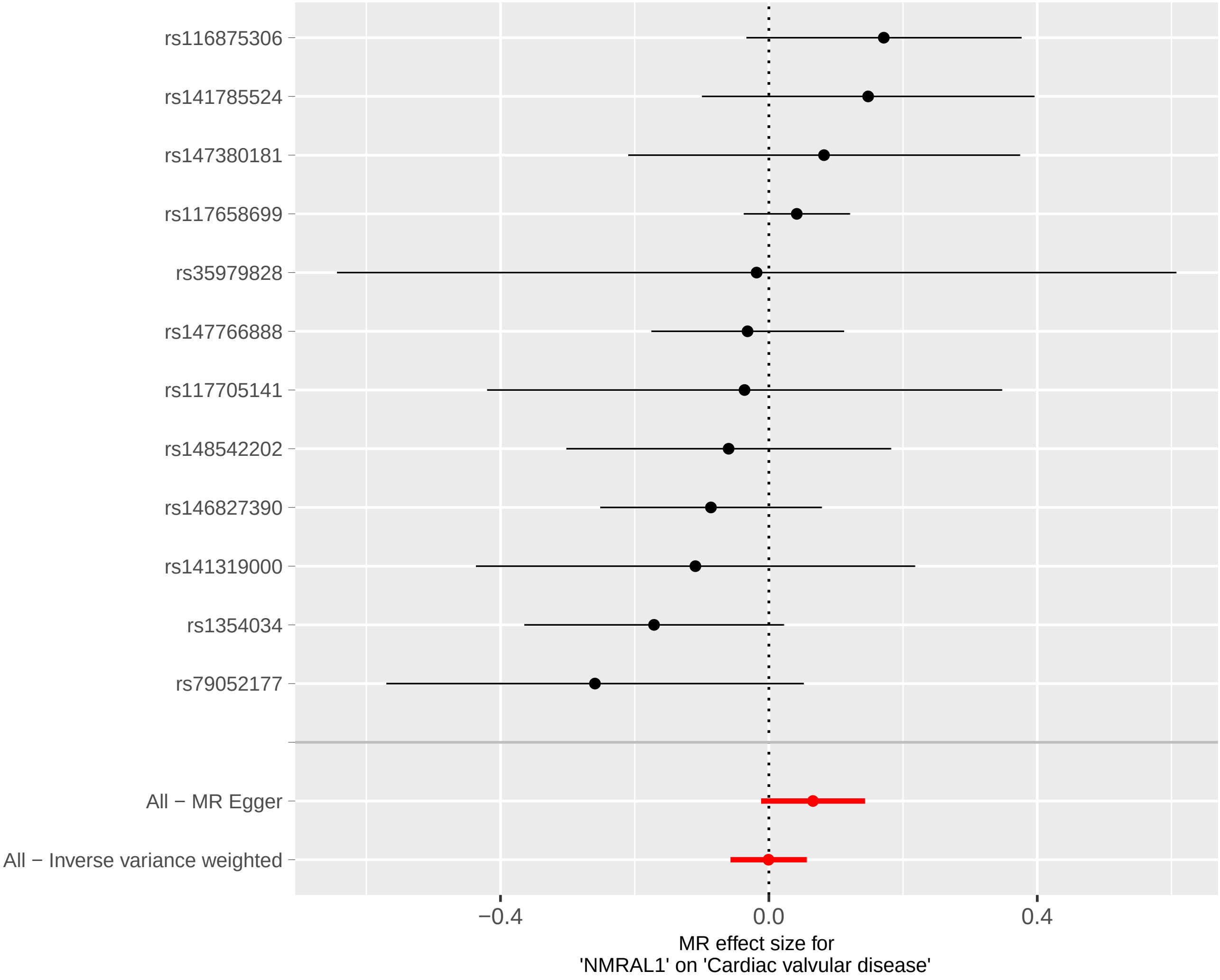
rs116875306
rs141785524
rs147380181
rs117658699
rs35979828
rs147766888
rs117705141
rs148542202
rs146827390
rs141319000
rs1354034
rs79052177
All − MR Egger
All − Inverse variance weighted
−0.4
0.0
0.4
MR effect size for
'NMRAL1' on 'Cardiac valvular disease'

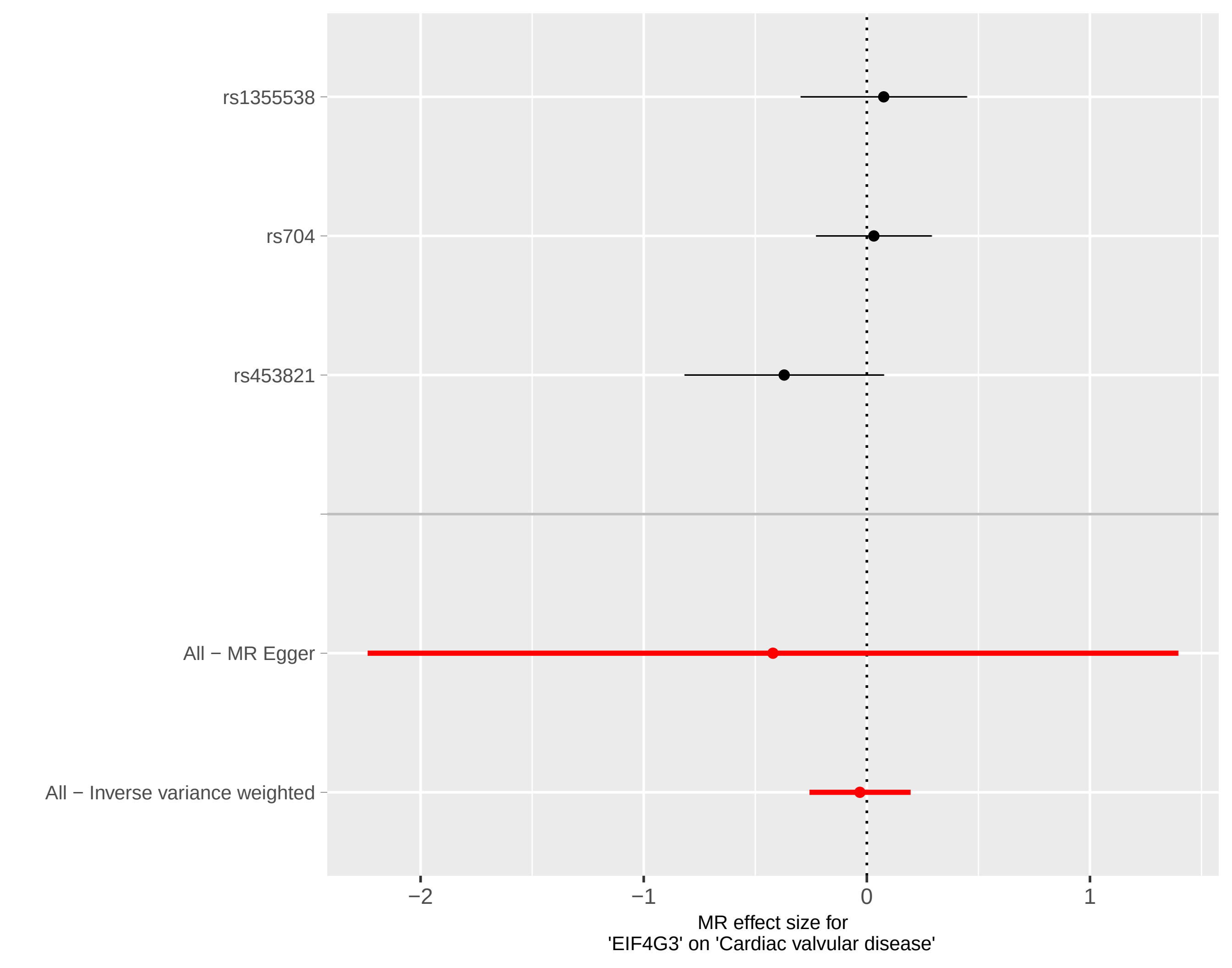
rs1355538
rs704
rs453821
All − MR Egger
All − Inverse variance weighted
−2
−1
0
1
MR effect size for
'EIF4G3' on 'Cardiac valvular disease'

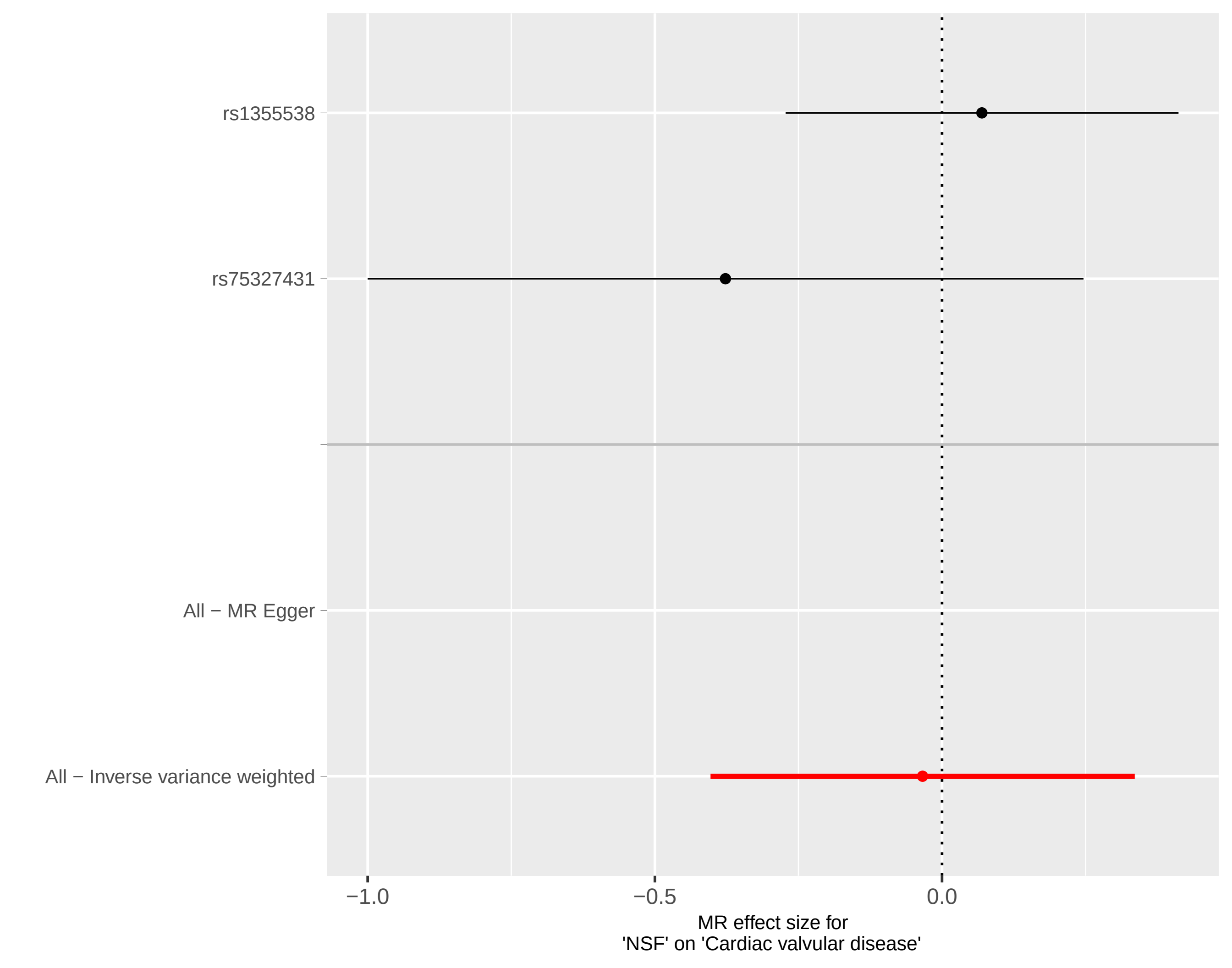
rs1355538
rs75327431
All − MR Egger
All − Inverse variance weighted
−1.0
−0.5
0.0
MR effect size for
'NSF' on 'Cardiac valvular disease'

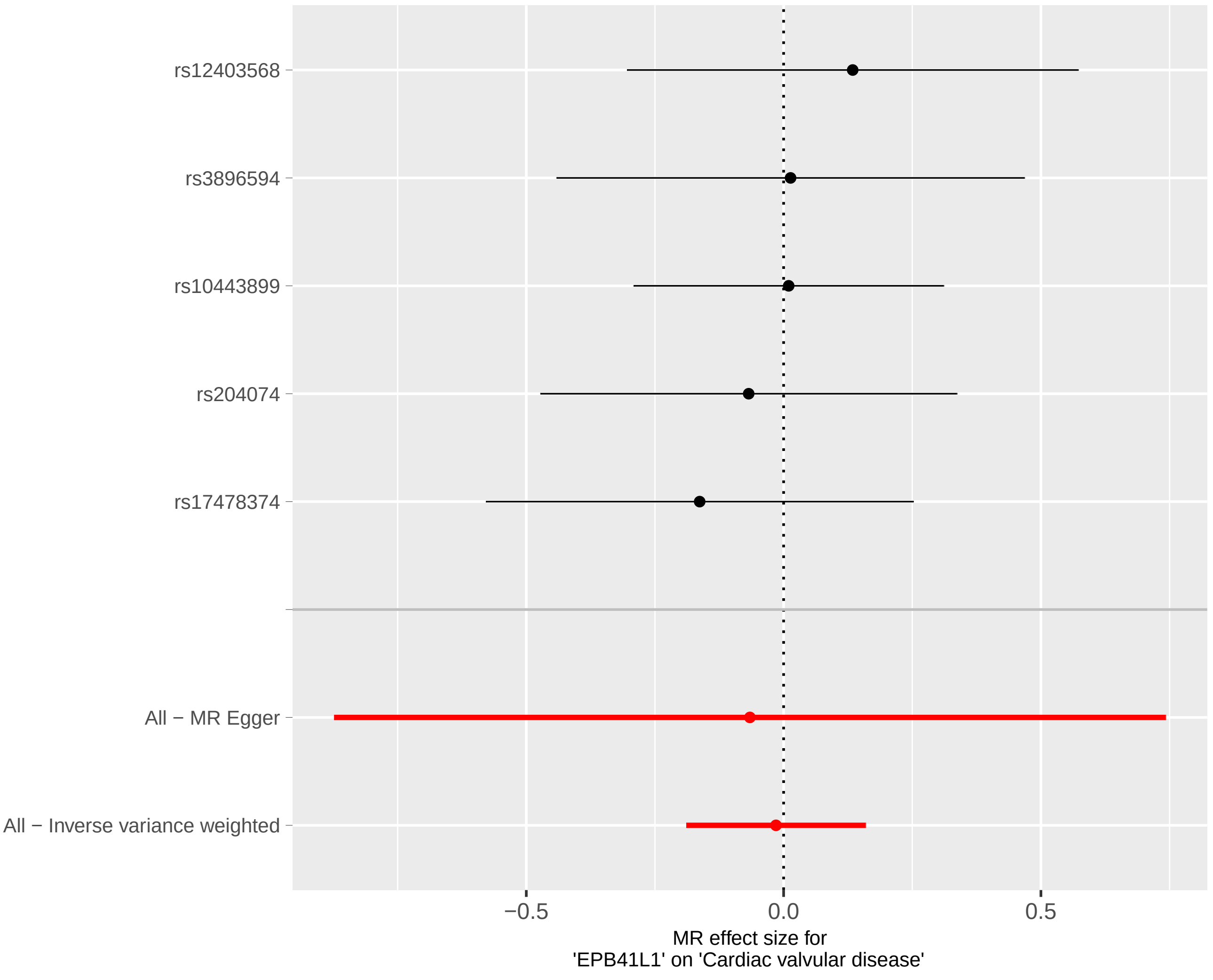
rs12403568
rs3896594
rs10443899
rs204074
rs17478374
All − MR Egger
All − Inverse variance weighted
−0.5
0.0
0.5
MR effect size for
'EPB41L1' on 'Cardiac valvular disease'

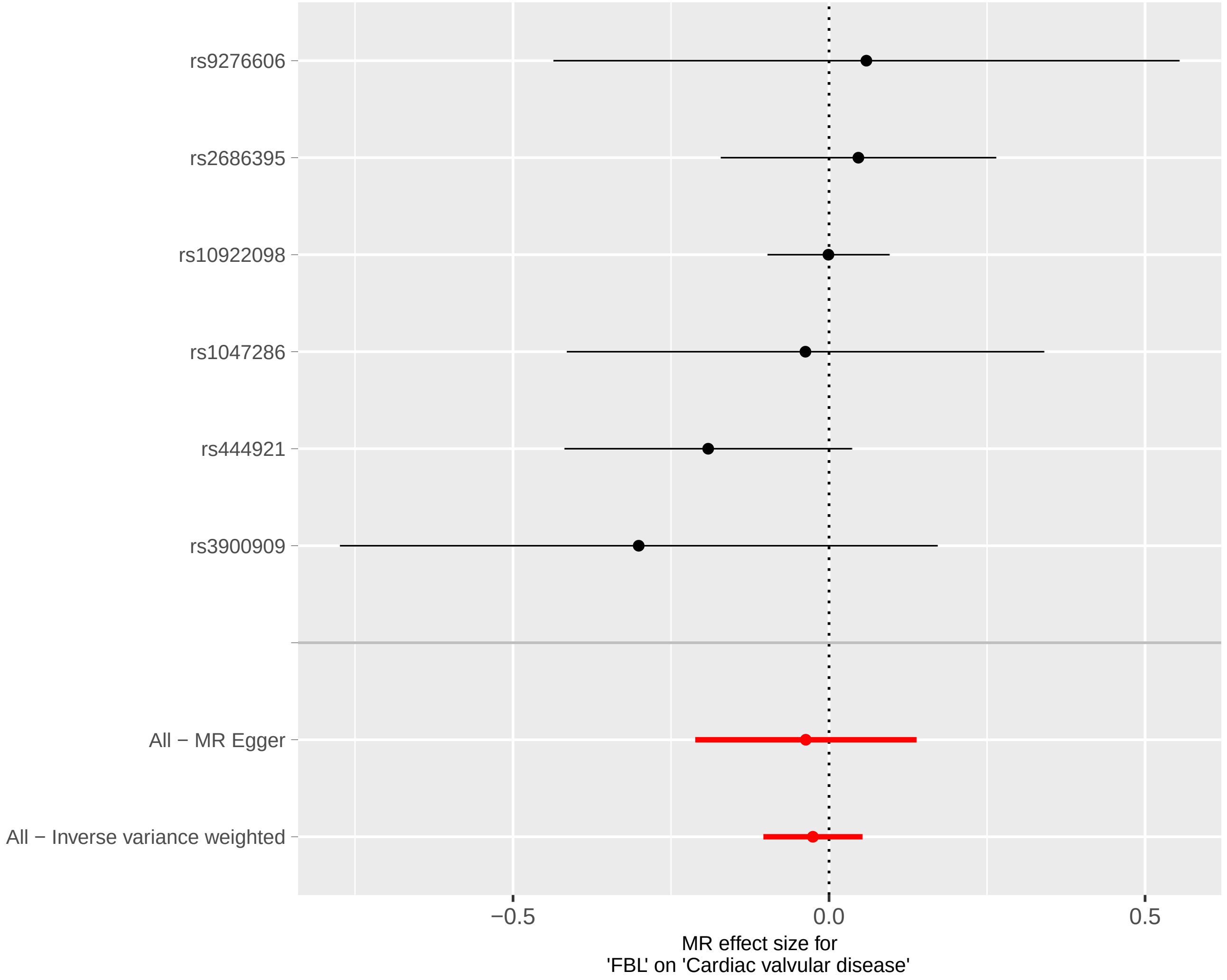

rs9276606
rs2686395
rs10922098
rs1047286
rs444921
rs3900909
All − MR Egger
All − Inverse variance weighted
−0.5
0.0
0.5
MR effect size for
'FBL' on 'Cardiac valvular disease'

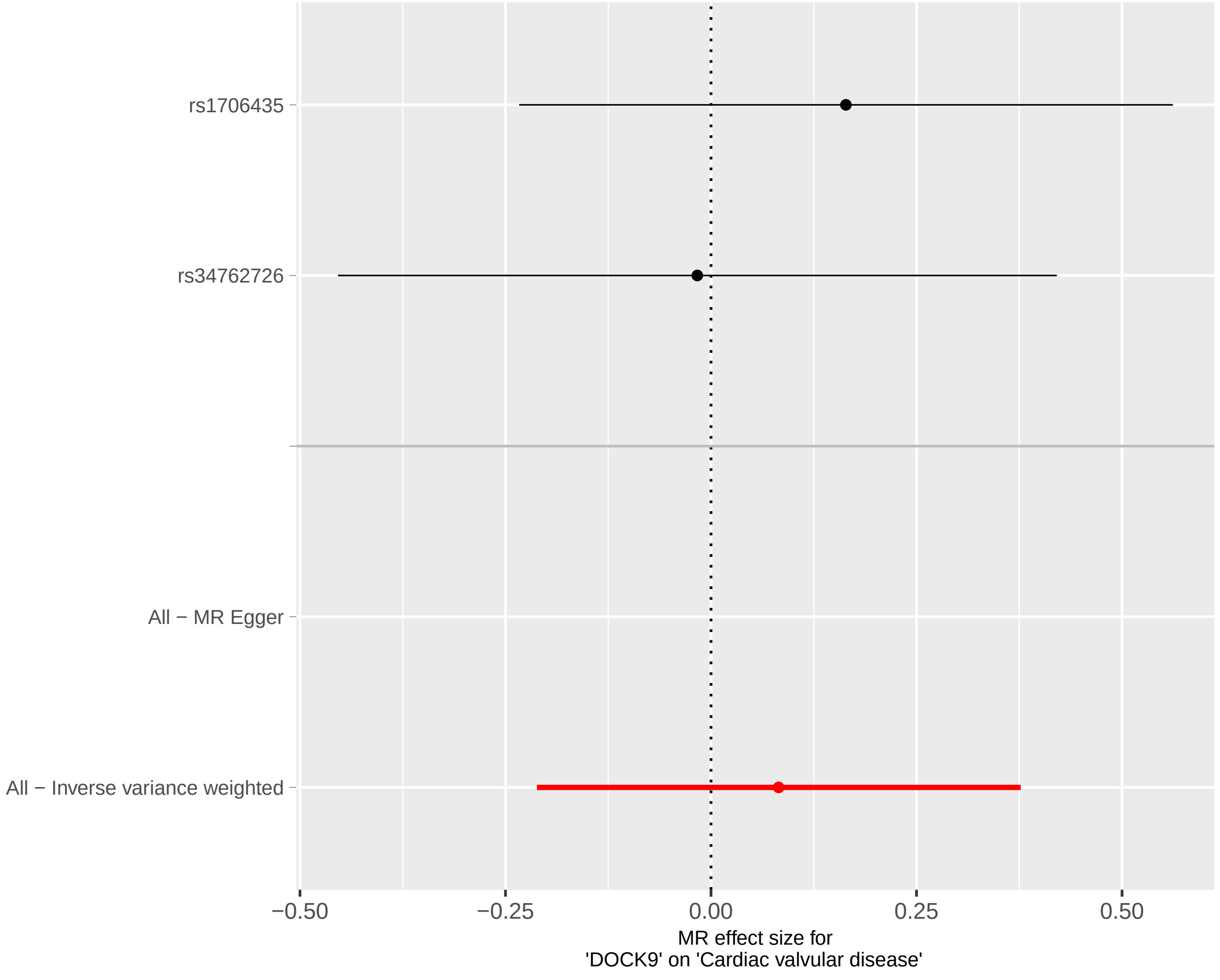

rs1706435
rs34762726
All − MR Egger
All − Inverse variance weighted
−0.50
−0.25
0.00
0.25
0.50
MR effect size for
'DOCK9' on 'Cardiac valvular disease'

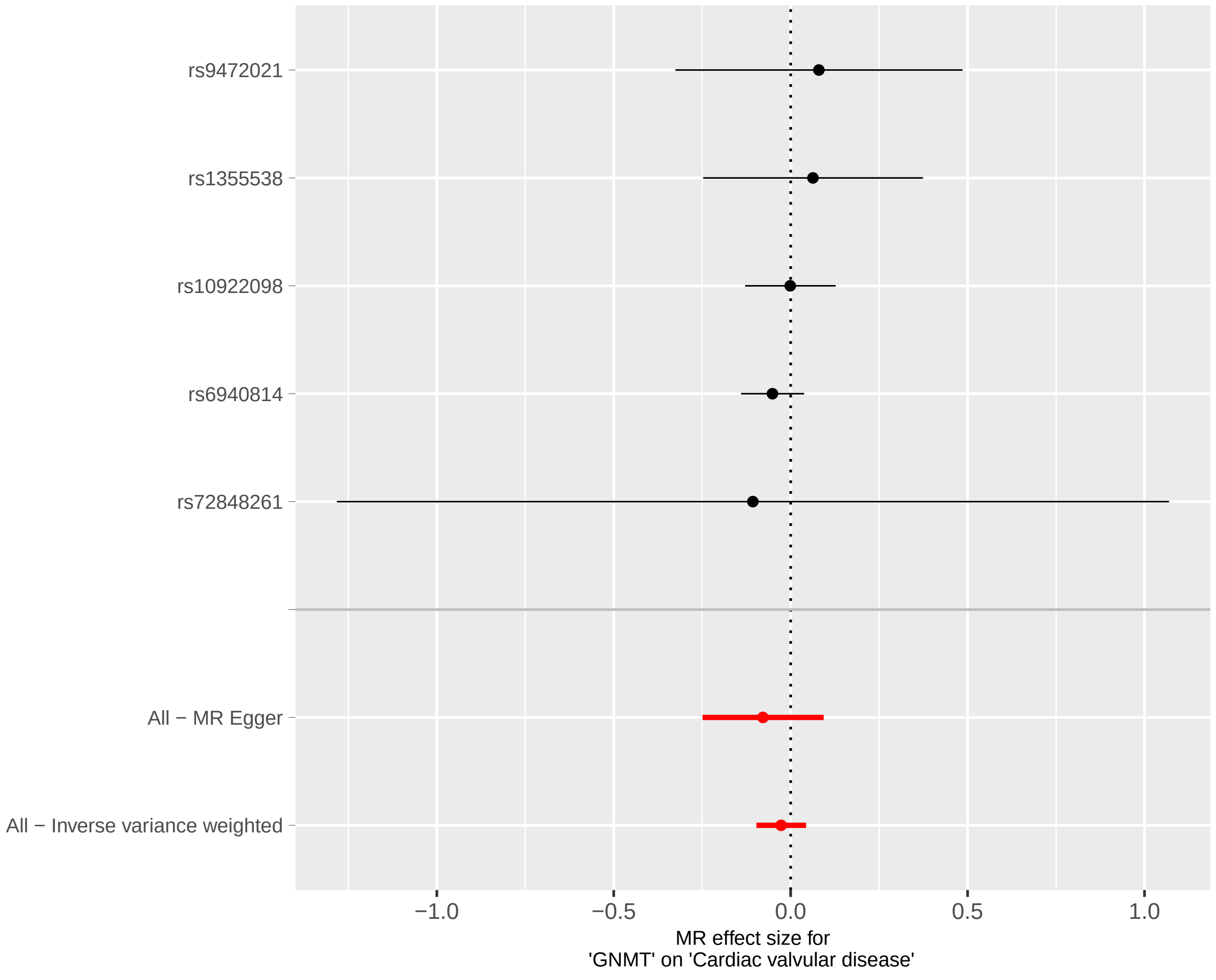
rs9472021
rs1355538
rs10922098
rs6940814
rs72848261
All − MR Egger
All − Inverse variance weighted
−1.0
−0.5
0.0
0.5
1.0
MR effect size for
'GNMT' on 'Cardiac valvular disease'

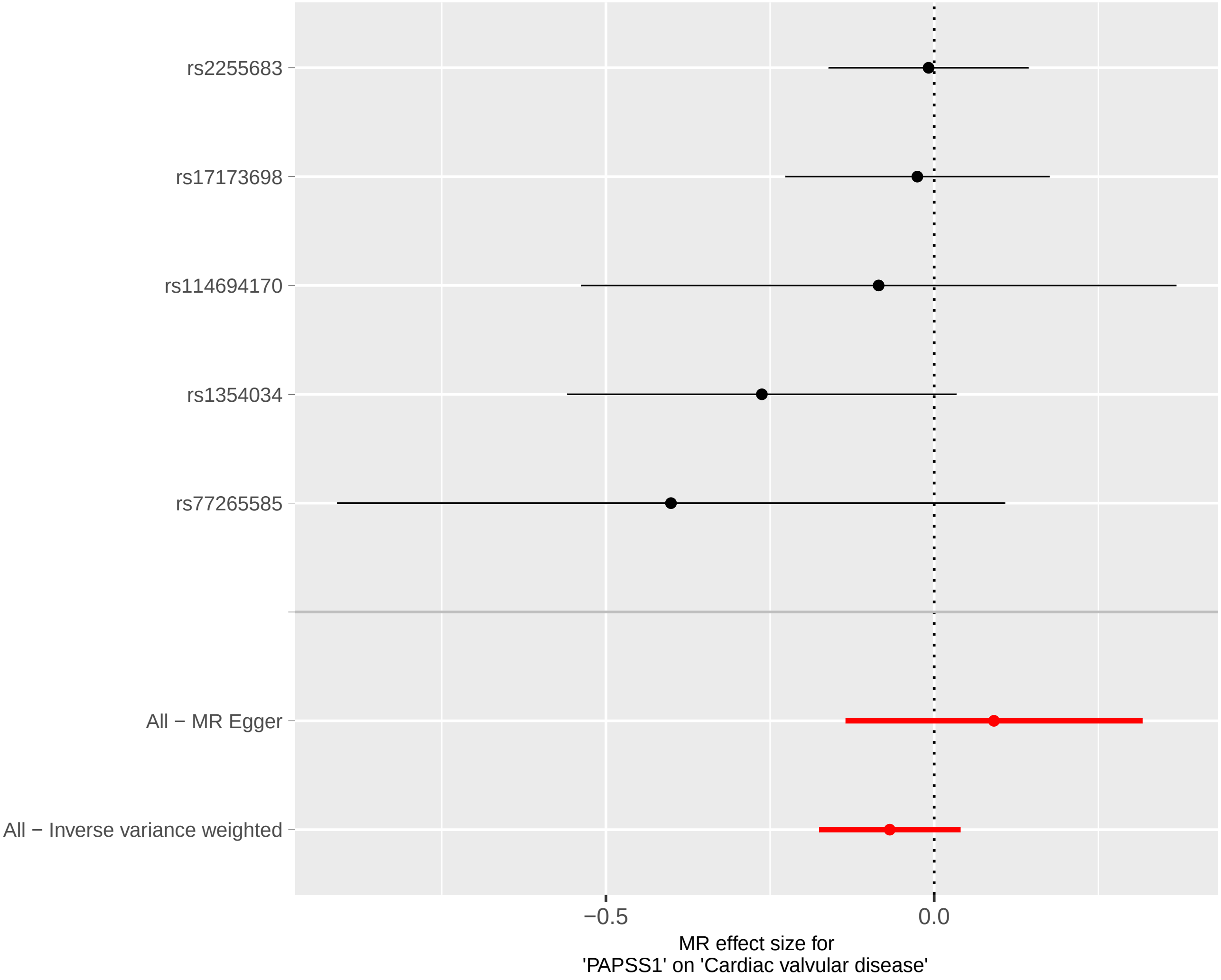

rs2255683
rs17173698
rs114694170
rs1354034
rs77265585
All − MR Egger
All − Inverse variance weighted
−0.5
0.0
MR effect size for
'PAPSS1' on 'Cardiac valvular disease'

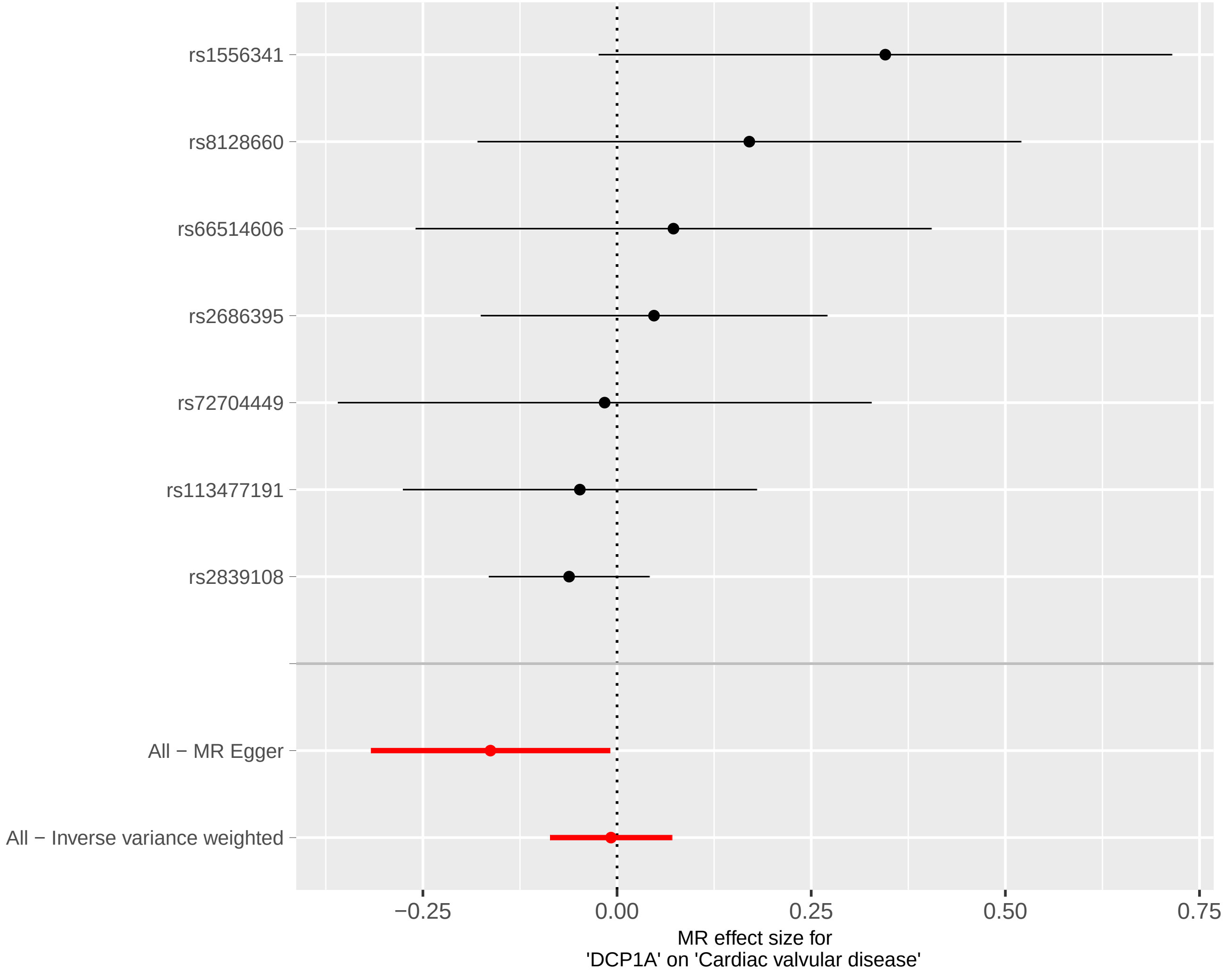
rs1556341
rs8128660
rs66514606
rs2686395
rs72704449
rs113477191
rs2839108
All − MR Egger
All − Inverse variance weighted
−0.25
0.00
0.25
0.50
0.75
MR effect size for
'DCP1A' on 'Cardiac valvular disease'

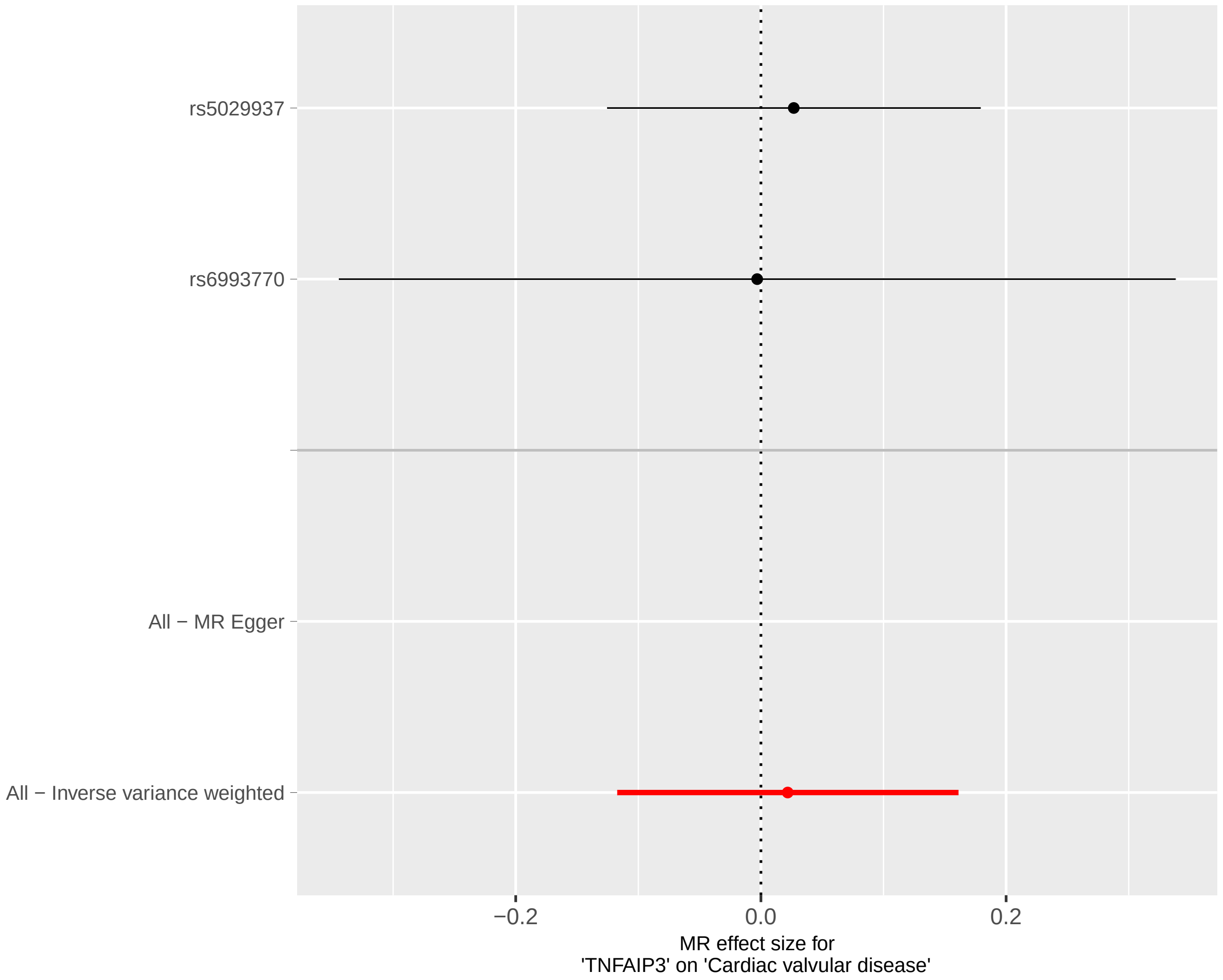

rs5029937
rs6993770
All − MR Egger
All − Inverse variance weighted
−0.2
0.0
0.2
MR effect size for
'TNFAIP3' on 'Cardiac valvular disease'

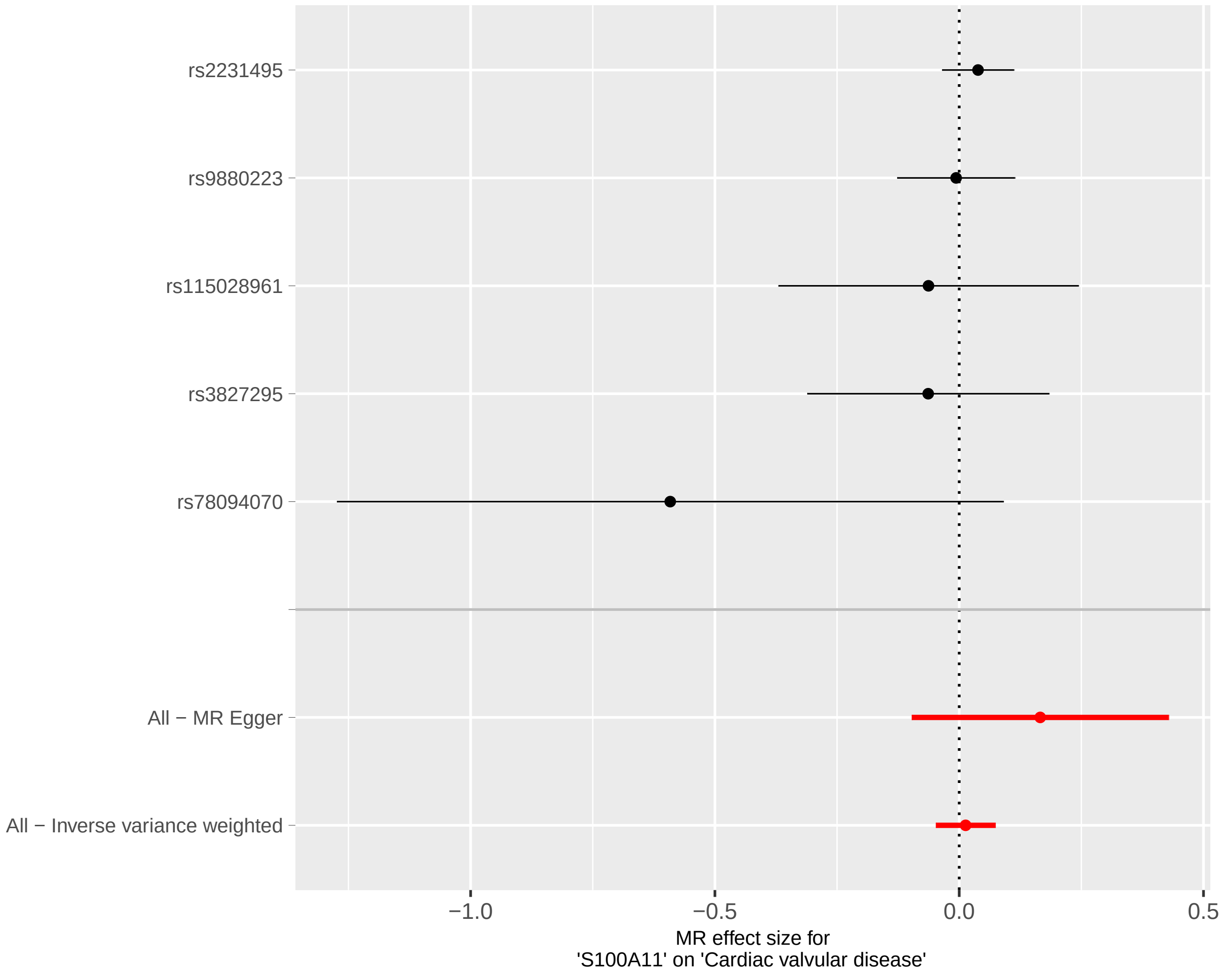

rs2231495
rs9880223
rs115028961
rs3827295
rs78094070
All − MR Egger
All − Inverse variance weighted
−1.0
−0.5
0.0
0.5
MR effect size for
'S100A11' on 'Cardiac valvular disease'

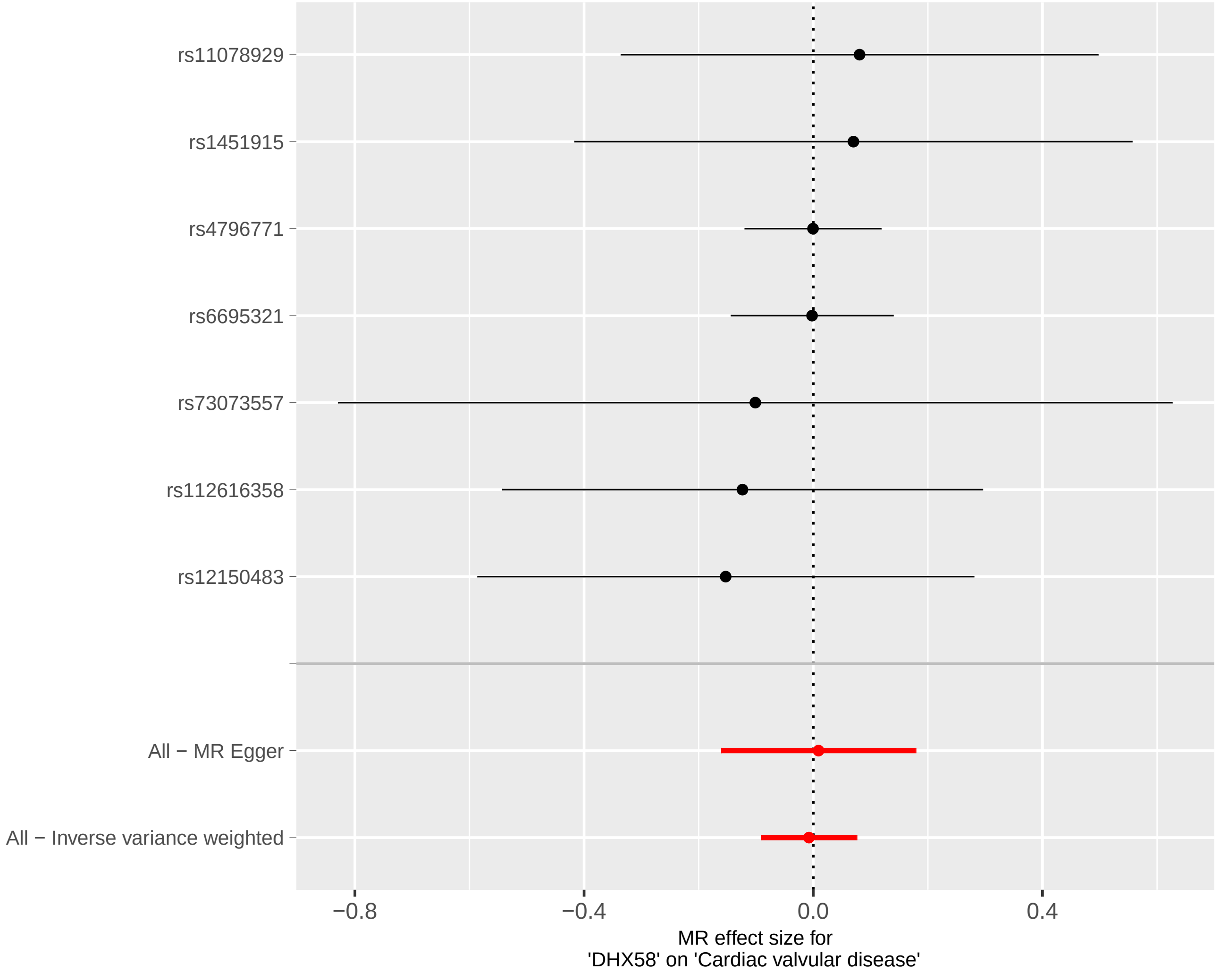
rs11078929
rs1451915
rs4796771
rs6695321
rs73073557
rs112616358
rs12150483
All − MR Egger
All − Inverse variance weighted
−0.8
−0.4
0.0
0.4
MR effect size for
'DHX58' on 'Cardiac valvular disease'

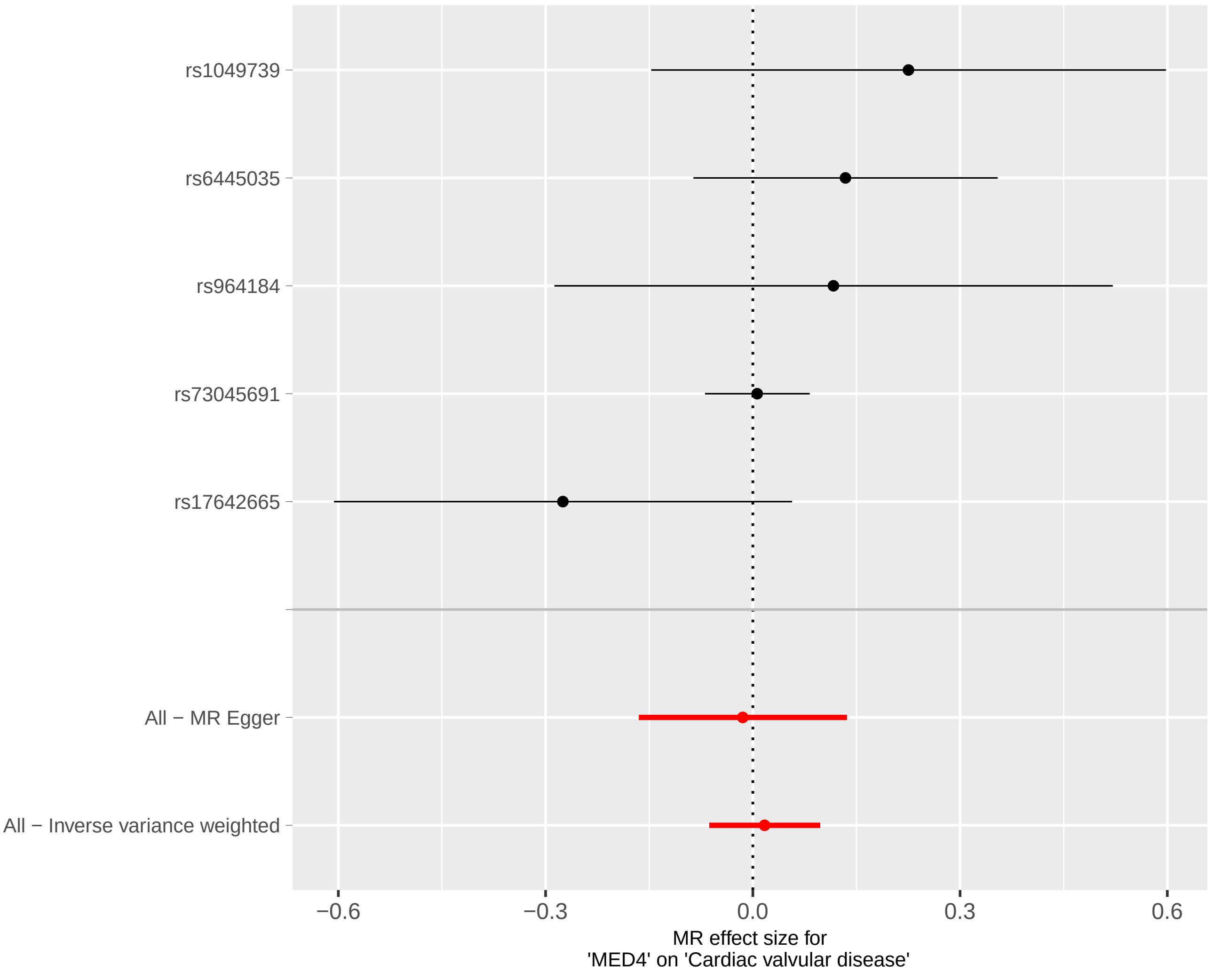

rs1049739
rs6445035
rs964184
rs73045691
rs17642665
All − MR Egger
All − Inverse variance weighted
−0.6
−0.3
0.0
0.3
0.6
MR effect size for
'MED4' on 'Cardiac valvular disease'

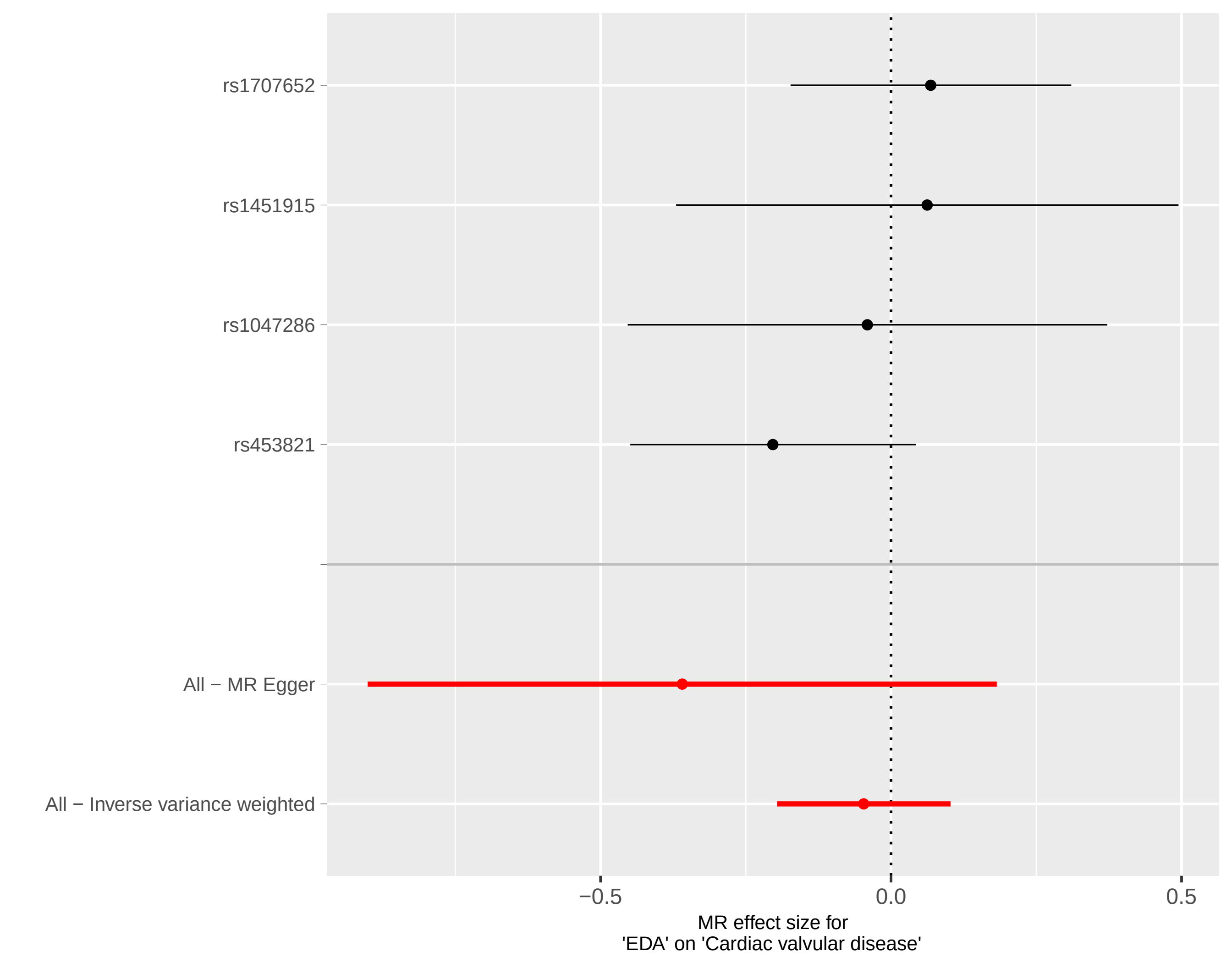
rs1707652
rs1451915
rs1047286
rs453821
All − MR Egger
All − Inverse variance weighted
−0.5
0.0
0.5
MR effect size for
'EDA' on 'Cardiac valvular disease'

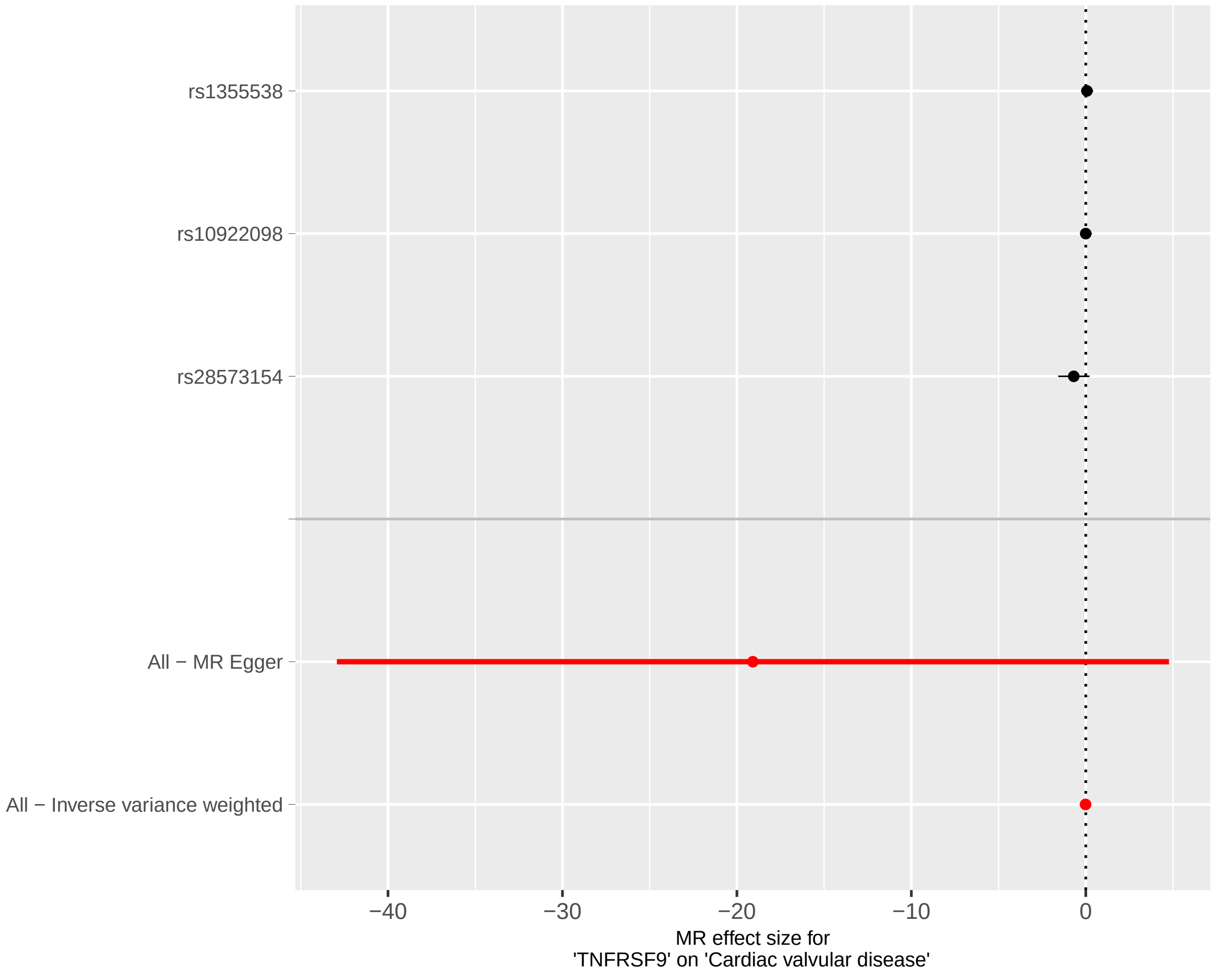
rs1355538
rs10922098
rs28573154
All − MR Egger
All − Inverse variance weighted
−40
−30
−20
−10
0
MR effect size for
'TNFRSF9' on 'Cardiac valvular disease'

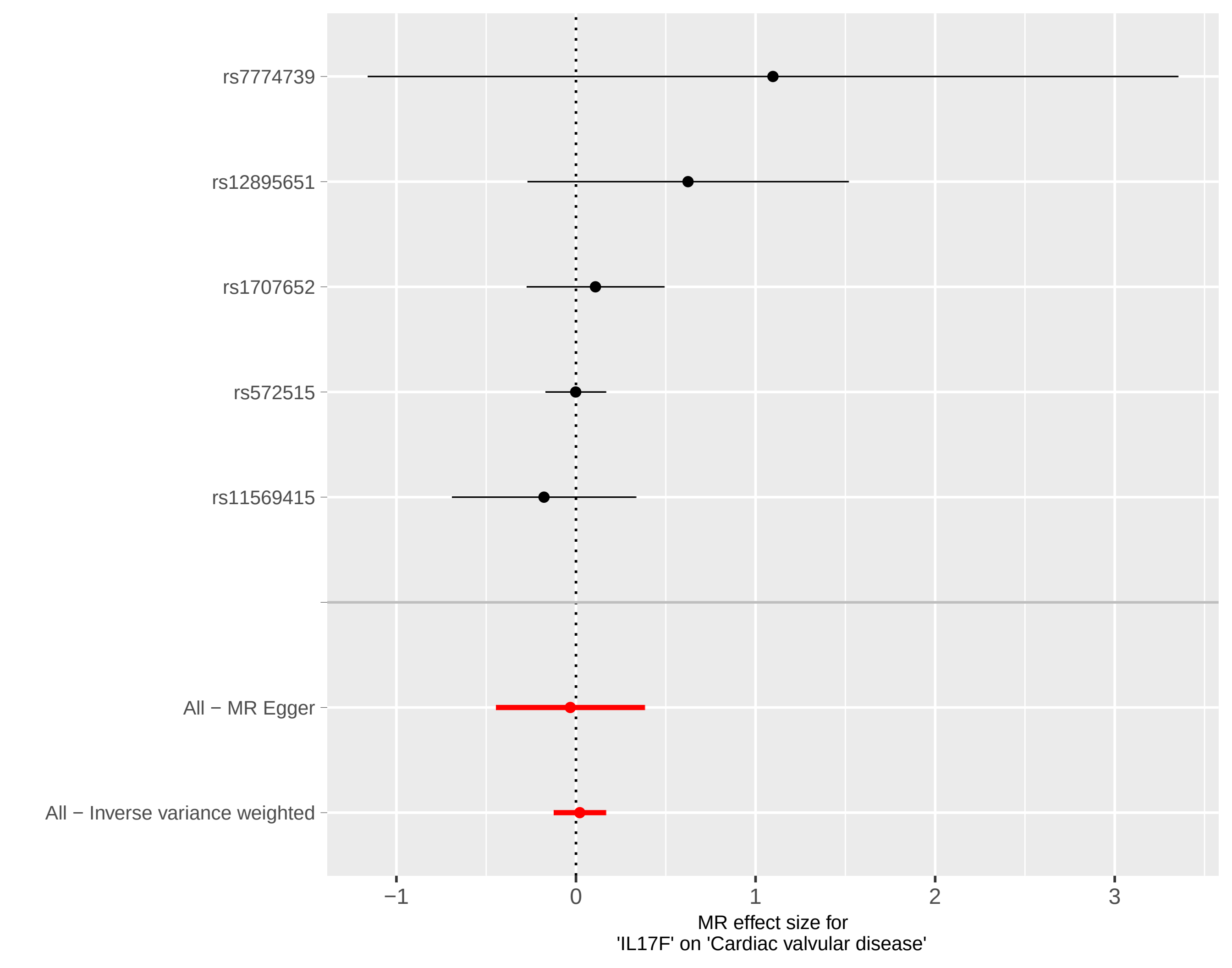
rs7774739
rs12895651
rs1707652
rs572515
rs11569415
All − MR Egger
All − Inverse variance weighted
−1
0
1
2
3
MR effect size for
'IL17F' on 'Cardiac valvular disease'

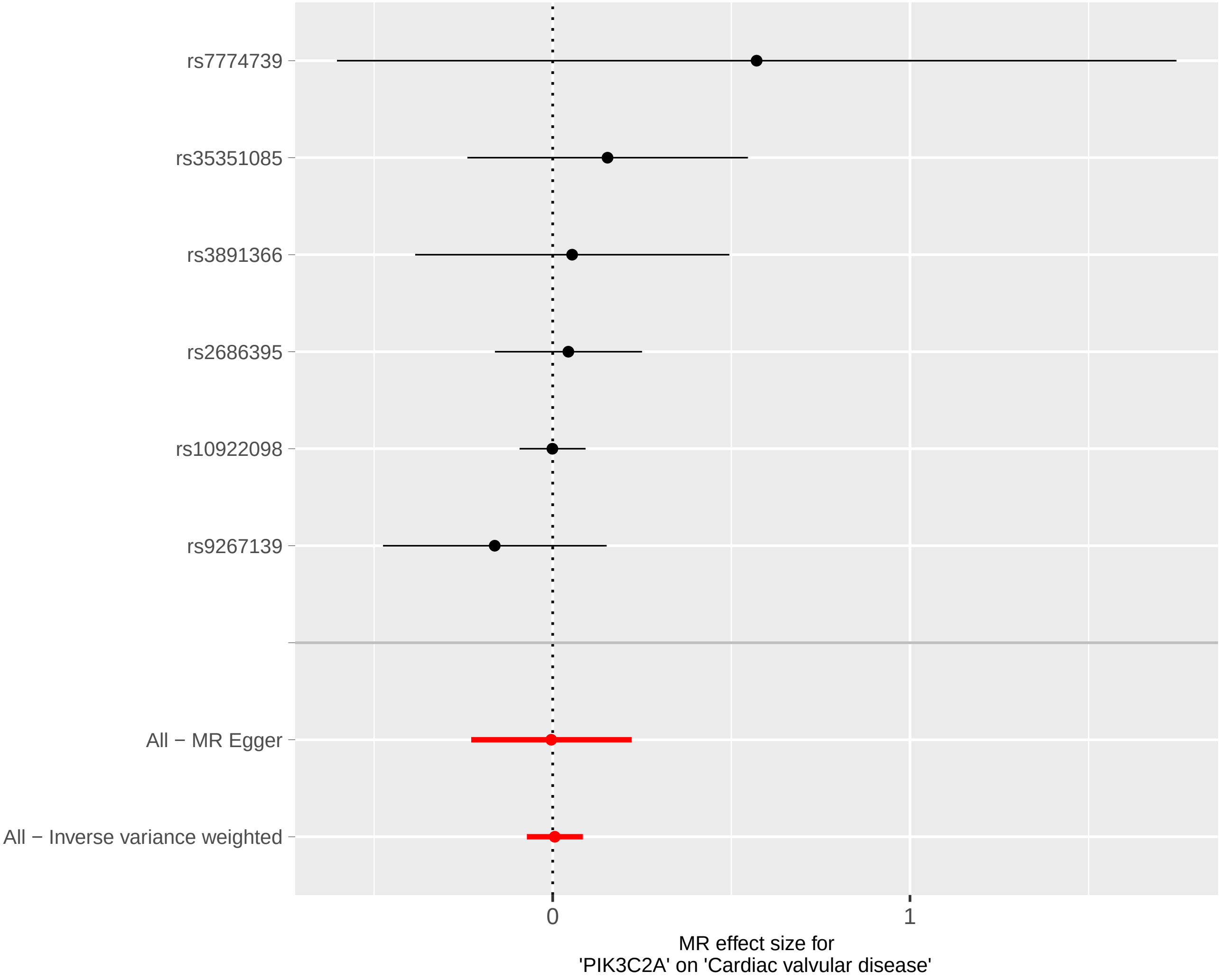
rs7774739
rs35351085
rs3891366
rs2686395
rs10922098
rs9267139
All − MR Egger
All − Inverse variance weighted
0
1
MR effect size for
'PIK3C2A' on 'Cardiac valvular disease'

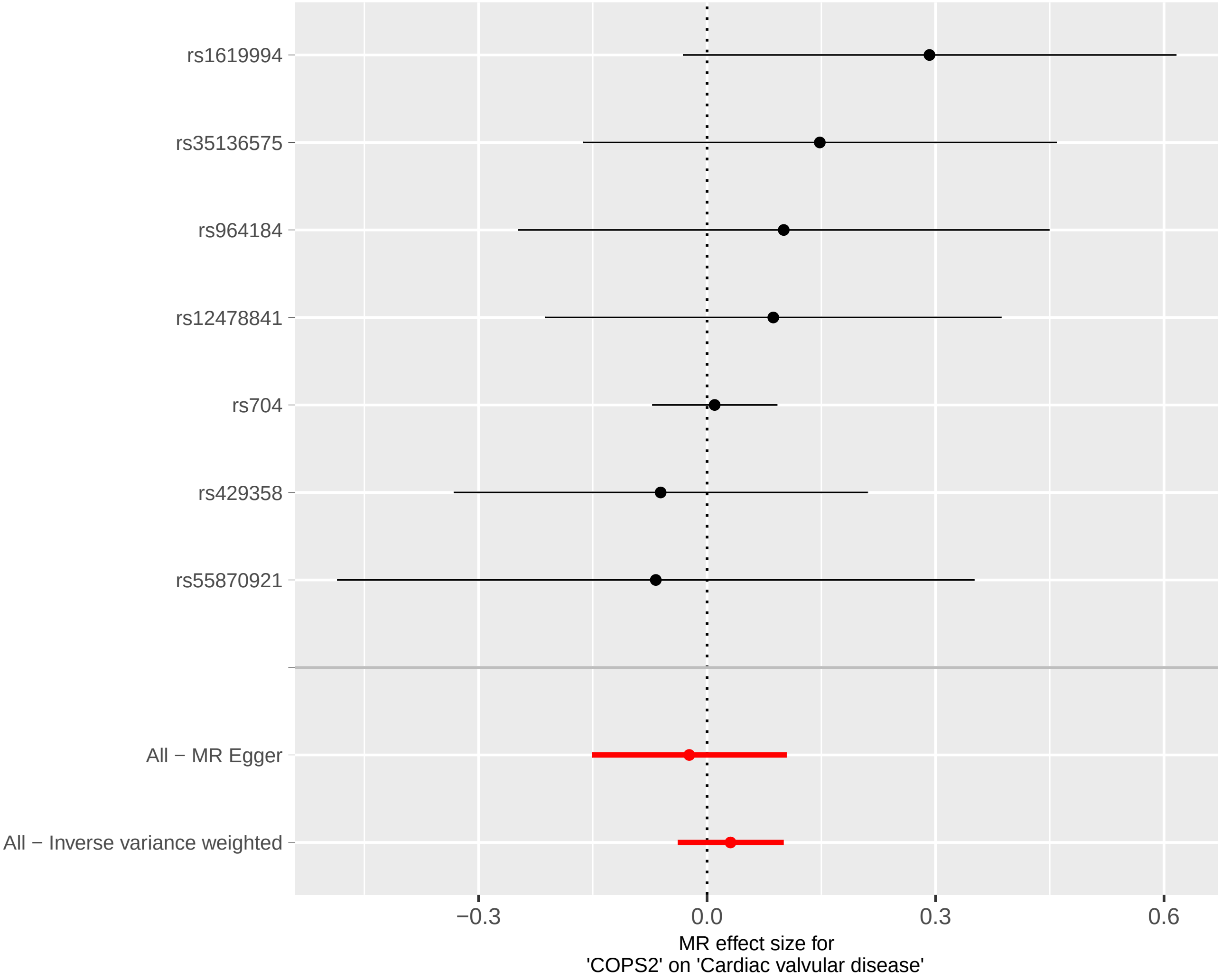

rs1619994
rs35136575
rs964184
rs12478841
rs704
rs429358
rs55870921
All − MR Egger
All − Inverse variance weighted
−0.3
0.0
0.3
0.6
MR effect size for
'COPS2' on 'Cardiac valvular disease'

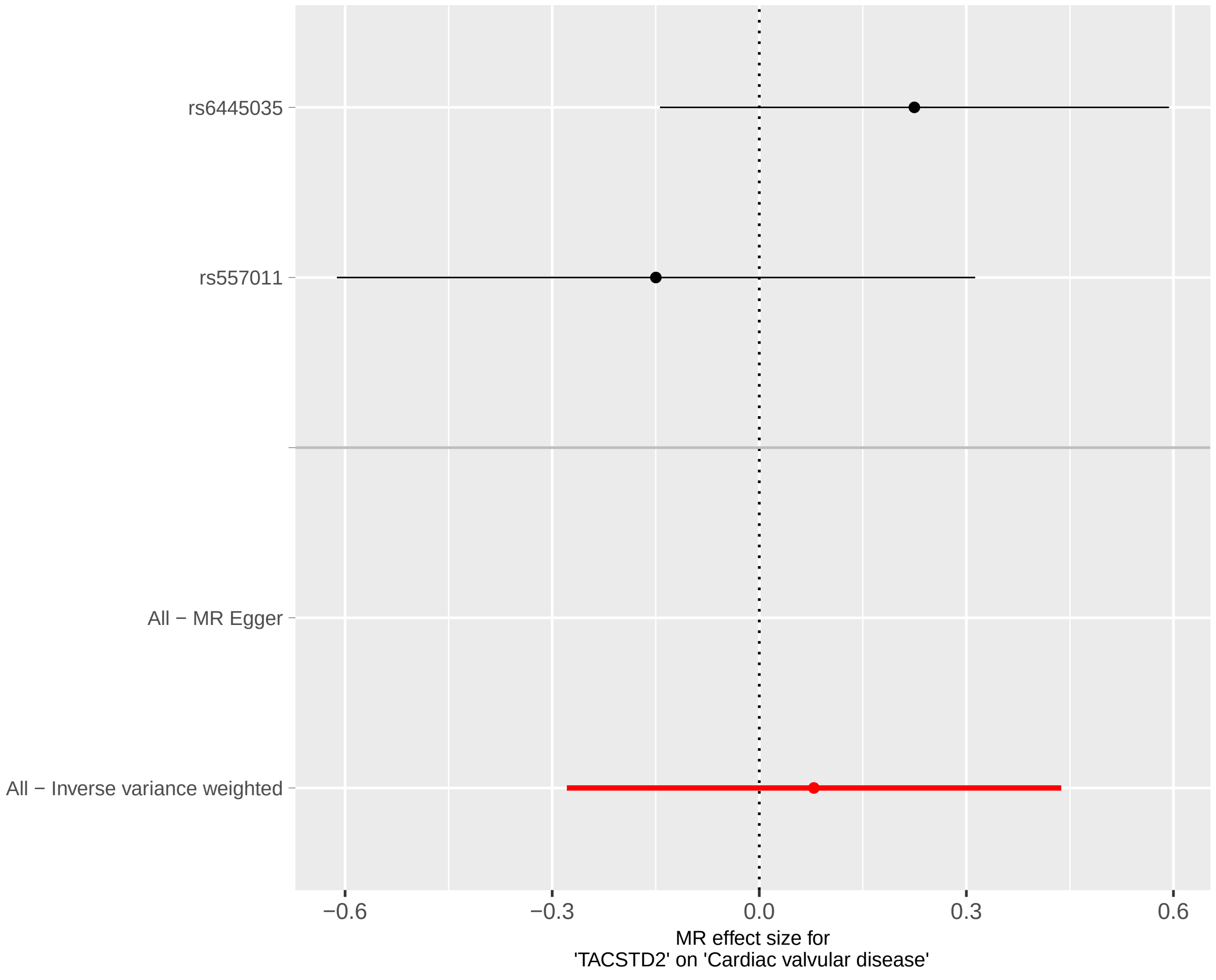
rs6445035
rs557011
All − MR Egger
All − Inverse variance weighted
−0.6
−0.3
0.0
0.3
0.6
MR effect size for
'TACSTD2' on 'Cardiac valvular disease'

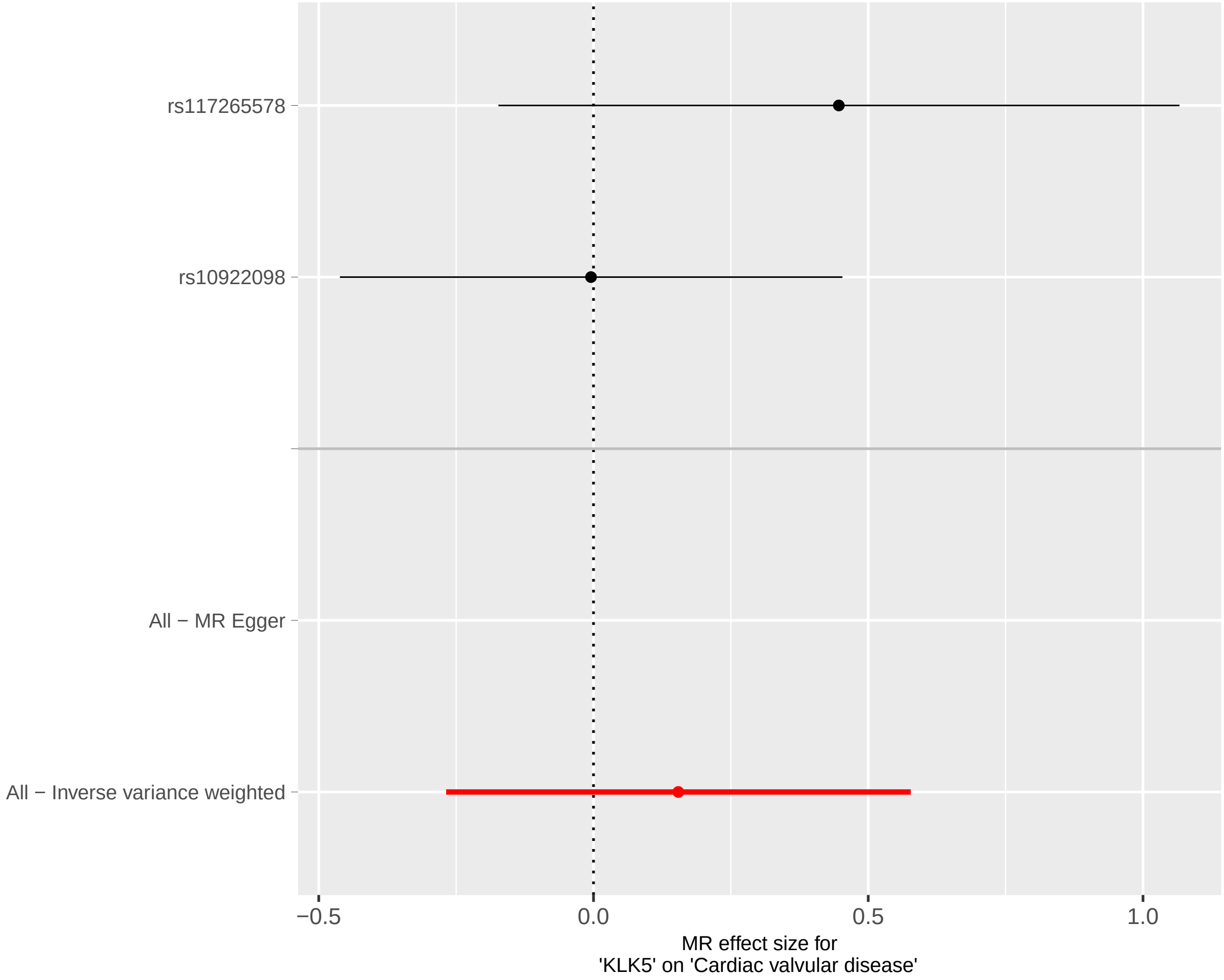

rs117265578
rs10922098
All − MR Egger
All − Inverse variance weighted
−0.5
0.0
0.5
1.0
MR effect size for
'KLK5' on 'Cardiac valvular disease'

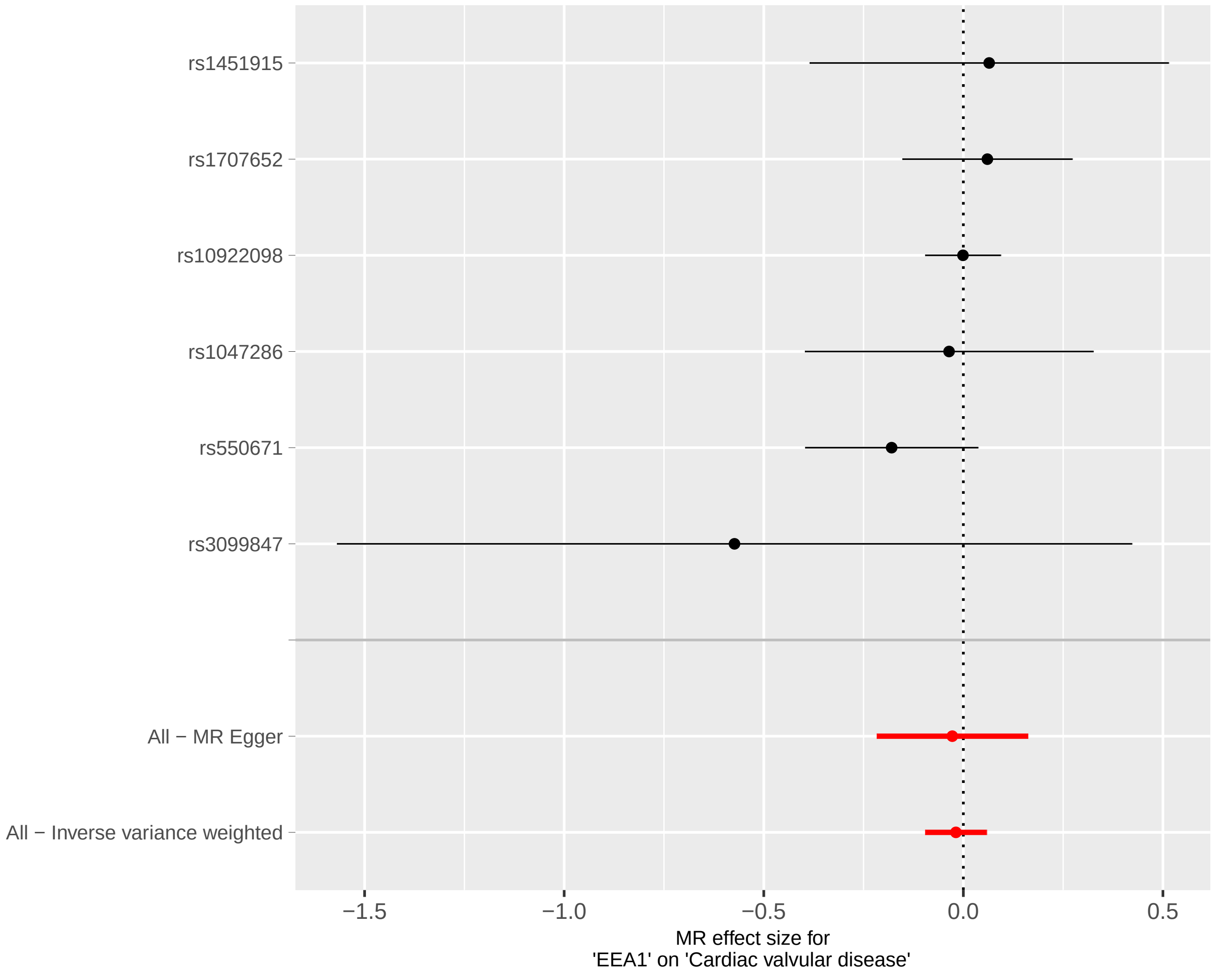
rs1451915
rs1707652
rs10922098
rs1047286
rs550671
rs3099847
All − MR Egger
All − Inverse variance weighted
−1.5
−1.0
−0.5
0.0
0.5
MR effect size for
'EEA1' on 'Cardiac valvular disease'

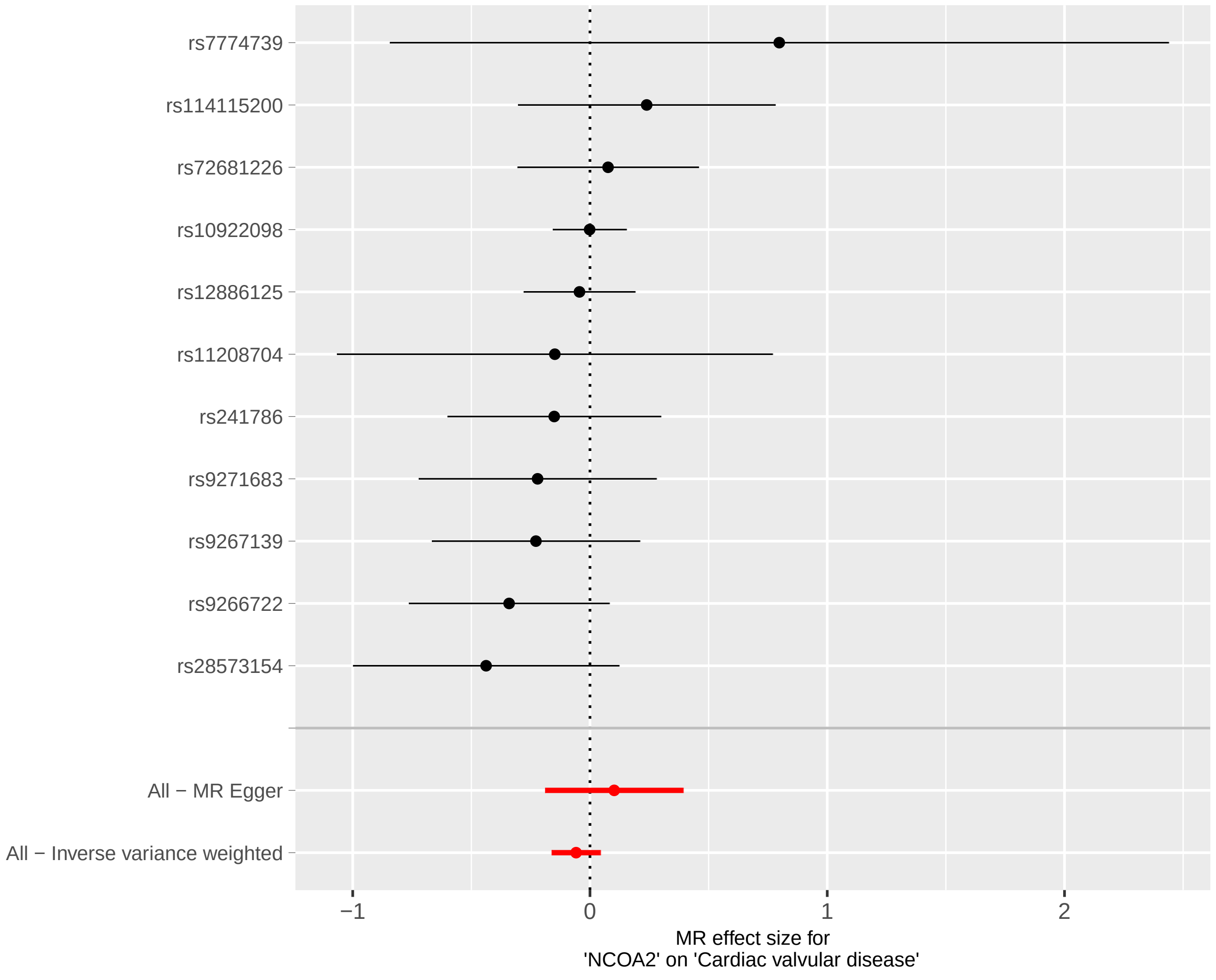
rs7774739
rs114115200
rs72681226
rs10922098
rs12886125
rs11208704
rs241786
rs9271683
rs9267139
rs9266722
rs28573154
All − MR Egger
All − Inverse variance weighted
−1
0
1
2
MR effect size for
'NCOA2' on 'Cardiac valvular disease'

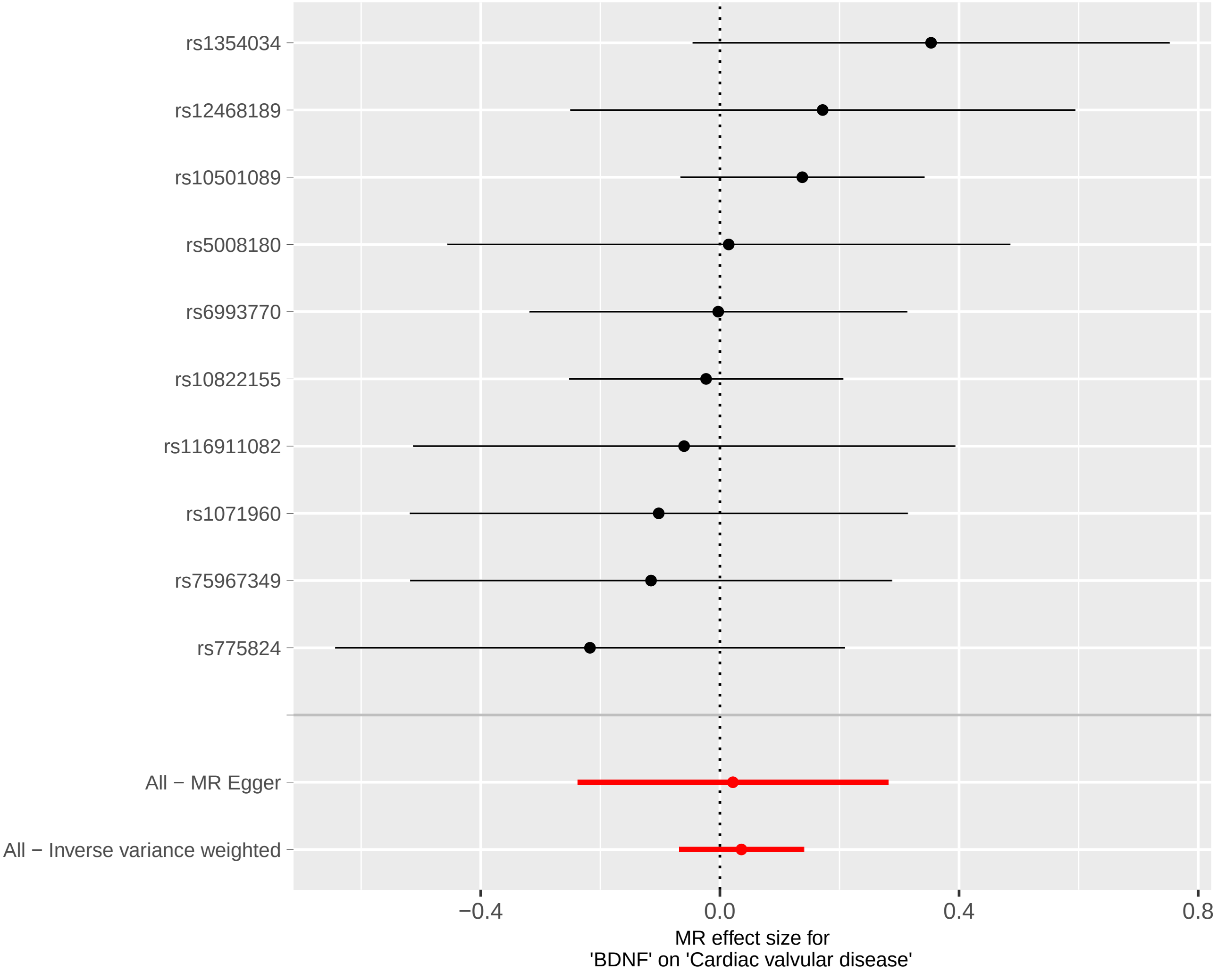
rs1354034
rs12468189
rs10501089
rs5008180
rs6993770
rs10822155
rs116911082
rs1071960
rs75967349
rs775824
All − MR Egger
All − Inverse variance weighted
−0.4
0.0
0.4
0.8
MR effect size for
'BDNF' on 'Cardiac valvular disease'

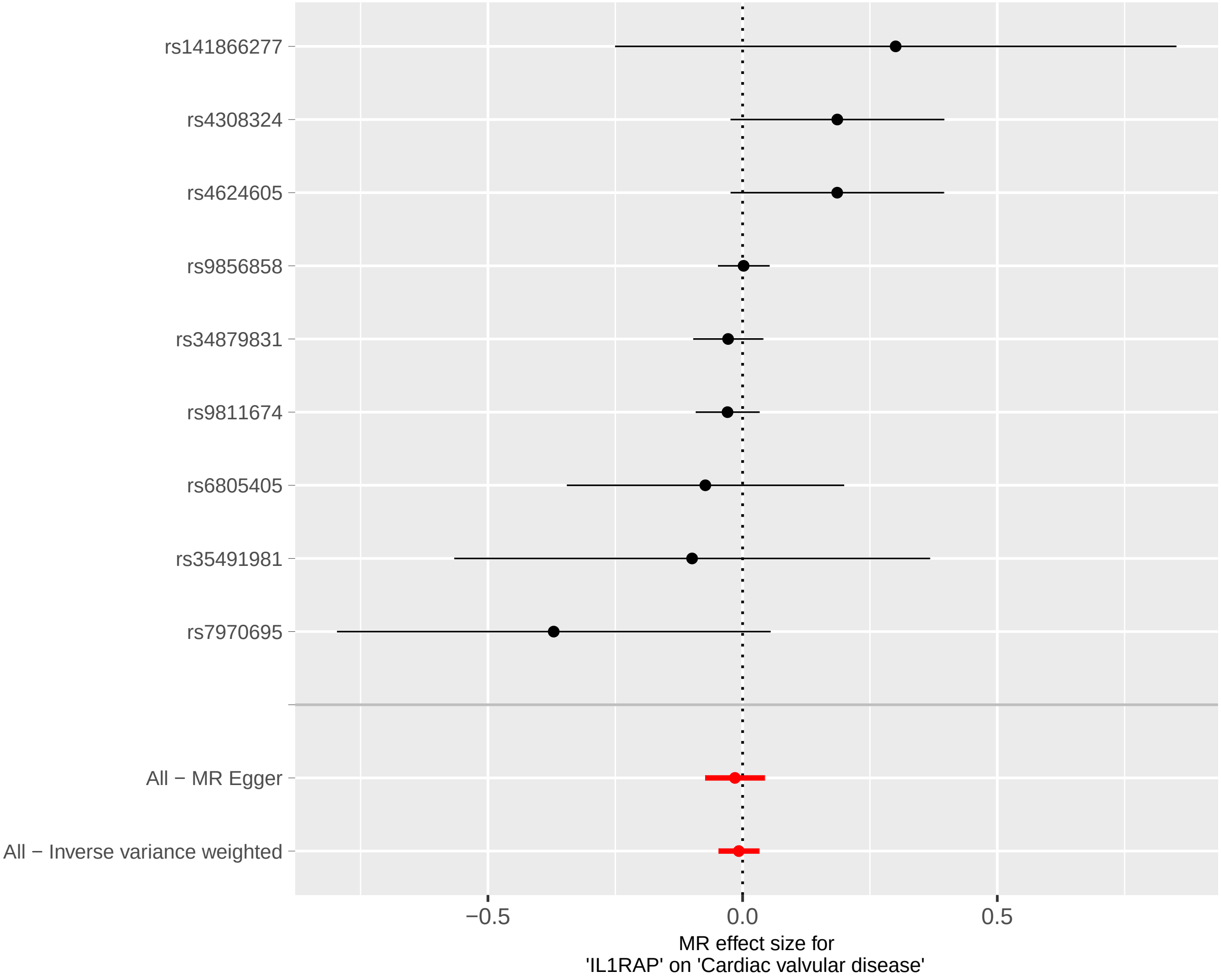
rs141866277
rs4308324
rs4624605
rs9856858
rs34879831
rs9811674
rs6805405
rs35491981
rs7970695
All − MR Egger
All − Inverse variance weighted
−0.5
0.0
0.5
MR effect size for
'IL1RAP' on 'Cardiac valvular disease'

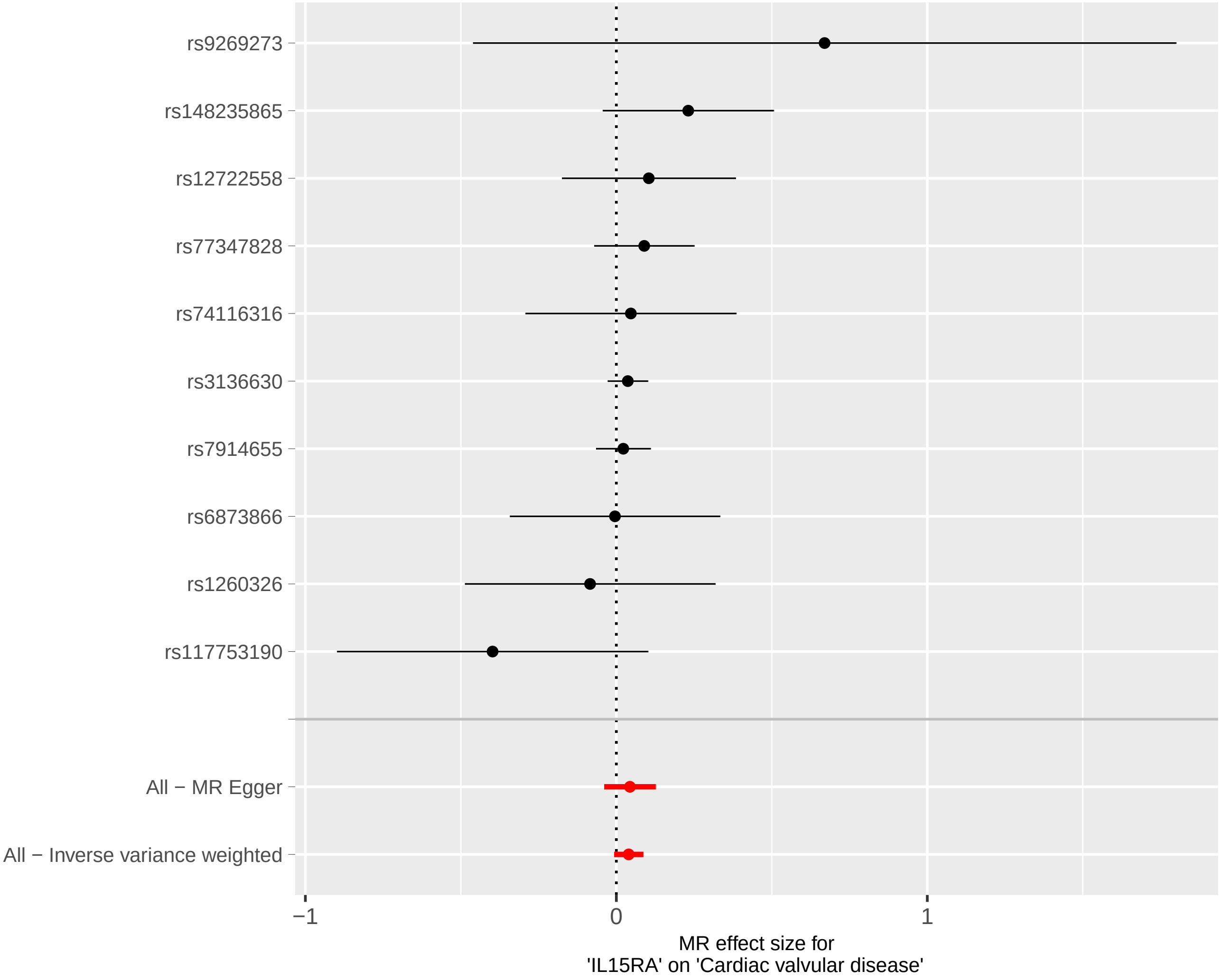

rs9269273
rs148235865
rs12722558
rs77347828
rs74116316
rs3136630
rs7914655
rs6873866
rs1260326
rs117753190
All − MR Egger
All − Inverse variance weighted
−1
0
1
MR effect size for
'IL15RA' on 'Cardiac valvular disease'

Insufficient number of SNPs

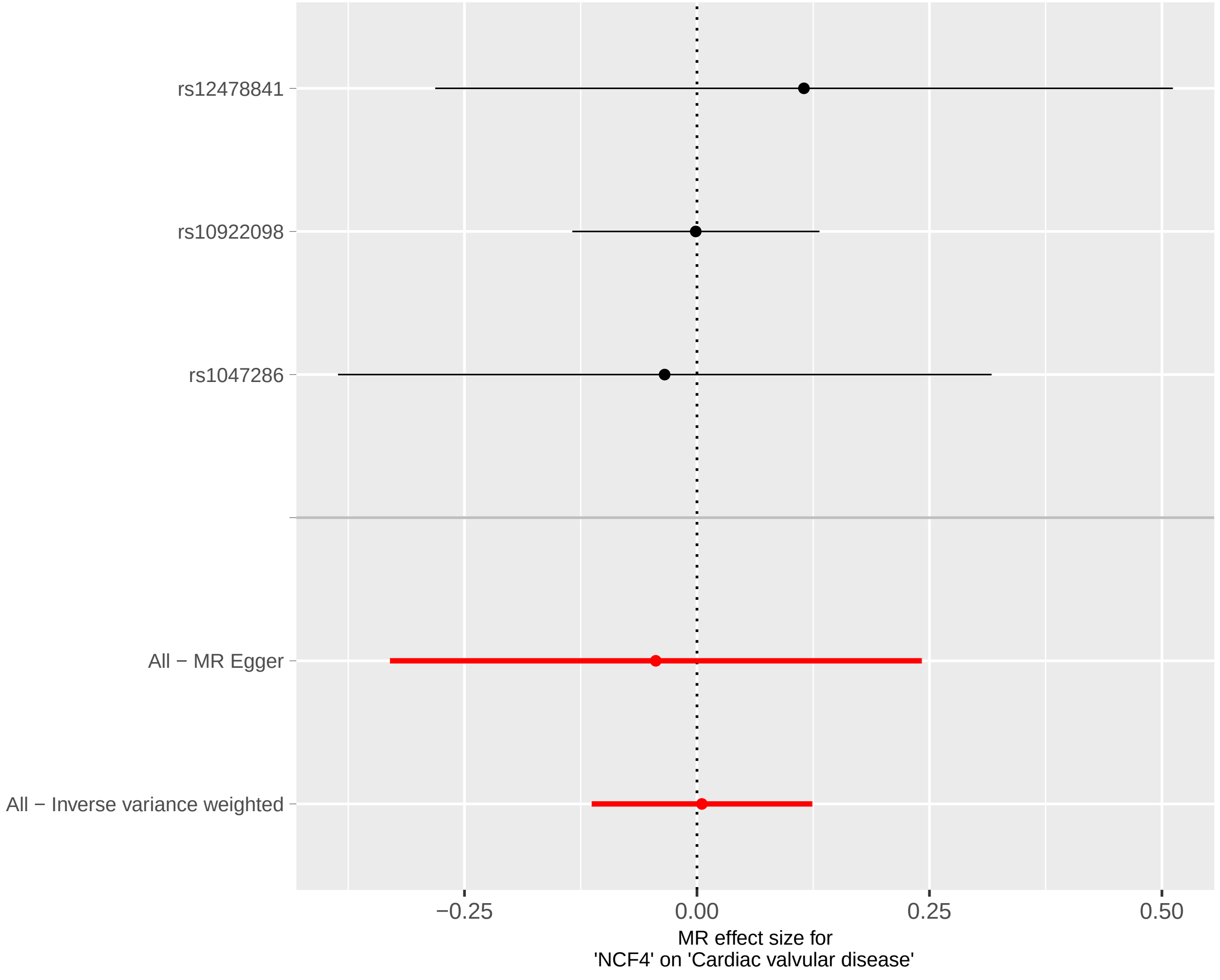
rs12478841
rs10922098
rs1047286
All − MR Egger
All − Inverse variance weighted
−0.25
0.00
0.25
0.50
MR effect size for
'NCF4' on 'Cardiac valvular disease'

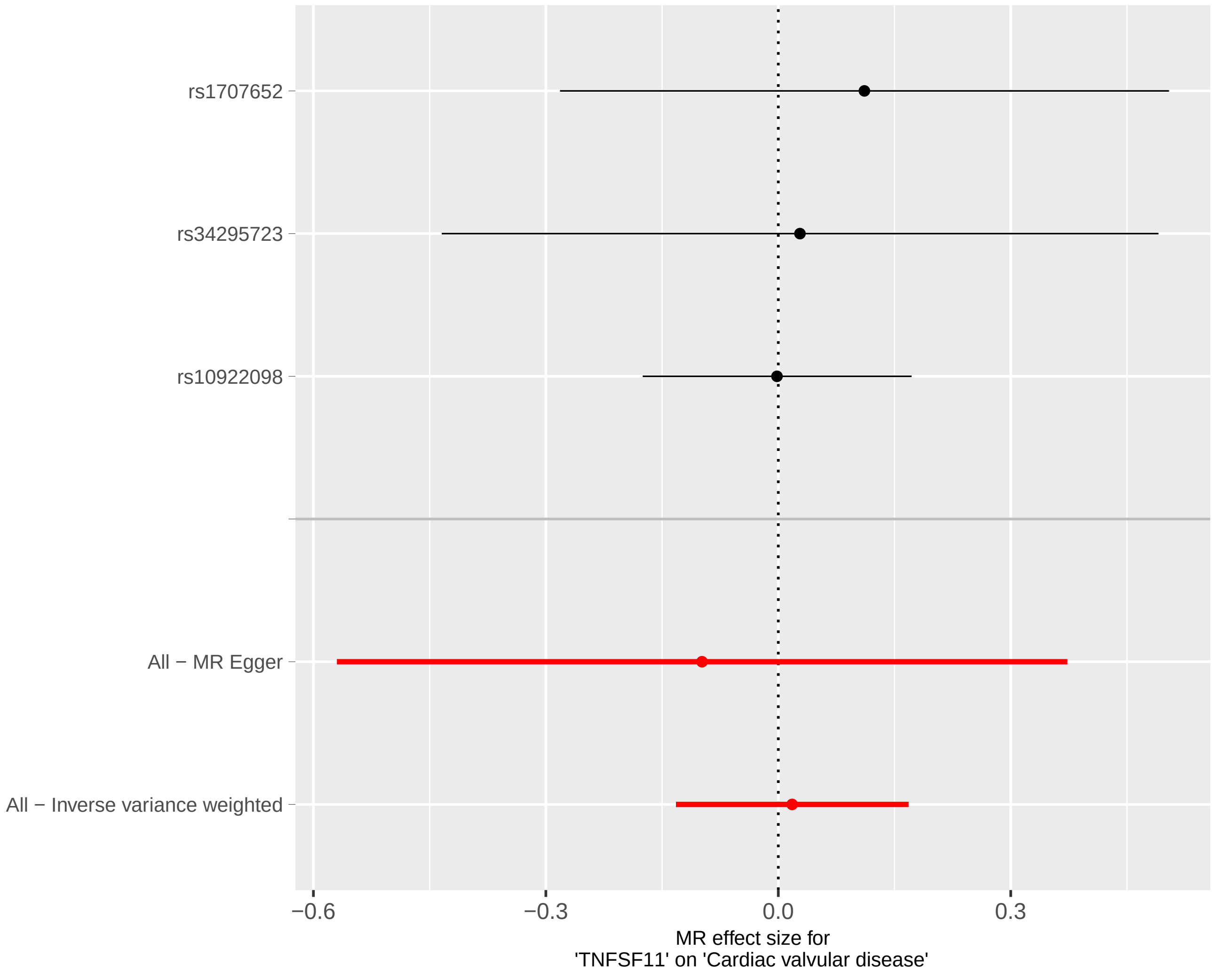
rs1707652
rs34295723
rs10922098
All − MR Egger
All − Inverse variance weighted
−0.6
−0.3
0.0
0.3
MR effect size for
'TNFSF11' on 'Cardiac valvular disease'

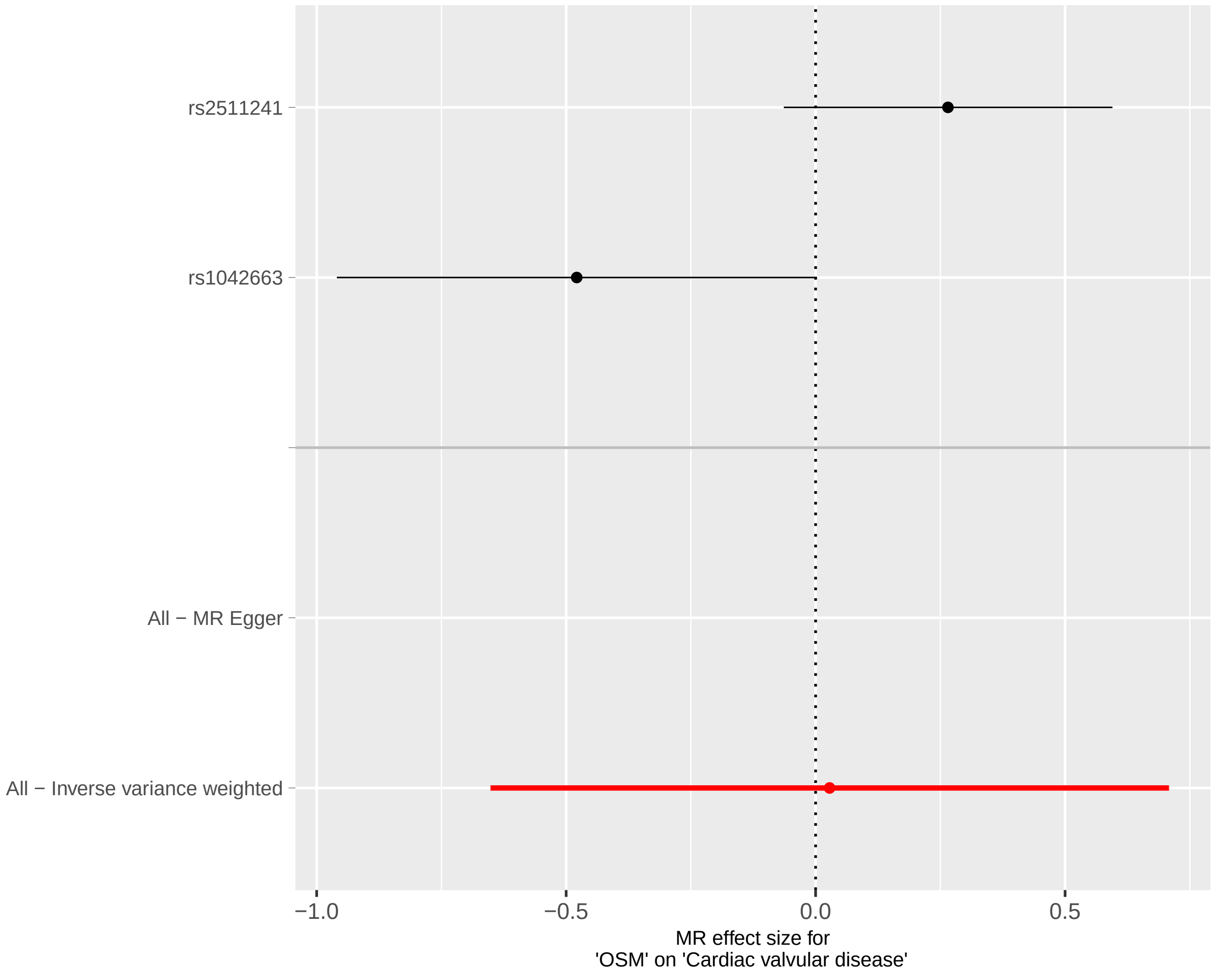
rs2511241
rs1042663
All − MR Egger
All − Inverse variance weighted
−1.0
−0.5
0.0
0.5
MR effect size for
'OSM' on 'Cardiac valvular disease'

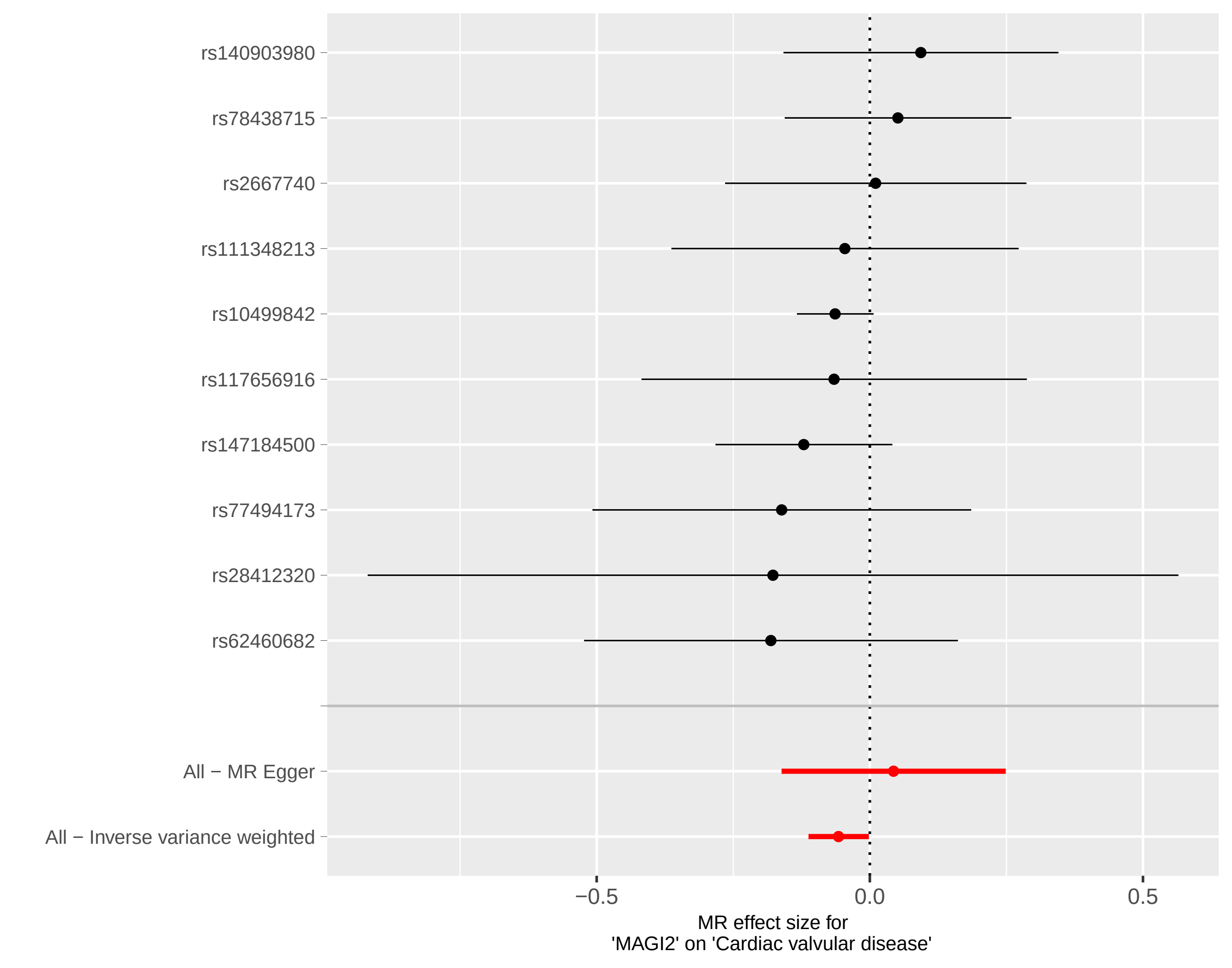
rs140903980
rs78438715
rs2667740
rs111348213
rs10499842
rs117656916
rs147184500
rs77494173
rs28412320
rs62460682
All − MR Egger
All − Inverse variance weighted
−0.5
0.0
0.5
MR effect size for
'MAGI2' on 'Cardiac valvular disease'

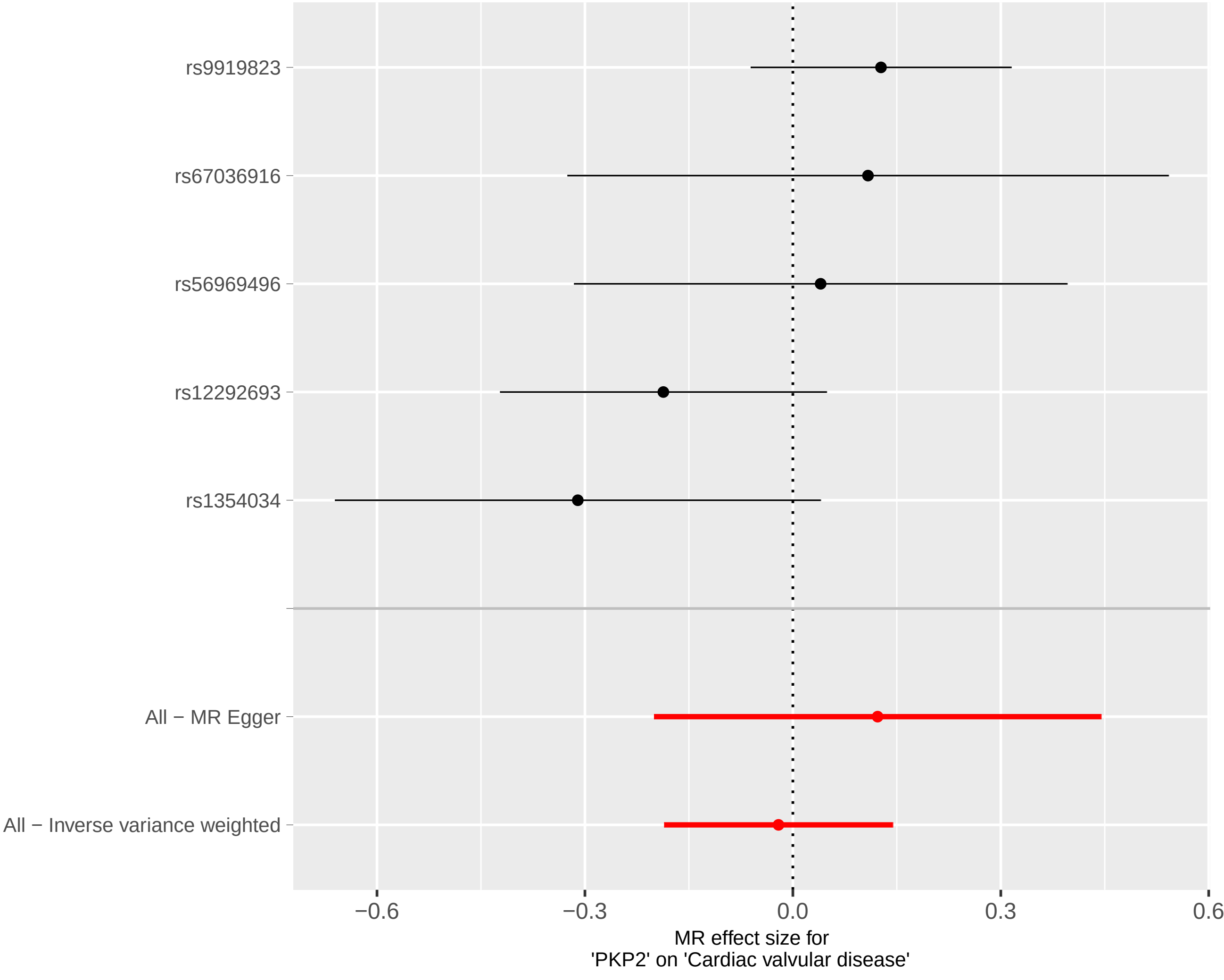
rs9919823
rs67036916
rs56969496
rs12292693
rs1354034
All − MR Egger
All − Inverse variance weighted
−0.6
−0.3
0.0
0.3
0.6
MR effect size for
'PKP2' on 'Cardiac valvular disease'

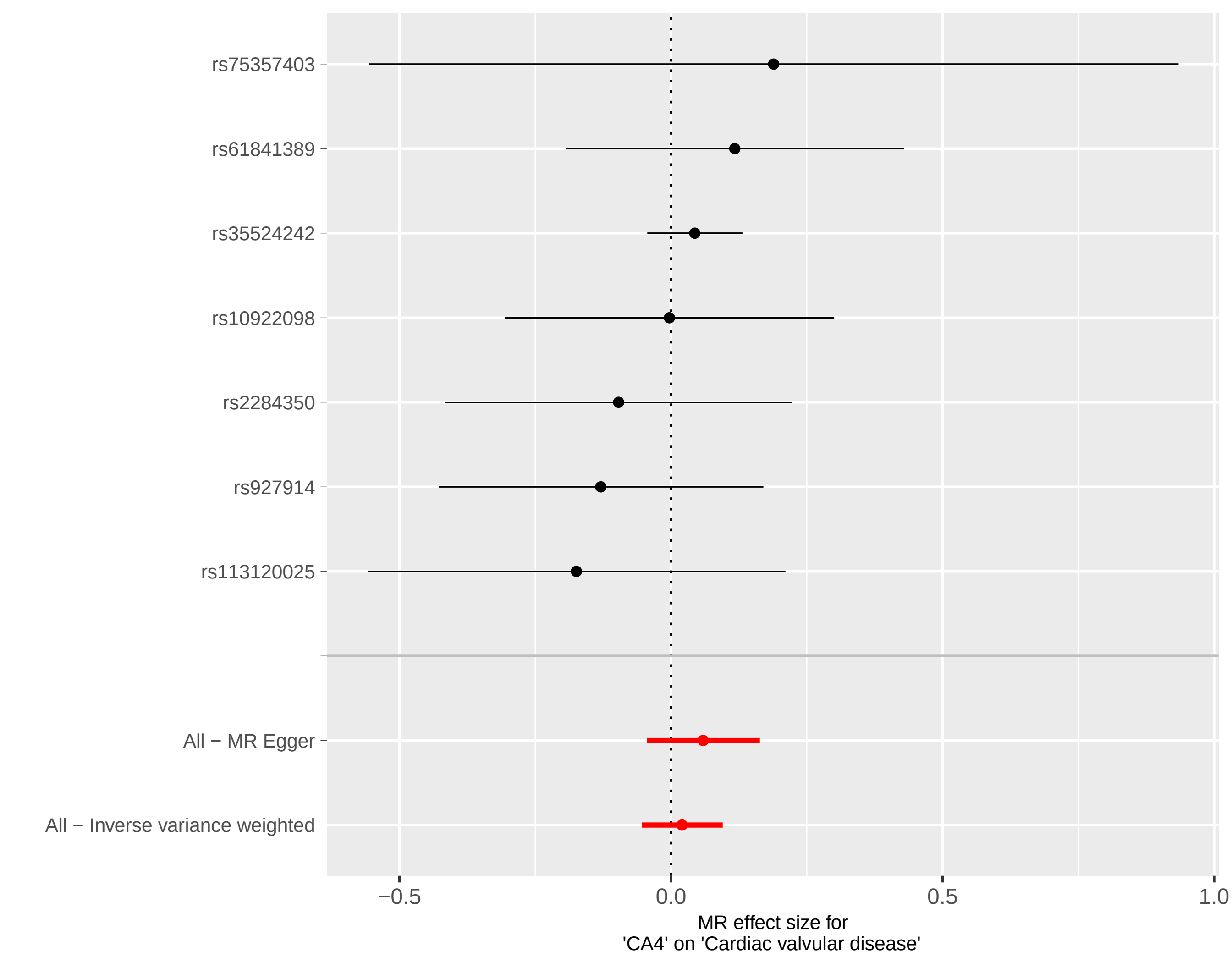
rs75357403
rs61841389
rs35524242
rs10922098
rs2284350
rs927914
rs113120025
All − MR Egger
All − Inverse variance weighted
−0.5
0.0
0.5
1.0
MR effect size for
'CA4' on 'Cardiac valvular disease'

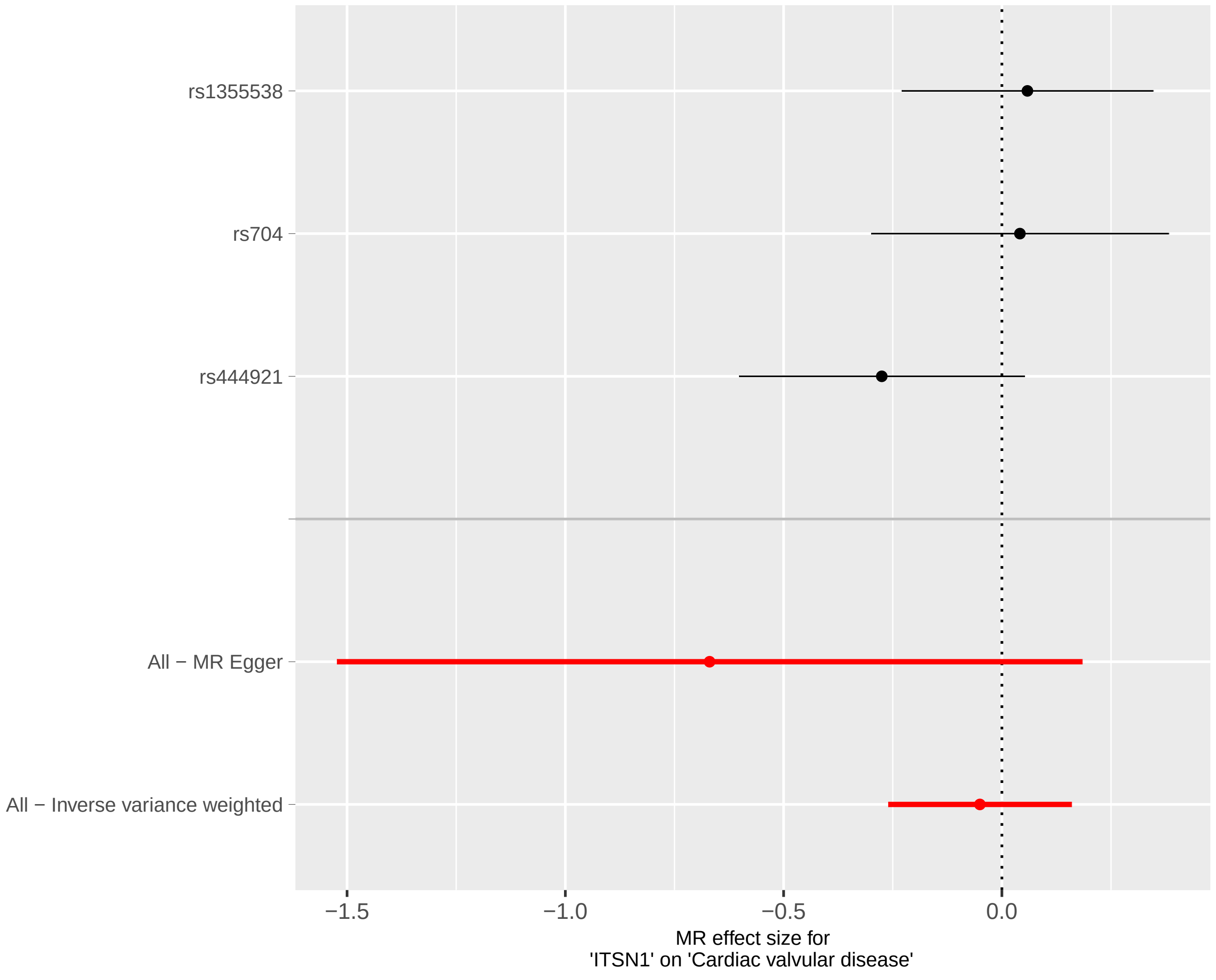
rs1355538
rs704
rs444921
All − MR Egger
All − Inverse variance weighted
−1.5
−1.0
−0.5
0.0
MR effect size for
'ITSN1' on 'Cardiac valvular disease'

Insufficient number of SNPs

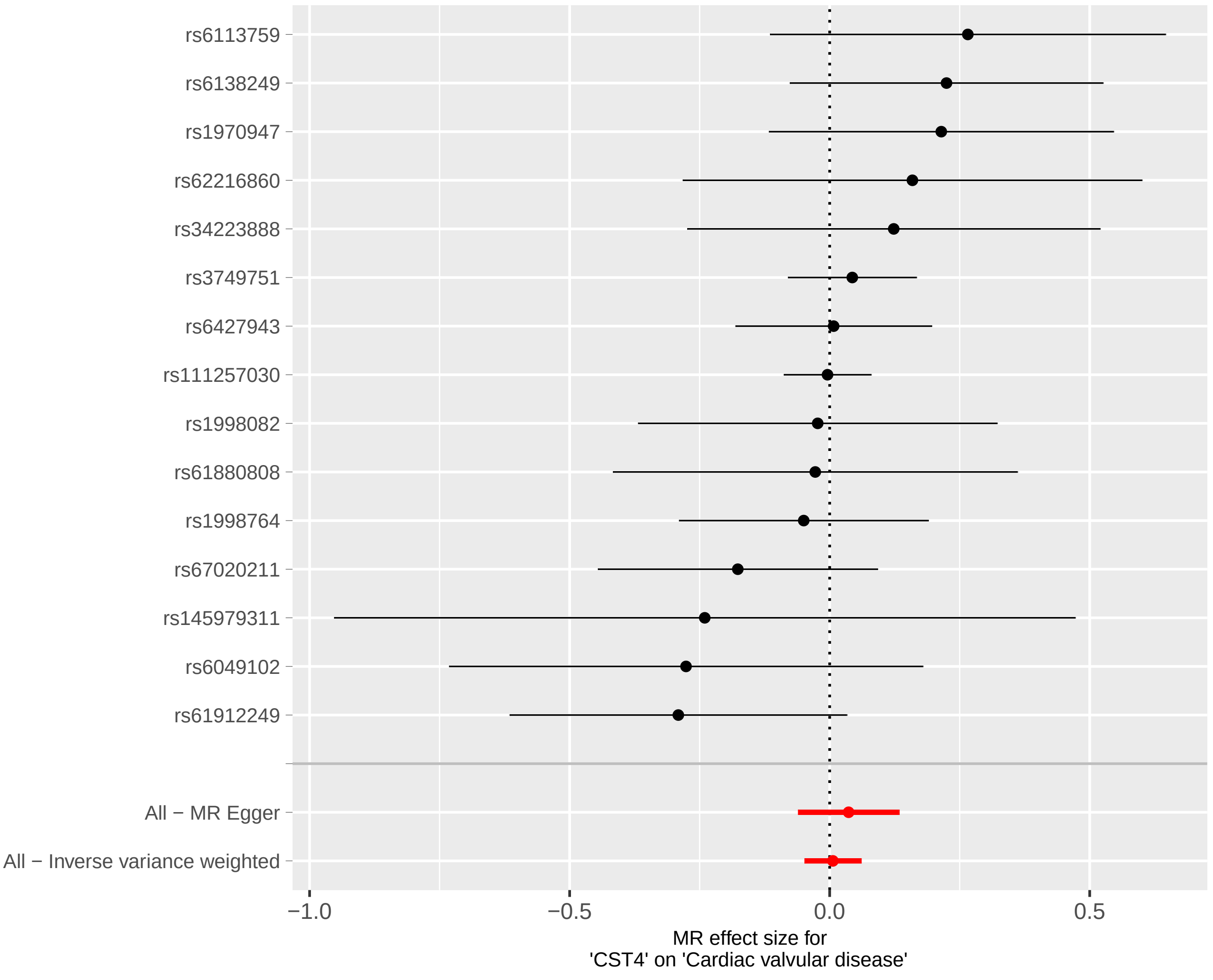
rs6113759
rs6138249
rs1970947
rs62216860
rs34223888
rs3749751
rs6427943
rs111257030
rs1998082
rs61880808
rs1998764
rs67020211
rs145979311
rs6049102
rs61912249
All − MR Egger
All − Inverse variance weighted
−1.0
−0.5
0.0
0.5
MR effect size for
'CST4' on 'Cardiac valvular disease'

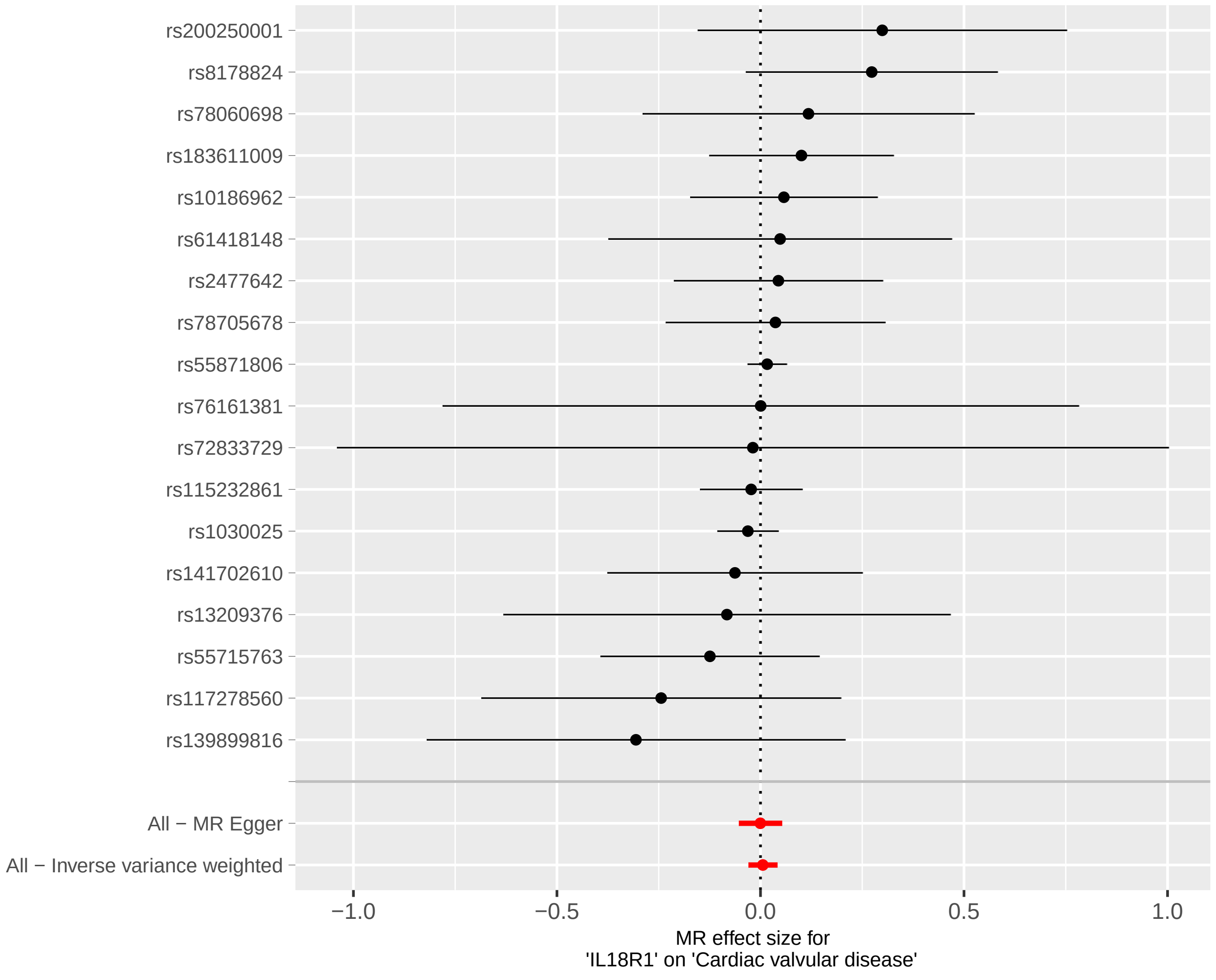

rs200250001
rs8178824
rs78060698
rs183611009
rs10186962
rs61418148
rs2477642
rs78705678
rs55871806
rs76161381
rs72833729
rs115232861
rs1030025
rs141702610
rs13209376
rs55715763
rs117278560
rs139899816
All − MR Egger
All − Inverse variance weighted
−1.0
−0.5
0.0
0.5
1.0
MR effect size for
'IL18R1' on 'Cardiac valvular disease'

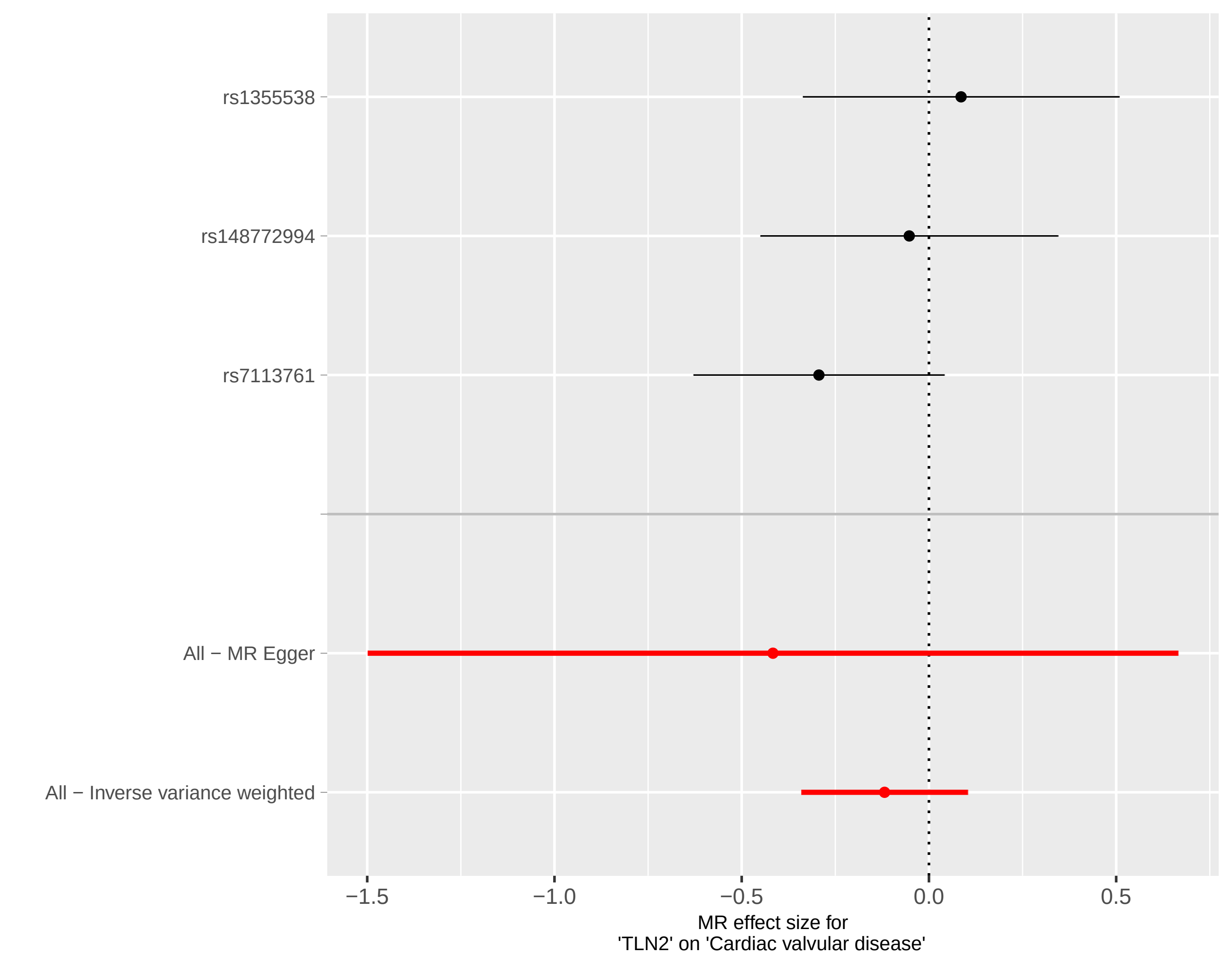
rs1355538
rs148772994
rs7113761
All − MR Egger
All − Inverse variance weighted
−1.5
−1.0
−0.5
0.0
0.5
MR effect size for
'TLN2' on 'Cardiac valvular disease'

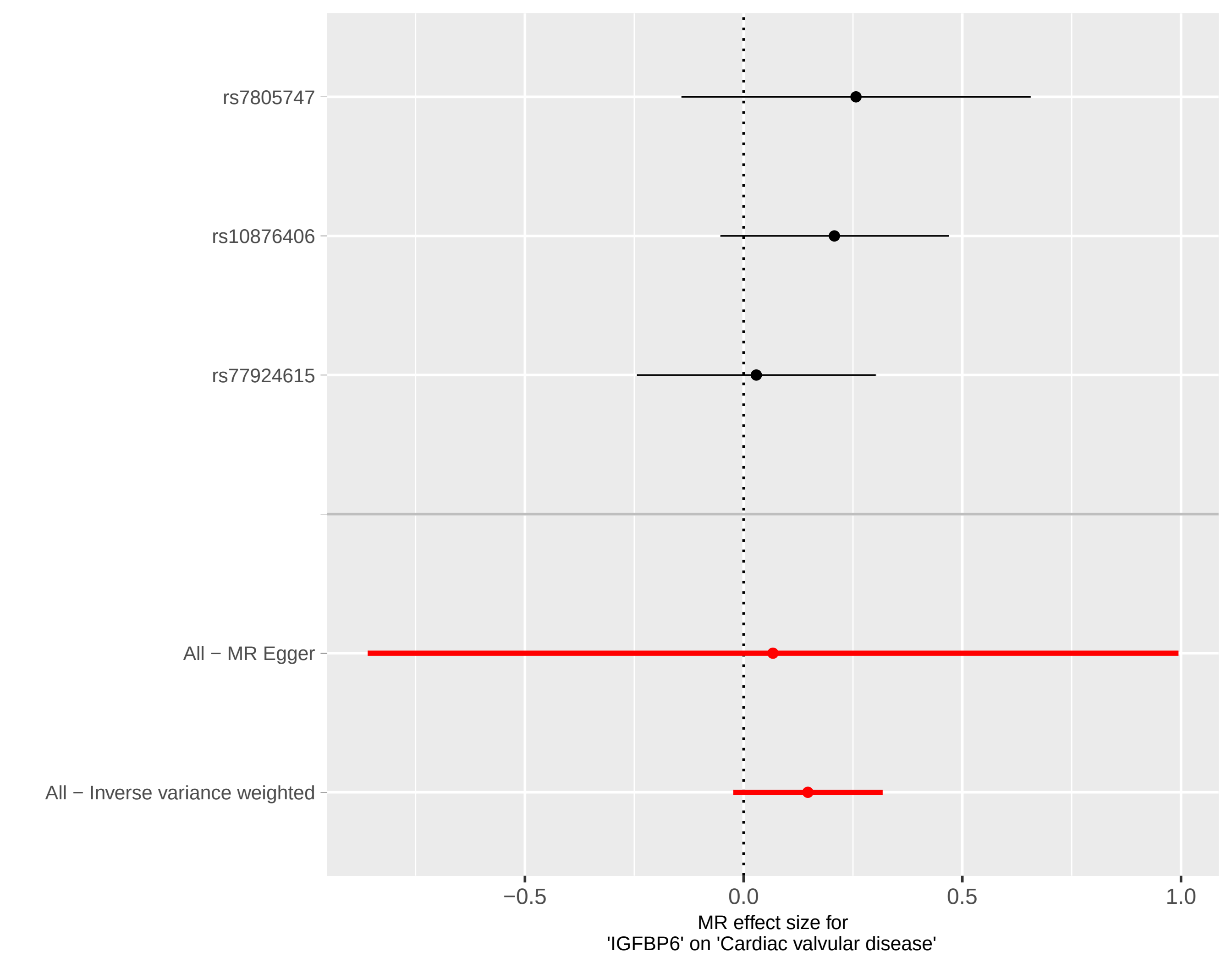

rs7805747
rs10876406
rs77924615
All − MR Egger
All − Inverse variance weighted
−0.5
0.0
0.5
1.0
MR effect size for
'IGFBP6' on 'Cardiac valvular disease'

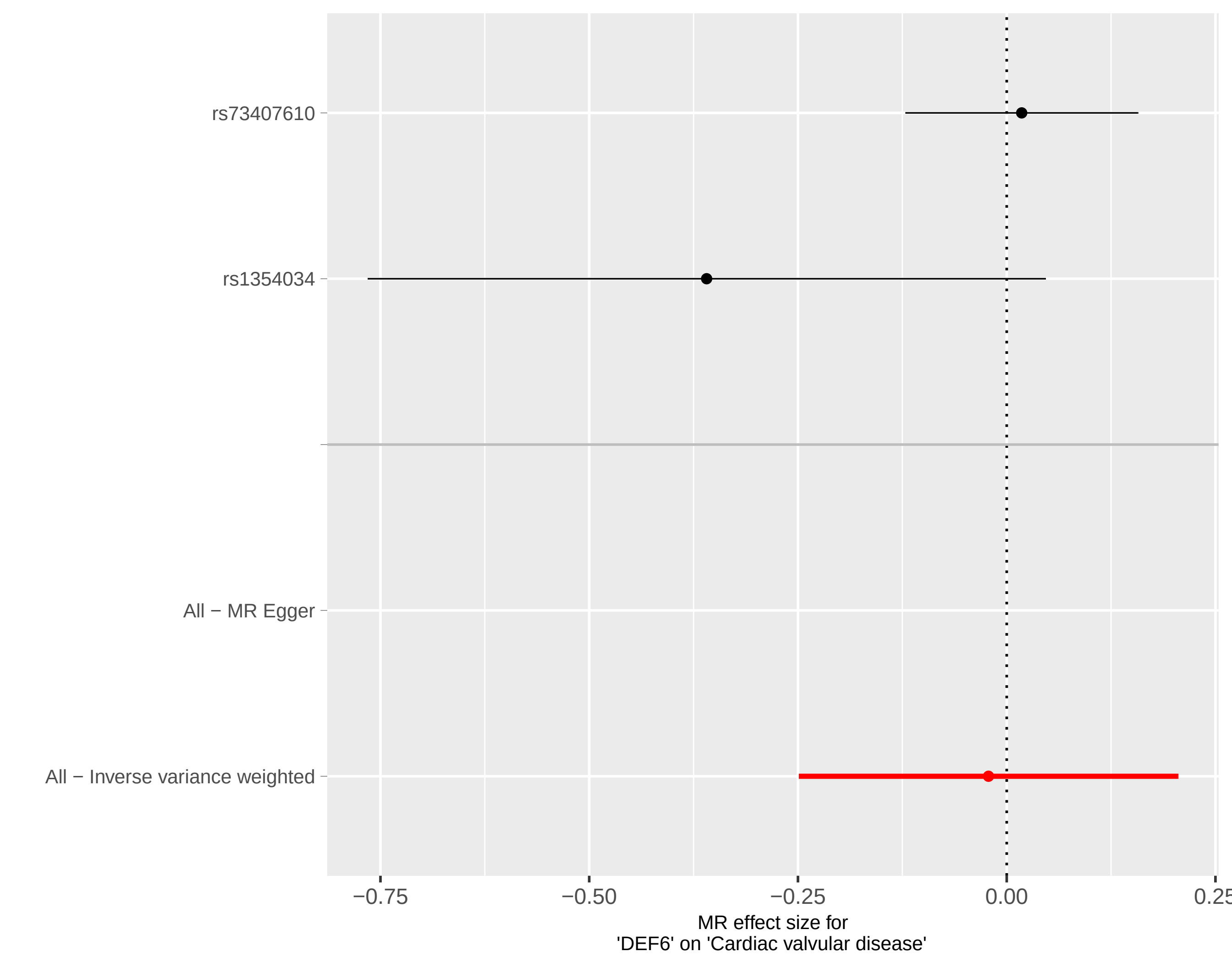

rs73407610
rs1354034
All − MR Egger
All − Inverse variance weighted
−0.75
−0.50
−0.25
0.00
0.25
MR effect size for
'DEF6' on 'Cardiac valvular disease'

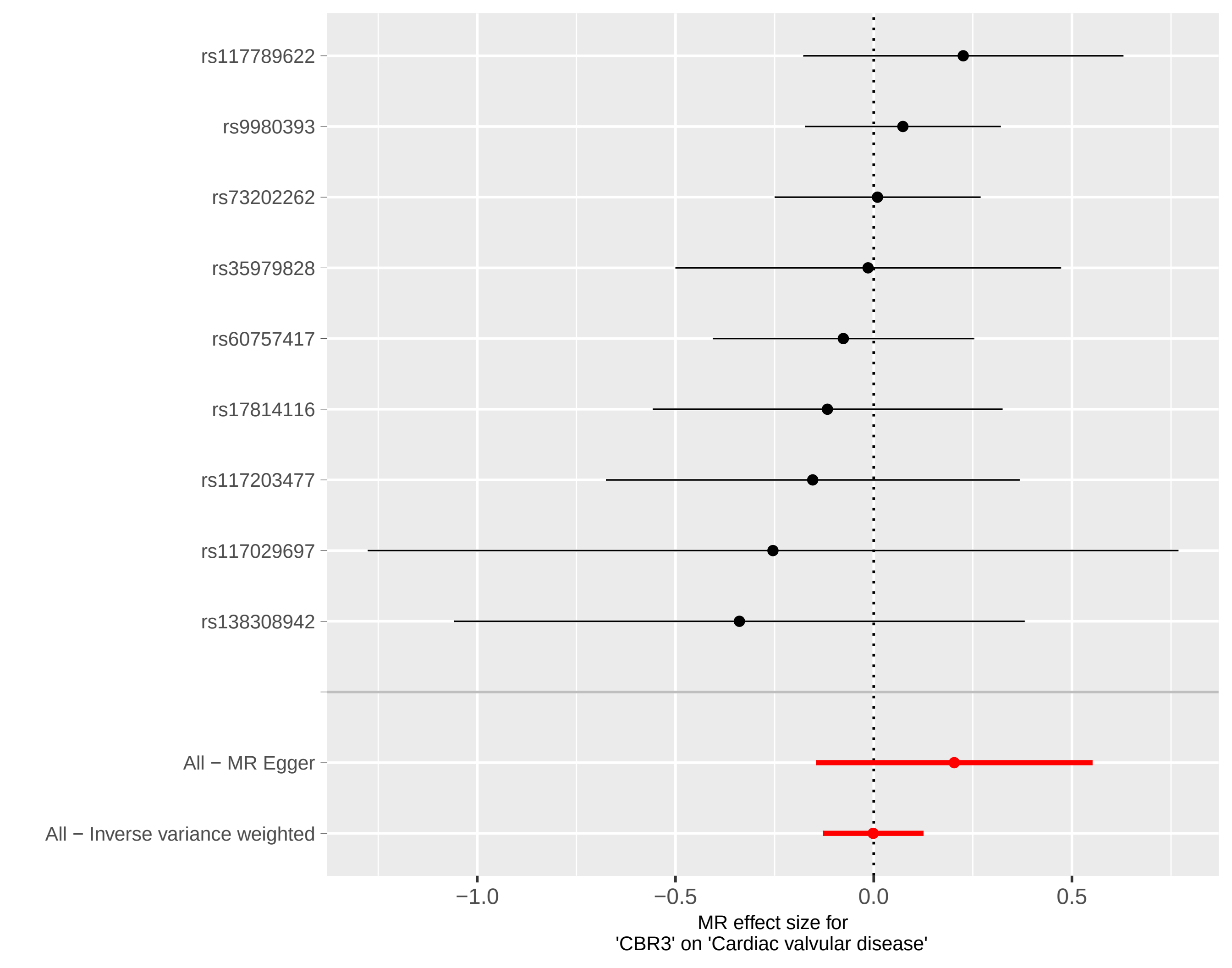

rs117789622
rs9980393
rs73202262
rs35979828
rs60757417
rs17814116
rs117203477
rs117029697
rs138308942
All − MR Egger
All − Inverse variance weighted
−1.0
−0.5
0.0
0.5
MR effect size for
'CBR3' on 'Cardiac valvular disease'

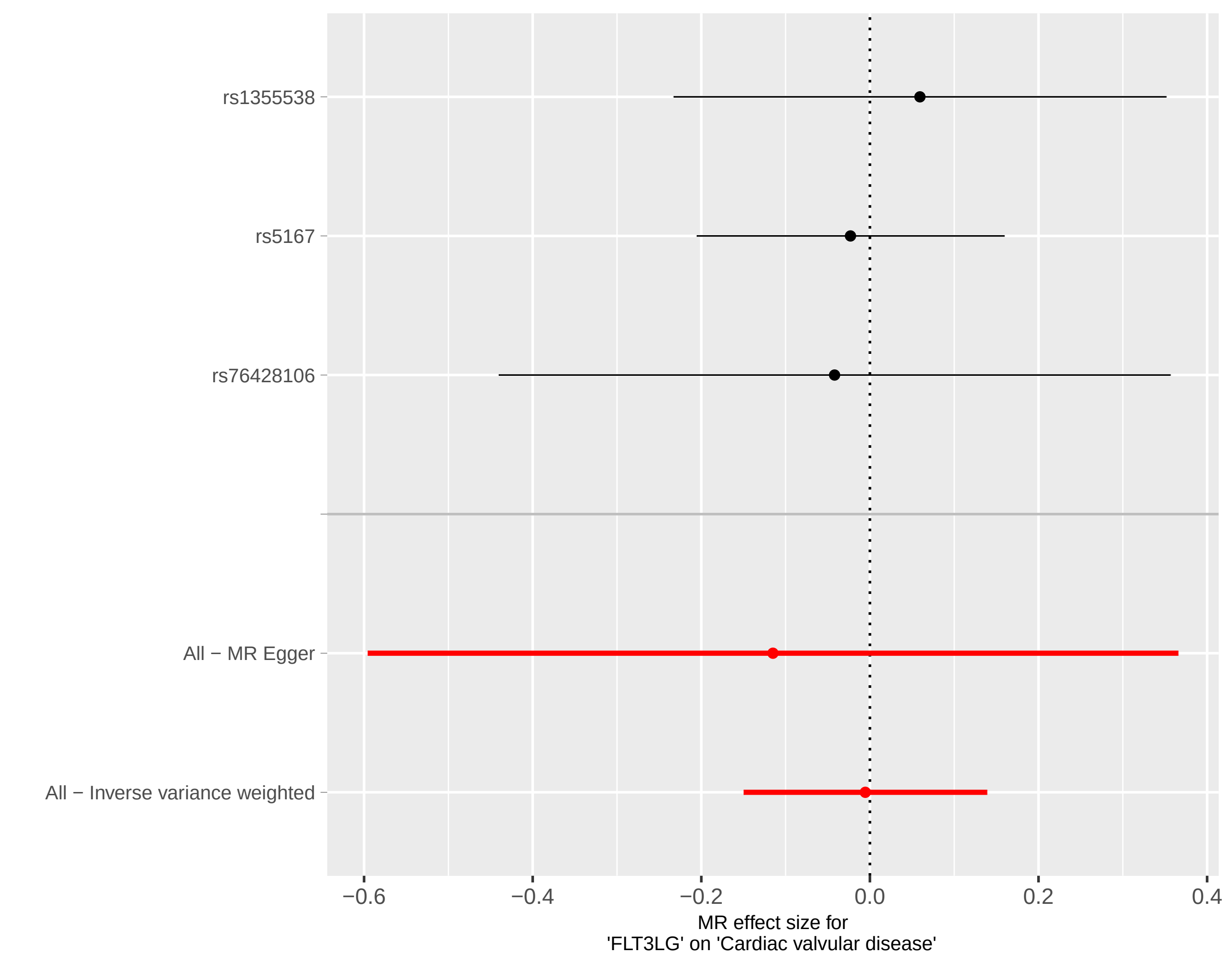

rs1355538
rs5167
rs76428106
All − MR Egger
All − Inverse variance weighted
−0.6
−0.4
−0.2
0.0
0.2
0.4
MR effect size for
'FLT3LG' on 'Cardiac valvular disease'

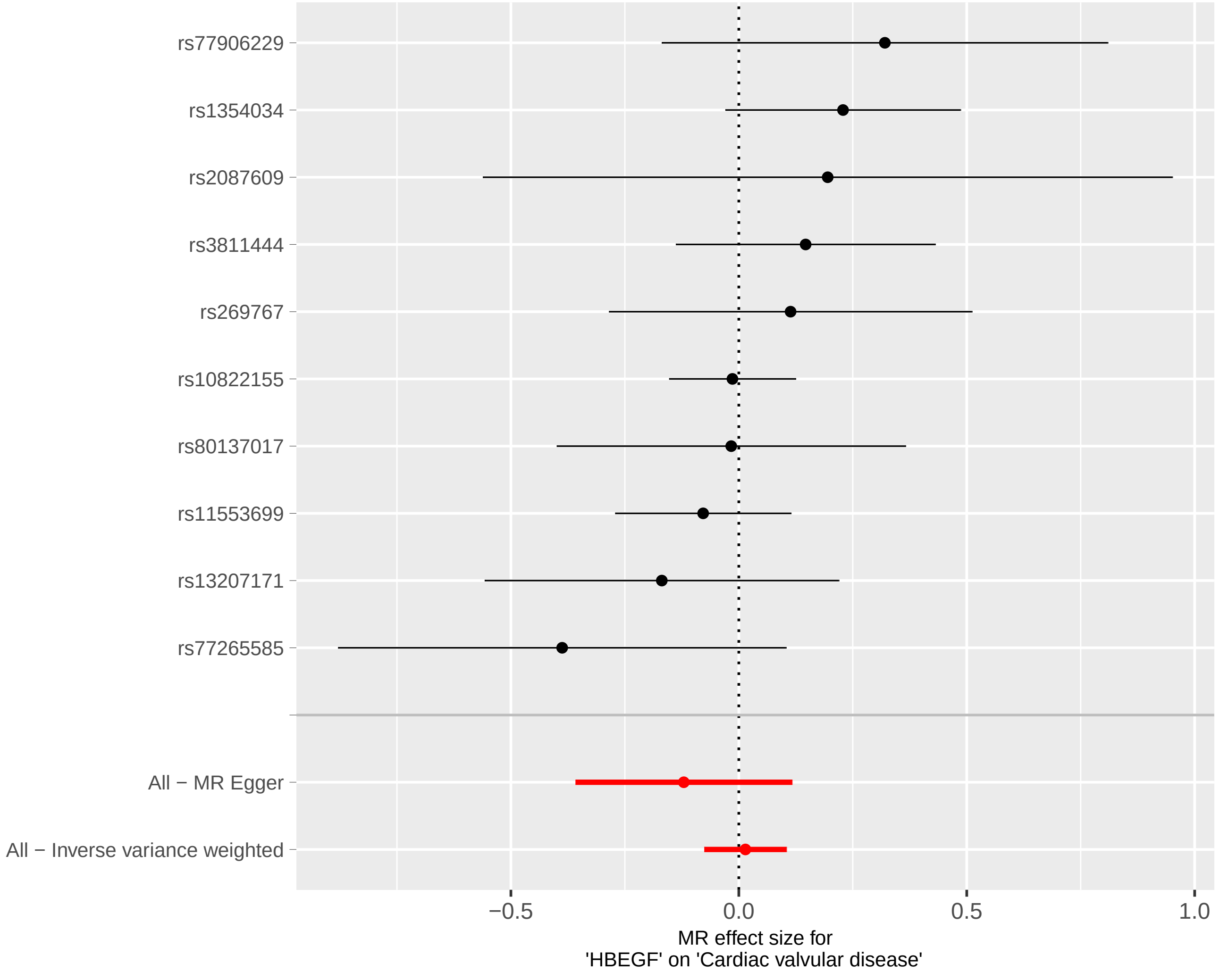
rs77906229
rs1354034
rs2087609
rs3811444
rs269767
rs10822155
rs80137017
rs11553699
rs13207171
rs77265585
All − MR Egger
All − Inverse variance weighted
−0.5
0.0
0.5
1.0
MR effect size for
'HBEGF' on 'Cardiac valvular disease'

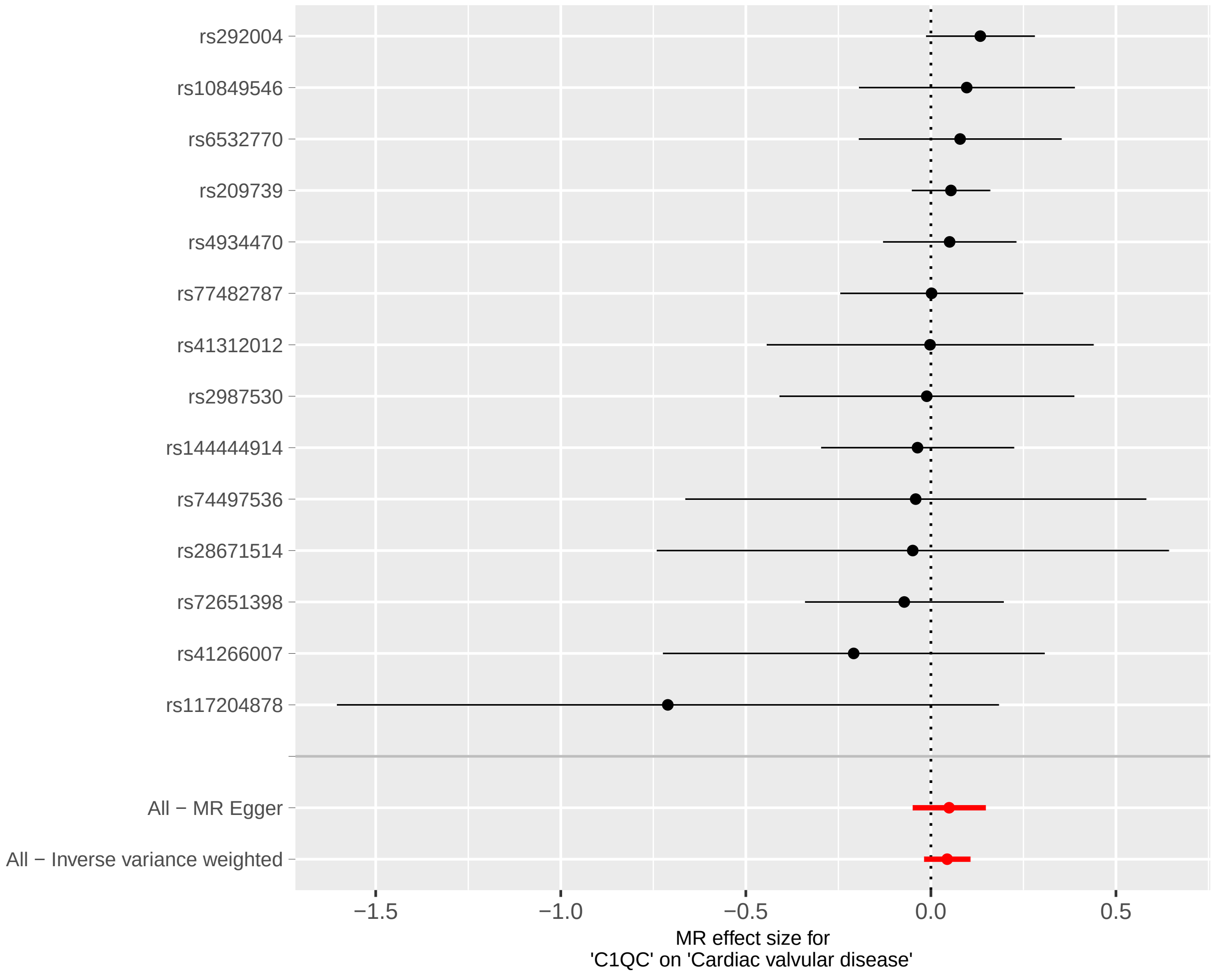
rs292004
rs10849546
rs6532770
rs209739
rs4934470
rs77482787
rs41312012
rs2987530
rs144444914
rs74497536
rs28671514
rs72651398
rs41266007
rs117204878
All − MR Egger
All − Inverse variance weighted
−1.5
−1.0
−0.5
0.0
0.5
MR effect size for
'C1QC' on 'Cardiac valvular disease'

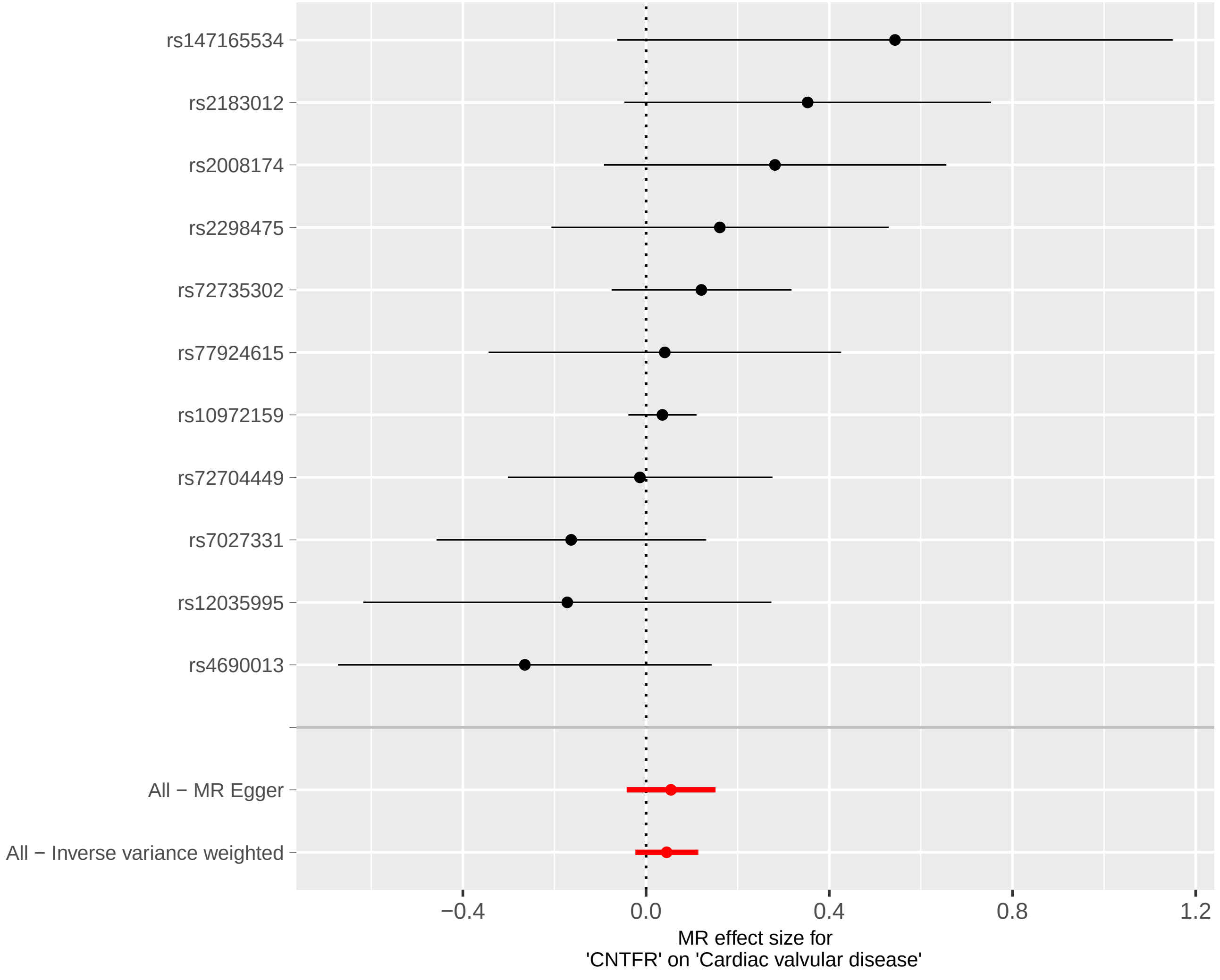

rs147165534
rs2183012
rs2008174
rs2298475
rs72735302
rs77924615
rs10972159
rs72704449
rs7027331
rs12035995
rs4690013
All − MR Egger
All − Inverse variance weighted
−0.4
0.0
0.4
0.8
1.2
MR effect size for
'CNTFR' on 'Cardiac valvular disease'

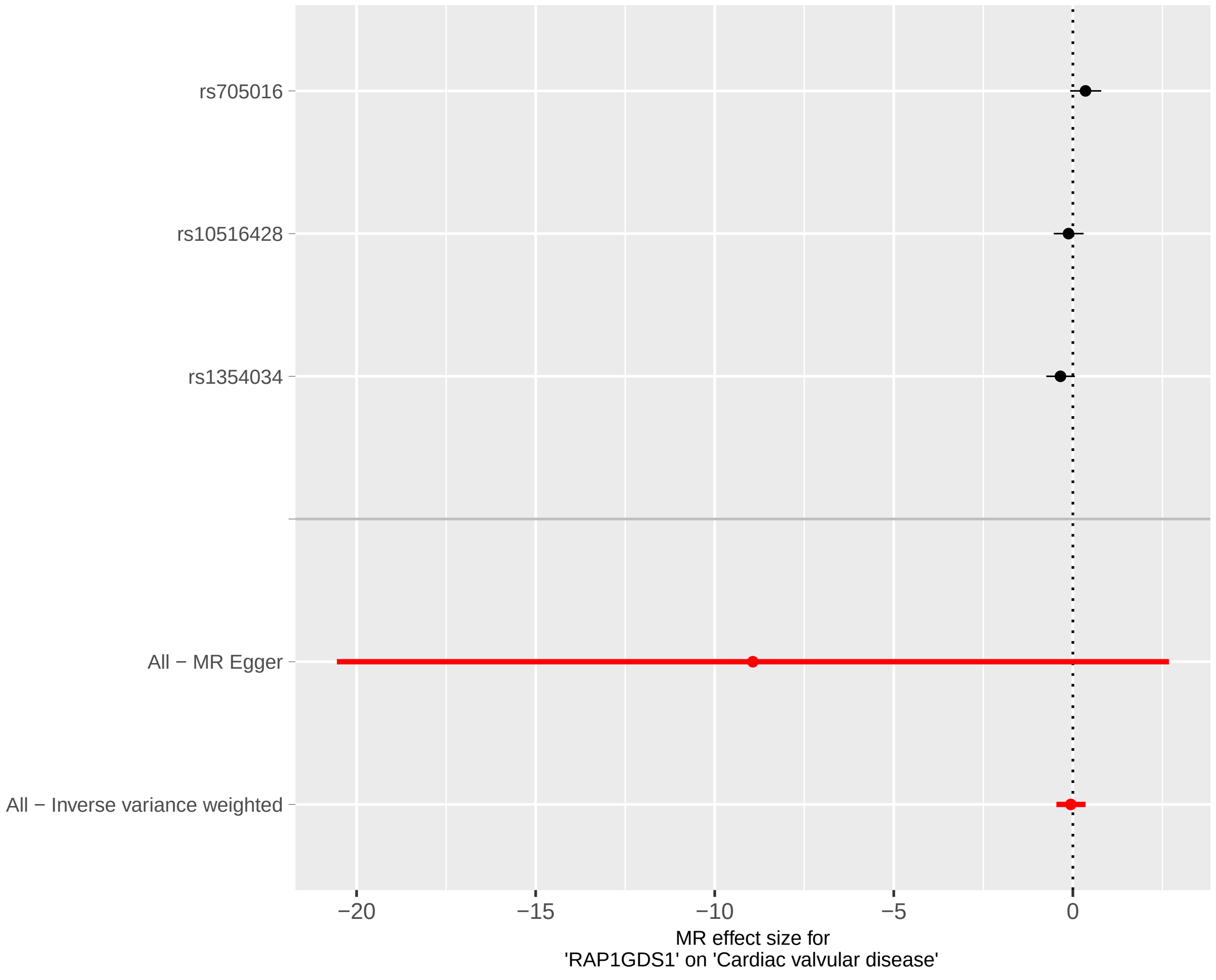
rs705016
rs10516428
rs1354034
All − MR Egger
All − Inverse variance weighted
−20
−15
−10
−5
0
MR effect size for
'RAP1GDS1' on 'Cardiac valvular disease'

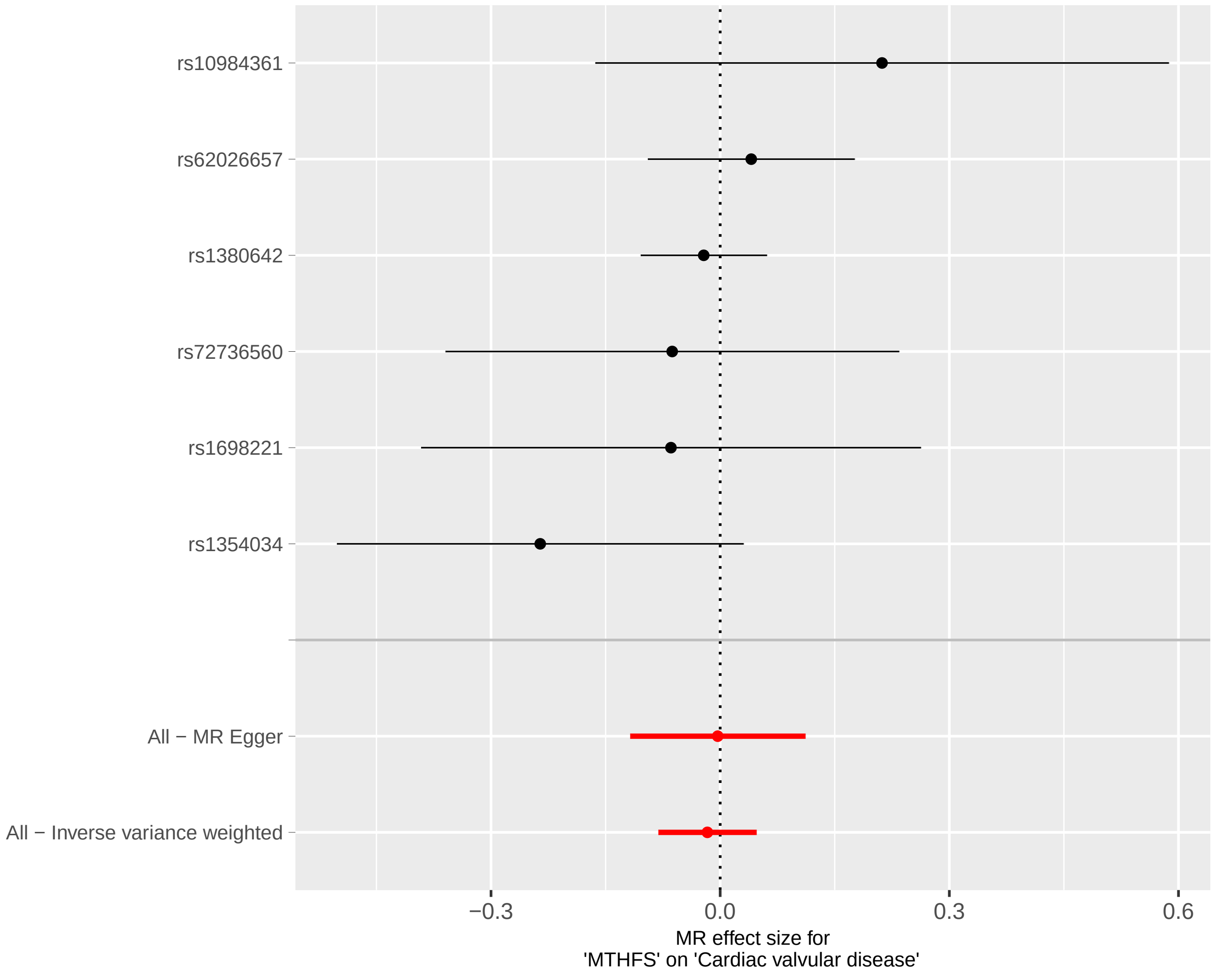
rs10984361
rs62026657
rs1380642
rs72736560
rs1698221
rs1354034
All − MR Egger
All − Inverse variance weighted
−0.3
0.0
0.3
0.6
MR effect size for
'MTHFS' on 'Cardiac valvular disease'

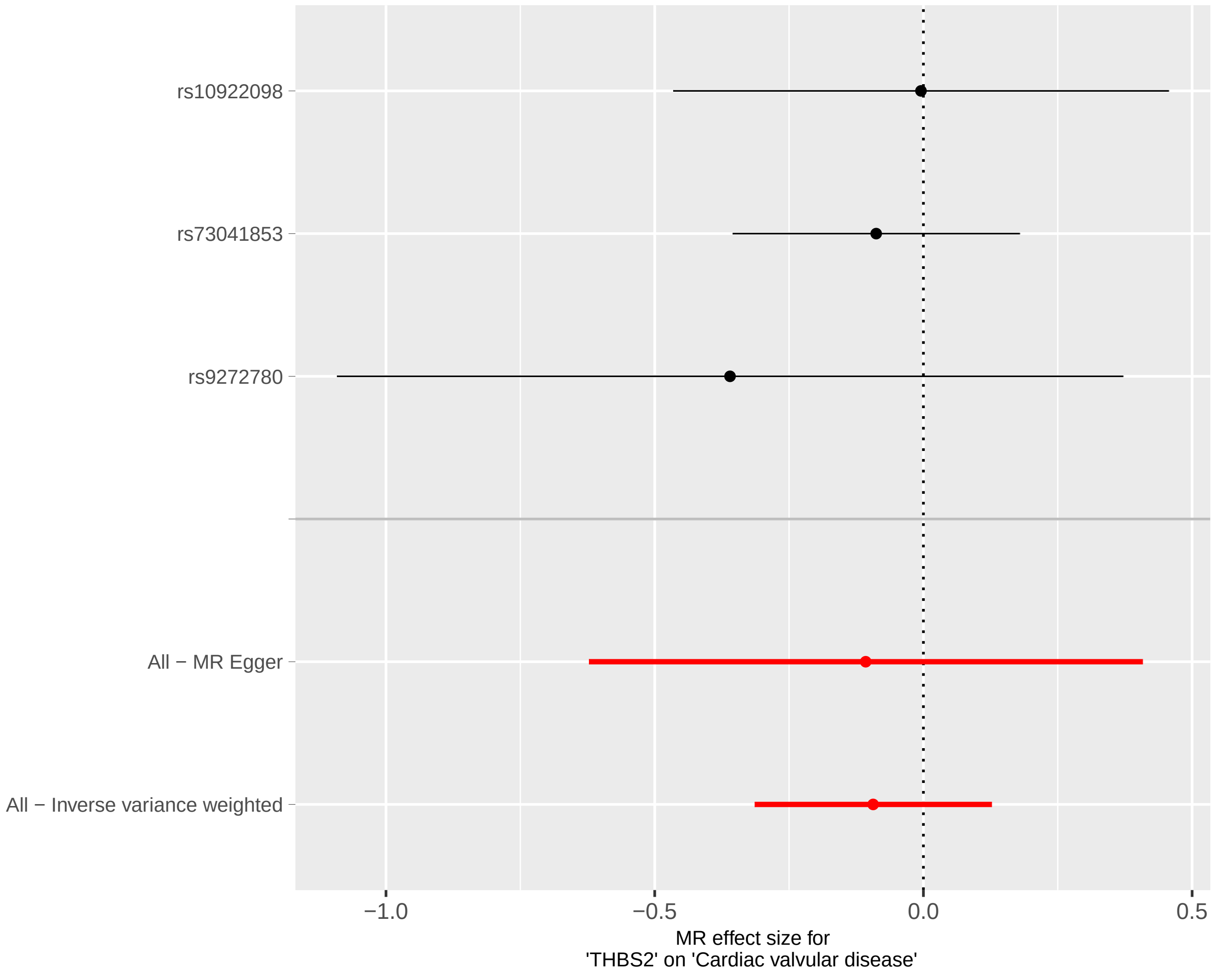
rs10922098
rs73041853
rs9272780
All − MR Egger
All − Inverse variance weighted
−1.0
−0.5
0.0
0.5
MR effect size for
'THBS2' on 'Cardiac valvular disease'

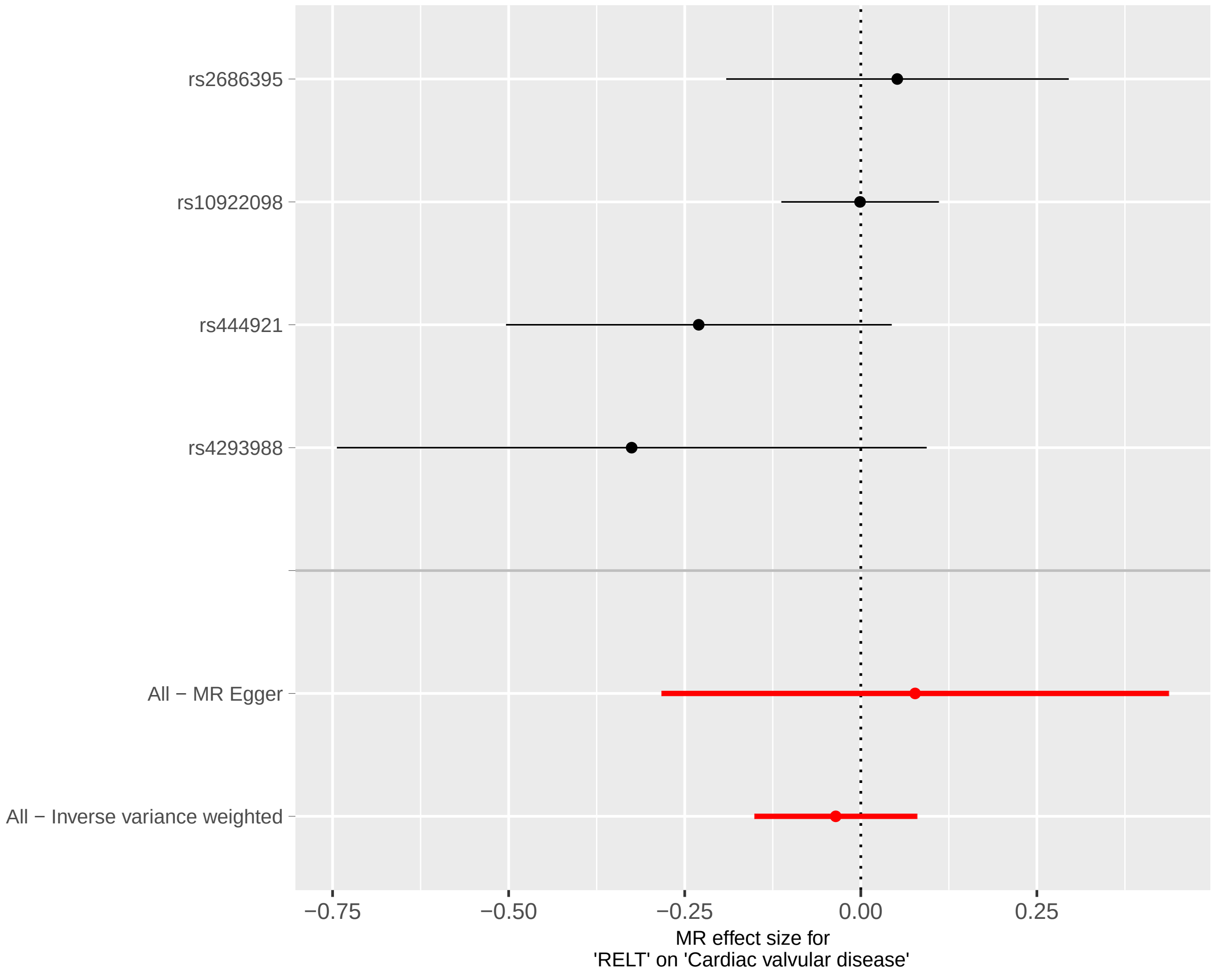
rs2686395
rs10922098
rs444921
rs4293988
All − MR Egger
All − Inverse variance weighted
−0.75
−0.50
−0.25
0.00
0.25
MR effect size for
'RELT' on 'Cardiac valvular disease'

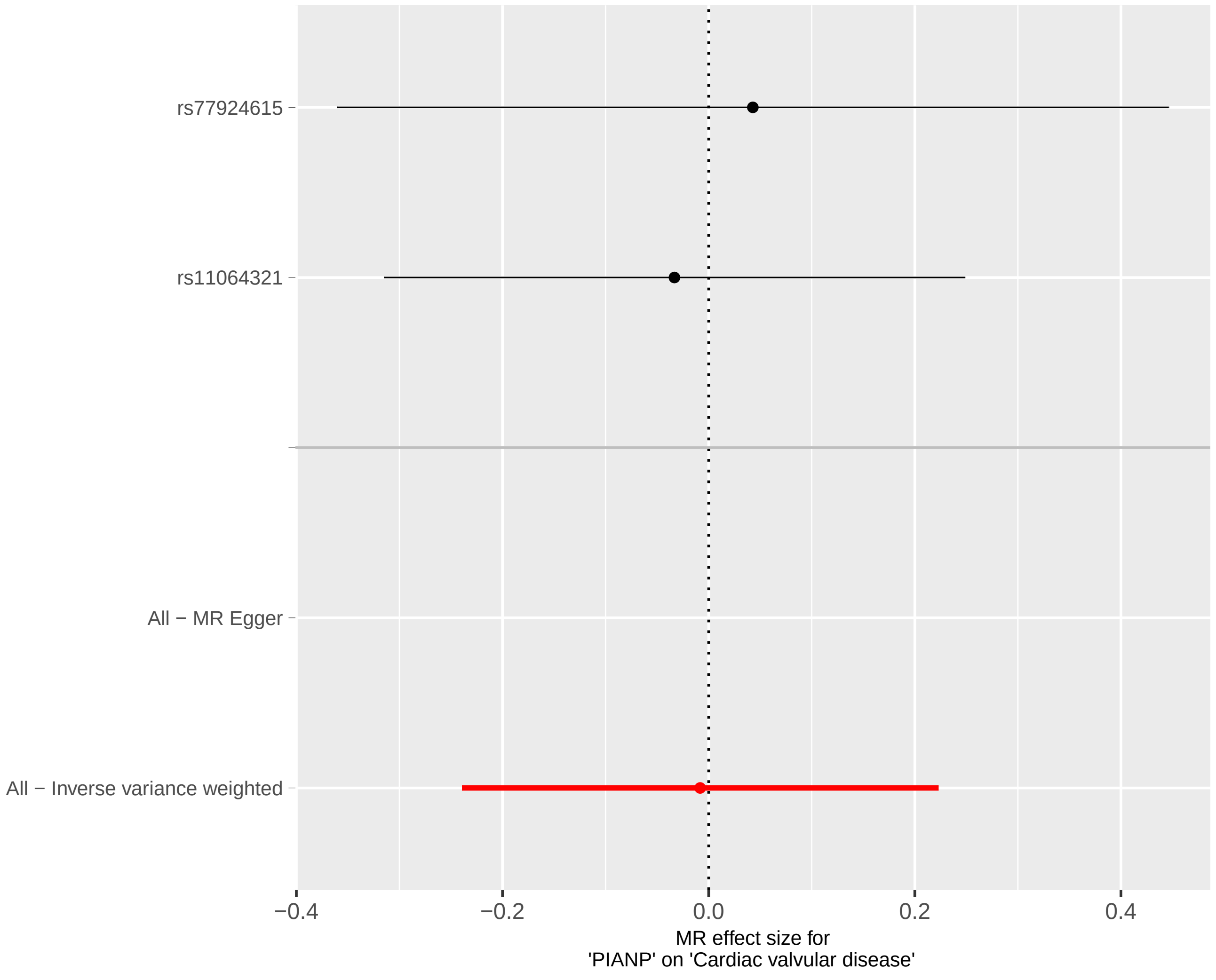
rs77924615
rs11064321
All − MR Egger
All − Inverse variance weighted
−0.4
−0.2
0.0
0.2
0.4
MR effect size for
'PIANP' on 'Cardiac valvular disease'

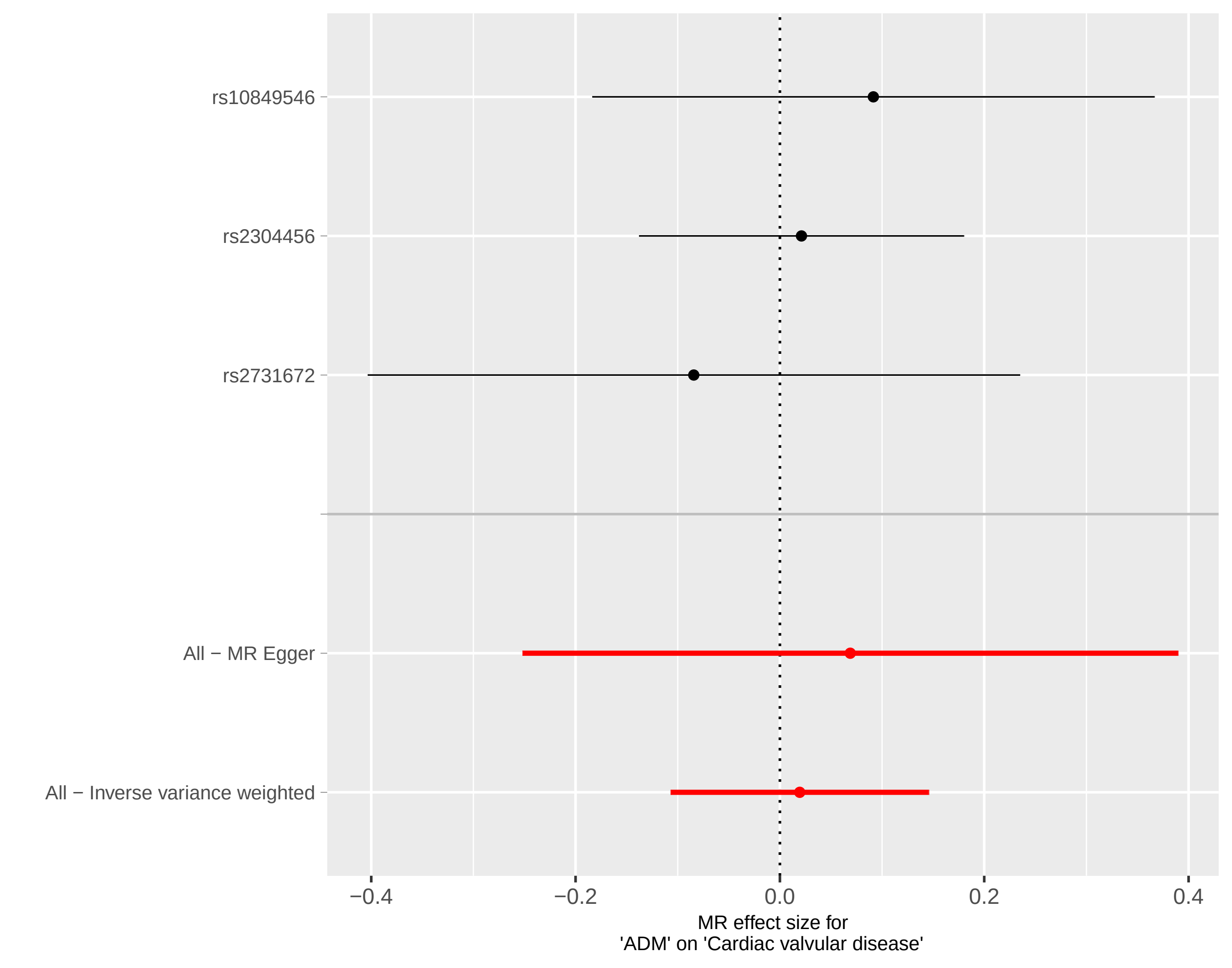

rs10849546
rs2304456
rs2731672
All − MR Egger
All − Inverse variance weighted
−0.4
−0.2
0.0
0.2
0.4
MR effect size for
'ADM' on 'Cardiac valvular disease'

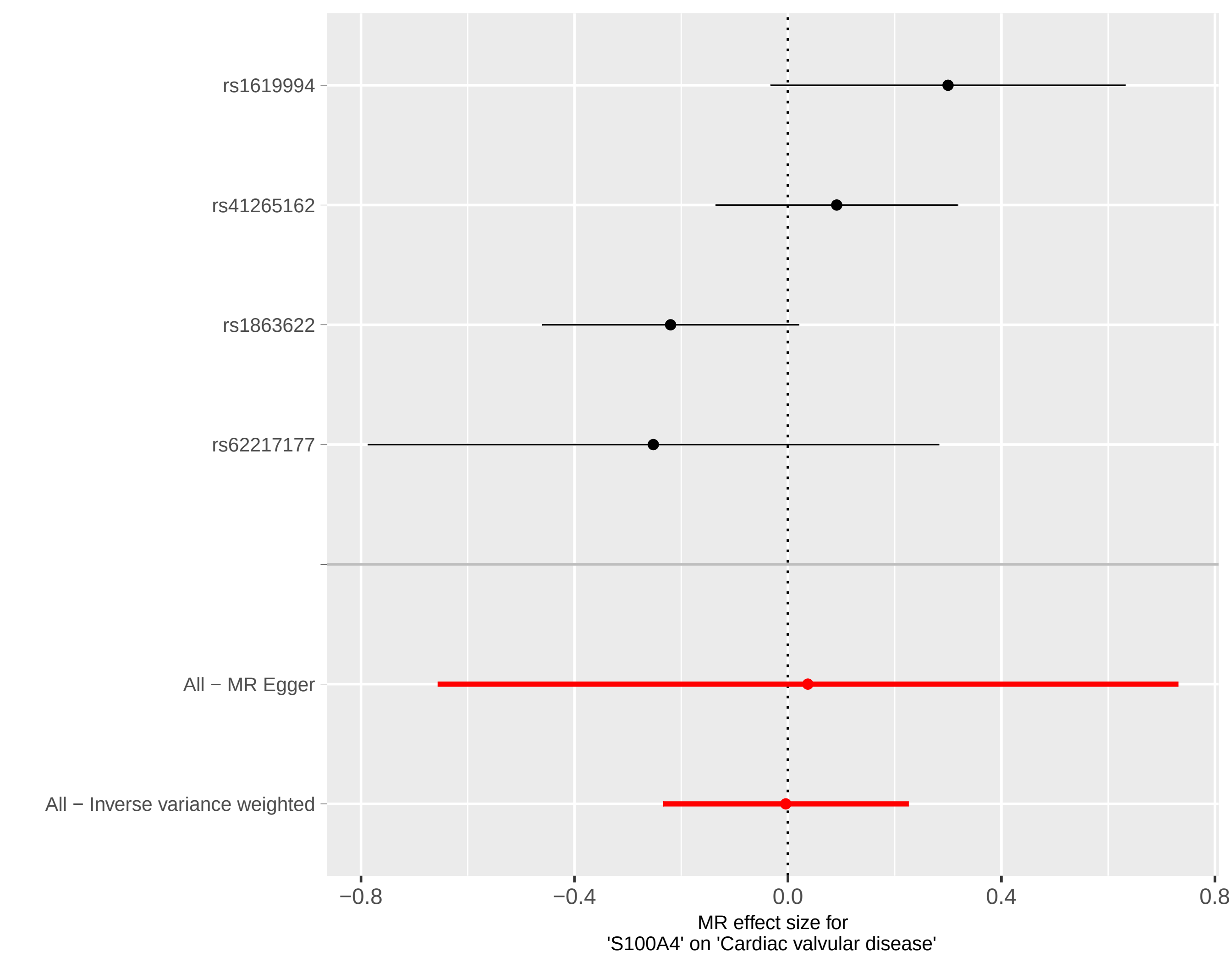
rs1619994
rs41265162
rs1863622
rs62217177
All − MR Egger
All − Inverse variance weighted
−0.8
−0.4
0.0
0.4
0.8
MR effect size for
'S100A4' on 'Cardiac valvular disease'

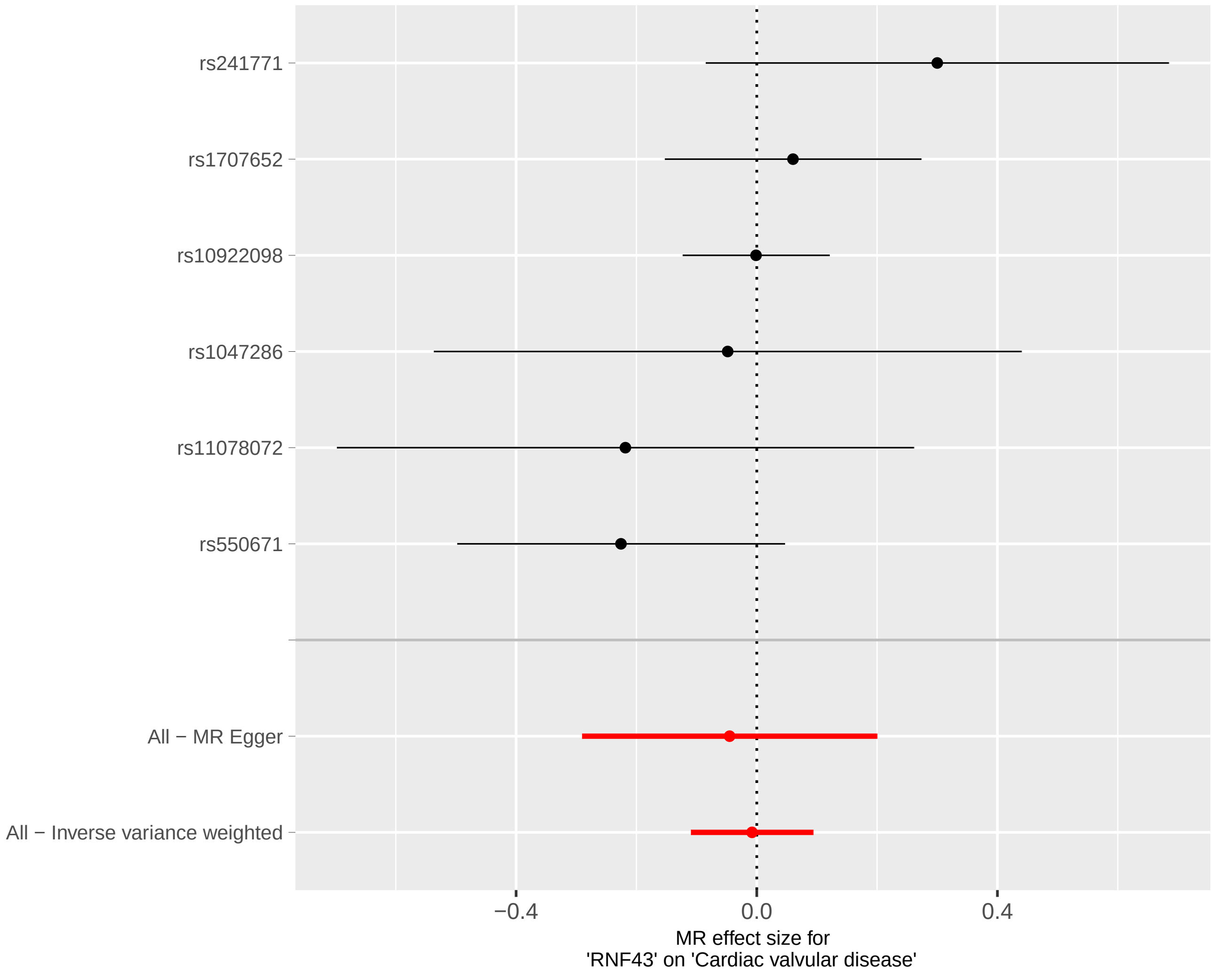
rs241771
rs1707652
rs10922098
rs1047286
rs11078072
rs550671
All − MR Egger
All − Inverse variance weighted
−0.4
0.0
0.4
MR effect size for
'RNF43' on 'Cardiac valvular disease'

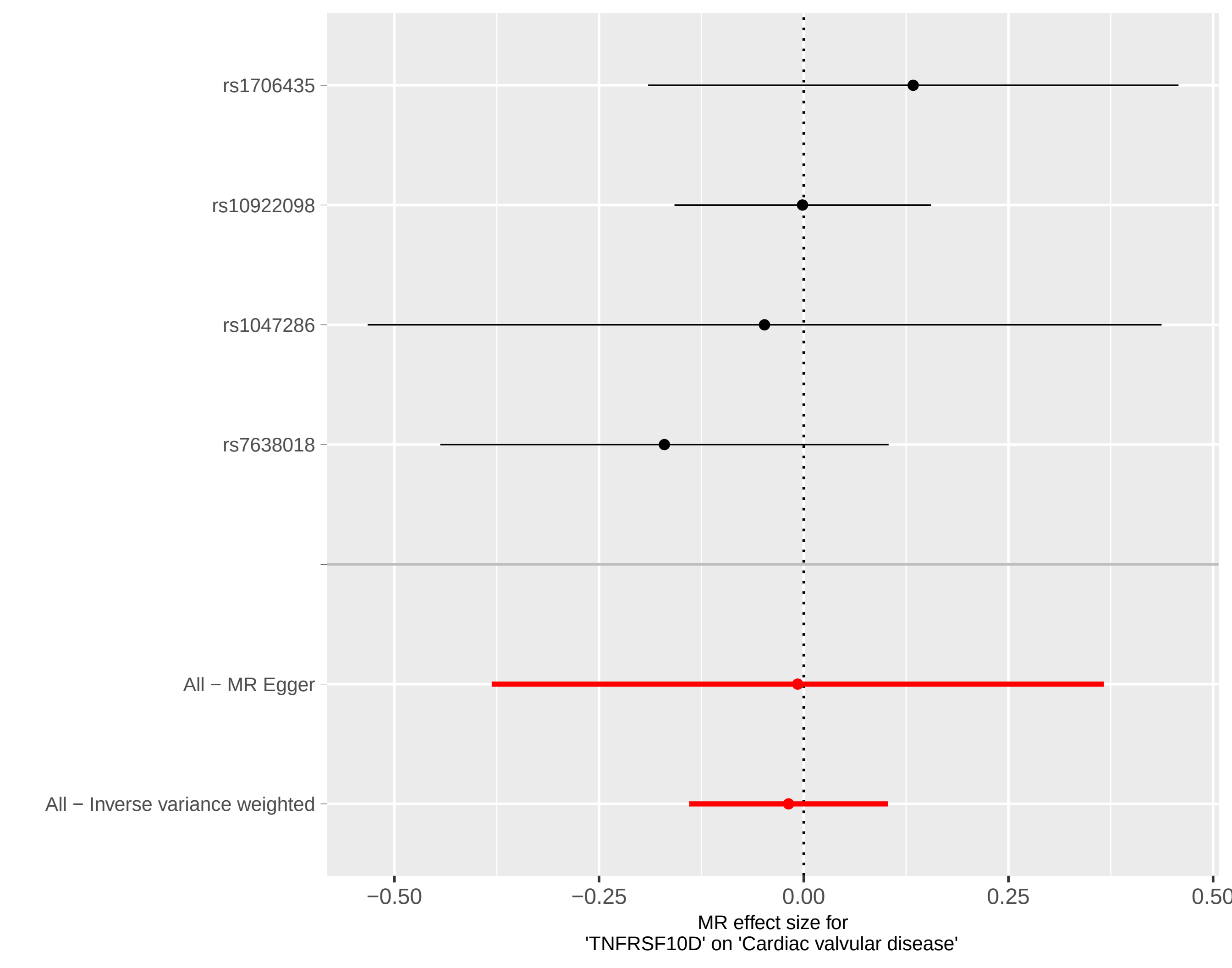
rs1706435
rs10922098
rs1047286
rs7638018
All − MR Egger
All − Inverse variance weighted
−0.50
−0.25
0.00
0.25
0.50
MR effect size for
'TNFRSF10D' on 'Cardiac valvular disease'

Insufficient number of SNPs

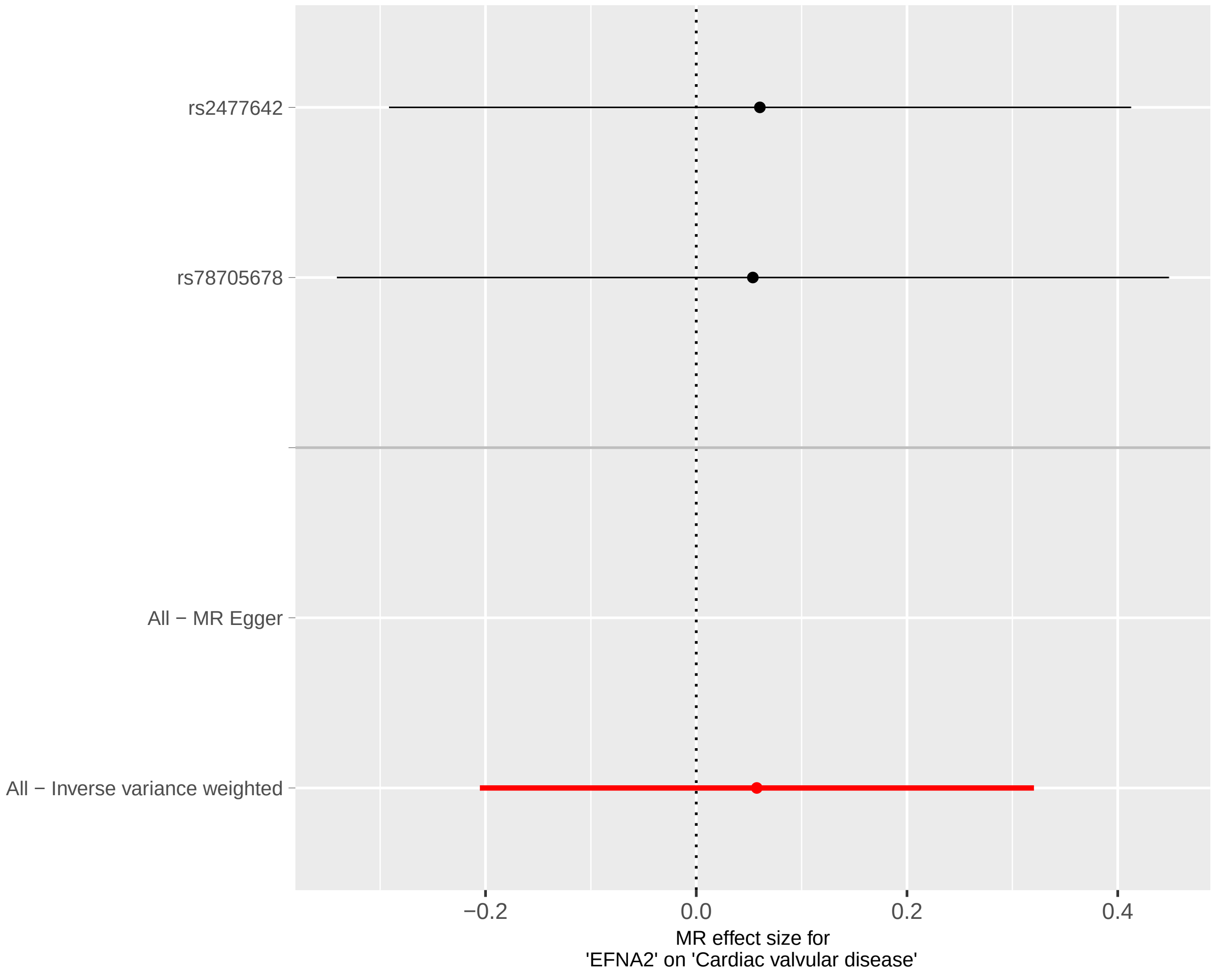
rs2477642
rs78705678
All − MR Egger
All − Inverse variance weighted
−0.2
0.0
0.2
0.4
MR effect size for
'EFNA2' on 'Cardiac valvular disease'

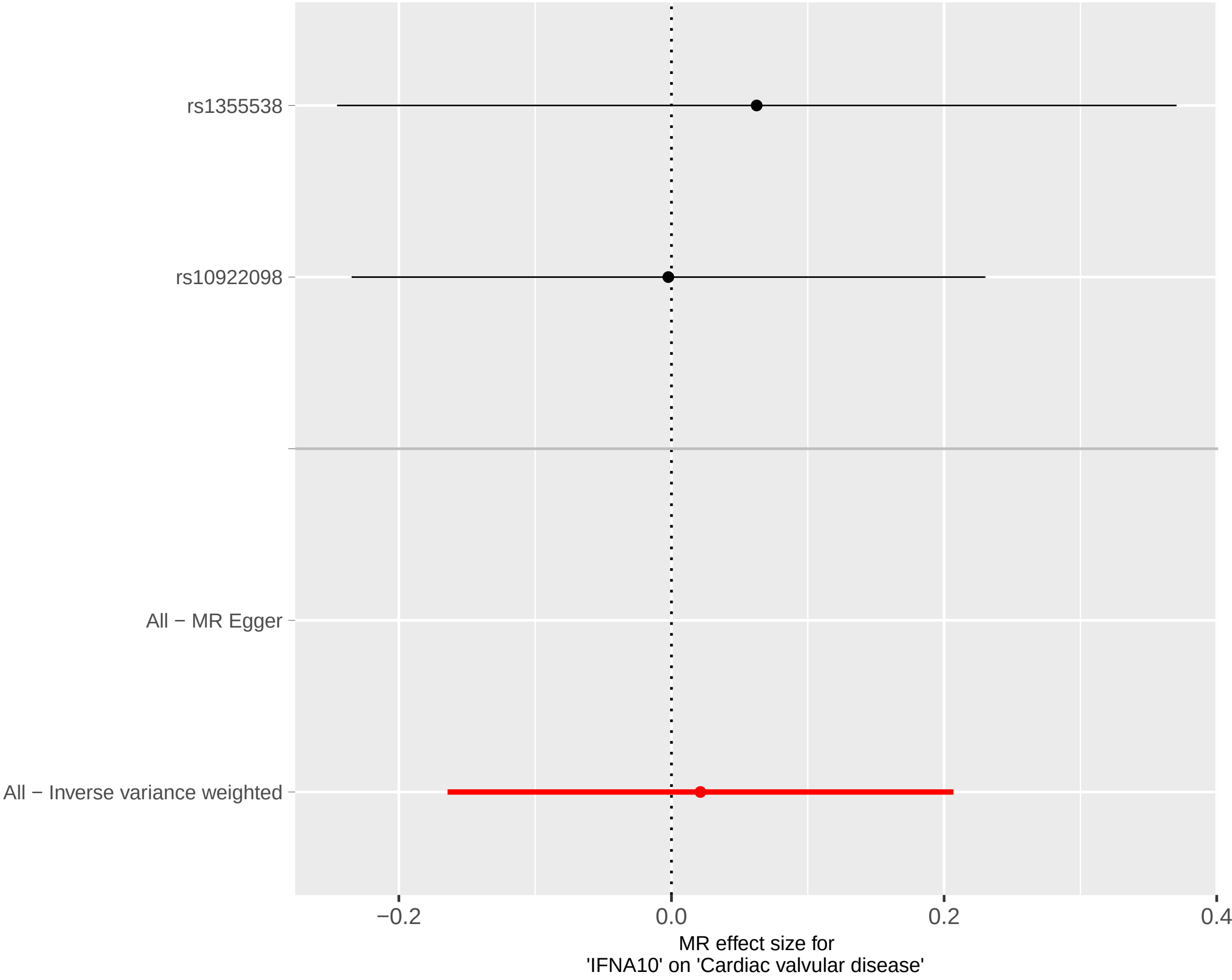

rs1355538
rs10922098
All − MR Egger
All − Inverse variance weighted
−0.2
0.0
0.2
0.4
MR effect size for
'IFNA10' on 'Cardiac valvular disease'

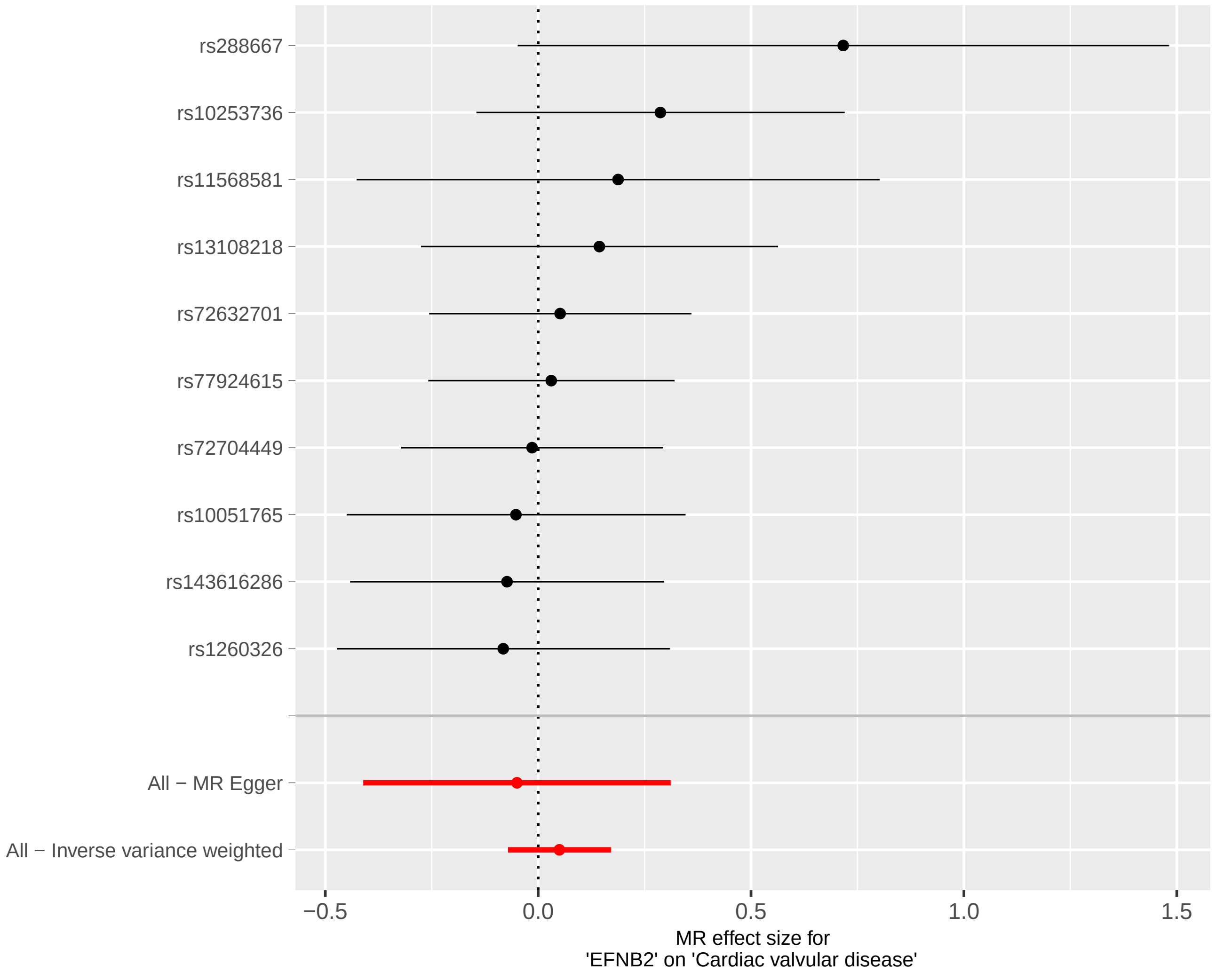
rs288667
rs10253736
rs11568581
rs13108218
rs72632701
rs77924615
rs72704449
rs10051765
rs143616286
rs1260326
All − MR Egger
All − Inverse variance weighted
−0.5
0.0
0.5
1.0
1.5
MR effect size for
'EFNB2' on 'Cardiac valvular disease'

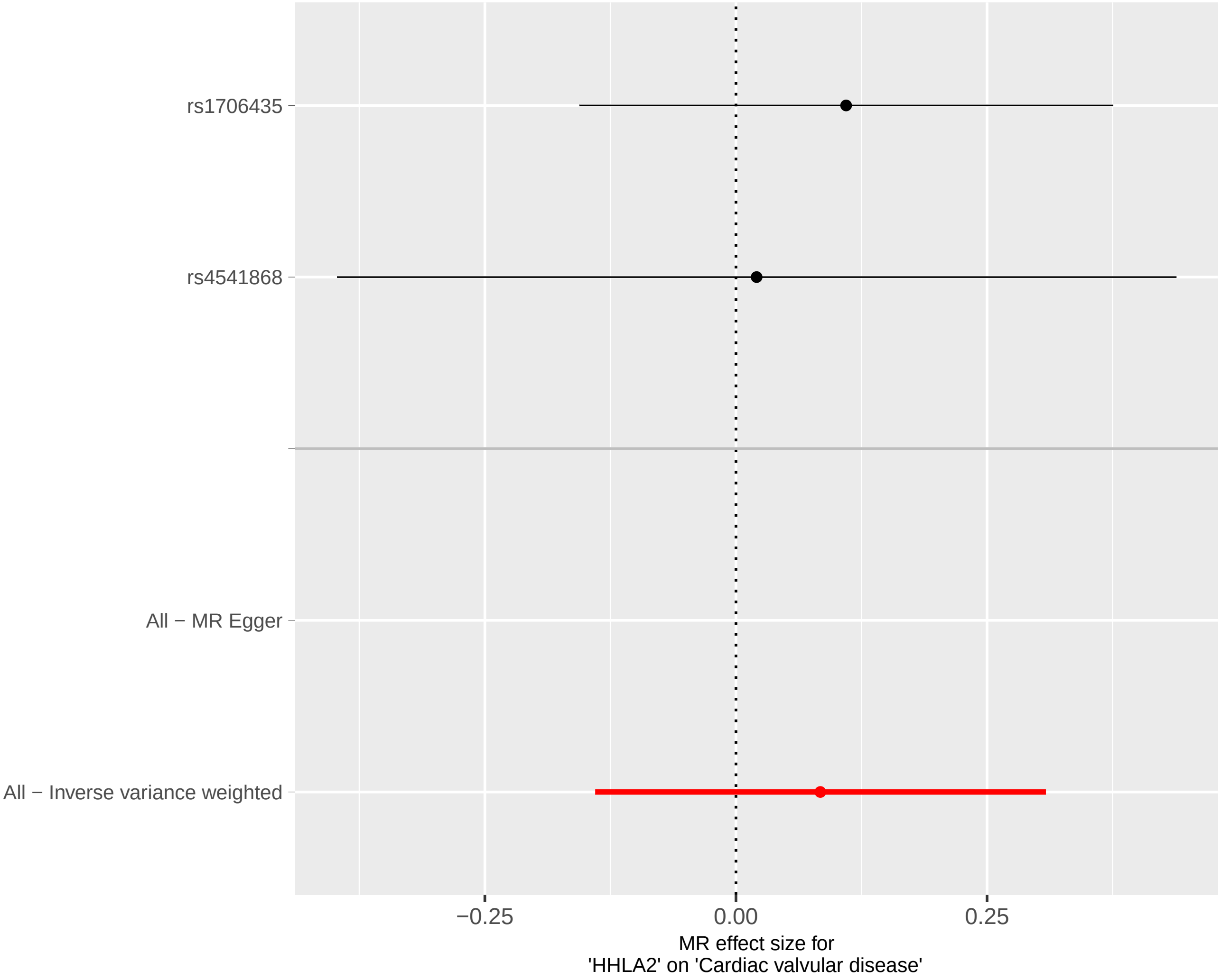
rs1706435
rs4541868
All − MR Egger
All − Inverse variance weighted
−0.25
0.00
0.25
MR effect size for
'HHLA2' on 'Cardiac valvular disease'

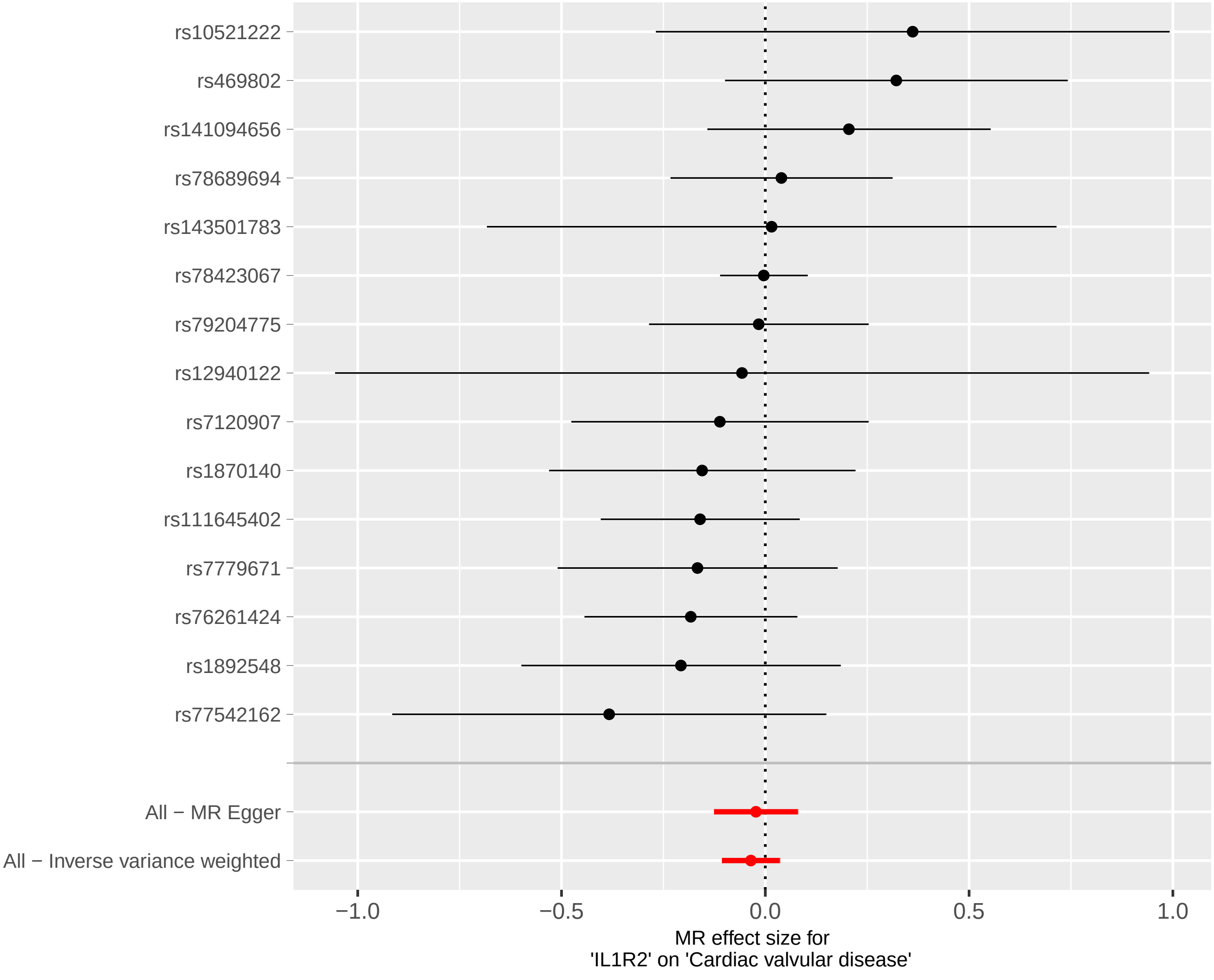
rs10521222
rs469802
rs141094656
rs78689694
rs143501783
rs78423067
rs79204775
rs12940122
rs7120907
rs1870140
rs111645402
rs7779671
rs76261424
rs1892548
rs77542162
All − MR Egger
All − Inverse variance weighted
−1.0
−0.5
0.0
0.5
1.0
MR effect size for
'IL1R2' on 'Cardiac valvular disease'

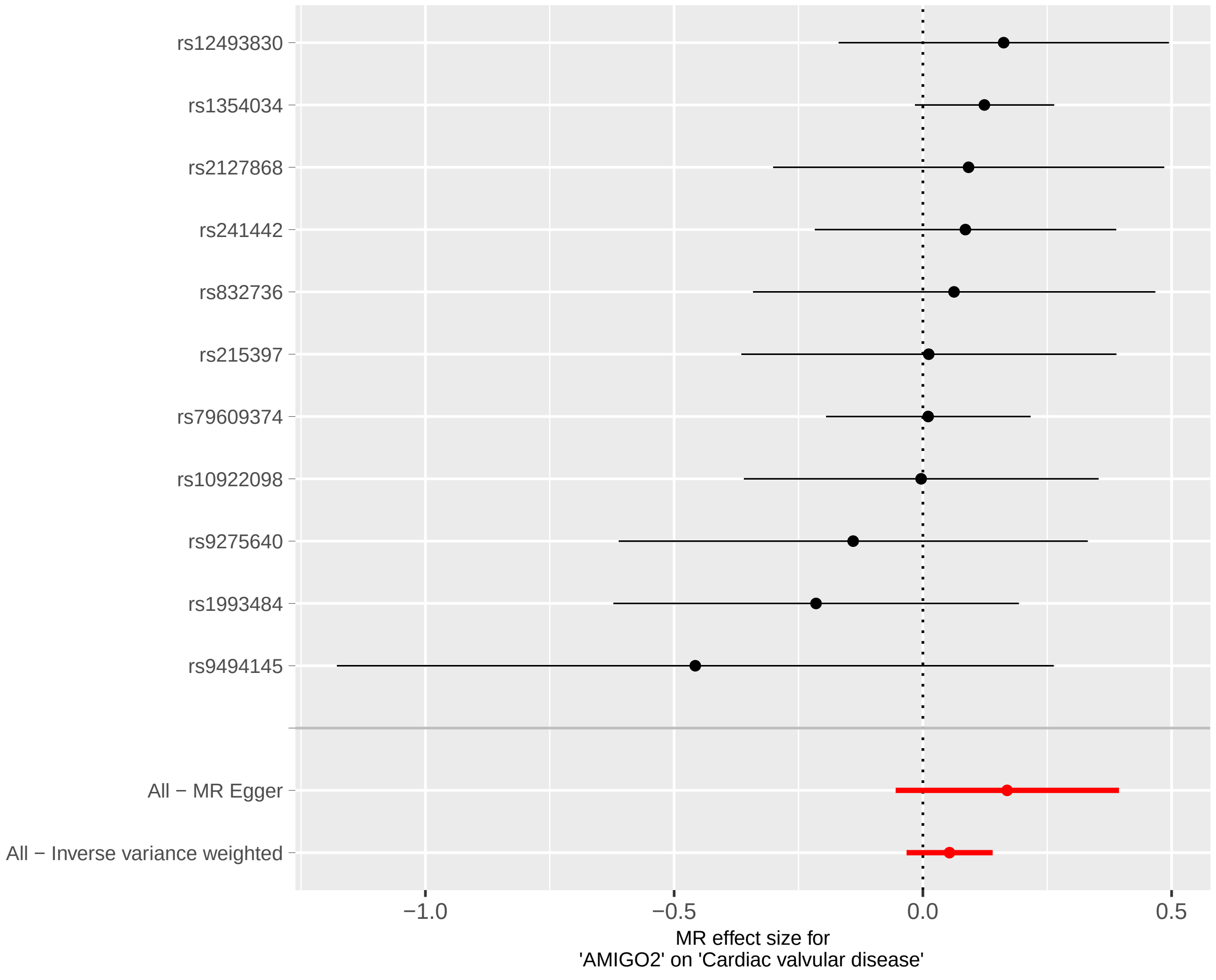

rs12493830
rs1354034
rs2127868
rs241442
rs832736
rs215397
rs79609374
rs10922098
rs9275640
rs1993484
rs9494145
All − MR Egger
All − Inverse variance weighted
−1.0
−0.5
0.0
0.5
MR effect size for
'AMIGO2' on 'Cardiac valvular disease'

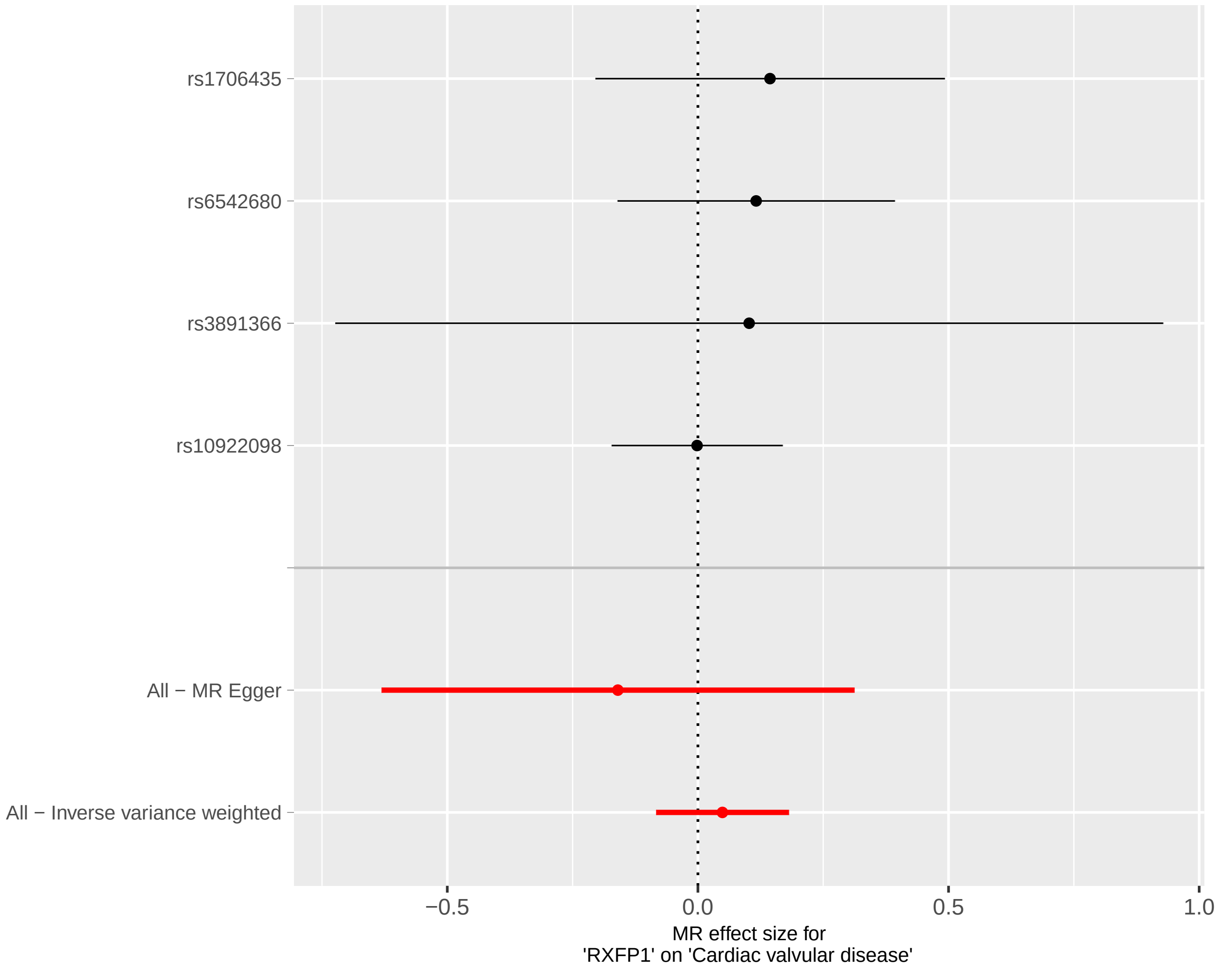
rs1706435
rs6542680
rs3891366
rs10922098
All − MR Egger
All − Inverse variance weighted
−0.5
0.0
0.5
1.0
MR effect size for
'RXFP1' on 'Cardiac valvular disease'

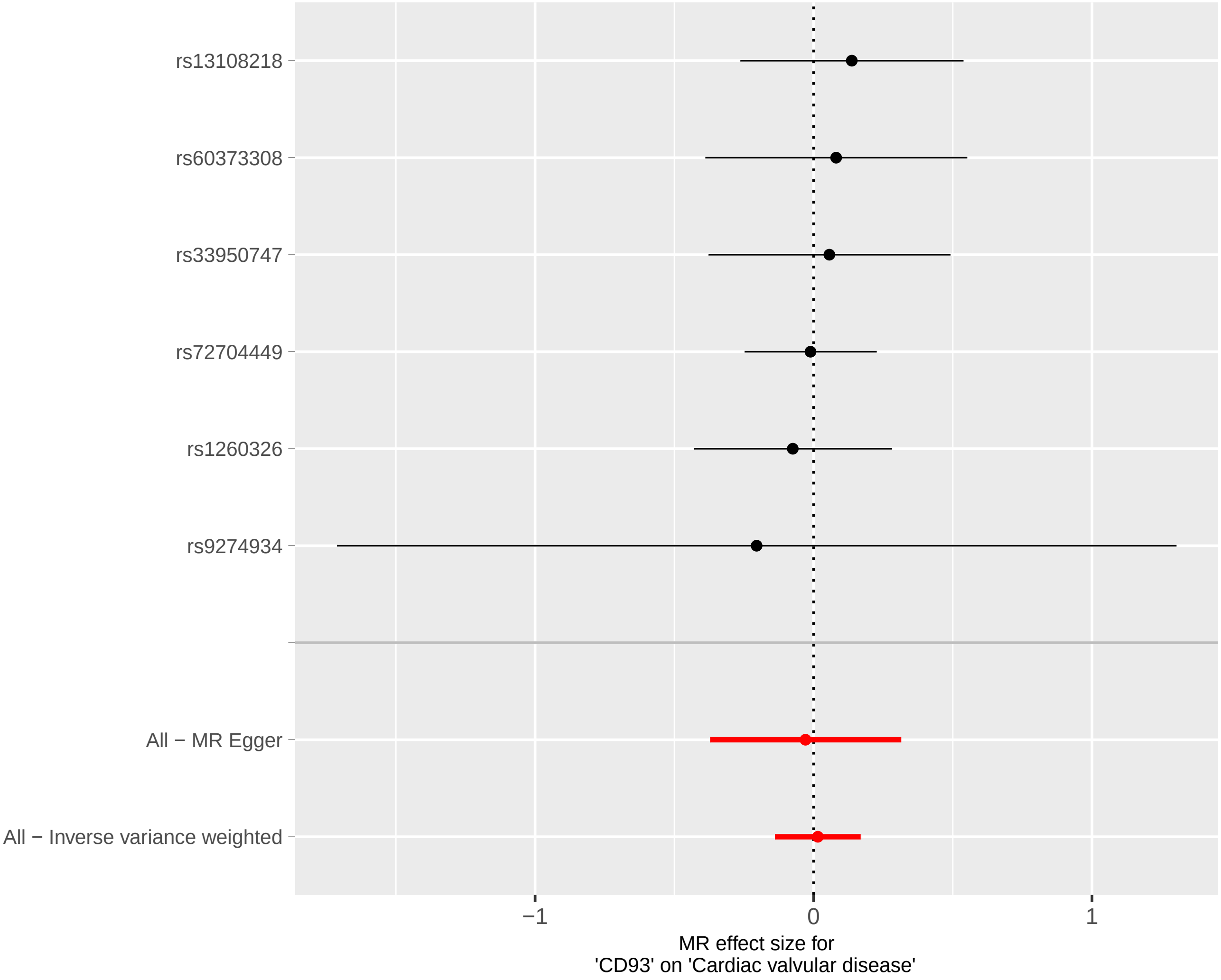

rs13108218
rs60373308
rs33950747
rs72704449
rs1260326
rs9274934
All − MR Egger
All − Inverse variance weighted
−1
0
1
MR effect size for
'CD93' on 'Cardiac valvular disease'

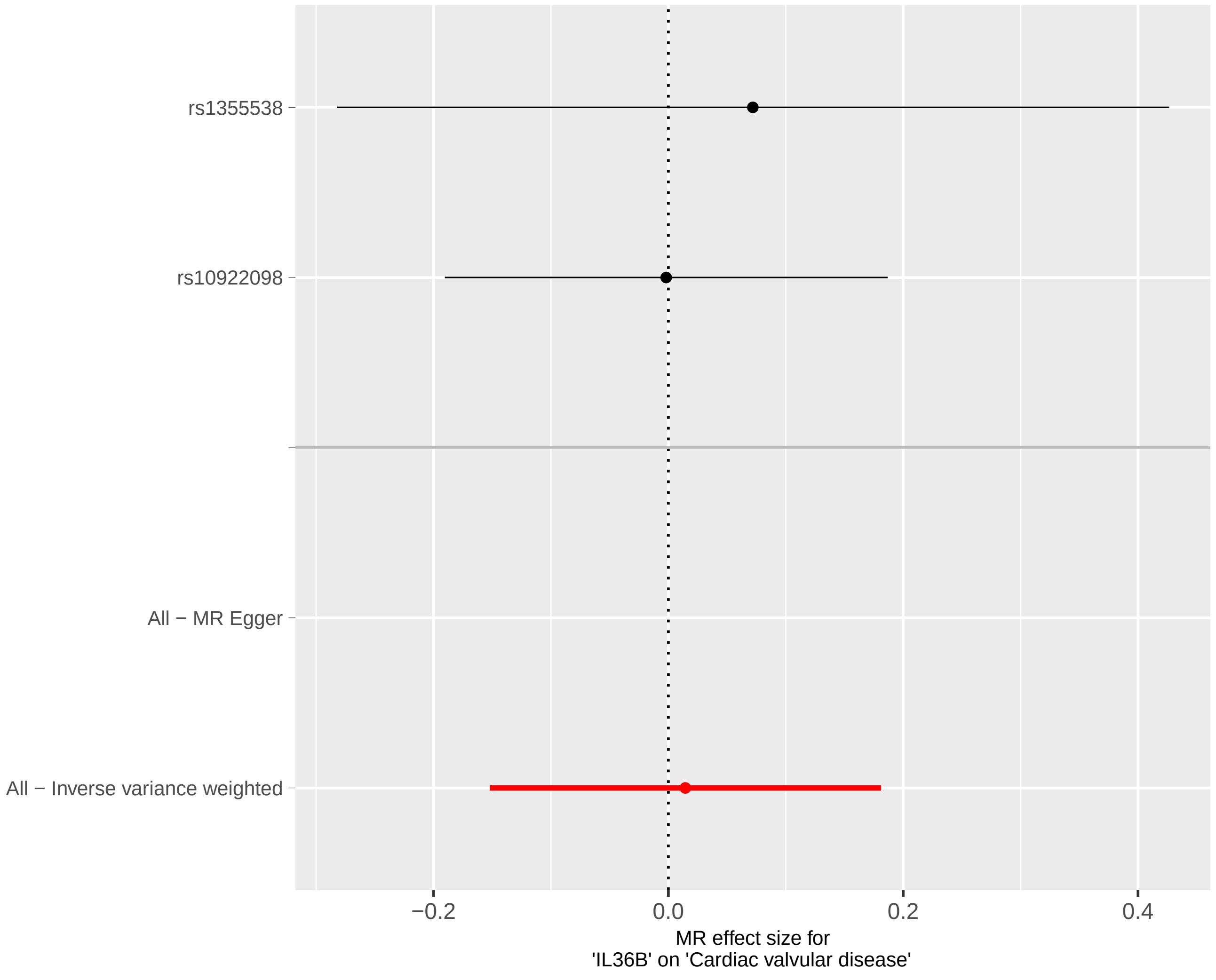
rs1355538
rs10922098
All − MR Egger
All − Inverse variance weighted
−0.2
0.0
0.2
0.4
MR effect size for
'IL36B' on 'Cardiac valvular disease'

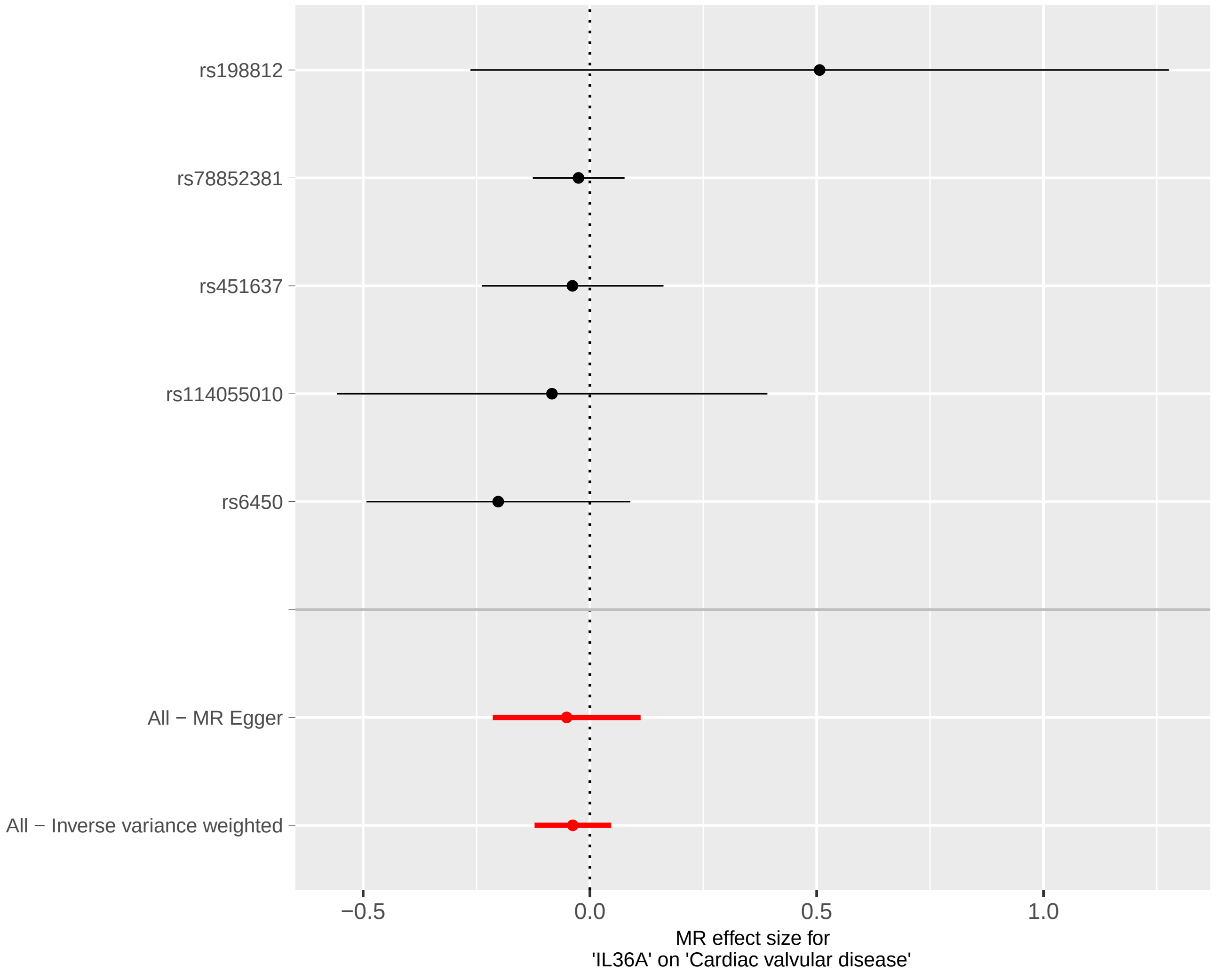

rs198812
rs78852381
rs451637
rs114055010
rs6450
All − MR Egger
All − Inverse variance weighted
−0.5
0.0
0.5
1.0
MR effect size for
'IL36A' on 'Cardiac valvular disease'

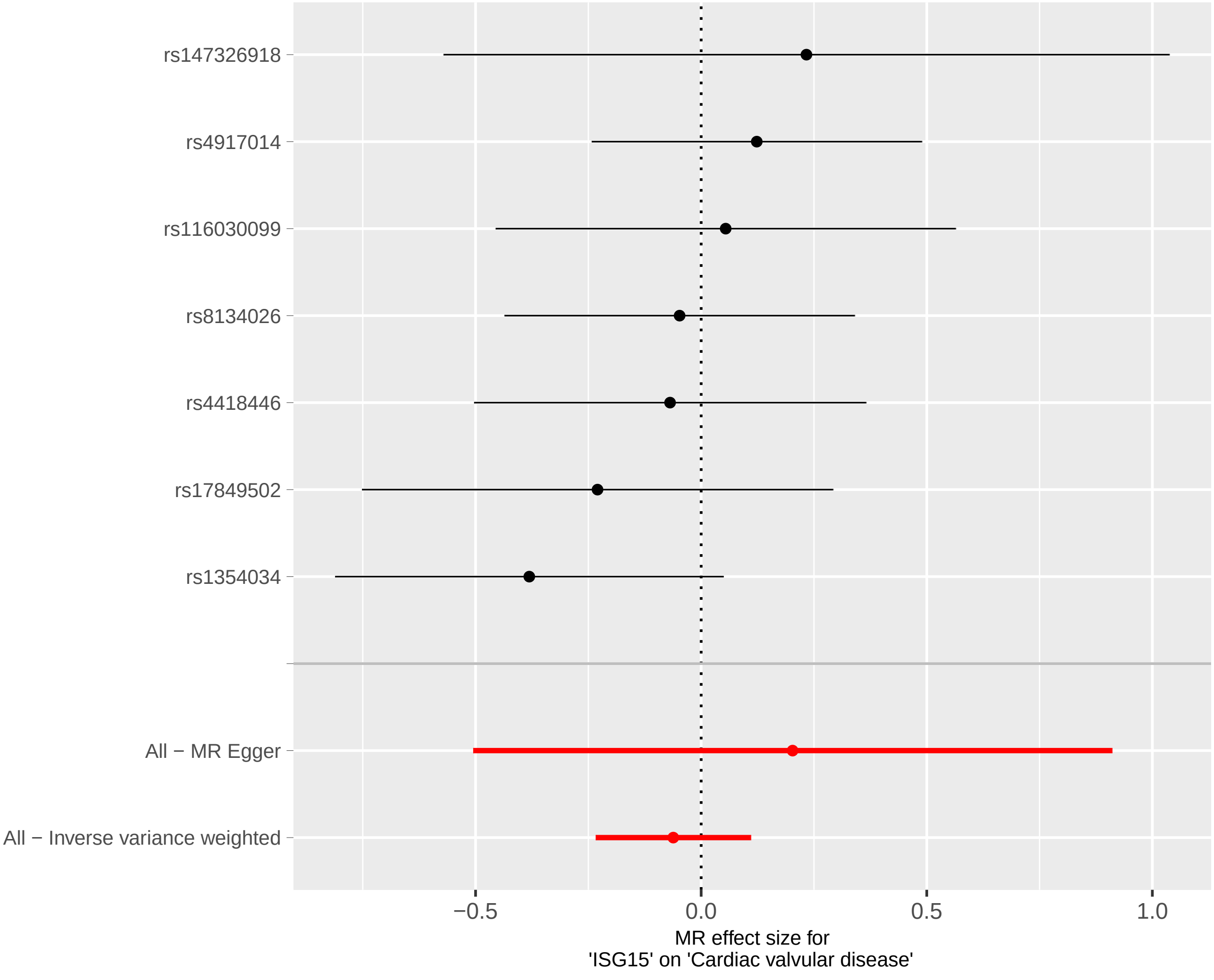
rs147326918
rs4917014
rs116030099
rs8134026
rs4418446
rs17849502
rs1354034
All − MR Egger
All − Inverse variance weighted
−0.5
0.0
0.5
1.0
MR effect size for
'ISG15' on 'Cardiac valvular disease'

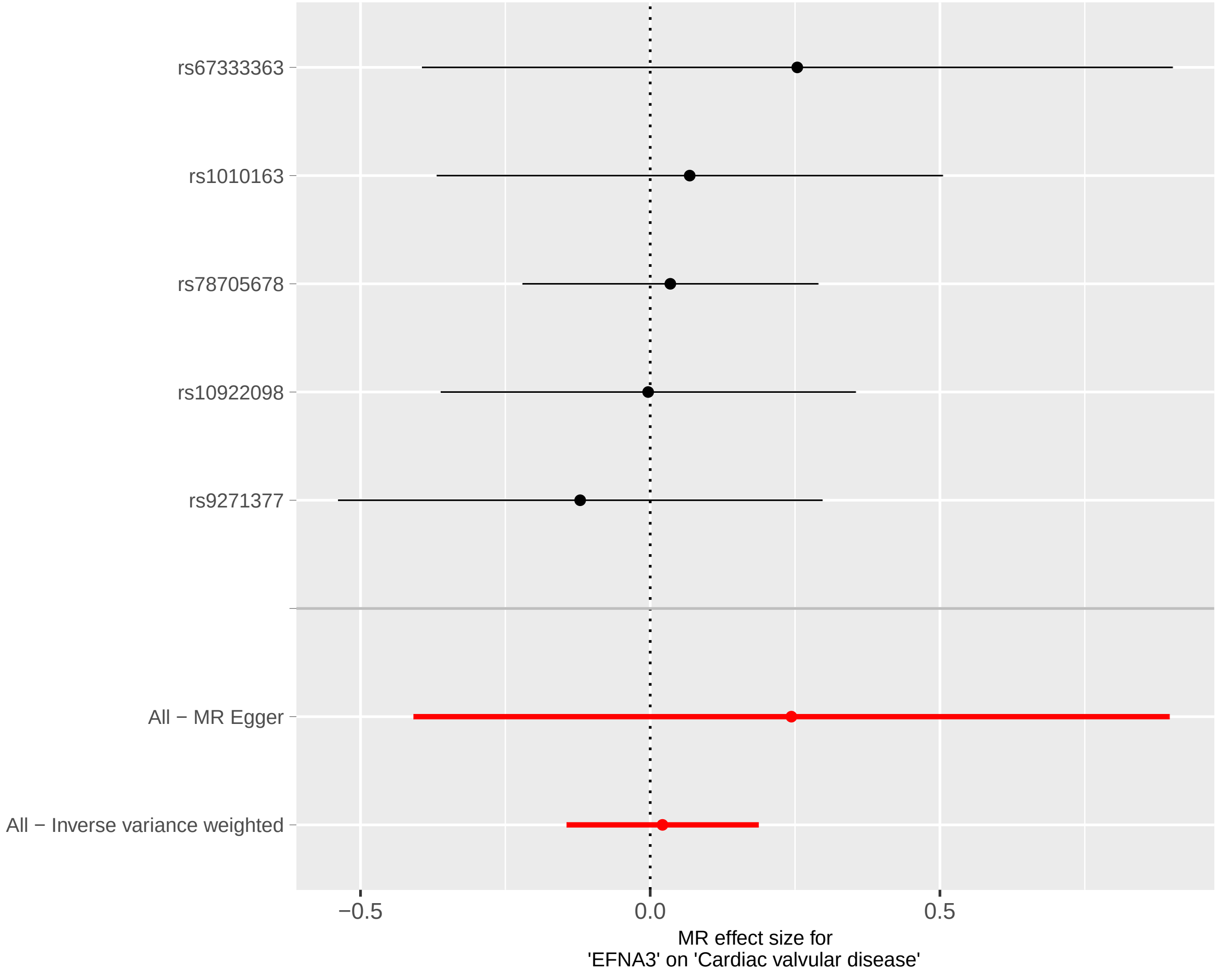
rs67333363
rs1010163
rs78705678
rs10922098
rs9271377
All − MR Egger
All − Inverse variance weighted
−0.5
0.0
0.5
MR effect size for
'EFNA3' on 'Cardiac valvular disease'

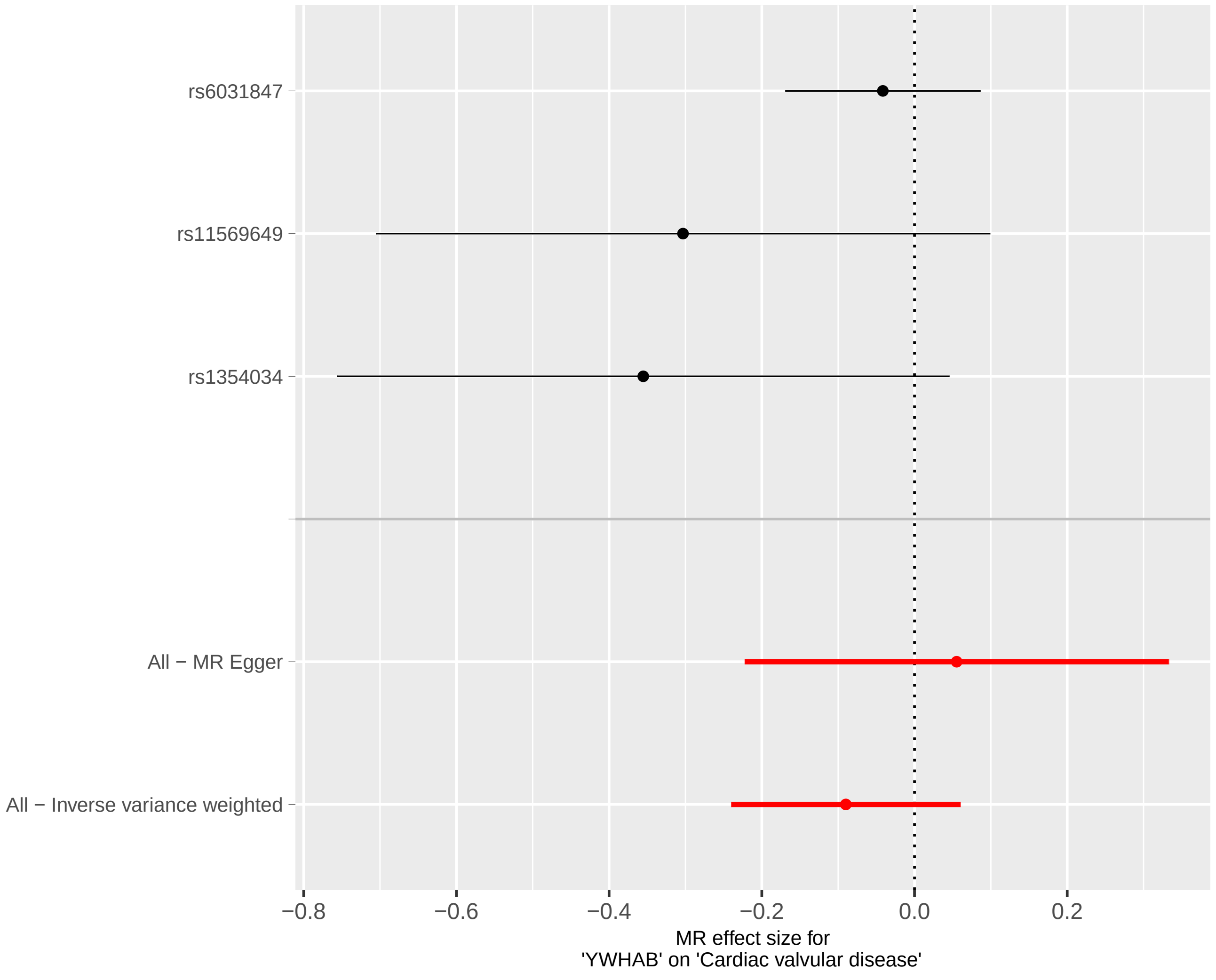
rs6031847
rs11569649
rs1354034
All − MR Egger
All − Inverse variance weighted
−0.8
−0.6
−0.4
−0.2
0.0
0.2
MR effect size for
'YWHAB' on 'Cardiac valvular disease'

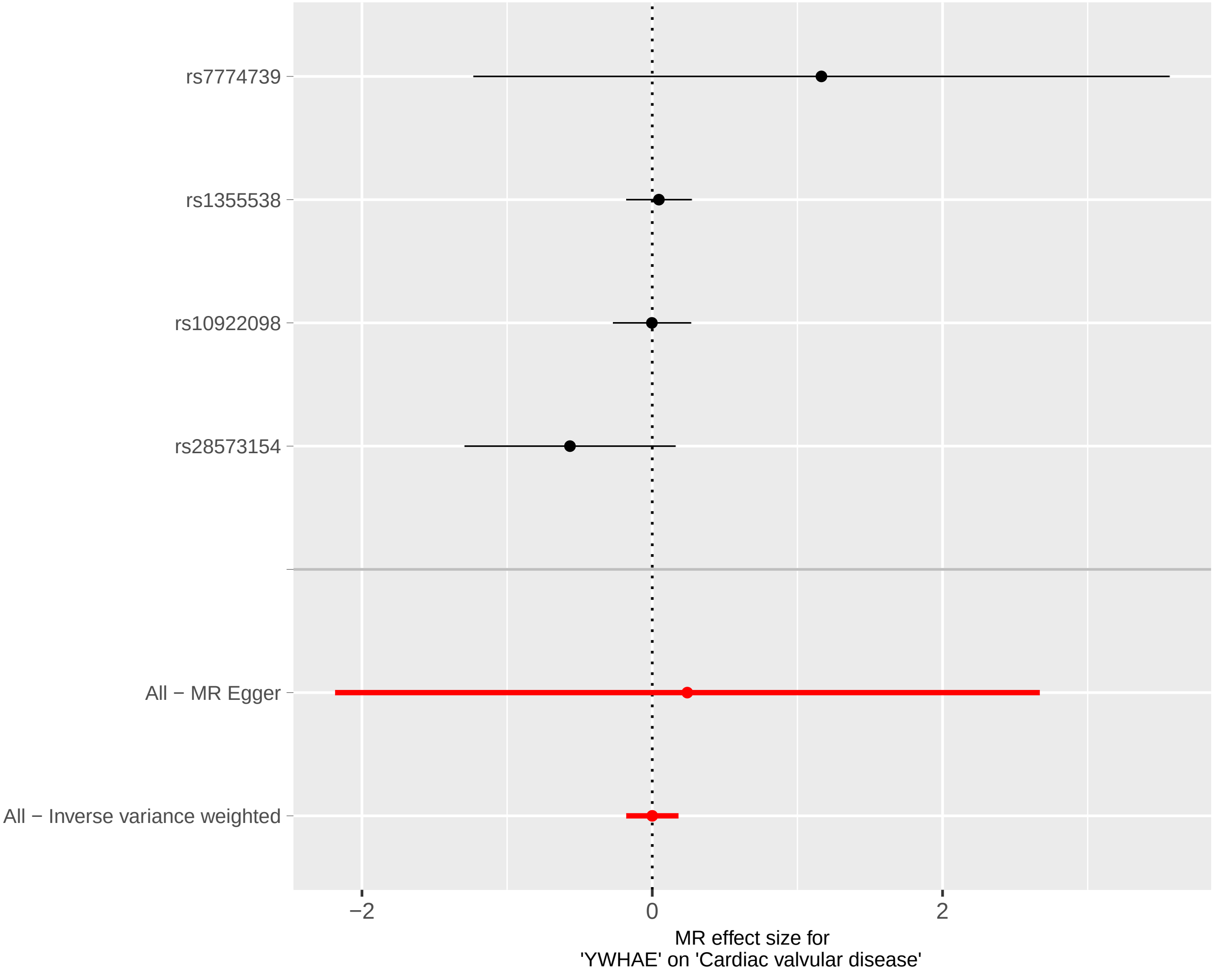
rs7774739
rs1355538
rs10922098
rs28573154
All − MR Egger
All − Inverse variance weighted
−2
0
2
MR effect size for
'YWHAE' on 'Cardiac valvular disease'

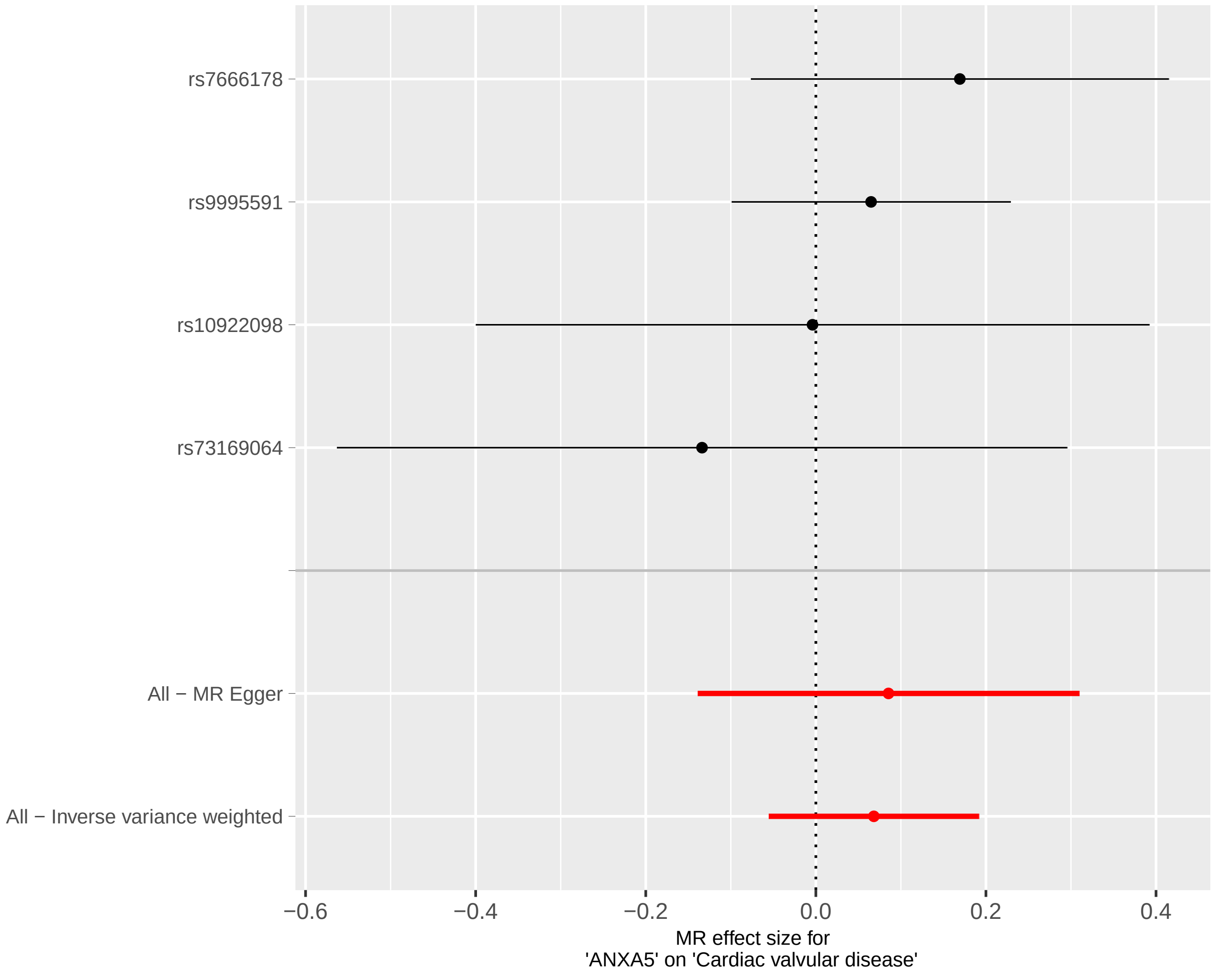

rs7666178
rs9995591
rs10922098
rs73169064
All − MR Egger
All − Inverse variance weighted
−0.6
−0.4
−0.2
0.0
0.2
0.4
MR effect size for
'ANXA5' on 'Cardiac valvular disease'

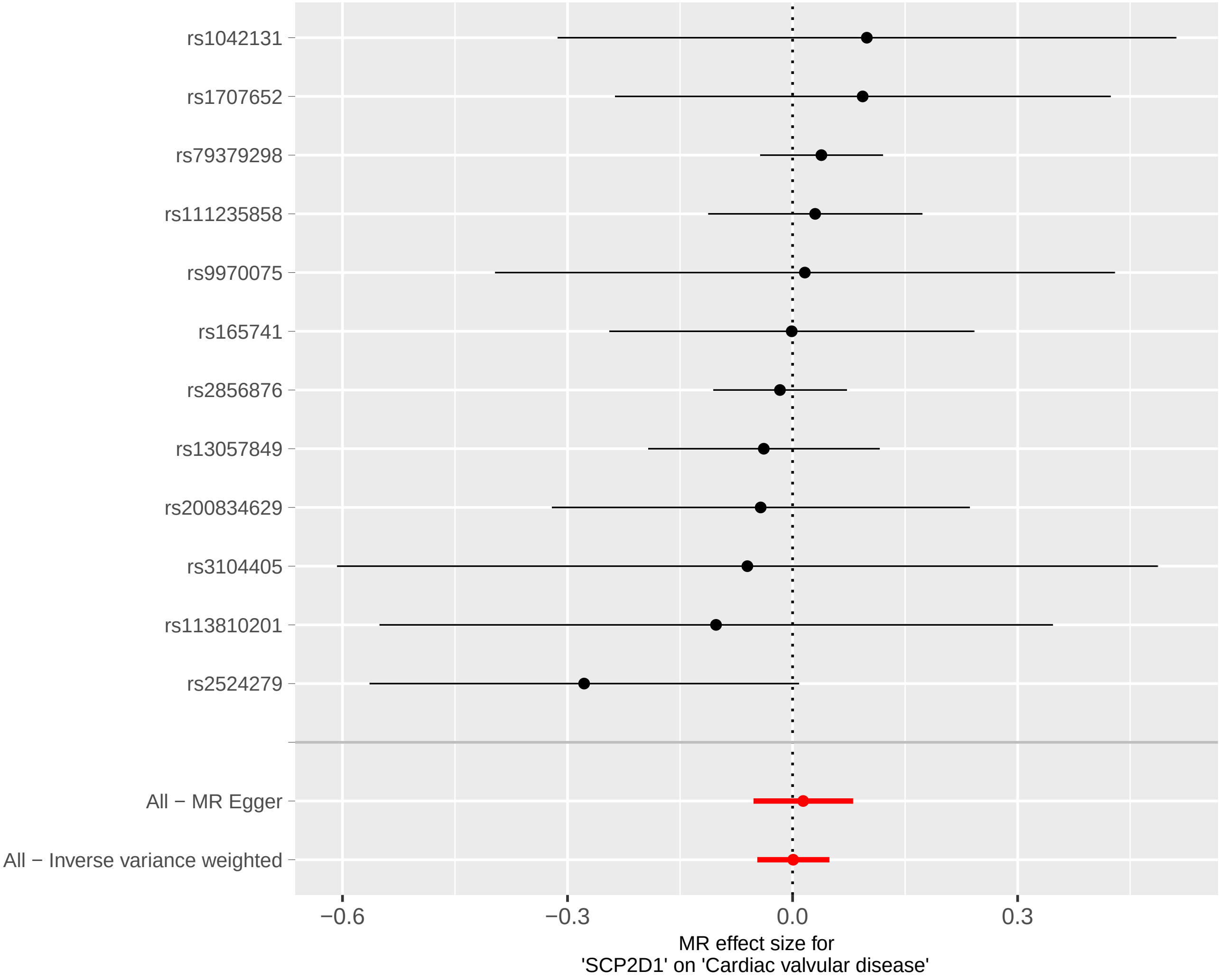

rs1042131
rs1707652
rs79379298
rs111235858
rs9970075
rs165741
rs2856876
rs13057849
rs200834629
rs3104405
rs113810201
rs2524279
All − MR Egger
All − Inverse variance weighted
−0.6
−0.3
0.0
0.3
MR effect size for
'SCP2D1' on 'Cardiac valvular disease'

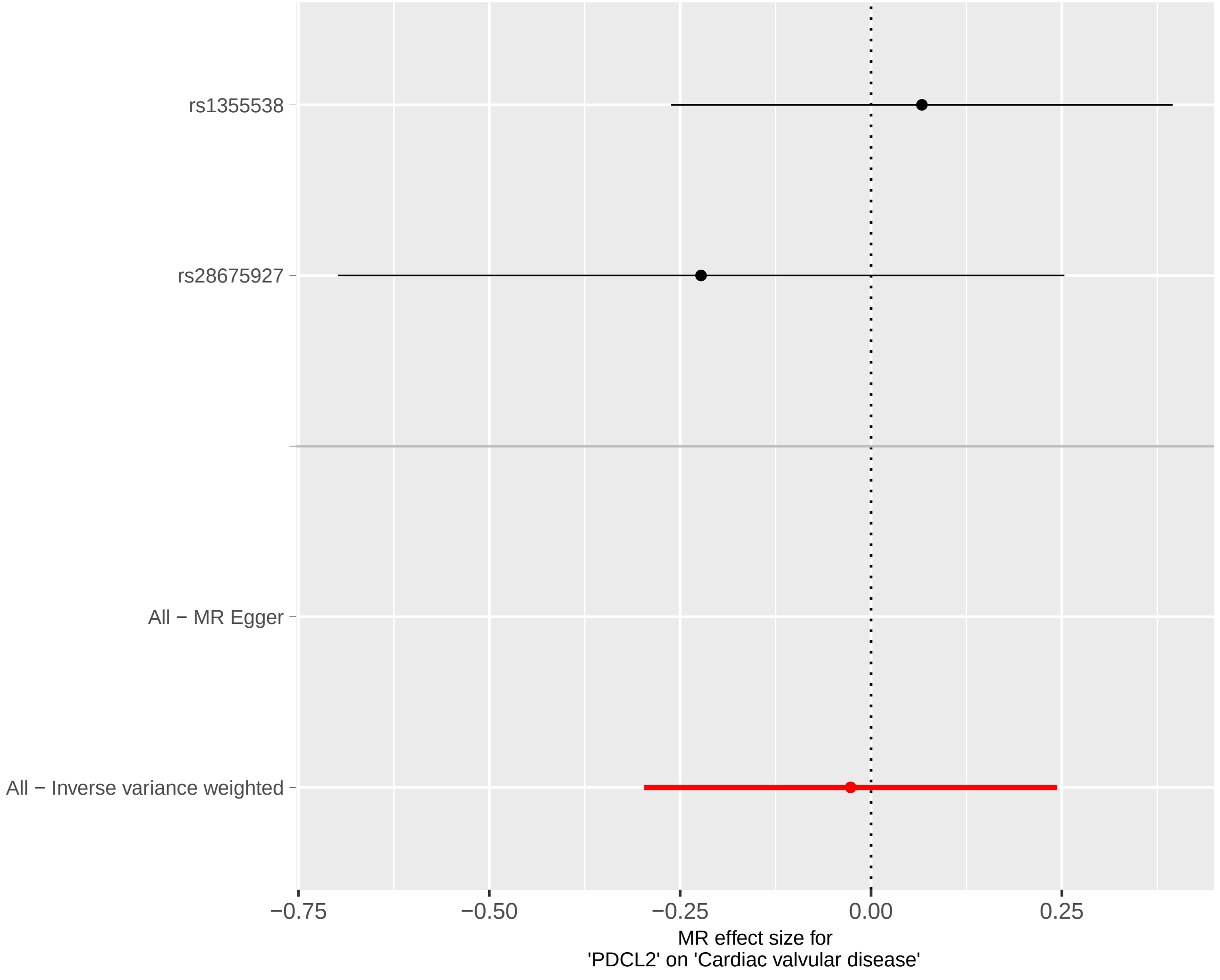

rs1355538
rs28675927
All − MR Egger
All − Inverse variance weighted
−0.75
−0.50
−0.25
0.00
0.25
MR effect size for
'PDCL2' on 'Cardiac valvular disease'

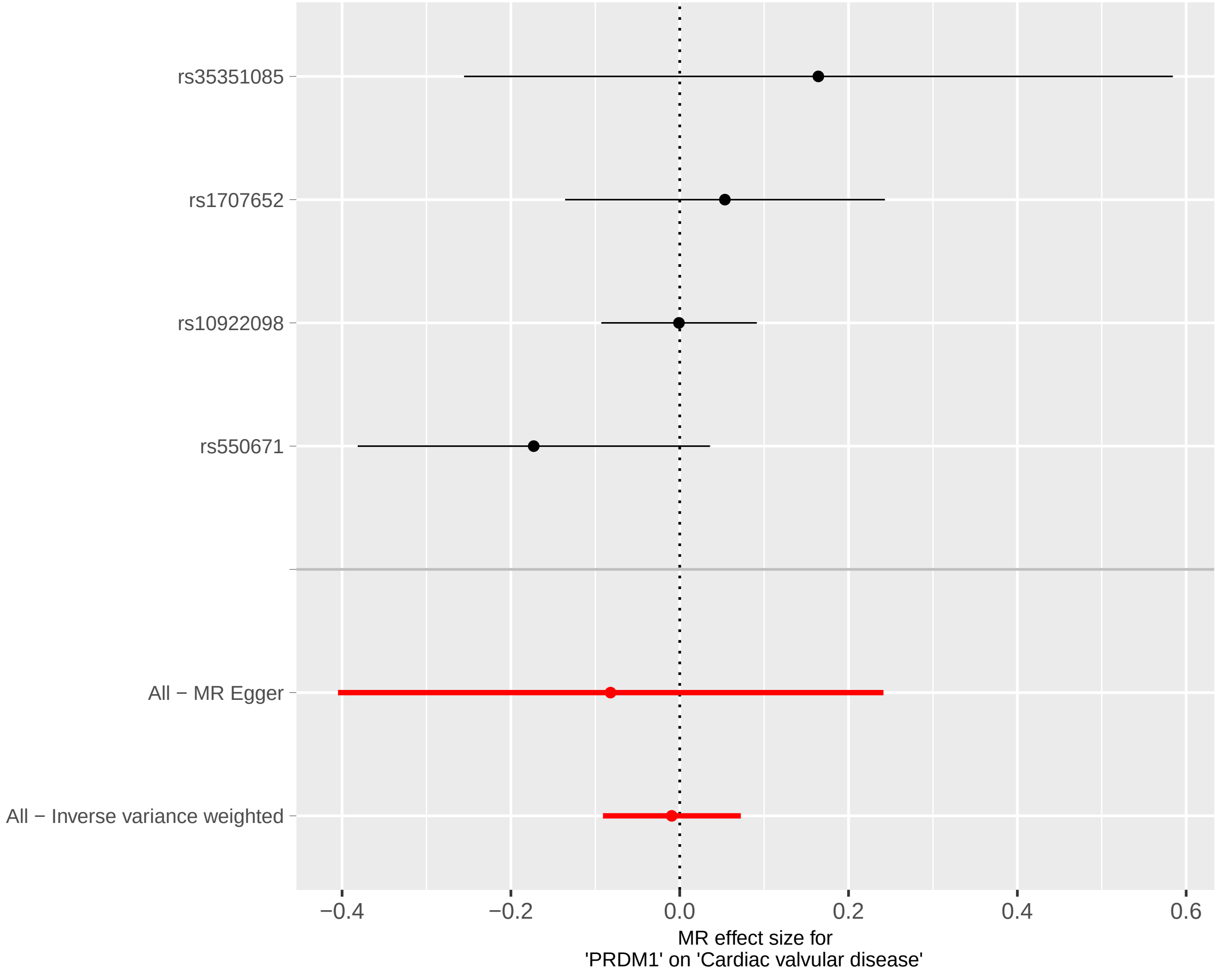
rs35351085
rs1707652
rs10922098
rs550671
All − MR Egger
All − Inverse variance weighted
−0.4
−0.2
0.0
0.2
0.4
0.6
MR effect size for
'PRDM1' on 'Cardiac valvular disease'

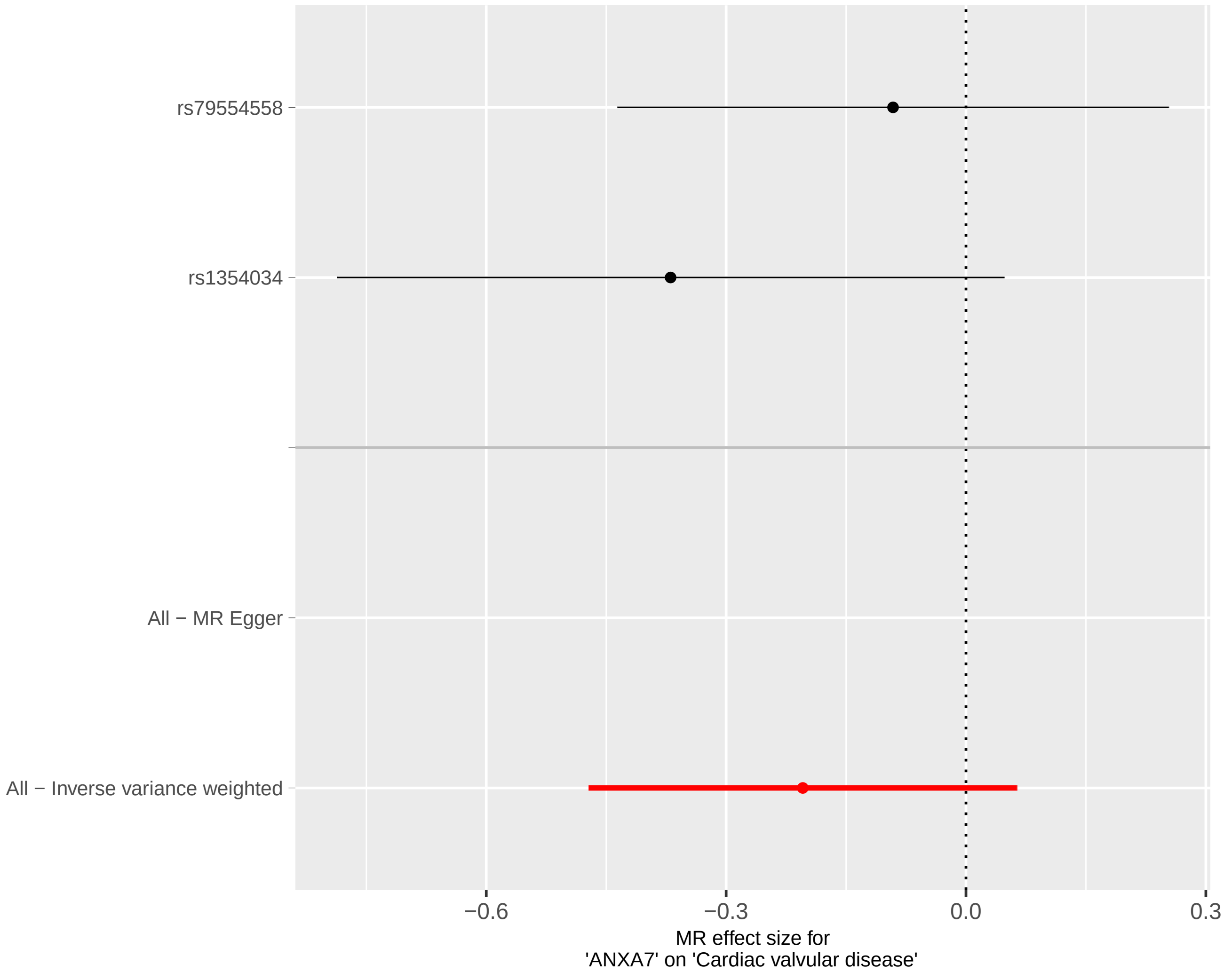

rs79554558
rs1354034
All − MR Egger
All − Inverse variance weighted
−0.6
−0.3
0.0
0.3
MR effect size for
'ANXA7' on 'Cardiac valvular disease'

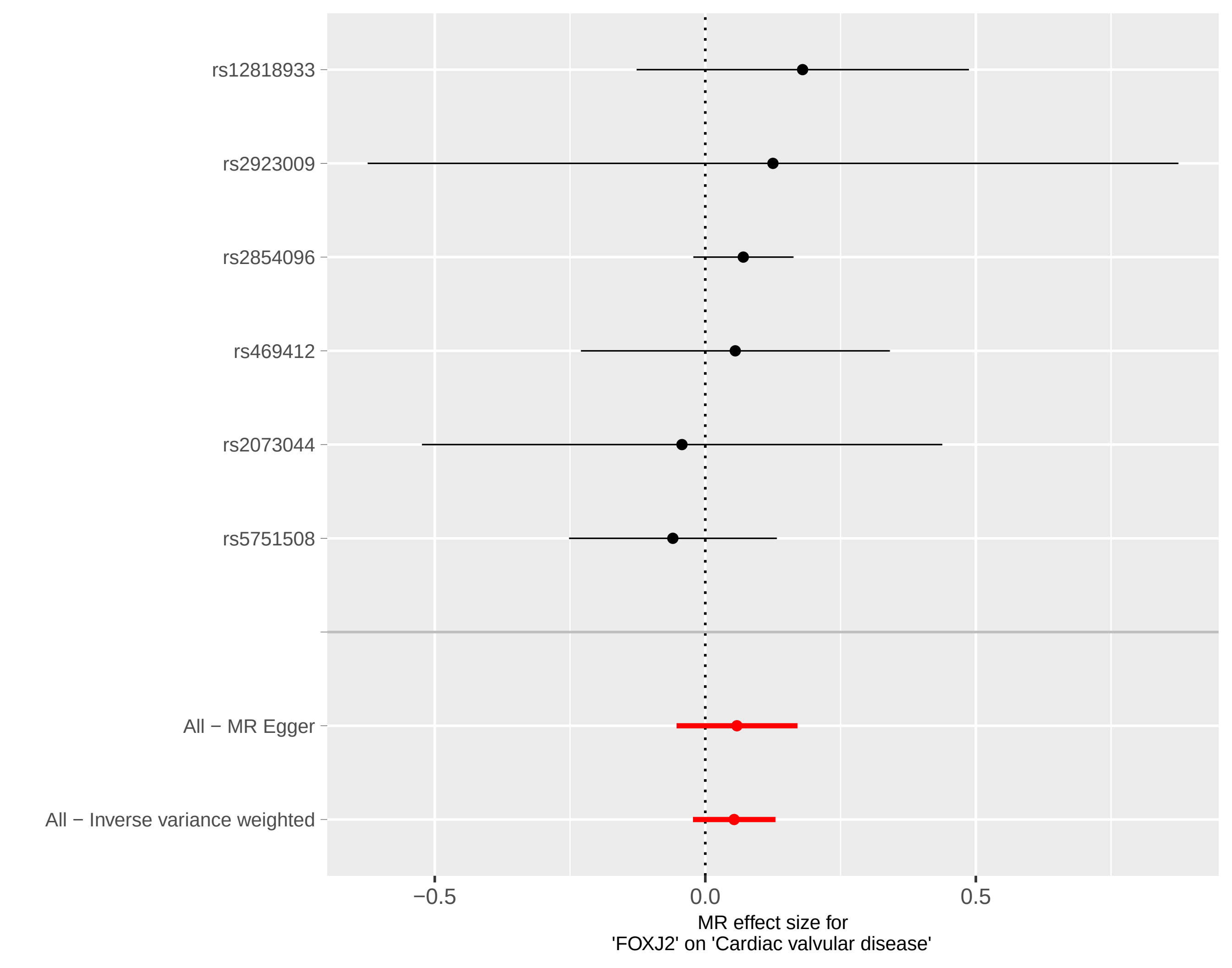
rs12818933
rs2923009
rs2854096
rs469412
rs2073044
rs5751508
All − MR Egger
All − Inverse variance weighted
−0.5
0.0
0.5
MR effect size for
'FOXJ2' on 'Cardiac valvular disease'

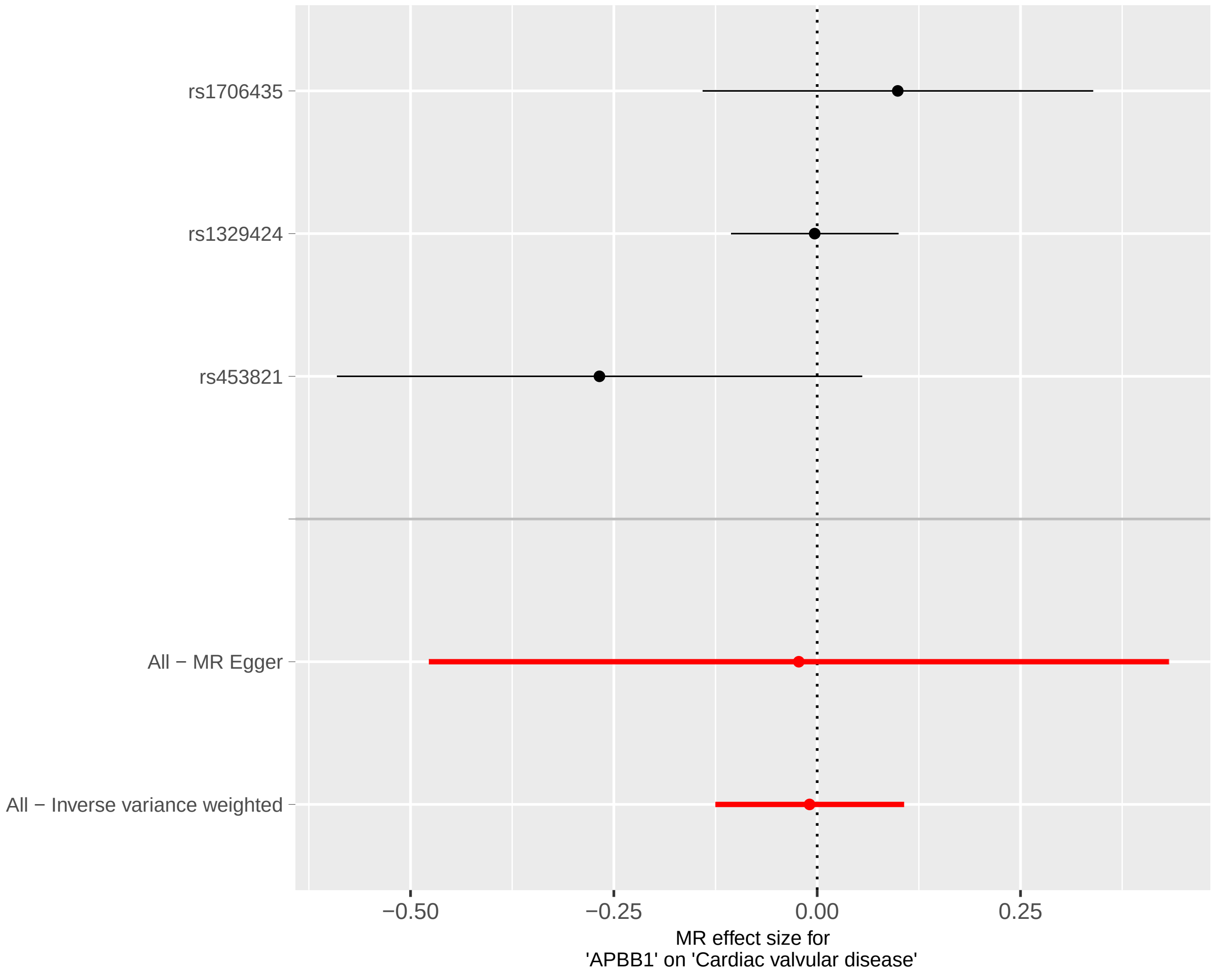

rs1706435
rs1329424
rs453821
All − MR Egger
All − Inverse variance weighted
−0.50
−0.25
0.00
0.25
MR effect size for
'APBB1' on 'Cardiac valvular disease'

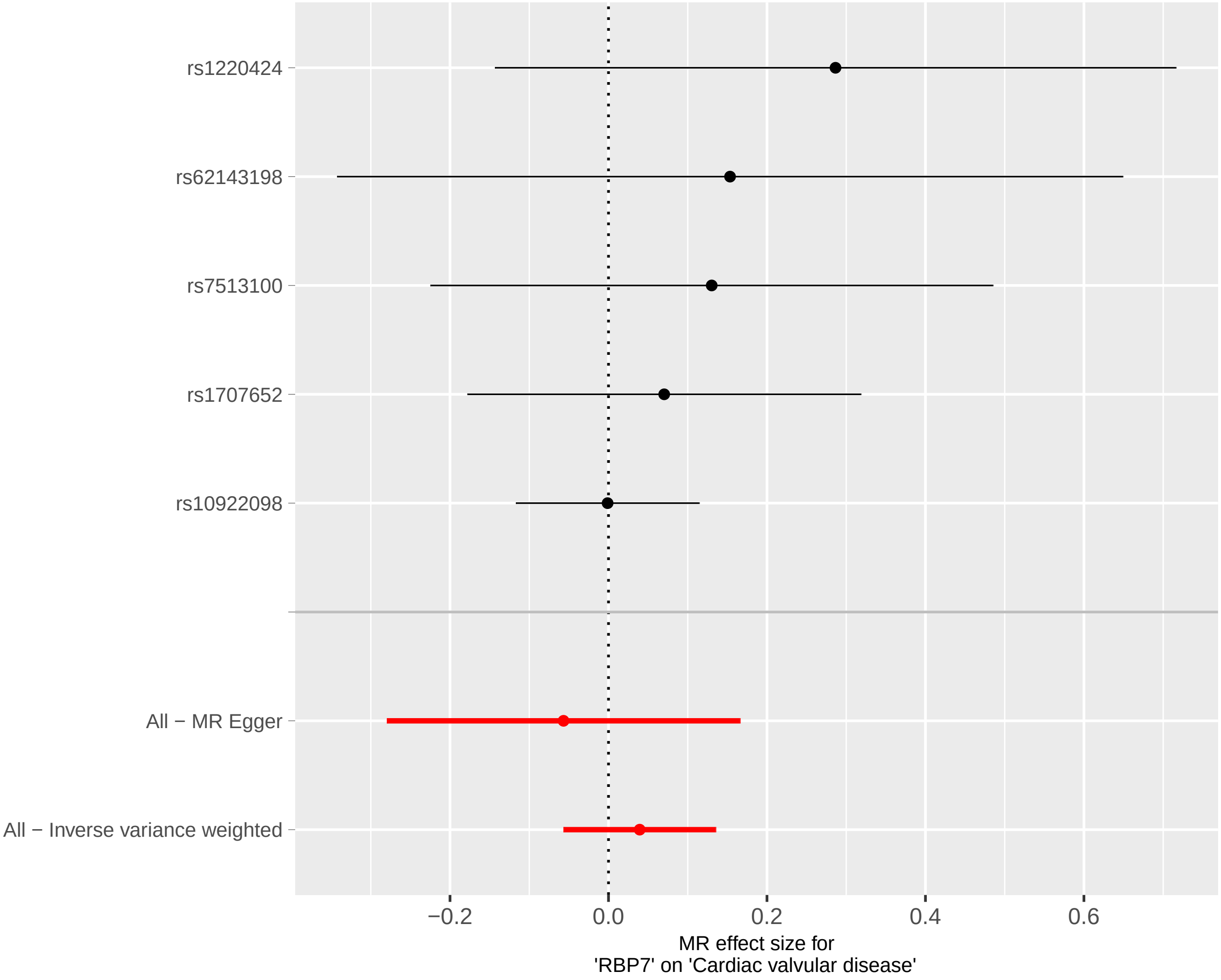
rs1220424
rs62143198
rs7513100
rs1707652
rs10922098
All − MR Egger
All − Inverse variance weighted
−0.2
0.0
0.2
0.4
0.6
MR effect size for
'RBP7' on 'Cardiac valvular disease'

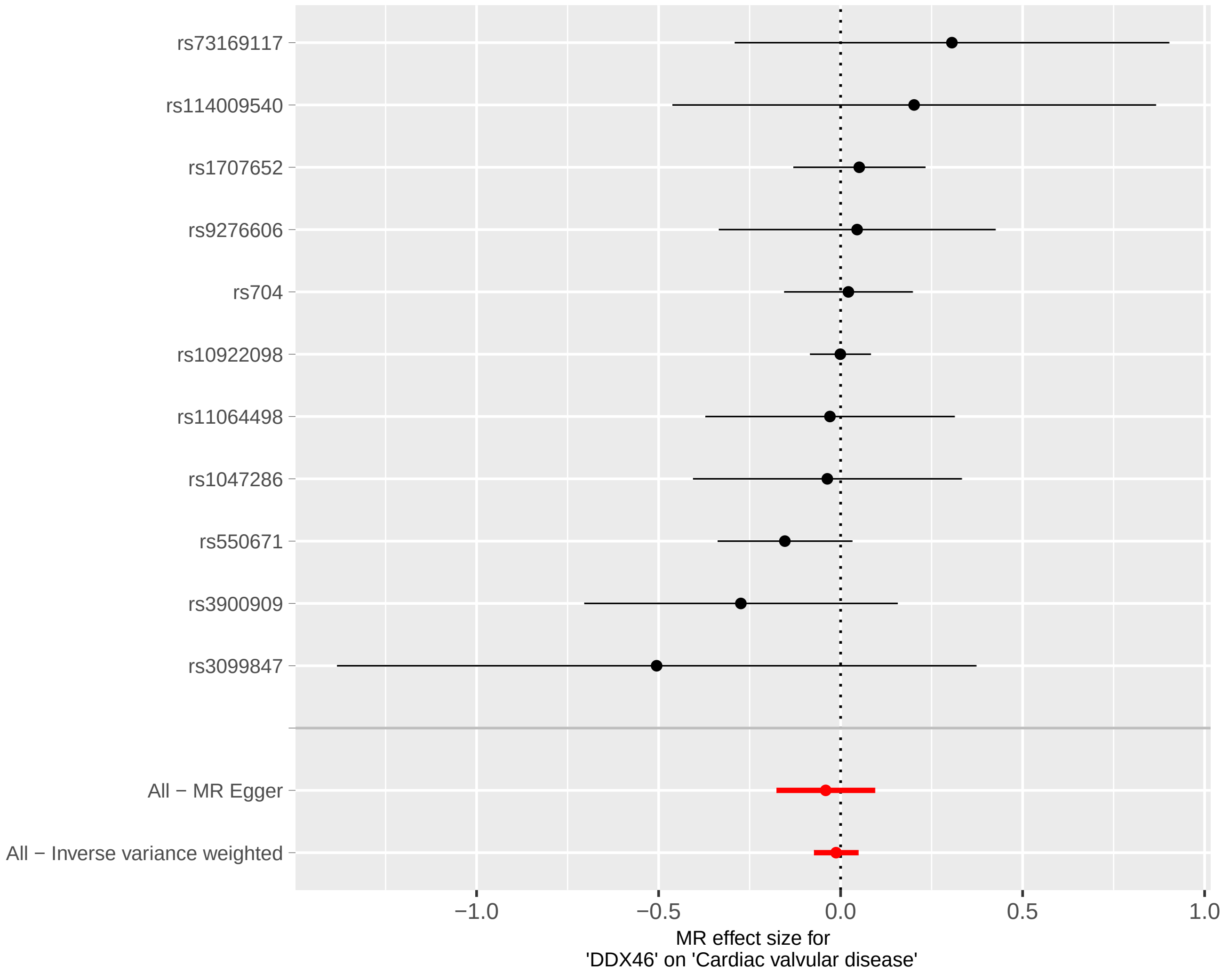
rs73169117
rs114009540
rs1707652
rs9276606
rs704
rs10922098
rs11064498
rs1047286
rs550671
rs3900909
rs3099847
All − MR Egger
All − Inverse variance weighted
−1.0
−0.5
0.0
0.5
1.0
MR effect size for
'DDX46' on 'Cardiac valvular disease'

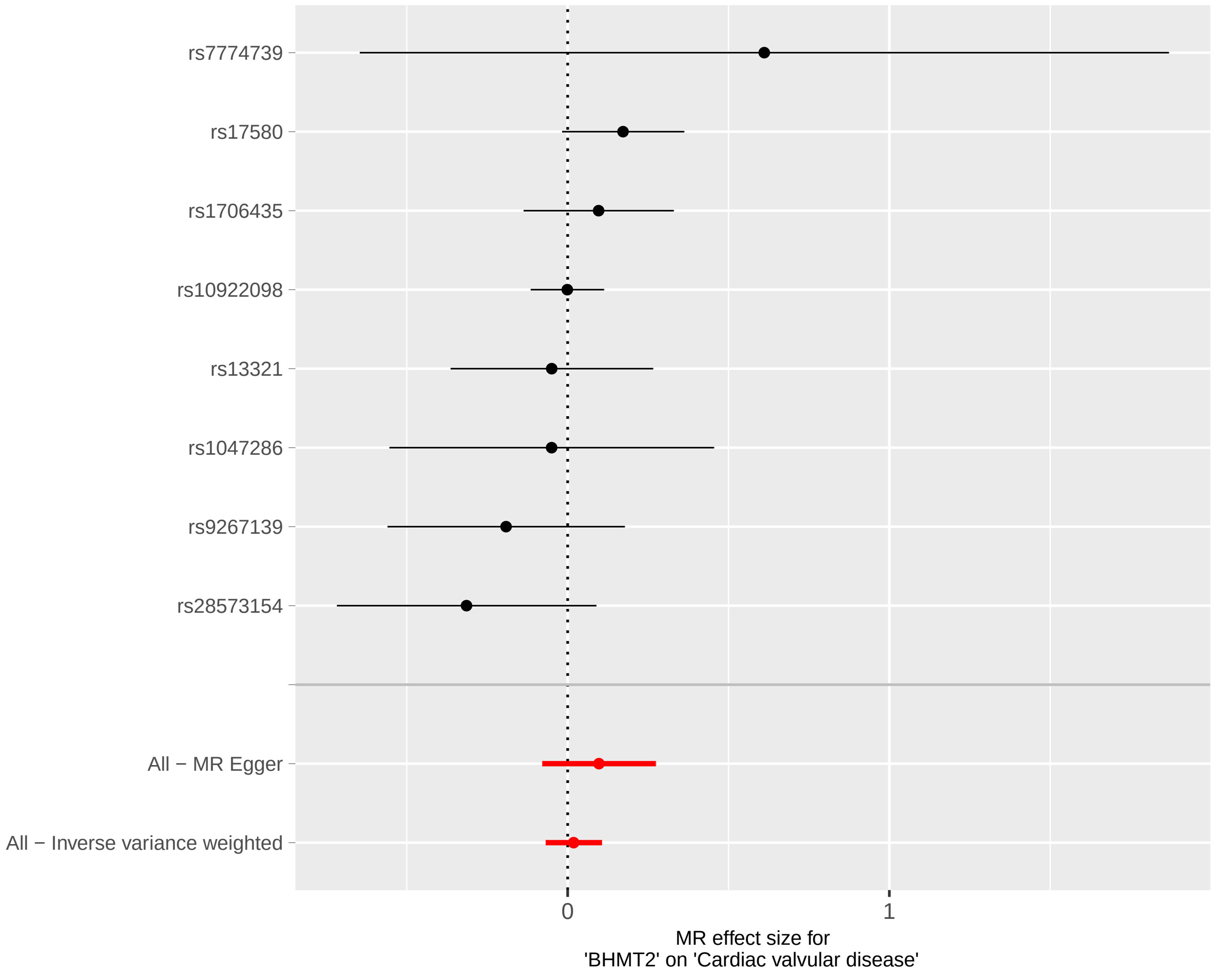
rs7774739
rs17580
rs1706435
rs10922098
rs13321
rs1047286
rs9267139
rs28573154
All − MR Egger
All − Inverse variance weighted
0
1
MR effect size for
'BHMT2' on 'Cardiac valvular disease'

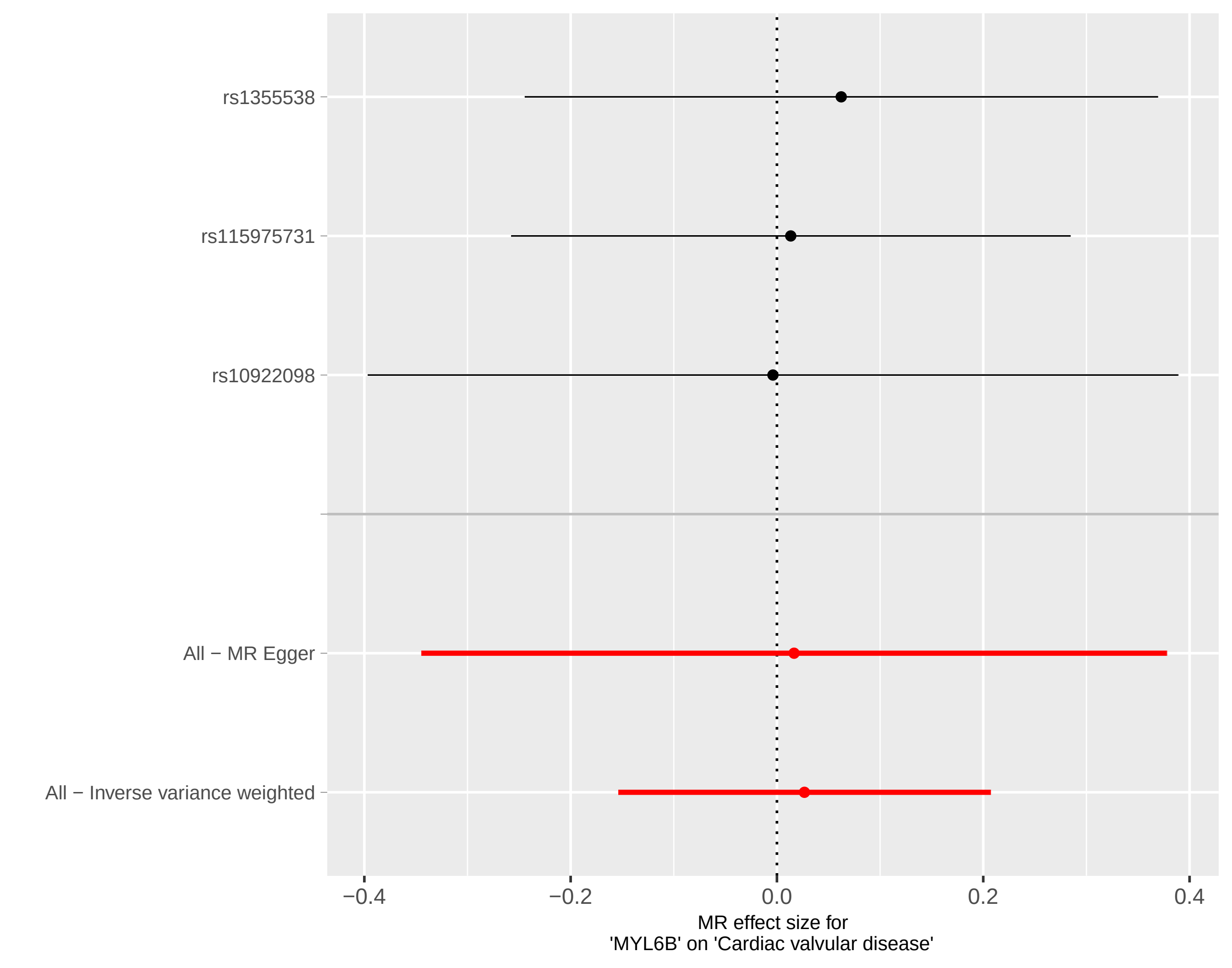
rs1355538
rs115975731
rs10922098
All − MR Egger
All − Inverse variance weighted
−0.4
−0.2
0.0
0.2
0.4
MR effect size for
'MYL6B' on 'Cardiac valvular disease'

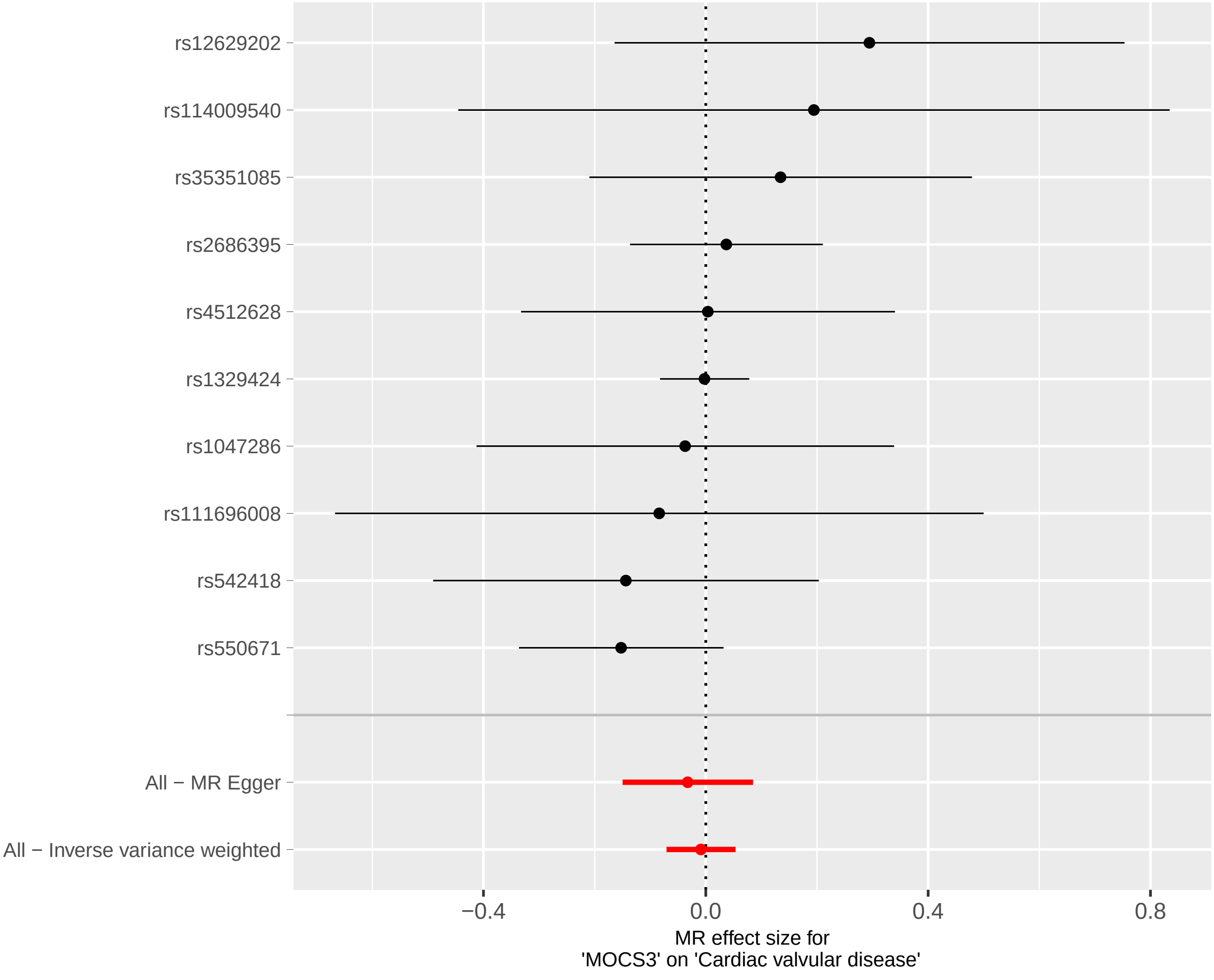
rs12629202
rs114009540
rs35351085
rs2686395
rs4512628
rs1329424
rs1047286
rs111696008
rs542418
rs550671
All − MR Egger
All − Inverse variance weighted
−0.4
0.0
0.4
0.8
MR effect size for
'MOCS3' on 'Cardiac valvular disease'

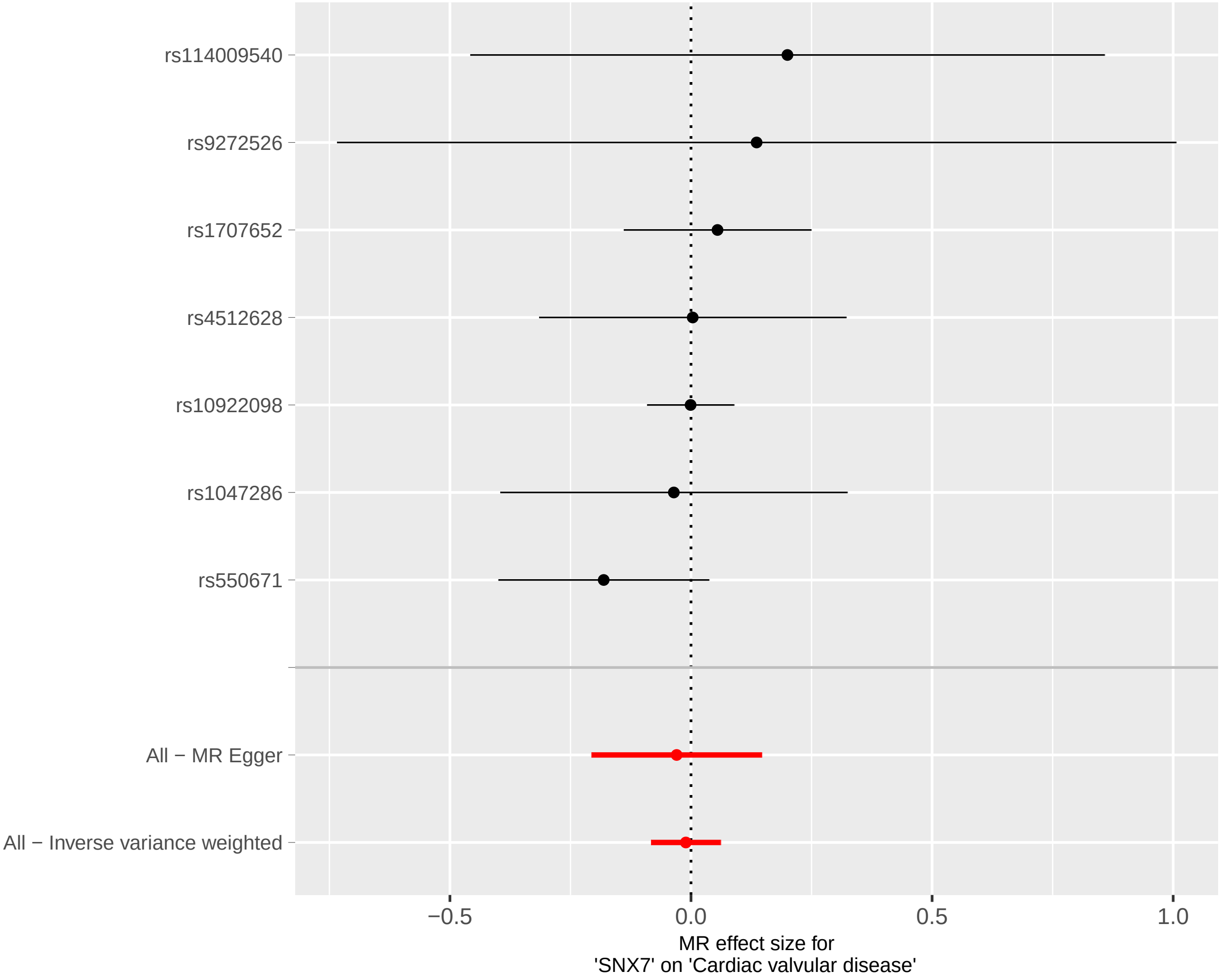

rs114009540
rs9272526
rs1707652
rs4512628
rs10922098
rs1047286
rs550671
All − MR Egger
All − Inverse variance weighted
−0.5
0.0
0.5
1.0
MR effect size for
'SNX7' on 'Cardiac valvular disease'

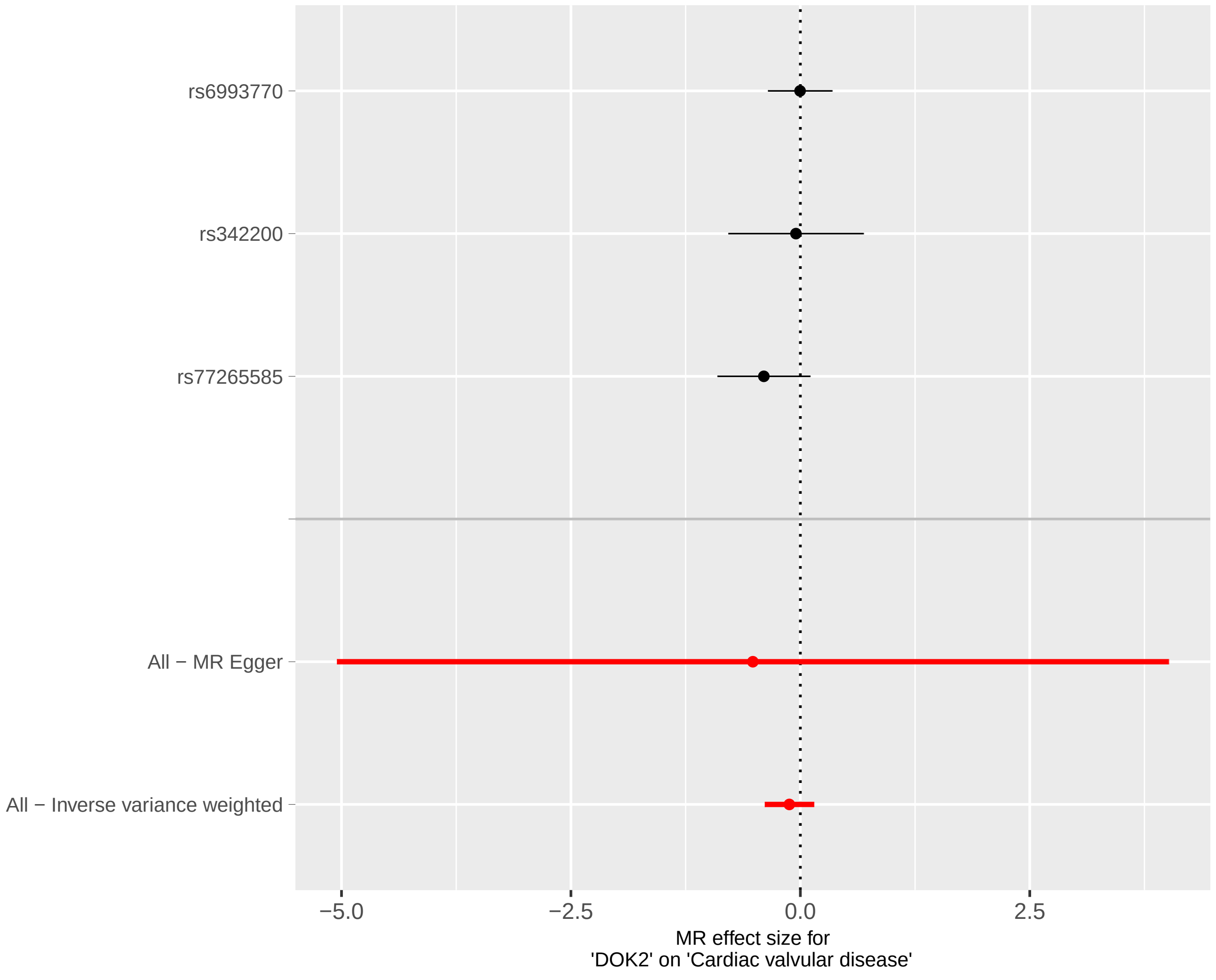
rs6993770
rs342200
rs77265585
All − MR Egger
All − Inverse variance weighted
−5.0
−2.5
0.0
2.5
MR effect size for
'DOK2' on 'Cardiac valvular disease'

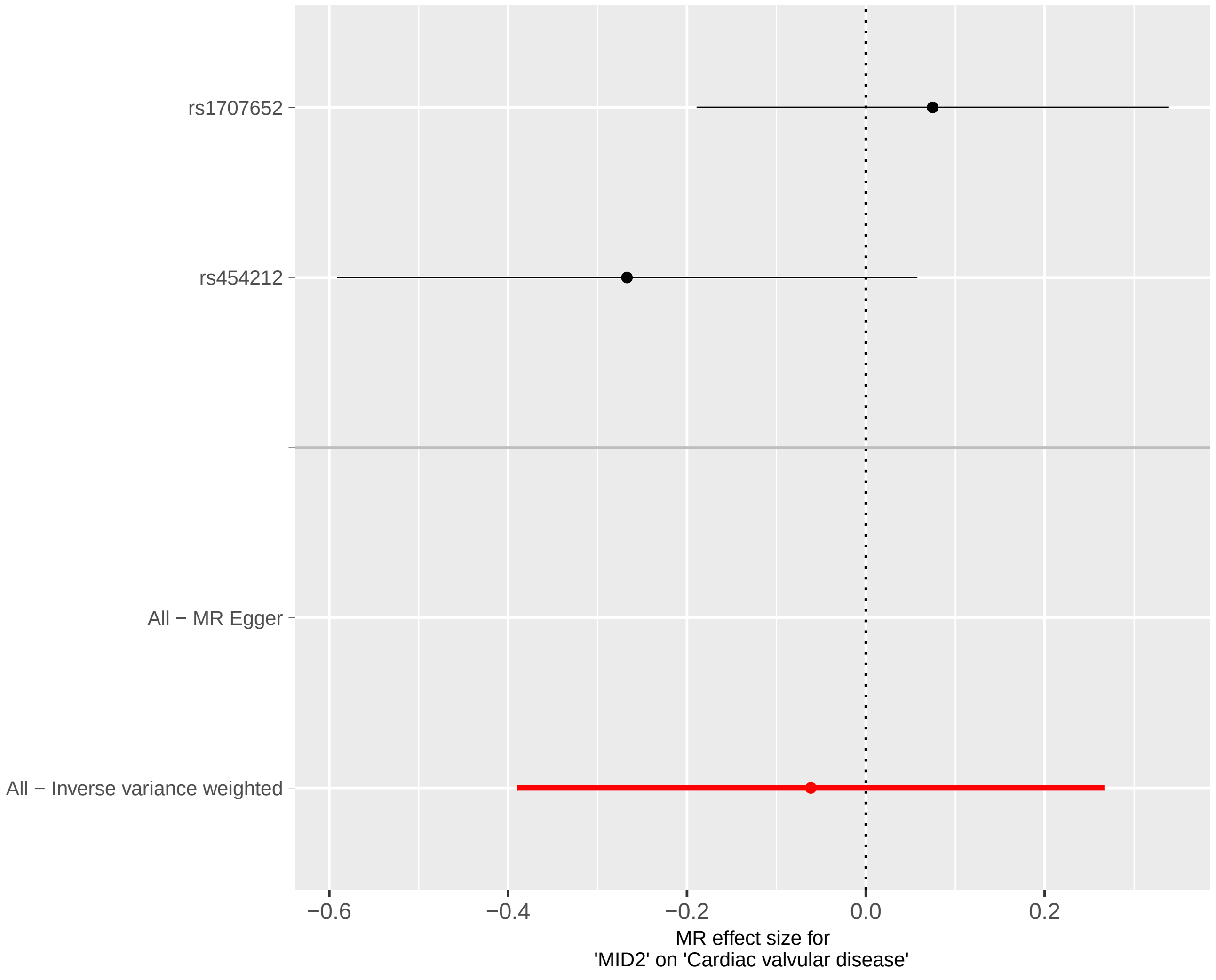

rs1707652
rs454212
All − MR Egger
All − Inverse variance weighted
−0.6
−0.4
−0.2
0.0
0.2
MR effect size for
'MID2' on 'Cardiac valvular disease'

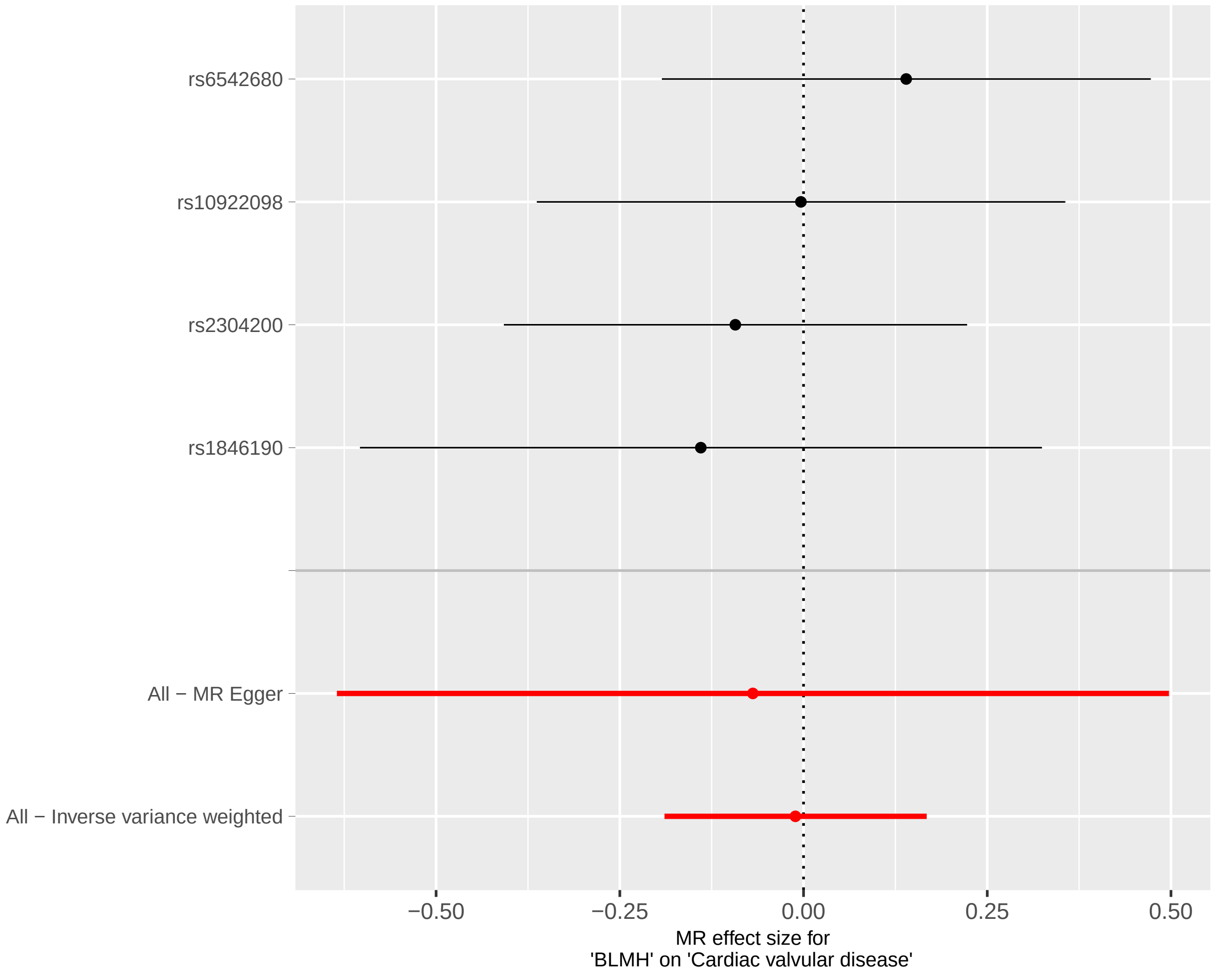

rs6542680
rs10922098
rs2304200
rs1846190
All − MR Egger
All − Inverse variance weighted
−0.50
−0.25
0.00
0.25
0.50
MR effect size for
'BLMH' on 'Cardiac valvular disease'

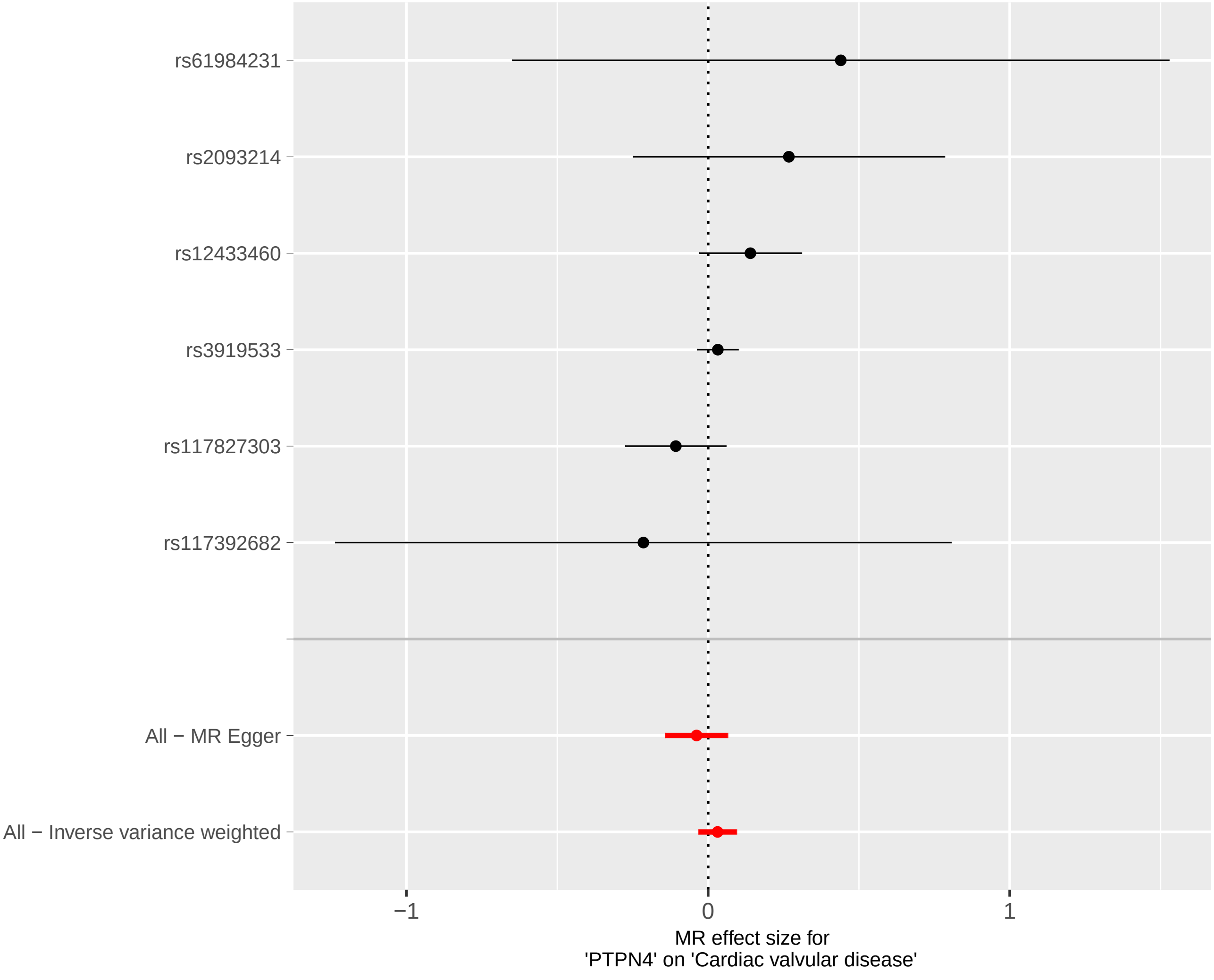
rs61984231
rs2093214
rs12433460
rs3919533
rs117827303
rs117392682
All − MR Egger
All − Inverse variance weighted
−1
0
1
MR effect size for
'PTPN4' on 'Cardiac valvular disease'

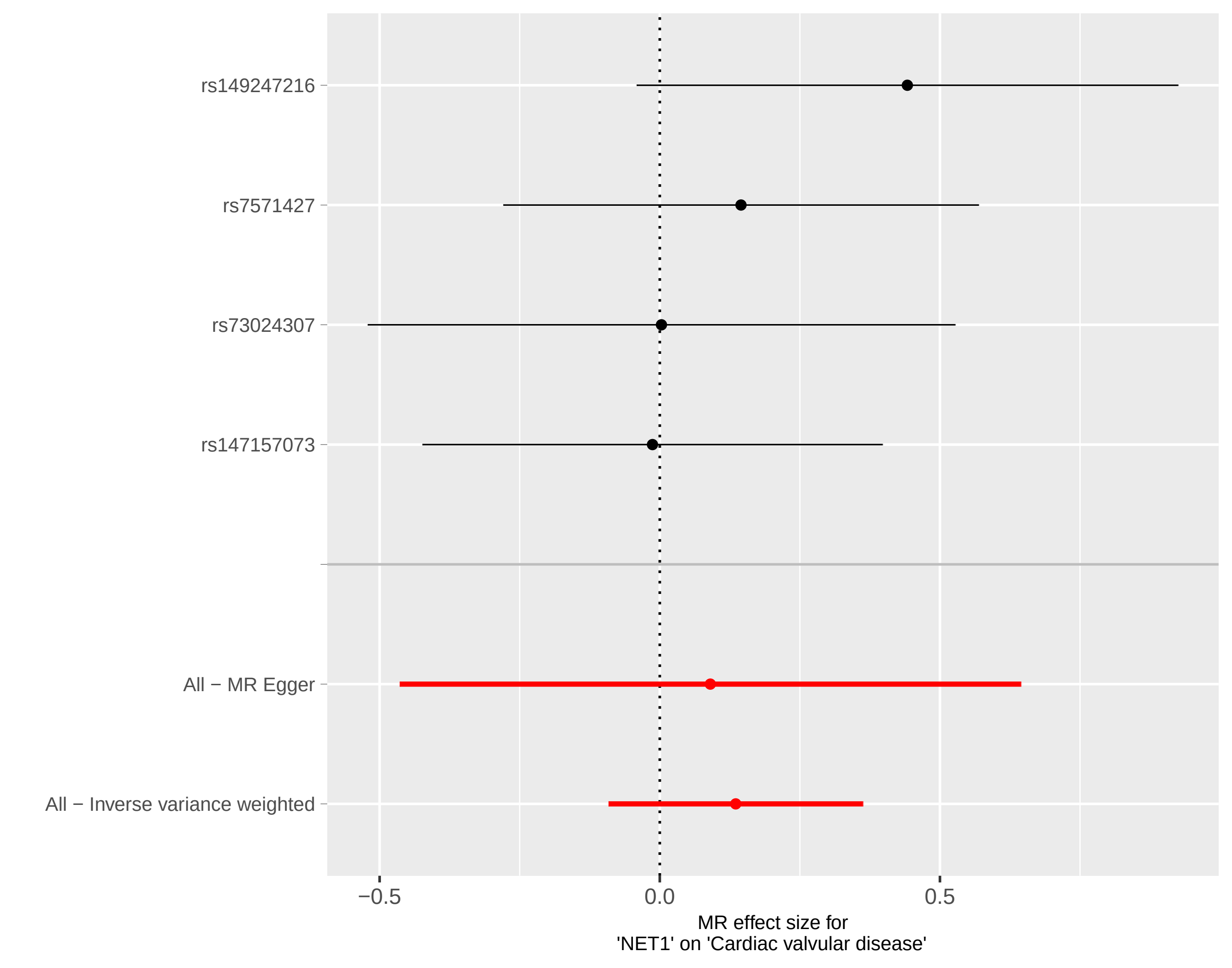
rs149247216
rs7571427
rs73024307
rs147157073
All − MR Egger
All − Inverse variance weighted
−0.5
0.0
0.5
MR effect size for
'NET1' on 'Cardiac valvular disease'

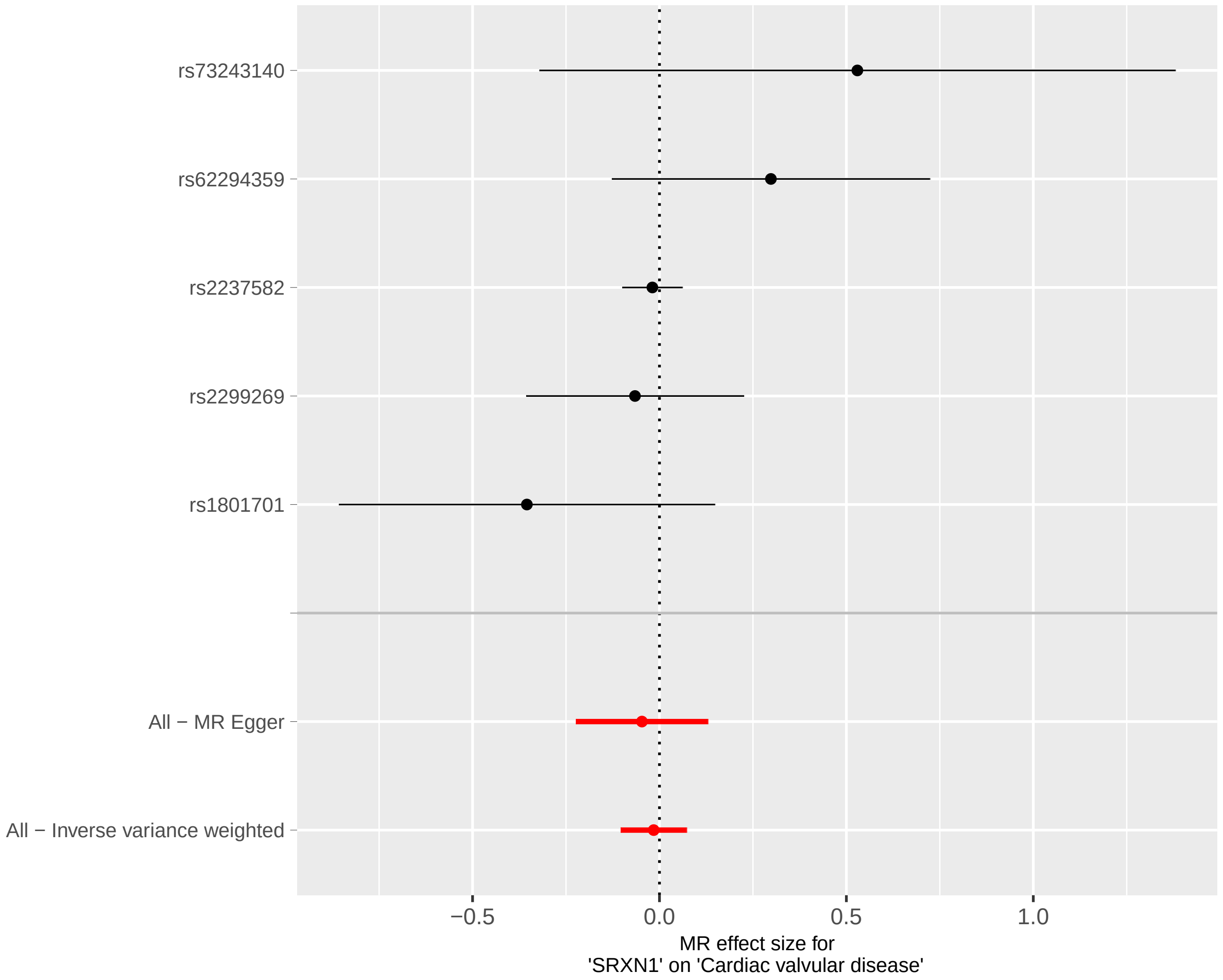
rs73243140
rs62294359
rs2237582
rs2299269
rs1801701
All − MR Egger
All − Inverse variance weighted
−0.5
0.0
0.5
1.0
MR effect size for
'SRXN1' on 'Cardiac valvular disease'

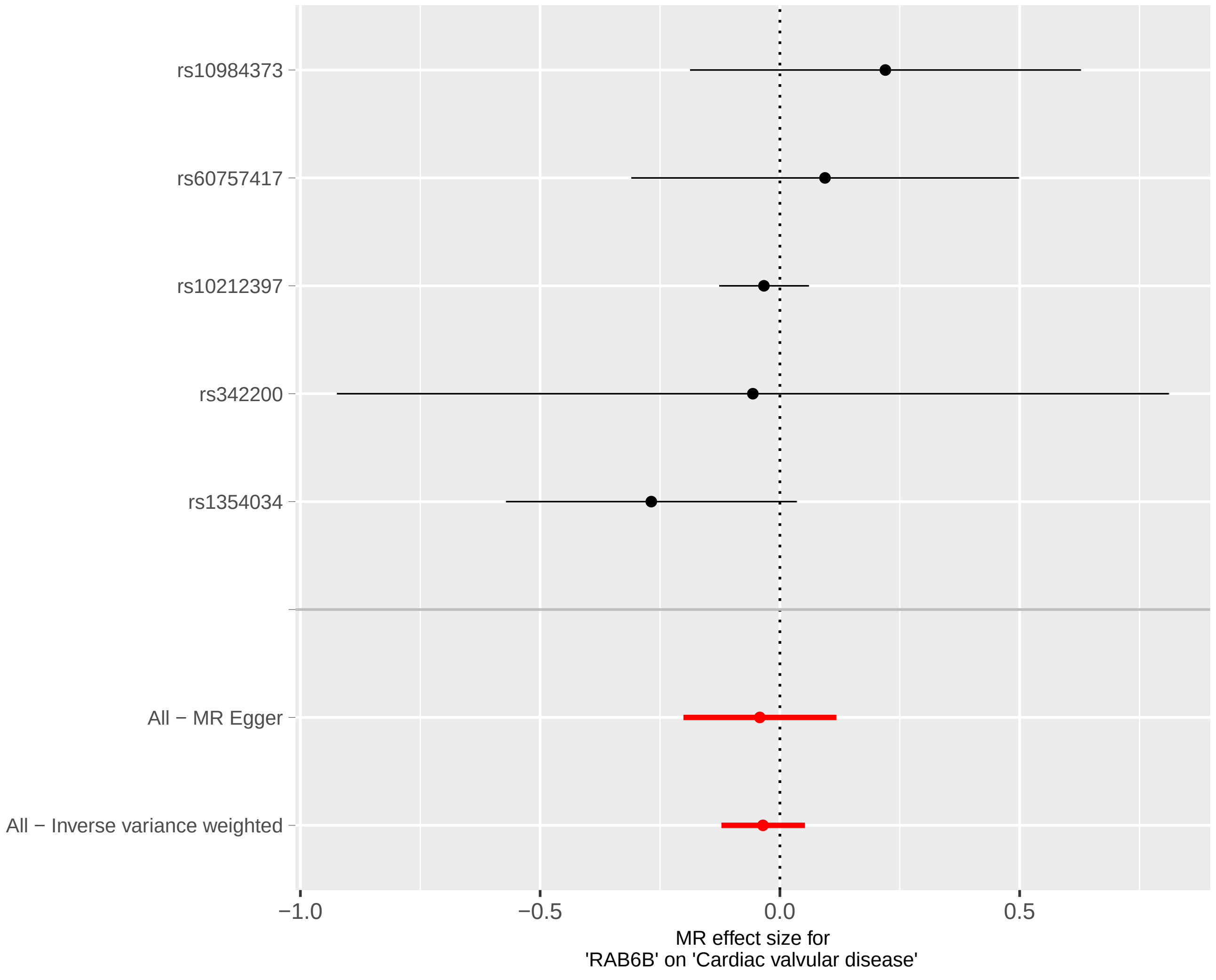
rs10984373
rs60757417
rs10212397
rs342200
rs1354034
All − MR Egger
All − Inverse variance weighted
−1.0
−0.5
0.0
0.5
MR effect size for
'RAB6B' on 'Cardiac valvular disease'

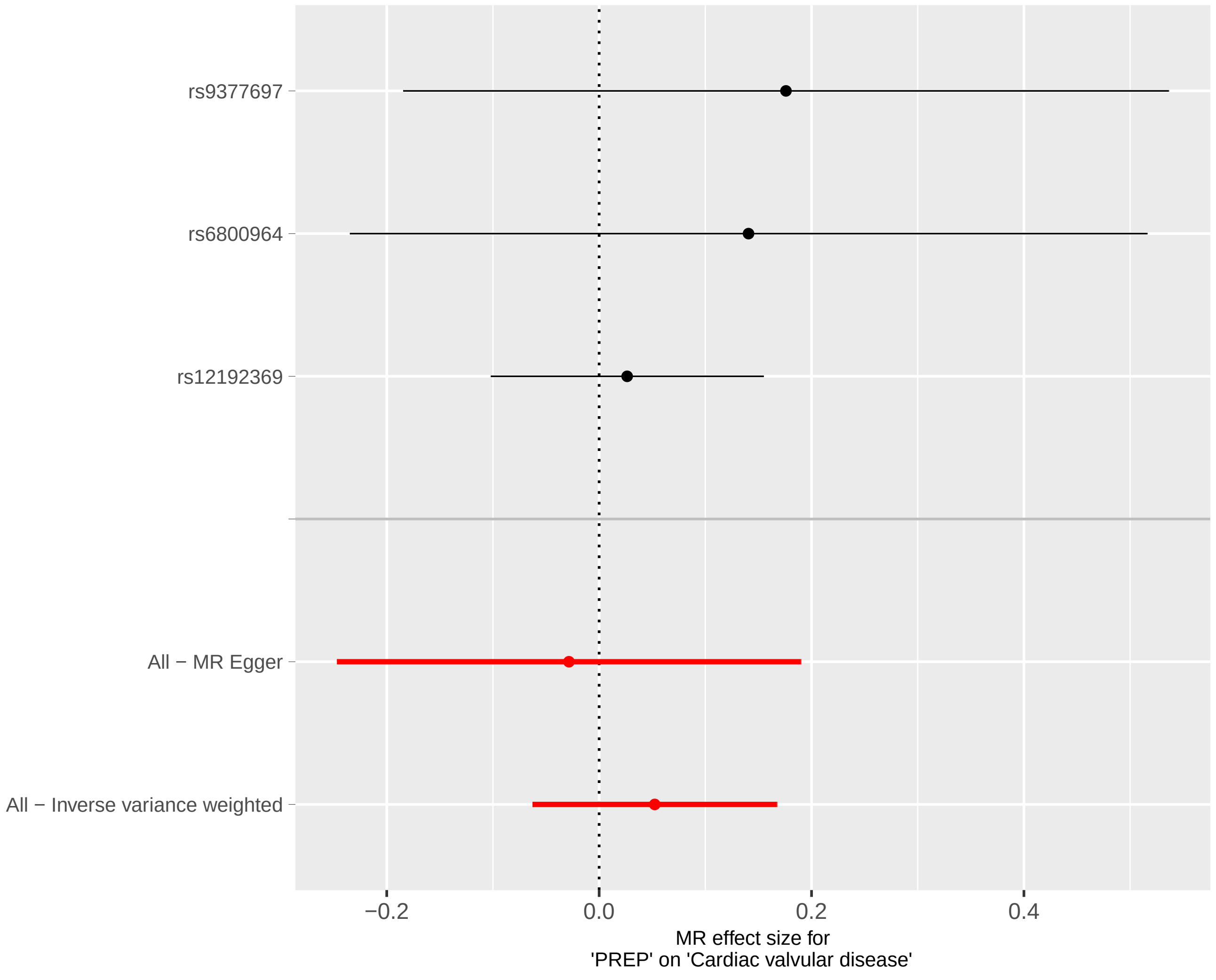
rs9377697
rs6800964
rs12192369
All − MR Egger
All − Inverse variance weighted
−0.2
0.0
0.2
0.4
MR effect size for
'PREP' on 'Cardiac valvular disease'

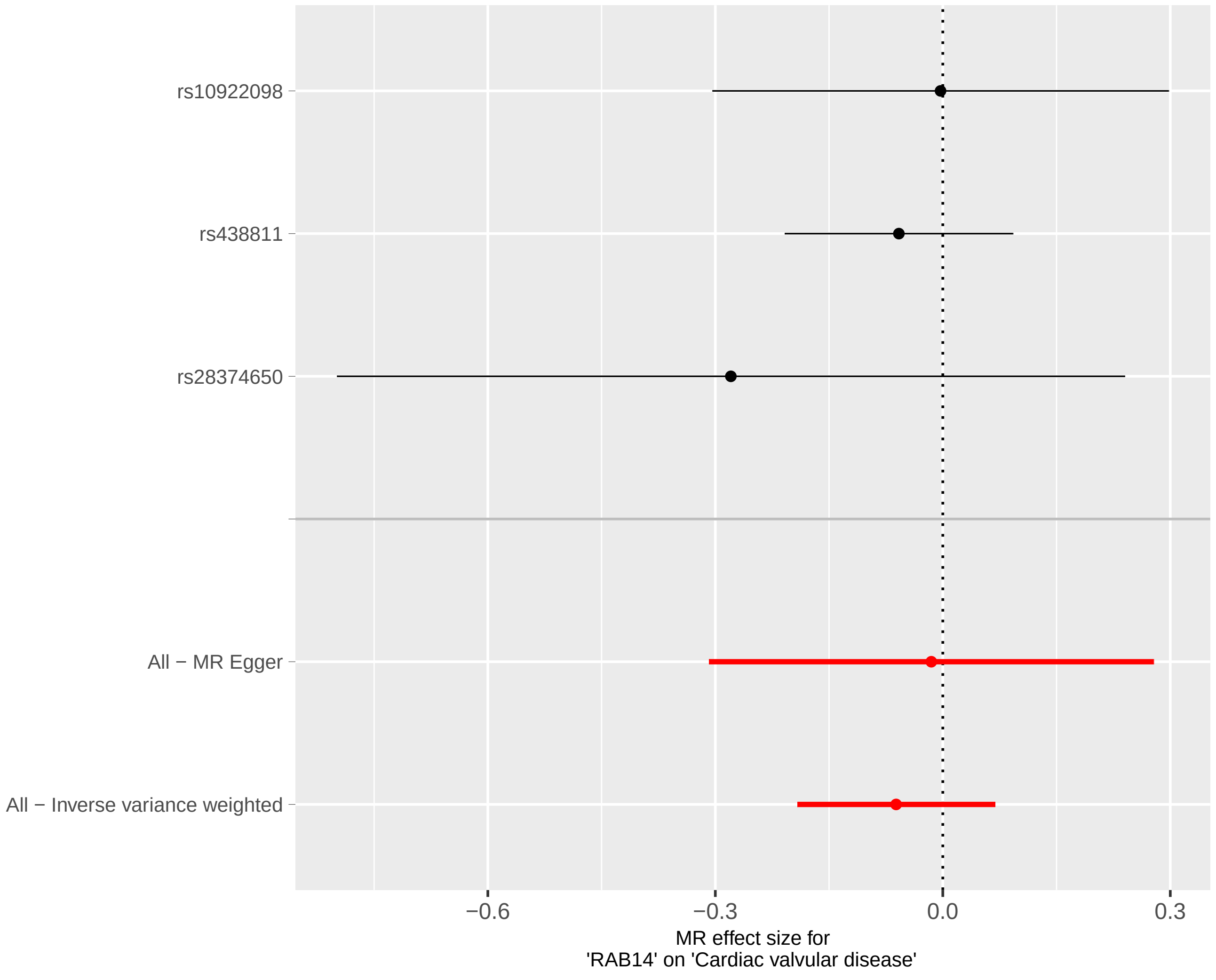
rs10922098
rs438811
rs28374650
All − MR Egger
All − Inverse variance weighted
−0.6
−0.3
0.0
0.3
MR effect size for
'RAB14' on 'Cardiac valvular disease'

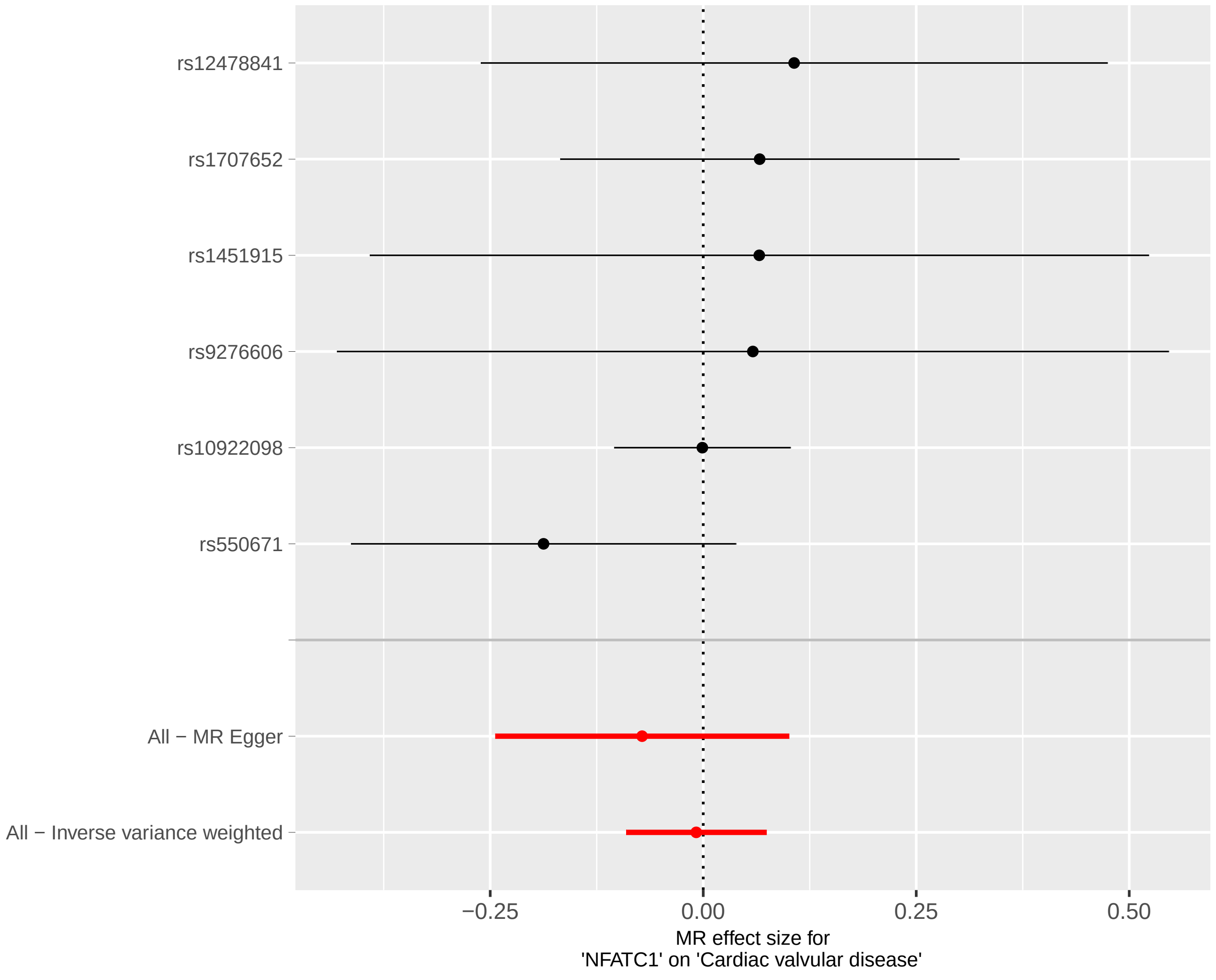
rs12478841
rs1707652
rs1451915
rs9276606
rs10922098
rs550671
All − MR Egger
All − Inverse variance weighted
−0.25
0.00
0.25
0.50
MR effect size for
'NFATC1' on 'Cardiac valvular disease'

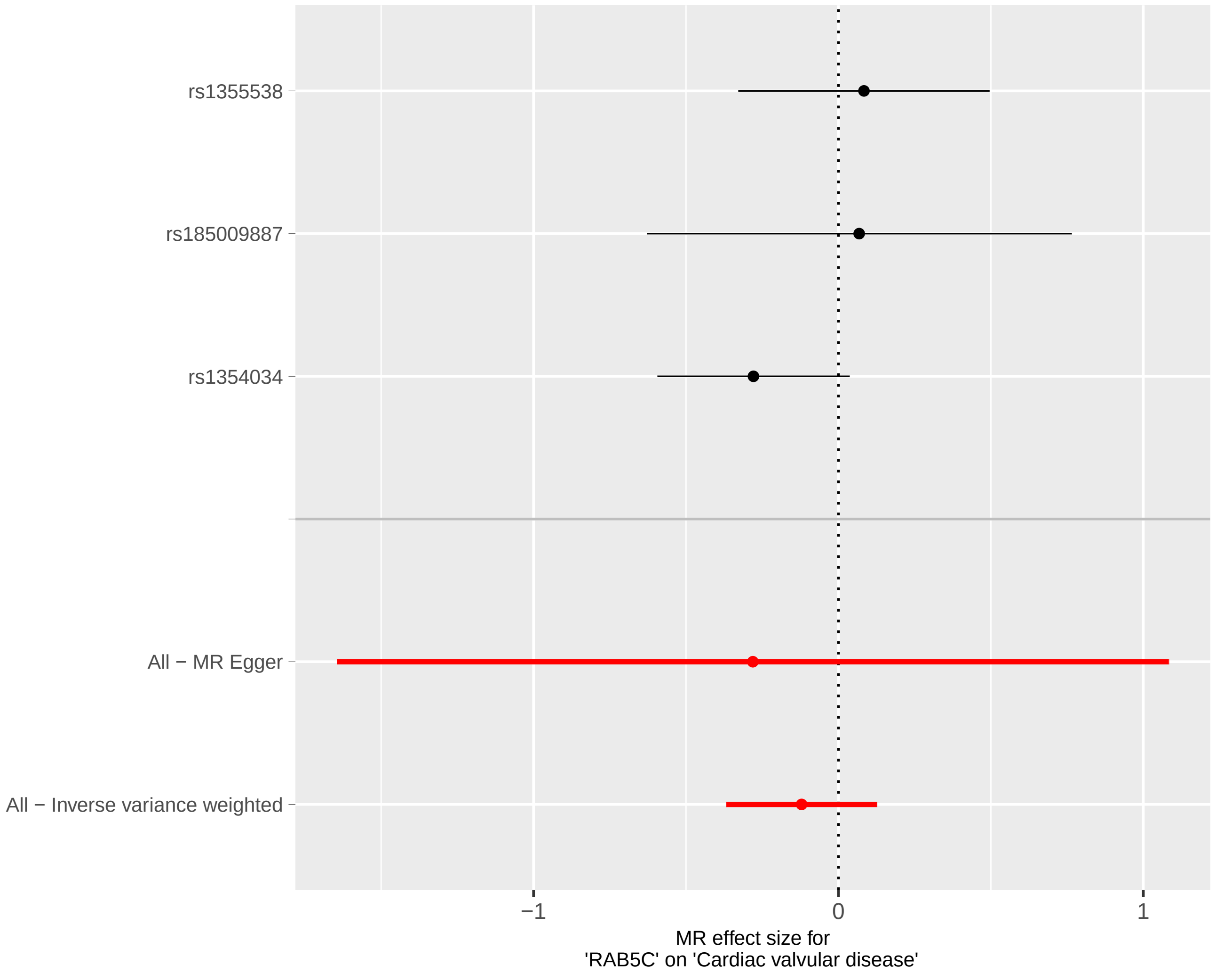

rs1355538
rs185009887
rs1354034
All − MR Egger
All − Inverse variance weighted
−1
0
1
MR effect size for
'RAB5C' on 'Cardiac valvular disease'

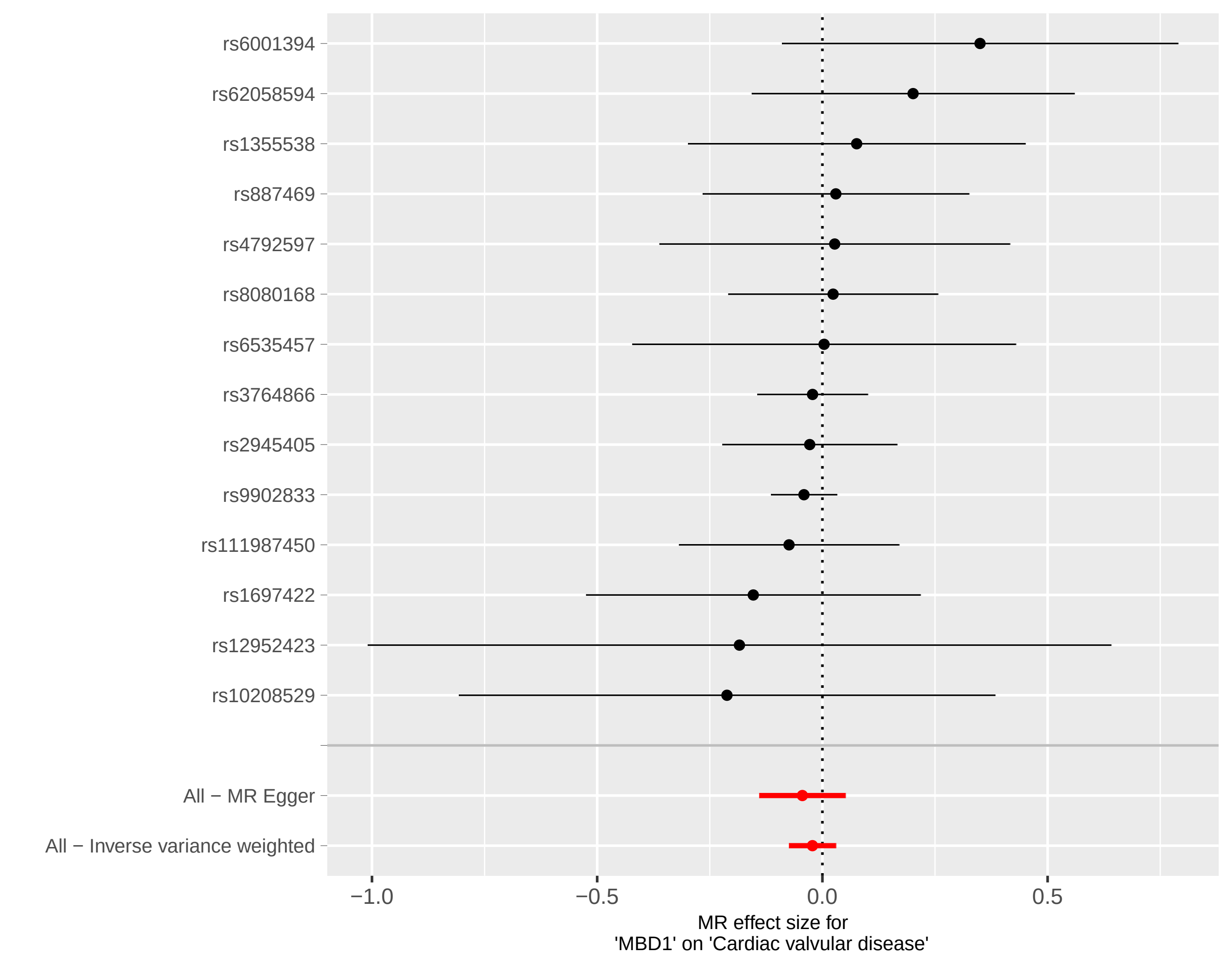
rs6001394
rs62058594
rs1355538
rs887469
rs4792597
rs8080168
rs6535457
rs3764866
rs2945405
rs9902833
rs111987450
rs1697422
rs12952423
rs10208529
All − MR Egger
All − Inverse variance weighted
−1.0
−0.5
0.0
0.5
MR effect size for
'MBD1' on 'Cardiac valvular disease'

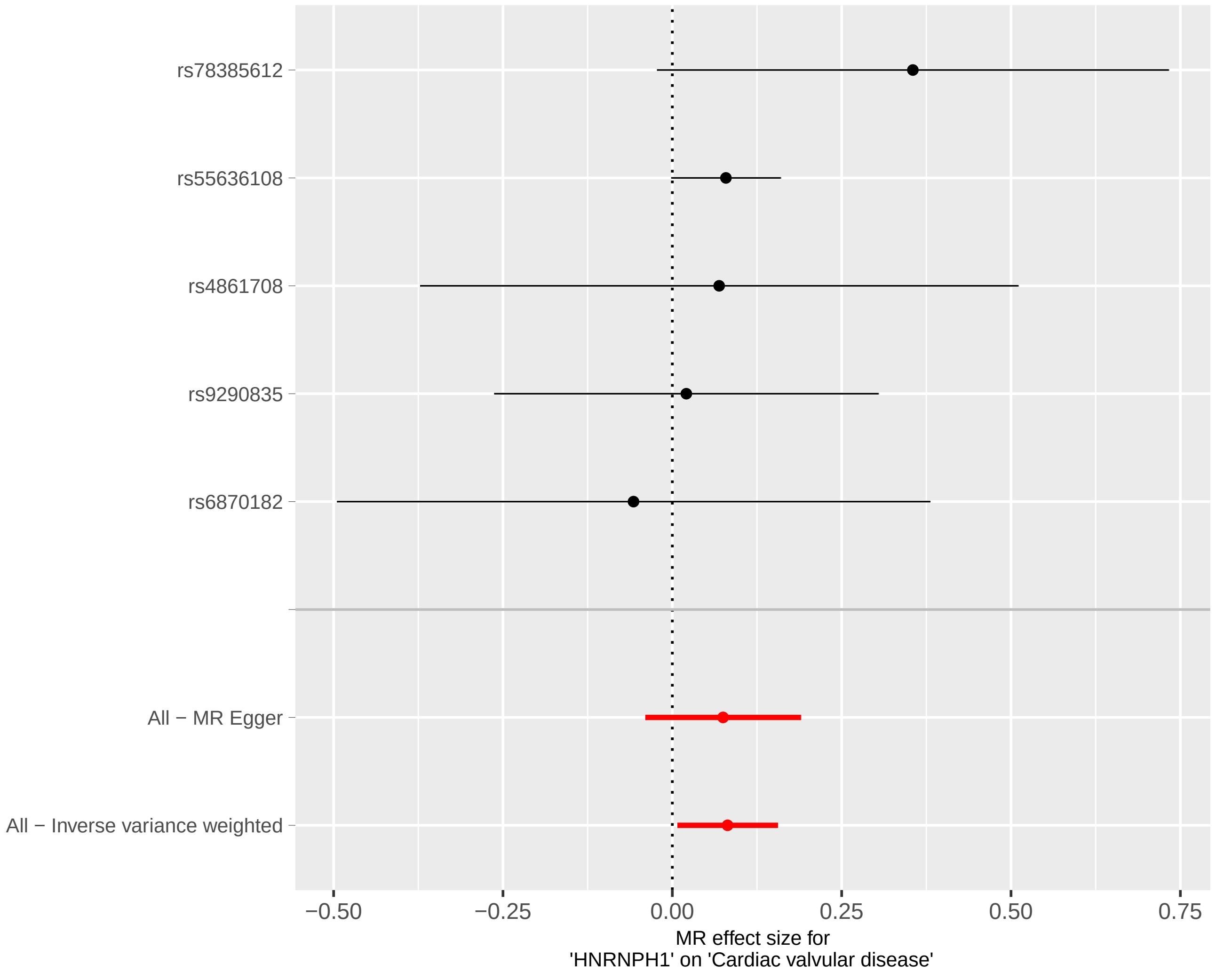
rs78385612
rs55636108
rs4861708
rs9290835
rs6870182
All − MR Egger
All − Inverse variance weighted
−0.50
−0.25
0.00
0.25
0.50
0.75
MR effect size for
'HNRNPH1' on 'Cardiac valvular disease'

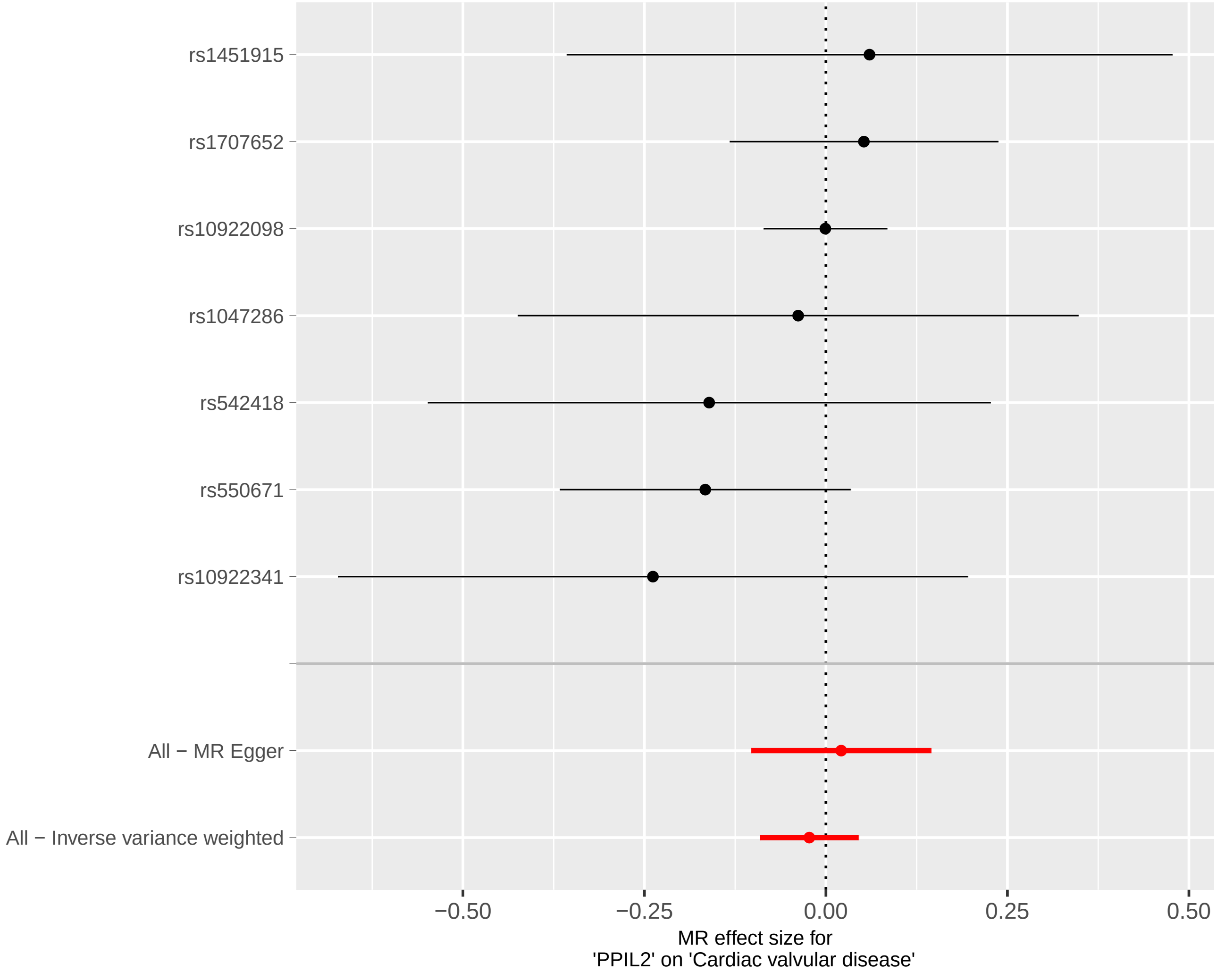

rs1451915
rs1707652
rs10922098
rs1047286
rs542418
rs550671
rs10922341
All − MR Egger
All − Inverse variance weighted
−0.50
−0.25
0.00
0.25
0.50
MR effect size for
'PPIL2' on 'Cardiac valvular disease'

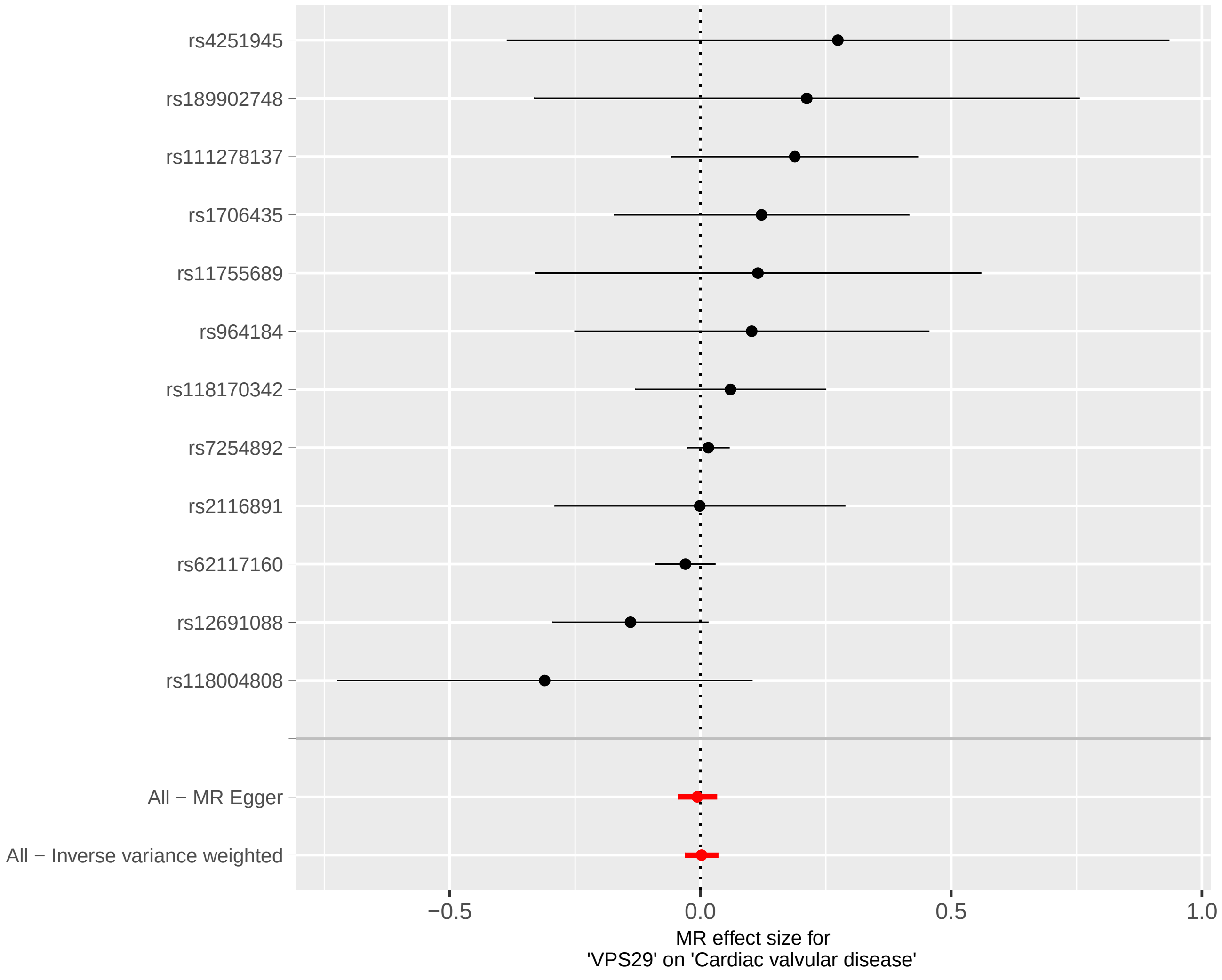

rs4251945
rs189902748
rs111278137
rs1706435
rs11755689
rs964184
rs118170342
rs7254892
rs2116891
rs62117160
rs12691088
rs118004808
All − MR Egger
All − Inverse variance weighted
−0.5
0.0
0.5
1.0
MR effect size for
'VPS29' on 'Cardiac valvular disease'

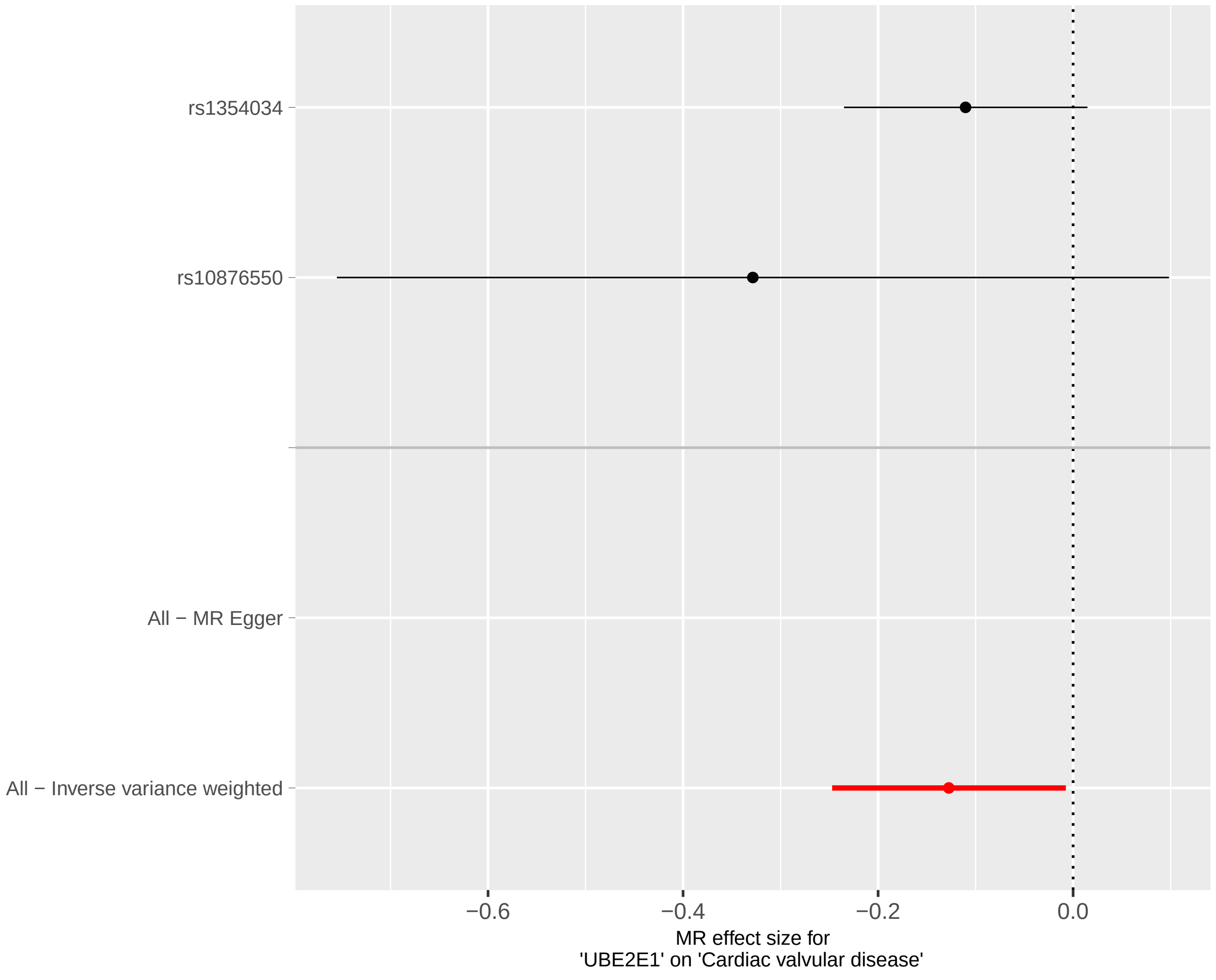
rs1354034
rs10876550
All − MR Egger
All − Inverse variance weighted
−0.6
−0.4
−0.2
0.0
MR effect size for
'UBE2E1' on 'Cardiac valvular disease'

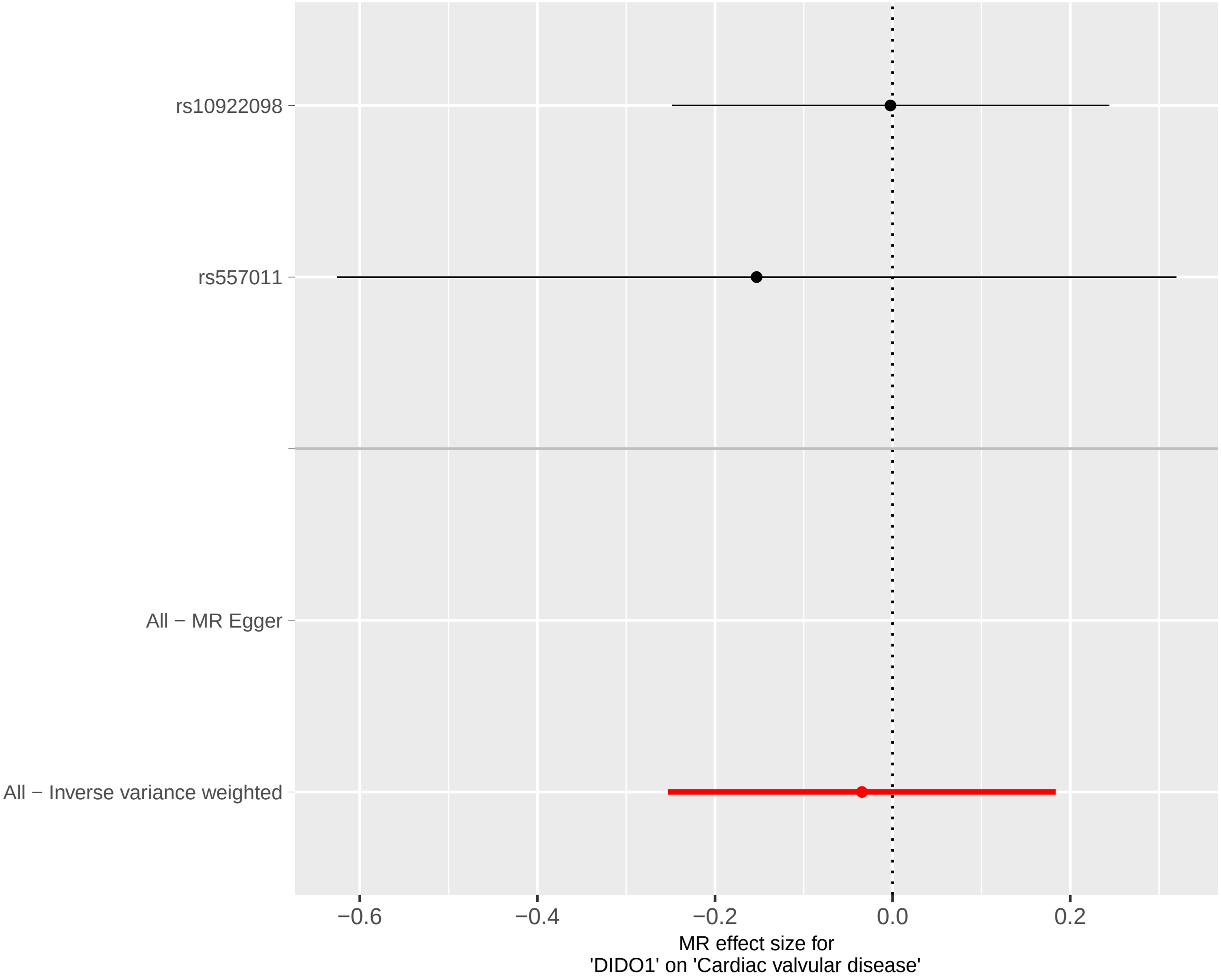
rs10922098
rs557011
All − MR Egger
All − Inverse variance weighted
−0.6
−0.4
−0.2
0.0
0.2
MR effect size for
'DIDO1' on 'Cardiac valvular disease'

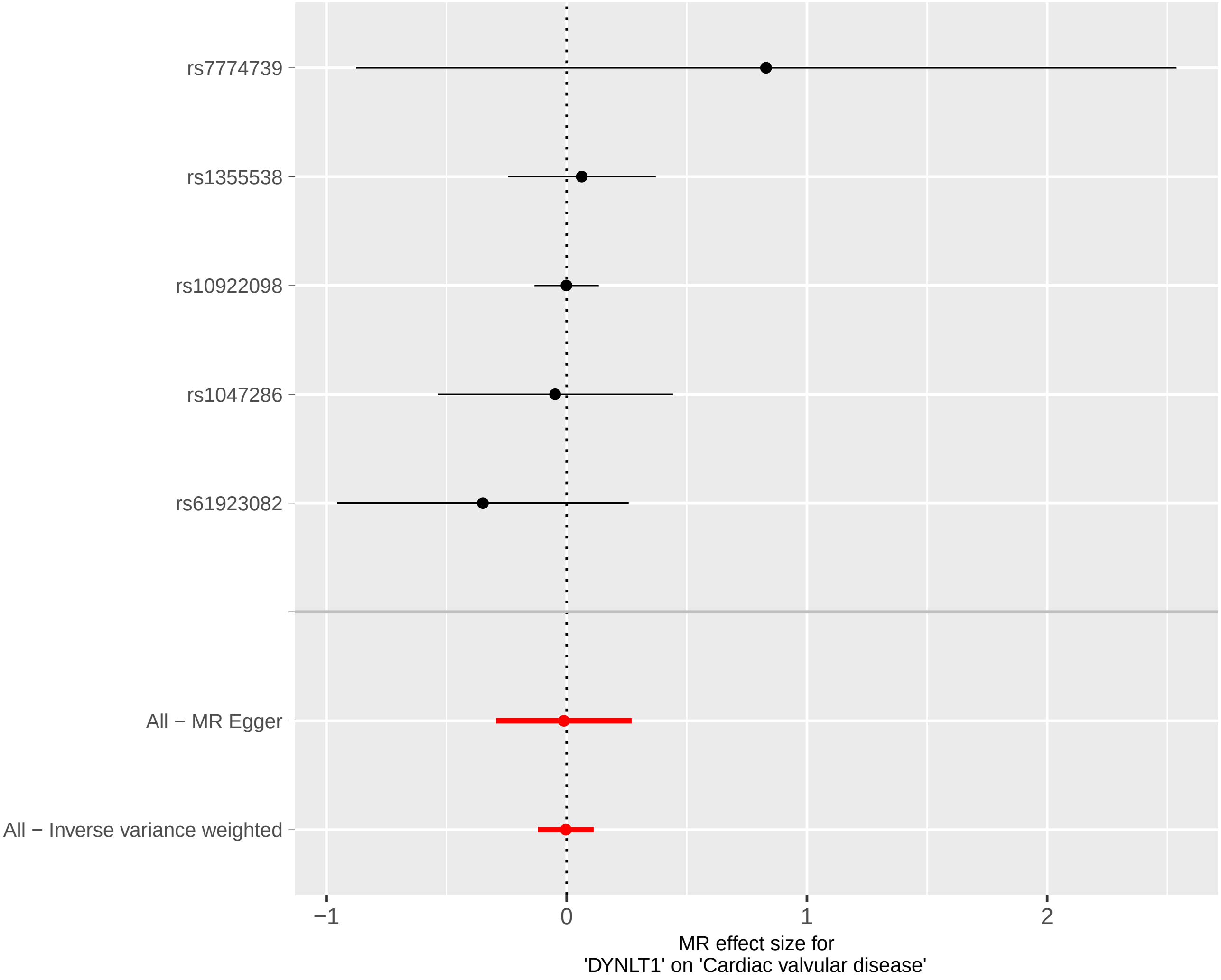
rs7774739
rs1355538
rs10922098
rs1047286
rs61923082
All − MR Egger
All − Inverse variance weighted
−1
0
1
2
MR effect size for
'DYNLT1' on 'Cardiac valvular disease'

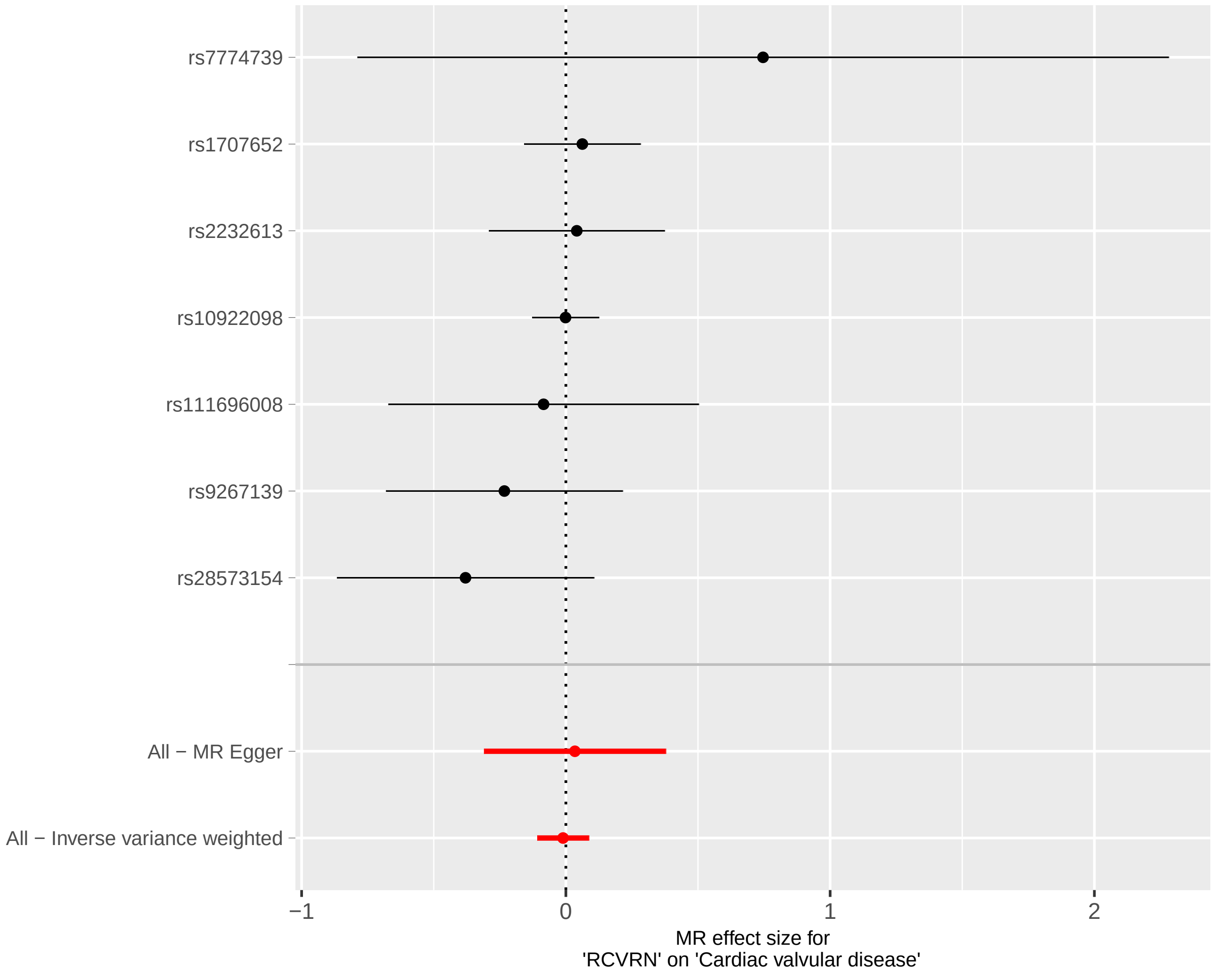
rs7774739
rs1707652
rs2232613
rs10922098
rs111696008
rs9267139
rs28573154
All − MR Egger
All − Inverse variance weighted
−1
0
1
2
MR effect size for
'RCVRN' on 'Cardiac valvular disease'

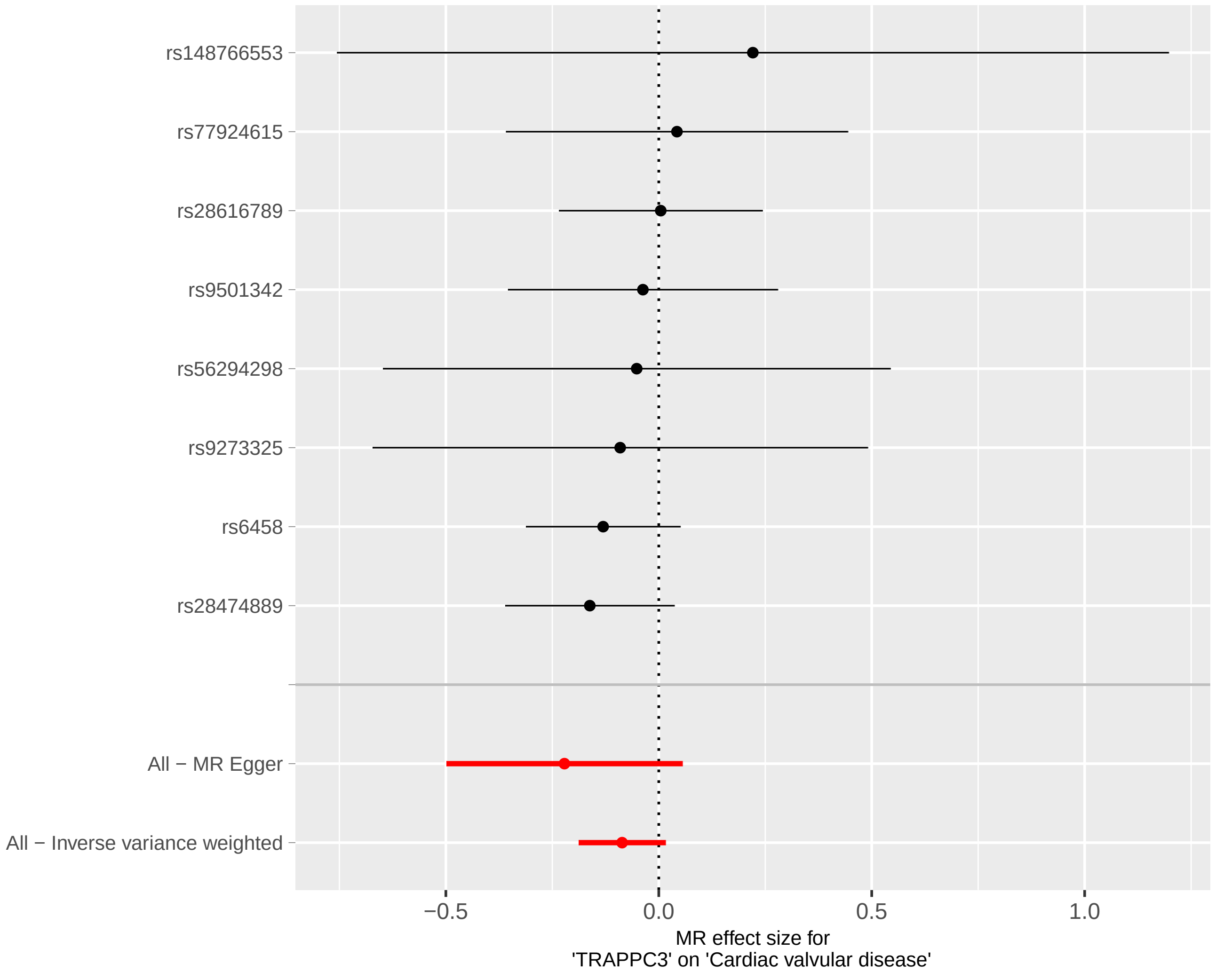
rs148766553
rs77924615
rs28616789
rs9501342
rs56294298
rs9273325
rs6458
rs28474889
All − MR Egger
All − Inverse variance weighted
−0.5
0.0
0.5
1.0
MR effect size for
'TRAPPC3' on 'Cardiac valvular disease'

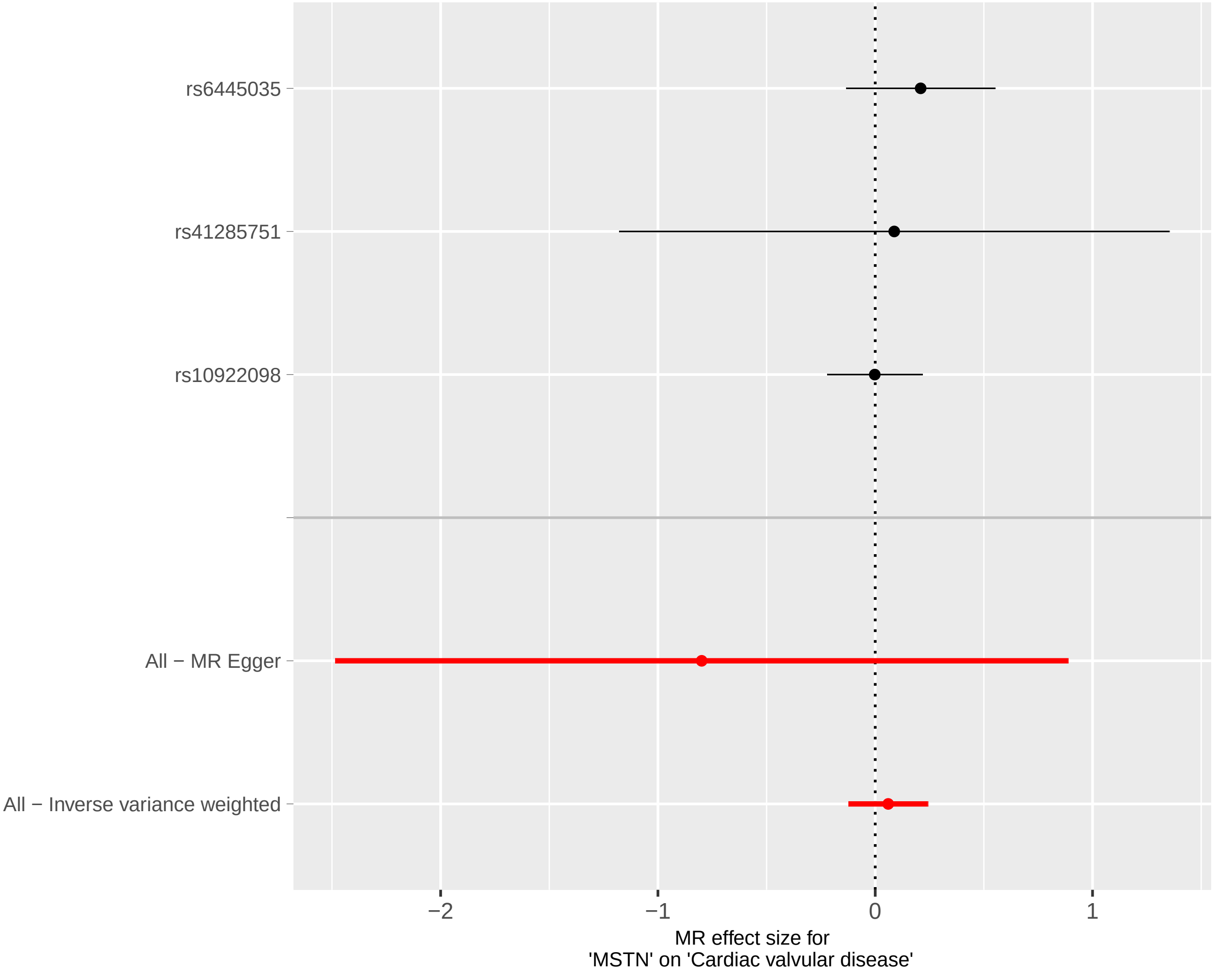
rs6445035
rs41285751
rs10922098
All − MR Egger
All − Inverse variance weighted
−2
−1
0
1
MR effect size for
'MSTN' on 'Cardiac valvular disease'

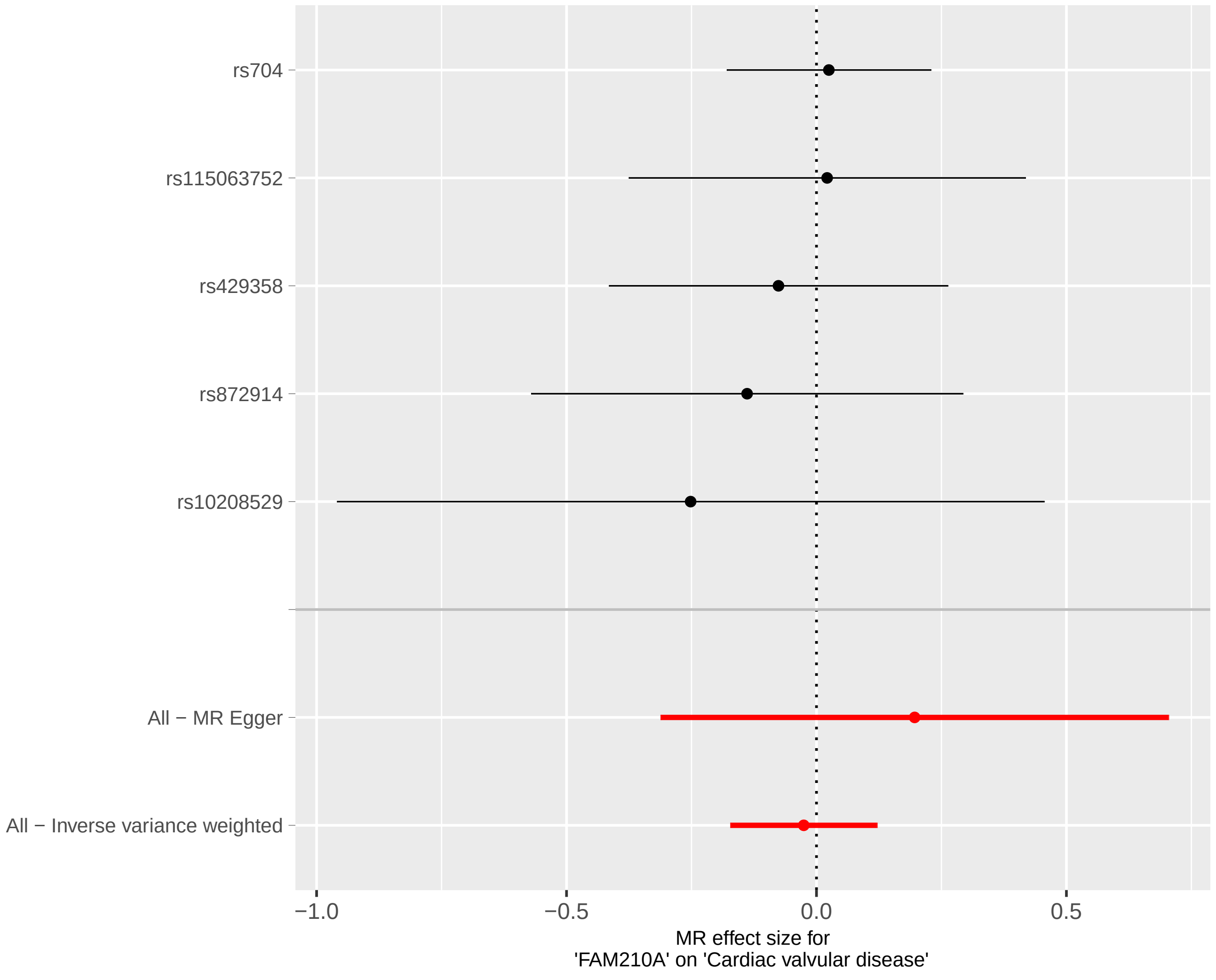
rs704
rs115063752
rs429358
rs872914
rs10208529
All − MR Egger
All − Inverse variance weighted
−1.0
−0.5
0.0
0.5
MR effect size for
'FAM210A' on 'Cardiac valvular disease'

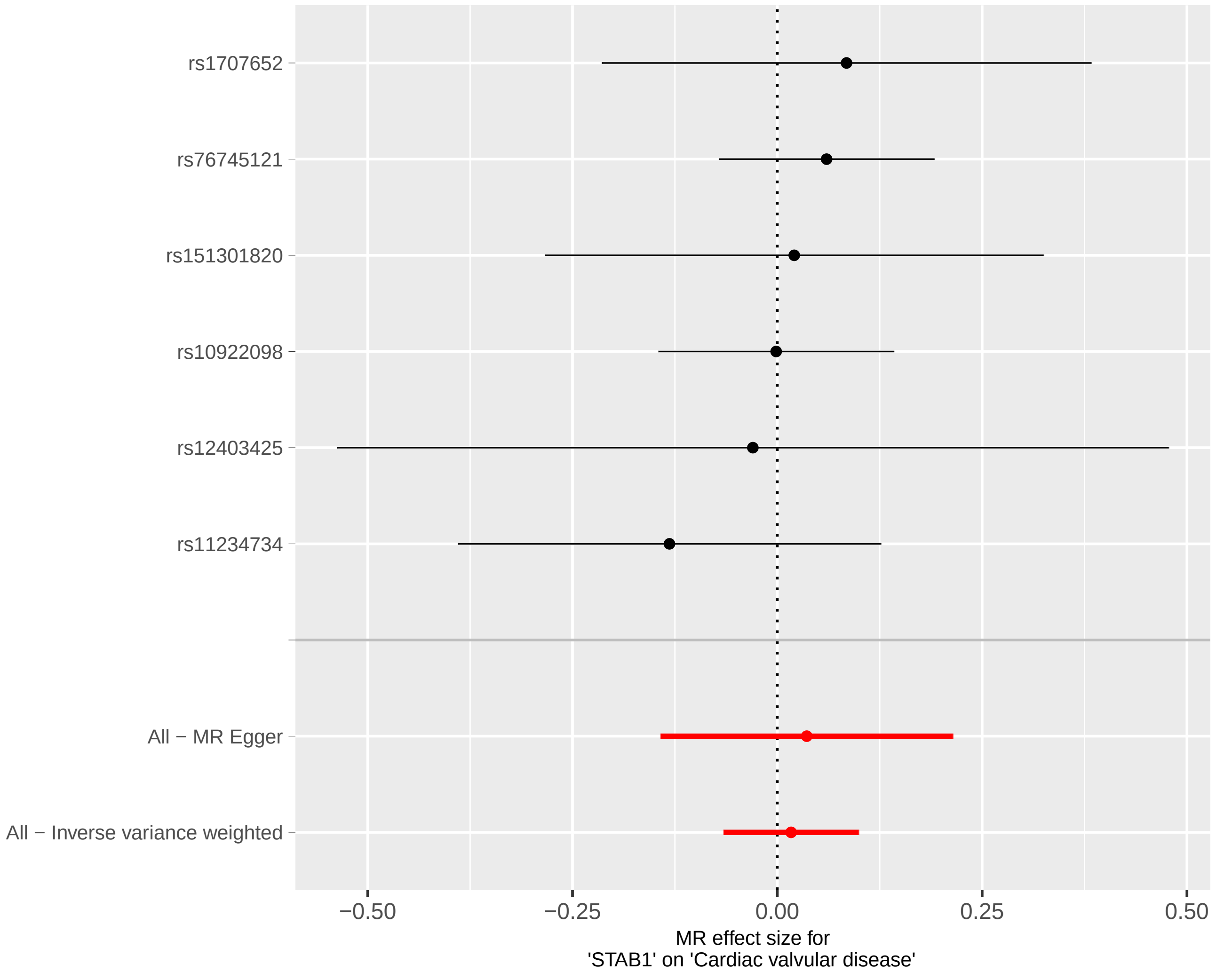

rs1707652
rs76745121
rs151301820
rs10922098
rs12403425
rs11234734
All − MR Egger
All − Inverse variance weighted
−0.50
−0.25
0.00
0.25
0.50
MR effect size for
'STAB1' on 'Cardiac valvular disease'

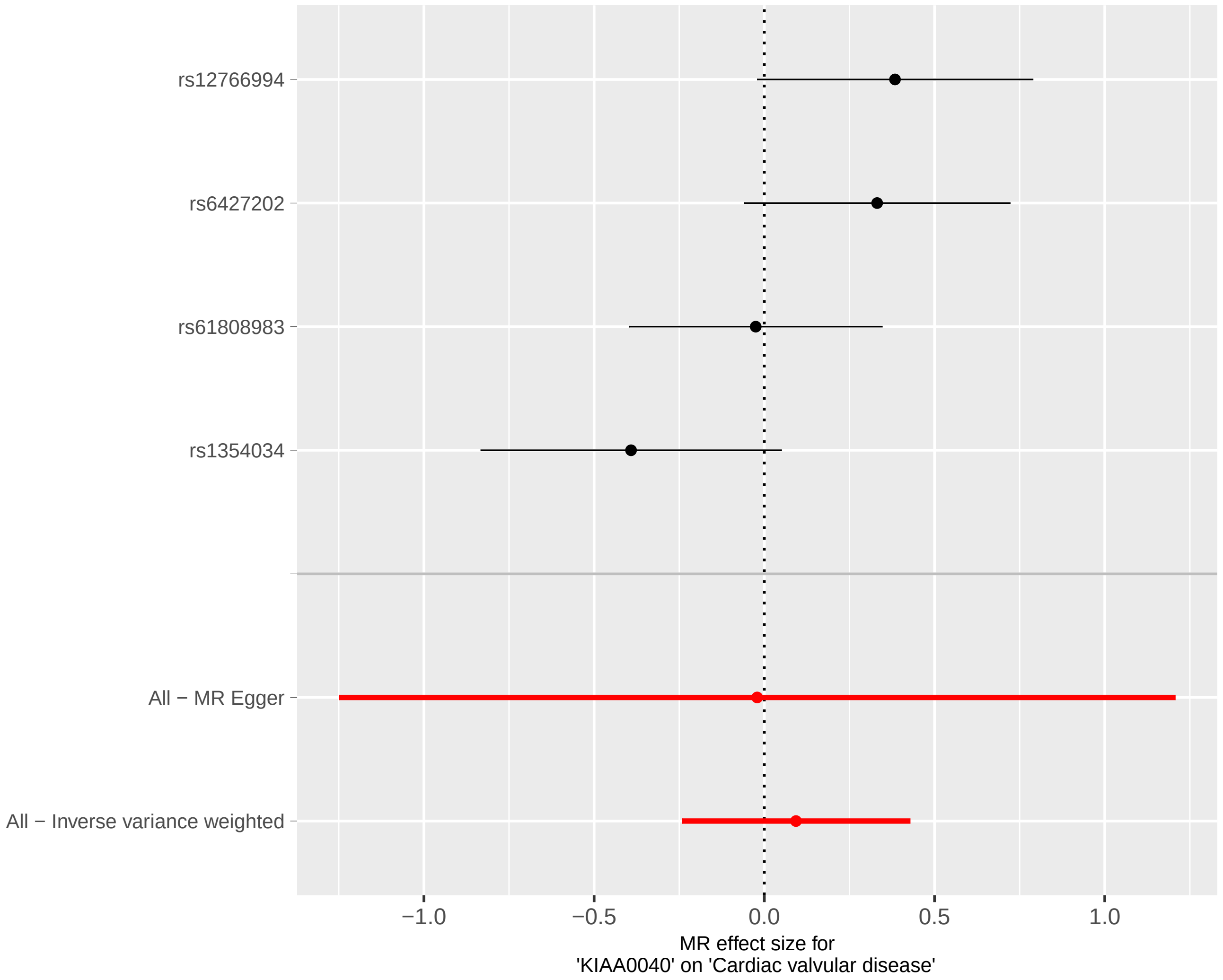
rs12766994
rs6427202
rs61808983
rs1354034
All − MR Egger
All − Inverse variance weighted
−1.0
−0.5
0.0
0.5
1.0
MR effect size for
'KIAA0040' on 'Cardiac valvular disease'

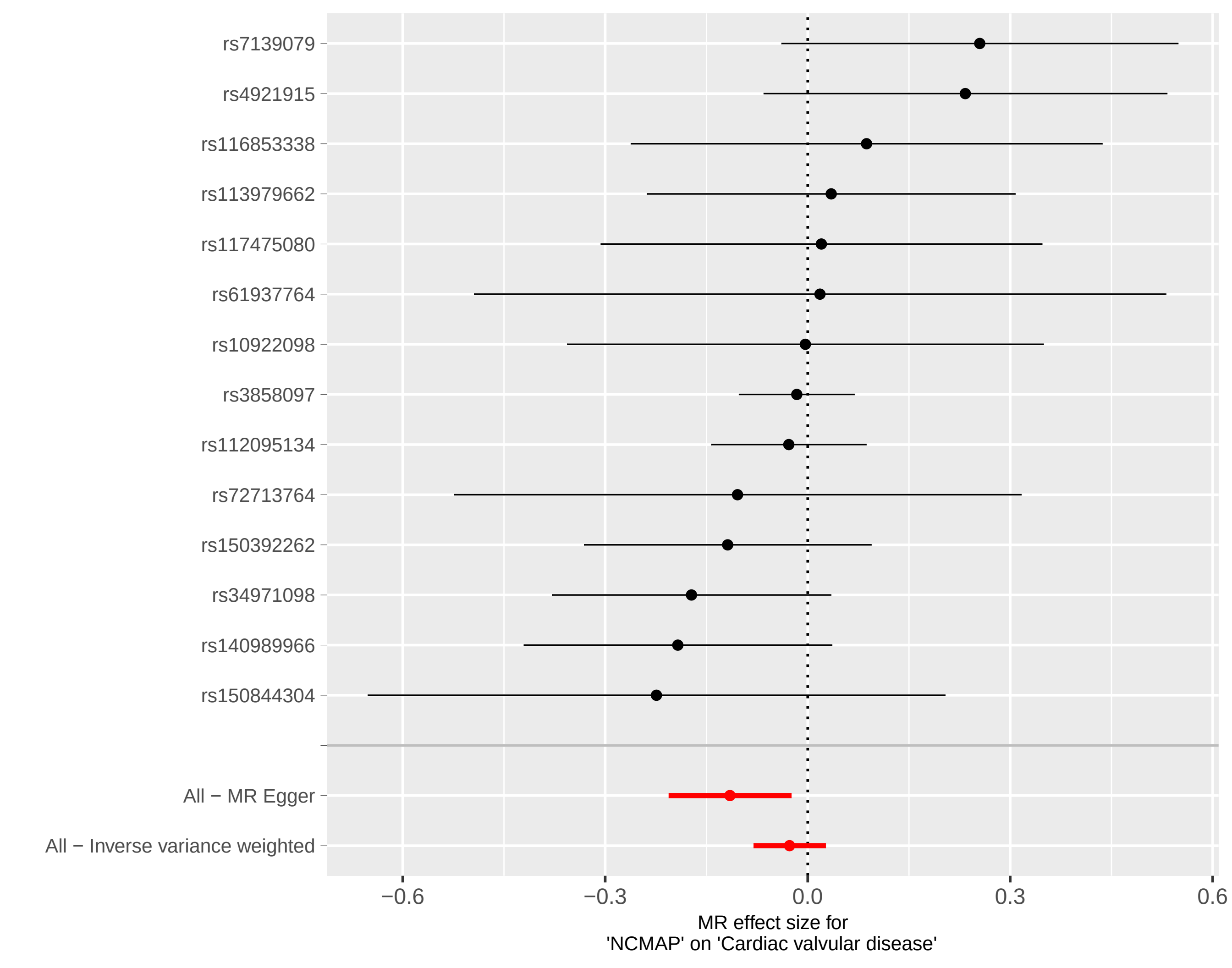
rs7139079
rs4921915
rs116853338
rs113979662
rs117475080
rs61937764
rs10922098
rs3858097
rs112095134
rs72713764
rs150392262
rs34971098
rs140989966
rs150844304
All − MR Egger
All − Inverse variance weighted
−0.6
−0.3
0.0
0.3
0.6
MR effect size for
'NCMAP' on 'Cardiac valvular disease'

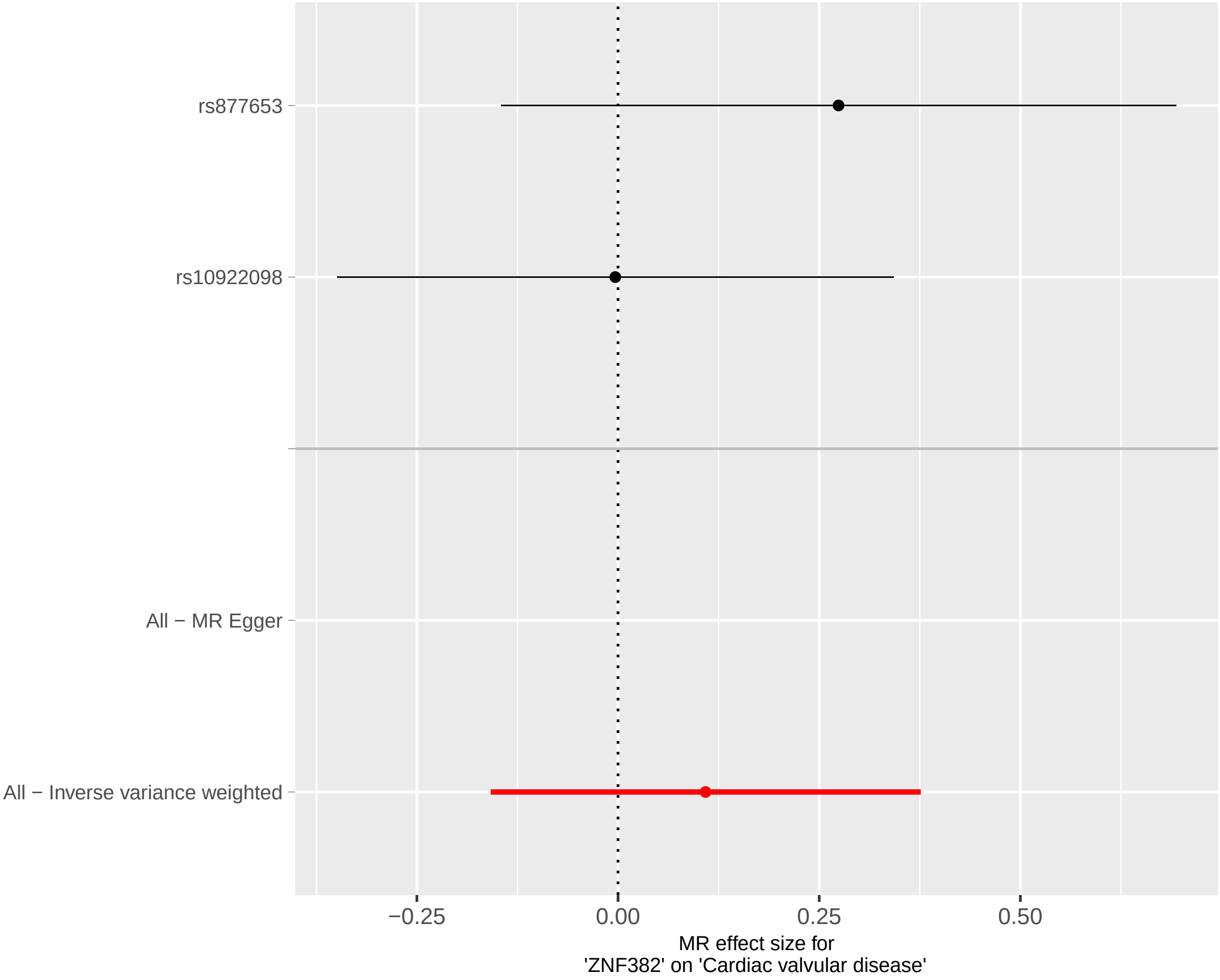
rs877653
rs10922098
All − MR Egger
All − Inverse variance weighted
−0.25
0.00
0.25
0.50
MR effect size for
'ZNF382' on 'Cardiac valvular disease'

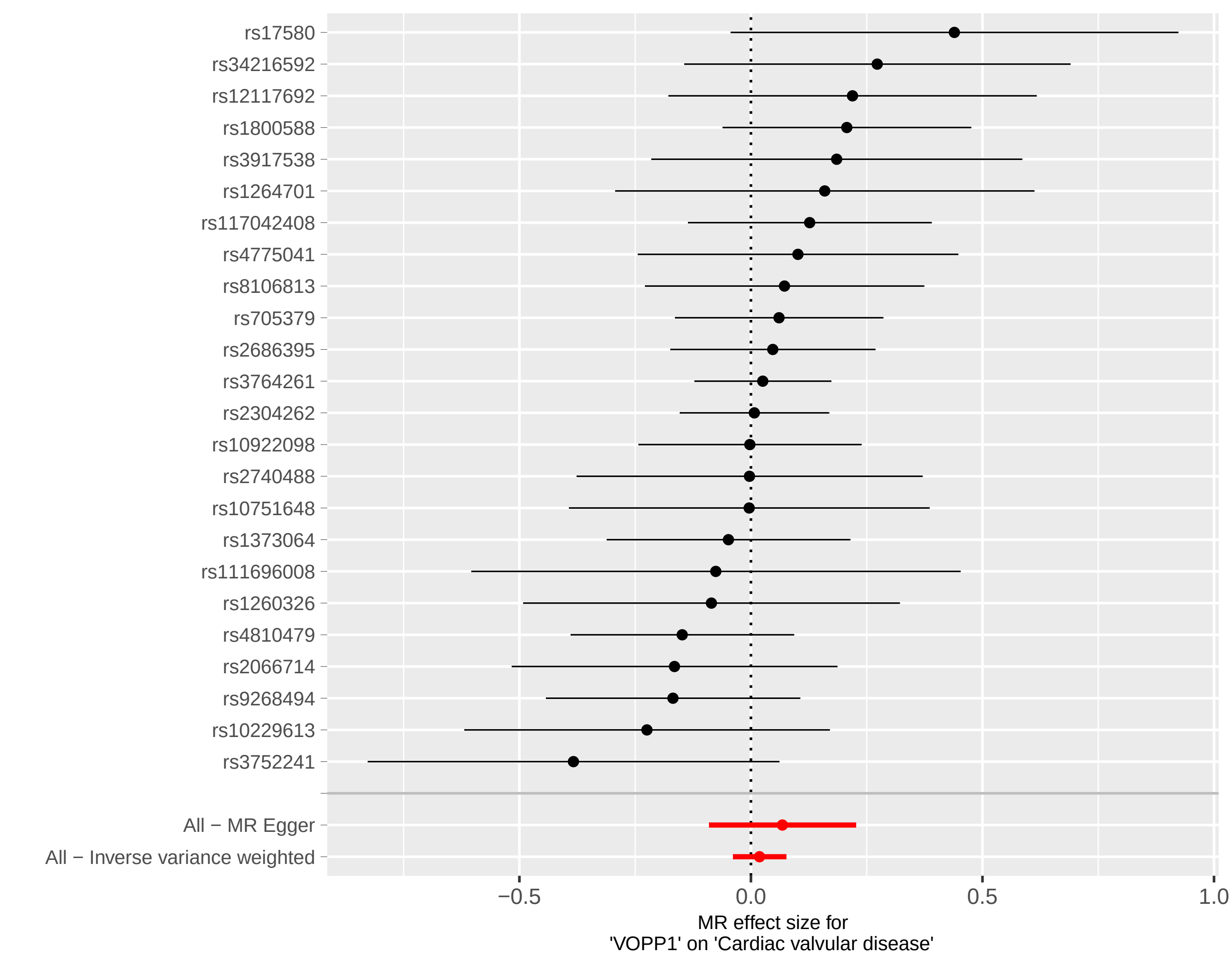

rs17580
rs34216592
rs12117692
rs1800588
rs3917538
rs1264701
rs117042408
rs4775041
rs8106813
rs705379
rs2686395
rs3764261
rs2304262
rs10922098
rs2740488
rs10751648
rs1373064
rs111696008
rs1260326
rs4810479
rs2066714
rs9268494
rs10229613
rs3752241
All − MR Egger
All − Inverse variance weighted
−0.5
0.0
0.5
1.0
MR effect size for
'VOPP1' on 'Cardiac valvular disease'

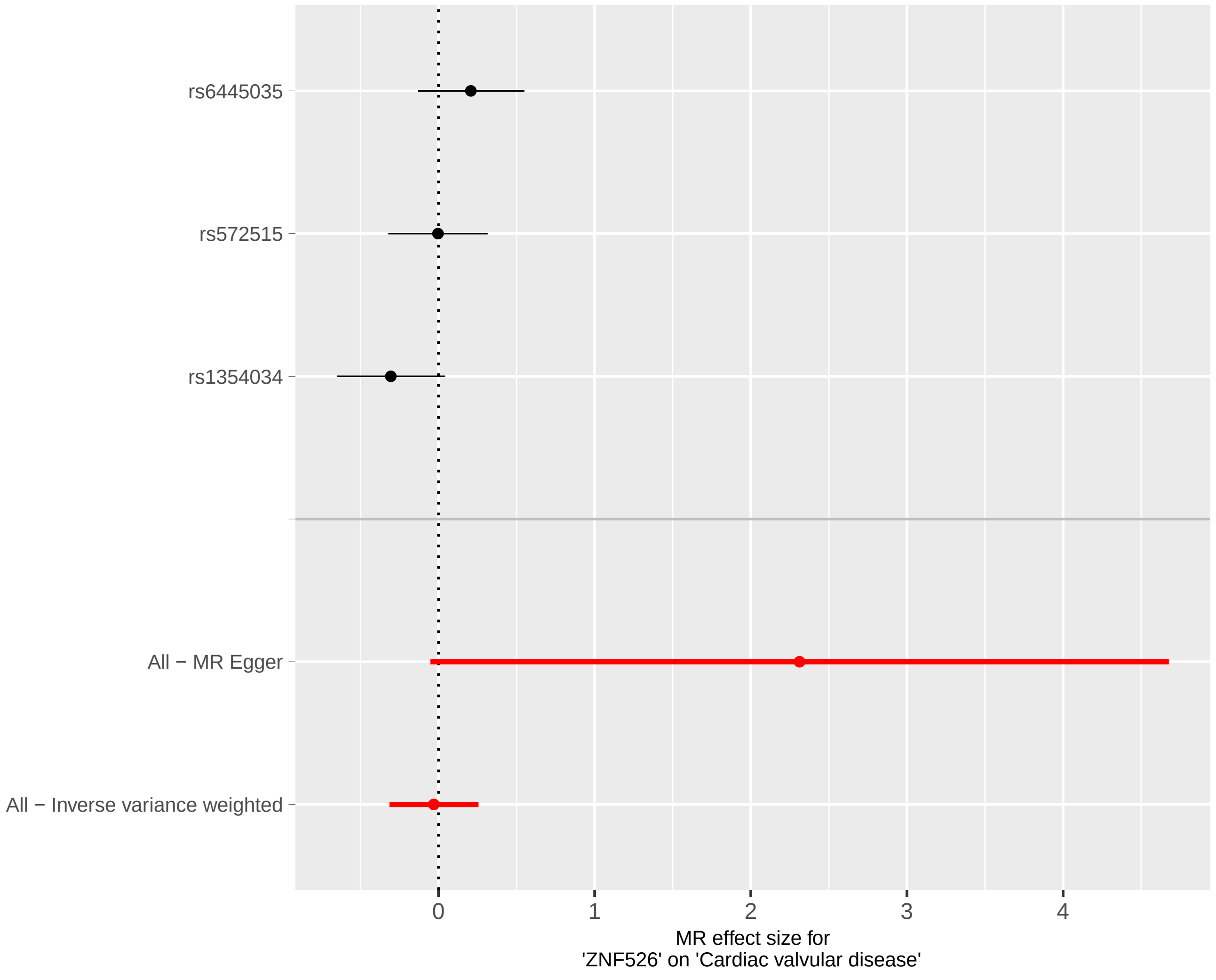
rs6445035
rs572515
rs1354034
All − MR Egger
All − Inverse variance weighted
0
1
2
3
4
MR effect size for
'ZNF526' on 'Cardiac valvular disease'

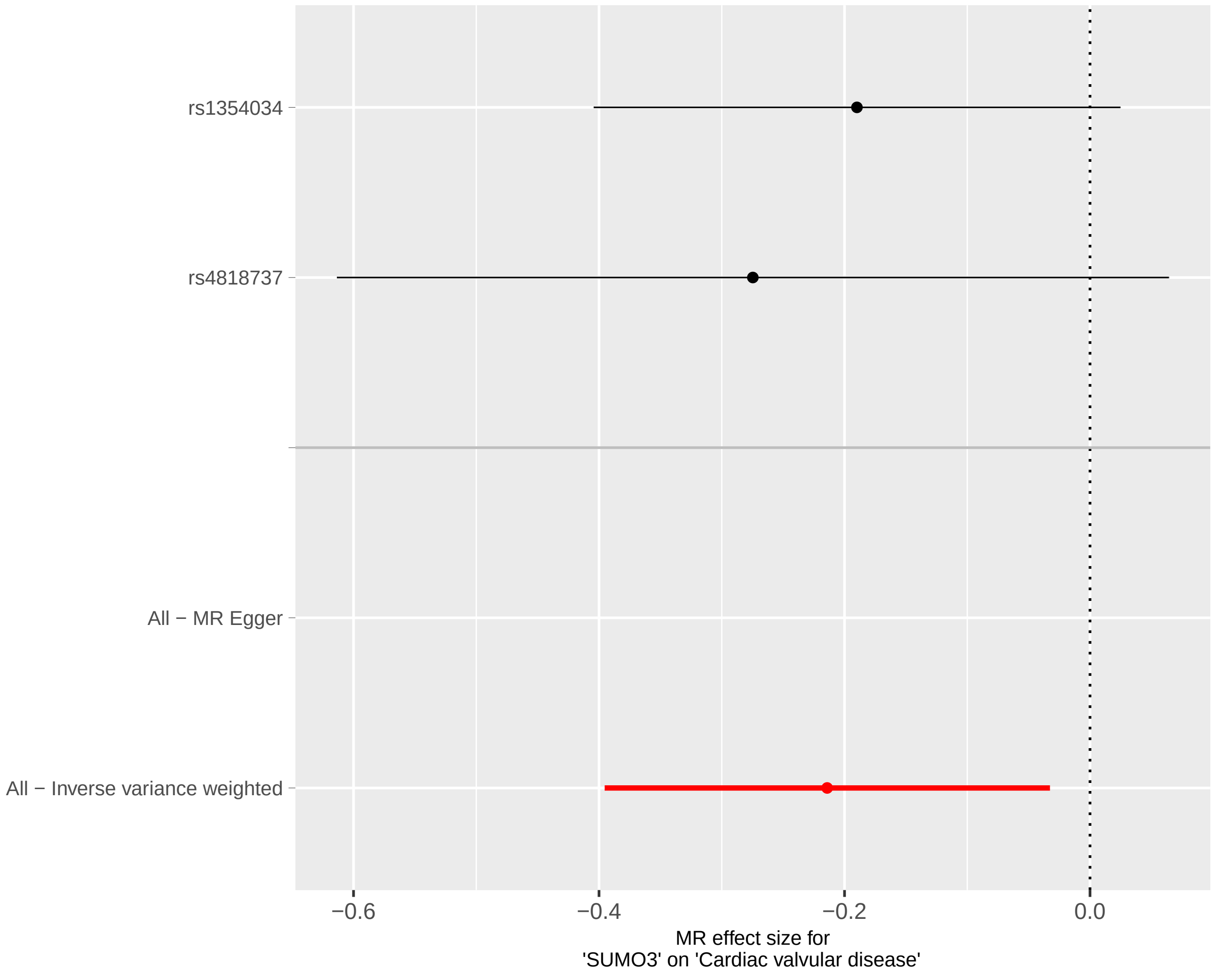

rs1354034
rs4818737
All − MR Egger
All − Inverse variance weighted
−0.6
−0.4
−0.2
0.0
MR effect size for
'SUMO3' on 'Cardiac valvular disease'

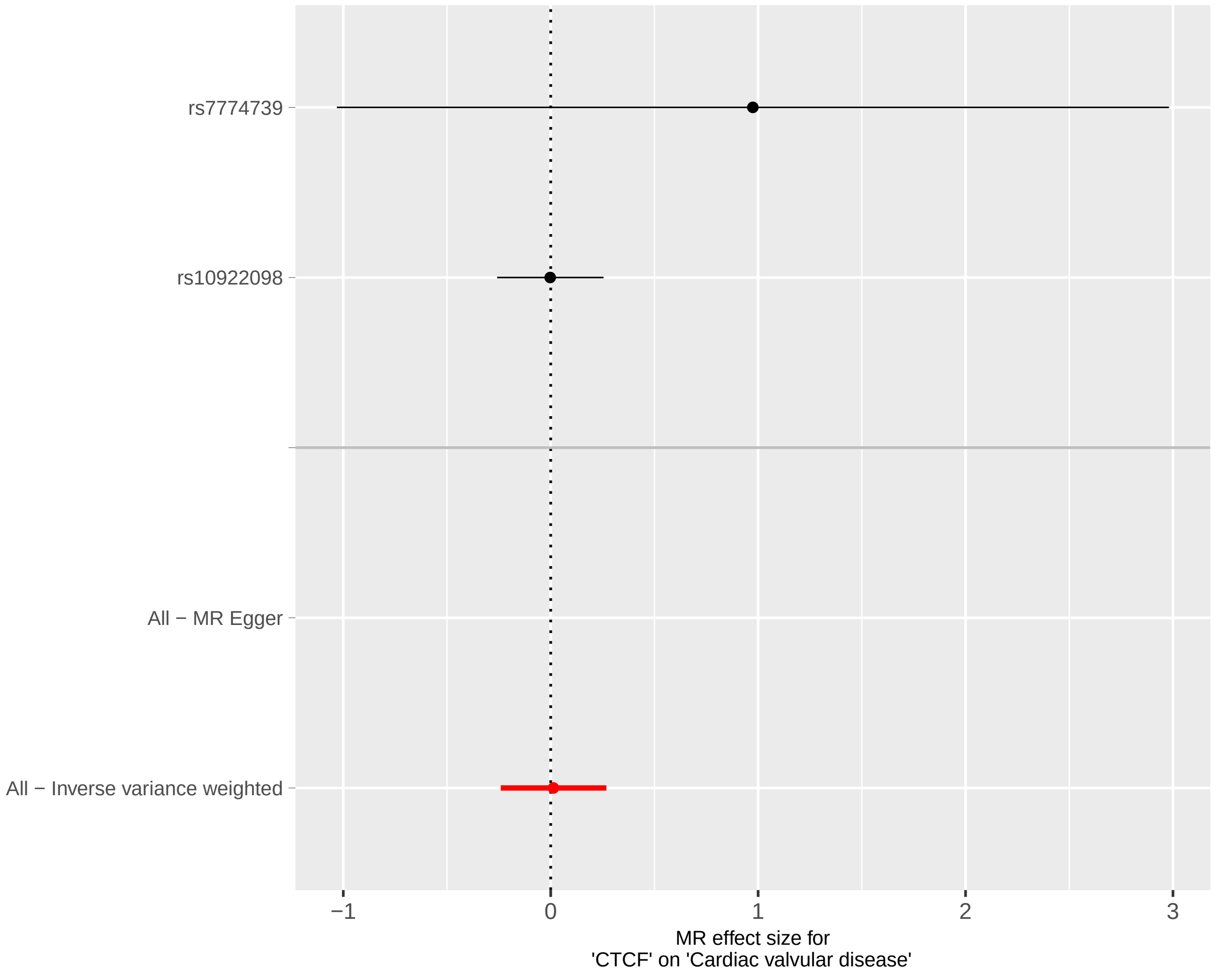
rs7774739
rs10922098
All − MR Egger
All − Inverse variance weighted
−1
0
1
2
3
MR effect size for
'CTCF' on 'Cardiac valvular disease'

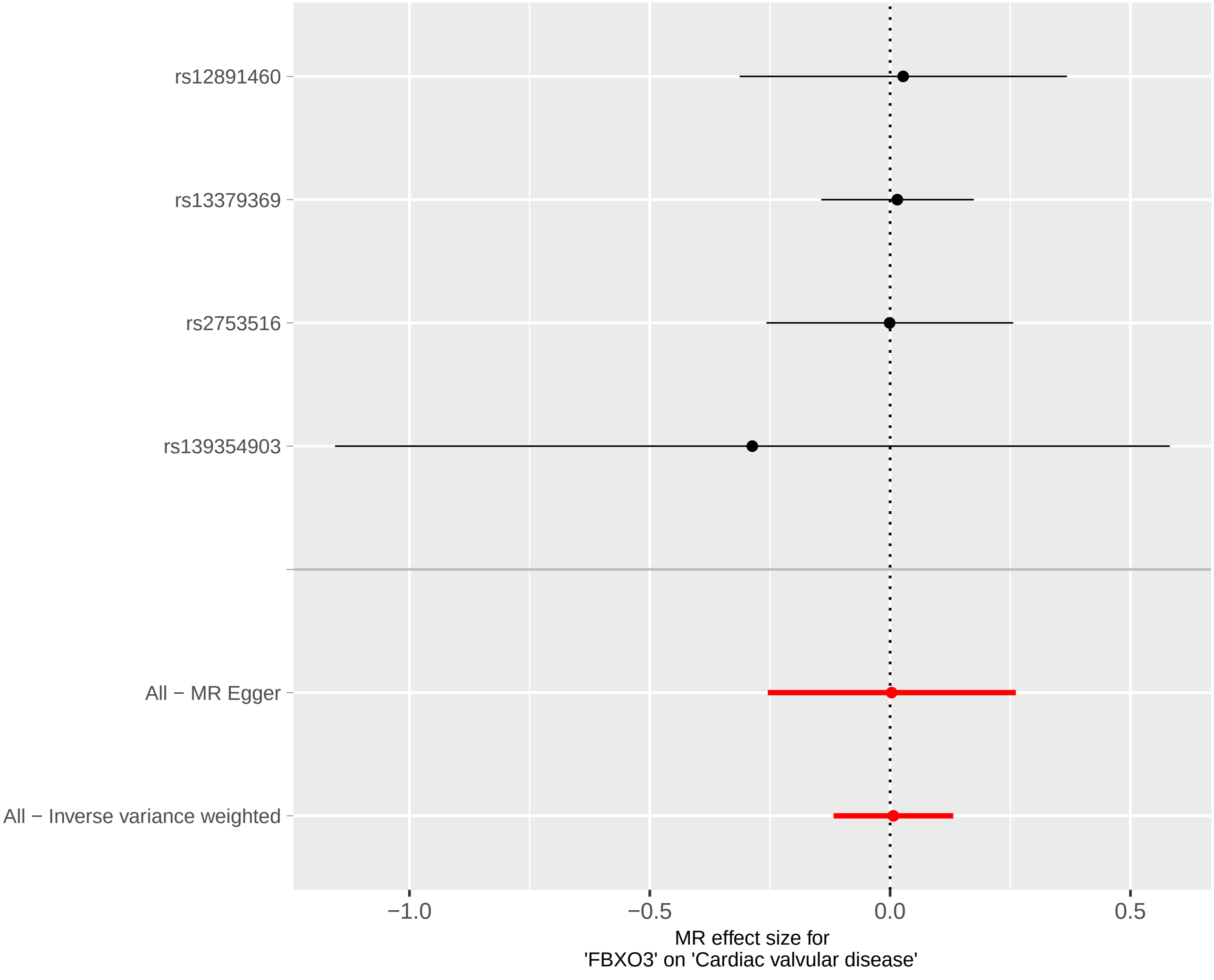
rs12891460
rs13379369
rs2753516
rs139354903
All − MR Egger
All − Inverse variance weighted
−1.0
−0.5
0.0
0.5
MR effect size for
'FBXO3' on 'Cardiac valvular disease'

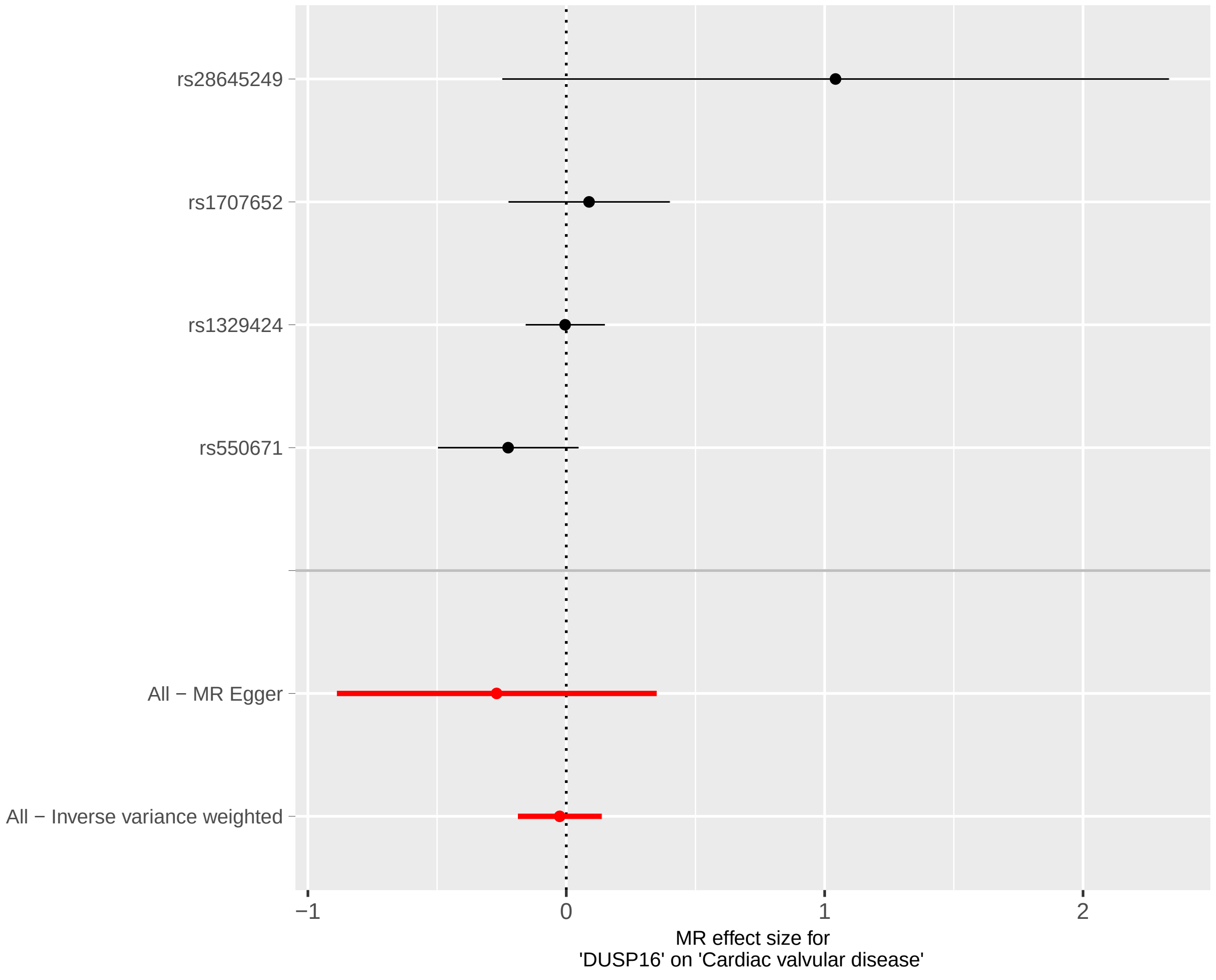
rs28645249
rs1707652
rs1329424
rs550671
All − MR Egger
All − Inverse variance weighted
−1
0
1
2
MR effect size for
'DUSP16' on 'Cardiac valvular disease'

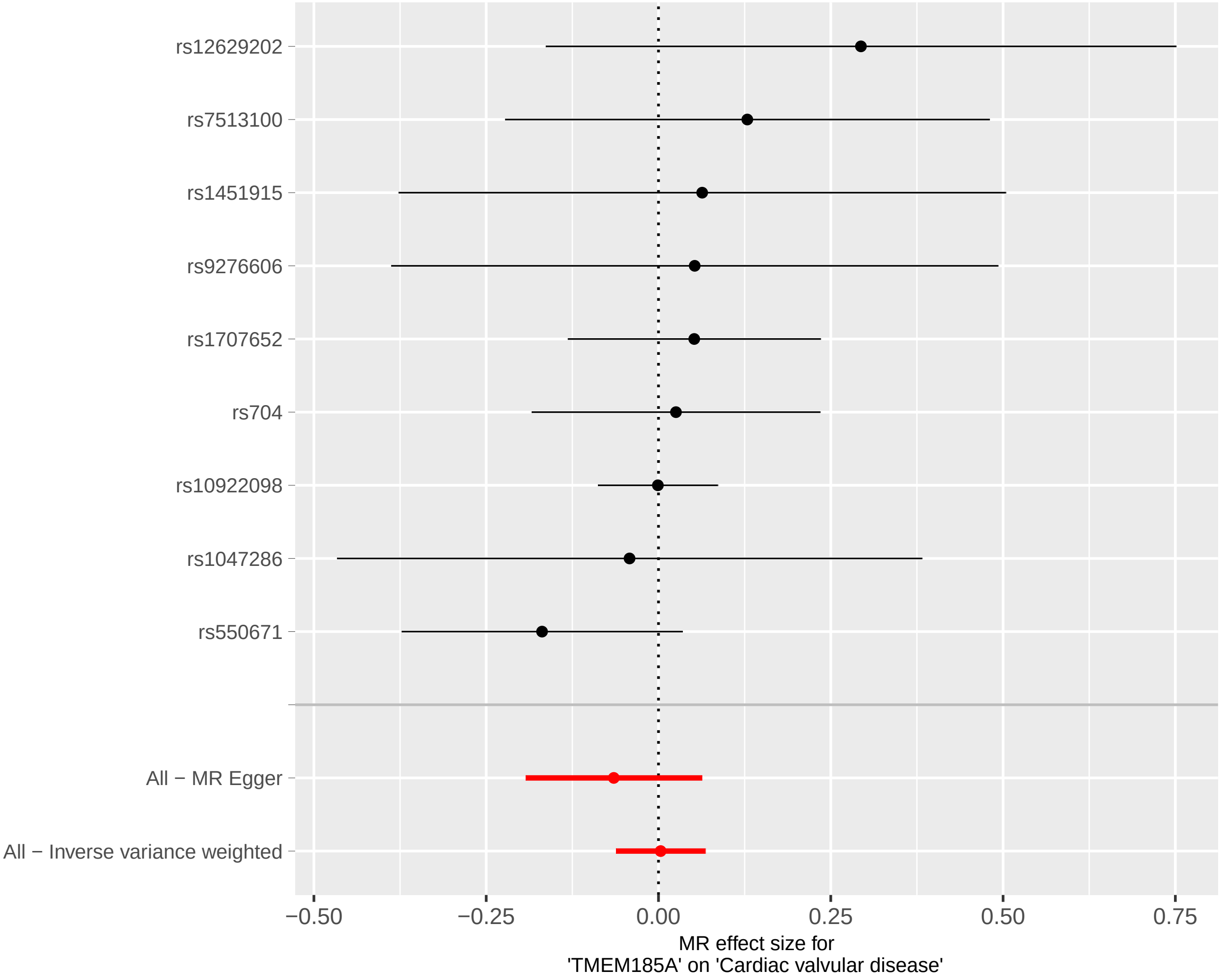
rs12629202
rs7513100
rs1451915
rs9276606
rs1707652
rs704
rs10922098
rs1047286
rs550671
All − MR Egger
All − Inverse variance weighted
−0.50
−0.25
0.00
0.25
0.50
0.75
MR effect size for
'TMEM185A' on 'Cardiac valvular disease'

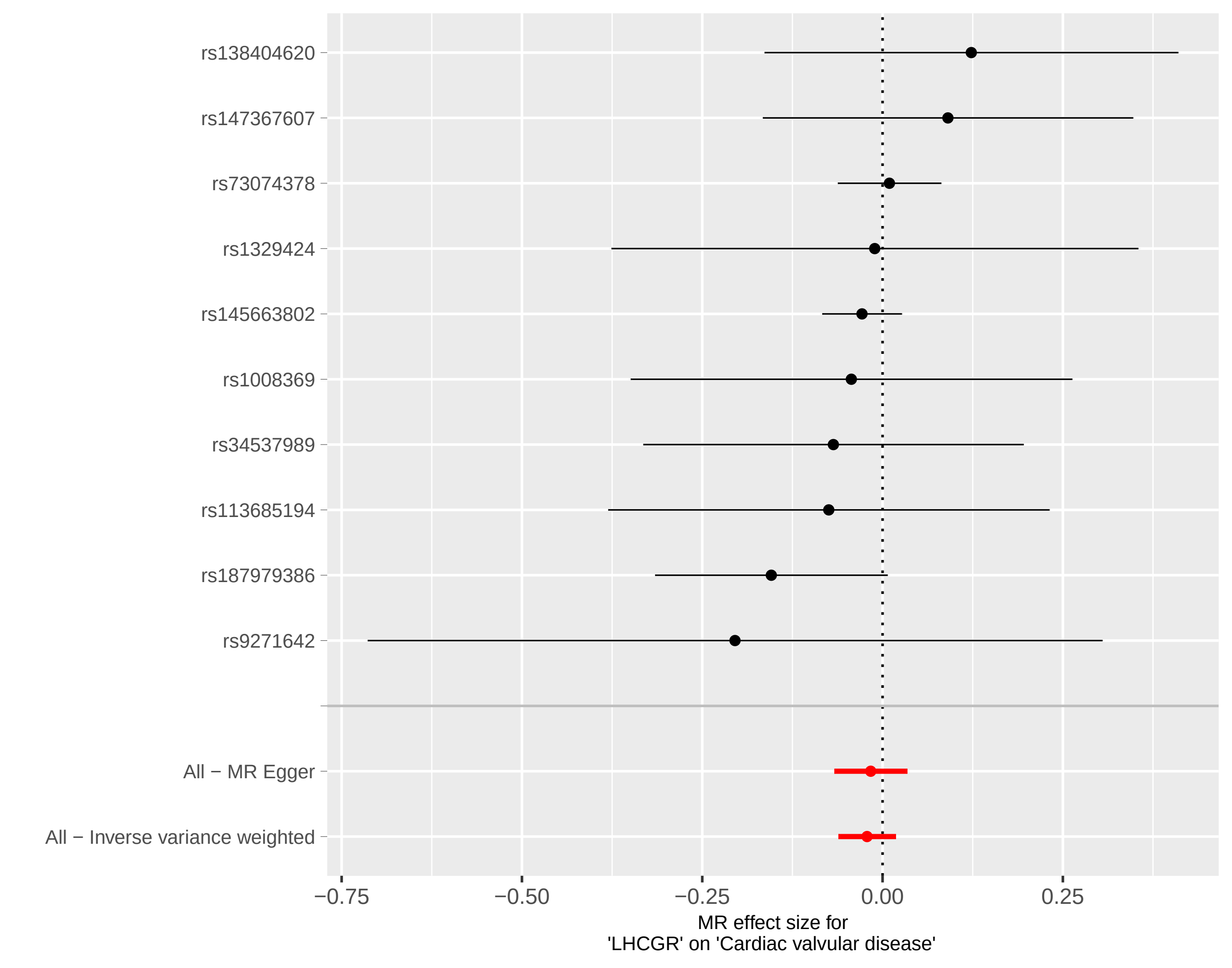

rs138404620
rs147367607
rs73074378
rs1329424
rs145663802
rs1008369
rs34537989
rs113685194
rs187979386
rs9271642
All − MR Egger
All − Inverse variance weighted
−0.75
−0.50
−0.25
0.00
0.25
MR effect size for
'LHCGR' on 'Cardiac valvular disease'

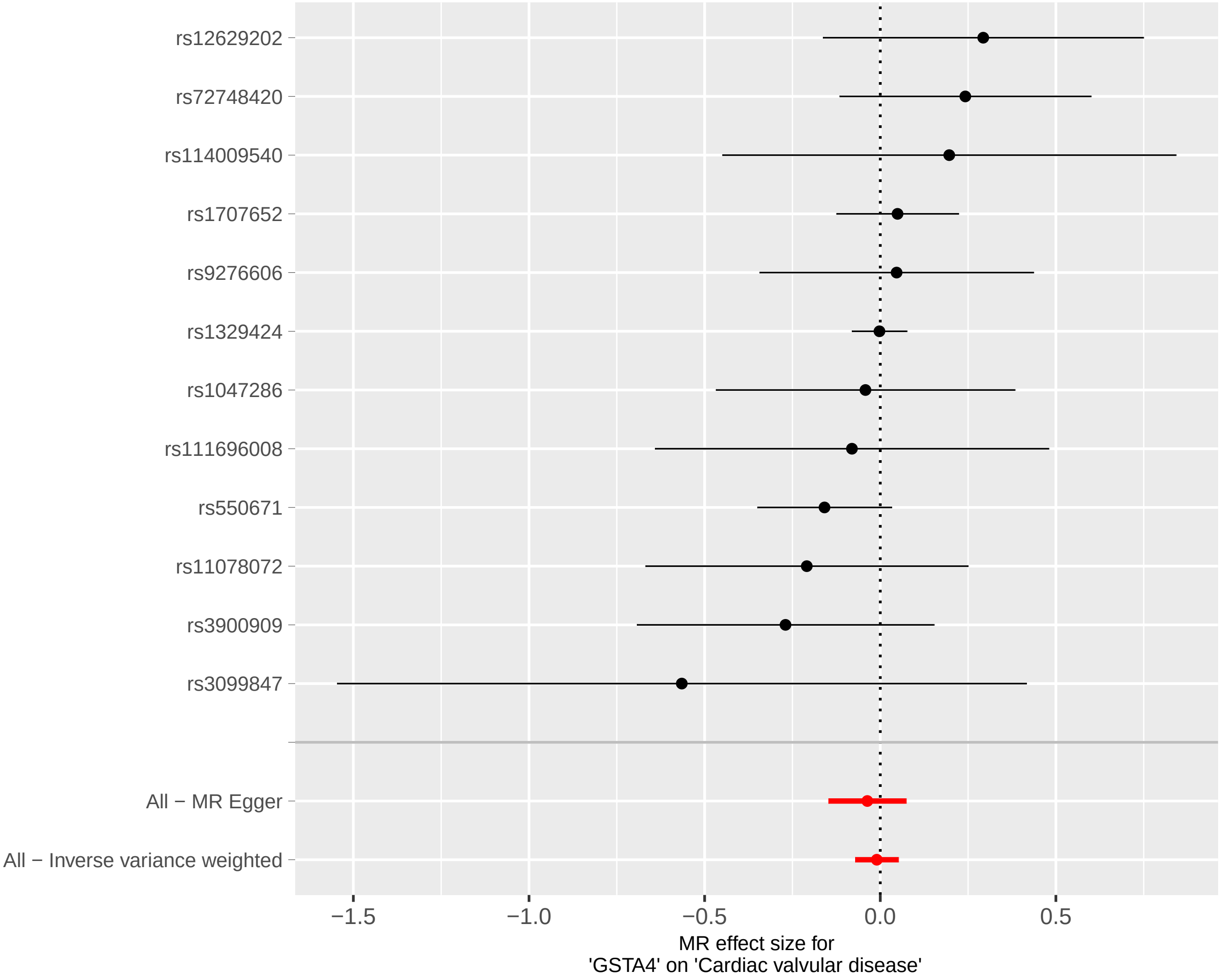
rs12629202
rs72748420
rs114009540
rs1707652
rs9276606
rs1329424
rs1047286
rs111696008
rs550671
rs11078072
rs3900909
rs3099847
All − MR Egger
All − Inverse variance weighted
−1.5
−1.0
−0.5
0.0
0.5
MR effect size for
'GSTA4' on 'Cardiac valvular disease'

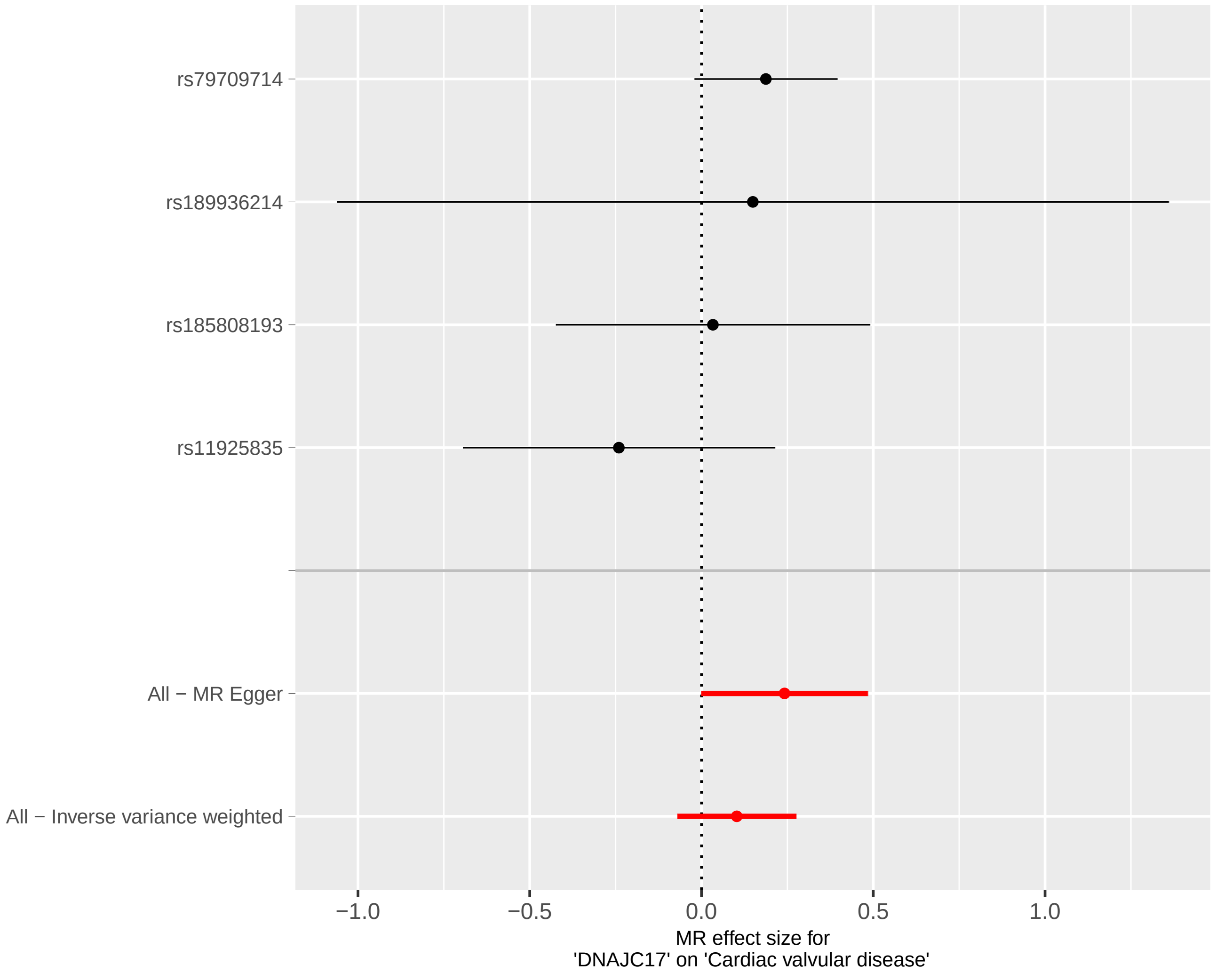
rs79709714
rs189936214
rs185808193
rs11925835
All − MR Egger
All − Inverse variance weighted
−1.0
−0.5
0.0
0.5
1.0
MR effect size for
'DNAJC17' on 'Cardiac valvular disease'

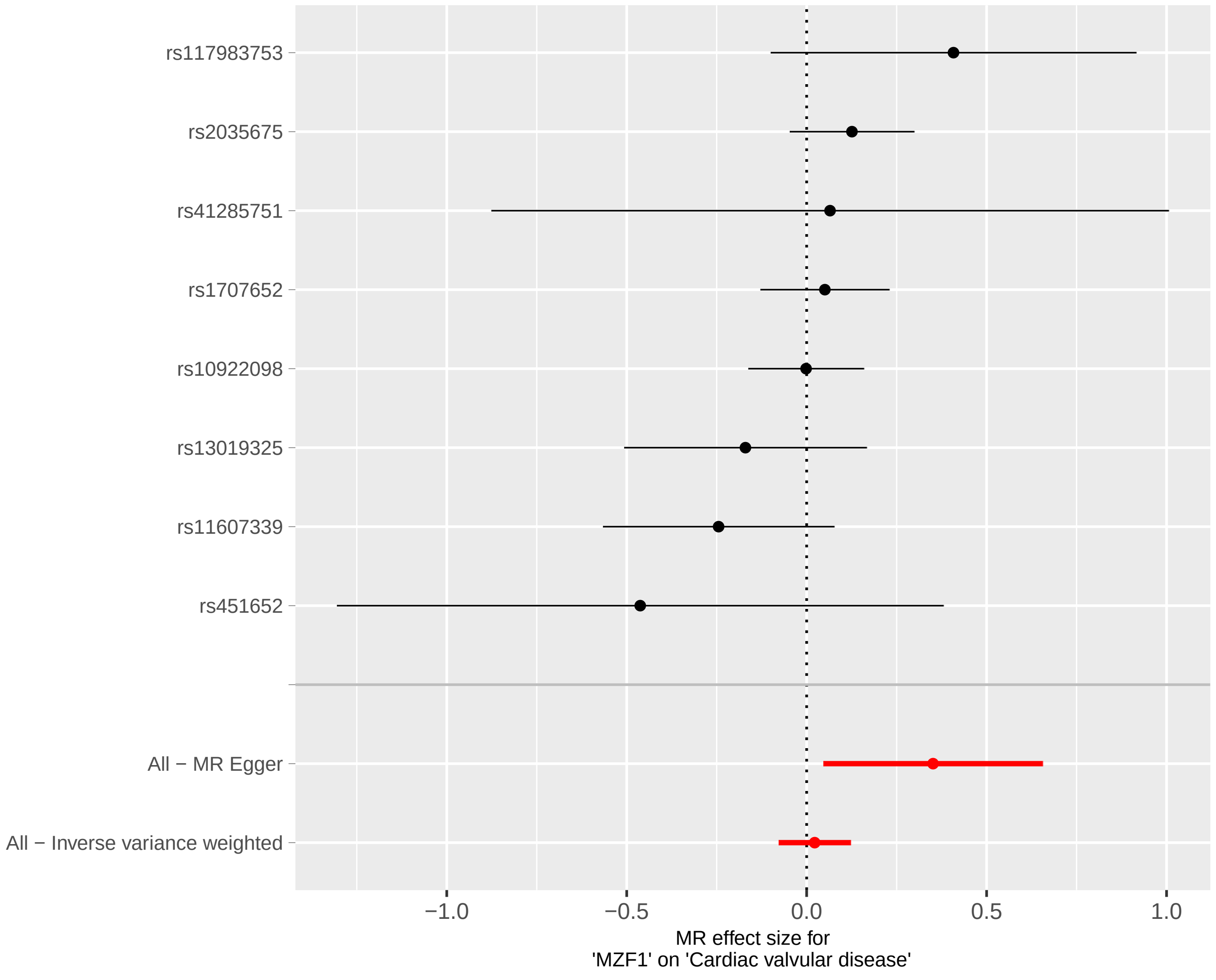

rs117983753
rs2035675
rs41285751
rs1707652
rs10922098
rs13019325
rs11607339
rs451652
All − MR Egger
All − Inverse variance weighted
−1.0
−0.5
0.0
0.5
1.0
MR effect size for
'MZF1' on 'Cardiac valvular disease'

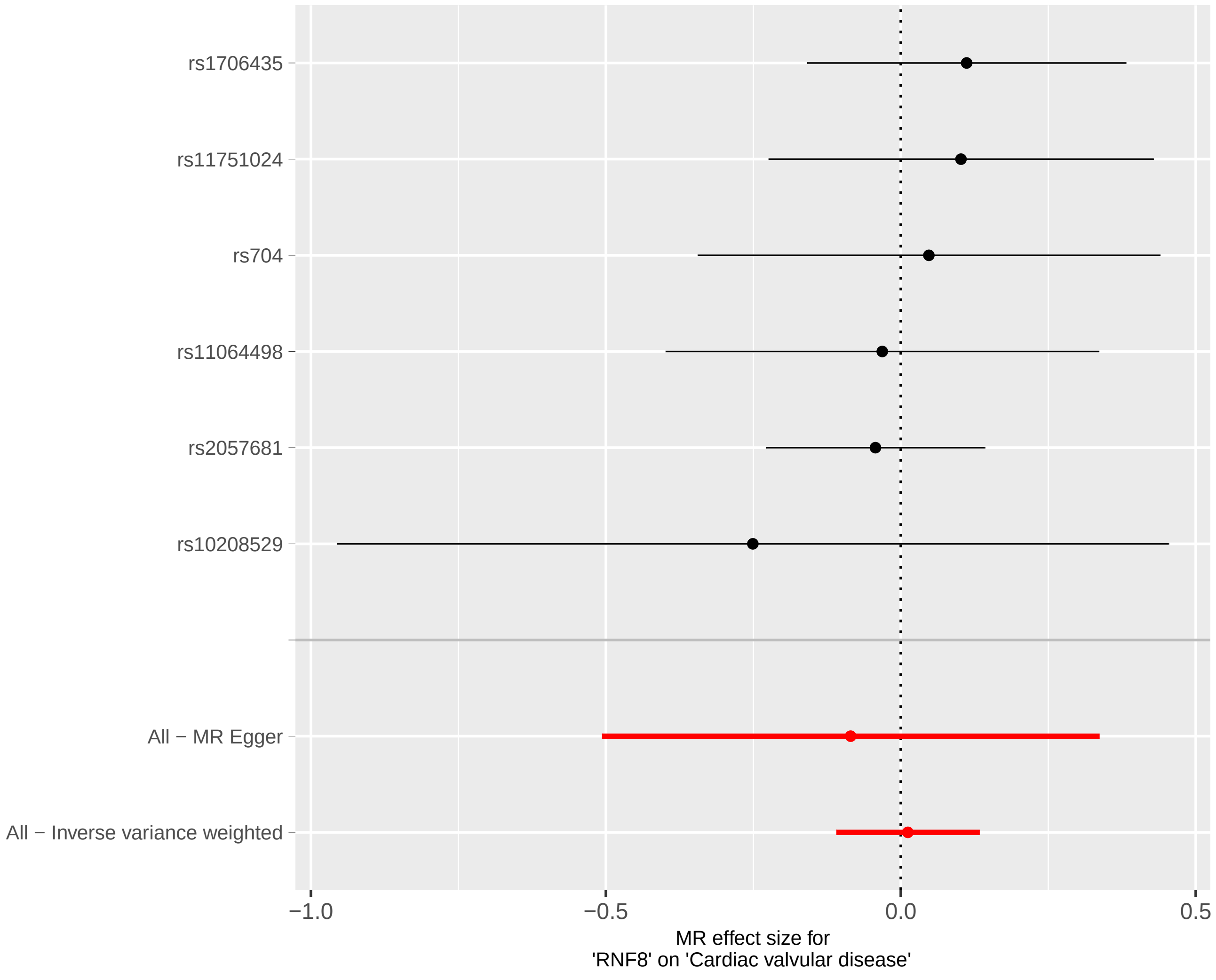

rs1706435
rs11751024
rs704
rs11064498
rs2057681
rs10208529
All − MR Egger
All − Inverse variance weighted
−1.0
−0.5
0.0
0.5
MR effect size for
'RNF8' on 'Cardiac valvular disease'

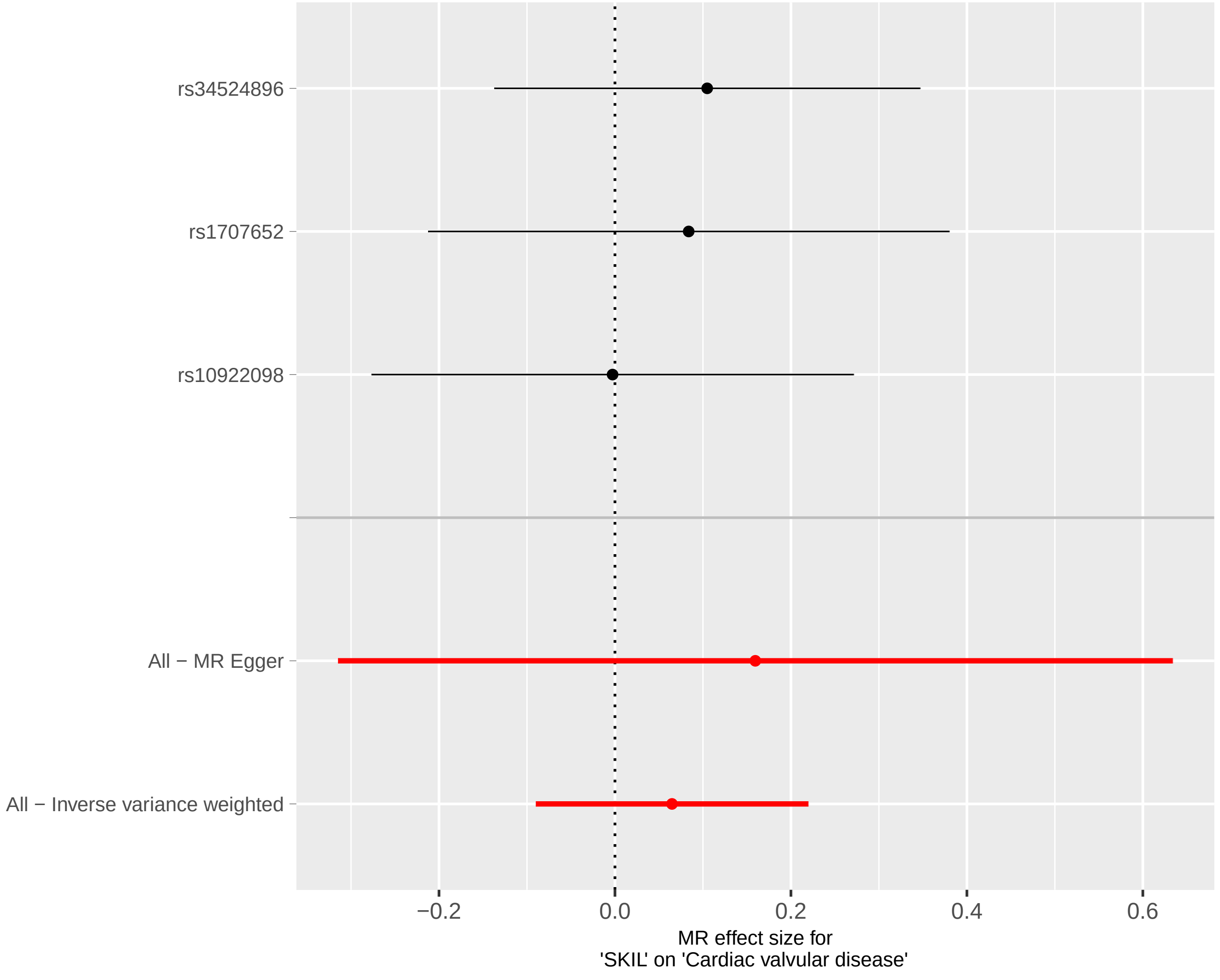

rs34524896
rs1707652
rs10922098
All − MR Egger
All − Inverse variance weighted
−0.2
0.0
0.2
0.4
0.6
MR effect size for
'SKIL' on 'Cardiac valvular disease'

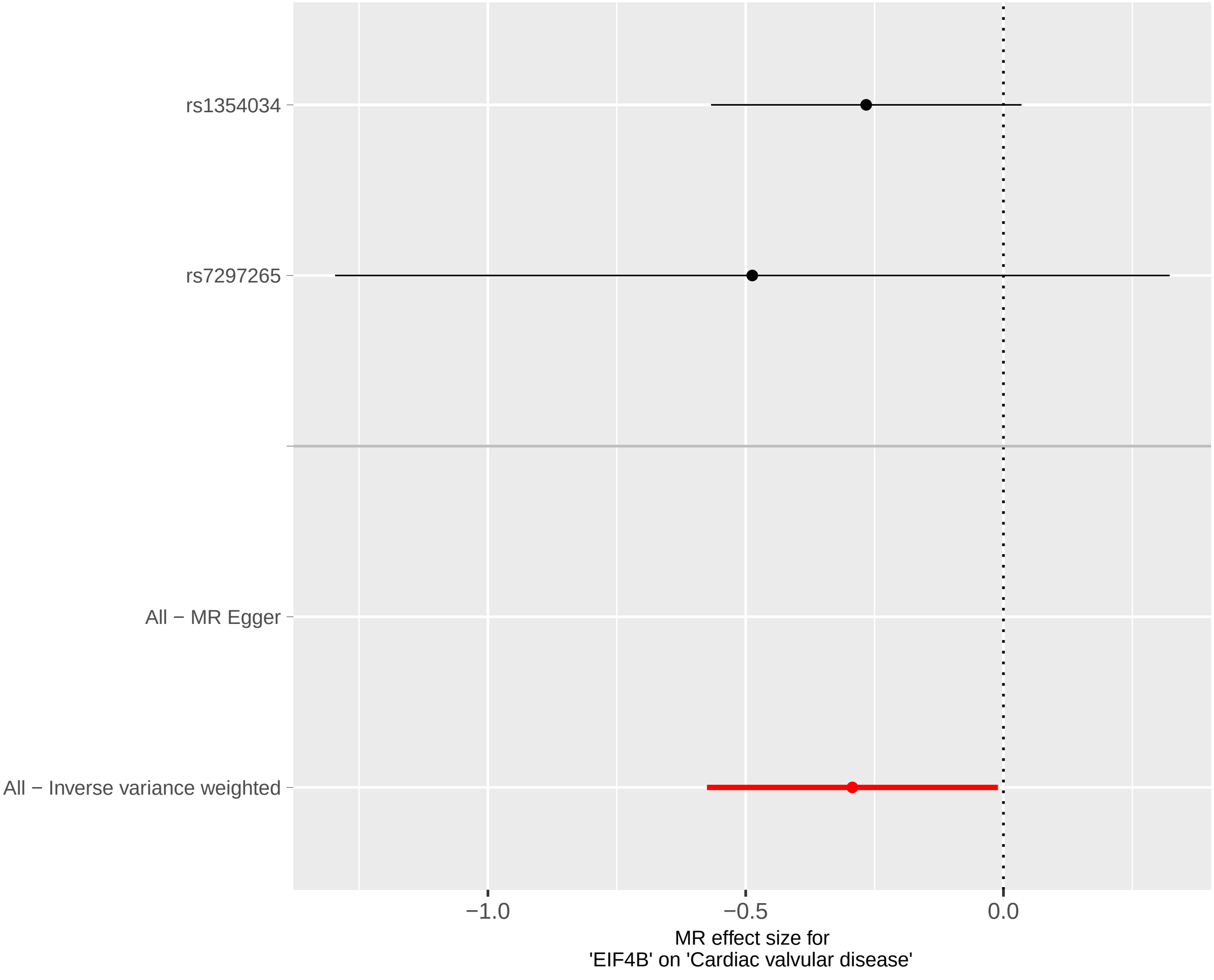
rs1354034
rs7297265
All − MR Egger
All − Inverse variance weighted
−1.0
−0.5
0.0
MR effect size for
'EIF4B' on 'Cardiac valvular disease'

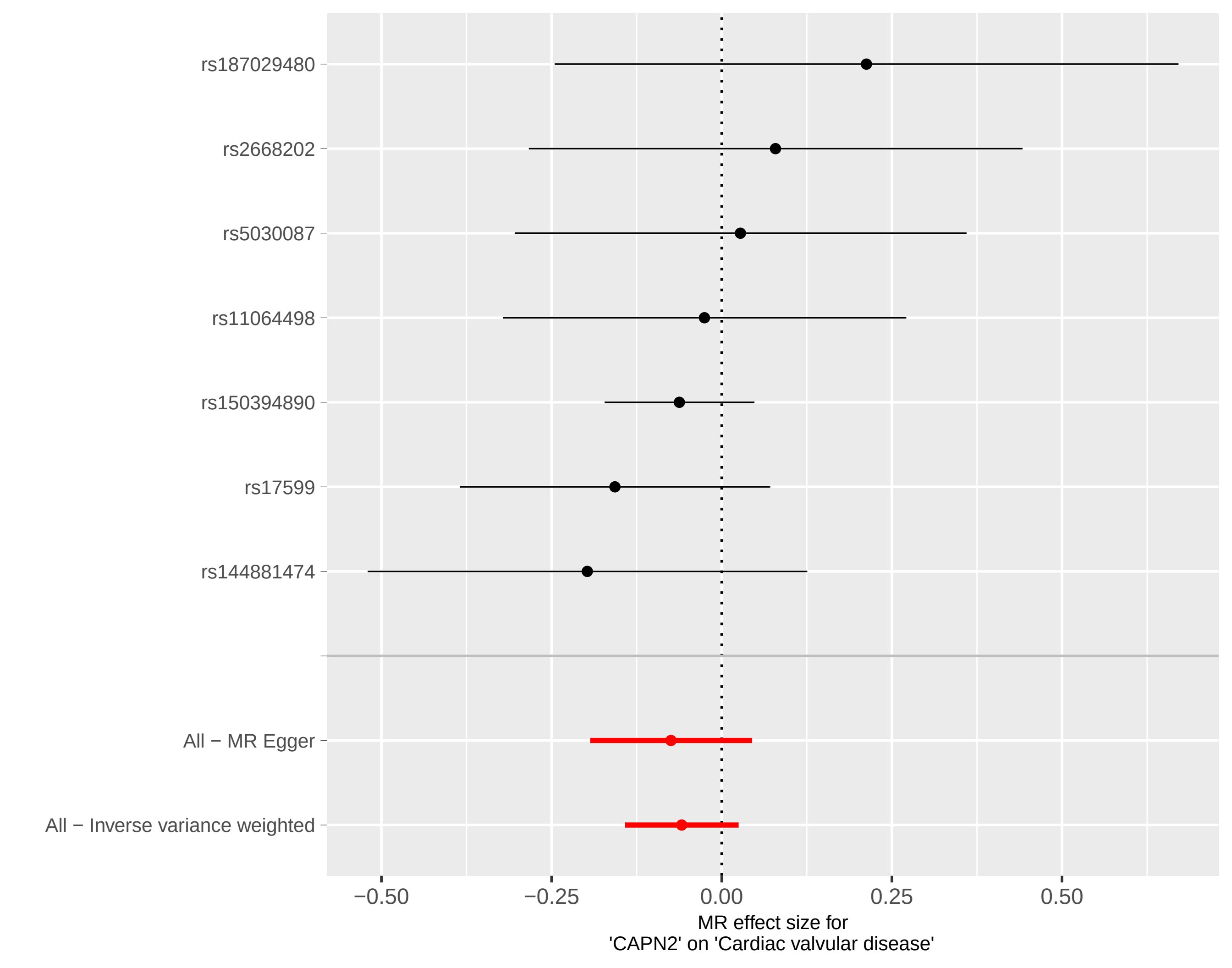
rs187029480
rs2668202
rs5030087
rs11064498
rs150394890
rs17599
rs144881474
All − MR Egger
All − Inverse variance weighted
−0.50
−0.25
0.00
0.25
0.50
MR effect size for
'CAPN2' on 'Cardiac valvular disease'

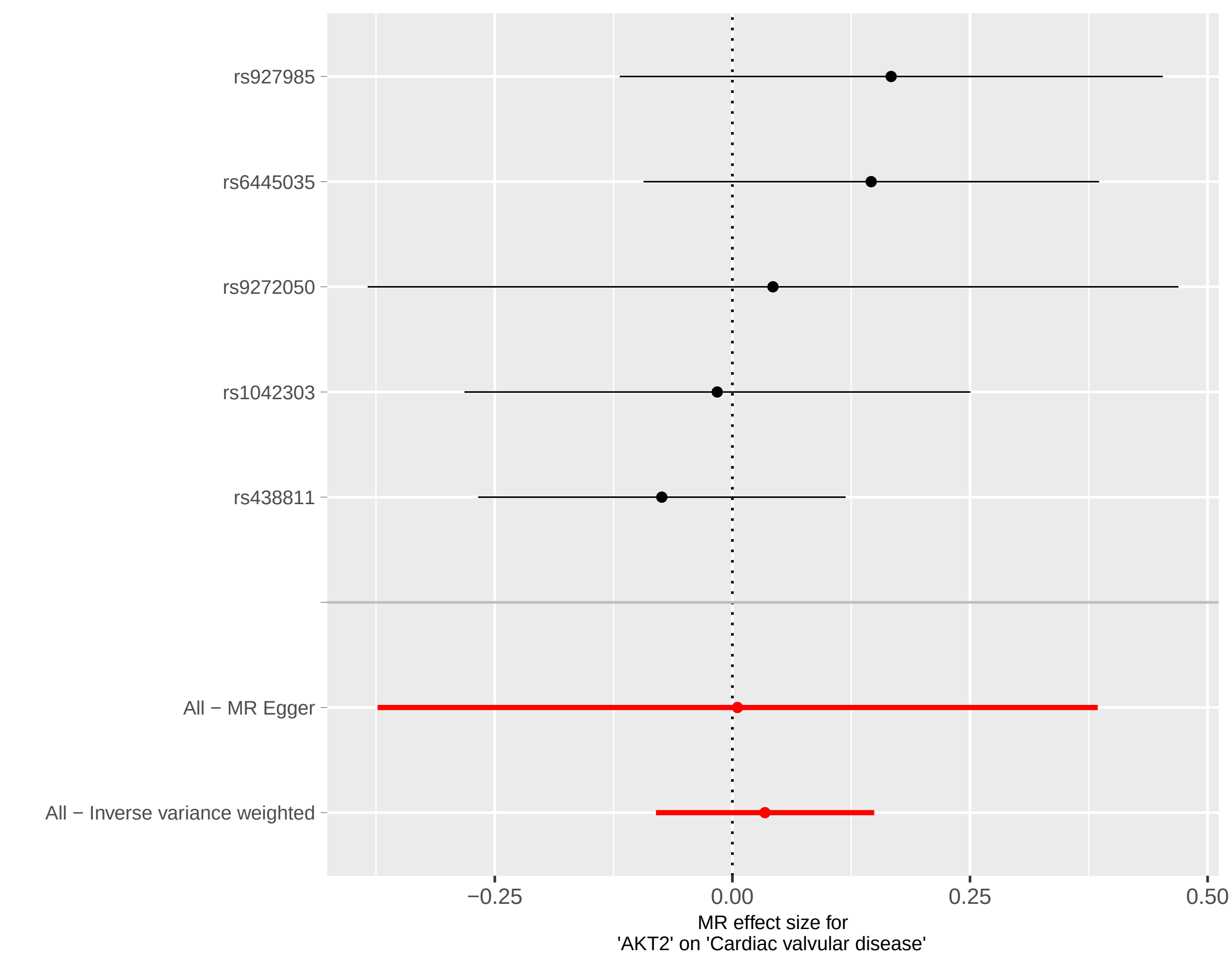

rs927985
rs6445035
rs9272050
rs1042303
rs438811
All − MR Egger
All − Inverse variance weighted
−0.25
0.00
0.25
0.50
MR effect size for
'AKT2' on 'Cardiac valvular disease'

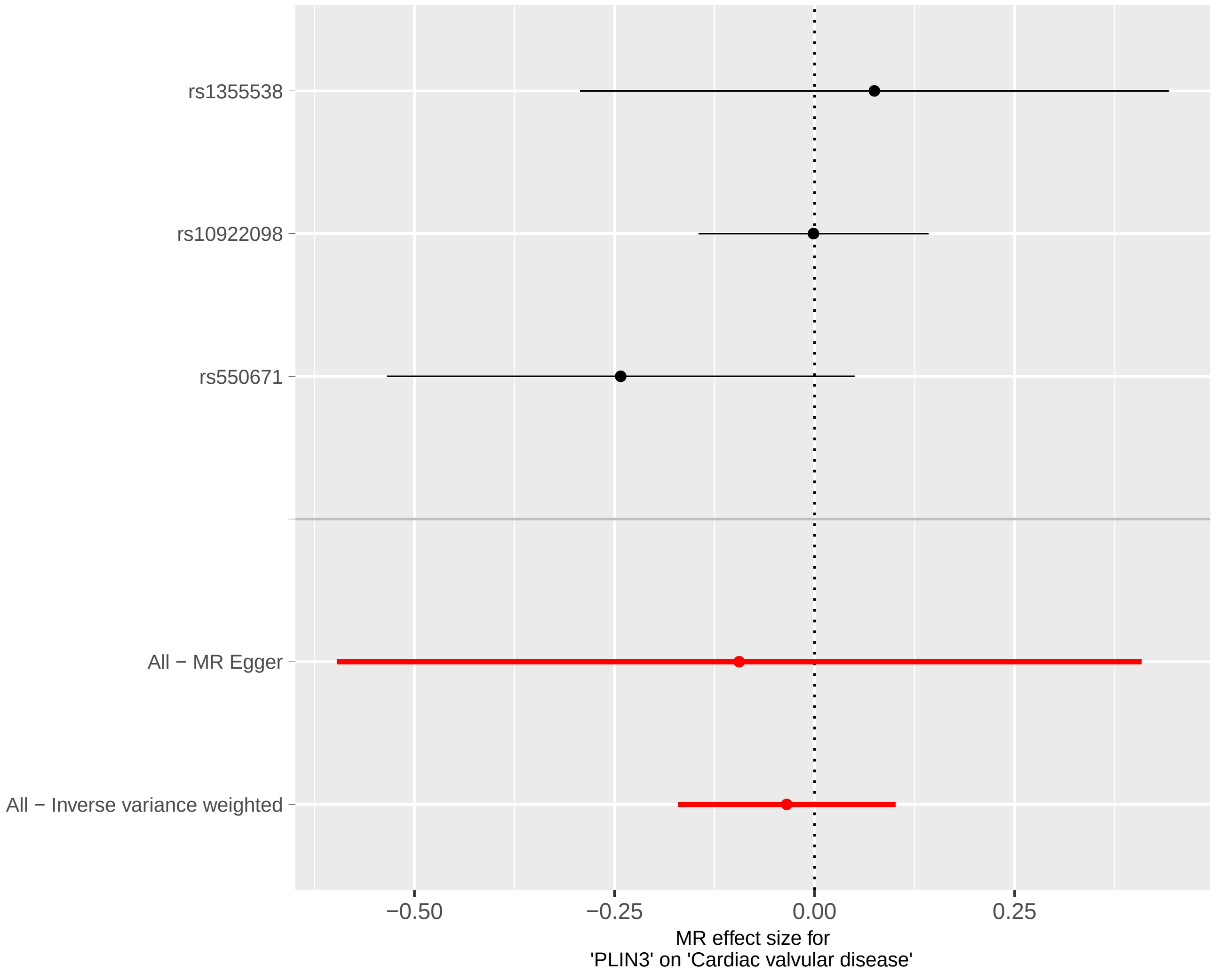

rs1355538
rs10922098
rs550671
All − MR Egger
All − Inverse variance weighted
−0.50
−0.25
0.00
0.25
MR effect size for
'PLIN3' on 'Cardiac valvular disease'

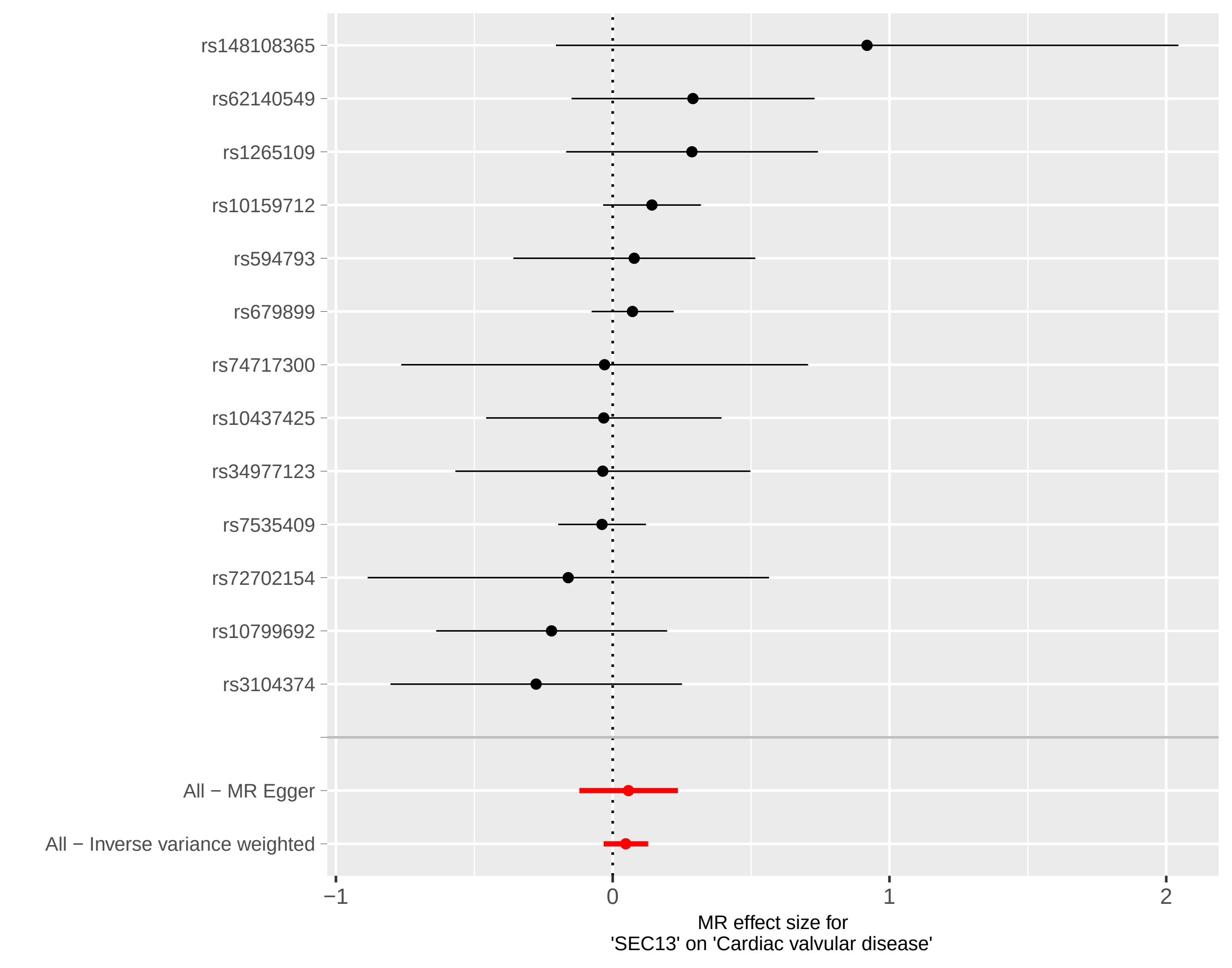
rs148108365
rs62140549
rs1265109
rs10159712
rs594793
rs679899
rs74717300
rs10437425
rs34977123
rs7535409
rs72702154
rs10799692
rs3104374
All − MR Egger
All − Inverse variance weighted
−1
0
1
2
MR effect size for
'SEC13' on 'Cardiac valvular disease'

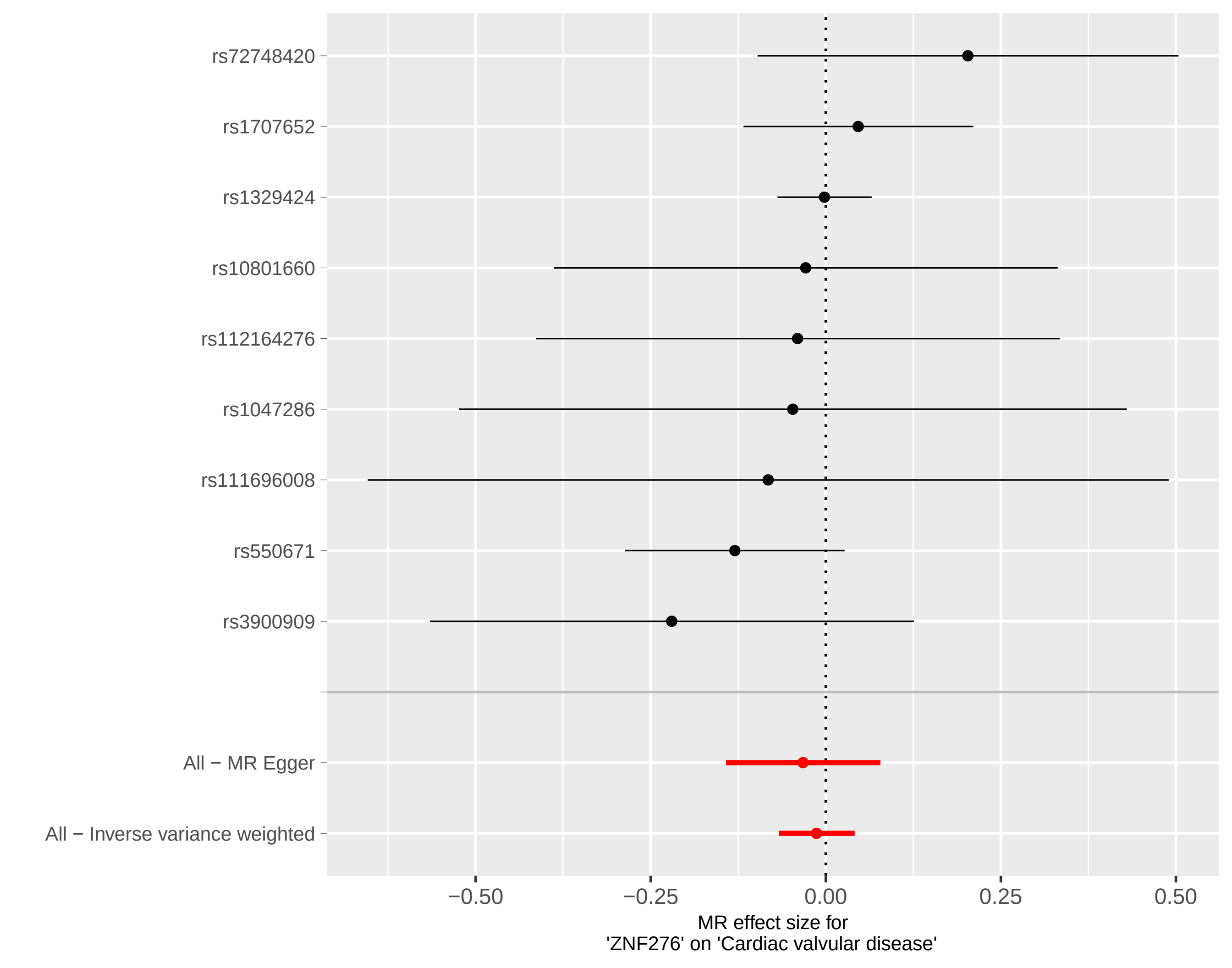
rs72748420
rs1707652
rs1329424
rs10801660
rs112164276
rs1047286
rs111696008
rs550671
rs3900909
All − MR Egger
All − Inverse variance weighted
−0.50
−0.25
0.00
0.25
0.50
MR effect size for
'ZNF276' on 'Cardiac valvular disease'

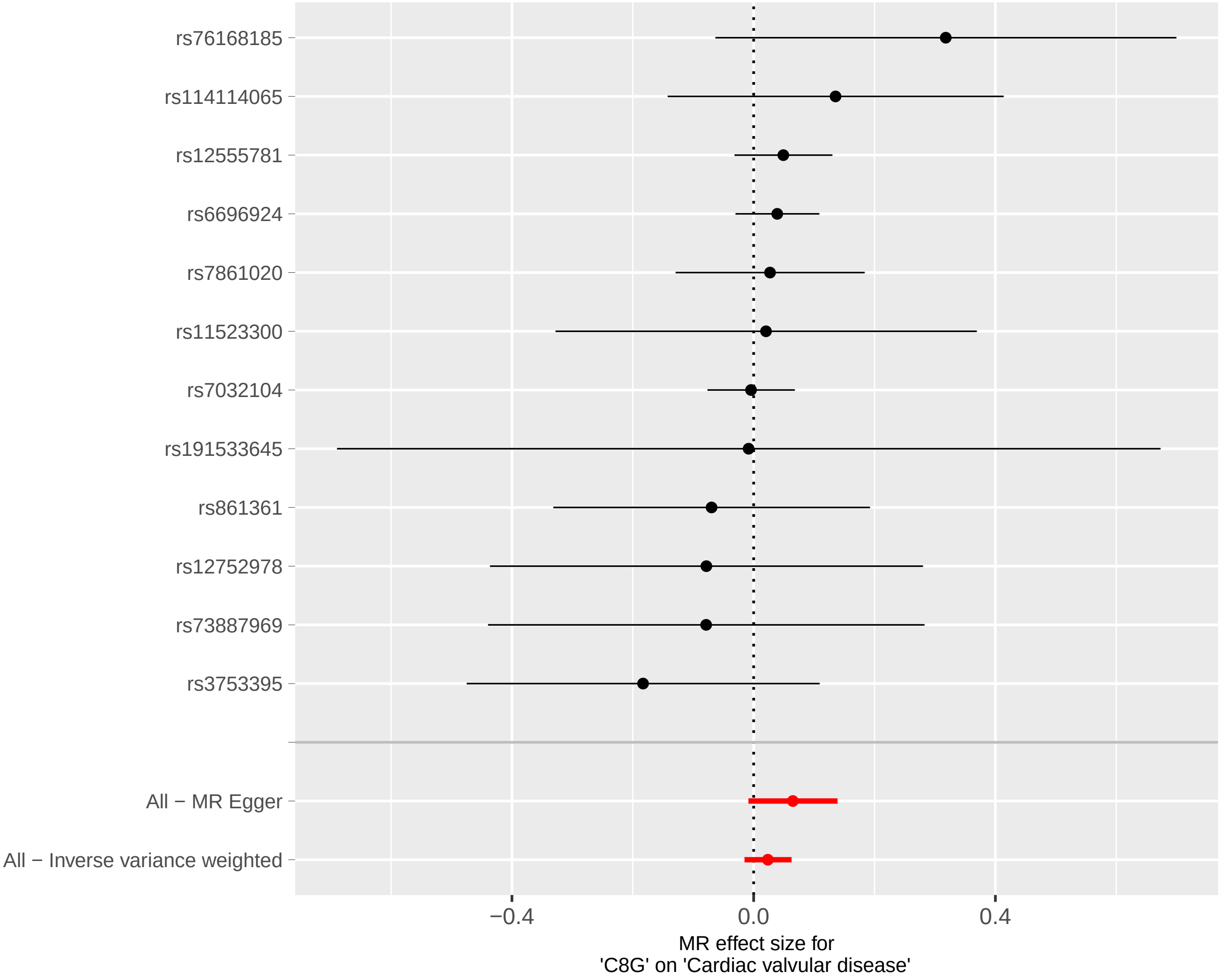
rs76168185
rs114114065
rs12555781
rs6696924
rs7861020
rs11523300
rs7032104
rs191533645
rs861361
rs12752978
rs73887969
rs3753395
All − MR Egger
All − Inverse variance weighted
−0.4
0.0
0.4
MR effect size for
'C8G' on 'Cardiac valvular disease'

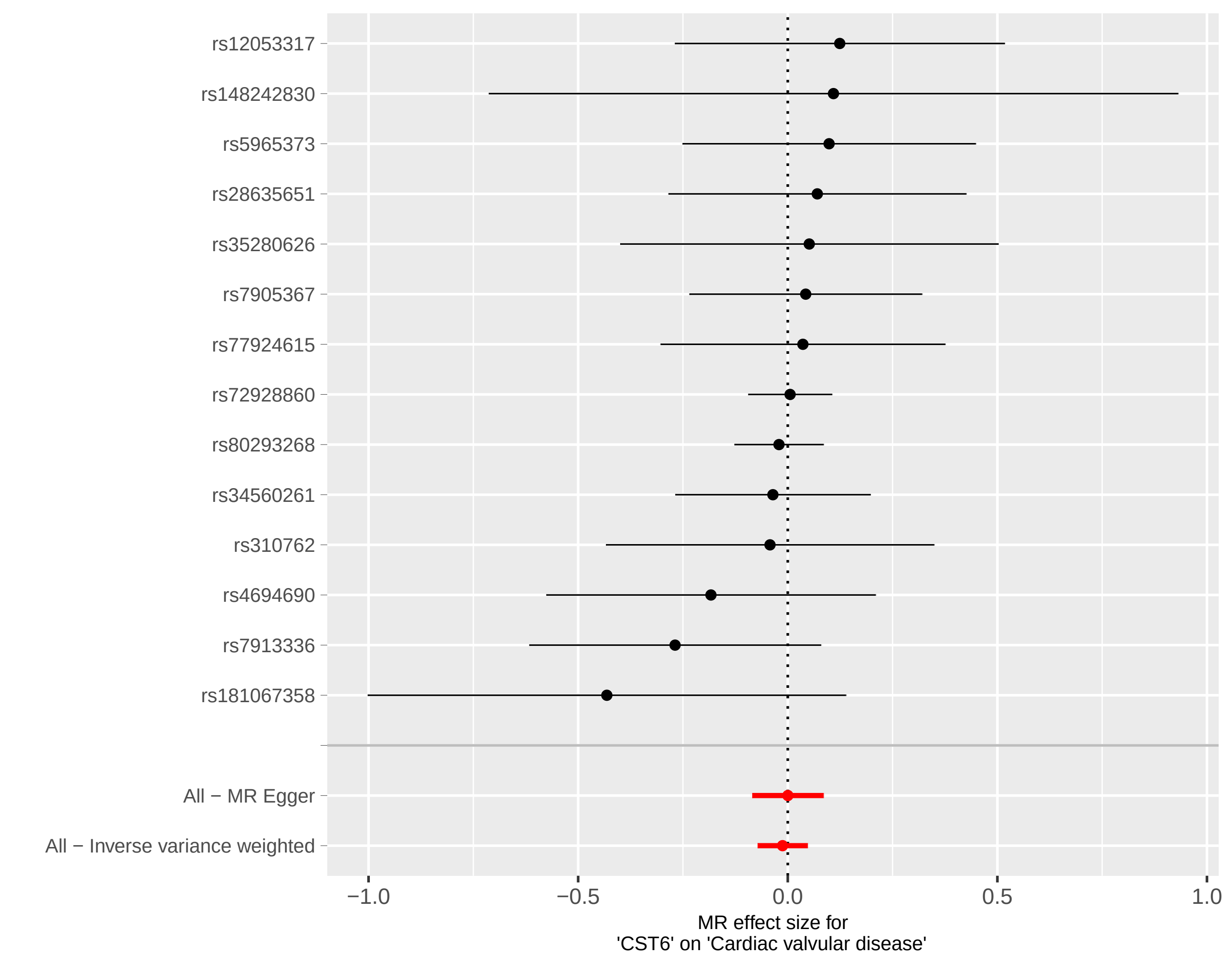
rs12053317
rs148242830
rs5965373
rs28635651
rs35280626
rs7905367
rs77924615
rs72928860
rs80293268
rs34560261
rs310762
rs4694690
rs7913336
rs181067358
All − MR Egger
All − Inverse variance weighted
−1.0
−0.5
0.0
0.5
1.0
MR effect size for
'CST6' on 'Cardiac valvular disease'

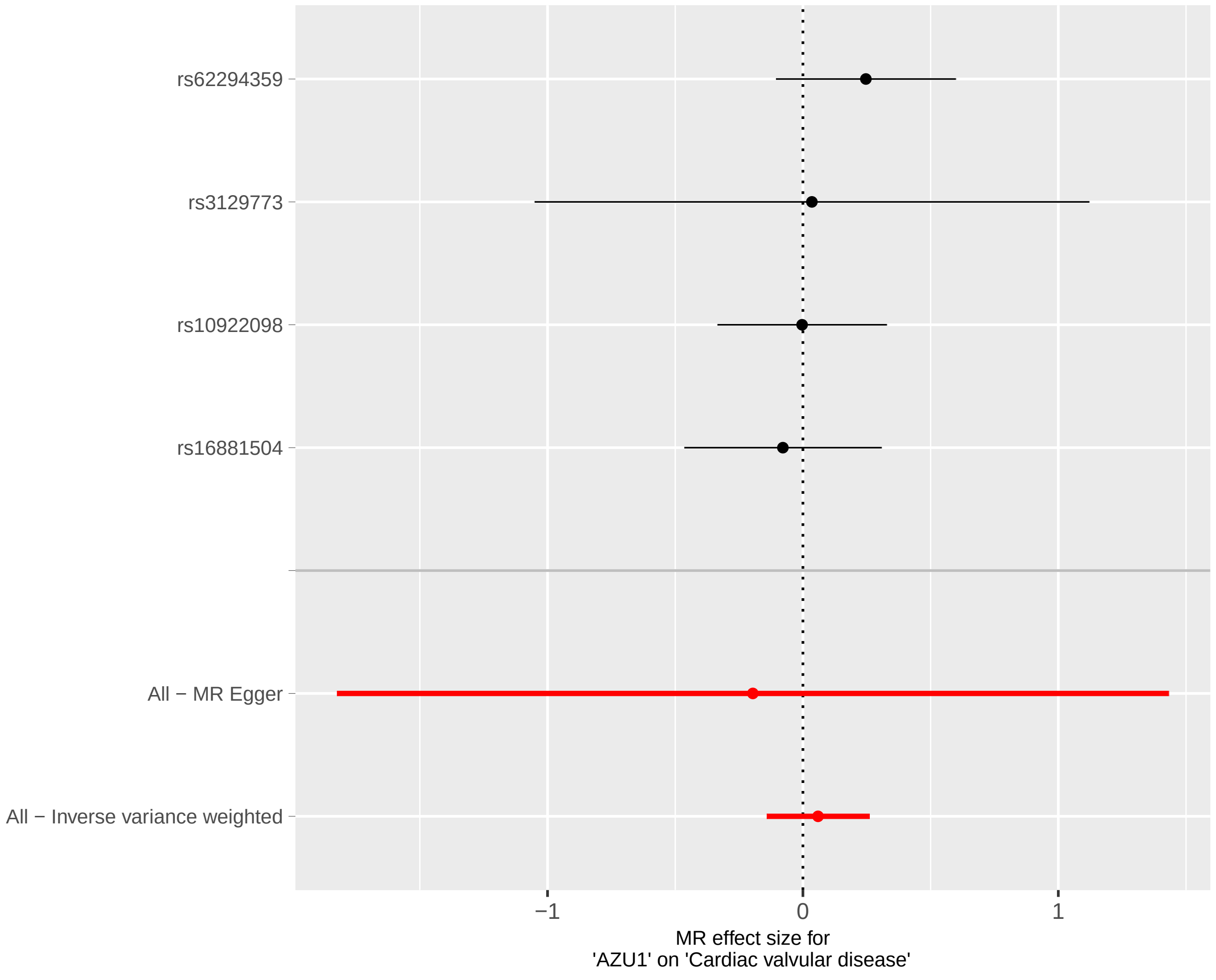

rs62294359
rs3129773
rs10922098
rs16881504
All − MR Egger
All − Inverse variance weighted
−1
0
1
MR effect size for
'AZU1' on 'Cardiac valvular disease'

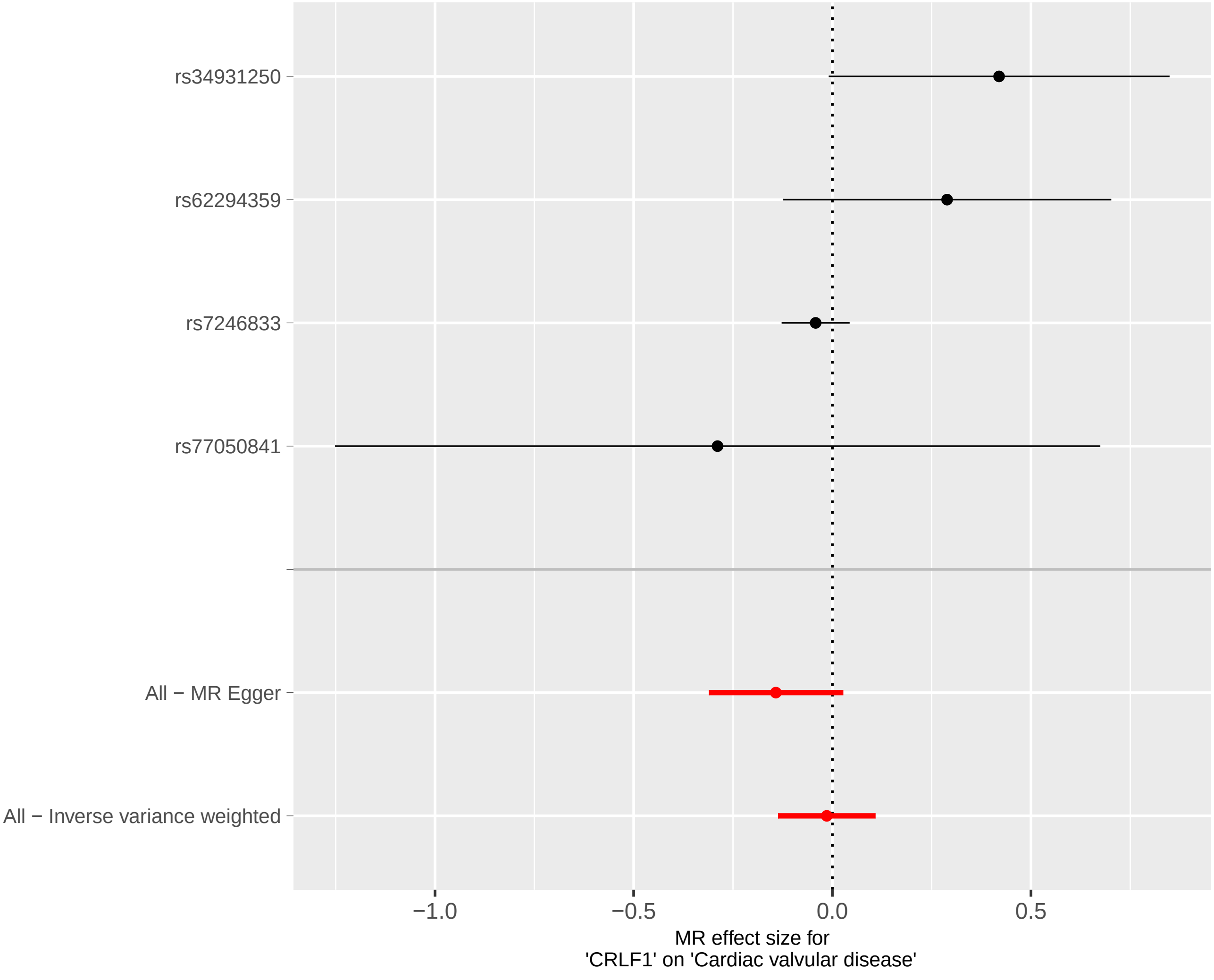
rs34931250
rs62294359
rs7246833
rs77050841
All − MR Egger
All − Inverse variance weighted
−1.0
−0.5
0.0
0.5
MR effect size for
'CRLF1' on 'Cardiac valvular disease'

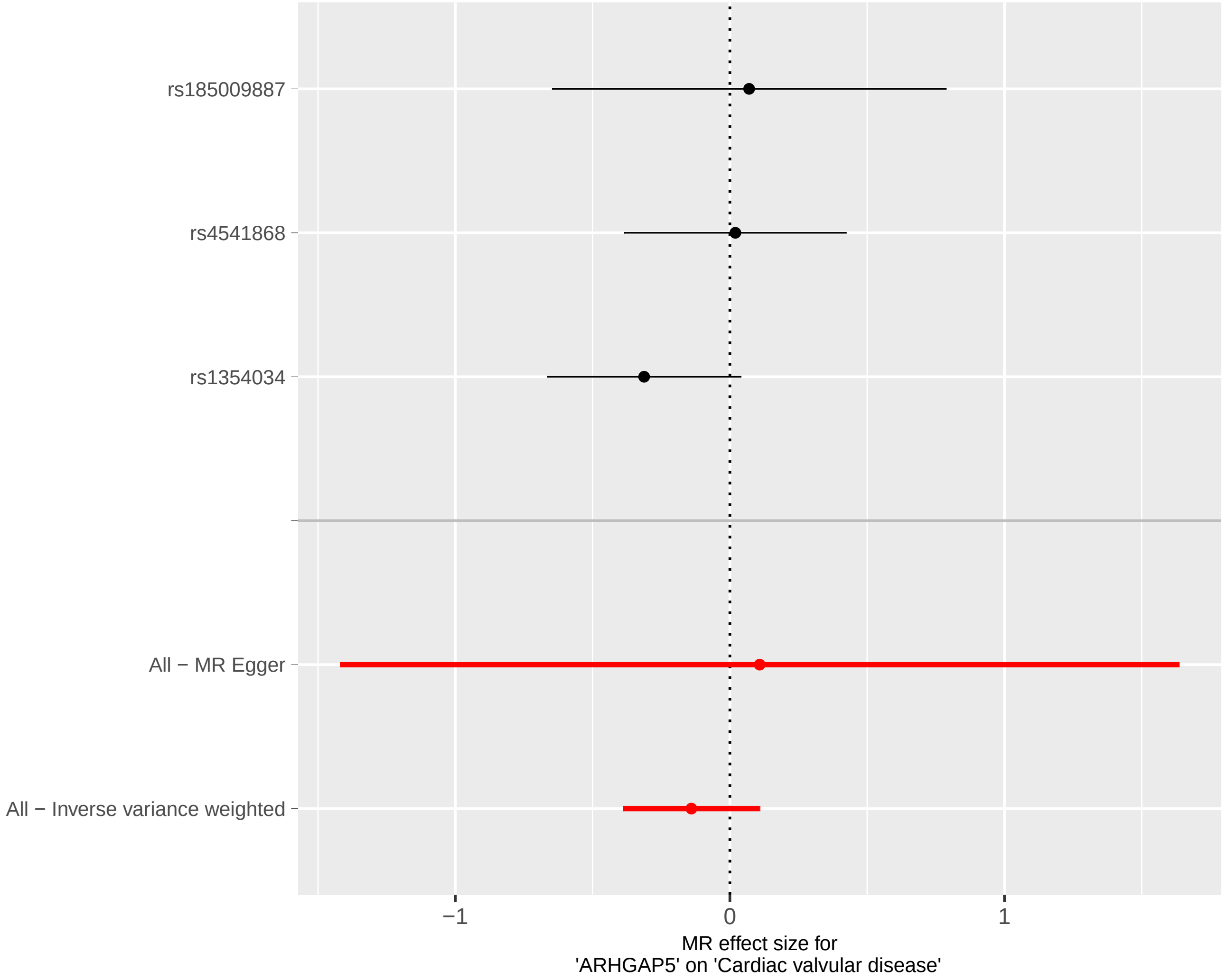

rs185009887
rs4541868
rs1354034
All − MR Egger
All − Inverse variance weighted
−1
0
1
MR effect size for
'ARHGAP5' on 'Cardiac valvular disease'

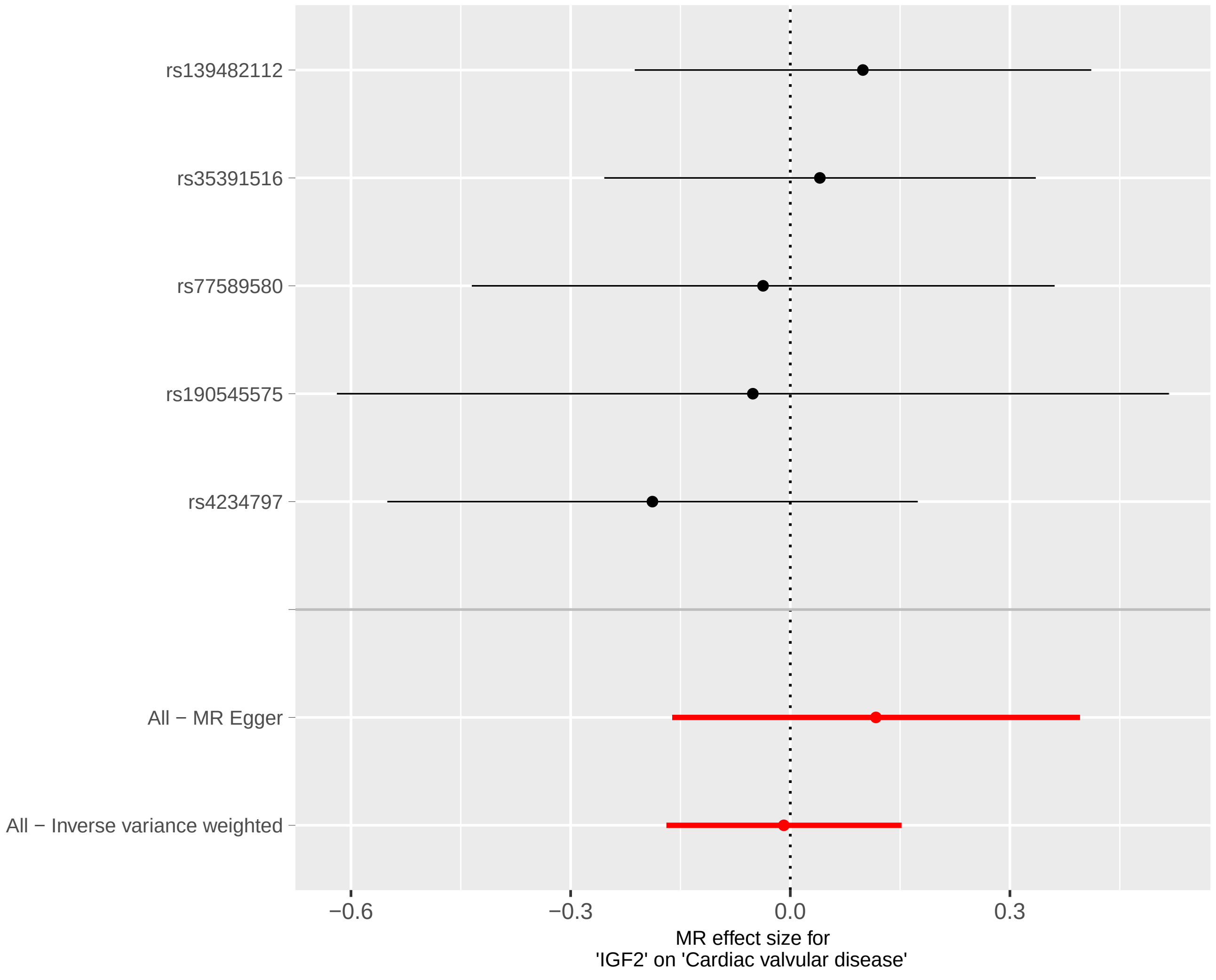

rs139482112
rs35391516
rs77589580
rs190545575
rs4234797
All − MR Egger
All − Inverse variance weighted
−0.6
−0.3
0.0
0.3
MR effect size for
'IGF2' on 'Cardiac valvular disease'

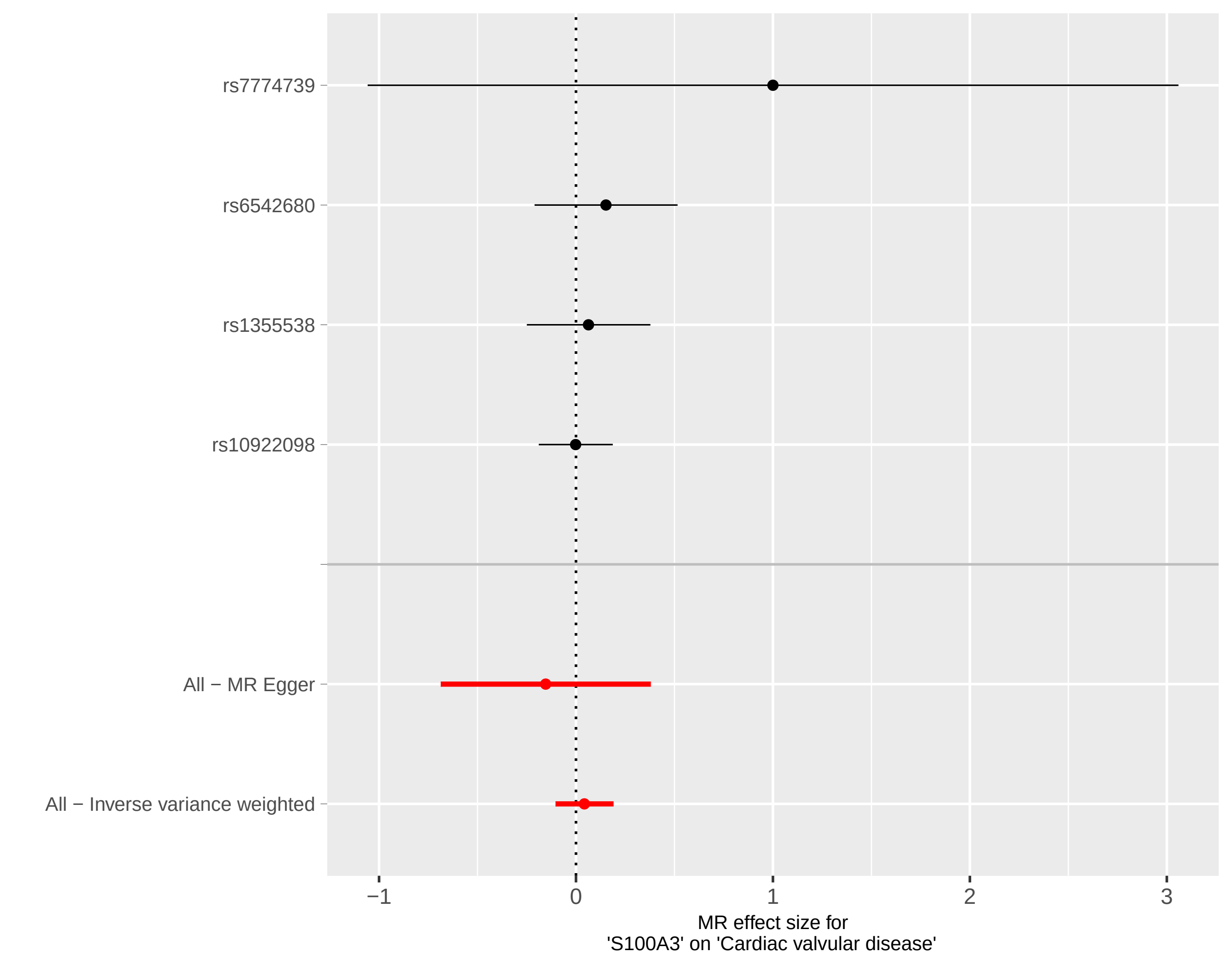

rs7774739
rs6542680
rs1355538
rs10922098
All − MR Egger
All − Inverse variance weighted
−1
0
1
2
3
MR effect size for
'S100A3' on 'Cardiac valvular disease'

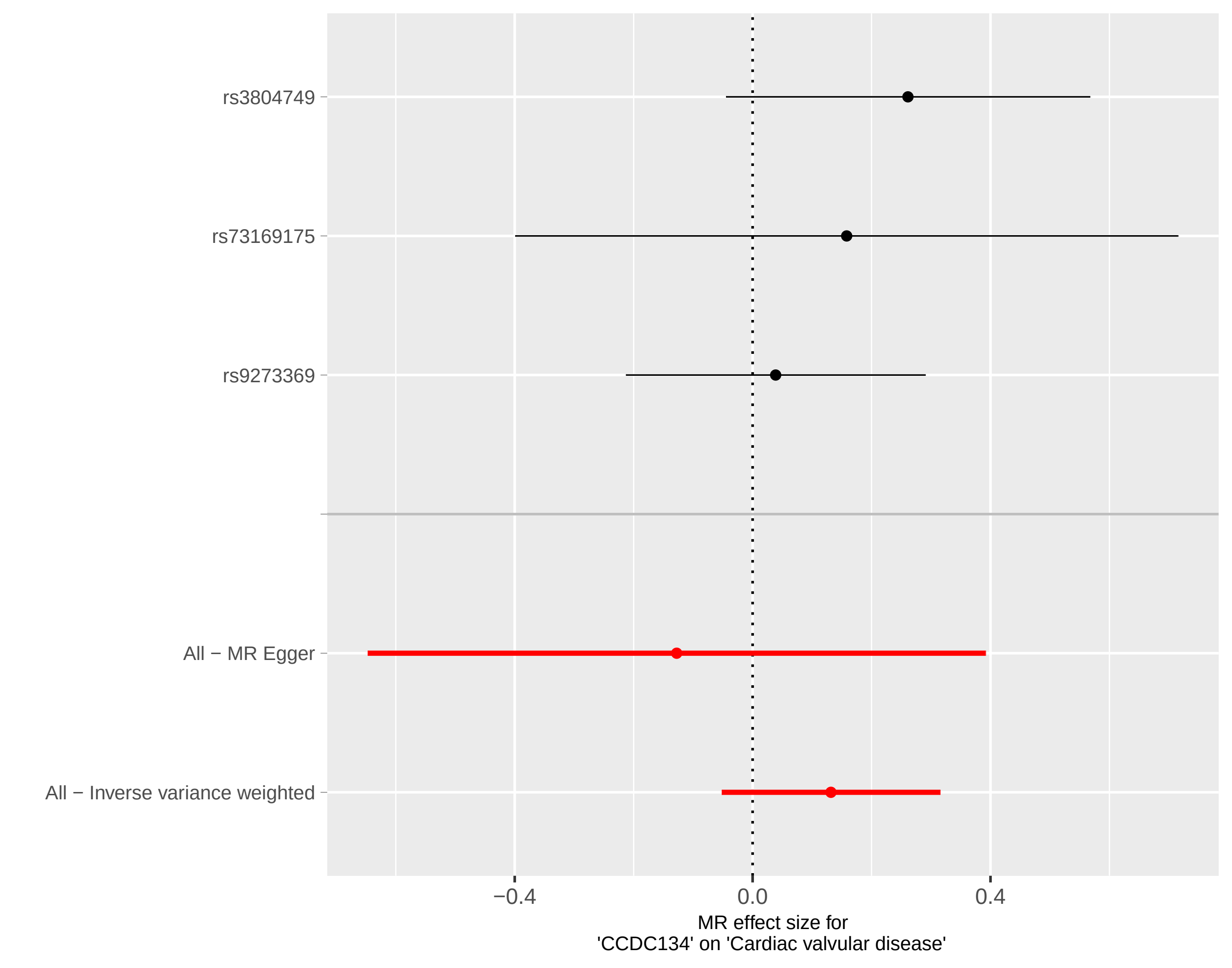
rs3804749
rs73169175
rs9273369
All − MR Egger
All − Inverse variance weighted
−0.4
0.0
0.4
MR effect size for
'CCDC134' on 'Cardiac valvular disease'

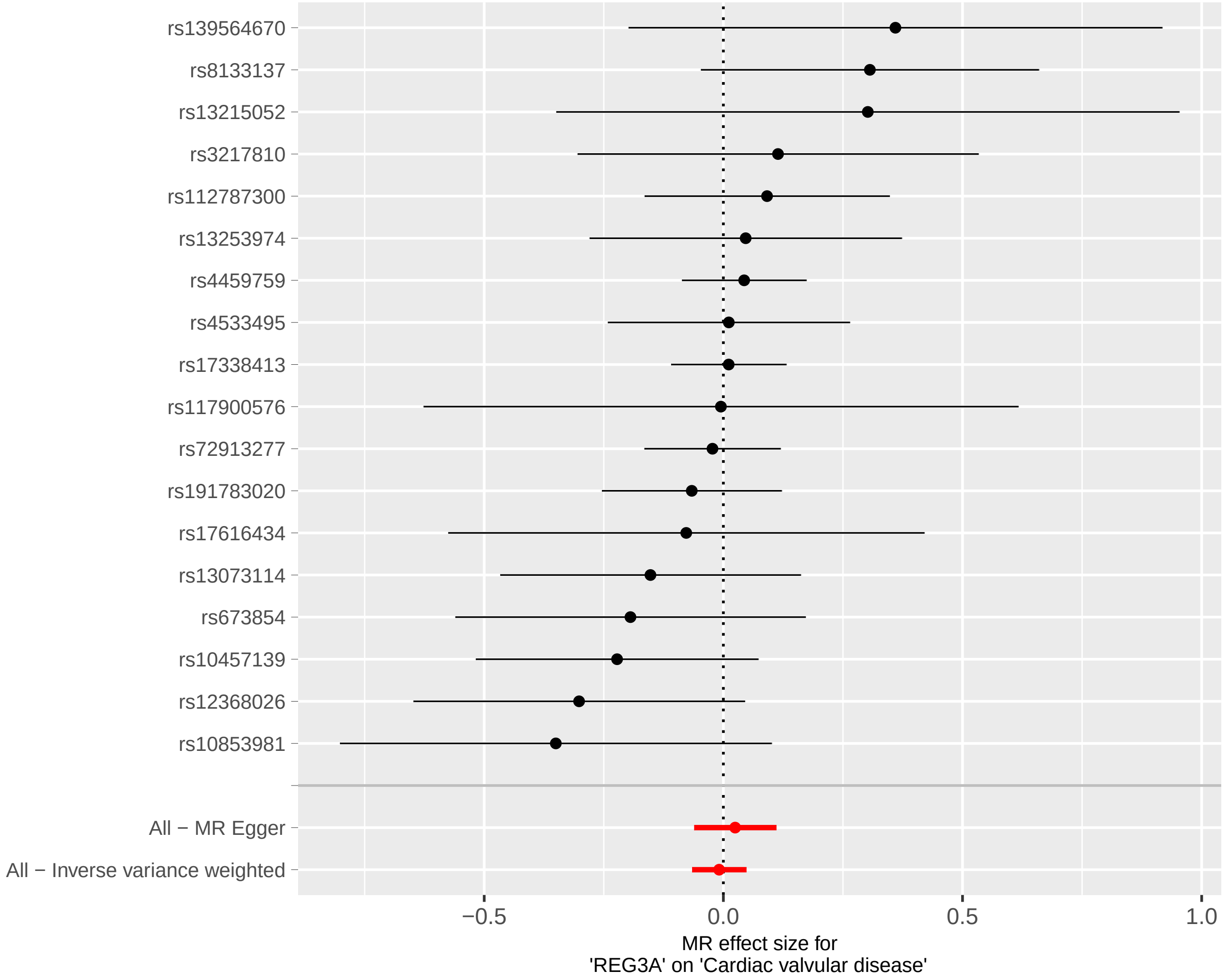
rs139564670
rs8133137
rs13215052
rs3217810
rs112787300
rs13253974
rs4459759
rs4533495
rs17338413
rs117900576
rs72913277
rs191783020
rs17616434
rs13073114
rs673854
rs10457139
rs12368026
rs10853981
All − MR Egger
All − Inverse variance weighted
−0.5
0.0
0.5
1.0
MR effect size for
'REG3A' on 'Cardiac valvular disease'

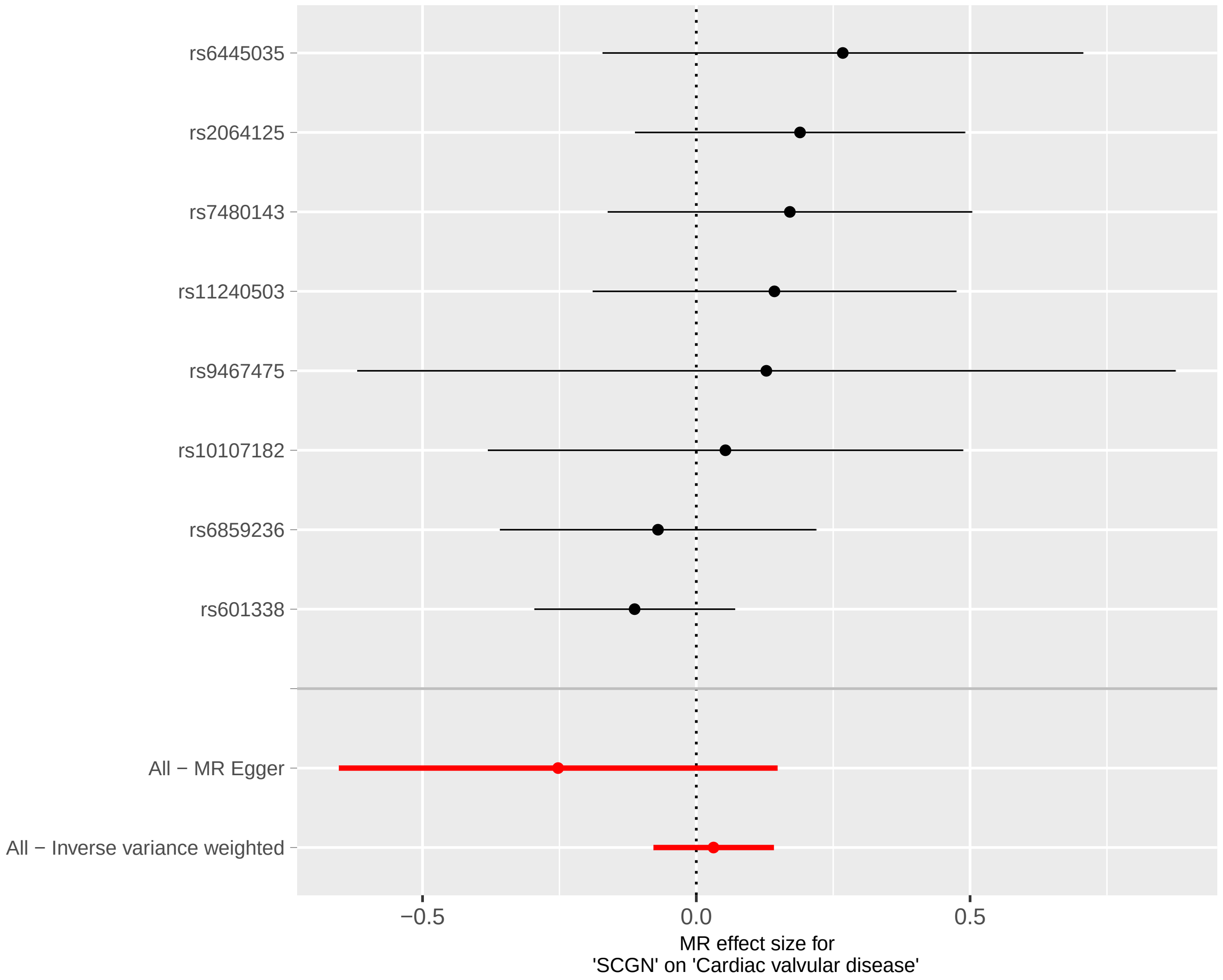
rs6445035
rs2064125
rs7480143
rs11240503
rs9467475
rs10107182
rs6859236
rs601338
All − MR Egger
All − Inverse variance weighted
−0.5
0.0
0.5
MR effect size for
'SCGN' on 'Cardiac valvular disease'

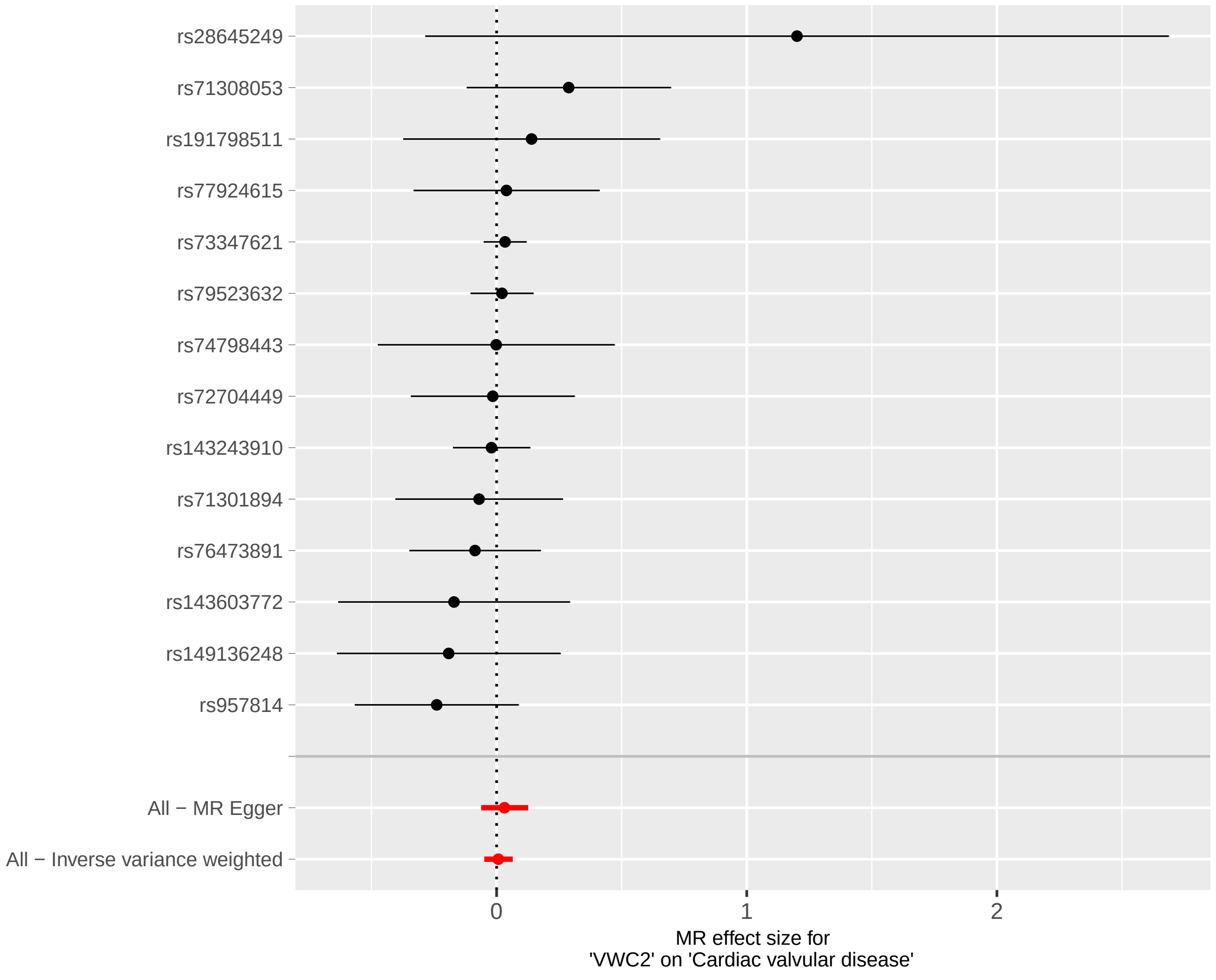
rs28645249
rs71308053
rs191798511
rs77924615
rs73347621
rs79523632
rs74798443
rs72704449
rs143243910
rs71301894
rs76473891
rs143603772
rs149136248
rs957814
All − MR Egger
All − Inverse variance weighted
0
1
2
MR effect size for
'VWC2' on 'Cardiac valvular disease'

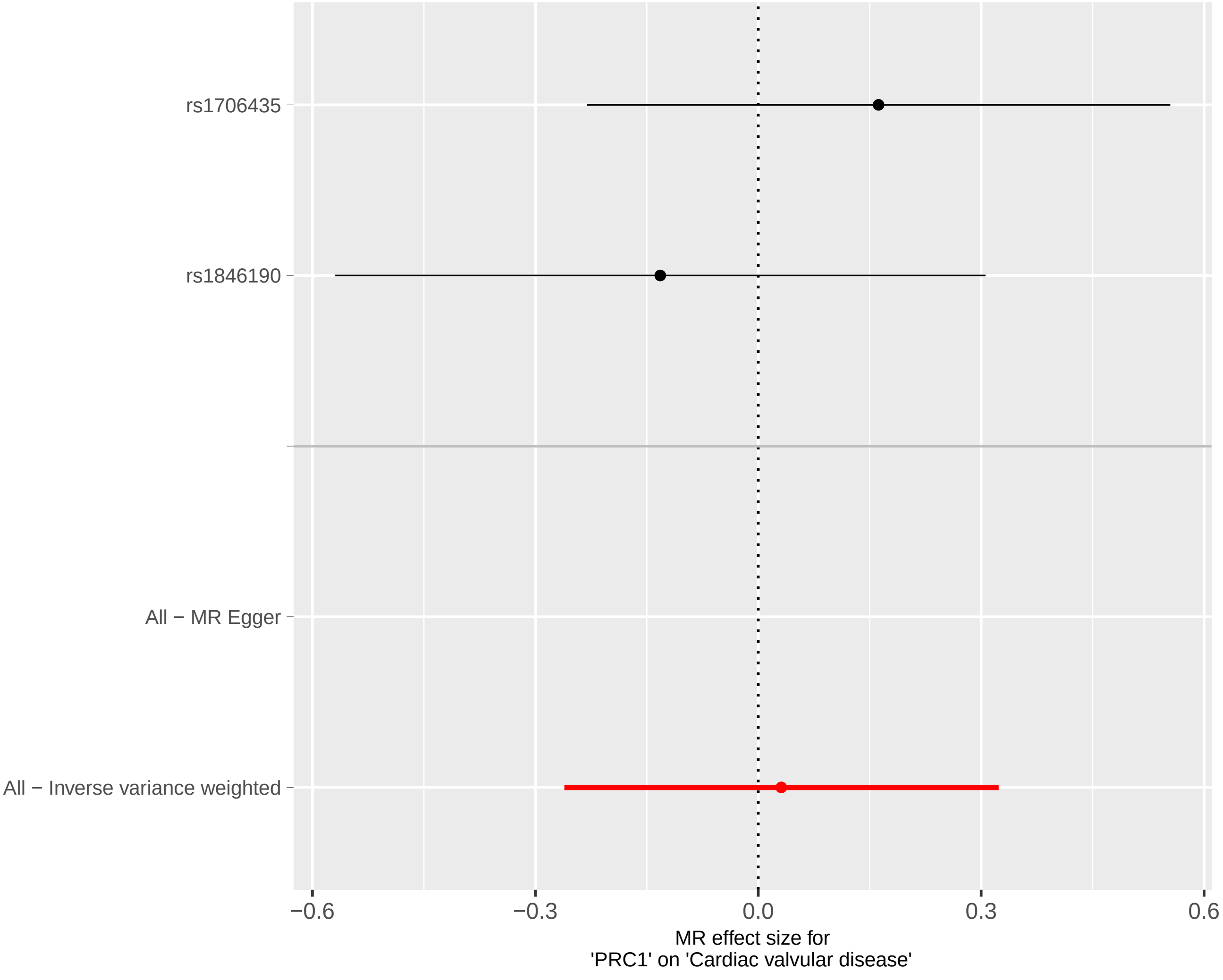
rs1706435
rs1846190
All − MR Egger
All − Inverse variance weighted
−0.6
−0.3
0.0
0.3
0.6
MR effect size for
'PRC1' on 'Cardiac valvular disease'

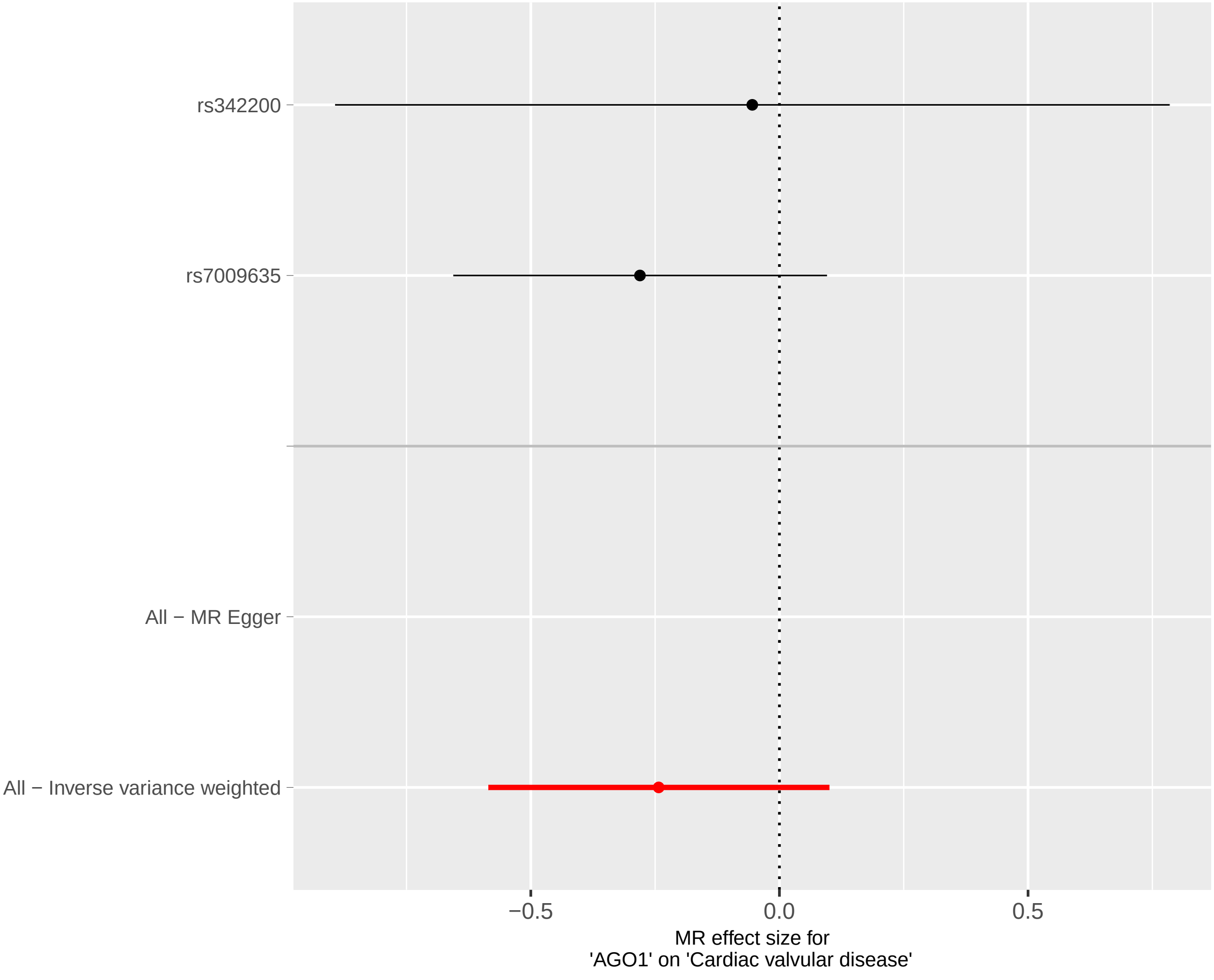

rs342200
rs7009635
All − MR Egger
All − Inverse variance weighted
−0.5
0.0
0.5
MR effect size for
'AGO1' on 'Cardiac valvular disease'

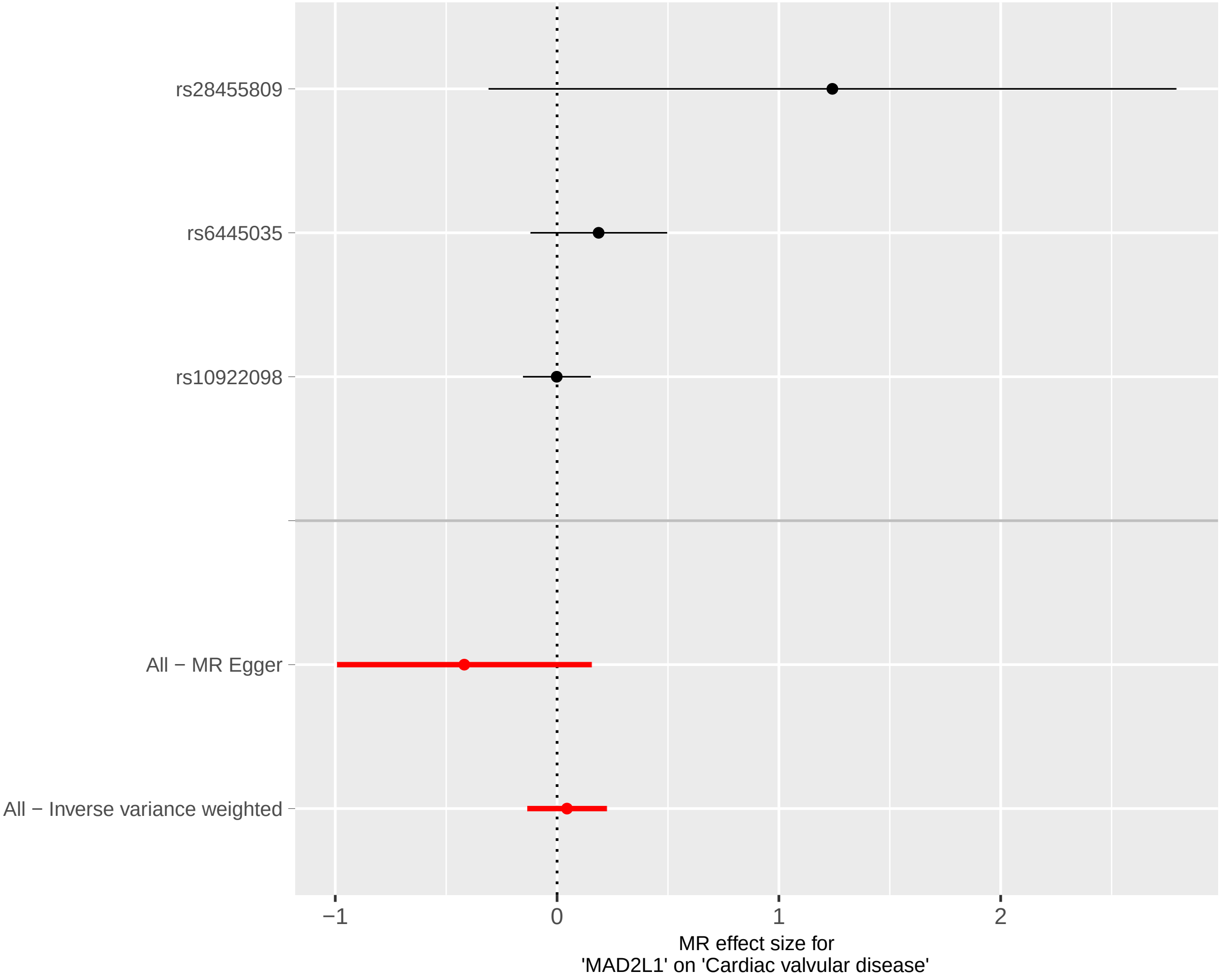
rs28455809
rs6445035
rs10922098
All − MR Egger
All − Inverse variance weighted
−1
0
1
2
MR effect size for
'MAD2L1' on 'Cardiac valvular disease'

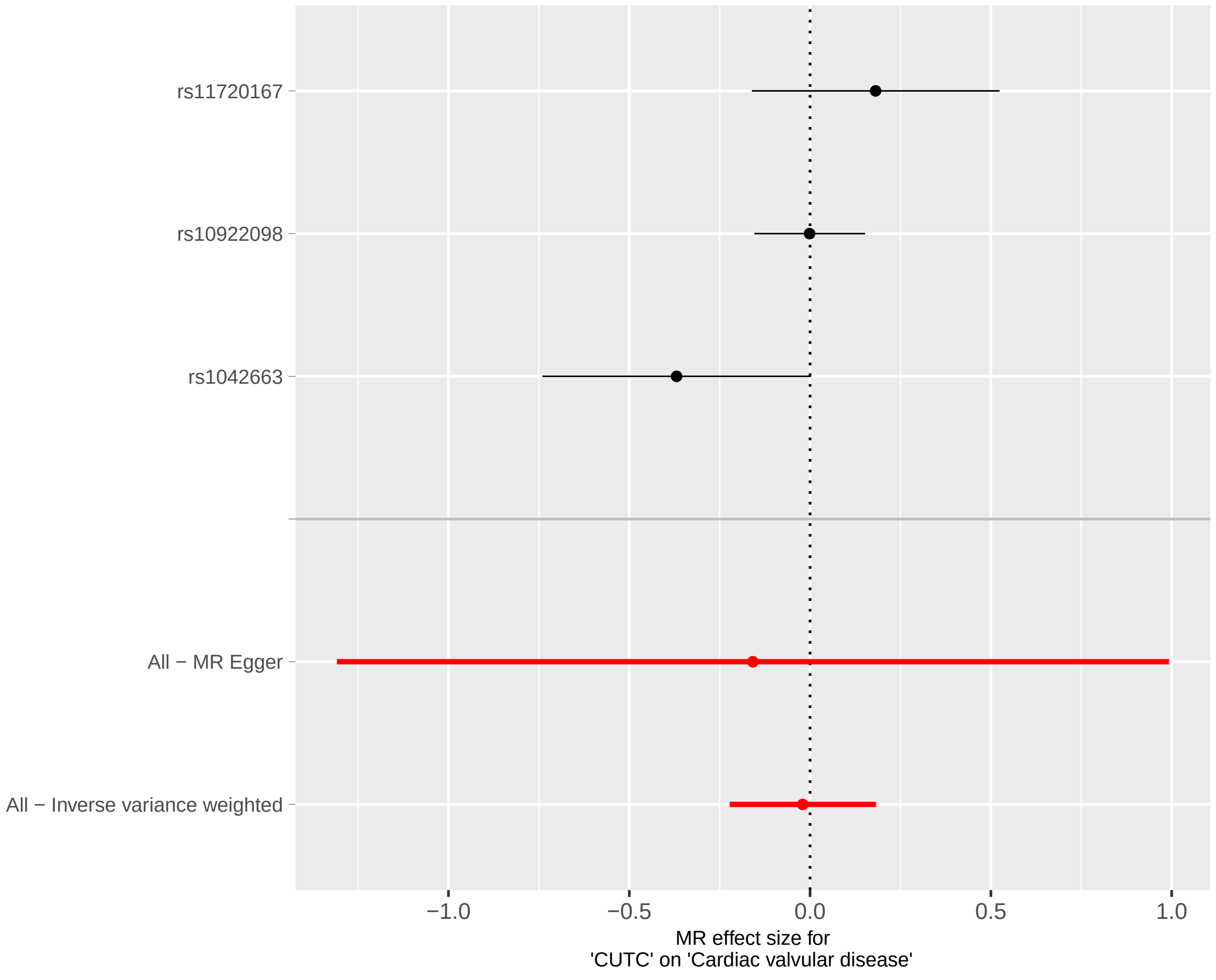
rs11720167
rs10922098
rs1042663
All − MR Egger
All − Inverse variance weighted
−1.0
−0.5
0.0
0.5
1.0
MR effect size for
'CUTC' on 'Cardiac valvular disease'

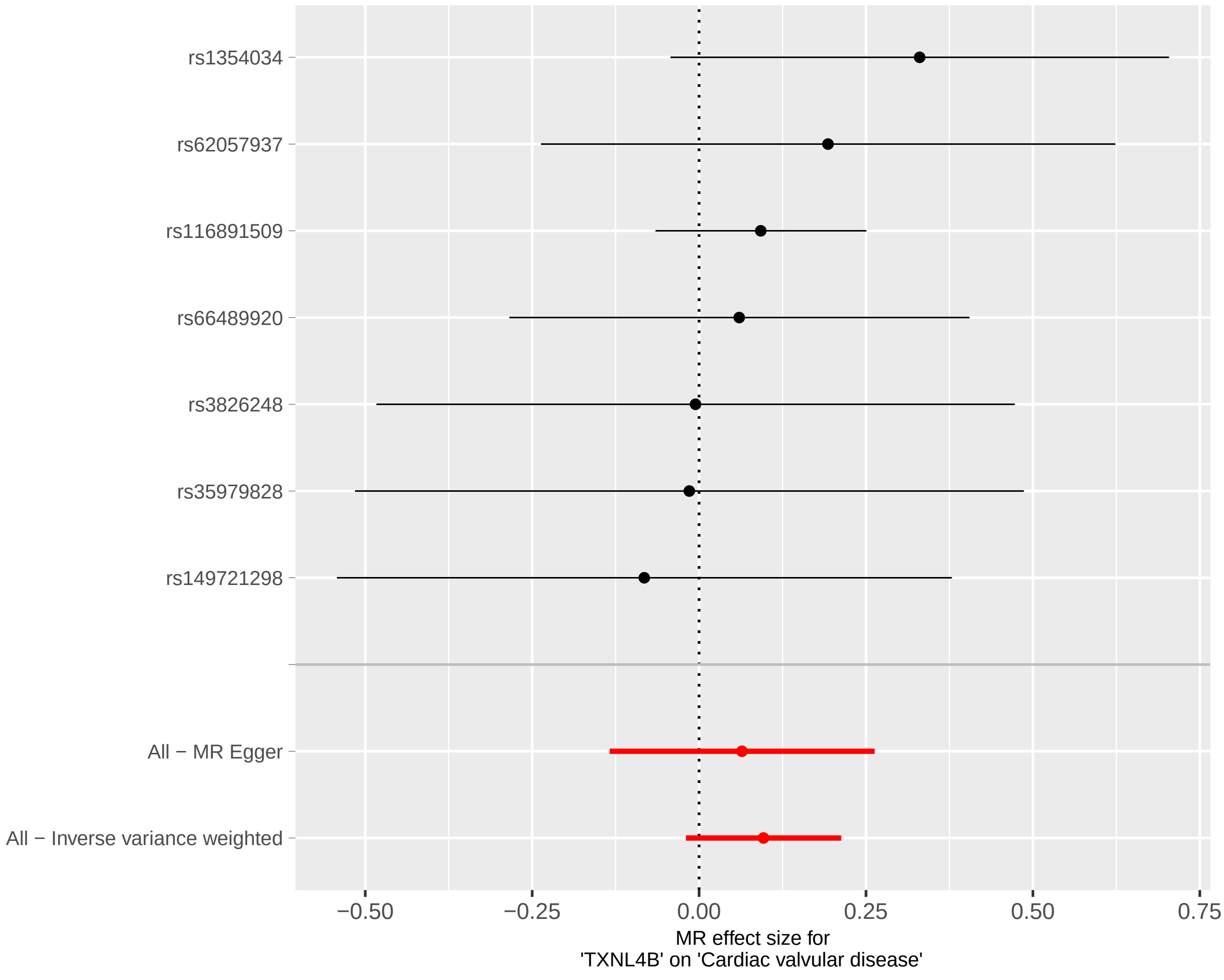
rs1354034
rs62057937
rs116891509
rs66489920
rs3826248
rs35979828
rs149721298
All − MR Egger
All − Inverse variance weighted
−0.50
−0.25
0.00
0.25
0.50
0.75
MR effect size for
'TXNL4B' on 'Cardiac valvular disease'

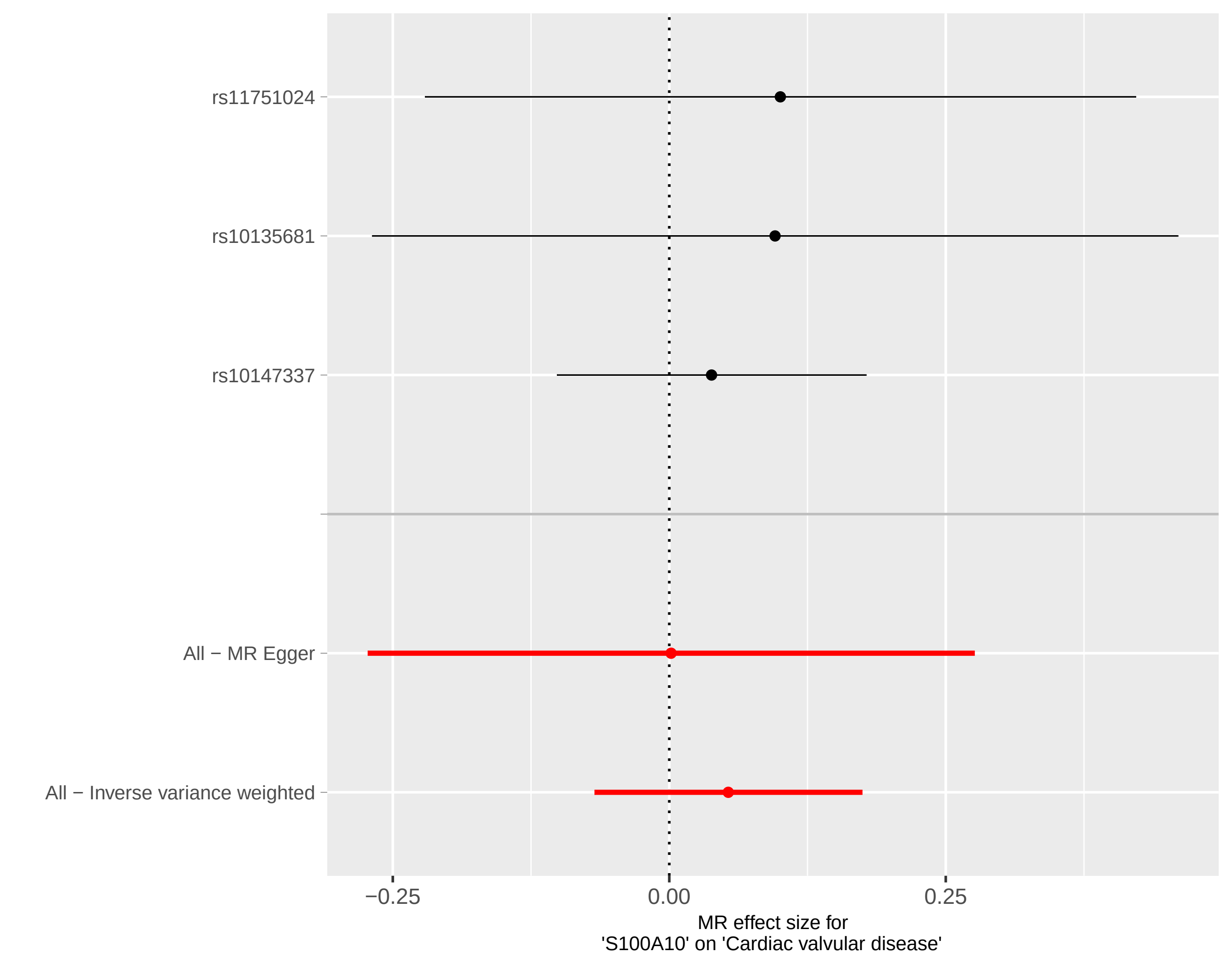

rs11751024
rs10135681
rs10147337
All − MR Egger
All − Inverse variance weighted
−0.25
0.00
0.25
MR effect size for
'S100A10' on 'Cardiac valvular disease'

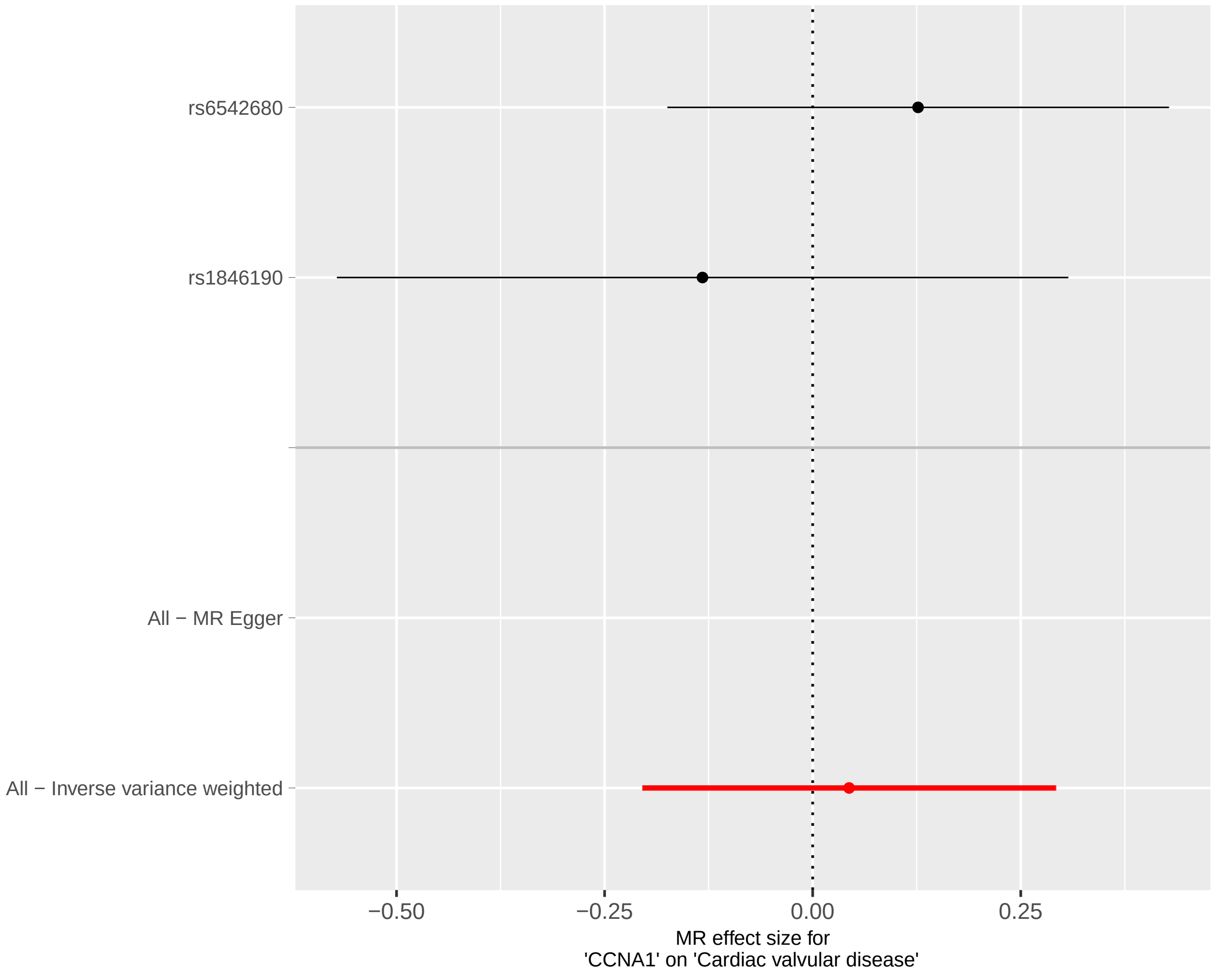
rs6542680
rs1846190
All − MR Egger
All − Inverse variance weighted
−0.50
−0.25
0.00
0.25
MR effect size for
'CCNA1' on 'Cardiac valvular disease'

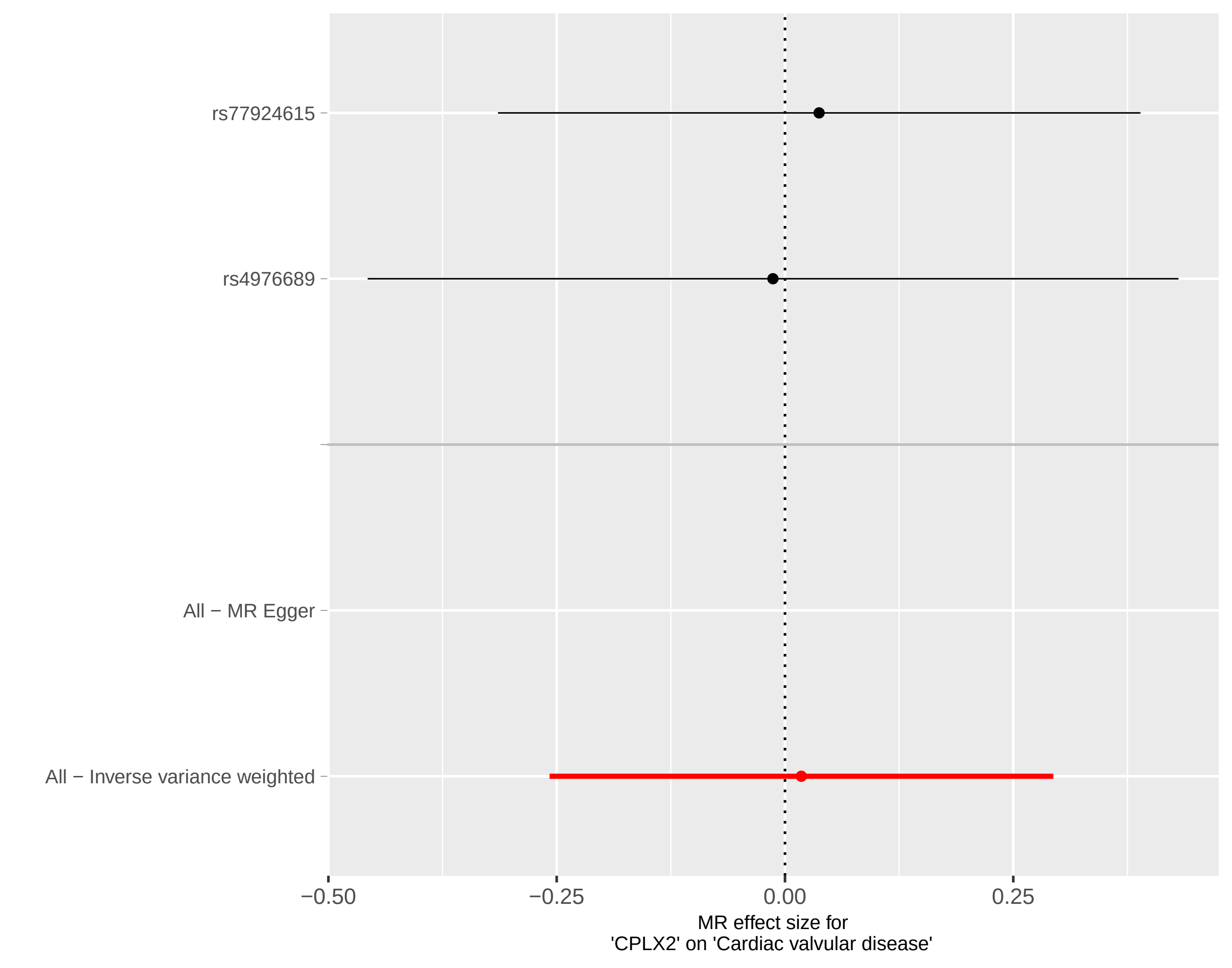
rs77924615
rs4976689
All − MR Egger
All − Inverse variance weighted
−0.50
−0.25
0.00
0.25
MR effect size for
'CPLX2' on 'Cardiac valvular disease'

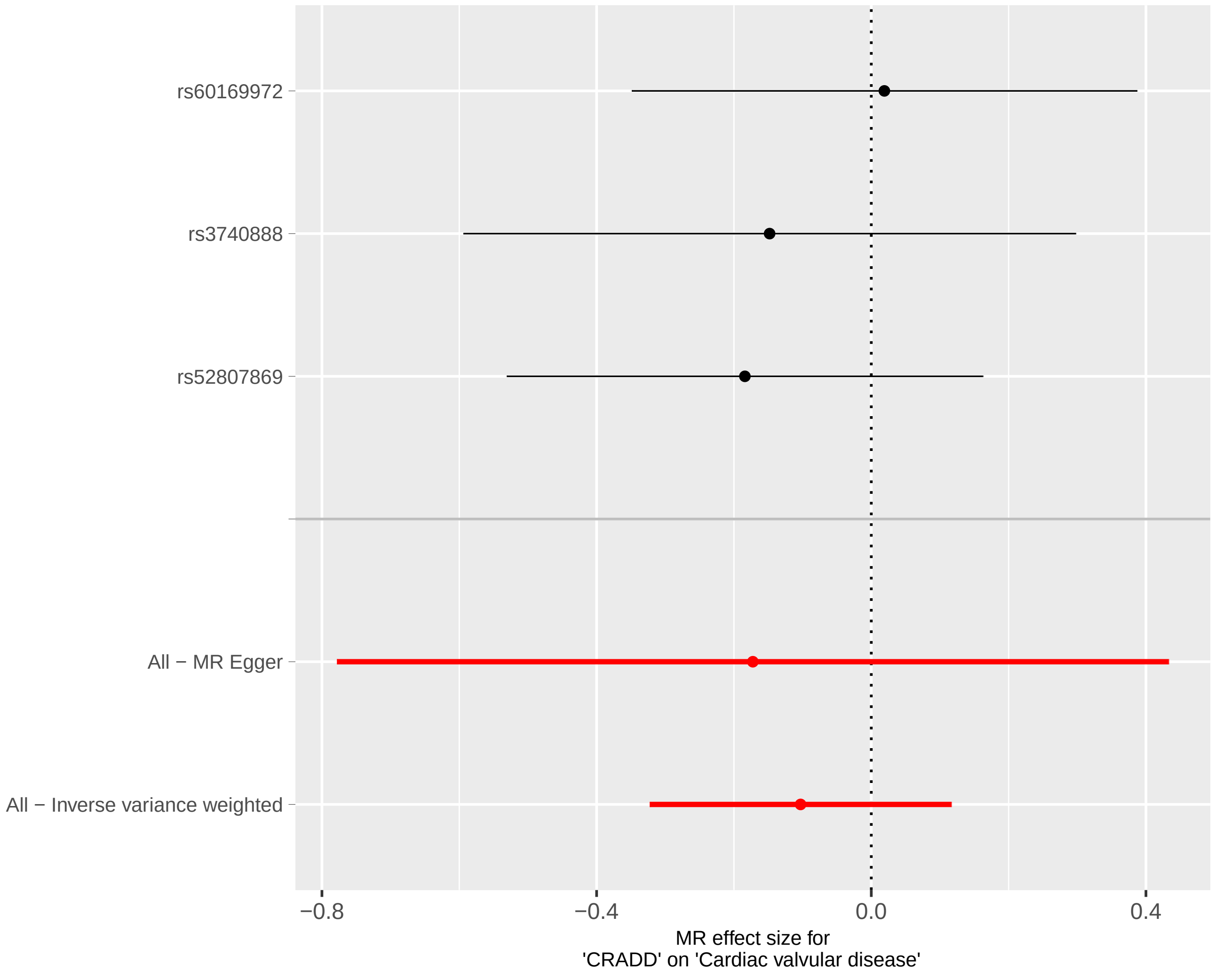
rs60169972
rs3740888
rs52807869
All − MR Egger
All − Inverse variance weighted
−0.8
−0.4
0.0
0.4
MR effect size for
'CRADD' on 'Cardiac valvular disease'

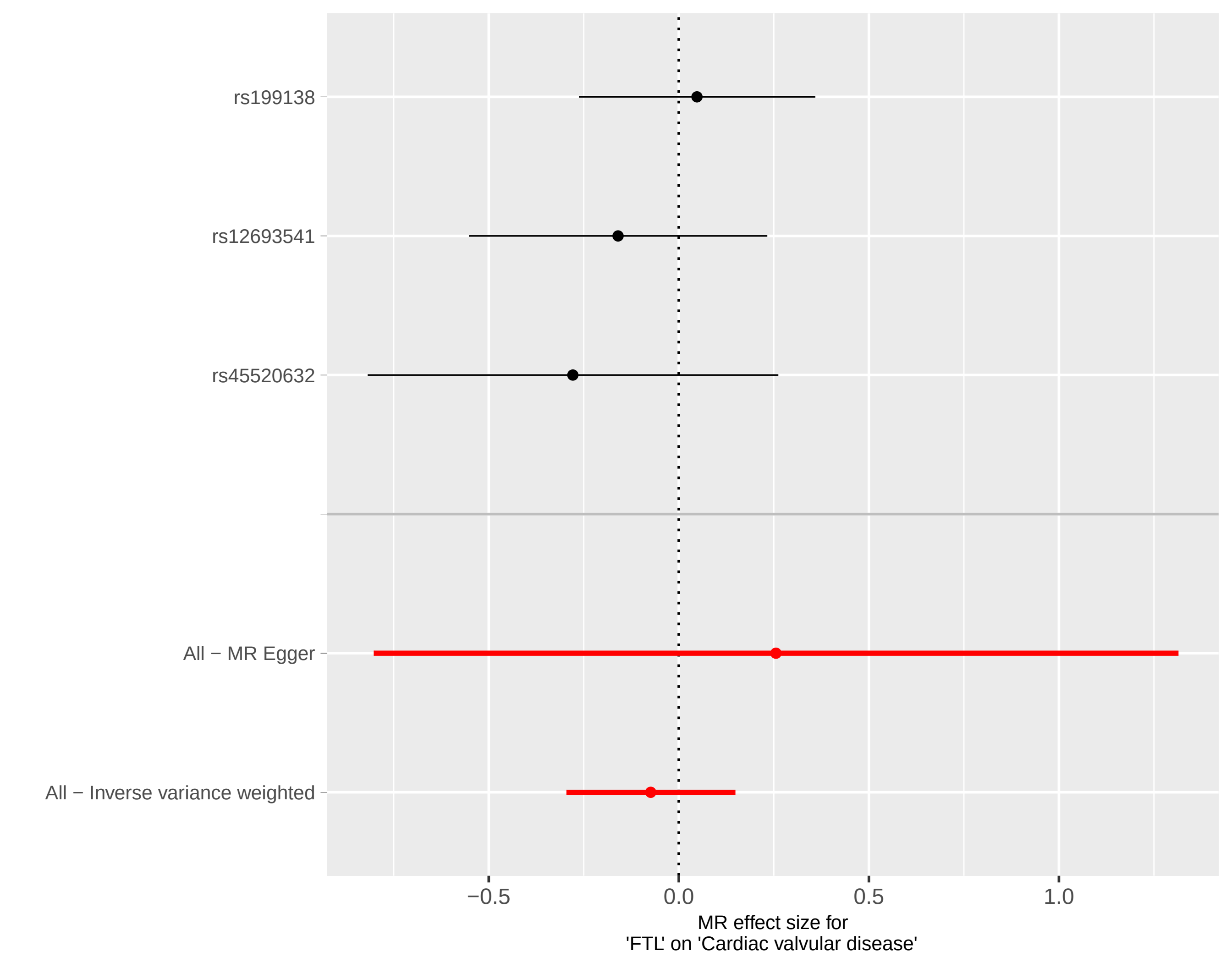
rs199138
rs12693541
rs45520632
All − MR Egger
All − Inverse variance weighted
−0.5
0.0
0.5
1.0
MR effect size for
'FTL' on 'Cardiac valvular disease'

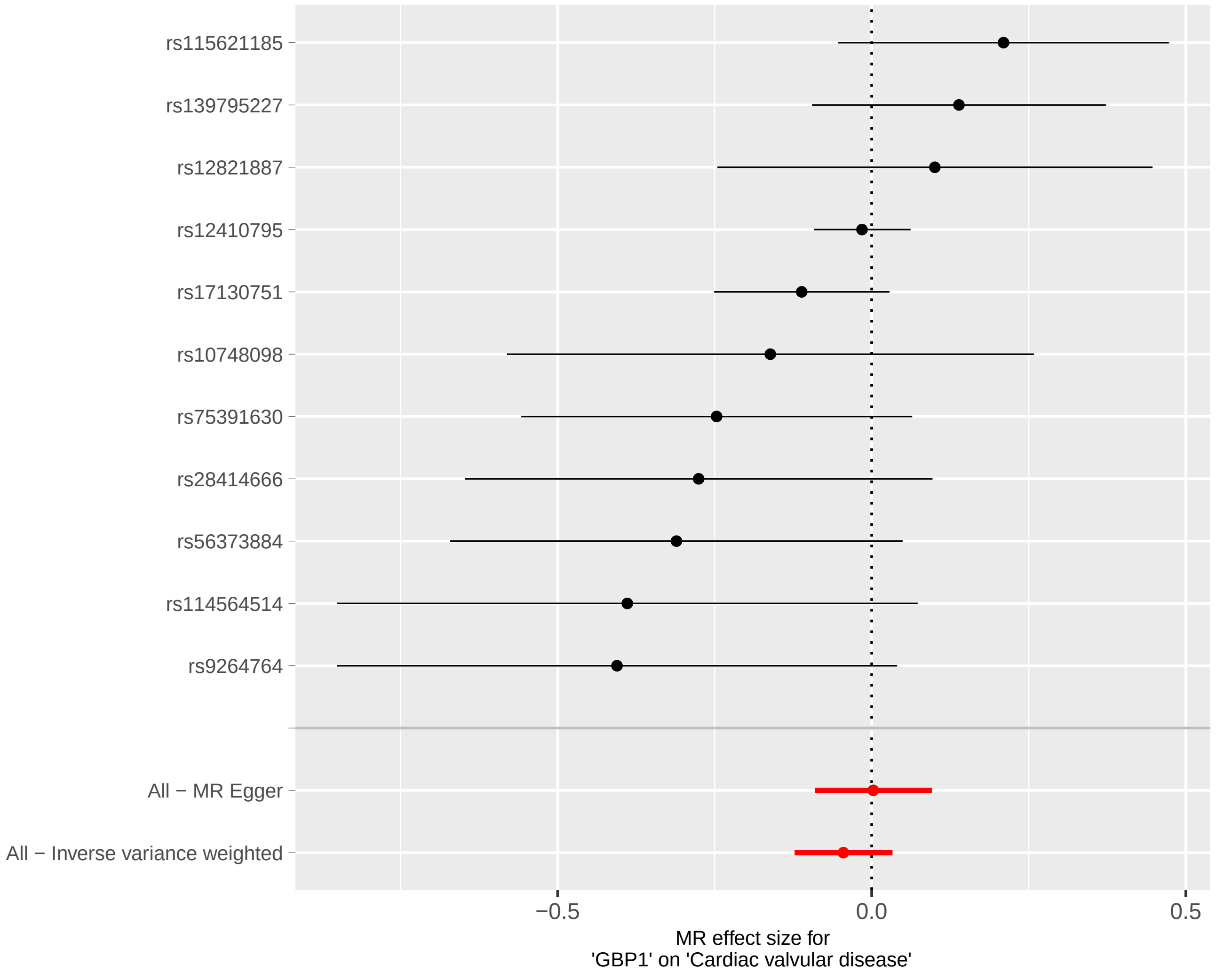

rs115621185
rs139795227
rs12821887
rs12410795
rs17130751
rs10748098
rs75391630
rs28414666
rs56373884
rs114564514
rs9264764
All − MR Egger
All − Inverse variance weighted
−0.5
0.0
0.5
MR effect size for
'GBP1' on 'Cardiac valvular disease'

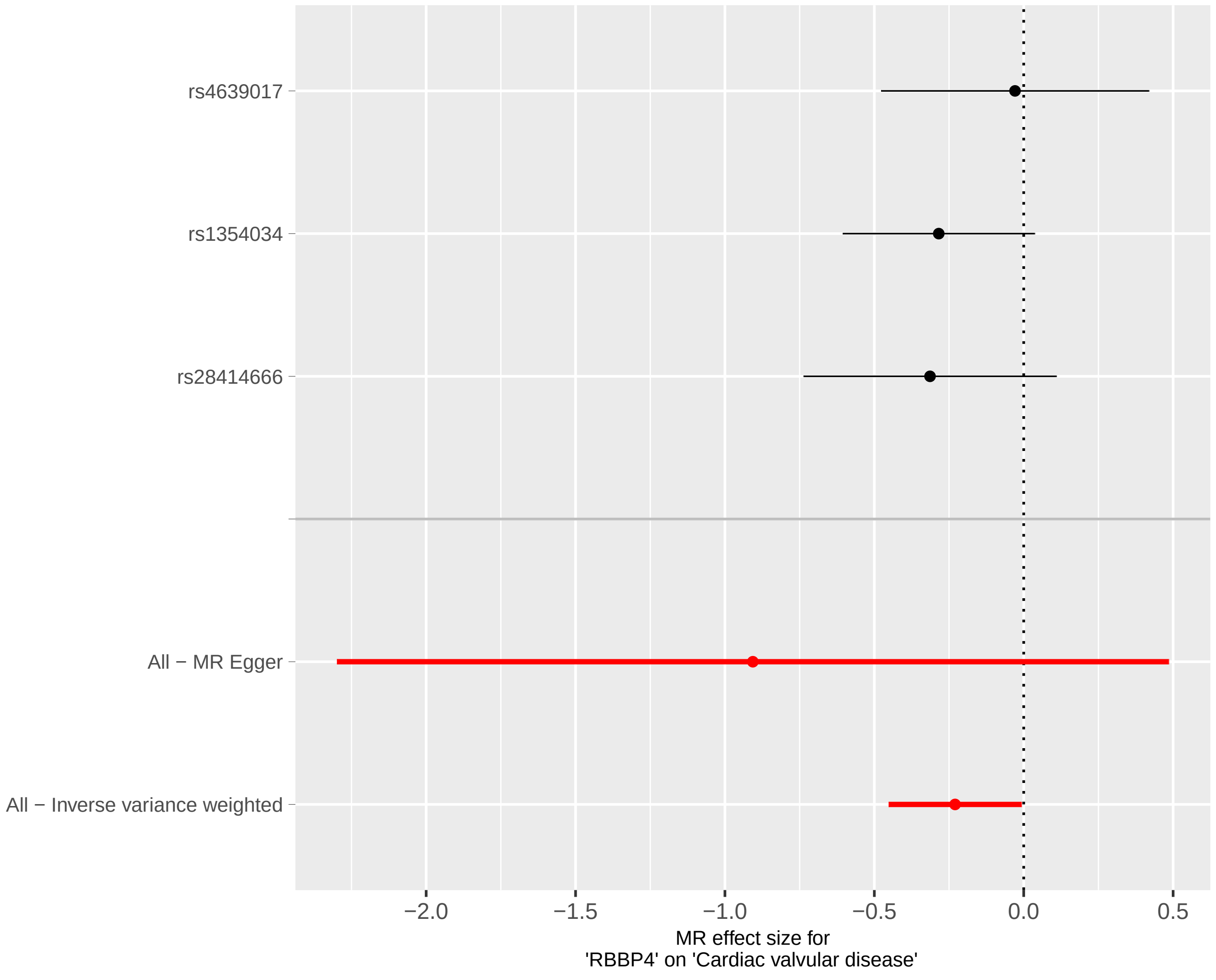
rs4639017
rs1354034
rs28414666
All − MR Egger
All − Inverse variance weighted
−2.0
−1.5
−1.0
−0.5
0.0
0.5
MR effect size for
'RBBP4' on 'Cardiac valvular disease'

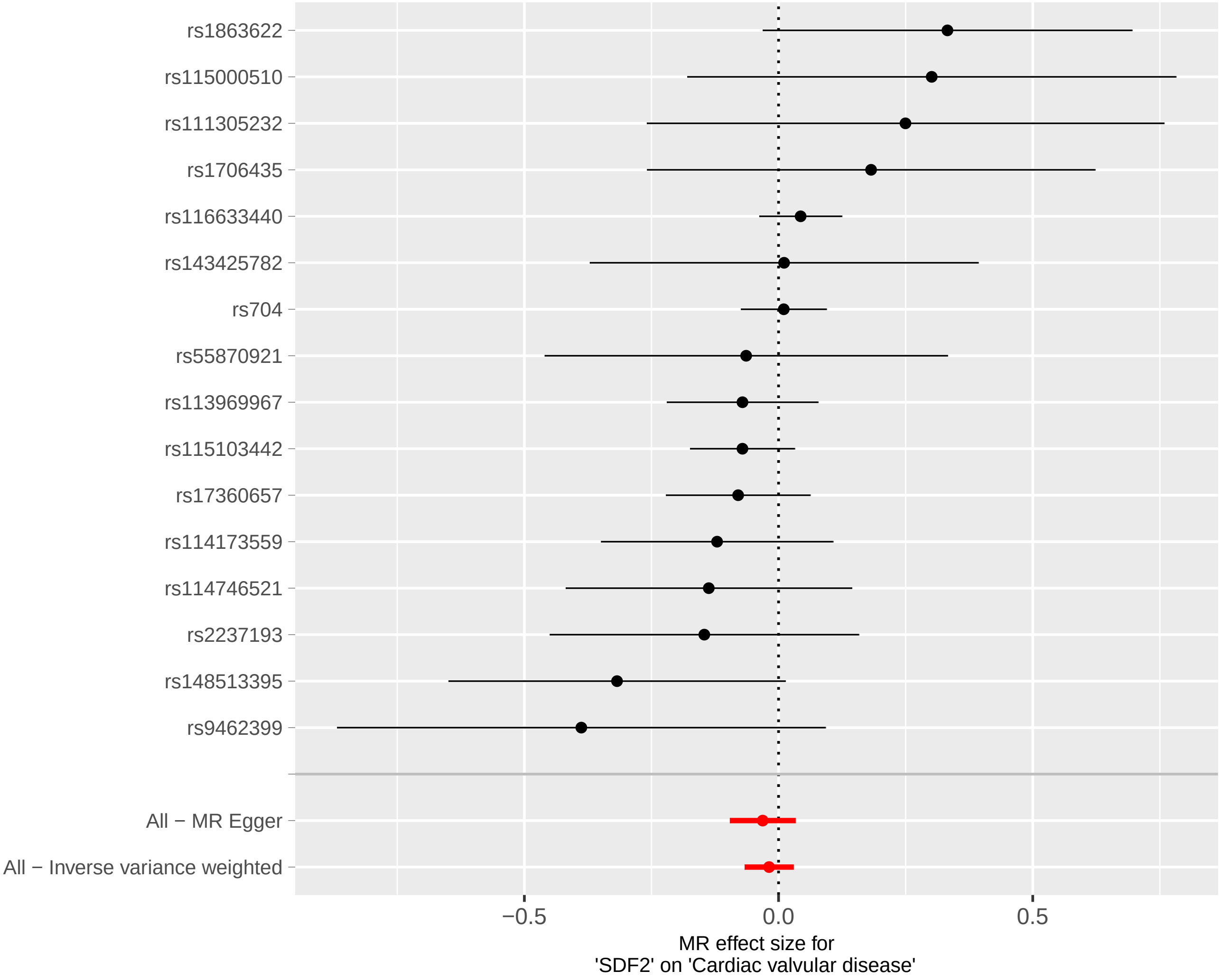
rs1863622
rs115000510
rs111305232
rs1706435
rs116633440
rs143425782
rs704
rs55870921
rs113969967
rs115103442
rs17360657
rs114173559
rs114746521
rs2237193
rs148513395
rs9462399
All − MR Egger
All − Inverse variance weighted
−0.5
0.0
0.5
MR effect size for
'SDF2' on 'Cardiac valvular disease'

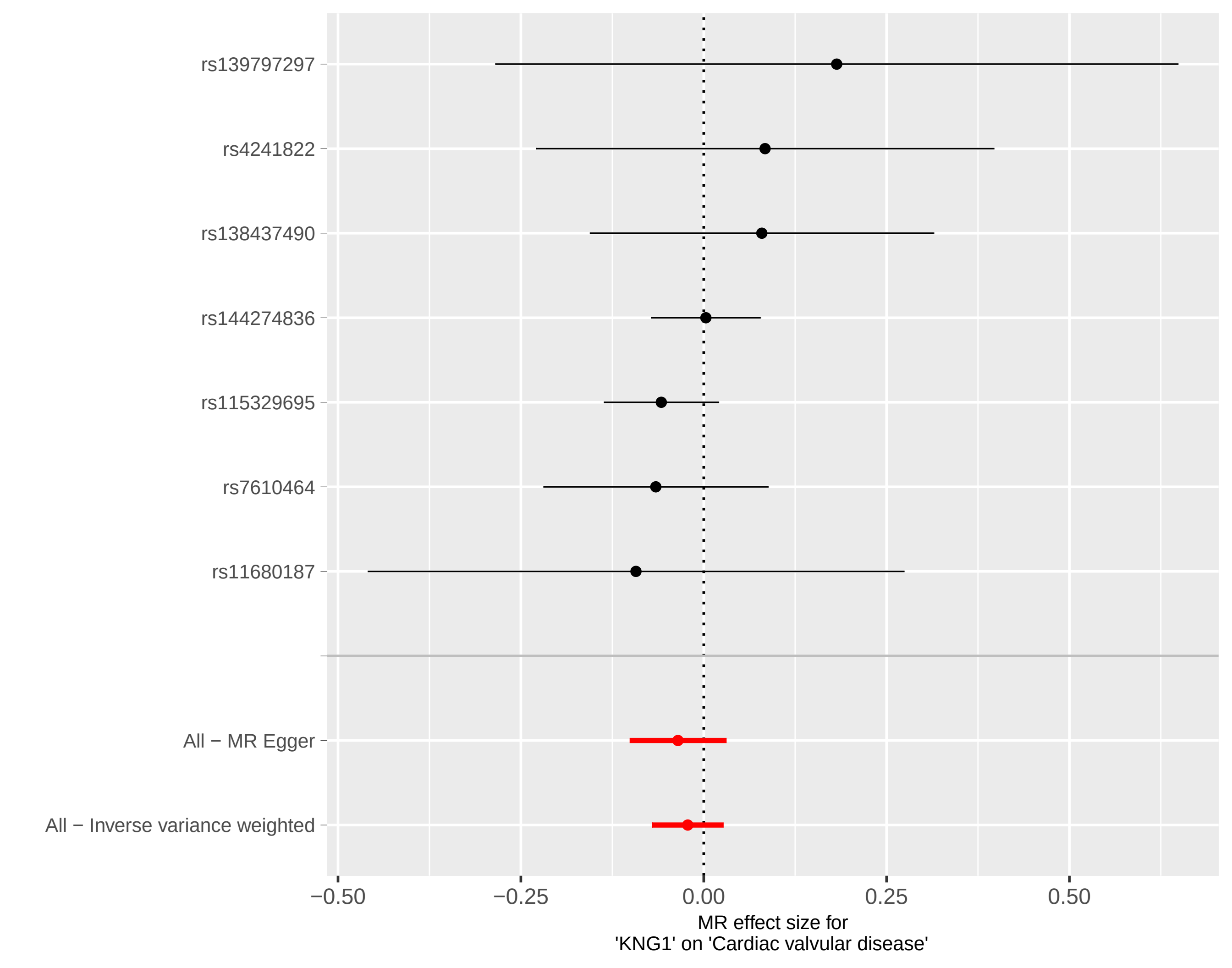
rs139797297
rs4241822
rs138437490
rs144274836
rs115329695
rs7610464
rs11680187
All − MR Egger
All − Inverse variance weighted
−0.50
−0.25
0.00
0.25
0.50
MR effect size for
'KNG1' on 'Cardiac valvular disease'

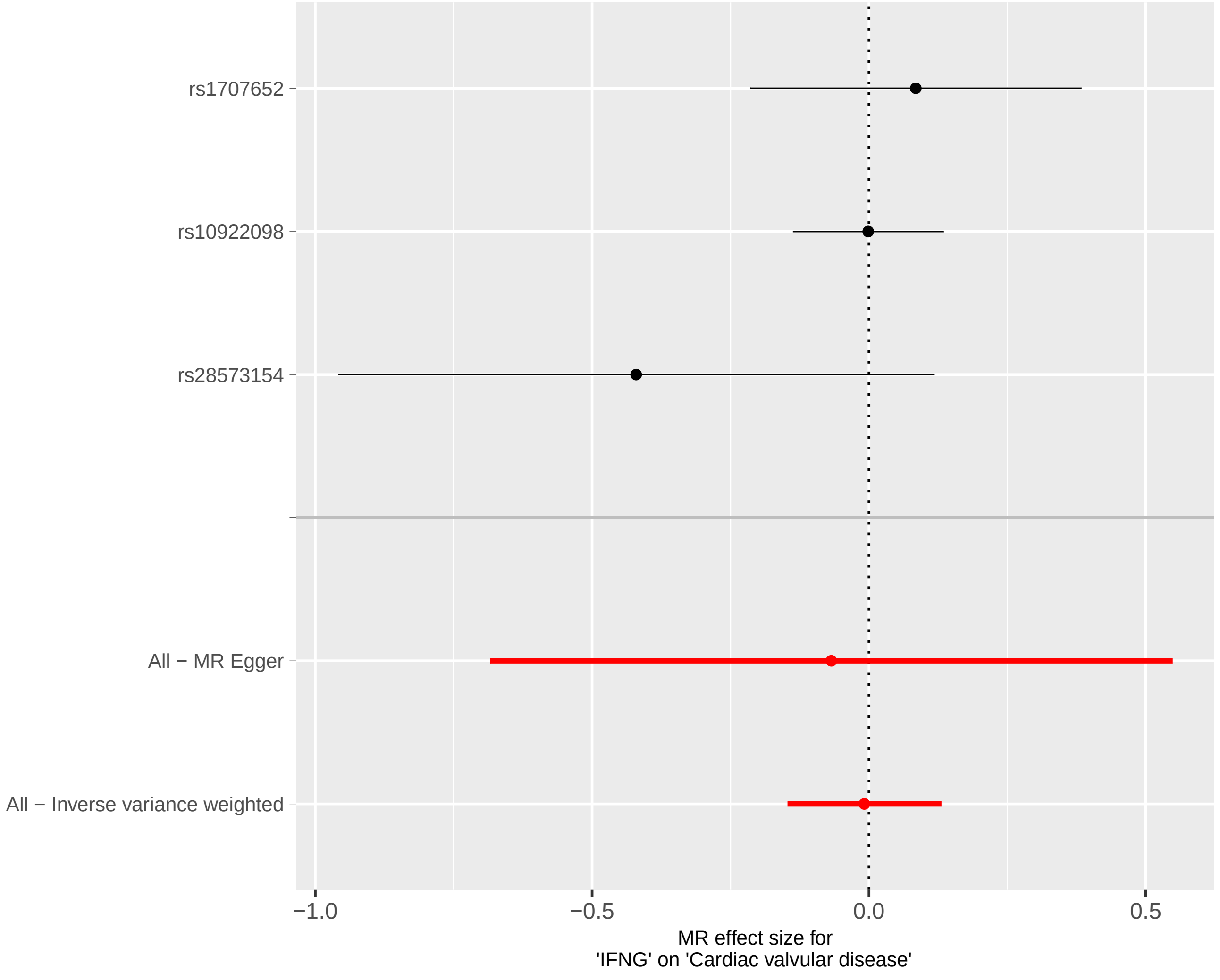

rs1707652
rs10922098
rs28573154
All − MR Egger
All − Inverse variance weighted
−1.0
−0.5
0.0
0.5
MR effect size for
'IFNG' on 'Cardiac valvular disease'

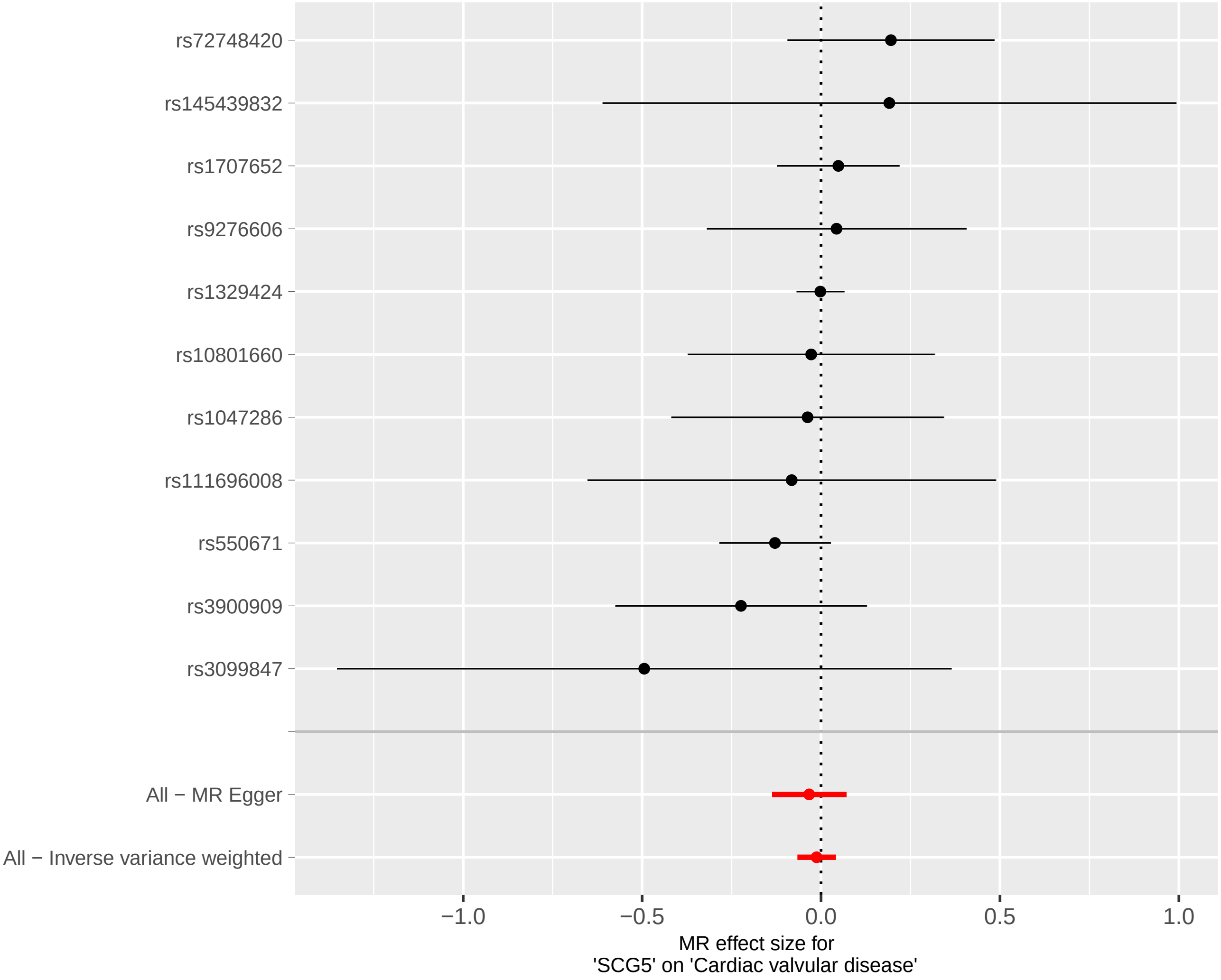
rs72748420
rs145439832
rs1707652
rs9276606
rs1329424
rs10801660
rs1047286
rs111696008
rs550671
rs3900909
rs3099847
All − MR Egger
All − Inverse variance weighted
−1.0
−0.5
0.0
0.5
1.0
MR effect size for
'SCG5' on 'Cardiac valvular disease'

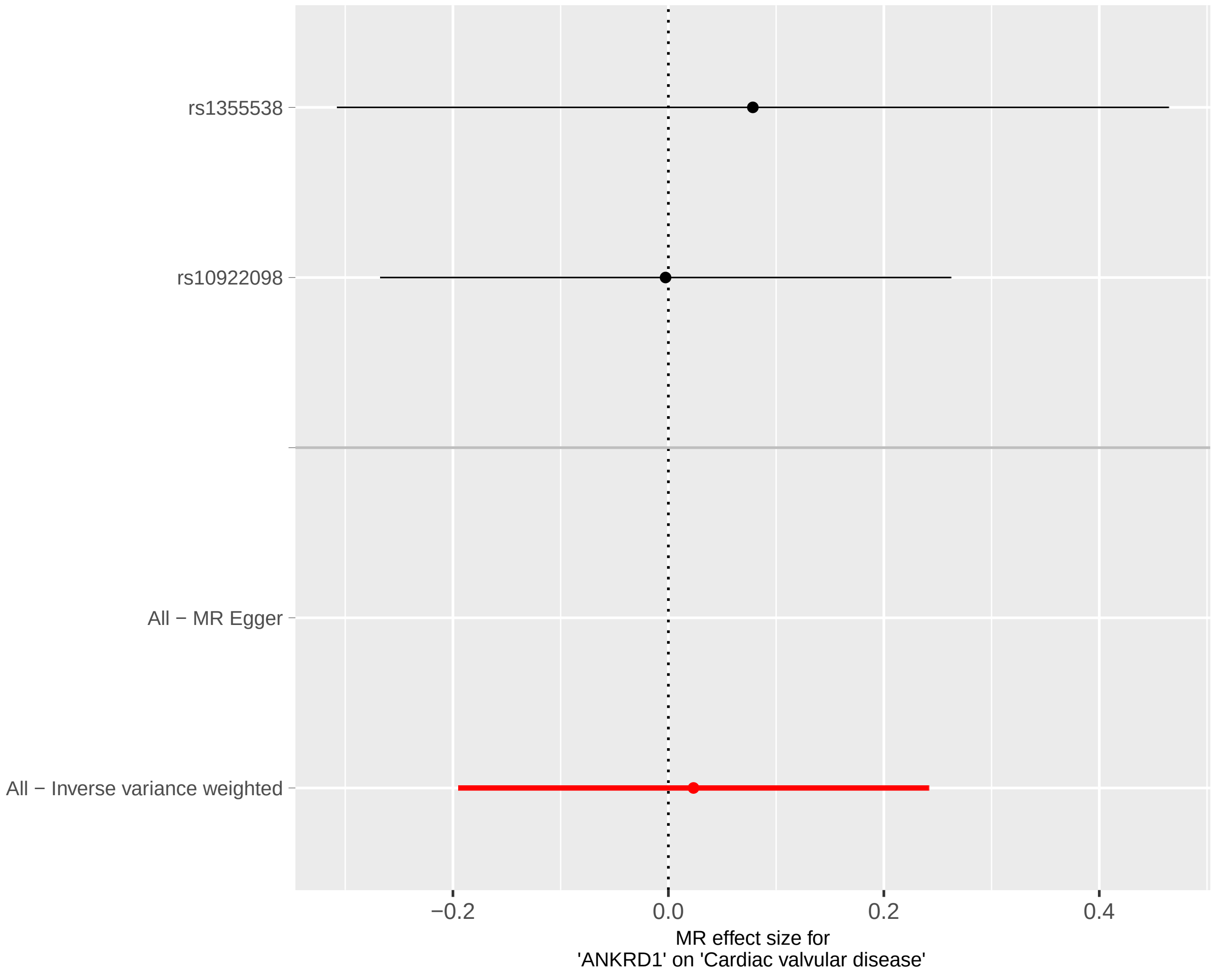
rs1355538
rs10922098
All − MR Egger
All − Inverse variance weighted
−0.2
0.0
0.2
0.4
MR effect size for
'ANKRD1' on 'Cardiac valvular disease'

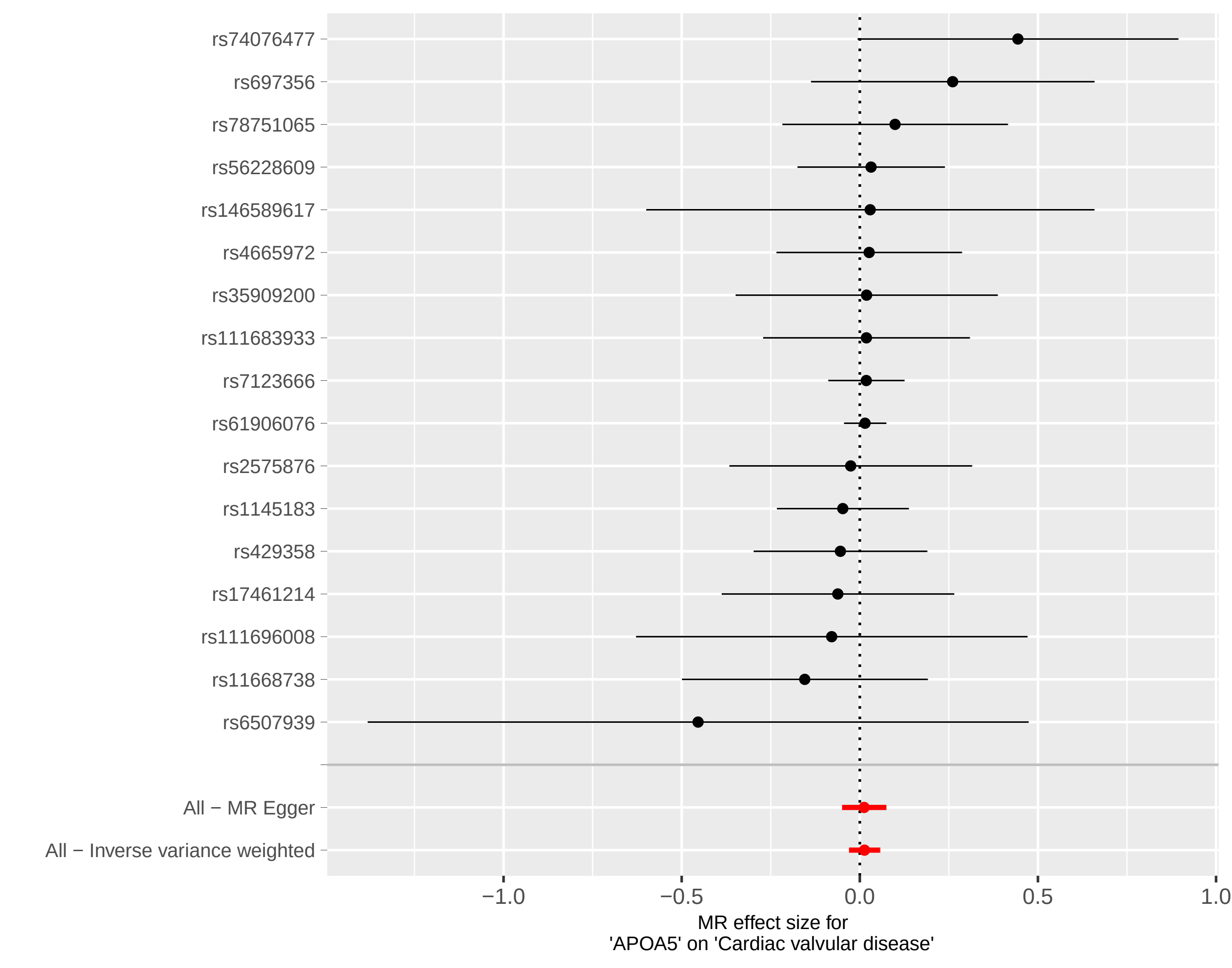

rs74076477
rs697356
rs78751065
rs56228609
rs146589617
rs4665972
rs35909200
rs111683933
rs7123666
rs61906076
rs2575876
rs1145183
rs429358
rs17461214
rs111696008
rs11668738
rs6507939
All − MR Egger
All − Inverse variance weighted
−1.0
−0.5
0.0
0.5
1.0
MR effect size for
'APOA5' on 'Cardiac valvular disease'

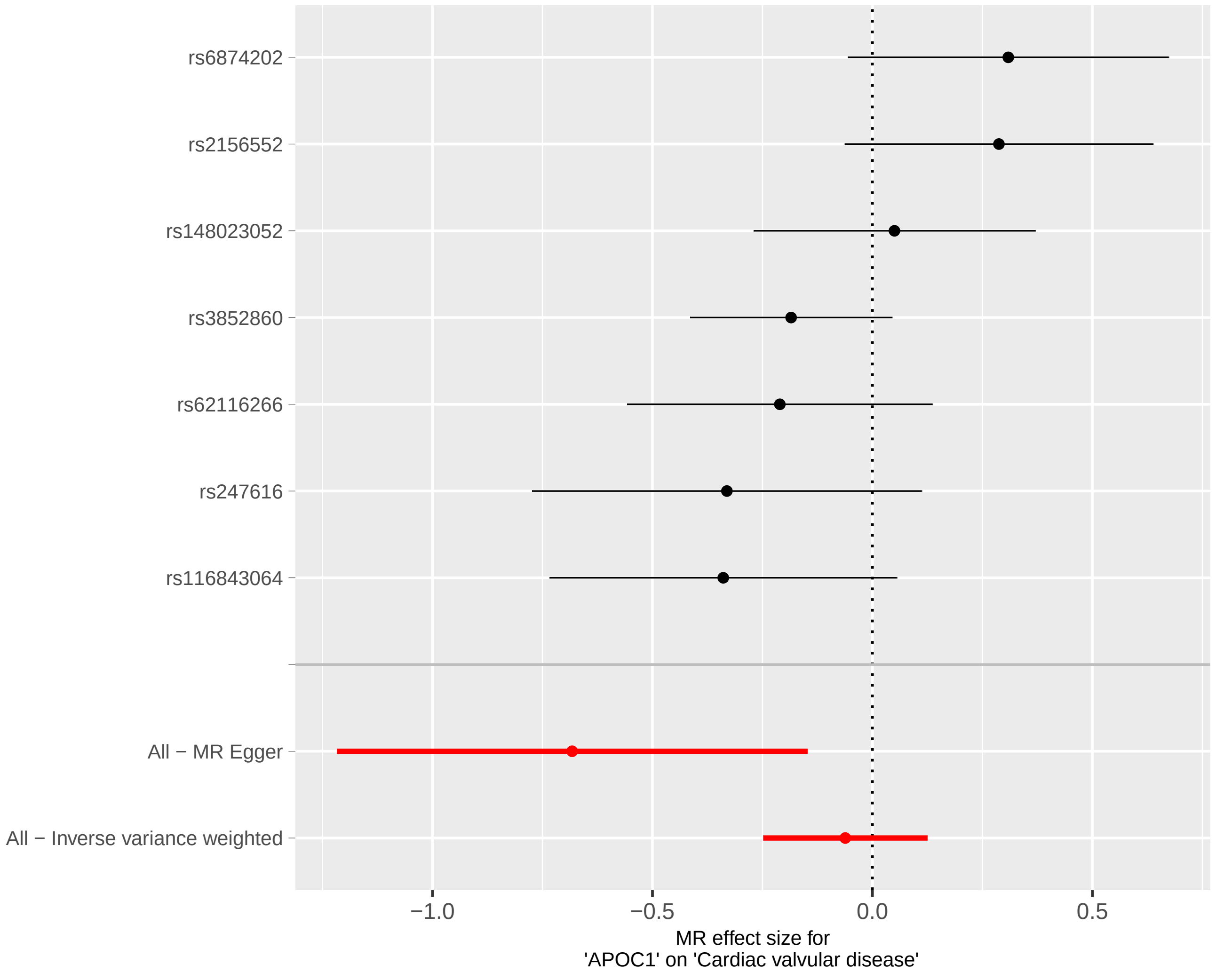

rs6874202
rs2156552
rs148023052
rs3852860
rs62116266
rs247616
rs116843064
All − MR Egger
All − Inverse variance weighted
−1.0
−0.5
0.0
0.5
MR effect size for
'APOC1' on 'Cardiac valvular disease'

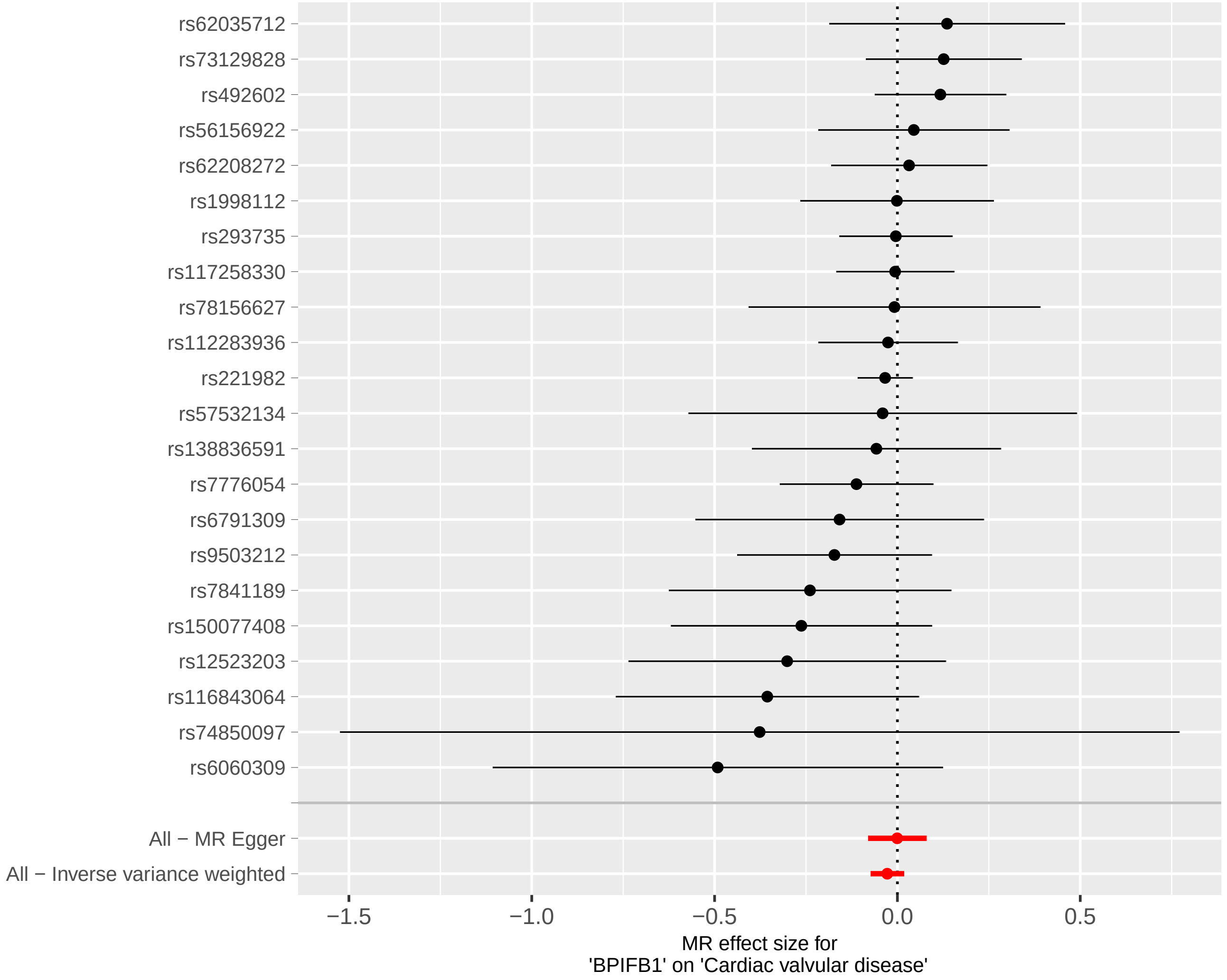

rs62035712
rs73129828
rs492602
rs56156922
rs62208272
rs1998112
rs293735
rs117258330
rs78156627
rs112283936
rs221982
rs57532134
rs138836591
rs7776054
rs6791309
rs9503212
rs7841189
rs150077408
rs12523203
rs116843064
rs74850097
rs6060309
All − MR Egger
All − Inverse variance weighted
−1.5
−1.0
−0.5
0.0
0.5
MR effect size for
'BPIFB1' on 'Cardiac valvular disease'

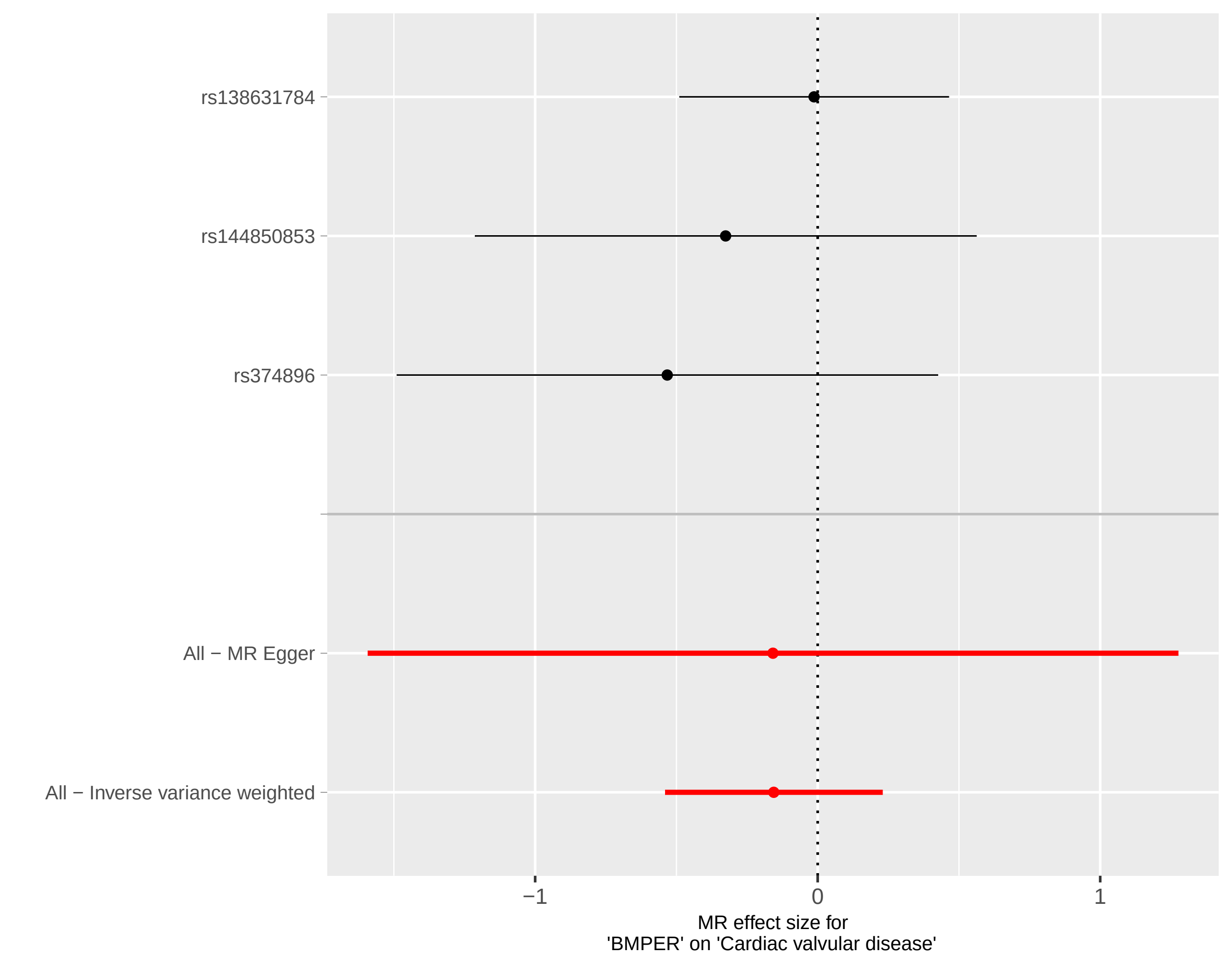

rs138631784
rs144850853
rs374896
All − MR Egger
All − Inverse variance weighted
−1
0
1
MR effect size for
'BMPER' on 'Cardiac valvular disease'

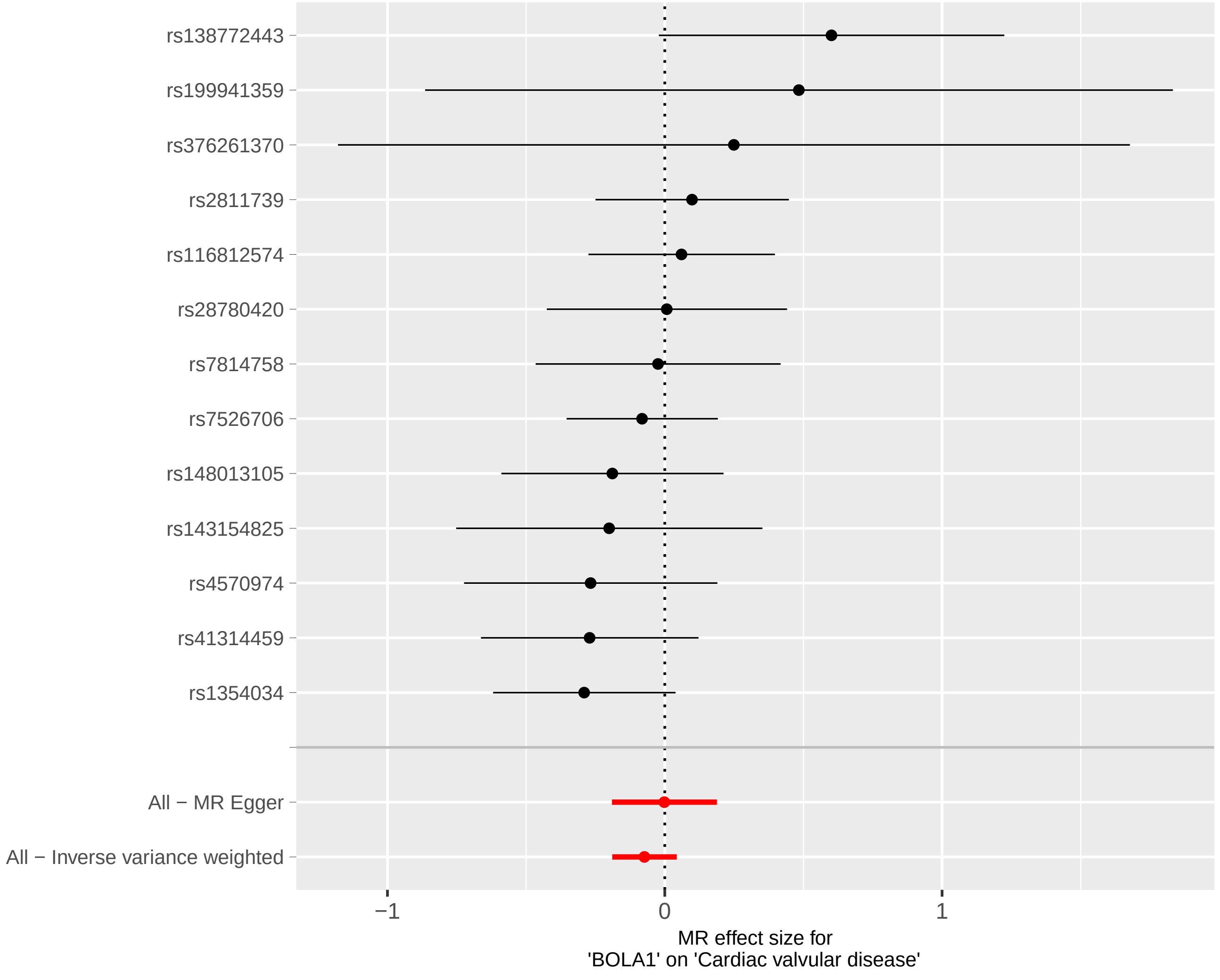

rs138772443
rs199941359
rs376261370
rs2811739
rs116812574
rs28780420
rs7814758
rs7526706
rs148013105
rs143154825
rs4570974
rs41314459
rs1354034
All − MR Egger
All − Inverse variance weighted
−1
0
1
MR effect size for
'BOLA1' on 'Cardiac valvular disease'

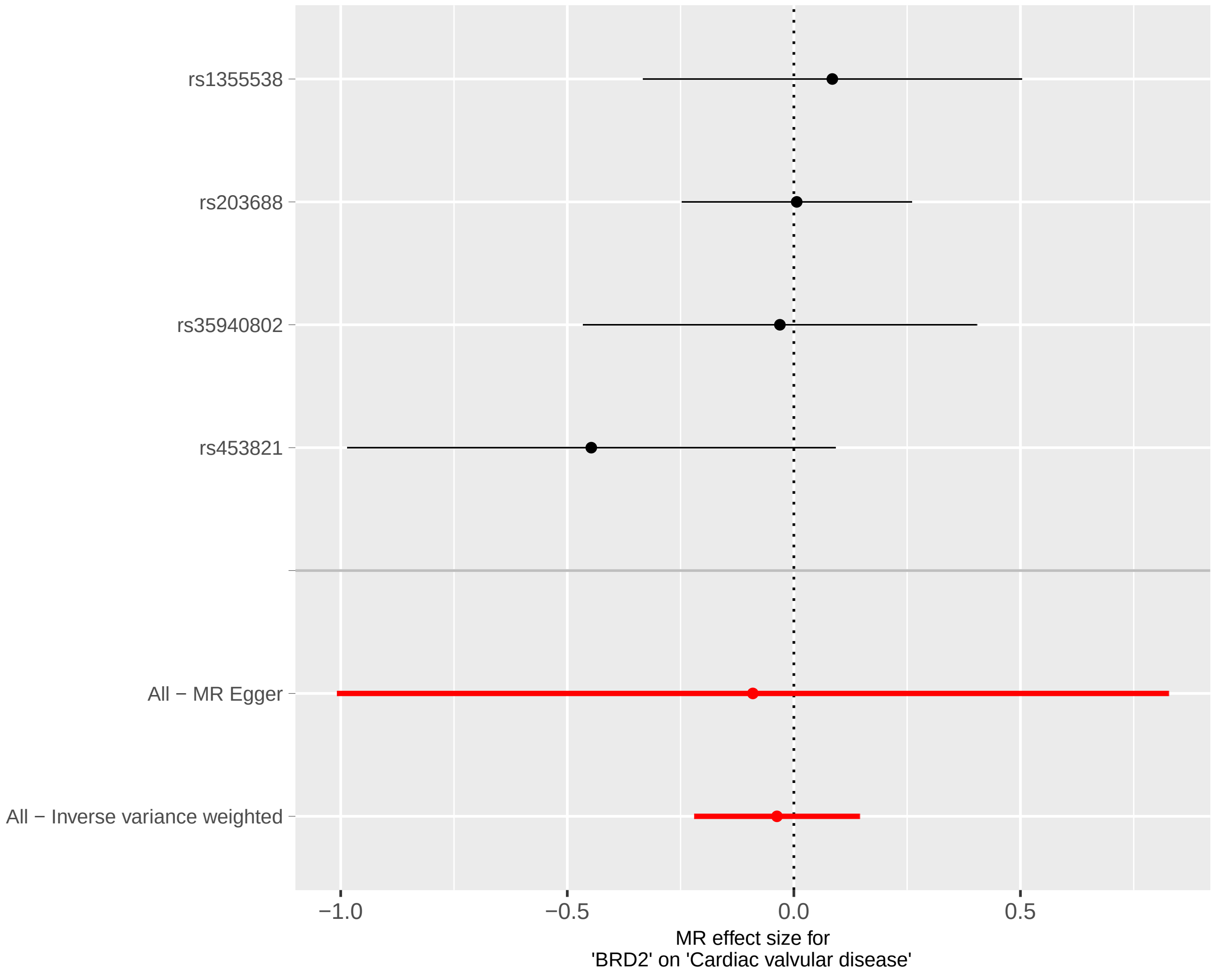
rs1355538
rs203688
rs35940802
rs453821
All − MR Egger
All − Inverse variance weighted
−1.0
−0.5
0.0
0.5
MR effect size for
'BRD2' on 'Cardiac valvular disease'

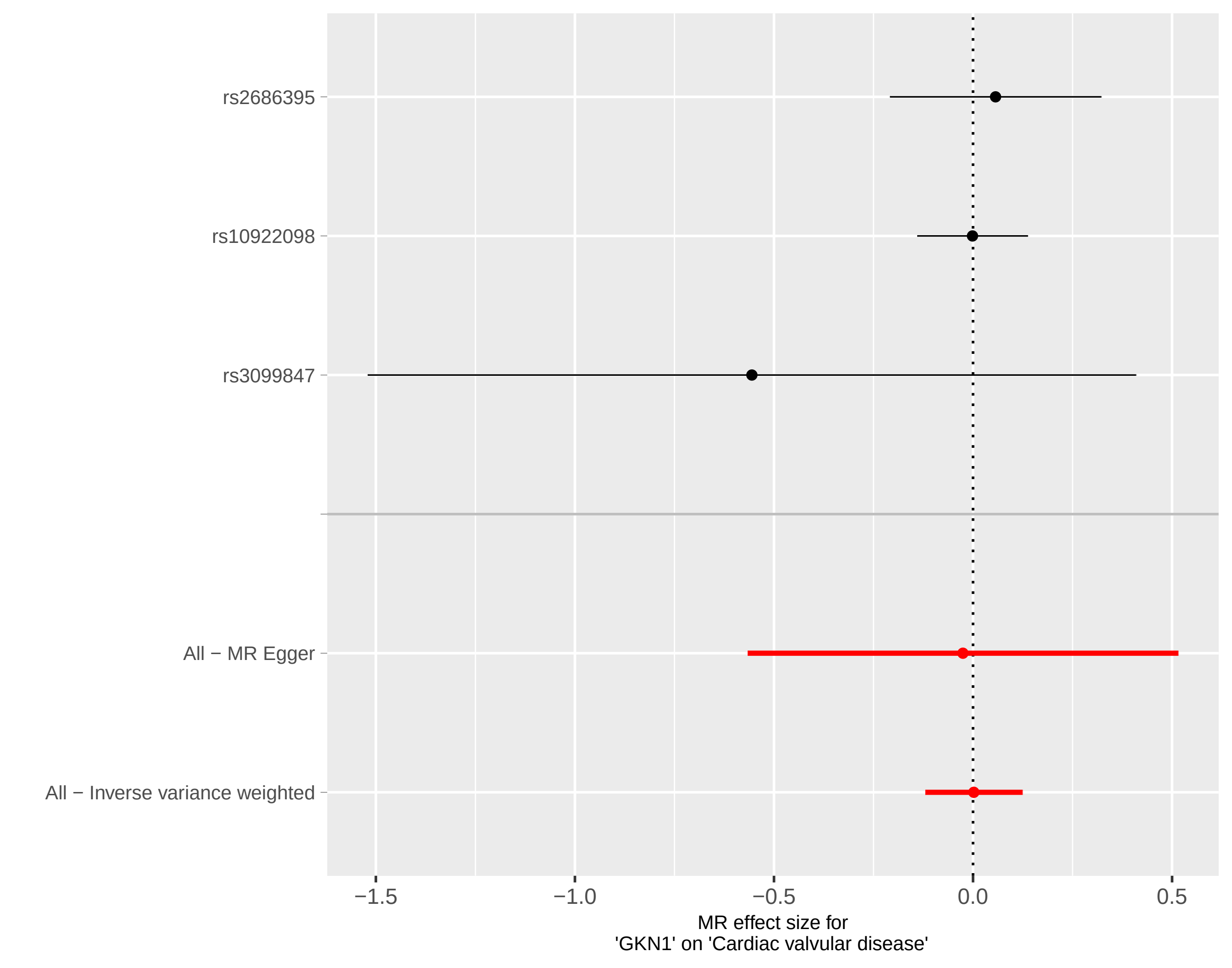

rs2686395
rs10922098
rs3099847
All − MR Egger
All − Inverse variance weighted
−1.5
−1.0
−0.5
0.0
0.5
MR effect size for
'GKN1' on 'Cardiac valvular disease'

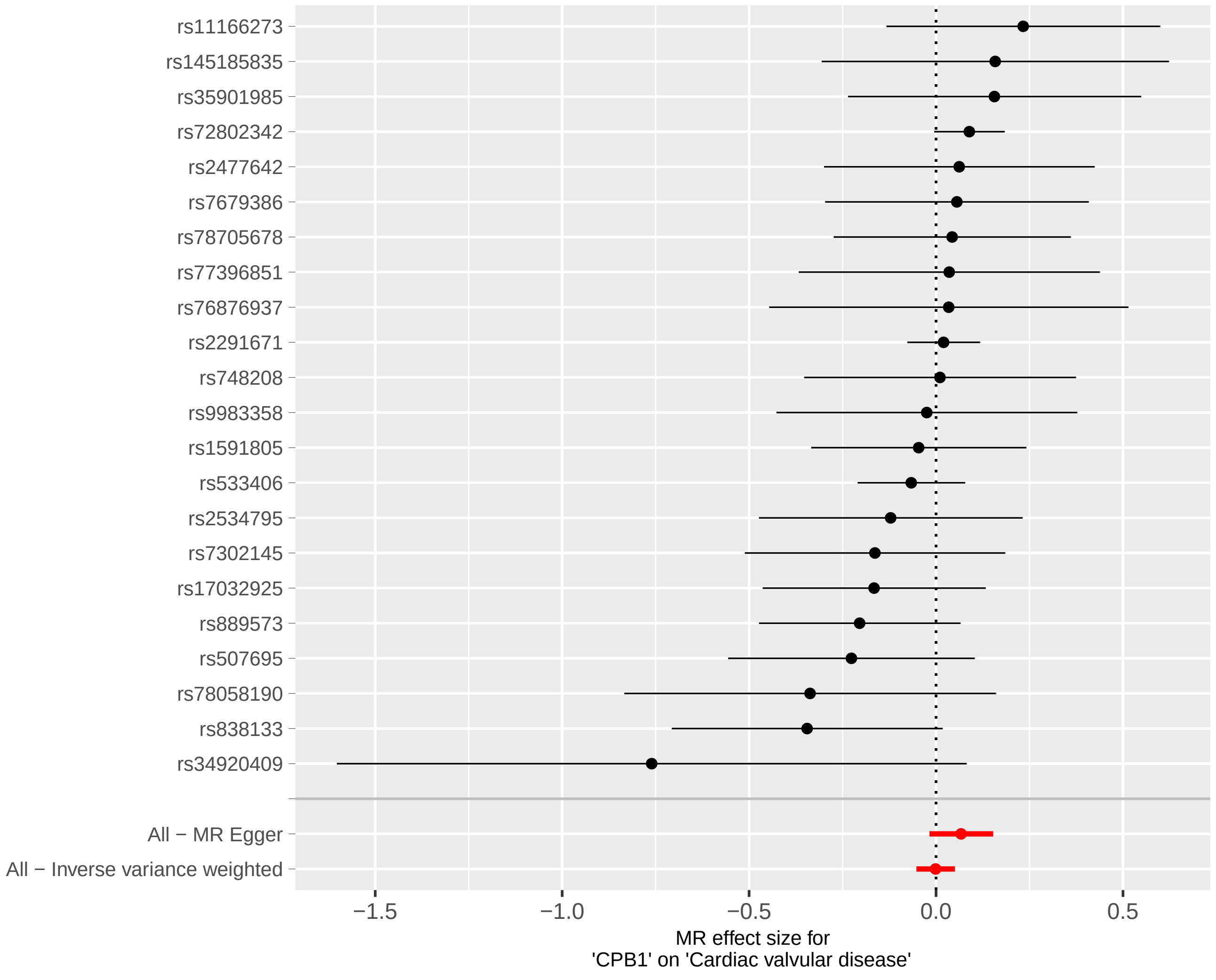
rs11166273
rs145185835
rs35901985
rs72802342
rs2477642
rs7679386
rs78705678
rs77396851
rs76876937
rs2291671
rs748208
rs9983358
rs1591805
rs533406
rs2534795
rs7302145
rs17032925
rs889573
rs507695
rs78058190
rs838133
rs34920409
All − MR Egger
All − Inverse variance weighted
−1.5
−1.0
−0.5
0.0
0.5
MR effect size for
'CPB1' on 'Cardiac valvular disease'

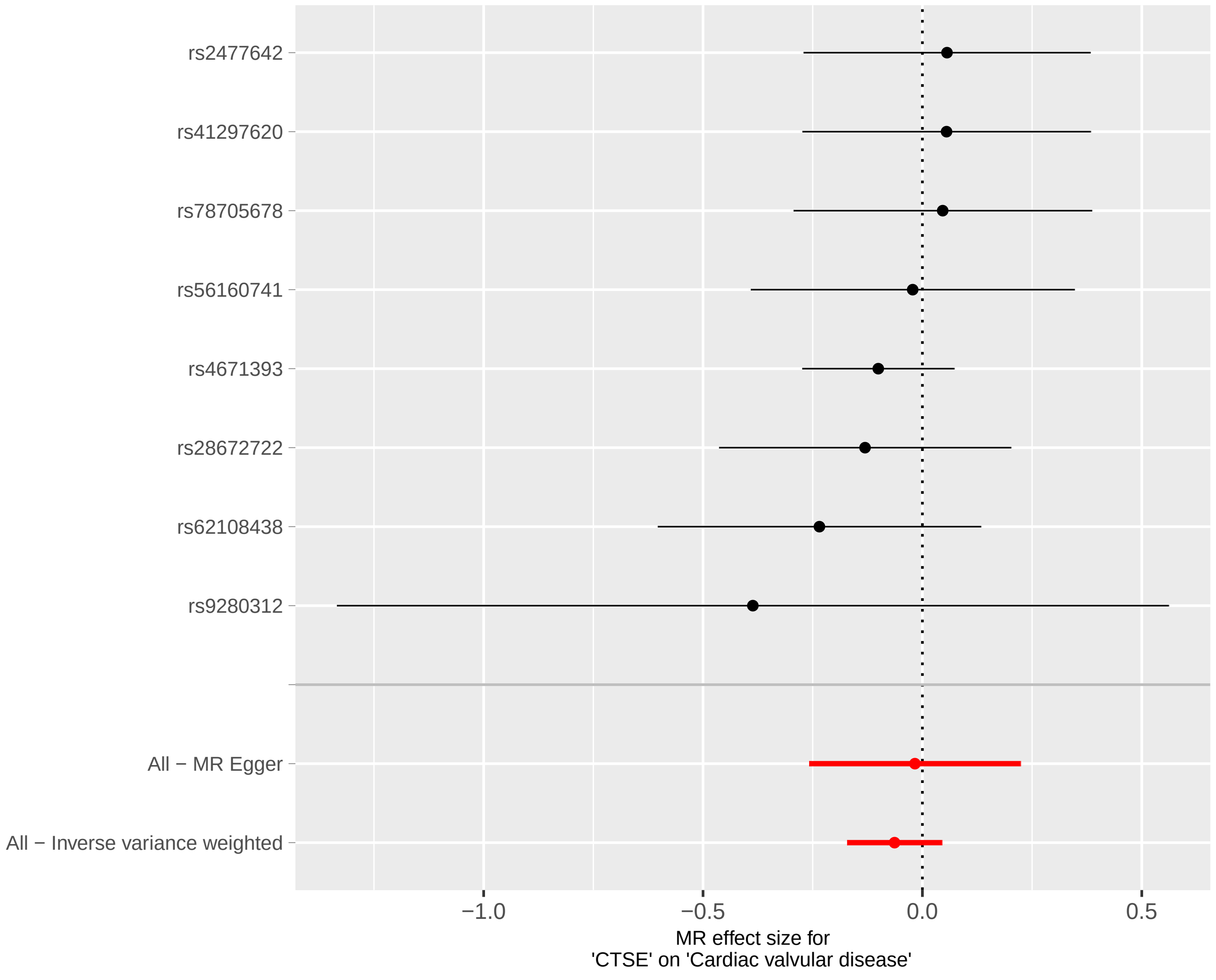
rs2477642
rs41297620
rs78705678
rs56160741
rs4671393
rs28672722
rs62108438
rs9280312
All − MR Egger
All − Inverse variance weighted
−1.0
−0.5
0.0
0.5
MR effect size for
'CTSE' on 'Cardiac valvular disease'

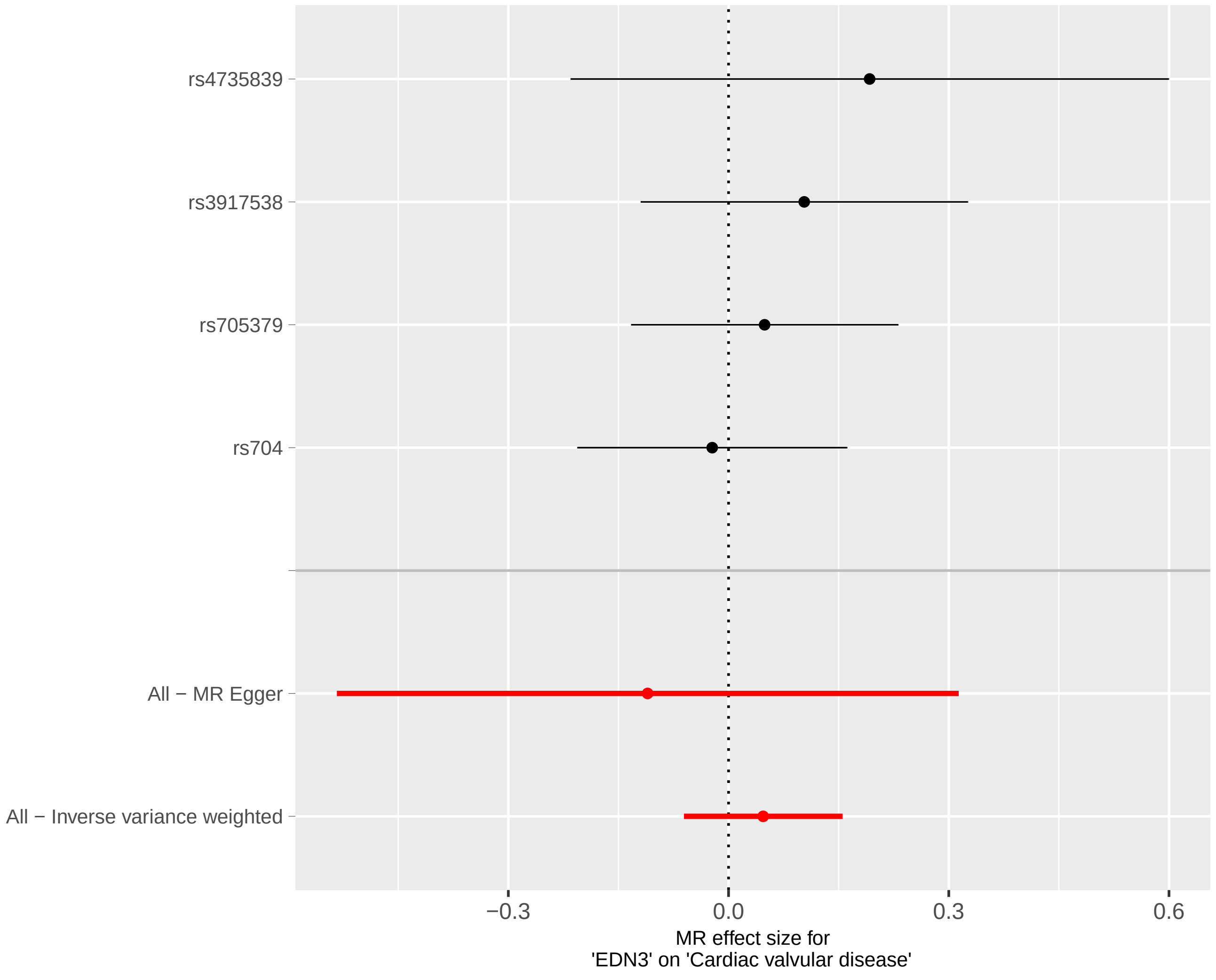
rs4735839
rs3917538
rs705379
rs704
All − MR Egger
All − Inverse variance weighted
−0.3
0.0
0.3
0.6
MR effect size for
'EDN3' on 'Cardiac valvular disease'

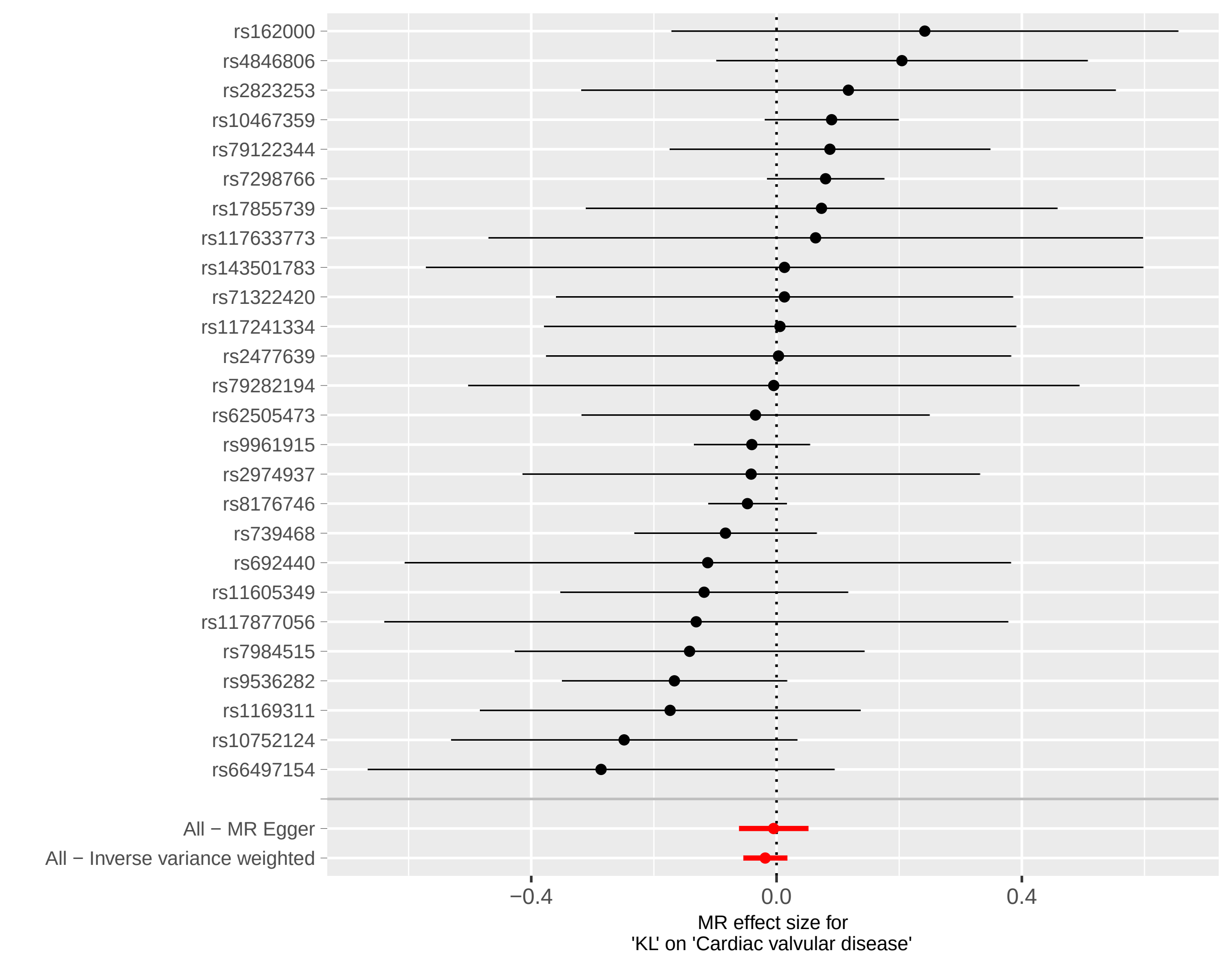
rs162000
rs4846806
rs2823253
rs10467359
rs79122344
rs7298766
rs17855739
rs117633773
rs143501783
rs71322420
rs117241334
rs2477639
rs79282194
rs62505473
rs9961915
rs2974937
rs8176746
rs739468
rs692440
rs11605349
rs117877056
rs7984515
rs9536282
rs1169311
rs10752124
rs66497154
All − MR Egger
All − Inverse variance weighted
−0.4
0.0
0.4
MR effect size for
'KL' on 'Cardiac valvular disease'

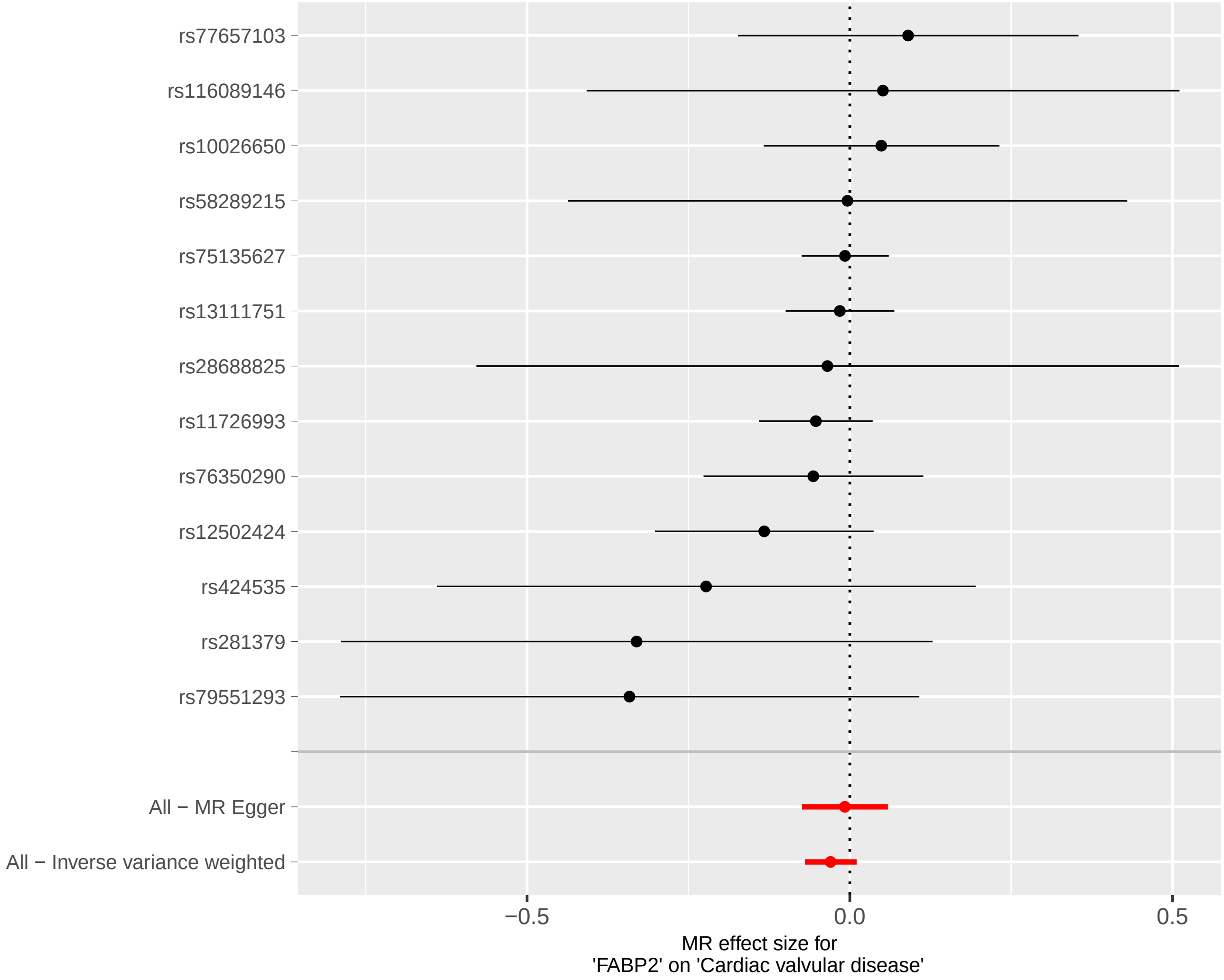
rs77657103
rs116089146
rs10026650
rs58289215
rs75135627
rs13111751
rs28688825
rs11726993
rs76350290
rs12502424
rs424535
rs281379
rs79551293
All − MR Egger
All − Inverse variance weighted
−0.5
0.0
0.5
MR effect size for
'FABP2' on 'Cardiac valvular disease'

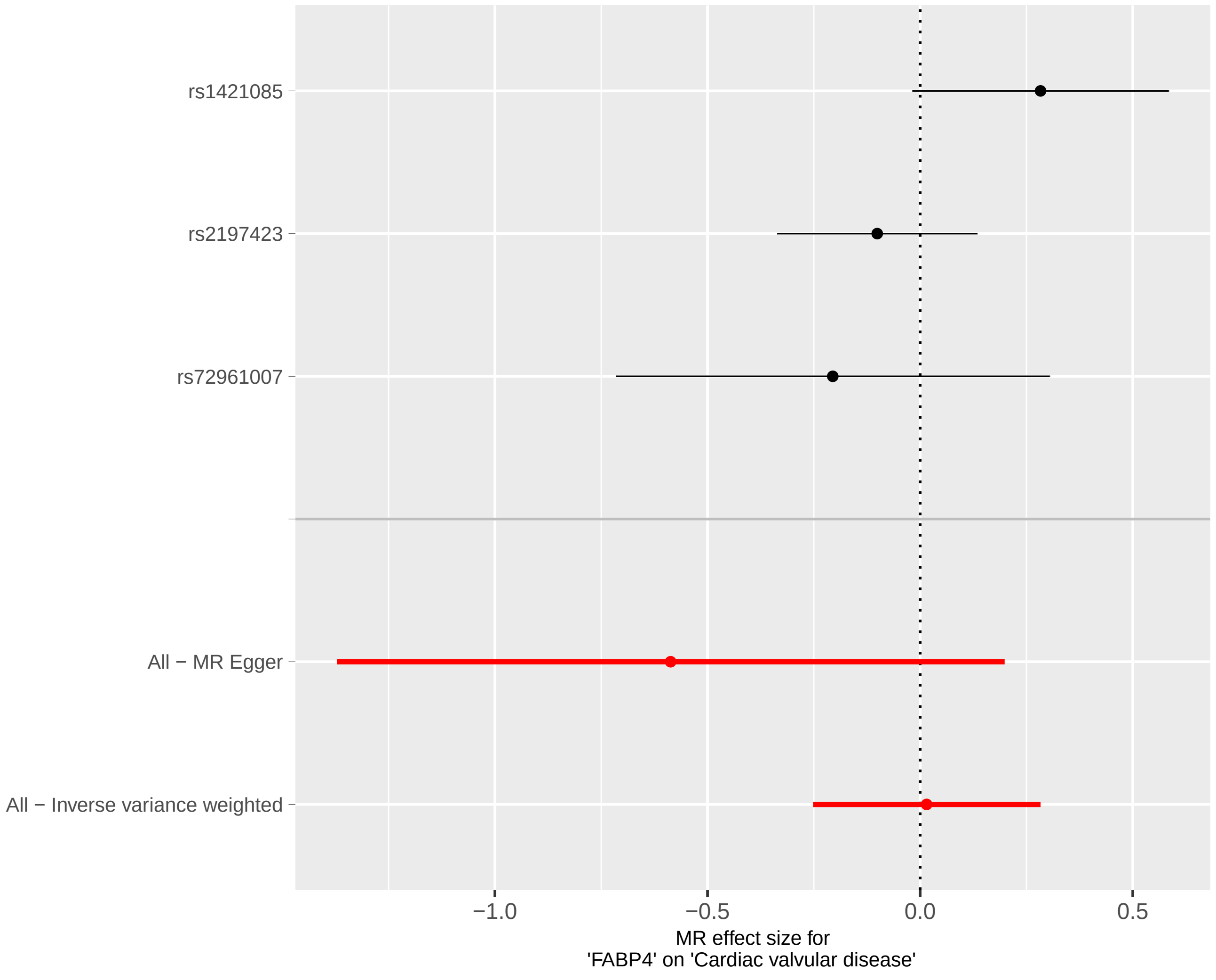
rs1421085
rs2197423
rs72961007
All − MR Egger
All − Inverse variance weighted
−1.0
−0.5
0.0
0.5
MR effect size for
'FABP4' on 'Cardiac valvular disease'

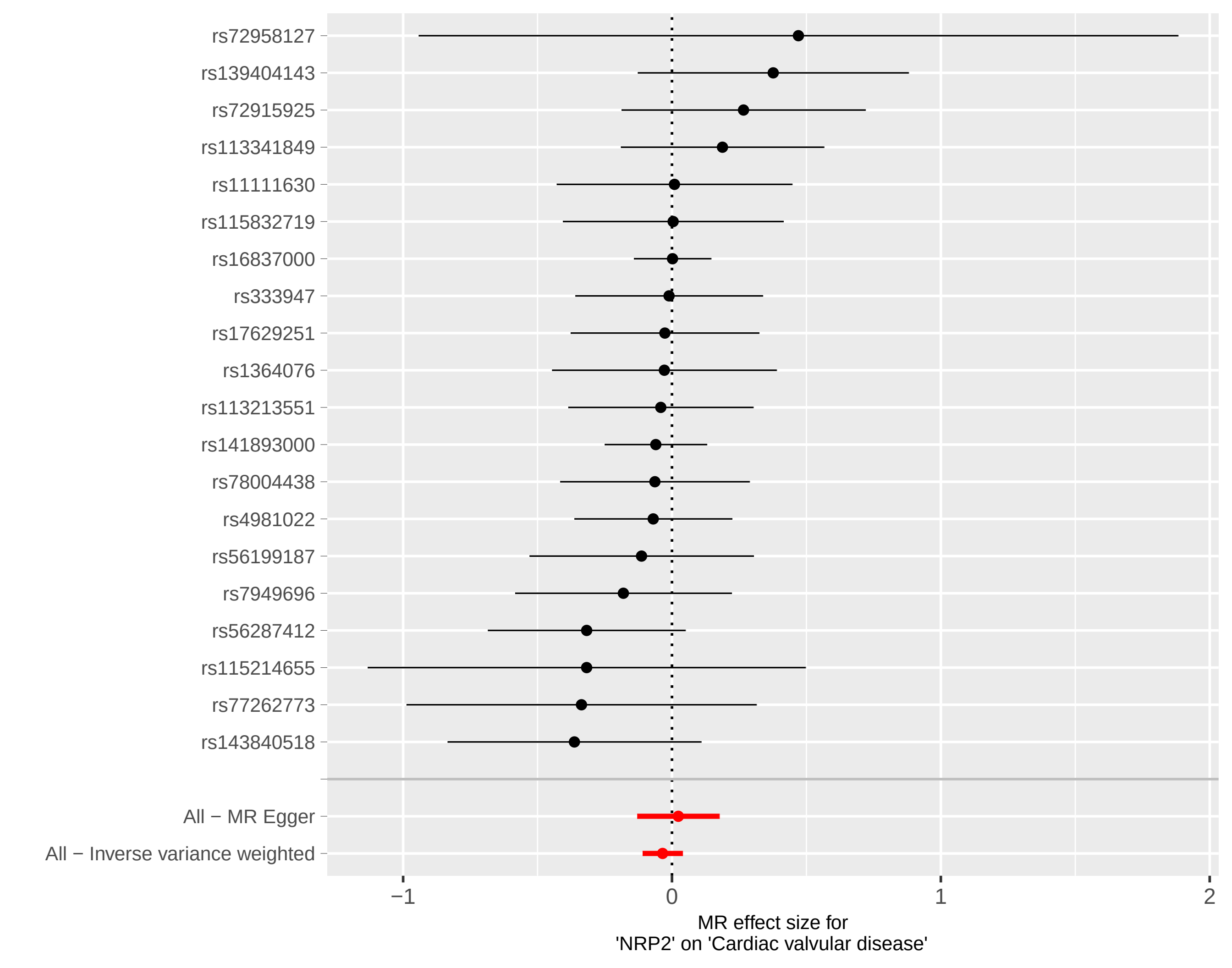

rs72958127
rs139404143
rs72915925
rs113341849
rs11111630
rs115832719
rs16837000
rs333947
rs17629251
rs1364076
rs113213551
rs141893000
rs78004438
rs4981022
rs56199187
rs7949696
rs56287412
rs115214655
rs77262773
rs143840518
All − MR Egger
All − Inverse variance weighted
−1
0
1
2
MR effect size for
'NRP2' on 'Cardiac valvular disease'

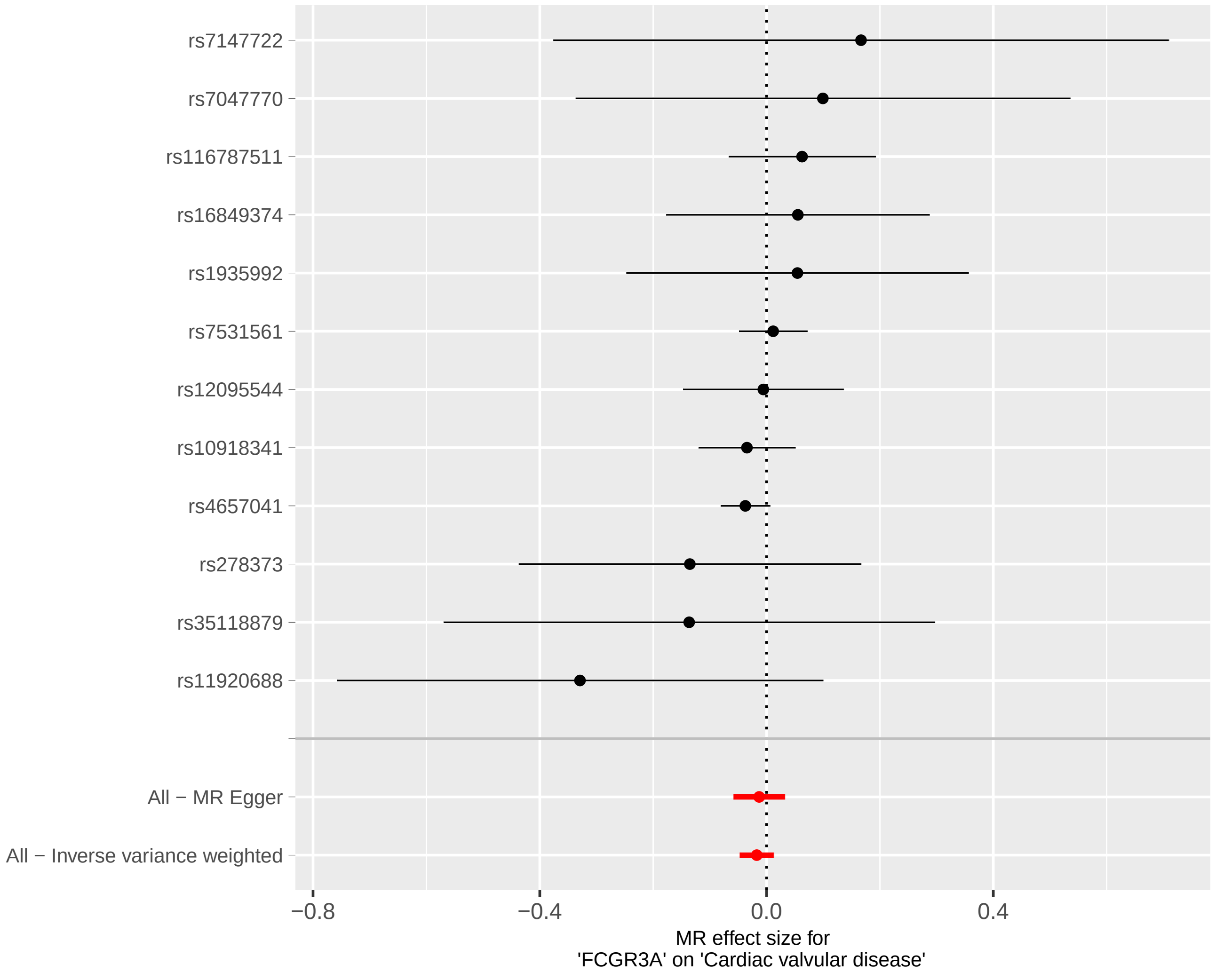
rs7147722
rs7047770
rs116787511
rs16849374
rs1935992
rs7531561
rs12095544
rs10918341
rs4657041
rs278373
rs35118879
rs11920688
All − MR Egger
All − Inverse variance weighted
−0.8
−0.4
0.0
0.4
MR effect size for
'FCGR3A' on 'Cardiac valvular disease'

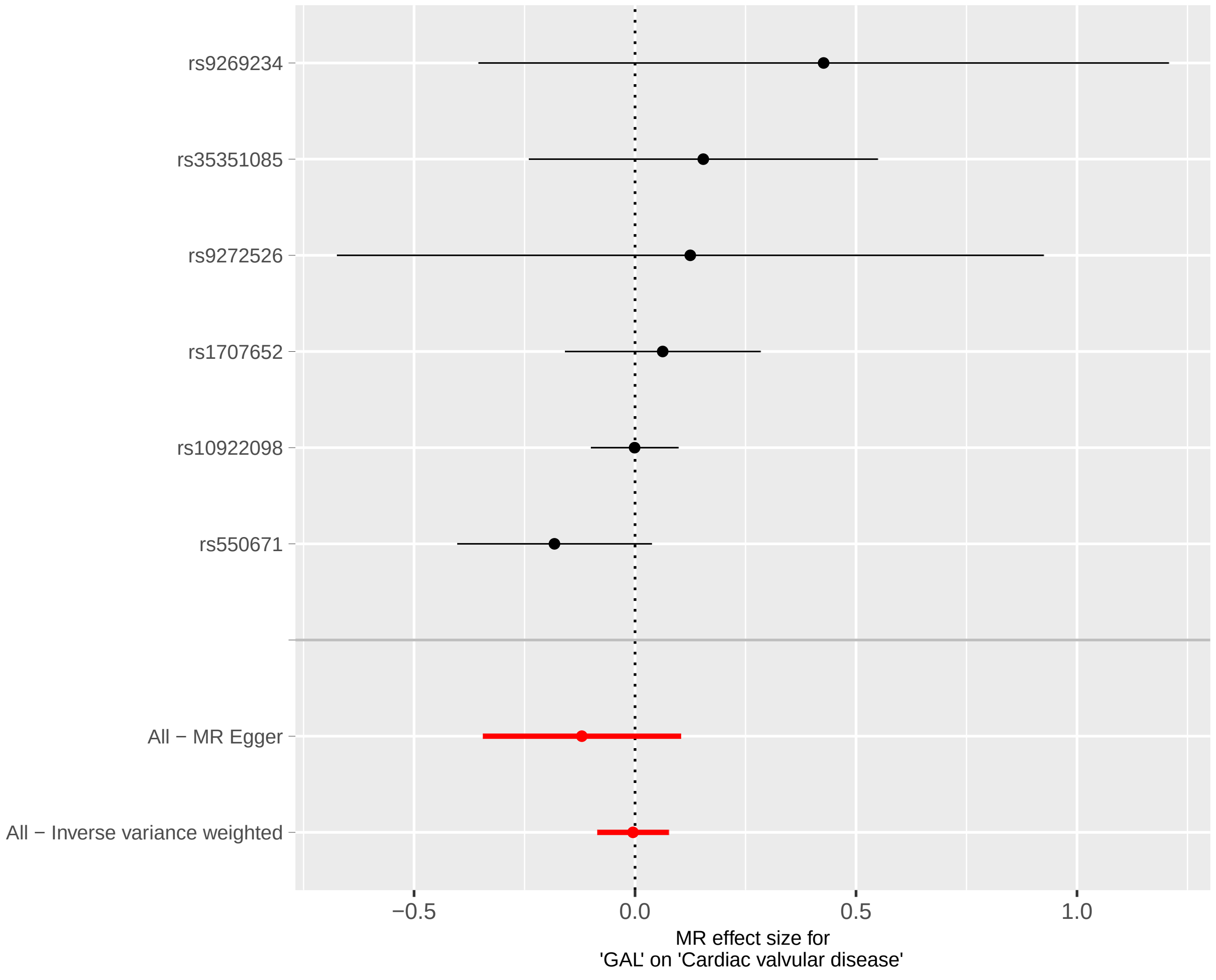

rs9269234
rs35351085
rs9272526
rs1707652
rs10922098
rs550671
All − MR Egger
All − Inverse variance weighted
−0.5
0.0
0.5
1.0
MR effect size for
'GAL' on 'Cardiac valvular disease'

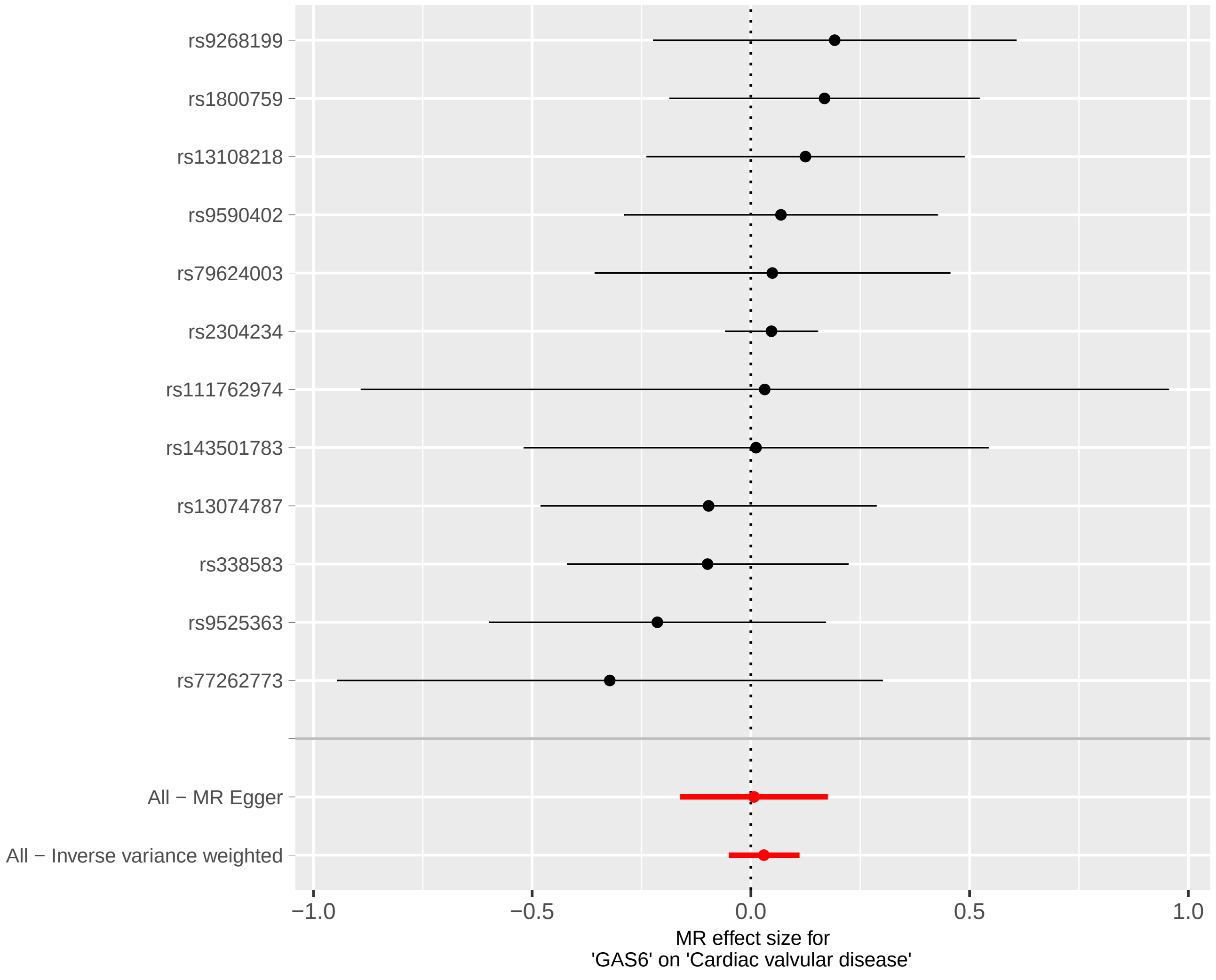
rs9268199
rs1800759
rs13108218
rs9590402
rs79624003
rs2304234
rs111762974
rs143501783
rs13074787
rs338583
rs9525363
rs77262773
All − MR Egger
All − Inverse variance weighted
−1.0
−0.5
0.0
0.5
1.0
MR effect size for
'GAS6' on 'Cardiac valvular disease'

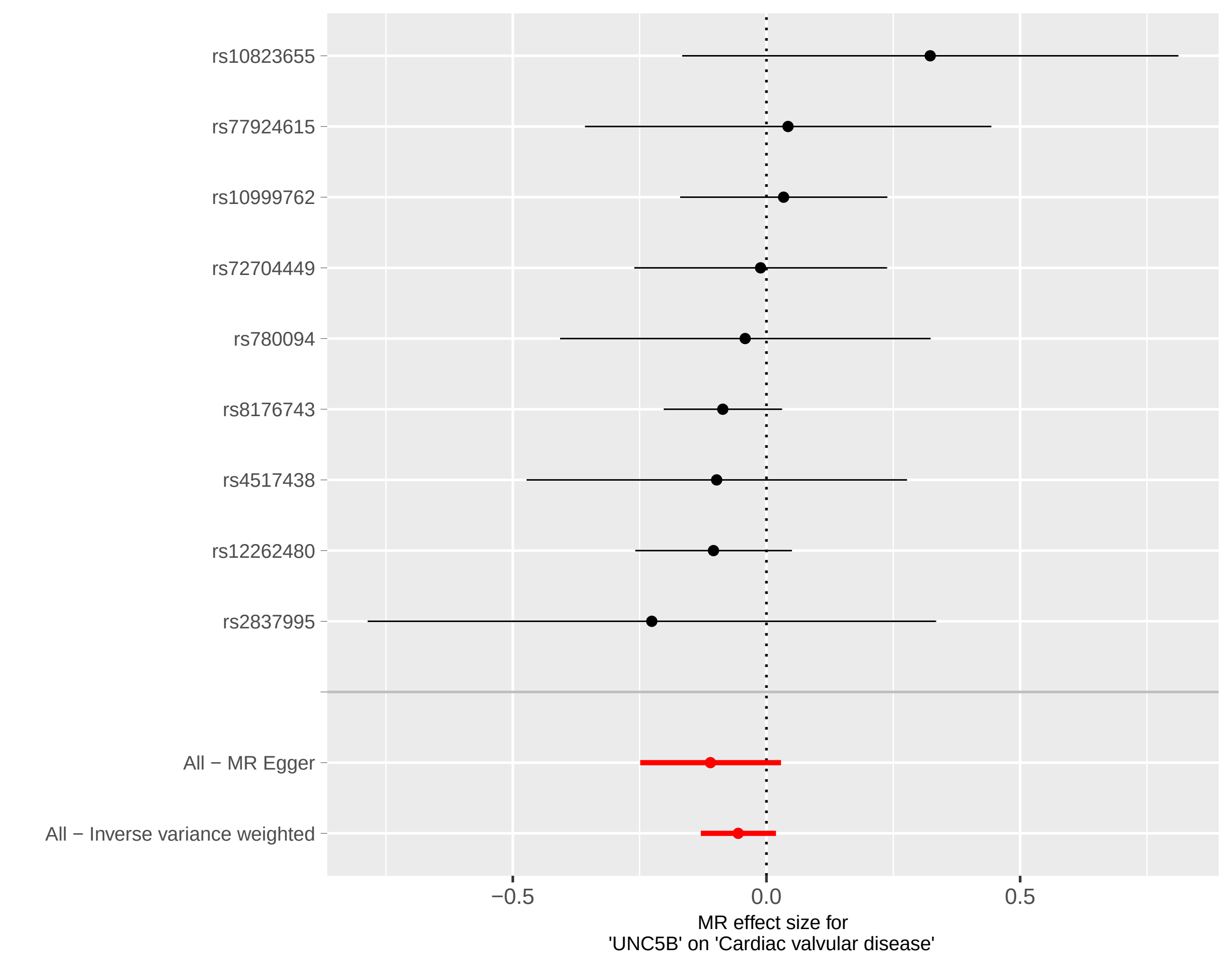

rs10823655
rs77924615
rs10999762
rs72704449
rs780094
rs8176743
rs4517438
rs12262480
rs2837995
All − MR Egger
All − Inverse variance weighted
−0.5
0.0
0.5
MR effect size for
'UNC5B' on 'Cardiac valvular disease'

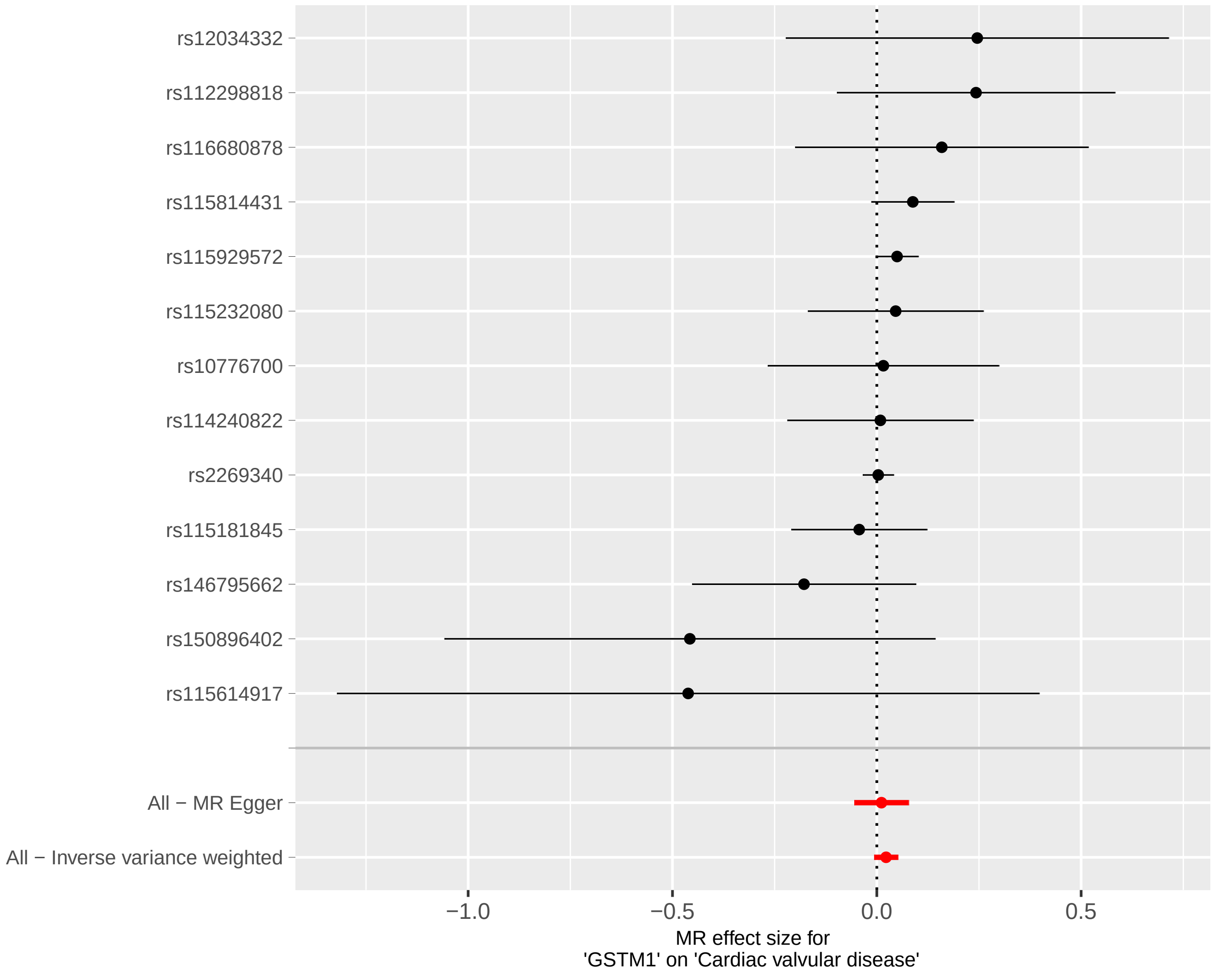
rs12034332
rs112298818
rs116680878
rs115814431
rs115929572
rs115232080
rs10776700
rs114240822
rs2269340
rs115181845
rs146795662
rs150896402
rs115614917
All − MR Egger
All − Inverse variance weighted
−1.0
−0.5
0.0
0.5
MR effect size for
'GSTM1' on 'Cardiac valvular disease'

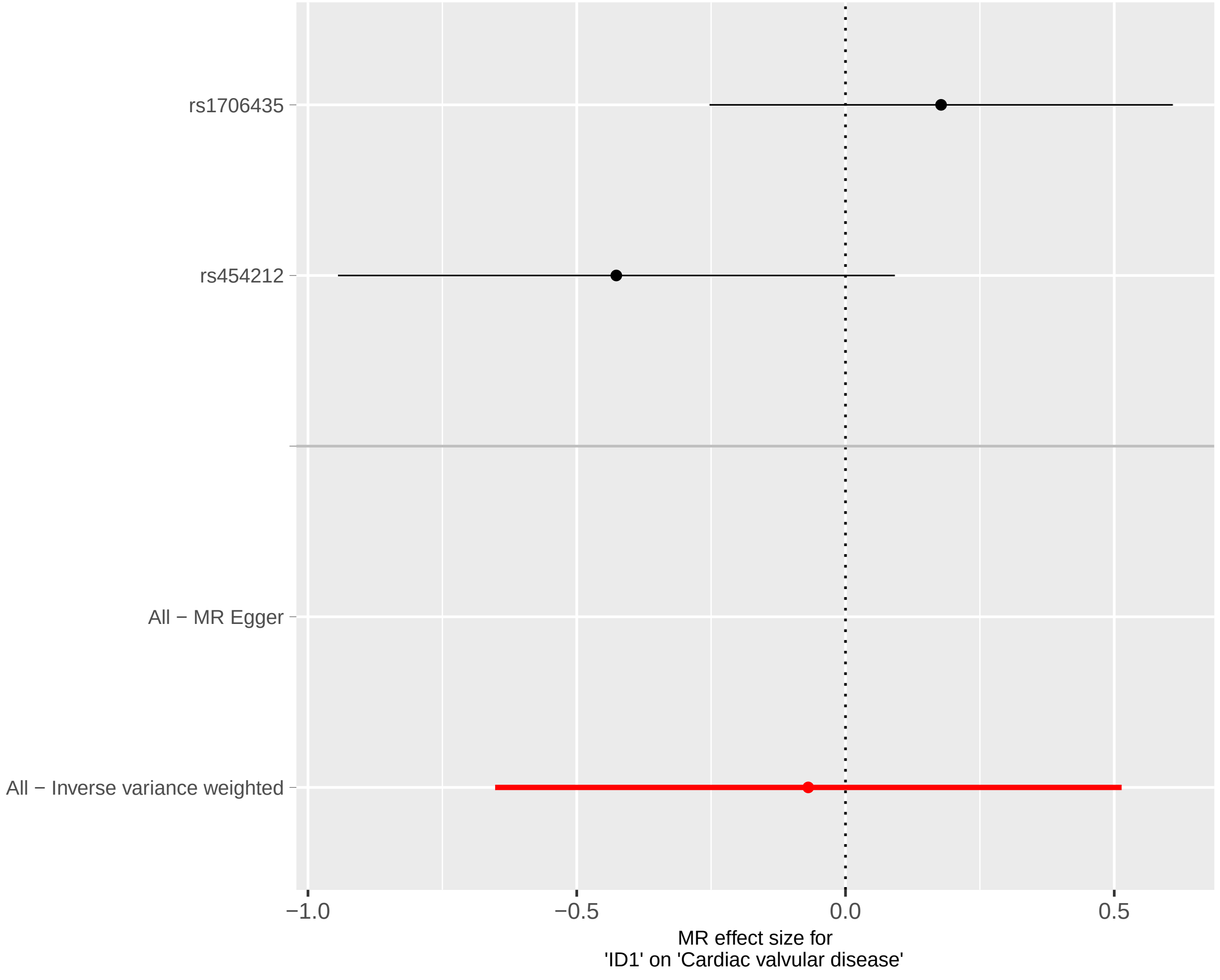
rs1706435
rs454212
All − MR Egger
All − Inverse variance weighted
−1.0
−0.5
0.0
0.5
MR effect size for
'ID1' on 'Cardiac valvular disease'

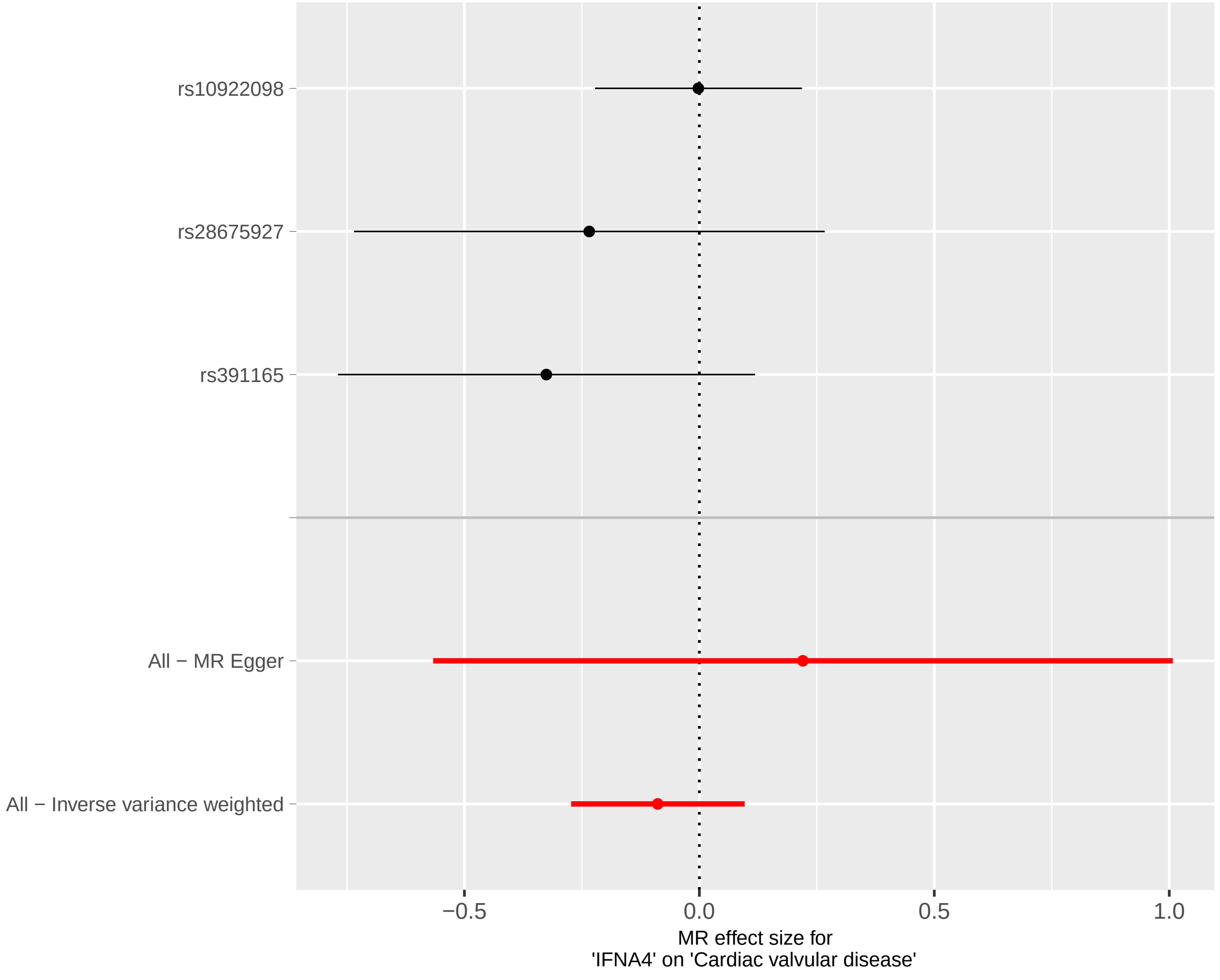
rs10922098
rs28675927
rs391165
All − MR Egger
All − Inverse variance weighted
−0.5
0.0
0.5
1.0
MR effect size for
'IFNA4' on 'Cardiac valvular disease'

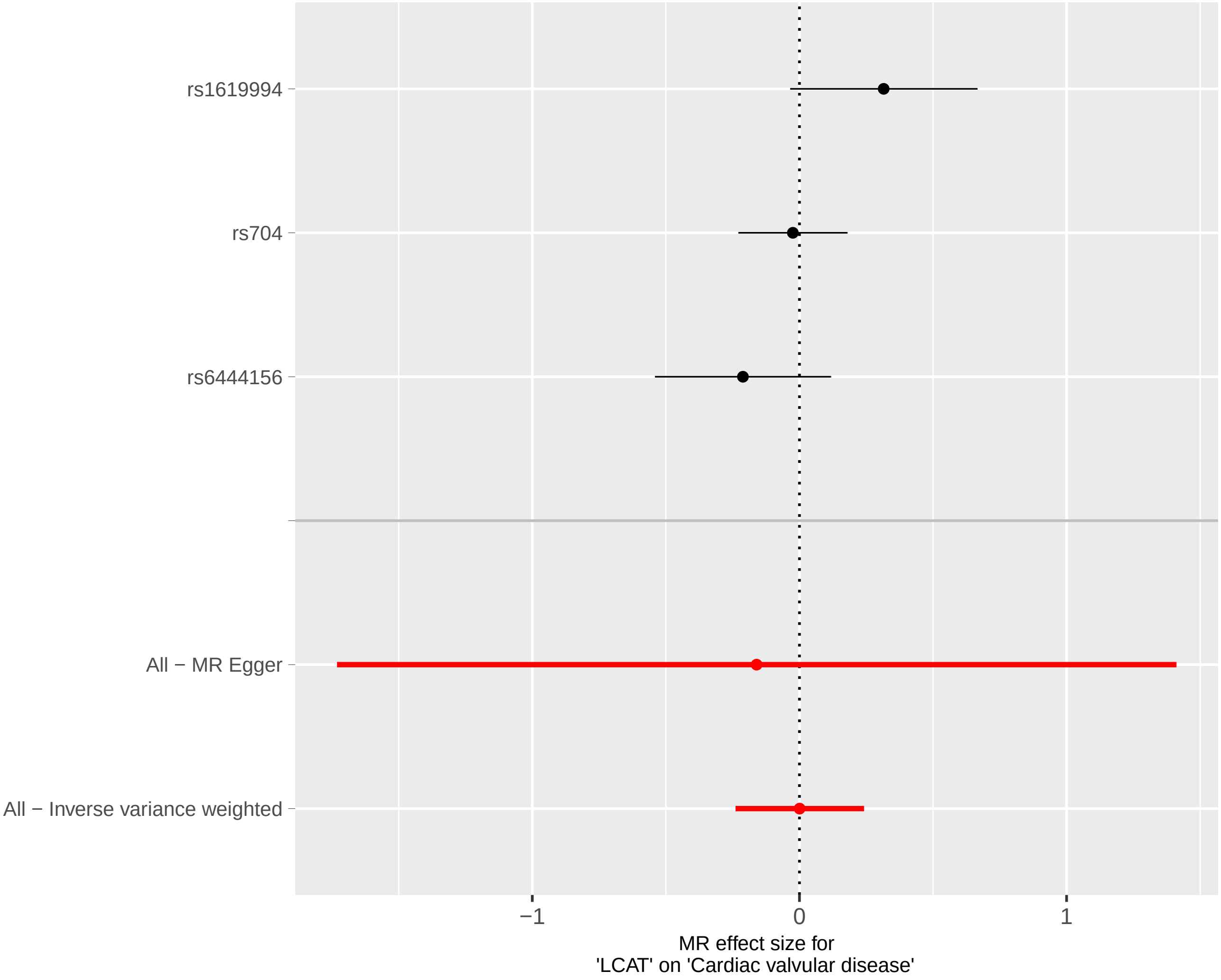
rs1619994
rs704
rs6444156
All − MR Egger
All − Inverse variance weighted
−1
0
1
MR effect size for
'LCAT' on 'Cardiac valvular disease'

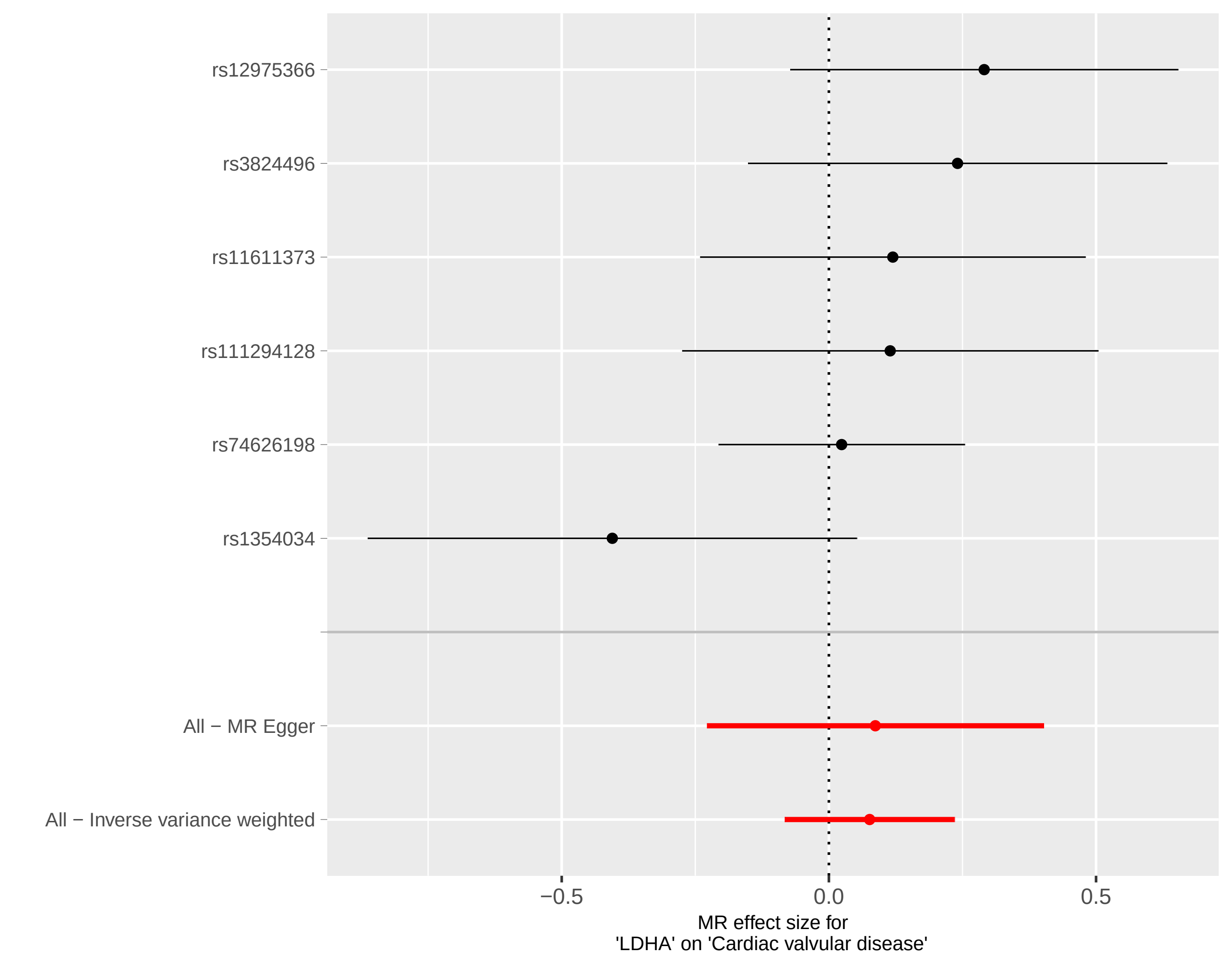
rs12975366
rs3824496
rs11611373
rs111294128
rs74626198
rs1354034
All − MR Egger
All − Inverse variance weighted
−0.5
0.0
0.5
MR effect size for
'LDHA' on 'Cardiac valvular disease'

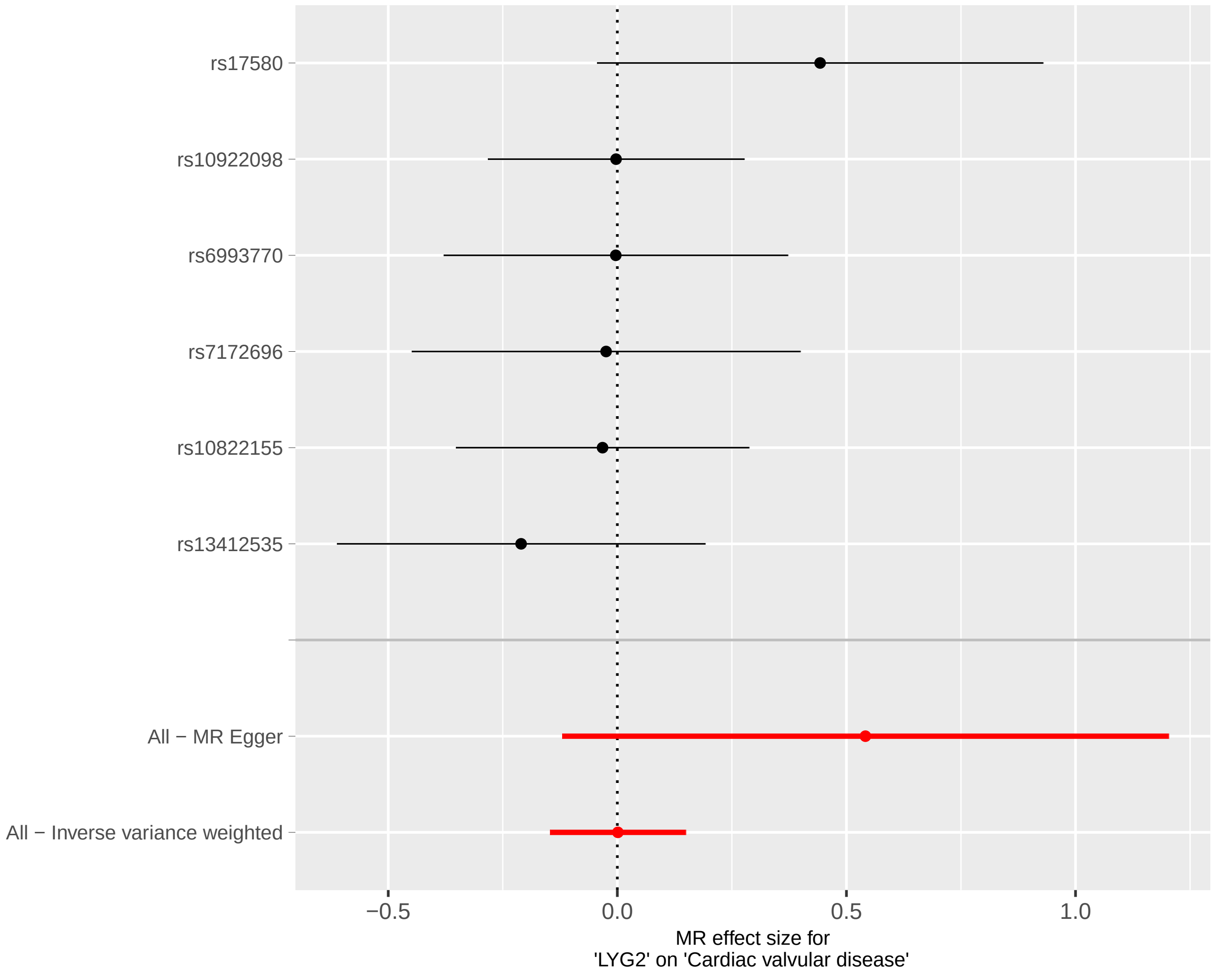
rs17580
rs10922098
rs6993770
rs7172696
rs10822155
rs13412535
All − MR Egger
All − Inverse variance weighted
−0.5
0.0
0.5
1.0
MR effect size for
'LYG2' on 'Cardiac valvular disease'

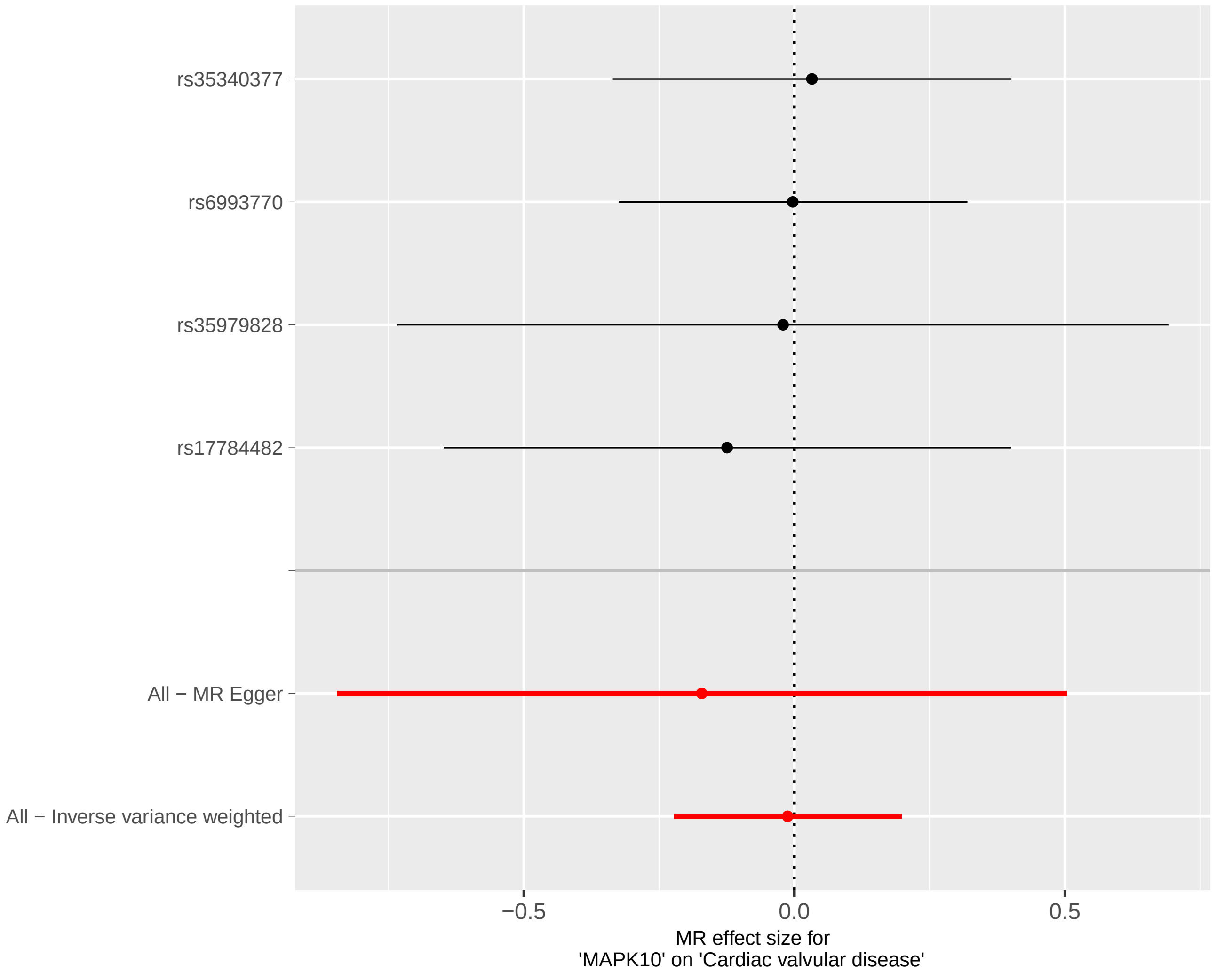
rs35340377
rs6993770
rs35979828
rs17784482
All − MR Egger
All − Inverse variance weighted
−0.5
0.0
0.5
MR effect size for
'MAPK10' on 'Cardiac valvular disease'

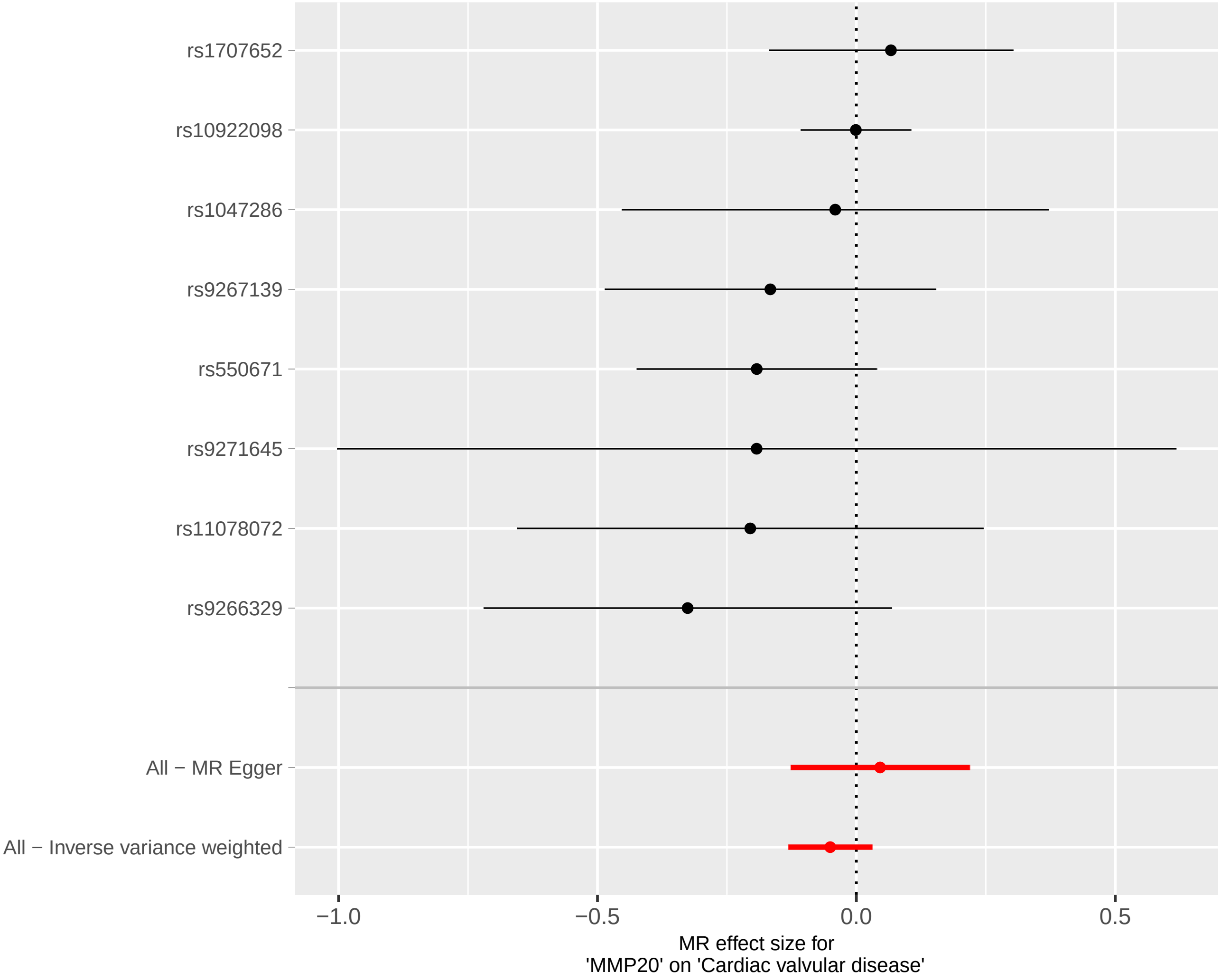
rs1707652
rs10922098
rs1047286
rs9267139
rs550671
rs9271645
rs11078072
rs9266329
All − MR Egger
All − Inverse variance weighted
−1.0
−0.5
0.0
0.5
MR effect size for
'MMP20' on 'Cardiac valvular disease'

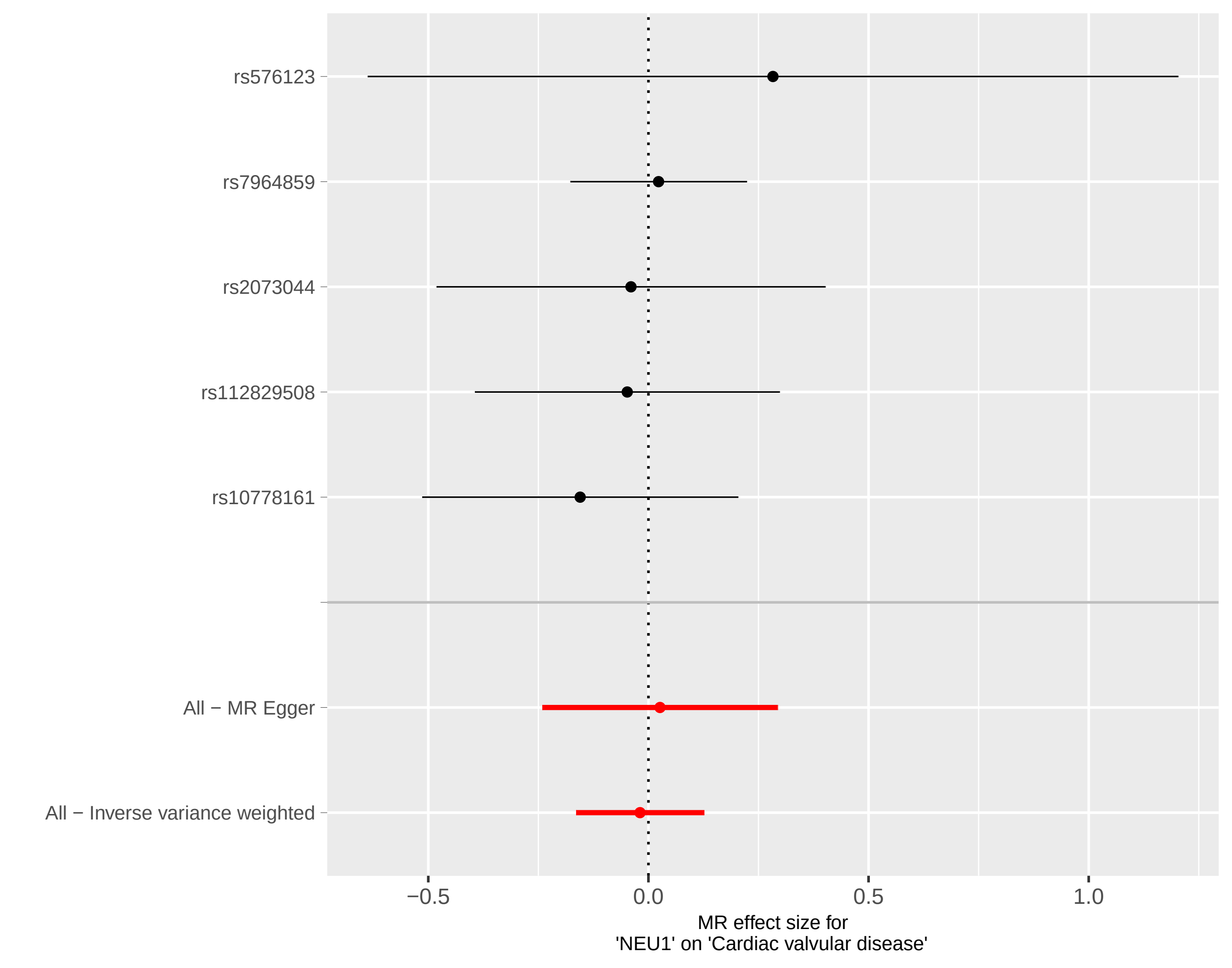
rs576123
rs7964859
rs2073044
rs112829508
rs10778161
All − MR Egger
All − Inverse variance weighted
−0.5
0.0
0.5
1.0
MR effect size for
'NEU1' on 'Cardiac valvular disease'

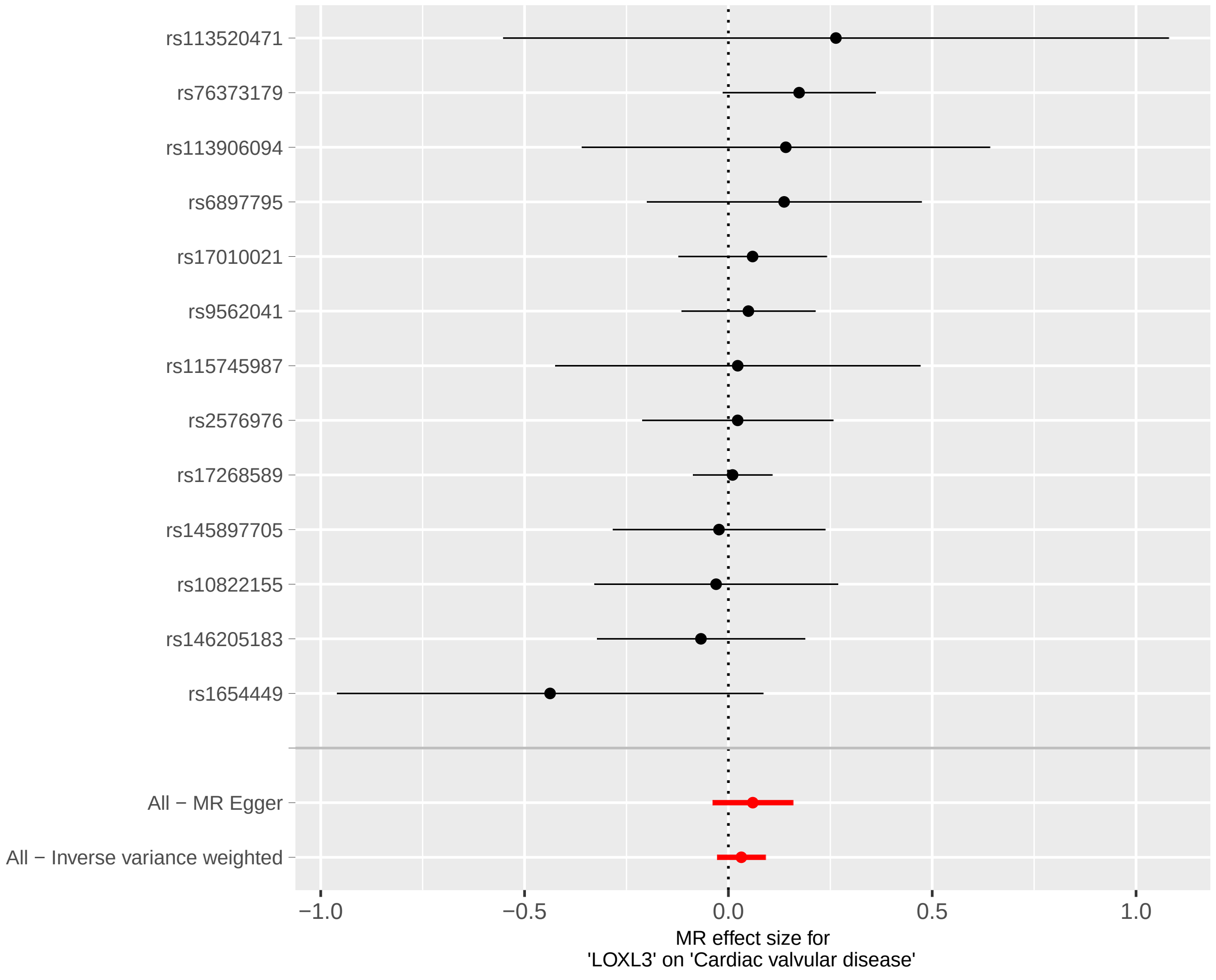

rs113520471
rs76373179
rs113906094
rs6897795
rs17010021
rs9562041
rs115745987
rs2576976
rs17268589
rs145897705
rs10822155
rs146205183
rs1654449
All − MR Egger
All − Inverse variance weighted
−1.0
−0.5
0.0
0.5
1.0
MR effect size for
'LOXL3' on 'Cardiac valvular disease'

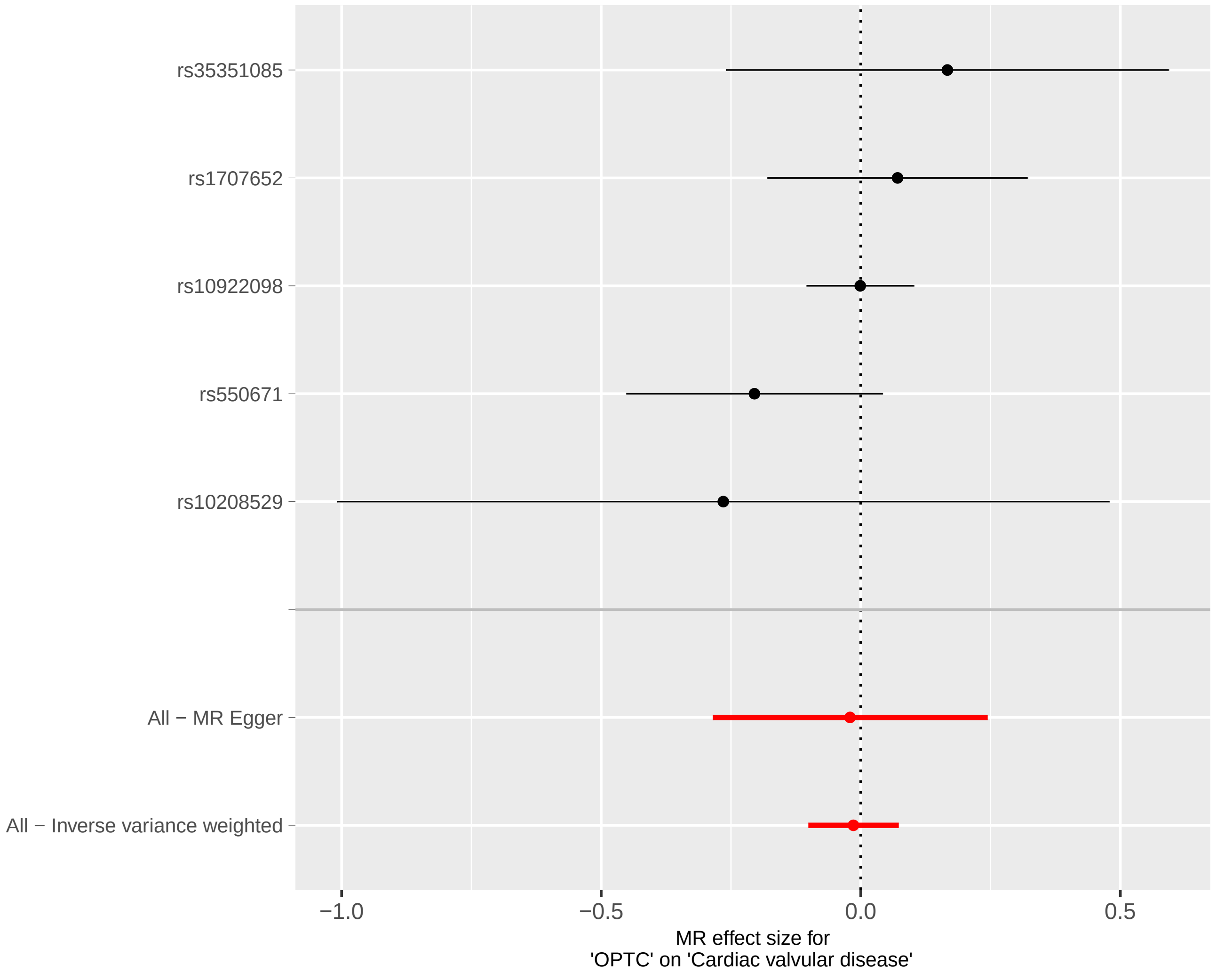

rs35351085
rs1707652
rs10922098
rs550671
rs10208529
All − MR Egger
All − Inverse variance weighted
−1.0
−0.5
0.0
0.5
MR effect size for
'OPTC' on 'Cardiac valvular disease'

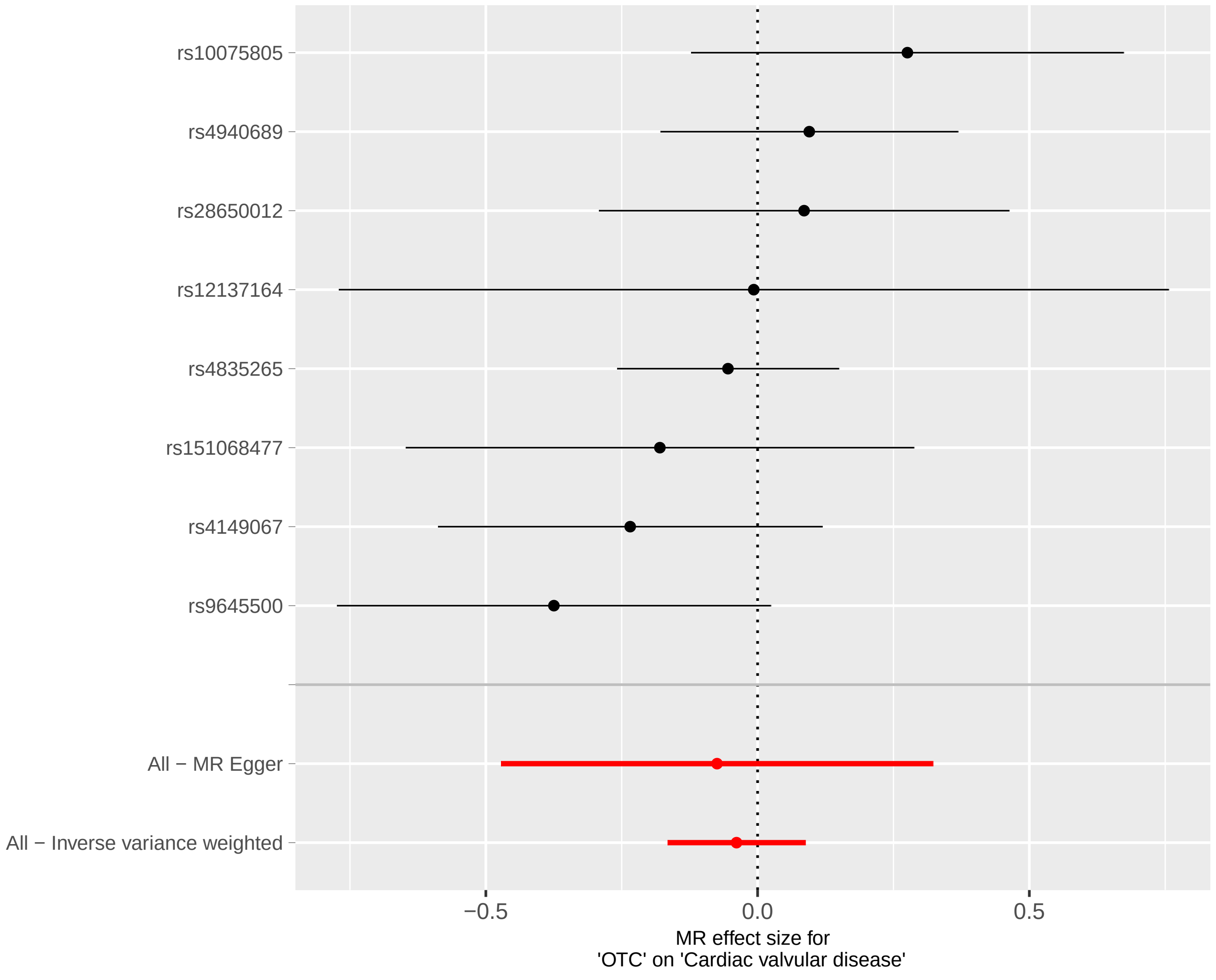
rs10075805
rs4940689
rs28650012
rs12137164
rs4835265
rs151068477
rs4149067
rs9645500
All − MR Egger
All − Inverse variance weighted
−0.5
0.0
0.5
MR effect size for
'OTC' on 'Cardiac valvular disease'

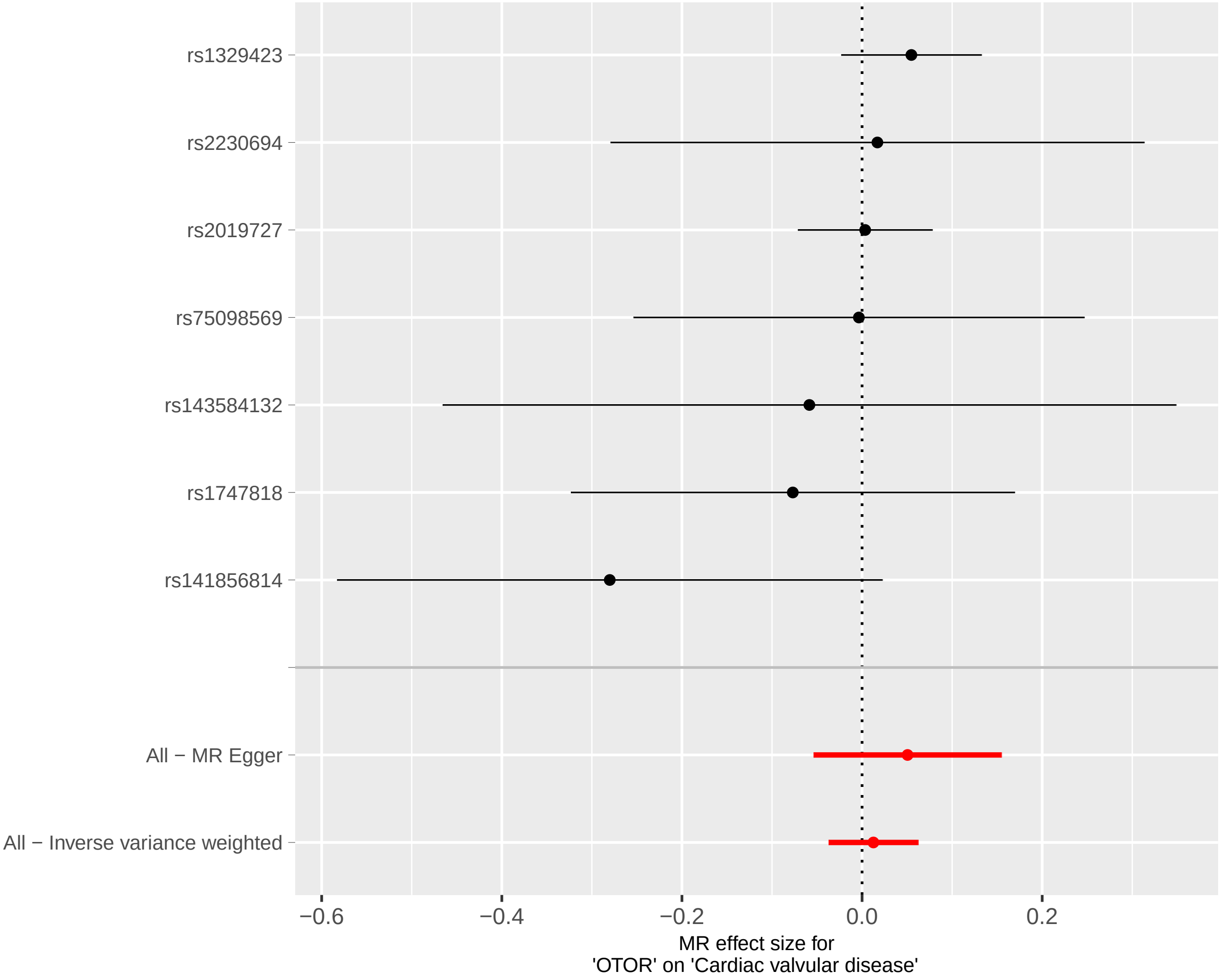

rs1329423
rs2230694
rs2019727
rs75098569
rs143584132
rs1747818
rs141856814
All − MR Egger
All − Inverse variance weighted
−0.6
−0.4
−0.2
0.0
0.2
MR effect size for
'OTOR' on 'Cardiac valvular disease'

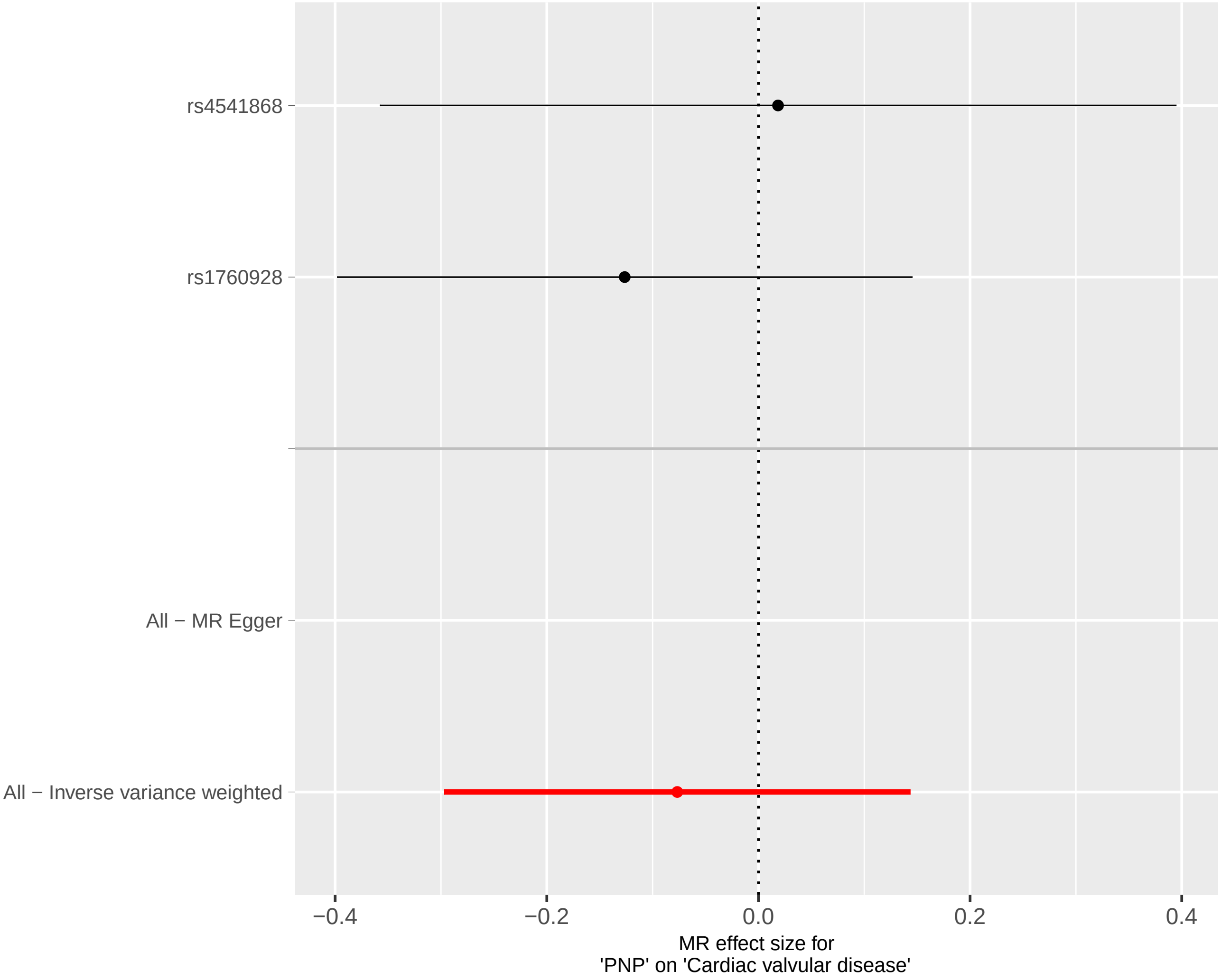
rs4541868
rs1760928
All − MR Egger
All − Inverse variance weighted
−0.4
−0.2
0.0
0.2
0.4
MR effect size for
'PNP' on 'Cardiac valvular disease'

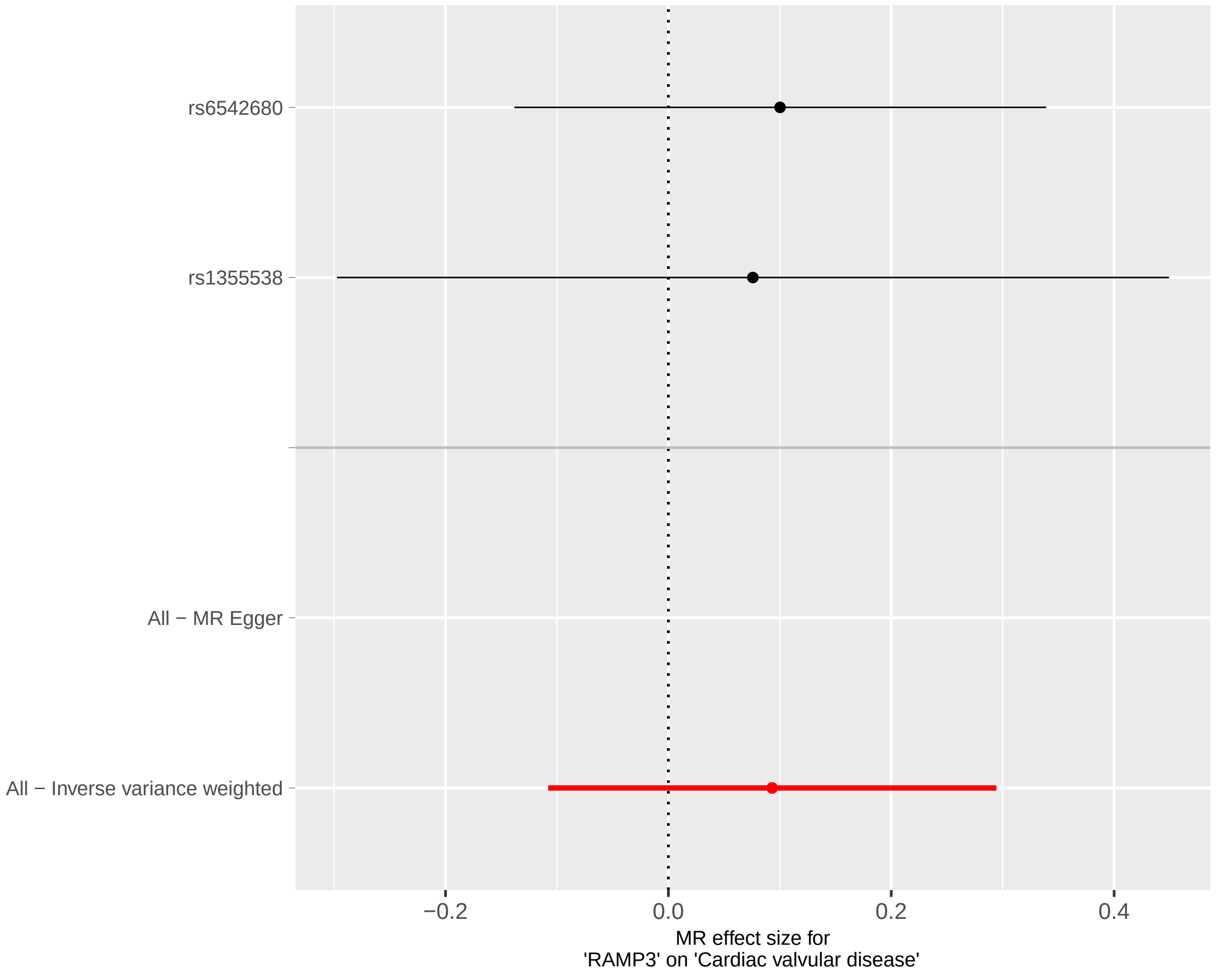

rs6542680
rs1355538
All − MR Egger
All − Inverse variance weighted
−0.2
0.0
0.2
0.4
MR effect size for
'RAMP3' on 'Cardiac valvular disease'

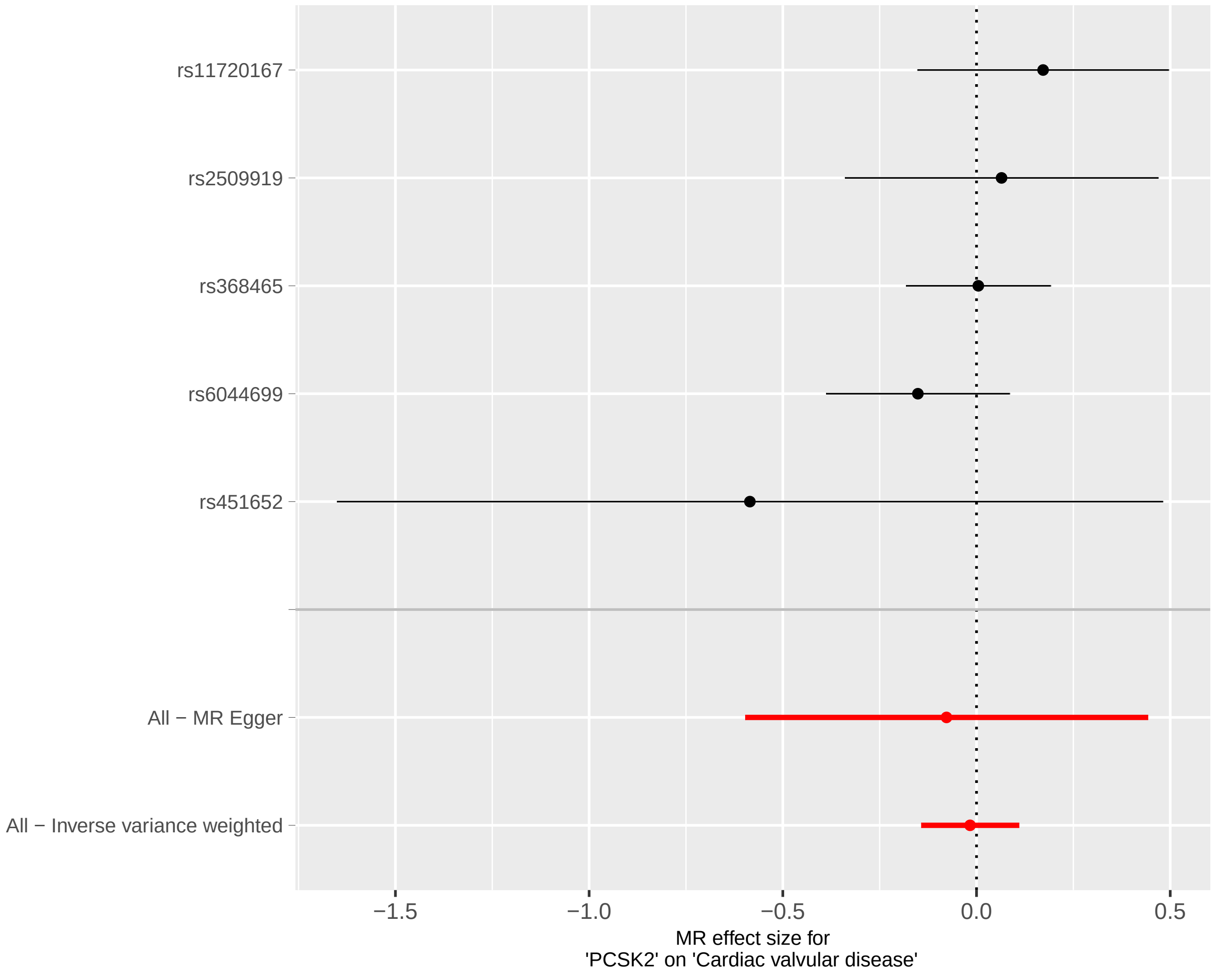
rs11720167
rs2509919
rs368465
rs6044699
rs451652
All − MR Egger
All − Inverse variance weighted
−1.5
−1.0
−0.5
0.0
0.5
MR effect size for
'PCSK2' on 'Cardiac valvular disease'

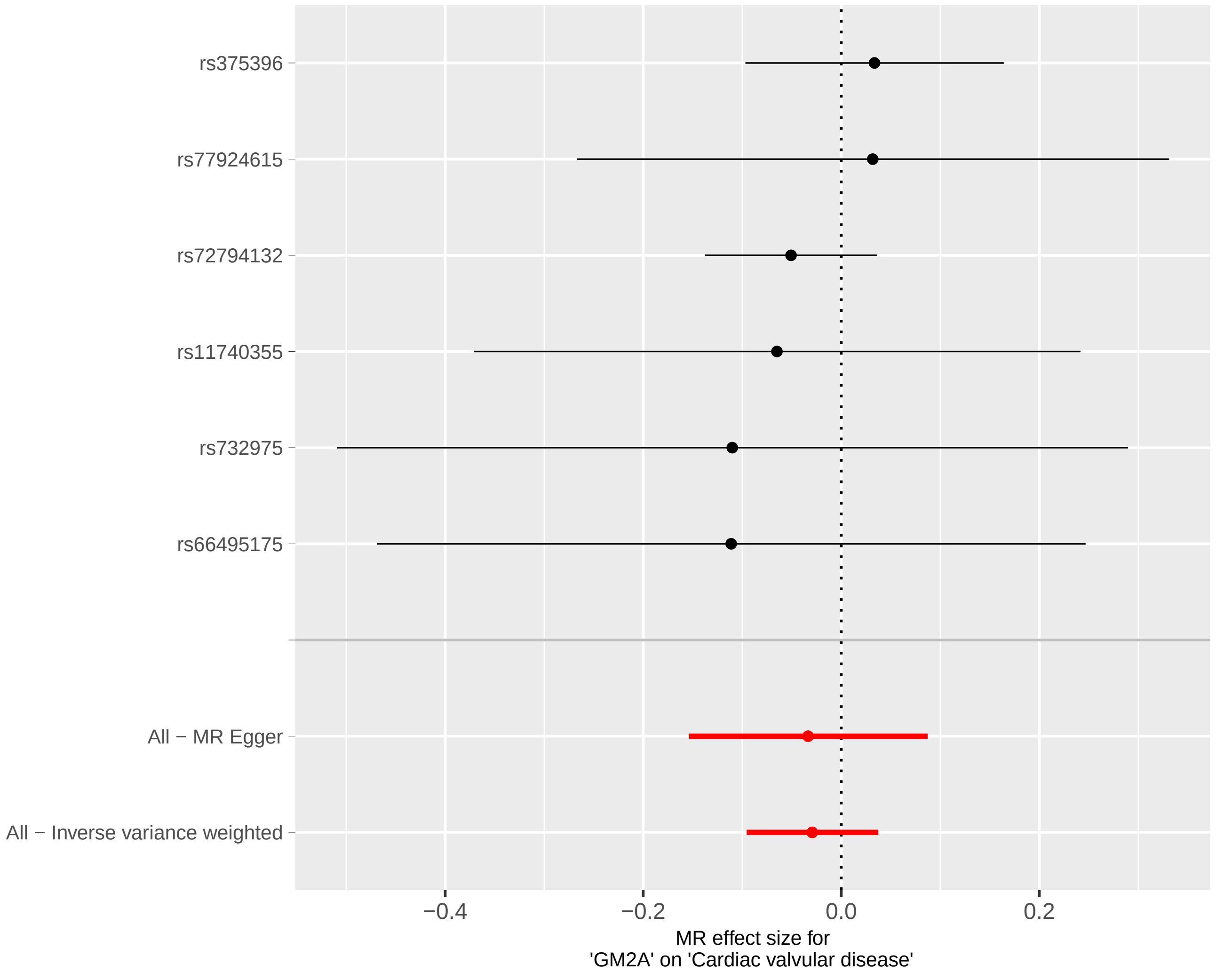

rs375396
rs77924615
rs72794132
rs11740355
rs732975
rs66495175
All − MR Egger
All − Inverse variance weighted
−0.4
−0.2
0.0
0.2
MR effect size for
'GM2A' on 'Cardiac valvular disease'

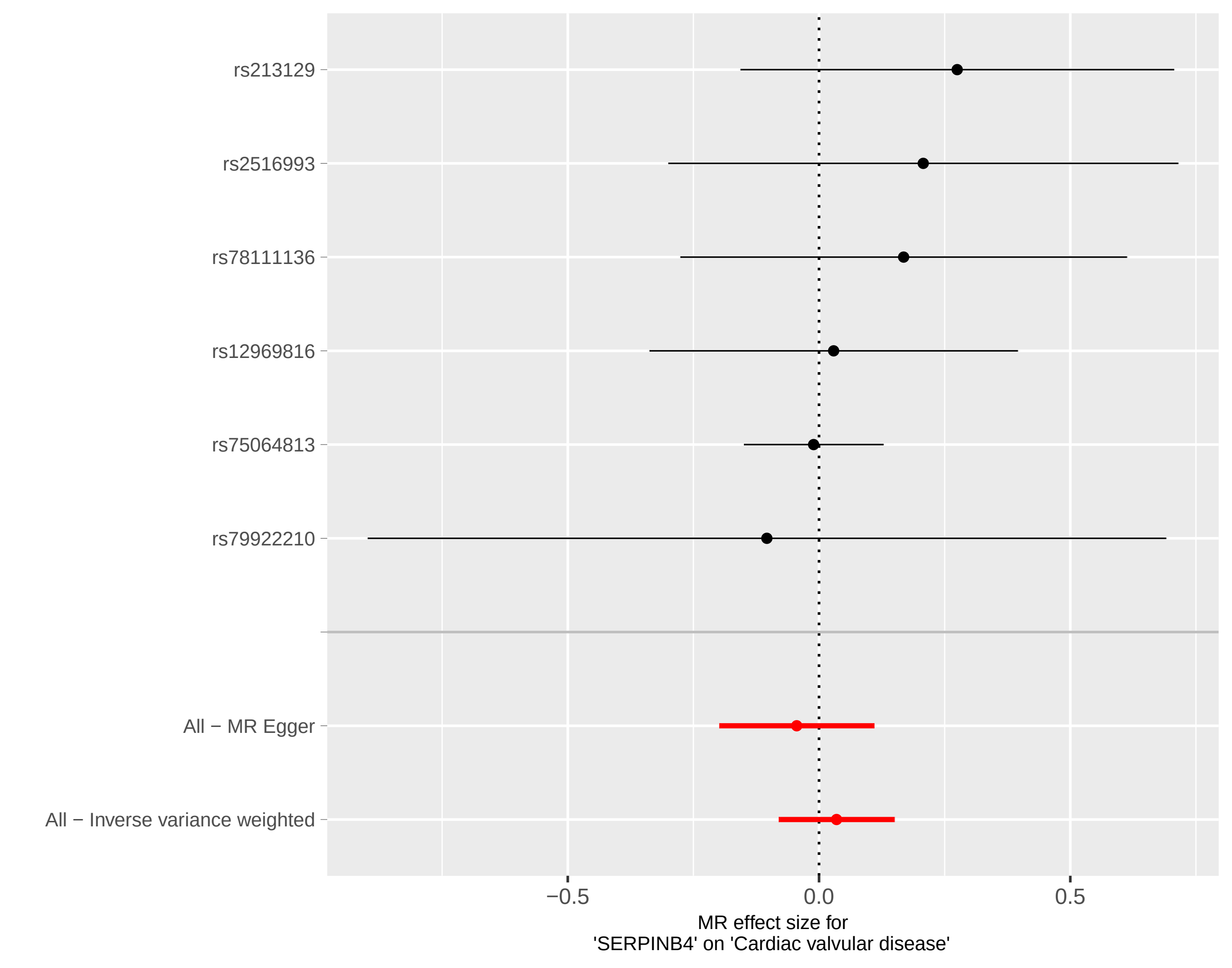
rs213129
rs2516993
rs78111136
rs12969816
rs75064813
rs79922210
All − MR Egger
All − Inverse variance weighted
−0.5
0.0
0.5
MR effect size for
'SERPINB4' on 'Cardiac valvular disease'

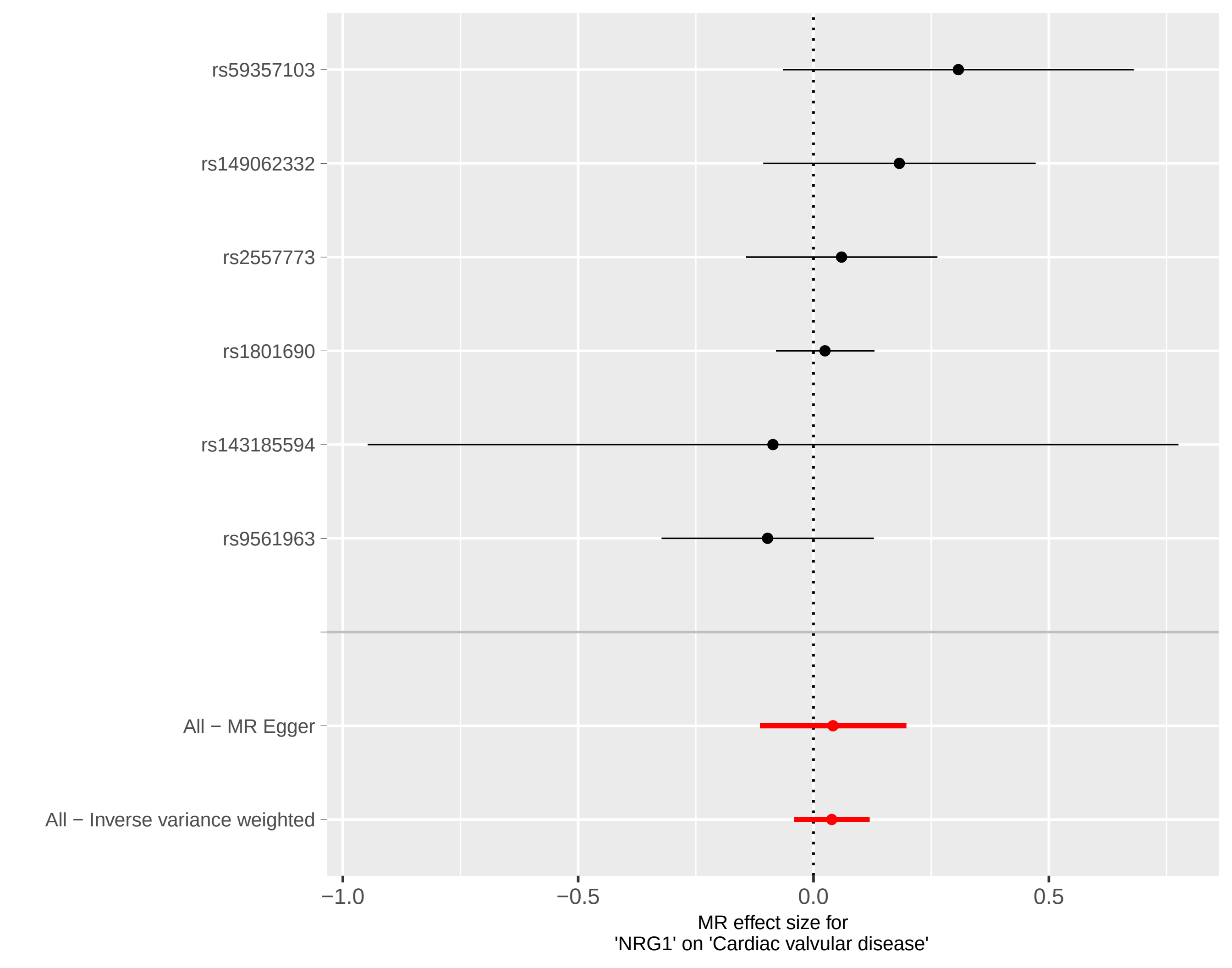

rs59357103
rs149062332
rs2557773
rs1801690
rs143185594
rs9561963
All − MR Egger
All − Inverse variance weighted
−1.0
−0.5
0.0
0.5
MR effect size for
'NRG1' on 'Cardiac valvular disease'

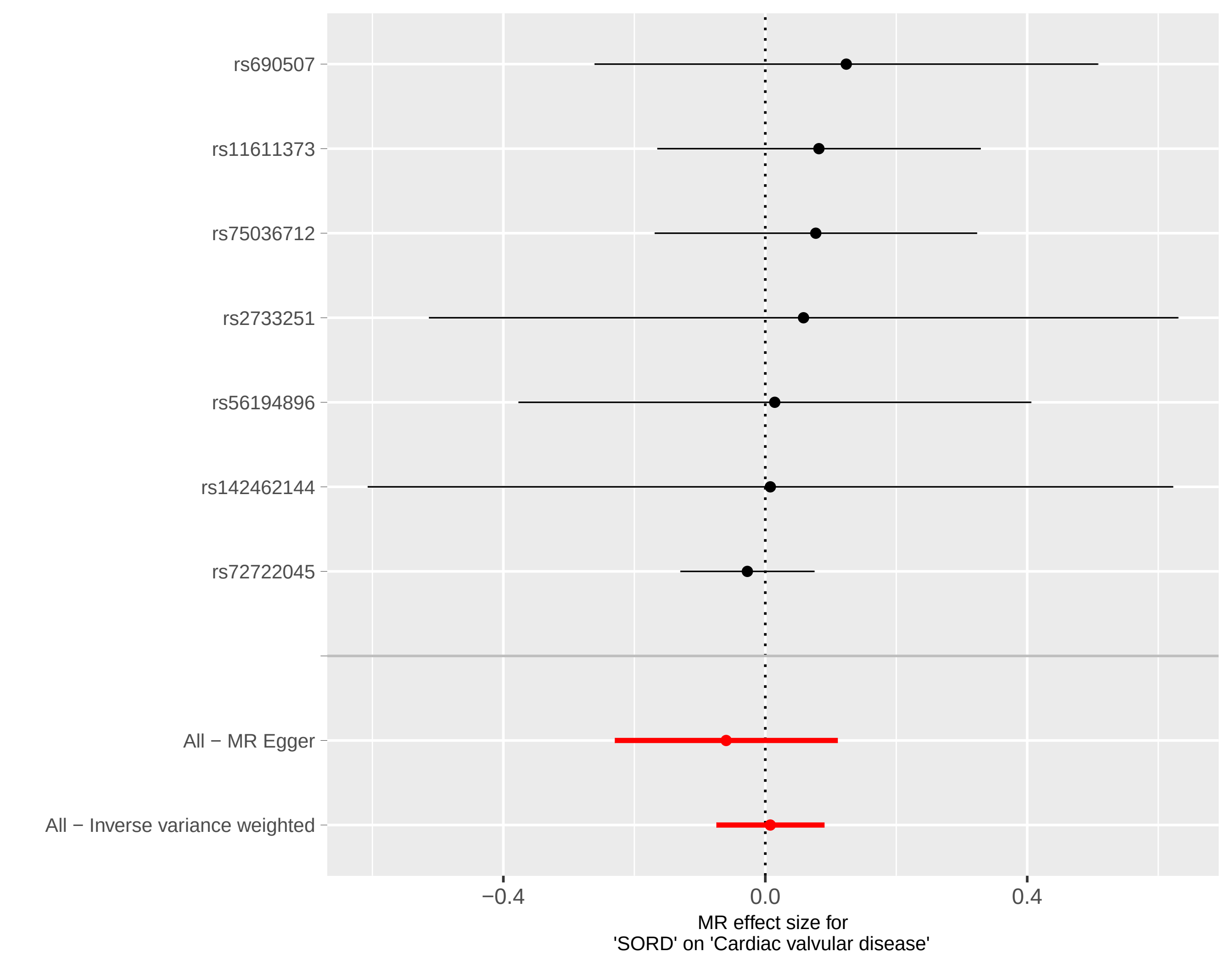
rs690507
rs11611373
rs75036712
rs2733251
rs56194896
rs142462144
rs72722045
All − MR Egger
All − Inverse variance weighted
−0.4
0.0
0.4
MR effect size for
'SORD' on 'Cardiac valvular disease'

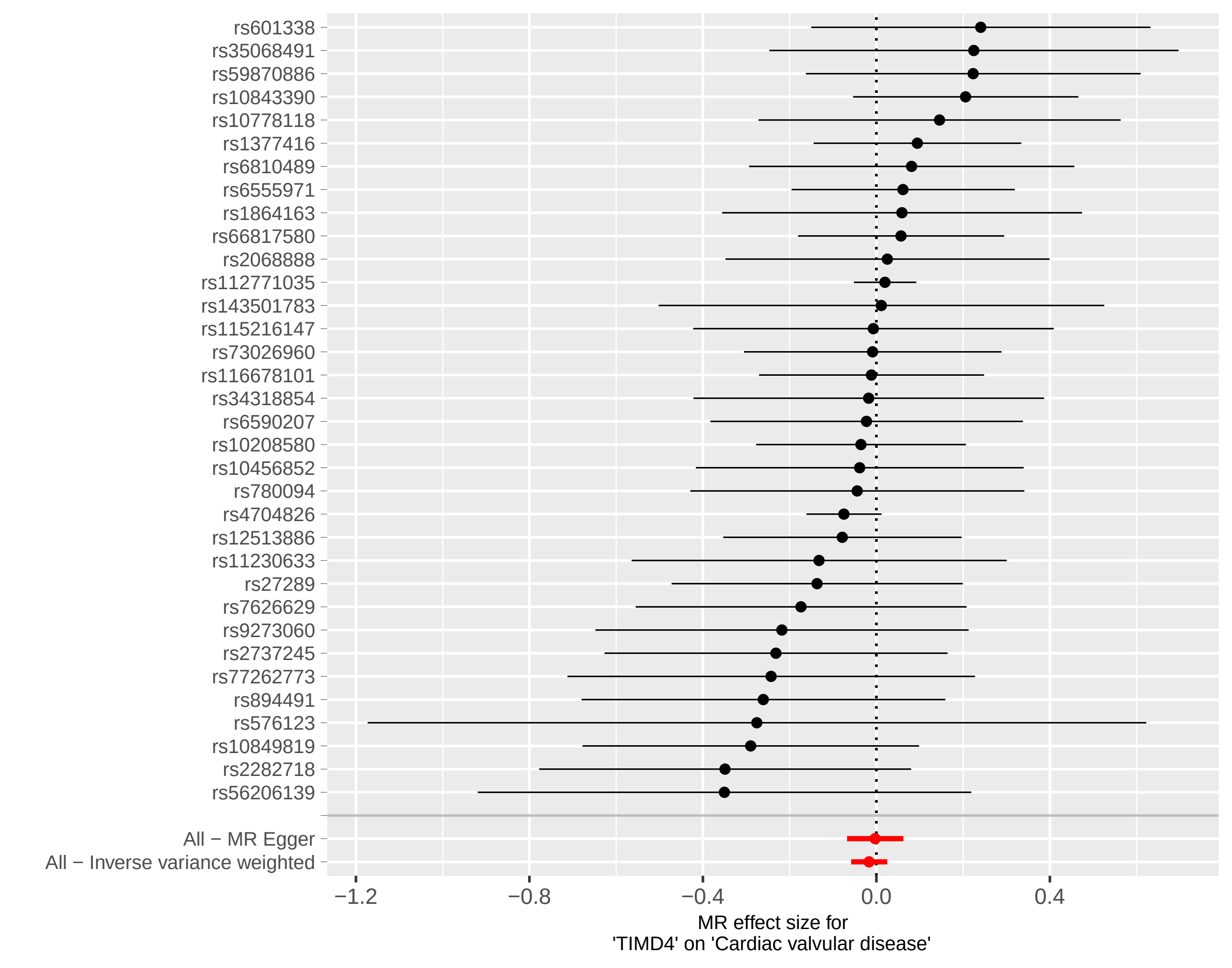
rs601338
rs35068491
rs59870886
rs10843390
rs10778118
rs1377416
rs6810489
rs6555971
rs1864163
rs66817580
rs2068888
rs112771035
rs143501783
rs115216147
rs73026960
rs116678101
rs34318854
rs6590207
rs10208580
rs10456852
rs780094
rs4704826
rs12513886
rs11230633
rs27289
rs7626629
rs9273060
rs2737245
rs77262773
rs894491
rs576123
rs10849819
rs2282718
rs56206139
All − MR Egger
All − Inverse variance weighted
−1.2
−0.8
−0.4
0.0
0.4
MR effect size for
'TIMD4' on 'Cardiac valvular disease'

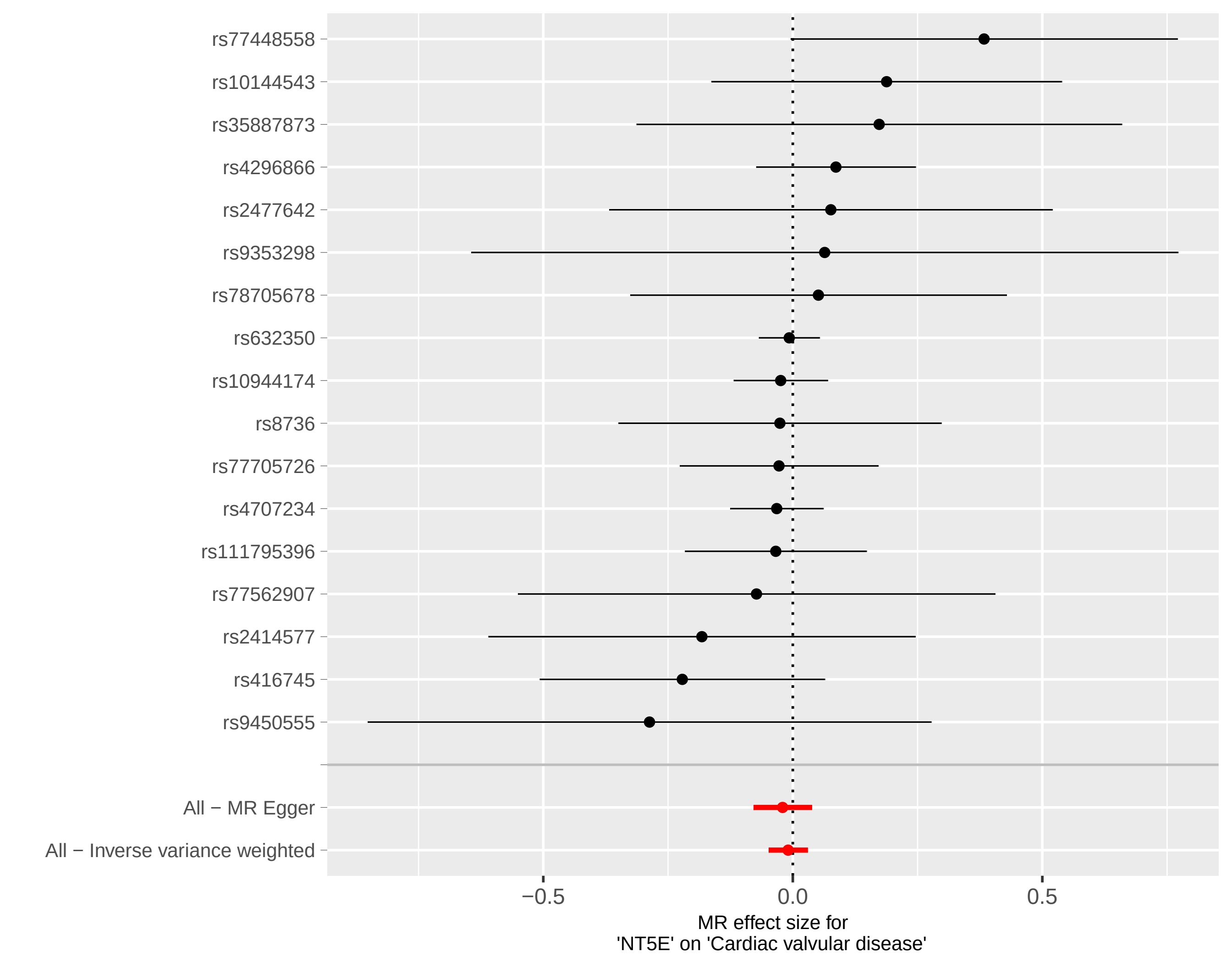
rs77448558
rs10144543
rs35887873
rs4296866
rs2477642
rs9353298
rs78705678
rs632350
rs10944174
rs8736
rs77705726
rs4707234
rs111795396
rs77562907
rs2414577
rs416745
rs9450555
All − MR Egger
All − Inverse variance weighted
−0.5
0.0
0.5
MR effect size for
'NT5E' on 'Cardiac valvular disease'

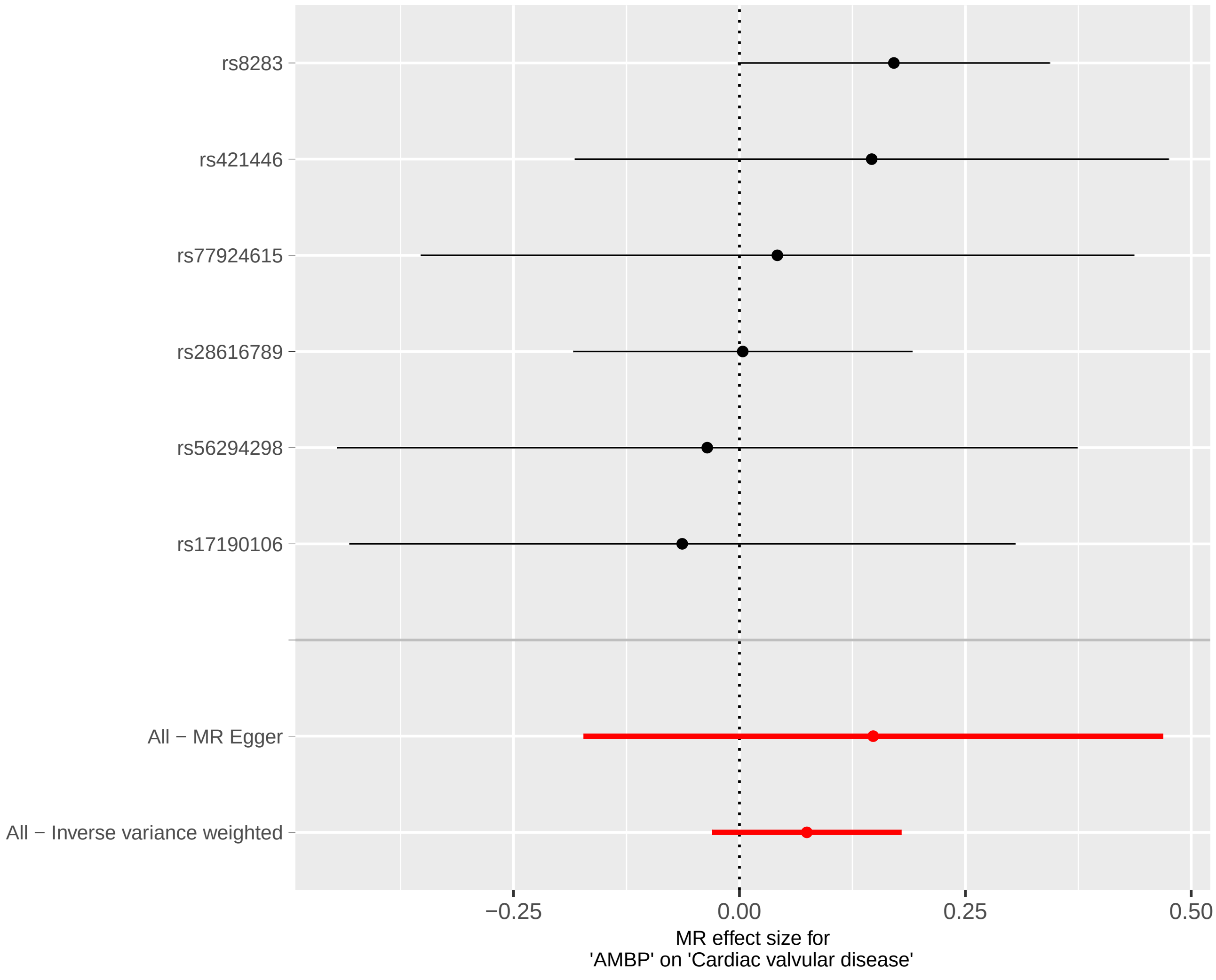
rs8283
rs421446
rs77924615
rs28616789
rs56294298
rs17190106
All − MR Egger
All − Inverse variance weighted
−0.25
0.00
0.25
0.50
MR effect size for
'AMBP' on 'Cardiac valvular disease'

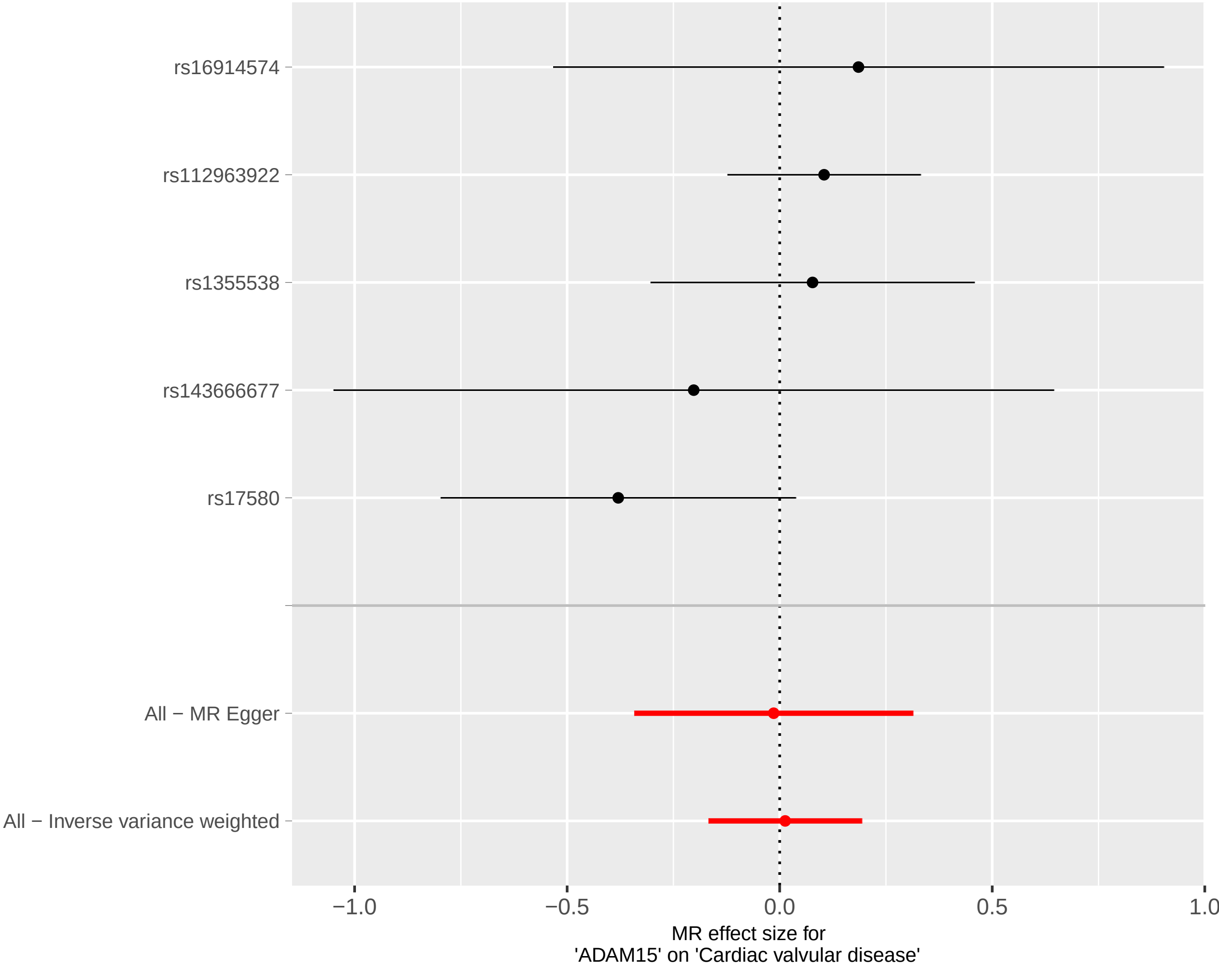

rs16914574
rs112963922
rs1355538
rs143666677
rs17580
All − MR Egger
All − Inverse variance weighted
−1.0
−0.5
0.0
0.5
1.0
MR effect size for
'ADAM15' on 'Cardiac valvular disease'

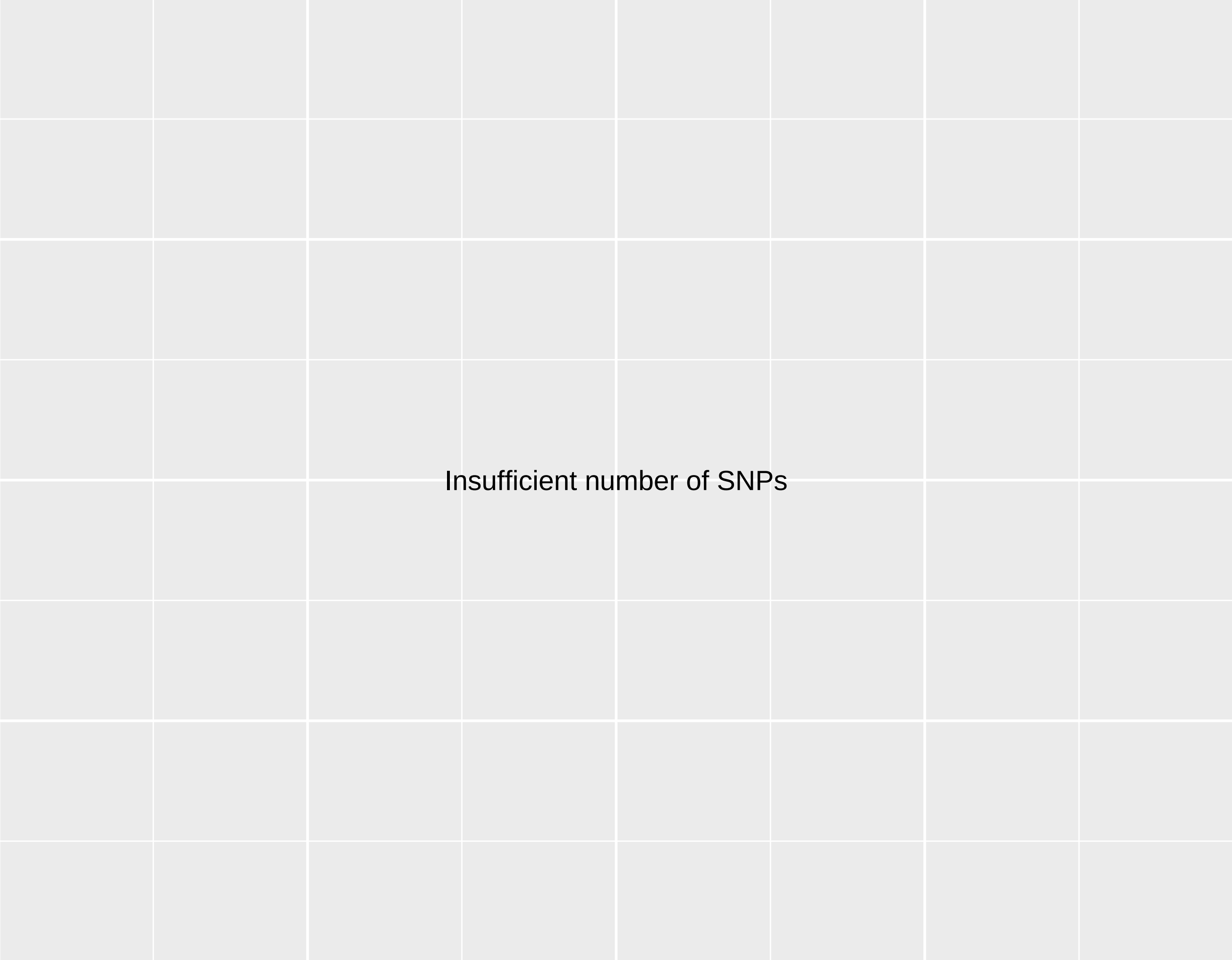
Insufficient number of SNPs

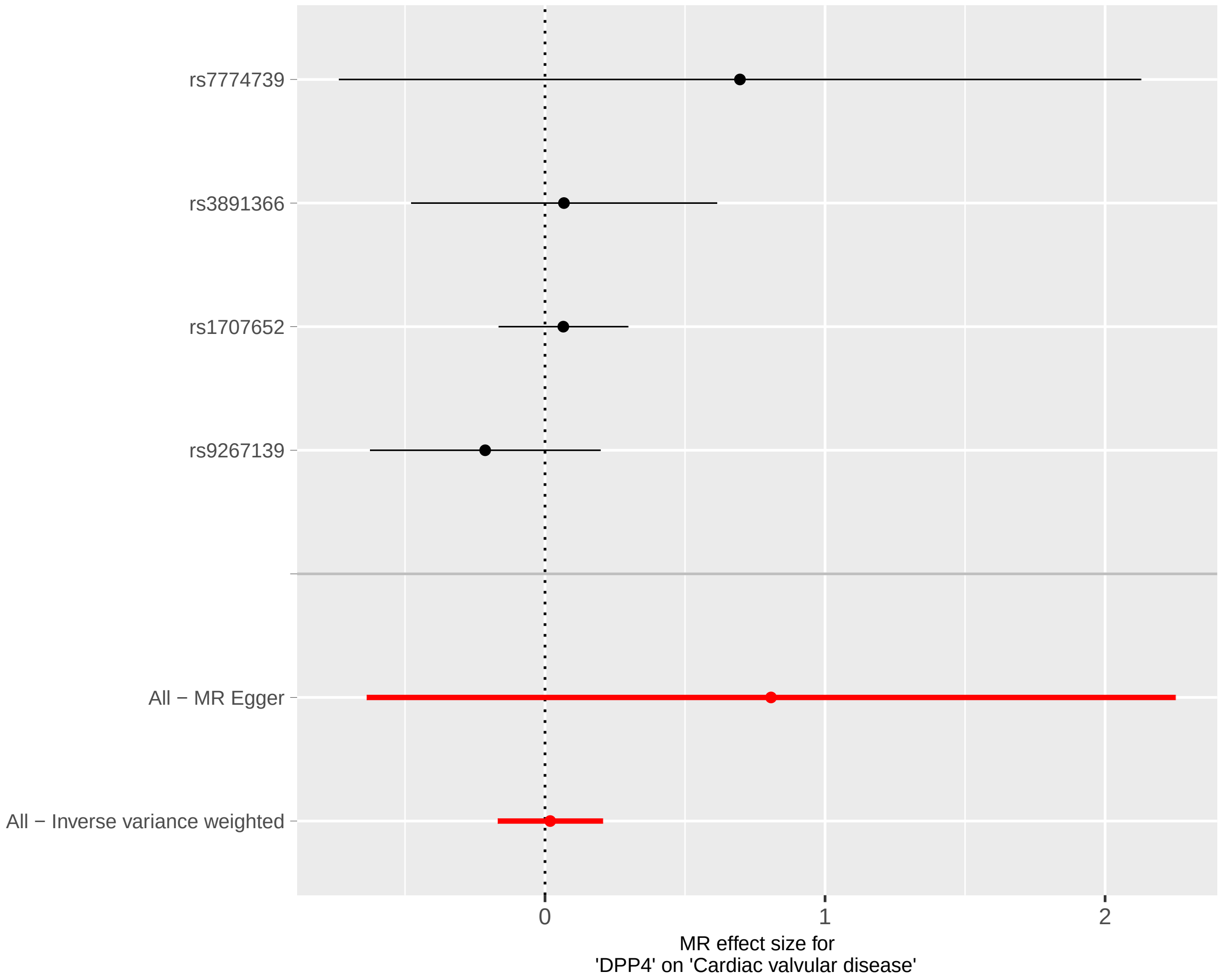
rs7774739
rs3891366
rs1707652
rs9267139
All − MR Egger
All − Inverse variance weighted
0
1
2
MR effect size for
'DPP4' on 'Cardiac valvular disease'

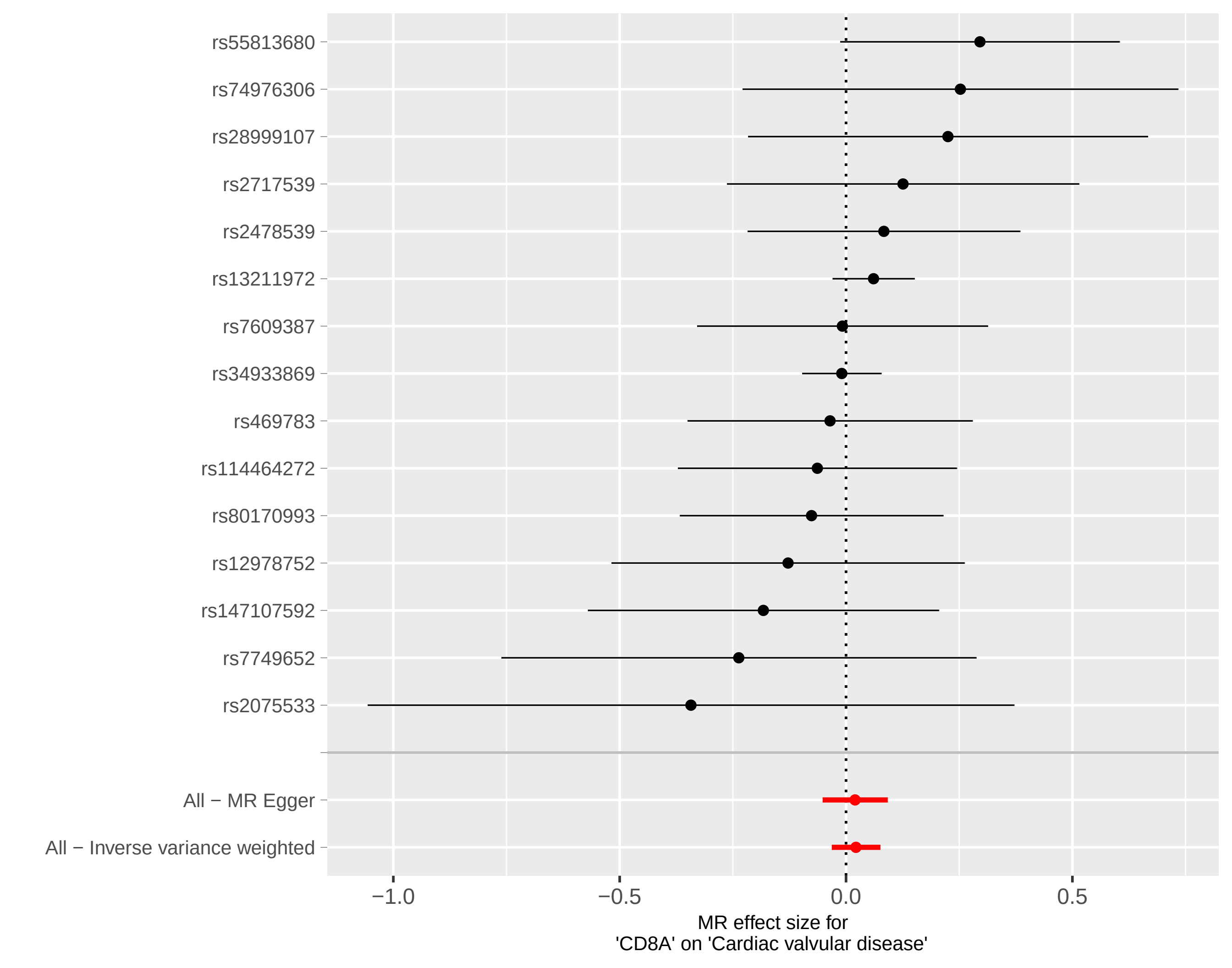
rs55813680
rs74976306
rs28999107
rs2717539
rs2478539
rs13211972
rs7609387
rs34933869
rs469783
rs114464272
rs80170993
rs12978752
rs147107592
rs7749652
rs2075533
All − MR Egger
All − Inverse variance weighted
−1.0
−0.5
0.0
0.5
MR effect size for
'CD8A' on 'Cardiac valvular disease'

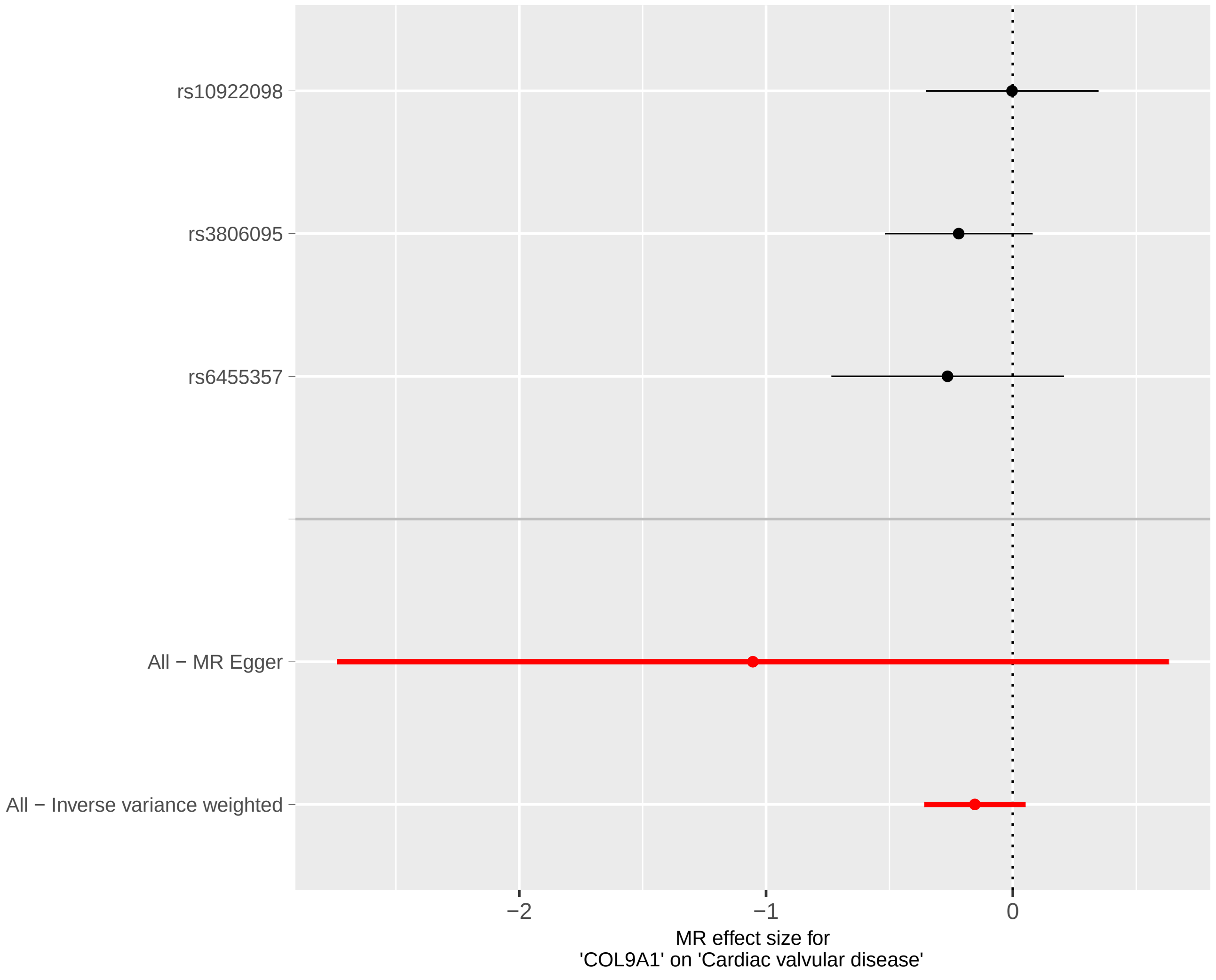
rs10922098
rs3806095
rs6455357
All − MR Egger
All − Inverse variance weighted
−2
−1
0
MR effect size for
'COL9A1' on 'Cardiac valvular disease'

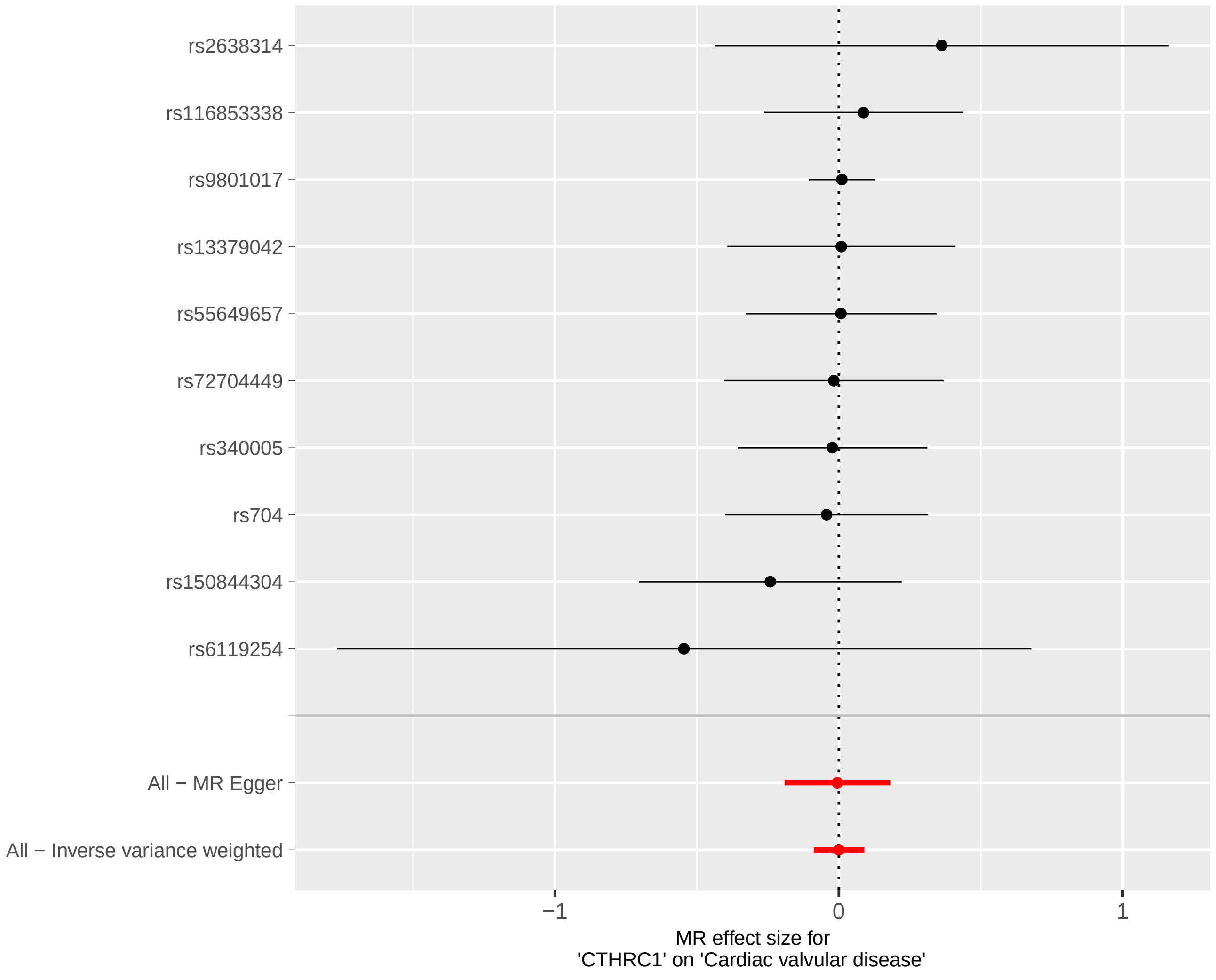
rs2638314
rs116853338
rs9801017
rs13379042
rs55649657
rs72704449
rs340005
rs704
rs150844304
rs6119254
All − MR Egger
All − Inverse variance weighted
−1
0
1
MR effect size for
'CTHRC1' on 'Cardiac valvular disease'

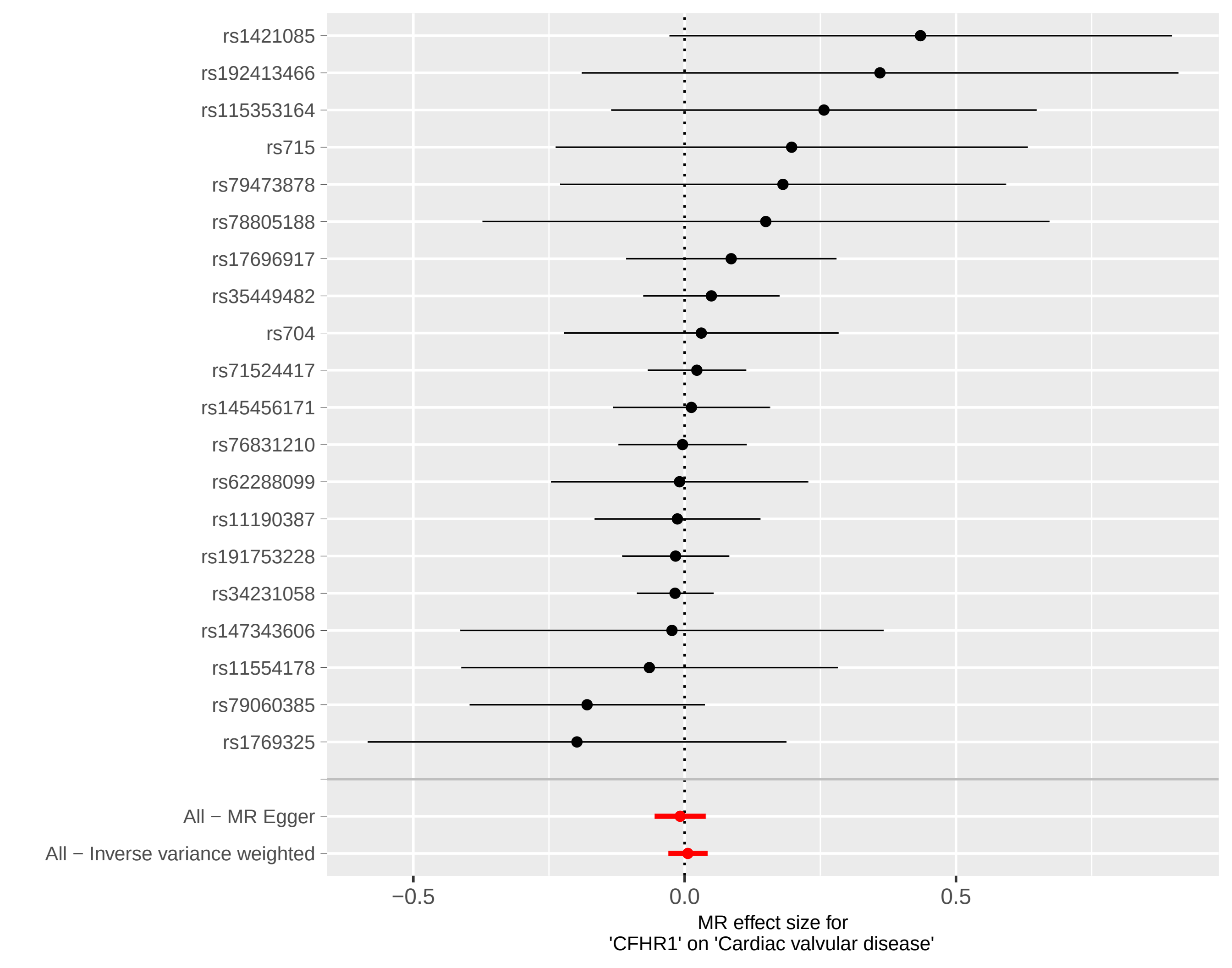
rs1421085
rs192413466
rs115353164
rs715
rs79473878
rs78805188
rs17696917
rs35449482
rs704
rs71524417
rs145456171
rs76831210
rs62288099
rs11190387
rs191753228
rs34231058
rs147343606
rs11554178
rs79060385
rs1769325
All − MR Egger
All − Inverse variance weighted
−0.5
0.0
0.5
MR effect size for
'CFHR1' on 'Cardiac valvular disease'

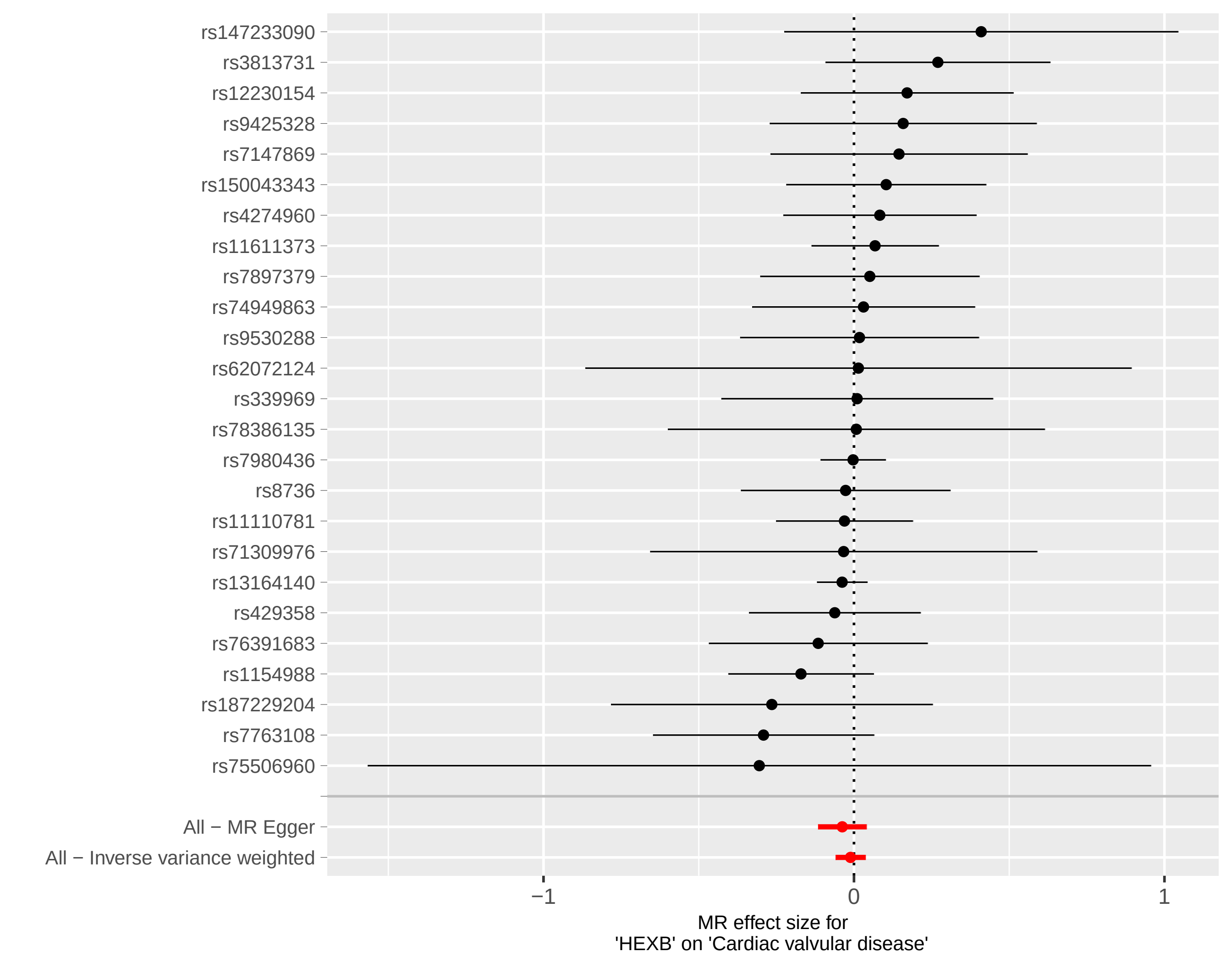
rs147233090
rs3813731
rs12230154
rs9425328
rs7147869
rs150043343
rs4274960
rs11611373
rs7897379
rs74949863
rs9530288
rs62072124
rs339969
rs78386135
rs7980436
rs8736
rs11110781
rs71309976
rs13164140
rs429358
rs76391683
rs1154988
rs187229204
rs7763108
rs75506960
All − MR Egger
All − Inverse variance weighted
−1
0
1
MR effect size for
'HEXB' on 'Cardiac valvular disease'

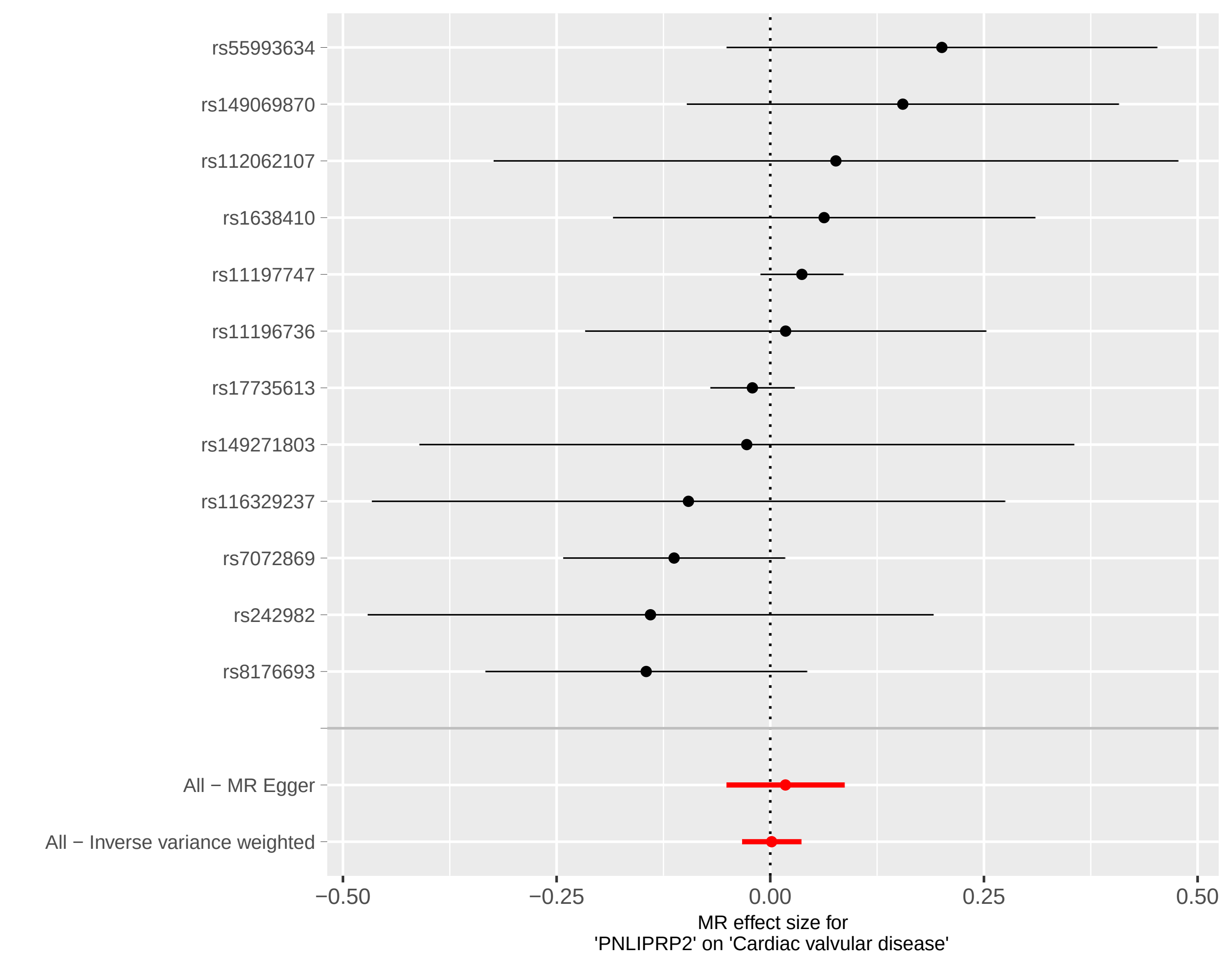

rs55993634
rs149069870
rs112062107
rs1638410
rs11197747
rs11196736
rs17735613
rs149271803
rs116329237
rs7072869
rs242982
rs8176693
All − MR Egger
All − Inverse variance weighted
−0.50
−0.25
0.00
0.25
0.50
MR effect size for
'PNLIPRP2' on 'Cardiac valvular disease'

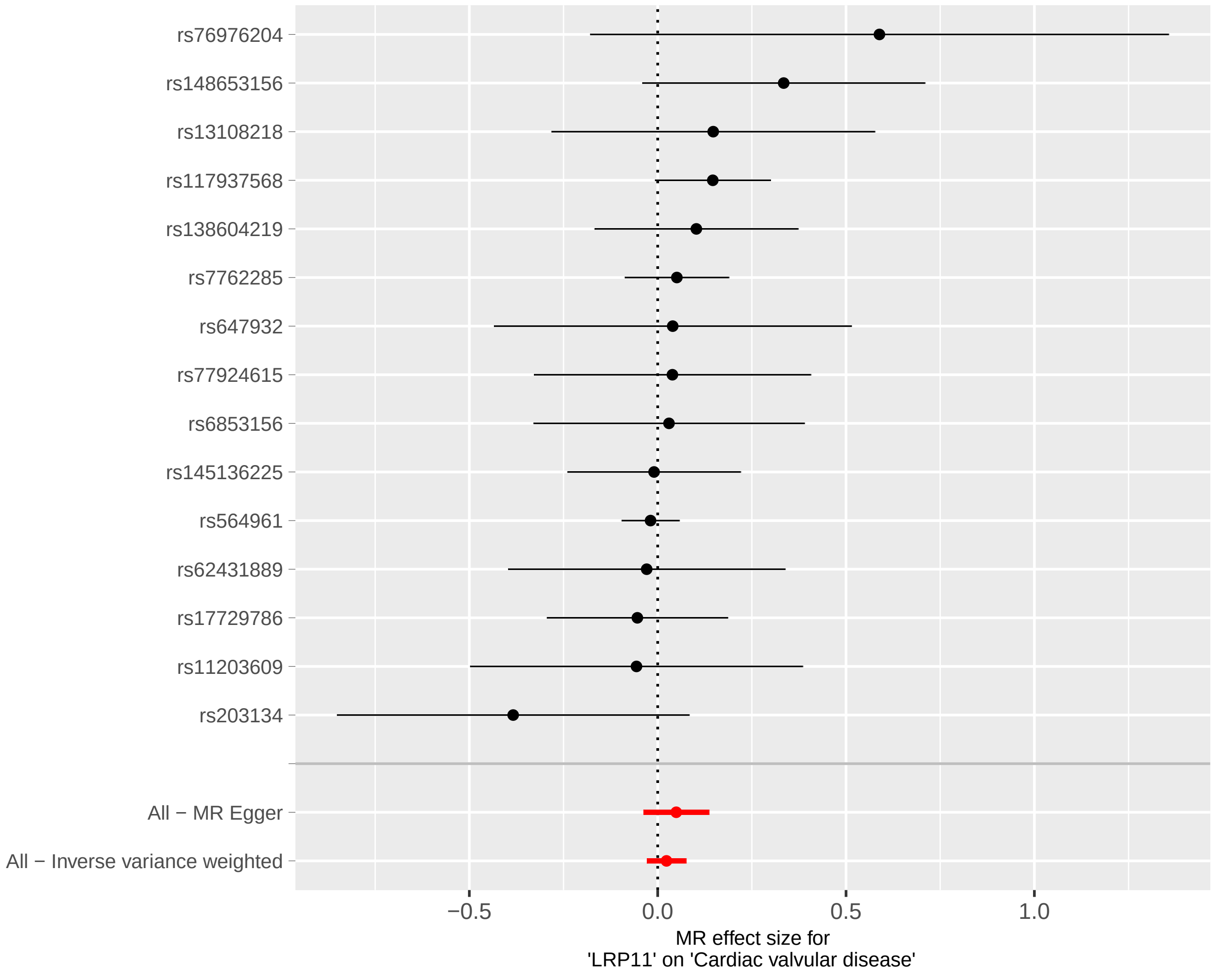
rs76976204
rs148653156
rs13108218
rs117937568
rs138604219
rs7762285
rs647932
rs77924615
rs6853156
rs145136225
rs564961
rs62431889
rs17729786
rs11203609
rs203134
All − MR Egger
All − Inverse variance weighted
−0.5
0.0
0.5
1.0
MR effect size for
'LRP11' on 'Cardiac valvular disease'

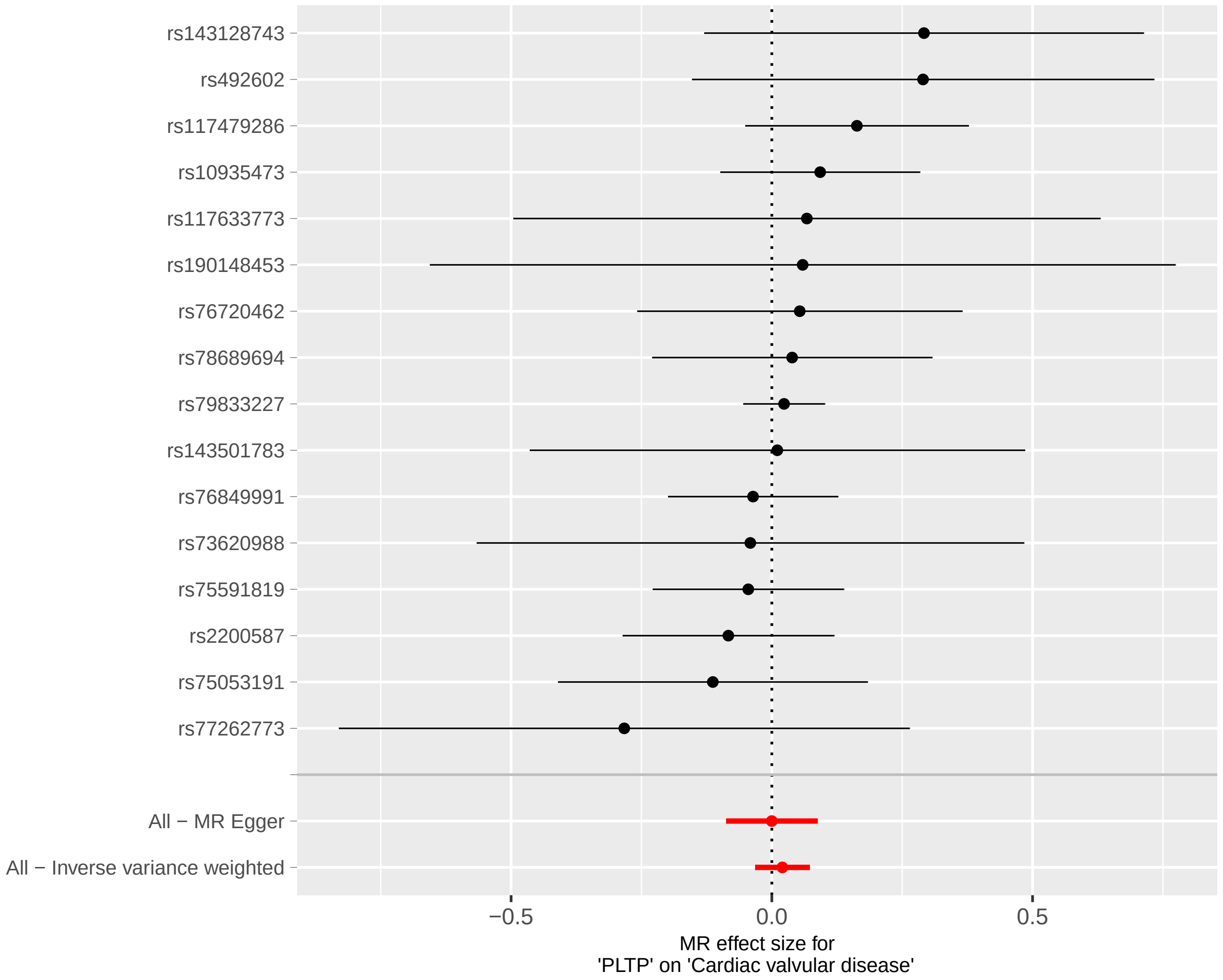

rs143128743
rs492602
rs117479286
rs10935473
rs117633773
rs190148453
rs76720462
rs78689694
rs79833227
rs143501783
rs76849991
rs73620988
rs75591819
rs2200587
rs75053191
rs77262773
All − MR Egger
All − Inverse variance weighted
−0.5
0.0
0.5
MR effect size for
'PLTP' on 'Cardiac valvular disease'

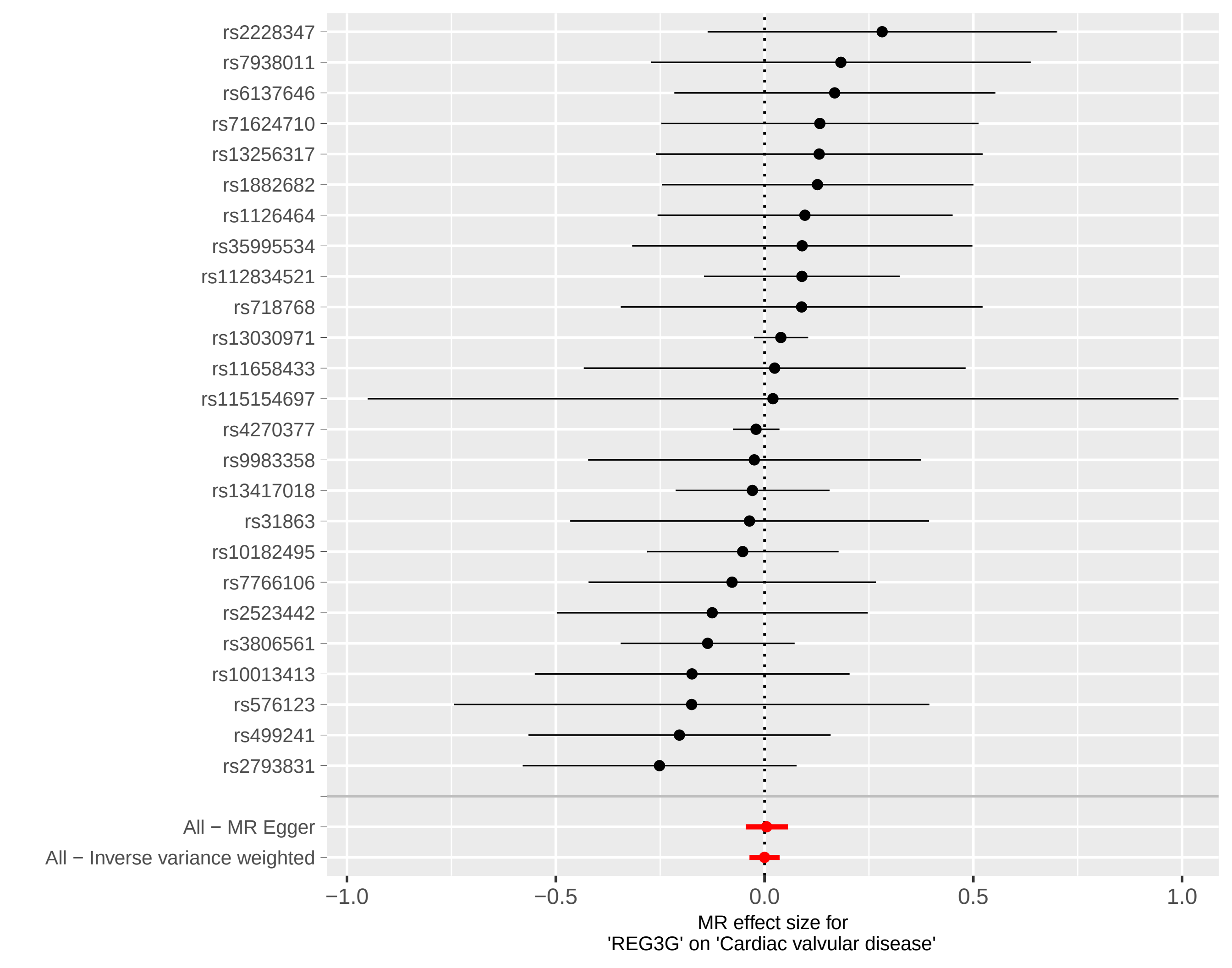

rs2228347
rs7938011
rs6137646
rs71624710
rs13256317
rs1882682
rs1126464
rs35995534
rs112834521
rs718768
rs13030971
rs11658433
rs115154697
rs4270377
rs9983358
rs13417018
rs31863
rs10182495
rs7766106
rs2523442
rs3806561
rs10013413
rs576123
rs499241
rs2793831
All − MR Egger
All − Inverse variance weighted
−1.0
−0.5
0.0
0.5
1.0
MR effect size for
'REG3G' on 'Cardiac valvular disease'

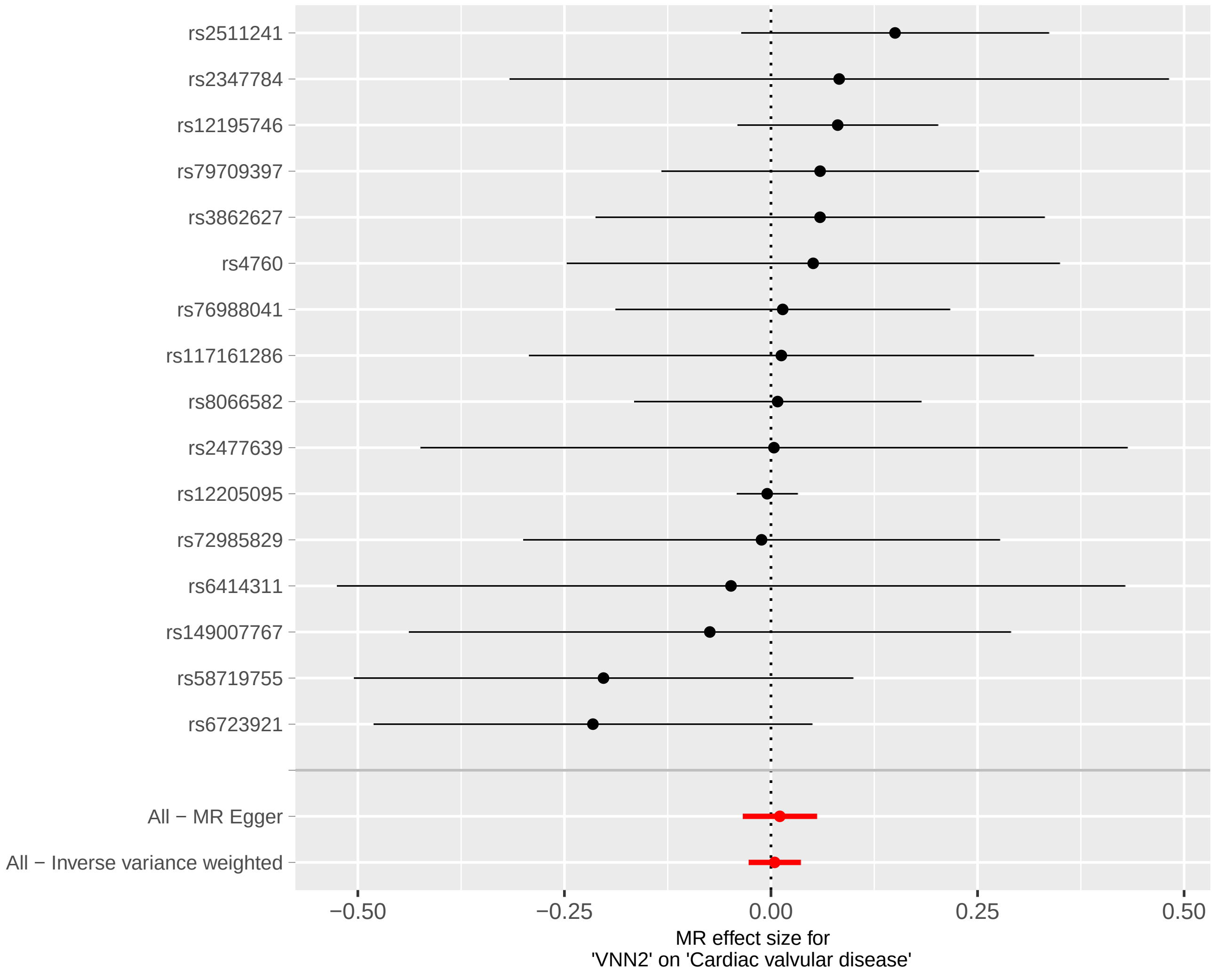

rs2511241
rs2347784
rs12195746
rs79709397
rs3862627
rs4760
rs76988041
rs117161286
rs8066582
rs2477639
rs12205095
rs72985829
rs6414311
rs149007767
rs58719755
rs6723921
All − MR Egger
All − Inverse variance weighted
−0.50
−0.25
0.00
0.25
0.50
MR effect size for
'VNN2' on 'Cardiac valvular disease'

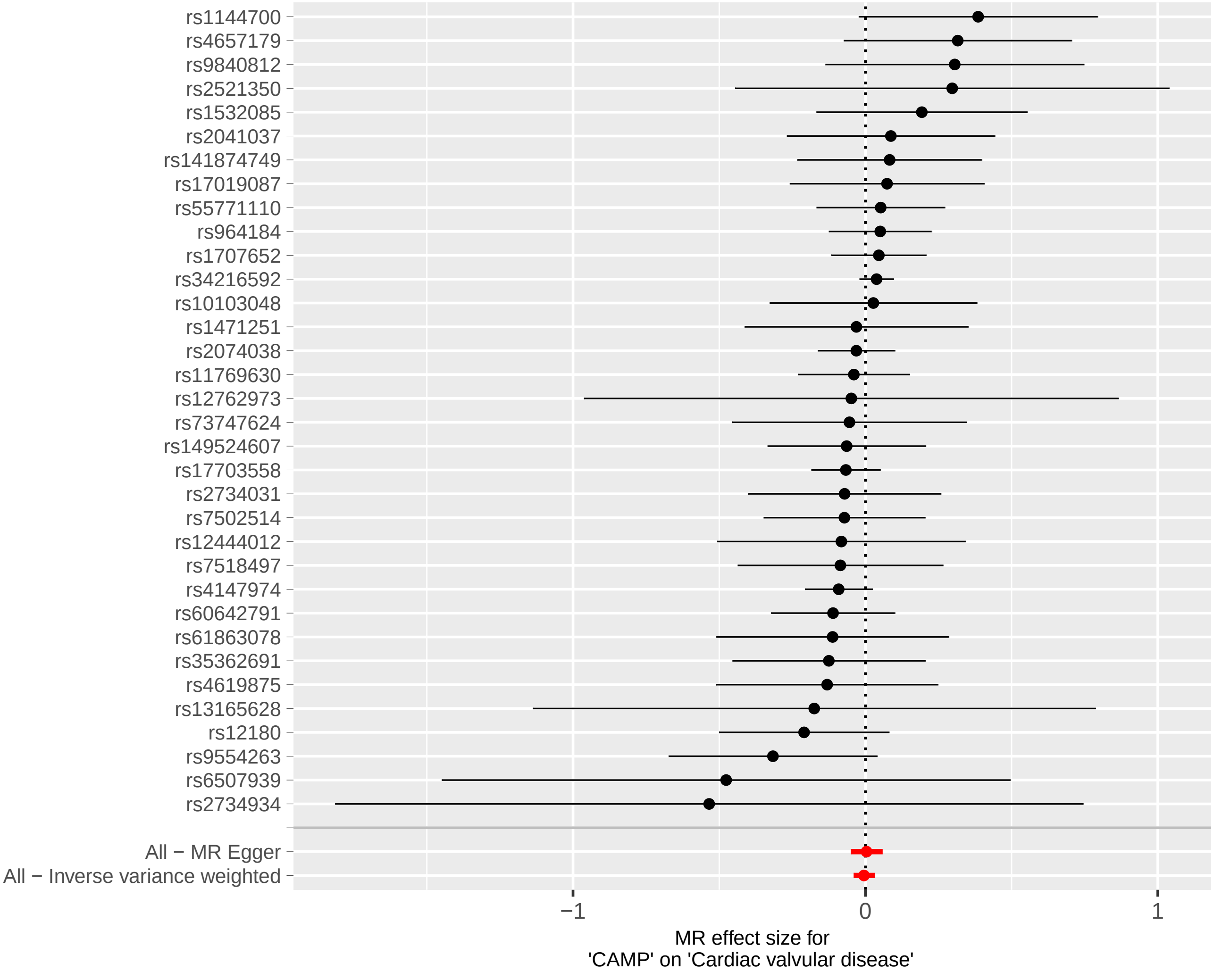
rs1144700
rs4657179
rs9840812
rs2521350
rs1532085
rs2041037
rs141874749
rs17019087
rs55771110
rs964184
rs1707652
rs34216592
rs10103048
rs1471251
rs2074038
rs11769630
rs12762973
rs73747624
rs149524607
rs17703558
rs2734031
rs7502514
rs12444012
rs7518497
rs4147974
rs60642791
rs61863078
rs35362691
rs4619875
rs13165628
rs12180
rs9554263
rs6507939
rs2734934
All − MR Egger
All − Inverse variance weighted
−1
0
1
MR effect size for
'CAMP' on 'Cardiac valvular disease'

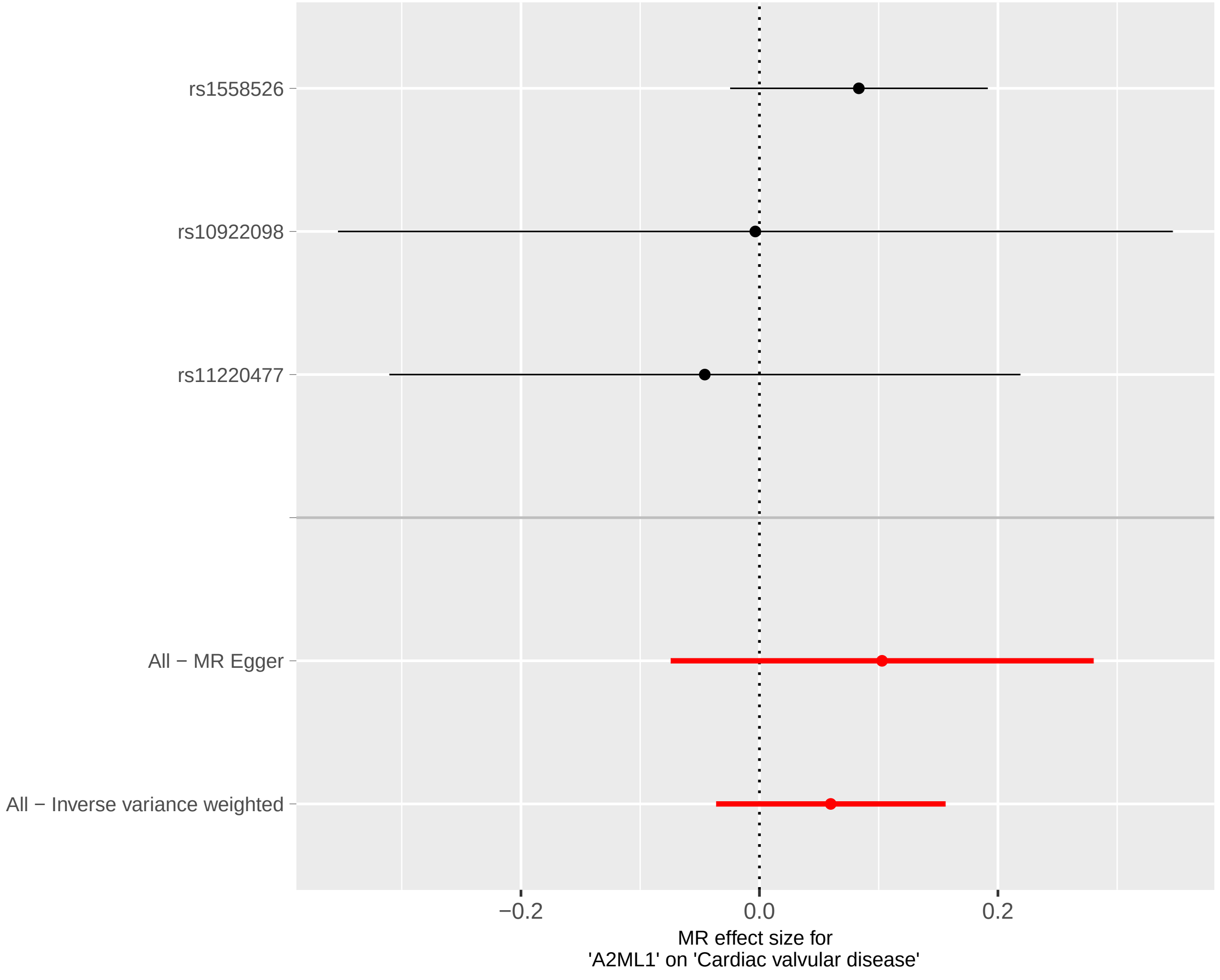
rs1558526
rs10922098
rs11220477
All − MR Egger
All − Inverse variance weighted
−0.2
0.0
0.2
MR effect size for
'A2ML1' on 'Cardiac valvular disease'

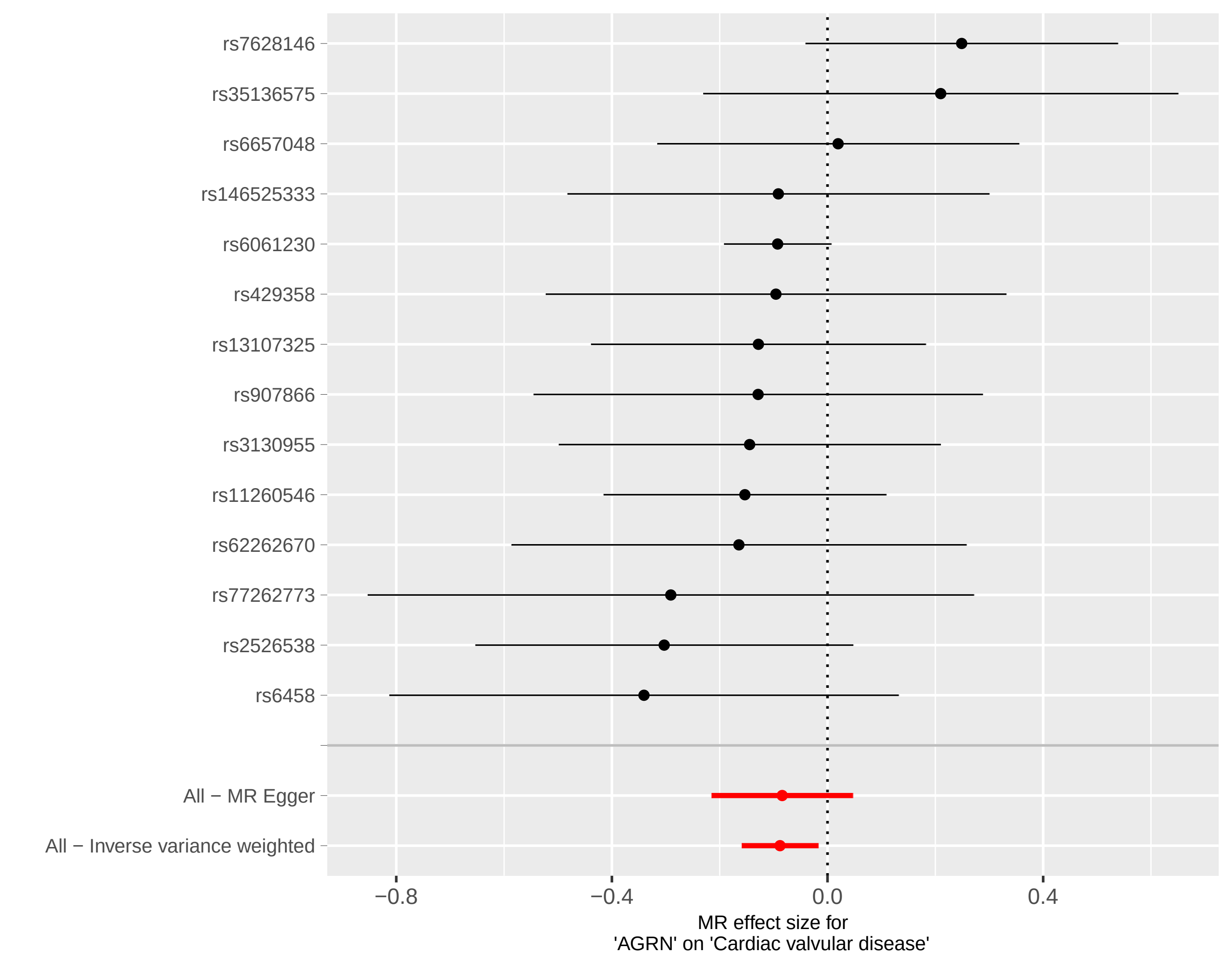
rs7628146
rs35136575
rs6657048
rs146525333
rs6061230
rs429358
rs13107325
rs907866
rs3130955
rs11260546
rs62262670
rs77262773
rs2526538
rs6458
All − MR Egger
All − Inverse variance weighted
−0.8
−0.4
0.0
0.4
MR effect size for
'AGRN' on 'Cardiac valvular disease'

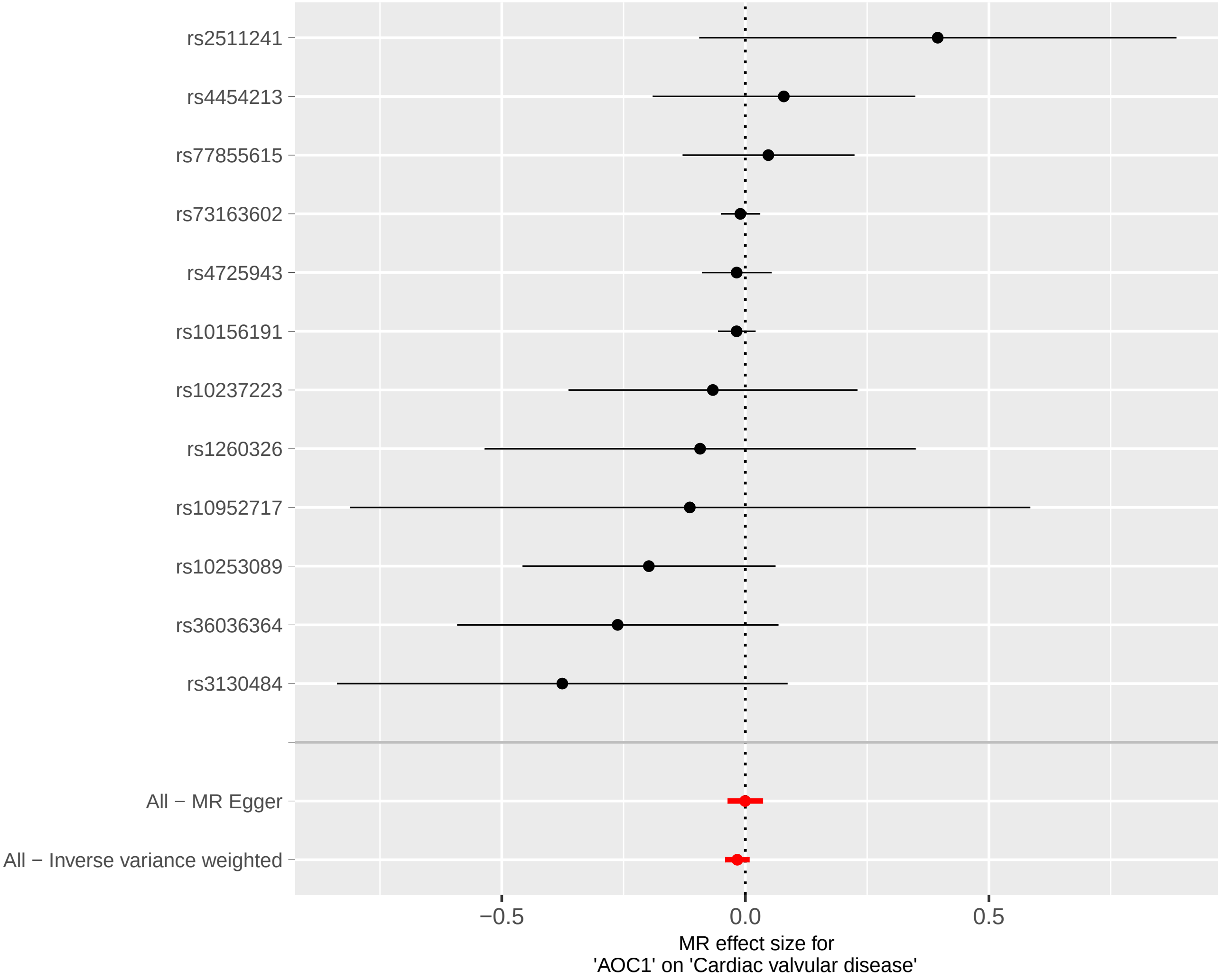
rs2511241
rs4454213
rs77855615
rs73163602
rs4725943
rs10156191
rs10237223
rs1260326
rs10952717
rs10253089
rs36036364
rs3130484
All − MR Egger
All − Inverse variance weighted
−0.5
0.0
0.5
MR effect size for
'AOC1' on 'Cardiac valvular disease'

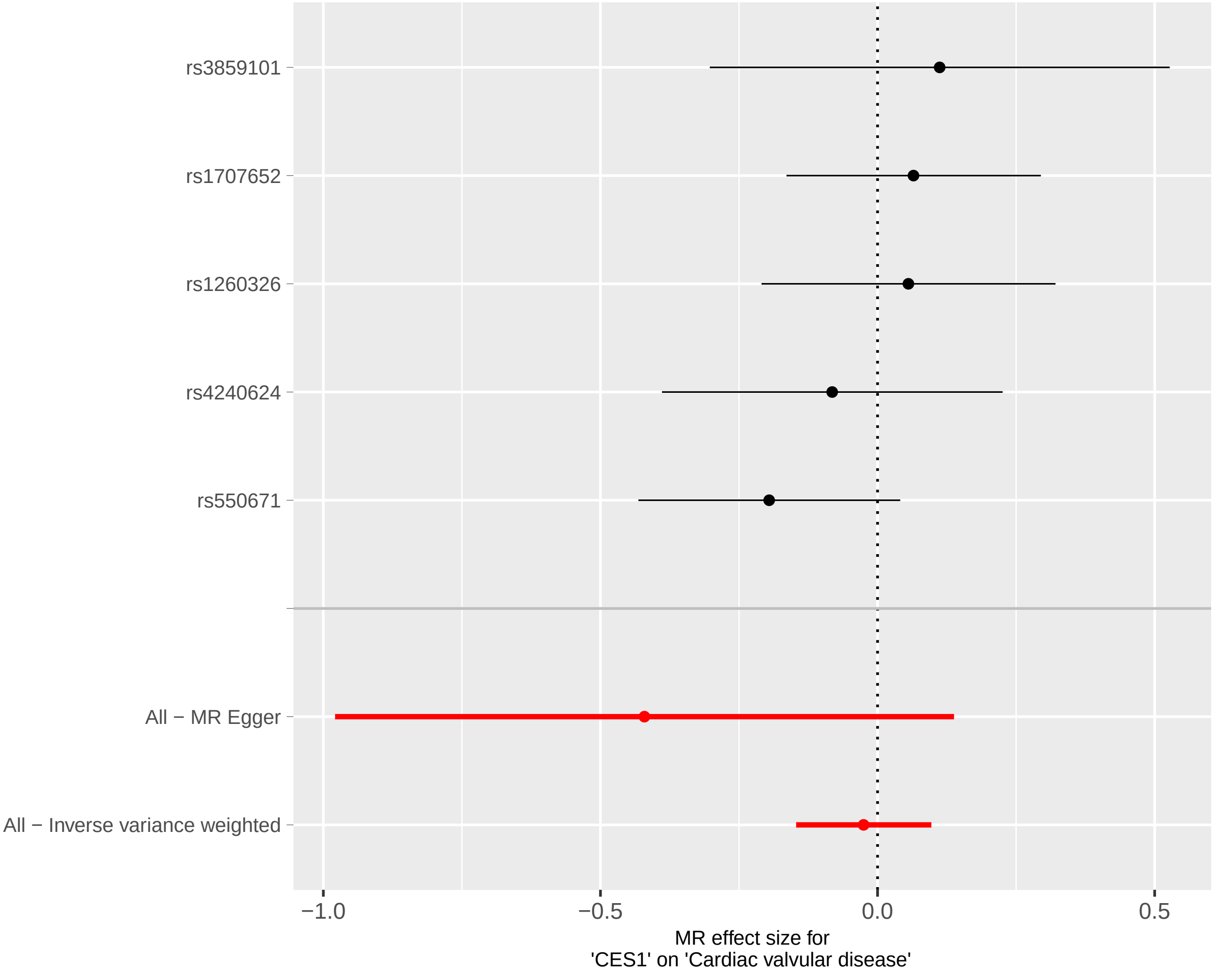
rs3859101
rs1707652
rs1260326
rs4240624
rs550671
All − MR Egger
All − Inverse variance weighted
−1.0
−0.5
0.0
0.5
MR effect size for
'CES1' on 'Cardiac valvular disease'

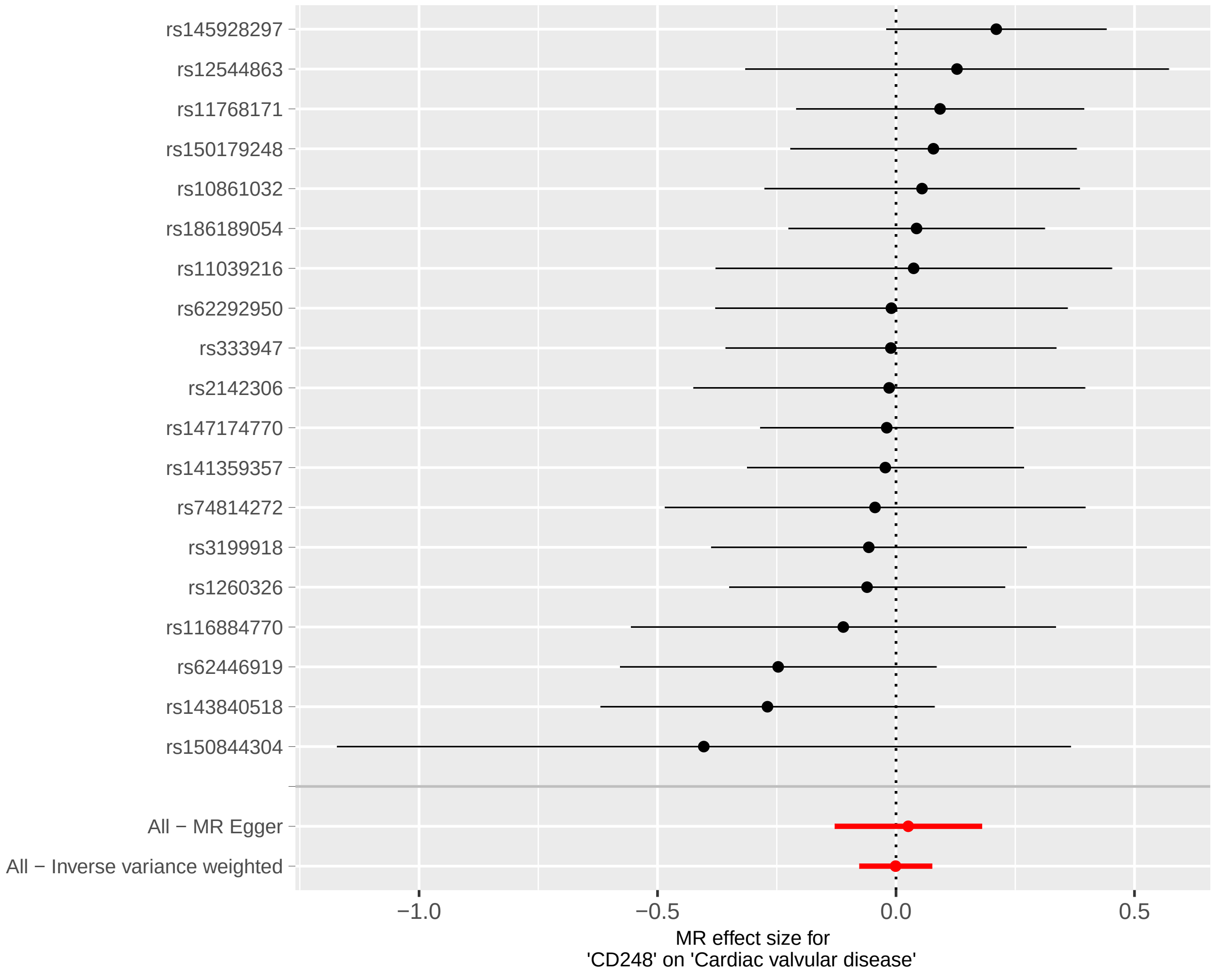
rs145928297
rs12544863
rs11768171
rs150179248
rs10861032
rs186189054
rs11039216
rs62292950
rs333947
rs2142306
rs147174770
rs141359357
rs74814272
rs3199918
rs1260326
rs116884770
rs62446919
rs143840518
rs150844304
All − MR Egger
All − Inverse variance weighted
−1.0
−0.5
0.0
0.5
MR effect size for
'CD248' on 'Cardiac valvular disease'

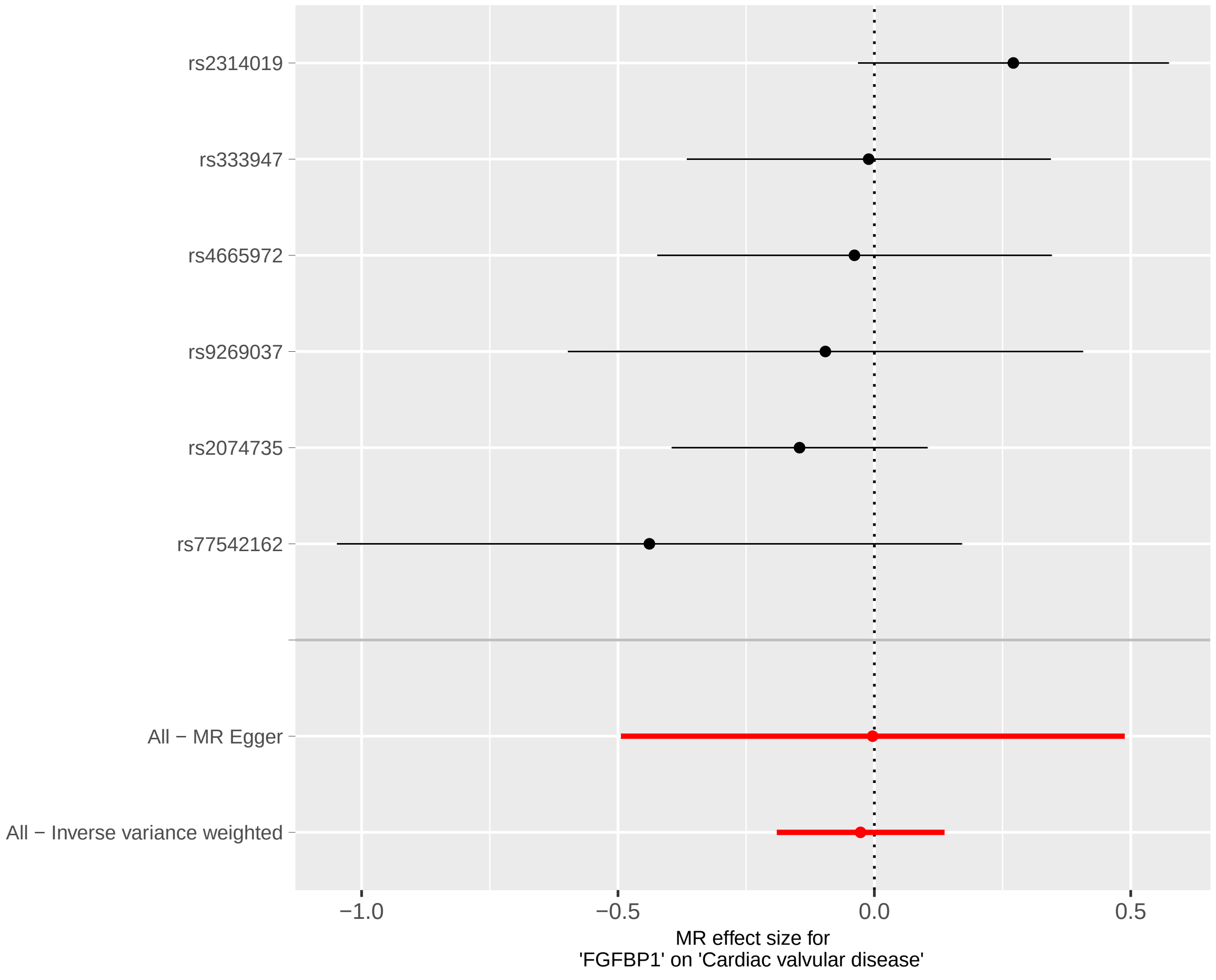
rs2314019
rs333947
rs4665972
rs9269037
rs2074735
rs77542162
All − MR Egger
All − Inverse variance weighted
−1.0
−0.5
0.0
0.5
MR effect size for
'FGFBP1' on 'Cardiac valvular disease'

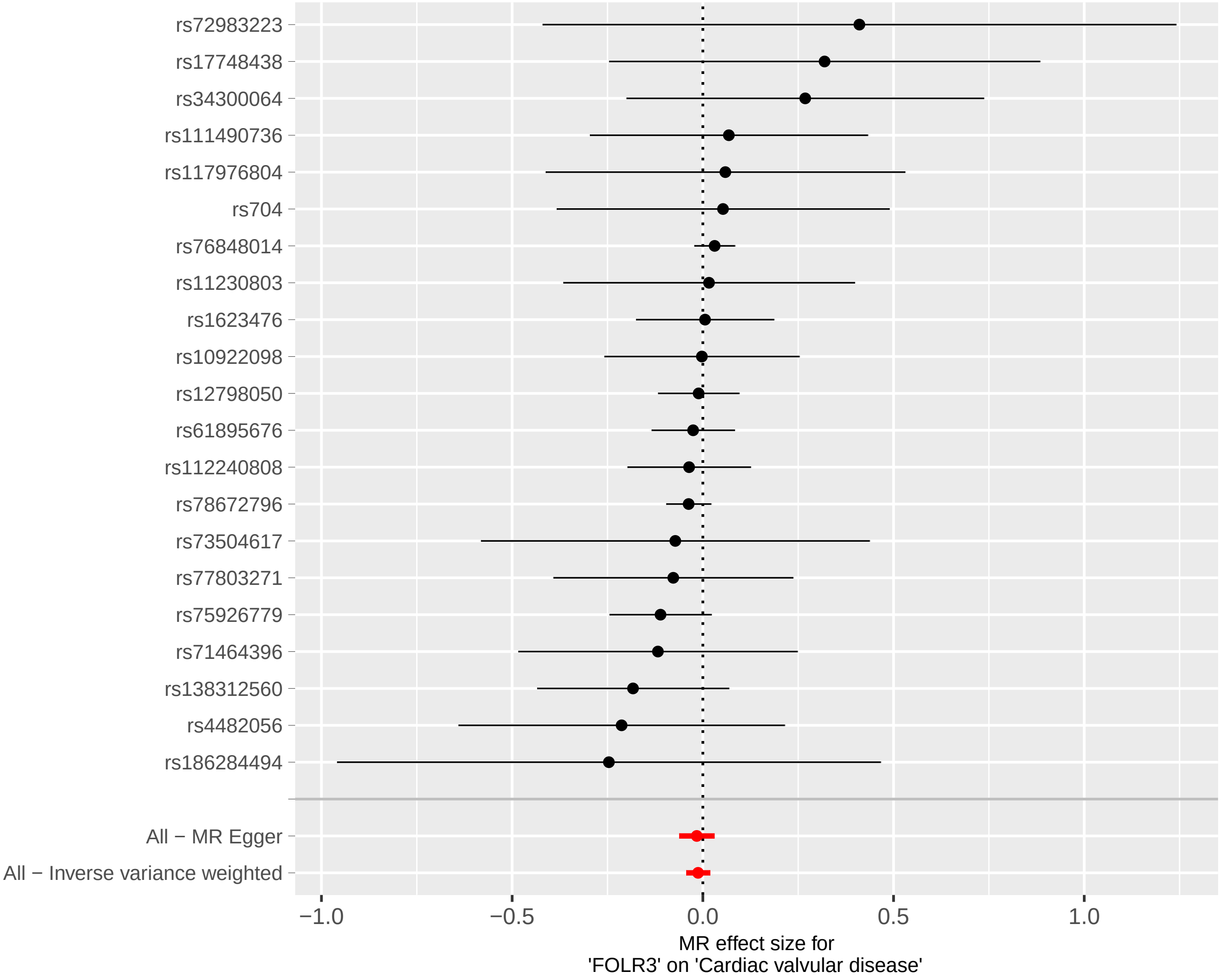

rs72983223
rs17748438
rs34300064
rs111490736
rs117976804
rs704
rs76848014
rs11230803
rs1623476
rs10922098
rs12798050
rs61895676
rs112240808
rs78672796
rs73504617
rs77803271
rs75926779
rs71464396
rs138312560
rs4482056
rs186284494
All − MR Egger
All − Inverse variance weighted
−1.0
−0.5
0.0
0.5
1.0
MR effect size for
'FOLR3' on 'Cardiac valvular disease'

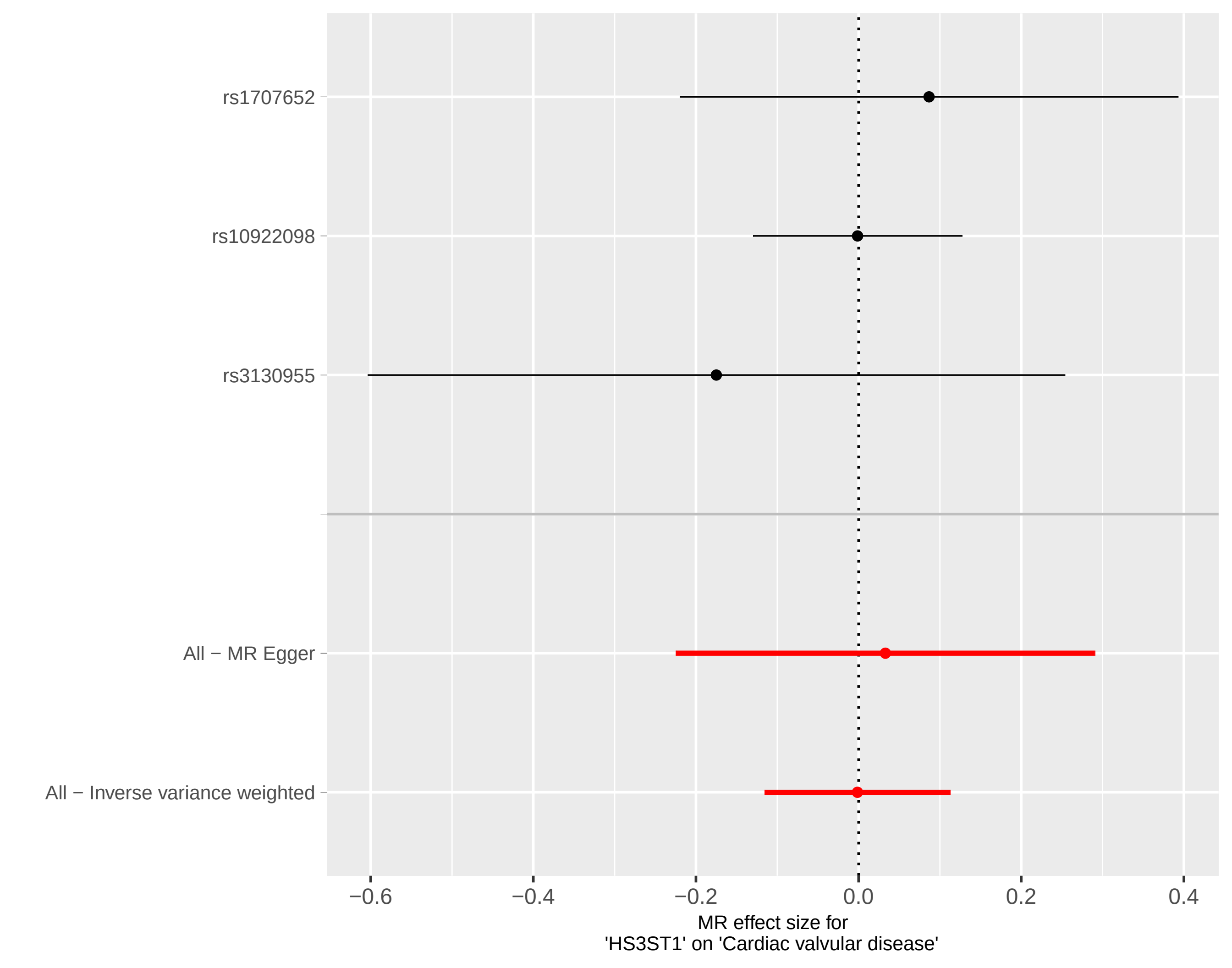

rs1707652
rs10922098
rs3130955
All − MR Egger
All − Inverse variance weighted
−0.6
−0.4
−0.2
0.0
0.2
0.4
MR effect size for
'HS3ST1' on 'Cardiac valvular disease'

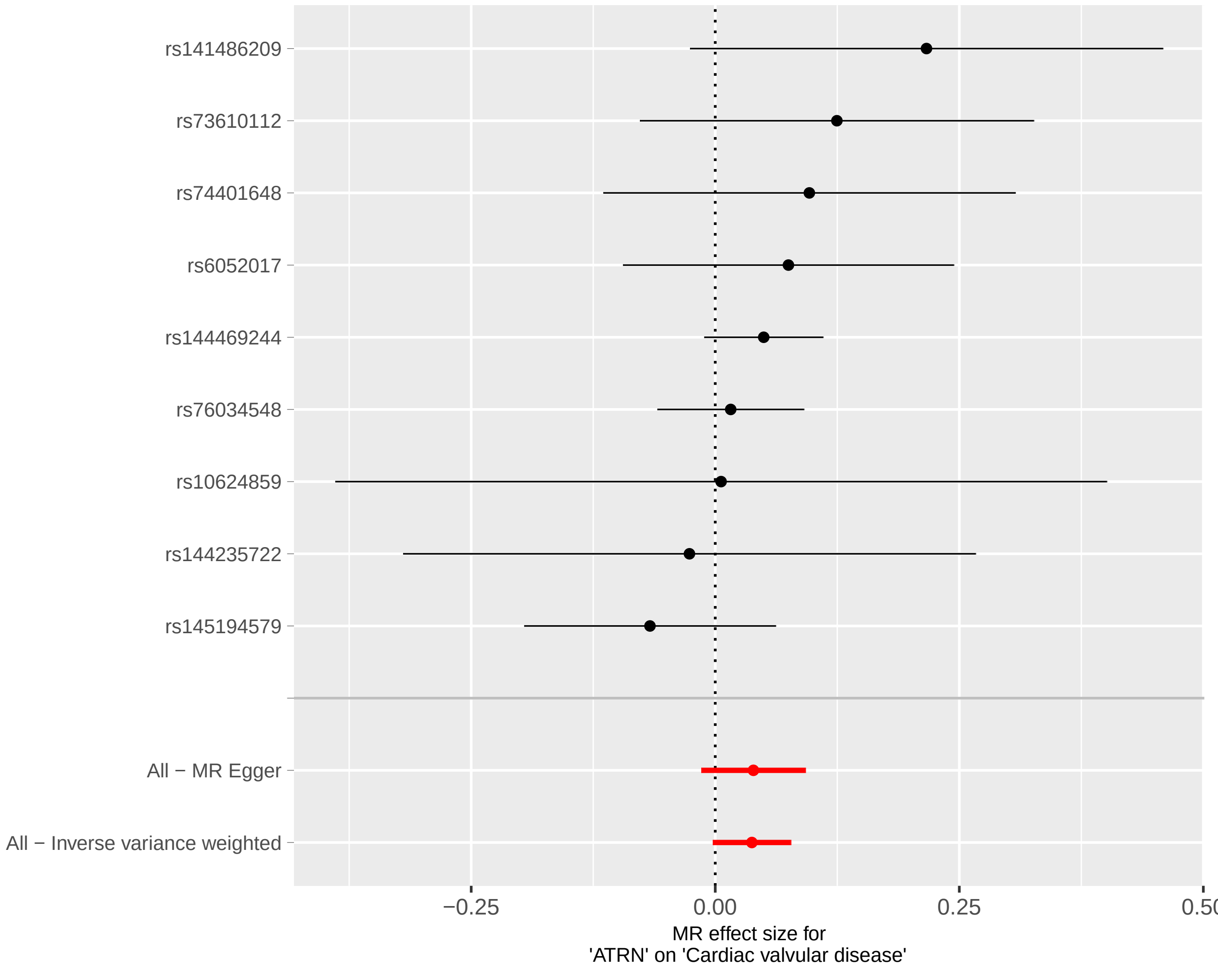
rs141486209
rs73610112
rs74401648
rs6052017
rs144469244
rs76034548
rs10624859
rs144235722
rs145194579
All − MR Egger
All − Inverse variance weighted
−0.25
0.00
0.25
0.50
MR effect size for
'ATRN' on 'Cardiac valvular disease'

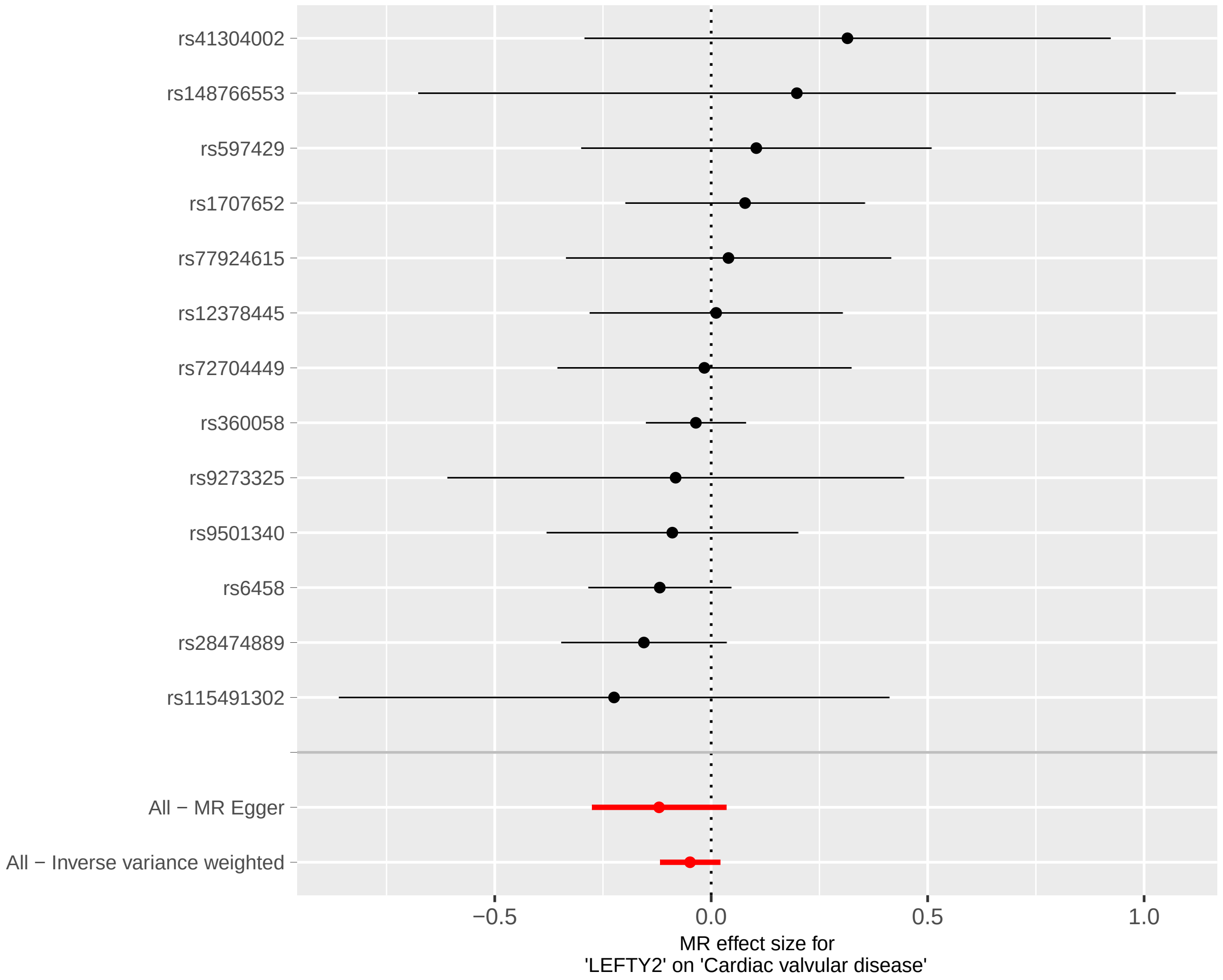
rs41304002
rs148766553
rs597429
rs1707652
rs77924615
rs12378445
rs72704449
rs360058
rs9273325
rs9501340
rs6458
rs28474889
rs115491302
All − MR Egger
All − Inverse variance weighted
−0.5
0.0
0.5
1.0
MR effect size for
'LEFTY2' on 'Cardiac valvular disease'

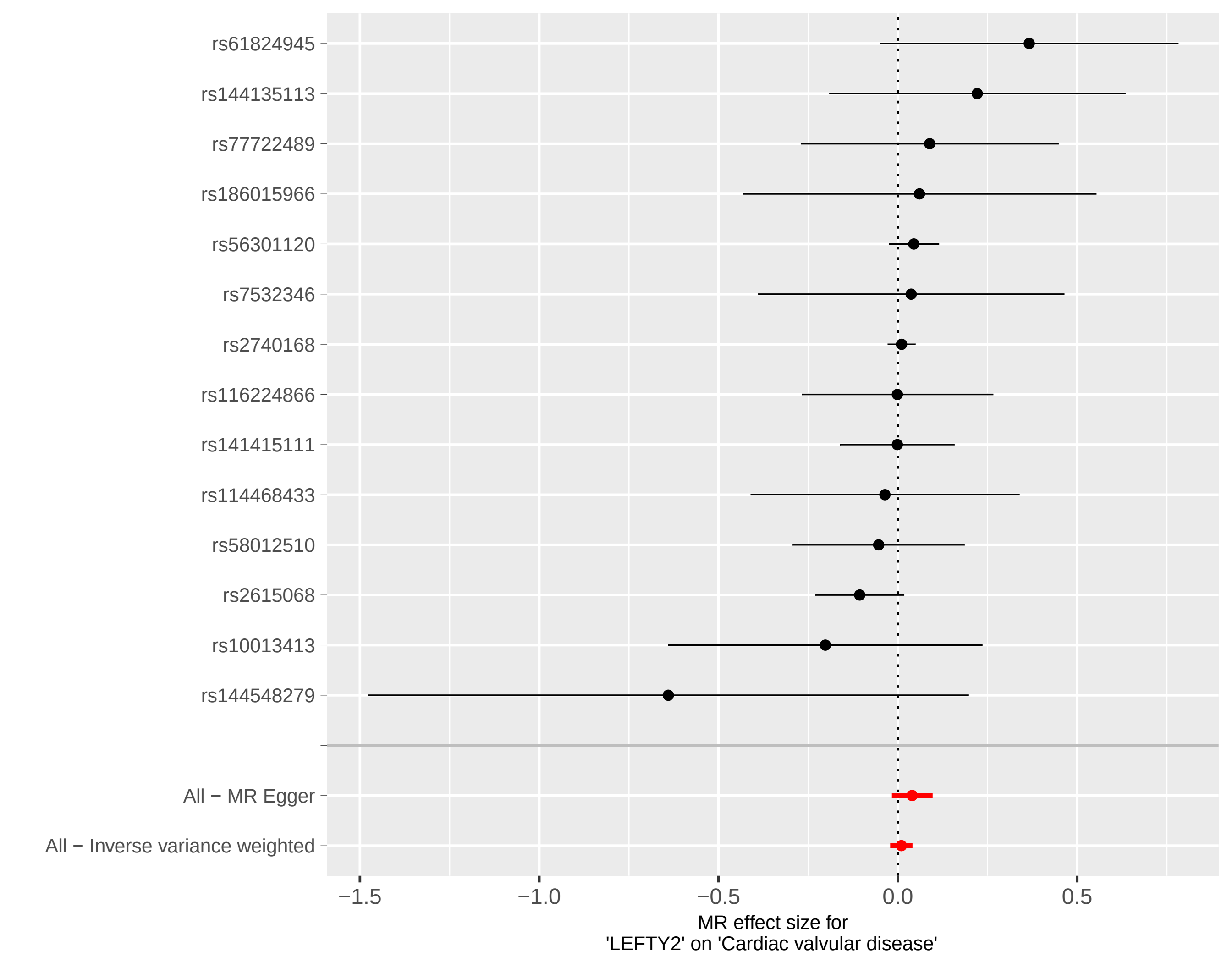
rs61824945
rs144135113
rs77722489
rs186015966
rs56301120
rs7532346
rs2740168
rs116224866
rs141415111
rs114468433
rs58012510
rs2615068
rs10013413
rs144548279
All − MR Egger
All − Inverse variance weighted
−1.5
−1.0
−0.5
0.0
0.5
MR effect size for
'LEFTY2' on 'Cardiac valvular disease'

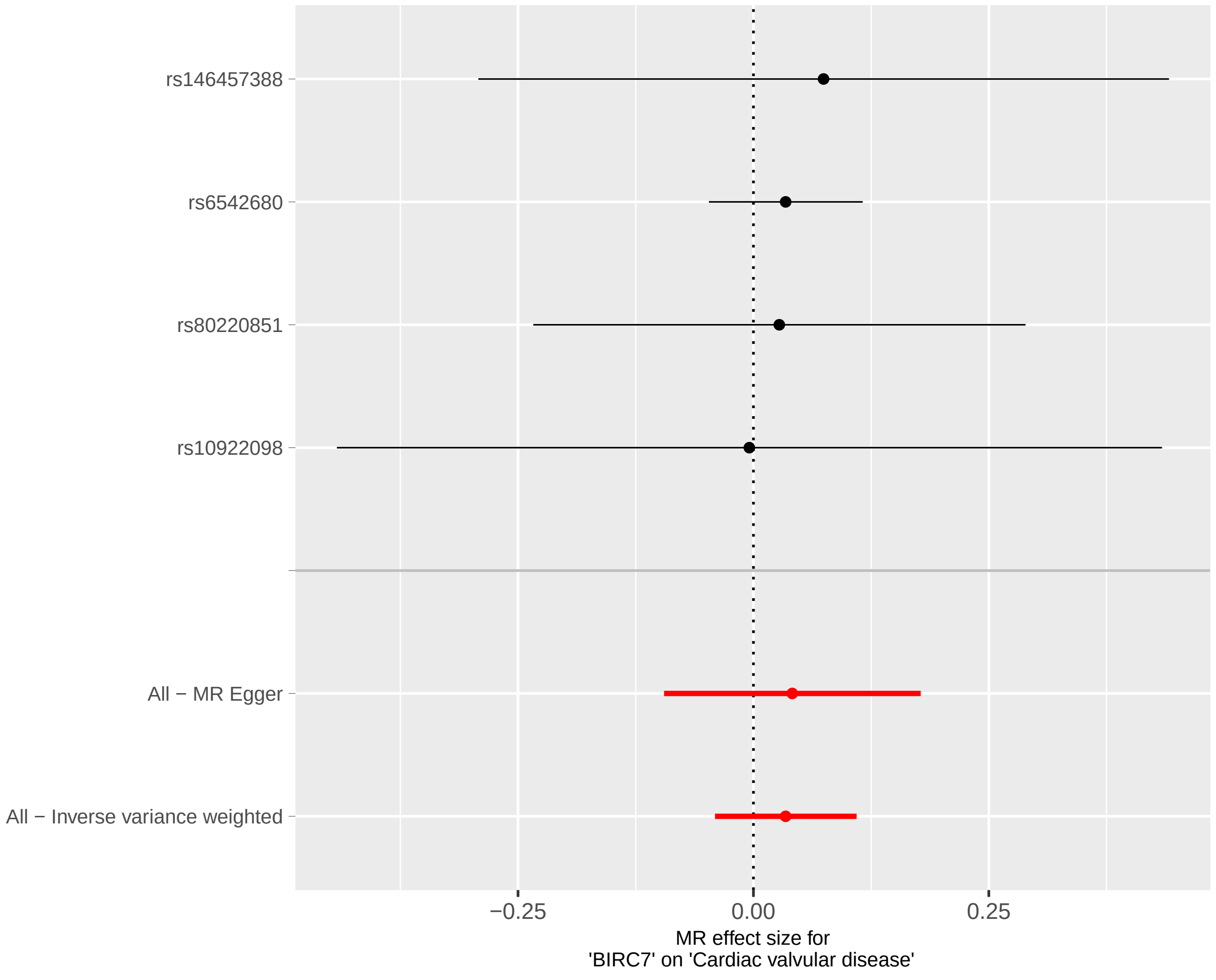
rs146457388
rs6542680
rs80220851
rs10922098
All − MR Egger
All − Inverse variance weighted
−0.25
0.00
0.25
MR effect size for
'BIRC7' on 'Cardiac valvular disease'

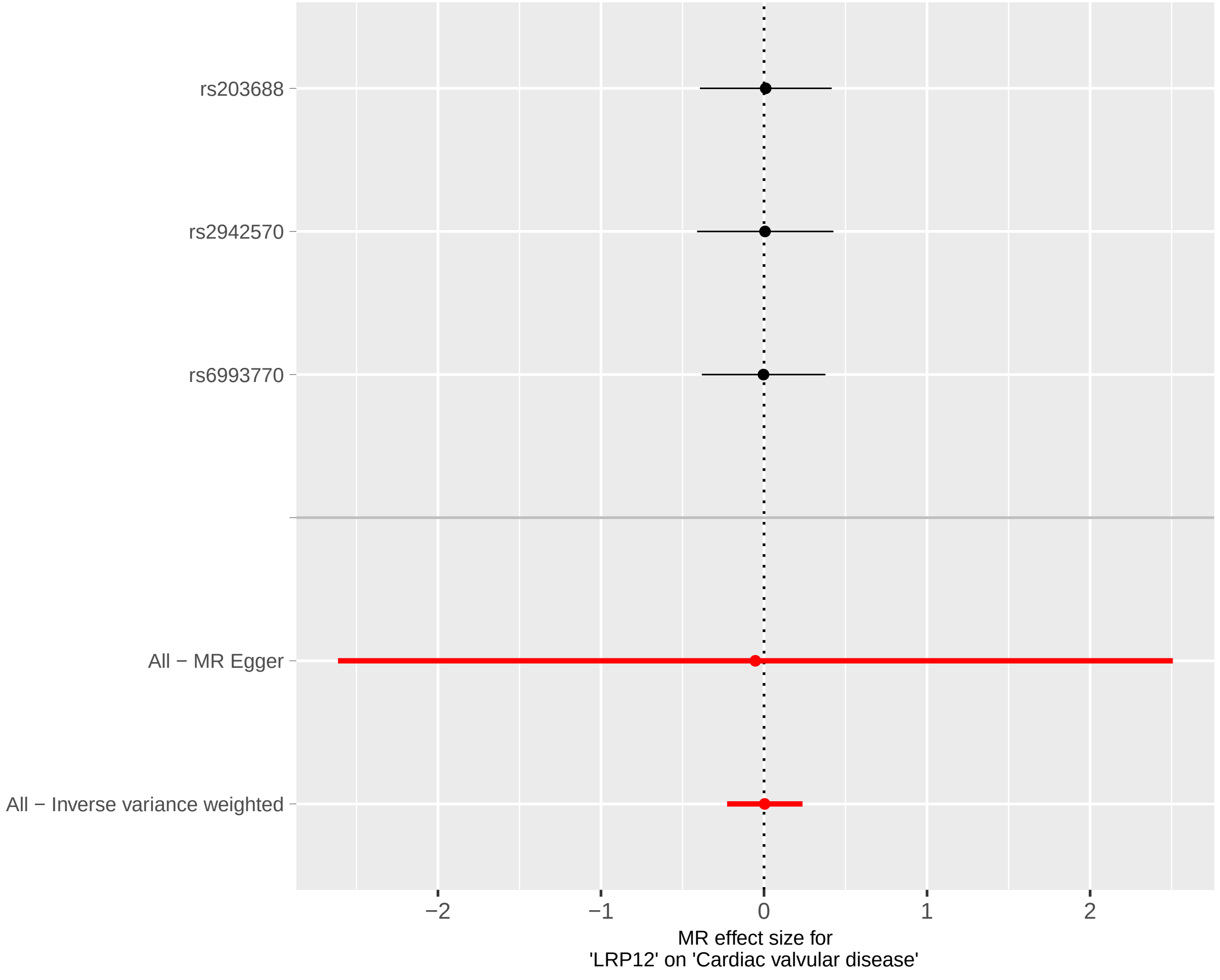
rs203688
rs2942570
rs6993770
All − MR Egger
All − Inverse variance weighted
−2
−1
0
1
2
MR effect size for
'LRP12' on 'Cardiac valvular disease'

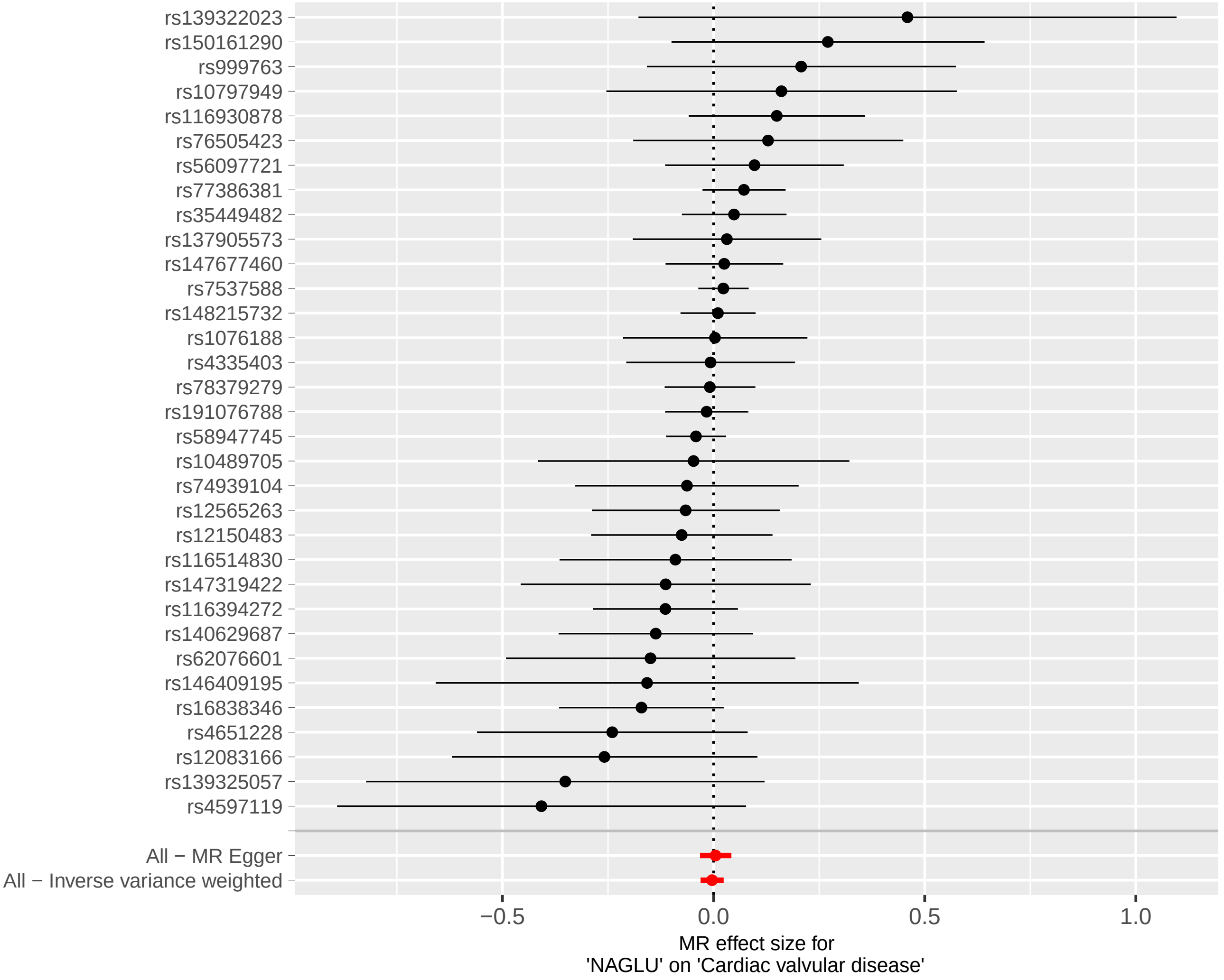
rs139322023
rs150161290
rs999763
rs10797949
rs116930878
rs76505423
rs56097721
rs77386381
rs35449482
rs137905573
rs147677460
rs7537588
rs148215732
rs1076188
rs4335403
rs78379279
rs191076788
rs58947745
rs10489705
rs74939104
rs12565263
rs12150483
rs116514830
rs147319422
rs116394272
rs140629687
rs62076601
rs146409195
rs16838346
rs4651228
rs12083166
rs139325057
rs4597119
All − MR Egger
All − Inverse variance weighted
−0.5
0.0
0.5
1.0
MR effect size for
'NAGLU' on 'Cardiac valvular disease'

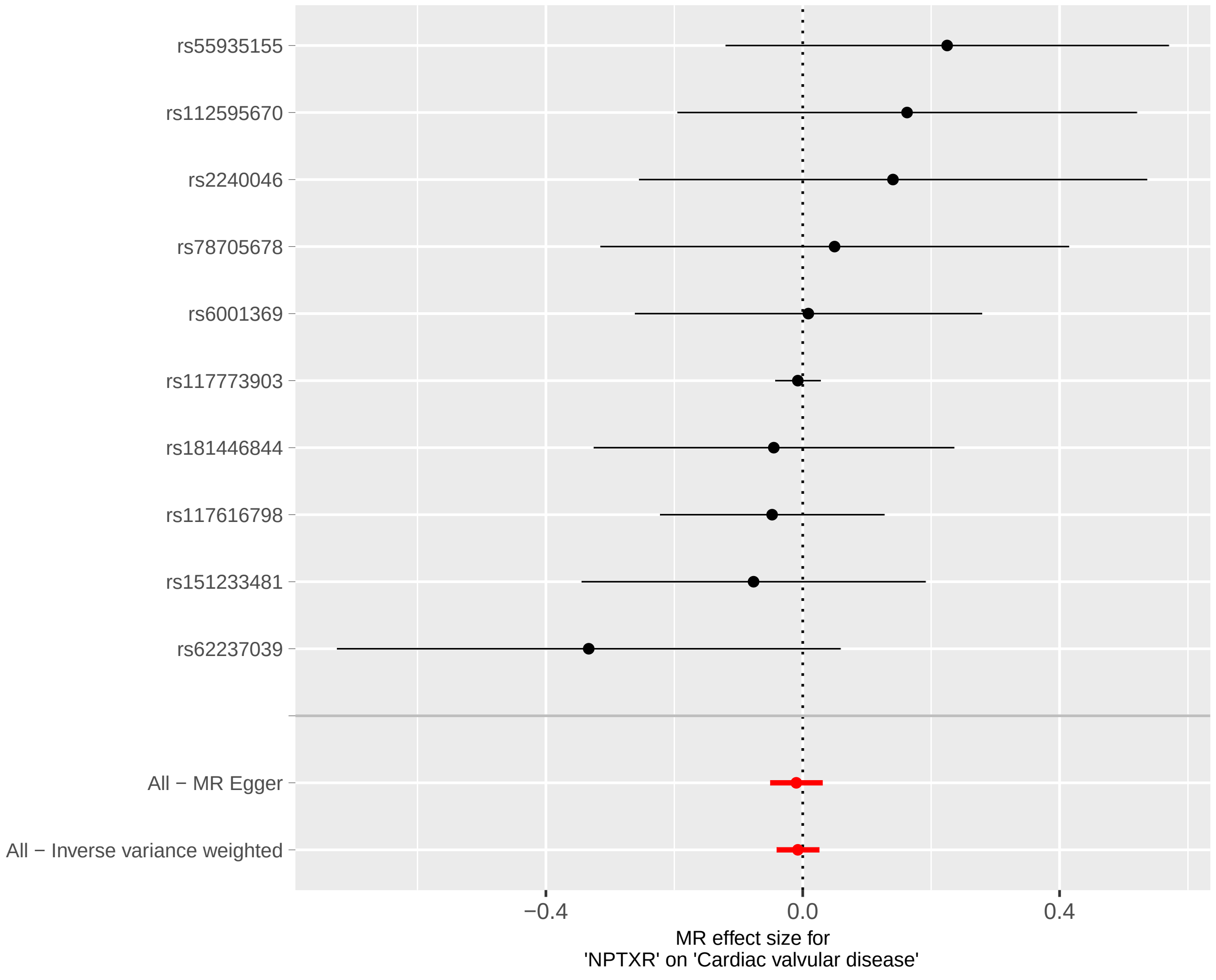
rs55935155
rs112595670
rs2240046
rs78705678
rs6001369
rs117773903
rs181446844
rs117616798
rs151233481
rs62237039
All − MR Egger
All − Inverse variance weighted
−0.4
0.0
0.4
MR effect size for
'NPTXR' on 'Cardiac valvular disease'

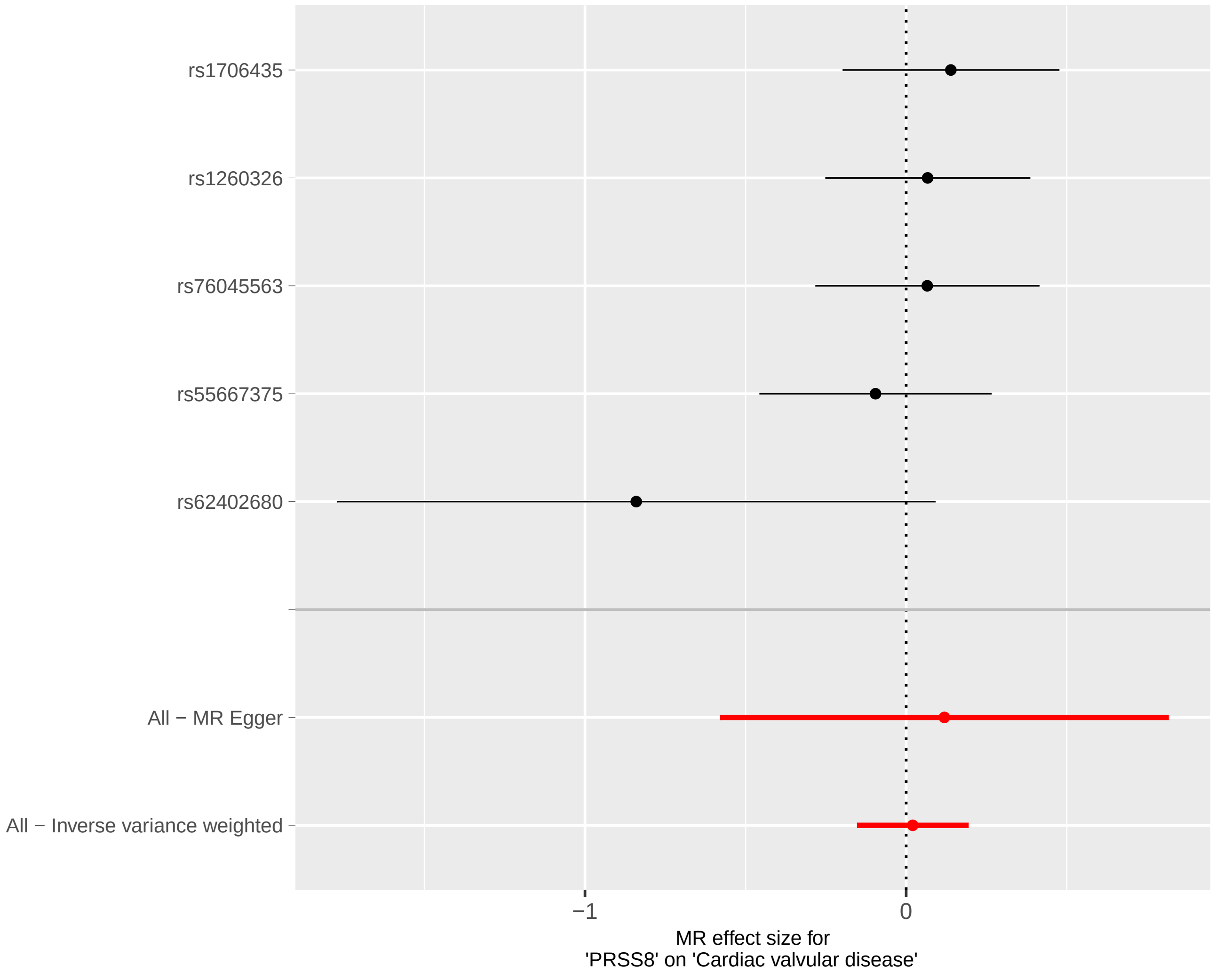
rs1706435
rs1260326
rs76045563
rs55667375
rs62402680
All − MR Egger
All − Inverse variance weighted
−1
0
MR effect size for
'PRSS8' on 'Cardiac valvular disease'

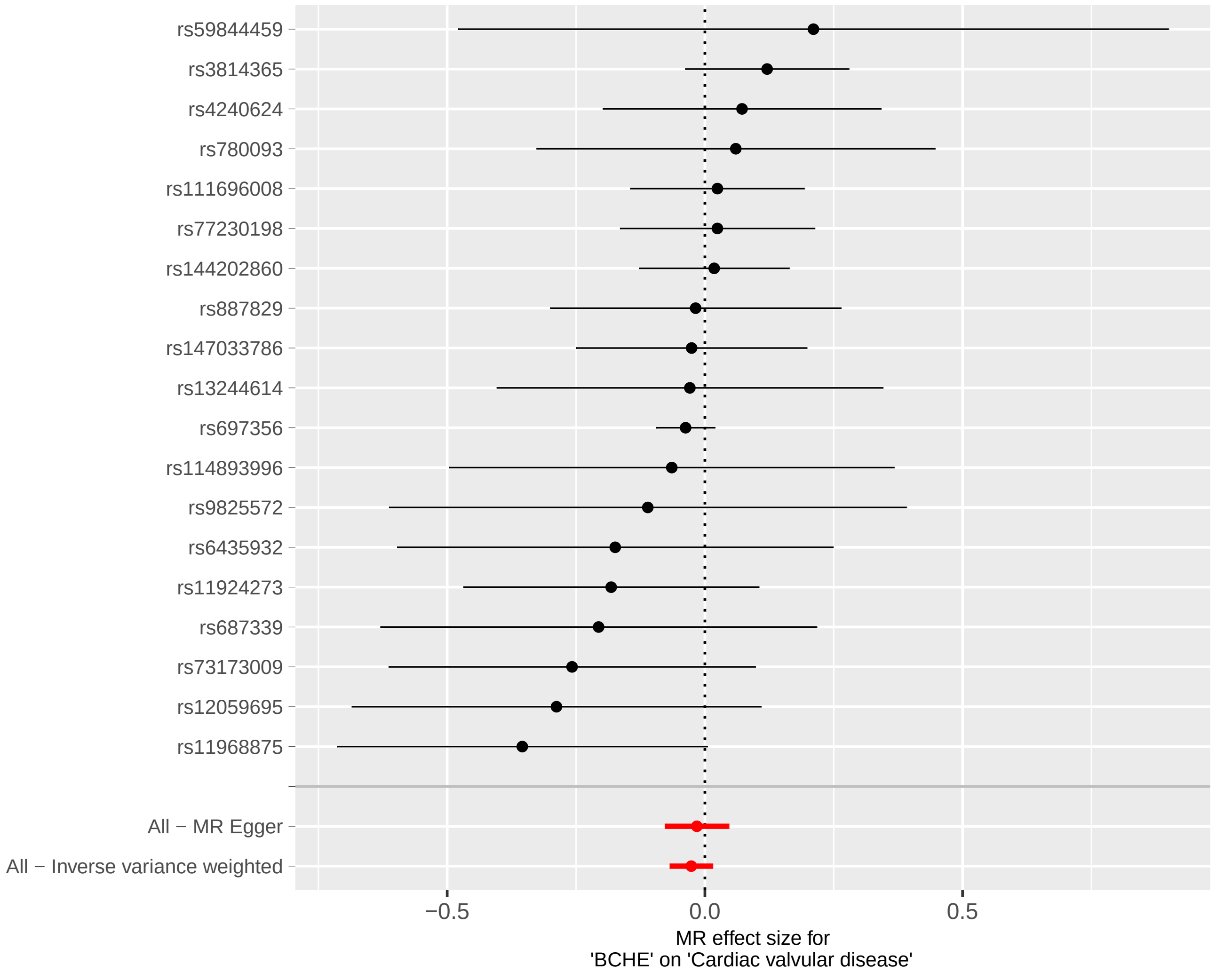

rs59844459
rs3814365
rs4240624
rs780093
rs111696008
rs77230198
rs144202860
rs887829
rs147033786
rs13244614
rs697356
rs114893996
rs9825572
rs6435932
rs11924273
rs687339
rs73173009
rs12059695
rs11968875
All − MR Egger
All − Inverse variance weighted
−0.5
0.0
0.5
MR effect size for
'BCHE' on 'Cardiac valvular disease'

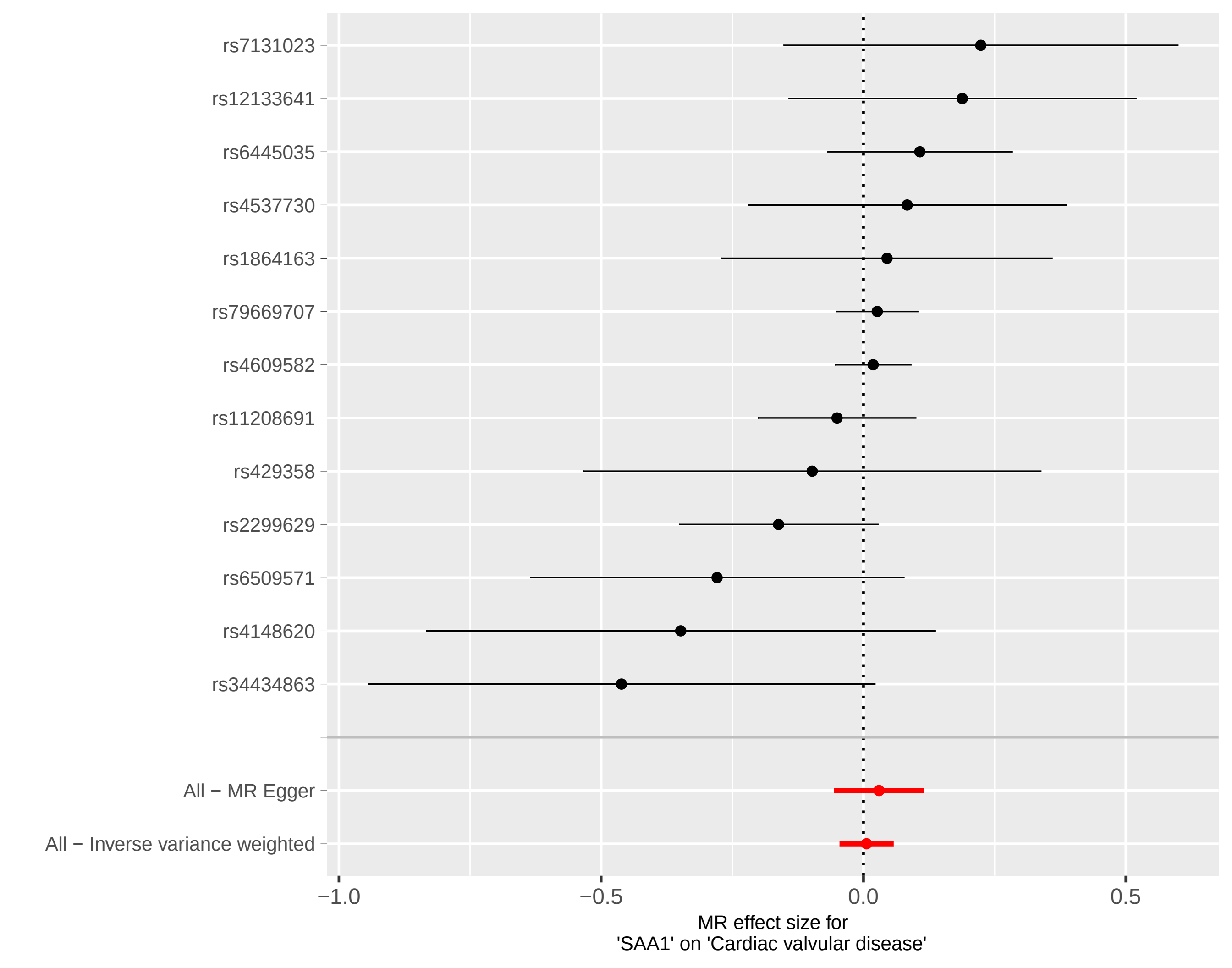
rs7131023
rs12133641
rs6445035
rs4537730
rs1864163
rs79669707
rs4609582
rs11208691
rs429358
rs2299629
rs6509571
rs4148620
rs34434863
All − MR Egger
All − Inverse variance weighted
−1.0
−0.5
0.0
0.5
MR effect size for
'SAA1' on 'Cardiac valvular disease'

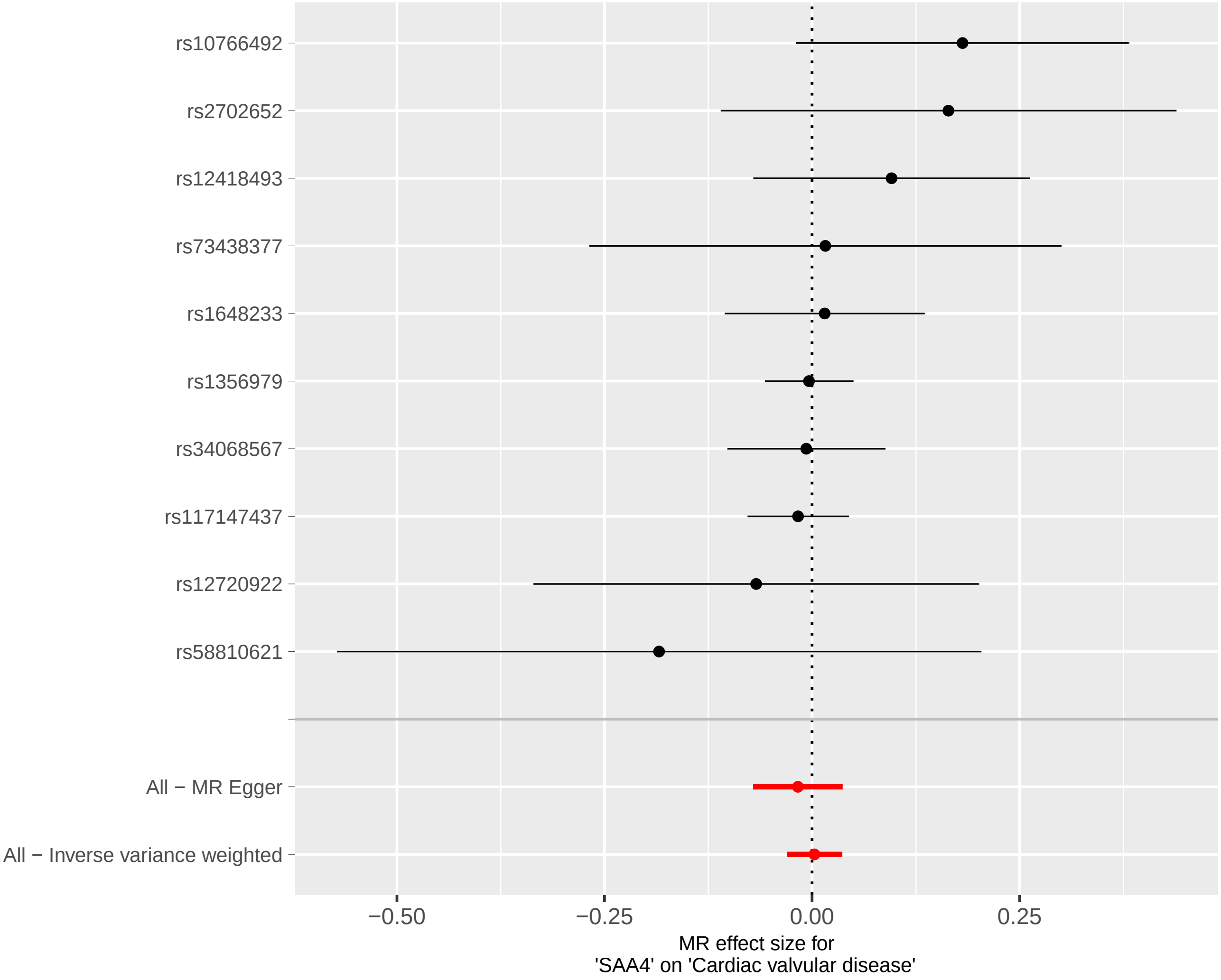
rs10766492
rs2702652
rs12418493
rs73438377
rs1648233
rs1356979
rs34068567
rs117147437
rs12720922
rs58810621
All − MR Egger
All − Inverse variance weighted
−0.50
−0.25
0.00
0.25
MR effect size for
'SAA4' on 'Cardiac valvular disease'

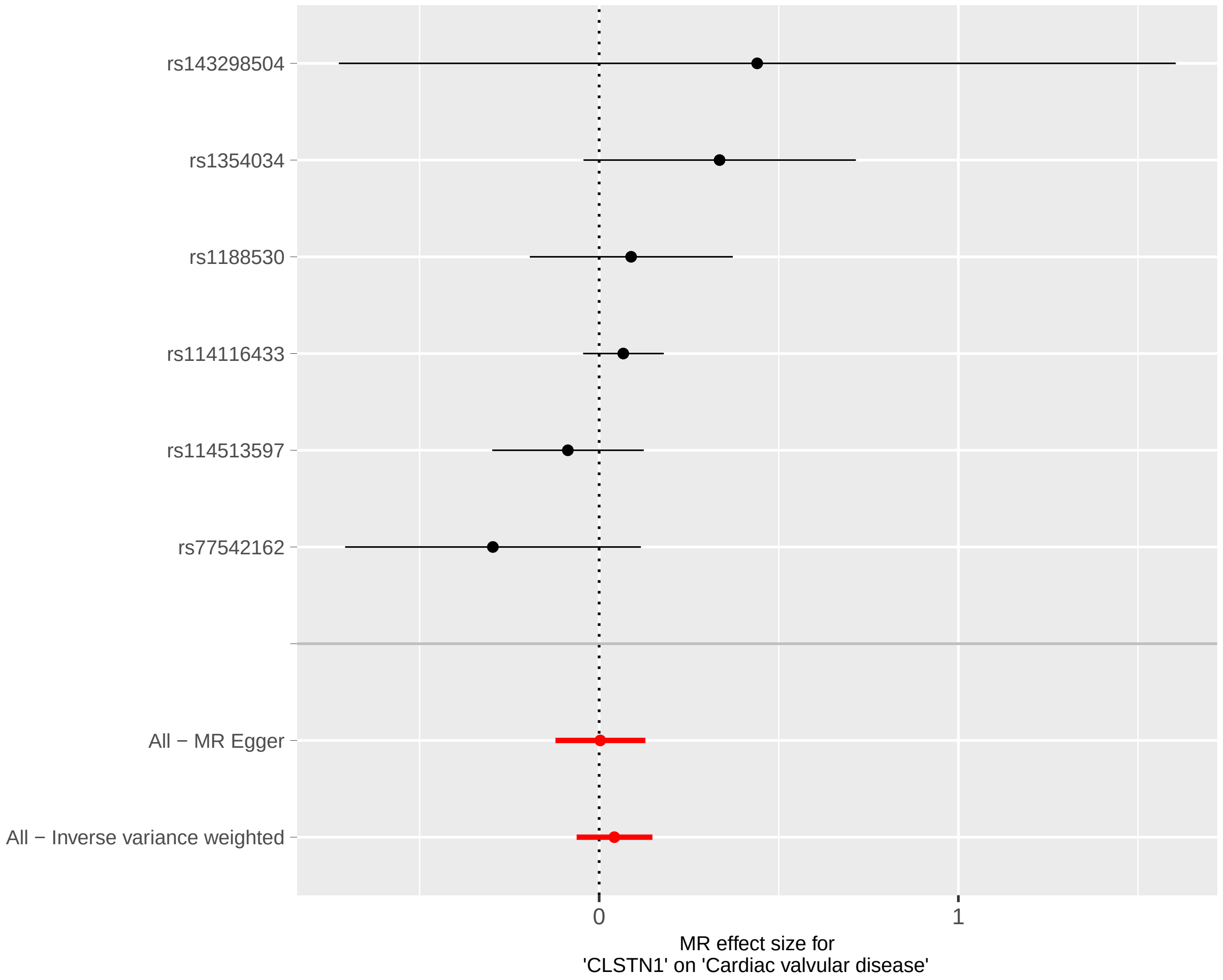
rs143298504
rs1354034
rs1188530
rs114116433
rs114513597
rs77542162
All − MR Egger
All − Inverse variance weighted
0
1
MR effect size for
'CLSTN1' on 'Cardiac valvular disease'

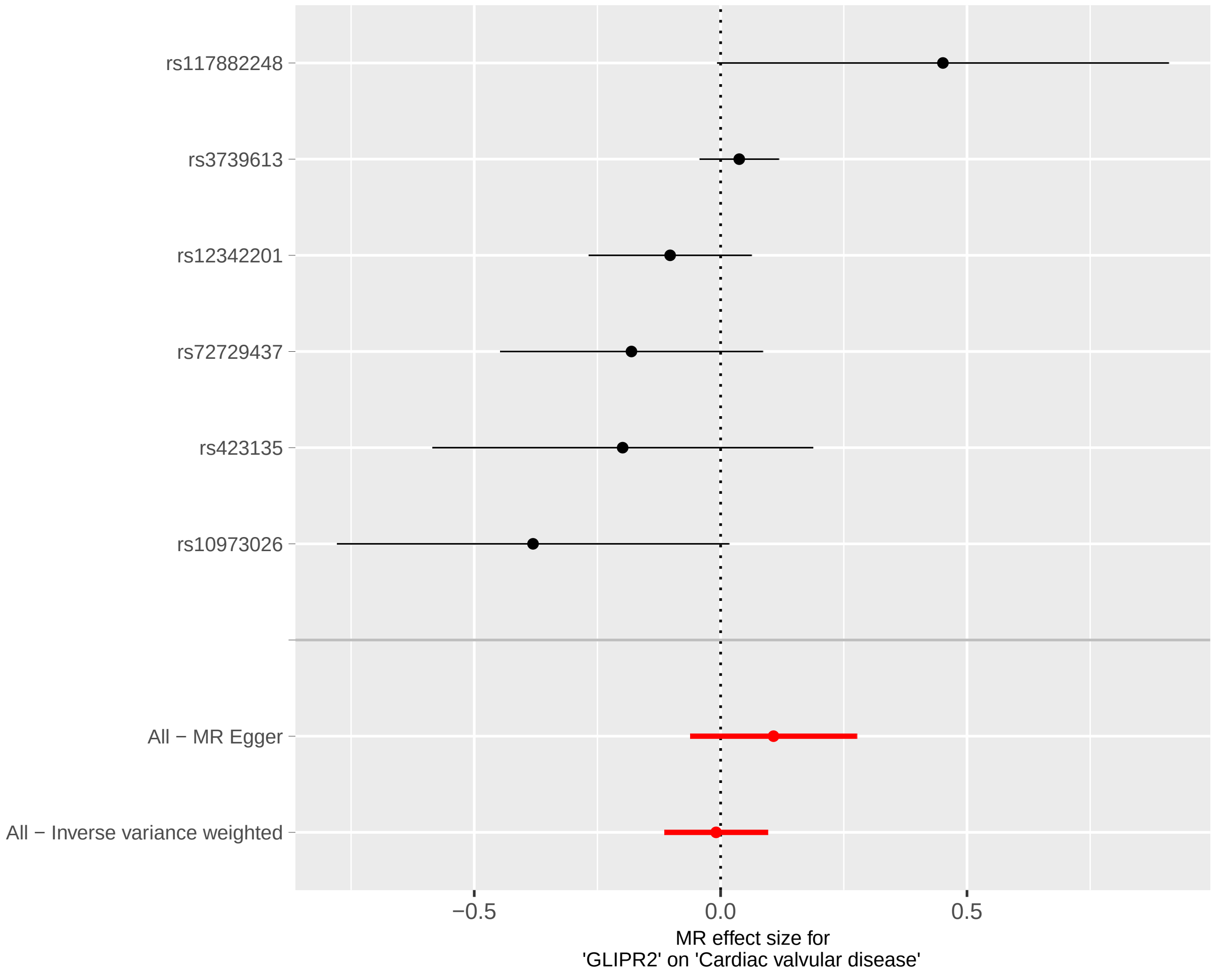
rs117882248
rs3739613
rs12342201
rs72729437
rs423135
rs10973026
All − MR Egger
All − Inverse variance weighted
−0.5
0.0
0.5
MR effect size for
'GLIPR2' on 'Cardiac valvular disease'

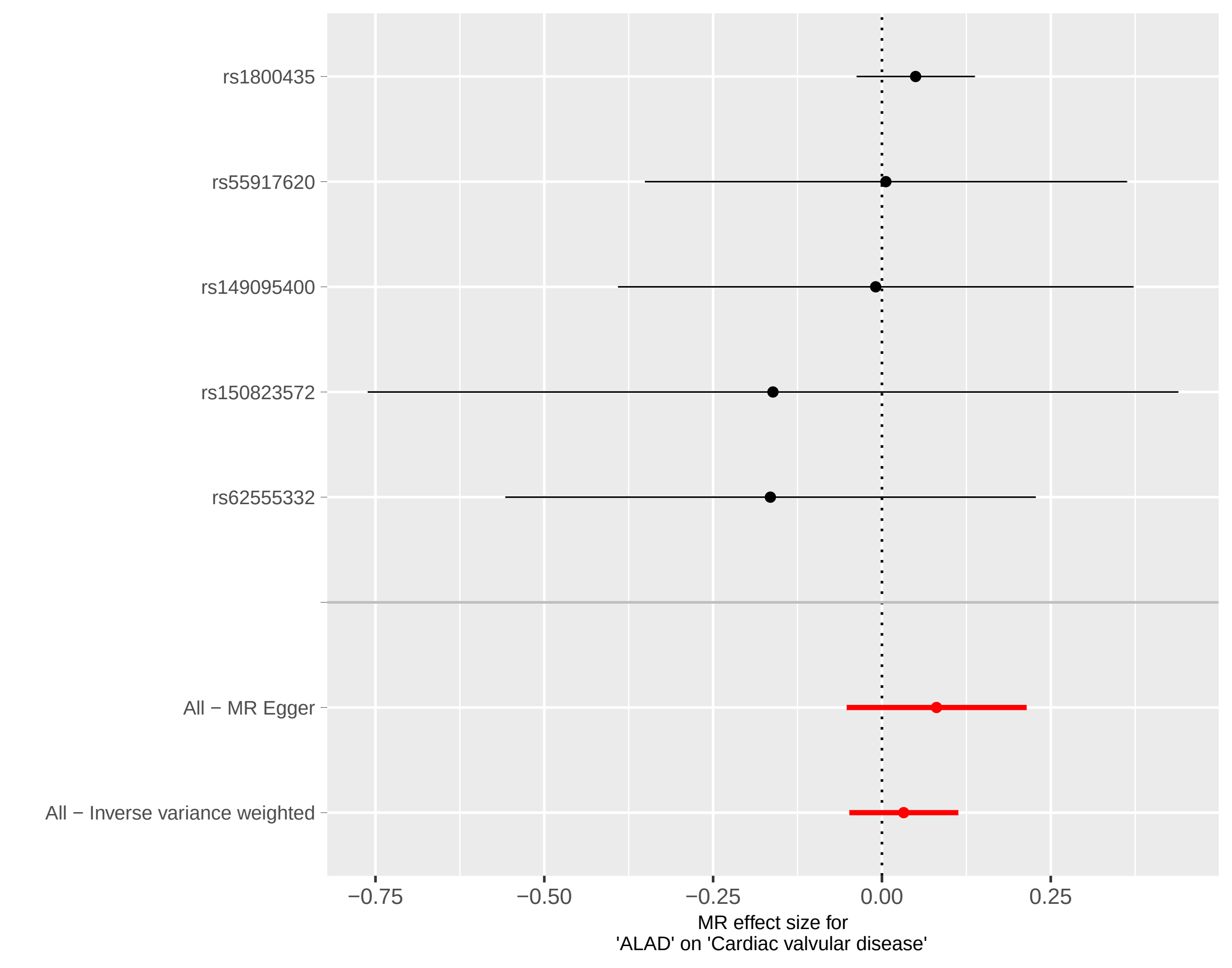
rs1800435
rs55917620
rs149095400
rs150823572
rs62555332
All − MR Egger
All − Inverse variance weighted
−0.75
−0.50
−0.25
0.00
0.25
MR effect size for
'ALAD' on 'Cardiac valvular disease'

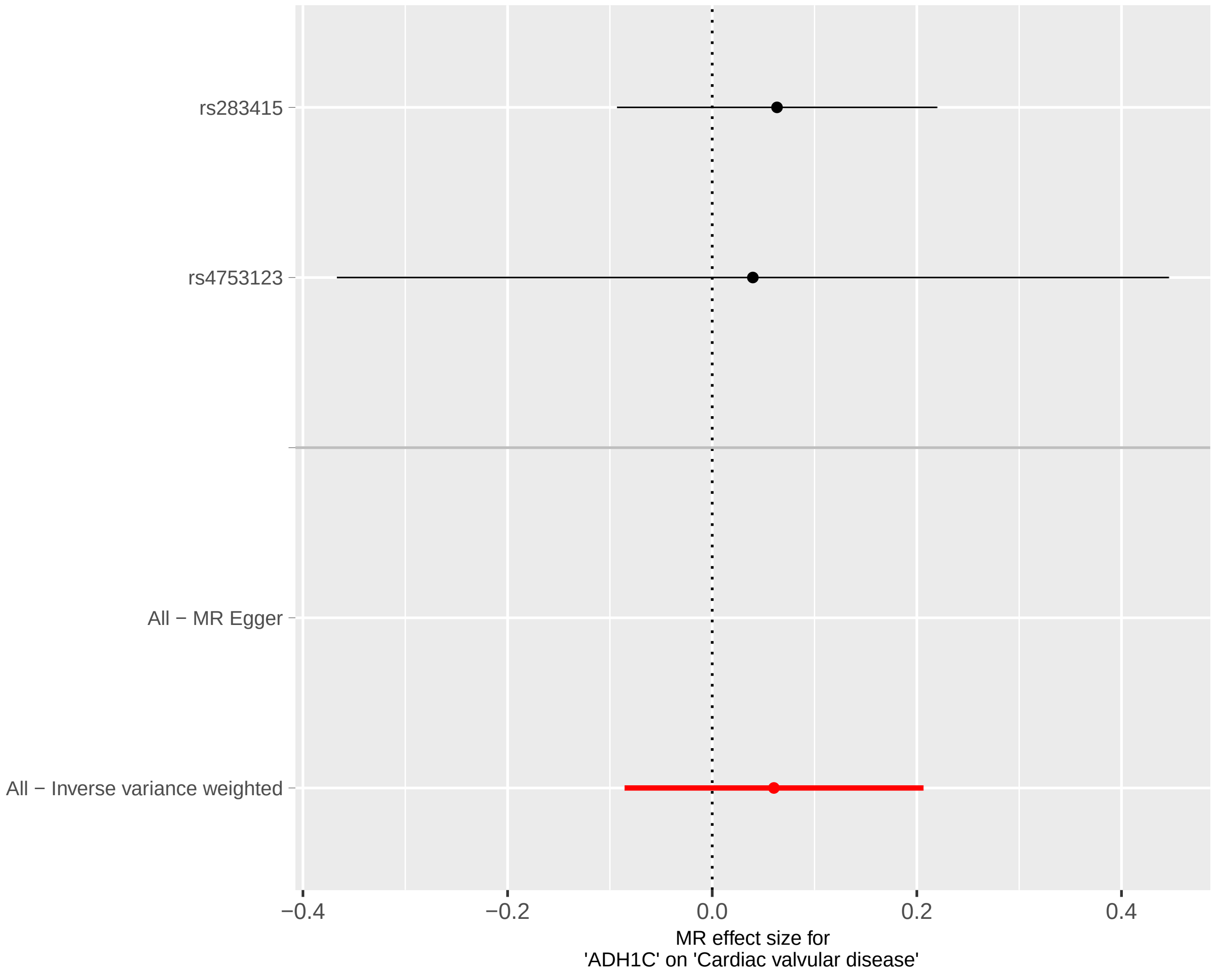
rs283415
rs4753123
All − MR Egger
All − Inverse variance weighted
−0.4
−0.2
0.0
0.2
0.4
MR effect size for
'ADH1C' on 'Cardiac valvular disease'

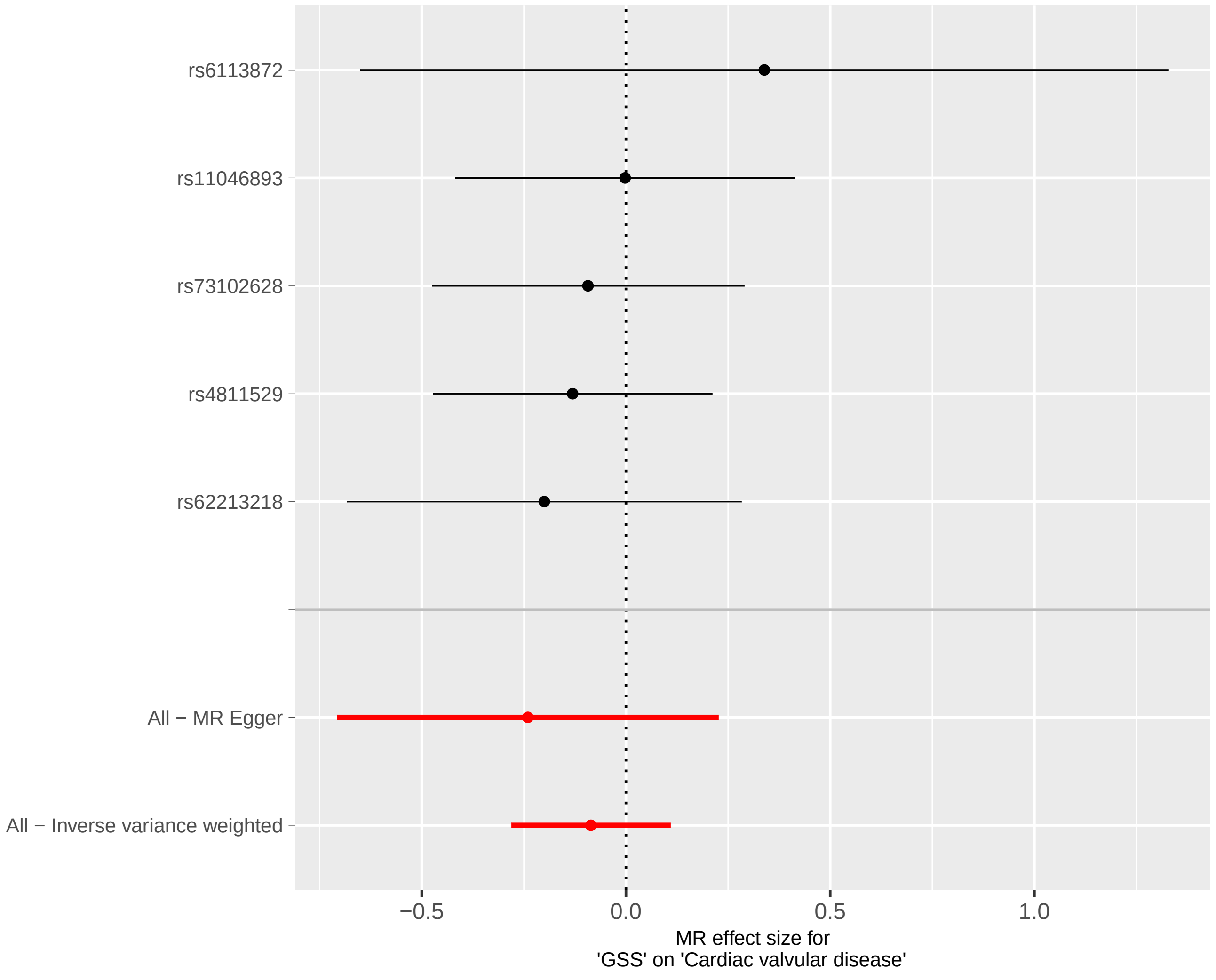
rs6113872
rs11046893
rs73102628
rs4811529
rs62213218
All − MR Egger
All − Inverse variance weighted
−0.5
0.0
0.5
1.0
MR effect size for
'GSS' on 'Cardiac valvular disease'

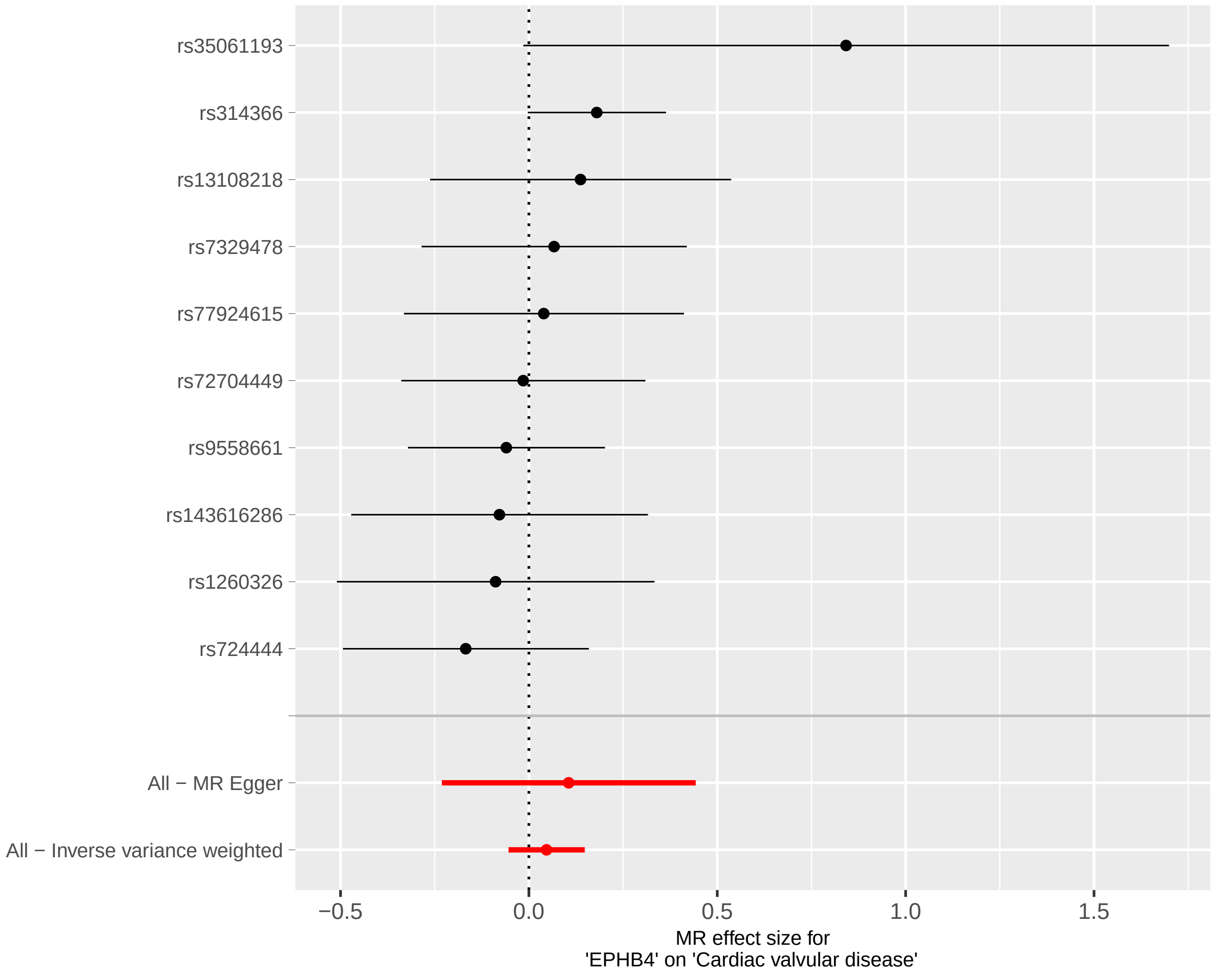

rs35061193
rs314366
rs13108218
rs7329478
rs77924615
rs72704449
rs9558661
rs143616286
rs1260326
rs724444
All − MR Egger
All − Inverse variance weighted
−0.5
0.0
0.5
1.0
1.5
MR effect size for
'EPHB4' on 'Cardiac valvular disease'

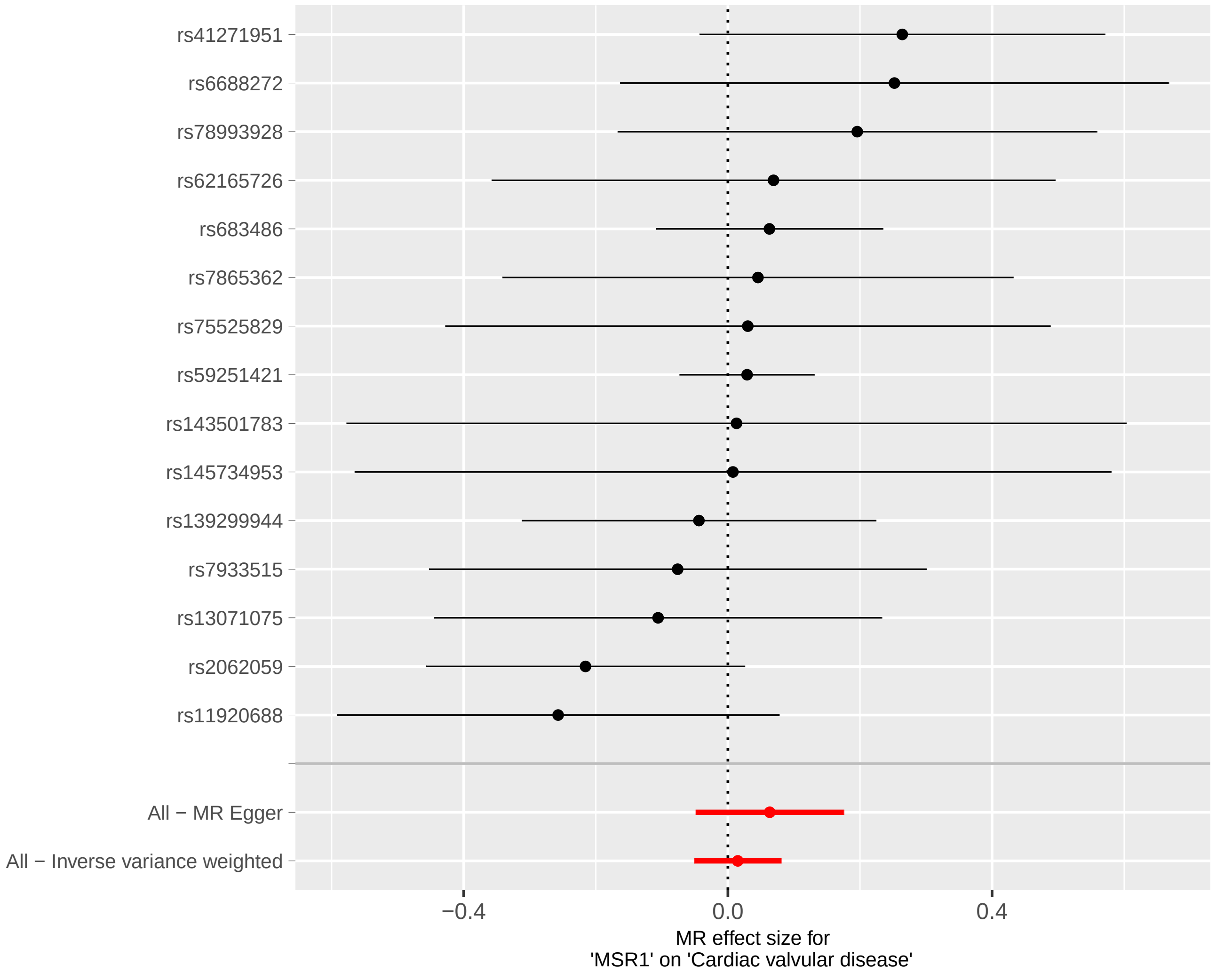
rs41271951
rs6688272
rs78993928
rs62165726
rs683486
rs7865362
rs75525829
rs59251421
rs143501783
rs145734953
rs139299944
rs7933515
rs13071075
rs2062059
rs11920688
All − MR Egger
All − Inverse variance weighted
−0.4
0.0
0.4
MR effect size for
'MSR1' on 'Cardiac valvular disease'

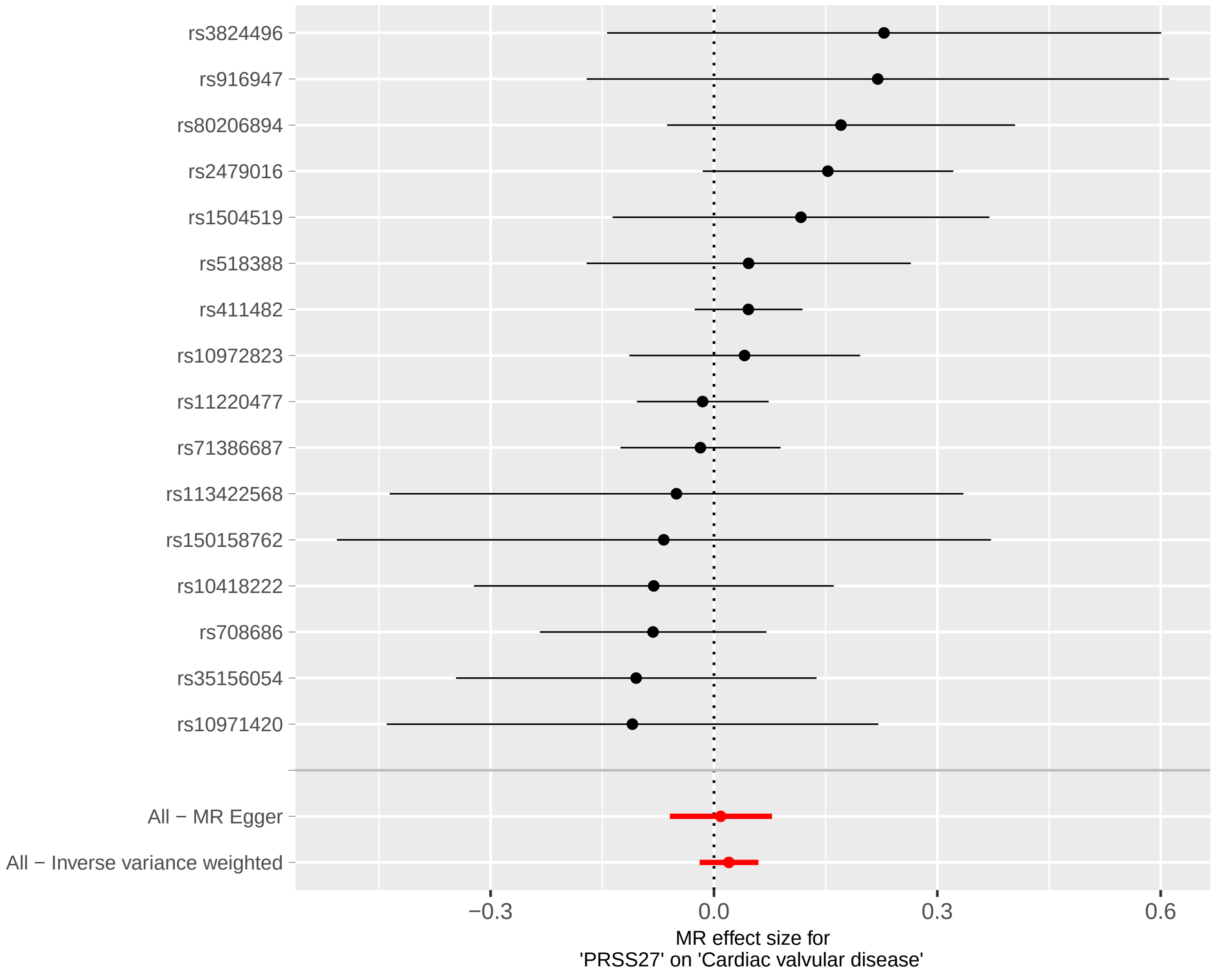
rs3824496
rs916947
rs80206894
rs2479016
rs1504519
rs518388
rs411482
rs10972823
rs11220477
rs71386687
rs113422568
rs150158762
rs10418222
rs708686
rs35156054
rs10971420
All − MR Egger
All − Inverse variance weighted
−0.3
0.0
0.3
0.6
MR effect size for
'PRSS27' on 'Cardiac valvular disease'

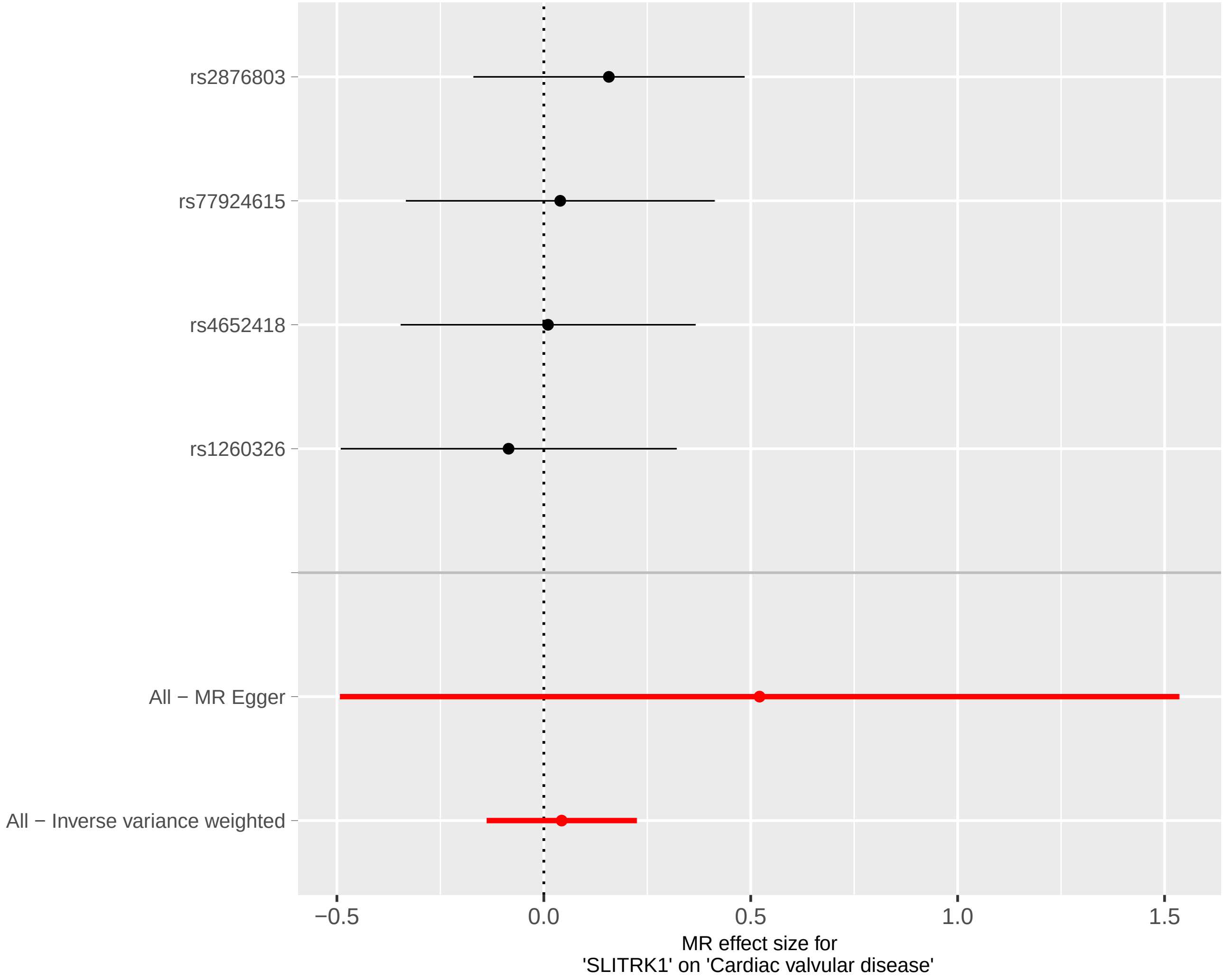
rs2876803
rs77924615
rs4652418
rs1260326
All − MR Egger
All − Inverse variance weighted
−0.5
0.0
0.5
1.0
1.5
MR effect size for
'SLITRK1' on 'Cardiac valvular disease'

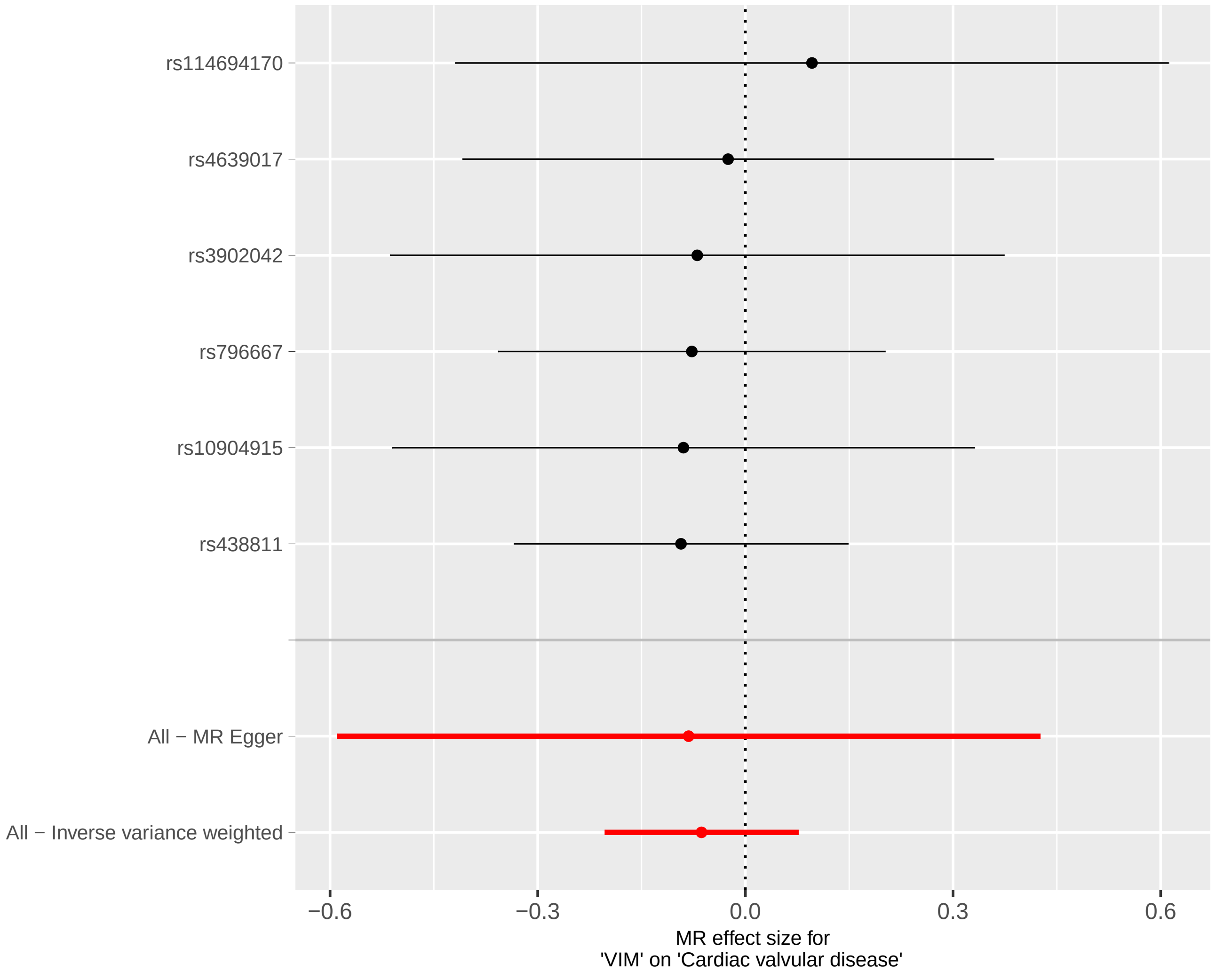

rs114694170
rs4639017
rs3902042
rs796667
rs10904915
rs438811
All − MR Egger
All − Inverse variance weighted
−0.6
−0.3
0.0
0.3
0.6
MR effect size for
'VIM' on 'Cardiac valvular disease'

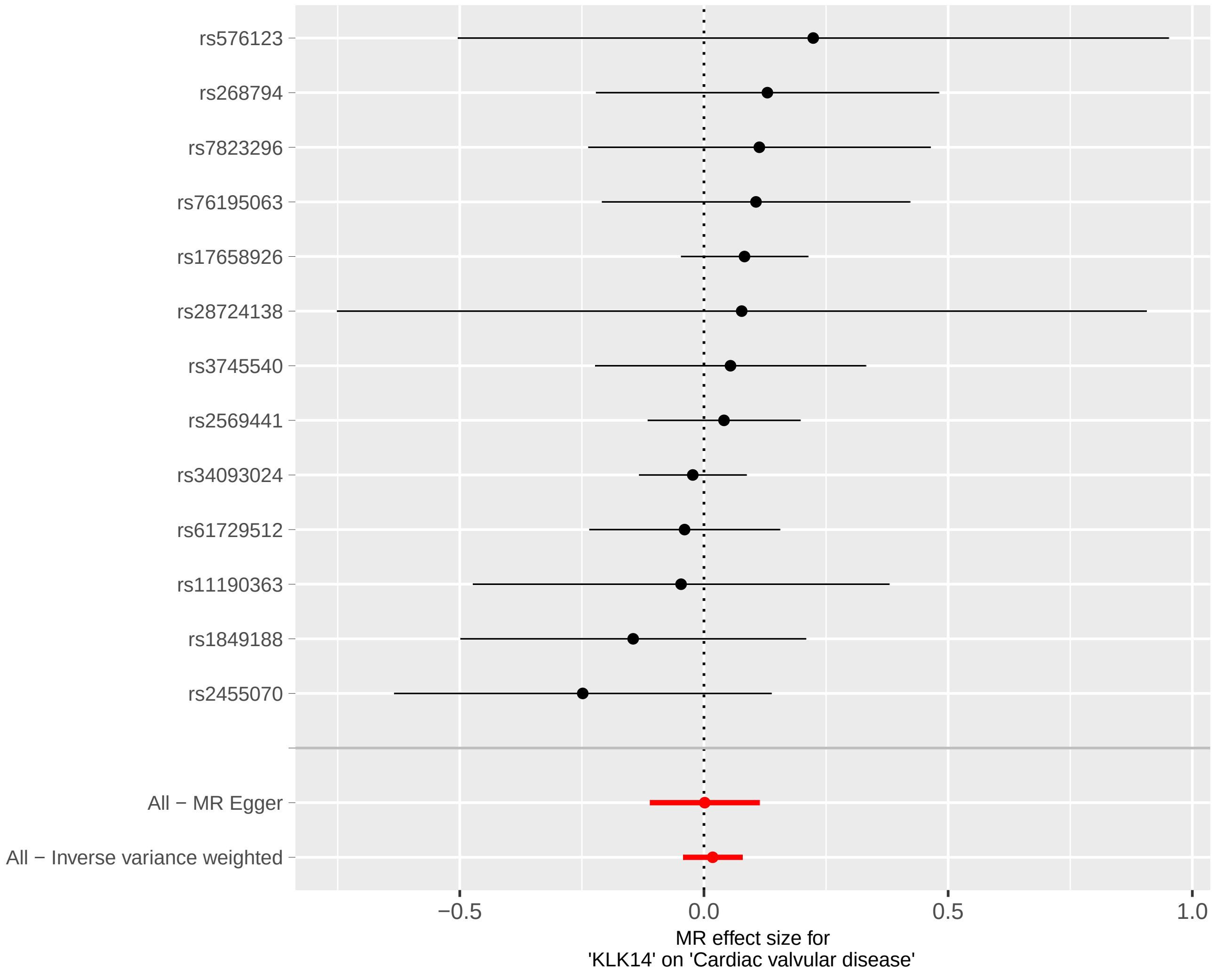
rs576123
rs268794
rs7823296
rs76195063
rs17658926
rs28724138
rs3745540
rs2569441
rs34093024
rs61729512
rs11190363
rs1849188
rs2455070
All − MR Egger
All − Inverse variance weighted
−0.5
0.0
0.5
1.0
MR effect size for
'KLK14' on 'Cardiac valvular disease'

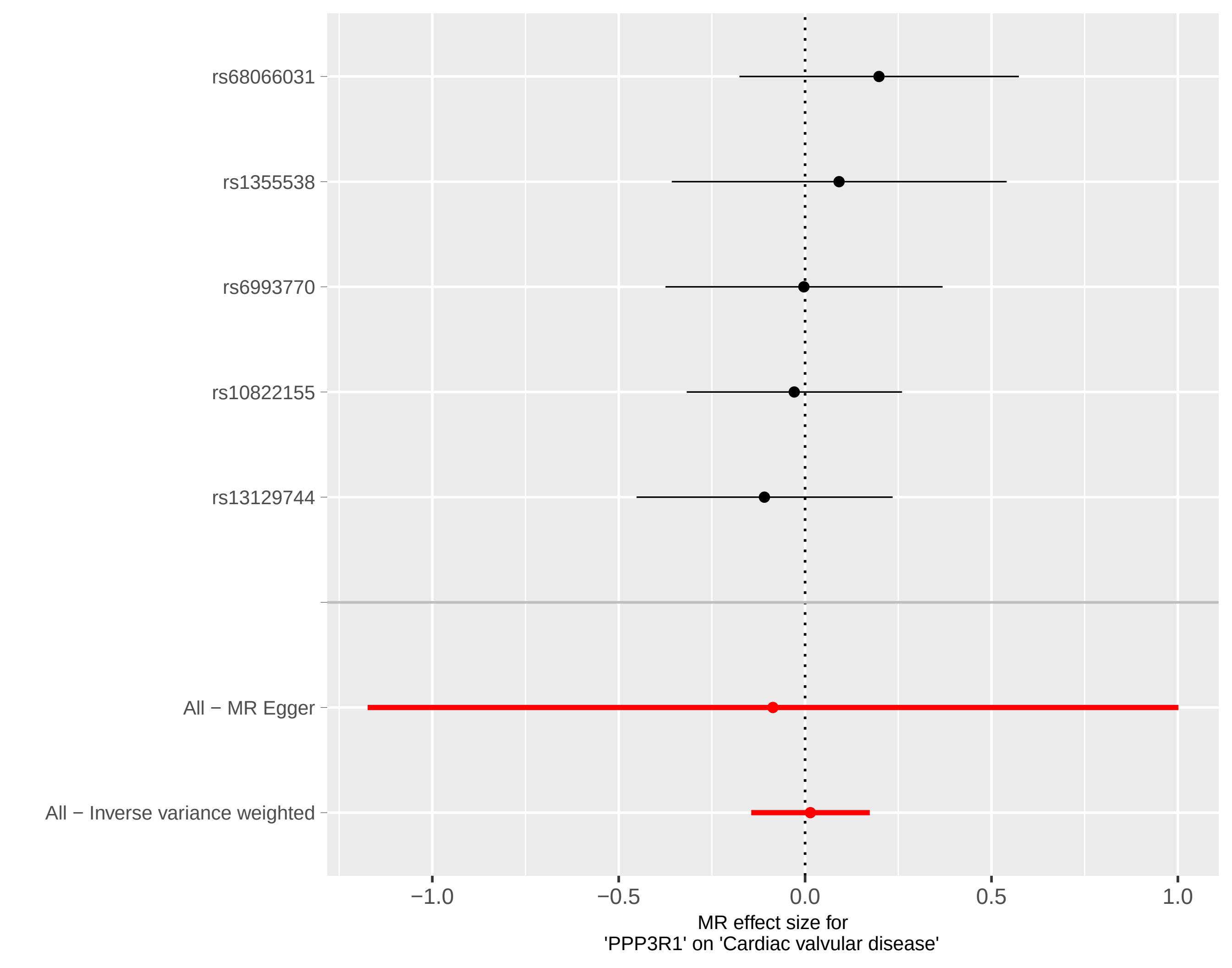
rs68066031
rs1355538
rs6993770
rs10822155
rs13129744
All − MR Egger
All − Inverse variance weighted
−1.0
−0.5
0.0
0.5
1.0
MR effect size for
'PPP3R1' on 'Cardiac valvular disease'

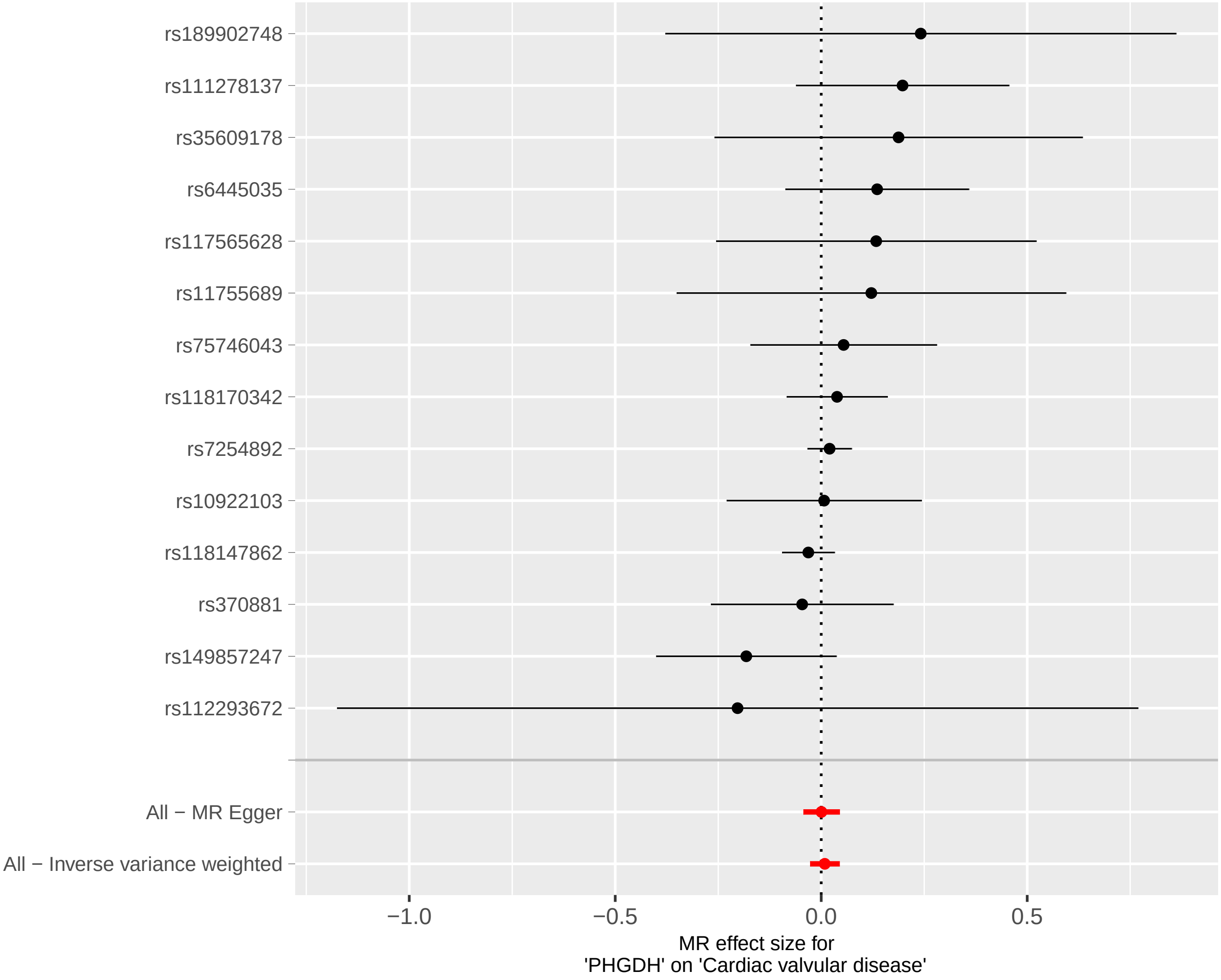
rs189902748
rs111278137
rs35609178
rs6445035
rs117565628
rs11755689
rs75746043
rs118170342
rs7254892
rs10922103
rs118147862
rs370881
rs149857247
rs112293672
All − MR Egger
All − Inverse variance weighted
−1.0
−0.5
0.0
0.5
MR effect size for
'PHGDH' on 'Cardiac valvular disease'

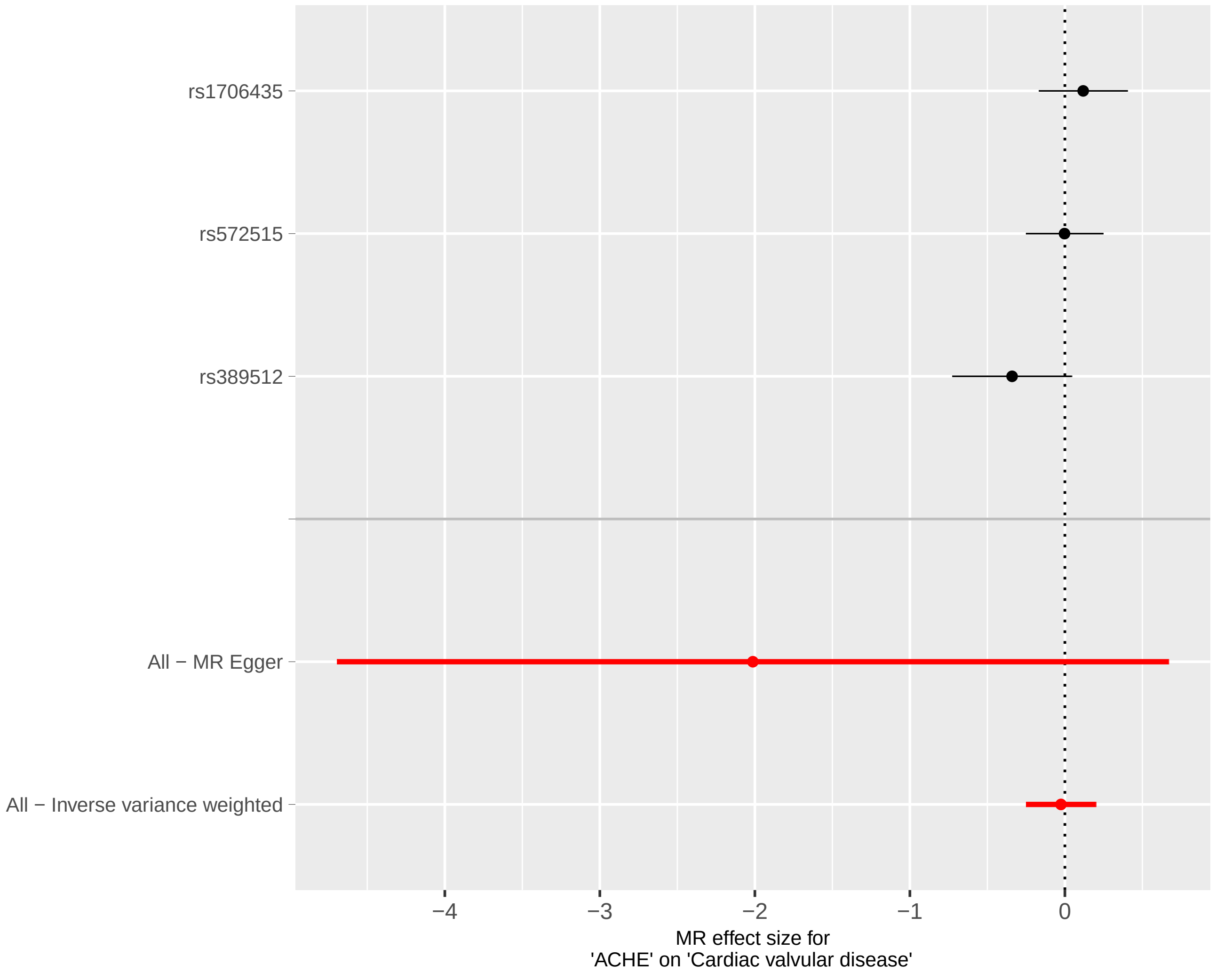

rs1706435
rs572515
rs389512
All − MR Egger
All − Inverse variance weighted
−4
−3
−2
−1
0
MR effect size for
'ACHE' on 'Cardiac valvular disease'

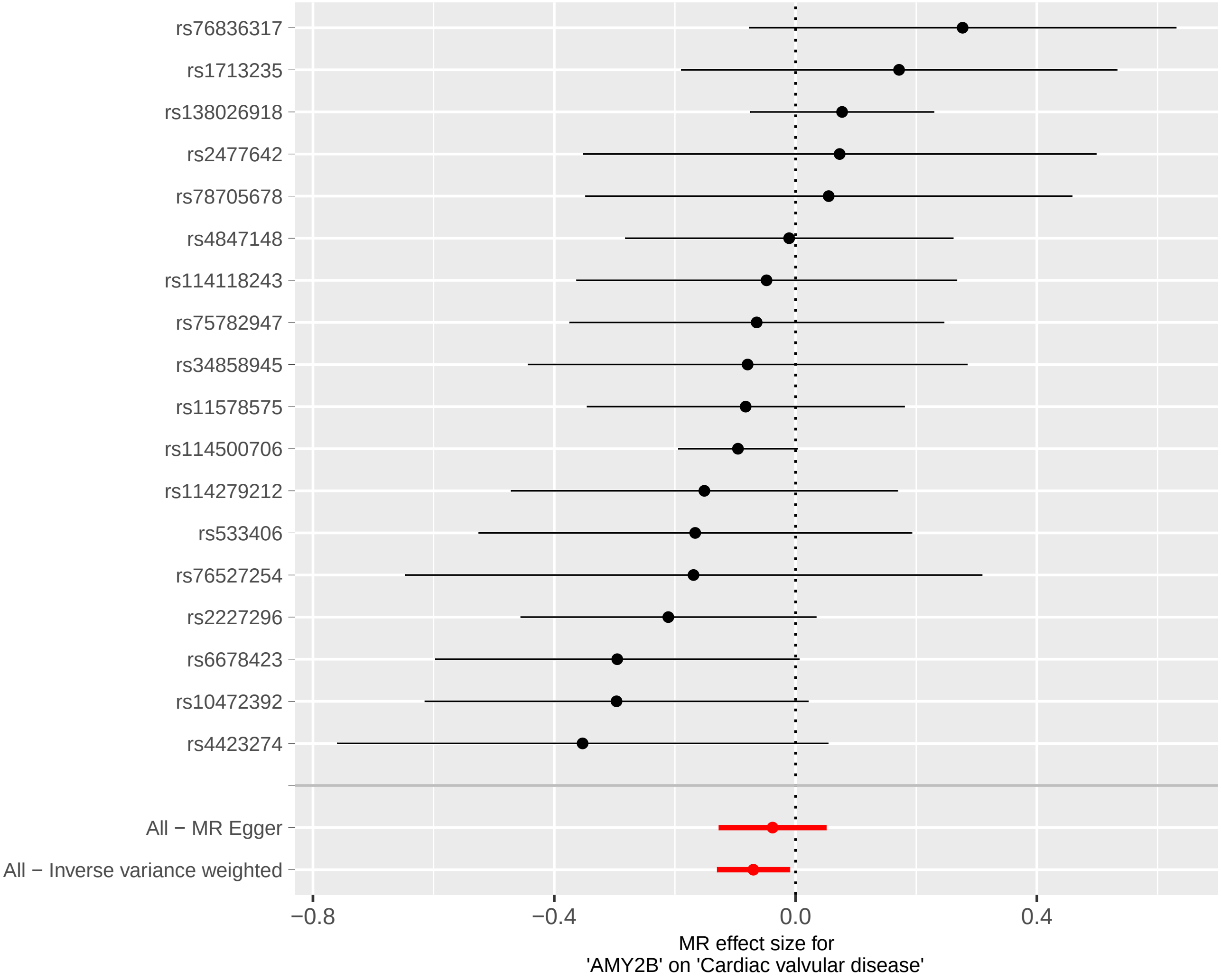
rs76836317
rs1713235
rs138026918
rs2477642
rs78705678
rs4847148
rs114118243
rs75782947
rs34858945
rs11578575
rs114500706
rs114279212
rs533406
rs76527254
rs2227296
rs6678423
rs10472392
rs4423274
All − MR Egger
All − Inverse variance weighted
−0.8
−0.4
0.0
0.4
MR effect size for
'AMY2B' on 'Cardiac valvular disease'

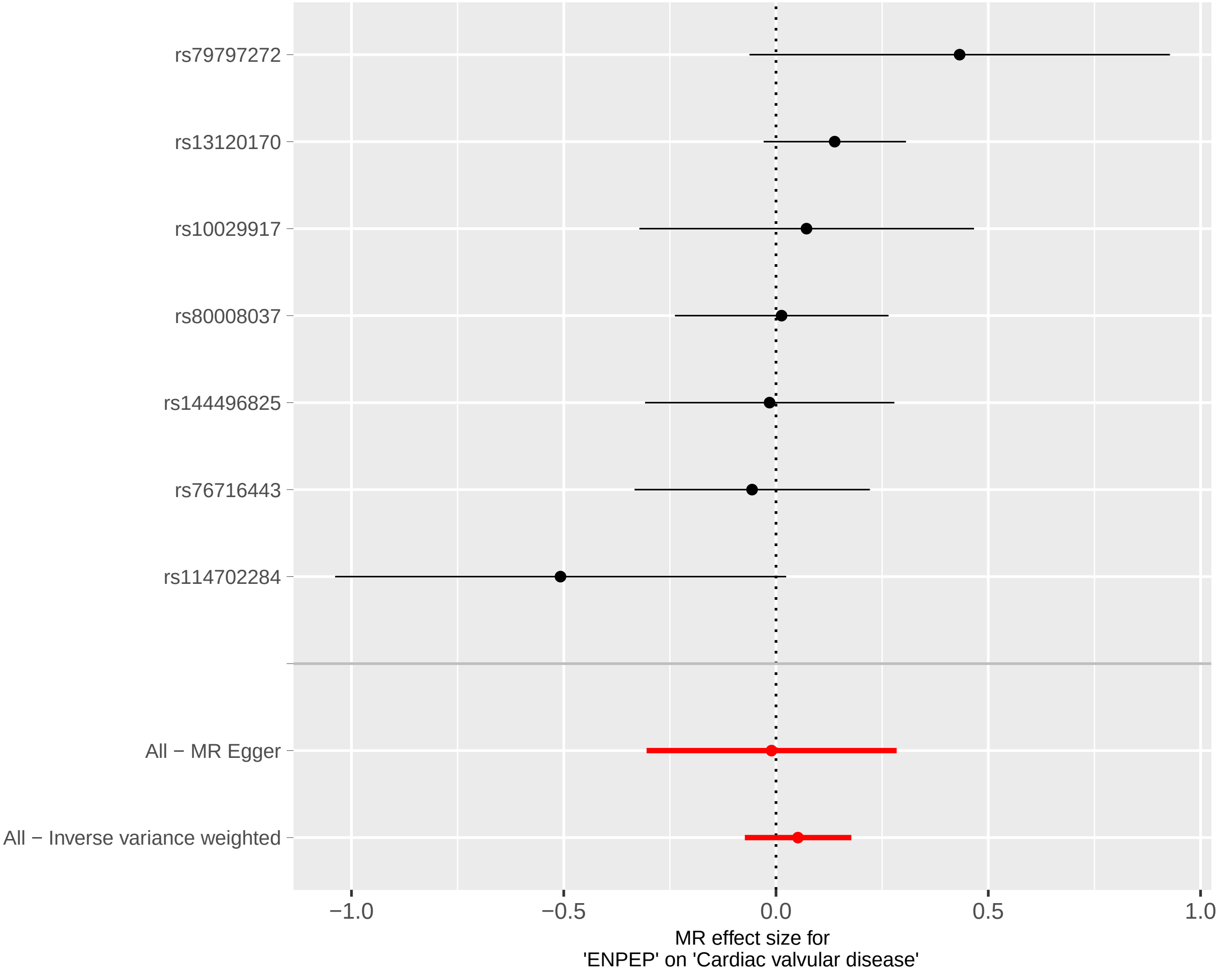

rs79797272
rs13120170
rs10029917
rs80008037
rs144496825
rs76716443
rs114702284
All − MR Egger
All − Inverse variance weighted
−1.0
−0.5
0.0
0.5
1.0
MR effect size for
'ENPEP' on 'Cardiac valvular disease'

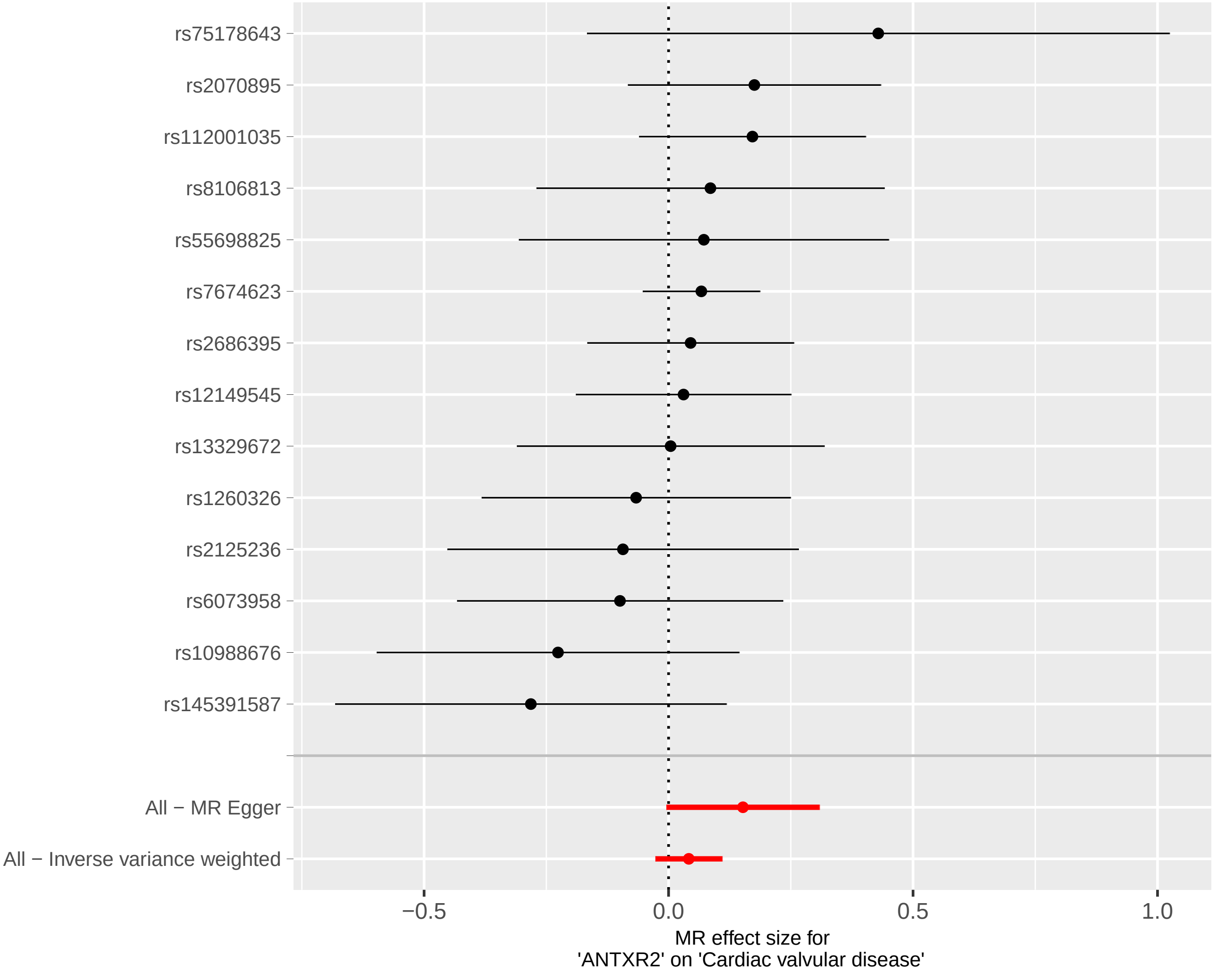
rs75178643
rs2070895
rs112001035
rs8106813
rs55698825
rs7674623
rs2686395
rs12149545
rs13329672
rs1260326
rs2125236
rs6073958
rs10988676
rs145391587
All − MR Egger
All − Inverse variance weighted
−0.5
0.0
0.5
1.0
MR effect size for
'ANTXR2' on 'Cardiac valvular disease'

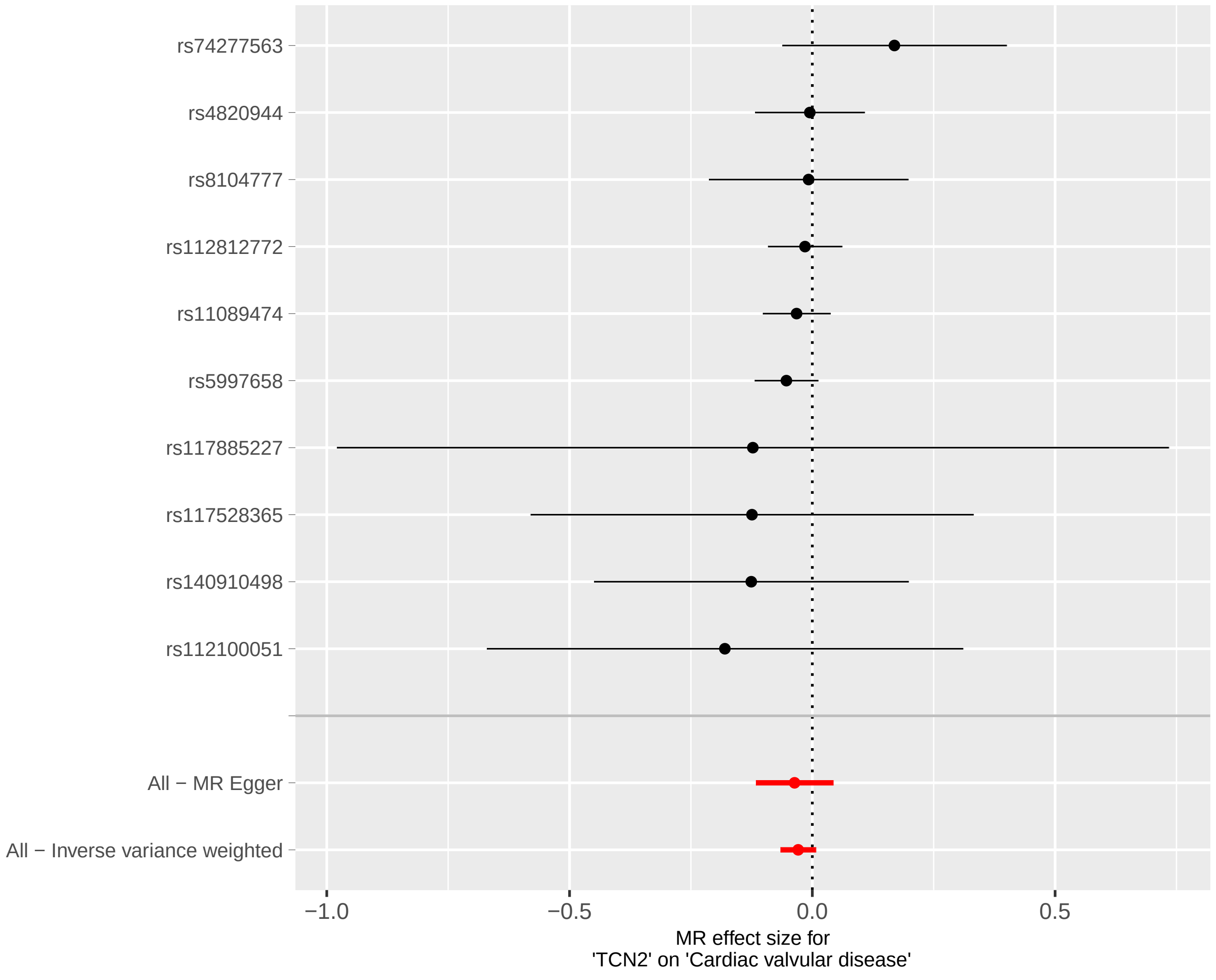
rs74277563
rs4820944
rs8104777
rs112812772
rs11089474
rs5997658
rs117885227
rs117528365
rs140910498
rs112100051
All − MR Egger
All − Inverse variance weighted
−1.0
−0.5
0.0
0.5
MR effect size for
'TCN2' on 'Cardiac valvular disease'

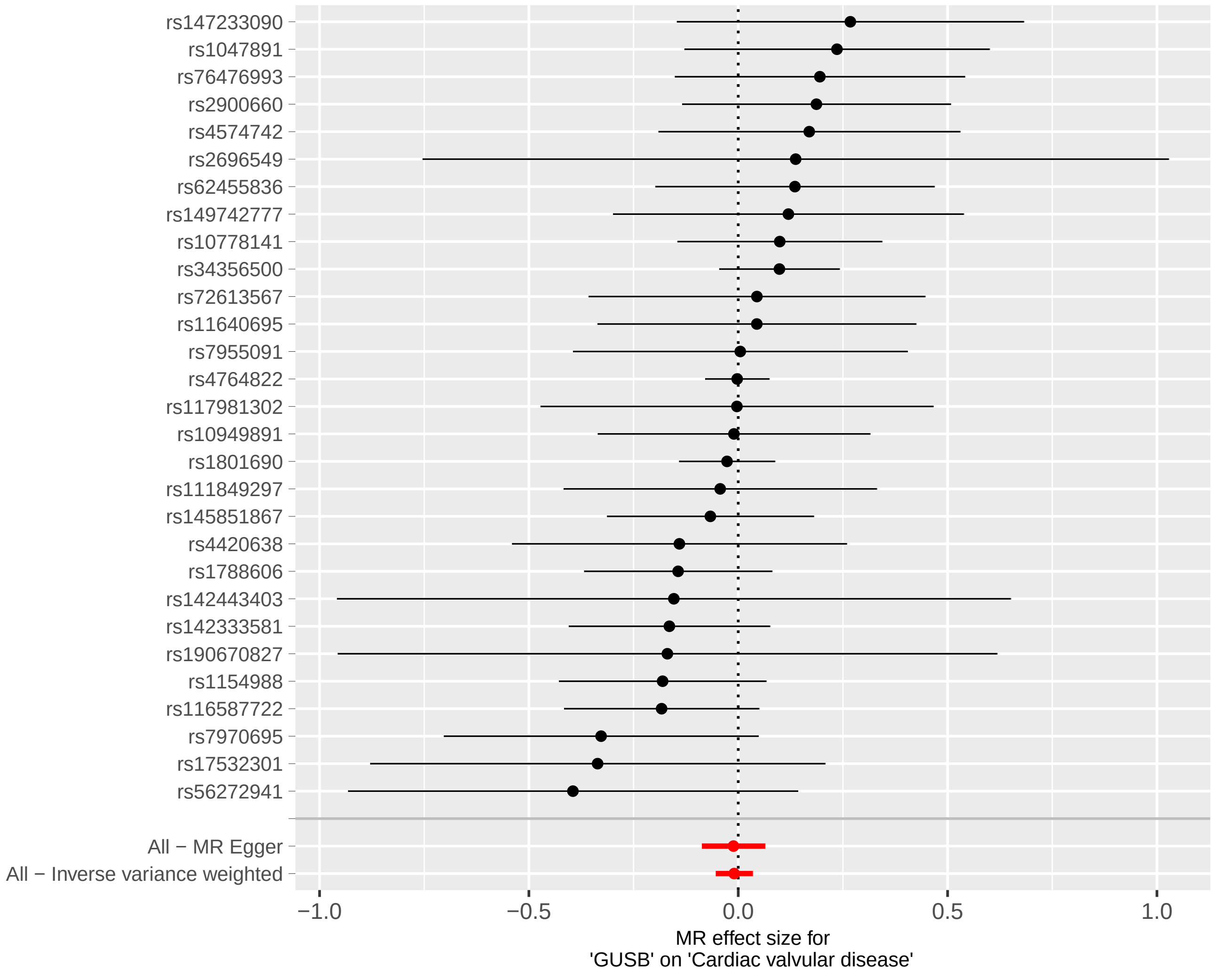
rs147233090
rs1047891
rs76476993
rs2900660
rs4574742
rs2696549
rs62455836
rs149742777
rs10778141
rs34356500
rs72613567
rs11640695
rs7955091
rs4764822
rs117981302
rs10949891
rs1801690
rs111849297
rs145851867
rs4420638
rs1788606
rs142443403
rs142333581
rs190670827
rs1154988
rs116587722
rs7970695
rs17532301
rs56272941
All − MR Egger
All − Inverse variance weighted
−1.0
−0.5
0.0
0.5
1.0
MR effect size for
'GUSB' on 'Cardiac valvular disease'

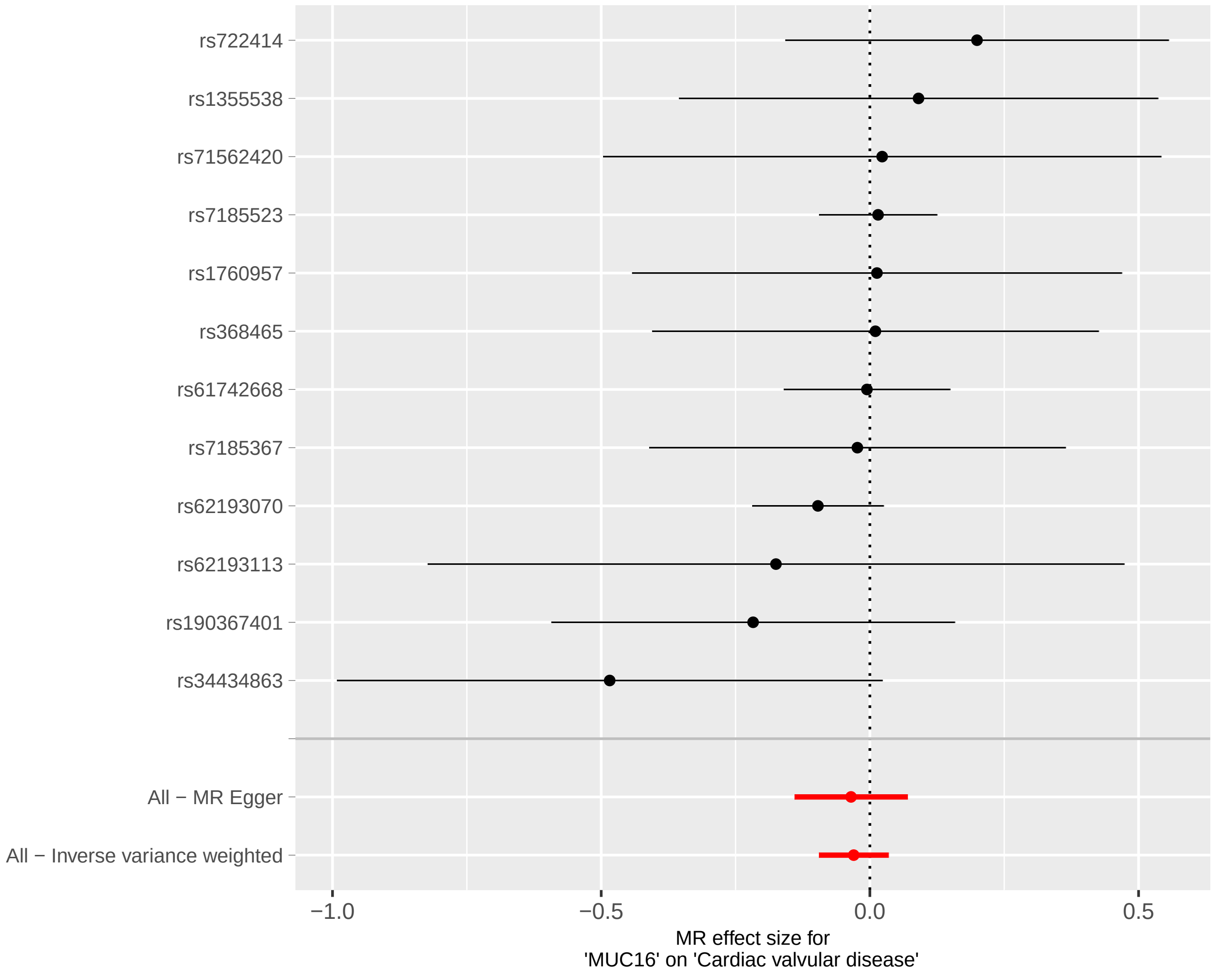
rs722414
rs1355538
rs71562420
rs7185523
rs1760957
rs368465
rs61742668
rs7185367
rs62193070
rs62193113
rs190367401
rs34434863
All − MR Egger
All − Inverse variance weighted
−1.0
−0.5
0.0
0.5
MR effect size for
'MUC16' on 'Cardiac valvular disease'

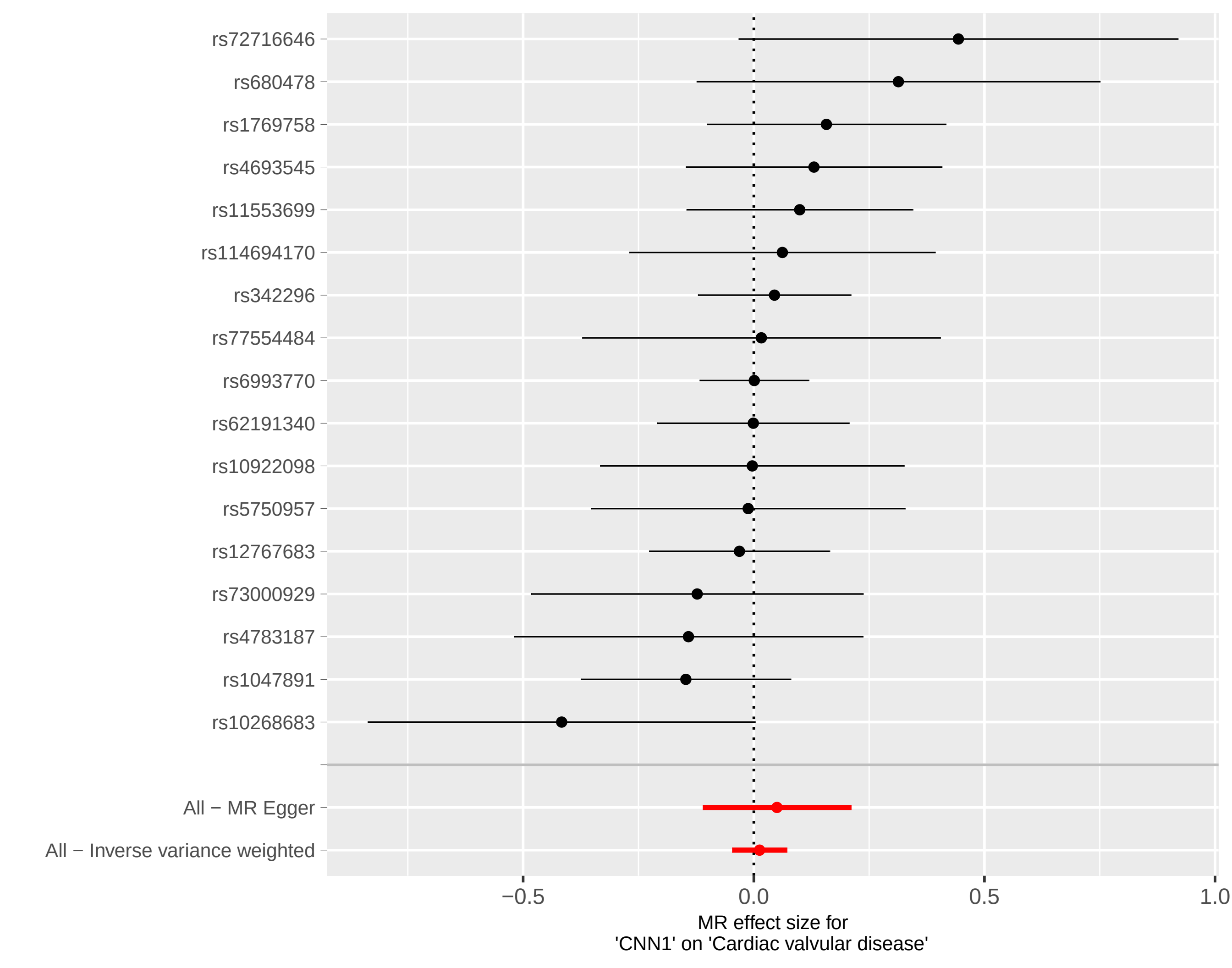
rs72716646
rs680478
rs1769758
rs4693545
rs11553699
rs114694170
rs342296
rs77554484
rs6993770
rs62191340
rs10922098
rs5750957
rs12767683
rs73000929
rs4783187
rs1047891
rs10268683
All − MR Egger
All − Inverse variance weighted
−0.5
0.0
0.5
1.0
MR effect size for
'CNN1' on 'Cardiac valvular disease'

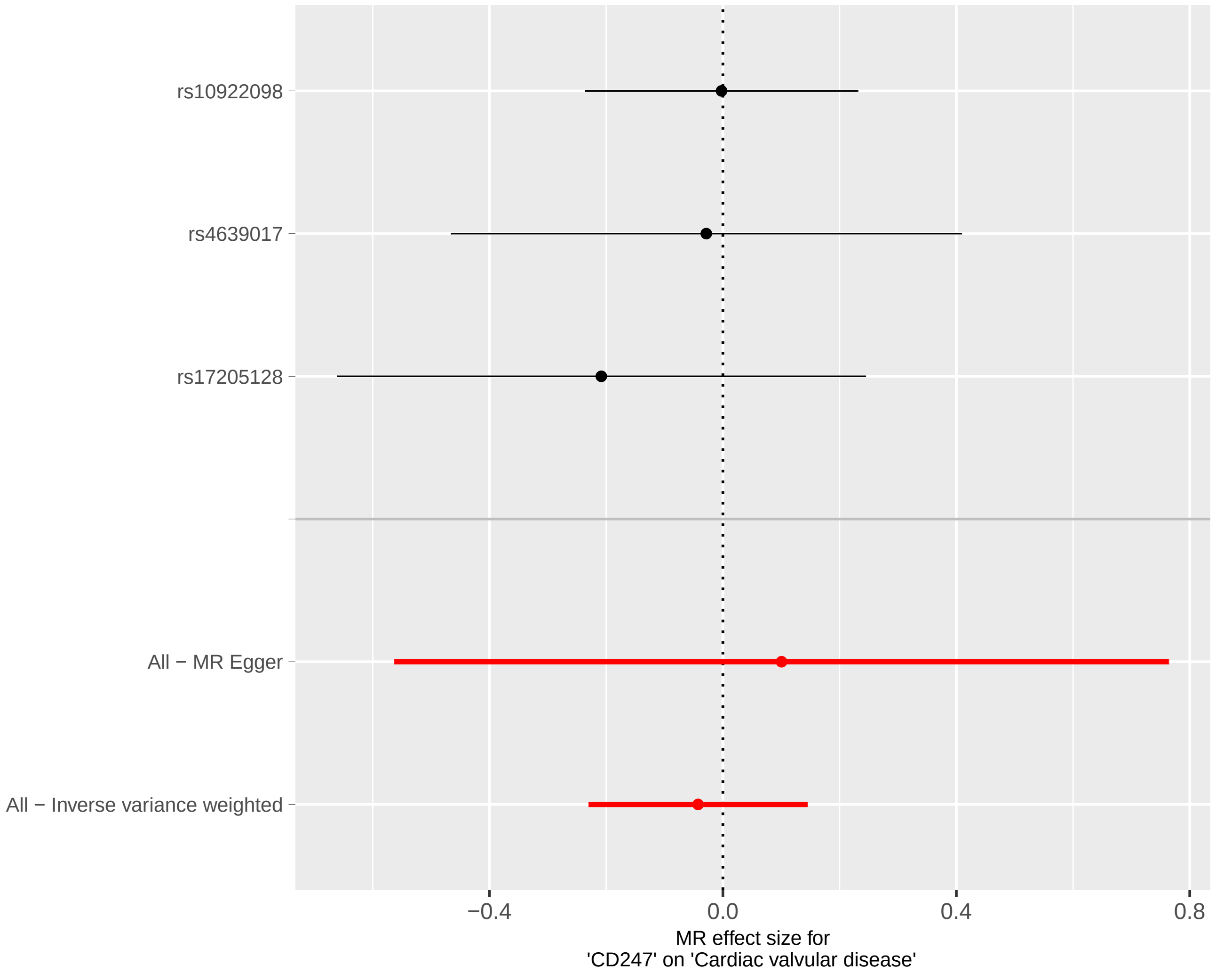
rs10922098
rs4639017
rs17205128
All − MR Egger
All − Inverse variance weighted
−0.4
0.0
0.4
0.8
MR effect size for
'CD247' on 'Cardiac valvular disease'

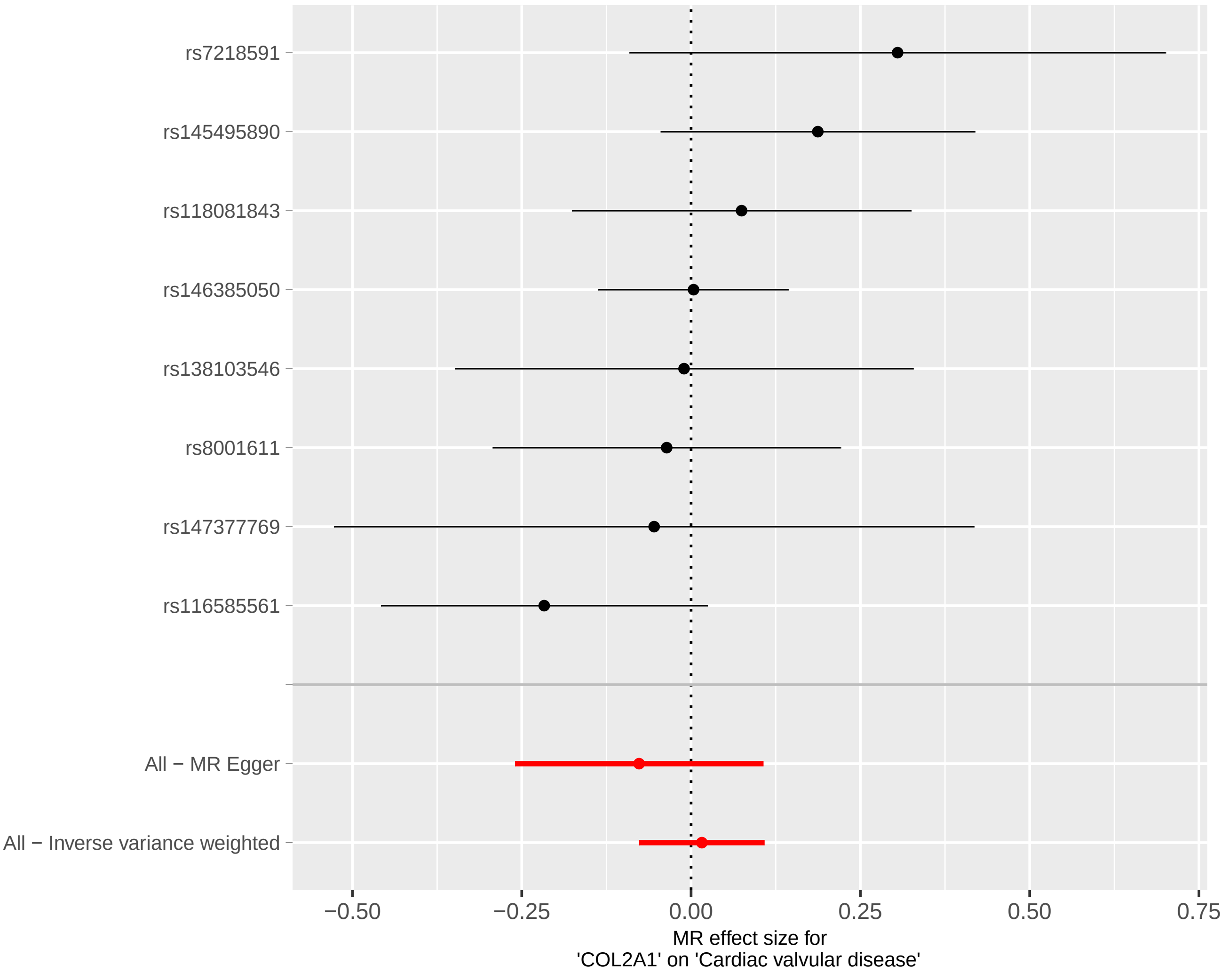
rs7218591
rs145495890
rs118081843
rs146385050
rs138103546
rs8001611
rs147377769
rs116585561
All − MR Egger
All − Inverse variance weighted
−0.50
−0.25
0.00
0.25
0.50
0.75
MR effect size for
'COL2A1' on 'Cardiac valvular disease'

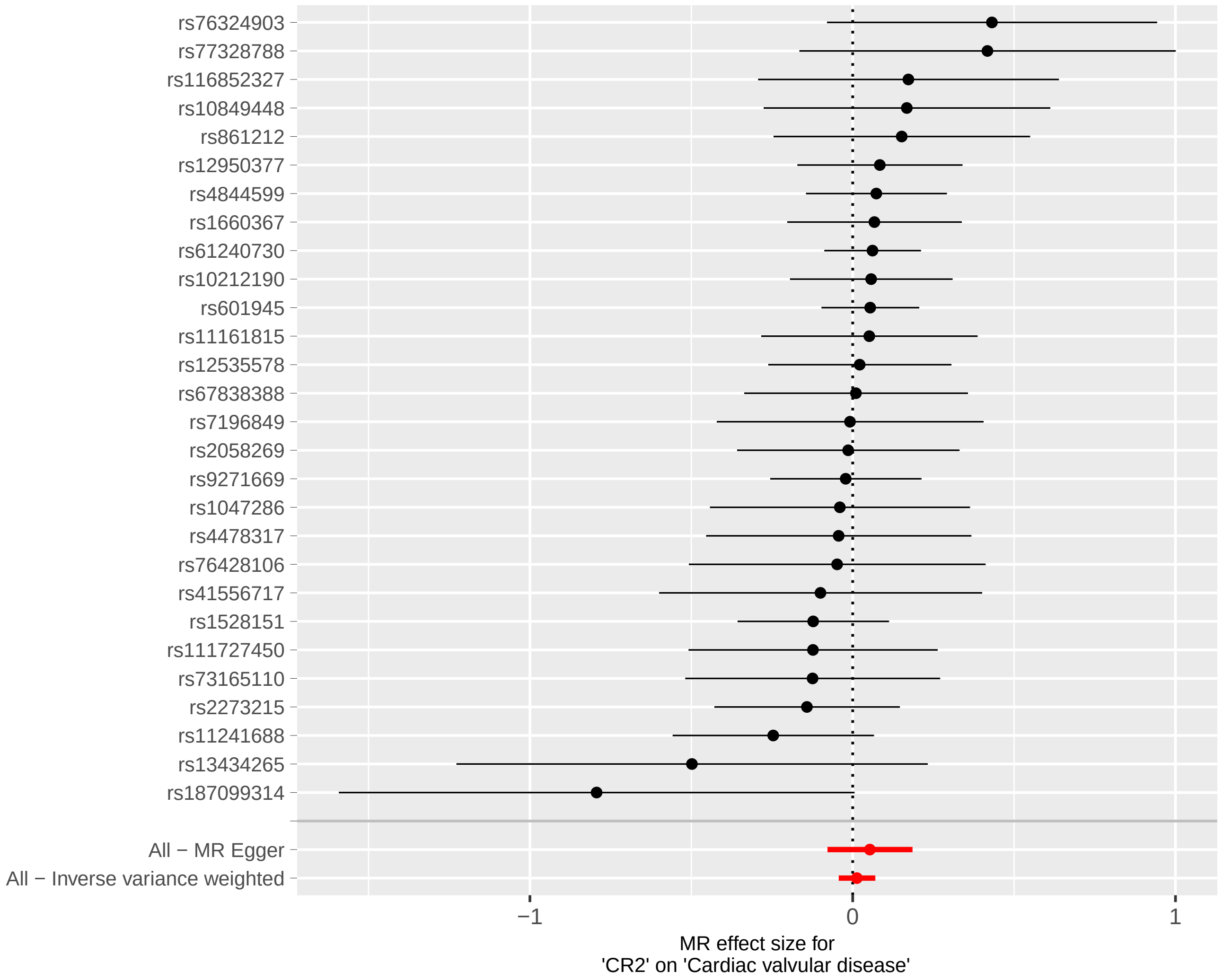

rs76324903
rs77328788
rs116852327
rs10849448
rs861212
rs12950377
rs4844599
rs1660367
rs61240730
rs10212190
rs601945
rs11161815
rs12535578
rs67838388
rs7196849
rs2058269
rs9271669
rs1047286
rs4478317
rs76428106
rs41556717
rs1528151
rs111727450
rs73165110
rs2273215
rs11241688
rs13434265
rs187099314
All − MR Egger
All − Inverse variance weighted
−1
0
1
MR effect size for
'CR2' on 'Cardiac valvular disease'

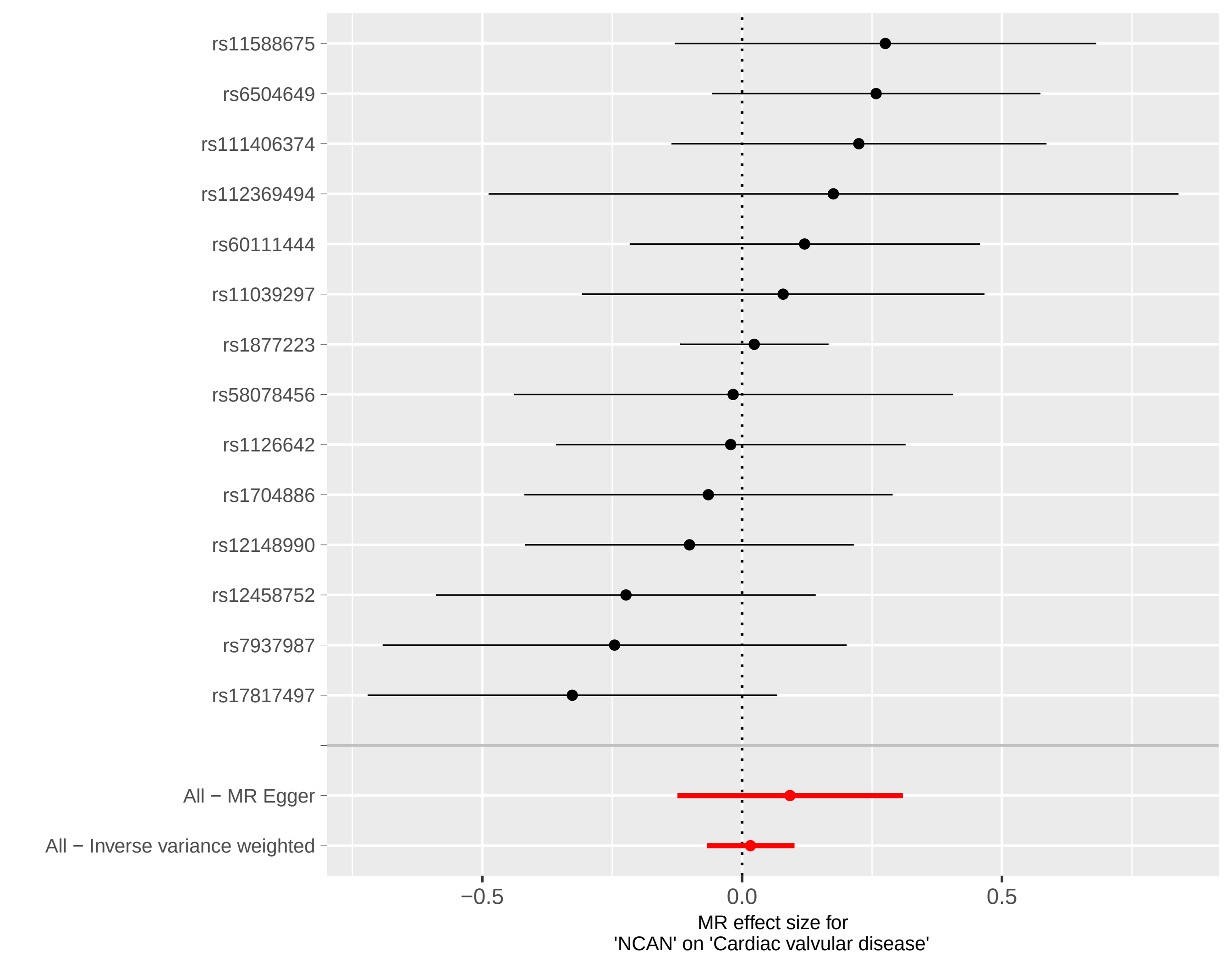
rs11588675
rs6504649
rs111406374
rs112369494
rs60111444
rs11039297
rs1877223
rs58078456
rs1126642
rs1704886
rs12148990
rs12458752
rs7937987
rs17817497
All − MR Egger
All − Inverse variance weighted
−0.5
0.0
0.5
MR effect size for
'NCAN' on 'Cardiac valvular disease'

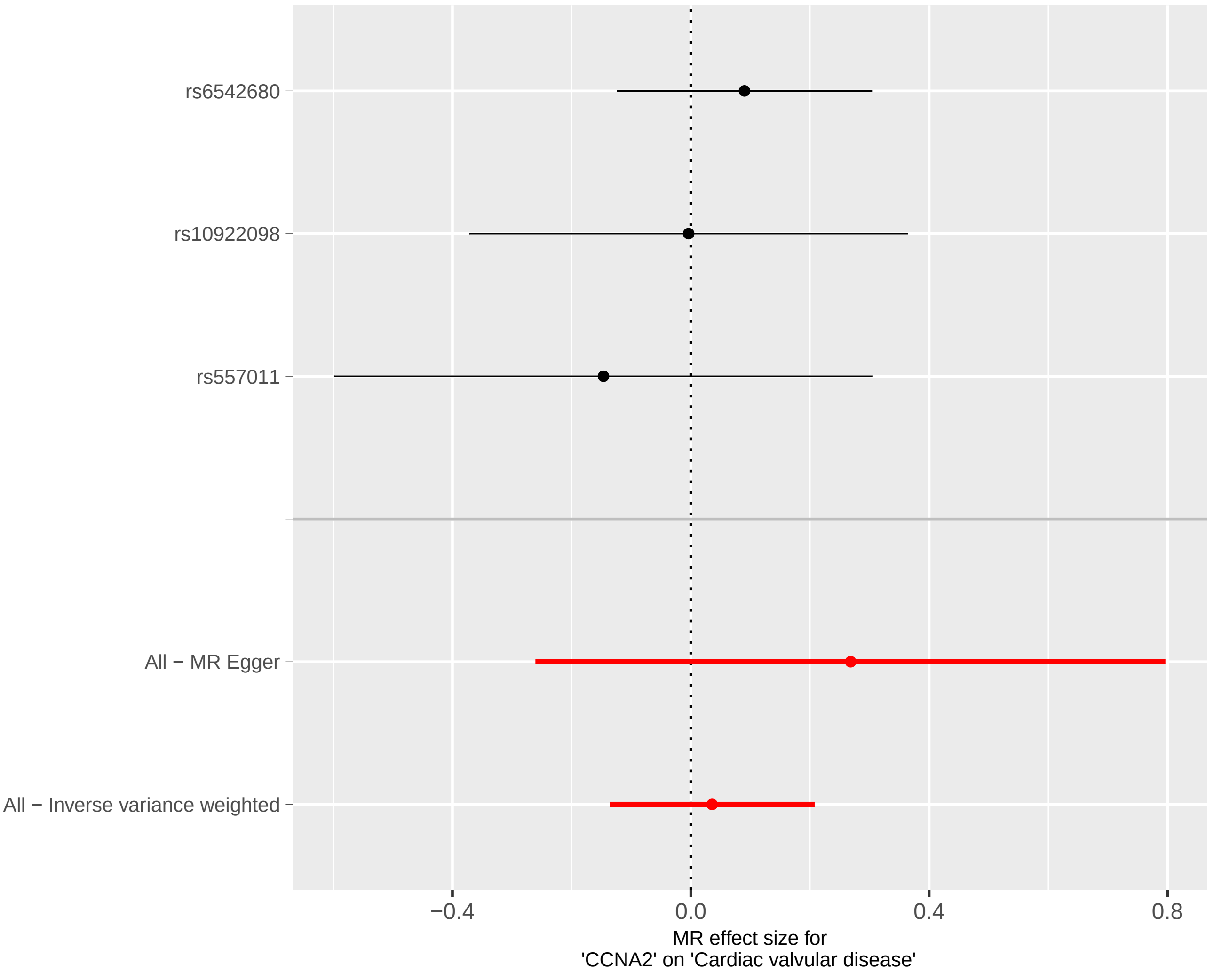
rs6542680
rs10922098
rs557011
All − MR Egger
All − Inverse variance weighted
−0.4
0.0
0.4
0.8
MR effect size for
'CCNA2' on 'Cardiac valvular disease'

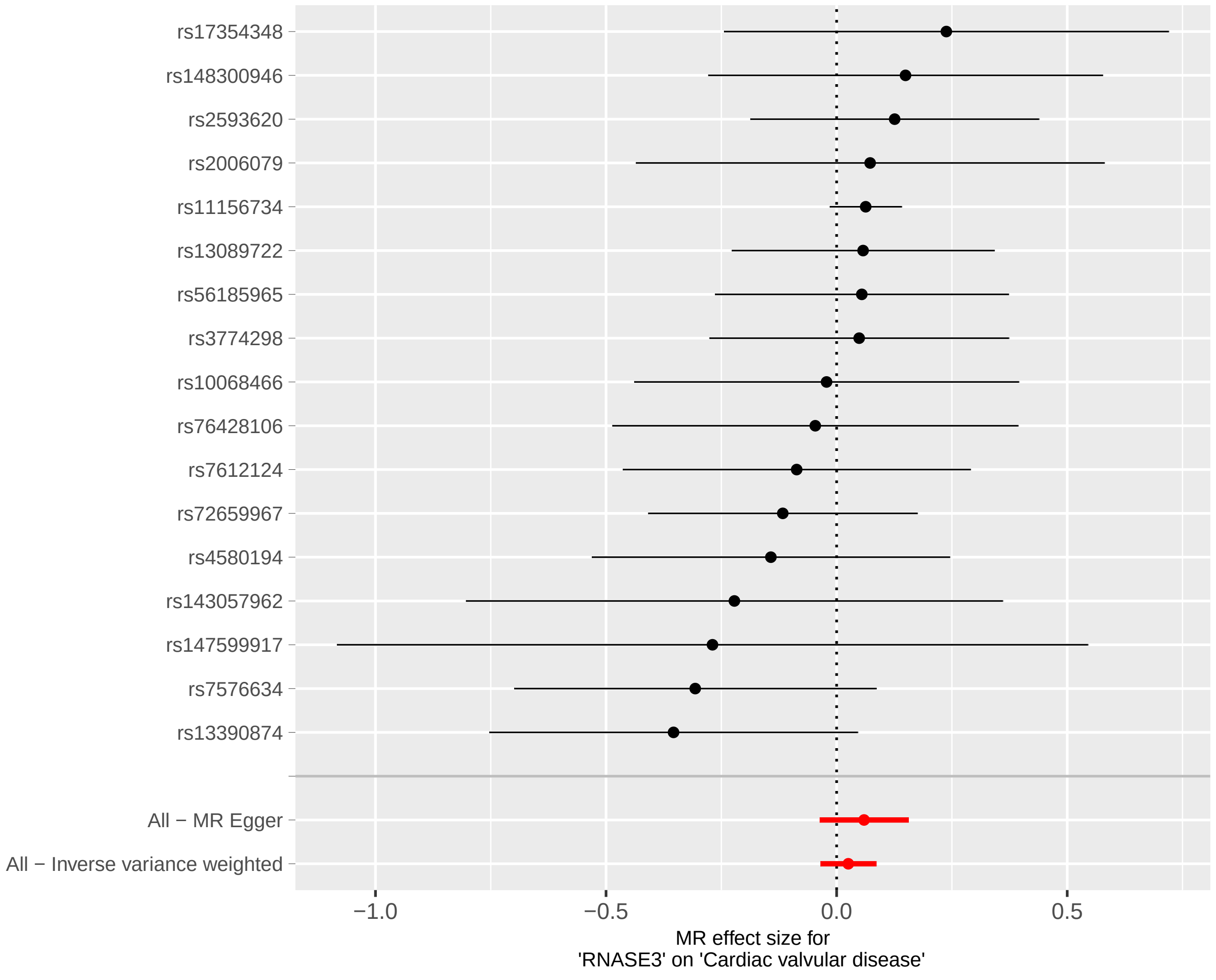

rs17354348
rs148300946
rs2593620
rs2006079
rs11156734
rs13089722
rs56185965
rs3774298
rs10068466
rs76428106
rs7612124
rs72659967
rs4580194
rs143057962
rs147599917
rs7576634
rs13390874
All − MR Egger
All − Inverse variance weighted
−1.0
−0.5
0.0
0.5
MR effect size for
'RNASE3' on 'Cardiac valvular disease'

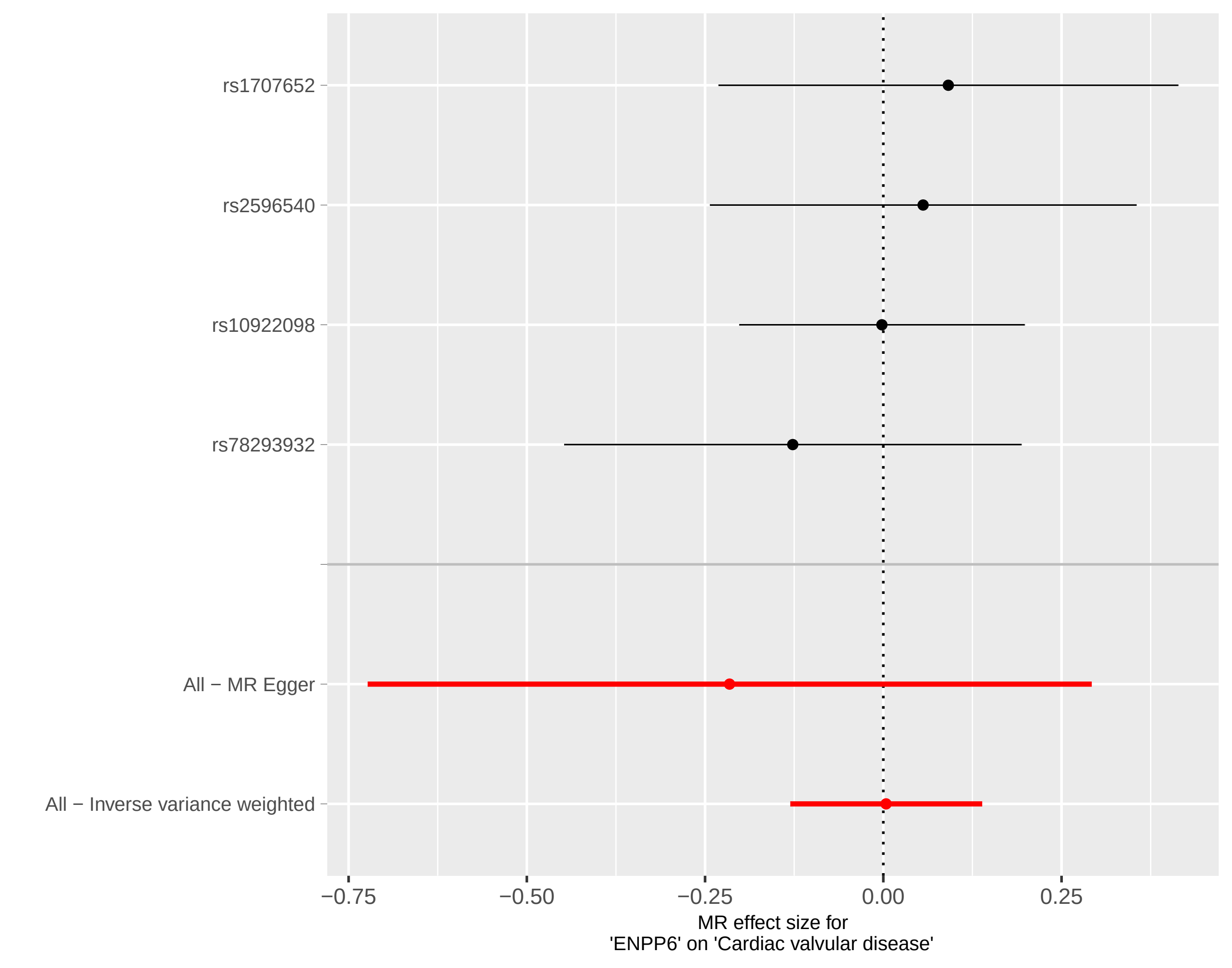

rs1707652
rs2596540
rs10922098
rs78293932
All − MR Egger
All − Inverse variance weighted
−0.75
−0.50
−0.25
0.00
0.25
MR effect size for
'ENPP6' on 'Cardiac valvular disease'

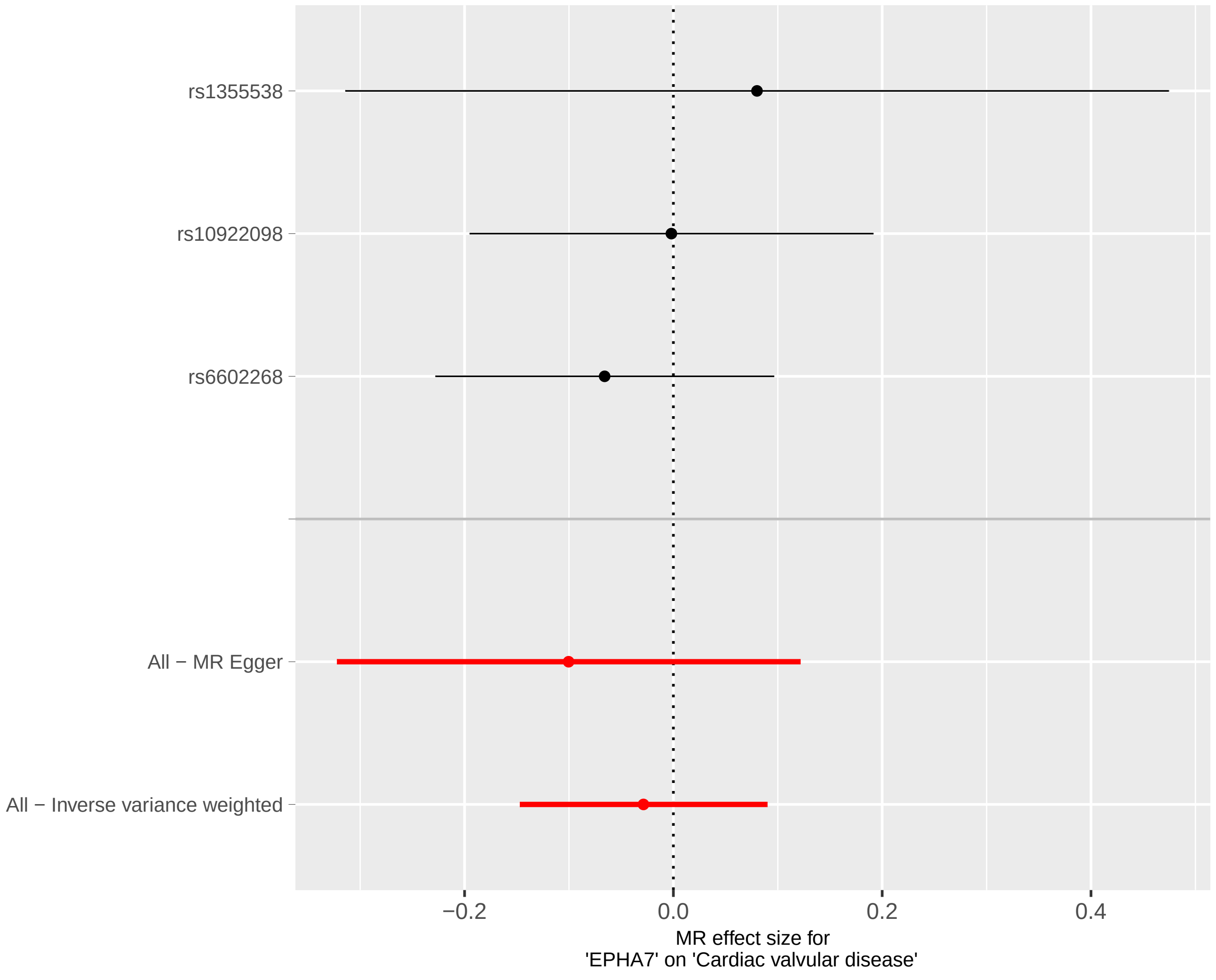
rs1355538
rs10922098
rs6602268
All − MR Egger
All − Inverse variance weighted
−0.2
0.0
0.2
0.4
MR effect size for
'EPHA7' on 'Cardiac valvular disease'

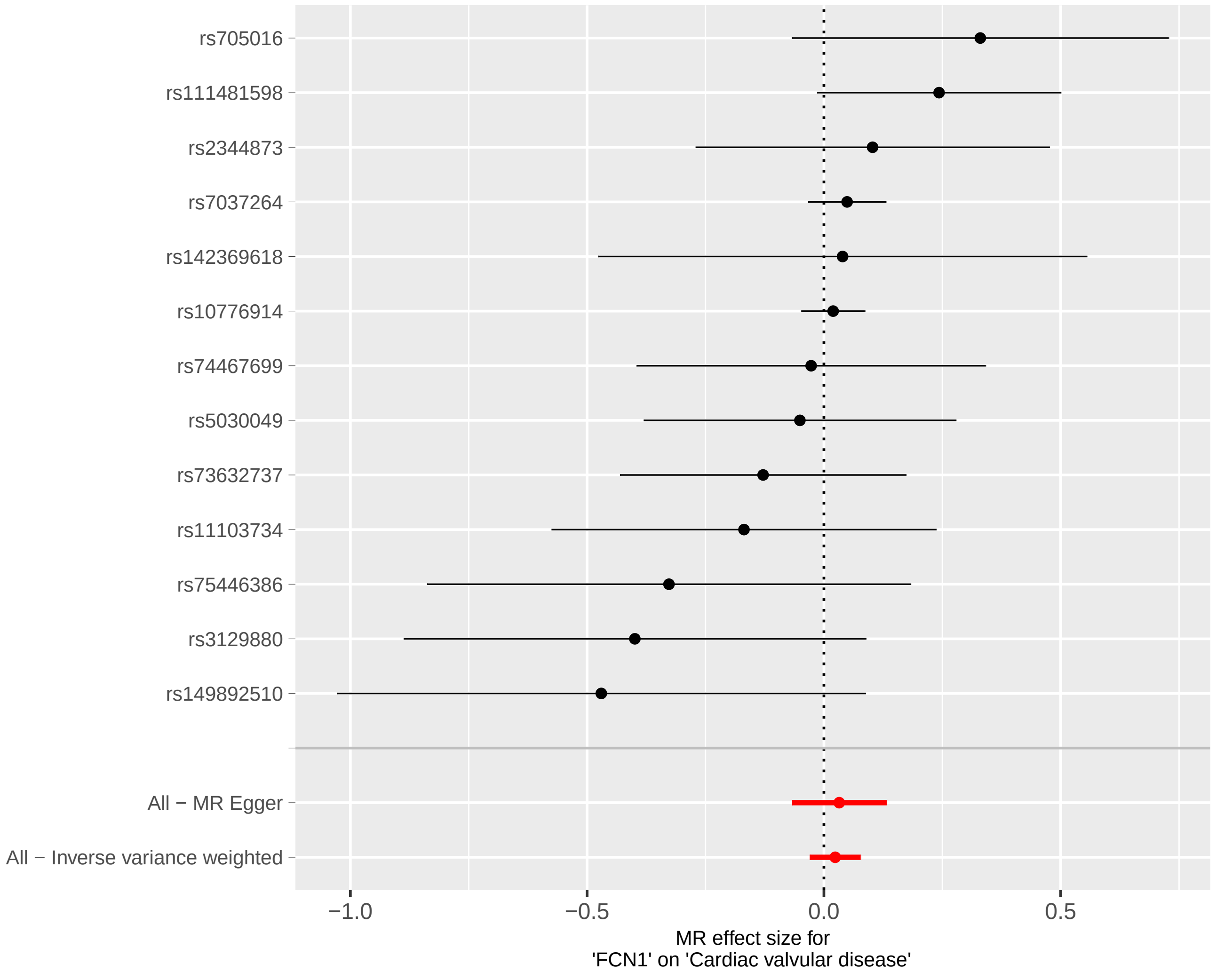
rs705016
rs111481598
rs2344873
rs7037264
rs142369618
rs10776914
rs74467699
rs5030049
rs73632737
rs11103734
rs75446386
rs3129880
rs149892510
All − MR Egger
All − Inverse variance weighted
−1.0
−0.5
0.0
0.5
MR effect size for
'FCN1' on 'Cardiac valvular disease'

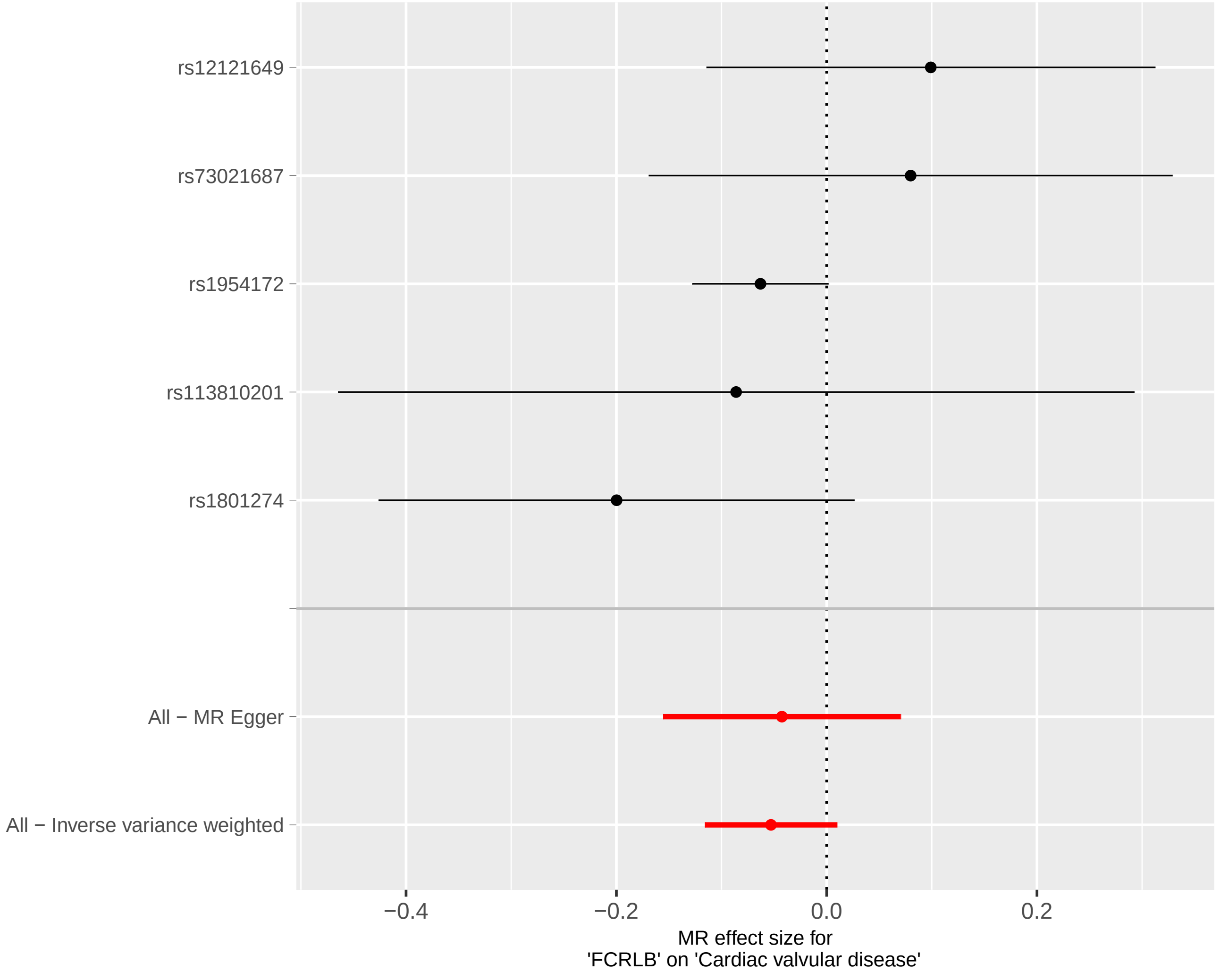

rs12121649
rs73021687
rs1954172
rs113810201
rs1801274
All − MR Egger
All − Inverse variance weighted
−0.4
−0.2
0.0
0.2
MR effect size for
'FCRLB' on 'Cardiac valvular disease'

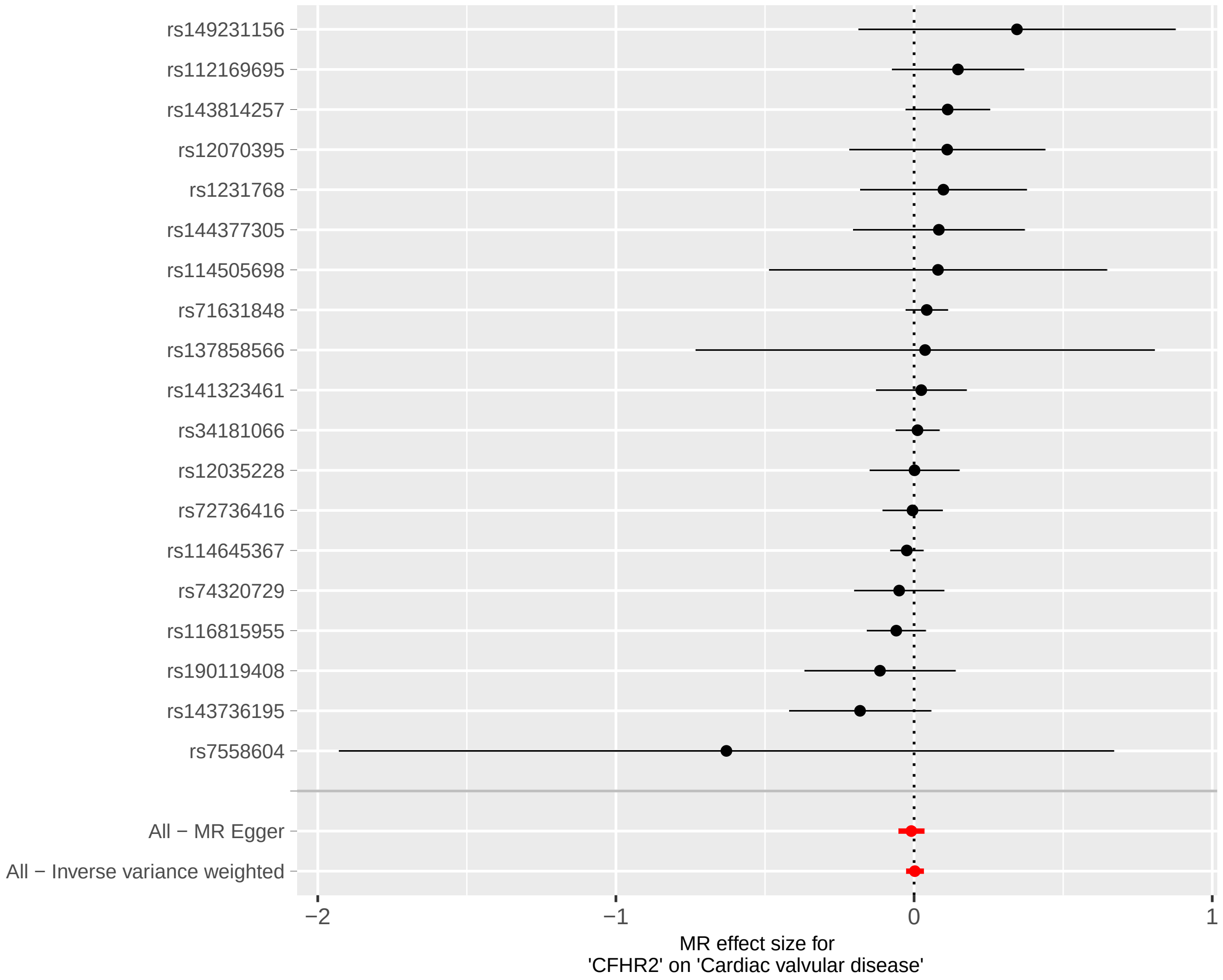
rs149231156
rs112169695
rs143814257
rs12070395
rs1231768
rs144377305
rs114505698
rs71631848
rs137858566
rs141323461
rs34181066
rs12035228
rs72736416
rs114645367
rs74320729
rs116815955
rs190119408
rs143736195
rs7558604
All − MR Egger
All − Inverse variance weighted
−2
−1
0
1
MR effect size for
'CFHR2' on 'Cardiac valvular disease'

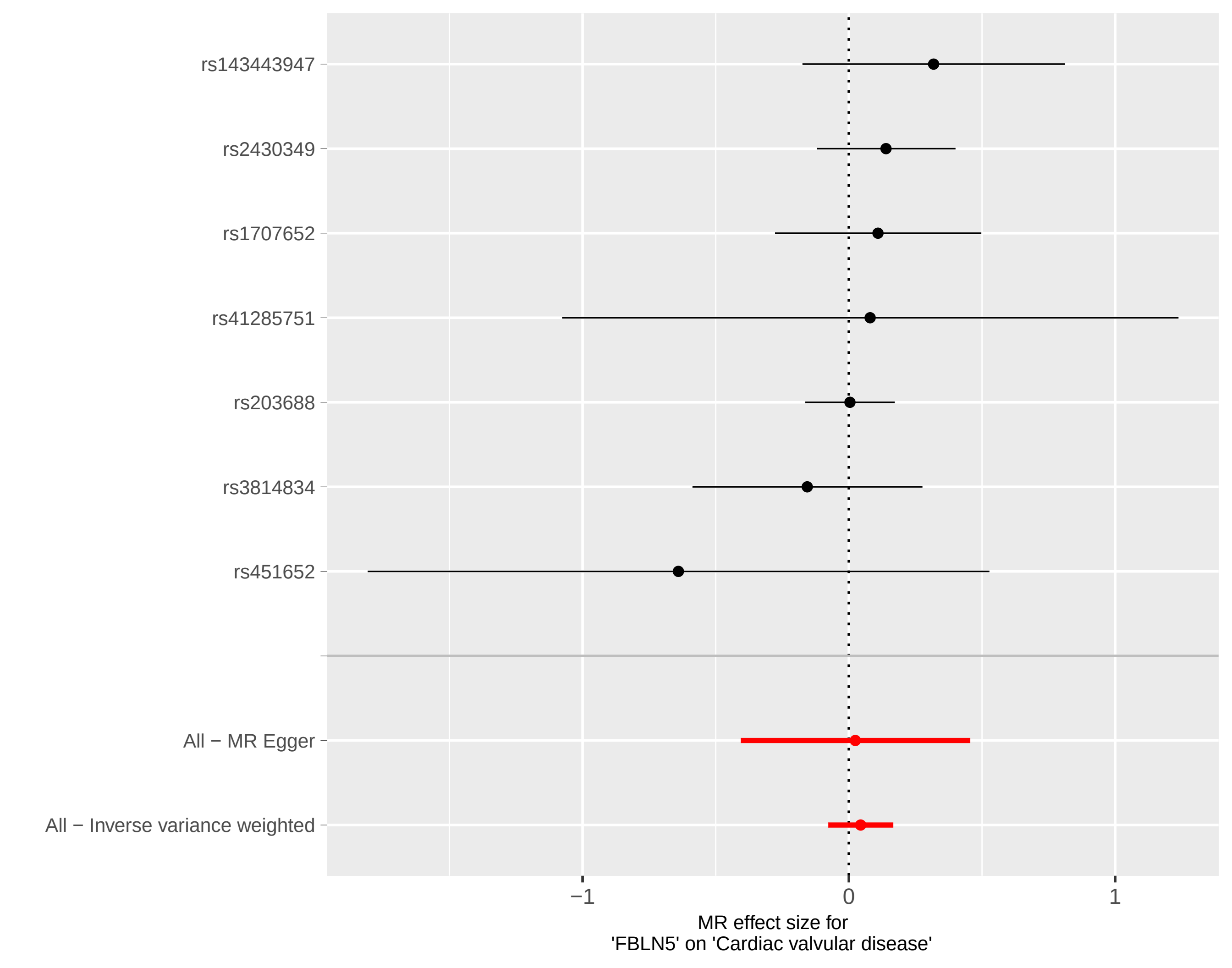
rs143443947
rs2430349
rs1707652
rs41285751
rs203688
rs3814834
rs451652
All − MR Egger
All − Inverse variance weighted
−1
0
1
MR effect size for
'FBLN5' on 'Cardiac valvular disease'

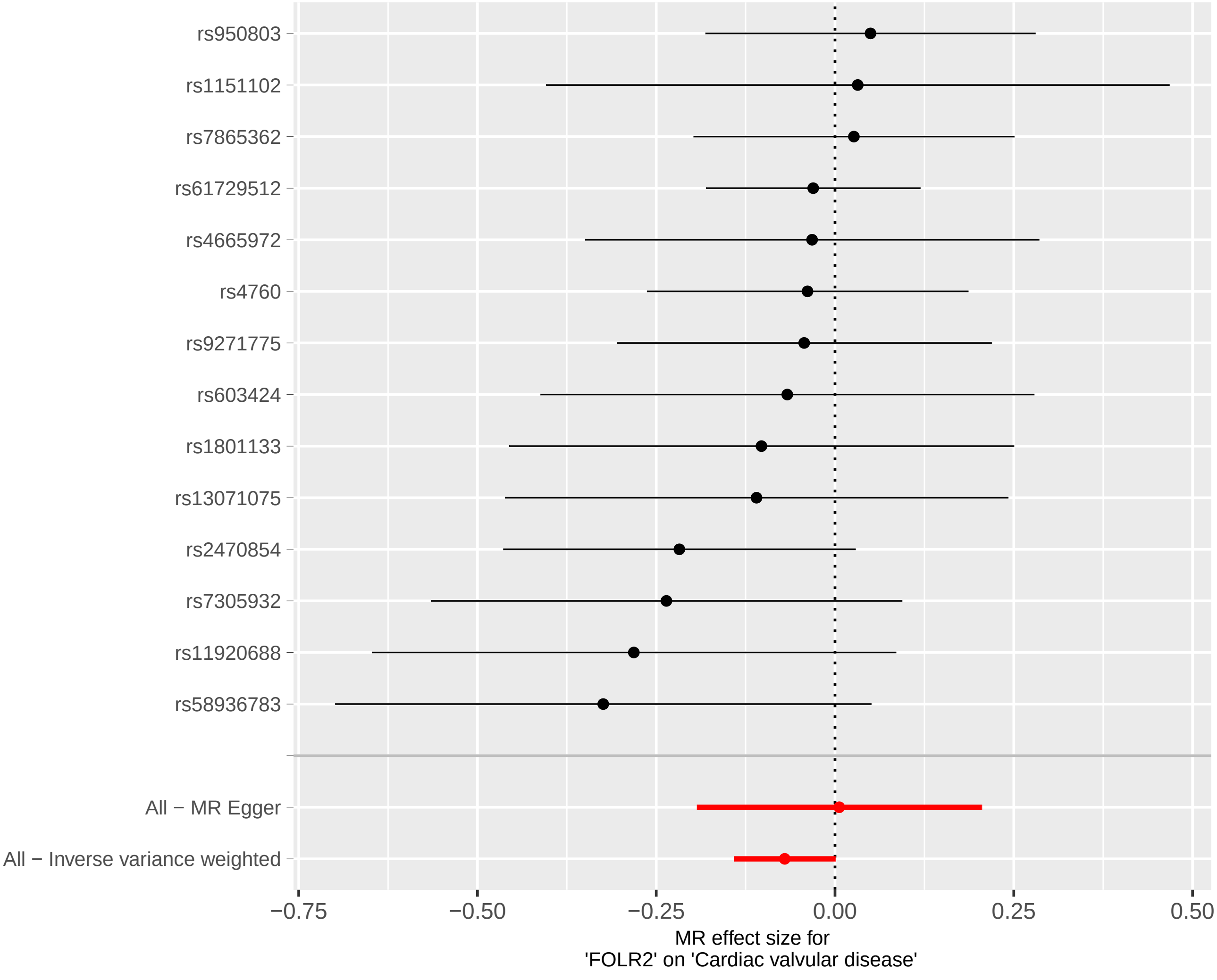
rs950803
rs1151102
rs7865362
rs61729512
rs4665972
rs4760
rs9271775
rs603424
rs1801133
rs13071075
rs2470854
rs7305932
rs11920688
rs58936783
All − MR Egger
All − Inverse variance weighted
−0.75
−0.50
−0.25
0.00
0.25
0.50
MR effect size for
'FOLR2' on 'Cardiac valvular disease'

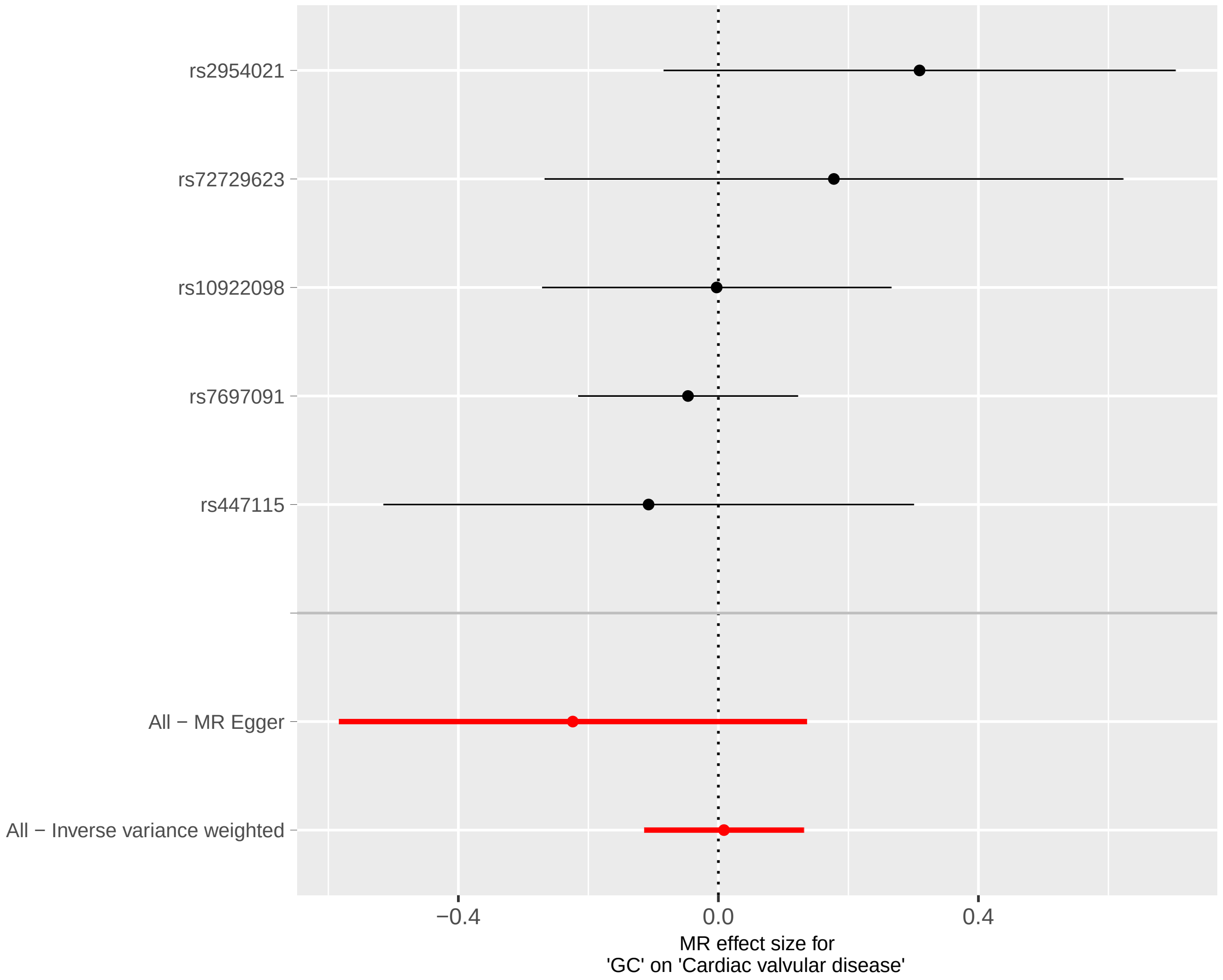
rs2954021
rs72729623
rs10922098
rs7697091
rs447115
All − MR Egger
All − Inverse variance weighted
−0.4
0.0
0.4
MR effect size for
'GC' on 'Cardiac valvular disease'

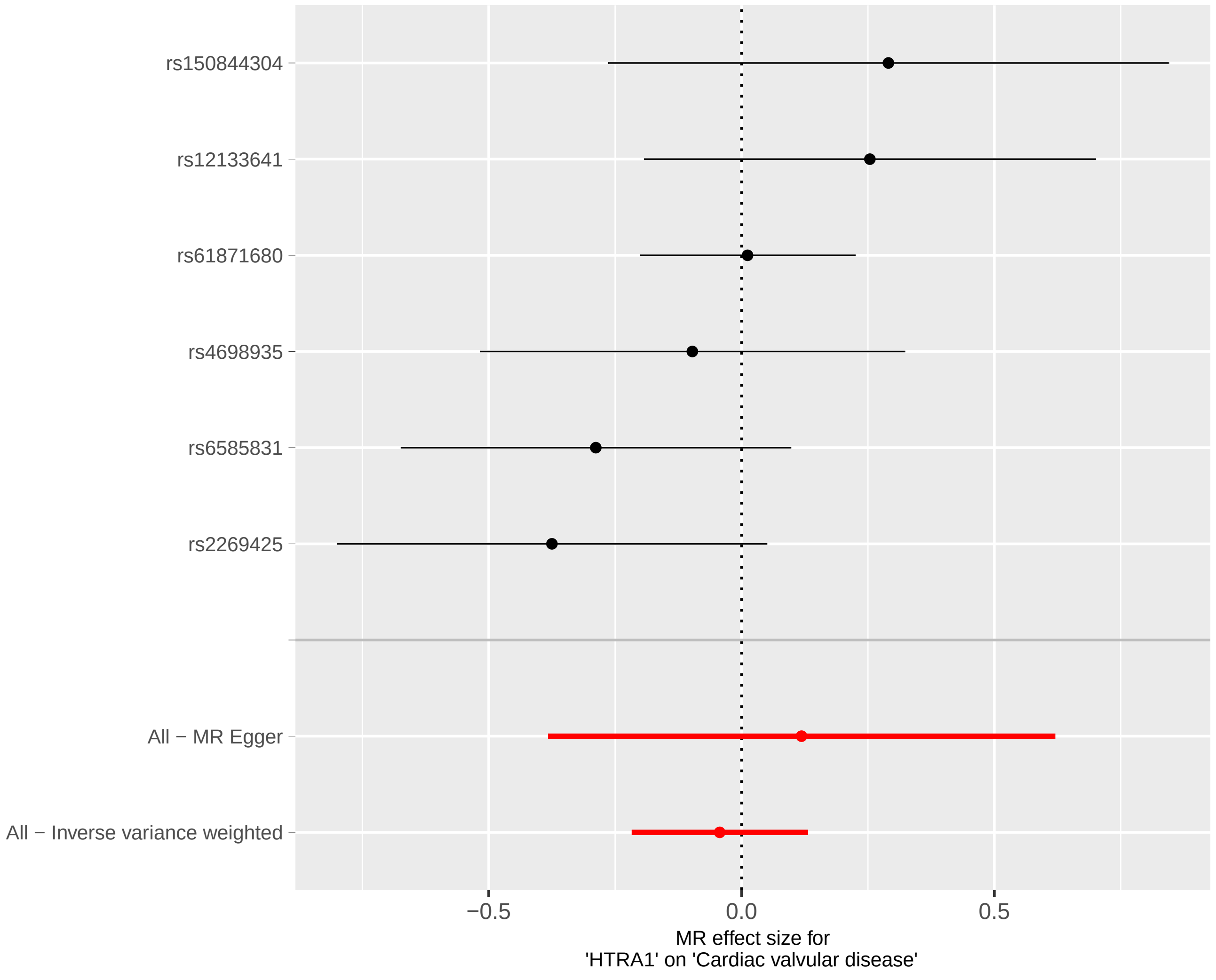

rs150844304
rs12133641
rs61871680
rs4698935
rs6585831
rs2269425
All − MR Egger
All − Inverse variance weighted
−0.5
0.0
0.5
MR effect size for
'HTRA1' on 'Cardiac valvular disease'

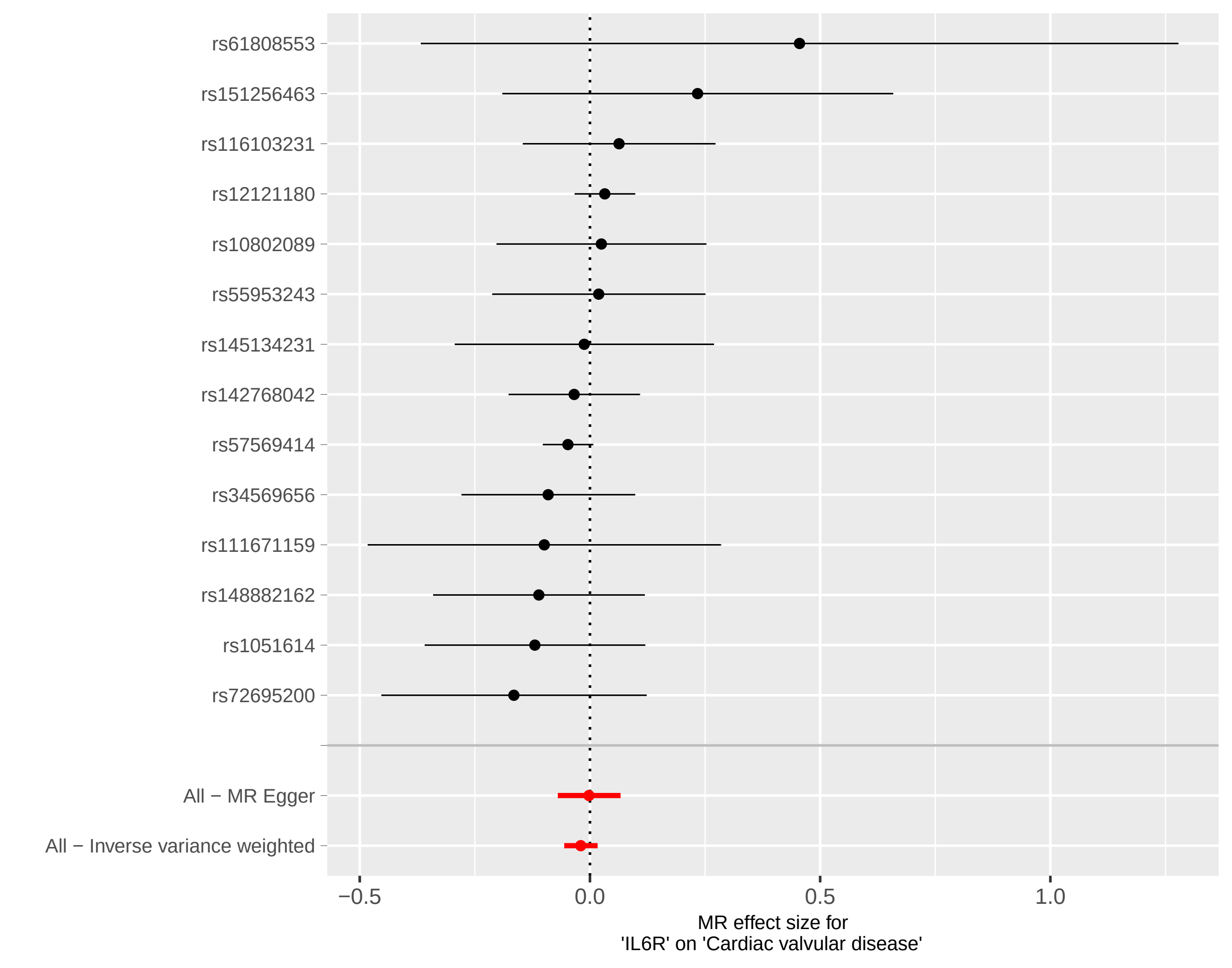
rs61808553
rs151256463
rs116103231
rs12121180
rs10802089
rs55953243
rs145134231
rs142768042
rs57569414
rs34569656
rs111671159
rs148882162
rs1051614
rs72695200
All − MR Egger
All − Inverse variance weighted
−0.5
0.0
0.5
1.0
MR effect size for
'IL6R' on 'Cardiac valvular disease'

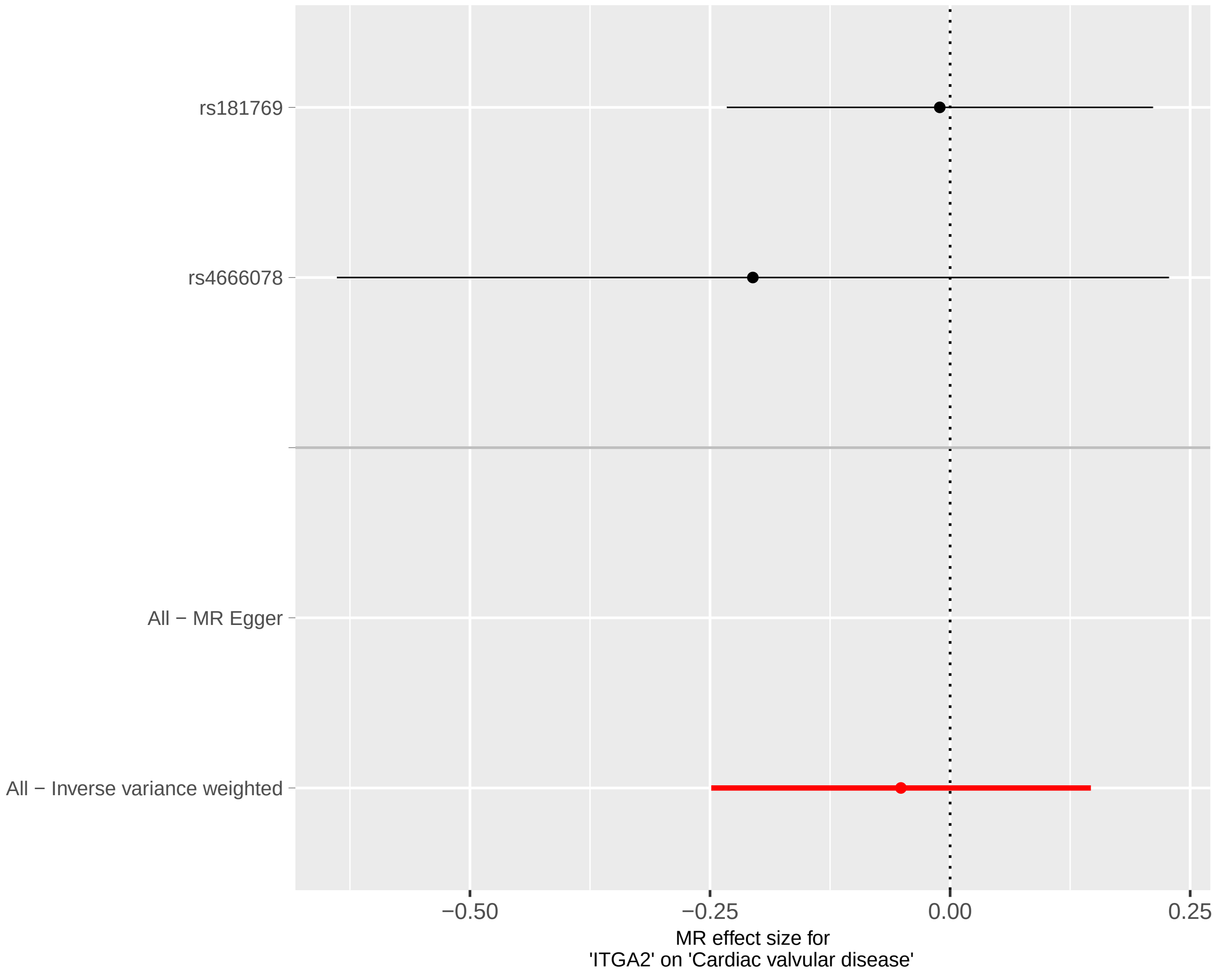
rs181769
rs4666078
All − MR Egger
All − Inverse variance weighted
−0.50
−0.25
0.00
0.25
MR effect size for
'ITGA2' on 'Cardiac valvular disease'

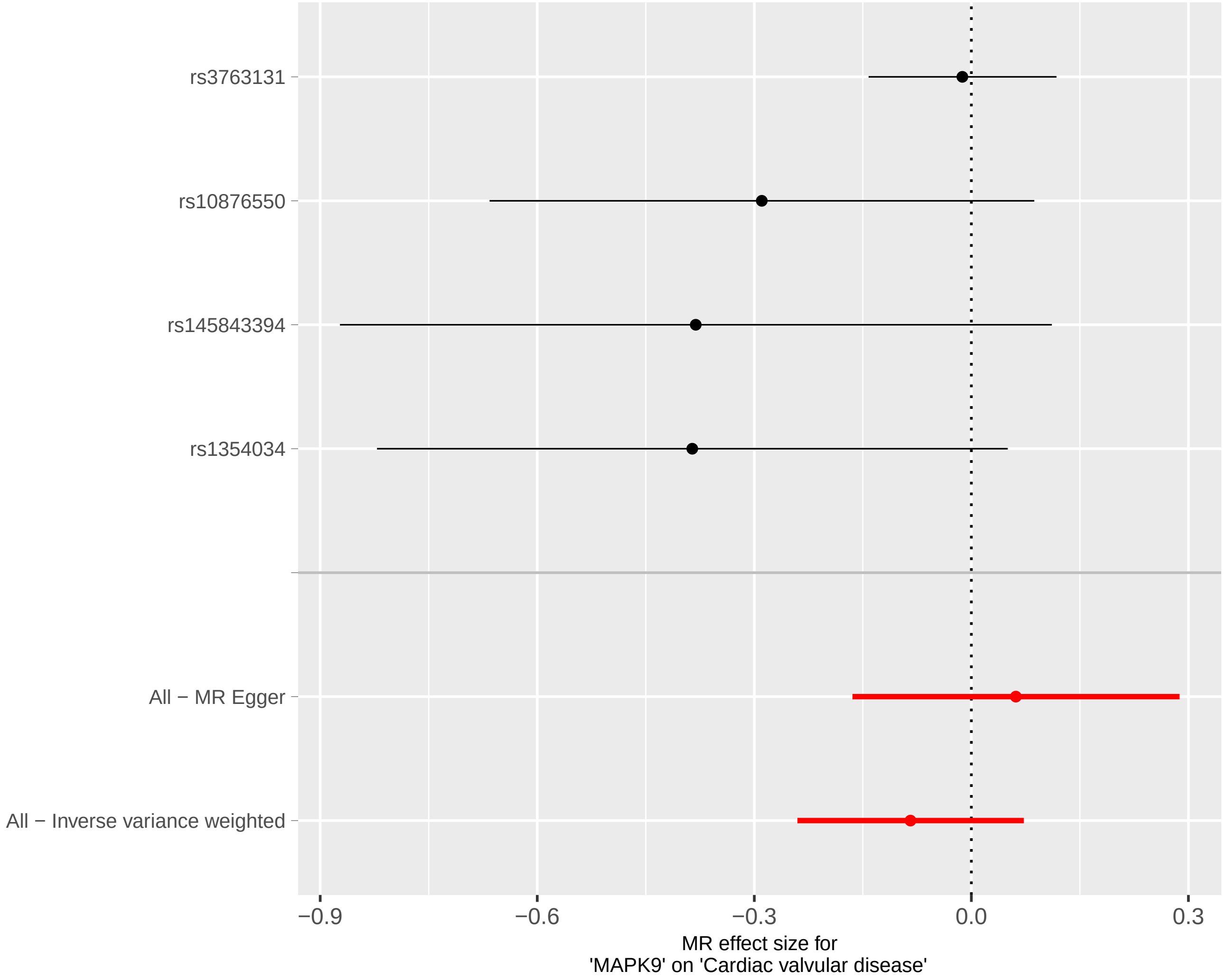
rs3763131
rs10876550
rs145843394
rs1354034
All − MR Egger
All − Inverse variance weighted
−0.9
−0.6
−0.3
0.0
0.3
MR effect size for
'MAPK9' on 'Cardiac valvular disease'

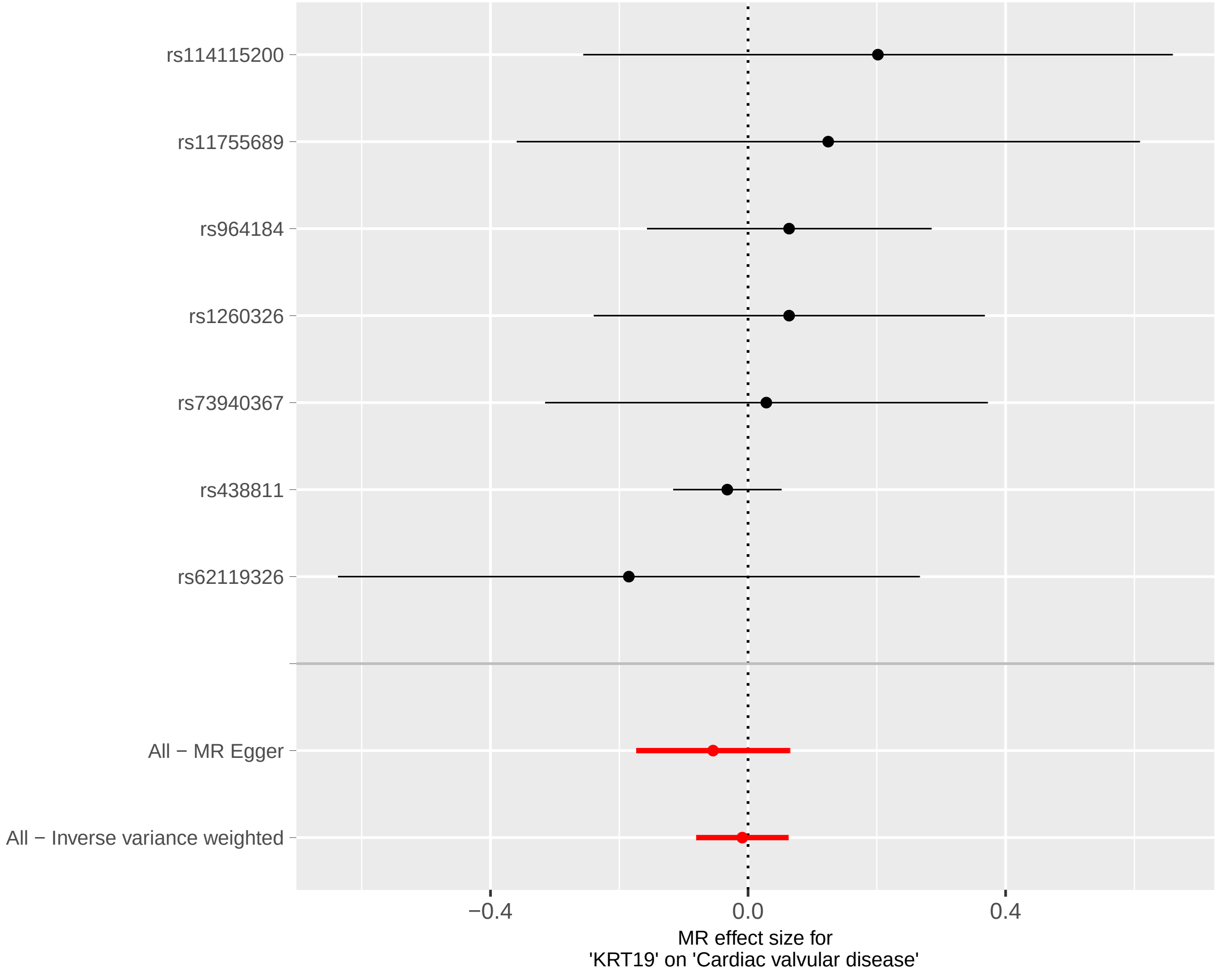
rs114115200
rs11755689
rs964184
rs1260326
rs73940367
rs438811
rs62119326
All − MR Egger
All − Inverse variance weighted
−0.4
0.0
0.4
MR effect size for
'KRT19' on 'Cardiac valvular disease'

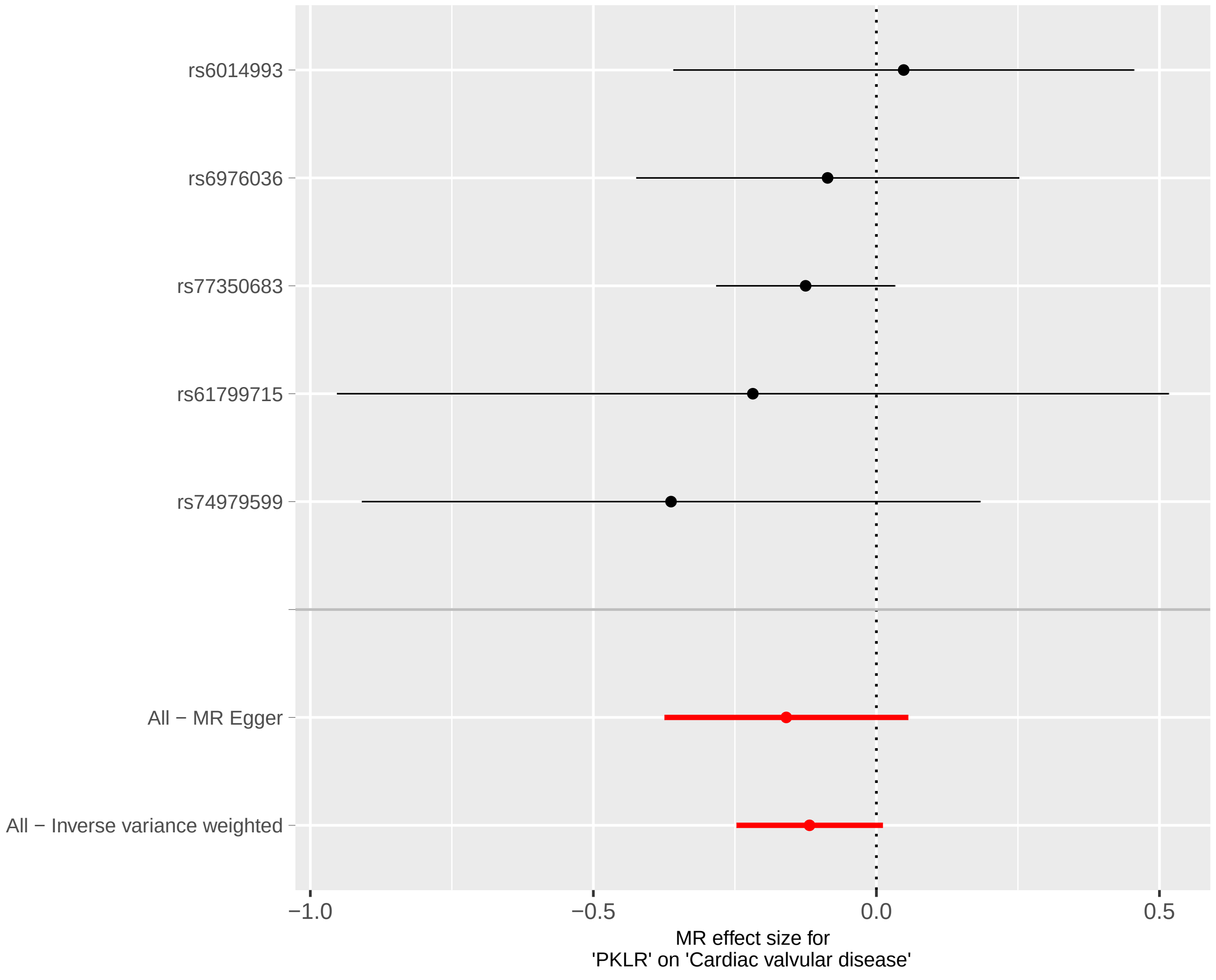
rs6014993
rs6976036
rs77350683
rs61799715
rs74979599
All − MR Egger
All − Inverse variance weighted
−1.0
−0.5
0.0
0.5
MR effect size for
'PKLR' on 'Cardiac valvular disease'

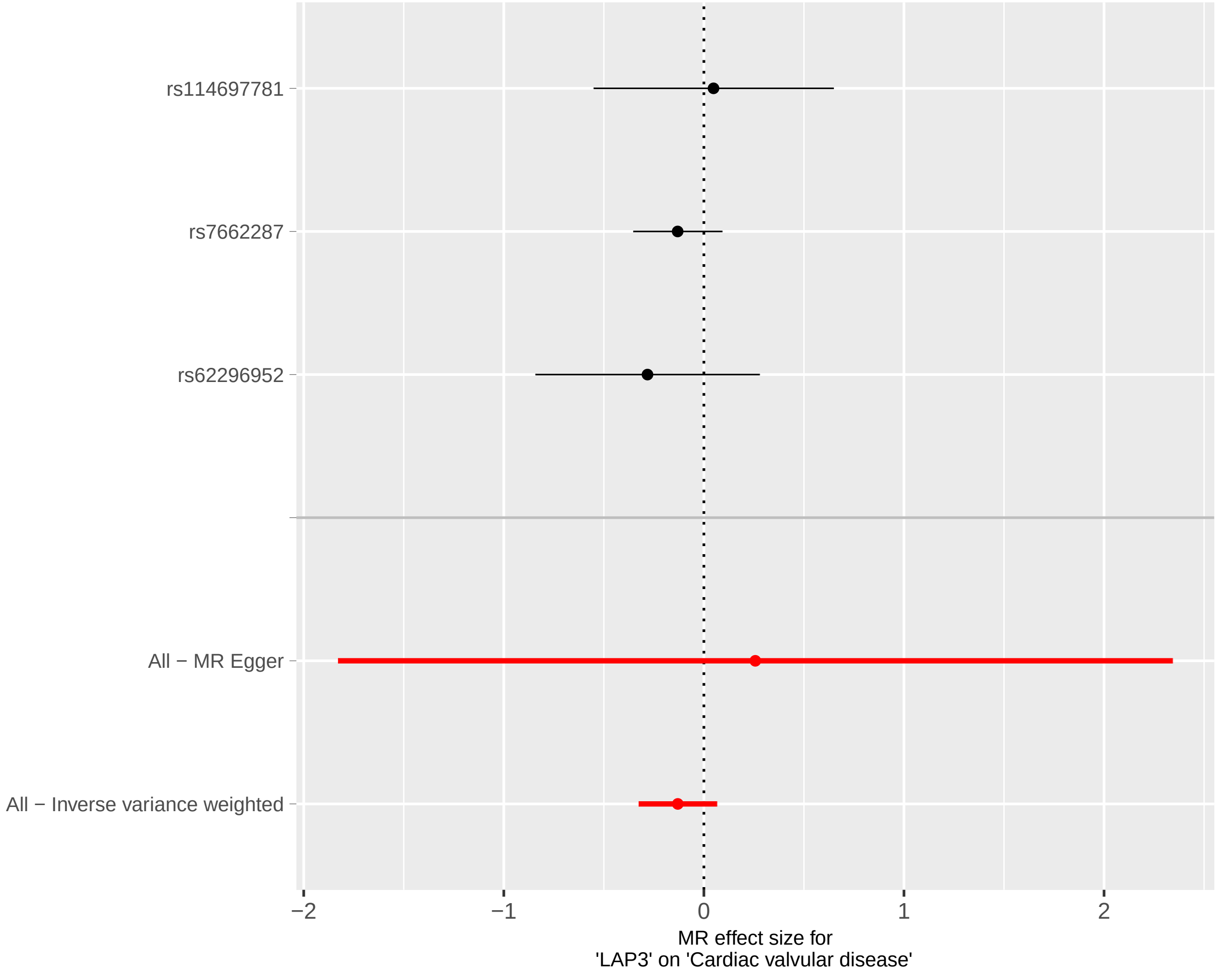
rs114697781
rs7662287
rs62296952
All − MR Egger
All − Inverse variance weighted
−2
−1
0
1
2
MR effect size for
'LAP3' on 'Cardiac valvular disease'

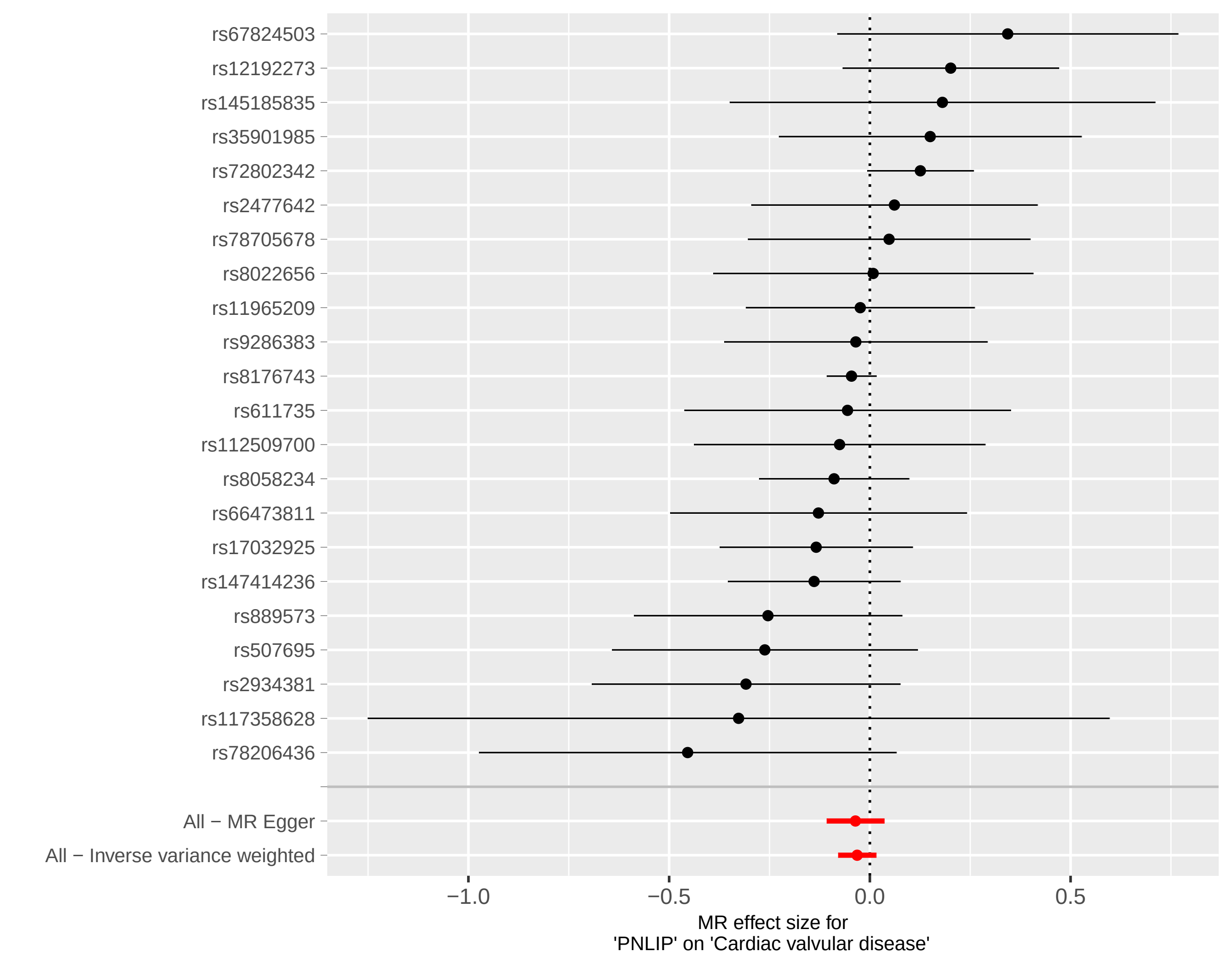
rs67824503
rs12192273
rs145185835
rs35901985
rs72802342
rs2477642
rs78705678
rs8022656
rs11965209
rs9286383
rs8176743
rs611735
rs112509700
rs8058234
rs66473811
rs17032925
rs147414236
rs889573
rs507695
rs2934381
rs117358628
rs78206436
All − MR Egger
All − Inverse variance weighted
−1.0
−0.5
0.0
0.5
MR effect size for
'PNLIP' on 'Cardiac valvular disease'

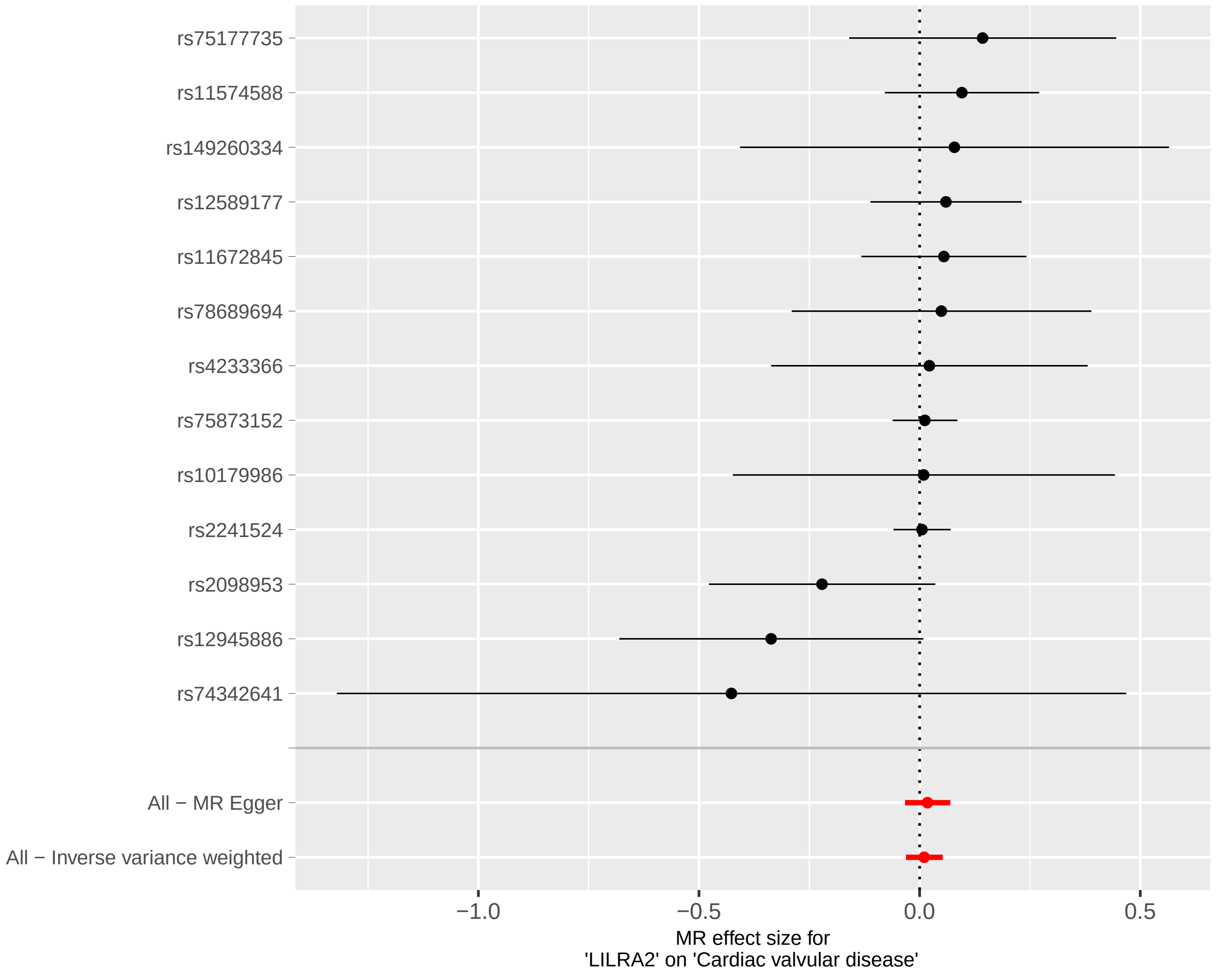

rs75177735
rs11574588
rs149260334
rs12589177
rs11672845
rs78689694
rs4233366
rs75873152
rs10179986
rs2241524
rs2098953
rs12945886
rs74342641
All − MR Egger
All − Inverse variance weighted
−1.0
−0.5
0.0
0.5
MR effect size for
'LILRA2' on 'Cardiac valvular disease'

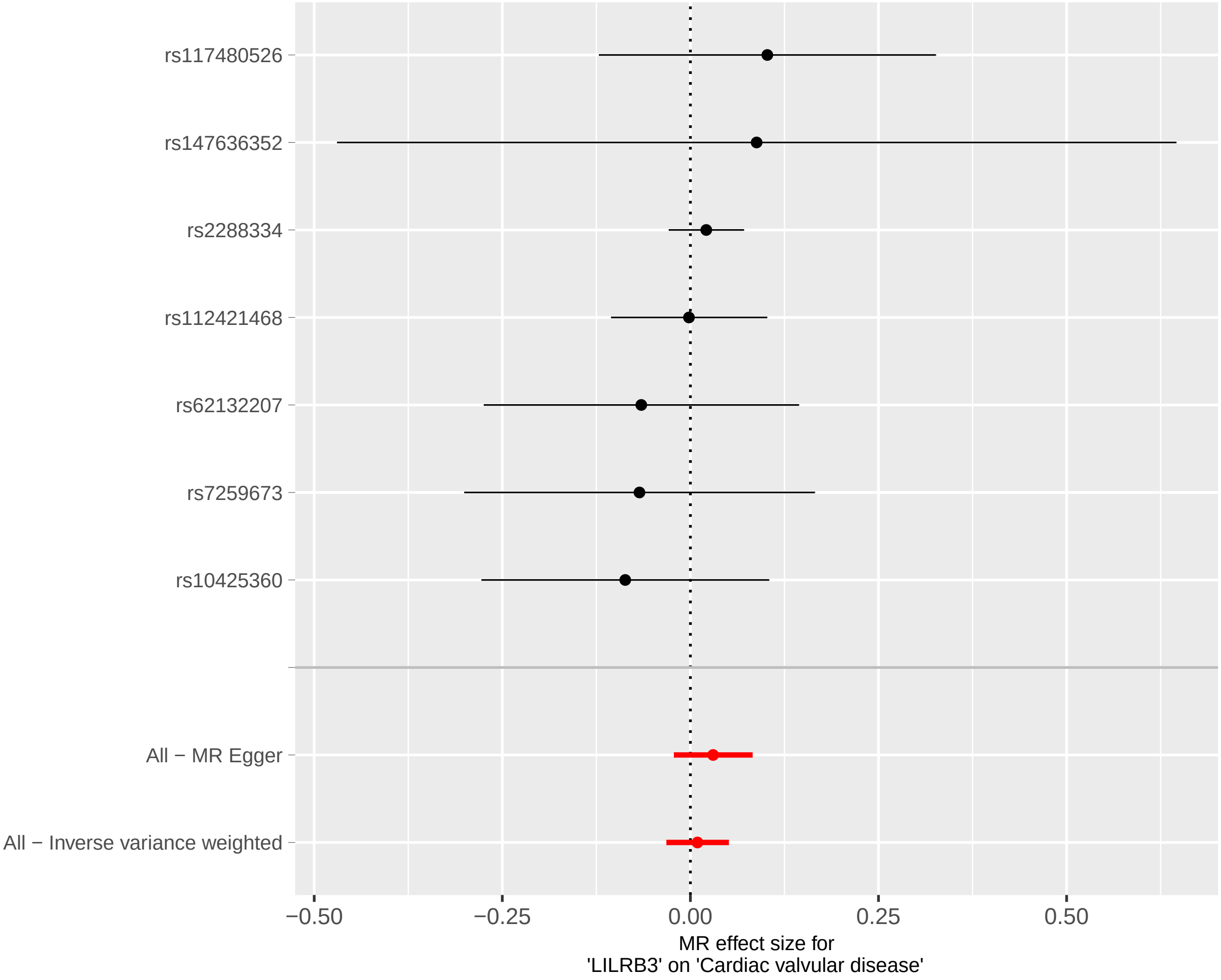

rs117480526
rs147636352
rs2288334
rs112421468
rs62132207
rs7259673
rs10425360
All − MR Egger
All − Inverse variance weighted
−0.50
−0.25
0.00
0.25
0.50
MR effect size for
'LILRB3' on 'Cardiac valvular disease'

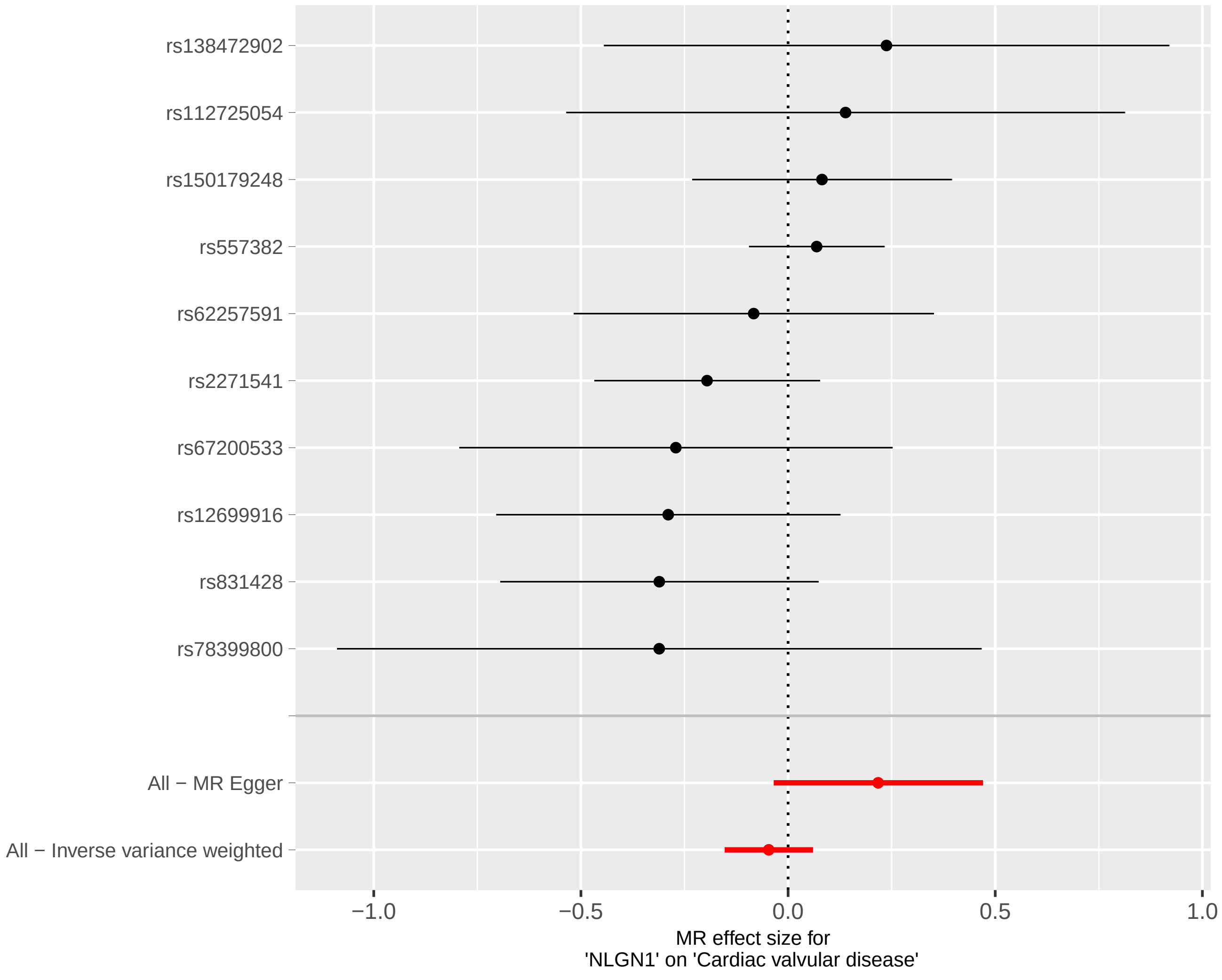
rs138472902
rs112725054
rs150179248
rs557382
rs62257591
rs2271541
rs67200533
rs12699916
rs831428
rs78399800
All − MR Egger
All − Inverse variance weighted
−1.0
−0.5
0.0
0.5
1.0
MR effect size for
'NLGN1' on 'Cardiac valvular disease'

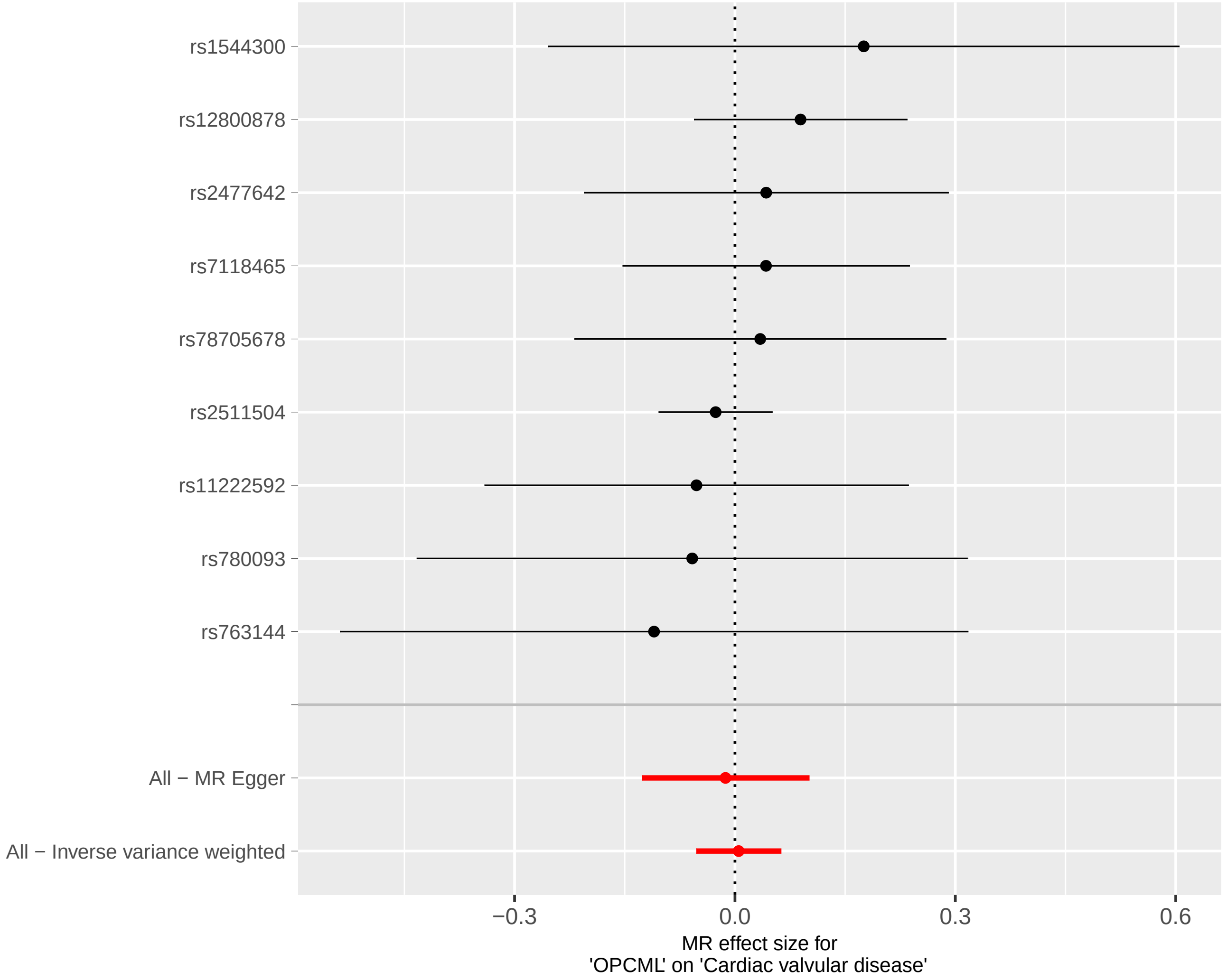

rs1544300
rs12800878
rs2477642
rs7118465
rs78705678
rs2511504
rs11222592
rs780093
rs763144
All − MR Egger
All − Inverse variance weighted
−0.3
0.0
0.3
0.6
MR effect size for
'OPCML' on 'Cardiac valvular disease'

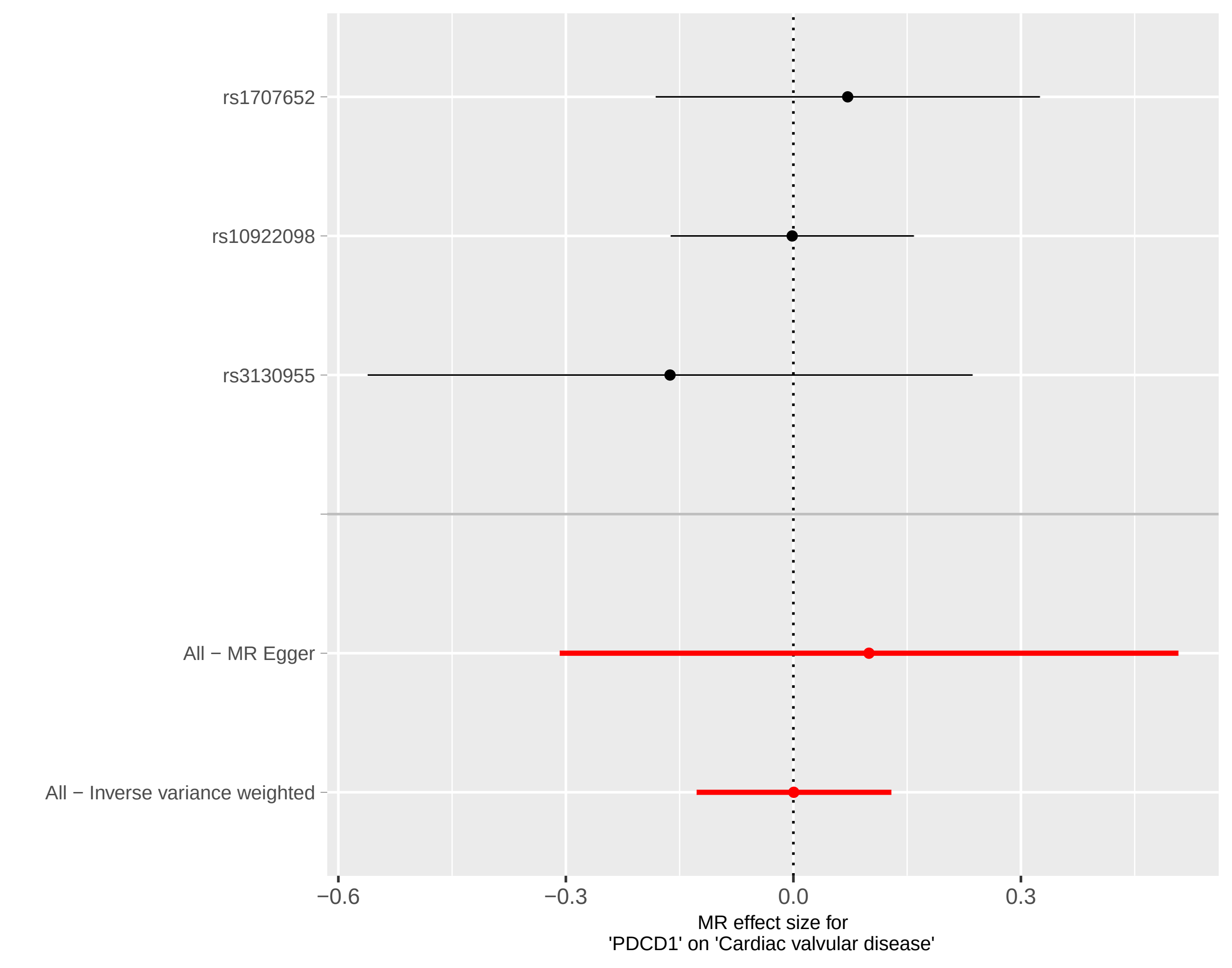
rs1707652
rs10922098
rs3130955
All − MR Egger
All − Inverse variance weighted
−0.6
−0.3
0.0
0.3
MR effect size for
'PDCD1' on 'Cardiac valvular disease'

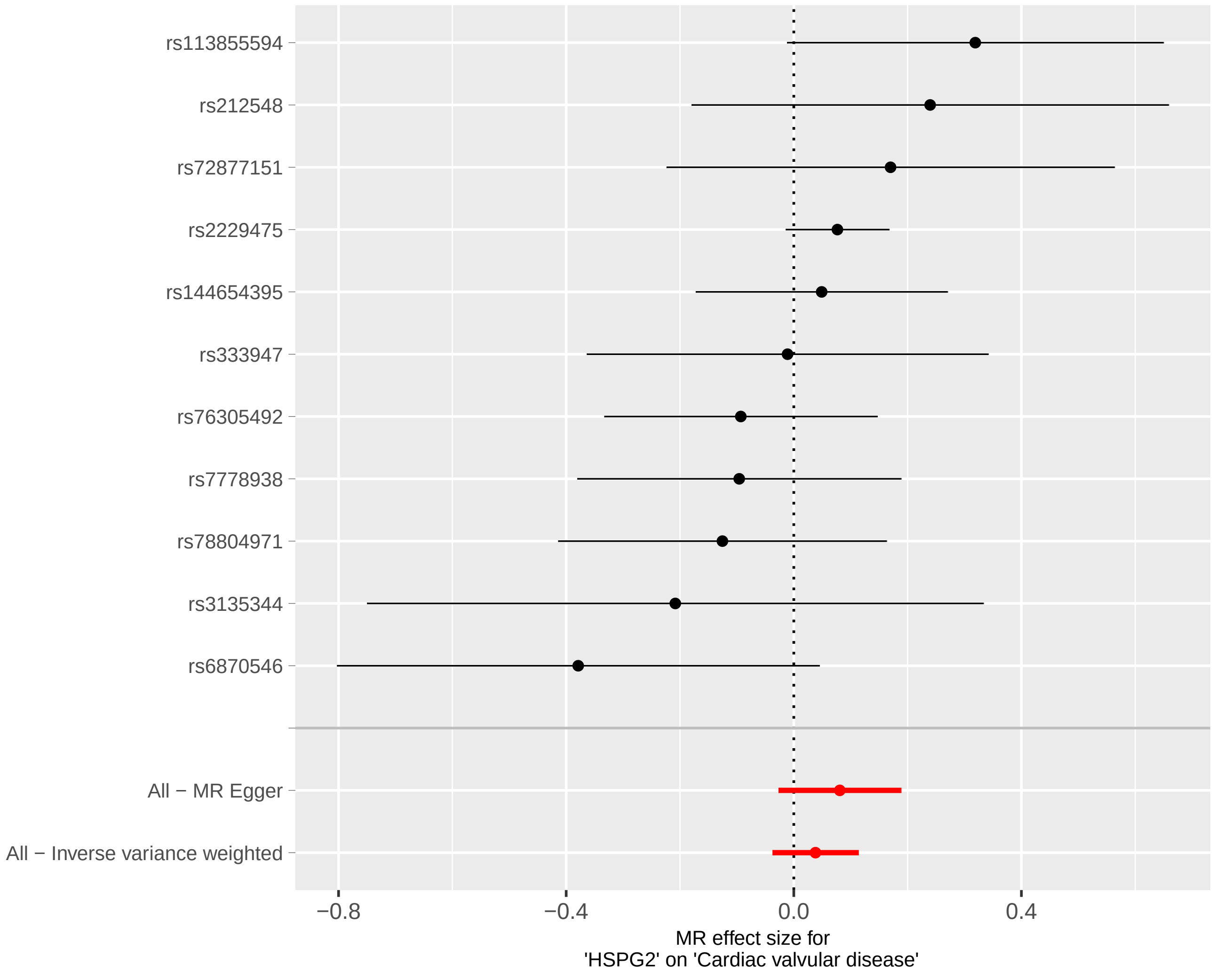
rs113855594
rs212548
rs72877151
rs2229475
rs144654395
rs333947
rs76305492
rs7778938
rs78804971
rs3135344
rs6870546
All − MR Egger
All − Inverse variance weighted
−0.8
−0.4
0.0
0.4
MR effect size for
'HSPG2' on 'Cardiac valvular disease'

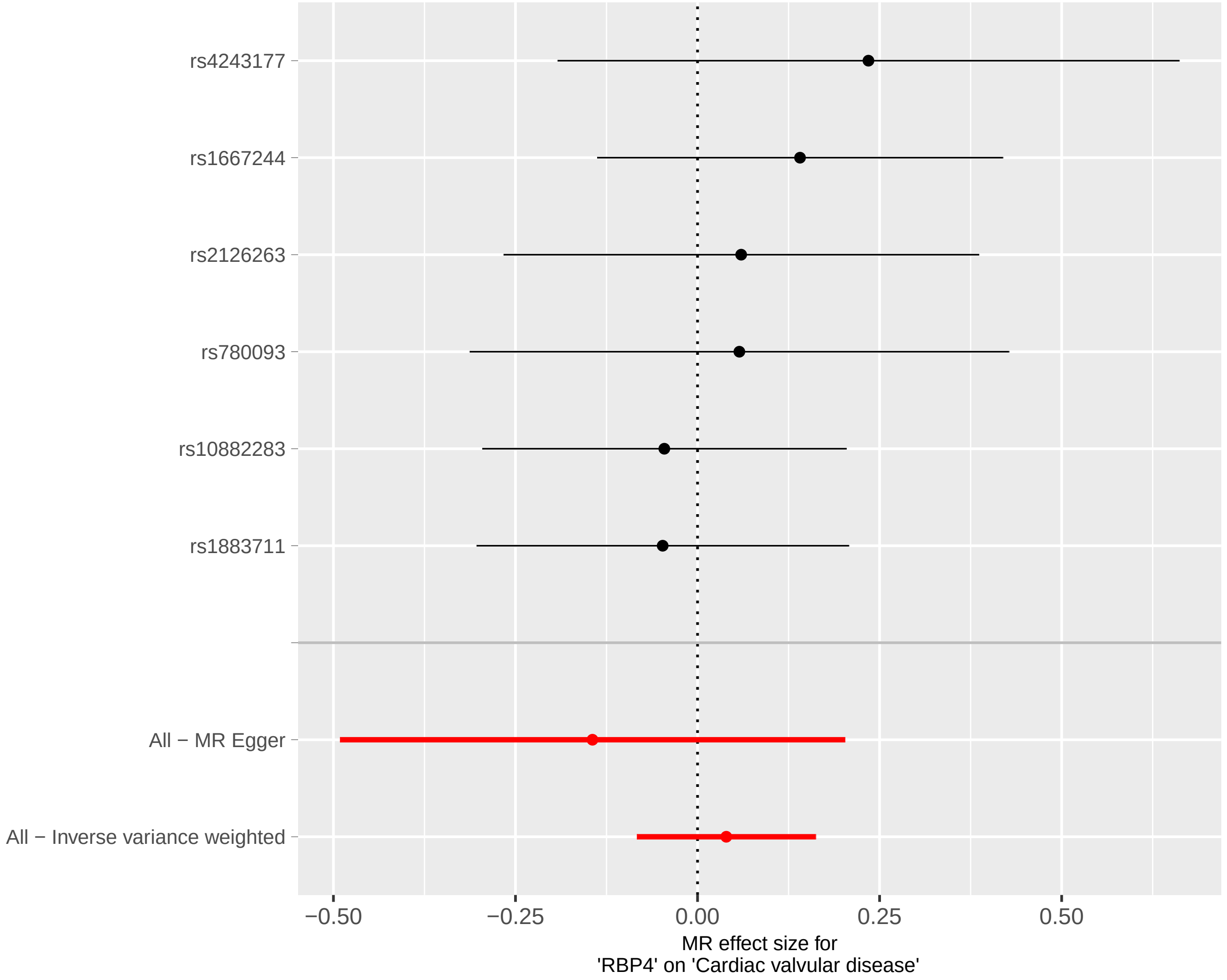
rs4243177
rs1667244
rs2126263
rs780093
rs10882283
rs1883711
All − MR Egger
All − Inverse variance weighted
−0.50
−0.25
0.00
0.25
0.50
MR effect size for
'RBP4' on 'Cardiac valvular disease'

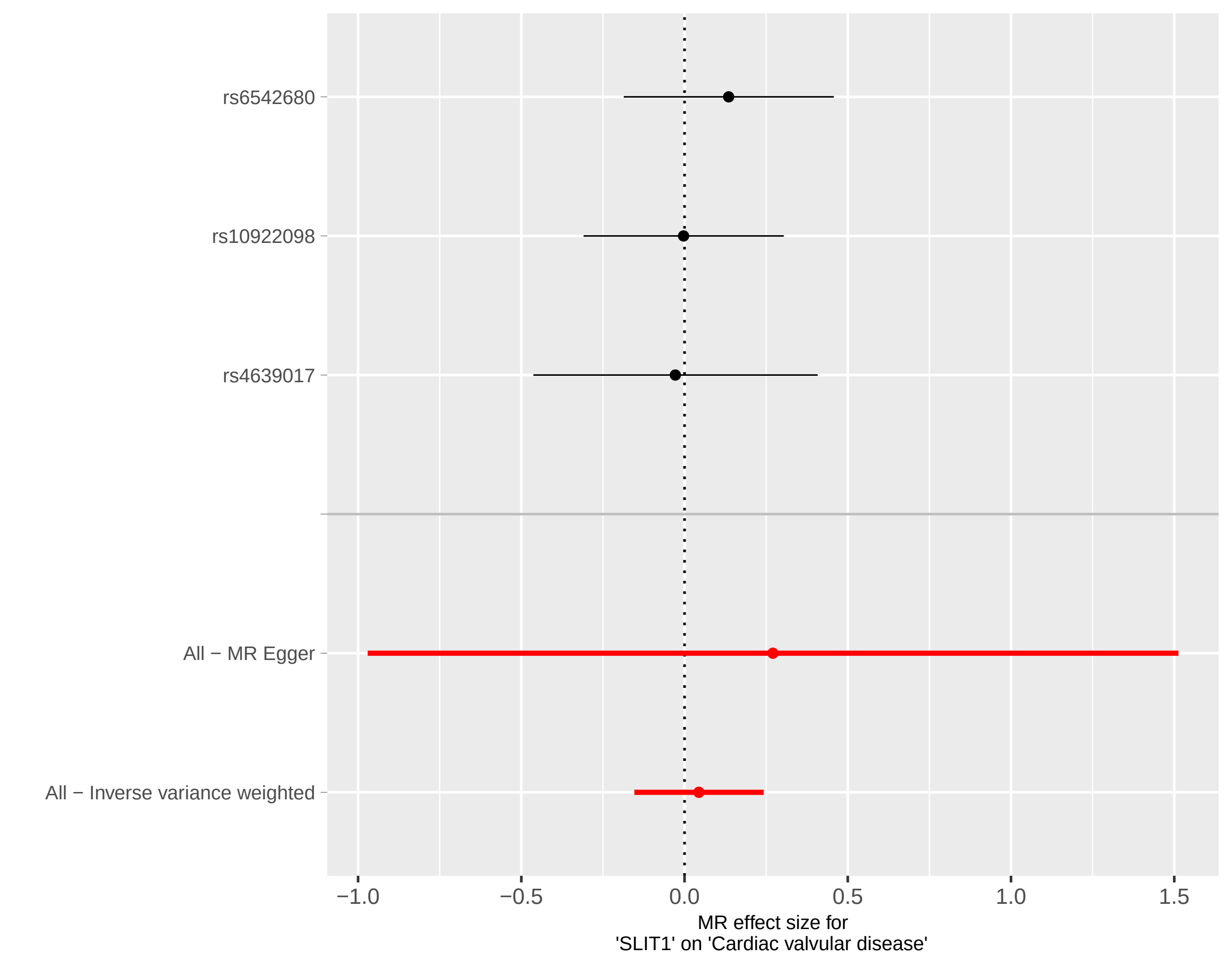

rs6542680
rs10922098
rs4639017
All − MR Egger
All − Inverse variance weighted
−1.0
−0.5
0.0
0.5
1.0
1.5
MR effect size for
'SLIT1' on 'Cardiac valvular disease'

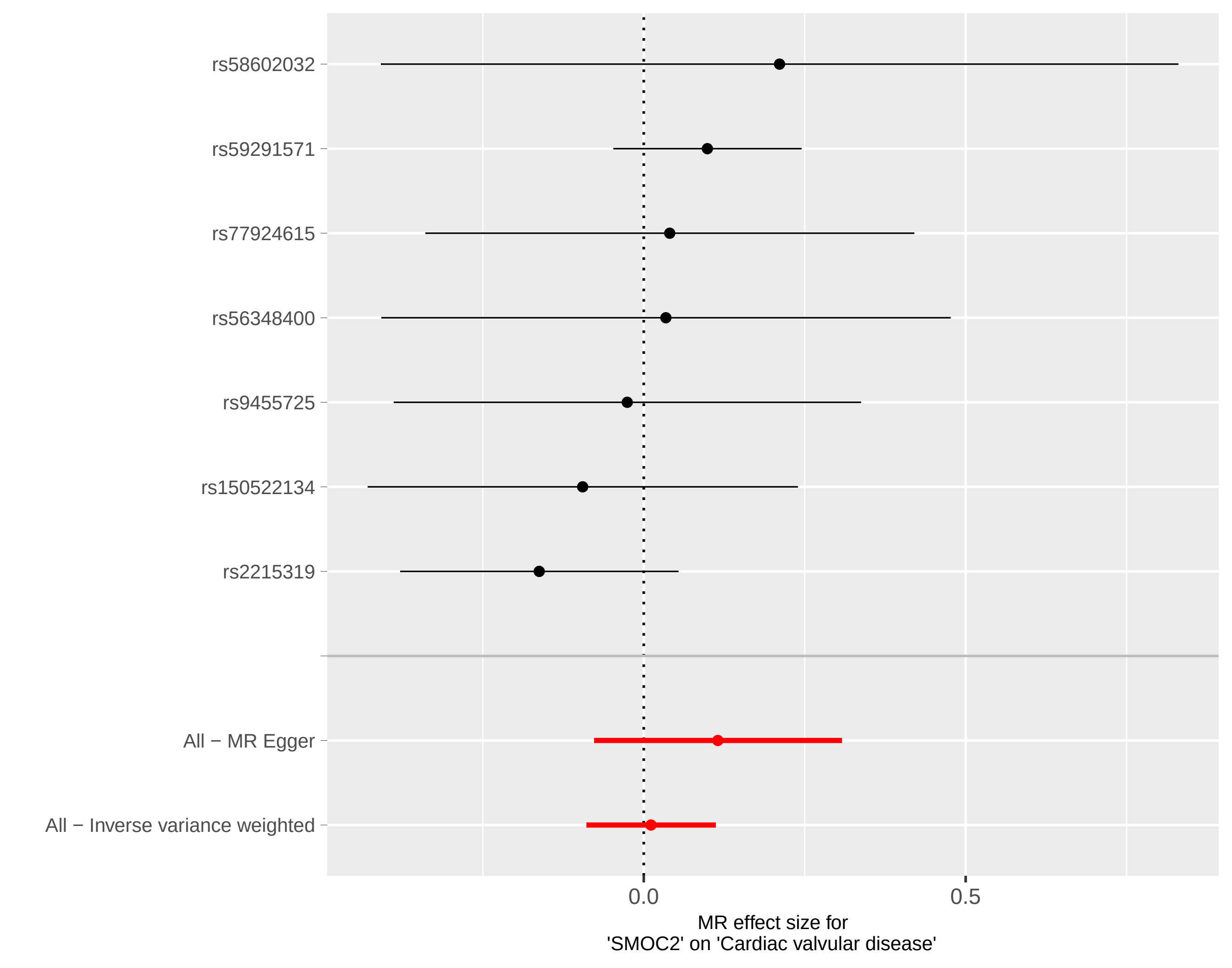
rs58602032
rs59291571
rs77924615
rs56348400
rs9455725
rs150522134
rs2215319
All − MR Egger
All − Inverse variance weighted
0.0
0.5
MR effect size for
'SMOC2' on 'Cardiac valvular disease'

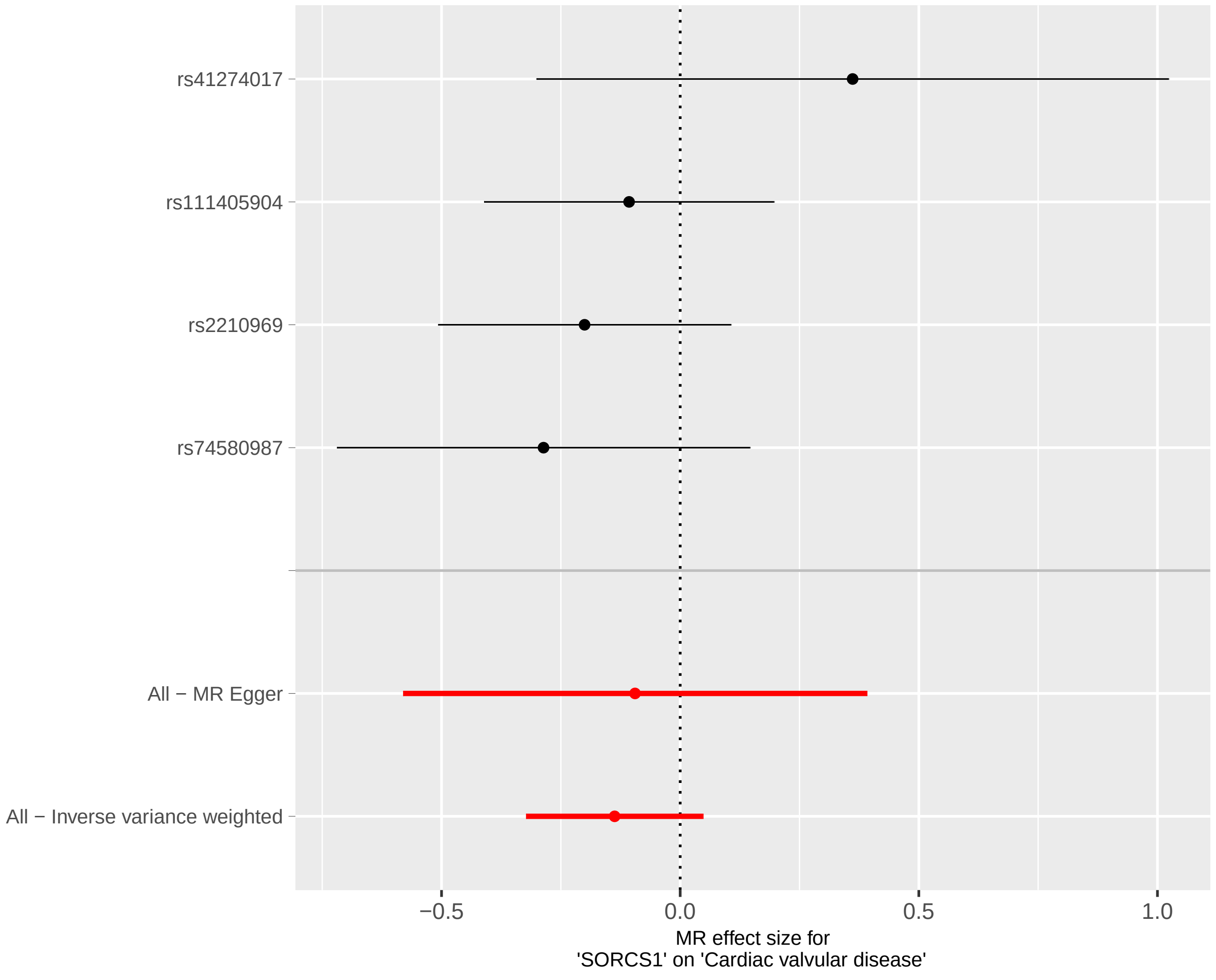

rs41274017
rs111405904
rs2210969
rs74580987
All − MR Egger
All − Inverse variance weighted
−0.5
0.0
0.5
1.0
MR effect size for
'SORCS1' on 'Cardiac valvular disease'

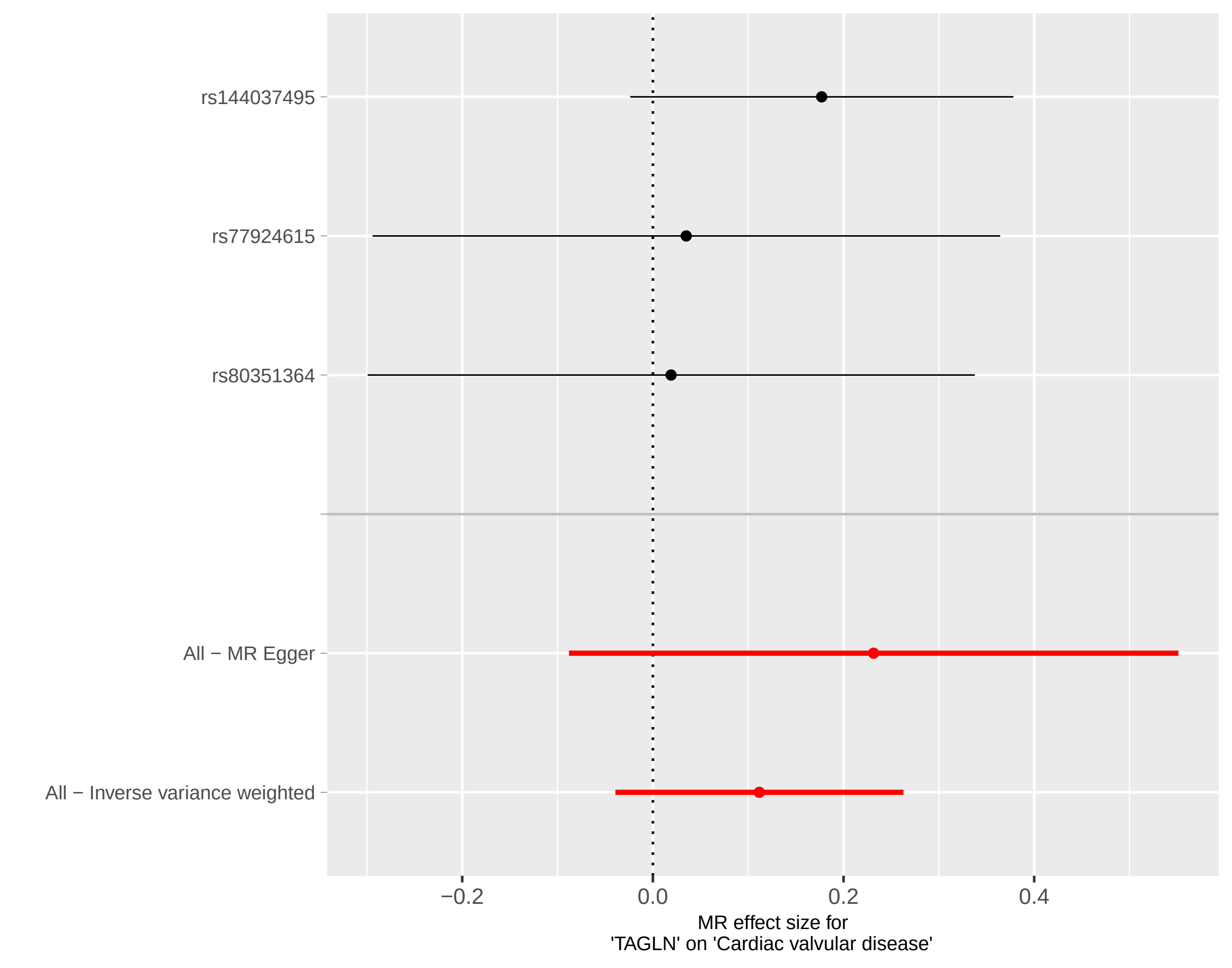
rs144037495
rs77924615
rs80351364
All − MR Egger
All − Inverse variance weighted
−0.2
0.0
0.2
0.4
MR effect size for
'TAGLN' on 'Cardiac valvular disease'

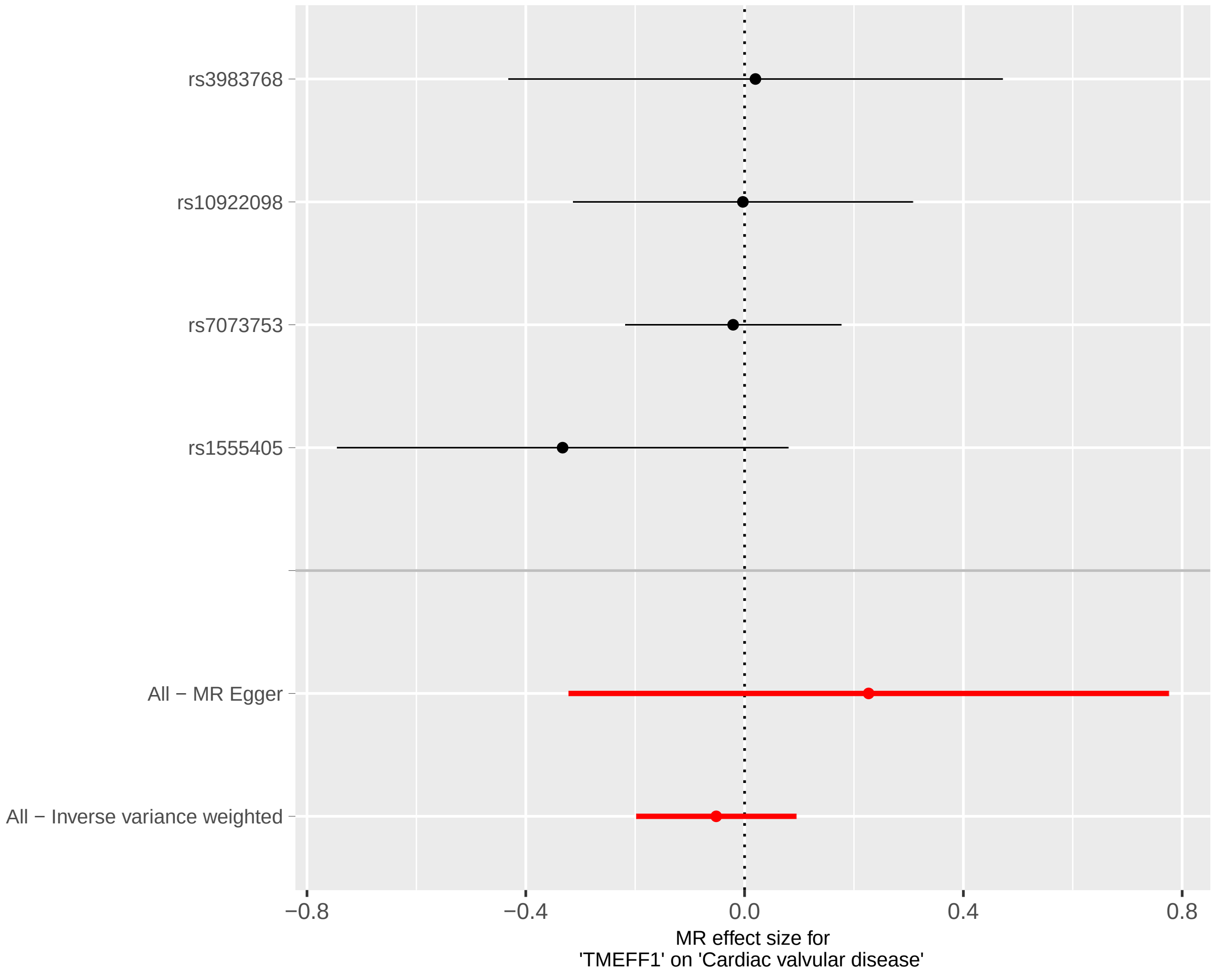
rs3983768
rs10922098
rs7073753
rs1555405
All − MR Egger
All − Inverse variance weighted
−0.8
−0.4
0.0
0.4
0.8
MR effect size for
'TMEFF1' on 'Cardiac valvular disease'

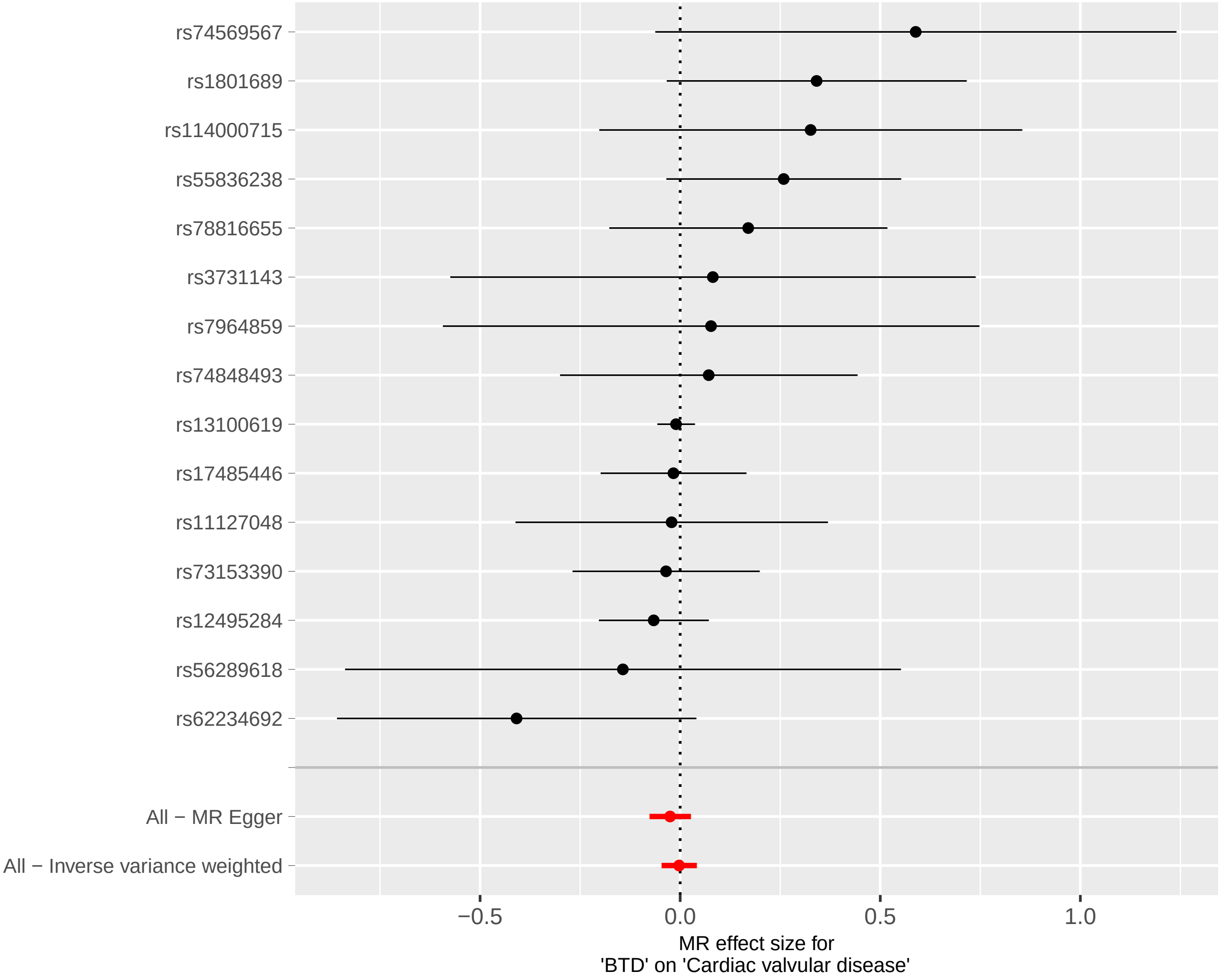

rs74569567
rs1801689
rs114000715
rs55836238
rs78816655
rs3731143
rs7964859
rs74848493
rs13100619
rs17485446
rs11127048
rs73153390
rs12495284
rs56289618
rs62234692
All − MR Egger
All − Inverse variance weighted
−0.5
0.0
0.5
1.0
MR effect size for
'BTD' on 'Cardiac valvular disease'

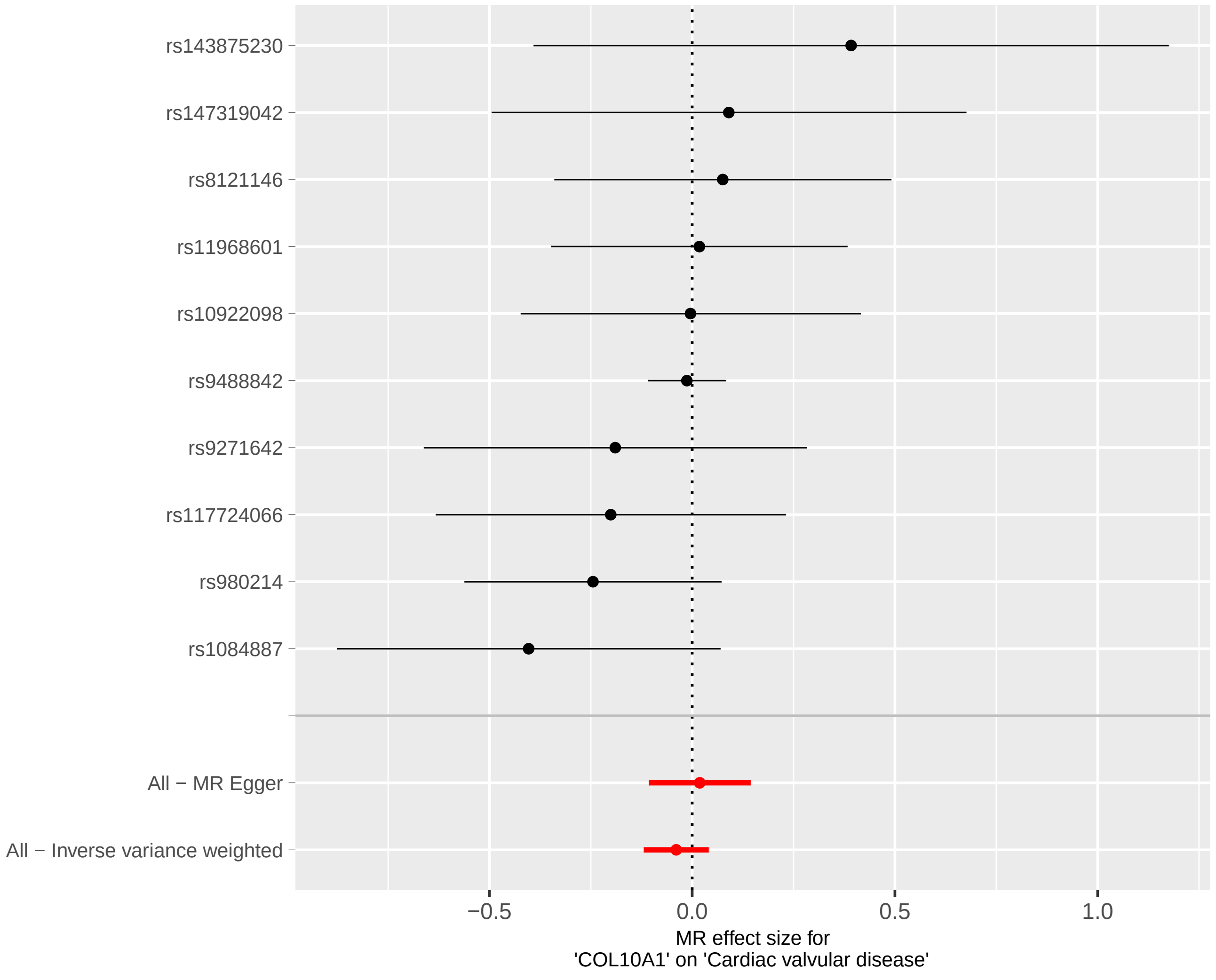
rs143875230
rs147319042
rs8121146
rs11968601
rs10922098
rs9488842
rs9271642
rs117724066
rs980214
rs1084887
All − MR Egger
All − Inverse variance weighted
−0.5
0.0
0.5
1.0
MR effect size for
'COL10A1' on 'Cardiac valvular disease'

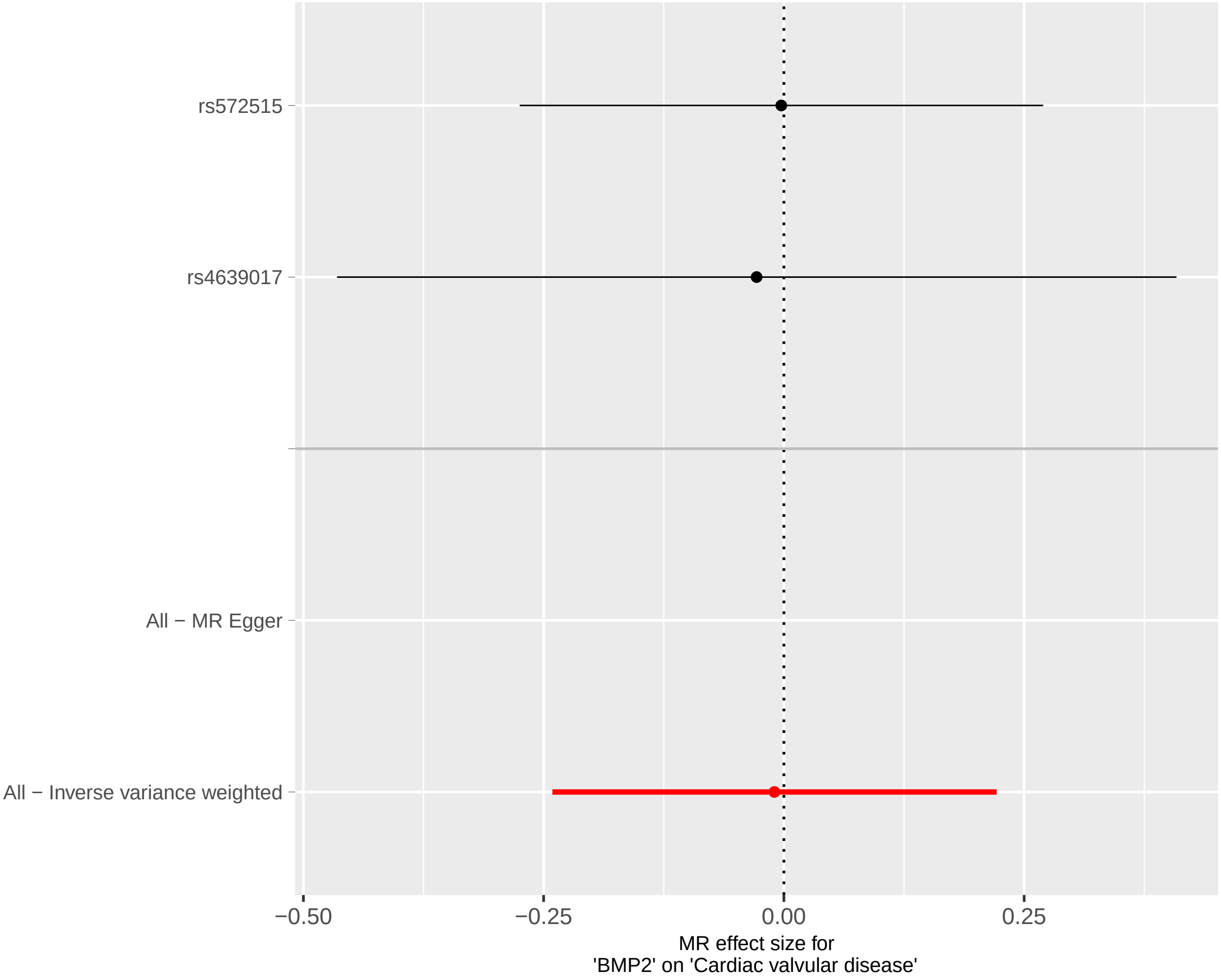
rs572515
rs4639017
All − MR Egger
All − Inverse variance weighted
−0.50
−0.25
0.00
0.25
MR effect size for
'BMP2' on 'Cardiac valvular disease'

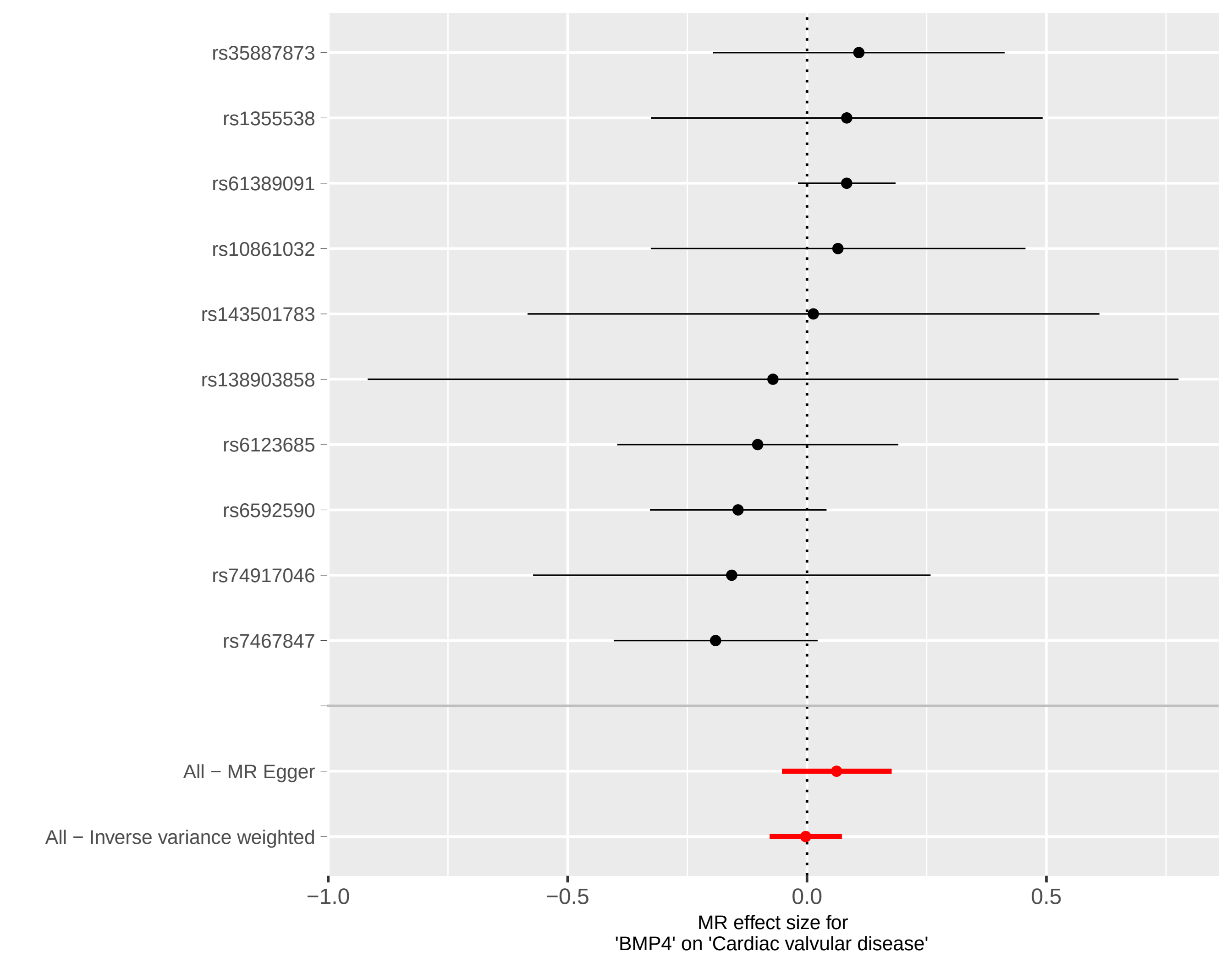
rs35887873
rs1355538
rs61389091
rs10861032
rs143501783
rs138903858
rs6123685
rs6592590
rs74917046
rs7467847
All − MR Egger
All − Inverse variance weighted
−1.0
−0.5
0.0
0.5
MR effect size for
'BMP4' on 'Cardiac valvular disease'

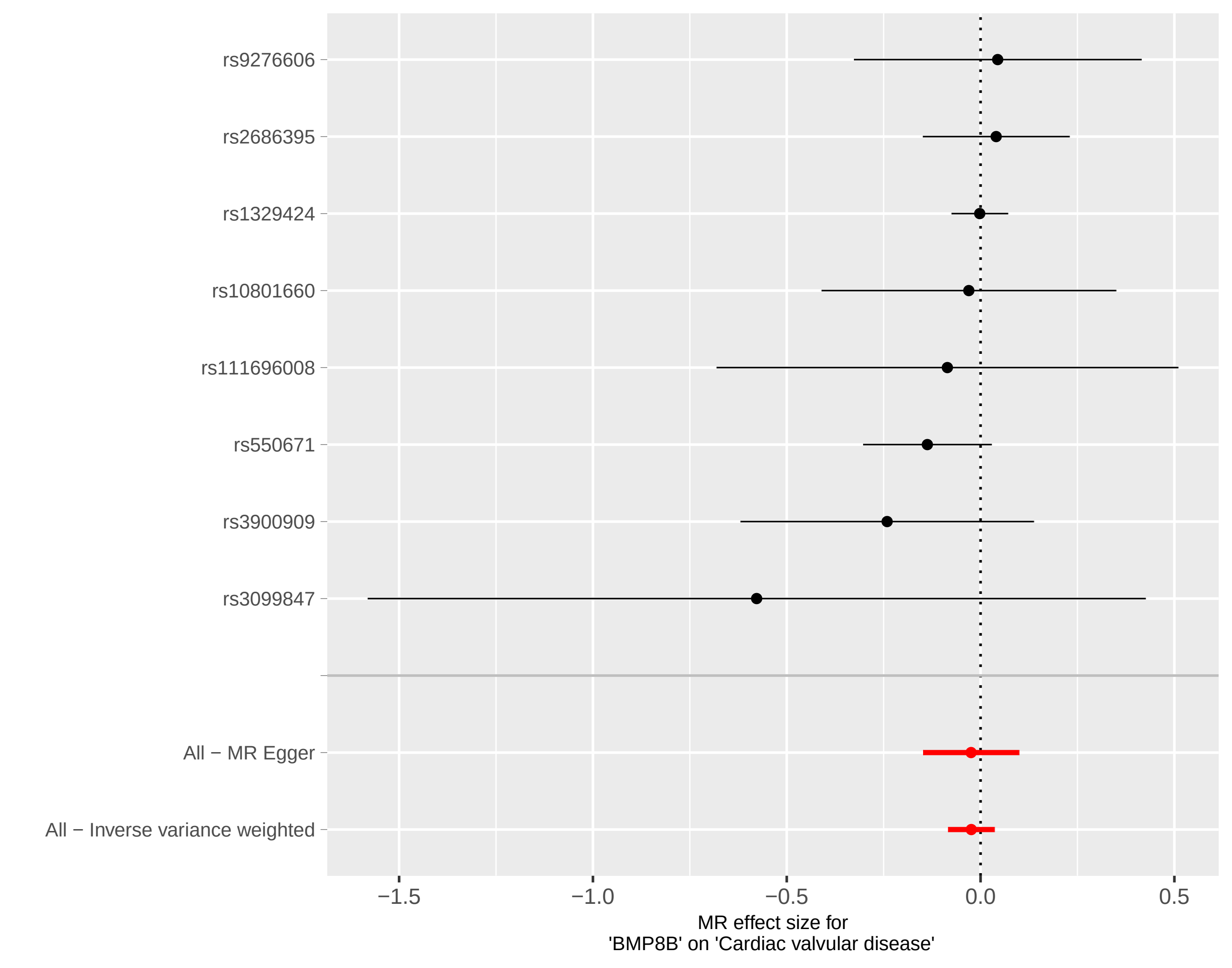
rs9276606
rs2686395
rs1329424
rs10801660
rs111696008
rs550671
rs3900909
rs3099847
All − MR Egger
All − Inverse variance weighted
−1.5
−1.0
−0.5
0.0
0.5
MR effect size for
'BMP8B' on 'Cardiac valvular disease'

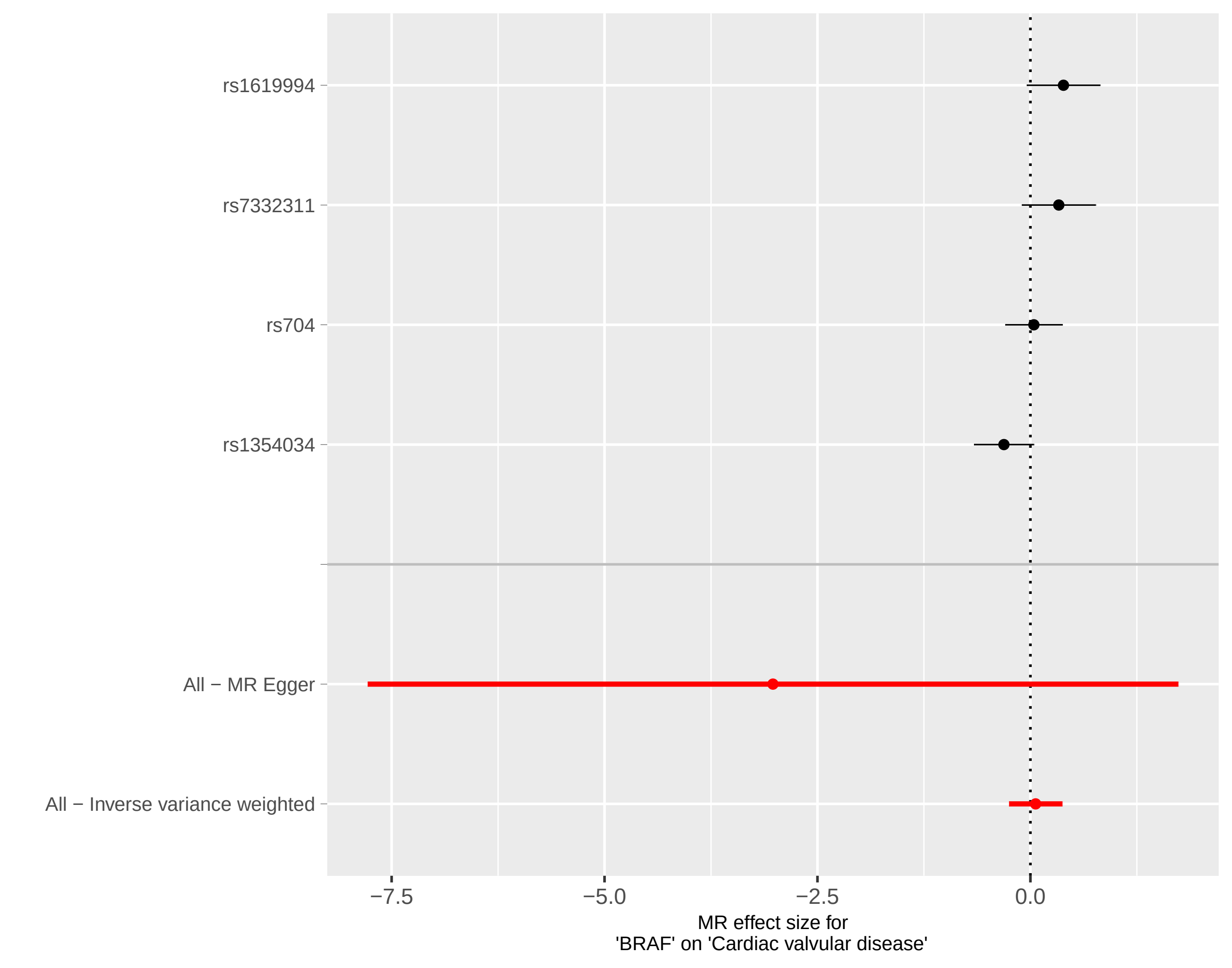

rs1619994
rs7332311
rs704
rs1354034
All − MR Egger
All − Inverse variance weighted
−7.5
−5.0
−2.5
0.0
MR effect size for
'BRAF' on 'Cardiac valvular disease'

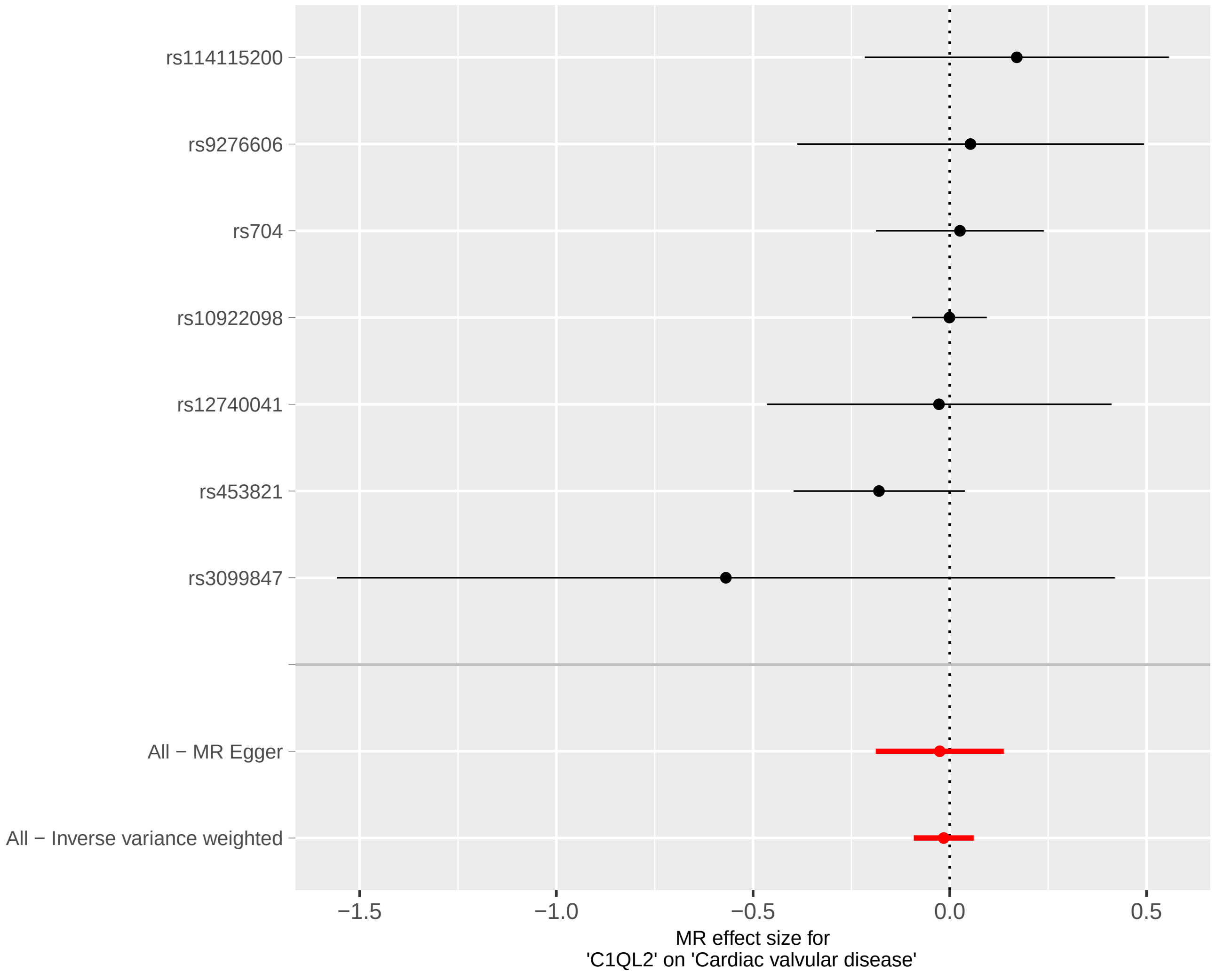
rs114115200
rs9276606
rs704
rs10922098
rs12740041
rs453821
rs3099847
All − MR Egger
All − Inverse variance weighted
−1.5
−1.0
−0.5
0.0
0.5
MR effect size for
'C1QL2' on 'Cardiac valvular disease'

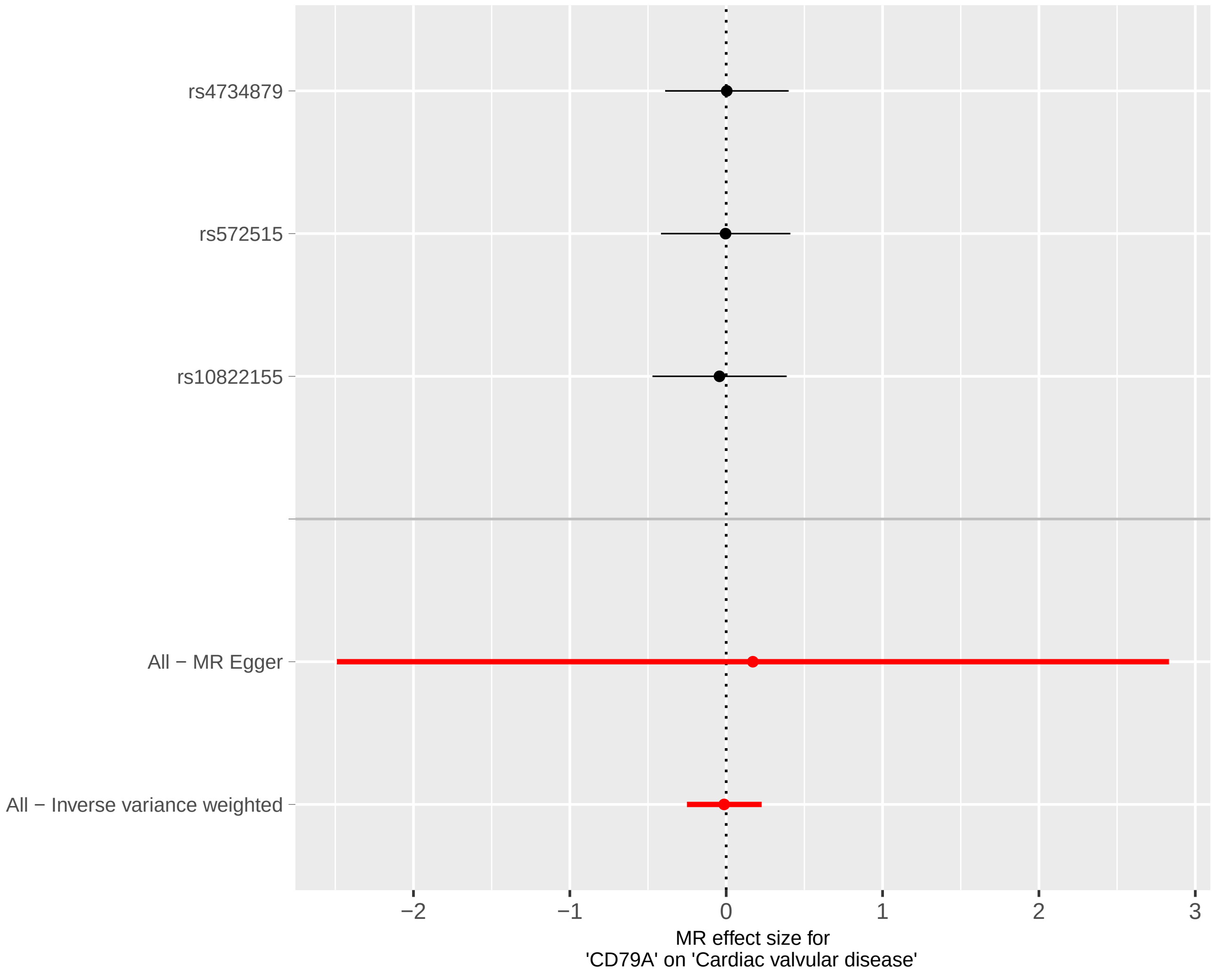
rs4734879
rs572515
rs10822155
All − MR Egger
All − Inverse variance weighted
−2
−1
0
1
2
3
MR effect size for
'CD79A' on 'Cardiac valvular disease'

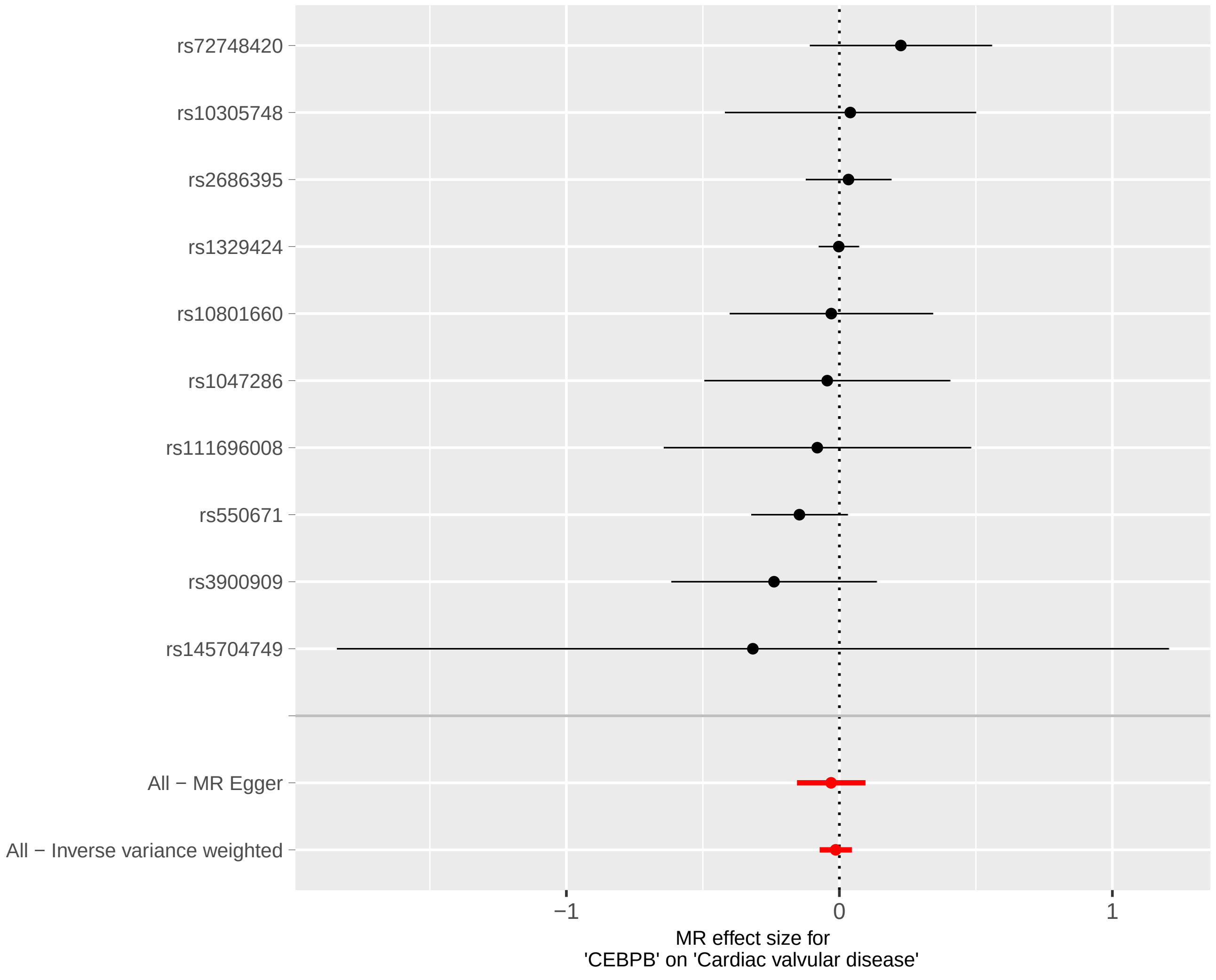
rs72748420
rs10305748
rs2686395
rs1329424
rs10801660
rs1047286
rs111696008
rs550671
rs3900909
rs145704749
All − MR Egger
All − Inverse variance weighted
−1
0
1
MR effect size for
'CEBPB' on 'Cardiac valvular disease'

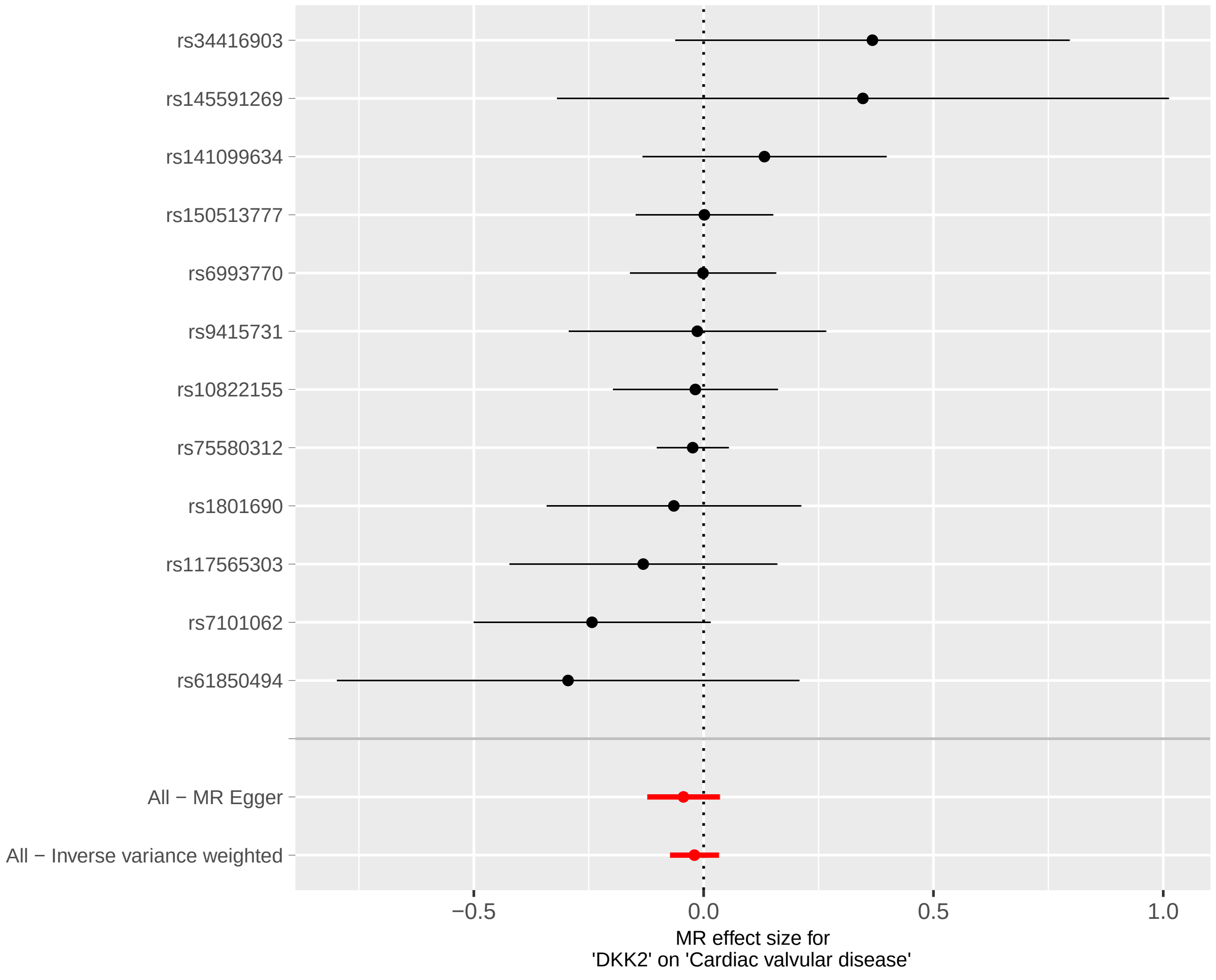
rs34416903
rs145591269
rs141099634
rs150513777
rs6993770
rs9415731
rs10822155
rs75580312
rs1801690
rs117565303
rs7101062
rs61850494
All − MR Egger
All − Inverse variance weighted
−0.5
0.0
0.5
1.0
MR effect size for
'DKK2' on 'Cardiac valvular disease'

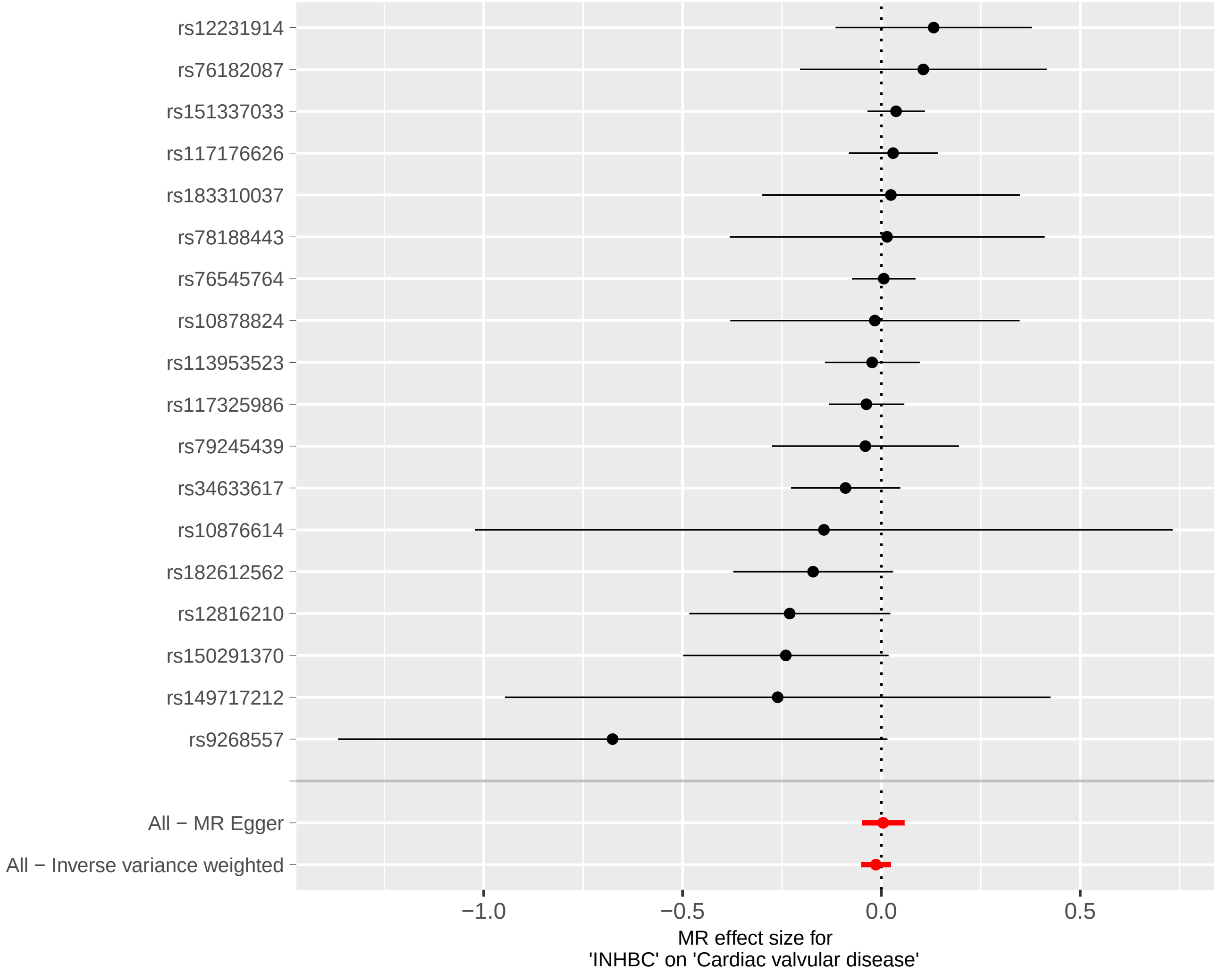

rs12231914
rs76182087
rs151337033
rs117176626
rs183310037
rs78188443
rs76545764
rs10878824
rs113953523
rs117325986
rs79245439
rs34633617
rs10876614
rs182612562
rs12816210
rs150291370
rs149717212
rs9268557
All − MR Egger
All − Inverse variance weighted
−1.0
−0.5
0.0
0.5
MR effect size for
'INHBC' on 'Cardiac valvular disease'

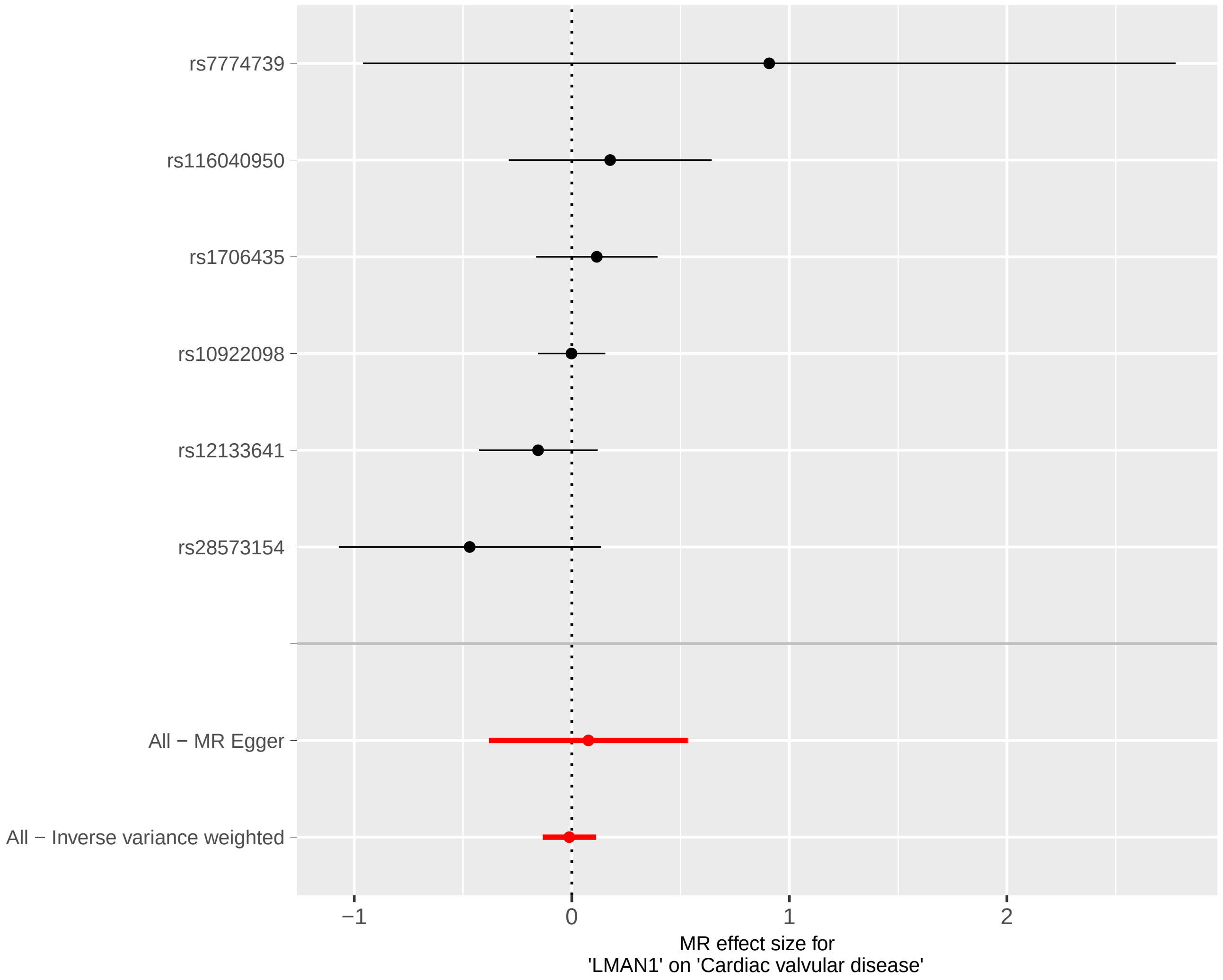

rs7774739
rs116040950
rs1706435
rs10922098
rs12133641
rs28573154
All − MR Egger
All − Inverse variance weighted
−1
0
1
2
MR effect size for
'LMAN1' on 'Cardiac valvular disease'

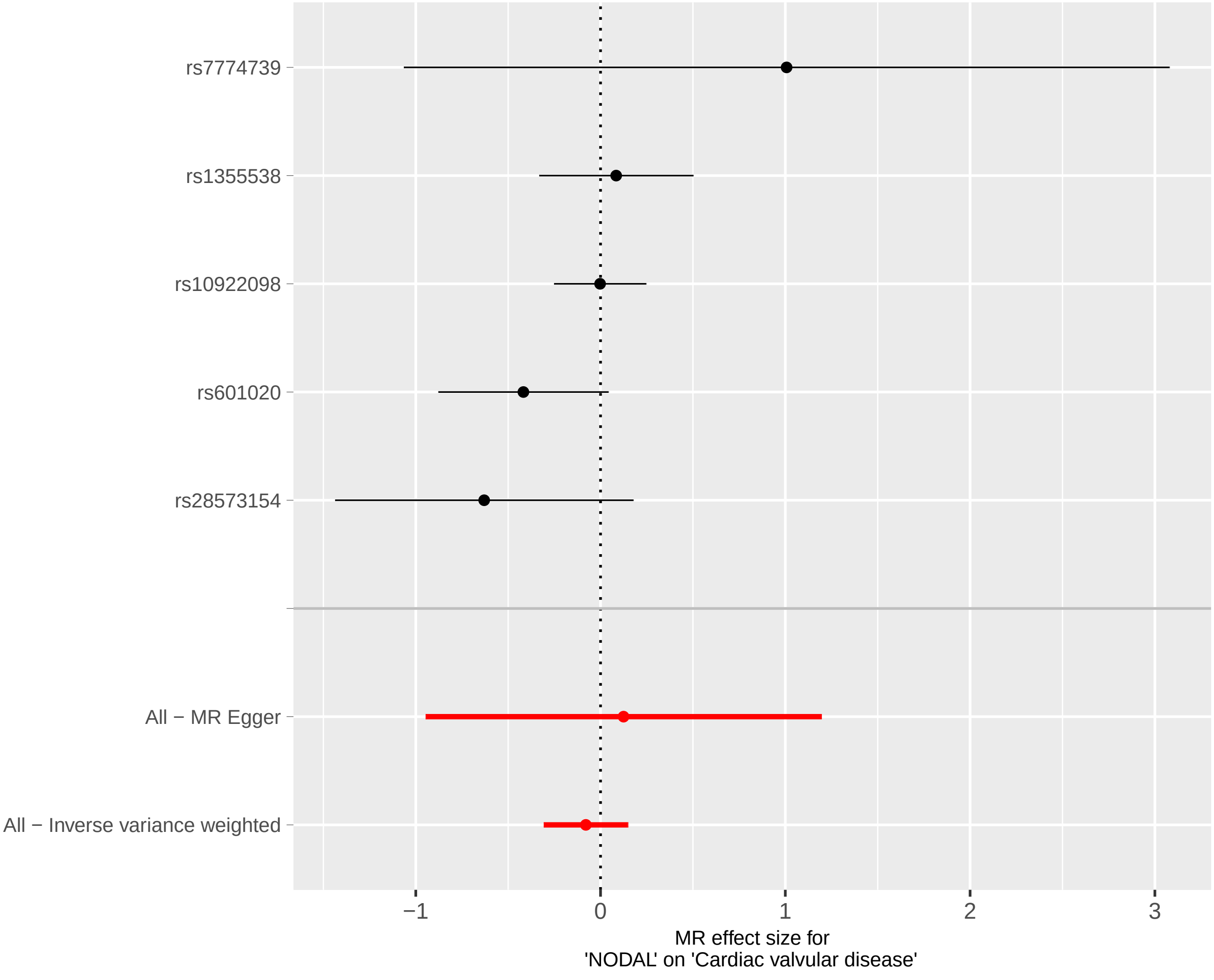
rs7774739
rs1355538
rs10922098
rs601020
rs28573154
All − MR Egger
All − Inverse variance weighted
−1
0
1
2
3
MR effect size for
'NODAL' on 'Cardiac valvular disease'

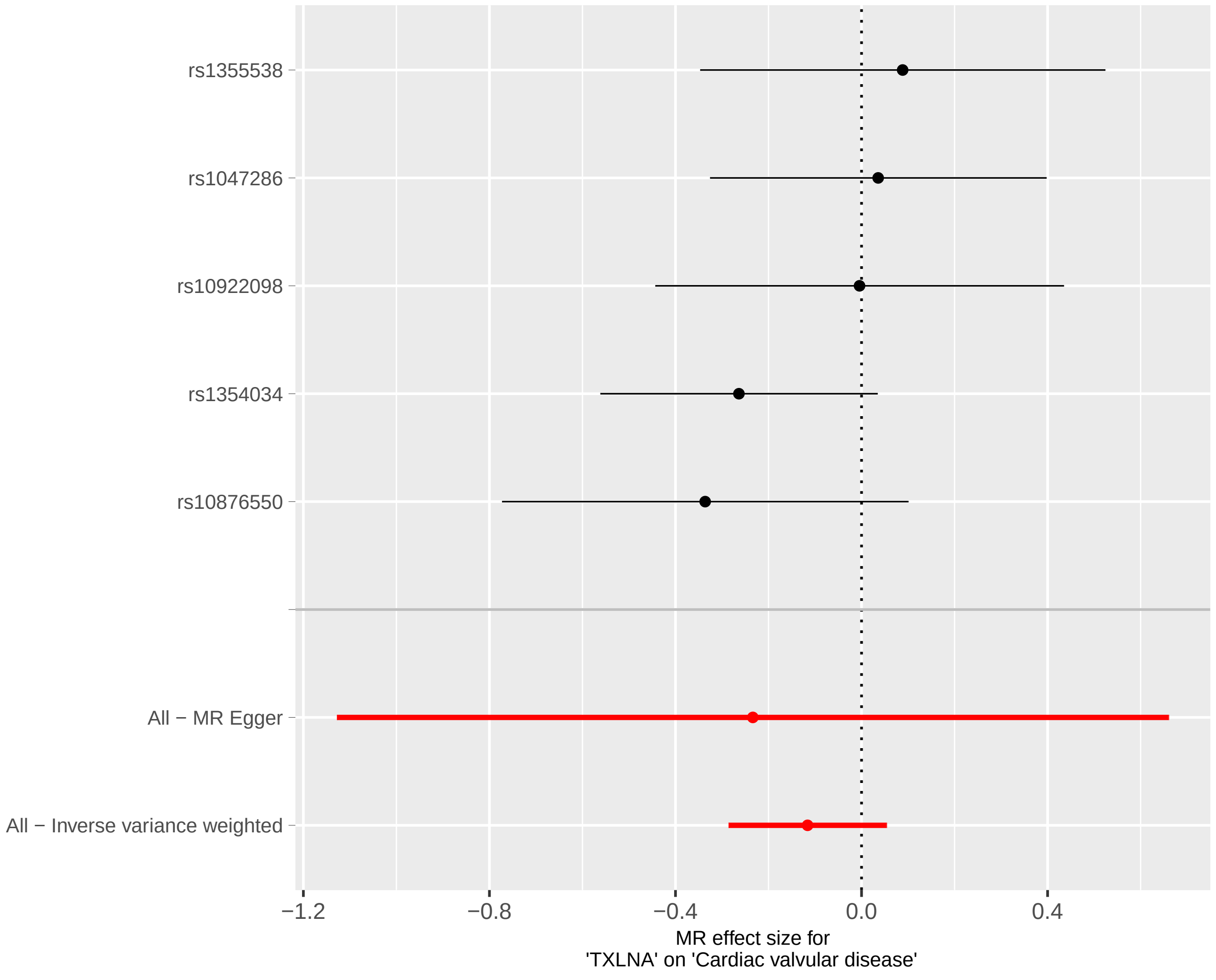
rs1355538
rs1047286
rs10922098
rs1354034
rs10876550
All − MR Egger
All − Inverse variance weighted
−1.2
−0.8
−0.4
0.0
0.4
MR effect size for
'TXLNA' on 'Cardiac valvular disease'

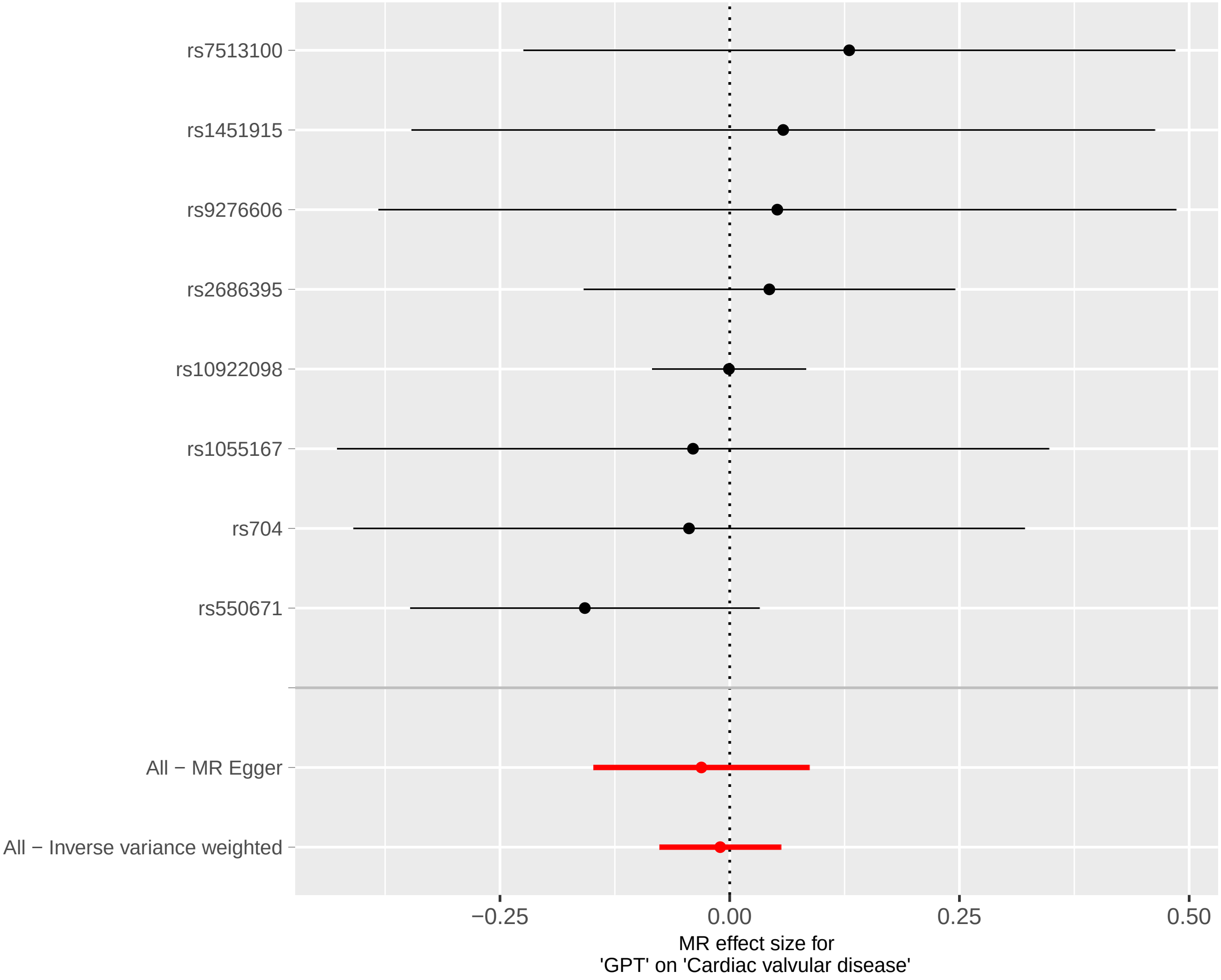
rs7513100
rs1451915
rs9276606
rs2686395
rs10922098
rs1055167
rs704
rs550671
All − MR Egger
All − Inverse variance weighted
−0.25
0.00
0.25
0.50
MR effect size for
'GPT' on 'Cardiac valvular disease'

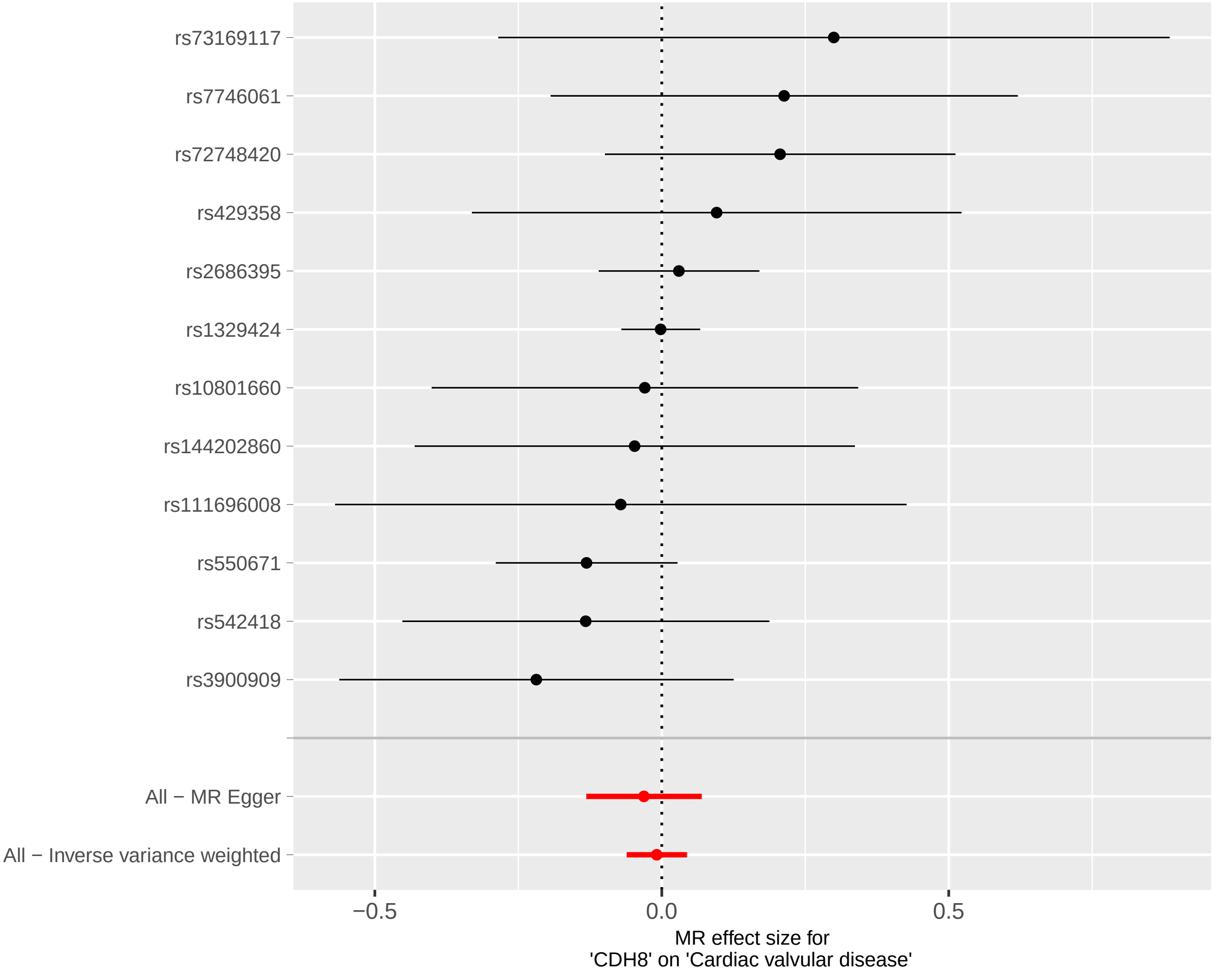
rs73169117
rs7746061
rs72748420
rs429358
rs2686395
rs1329424
rs10801660
rs144202860
rs111696008
rs550671
rs542418
rs3900909
All − MR Egger
All − Inverse variance weighted
−0.5
0.0
0.5
MR effect size for
'CDH8' on 'Cardiac valvular disease'

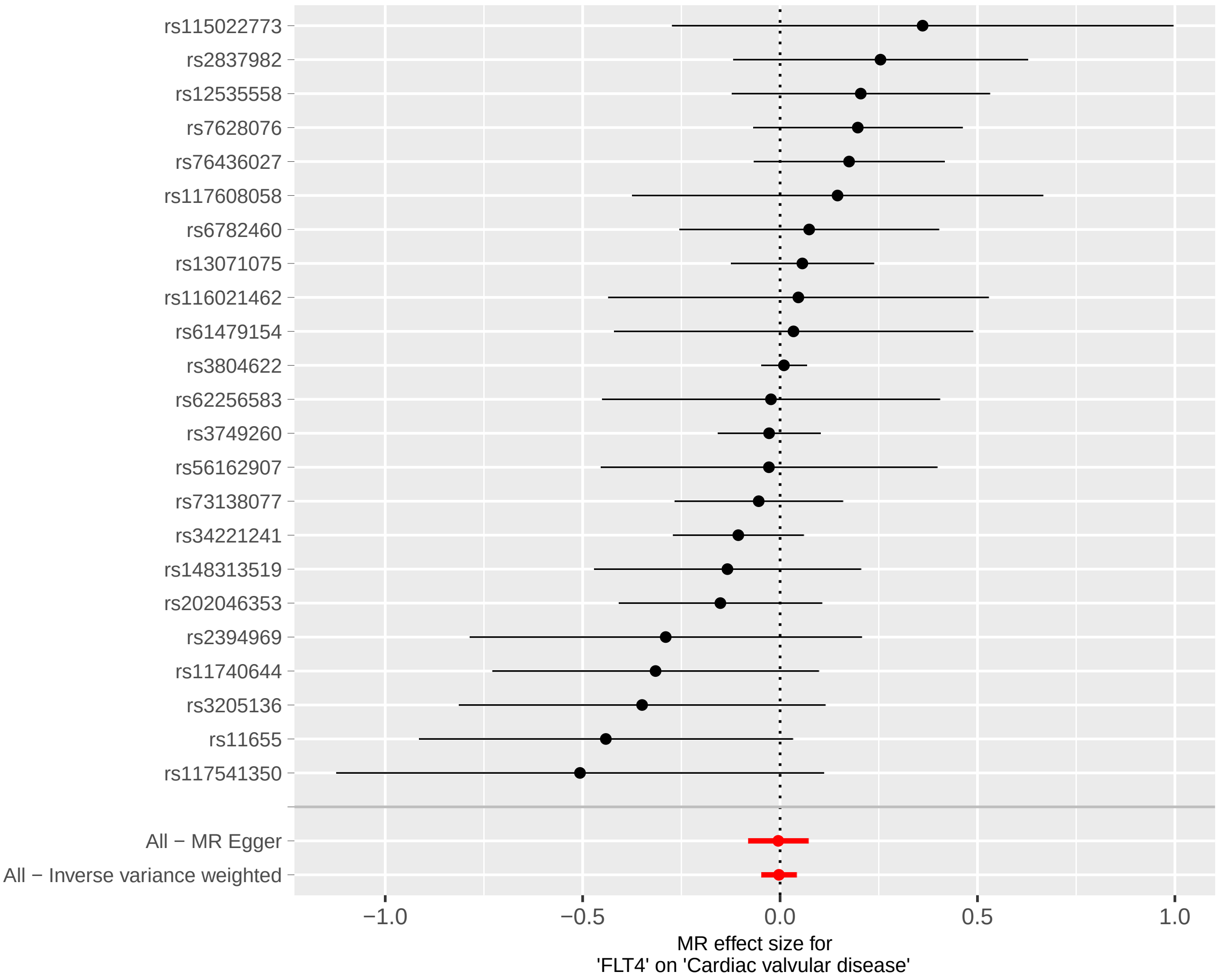
rs115022773
rs2837982
rs12535558
rs7628076
rs76436027
rs117608058
rs6782460
rs13071075
rs116021462
rs61479154
rs3804622
rs62256583
rs3749260
rs56162907
rs73138077
rs34221241
rs148313519
rs202046353
rs2394969
rs11740644
rs3205136
rs11655
rs117541350
All − MR Egger
All − Inverse variance weighted
−1.0
−0.5
0.0
0.5
1.0
MR effect size for
'FLT4' on 'Cardiac valvular disease'

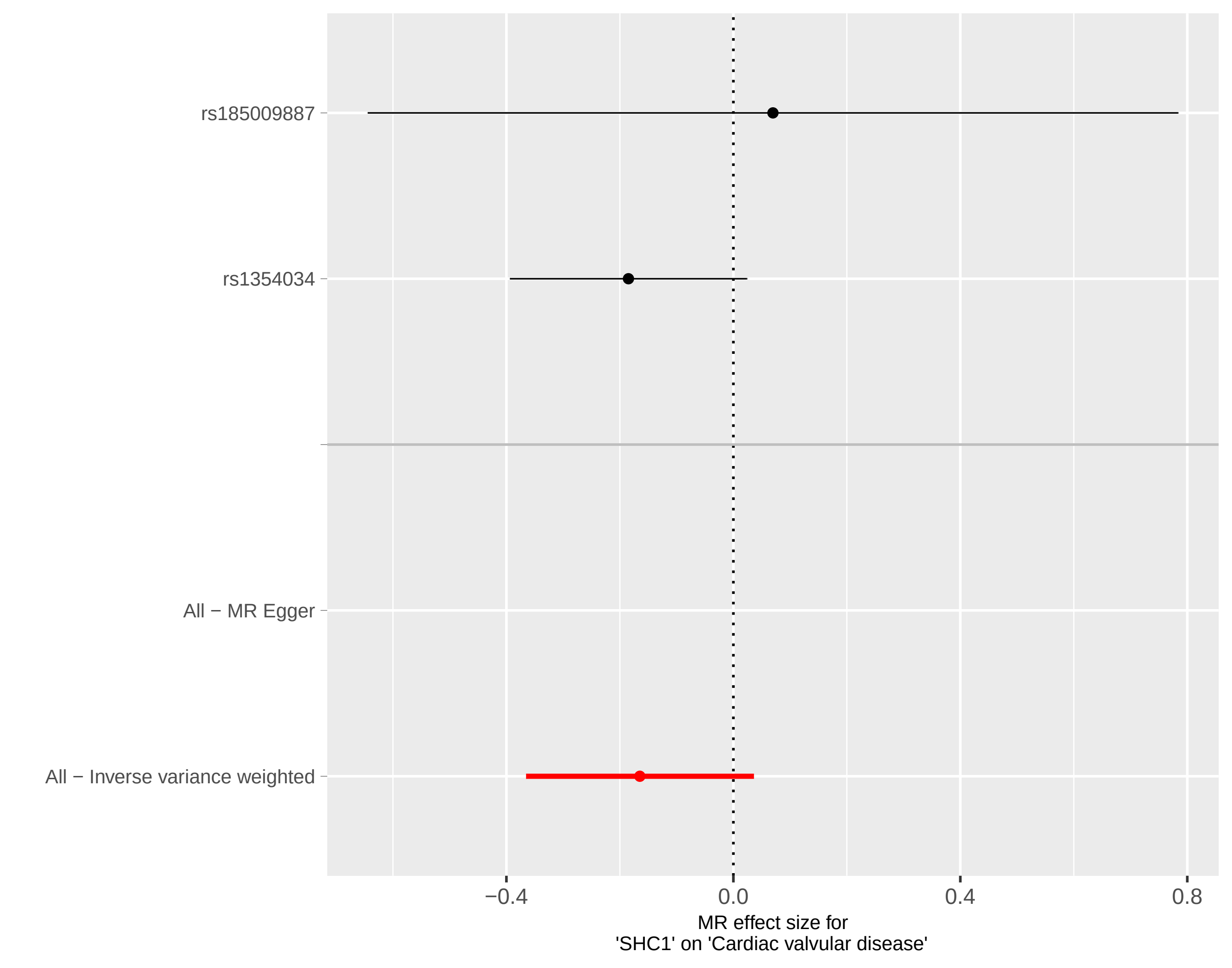
rs185009887
rs1354034
All − MR Egger
All − Inverse variance weighted
−0.4
0.0
0.4
0.8
MR effect size for
'SHC1' on 'Cardiac valvular disease'

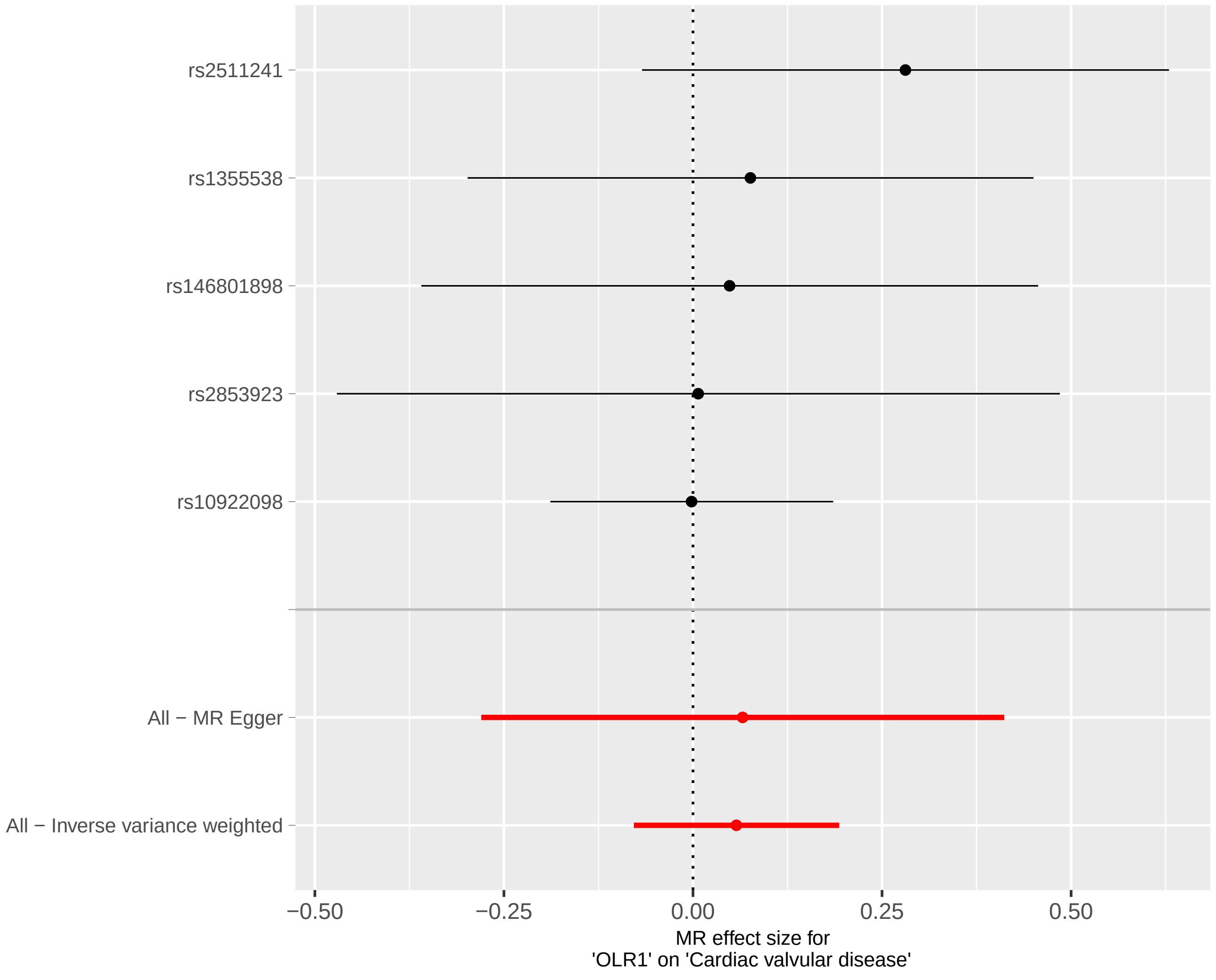
rs2511241
rs1355538
rs146801898
rs2853923
rs10922098
All − MR Egger
All − Inverse variance weighted
−0.50
−0.25
0.00
0.25
0.50
MR effect size for
'OLR1' on 'Cardiac valvular disease'

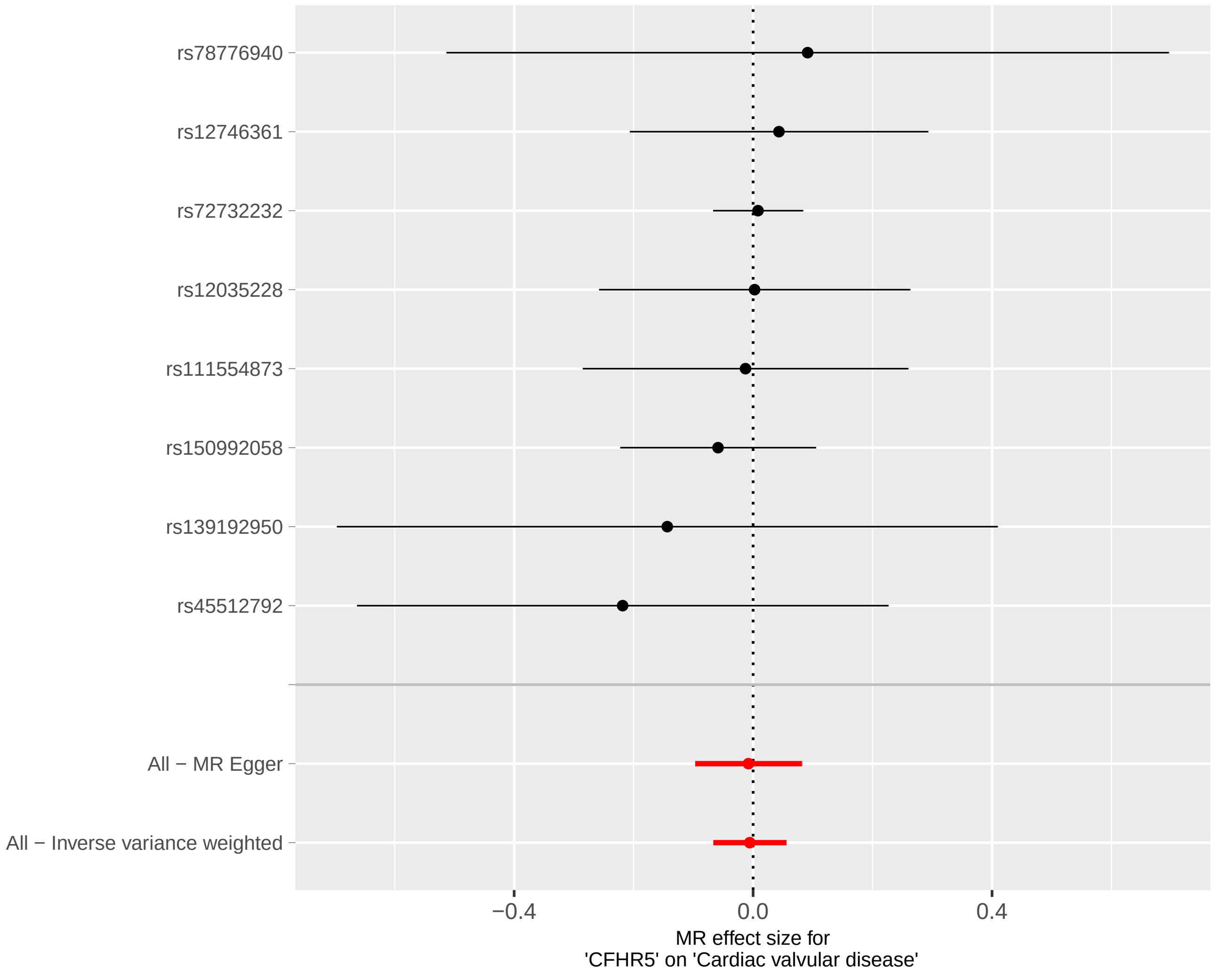

rs78776940
rs12746361
rs72732232
rs12035228
rs111554873
rs150992058
rs139192950
rs45512792
All − MR Egger
All − Inverse variance weighted
−0.4
0.0
0.4
MR effect size for
'CFHR5' on 'Cardiac valvular disease'

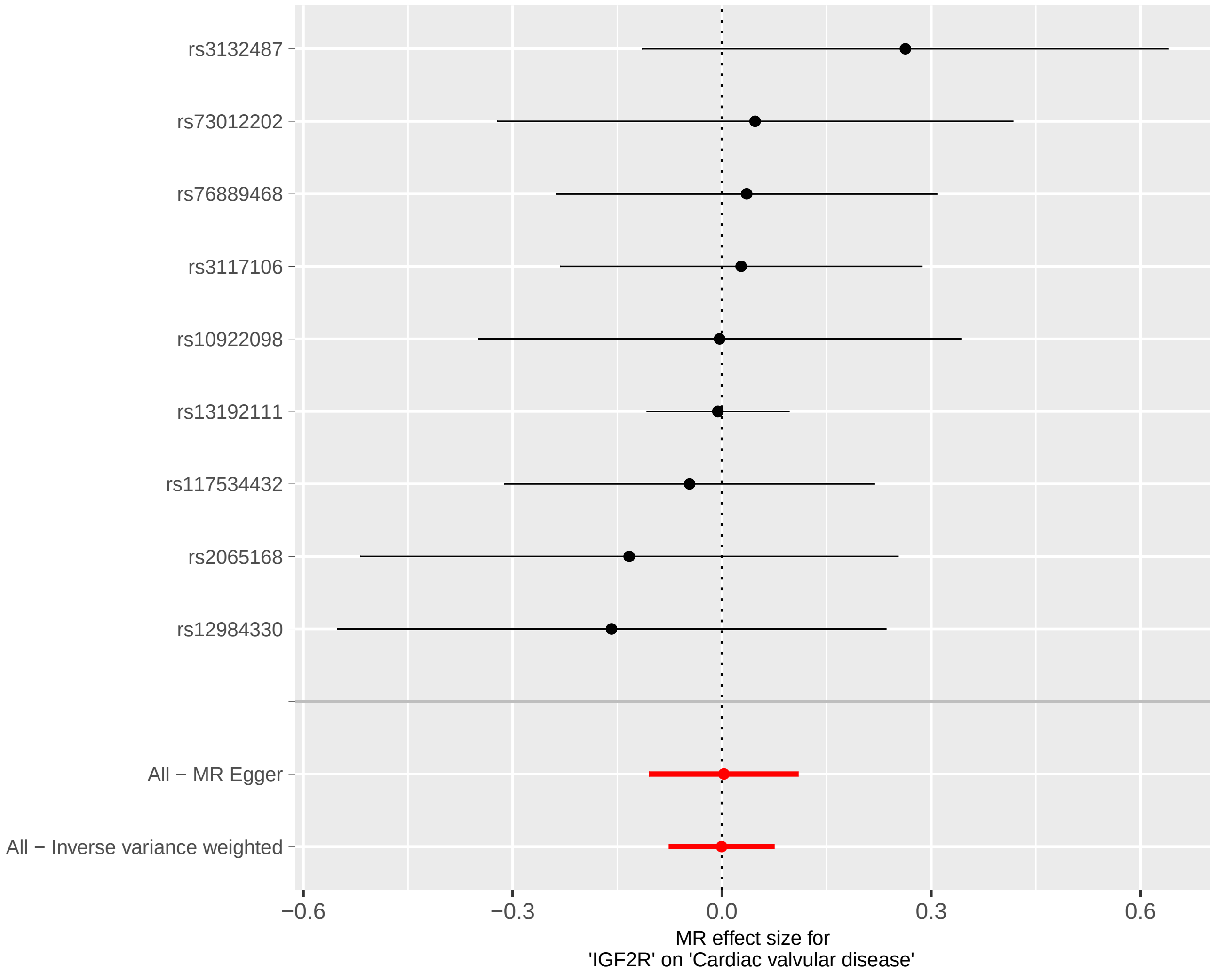
rs3132487
rs73012202
rs76889468
rs3117106
rs10922098
rs13192111
rs117534432
rs2065168
rs12984330
All − MR Egger
All − Inverse variance weighted
−0.6
−0.3
0.0
0.3
0.6
MR effect size for
'IGF2R' on 'Cardiac valvular disease'

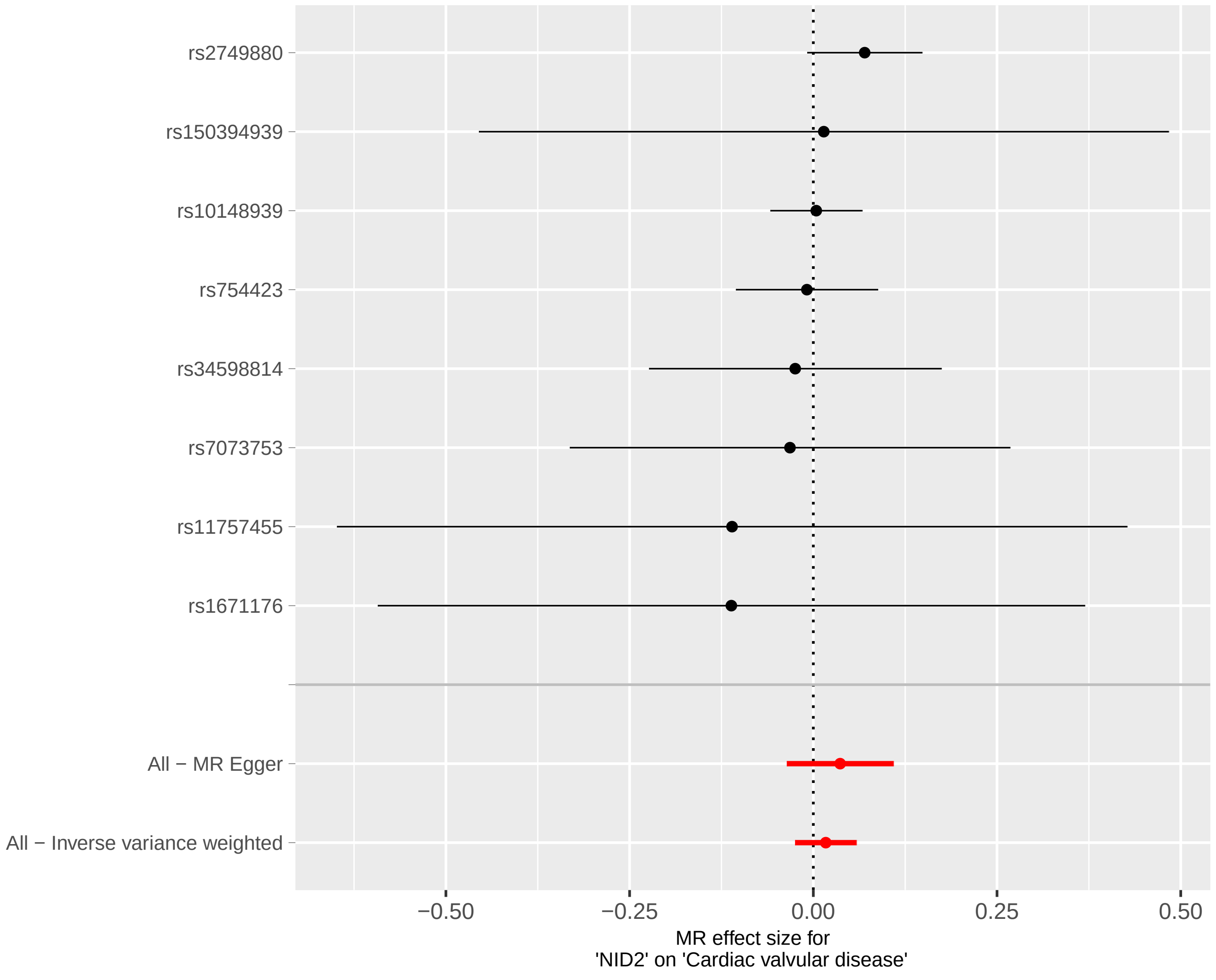
rs2749880
rs150394939
rs10148939
rs754423
rs34598814
rs7073753
rs11757455
rs1671176
All − MR Egger
All − Inverse variance weighted
−0.50
−0.25
0.00
0.25
0.50
MR effect size for
'NID2' on 'Cardiac valvular disease'

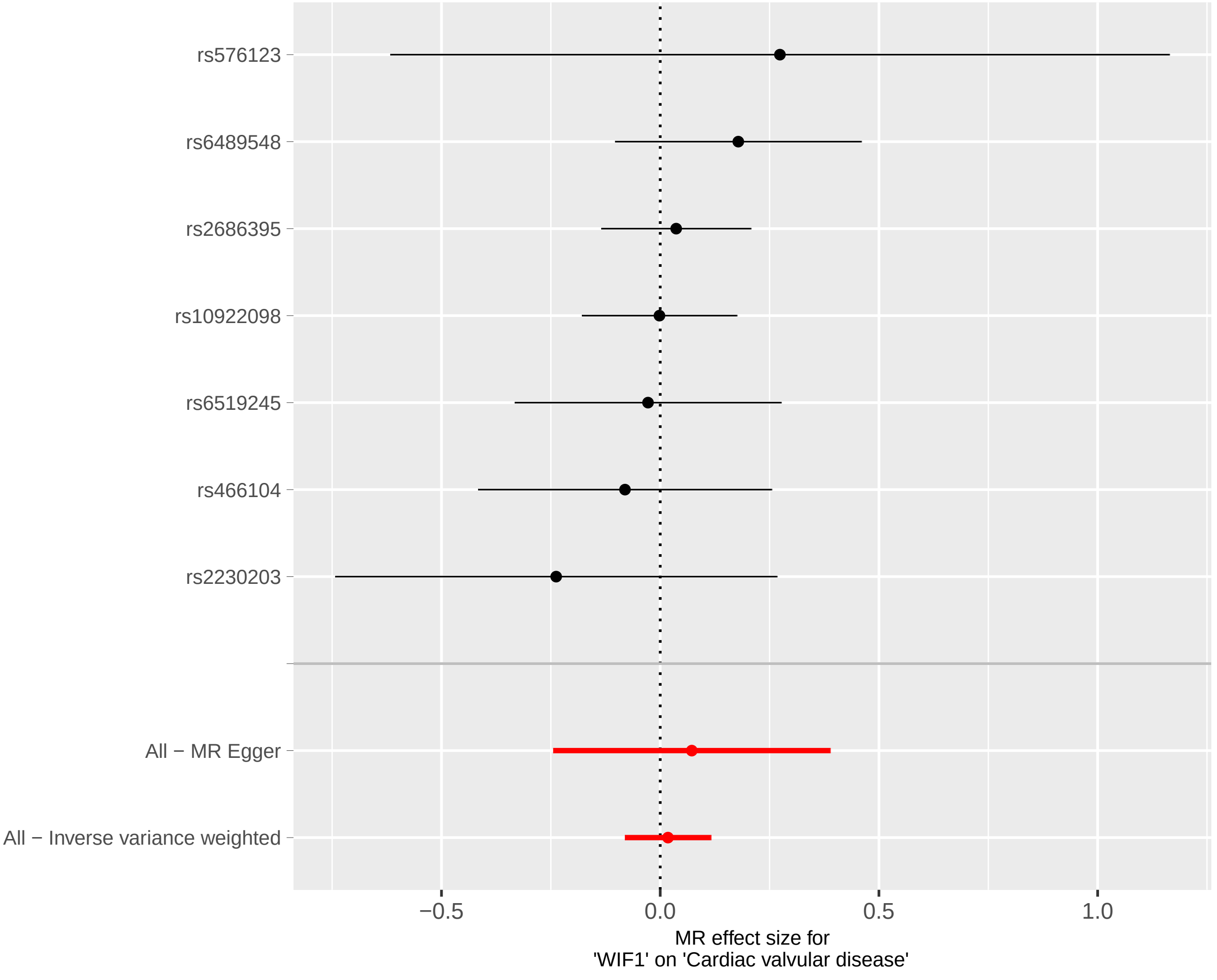
rs576123
rs6489548
rs2686395
rs10922098
rs6519245
rs466104
rs2230203
All − MR Egger
All − Inverse variance weighted
−0.5
0.0
0.5
1.0
MR effect size for
'WIF1' on 'Cardiac valvular disease'

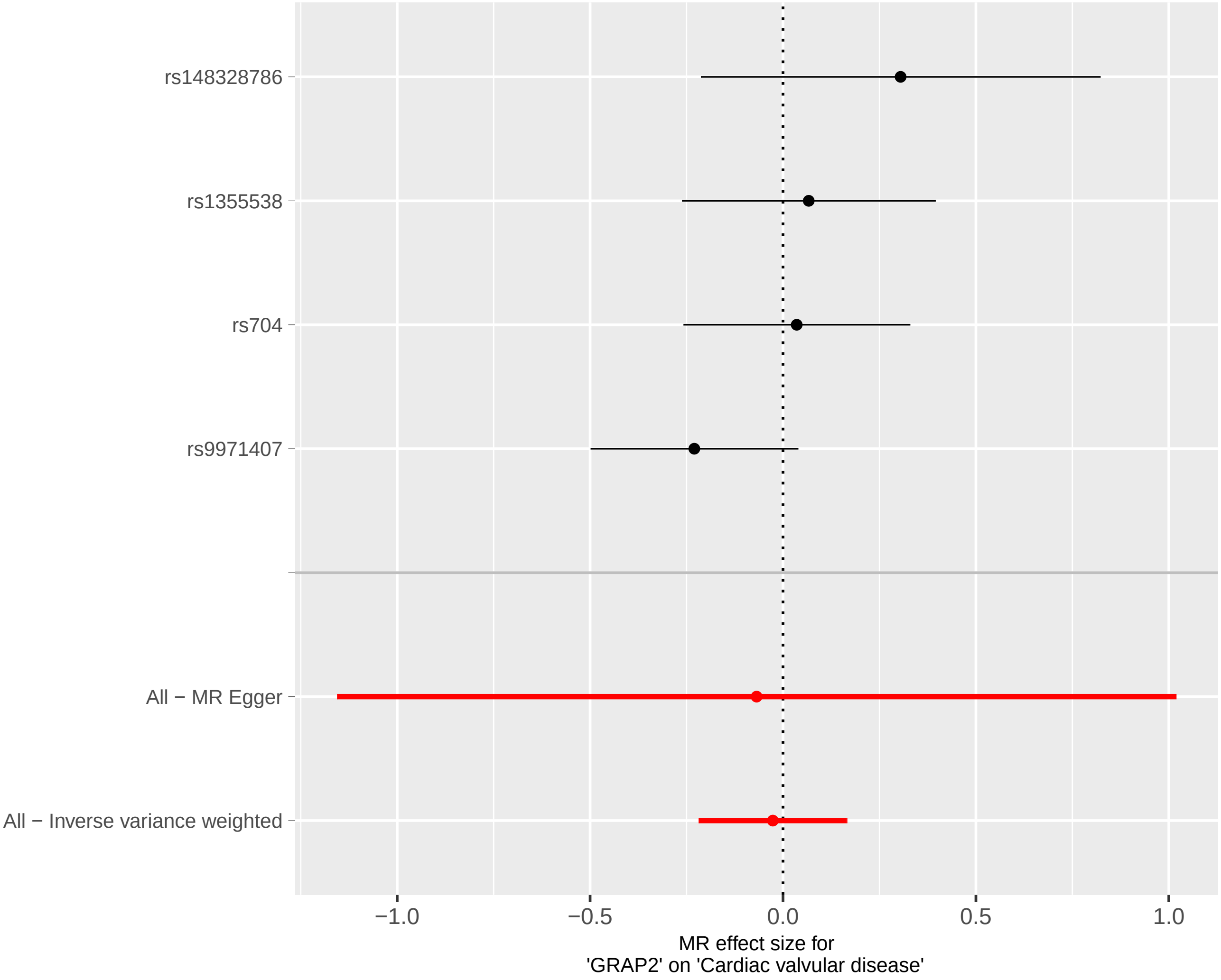
rs148328786
rs1355538
rs704
rs9971407
All − MR Egger
All − Inverse variance weighted
−1.0
−0.5
0.0
0.5
1.0
MR effect size for
'GRAP2' on 'Cardiac valvular disease'

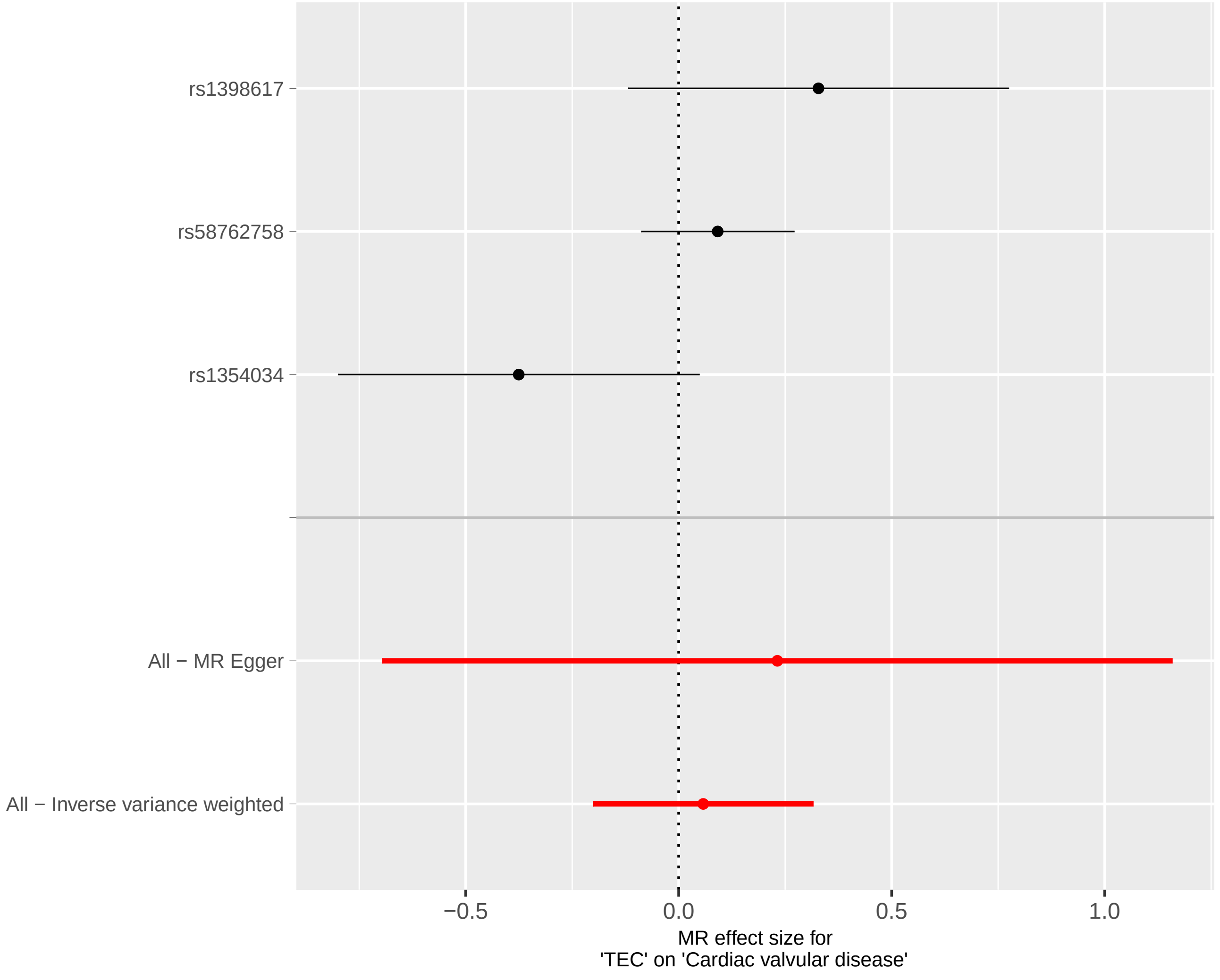

rs1398617
rs58762758
rs1354034
All − MR Egger
All − Inverse variance weighted
−0.5
0.0
0.5
1.0
MR effect size for
'TEC' on 'Cardiac valvular disease'

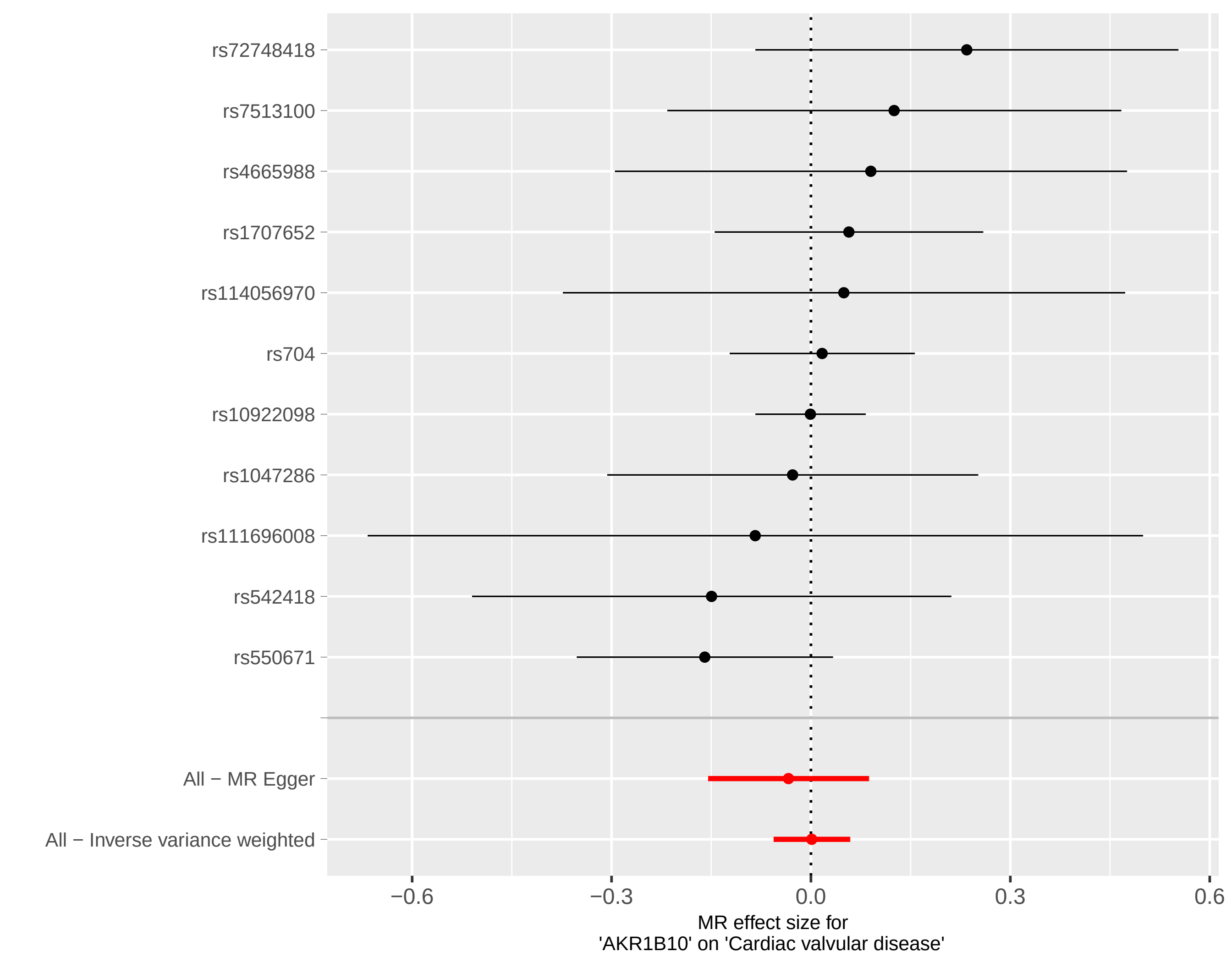
rs72748418
rs7513100
rs4665988
rs1707652
rs114056970
rs704
rs10922098
rs1047286
rs111696008
rs542418
rs550671
All − MR Egger
All − Inverse variance weighted
−0.6
−0.3
0.0
0.3
0.6
MR effect size for
'AKR1B10' on 'Cardiac valvular disease'

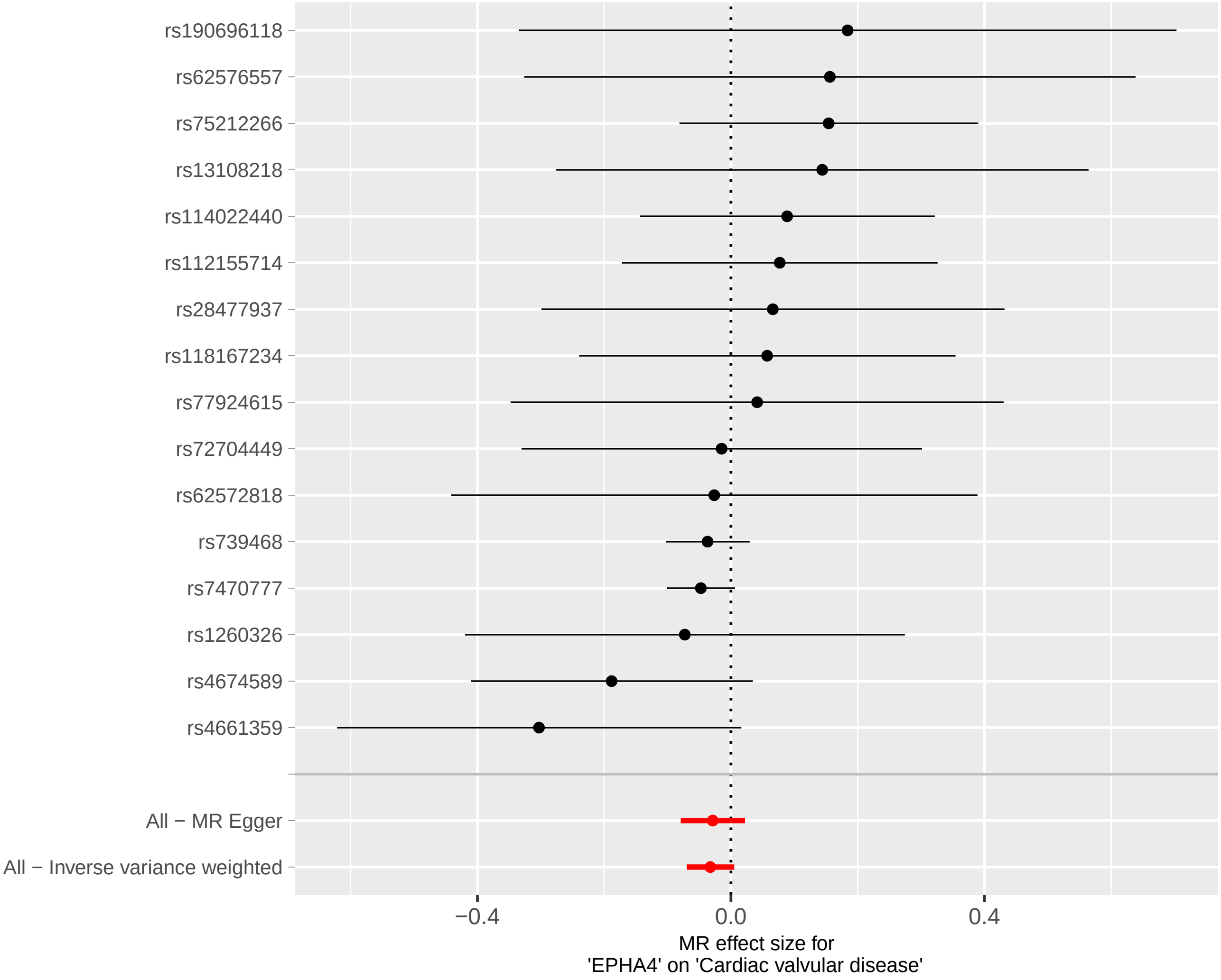
rs190696118
rs62576557
rs75212266
rs13108218
rs114022440
rs112155714
rs28477937
rs118167234
rs77924615
rs72704449
rs62572818
rs739468
rs7470777
rs1260326
rs4674589
rs4661359
All − MR Egger
All − Inverse variance weighted
−0.4
0.0
0.4
MR effect size for
'EPHA4' on 'Cardiac valvular disease'

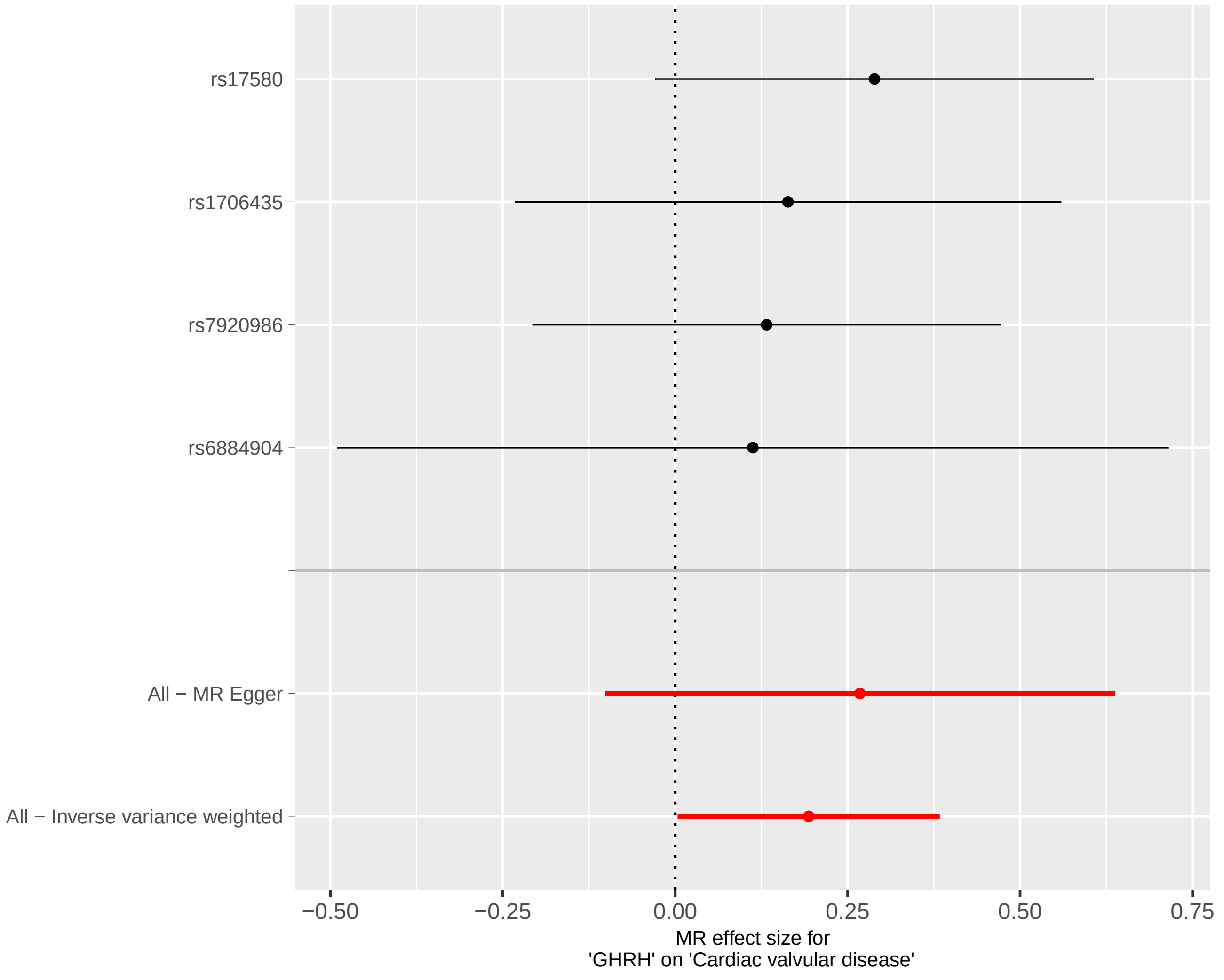
rs17580
rs1706435
rs7920986
rs6884904
All − MR Egger
All − Inverse variance weighted
−0.50
−0.25
0.00
0.25
0.50
0.75
MR effect size for
'GHRH' on 'Cardiac valvular disease'

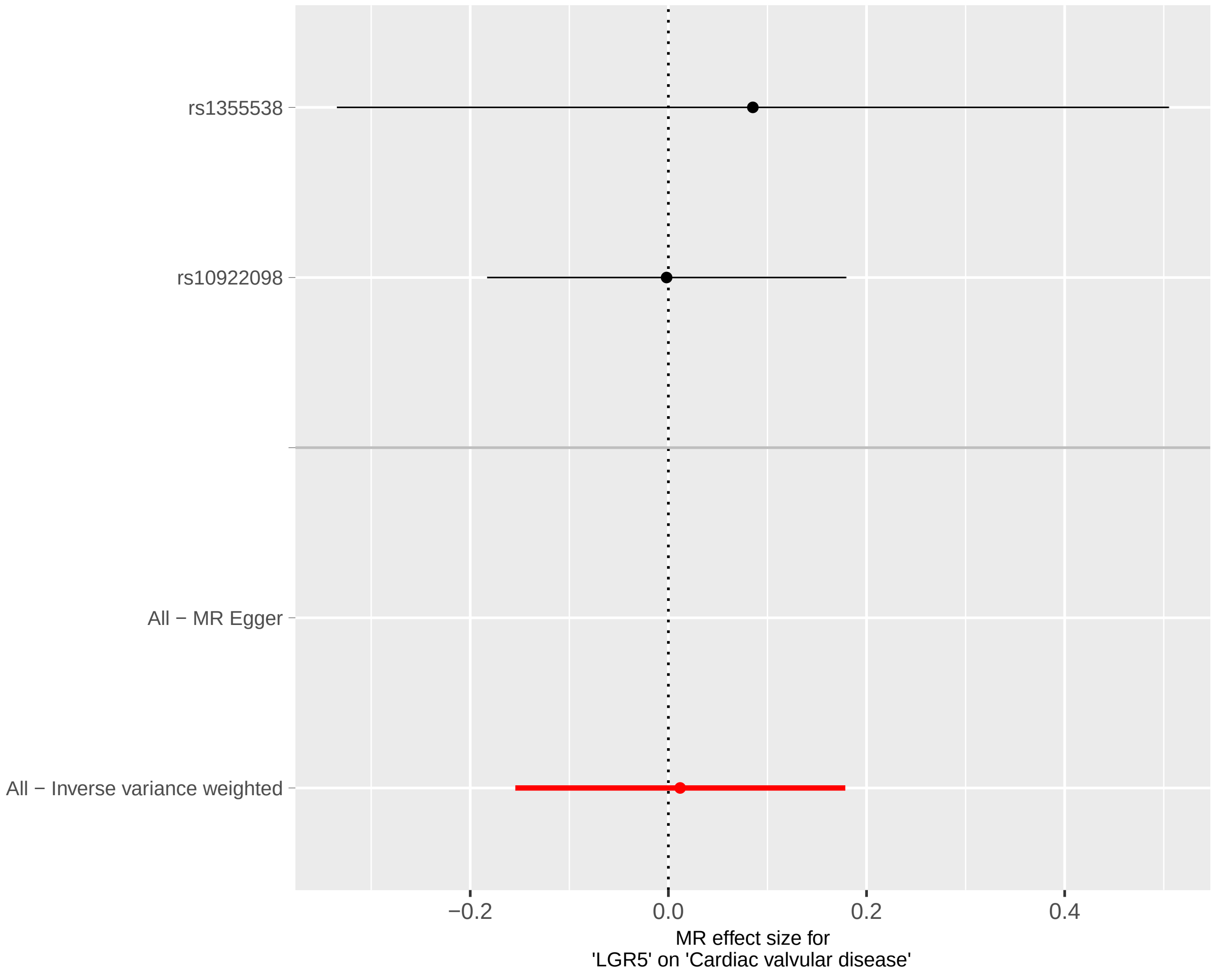
rs1355538
rs10922098
All − MR Egger
All − Inverse variance weighted
−0.2
0.0
0.2
0.4
MR effect size for
'LGR5' on 'Cardiac valvular disease'

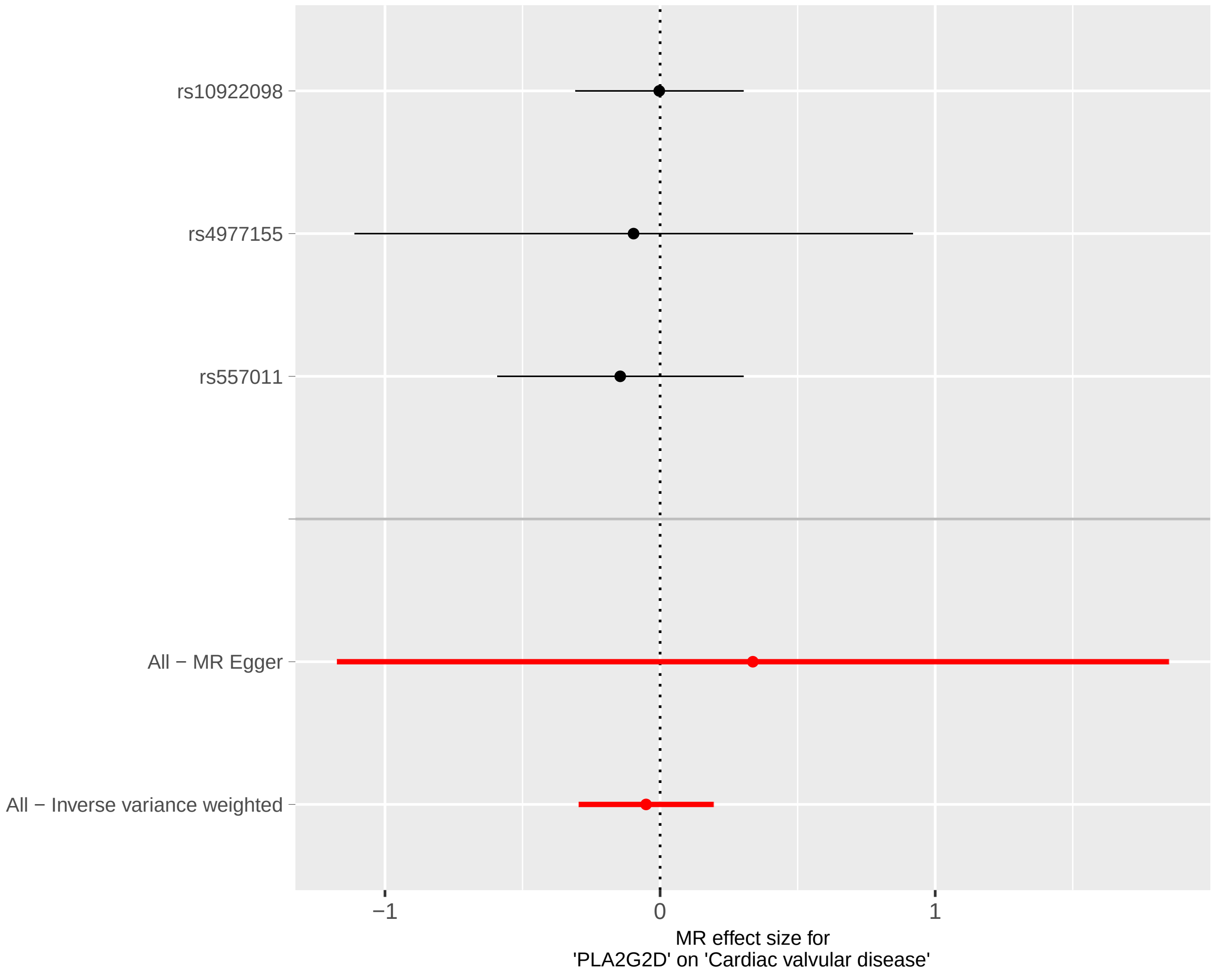
rs10922098
rs4977155
rs557011
All − MR Egger
All − Inverse variance weighted
−1
0
1
MR effect size for
'PLA2G2D' on 'Cardiac valvular disease'

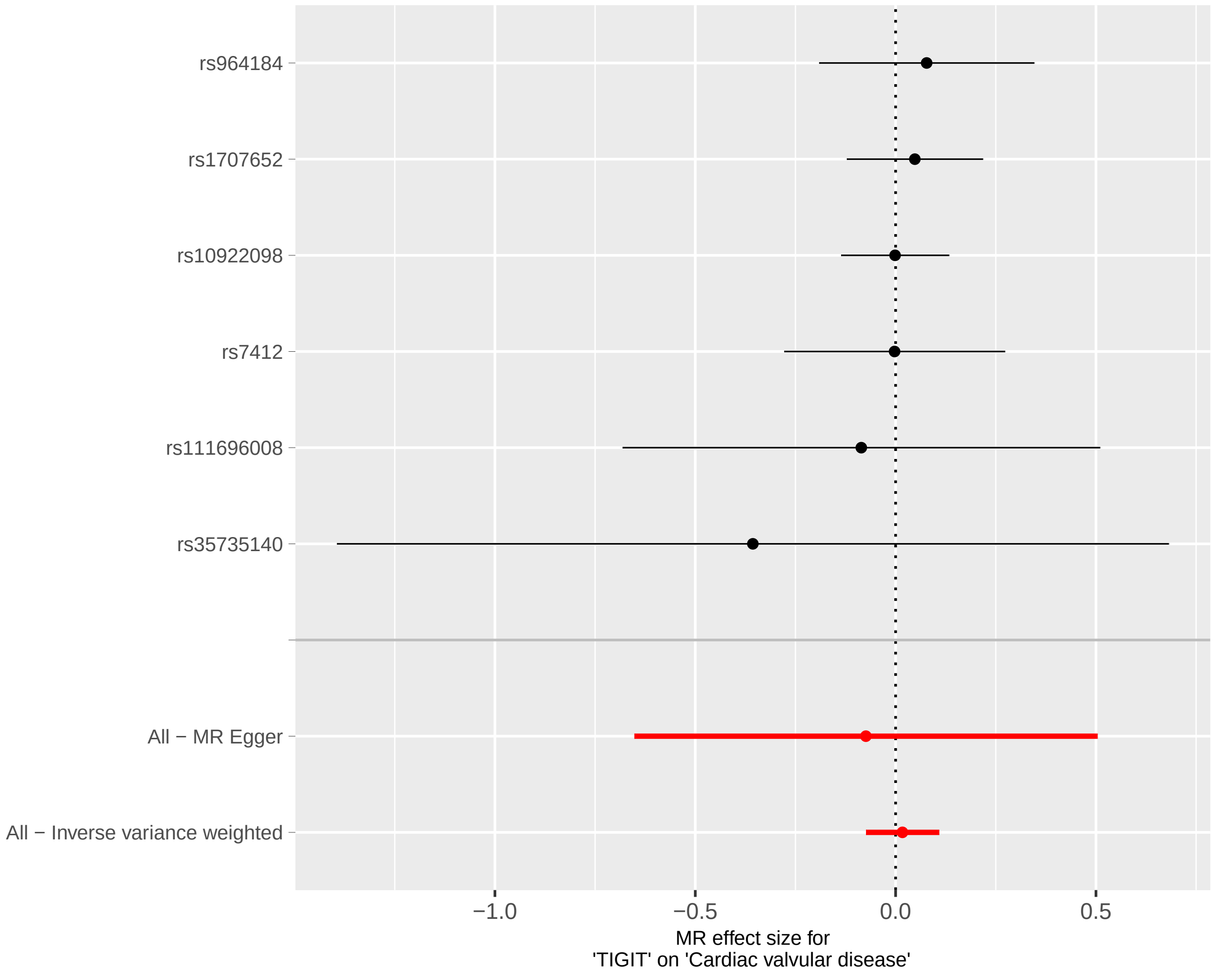

rs964184
rs1707652
rs10922098
rs7412
rs111696008
rs35735140
All − MR Egger
All − Inverse variance weighted
−1.0
−0.5
0.0
0.5
MR effect size for
'TIGIT' on 'Cardiac valvular disease'

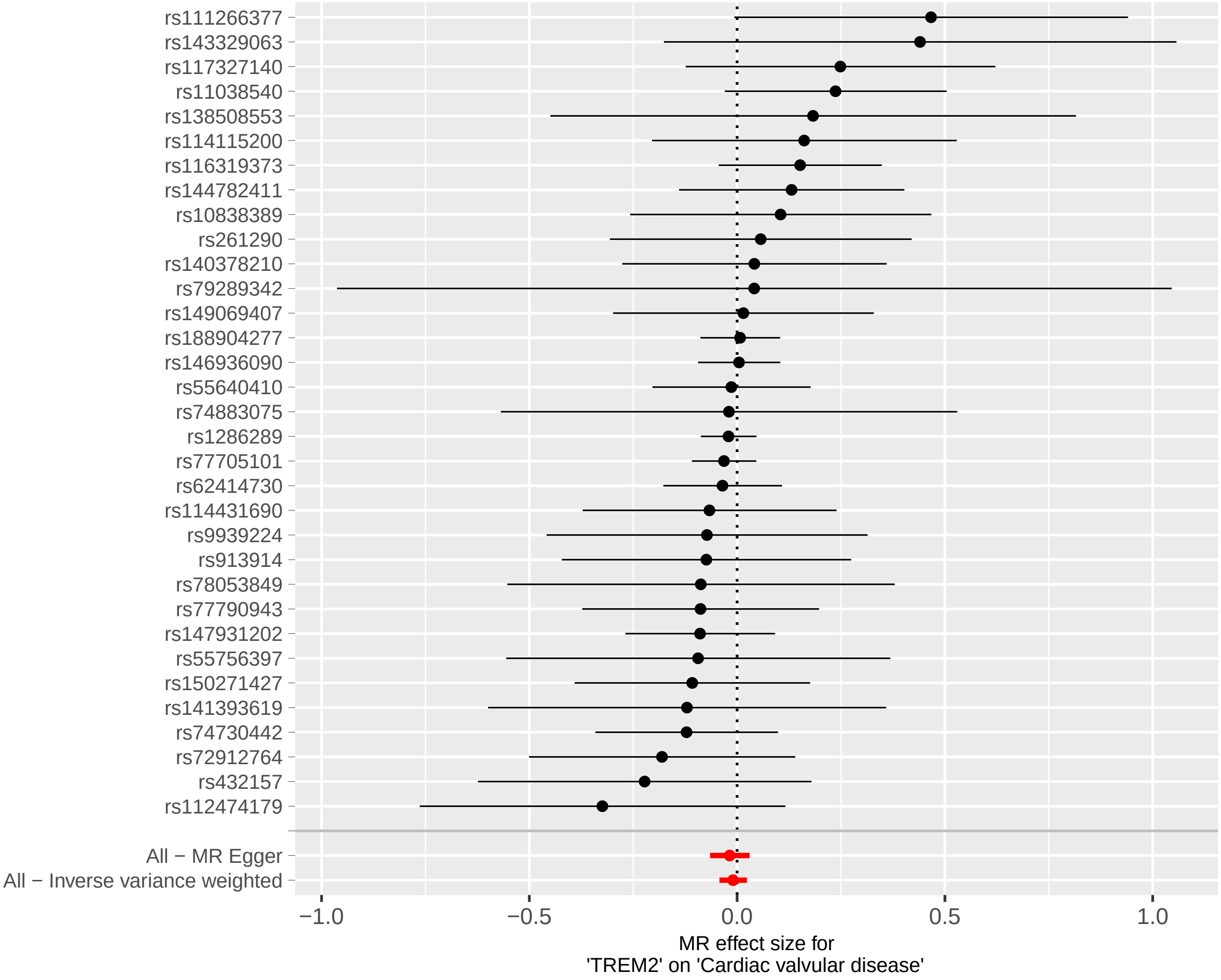
rs111266377
rs143329063
rs117327140
rs11038540
rs138508553
rs114115200
rs116319373
rs144782411
rs10838389
rs261290
rs140378210
rs79289342
rs149069407
rs188904277
rs146936090
rs55640410
rs74883075
rs1286289
rs77705101
rs62414730
rs114431690
rs9939224
rs913914
rs78053849
rs77790943
rs147931202
rs55756397
rs150271427
rs141393619
rs74730442
rs72912764
rs432157
rs112474179
All − MR Egger
All − Inverse variance weighted
−1.0
−0.5
0.0
0.5
1.0
MR effect size for
'TREM2' on 'Cardiac valvular disease'

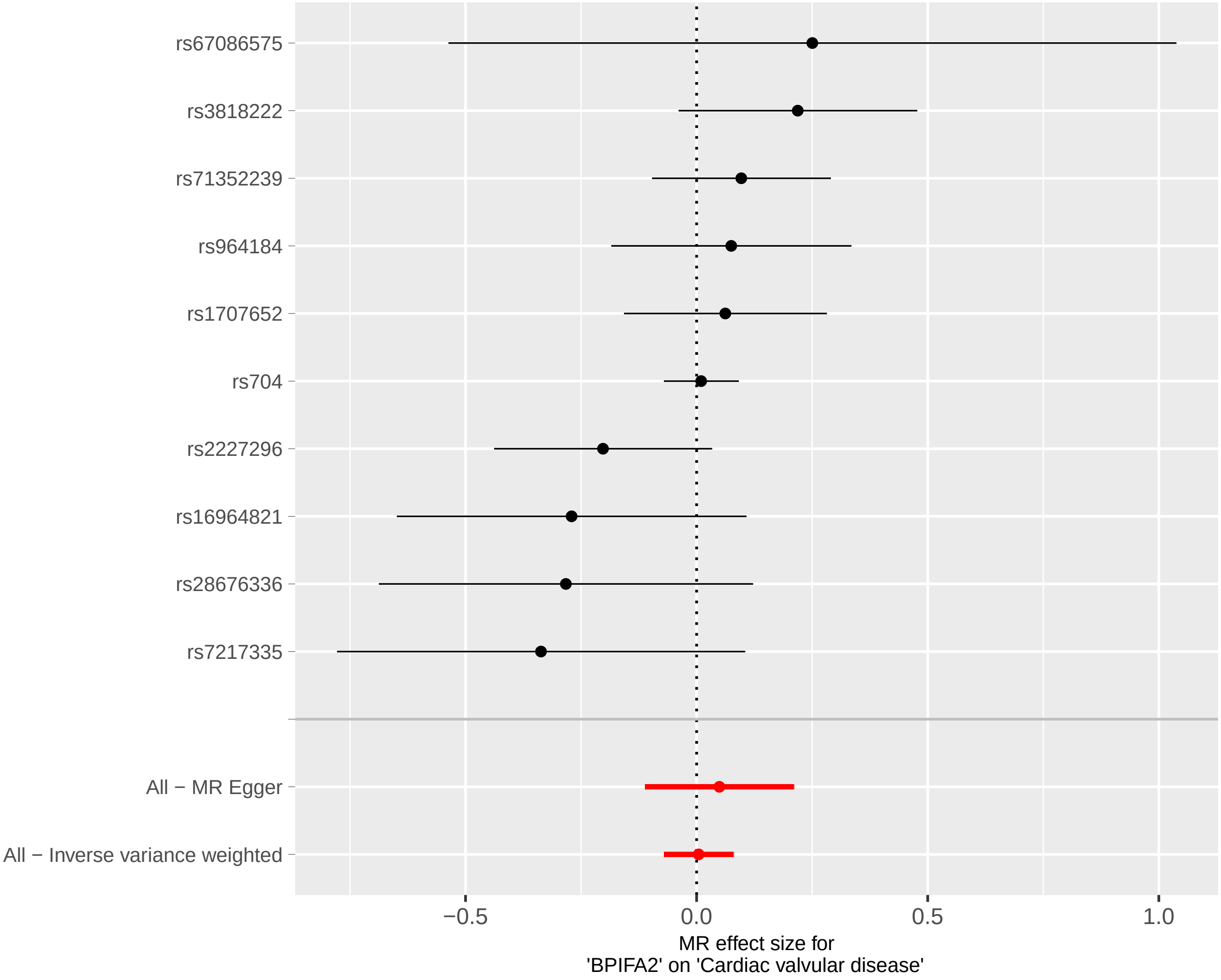

rs67086575
rs3818222
rs71352239
rs964184
rs1707652
rs704
rs2227296
rs16964821
rs28676336
rs7217335
All − MR Egger
All − Inverse variance weighted
−0.5
0.0
0.5
1.0
MR effect size for
'BPIFA2' on 'Cardiac valvular disease'

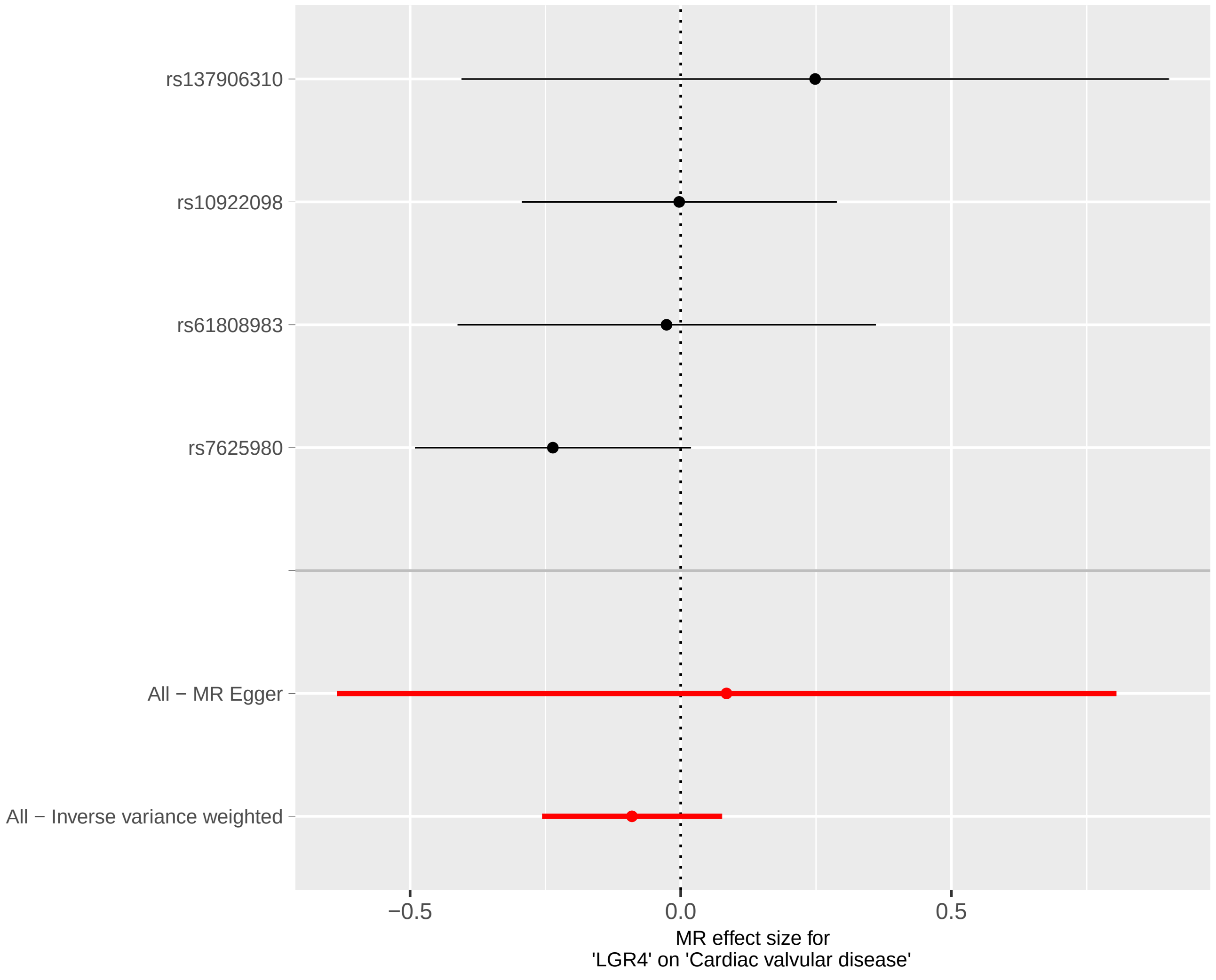
rs137906310
rs10922098
rs61808983
rs7625980
All − MR Egger
All − Inverse variance weighted
−0.5
0.0
0.5
MR effect size for
'LGR4' on 'Cardiac valvular disease'

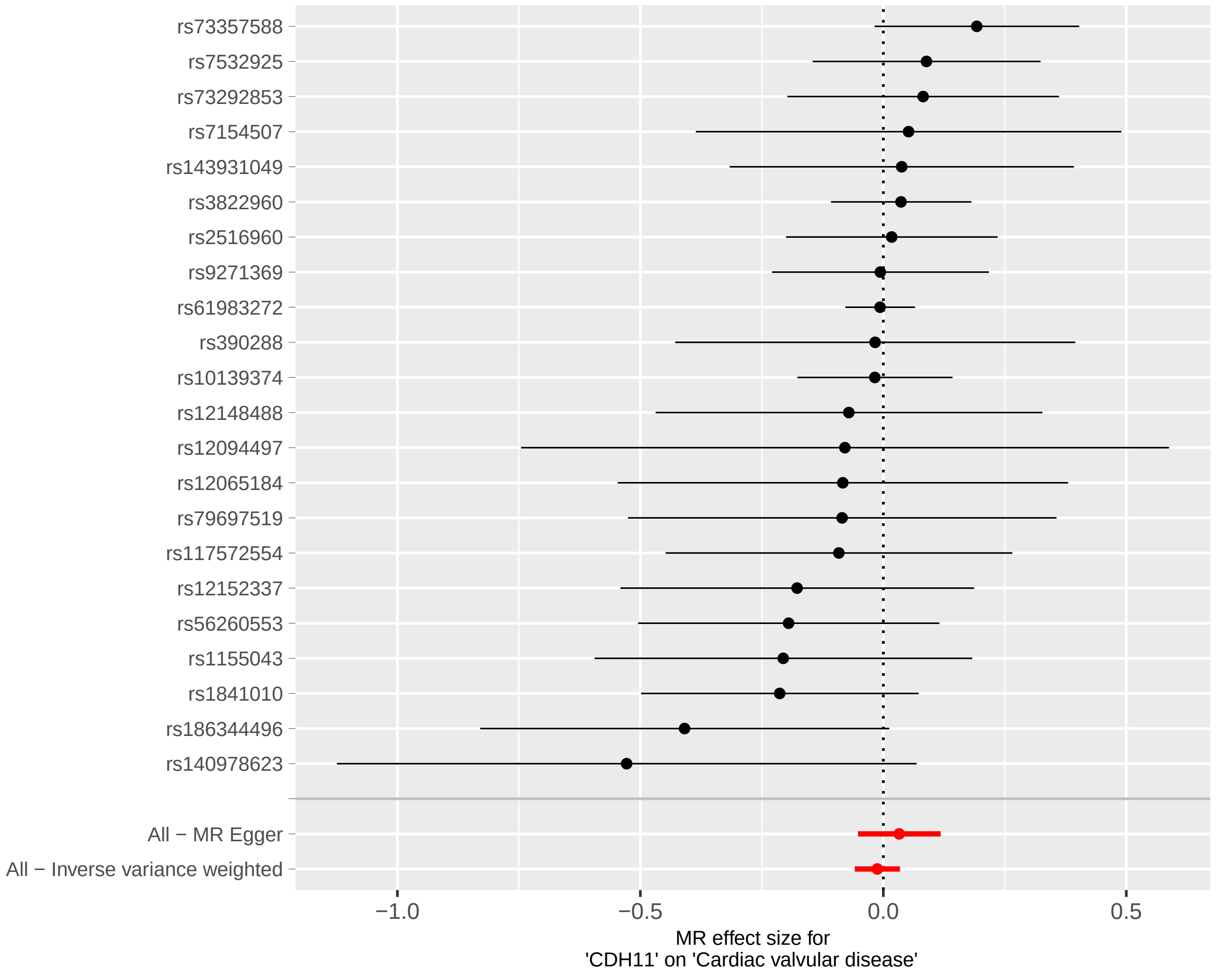
rs73357588
rs7532925
rs73292853
rs7154507
rs143931049
rs3822960
rs2516960
rs9271369
rs61983272
rs390288
rs10139374
rs12148488
rs12094497
rs12065184
rs79697519
rs117572554
rs12152337
rs56260553
rs1155043
rs1841010
rs186344496
rs140978623
All − MR Egger
All − Inverse variance weighted
−1.0
−0.5
0.0
0.5
MR effect size for
'CDH11' on 'Cardiac valvular disease'

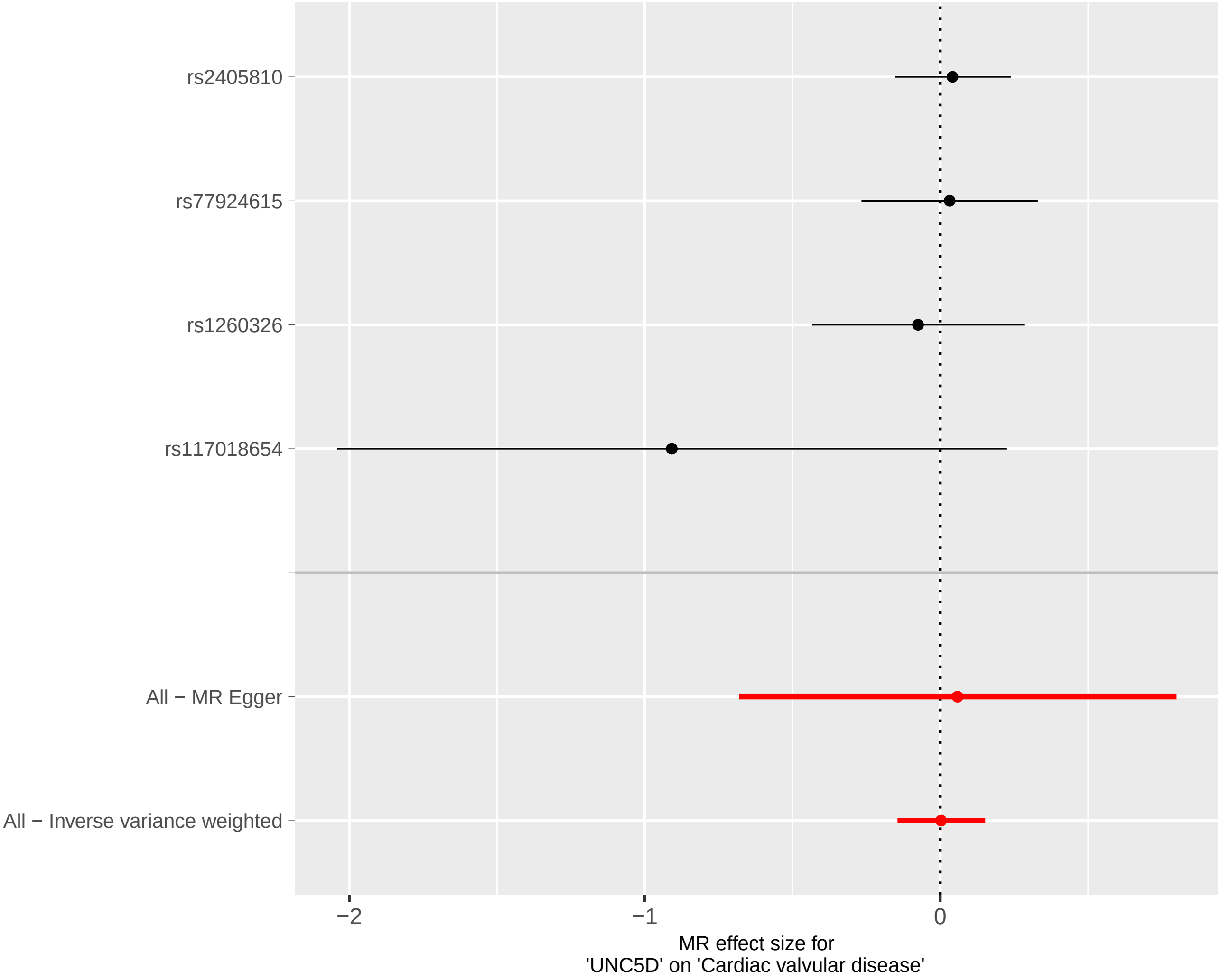
rs2405810
rs77924615
rs1260326
rs117018654
All − MR Egger
All − Inverse variance weighted
−2
−1
0
MR effect size for
'UNC5D' on 'Cardiac valvular disease'

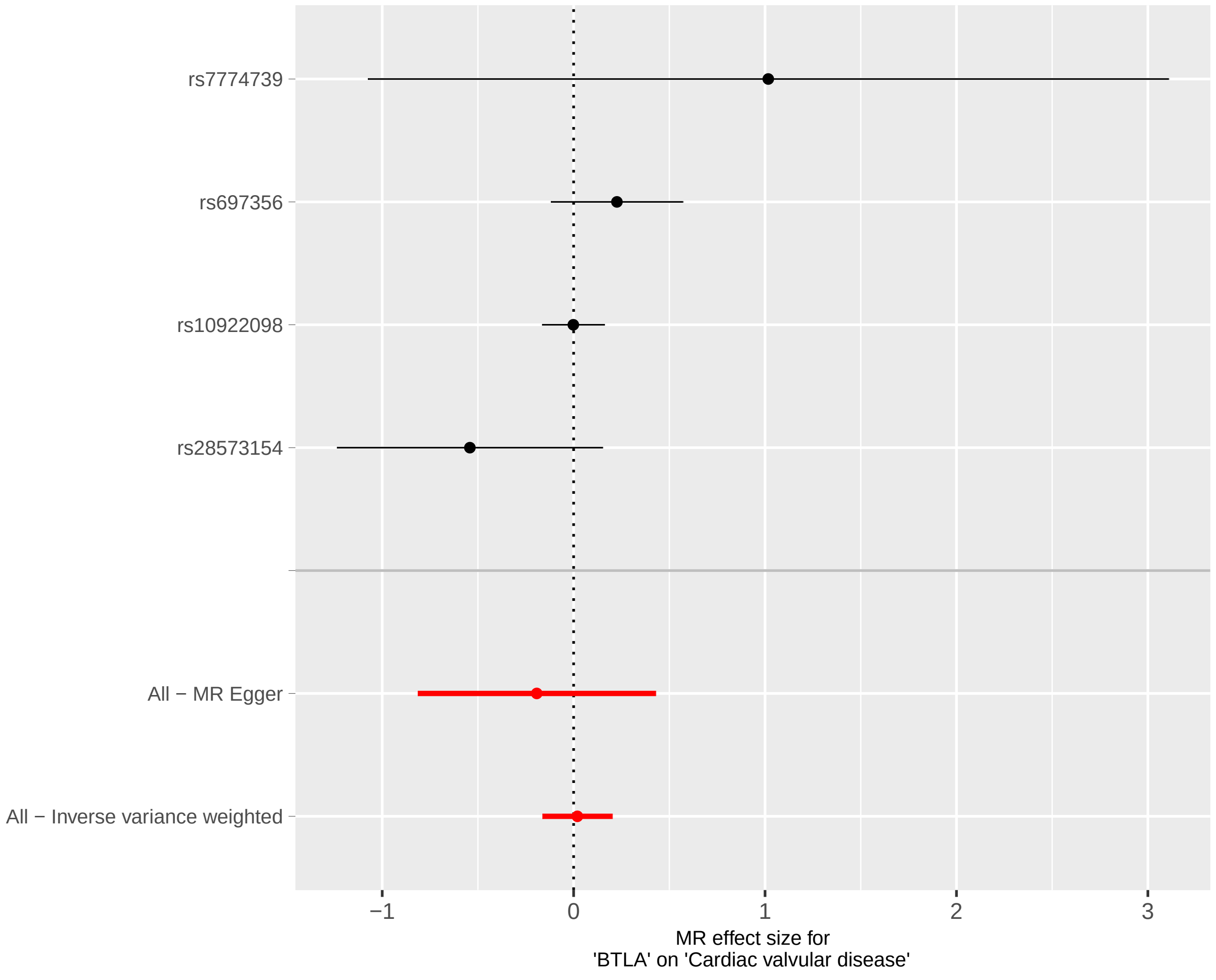

rs7774739
rs697356
rs10922098
rs28573154
All − MR Egger
All − Inverse variance weighted
−1
0
1
2
3
MR effect size for
'BTLA' on 'Cardiac valvular disease'

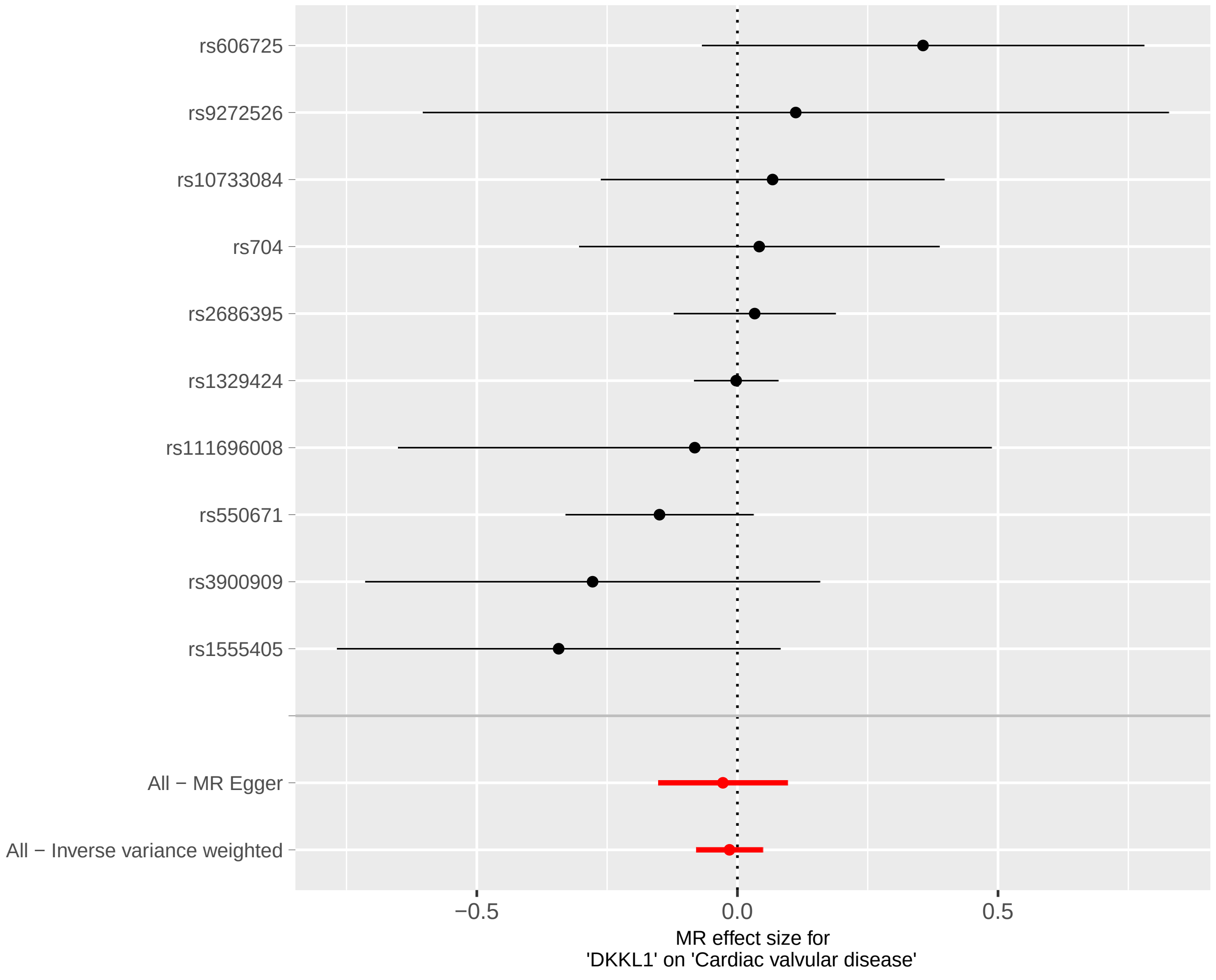

rs606725
rs9272526
rs10733084
rs704
rs2686395
rs1329424
rs111696008
rs550671
rs3900909
rs1555405
All − MR Egger
All − Inverse variance weighted
−0.5
0.0
0.5
MR effect size for
'DKKL1' on 'Cardiac valvular disease'

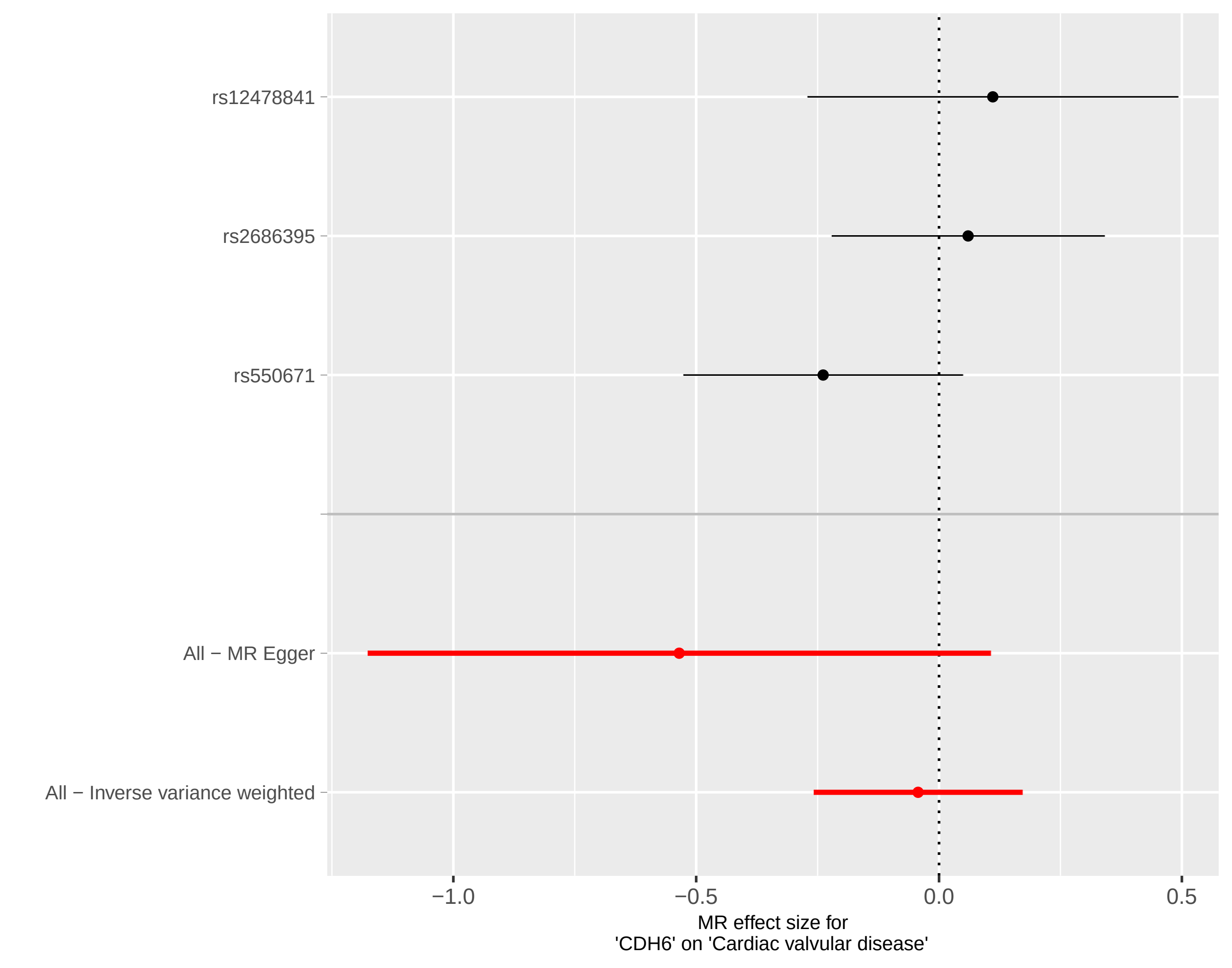
rs12478841
rs2686395
rs550671
All − MR Egger
All − Inverse variance weighted
−1.0
−0.5
0.0
0.5
MR effect size for
'CDH6' on 'Cardiac valvular disease'

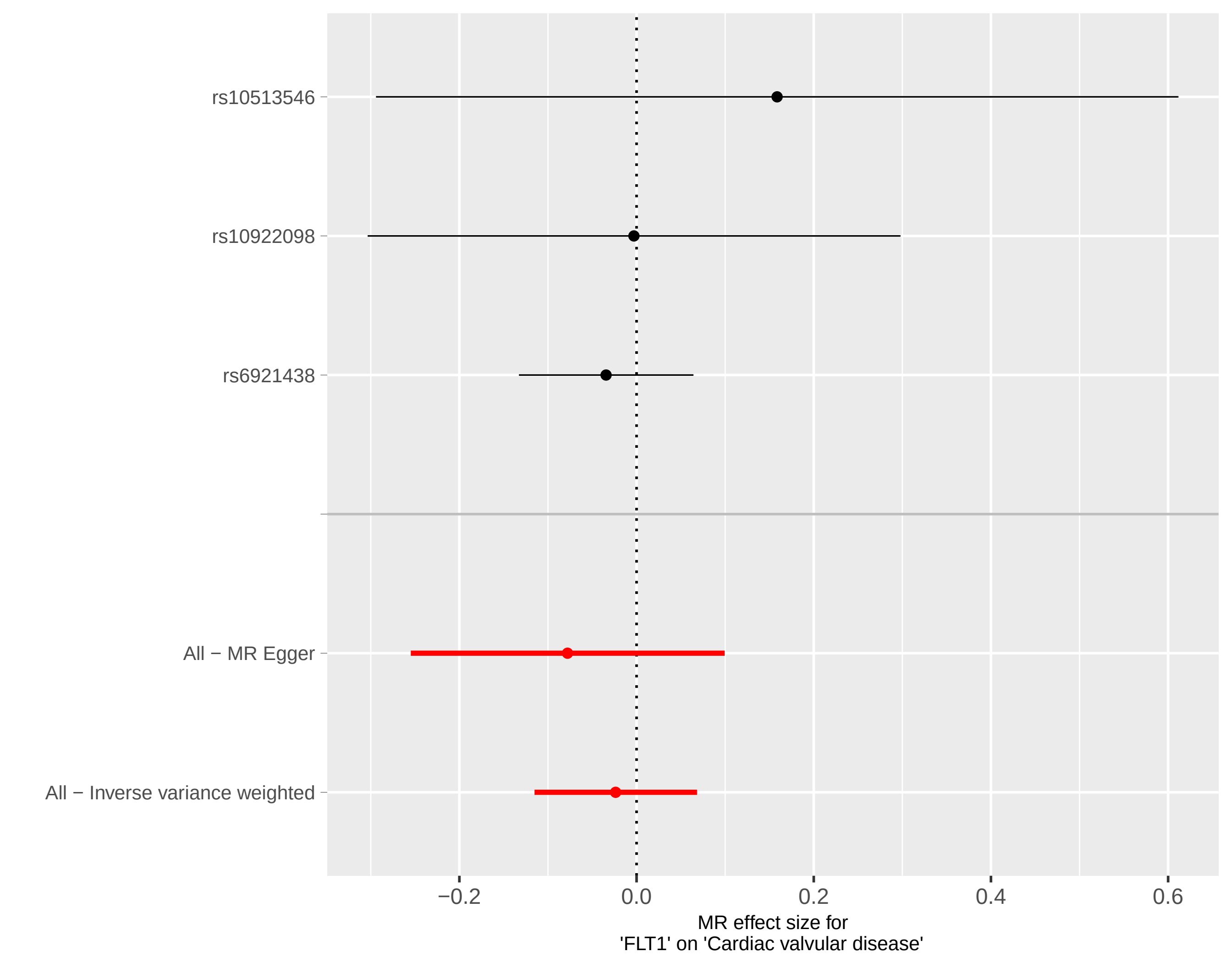

rs10513546
rs10922098
rs6921438
All − MR Egger
All − Inverse variance weighted
−0.2
0.0
0.2
0.4
0.6
MR effect size for
'FLT1' on 'Cardiac valvular disease'

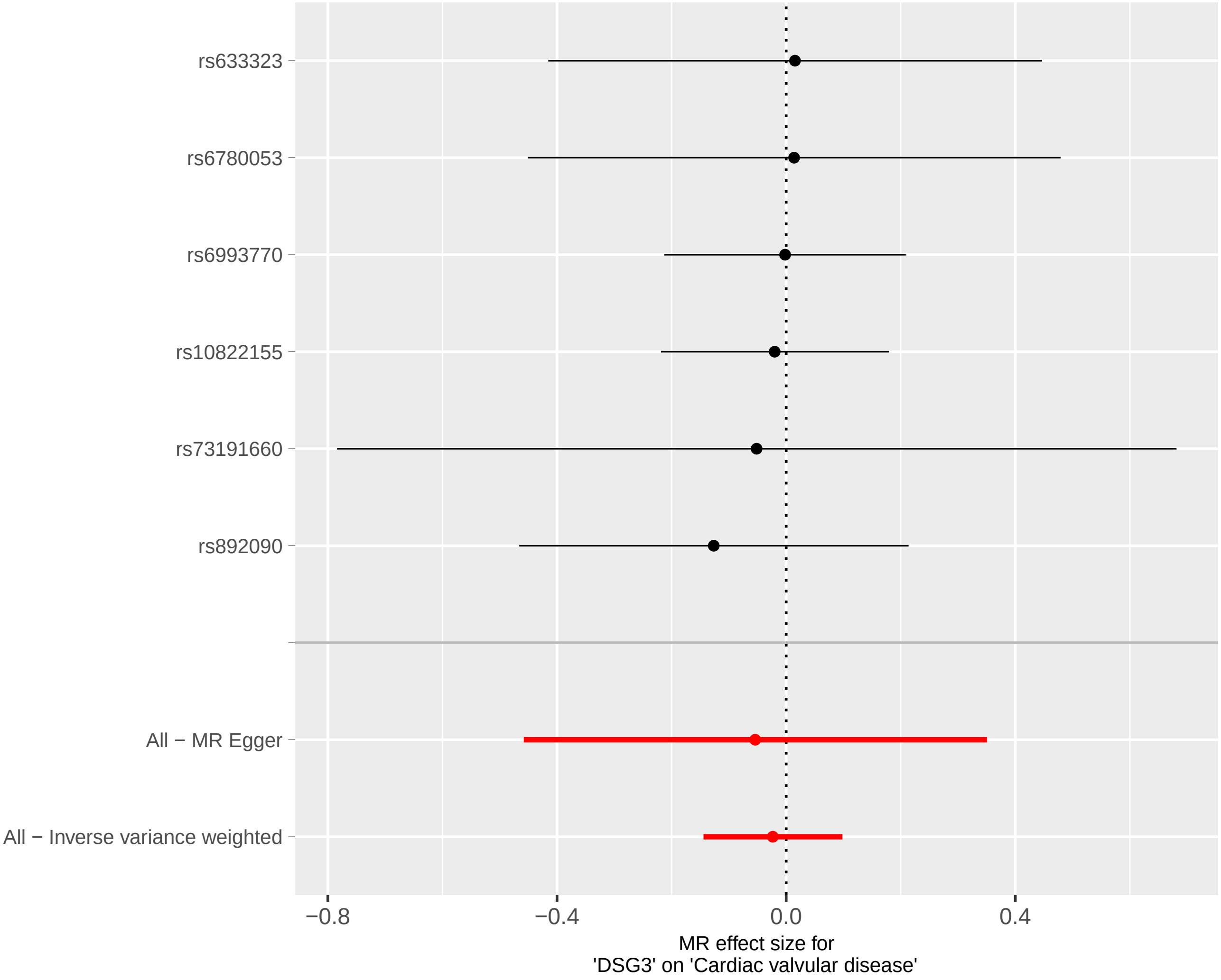

rs633323
rs6780053
rs6993770
rs10822155
rs73191660
rs892090
All − MR Egger
All − Inverse variance weighted
−0.8
−0.4
0.0
0.4
MR effect size for
'DSG3' on 'Cardiac valvular disease'

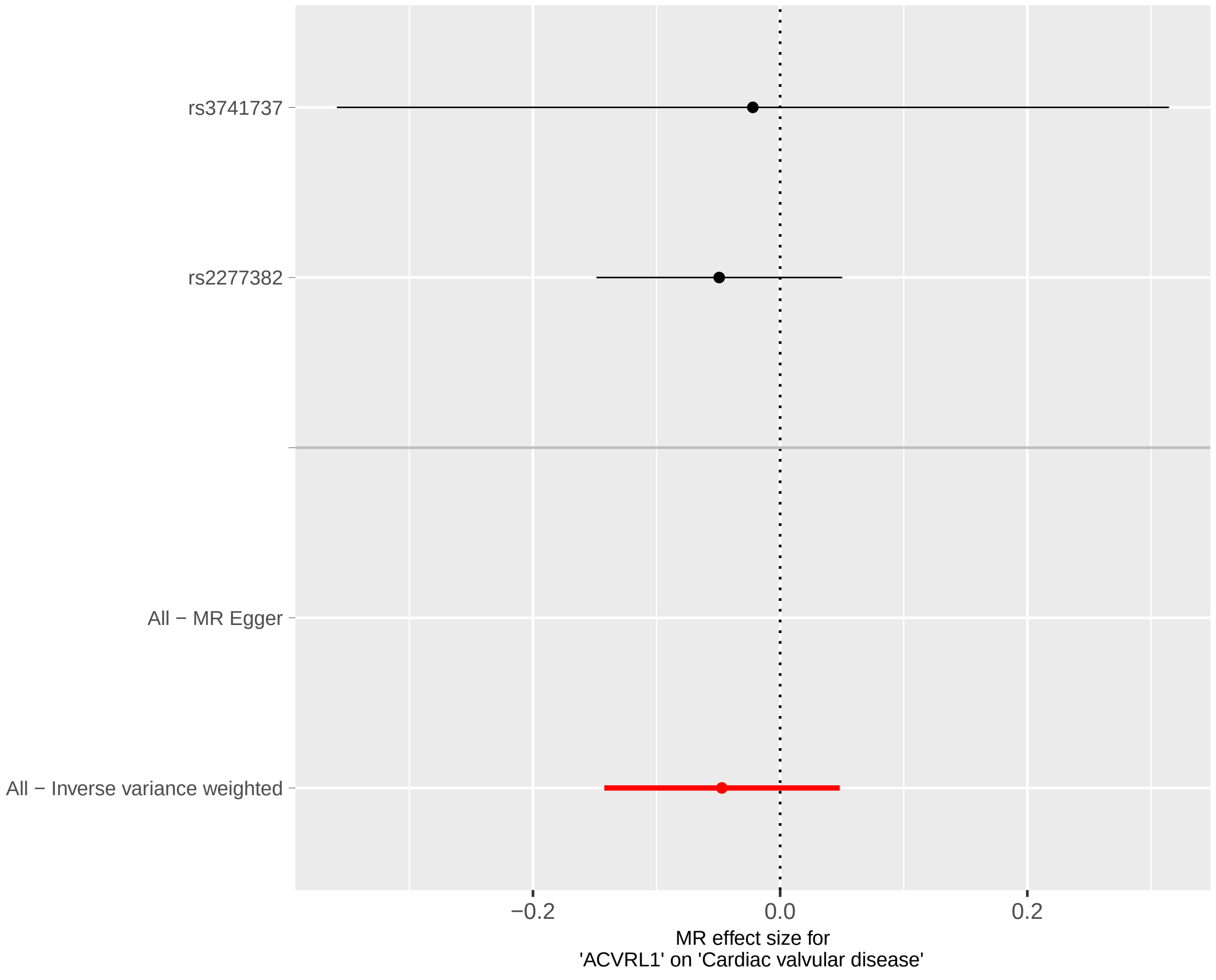
rs3741737
rs2277382
All − MR Egger
All − Inverse variance weighted
−0.2
0.0
0.2
MR effect size for
'ACVRL1' on 'Cardiac valvular disease'

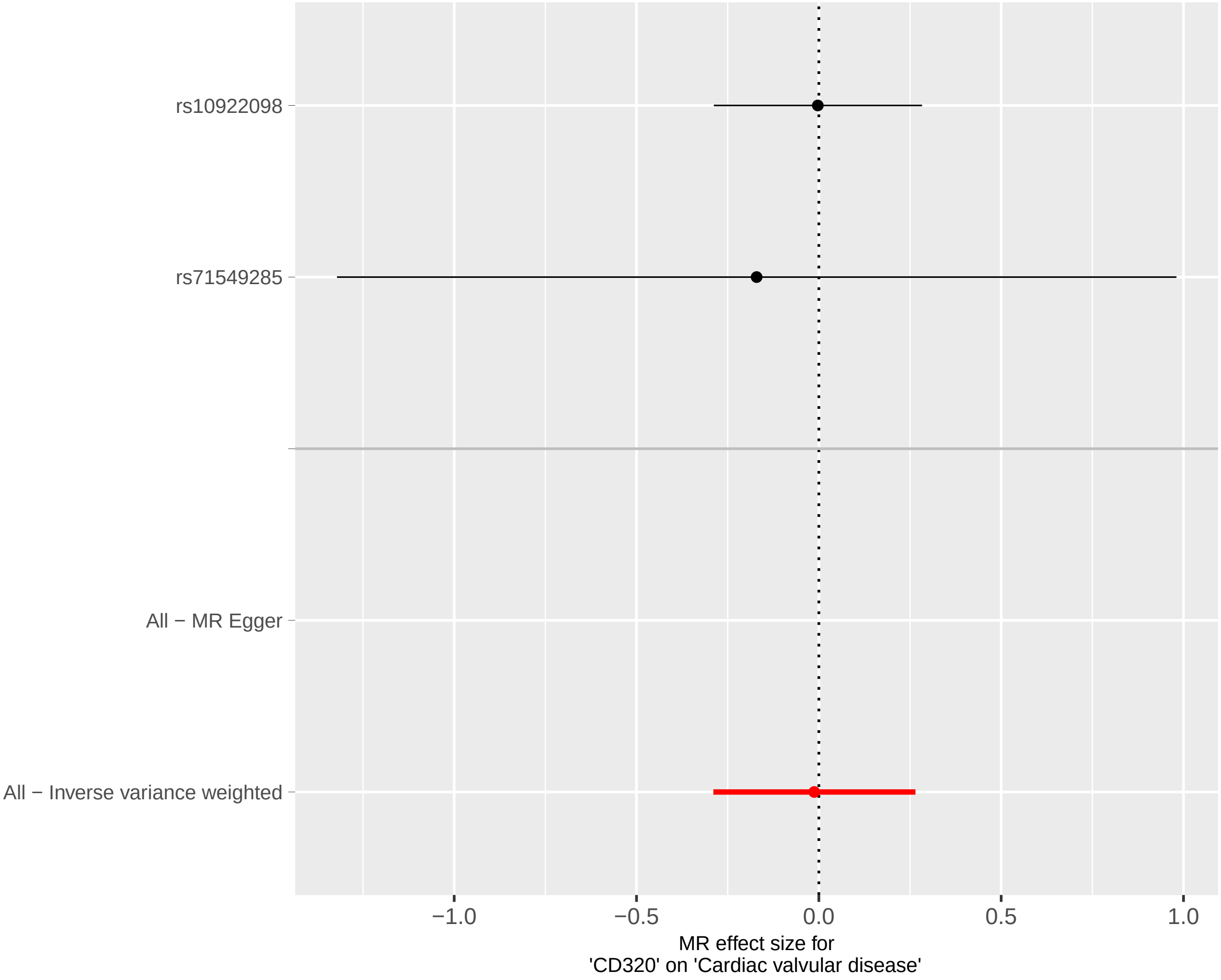
rs10922098
rs71549285
All − MR Egger
All − Inverse variance weighted
−1.0
−0.5
0.0
0.5
1.0
MR effect size for
'CD320' on 'Cardiac valvular disease'

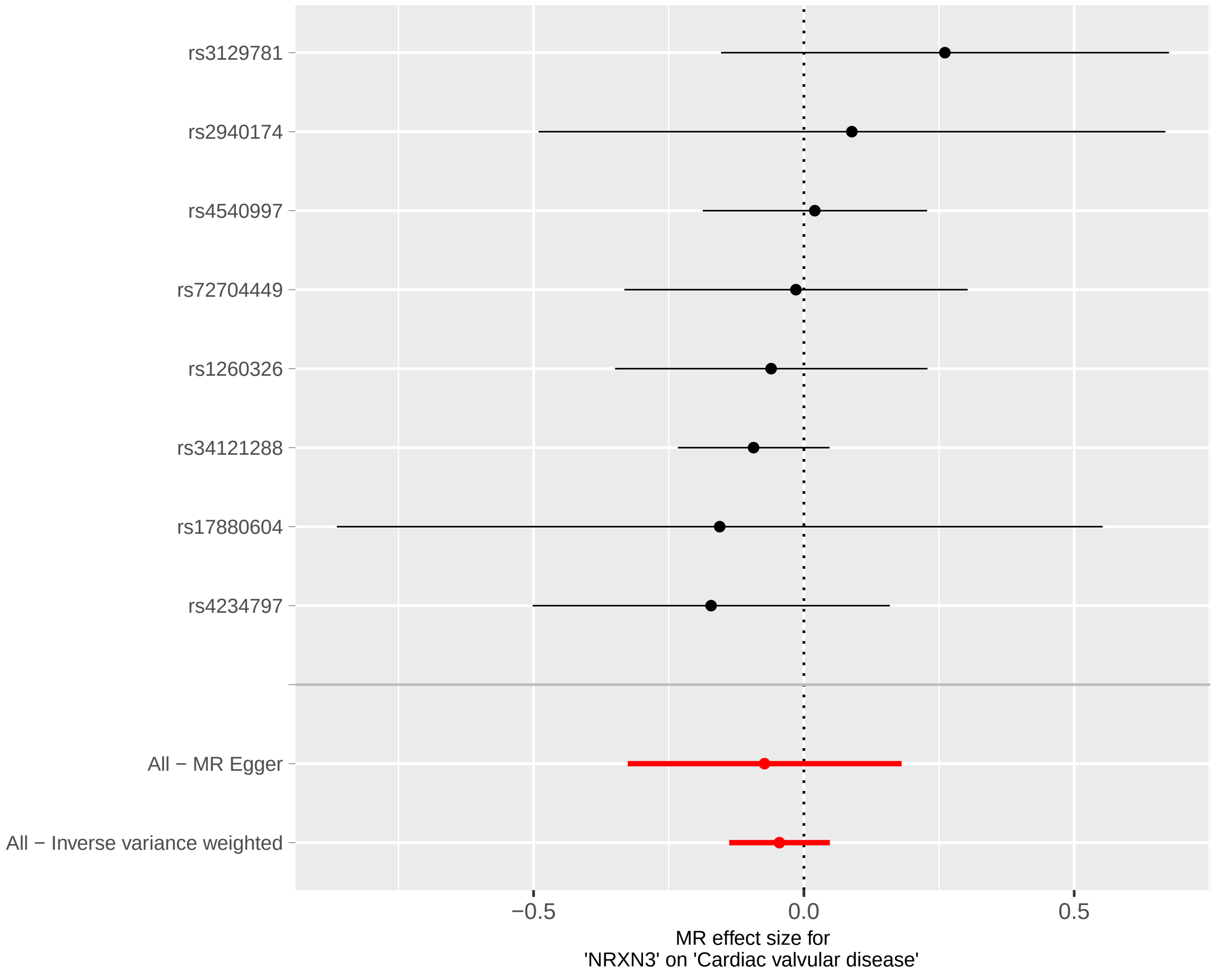
rs3129781
rs2940174
rs4540997
rs72704449
rs1260326
rs34121288
rs17880604
rs4234797
All − MR Egger
All − Inverse variance weighted
−0.5
0.0
0.5
MR effect size for
'NRXN3' on 'Cardiac valvular disease'

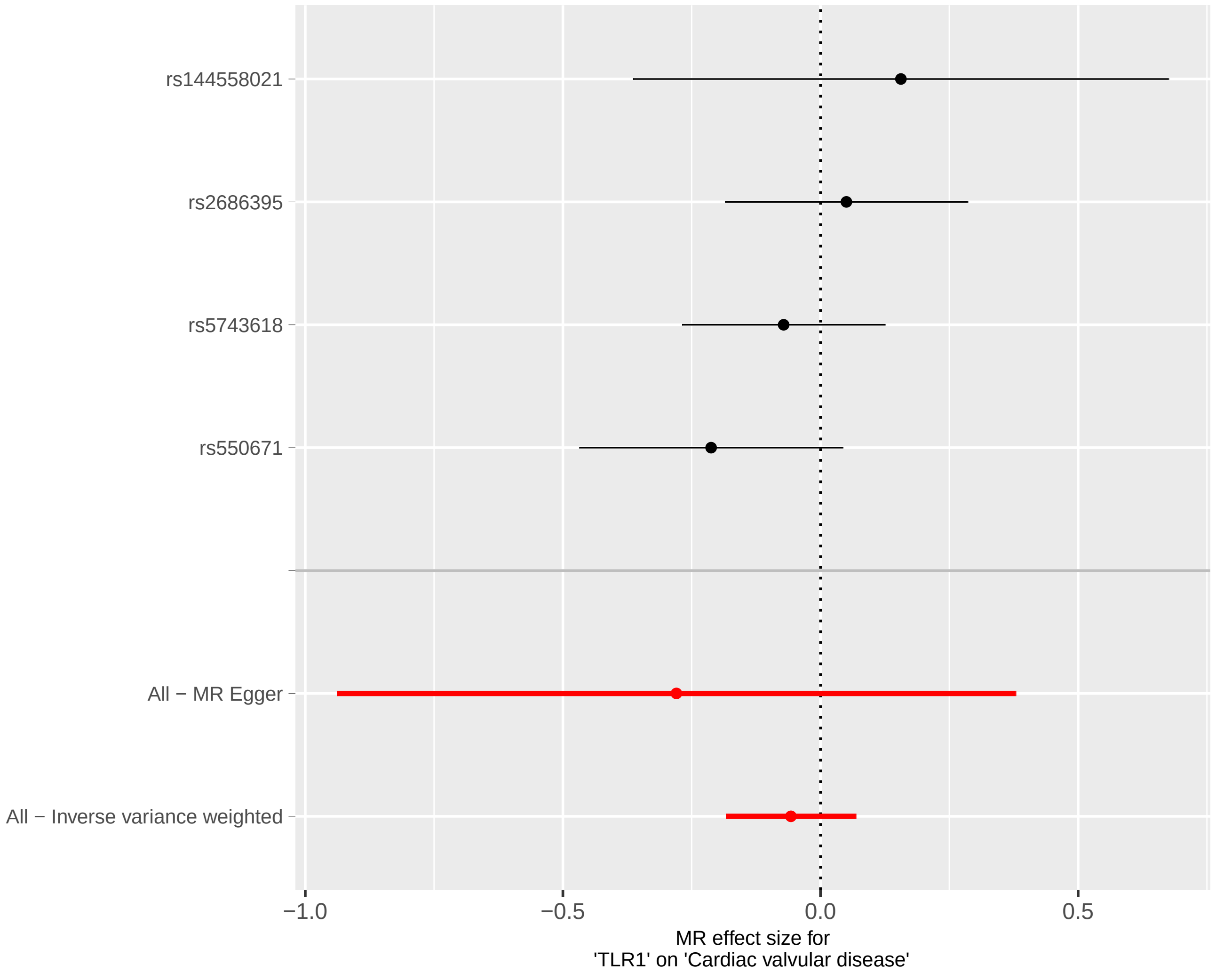
rs144558021
rs2686395
rs5743618
rs550671
All − MR Egger
All − Inverse variance weighted
−1.0
−0.5
0.0
0.5
MR effect size for
'TLR1' on 'Cardiac valvular disease'

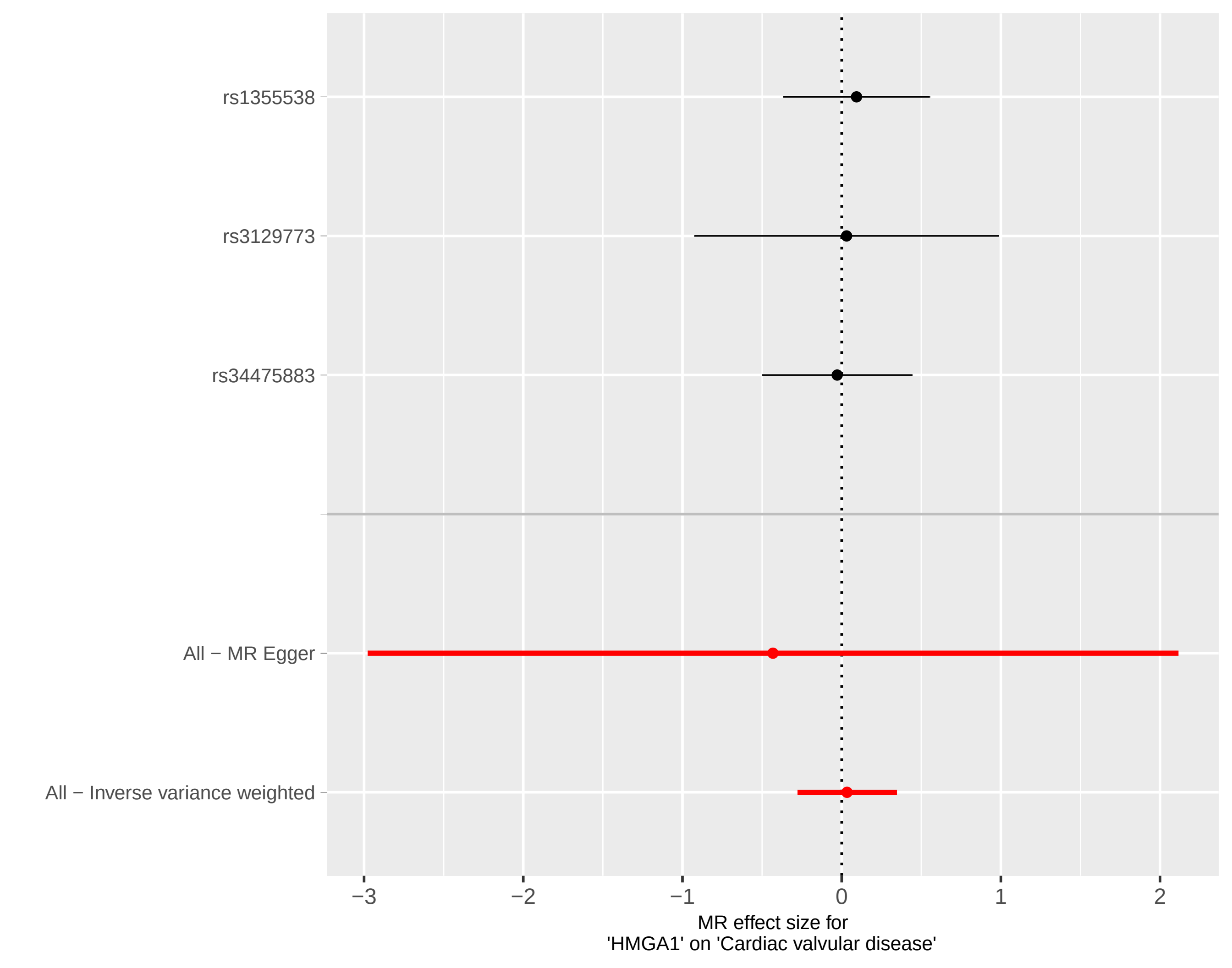
rs1355538
rs3129773
rs34475883
All − MR Egger
All − Inverse variance weighted
−3
−2
−1
0
1
2
MR effect size for
'HMGA1' on 'Cardiac valvular disease'

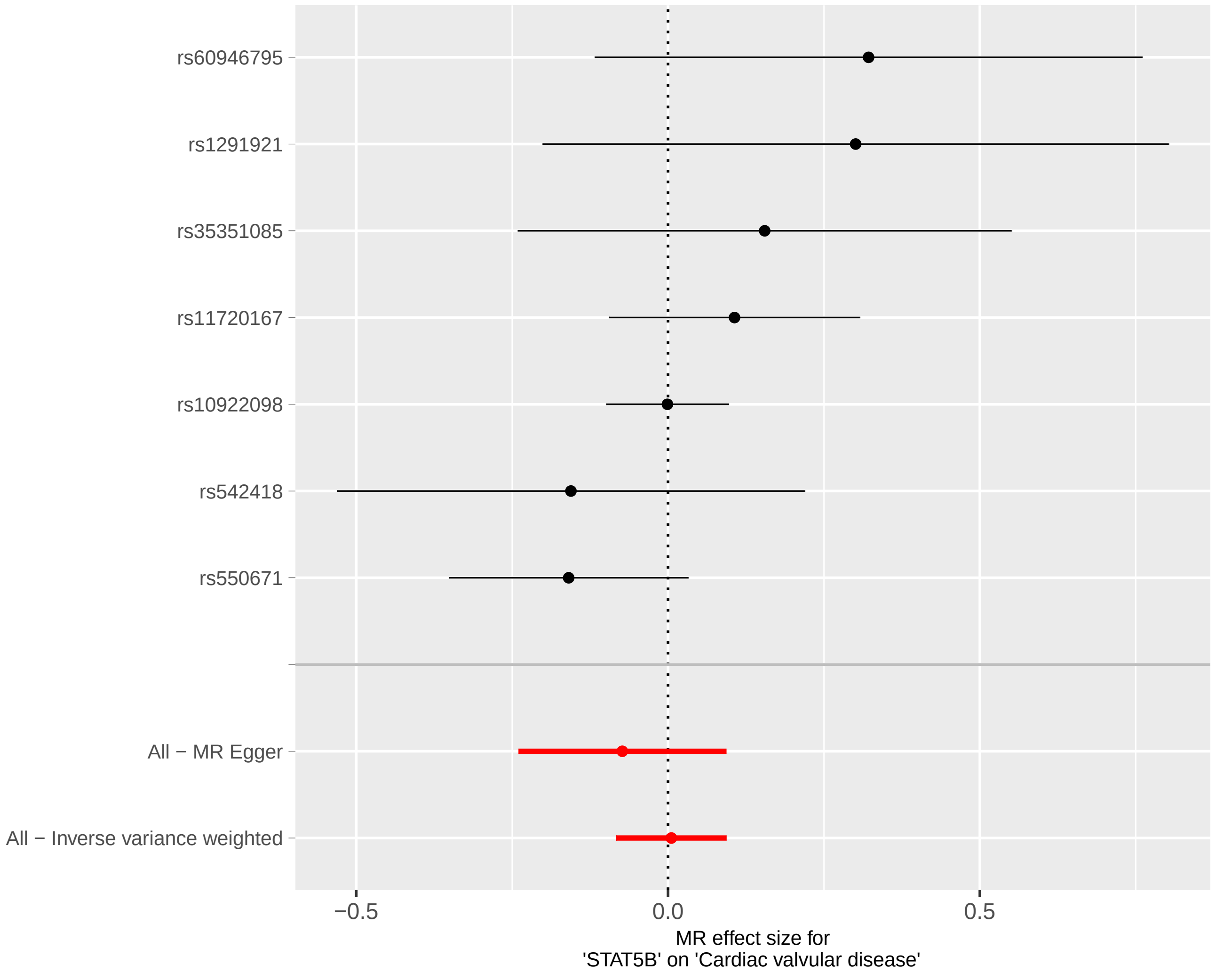

rs60946795
rs1291921
rs35351085
rs11720167
rs10922098
rs542418
rs550671
All − MR Egger
All − Inverse variance weighted
−0.5
0.0
0.5
MR effect size for
'STAT5B' on 'Cardiac valvular disease'

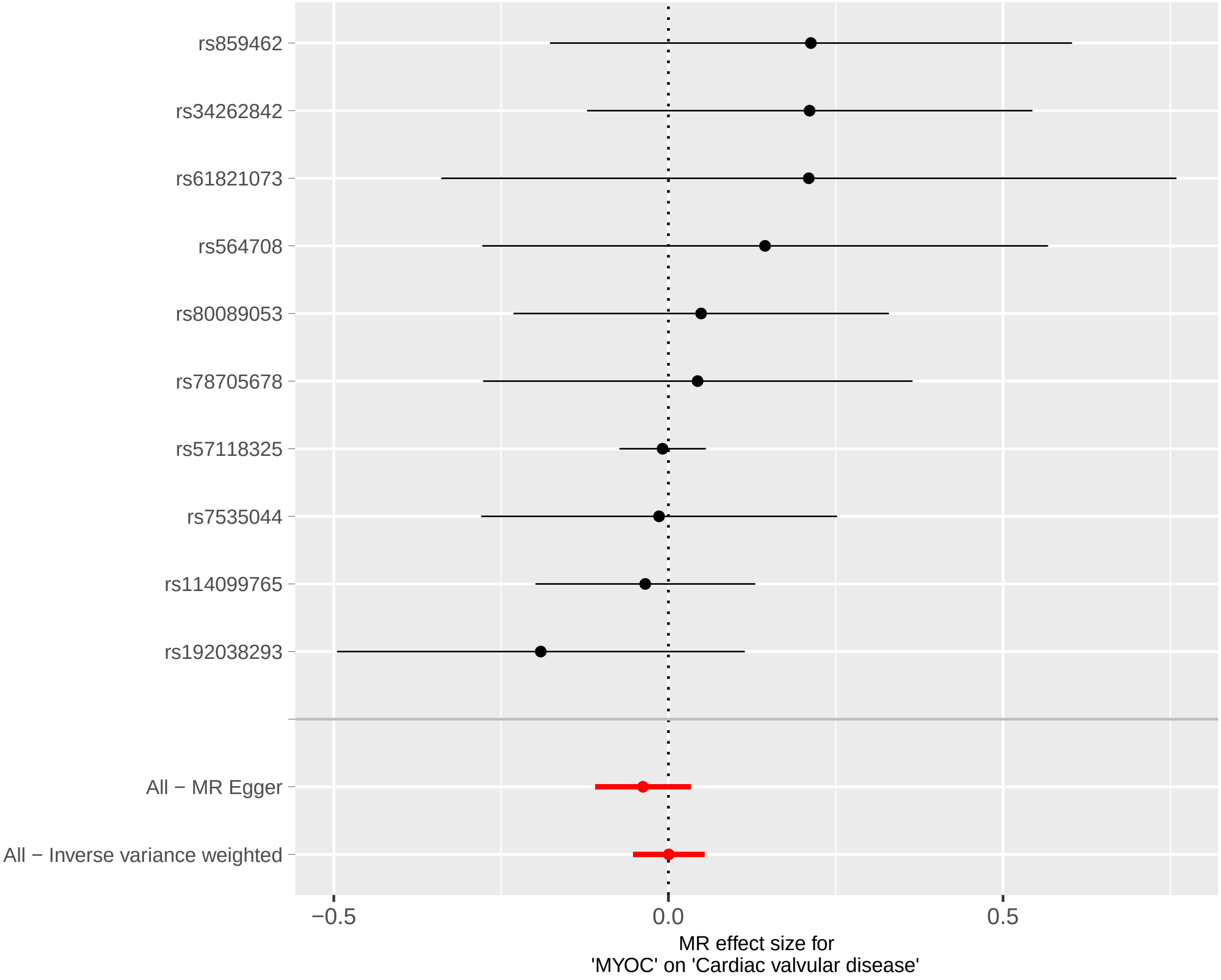
rs859462
rs34262842
rs61821073
rs564708
rs80089053
rs78705678
rs57118325
rs7535044
rs114099765
rs192038293
All − MR Egger
All − Inverse variance weighted
−0.5
0.0
0.5
MR effect size for
'MYOC' on 'Cardiac valvular disease'

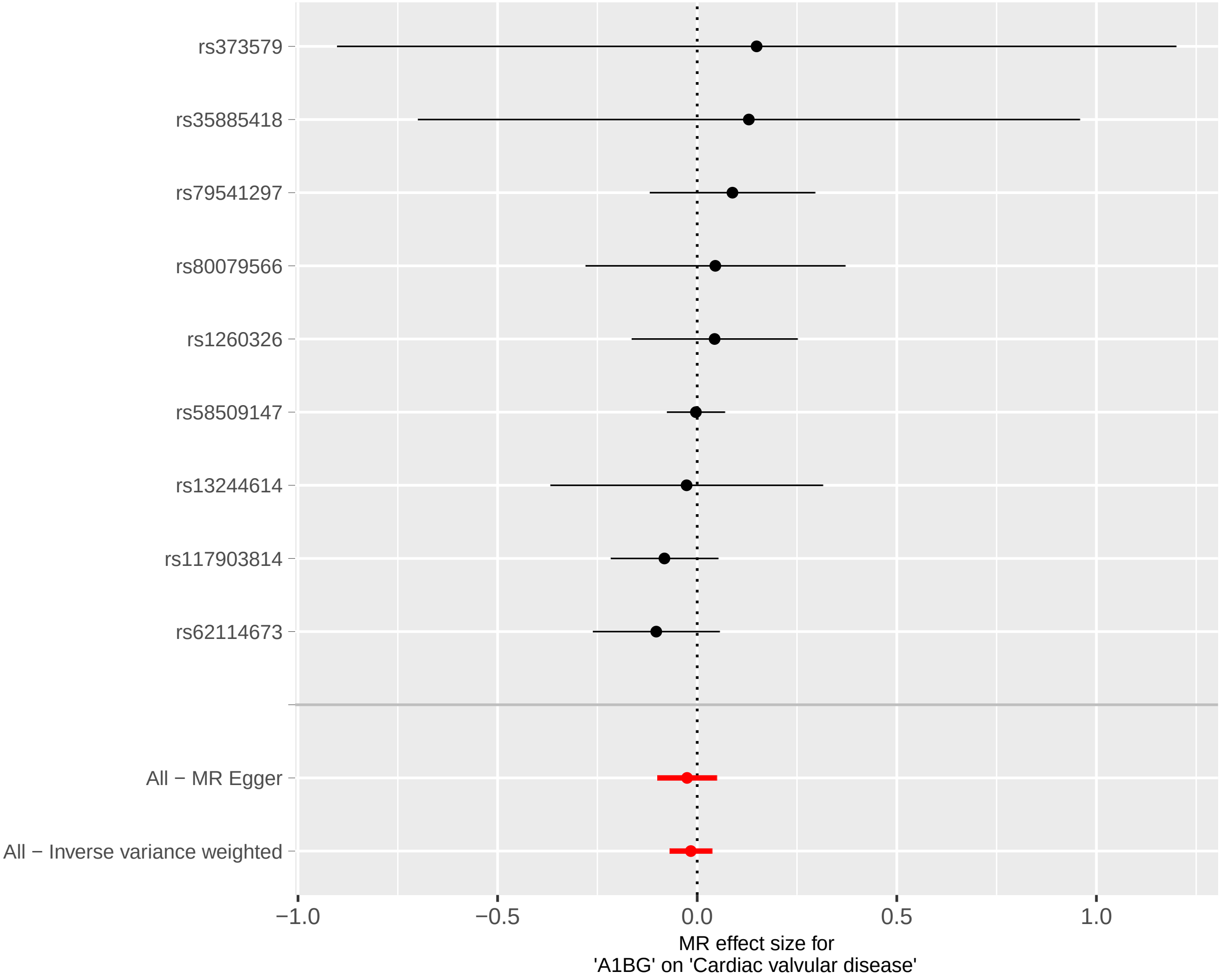
rs373579
rs35885418
rs79541297
rs80079566
rs1260326
rs58509147
rs13244614
rs117903814
rs62114673
All − MR Egger
All − Inverse variance weighted
−1.0
−0.5
0.0
0.5
1.0
MR effect size for
'A1BG' on 'Cardiac valvular disease'

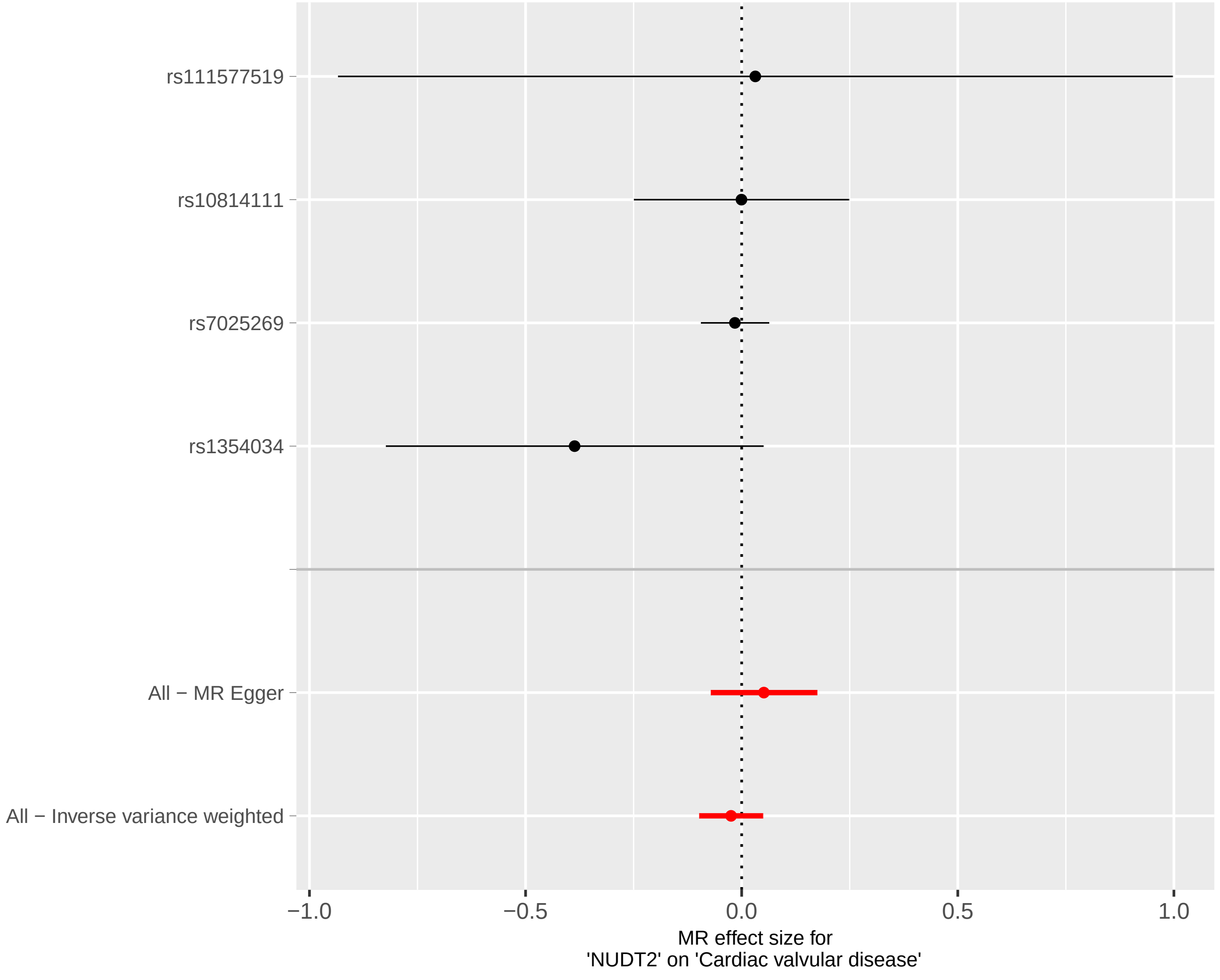
rs111577519
rs10814111
rs7025269
rs1354034
All − MR Egger
All − Inverse variance weighted
−1.0
−0.5
0.0
0.5
1.0
MR effect size for
'NUDT2' on 'Cardiac valvular disease'

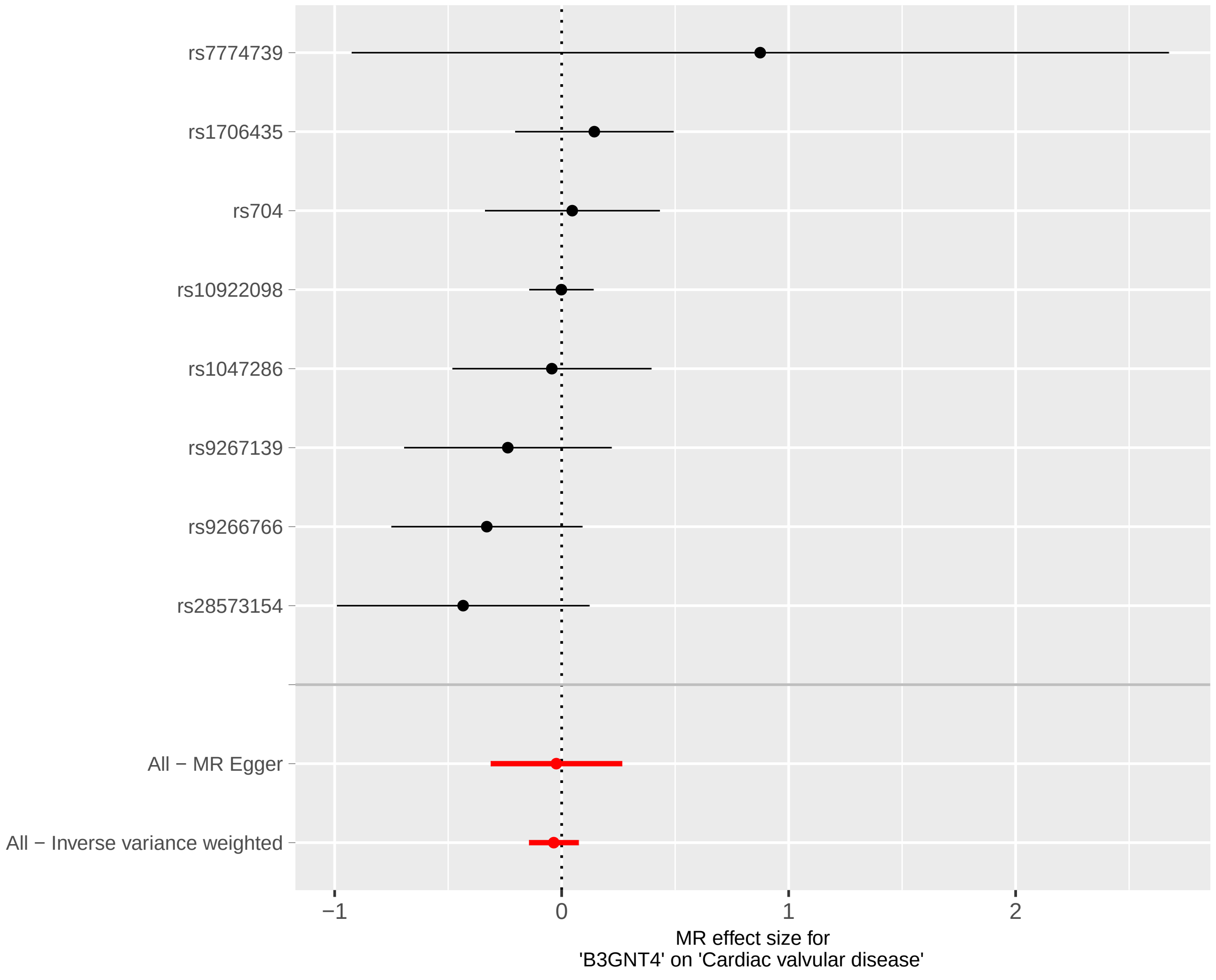
rs7774739
rs1706435
rs704
rs10922098
rs1047286
rs9267139
rs9266766
rs28573154
All − MR Egger
All − Inverse variance weighted
−1
0
1
2
MR effect size for
'B3GNT4' on 'Cardiac valvular disease'

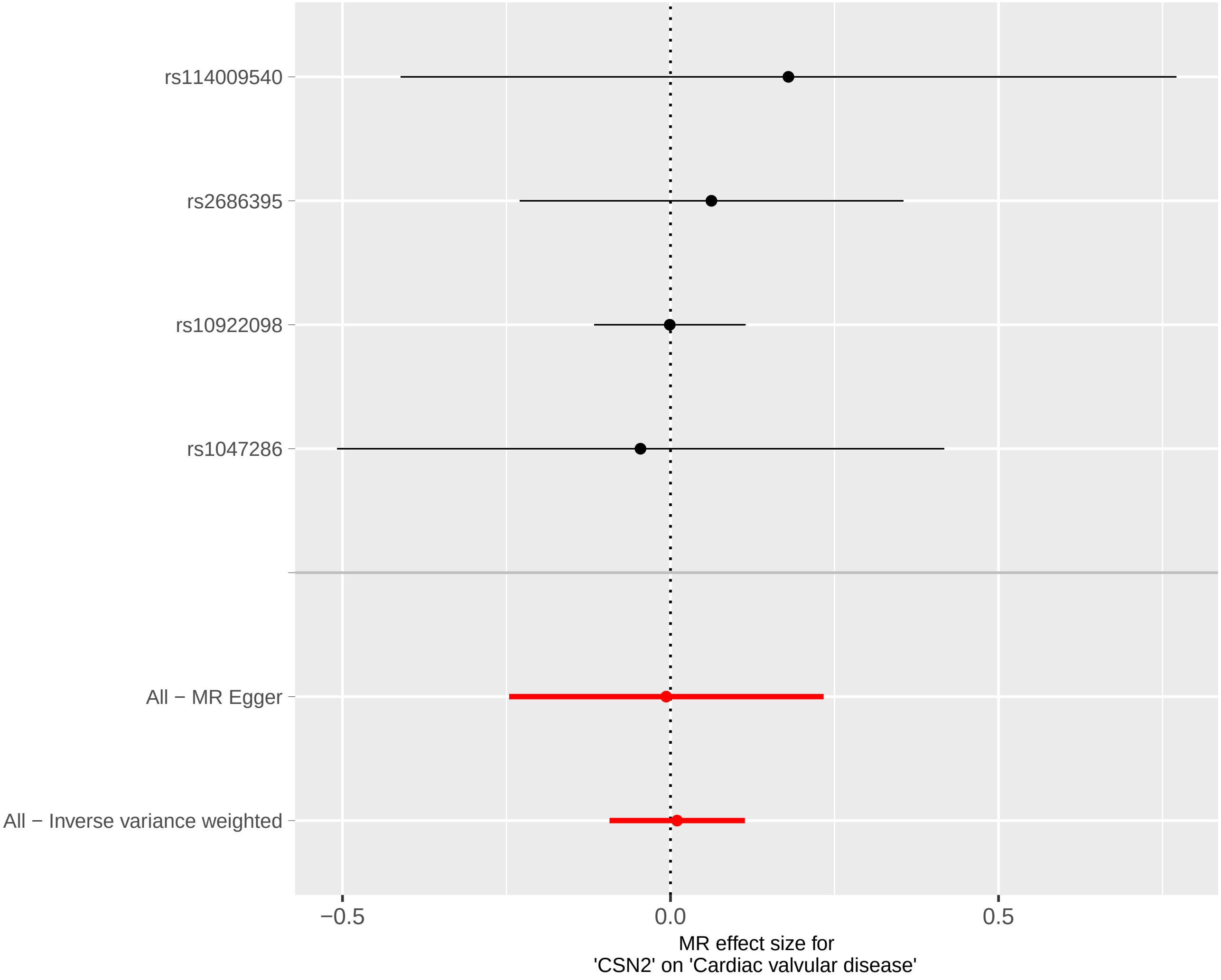
rs114009540
rs2686395
rs10922098
rs1047286
All − MR Egger
All − Inverse variance weighted
−0.5
0.0
0.5
MR effect size for
'CSN2' on 'Cardiac valvular disease'

Insufficient number of SNPs

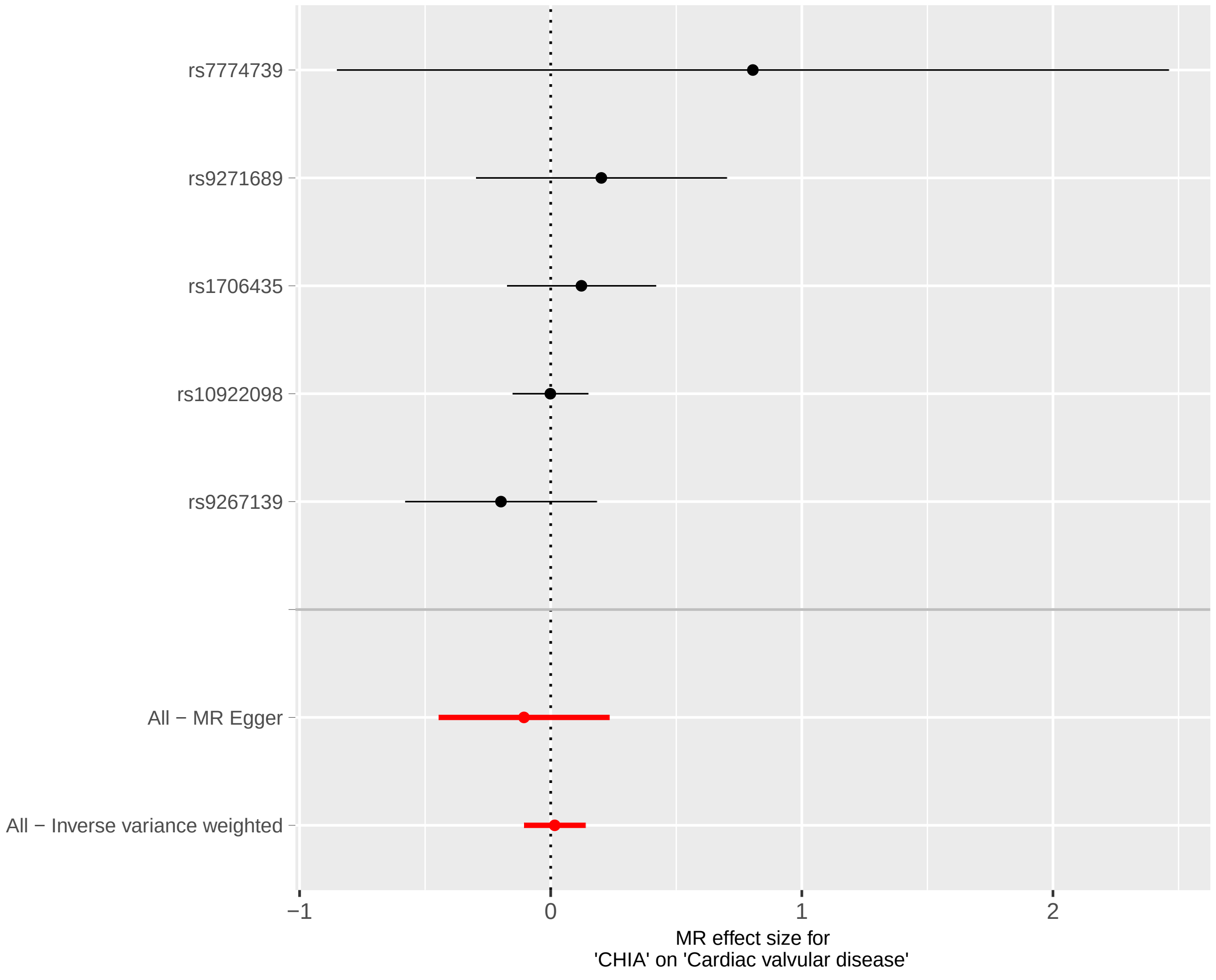
rs7774739
rs9271689
rs1706435
rs10922098
rs9267139
All − MR Egger
All − Inverse variance weighted
−1
0
1
2
MR effect size for
'CHIA' on 'Cardiac valvular disease'

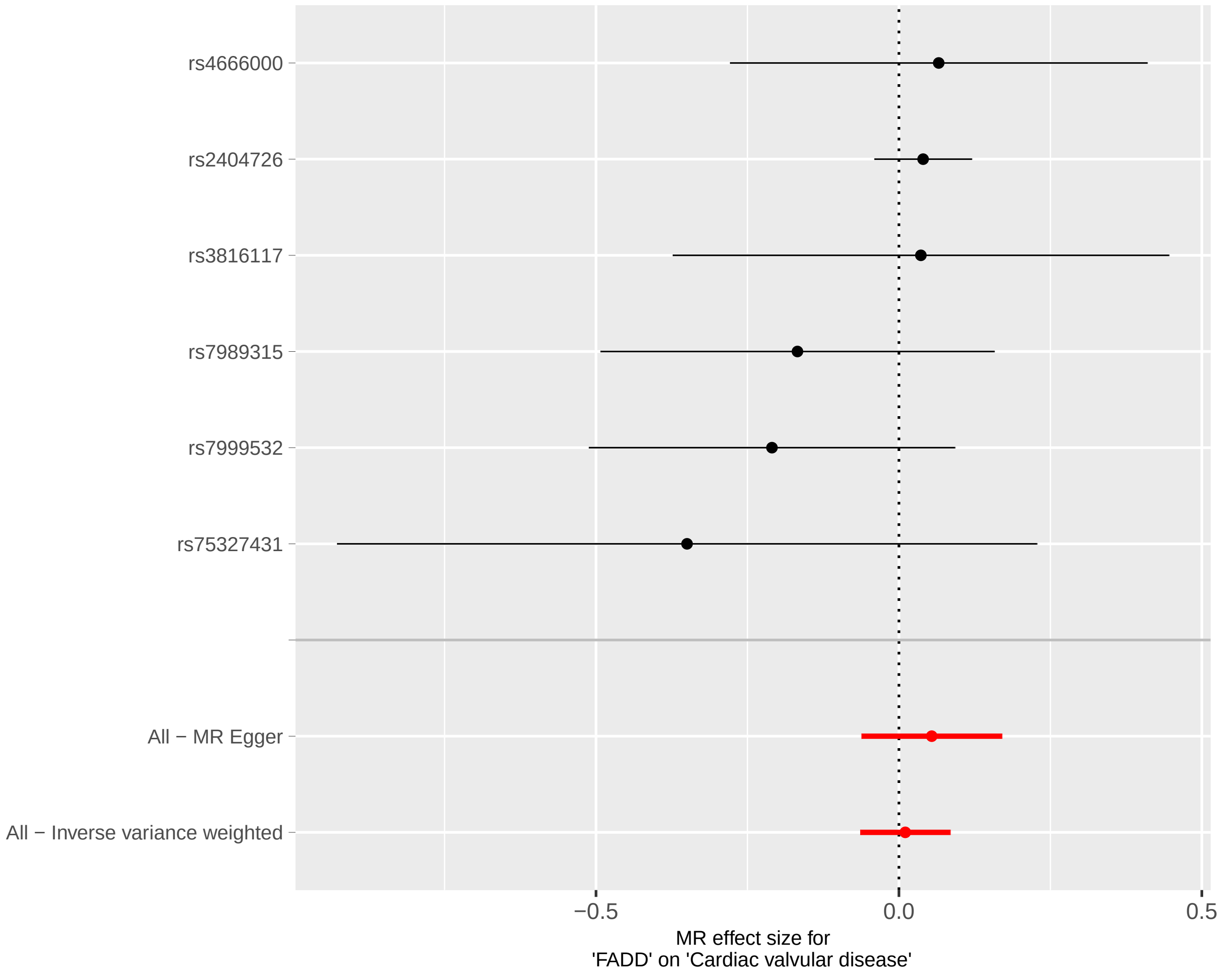

rs4666000
rs2404726
rs3816117
rs7989315
rs7999532
rs75327431
All − MR Egger
All − Inverse variance weighted
−0.5
0.0
0.5
MR effect size for
'FADD' on 'Cardiac valvular disease'

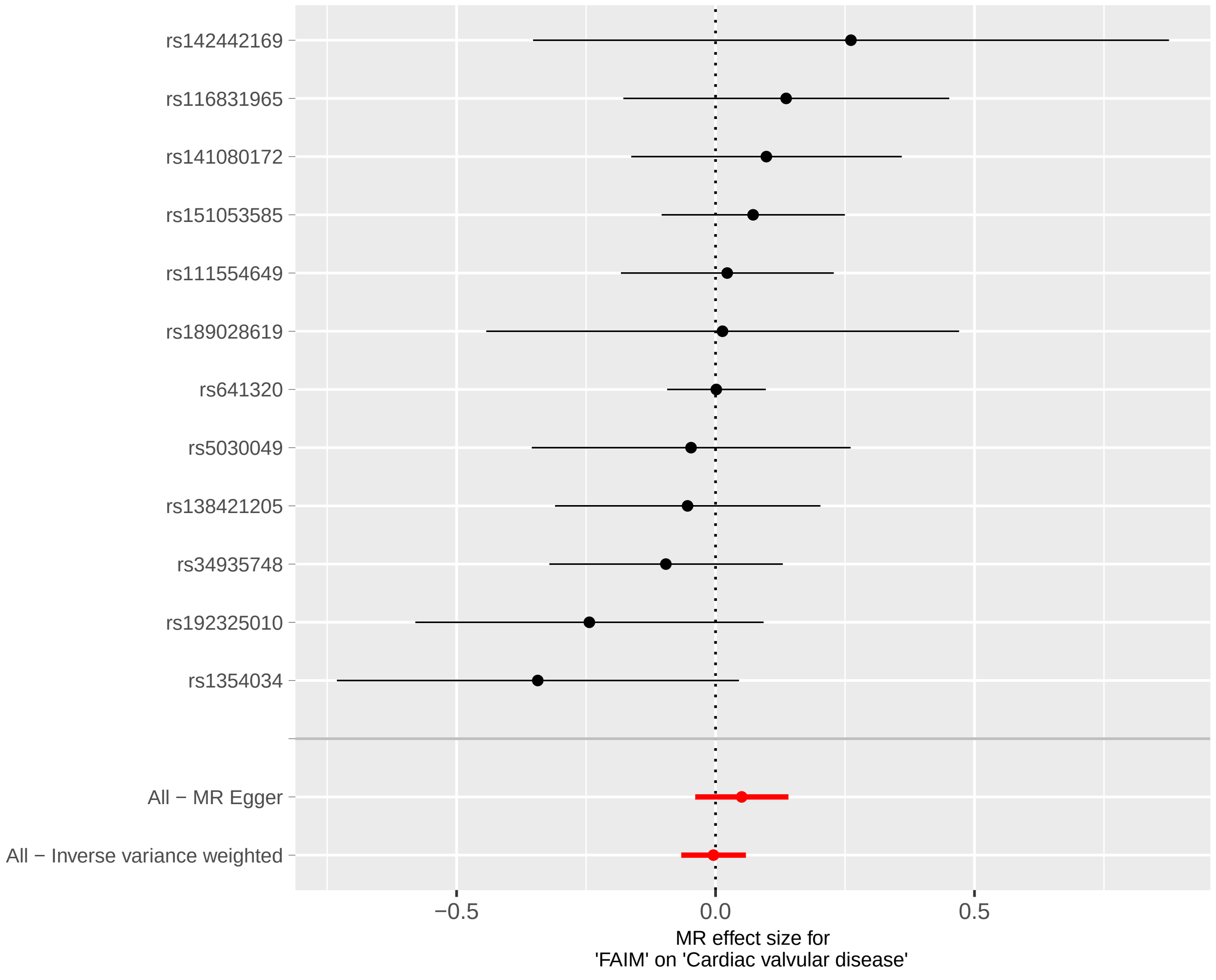
rs142442169
rs116831965
rs141080172
rs151053585
rs111554649
rs189028619
rs641320
rs5030049
rs138421205
rs34935748
rs192325010
rs1354034
All − MR Egger
All − Inverse variance weighted
−0.5
0.0
0.5
MR effect size for
'FAIM' on 'Cardiac valvular disease'

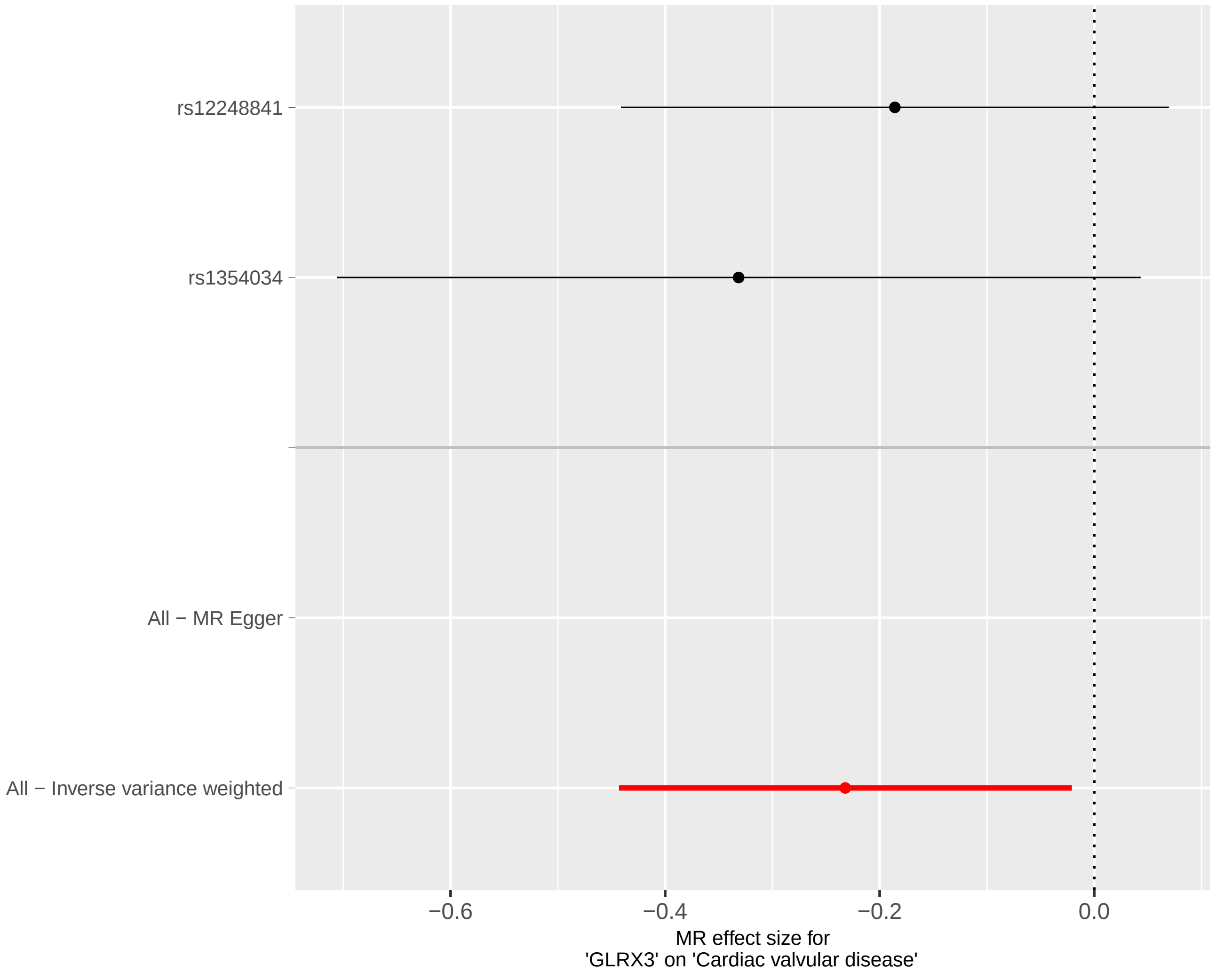
rs12248841
rs1354034
All − MR Egger
All − Inverse variance weighted
−0.6
−0.4
−0.2
0.0
MR effect size for
'GLRX3' on 'Cardiac valvular disease'

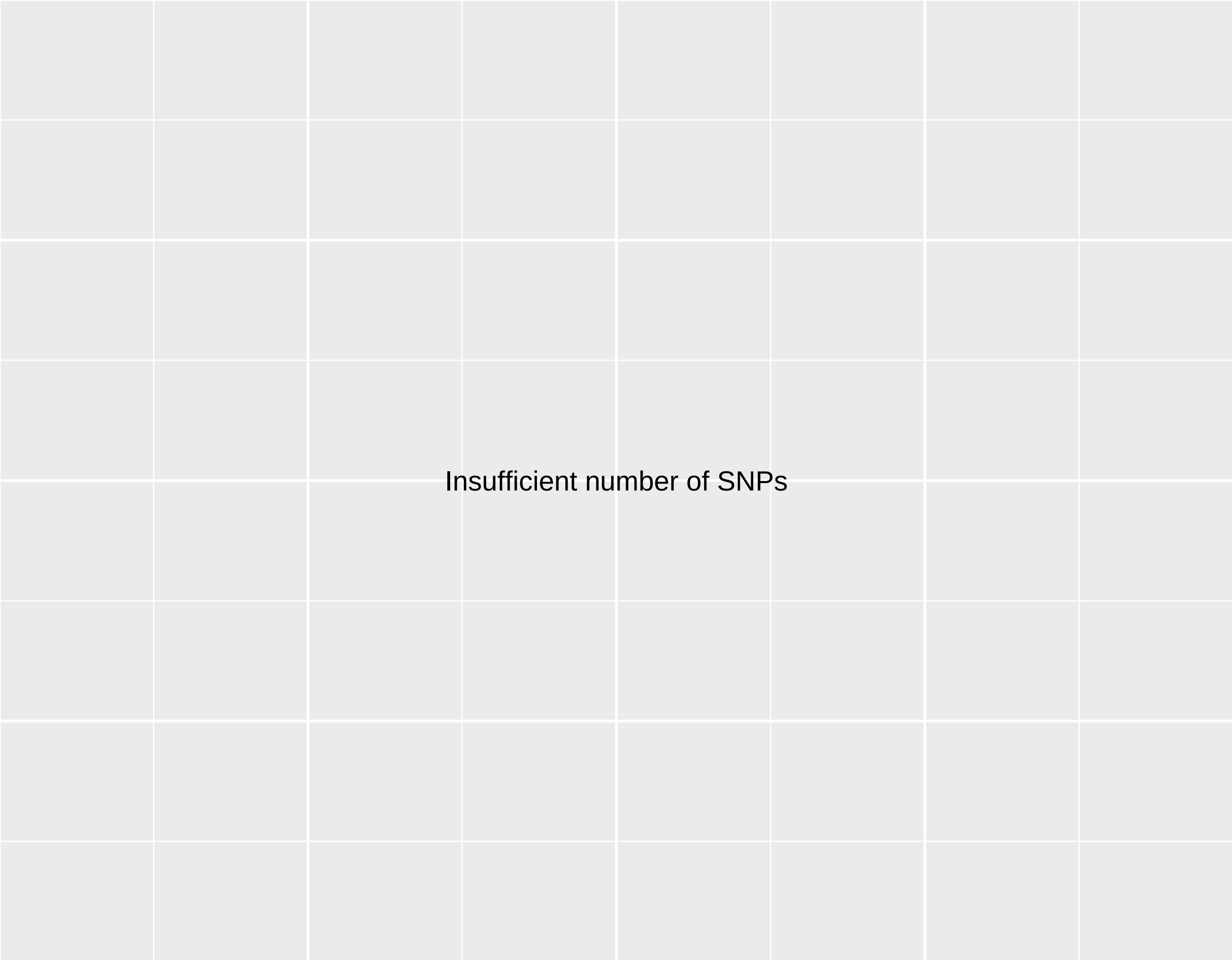
Insufficient number of SNPs

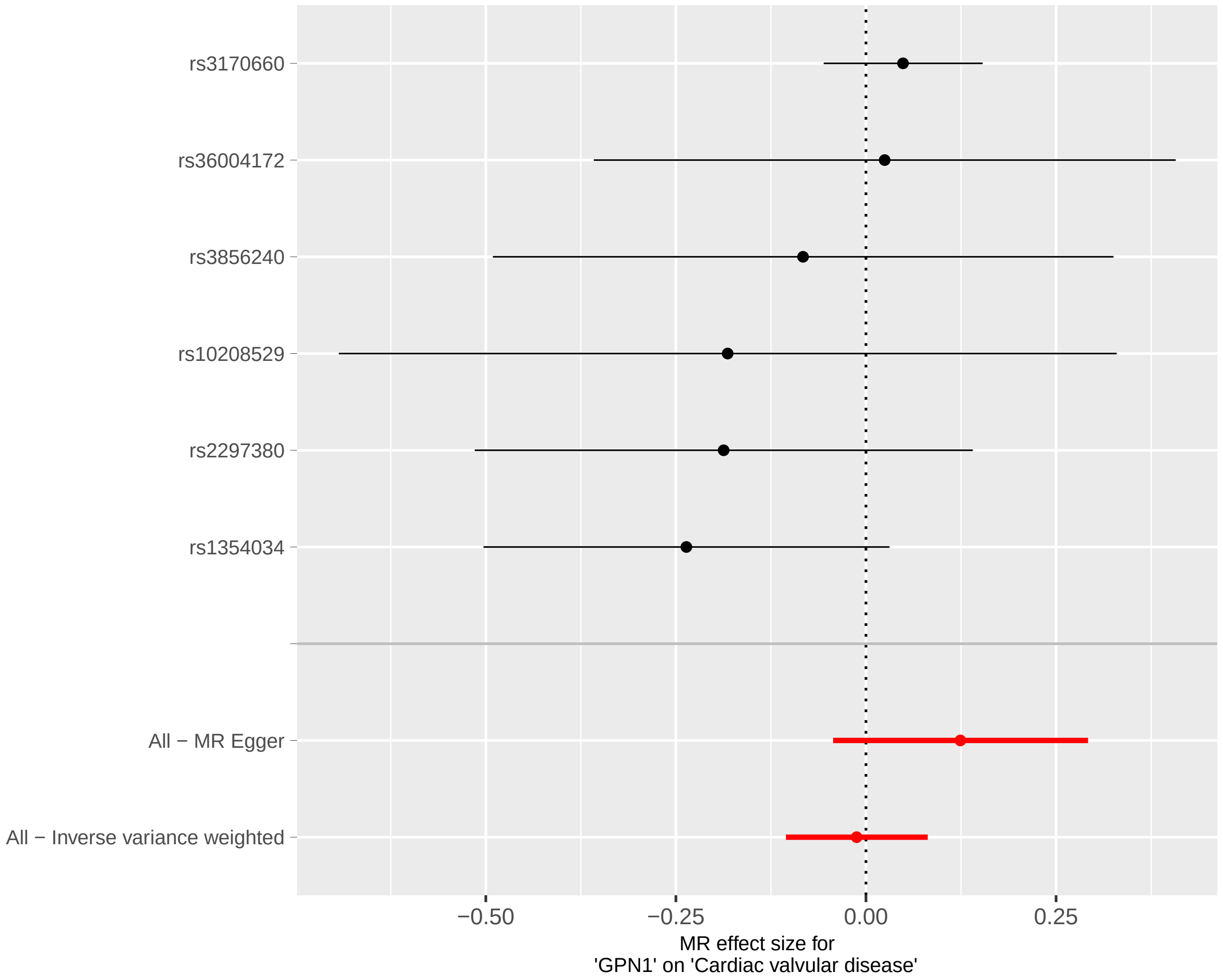
rs3170660
rs36004172
rs3856240
rs10208529
rs2297380
rs1354034
All − MR Egger
All − Inverse variance weighted
−0.50
−0.25
0.00
0.25
MR effect size for
'GPN1' on 'Cardiac valvular disease'

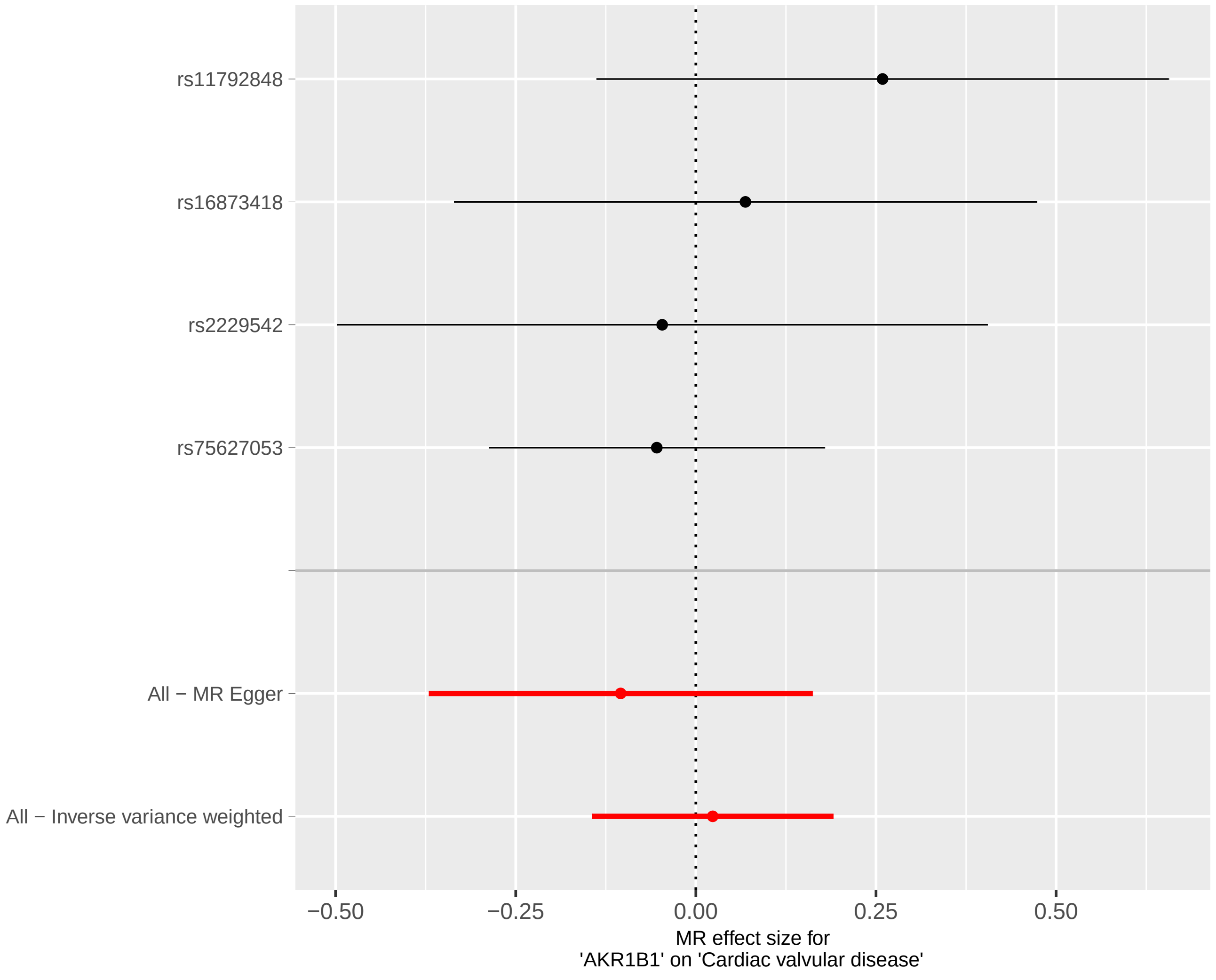
rs11792848
rs16873418
rs2229542
rs75627053
All − MR Egger
All − Inverse variance weighted
−0.50
−0.25
0.00
0.25
0.50
MR effect size for
'AKR1B1' on 'Cardiac valvular disease'

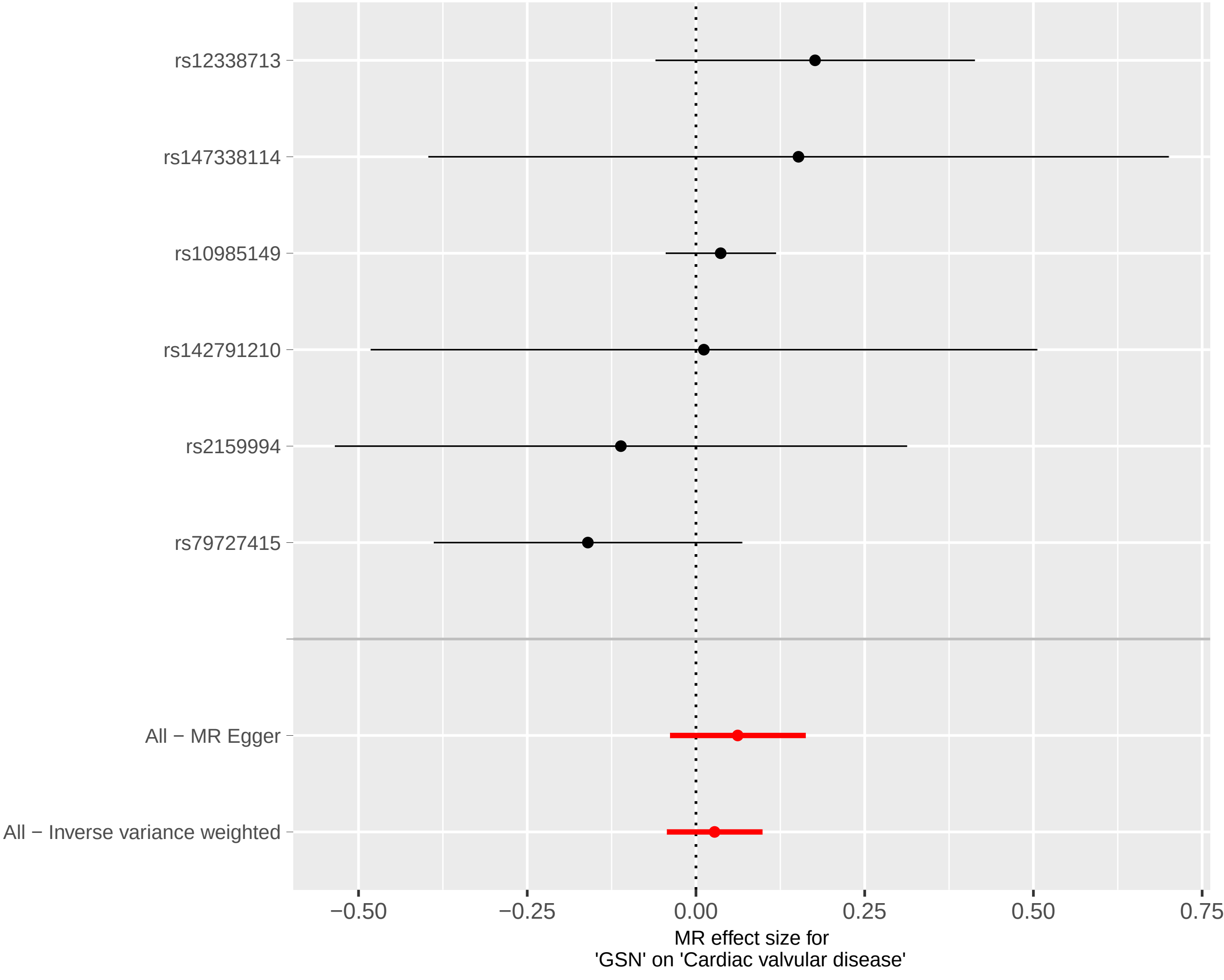
rs12338713
rs147338114
rs10985149
rs142791210
rs2159994
rs79727415
All − MR Egger
All − Inverse variance weighted
−0.50
−0.25
0.00
0.25
0.50
0.75
MR effect size for
'GSN' on 'Cardiac valvular disease'

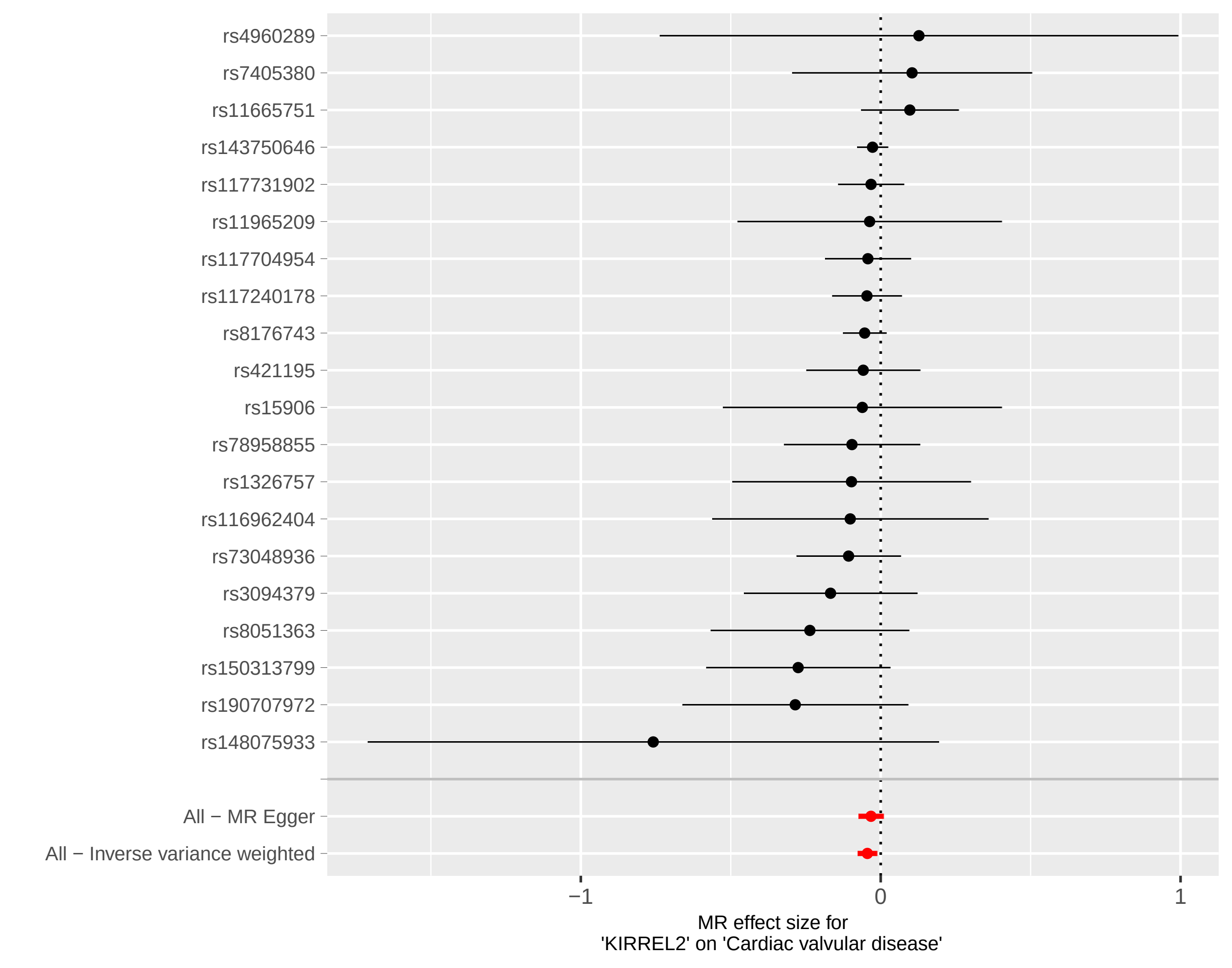
rs4960289
rs7405380
rs11665751
rs143750646
rs117731902
rs11965209
rs117704954
rs117240178
rs8176743
rs421195
rs15906
rs78958855
rs1326757
rs116962404
rs73048936
rs3094379
rs8051363
rs150313799
rs190707972
rs148075933
All − MR Egger
All − Inverse variance weighted
−1
0
1
MR effect size for
'KIRREL2' on 'Cardiac valvular disease'

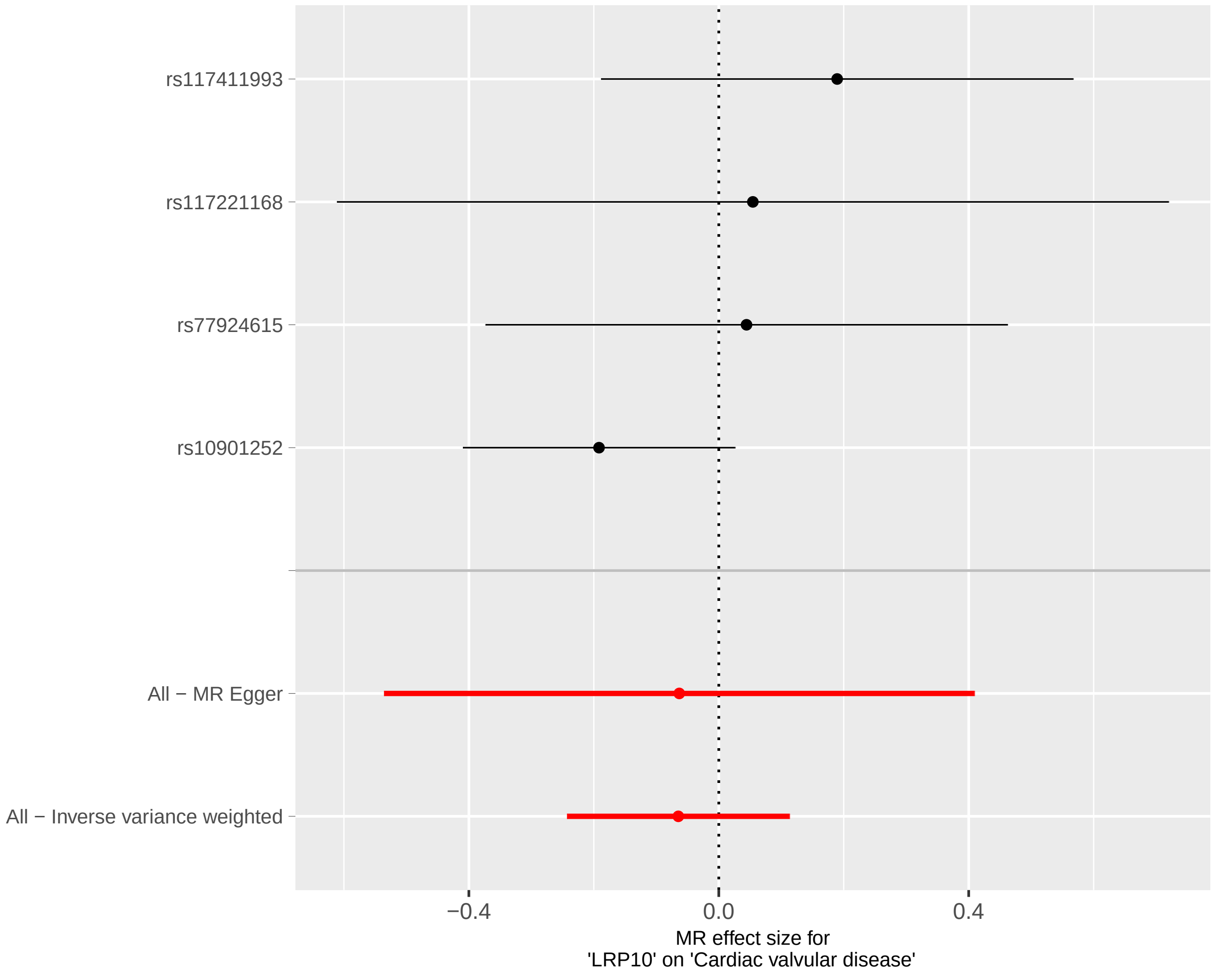
rs117411993
rs117221168
rs77924615
rs10901252
All − MR Egger
All − Inverse variance weighted
−0.4
0.0
0.4
MR effect size for
'LRP10' on 'Cardiac valvular disease'

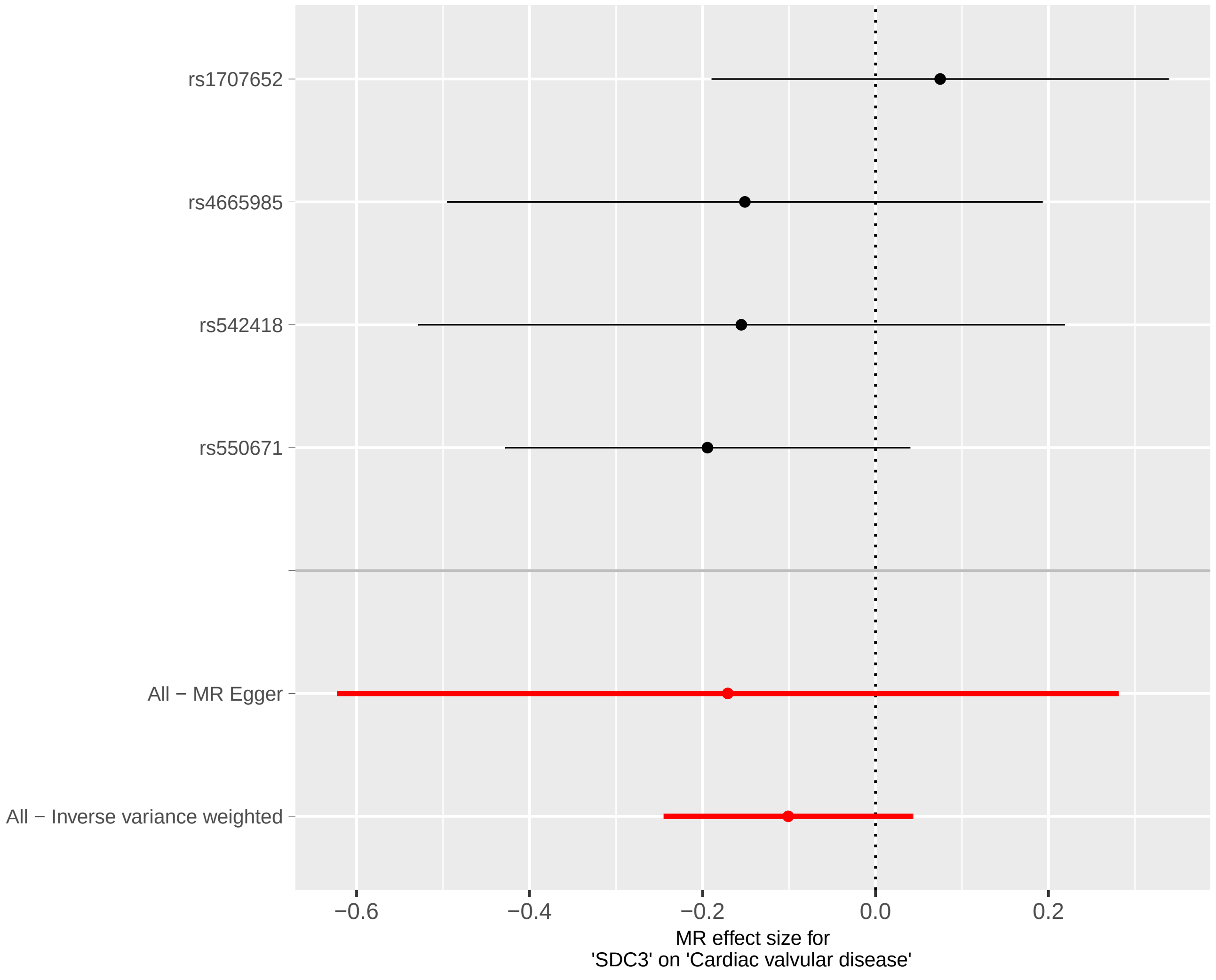

rs1707652
rs4665985
rs542418
rs550671
All − MR Egger
All − Inverse variance weighted
−0.6
−0.4
−0.2
0.0
0.2
MR effect size for
'SDC3' on 'Cardiac valvular disease'

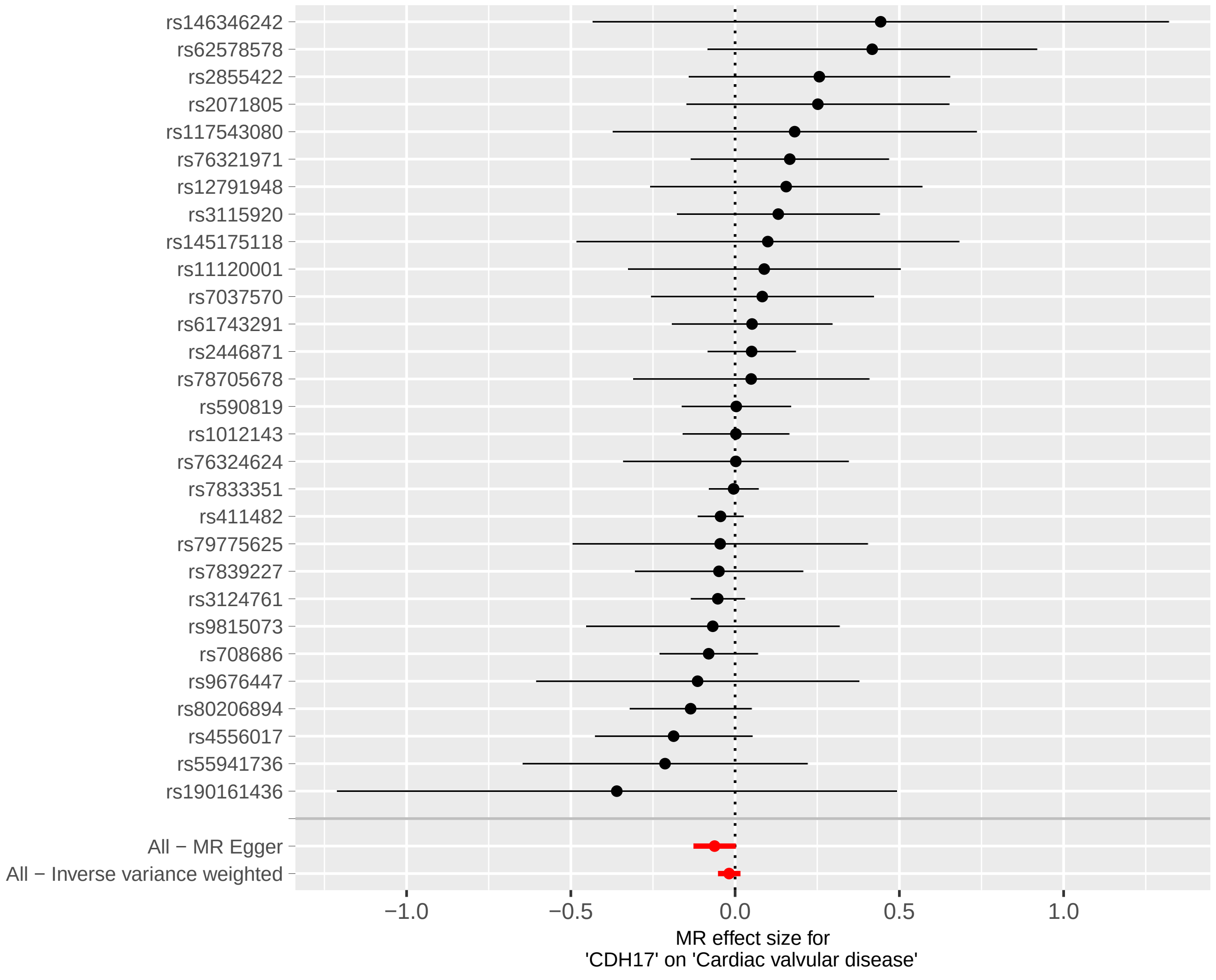
rs146346242
rs62578578
rs2855422
rs2071805
rs117543080
rs76321971
rs12791948
rs3115920
rs145175118
rs11120001
rs7037570
rs61743291
rs2446871
rs78705678
rs590819
rs1012143
rs76324624
rs7833351
rs411482
rs79775625
rs7839227
rs3124761
rs9815073
rs708686
rs9676447
rs80206894
rs4556017
rs55941736
rs190161436
All − MR Egger
All − Inverse variance weighted
−1.0
−0.5
0.0
0.5
1.0
MR effect size for
'CDH17' on 'Cardiac valvular disease'

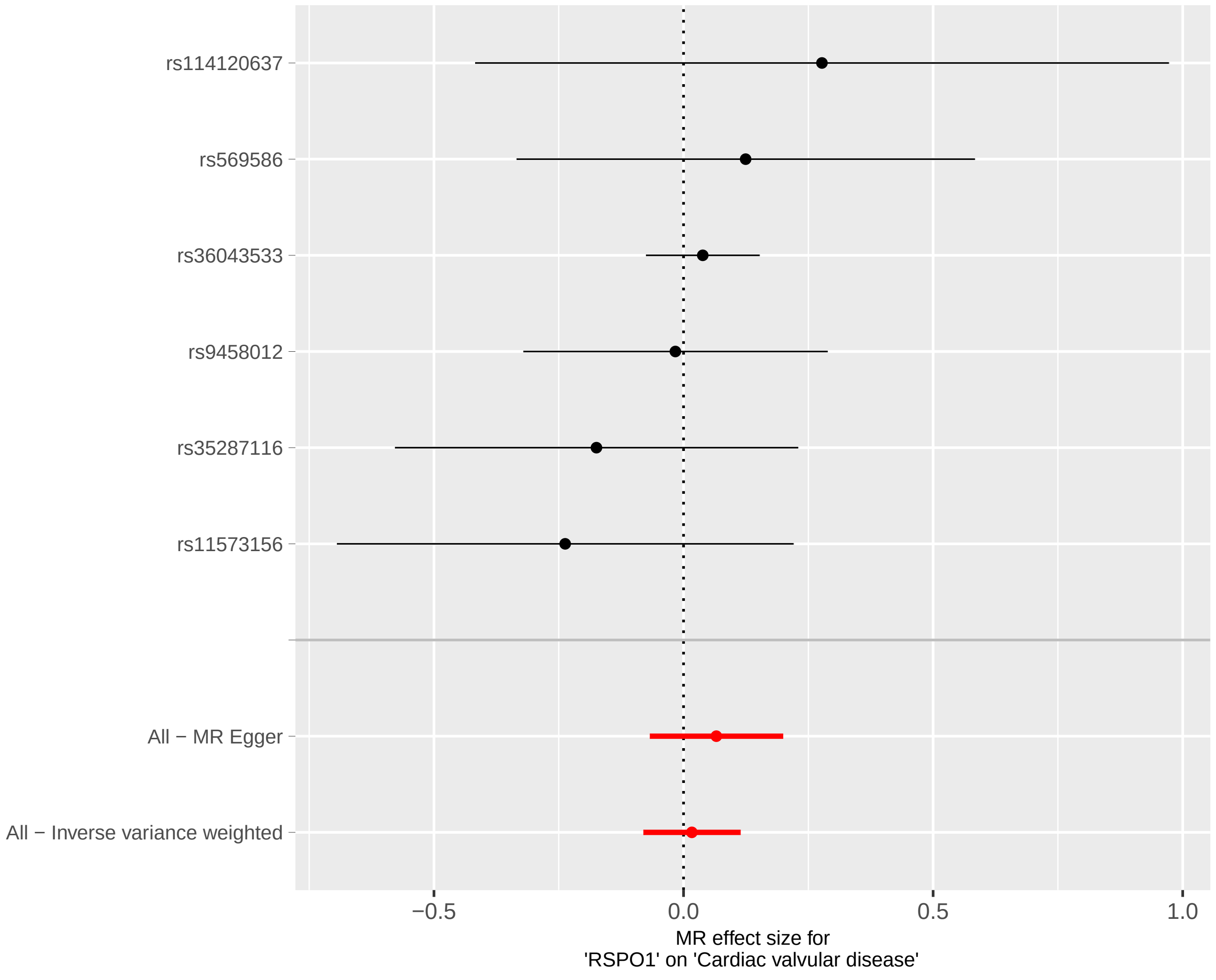
rs114120637
rs569586
rs36043533
rs9458012
rs35287116
rs11573156
All − MR Egger
All − Inverse variance weighted
−0.5
0.0
0.5
1.0
MR effect size for
'RSPO1' on 'Cardiac valvular disease'

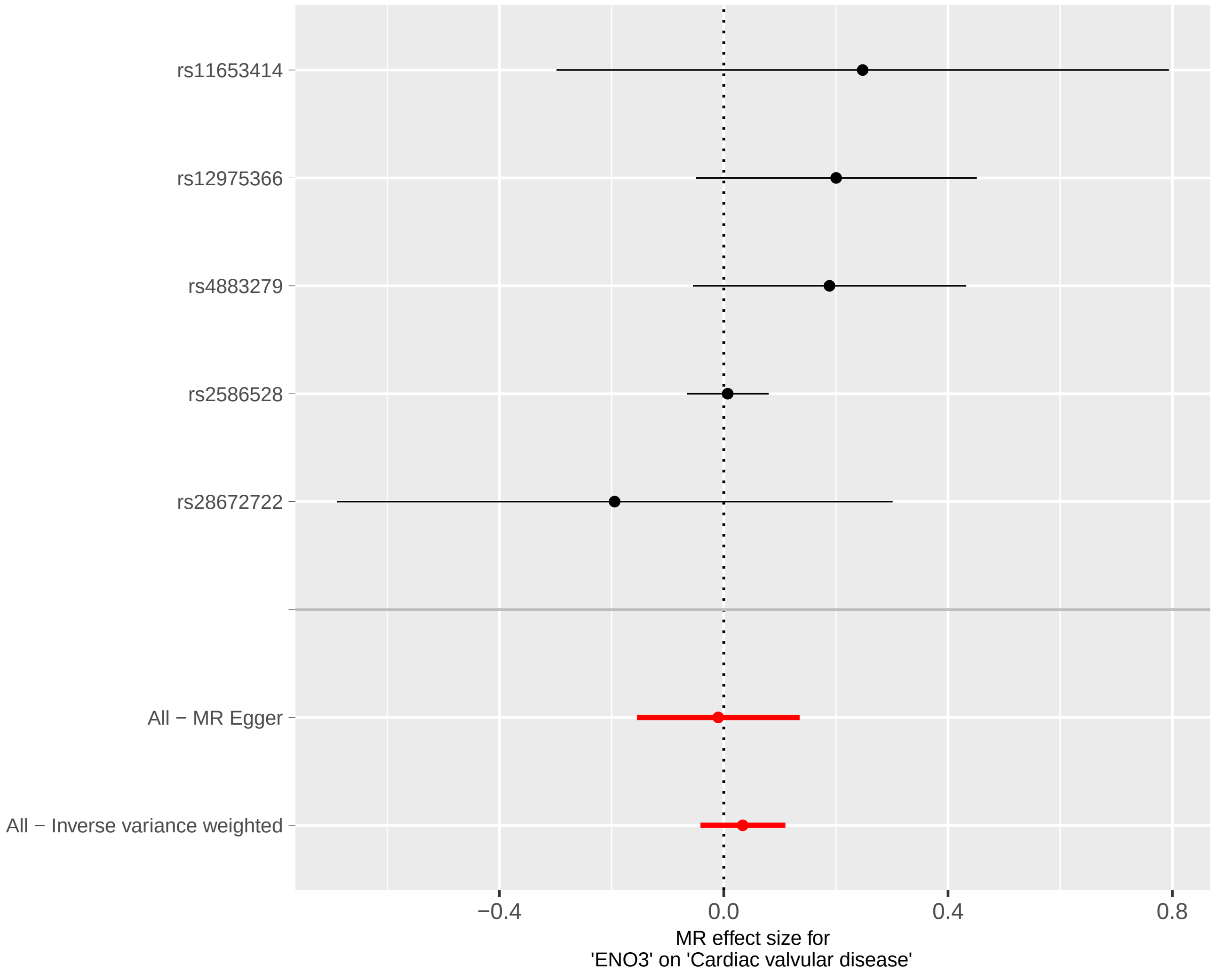
rs11653414
rs12975366
rs4883279
rs2586528
rs28672722
All − MR Egger
All − Inverse variance weighted
−0.4
0.0
0.4
0.8
MR effect size for
'ENO3' on 'Cardiac valvular disease'

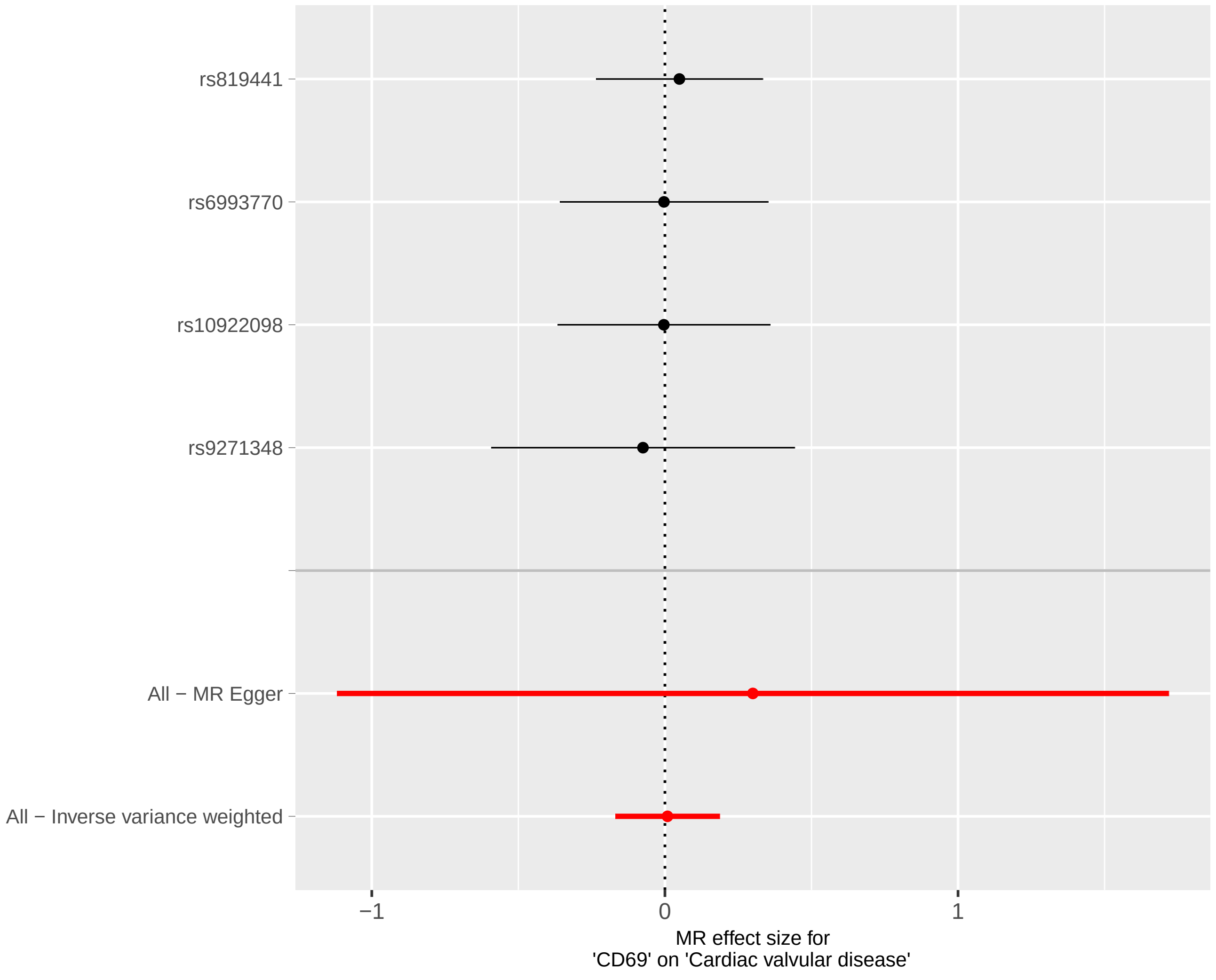

rs819441
rs6993770
rs10922098
rs9271348
All − MR Egger
All − Inverse variance weighted
−1
0
1
MR effect size for
'CD69' on 'Cardiac valvular disease'

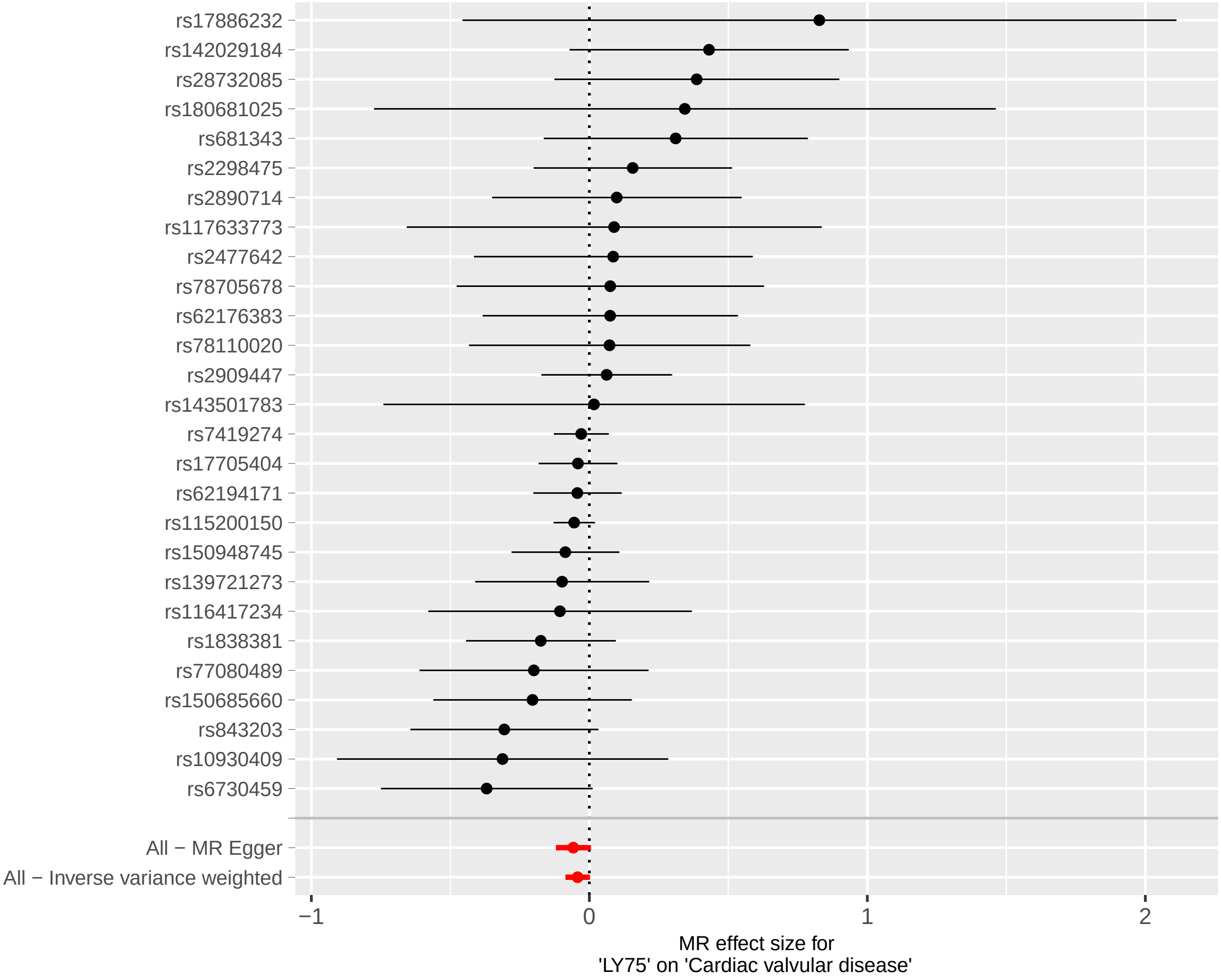

rs17886232
rs142029184
rs28732085
rs180681025
rs681343
rs2298475
rs2890714
rs117633773
rs2477642
rs78705678
rs62176383
rs78110020
rs2909447
rs143501783
rs7419274
rs17705404
rs62194171
rs115200150
rs150948745
rs139721273
rs116417234
rs1838381
rs77080489
rs150685660
rs843203
rs10930409
rs6730459
All − MR Egger
All − Inverse variance weighted
−1
0
1
2
MR effect size for
'LY75' on 'Cardiac valvular disease'

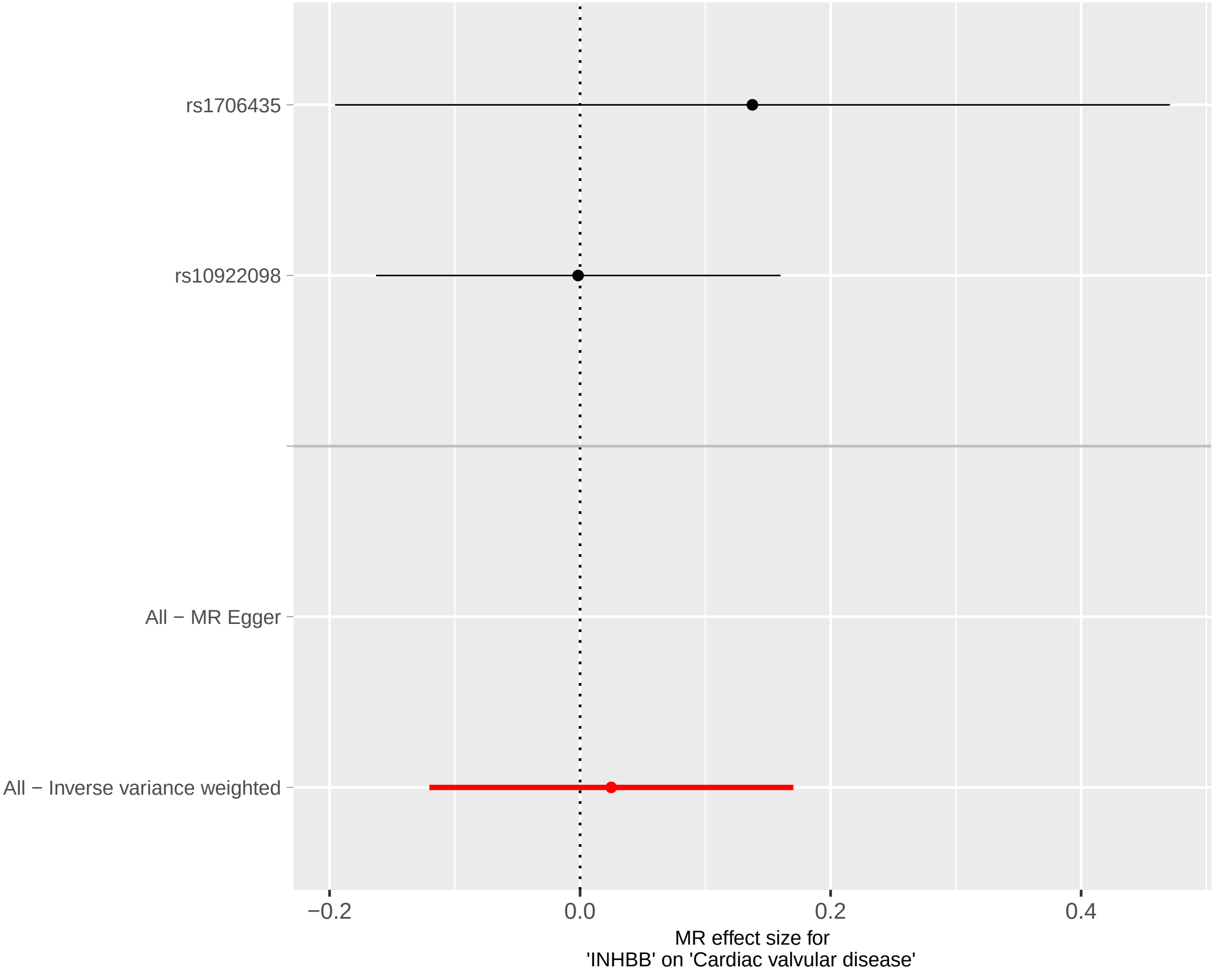
rs1706435
rs10922098
All − MR Egger
All − Inverse variance weighted
−0.2
0.0
0.2
0.4
MR effect size for
'INHBB' on 'Cardiac valvular disease'

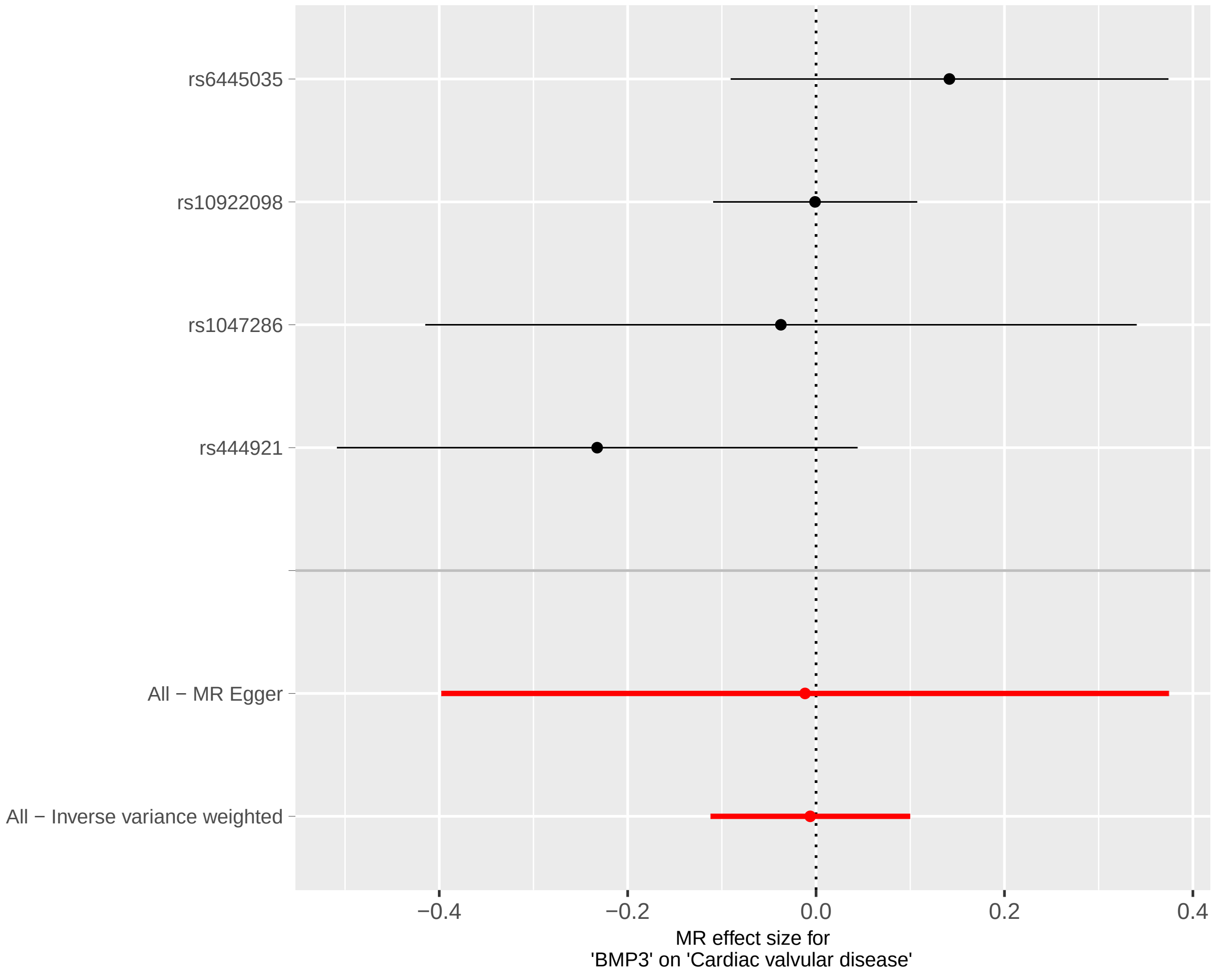
rs6445035
rs10922098
rs1047286
rs444921
All − MR Egger
All − Inverse variance weighted
−0.4
−0.2
0.0
0.2
0.4
MR effect size for
'BMP3' on 'Cardiac valvular disease'

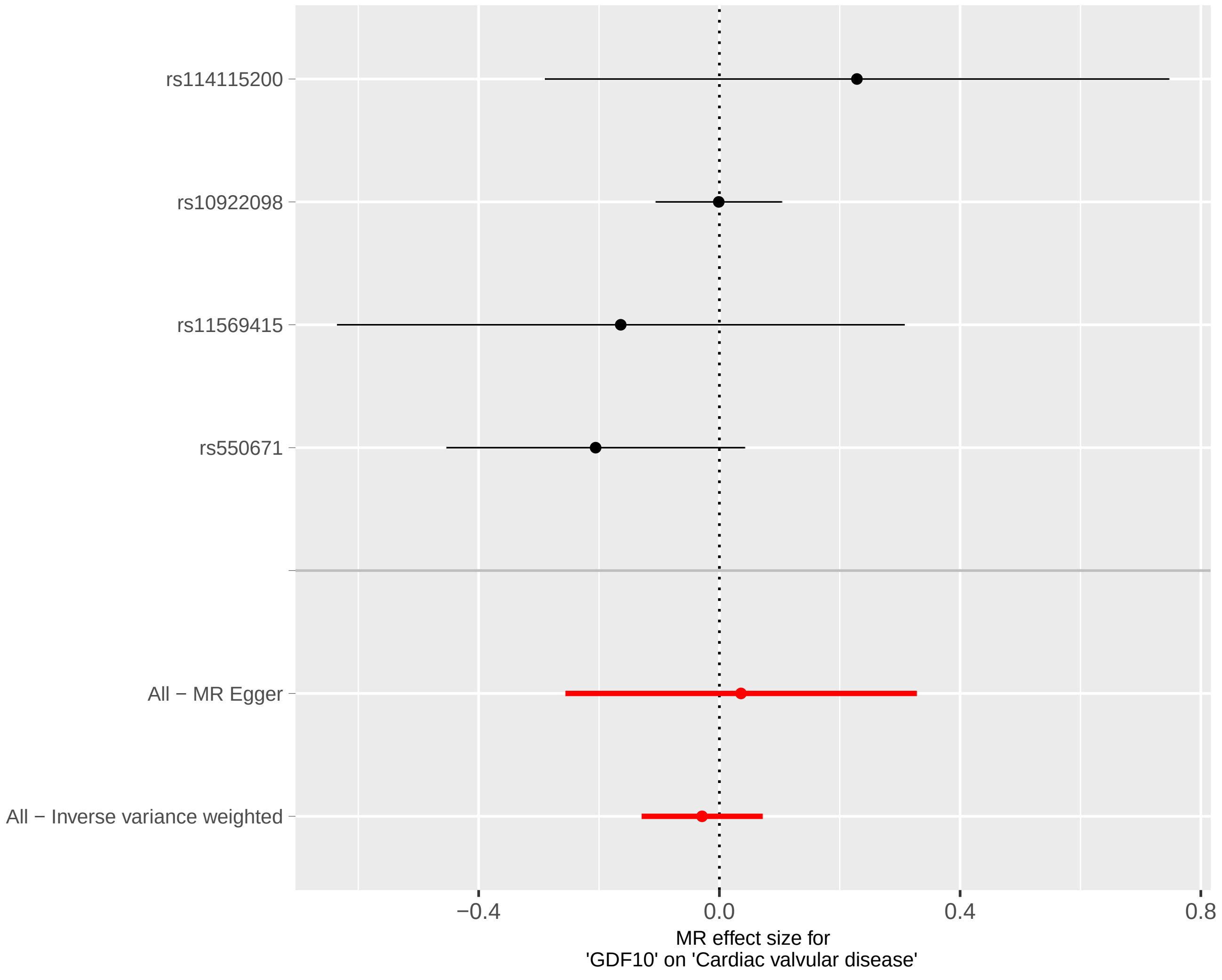

rs114115200
rs10922098
rs11569415
rs550671
All − MR Egger
All − Inverse variance weighted
−0.4
0.0
0.4
0.8
MR effect size for
'GDF10' on 'Cardiac valvular disease'

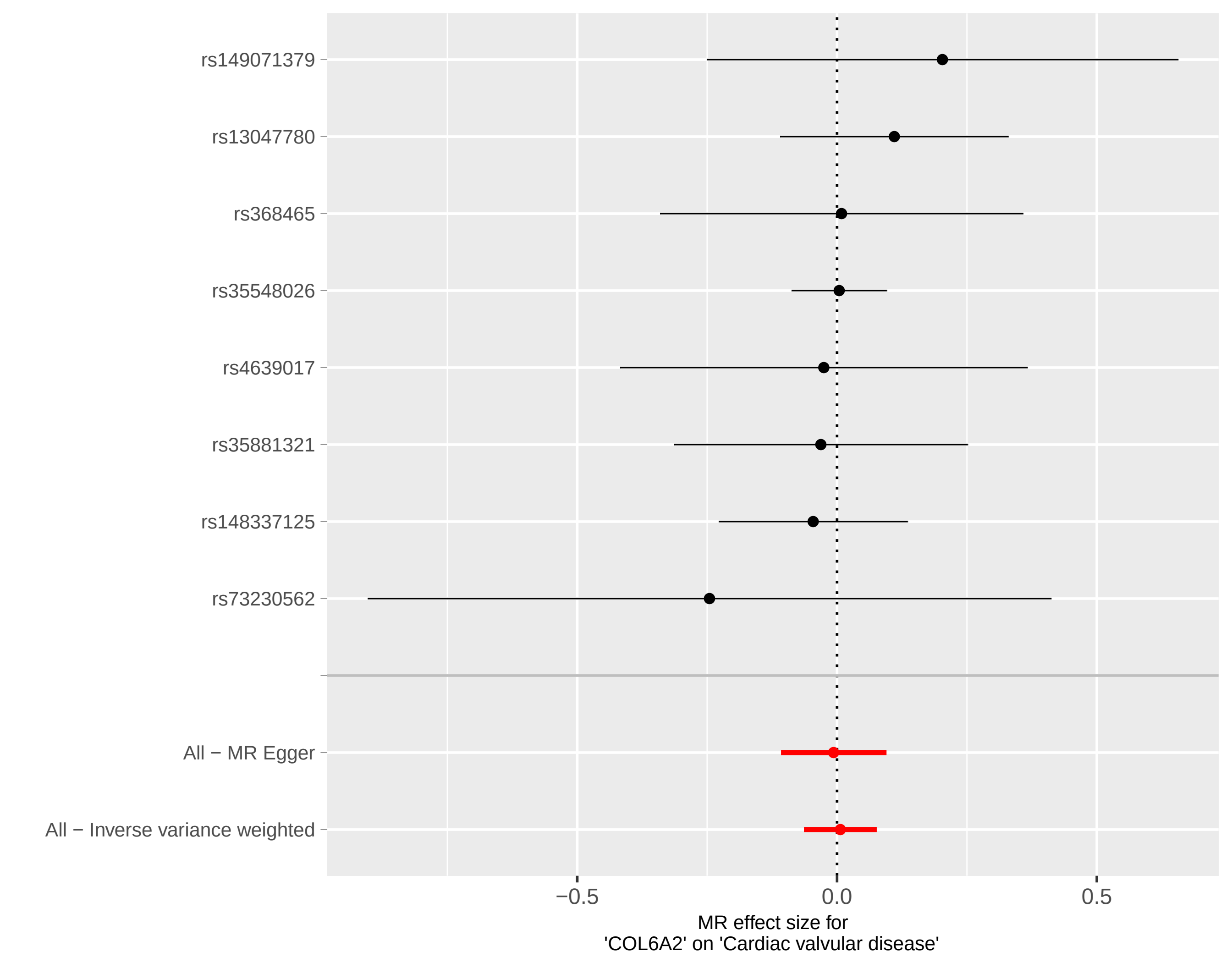

rs149071379
rs13047780
rs368465
rs35548026
rs4639017
rs35881321
rs148337125
rs73230562
All − MR Egger
All − Inverse variance weighted
−0.5
0.0
0.5
MR effect size for
'COL6A2' on 'Cardiac valvular disease'

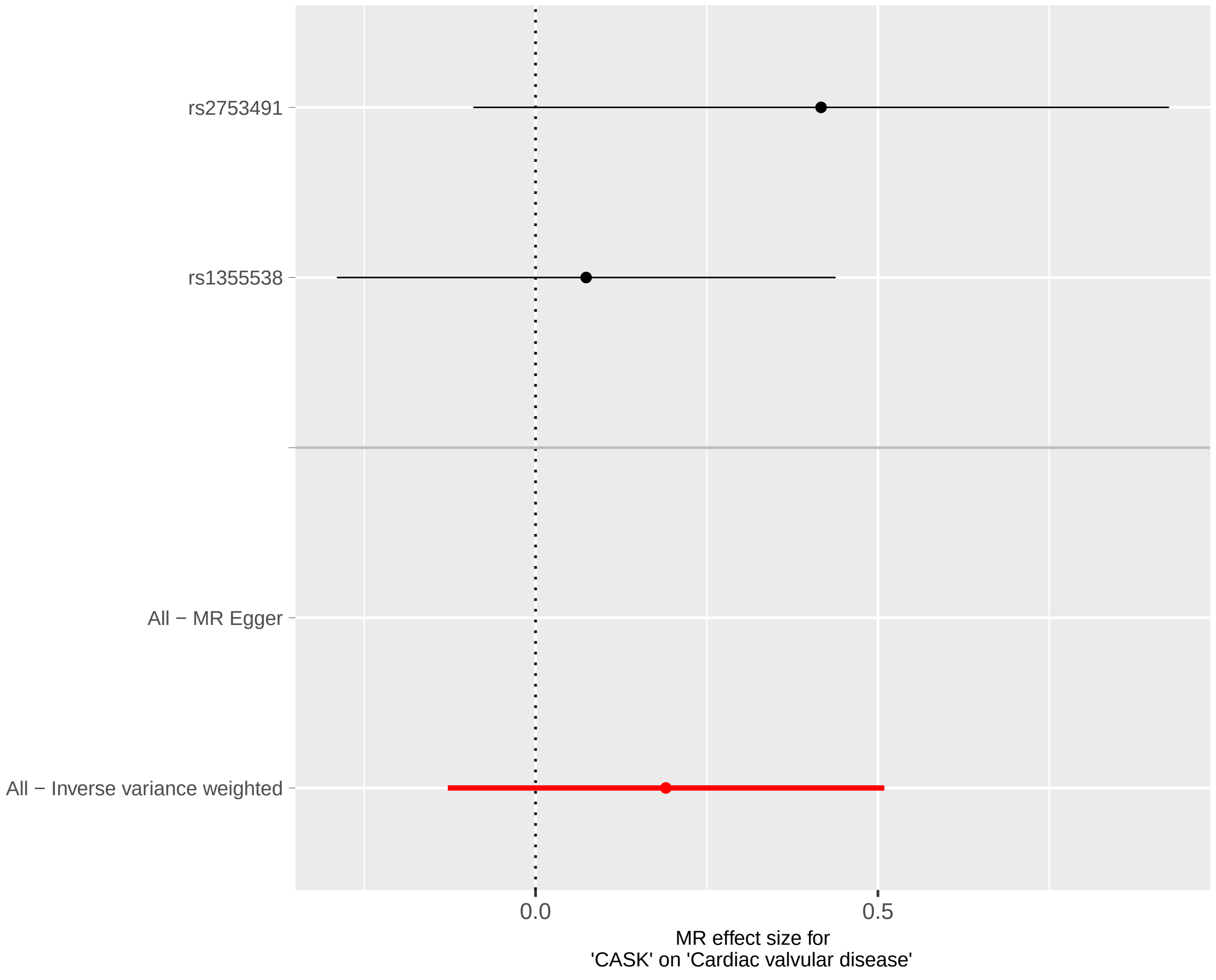
rs2753491
rs1355538
All − MR Egger
All − Inverse variance weighted
0.0
0.5
MR effect size for
'CASK' on 'Cardiac valvular disease'

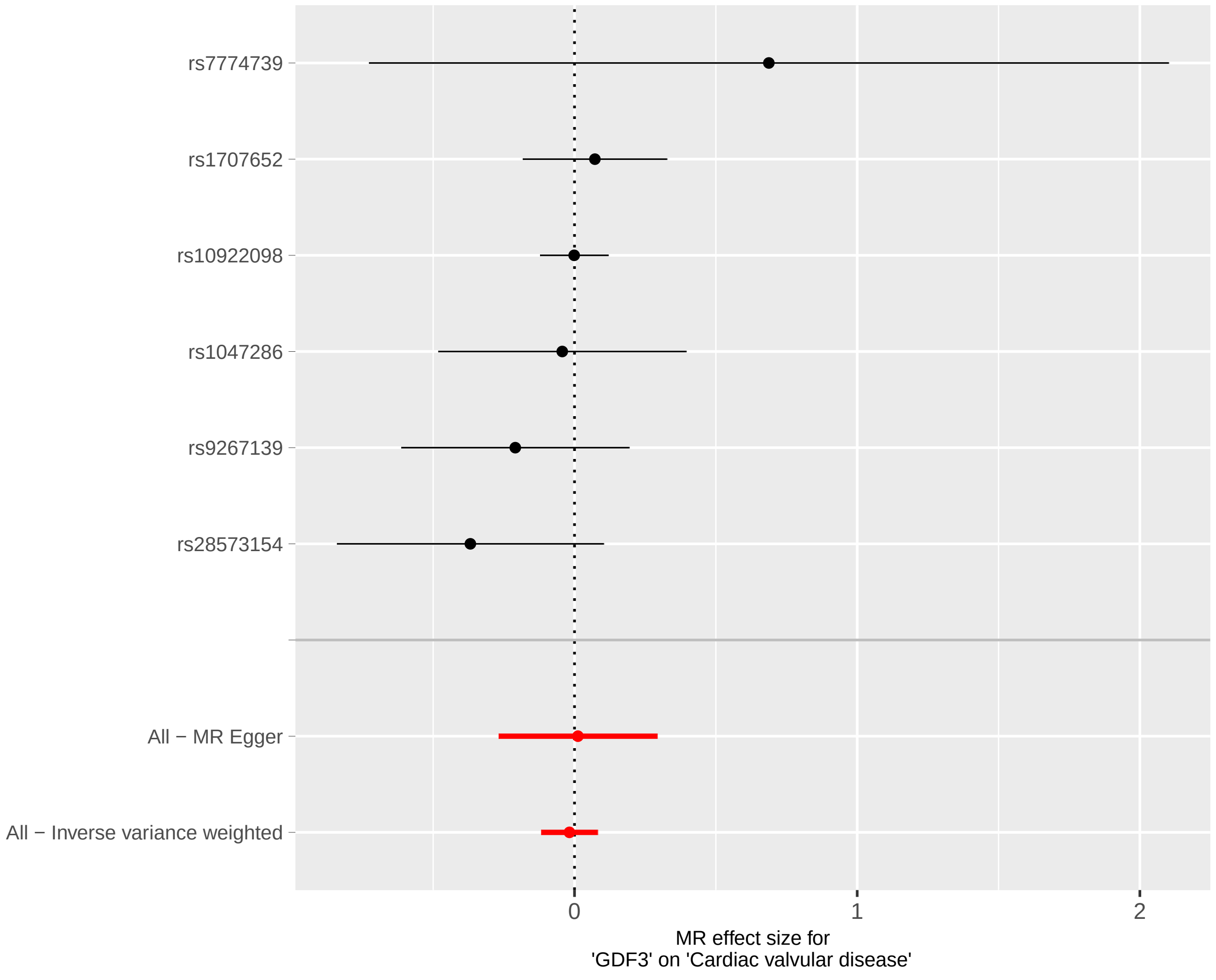
rs7774739
rs1707652
rs10922098
rs1047286
rs9267139
rs28573154
All − MR Egger
All − Inverse variance weighted
0
1
2
MR effect size for
'GDF3' on 'Cardiac valvular disease'

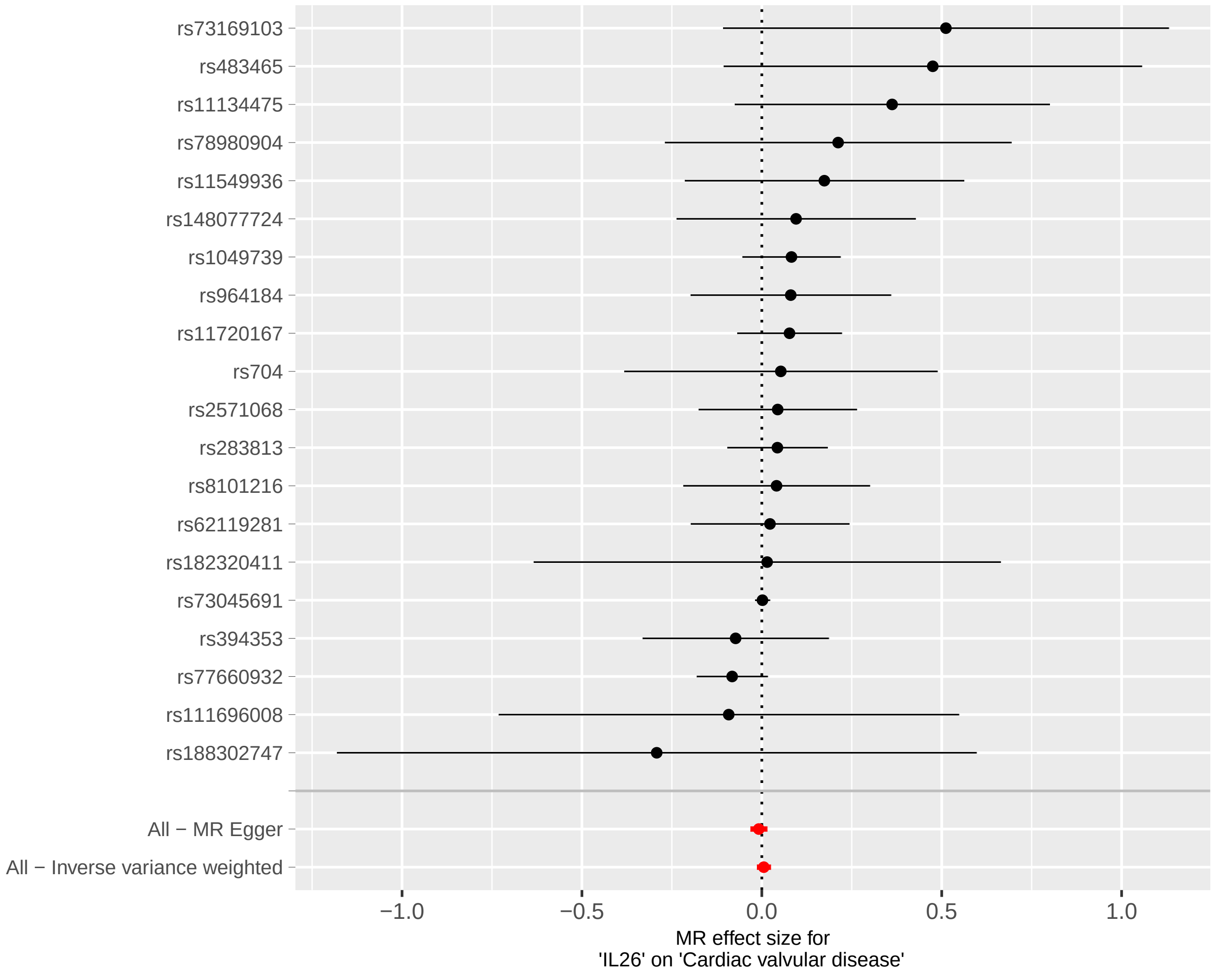
rs73169103
rs483465
rs11134475
rs78980904
rs11549936
rs148077724
rs1049739
rs964184
rs11720167
rs704
rs2571068
rs283813
rs8101216
rs62119281
rs182320411
rs73045691
rs394353
rs77660932
rs111696008
rs188302747
All − MR Egger
All − Inverse variance weighted
−1.0
−0.5
0.0
0.5
1.0
MR effect size for
'IL26' on 'Cardiac valvular disease'

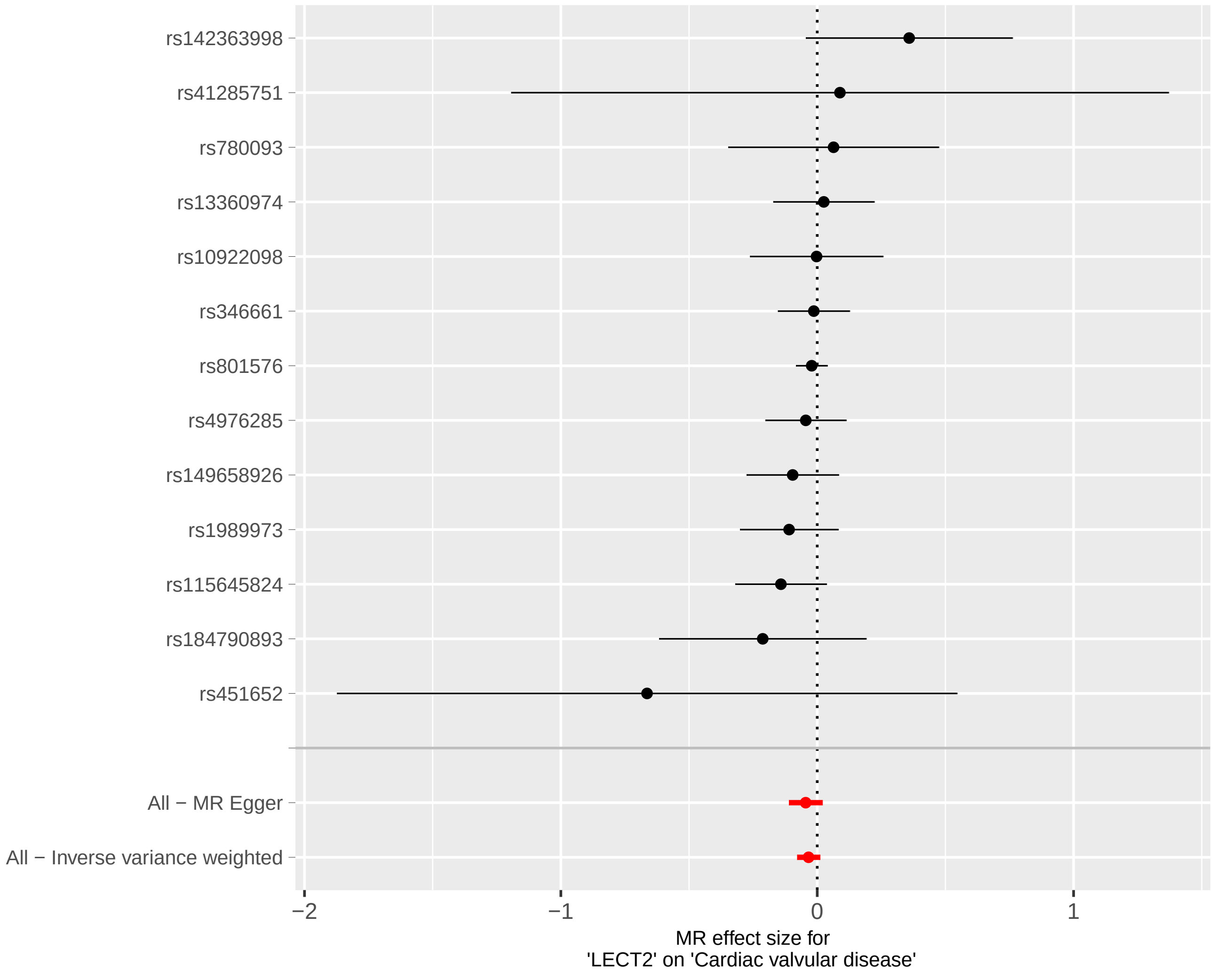
rs142363998
rs41285751
rs780093
rs13360974
rs10922098
rs346661
rs801576
rs4976285
rs149658926
rs1989973
rs115645824
rs184790893
rs451652
All − MR Egger
All − Inverse variance weighted
−2
−1
0
1
MR effect size for
'LECT2' on 'Cardiac valvular disease'

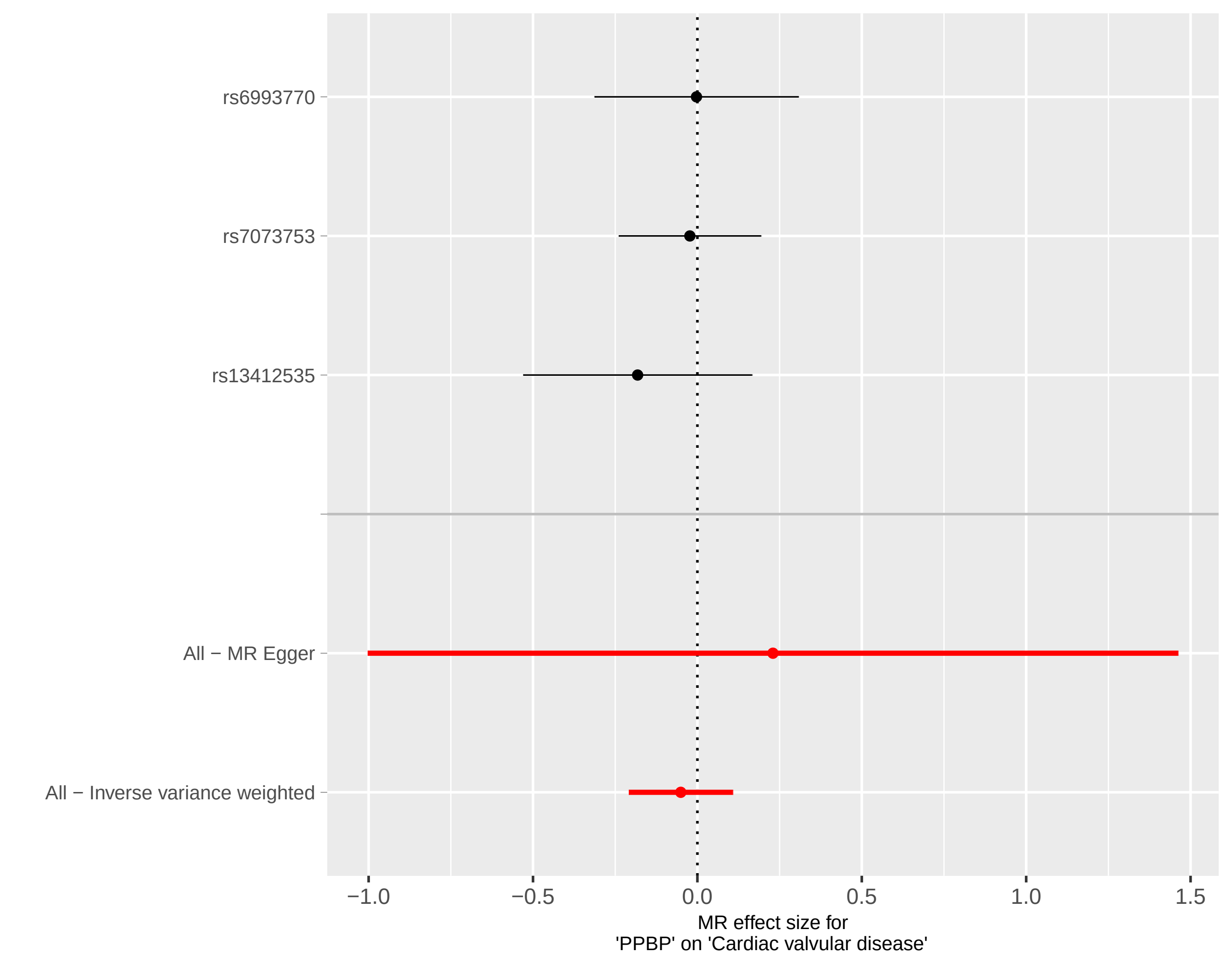
rs6993770
rs7073753
rs13412535
All − MR Egger
All − Inverse variance weighted
−1.0
−0.5
0.0
0.5
1.0
1.5
MR effect size for
'PPBP' on 'Cardiac valvular disease'

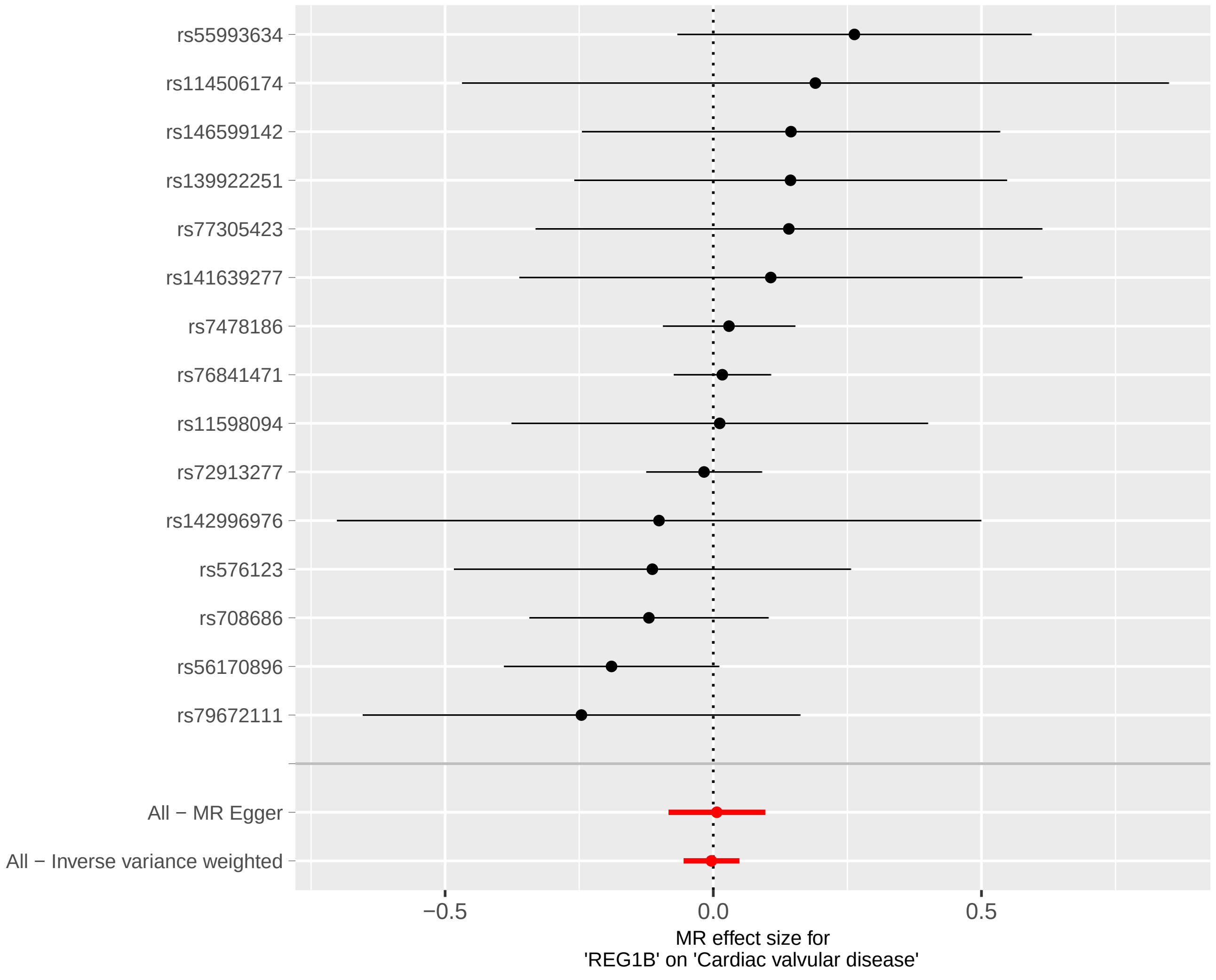
rs55993634
rs114506174
rs146599142
rs139922251
rs77305423
rs141639277
rs7478186
rs76841471
rs11598094
rs72913277
rs142996976
rs576123
rs708686
rs56170896
rs79672111
All − MR Egger
All − Inverse variance weighted
−0.5
0.0
0.5
MR effect size for
'REG1B' on 'Cardiac valvular disease'

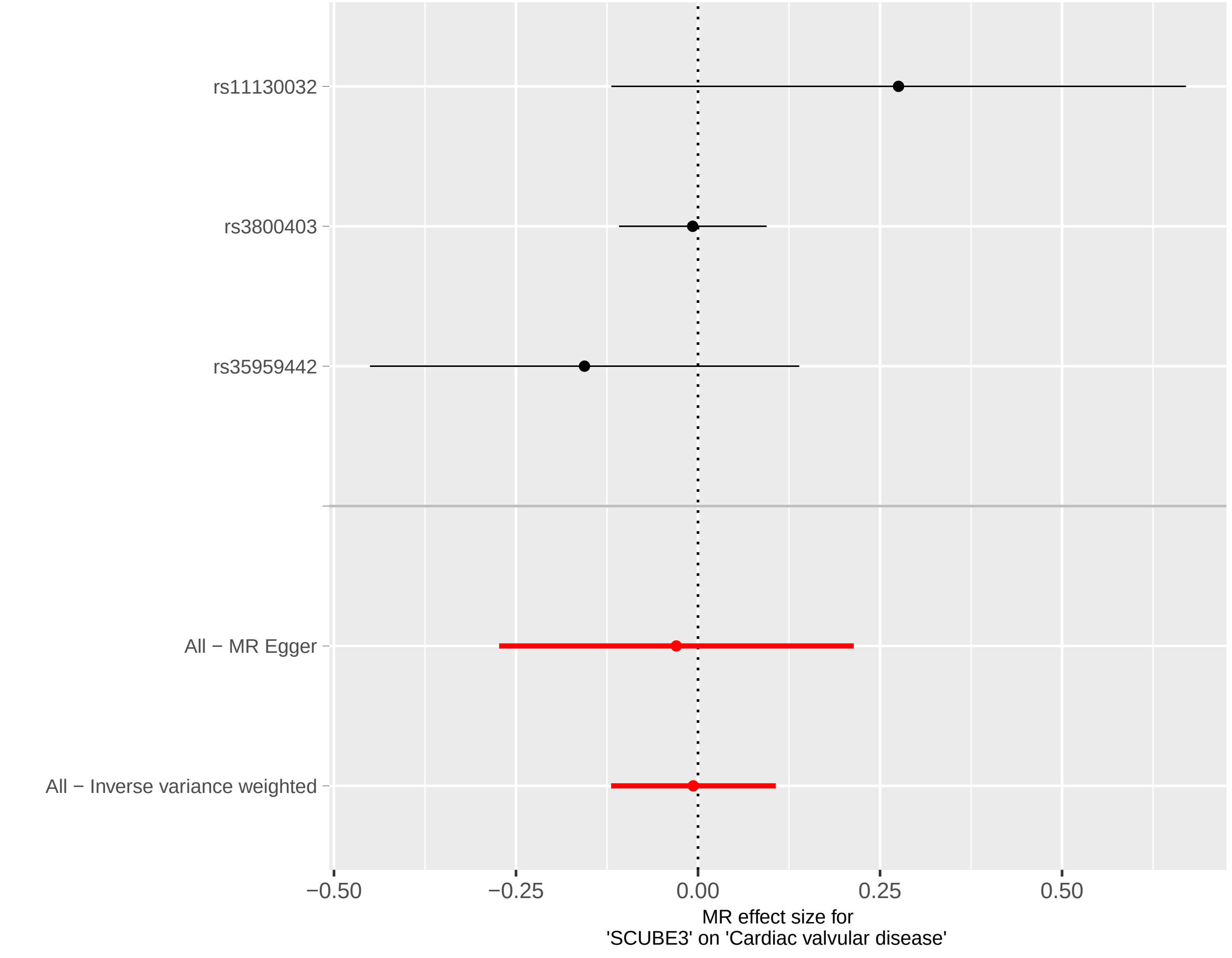

rs11130032
rs3800403
rs35959442
All − MR Egger
All − Inverse variance weighted
−0.50
−0.25
0.00
0.25
0.50
MR effect size for
'SCUBE3' on 'Cardiac valvular disease'

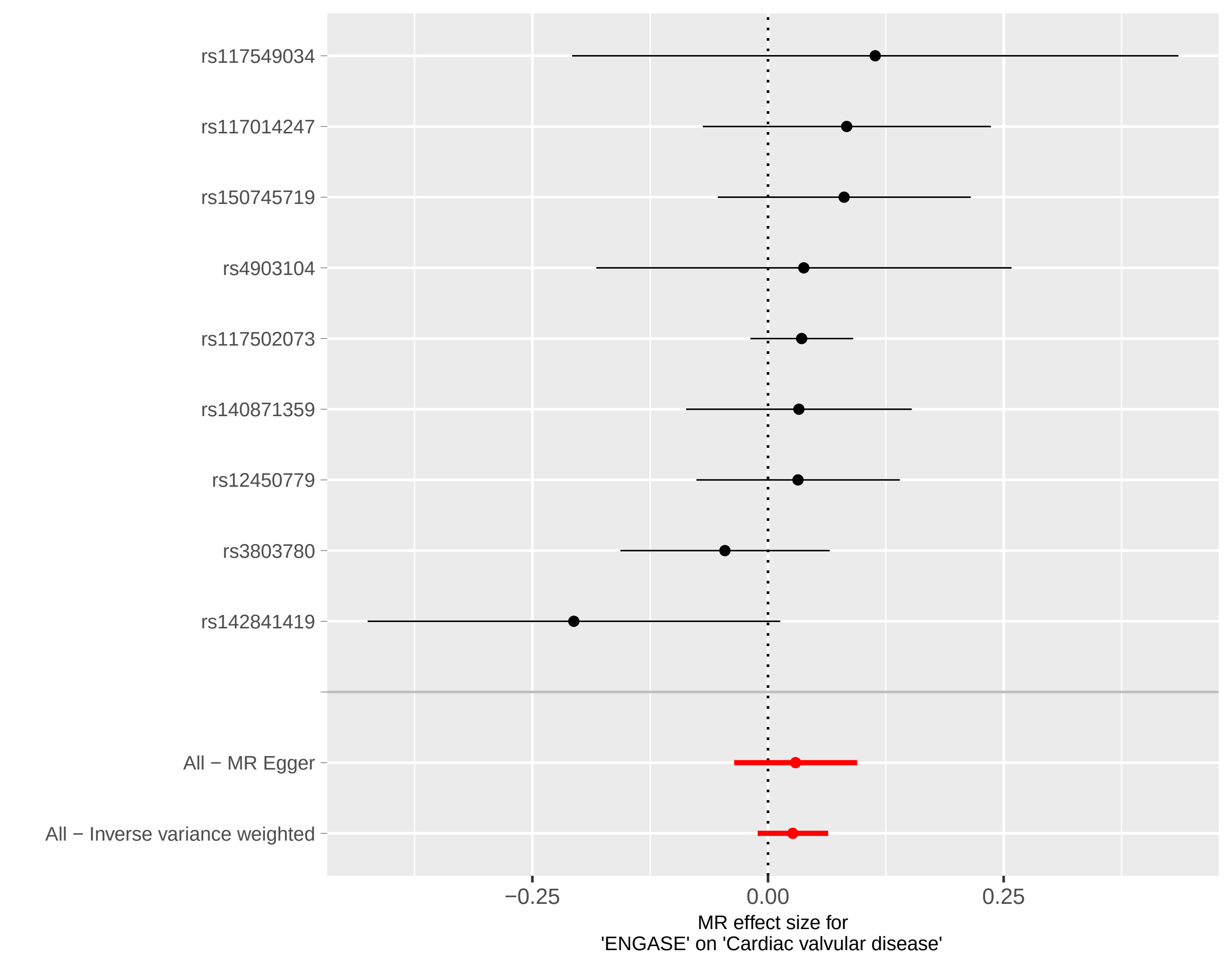
rs117549034
rs117014247
rs150745719
rs4903104
rs117502073
rs140871359
rs12450779
rs3803780
rs142841419
All − MR Egger
All − Inverse variance weighted
−0.25
0.00
0.25
MR effect size for
'ENGASE' on 'Cardiac valvular disease'

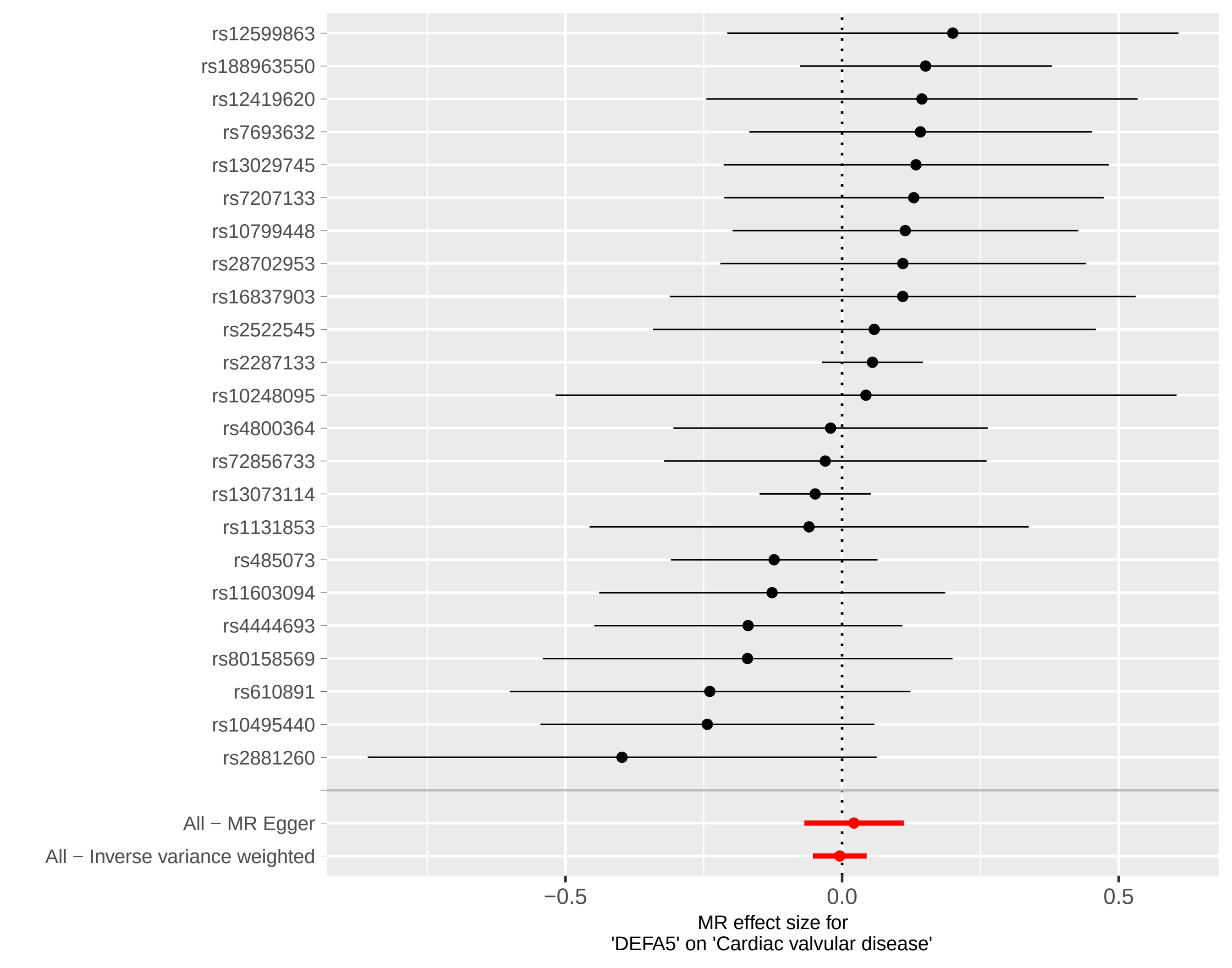
rs12599863
rs188963550
rs12419620
rs7693632
rs13029745
rs7207133
rs10799448
rs28702953
rs16837903
rs2522545
rs2287133
rs10248095
rs4800364
rs72856733
rs13073114
rs1131853
rs485073
rs11603094
rs4444693
rs80158569
rs610891
rs10495440
rs2881260
All − MR Egger
All − Inverse variance weighted
−0.5
0.0
0.5
MR effect size for
'DEFA5' on 'Cardiac valvular disease'

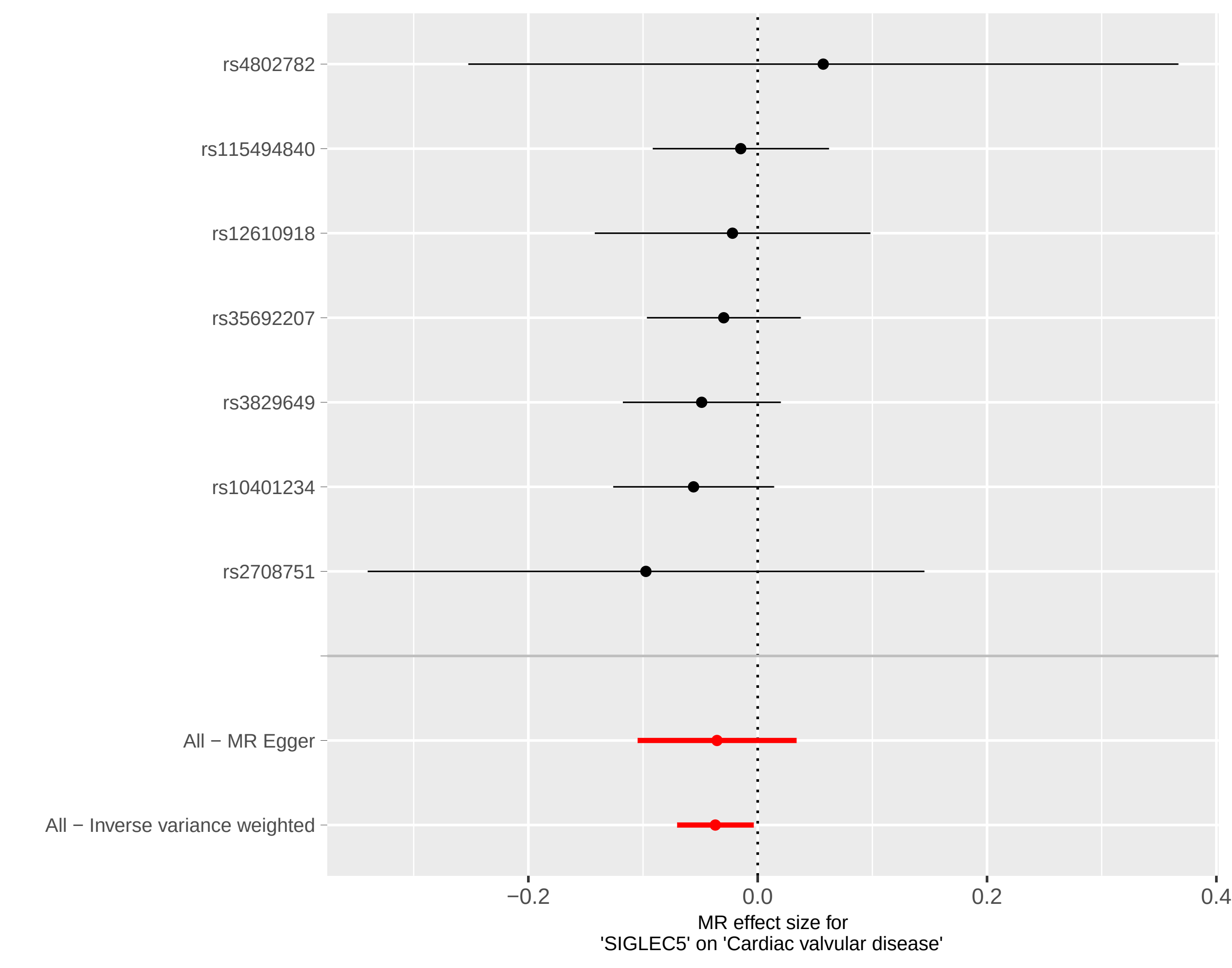
rs4802782
rs115494840
rs12610918
rs35692207
rs3829649
rs10401234
rs2708751
All − MR Egger
All − Inverse variance weighted
−0.2
0.0
0.2
0.4
MR effect size for
'SIGLEC5' on 'Cardiac valvular disease'

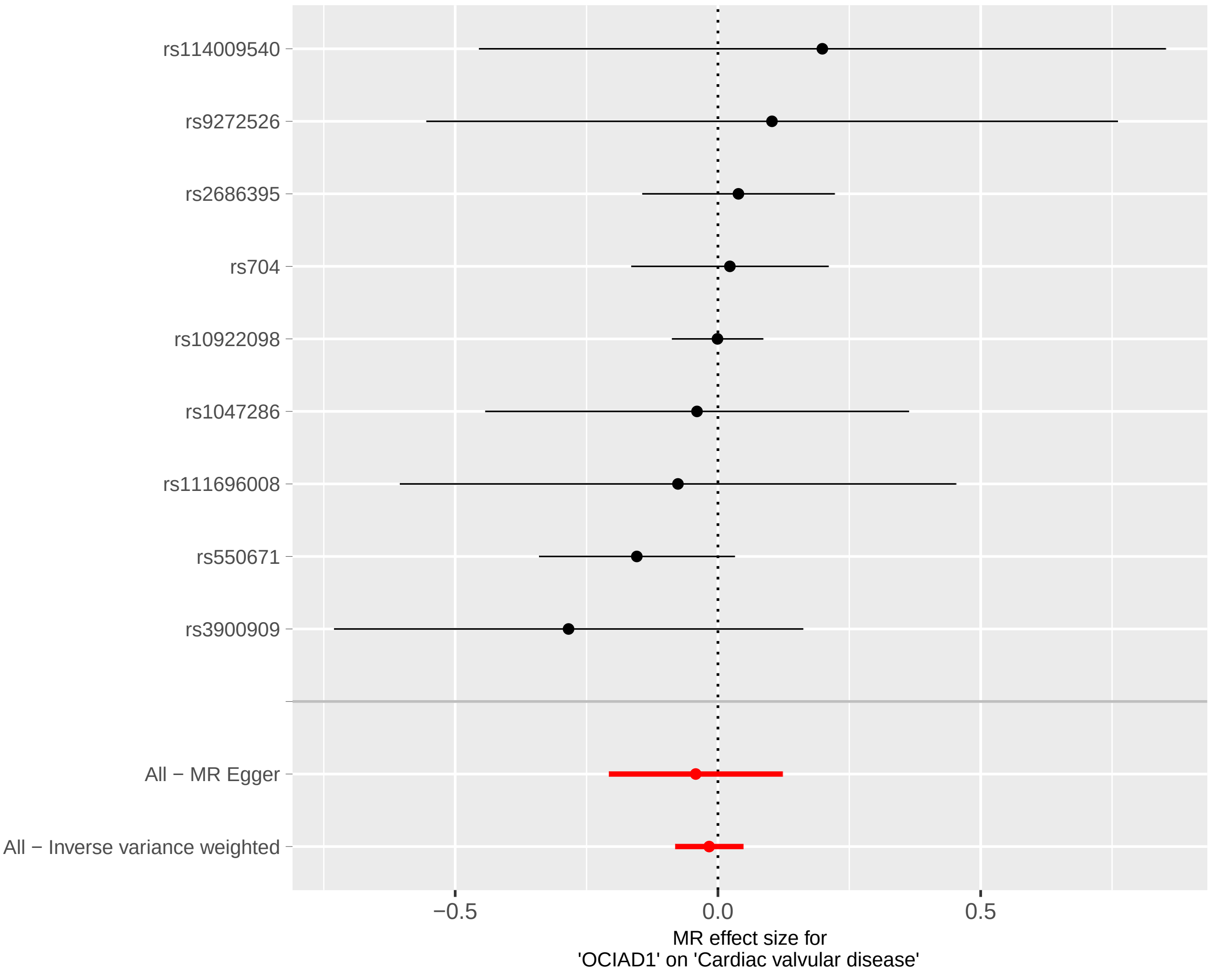
rs114009540
rs9272526
rs2686395
rs704
rs10922098
rs1047286
rs111696008
rs550671
rs3900909
All − MR Egger
All − Inverse variance weighted
−0.5
0.0
0.5
MR effect size for
'OCIAD1' on 'Cardiac valvular disease'

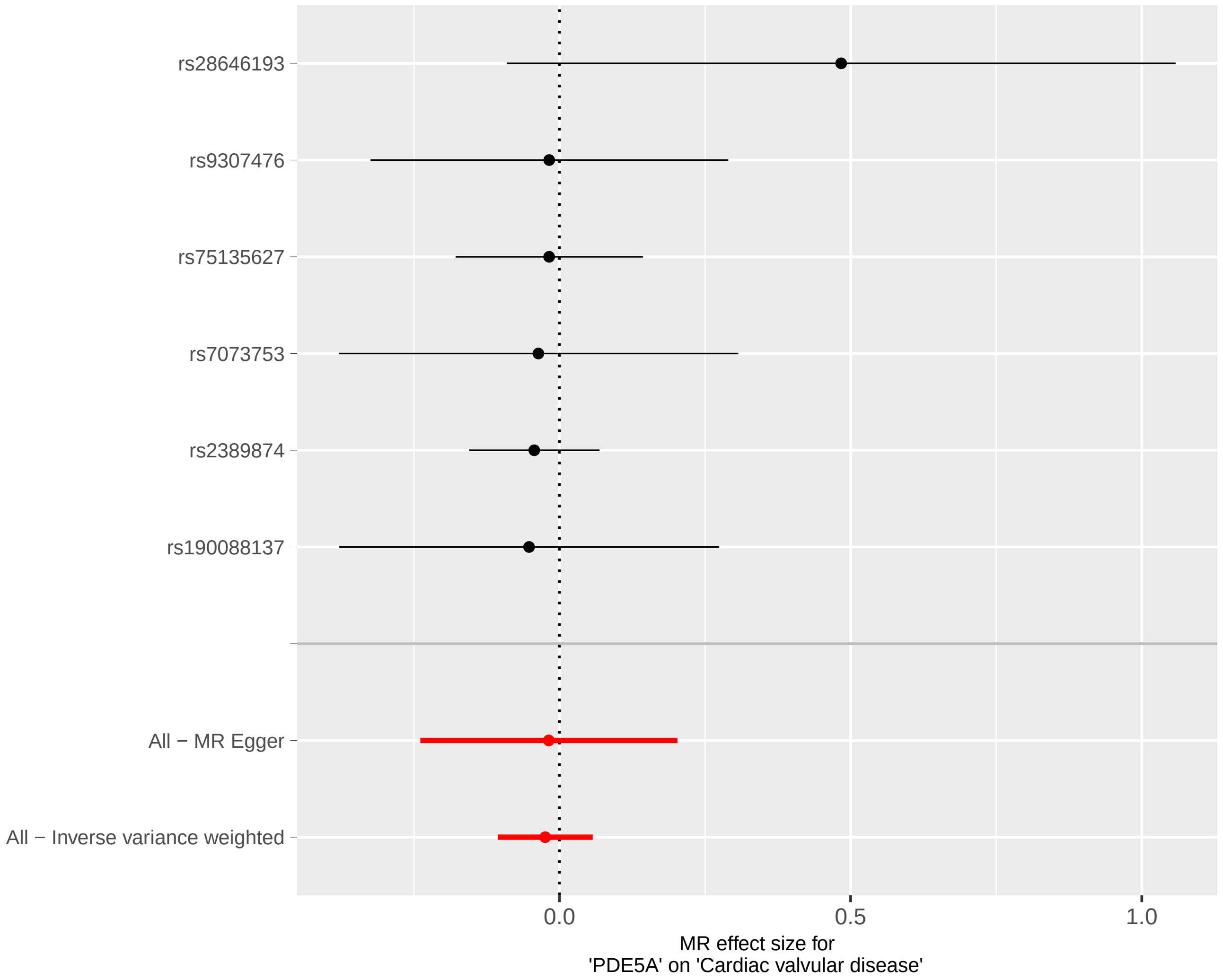
rs28646193
rs9307476
rs75135627
rs7073753
rs2389874
rs190088137
All − MR Egger
All − Inverse variance weighted
0.0
0.5
1.0
MR effect size for
'PDE5A' on 'Cardiac valvular disease'

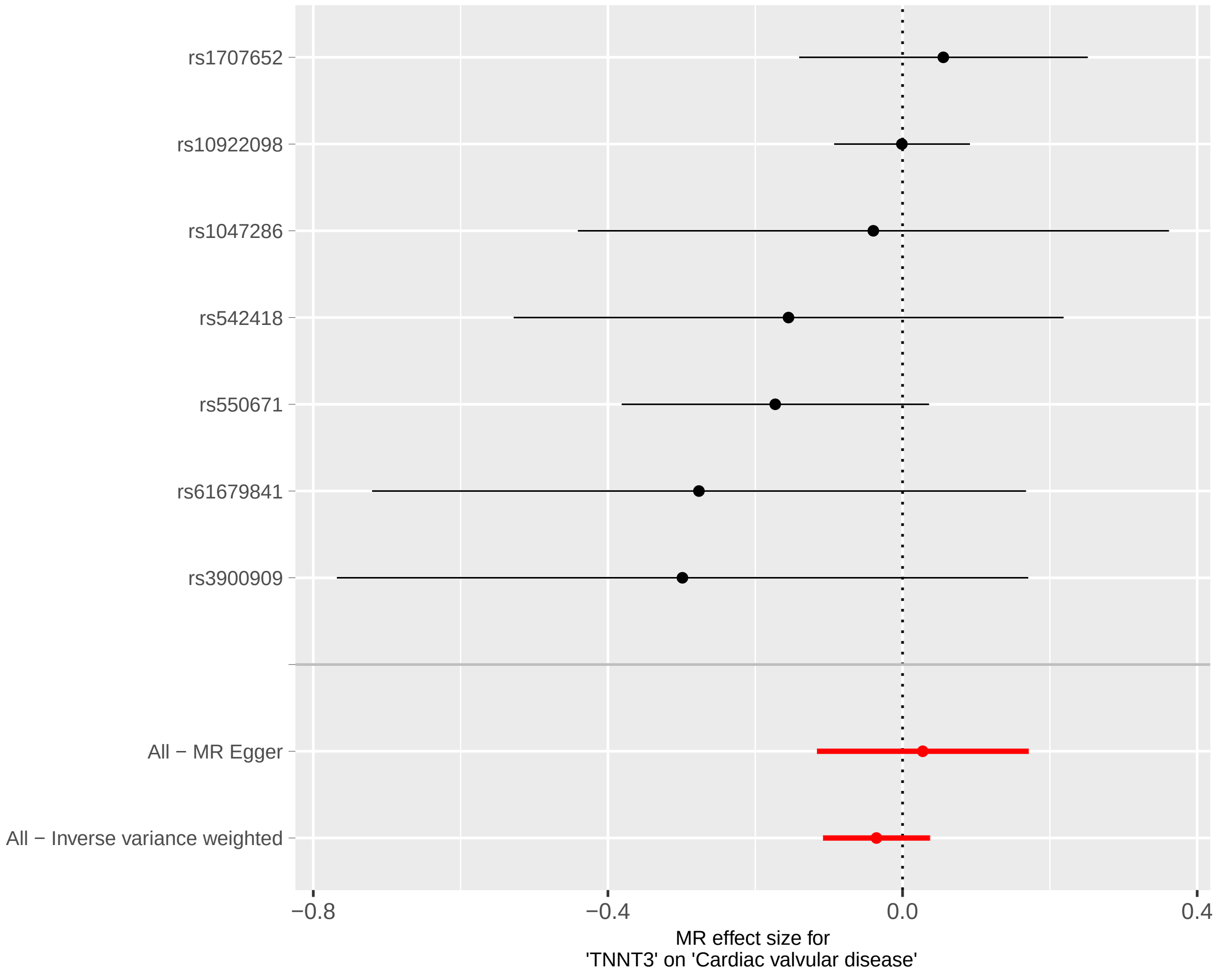
rs1707652
rs10922098
rs1047286
rs542418
rs550671
rs61679841
rs3900909
All − MR Egger
All − Inverse variance weighted
−0.8
−0.4
0.0
0.4
MR effect size for
'TNNT3' on 'Cardiac valvular disease'

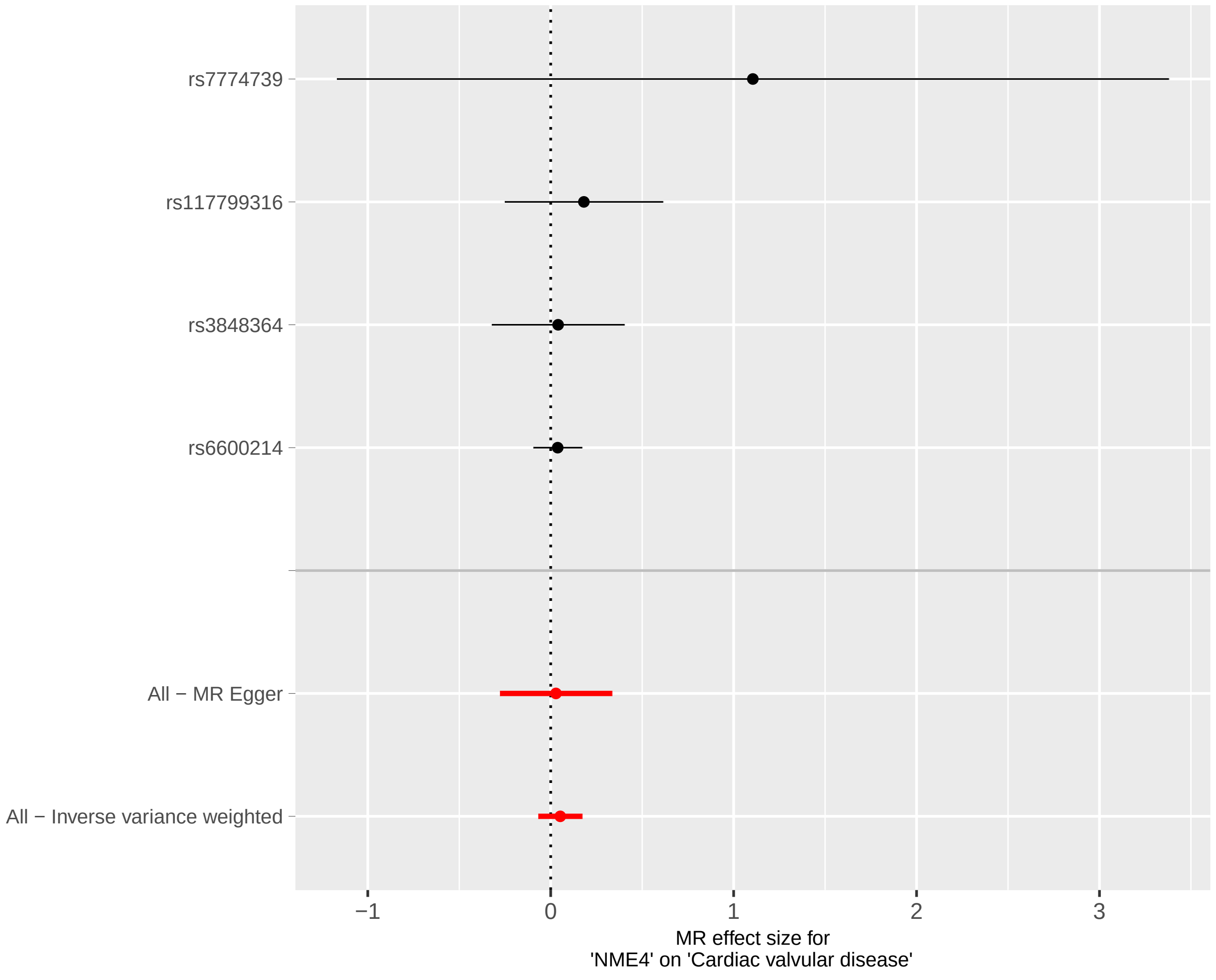
rs7774739
rs117799316
rs3848364
rs6600214
All − MR Egger
All − Inverse variance weighted
−1
0
1
2
3
MR effect size for
'NME4' on 'Cardiac valvular disease'

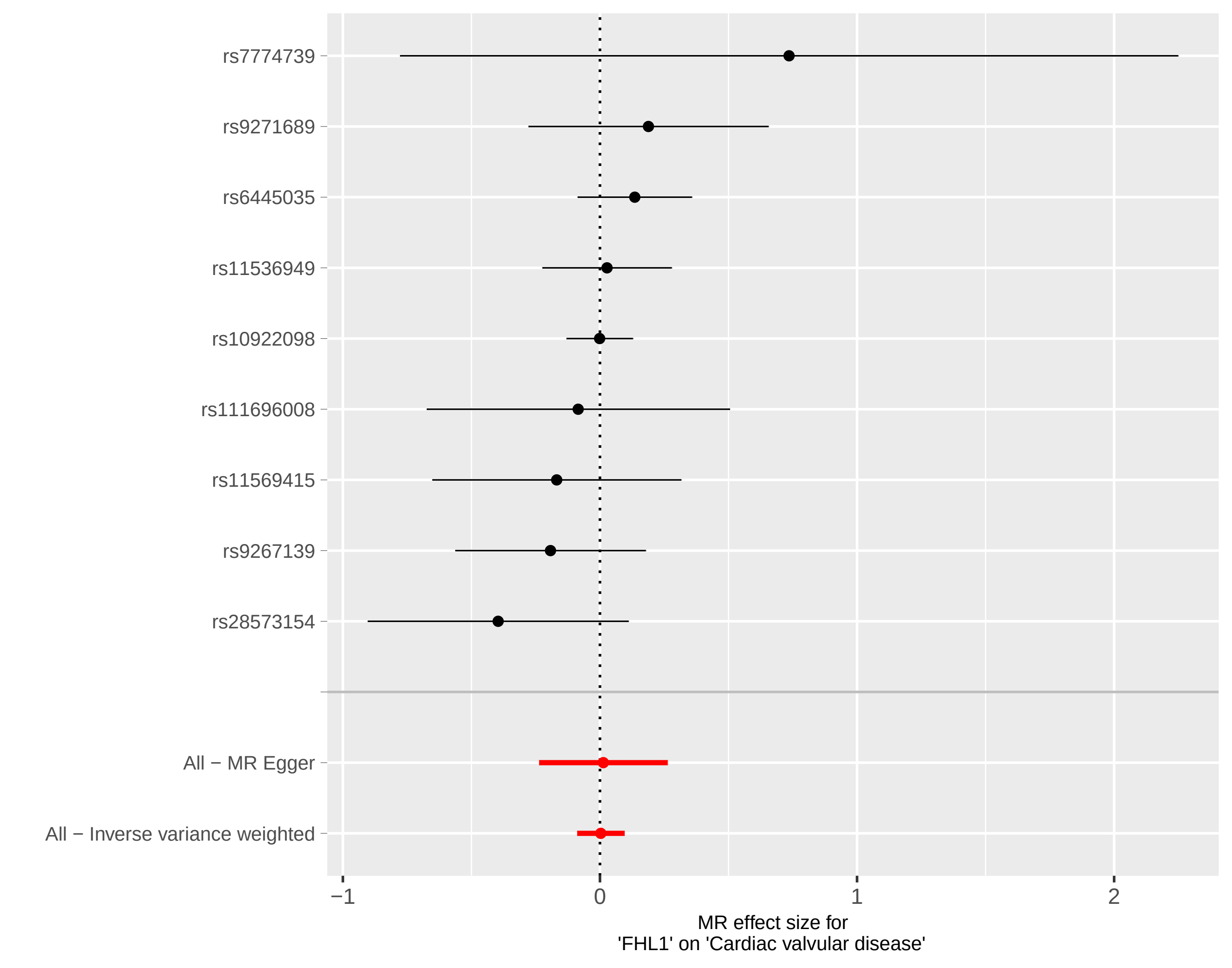
rs7774739
rs9271689
rs6445035
rs11536949
rs10922098
rs111696008
rs11569415
rs9267139
rs28573154
All − MR Egger
All − Inverse variance weighted
−1
0
1
2
MR effect size for
'FHL1' on 'Cardiac valvular disease'

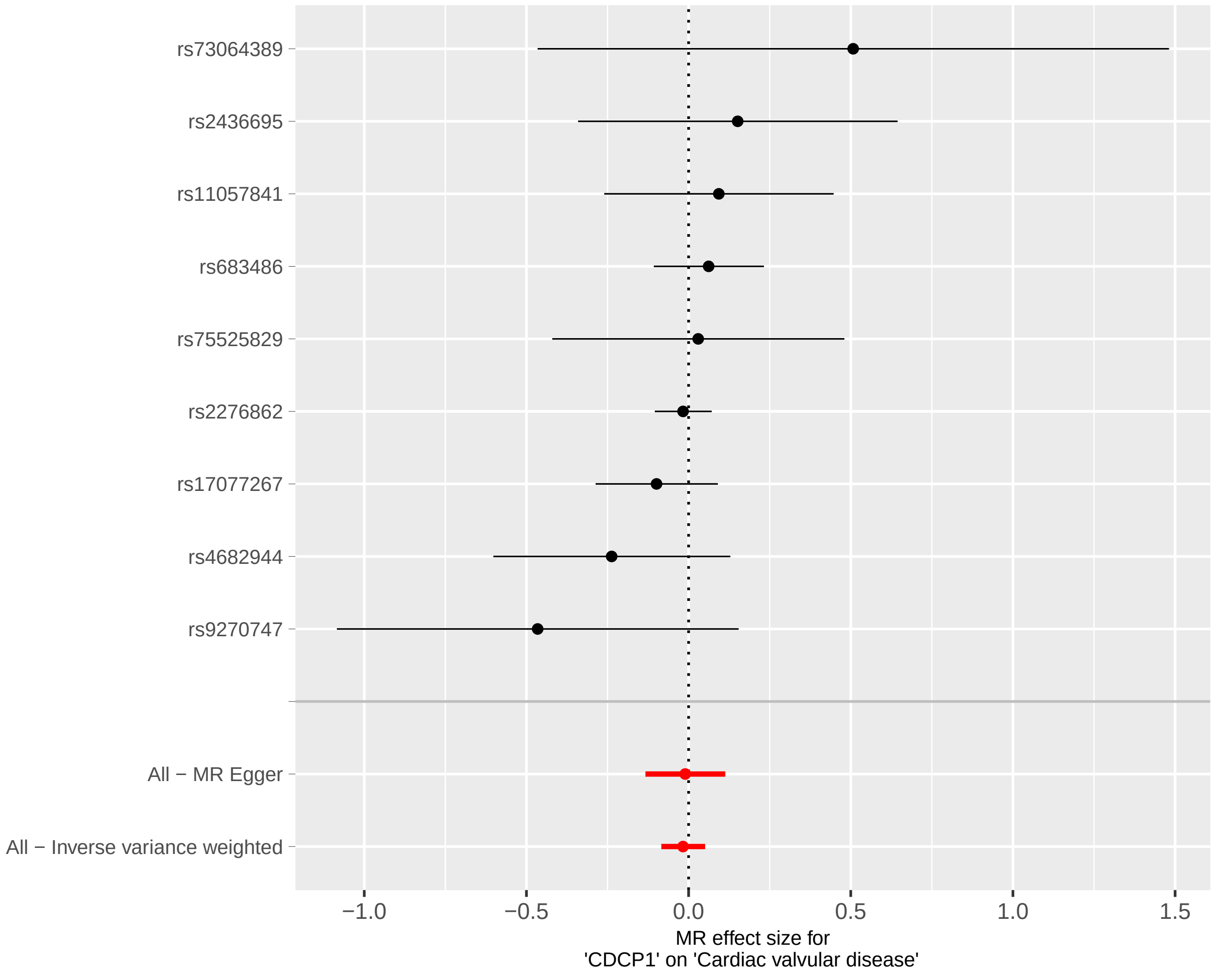
rs73064389
rs2436695
rs11057841
rs683486
rs75525829
rs2276862
rs17077267
rs4682944
rs9270747
All − MR Egger
All − Inverse variance weighted
−1.0
−0.5
0.0
0.5
1.0
1.5
MR effect size for
'CDCP1' on 'Cardiac valvular disease'

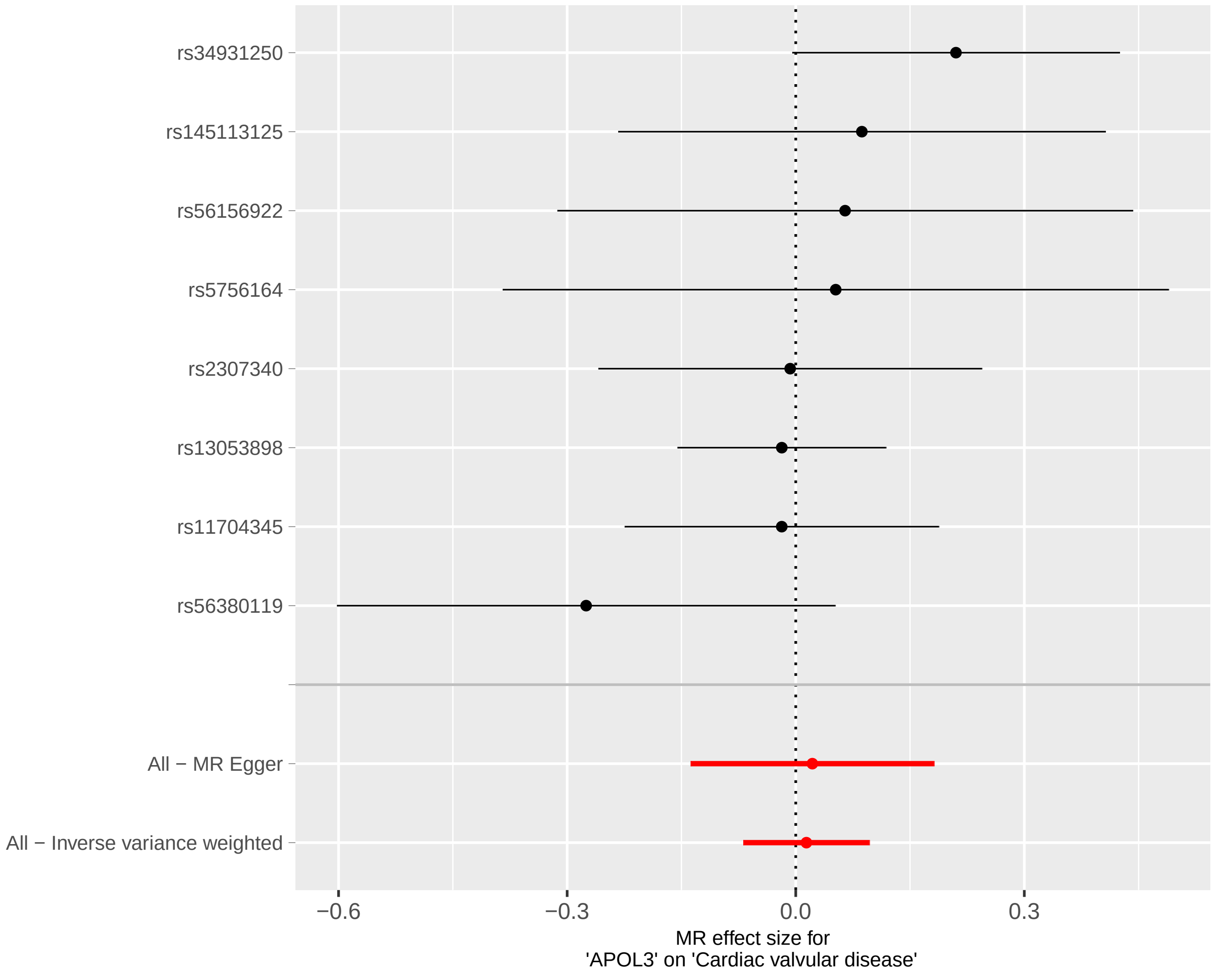

rs34931250
rs145113125
rs56156922
rs5756164
rs2307340
rs13053898
rs11704345
rs56380119
All − MR Egger
All − Inverse variance weighted
−0.6
−0.3
0.0
0.3
MR effect size for
'APOL3' on 'Cardiac valvular disease'

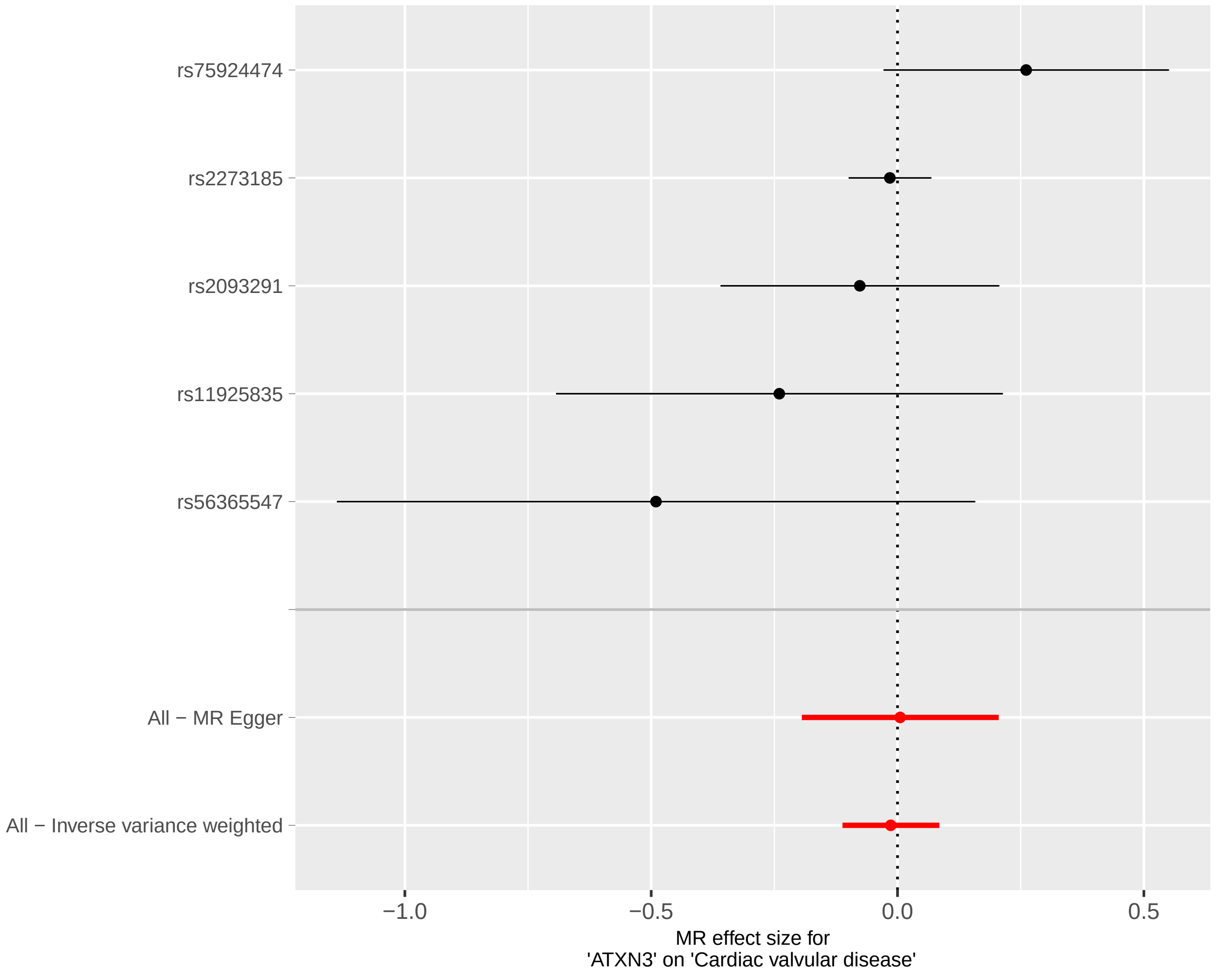
rs75924474
rs2273185
rs2093291
rs11925835
rs56365547
All − MR Egger
All − Inverse variance weighted
−1.0
−0.5
0.0
0.5
MR effect size for
'ATXN3' on 'Cardiac valvular disease'

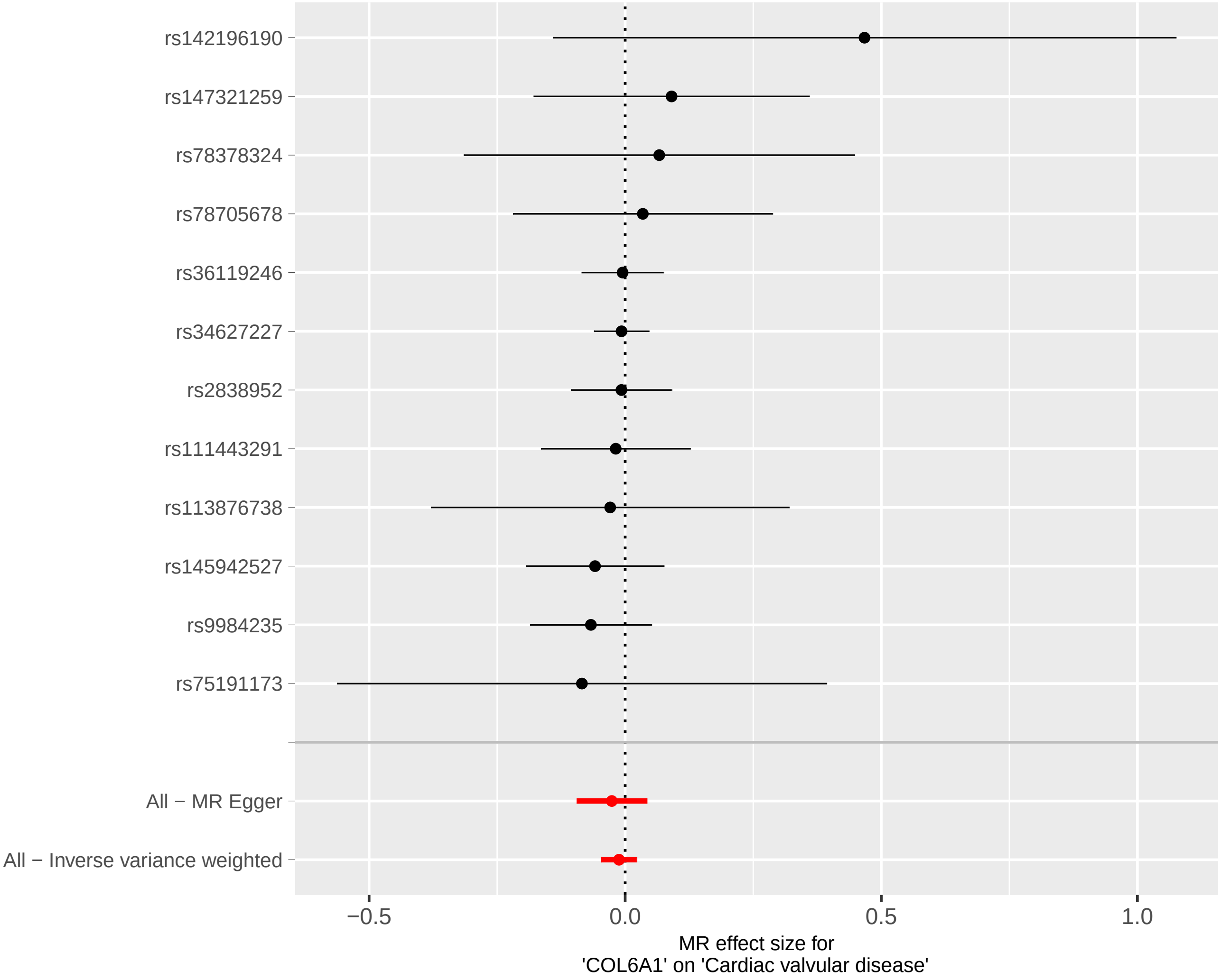
rs142196190
rs147321259
rs78378324
rs78705678
rs36119246
rs34627227
rs2838952
rs111443291
rs113876738
rs145942527
rs9984235
rs75191173
All − MR Egger
All − Inverse variance weighted
−0.5
0.0
0.5
1.0
MR effect size for
'COL6A1' on 'Cardiac valvular disease'

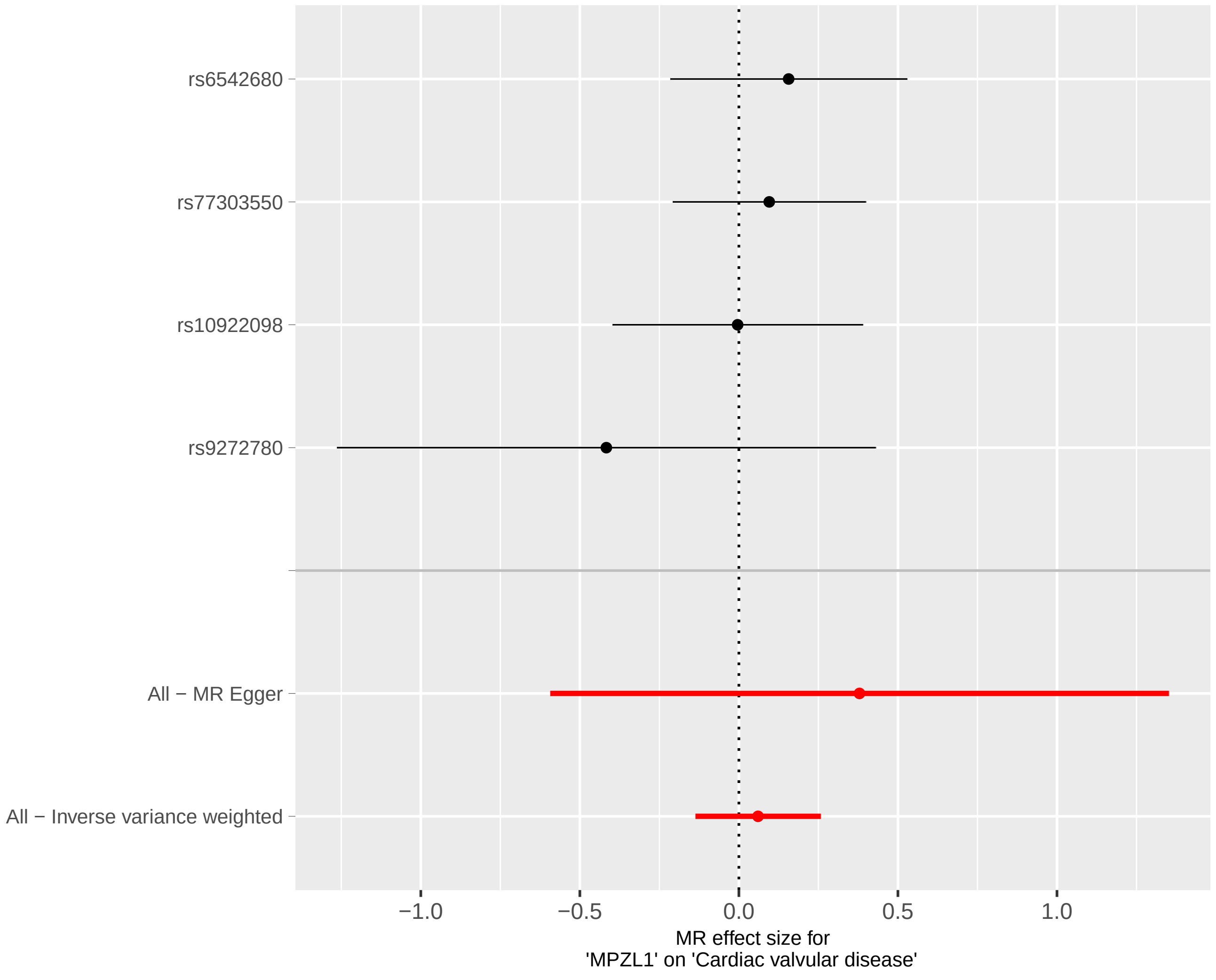

rs6542680
rs77303550
rs10922098
rs9272780
All − MR Egger
All − Inverse variance weighted
−1.0
−0.5
0.0
0.5
1.0
MR effect size for
'MPZL1' on 'Cardiac valvular disease'

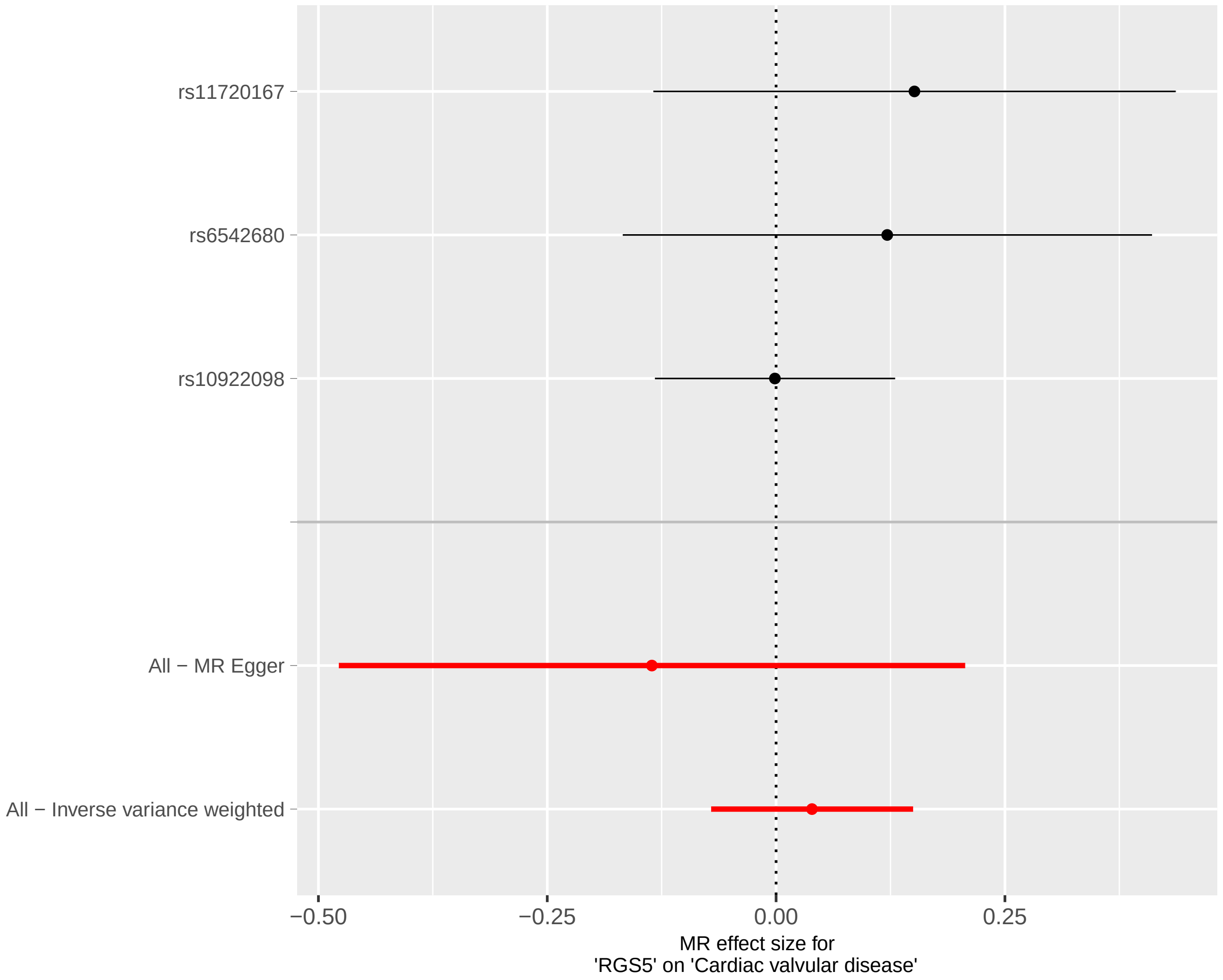
rs11720167
rs6542680
rs10922098
All − MR Egger
All − Inverse variance weighted
−0.50
−0.25
0.00
0.25
MR effect size for
'RGS5' on 'Cardiac valvular disease'

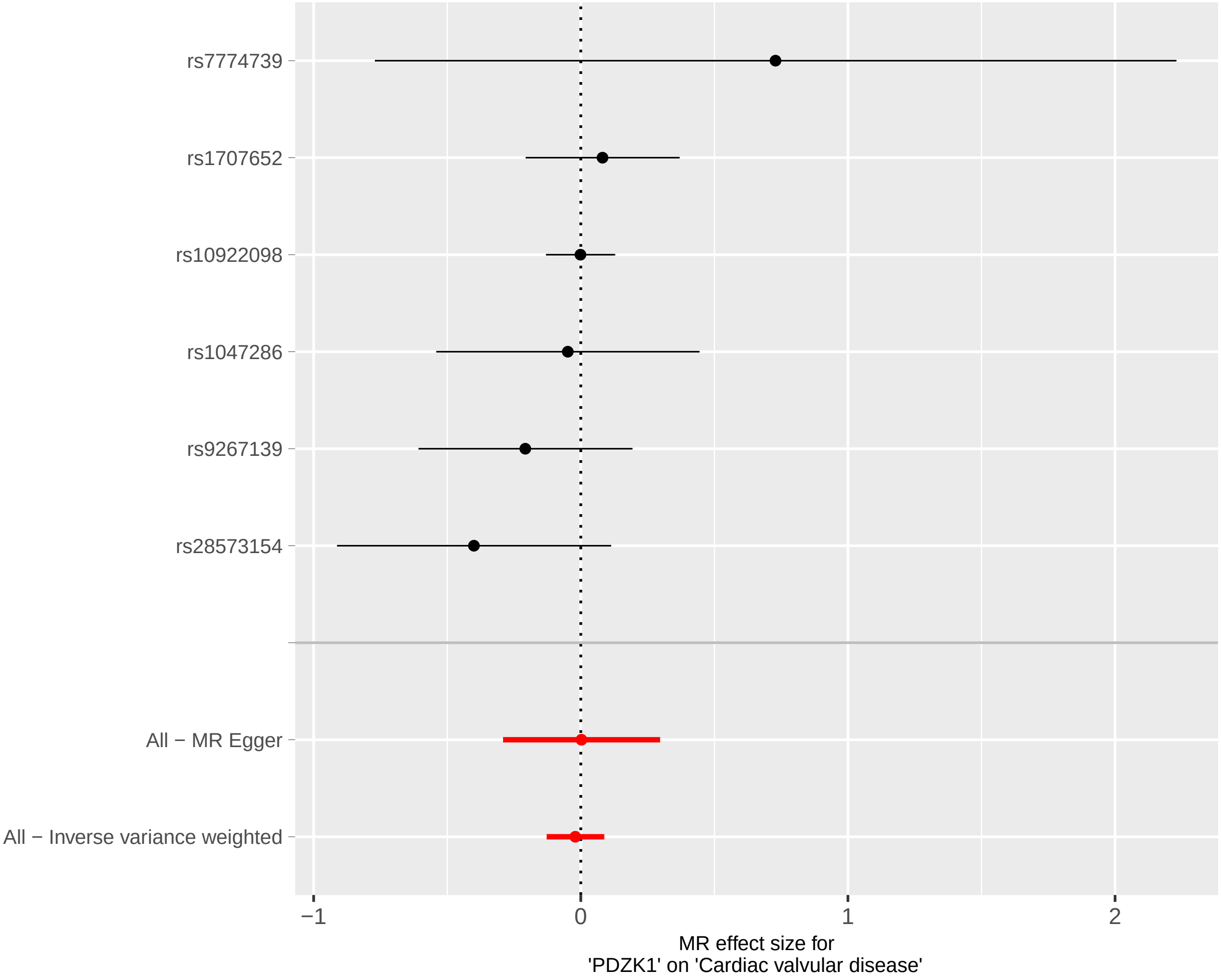
rs7774739
rs1707652
rs10922098
rs1047286
rs9267139
rs28573154
All − MR Egger
All − Inverse variance weighted
−1
0
1
2
MR effect size for
'PDZK1' on 'Cardiac valvular disease'

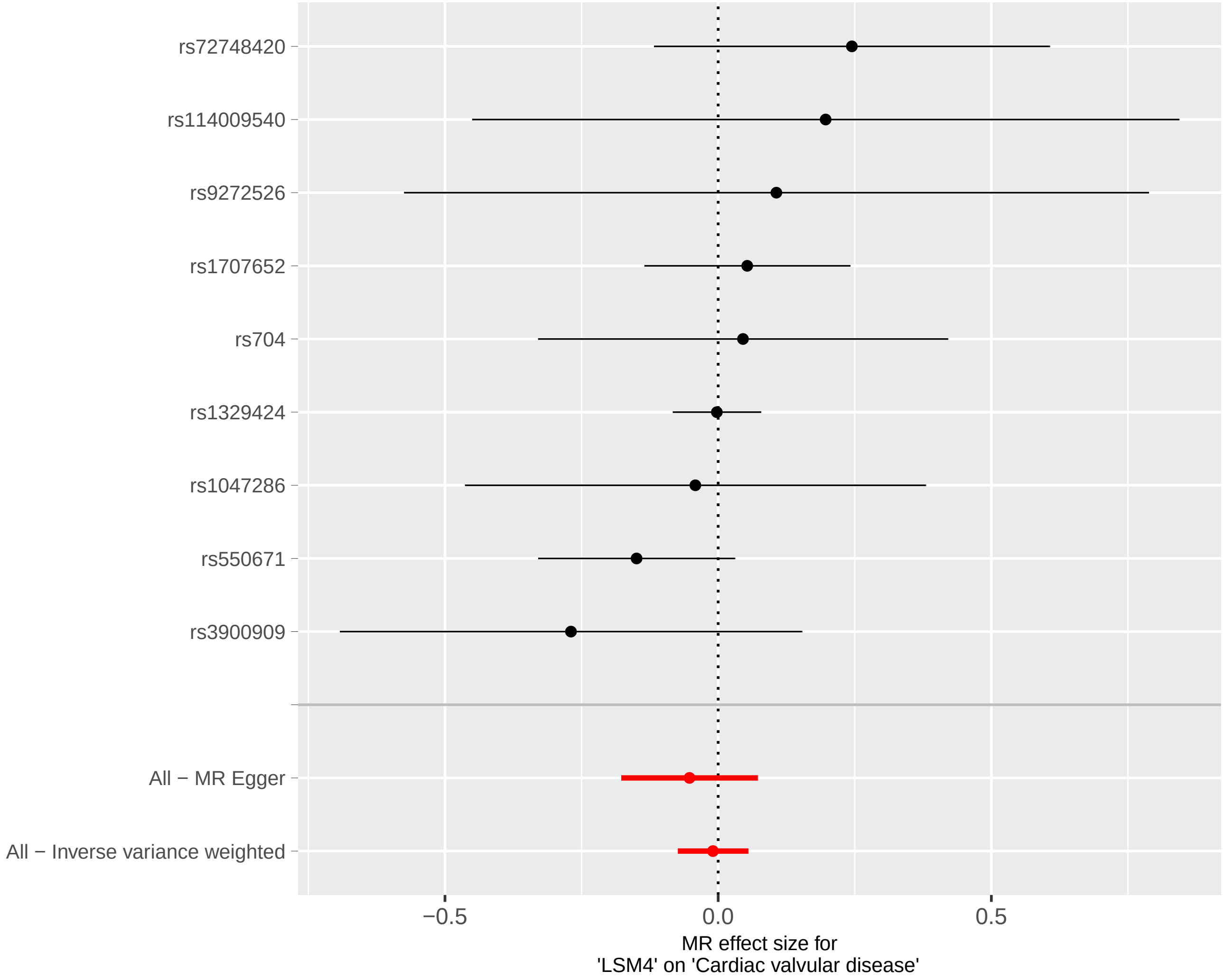
rs72748420
rs114009540
rs9272526
rs1707652
rs704
rs1329424
rs1047286
rs550671
rs3900909
All − MR Egger
All − Inverse variance weighted
−0.5
0.0
0.5
MR effect size for
'LSM4' on 'Cardiac valvular disease'

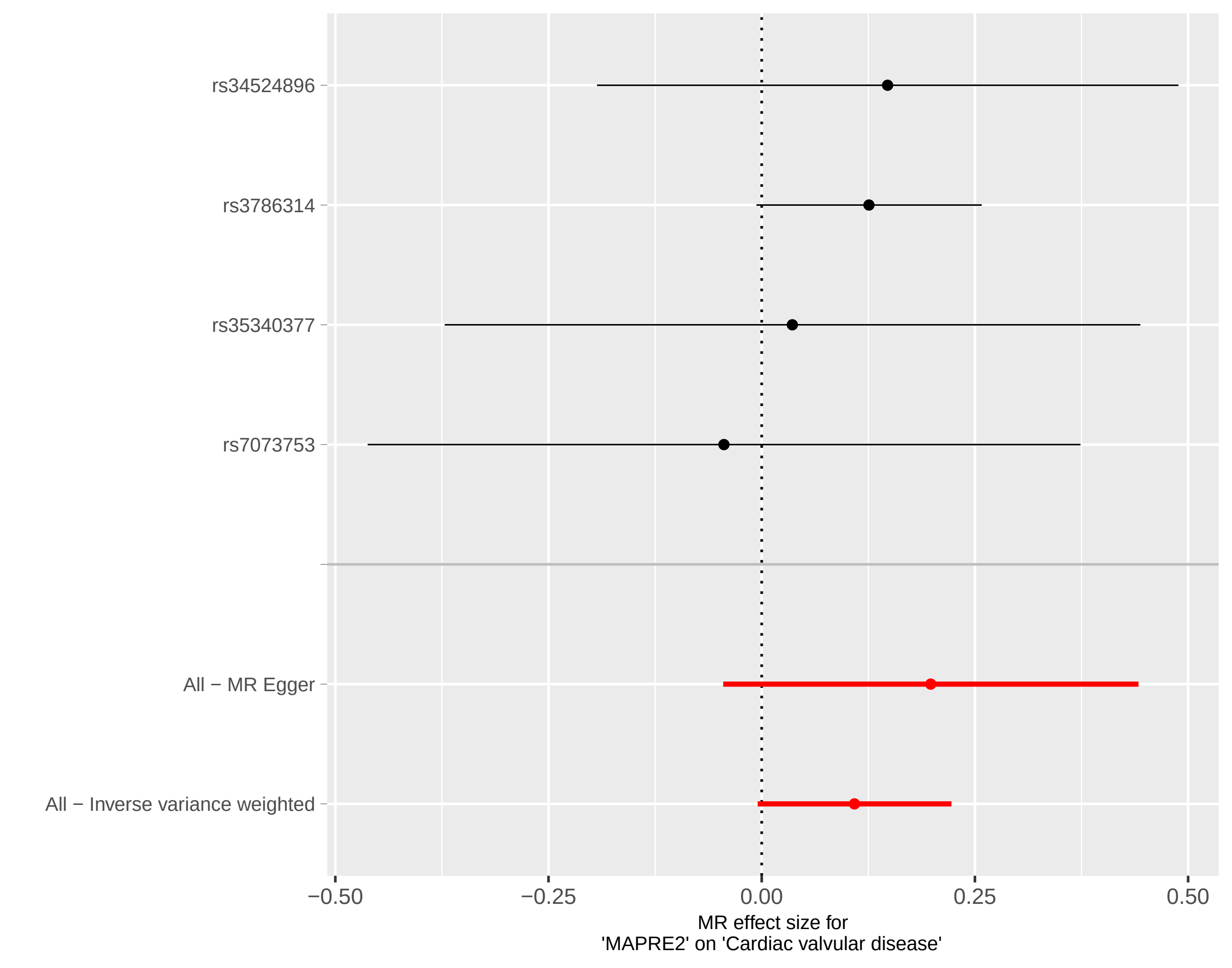
rs34524896
rs3786314
rs35340377
rs7073753
All − MR Egger
All − Inverse variance weighted
−0.50
−0.25
0.00
0.25
0.50
MR effect size for
'MAPRE2' on 'Cardiac valvular disease'

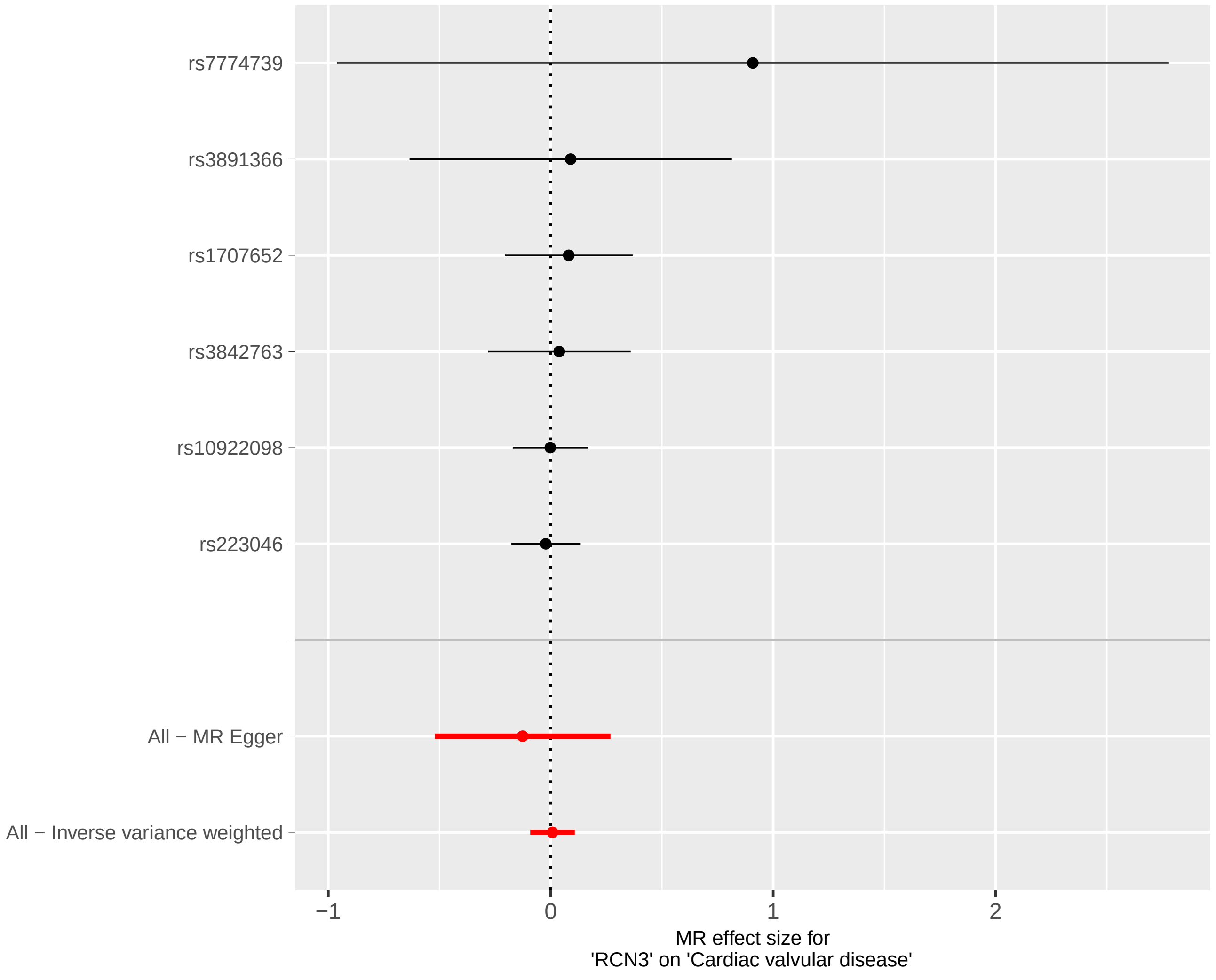

rs7774739
rs3891366
rs1707652
rs3842763
rs10922098
rs223046
All − MR Egger
All − Inverse variance weighted
−1
0
1
2
MR effect size for
'RCN3' on 'Cardiac valvular disease'

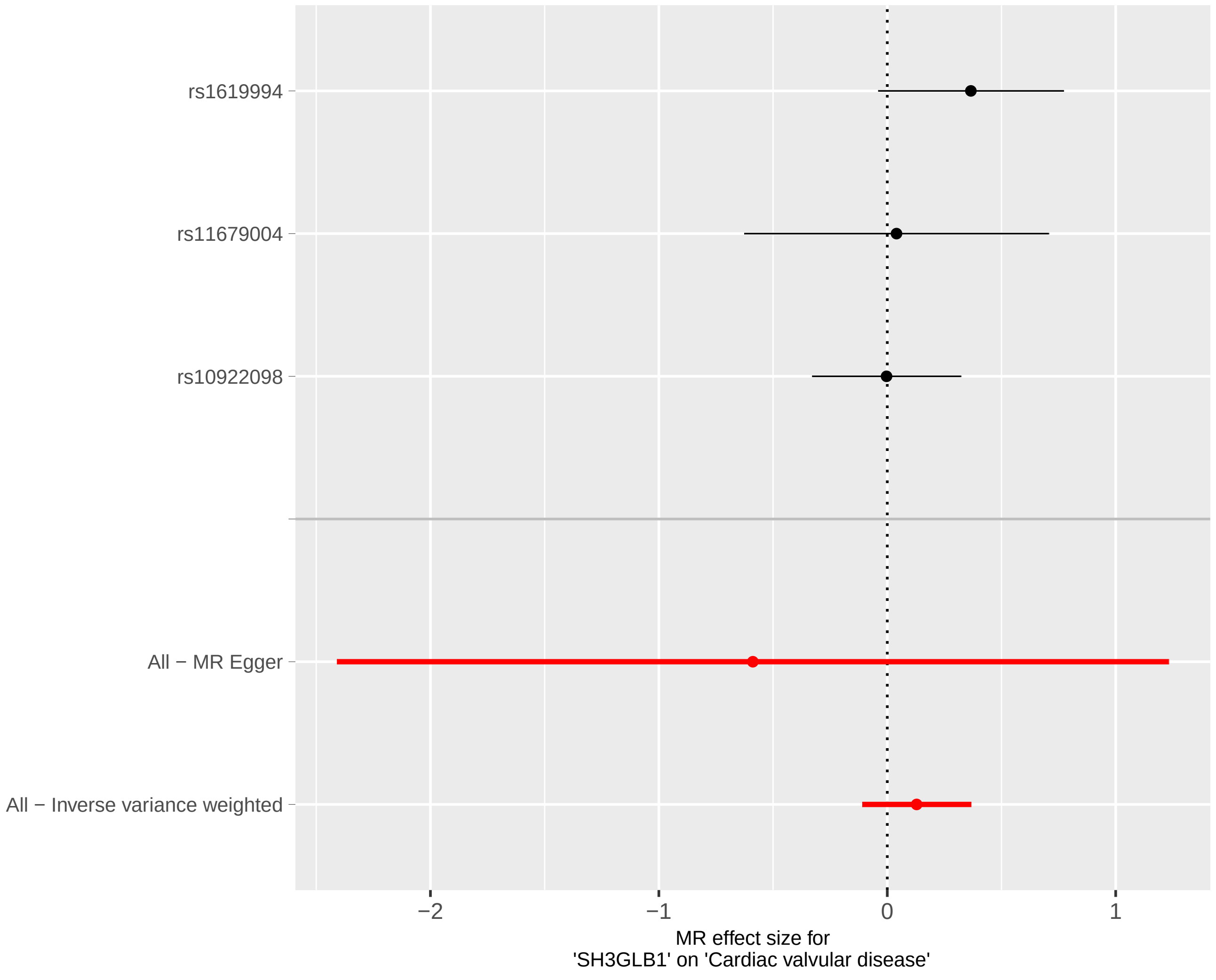
rs1619994
rs11679004
rs10922098
All − MR Egger
All − Inverse variance weighted
−2
−1
0
1
MR effect size for
'SH3GLB1' on 'Cardiac valvular disease'

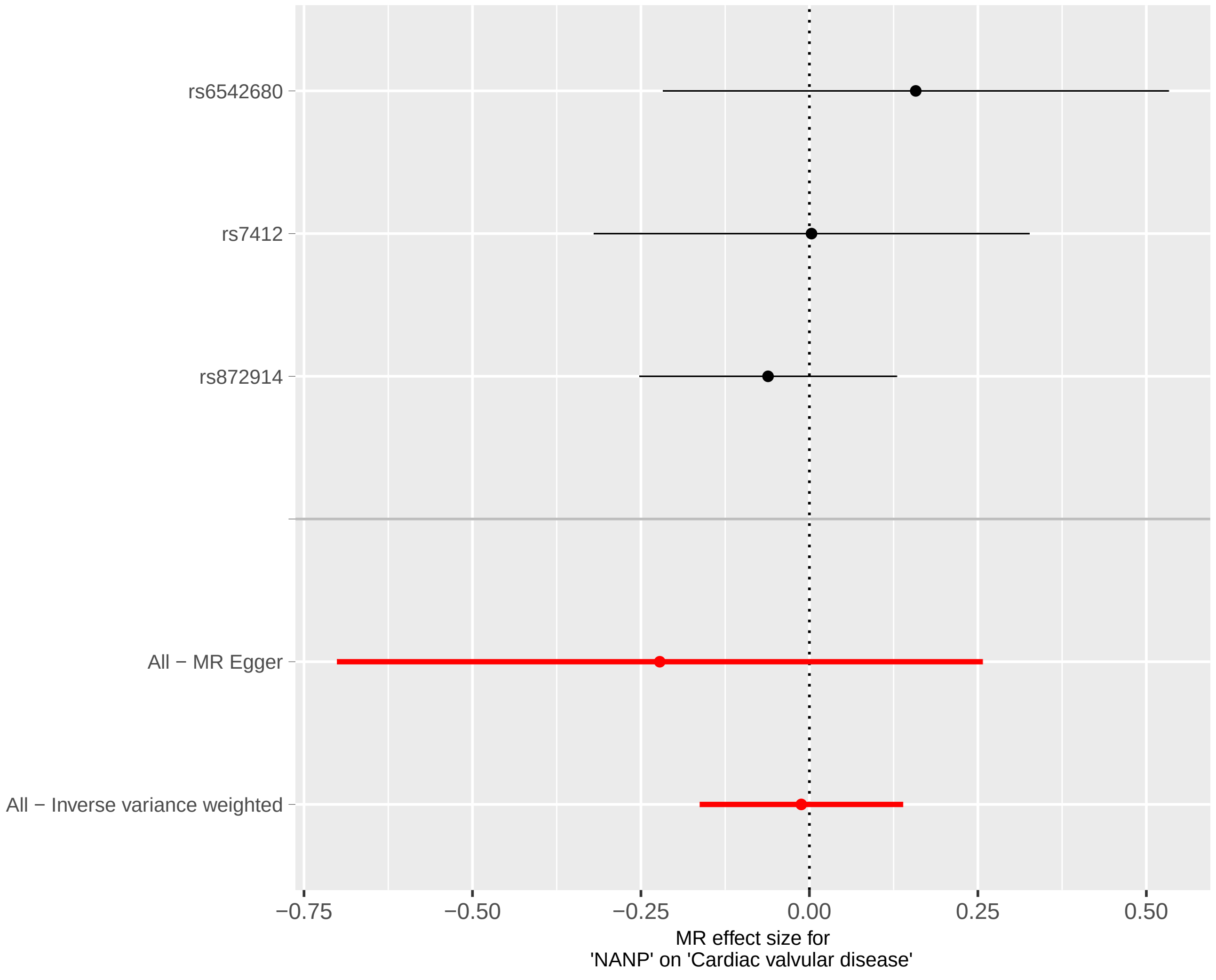
rs6542680
rs7412
rs872914
All − MR Egger
All − Inverse variance weighted
−0.75
−0.50
−0.25
0.00
0.25
0.50
MR effect size for
'NANP' on 'Cardiac valvular disease'

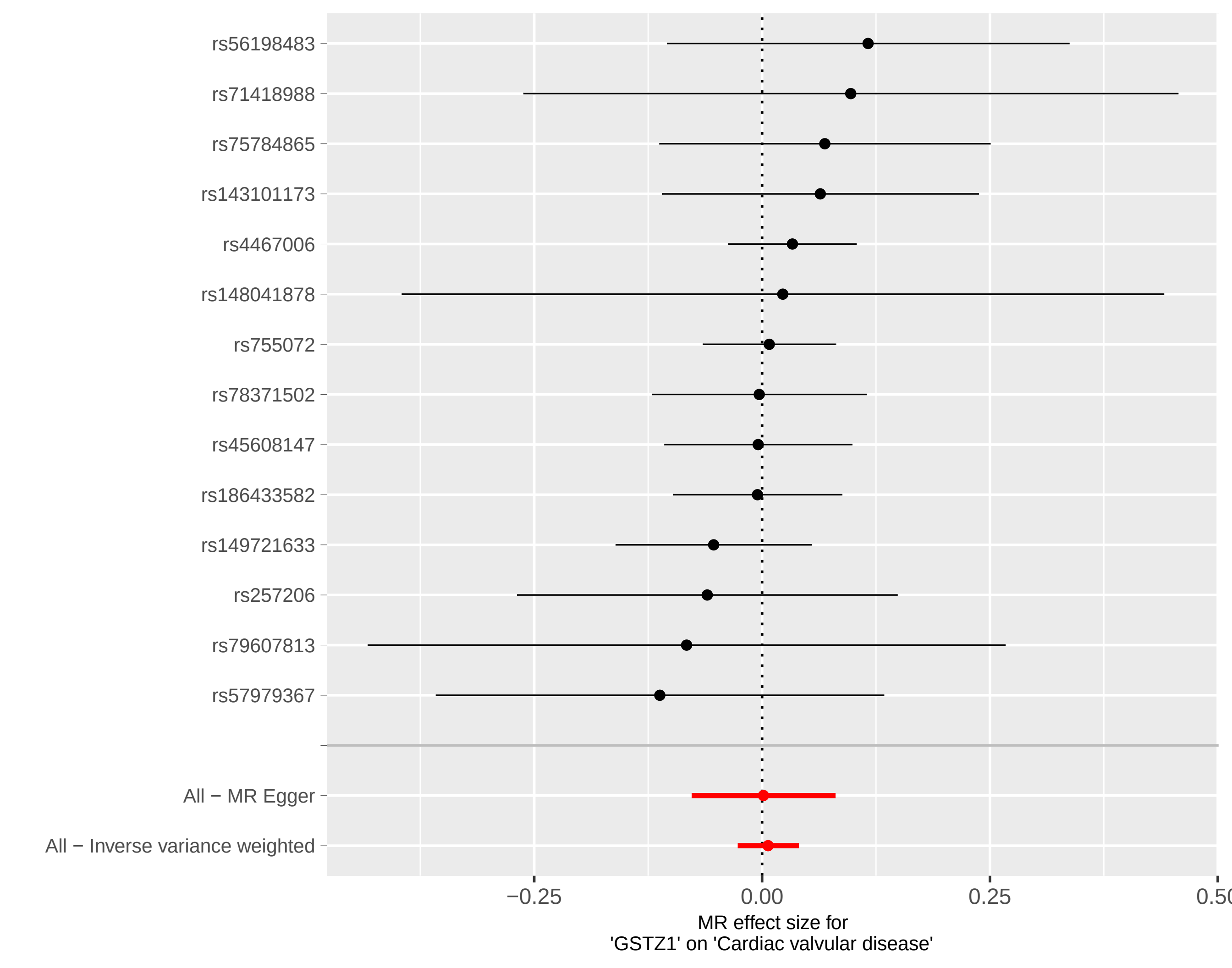
rs56198483
rs71418988
rs75784865
rs143101173
rs4467006
rs148041878
rs755072
rs78371502
rs45608147
rs186433582
rs149721633
rs257206
rs79607813
rs57979367
All − MR Egger
All − Inverse variance weighted
−0.25
0.00
0.25
0.50
MR effect size for
'GSTZ1' on 'Cardiac valvular disease'

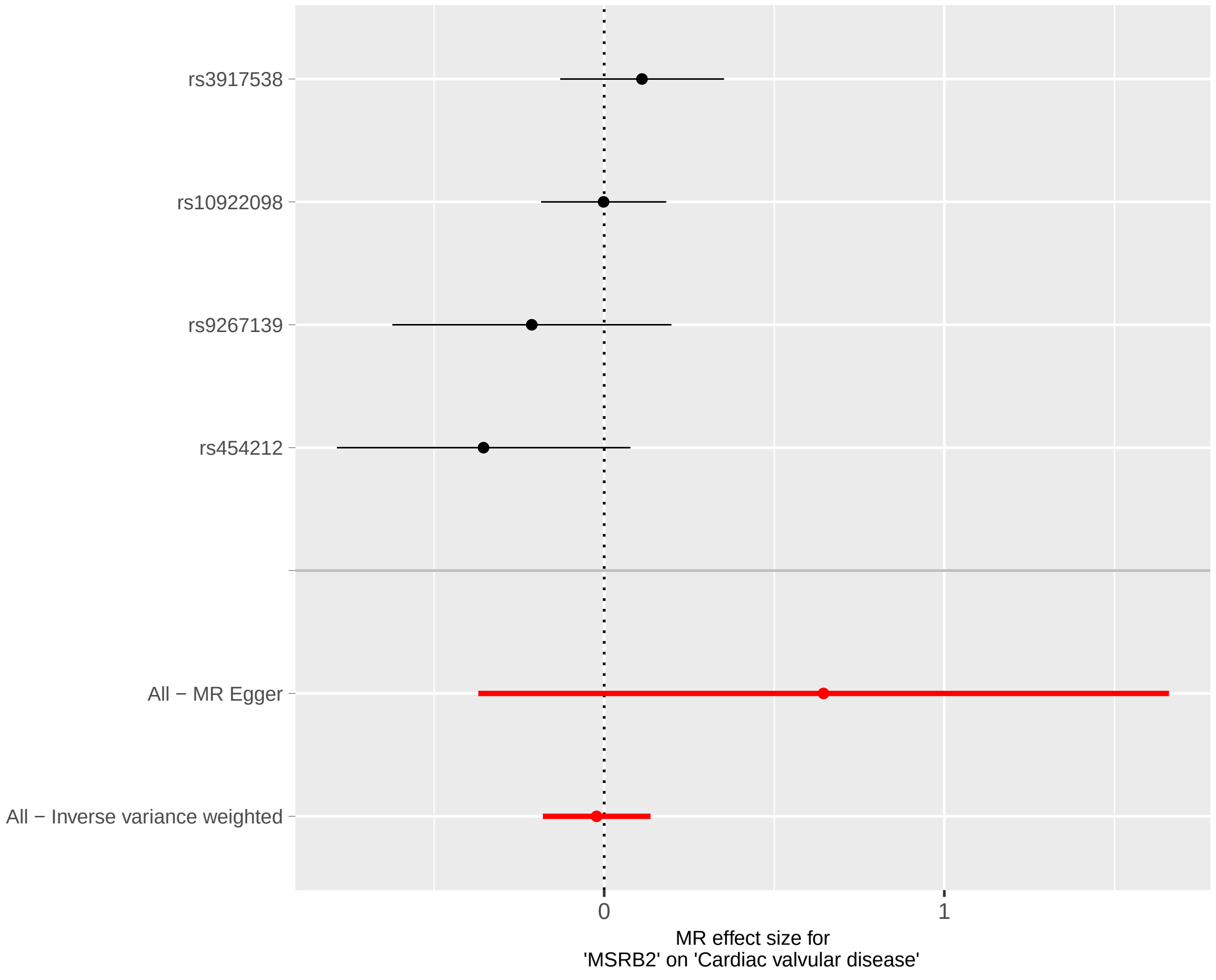
rs3917538
rs10922098
rs9267139
rs454212
All − MR Egger
All − Inverse variance weighted
0
1
MR effect size for
'MSRB2' on 'Cardiac valvular disease'

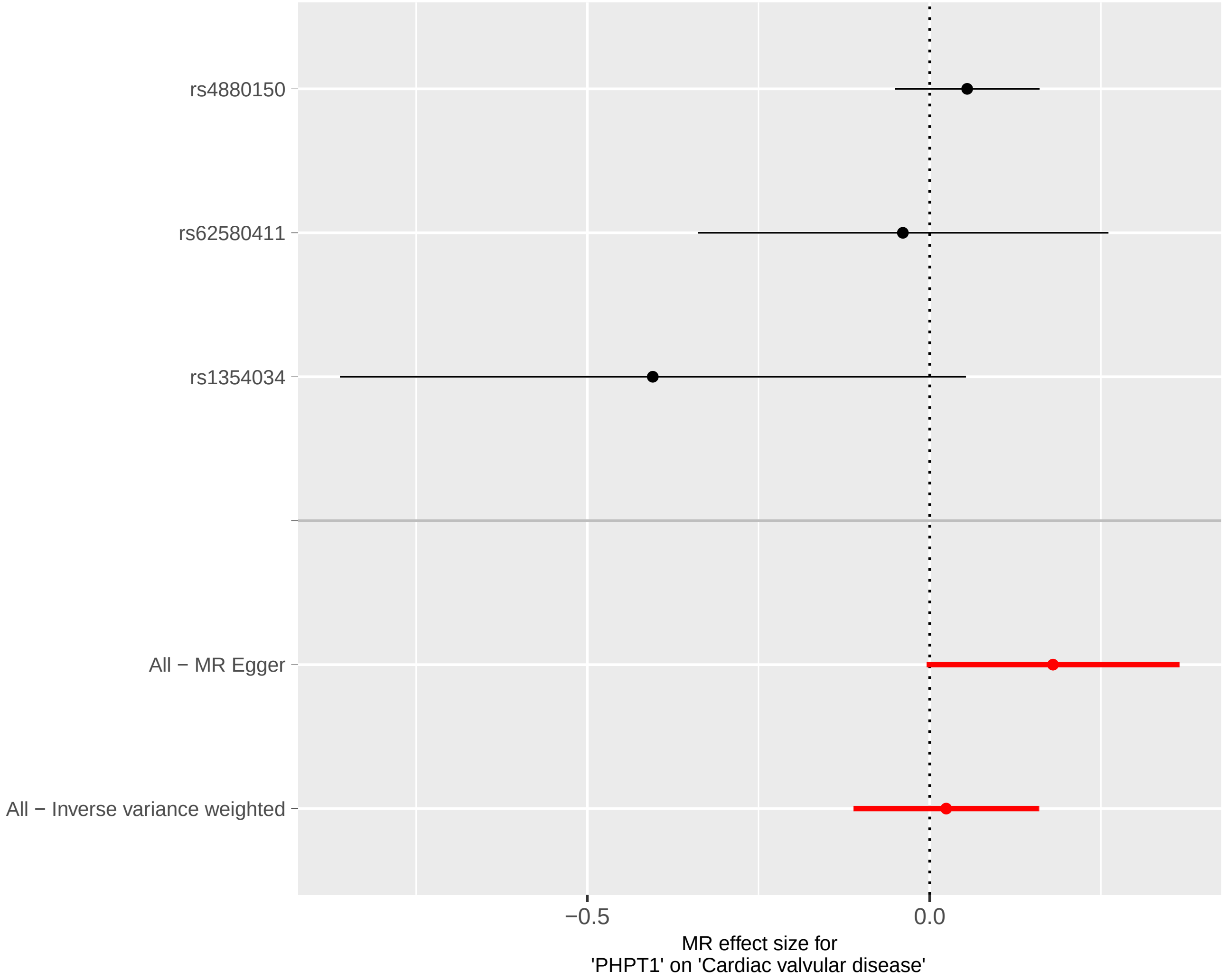
rs4880150
rs62580411
rs1354034
All − MR Egger
All − Inverse variance weighted
−0.5
0.0
MR effect size for
'PHPT1' on 'Cardiac valvular disease'

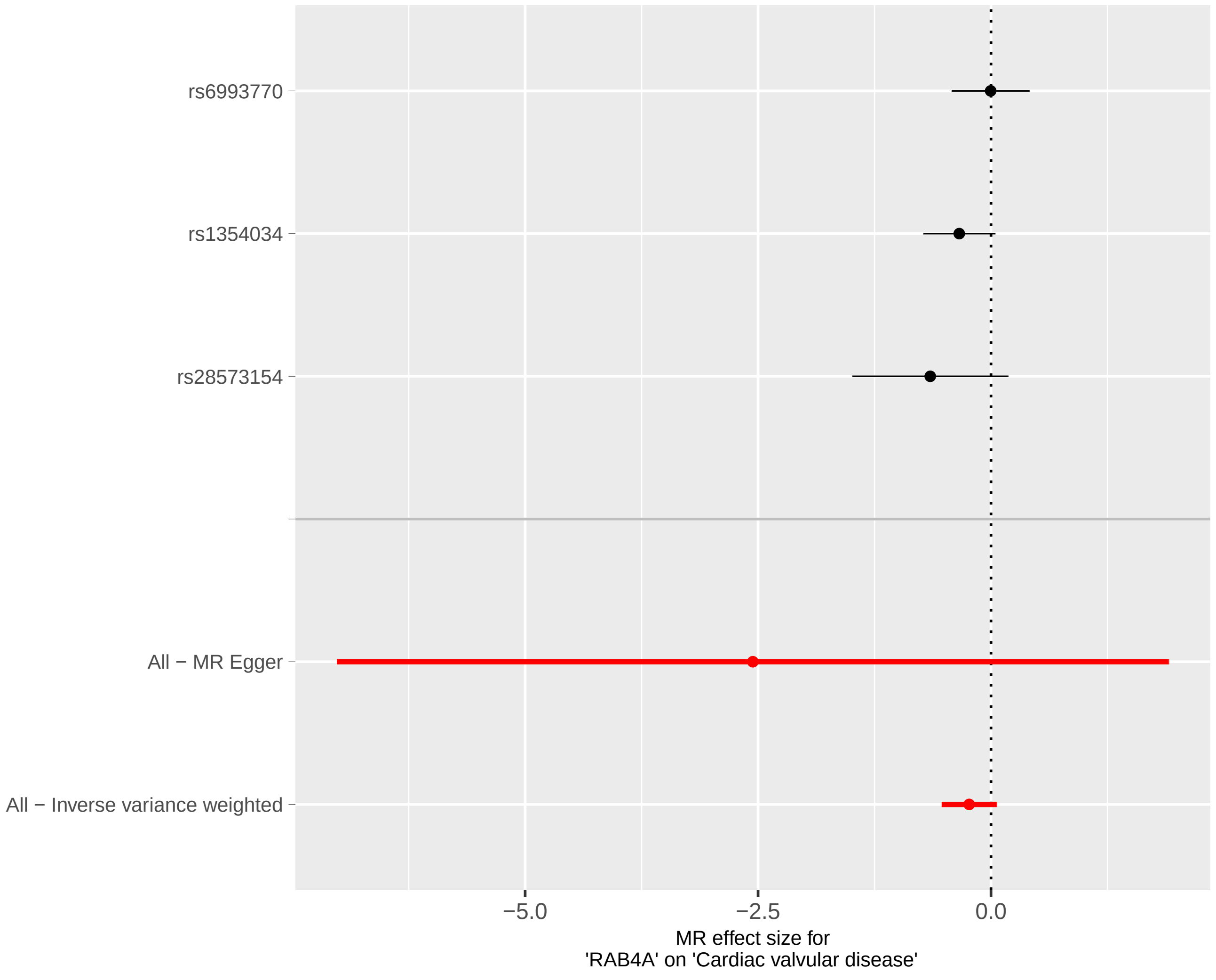
rs6993770
rs1354034
rs28573154
All − MR Egger
All − Inverse variance weighted
−5.0
−2.5
0.0
MR effect size for
'RAB4A' on 'Cardiac valvular disease'

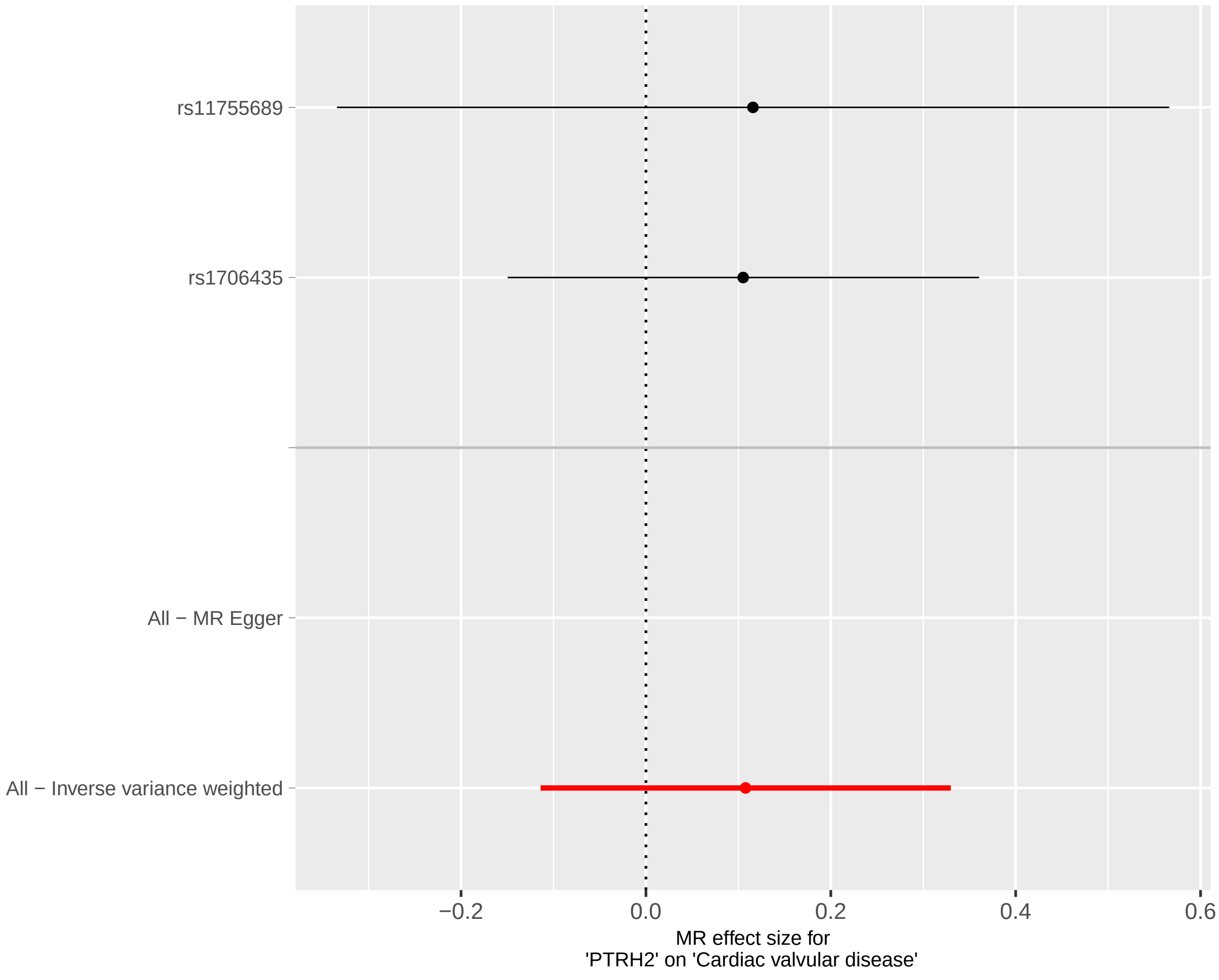

rs11755689
rs1706435
All − MR Egger
All − Inverse variance weighted
−0.2
0.0
0.2
0.4
0.6
MR effect size for
'PTRH2' on 'Cardiac valvular disease'

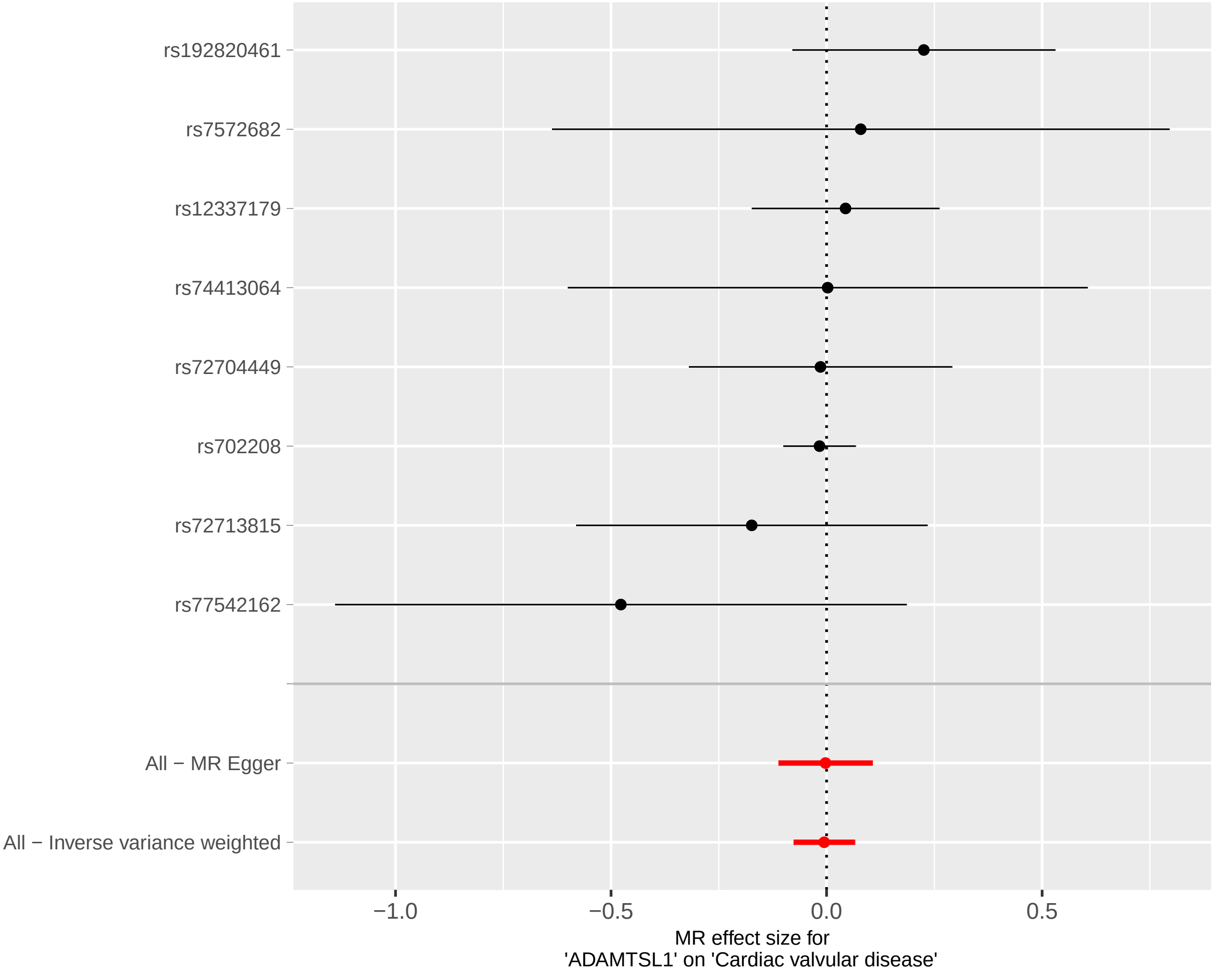
rs192820461
rs7572682
rs12337179
rs74413064
rs72704449
rs702208
rs72713815
rs77542162
All − MR Egger
All − Inverse variance weighted
−1.0
−0.5
0.0
0.5
MR effect size for
'ADAMTSL1' on 'Cardiac valvular disease'

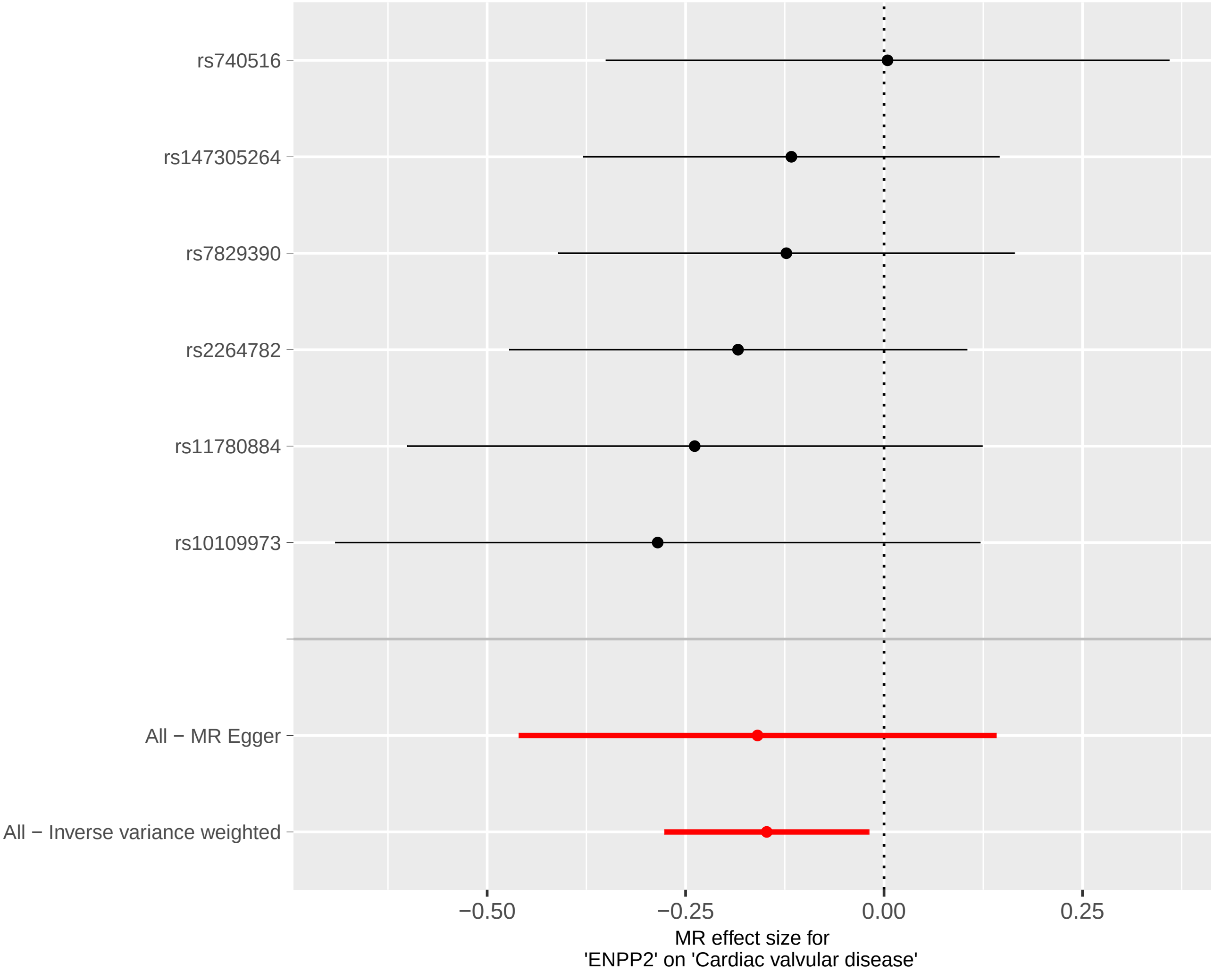

rs740516
rs147305264
rs7829390
rs2264782
rs11780884
rs10109973
All − MR Egger
All − Inverse variance weighted
−0.50
−0.25
0.00
0.25
MR effect size for
'ENPP2' on 'Cardiac valvular disease'

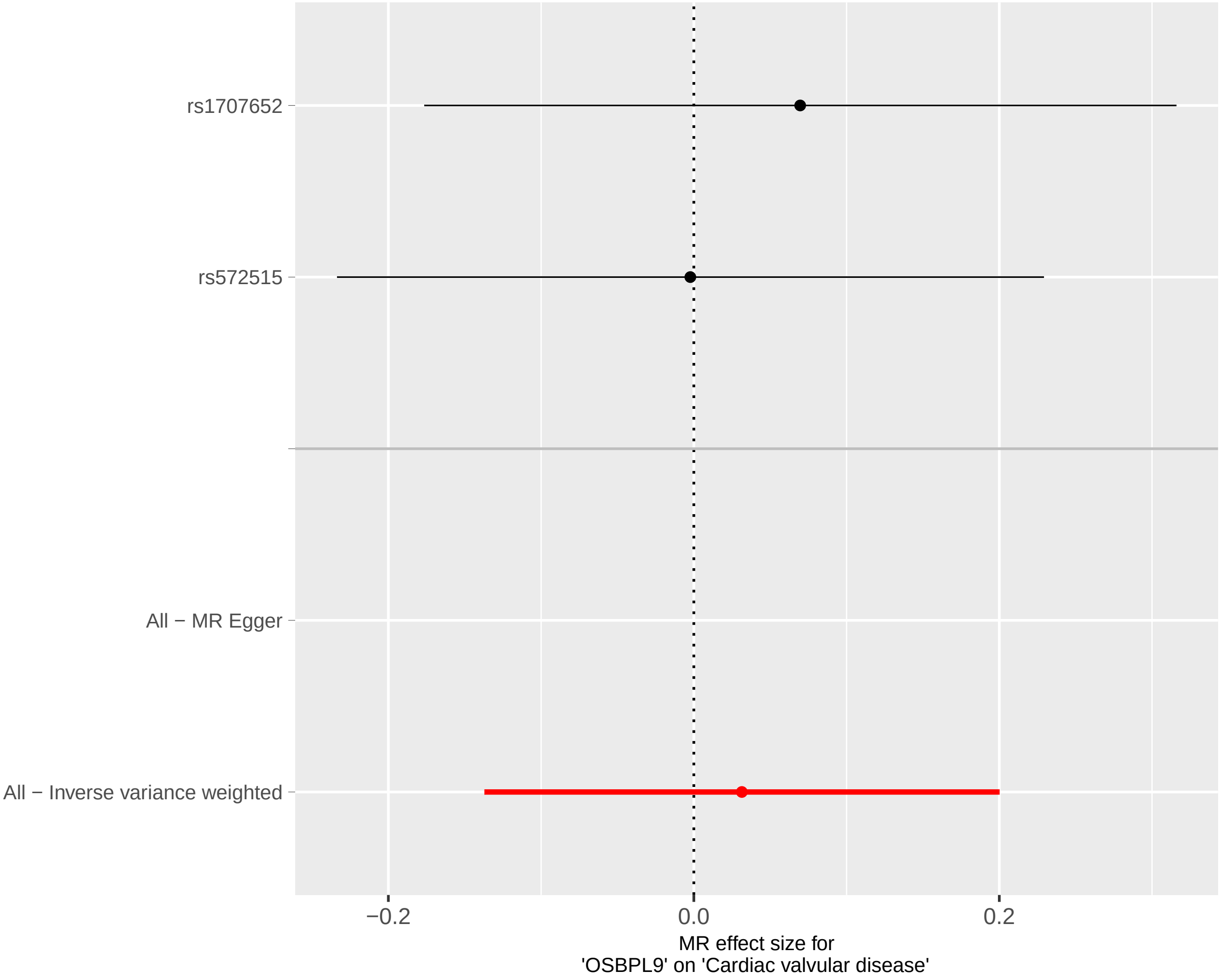

rs1707652
rs572515
All − MR Egger
All − Inverse variance weighted
−0.2
0.0
0.2
MR effect size for
'OSBPL9' on 'Cardiac valvular disease'

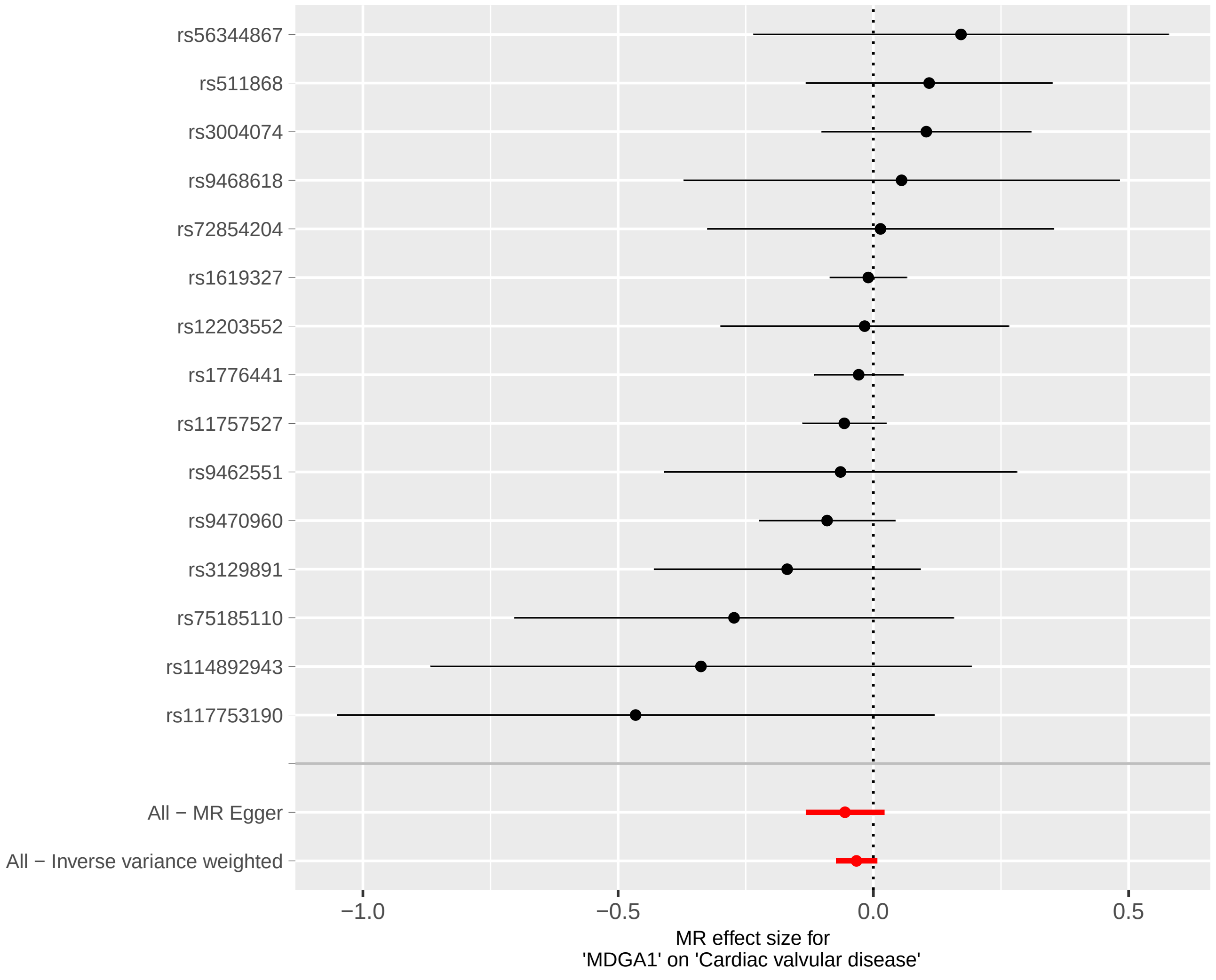

rs56344867
rs511868
rs3004074
rs9468618
rs72854204
rs1619327
rs12203552
rs1776441
rs11757527
rs9462551
rs9470960
rs3129891
rs75185110
rs114892943
rs117753190
All − MR Egger
All − Inverse variance weighted
−1.0
−0.5
0.0
0.5
MR effect size for
'MDGA1' on 'Cardiac valvular disease'

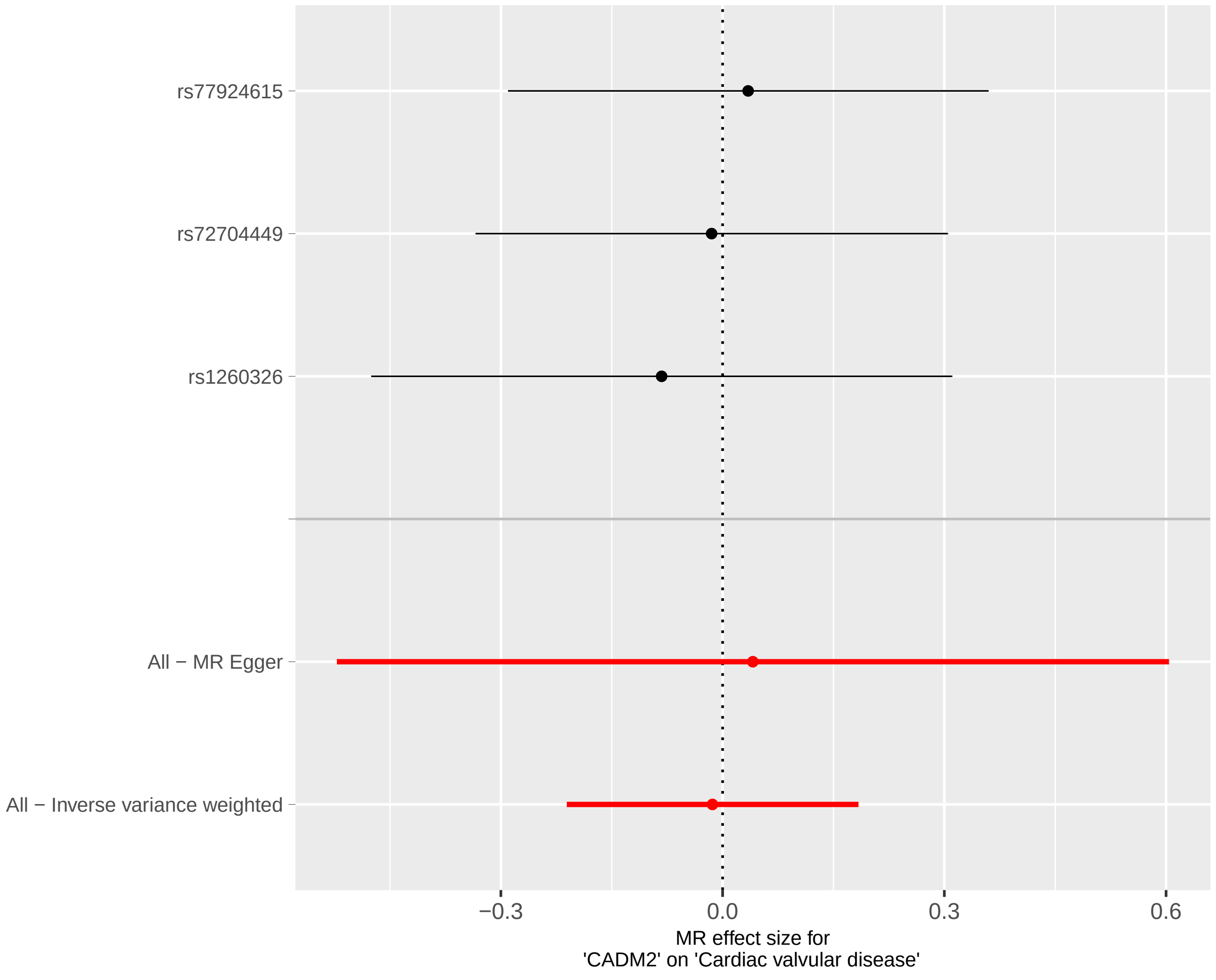
rs77924615
rs72704449
rs1260326
All − MR Egger
All − Inverse variance weighted
−0.3
0.0
0.3
0.6
MR effect size for
'CADM2' on 'Cardiac valvular disease'

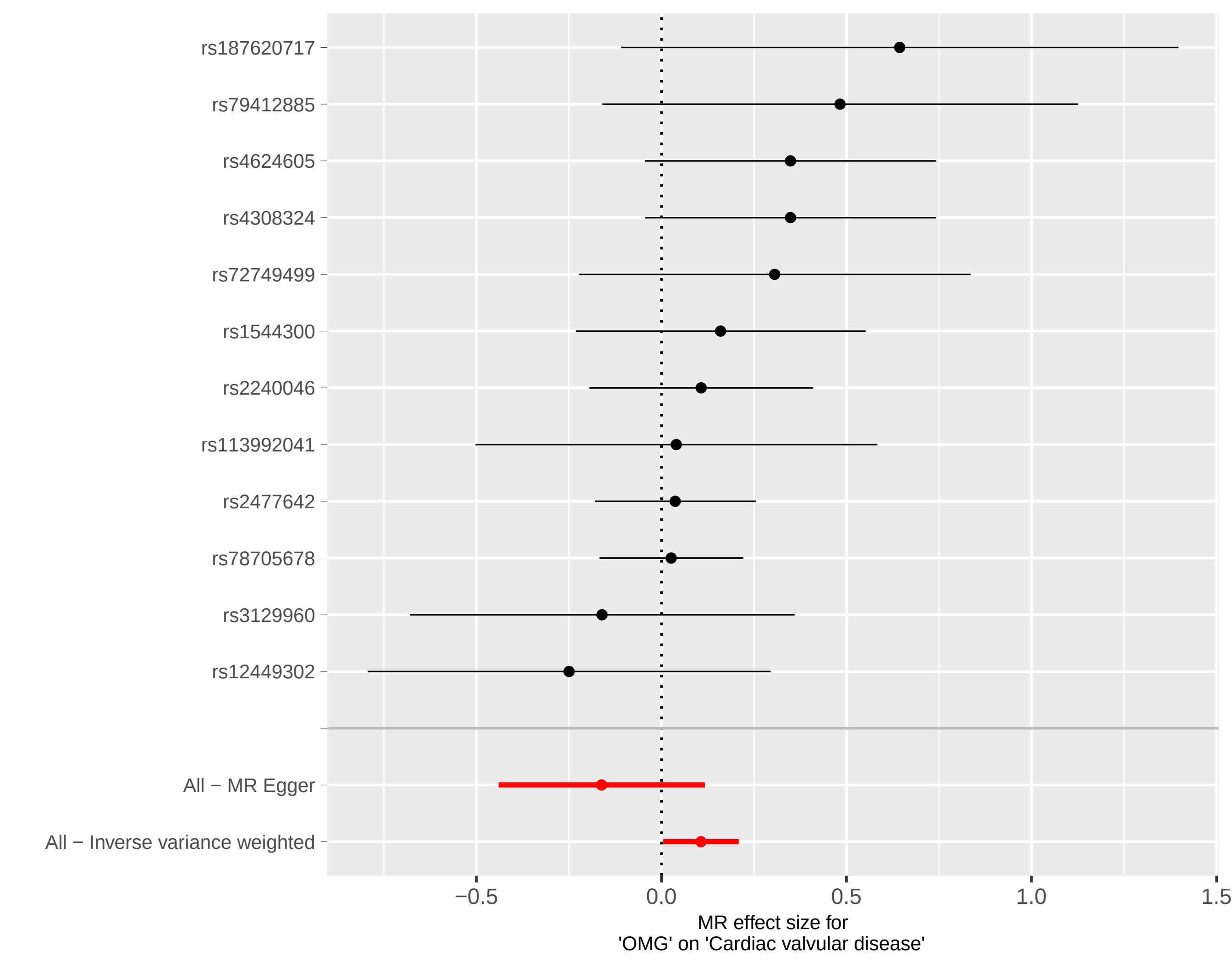
rs187620717
rs79412885
rs4624605
rs4308324
rs72749499
rs1544300
rs2240046
rs113992041
rs2477642
rs78705678
rs3129960
rs12449302
All − MR Egger
All − Inverse variance weighted
−0.5
0.0
0.5
1.0
1.5
MR effect size for
'OMG' on 'Cardiac valvular disease'

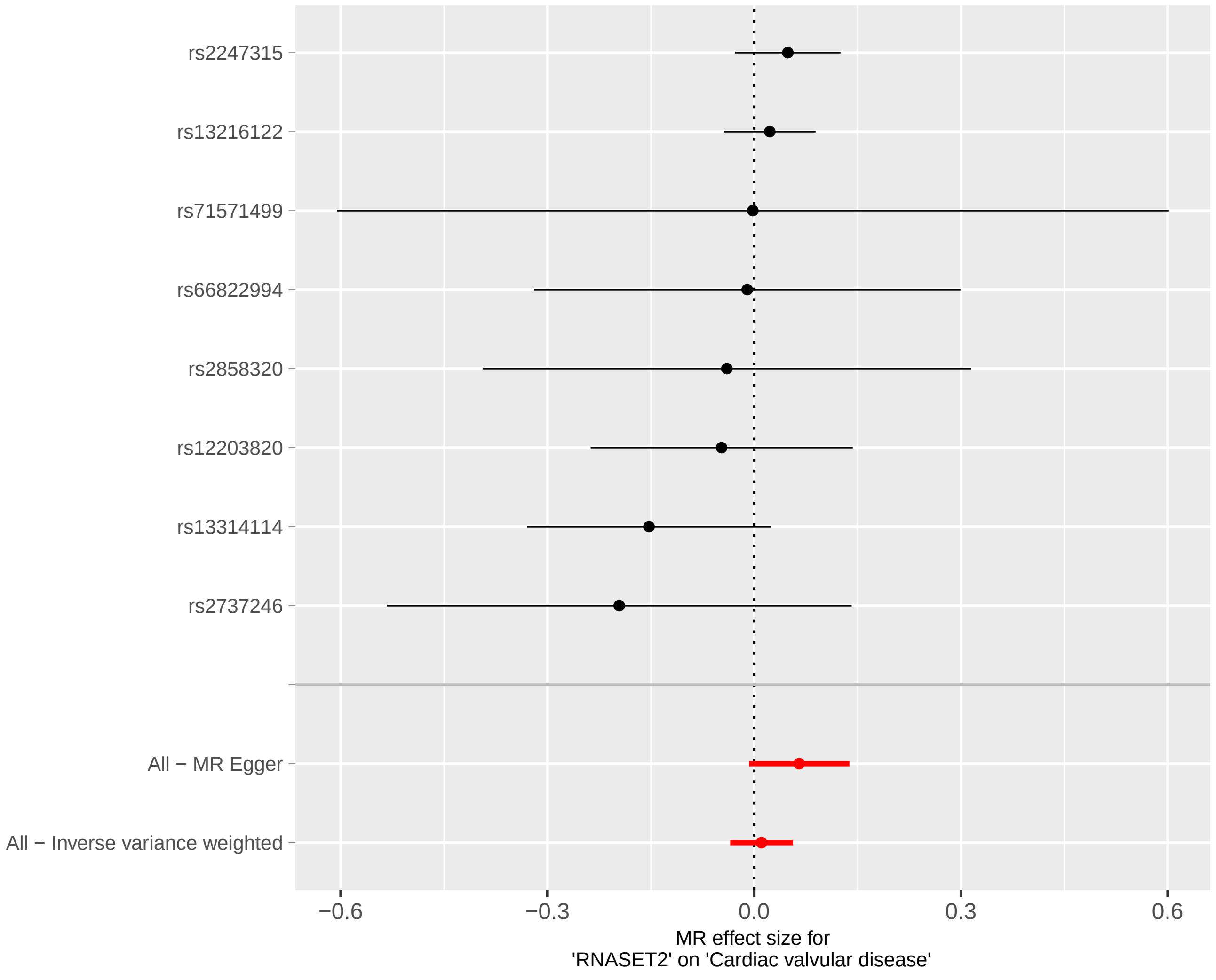
rs2247315
rs13216122
rs71571499
rs66822994
rs2858320
rs12203820
rs13314114
rs2737246
All − MR Egger
All − Inverse variance weighted
−0.6
−0.3
0.0
0.3
0.6
MR effect size for
'RNASET2' on 'Cardiac valvular disease'

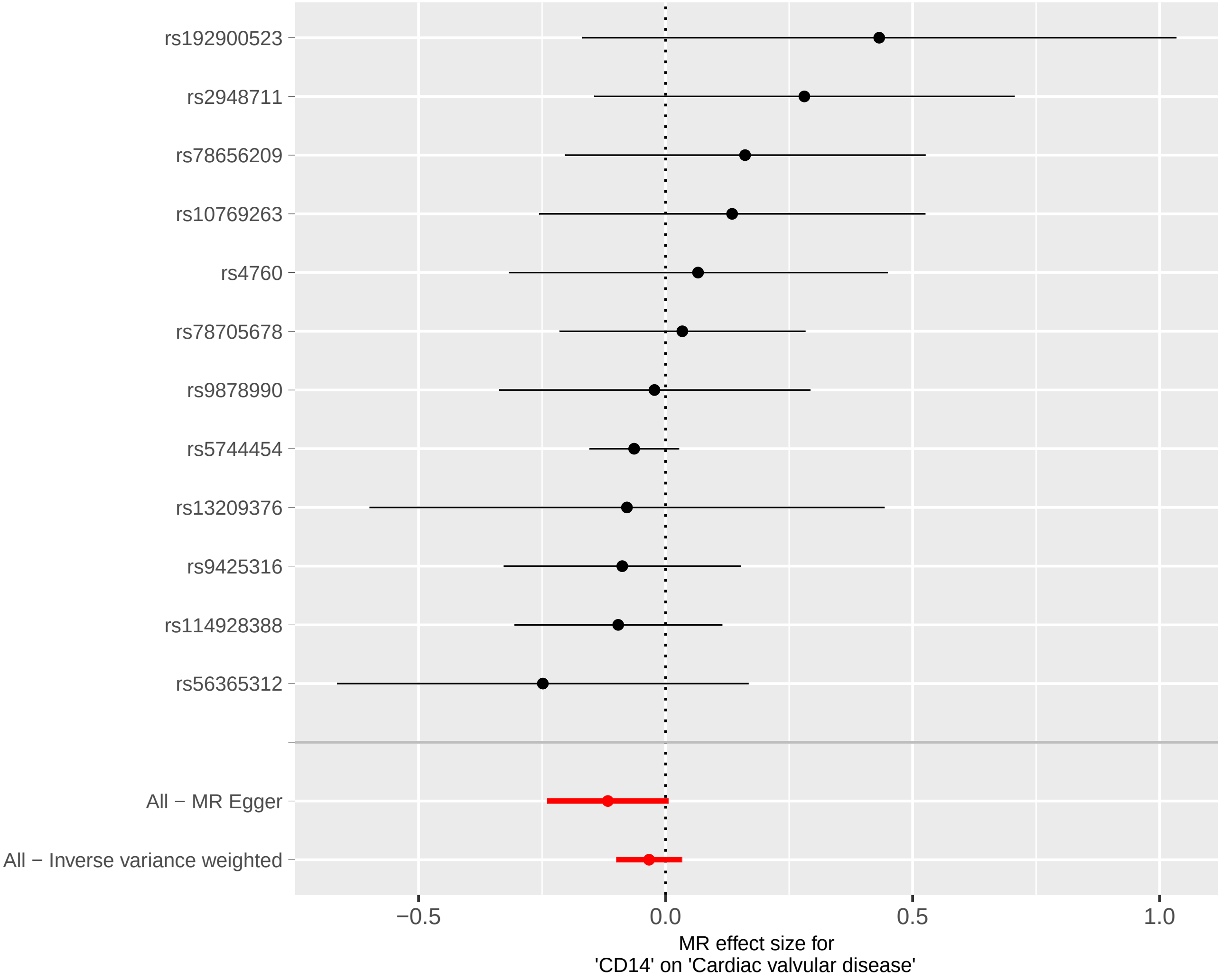
rs192900523
rs2948711
rs78656209
rs10769263
rs4760
rs78705678
rs9878990
rs5744454
rs13209376
rs9425316
rs114928388
rs56365312
All − MR Egger
All − Inverse variance weighted
−0.5
0.0
0.5
1.0
MR effect size for
'CD14' on 'Cardiac valvular disease'

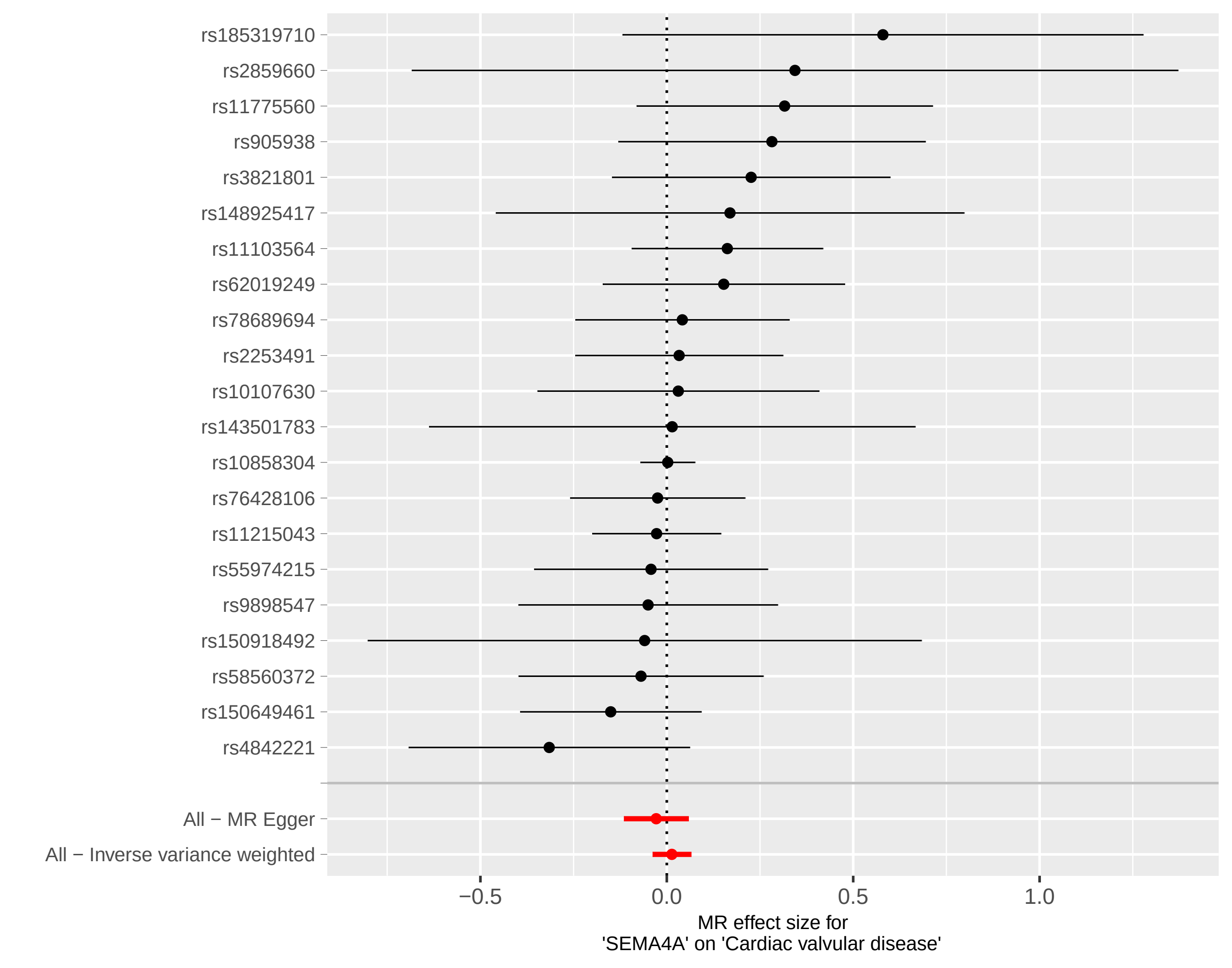
rs185319710
rs2859660
rs11775560
rs905938
rs3821801
rs148925417
rs11103564
rs62019249
rs78689694
rs2253491
rs10107630
rs143501783
rs10858304
rs76428106
rs11215043
rs55974215
rs9898547
rs150918492
rs58560372
rs150649461
rs4842221
All − MR Egger
All − Inverse variance weighted
−0.5
0.0
0.5
1.0
MR effect size for
'SEMA4A' on 'Cardiac valvular disease'

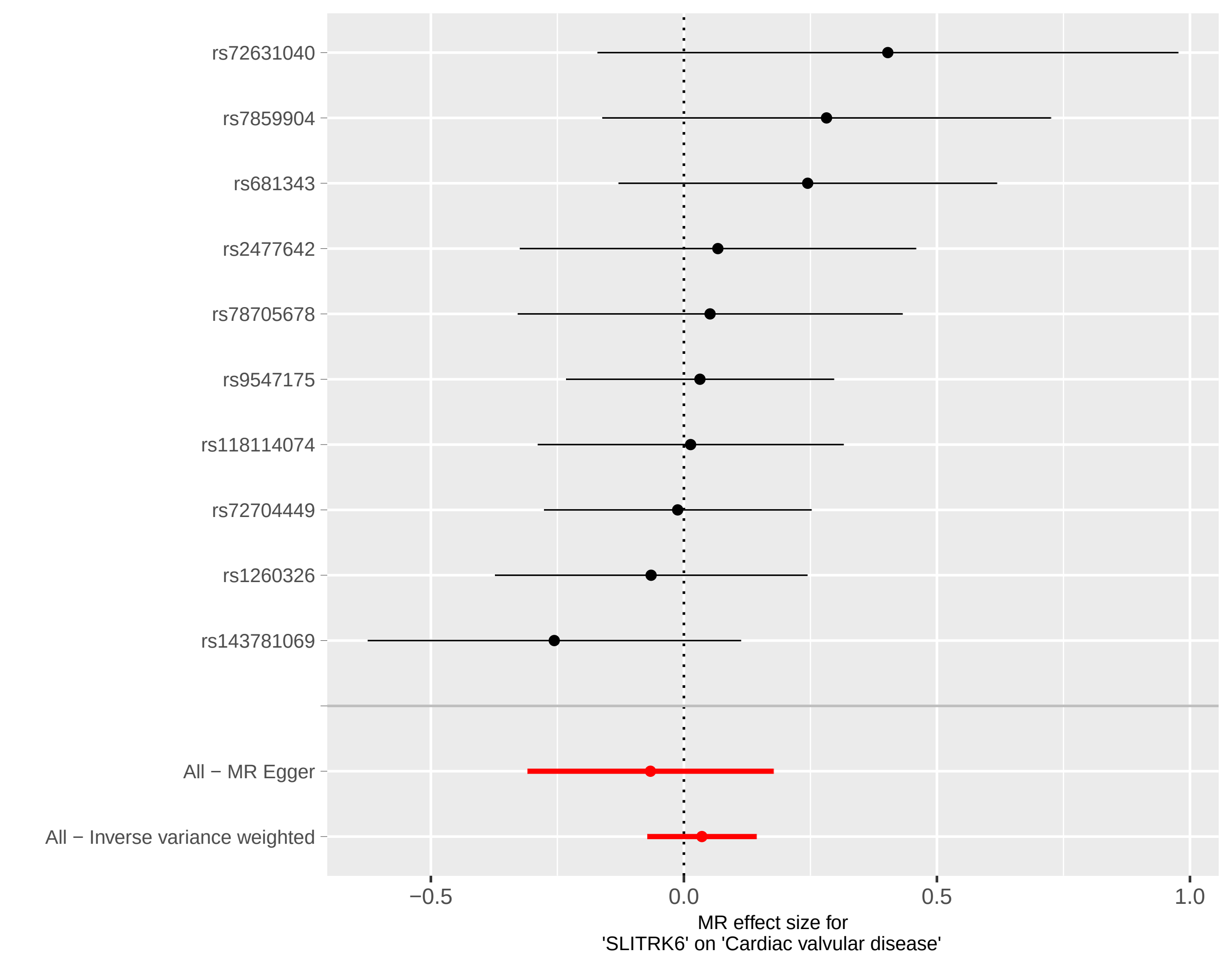
rs72631040
rs7859904
rs681343
rs2477642
rs78705678
rs9547175
rs118114074
rs72704449
rs1260326
rs143781069
All − MR Egger
All − Inverse variance weighted
−0.5
0.0
0.5
1.0
MR effect size for
'SLITRK6' on 'Cardiac valvular disease'

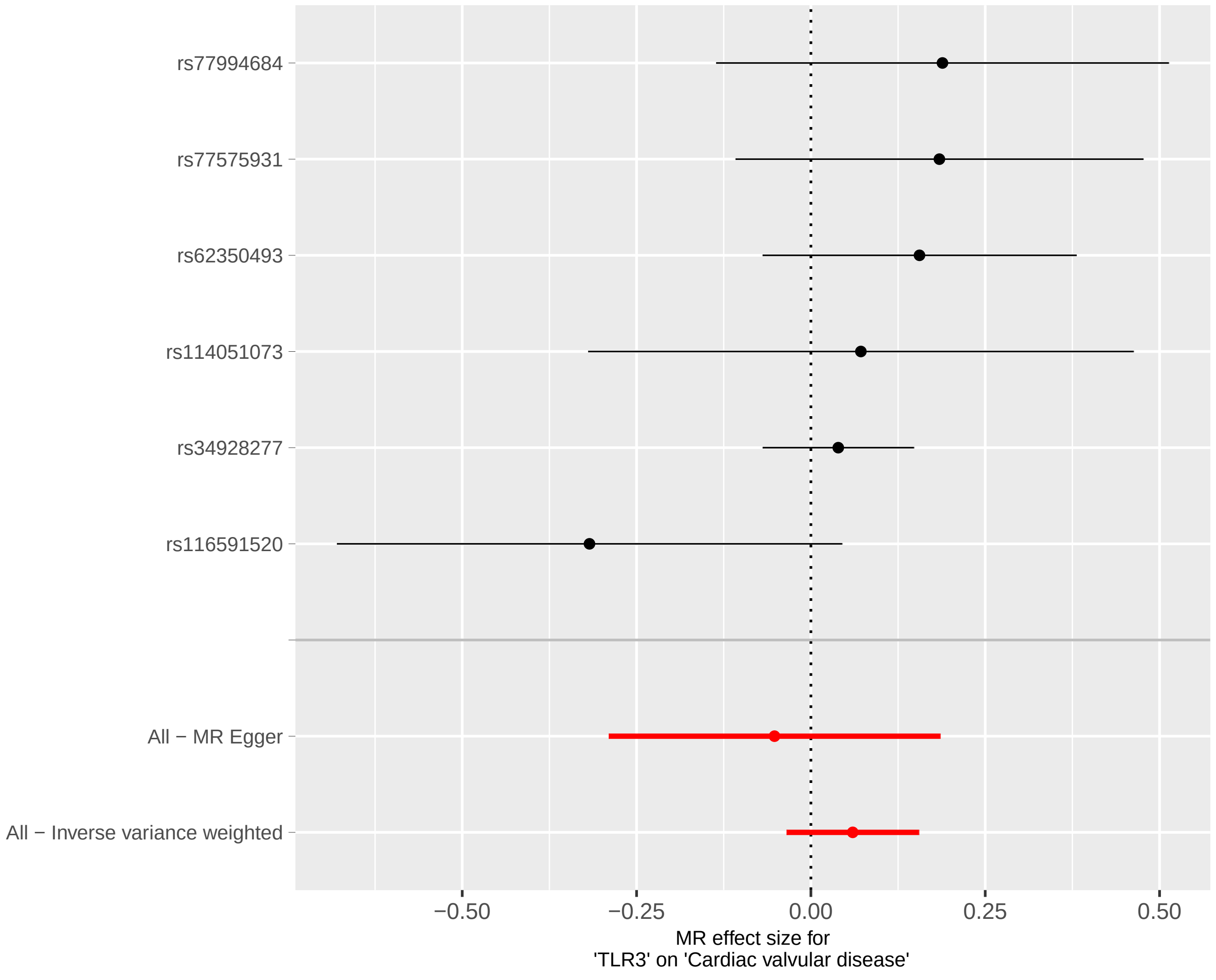
rs77994684
rs77575931
rs62350493
rs114051073
rs34928277
rs116591520
All − MR Egger
All − Inverse variance weighted
−0.50
−0.25
0.00
0.25
0.50
MR effect size for
'TLR3' on 'Cardiac valvular disease'

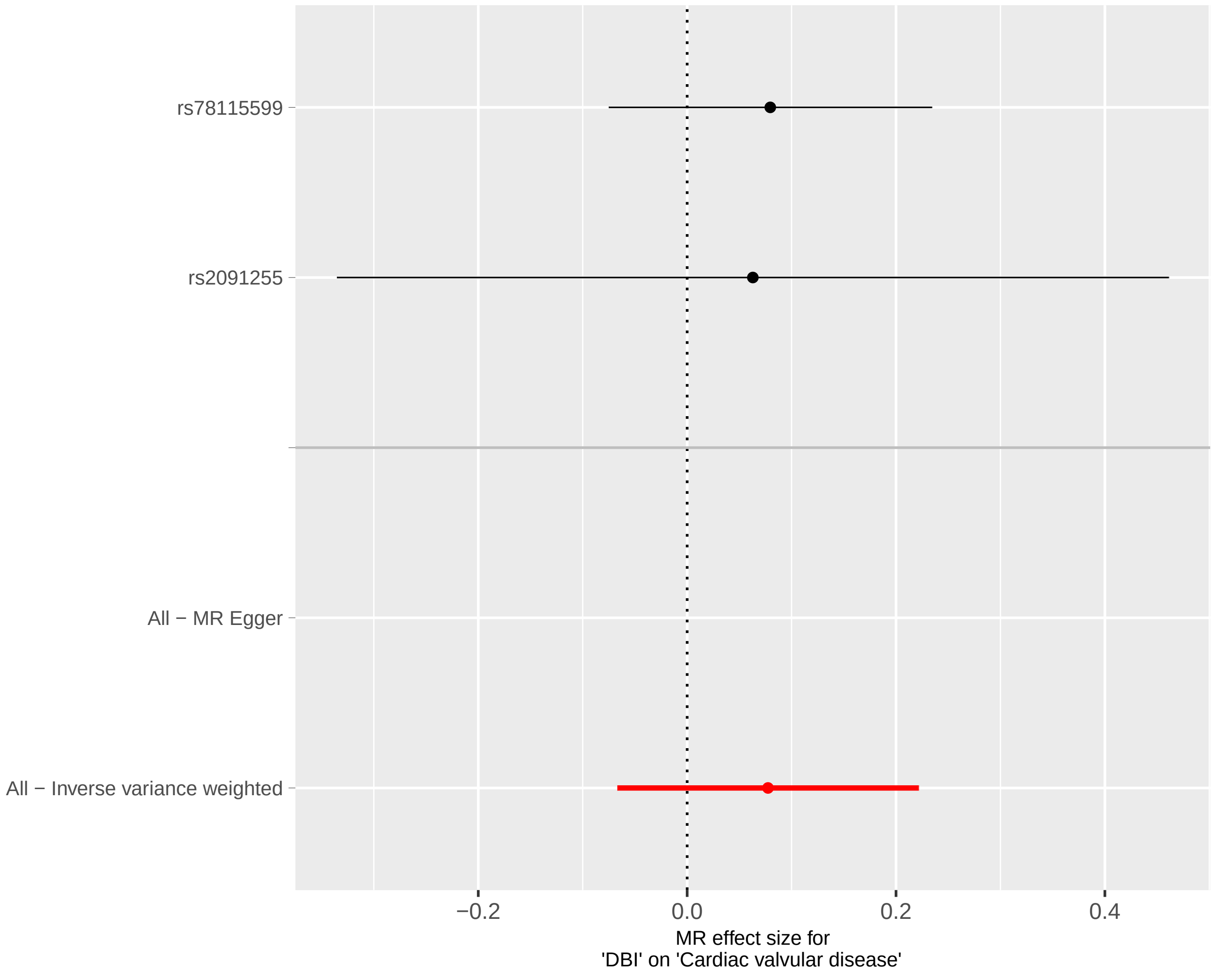
rs78115599
rs2091255
All − MR Egger
All − Inverse variance weighted
−0.2
0.0
0.2
0.4
MR effect size for
'DBI' on 'Cardiac valvular disease'

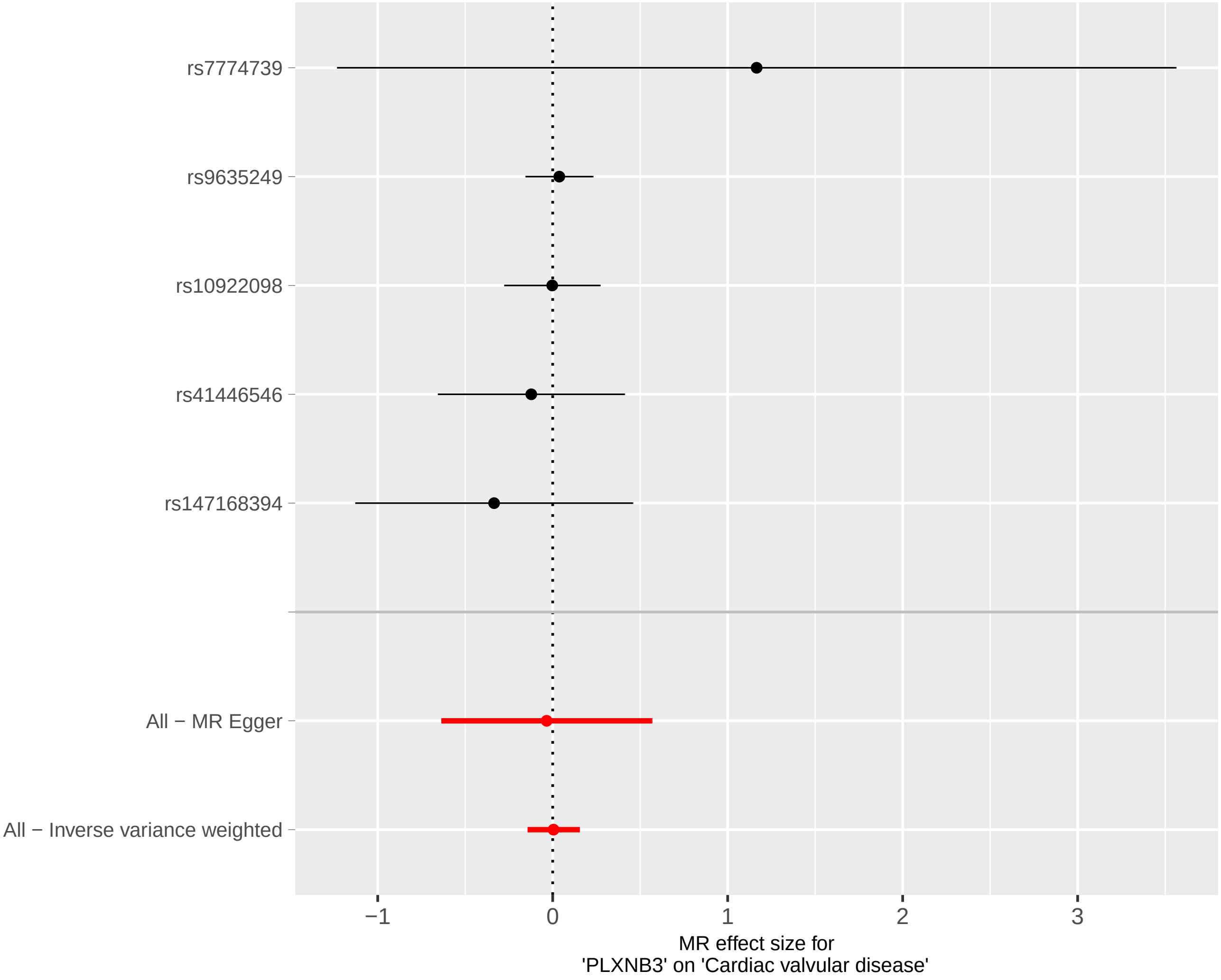

rs7774739
rs9635249
rs10922098
rs41446546
rs147168394
All − MR Egger
All − Inverse variance weighted
−1
0
1
2
3
MR effect size for
'PLXNB3' on 'Cardiac valvular disease'

Insufficient number of SNPs

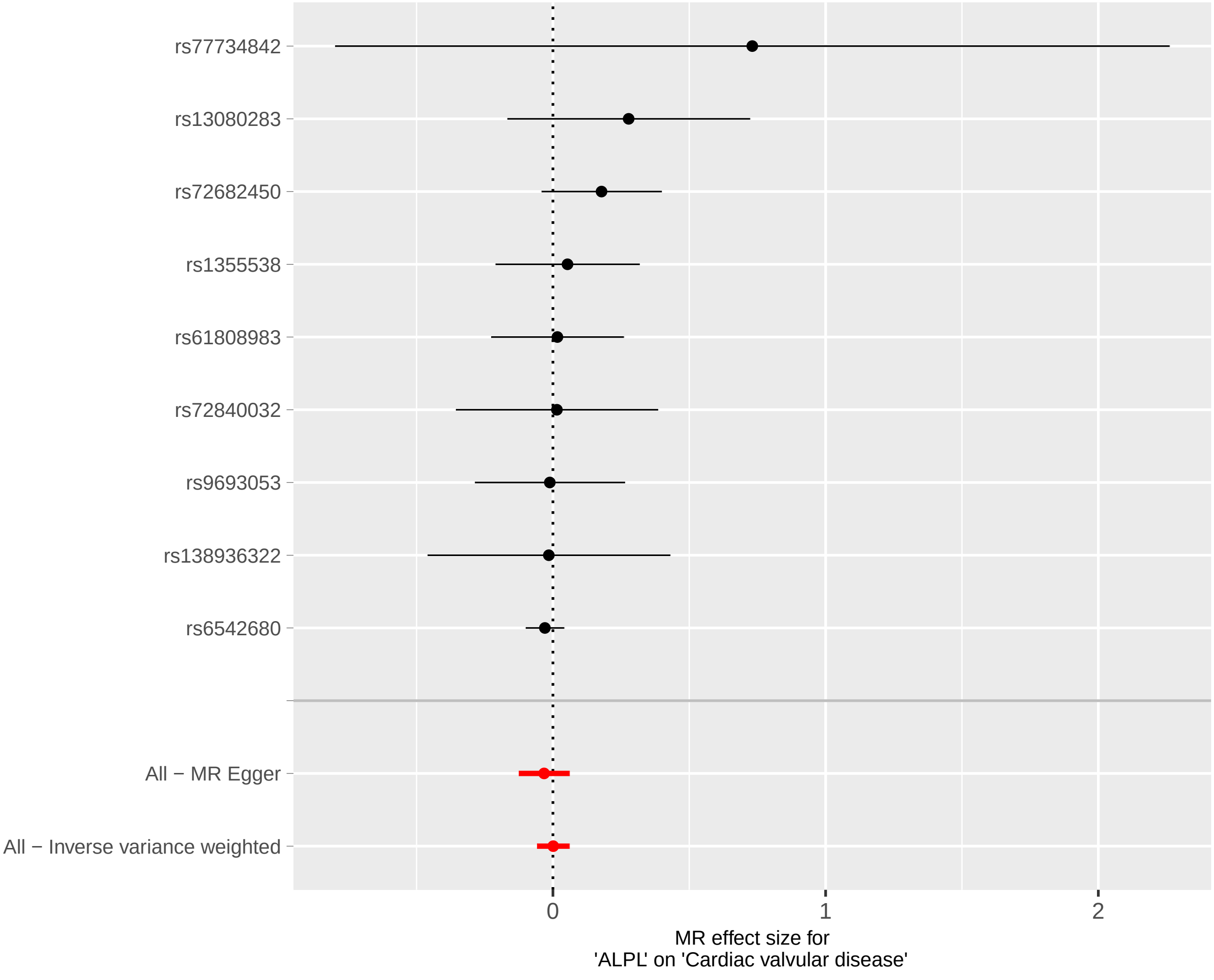
rs77734842
rs13080283
rs72682450
rs1355538
rs61808983
rs72840032
rs9693053
rs138936322
rs6542680
All − MR Egger
All − Inverse variance weighted
0
1
2
MR effect size for
'ALPL' on 'Cardiac valvular disease'

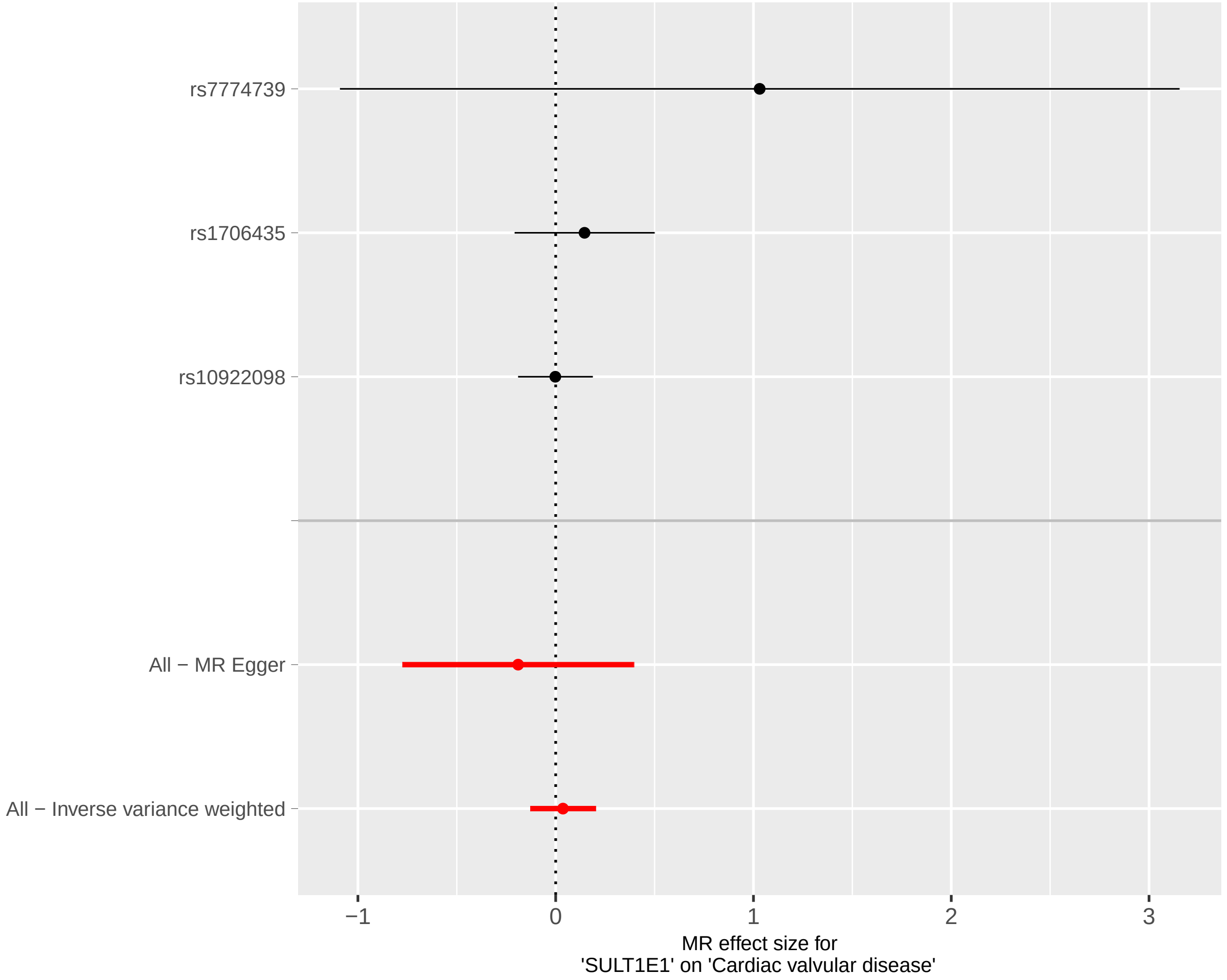
rs7774739
rs1706435
rs10922098
All − MR Egger
All − Inverse variance weighted
−1
0
1
2
3
MR effect size for
'SULT1E1' on 'Cardiac valvular disease'

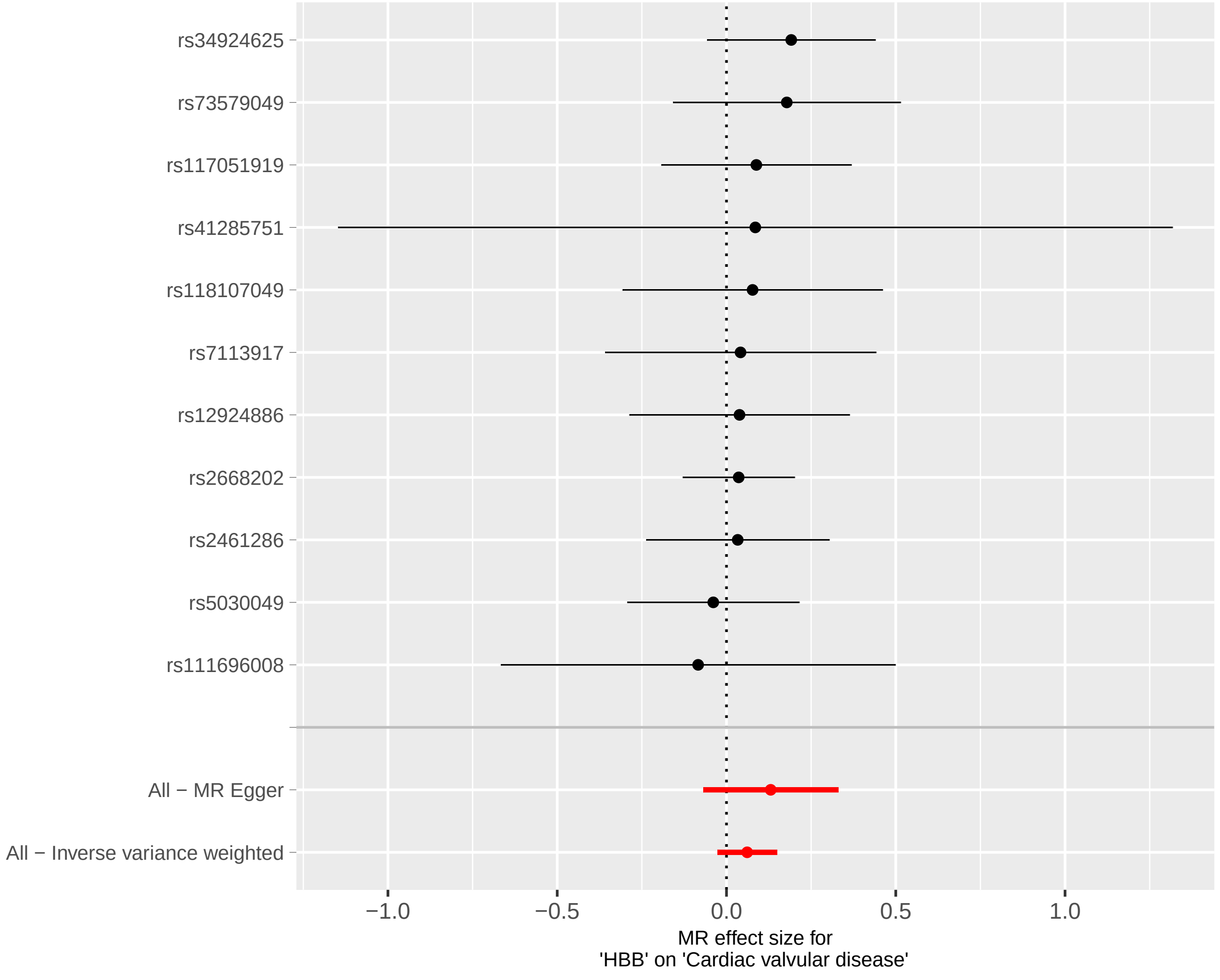
rs34924625
rs73579049
rs117051919
rs41285751
rs118107049
rs7113917
rs12924886
rs2668202
rs2461286
rs5030049
rs111696008
All − MR Egger
All − Inverse variance weighted
−1.0
−0.5
0.0
0.5
1.0
MR effect size for
'HBB' on 'Cardiac valvular disease'

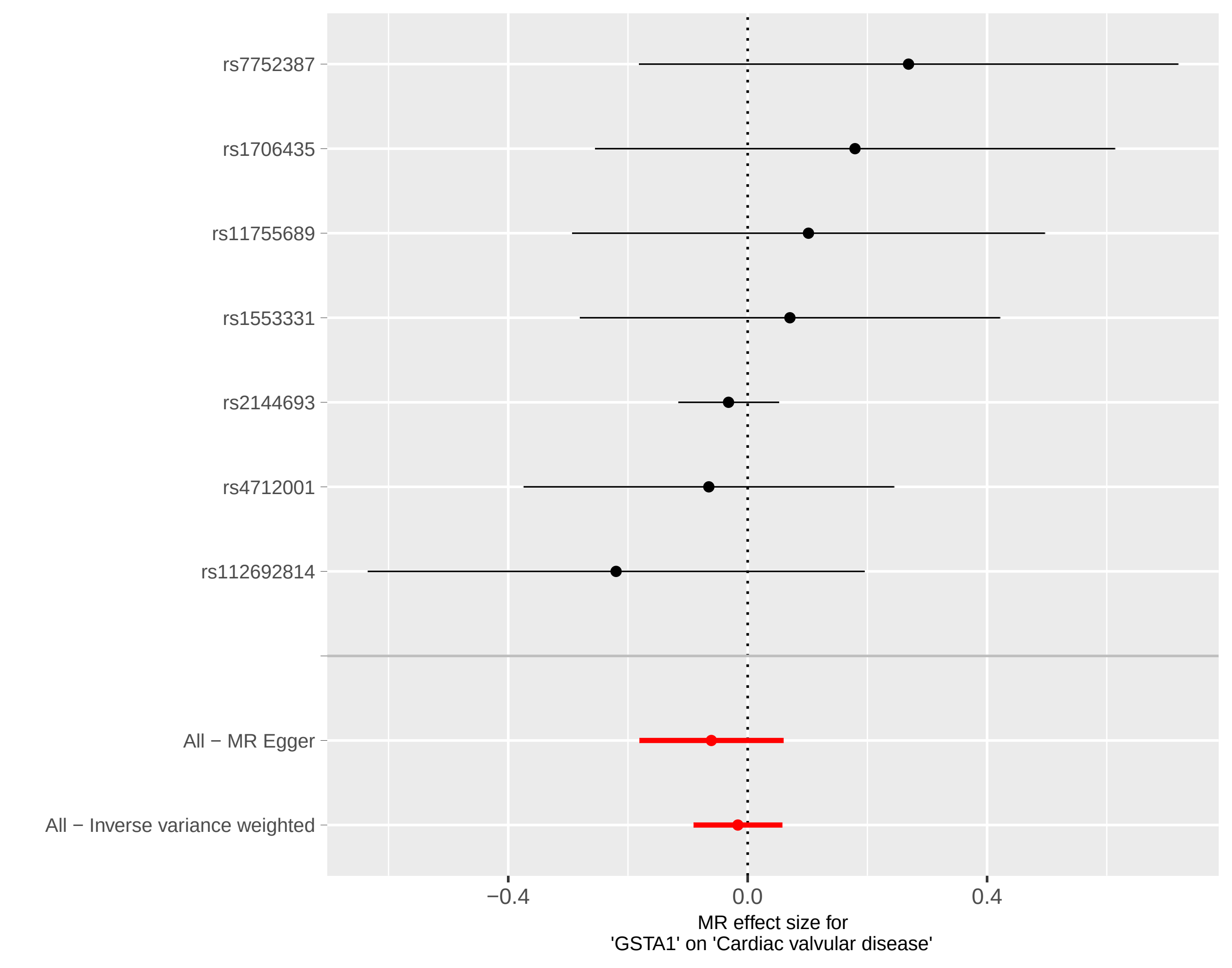

rs7752387
rs1706435
rs11755689
rs1553331
rs2144693
rs4712001
rs112692814
All − MR Egger
All − Inverse variance weighted
−0.4
0.0
0.4
MR effect size for
'GSTA1' on 'Cardiac valvular disease'

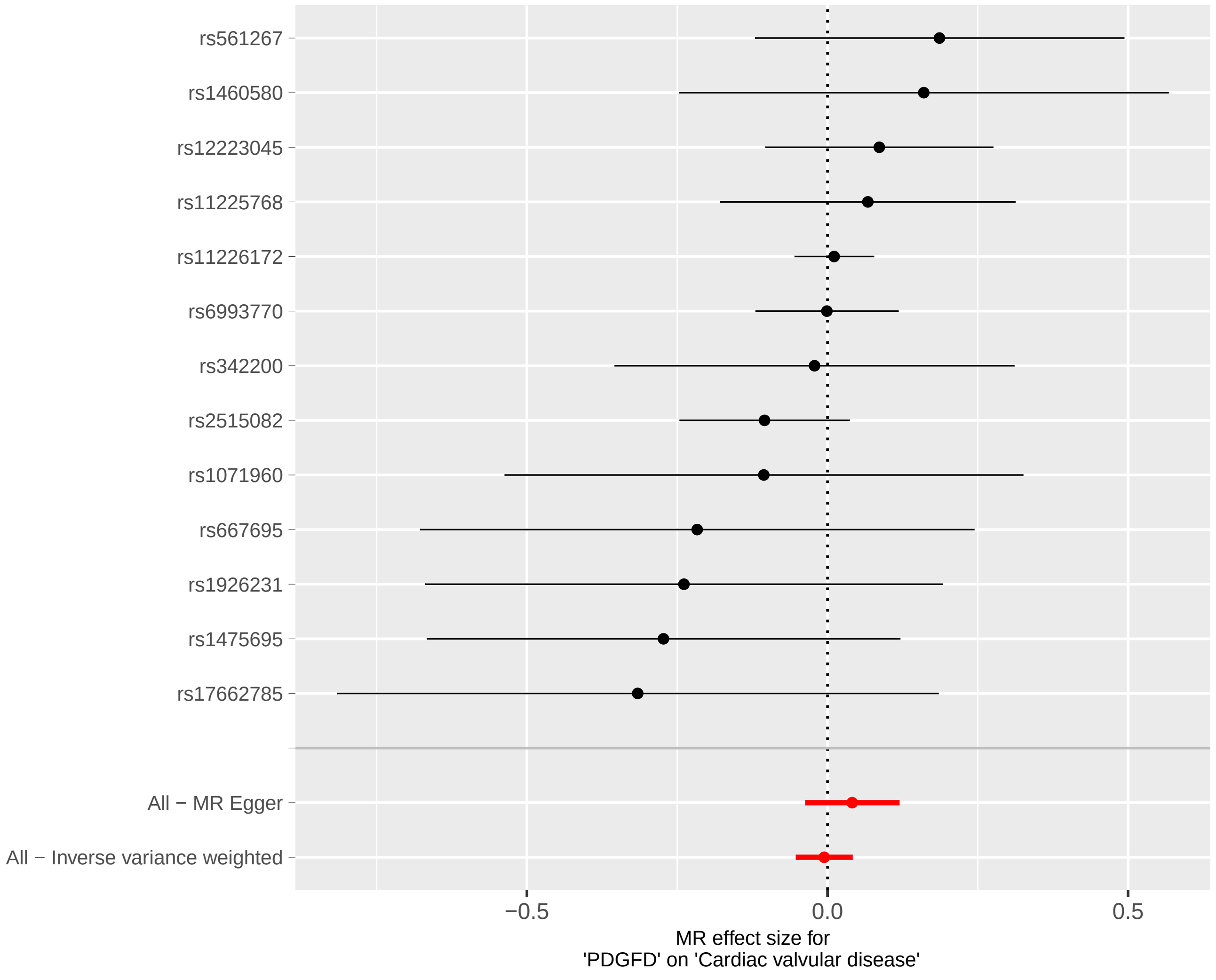
rs561267
rs1460580
rs12223045
rs11225768
rs11226172
rs6993770
rs342200
rs2515082
rs1071960
rs667695
rs1926231
rs1475695
rs17662785
All − MR Egger
All − Inverse variance weighted
−0.5
0.0
0.5
MR effect size for
'PDGFD' on 'Cardiac valvular disease'

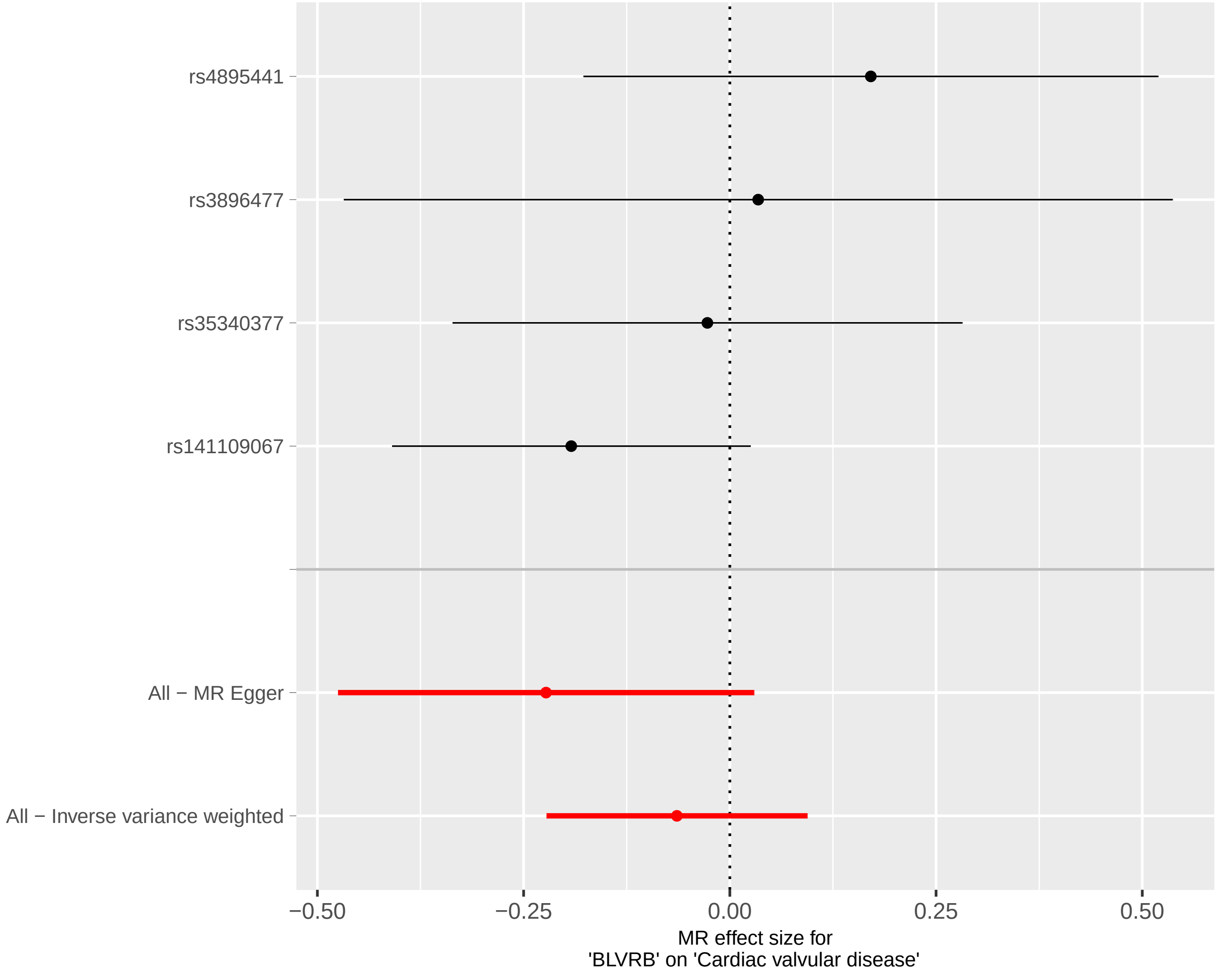
rs4895441
rs3896477
rs35340377
rs141109067
All − MR Egger
All − Inverse variance weighted
−0.50
−0.25
0.00
0.25
0.50
MR effect size for
'BLVRB' on 'Cardiac valvular disease'

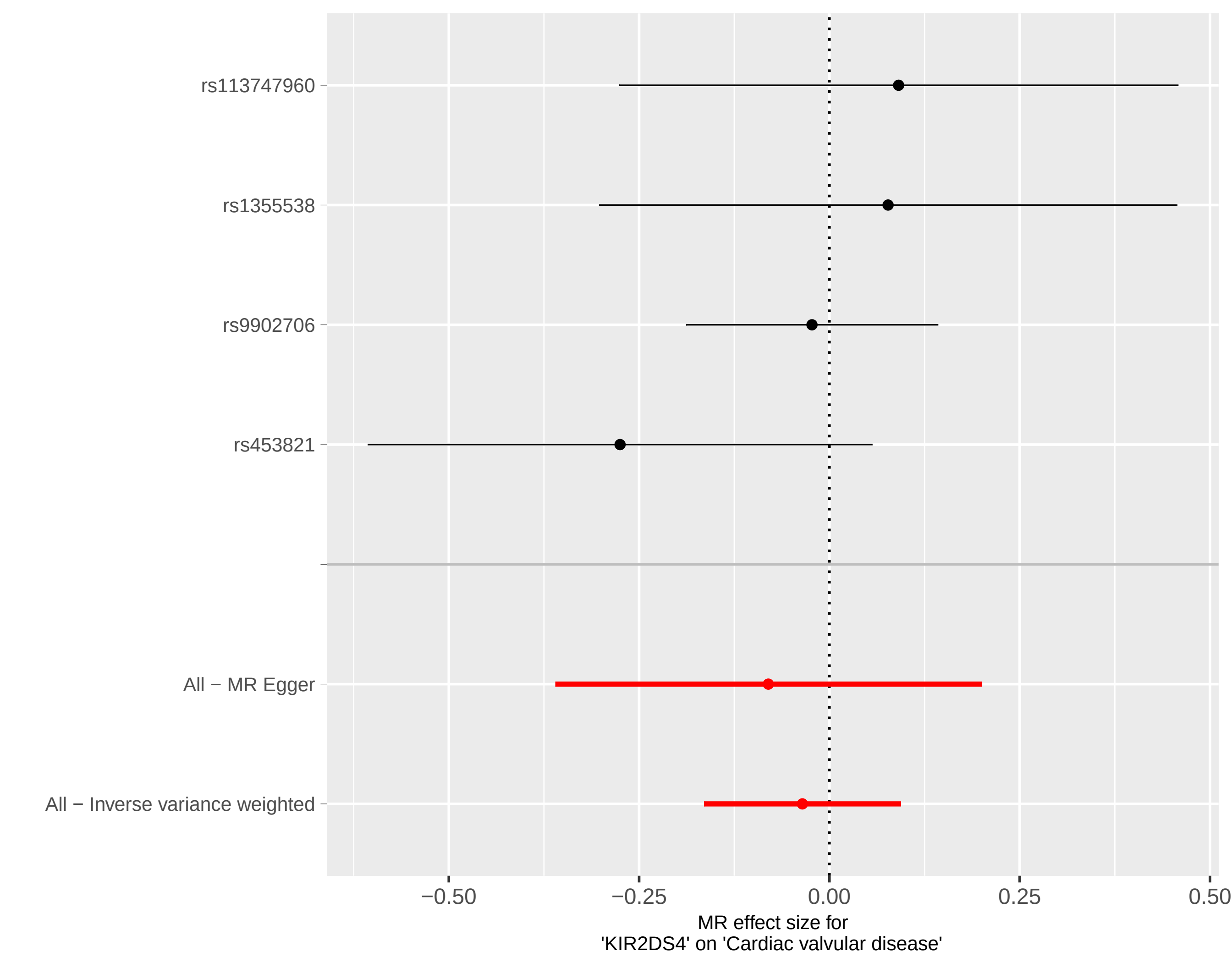

rs113747960
rs1355538
rs9902706
rs453821
All − MR Egger
All − Inverse variance weighted
−0.50
−0.25
0.00
0.25
0.50
MR effect size for
'KIR2DS4' on 'Cardiac valvular disease'

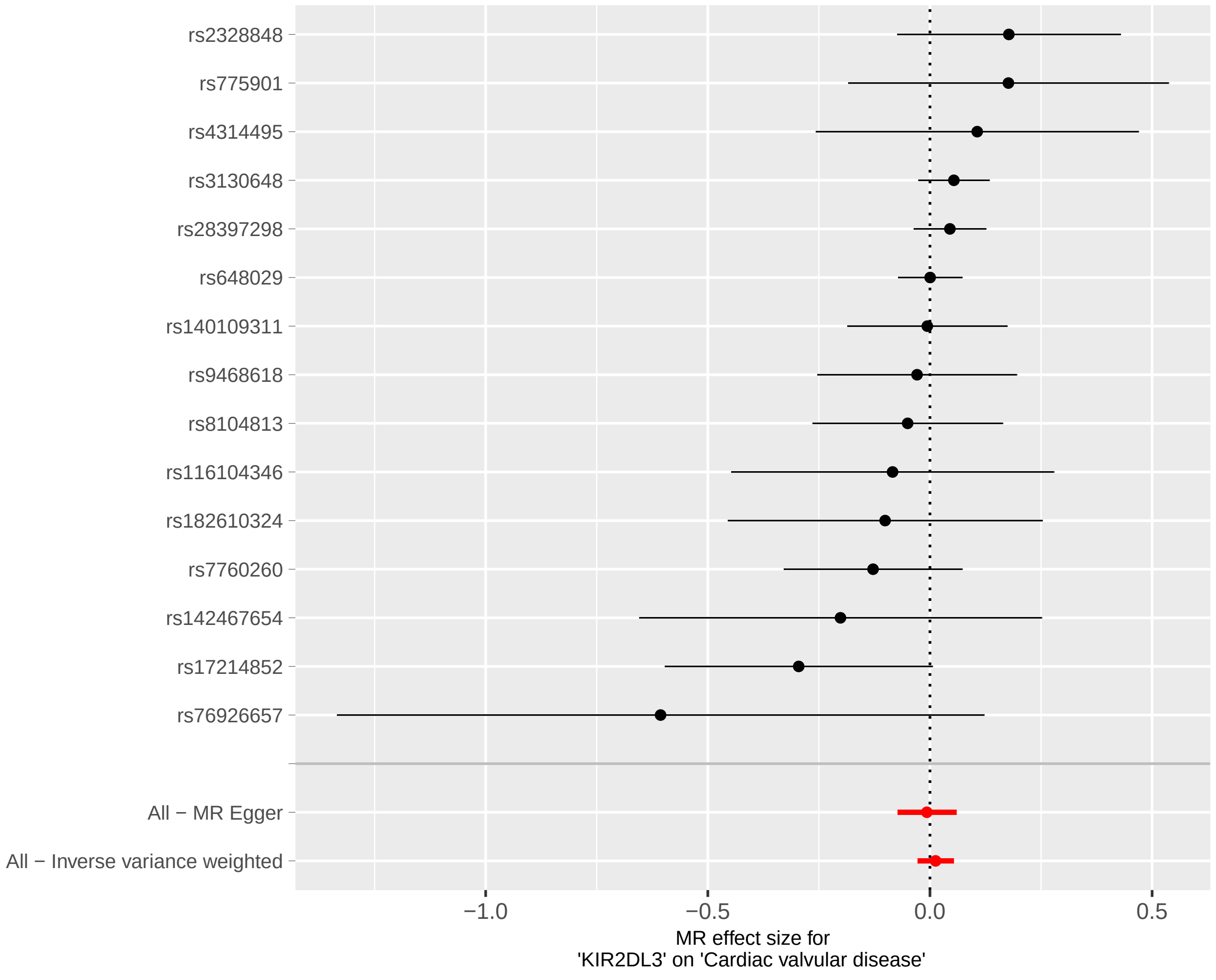
rs2328848
rs775901
rs4314495
rs3130648
rs28397298
rs648029
rs140109311
rs9468618
rs8104813
rs116104346
rs182610324
rs7760260
rs142467654
rs17214852
rs76926657
All − MR Egger
All − Inverse variance weighted
−1.0
−0.5
0.0
0.5
MR effect size for
'KIR2DL3' on 'Cardiac valvular disease'

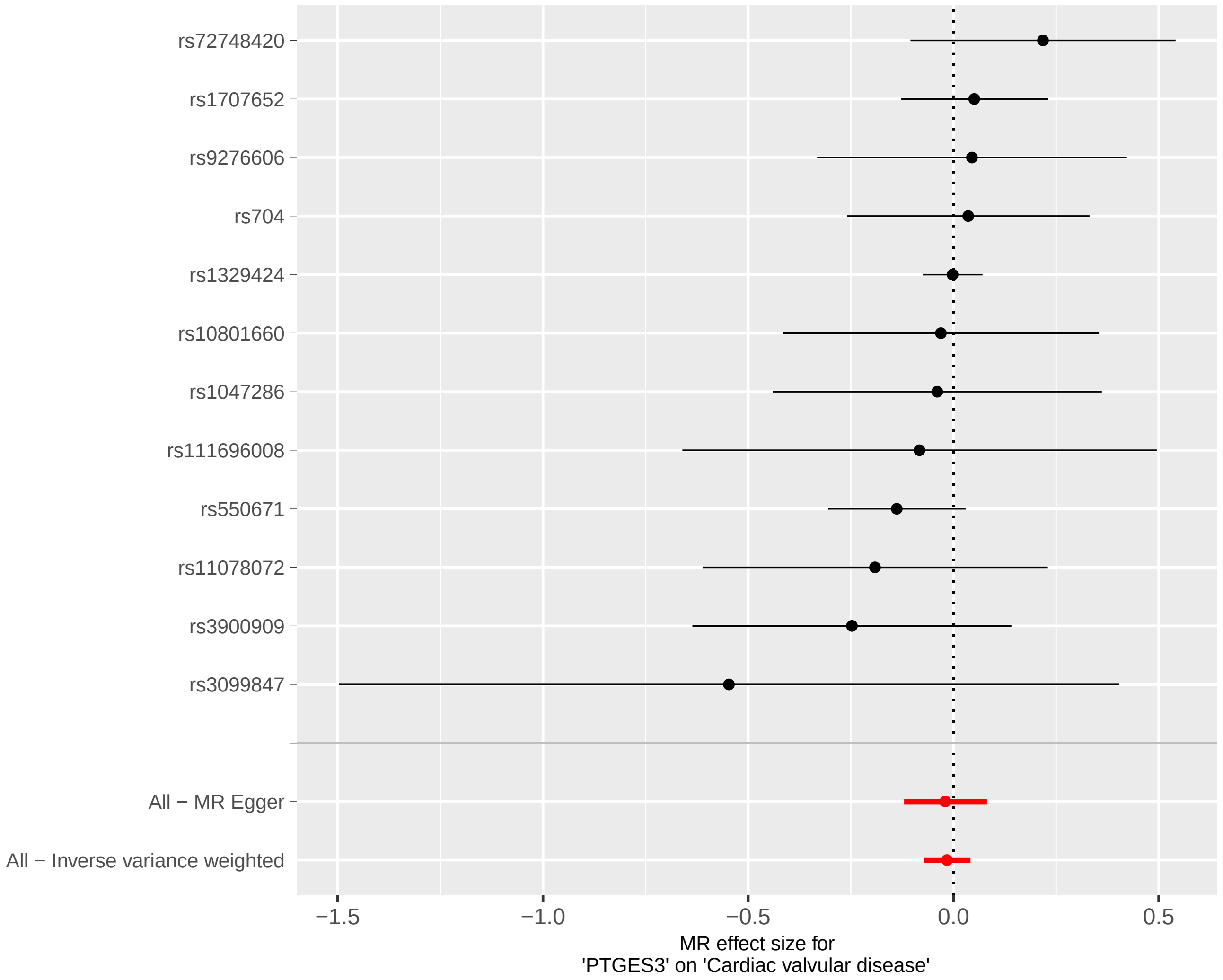
rs72748420
rs1707652
rs9276606
rs704
rs1329424
rs10801660
rs1047286
rs111696008
rs550671
rs11078072
rs3900909
rs3099847
All − MR Egger
All − Inverse variance weighted
−1.5
−1.0
−0.5
0.0
0.5
MR effect size for
'PTGES3' on 'Cardiac valvular disease'

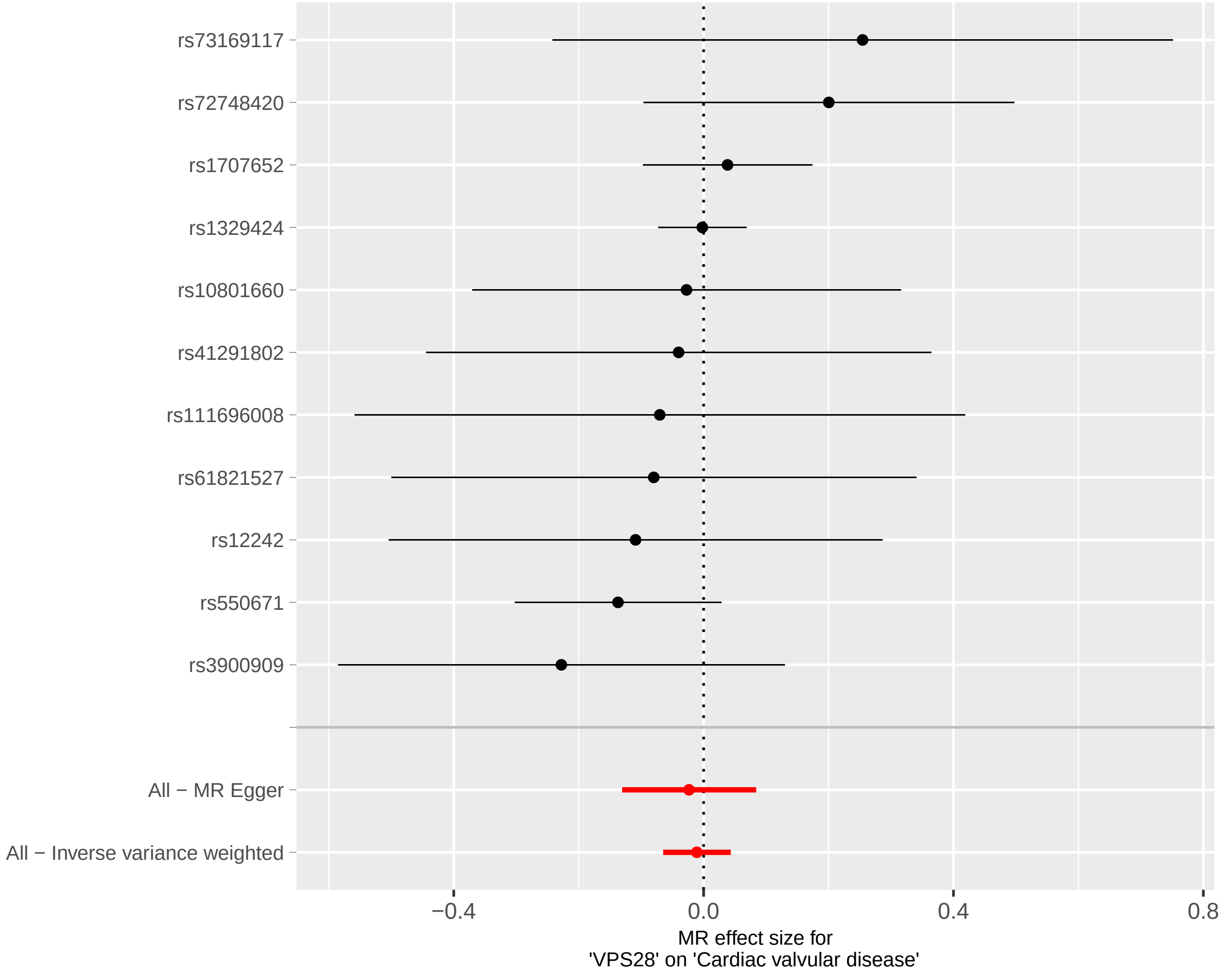

rs73169117
rs72748420
rs1707652
rs1329424
rs10801660
rs41291802
rs111696008
rs61821527
rs12242
rs550671
rs3900909
All − MR Egger
All − Inverse variance weighted
−0.4
0.0
0.4
0.8
MR effect size for
'VPS28' on 'Cardiac valvular disease'

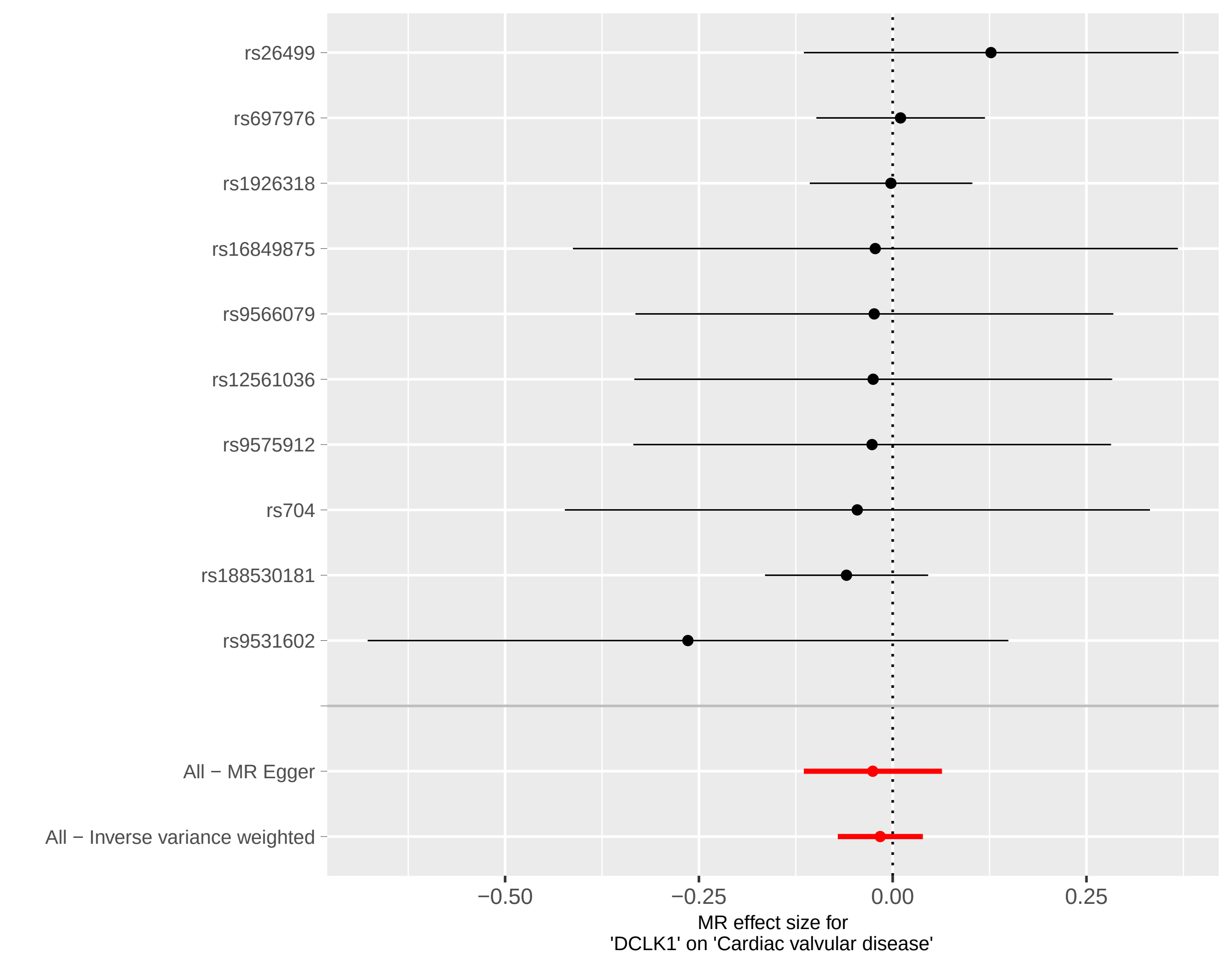

rs26499
rs697976
rs1926318
rs16849875
rs9566079
rs12561036
rs9575912
rs704
rs188530181
rs9531602
All − MR Egger
All − Inverse variance weighted
−0.50
−0.25
0.00
0.25
MR effect size for
'DCLK1' on 'Cardiac valvular disease'

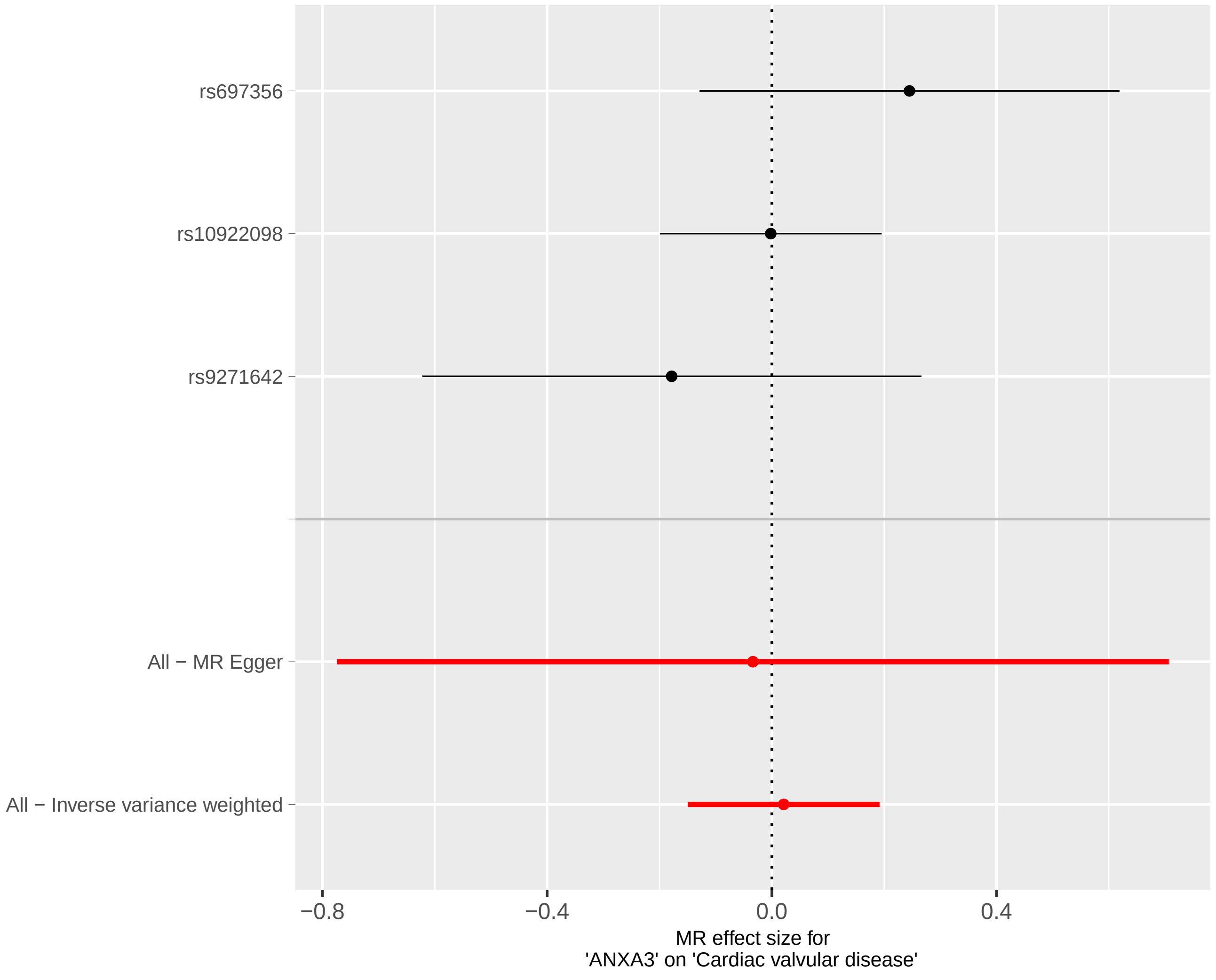

rs697356
rs10922098
rs9271642
All − MR Egger
All − Inverse variance weighted
−0.8
−0.4
0.0
0.4
MR effect size for
'ANXA3' on 'Cardiac valvular disease'

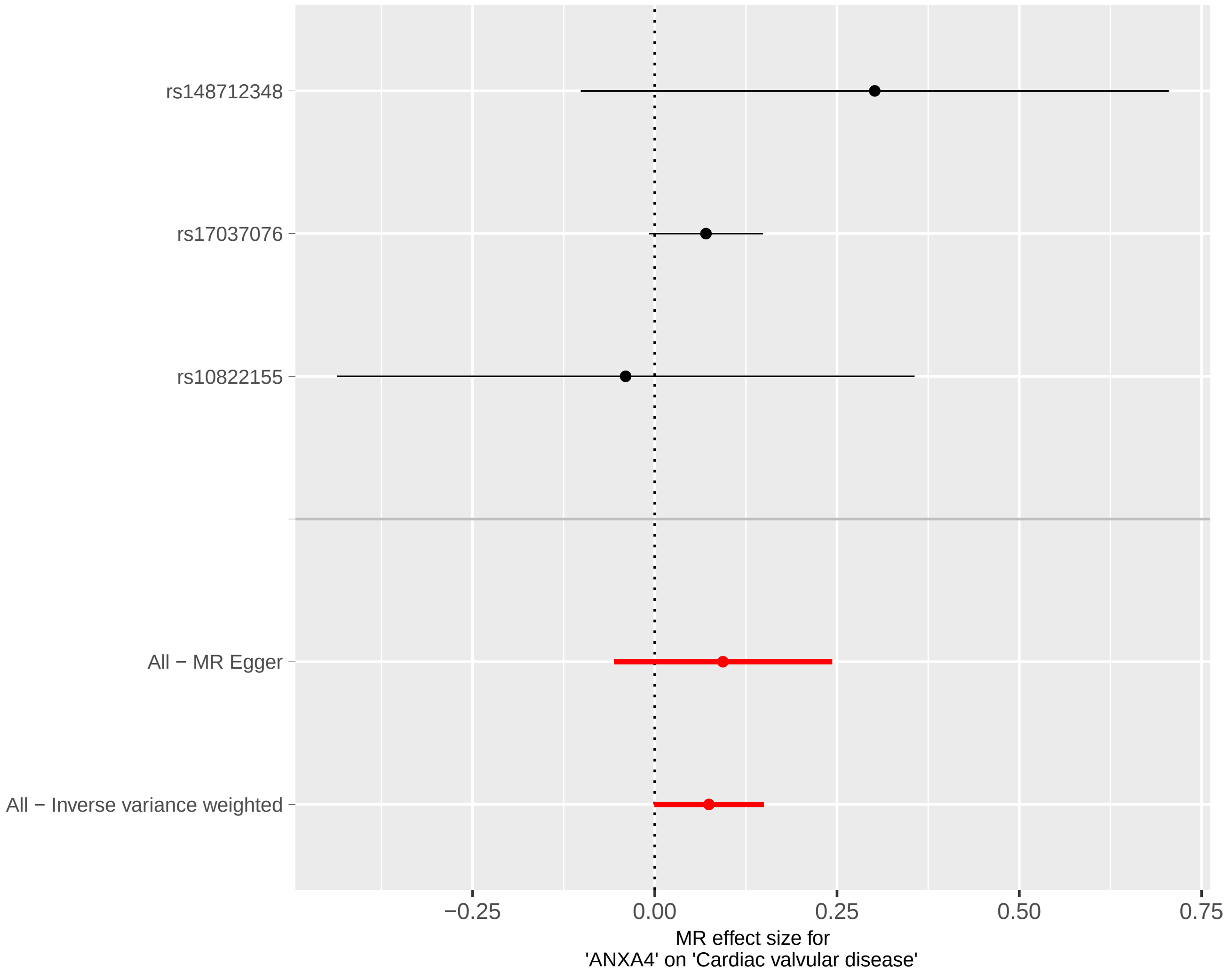
rs148712348
rs17037076
rs10822155
All − MR Egger
All − Inverse variance weighted
−0.25
0.00
0.25
0.50
0.75
MR effect size for
'ANXA4' on 'Cardiac valvular disease'

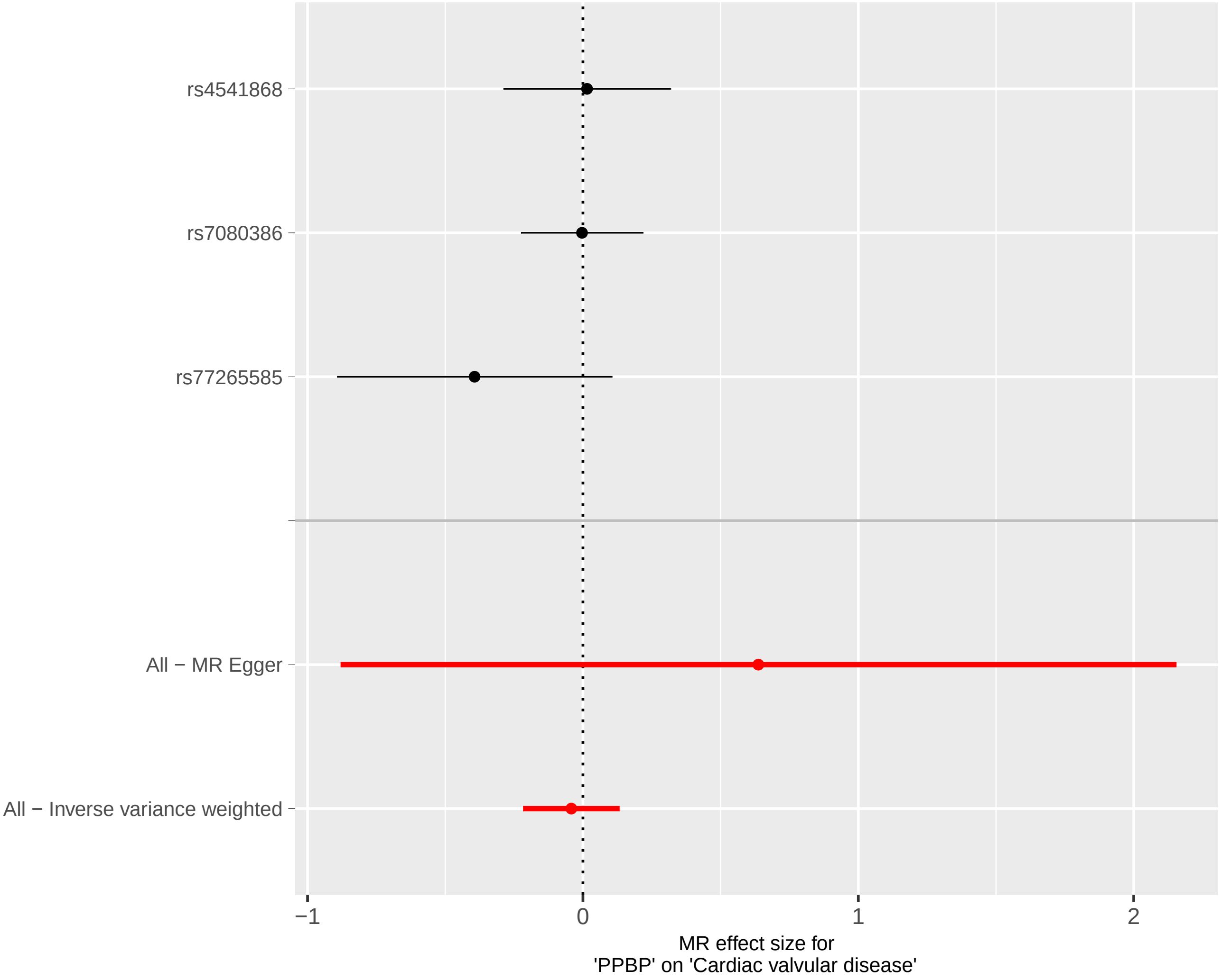
rs4541868
rs7080386
rs77265585
All − MR Egger
All − Inverse variance weighted
−1
0
1
2
MR effect size for
'PPBP' on 'Cardiac valvular disease'

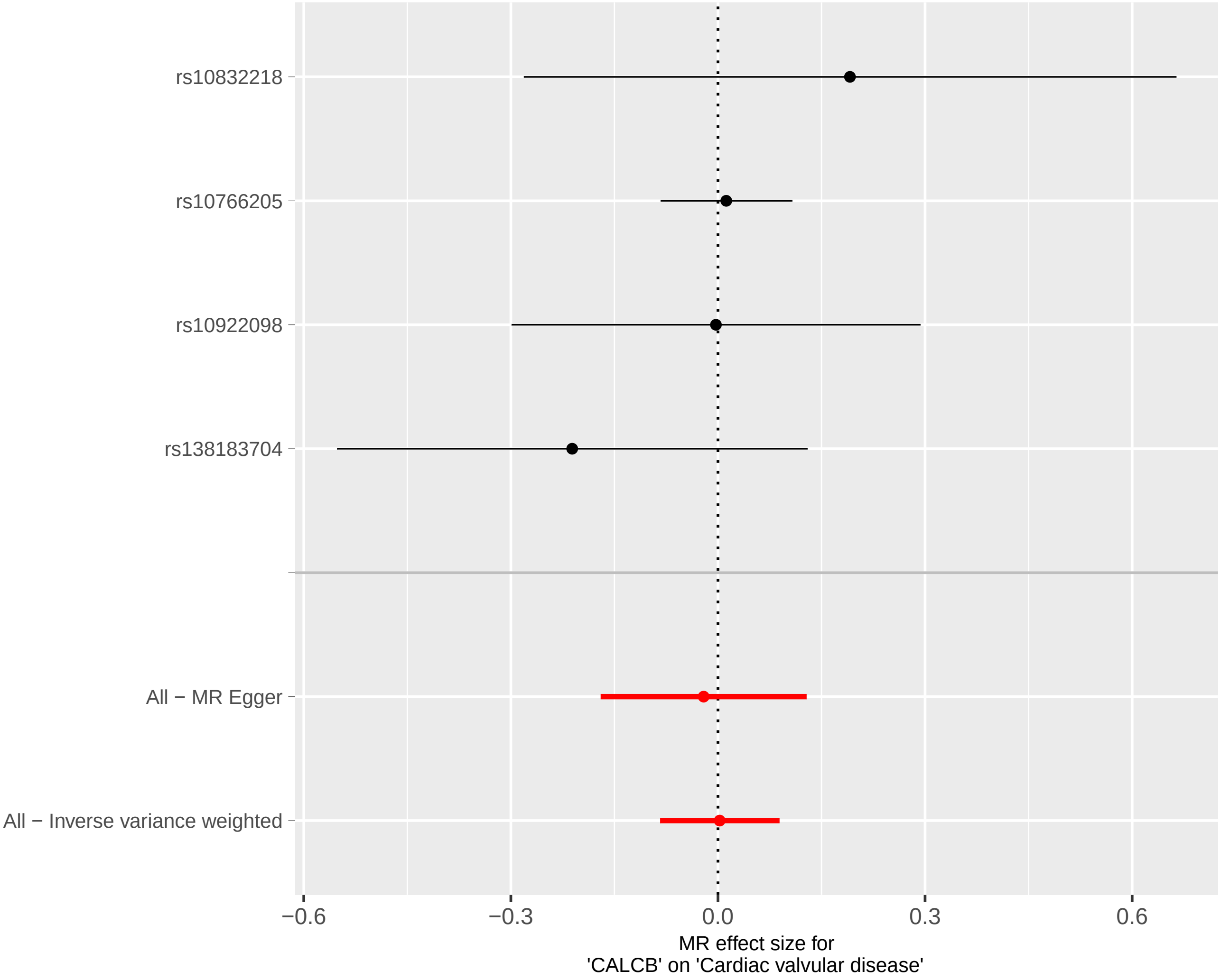

rs10832218
rs10766205
rs10922098
rs138183704
All − MR Egger
All − Inverse variance weighted
−0.6
−0.3
0.0
0.3
0.6
MR effect size for
'CALCB' on 'Cardiac valvular disease'

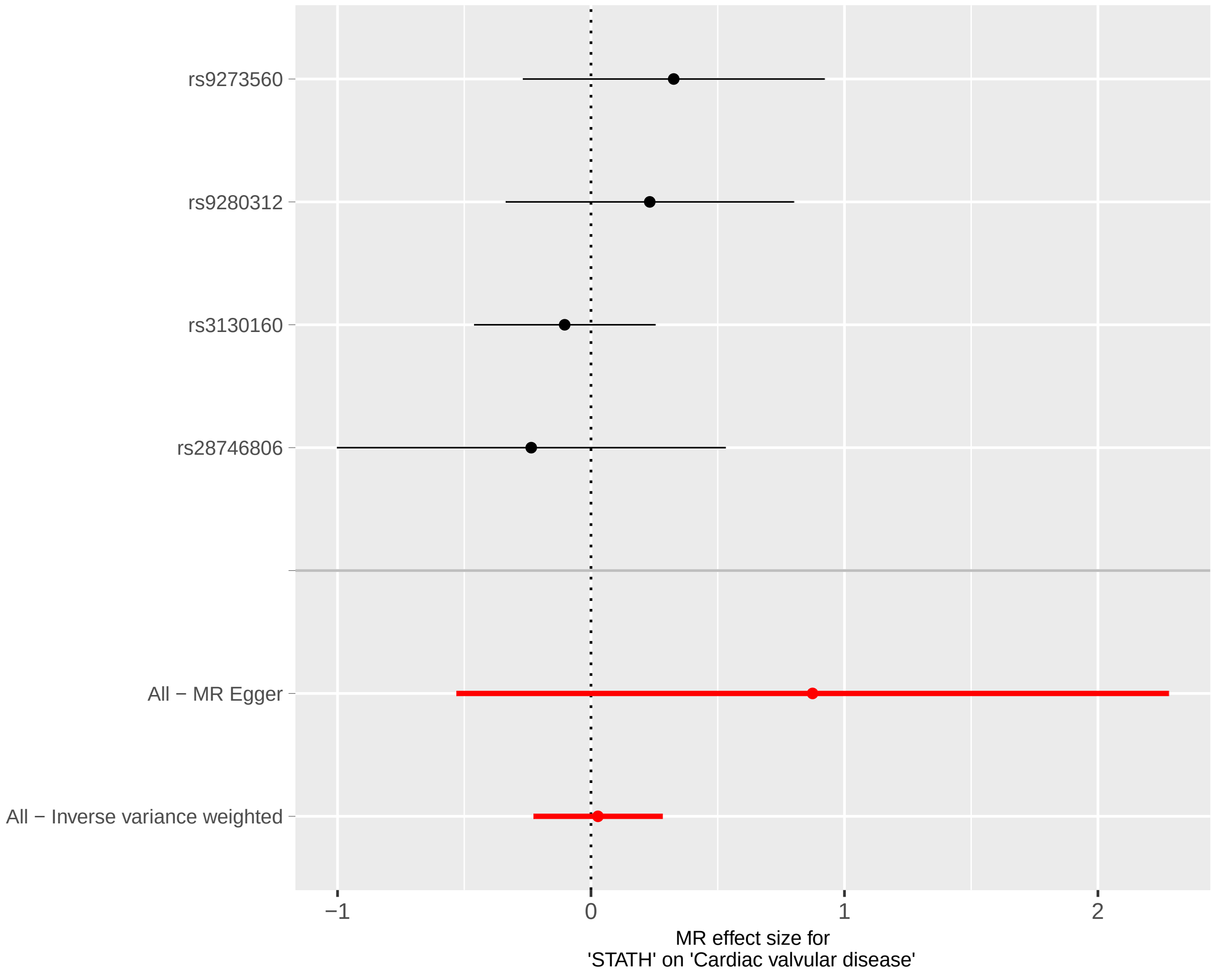

rs9273560
rs9280312
rs3130160
rs28746806
All − MR Egger
All − Inverse variance weighted
−1
0
1
2
MR effect size for
'STATH' on 'Cardiac valvular disease'

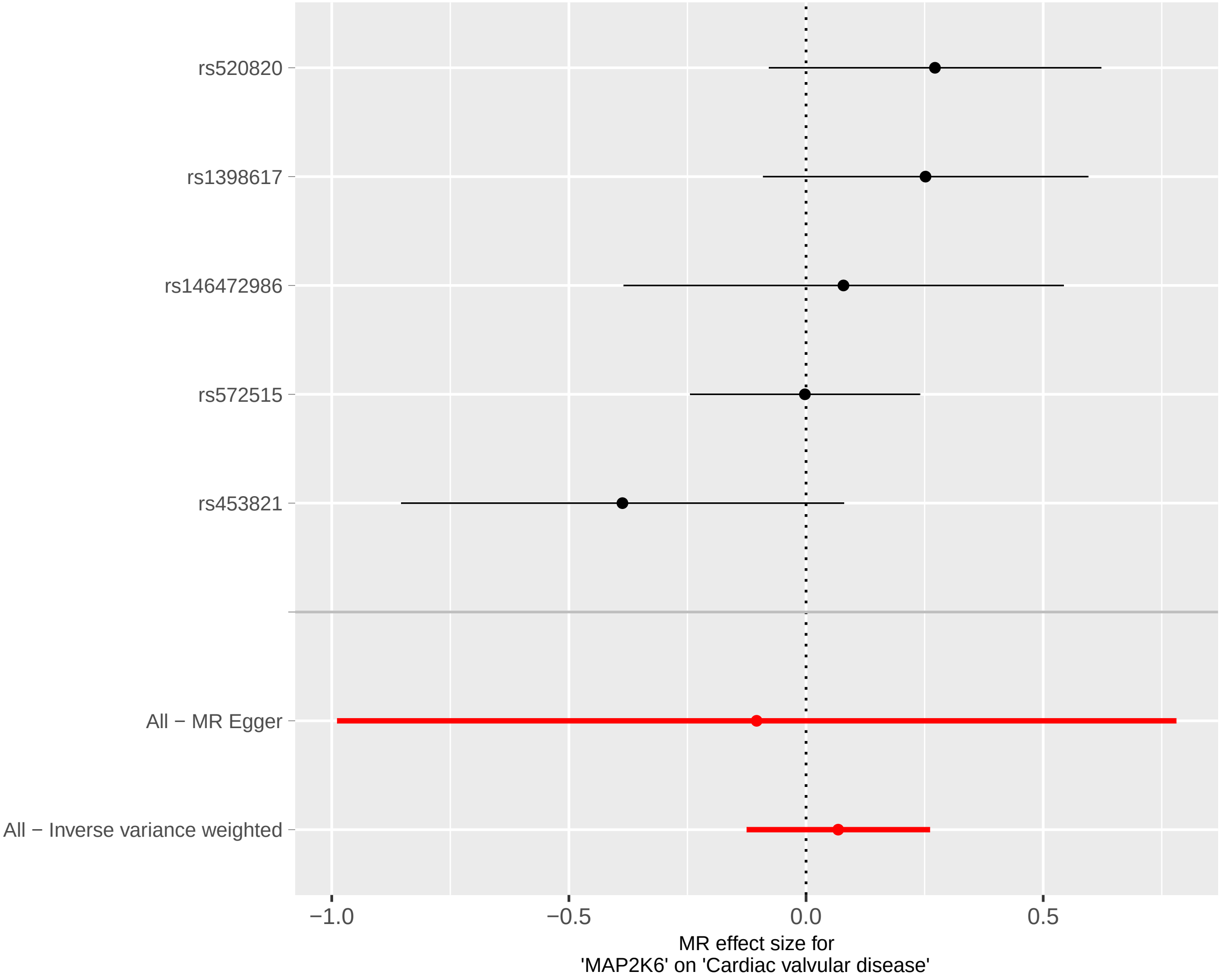
rs520820
rs1398617
rs146472986
rs572515
rs453821
All − MR Egger
All − Inverse variance weighted
−1.0
−0.5
0.0
0.5
MR effect size for
'MAP2K6' on 'Cardiac valvular disease'

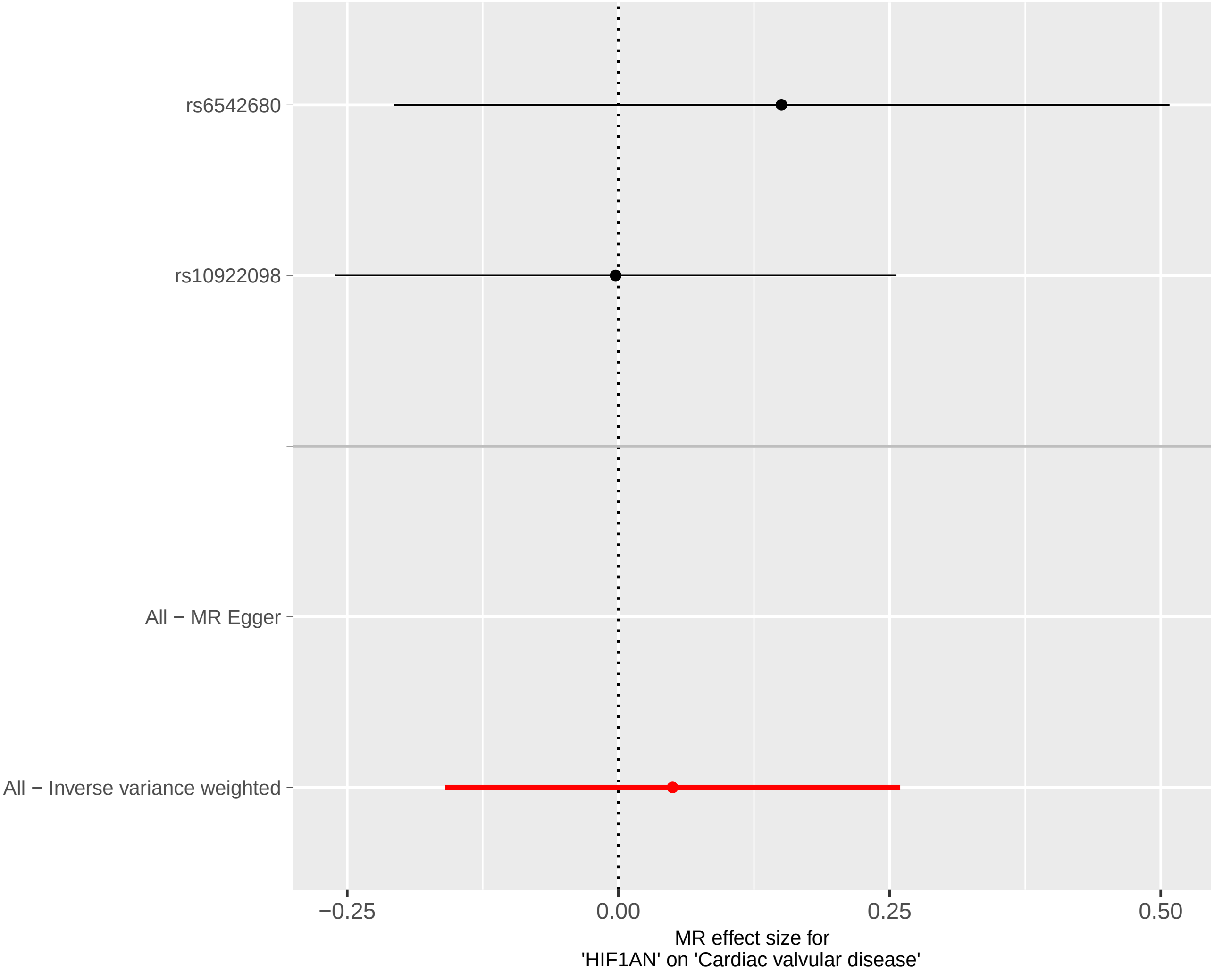

rs6542680
rs10922098
All − MR Egger
All − Inverse variance weighted
−0.25
0.00
0.25
0.50
MR effect size for
'HIF1AN' on 'Cardiac valvular disease'

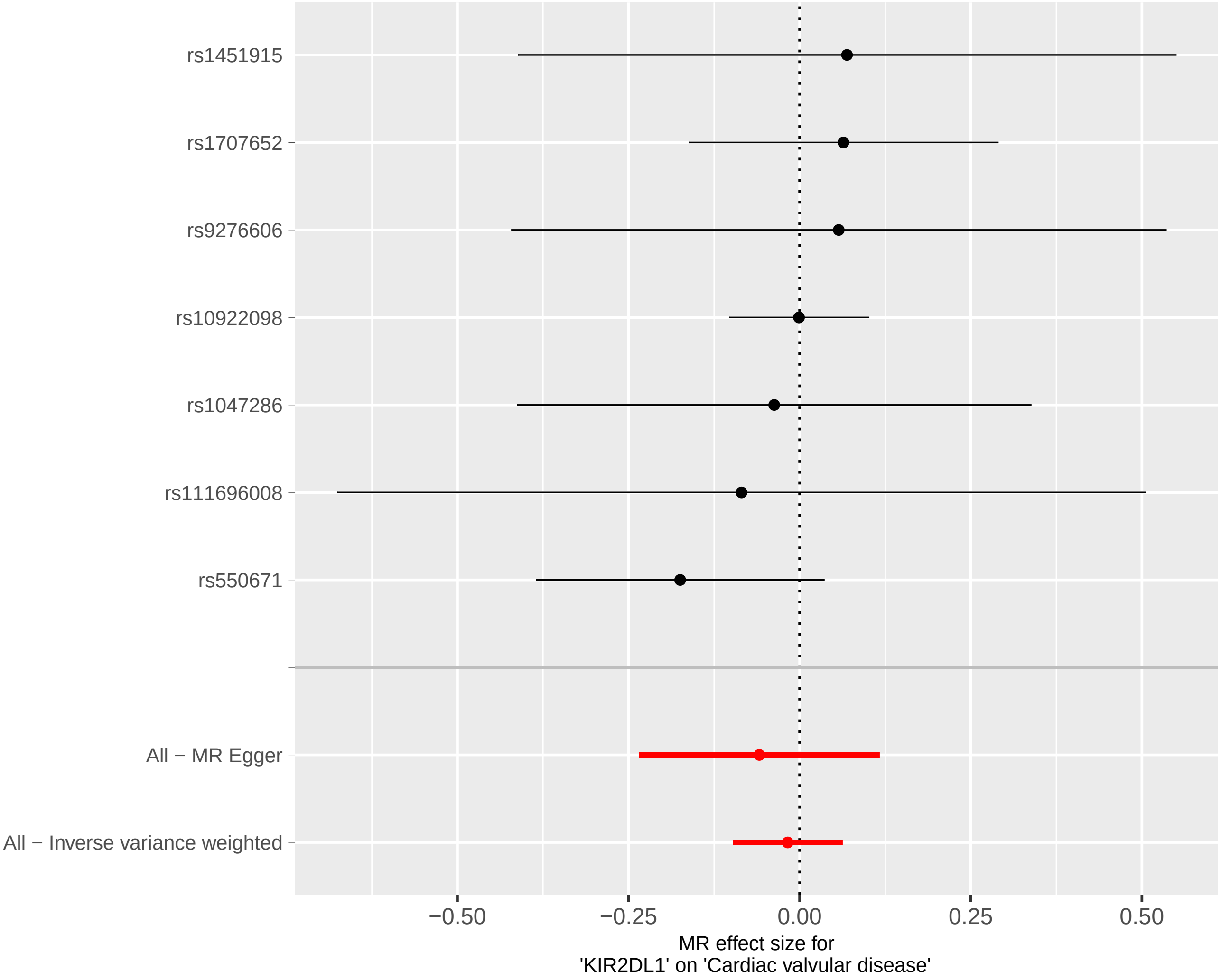

rs1451915
rs1707652
rs9276606
rs10922098
rs1047286
rs111696008
rs550671
All − MR Egger
All − Inverse variance weighted
−0.50
−0.25
0.00
0.25
0.50
MR effect size for
'KIR2DL1' on 'Cardiac valvular disease'

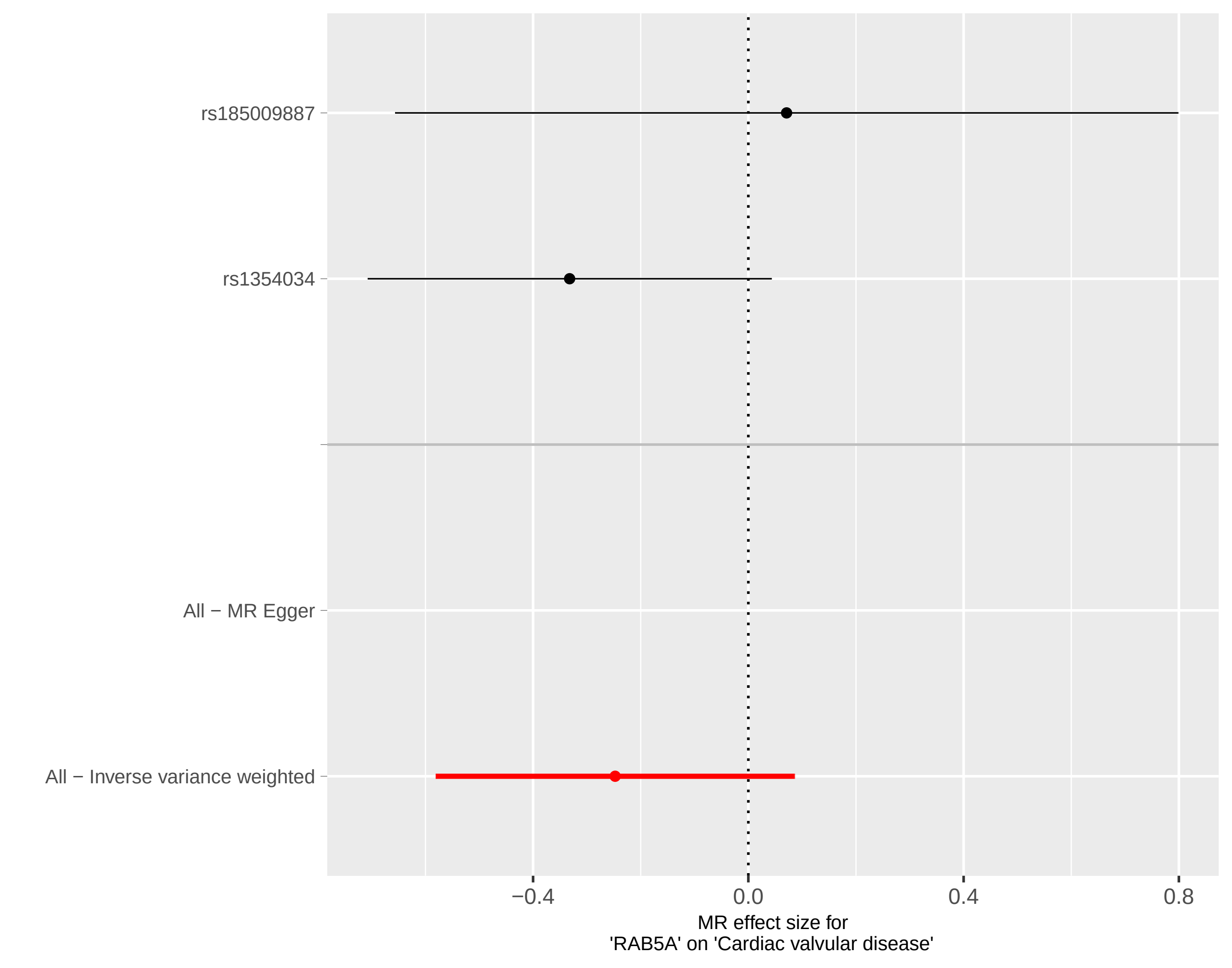
rs185009887
rs1354034
All − MR Egger
All − Inverse variance weighted
−0.4
0.0
0.4
0.8
MR effect size for
'RAB5A' on 'Cardiac valvular disease'

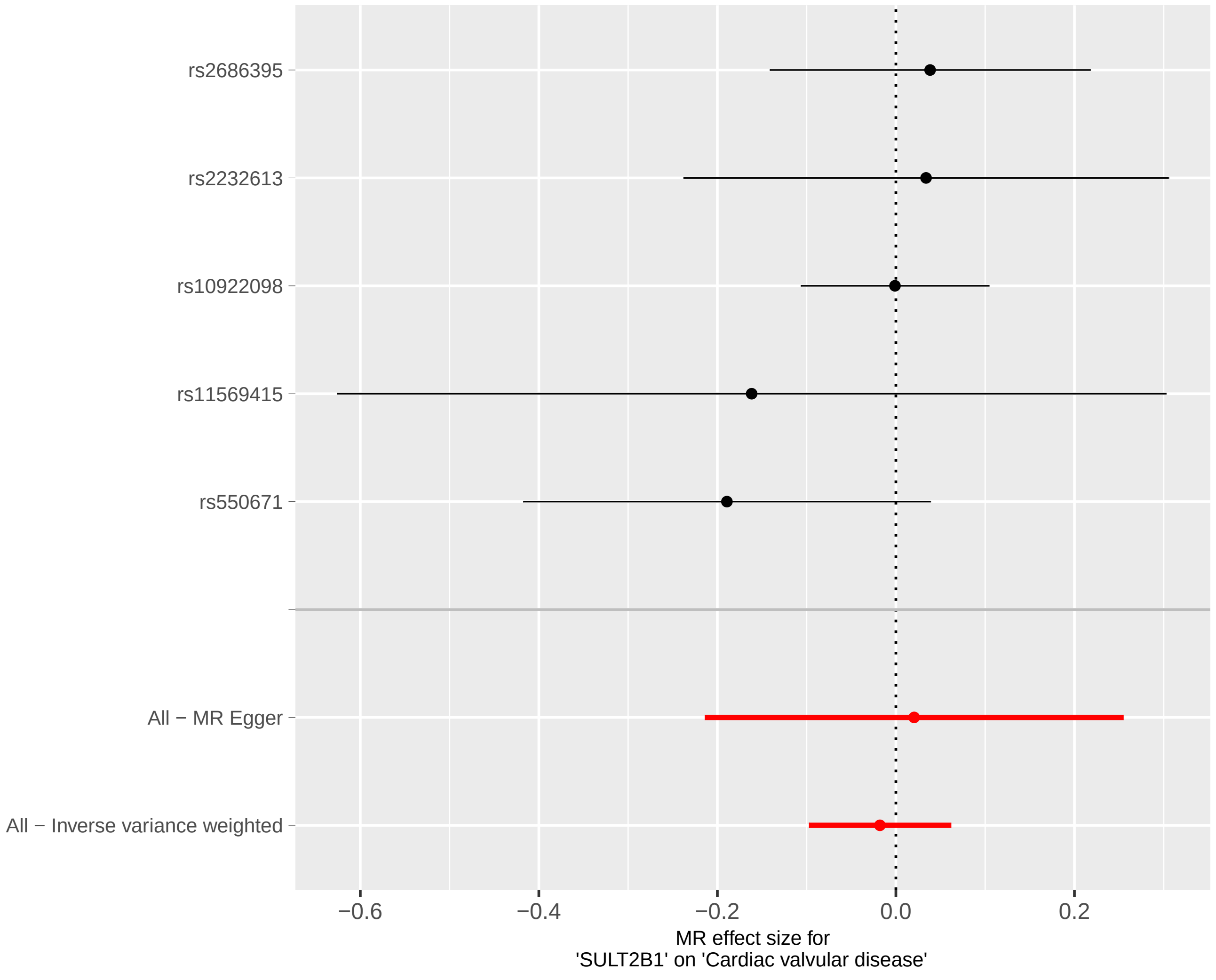
rs2686395
rs2232613
rs10922098
rs11569415
rs550671
All − MR Egger
All − Inverse variance weighted
−0.6
−0.4
−0.2
0.0
0.2
MR effect size for
'SULT2B1' on 'Cardiac valvular disease'

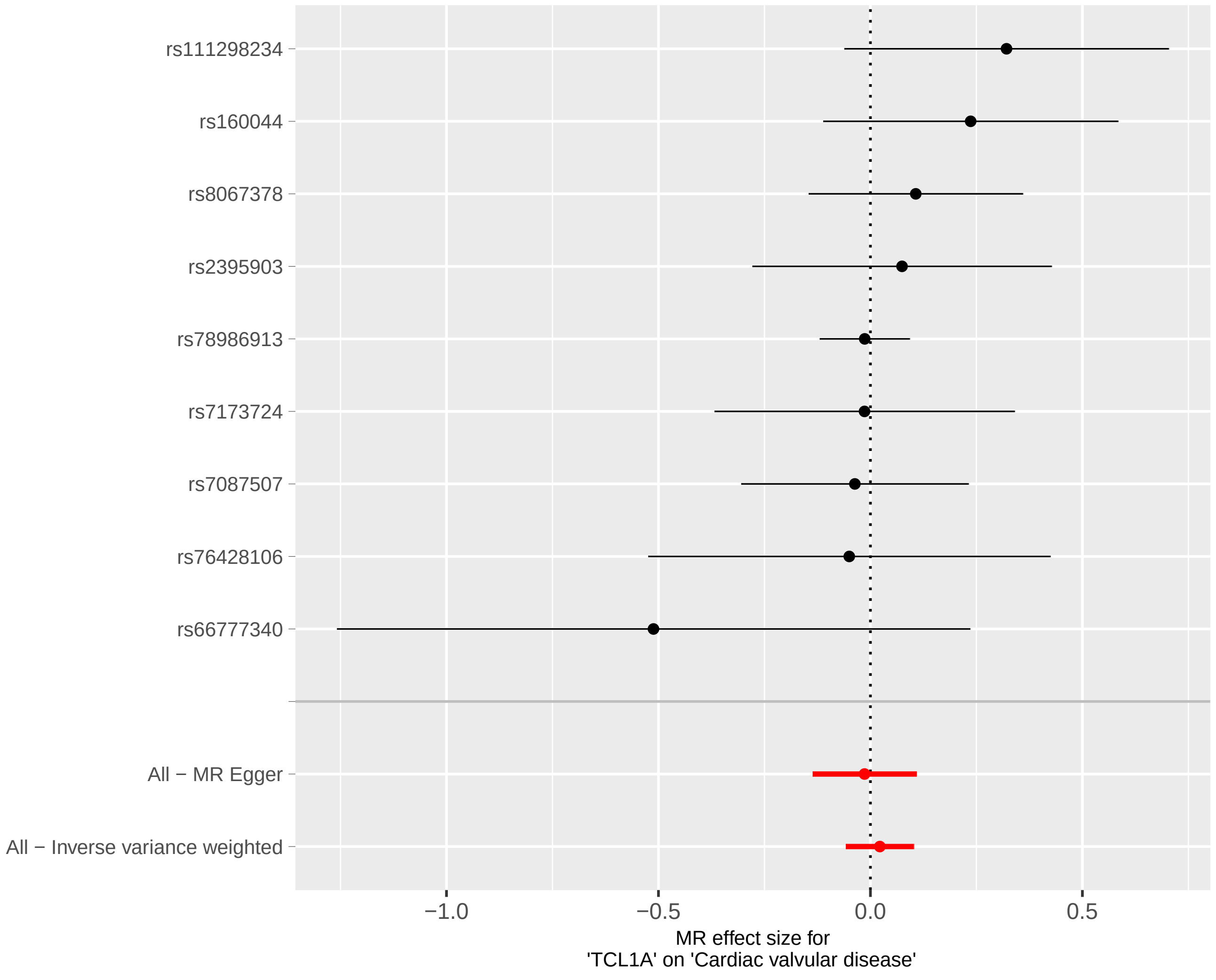

rs111298234
rs160044
rs8067378
rs2395903
rs78986913
rs7173724
rs7087507
rs76428106
rs66777340
All − MR Egger
All − Inverse variance weighted
−1.0
−0.5
0.0
0.5
MR effect size for
'TCL1A' on 'Cardiac valvular disease'

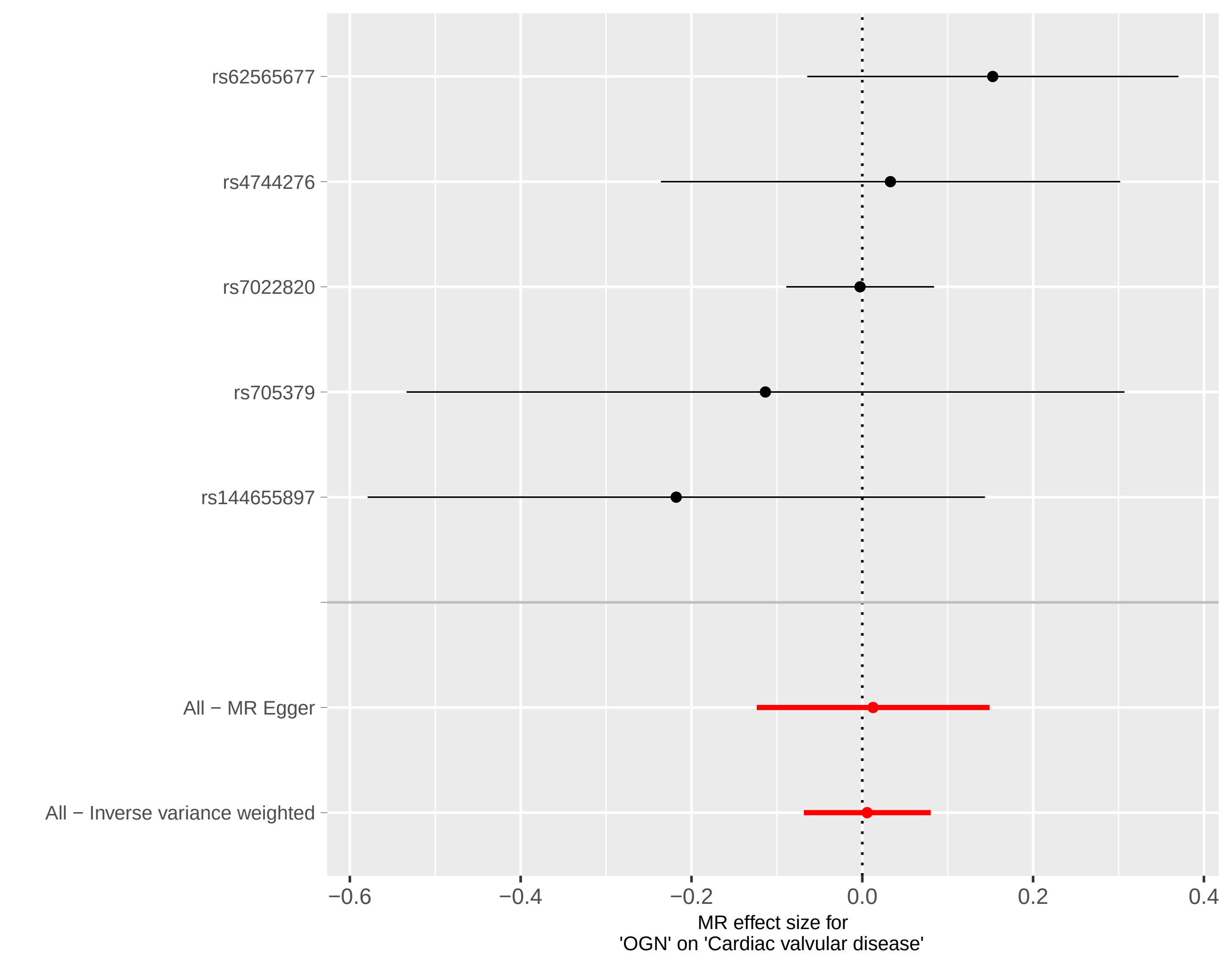
rs62565677
rs4744276
rs7022820
rs705379
rs144655897
All − MR Egger
All − Inverse variance weighted
−0.6
−0.4
−0.2
0.0
0.2
0.4
MR effect size for
'OGN' on 'Cardiac valvular disease'

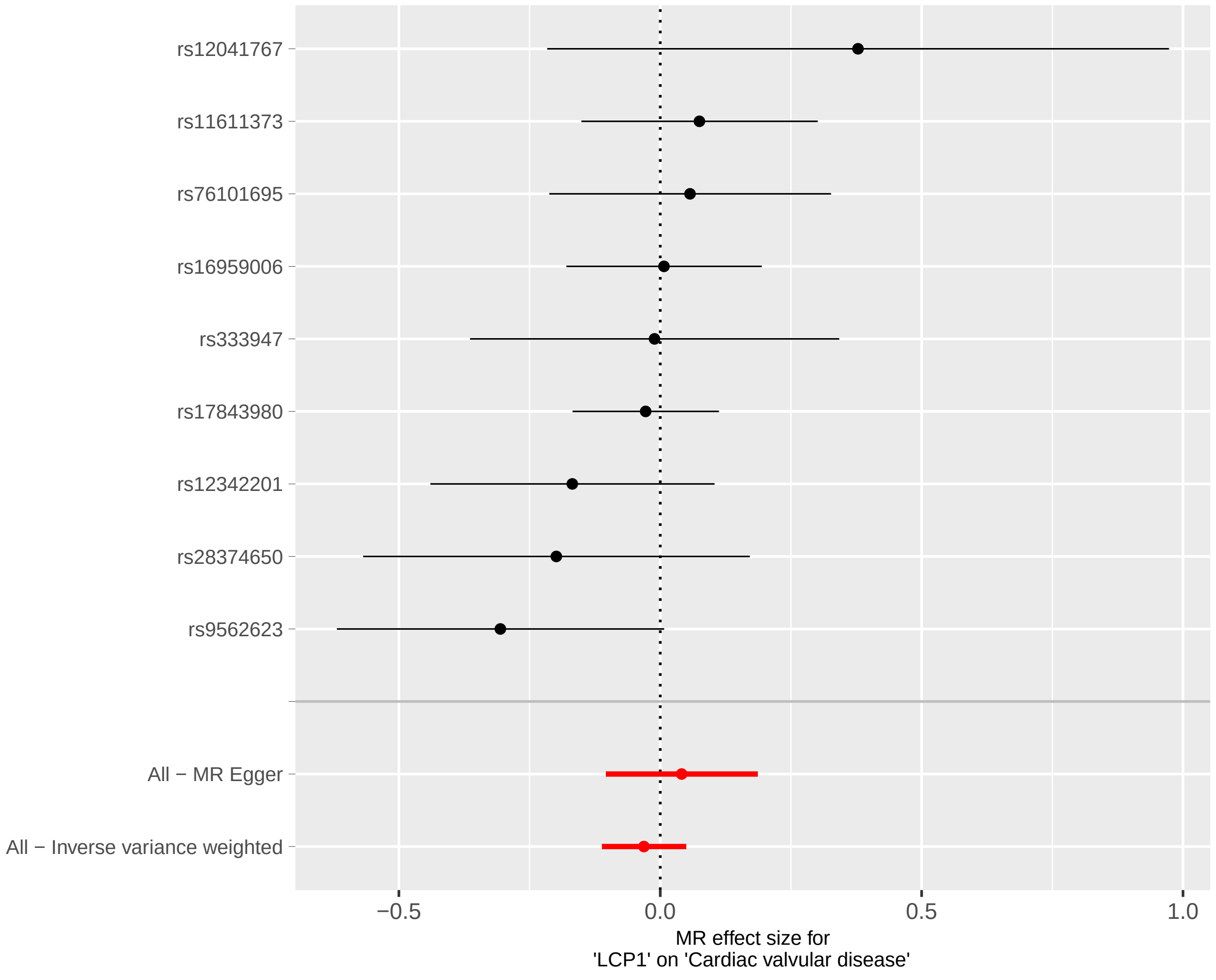
rs12041767
rs11611373
rs76101695
rs16959006
rs333947
rs17843980
rs12342201
rs28374650
rs9562623
All − MR Egger
All − Inverse variance weighted
−0.5
0.0
0.5
1.0
MR effect size for
'LCP1' on 'Cardiac valvular disease'

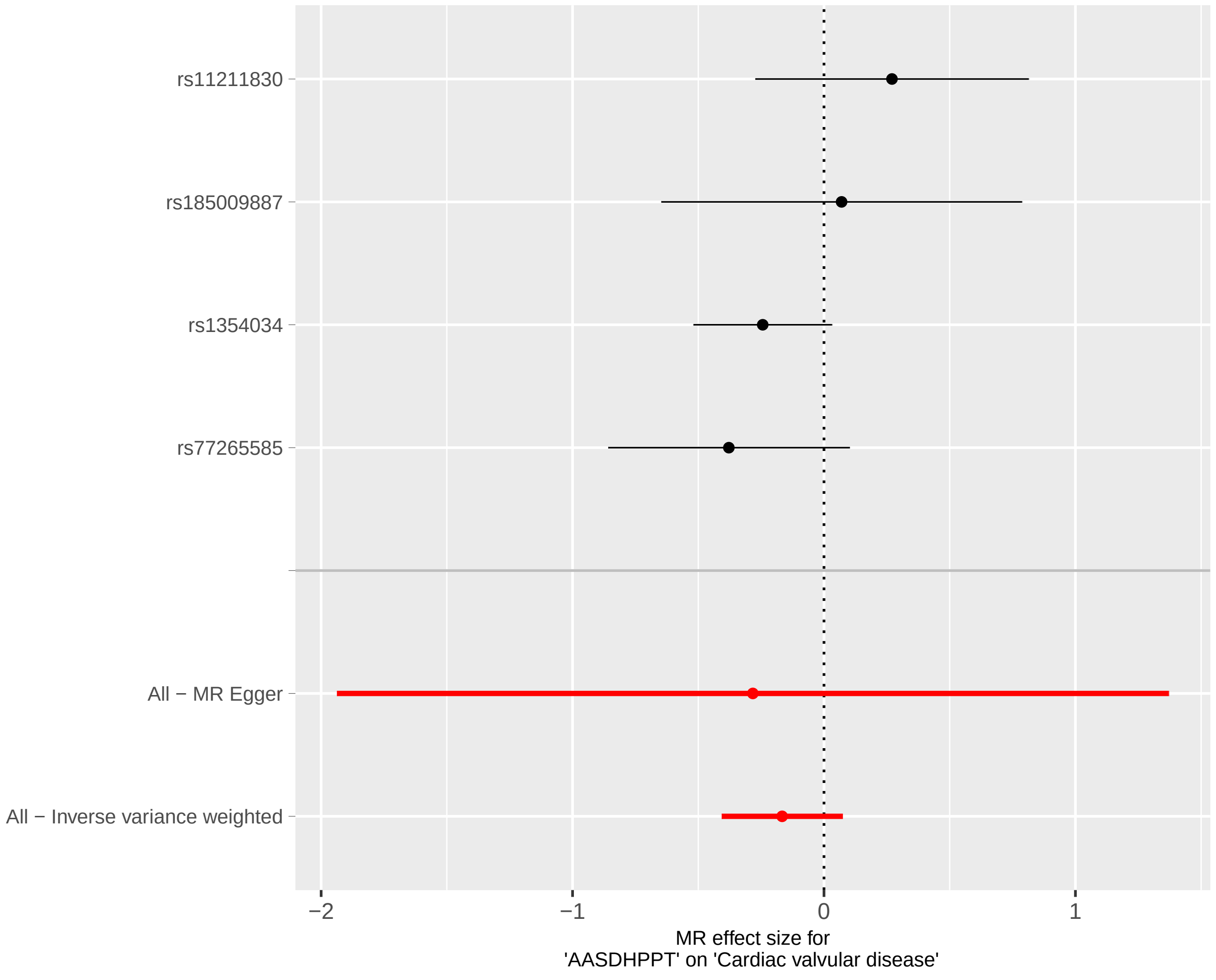

rs11211830
rs185009887
rs1354034
rs77265585
All − MR Egger
All − Inverse variance weighted
−2
−1
0
1
MR effect size for
'AASDHPPT' on 'Cardiac valvular disease'

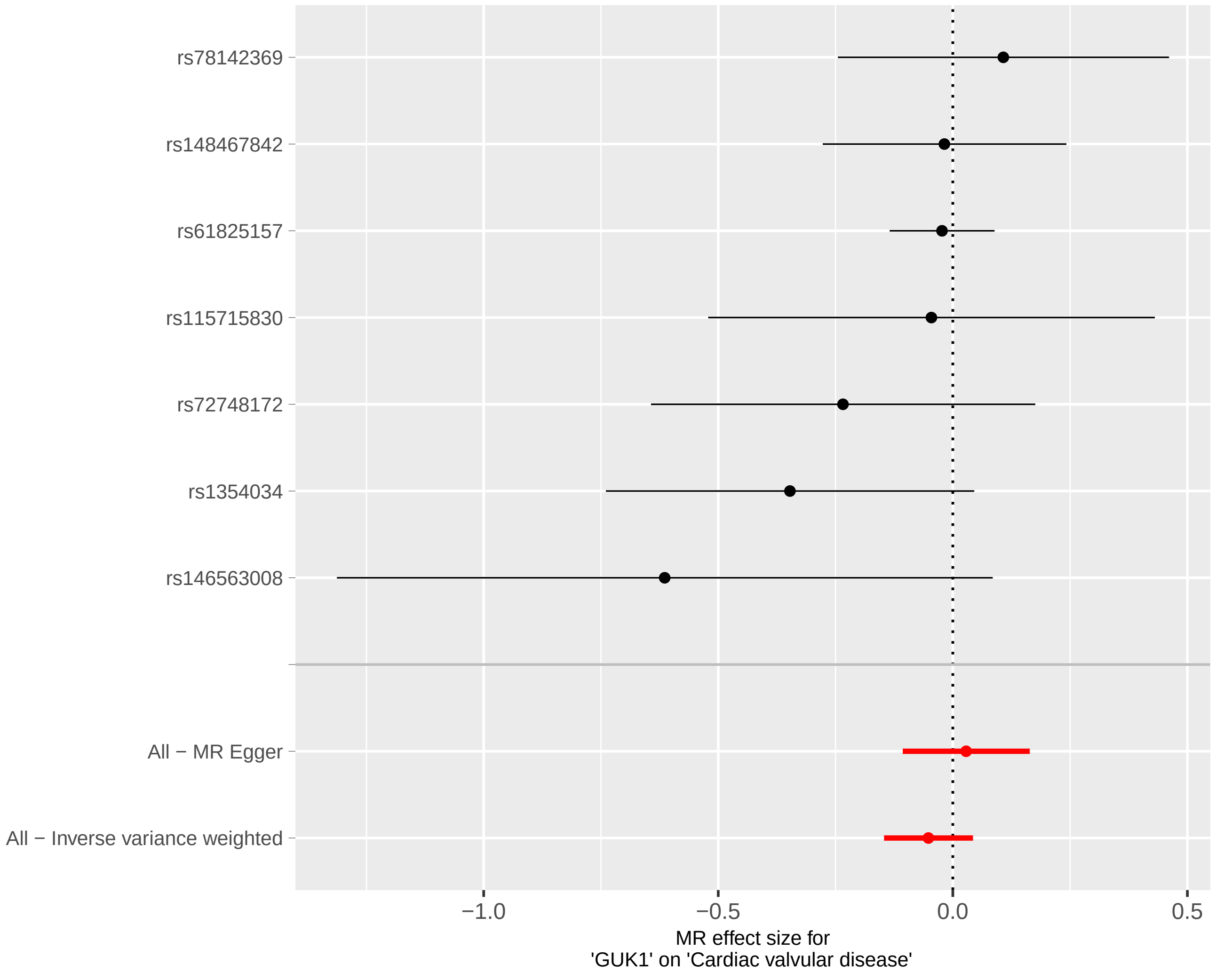
rs78142369
rs148467842
rs61825157
rs115715830
rs72748172
rs1354034
rs146563008
All − MR Egger
All − Inverse variance weighted
−1.0
−0.5
0.0
0.5
MR effect size for
'GUK1' on 'Cardiac valvular disease'

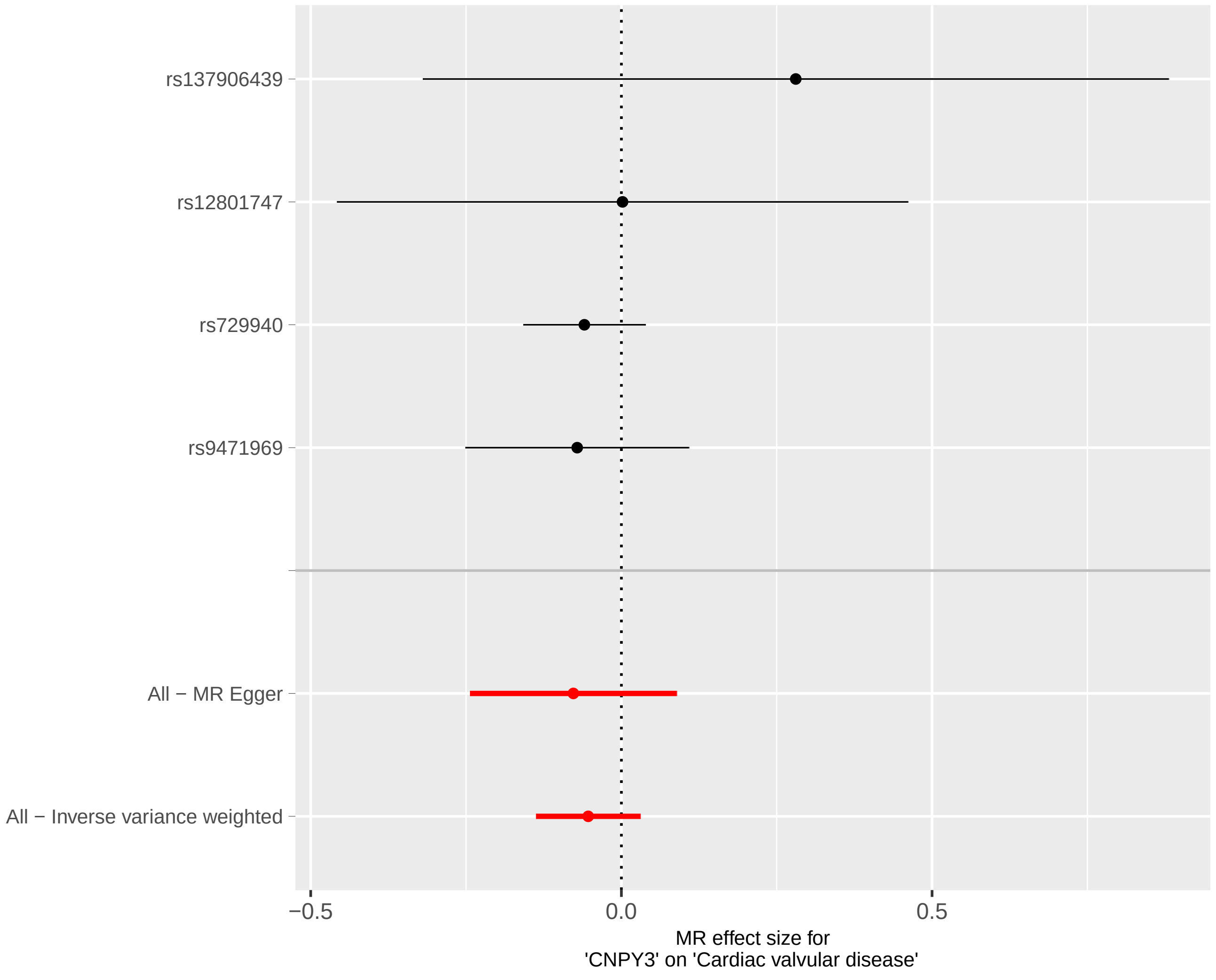
rs137906439
rs12801747
rs729940
rs9471969
All − MR Egger
All − Inverse variance weighted
−0.5
0.0
0.5
MR effect size for
'CNPY3' on 'Cardiac valvular disease'

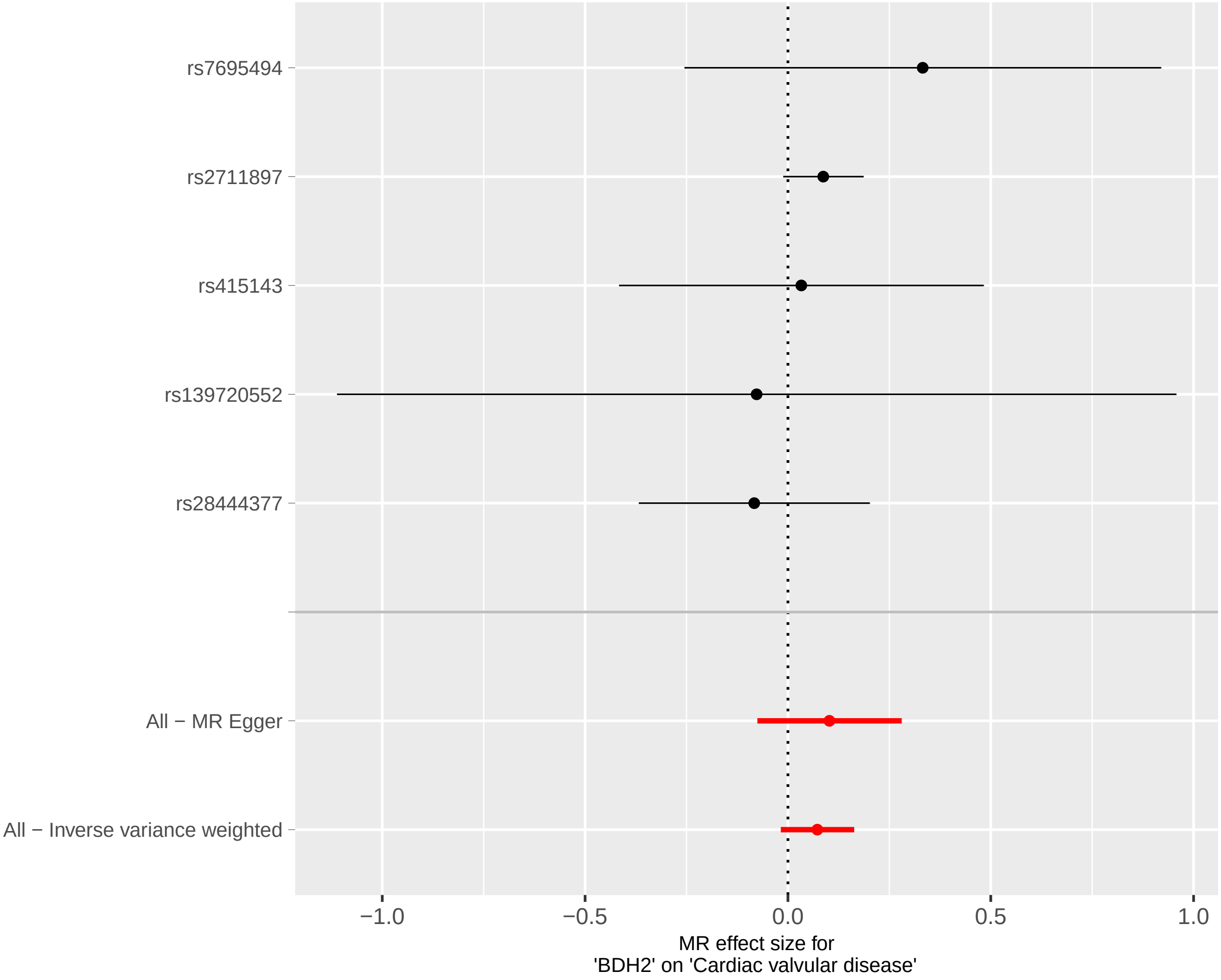
rs7695494
rs2711897
rs415143
rs139720552
rs28444377
All − MR Egger
All − Inverse variance weighted
−1.0
−0.5
0.0
0.5
1.0
MR effect size for
'BDH2' on 'Cardiac valvular disease'

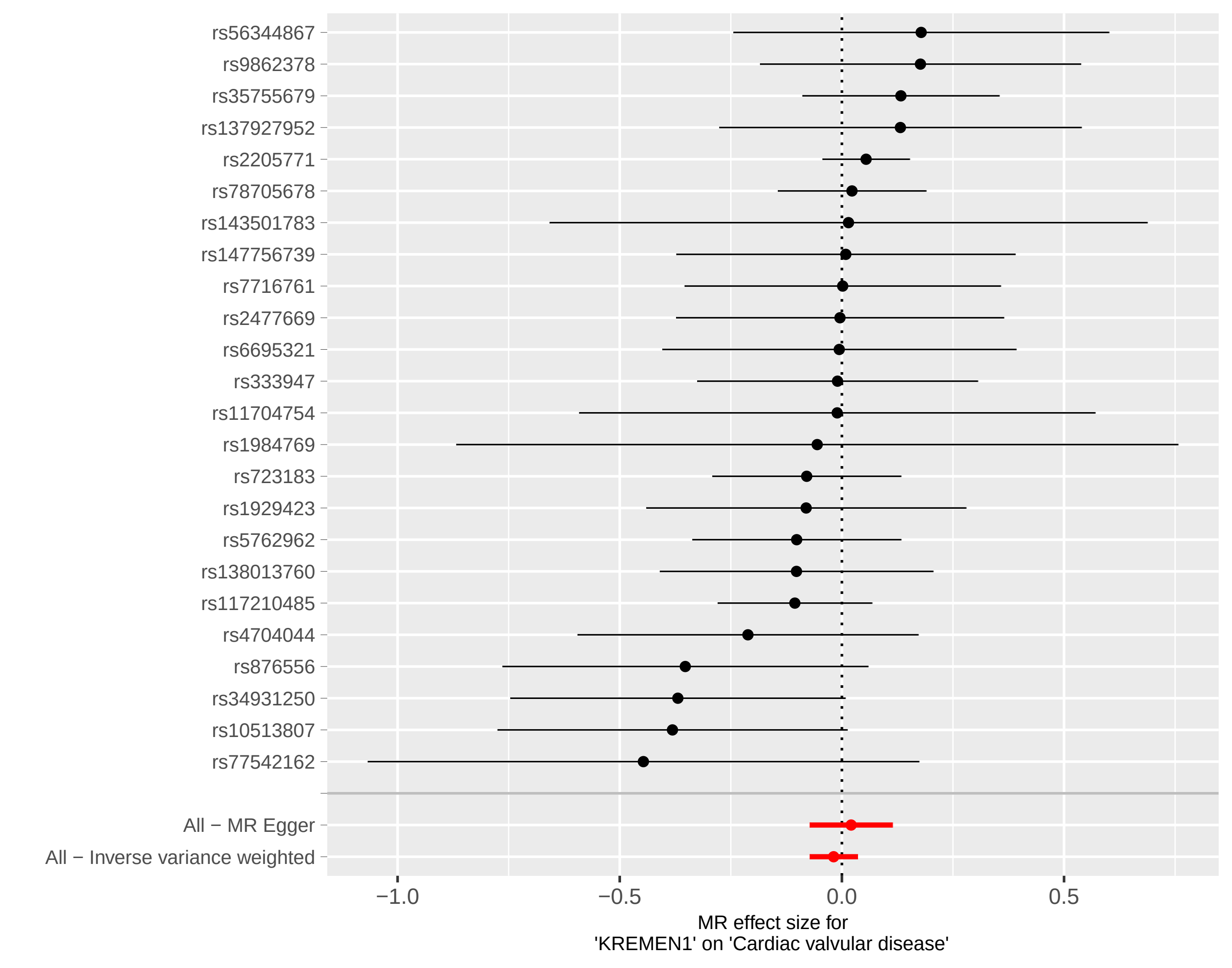

rs56344867
rs9862378
rs35755679
rs137927952
rs2205771
rs78705678
rs143501783
rs147756739
rs7716761
rs2477669
rs6695321
rs333947
rs11704754
rs1984769
rs723183
rs1929423
rs5762962
rs138013760
rs117210485
rs4704044
rs876556
rs34931250
rs10513807
rs77542162
All − MR Egger
All − Inverse variance weighted
−1.0
−0.5
0.0
0.5
MR effect size for
'KREMEN1' on 'Cardiac valvular disease'

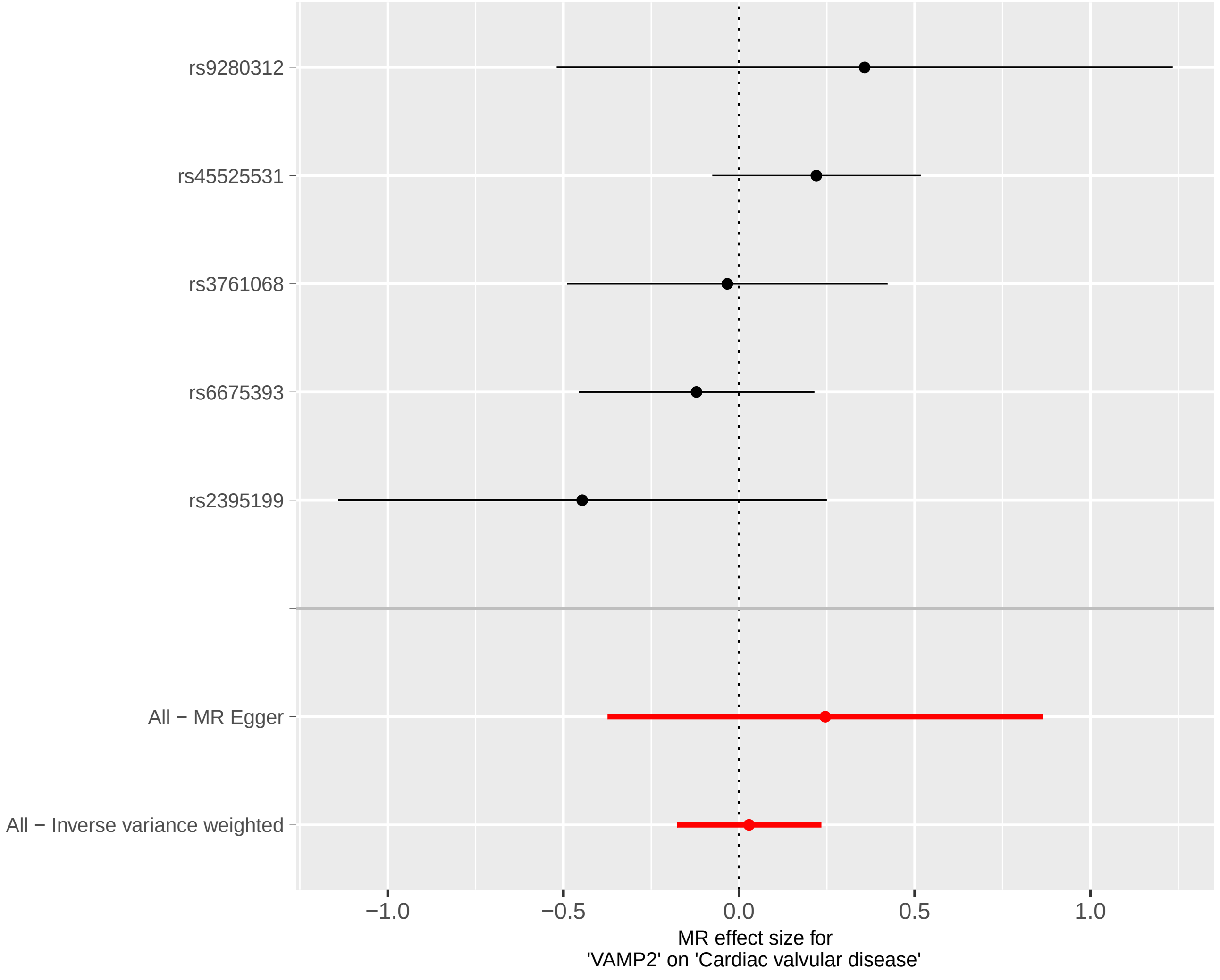
rs9280312
rs45525531
rs3761068
rs6675393
rs2395199
All − MR Egger
All − Inverse variance weighted
−1.0
−0.5
0.0
0.5
1.0
MR effect size for
'VAMP2' on 'Cardiac valvular disease'

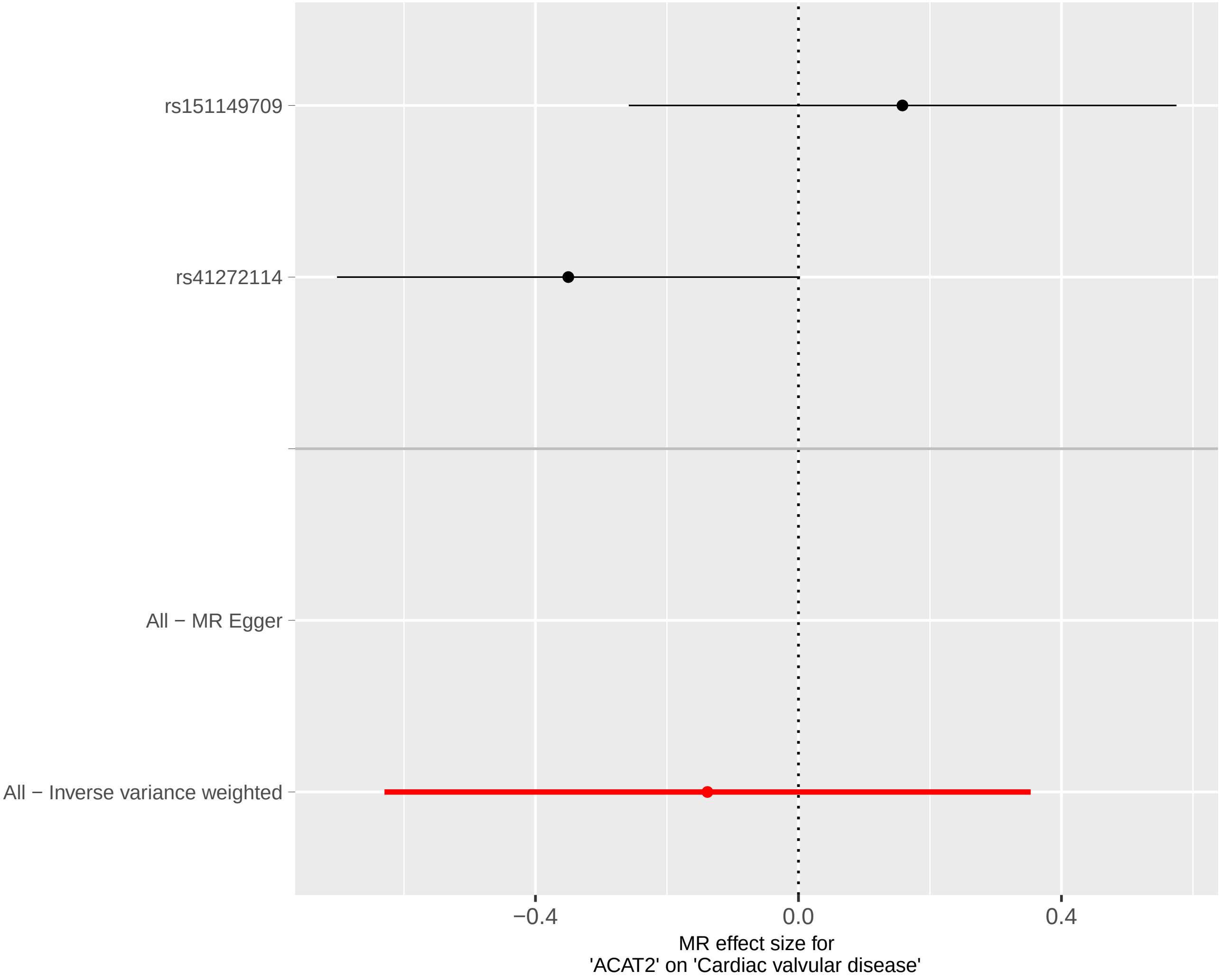

rs151149709
rs41272114
All − MR Egger
All − Inverse variance weighted
−0.4
0.0
0.4
MR effect size for
'ACAT2' on 'Cardiac valvular disease'

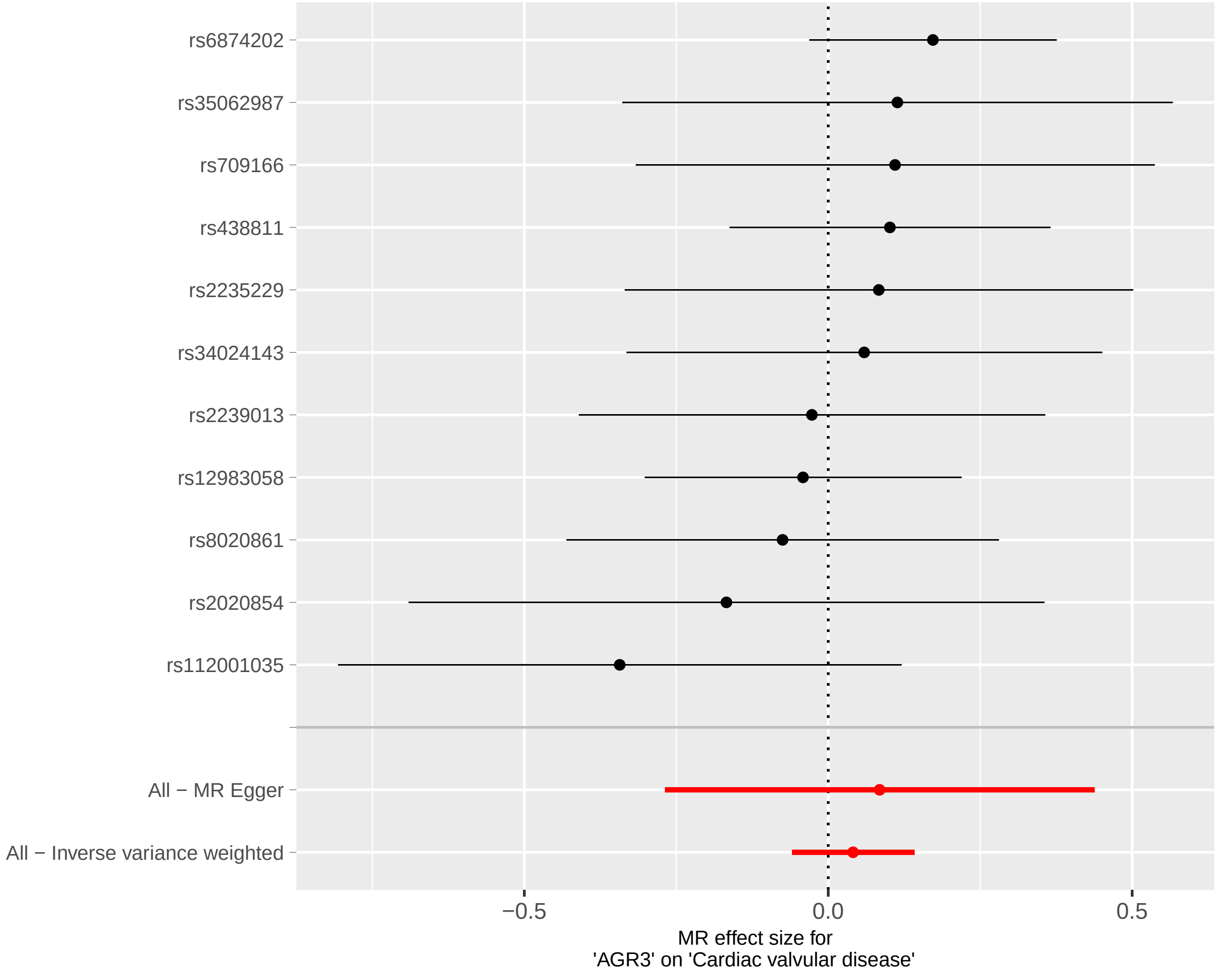
rs6874202
rs35062987
rs709166
rs438811
rs2235229
rs34024143
rs2239013
rs12983058
rs8020861
rs2020854
rs112001035
All − MR Egger
All − Inverse variance weighted
−0.5
0.0
0.5
MR effect size for
'AGR3' on 'Cardiac valvular disease'

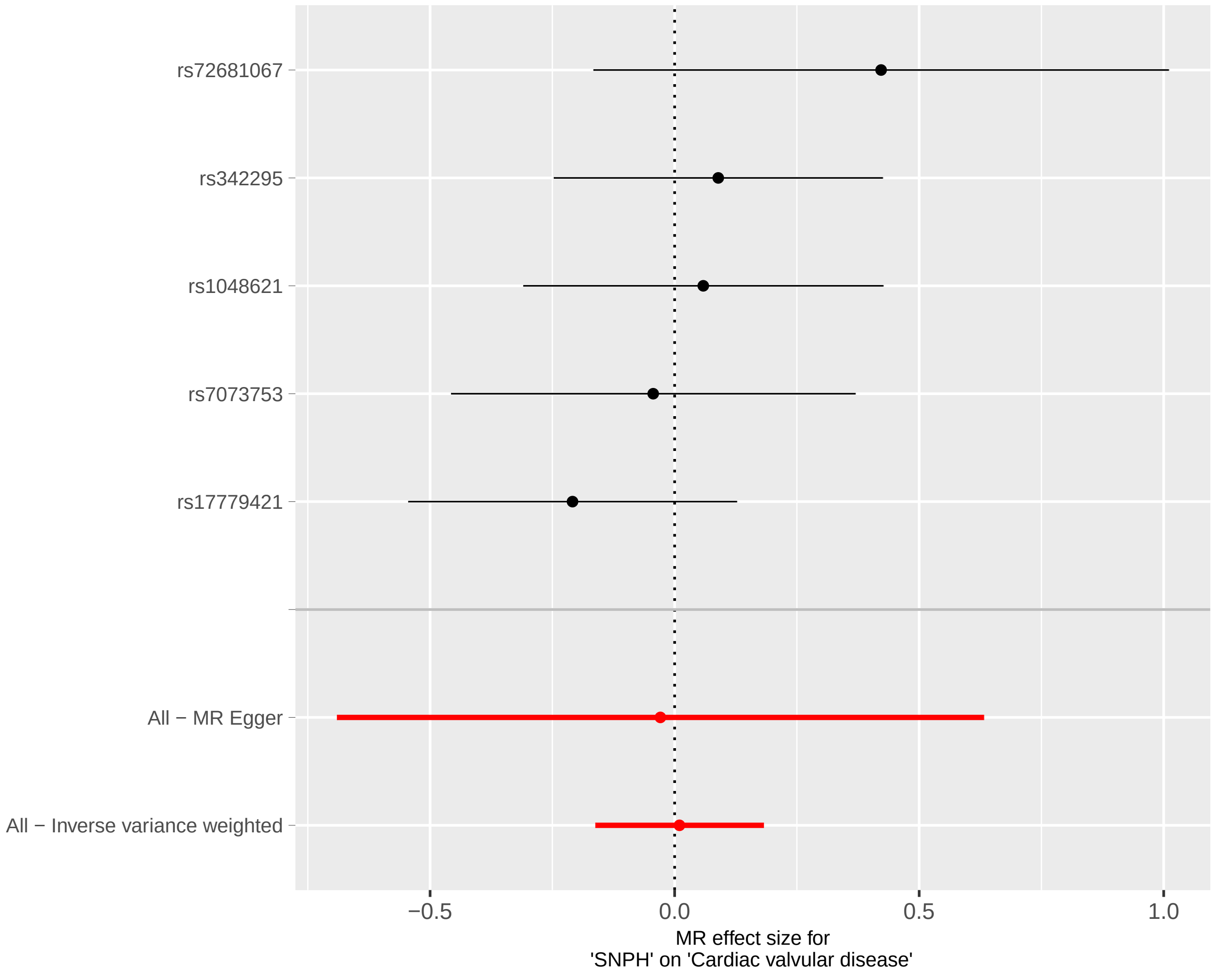
rs72681067
rs342295
rs1048621
rs7073753
rs17779421
All − MR Egger
All − Inverse variance weighted
−0.5
0.0
0.5
1.0
MR effect size for
'SNPH' on 'Cardiac valvular disease'

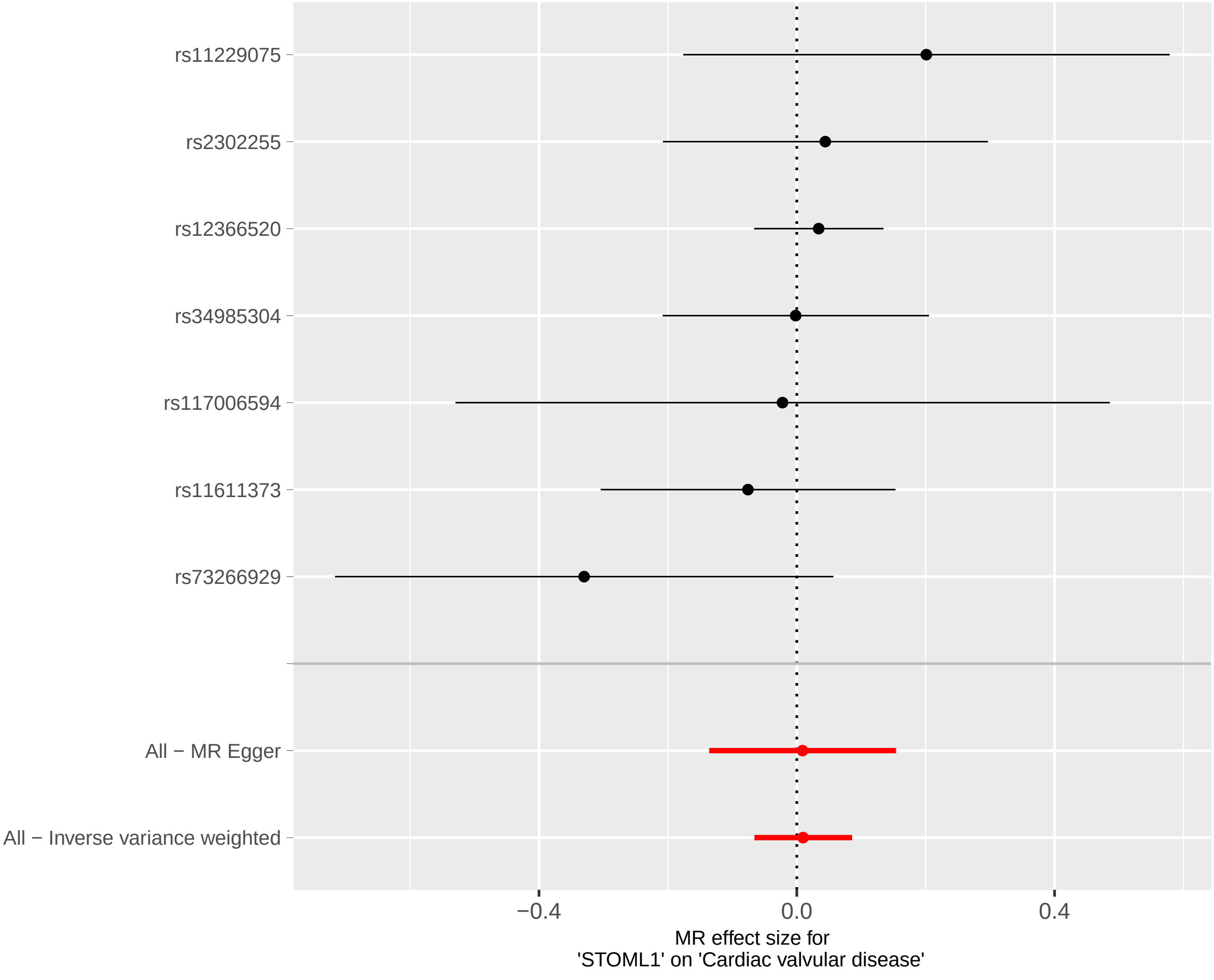
rs11229075
rs2302255
rs12366520
rs34985304
rs117006594
rs11611373
rs73266929
All − MR Egger
All − Inverse variance weighted
−0.4
0.0
0.4
MR effect size for
'STOML1' on 'Cardiac valvular disease'

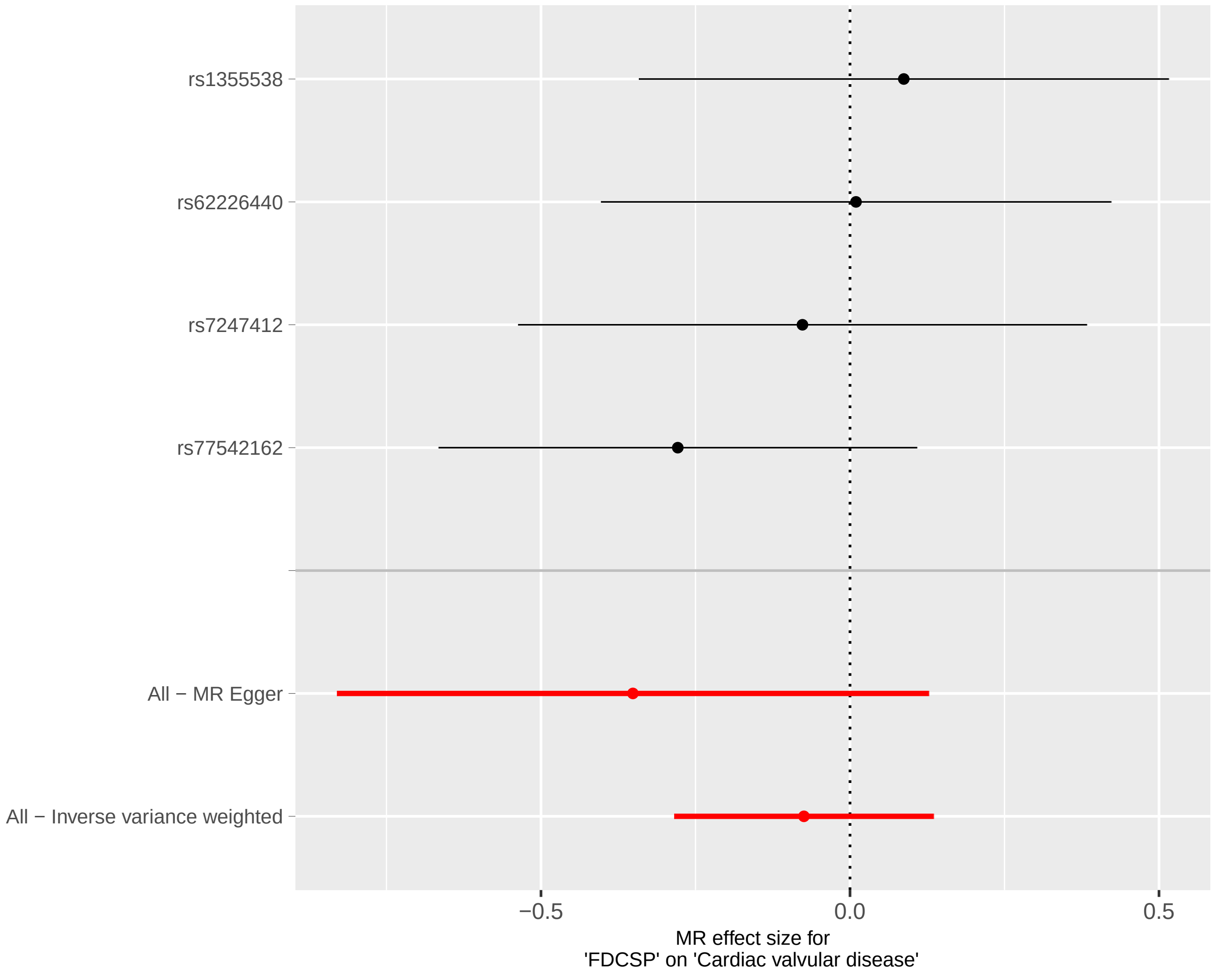
rs1355538
rs62226440
rs7247412
rs77542162
All − MR Egger
All − Inverse variance weighted
−0.5
0.0
0.5
MR effect size for
'FDCSP' on 'Cardiac valvular disease'

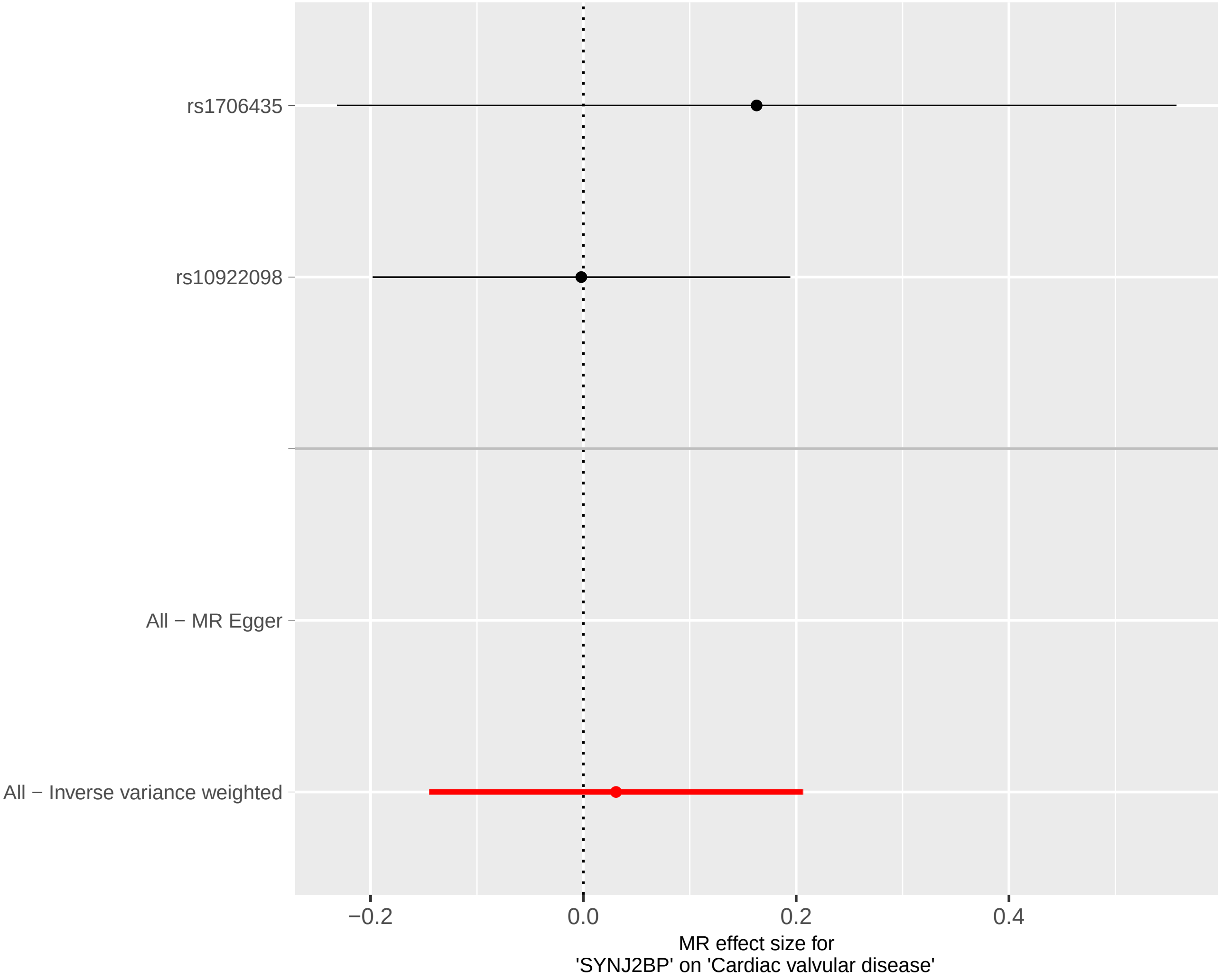
rs1706435
rs10922098
All − MR Egger
All − Inverse variance weighted
−0.2
0.0
0.2
0.4
MR effect size for
'SYNJ2BP' on 'Cardiac valvular disease'

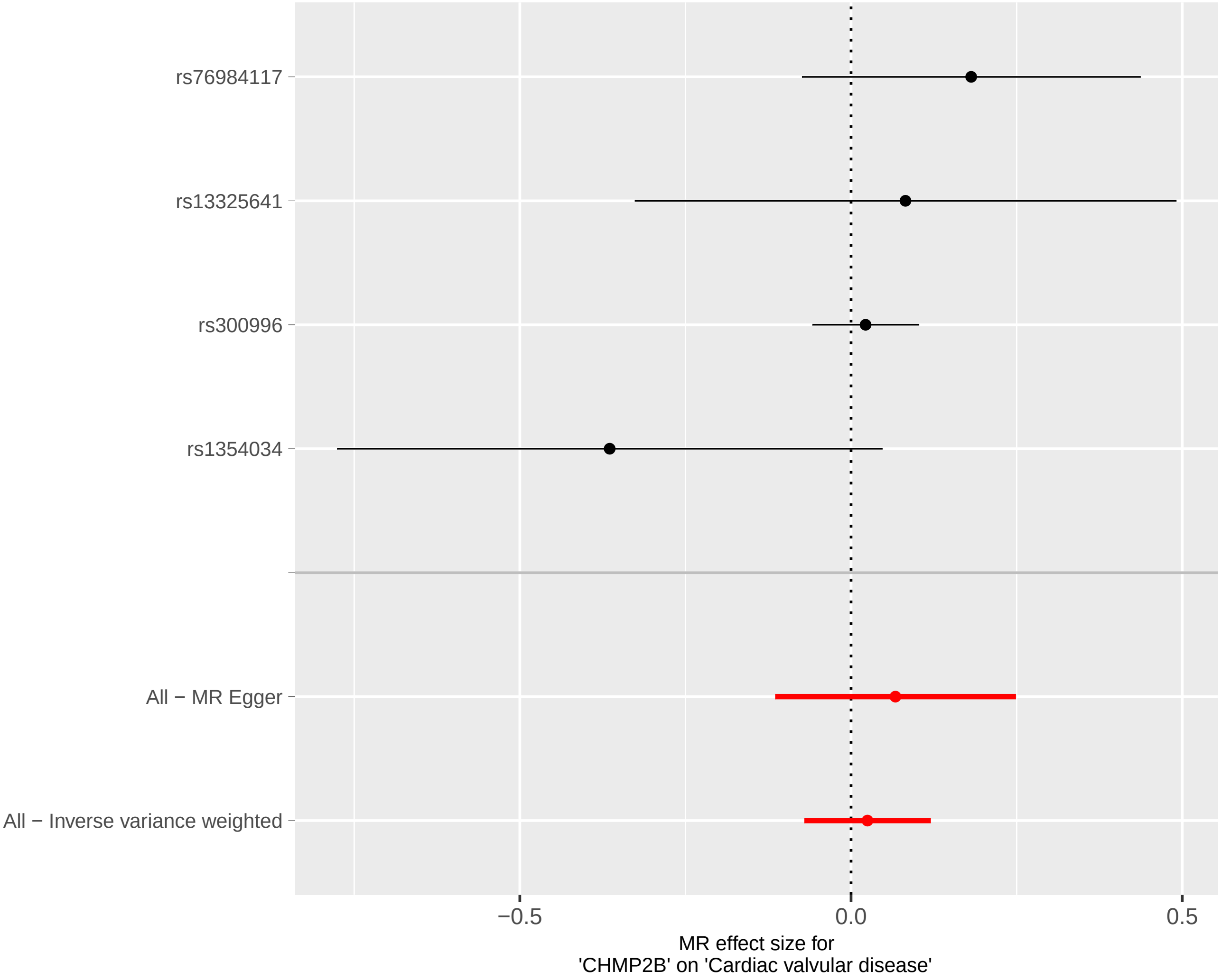
rs76984117
rs13325641
rs300996
rs1354034
All − MR Egger
All − Inverse variance weighted
−0.5
0.0
0.5
MR effect size for
'CHMP2B' on 'Cardiac valvular disease'

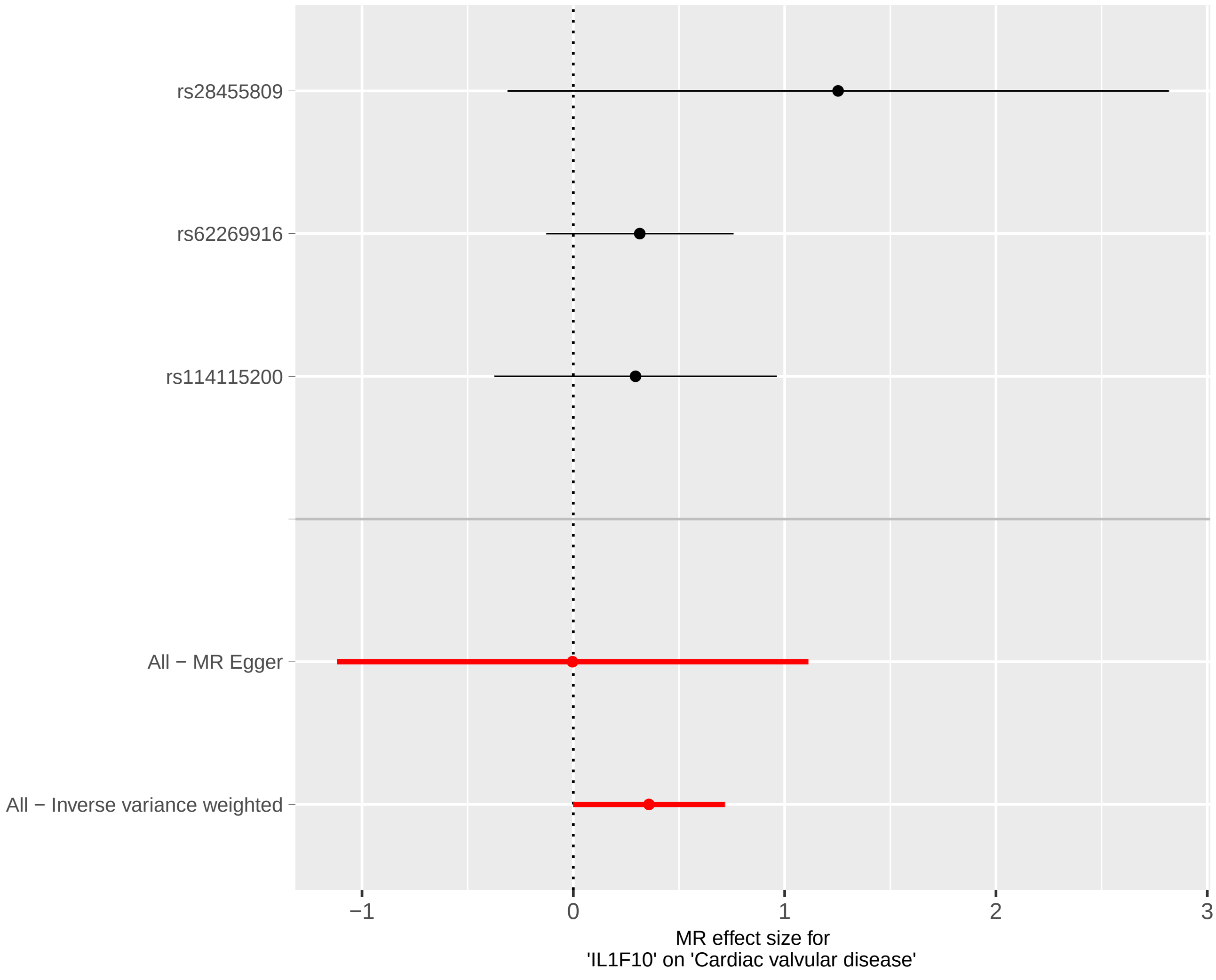
rs28455809
rs62269916
rs114115200
All − MR Egger
All − Inverse variance weighted
−1
0
1
2
3
MR effect size for
'IL1F10' on 'Cardiac valvular disease'

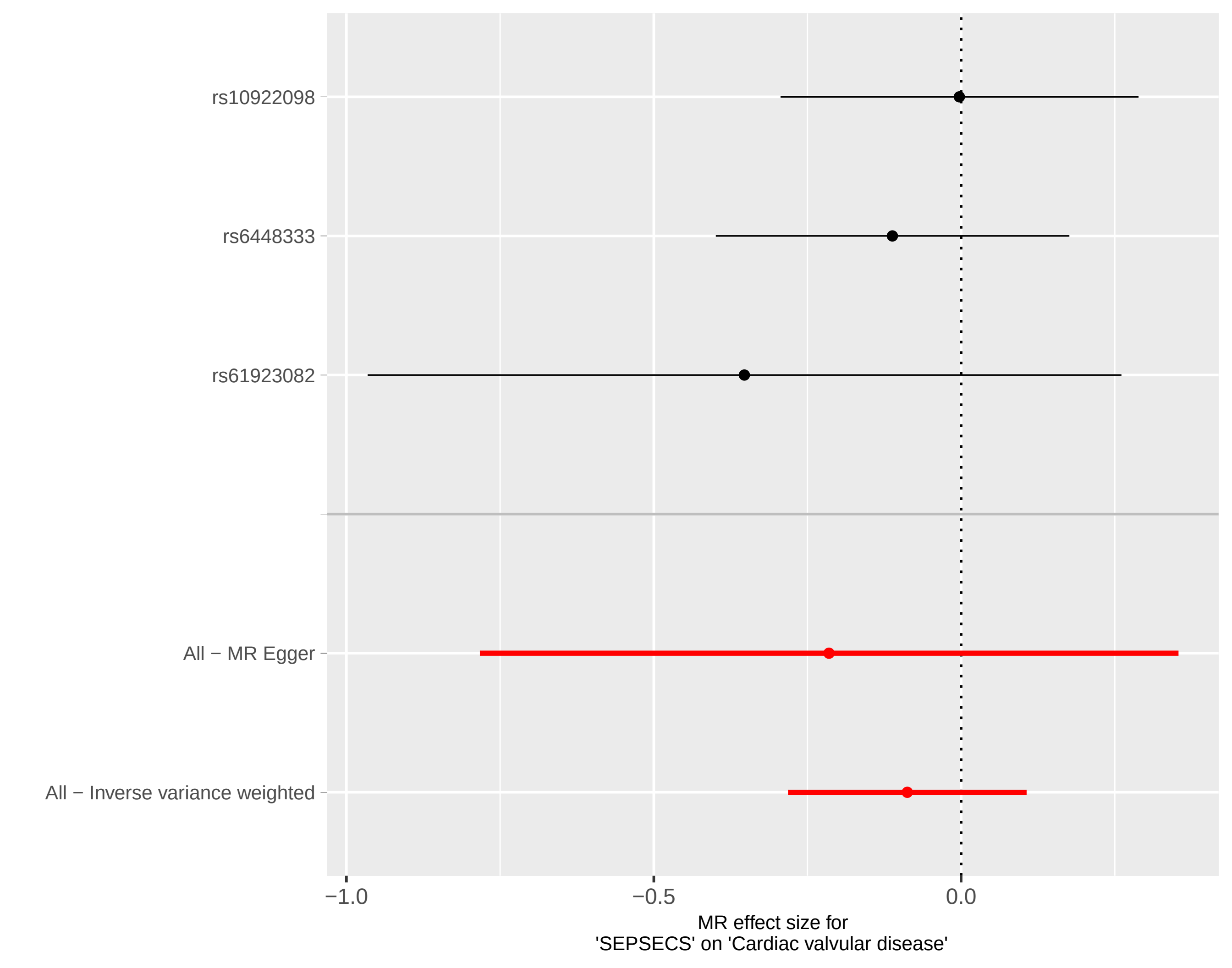
rs10922098
rs6448333
rs61923082
All − MR Egger
All − Inverse variance weighted
−1.0
−0.5
0.0
MR effect size for
'SEPSECS' on 'Cardiac valvular disease'

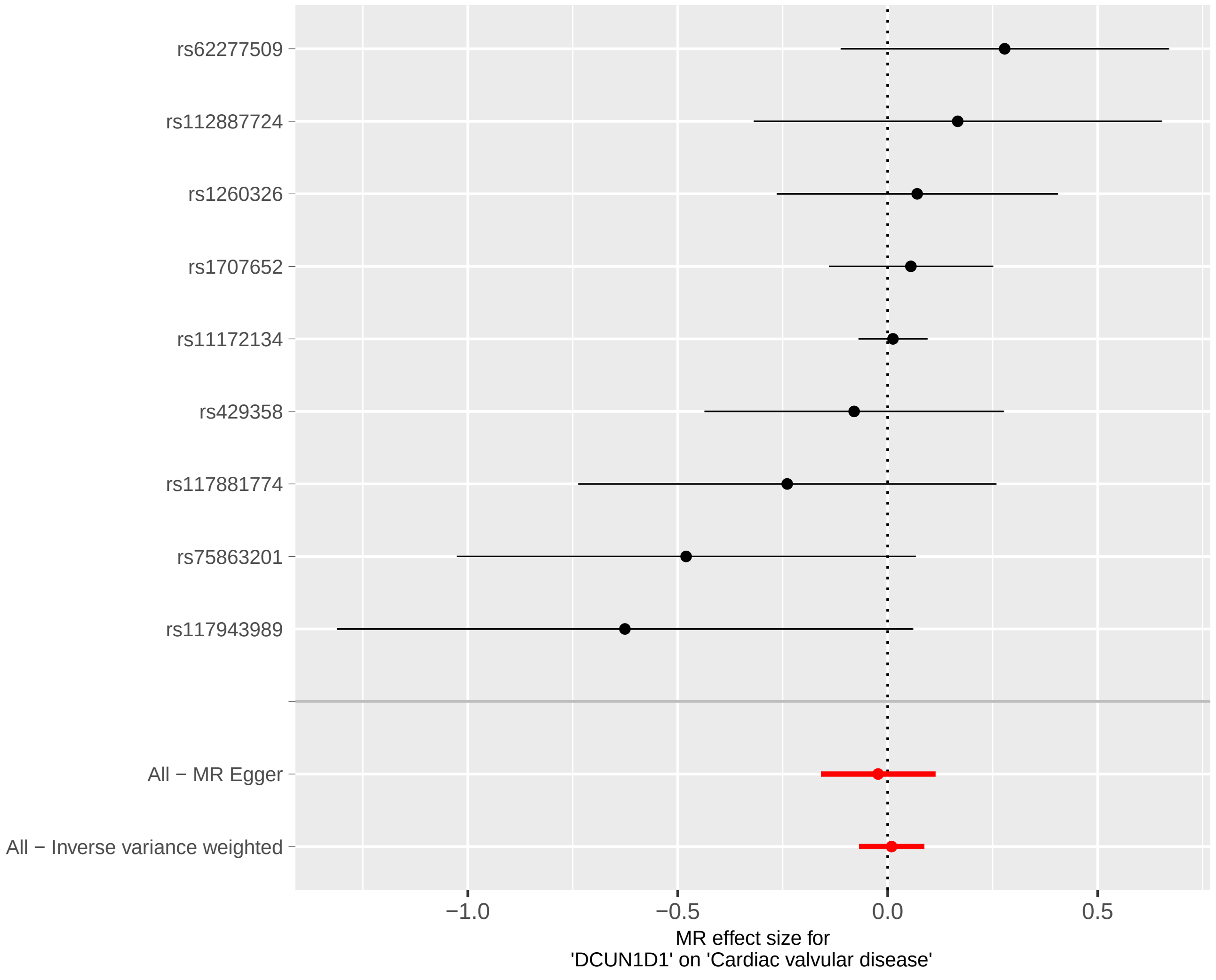
rs62277509
rs112887724
rs1260326
rs1707652
rs11172134
rs429358
rs117881774
rs75863201
rs117943989
All − MR Egger
All − Inverse variance weighted
−1.0
−0.5
0.0
0.5
MR effect size for
'DCUN1D1' on 'Cardiac valvular disease'

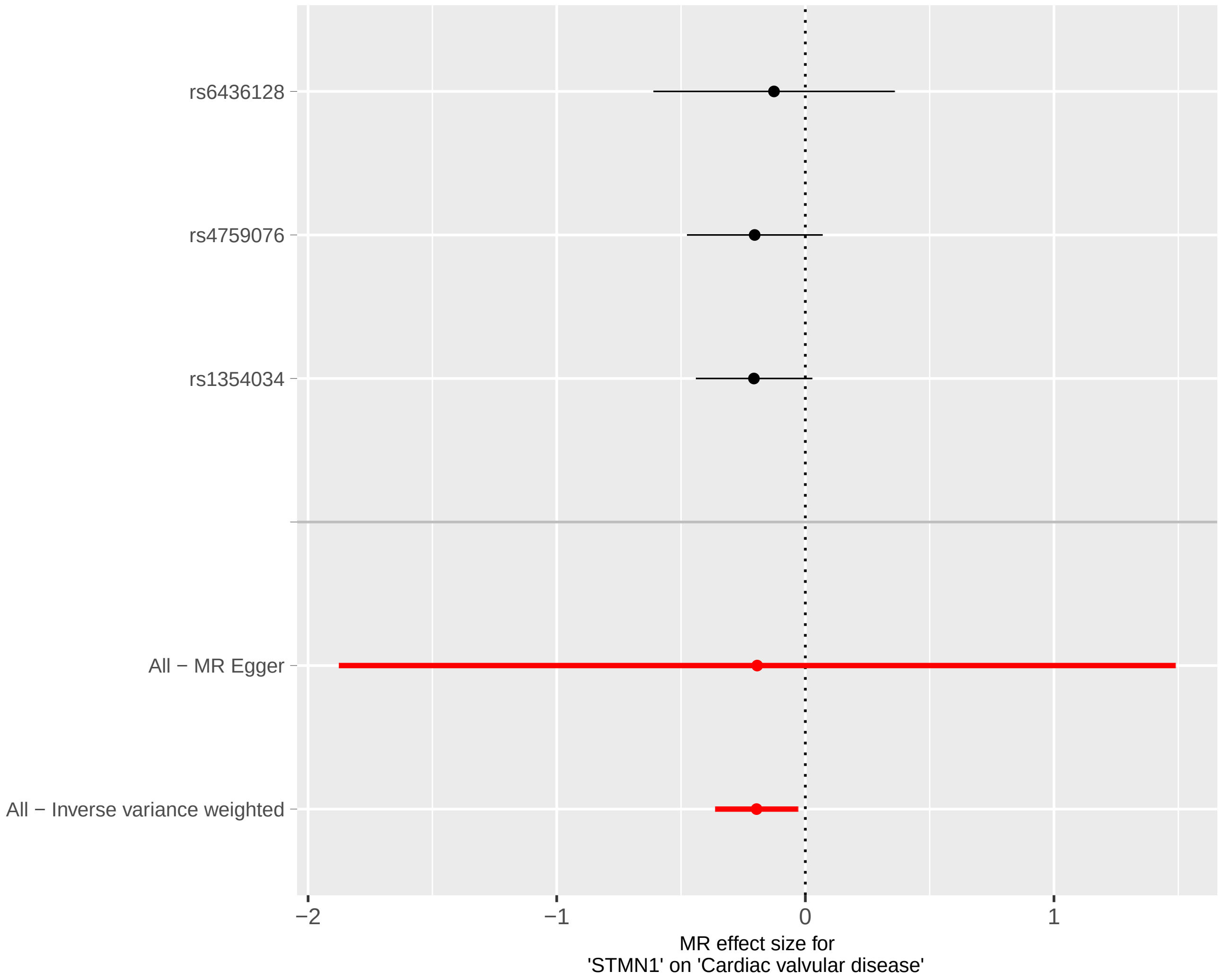

rs6436128
rs4759076
rs1354034
All − MR Egger
All − Inverse variance weighted
−2
−1
0
1
MR effect size for
'STMN1' on 'Cardiac valvular disease'

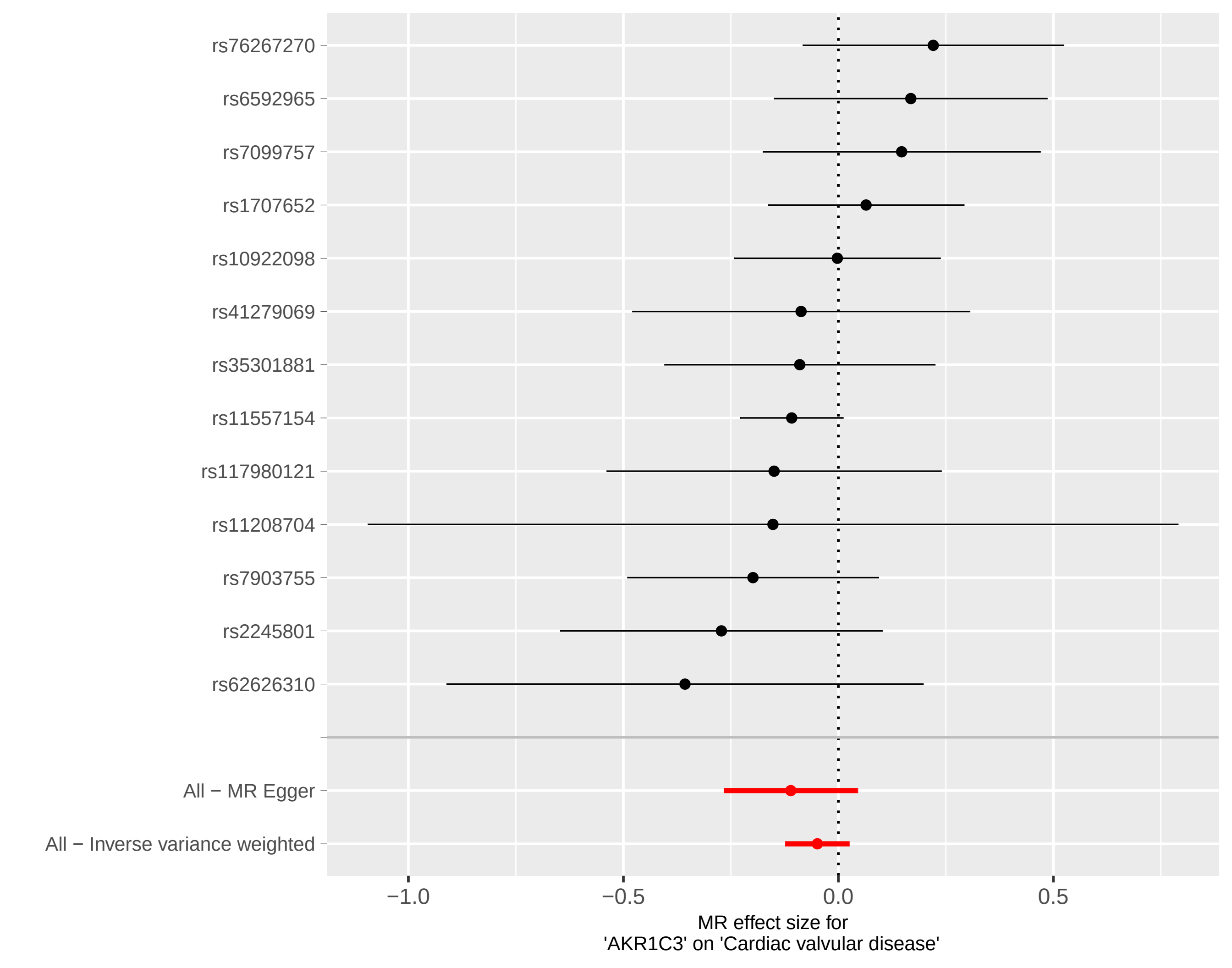

rs76267270
rs6592965
rs7099757
rs1707652
rs10922098
rs41279069
rs35301881
rs11557154
rs117980121
rs11208704
rs7903755
rs2245801
rs62626310
All − MR Egger
All − Inverse variance weighted
−1.0
−0.5
0.0
0.5
MR effect size for
'AKR1C3' on 'Cardiac valvular disease'

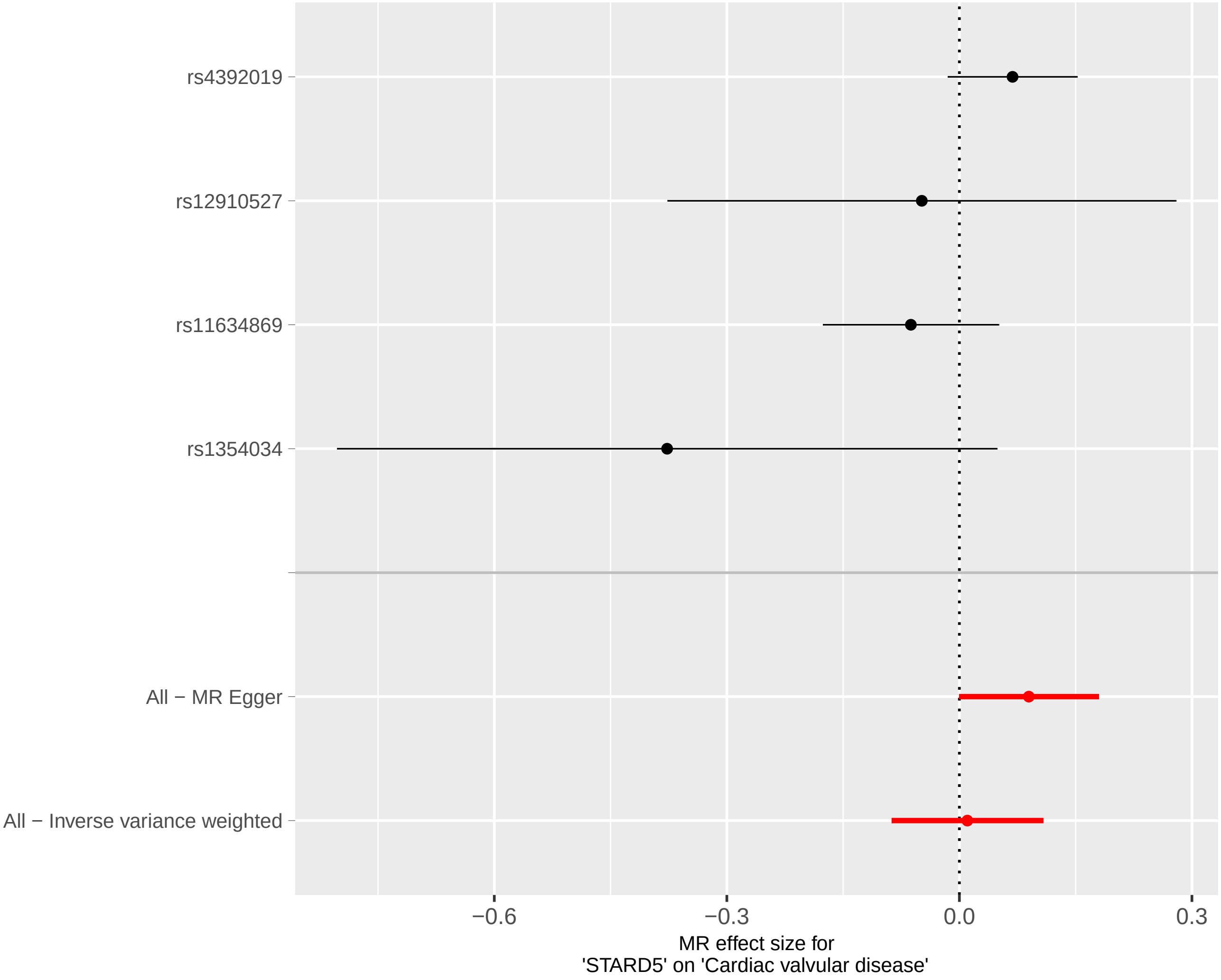
rs4392019
rs12910527
rs11634869
rs1354034
All − MR Egger
All − Inverse variance weighted
−0.6
−0.3
0.0
0.3
MR effect size for
'STARD5' on 'Cardiac valvular disease'

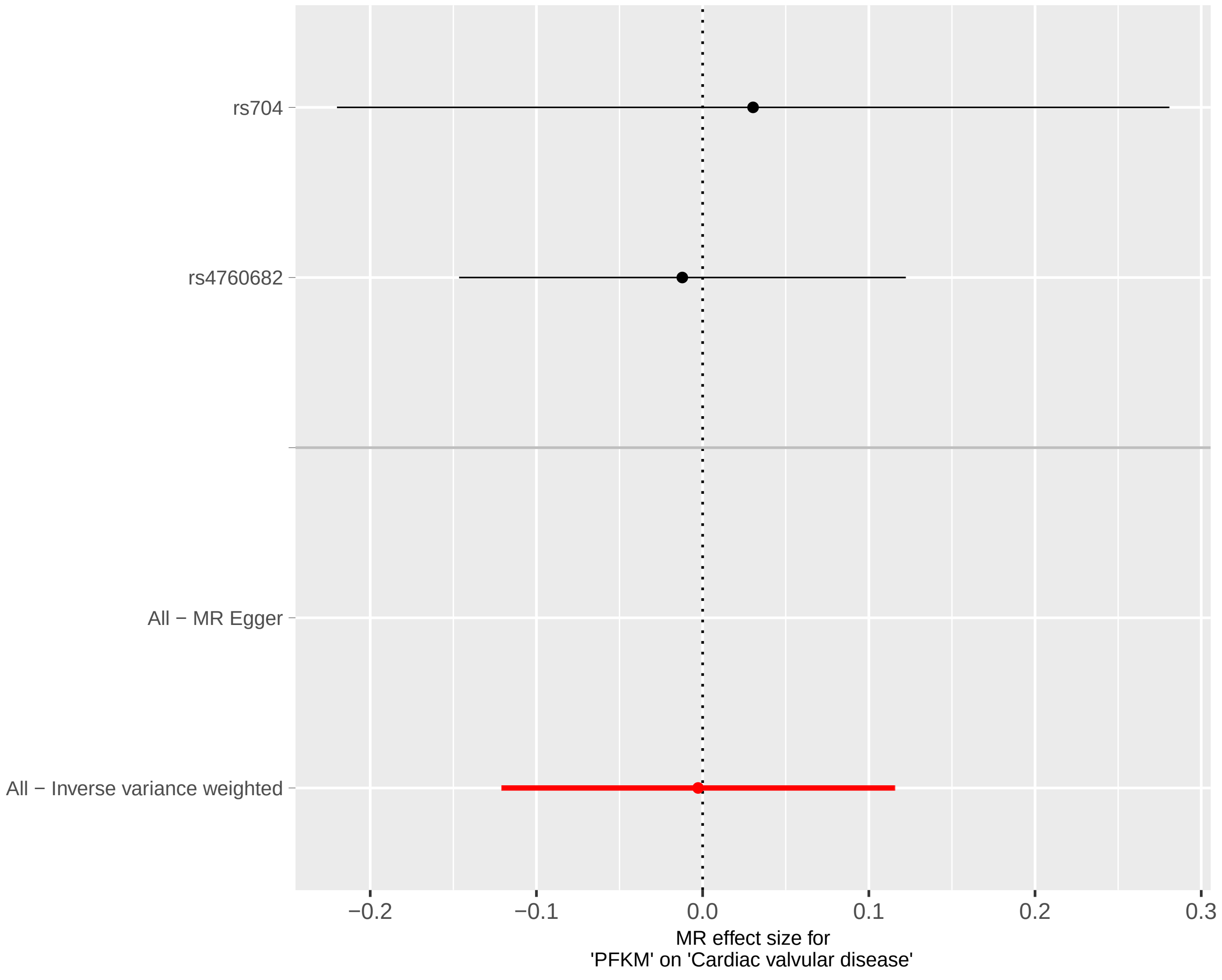
rs704
rs4760682
All − MR Egger
All − Inverse variance weighted
−0.2
−0.1
0.0
0.1
0.2
0.3
MR effect size for
'PFKM' on 'Cardiac valvular disease'

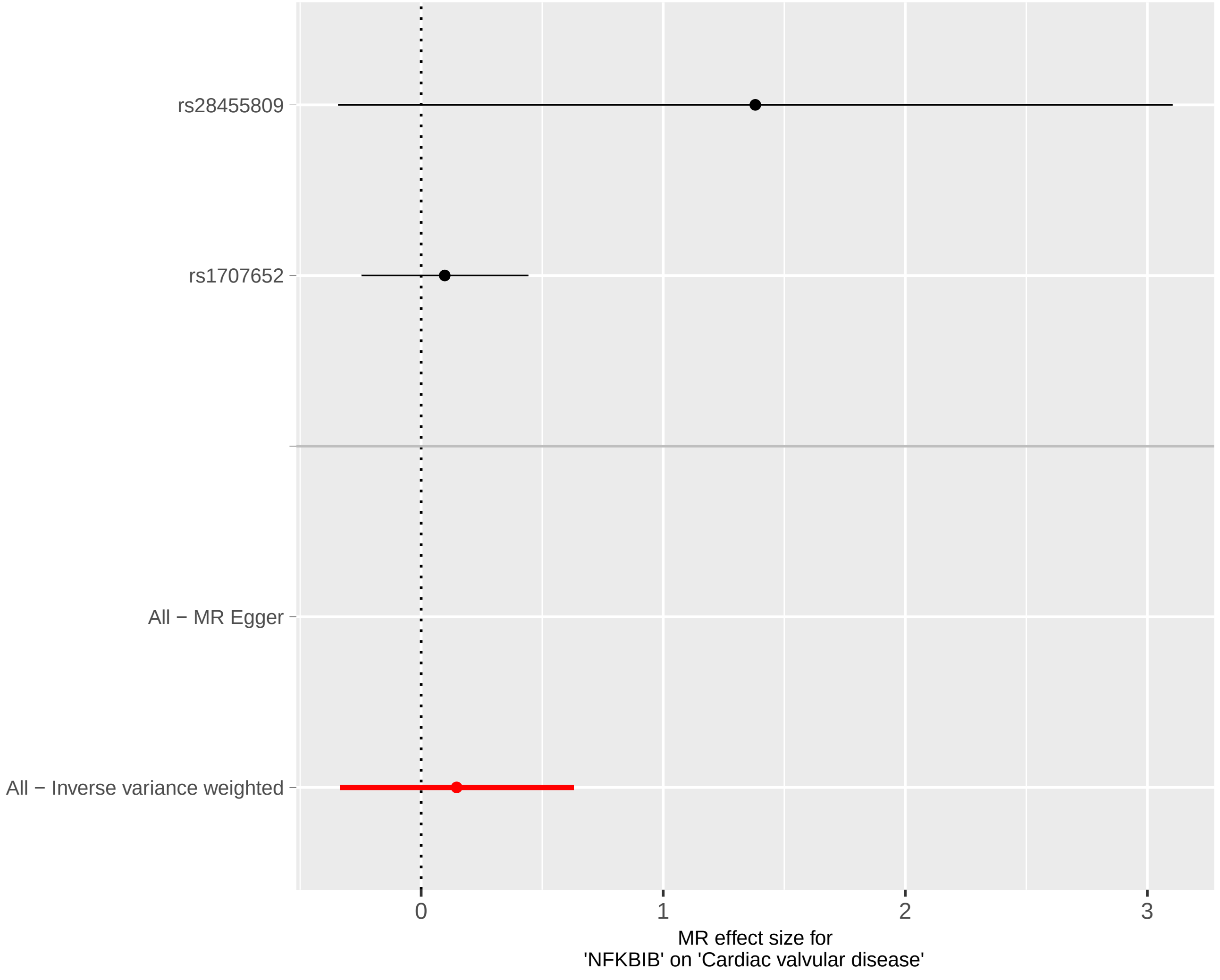
rs28455809
rs1707652
All − MR Egger
All − Inverse variance weighted
0
1
2
3
MR effect size for
'NFKBIB' on 'Cardiac valvular disease'

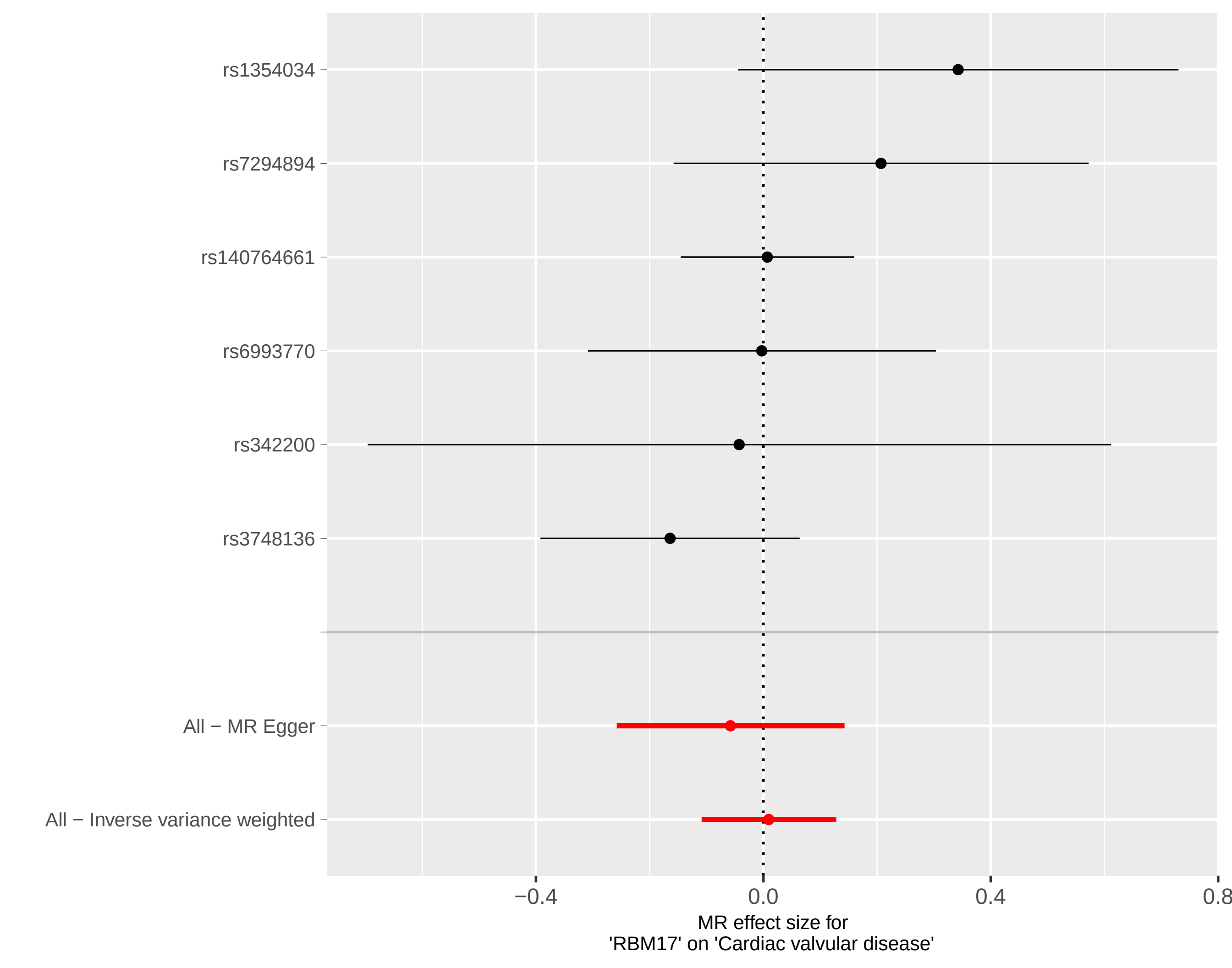
rs1354034
rs7294894
rs140764661
rs6993770
rs342200
rs3748136
All − MR Egger
All − Inverse variance weighted
−0.4
0.0
0.4
0.8
MR effect size for
'RBM17' on 'Cardiac valvular disease'

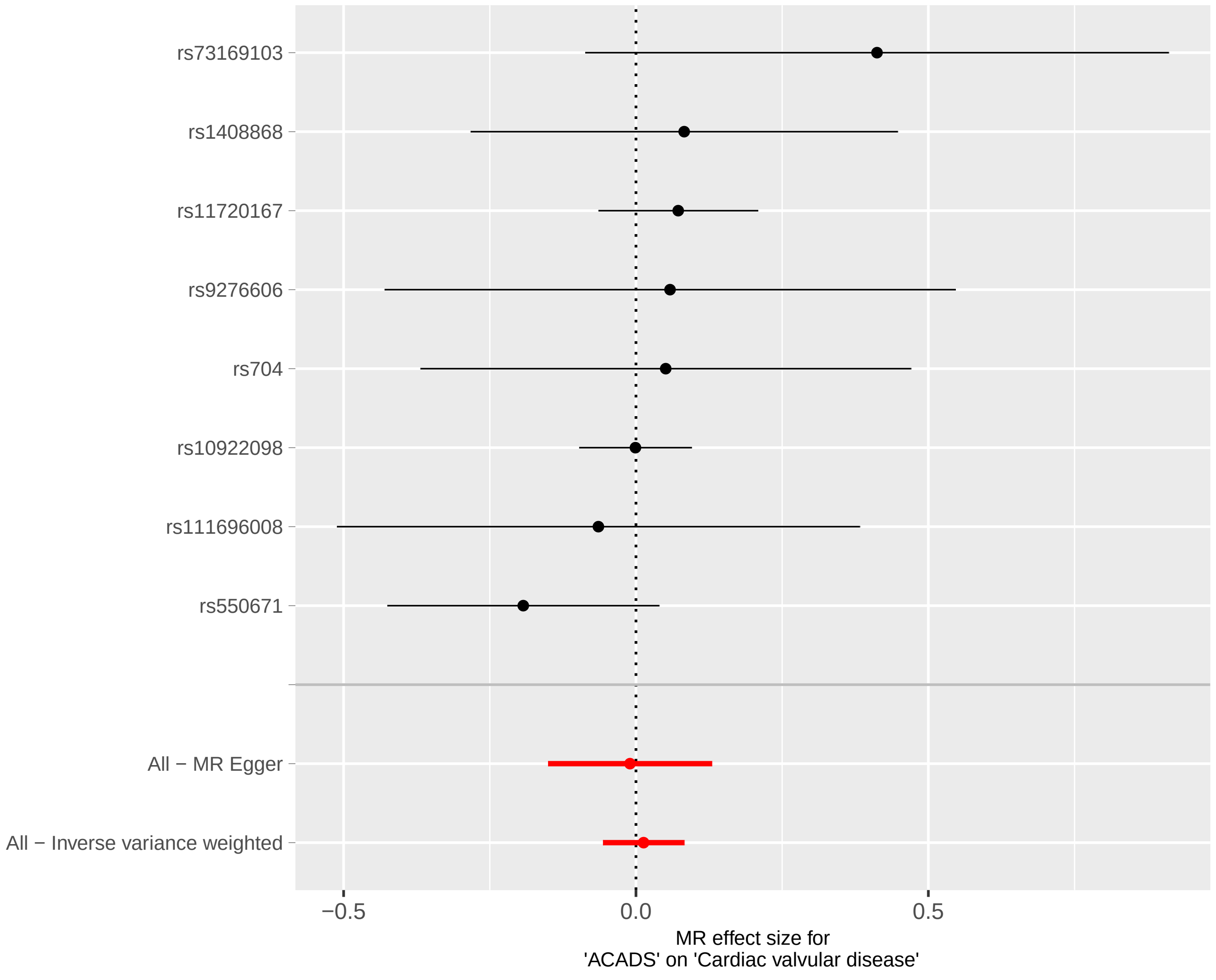
rs73169103
rs1408868
rs11720167
rs9276606
rs704
rs10922098
rs111696008
rs550671
All − MR Egger
All − Inverse variance weighted
−0.5
0.0
0.5
MR effect size for
'ACADS' on 'Cardiac valvular disease'

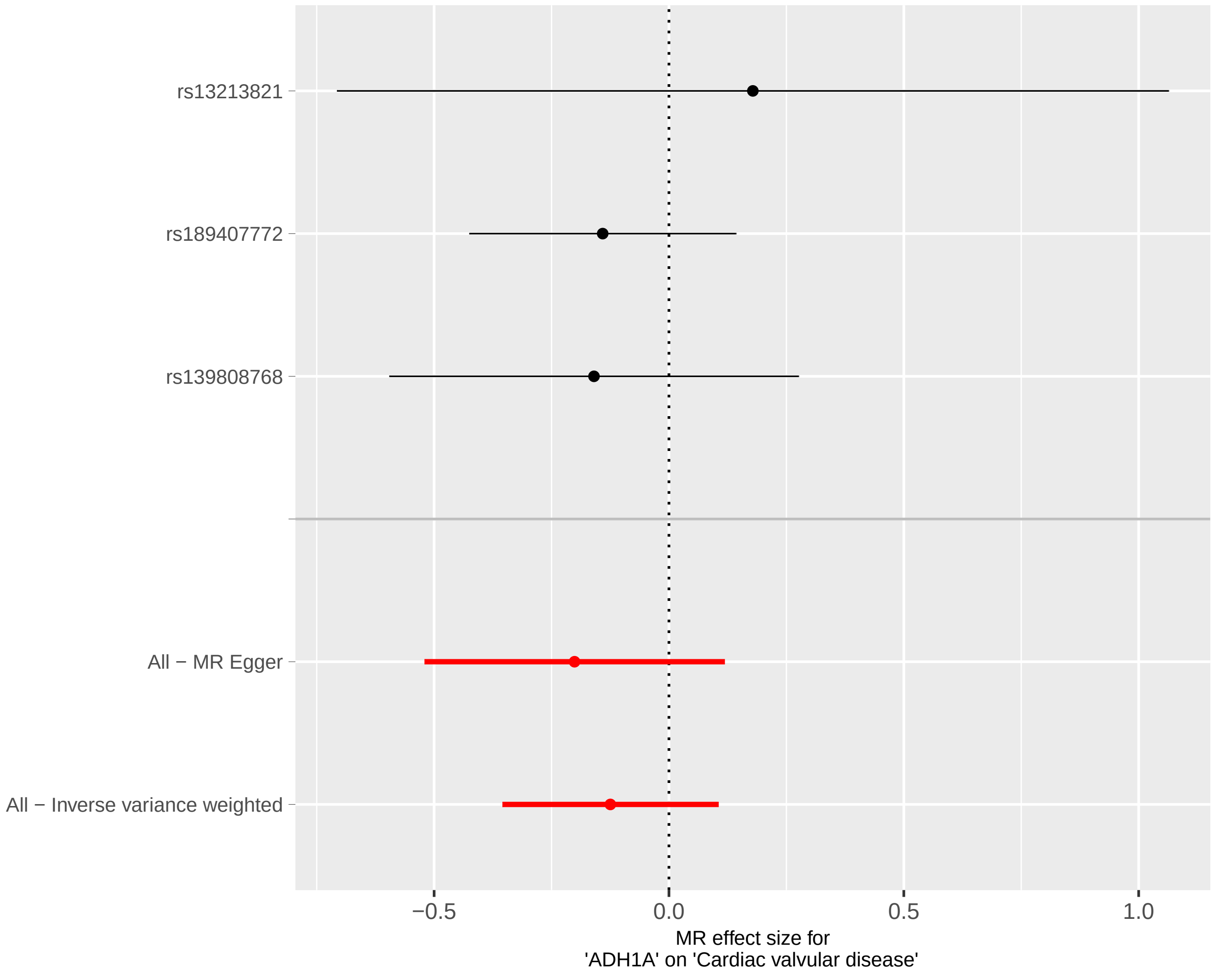

rs13213821
rs189407772
rs139808768
All − MR Egger
All − Inverse variance weighted
−0.5
0.0
0.5
1.0
MR effect size for
'ADH1A' on 'Cardiac valvular disease'

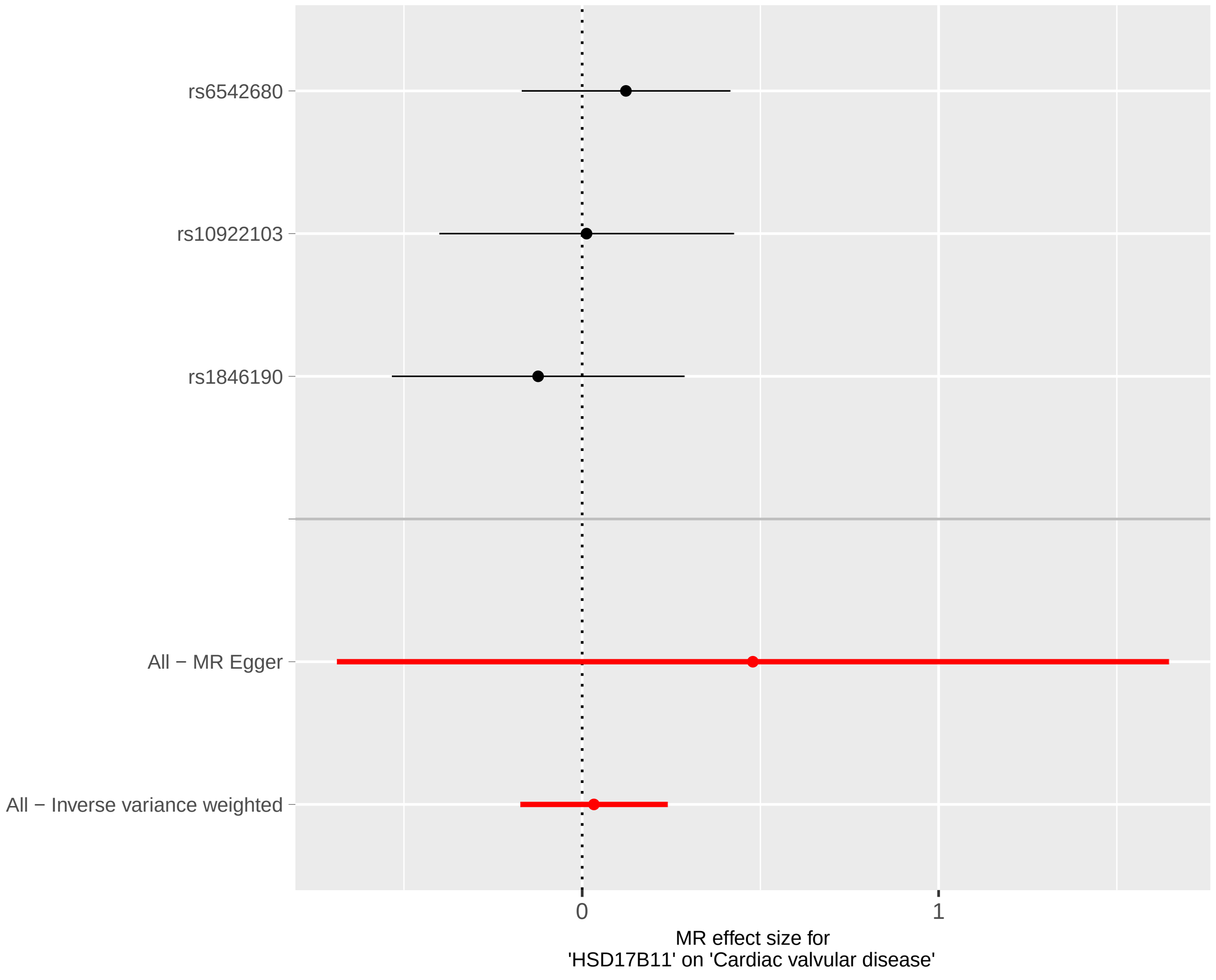
rs6542680
rs10922103
rs1846190
All − MR Egger
All − Inverse variance weighted
0
1
MR effect size for
'HSD17B11' on 'Cardiac valvular disease'

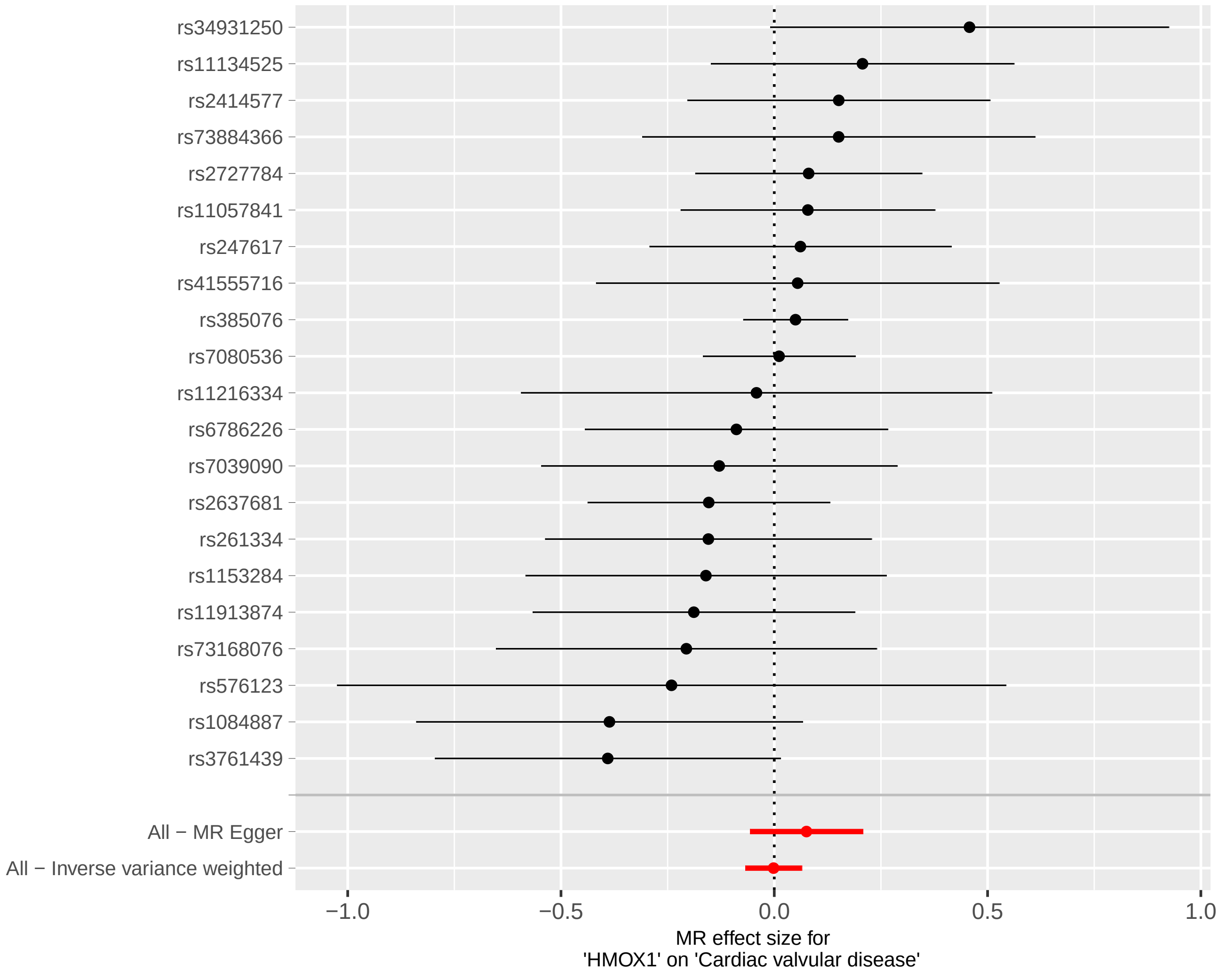

rs34931250
rs11134525
rs2414577
rs73884366
rs2727784
rs11057841
rs247617
rs41555716
rs385076
rs7080536
rs11216334
rs6786226
rs7039090
rs2637681
rs261334
rs1153284
rs11913874
rs73168076
rs576123
rs1084887
rs3761439
All − MR Egger
All − Inverse variance weighted
−1.0
−0.5
0.0
0.5
1.0
MR effect size for
'HMOX1' on 'Cardiac valvular disease'

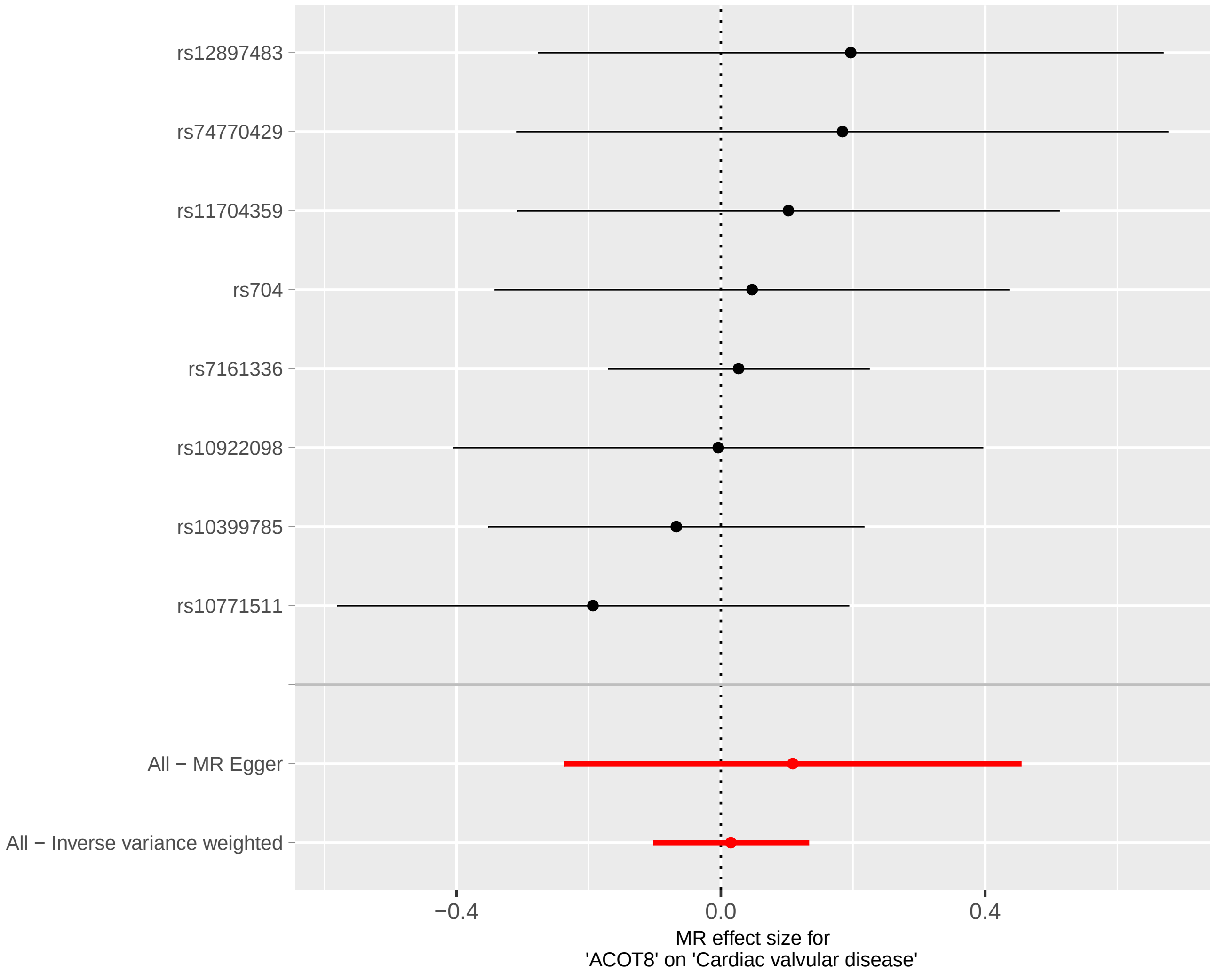
rs12897483
rs74770429
rs11704359
rs704
rs7161336
rs10922098
rs10399785
rs10771511
All − MR Egger
All − Inverse variance weighted
−0.4
0.0
0.4
MR effect size for
'ACOT8' on 'Cardiac valvular disease'

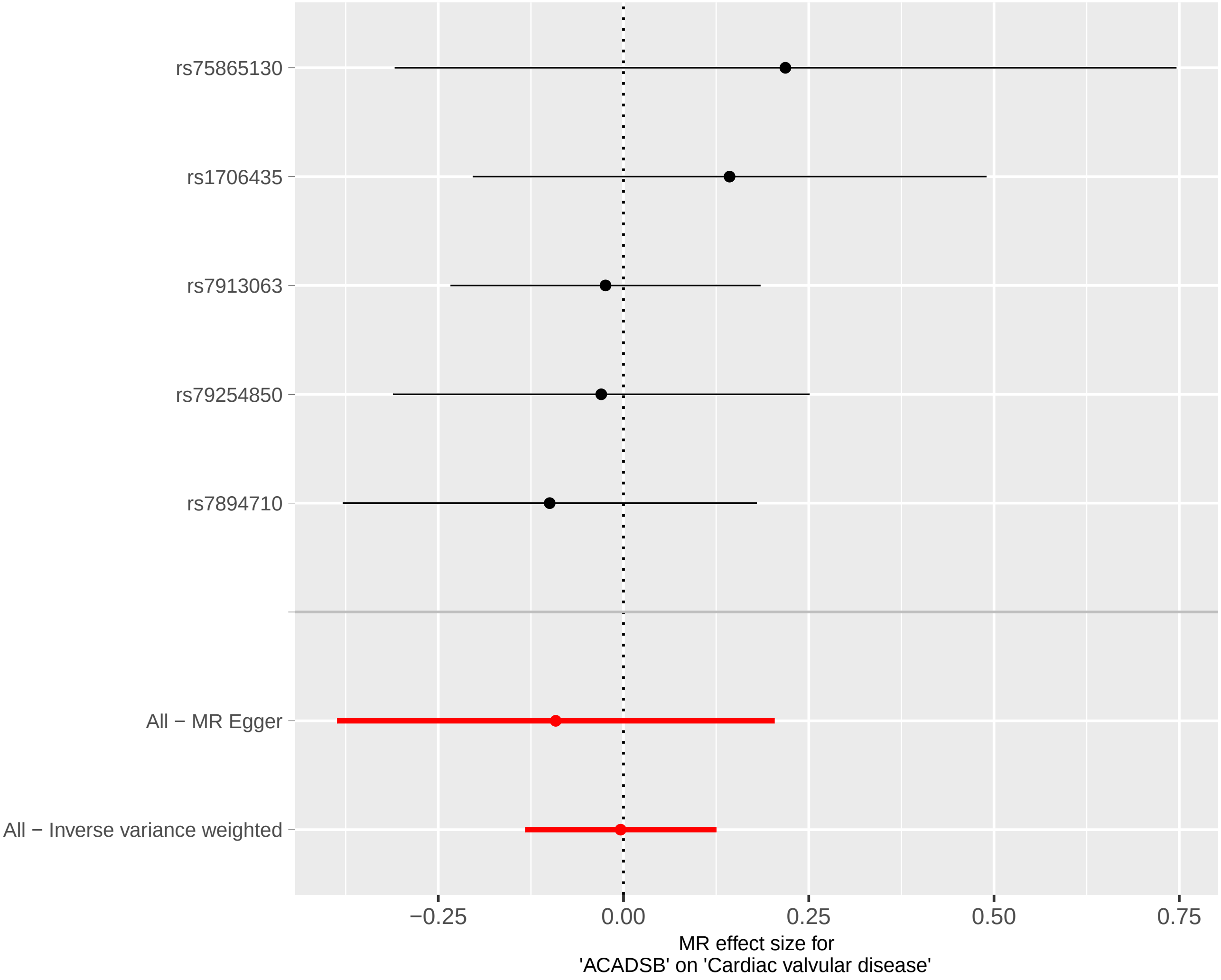
rs75865130
rs1706435
rs7913063
rs79254850
rs7894710
All − MR Egger
All − Inverse variance weighted
−0.25
0.00
0.25
0.50
0.75
MR effect size for
'ACADSB' on 'Cardiac valvular disease'

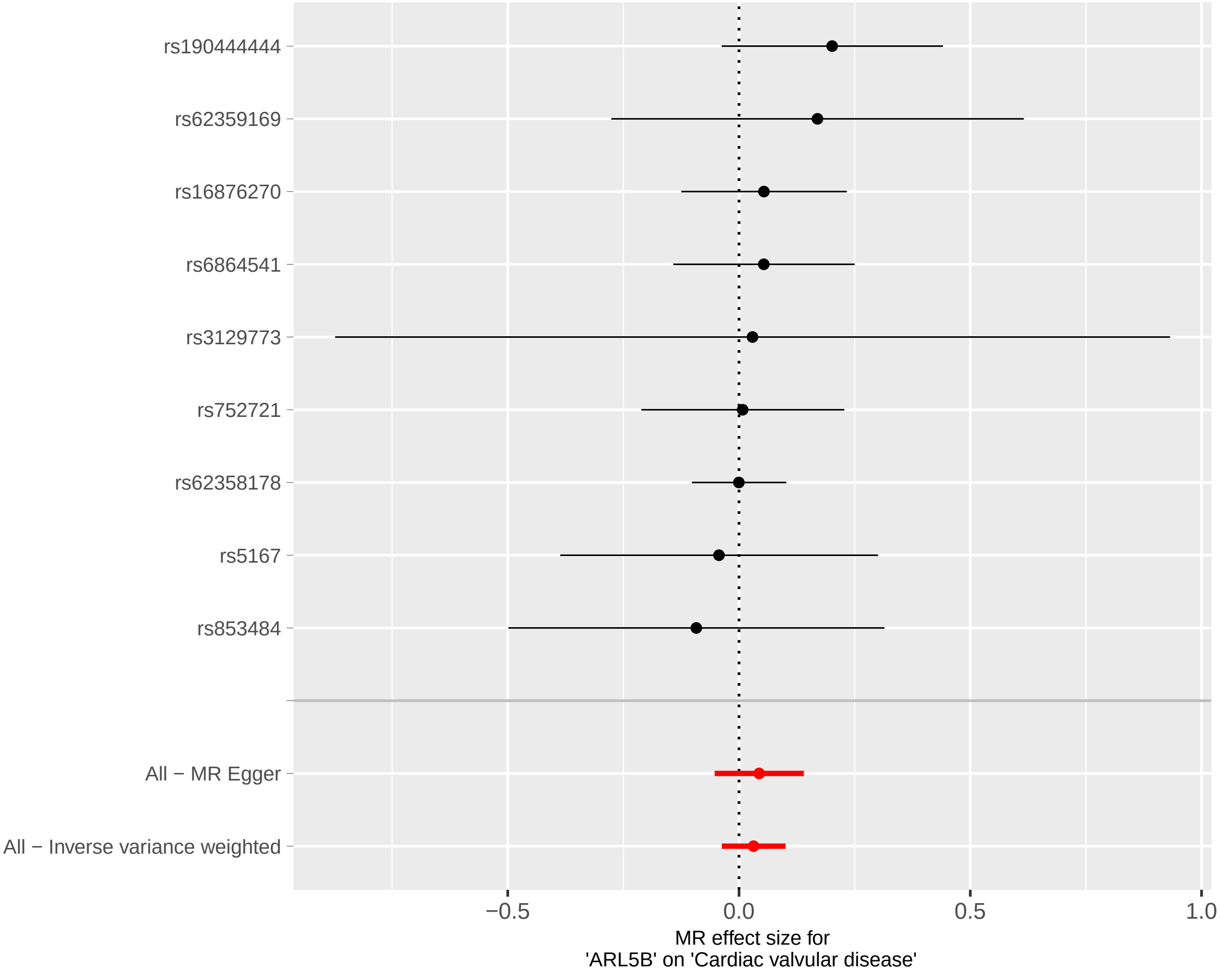
rs190444444
rs62359169
rs16876270
rs6864541
rs3129773
rs752721
rs62358178
rs5167
rs853484
All − MR Egger
All − Inverse variance weighted
−0.5
0.0
0.5
1.0
MR effect size for
'ARL5B' on 'Cardiac valvular disease'

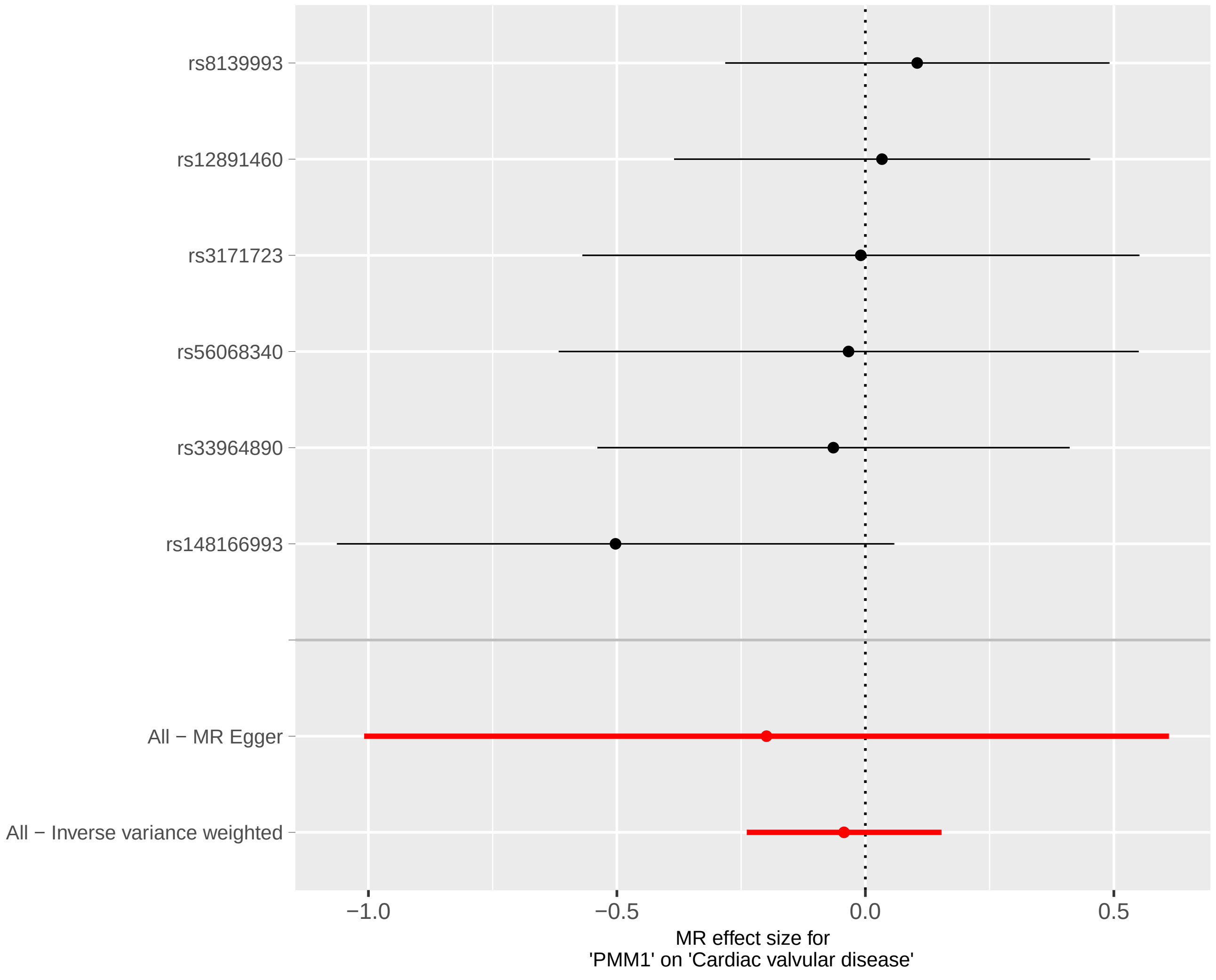
rs8139993
rs12891460
rs3171723
rs56068340
rs33964890
rs148166993
All − MR Egger
All − Inverse variance weighted
−1.0
−0.5
0.0
0.5
MR effect size for
'PMM1' on 'Cardiac valvular disease'

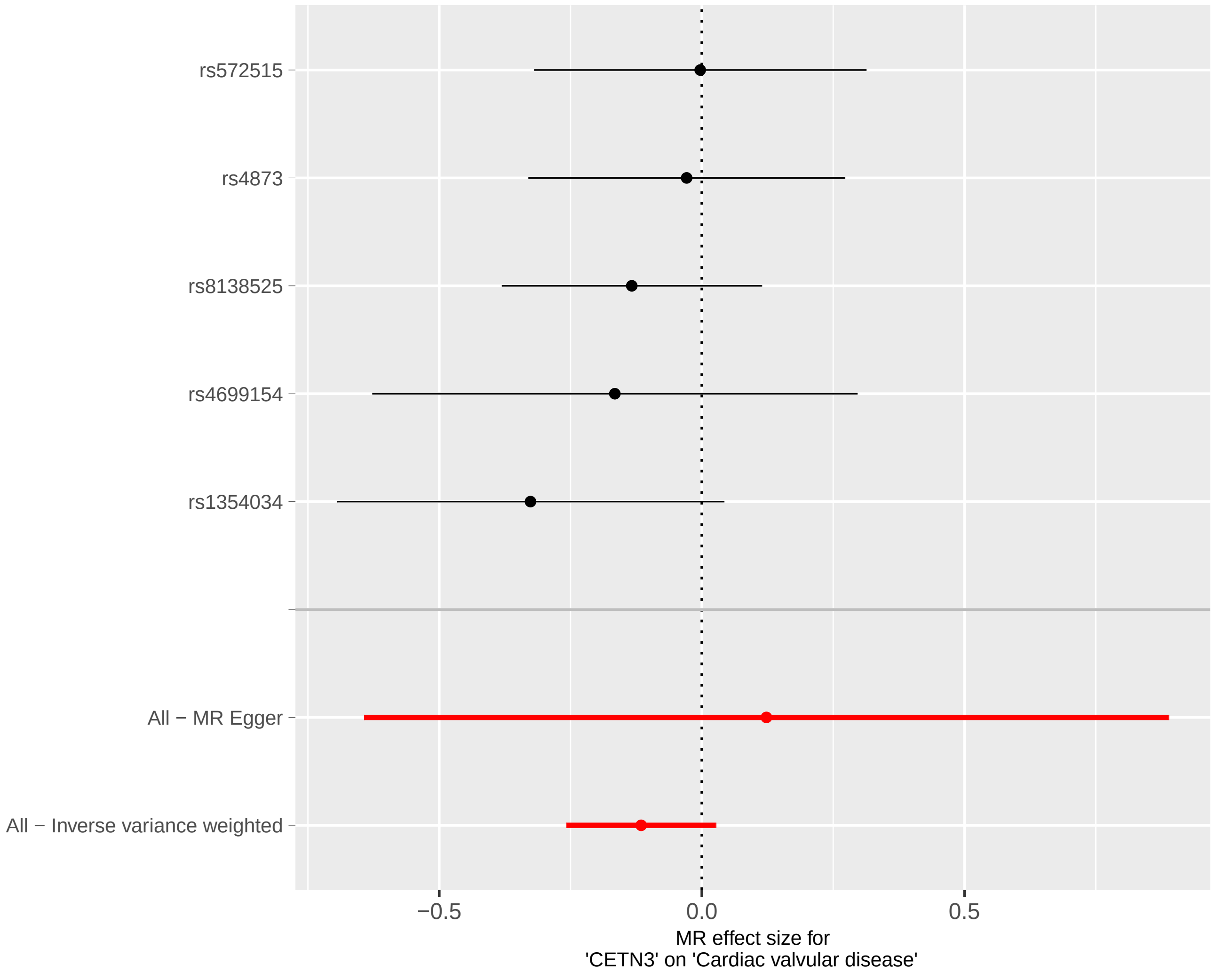
rs572515
rs4873
rs8138525
rs4699154
rs1354034
All − MR Egger
All − Inverse variance weighted
−0.5
0.0
0.5
MR effect size for
'CETN3' on 'Cardiac valvular disease'

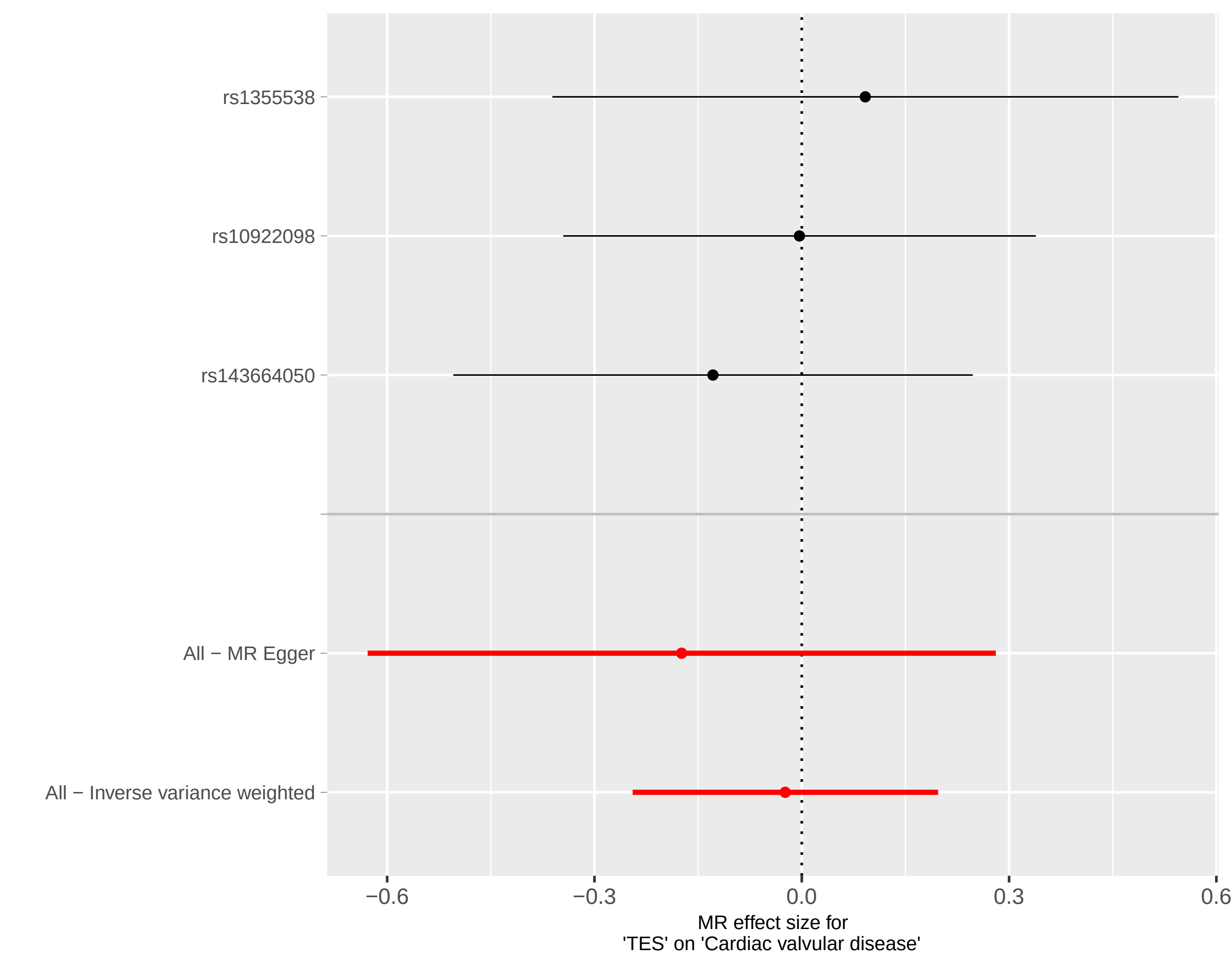
rs1355538
rs10922098
rs143664050
All − MR Egger
All − Inverse variance weighted
−0.6
−0.3
0.0
0.3
0.6
MR effect size for
'TES' on 'Cardiac valvular disease'

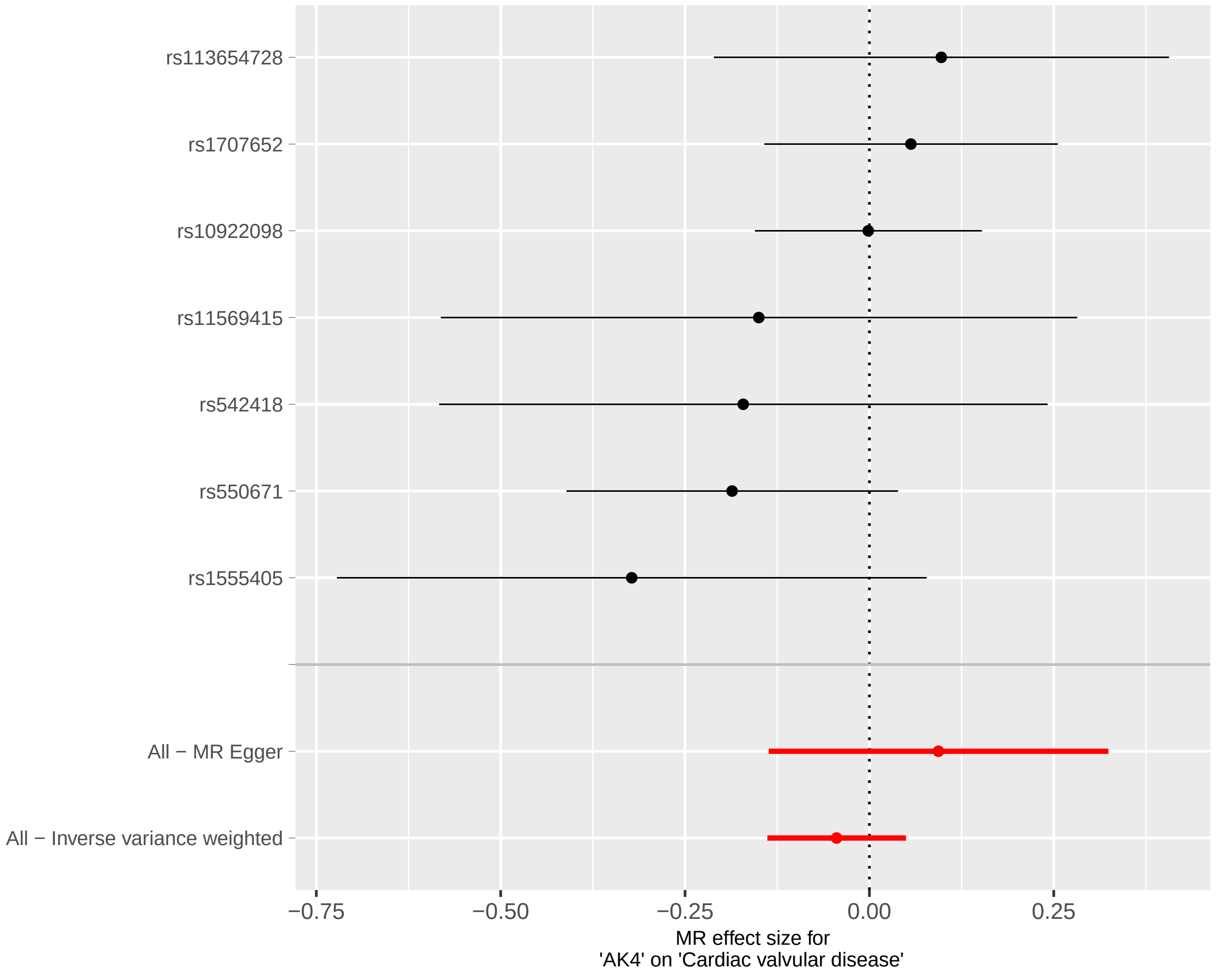
rs113654728
rs1707652
rs10922098
rs11569415
rs542418
rs550671
rs1555405
All − MR Egger
All − Inverse variance weighted
−0.75
−0.50
−0.25
0.00
0.25
MR effect size for
'AK4' on 'Cardiac valvular disease'

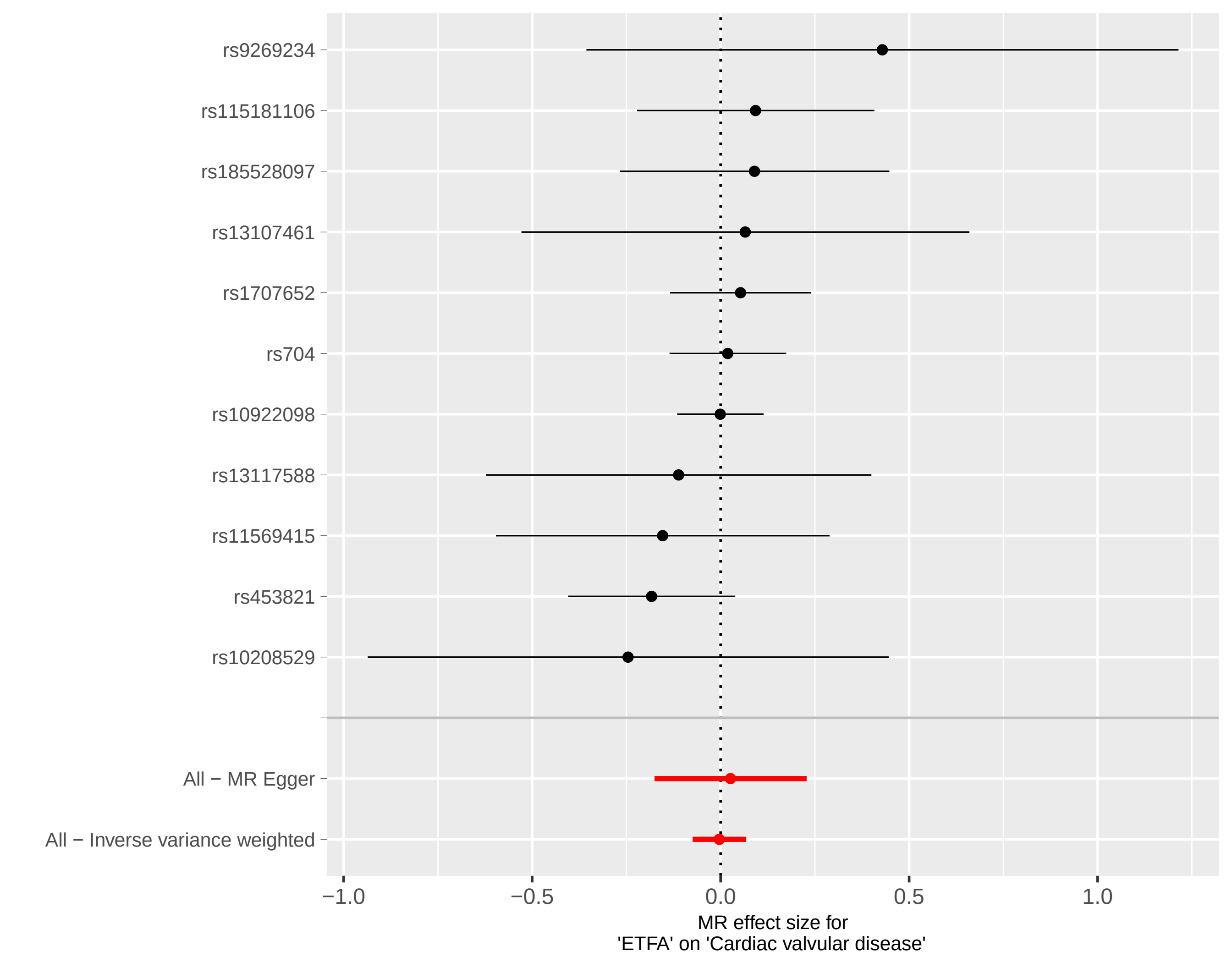
rs9269234
rs115181106
rs185528097
rs13107461
rs1707652
rs704
rs10922098
rs13117588
rs11569415
rs453821
rs10208529
All − MR Egger
All − Inverse variance weighted
−1.0
−0.5
0.0
0.5
1.0
MR effect size for
'ETFA' on 'Cardiac valvular disease'

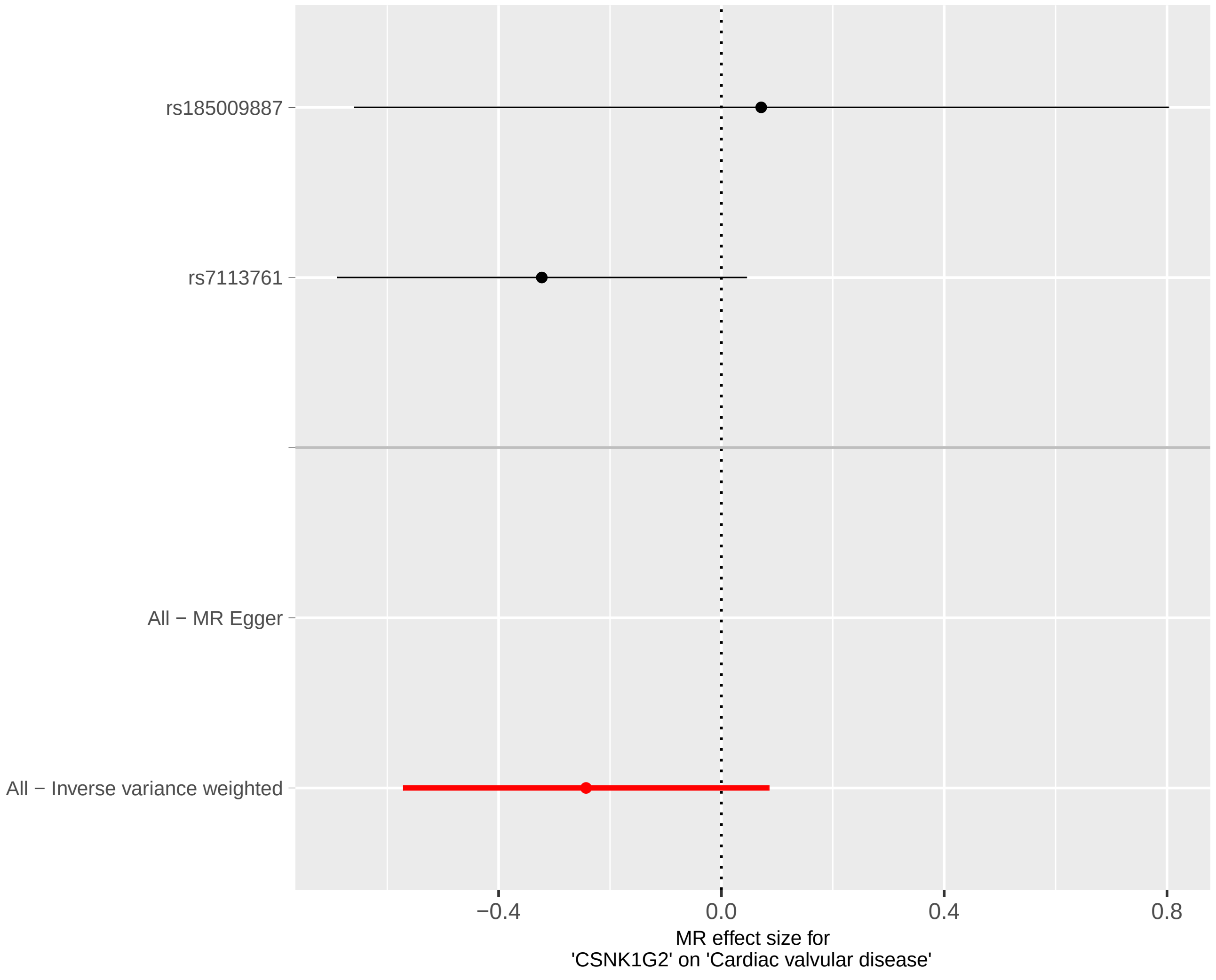
rs185009887
rs7113761
All − MR Egger
All − Inverse variance weighted
−0.4
0.0
0.4
0.8
MR effect size for
'CSNK1G2' on 'Cardiac valvular disease'

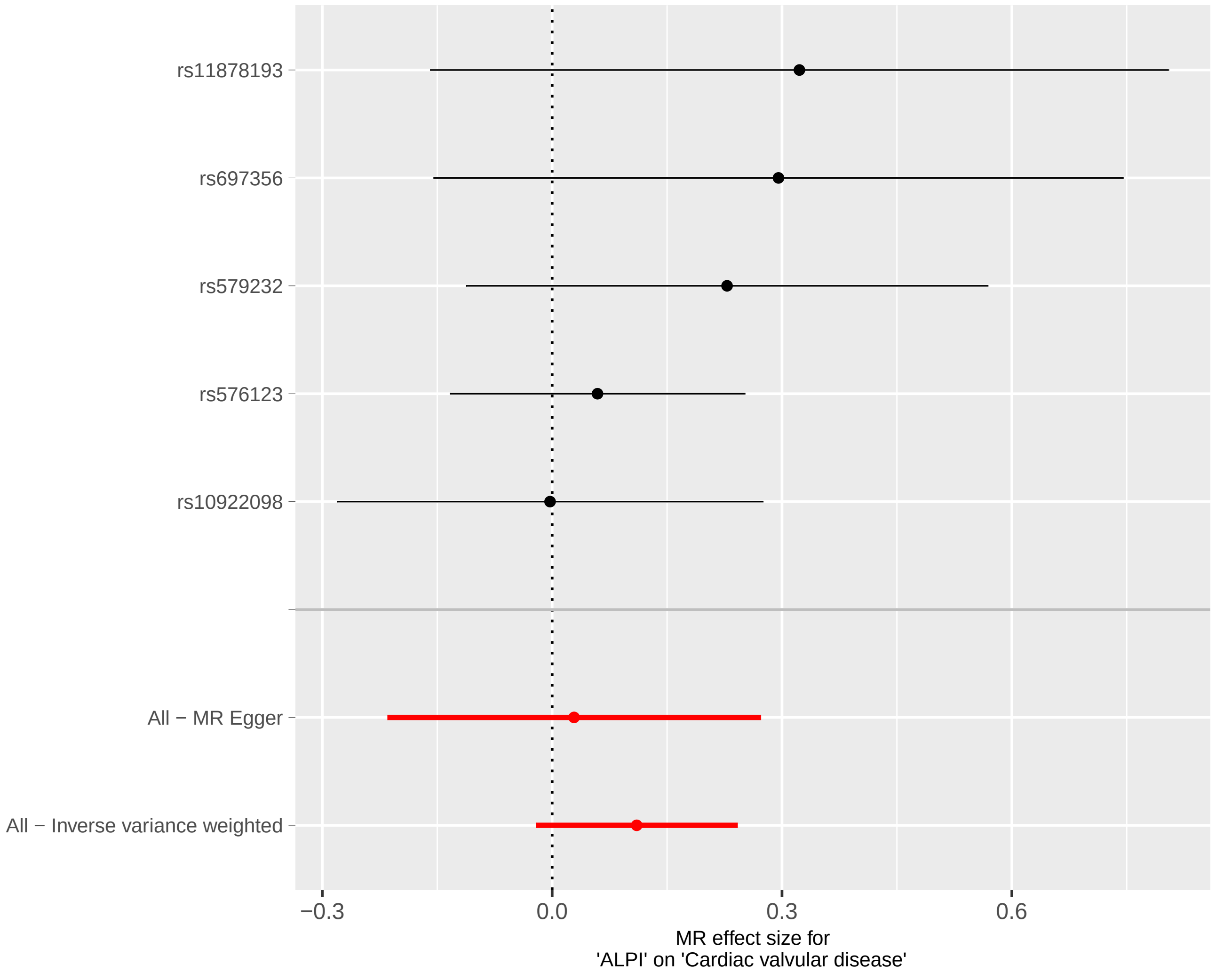
rs11878193
rs697356
rs579232
rs576123
rs10922098
All − MR Egger
All − Inverse variance weighted
−0.3
0.0
0.3
0.6
MR effect size for
'ALPI' on 'Cardiac valvular disease'

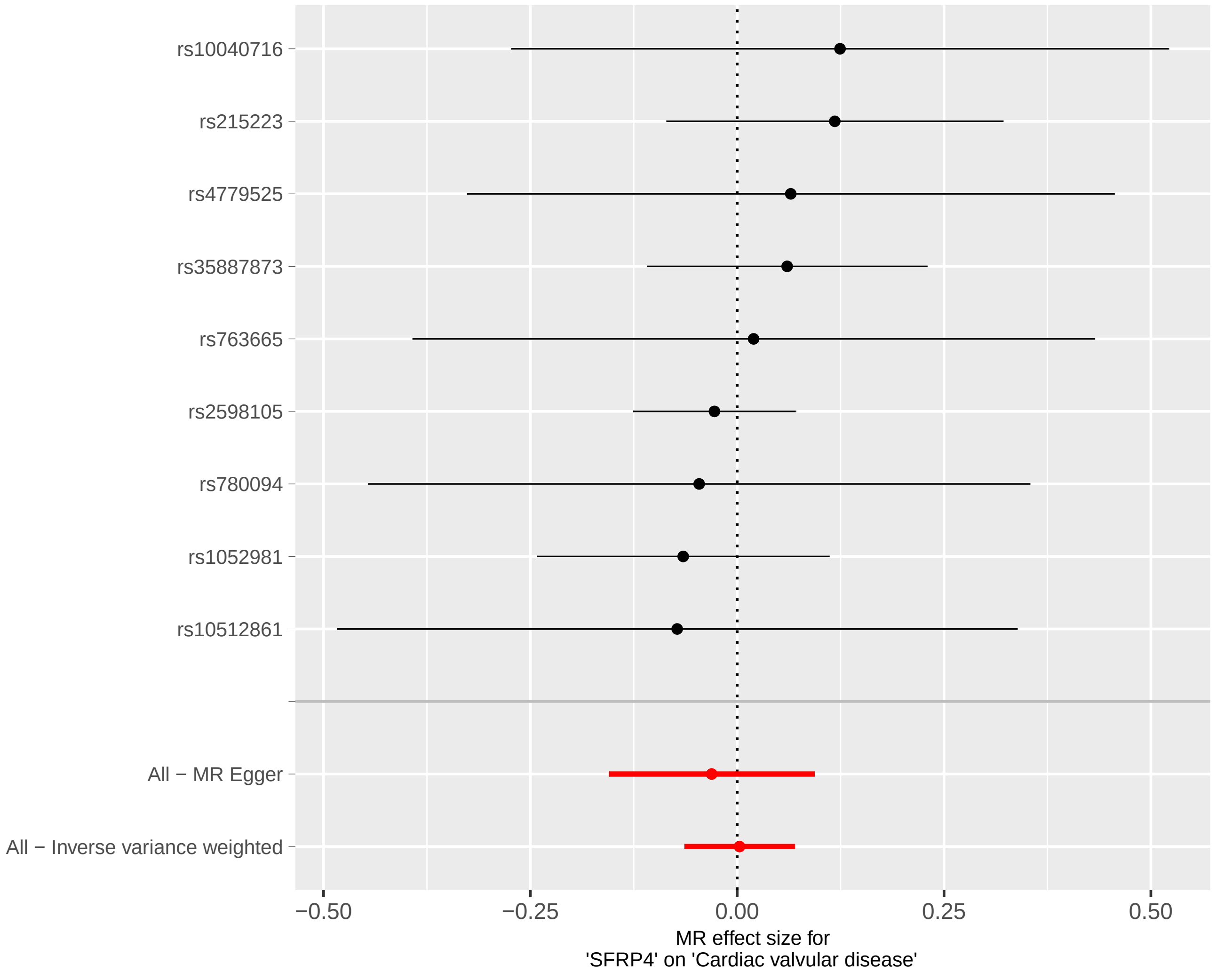
rs10040716
rs215223
rs4779525
rs35887873
rs763665
rs2598105
rs780094
rs1052981
rs10512861
All − MR Egger
All − Inverse variance weighted
−0.50
−0.25
0.00
0.25
0.50
MR effect size for
'SFRP4' on 'Cardiac valvular disease'

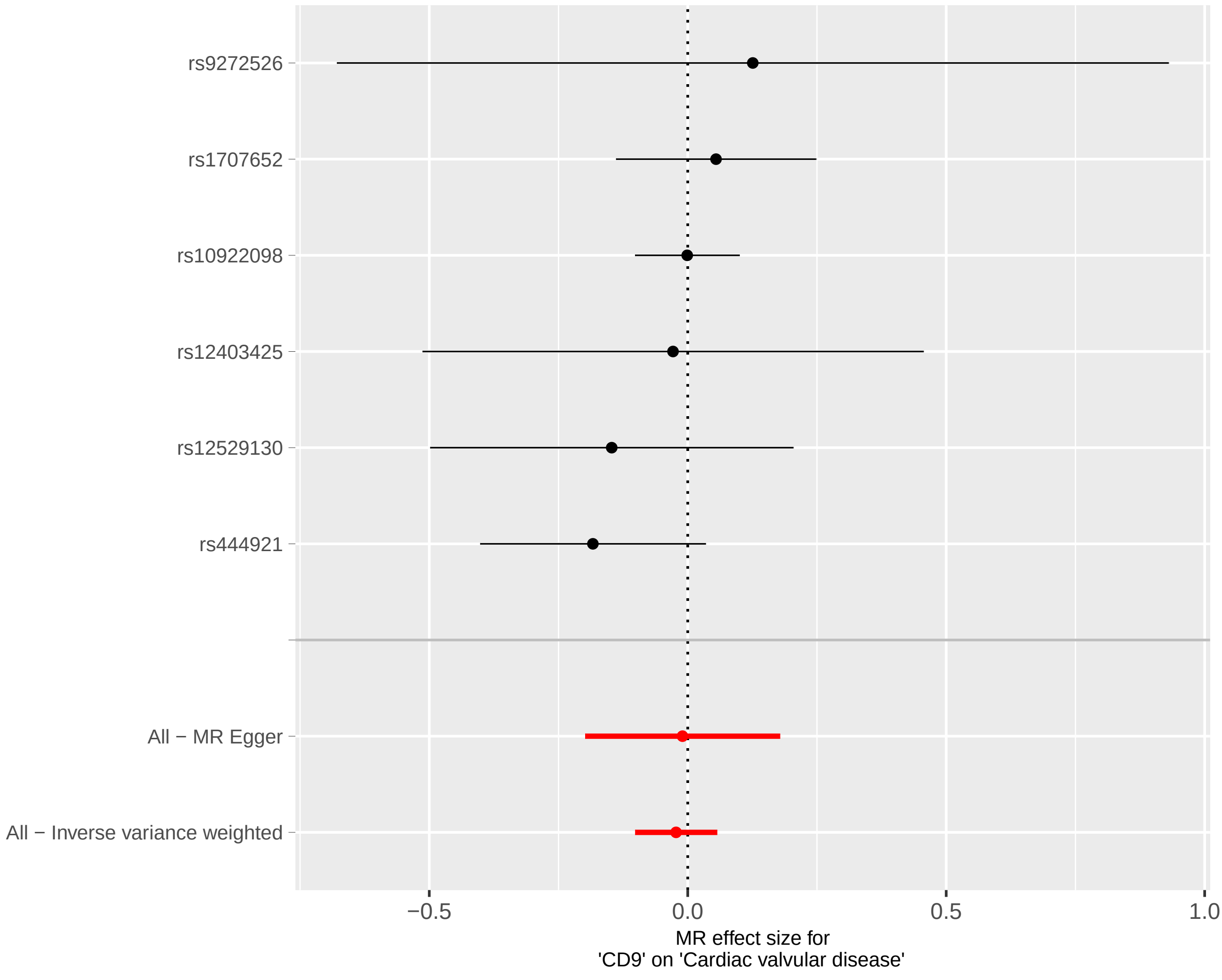
rs9272526
rs1707652
rs10922098
rs12403425
rs12529130
rs444921
All − MR Egger
All − Inverse variance weighted
−0.5
0.0
0.5
1.0
MR effect size for
'CD9' on 'Cardiac valvular disease'

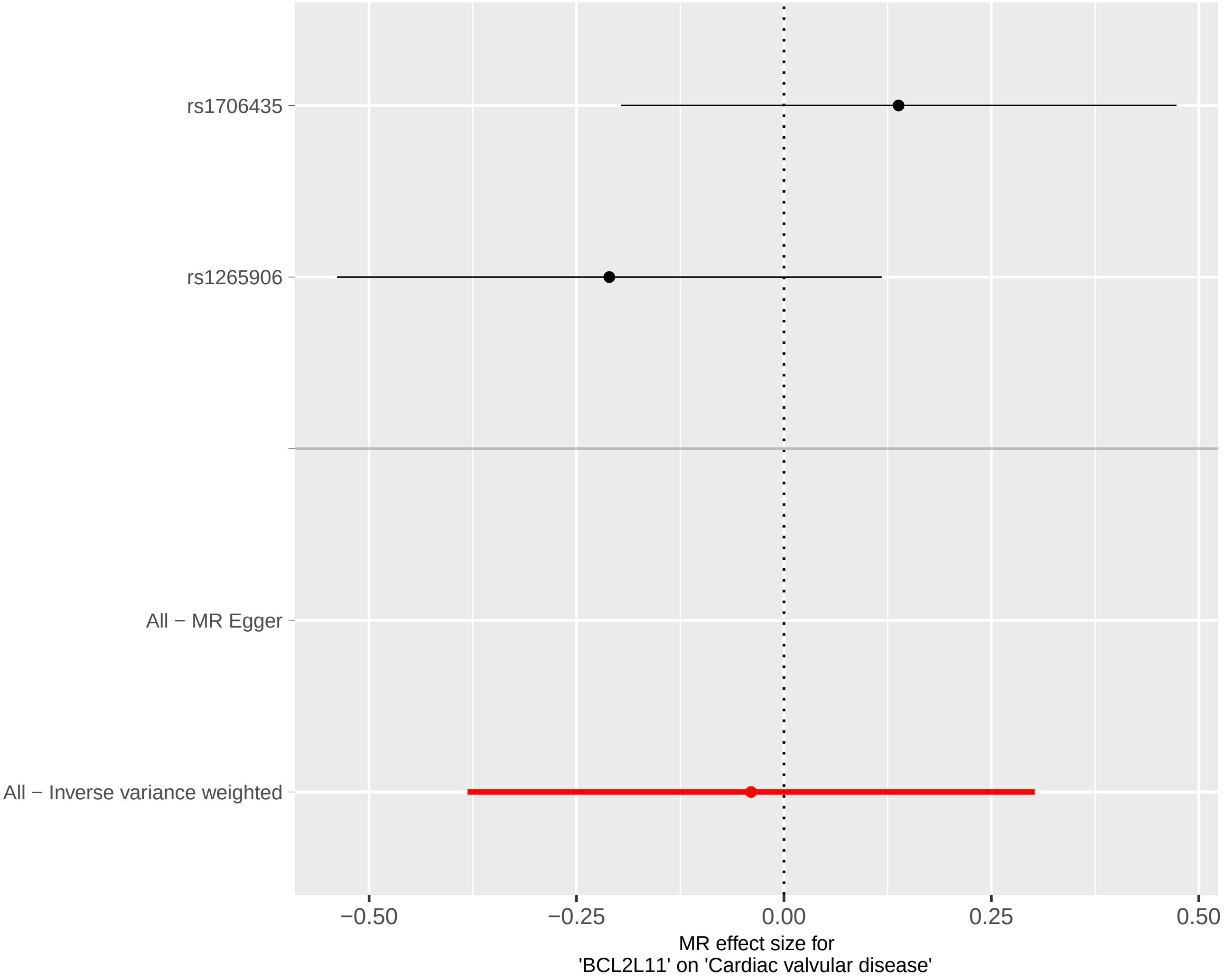

rs1706435
rs1265906
All − MR Egger
All − Inverse variance weighted
−0.50
−0.25
0.00
0.25
0.50
MR effect size for
'BCL2L11' on 'Cardiac valvular disease'

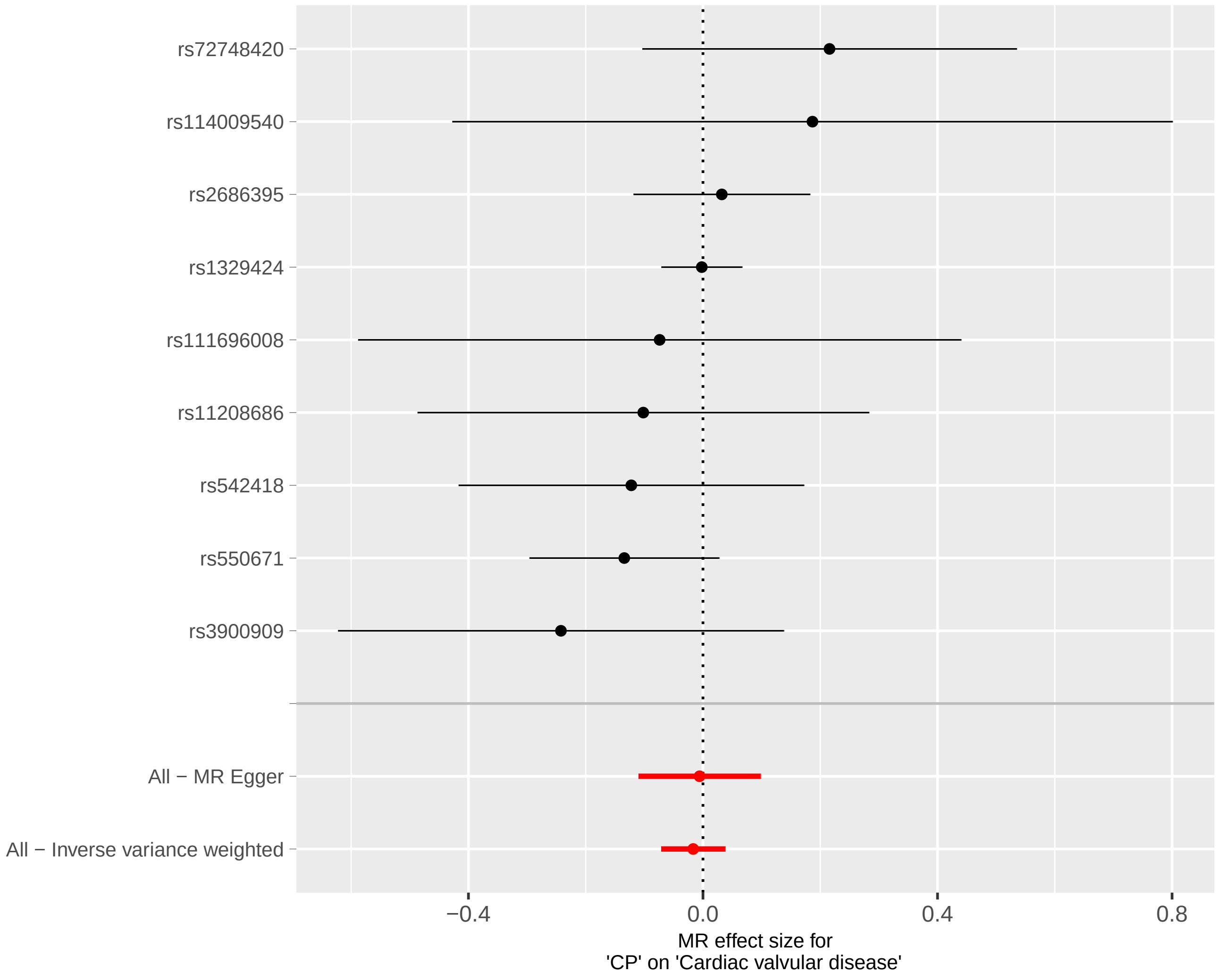
rs72748420
rs114009540
rs2686395
rs1329424
rs111696008
rs11208686
rs542418
rs550671
rs3900909
All − MR Egger
All − Inverse variance weighted
−0.4
0.0
0.4
0.8
MR effect size for
'CP' on 'Cardiac valvular disease'

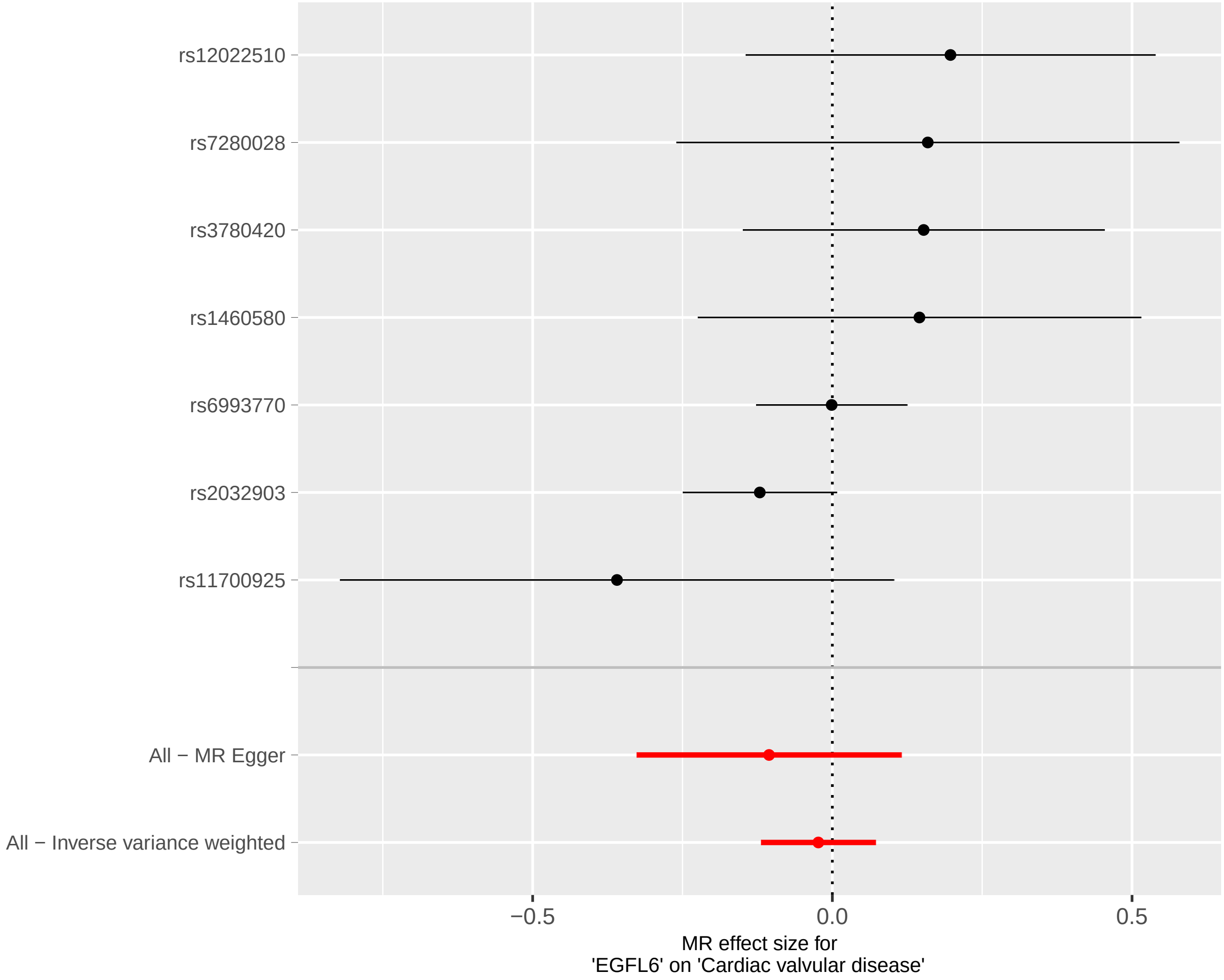
rs12022510
rs7280028
rs3780420
rs1460580
rs6993770
rs2032903
rs11700925
All − MR Egger
All − Inverse variance weighted
−0.5
0.0
0.5
MR effect size for
'EGFL6' on 'Cardiac valvular disease'

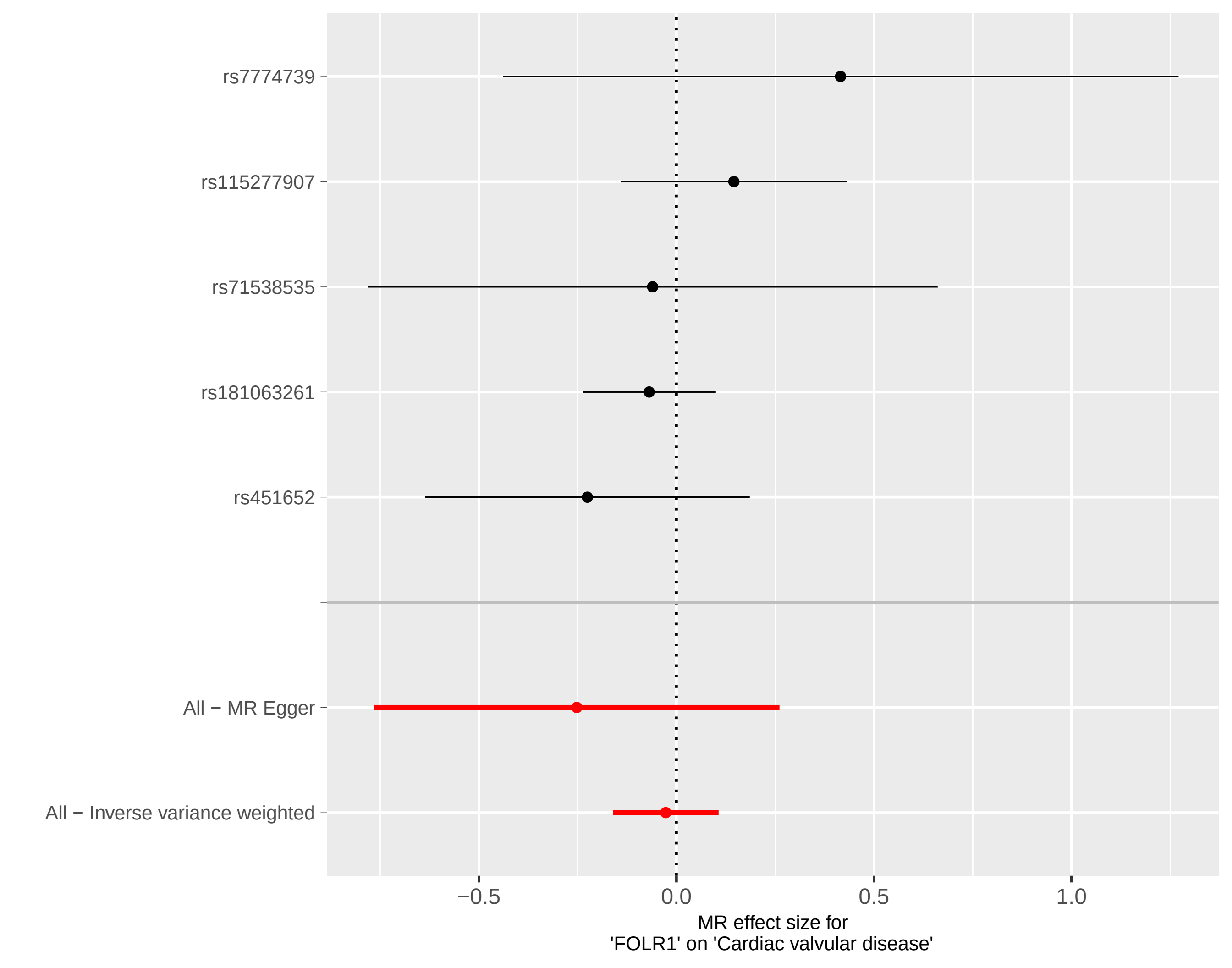

rs7774739
rs115277907
rs71538535
rs181063261
rs451652
All − MR Egger
All − Inverse variance weighted
−0.5
0.0
0.5
1.0
MR effect size for
'FOLR1' on 'Cardiac valvular disease'

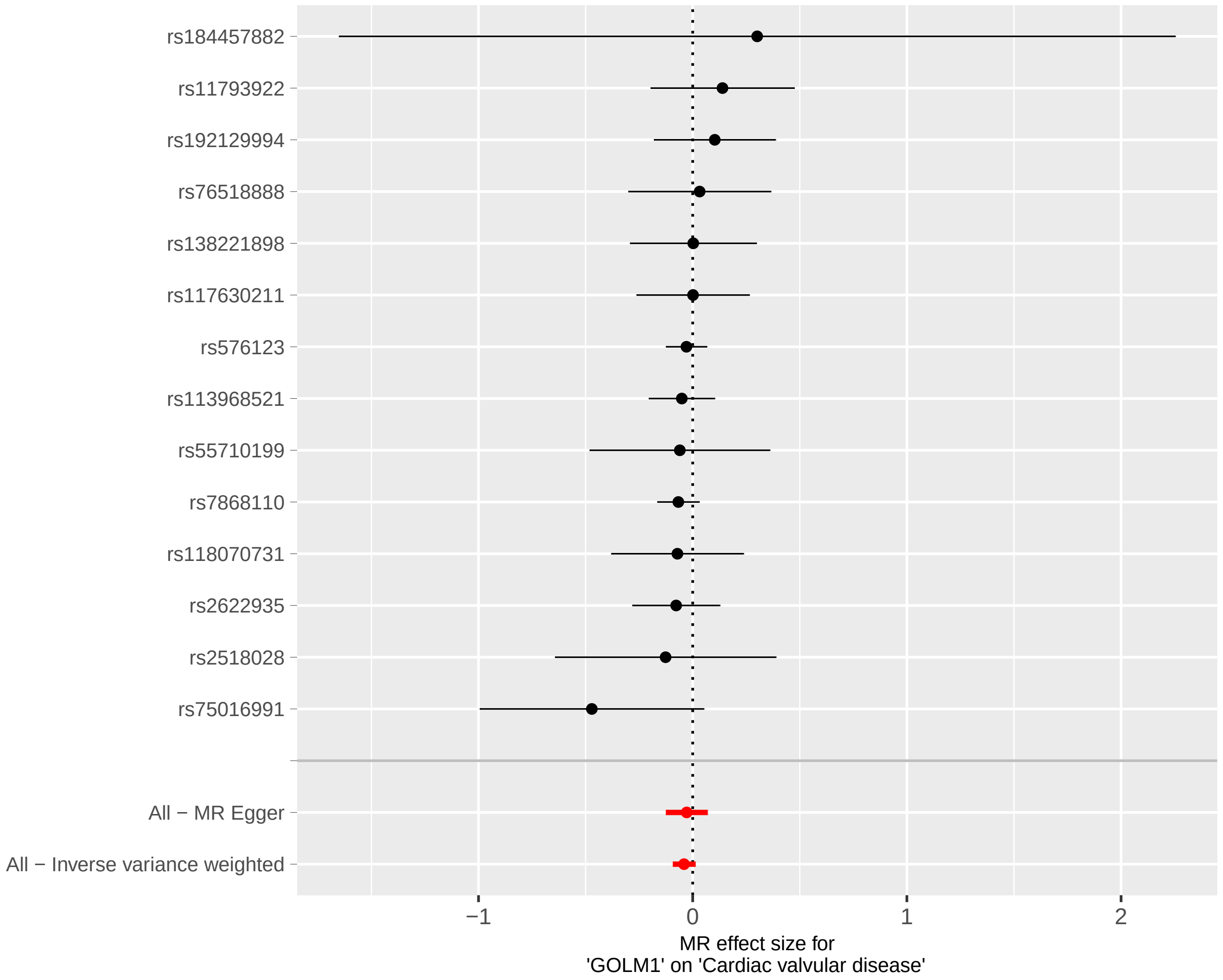
rs184457882
rs11793922
rs192129994
rs76518888
rs138221898
rs117630211
rs576123
rs113968521
rs55710199
rs7868110
rs118070731
rs2622935
rs2518028
rs75016991
All − MR Egger
All − Inverse variance weighted
−1
0
1
2
MR effect size for
'GOLM1' on 'Cardiac valvular disease'

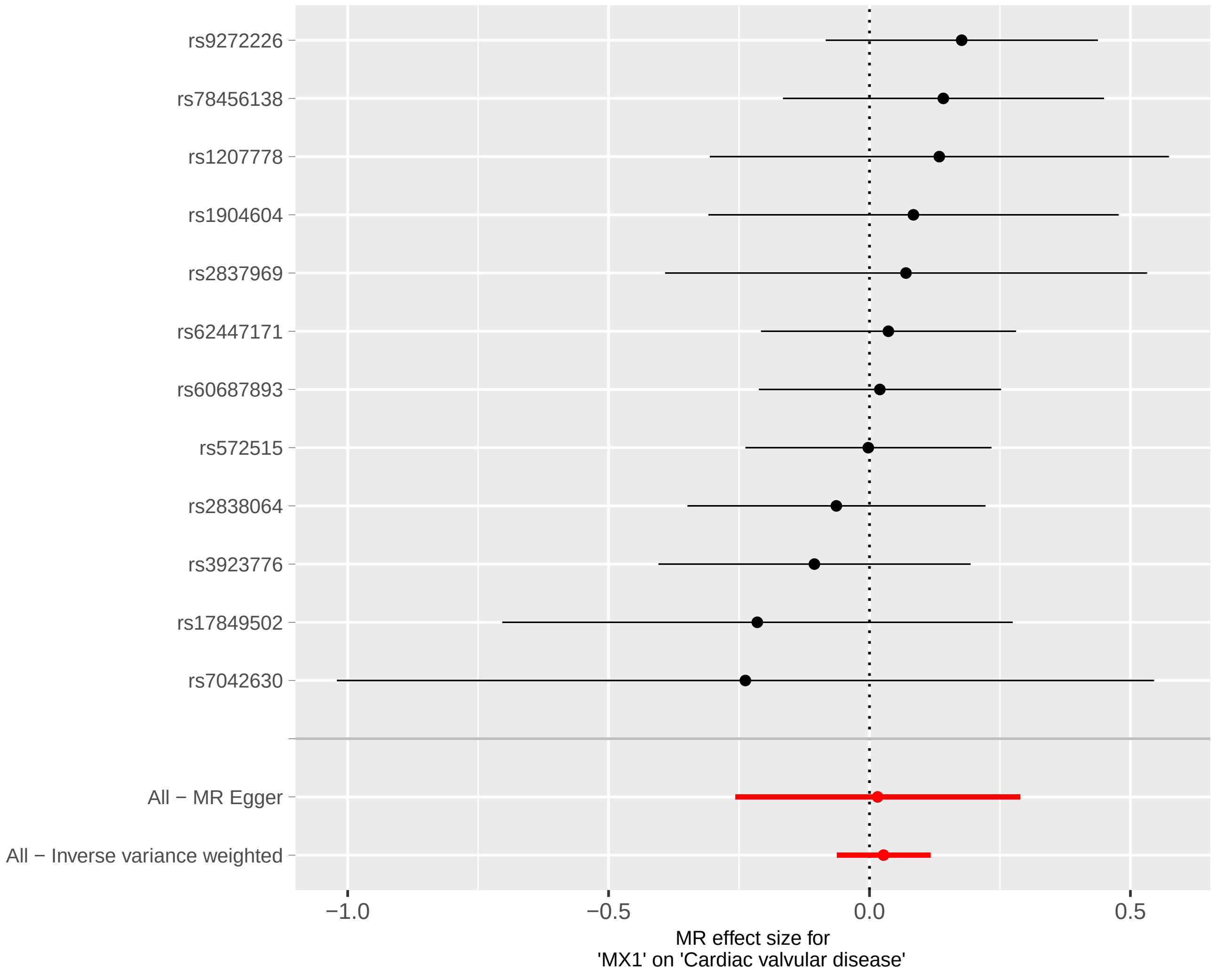
rs9272226
rs78456138
rs1207778
rs1904604
rs2837969
rs62447171
rs60687893
rs572515
rs2838064
rs3923776
rs17849502
rs7042630
All − MR Egger
All − Inverse variance weighted
−1.0
−0.5
0.0
0.5
MR effect size for
'MX1' on 'Cardiac valvular disease'

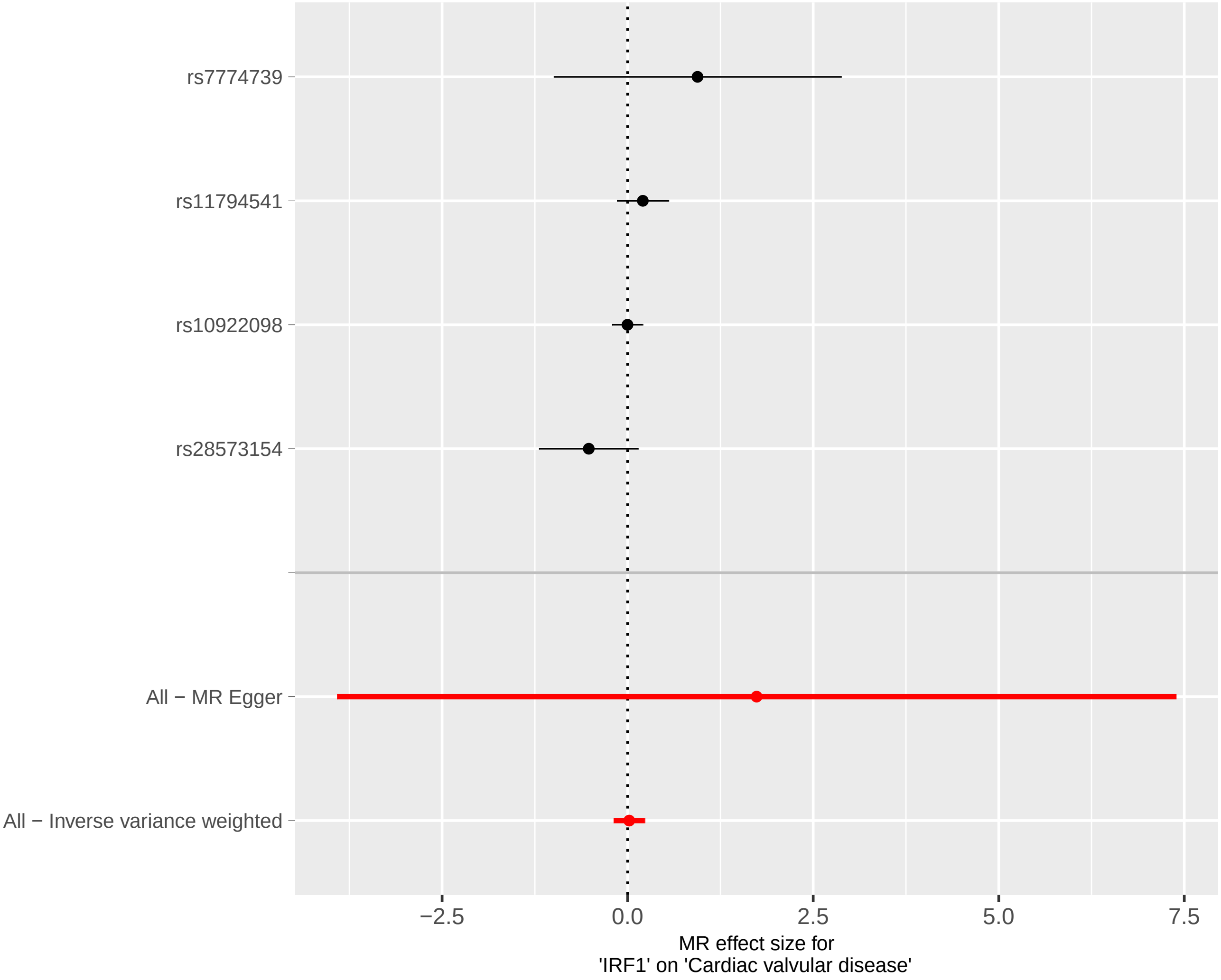
rs7774739
rs11794541
rs10922098
rs28573154
All − MR Egger
All − Inverse variance weighted
−2.5
0.0
2.5
5.0
7.5
MR effect size for
'IRF1' on 'Cardiac valvular disease'

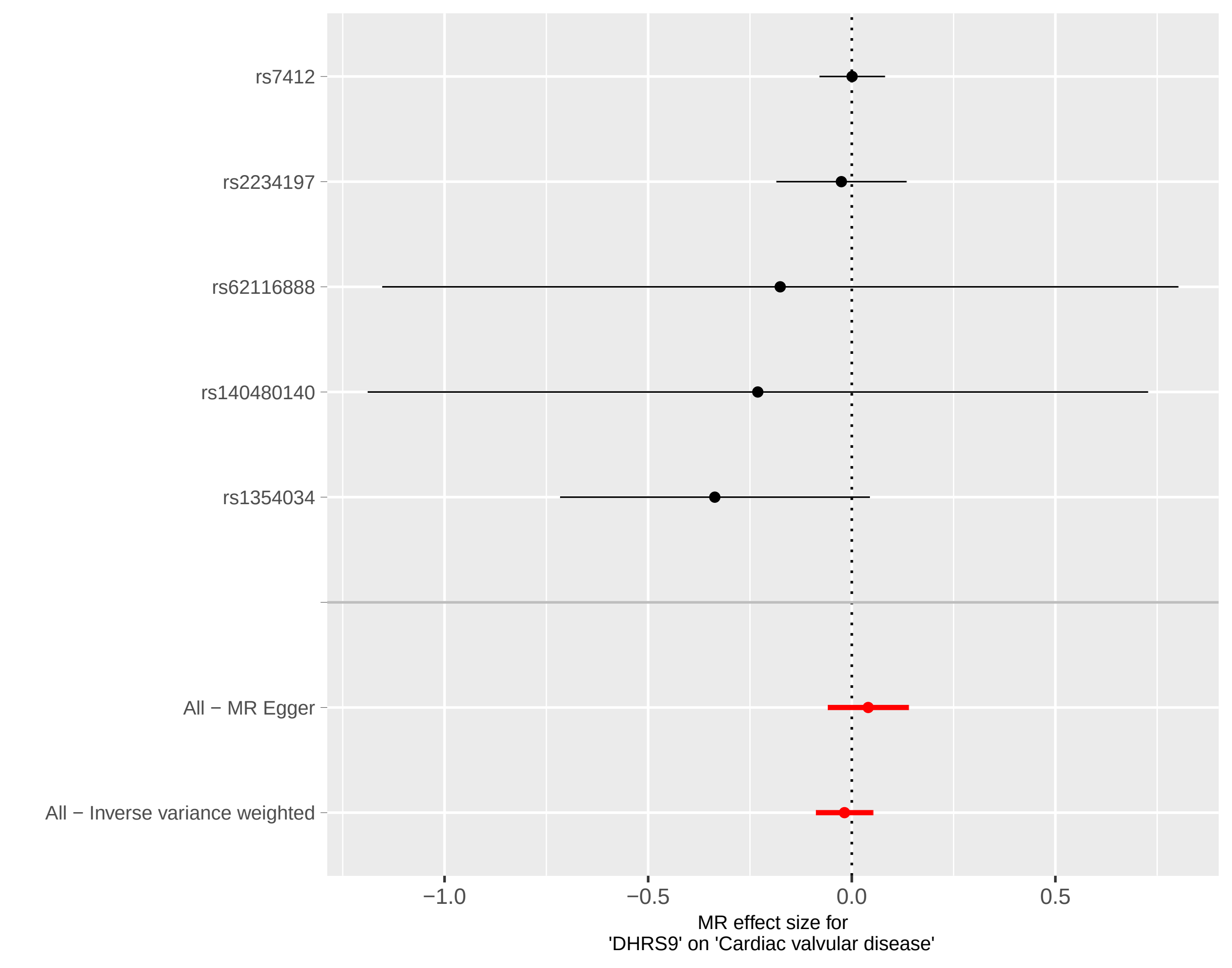

rs7412
rs2234197
rs62116888
rs140480140
rs1354034
All − MR Egger
All − Inverse variance weighted
−1.0
−0.5
0.0
0.5
MR effect size for
'DHRS9' on 'Cardiac valvular disease'

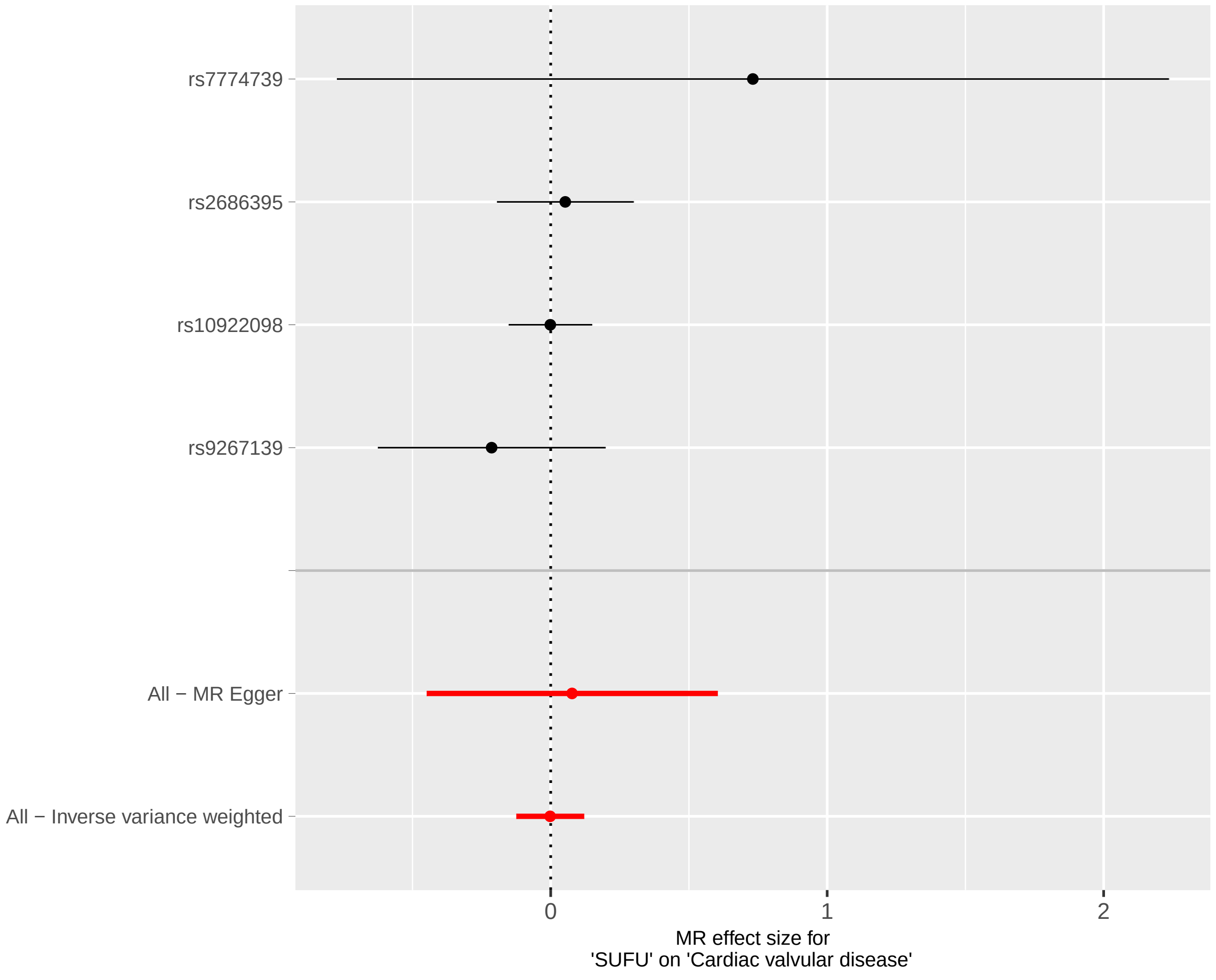

rs7774739
rs2686395
rs10922098
rs9267139
All − MR Egger
All − Inverse variance weighted
0
1
2
MR effect size for
'SUFU' on 'Cardiac valvular disease'

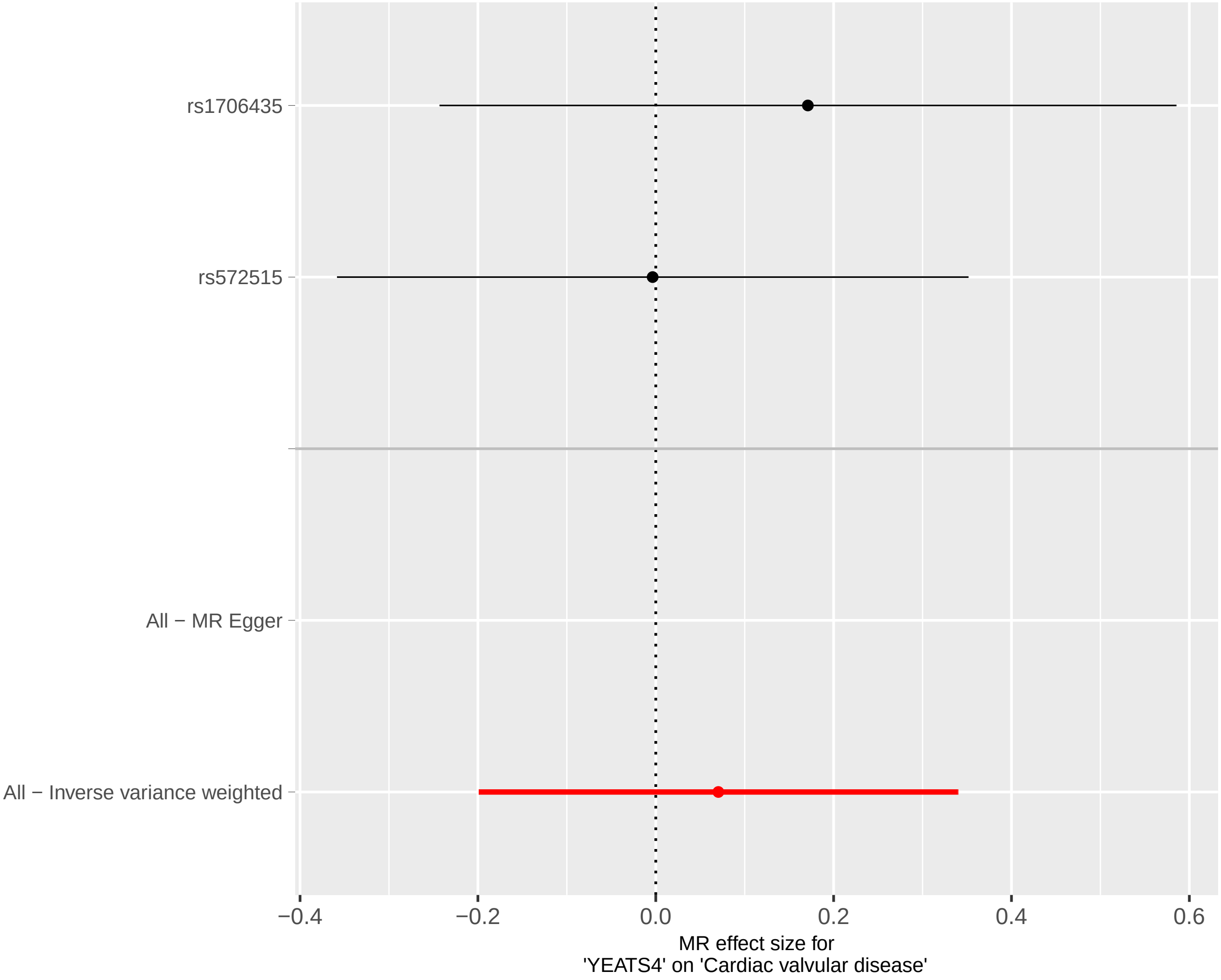
rs1706435
rs572515
All − MR Egger
All − Inverse variance weighted
−0.4
−0.2
0.0
0.2
0.4
0.6
MR effect size for
'YEATS4' on 'Cardiac valvular disease'

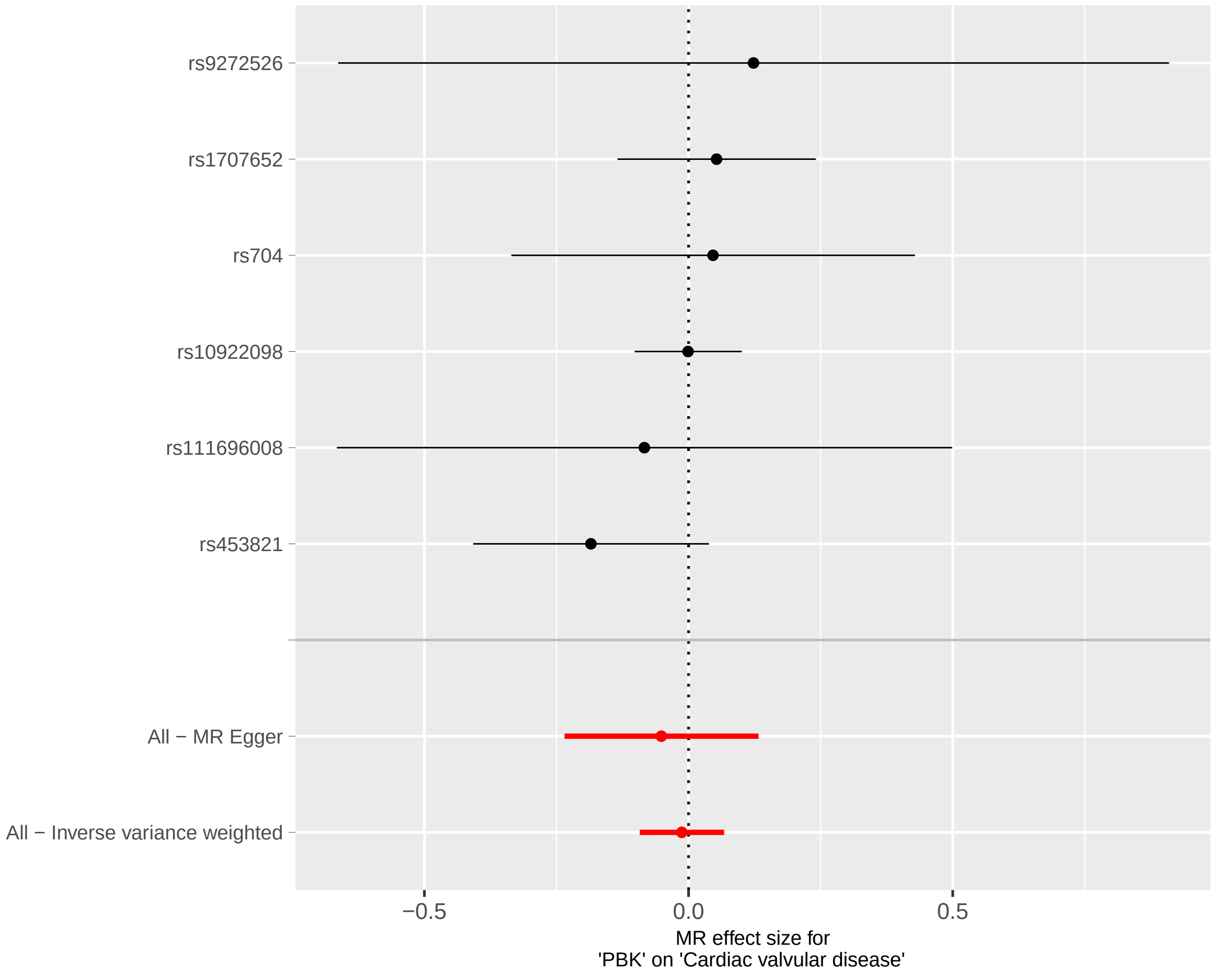
rs9272526
rs1707652
rs704
rs10922098
rs111696008
rs453821
All − MR Egger
All − Inverse variance weighted
−0.5
0.0
0.5
MR effect size for
'PBK' on 'Cardiac valvular disease'

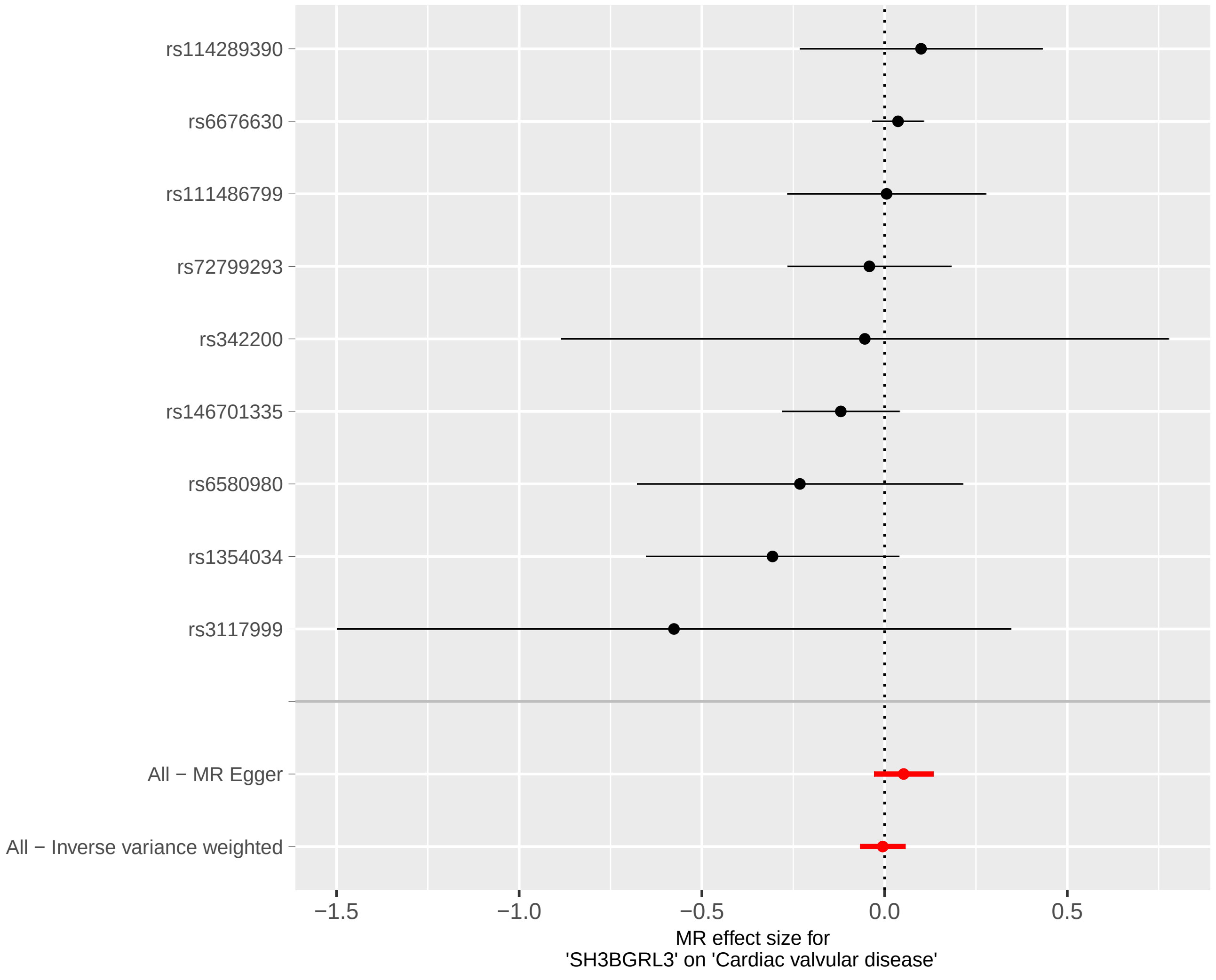

rs114289390
rs6676630
rs111486799
rs72799293
rs342200
rs146701335
rs6580980
rs1354034
rs3117999
All − MR Egger
All − Inverse variance weighted
−1.5
−1.0
−0.5
0.0
0.5
MR effect size for
'SH3BGRL3' on 'Cardiac valvular disease'

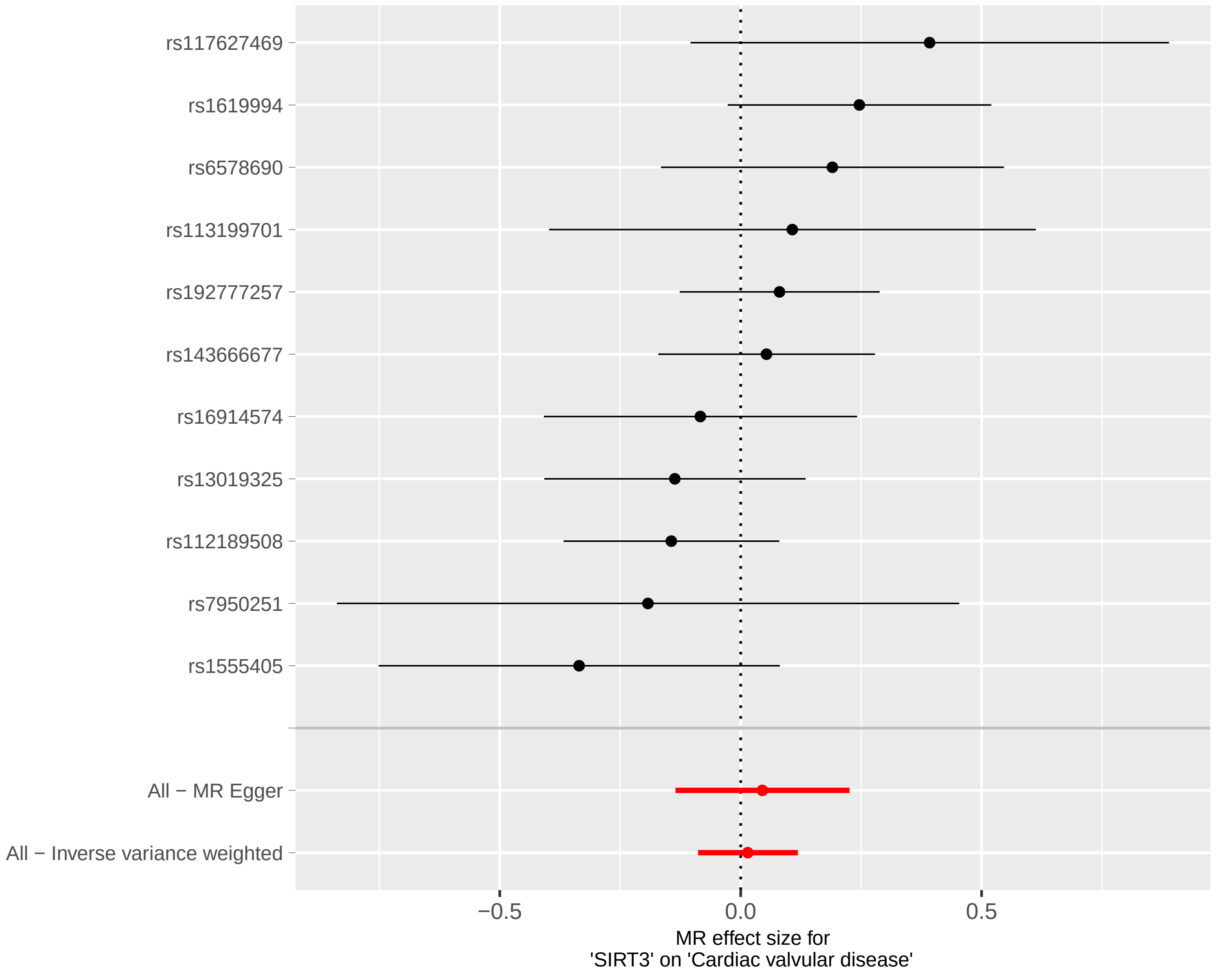
rs117627469
rs1619994
rs6578690
rs113199701
rs192777257
rs143666677
rs16914574
rs13019325
rs112189508
rs7950251
rs1555405
All − MR Egger
All − Inverse variance weighted
−0.5
0.0
0.5
MR effect size for
'SIRT3' on 'Cardiac valvular disease'

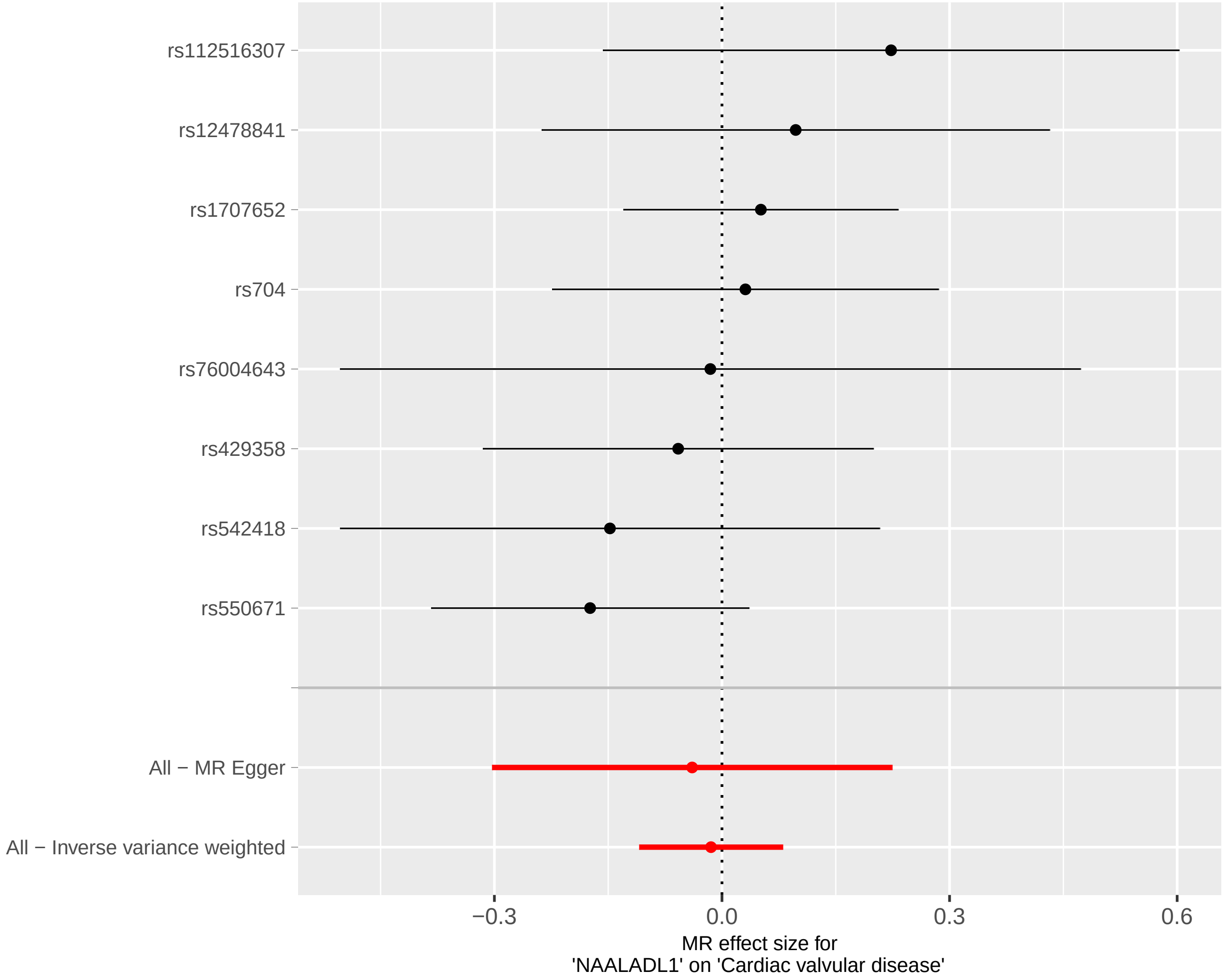
rs112516307
rs12478841
rs1707652
rs704
rs76004643
rs429358
rs542418
rs550671
All − MR Egger
All − Inverse variance weighted
−0.3
0.0
0.3
0.6
MR effect size for
'NAALADL1' on 'Cardiac valvular disease'

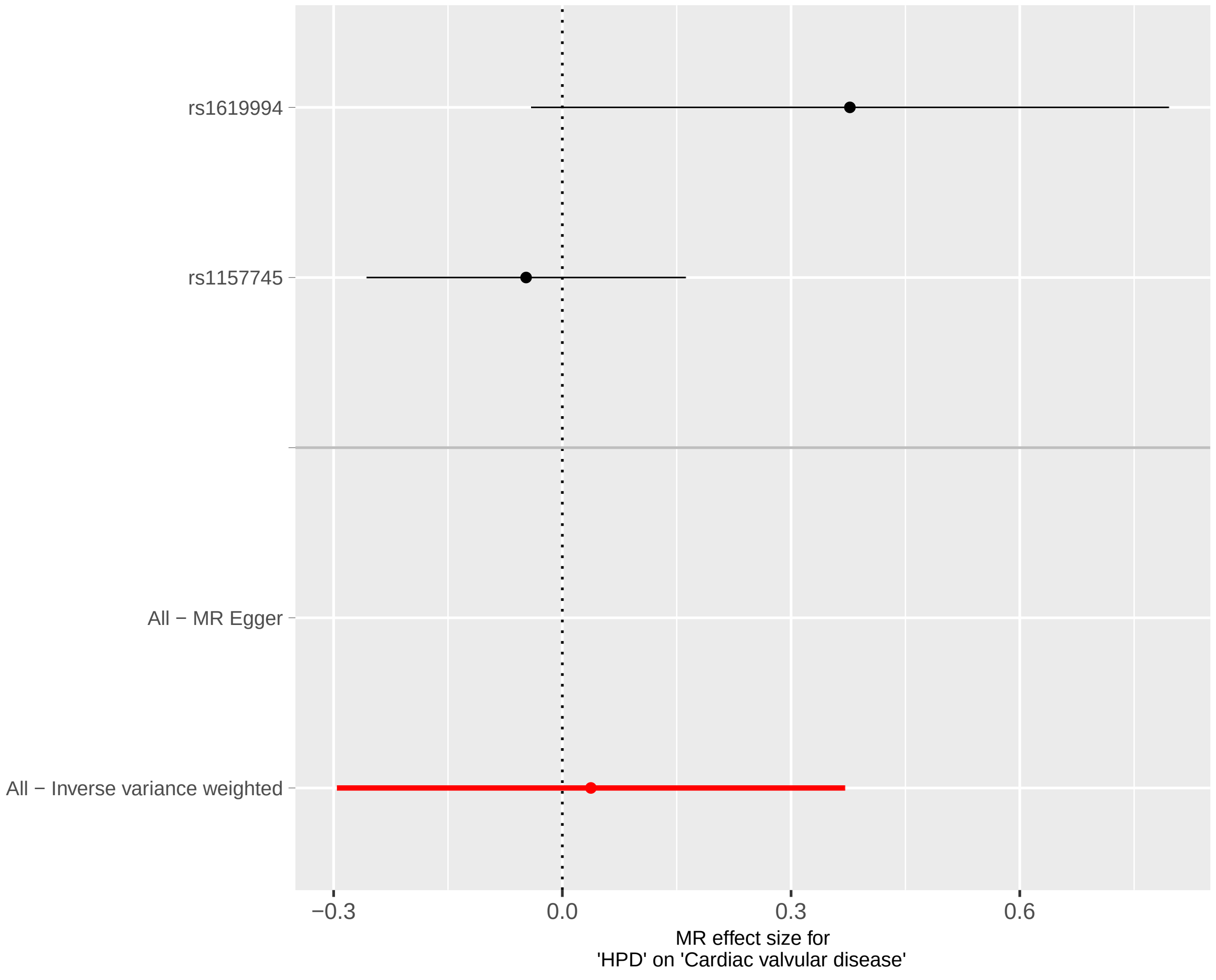

rs1619994
rs1157745
All − MR Egger
All − Inverse variance weighted
−0.3
0.0
0.3
0.6
MR effect size for
'HPD' on 'Cardiac valvular disease'

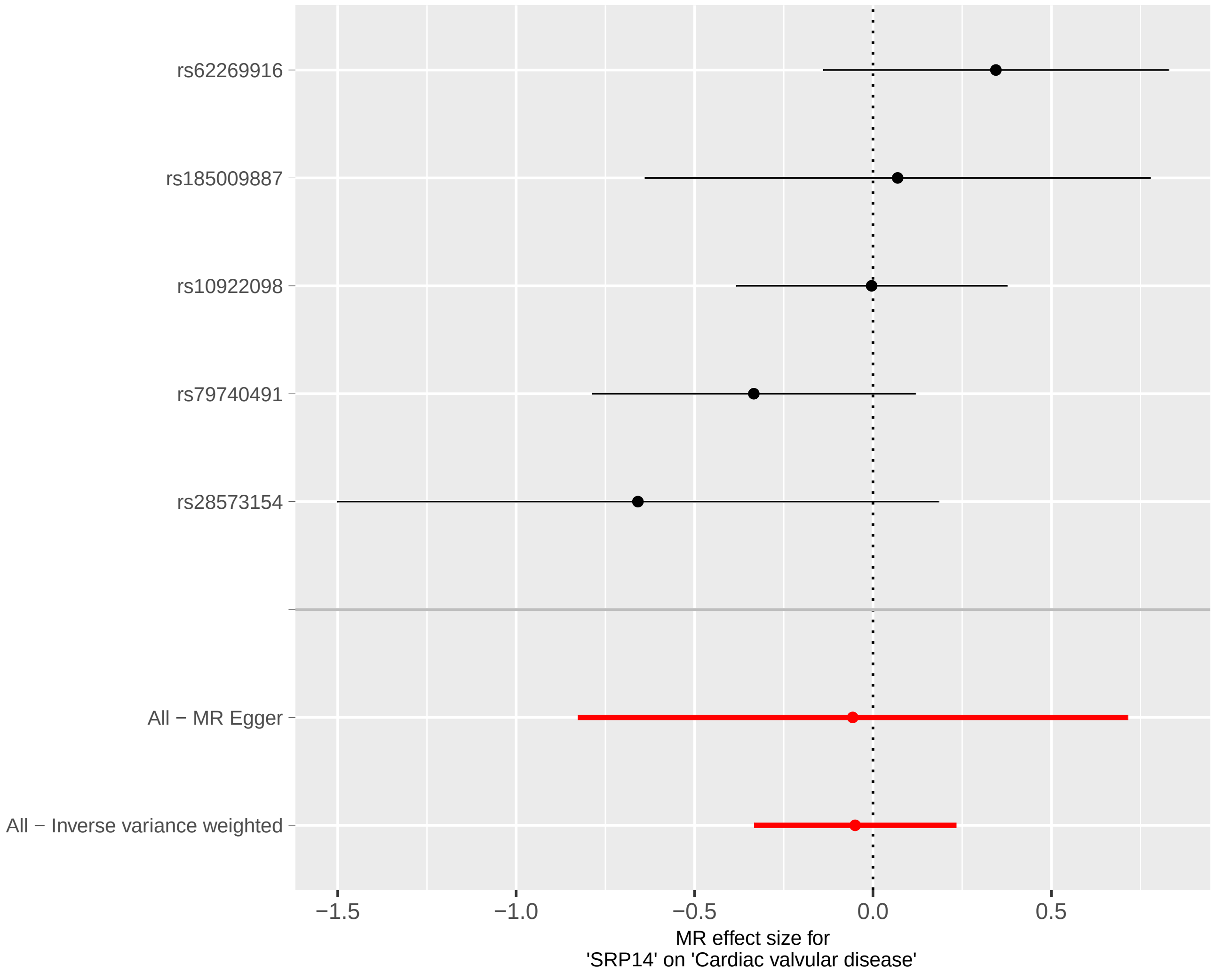

rs62269916
rs185009887
rs10922098
rs79740491
rs28573154
All − MR Egger
All − Inverse variance weighted
−1.5
−1.0
−0.5
0.0
0.5
MR effect size for
'SRP14' on 'Cardiac valvular disease'

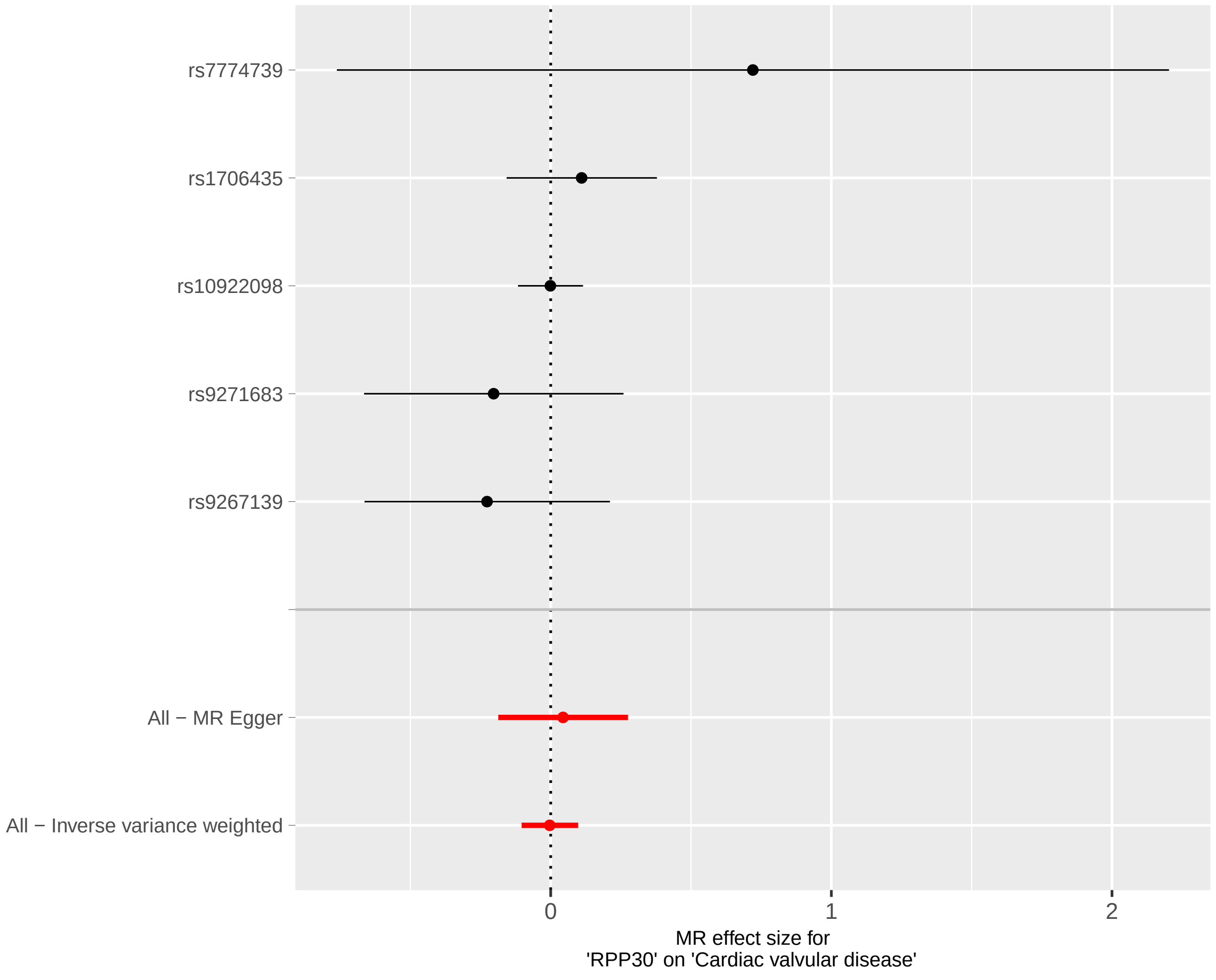
rs7774739
rs1706435
rs10922098
rs9271683
rs9267139
All − MR Egger
All − Inverse variance weighted
0
1
2
MR effect size for
'RPP30' on 'Cardiac valvular disease'

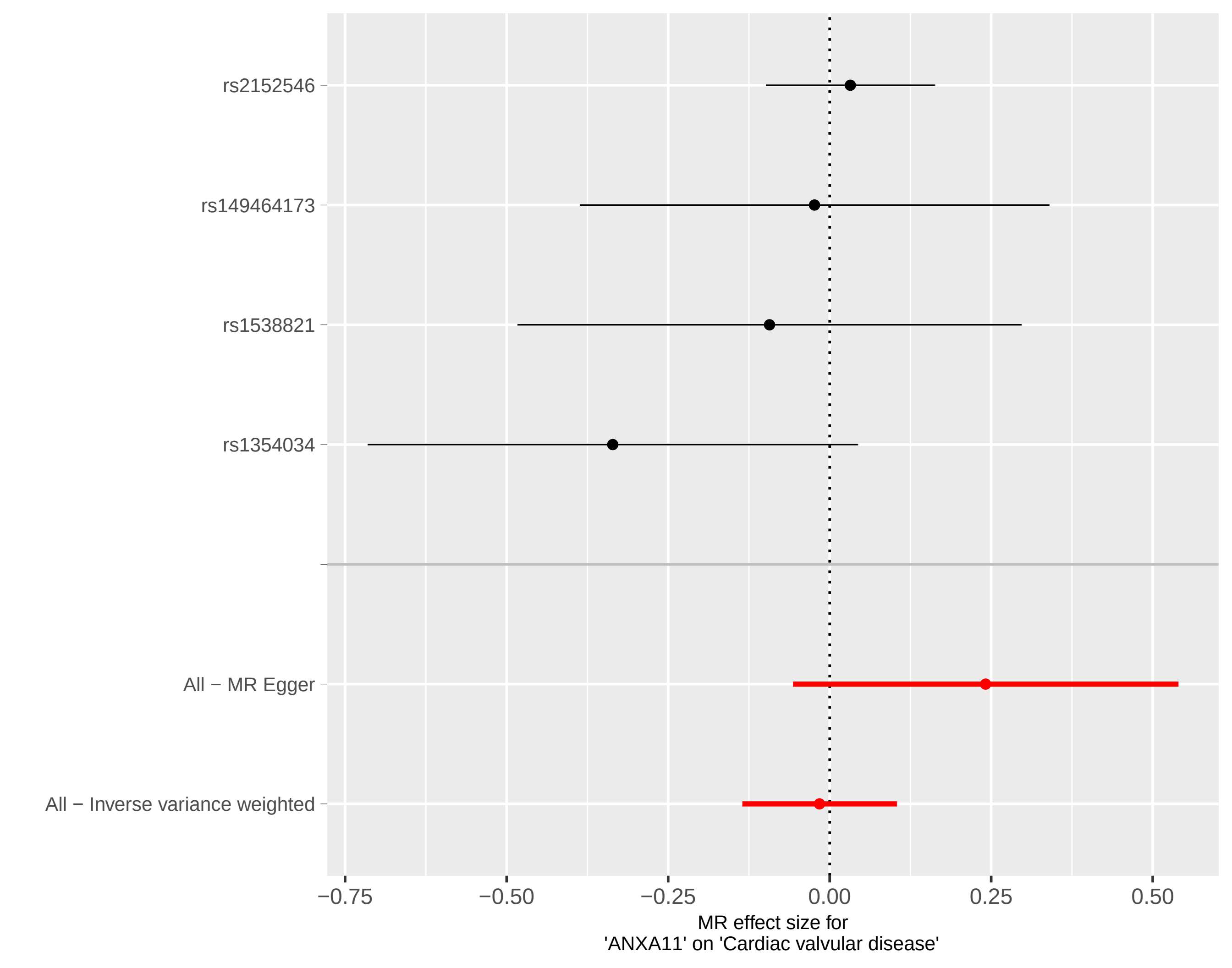
rs2152546
rs149464173
rs1538821
rs1354034
All − MR Egger
All − Inverse variance weighted
−0.75
−0.50
−0.25
0.00
0.25
0.50
MR effect size for
'ANXA11' on 'Cardiac valvular disease'

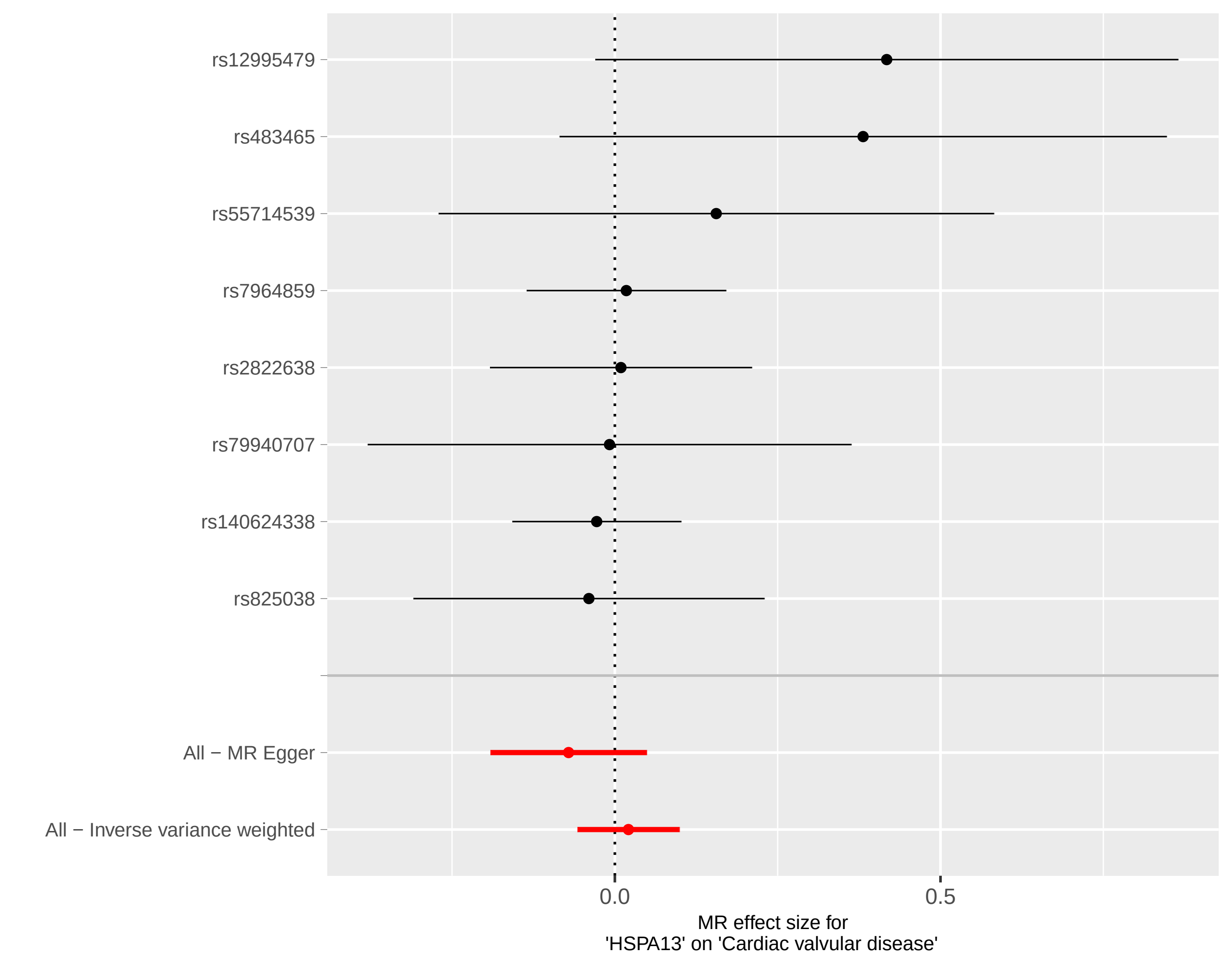
rs12995479
rs483465
rs55714539
rs7964859
rs2822638
rs79940707
rs140624338
rs825038
All − MR Egger
All − Inverse variance weighted
0.0
0.5
MR effect size for
'HSPA13' on 'Cardiac valvular disease'

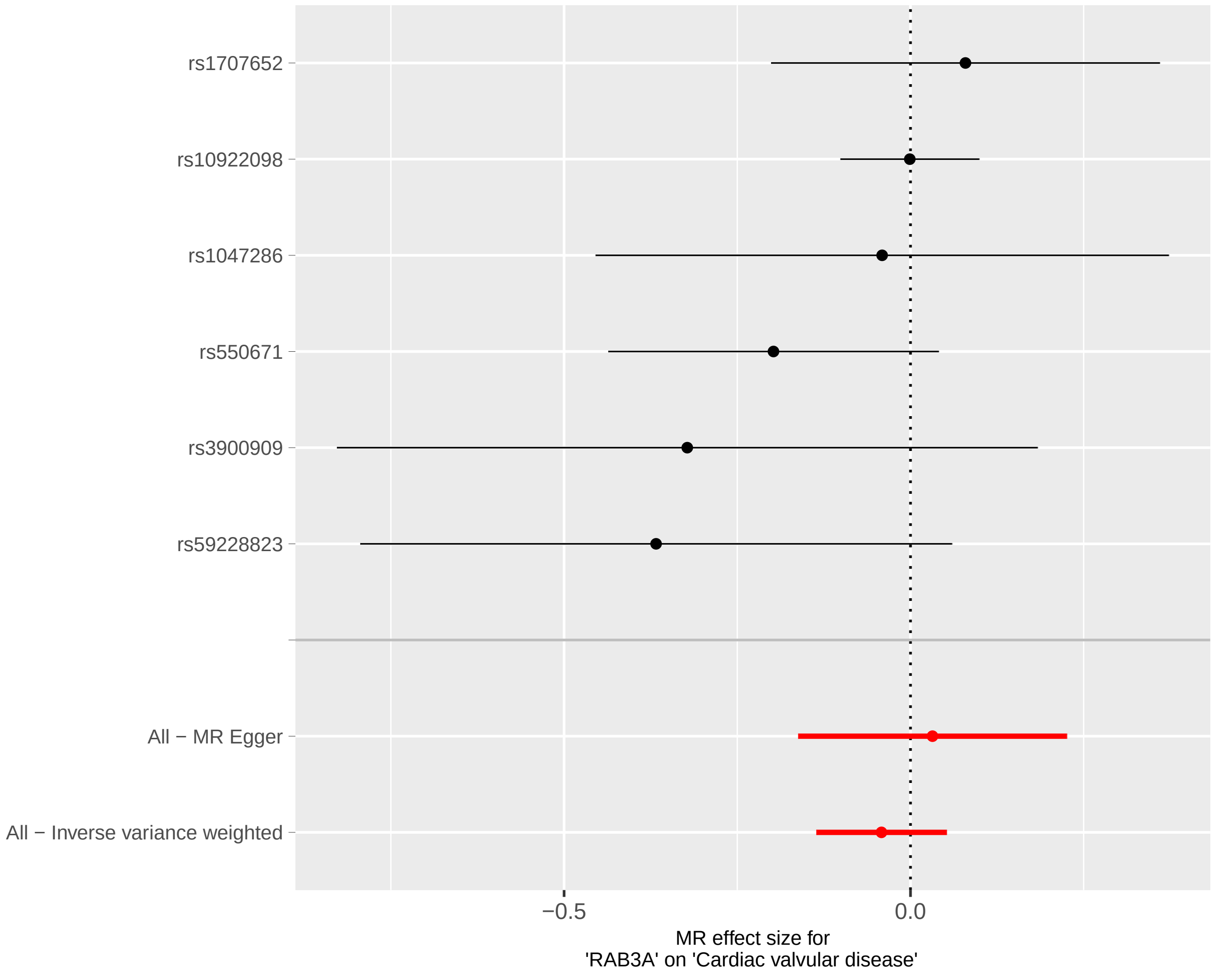
rs1707652
rs10922098
rs1047286
rs550671
rs3900909
rs59228823
All − MR Egger
All − Inverse variance weighted
−0.5
0.0
MR effect size for
'RAB3A' on 'Cardiac valvular disease'

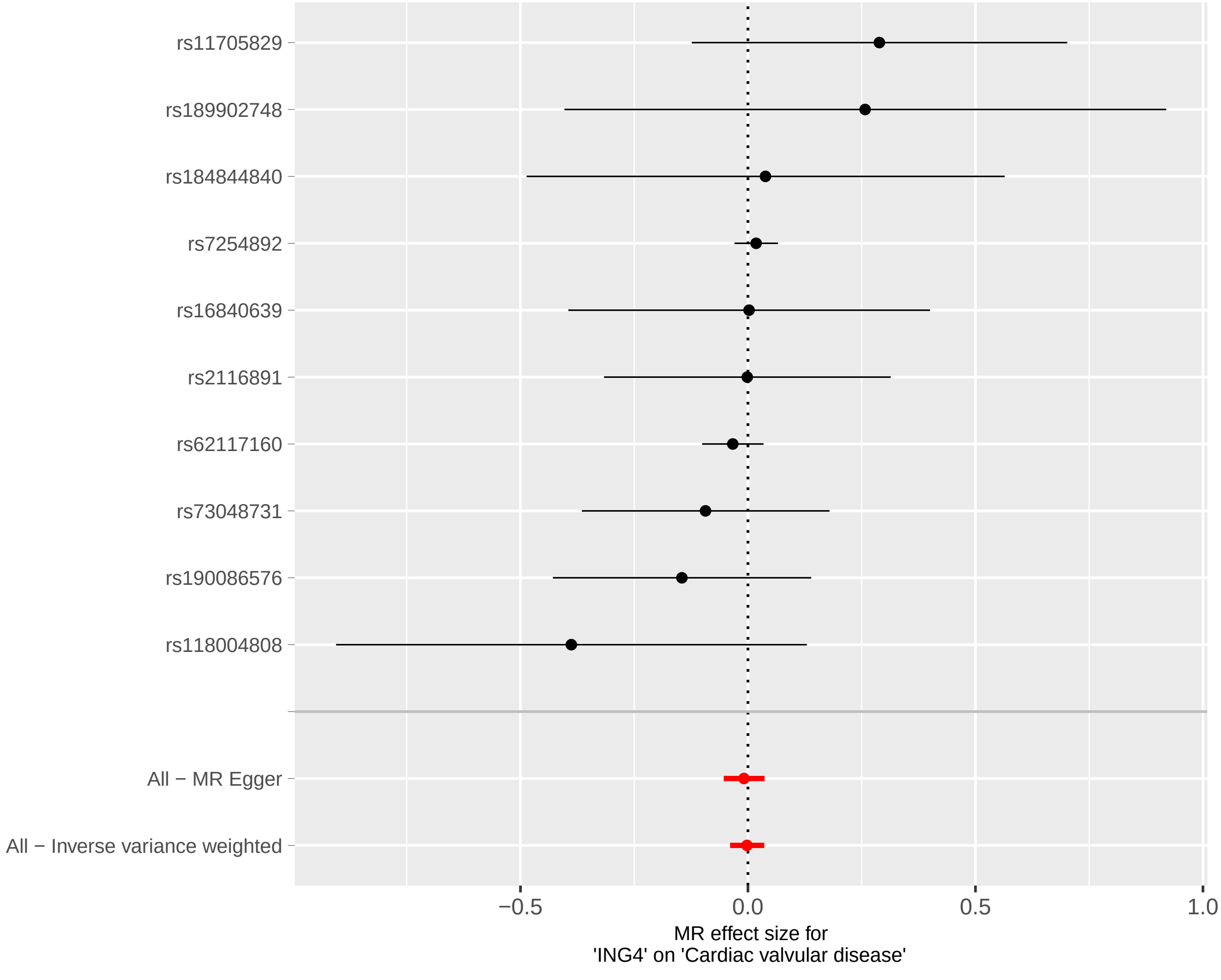
rs11705829
rs189902748
rs184844840
rs7254892
rs16840639
rs2116891
rs62117160
rs73048731
rs190086576
rs118004808
All − MR Egger
All − Inverse variance weighted
−0.5
0.0
0.5
1.0
MR effect size for
'ING4' on 'Cardiac valvular disease'

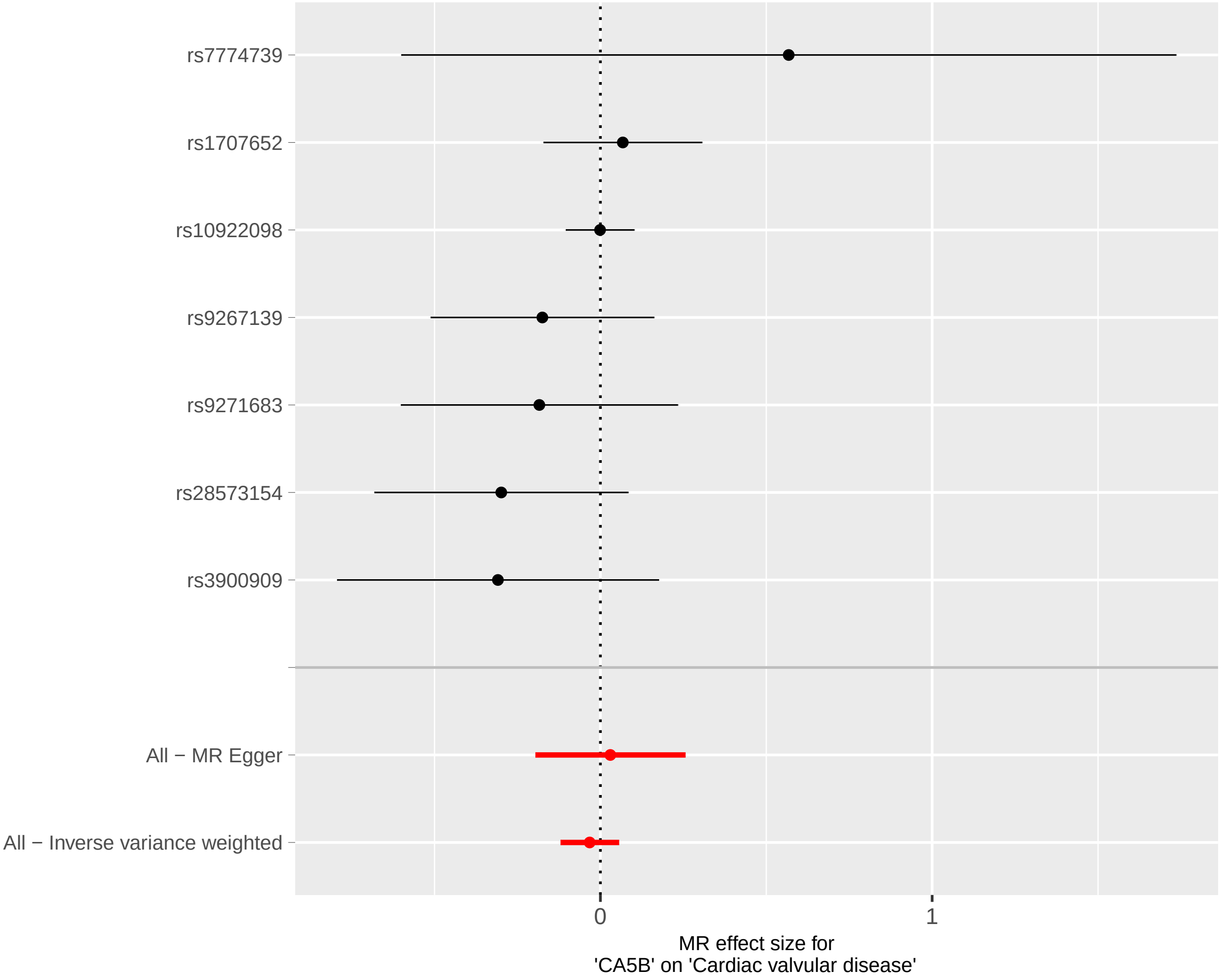
rs7774739
rs1707652
rs10922098
rs9267139
rs9271683
rs28573154
rs3900909
All − MR Egger
All − Inverse variance weighted
0
1
MR effect size for
'CA5B' on 'Cardiac valvular disease'

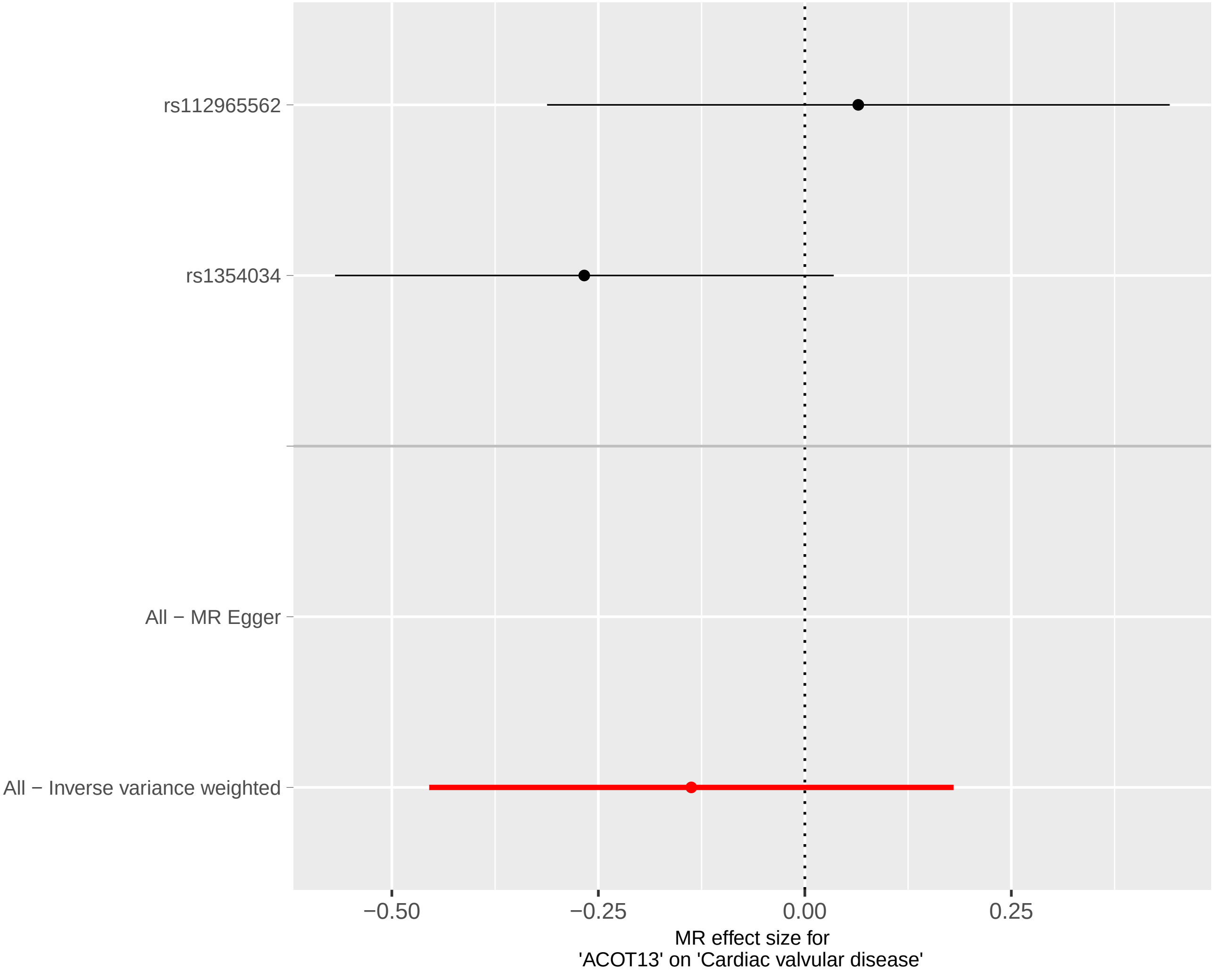

rs112965562
rs1354034
All − MR Egger
All − Inverse variance weighted
−0.50
−0.25
0.00
0.25
MR effect size for
'ACOT13' on 'Cardiac valvular disease'

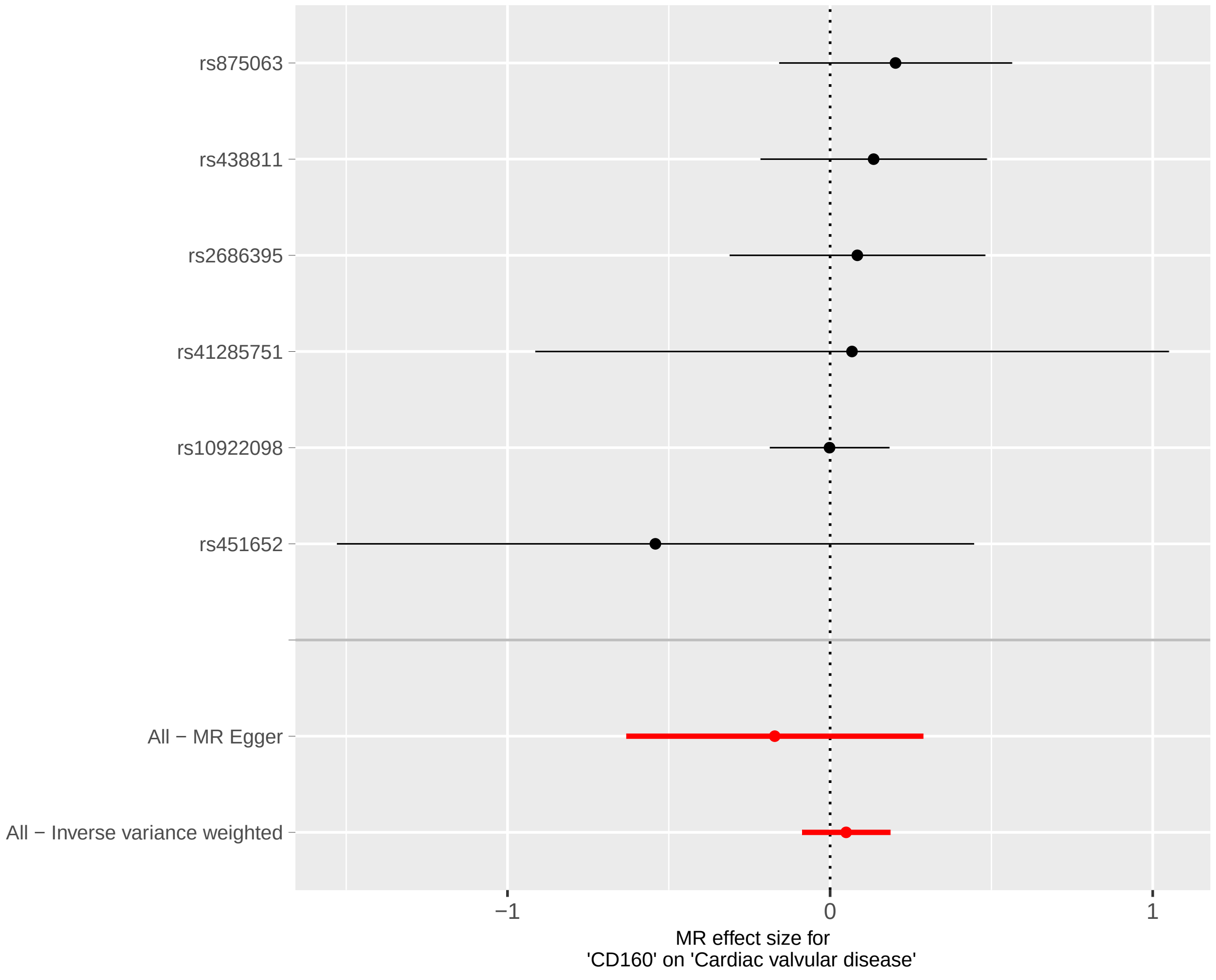
rs875063
rs438811
rs2686395
rs41285751
rs10922098
rs451652
All − MR Egger
All − Inverse variance weighted
−1
0
1
MR effect size for
'CD160' on 'Cardiac valvular disease'

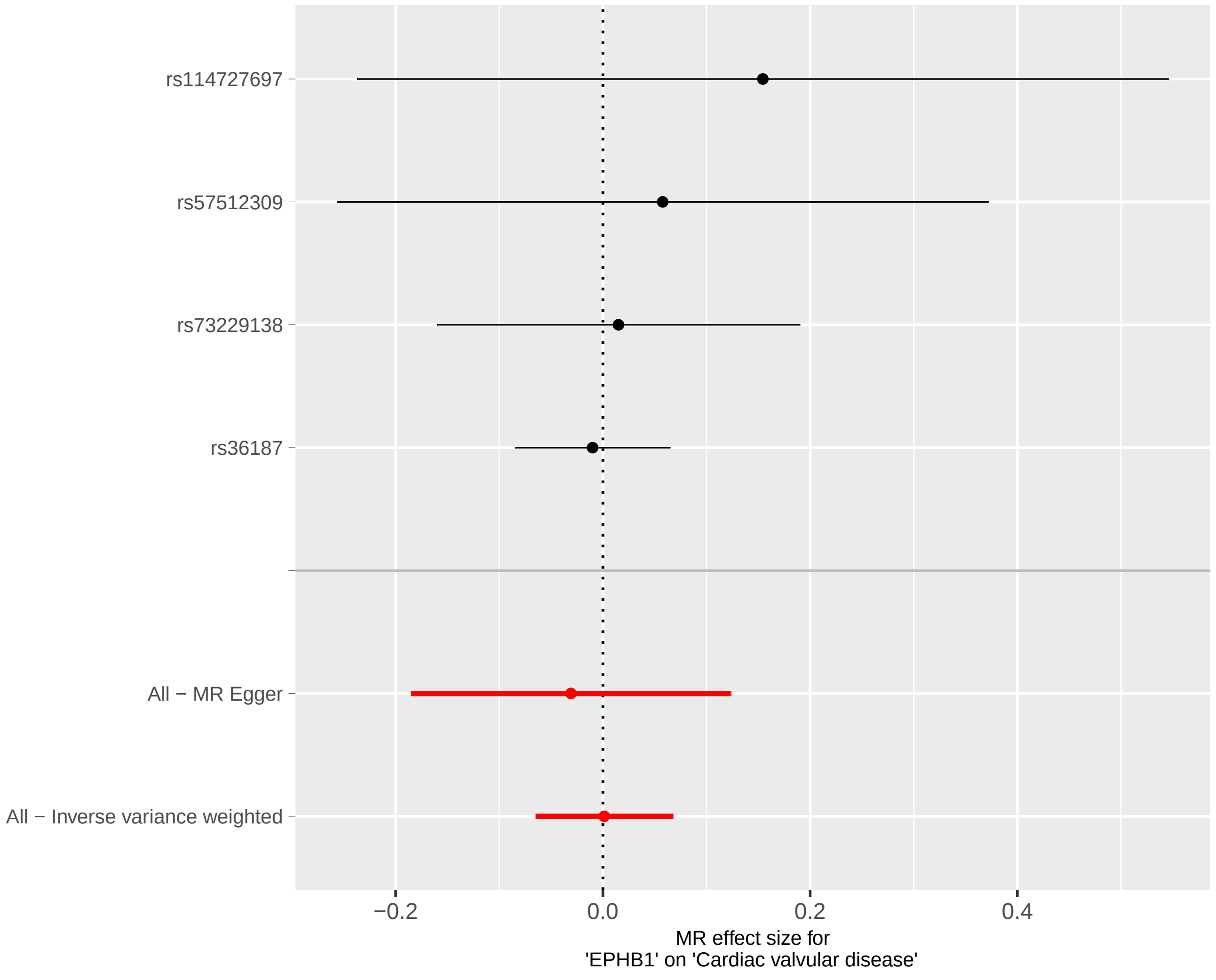

rs114727697
rs57512309
rs73229138
rs36187
All − MR Egger
All − Inverse variance weighted
−0.2
0.0
0.2
0.4
MR effect size for
'EPHB1' on 'Cardiac valvular disease'

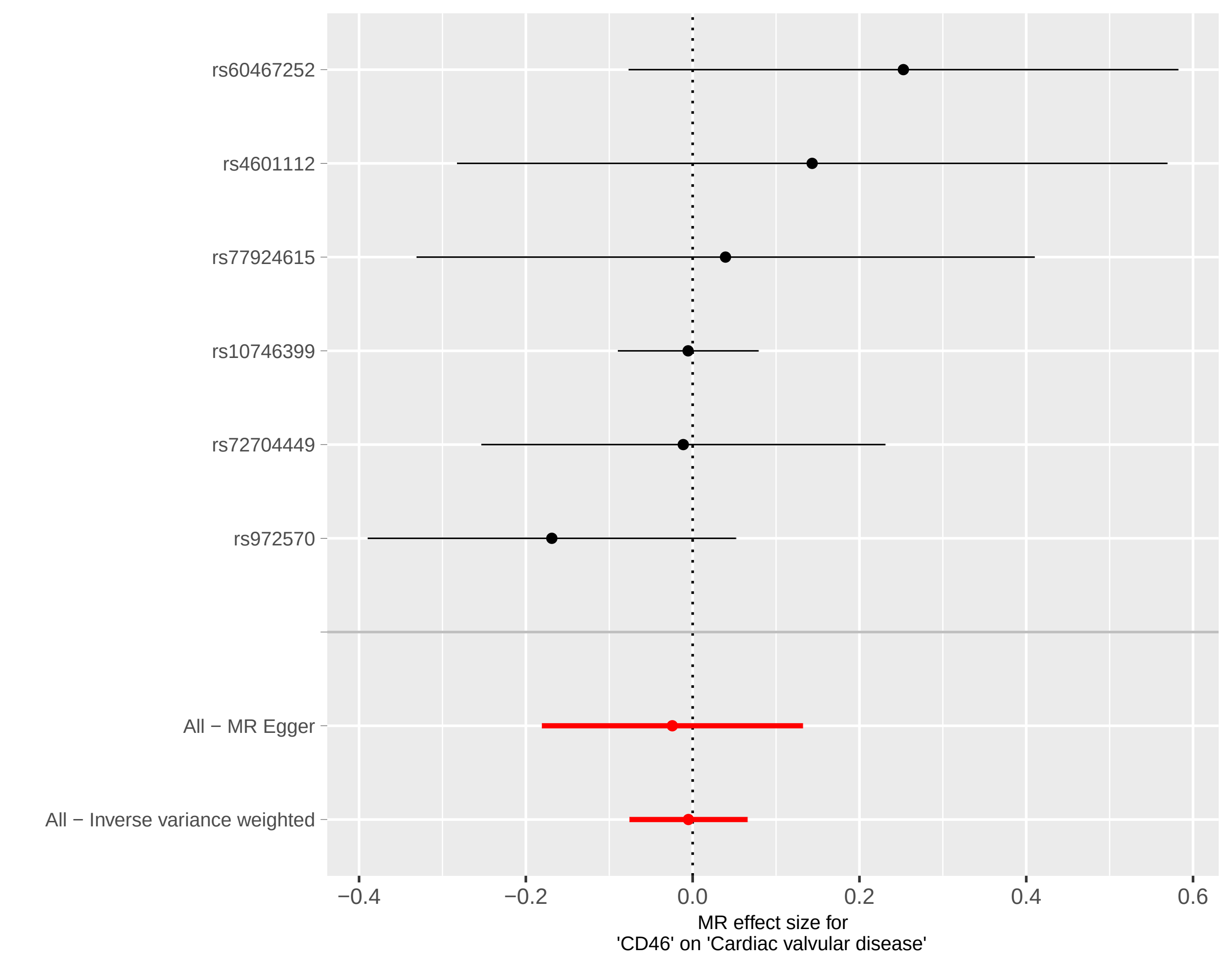
rs60467252
rs4601112
rs77924615
rs10746399
rs72704449
rs972570
All − MR Egger
All − Inverse variance weighted
−0.4
−0.2
0.0
0.2
0.4
0.6
MR effect size for
'CD46' on 'Cardiac valvular disease'

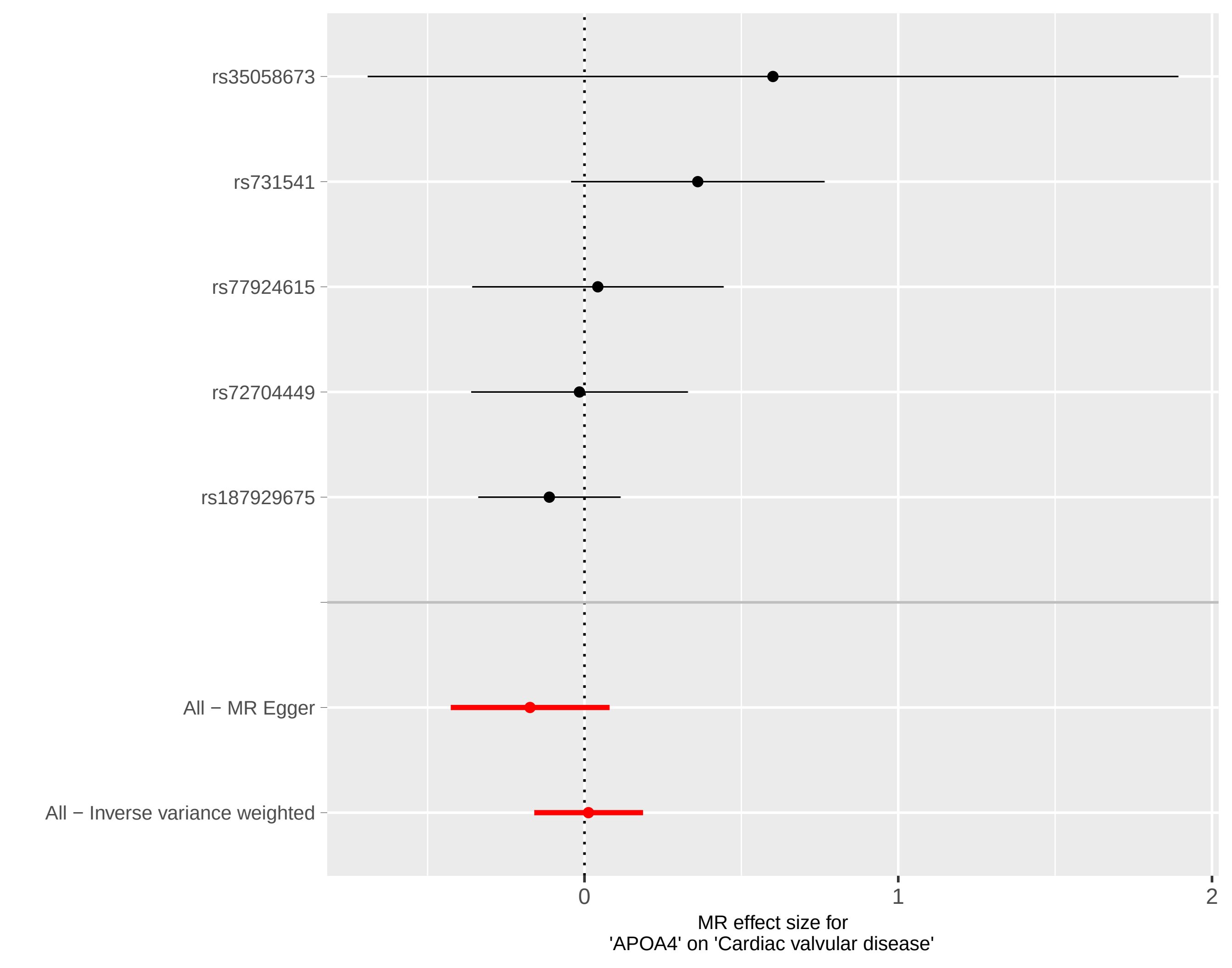
rs35058673
rs731541
rs77924615
rs72704449
rs187929675
All − MR Egger
All − Inverse variance weighted
0
1
2
MR effect size for
'APOA4' on 'Cardiac valvular disease'

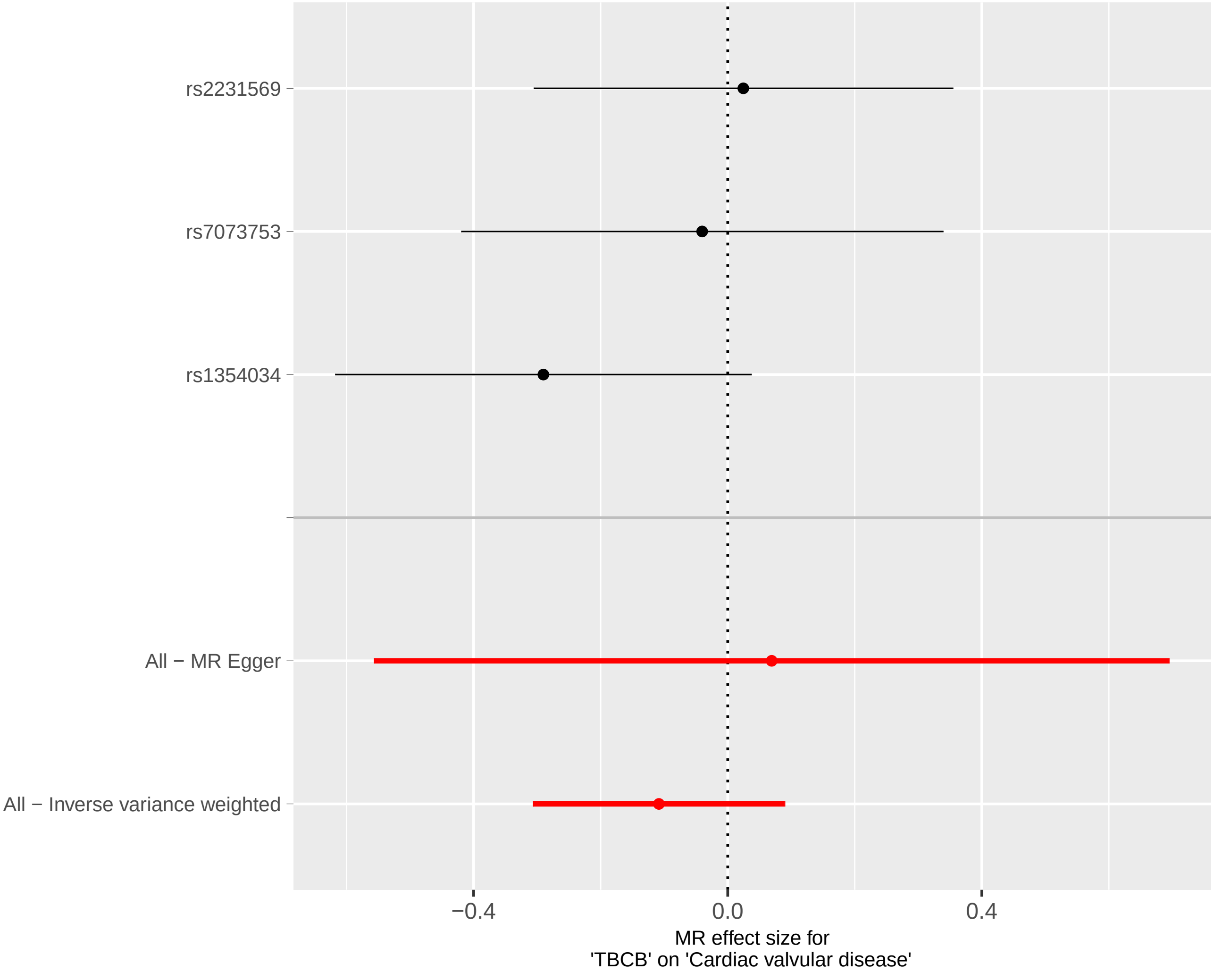

rs2231569
rs7073753
rs1354034
All − MR Egger
All − Inverse variance weighted
−0.4
0.0
0.4
MR effect size for
'TBCB' on 'Cardiac valvular disease'

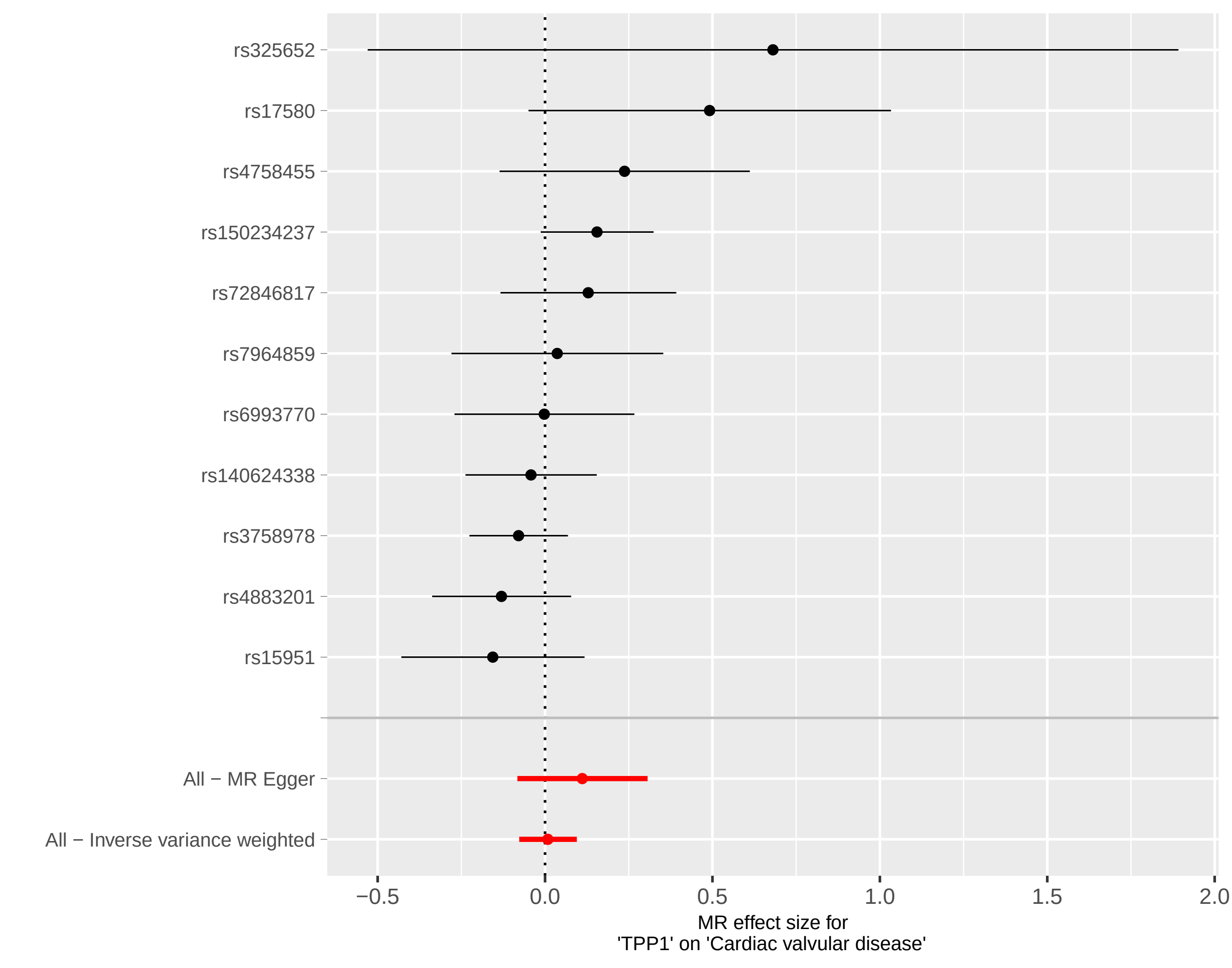

rs325652
rs17580
rs4758455
rs150234237
rs72846817
rs7964859
rs6993770
rs140624338
rs3758978
rs4883201
rs15951
All − MR Egger
All − Inverse variance weighted
−0.5
0.0
0.5
1.0
1.5
2.0
MR effect size for
'TPP1' on 'Cardiac valvular disease'

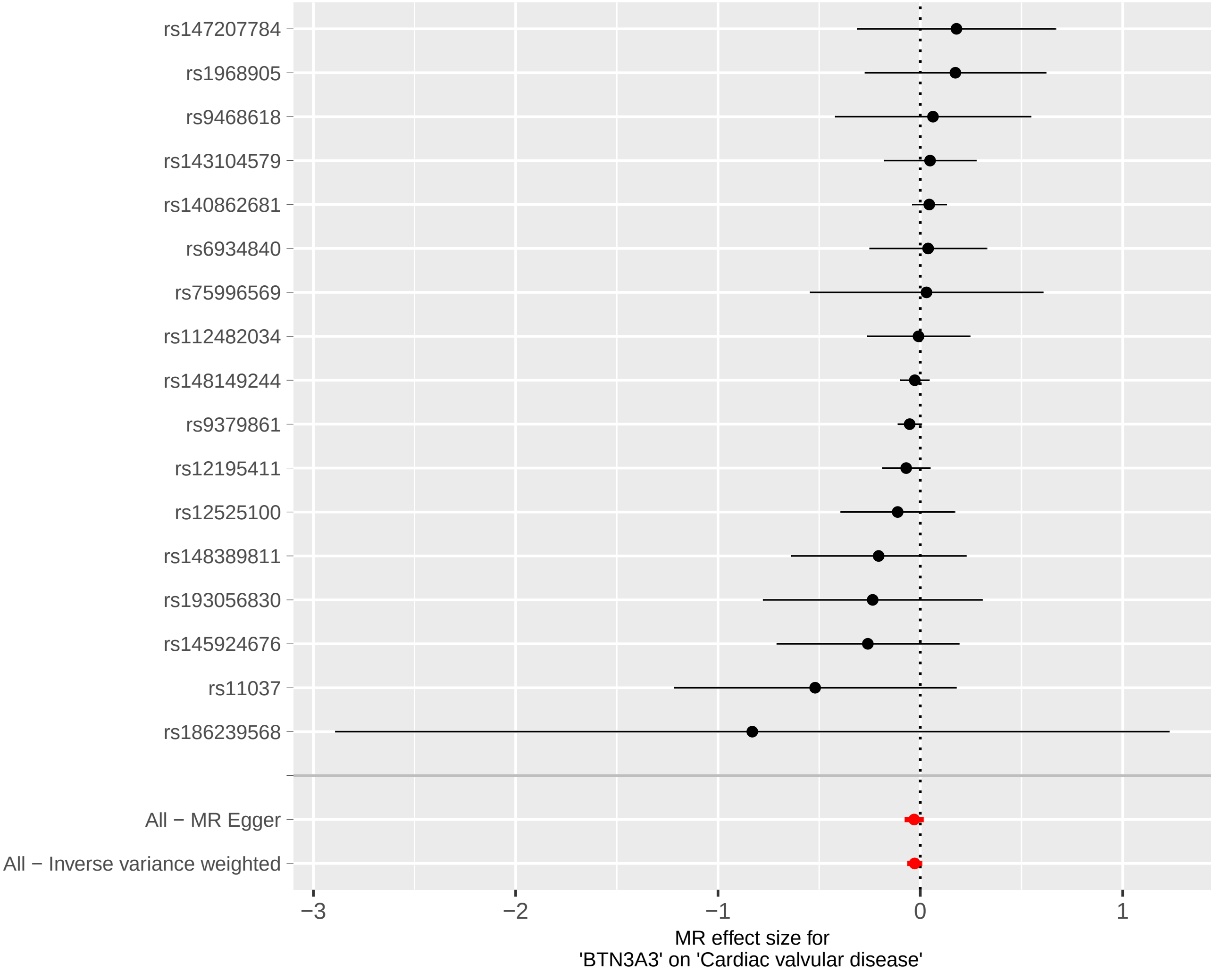

rs147207784
rs1968905
rs9468618
rs143104579
rs140862681
rs6934840
rs75996569
rs112482034
rs148149244
rs9379861
rs12195411
rs12525100
rs148389811
rs193056830
rs145924676
rs11037
rs186239568
All − MR Egger
All − Inverse variance weighted
−3
−2
−1
0
1
MR effect size for
'BTN3A3' on 'Cardiac valvular disease'

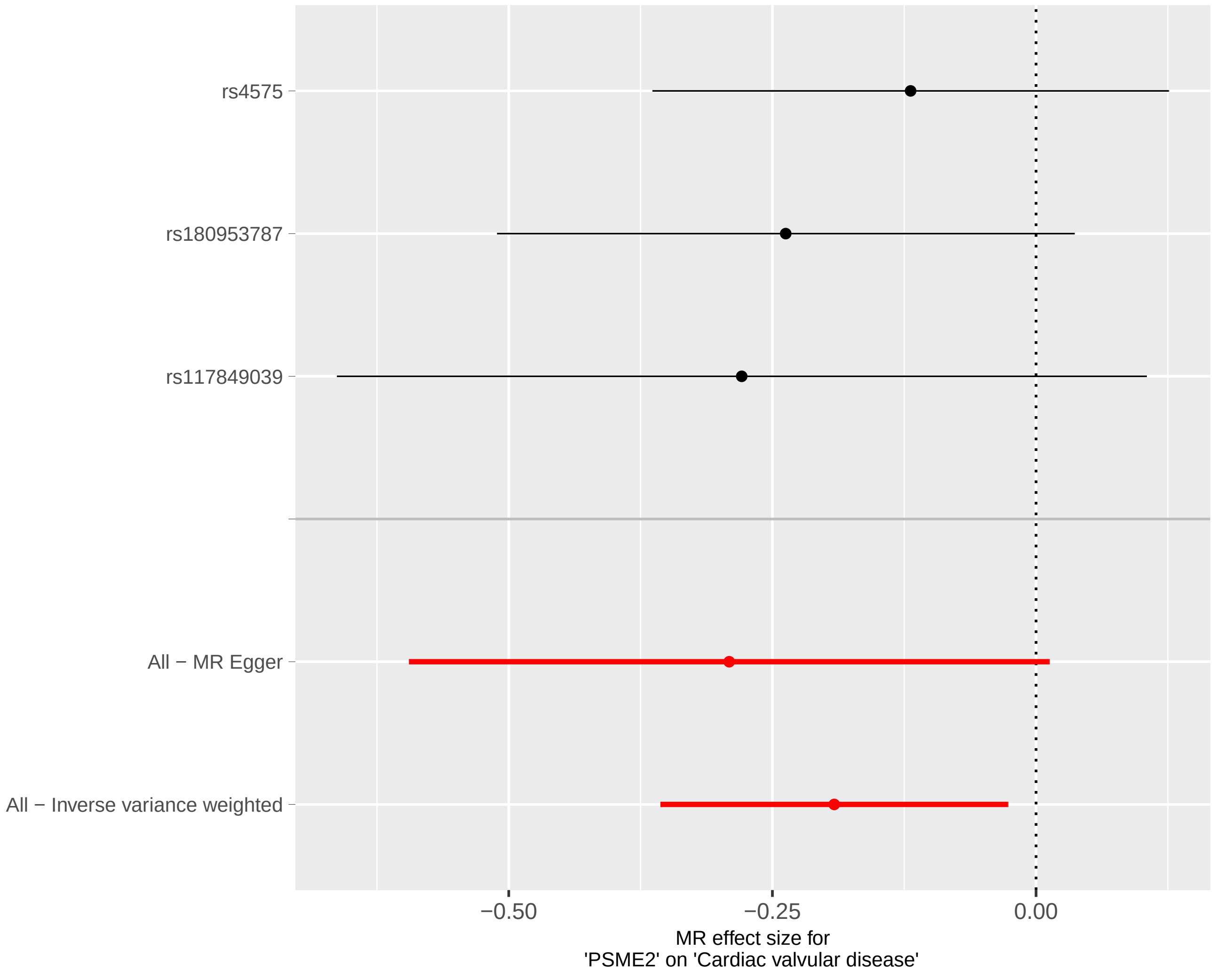
rs4575
rs180953787
rs117849039
All − MR Egger
All − Inverse variance weighted
−0.50
−0.25
0.00
MR effect size for
'PSME2' on 'Cardiac valvular disease'

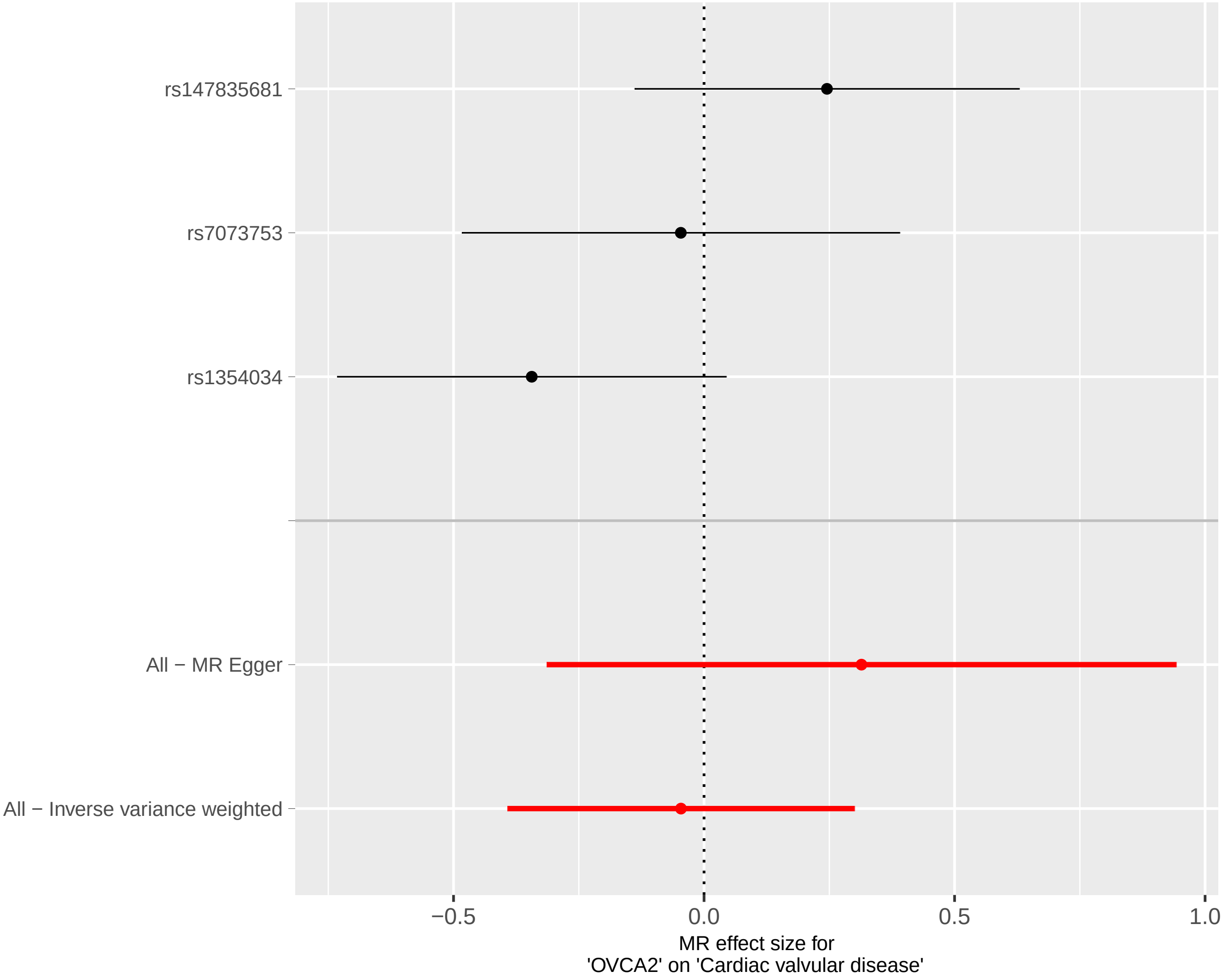
rs147835681
rs7073753
rs1354034
All − MR Egger
All − Inverse variance weighted
−0.5
0.0
0.5
1.0
MR effect size for
'OVCA2' on 'Cardiac valvular disease'

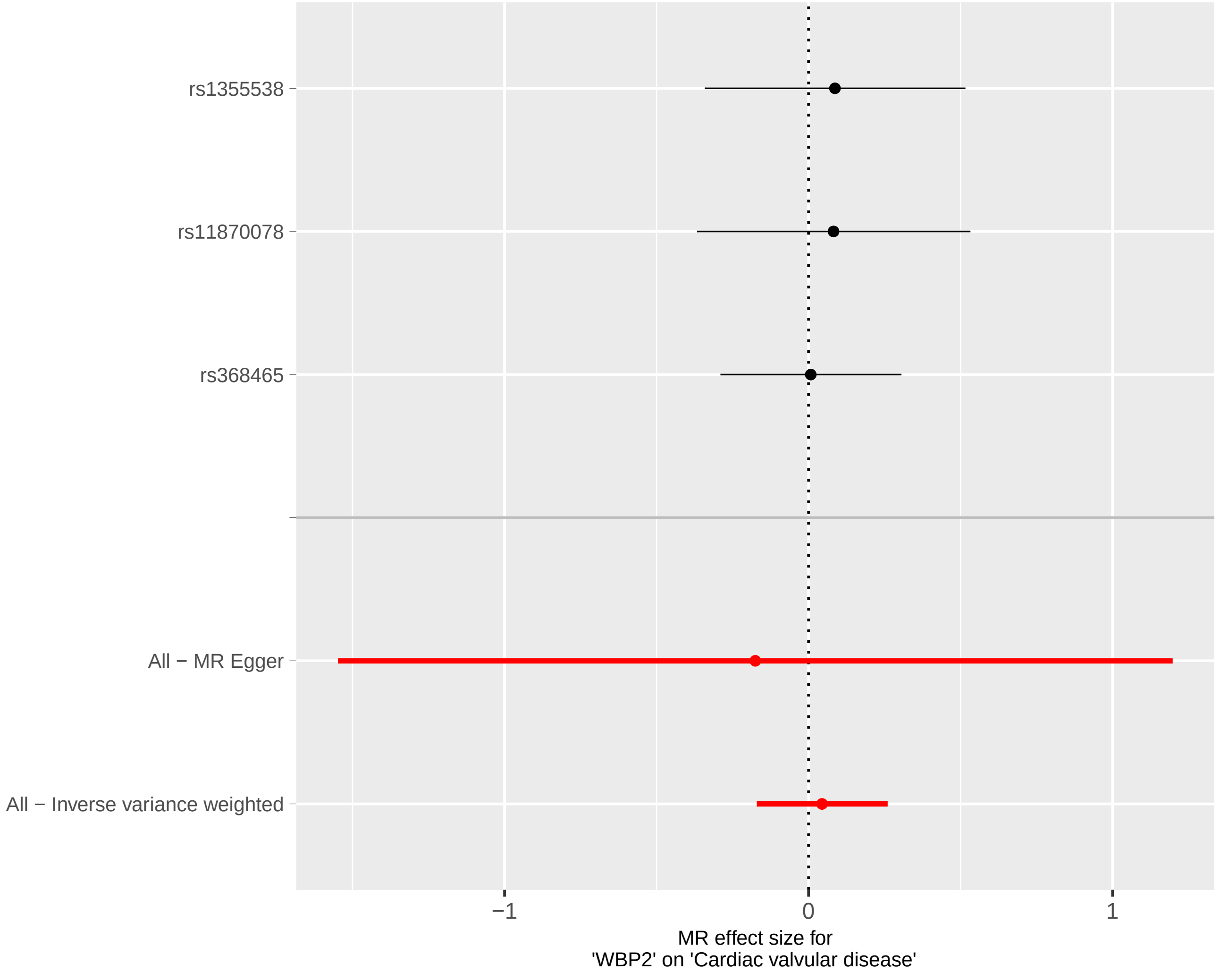
rs1355538
rs11870078
rs368465
All − MR Egger
All − Inverse variance weighted
−1
0
1
MR effect size for
'WBP2' on 'Cardiac valvular disease'

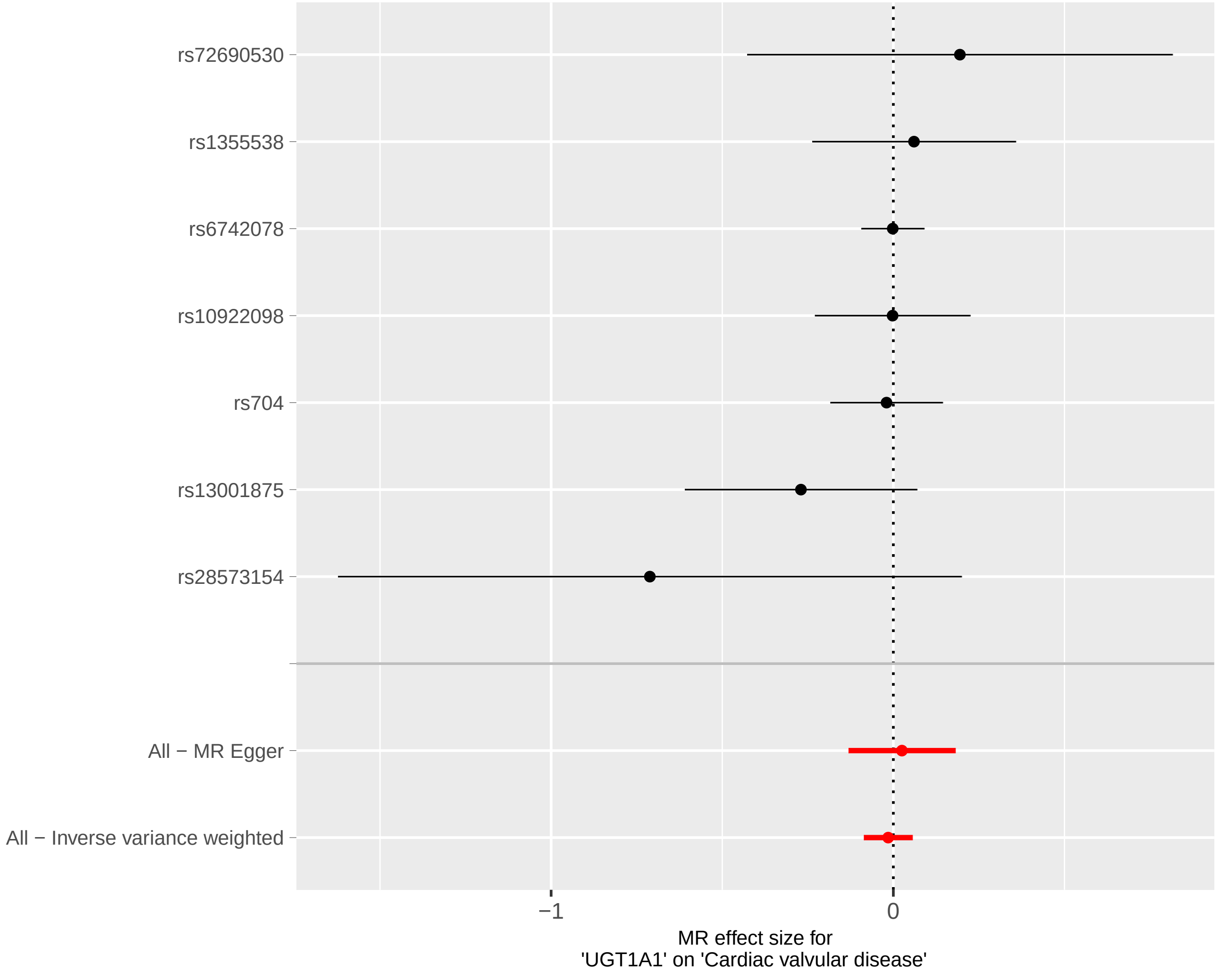
rs72690530
rs1355538
rs6742078
rs10922098
rs704
rs13001875
rs28573154
All − MR Egger
All − Inverse variance weighted
−1
0
MR effect size for
'UGT1A1' on 'Cardiac valvular disease'

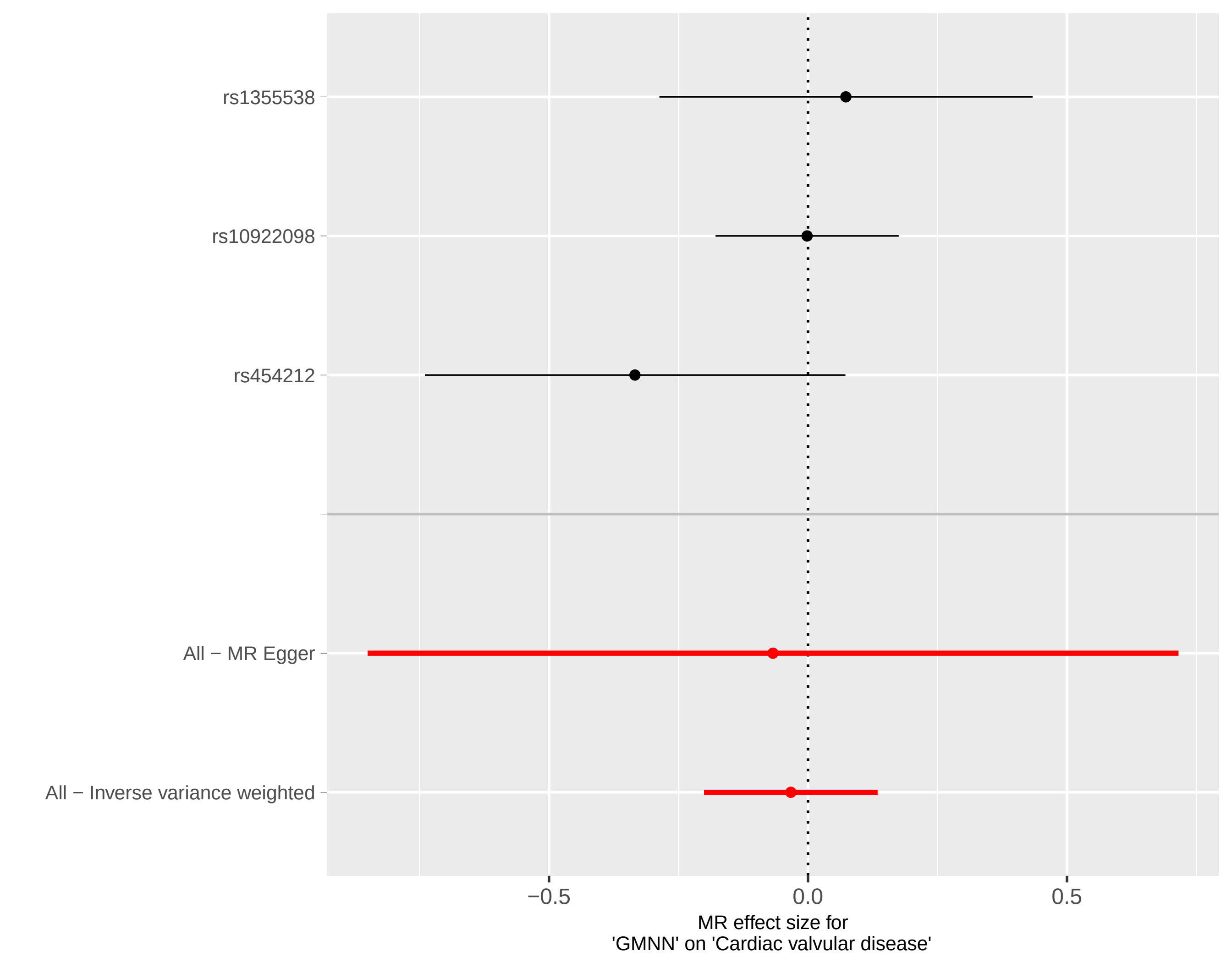
rs1355538
rs10922098
rs454212
All − MR Egger
All − Inverse variance weighted
−0.5
0.0
0.5
MR effect size for
'GMNN' on 'Cardiac valvular disease'

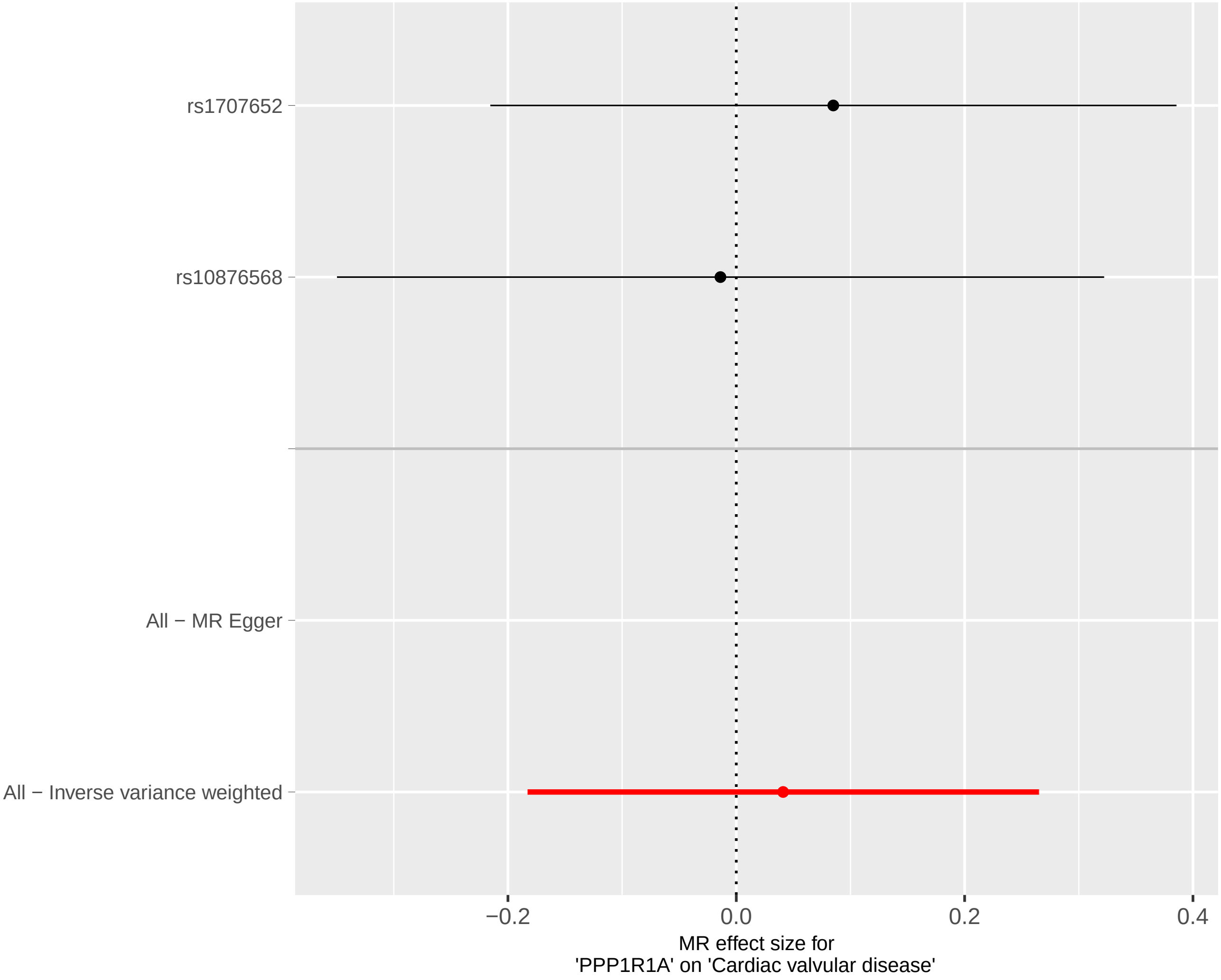
rs1707652
rs10876568
All − MR Egger
All − Inverse variance weighted
−0.2
0.0
0.2
0.4
MR effect size for
'PPP1R1A' on 'Cardiac valvular disease'

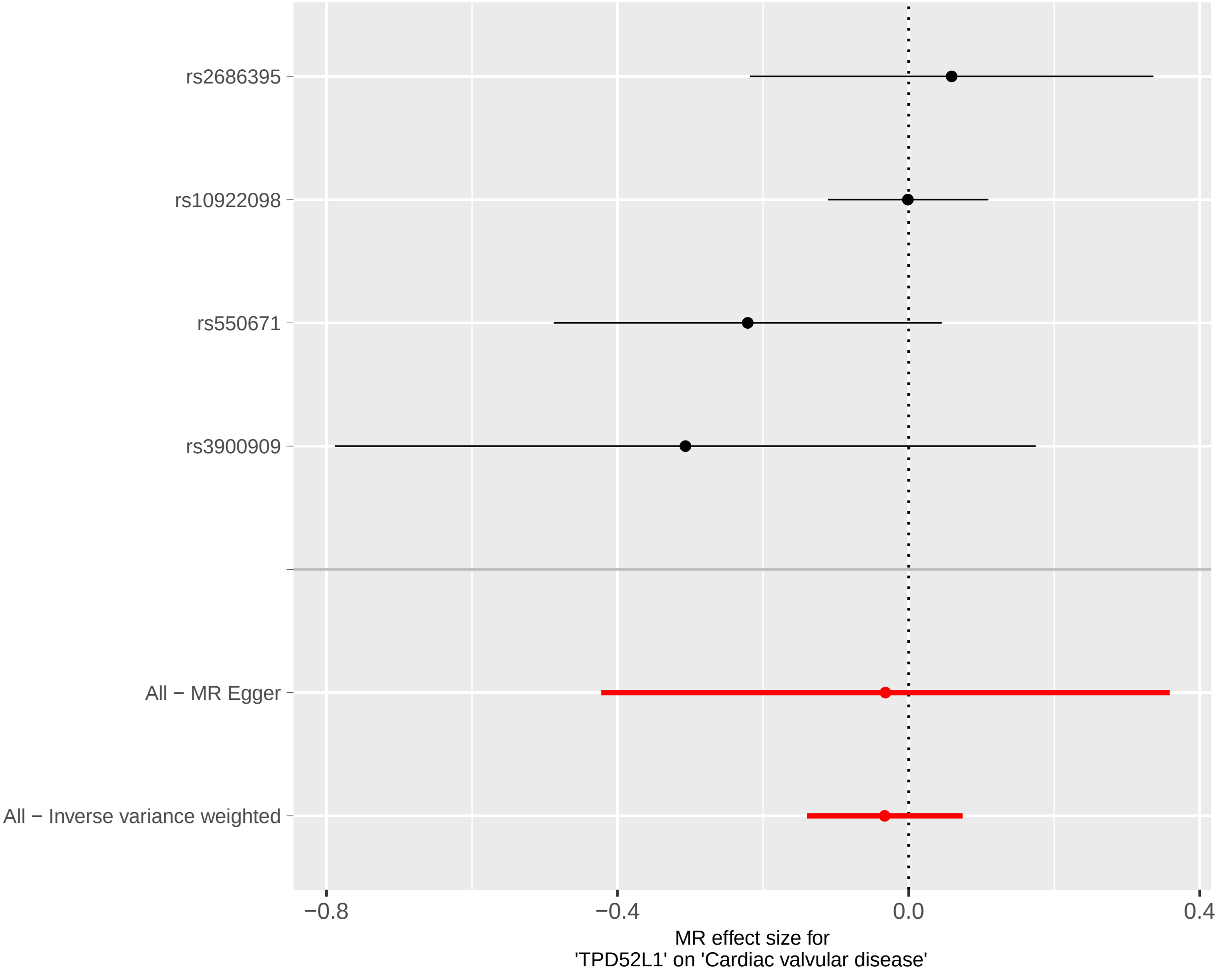
rs2686395
rs10922098
rs550671
rs3900909
All − MR Egger
All − Inverse variance weighted
−0.8
−0.4
0.0
0.4
MR effect size for
'TPD52L1' on 'Cardiac valvular disease'

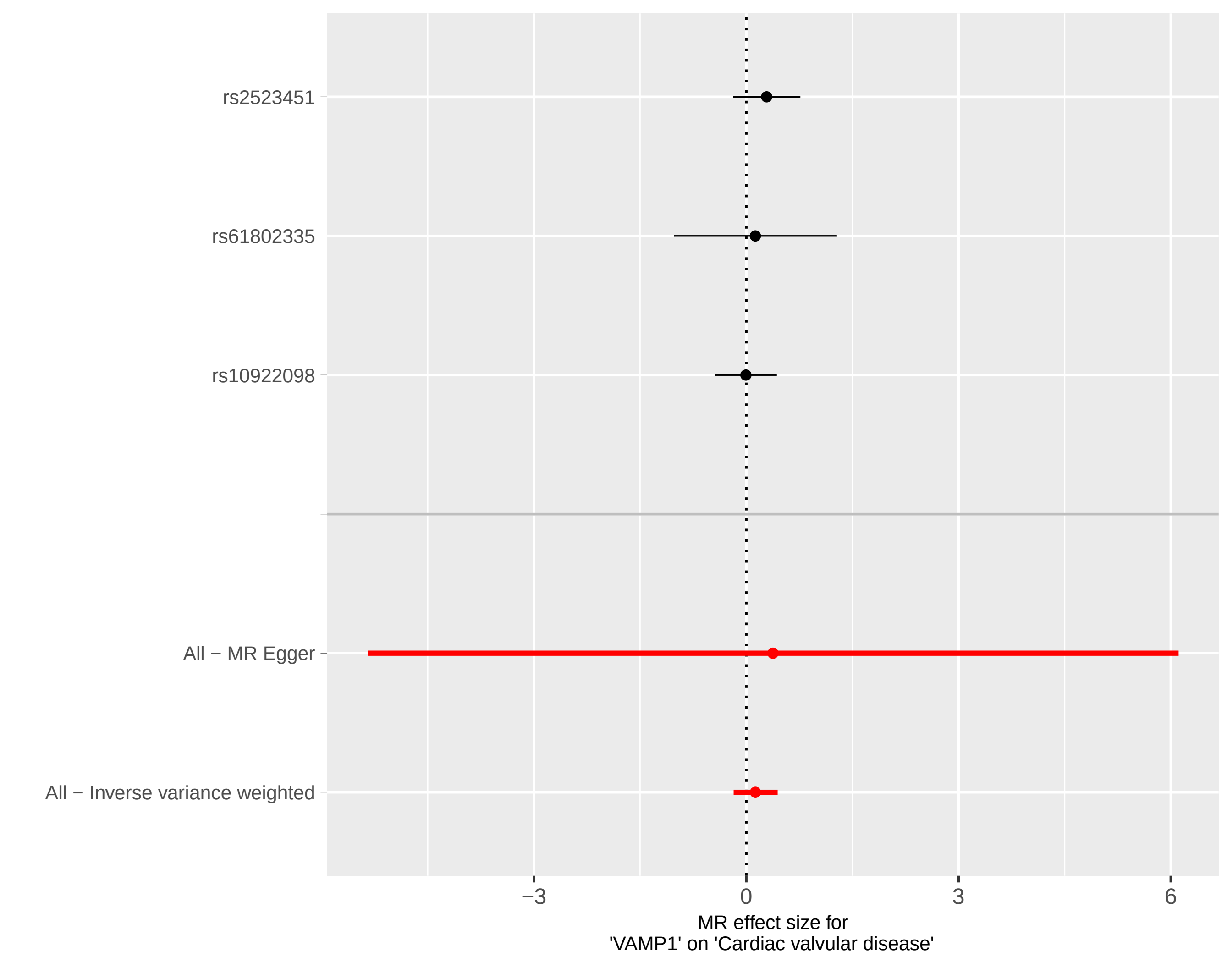
rs2523451
rs61802335
rs10922098
All − MR Egger
All − Inverse variance weighted
−3
0
3
6
MR effect size for
'VAMP1' on 'Cardiac valvular disease'

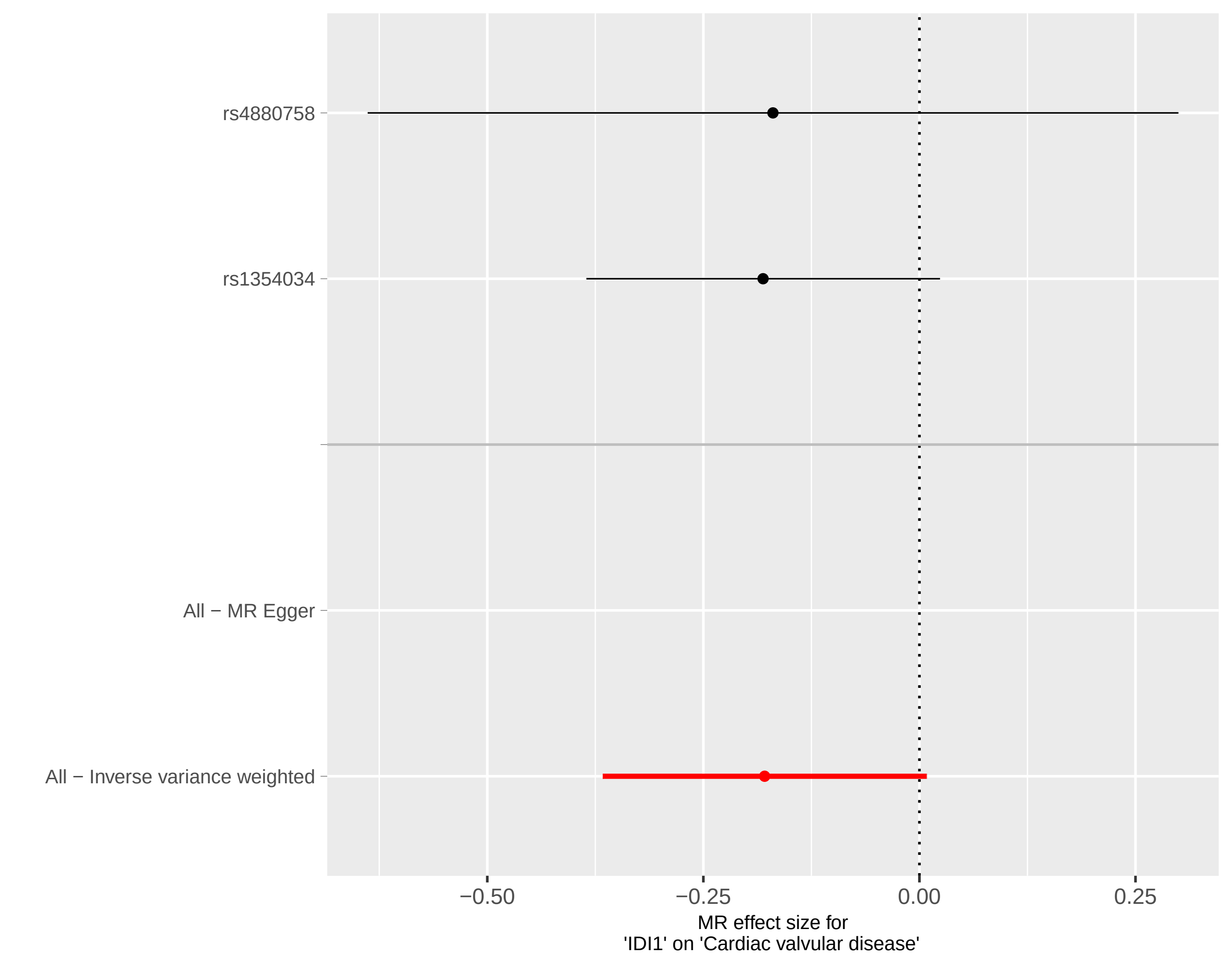
rs4880758
rs1354034
All − MR Egger
All − Inverse variance weighted
−0.50
−0.25
0.00
0.25
MR effect size for
'IDI1' on 'Cardiac valvular disease'

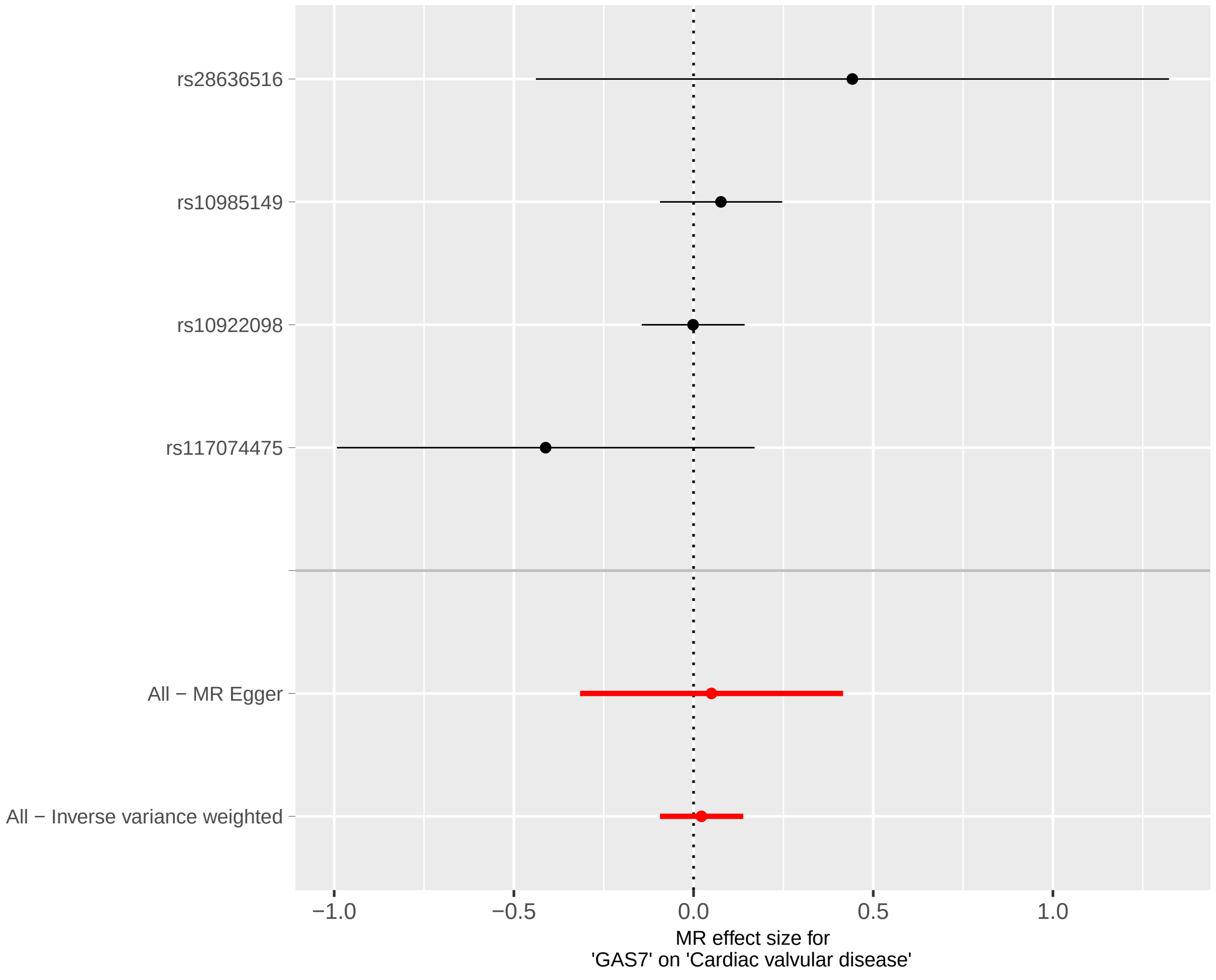

rs28636516
rs10985149
rs10922098
rs117074475
All − MR Egger
All − Inverse variance weighted
−1.0
−0.5
0.0
0.5
1.0
MR effect size for
'GAS7' on 'Cardiac valvular disease'

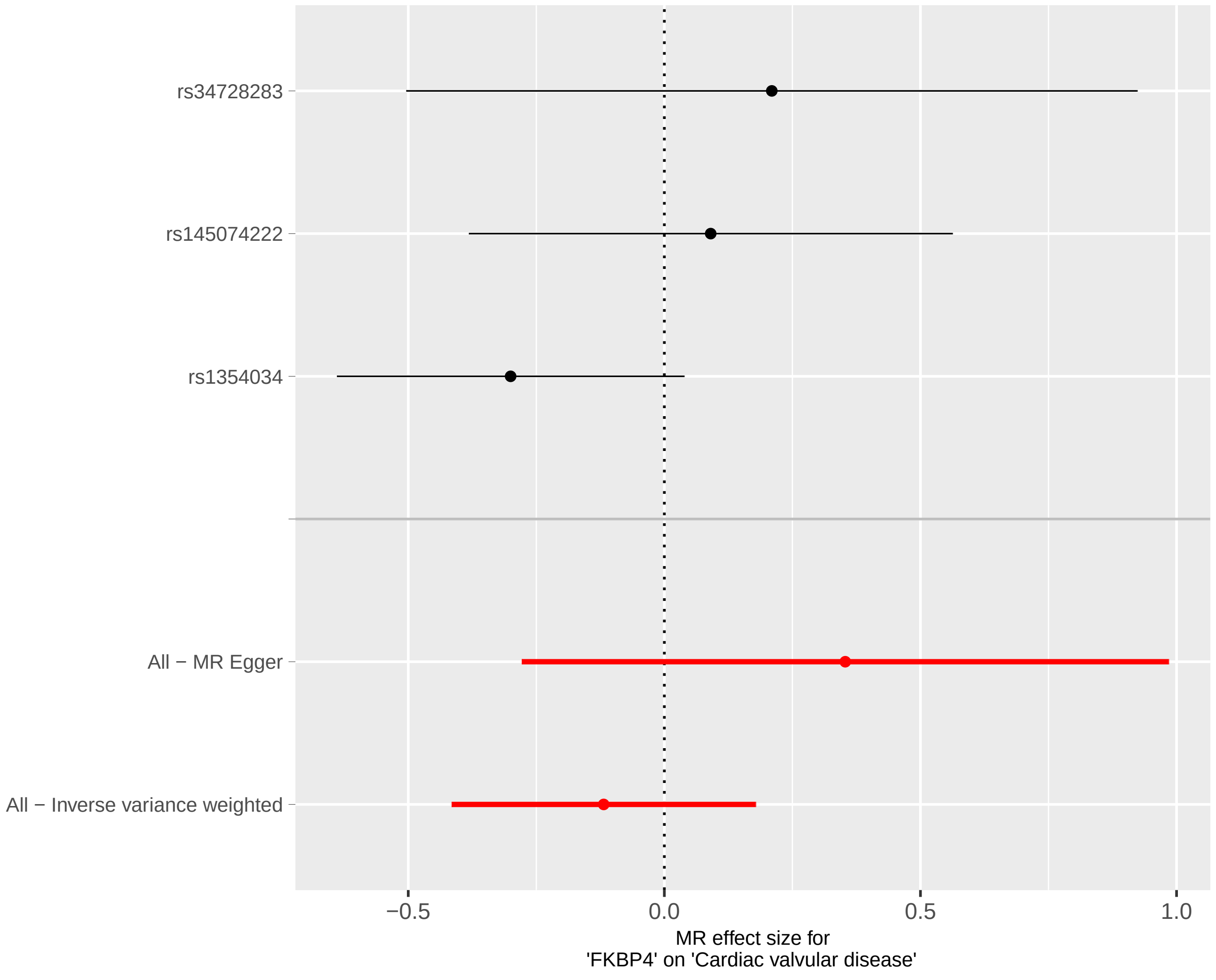
rs34728283
rs145074222
rs1354034
All − MR Egger
All − Inverse variance weighted
−0.5
0.0
0.5
1.0
MR effect size for
'FKBP4' on 'Cardiac valvular disease'

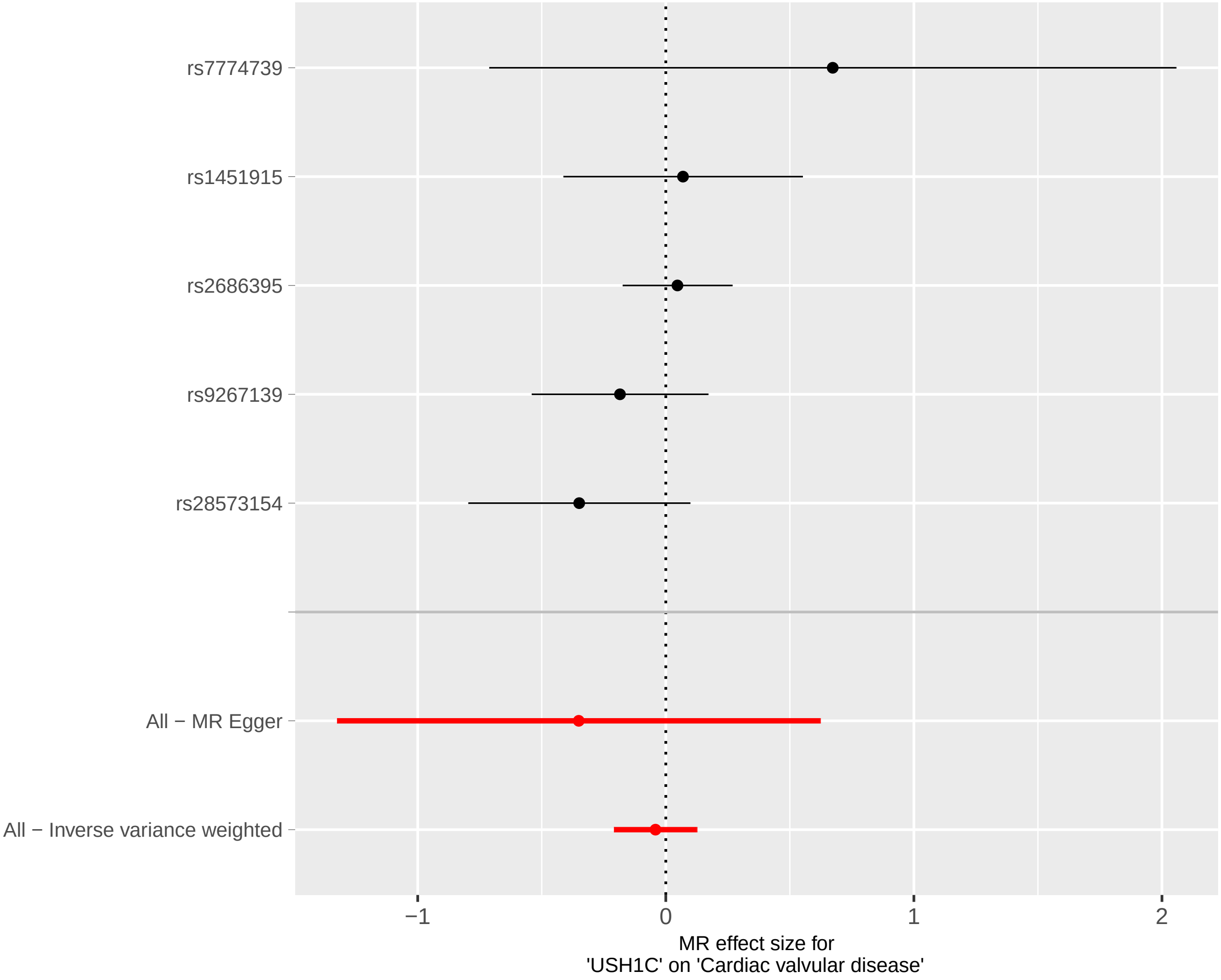
rs7774739
rs1451915
rs2686395
rs9267139
rs28573154
All − MR Egger
All − Inverse variance weighted
−1
0
1
2
MR effect size for
'USH1C' on 'Cardiac valvular disease'

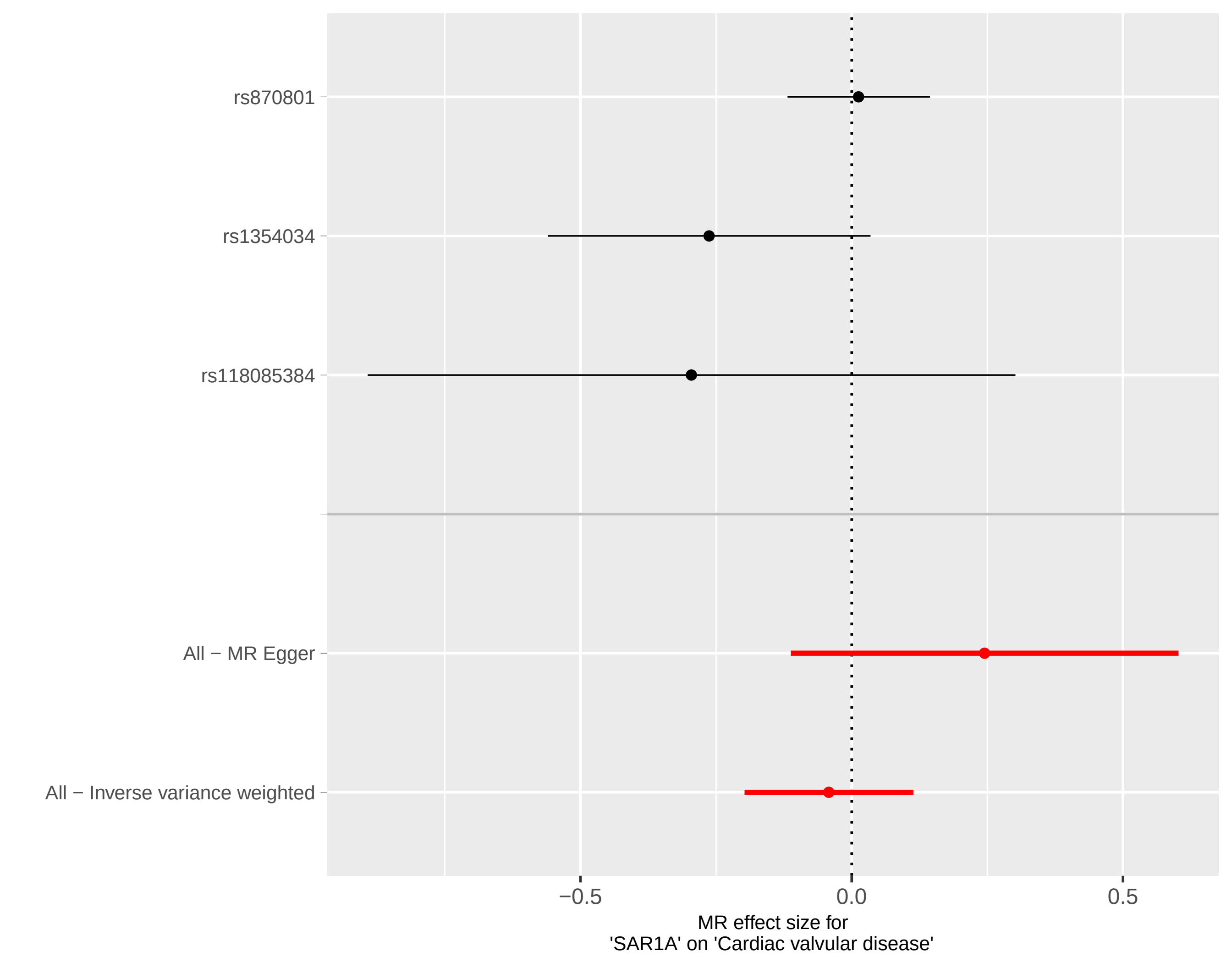
rs870801
rs1354034
rs118085384
All − MR Egger
All − Inverse variance weighted
−0.5
0.0
0.5
MR effect size for
'SAR1A' on 'Cardiac valvular disease'

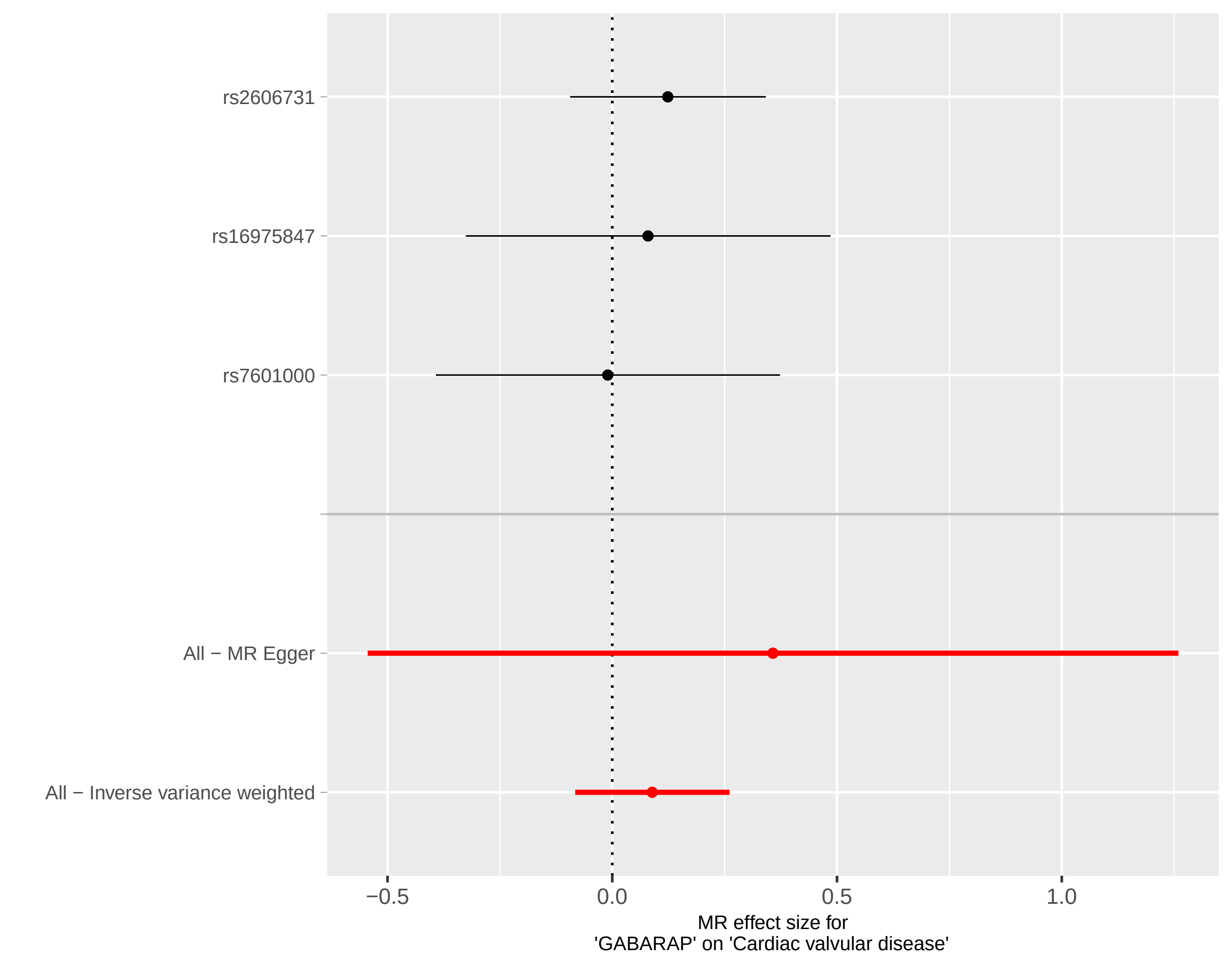

rs2606731
rs16975847
rs7601000
All − MR Egger
All − Inverse variance weighted
−0.5
0.0
0.5
1.0
MR effect size for
'GABARAP' on 'Cardiac valvular disease'

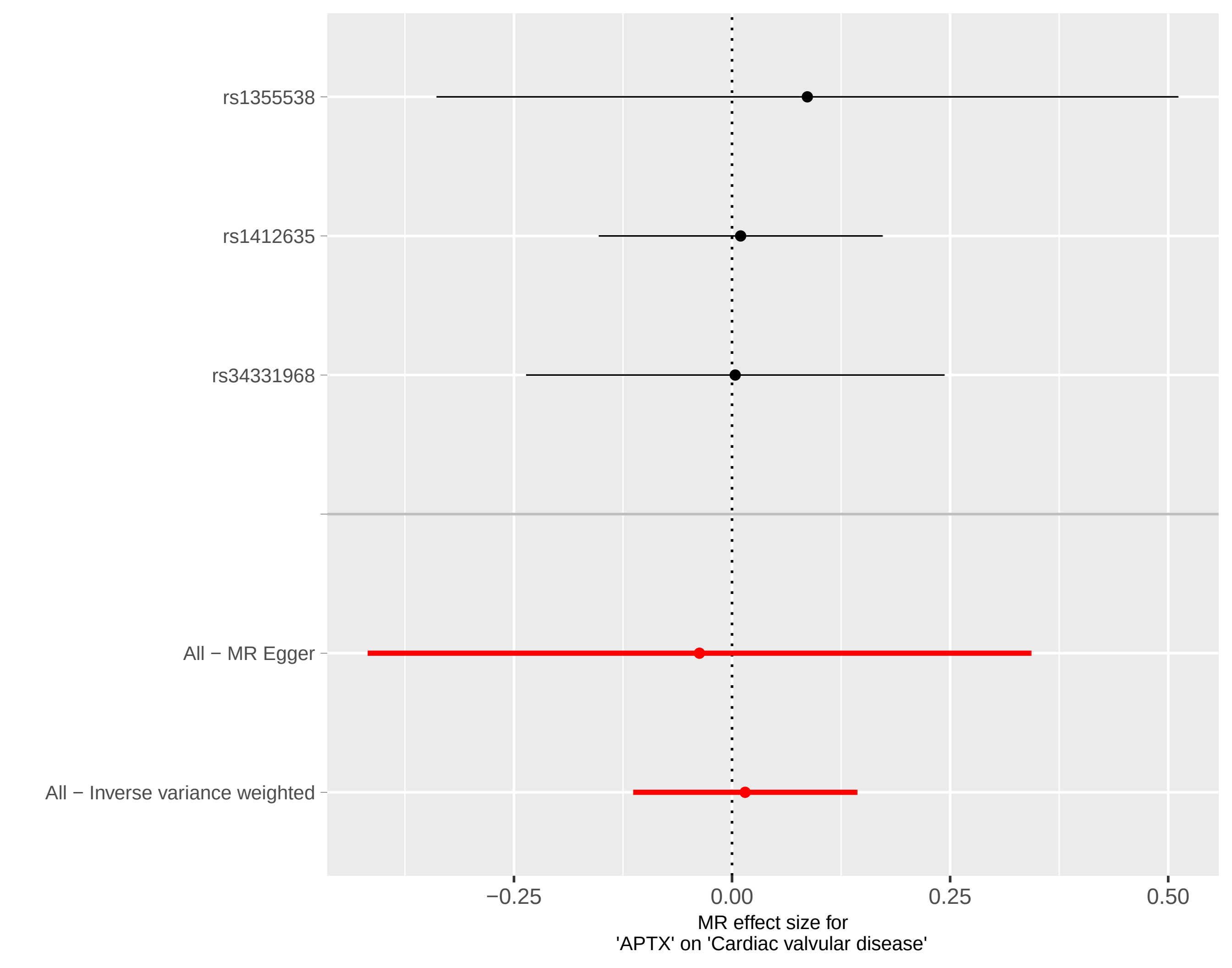
rs1355538
rs1412635
rs34331968
All − MR Egger
All − Inverse variance weighted
−0.25
0.00
0.25
0.50
MR effect size for
'APTX' on 'Cardiac valvular disease'

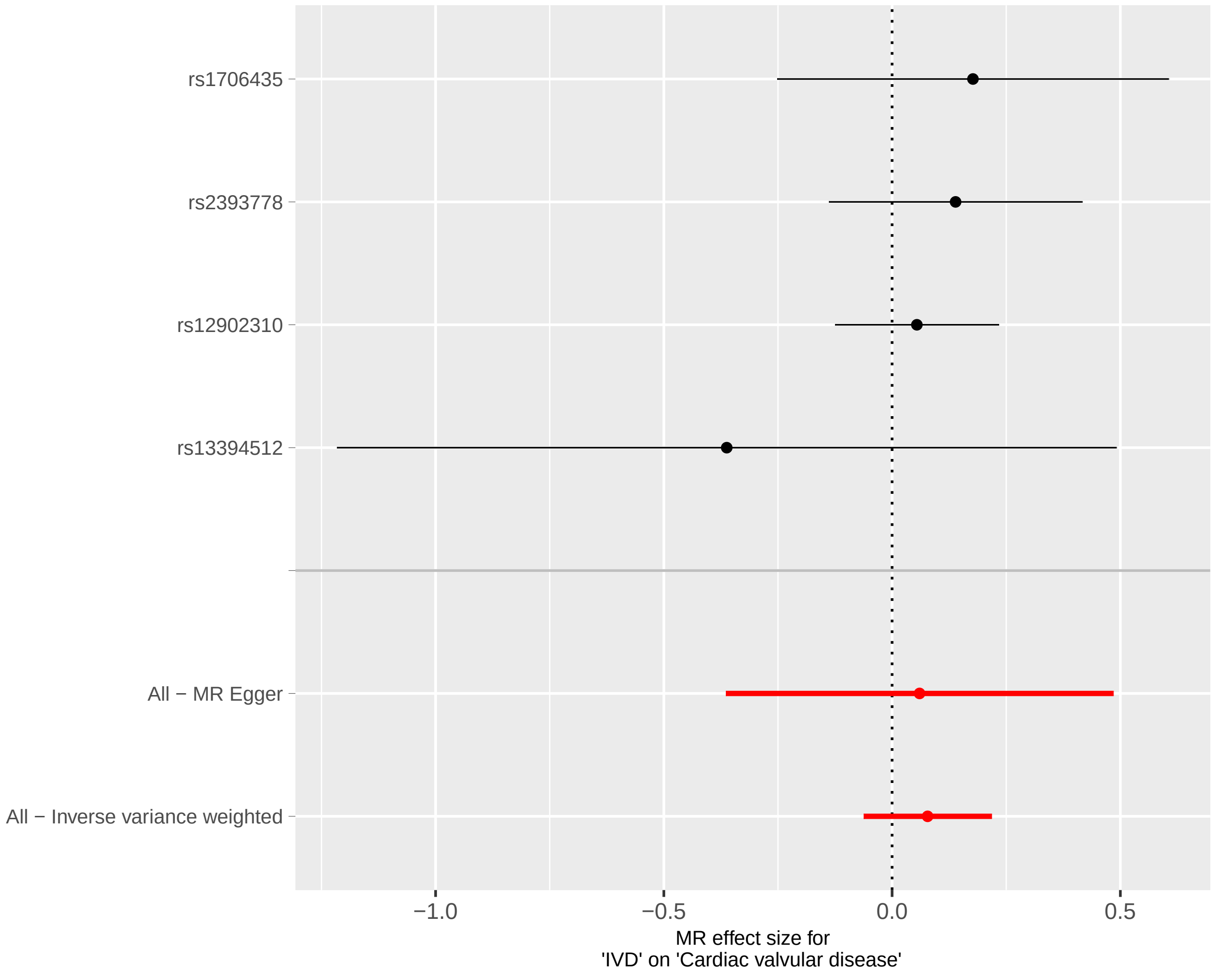
rs1706435
rs2393778
rs12902310
rs13394512
All − MR Egger
All − Inverse variance weighted
−1.0
−0.5
0.0
0.5
MR effect size for
'IVD' on 'Cardiac valvular disease'

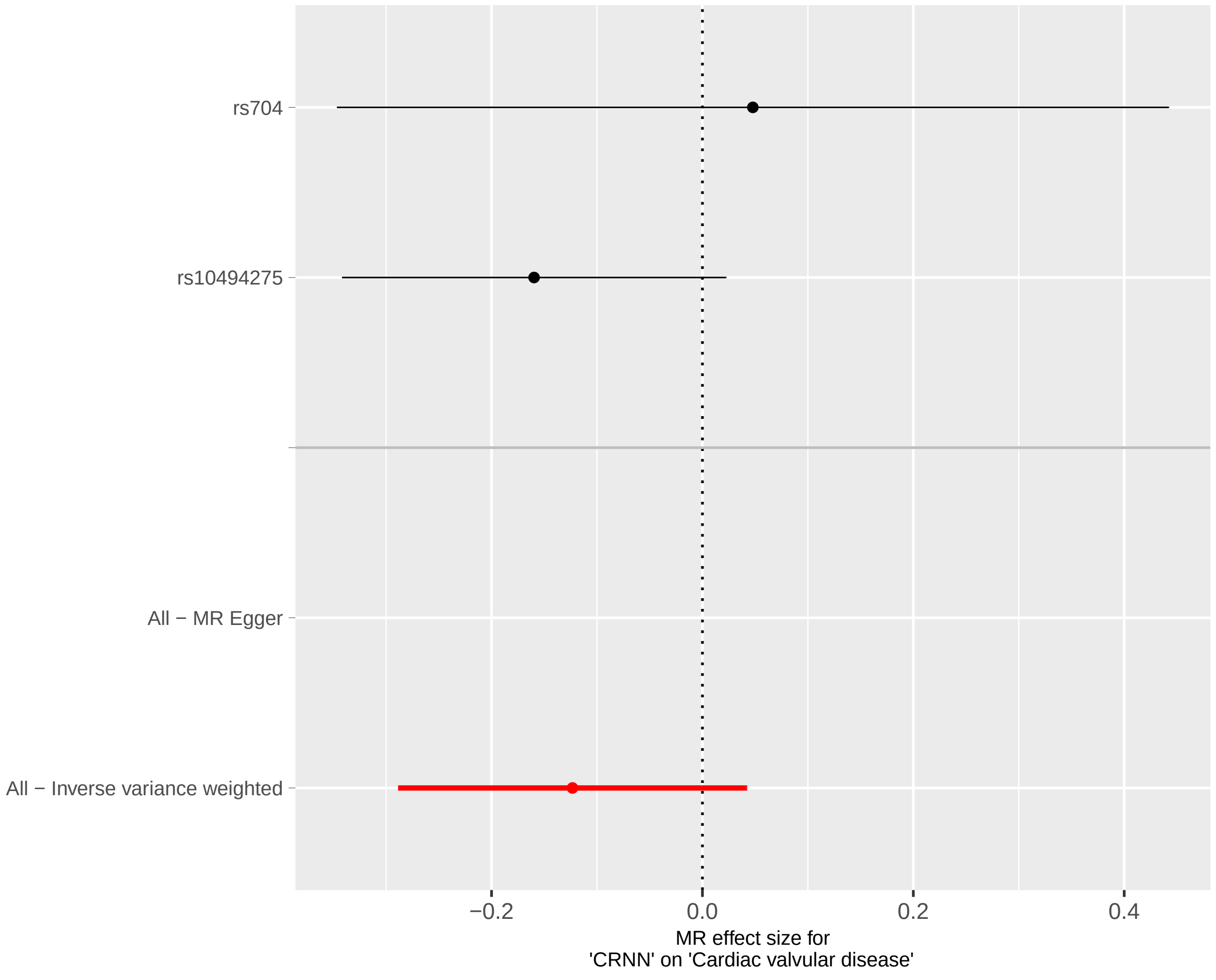
rs704
rs10494275
All − MR Egger
All − Inverse variance weighted
−0.2
0.0
0.2
0.4
MR effect size for
'CRNN' on 'Cardiac valvular disease'

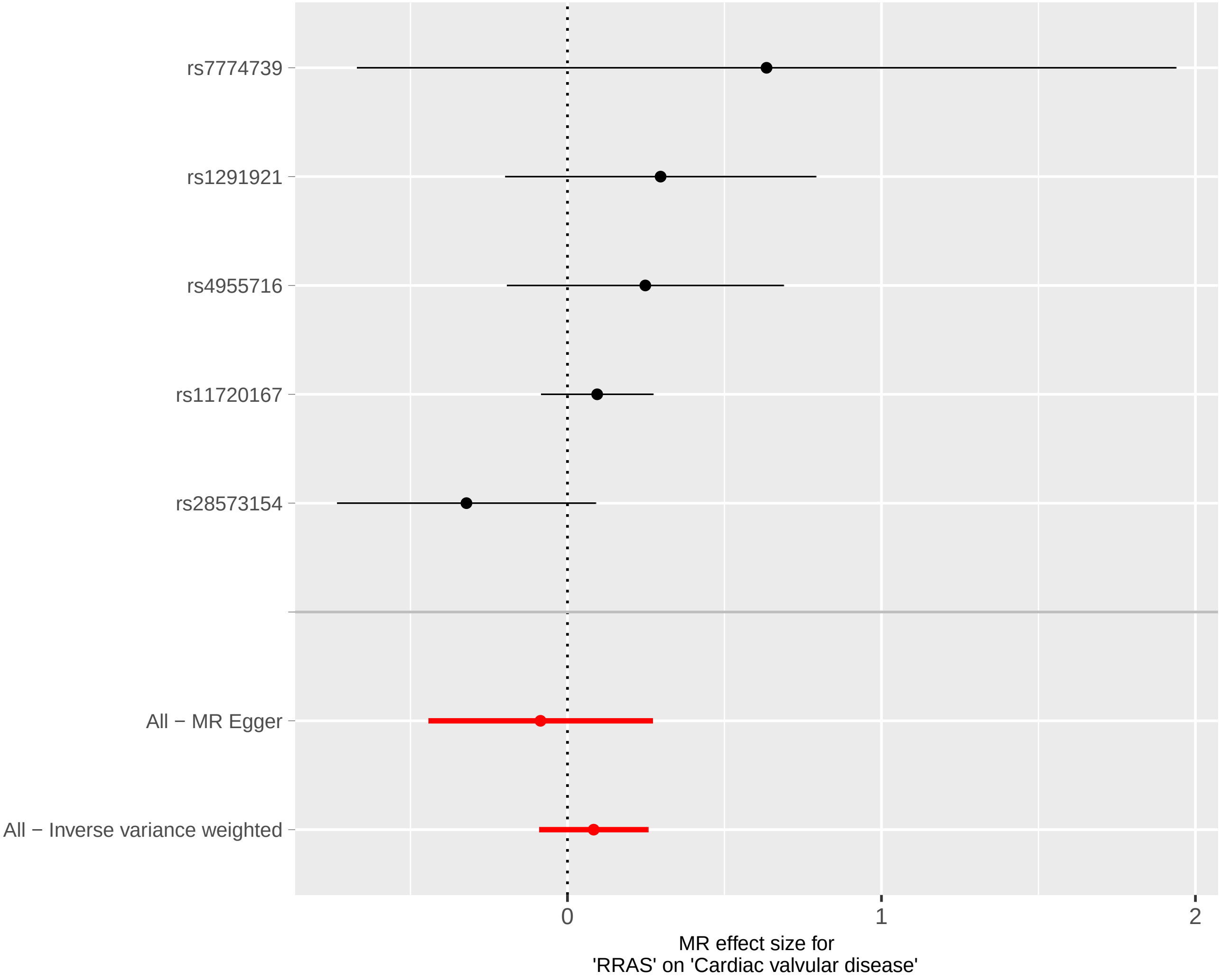
rs7774739
rs1291921
rs4955716
rs11720167
rs28573154
All − MR Egger
All − Inverse variance weighted
0
1
2
MR effect size for
'RRAS' on 'Cardiac valvular disease'

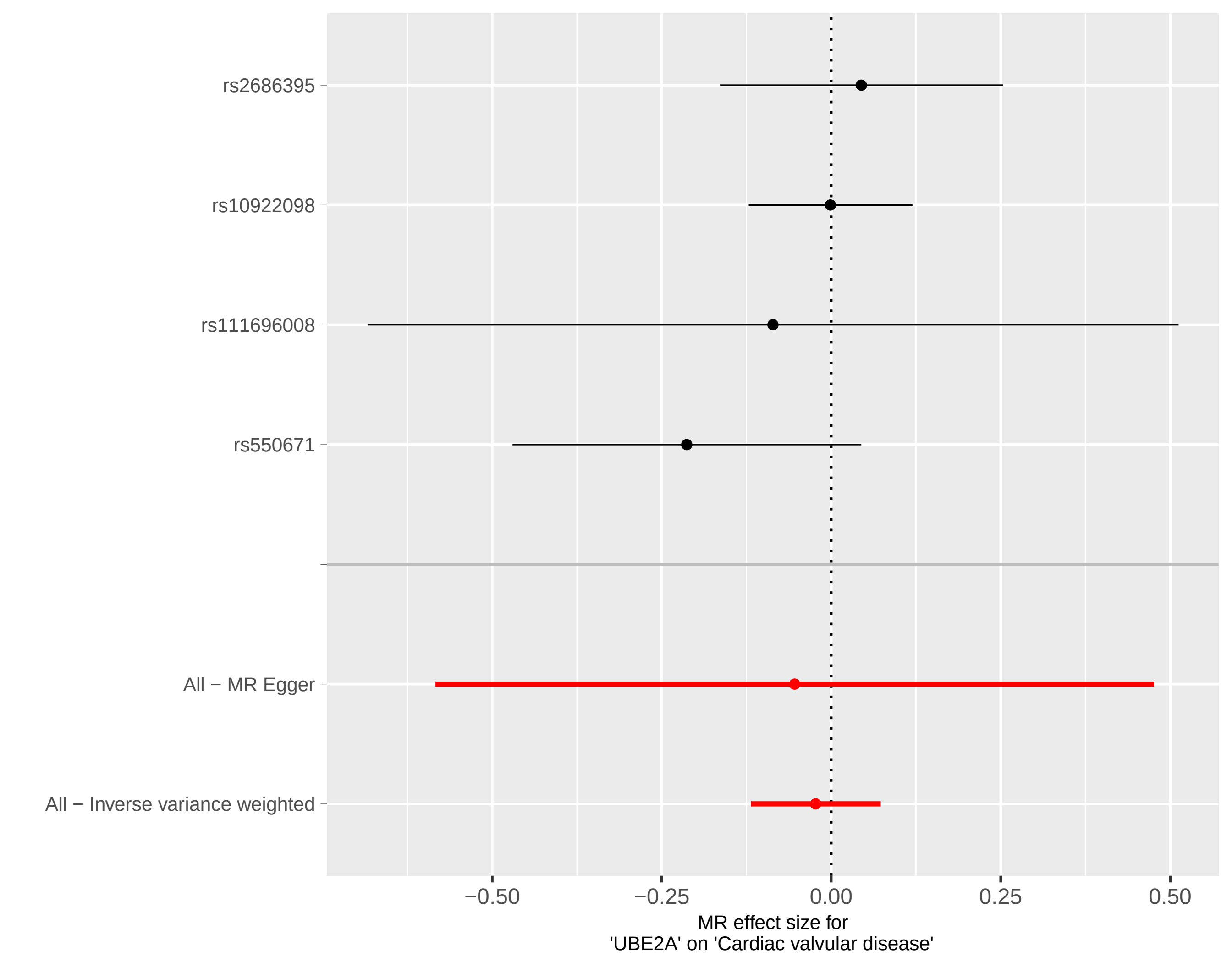
rs2686395
rs10922098
rs111696008
rs550671
All − MR Egger
All − Inverse variance weighted
−0.50
−0.25
0.00
0.25
0.50
MR effect size for
'UBE2A' on 'Cardiac valvular disease'

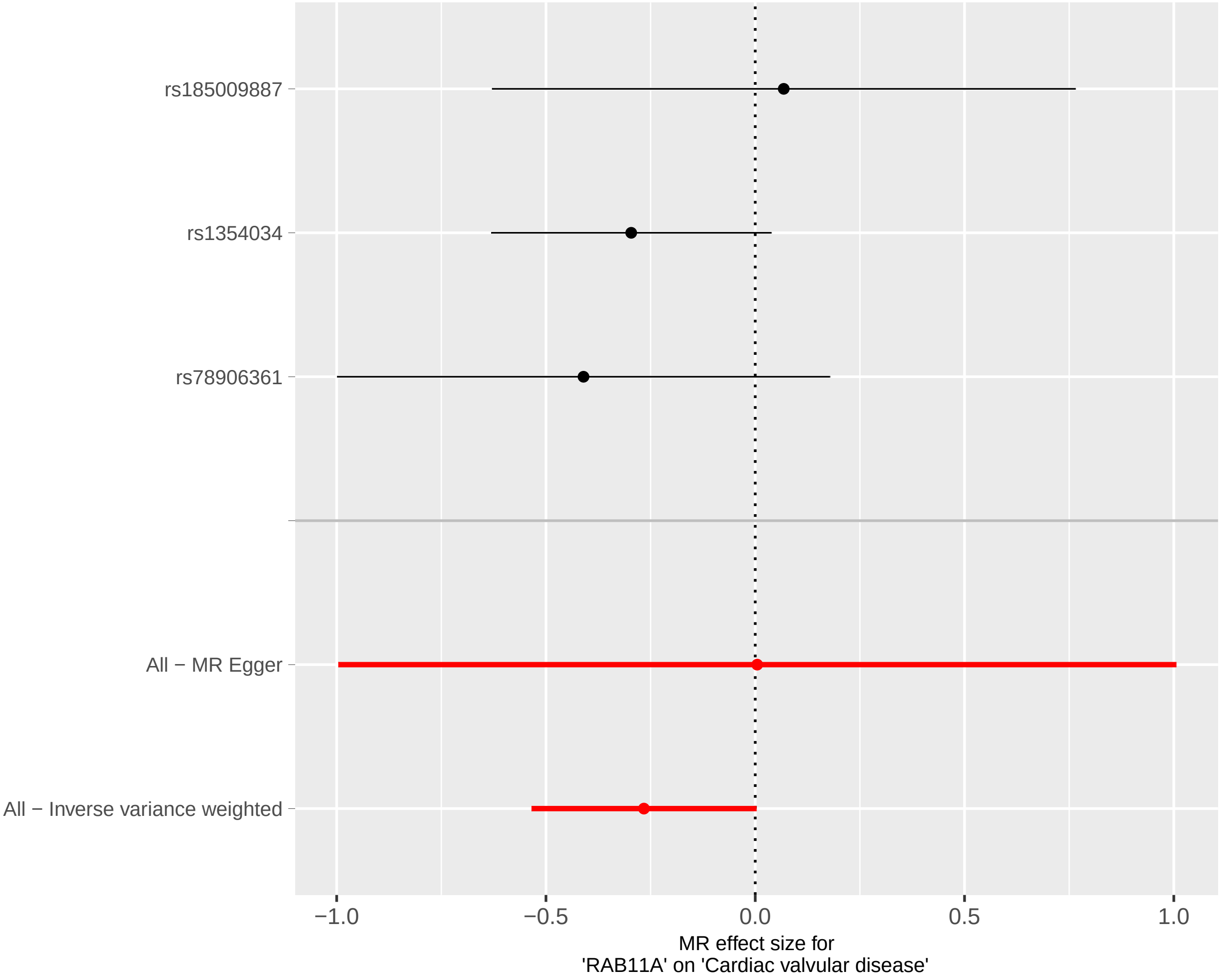

rs185009887
rs1354034
rs78906361
All − MR Egger
All − Inverse variance weighted
−1.0
−0.5
0.0
0.5
1.0
MR effect size for
'RAB11A' on 'Cardiac valvular disease'

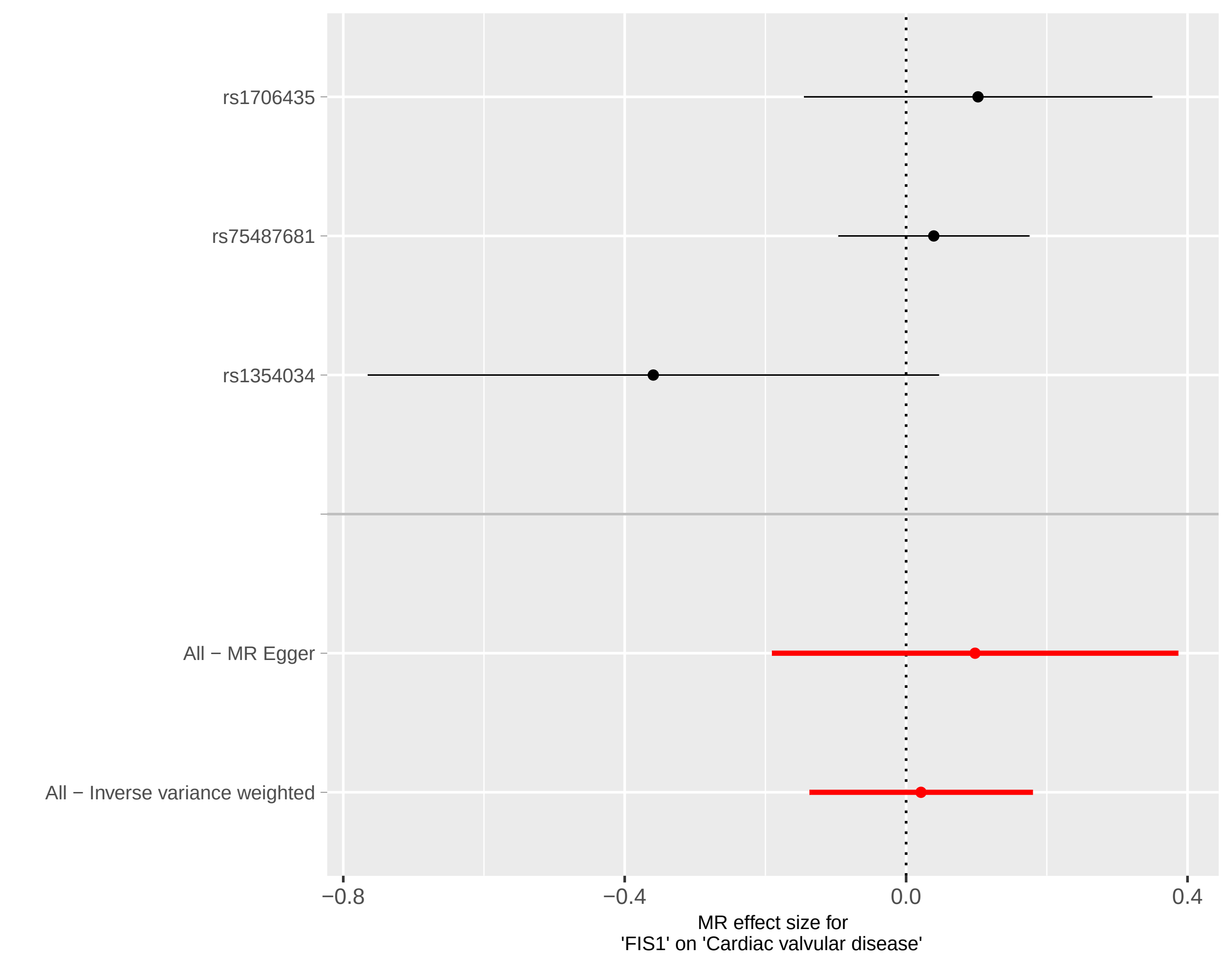
rs1706435
rs75487681
rs1354034
All − MR Egger
All − Inverse variance weighted
−0.8
−0.4
0.0
0.4
MR effect size for
'FIS1' on 'Cardiac valvular disease'

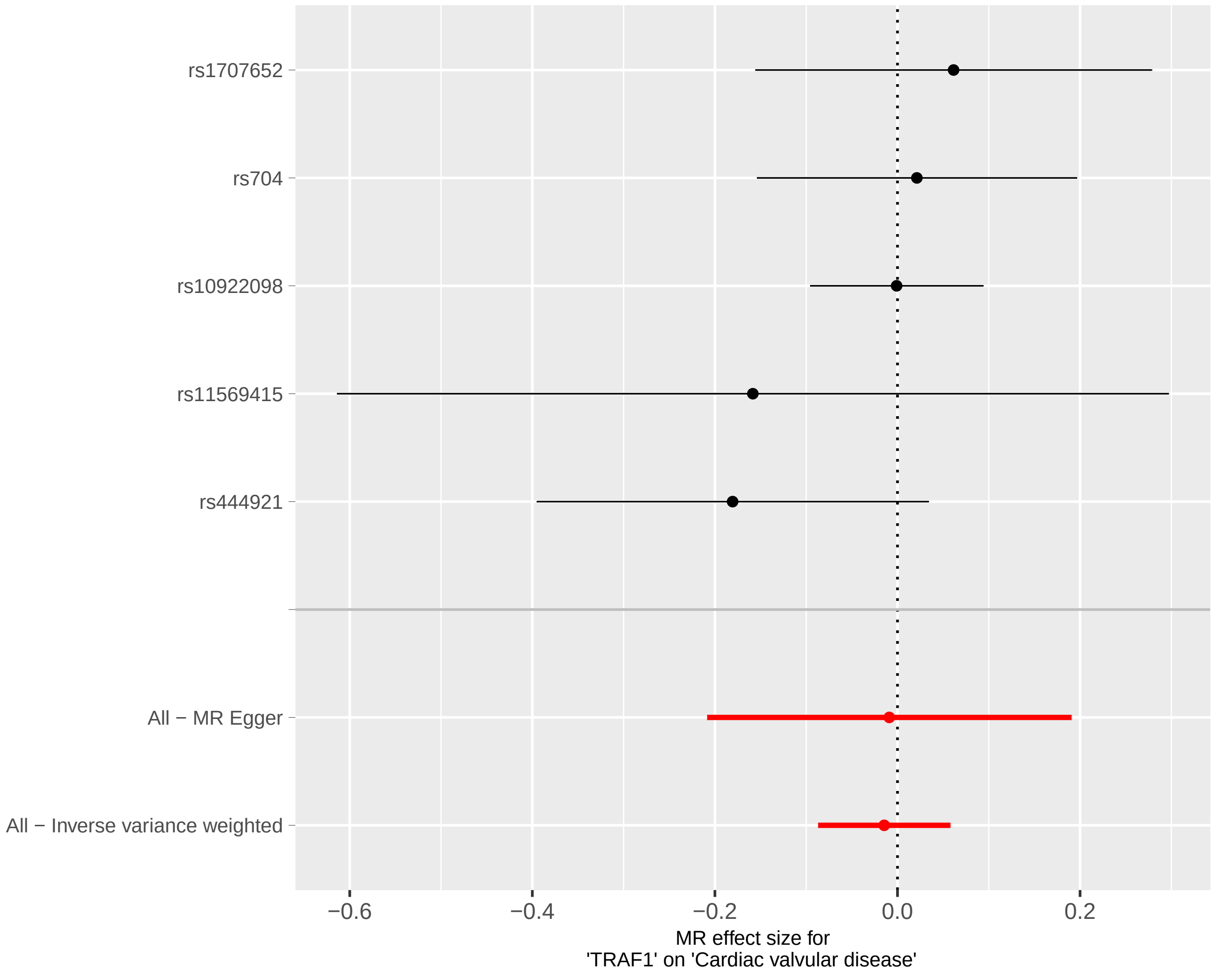
rs1707652
rs704
rs10922098
rs11569415
rs444921
All − MR Egger
All − Inverse variance weighted
−0.6
−0.4
−0.2
0.0
0.2
MR effect size for
'TRAF1' on 'Cardiac valvular disease'

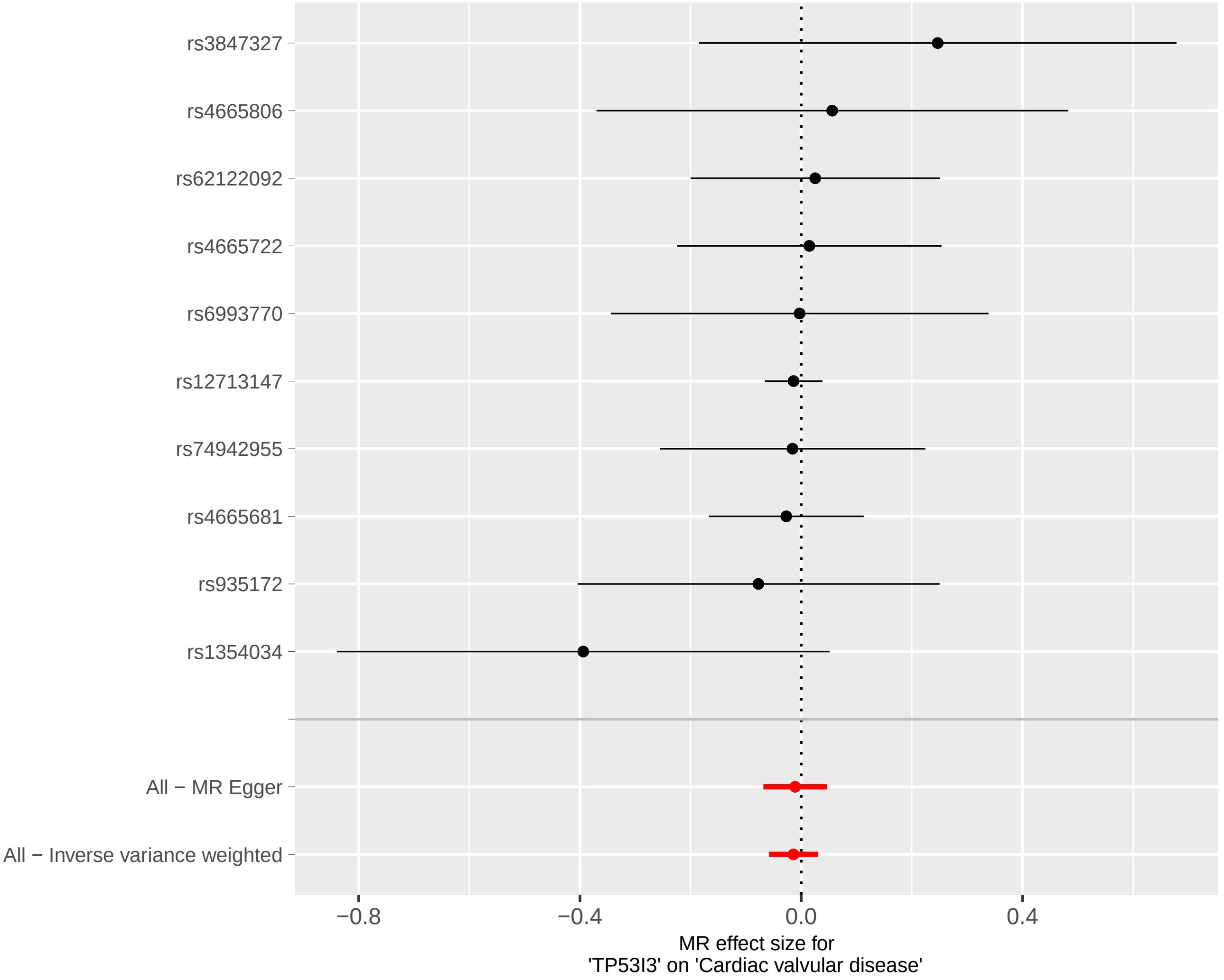
rs3847327
rs4665806
rs62122092
rs4665722
rs6993770
rs12713147
rs74942955
rs4665681
rs935172
rs1354034
All − MR Egger
All − Inverse variance weighted
−0.8
−0.4
0.0
0.4
MR effect size for
'TP53I3' on 'Cardiac valvular disease'

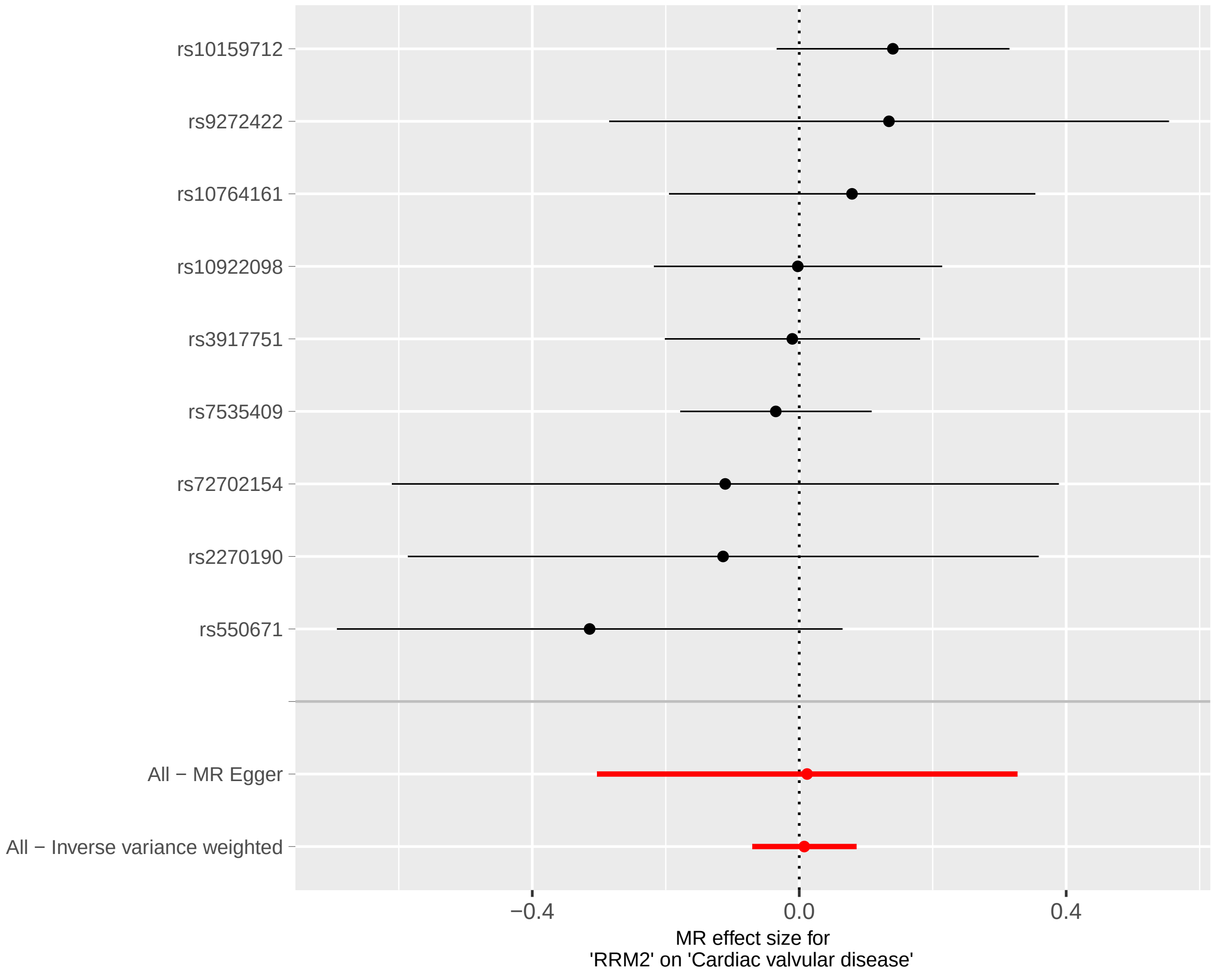
rs10159712
rs9272422
rs10764161
rs10922098
rs3917751
rs7535409
rs72702154
rs2270190
rs550671
All − MR Egger
All − Inverse variance weighted
−0.4
0.0
0.4
MR effect size for
'RRM2' on 'Cardiac valvular disease'

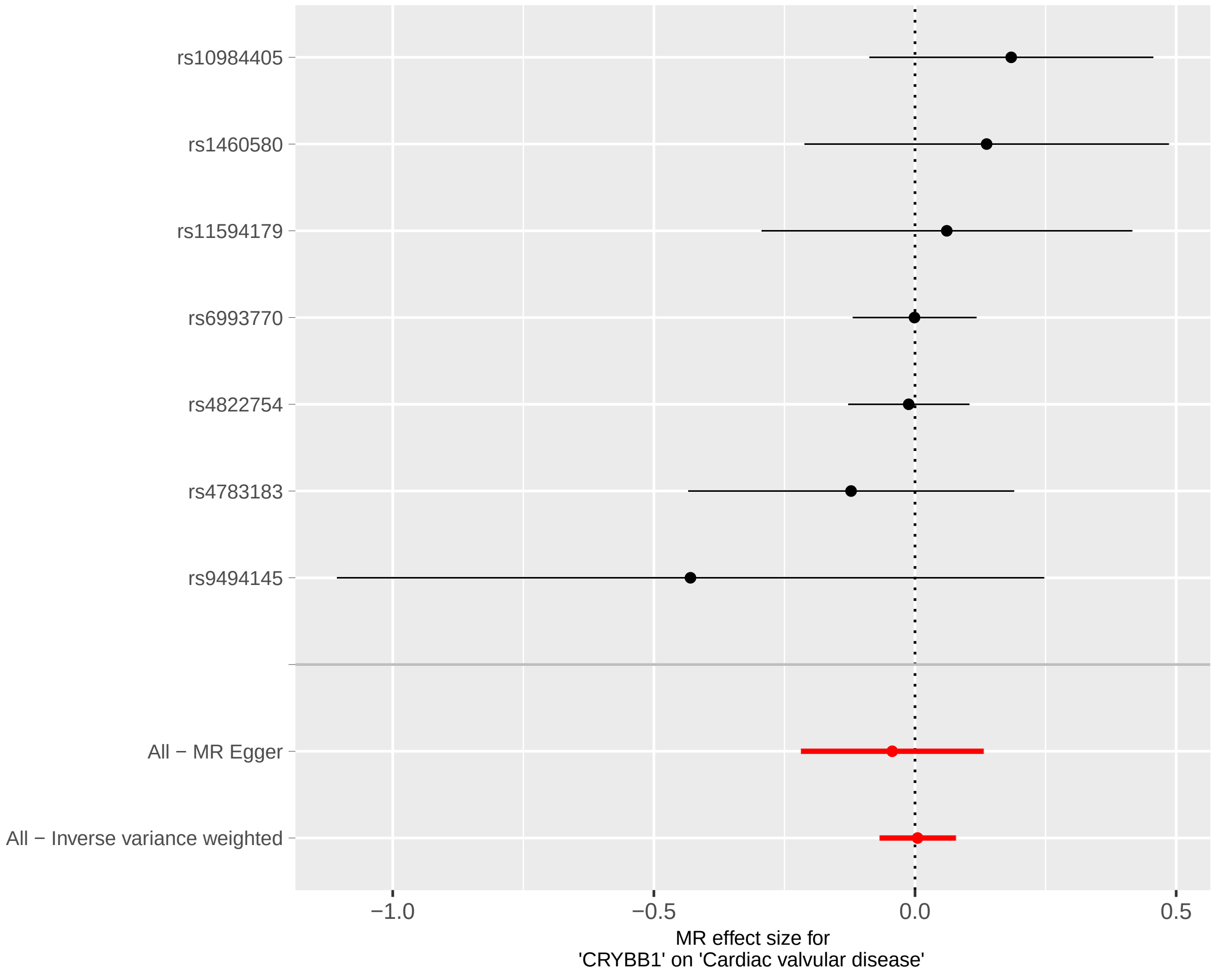

rs10984405
rs1460580
rs11594179
rs6993770
rs4822754
rs4783183
rs9494145
All − MR Egger
All − Inverse variance weighted
−1.0
−0.5
0.0
0.5
MR effect size for
'CRYBB1' on 'Cardiac valvular disease'

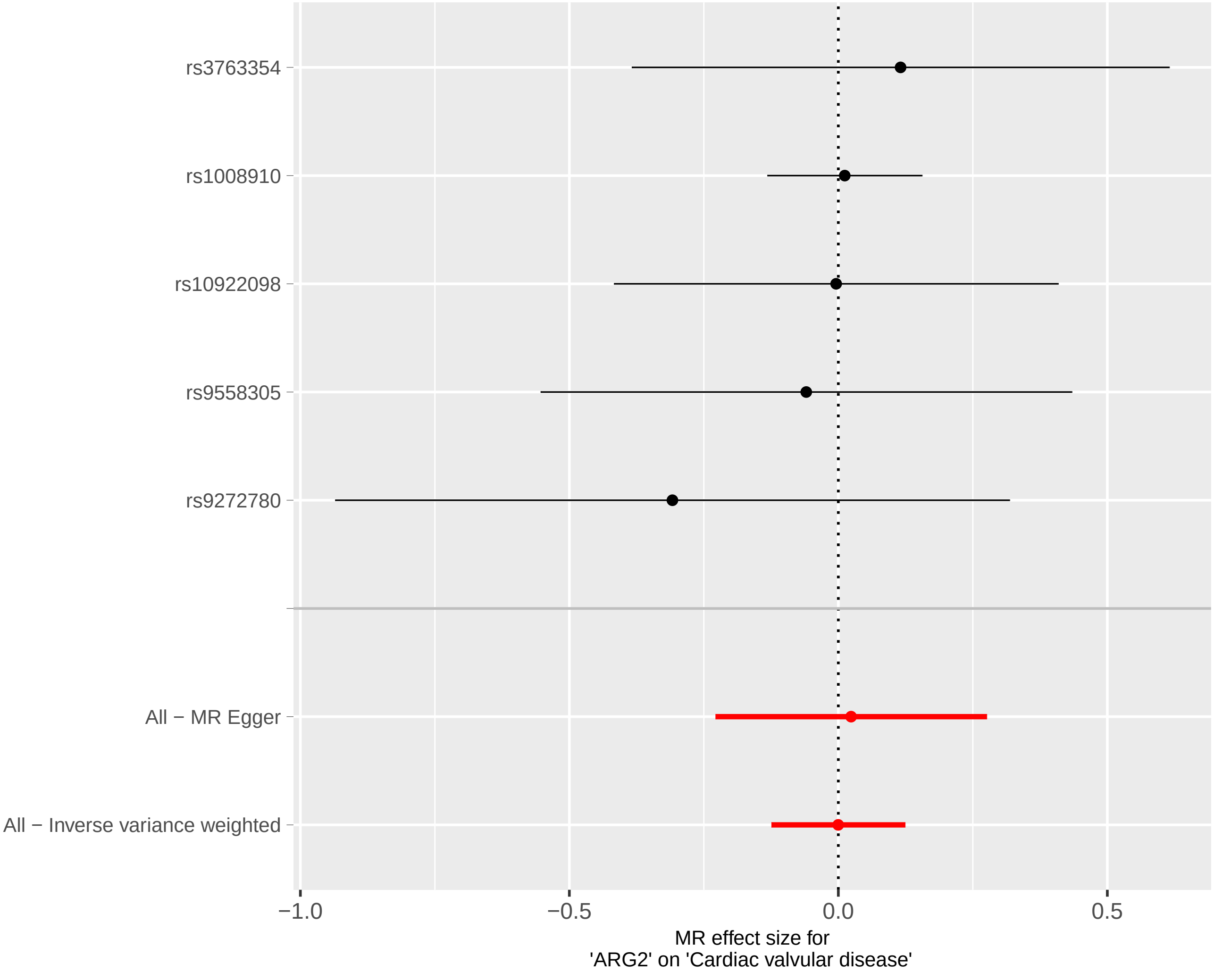
rs3763354
rs1008910
rs10922098
rs9558305
rs9272780
All − MR Egger
All − Inverse variance weighted
−1.0
−0.5
0.0
0.5
MR effect size for
'ARG2' on 'Cardiac valvular disease'

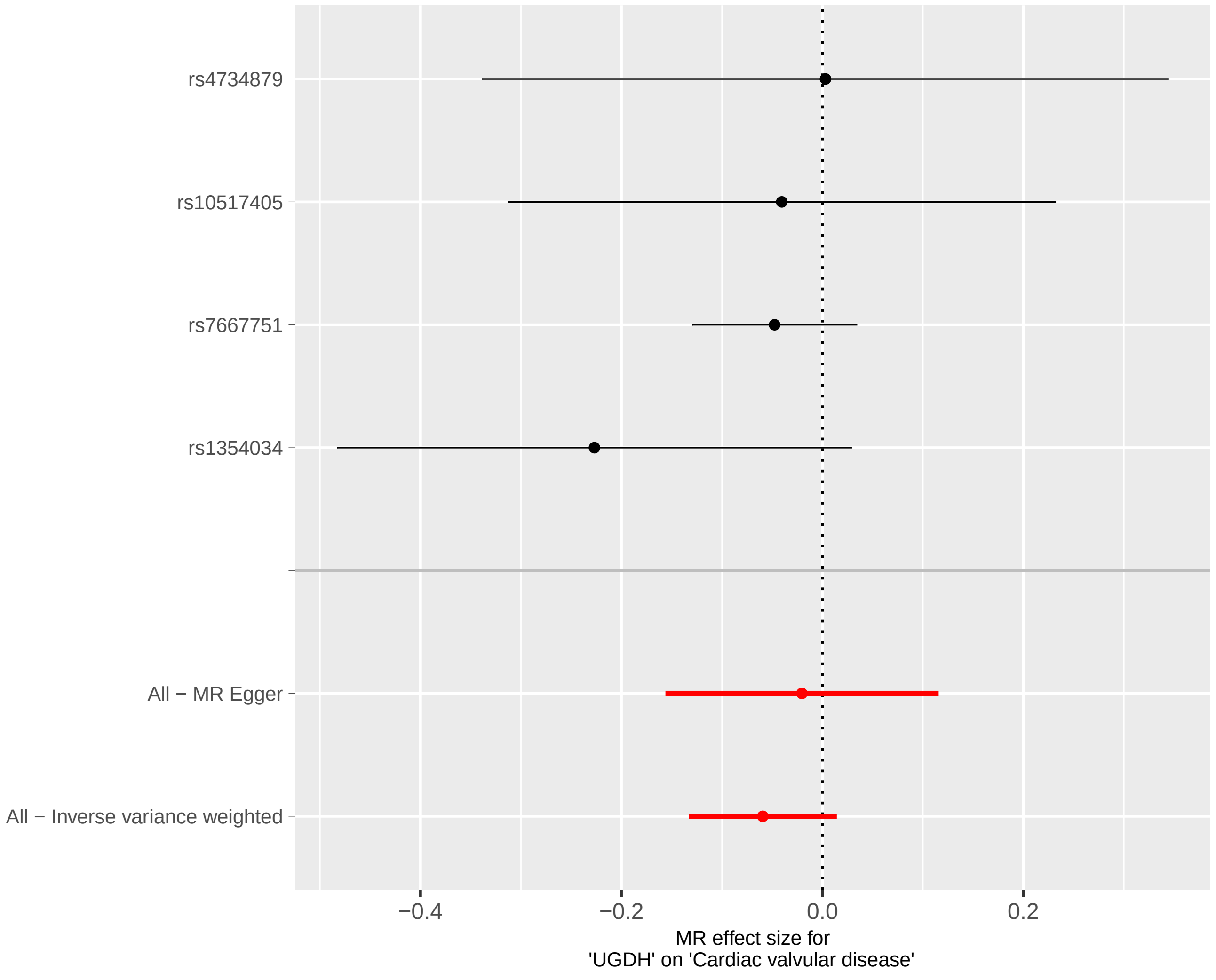
rs4734879
rs10517405
rs7667751
rs1354034
All − MR Egger
All − Inverse variance weighted
−0.4
−0.2
0.0
0.2
MR effect size for
'UGDH' on 'Cardiac valvular disease'

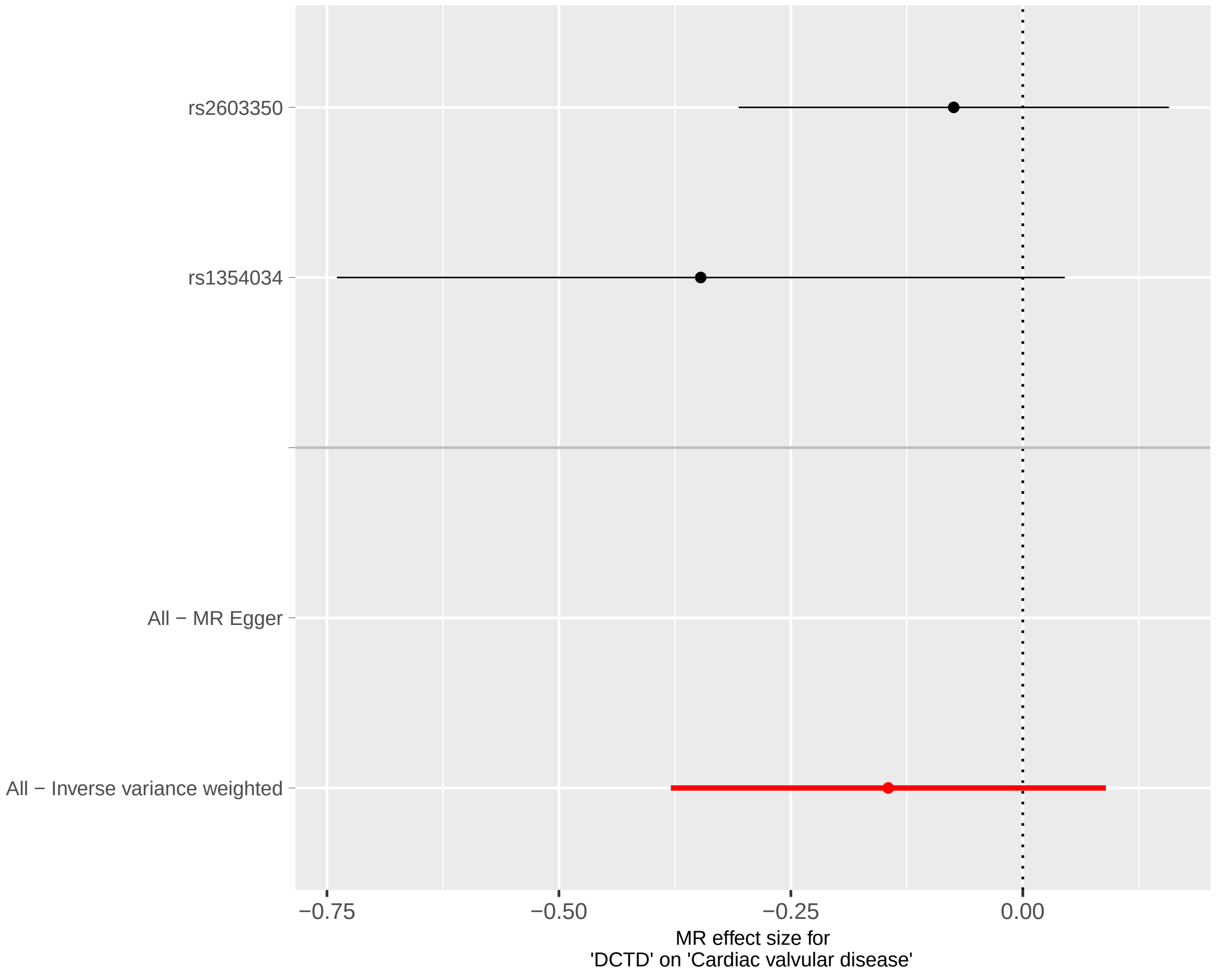
rs2603350
rs1354034
All − MR Egger
All − Inverse variance weighted
−0.75
−0.50
−0.25
0.00
MR effect size for
'DCTD' on 'Cardiac valvular disease'

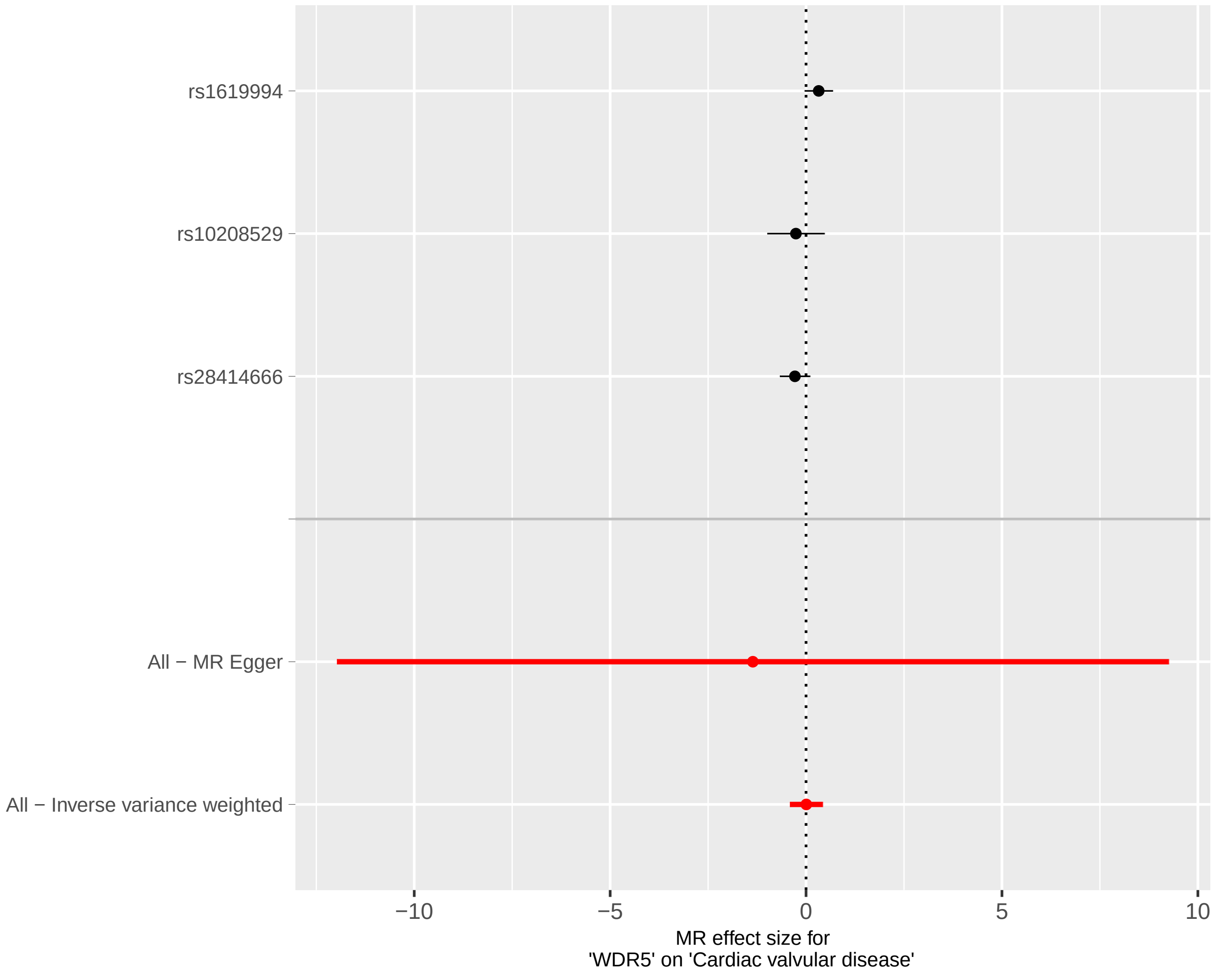

rs1619994
rs10208529
rs28414666
All − MR Egger
All − Inverse variance weighted
−10
−5
0
5
10
MR effect size for
'WDR5' on 'Cardiac valvular disease'

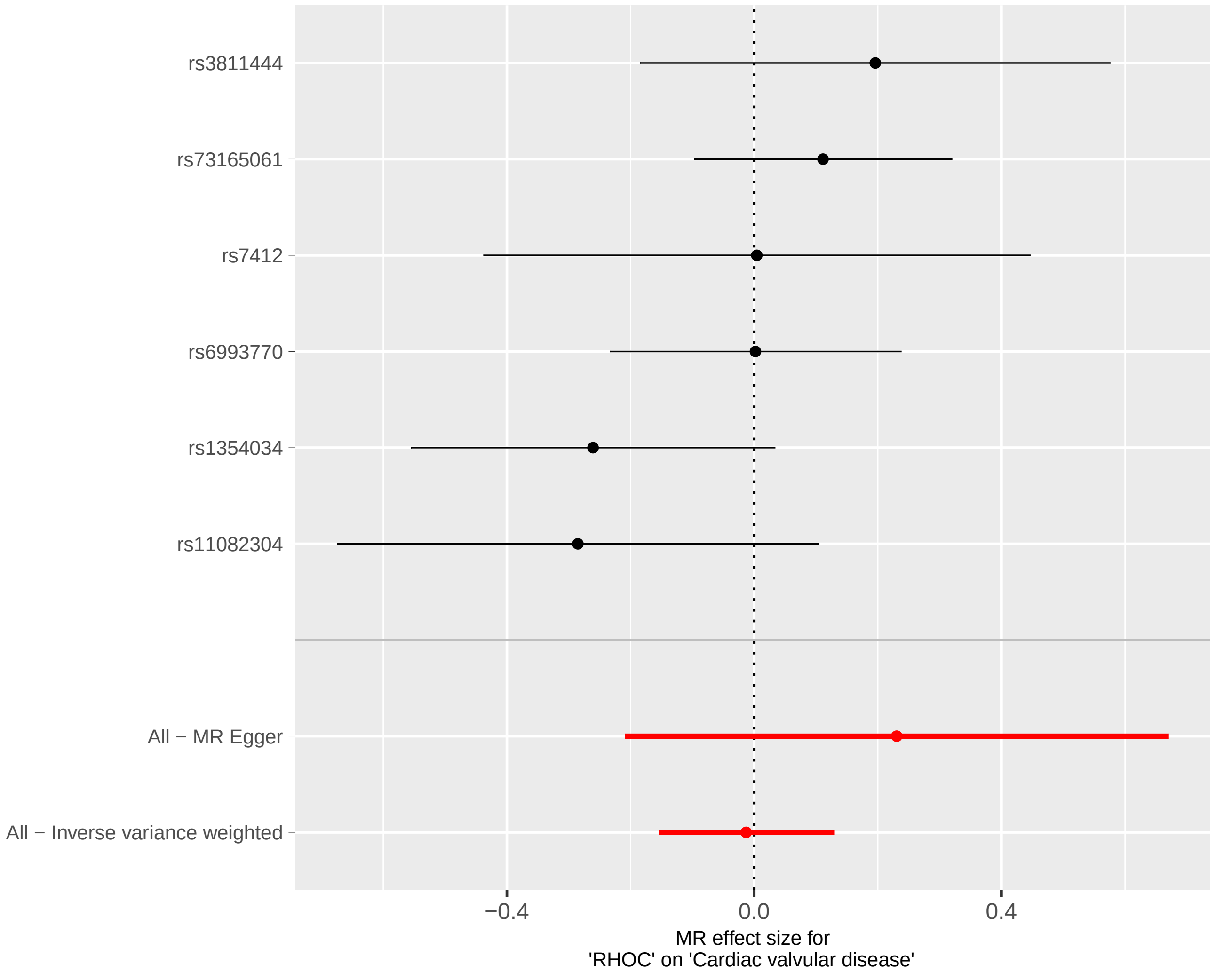

rs3811444
rs73165061
rs7412
rs6993770
rs1354034
rs11082304
All − MR Egger
All − Inverse variance weighted
−0.4
0.0
0.4
MR effect size for
'RHOC' on 'Cardiac valvular disease'

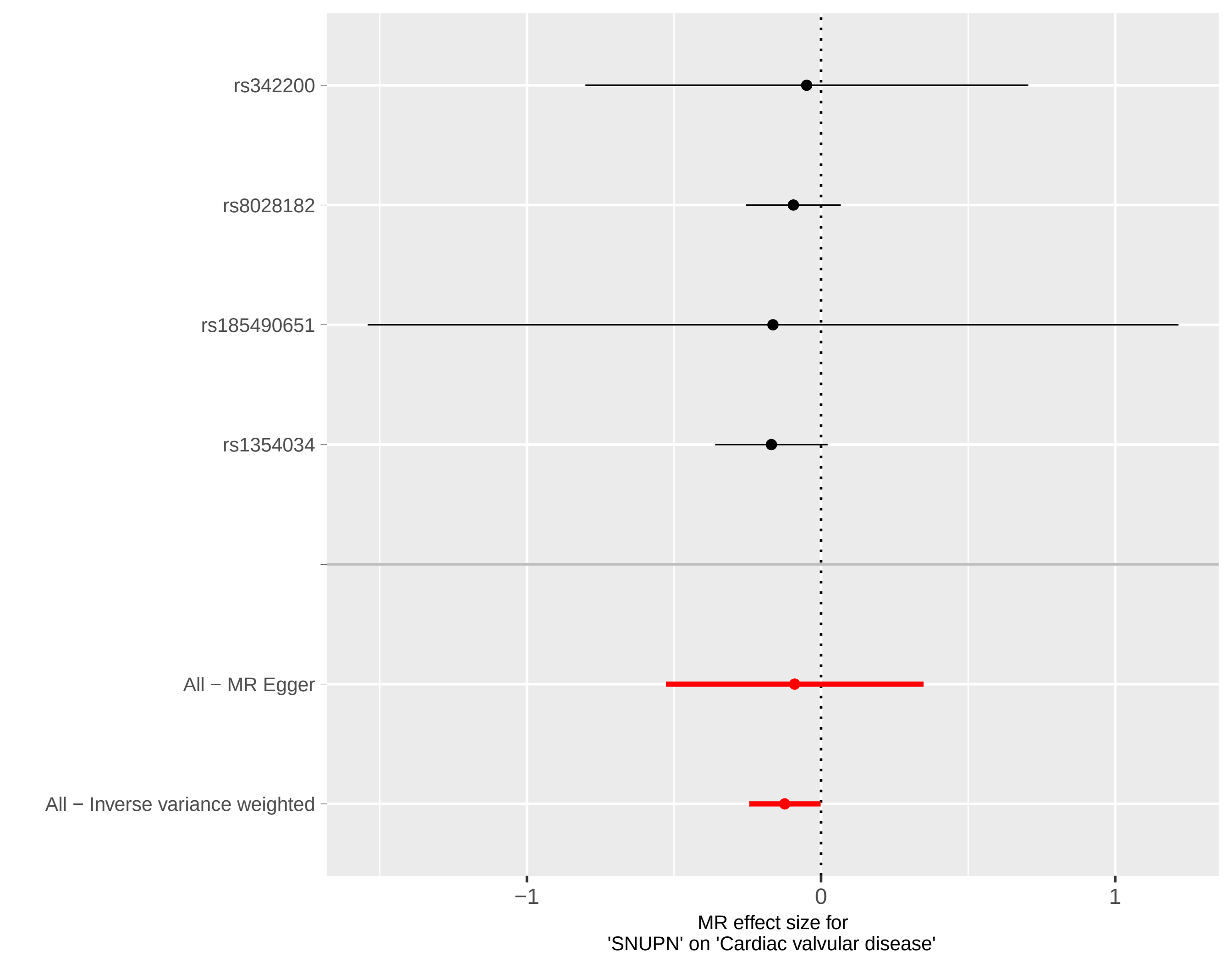
rs342200
rs8028182
rs185490651
rs1354034
All − MR Egger
All − Inverse variance weighted
−1
0
1
MR effect size for
'SNUPN' on 'Cardiac valvular disease'

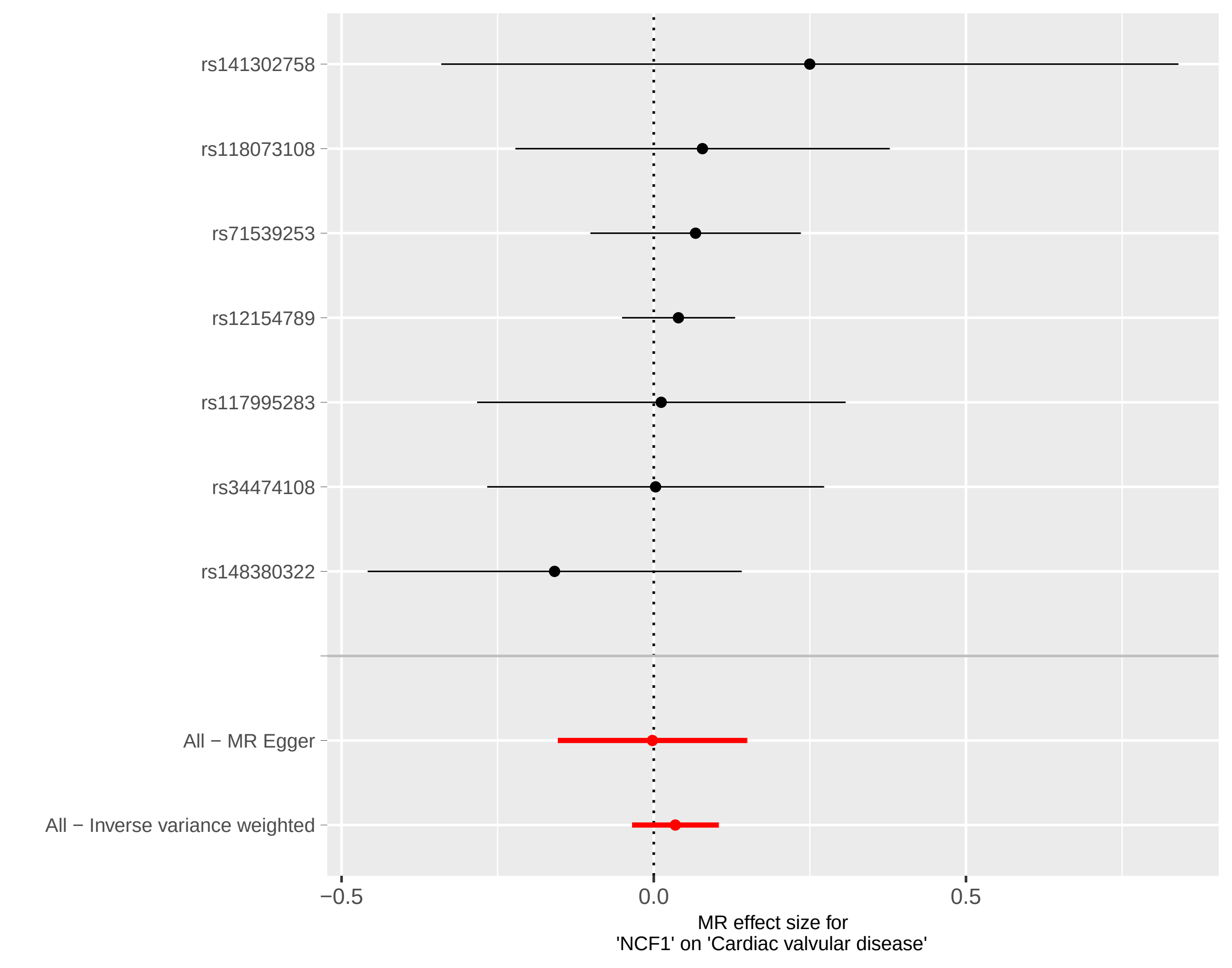
rs141302758
rs118073108
rs71539253
rs12154789
rs117995283
rs34474108
rs148380322
All − MR Egger
All − Inverse variance weighted
−0.5
0.0
0.5
MR effect size for
'NCF1' on 'Cardiac valvular disease'

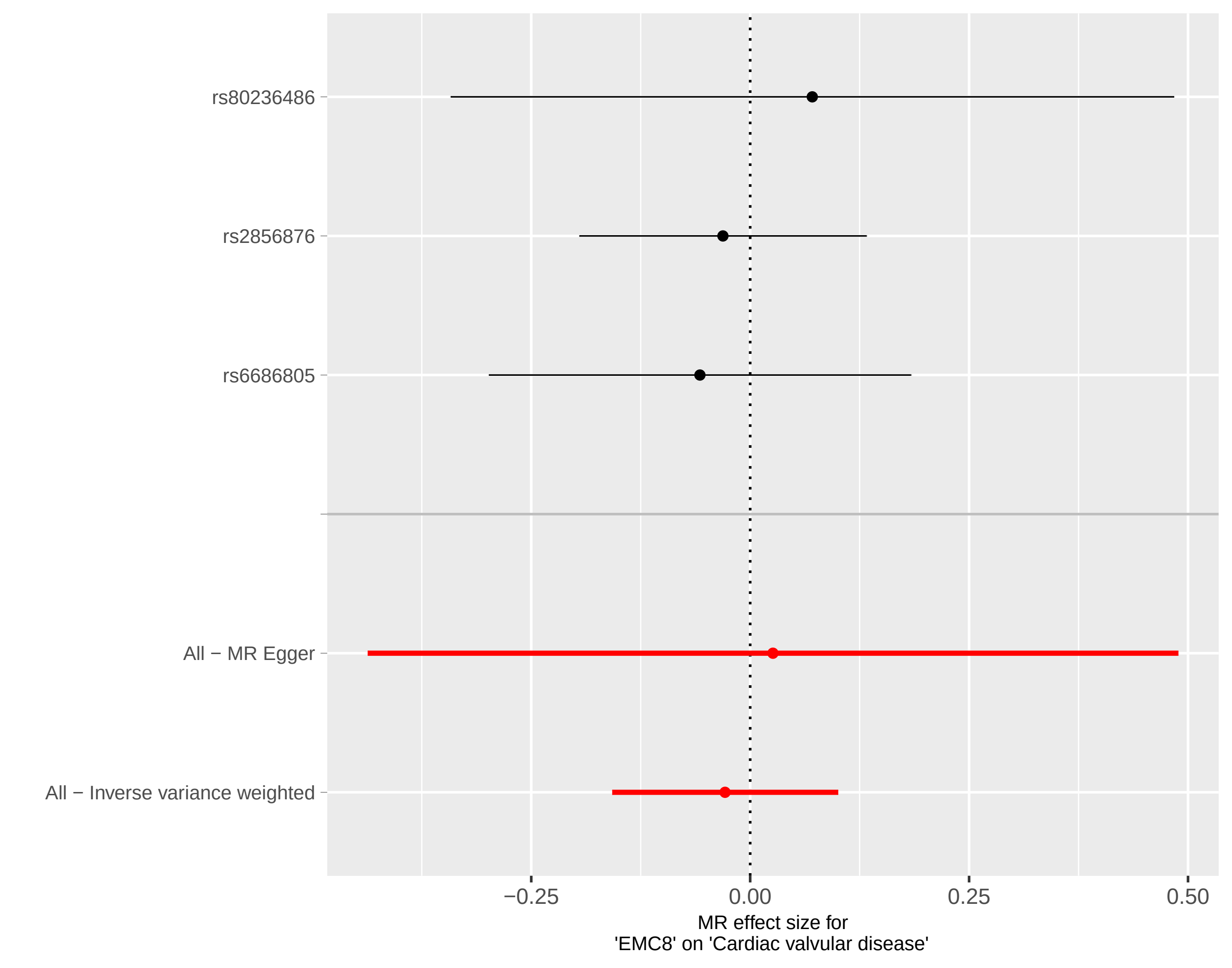
rs80236486
rs2856876
rs6686805
All − MR Egger
All − Inverse variance weighted
−0.25
0.00
0.25
0.50
MR effect size for
'EMC8' on 'Cardiac valvular disease'

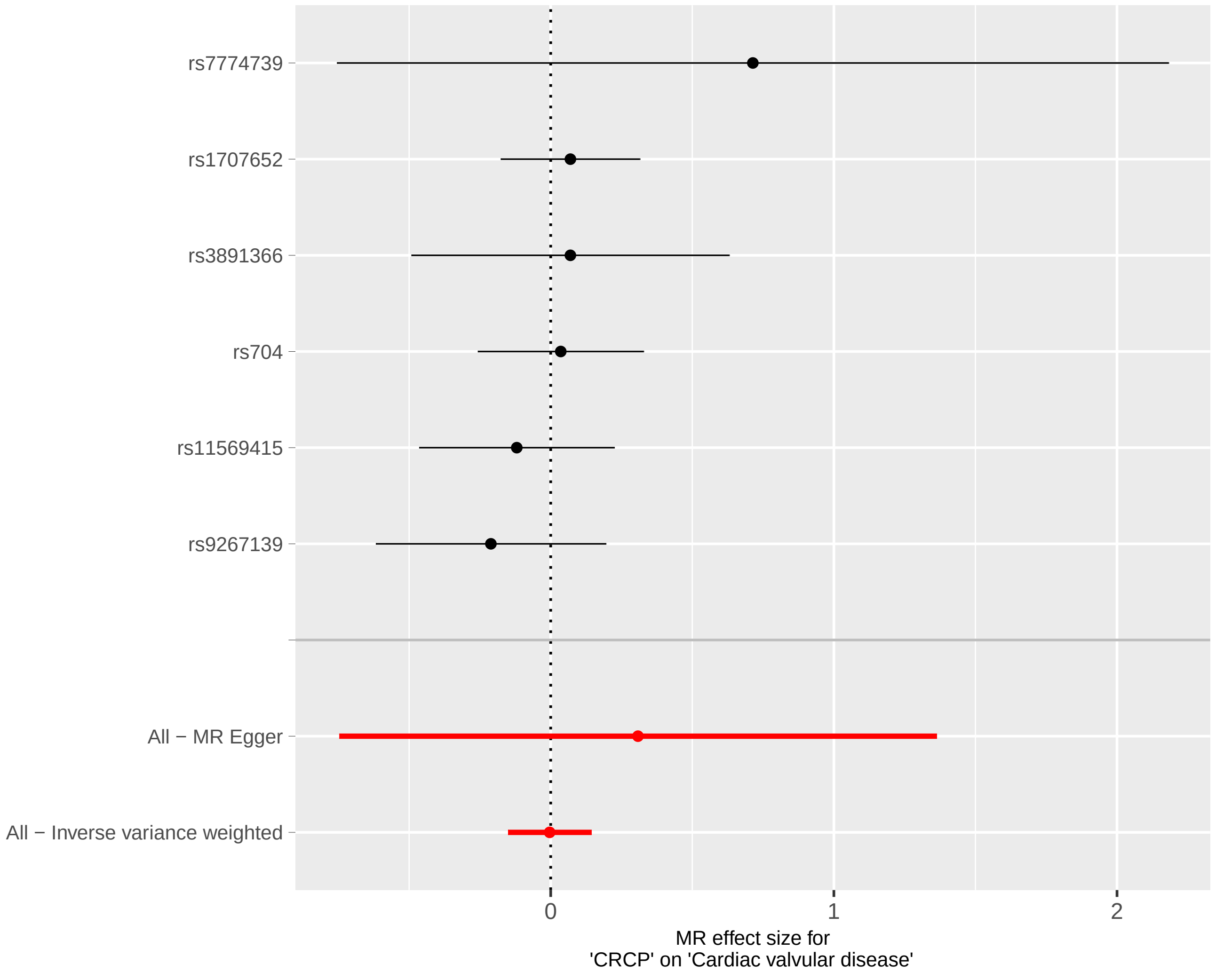
rs7774739
rs1707652
rs3891366
rs704
rs11569415
rs9267139
All − MR Egger
All − Inverse variance weighted
0
1
2
MR effect size for
'CRCP' on 'Cardiac valvular disease'

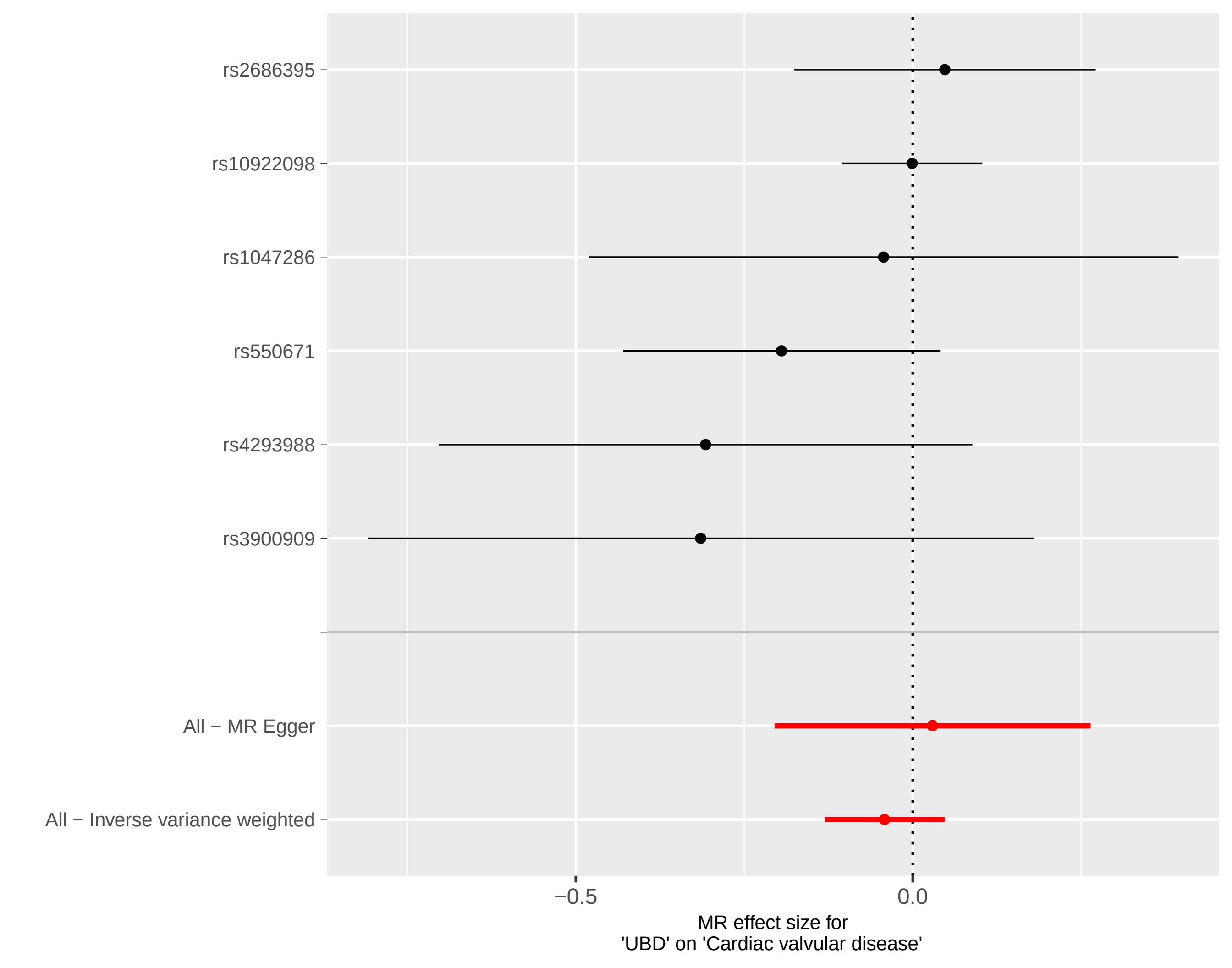
rs2686395
rs10922098
rs1047286
rs550671
rs4293988
rs3900909
All − MR Egger
All − Inverse variance weighted
−0.5
0.0
MR effect size for
'UBD' on 'Cardiac valvular disease'

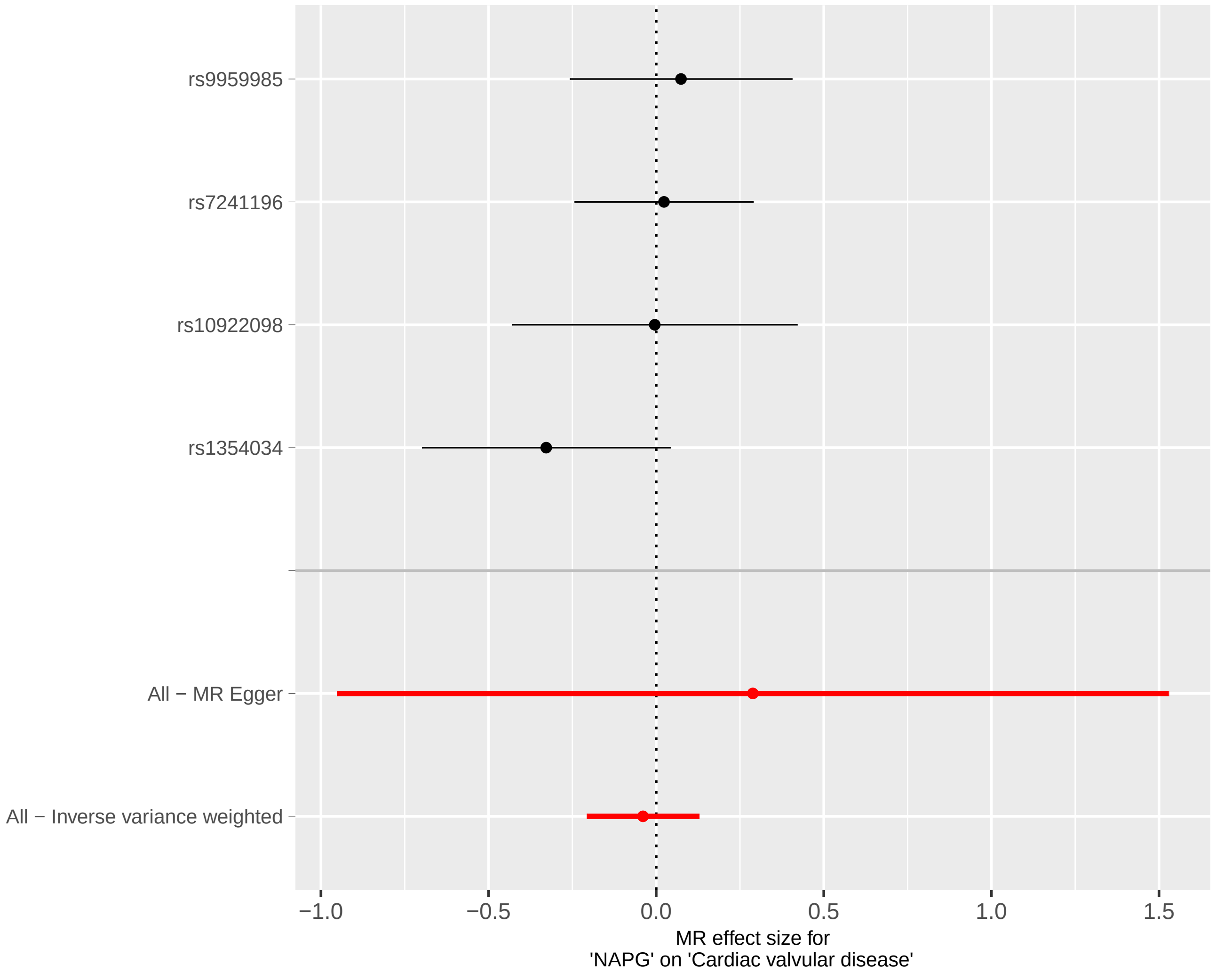
rs9959985
rs7241196
rs10922098
rs1354034
All − MR Egger
All − Inverse variance weighted
−1.0
−0.5
0.0
0.5
1.0
1.5
MR effect size for
'NAPG' on 'Cardiac valvular disease'

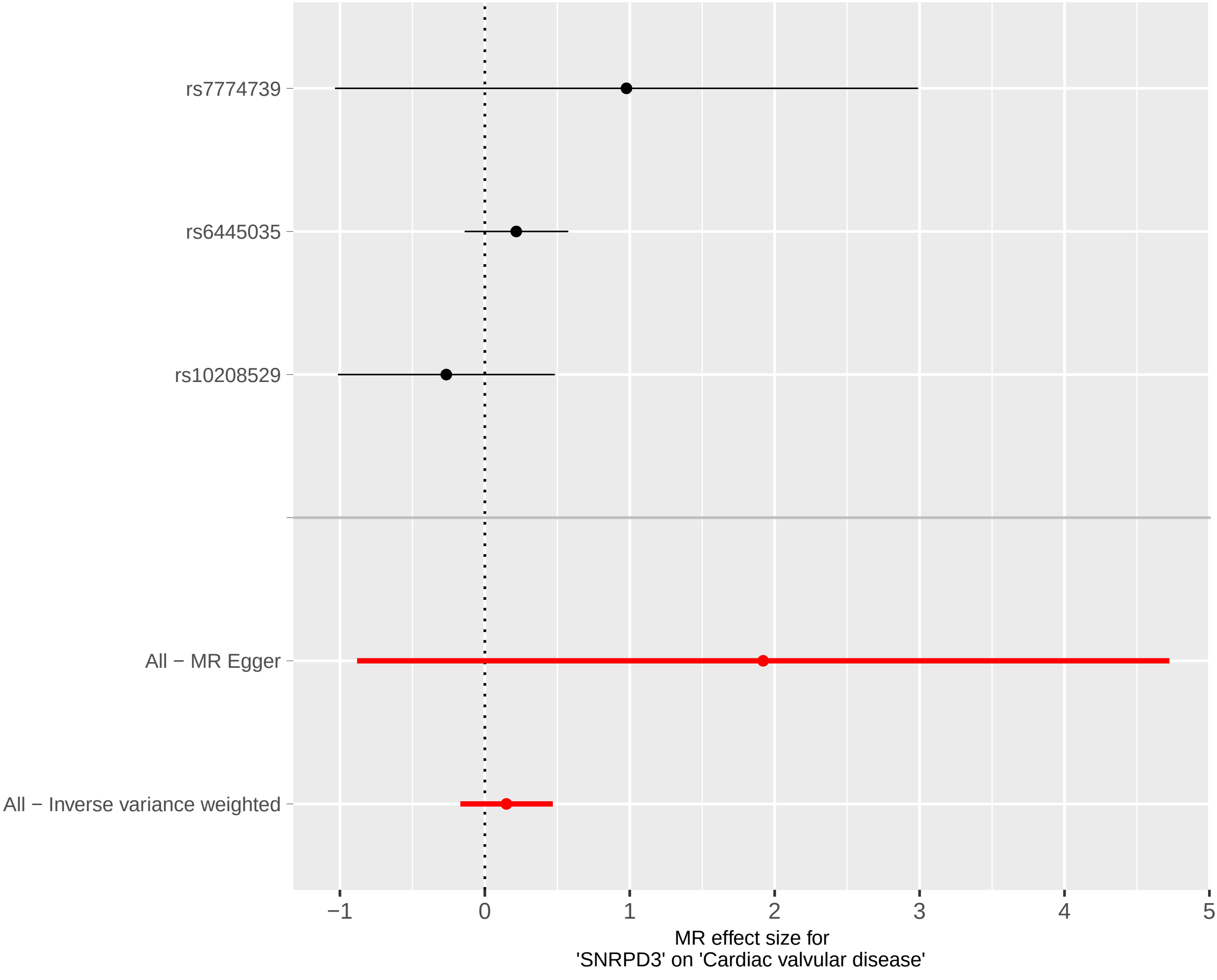
rs7774739
rs6445035
rs10208529
All − MR Egger
All − Inverse variance weighted
−1
0
1
2
3
4
5
MR effect size for
'SNRPD3' on 'Cardiac valvular disease'

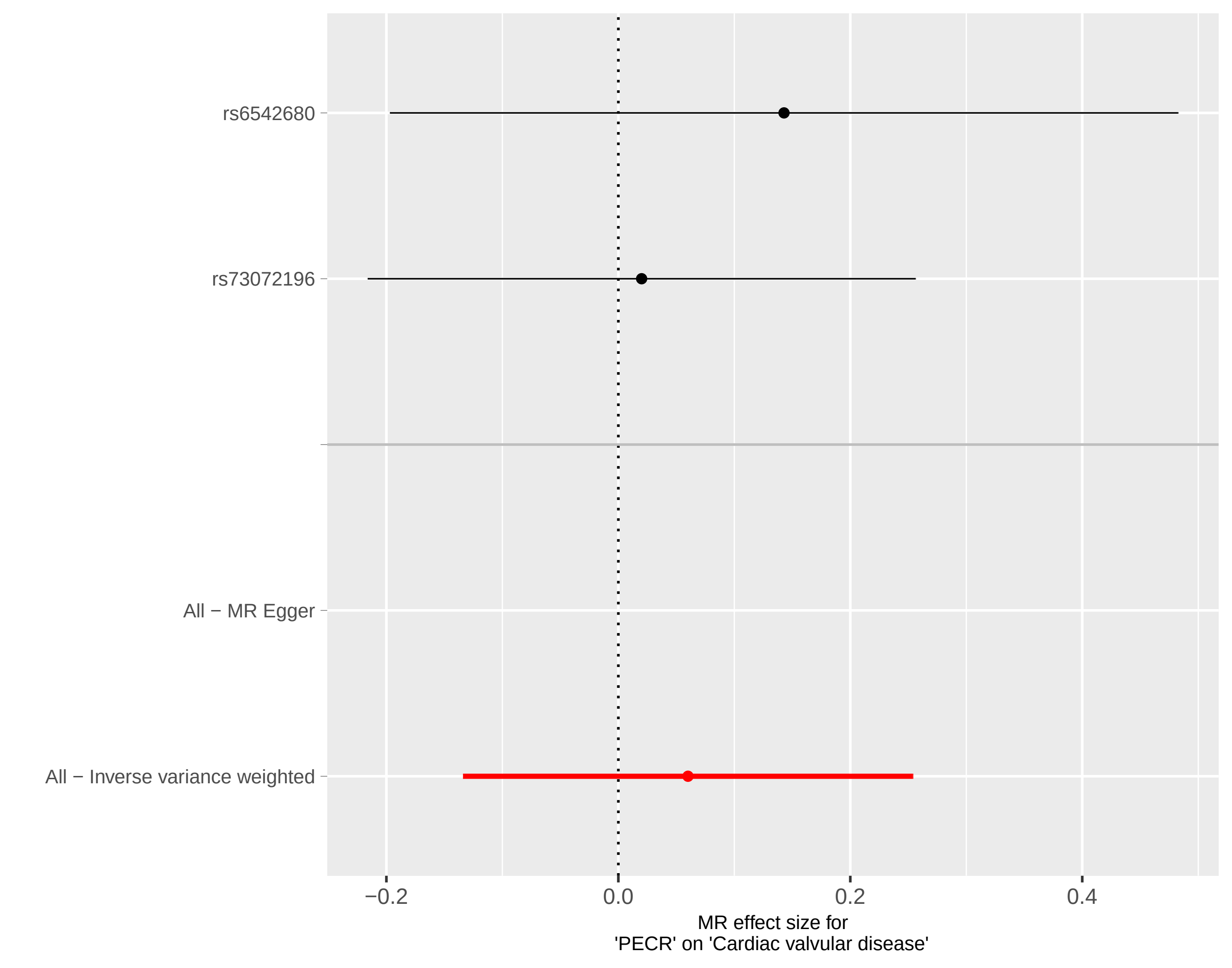
rs6542680
rs73072196
All − MR Egger
All − Inverse variance weighted
−0.2
0.0
0.2
0.4
MR effect size for
'PECR' on 'Cardiac valvular disease'

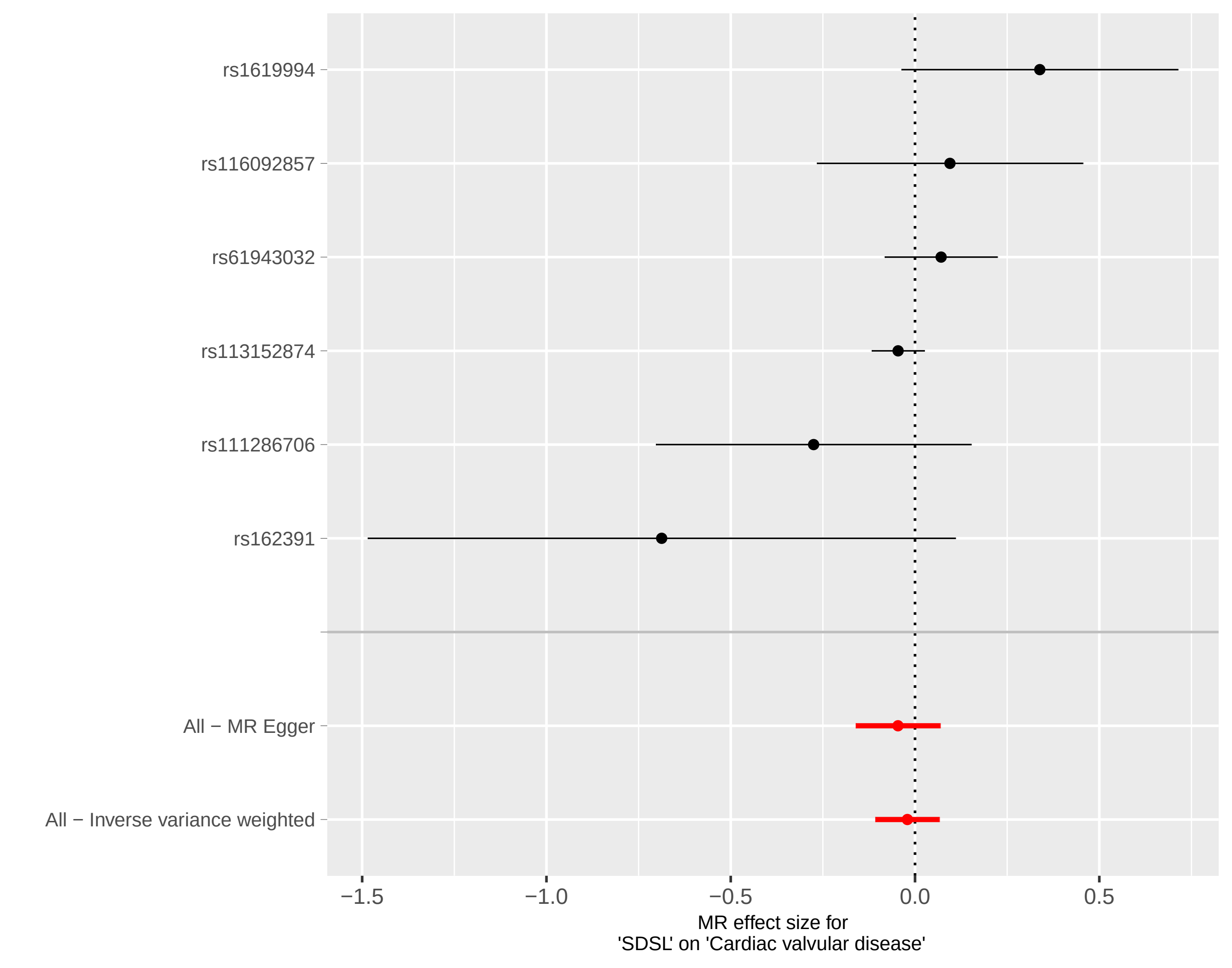
rs1619994
rs116092857
rs61943032
rs113152874
rs111286706
rs162391
All − MR Egger
All − Inverse variance weighted
−1.5
−1.0
−0.5
0.0
0.5
MR effect size for
'SDSL' on 'Cardiac valvular disease'

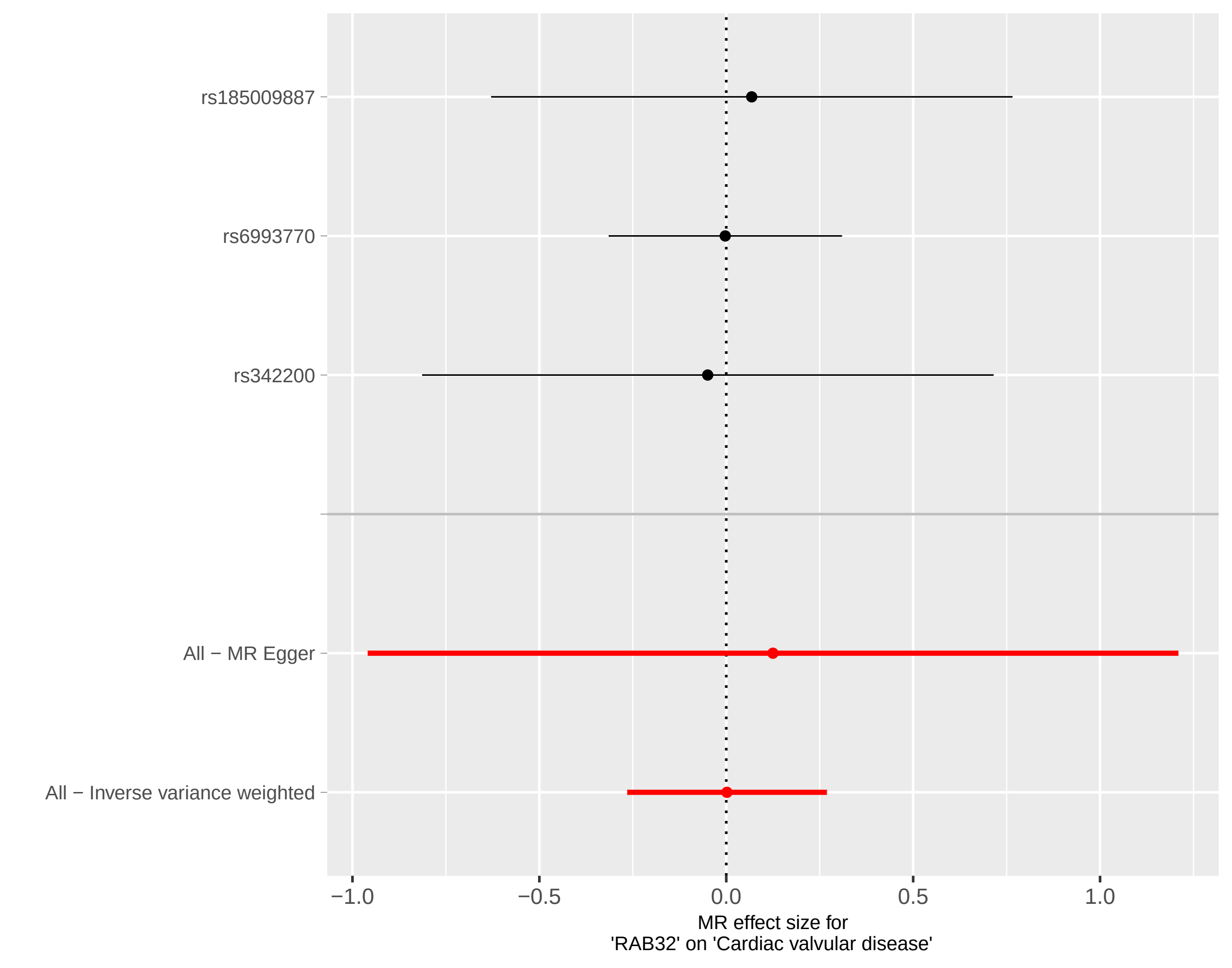
rs185009887
rs6993770
rs342200
All − MR Egger
All − Inverse variance weighted
−1.0
−0.5
0.0
0.5
1.0
MR effect size for
'RAB32' on 'Cardiac valvular disease'

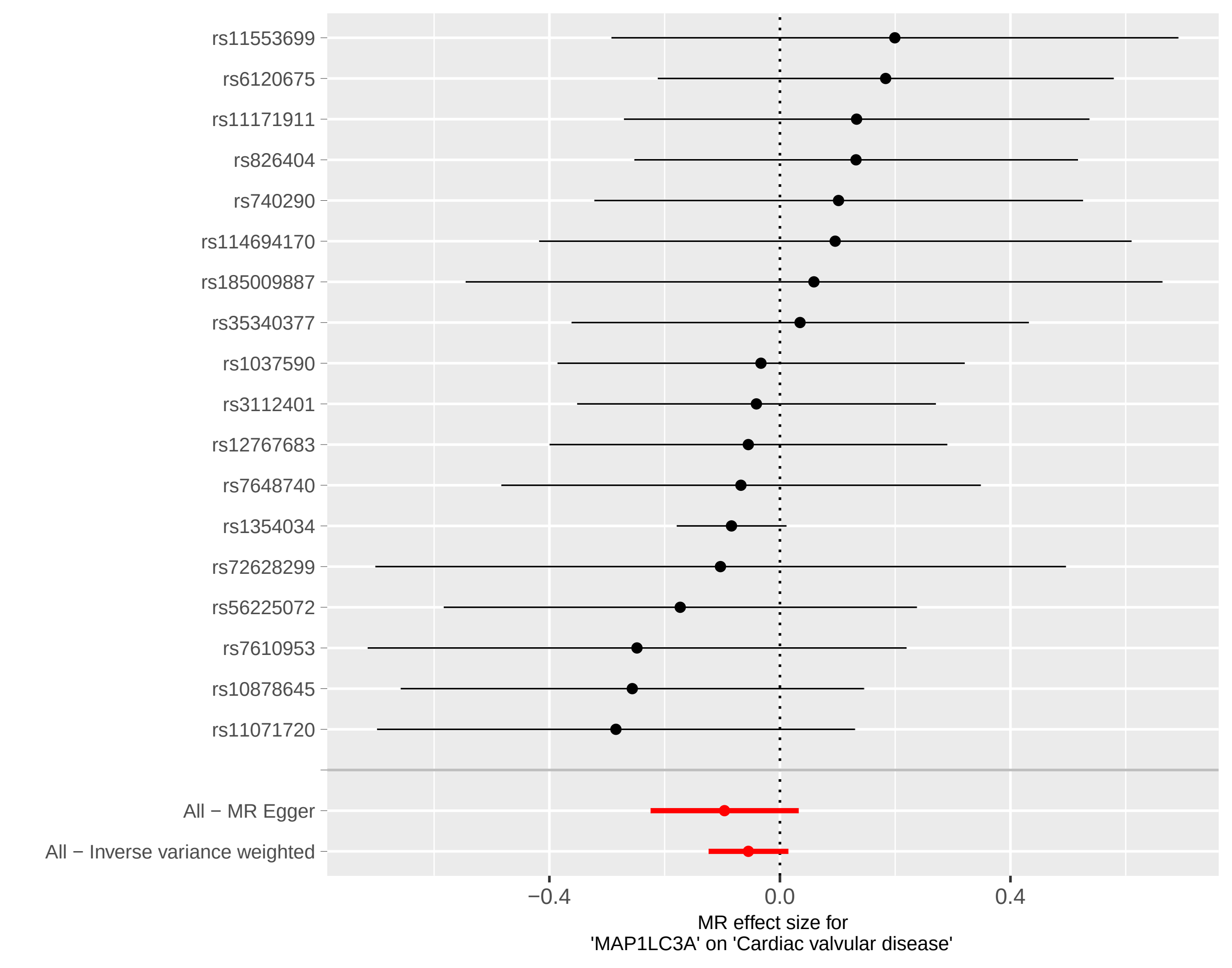
rs11553699
rs6120675
rs11171911
rs826404
rs740290
rs114694170
rs185009887
rs35340377
rs1037590
rs3112401
rs12767683
rs7648740
rs1354034
rs72628299
rs56225072
rs7610953
rs10878645
rs11071720
All − MR Egger
All − Inverse variance weighted
−0.4
0.0
0.4
MR effect size for
'MAP1LC3A' on 'Cardiac valvular disease'

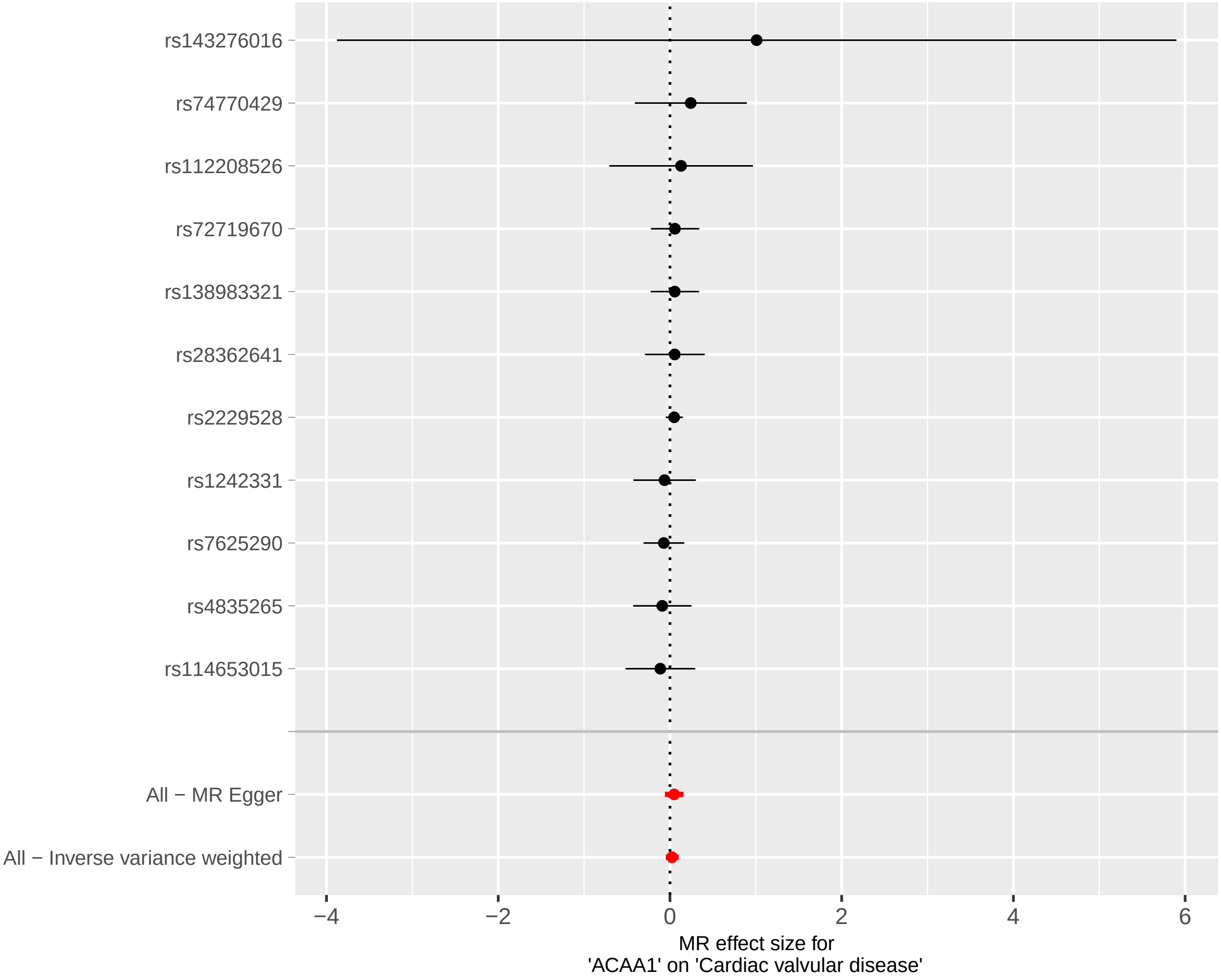

rs143276016
rs74770429
rs112208526
rs72719670
rs138983321
rs28362641
rs2229528
rs1242331
rs7625290
rs4835265
rs114653015
All − MR Egger
All − Inverse variance weighted
−4
−2
0
2
4
6
MR effect size for
'ACAA1' on 'Cardiac valvular disease'

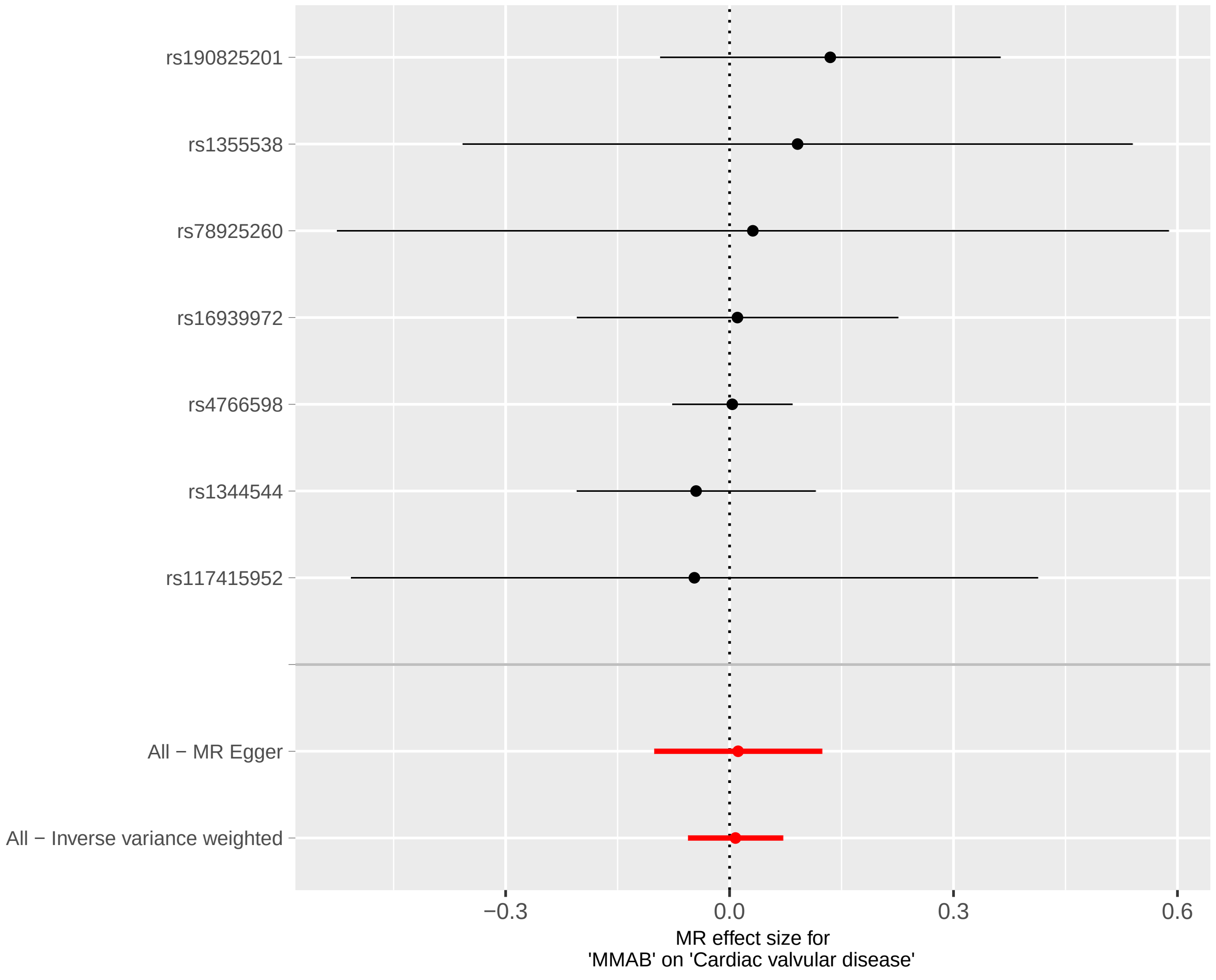
rs190825201
rs1355538
rs78925260
rs16939972
rs4766598
rs1344544
rs117415952
All − MR Egger
All − Inverse variance weighted
−0.3
0.0
0.3
0.6
MR effect size for
'MMAB' on 'Cardiac valvular disease'

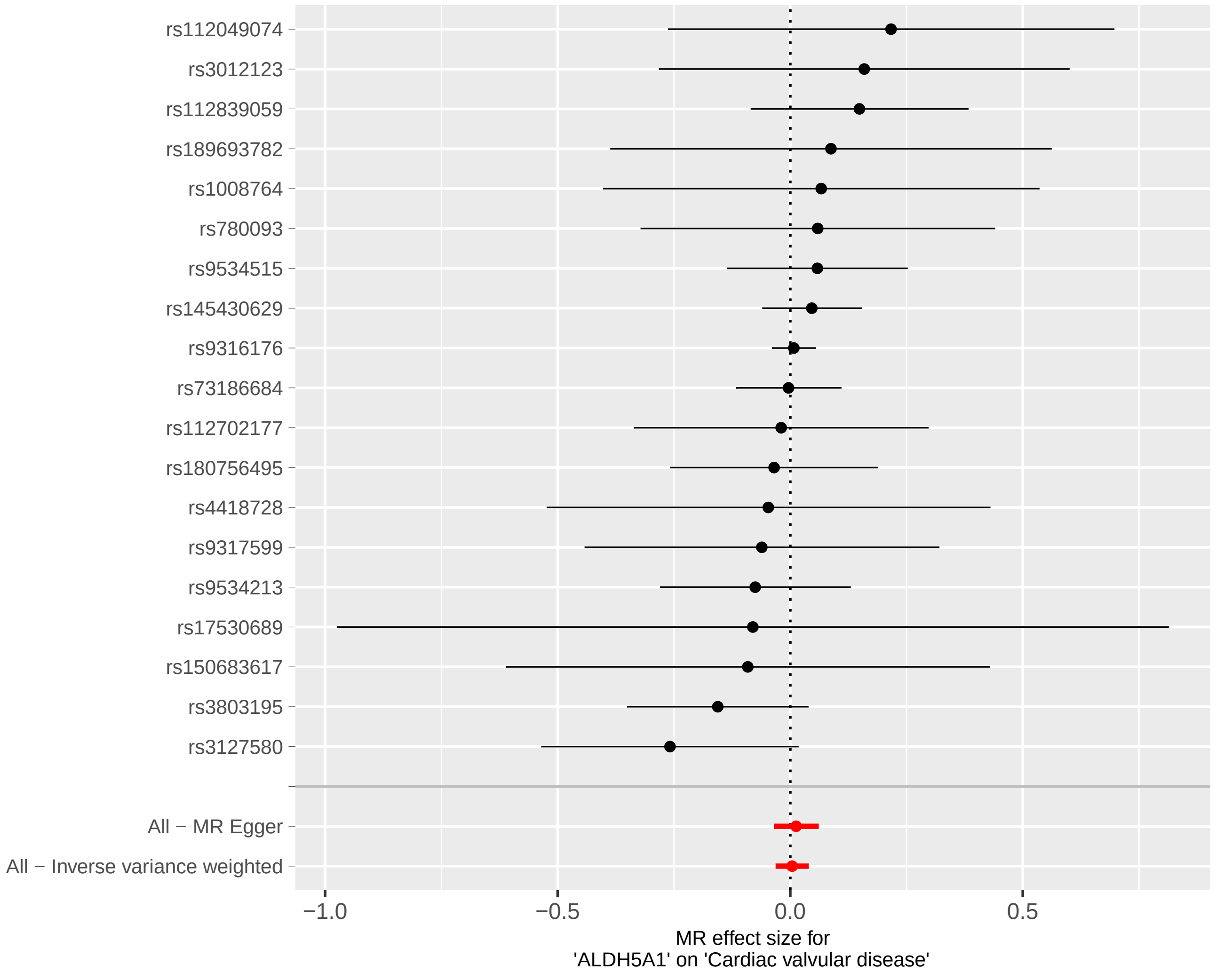
rs112049074
rs3012123
rs112839059
rs189693782
rs1008764
rs780093
rs9534515
rs145430629
rs9316176
rs73186684
rs112702177
rs180756495
rs4418728
rs9317599
rs9534213
rs17530689
rs150683617
rs3803195
rs3127580
All − MR Egger
All − Inverse variance weighted
−1.0
−0.5
0.0
0.5
MR effect size for
'ALDH5A1' on 'Cardiac valvular disease'

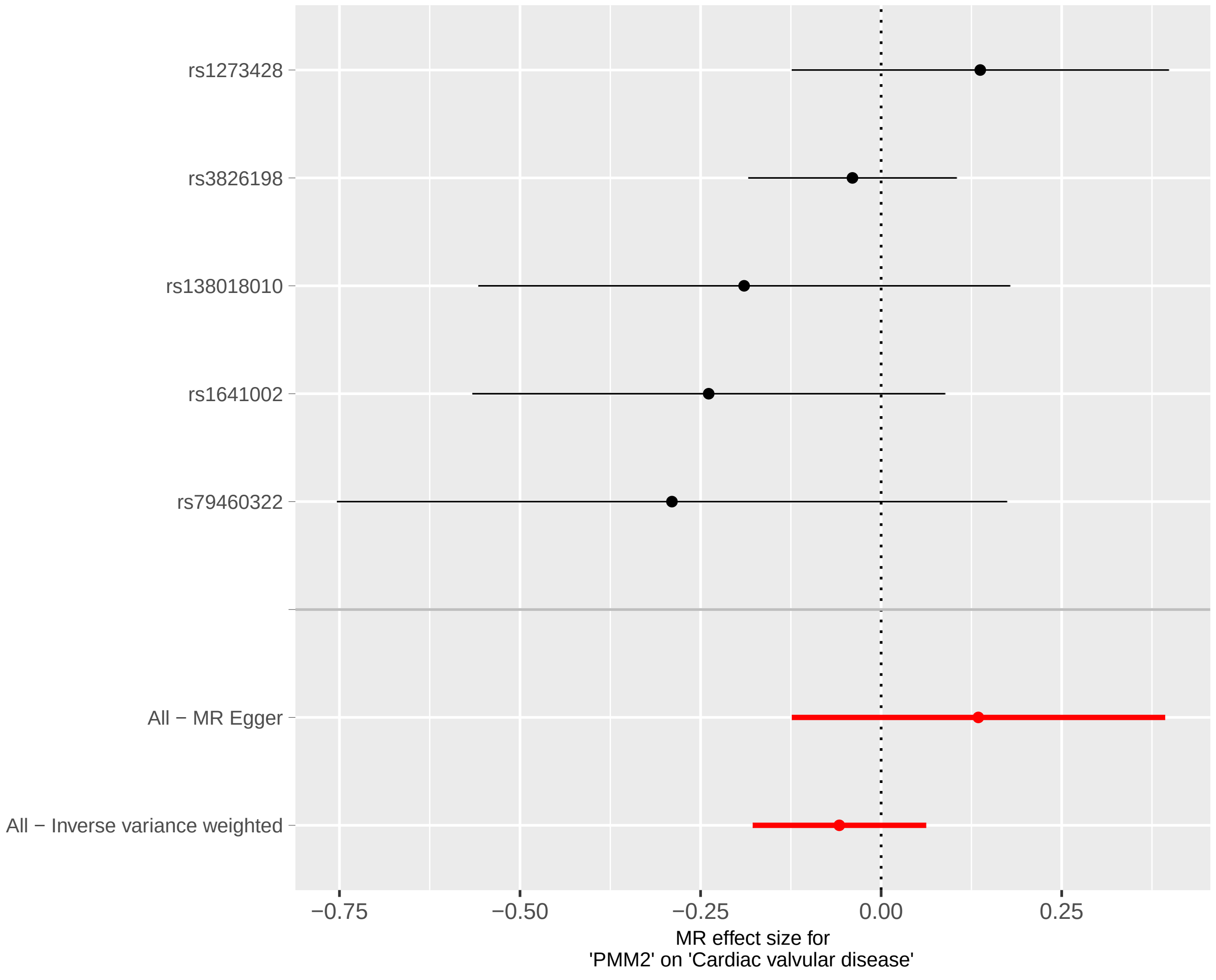
rs1273428
rs3826198
rs138018010
rs1641002
rs79460322
All − MR Egger
All − Inverse variance weighted
−0.75
−0.50
−0.25
0.00
0.25
MR effect size for
'PMM2' on 'Cardiac valvular disease'

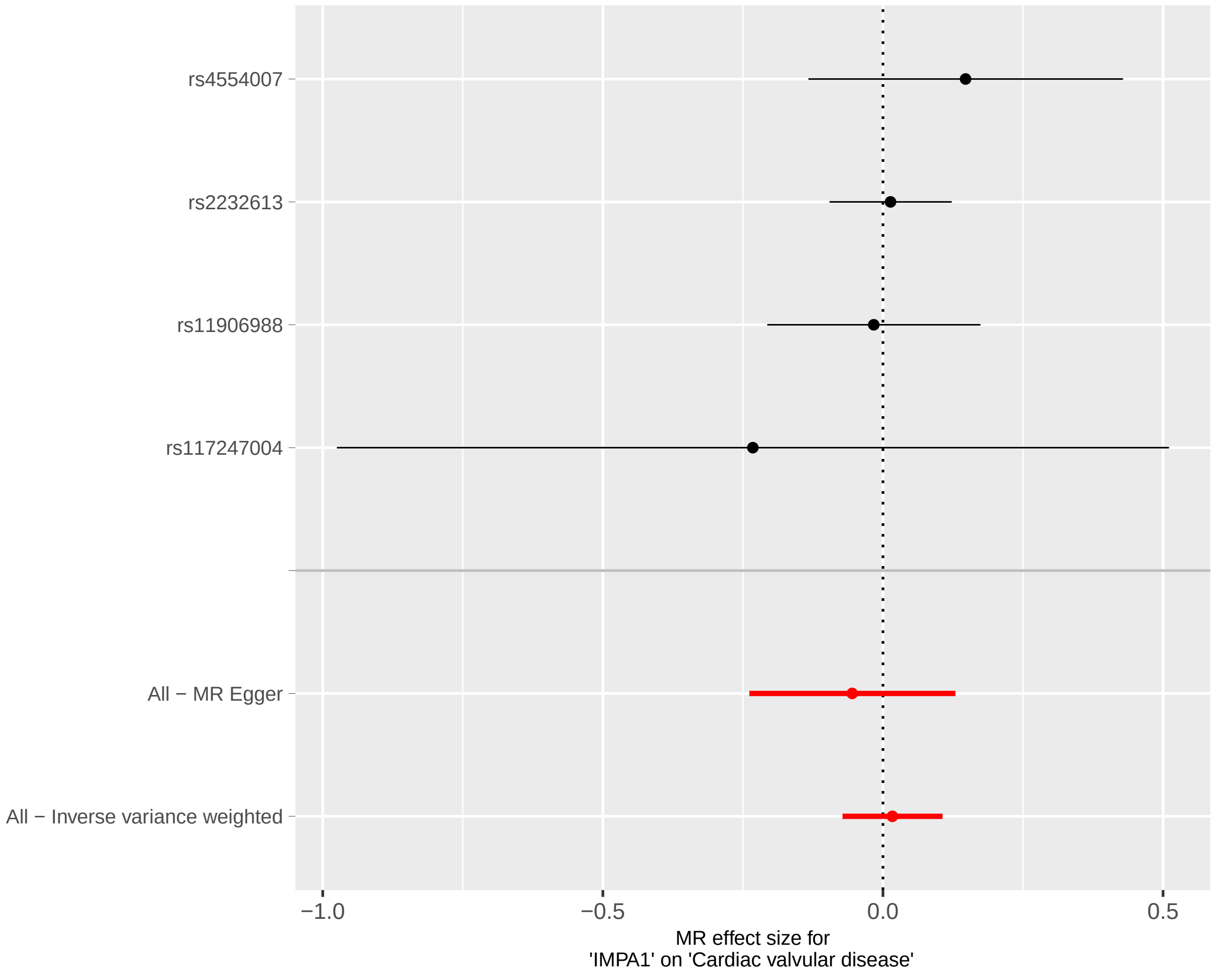
rs4554007
rs2232613
rs11906988
rs117247004
All − MR Egger
All − Inverse variance weighted
−1.0
−0.5
0.0
0.5
MR effect size for
'IMPA1' on 'Cardiac valvular disease'

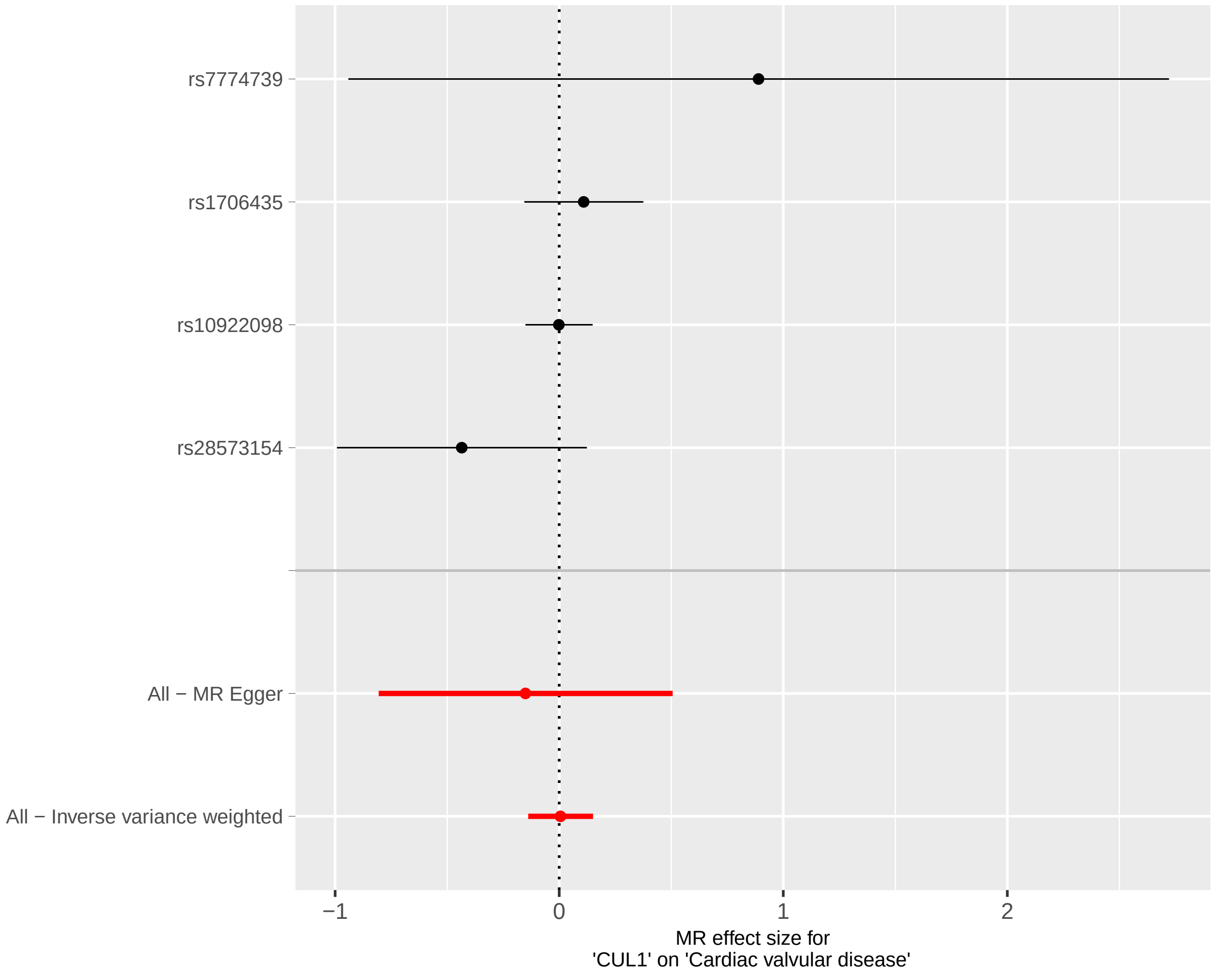

rs7774739
rs1706435
rs10922098
rs28573154
All − MR Egger
All − Inverse variance weighted
−1
0
1
2
MR effect size for
'CUL1' on 'Cardiac valvular disease'

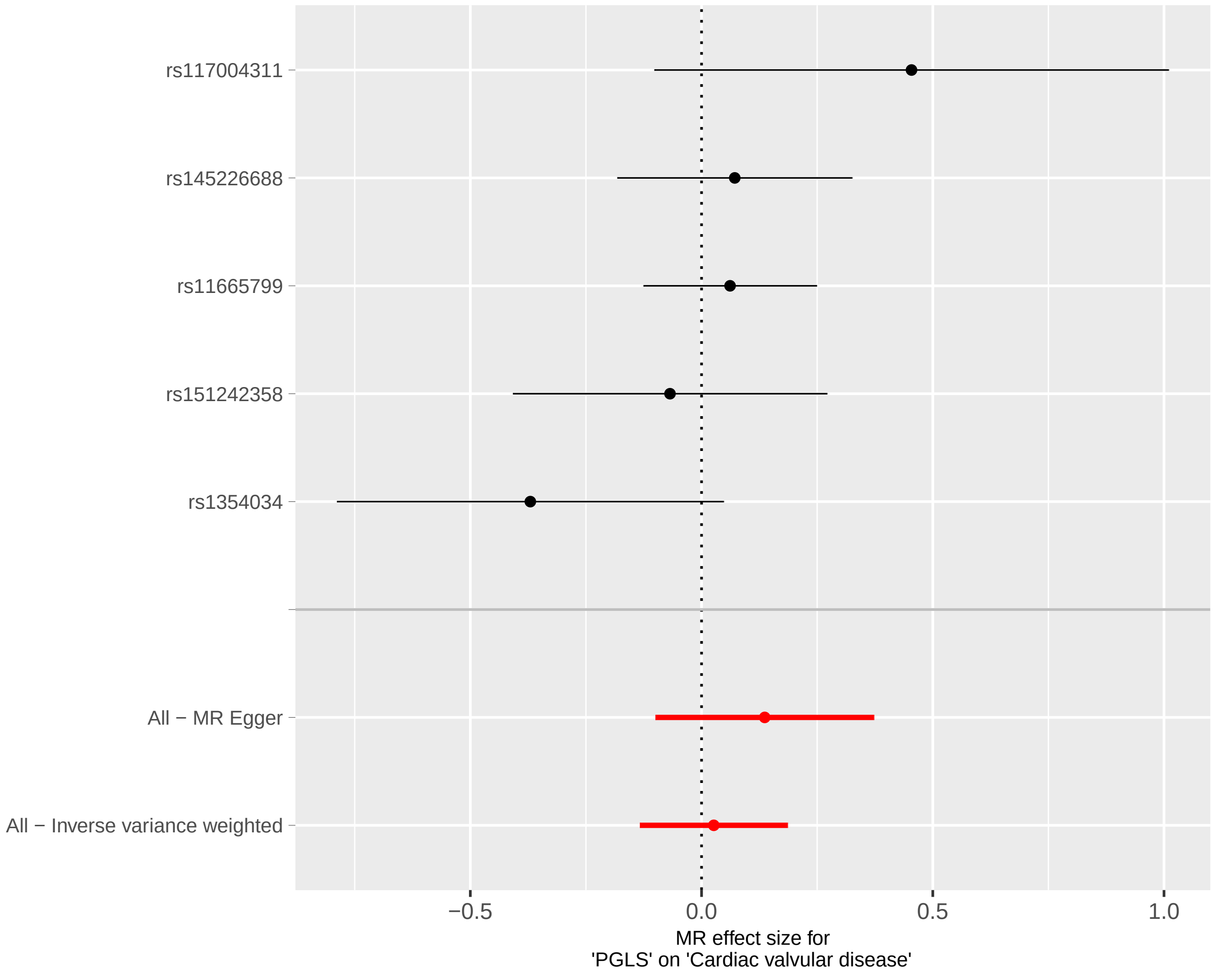
rs117004311
rs145226688
rs11665799
rs151242358
rs1354034
All − MR Egger
All − Inverse variance weighted
−0.5
0.0
0.5
1.0
MR effect size for
'PGLS' on 'Cardiac valvular disease'

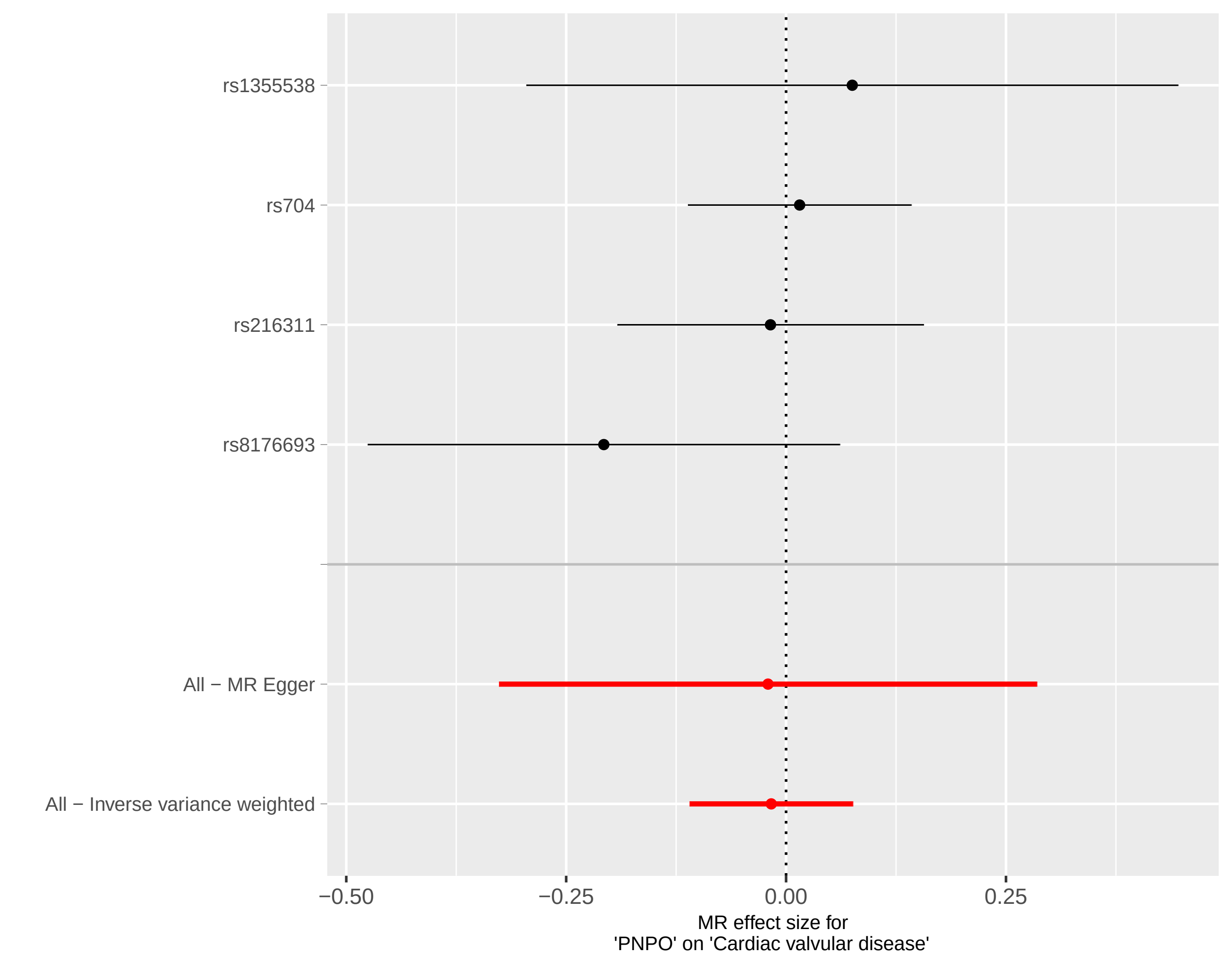
rs1355538
rs704
rs216311
rs8176693
All − MR Egger
All − Inverse variance weighted
−0.50
−0.25
0.00
0.25
MR effect size for
'PNPO' on 'Cardiac valvular disease'

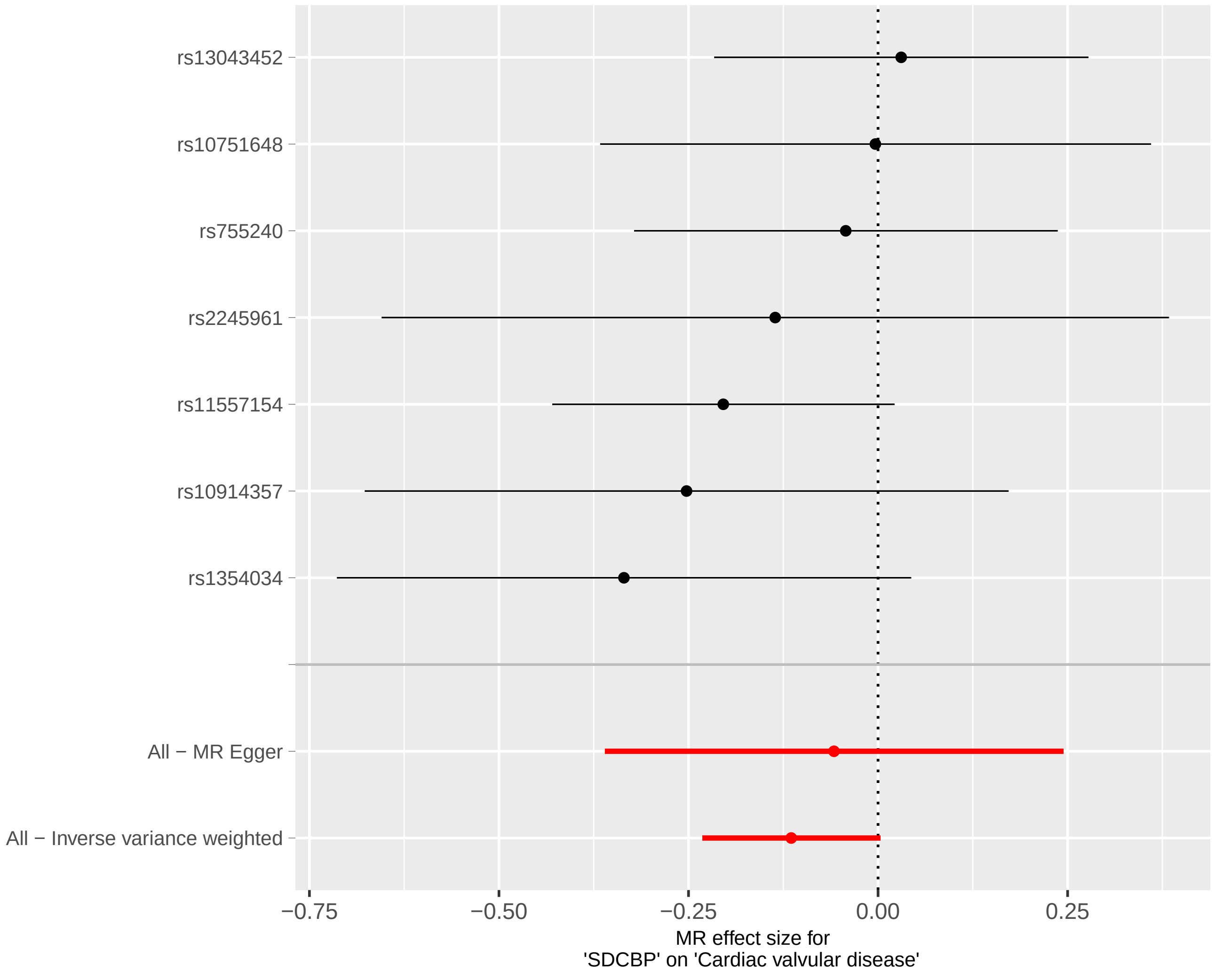

rs13043452
rs10751648
rs755240
rs2245961
rs11557154
rs10914357
rs1354034
All − MR Egger
All − Inverse variance weighted
−0.75
−0.50
−0.25
0.00
0.25
MR effect size for
'SDCBP' on 'Cardiac valvular disease'

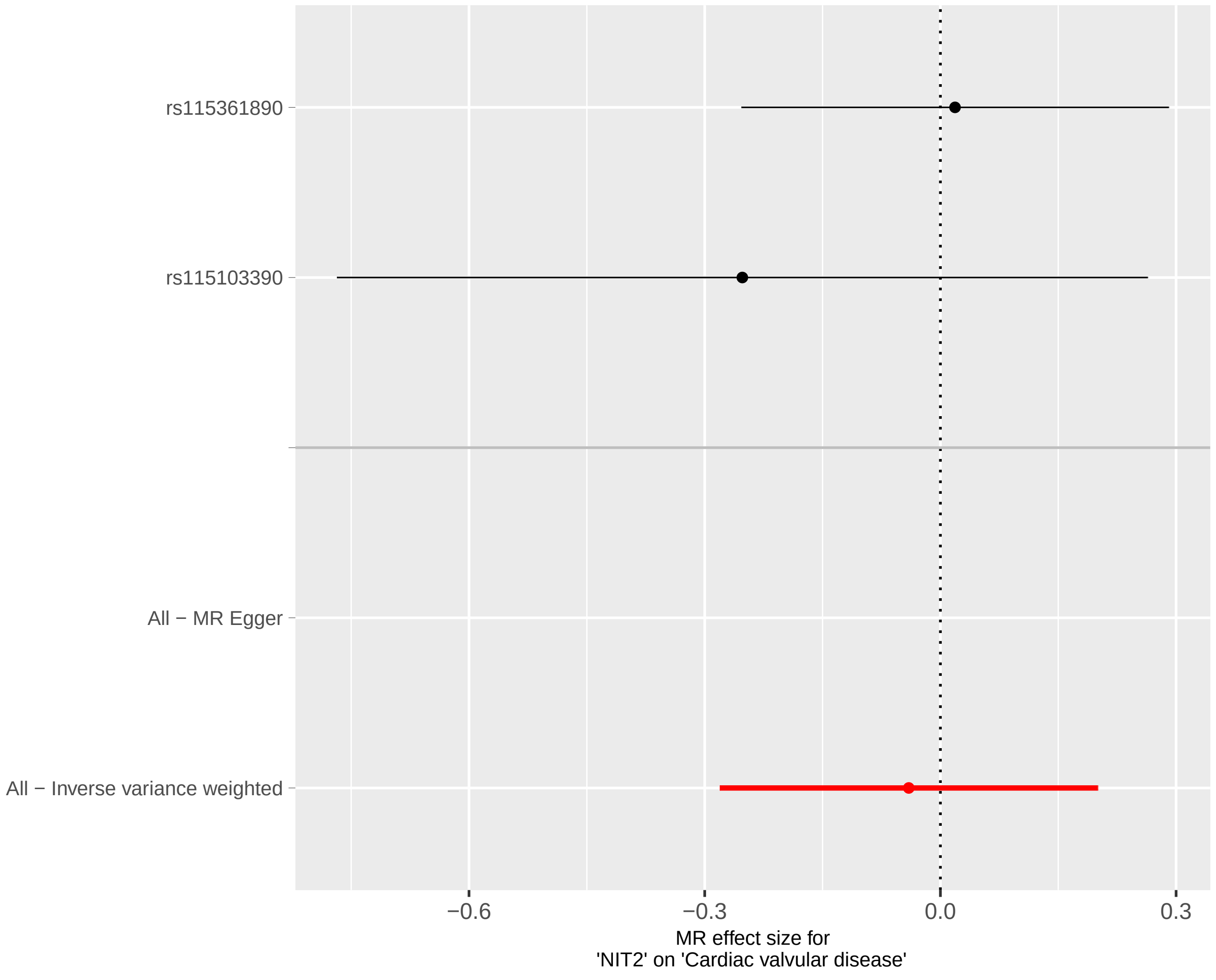

rs115361890
rs115103390
All − MR Egger
All − Inverse variance weighted
−0.6
−0.3
0.0
0.3
MR effect size for
'NIT2' on 'Cardiac valvular disease'

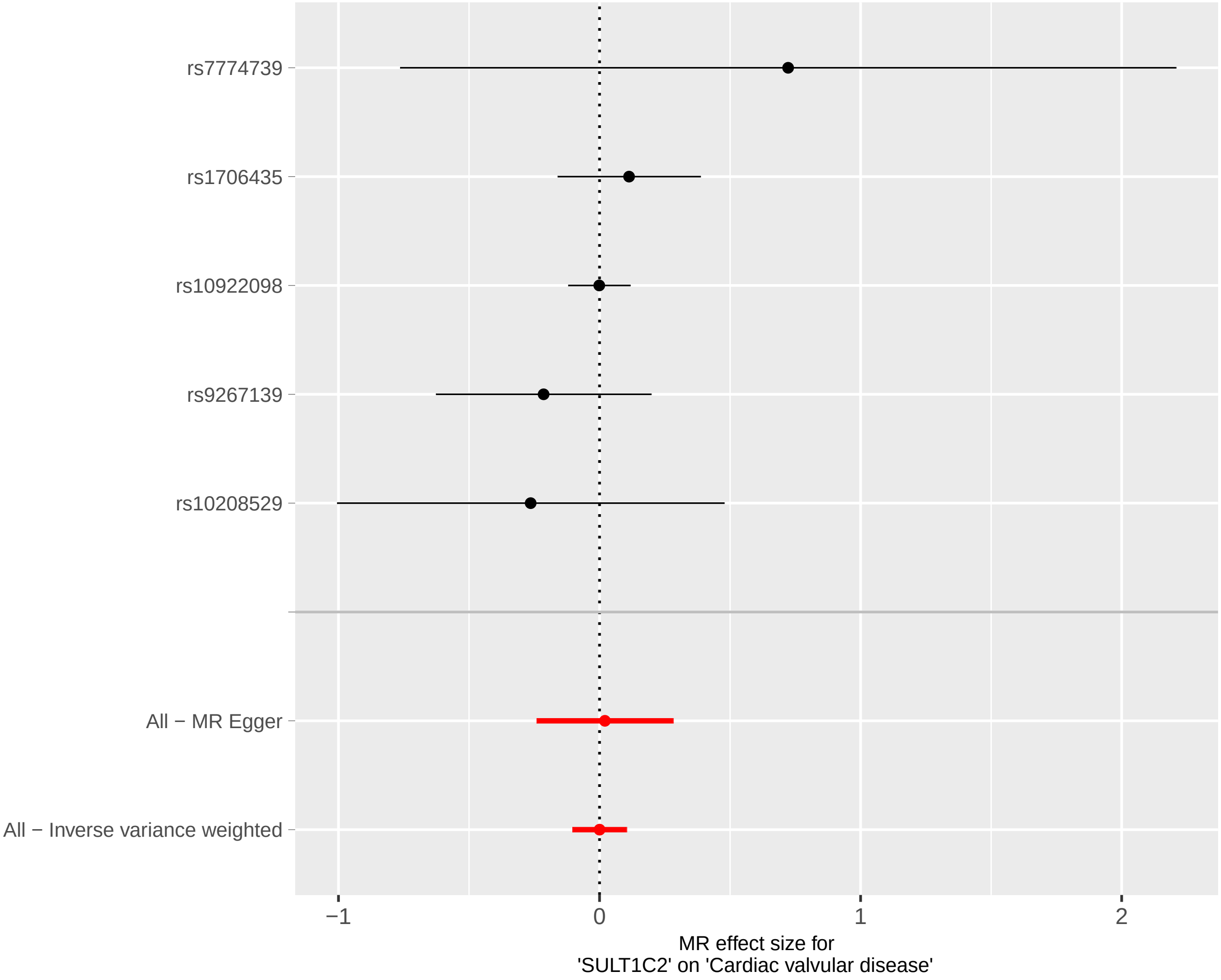
rs7774739
rs1706435
rs10922098
rs9267139
rs10208529
All − MR Egger
All − Inverse variance weighted
−1
0
1
2
MR effect size for
'SULT1C2' on 'Cardiac valvular disease'

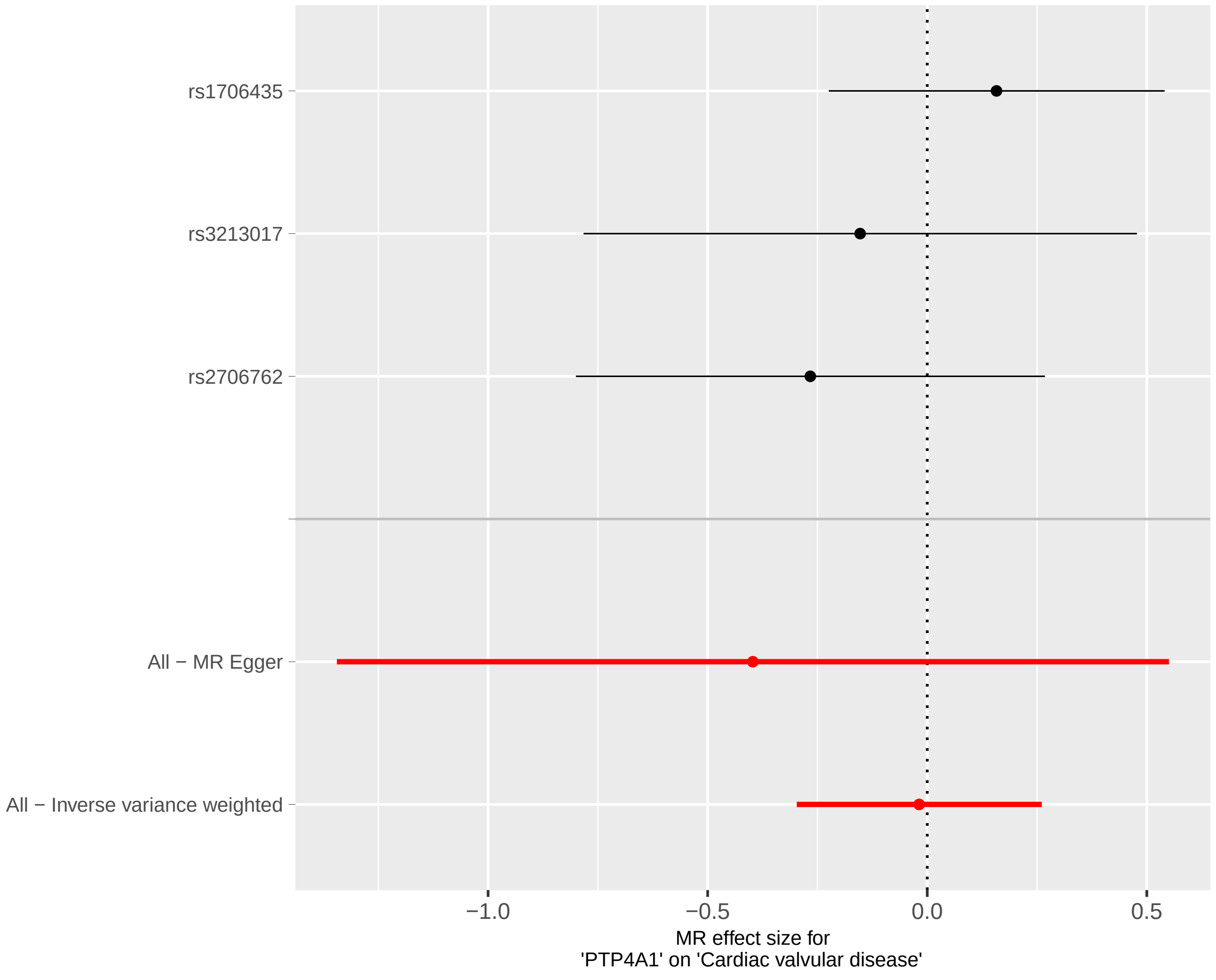
rs1706435
rs3213017
rs2706762
All − MR Egger
All − Inverse variance weighted
−1.0
−0.5
0.0
0.5
MR effect size for
'PTP4A1' on 'Cardiac valvular disease'

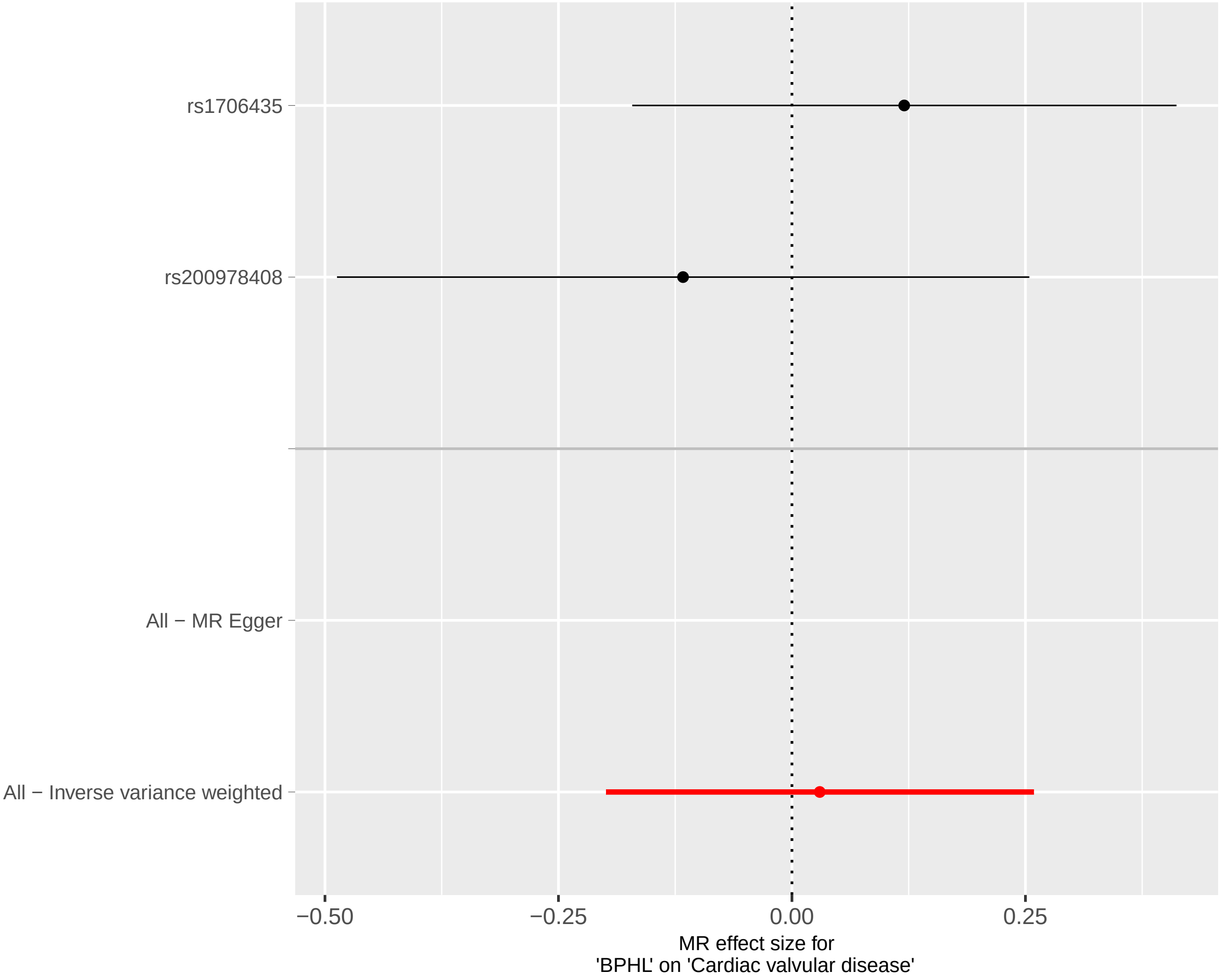
rs1706435
rs200978408
All − MR Egger
All − Inverse variance weighted
−0.50
−0.25
0.00
0.25
MR effect size for
'BPHL' on 'Cardiac valvular disease'

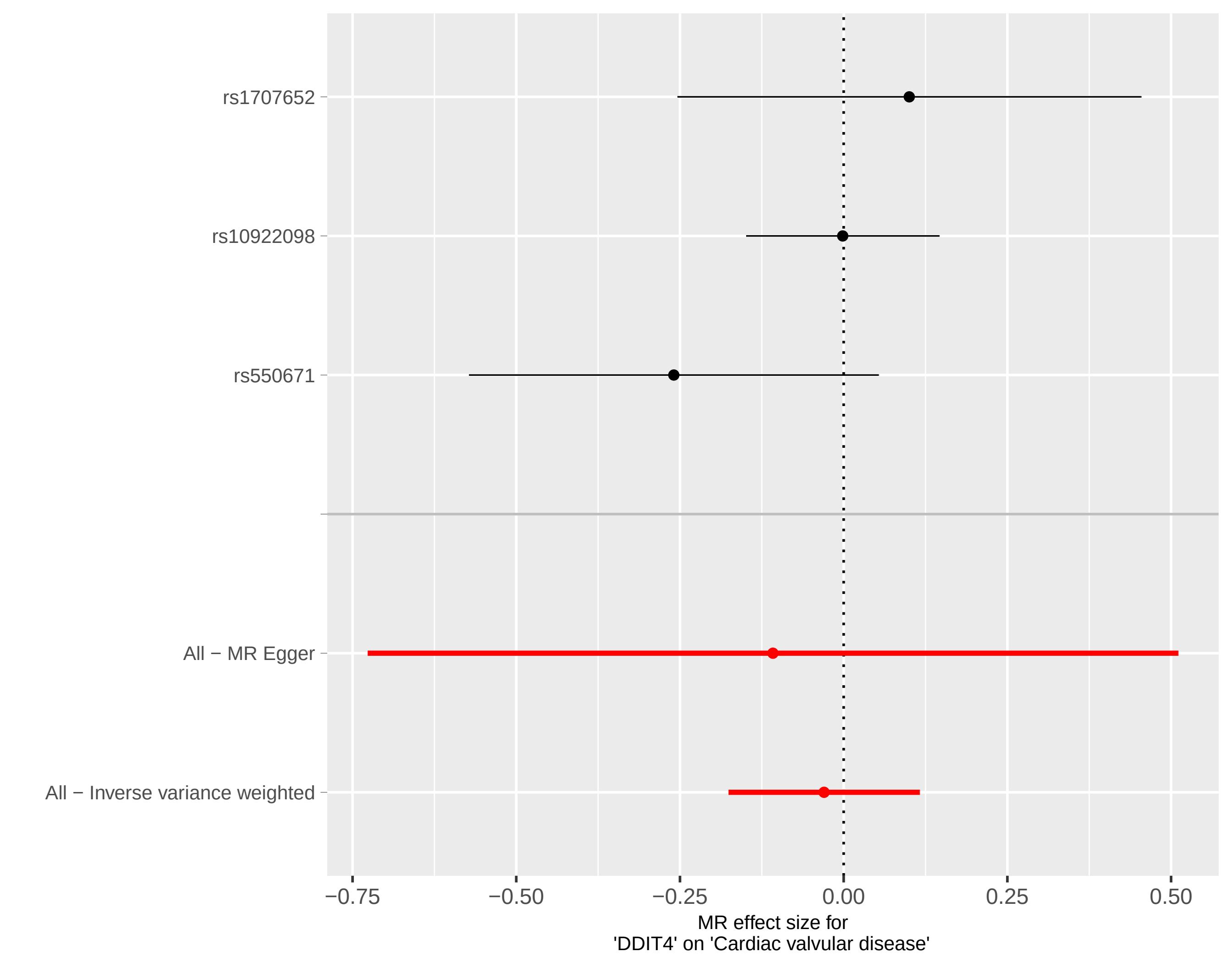
rs1707652
rs10922098
rs550671
All − MR Egger
All − Inverse variance weighted
−0.75
−0.50
−0.25
0.00
0.25
0.50
MR effect size for
'DDIT4' on 'Cardiac valvular disease'

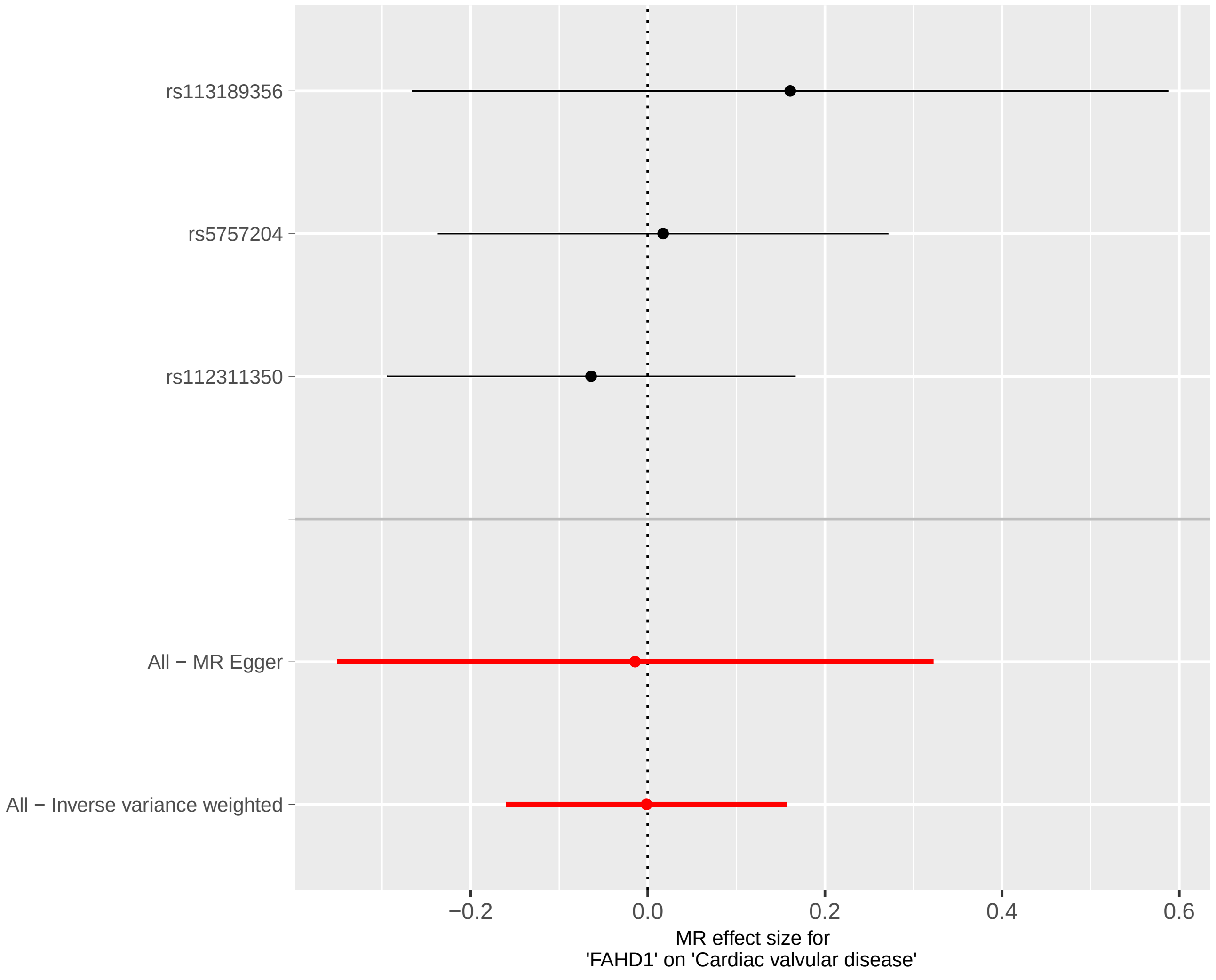
rs113189356
rs5757204
rs112311350
All − MR Egger
All − Inverse variance weighted
−0.2
0.0
0.2
0.4
0.6
MR effect size for
'FAHD1' on 'Cardiac valvular disease'

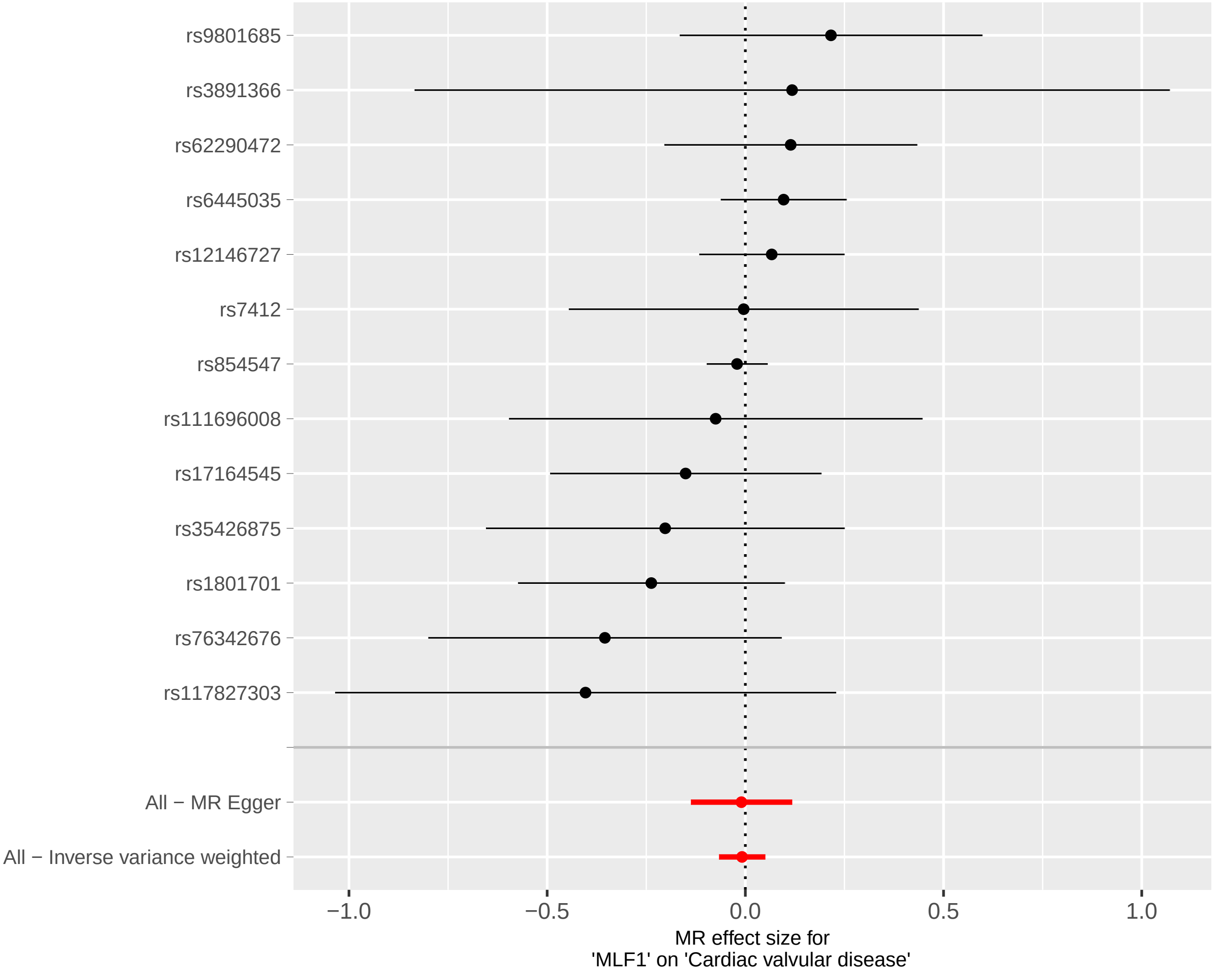
rs9801685
rs3891366
rs62290472
rs6445035
rs12146727
rs7412
rs854547
rs111696008
rs17164545
rs35426875
rs1801701
rs76342676
rs117827303
All − MR Egger
All − Inverse variance weighted
−1.0
−0.5
0.0
0.5
1.0
MR effect size for
'MLF1' on 'Cardiac valvular disease'

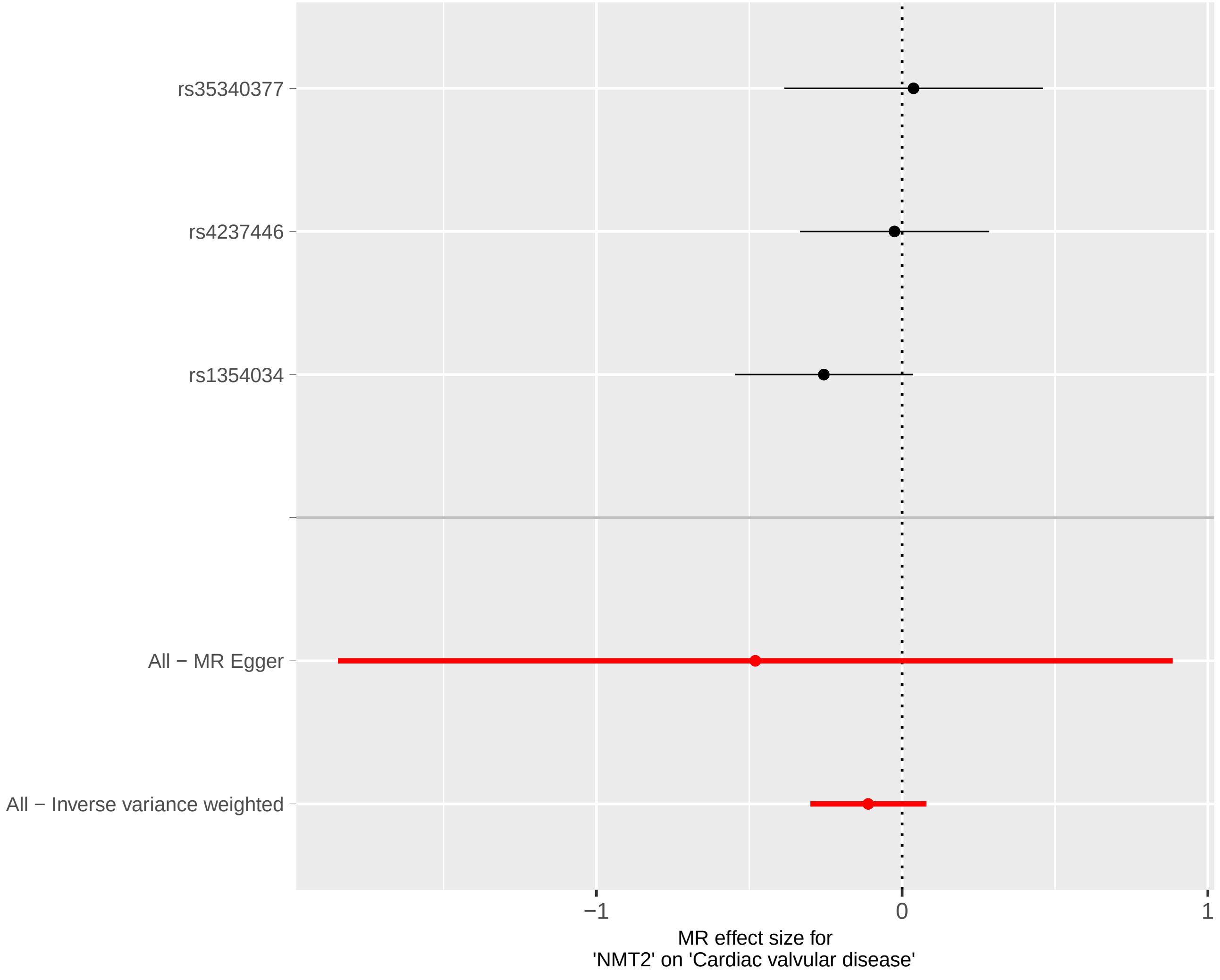

rs35340377
rs4237446
rs1354034
All − MR Egger
All − Inverse variance weighted
−1
0
1
MR effect size for
'NMT2' on 'Cardiac valvular disease'

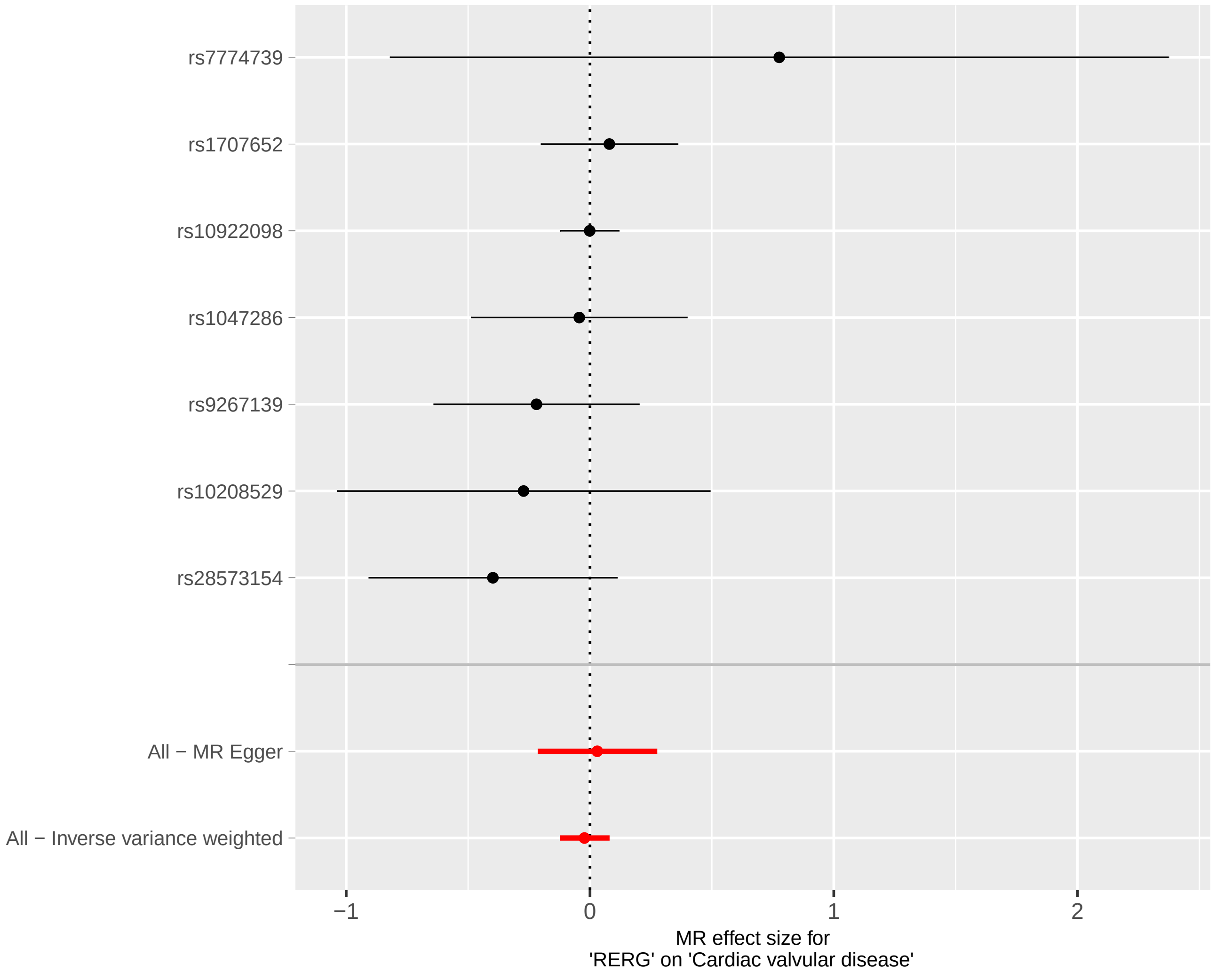
rs7774739
rs1707652
rs10922098
rs1047286
rs9267139
rs10208529
rs28573154
All − MR Egger
All − Inverse variance weighted
−1
0
1
2
MR effect size for
'RERG' on 'Cardiac valvular disease'

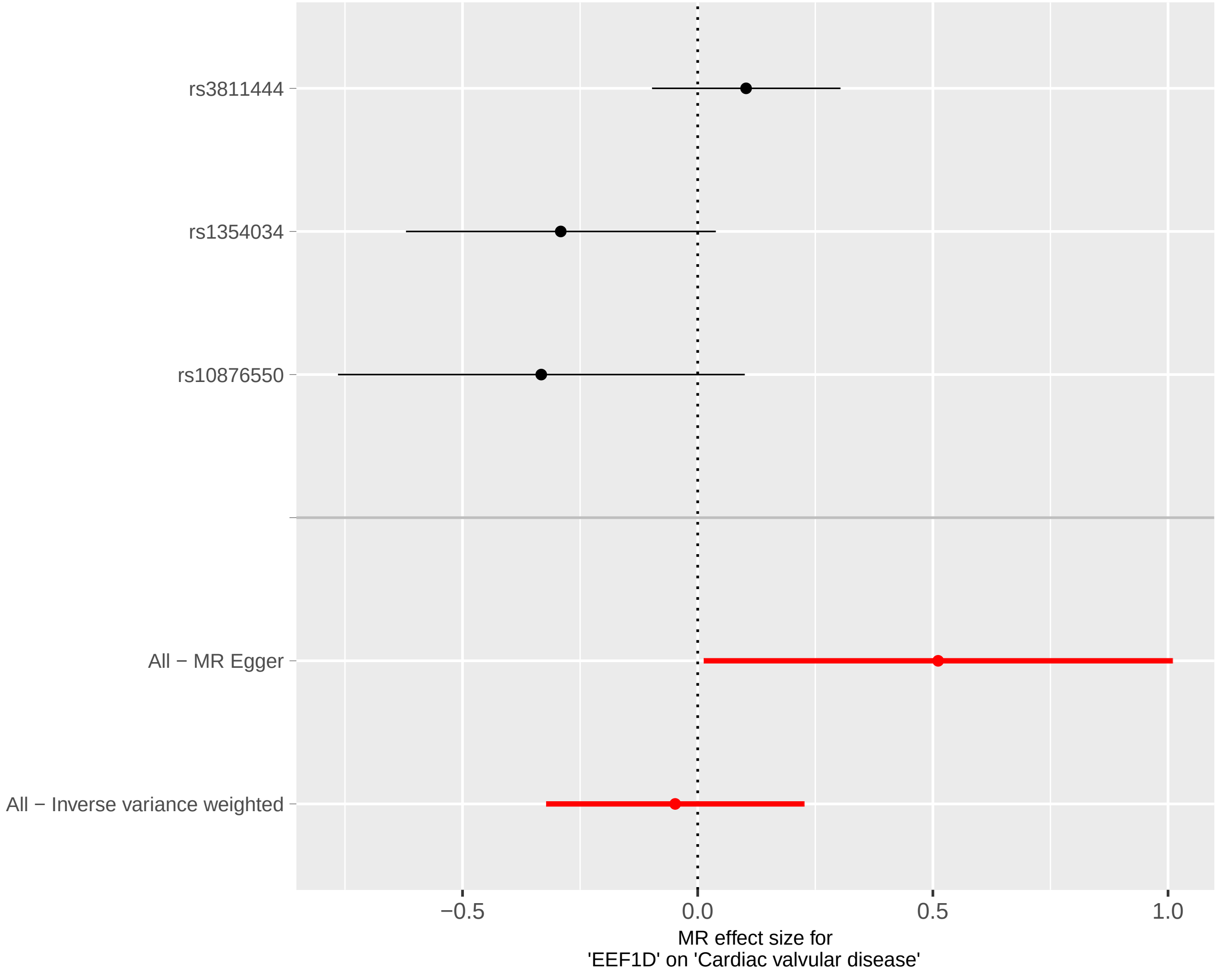
rs3811444
rs1354034
rs10876550
All − MR Egger
All − Inverse variance weighted
−0.5
0.0
0.5
1.0
MR effect size for
'EEF1D' on 'Cardiac valvular disease'

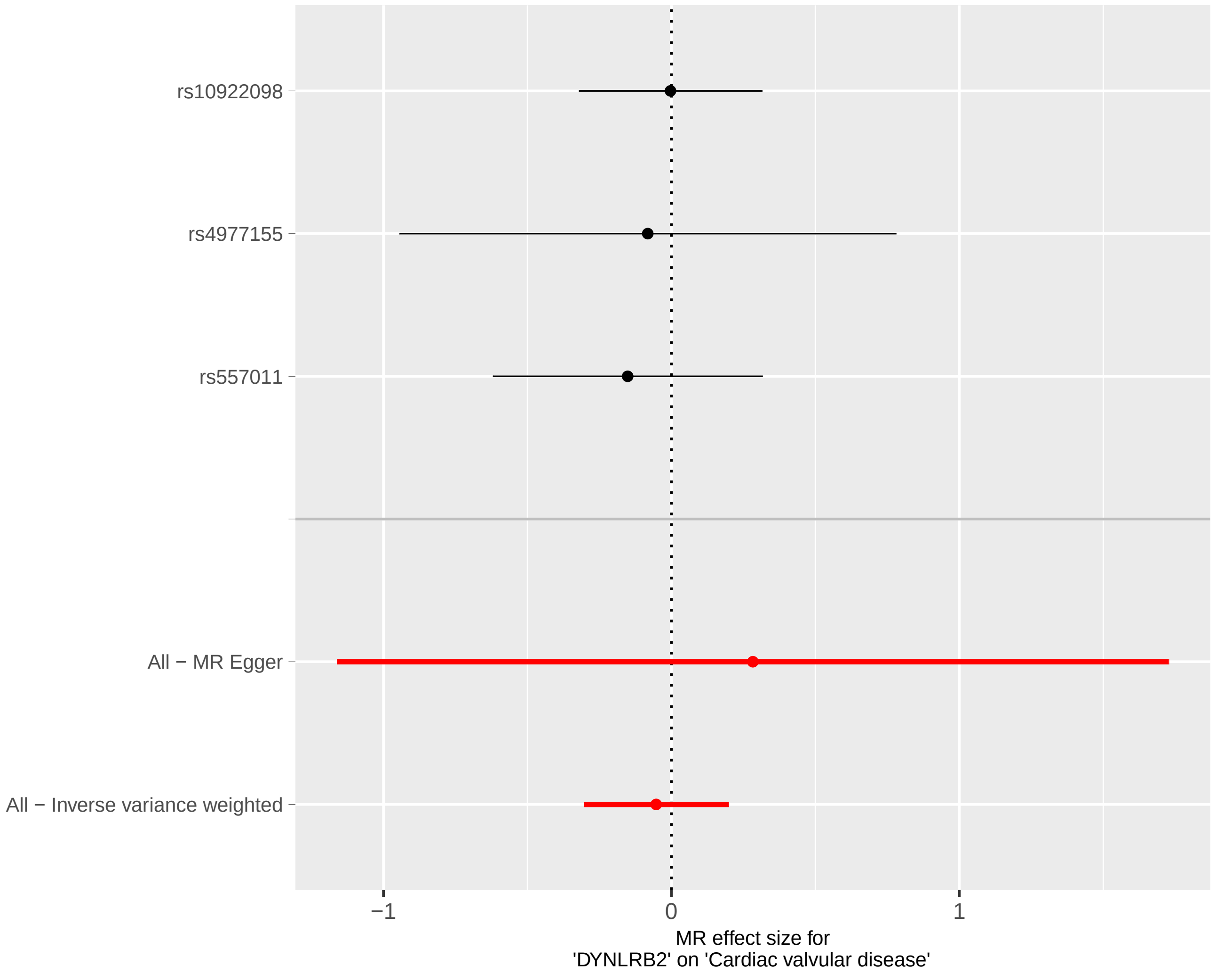
rs10922098
rs4977155
rs557011
All − MR Egger
All − Inverse variance weighted
−1
0
1
MR effect size for
'DYNLRB2' on 'Cardiac valvular disease'

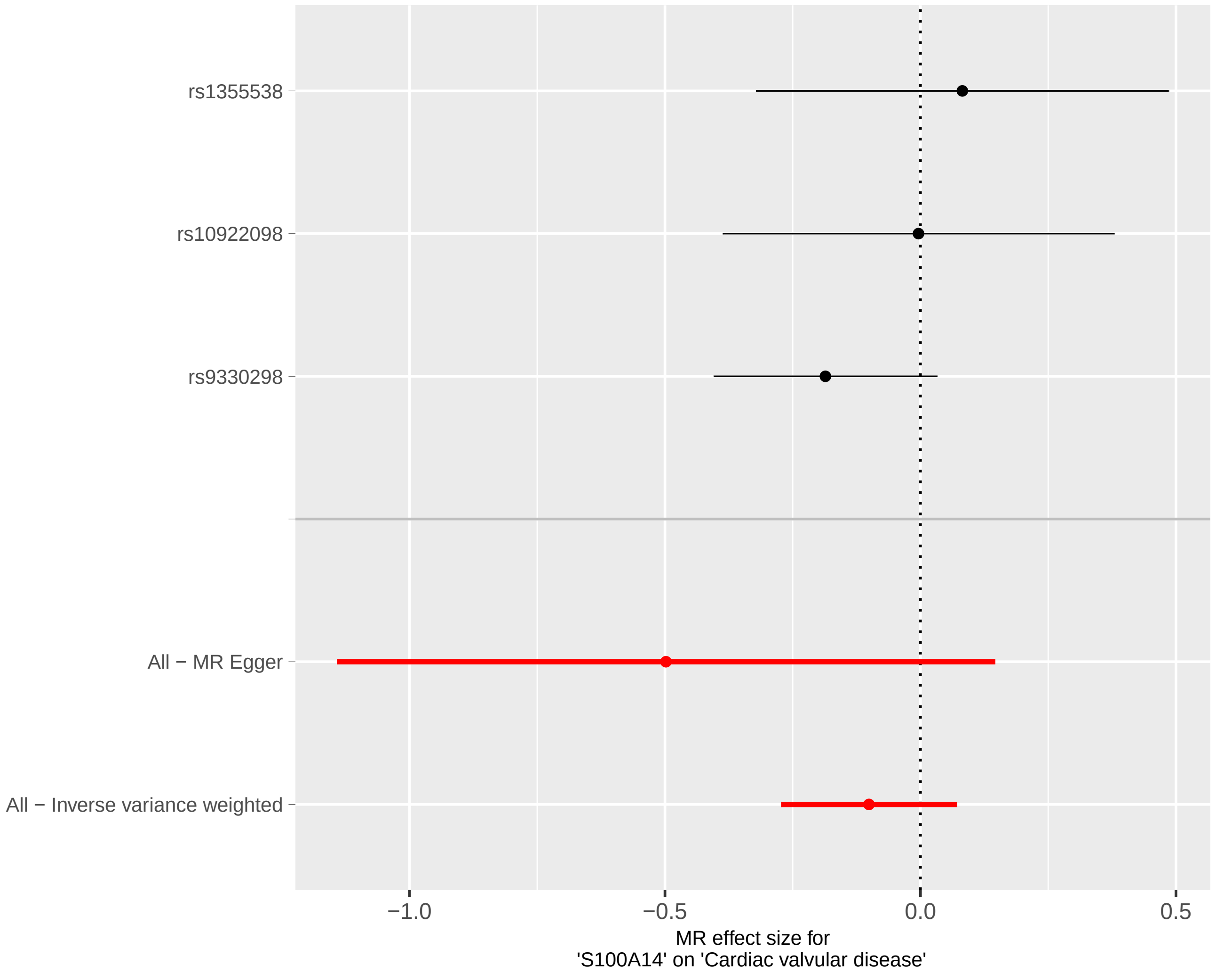
rs1355538
rs10922098
rs9330298
All − MR Egger
All − Inverse variance weighted
−1.0
−0.5
0.0
0.5
MR effect size for
'S100A14' on 'Cardiac valvular disease'

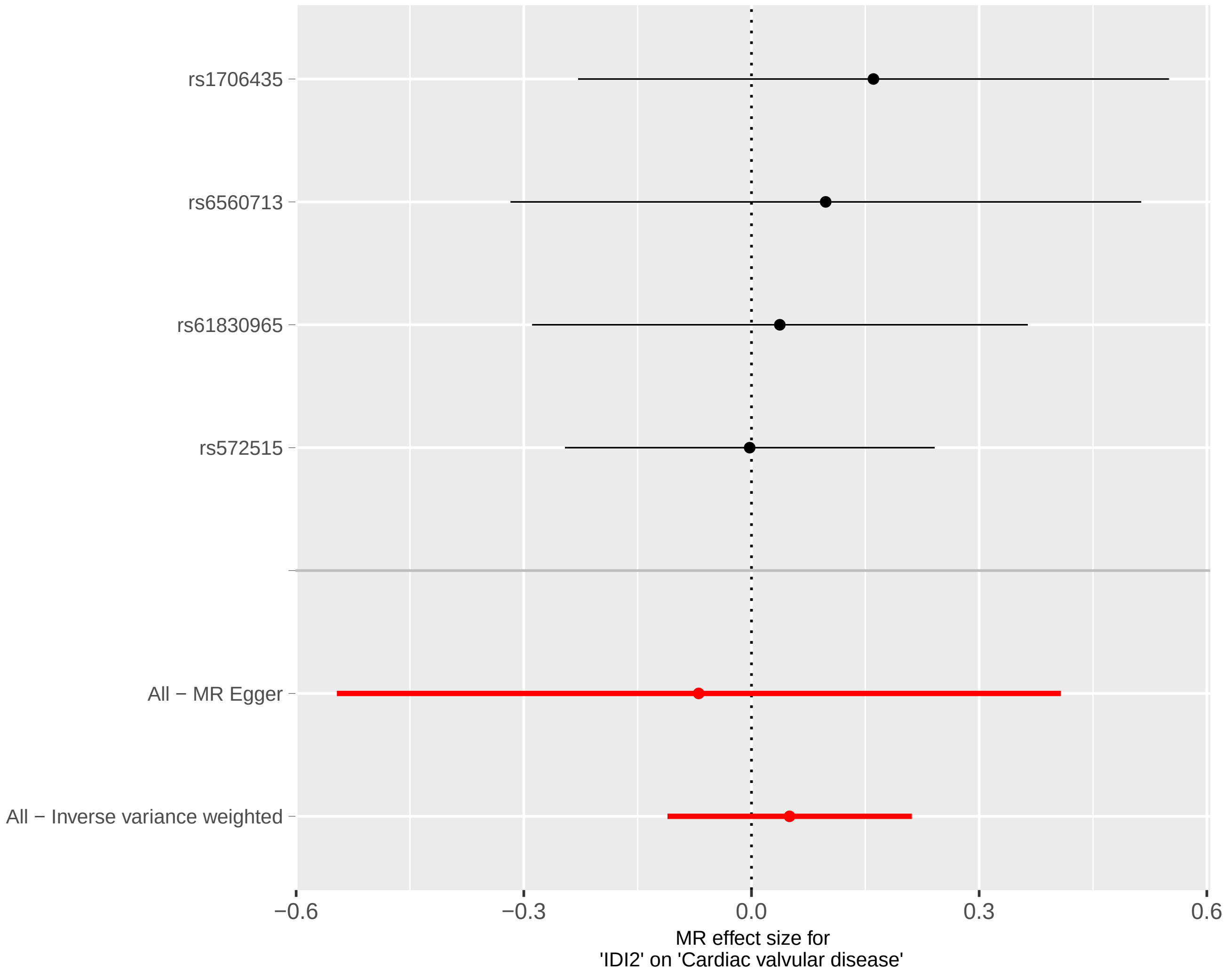

rs1706435
rs6560713
rs61830965
rs572515
All − MR Egger
All − Inverse variance weighted
−0.6
−0.3
0.0
0.3
0.6
MR effect size for
'IDI2' on 'Cardiac valvular disease'

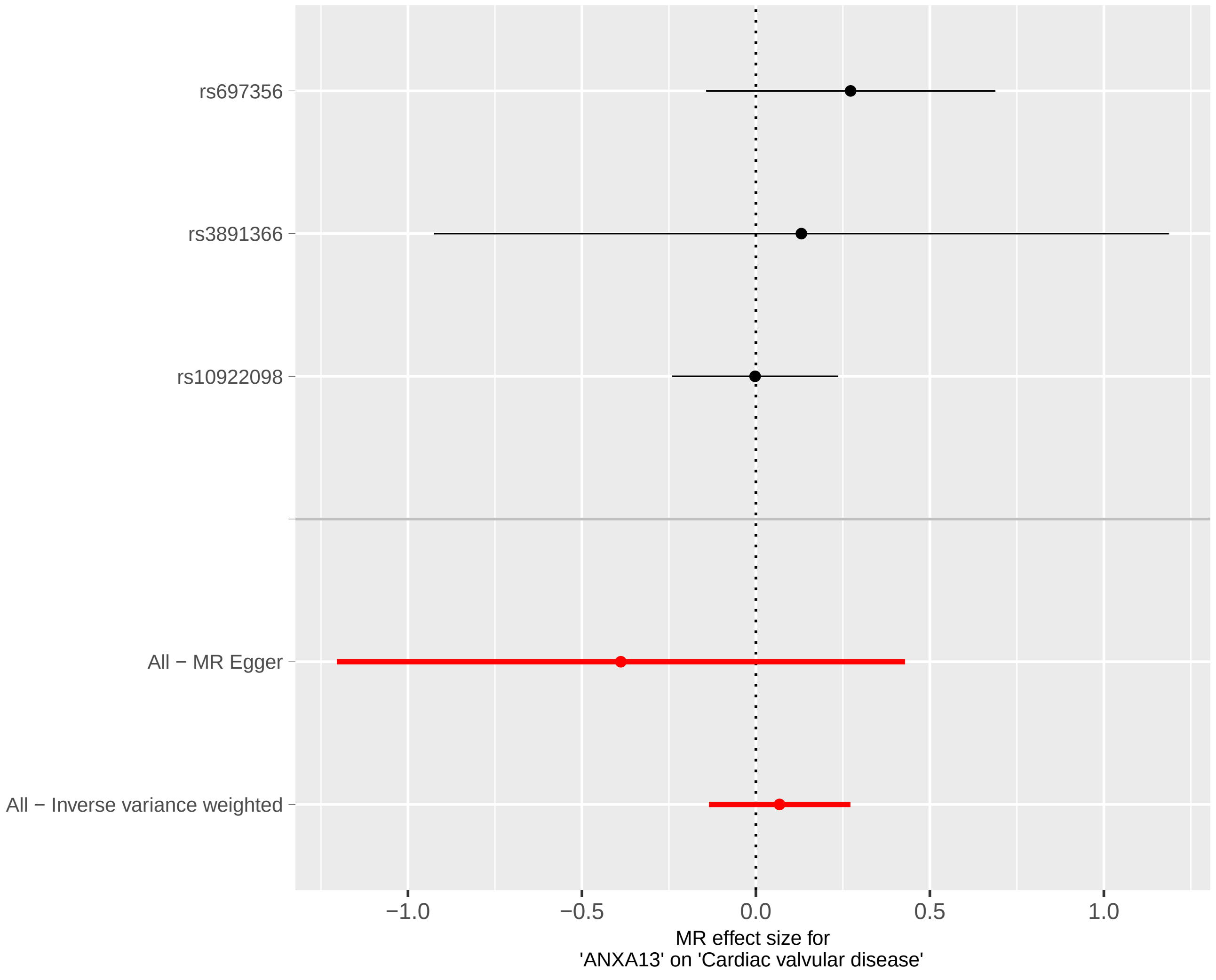
rs697356
rs3891366
rs10922098
All − MR Egger
All − Inverse variance weighted
−1.0
−0.5
0.0
0.5
1.0
MR effect size for
'ANXA13' on 'Cardiac valvular disease'

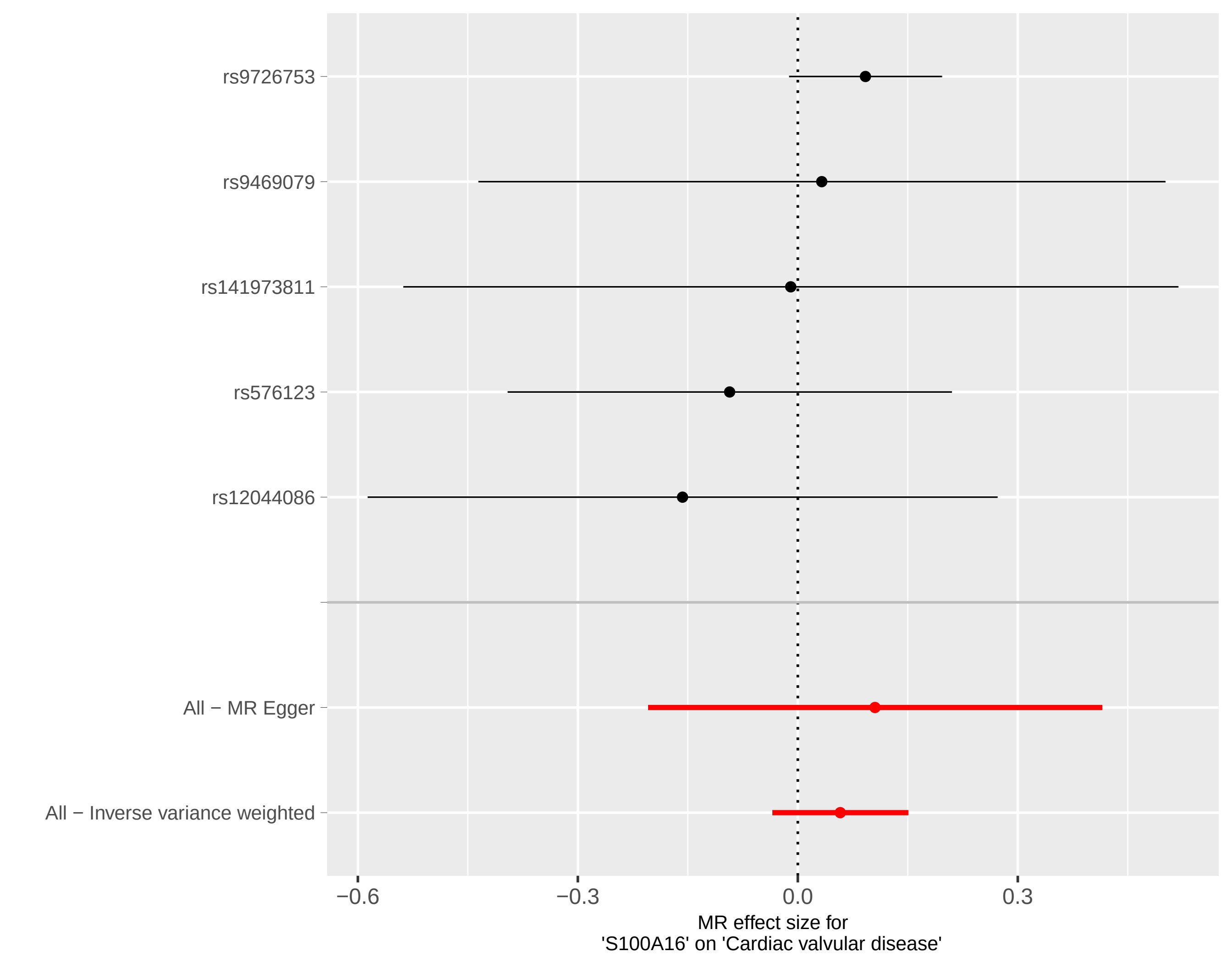
rs9726753
rs9469079
rs141973811
rs576123
rs12044086
All − MR Egger
All − Inverse variance weighted
−0.6
−0.3
0.0
0.3
MR effect size for
'S100A16' on 'Cardiac valvular disease'

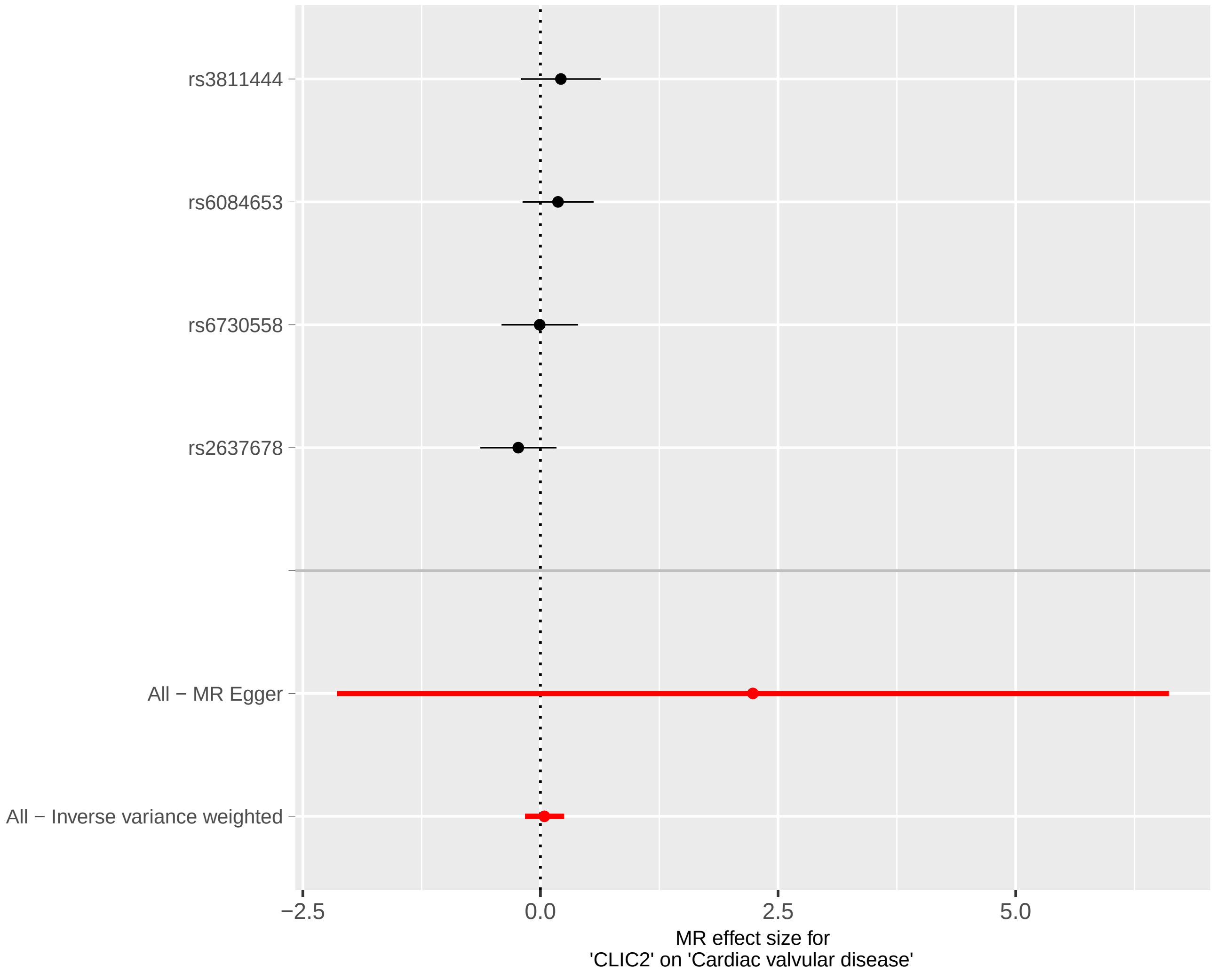
rs3811444
rs6084653
rs6730558
rs2637678
All − MR Egger
All − Inverse variance weighted
−2.5
0.0
2.5
5.0
MR effect size for
'CLIC2' on 'Cardiac valvular disease'

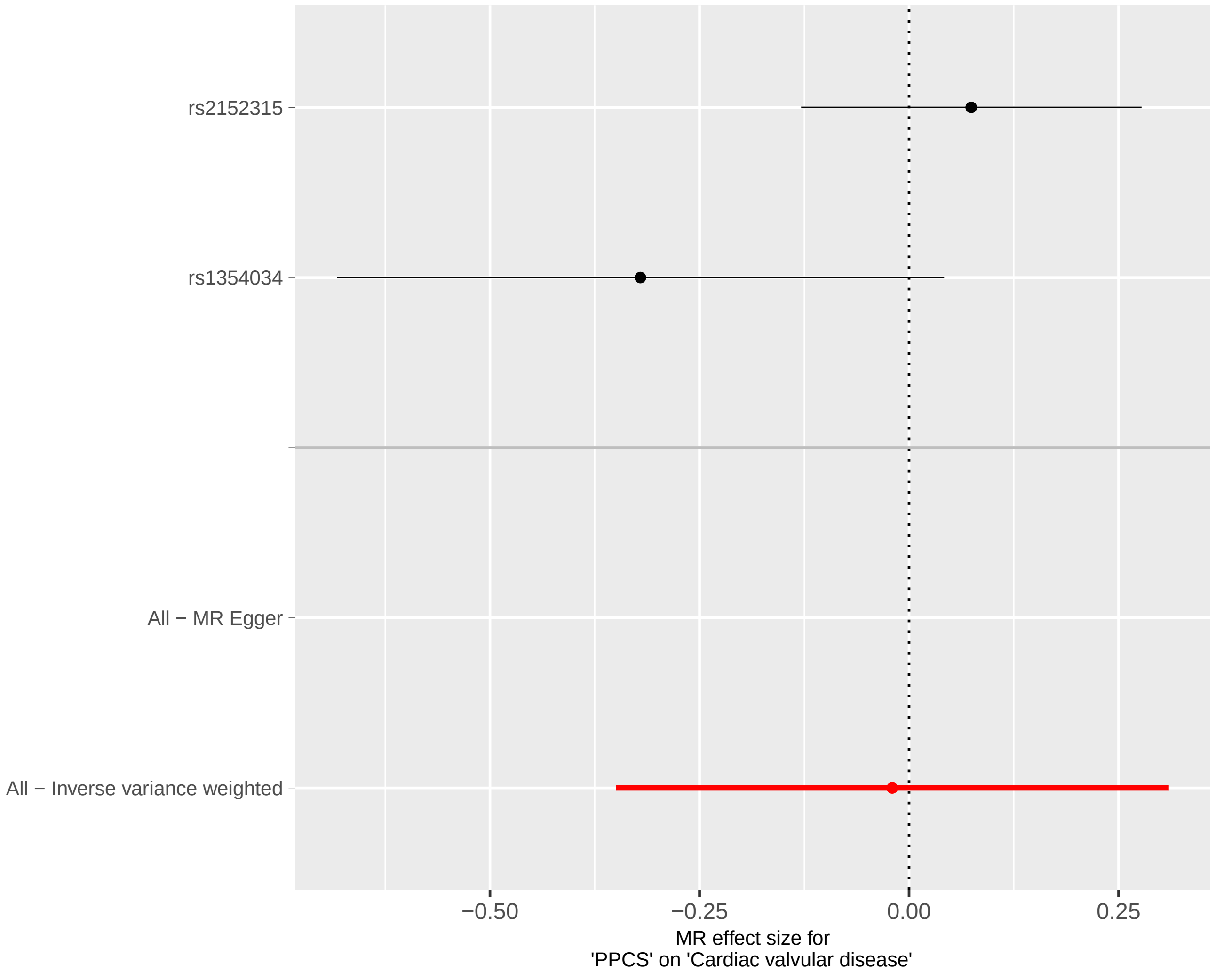
rs2152315
rs1354034
All − MR Egger
All − Inverse variance weighted
−0.50
−0.25
0.00
0.25
MR effect size for
'PPCS' on 'Cardiac valvular disease'

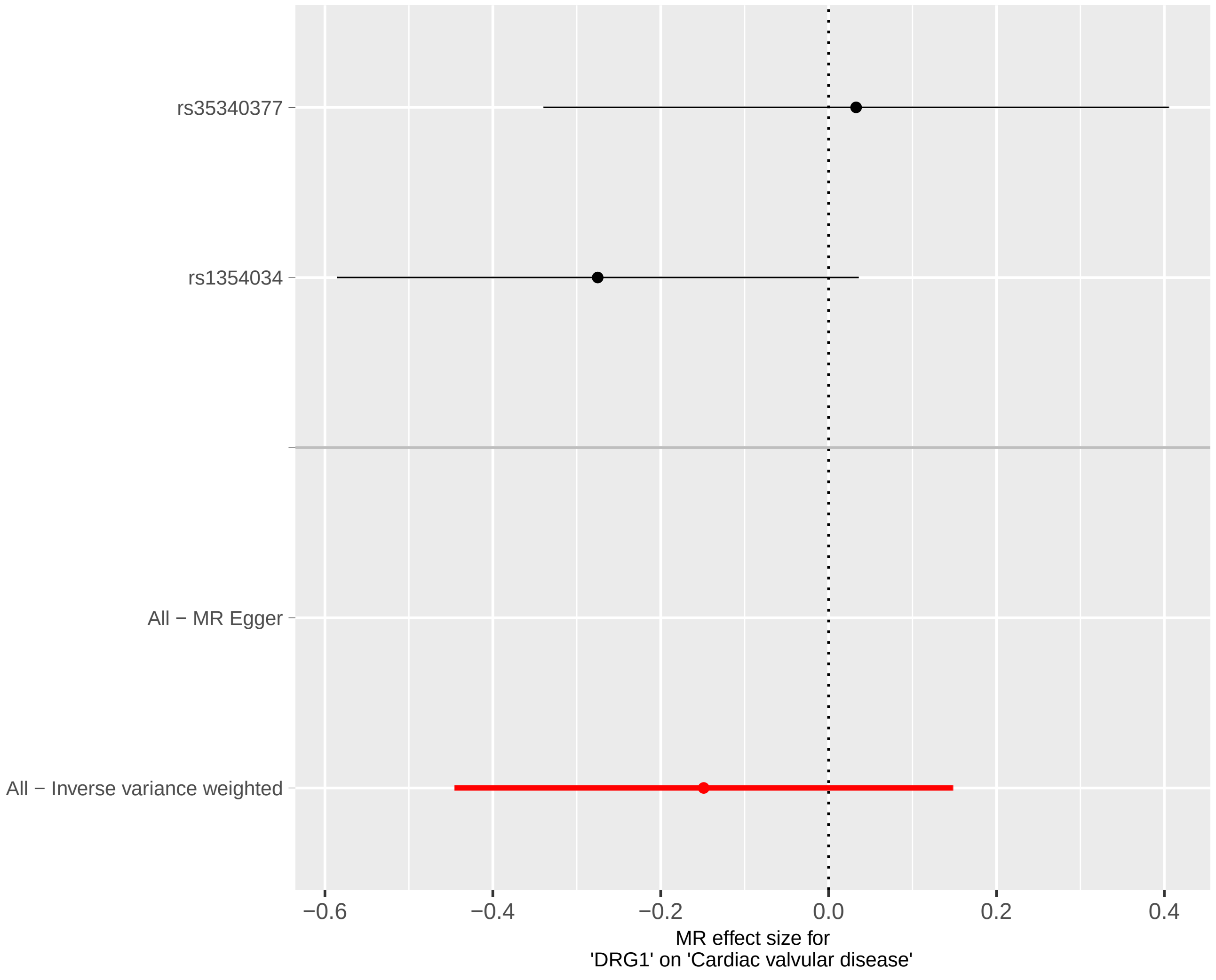

rs35340377
rs1354034
All − MR Egger
All − Inverse variance weighted
−0.6
−0.4
−0.2
0.0
0.2
0.4
MR effect size for
'DRG1' on 'Cardiac valvular disease'

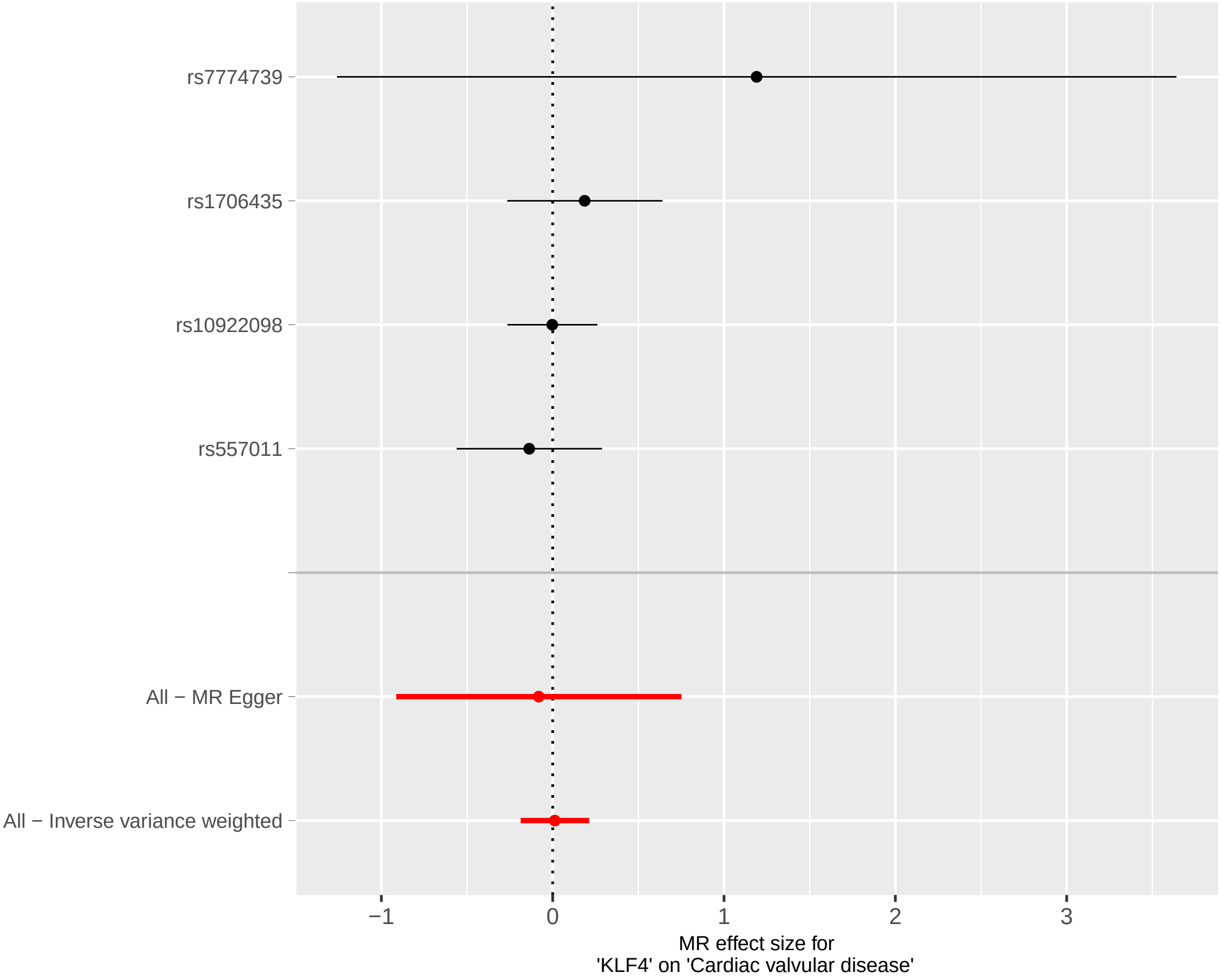
rs7774739
rs1706435
rs10922098
rs557011
All − MR Egger
All − Inverse variance weighted
−1
0
1
2
3
MR effect size for
'KLF4' on 'Cardiac valvular disease'

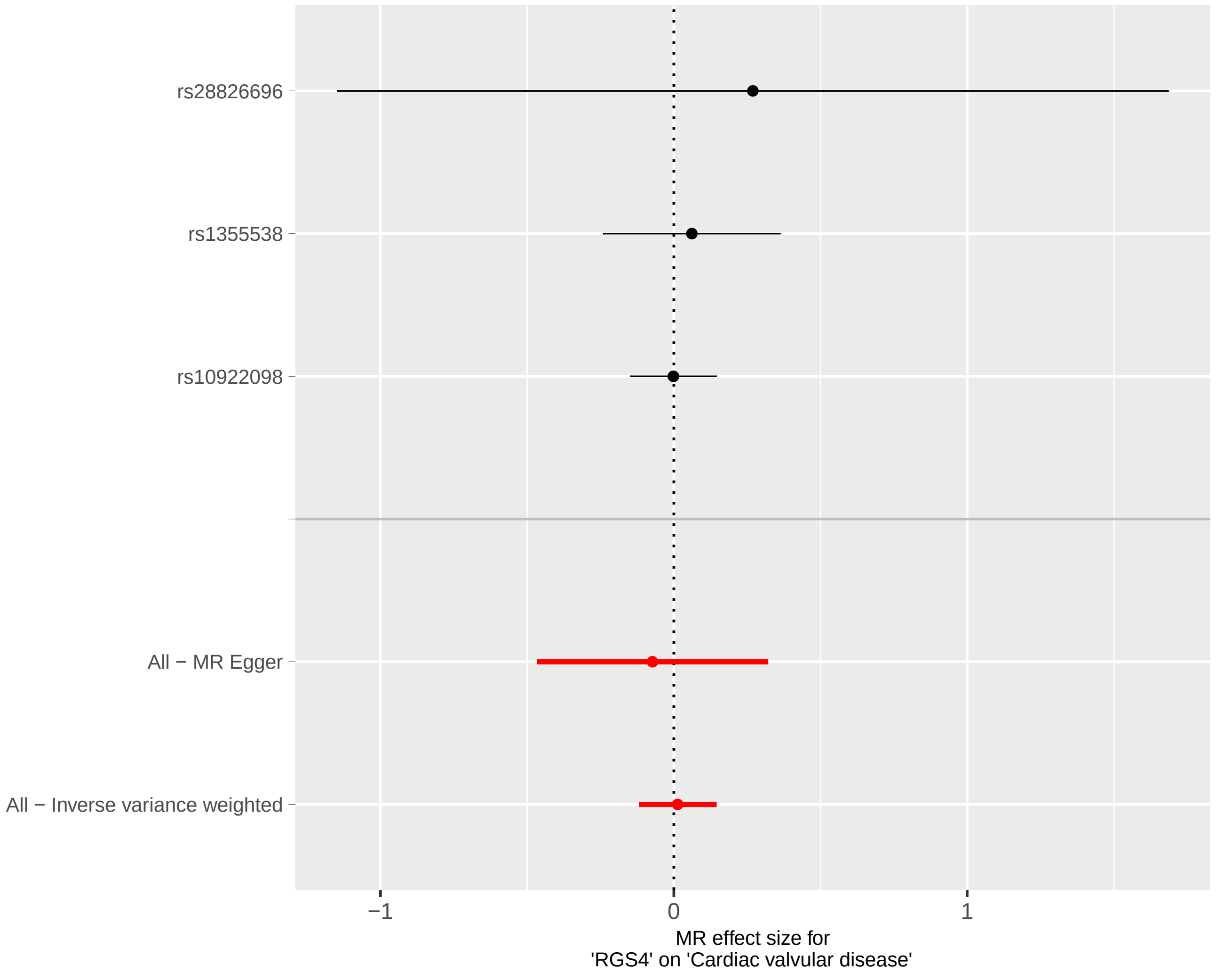

rs28826696
rs1355538
rs10922098
All − MR Egger
All − Inverse variance weighted
−1
0
1
MR effect size for
'RGS4' on 'Cardiac valvular disease'

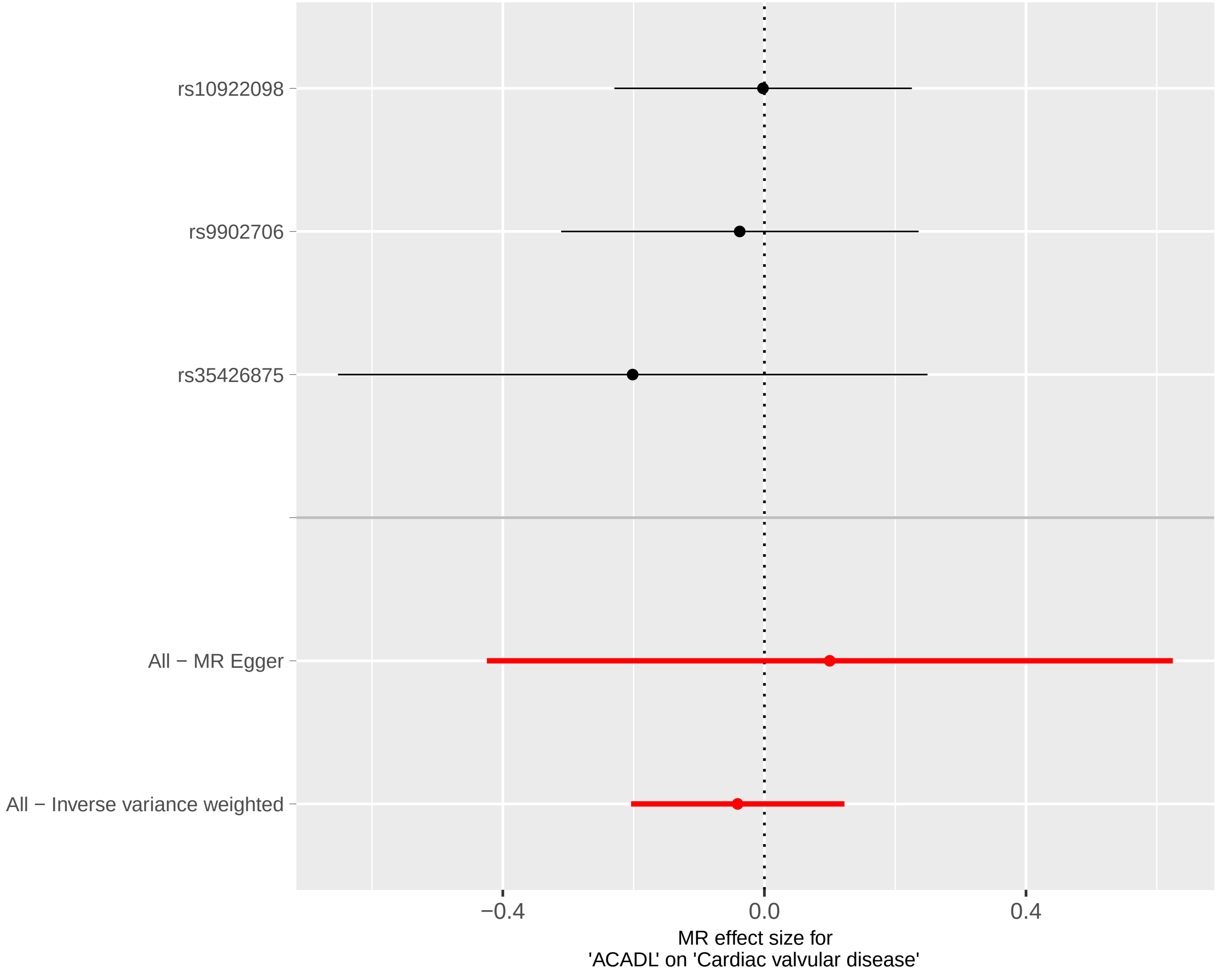
rs10922098
rs9902706
rs35426875
All − MR Egger
All − Inverse variance weighted
−0.4
0.0
0.4
MR effect size for
'ACADL' on 'Cardiac valvular disease'

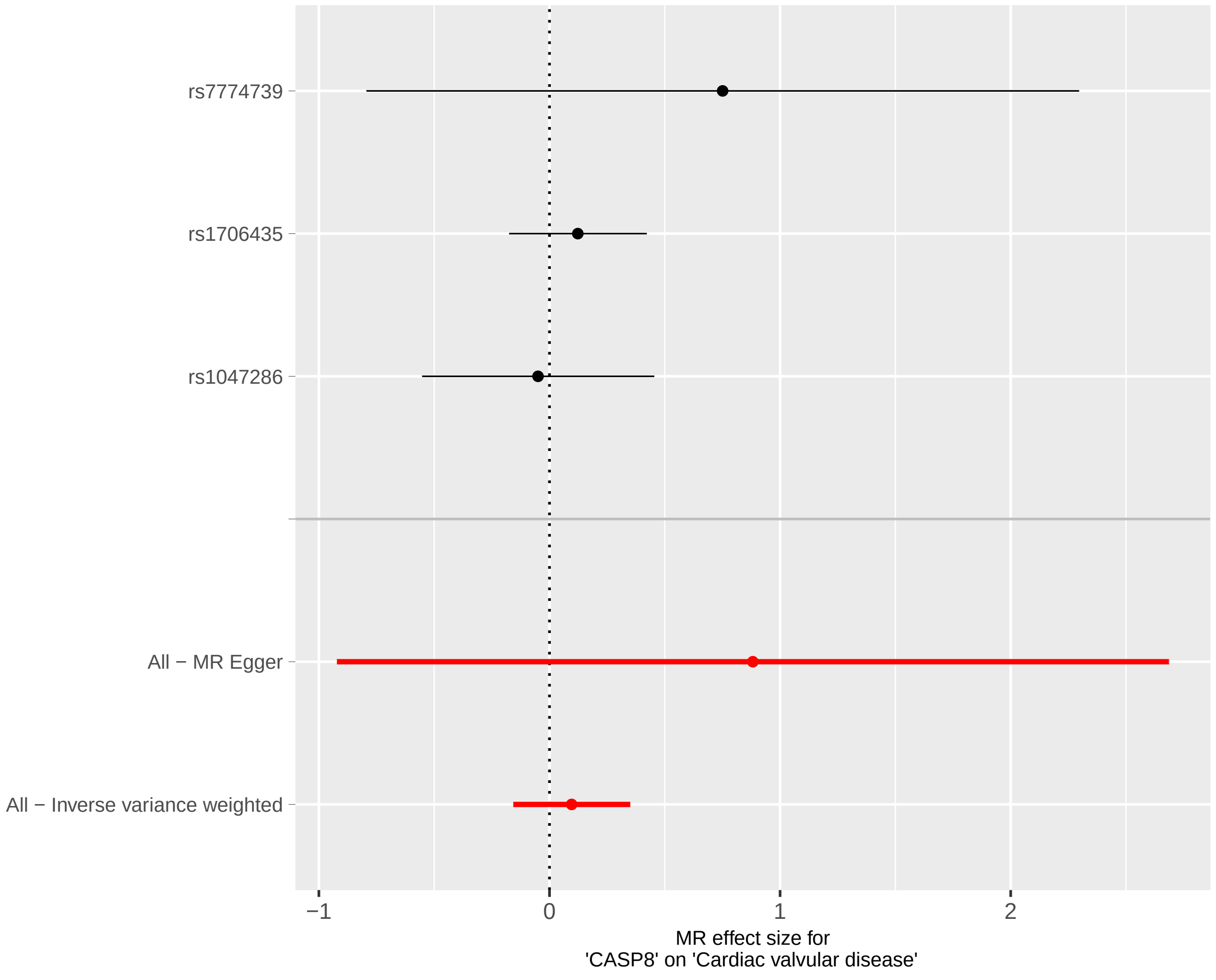
rs7774739
rs1706435
rs1047286
All − MR Egger
All − Inverse variance weighted
−1
0
1
2
MR effect size for
'CASP8' on 'Cardiac valvular disease'

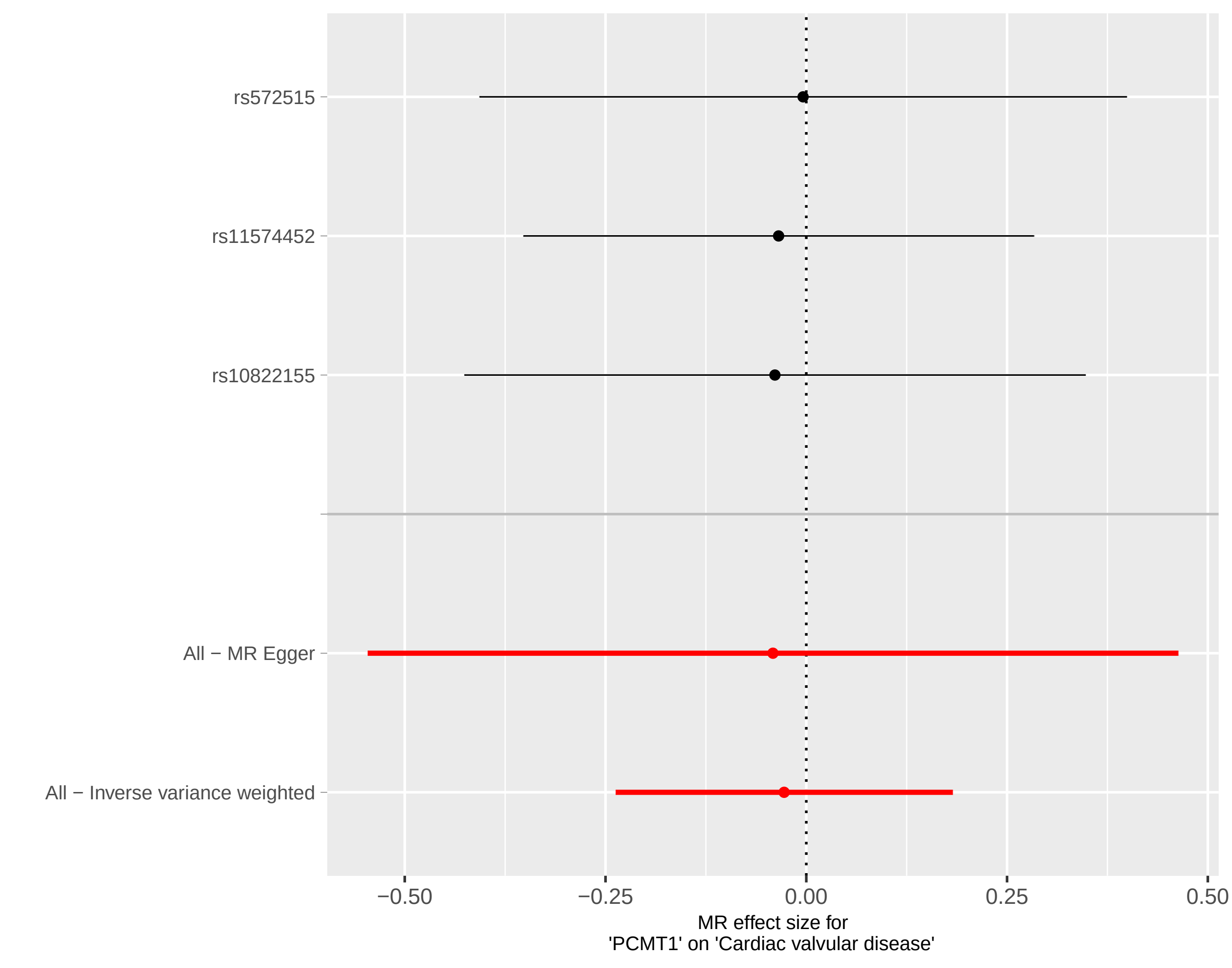
rs572515
rs11574452
rs10822155
All − MR Egger
All − Inverse variance weighted
−0.50
−0.25
0.00
0.25
0.50
MR effect size for
'PCMT1' on 'Cardiac valvular disease'

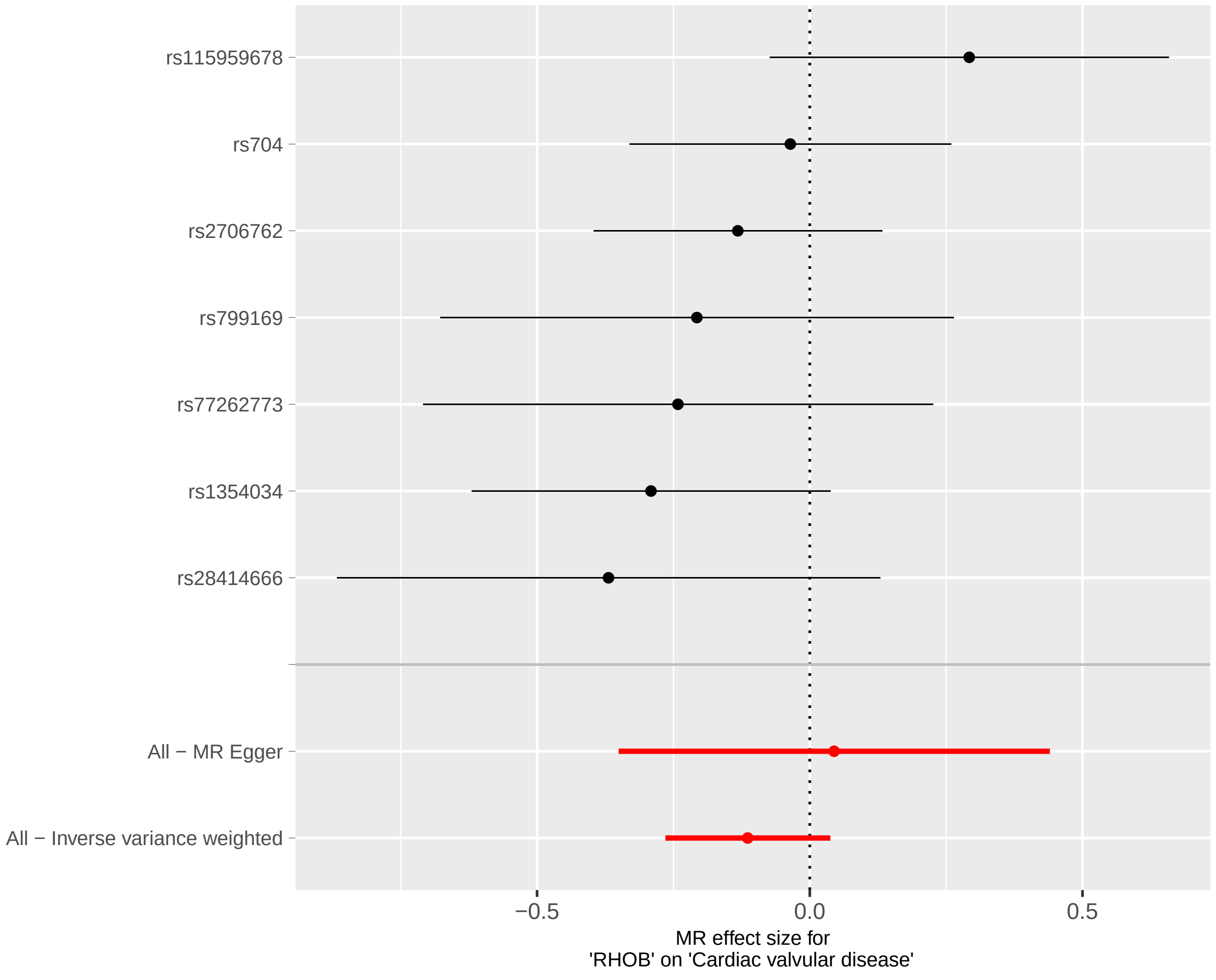
rs115959678
rs704
rs2706762
rs799169
rs77262773
rs1354034
rs28414666
All − MR Egger
All − Inverse variance weighted
−0.5
0.0
0.5
MR effect size for
'RHOB' on 'Cardiac valvular disease'

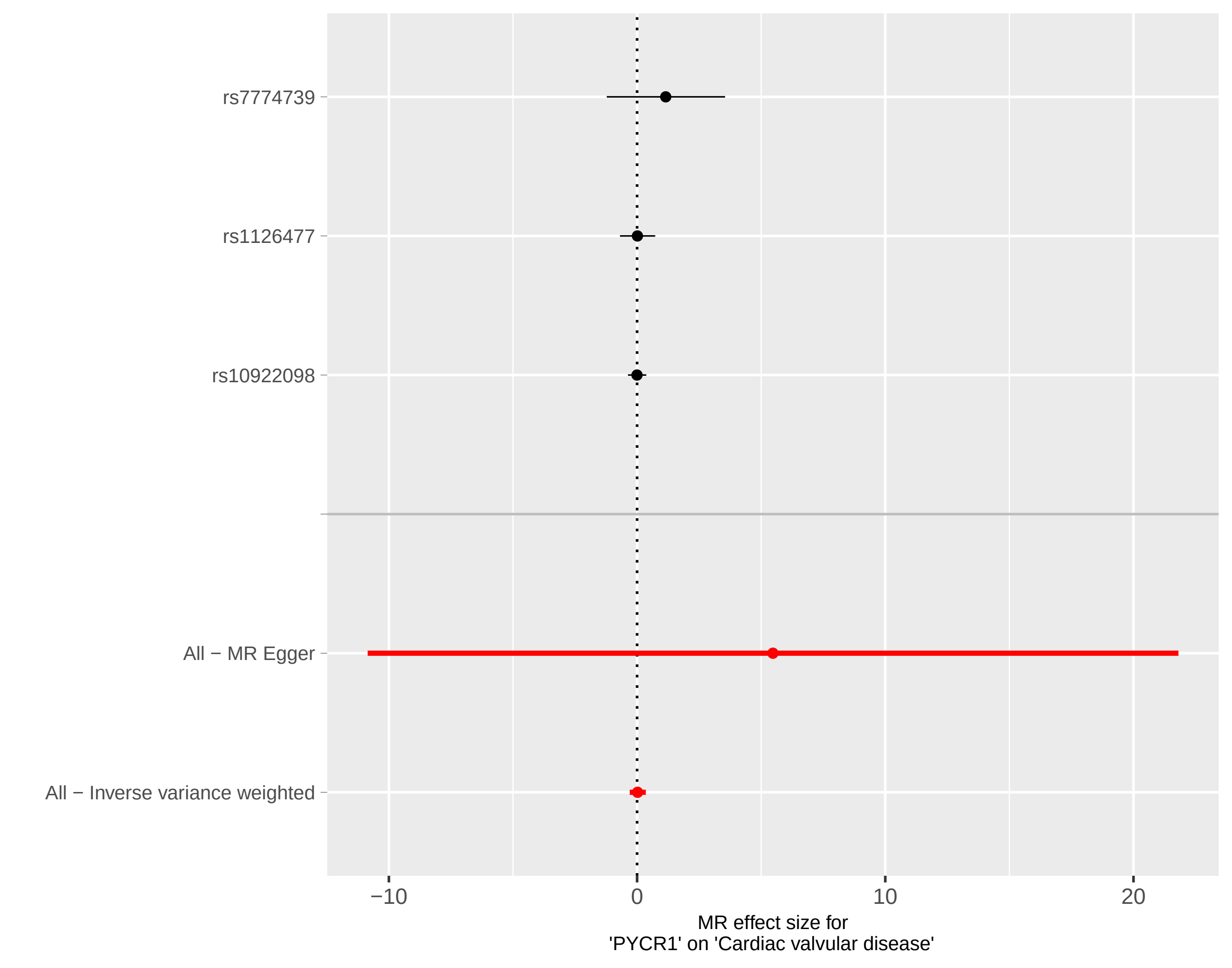

rs7774739
rs1126477
rs10922098
All − MR Egger
All − Inverse variance weighted
−10
0
10
20
MR effect size for
'PYCR1' on 'Cardiac valvular disease'

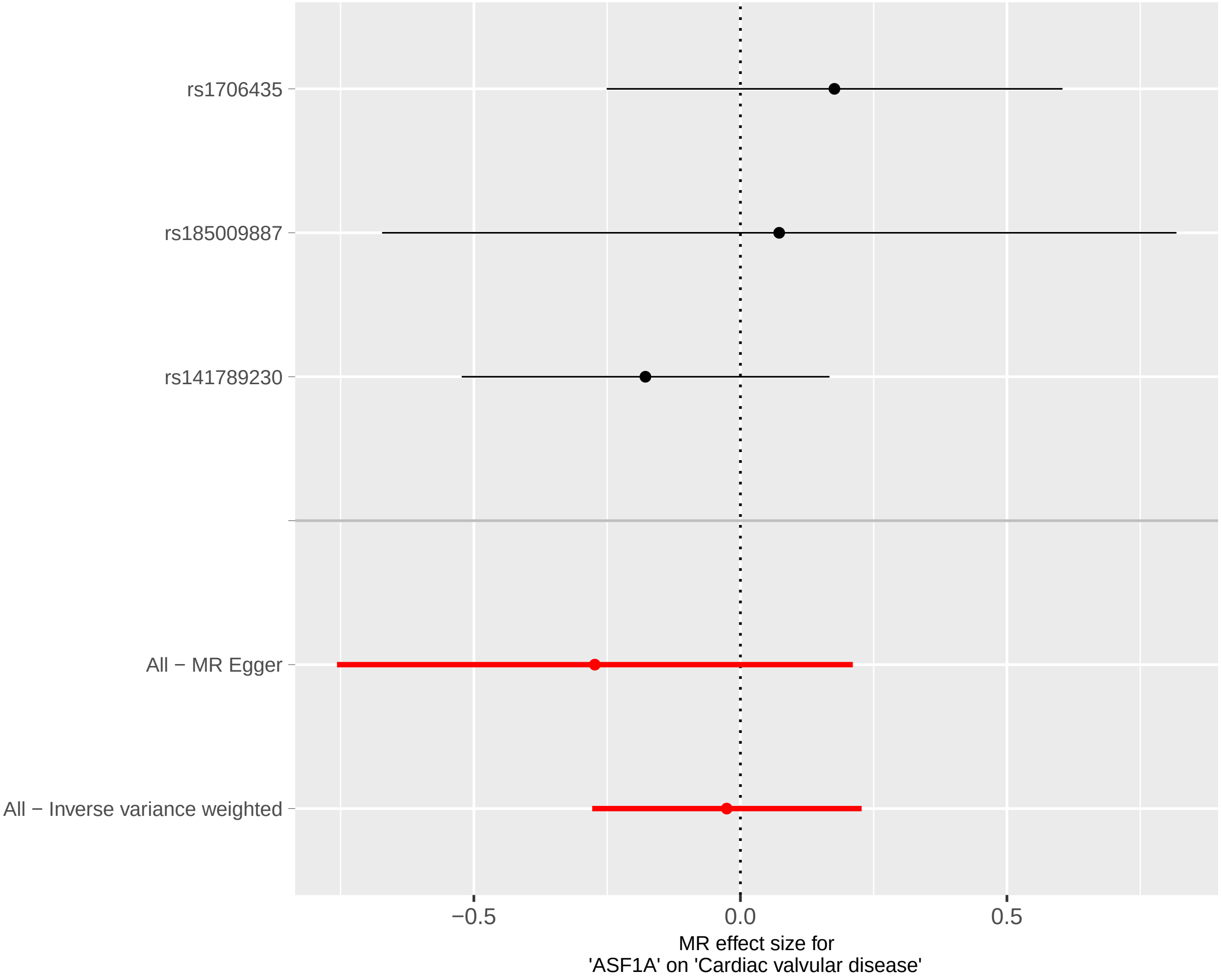
rs1706435
rs185009887
rs141789230
All − MR Egger
All − Inverse variance weighted
−0.5
0.0
0.5
MR effect size for
'ASF1A' on 'Cardiac valvular disease'

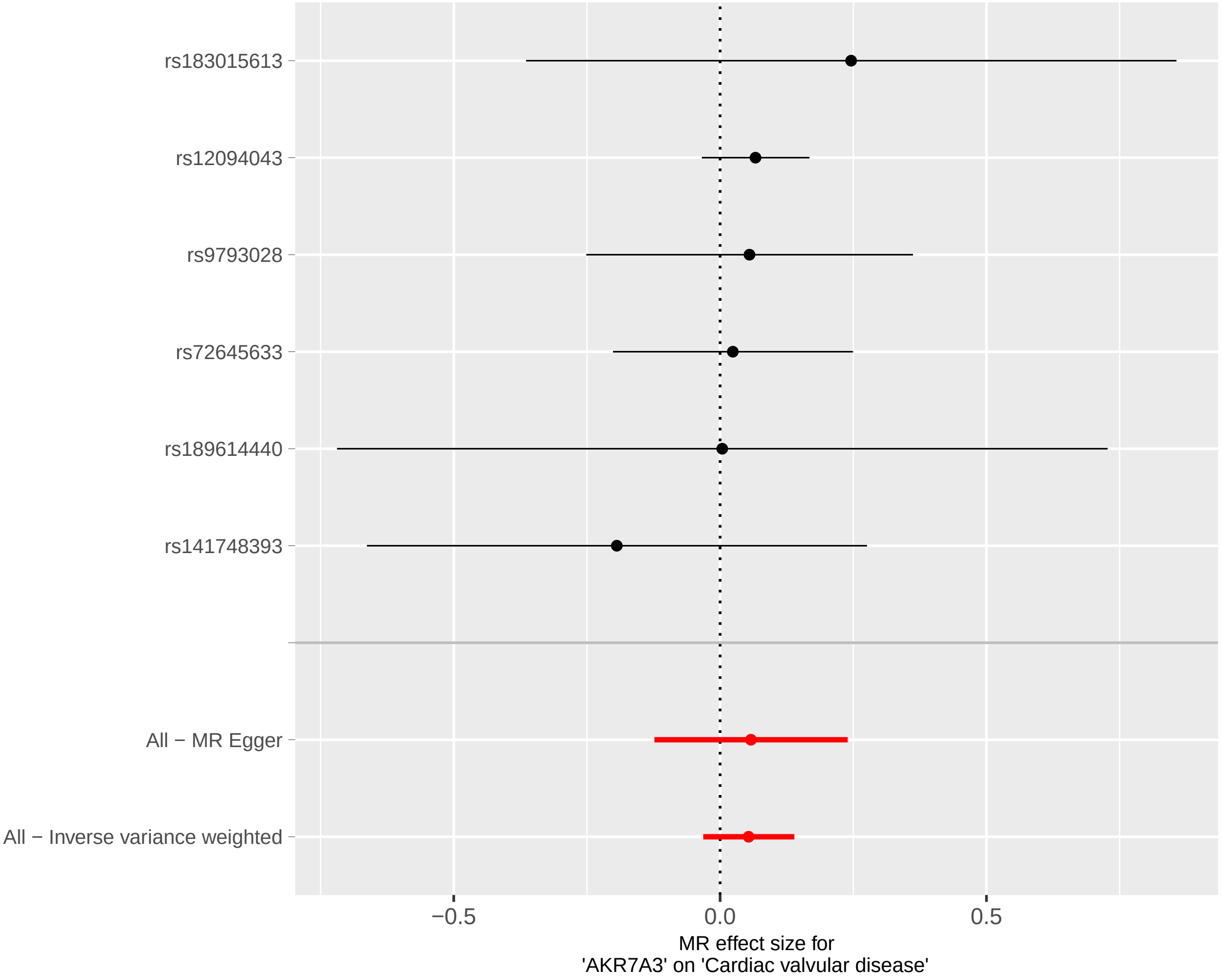
rs183015613
rs12094043
rs9793028
rs72645633
rs189614440
rs141748393
All − MR Egger
All − Inverse variance weighted
−0.5
0.0
0.5
MR effect size for
'AKR7A3' on 'Cardiac valvular disease'

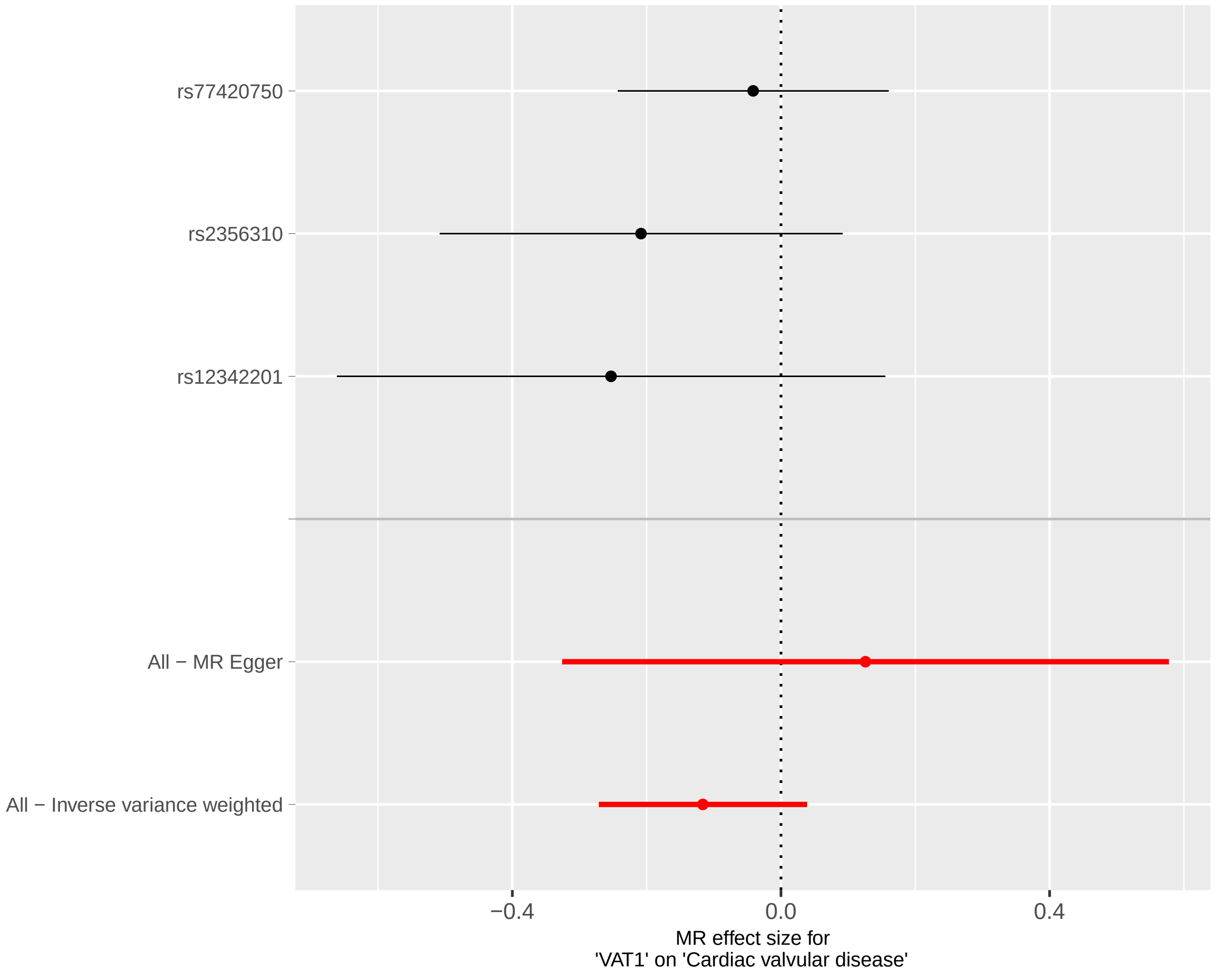

rs77420750
rs2356310
rs12342201
All − MR Egger
All − Inverse variance weighted
−0.4
0.0
0.4
MR effect size for
'VAT1' on 'Cardiac valvular disease'

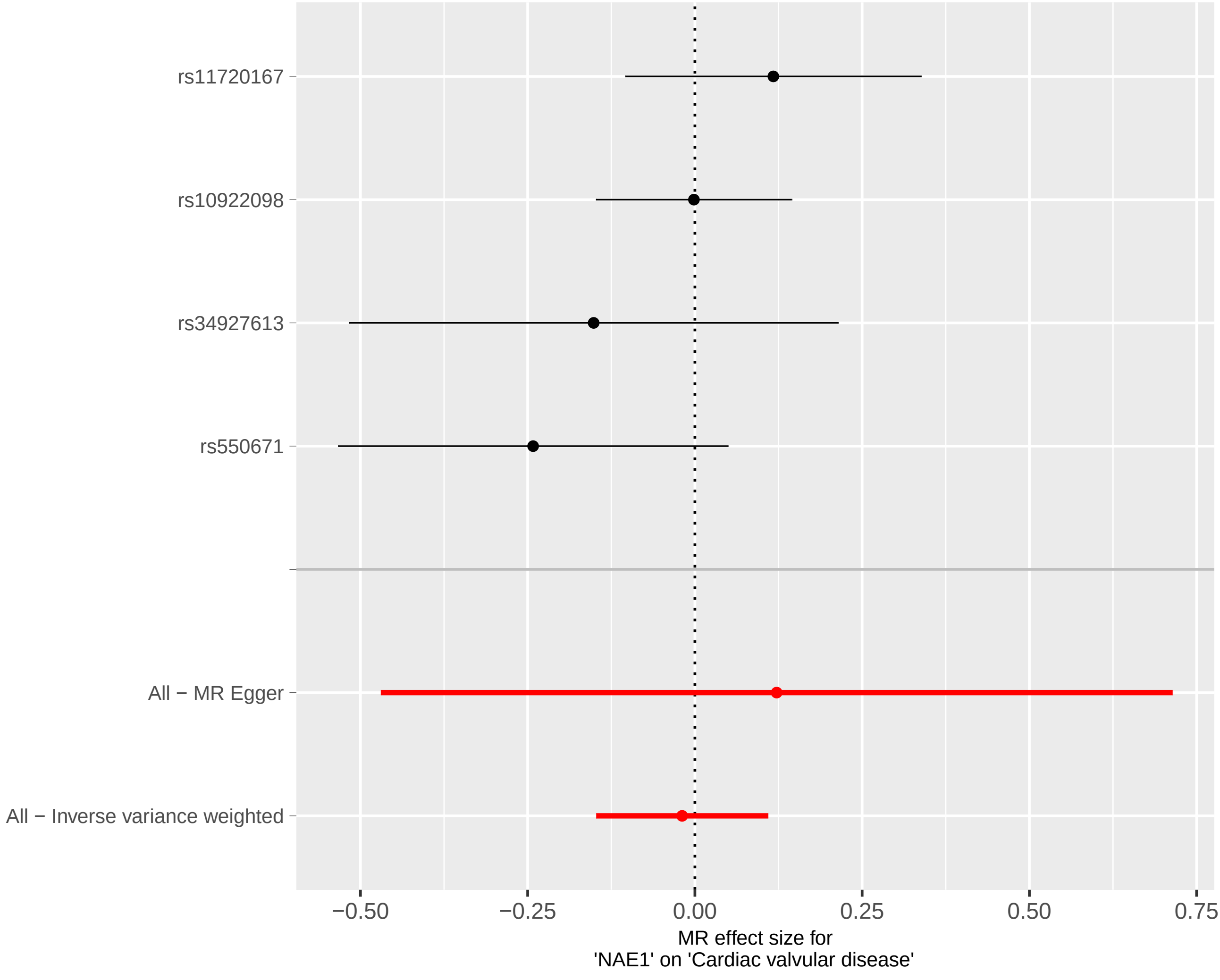
rs11720167
rs10922098
rs34927613
rs550671
All − MR Egger
All − Inverse variance weighted
−0.50
−0.25
0.00
0.25
0.50
0.75
MR effect size for
'NAE1' on 'Cardiac valvular disease'

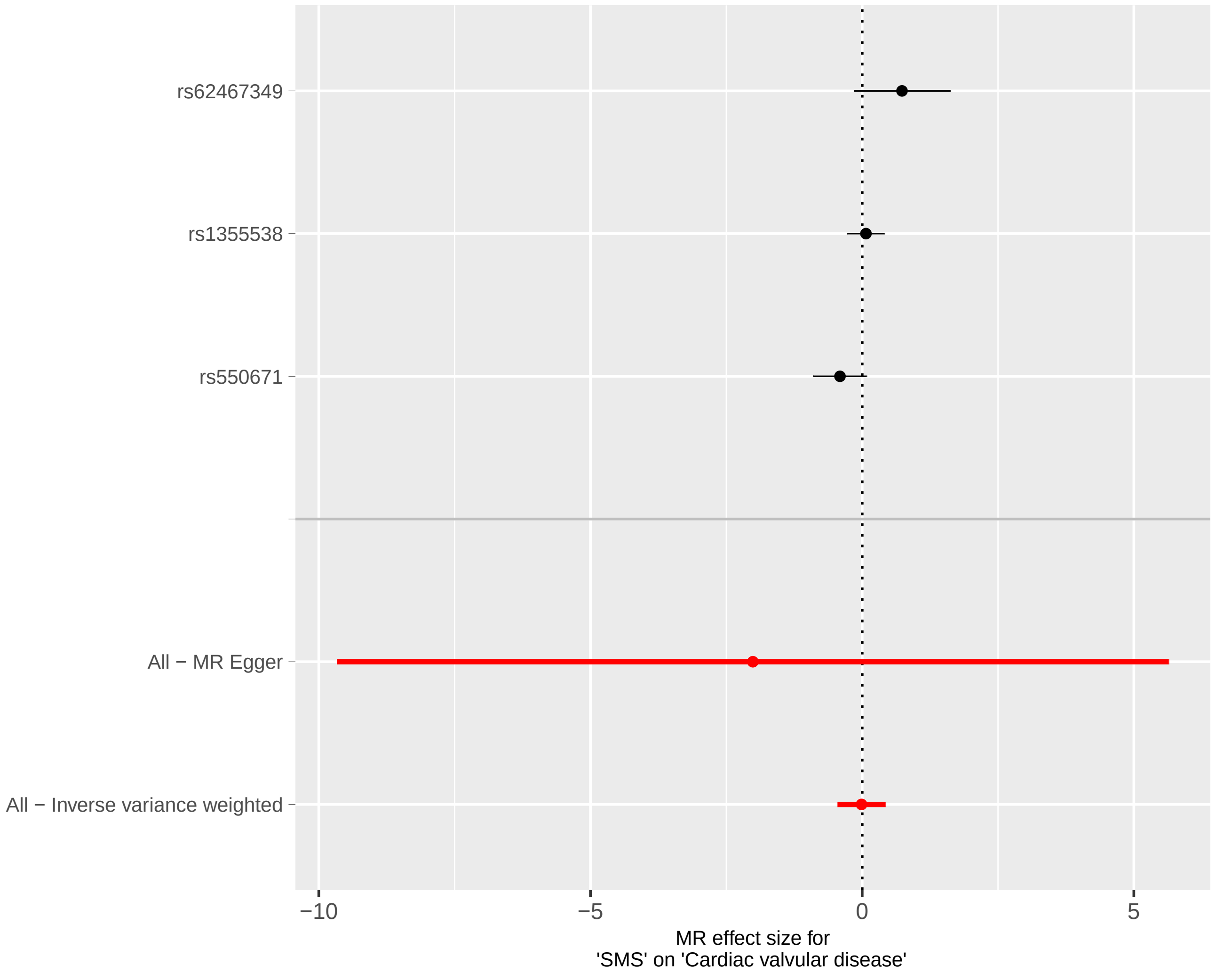
rs62467349
rs1355538
rs550671
All − MR Egger
All − Inverse variance weighted
−10
−5
0
5
MR effect size for
'SMS' on 'Cardiac valvular disease'

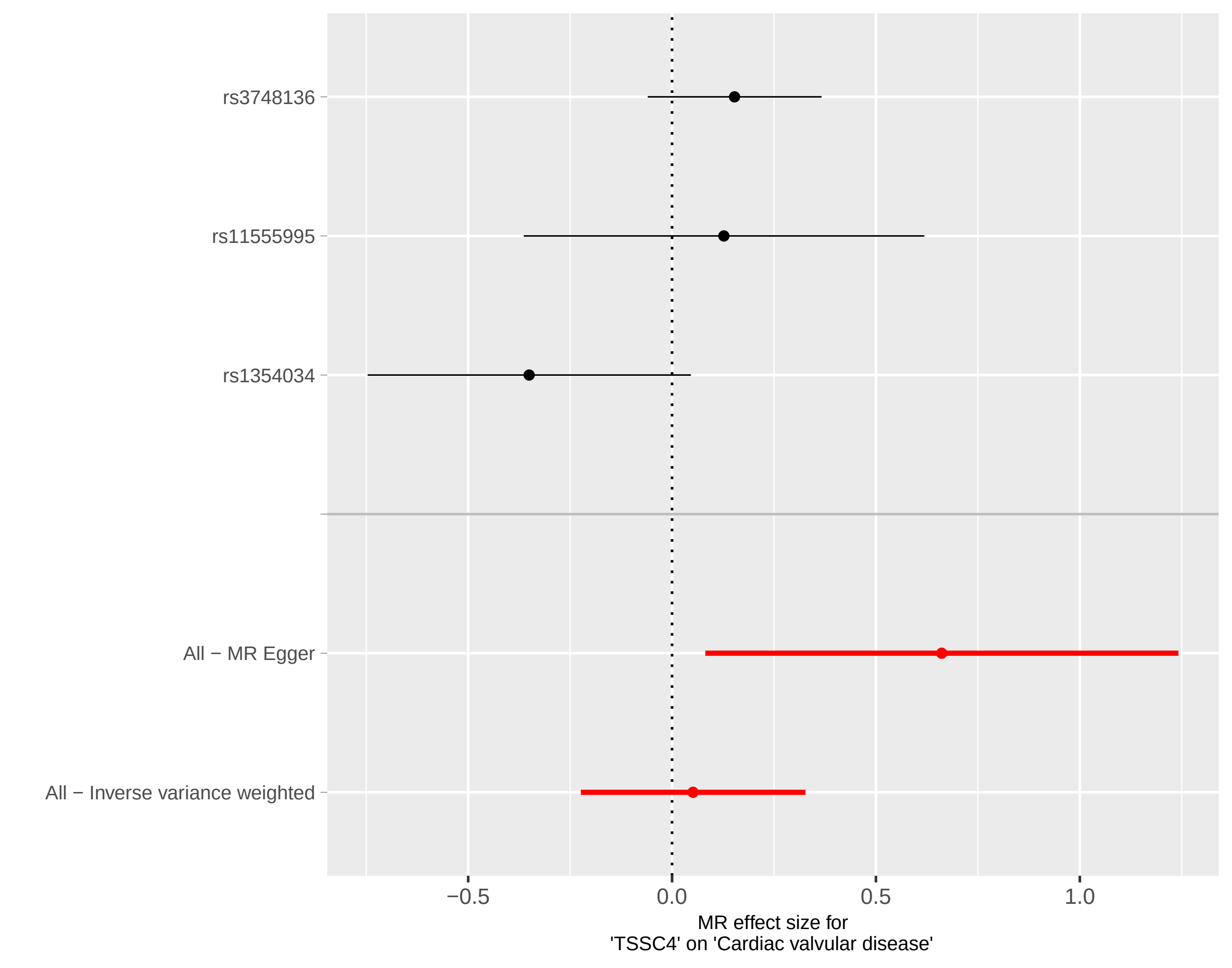

rs3748136
rs11555995
rs1354034
All − MR Egger
All − Inverse variance weighted
−0.5
0.0
0.5
1.0
MR effect size for
'TSSC4' on 'Cardiac valvular disease'

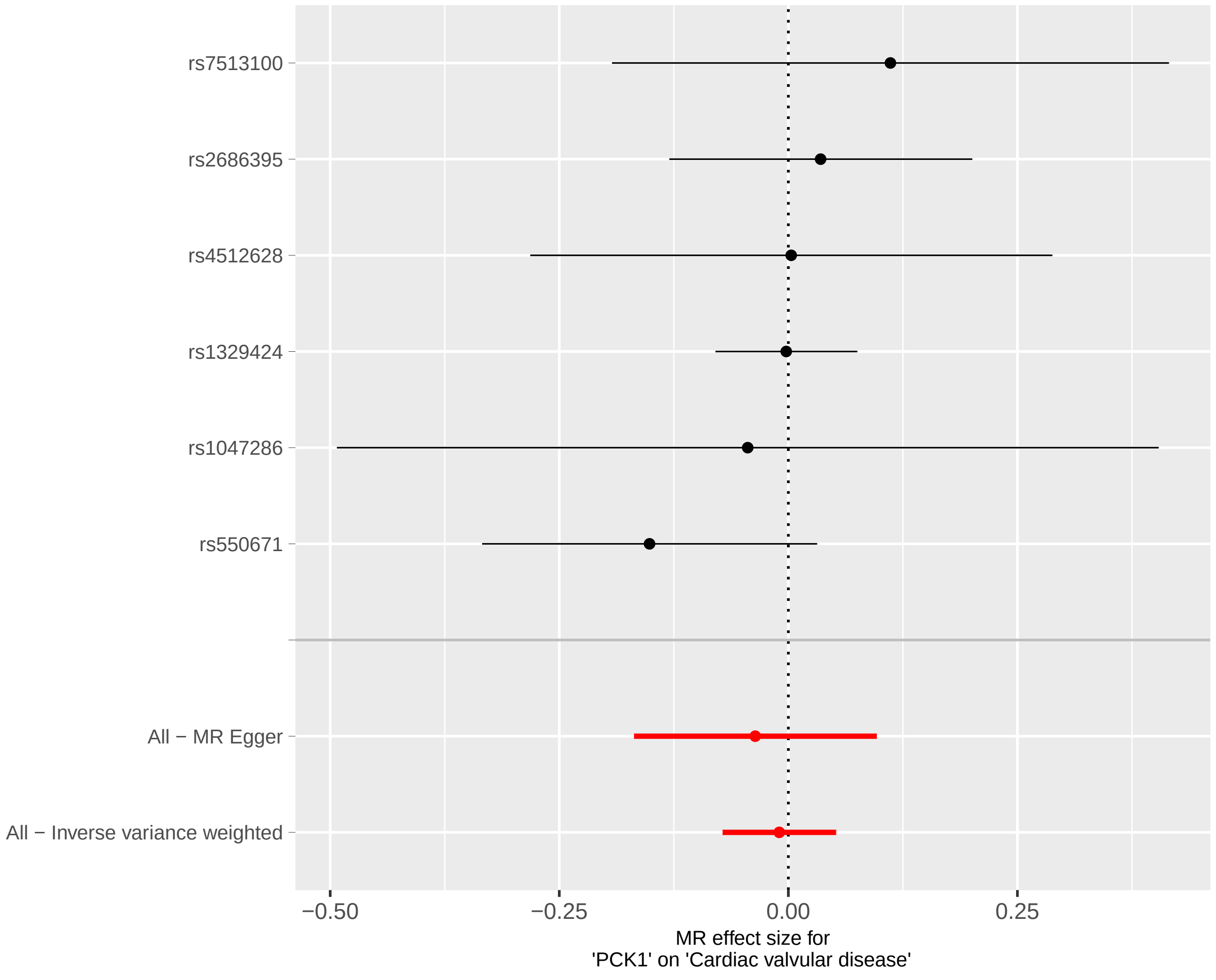

rs7513100
rs2686395
rs4512628
rs1329424
rs1047286
rs550671
All − MR Egger
All − Inverse variance weighted
−0.50
−0.25
0.00
0.25
MR effect size for
'PCK1' on 'Cardiac valvular disease'

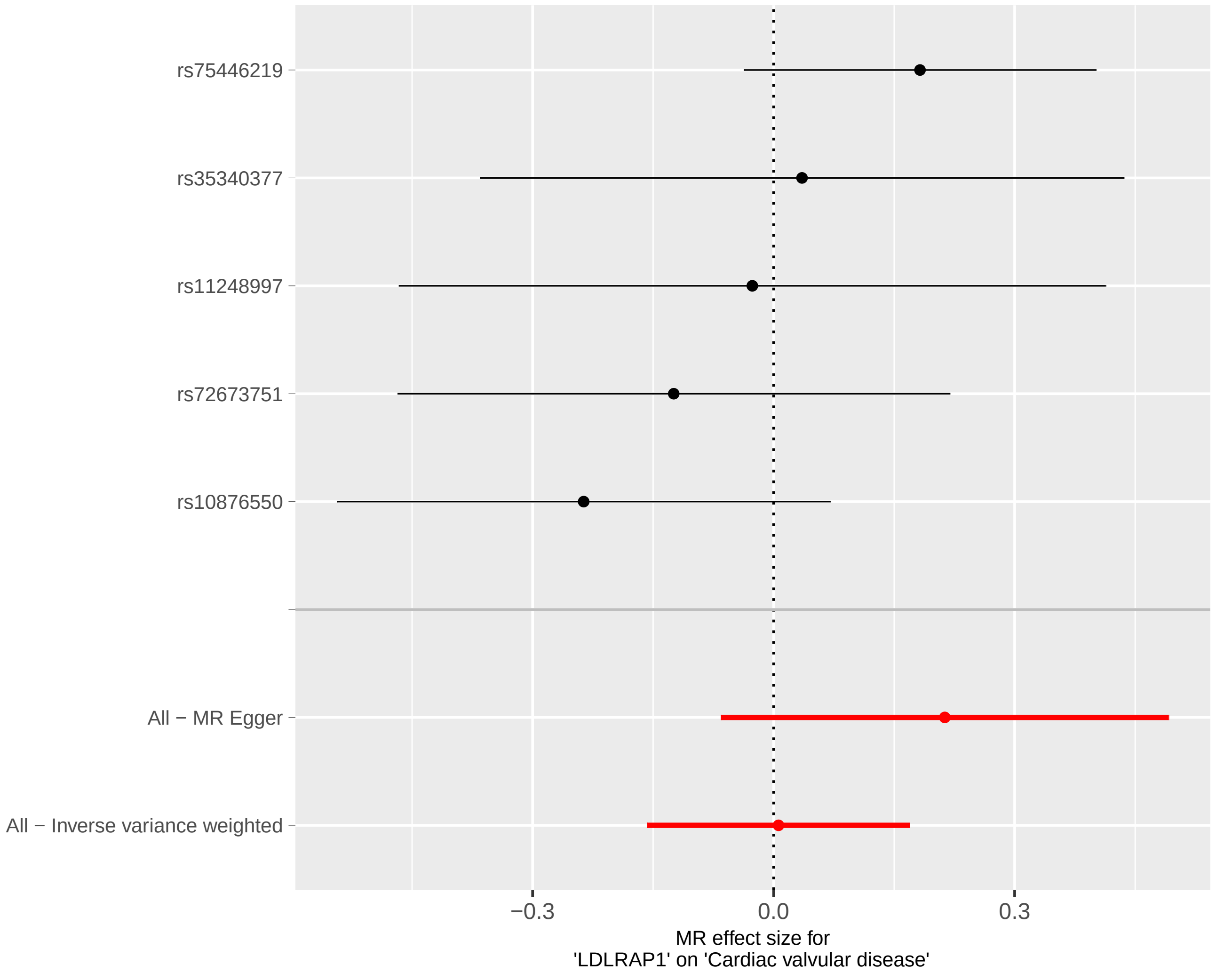
rs75446219
rs35340377
rs11248997
rs72673751
rs10876550
All − MR Egger
All − Inverse variance weighted
−0.3
0.0
0.3
MR effect size for
'LDLRAP1' on 'Cardiac valvular disease'

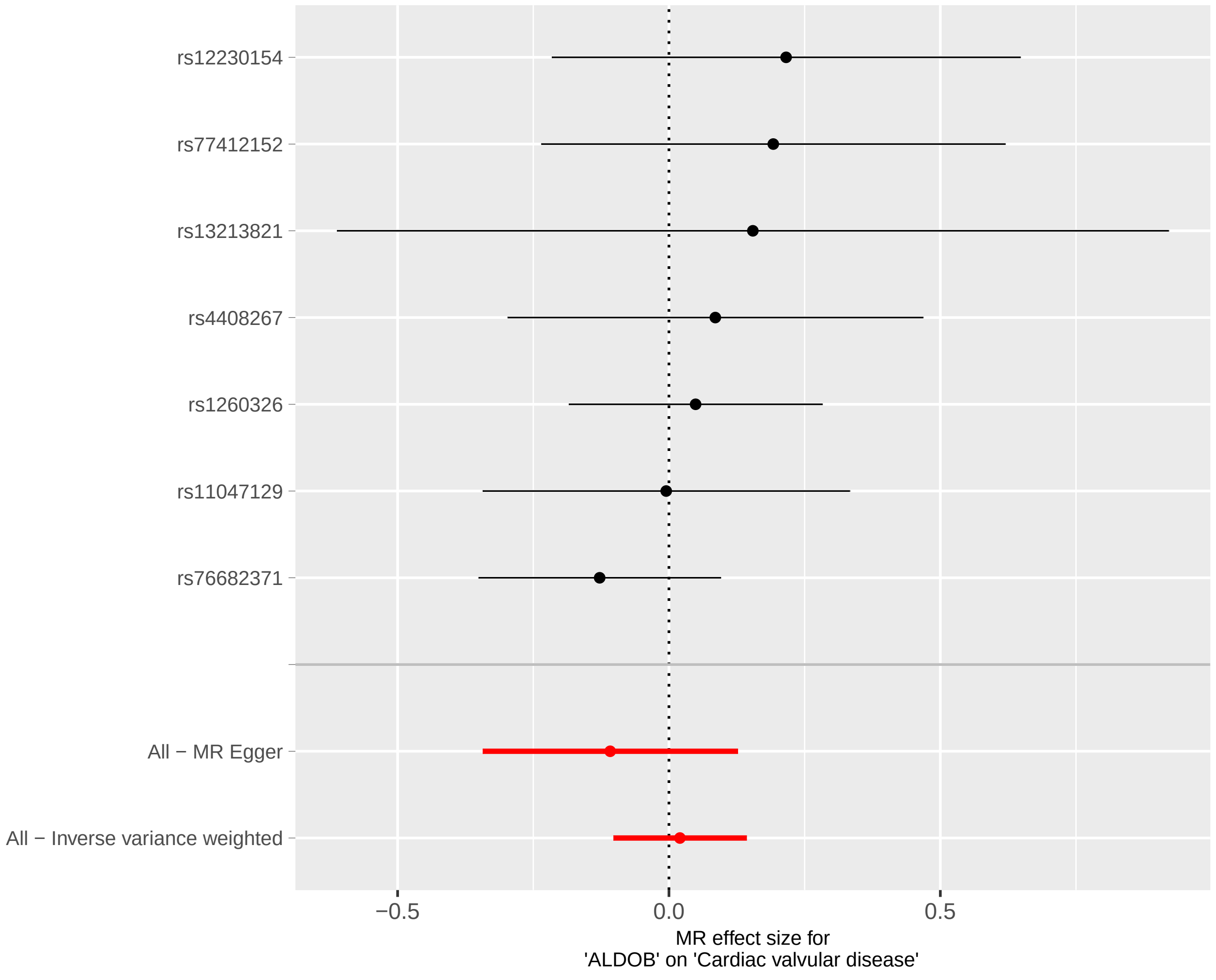
rs12230154
rs77412152
rs13213821
rs4408267
rs1260326
rs11047129
rs76682371
All − MR Egger
All − Inverse variance weighted
−0.5
0.0
0.5
MR effect size for
'ALDOB' on 'Cardiac valvular disease'

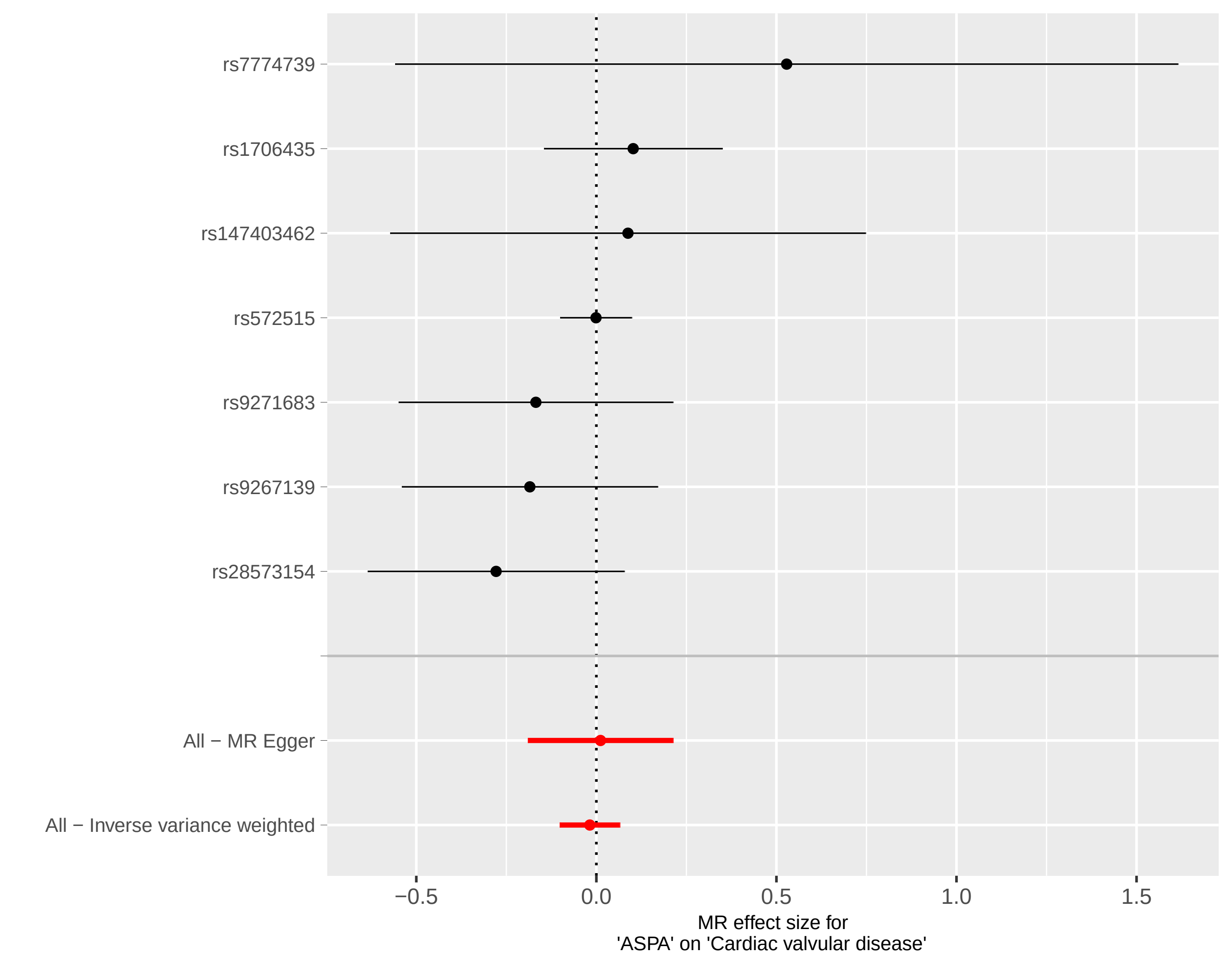
rs7774739
rs1706435
rs147403462
rs572515
rs9271683
rs9267139
rs28573154
All − MR Egger
All − Inverse variance weighted
−0.5
0.0
0.5
1.0
1.5
MR effect size for
'ASPA' on 'Cardiac valvular disease'

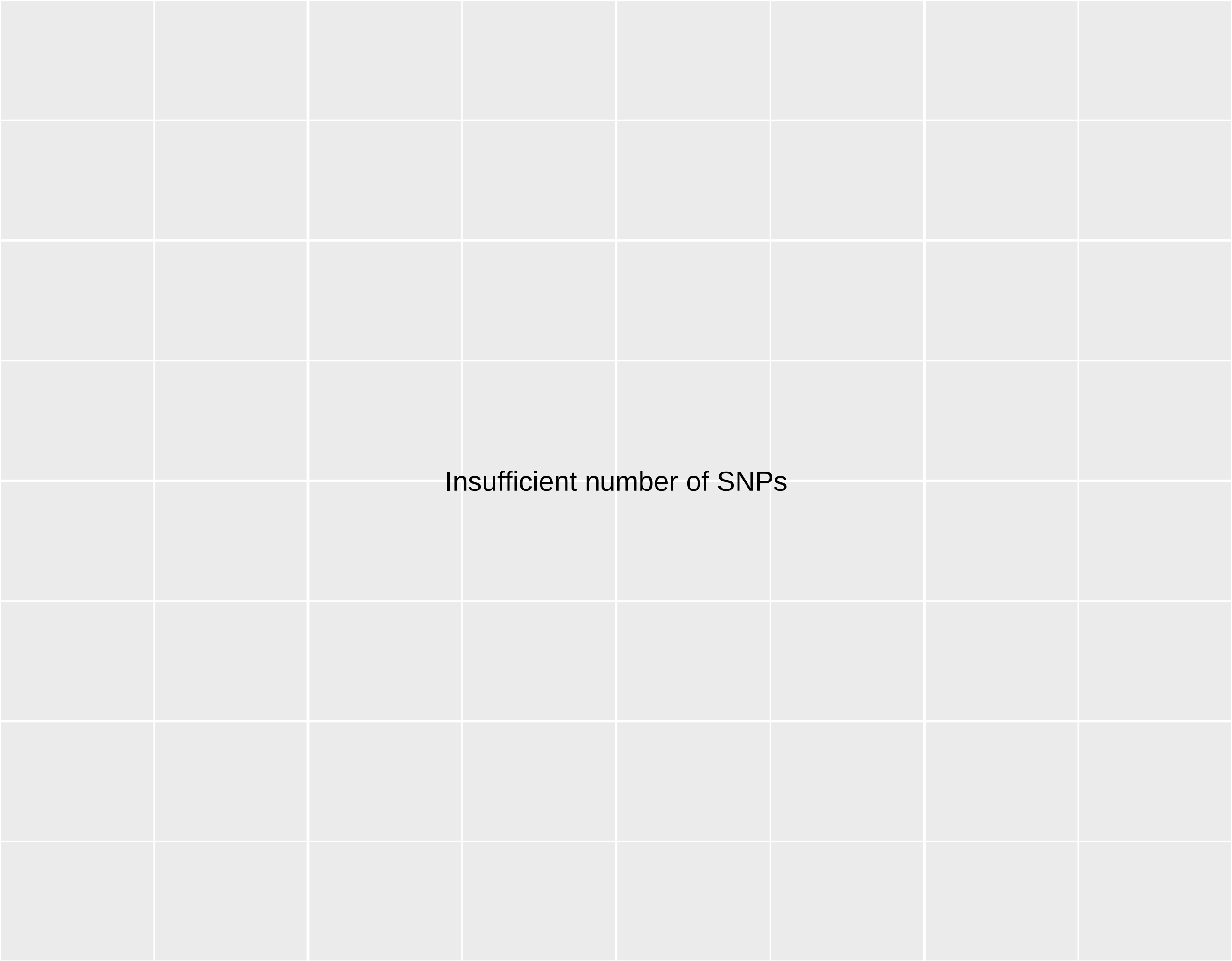
Insufficient number of SNPs

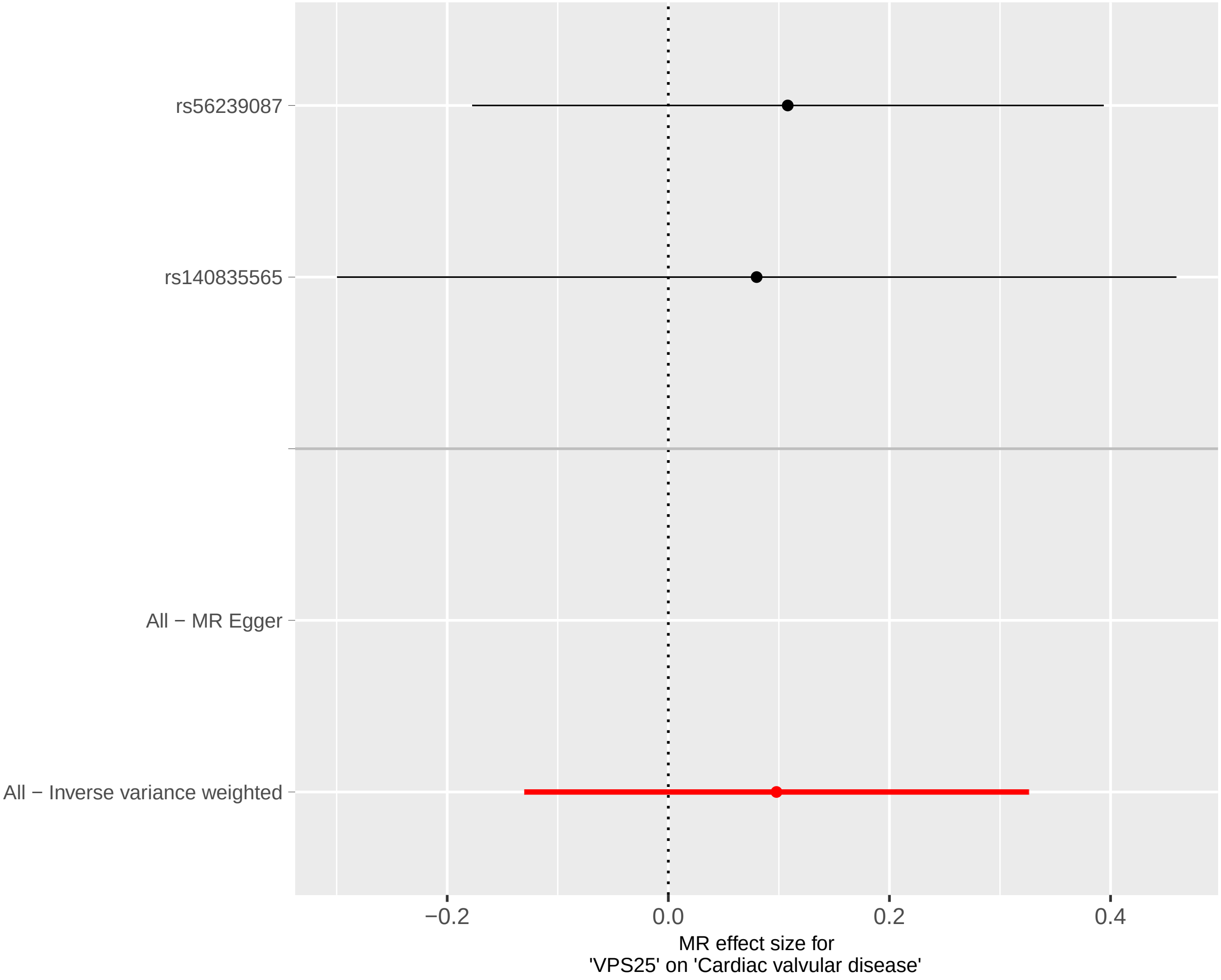
rs56239087
rs140835565
All − MR Egger
All − Inverse variance weighted
−0.2
0.0
0.2
0.4
MR effect size for
'VPS25' on 'Cardiac valvular disease'

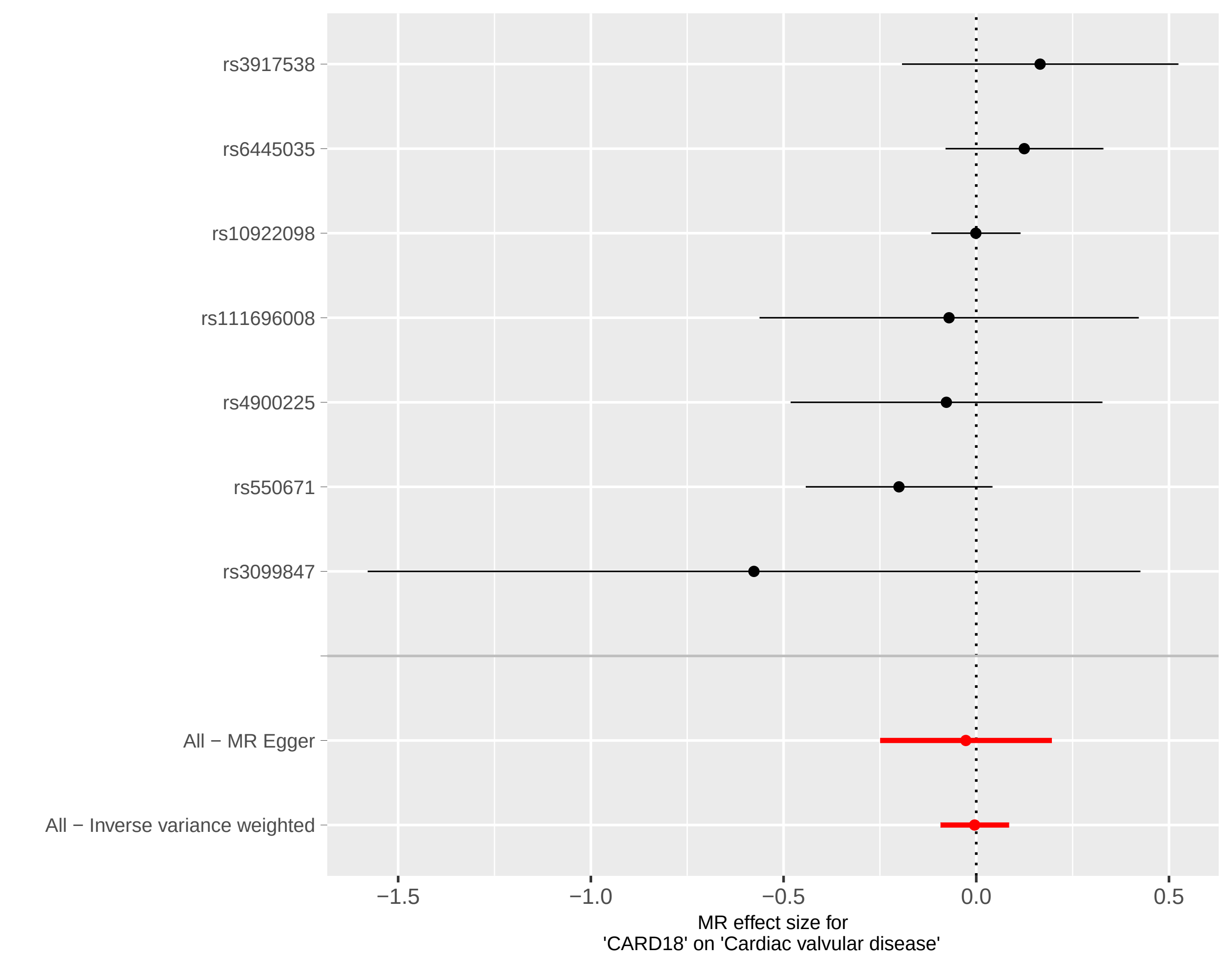
rs3917538
rs6445035
rs10922098
rs111696008
rs4900225
rs550671
rs3099847
All − MR Egger
All − Inverse variance weighted
−1.5
−1.0
−0.5
0.0
0.5
MR effect size for
'CARD18' on 'Cardiac valvular disease'

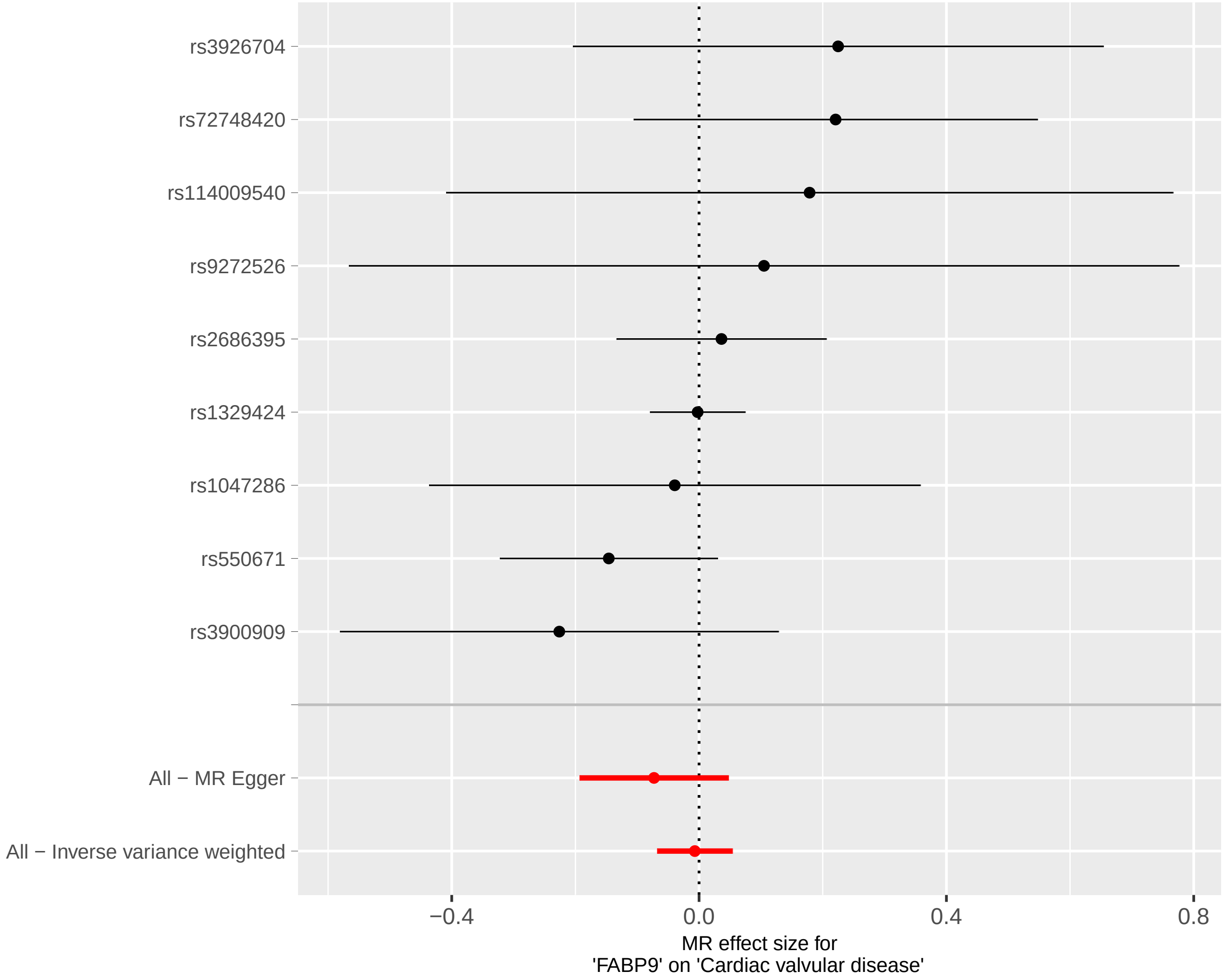
rs3926704
rs72748420
rs114009540
rs9272526
rs2686395
rs1329424
rs1047286
rs550671
rs3900909
All − MR Egger
All − Inverse variance weighted
−0.4
0.0
0.4
0.8
MR effect size for
'FABP9' on 'Cardiac valvular disease'

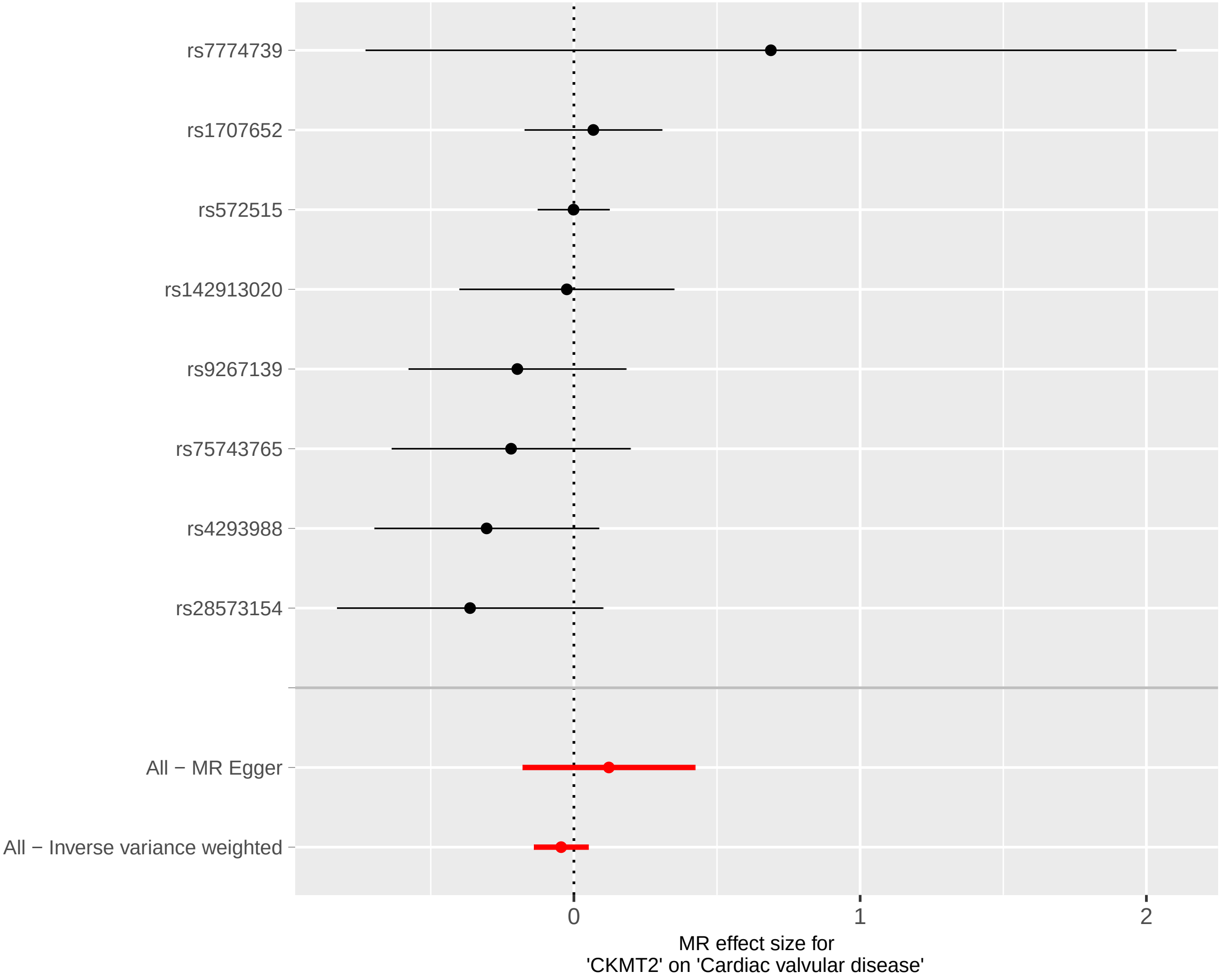

rs7774739
rs1707652
rs572515
rs142913020
rs9267139
rs75743765
rs4293988
rs28573154
All − MR Egger
All − Inverse variance weighted
0
1
2
MR effect size for
'CKMT2' on 'Cardiac valvular disease'

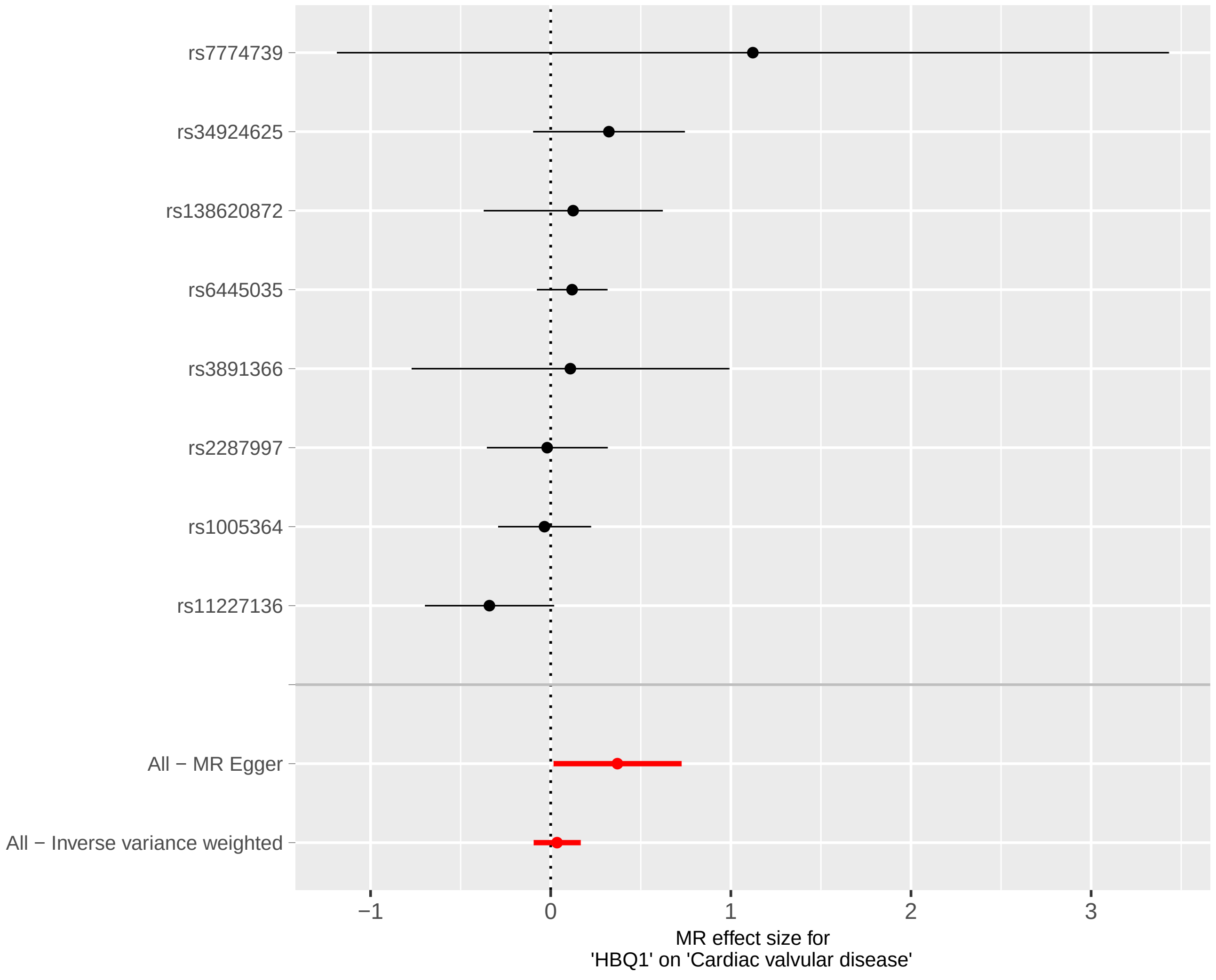
rs7774739
rs34924625
rs138620872
rs6445035
rs3891366
rs2287997
rs1005364
rs11227136
All − MR Egger
All − Inverse variance weighted
−1
0
1
2
3
MR effect size for
'HBQ1' on 'Cardiac valvular disease'

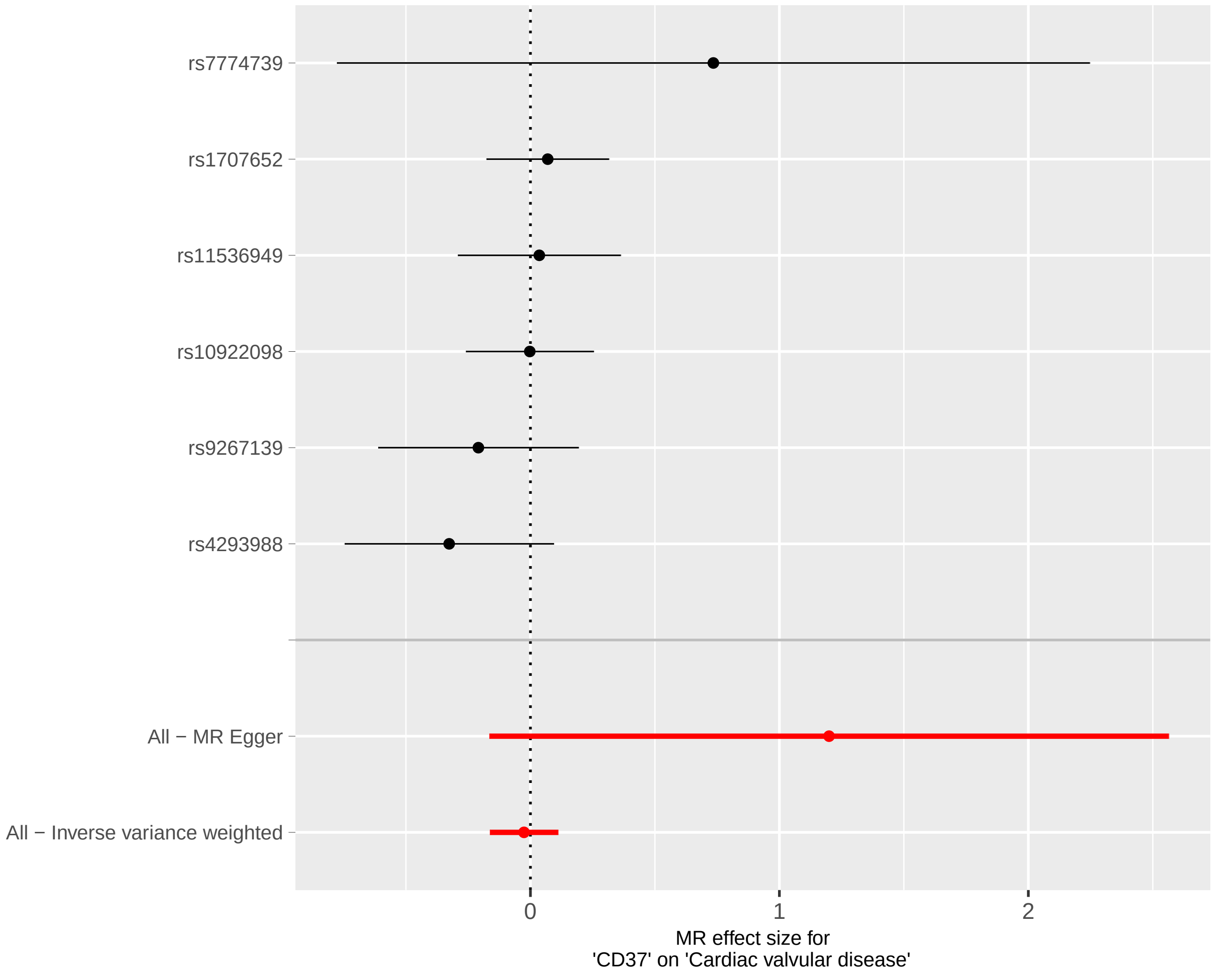

rs7774739
rs1707652
rs11536949
rs10922098
rs9267139
rs4293988
All − MR Egger
All − Inverse variance weighted
0
1
2
MR effect size for
'CD37' on 'Cardiac valvular disease'

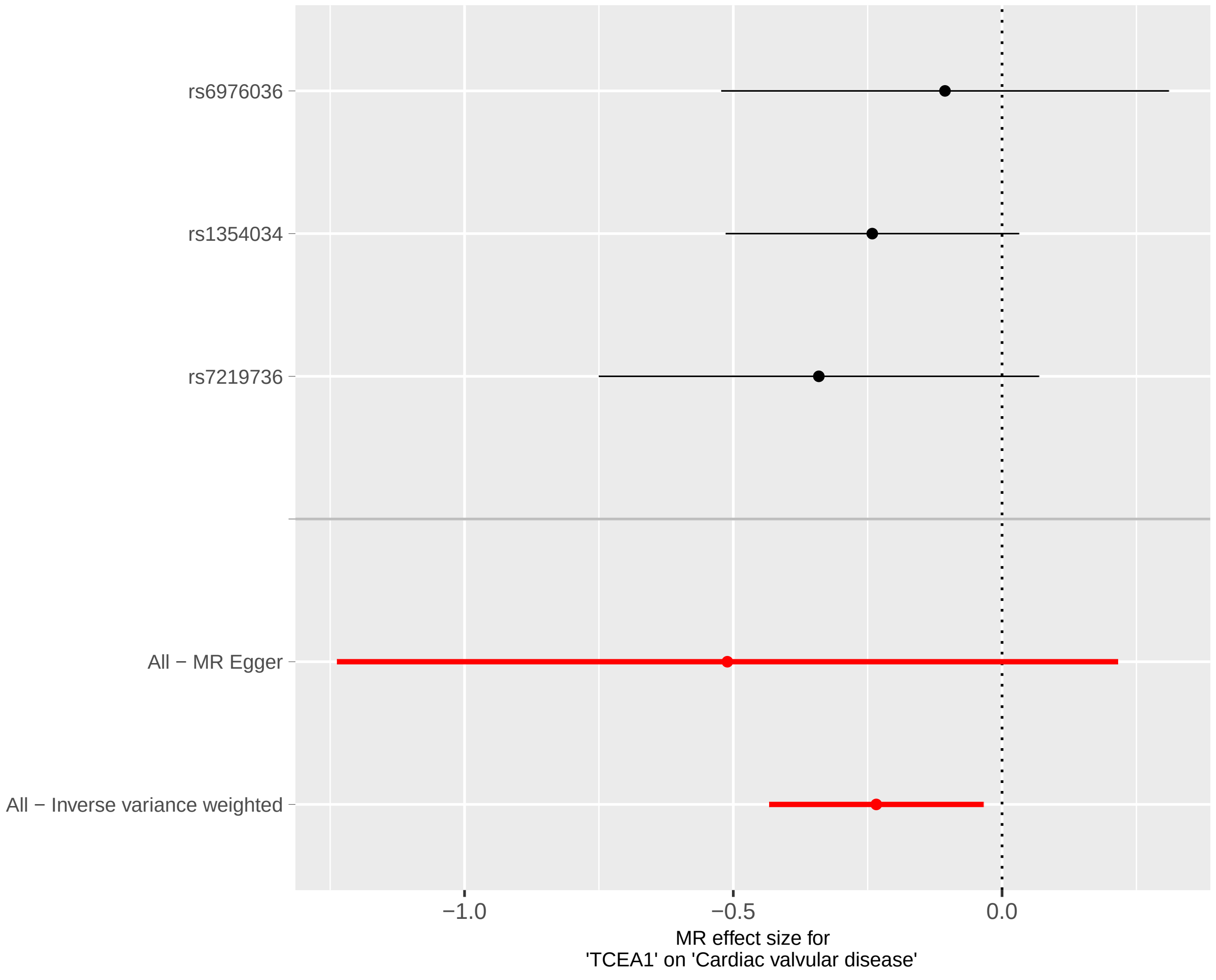
rs6976036
rs1354034
rs7219736
All − MR Egger
All − Inverse variance weighted
−1.0
−0.5
0.0
MR effect size for
'TCEA1' on 'Cardiac valvular disease'

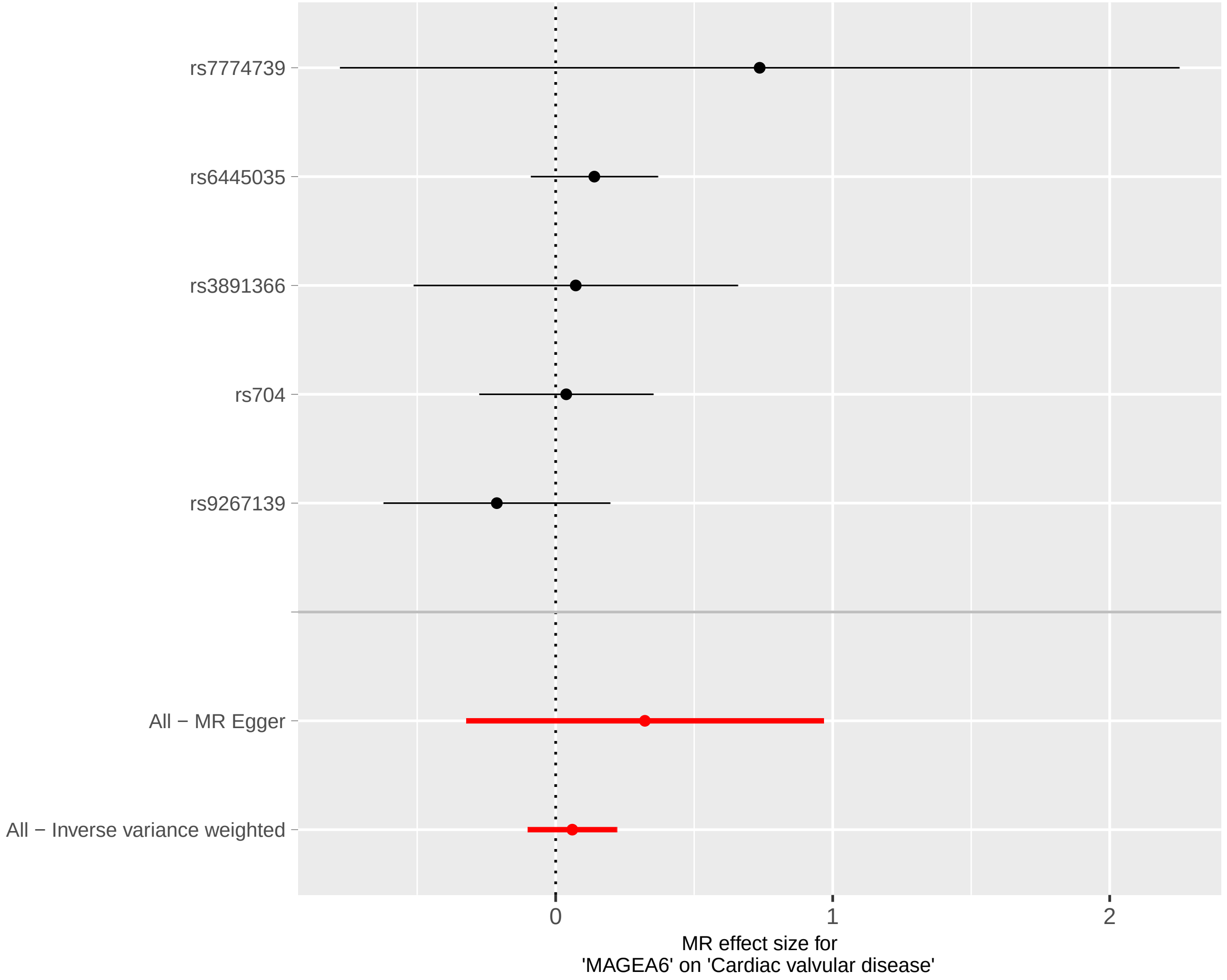

rs7774739
rs6445035
rs3891366
rs704
rs9267139
All − MR Egger
All − Inverse variance weighted
0
1
2
MR effect size for
'MAGEA6' on 'Cardiac valvular disease'

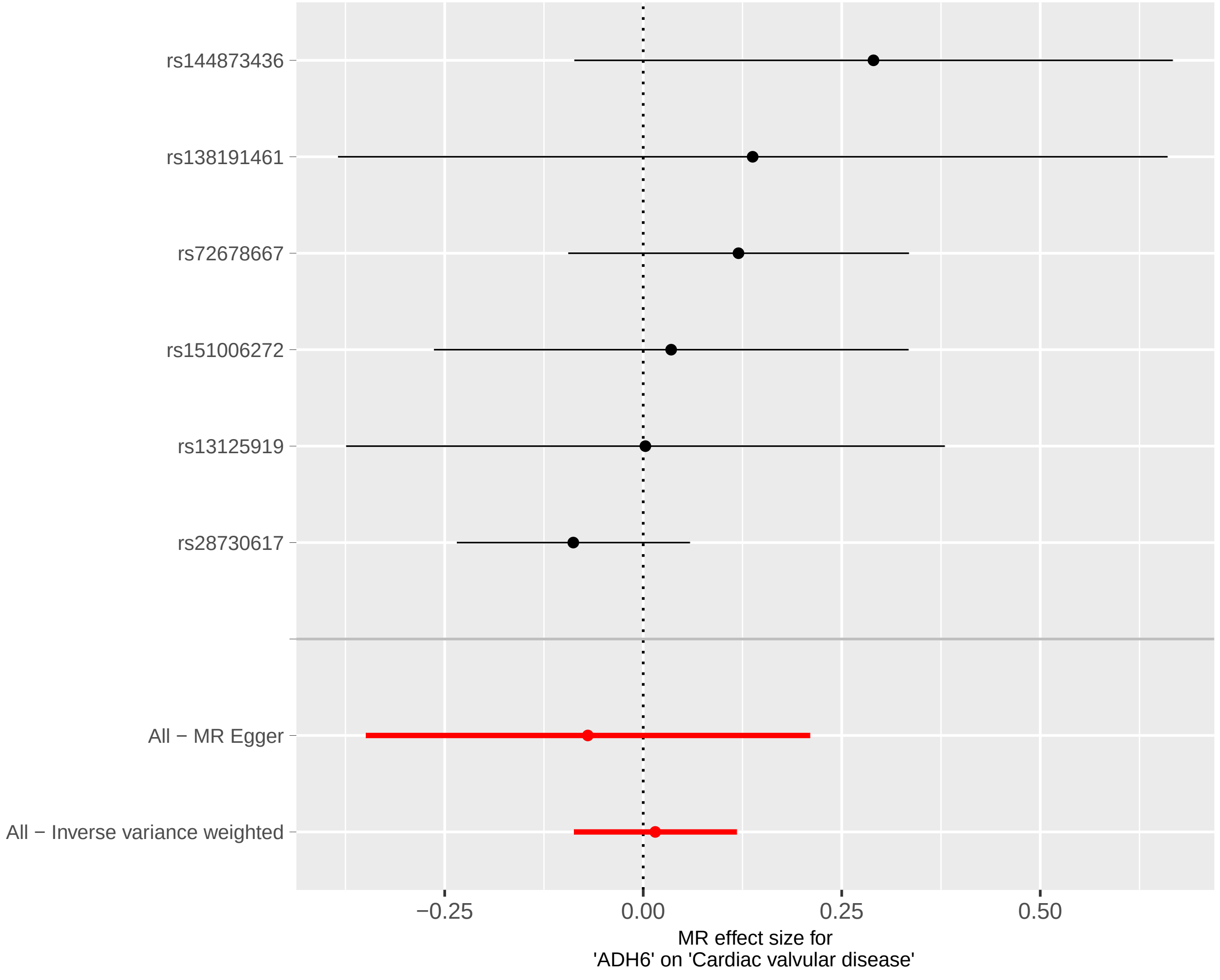
rs144873436
rs138191461
rs72678667
rs151006272
rs13125919
rs28730617
All − MR Egger
All − Inverse variance weighted
−0.25
0.00
0.25
0.50
MR effect size for
'ADH6' on 'Cardiac valvular disease'

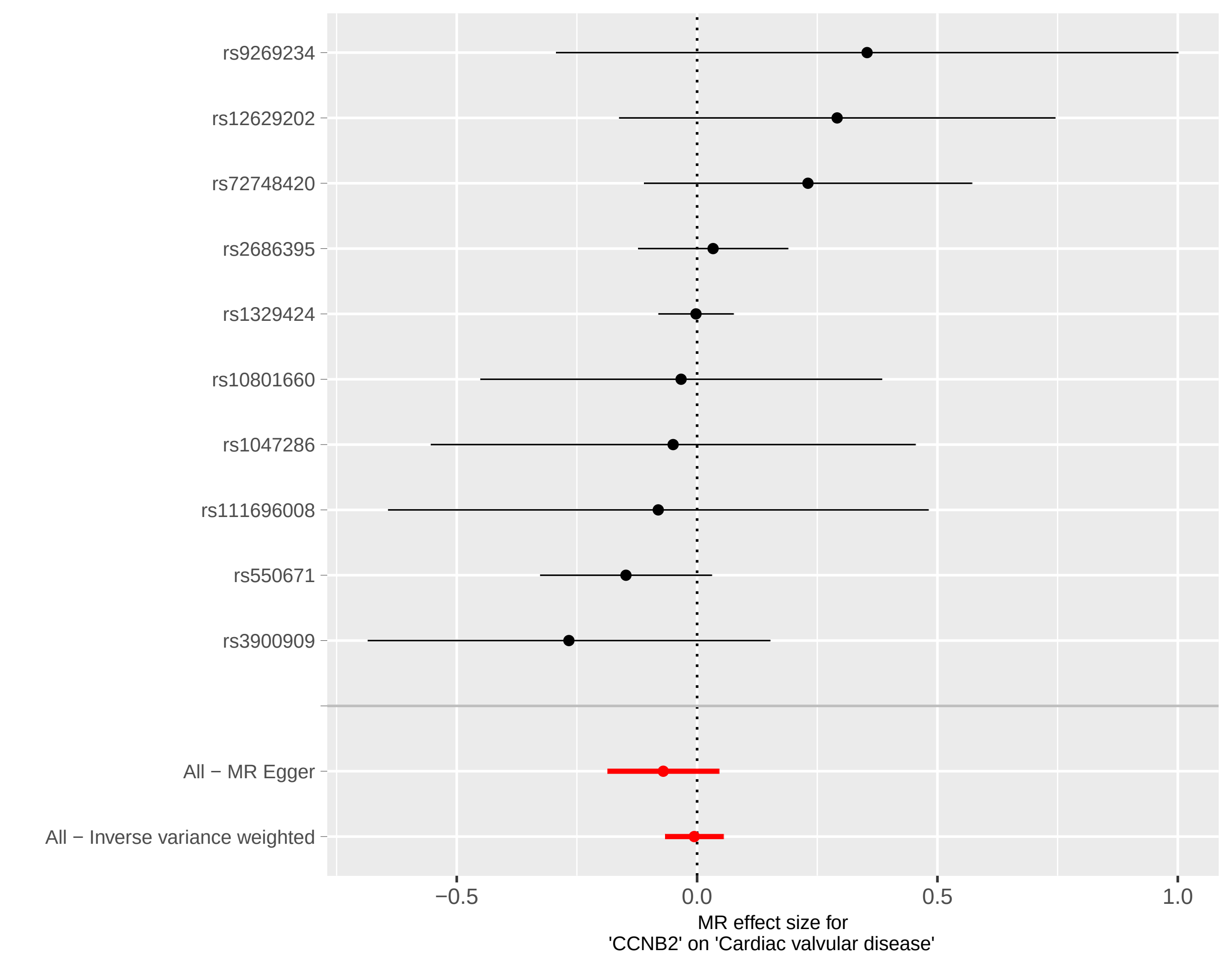
rs9269234
rs12629202
rs72748420
rs2686395
rs1329424
rs10801660
rs1047286
rs111696008
rs550671
rs3900909
All − MR Egger
All − Inverse variance weighted
−0.5
0.0
0.5
1.0
MR effect size for
'CCNB2' on 'Cardiac valvular disease'

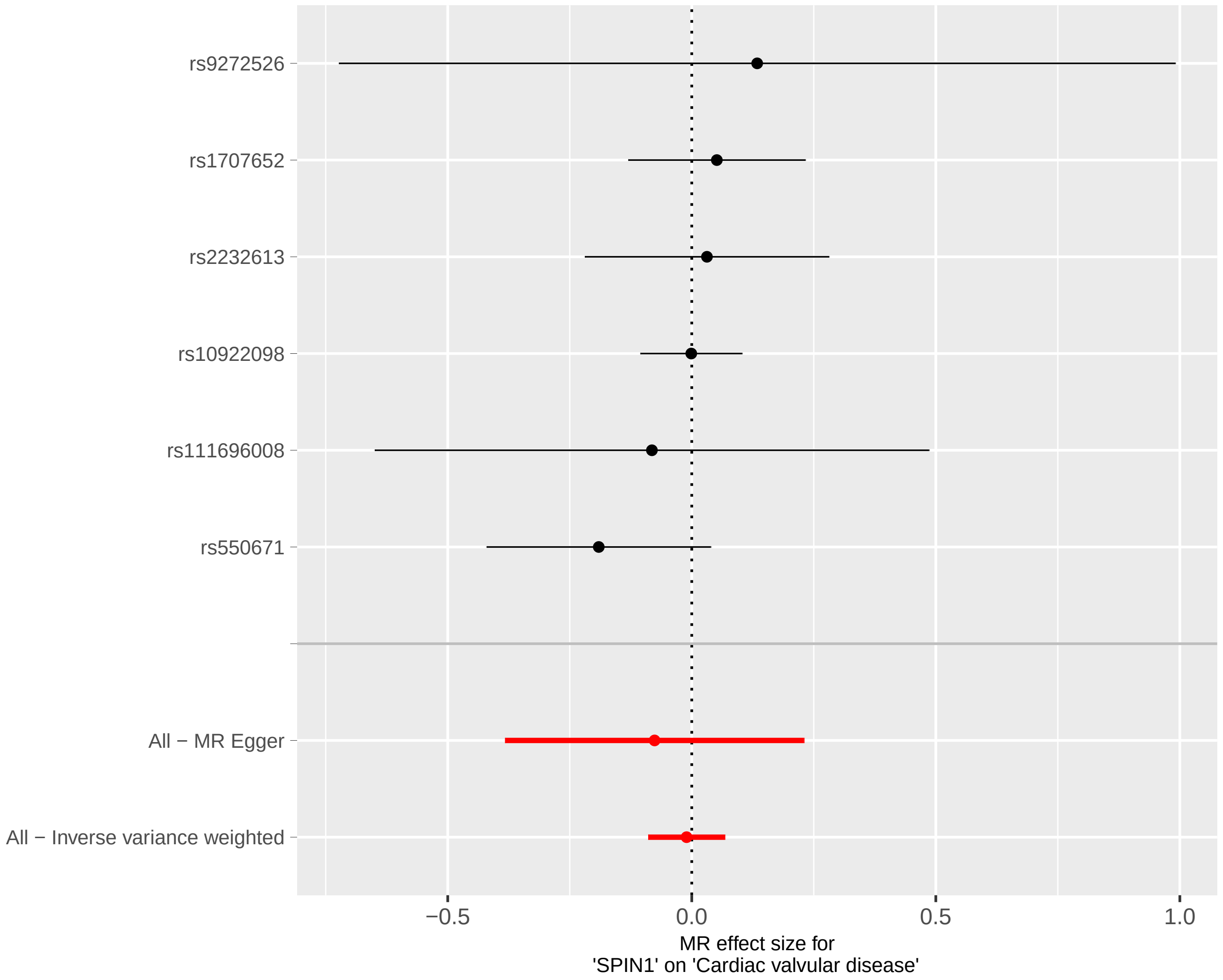

rs9272526
rs1707652
rs2232613
rs10922098
rs11696008
rs550671
All − MR Egger
All − Inverse variance weighted
−0.5
0.0
0.5
1.0
MR effect size for
'SPIN1' on 'Cardiac valvular disease'

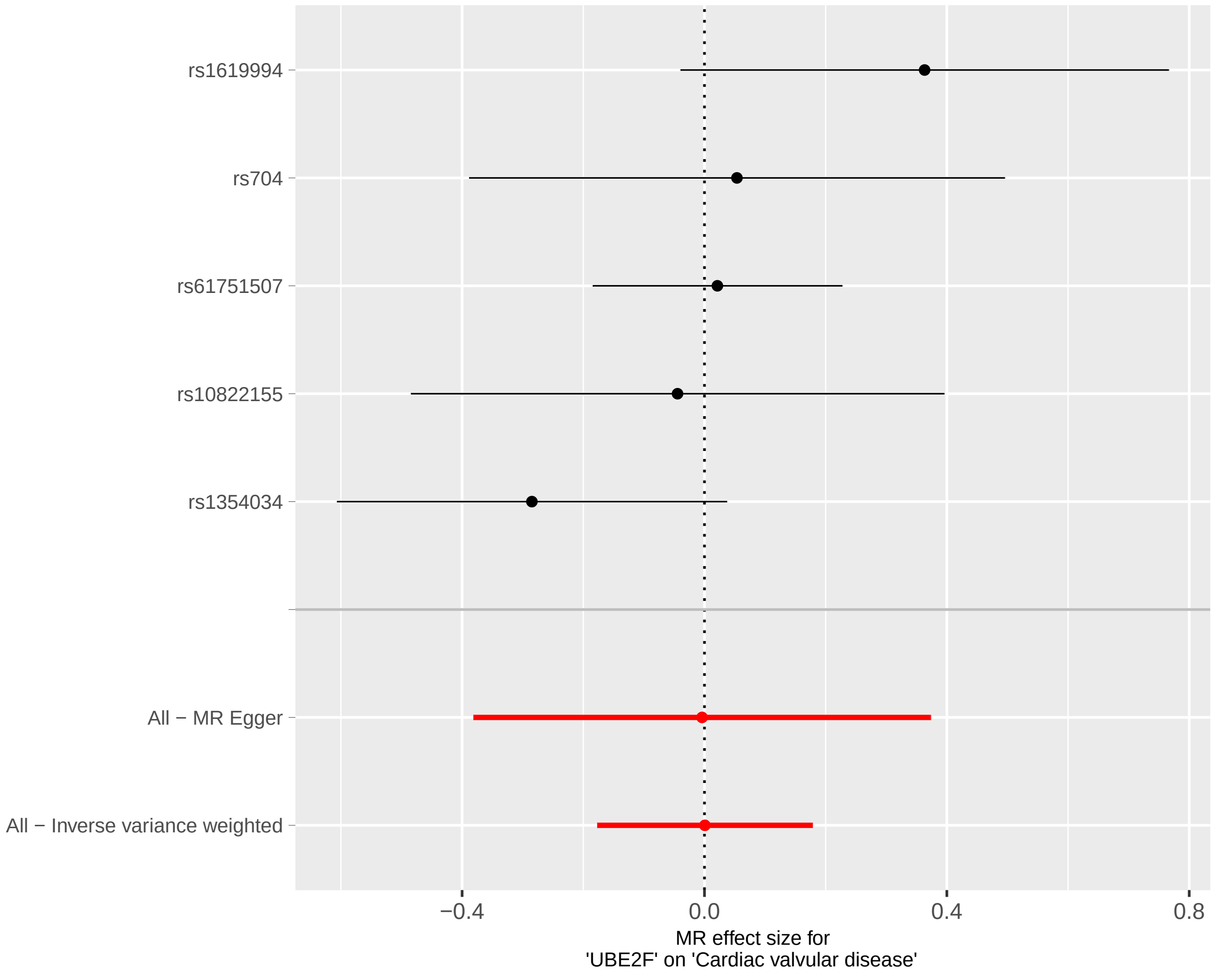
rs1619994
rs704
rs61751507
rs10822155
rs1354034
All − MR Egger
All − Inverse variance weighted
−0.4
0.0
0.4
0.8
MR effect size for
'UBE2F' on 'Cardiac valvular disease'

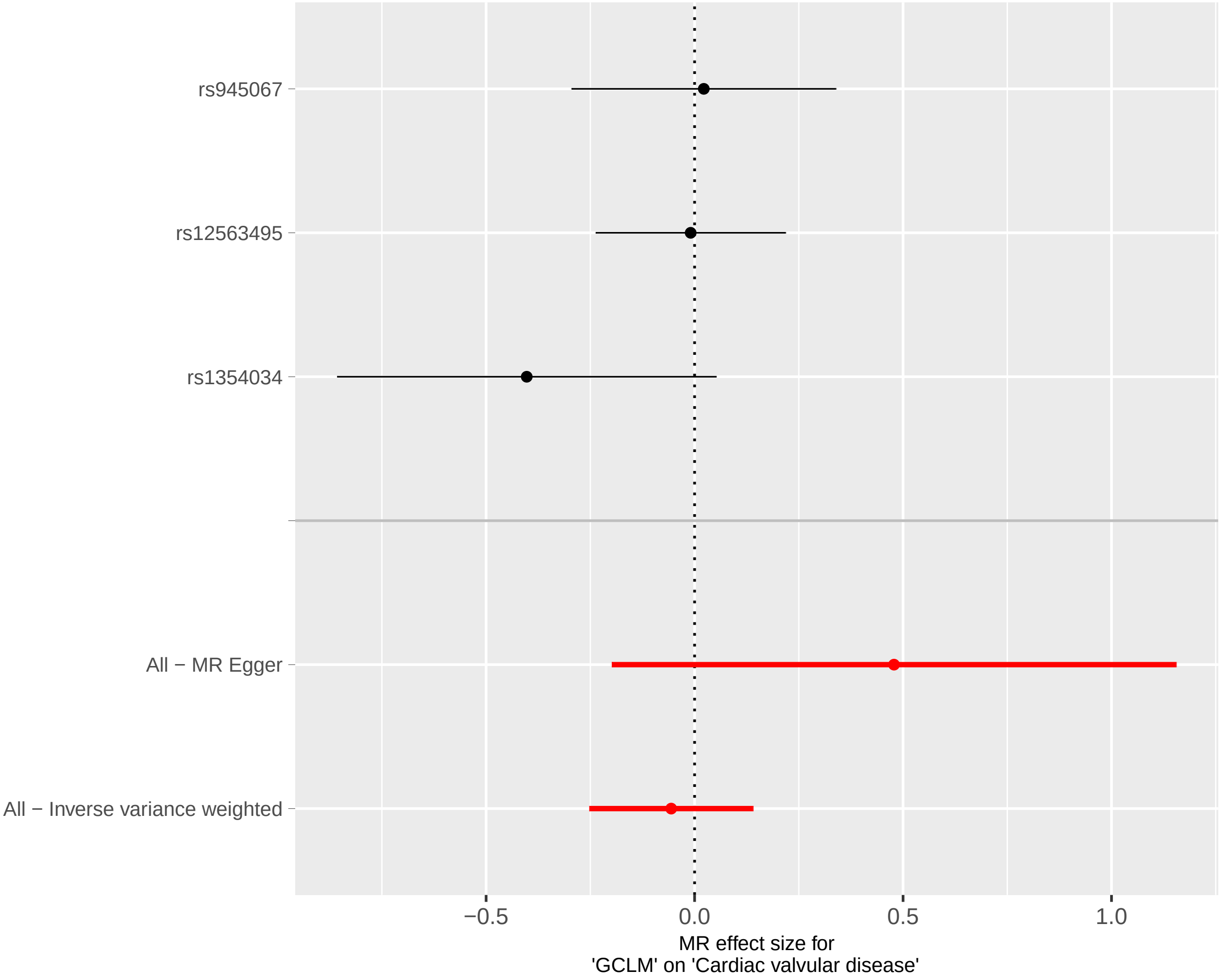
rs945067
rs12563495
rs1354034
All − MR Egger
All − Inverse variance weighted
−0.5
0.0
0.5
1.0
MR effect size for
'GCLM' on 'Cardiac valvular disease'

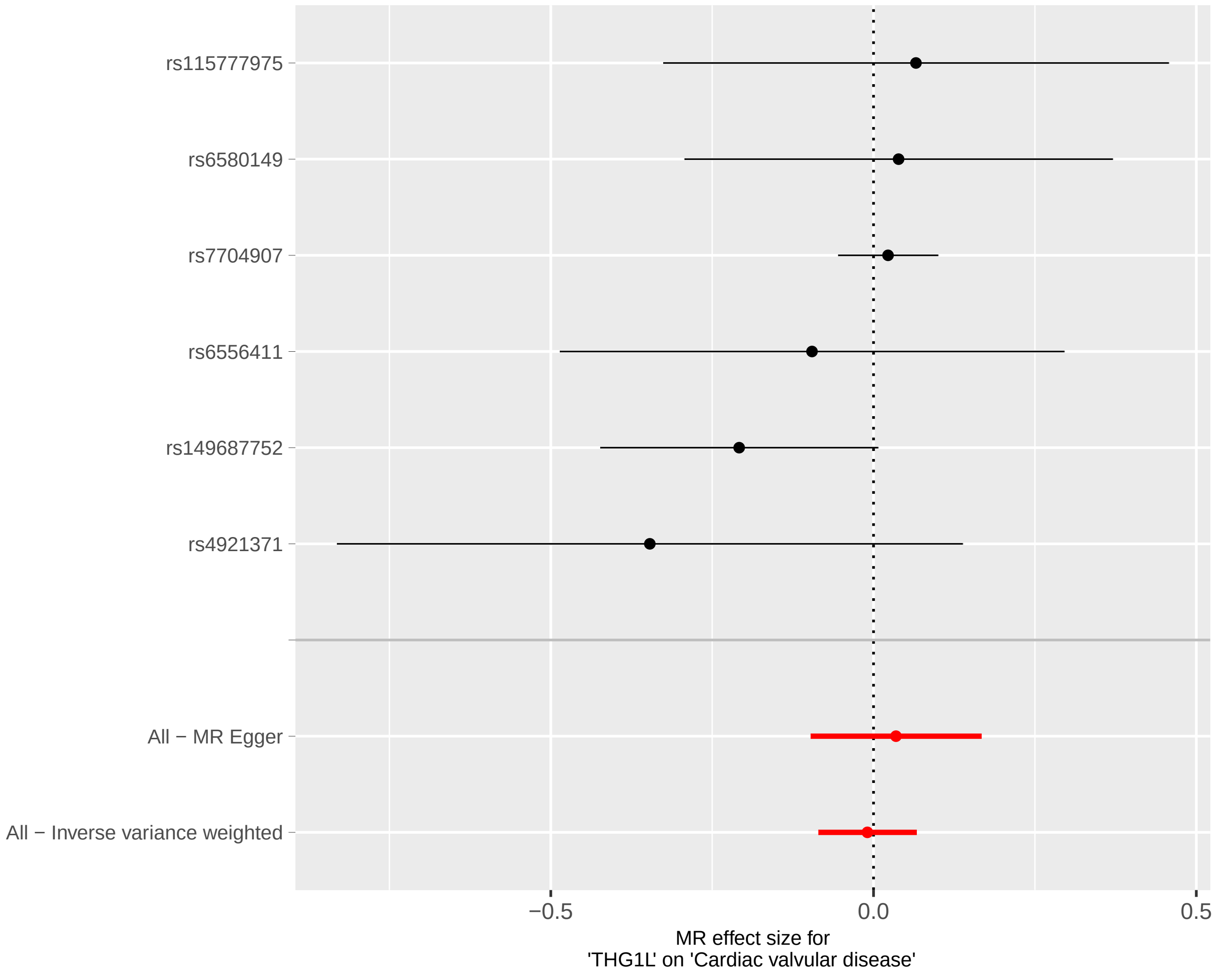
rs115777975
rs6580149
rs7704907
rs6556411
rs149687752
rs4921371
All − MR Egger
All − Inverse variance weighted
−0.5
0.0
0.5
MR effect size for
'THG1L' on 'Cardiac valvular disease'

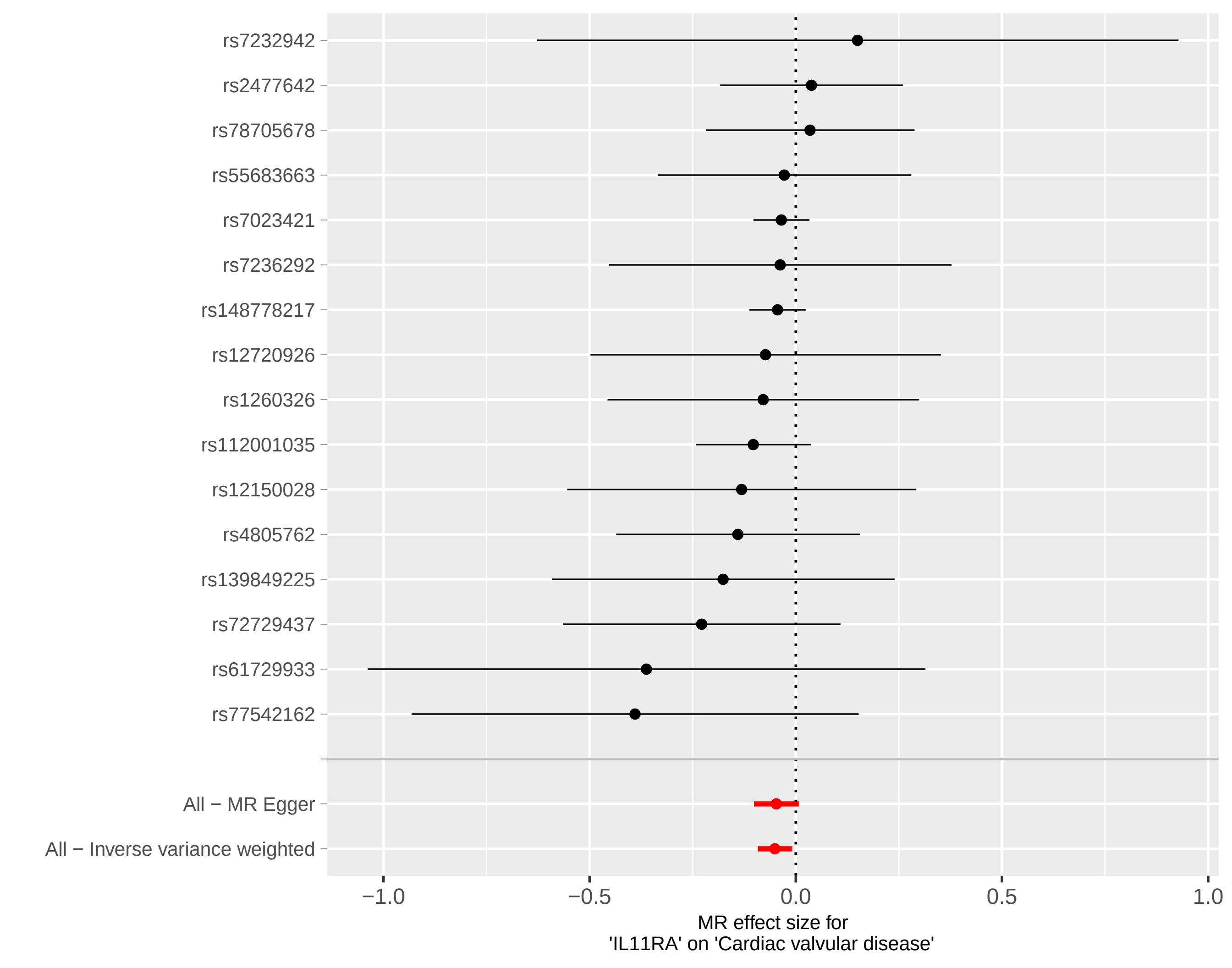
rs7232942
rs2477642
rs78705678
rs55683663
rs7023421
rs7236292
rs148778217
rs12720926
rs1260326
rs112001035
rs12150028
rs4805762
rs139849225
rs72729437
rs61729933
rs77542162
All − MR Egger
All − Inverse variance weighted
−1.0
−0.5
0.0
0.5
1.0
MR effect size for
'IL11RA' on 'Cardiac valvular disease'

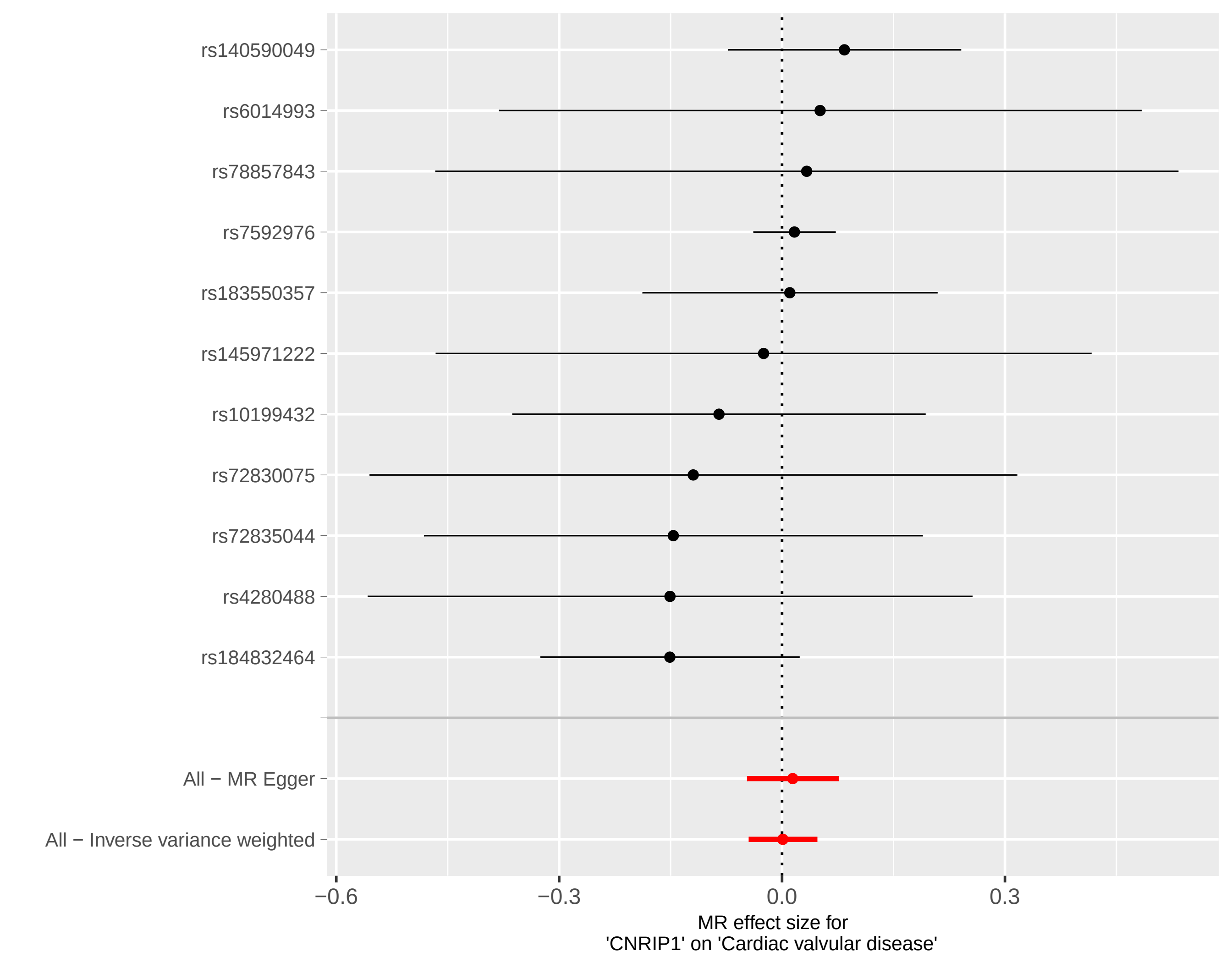
rs140590049
rs6014993
rs78857843
rs7592976
rs183550357
rs145971222
rs10199432
rs72830075
rs72835044
rs4280488
rs184832464
All − MR Egger
All − Inverse variance weighted
−0.6
−0.3
0.0
0.3
MR effect size for
'CNRIP1' on 'Cardiac valvular disease'

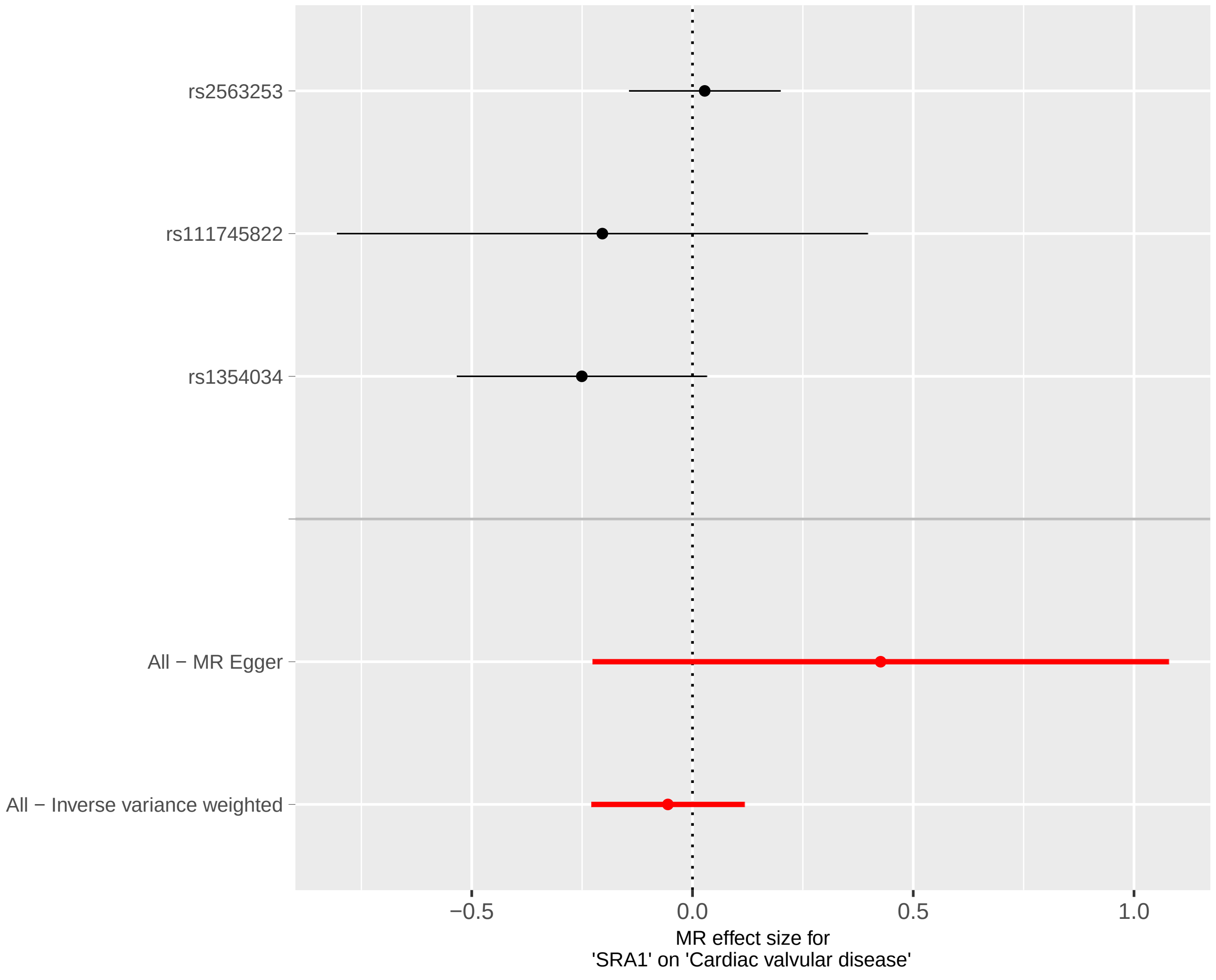
rs2563253
rs111745822
rs1354034
All − MR Egger
All − Inverse variance weighted
−0.5
0.0
0.5
1.0
MR effect size for
'SRA1' on 'Cardiac valvular disease'

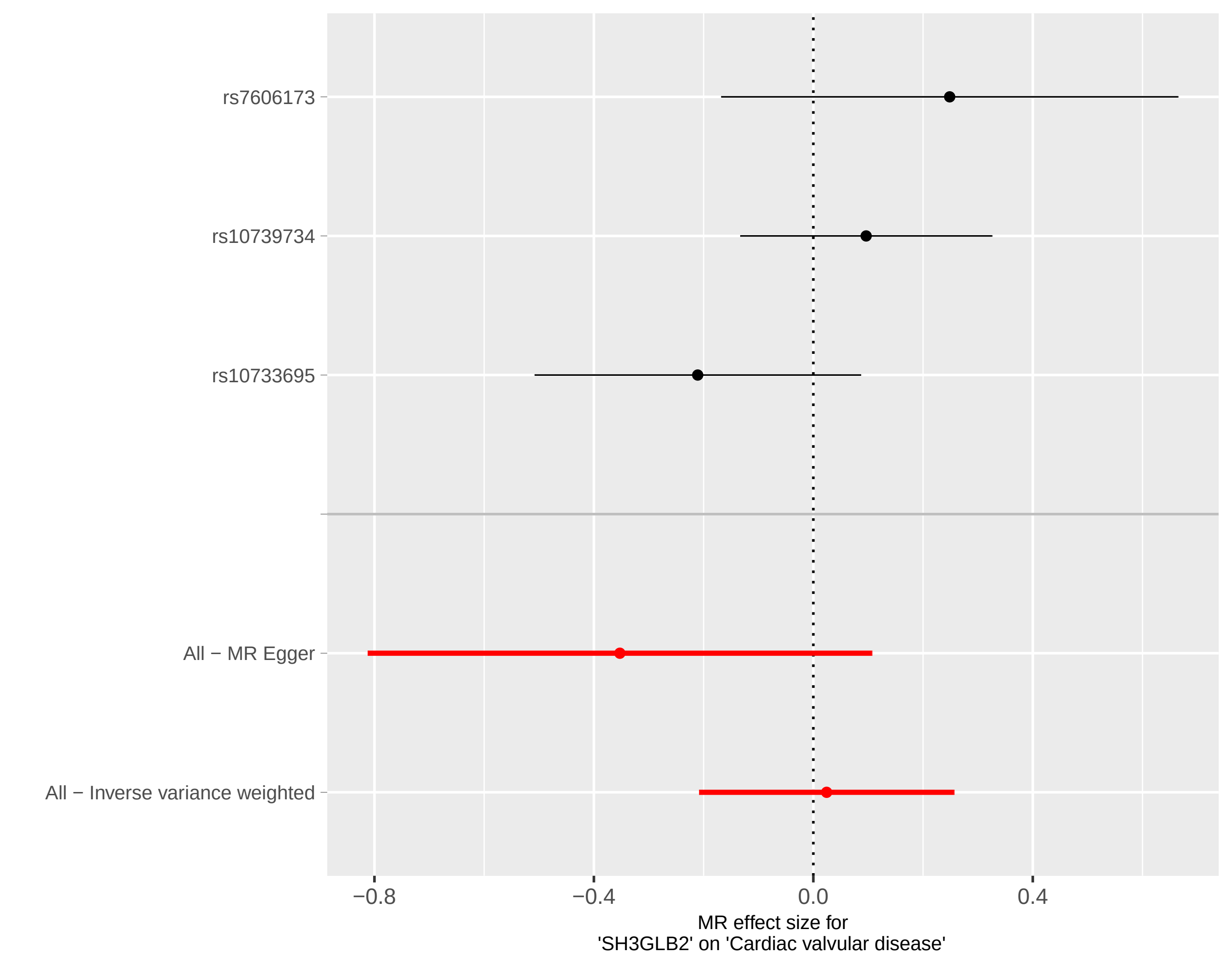

rs7606173
rs10739734
rs10733695
All − MR Egger
All − Inverse variance weighted
−0.8
−0.4
0.0
0.4
MR effect size for
'SH3GLB2' on 'Cardiac valvular disease'

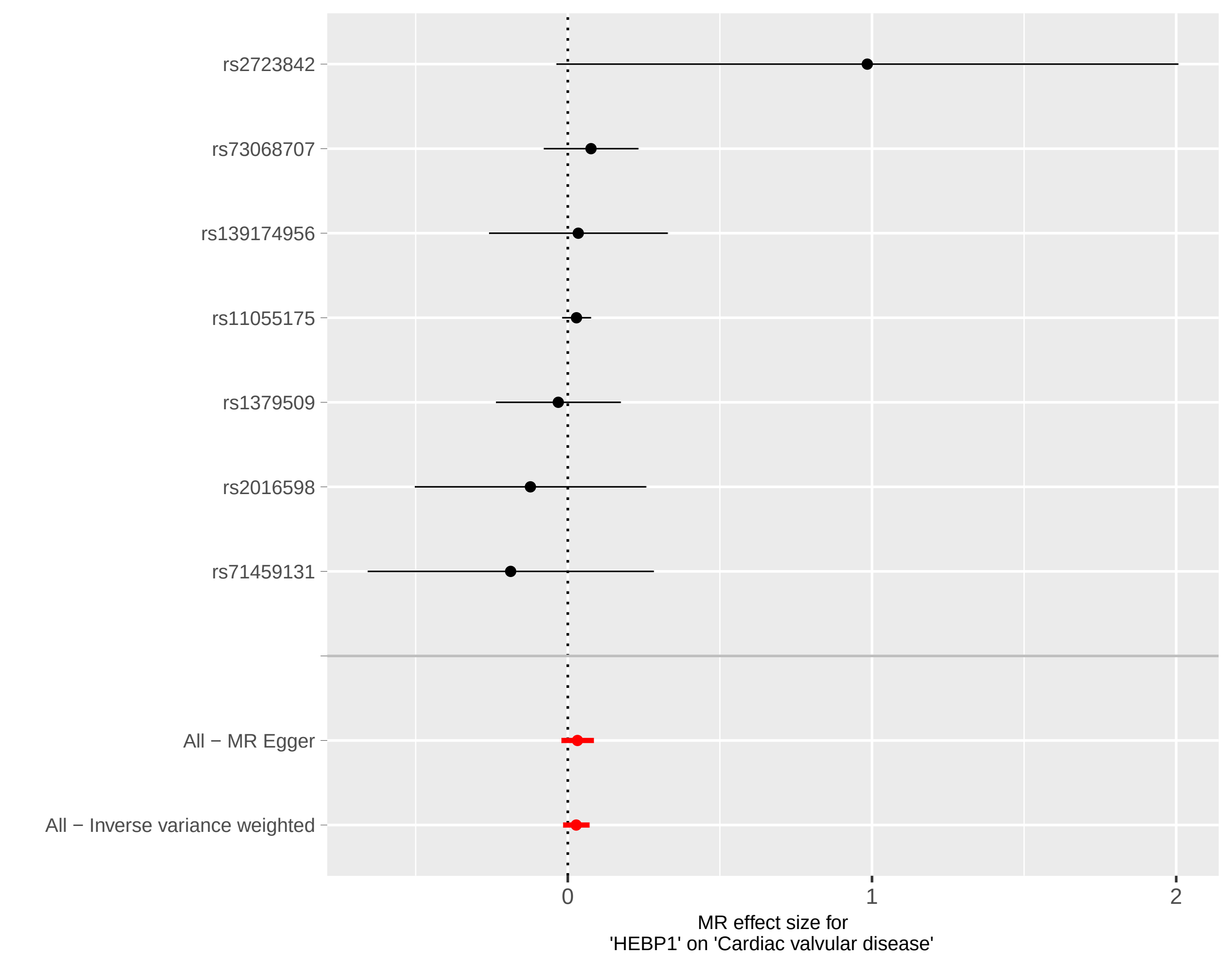
rs2723842
rs73068707
rs139174956
rs11055175
rs1379509
rs2016598
rs71459131
All − MR Egger
All − Inverse variance weighted
0
1
2
MR effect size for
'HEBP1' on 'Cardiac valvular disease'

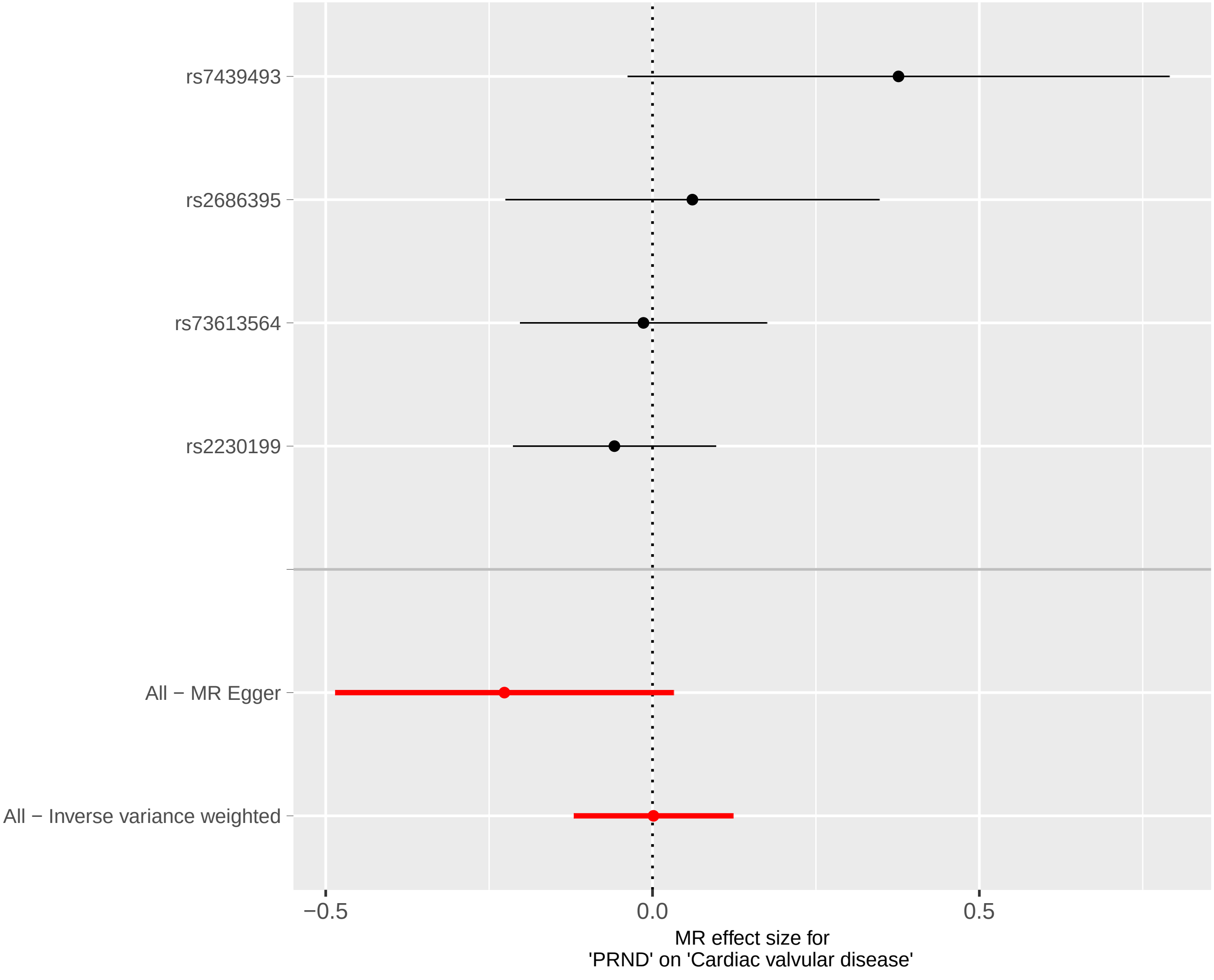
rs7439493
rs2686395
rs73613564
rs2230199
All − MR Egger
All − Inverse variance weighted
−0.5
0.0
0.5
MR effect size for
'PRND' on 'Cardiac valvular disease'

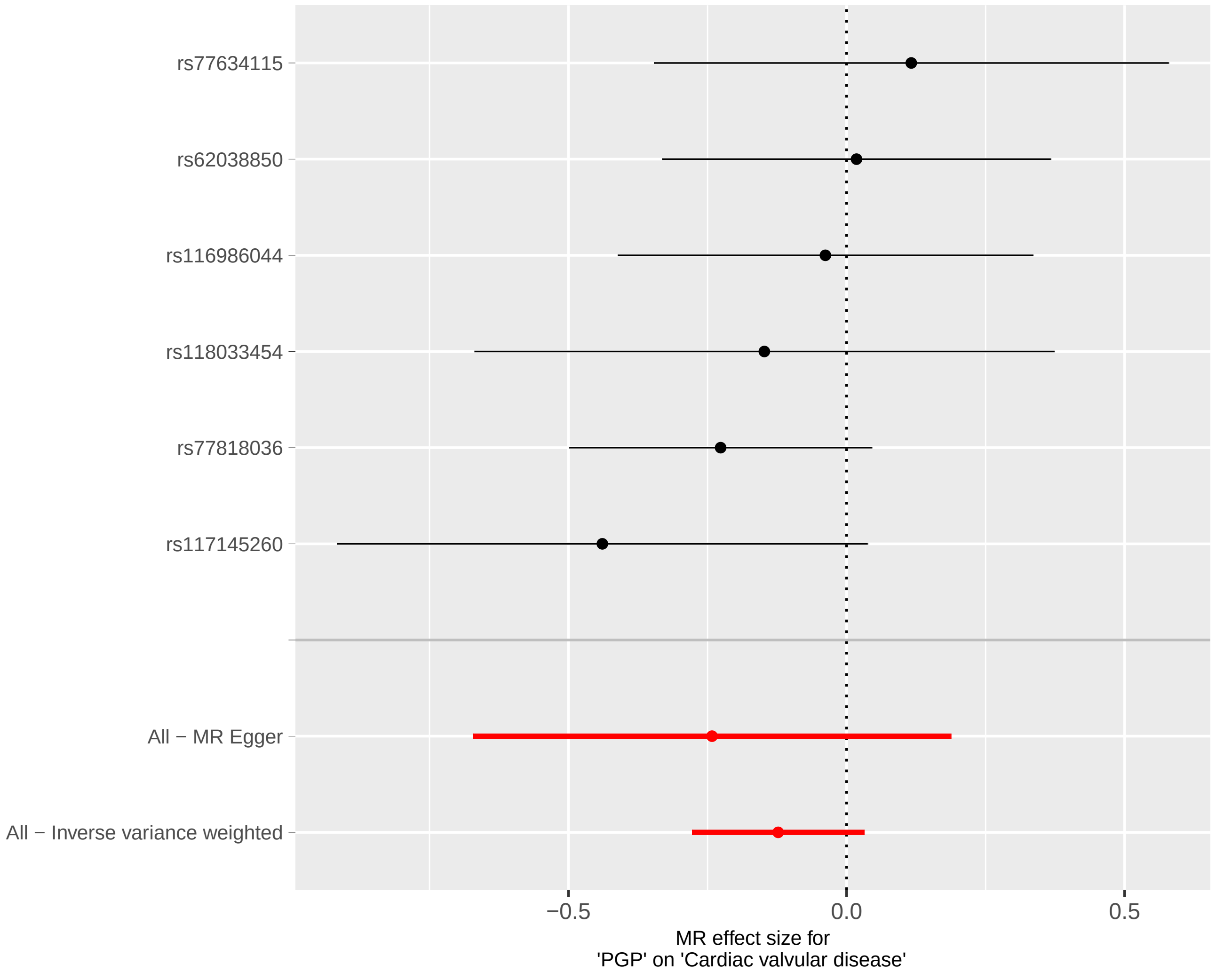
rs77634115
rs62038850
rs116986044
rs118033454
rs77818036
rs117145260
All − MR Egger
All − Inverse variance weighted
−0.5
0.0
0.5
MR effect size for
'PGP' on 'Cardiac valvular disease'

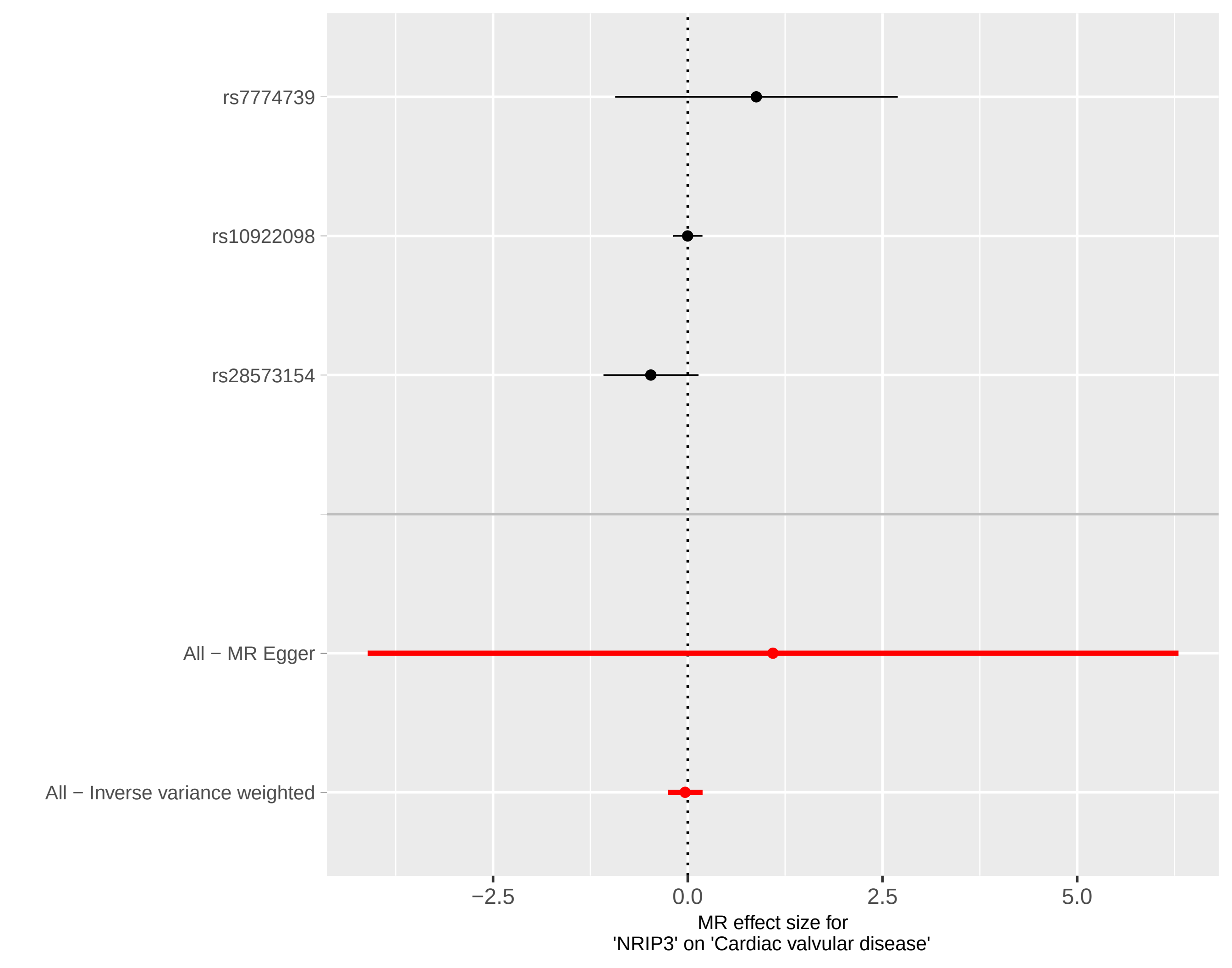

rs7774739
rs10922098
rs28573154
All − MR Egger
All − Inverse variance weighted
−2.5
0.0
2.5
5.0
MR effect size for
'NRIP3' on 'Cardiac valvular disease'

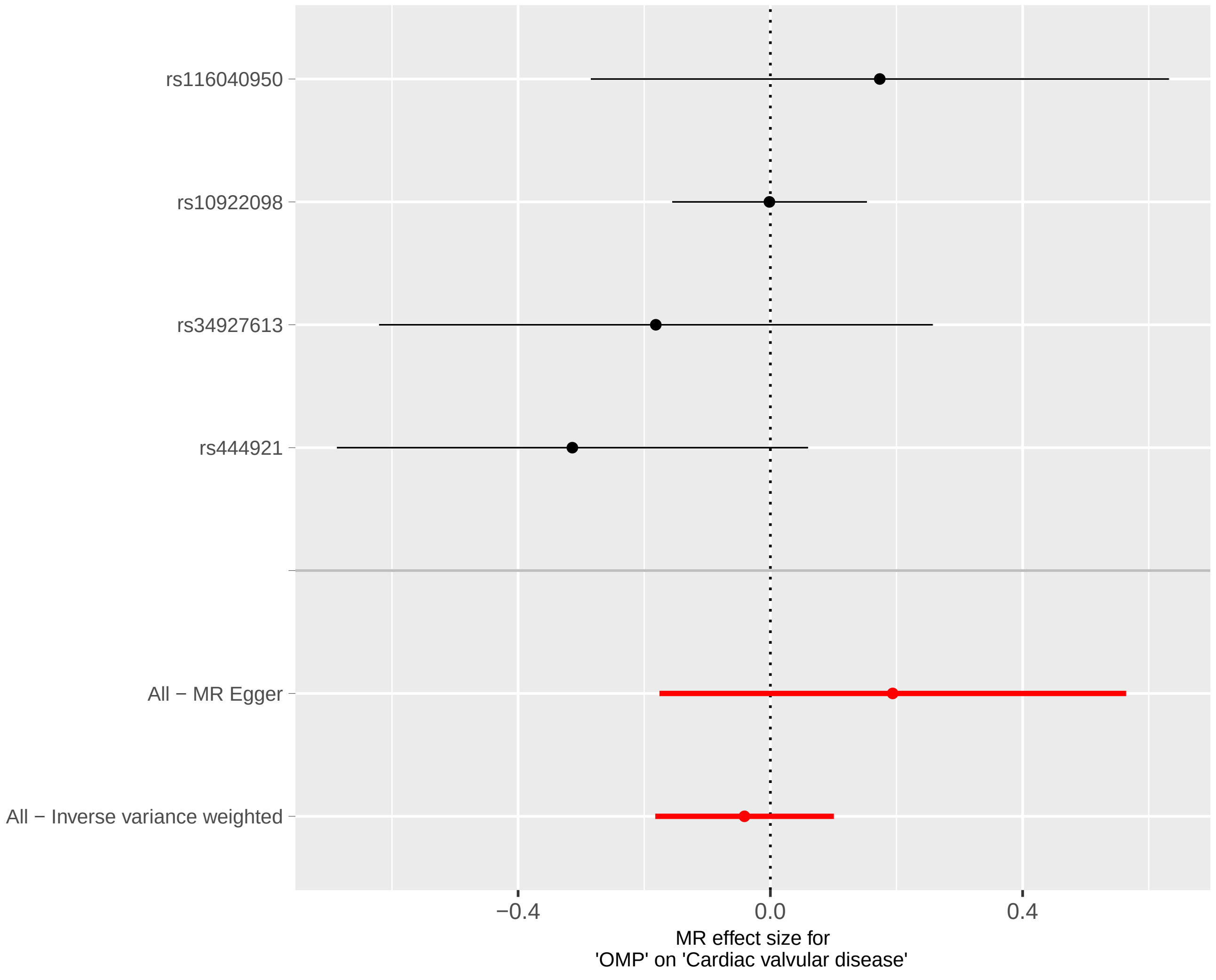
rs116040950
rs10922098
rs34927613
rs444921
All − MR Egger
All − Inverse variance weighted
−0.4
0.0
0.4
MR effect size for
'OMP' on 'Cardiac valvular disease'

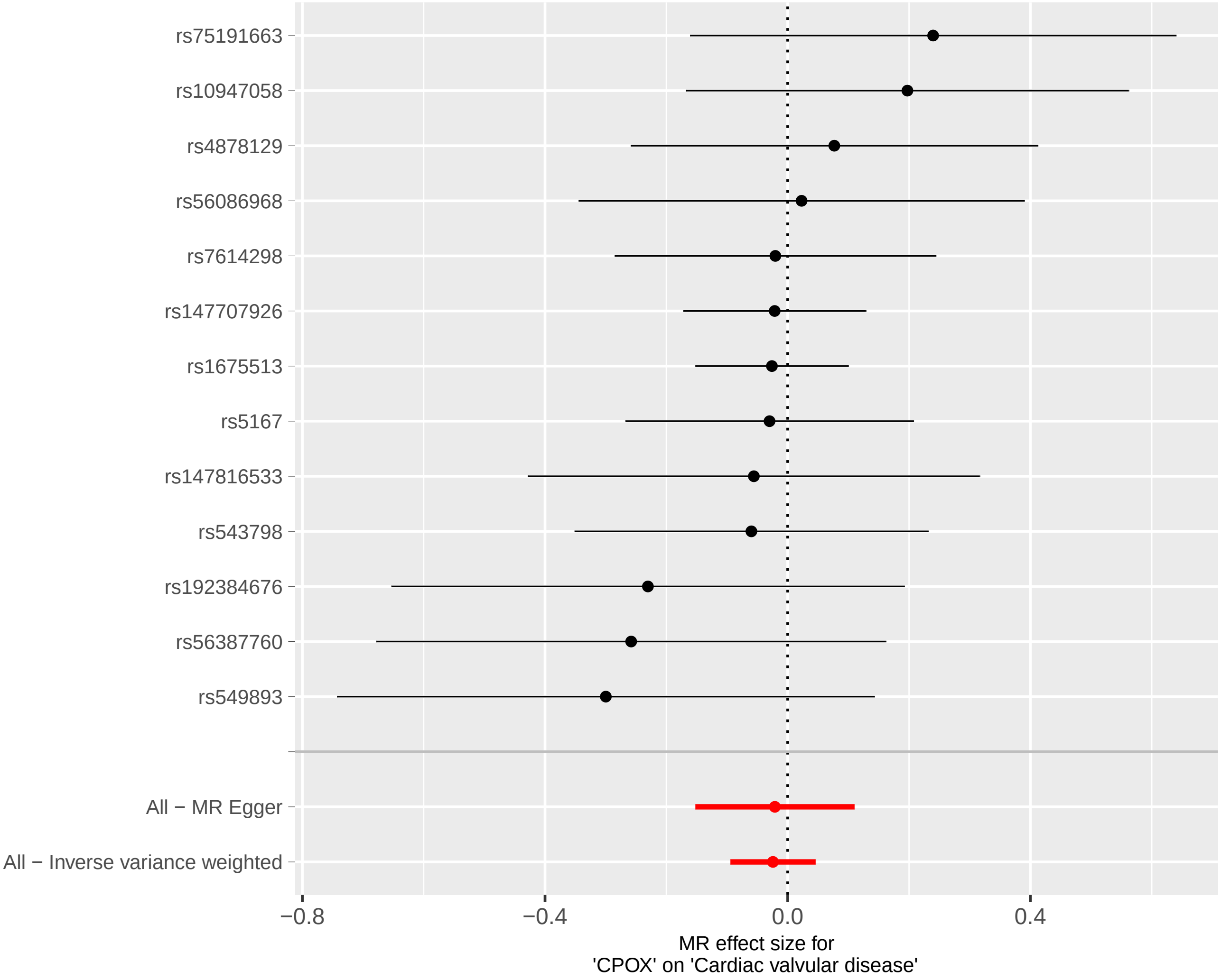
rs75191663
rs10947058
rs4878129
rs56086968
rs7614298
rs147707926
rs1675513
rs5167
rs147816533
rs543798
rs192384676
rs56387760
rs549893
All − MR Egger
All − Inverse variance weighted
−0.8
−0.4
0.0
0.4
MR effect size for
'CPOX' on 'Cardiac valvular disease'

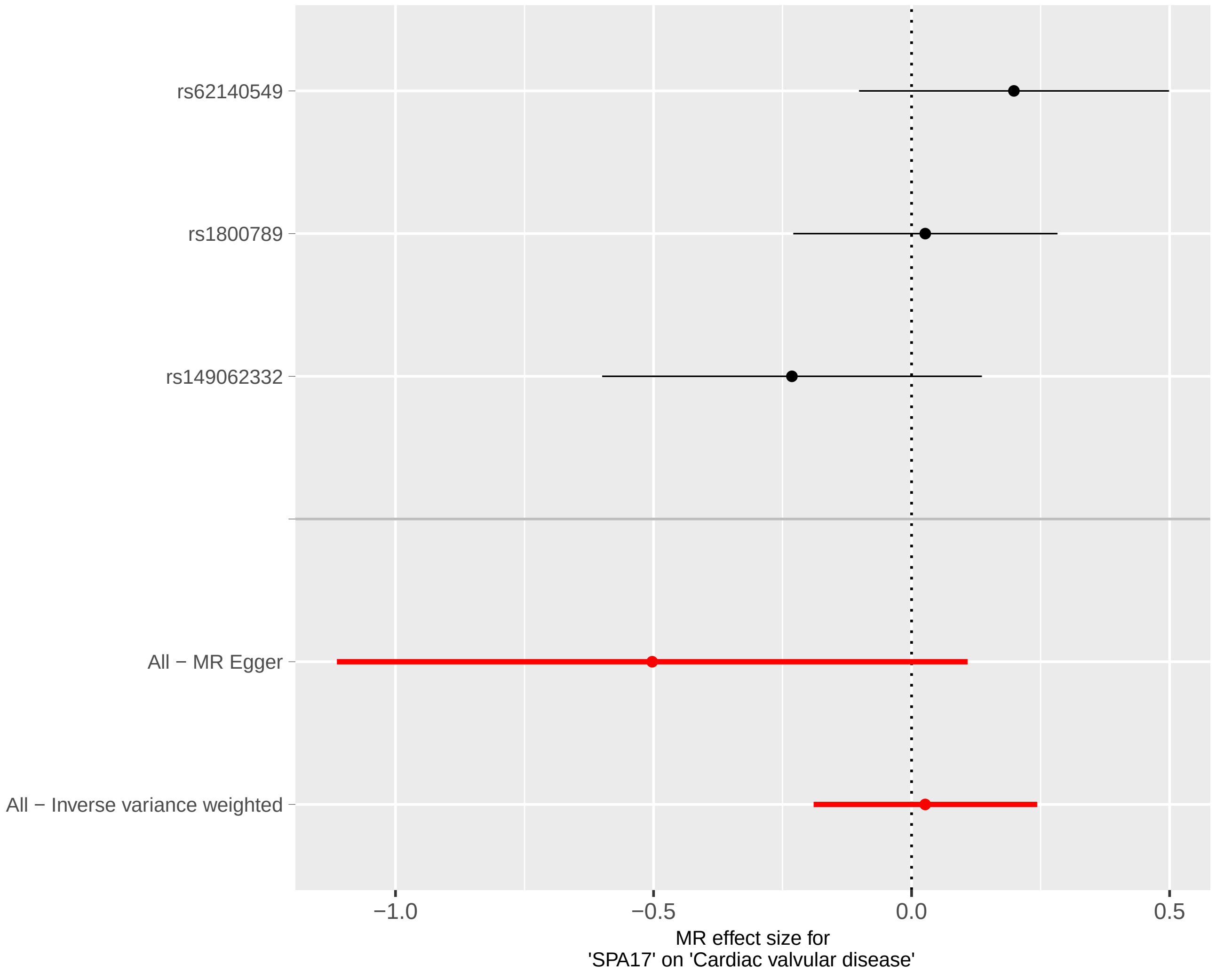

rs62140549
rs1800789
rs149062332
All − MR Egger
All − Inverse variance weighted
−1.0
−0.5
0.0
0.5
MR effect size for
'SPA17' on 'Cardiac valvular disease'

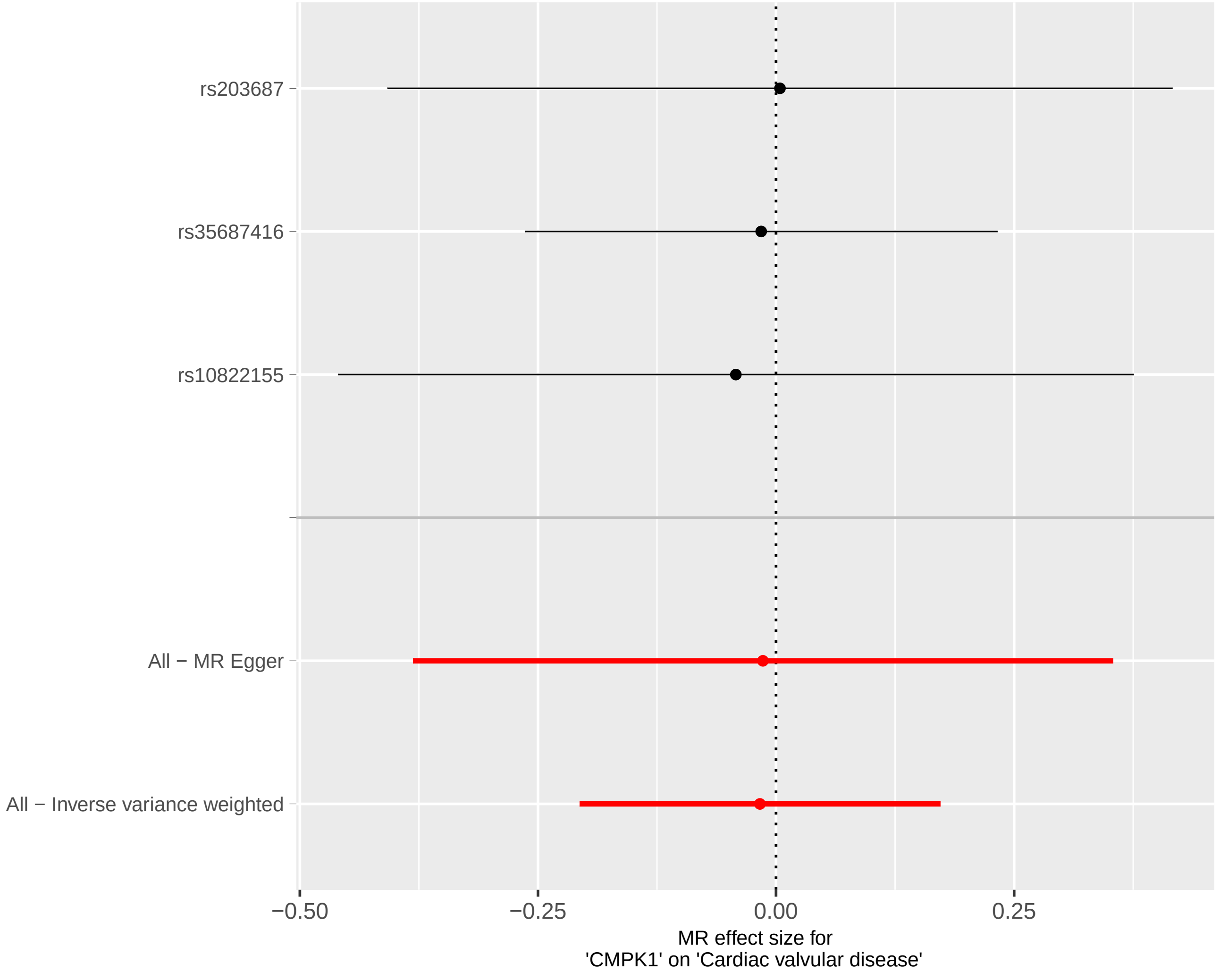
rs203687
rs35687416
rs10822155
All − MR Egger
All − Inverse variance weighted
−0.50
−0.25
0.00
0.25
MR effect size for
'CMPK1' on 'Cardiac valvular disease'

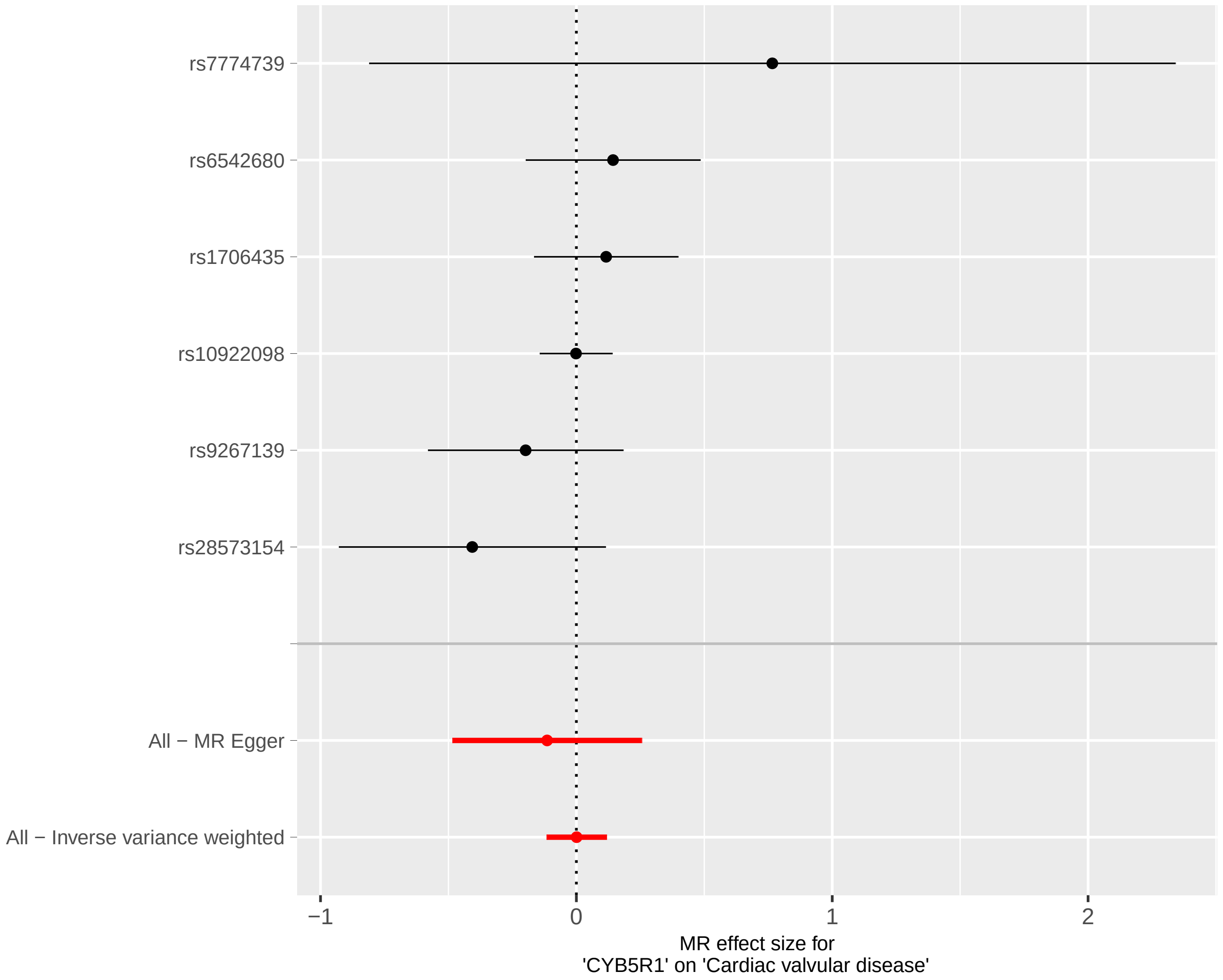

rs7774739
rs6542680
rs1706435
rs10922098
rs9267139
rs28573154
All − MR Egger
All − Inverse variance weighted
−1
0
1
2
MR effect size for
'CYB5R1' on 'Cardiac valvular disease'

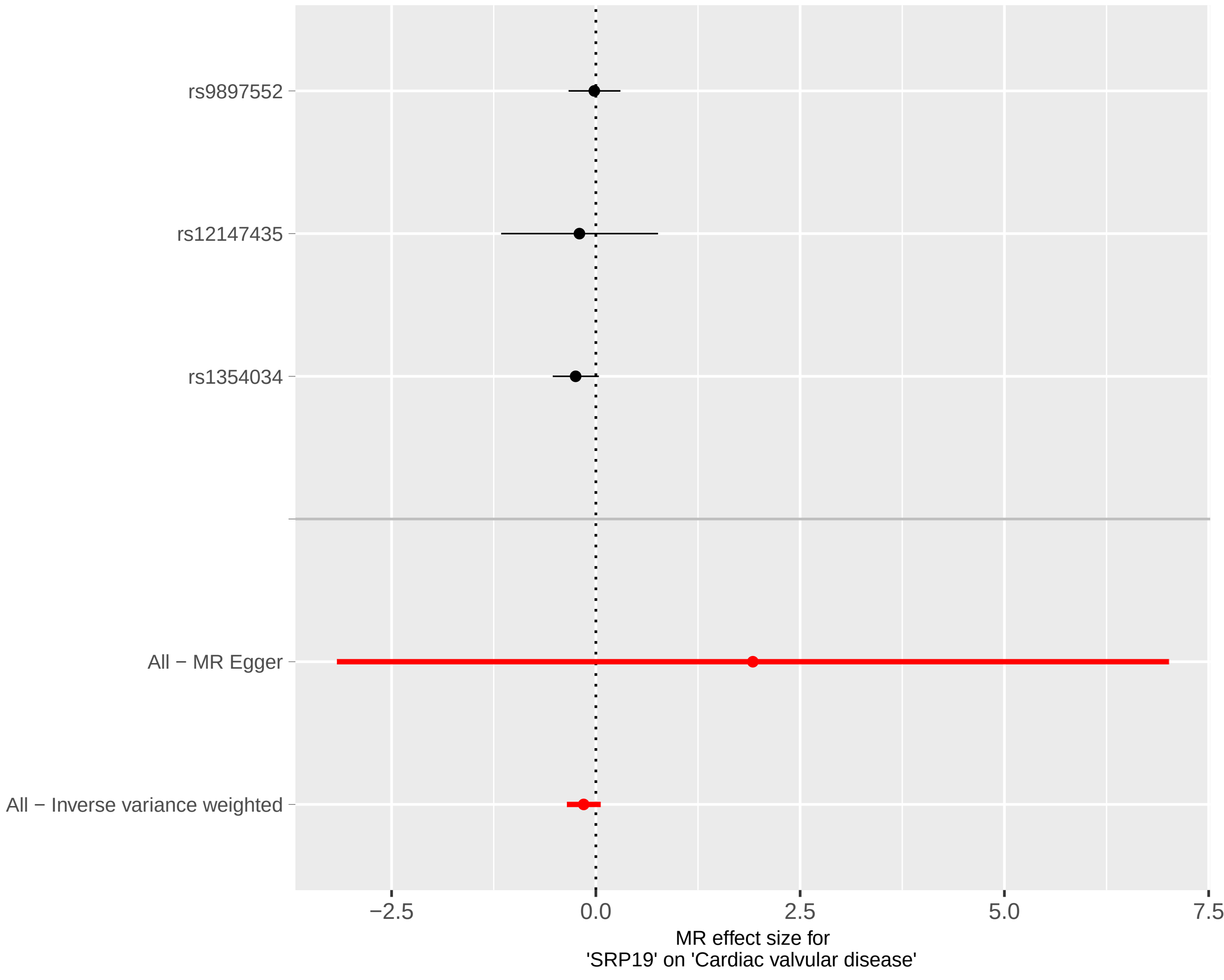

rs9897552
rs12147435
rs1354034
All − MR Egger
All − Inverse variance weighted
−2.5
0.0
2.5
5.0
7.5
MR effect size for
'SRP19' on 'Cardiac valvular disease'

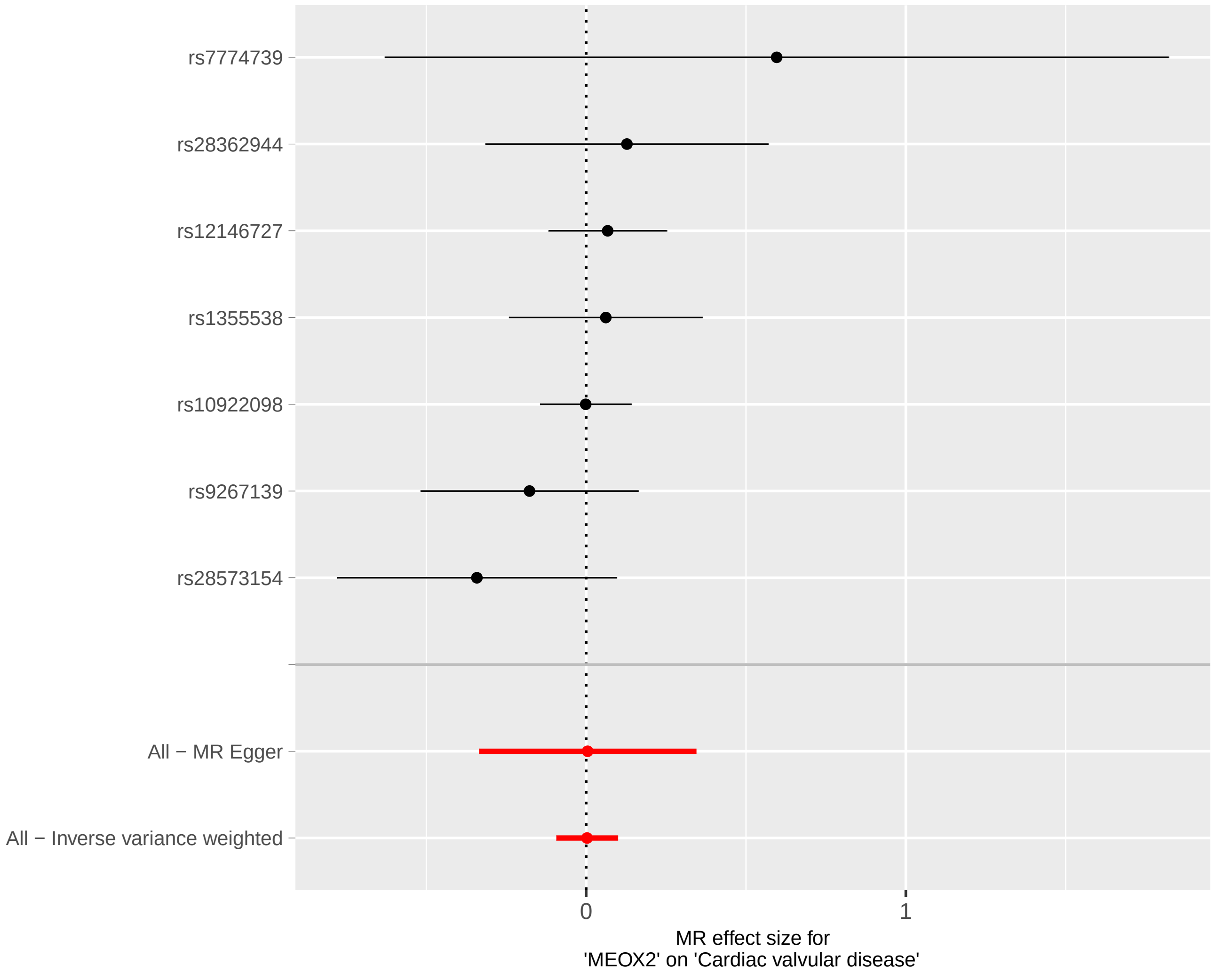
rs7774739
rs28362944
rs12146727
rs1355538
rs10922098
rs9267139
rs28573154
All − MR Egger
All − Inverse variance weighted
0
1
MR effect size for
'MEOX2' on 'Cardiac valvular disease'

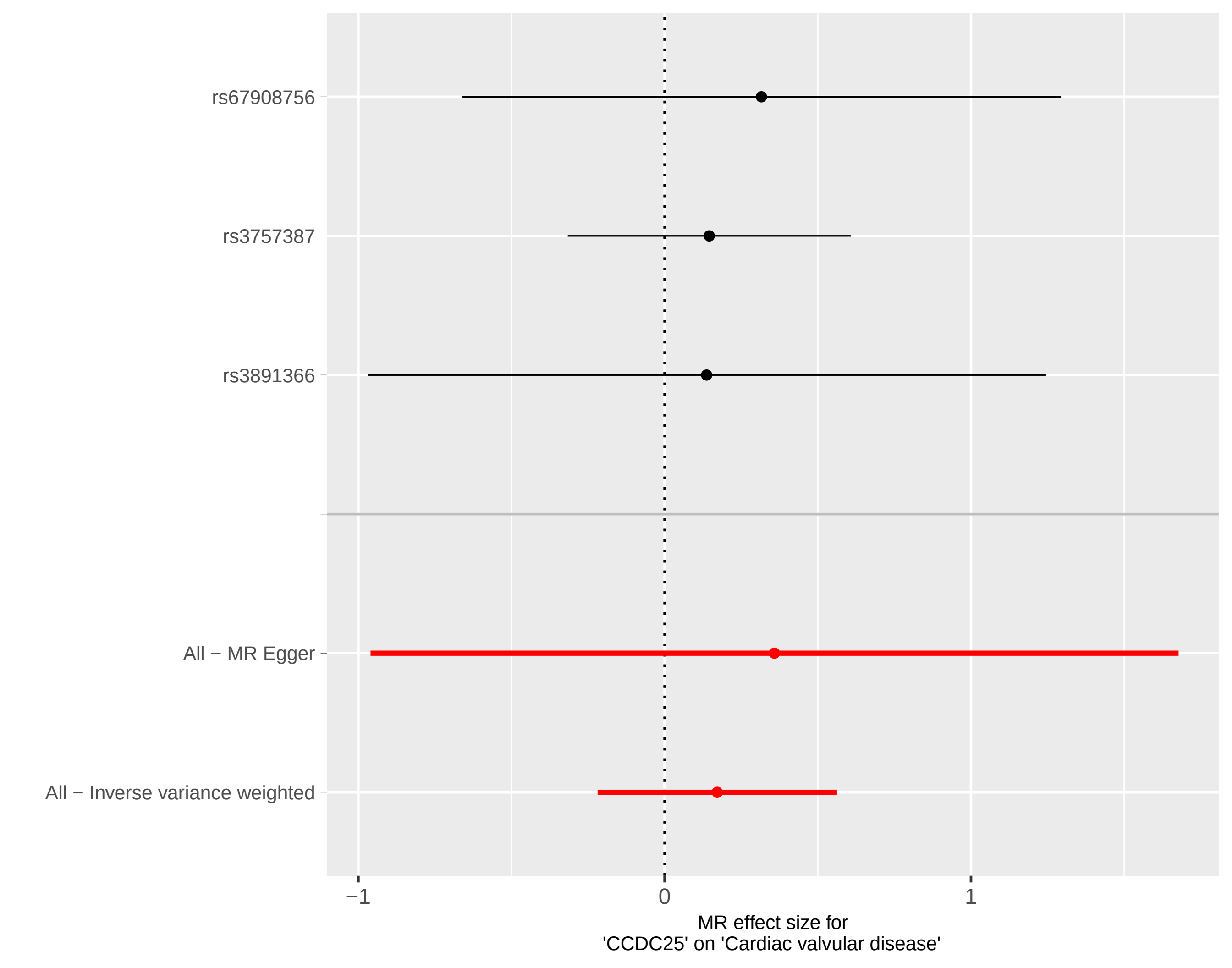

rs67908756
rs3757387
rs3891366
All − MR Egger
All − Inverse variance weighted
−1
0
1
MR effect size for
'CCDC25' on 'Cardiac valvular disease'

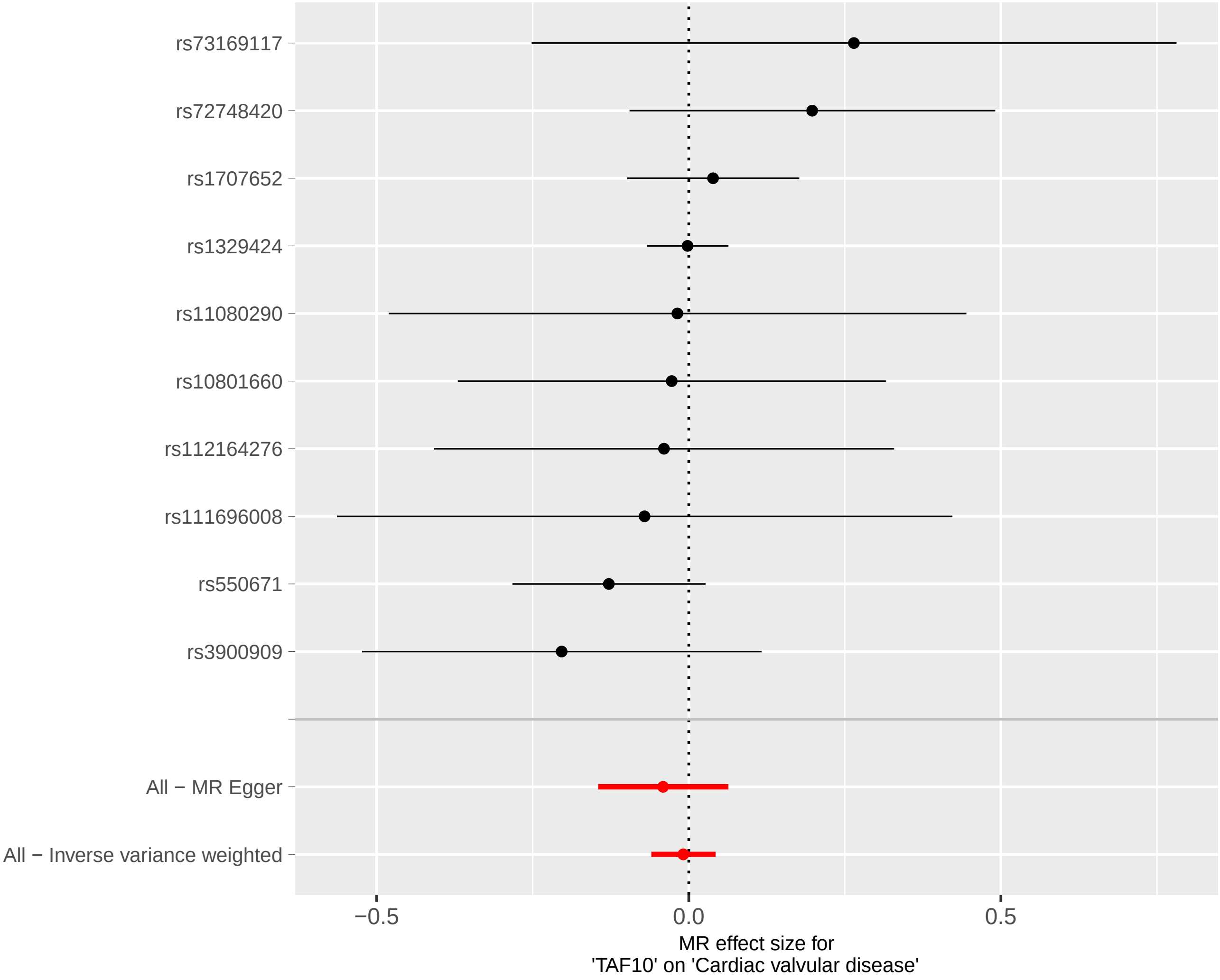
rs73169117
rs72748420
rs1707652
rs1329424
rs11080290
rs10801660
rs112164276
rs111696008
rs550671
rs3900909
All − MR Egger
All − Inverse variance weighted
−0.5
0.0
0.5
MR effect size for
'TAF10' on 'Cardiac valvular disease'

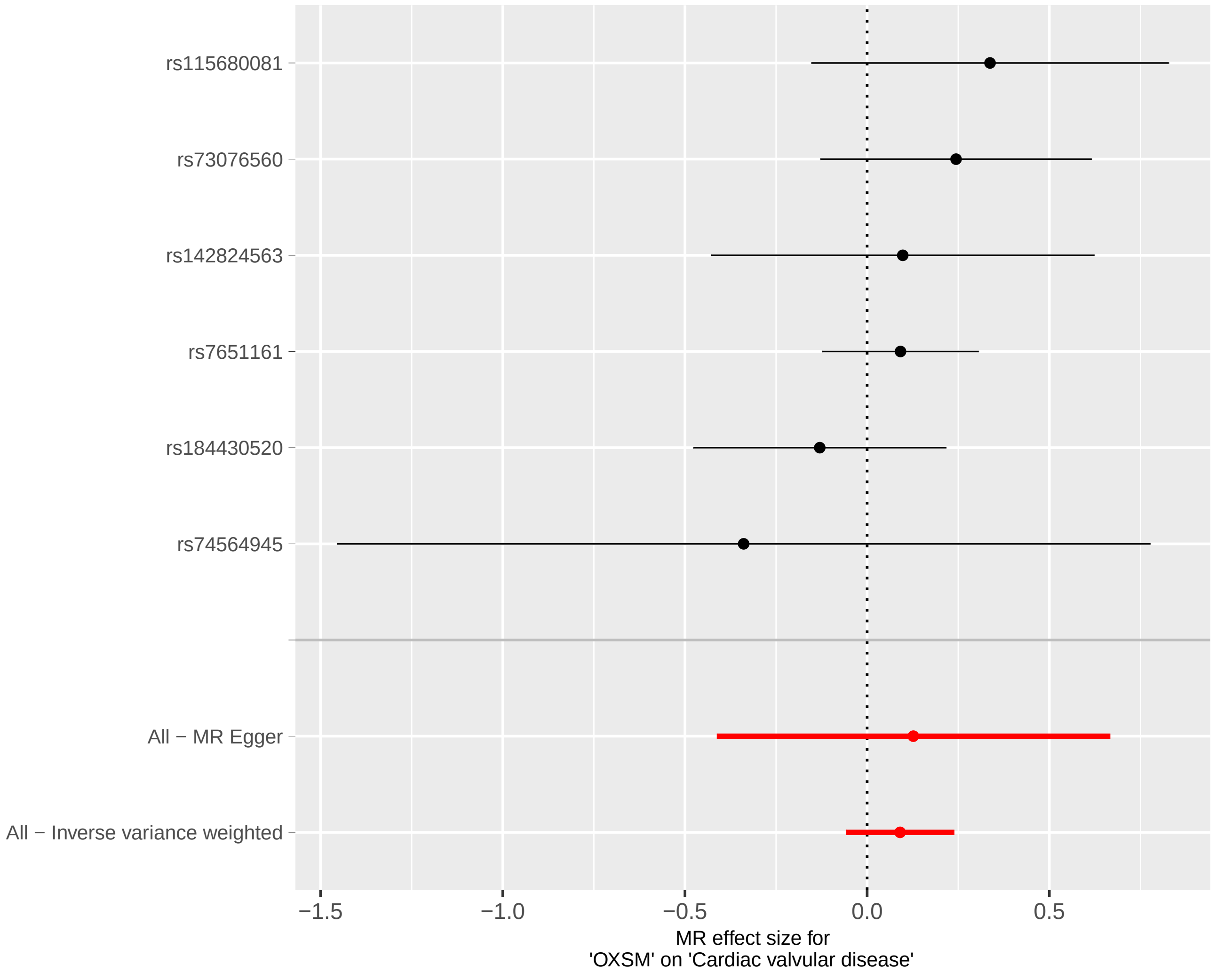
rs115680081
rs73076560
rs142824563
rs7651161
rs184430520
rs74564945
All − MR Egger
All − Inverse variance weighted
−1.5
−1.0
−0.5
0.0
0.5
MR effect size for
'OXSM' on 'Cardiac valvular disease'

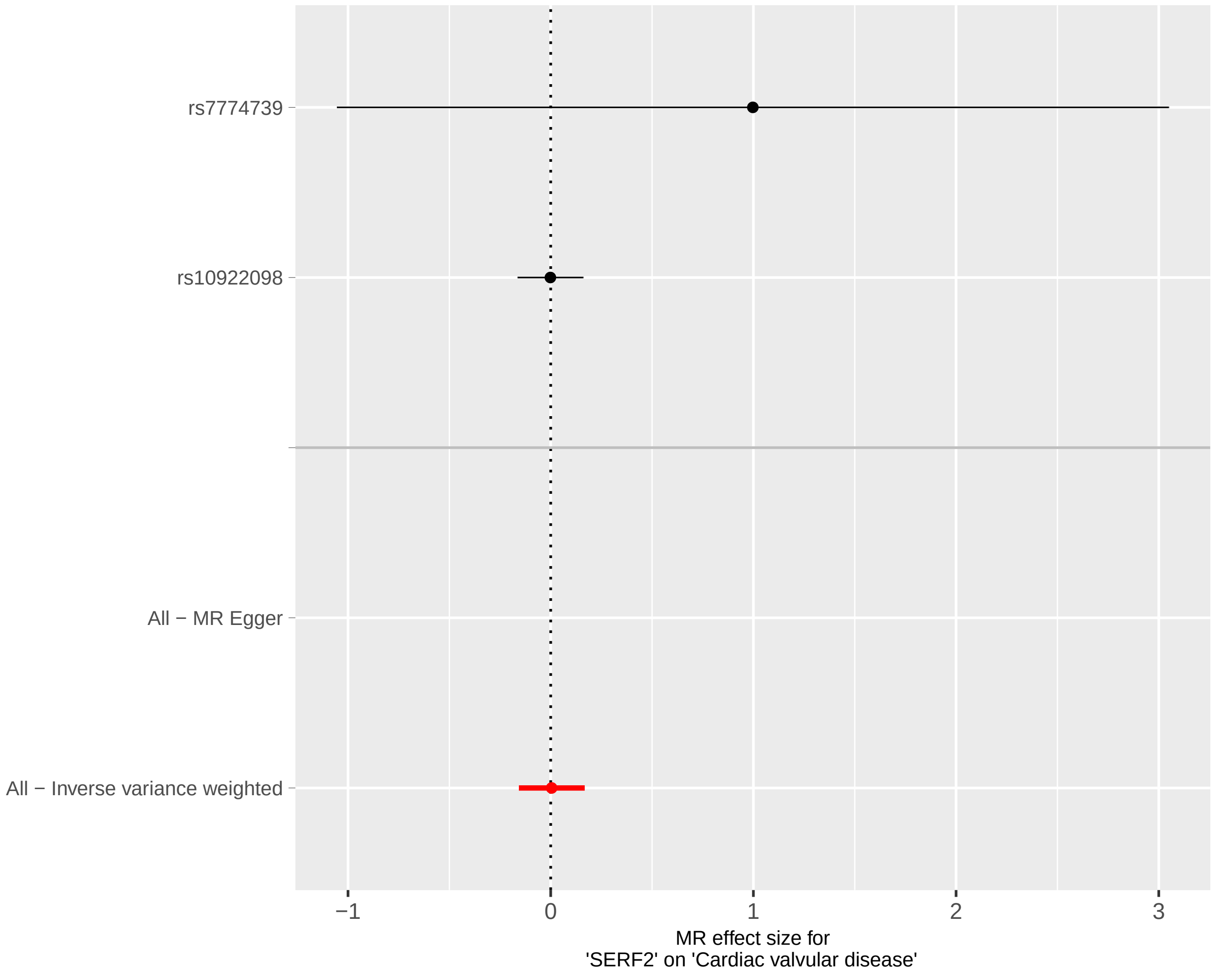

rs7774739
rs10922098
All − MR Egger
All − Inverse variance weighted
−1
0
1
2
3
MR effect size for
'SERF2' on 'Cardiac valvular disease'

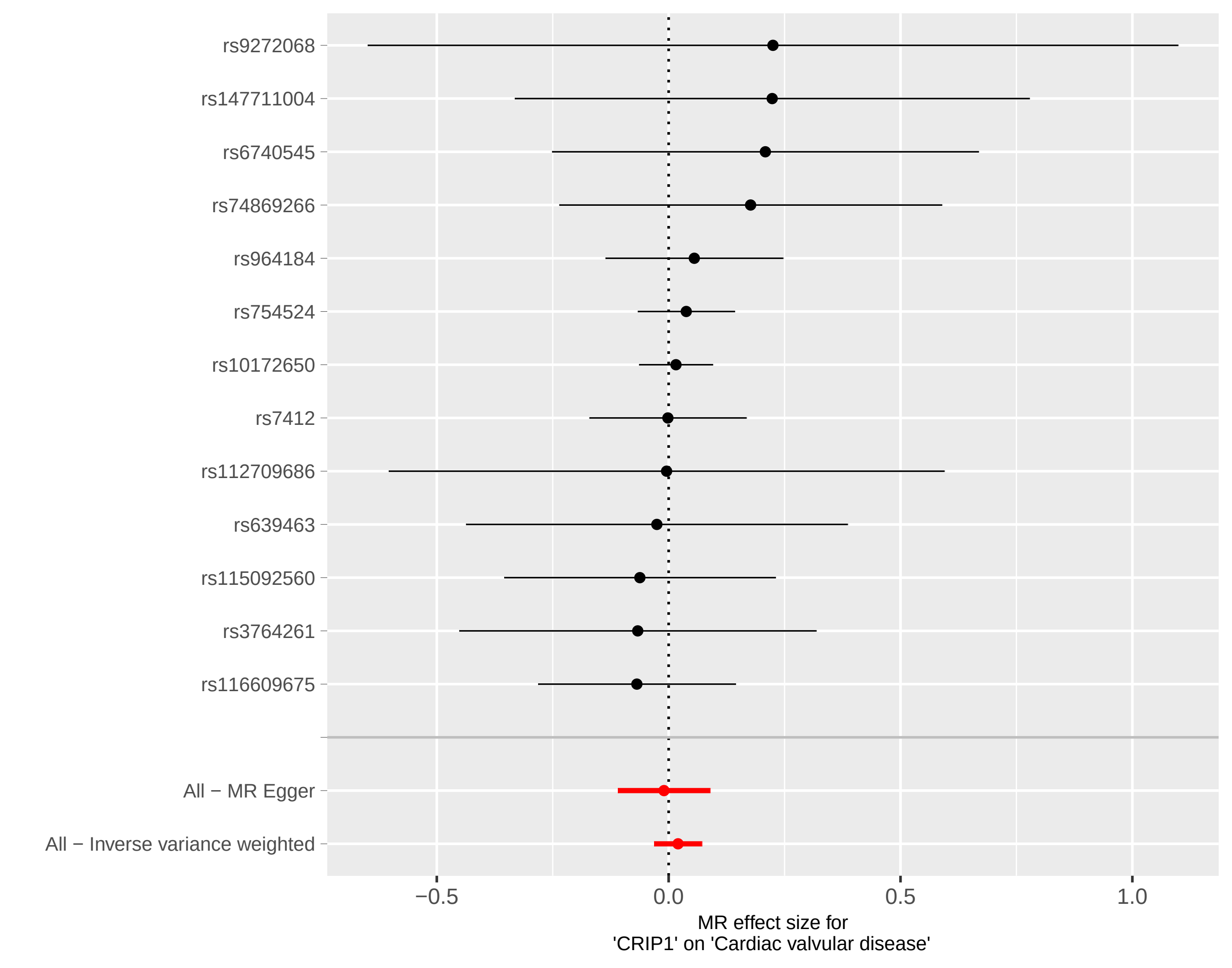
rs9272068
rs147711004
rs6740545
rs74869266
rs964184
rs754524
rs10172650
rs7412
rs112709686
rs639463
rs115092560
rs3764261
rs116609675
All − MR Egger
All − Inverse variance weighted
−0.5
0.0
0.5
1.0
MR effect size for
'CRIP1' on 'Cardiac valvular disease'

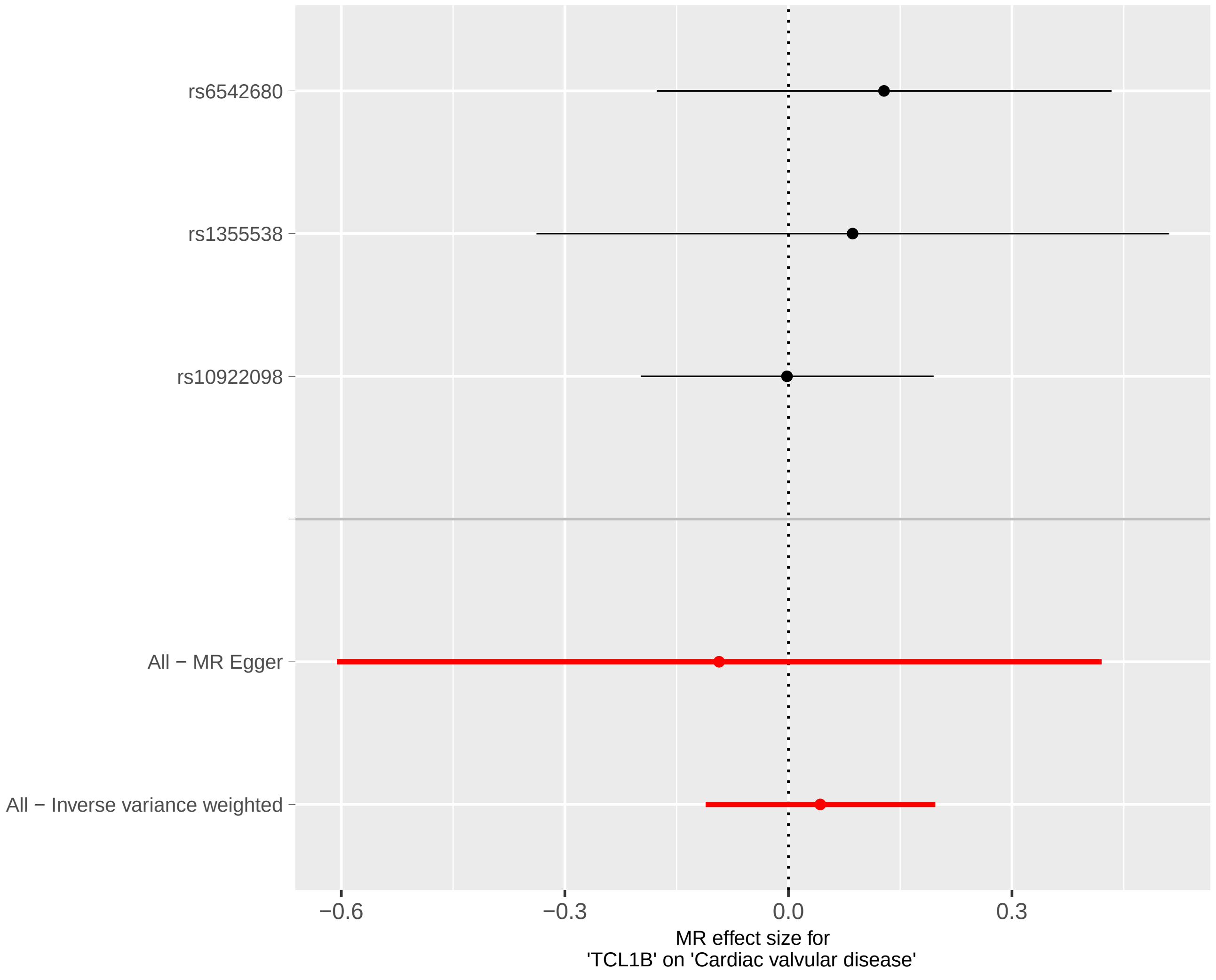
rs6542680
rs1355538
rs10922098
All − MR Egger
All − Inverse variance weighted
−0.6
−0.3
0.0
0.3
MR effect size for
'TCL1B' on 'Cardiac valvular disease'

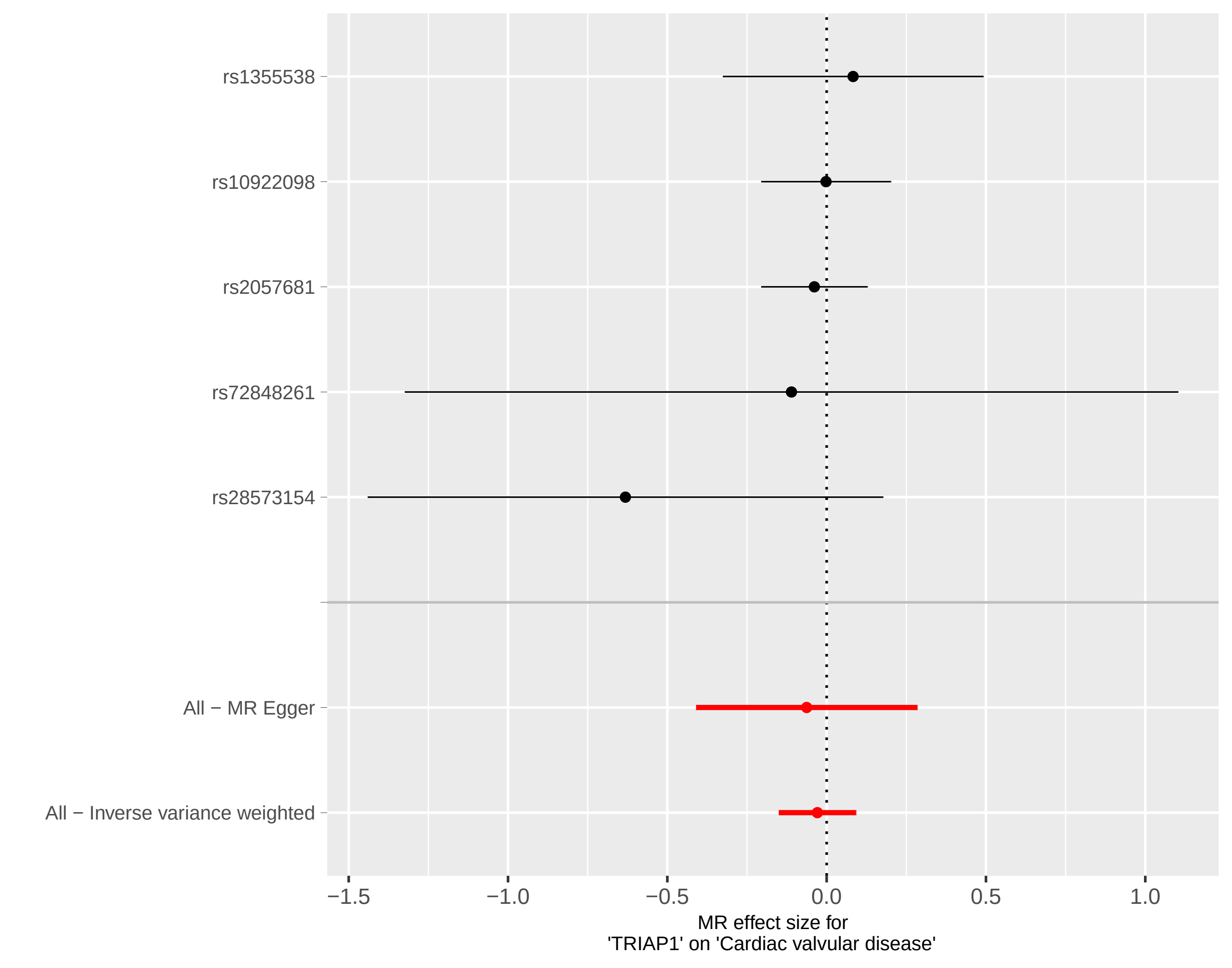

rs1355538
rs10922098
rs2057681
rs72848261
rs28573154
All − MR Egger
All − Inverse variance weighted
−1.5
−1.0
−0.5
0.0
0.5
1.0
MR effect size for
'TRIAP1' on 'Cardiac valvular disease'

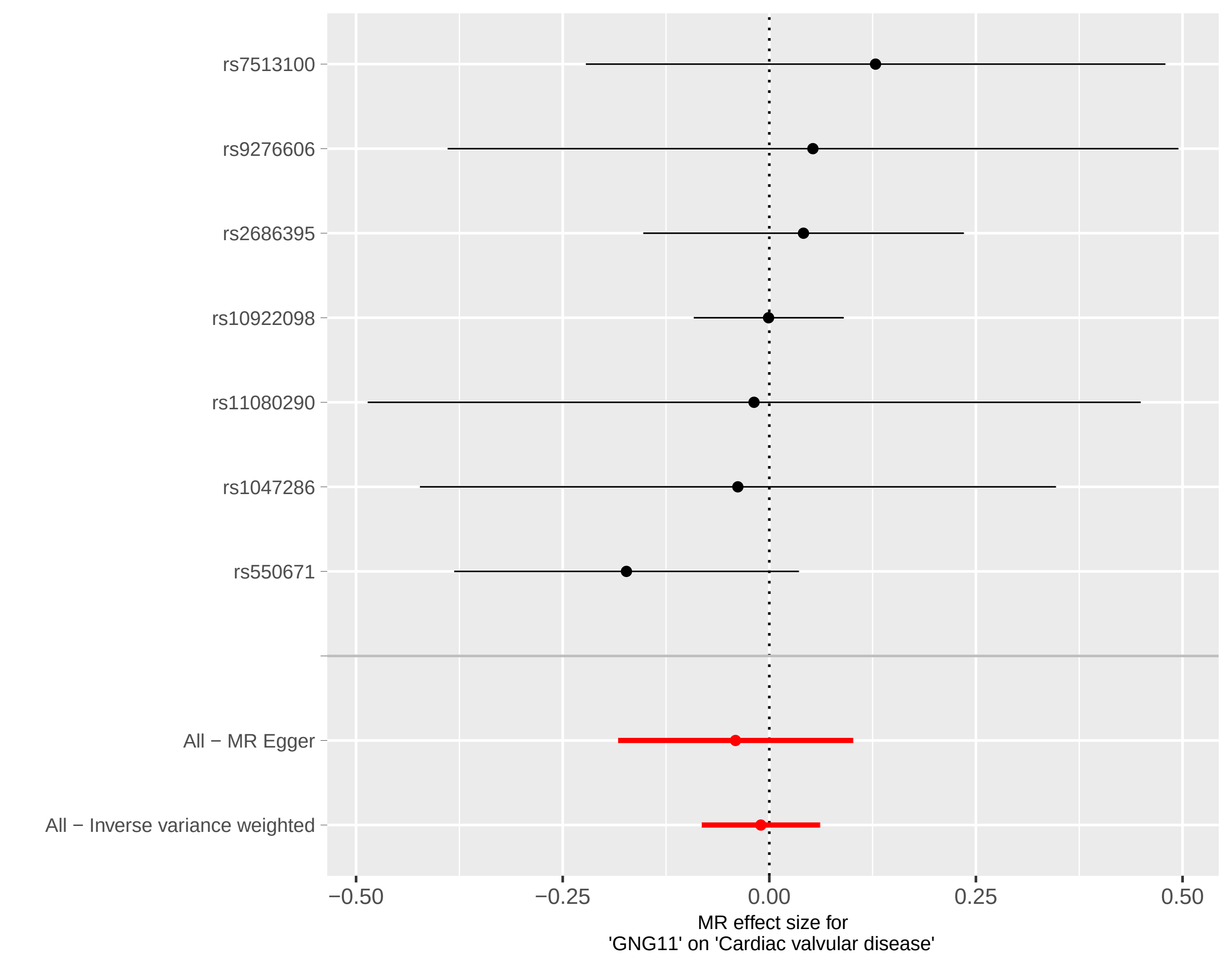

rs7513100
rs9276606
rs2686395
rs10922098
rs11080290
rs1047286
rs550671
All − MR Egger
All − Inverse variance weighted
−0.50
−0.25
0.00
0.25
0.50
MR effect size for
'GNG11' on 'Cardiac valvular disease'

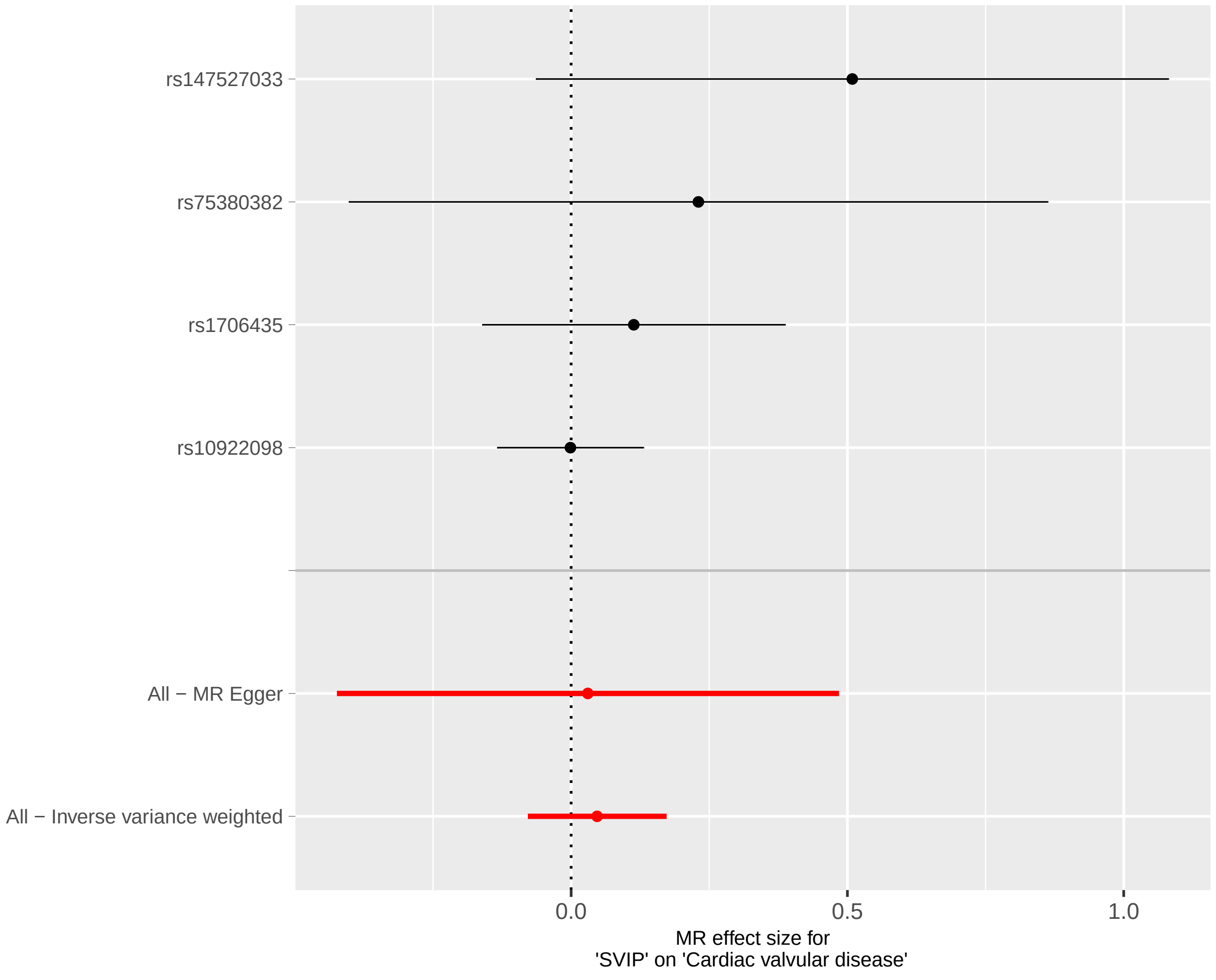

rs147527033
rs75380382
rs1706435
rs10922098
All − MR Egger
All − Inverse variance weighted
0.0
0.5
1.0
MR effect size for
'SVIP' on 'Cardiac valvular disease'

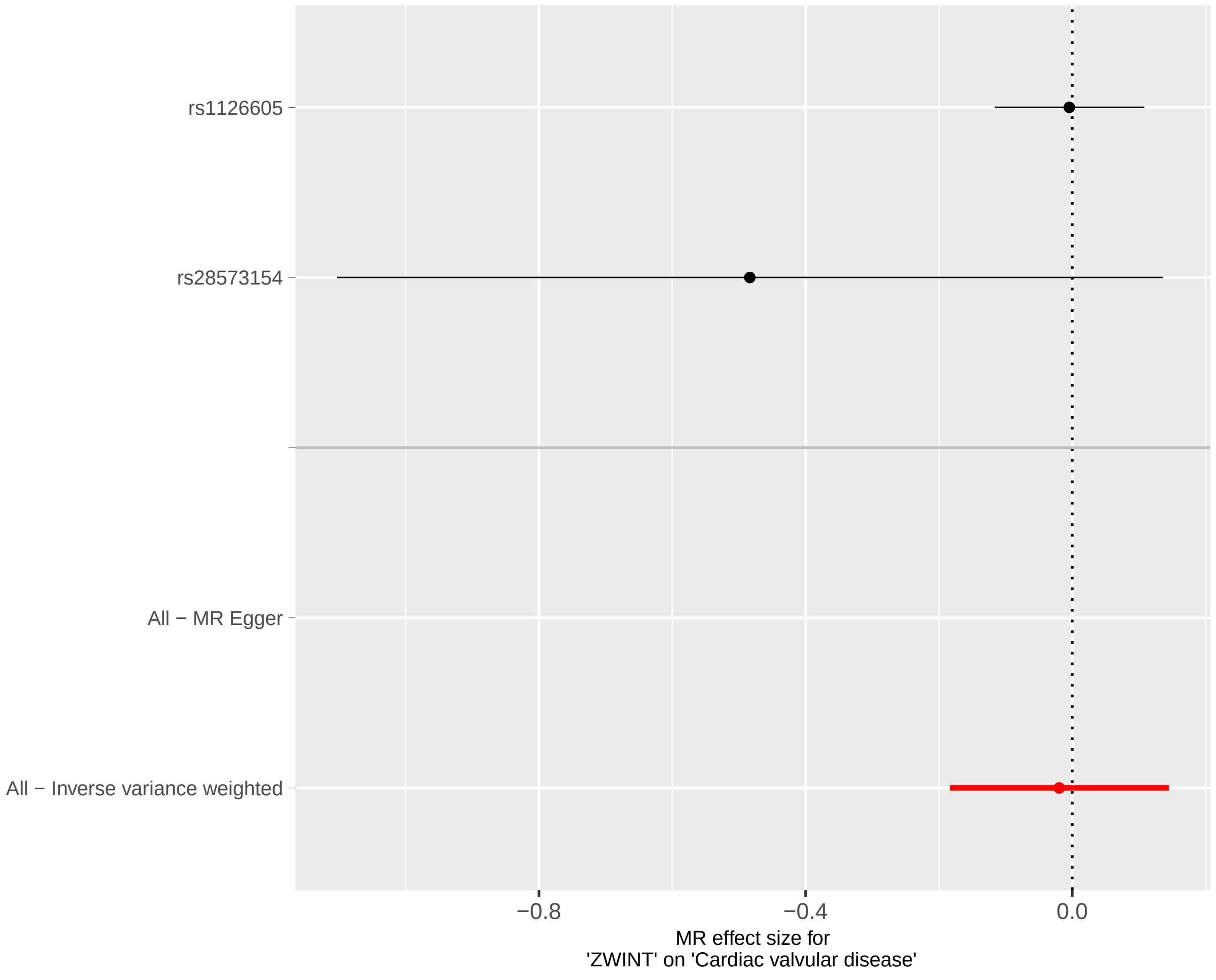

rs1126605
rs28573154
All − MR Egger
All − Inverse variance weighted
−0.8
−0.4
0.0
MR effect size for
'ZWINT' on 'Cardiac valvular disease'

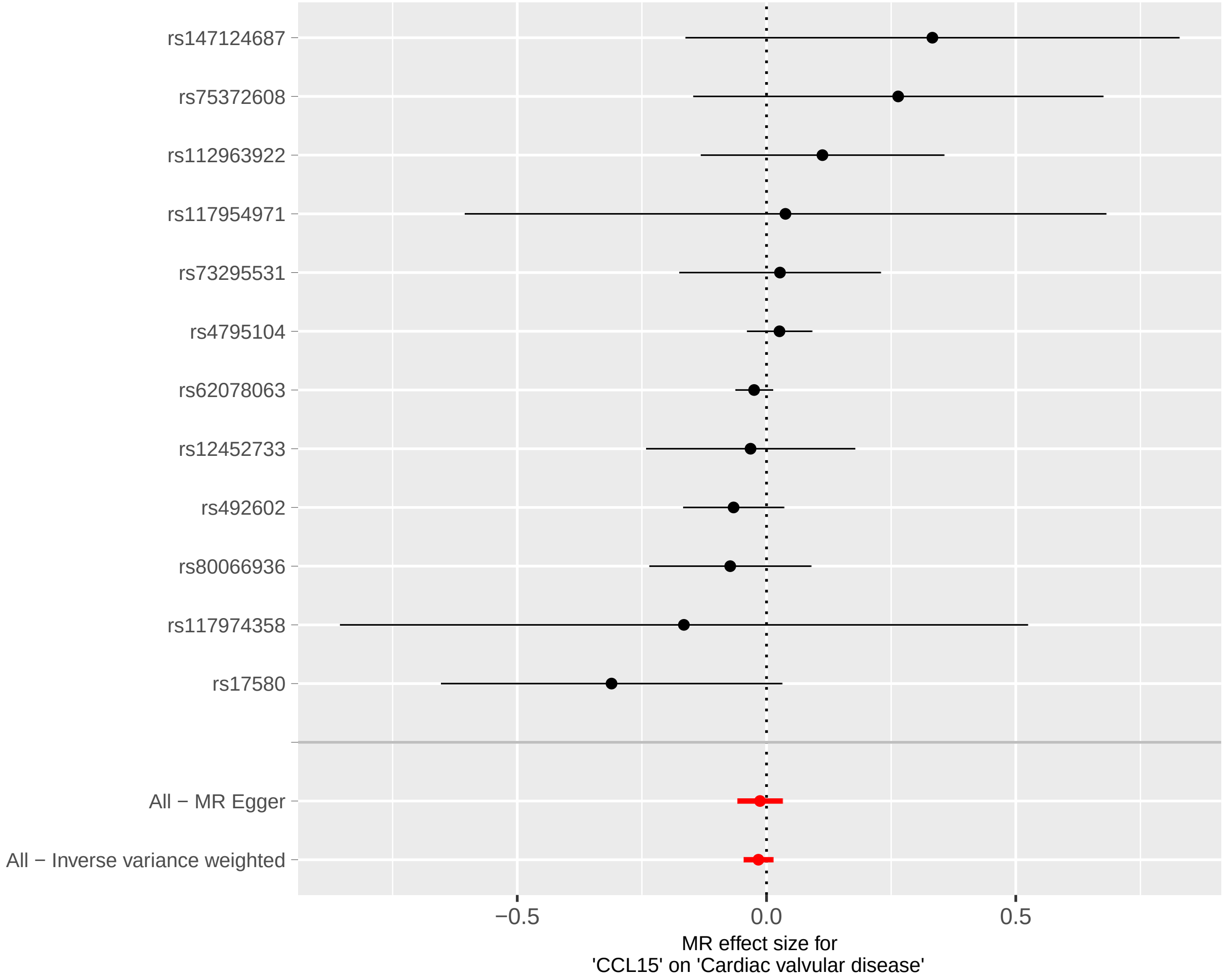
rs147124687
rs75372608
rs112963922
rs117954971
rs73295531
rs4795104
rs62078063
rs12452733
rs492602
rs80066936
rs117974358
rs17580
All − MR Egger
All − Inverse variance weighted
−0.5
0.0
0.5
MR effect size for
'CCL15' on 'Cardiac valvular disease'

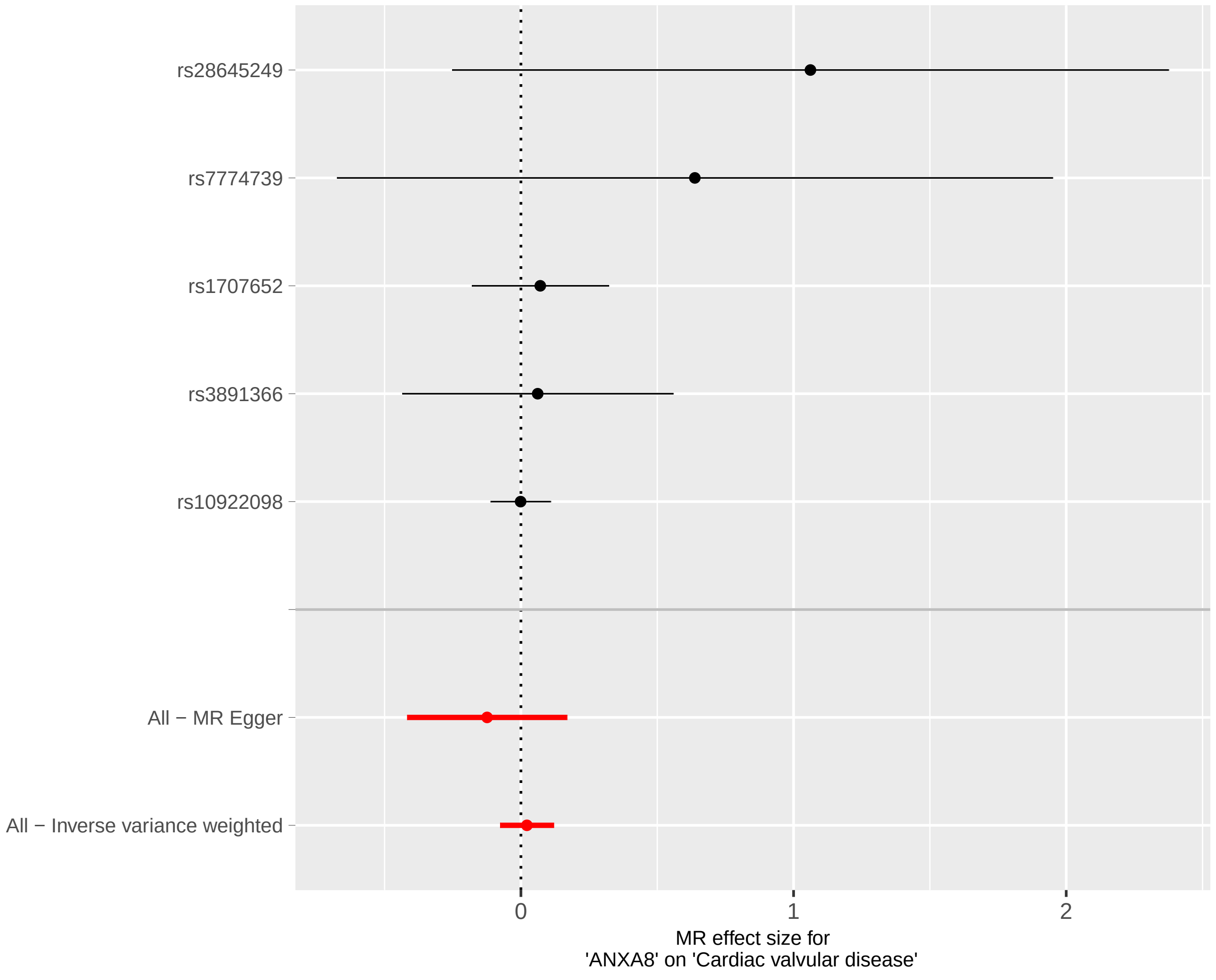
rs28645249
rs7774739
rs1707652
rs3891366
rs10922098
All − MR Egger
All − Inverse variance weighted
0
1
2
MR effect size for
'ANXA8' on 'Cardiac valvular disease'

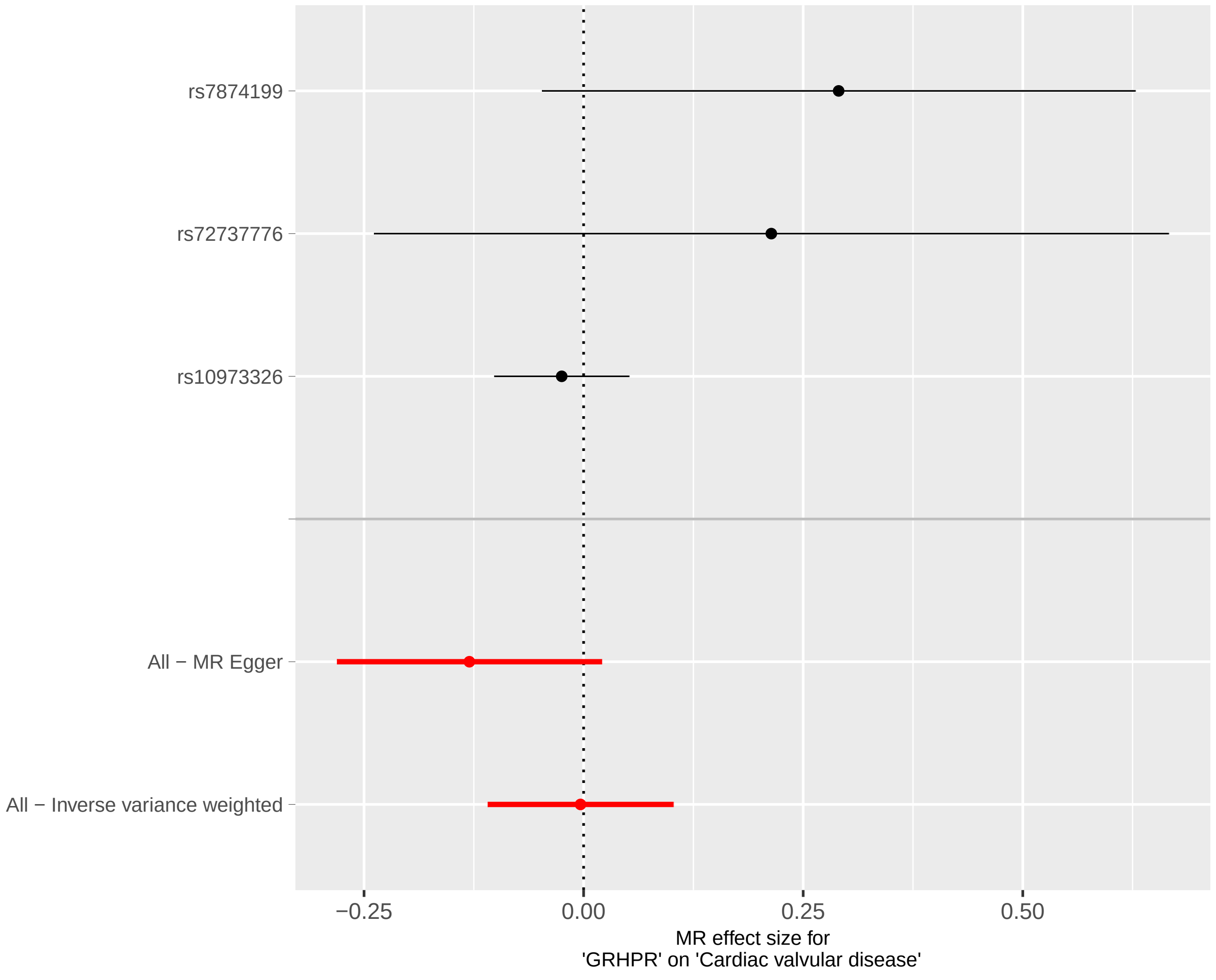

rs7874199
rs72737776
rs10973326
All − MR Egger
All − Inverse variance weighted
−0.25
0.00
0.25
0.50
MR effect size for
'GRHPR' on 'Cardiac valvular disease'

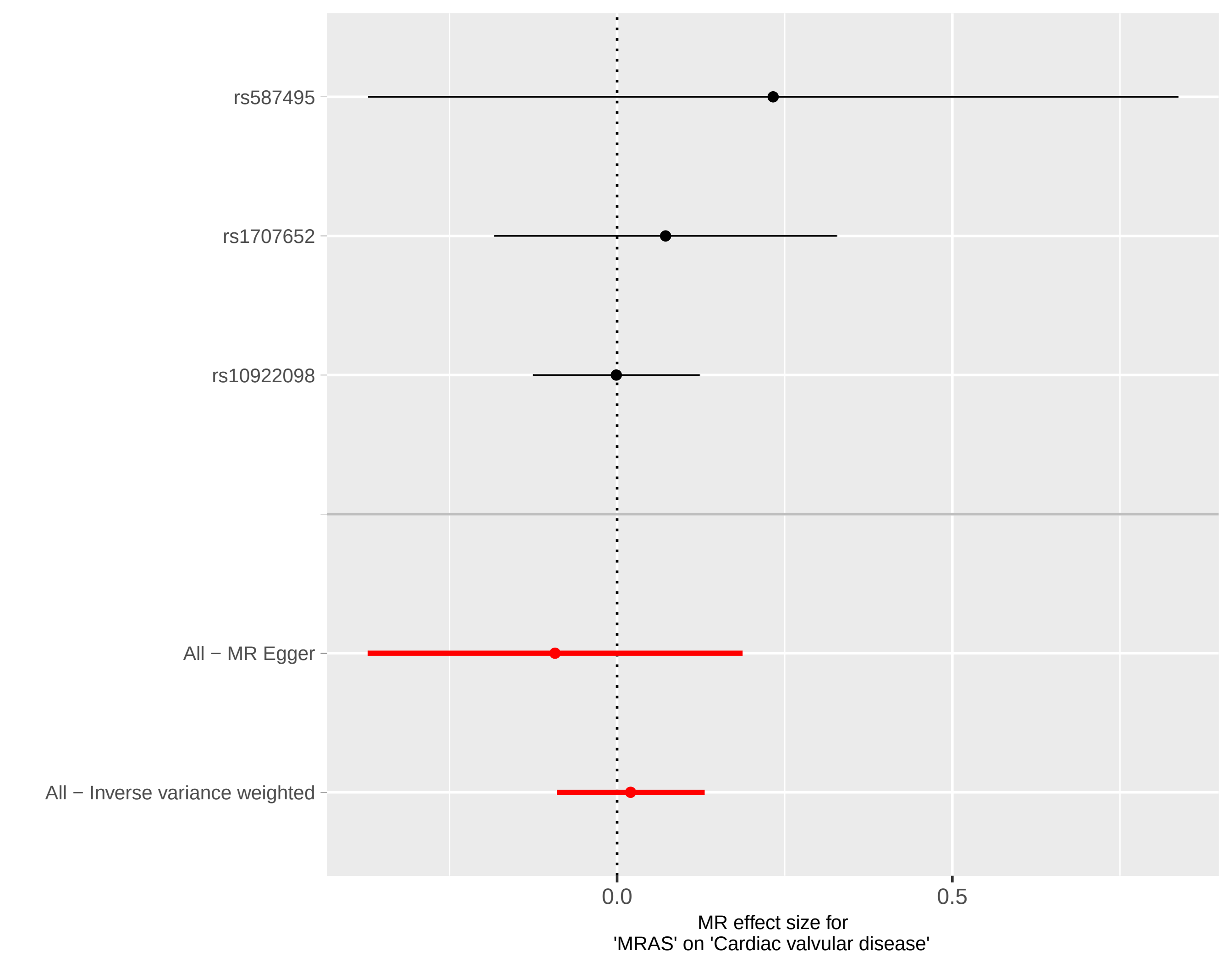
rs587495
rs1707652
rs10922098
All − MR Egger
All − Inverse variance weighted
0.0
0.5
MR effect size for
'MRAS' on 'Cardiac valvular disease'

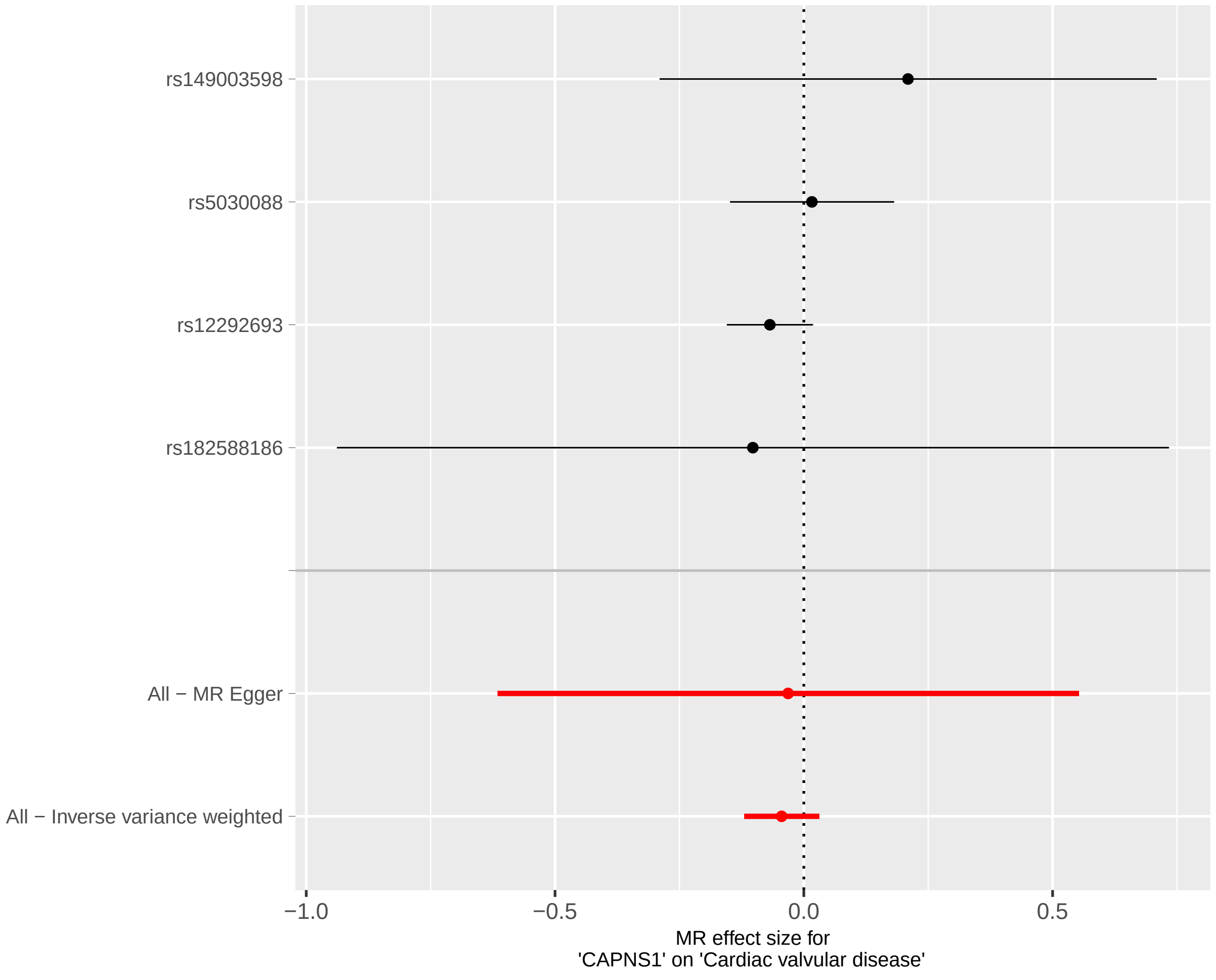

rs149003598
rs5030088
rs12292693
rs182588186
All − MR Egger
All − Inverse variance weighted
−1.0
−0.5
0.0
0.5
MR effect size for
'CAPNS1' on 'Cardiac valvular disease'

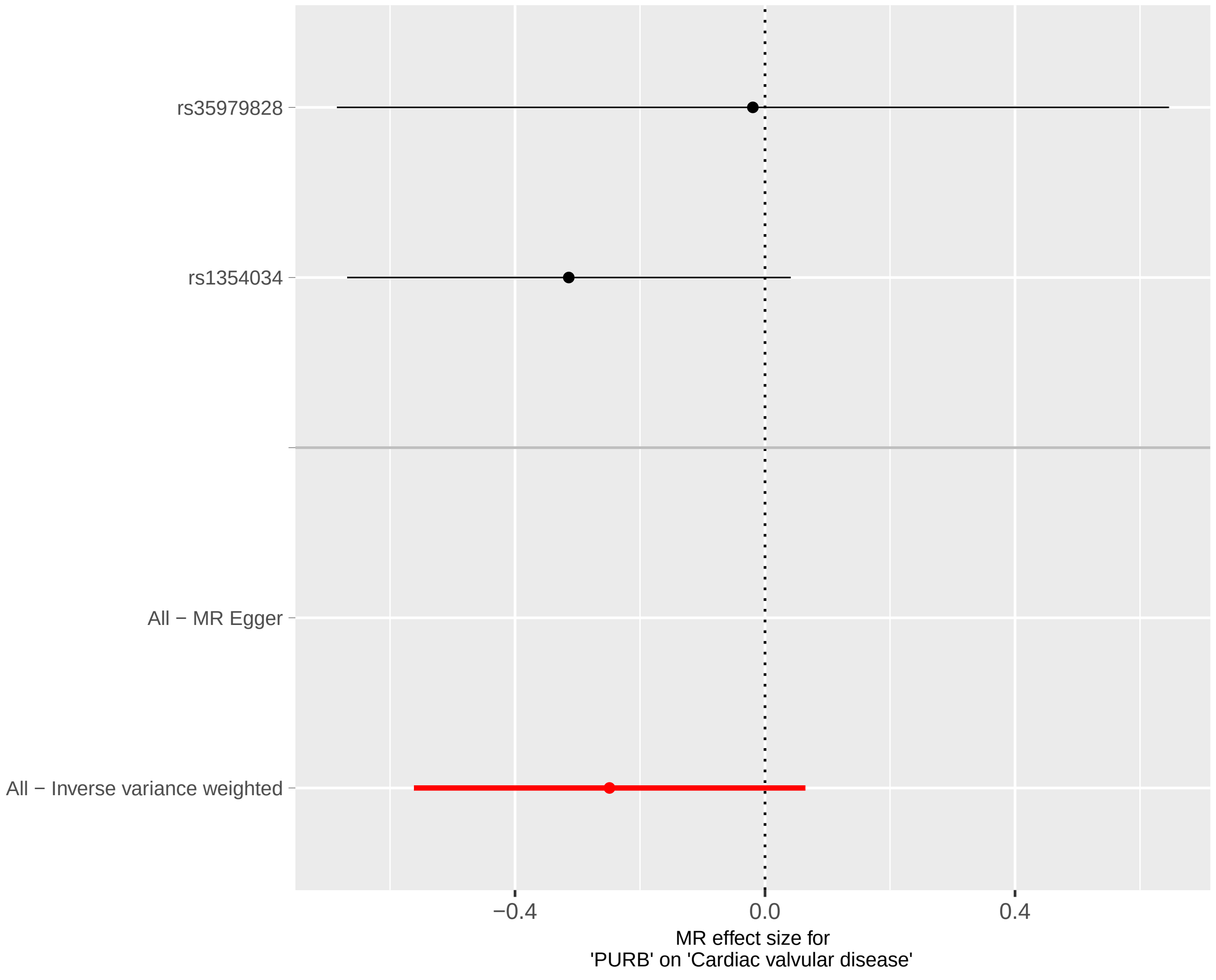
rs35979828
rs1354034
All − MR Egger
All − Inverse variance weighted
−0.4
0.0
0.4
MR effect size for
'PURB' on 'Cardiac valvular disease'

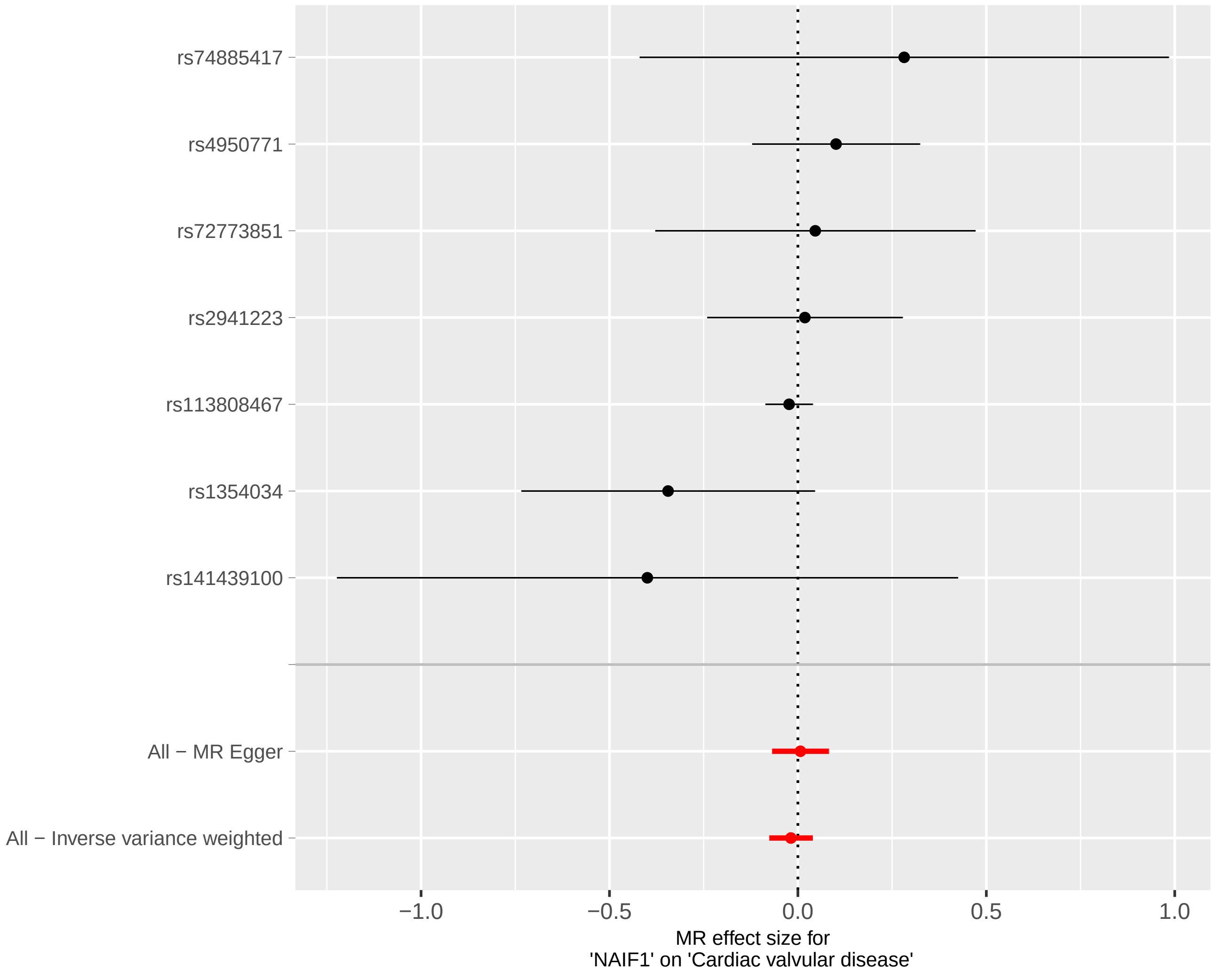
rs74885417
rs4950771
rs72773851
rs2941223
rs113808467
rs1354034
rs141439100
All − MR Egger
All − Inverse variance weighted
−1.0
−0.5
0.0
0.5
1.0
MR effect size for
'NAIF1' on 'Cardiac valvular disease'

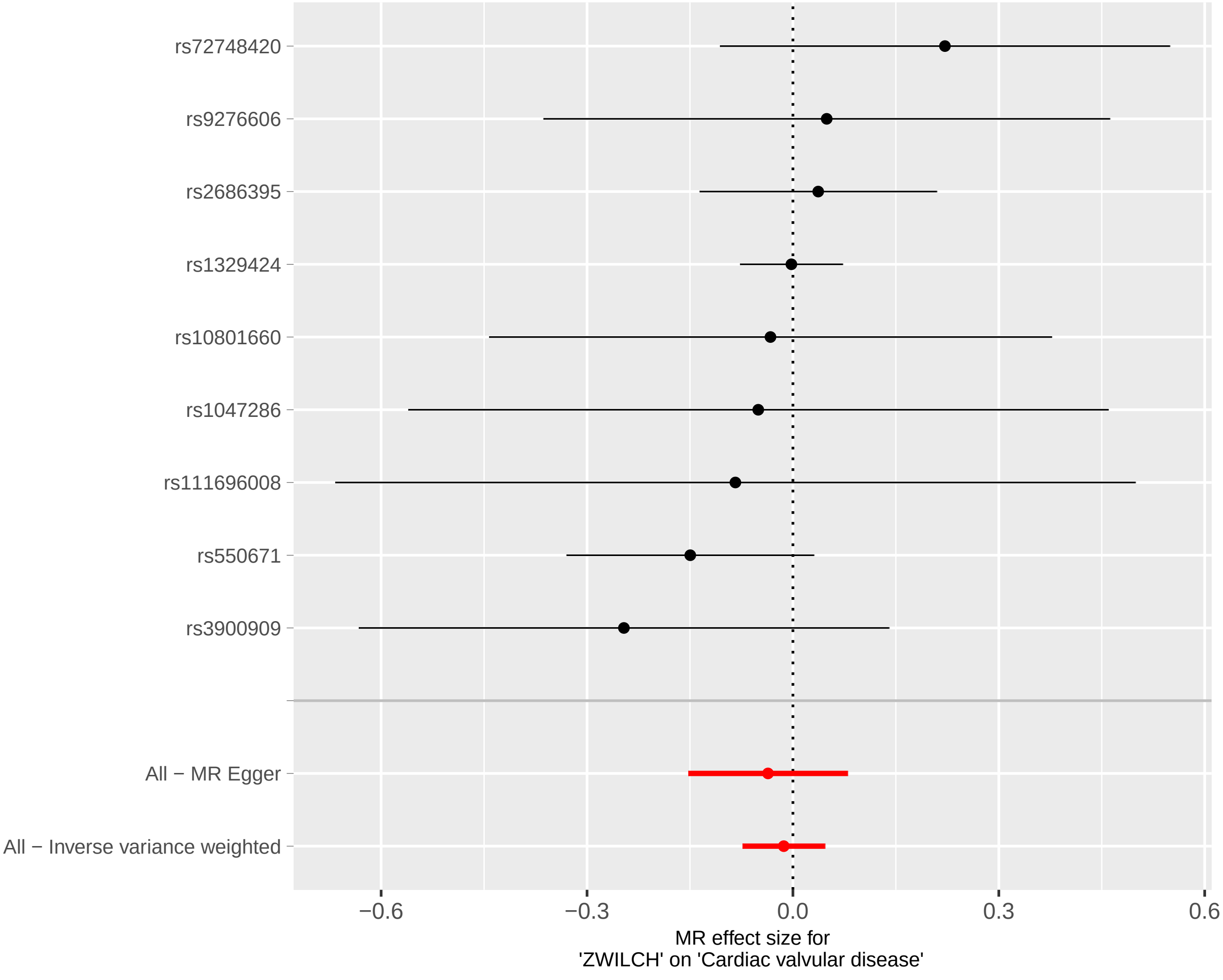
rs72748420
rs9276606
rs2686395
rs1329424
rs10801660
rs1047286
rs111696008
rs550671
rs3900909
All − MR Egger
All − Inverse variance weighted
−0.6
−0.3
0.0
0.3
0.6
MR effect size for
'ZWILCH' on 'Cardiac valvular disease'

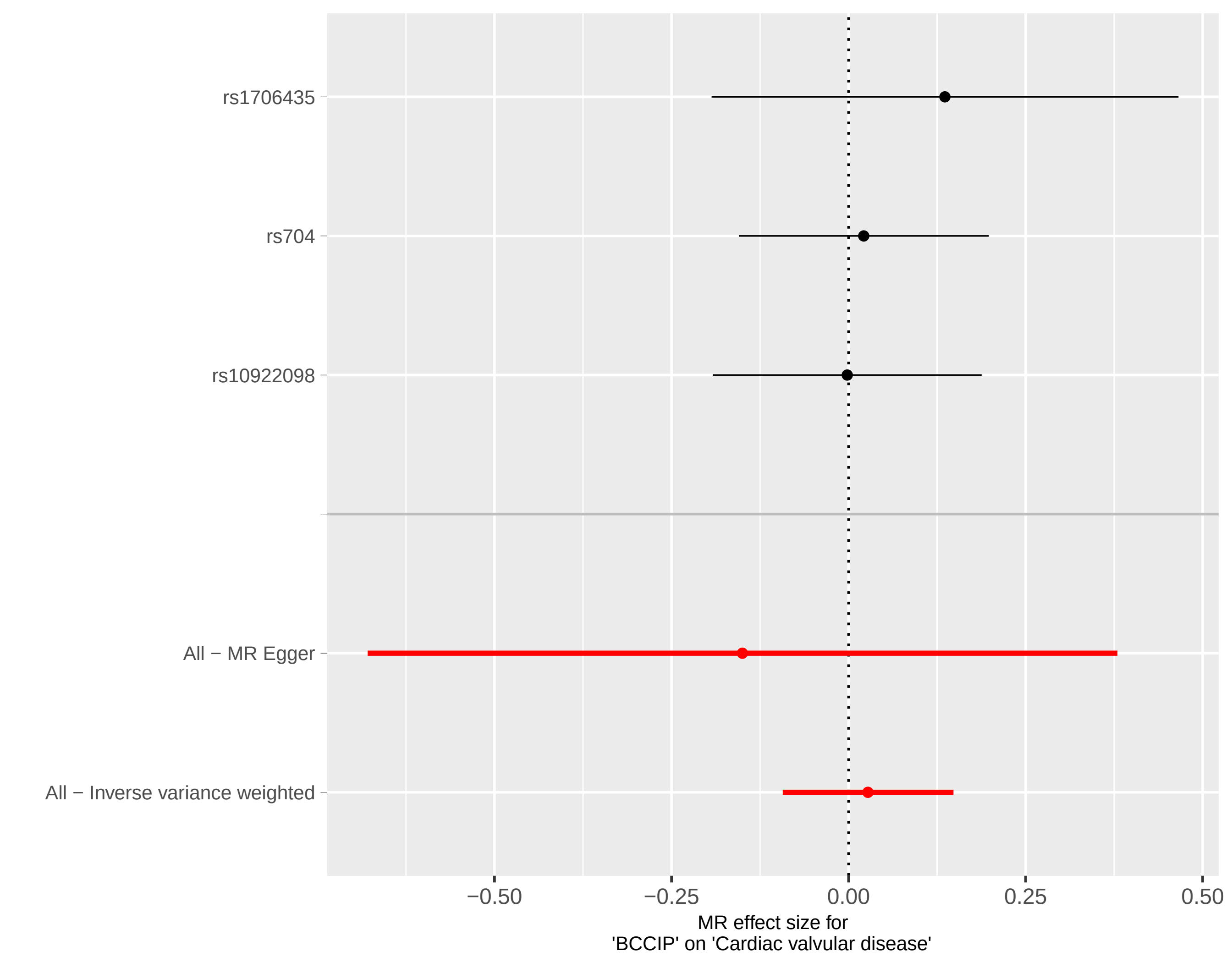
rs1706435
rs704
rs10922098
All − MR Egger
All − Inverse variance weighted
−0.50
−0.25
0.00
0.25
0.50
MR effect size for
'BCCIP' on 'Cardiac valvular disease'

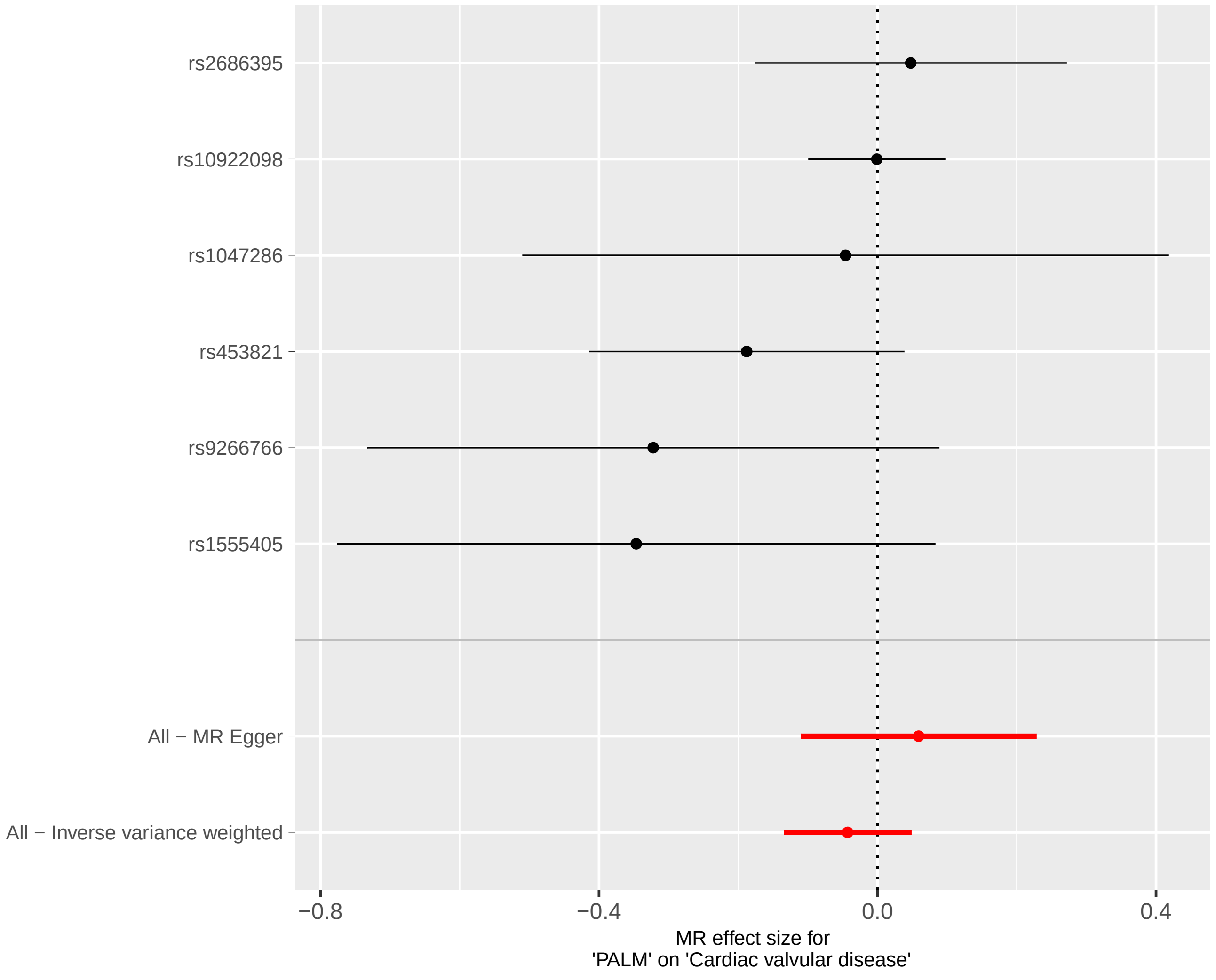
rs2686395
rs10922098
rs1047286
rs453821
rs9266766
rs1555405
All − MR Egger
All − Inverse variance weighted
−0.8
−0.4
0.0
0.4
MR effect size for
'PALM' on 'Cardiac valvular disease'

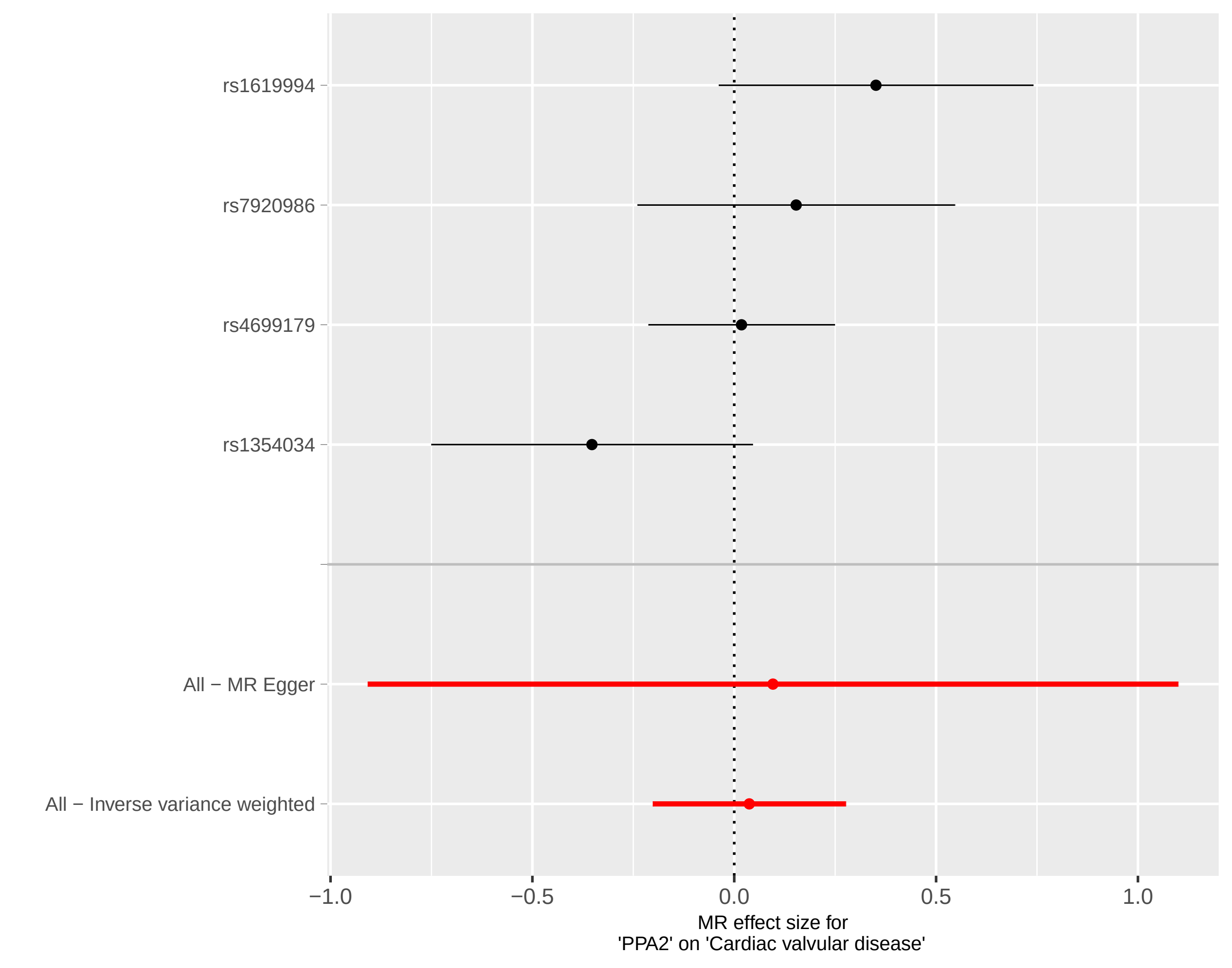
rs1619994
rs7920986
rs4699179
rs1354034
All − MR Egger
All − Inverse variance weighted
−1.0
−0.5
0.0
0.5
1.0
MR effect size for
'PPA2' on 'Cardiac valvular disease'

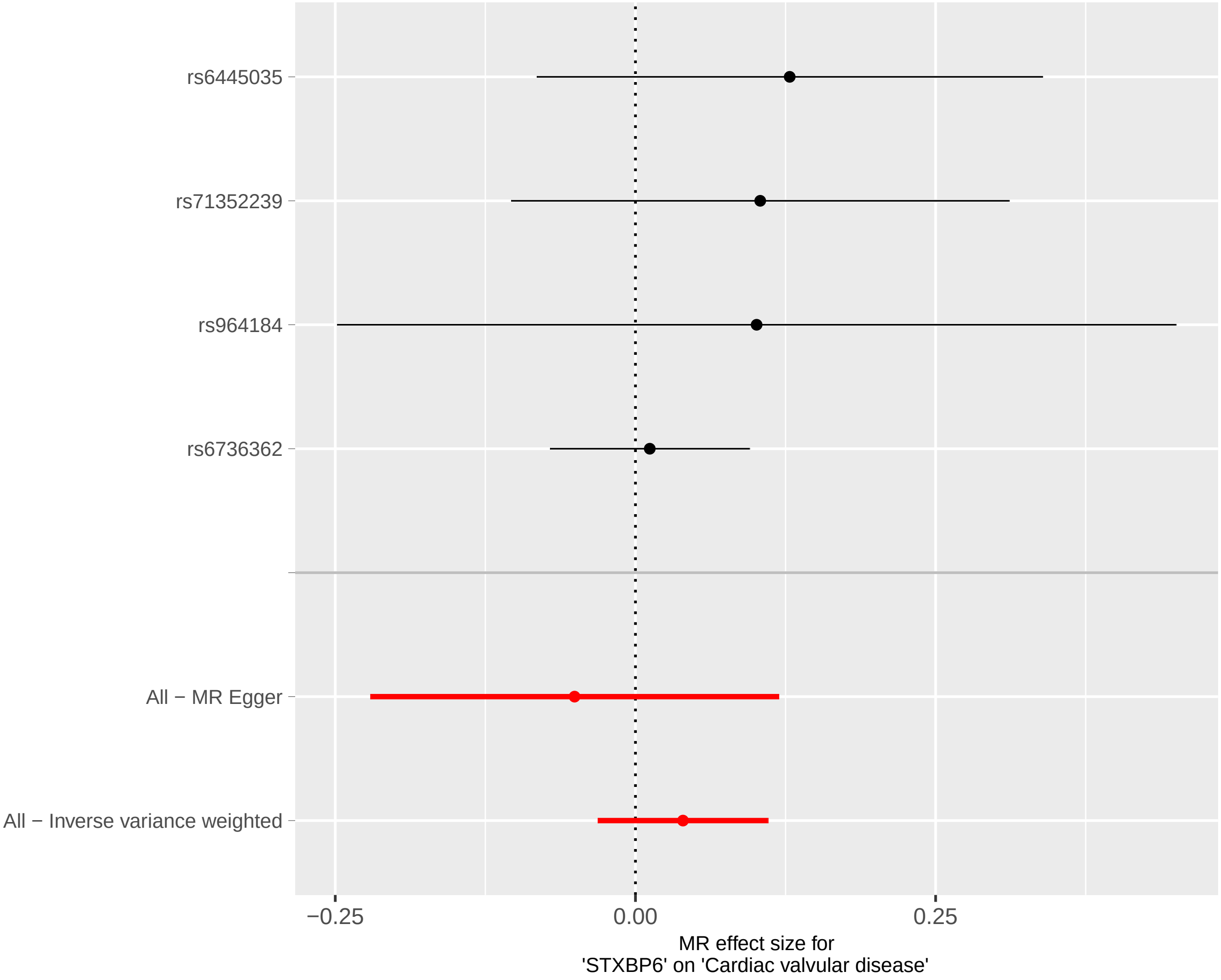
rs6445035
rs71352239
rs964184
rs6736362
All − MR Egger
All − Inverse variance weighted
−0.25
0.00
0.25
MR effect size for
'STXBP6' on 'Cardiac valvular disease'

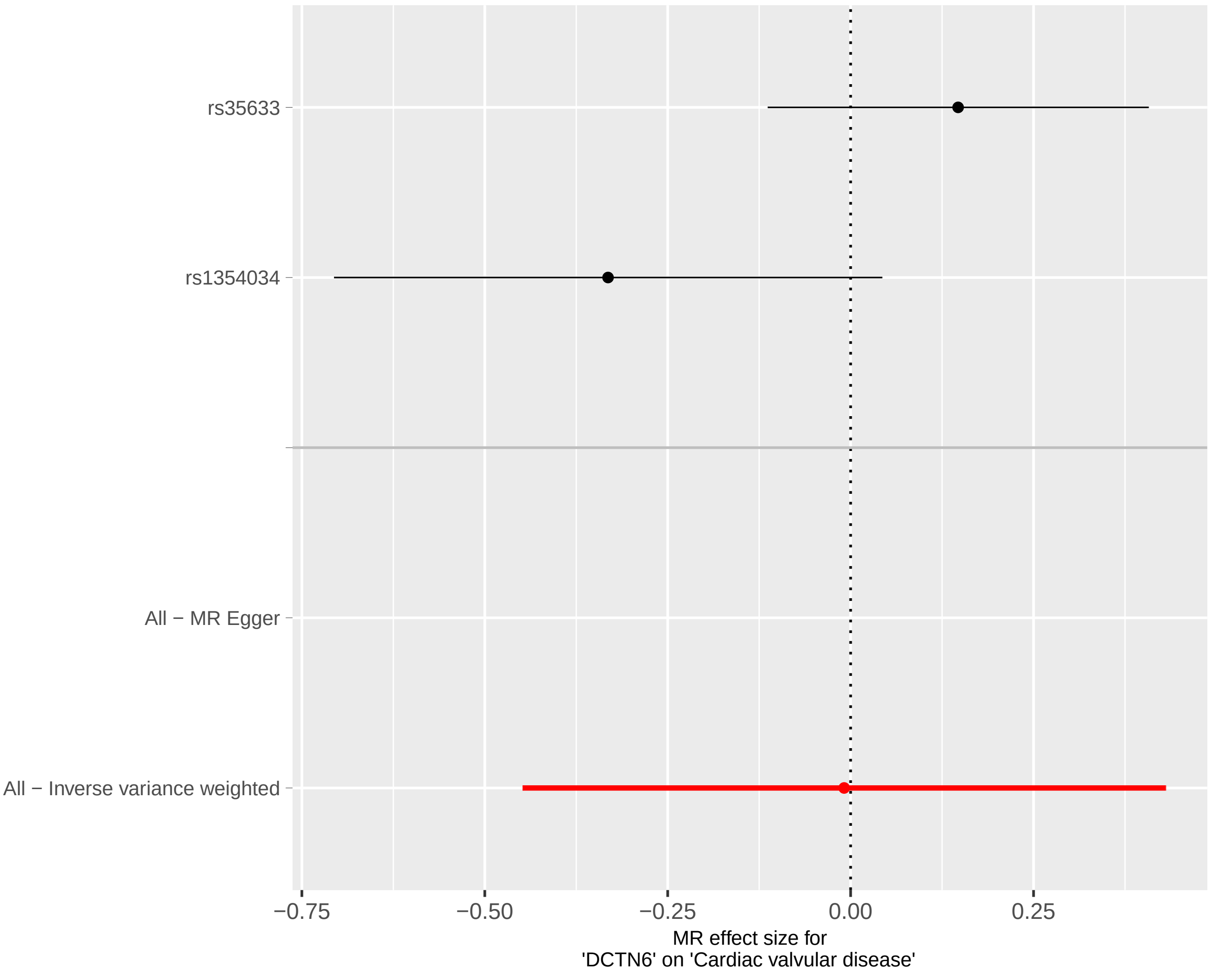
rs35633
rs1354034
All − MR Egger
All − Inverse variance weighted
−0.75
−0.50
−0.25
0.00
0.25
MR effect size for
'DCTN6' on 'Cardiac valvular disease'

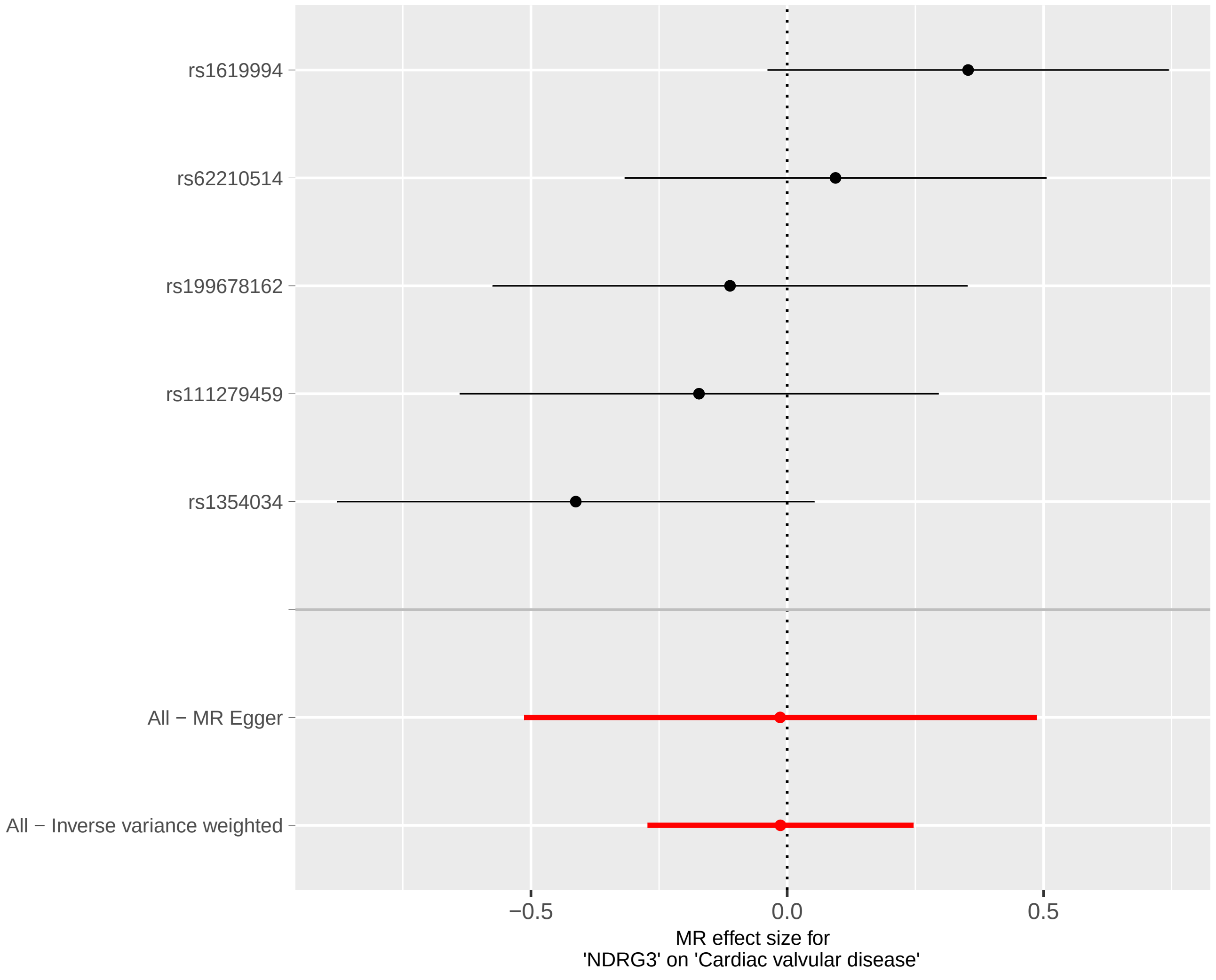
rs1619994
rs62210514
rs199678162
rs111279459
rs1354034
All − MR Egger
All − Inverse variance weighted
−0.5
0.0
0.5
MR effect size for
'NDRG3' on 'Cardiac valvular disease'

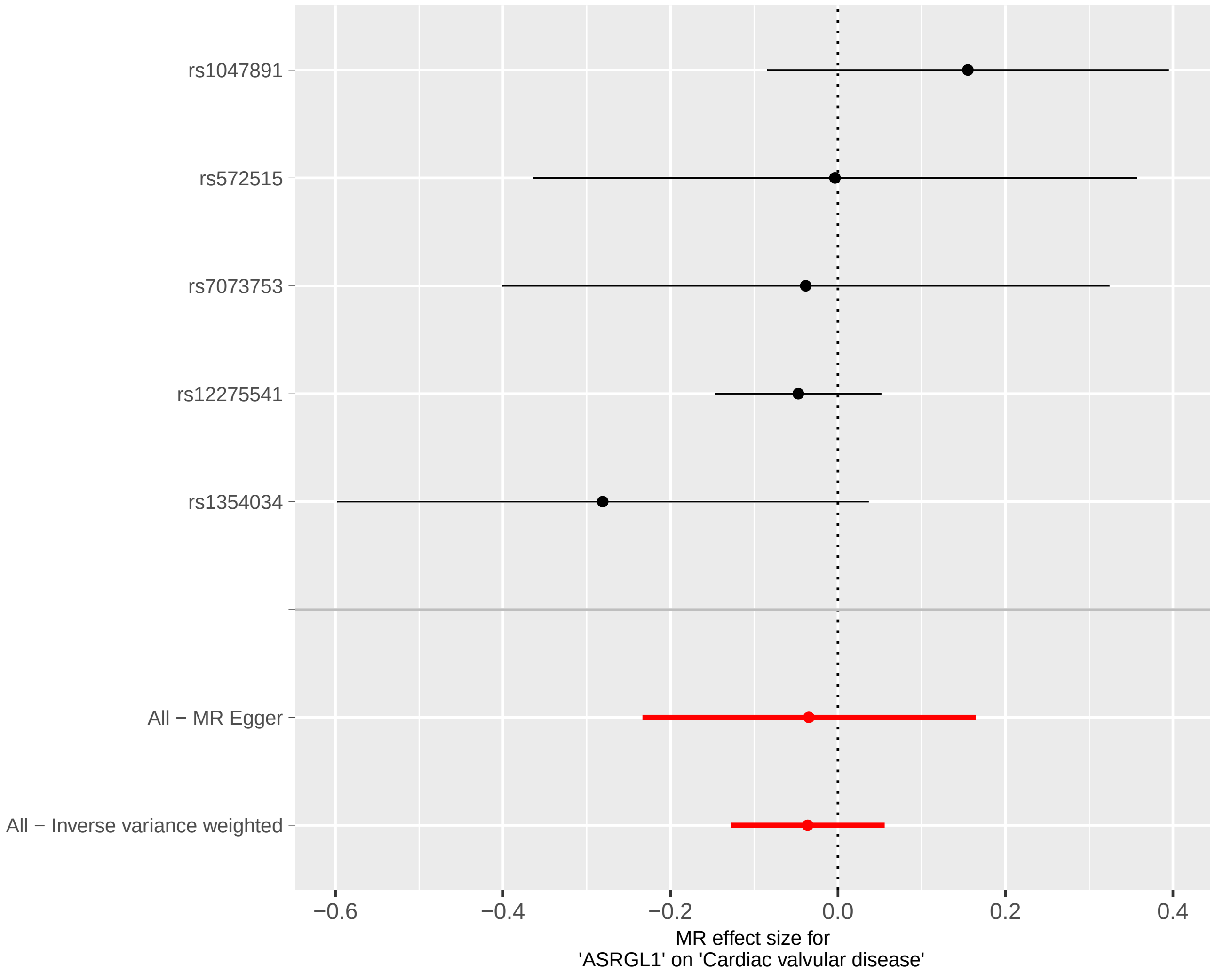
rs1047891
rs572515
rs7073753
rs12275541
rs1354034
All − MR Egger
All − Inverse variance weighted
−0.6
−0.4
−0.2
0.0
0.2
0.4
MR effect size for
'ASRGL1' on 'Cardiac valvular disease'

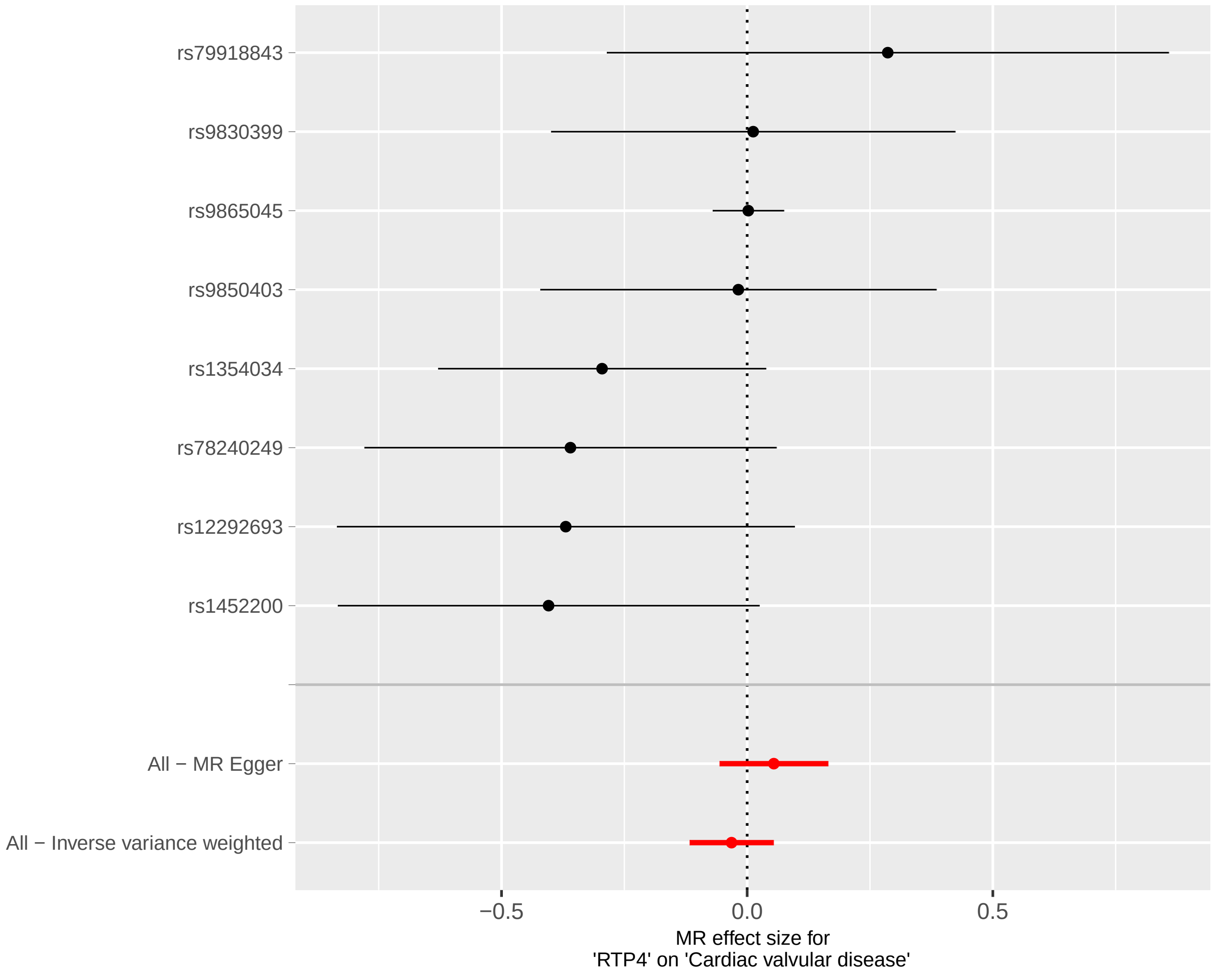
rs79918843
rs9830399
rs9865045
rs9850403
rs1354034
rs78240249
rs12292693
rs1452200
All − MR Egger
All − Inverse variance weighted
−0.5
0.0
0.5
MR effect size for
'RTP4' on 'Cardiac valvular disease'

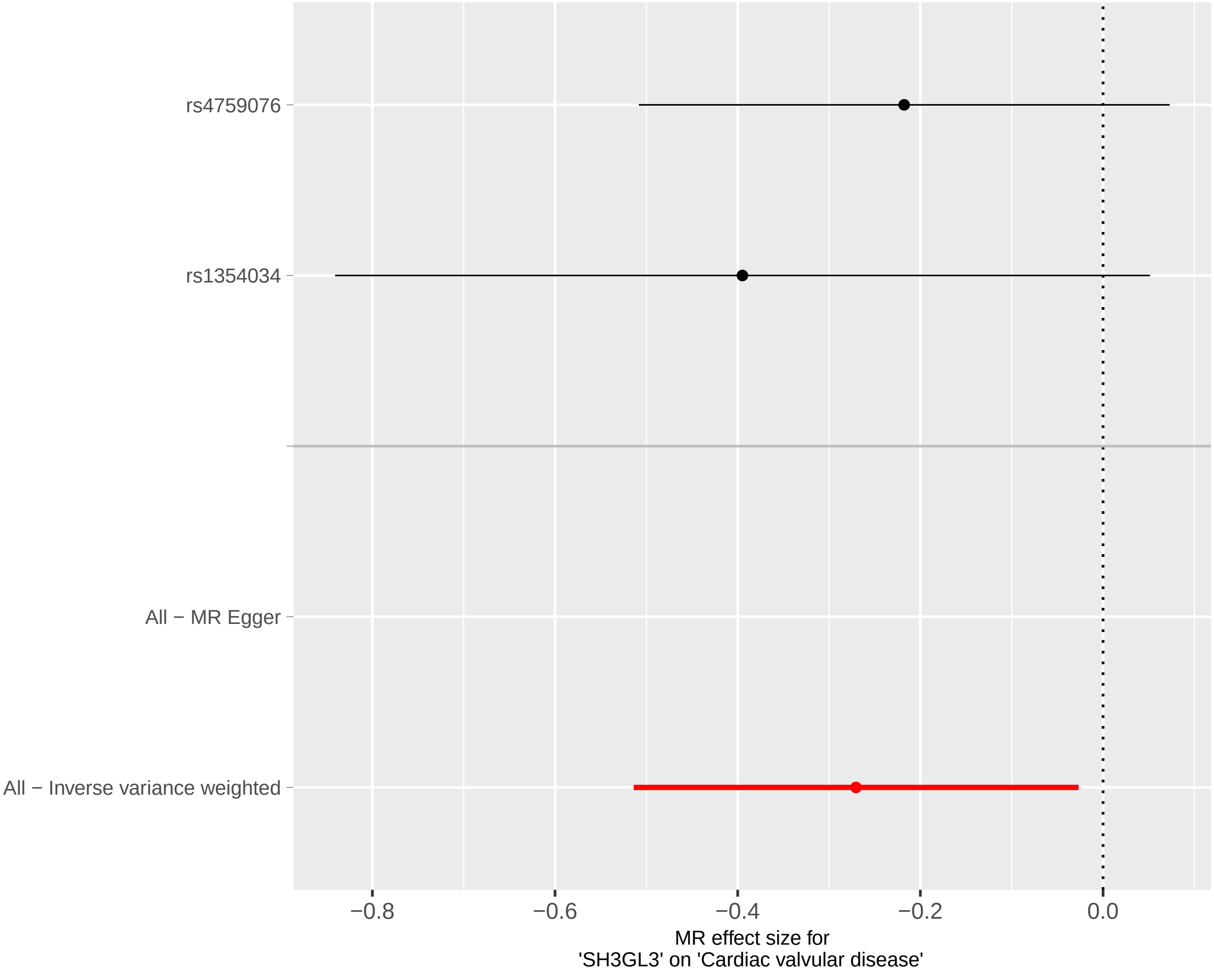
rs4759076
rs1354034
All − MR Egger
All − Inverse variance weighted
−0.8
−0.6
−0.4
−0.2
0.0
MR effect size for
'SH3GL3' on 'Cardiac valvular disease'

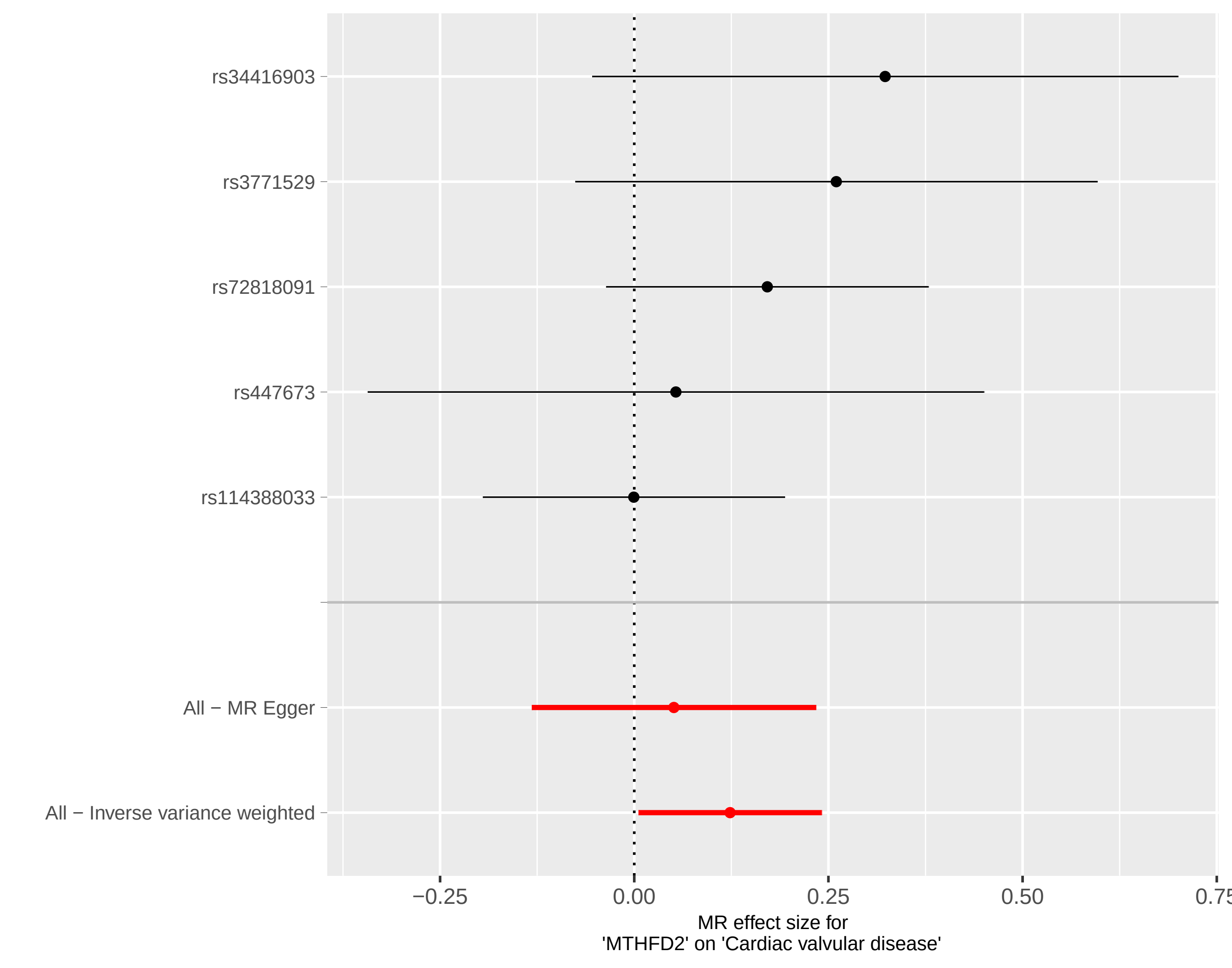
rs34416903
rs3771529
rs72818091
rs447673
rs114388033
All − MR Egger
All − Inverse variance weighted
−0.25
0.00
0.25
0.50
0.75
MR effect size for
'MTHFD2' on 'Cardiac valvular disease'

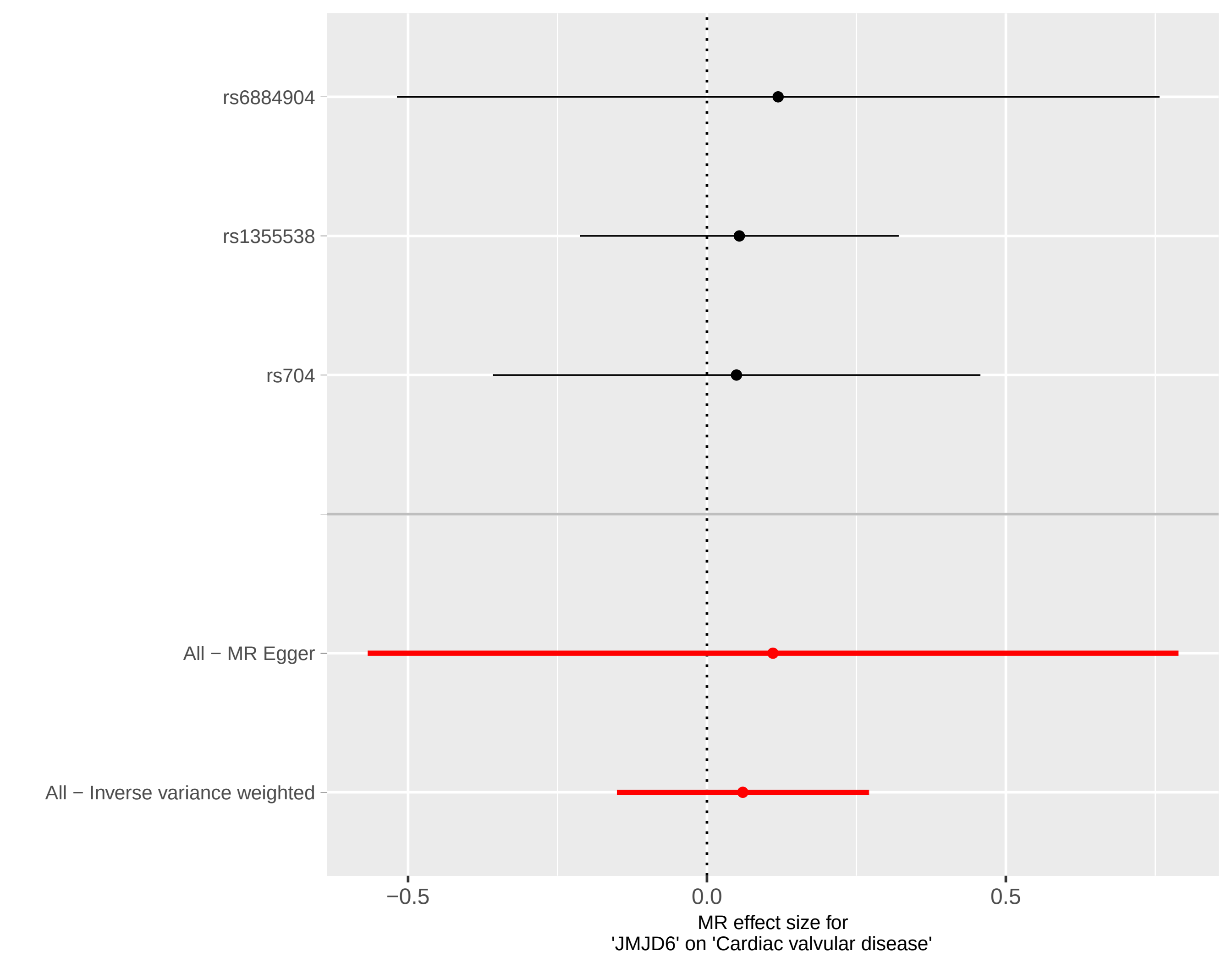
rs6884904
rs1355538
rs704
All − MR Egger
All − Inverse variance weighted
−0.5
0.0
0.5
MR effect size for
'JMJD6' on 'Cardiac valvular disease'

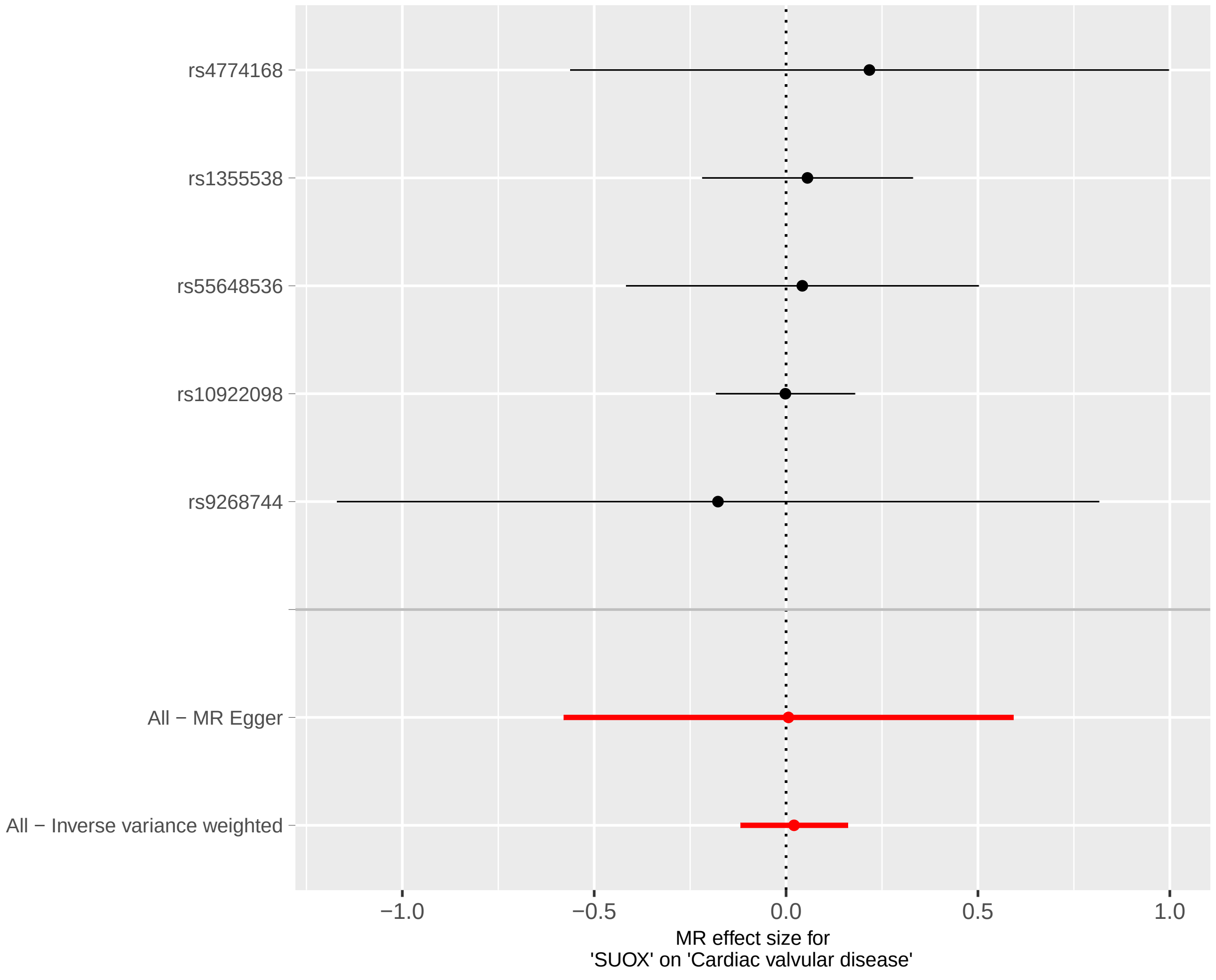

rs4774168
rs1355538
rs55648536
rs10922098
rs9268744
All − MR Egger
All − Inverse variance weighted
−1.0
−0.5
0.0
0.5
1.0
MR effect size for
'SUOX' on 'Cardiac valvular disease'

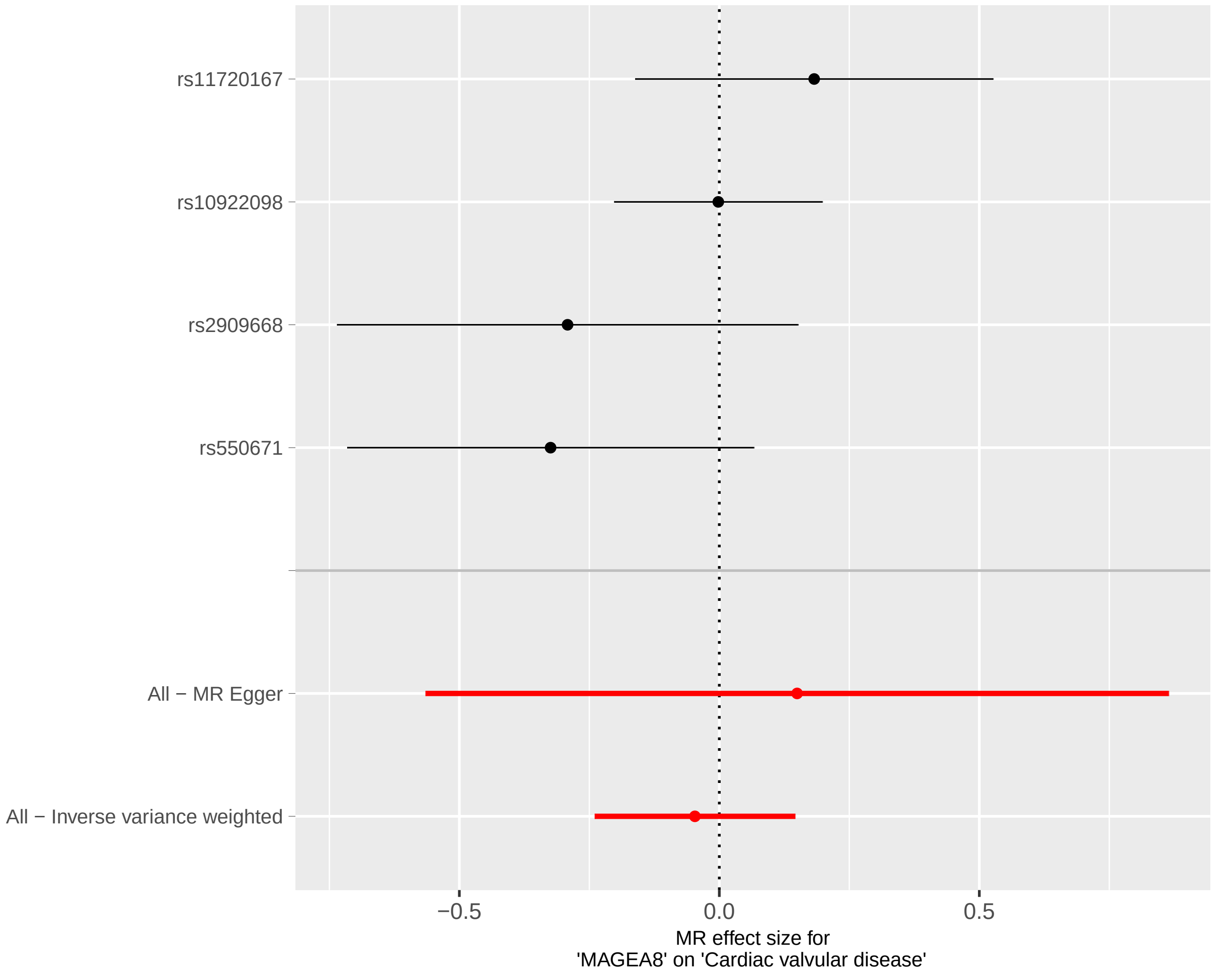
rs11720167
rs10922098
rs2909668
rs550671
All − MR Egger
All − Inverse variance weighted
−0.5
0.0
0.5
MR effect size for
'MAGEA8' on 'Cardiac valvular disease'

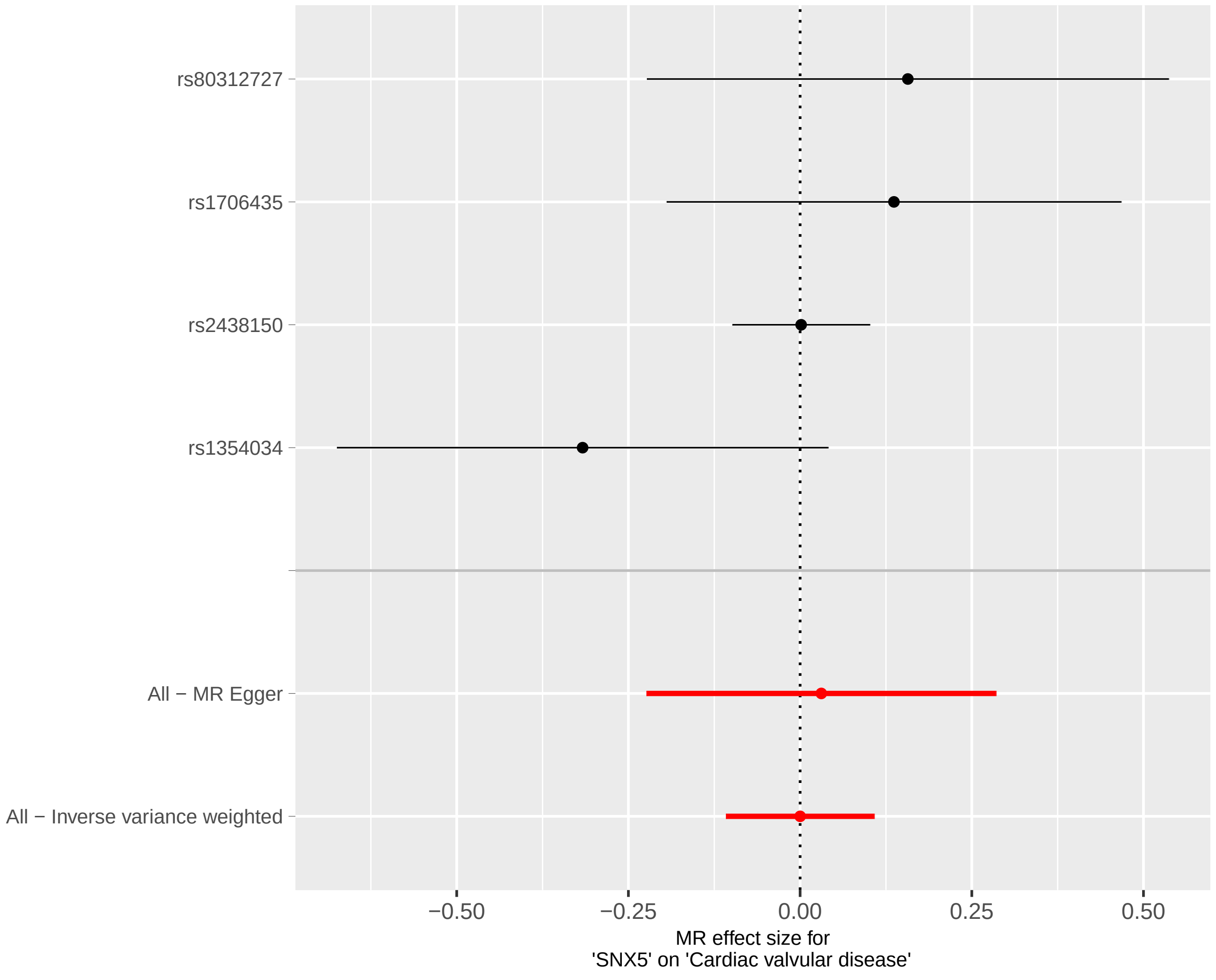
rs80312727
rs1706435
rs2438150
rs1354034
All − MR Egger
All − Inverse variance weighted
−0.50
−0.25
0.00
0.25
0.50
MR effect size for
'SNX5' on 'Cardiac valvular disease'

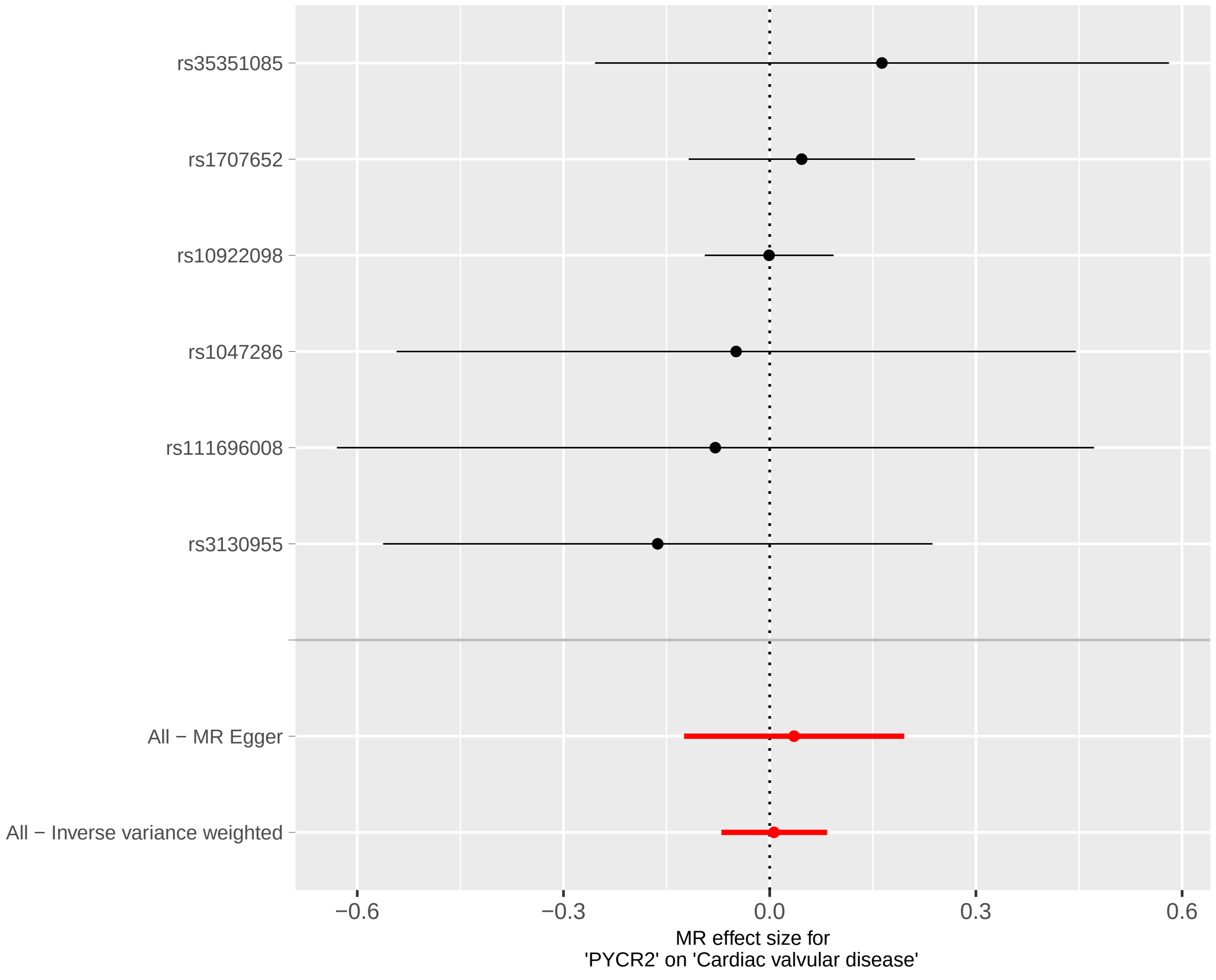
rs35351085
rs1707652
rs10922098
rs1047286
rs111696008
rs3130955
All − MR Egger
All − Inverse variance weighted
−0.6
−0.3
0.0
0.3
0.6
MR effect size for
'PYCR2' on 'Cardiac valvular disease'

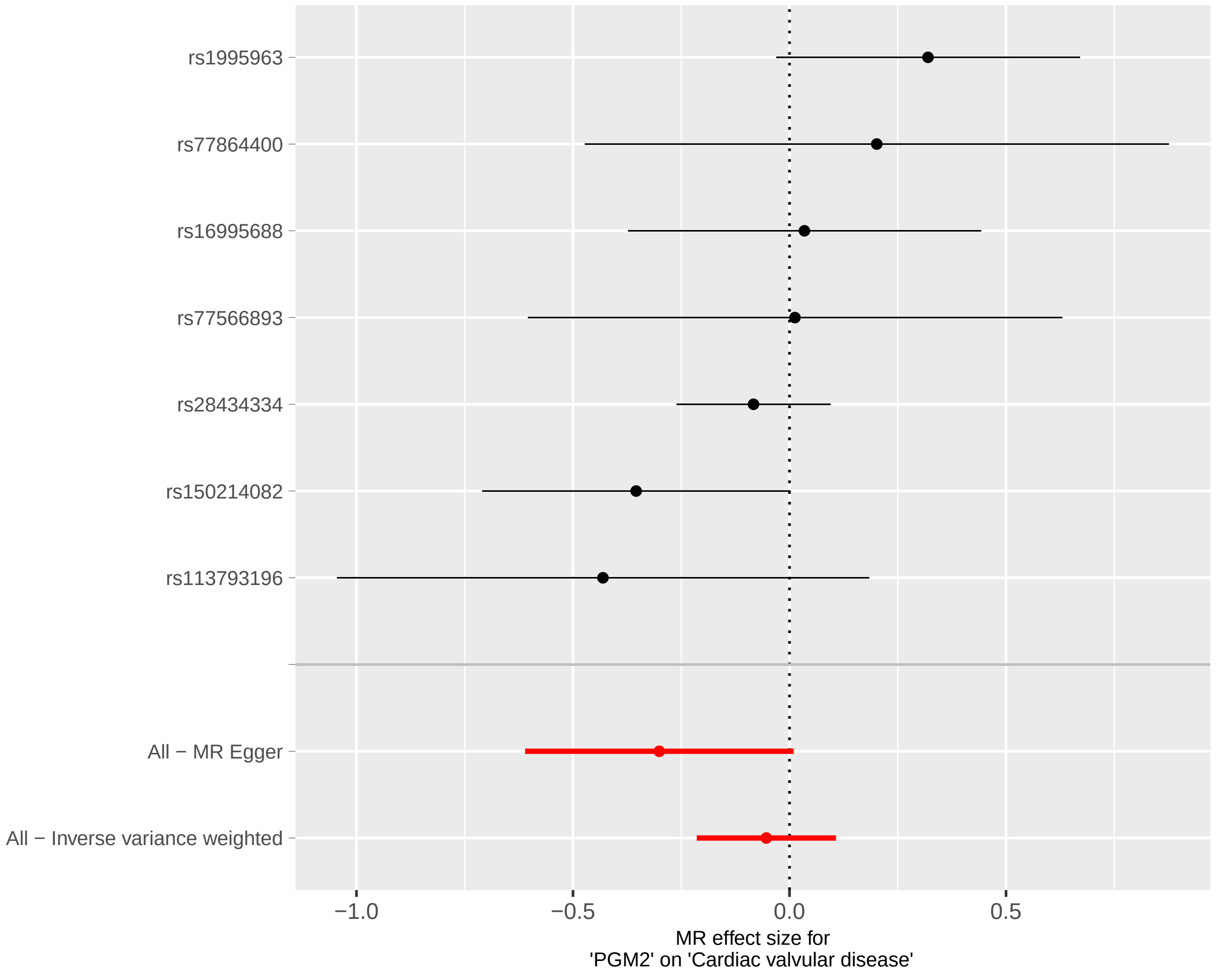

rs1995963
rs77864400
rs16995688
rs77566893
rs28434334
rs150214082
rs113793196
All − MR Egger
All − Inverse variance weighted
−1.0
−0.5
0.0
0.5
MR effect size for
'PGM2' on 'Cardiac valvular disease'

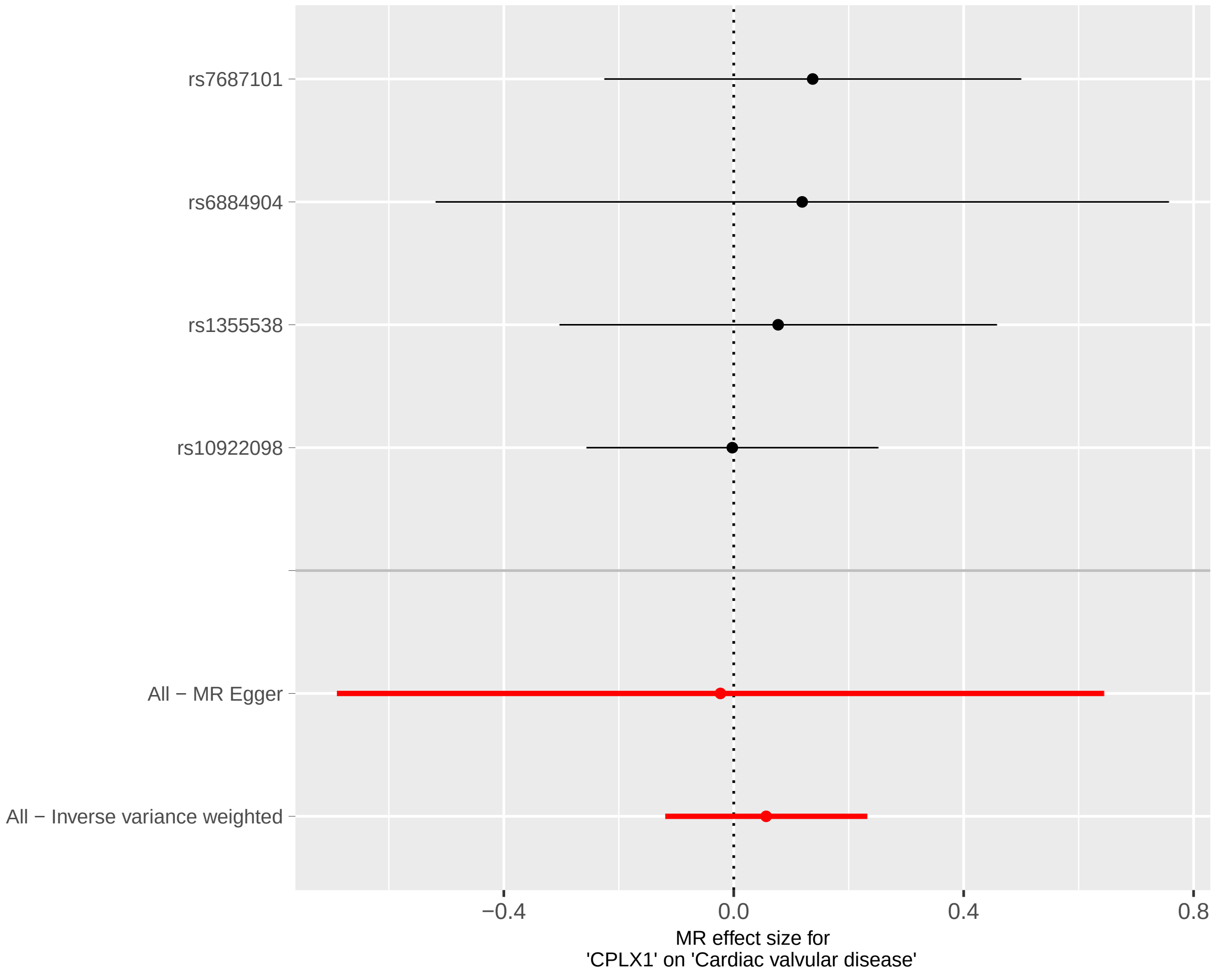

rs7687101
rs6884904
rs1355538
rs10922098
All − MR Egger
All − Inverse variance weighted
−0.4
0.0
0.4
0.8
MR effect size for
'CPLX1' on 'Cardiac valvular disease'

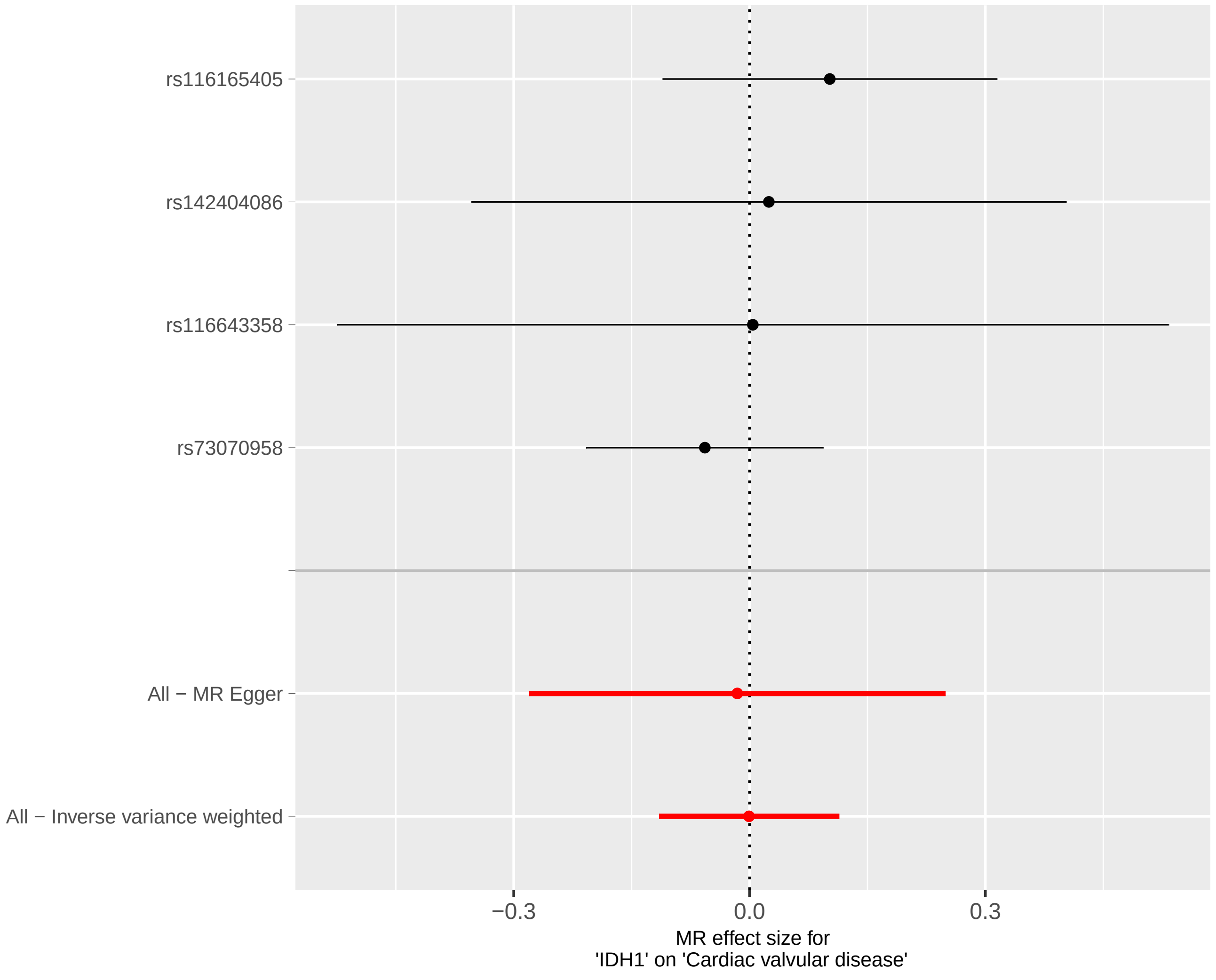
rs116165405
rs142404086
rs116643358
rs73070958
All − MR Egger
All − Inverse variance weighted
−0.3
0.0
0.3
MR effect size for
'IDH1' on 'Cardiac valvular disease'

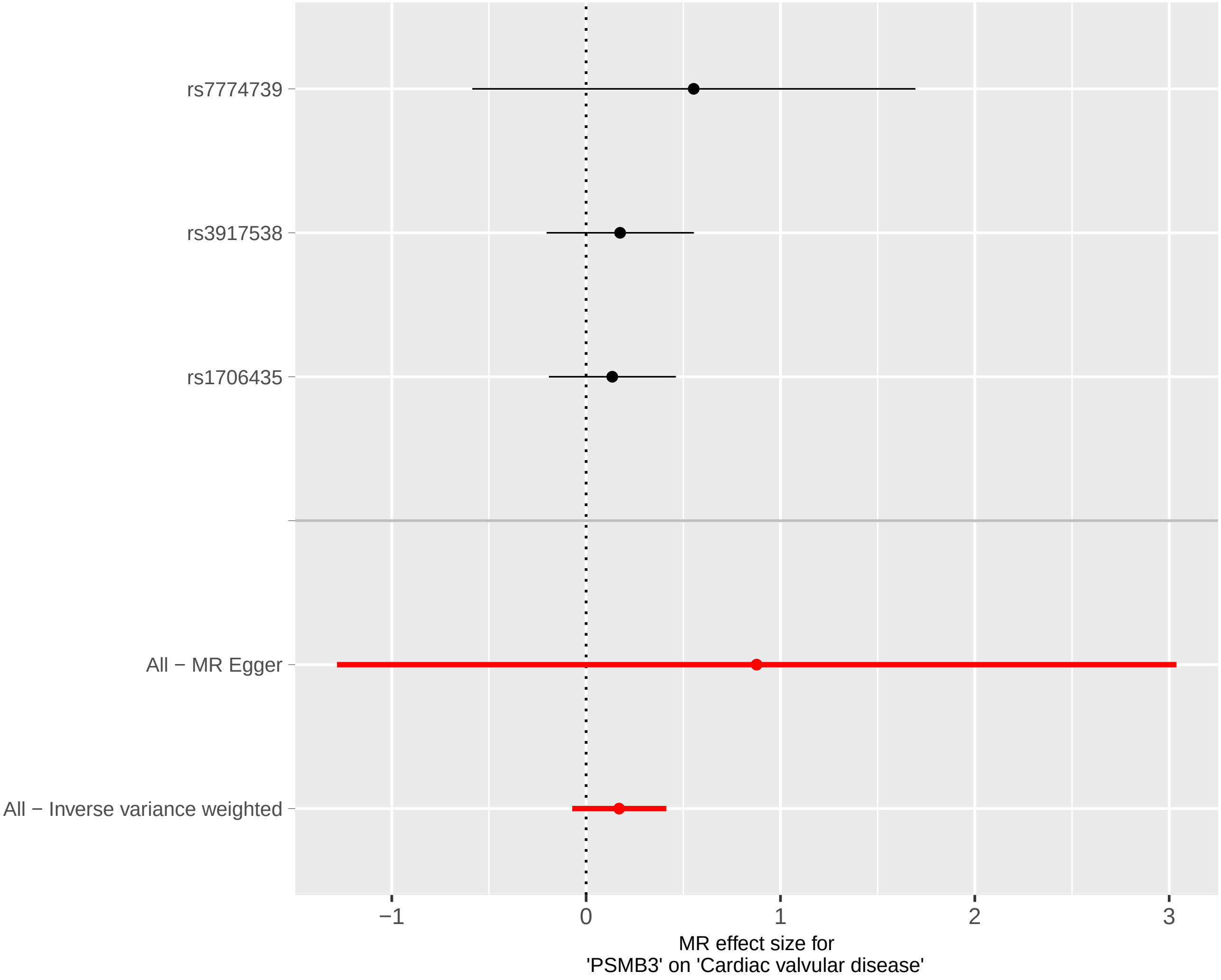

rs7774739
rs3917538
rs1706435
All − MR Egger
All − Inverse variance weighted
−1
0
1
2
3
MR effect size for
'PSMB3' on 'Cardiac valvular disease'

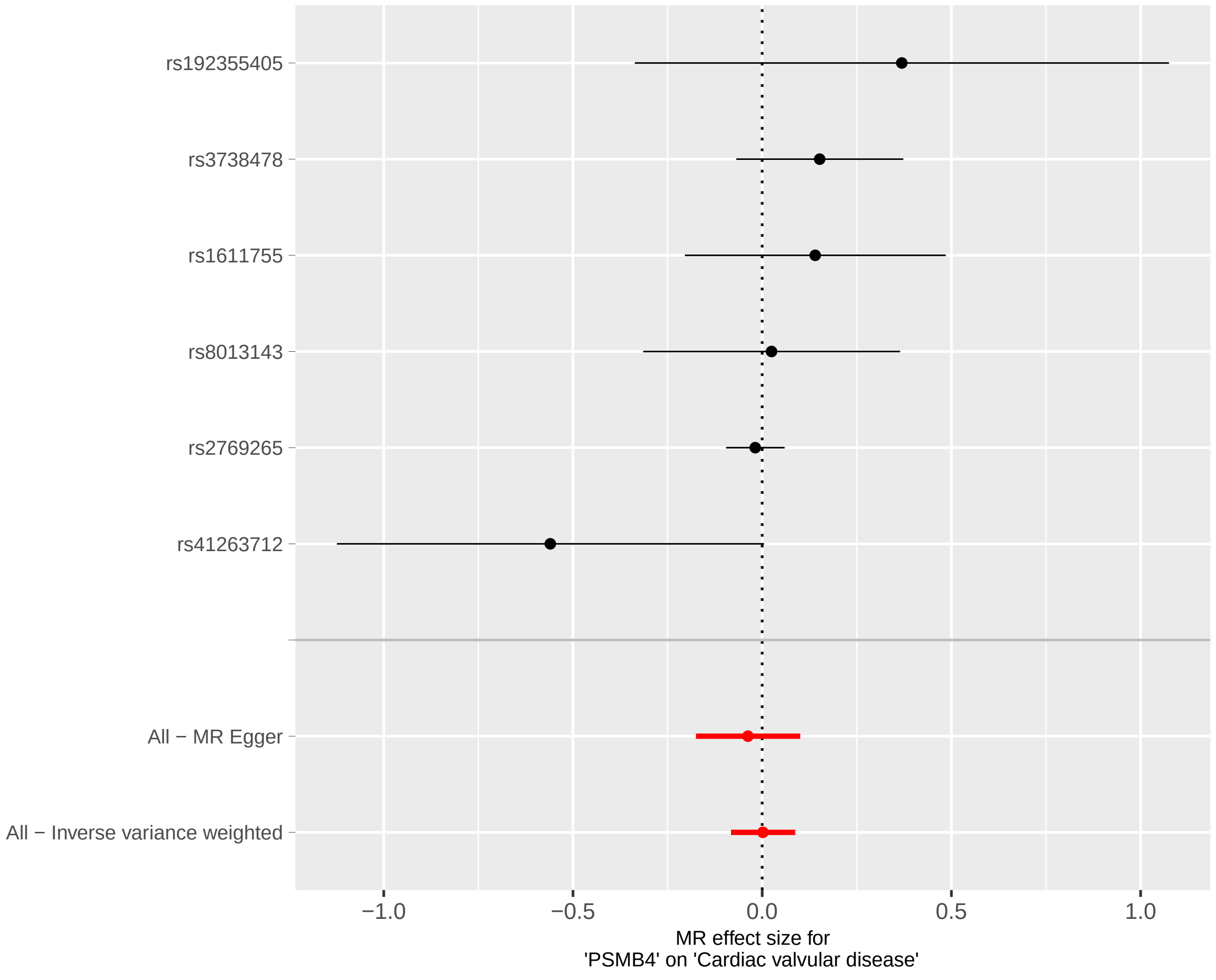

rs192355405
rs3738478
rs1611755
rs8013143
rs2769265
rs41263712
All − MR Egger
All − Inverse variance weighted
−1.0
−0.5
0.0
0.5
1.0
MR effect size for
'PSMB4' on 'Cardiac valvular disease'

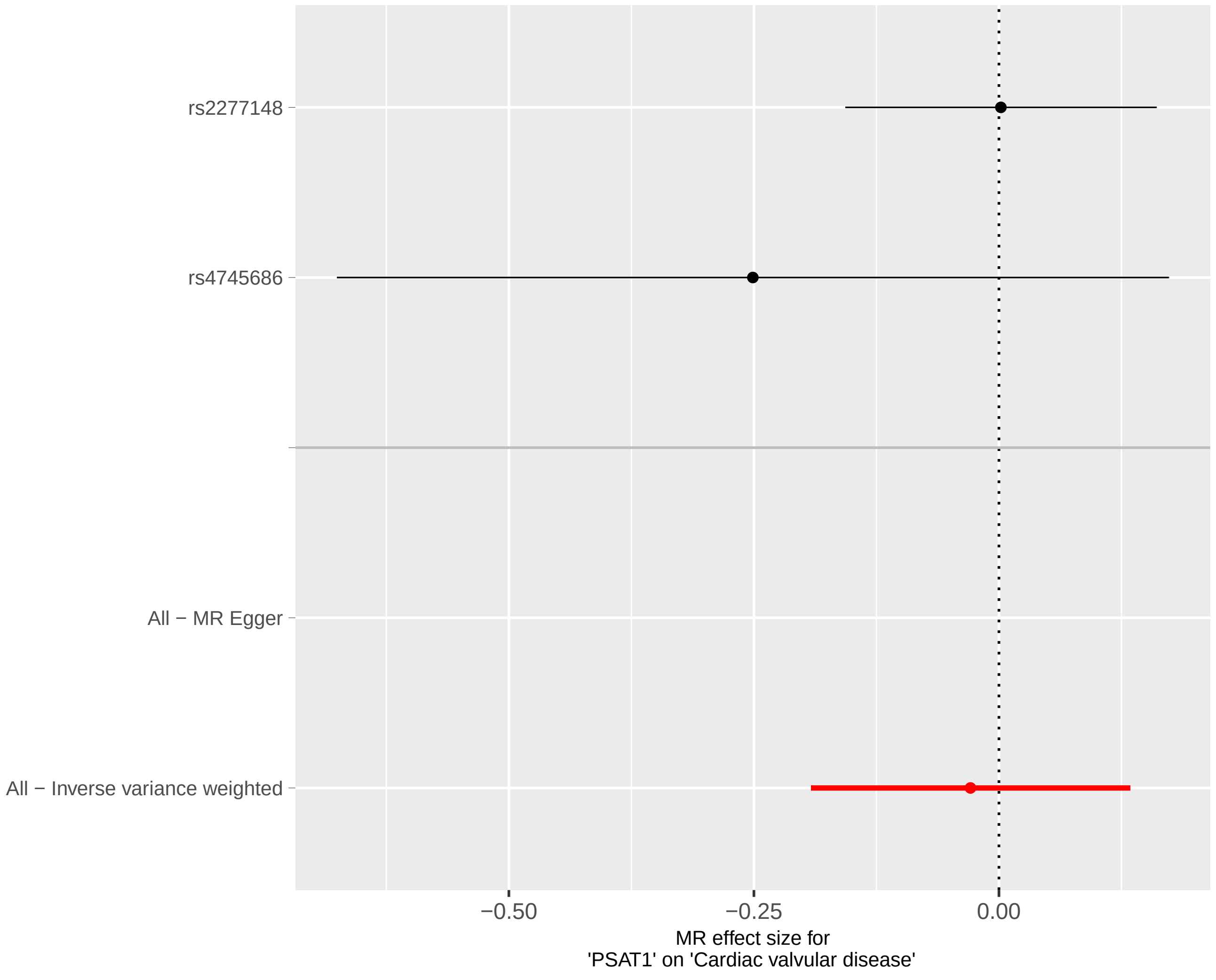

rs2277148
rs4745686
All − MR Egger
All − Inverse variance weighted
−0.50
−0.25
0.00
MR effect size for
'PSAT1' on 'Cardiac valvular disease'

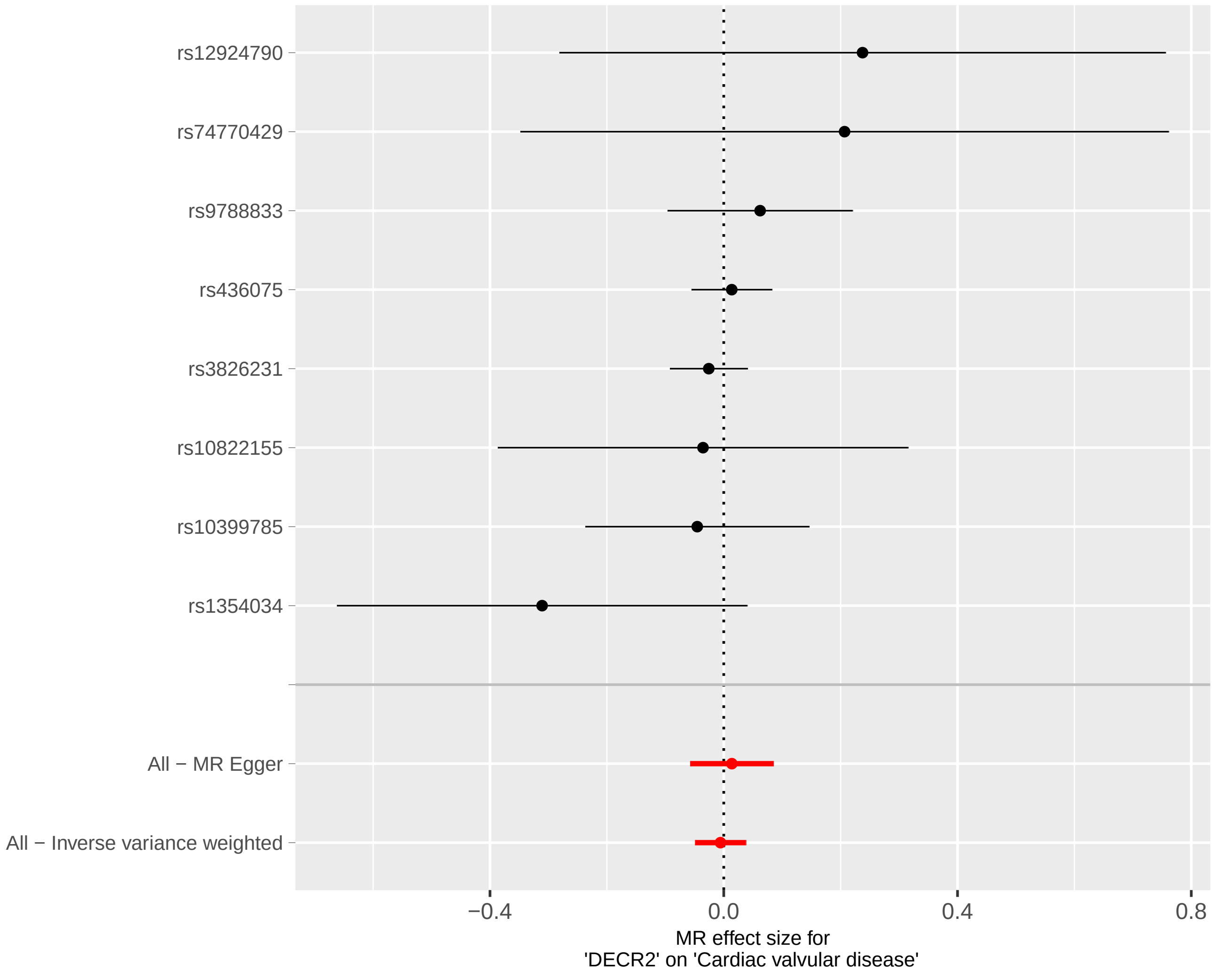
rs12924790
rs74770429
rs9788833
rs436075
rs3826231
rs10822155
rs10399785
rs1354034
All − MR Egger
All − Inverse variance weighted
−0.4
0.0
0.4
0.8
MR effect size for
'DECR2' on 'Cardiac valvular disease'

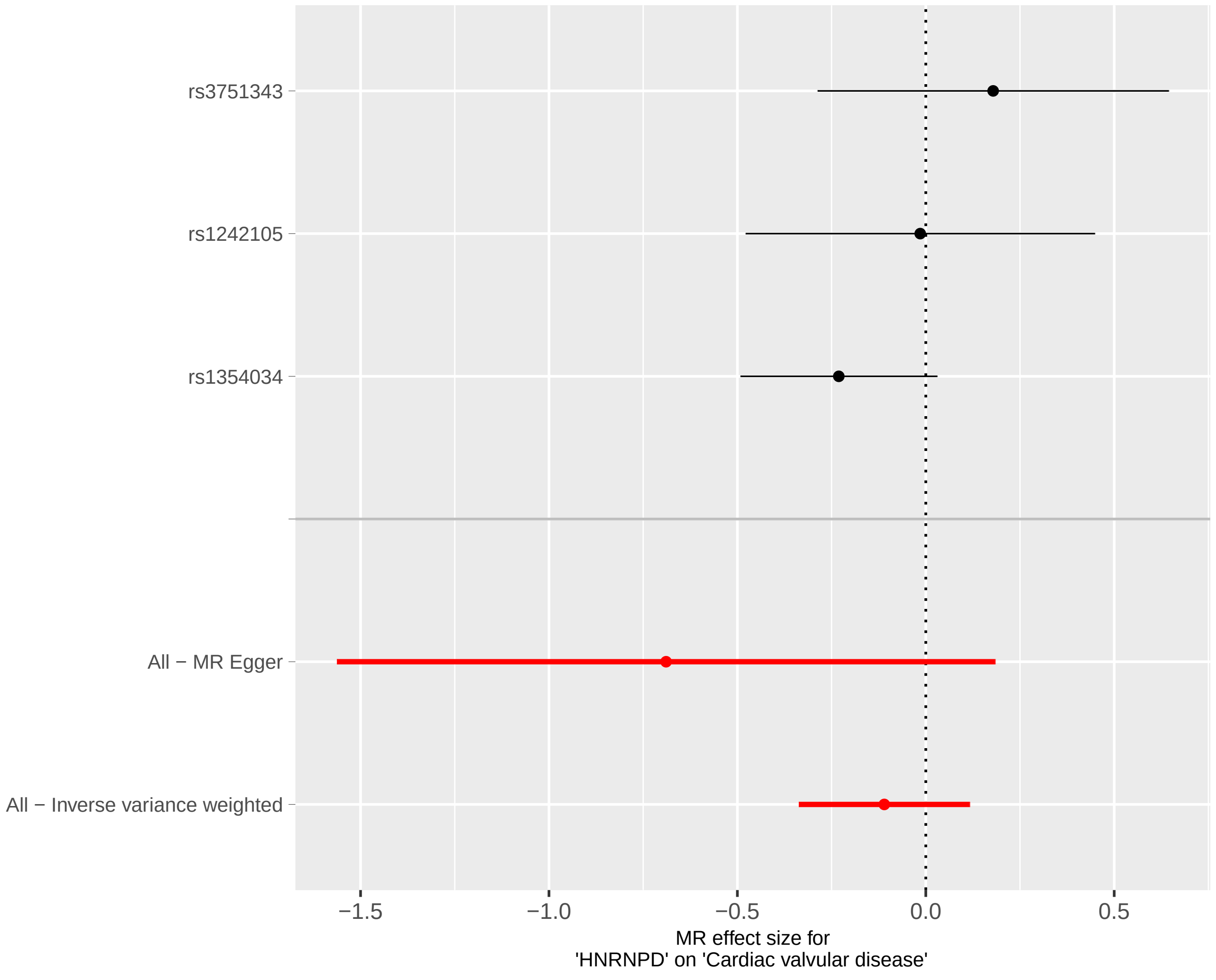
rs3751343
rs1242105
rs1354034
All − MR Egger
All − Inverse variance weighted
−1.5
−1.0
−0.5
0.0
0.5
MR effect size for
'HNRNPD' on 'Cardiac valvular disease'

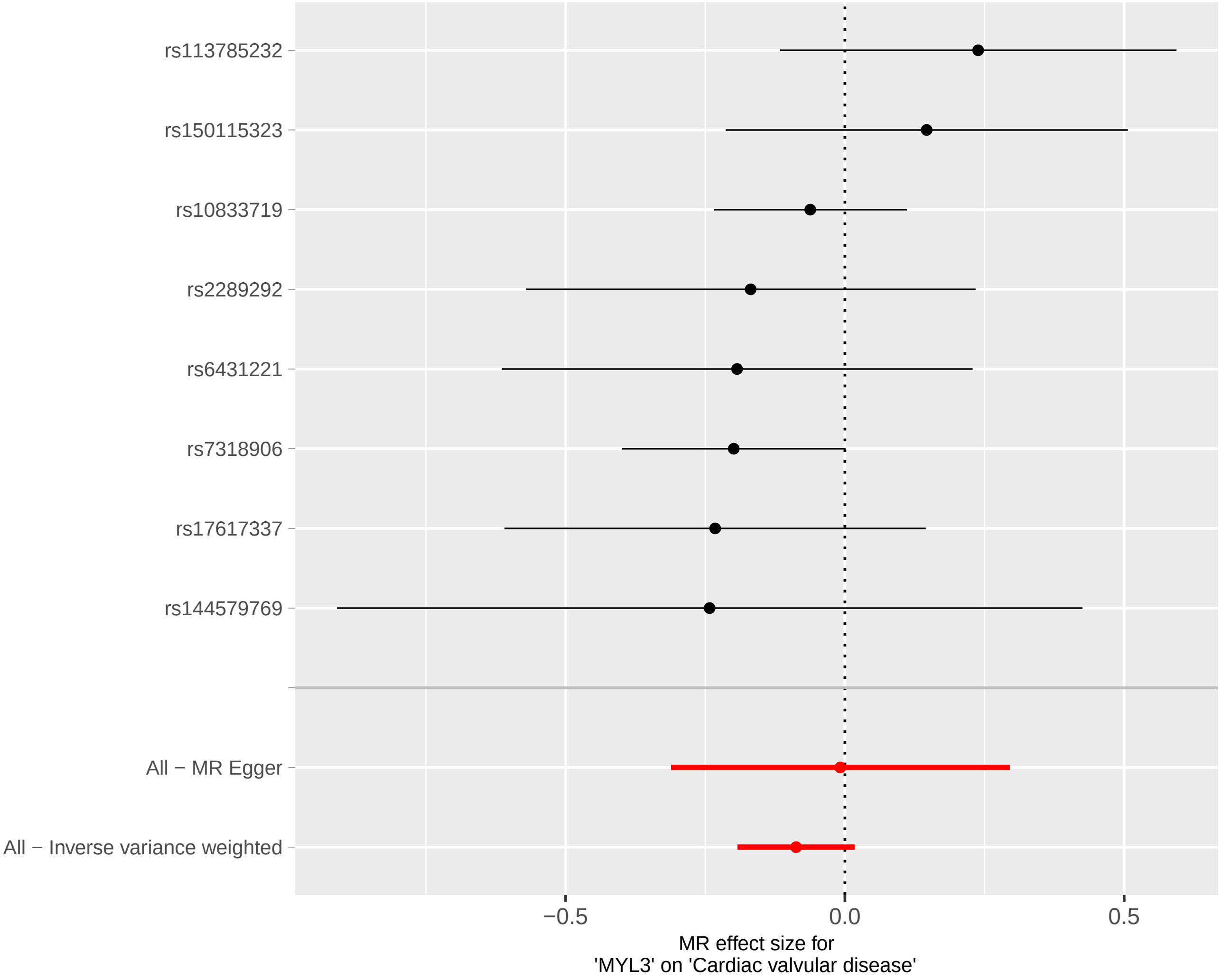
rs113785232
rs150115323
rs10833719
rs2289292
rs6431221
rs7318906
rs17617337
rs144579769
All − MR Egger
All − Inverse variance weighted
−0.5
0.0
0.5
MR effect size for
'MYL3' on 'Cardiac valvular disease'

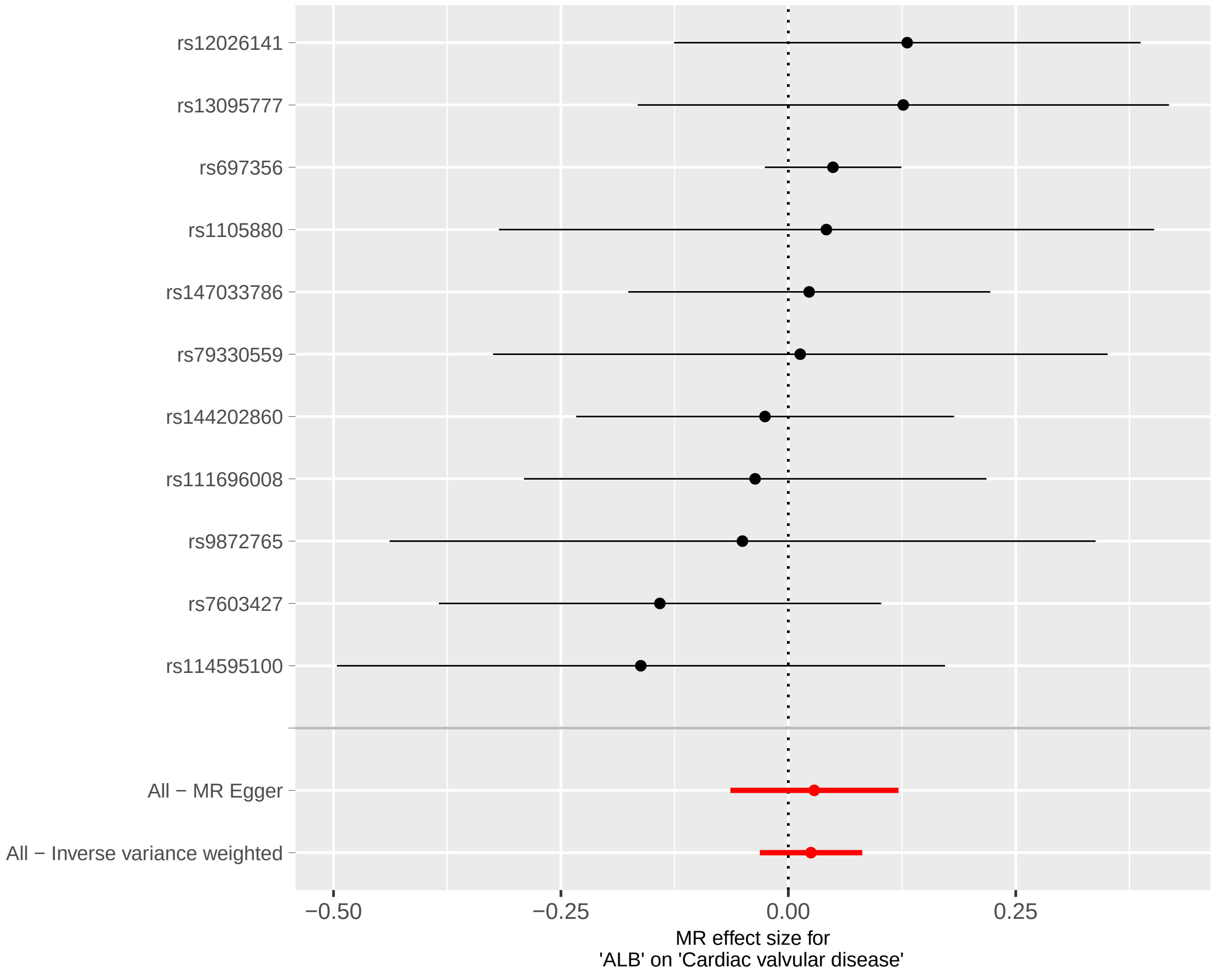
rs12026141
rs13095777
rs697356
rs1105880
rs147033786
rs79330559
rs144202860
rs111696008
rs9872765
rs7603427
rs114595100
All − MR Egger
All − Inverse variance weighted
−0.50
−0.25
0.00
0.25
MR effect size for
'ALB' on 'Cardiac valvular disease'

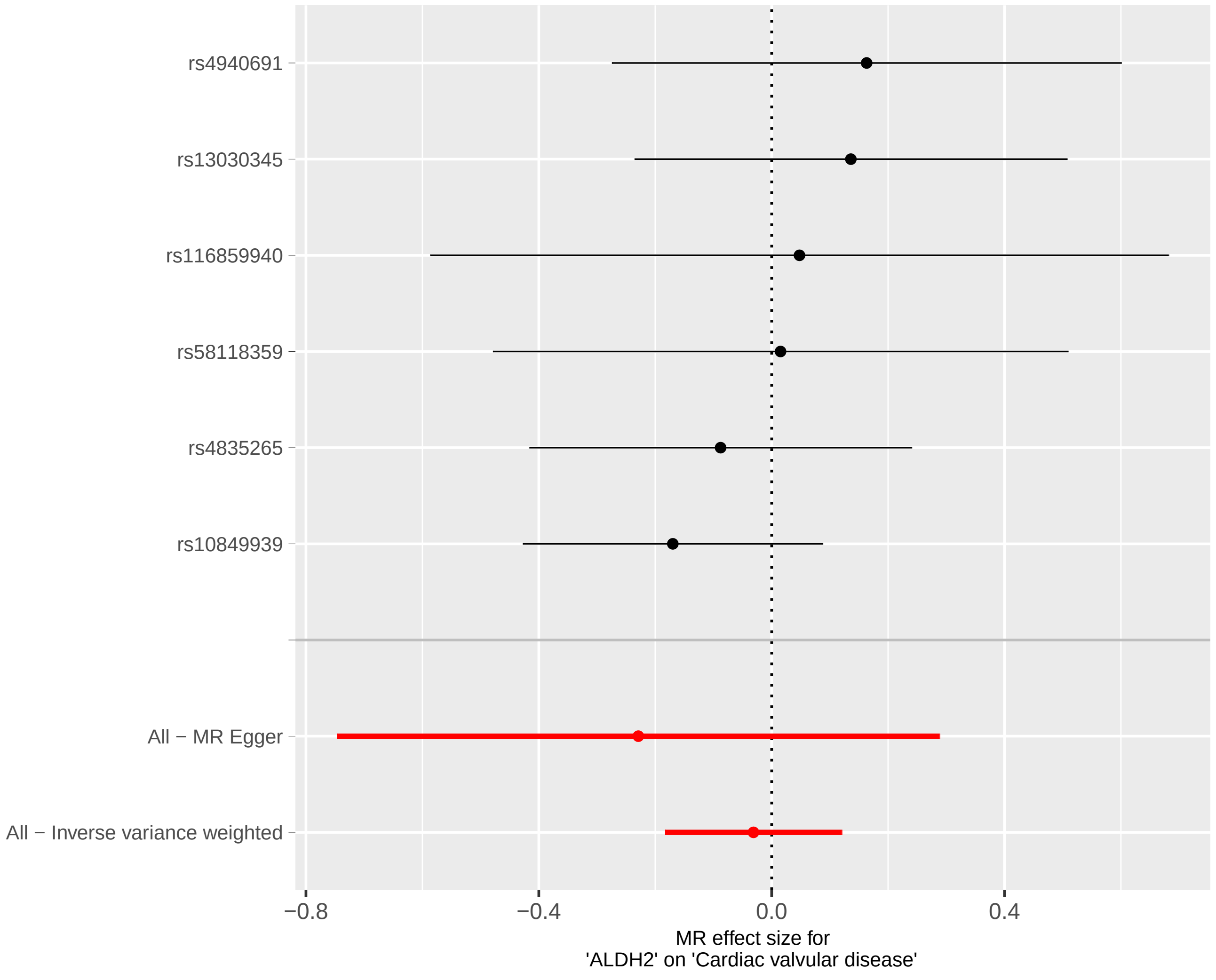
rs4940691
rs13030345
rs116859940
rs58118359
rs4835265
rs10849939
All − MR Egger
All − Inverse variance weighted
−0.8
−0.4
0.0
0.4
MR effect size for
'ALDH2' on 'Cardiac valvular disease'

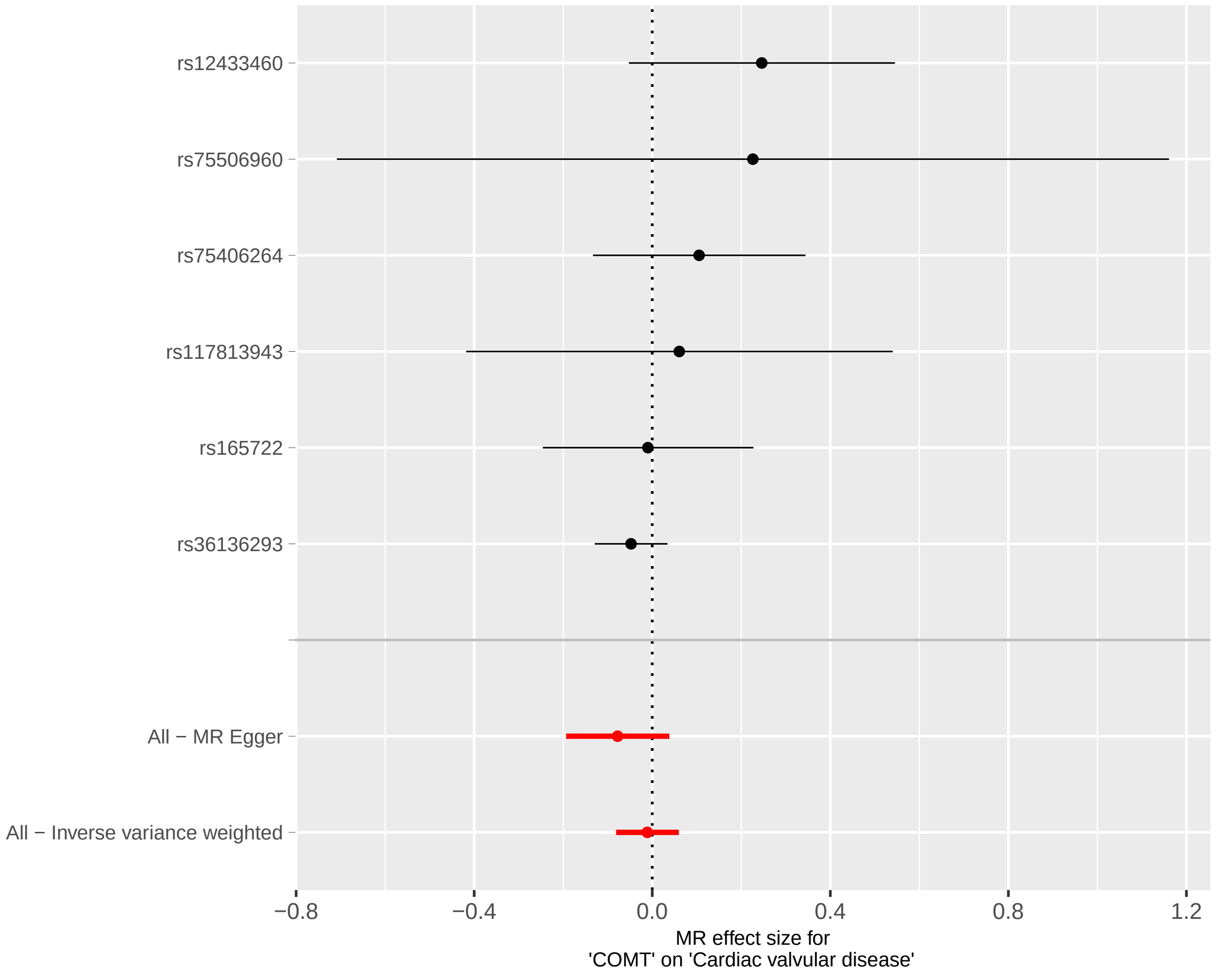
rs12433460
rs75506960
rs75406264
rs117813943
rs165722
rs36136293
All − MR Egger
All − Inverse variance weighted
−0.8
−0.4
0.0
0.4
0.8
1.2
MR effect size for
'COMT' on 'Cardiac valvular disease'

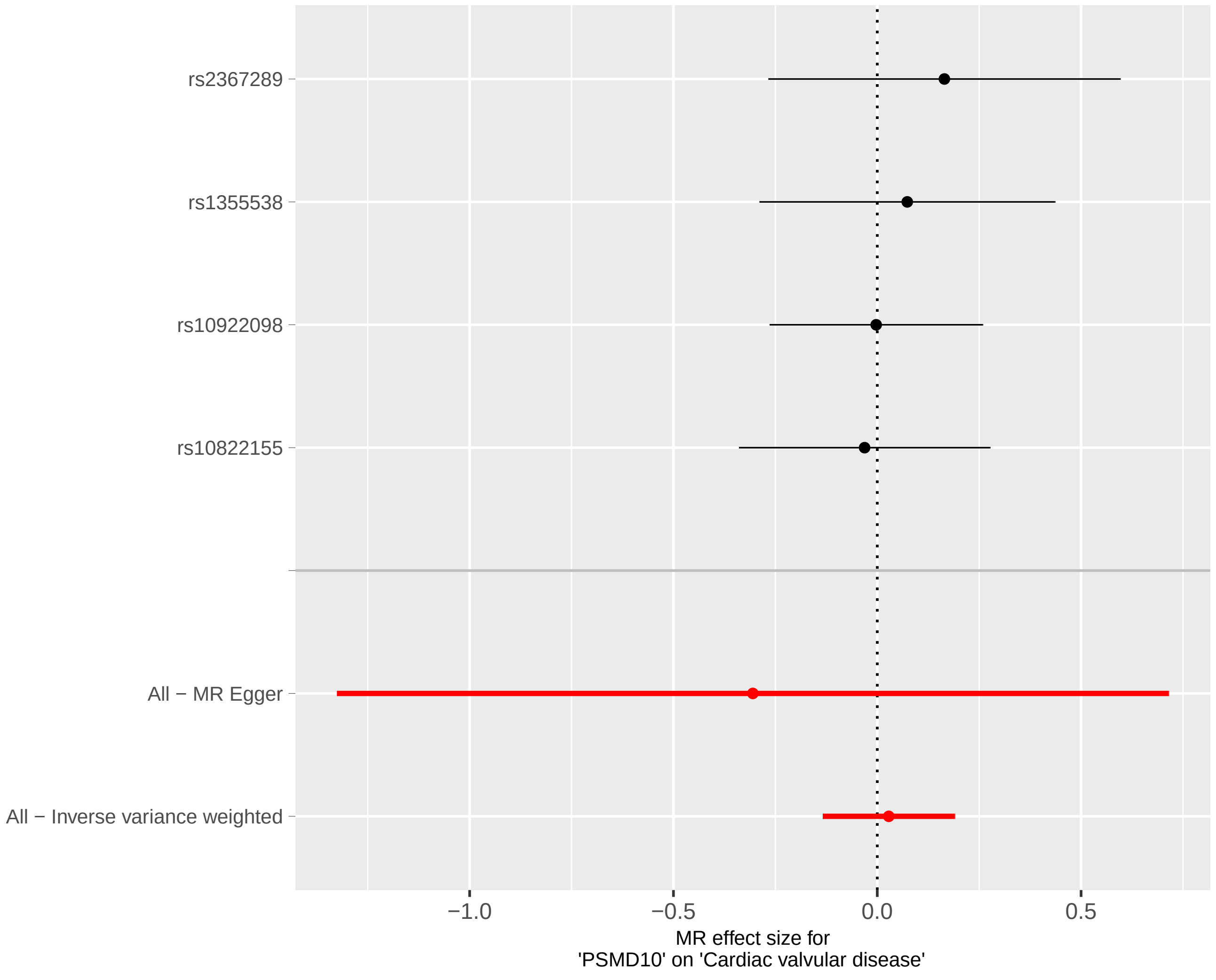
rs2367289
rs1355538
rs10922098
rs10822155
All − MR Egger
All − Inverse variance weighted
−1.0
−0.5
0.0
0.5
MR effect size for
'PSMD10' on 'Cardiac valvular disease'

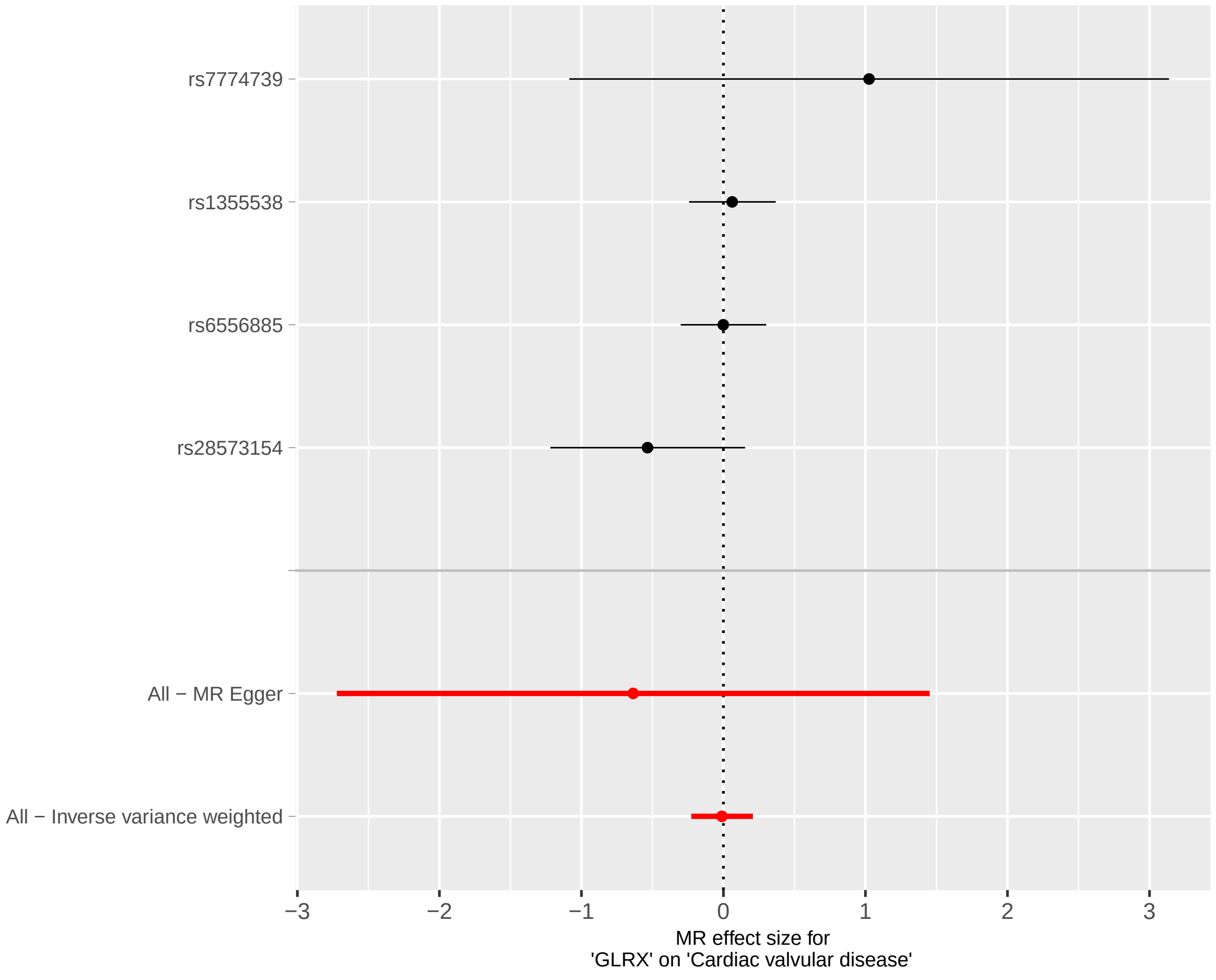

rs7774739
rs1355538
rs6556885
rs28573154
All − MR Egger
All − Inverse variance weighted
−3
−2
−1
0
1
2
3
MR effect size for
'GLRX' on 'Cardiac valvular disease'

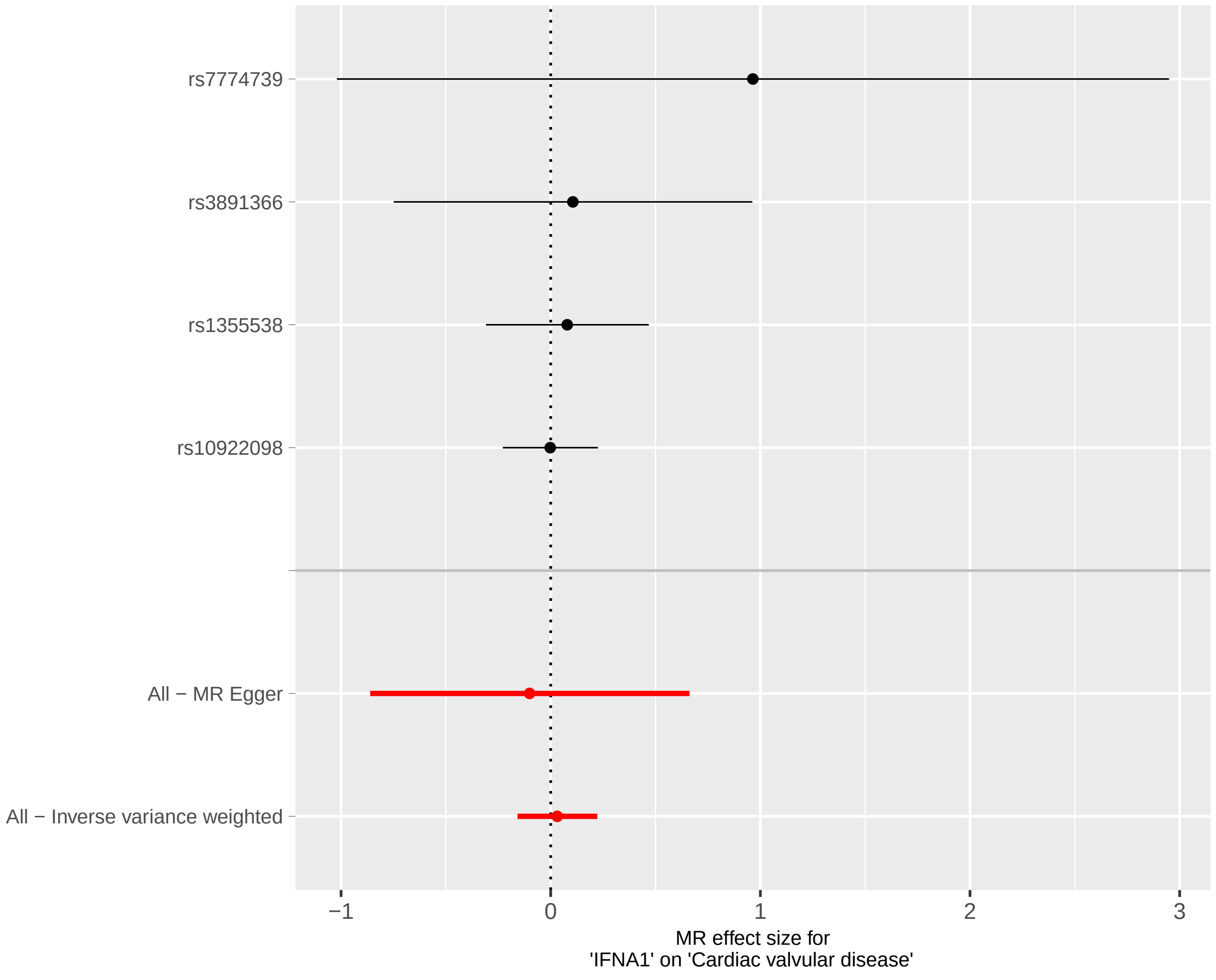
rs7774739
rs3891366
rs1355538
rs10922098
All − MR Egger
All − Inverse variance weighted
−1
0
1
2
3
MR effect size for
'IFNA1' on 'Cardiac valvular disease'

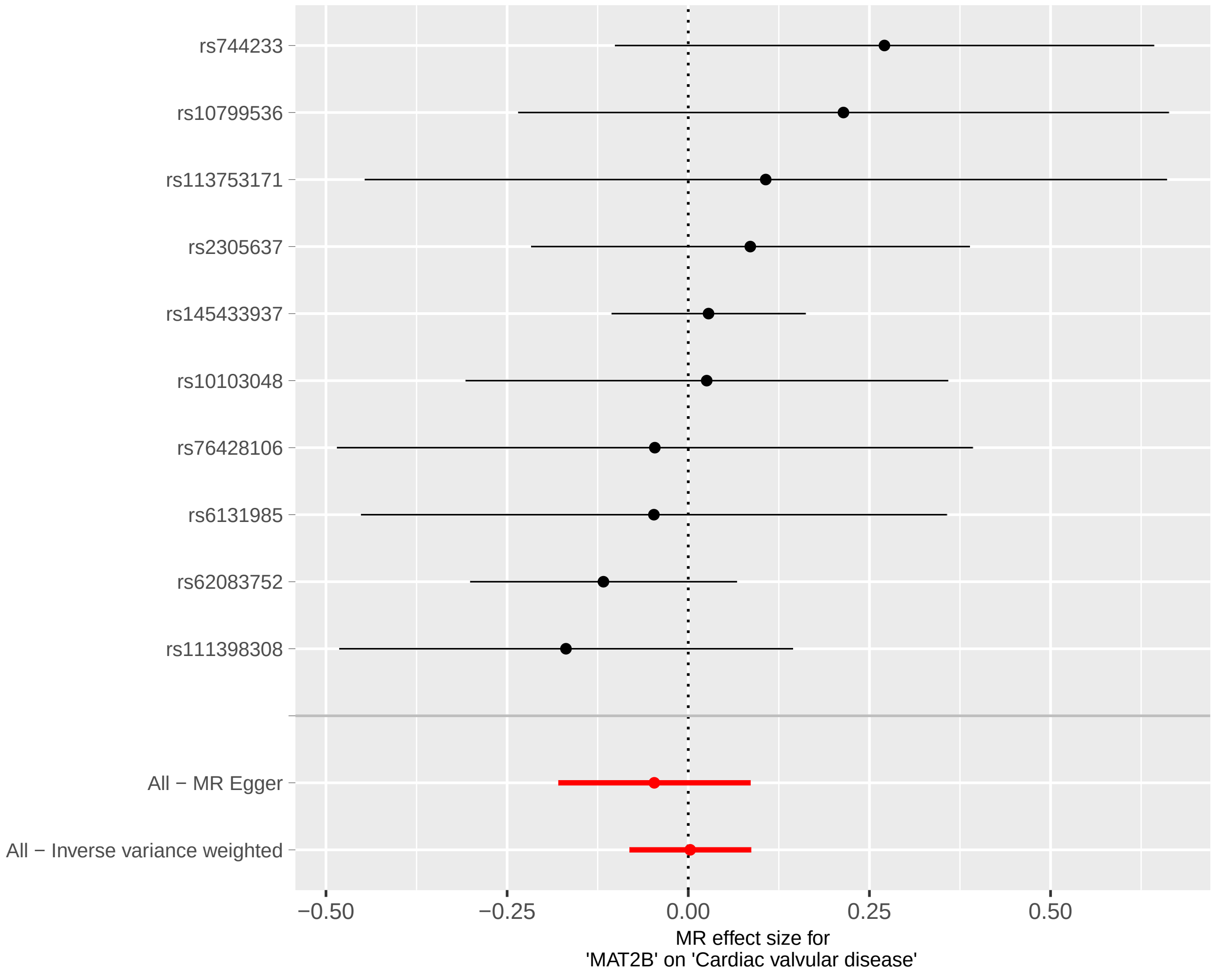
rs744233
rs10799536
rs113753171
rs2305637
rs145433937
rs10103048
rs76428106
rs6131985
rs62083752
rs111398308
All − MR Egger
All − Inverse variance weighted
−0.50
−0.25
0.00
0.25
0.50
MR effect size for
'MAT2B' on 'Cardiac valvular disease'

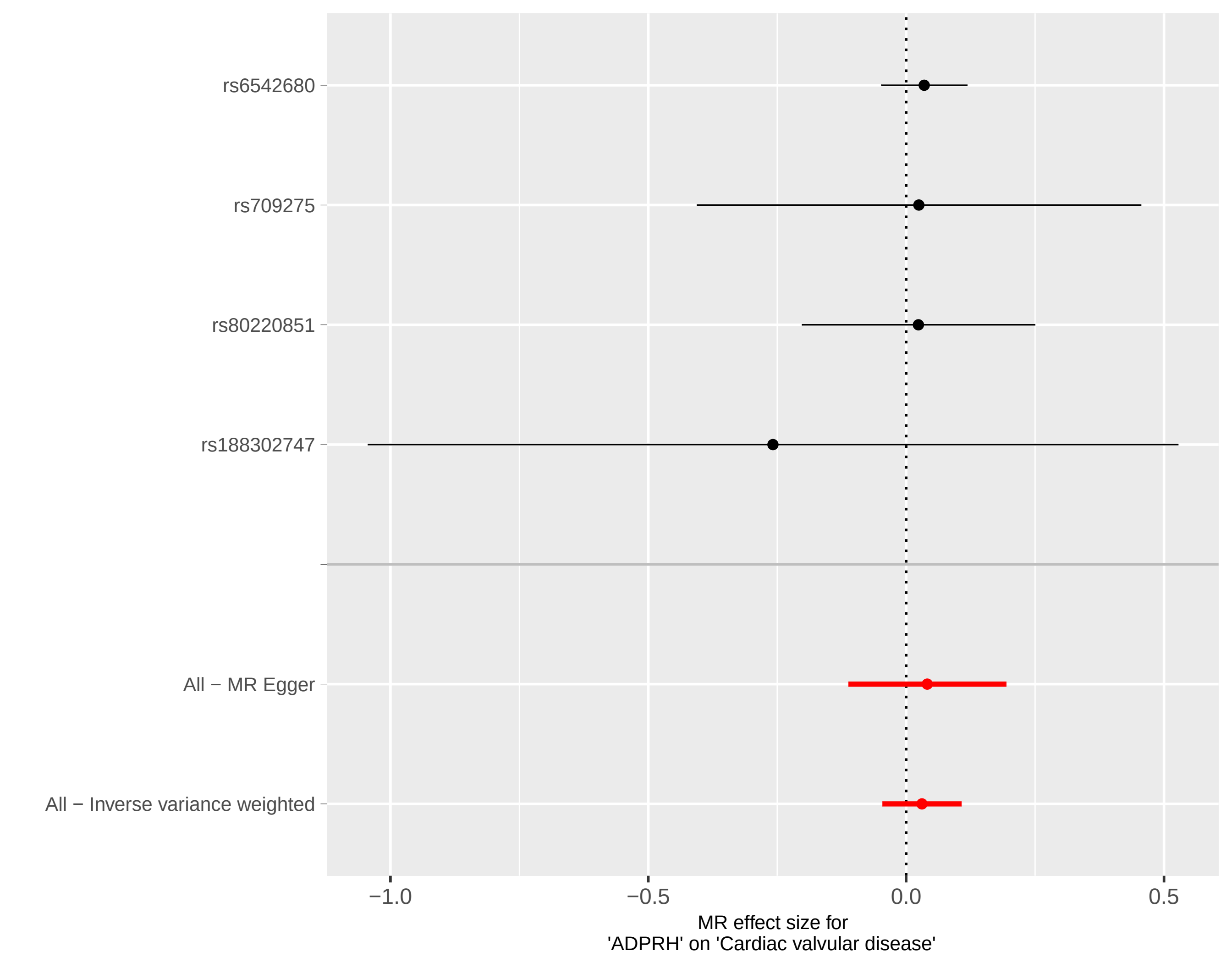
rs6542680
rs709275
rs80220851
rs188302747
All − MR Egger
All − Inverse variance weighted
−1.0
−0.5
0.0
0.5
MR effect size for
'ADPRH' on 'Cardiac valvular disease'

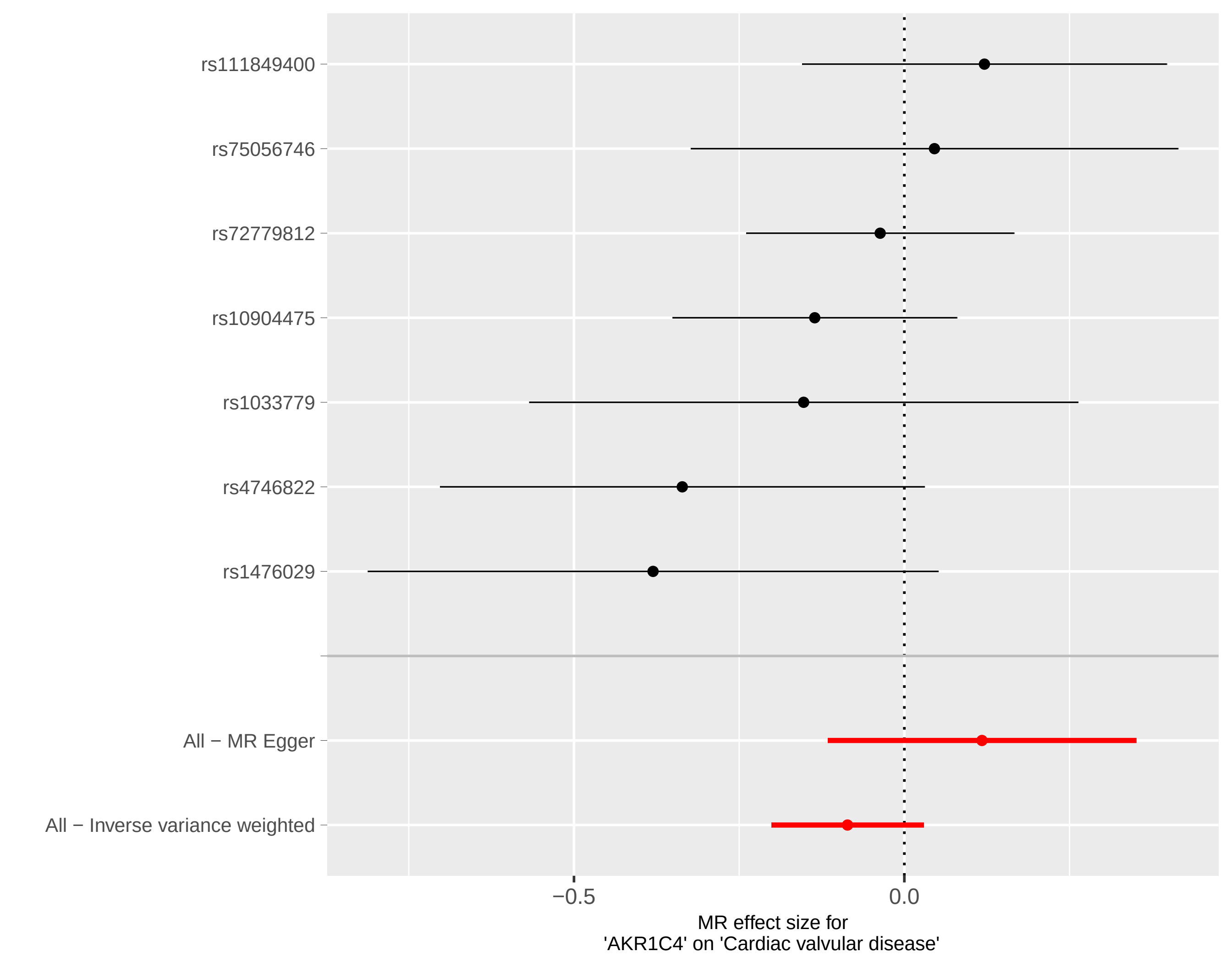
rs111849400
rs75056746
rs72779812
rs10904475
rs1033779
rs4746822
rs1476029
All − MR Egger
All − Inverse variance weighted
−0.5
0.0
MR effect size for
'AKR1C4' on 'Cardiac valvular disease'

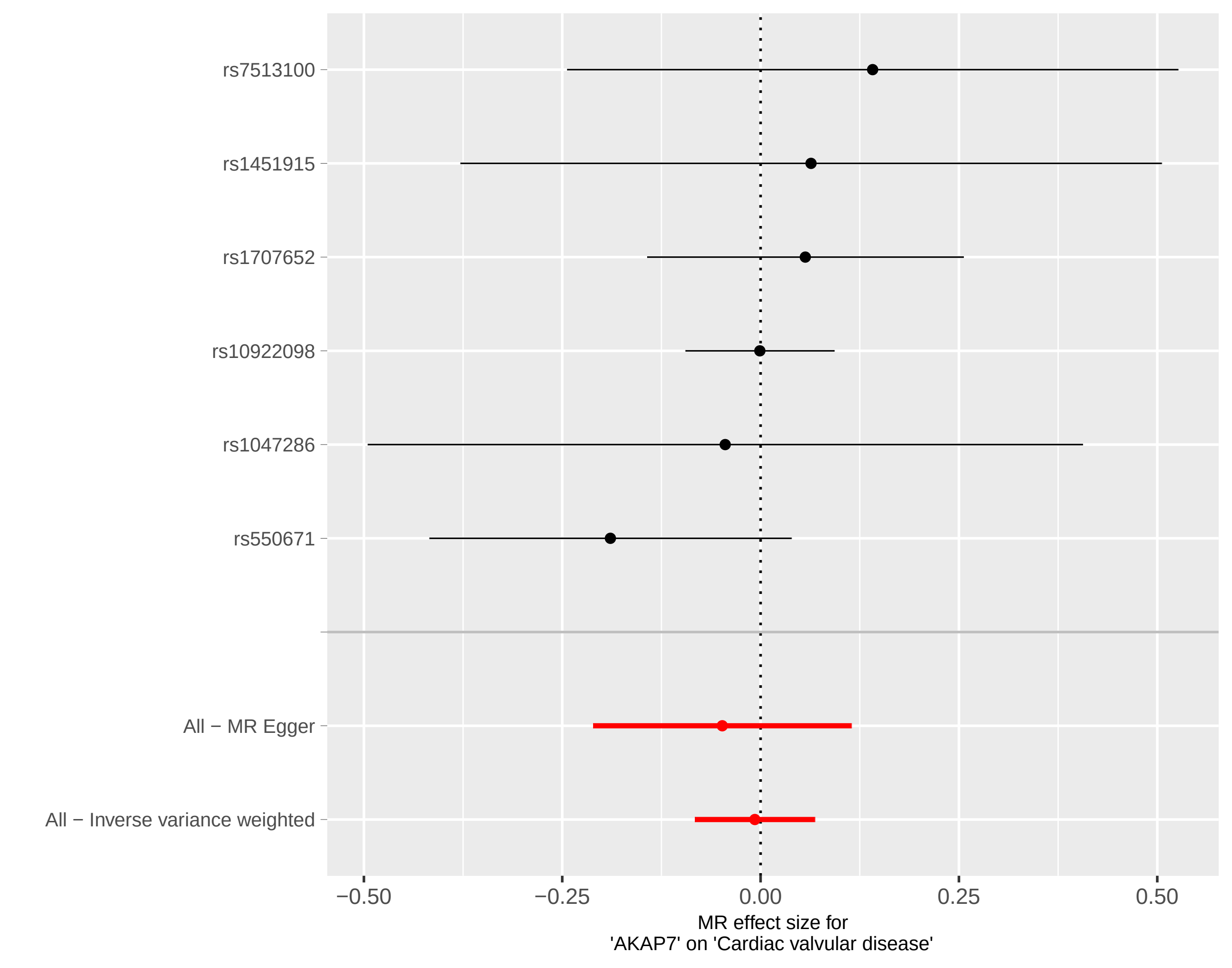
rs7513100
rs1451915
rs1707652
rs10922098
rs1047286
rs550671
All − MR Egger
All − Inverse variance weighted
−0.50
−0.25
0.00
0.25
0.50
MR effect size for
'AKAP7' on 'Cardiac valvular disease'

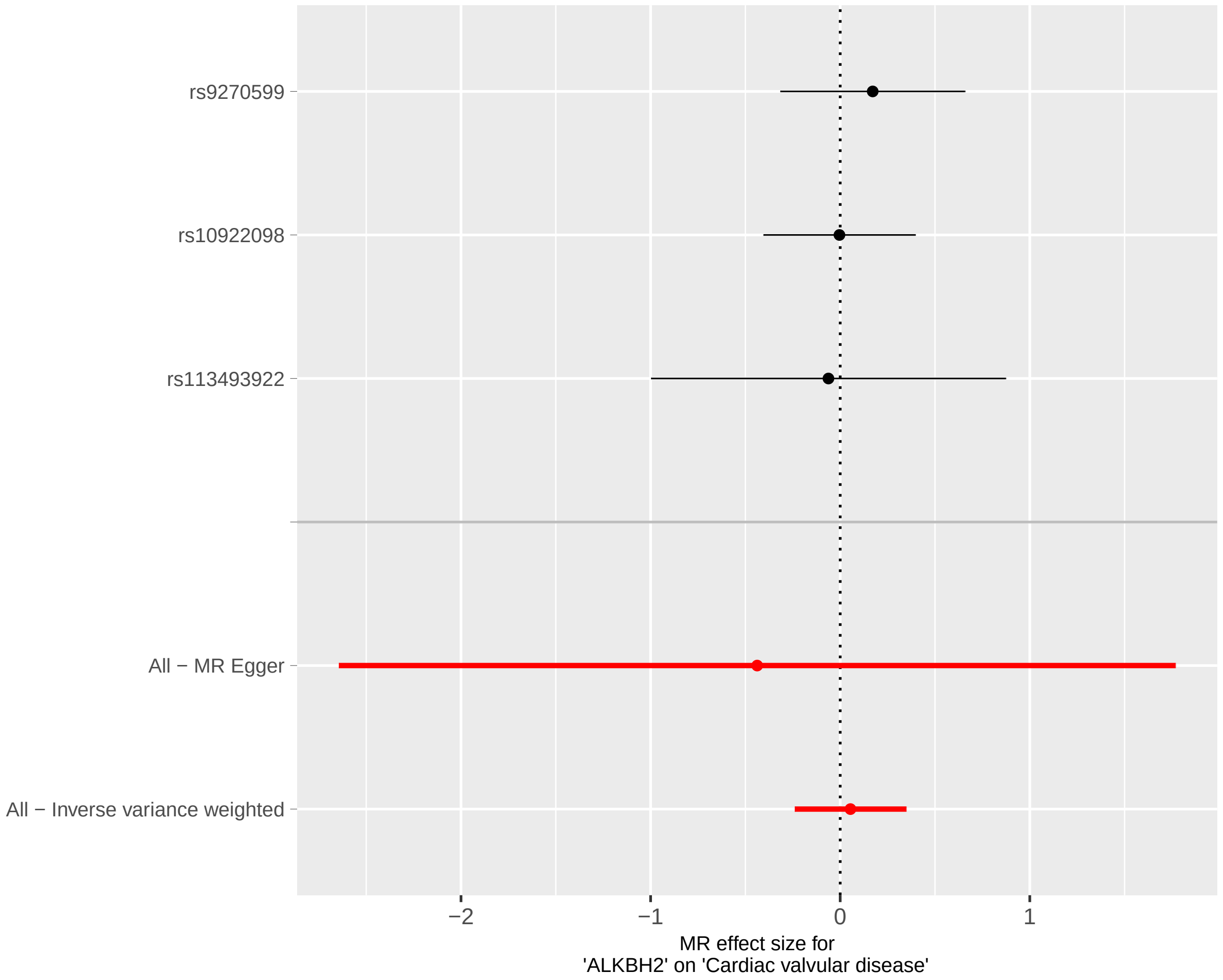
rs9270599
rs10922098
rs113493922
All − MR Egger
All − Inverse variance weighted
−2
−1
0
1
MR effect size for
'ALKBH2' on 'Cardiac valvular disease'

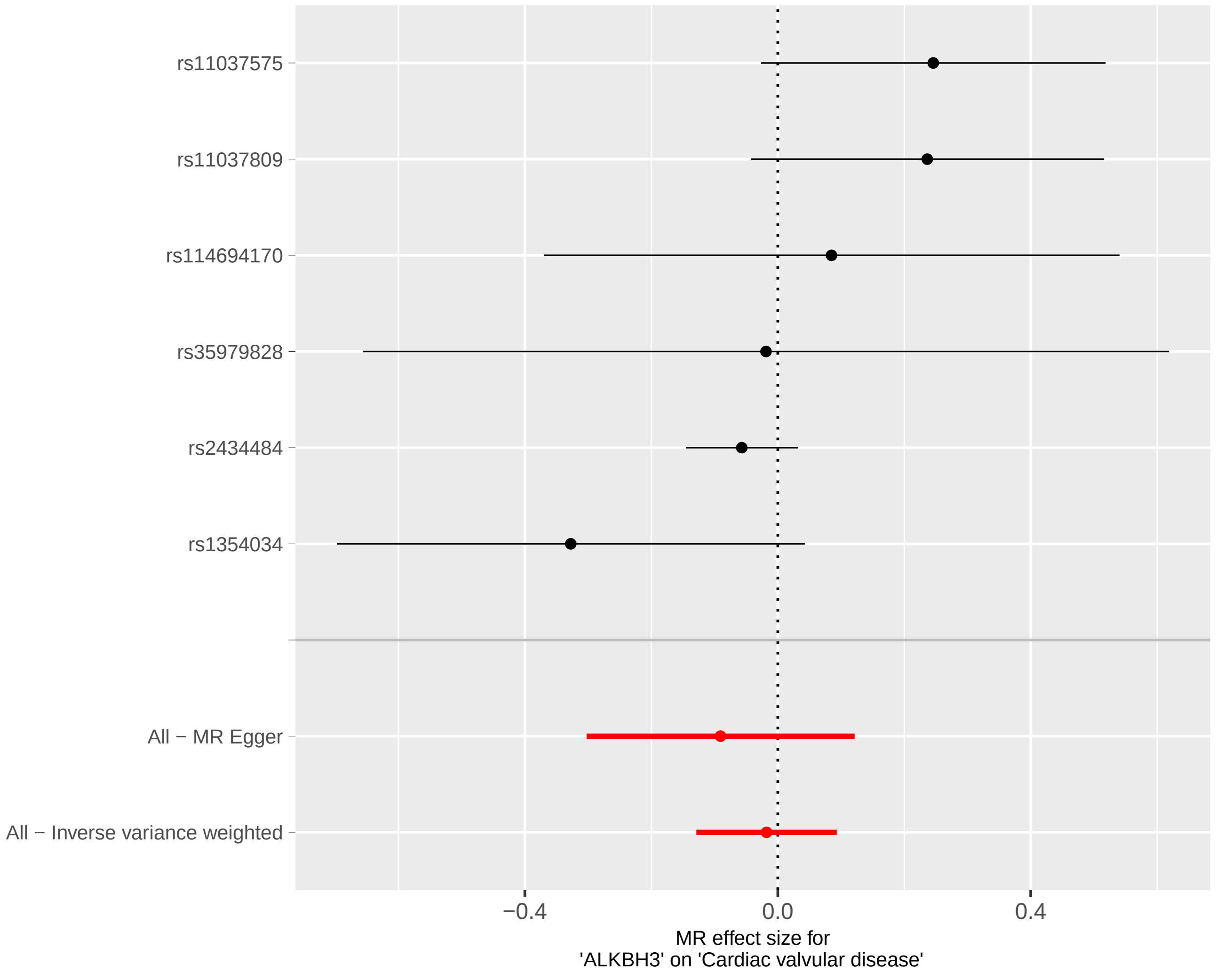
rs11037575
rs11037809
rs114694170
rs35979828
rs2434484
rs1354034
All − MR Egger
All − Inverse variance weighted
−0.4
0.0
0.4
MR effect size for
'ALKBH3' on 'Cardiac valvular disease'

Insufficient number of SNPs

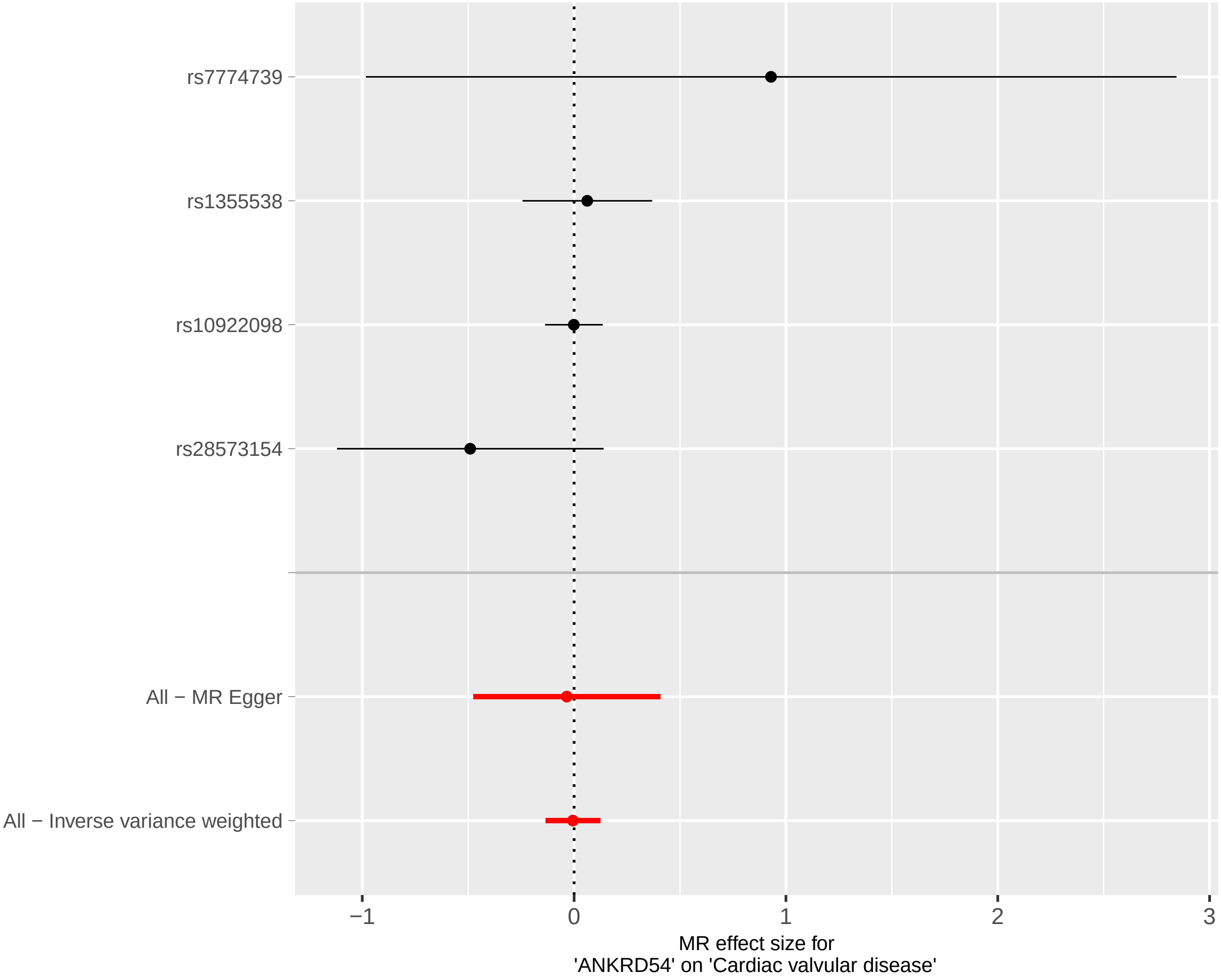
rs7774739
rs1355538
rs10922098
rs28573154
All − MR Egger
All − Inverse variance weighted
−1
0
1
2
3
MR effect size for
'ANKRD54' on 'Cardiac valvular disease'

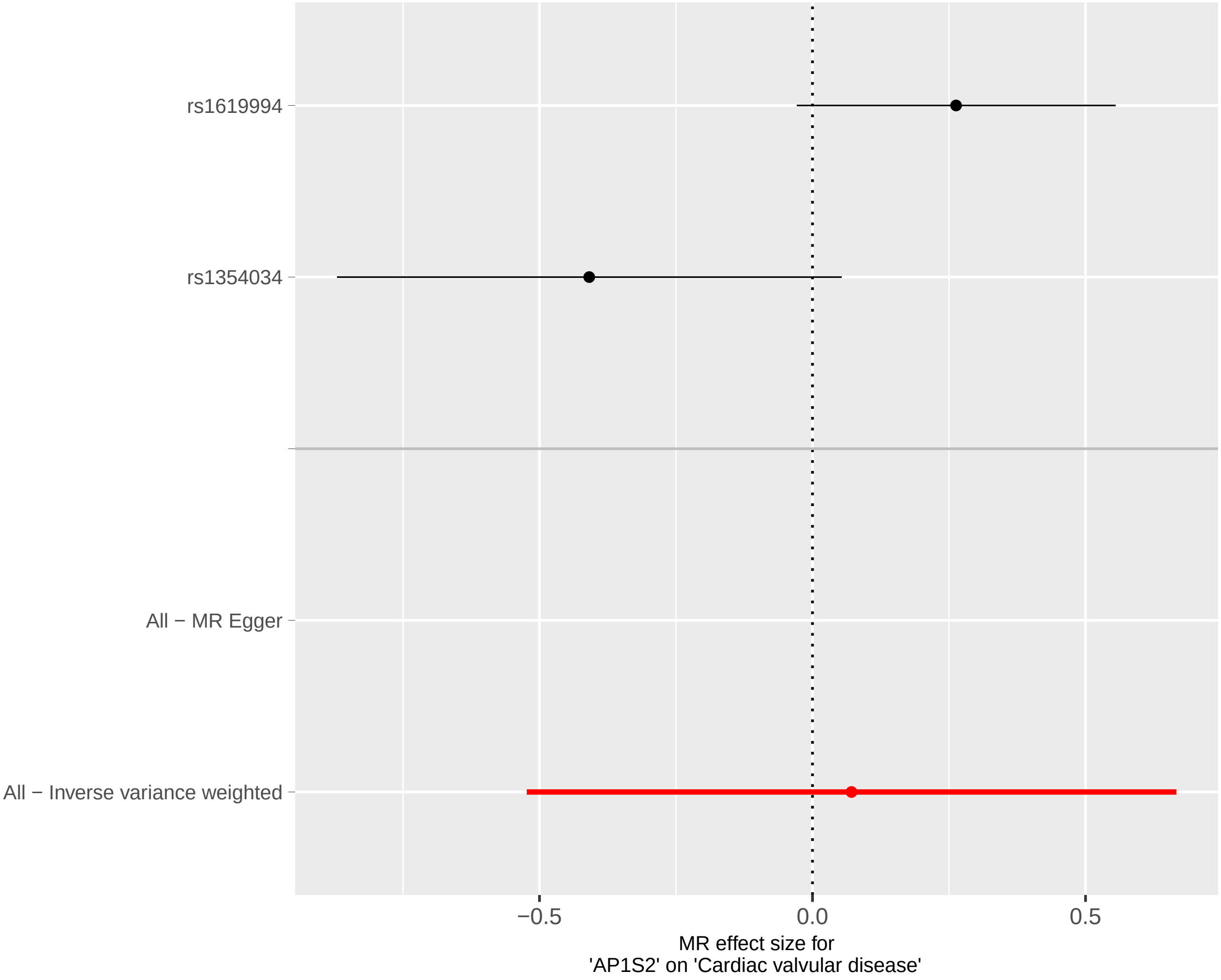
rs1619994
rs1354034
All − MR Egger
All − Inverse variance weighted
−0.5
0.0
0.5
MR effect size for
'AP1S2' on 'Cardiac valvular disease'

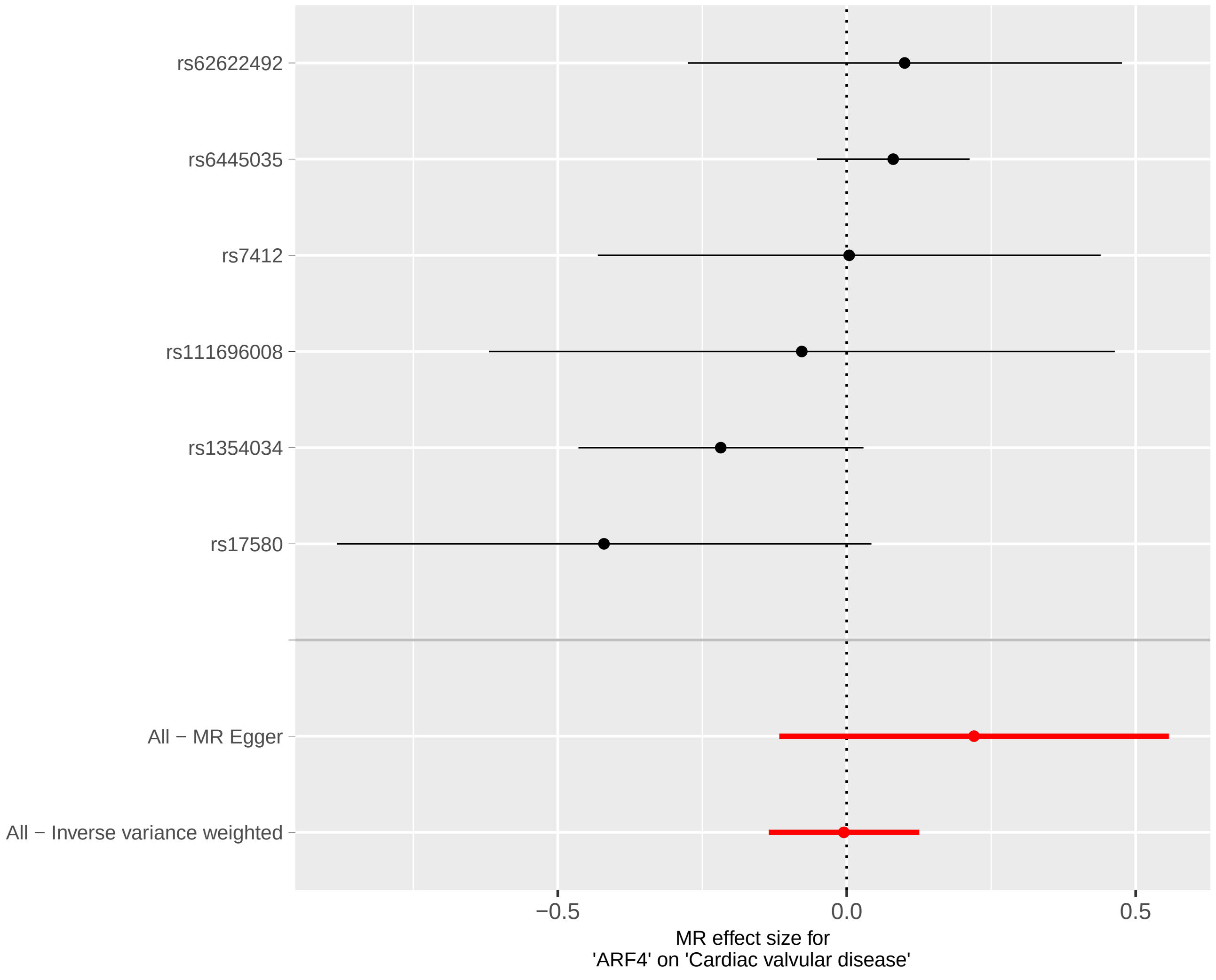
rs62622492
rs6445035
rs7412
rs111696008
rs1354034
rs17580
All − MR Egger
All − Inverse variance weighted
−0.5
0.0
0.5
MR effect size for
'ARF4' on 'Cardiac valvular disease'

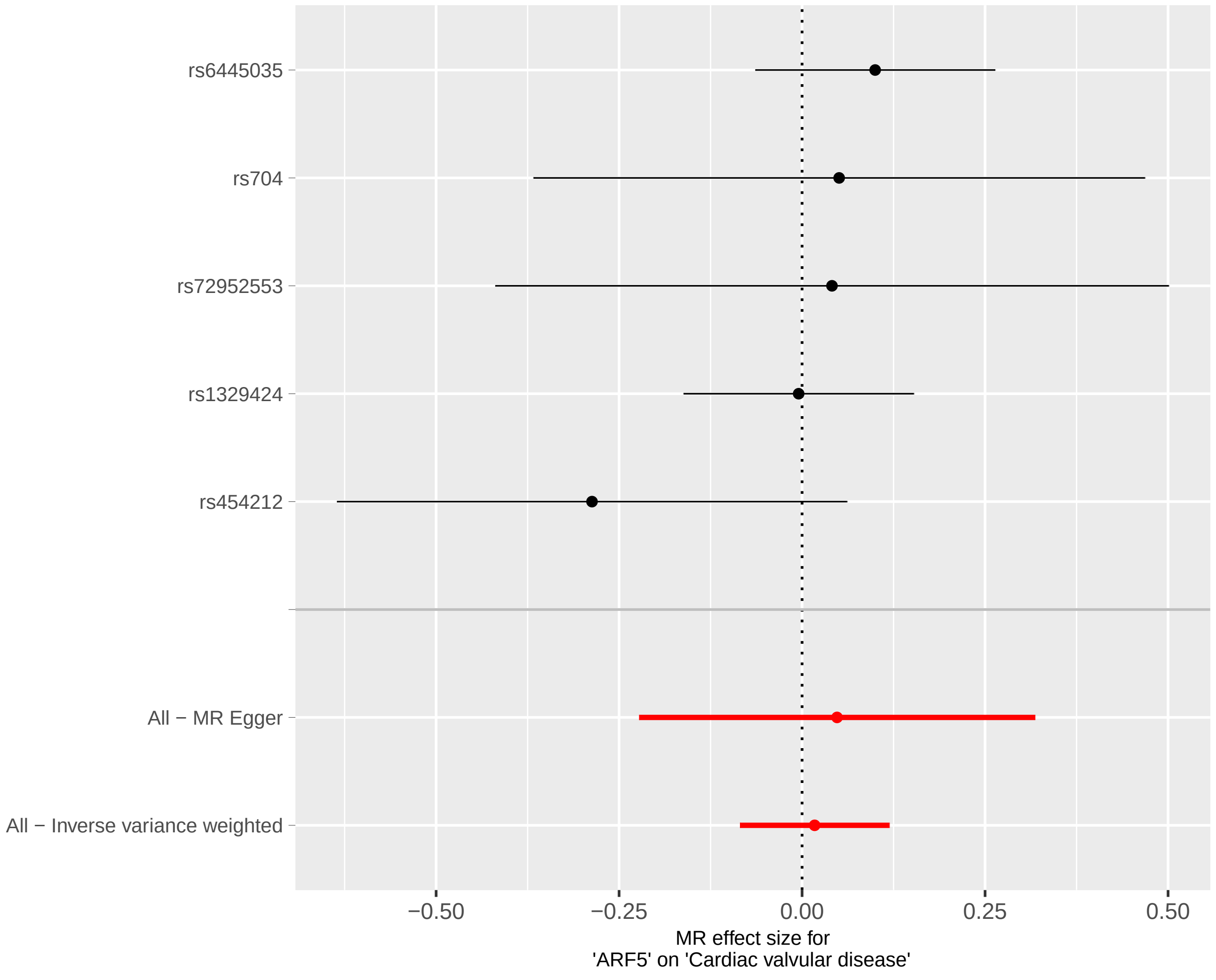
rs6445035
rs704
rs72952553
rs1329424
rs454212
All − MR Egger
All − Inverse variance weighted
−0.50
−0.25
0.00
0.25
0.50
MR effect size for
'ARF5' on 'Cardiac valvular disease'

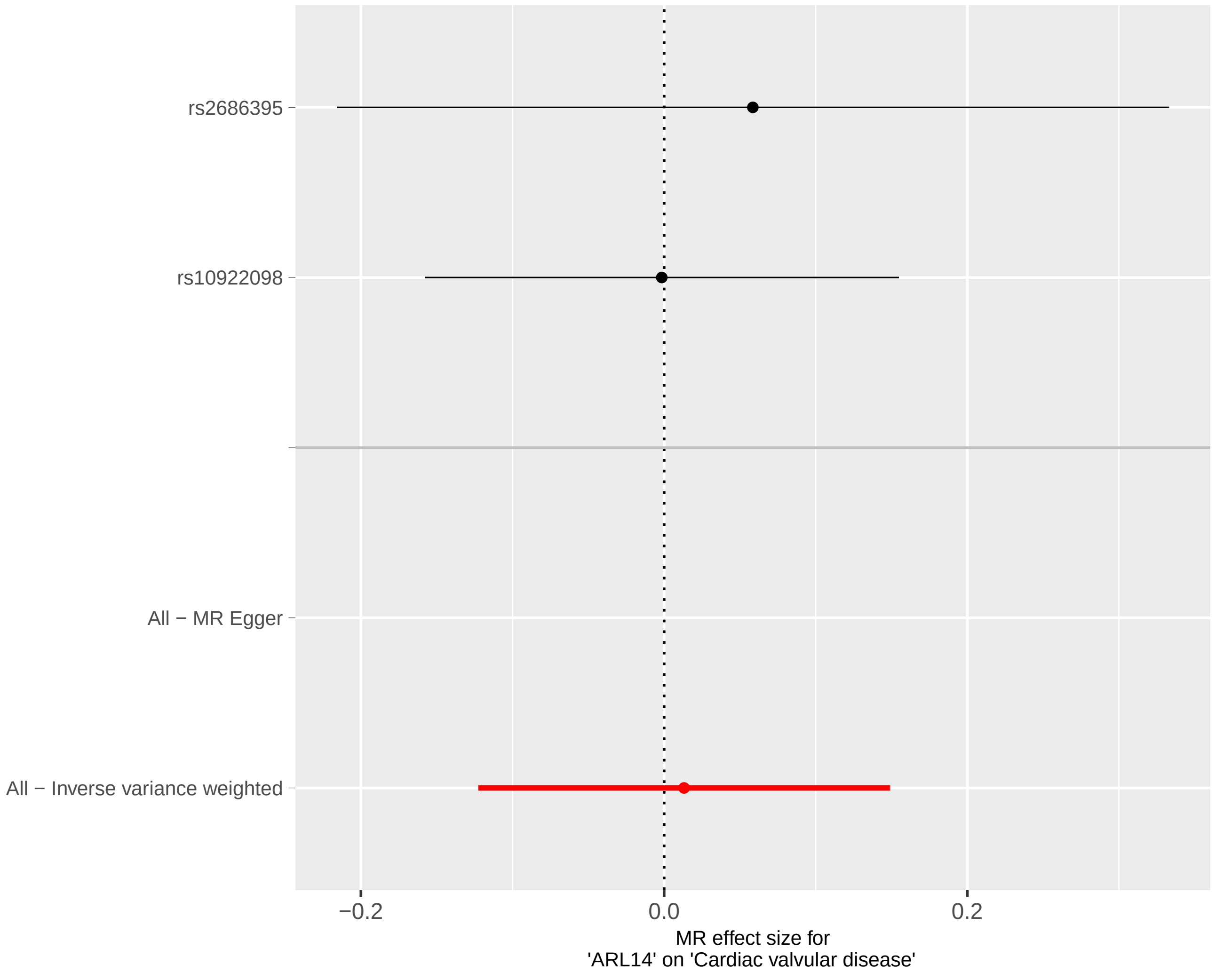

rs2686395
rs10922098
All − MR Egger
All − Inverse variance weighted
−0.2
0.0
0.2
MR effect size for
'ARL14' on 'Cardiac valvular disease'

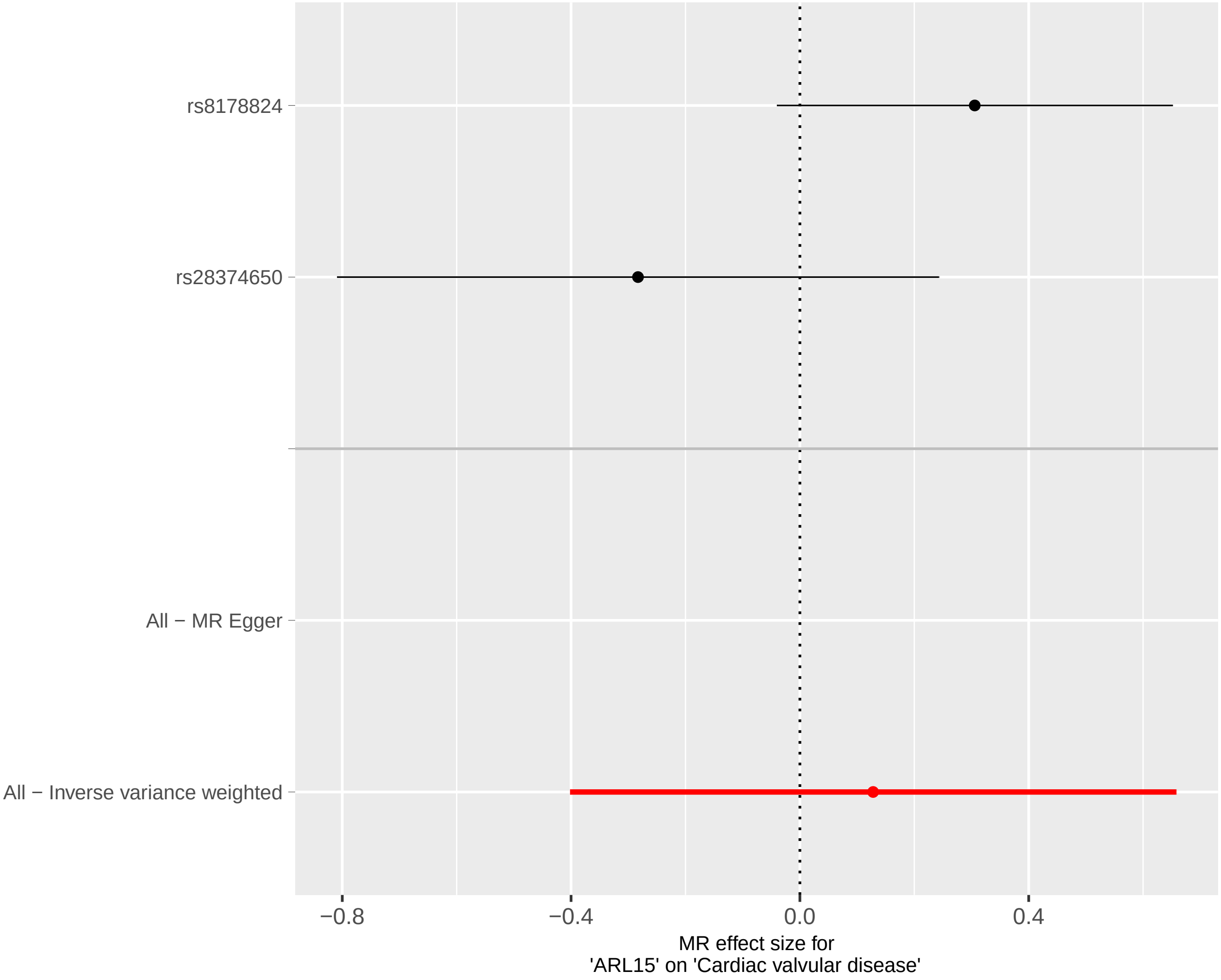
rs8178824
rs28374650
All − MR Egger
All − Inverse variance weighted
−0.8
−0.4
0.0
0.4
MR effect size for
'ARL15' on 'Cardiac valvular disease'

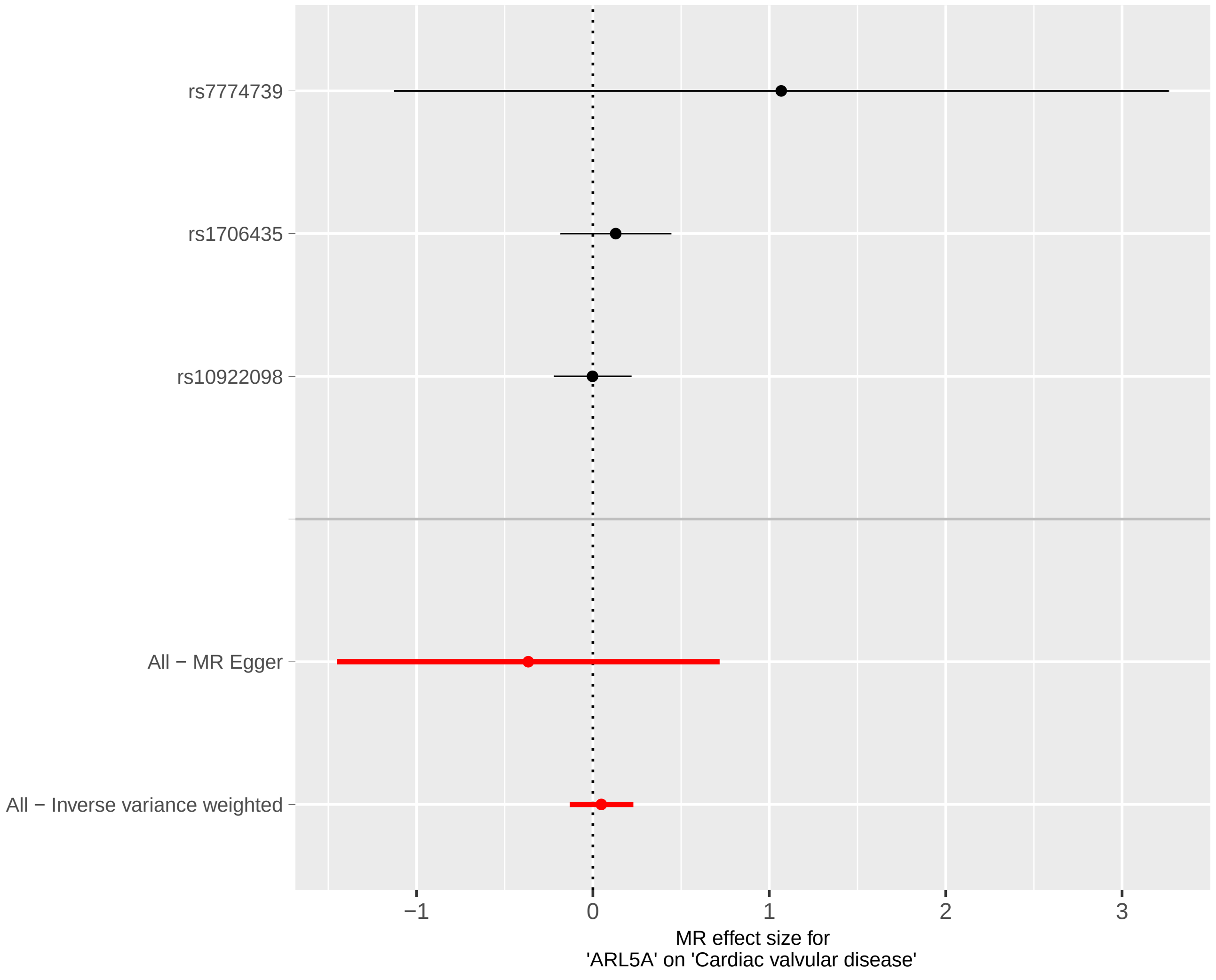

rs7774739
rs1706435
rs10922098
All − MR Egger
All − Inverse variance weighted
−1
0
1
2
3
MR effect size for
'ARL5A' on 'Cardiac valvular disease'

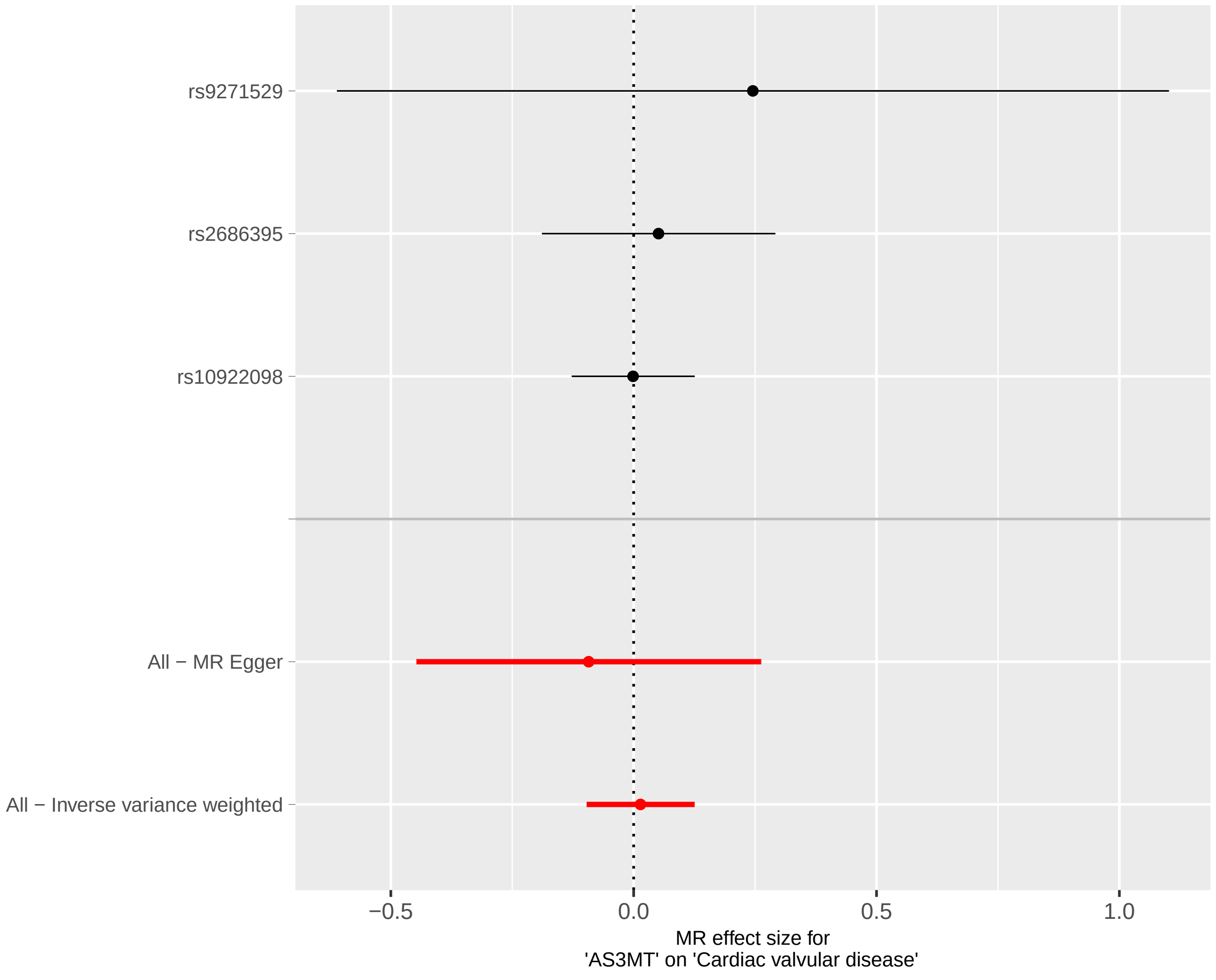
rs9271529
rs2686395
rs10922098
All − MR Egger
All − Inverse variance weighted
−0.5
0.0
0.5
1.0
MR effect size for
'AS3MT' on 'Cardiac valvular disease'

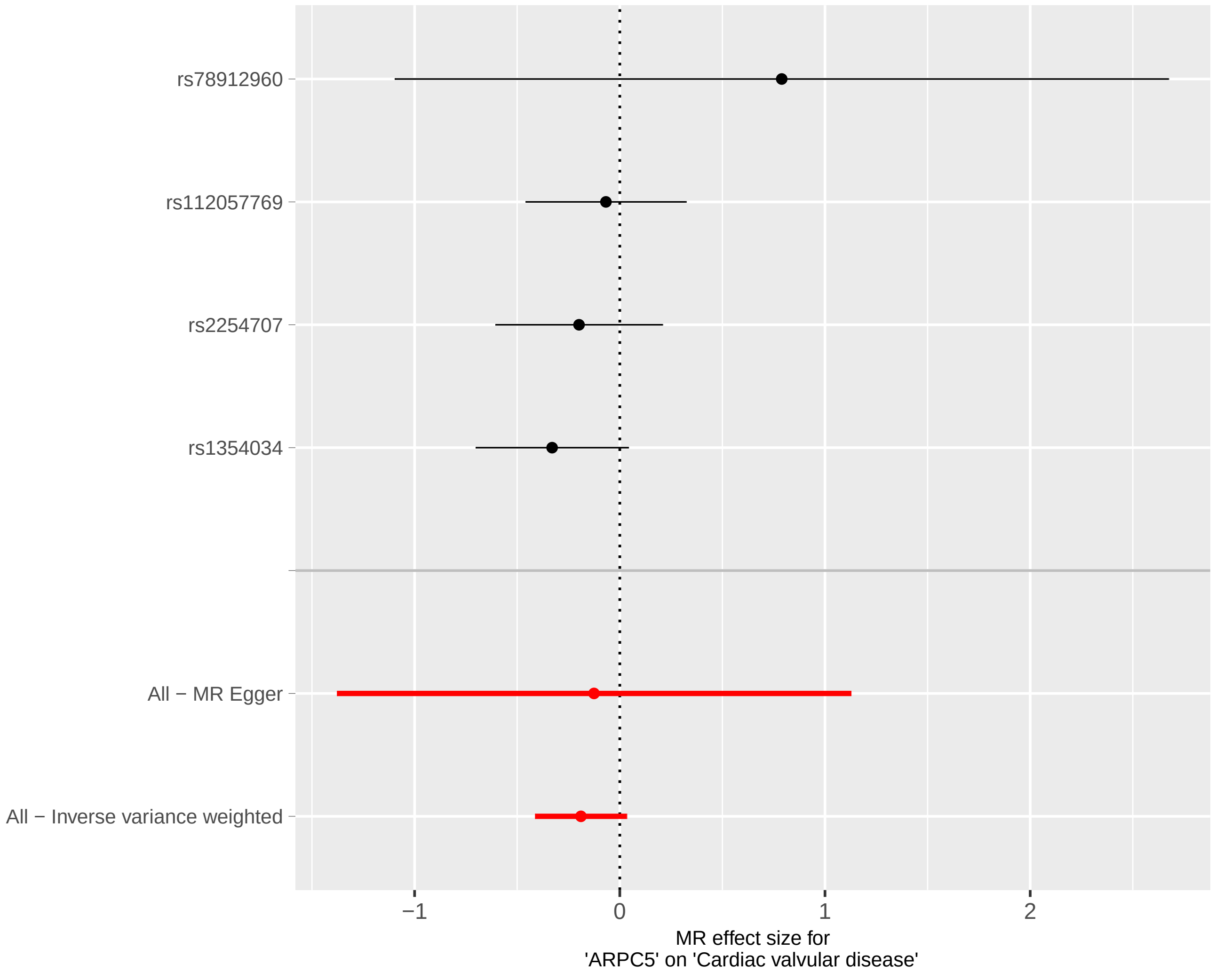
rs78912960
rs112057769
rs2254707
rs1354034
All − MR Egger
All − Inverse variance weighted
−1
0
1
2
MR effect size for
'ARPC5' on 'Cardiac valvular disease'

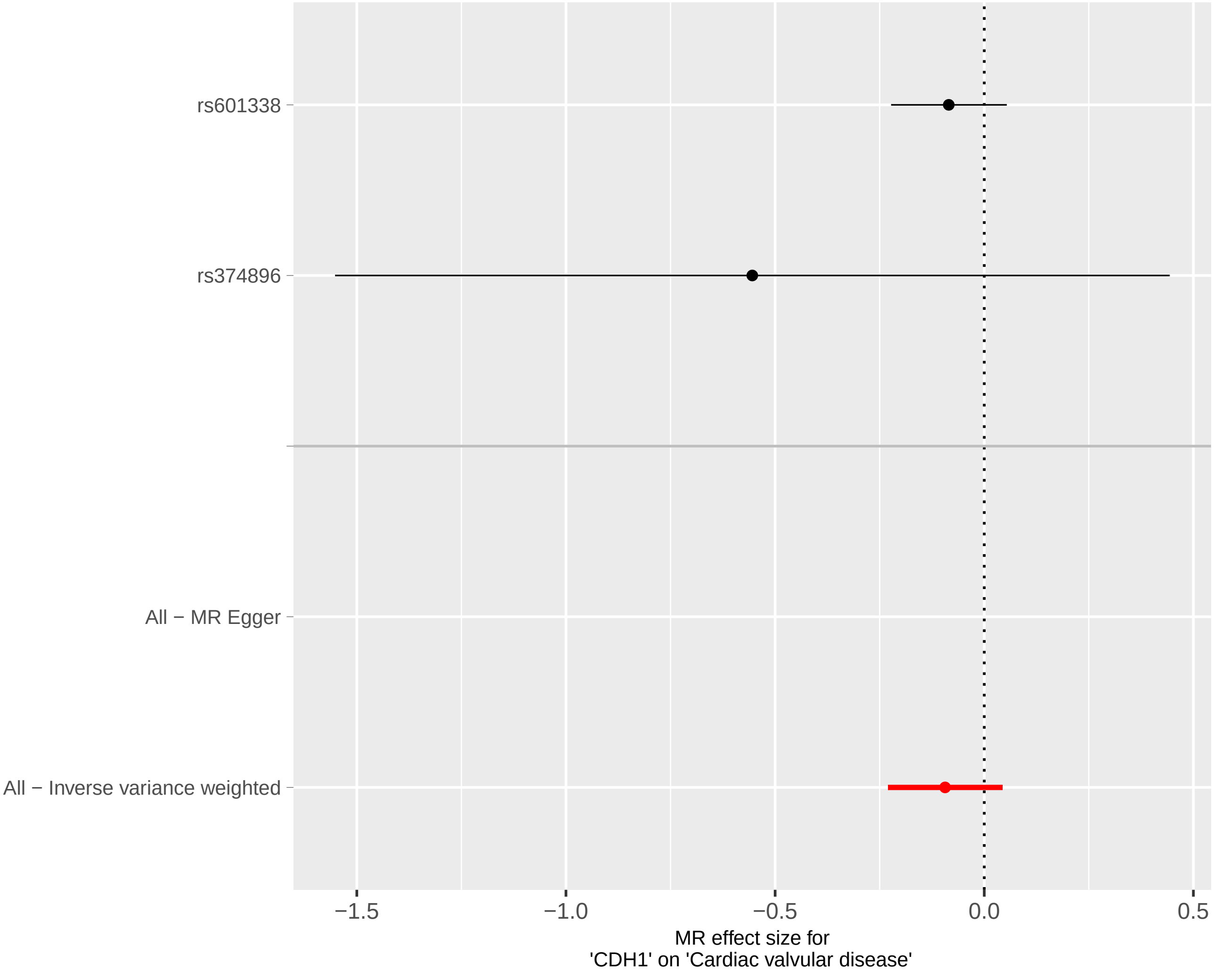
rs601338
rs374896
All − MR Egger
All − Inverse variance weighted
−1.5
−1.0
−0.5
0.0
0.5
MR effect size for
'CDH1' on 'Cardiac valvular disease'

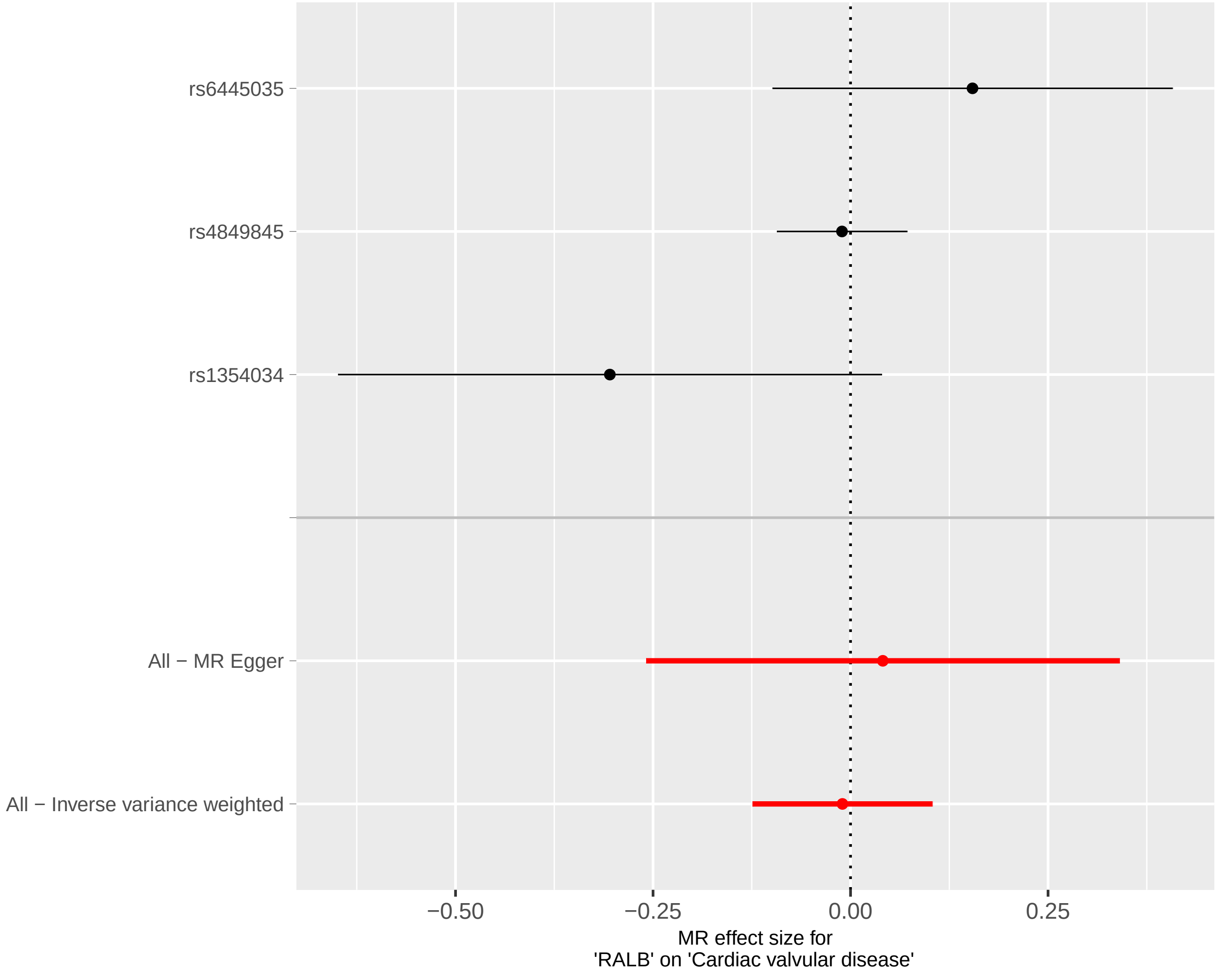
rs6445035
rs4849845
rs1354034
All − MR Egger
All − Inverse variance weighted
−0.50
−0.25
0.00
0.25
MR effect size for
'RALB' on 'Cardiac valvular disease'

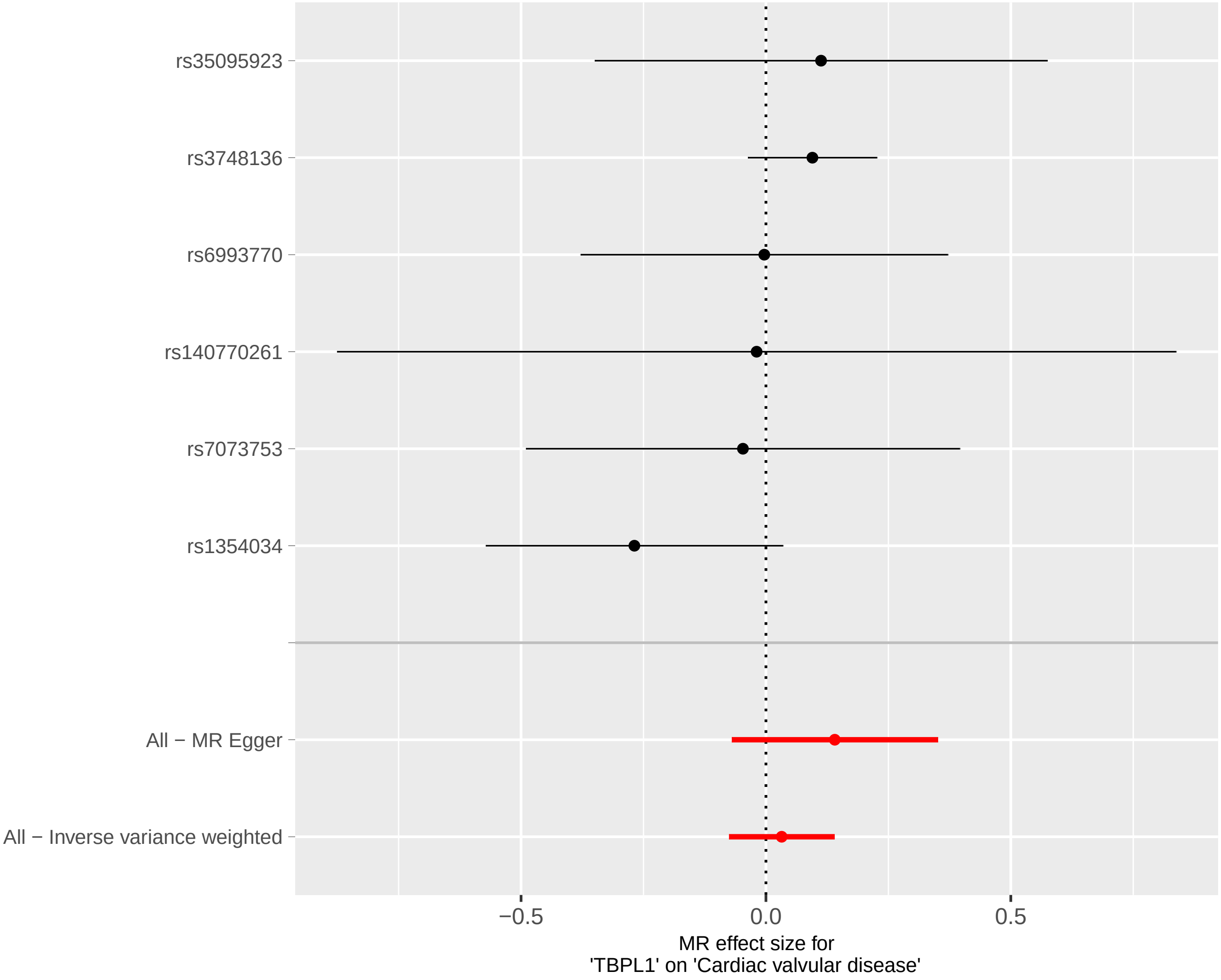
rs35095923
rs3748136
rs6993770
rs140770261
rs7073753
rs1354034
All − MR Egger
All − Inverse variance weighted
−0.5
0.0
0.5
MR effect size for
'TBPL1' on 'Cardiac valvular disease'

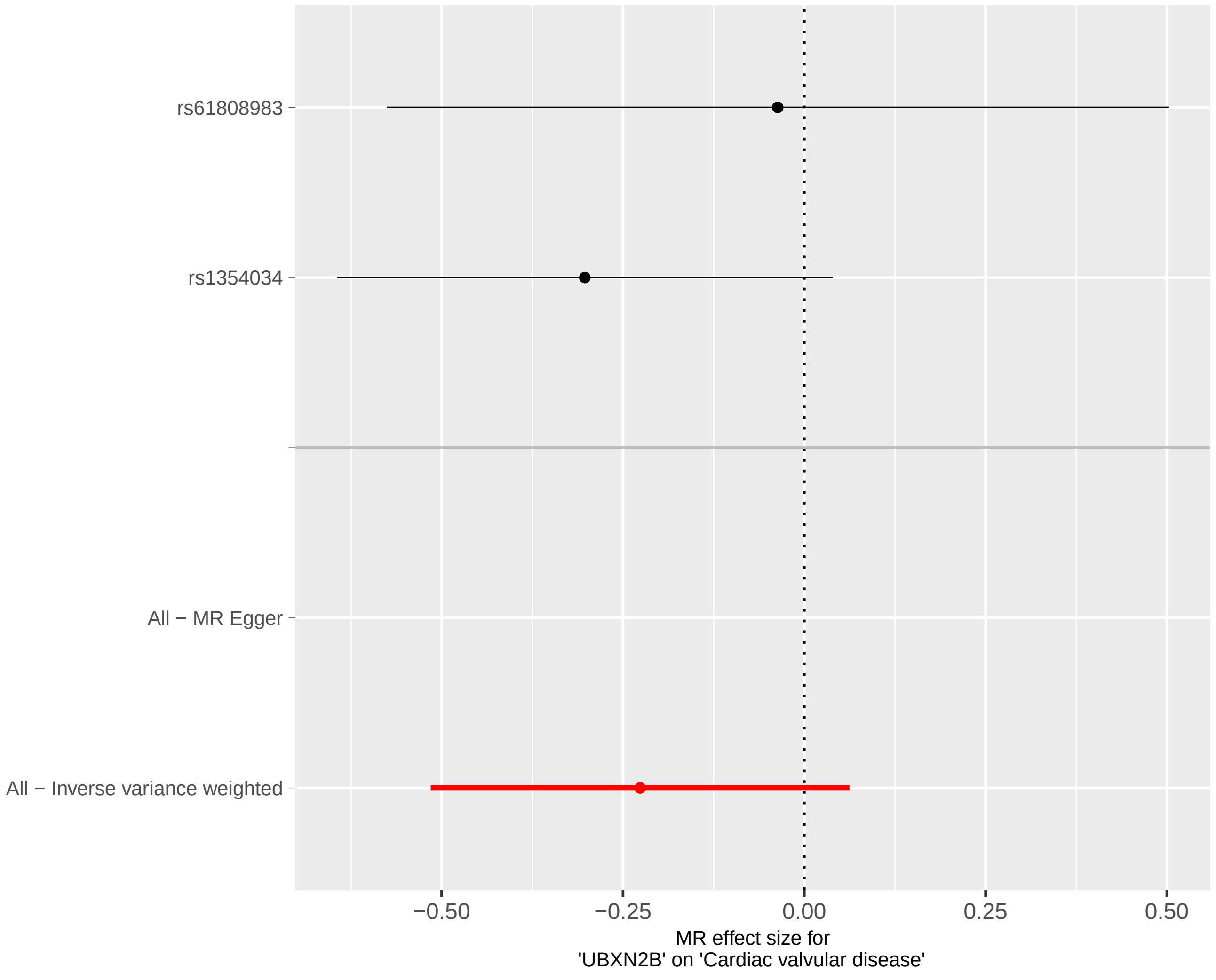
rs61808983
rs1354034
All − MR Egger
All − Inverse variance weighted
−0.50
−0.25
0.00
0.25
0.50
MR effect size for
'UBXN2B' on 'Cardiac valvular disease'

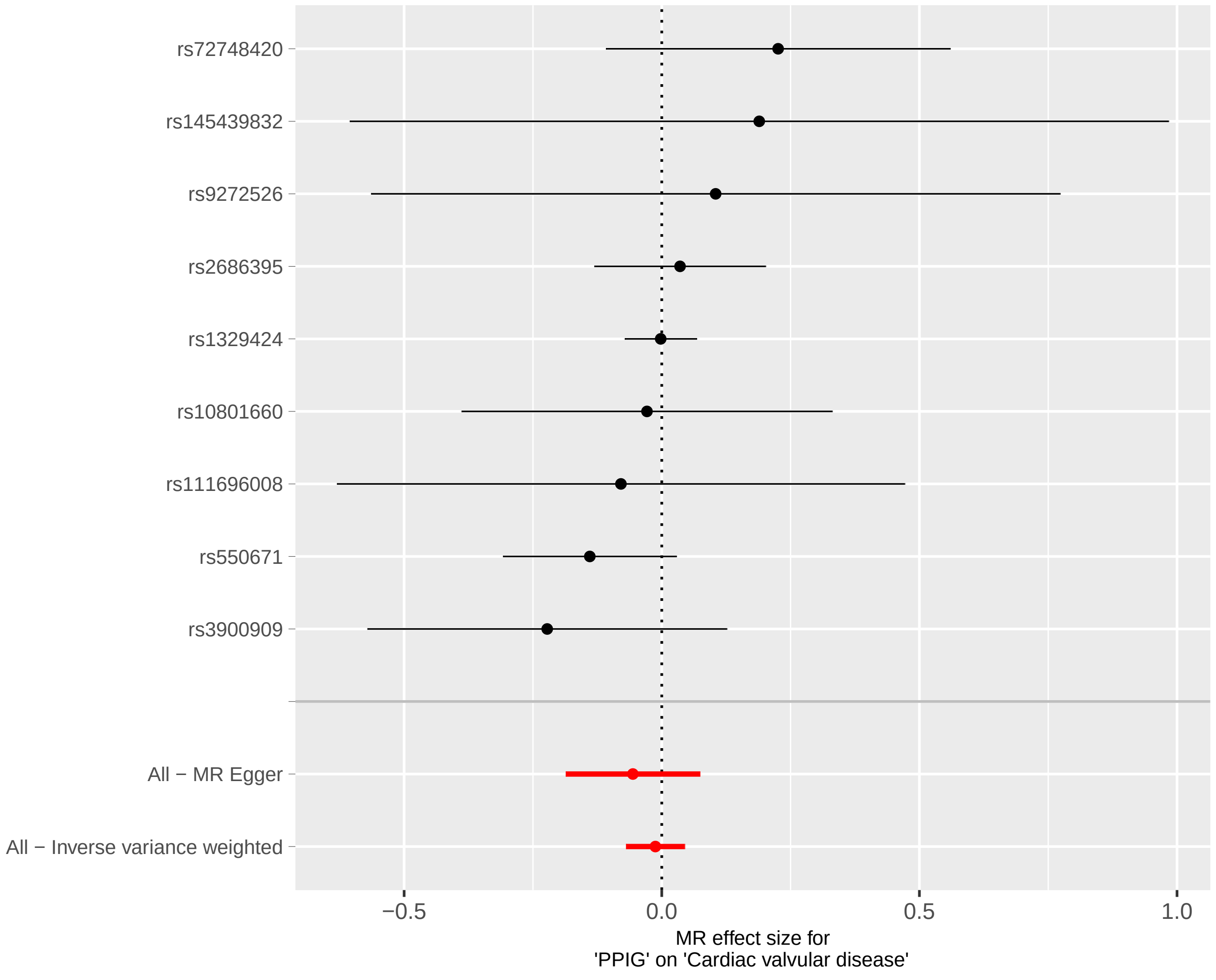
rs72748420
rs145439832
rs9272526
rs2686395
rs1329424
rs10801660
rs111696008
rs550671
rs3900909
All − MR Egger
All − Inverse variance weighted
−0.5
0.0
0.5
1.0
MR effect size for
'PPIG' on 'Cardiac valvular disease'

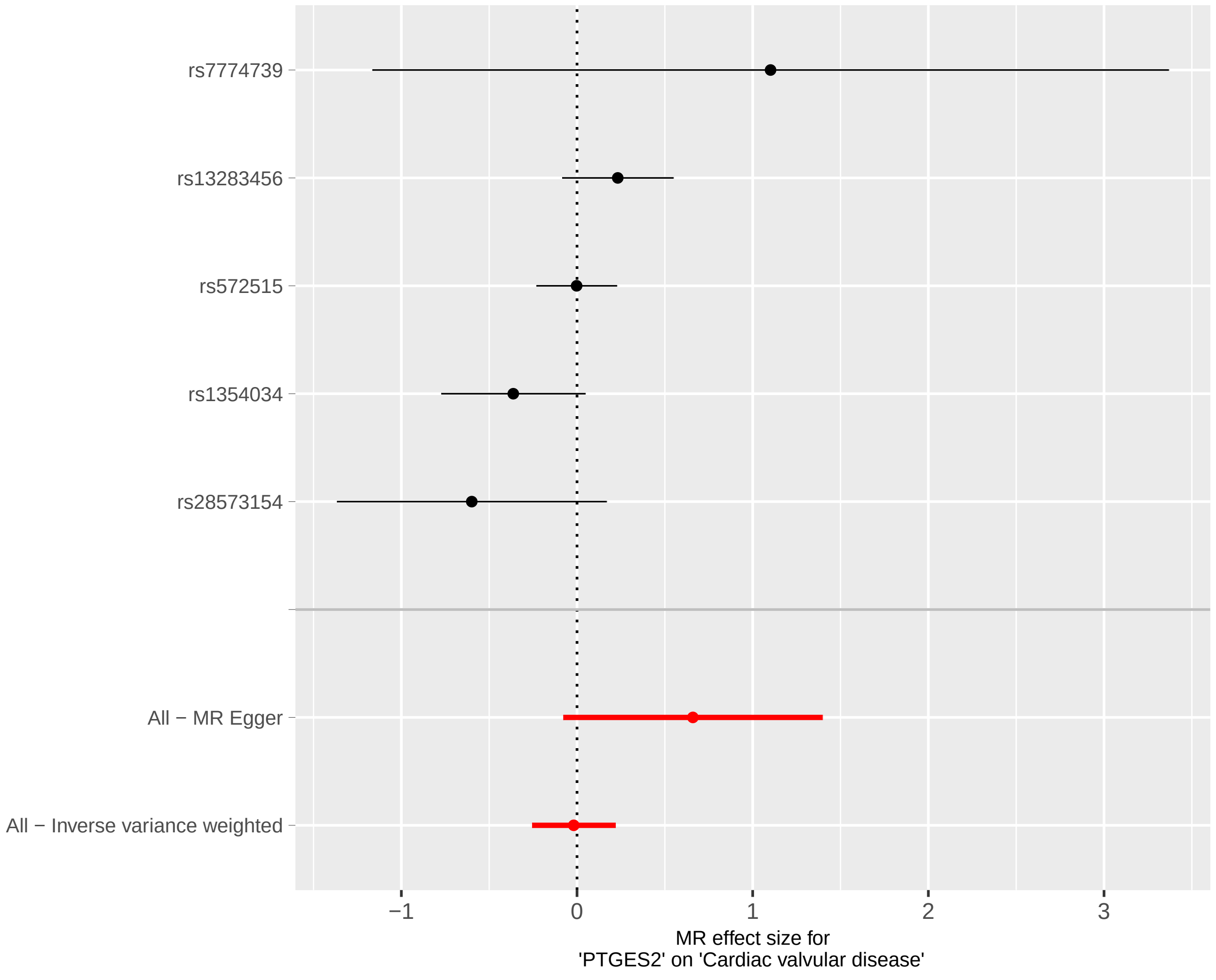
rs7774739
rs13283456
rs572515
rs1354034
rs28573154
All − MR Egger
All − Inverse variance weighted
−1
0
1
2
3
MR effect size for
'PTGES2' on 'Cardiac valvular disease'

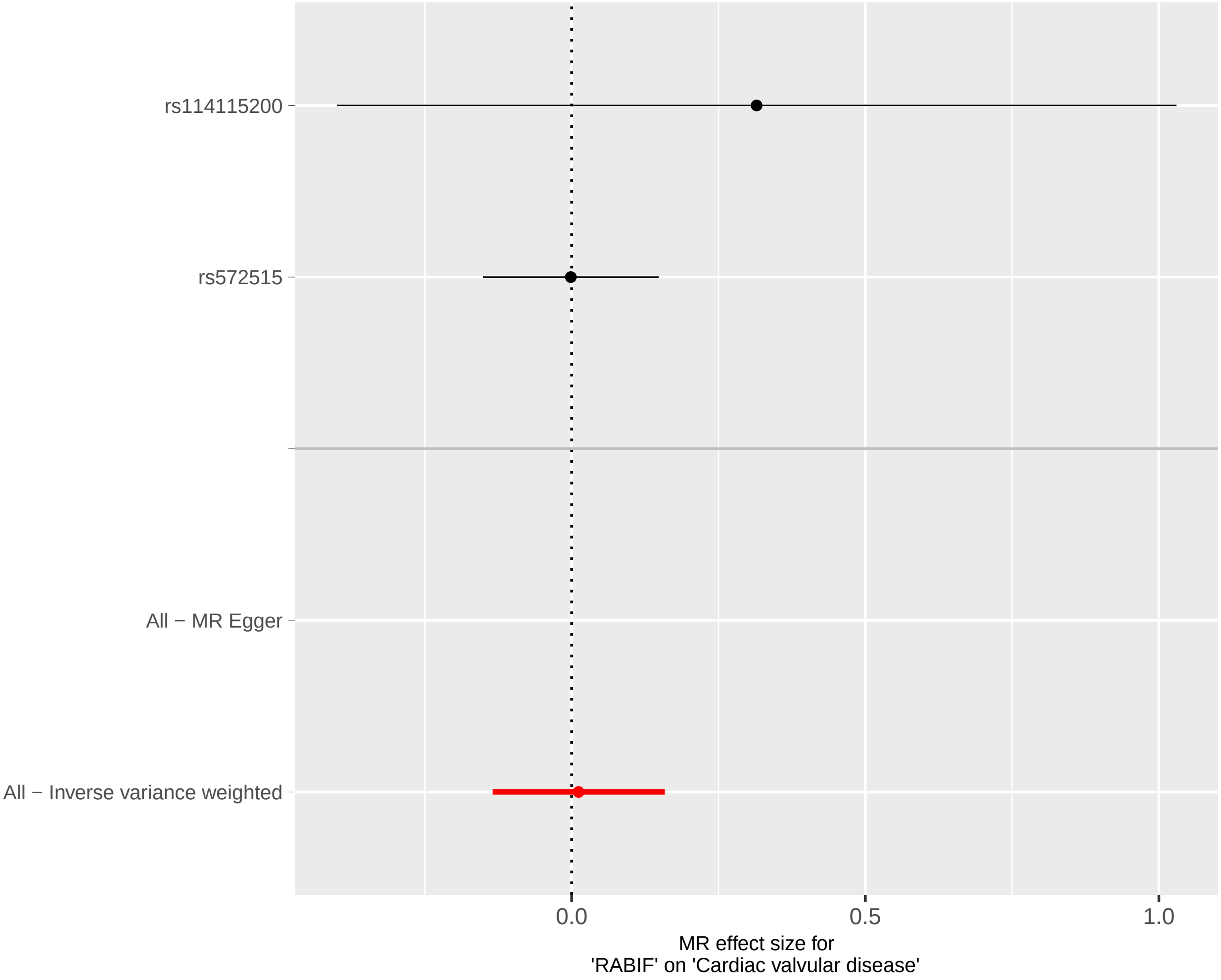
rs114115200
rs572515
All − MR Egger
All − Inverse variance weighted
0.0
0.5
1.0
MR effect size for
'RABIF' on 'Cardiac valvular disease'

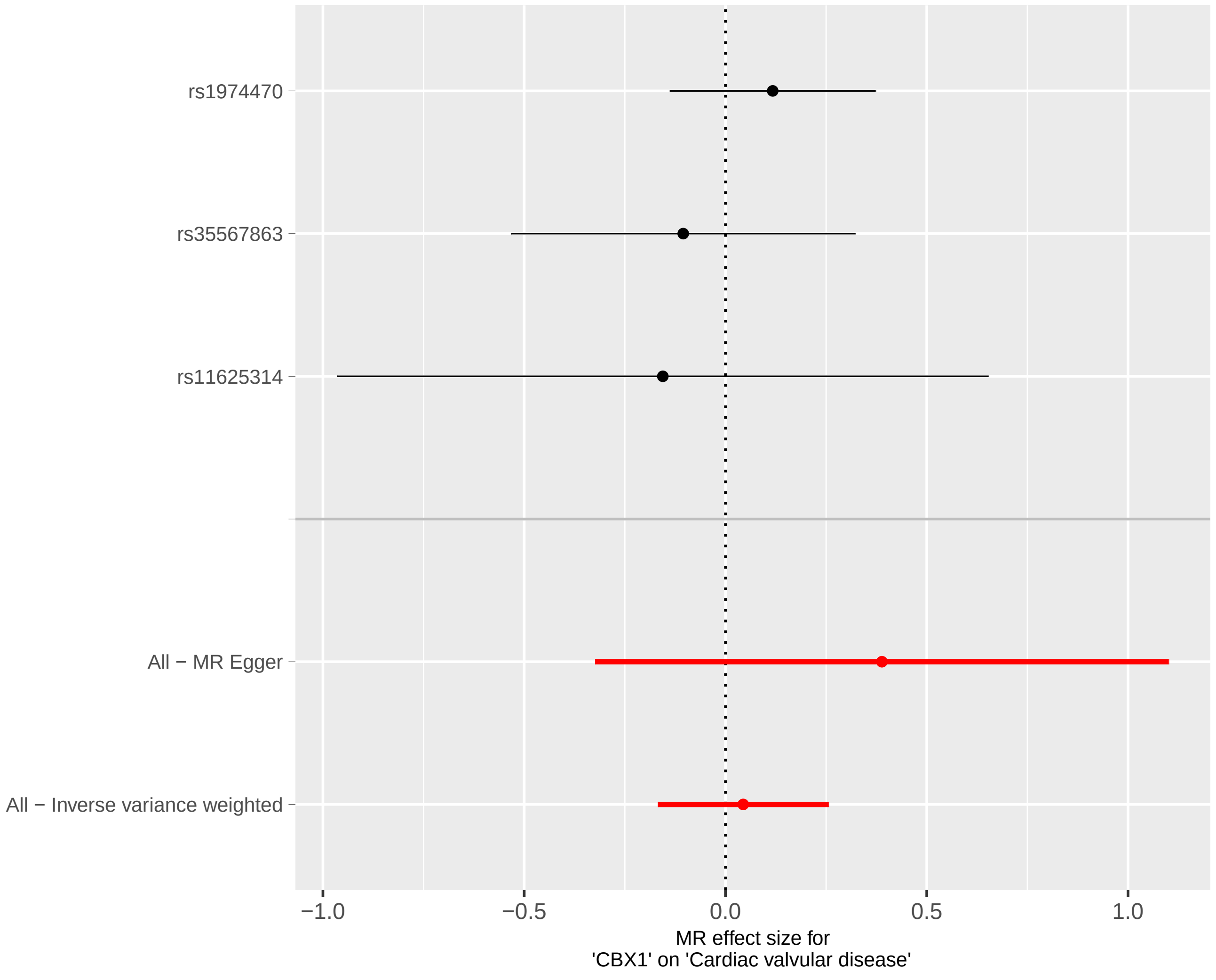
rs1974470
rs35567863
rs11625314
All − MR Egger
All − Inverse variance weighted
−1.0
−0.5
0.0
0.5
1.0
MR effect size for
'CBX1' on 'Cardiac valvular disease'

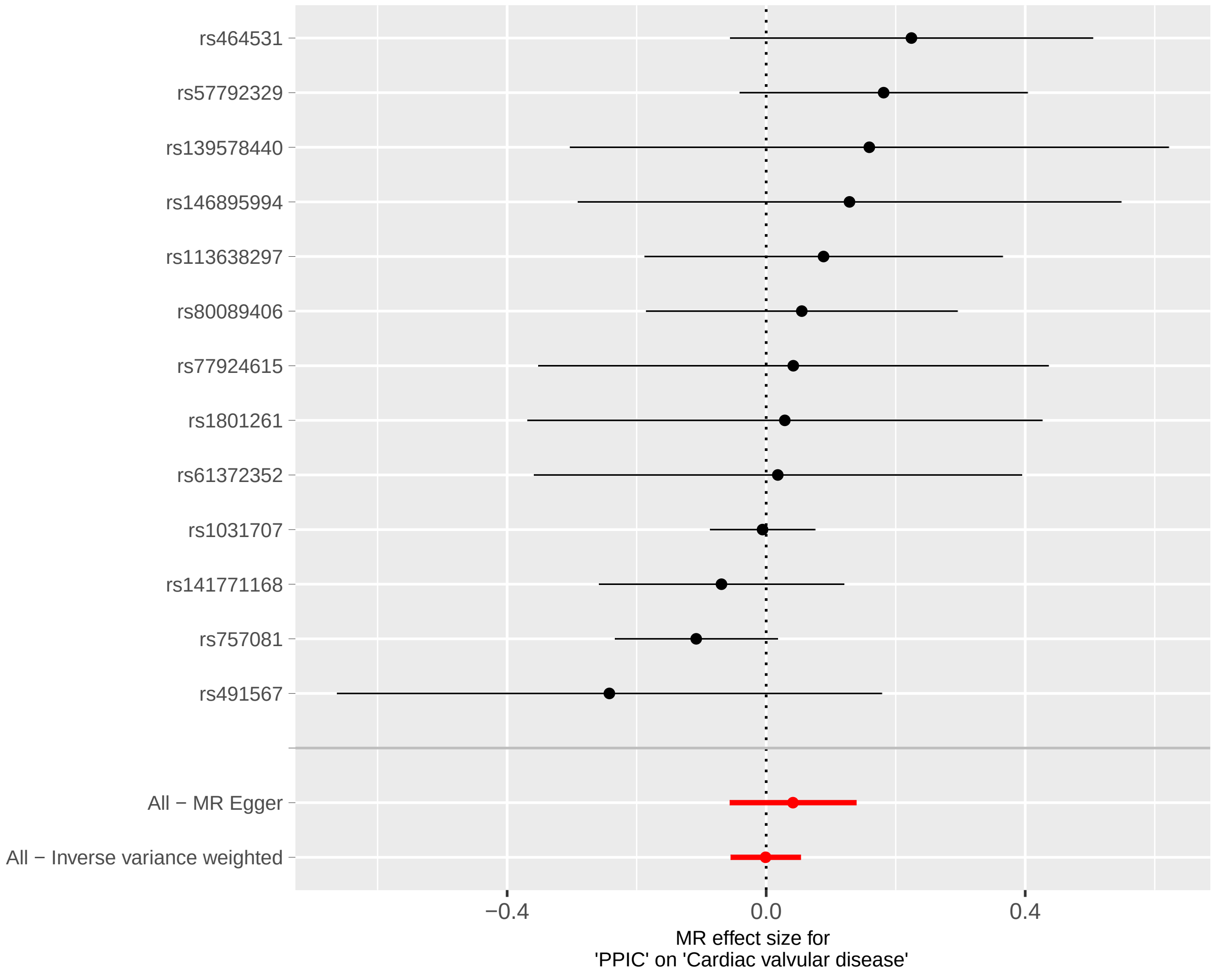
rs464531
rs57792329
rs139578440
rs146895994
rs113638297
rs80089406
rs77924615
rs1801261
rs61372352
rs1031707
rs141771168
rs757081
rs491567
All − MR Egger
All − Inverse variance weighted
−0.4
0.0
0.4
MR effect size for
'PPIC' on 'Cardiac valvular disease'

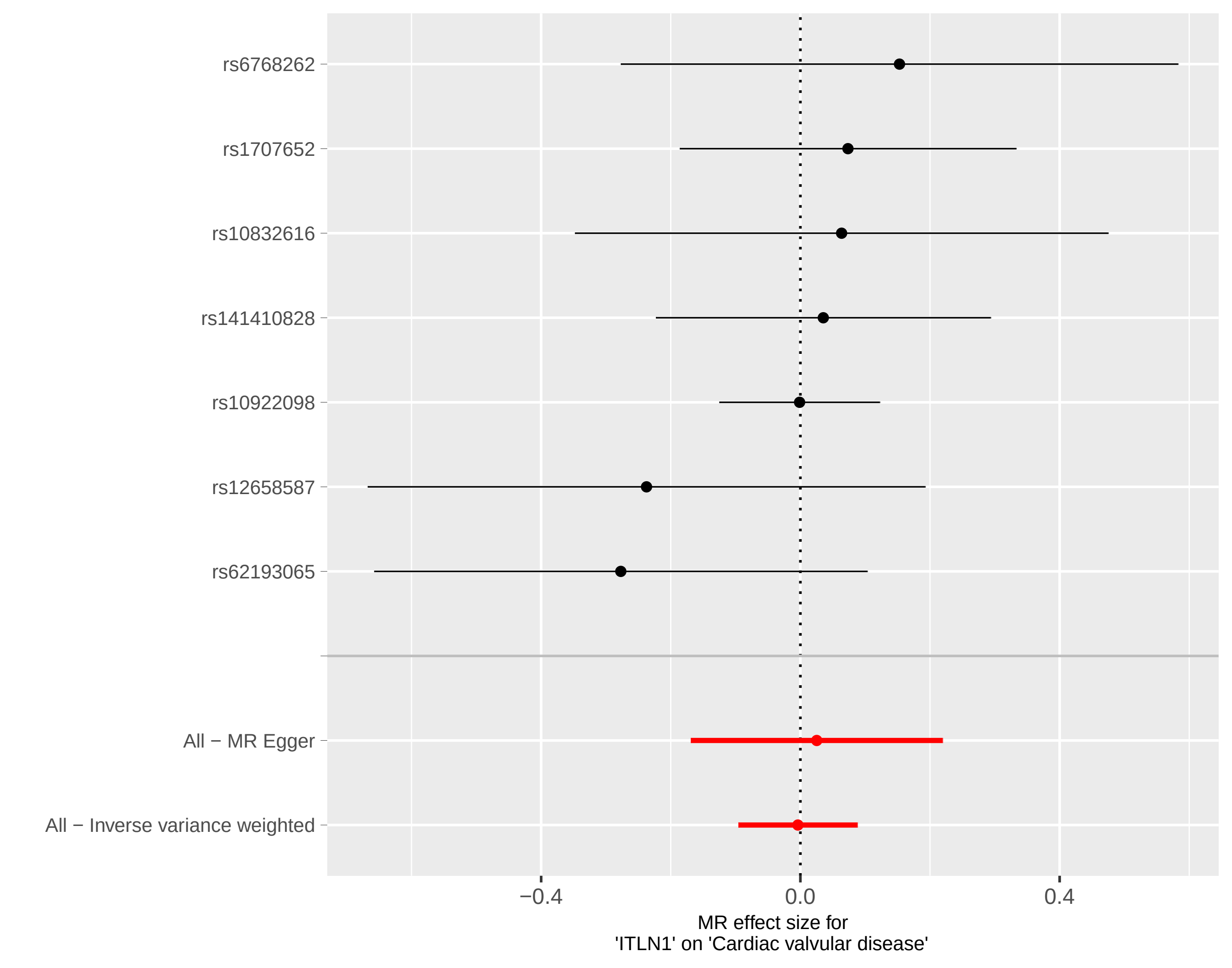

rs6768262
rs1707652
rs10832616
rs141410828
rs10922098
rs12658587
rs62193065
All − MR Egger
All − Inverse variance weighted
−0.4
0.0
0.4
MR effect size for
'ITLN1' on 'Cardiac valvular disease'

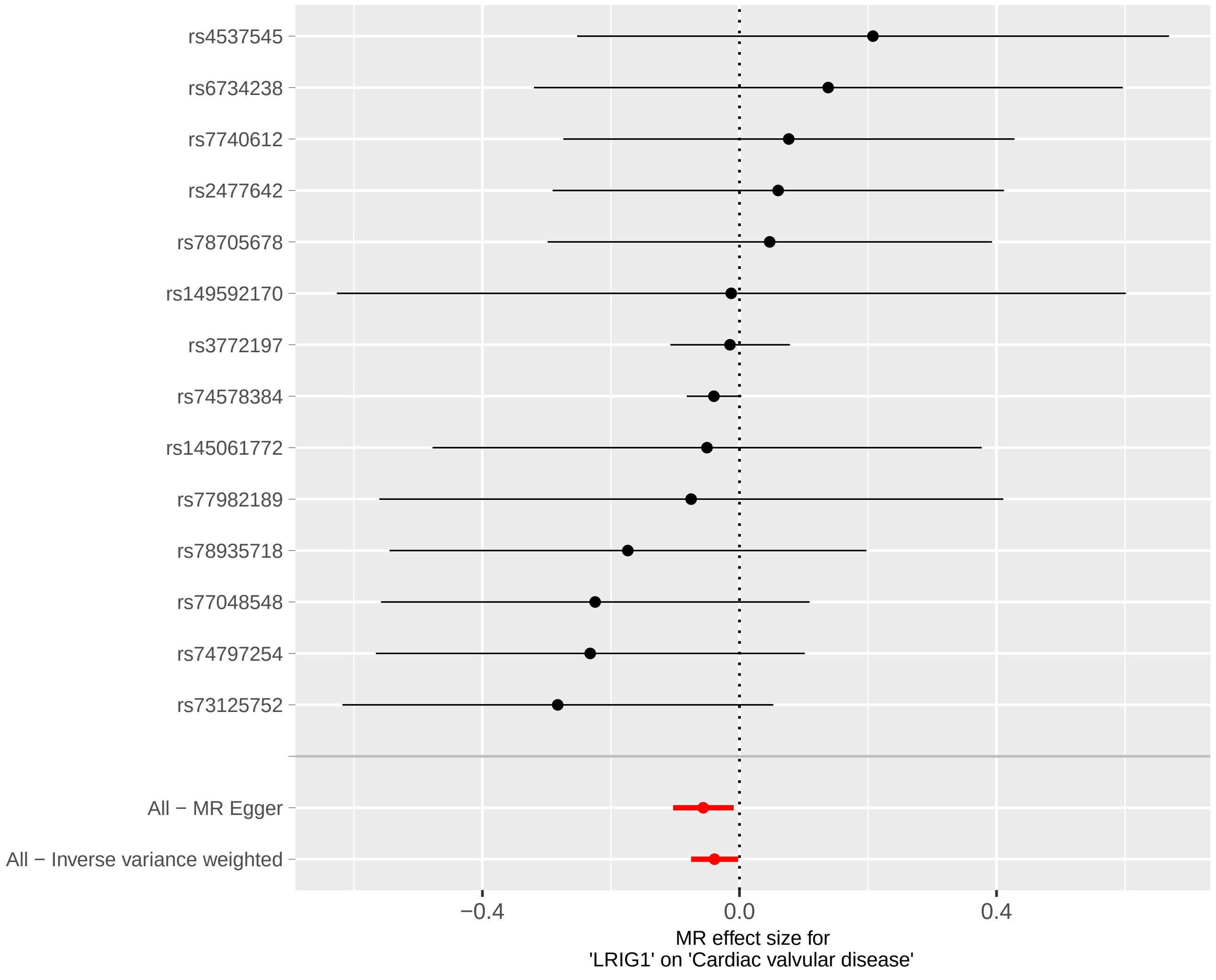

rs4537545
rs6734238
rs7740612
rs2477642
rs78705678
rs149592170
rs3772197
rs74578384
rs145061772
rs77982189
rs78935718
rs77048548
rs74797254
rs73125752
All − MR Egger
All − Inverse variance weighted
−0.4
0.0
0.4
MR effect size for
'LRIG1' on 'Cardiac valvular disease'

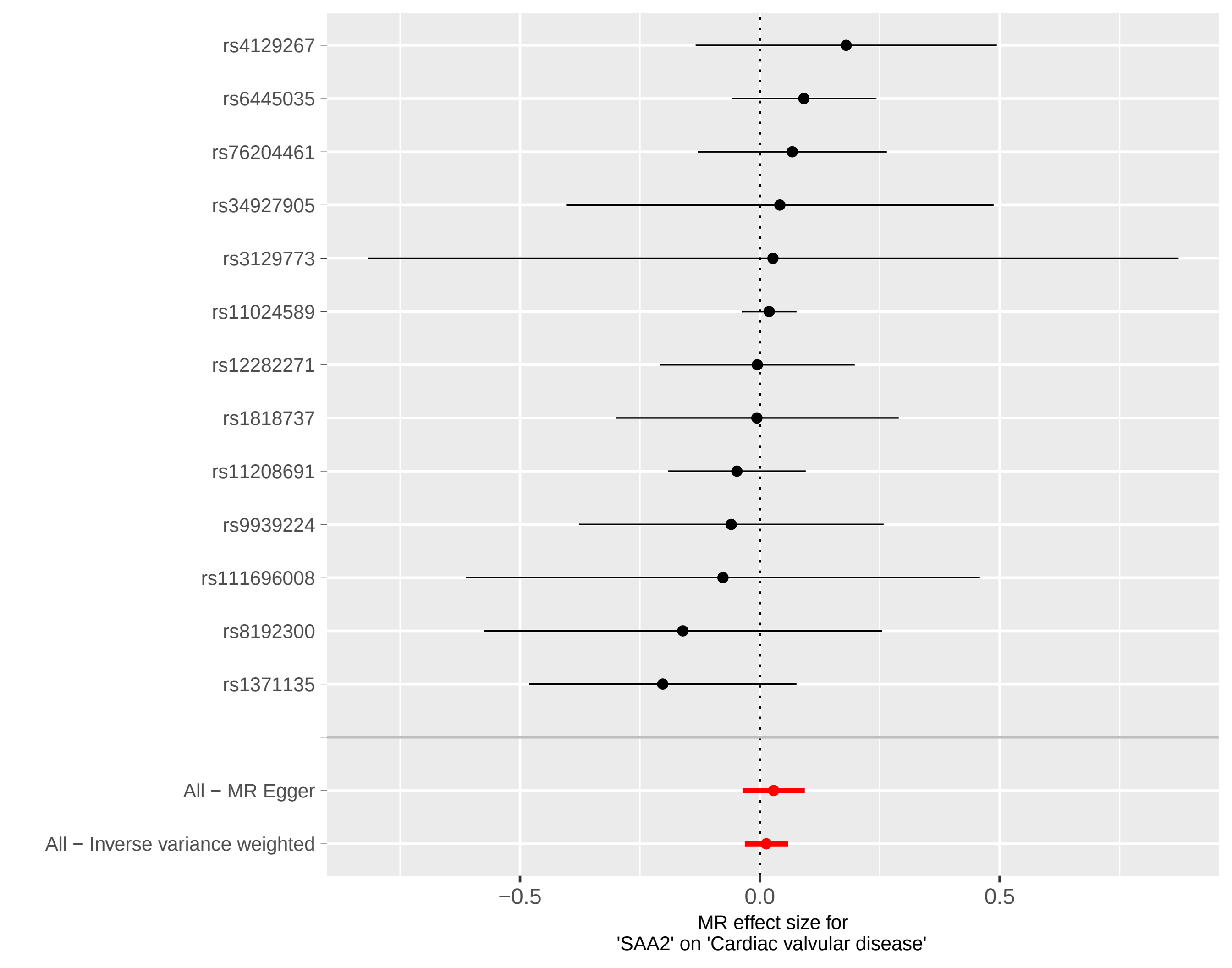
rs4129267
rs6445035
rs76204461
rs34927905
rs3129773
rs11024589
rs12282271
rs1818737
rs11208691
rs9939224
rs111696008
rs8192300
rs1371135
All − MR Egger
All − Inverse variance weighted
−0.5
0.0
0.5
MR effect size for
'SAA2' on 'Cardiac valvular disease'

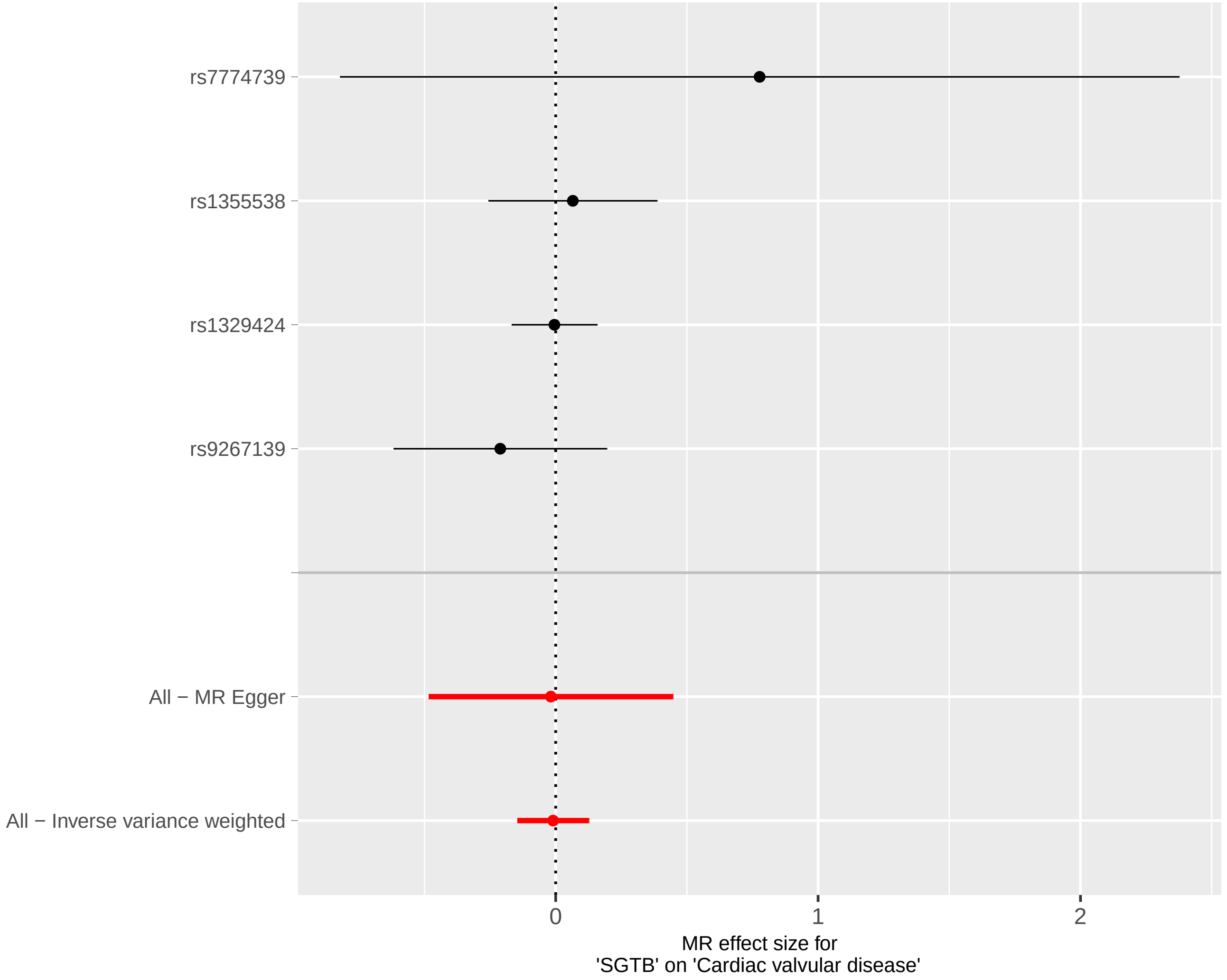

rs7774739
rs1355538
rs1329424
rs9267139
All − MR Egger
All − Inverse variance weighted
0
1
2
MR effect size for
'SGTB' on 'Cardiac valvular disease'

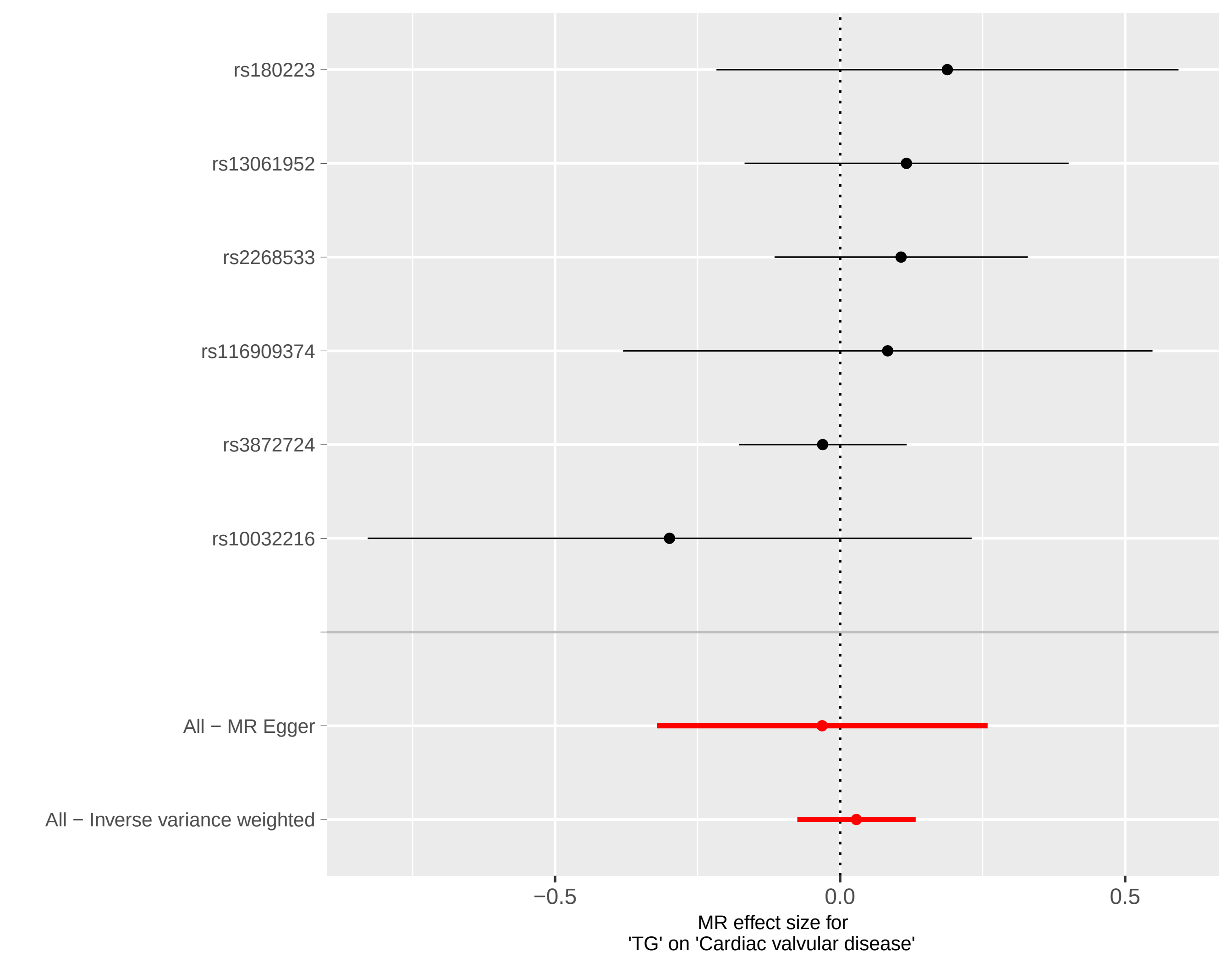
rs180223
rs13061952
rs2268533
rs116909374
rs3872724
rs10032216
All − MR Egger
All − Inverse variance weighted
−0.5
0.0
0.5
MR effect size for
'TG' on 'Cardiac valvular disease'

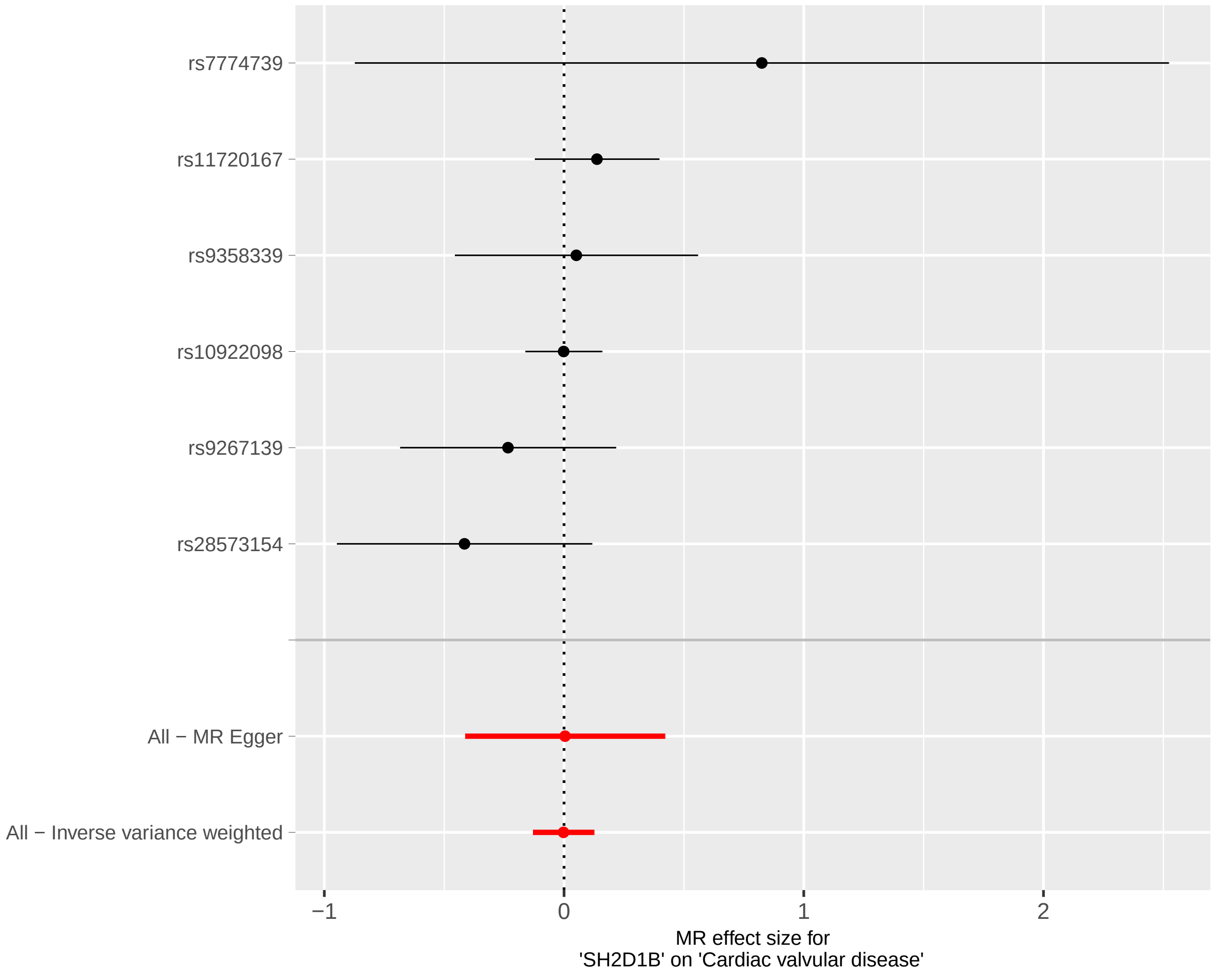
rs7774739
rs11720167
rs9358339
rs10922098
rs9267139
rs28573154
All − MR Egger
All − Inverse variance weighted
−1
0
1
2
MR effect size for
'SH2D1B' on 'Cardiac valvular disease'

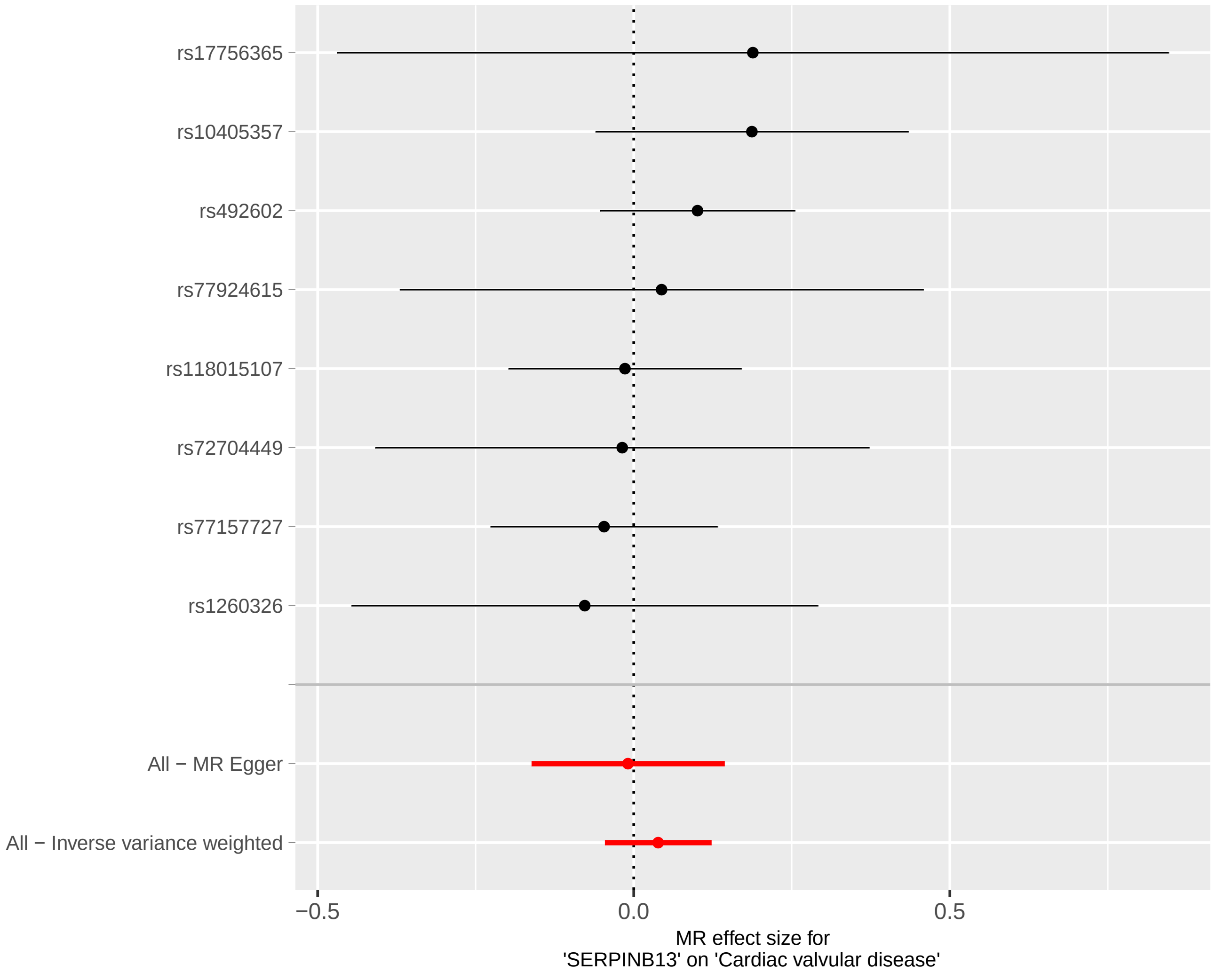
rs17756365
rs10405357
rs492602
rs77924615
rs118015107
rs72704449
rs77157727
rs1260326
All − MR Egger
All − Inverse variance weighted
−0.5
0.0
0.5
MR effect size for
'SERPINB13' on 'Cardiac valvular disease'

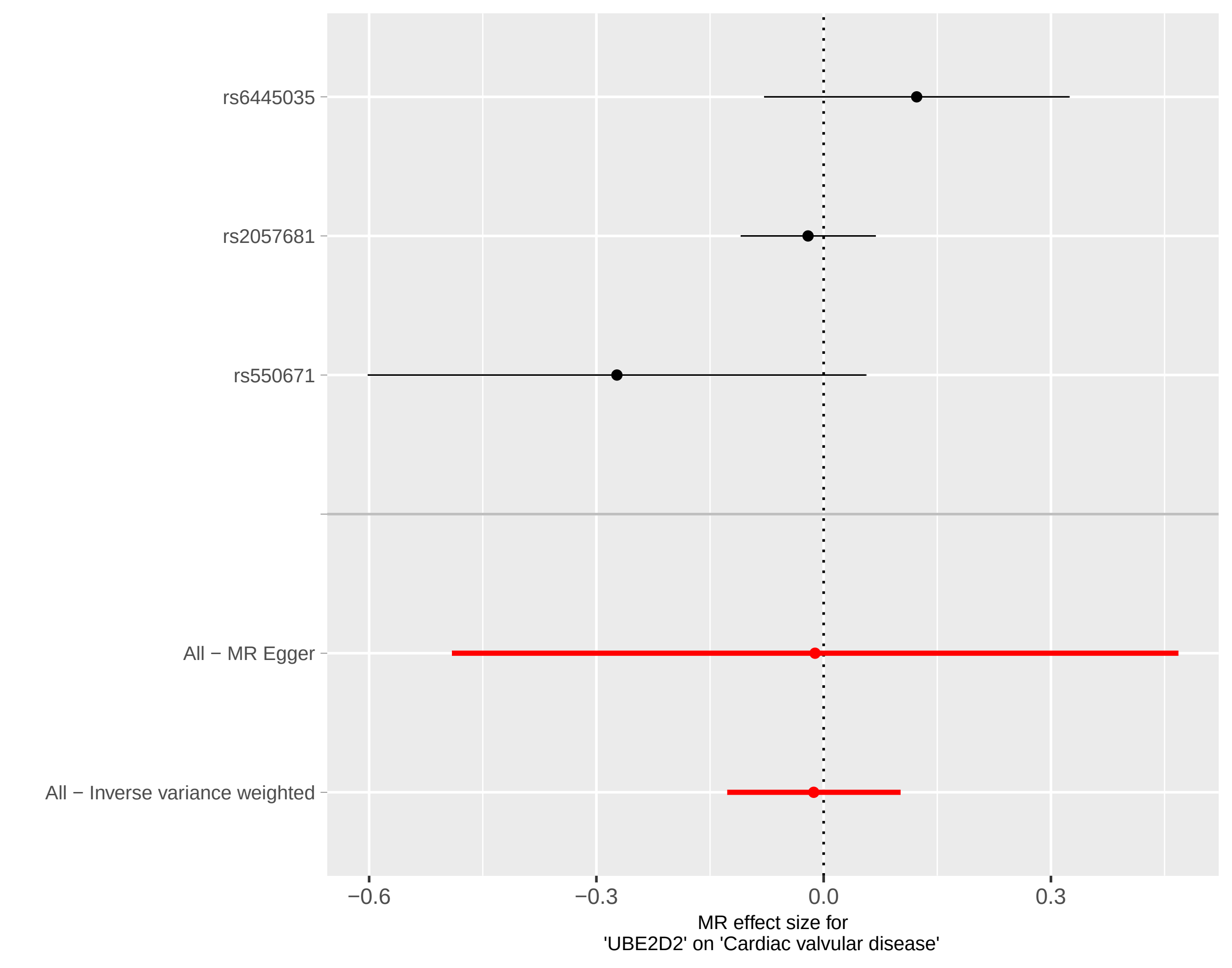

rs6445035
rs2057681
rs550671
All − MR Egger
All − Inverse variance weighted
−0.6
−0.3
0.0
0.3
MR effect size for
'UBE2D2' on 'Cardiac valvular disease'

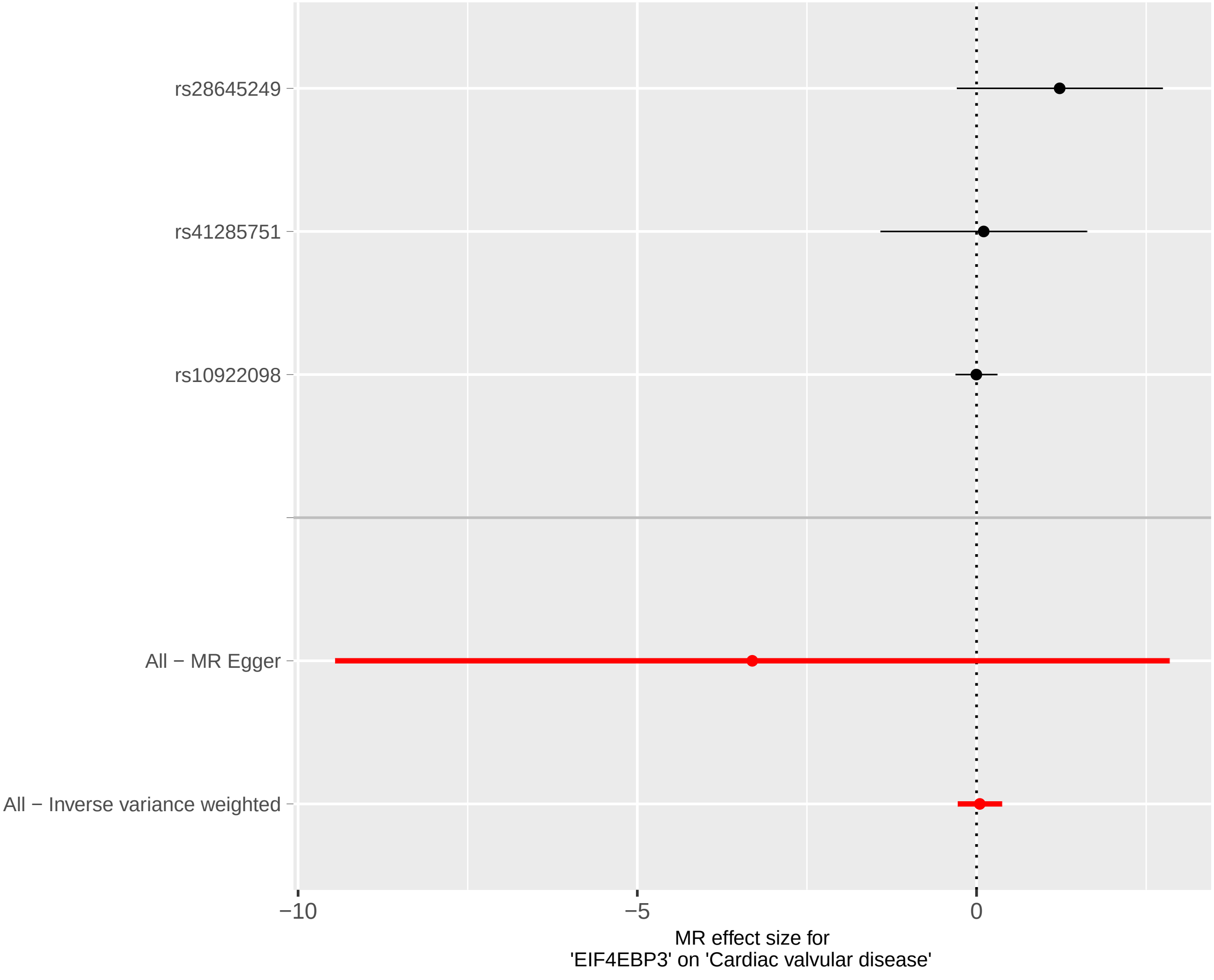
rs28645249
rs41285751
rs10922098
All − MR Egger
All − Inverse variance weighted
−10
−5
0
MR effect size for
'EIF4EBP3' on 'Cardiac valvular disease'

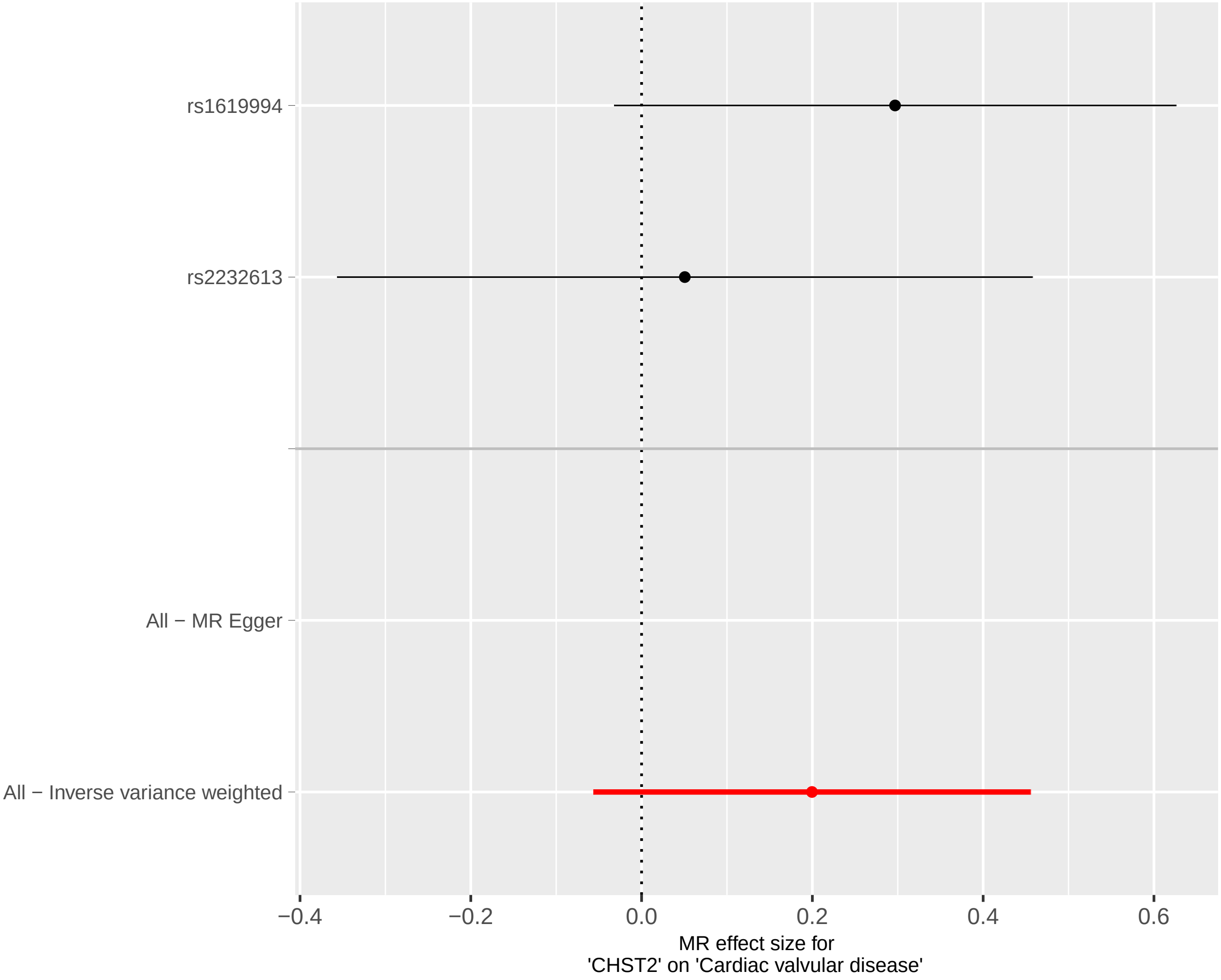
rs1619994
rs2232613
All − MR Egger
All − Inverse variance weighted
−0.4
−0.2
0.0
0.2
0.4
0.6
MR effect size for
'CHST2' on 'Cardiac valvular disease'

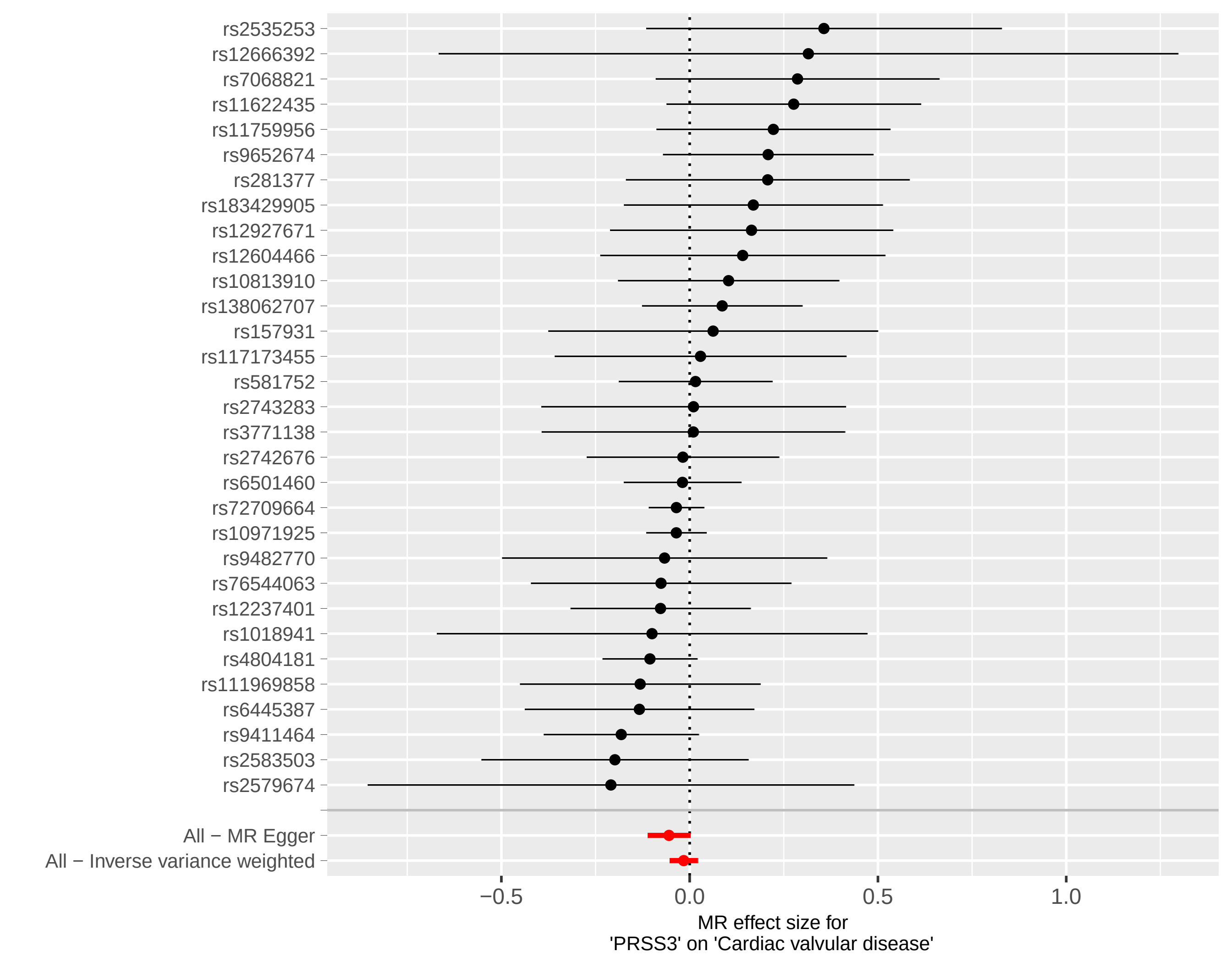

rs2535253
rs12666392
rs7068821
rs11622435
rs11759956
rs9652674
rs281377
rs183429905
rs12927671
rs12604466
rs10813910
rs138062707
rs157931
rs117173455
rs581752
rs2743283
rs3771138
rs2742676
rs6501460
rs72709664
rs10971925
rs9482770
rs76544063
rs12237401
rs1018941
rs4804181
rs111969858
rs6445387
rs9411464
rs2583503
rs2579674
All − MR Egger
All − Inverse variance weighted
−0.5
0.0
0.5
1.0
MR effect size for
'PRSS3' on 'Cardiac valvular disease'

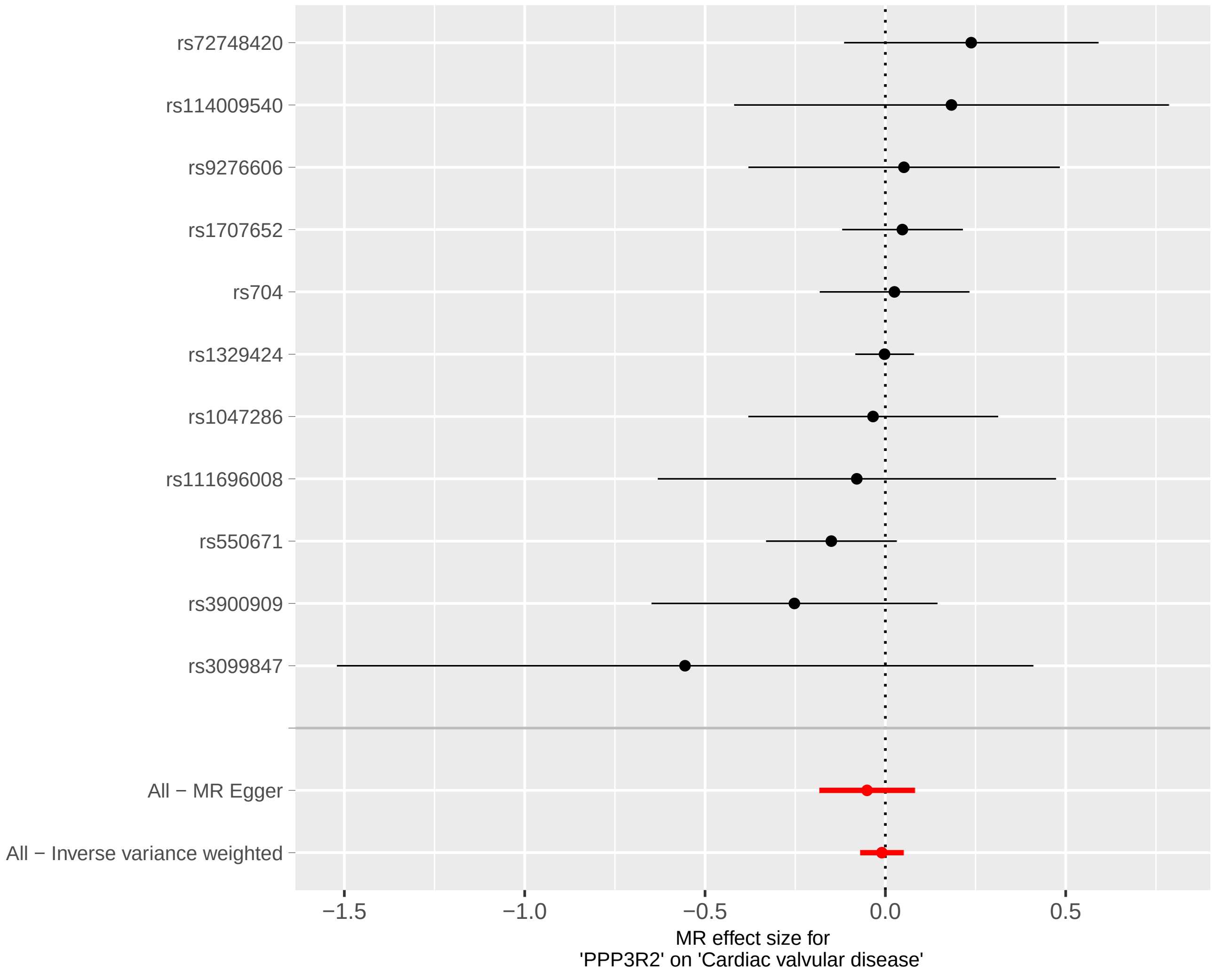
rs72748420
rs114009540
rs9276606
rs1707652
rs704
rs1329424
rs1047286
rs111696008
rs550671
rs3900909
rs3099847
All − MR Egger
All − Inverse variance weighted
−1.5
−1.0
−0.5
0.0
0.5
MR effect size for
'PPP3R2' on 'Cardiac valvular disease'

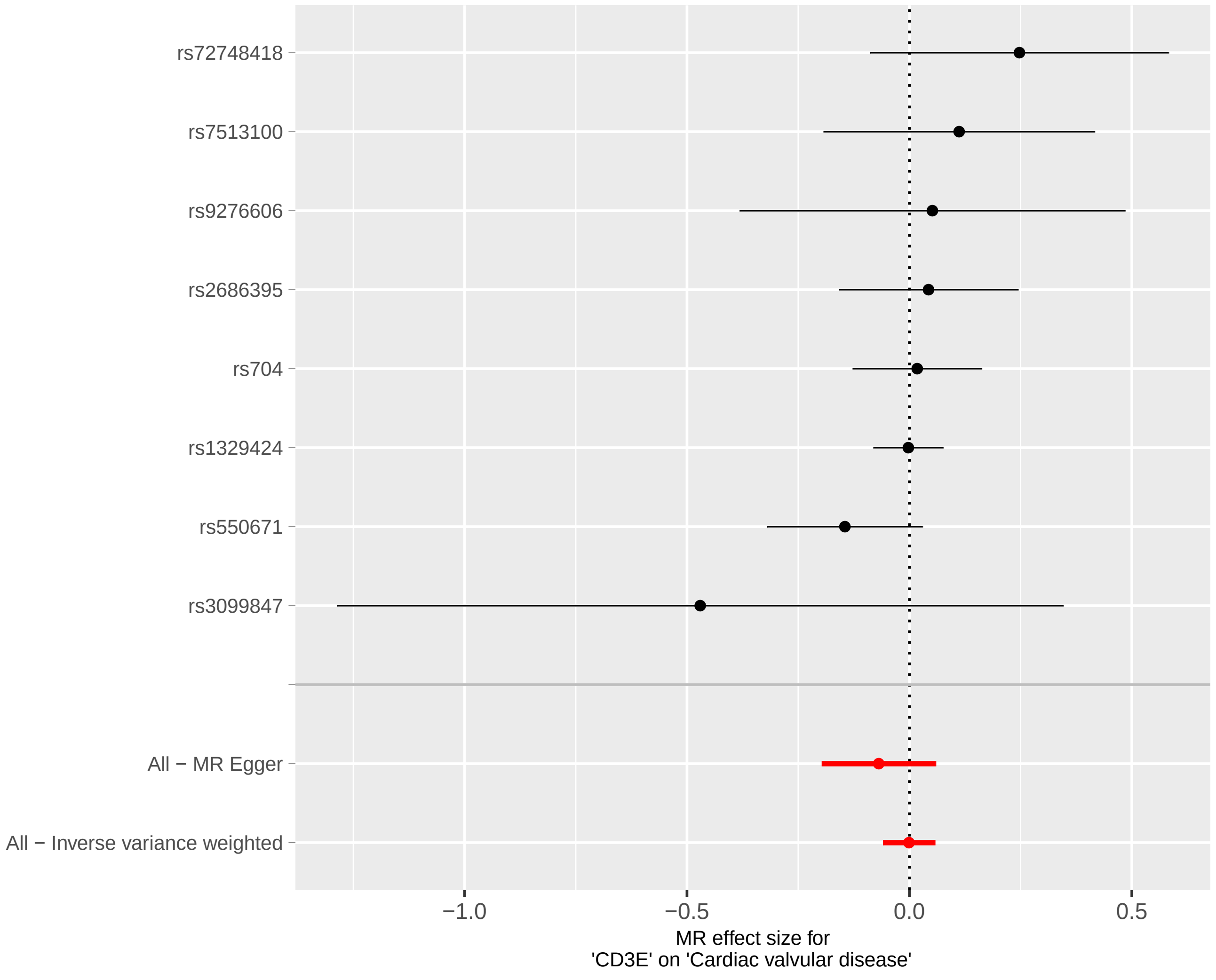
rs72748418
rs7513100
rs9276606
rs2686395
rs704
rs1329424
rs550671
rs3099847
All − MR Egger
All − Inverse variance weighted
−1.0
−0.5
0.0
0.5
MR effect size for
'CD3E' on 'Cardiac valvular disease'

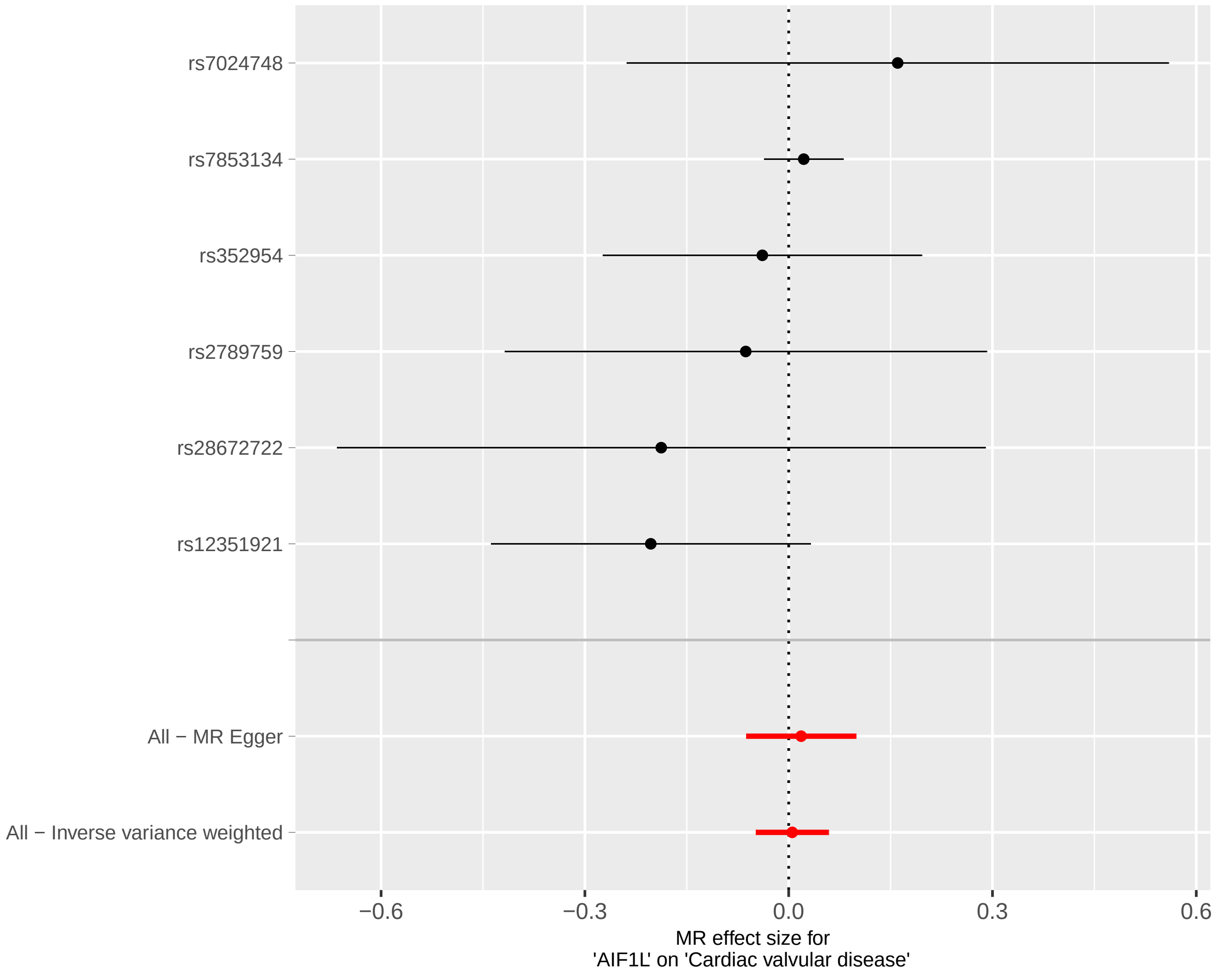

rs7024748
rs7853134
rs352954
rs2789759
rs28672722
rs12351921
All − MR Egger
All − Inverse variance weighted
−0.6
−0.3
0.0
0.3
0.6
MR effect size for
'AIF1L' on 'Cardiac valvular disease'

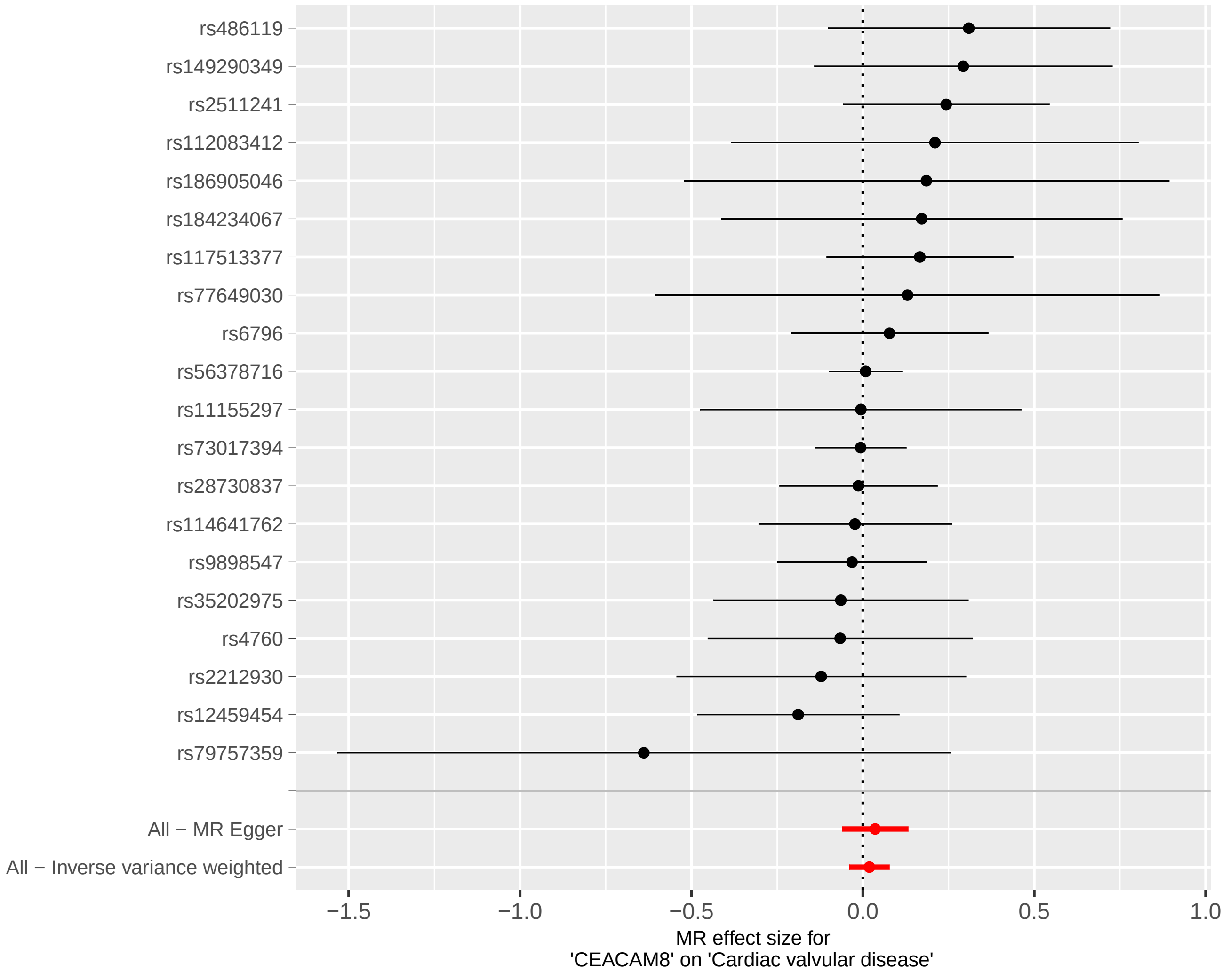
rs486119
rs149290349
rs2511241
rs112083412
rs186905046
rs184234067
rs117513377
rs77649030
rs6796
rs56378716
rs11155297
rs73017394
rs28730837
rs114641762
rs9898547
rs35202975
rs4760
rs2212930
rs12459454
rs79757359
All − MR Egger
All − Inverse variance weighted
−1.5
−1.0
−0.5
0.0
0.5
1.0
MR effect size for
'CEACAM8' on 'Cardiac valvular disease'

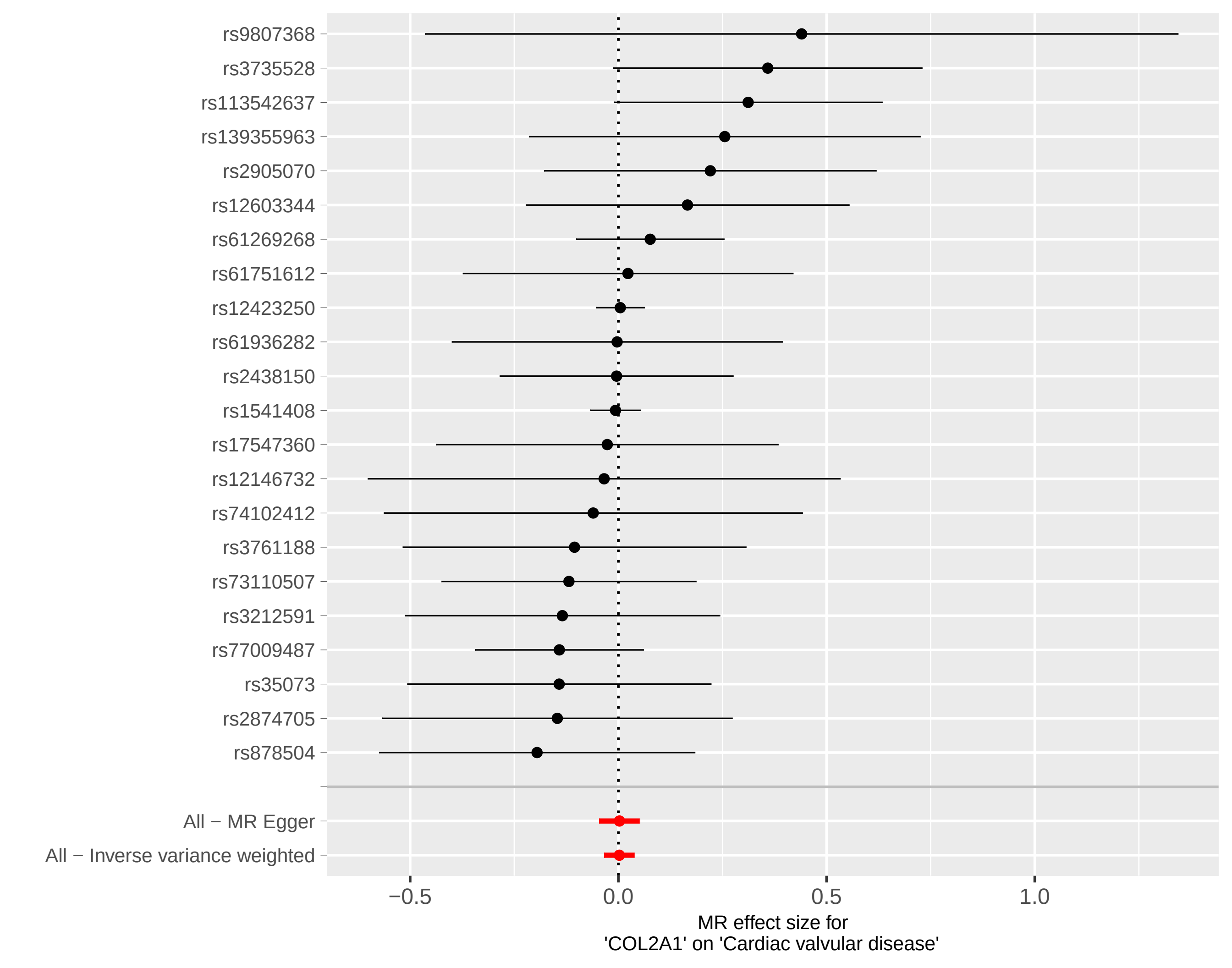

rs9807368
rs3735528
rs113542637
rs139355963
rs2905070
rs12603344
rs61269268
rs61751612
rs12423250
rs61936282
rs2438150
rs1541408
rs17547360
rs12146732
rs74102412
rs3761188
rs73110507
rs3212591
rs77009487
rs35073
rs2874705
rs878504
All − MR Egger
All − Inverse variance weighted
−0.5
0.0
0.5
1.0
MR effect size for
'COL2A1' on 'Cardiac valvular disease'

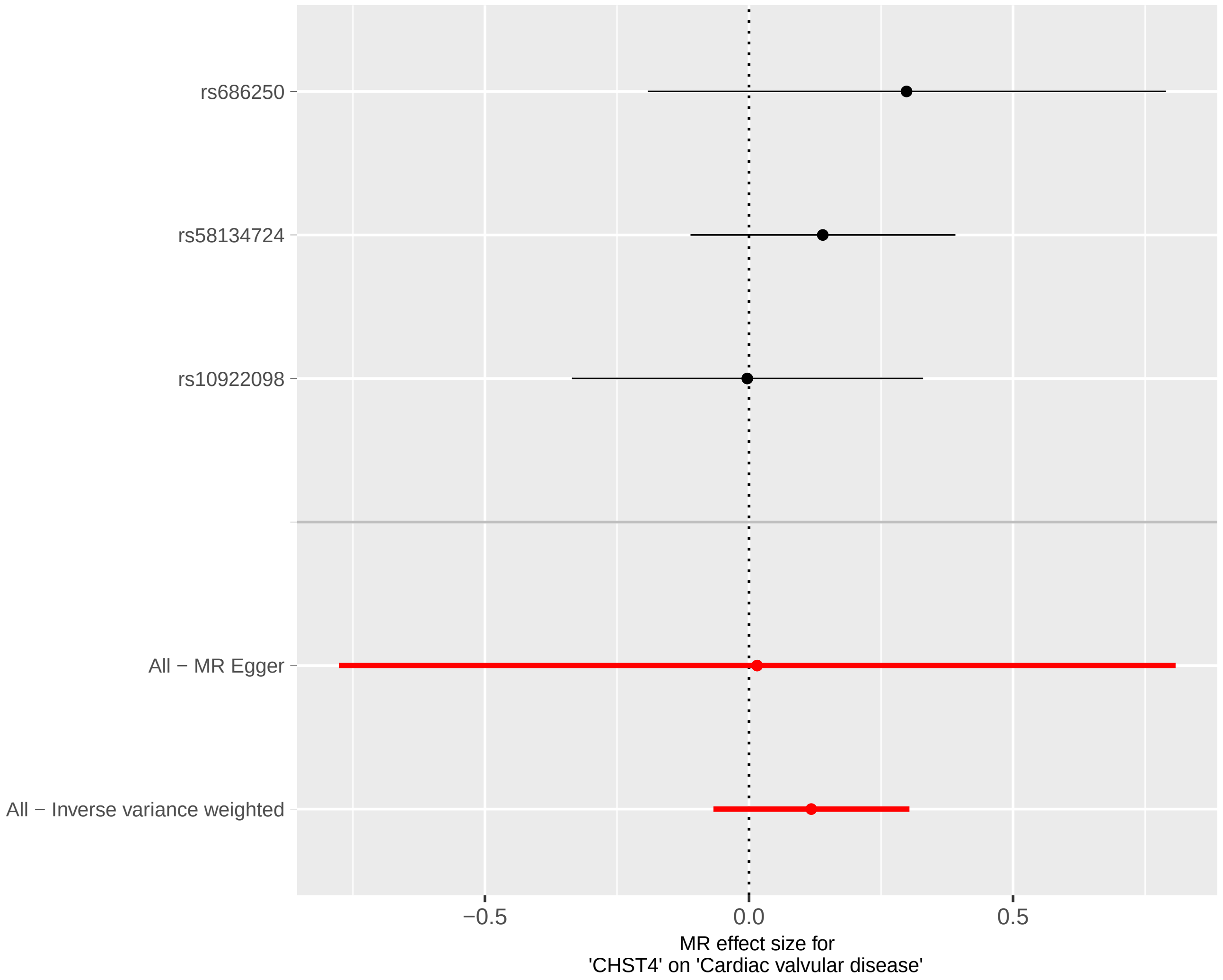

rs686250
rs58134724
rs10922098
All − MR Egger
All − Inverse variance weighted
−0.5
0.0
0.5
MR effect size for
'CHST4' on 'Cardiac valvular disease'

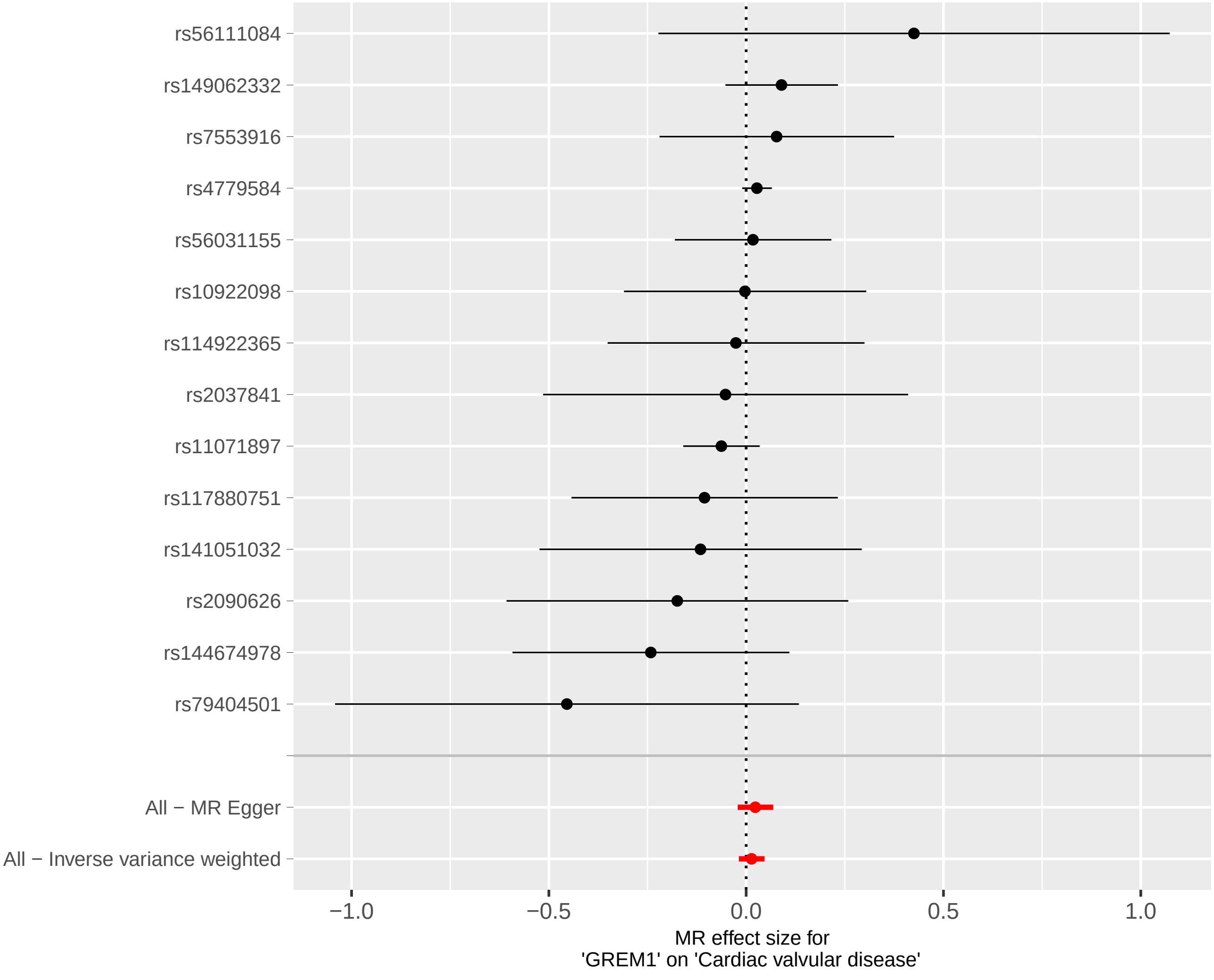
rs56111084
rs149062332
rs7553916
rs4779584
rs56031155
rs10922098
rs114922365
rs2037841
rs11071897
rs117880751
rs141051032
rs2090626
rs144674978
rs79404501
All − MR Egger
All − Inverse variance weighted
−1.0
−0.5
0.0
0.5
1.0
MR effect size for
'GREM1' on 'Cardiac valvular disease'

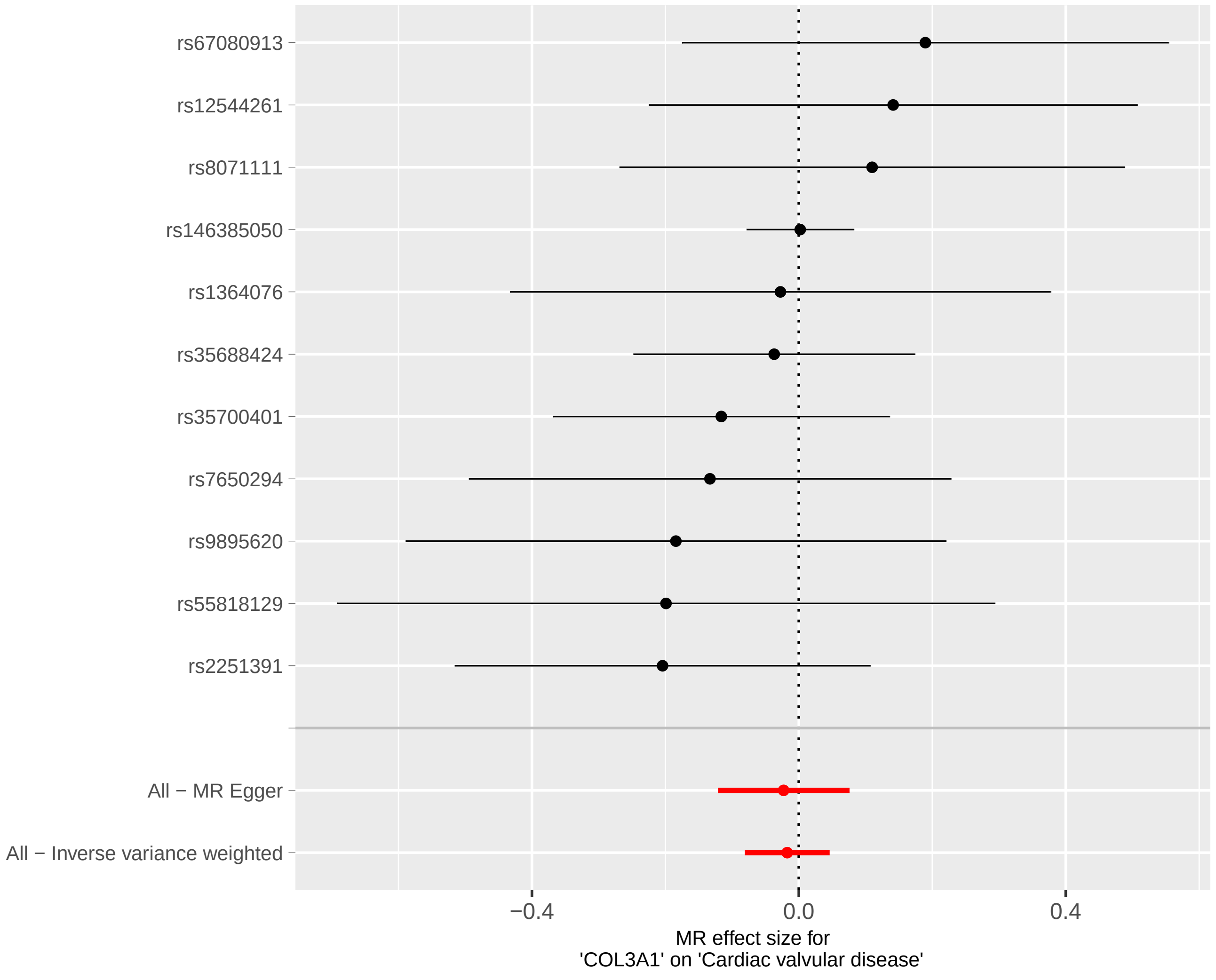
rs67080913
rs12544261
rs8071111
rs146385050
rs1364076
rs35688424
rs35700401
rs7650294
rs9895620
rs55818129
rs2251391
All − MR Egger
All − Inverse variance weighted
−0.4
0.0
0.4
MR effect size for
'COL3A1' on 'Cardiac valvular disease'

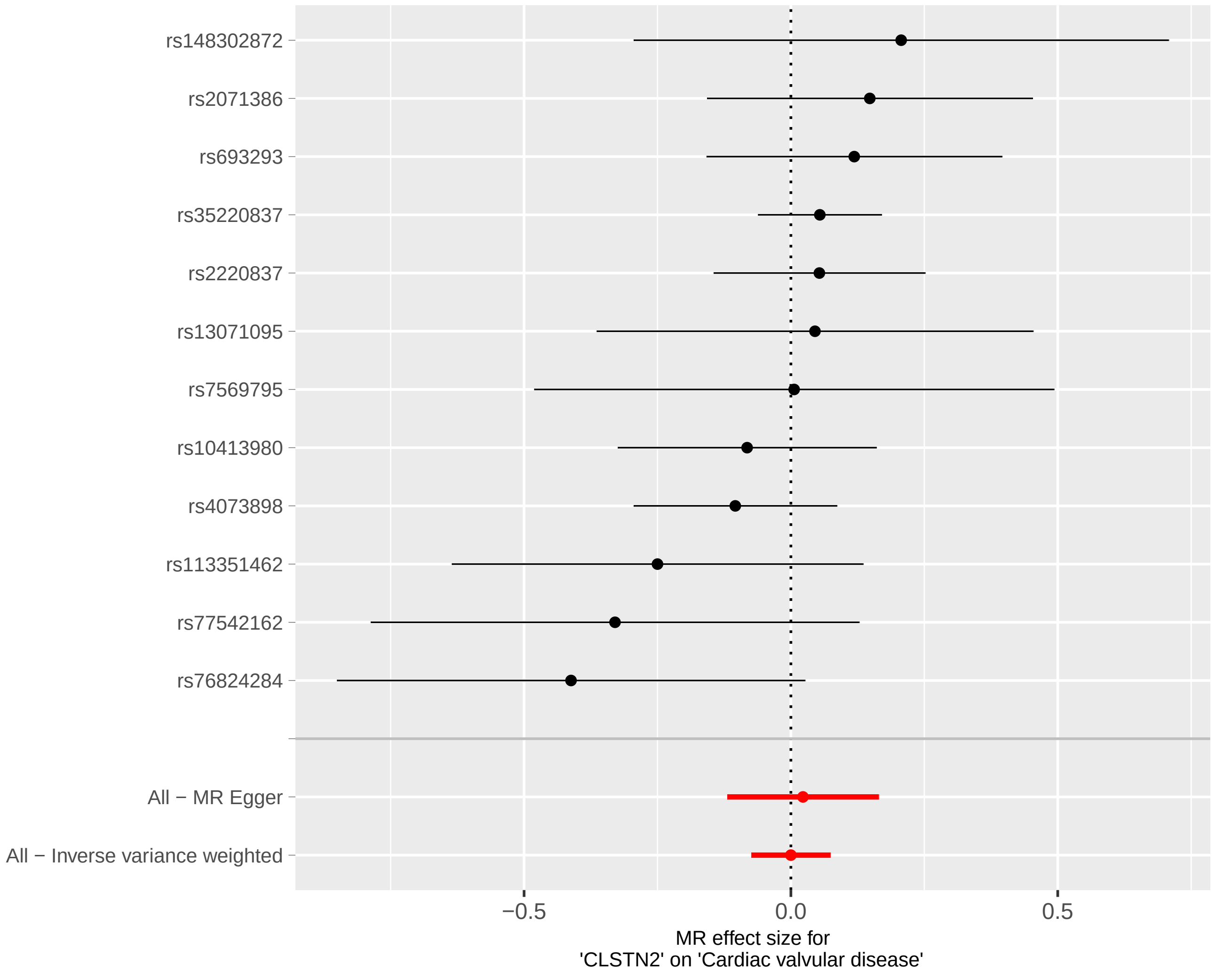
rs148302872
rs2071386
rs693293
rs35220837
rs2220837
rs13071095
rs7569795
rs10413980
rs4073898
rs113351462
rs77542162
rs76824284
All − MR Egger
All − Inverse variance weighted
−0.5
0.0
0.5
MR effect size for
'CLSTN2' on 'Cardiac valvular disease'

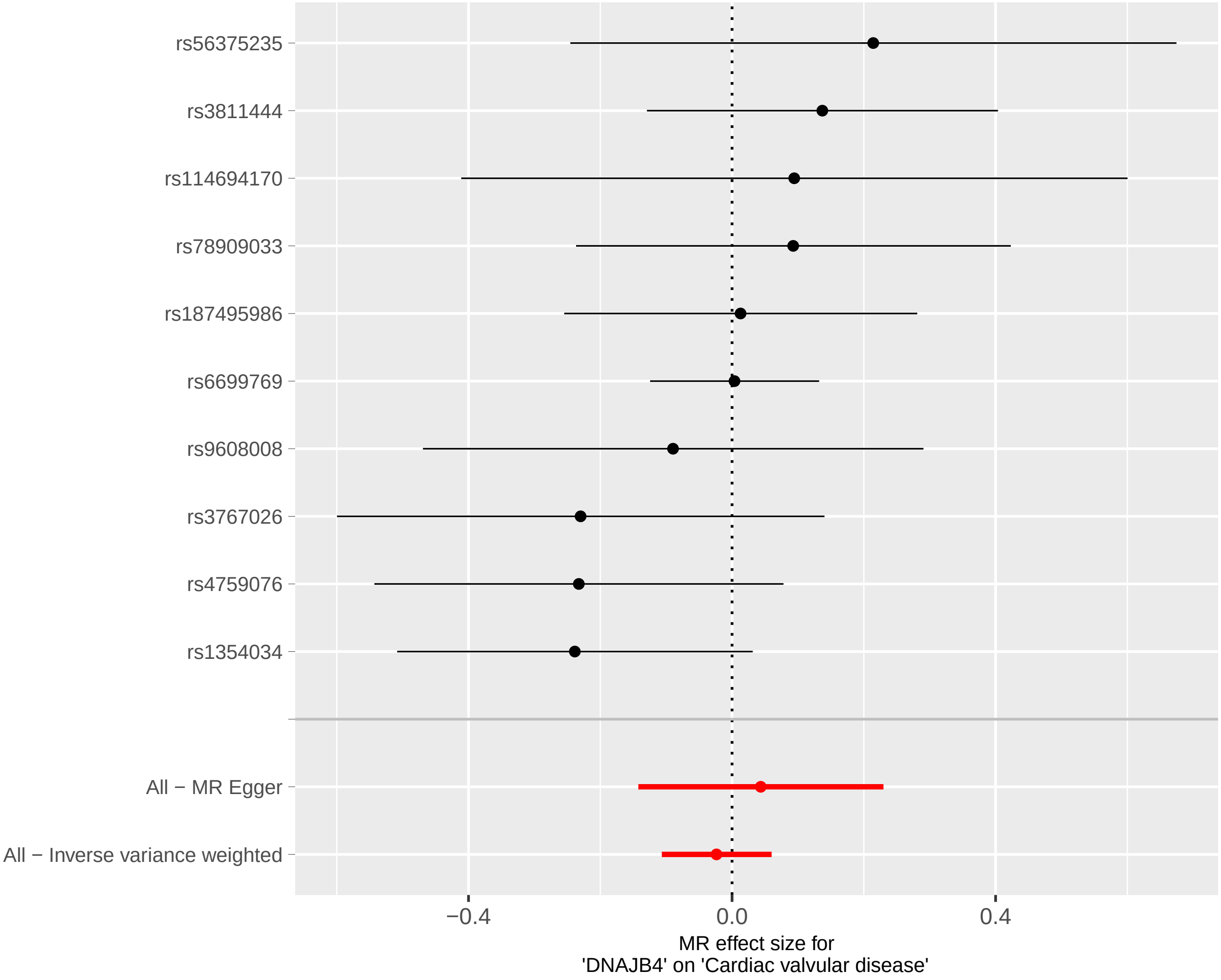

rs56375235
rs3811444
rs114694170
rs78909033
rs187495986
rs6699769
rs9608008
rs3767026
rs4759076
rs1354034
All − MR Egger
All − Inverse variance weighted
−0.4
0.0
0.4
MR effect size for
'DNAJB4' on 'Cardiac valvular disease'

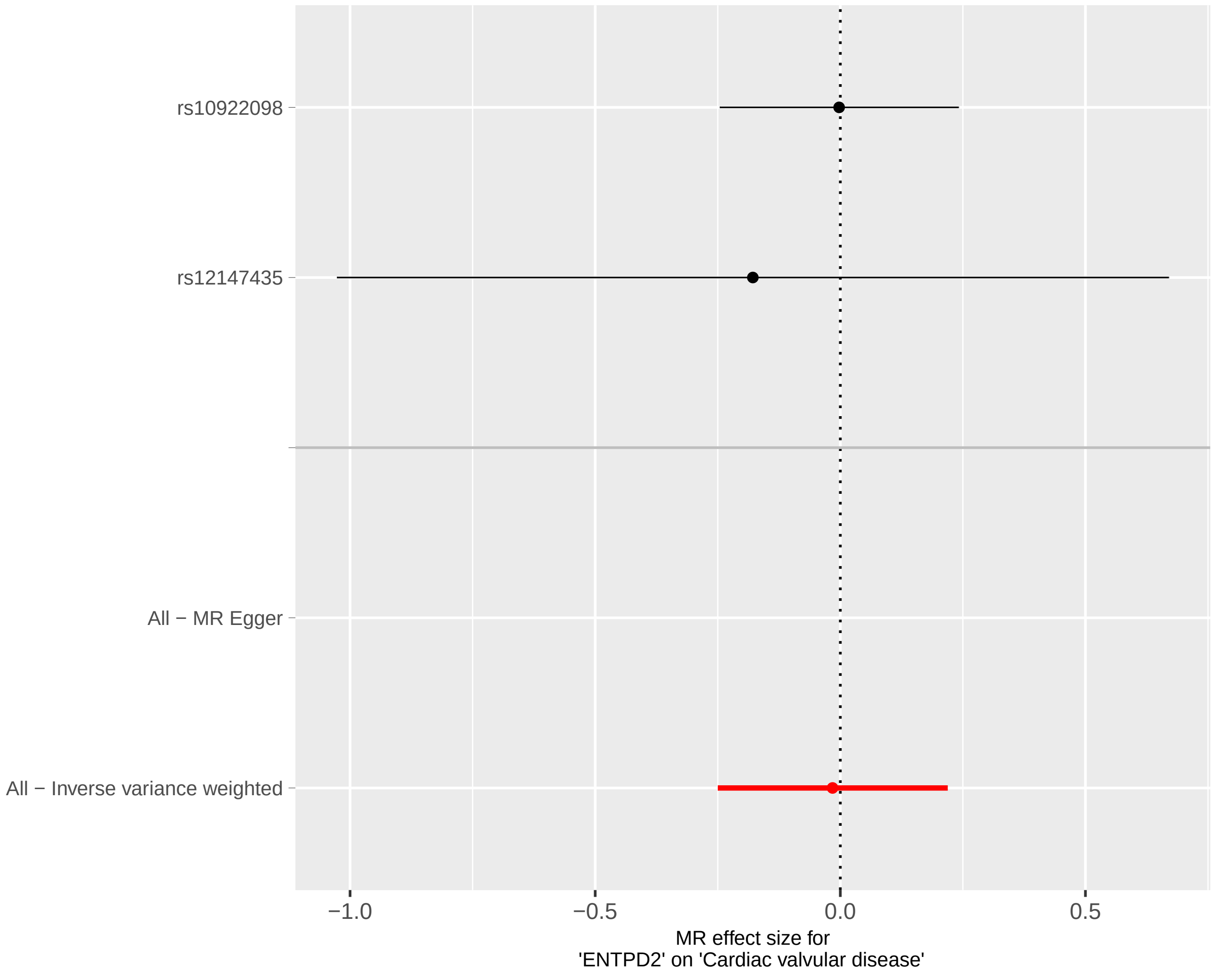
rs10922098
rs12147435
All − MR Egger
All − Inverse variance weighted
−1.0
−0.5
0.0
0.5
MR effect size for
'ENTPD2' on 'Cardiac valvular disease'

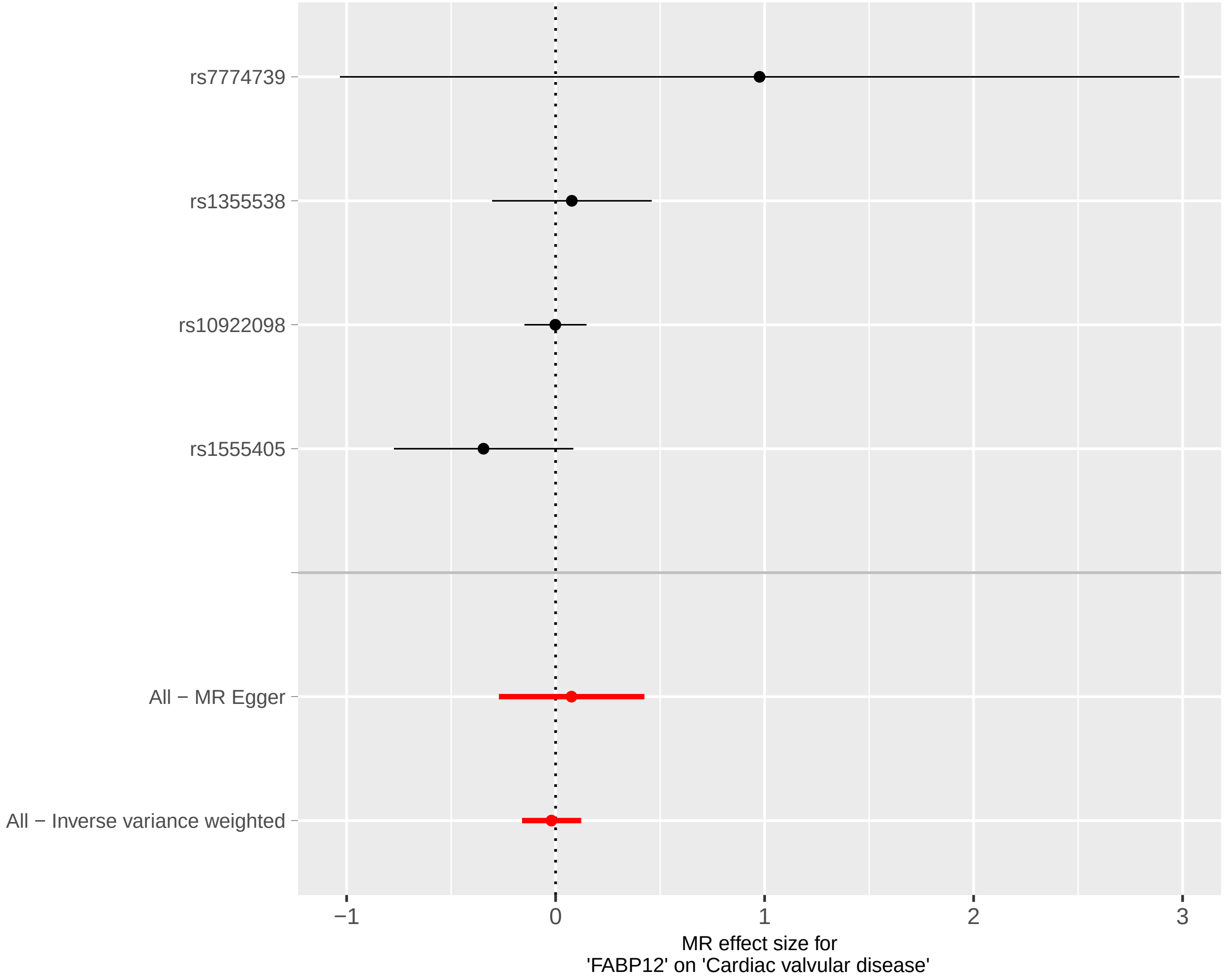

rs7774739
rs1355538
rs10922098
rs1555405
All − MR Egger
All − Inverse variance weighted
−1
0
1
2
3
MR effect size for
'FABP12' on 'Cardiac valvular disease'

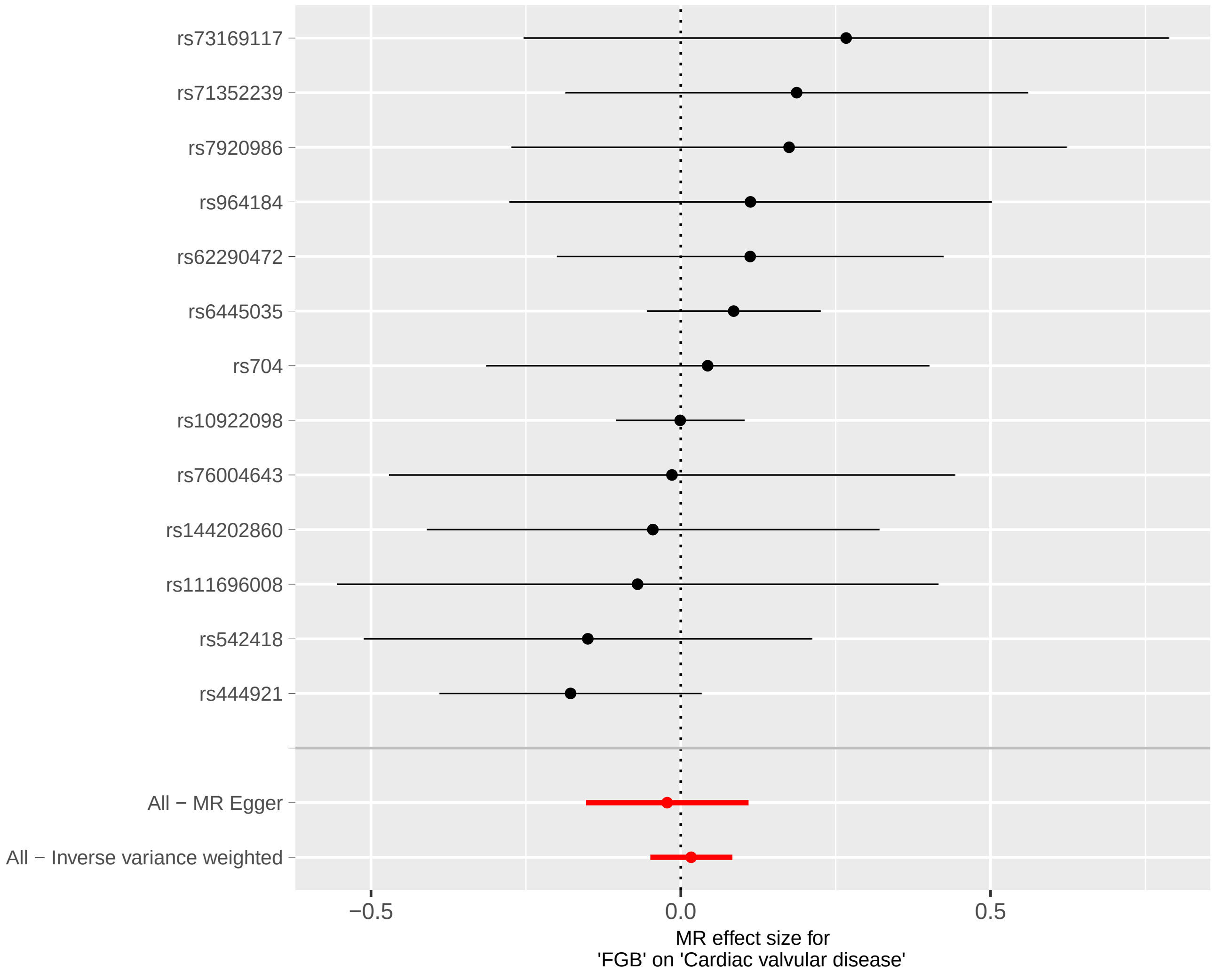
rs73169117
rs71352239
rs7920986
rs964184
rs62290472
rs6445035
rs704
rs10922098
rs76004643
rs144202860
rs111696008
rs542418
rs444921
All − MR Egger
All − Inverse variance weighted
−0.5
0.0
0.5
MR effect size for
'FGB' on 'Cardiac valvular disease'

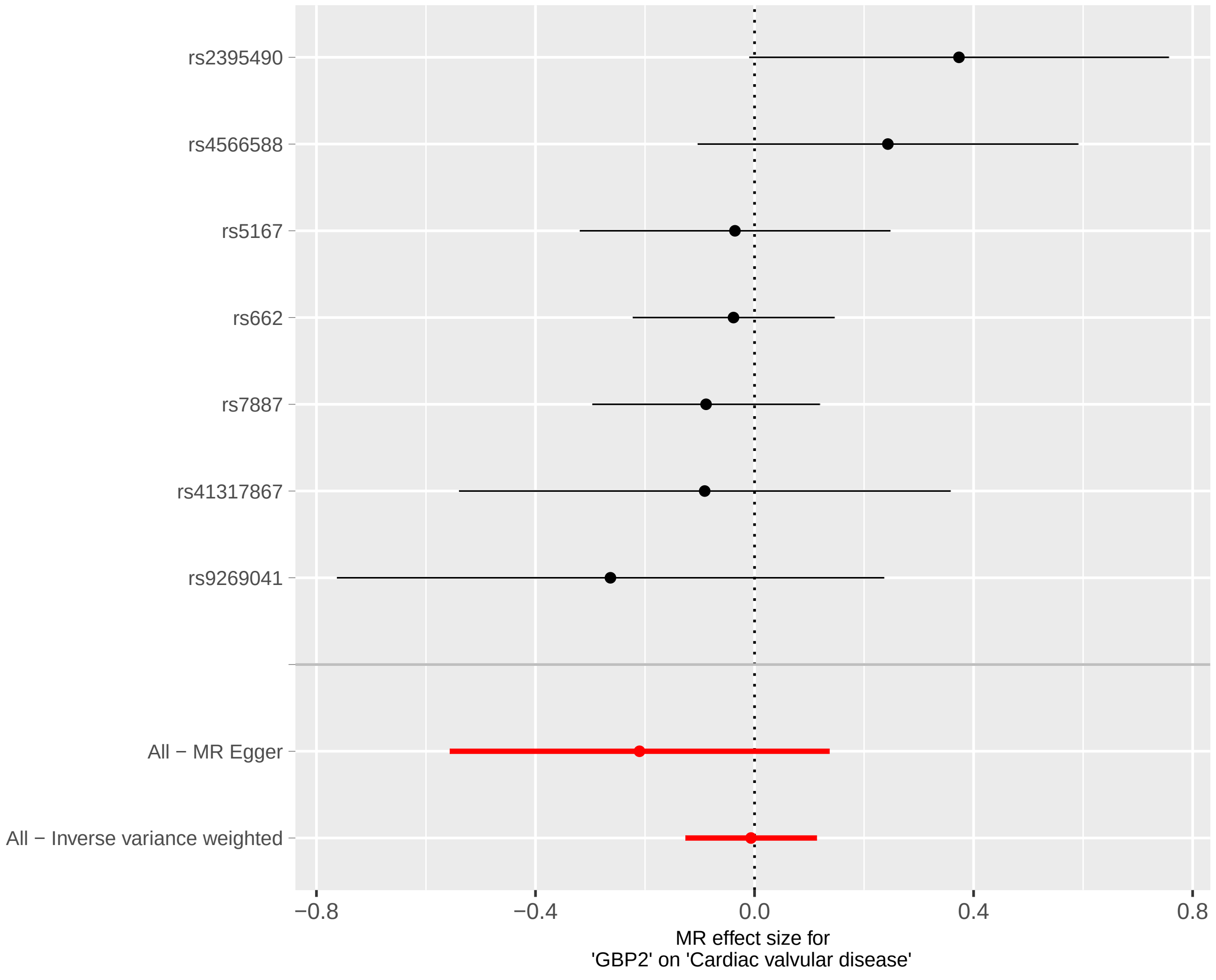

rs2395490
rs4566588
rs5167
rs662
rs7887
rs41317867
rs9269041
All − MR Egger
All − Inverse variance weighted
−0.8
−0.4
0.0
0.4
0.8
MR effect size for
'GBP2' on 'Cardiac valvular disease'

Insufficient number of SNPs

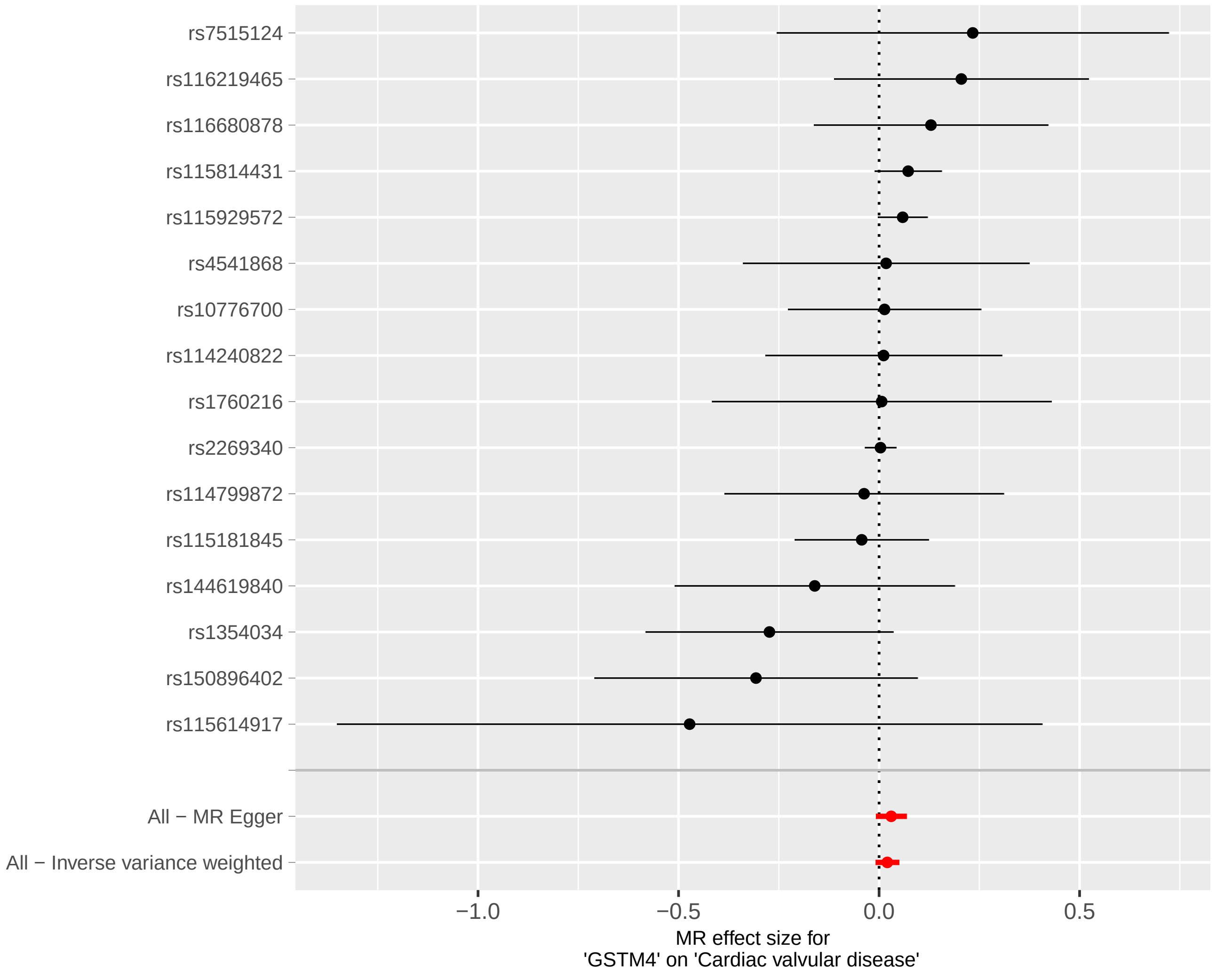

rs7515124
rs116219465
rs116680878
rs115814431
rs115929572
rs4541868
rs10776700
rs114240822
rs1760216
rs2269340
rs114799872
rs115181845
rs144619840
rs1354034
rs150896402
rs115614917
All − MR Egger
All − Inverse variance weighted
−1.0
−0.5
0.0
0.5
MR effect size for
'GSTM4' on 'Cardiac valvular disease'

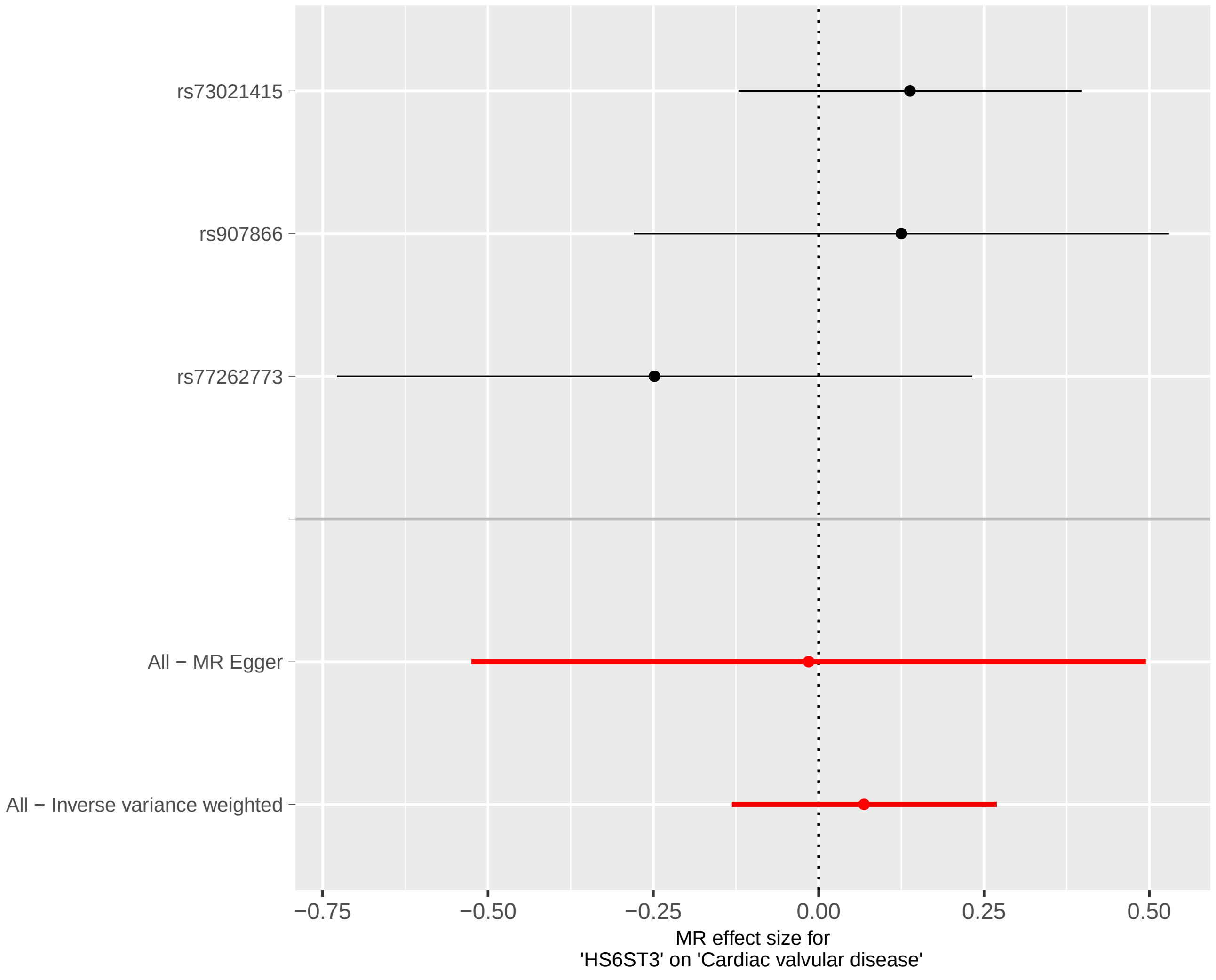
rs73021415
rs907866
rs77262773
All − MR Egger
All − Inverse variance weighted
−0.75
−0.50
−0.25
0.00
0.25
0.50
MR effect size for
'HS6ST3' on 'Cardiac valvular disease'

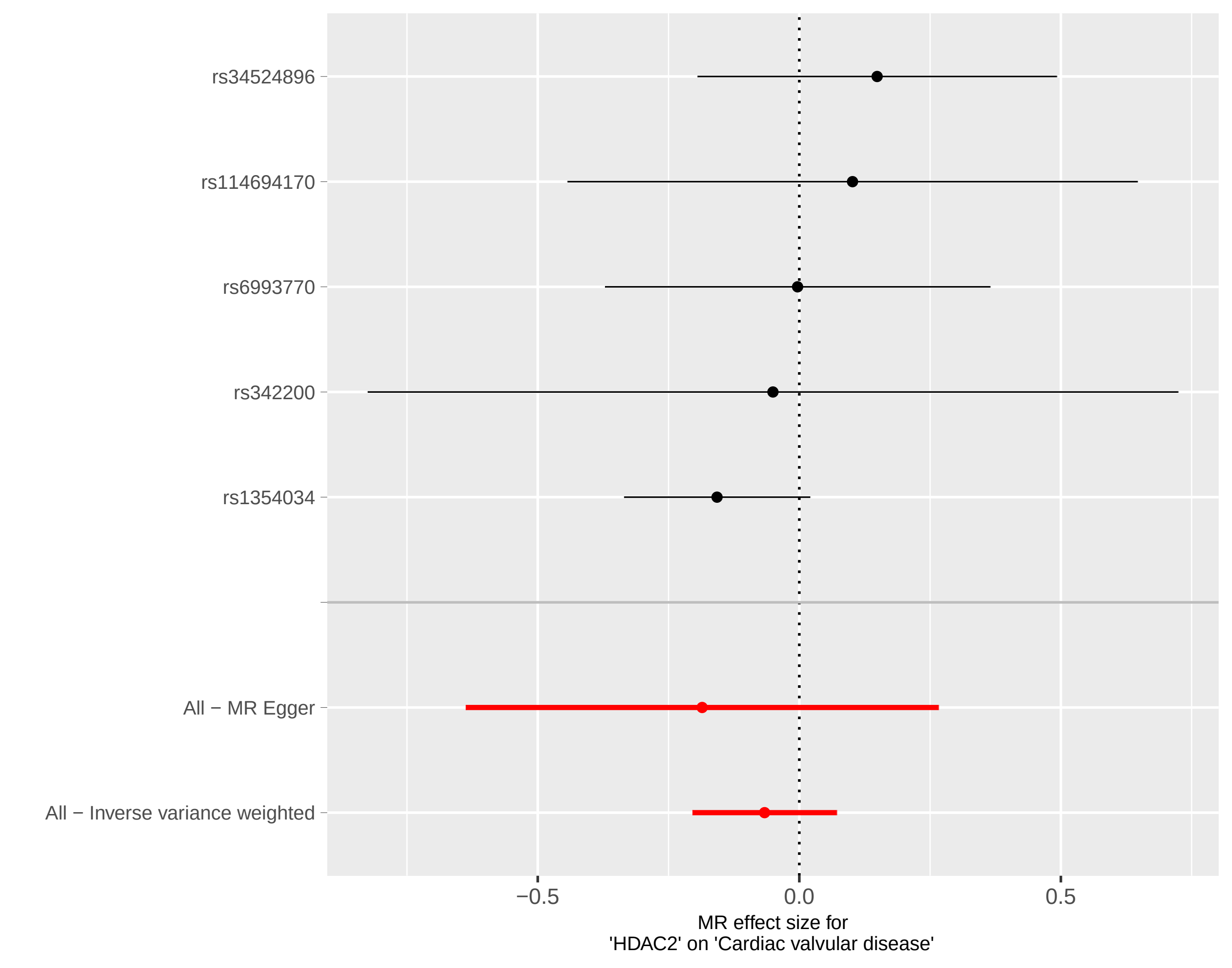
rs34524896
rs114694170
rs6993770
rs342200
rs1354034
All − MR Egger
All − Inverse variance weighted
−0.5
0.0
0.5
MR effect size for
'HDAC2' on 'Cardiac valvular disease'

Insufficient number of SNPs

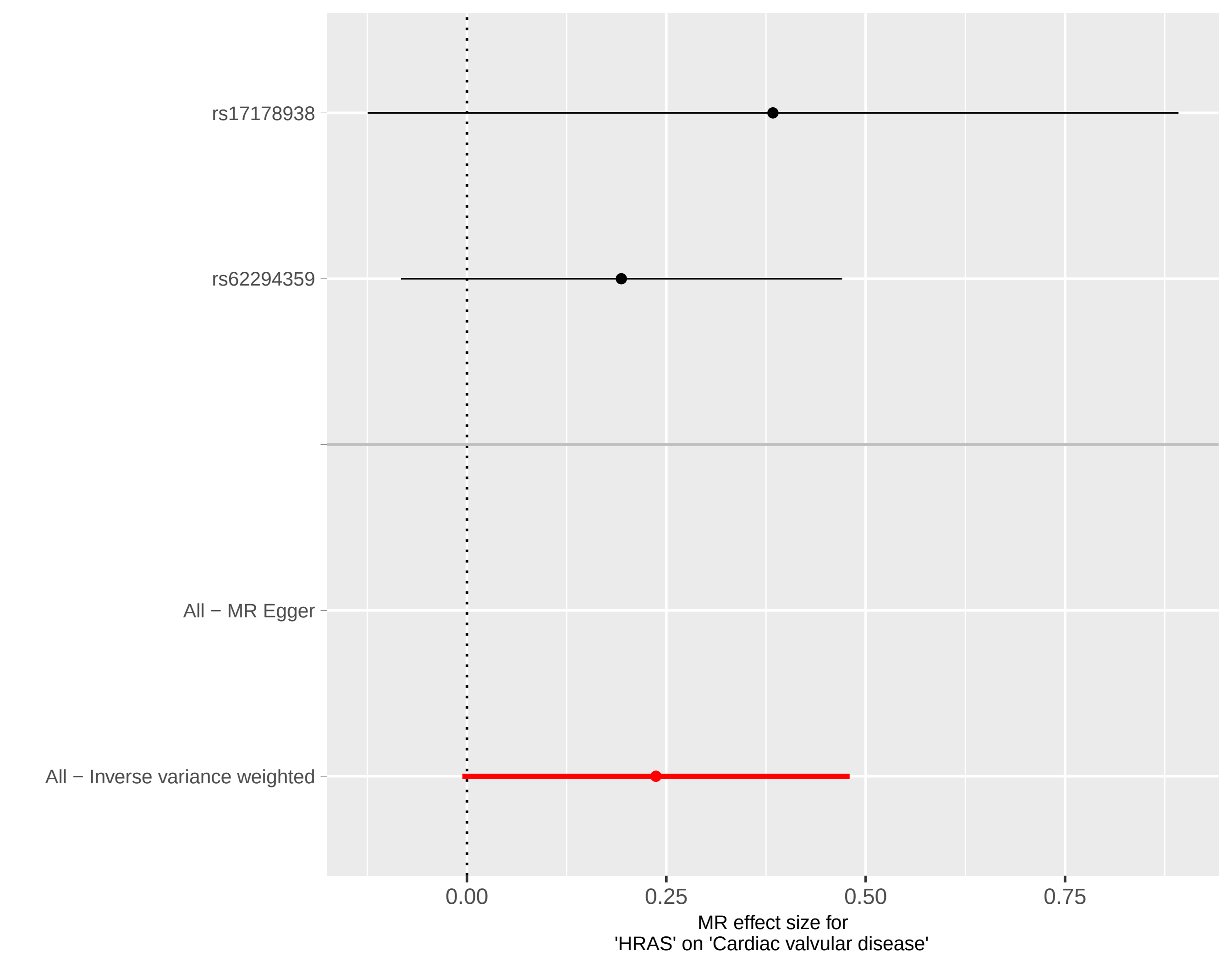
rs17178938
rs62294359
All − MR Egger
All − Inverse variance weighted
0.00
0.25
0.50
0.75
MR effect size for
'HRAS' on 'Cardiac valvular disease'

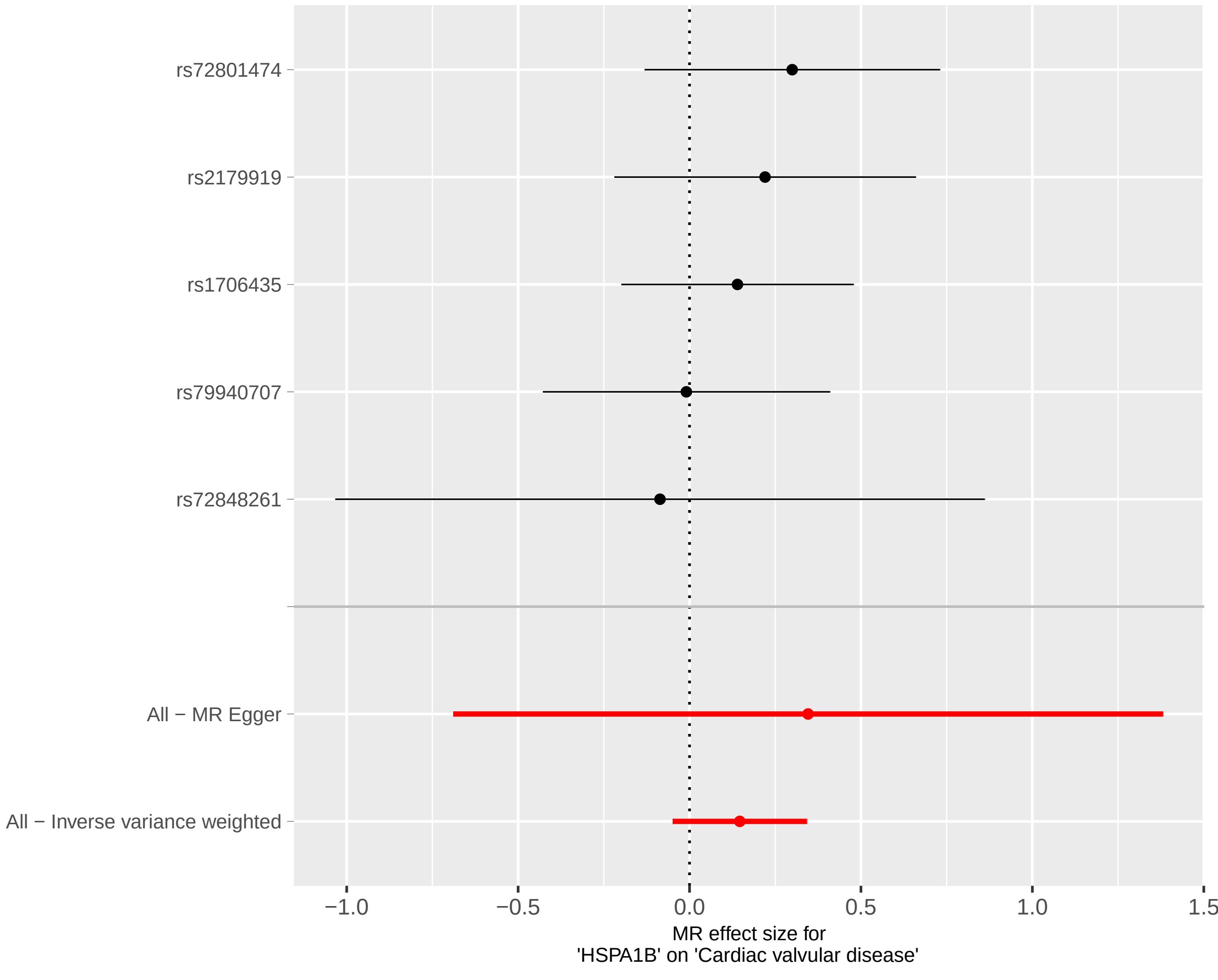
rs72801474
rs2179919
rs1706435
rs79940707
rs72848261
All − MR Egger
All − Inverse variance weighted
−1.0
−0.5
0.0
0.5
1.0
1.5
MR effect size for
'HSPA1B' on 'Cardiac valvular disease'

Insufficient number of SNPs

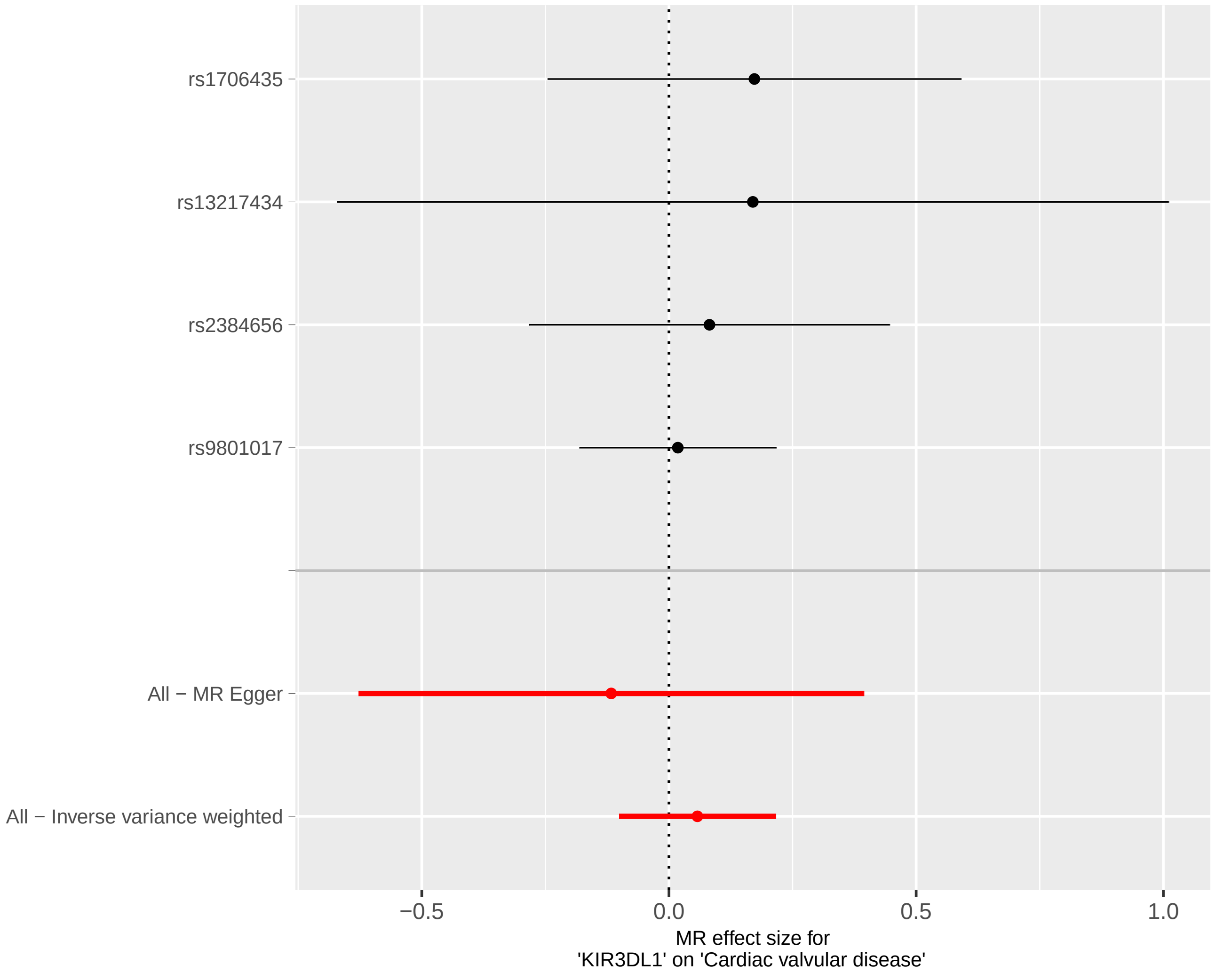
rs1706435
rs13217434
rs2384656
rs9801017
All − MR Egger
All − Inverse variance weighted
−0.5
0.0
0.5
1.0
MR effect size for
'KIR3DL1' on 'Cardiac valvular disease'

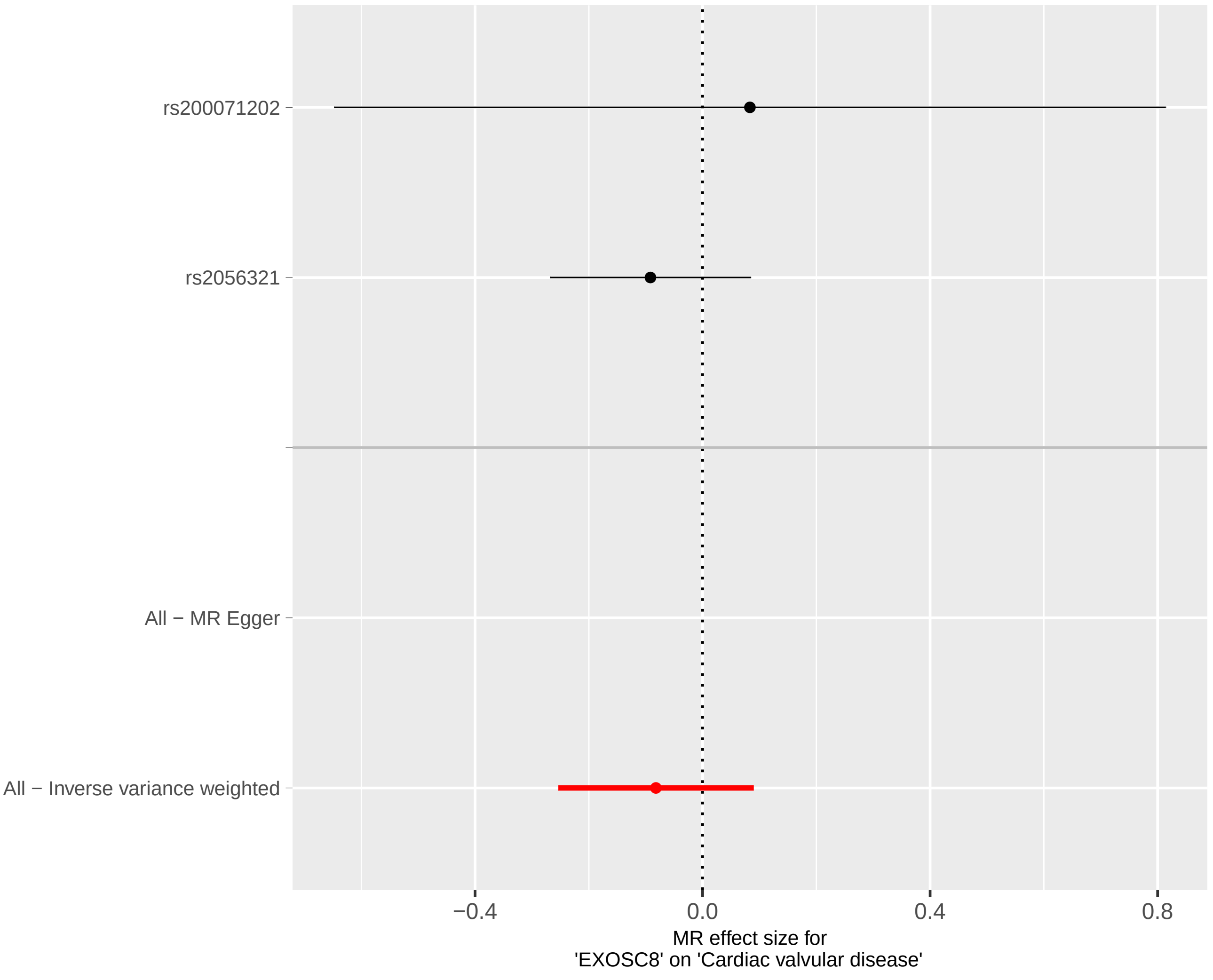
rs200071202
rs2056321
All − MR Egger
All − Inverse variance weighted
−0.4
0.0
0.4
0.8
MR effect size for
'EXOSC8' on 'Cardiac valvular disease'

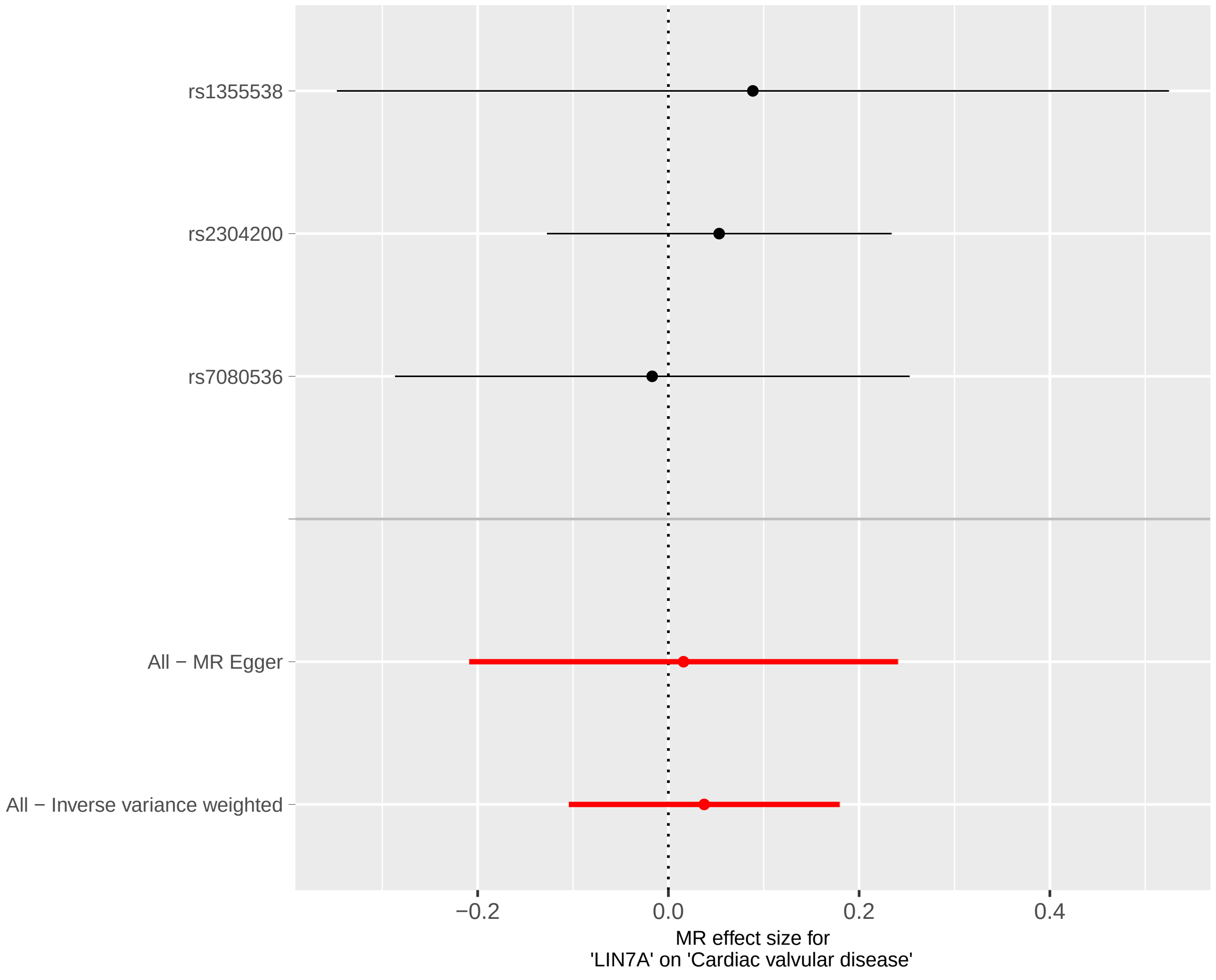
rs1355538
rs2304200
rs7080536
All − MR Egger
All − Inverse variance weighted
−0.2
0.0
0.2
0.4
MR effect size for
'LIN7A' on 'Cardiac valvular disease'

Insufficient number of SNPs

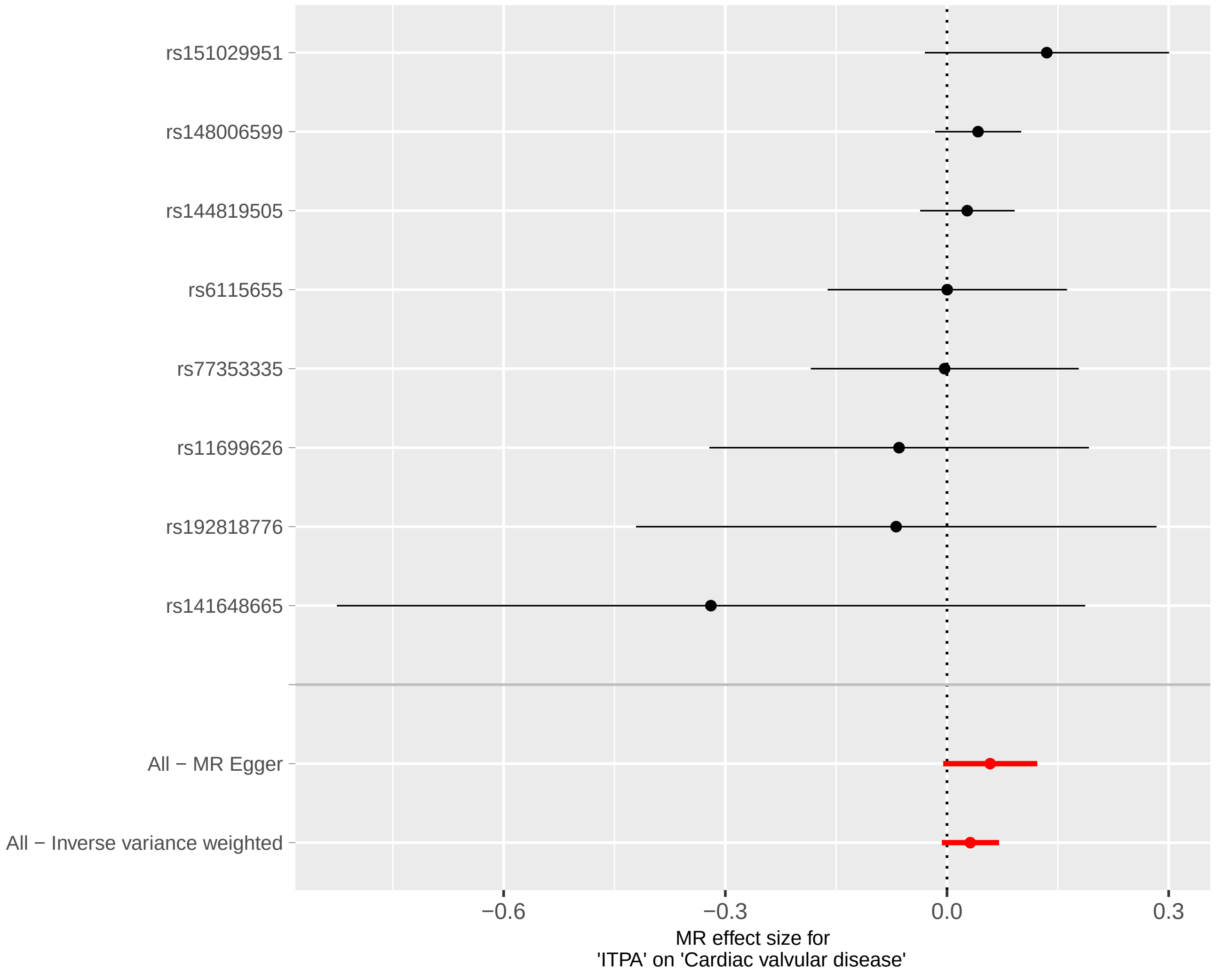
rs151029951
rs148006599
rs144819505
rs6115655
rs77353335
rs11699626
rs192818776
rs141648665
All − MR Egger
All − Inverse variance weighted
−0.6
−0.3
0.0
0.3
MR effect size for
'ITPA' on 'Cardiac valvular disease'

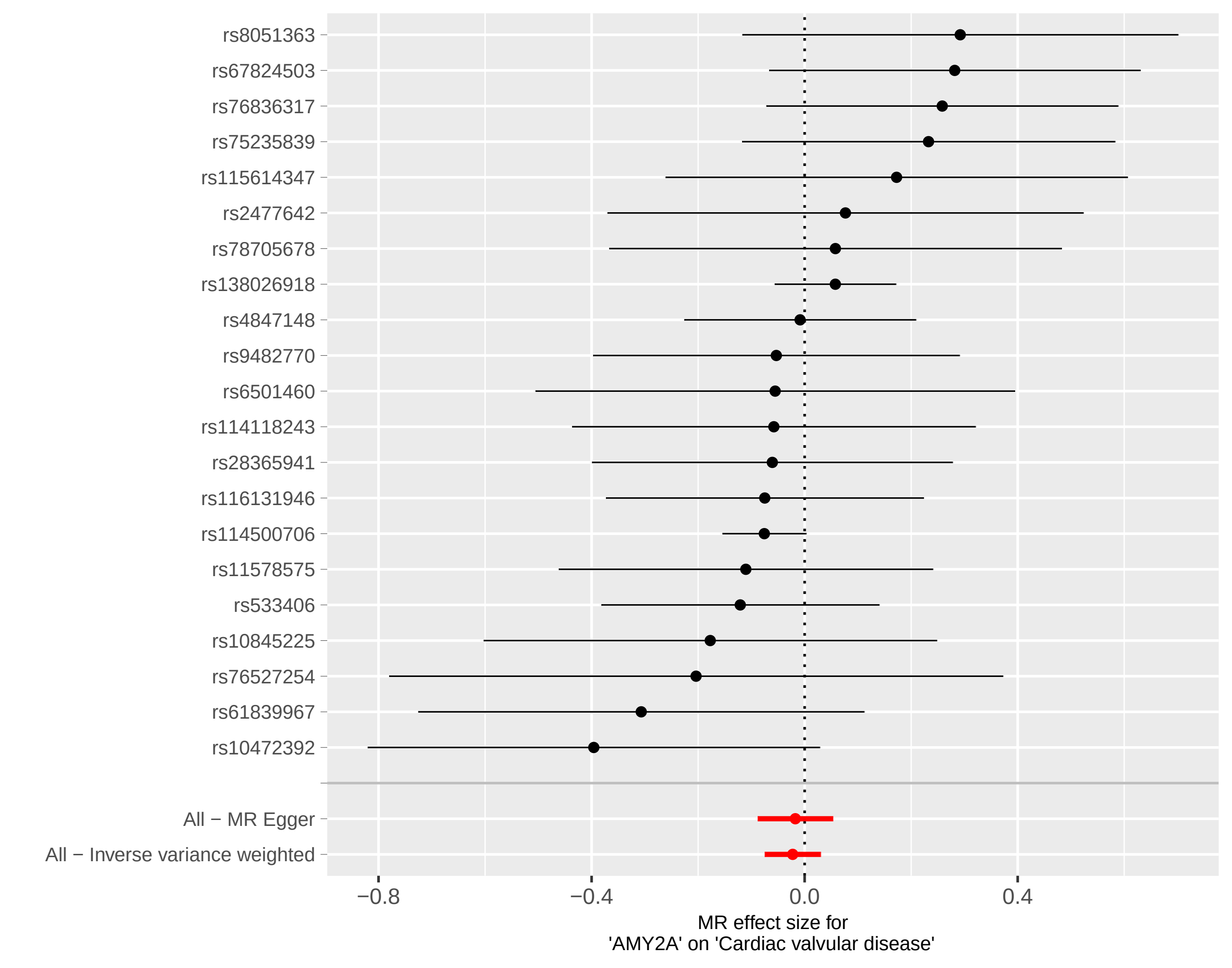
rs8051363
rs67824503
rs76836317
rs75235839
rs115614347
rs2477642
rs78705678
rs138026918
rs4847148
rs9482770
rs6501460
rs114118243
rs28365941
rs116131946
rs114500706
rs11578575
rs533406
rs10845225
rs76527254
rs61839967
rs10472392
All − MR Egger
All − Inverse variance weighted
−0.8
−0.4
0.0
0.4
MR effect size for
'AMY2A' on 'Cardiac valvular disease'

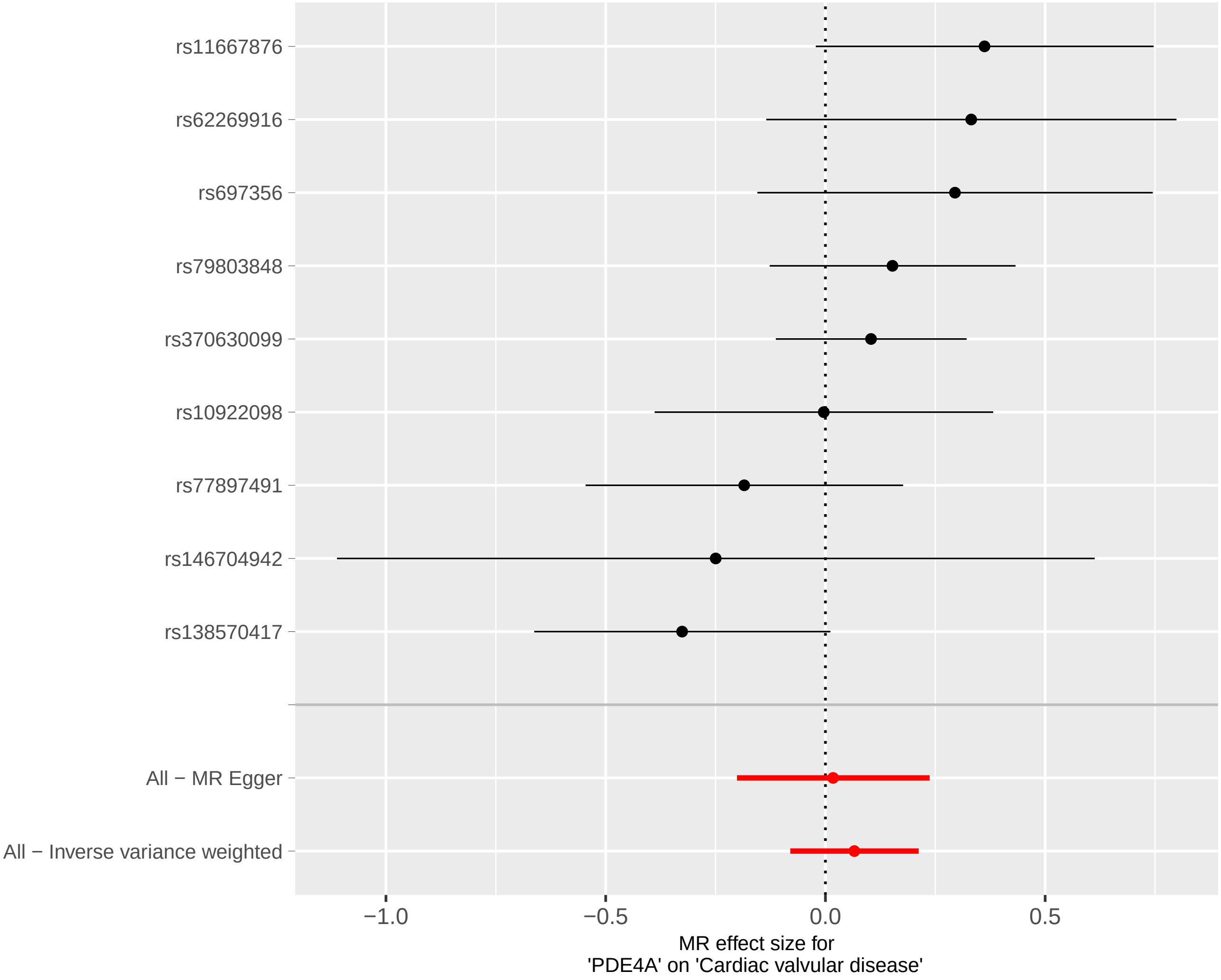
rs11667876
rs62269916
rs697356
rs79803848
rs370630099
rs10922098
rs77897491
rs146704942
rs138570417
All − MR Egger
All − Inverse variance weighted
−1.0
−0.5
0.0
0.5
MR effect size for
'PDE4A' on 'Cardiac valvular disease'

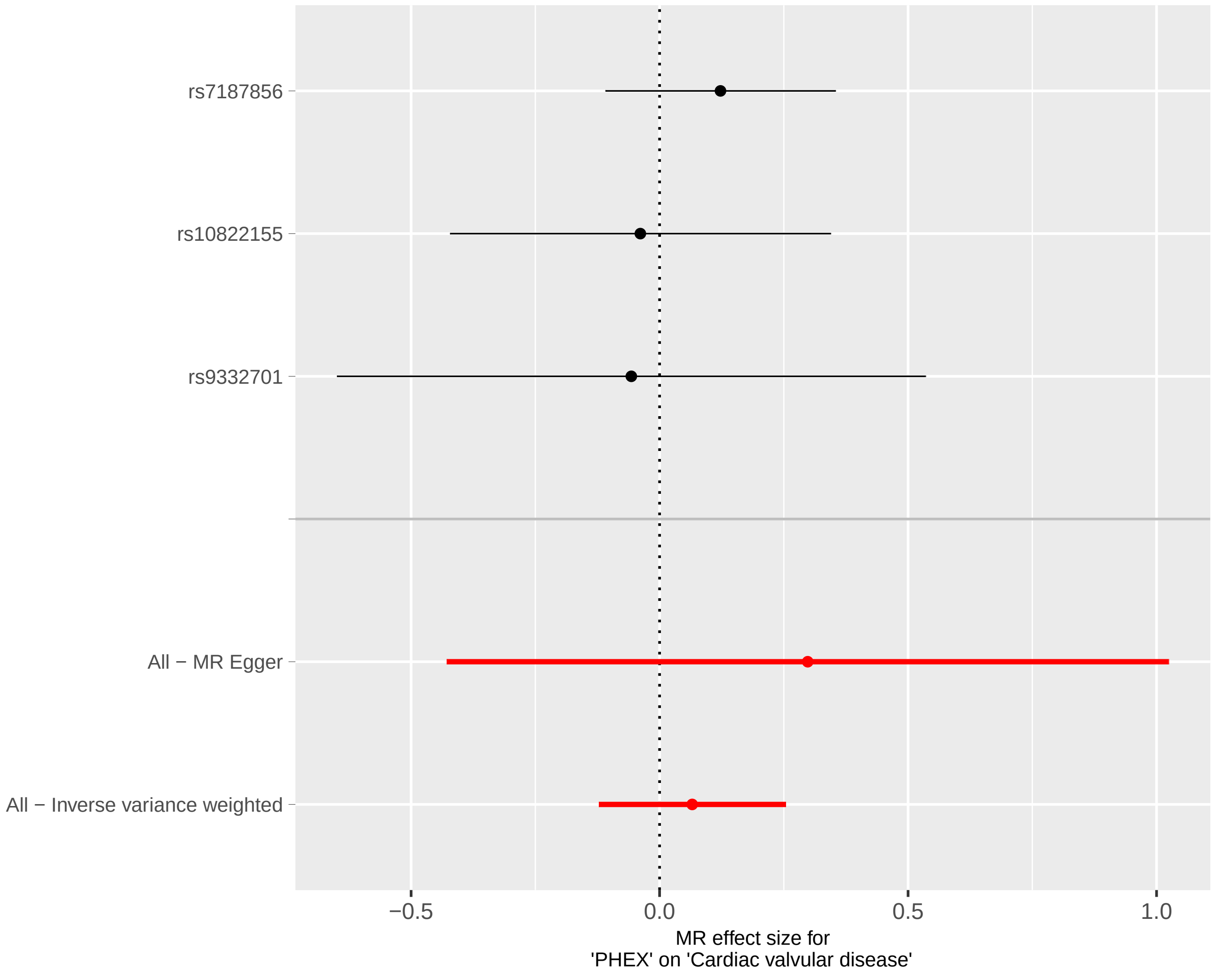
rs7187856
rs10822155
rs9332701
All − MR Egger
All − Inverse variance weighted
−0.5
0.0
0.5
1.0
MR effect size for
'PHEX' on 'Cardiac valvular disease'

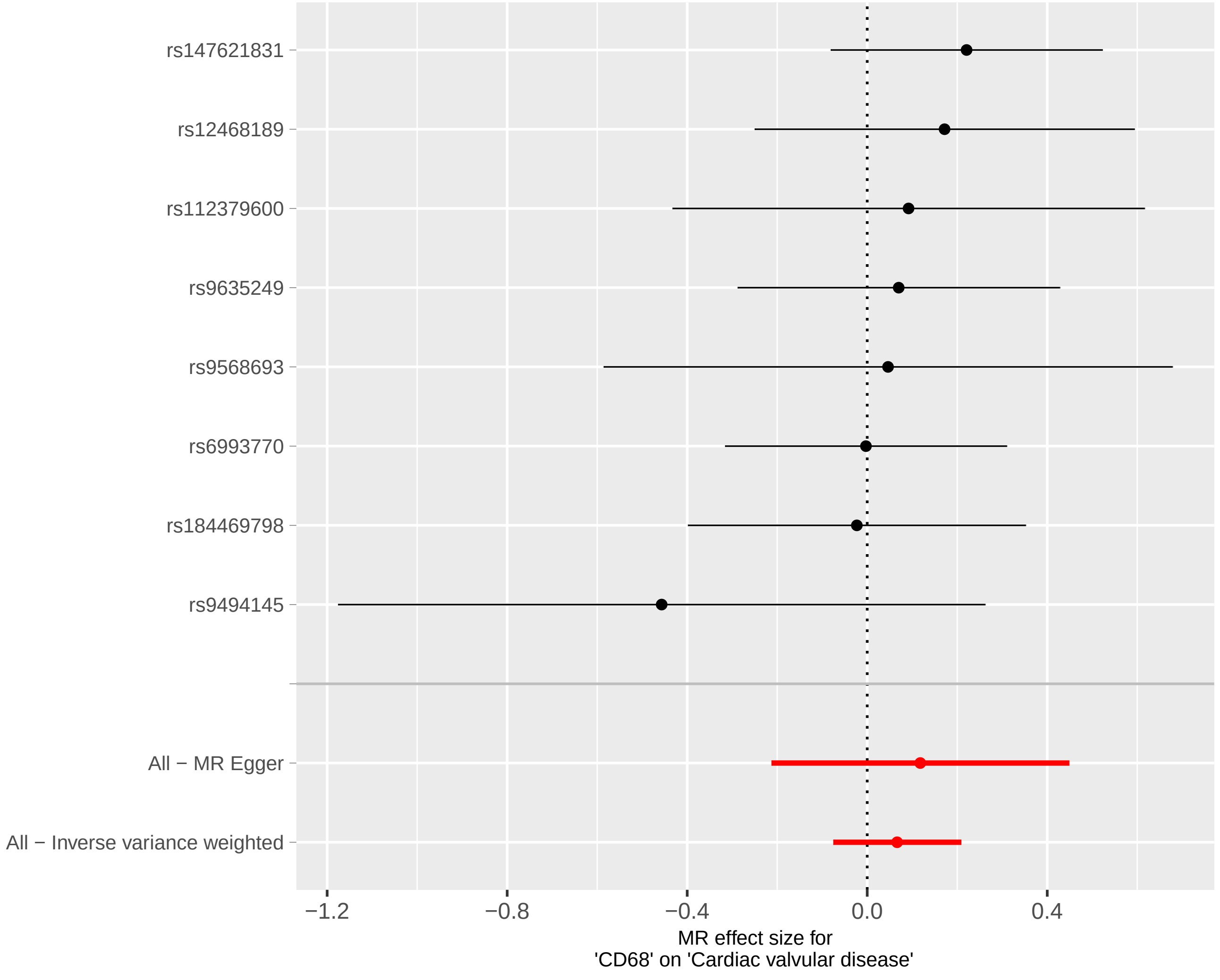
rs147621831
rs12468189
rs112379600
rs9635249
rs9568693
rs6993770
rs184469798
rs9494145
All − MR Egger
All − Inverse variance weighted
−1.2
−0.8
−0.4
0.0
0.4
MR effect size for
'CD68' on 'Cardiac valvular disease'

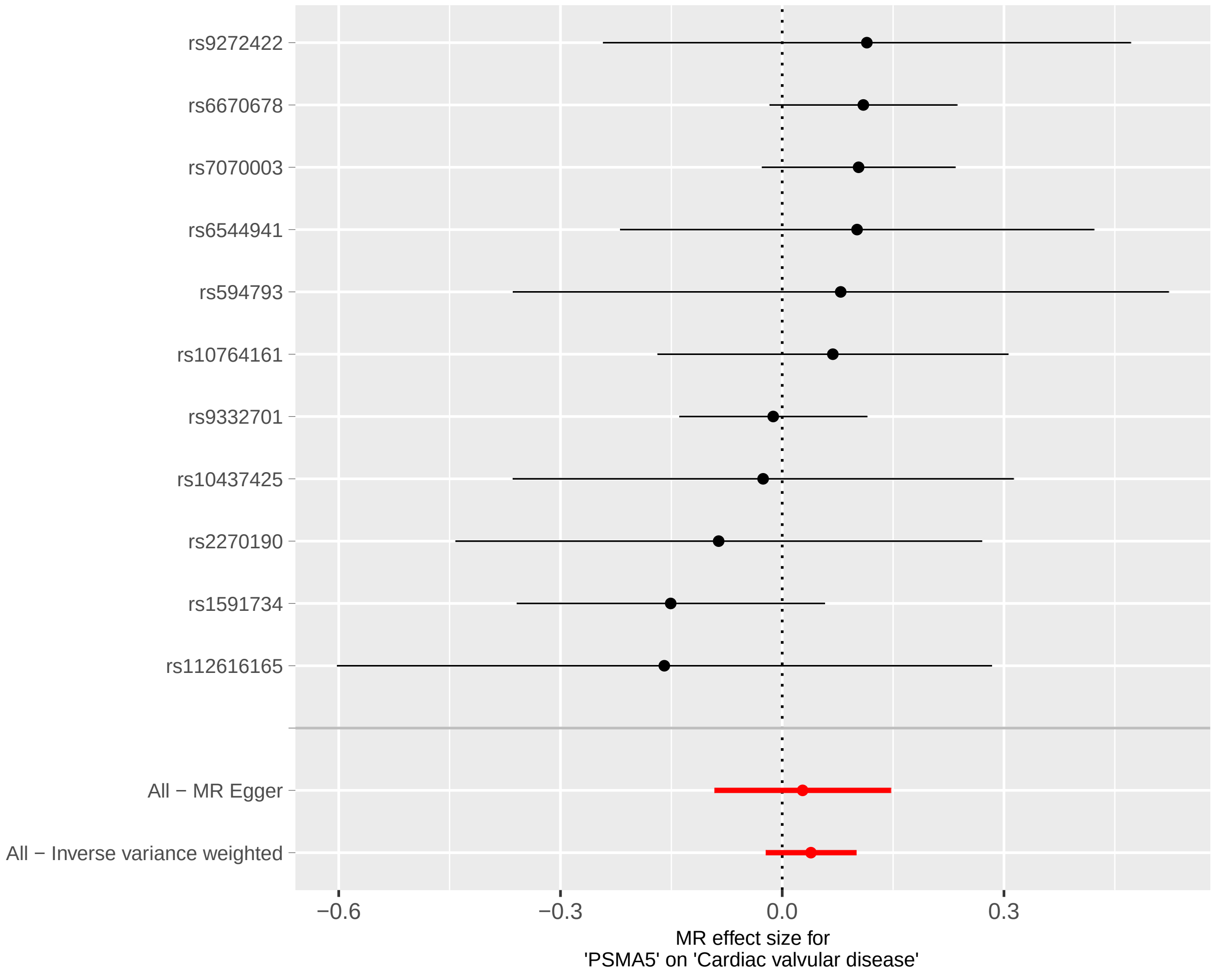

rs9272422
rs6670678
rs7070003
rs6544941
rs594793
rs10764161
rs9332701
rs10437425
rs2270190
rs1591734
rs112616165
All − MR Egger
All − Inverse variance weighted
−0.6
−0.3
0.0
0.3
MR effect size for
'PSMA5' on 'Cardiac valvular disease'

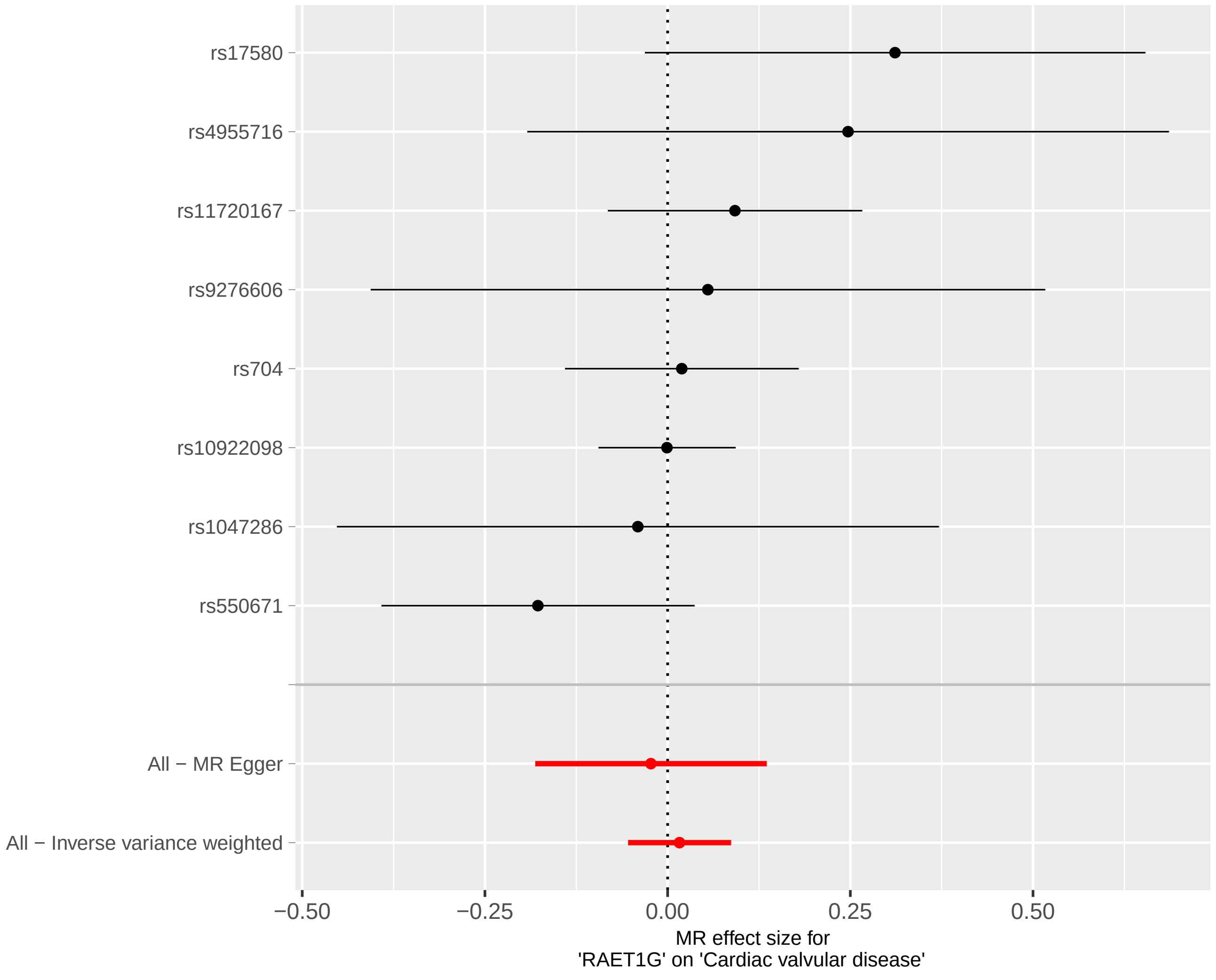

rs17580
rs4955716
rs11720167
rs9276606
rs704
rs10922098
rs1047286
rs550671
All − MR Egger
All − Inverse variance weighted
−0.50
−0.25
0.00
0.25
0.50
MR effect size for
'RAET1G' on 'Cardiac valvular disease'

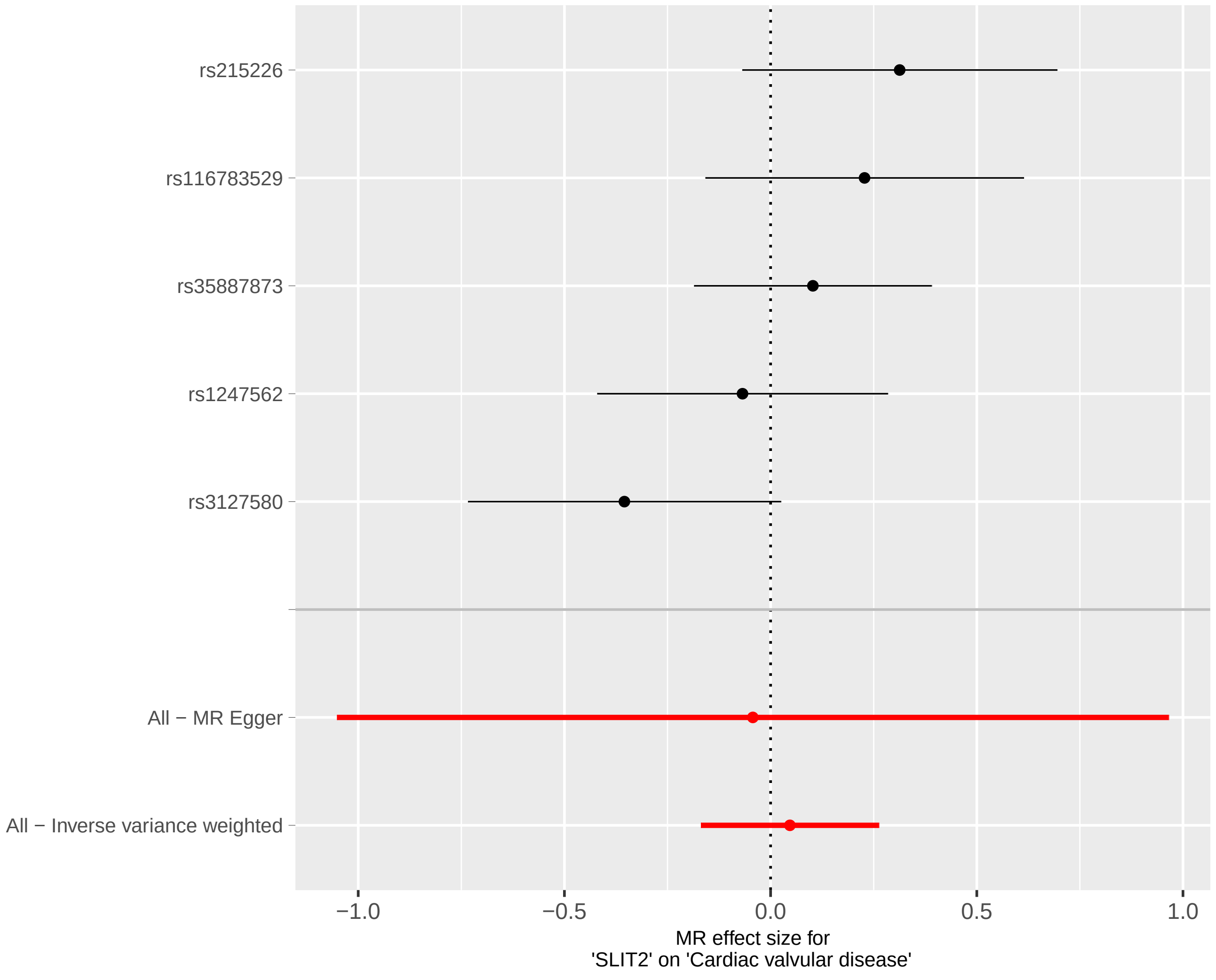
rs215226
rs116783529
rs35887873
rs1247562
rs3127580
All − MR Egger
All − Inverse variance weighted
−1.0
−0.5
0.0
0.5
1.0
MR effect size for
'SLIT2' on 'Cardiac valvular disease'

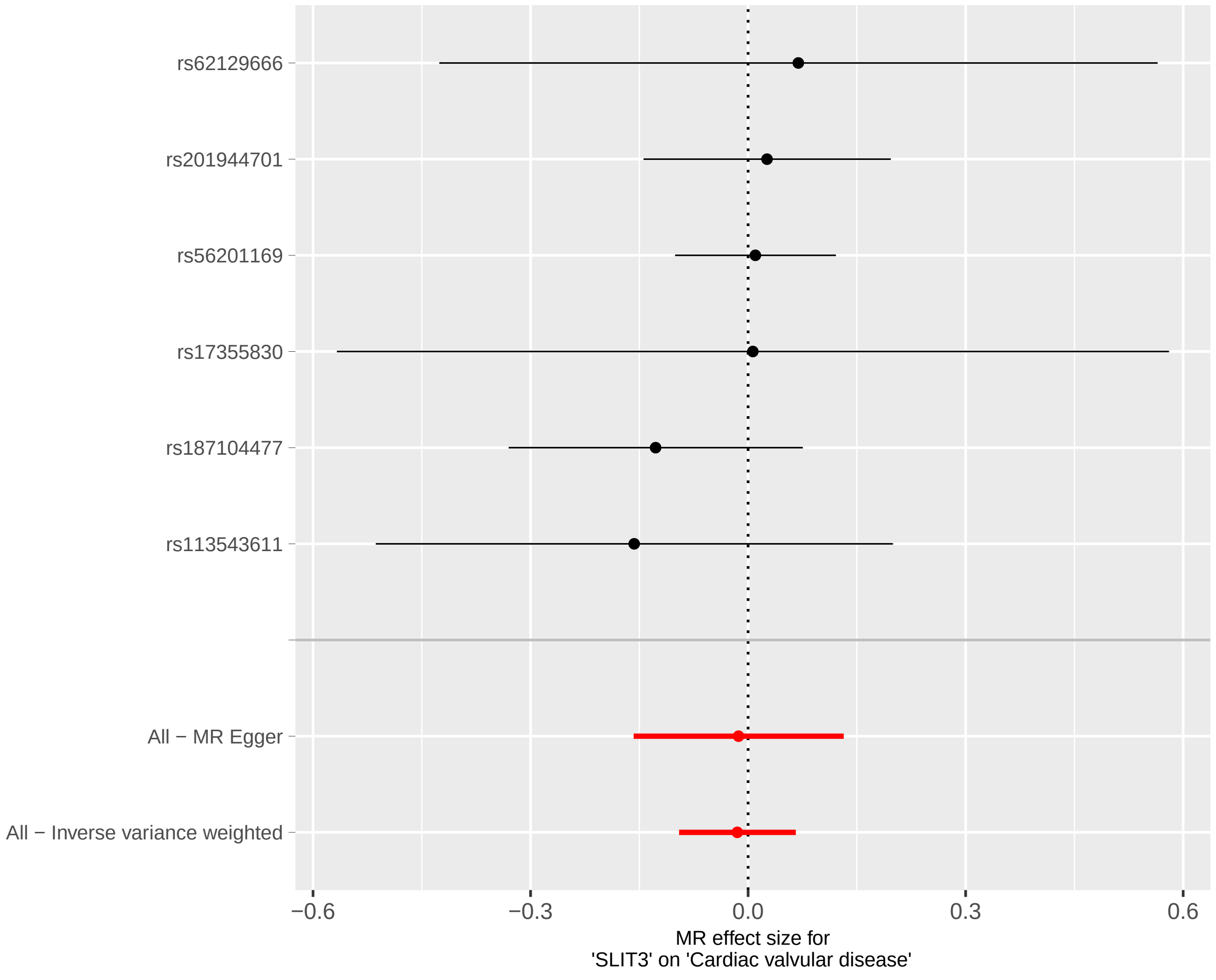
rs62129666
rs201944701
rs56201169
rs17355830
rs187104477
rs113543611
All − MR Egger
All − Inverse variance weighted
−0.6
−0.3
0.0
0.3
0.6
MR effect size for
'SLIT3' on 'Cardiac valvular disease'

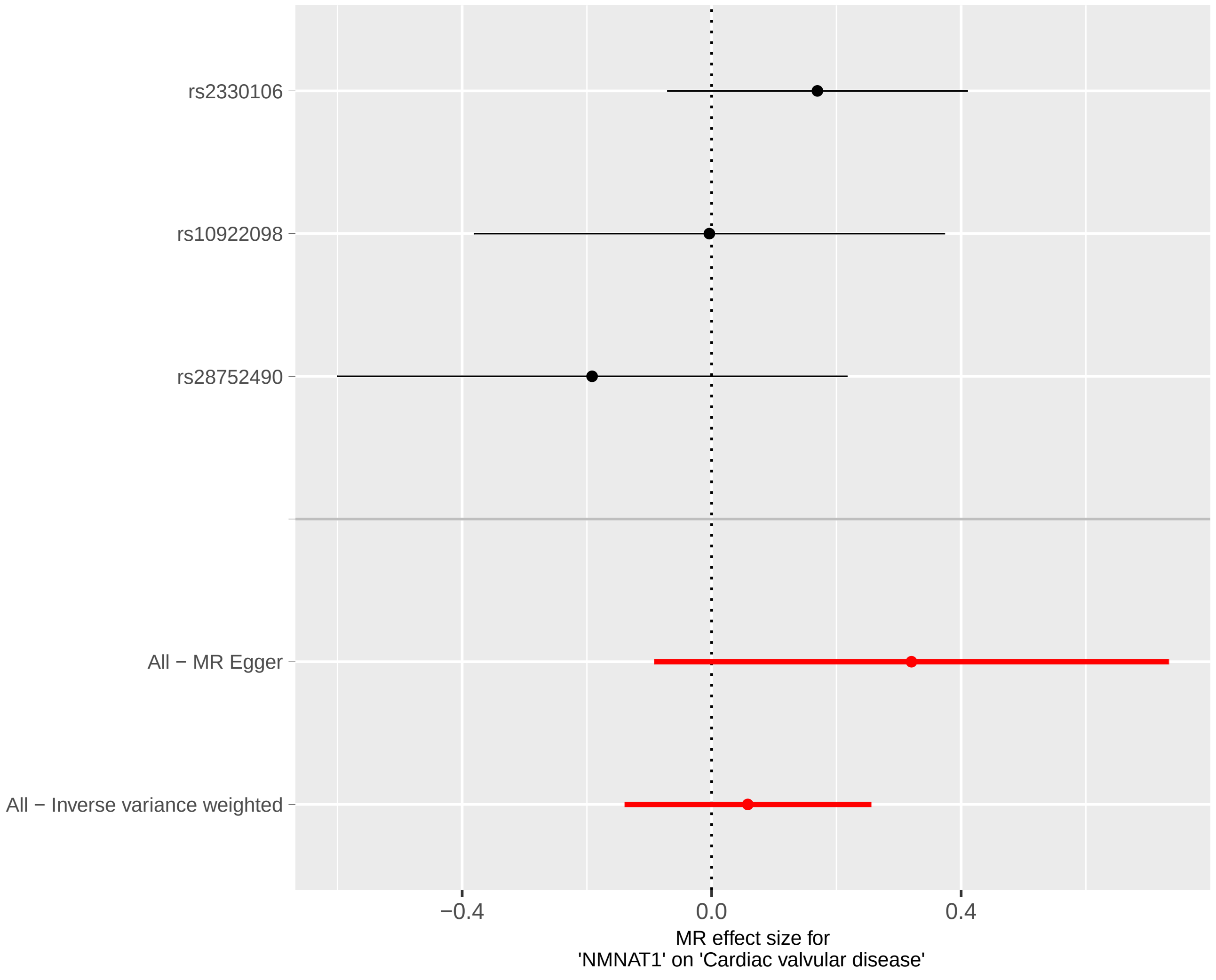
rs2330106
rs10922098
rs28752490
All − MR Egger
All − Inverse variance weighted
−0.4
0.0
0.4
MR effect size for
'NMNAT1' on 'Cardiac valvular disease'

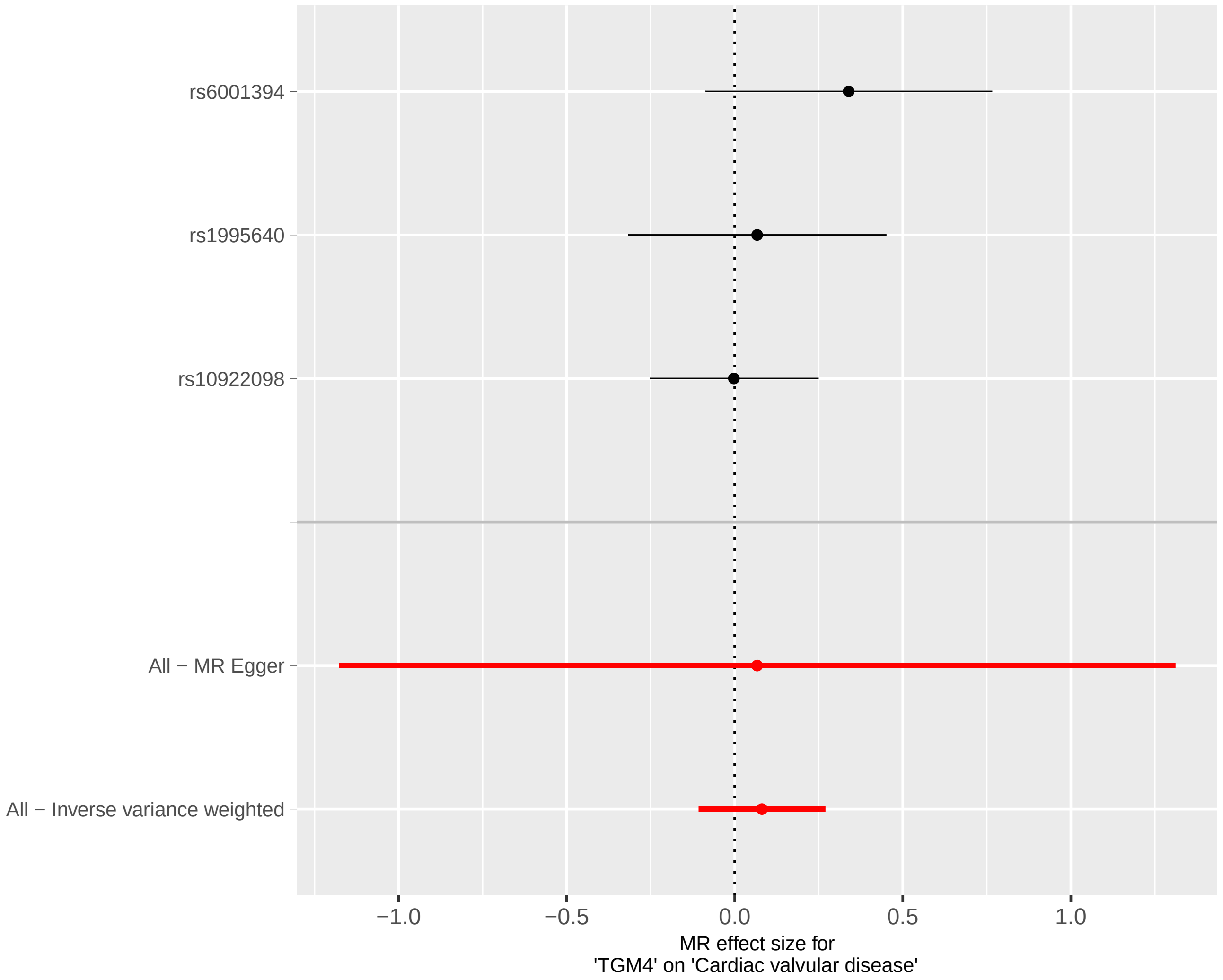

rs6001394
rs1995640
rs10922098
All − MR Egger
All − Inverse variance weighted
−1.0
−0.5
0.0
0.5
1.0
MR effect size for
'TGM4' on 'Cardiac valvular disease'

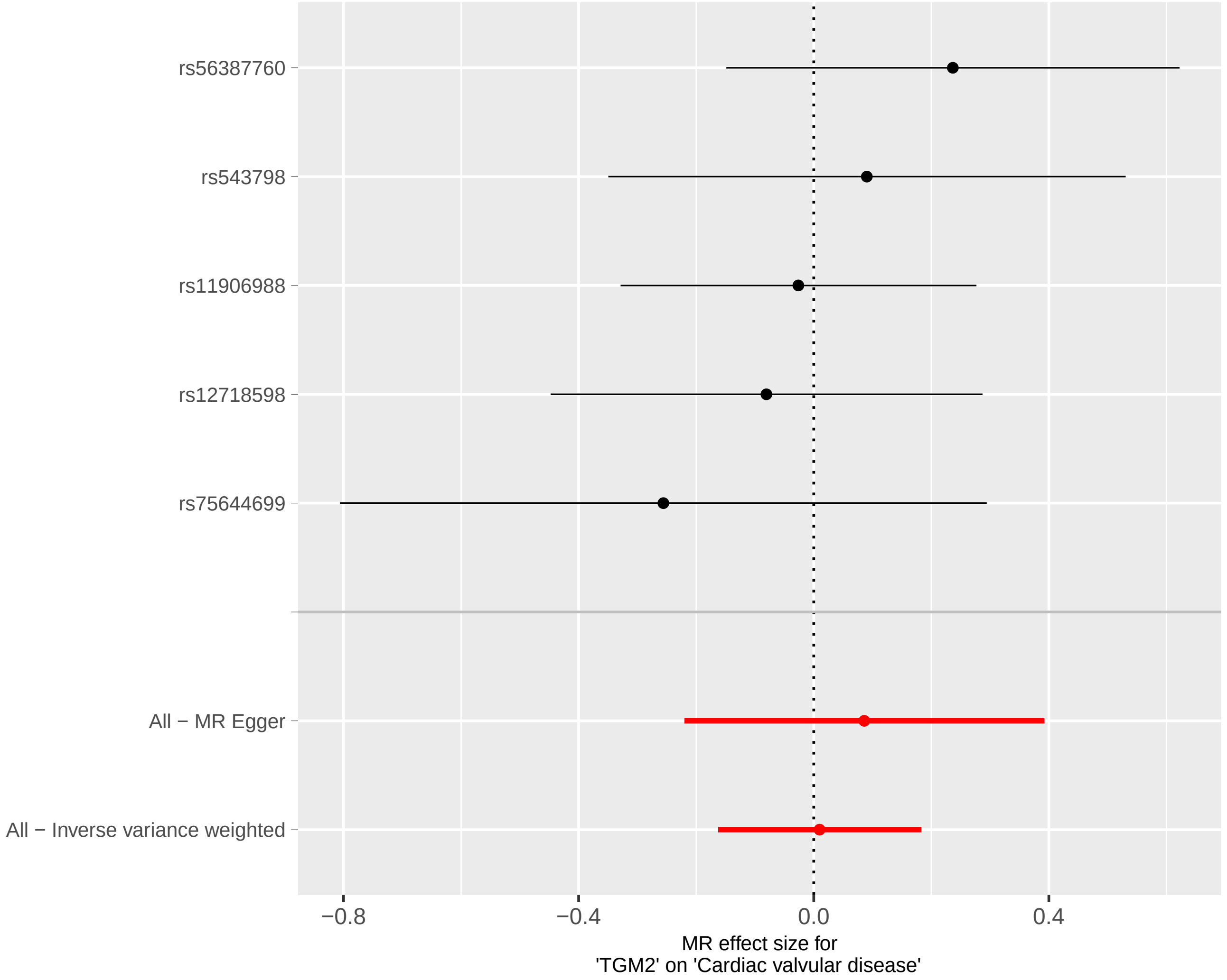
rs56387760
rs543798
rs11906988
rs12718598
rs75644699
All − MR Egger
All − Inverse variance weighted
−0.8
−0.4
0.0
0.4
MR effect size for
'TGM2' on 'Cardiac valvular disease'

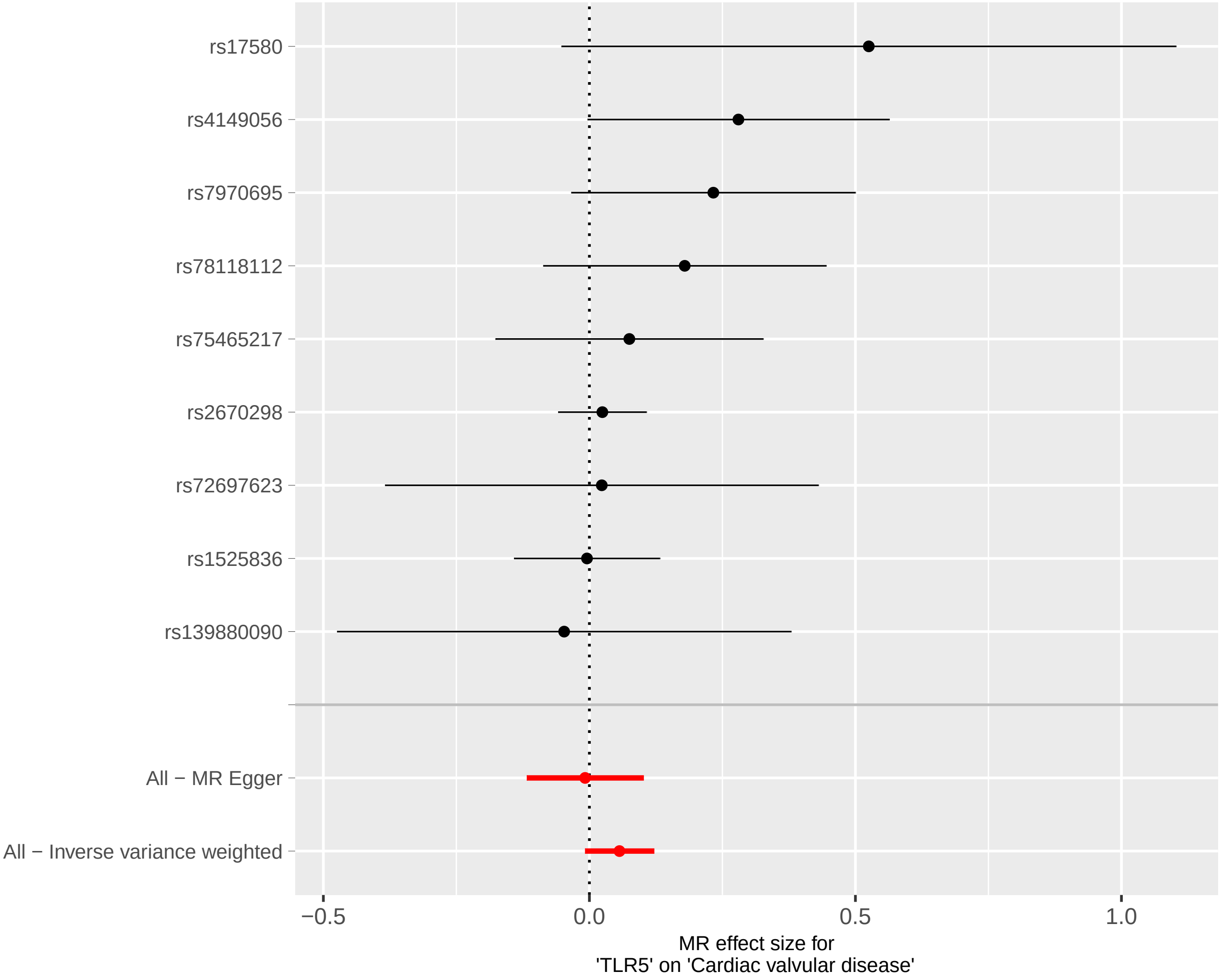

rs17580
rs4149056
rs7970695
rs78118112
rs75465217
rs2670298
rs72697623
rs1525836
rs139880090
All − MR Egger
All − Inverse variance weighted
−0.5
0.0
0.5
1.0
MR effect size for
'TLR5' on 'Cardiac valvular disease'

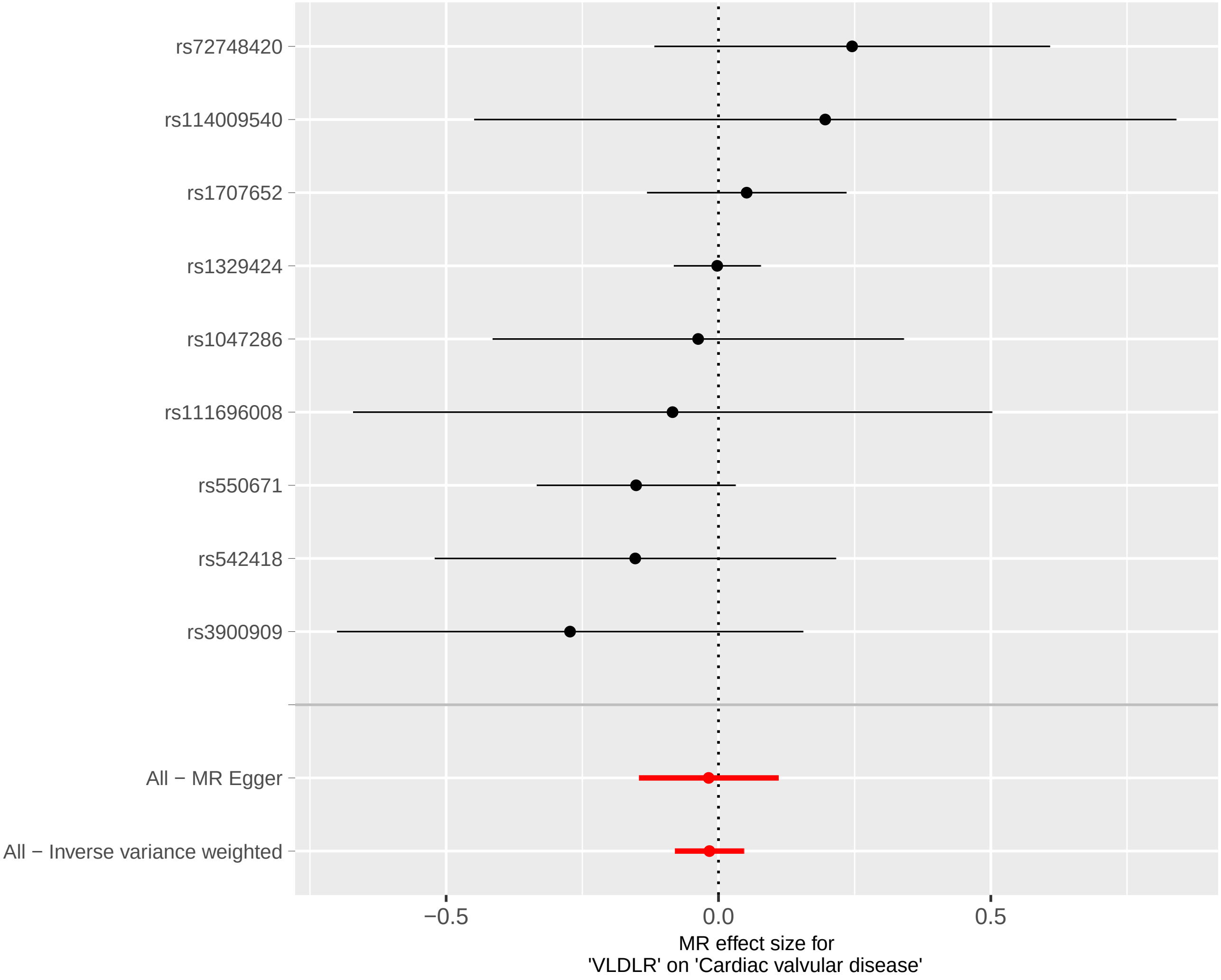

rs72748420
rs114009540
rs1707652
rs1329424
rs1047286
rs111696008
rs550671
rs542418
rs3900909
All − MR Egger
All − Inverse variance weighted
−0.5
0.0
0.5
MR effect size for
'VLDLR' on 'Cardiac valvular disease'

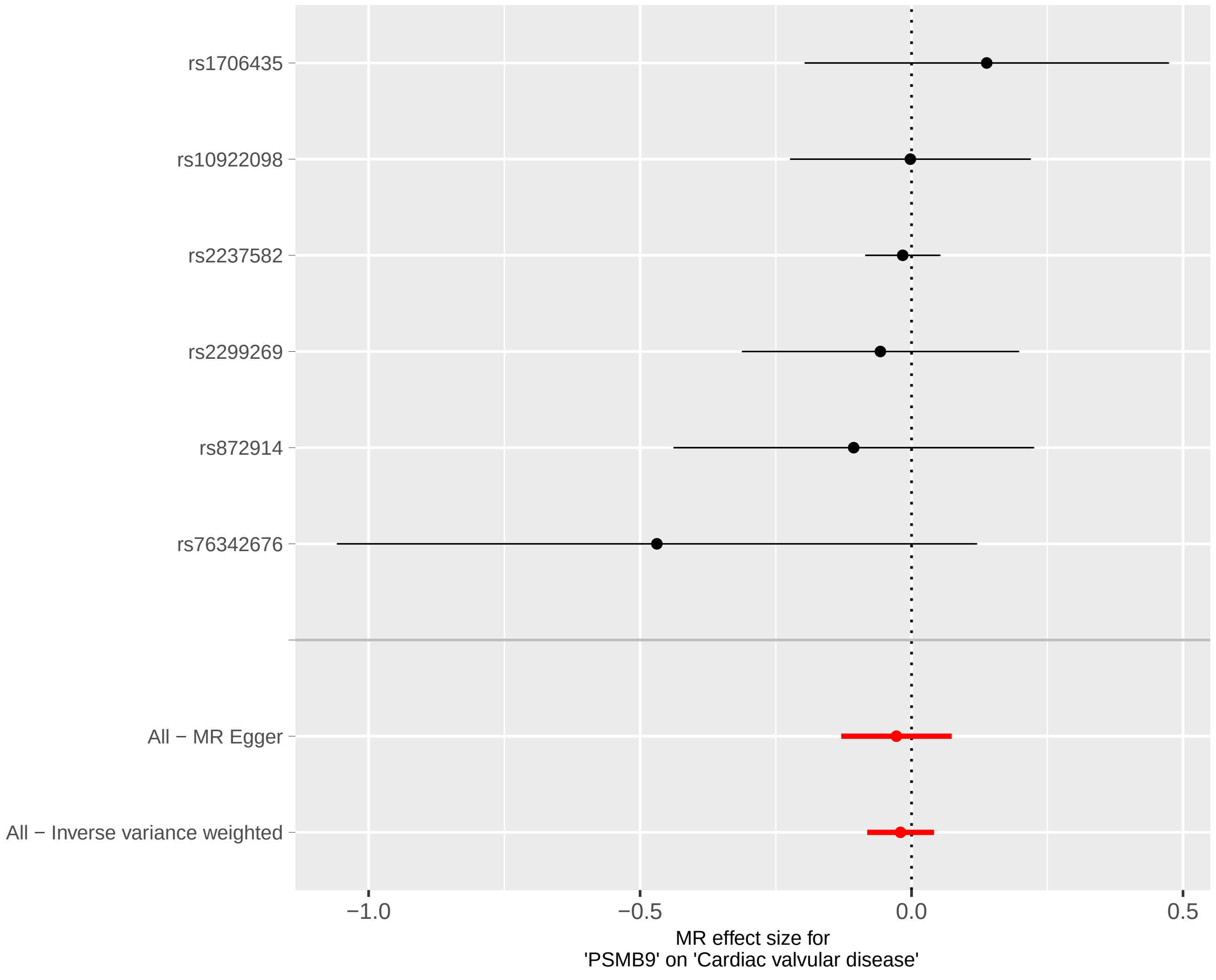
rs1706435
rs10922098
rs2237582
rs2299269
rs872914
rs76342676
All − MR Egger
All − Inverse variance weighted
−1.0
−0.5
0.0
0.5
MR effect size for
'PSMB9' on 'Cardiac valvular disease'

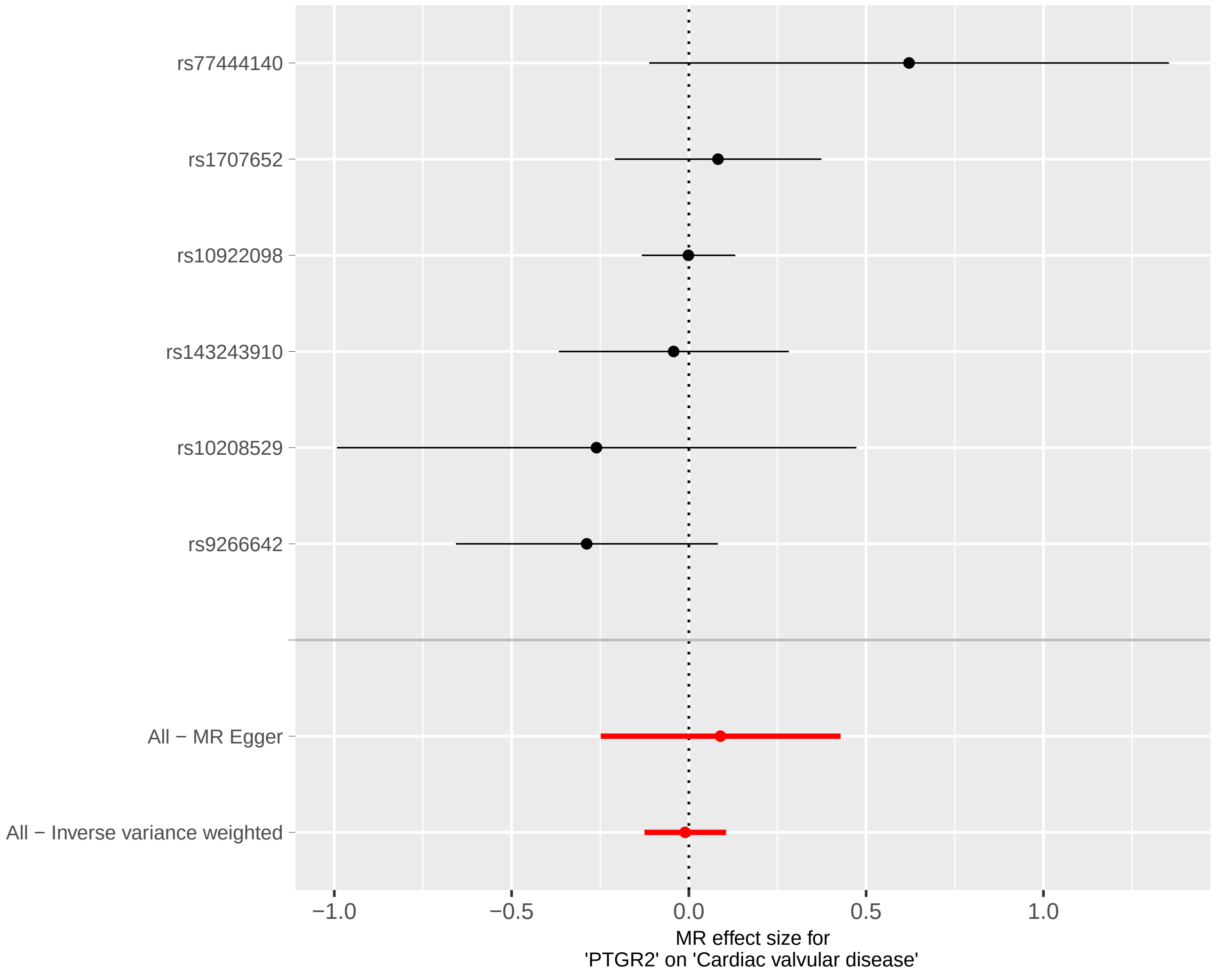
rs77444140
rs1707652
rs10922098
rs143243910
rs10208529
rs9266642
All − MR Egger
All − Inverse variance weighted
−1.0
−0.5
0.0
0.5
1.0
MR effect size for
'PTGR2' on 'Cardiac valvular disease'

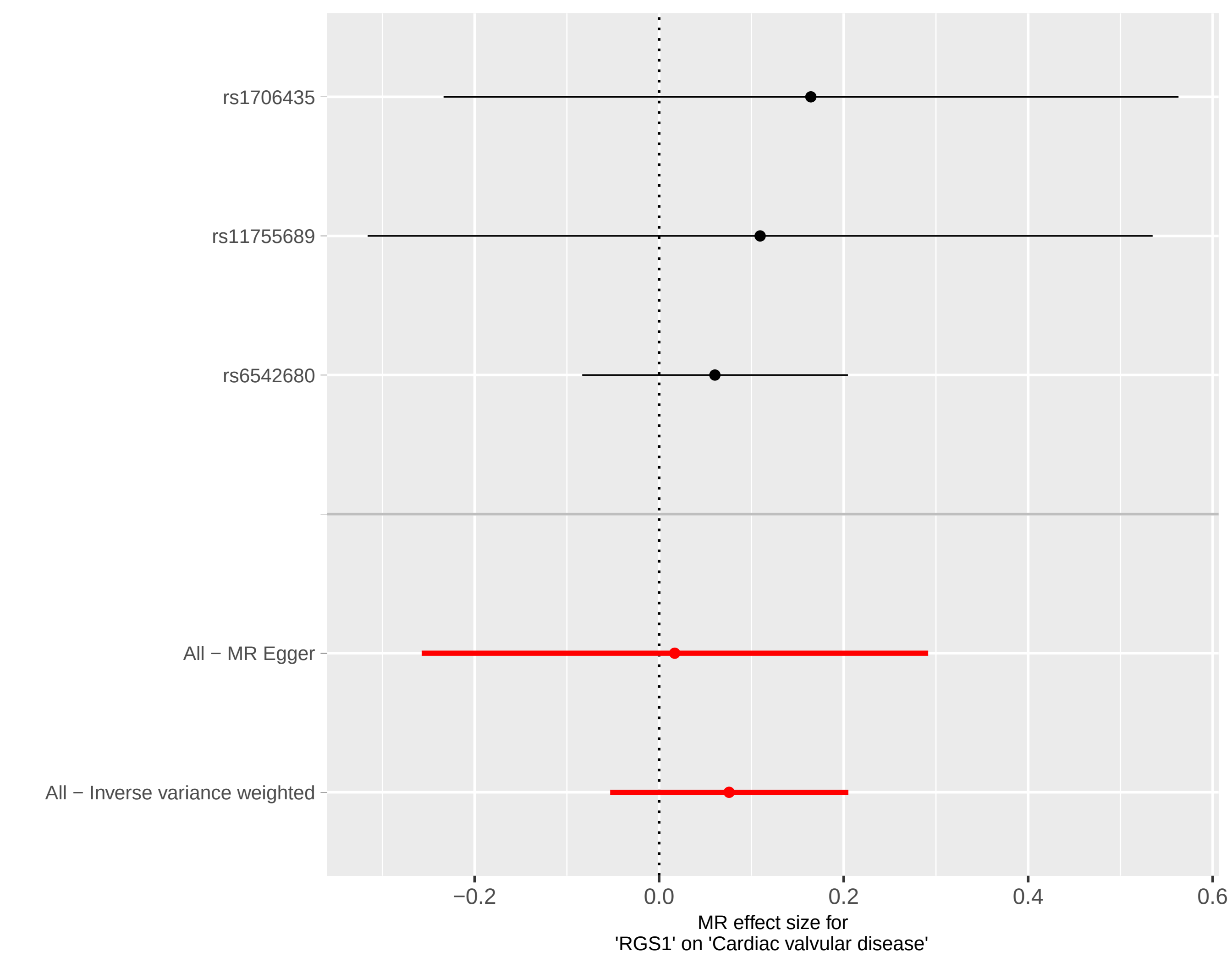
rs1706435
rs11755689
rs6542680
All − MR Egger
All − Inverse variance weighted
−0.2
0.0
0.2
0.4
0.6
MR effect size for
'RGS1' on 'Cardiac valvular disease'

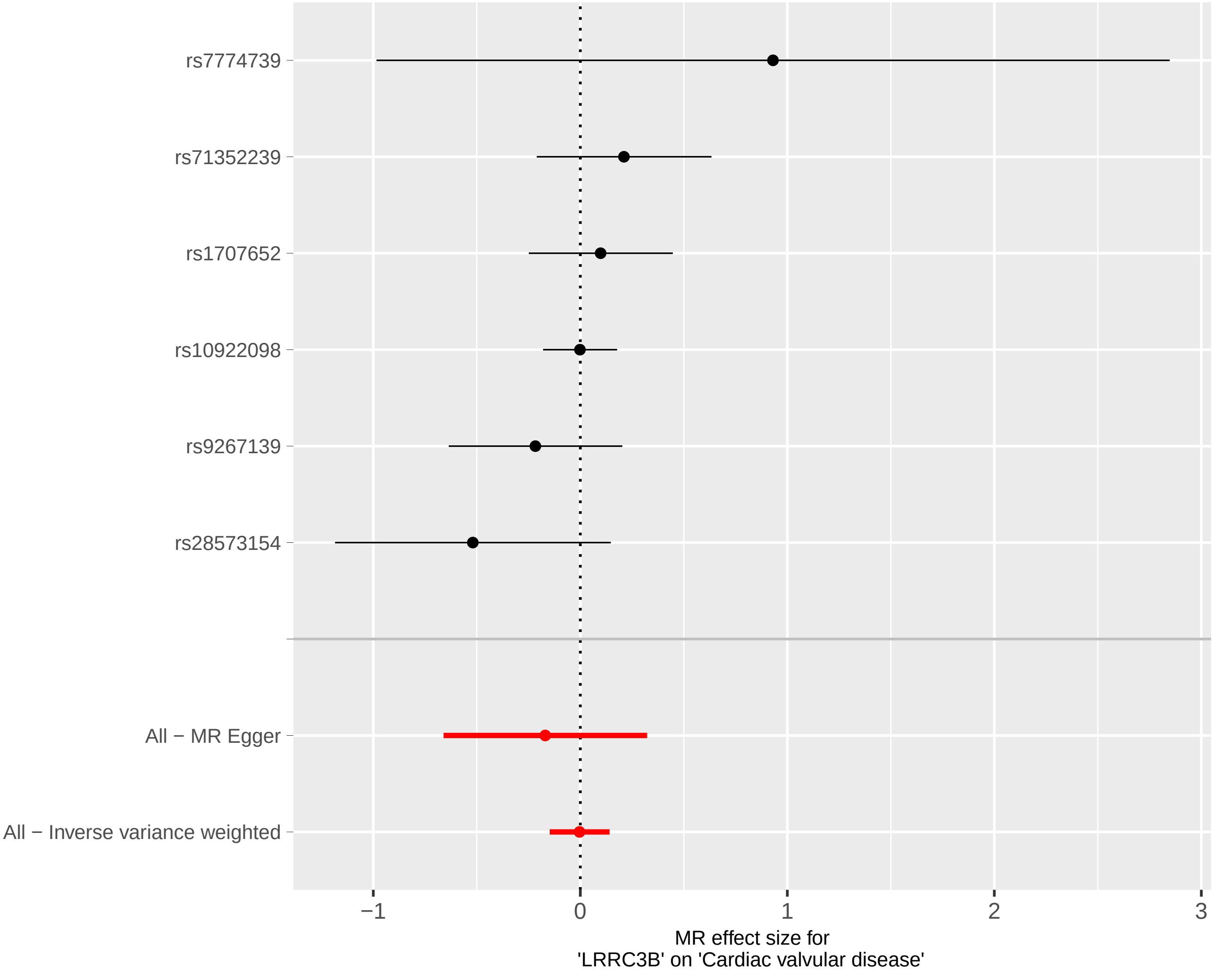
rs7774739
rs71352239
rs1707652
rs10922098
rs9267139
rs28573154
All − MR Egger
All − Inverse variance weighted
−1
0
1
2
3
MR effect size for
'LRRC3B' on 'Cardiac valvular disease'

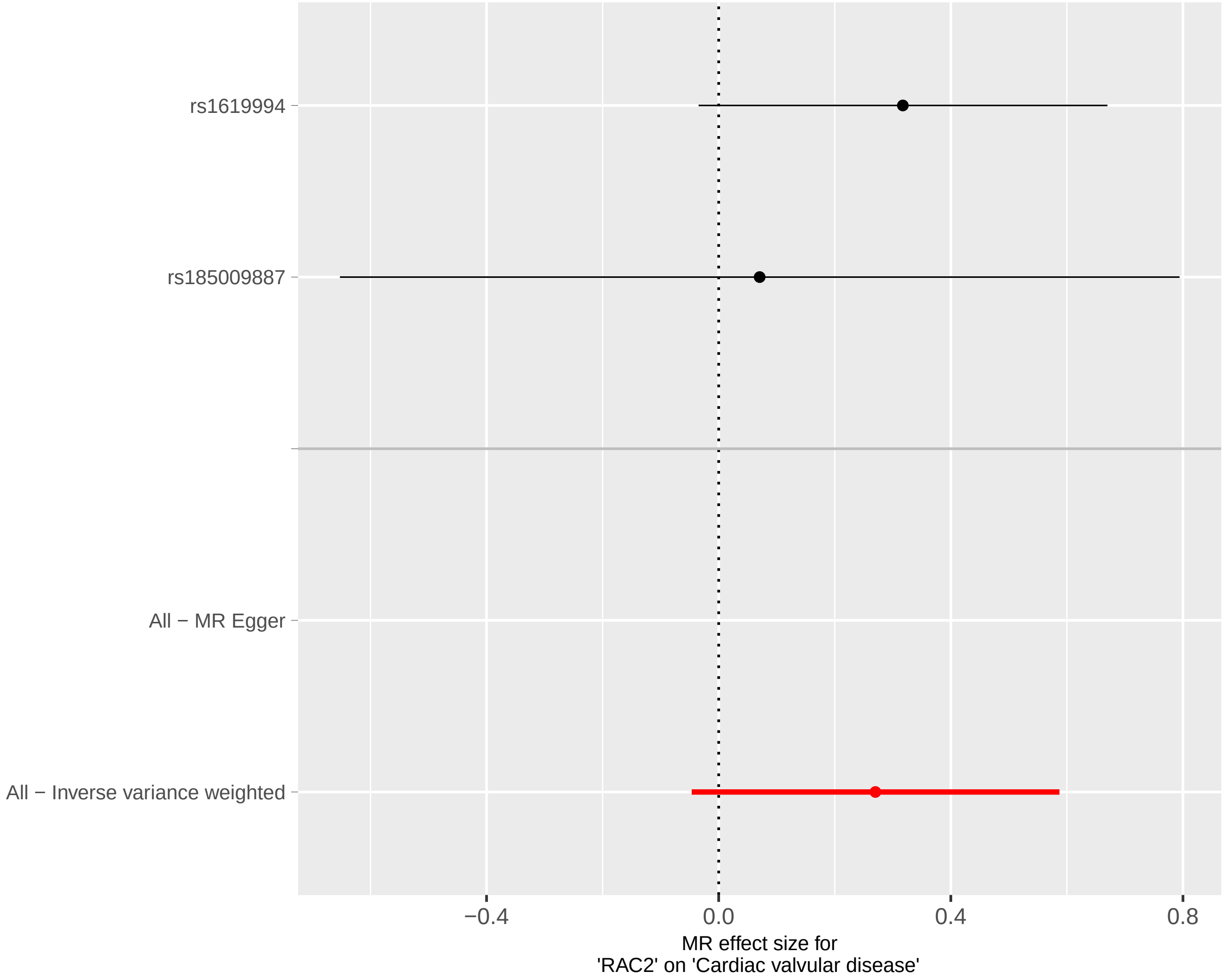
rs1619994
rs185009887
All − MR Egger
All − Inverse variance weighted
−0.4
0.0
0.4
0.8
MR effect size for
'RAC2' on 'Cardiac valvular disease'

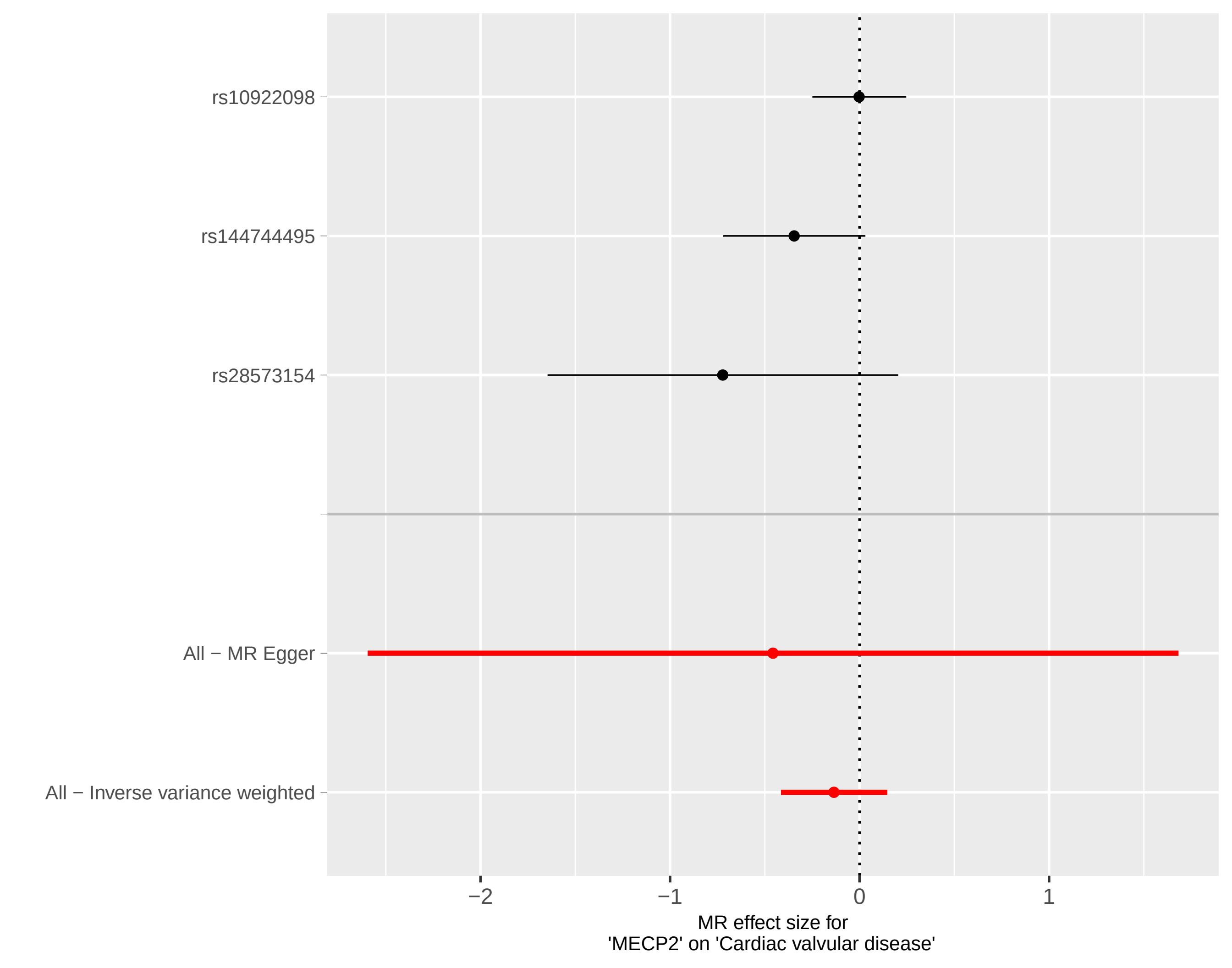

rs10922098
rs144744495
rs28573154
All − MR Egger
All − Inverse variance weighted
−2
−1
0
1
MR effect size for
'MECP2' on 'Cardiac valvular disease'

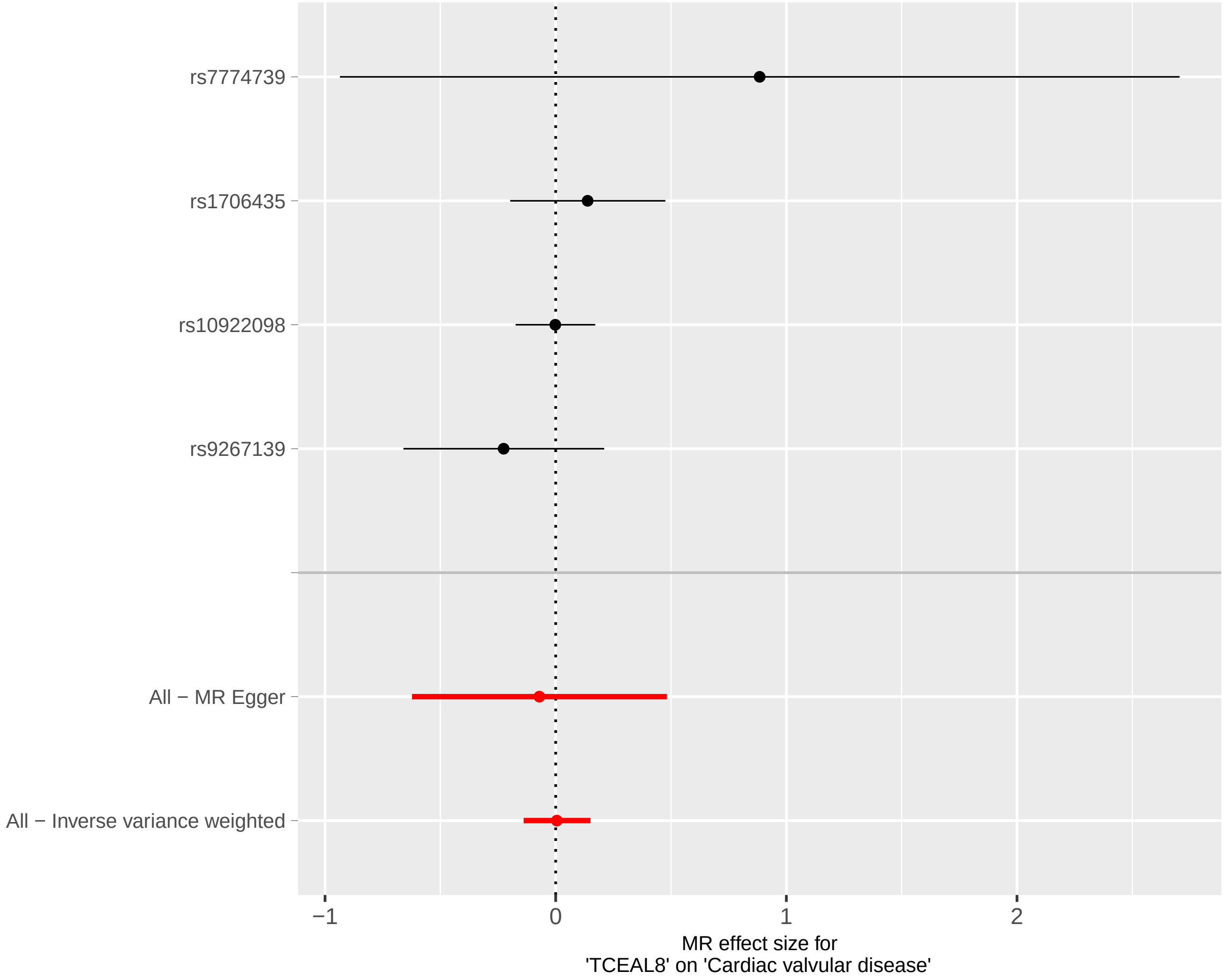
rs7774739
rs1706435
rs10922098
rs9267139
All − MR Egger
All − Inverse variance weighted
−1
0
1
2
MR effect size for
'TCEAL8' on 'Cardiac valvular disease'

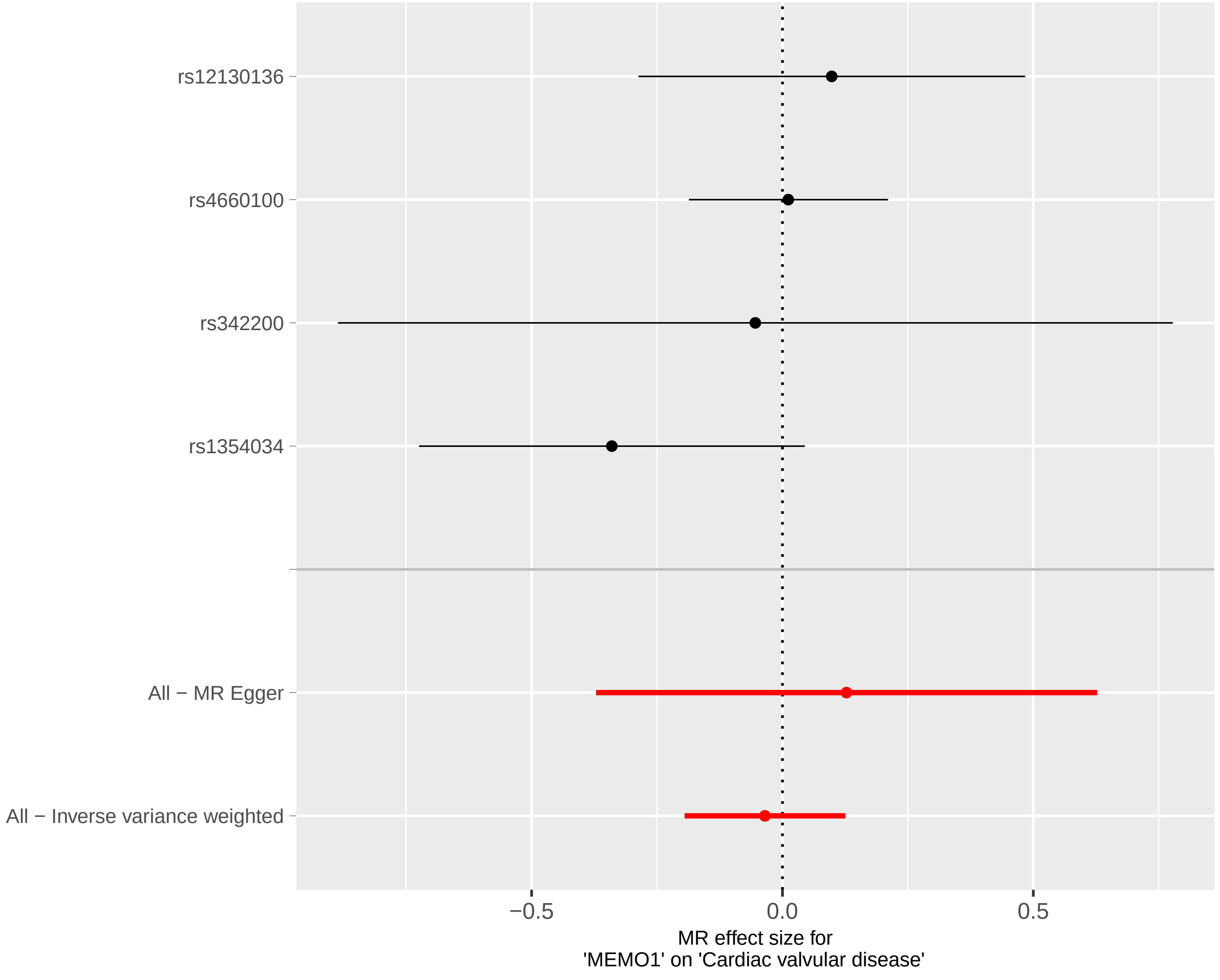

rs12130136
rs4660100
rs342200
rs1354034
All − MR Egger
All − Inverse variance weighted
−0.5
0.0
0.5
MR effect size for
'MEMO1' on 'Cardiac valvular disease'

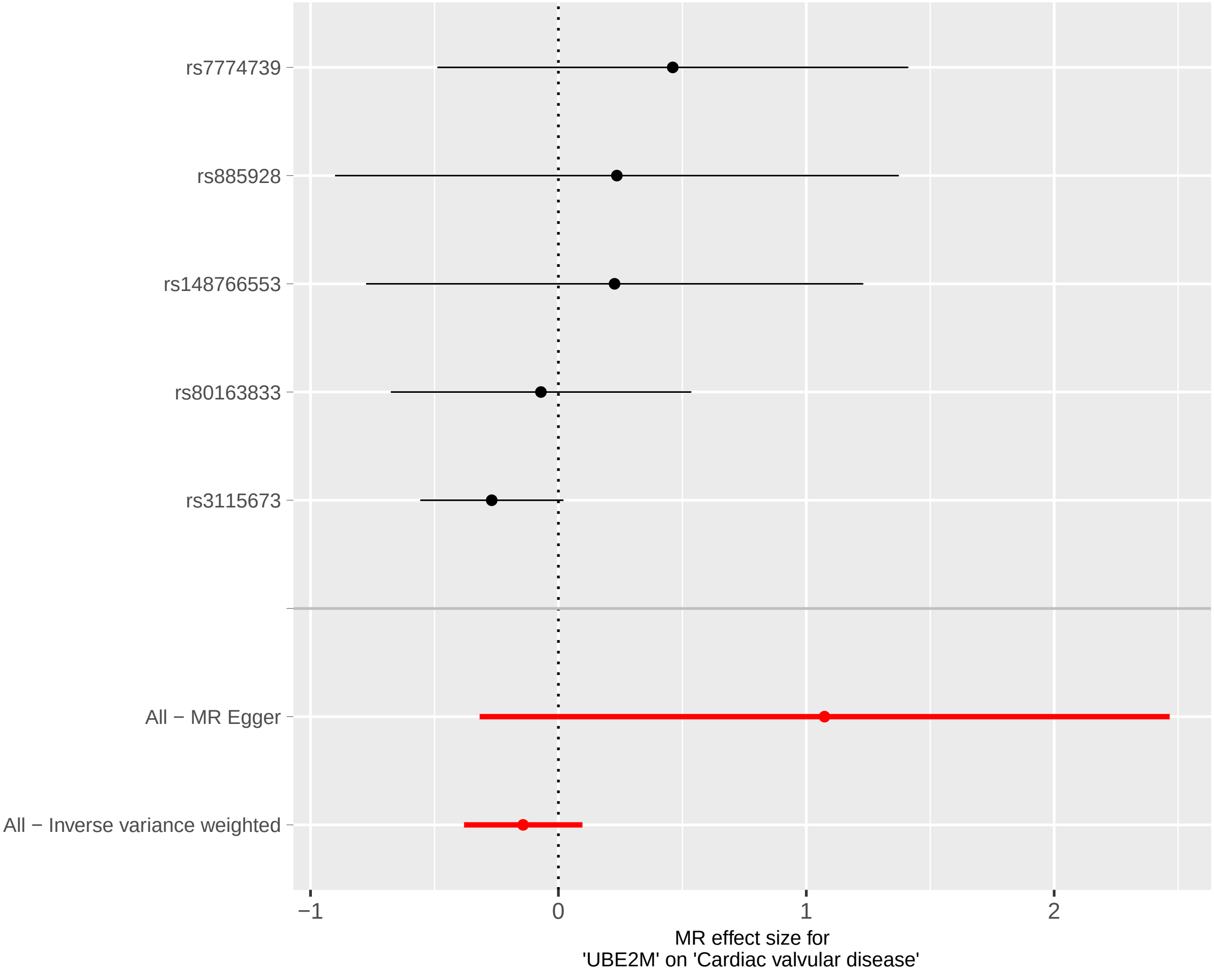

rs7774739
rs885928
rs148766553
rs80163833
rs3115673
All − MR Egger
All − Inverse variance weighted
−1
0
1
2
MR effect size for
'UBE2M' on 'Cardiac valvular disease'

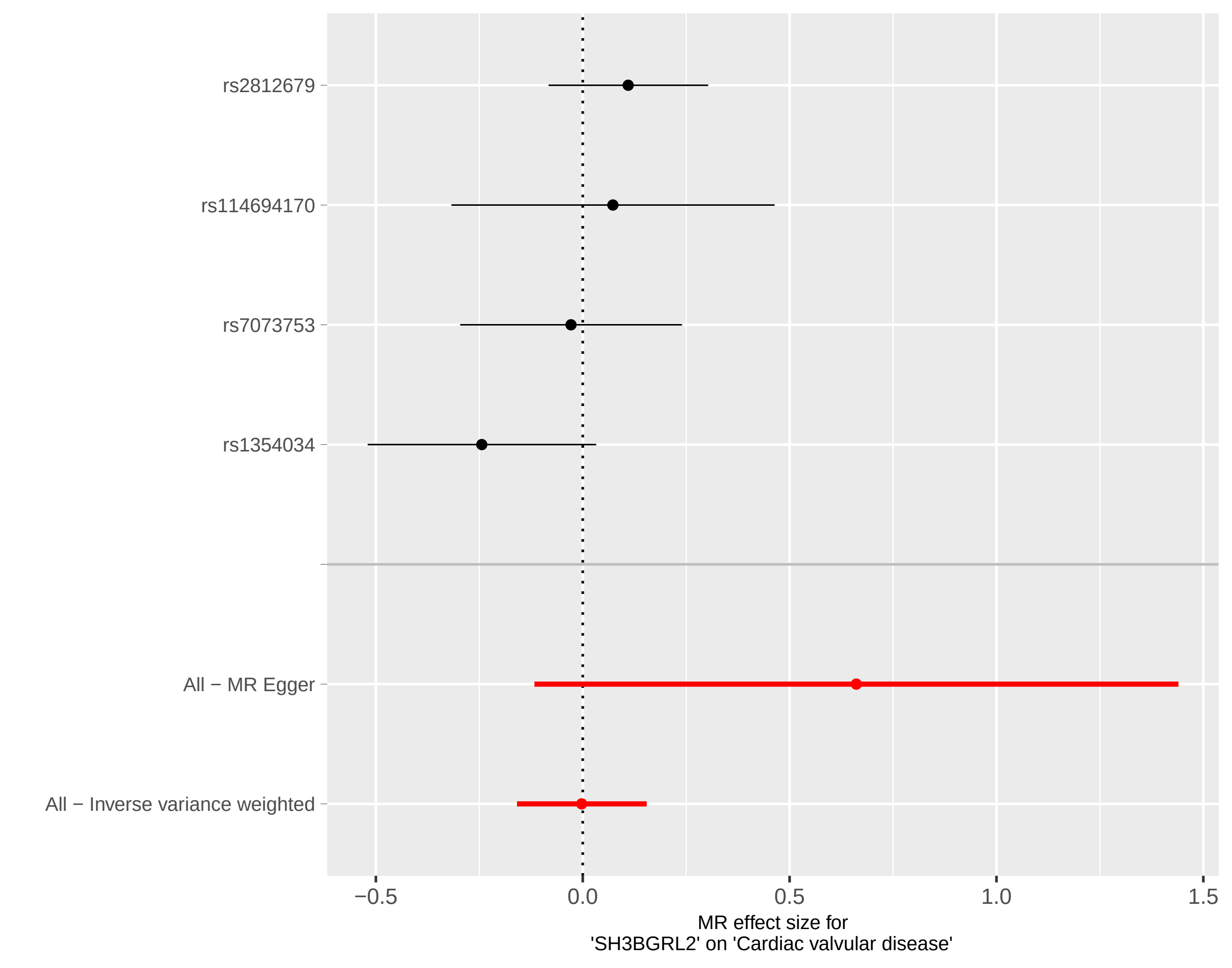
rs2812679
rs114694170
rs7073753
rs1354034
All − MR Egger
All − Inverse variance weighted
−0.5
0.0
0.5
1.0
1.5
MR effect size for
'SH3BGRL2' on 'Cardiac valvular disease'

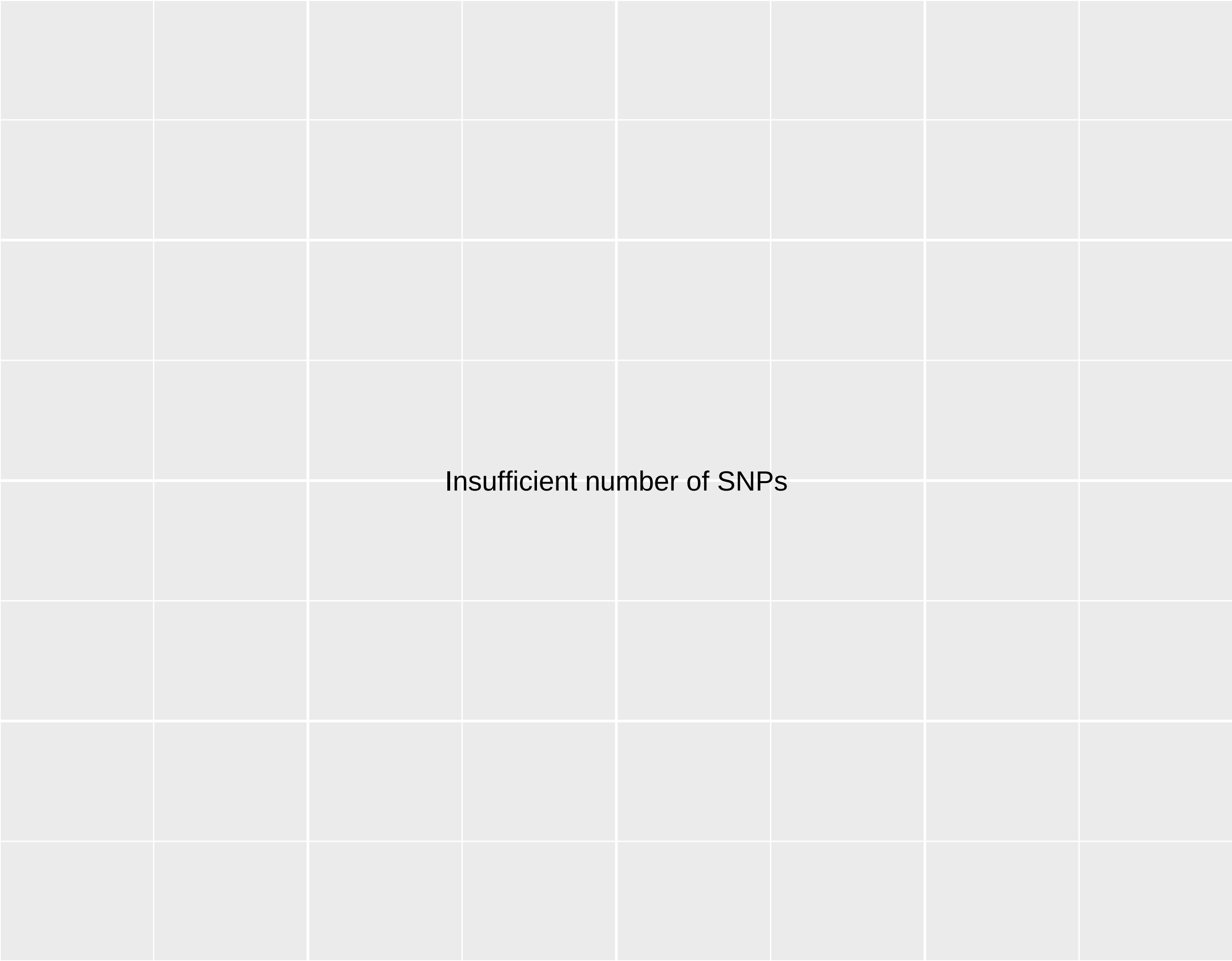
Insufficient number of SNPs

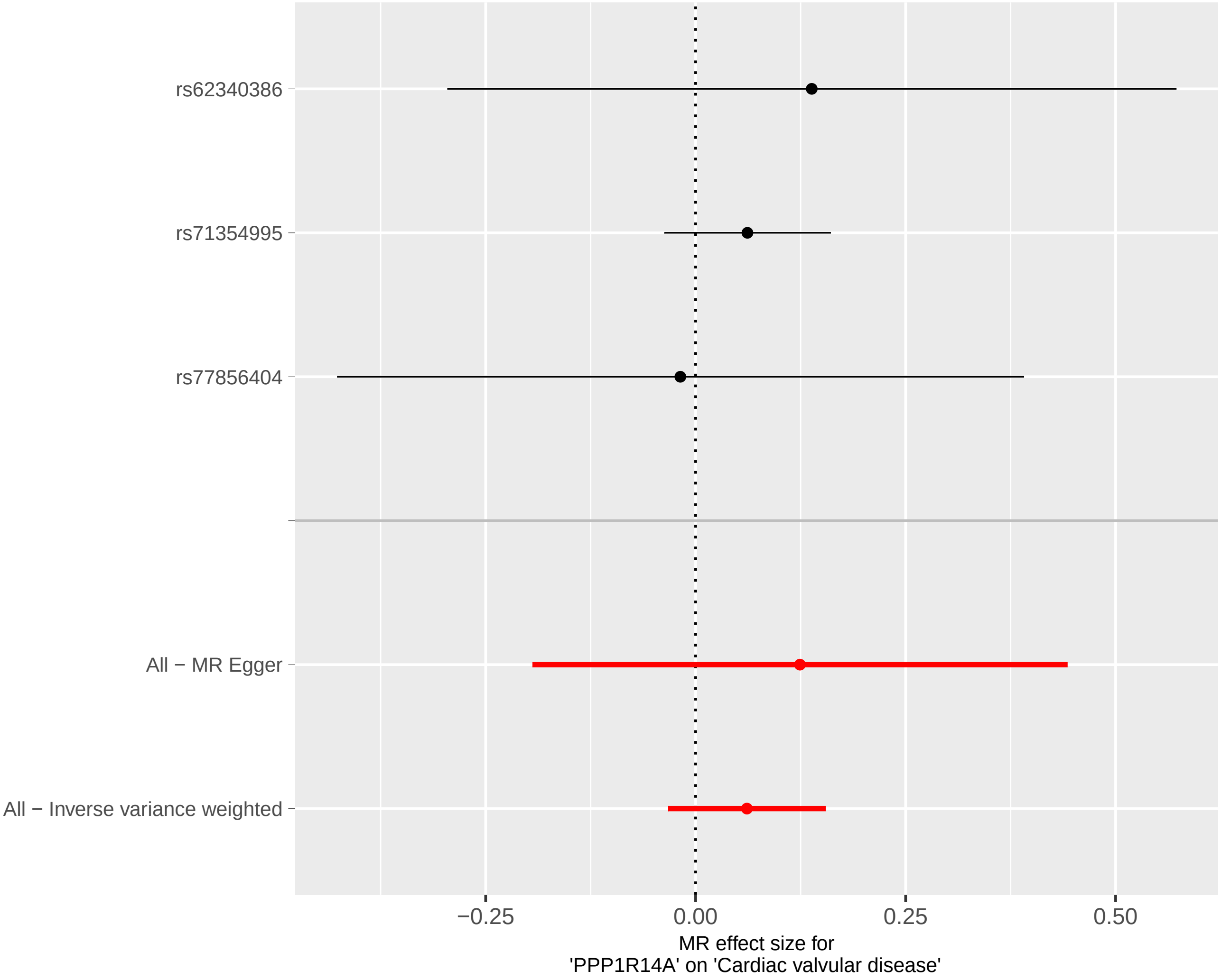
rs62340386
rs71354995
rs77856404
All − MR Egger
All − Inverse variance weighted
−0.25
0.00
0.25
0.50
MR effect size for
'PPP1R14A' on 'Cardiac valvular disease'

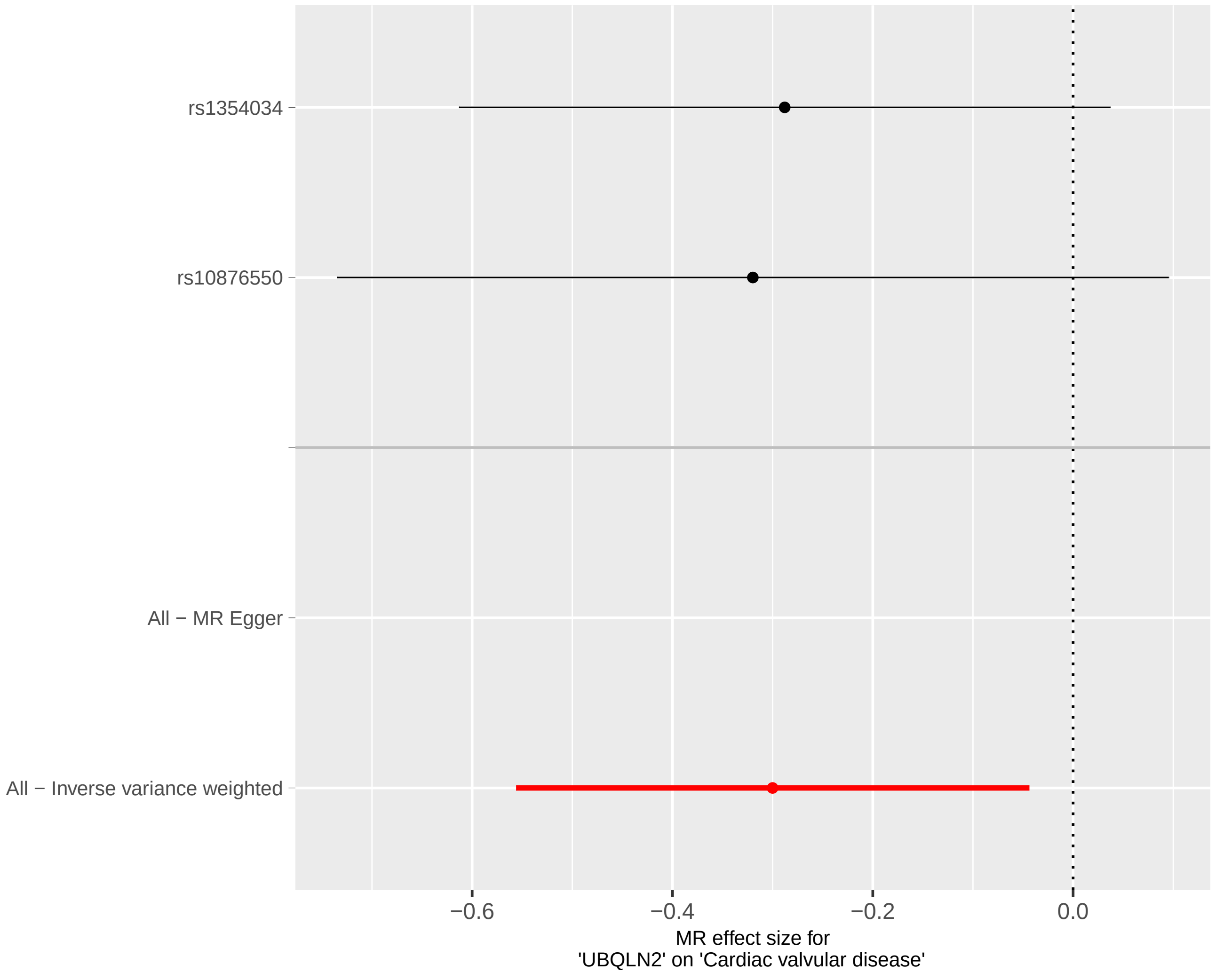

rs1354034
rs10876550
All − MR Egger
All − Inverse variance weighted
−0.6
−0.4
−0.2
0.0
MR effect size for
'UBQLN2' on 'Cardiac valvular disease'

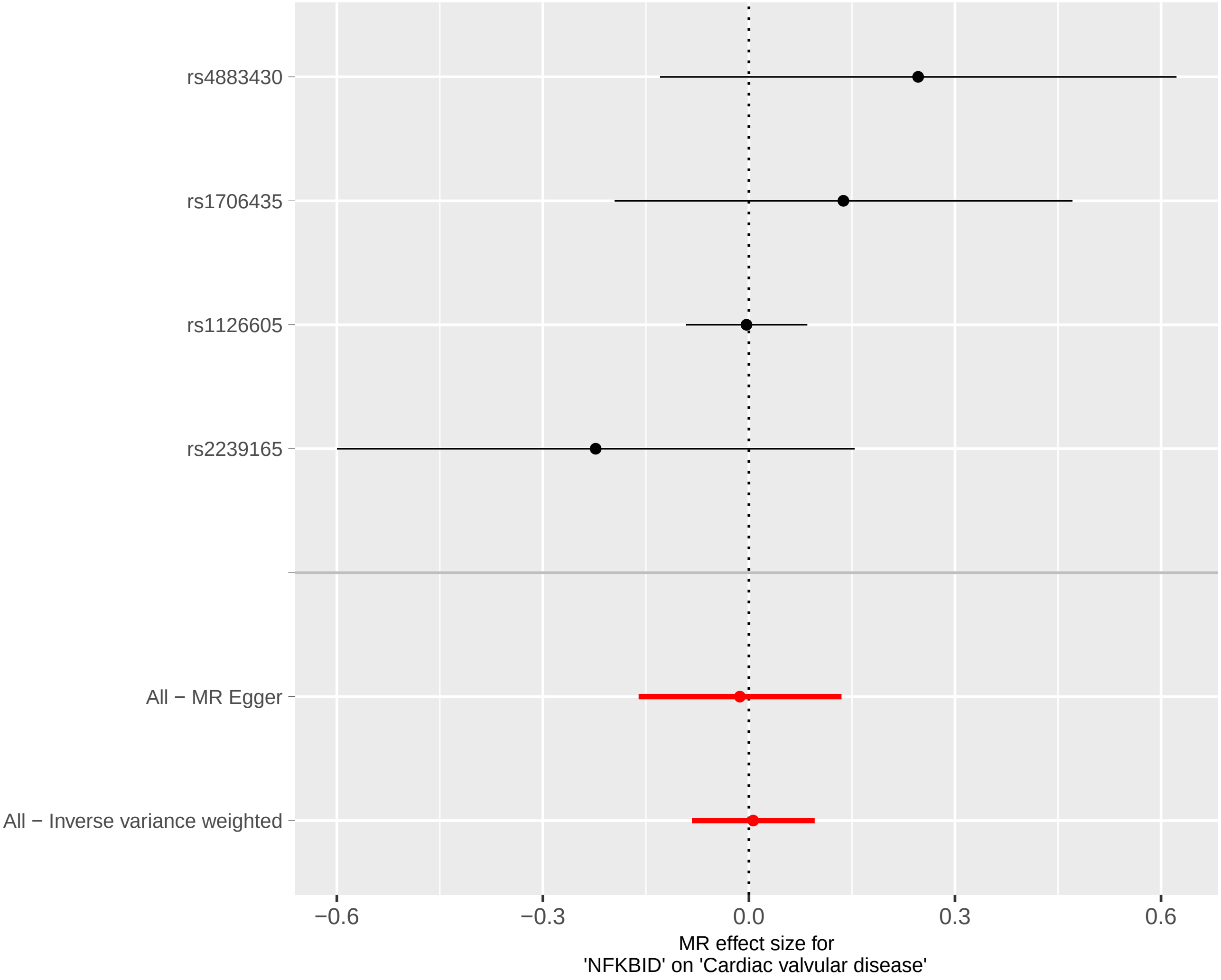
rs4883430
rs1706435
rs1126605
rs2239165
All − MR Egger
All − Inverse variance weighted
−0.6
−0.3
0.0
0.3
0.6
MR effect size for
'NFKBID' on 'Cardiac valvular disease'

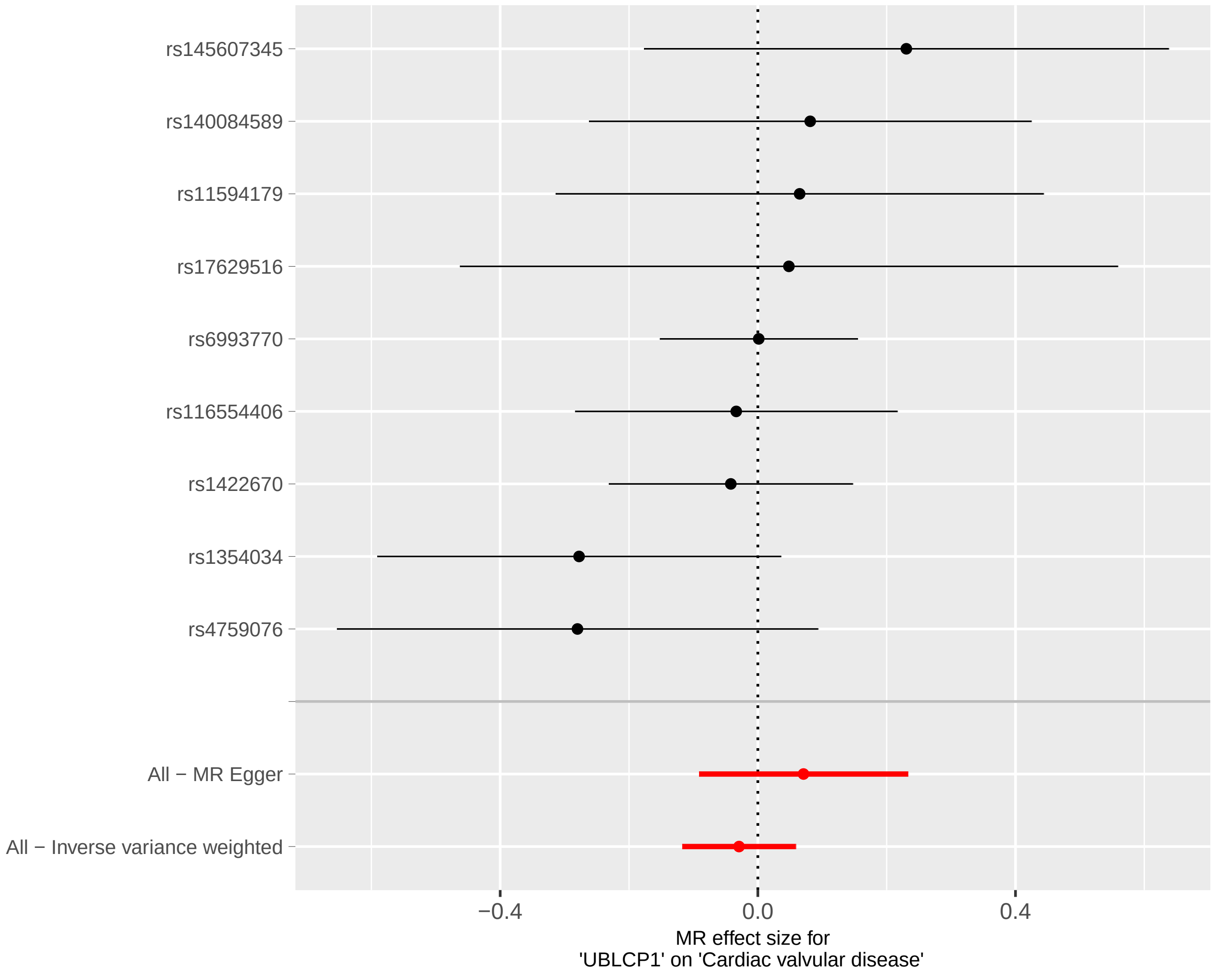
rs145607345
rs140084589
rs11594179
rs17629516
rs6993770
rs116554406
rs1422670
rs1354034
rs4759076
All − MR Egger
All − Inverse variance weighted
−0.4
0.0
0.4
MR effect size for
'UBLCP1' on 'Cardiac valvular disease'

Insufficient number of SNPs

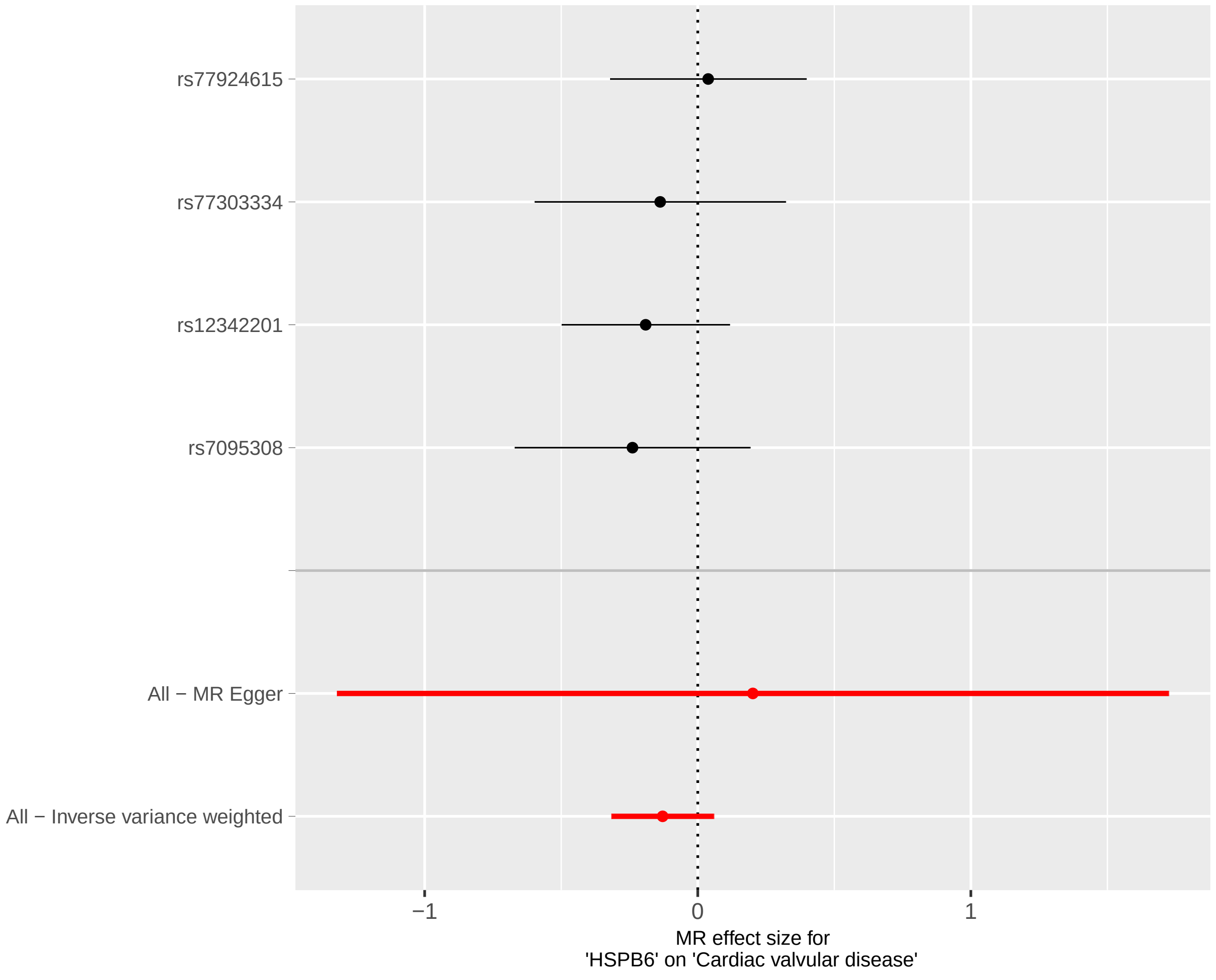
rs77924615
rs77303334
rs12342201
rs7095308
All − MR Egger
All − Inverse variance weighted
−1
0
1
MR effect size for
'HSPB6' on 'Cardiac valvular disease'

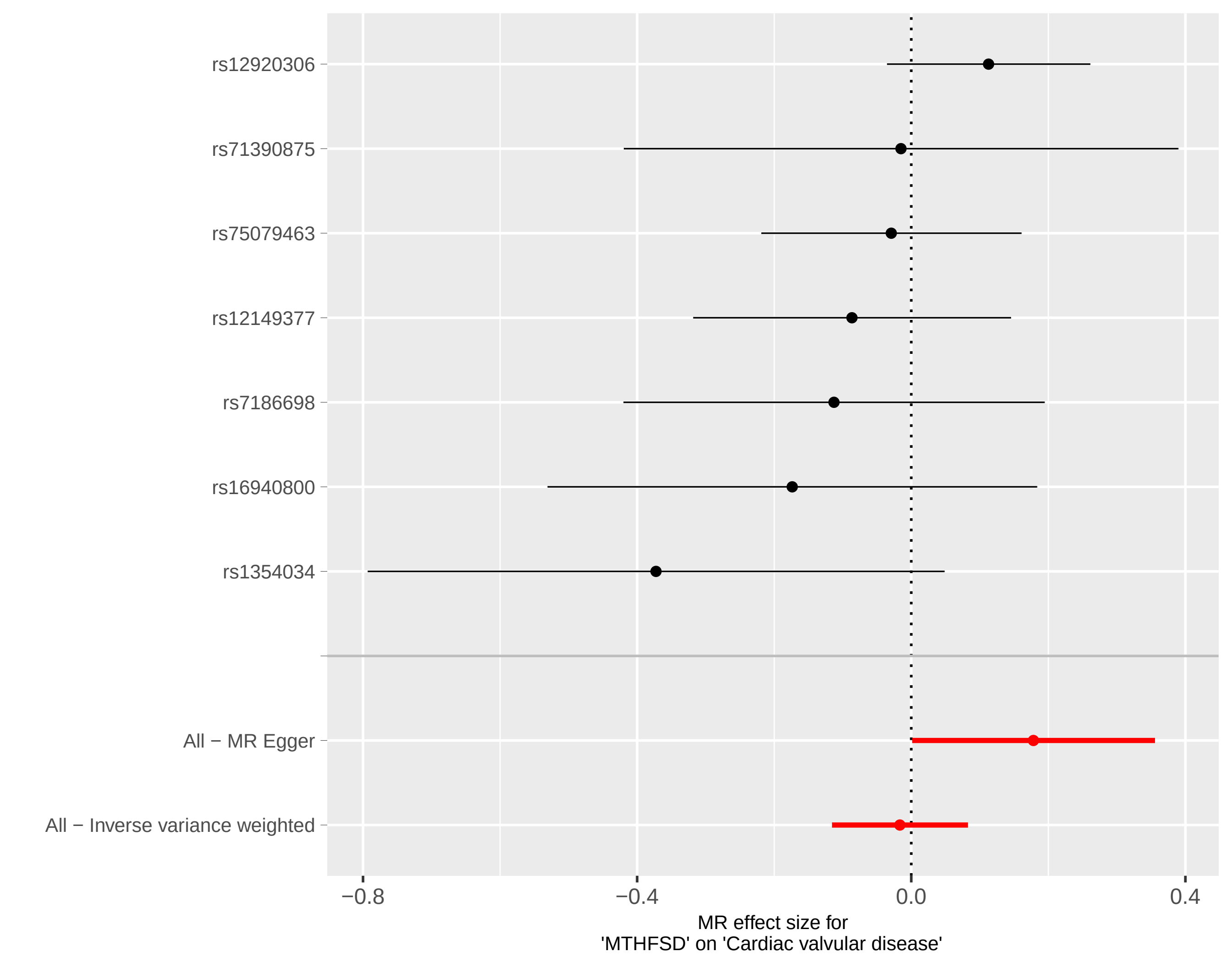
rs12920306
rs71390875
rs75079463
rs12149377
rs7186698
rs16940800
rs1354034
All − MR Egger
All − Inverse variance weighted
−0.8
−0.4
0.0
0.4
MR effect size for
'MTHFSD' on 'Cardiac valvular disease'

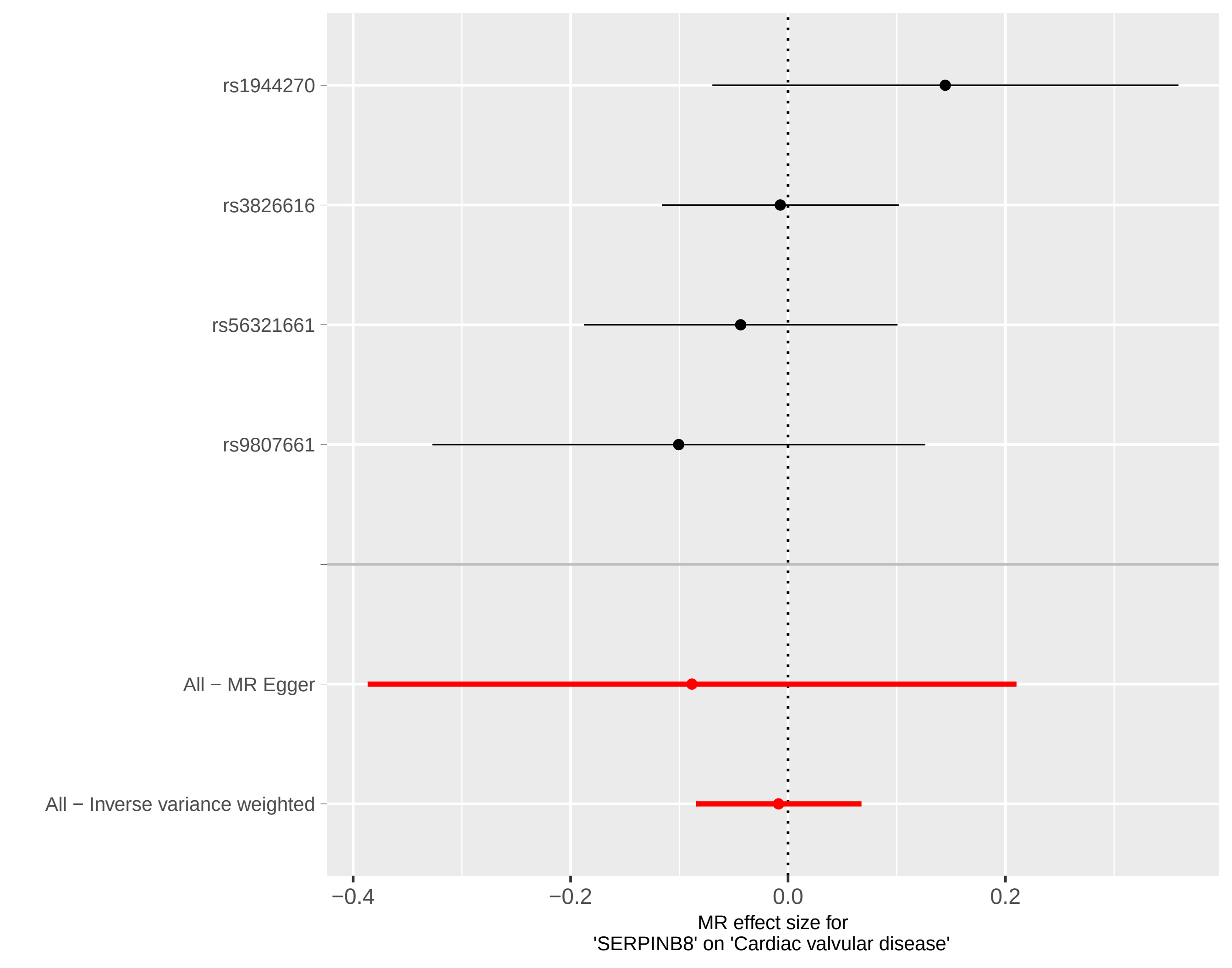
rs1944270
rs3826616
rs56321661
rs9807661
All − MR Egger
All − Inverse variance weighted
−0.4
−0.2
0.0
0.2
MR effect size for
'SERPINB8' on 'Cardiac valvular disease'

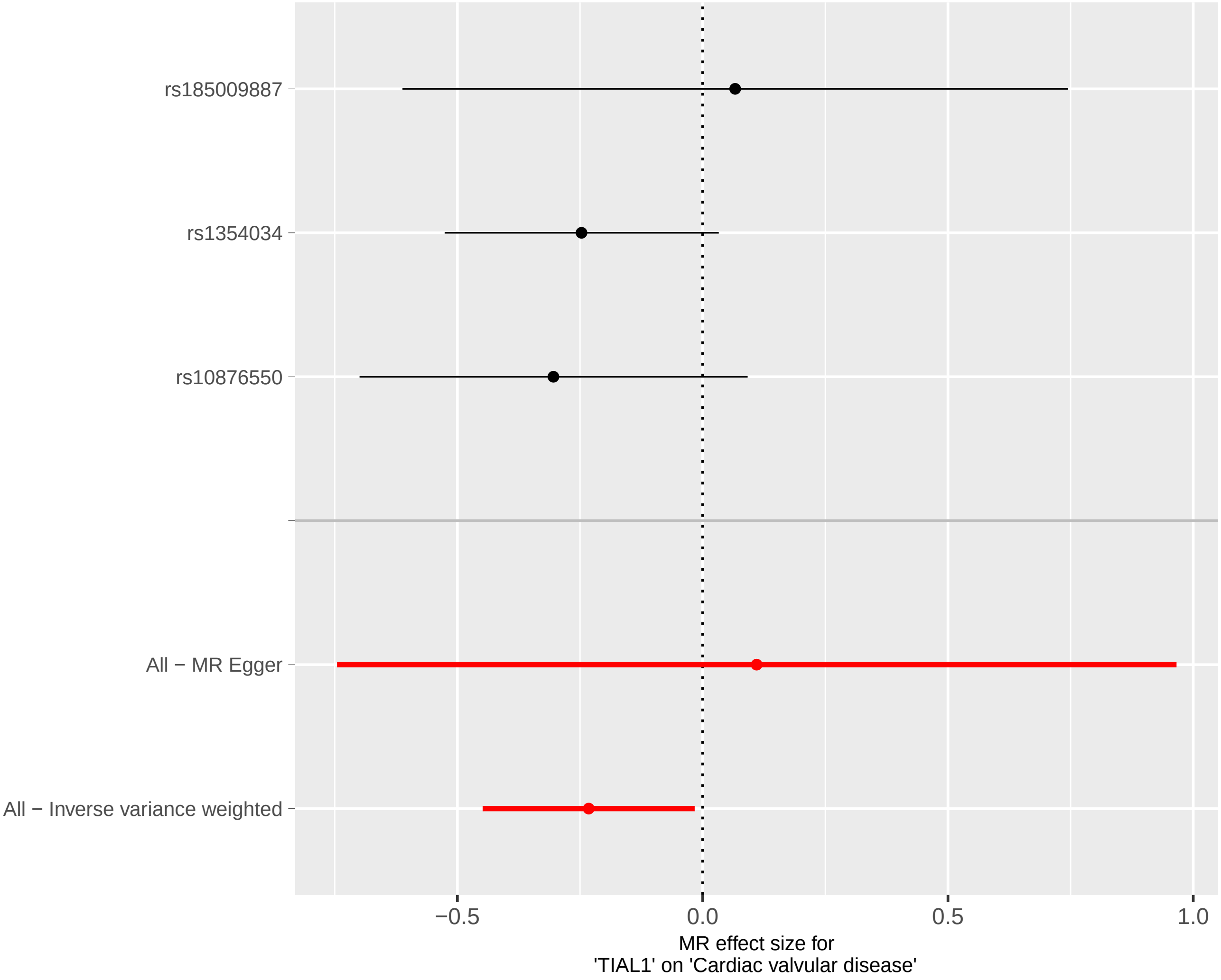
rs185009887
rs1354034
rs10876550
All − MR Egger
All − Inverse variance weighted
−0.5
0.0
0.5
1.0
MR effect size for
'TIAL1' on 'Cardiac valvular disease'

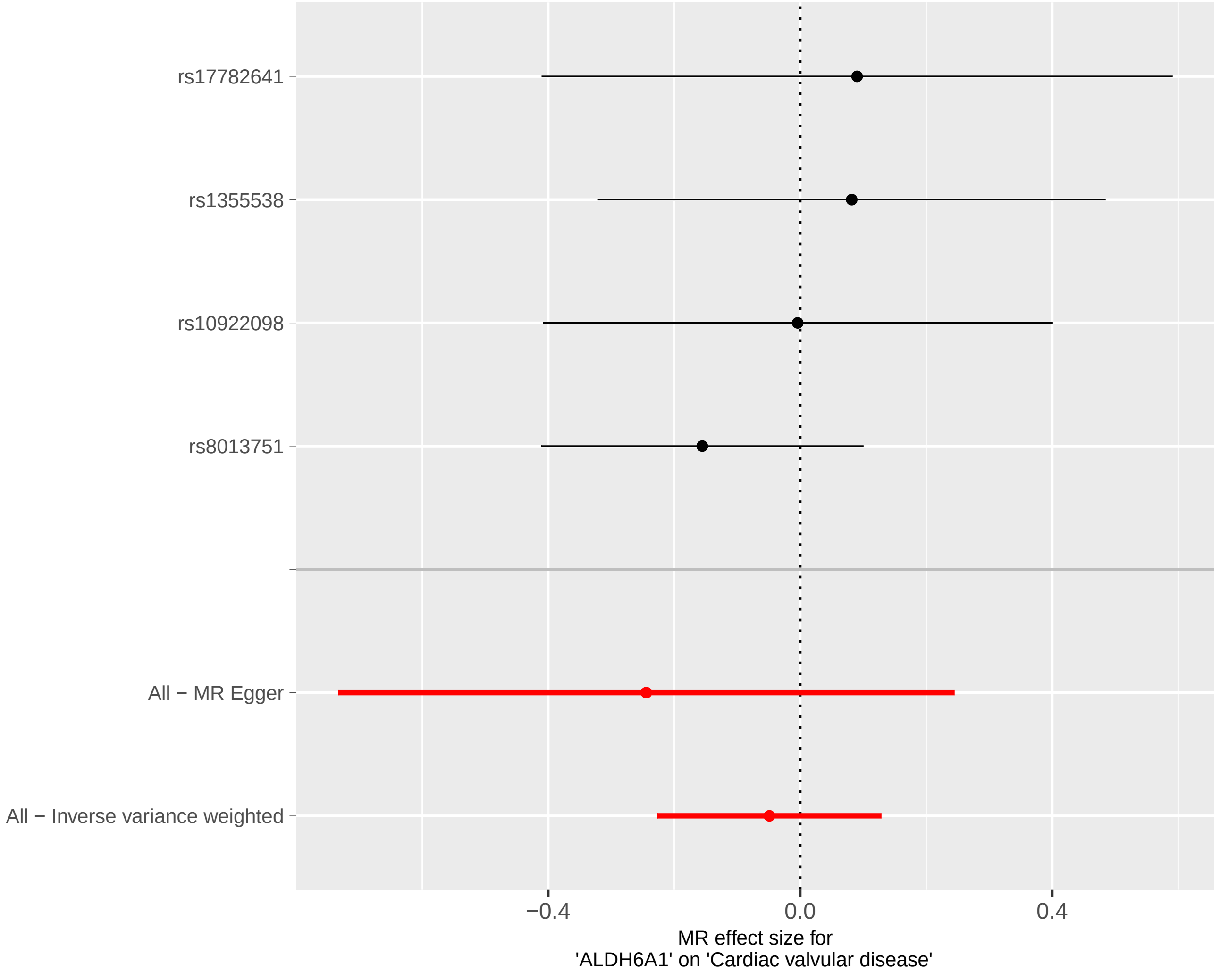
rs17782641
rs1355538
rs10922098
rs8013751
All − MR Egger
All − Inverse variance weighted
−0.4
0.0
0.4
MR effect size for
'ALDH6A1' on 'Cardiac valvular disease'

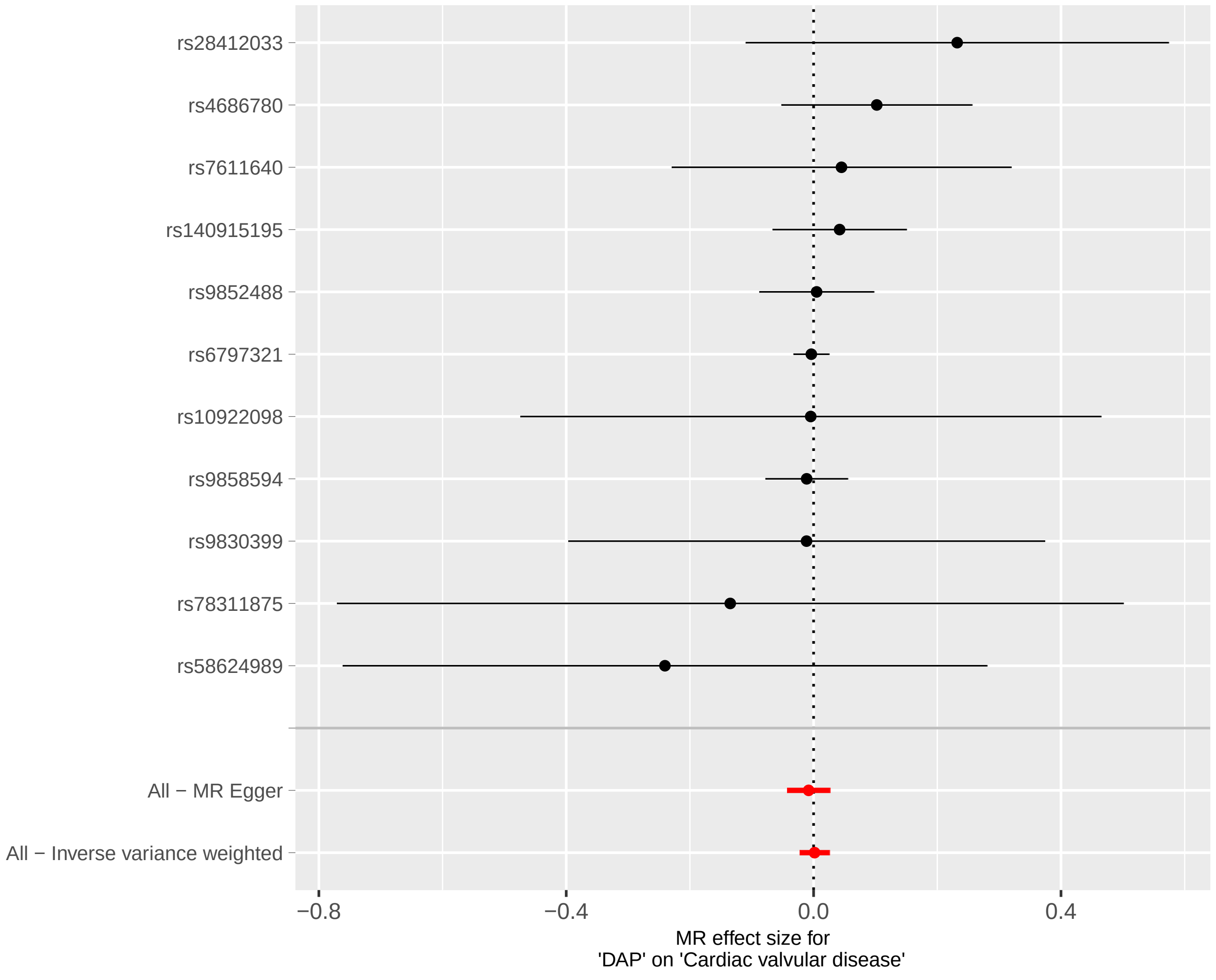
rs28412033
rs4686780
rs7611640
rs140915195
rs9852488
rs6797321
rs10922098
rs9858594
rs9830399
rs78311875
rs58624989
All − MR Egger
All − Inverse variance weighted
−0.8
−0.4
0.0
0.4
MR effect size for
'DAP' on 'Cardiac valvular disease'

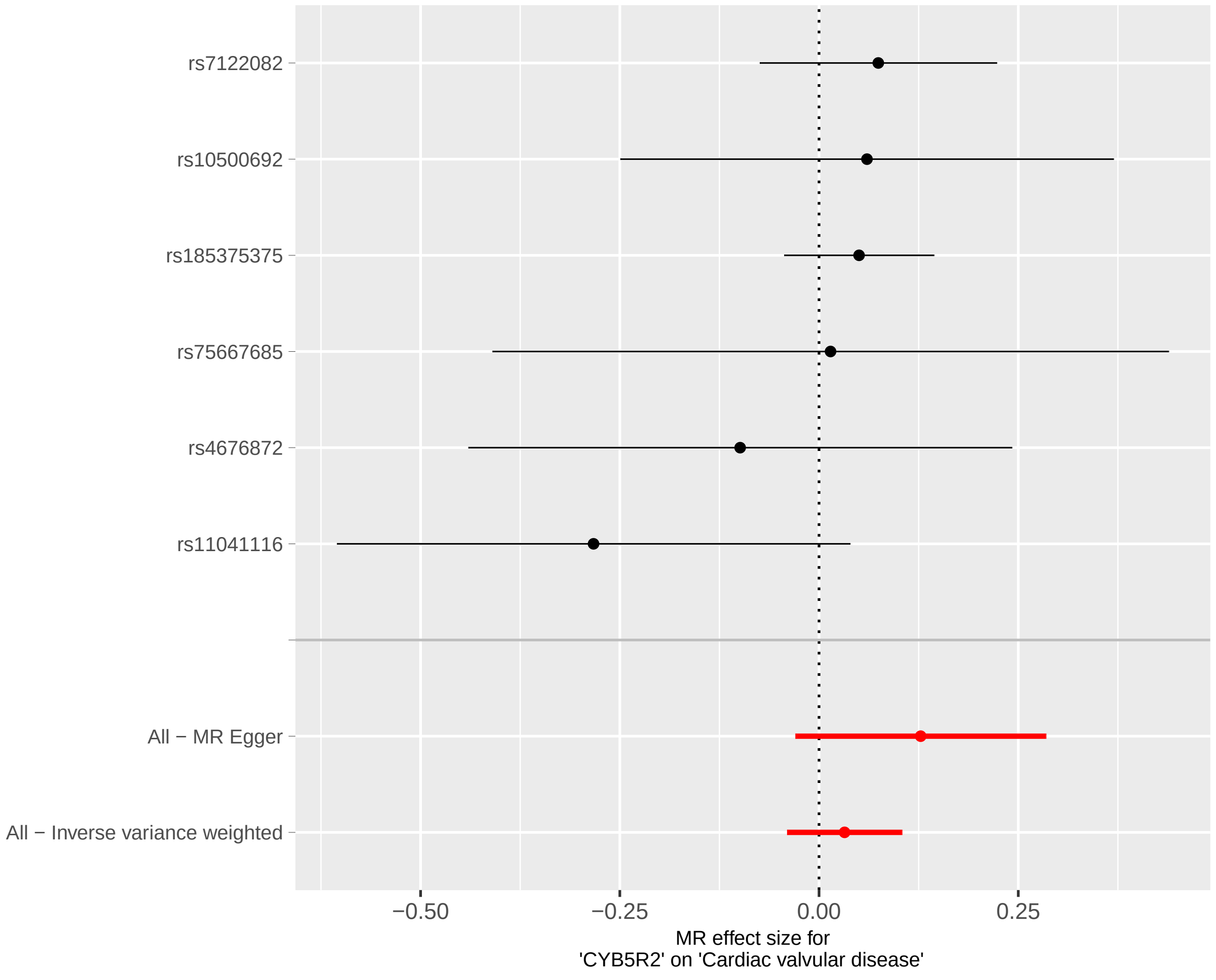
rs7122082
rs10500692
rs185375375
rs75667685
rs4676872
rs11041116
All − MR Egger
All − Inverse variance weighted
−0.50
−0.25
0.00
0.25
MR effect size for
'CYB5R2' on 'Cardiac valvular disease'

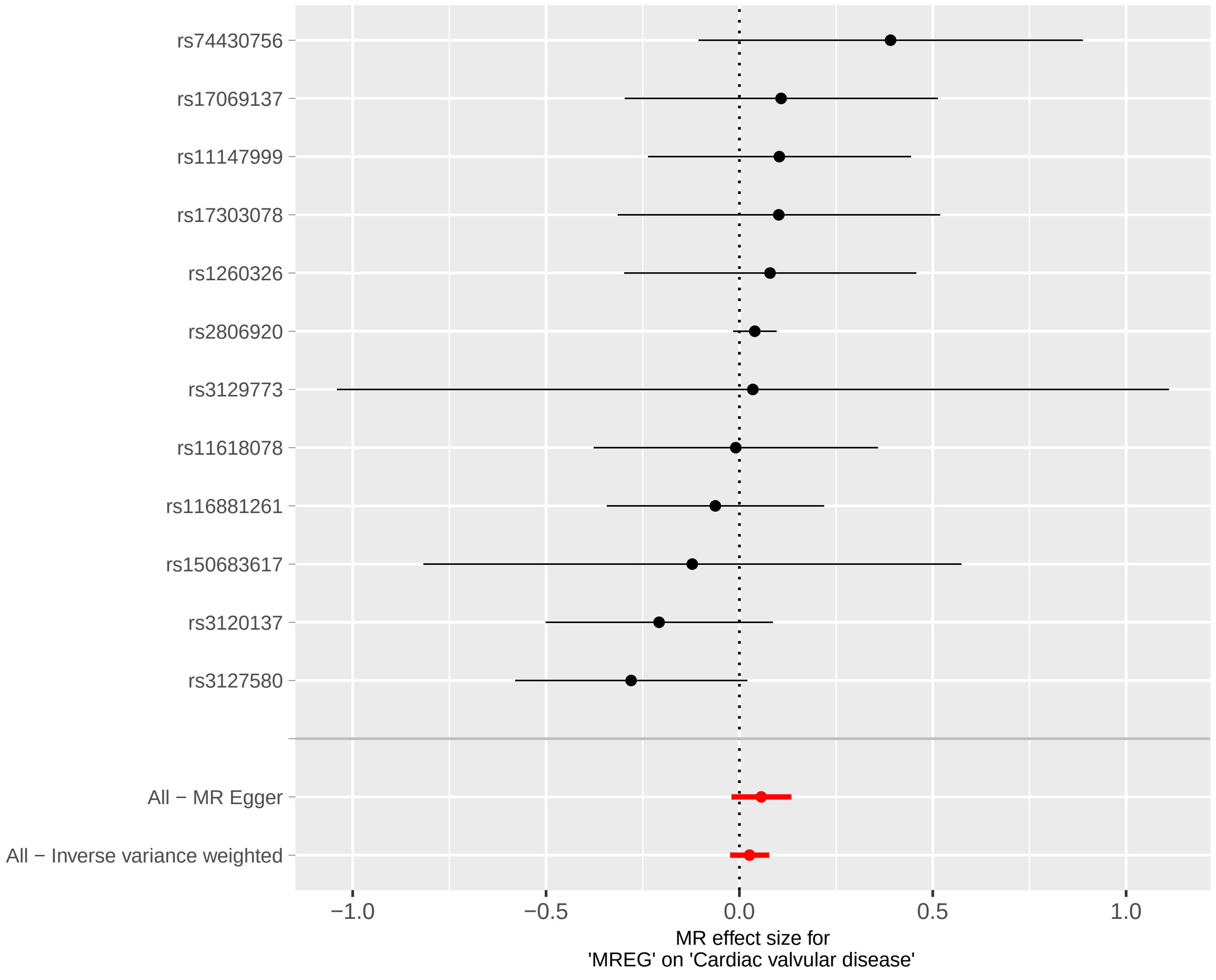
rs74430756
rs17069137
rs11147999
rs17303078
rs1260326
rs2806920
rs3129773
rs11618078
rs116881261
rs150683617
rs3120137
rs3127580
All − MR Egger
All − Inverse variance weighted
−1.0
−0.5
0.0
0.5
1.0
MR effect size for
'MREG' on 'Cardiac valvular disease'

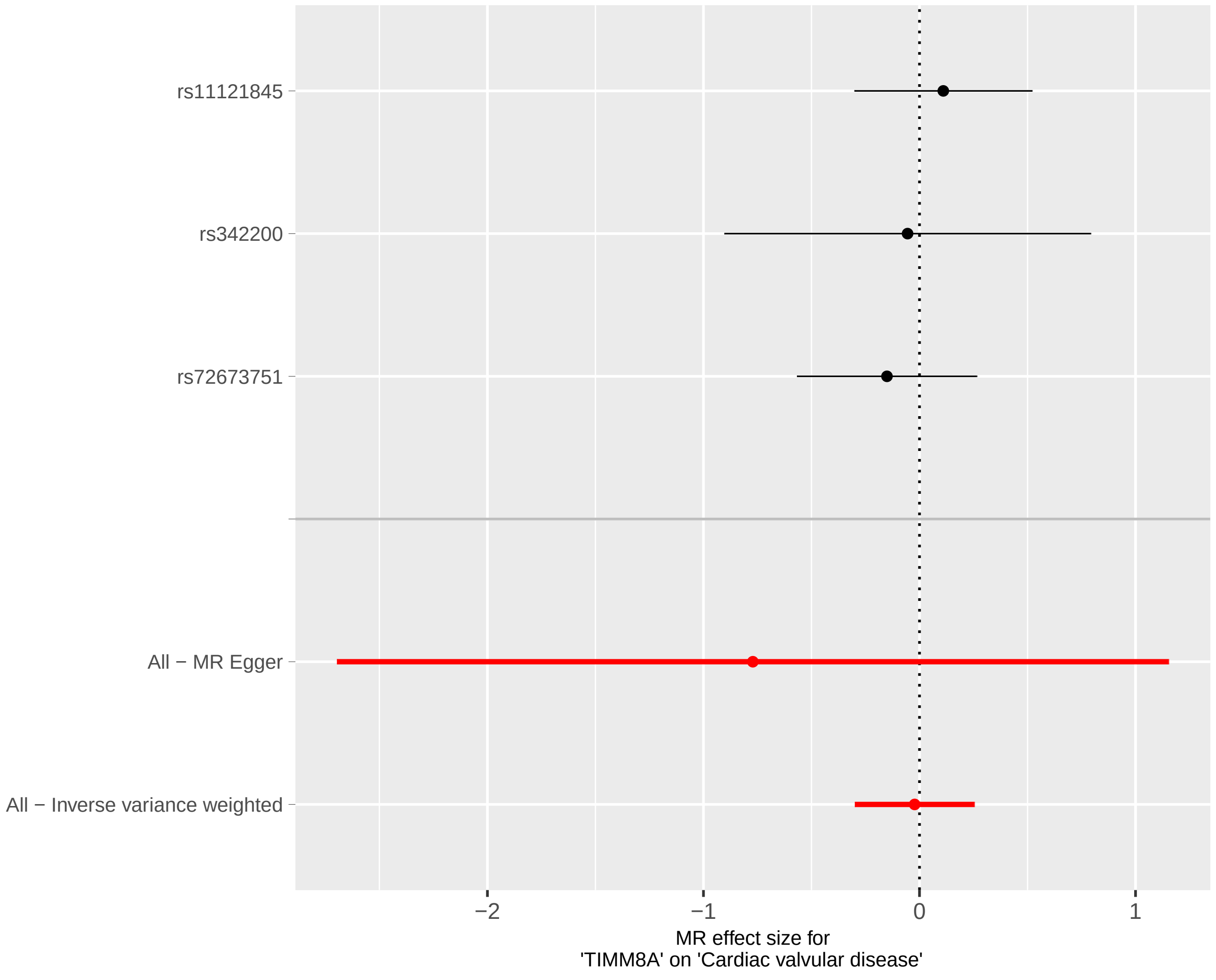
rs11121845
rs342200
rs72673751
All − MR Egger
All − Inverse variance weighted
−2
−1
0
1
MR effect size for
'TIMM8A' on 'Cardiac valvular disease'

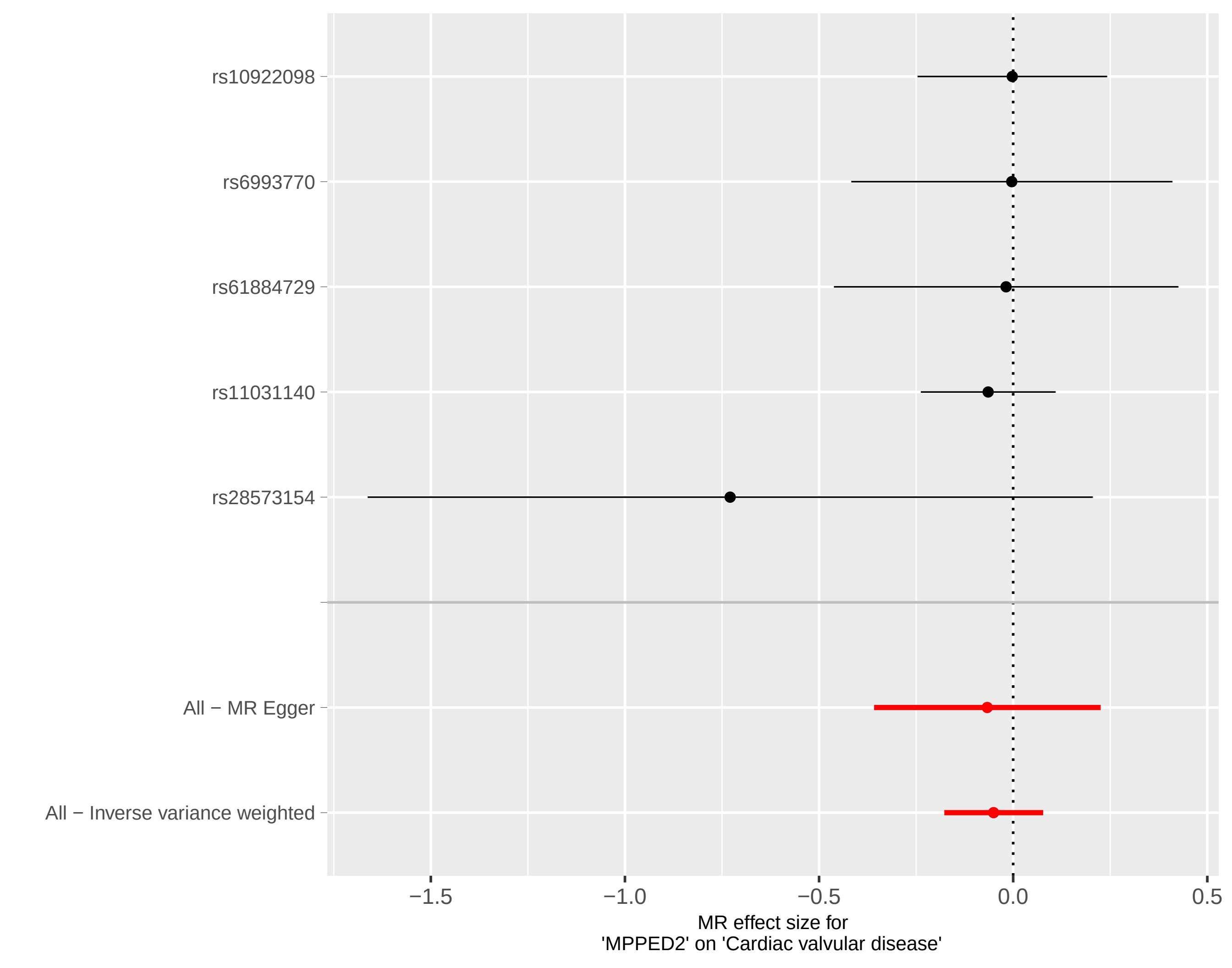

rs10922098
rs6993770
rs61884729
rs11031140
rs28573154
All − MR Egger
All − Inverse variance weighted
−1.5
−1.0
−0.5
0.0
0.5
MR effect size for
'MPPED2' on 'Cardiac valvular disease'

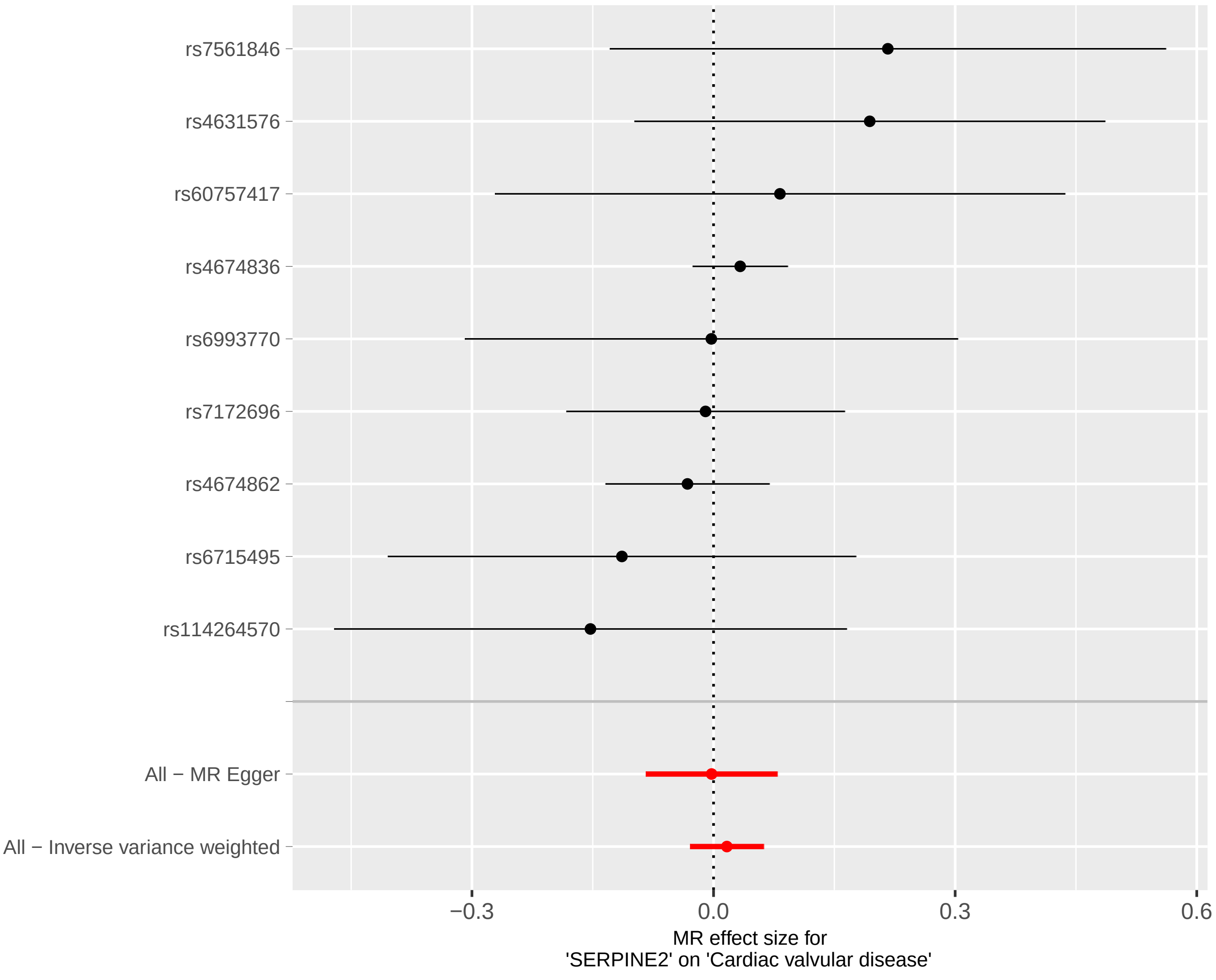
rs7561846
rs4631576
rs60757417
rs4674836
rs6993770
rs7172696
rs4674862
rs6715495
rs114264570
All − MR Egger
All − Inverse variance weighted
−0.3
0.0
0.3
0.6
MR effect size for
'SERPINE2' on 'Cardiac valvular disease'

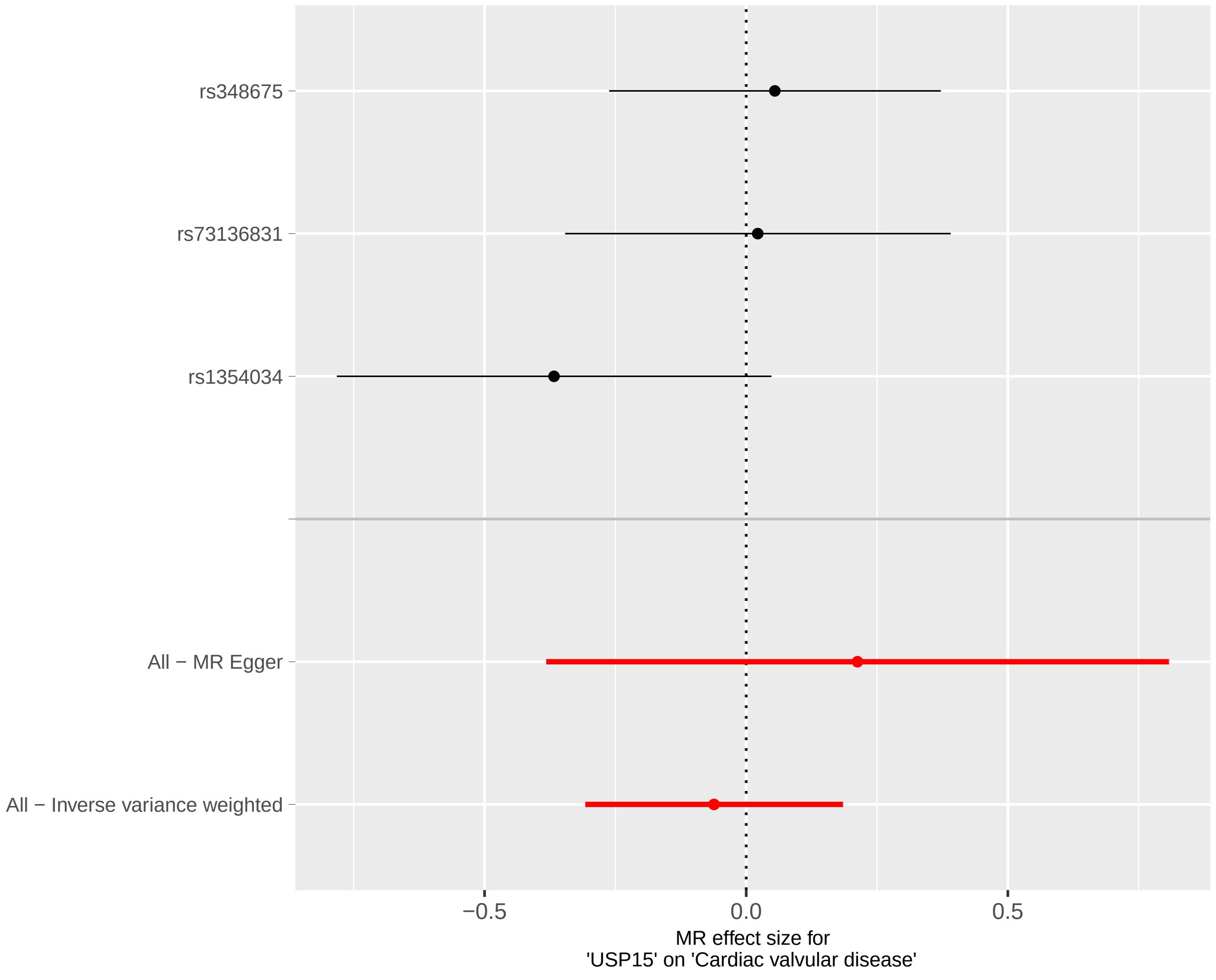
rs348675
rs73136831
rs1354034
All − MR Egger
All − Inverse variance weighted
−0.5
0.0
0.5
MR effect size for
'USP15' on 'Cardiac valvular disease'

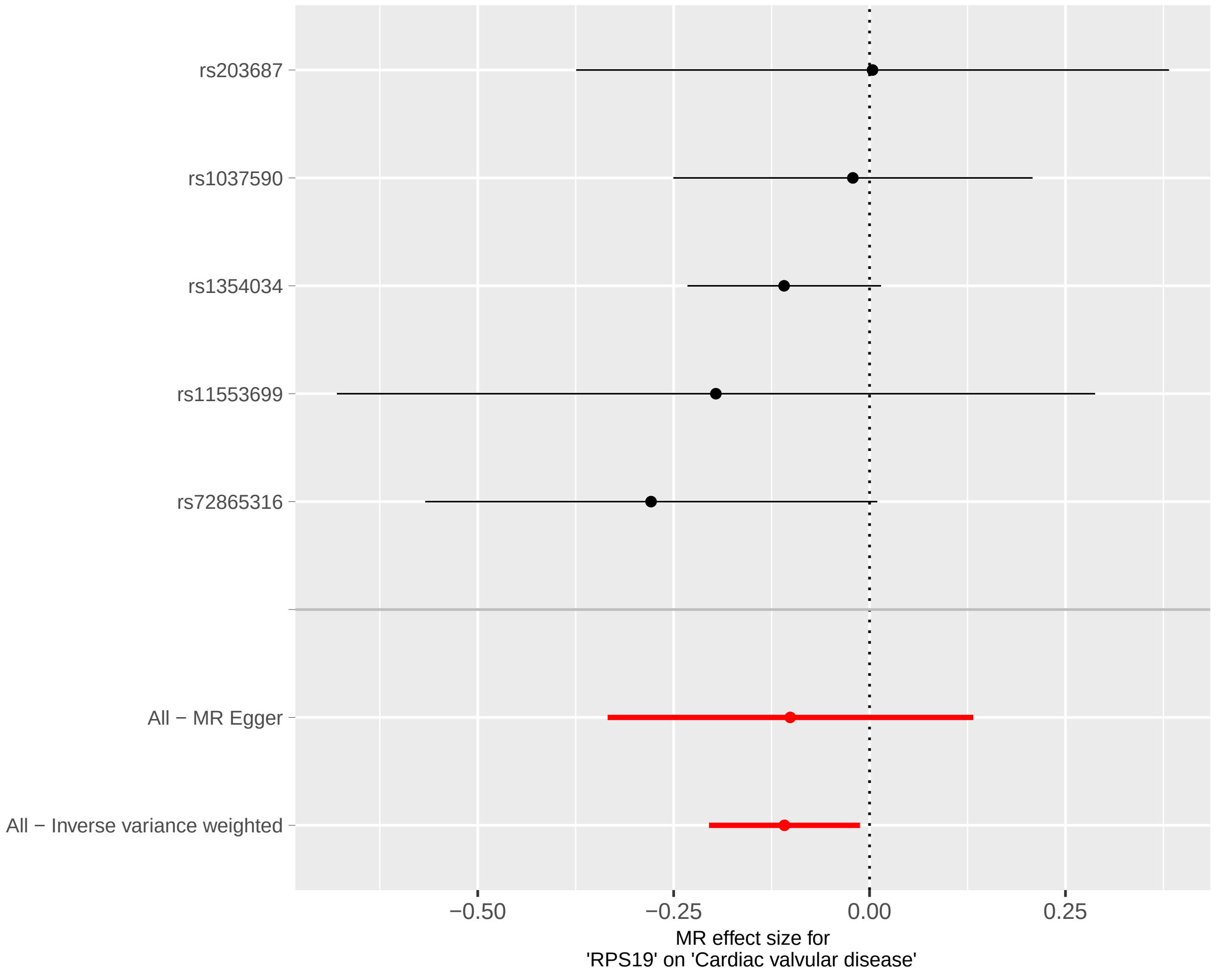
rs203687
rs1037590
rs1354034
rs11553699
rs72865316
All − MR Egger
All − Inverse variance weighted
−0.50
−0.25
0.00
0.25
MR effect size for
'RPS19' on 'Cardiac valvular disease'

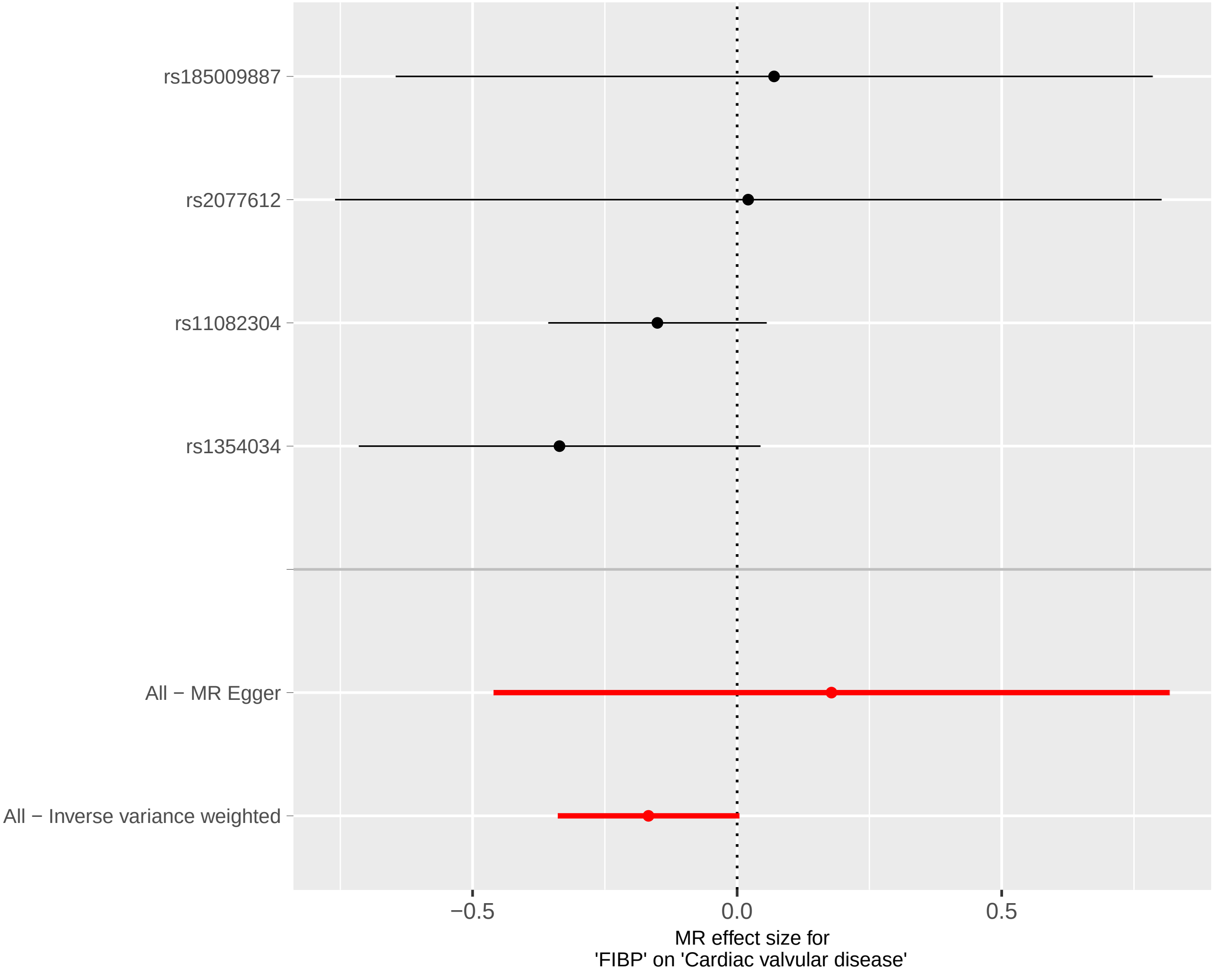
rs185009887
rs2077612
rs11082304
rs1354034
All − MR Egger
All − Inverse variance weighted
−0.5
0.0
0.5
MR effect size for
'FIBP' on 'Cardiac valvular disease'

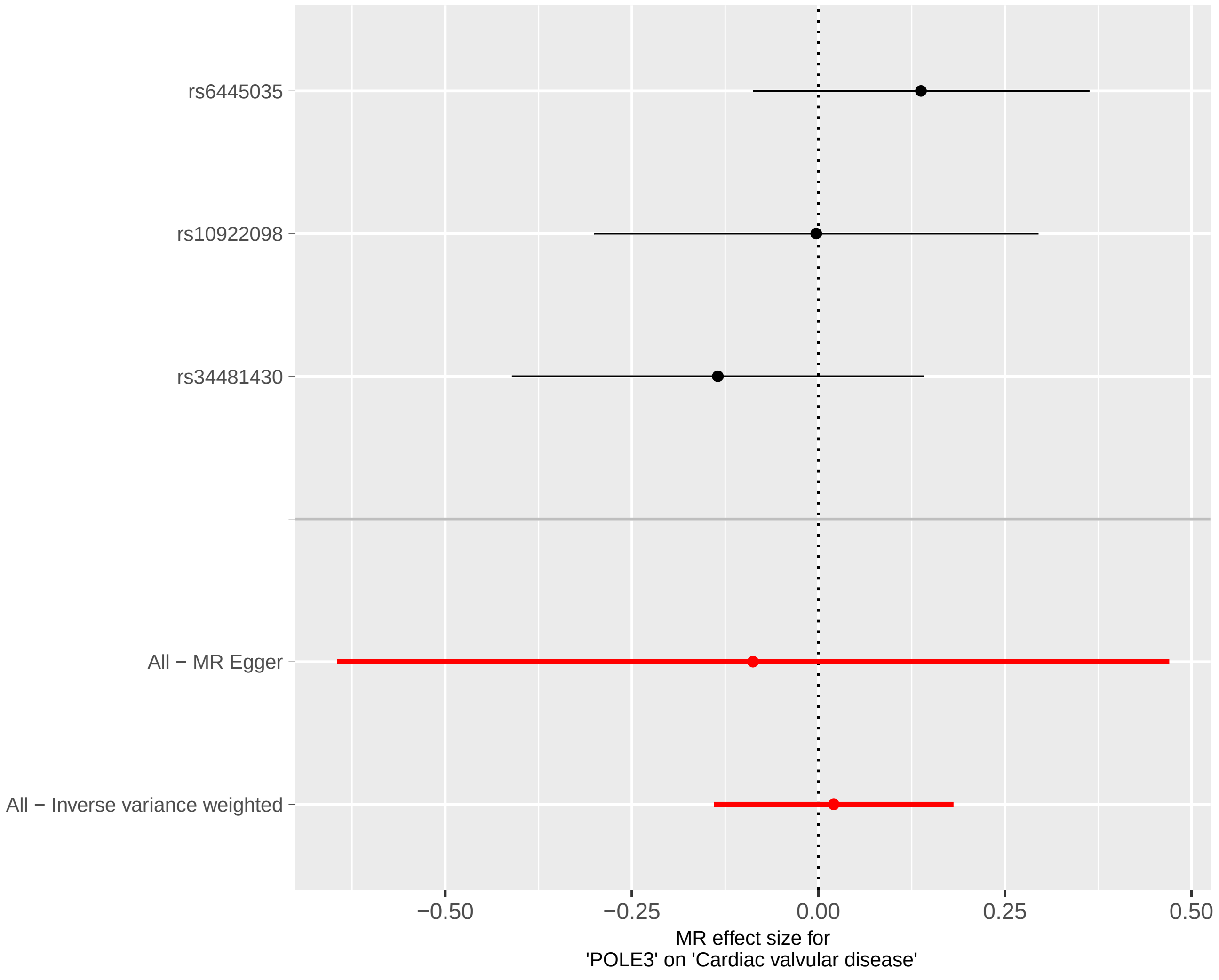

rs6445035
rs10922098
rs34481430
All − MR Egger
All − Inverse variance weighted
−0.50
−0.25
0.00
0.25
0.50
MR effect size for
'POLE3' on 'Cardiac valvular disease'

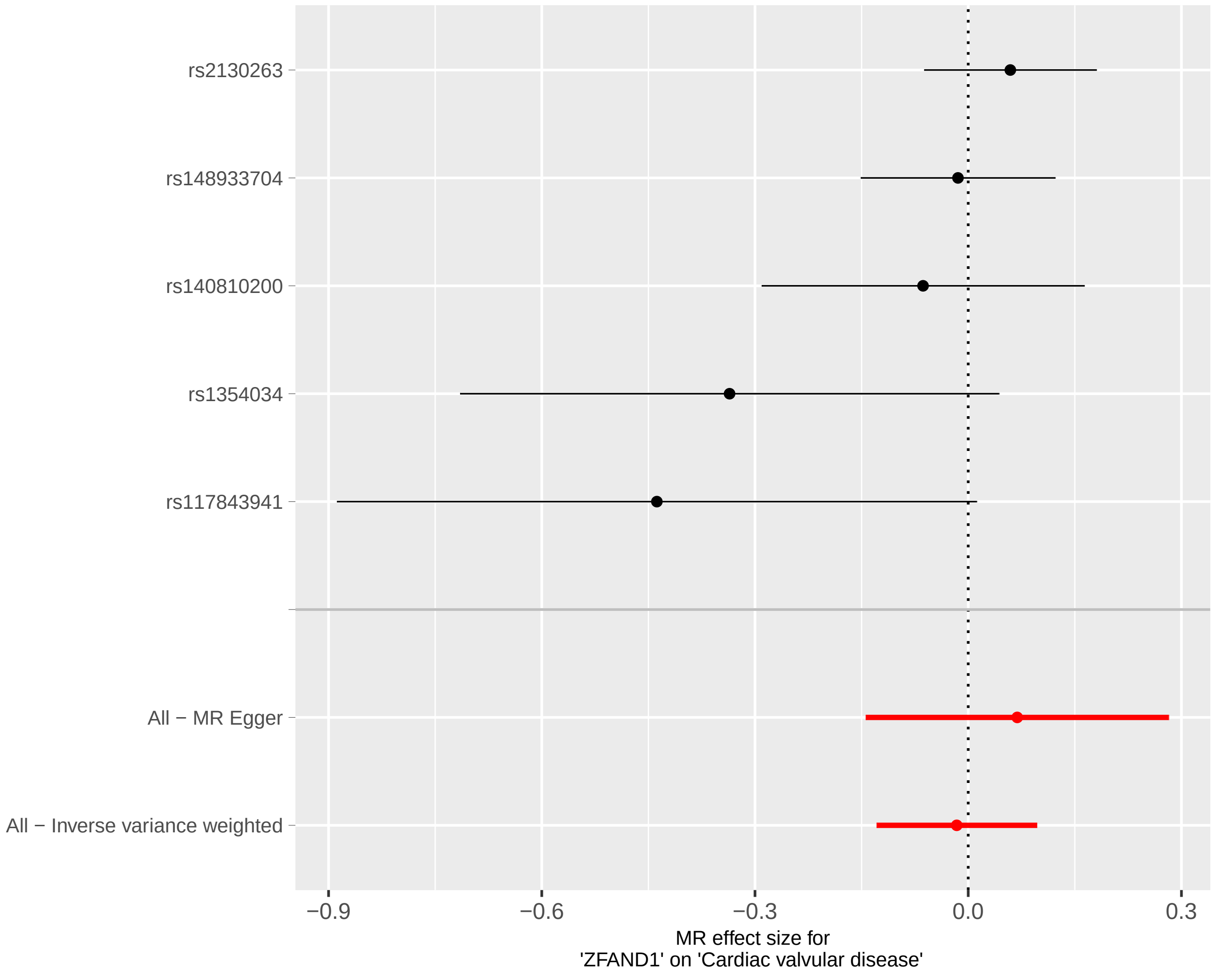
rs2130263
rs148933704
rs140810200
rs1354034
rs117843941
All − MR Egger
All − Inverse variance weighted
−0.9
−0.6
−0.3
0.0
0.3
MR effect size for
'ZFAND1' on 'Cardiac valvular disease'

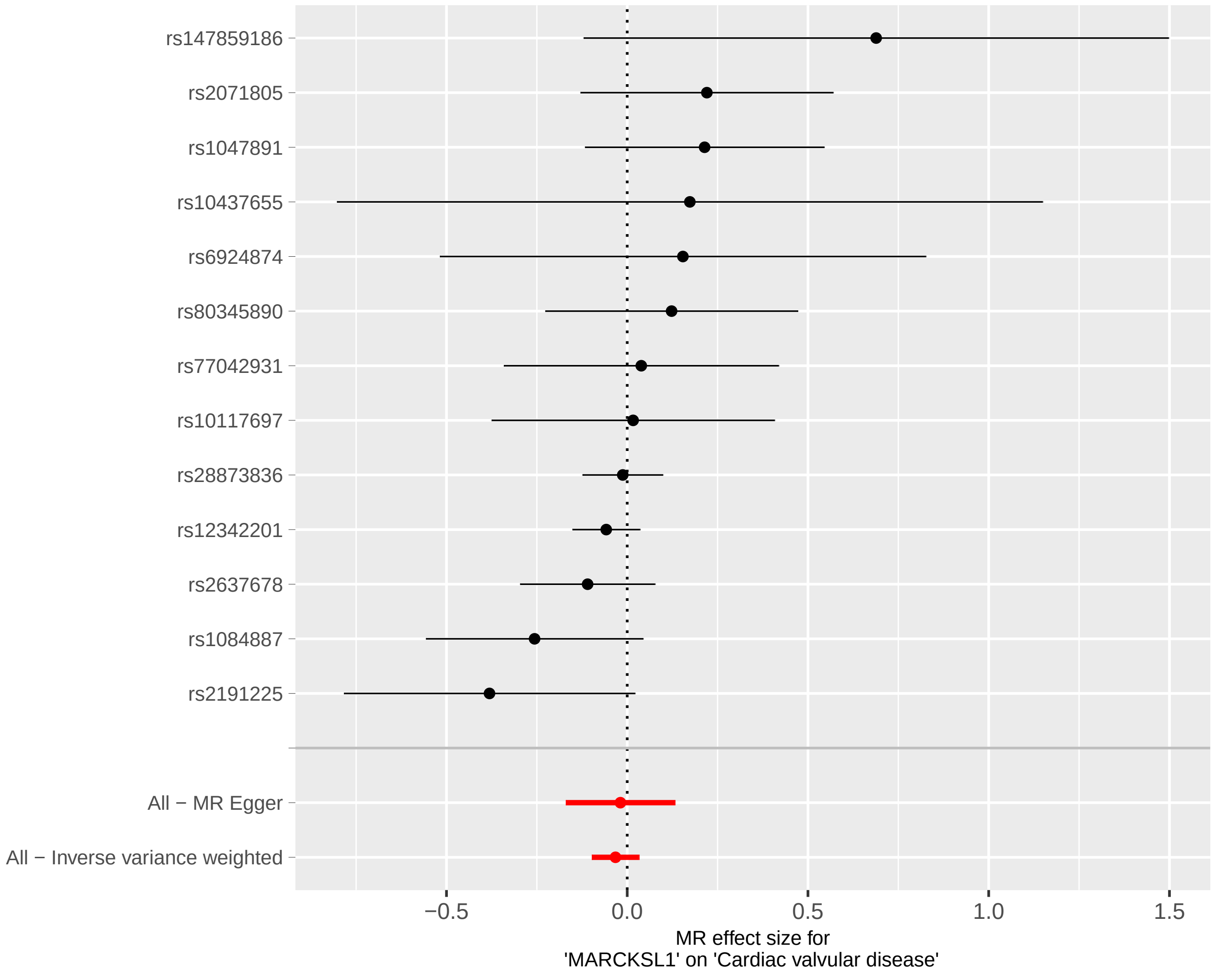
rs147859186
rs2071805
rs1047891
rs10437655
rs6924874
rs80345890
rs77042931
rs10117697
rs28873836
rs12342201
rs2637678
rs1084887
rs2191225
All − MR Egger
All − Inverse variance weighted
−0.5
0.0
0.5
1.0
1.5
MR effect size for
'MARCKSL1' on 'Cardiac valvular disease'

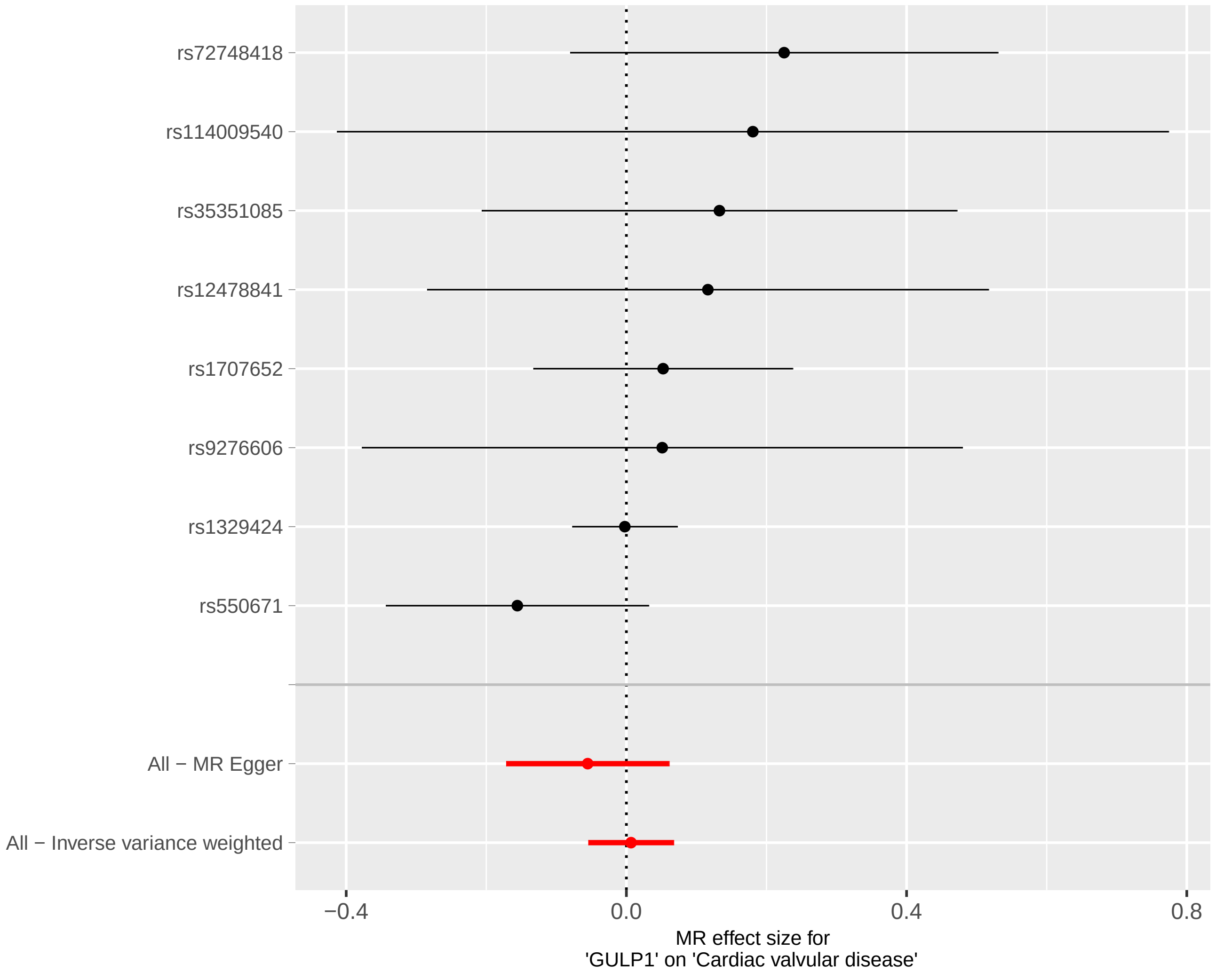
rs72748418
rs114009540
rs35351085
rs12478841
rs1707652
rs9276606
rs1329424
rs550671
All − MR Egger
All − Inverse variance weighted
−0.4
0.0
0.4
0.8
MR effect size for
'GULP1' on 'Cardiac valvular disease'

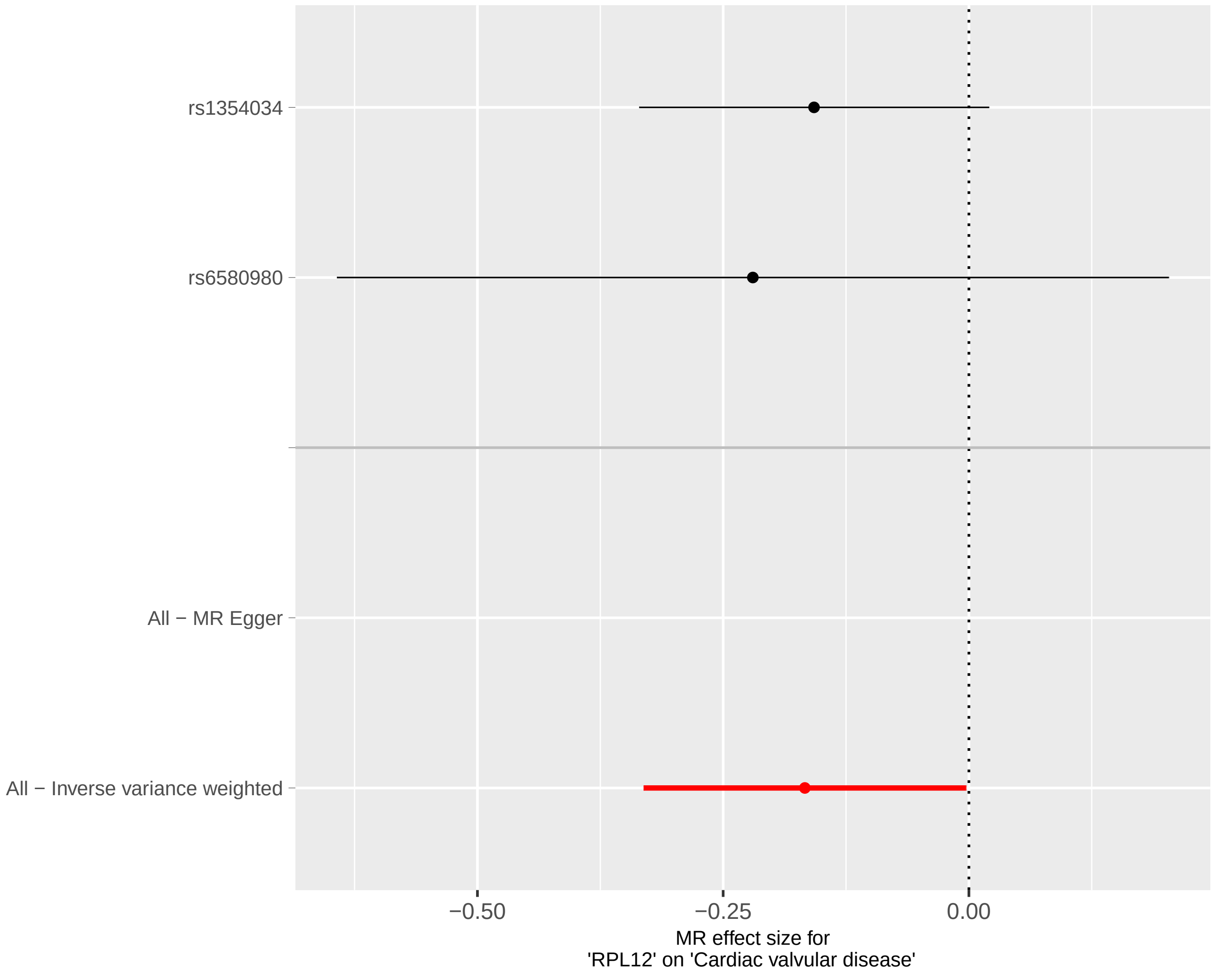
rs1354034
rs6580980
All − MR Egger
All − Inverse variance weighted
−0.50
−0.25
0.00
MR effect size for
'RPL12' on 'Cardiac valvular disease'

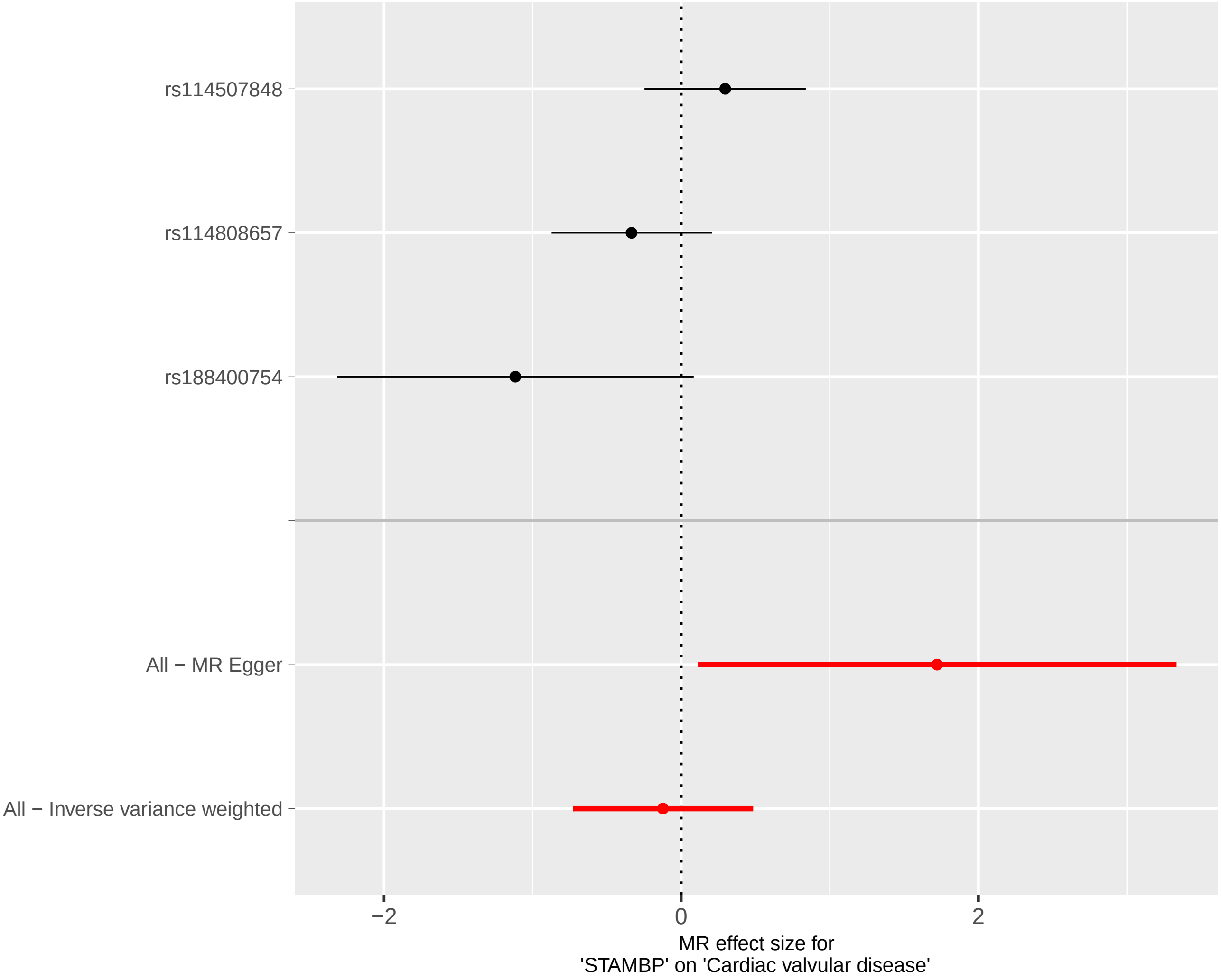

rs114507848
rs114808657
rs188400754
All − MR Egger
All − Inverse variance weighted
−2
0
2
MR effect size for
'STAMBP' on 'Cardiac valvular disease'

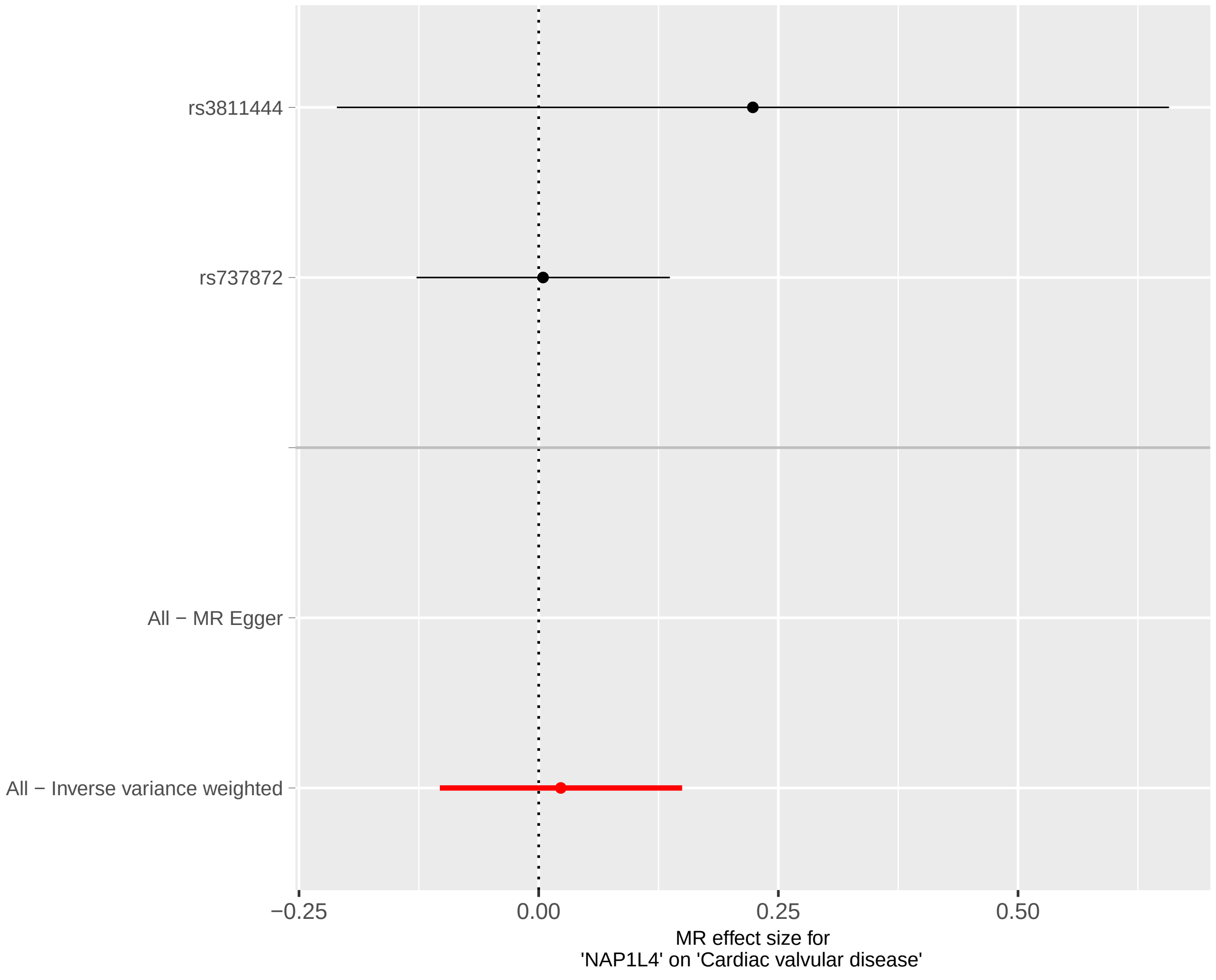
rs3811444
rs737872
All − MR Egger
All − Inverse variance weighted
−0.25
0.00
0.25
0.50
MR effect size for
'NAP1L4' on 'Cardiac valvular disease'

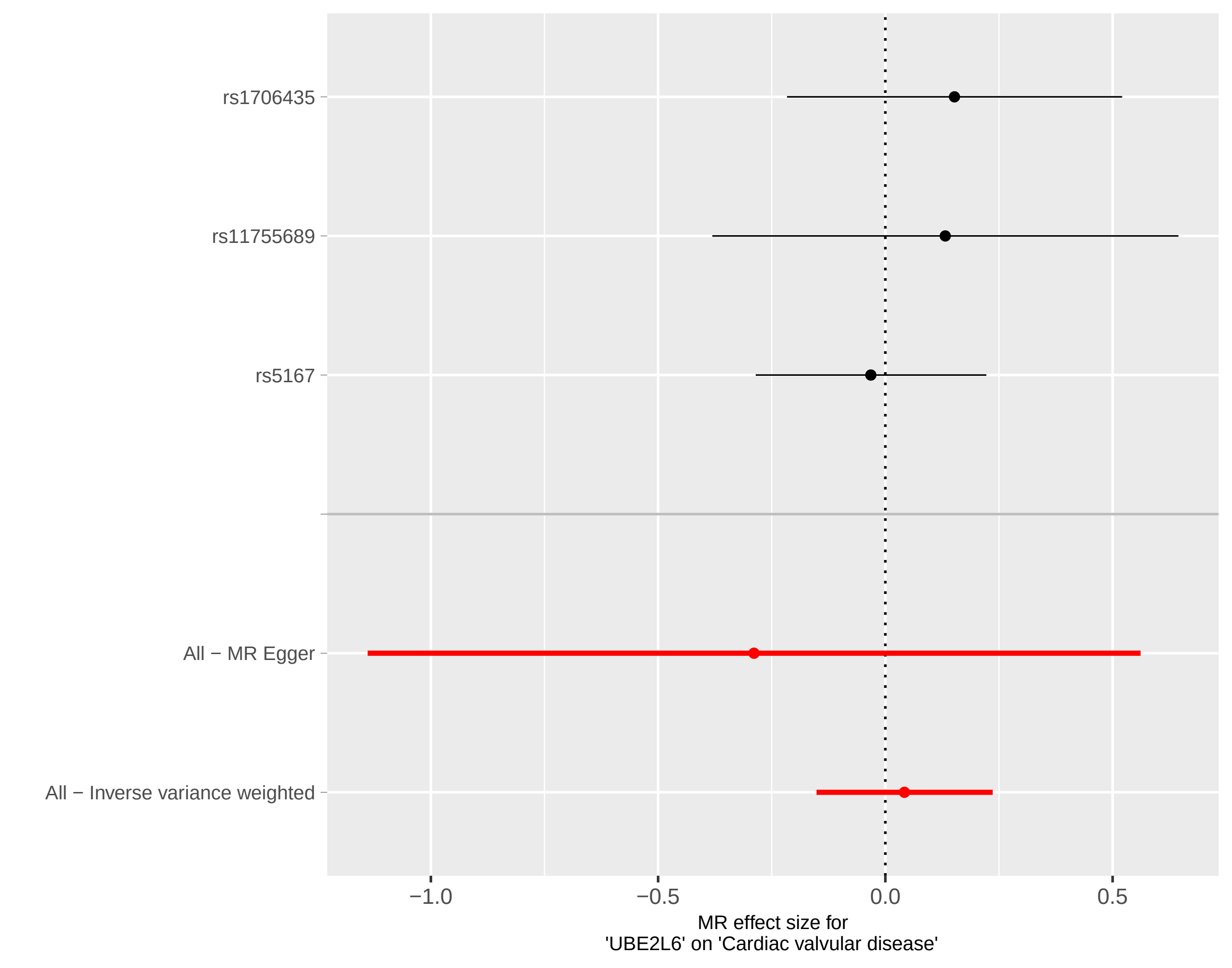
rs1706435
rs11755689
rs5167
All − MR Egger
All − Inverse variance weighted
−1.0
−0.5
0.0
0.5
MR effect size for
'UBE2L6' on 'Cardiac valvular disease'

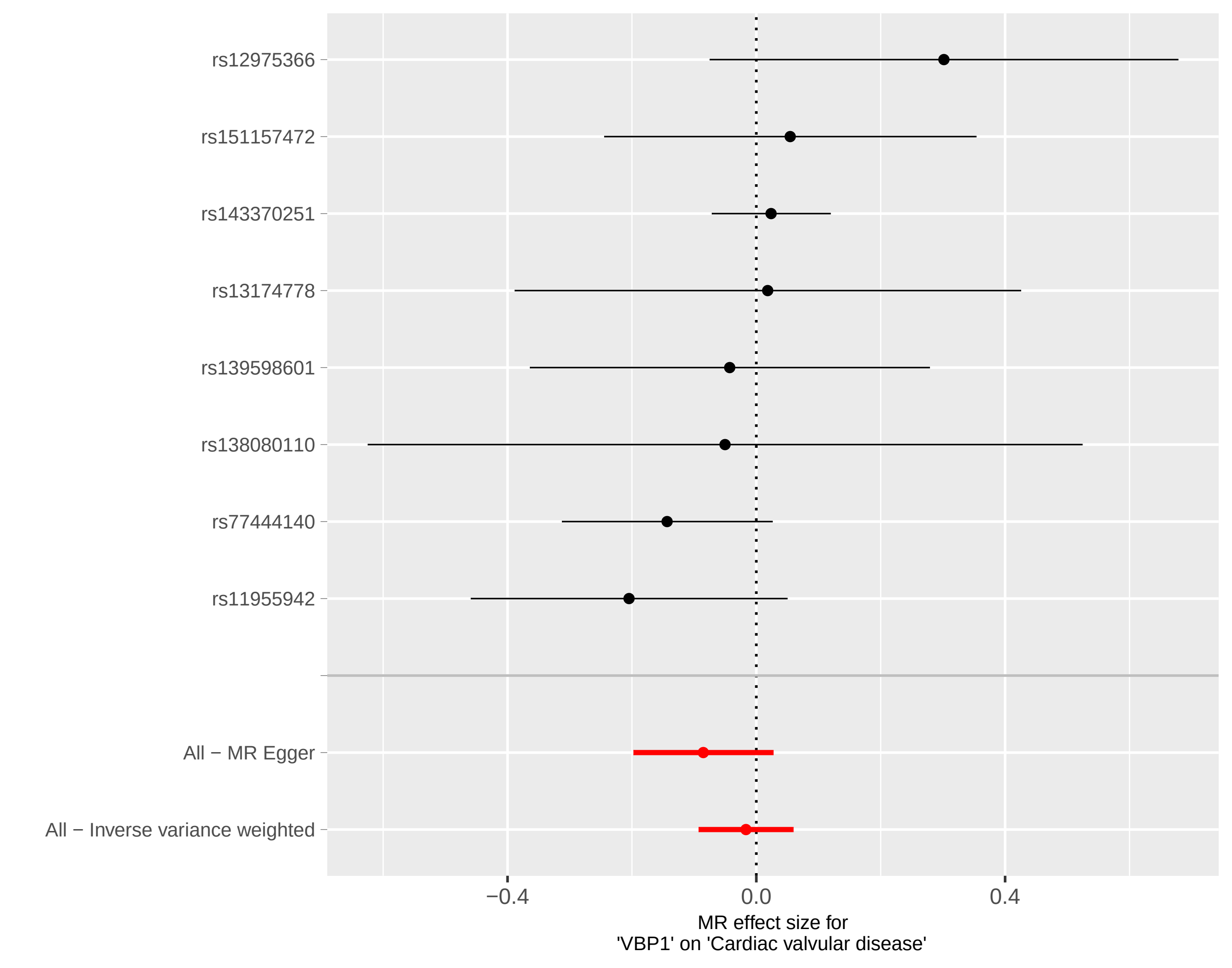

rs12975366
rs151157472
rs143370251
rs13174778
rs139598601
rs138080110
rs77444140
rs11955942
All − MR Egger
All − Inverse variance weighted
−0.4
0.0
0.4
MR effect size for
'VBP1' on 'Cardiac valvular disease'

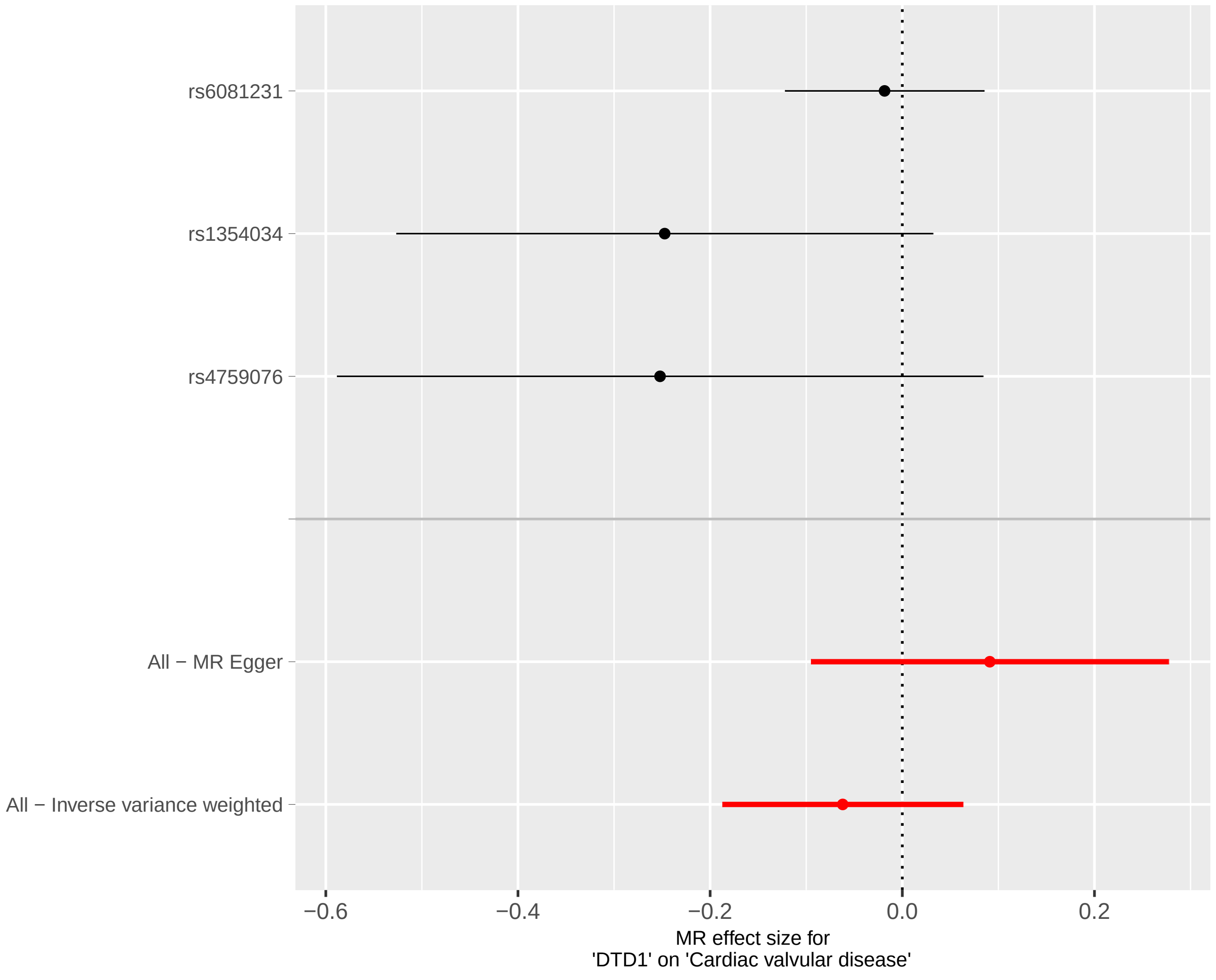

rs6081231
rs1354034
rs4759076
All − MR Egger
All − Inverse variance weighted
−0.6
−0.4
−0.2
0.0
0.2
MR effect size for
'DTD1' on 'Cardiac valvular disease'

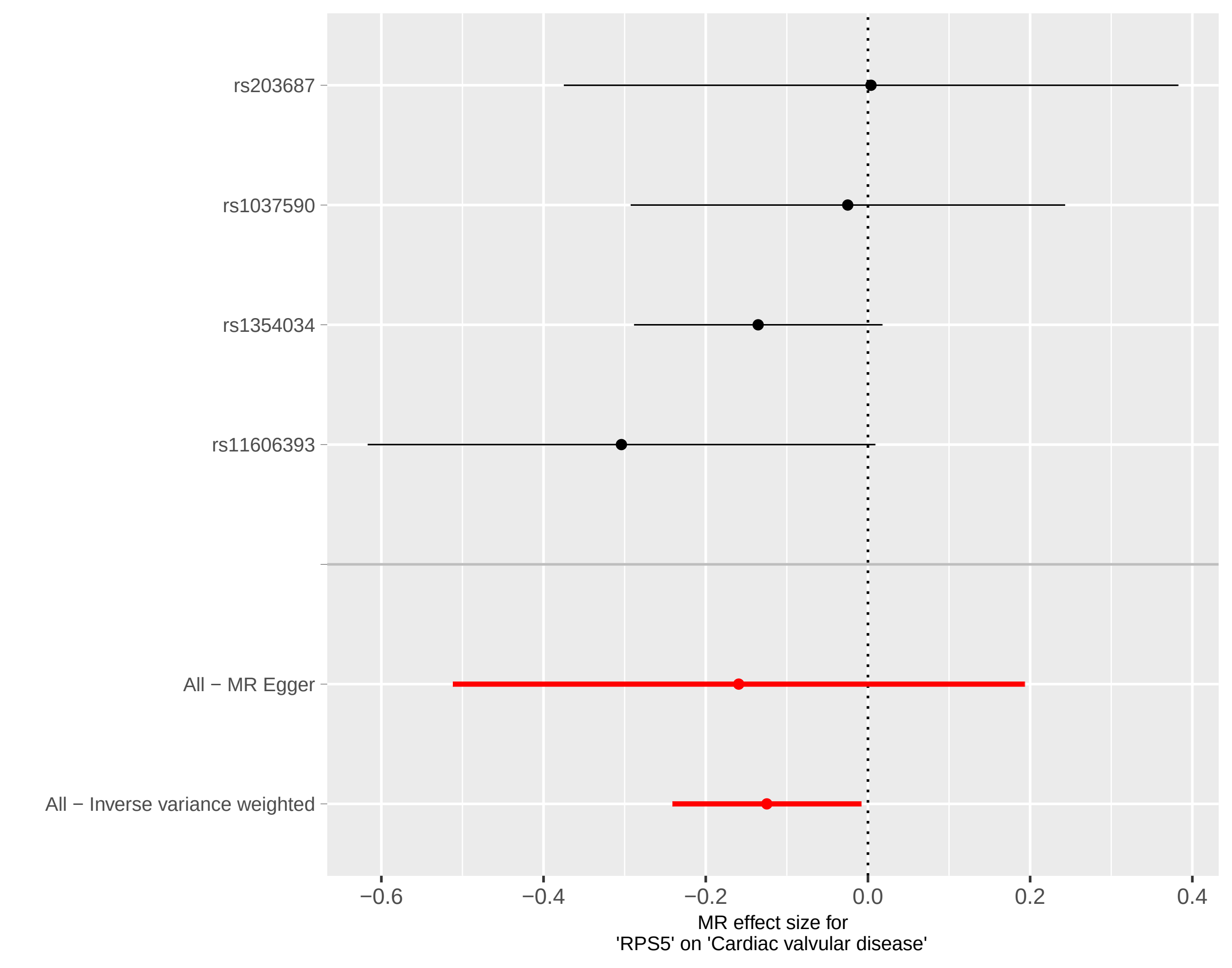
rs203687
rs1037590
rs1354034
rs11606393
All − MR Egger
All − Inverse variance weighted
−0.6
−0.4
−0.2
0.0
0.2
0.4
MR effect size for
'RPS5' on 'Cardiac valvular disease'

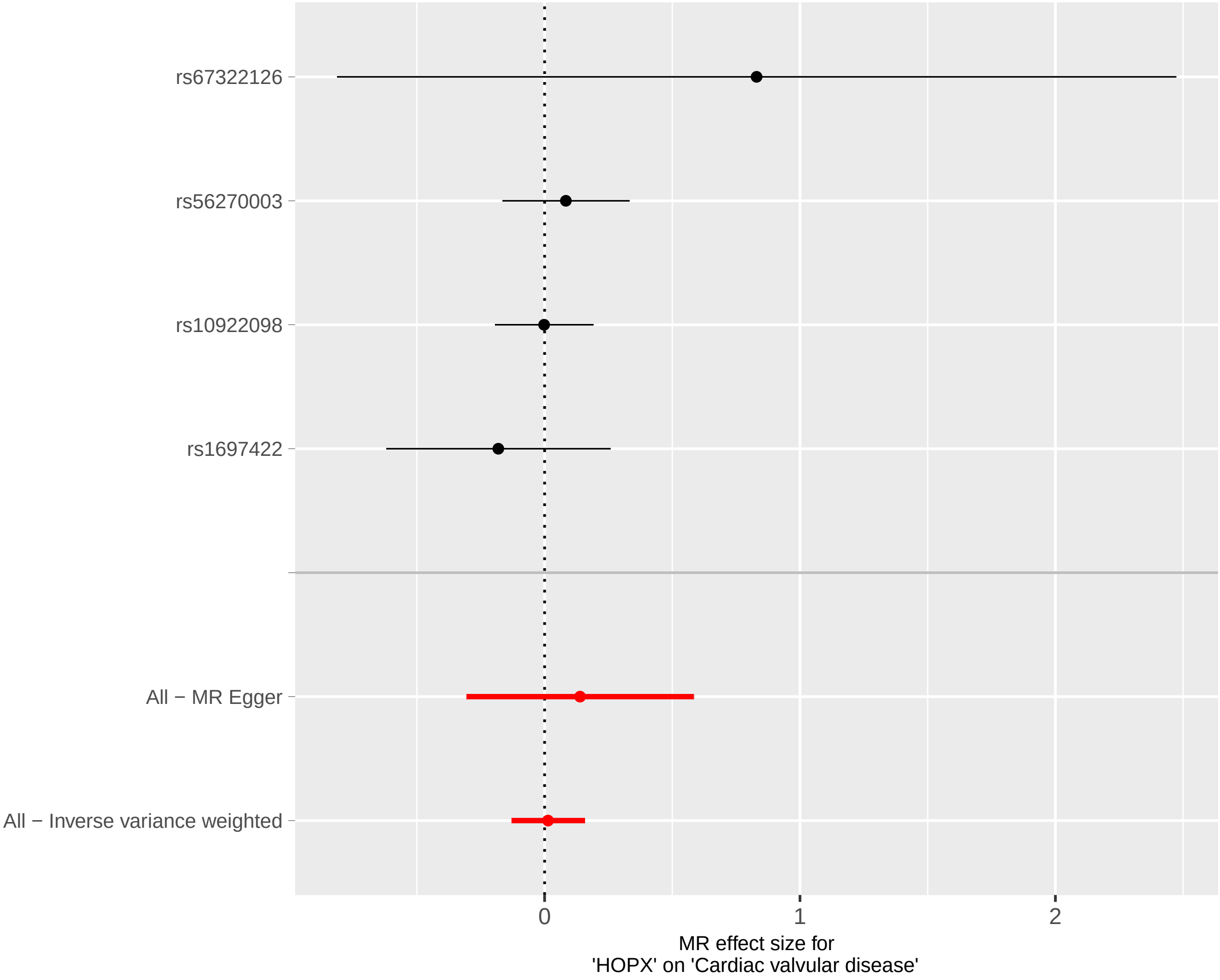
rs67322126
rs56270003
rs10922098
rs1697422
All − MR Egger
All − Inverse variance weighted
0
1
2
MR effect size for
'HOPX' on 'Cardiac valvular disease'

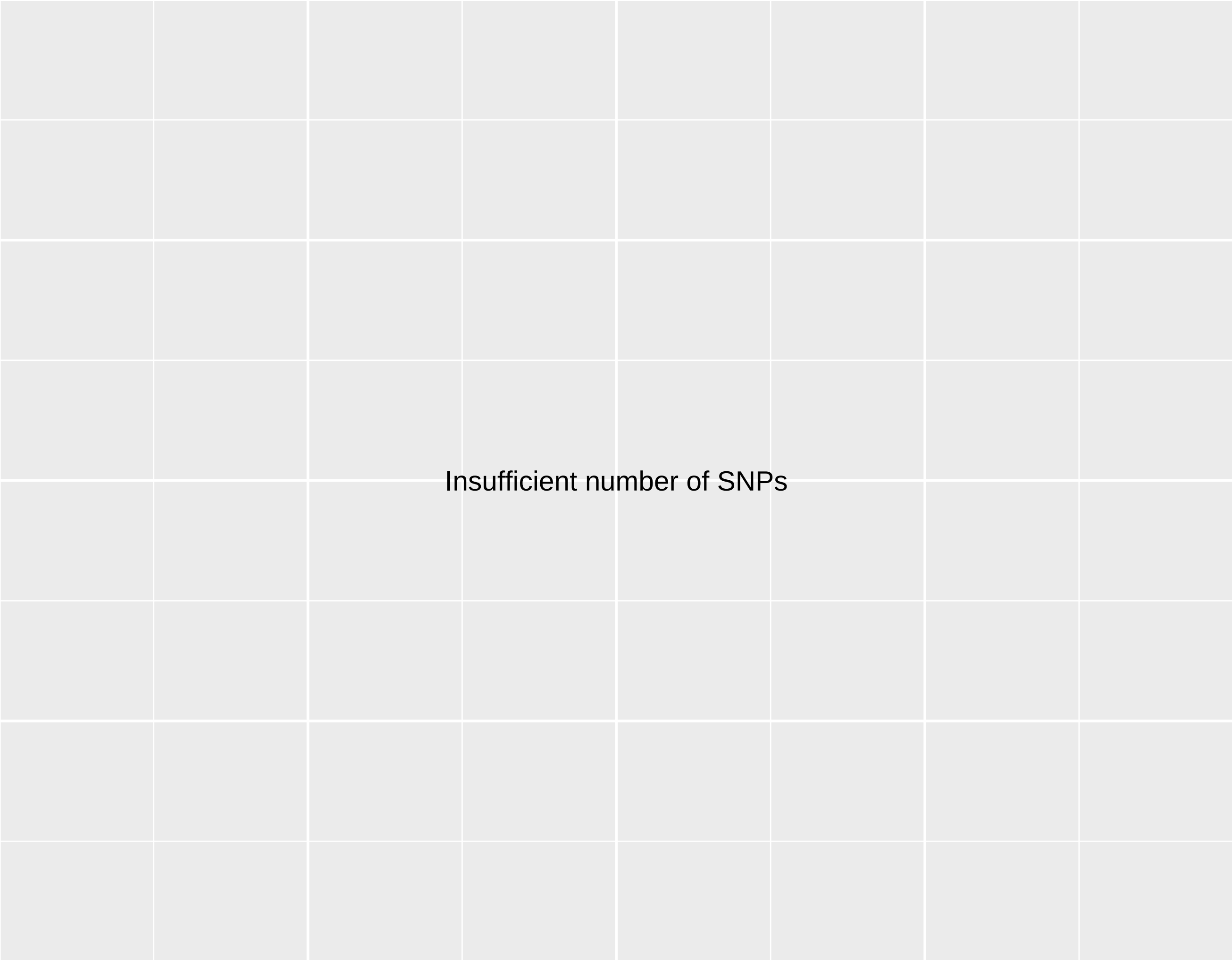
Insufficient number of SNPs

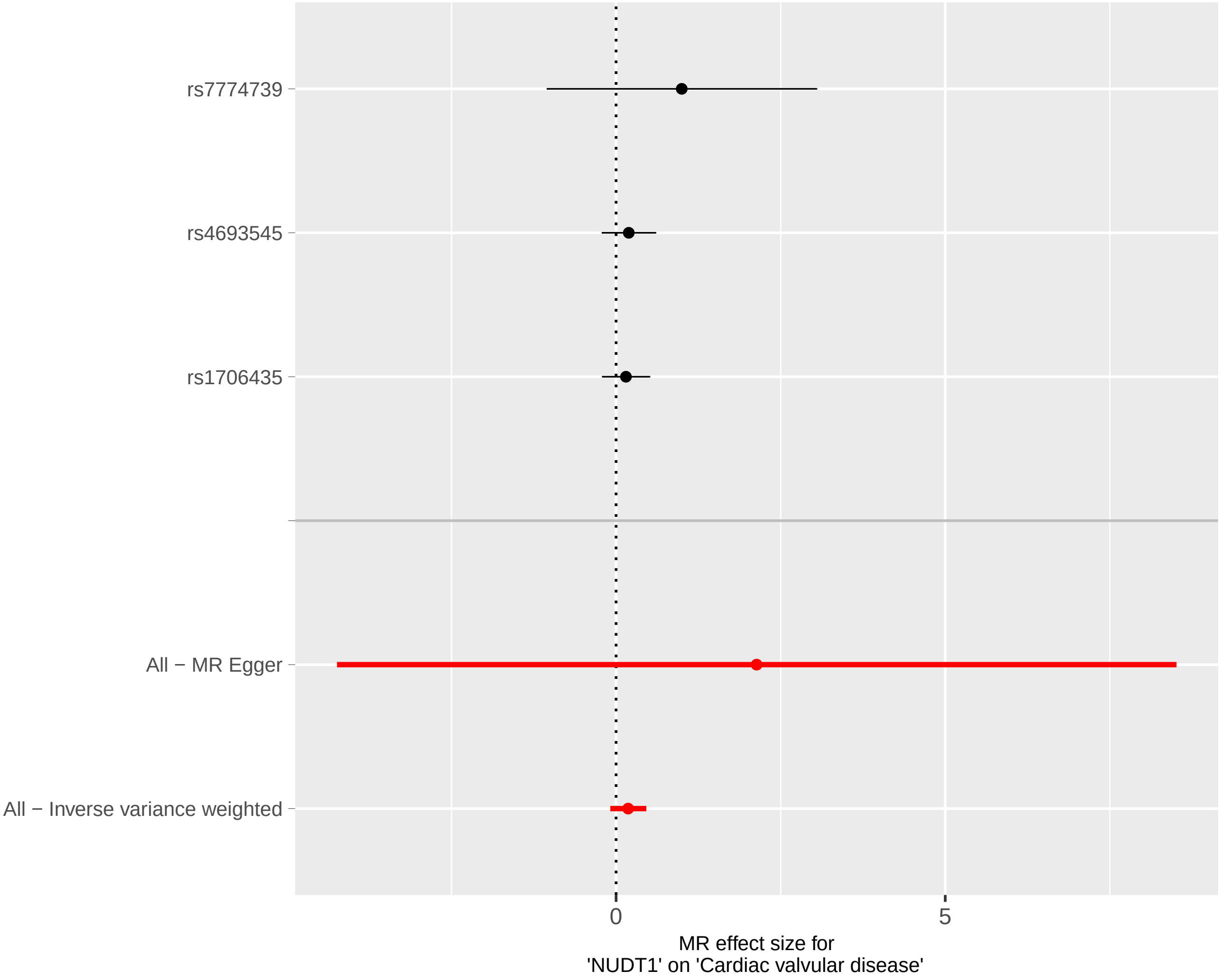
rs7774739
rs4693545
rs1706435
All − MR Egger
All − Inverse variance weighted
0
5
MR effect size for
'NUDT1' on 'Cardiac valvular disease'

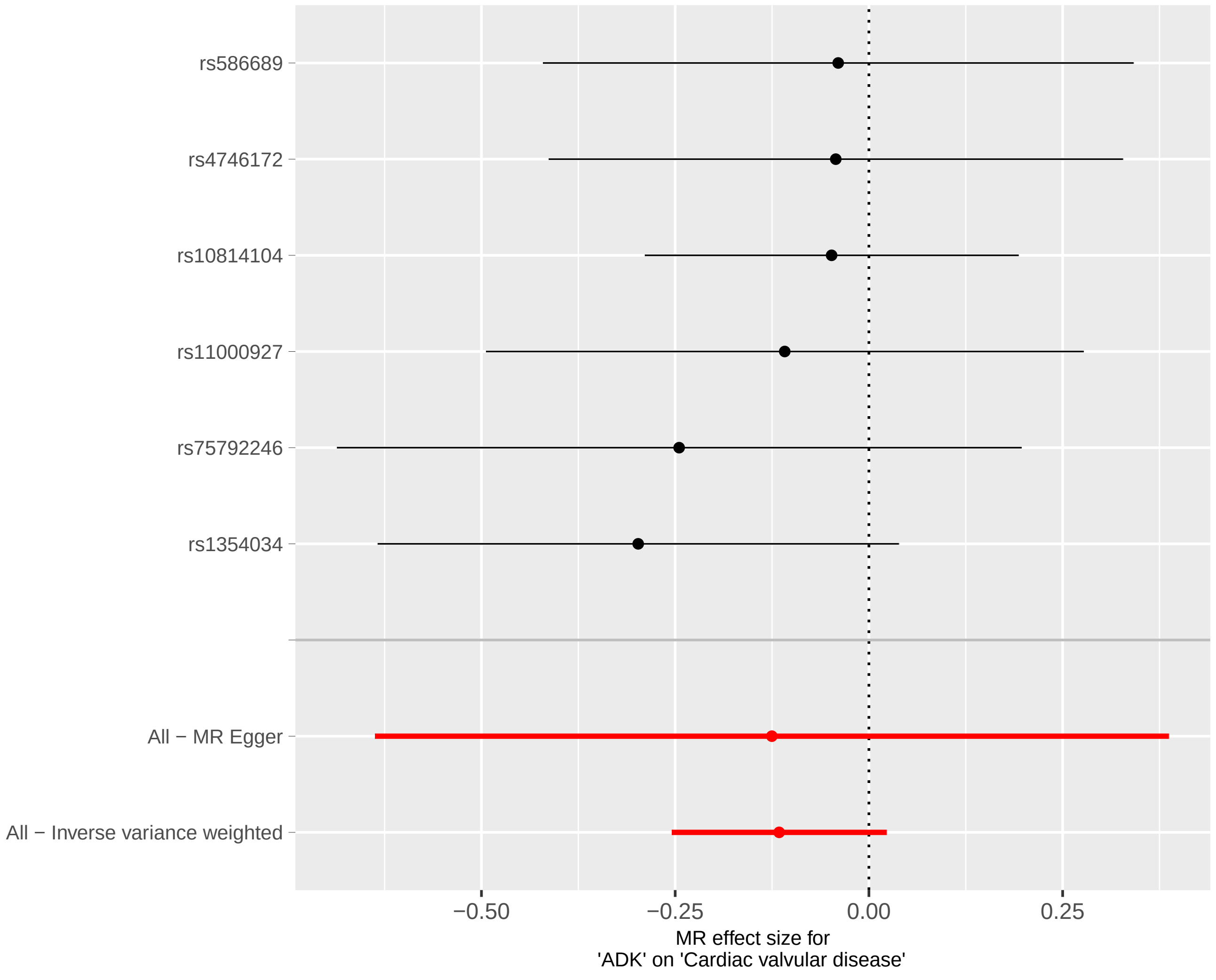
rs586689
rs4746172
rs10814104
rs11000927
rs75792246
rs1354034
All − MR Egger
All − Inverse variance weighted
−0.50
−0.25
0.00
0.25
MR effect size for
'ADK' on 'Cardiac valvular disease'

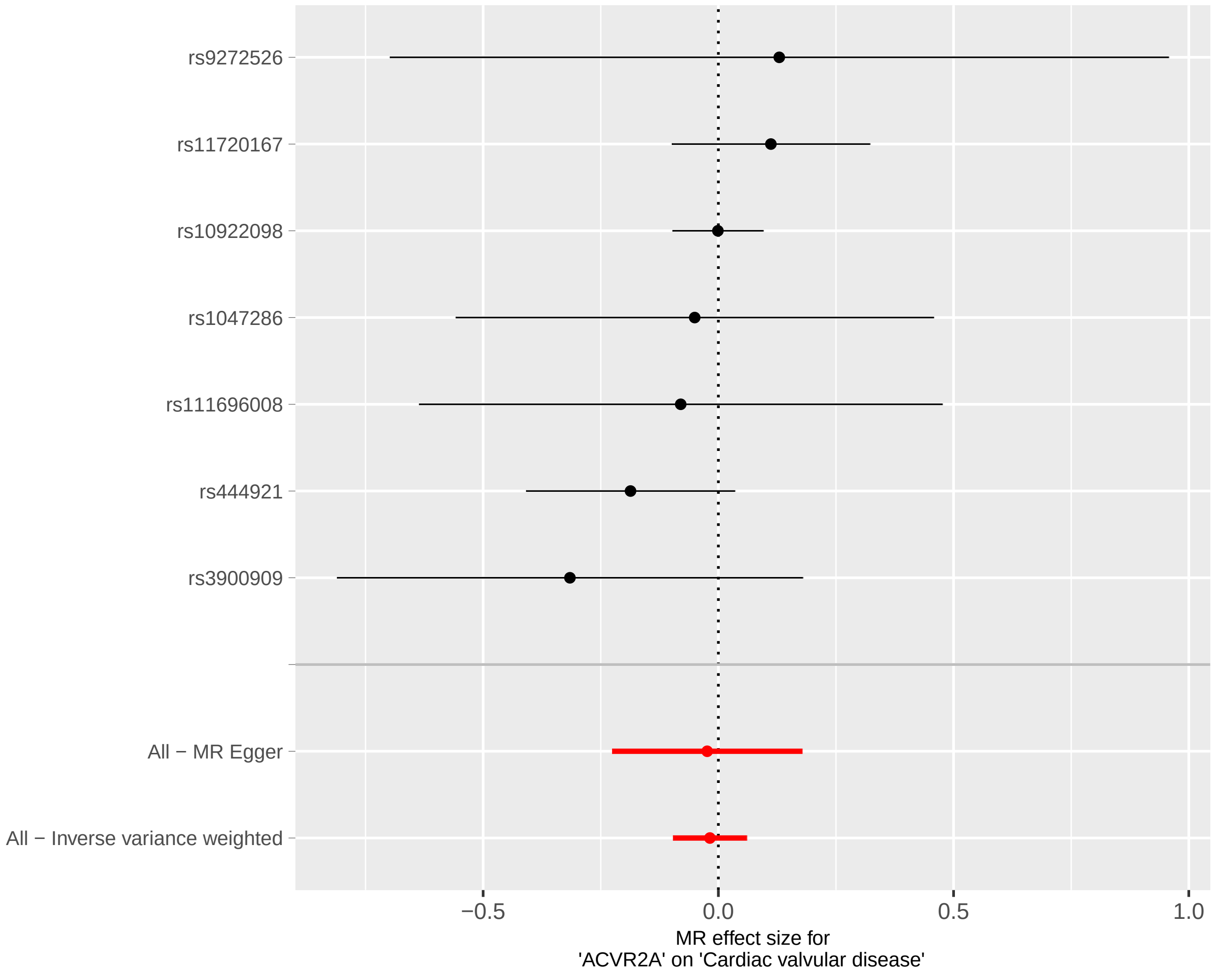
rs9272526
rs11720167
rs10922098
rs1047286
rs111696008
rs444921
rs3900909
All − MR Egger
All − Inverse variance weighted
−0.5
0.0
0.5
1.0
MR effect size for
'ACVR2A' on 'Cardiac valvular disease'

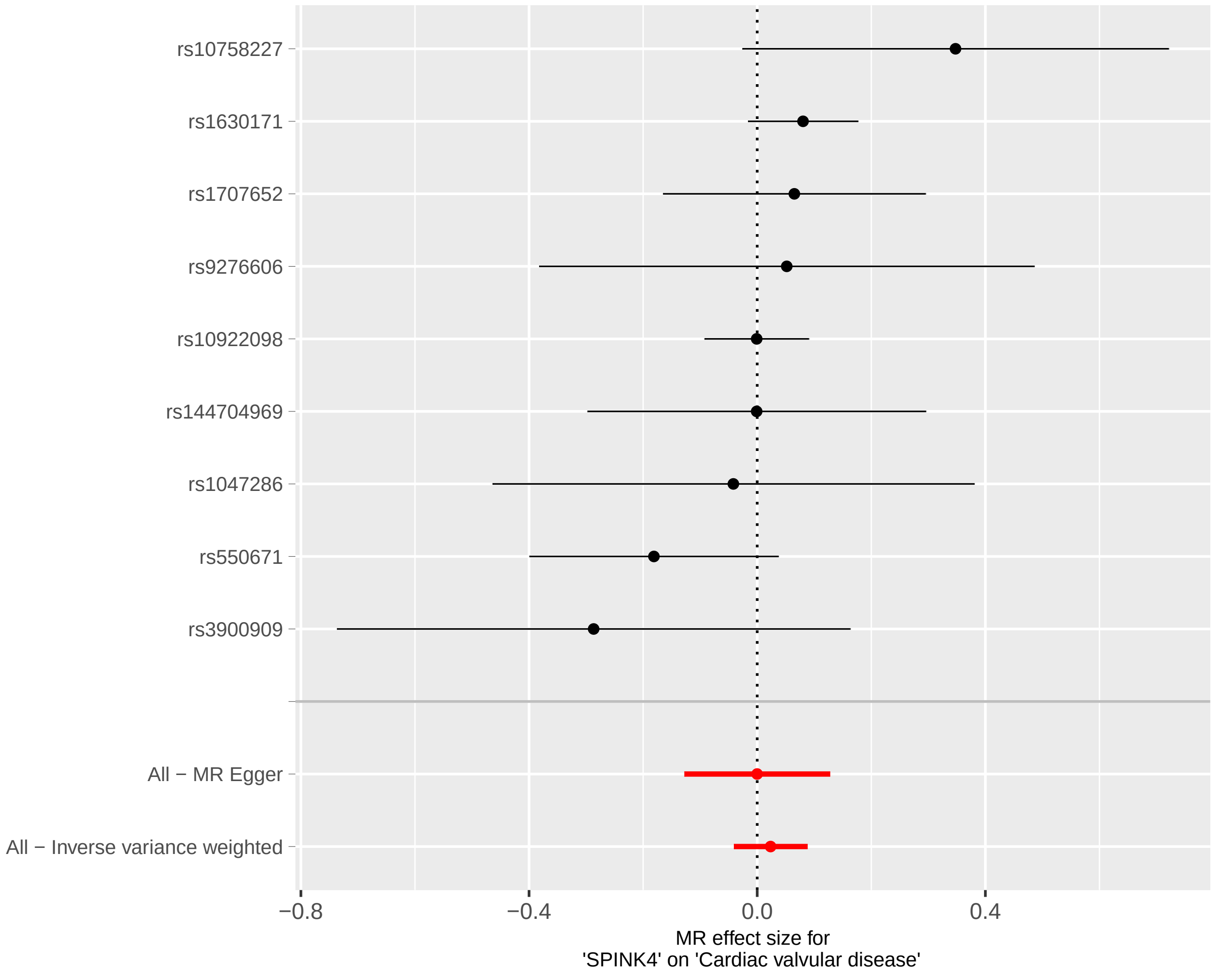
rs10758227
rs1630171
rs1707652
rs9276606
rs10922098
rs144704969
rs1047286
rs550671
rs3900909
All − MR Egger
All − Inverse variance weighted
−0.8
−0.4
0.0
0.4
MR effect size for
'SPINK4' on 'Cardiac valvular disease'

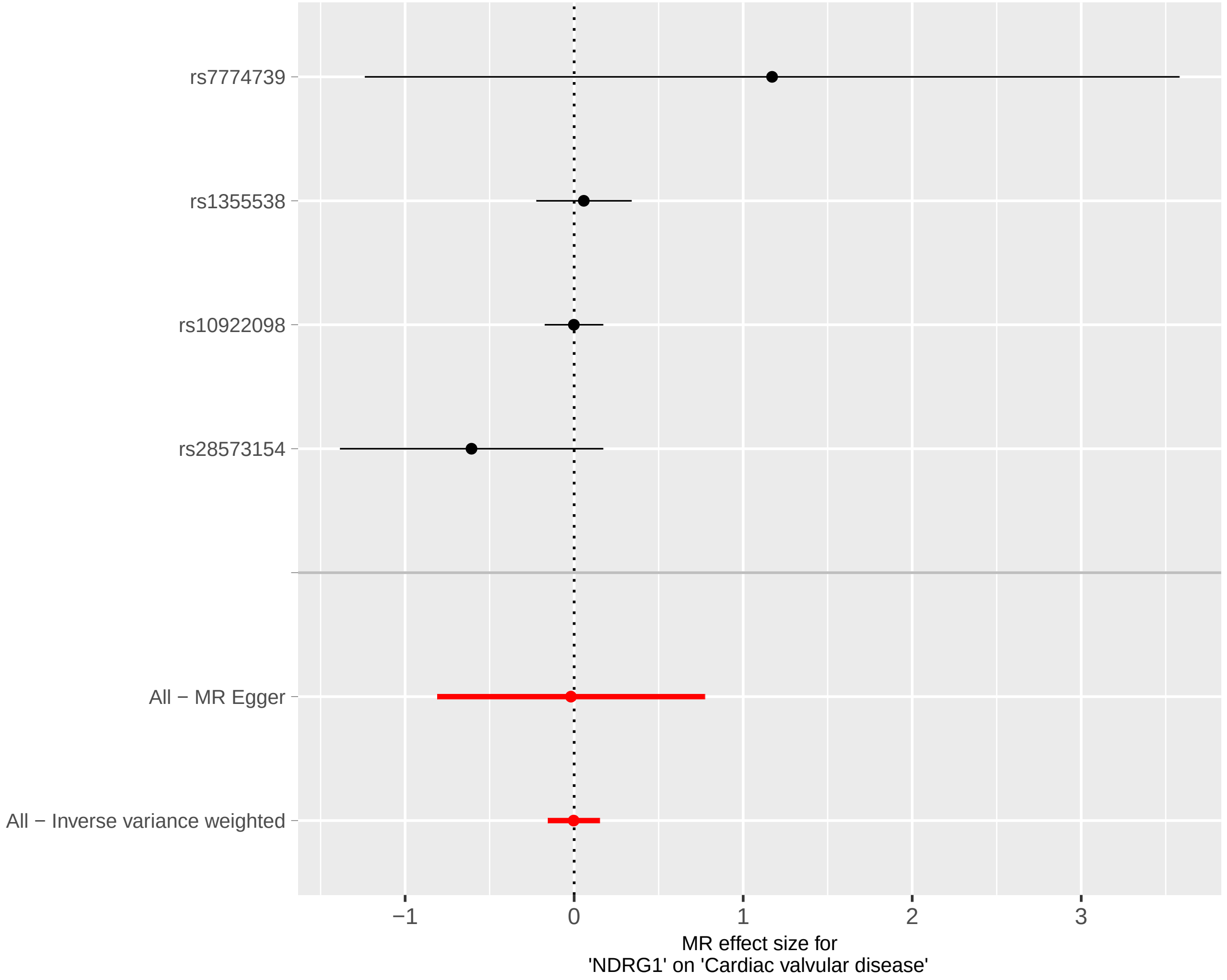

rs7774739
rs1355538
rs10922098
rs28573154
All − MR Egger
All − Inverse variance weighted
−1
0
1
2
3
MR effect size for
'NDRG1' on 'Cardiac valvular disease'

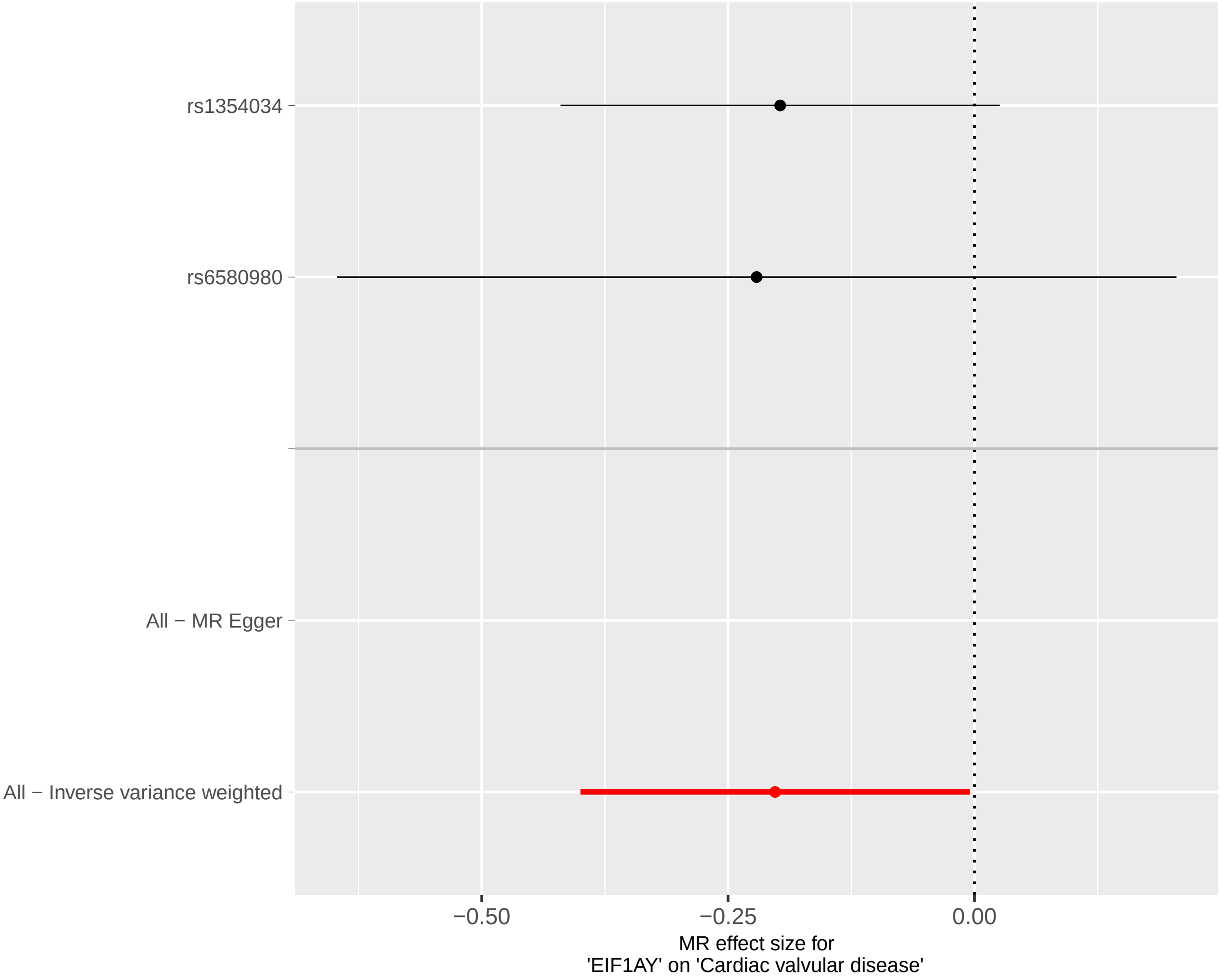
rs1354034
rs6580980
All − MR Egger
All − Inverse variance weighted
−0.50
−0.25
0.00
MR effect size for
'EIF1AY' on 'Cardiac valvular disease'

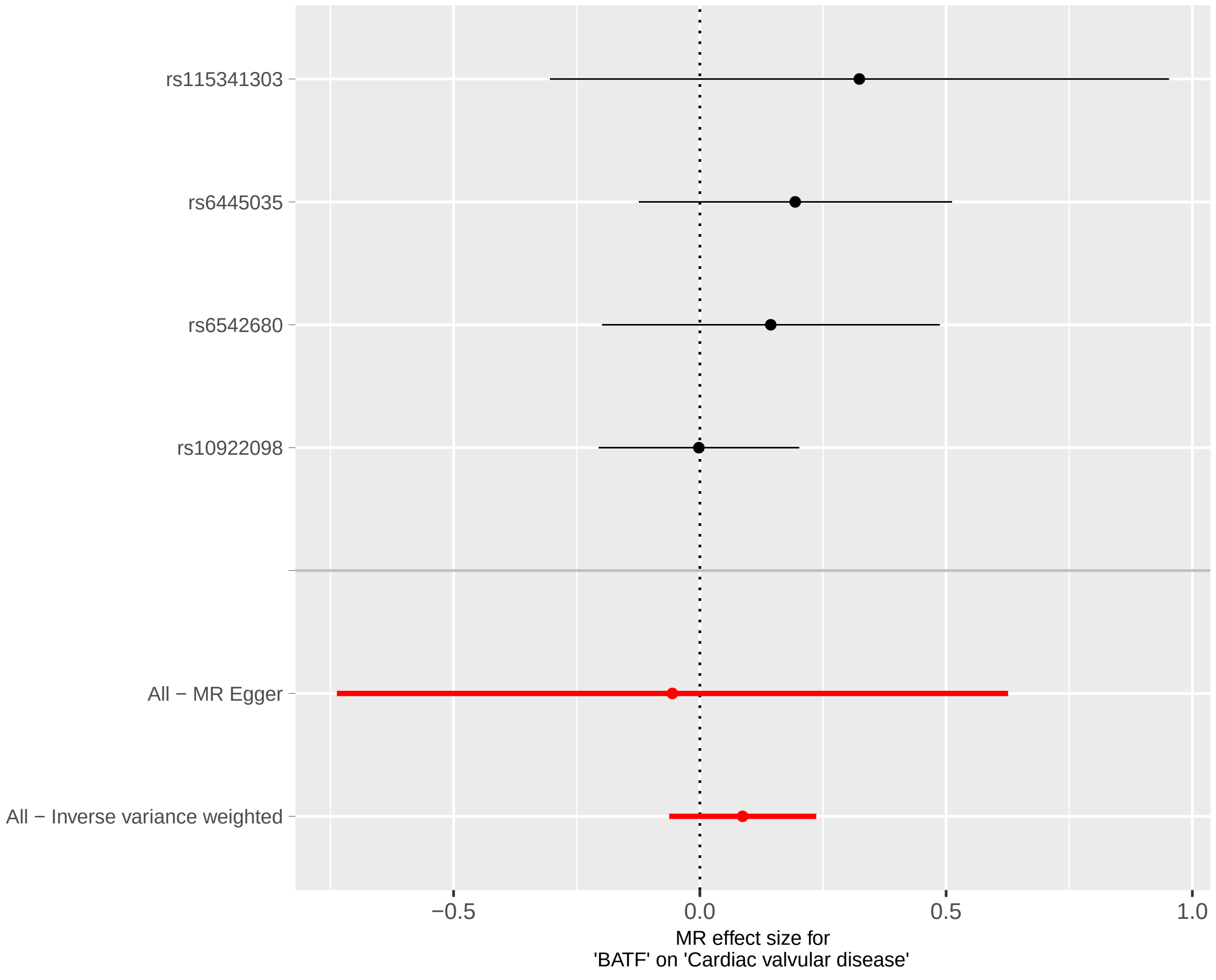
rs115341303
rs6445035
rs6542680
rs10922098
All − MR Egger
All − Inverse variance weighted
−0.5
0.0
0.5
1.0
MR effect size for
'BATF' on 'Cardiac valvular disease'

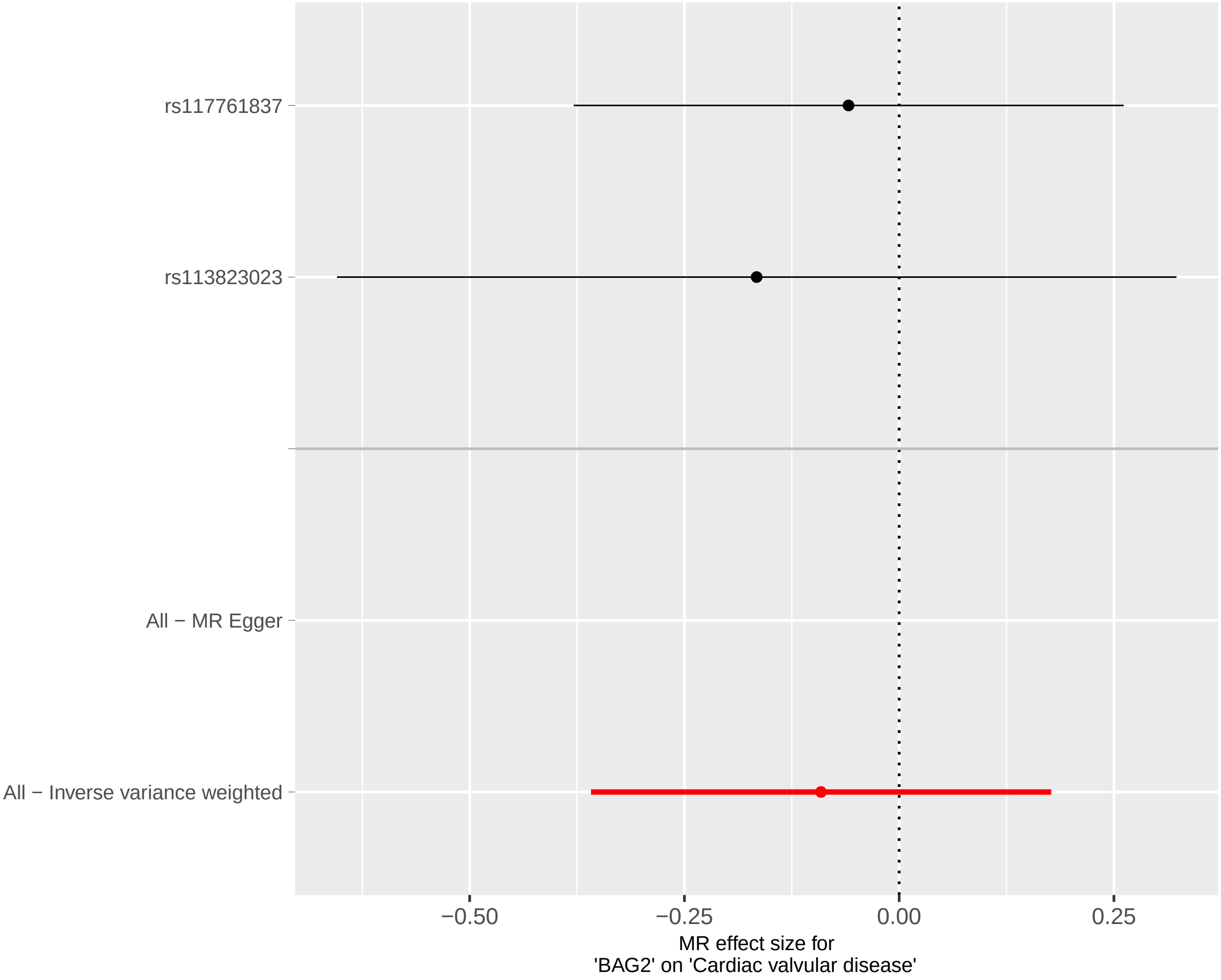
rs117761837
rs113823023
All − MR Egger
All − Inverse variance weighted
−0.50
−0.25
0.00
0.25
MR effect size for
'BAG2' on 'Cardiac valvular disease'

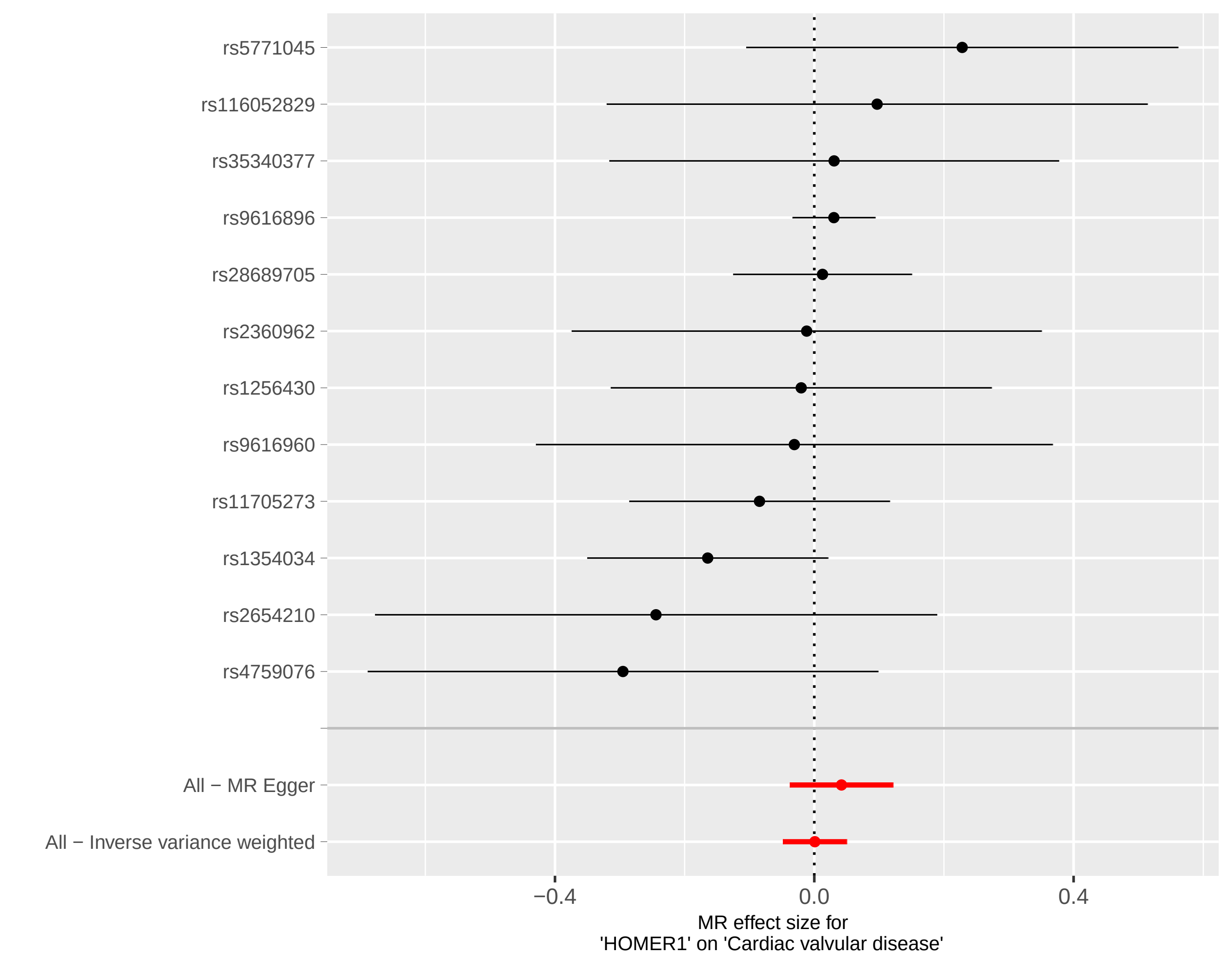
rs5771045
rs116052829
rs35340377
rs9616896
rs28689705
rs2360962
rs1256430
rs9616960
rs11705273
rs1354034
rs2654210
rs4759076
All − MR Egger
All − Inverse variance weighted
−0.4
0.0
0.4
MR effect size for
'HOMER1' on 'Cardiac valvular disease'

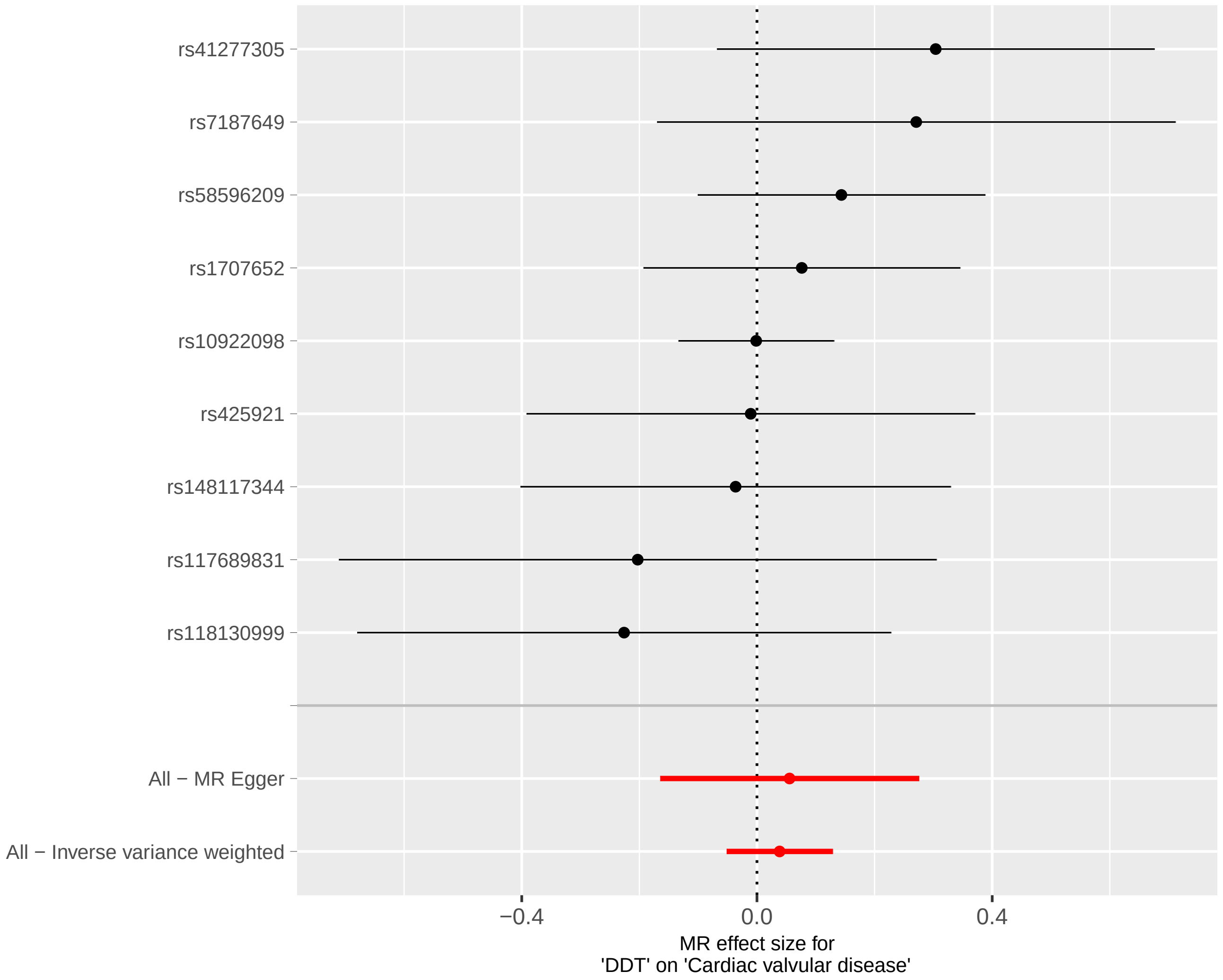
rs41277305
rs7187649
rs58596209
rs1707652
rs10922098
rs425921
rs148117344
rs117689831
rs118130999
All − MR Egger
All − Inverse variance weighted
−0.4
0.0
0.4
MR effect size for
'DDT' on 'Cardiac valvular disease'

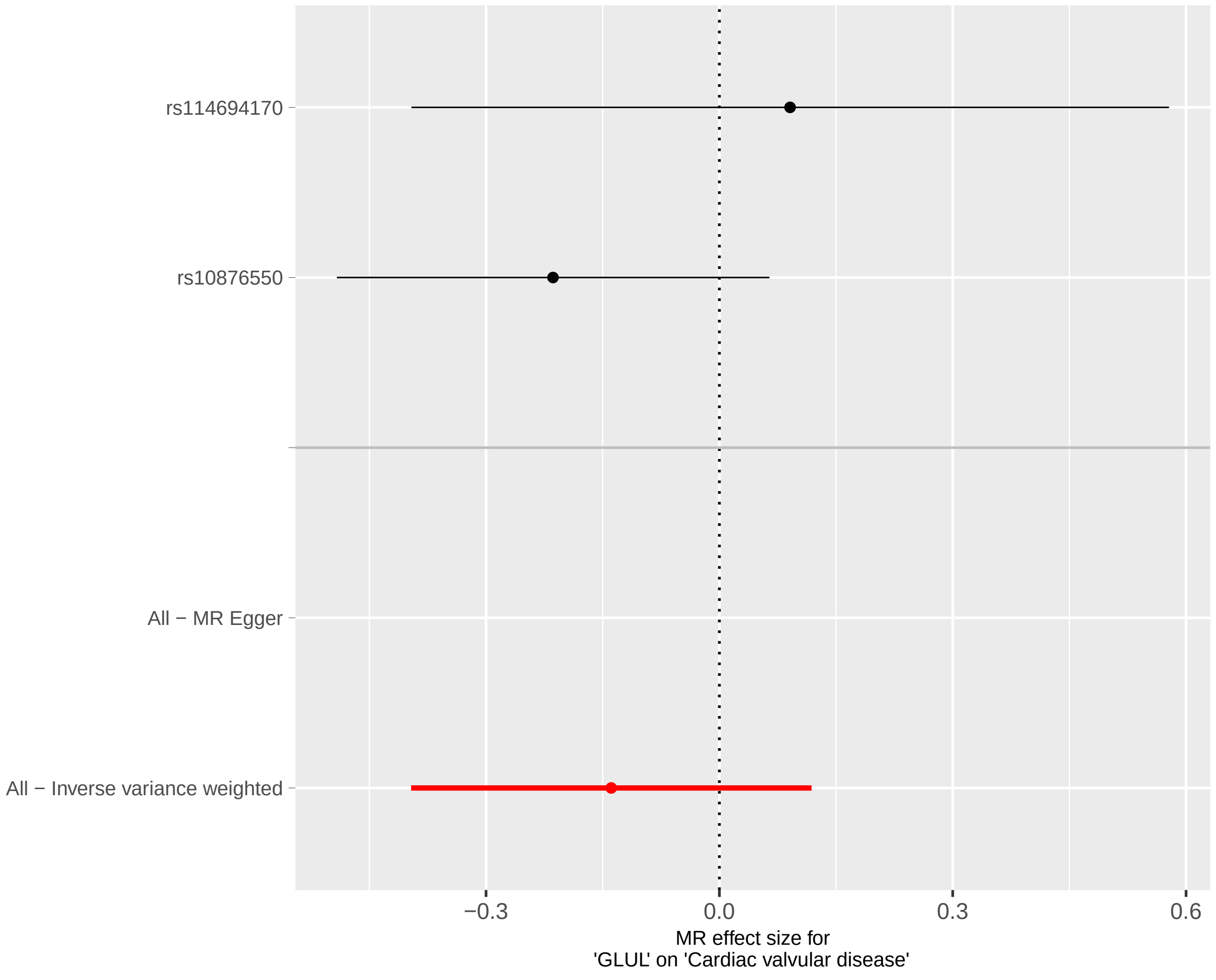
rs114694170
rs10876550
All − MR Egger
All − Inverse variance weighted
−0.3
0.0
0.3
0.6
MR effect size for
'GLUL' on 'Cardiac valvular disease'

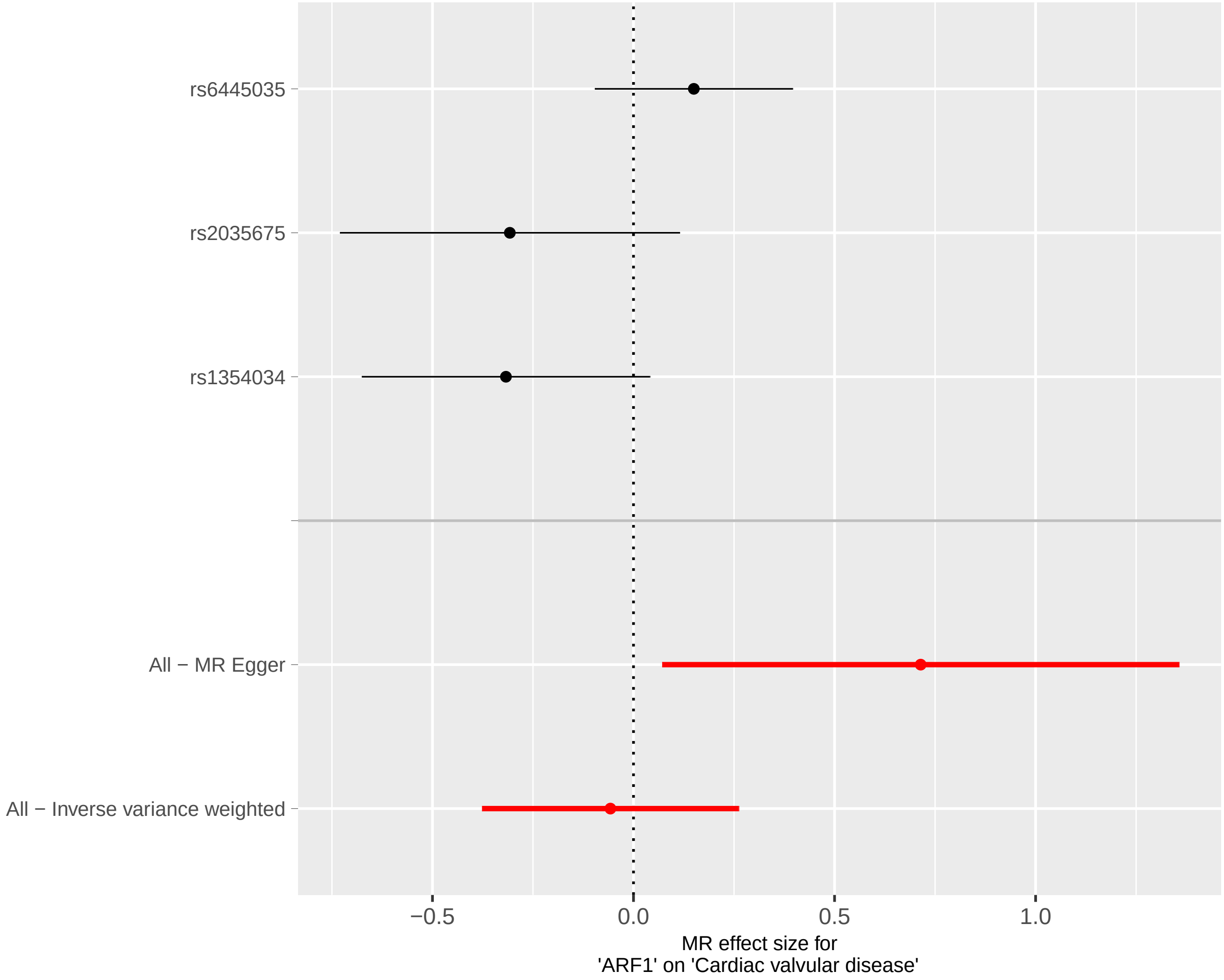

rs6445035
rs2035675
rs1354034
All − MR Egger
All − Inverse variance weighted
−0.5
0.0
0.5
1.0
MR effect size for
'ARF1' on 'Cardiac valvular disease'

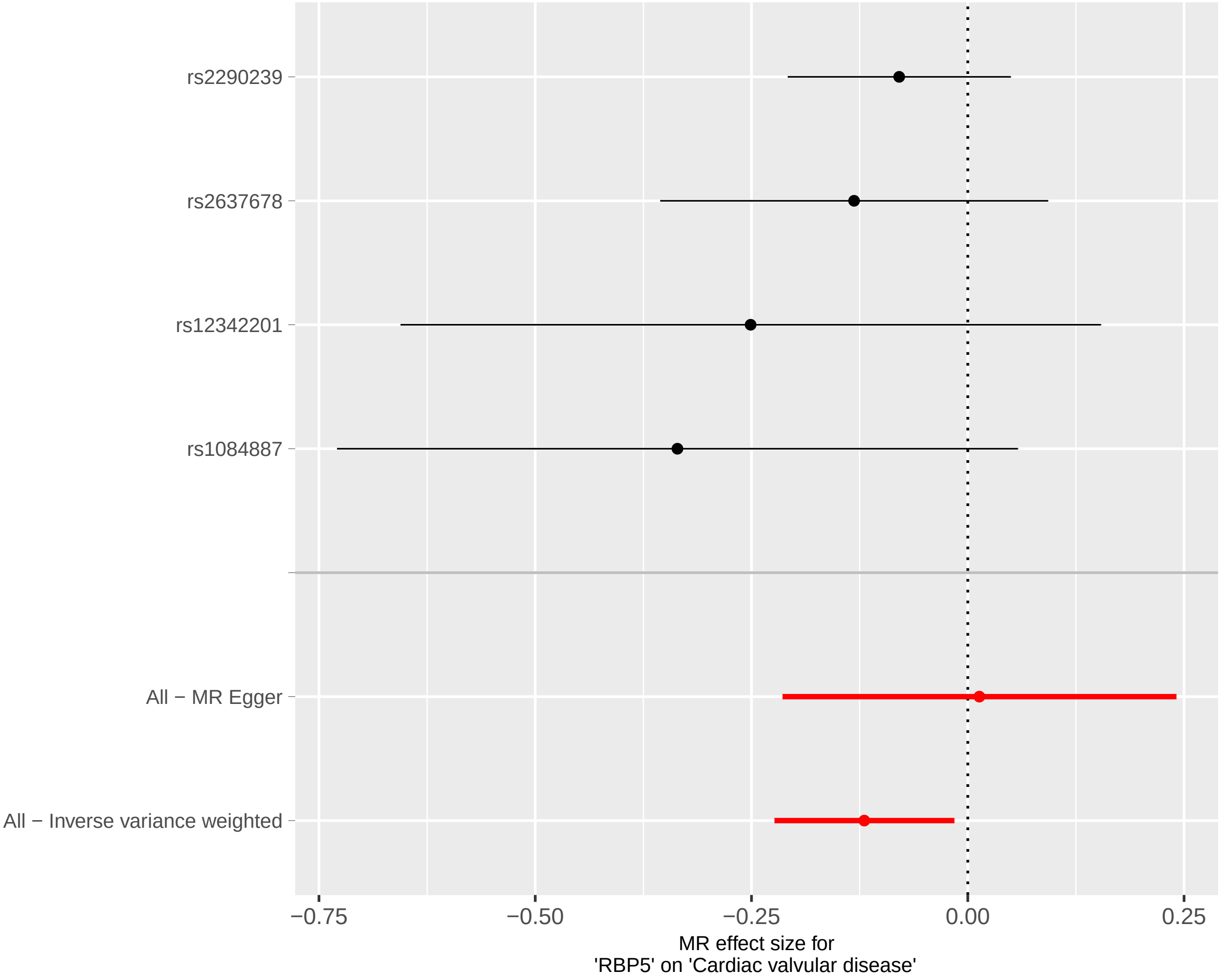
rs2290239
rs2637678
rs12342201
rs1084887
All − MR Egger
All − Inverse variance weighted
−0.75
−0.50
−0.25
0.00
0.25
MR effect size for
'RBP5' on 'Cardiac valvular disease'

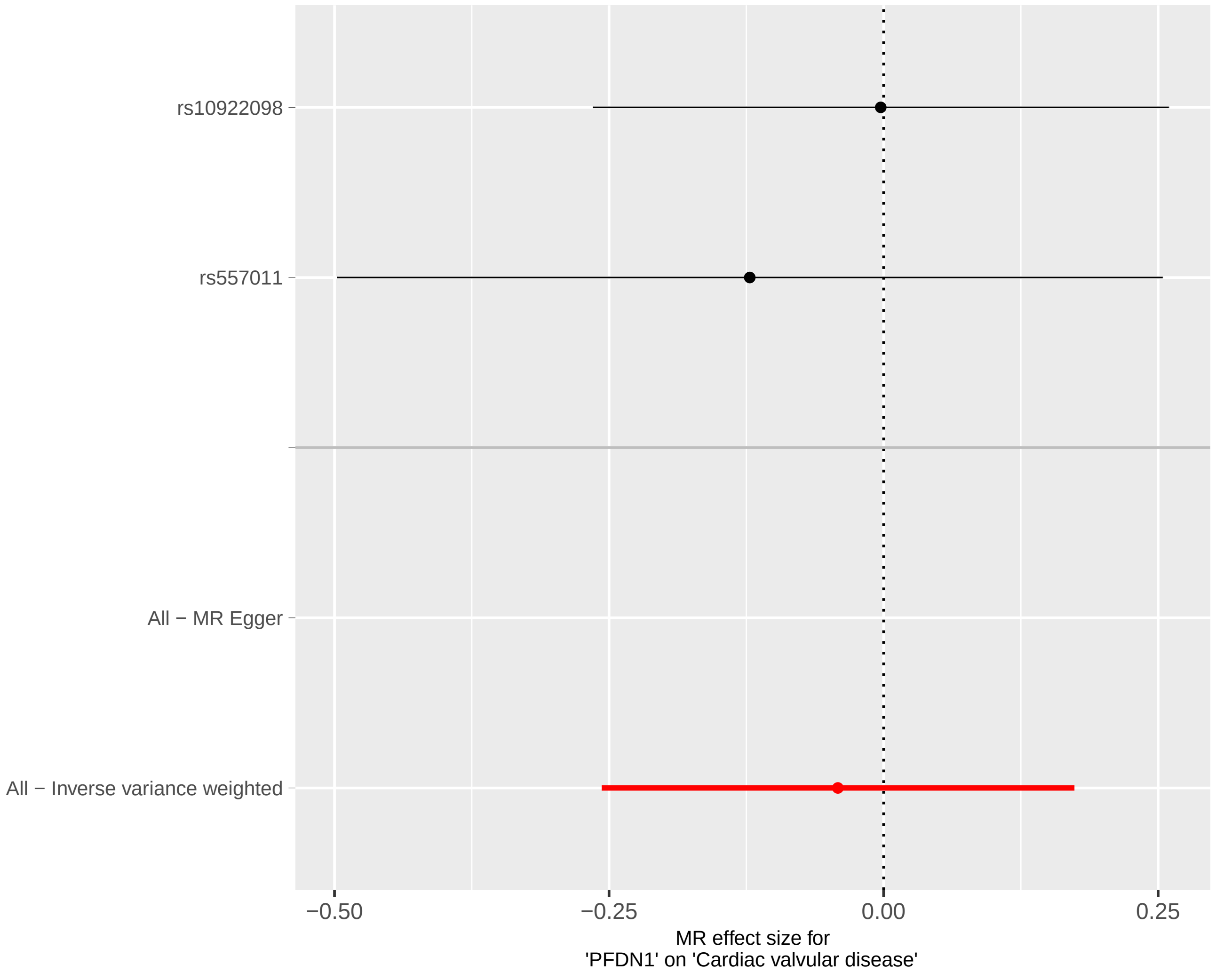
rs10922098
rs557011
All − MR Egger
All − Inverse variance weighted
−0.50
−0.25
0.00
0.25
MR effect size for
'PFDN1' on 'Cardiac valvular disease'

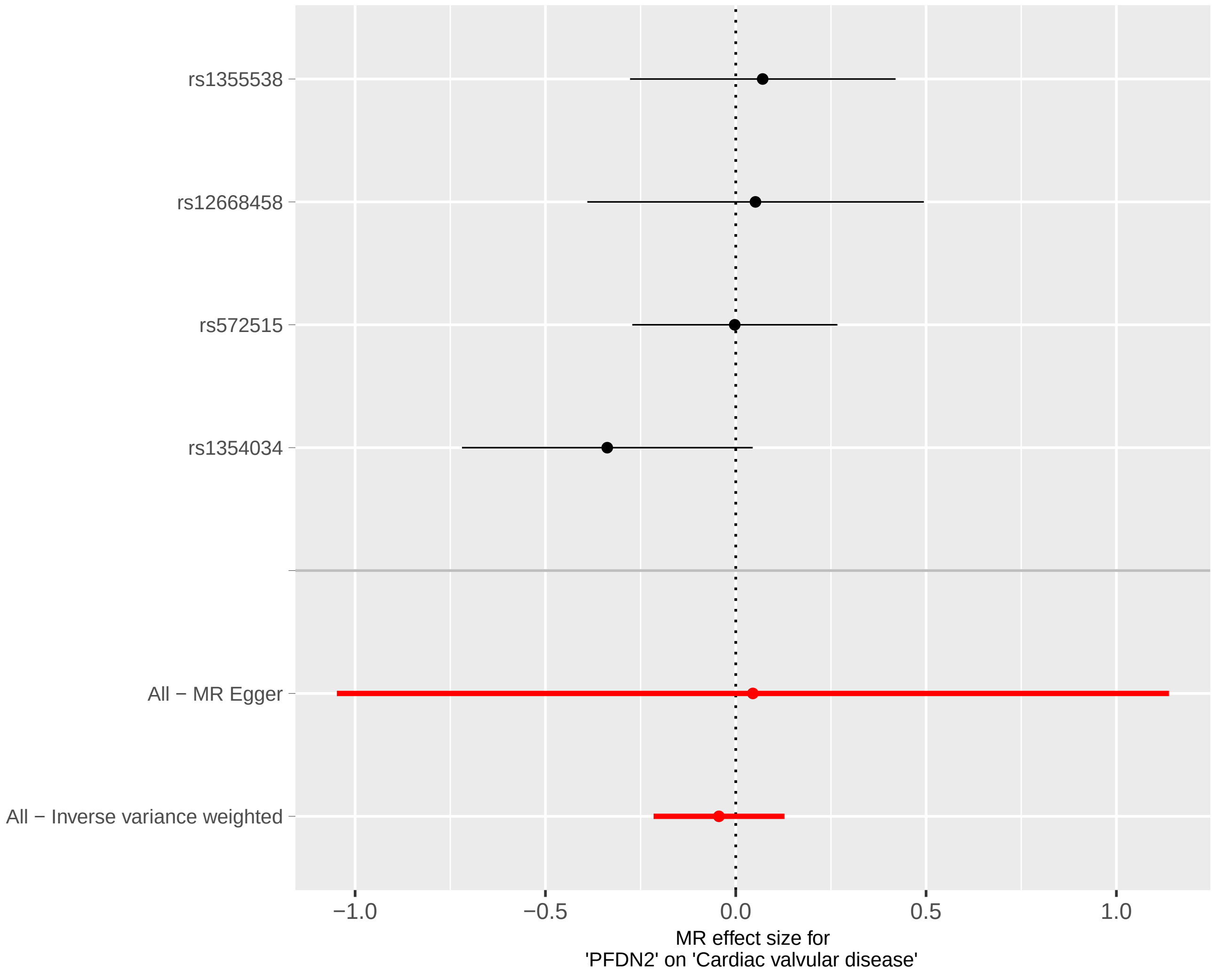
rs1355538
rs12668458
rs572515
rs1354034
All − MR Egger
All − Inverse variance weighted
−1.0
−0.5
0.0
0.5
1.0
MR effect size for
'PFDN2' on 'Cardiac valvular disease'

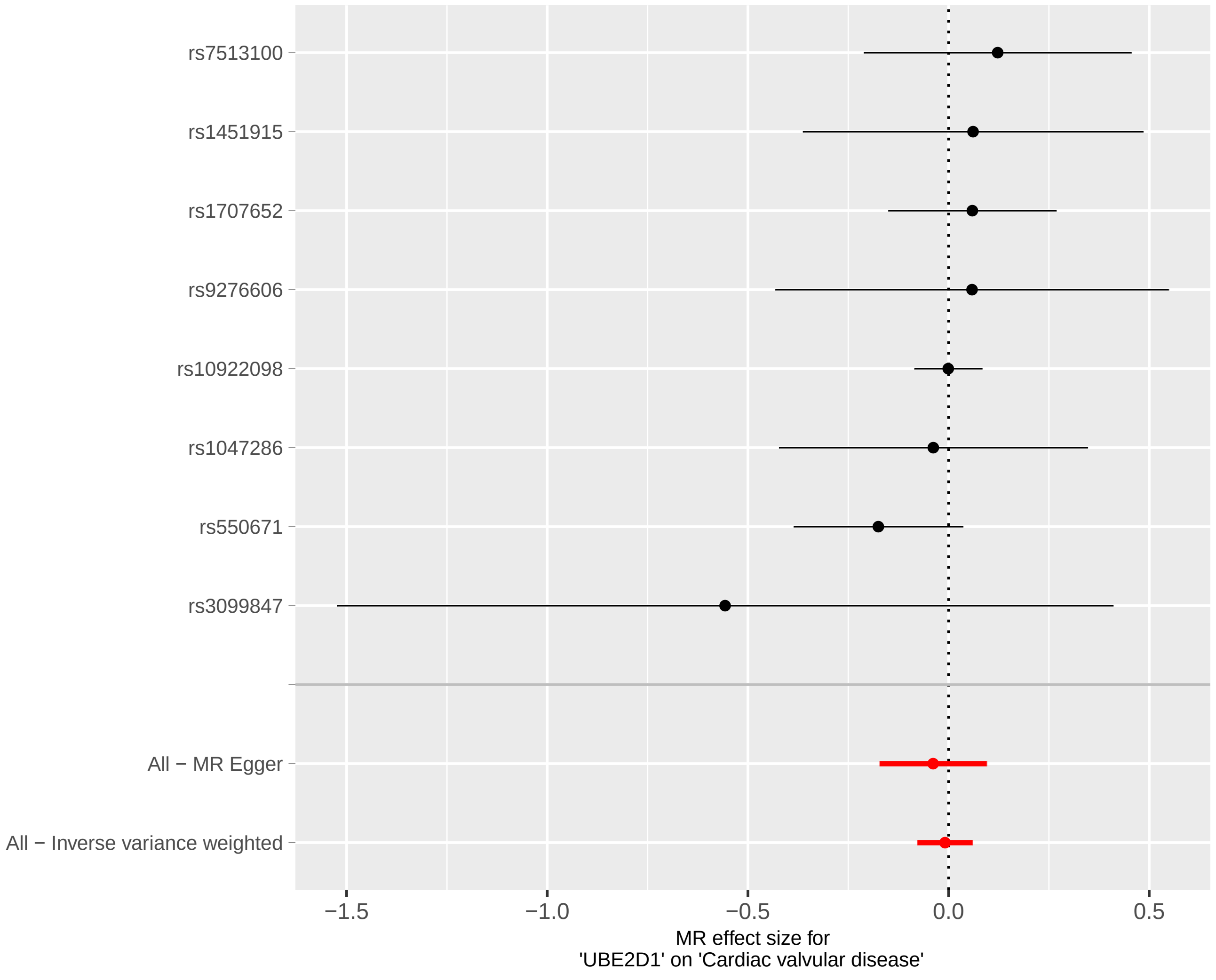
rs7513100
rs1451915
rs1707652
rs9276606
rs10922098
rs1047286
rs550671
rs3099847
All − MR Egger
All − Inverse variance weighted
−1.5
−1.0
−0.5
0.0
0.5
MR effect size for
'UBE2D1' on 'Cardiac valvular disease'

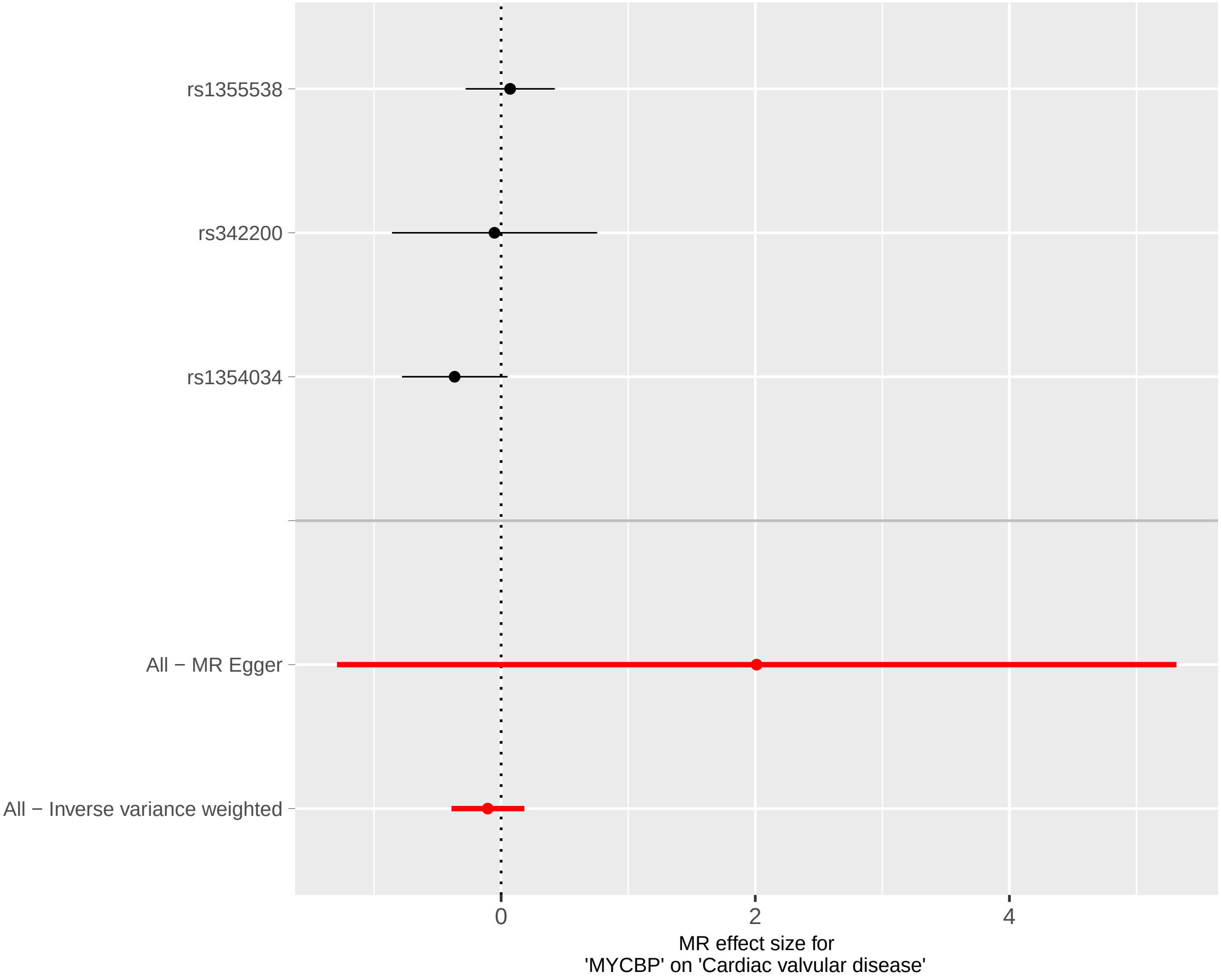
rs1355538
rs342200
rs1354034
All − MR Egger
All − Inverse variance weighted
0
2
4
MR effect size for
'MYCBP' on 'Cardiac valvular disease'

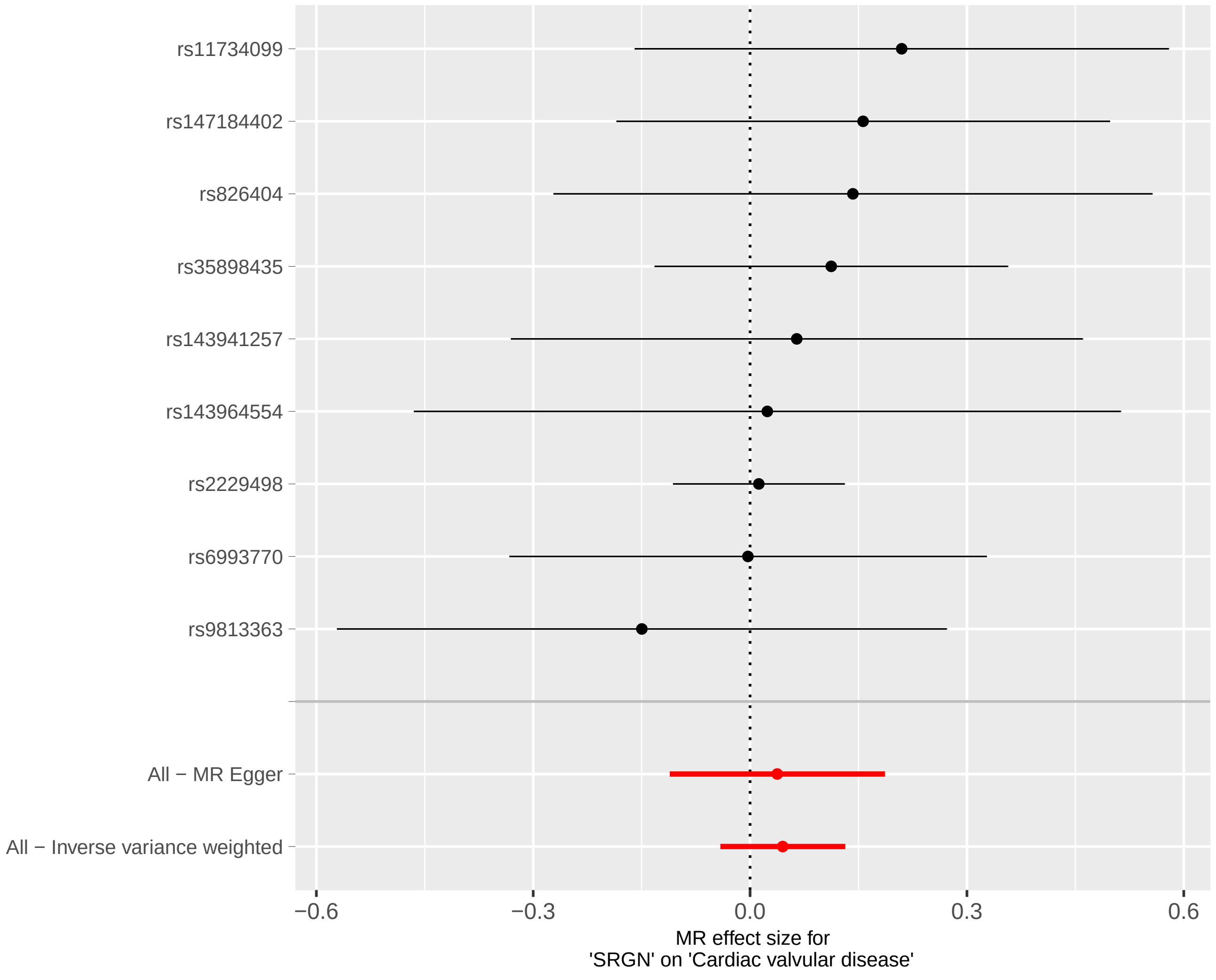
rs11734099
rs147184402
rs826404
rs35898435
rs143941257
rs143964554
rs2229498
rs6993770
rs9813363
All − MR Egger
All − Inverse variance weighted
−0.6
−0.3
0.0
0.3
0.6
MR effect size for
'SRGN' on 'Cardiac valvular disease'

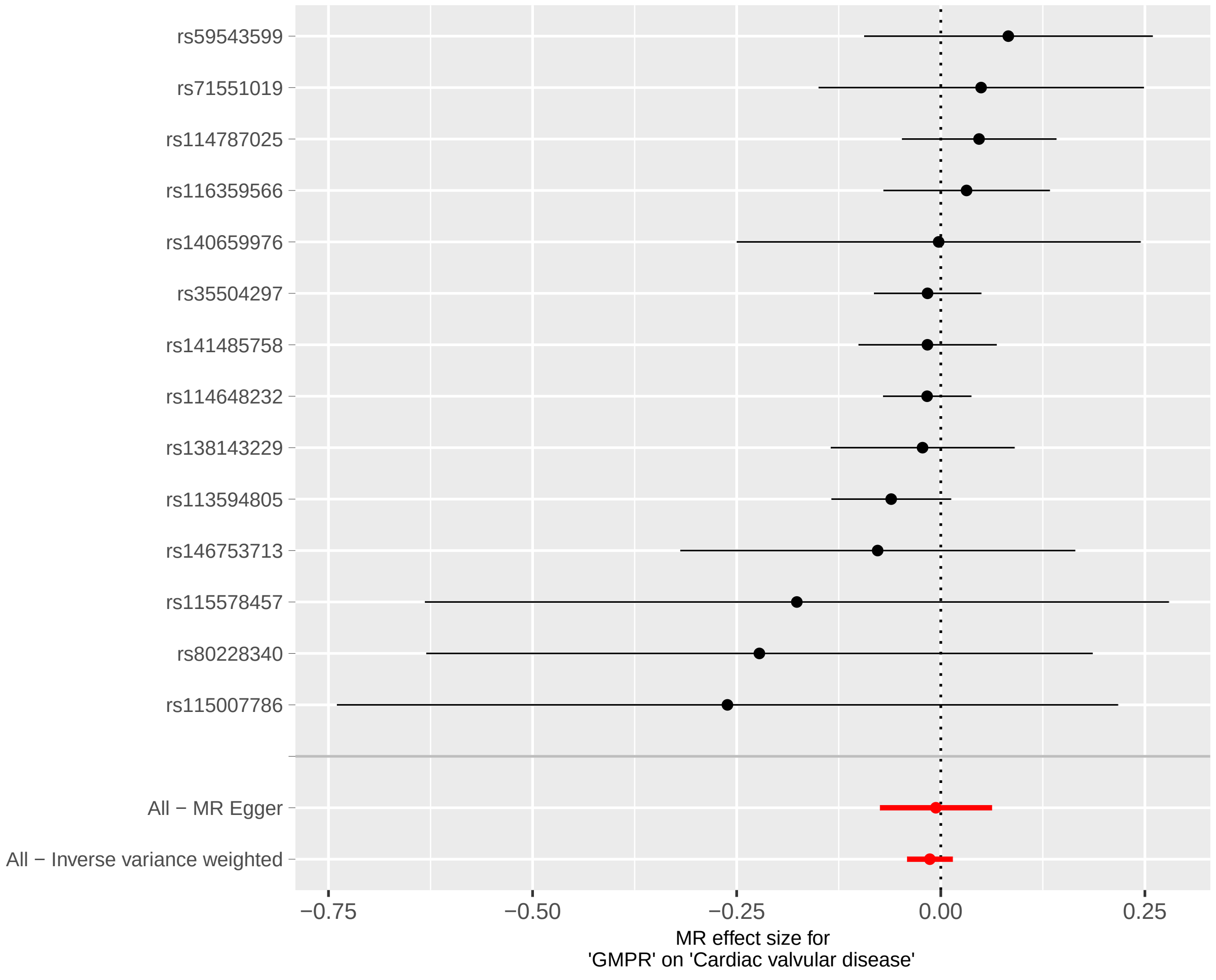

rs59543599
rs71551019
rs114787025
rs116359566
rs140659976
rs35504297
rs141485758
rs114648232
rs138143229
rs113594805
rs146753713
rs115578457
rs80228340
rs115007786
All − MR Egger
All − Inverse variance weighted
−0.75
−0.50
−0.25
0.00
0.25
MR effect size for
'GMPR' on 'Cardiac valvular disease'

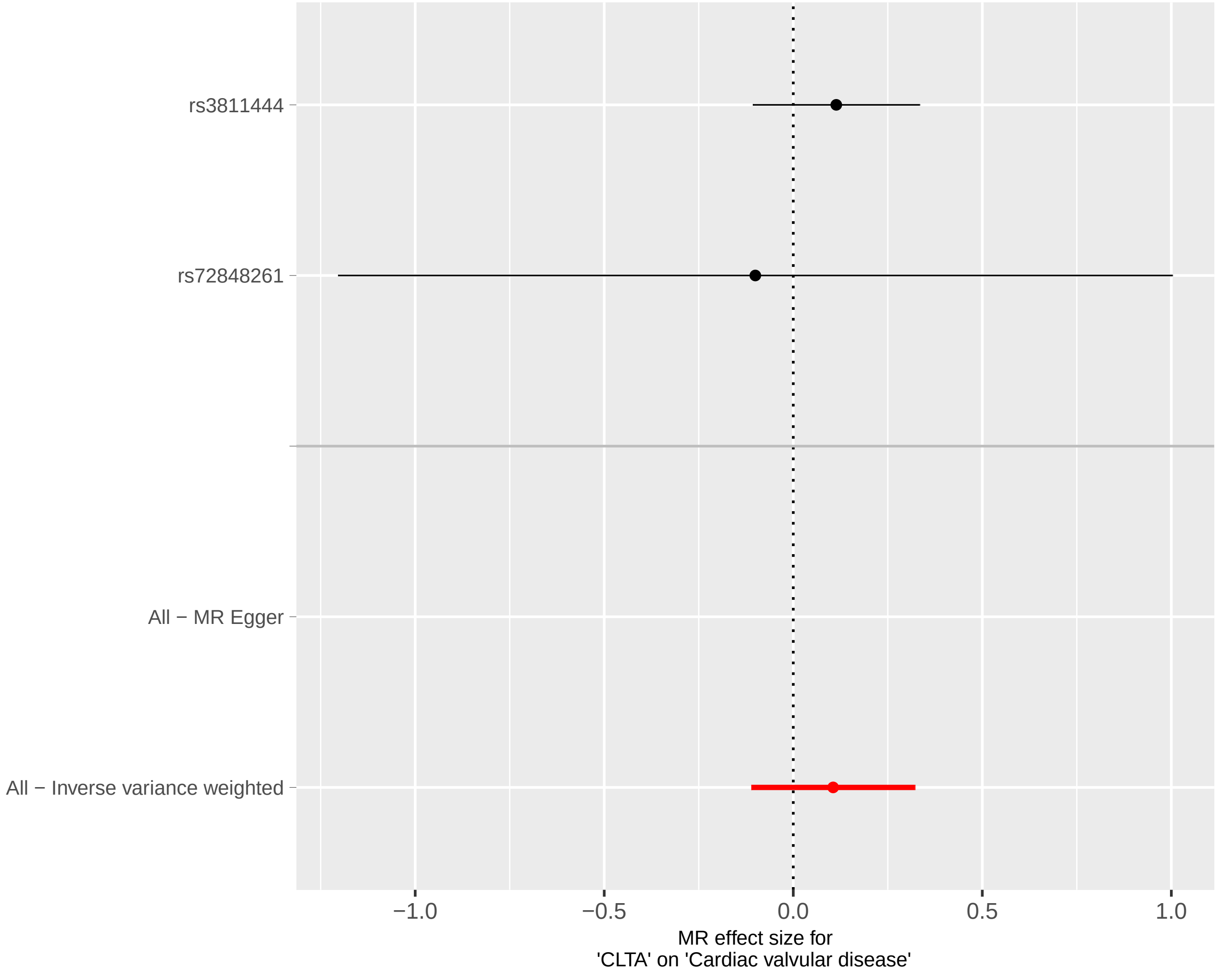

rs3811444
rs72848261
All − MR Egger
All − Inverse variance weighted
−1.0
−0.5
0.0
0.5
1.0
MR effect size for
'CLTA' on 'Cardiac valvular disease'

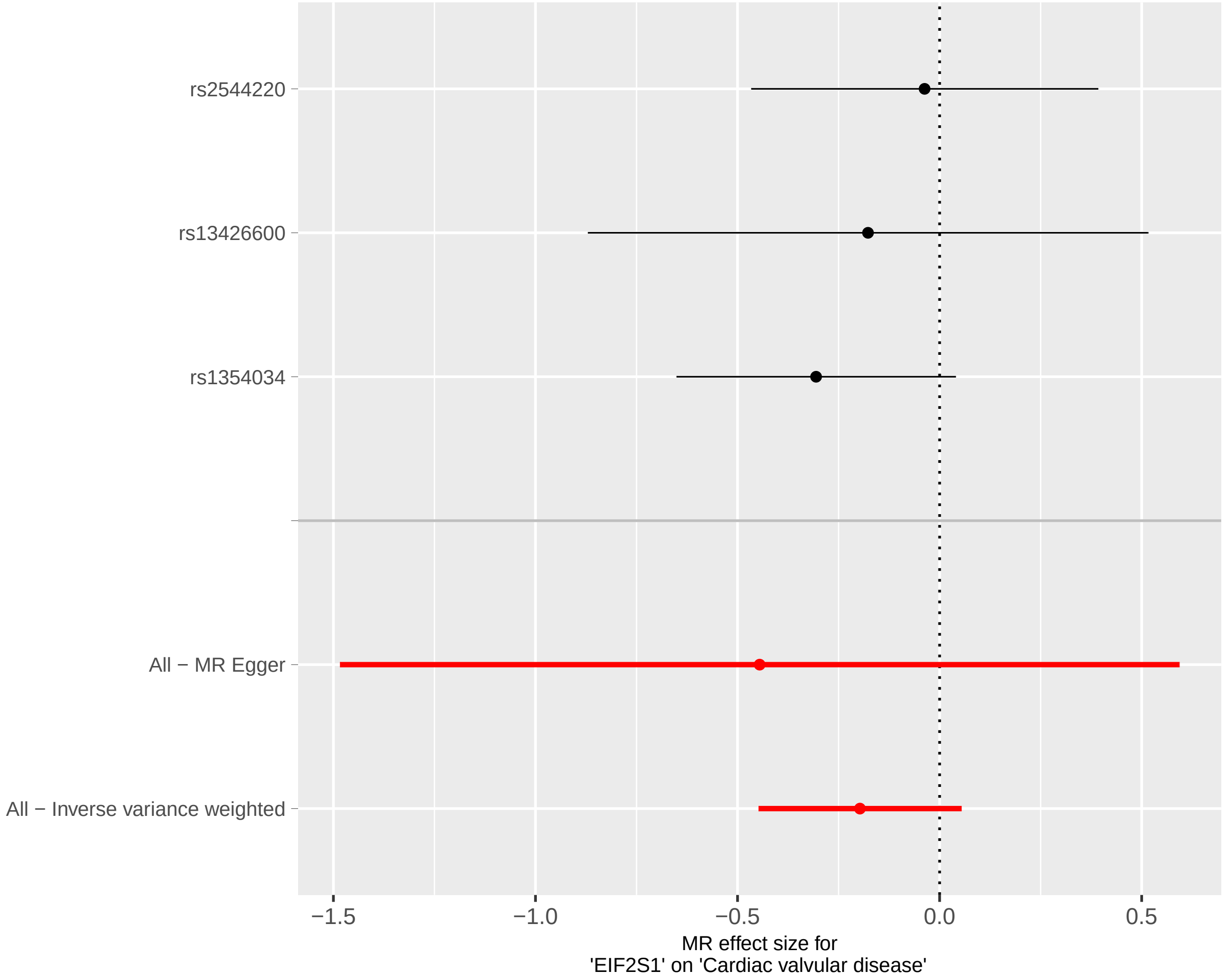
rs2544220
rs13426600
rs1354034
All − MR Egger
All − Inverse variance weighted
−1.5
−1.0
−0.5
0.0
0.5
MR effect size for
'EIF2S1' on 'Cardiac valvular disease'

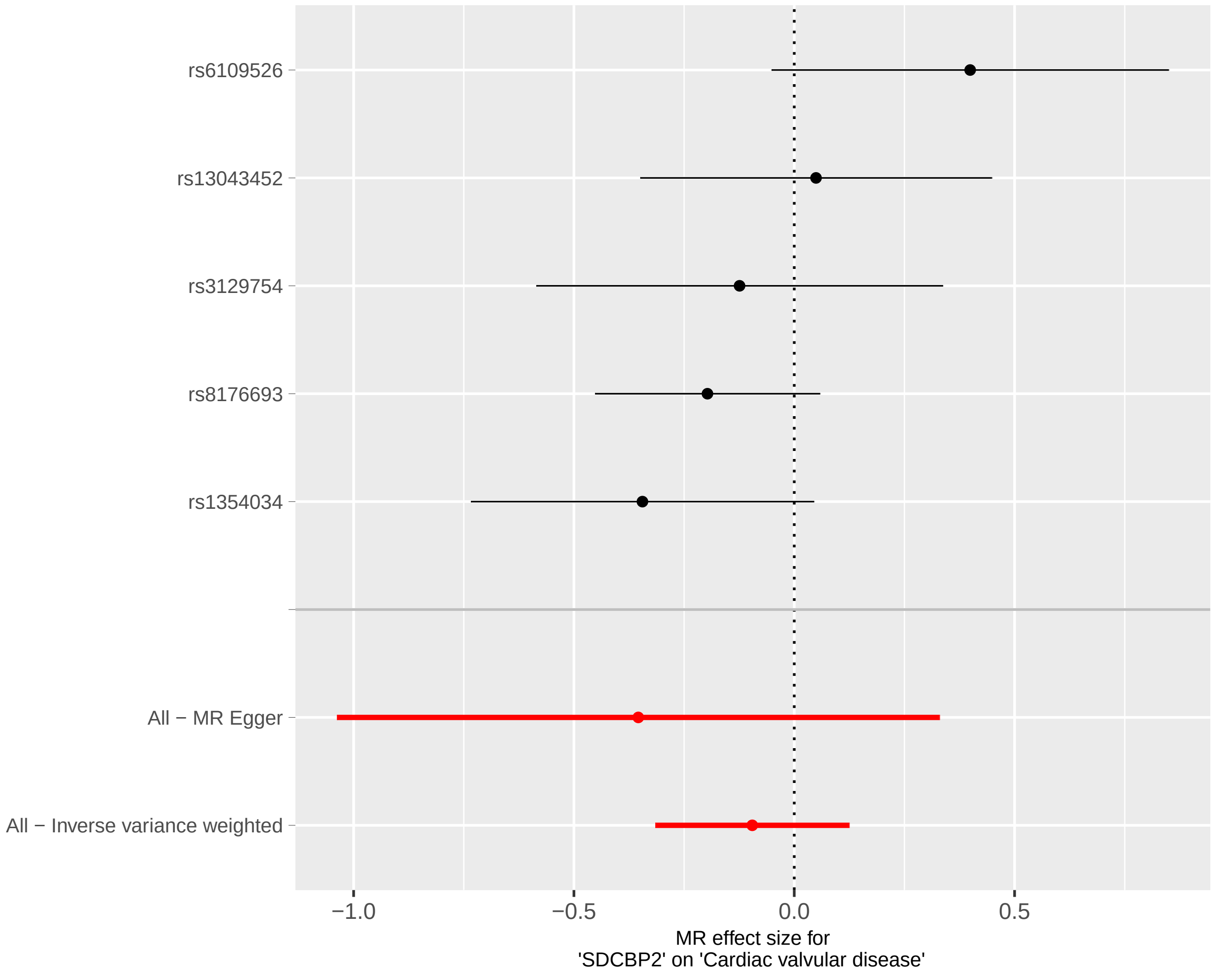
rs6109526
rs13043452
rs3129754
rs8176693
rs1354034
All − MR Egger
All − Inverse variance weighted
−1.0
−0.5
0.0
0.5
MR effect size for
'SDCBP2' on 'Cardiac valvular disease'

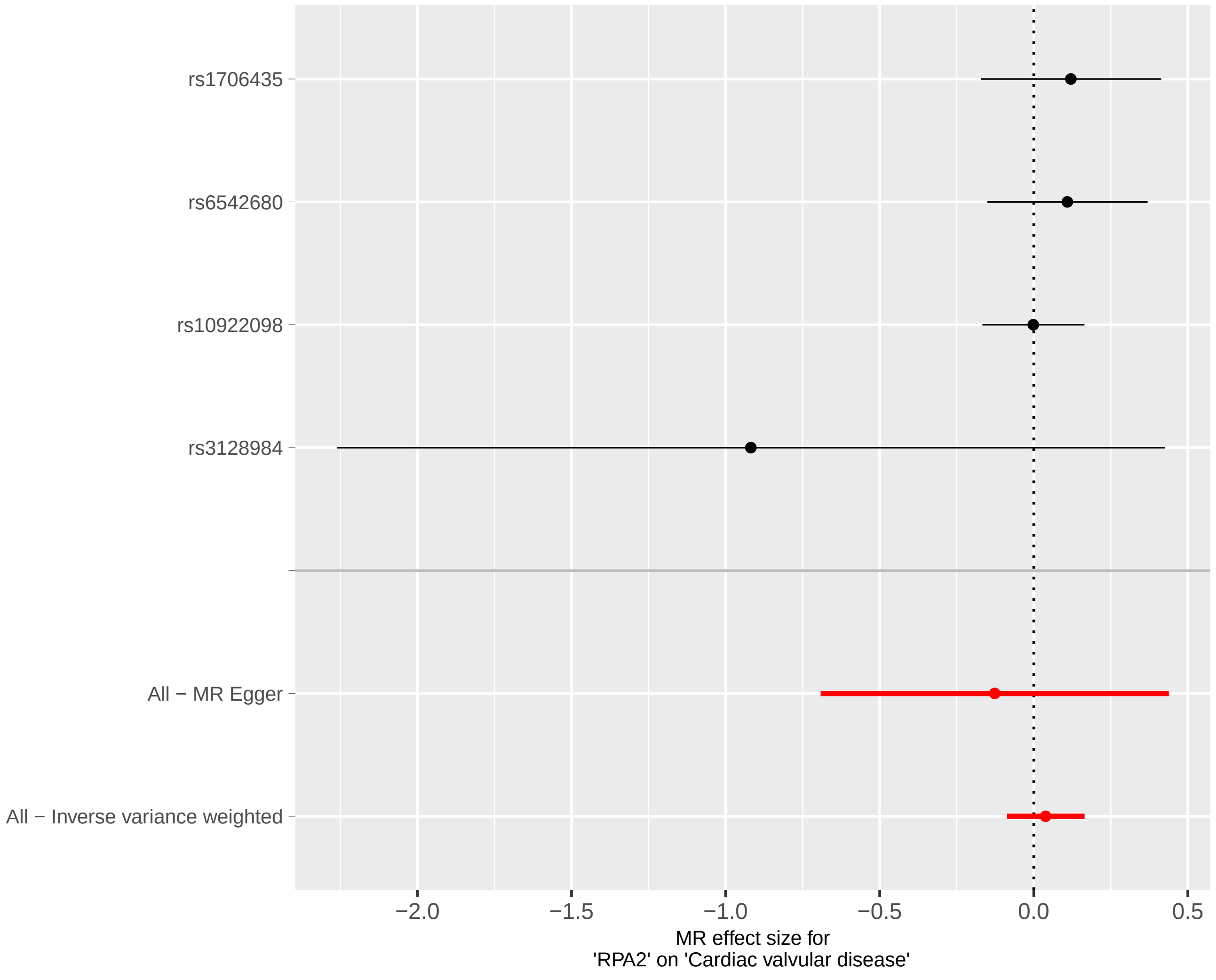
rs1706435
rs6542680
rs10922098
rs3128984
All − MR Egger
All − Inverse variance weighted
−2.0
−1.5
−1.0
−0.5
0.0
0.5
MR effect size for
'RPA2' on 'Cardiac valvular disease'

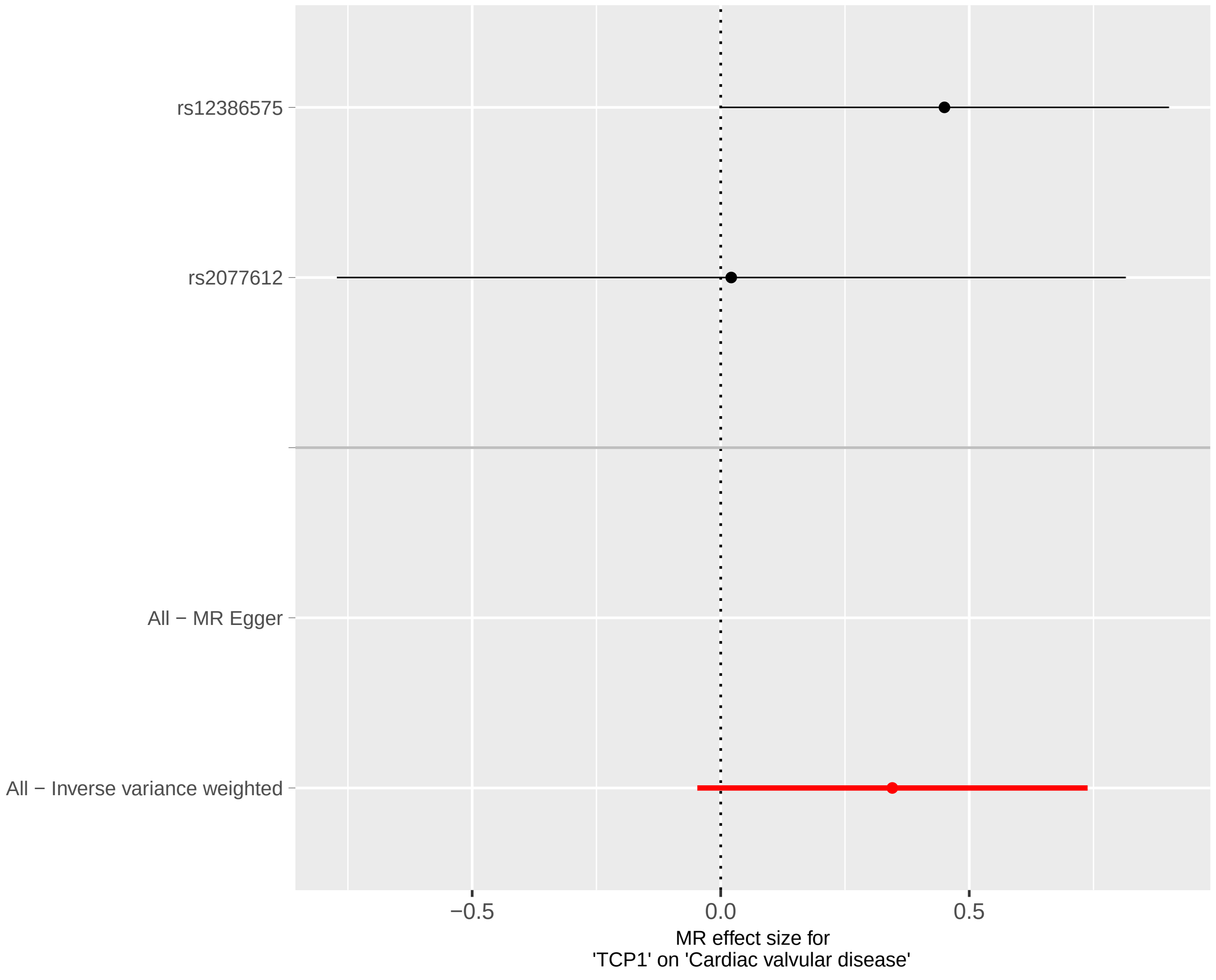

rs12386575
rs2077612
All − MR Egger
All − Inverse variance weighted
−0.5
0.0
0.5
MR effect size for
'TCP1' on 'Cardiac valvular disease'

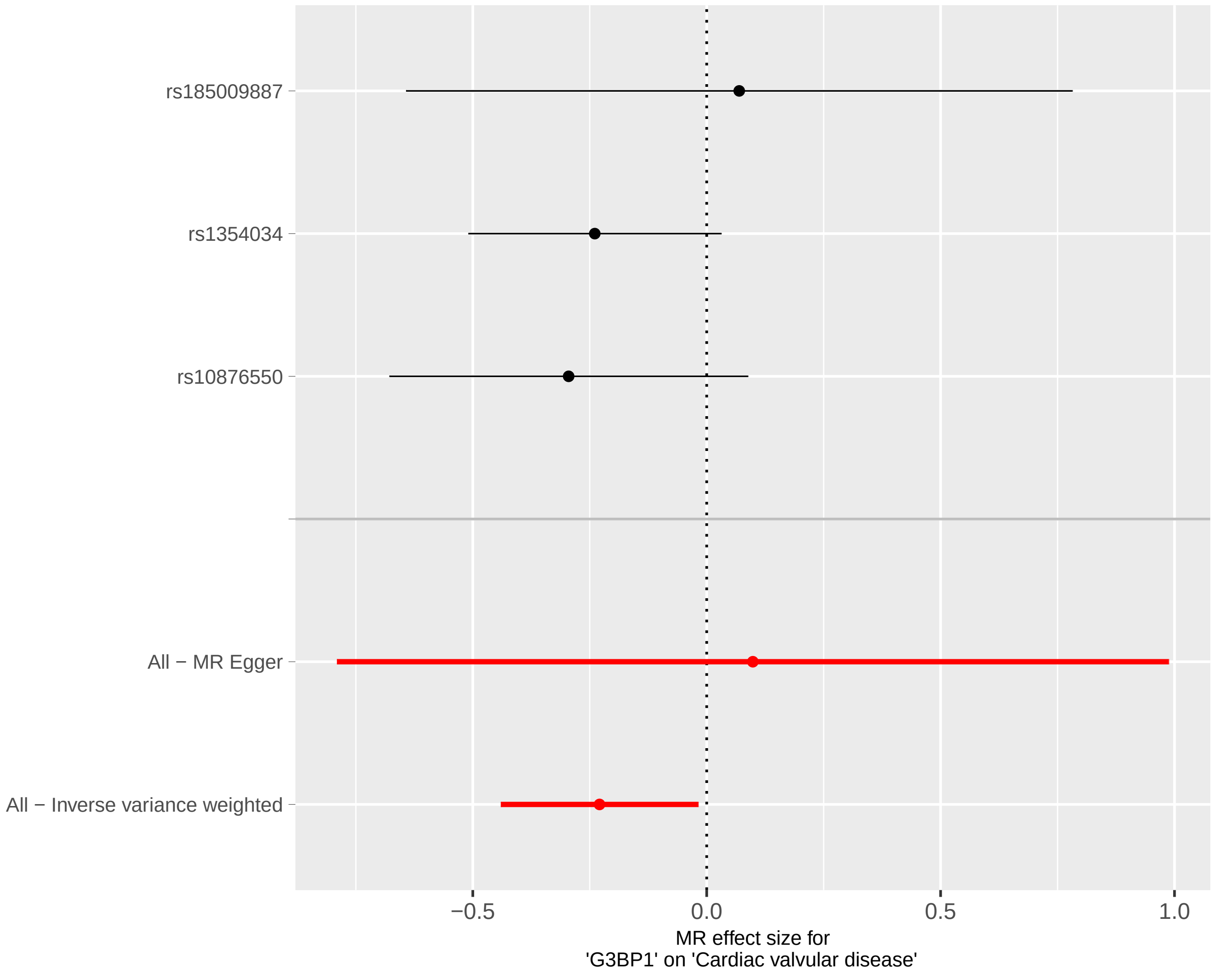
rs185009887
rs1354034
rs10876550
All − MR Egger
All − Inverse variance weighted
−0.5
0.0
0.5
1.0
MR effect size for
'G3BP1' on 'Cardiac valvular disease'

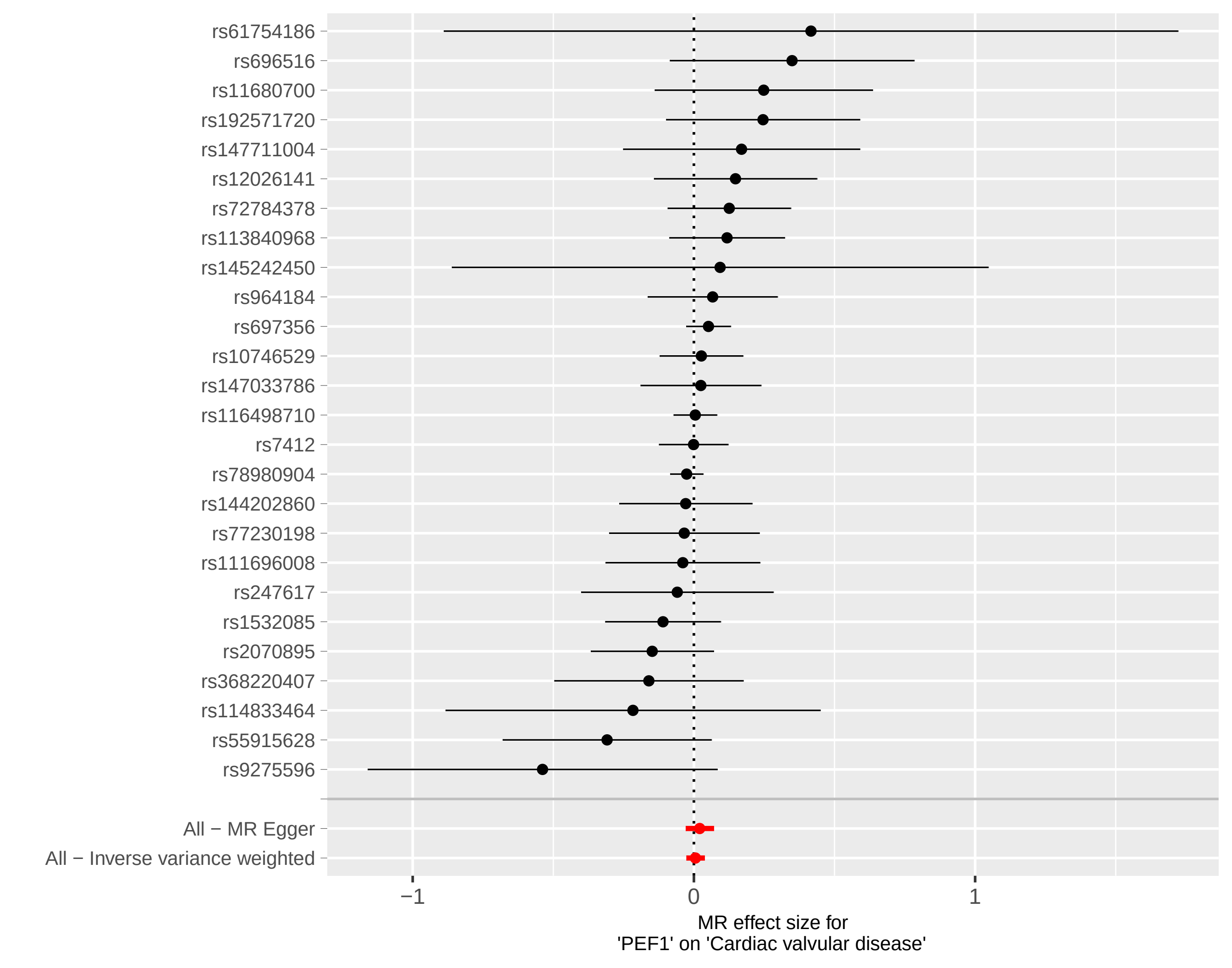
rs61754186
rs696516
rs11680700
rs192571720
rs147711004
rs12026141
rs72784378
rs113840968
rs145242450
rs964184
rs697356
rs10746529
rs147033786
rs116498710
rs7412
rs78980904
rs144202860
rs77230198
rs111696008
rs247617
rs1532085
rs2070895
rs368220407
rs114833464
rs55915628
rs9275596
All − MR Egger
All − Inverse variance weighted
−1
0
1
MR effect size for
'PEF1' on 'Cardiac valvular disease'

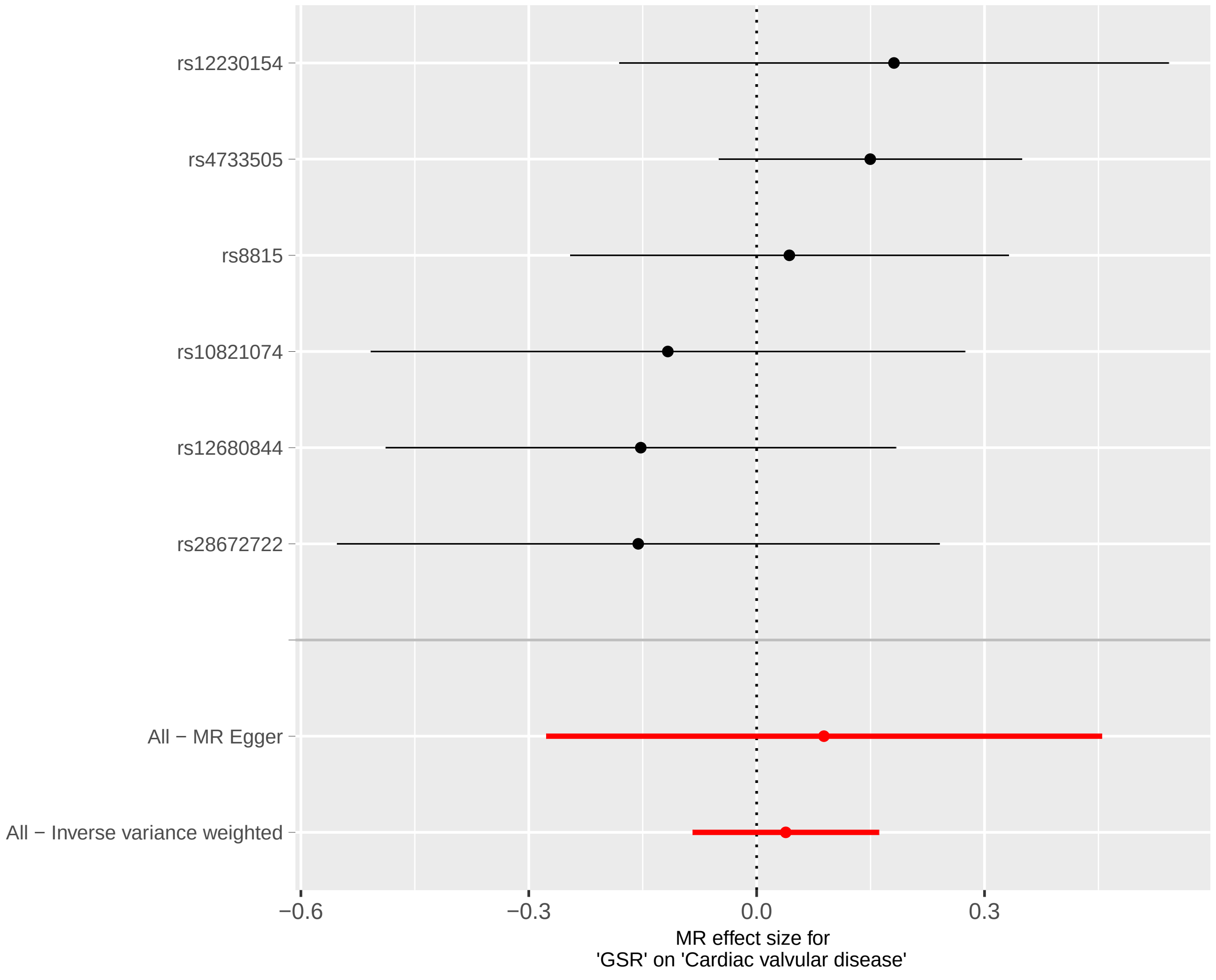
rs12230154
rs4733505
rs8815
rs10821074
rs12680844
rs28672722
All − MR Egger
All − Inverse variance weighted
−0.6
−0.3
0.0
0.3
MR effect size for
'GSR' on 'Cardiac valvular disease'

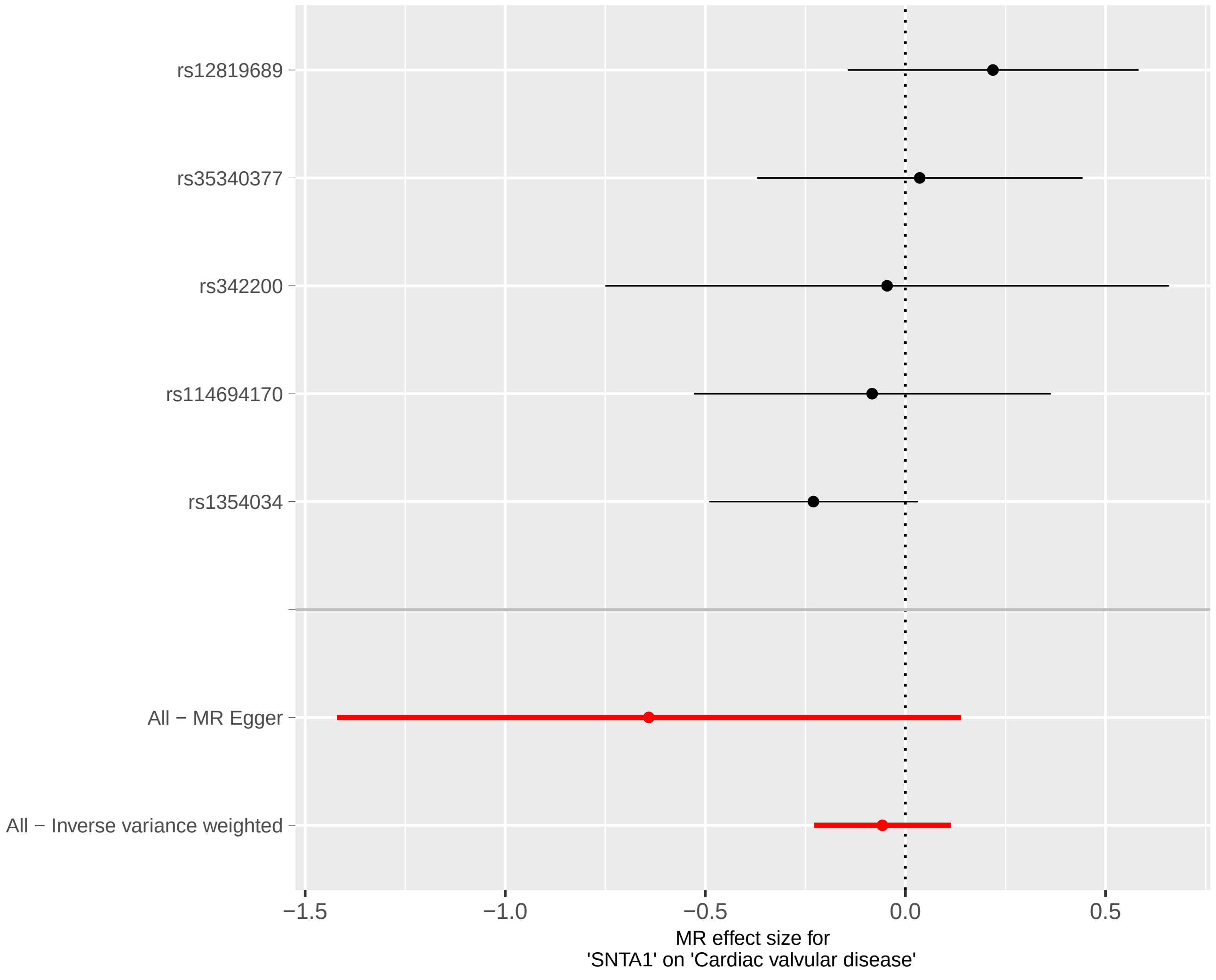
rs12819689
rs35340377
rs342200
rs114694170
rs1354034
All − MR Egger
All − Inverse variance weighted
−1.5
−1.0
−0.5
0.0
0.5
MR effect size for
'SNTA1' on 'Cardiac valvular disease'

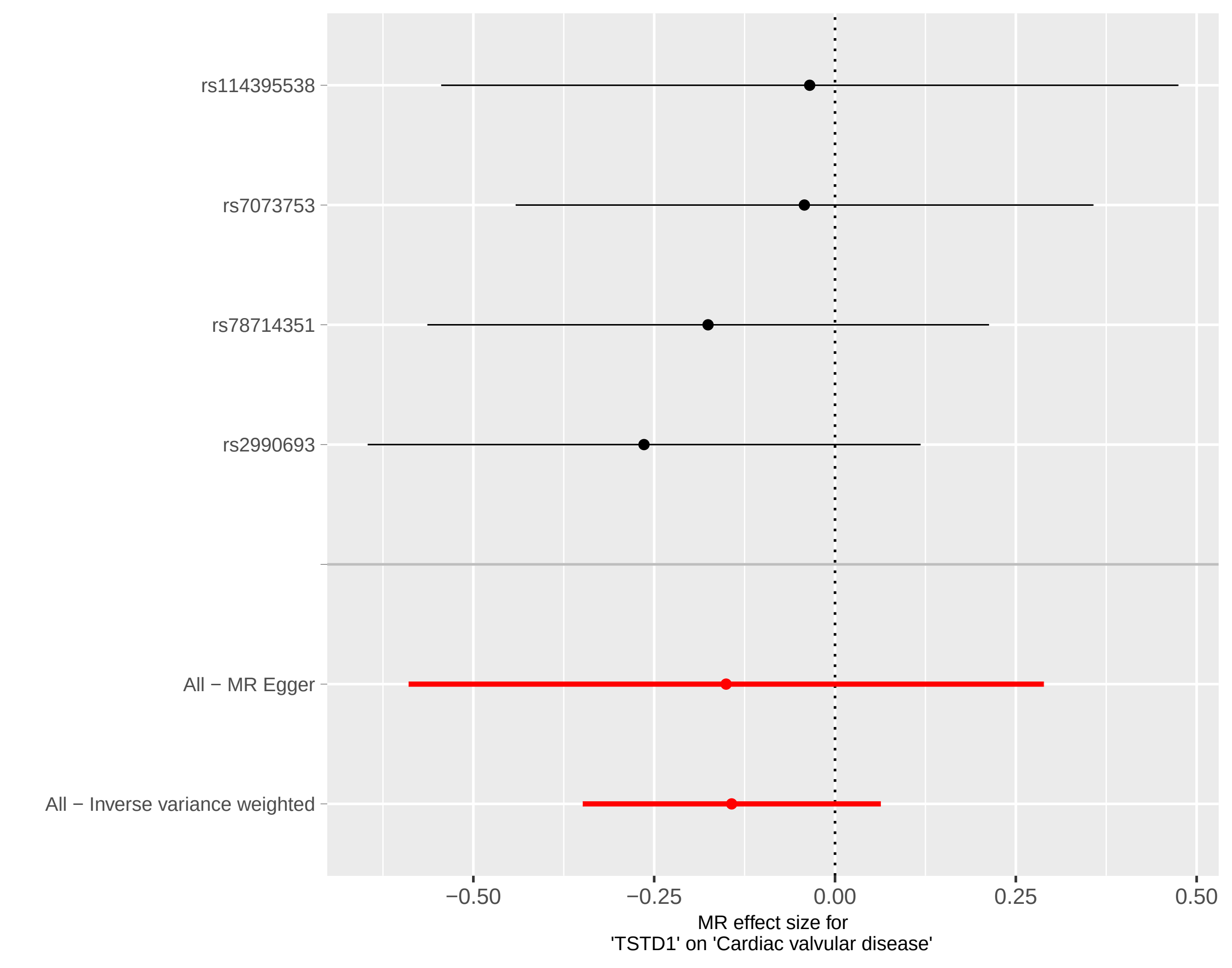
rs114395538
rs7073753
rs78714351
rs2990693
All − MR Egger
All − Inverse variance weighted
−0.50
−0.25
0.00
0.25
0.50
MR effect size for
'TSTD1' on 'Cardiac valvular disease'

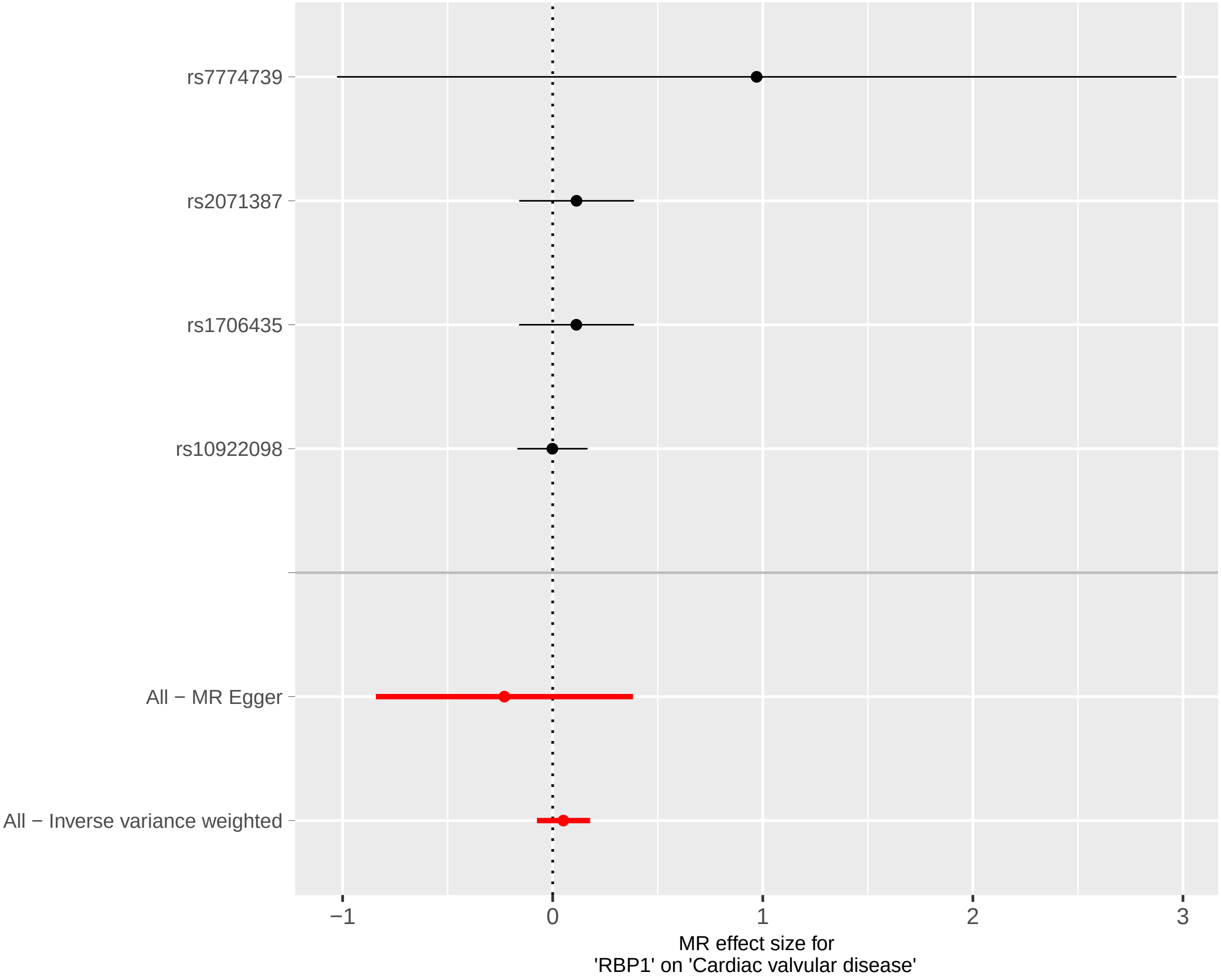
rs7774739
rs2071387
rs1706435
rs10922098
All − MR Egger
All − Inverse variance weighted
−1
0
1
2
3
MR effect size for
'RBP1' on 'Cardiac valvular disease'

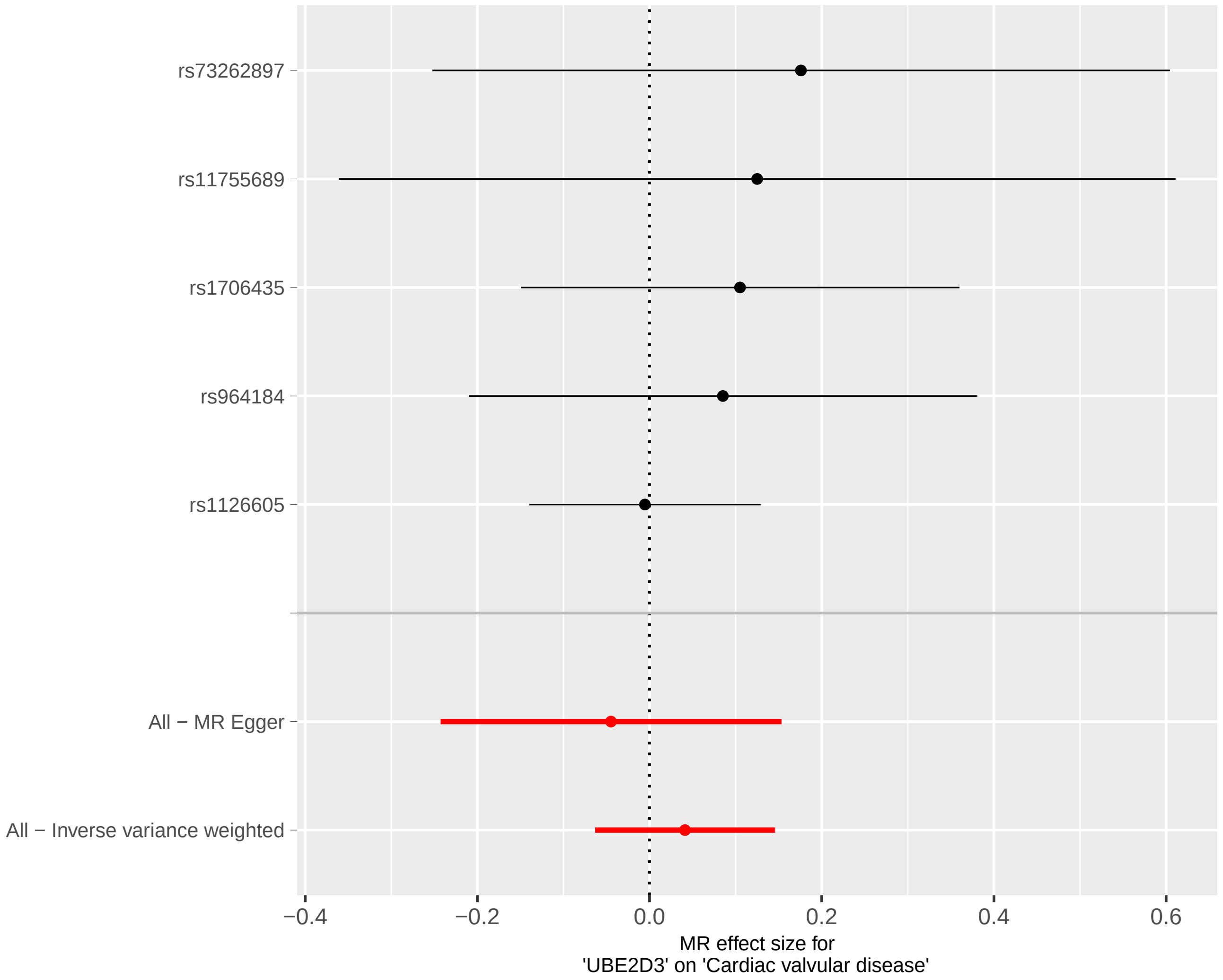

rs73262897
rs11755689
rs1706435
rs964184
rs1126605
All − MR Egger
All − Inverse variance weighted
−0.4
−0.2
0.0
0.2
0.4
0.6
MR effect size for
'UBE2D3' on 'Cardiac valvular disease'

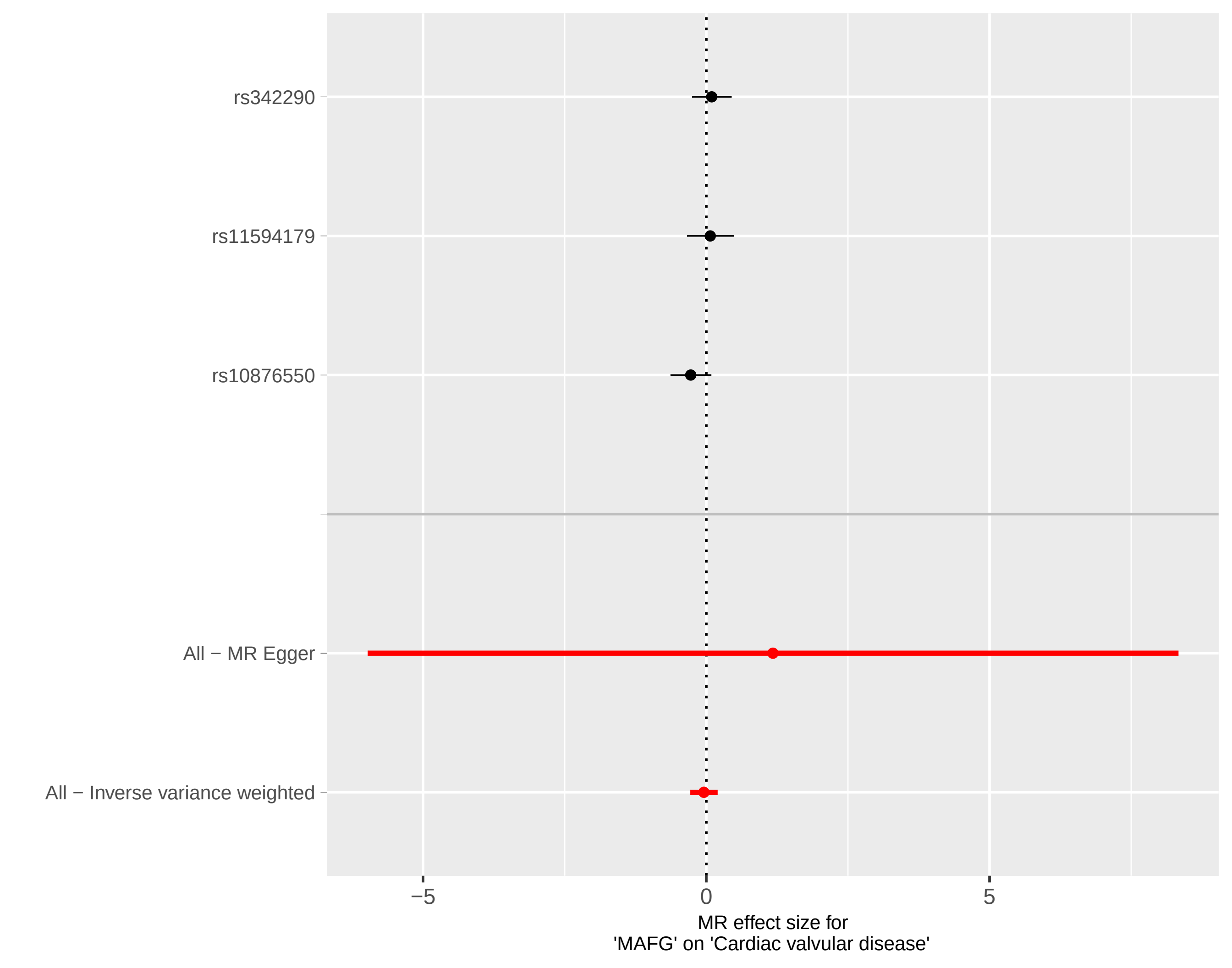
rs342290
rs11594179
rs10876550
All − MR Egger
All − Inverse variance weighted
−5
0
5
MR effect size for
'MAFG' on 'Cardiac valvular disease'

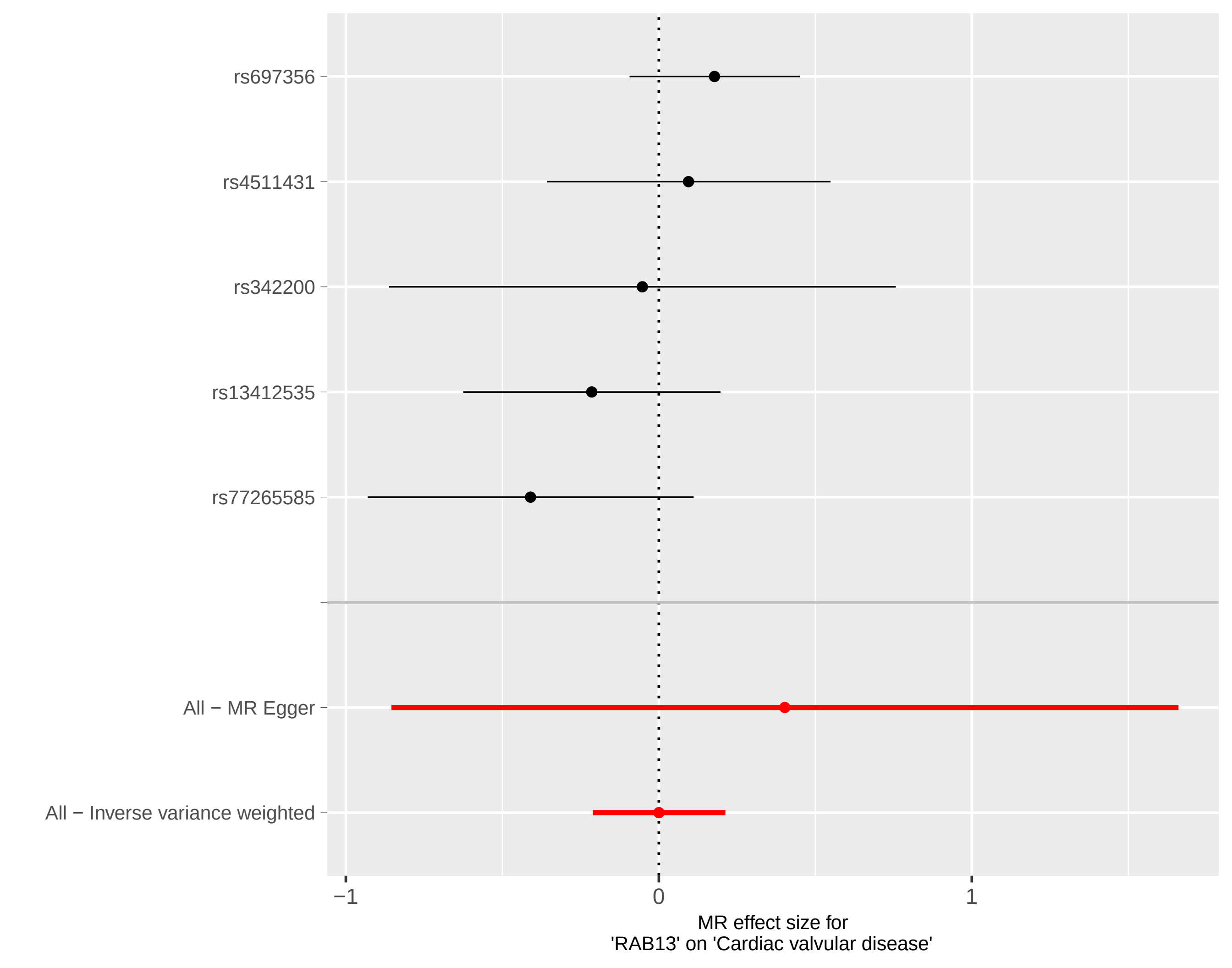

rs697356
rs4511431
rs342200
rs13412535
rs77265585
All − MR Egger
All − Inverse variance weighted
−1
0
1
MR effect size for
'RAB13' on 'Cardiac valvular disease'

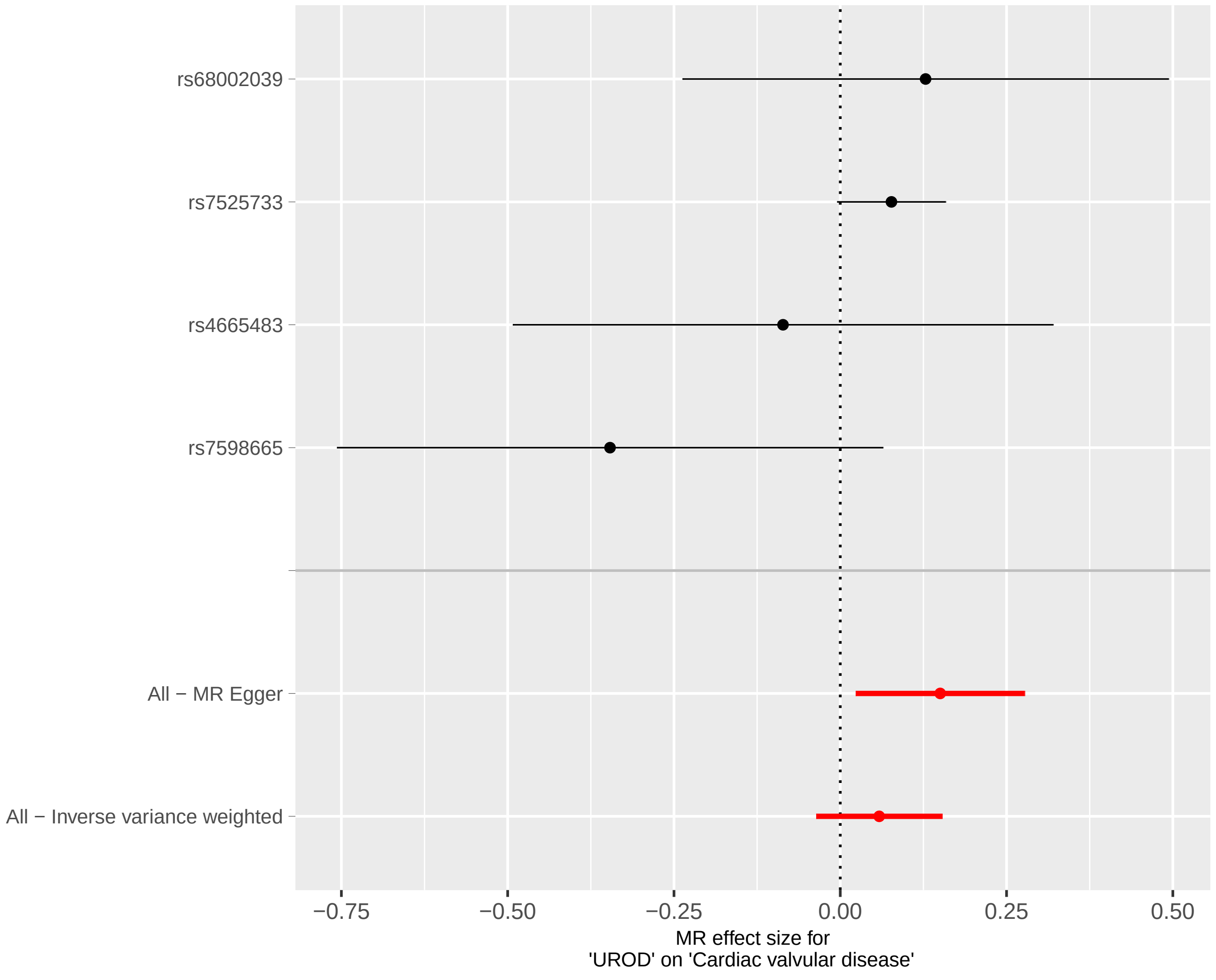
rs68002039
rs7525733
rs4665483
rs7598665
All − MR Egger
All − Inverse variance weighted
−0.75
−0.50
−0.25
0.00
0.25
0.50
MR effect size for
'UROD' on 'Cardiac valvular disease'

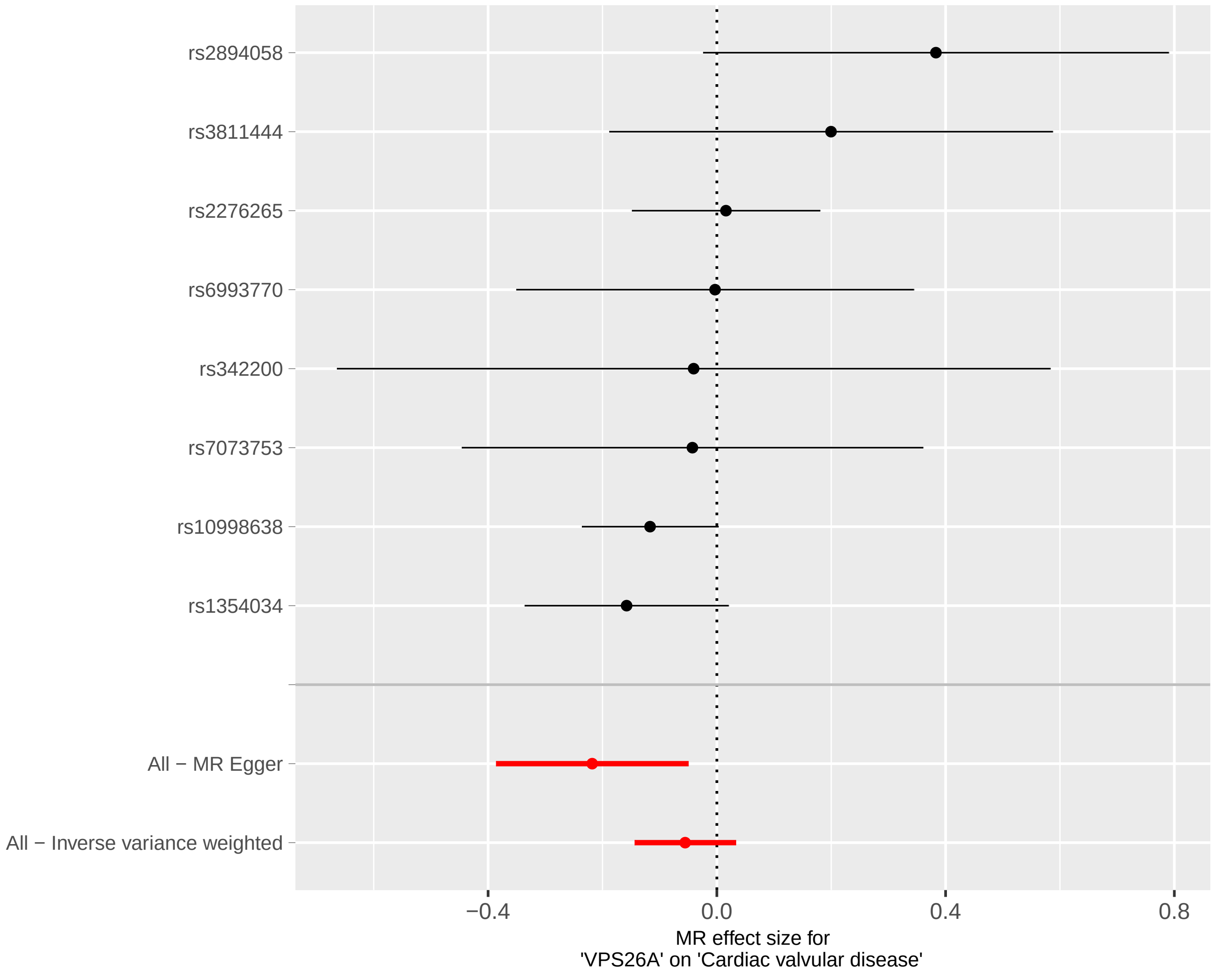
rs2894058
rs3811444
rs2276265
rs6993770
rs342200
rs7073753
rs10998638
rs1354034
All − MR Egger
All − Inverse variance weighted
−0.4
0.0
0.4
0.8
MR effect size for
'VPS26A' on 'Cardiac valvular disease'

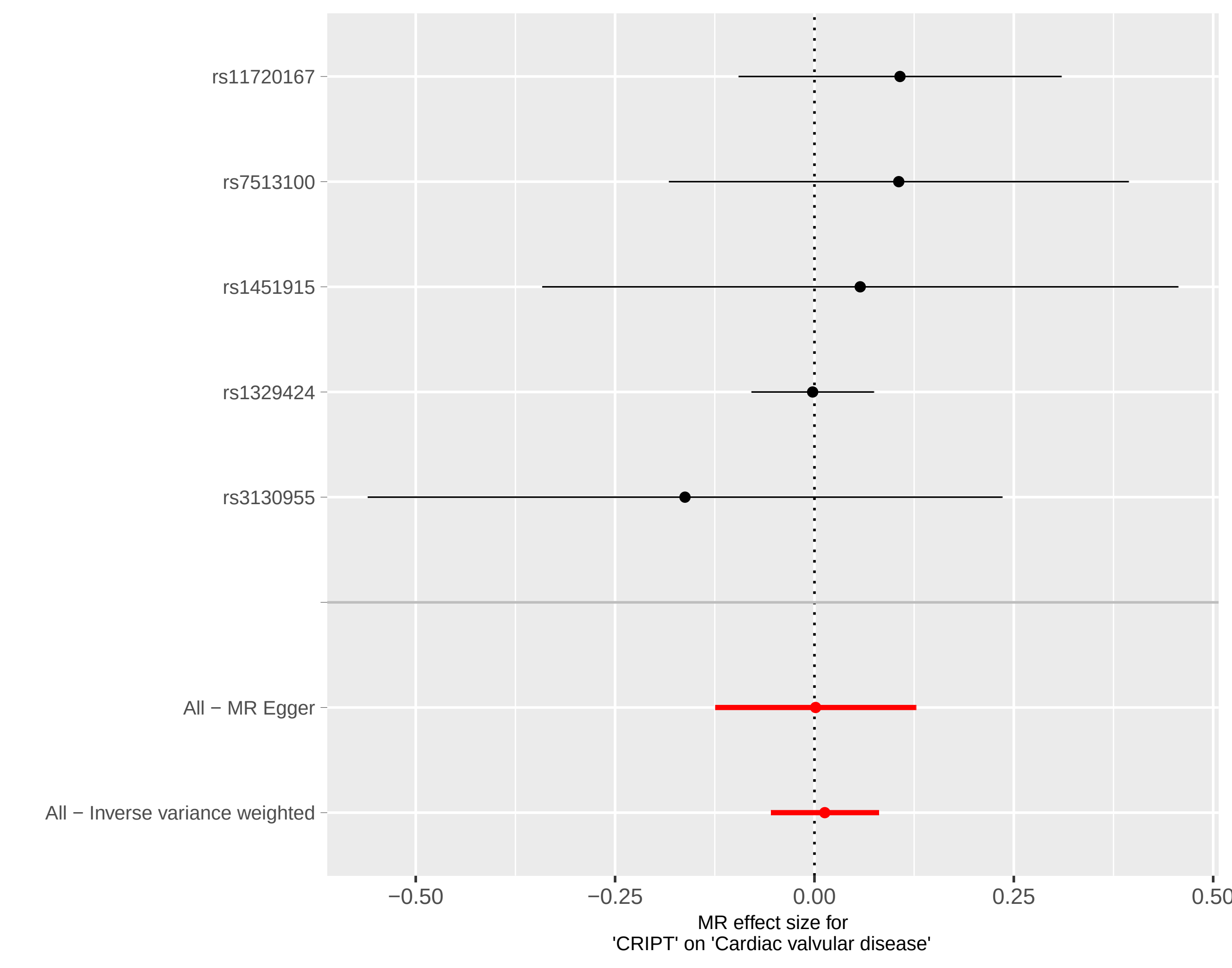
rs11720167
rs7513100
rs1451915
rs1329424
rs3130955
All − MR Egger
All − Inverse variance weighted
−0.50
−0.25
0.00
0.25
0.50
MR effect size for
'CRIPT' on 'Cardiac valvular disease'

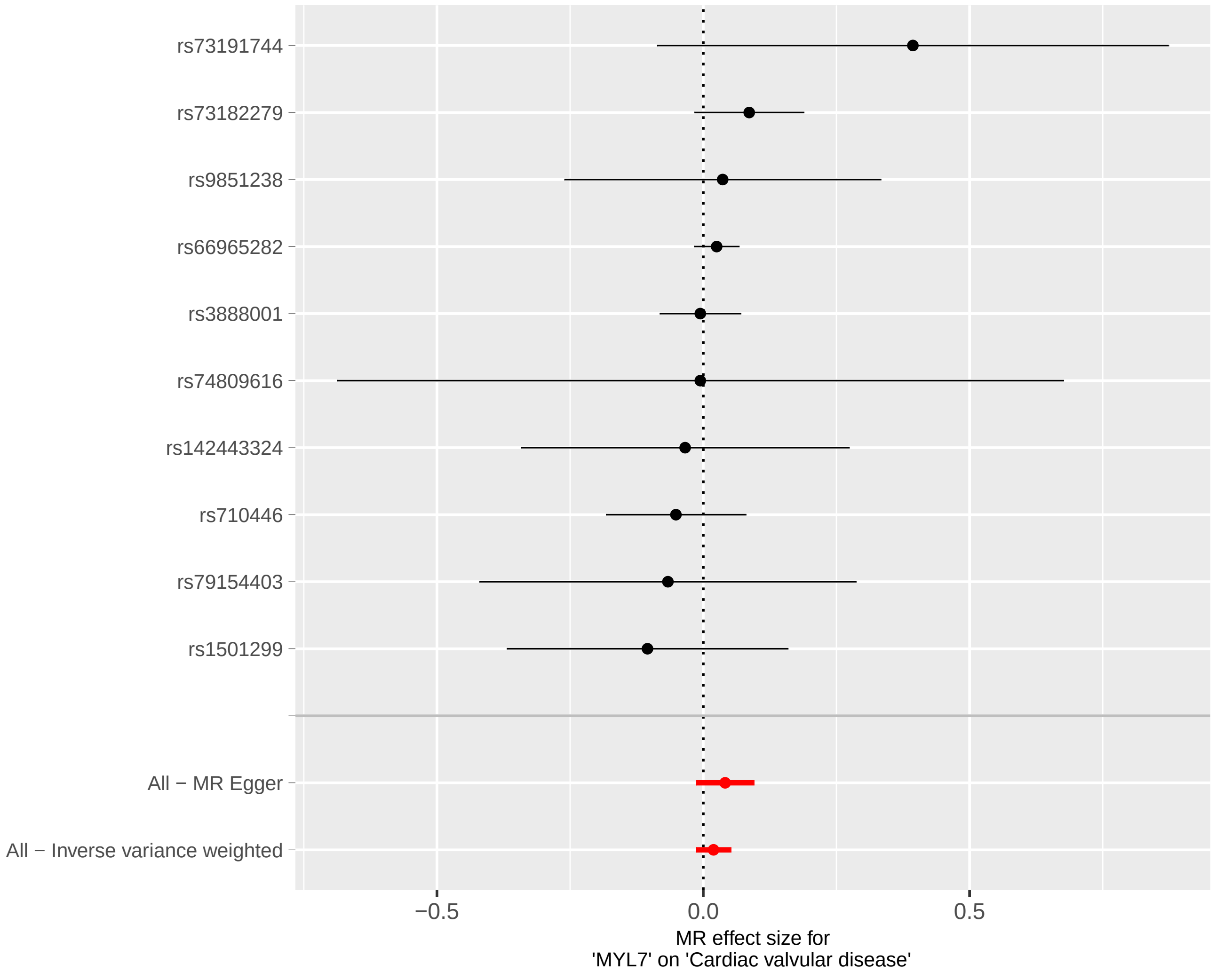

rs73191744
rs73182279
rs9851238
rs66965282
rs3888001
rs74809616
rs142443324
rs710446
rs79154403
rs1501299
All − MR Egger
All − Inverse variance weighted
−0.5
0.0
0.5
MR effect size for
'MYL7' on 'Cardiac valvular disease'

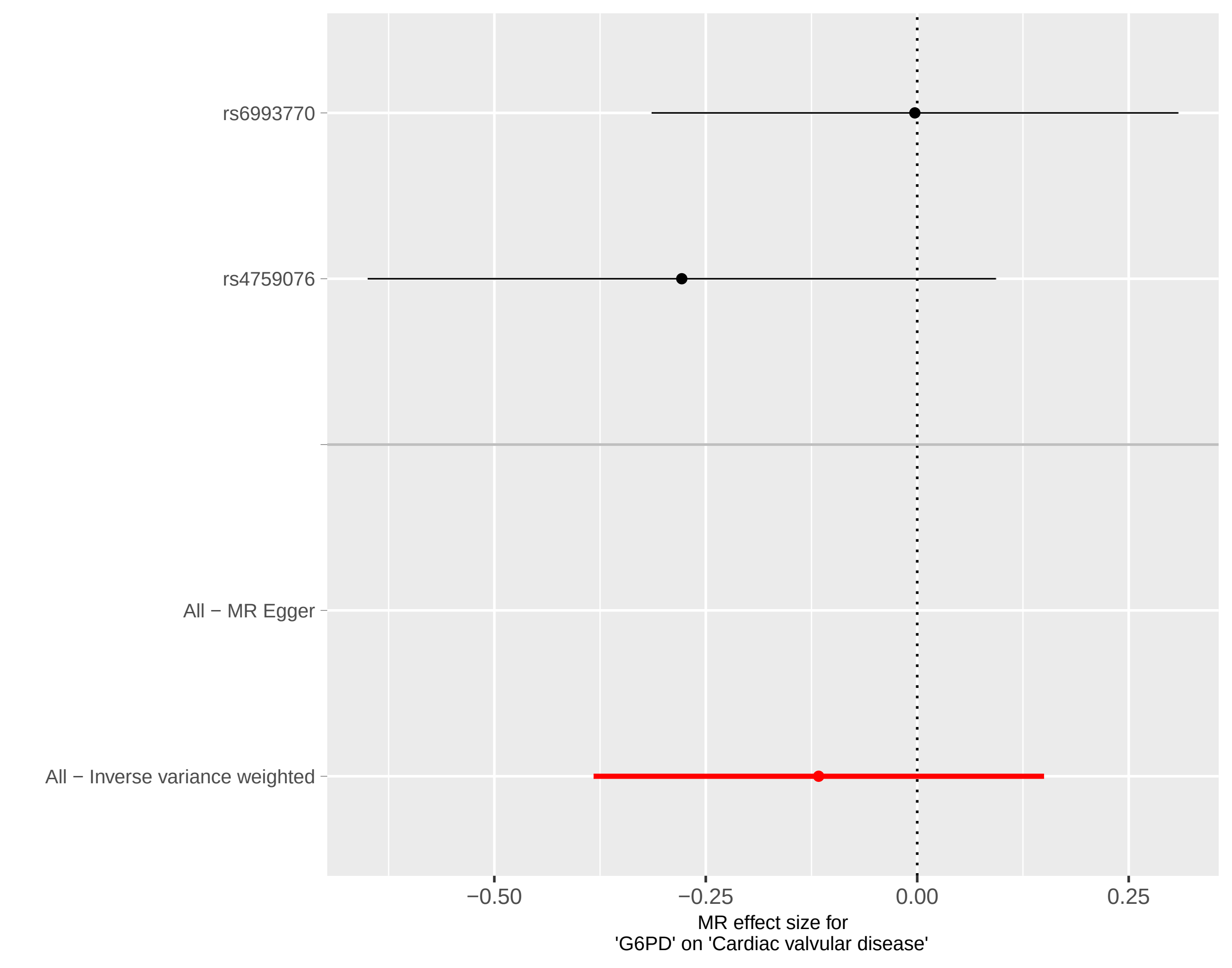

rs6993770
rs4759076
All − MR Egger
All − Inverse variance weighted
−0.50
−0.25
0.00
0.25
MR effect size for
'G6PD' on 'Cardiac valvular disease'

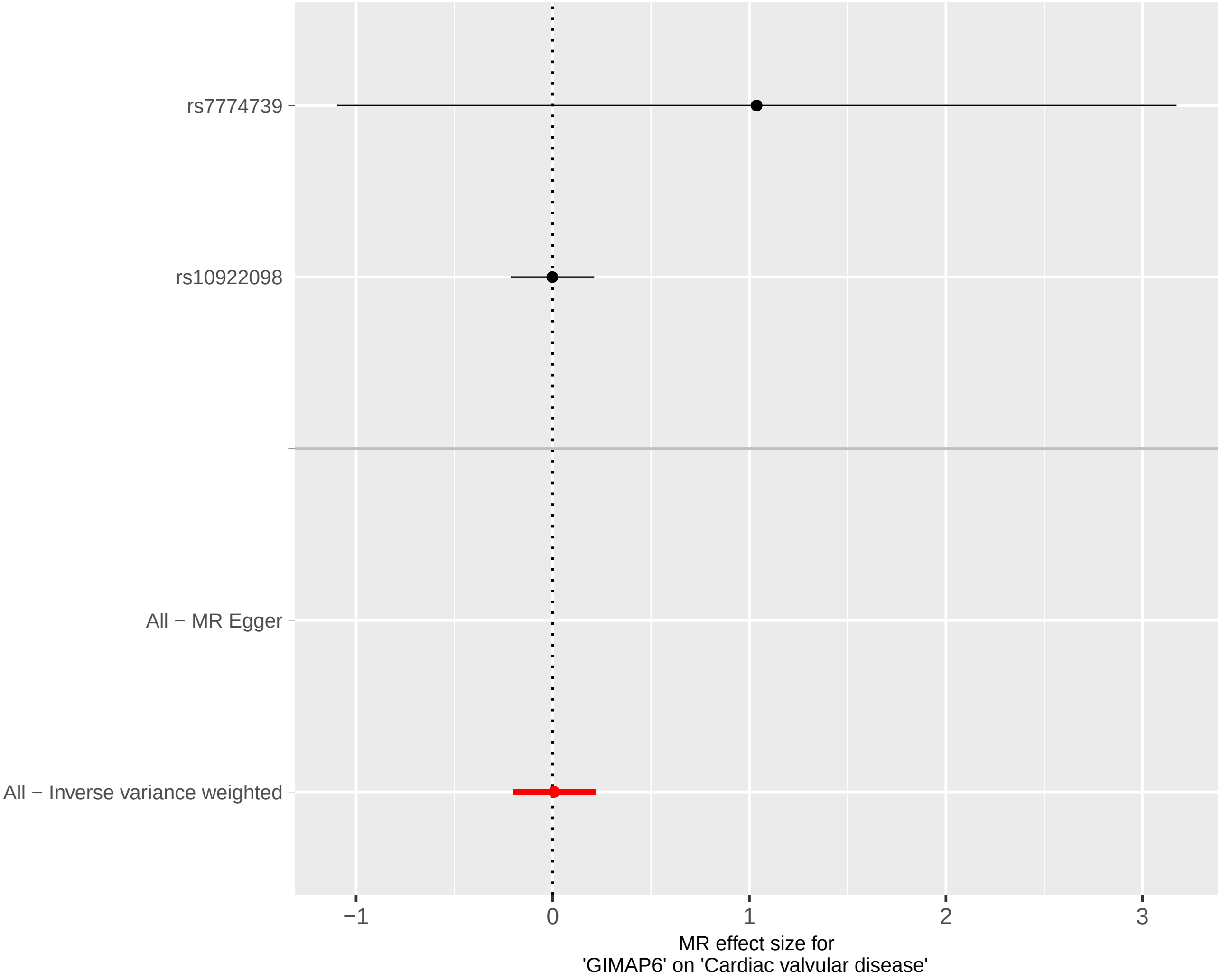
rs7774739
rs10922098
All − MR Egger
All − Inverse variance weighted
−1
0
1
2
3
MR effect size for
'GIMAP6' on 'Cardiac valvular disease'

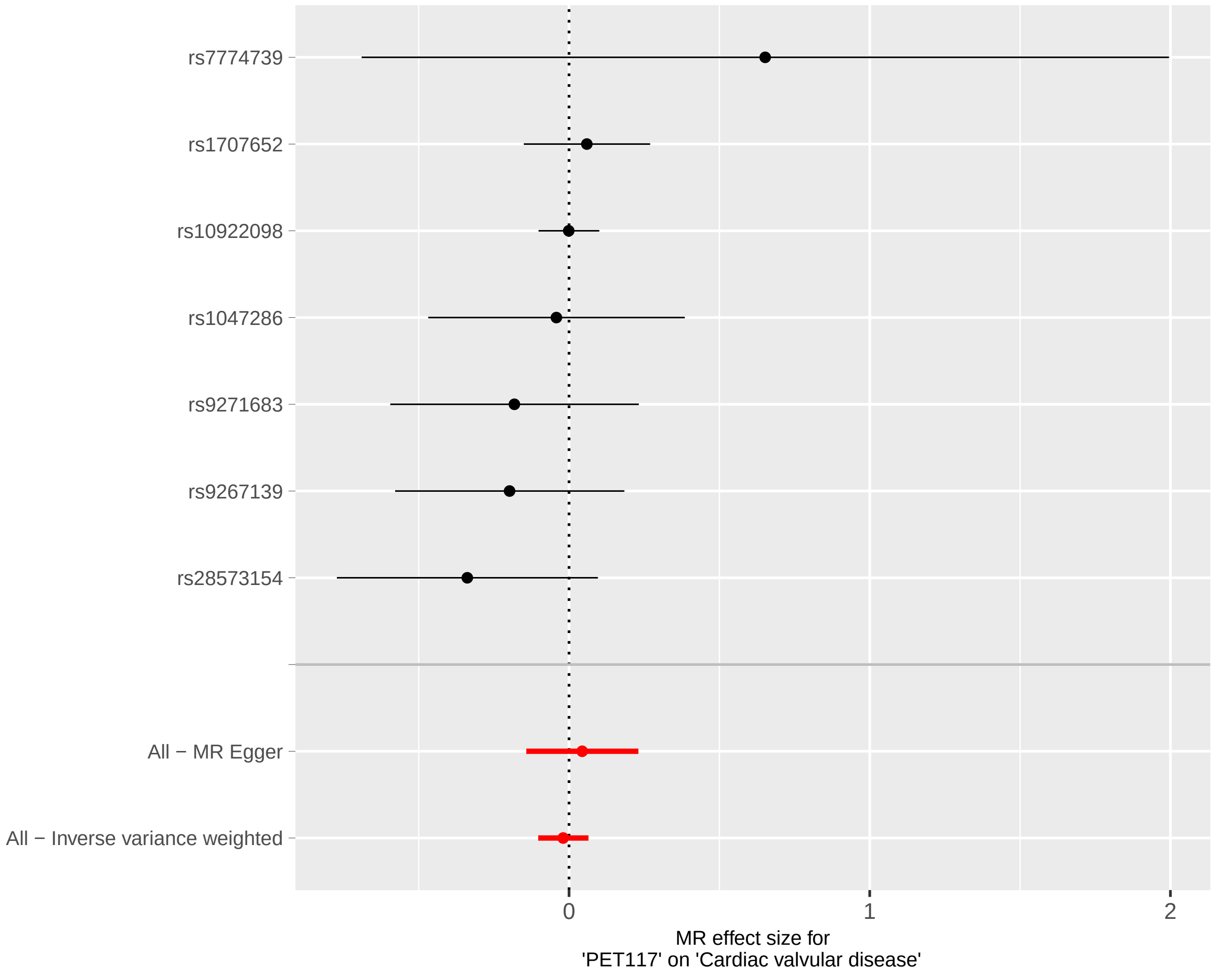

rs7774739
rs1707652
rs10922098
rs1047286
rs9271683
rs9267139
rs28573154
All − MR Egger
All − Inverse variance weighted
0
1
2
MR effect size for
'PET117' on 'Cardiac valvular disease'

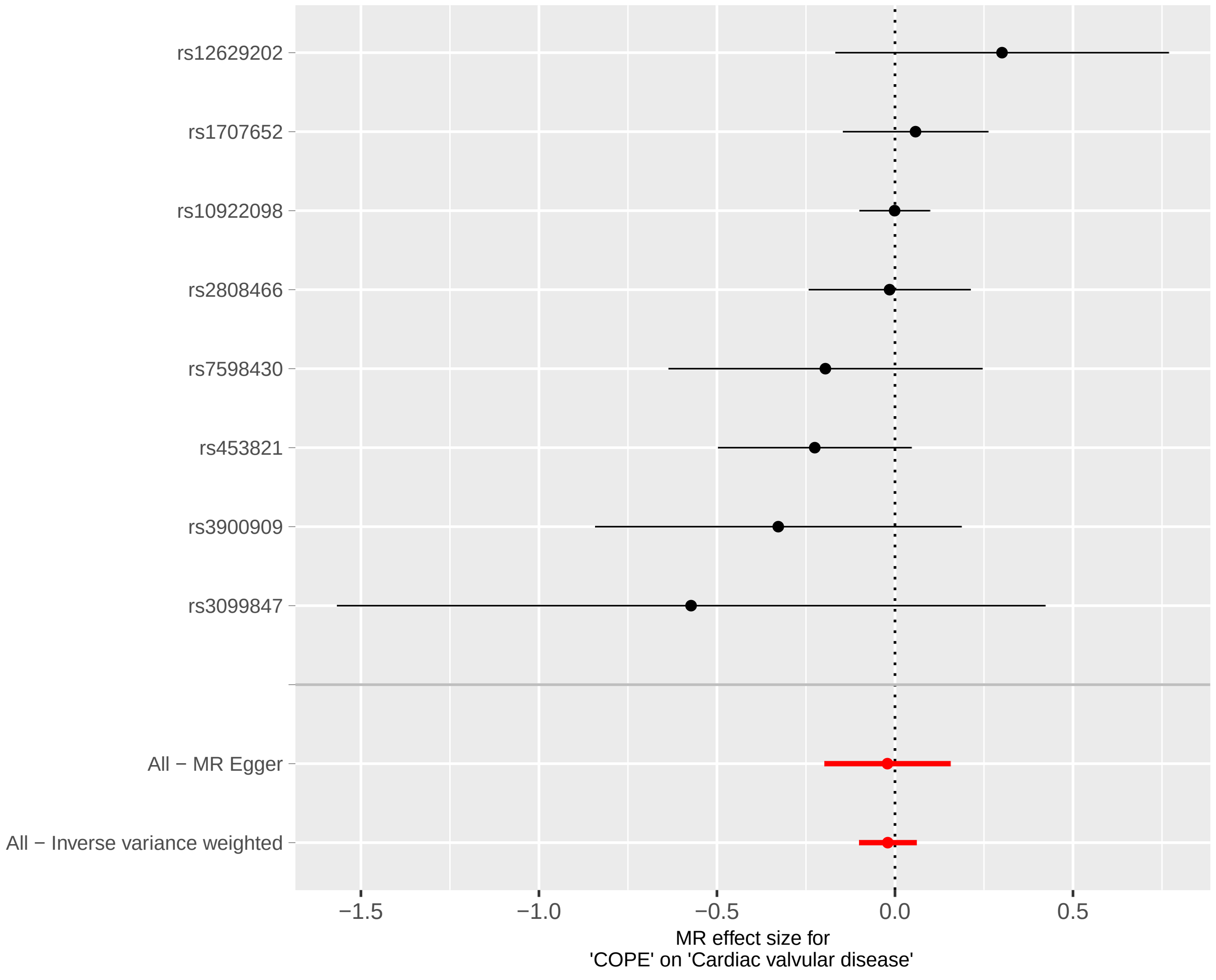
rs12629202
rs1707652
rs10922098
rs2808466
rs7598430
rs453821
rs3900909
rs3099847
All − MR Egger
All − Inverse variance weighted
−1.5
−1.0
−0.5
0.0
0.5
MR effect size for
'COPE' on 'Cardiac valvular disease'

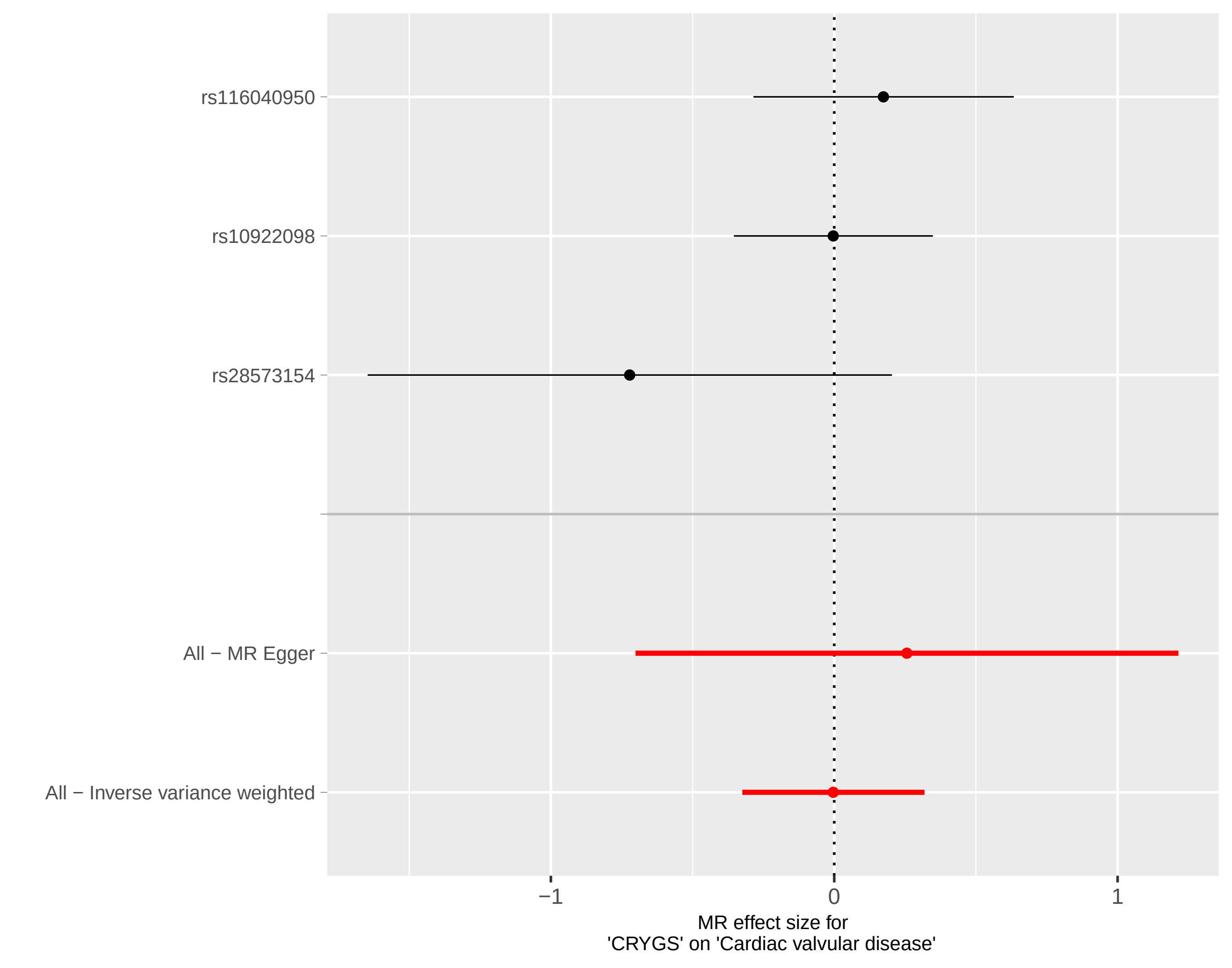
rs116040950
rs10922098
rs28573154
All − MR Egger
All − Inverse variance weighted
−1
0
1
MR effect size for
'CRYGS' on 'Cardiac valvular disease'

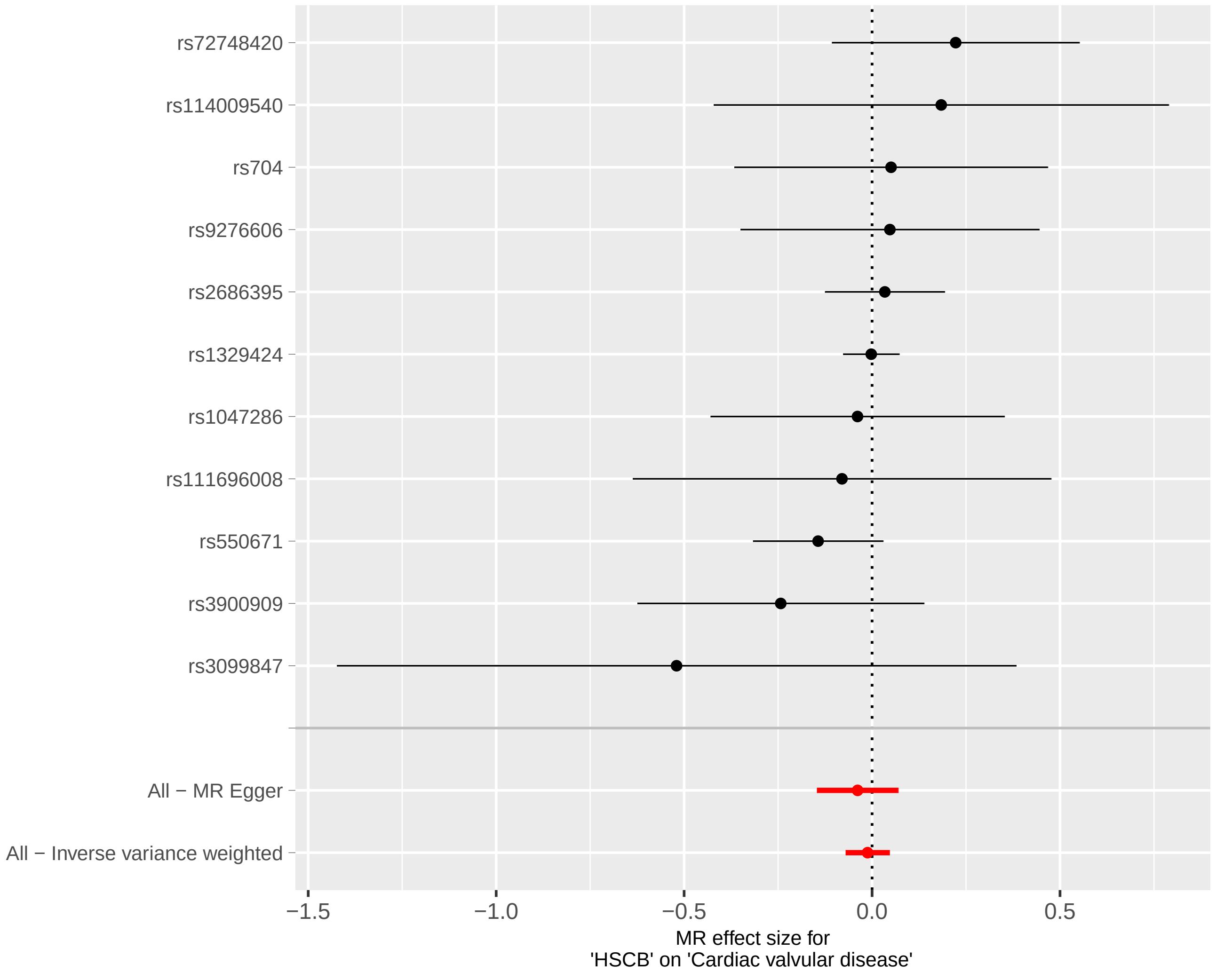
rs72748420
rs114009540
rs704
rs9276606
rs2686395
rs1329424
rs1047286
rs111696008
rs550671
rs3900909
rs3099847
All − MR Egger
All − Inverse variance weighted
−1.5
−1.0
−0.5
0.0
0.5
MR effect size for
'HSCB' on 'Cardiac valvular disease'

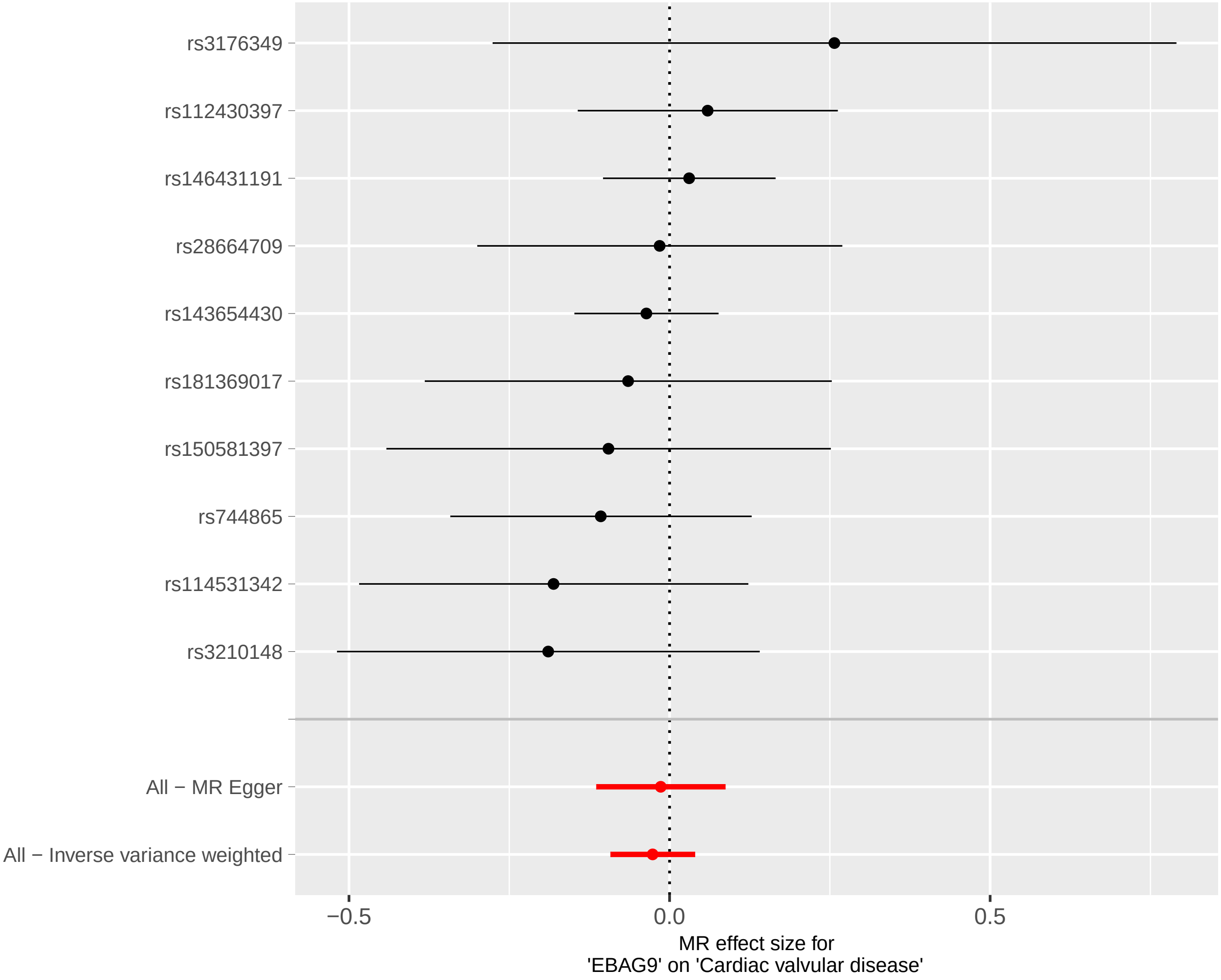

rs3176349
rs112430397
rs146431191
rs28664709
rs143654430
rs181369017
rs150581397
rs744865
rs114531342
rs3210148
All − MR Egger
All − Inverse variance weighted
−0.5
0.0
0.5
MR effect size for
'EBAG9' on 'Cardiac valvular disease'

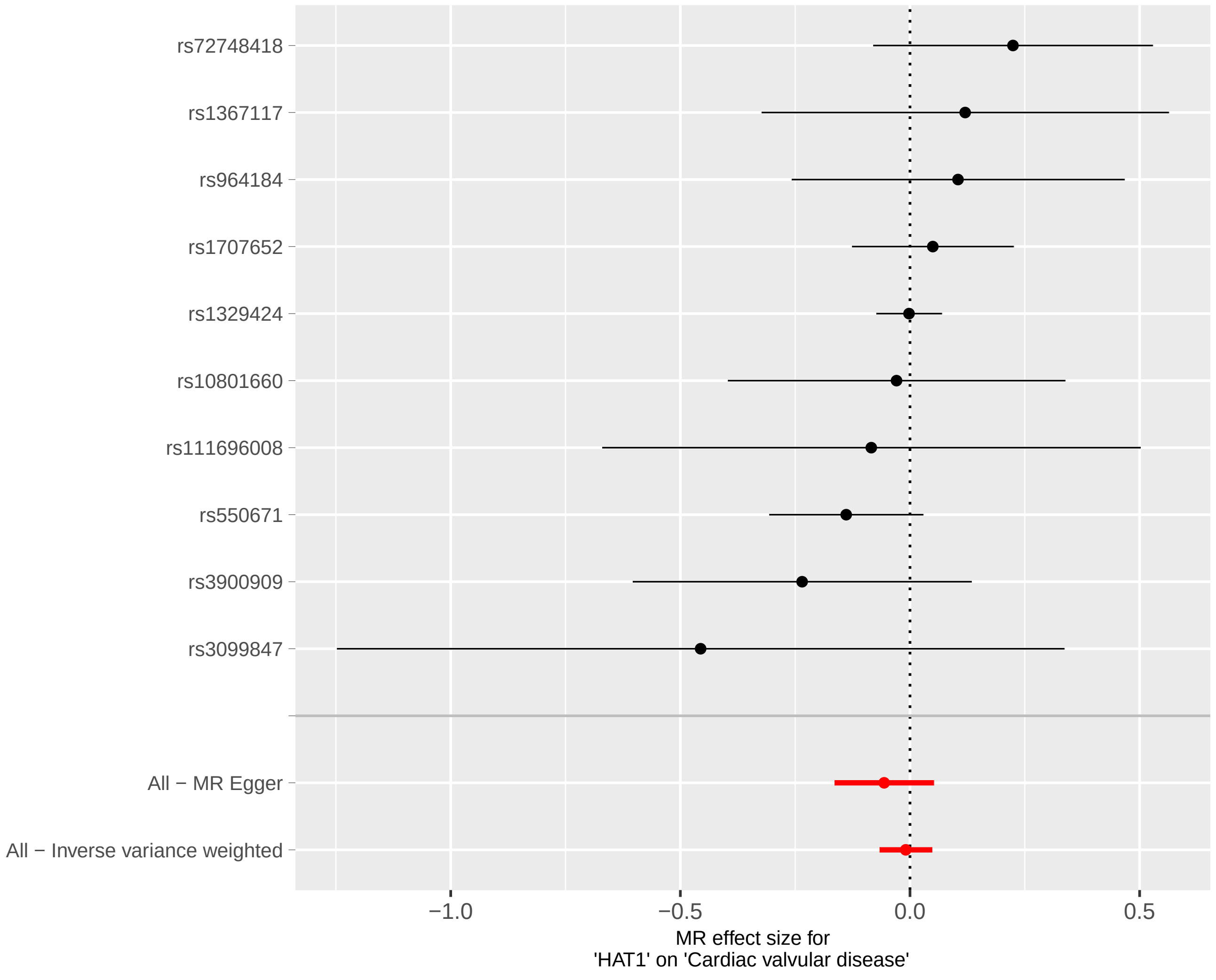

rs72748418
rs1367117
rs964184
rs1707652
rs1329424
rs10801660
rs111696008
rs550671
rs3900909
rs3099847
All − MR Egger
All − Inverse variance weighted
−1.0
−0.5
0.0
0.5
MR effect size for
'HAT1' on 'Cardiac valvular disease'

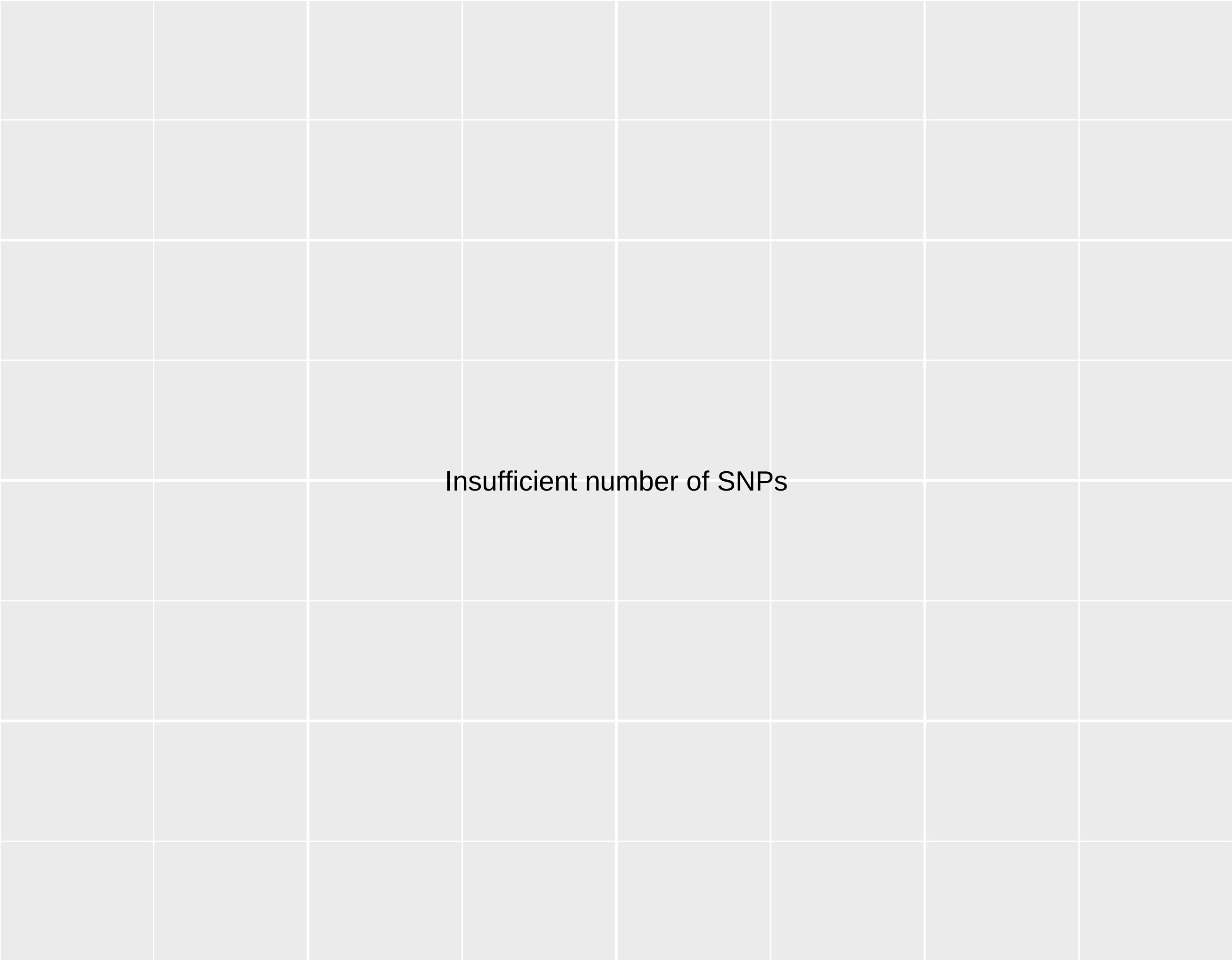
Insufficient number of SNPs

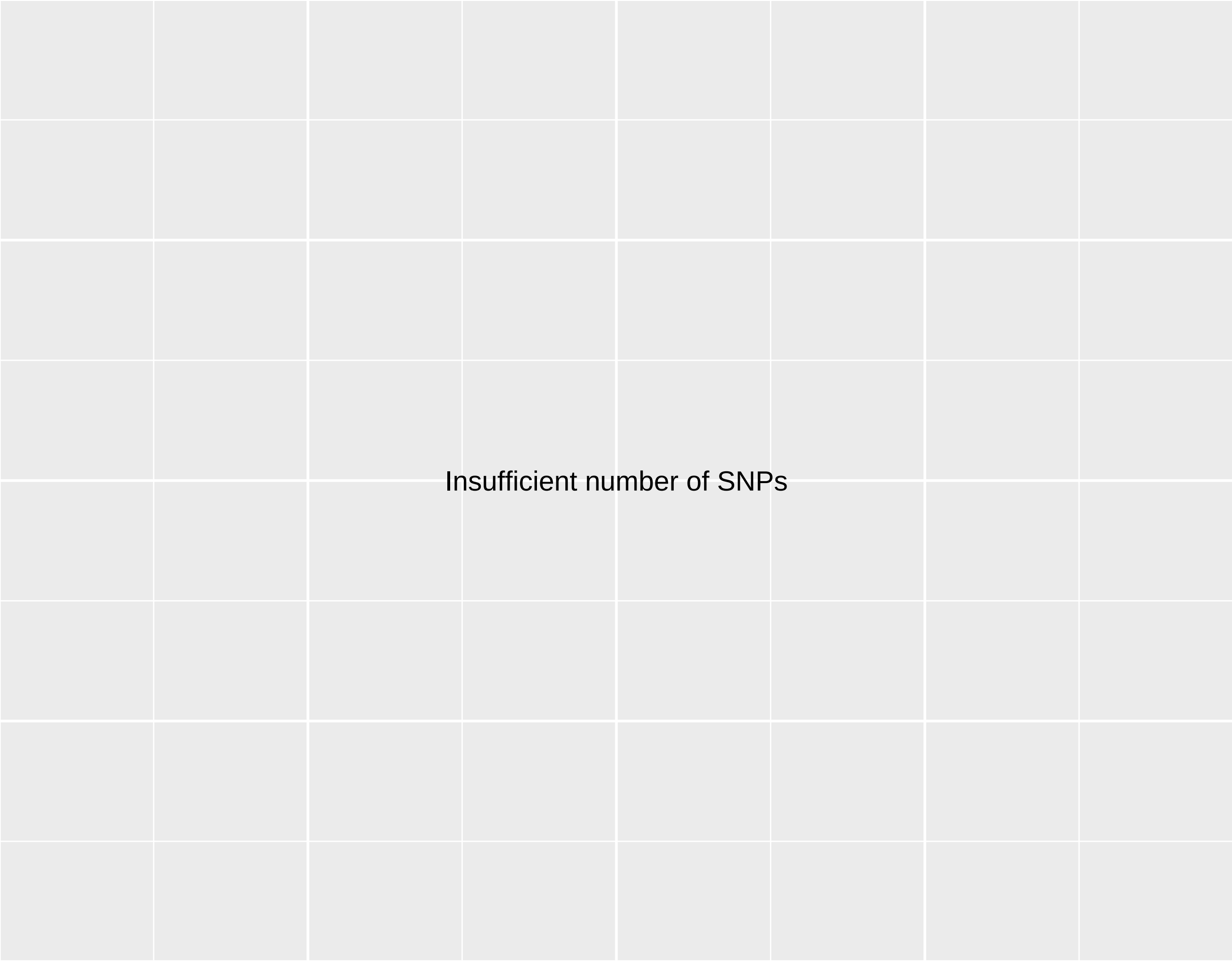
Insufficient number of SNPs

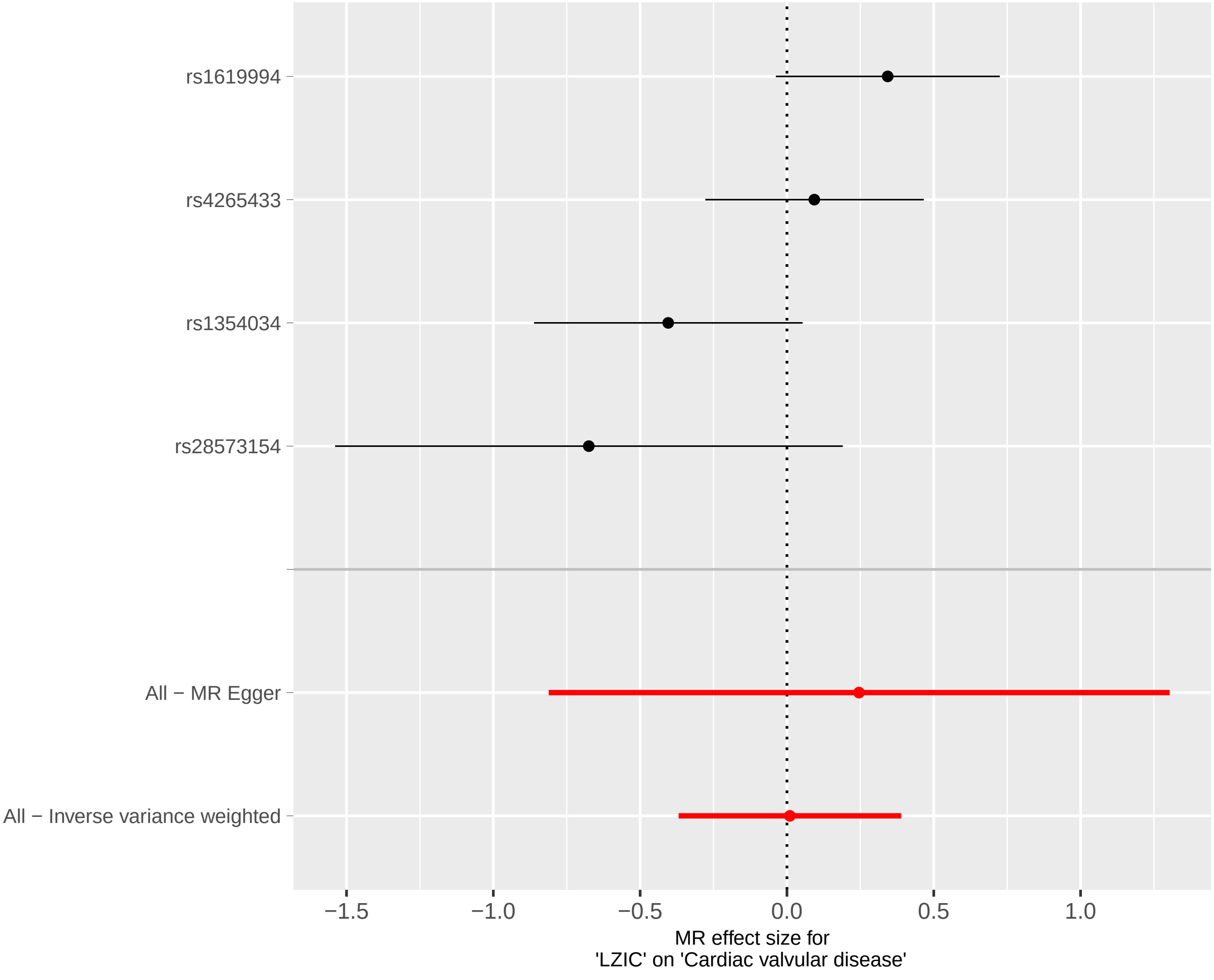
rs1619994
rs4265433
rs1354034
rs28573154
All − MR Egger
All − Inverse variance weighted
−1.5
−1.0
−0.5
0.0
0.5
1.0
MR effect size for
'LZIC' on 'Cardiac valvular disease'

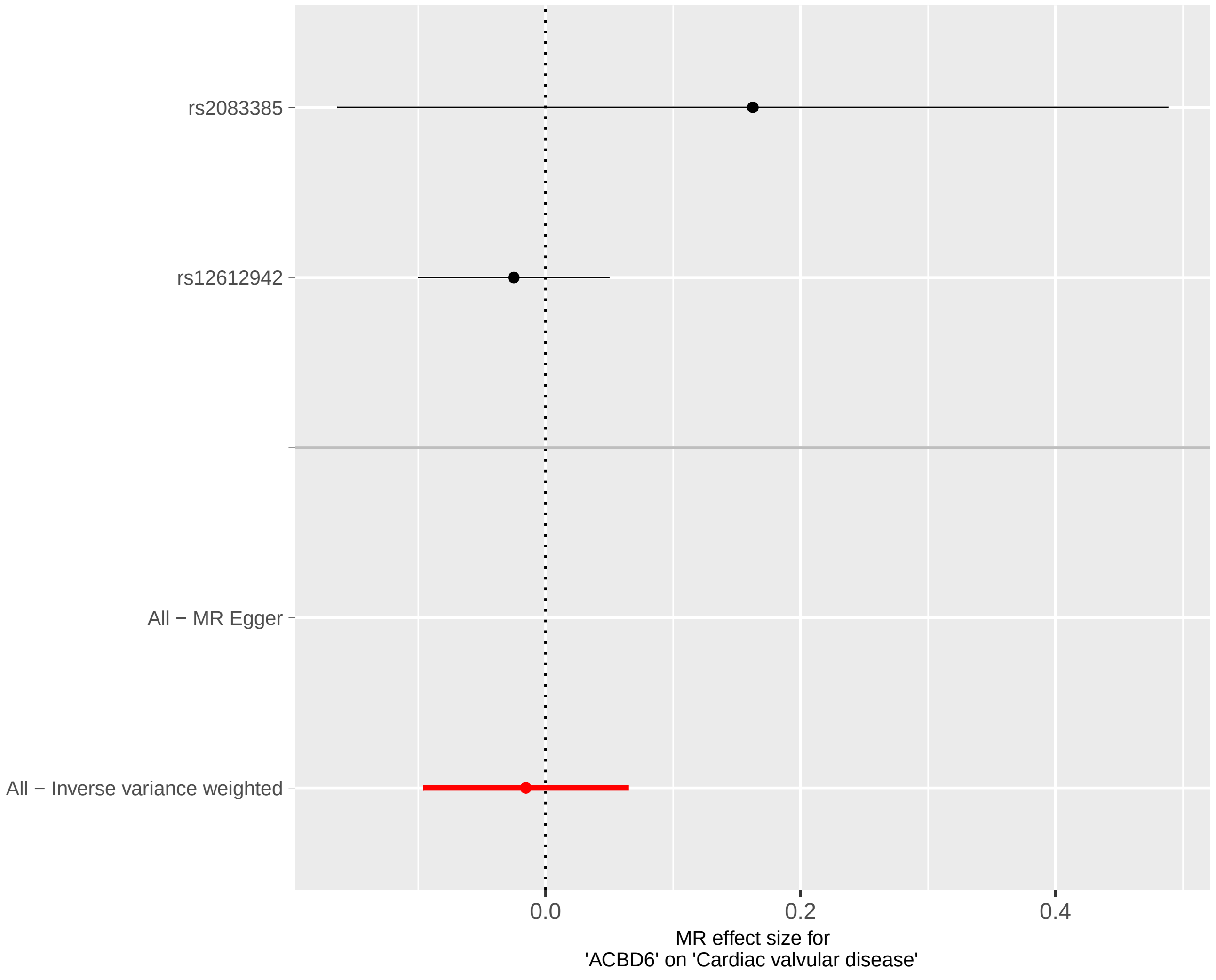
rs2083385
rs12612942
All − MR Egger
All − Inverse variance weighted
0.0
0.2
0.4
MR effect size for
'ACBD6' on 'Cardiac valvular disease'

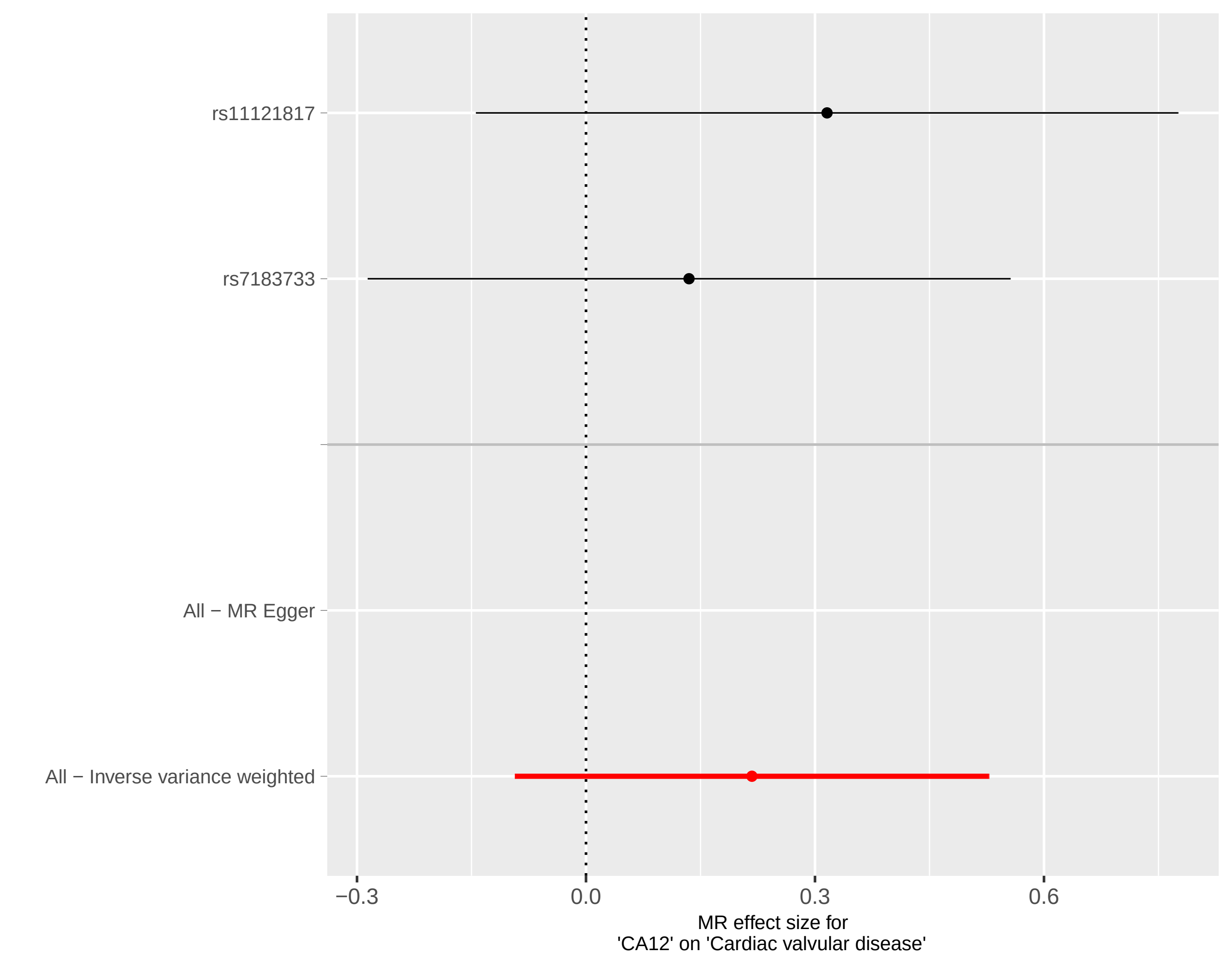
rs11121817
rs7183733
All − MR Egger
All − Inverse variance weighted
−0.3
0.0
0.3
0.6
MR effect size for
'CA12' on 'Cardiac valvular disease'

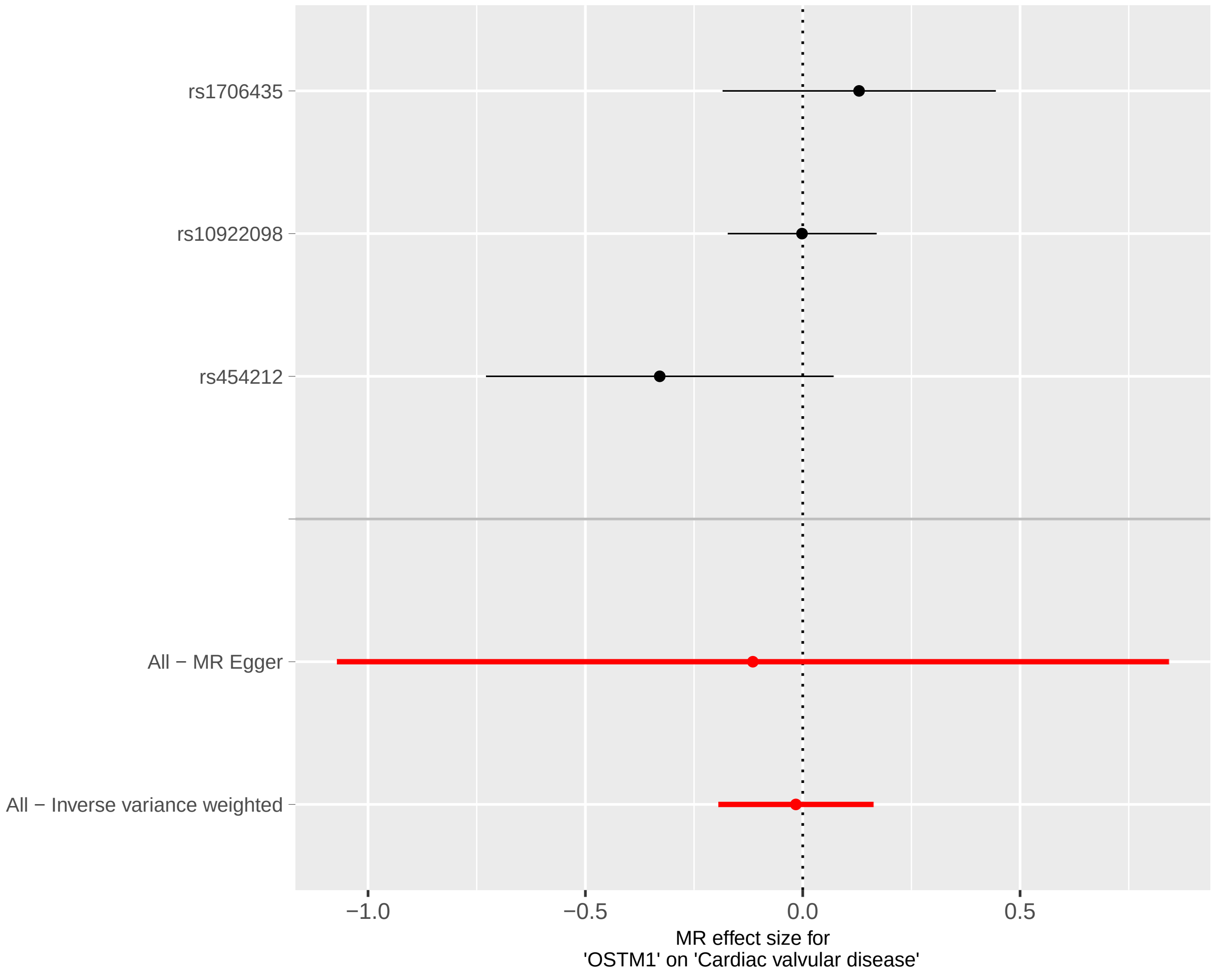
rs1706435
rs10922098
rs454212
All − MR Egger
All − Inverse variance weighted
−1.0
−0.5
0.0
0.5
MR effect size for
'OSTM1' on 'Cardiac valvular disease'

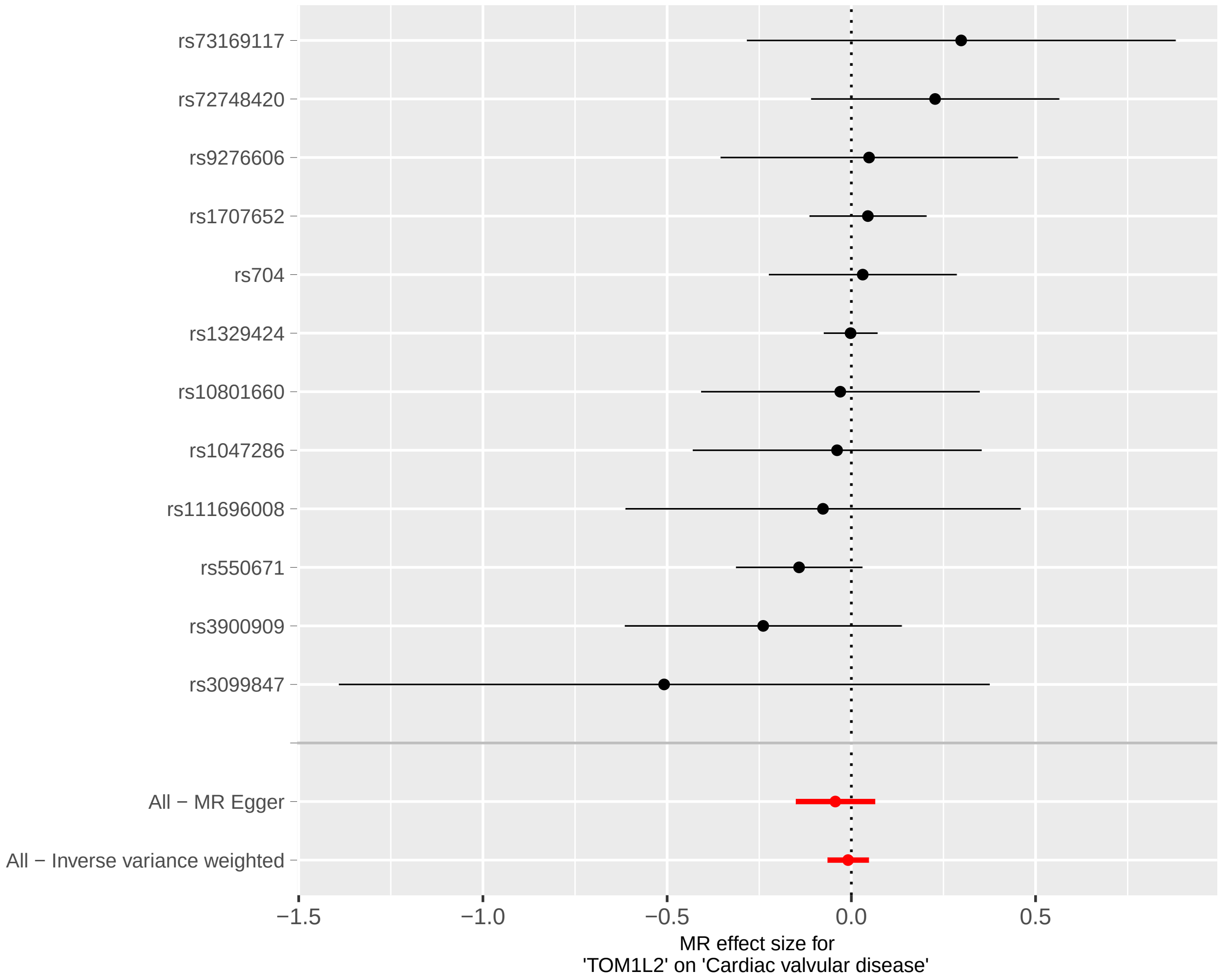

rs73169117
rs72748420
rs9276606
rs1707652
rs704
rs1329424
rs10801660
rs1047286
rs111696008
rs550671
rs3900909
rs3099847
All − MR Egger
All − Inverse variance weighted
−1.5
−1.0
−0.5
0.0
0.5
MR effect size for
'TOM1L2' on 'Cardiac valvular disease'

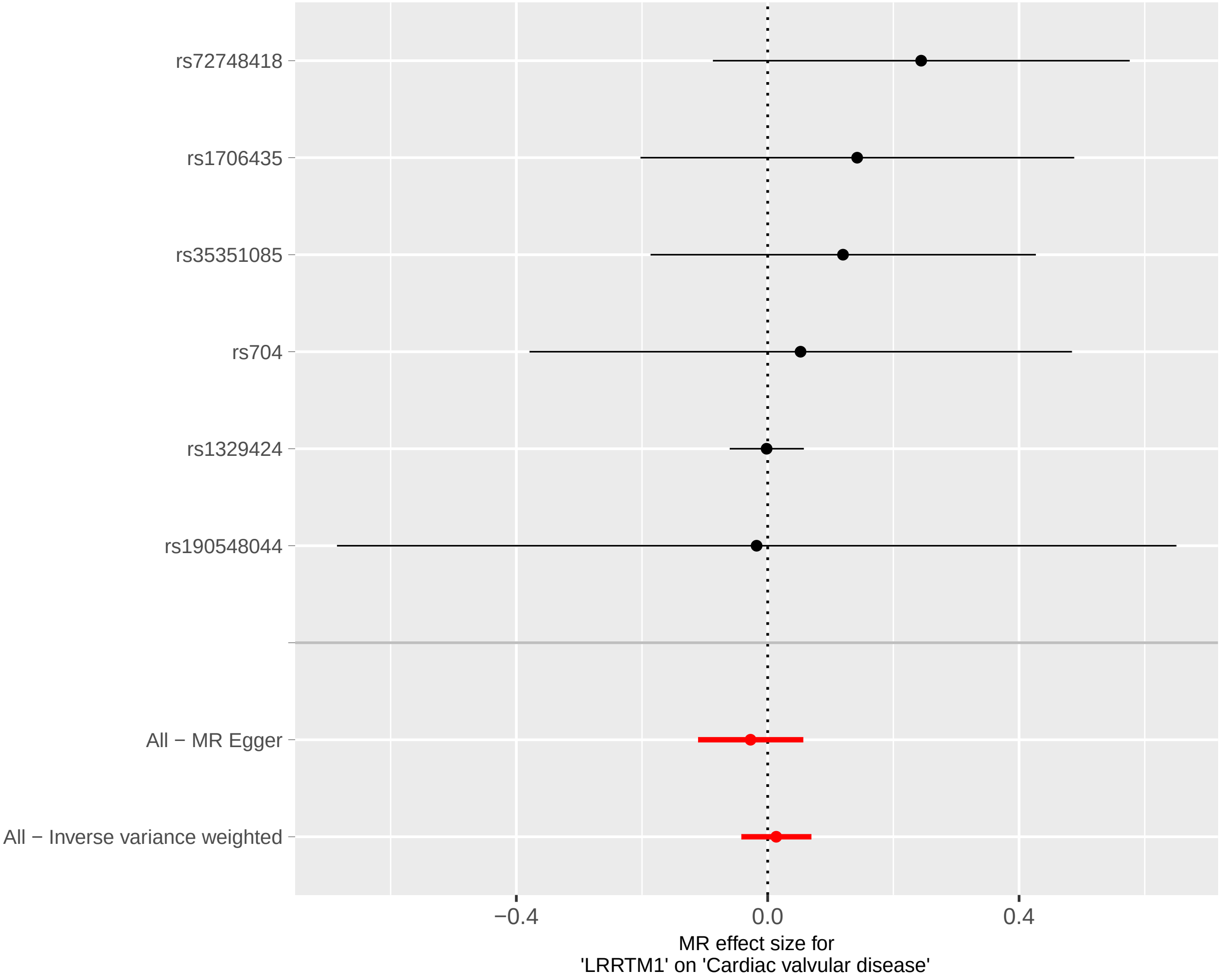

rs72748418
rs1706435
rs35351085
rs704
rs1329424
rs190548044
All − MR Egger
All − Inverse variance weighted
−0.4
0.0
0.4
MR effect size for
'LRRTM1' on 'Cardiac valvular disease'

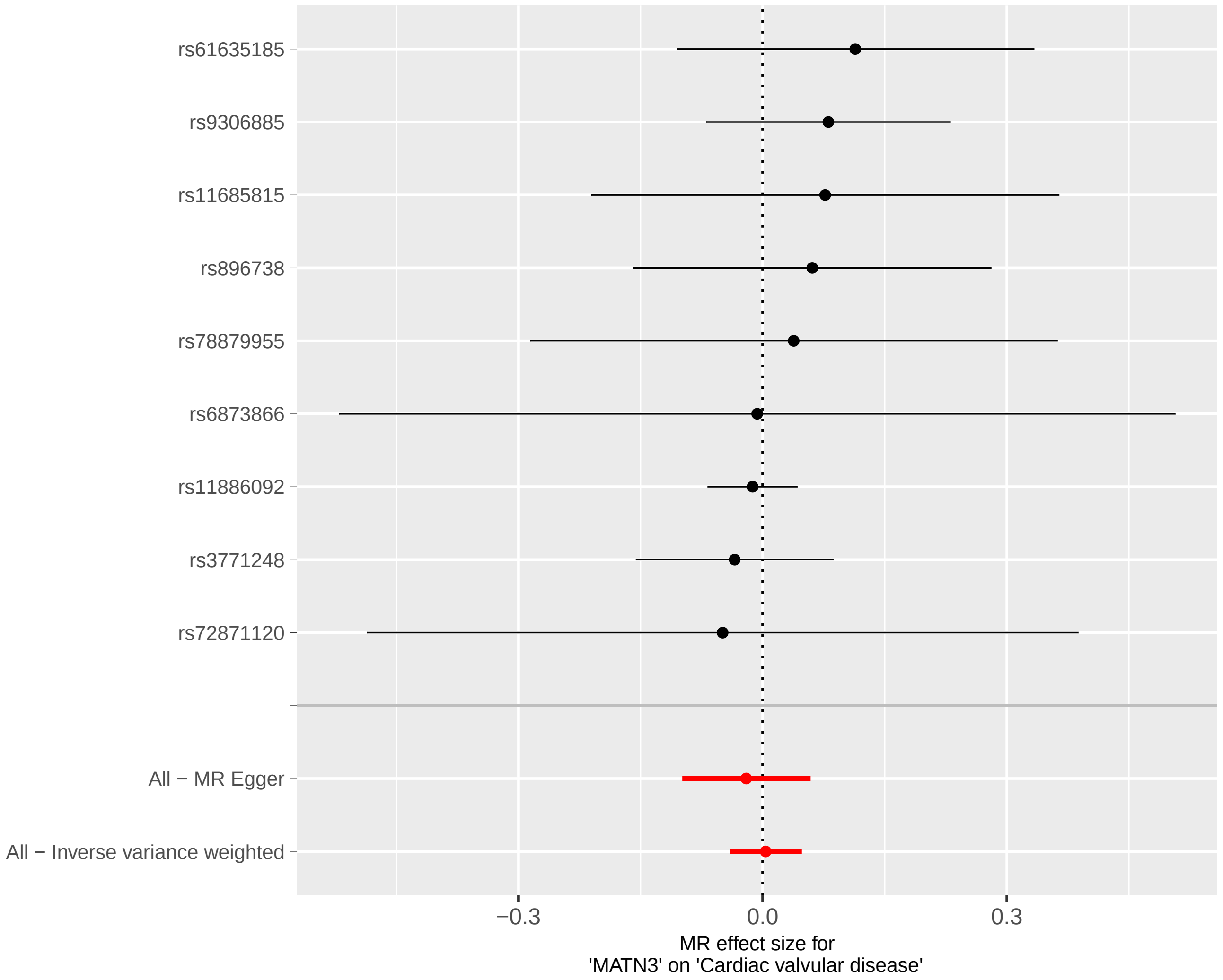
rs61635185
rs9306885
rs11685815
rs896738
rs78879955
rs6873866
rs11886092
rs3771248
rs72871120
All − MR Egger
All − Inverse variance weighted
−0.3
0.0
0.3
MR effect size for
'MATN3' on 'Cardiac valvular disease'

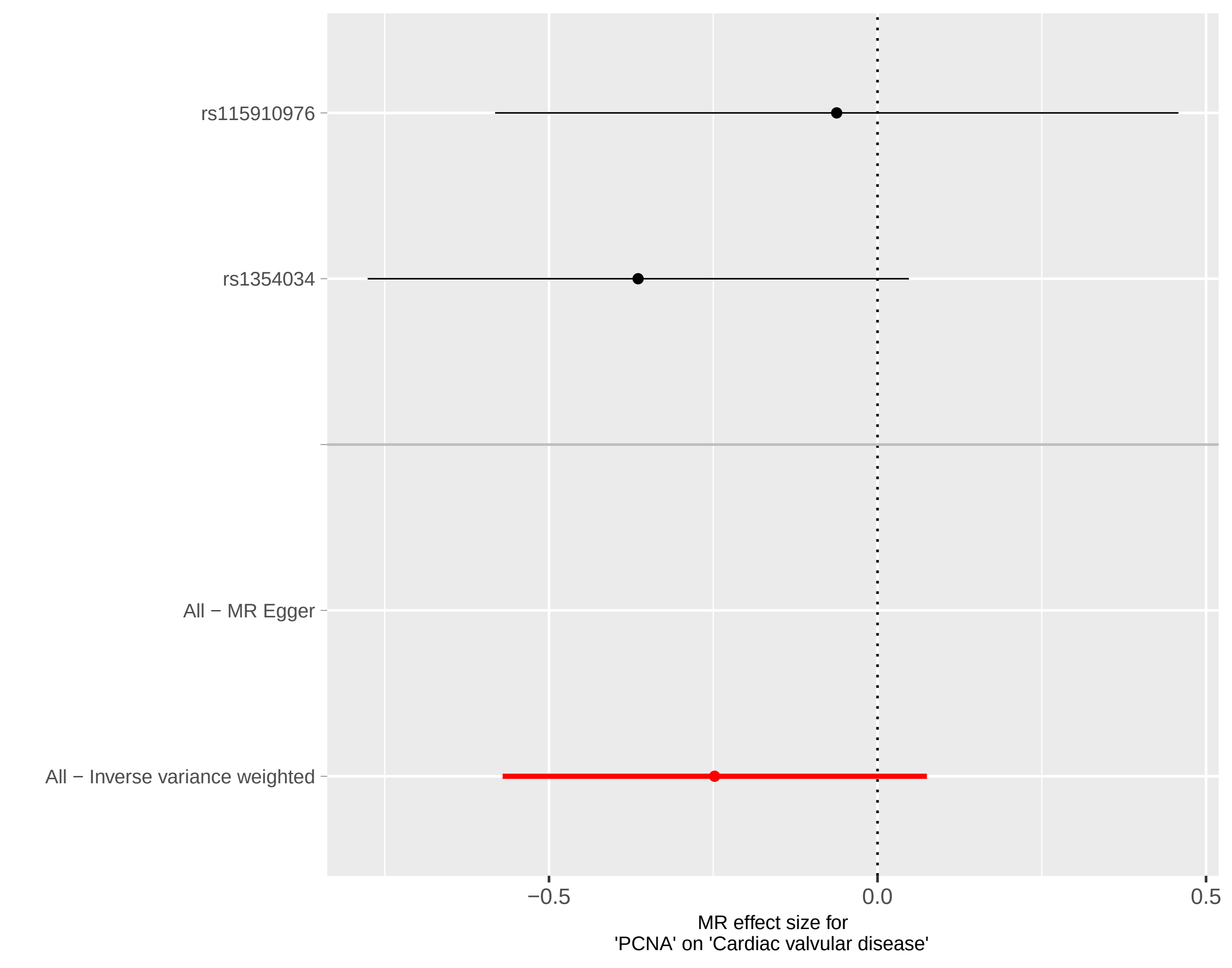
rs115910976
rs1354034
All − MR Egger
All − Inverse variance weighted
−0.5
0.0
0.5
MR effect size for
'PCNA' on 'Cardiac valvular disease'

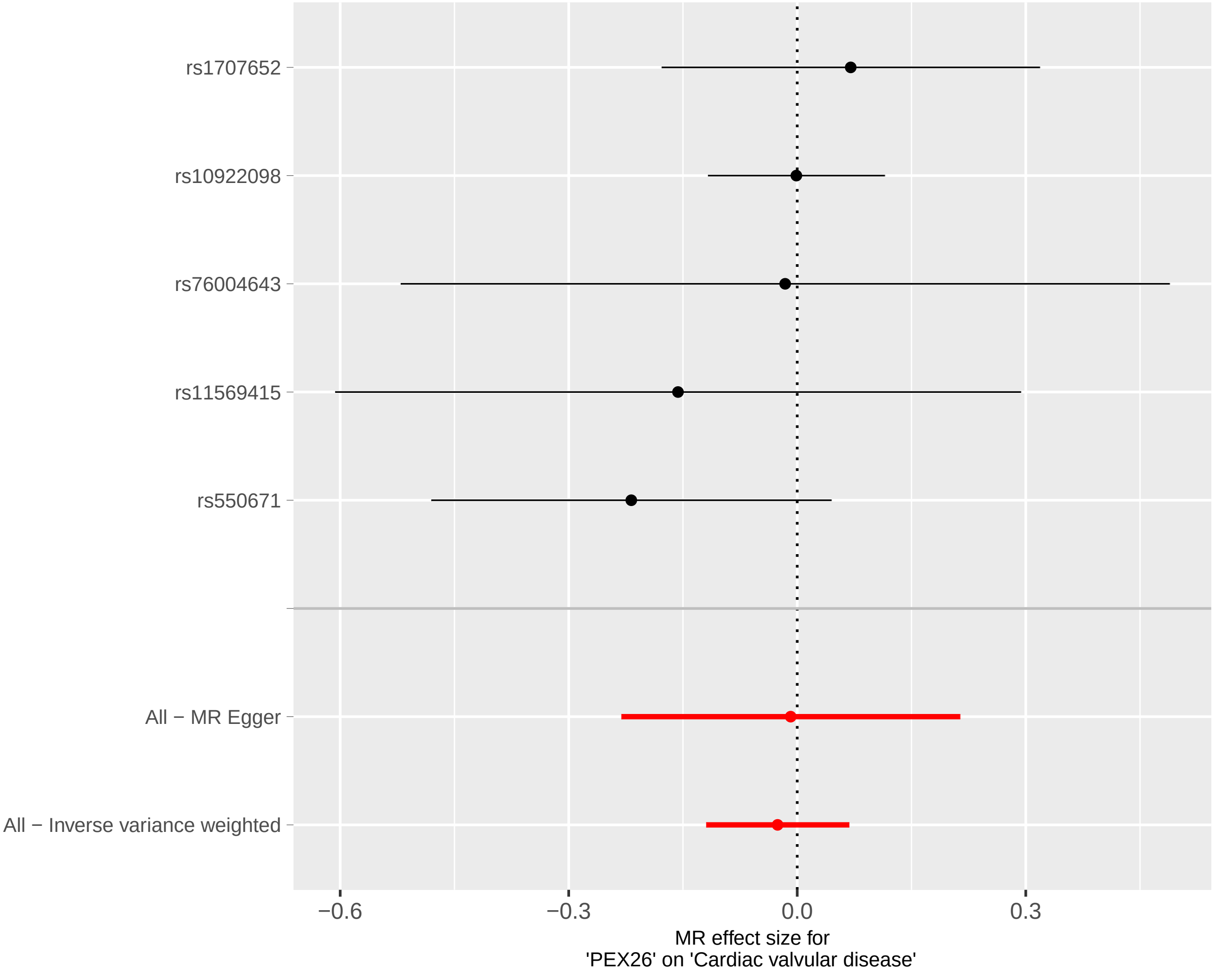

rs1707652
rs10922098
rs76004643
rs11569415
rs550671
All − MR Egger
All − Inverse variance weighted
−0.6
−0.3
0.0
0.3
MR effect size for
'PEX26' on 'Cardiac valvular disease'

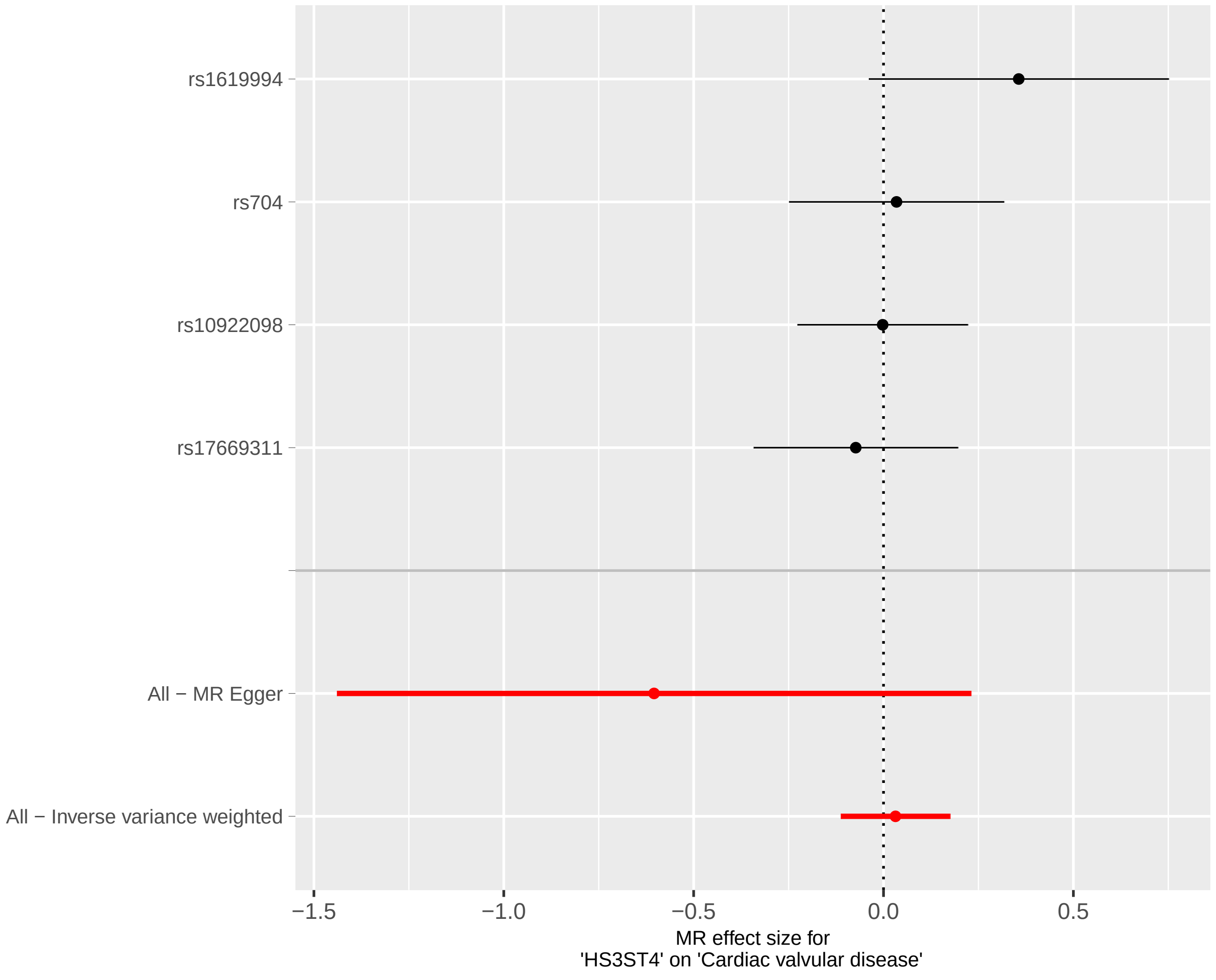

rs1619994
rs704
rs10922098
rs17669311
All − MR Egger
All − Inverse variance weighted
−1.5
−1.0
−0.5
0.0
0.5
MR effect size for
'HS3ST4' on 'Cardiac valvular disease'

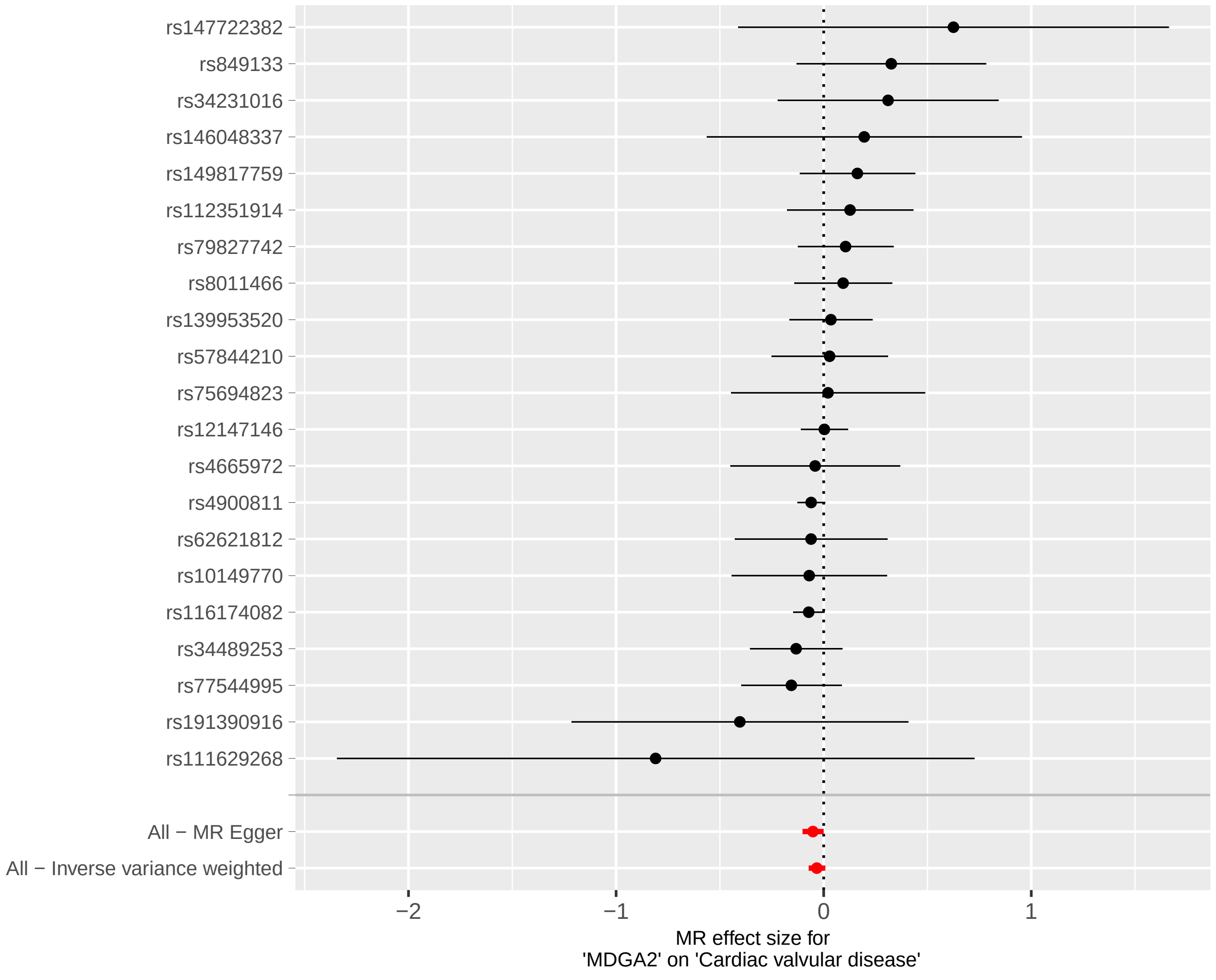
rs147722382
rs849133
rs34231016
rs146048337
rs149817759
rs112351914
rs79827742
rs8011466
rs139953520
rs57844210
rs75694823
rs12147146
rs4665972
rs4900811
rs62621812
rs10149770
rs116174082
rs34489253
rs77544995
rs191390916
rs111629268
All − MR Egger
All − Inverse variance weighted
−2
−1
0
1
MR effect size for
'MDGA2' on 'Cardiac valvular disease'

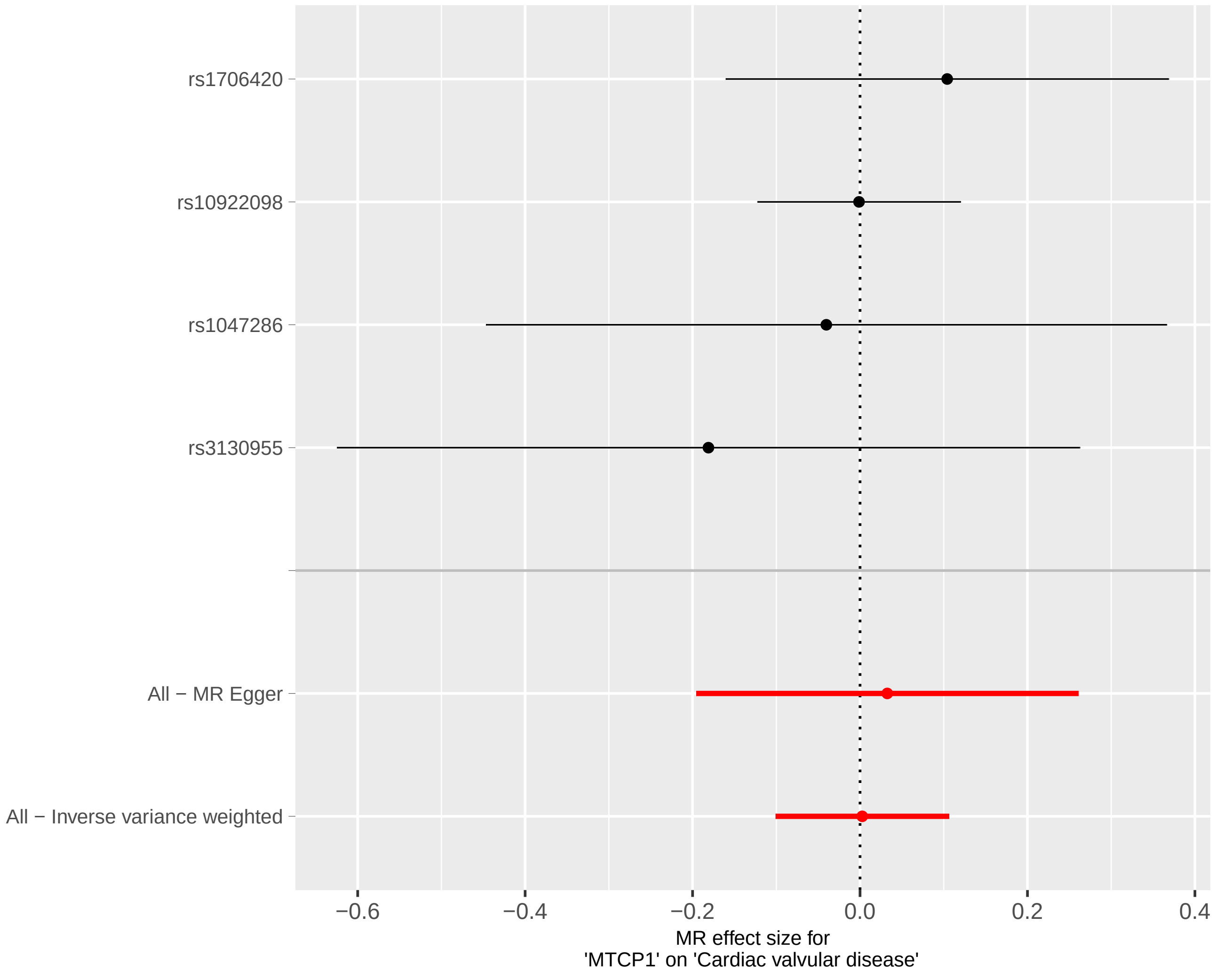
rs1706420
rs10922098
rs1047286
rs3130955
All − MR Egger
All − Inverse variance weighted
−0.6
−0.4
−0.2
0.0
0.2
0.4
MR effect size for
'MTCP1' on 'Cardiac valvular disease'

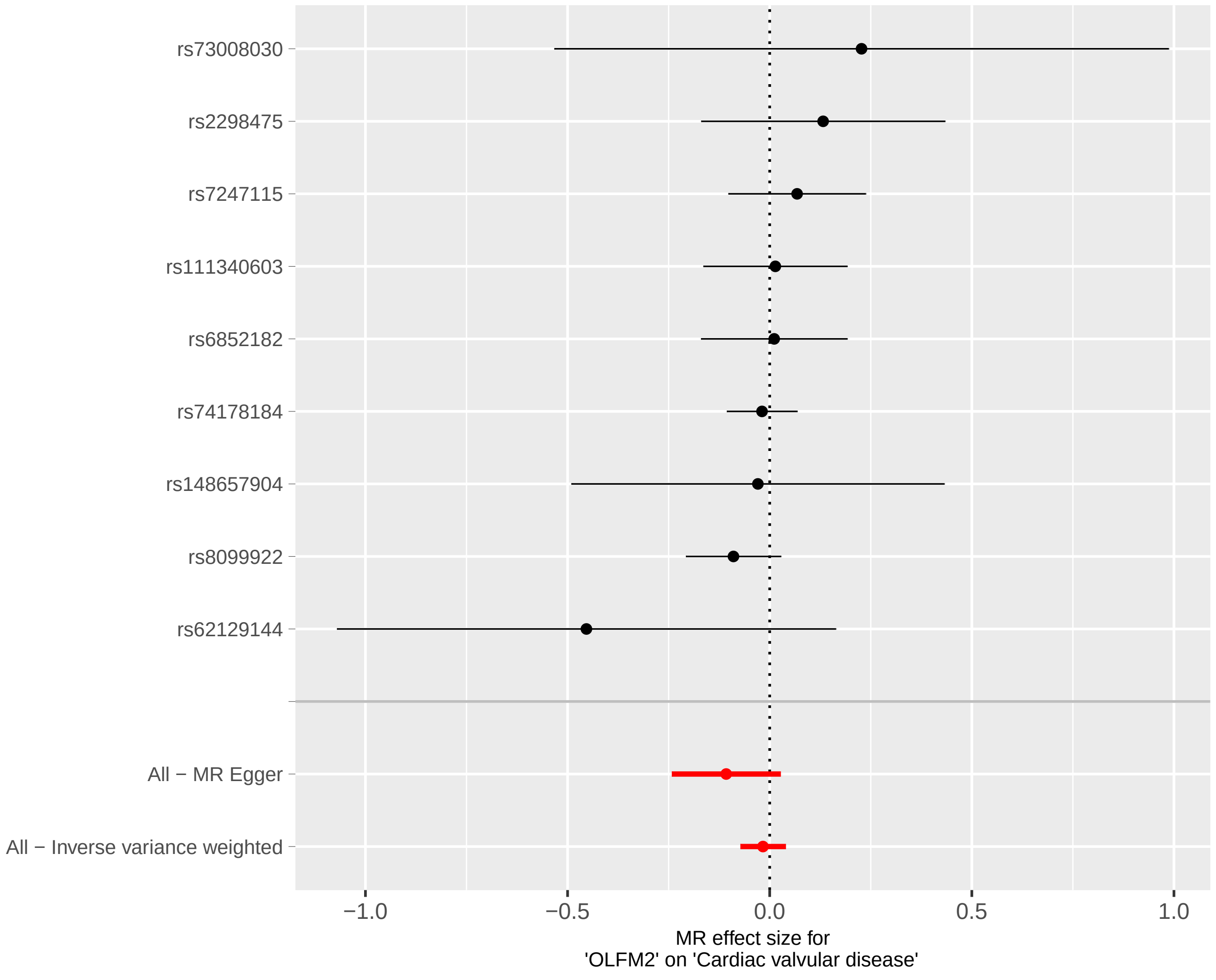

rs73008030
rs2298475
rs7247115
rs111340603
rs6852182
rs74178184
rs148657904
rs8099922
rs62129144
All − MR Egger
All − Inverse variance weighted
−1.0
−0.5
0.0
0.5
1.0
MR effect size for
'OLFM2' on 'Cardiac valvular disease'

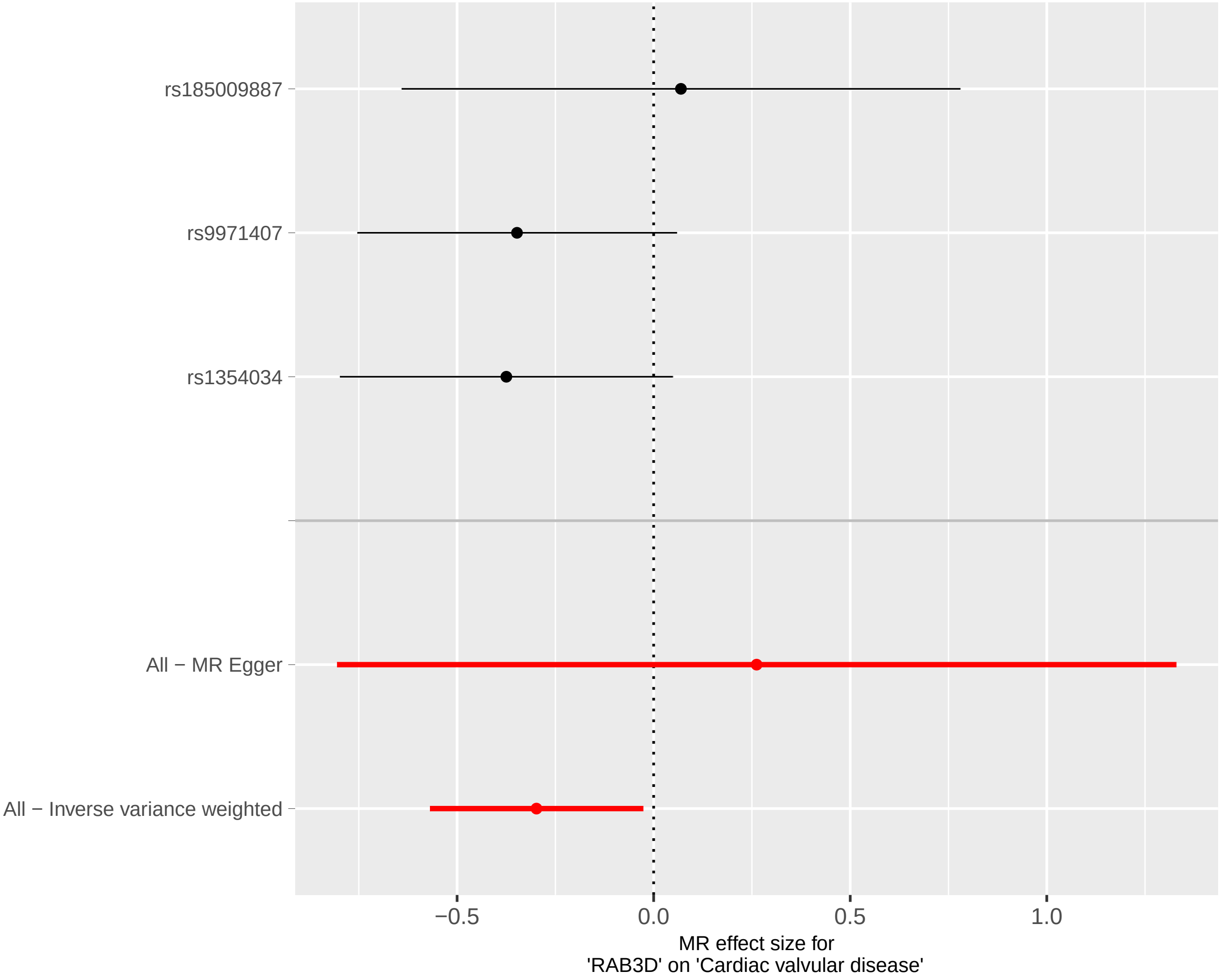
rs185009887
rs9971407
rs1354034
All − MR Egger
All − Inverse variance weighted
−0.5
0.0
0.5
1.0
MR effect size for
'RAB3D' on 'Cardiac valvular disease'

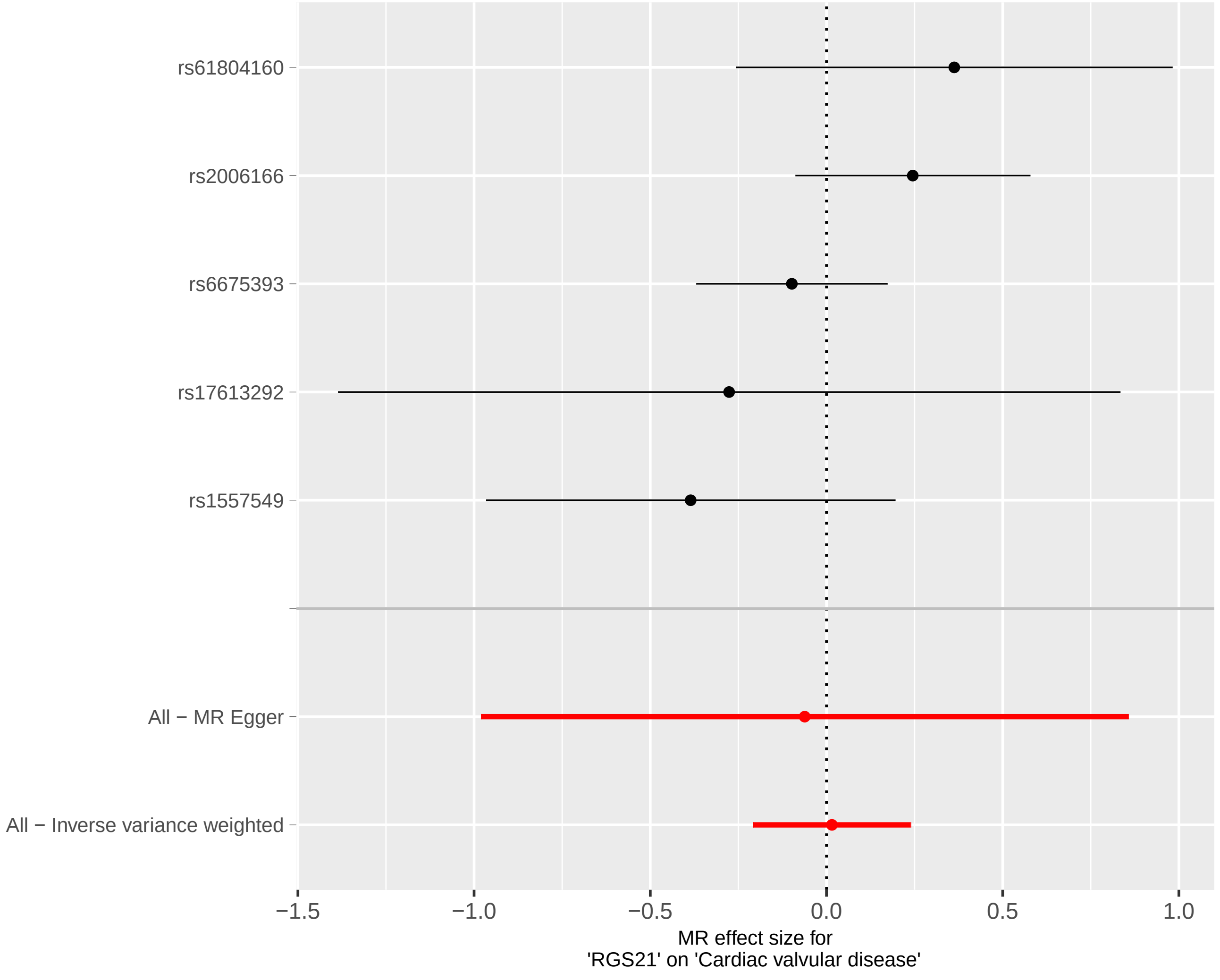
rs61804160
rs2006166
rs6675393
rs17613292
rs1557549
All − MR Egger
All − Inverse variance weighted
−1.5
−1.0
−0.5
0.0
0.5
1.0
MR effect size for
'RGS21' on 'Cardiac valvular disease'

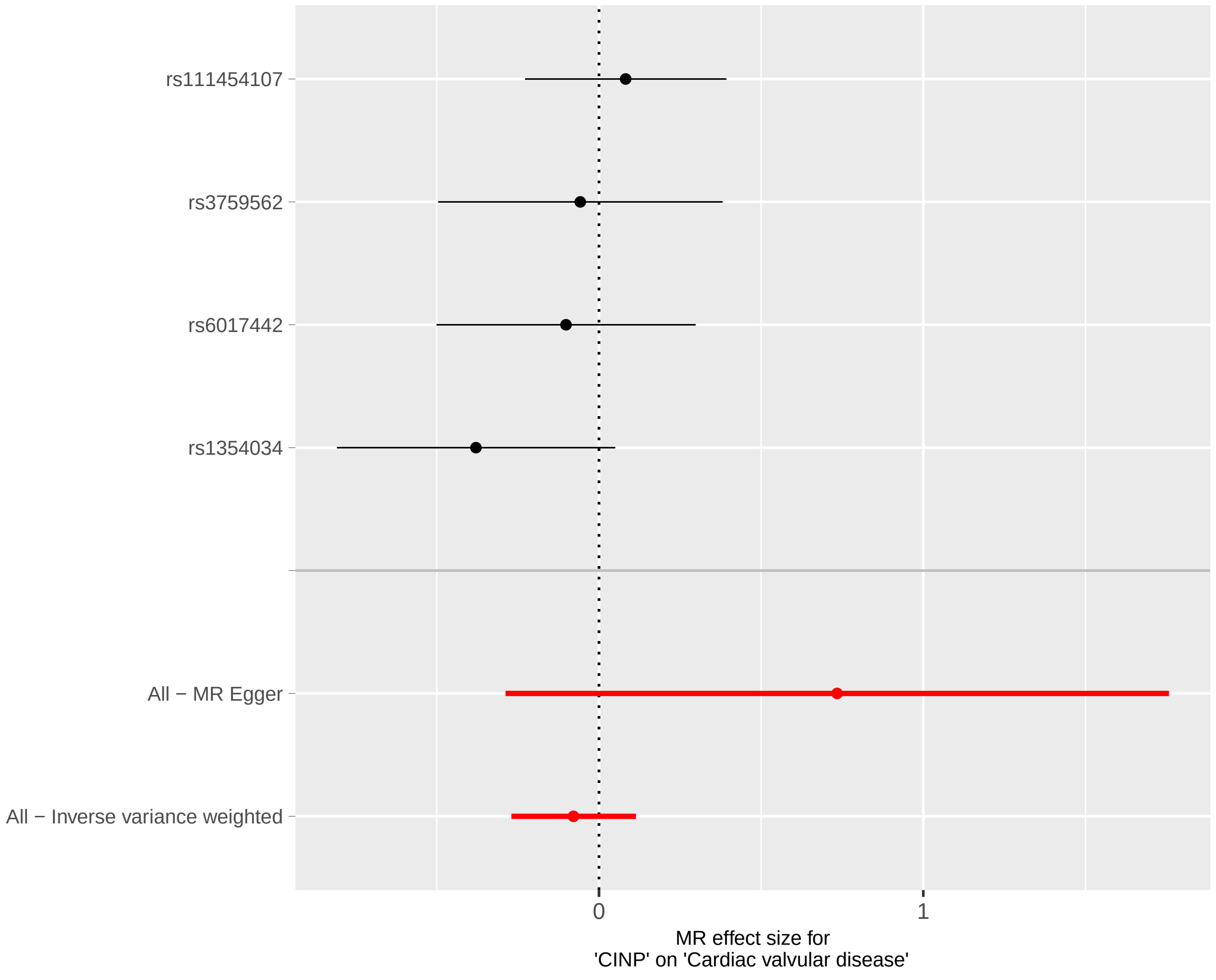
rs111454107
rs3759562
rs6017442
rs1354034
All − MR Egger
All − Inverse variance weighted
0
1
MR effect size for
'CINP' on 'Cardiac valvular disease'

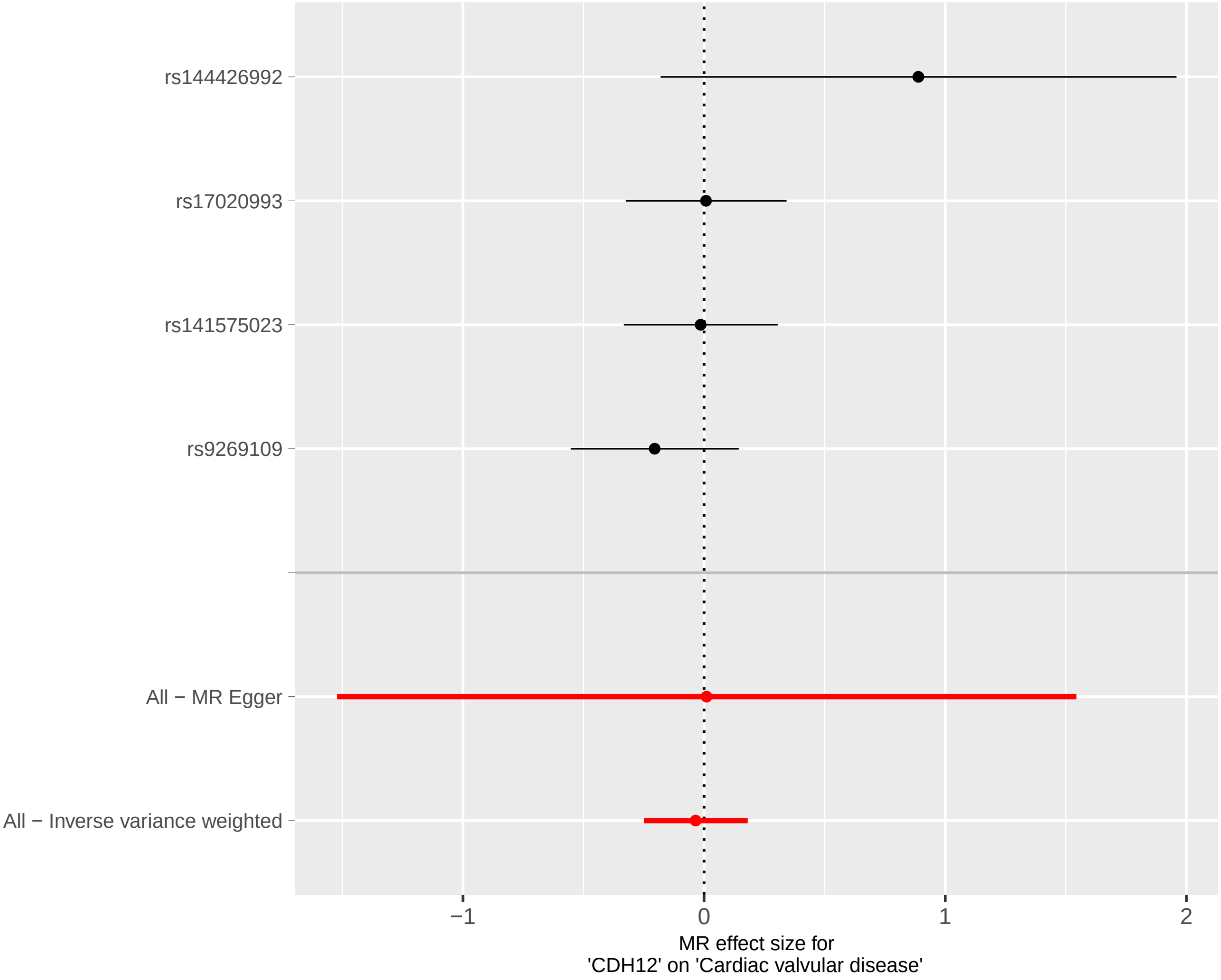

rs144426992
rs17020993
rs141575023
rs9269109
All − MR Egger
All − Inverse variance weighted
−1
0
1
2
MR effect size for
'CDH12' on 'Cardiac valvular disease'

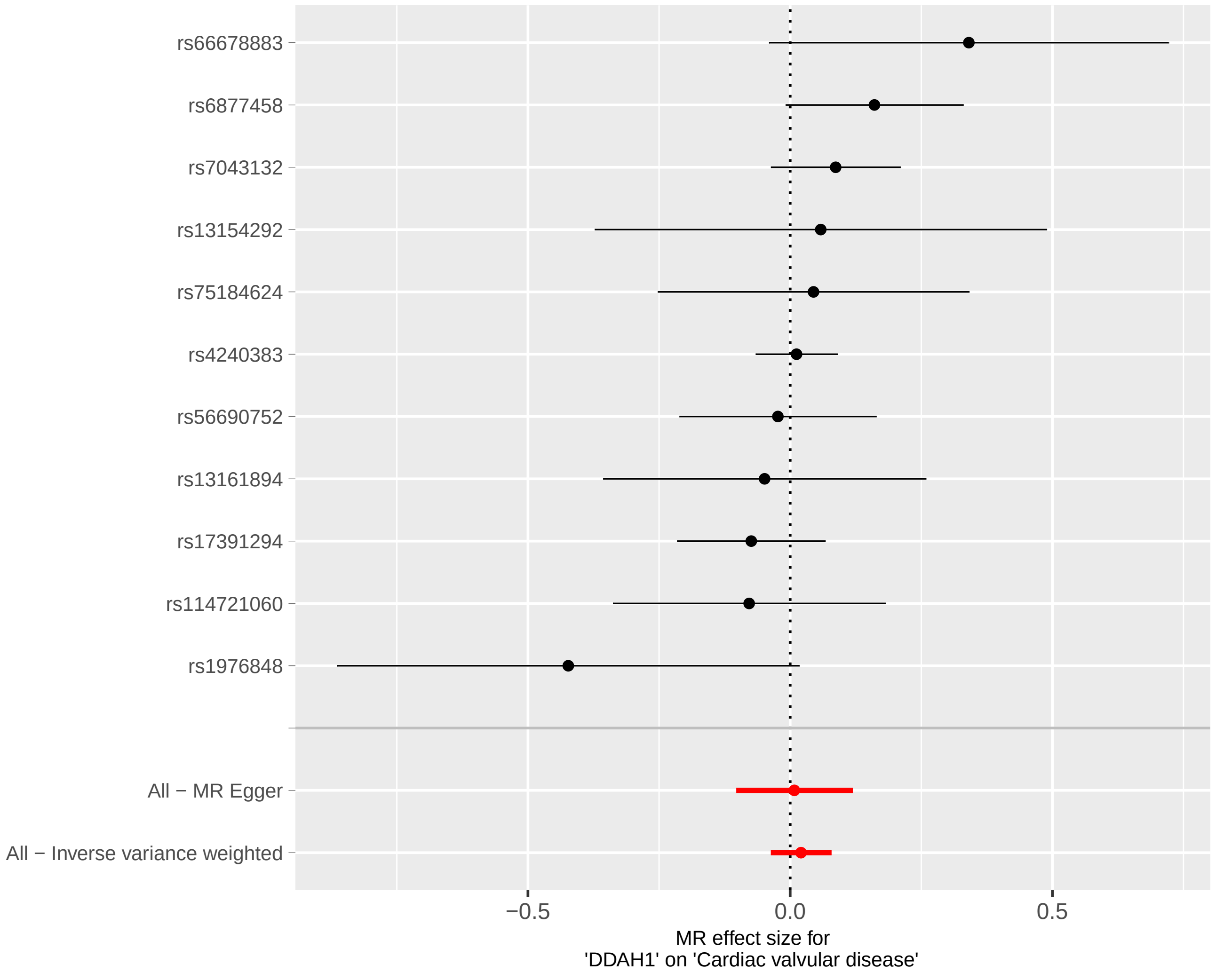

rs66678883
rs6877458
rs7043132
rs13154292
rs75184624
rs4240383
rs56690752
rs13161894
rs17391294
rs114721060
rs1976848
All − MR Egger
All − Inverse variance weighted
−0.5
0.0
0.5
MR effect size for
'DDAH1' on 'Cardiac valvular disease'

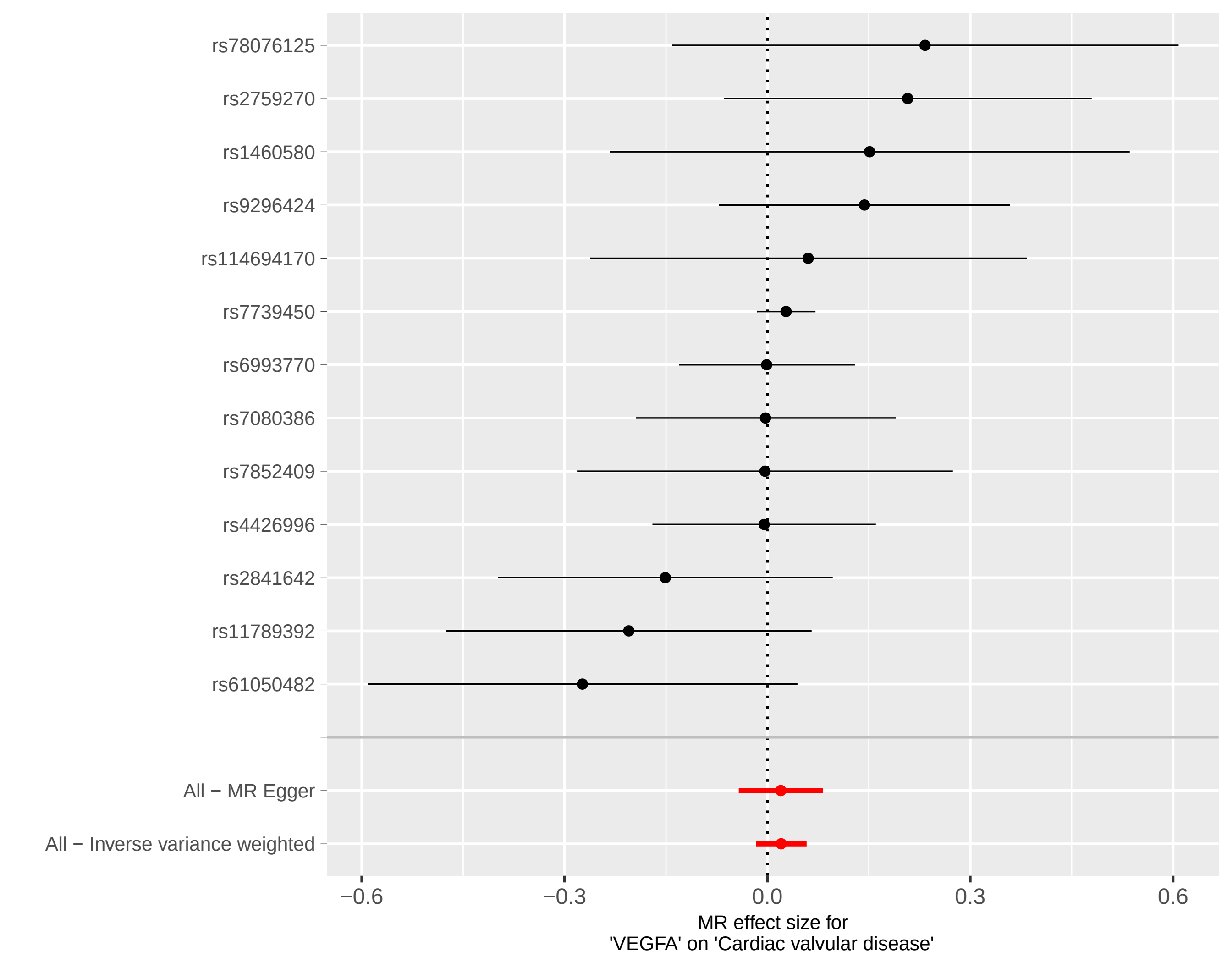
rs78076125
rs2759270
rs1460580
rs9296424
rs114694170
rs7739450
rs6993770
rs7080386
rs7852409
rs4426996
rs2841642
rs11789392
rs61050482
All − MR Egger
All − Inverse variance weighted
−0.6
−0.3
0.0
0.3
0.6
MR effect size for
'VEGFA' on 'Cardiac valvular disease'

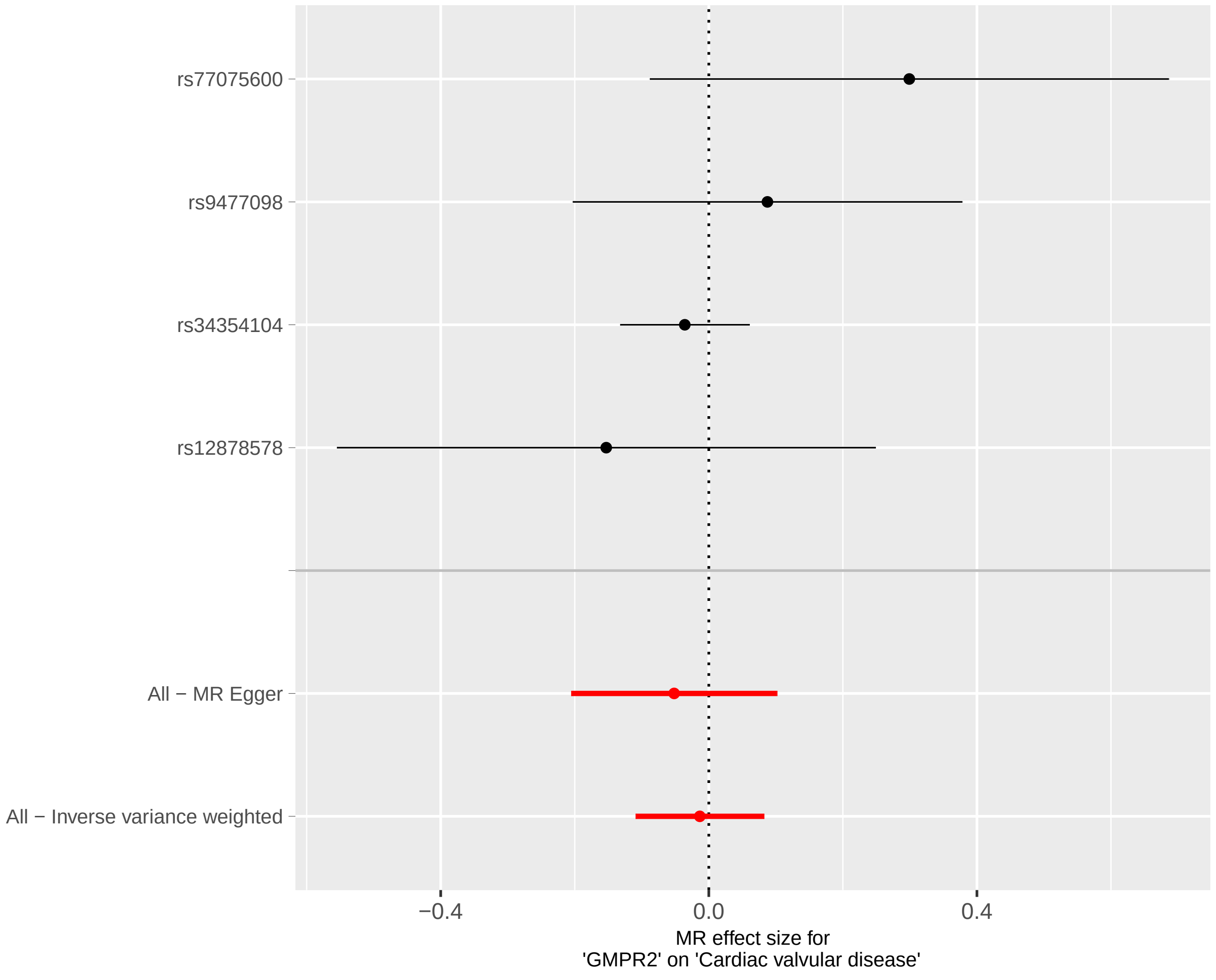

rs77075600
rs9477098
rs34354104
rs12878578
All − MR Egger
All − Inverse variance weighted
−0.4
0.0
0.4
MR effect size for
'GMPR2' on 'Cardiac valvular disease'

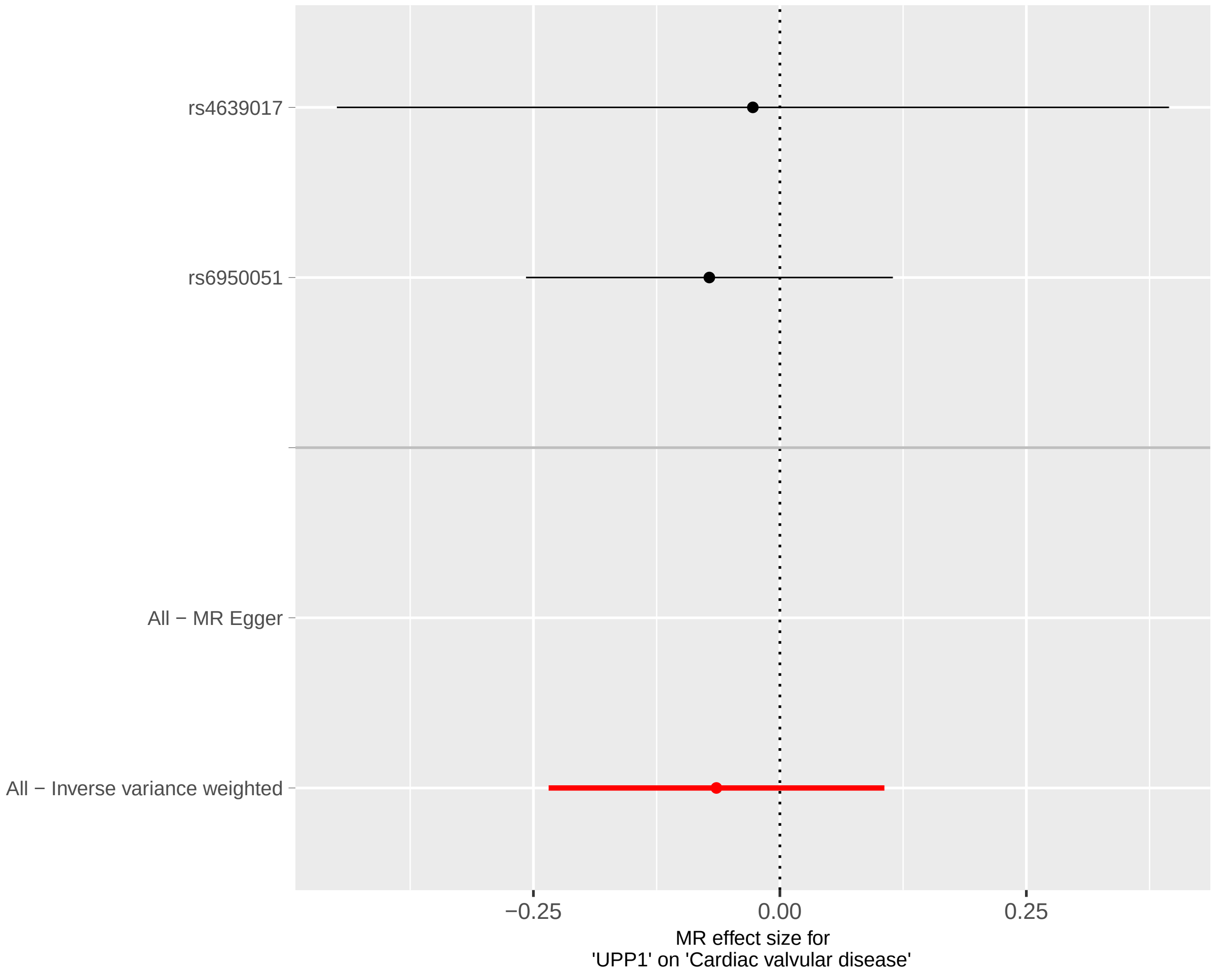
rs4639017
rs6950051
All − MR Egger
All − Inverse variance weighted
−0.25
0.00
0.25
MR effect size for
'UPP1' on 'Cardiac valvular disease'

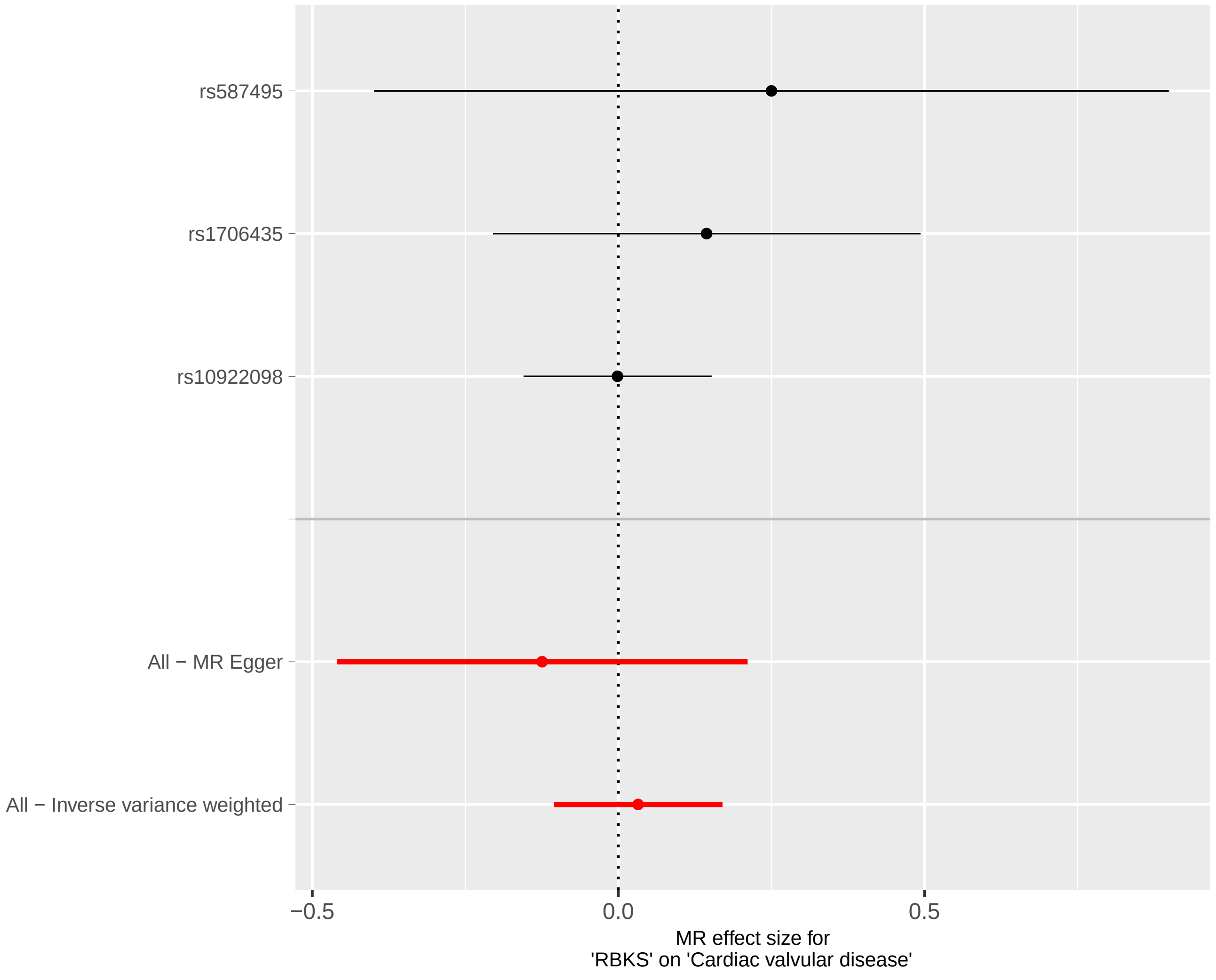
rs587495
rs1706435
rs10922098
All − MR Egger
All − Inverse variance weighted
−0.5
0.0
0.5
MR effect size for
'RBKS' on 'Cardiac valvular disease'

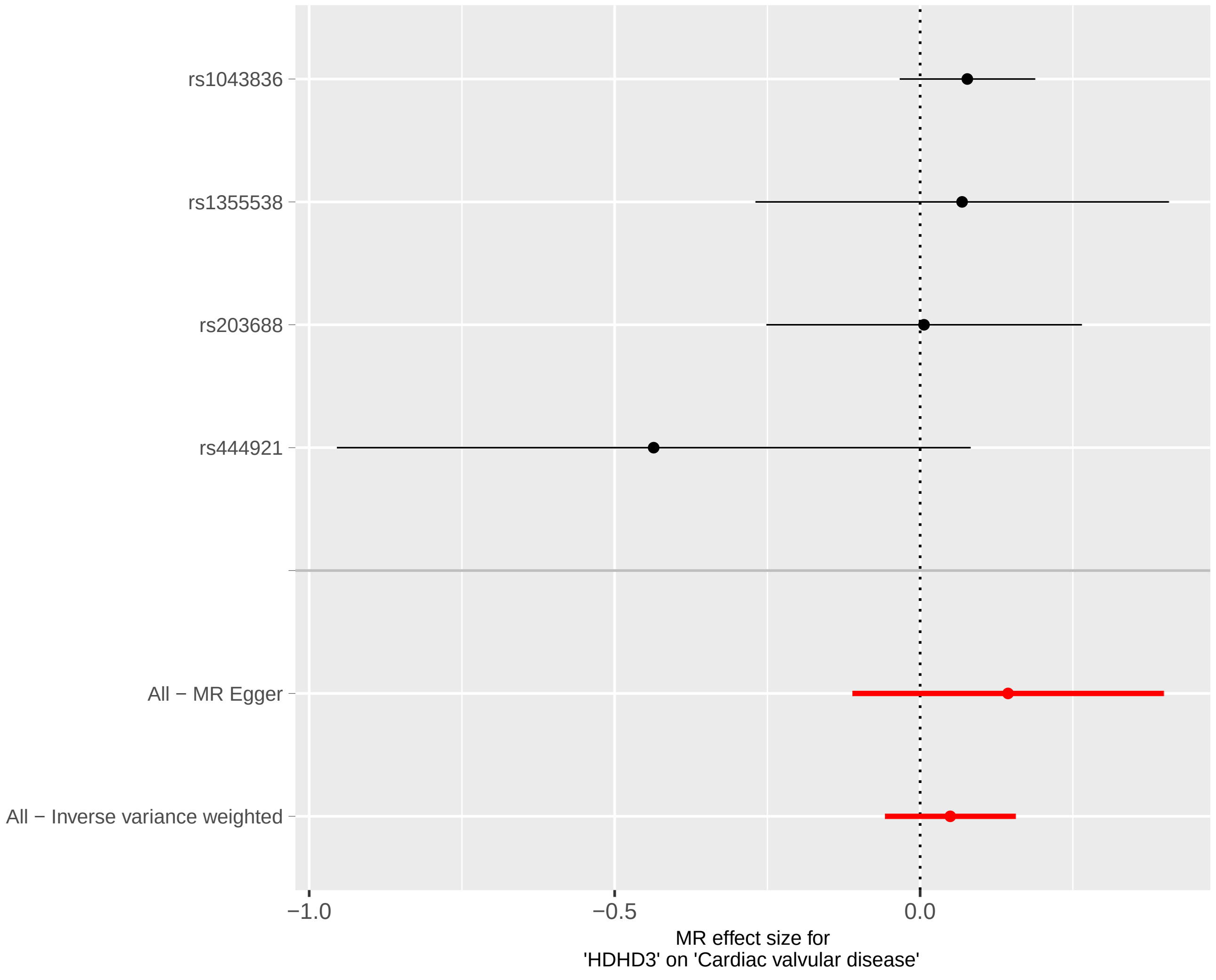
rs1043836
rs1355538
rs203688
rs444921
All − MR Egger
All − Inverse variance weighted
−1.0
−0.5
0.0
MR effect size for
'HDHD3' on 'Cardiac valvular disease'

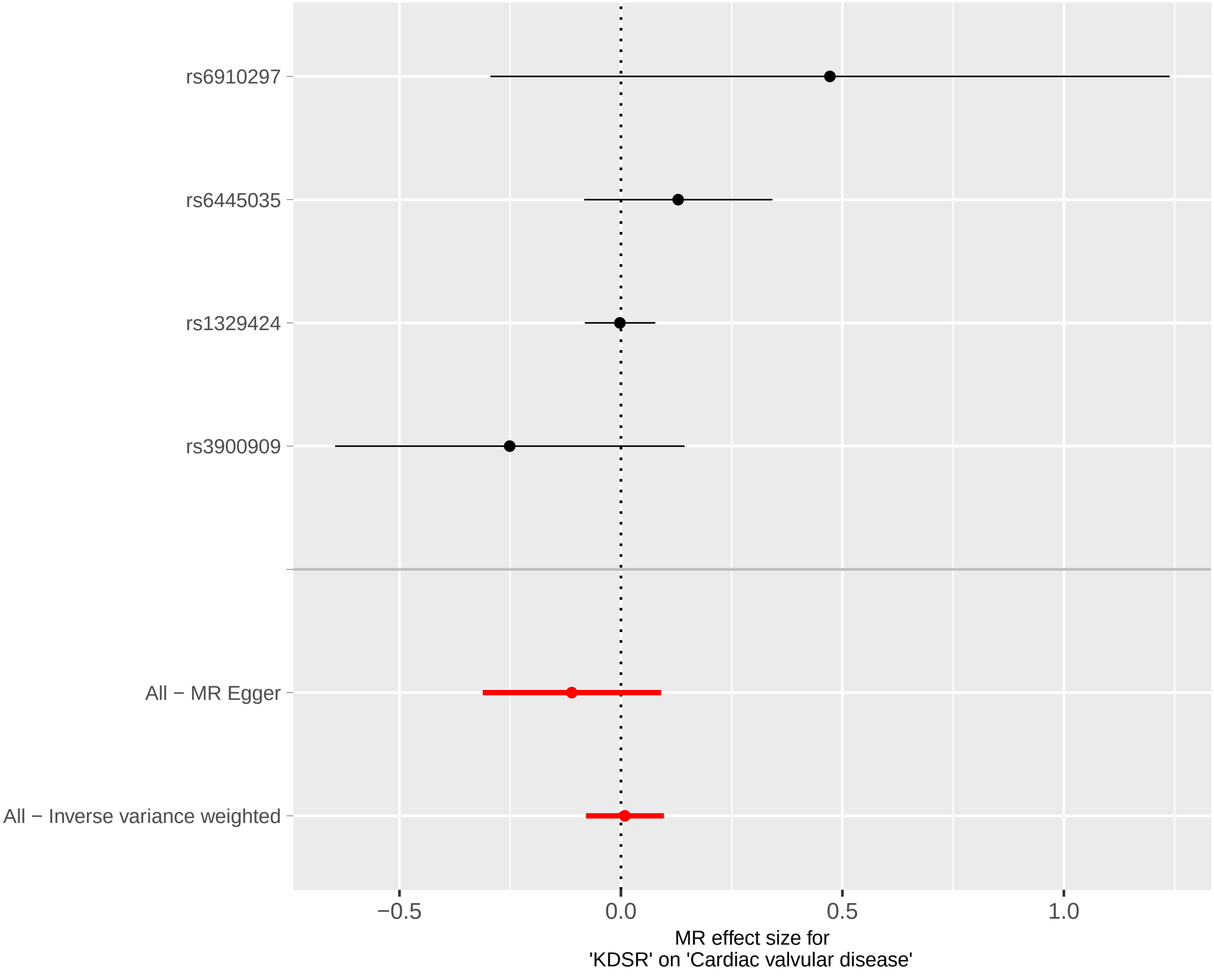
rs6910297
rs6445035
rs1329424
rs3900909
All − MR Egger
All − Inverse variance weighted
−0.5
0.0
0.5
1.0
MR effect size for
'KDSR' on 'Cardiac valvular disease'

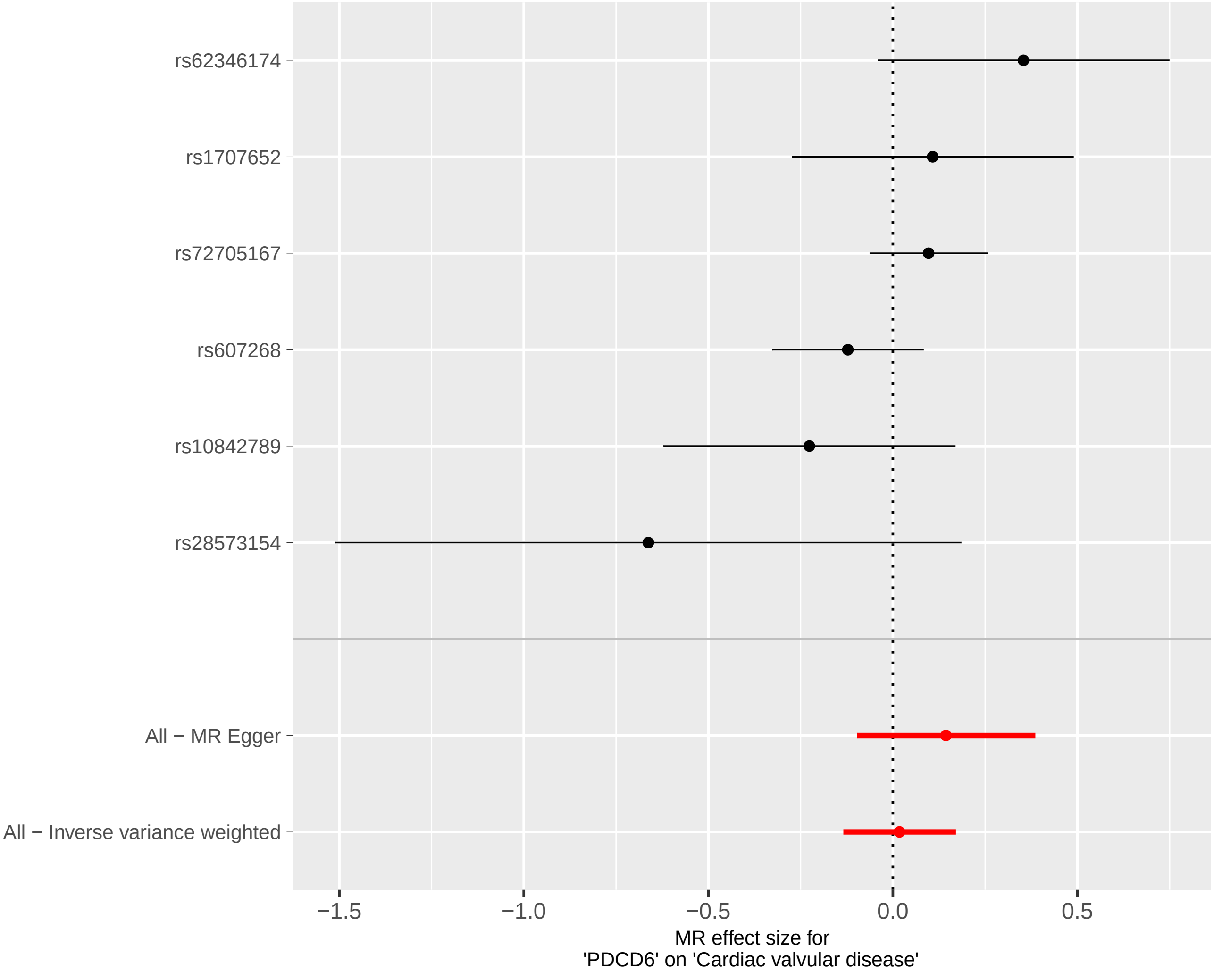
rs62346174
rs1707652
rs72705167
rs607268
rs10842789
rs28573154
All − MR Egger
All − Inverse variance weighted
−1.5
−1.0
−0.5
0.0
0.5
MR effect size for
'PDCD6' on 'Cardiac valvular disease'

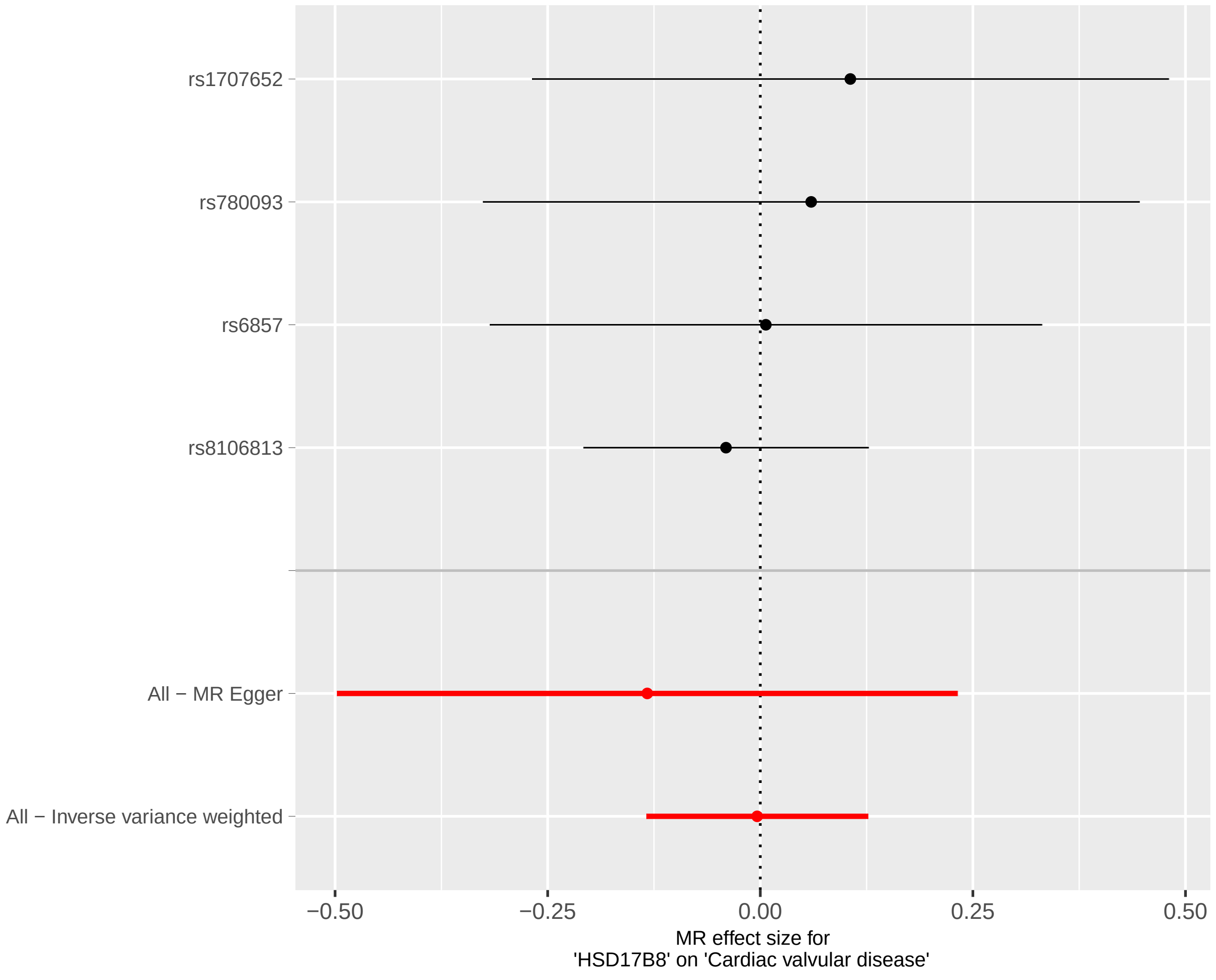
rs1707652
rs780093
rs6857
rs8106813
All − MR Egger
All − Inverse variance weighted
−0.50
−0.25
0.00
0.25
0.50
MR effect size for
'HSD17B8' on 'Cardiac valvular disease'

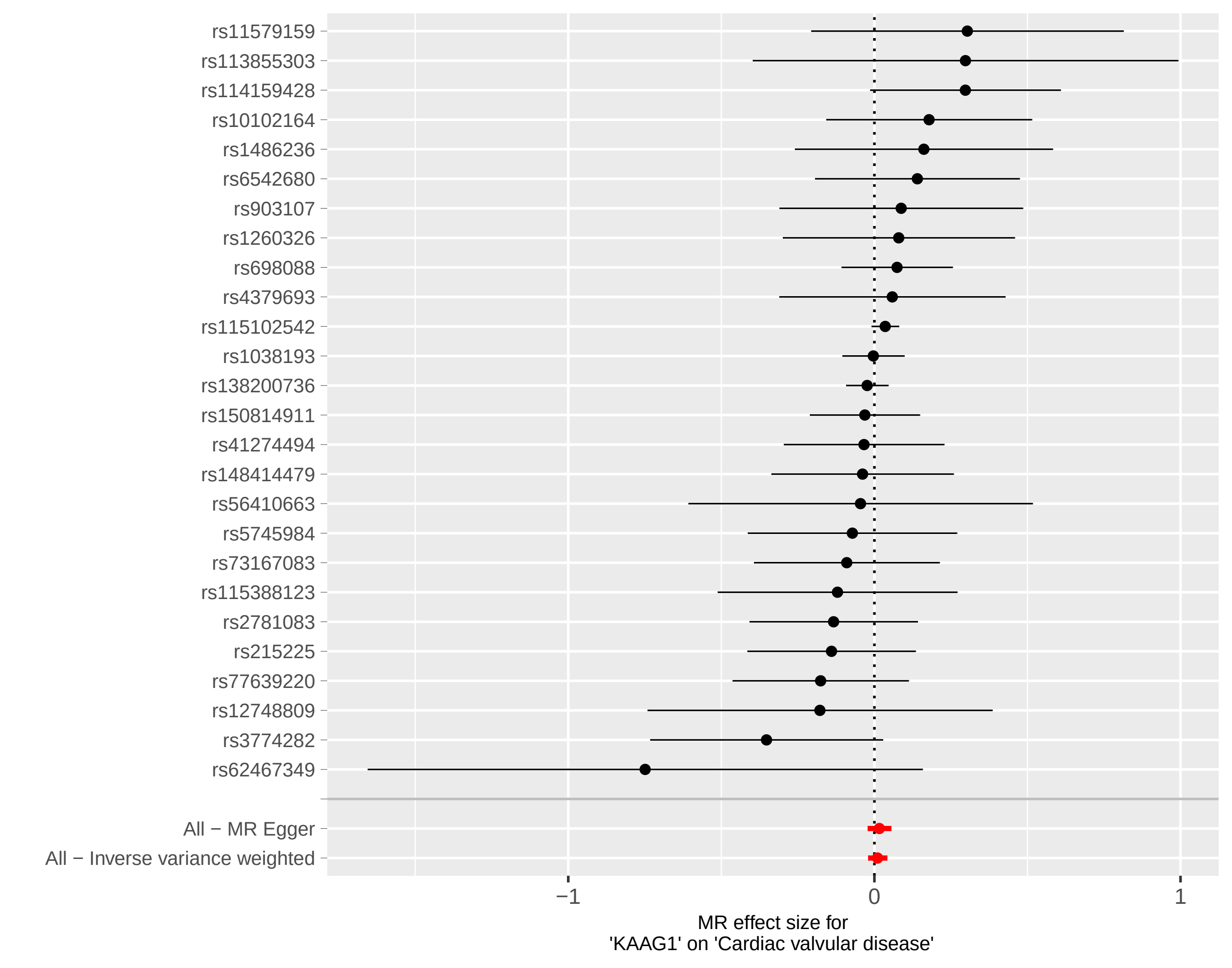

rs11579159
rs113855303
rs114159428
rs10102164
rs1486236
rs6542680
rs903107
rs1260326
rs698088
rs4379693
rs115102542
rs1038193
rs138200736
rs150814911
rs41274494
rs148414479
rs56410663
rs5745984
rs73167083
rs115388123
rs2781083
rs215225
rs77639220
rs12748809
rs3774282
rs62467349
All − MR Egger
All − Inverse variance weighted
−1
0
1
MR effect size for
'KAAG1' on 'Cardiac valvular disease'

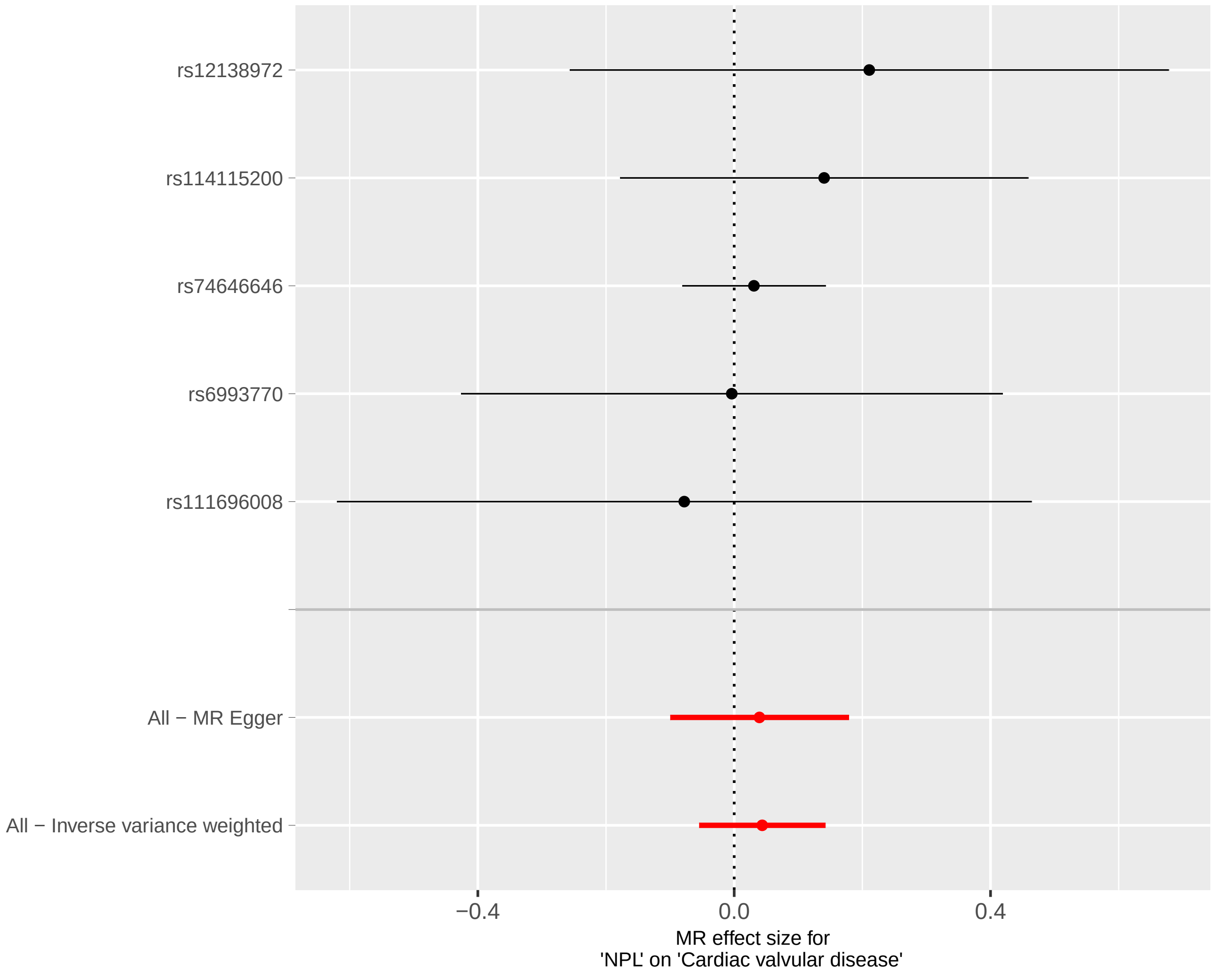
rs12138972
rs114115200
rs74646646
rs6993770
rs111696008
All − MR Egger
All − Inverse variance weighted
−0.4
0.0
0.4
MR effect size for
'NPL' on 'Cardiac valvular disease'

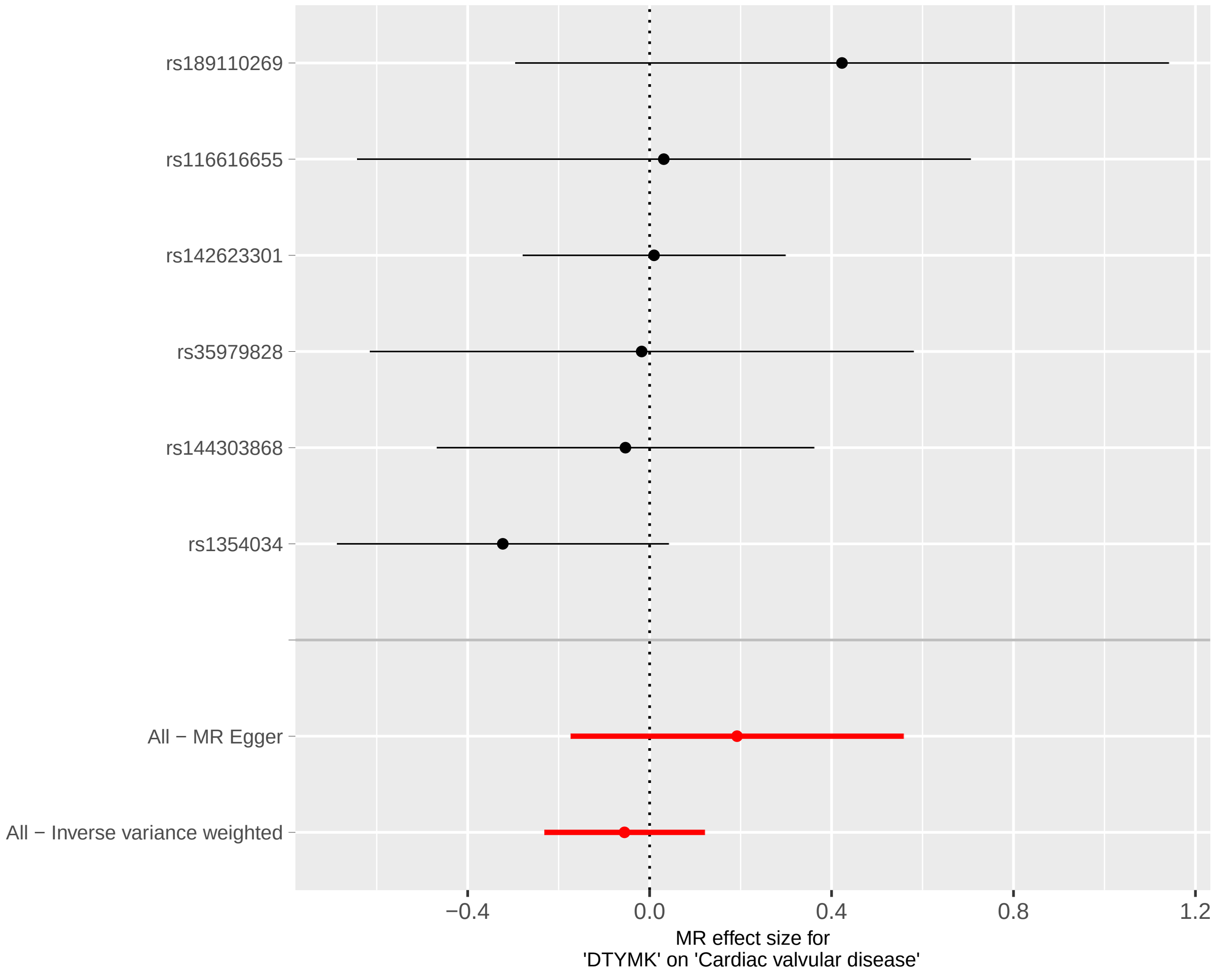
rs189110269
rs116616655
rs142623301
rs35979828
rs144303868
rs1354034
All − MR Egger
All − Inverse variance weighted
−0.4
0.0
0.4
0.8
1.2
MR effect size for
'DTYMK' on 'Cardiac valvular disease'

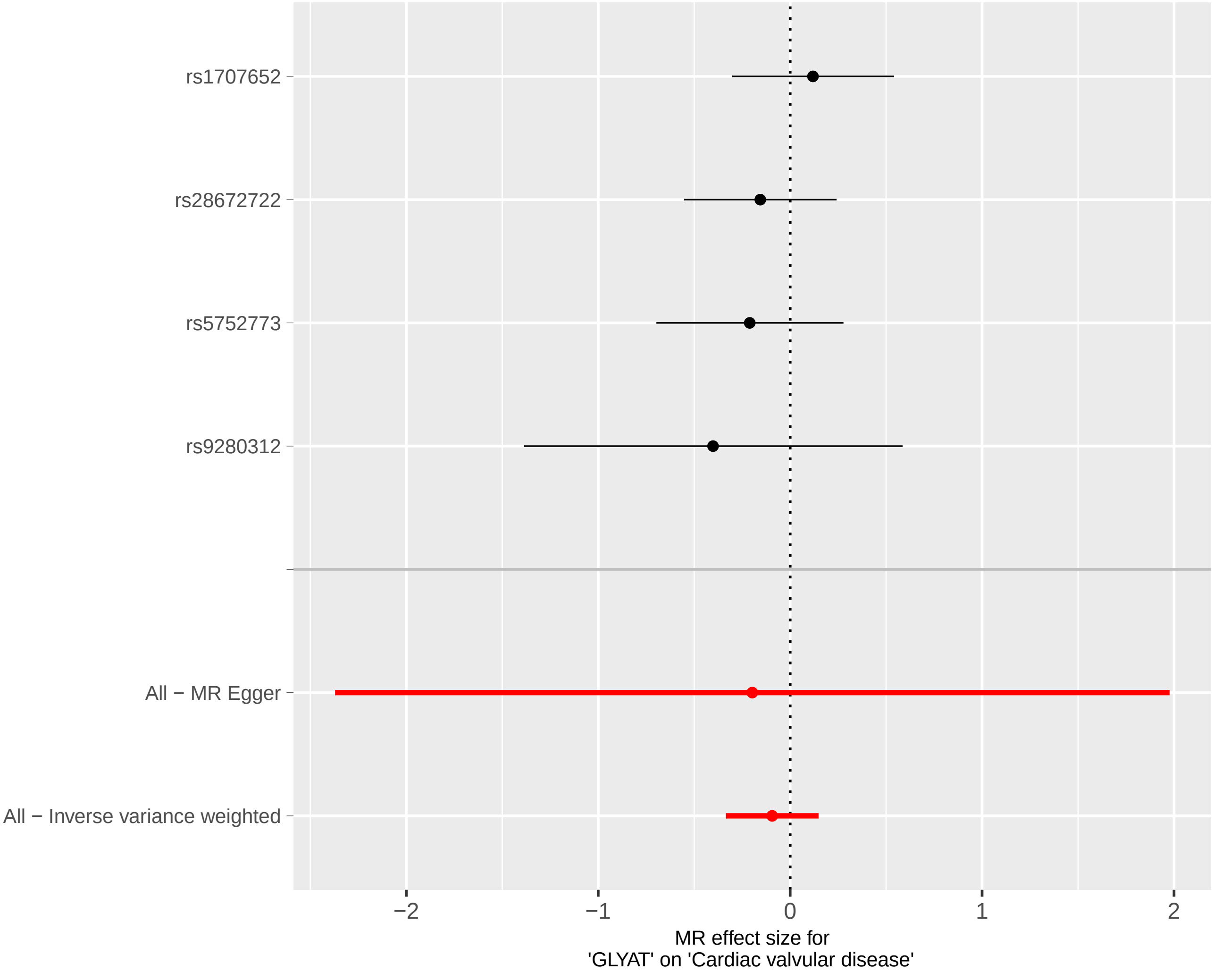
rs1707652
rs28672722
rs5752773
rs9280312
All − MR Egger
All − Inverse variance weighted
−2
−1
0
1
2
MR effect size for
'GLYAT' on 'Cardiac valvular disease'

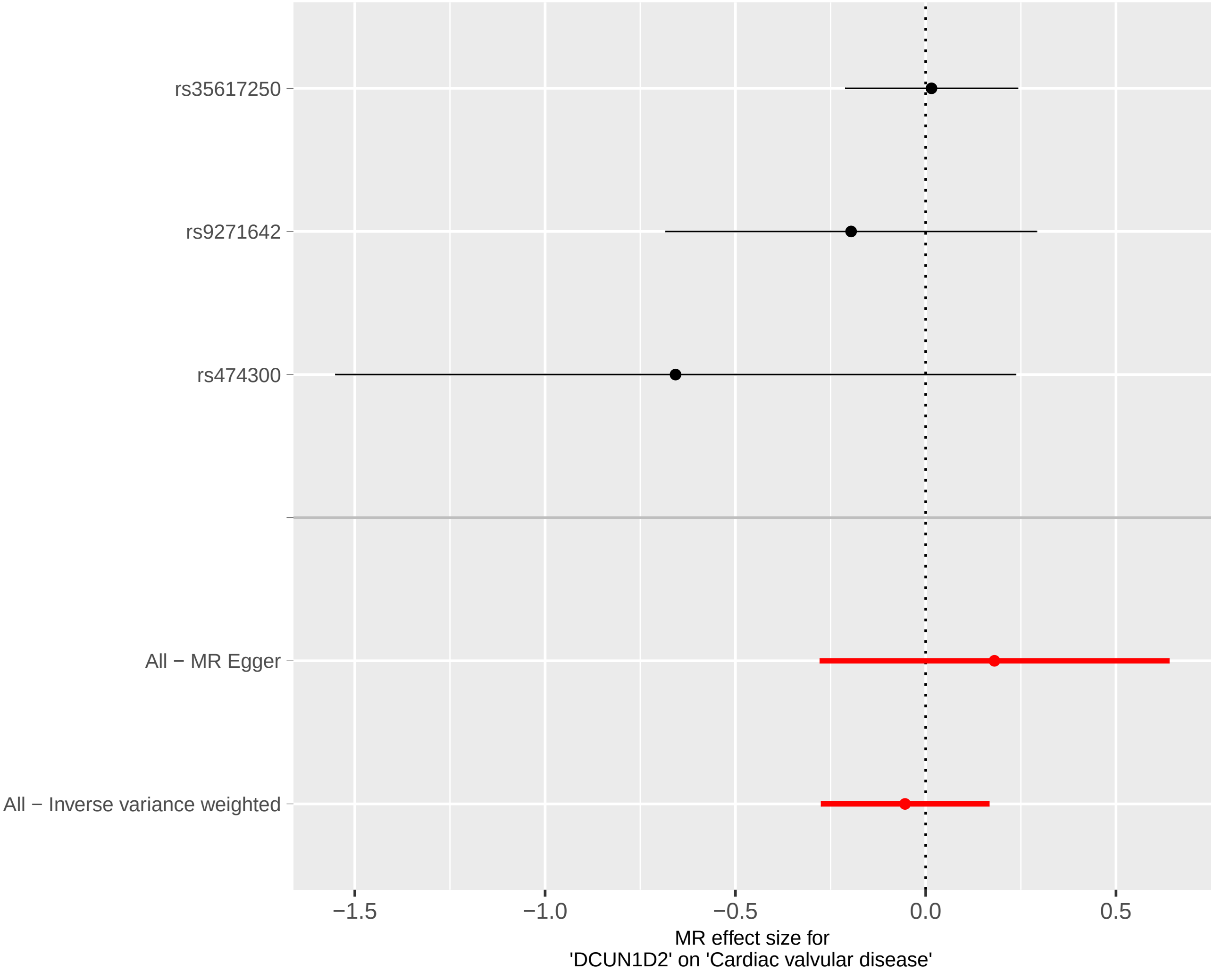
rs35617250
rs9271642
rs474300
All − MR Egger
All − Inverse variance weighted
−1.5
−1.0
−0.5
0.0
0.5
MR effect size for
'DCUN1D2' on 'Cardiac valvular disease'

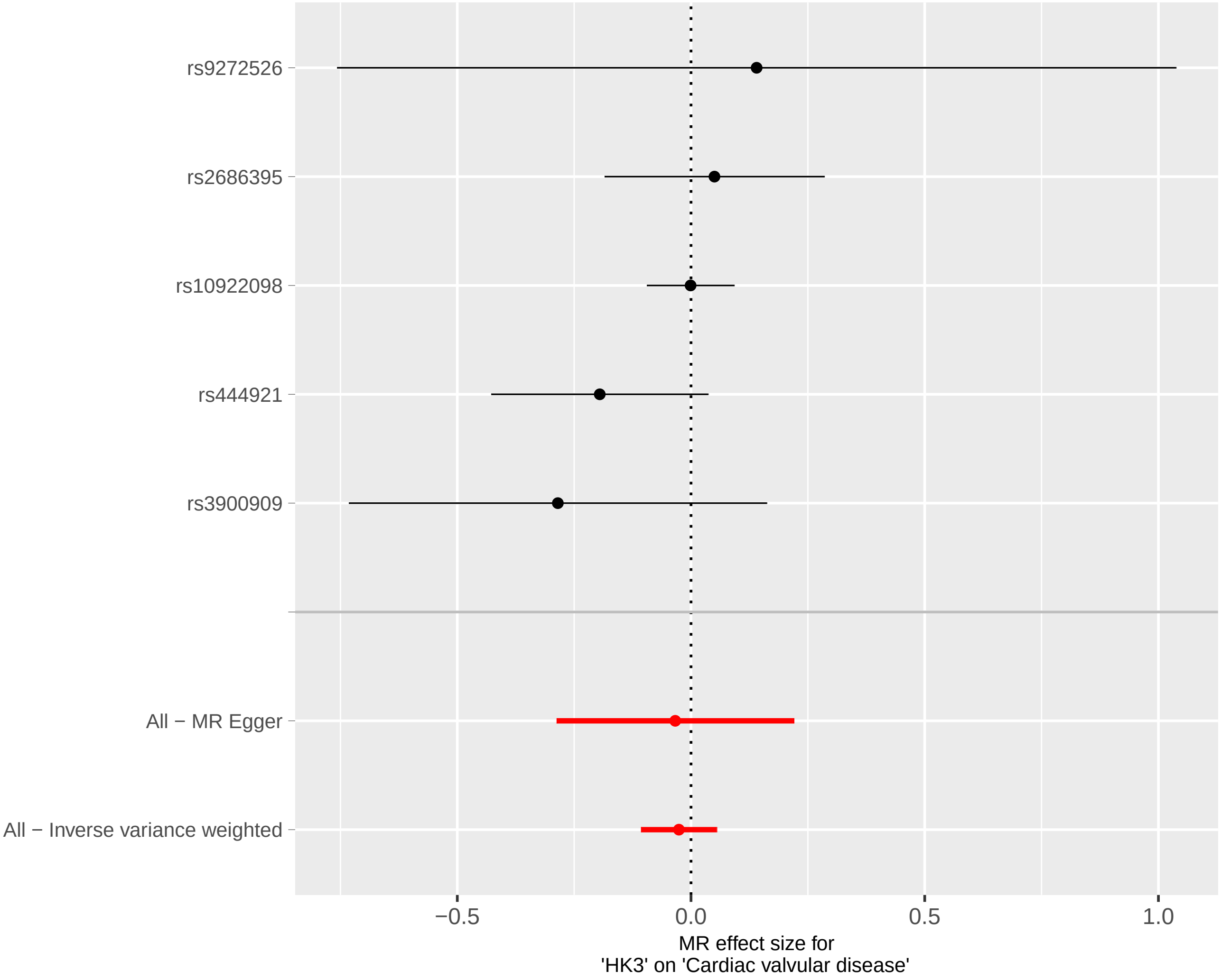
rs9272526
rs2686395
rs10922098
rs444921
rs3900909
All − MR Egger
All − Inverse variance weighted
−0.5
0.0
0.5
1.0
MR effect size for
'HK3' on 'Cardiac valvular disease'

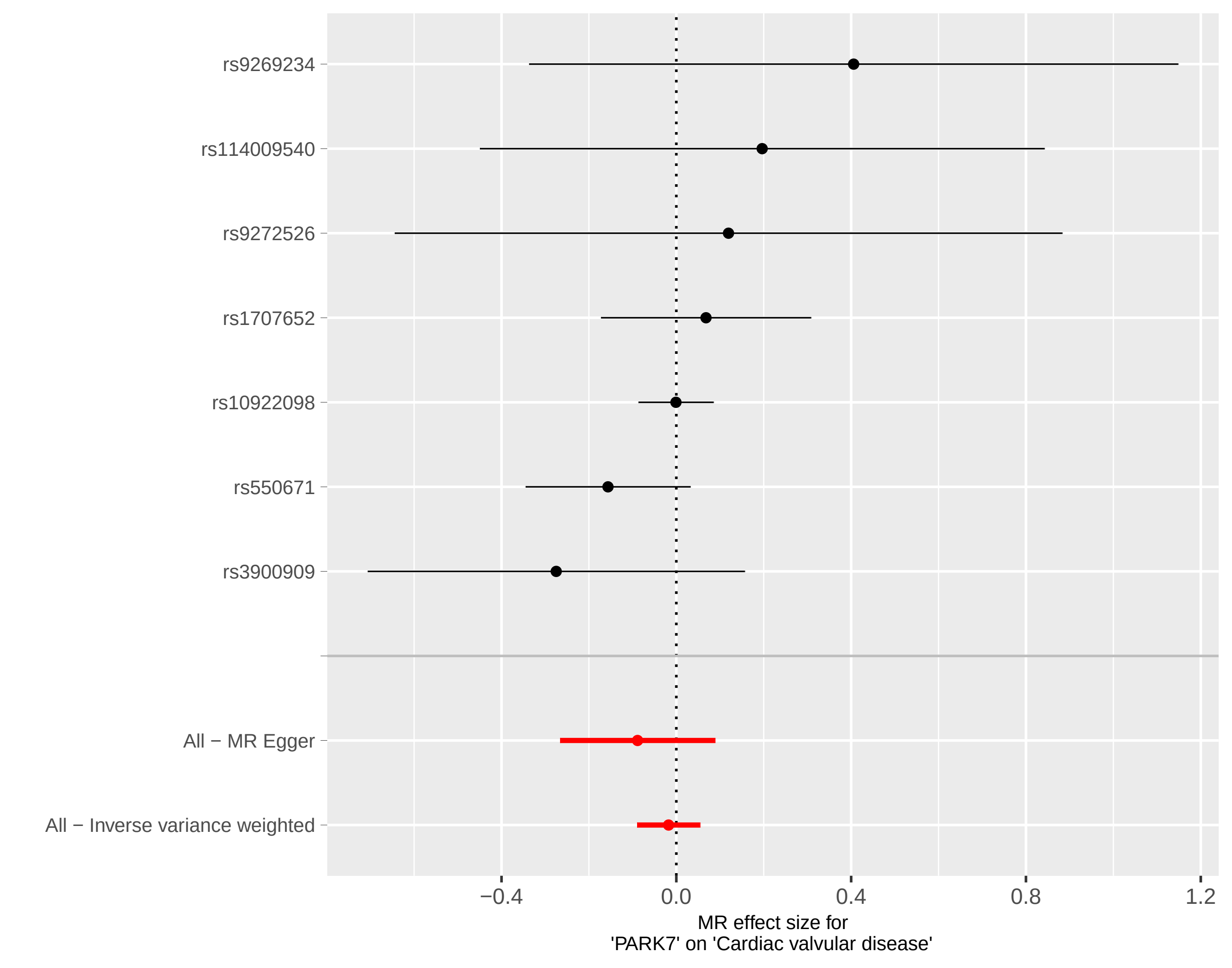

rs9269234
rs114009540
rs9272526
rs1707652
rs10922098
rs550671
rs3900909
All − MR Egger
All − Inverse variance weighted
−0.4
0.0
0.4
0.8
1.2
MR effect size for
'PARK7' on 'Cardiac valvular disease'

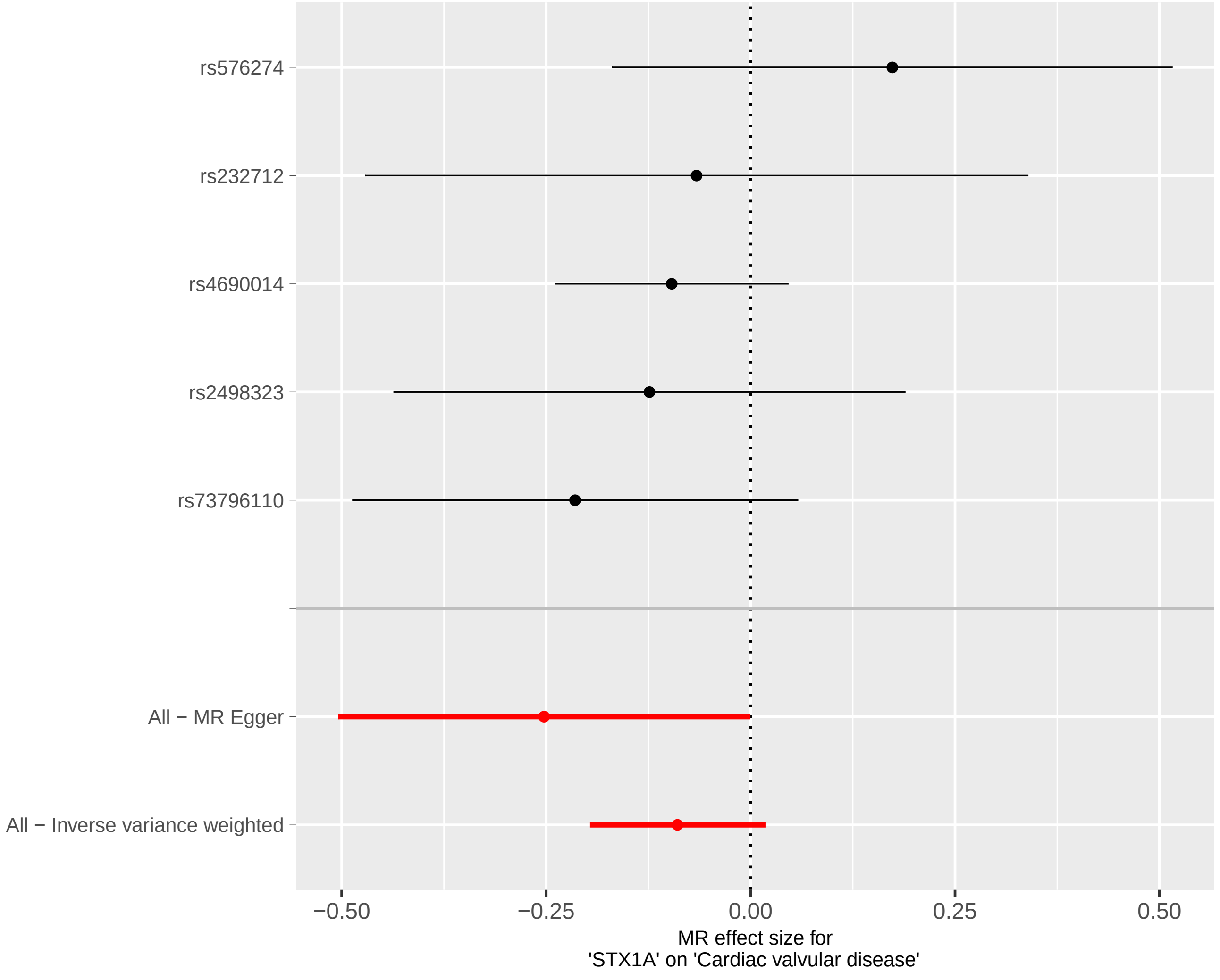
rs576274
rs232712
rs4690014
rs2498323
rs73796110
All − MR Egger
All − Inverse variance weighted
−0.50
−0.25
0.00
0.25
0.50
MR effect size for
'STX1A' on 'Cardiac valvular disease'

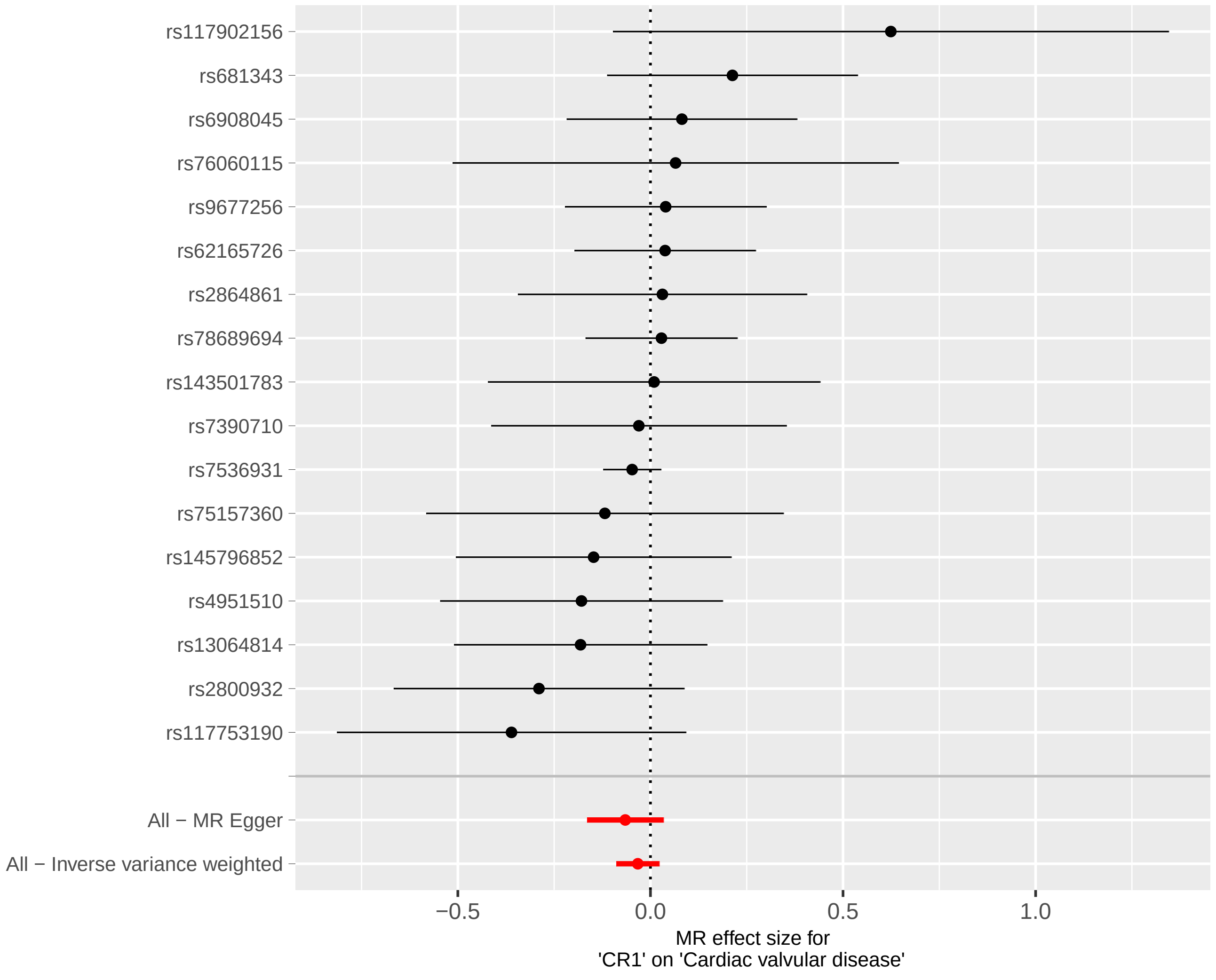
rs117902156
rs681343
rs6908045
rs76060115
rs9677256
rs62165726
rs2864861
rs78689694
rs143501783
rs7390710
rs7536931
rs75157360
rs145796852
rs4951510
rs13064814
rs2800932
rs117753190
All − MR Egger
All − Inverse variance weighted
−0.5
0.0
0.5
1.0
MR effect size for
'CR1' on 'Cardiac valvular disease'

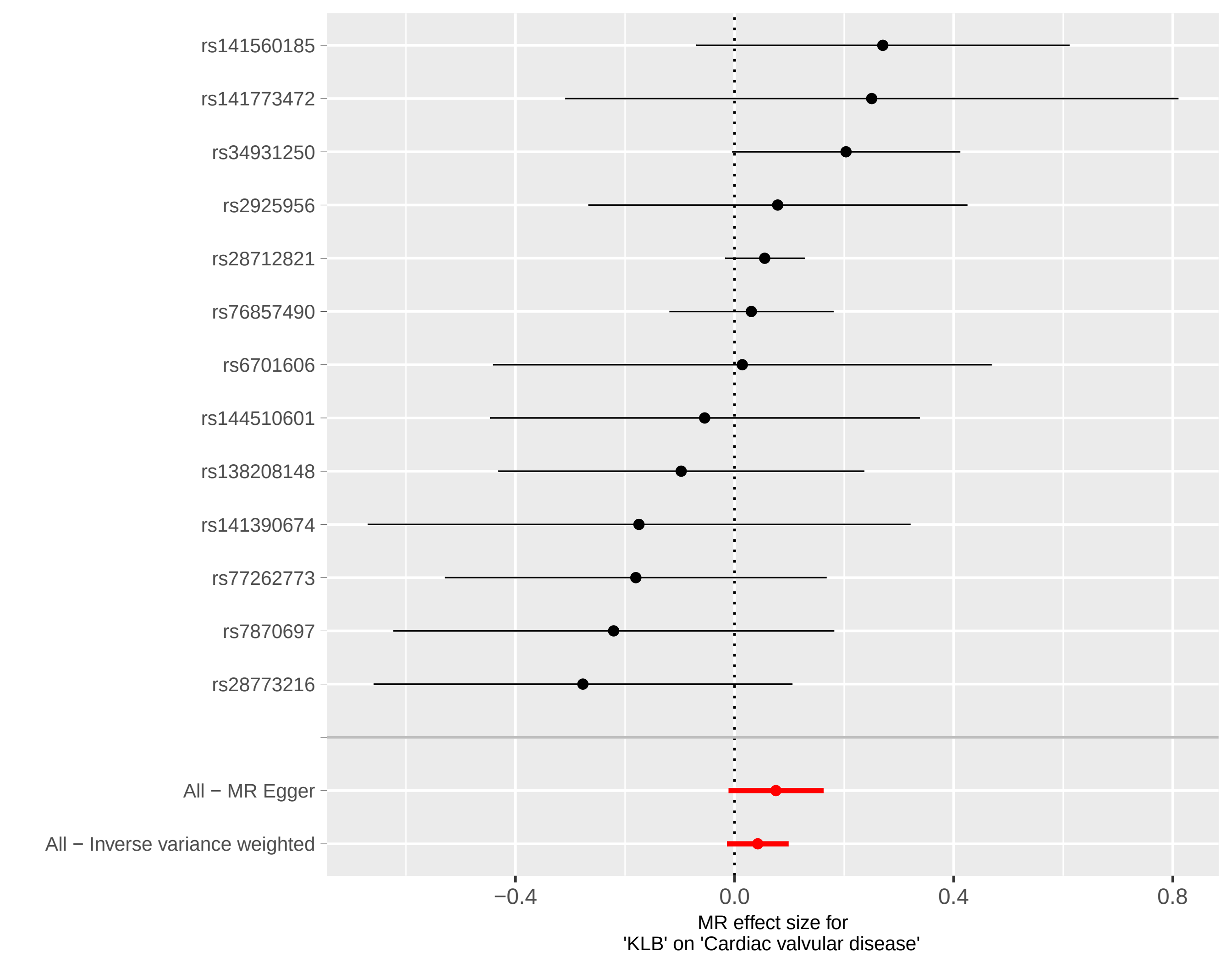

rs141560185
rs141773472
rs34931250
rs2925956
rs28712821
rs76857490
rs6701606
rs144510601
rs138208148
rs141390674
rs77262773
rs7870697
rs28773216
All − MR Egger
All − Inverse variance weighted
−0.4
0.0
0.4
0.8
MR effect size for
'KLB' on 'Cardiac valvular disease'

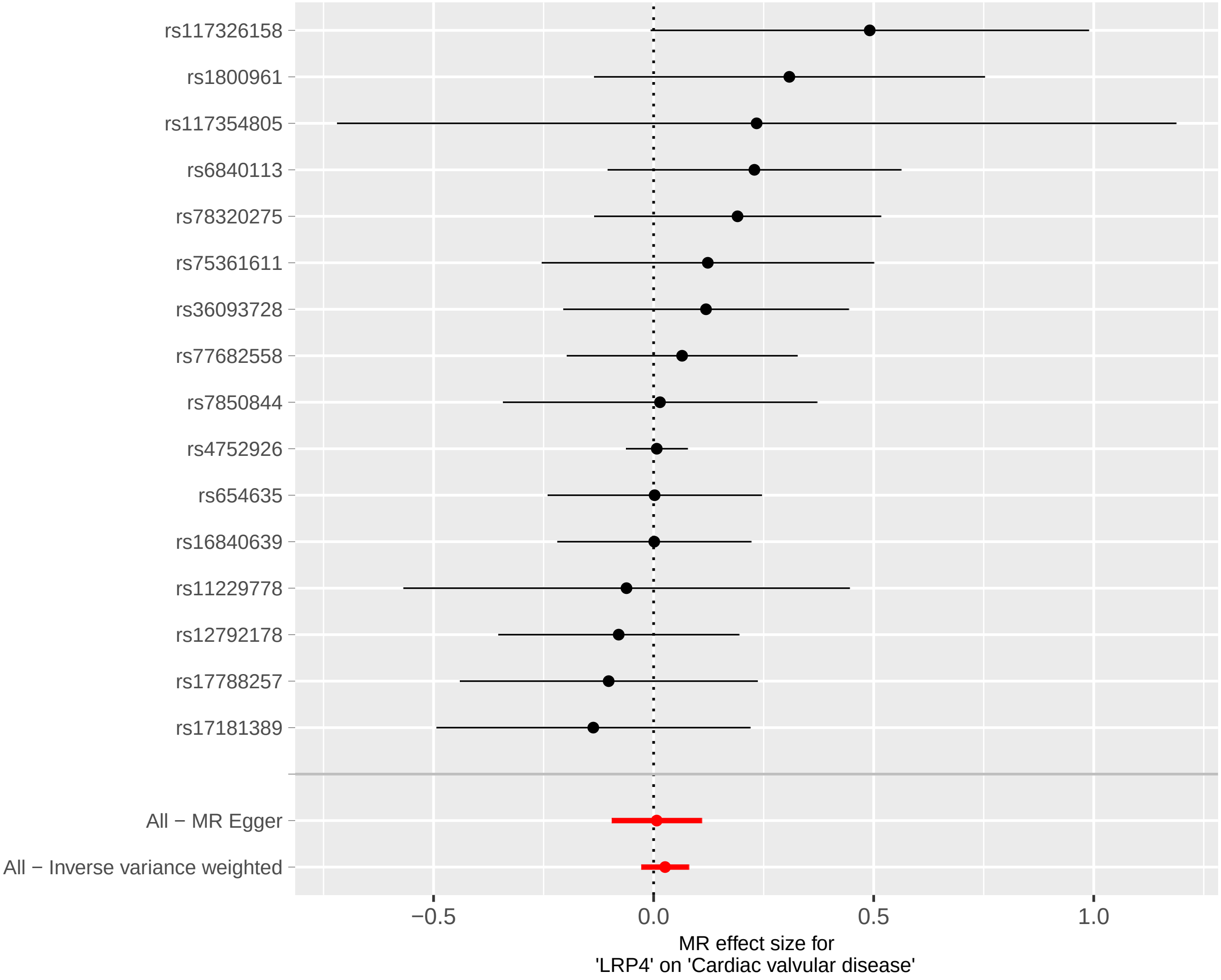
rs117326158
rs1800961
rs117354805
rs6840113
rs78320275
rs75361611
rs36093728
rs77682558
rs7850844
rs4752926
rs654635
rs16840639
rs11229778
rs12792178
rs17788257
rs17181389
All − MR Egger
All − Inverse variance weighted
−0.5
0.0
0.5
1.0
MR effect size for
'LRP4' on 'Cardiac valvular disease'

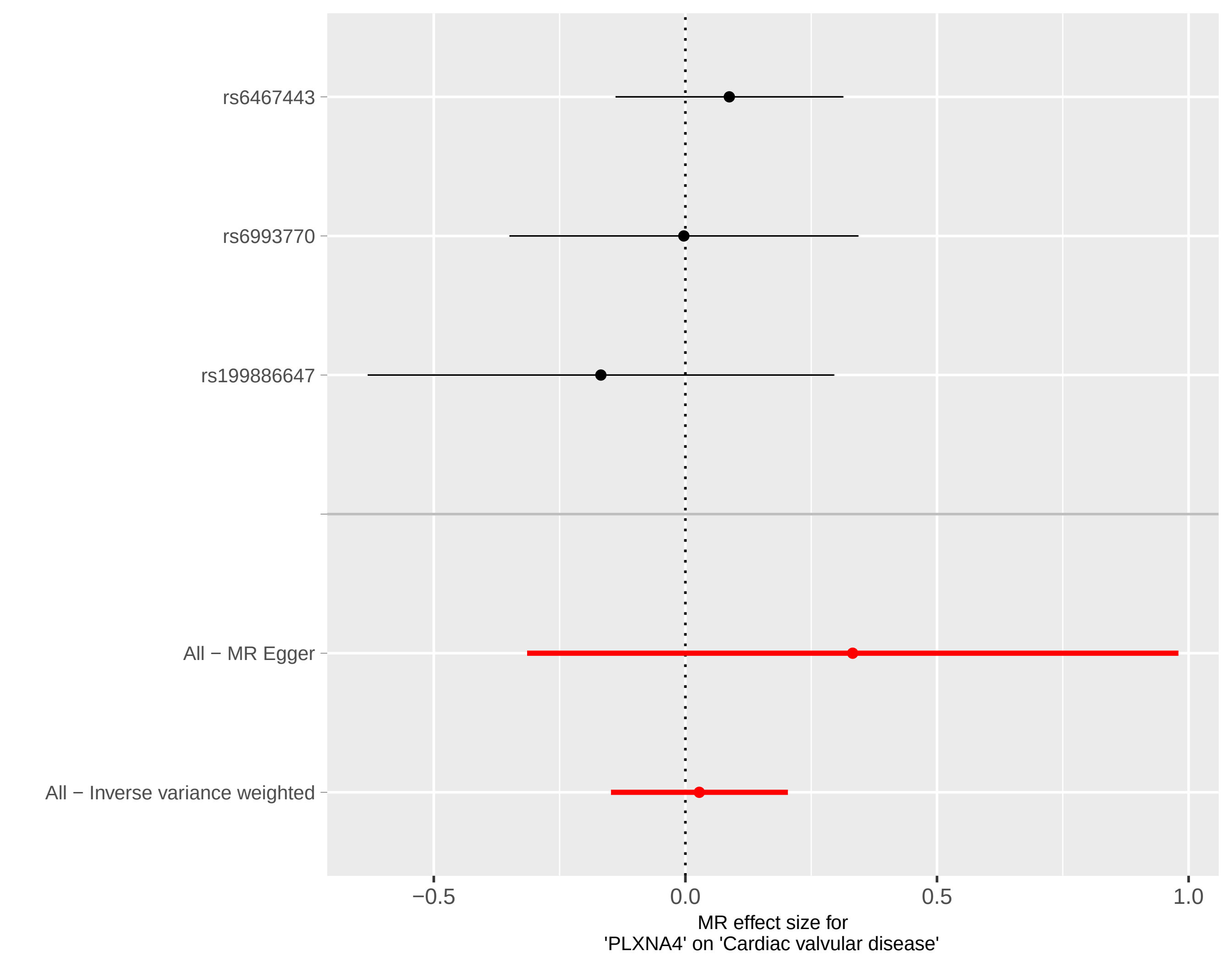
rs6467443
rs6993770
rs199886647
All − MR Egger
All − Inverse variance weighted
−0.5
0.0
0.5
1.0
MR effect size for
'PLXNA4' on 'Cardiac valvular disease'

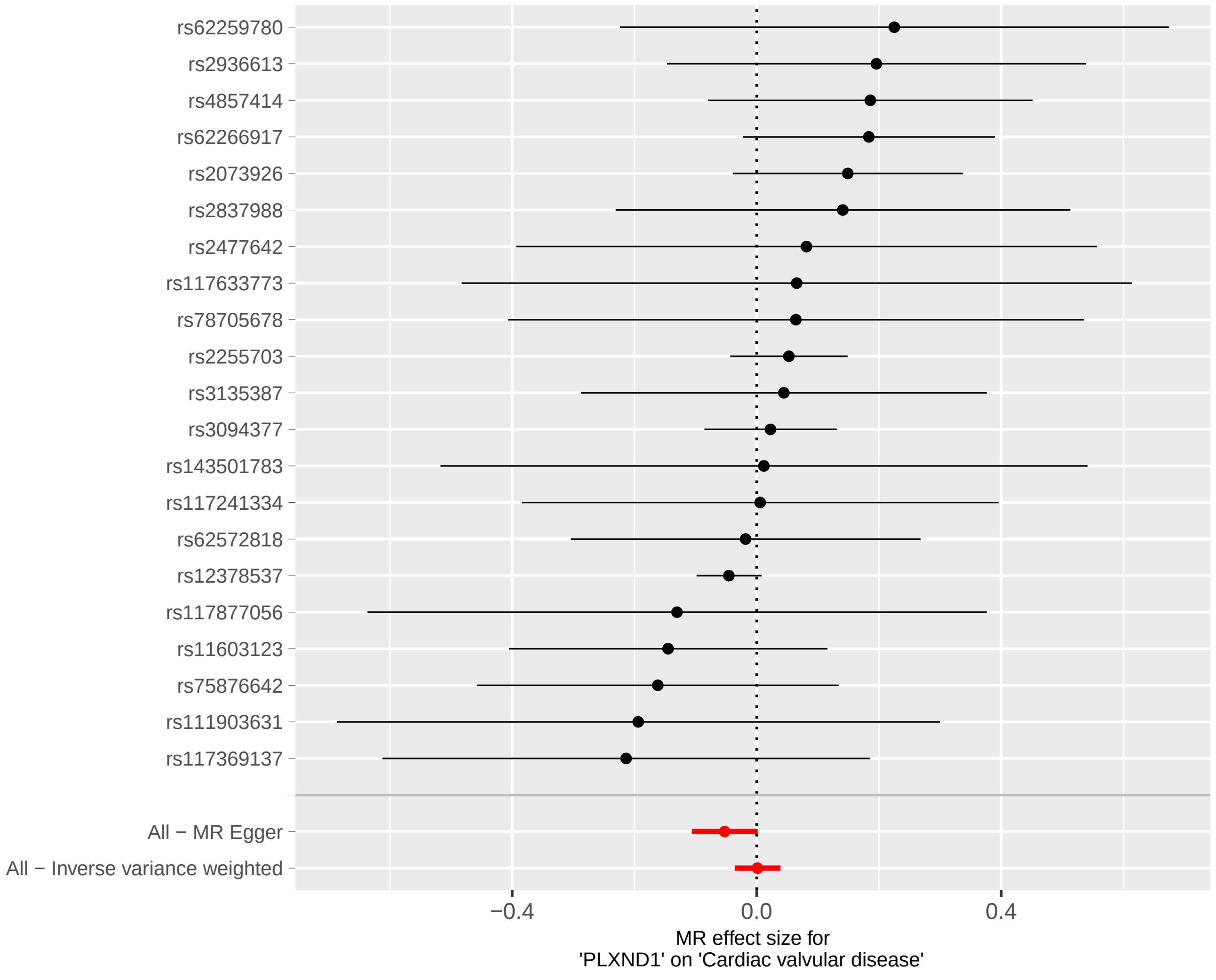

rs62259780
rs2936613
rs4857414
rs62266917
rs2073926
rs2837988
rs2477642
rs117633773
rs78705678
rs2255703
rs3135387
rs3094377
rs143501783
rs117241334
rs62572818
rs12378537
rs117877056
rs11603123
rs75876642
rs111903631
rs117369137
All − MR Egger
All − Inverse variance weighted
−0.4
0.0
0.4
MR effect size for
'PLXND1' on 'Cardiac valvular disease'

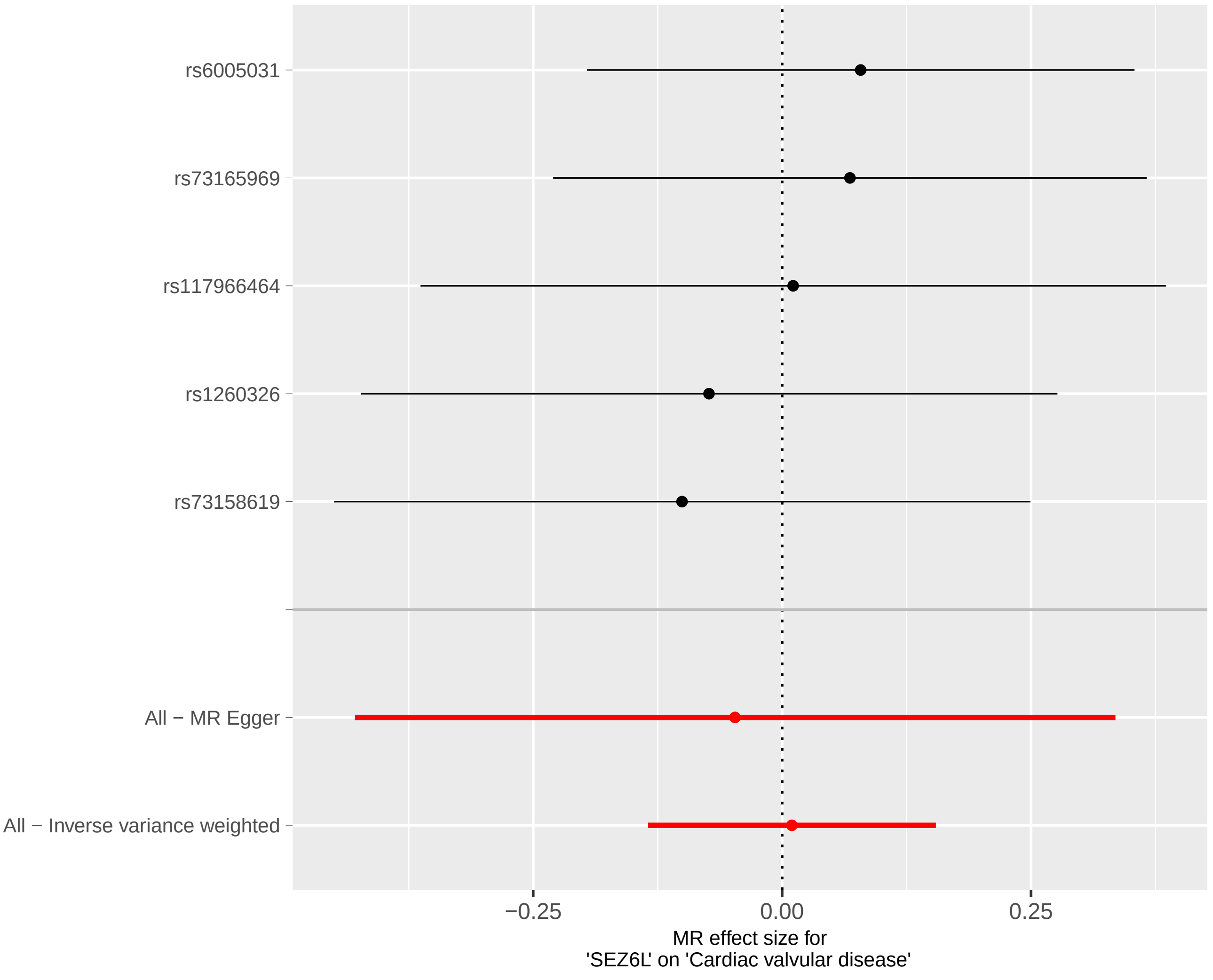
rs6005031
rs73165969
rs117966464
rs1260326
rs73158619
All − MR Egger
All − Inverse variance weighted
−0.25
0.00
0.25
MR effect size for
'SEZ6L' on 'Cardiac valvular disease'

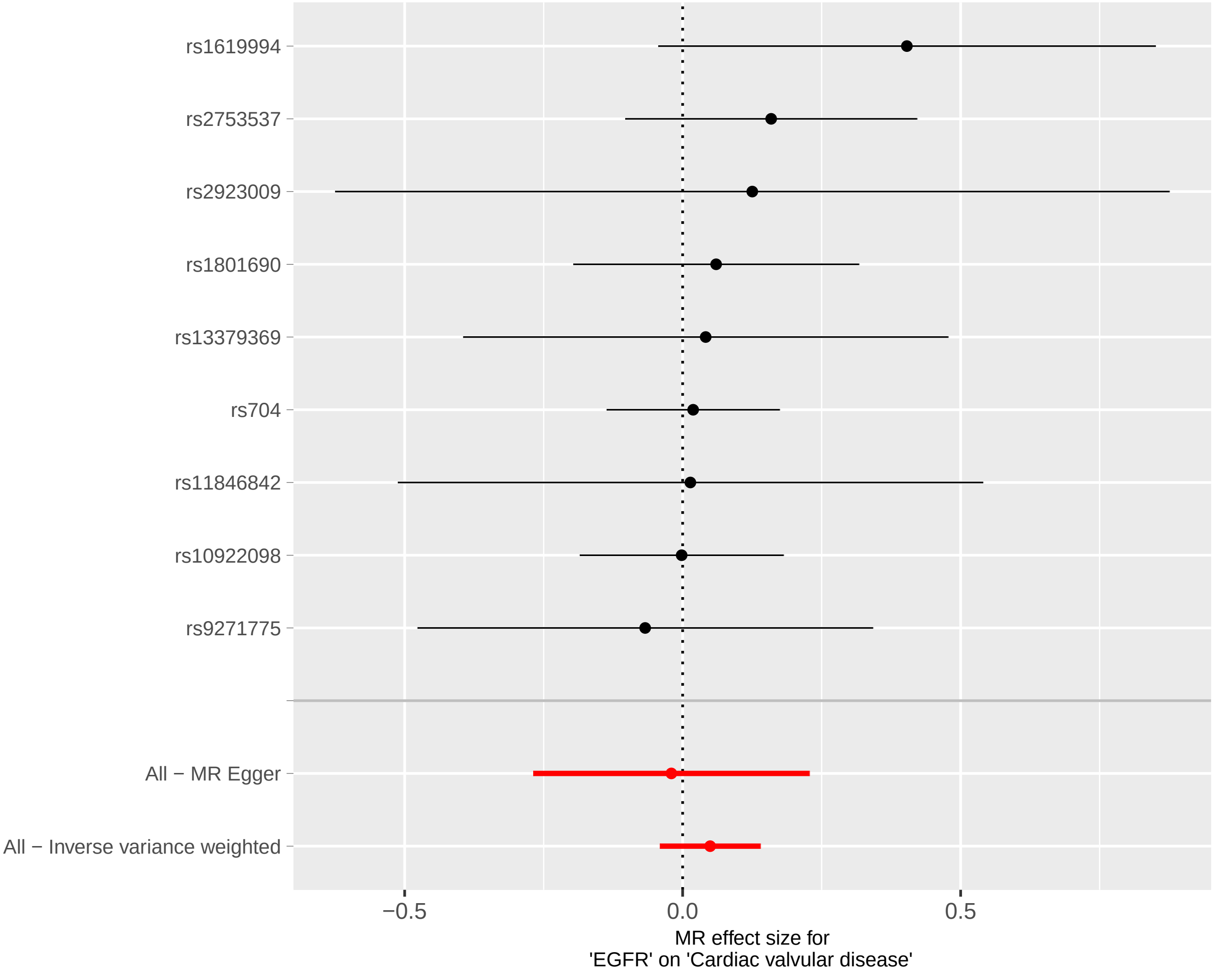
rs1619994
rs2753537
rs2923009
rs1801690
rs13379369
rs704
rs11846842
rs10922098
rs9271775
All − MR Egger
All − Inverse variance weighted
−0.5
0.0
0.5
MR effect size for
'EGFR' on 'Cardiac valvular disease'

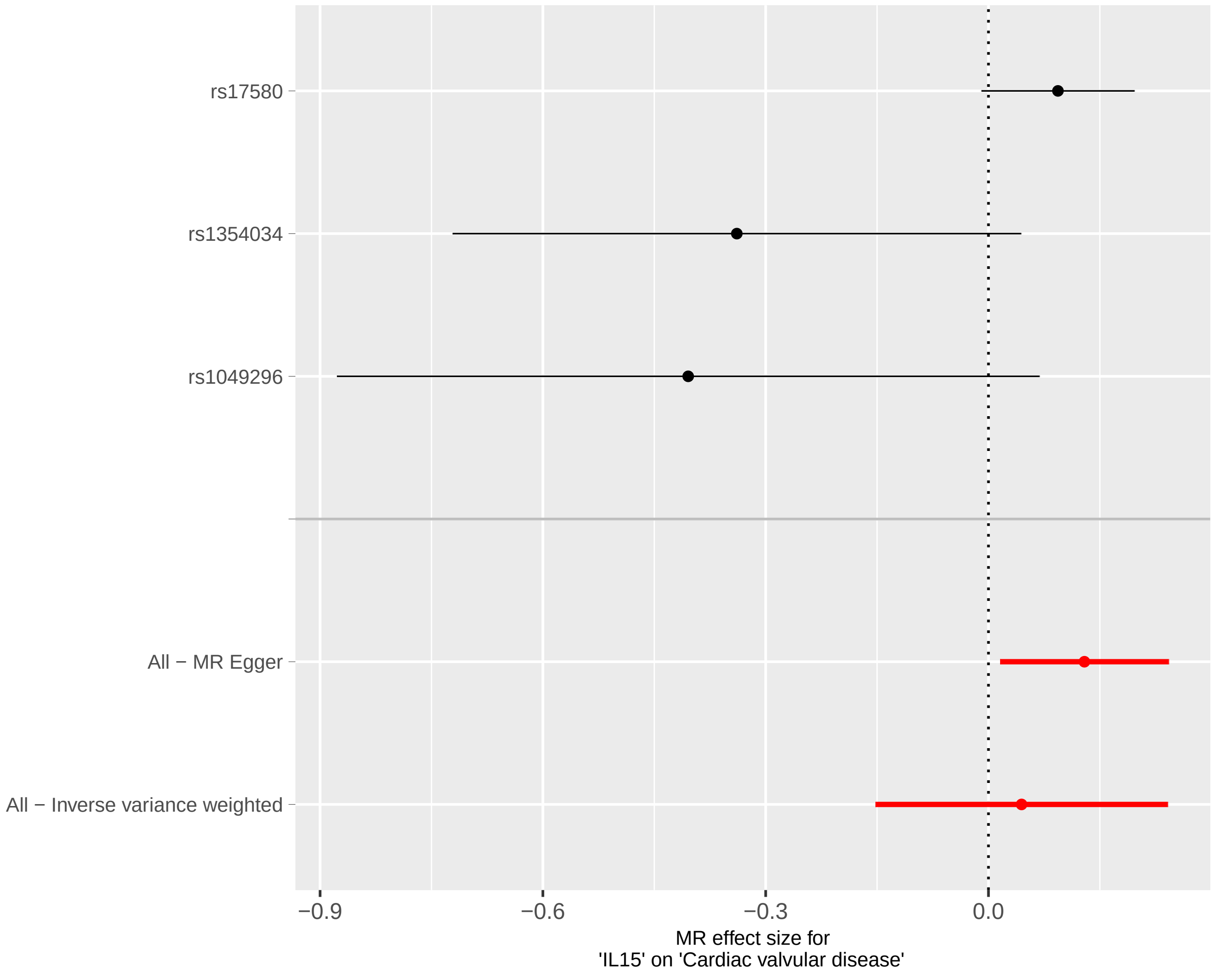
rs17580
rs1354034
rs1049296
All − MR Egger
All − Inverse variance weighted
−0.9
−0.6
−0.3
0.0
MR effect size for
'IL15' on 'Cardiac valvular disease'

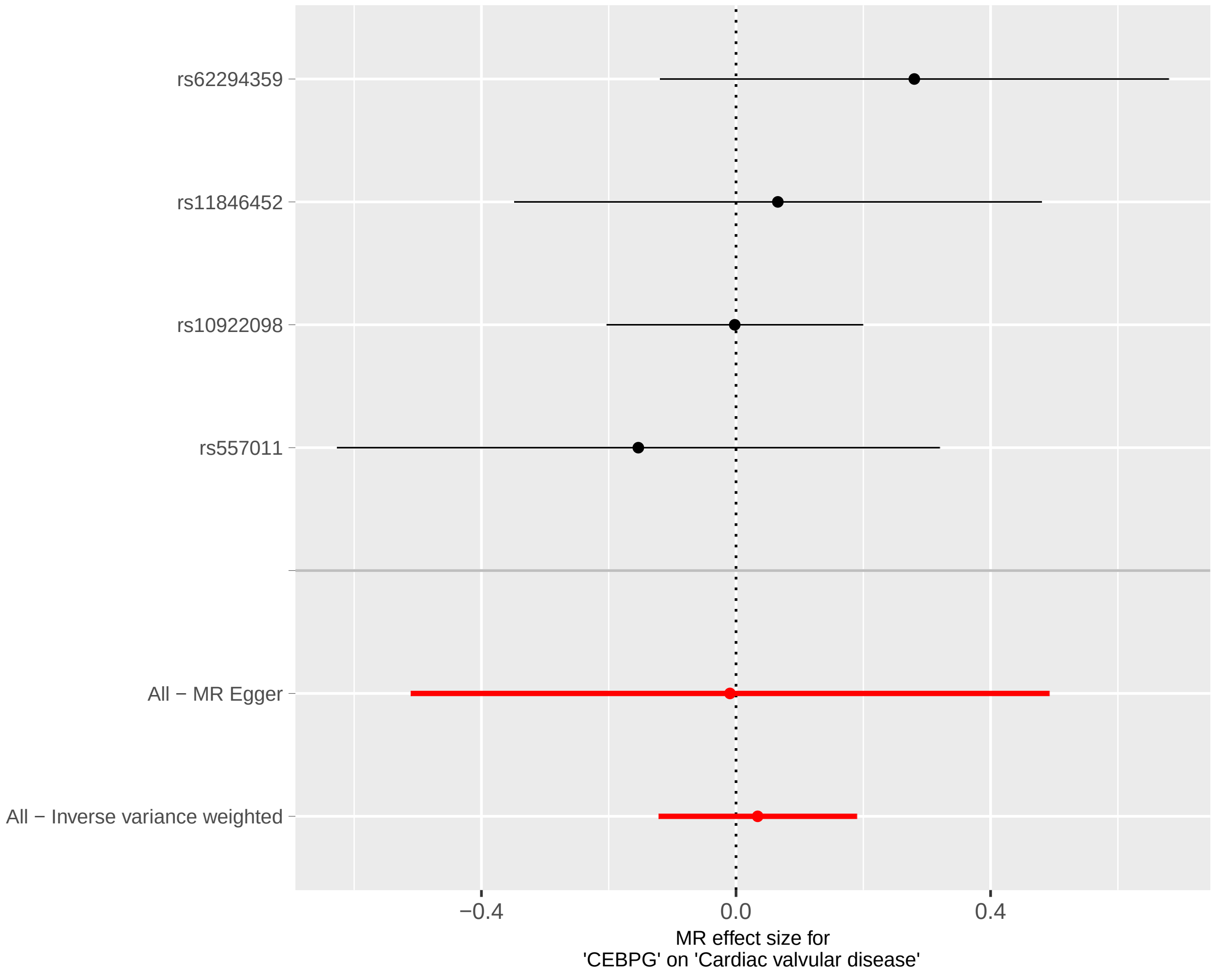
rs62294359
rs11846452
rs10922098
rs557011
All − MR Egger
All − Inverse variance weighted
−0.4
0.0
0.4
MR effect size for
'CEBPG' on 'Cardiac valvular disease'

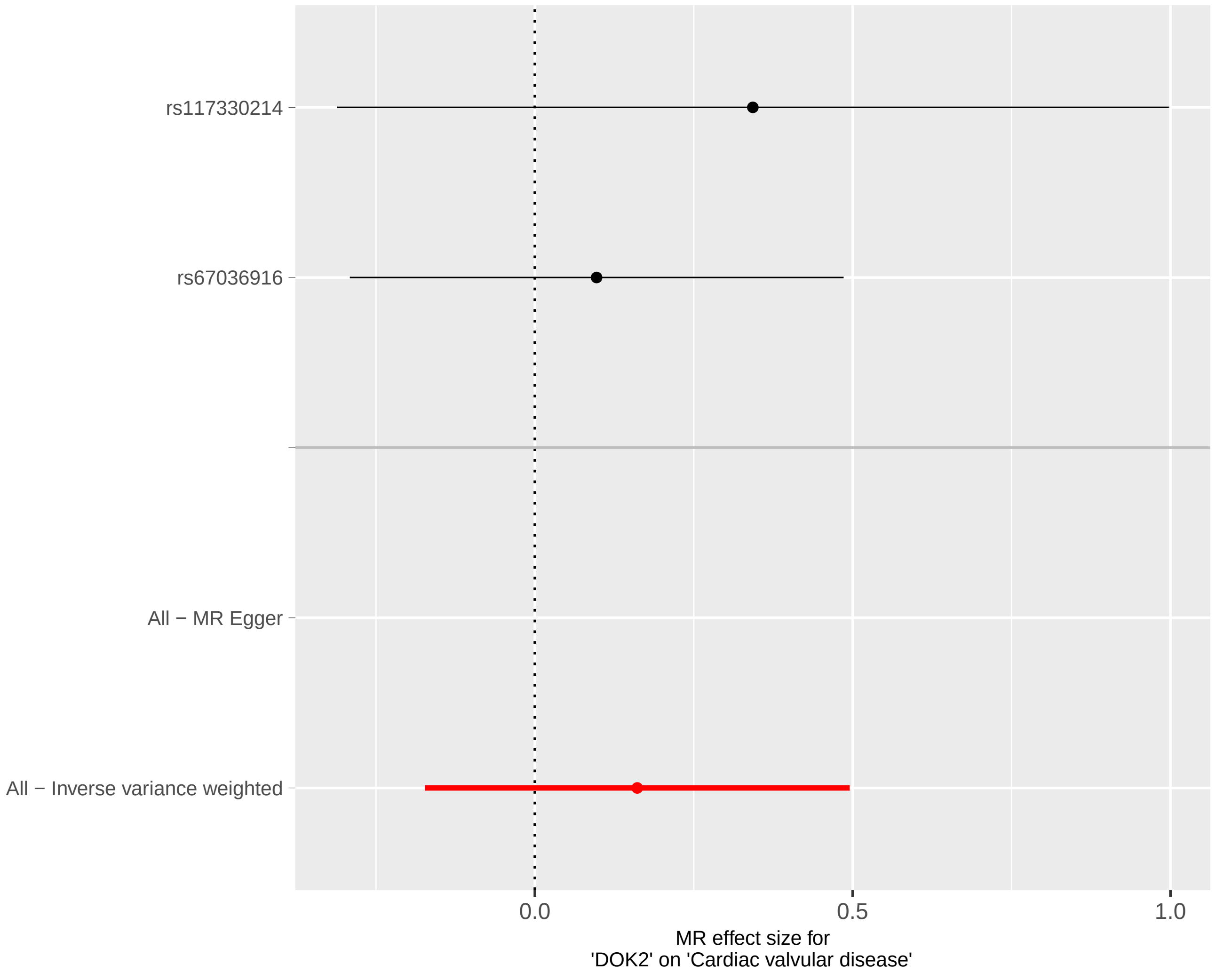
rs117330214
rs67036916
All − MR Egger
All − Inverse variance weighted
0.0
0.5
1.0
MR effect size for
'DOK2' on 'Cardiac valvular disease'

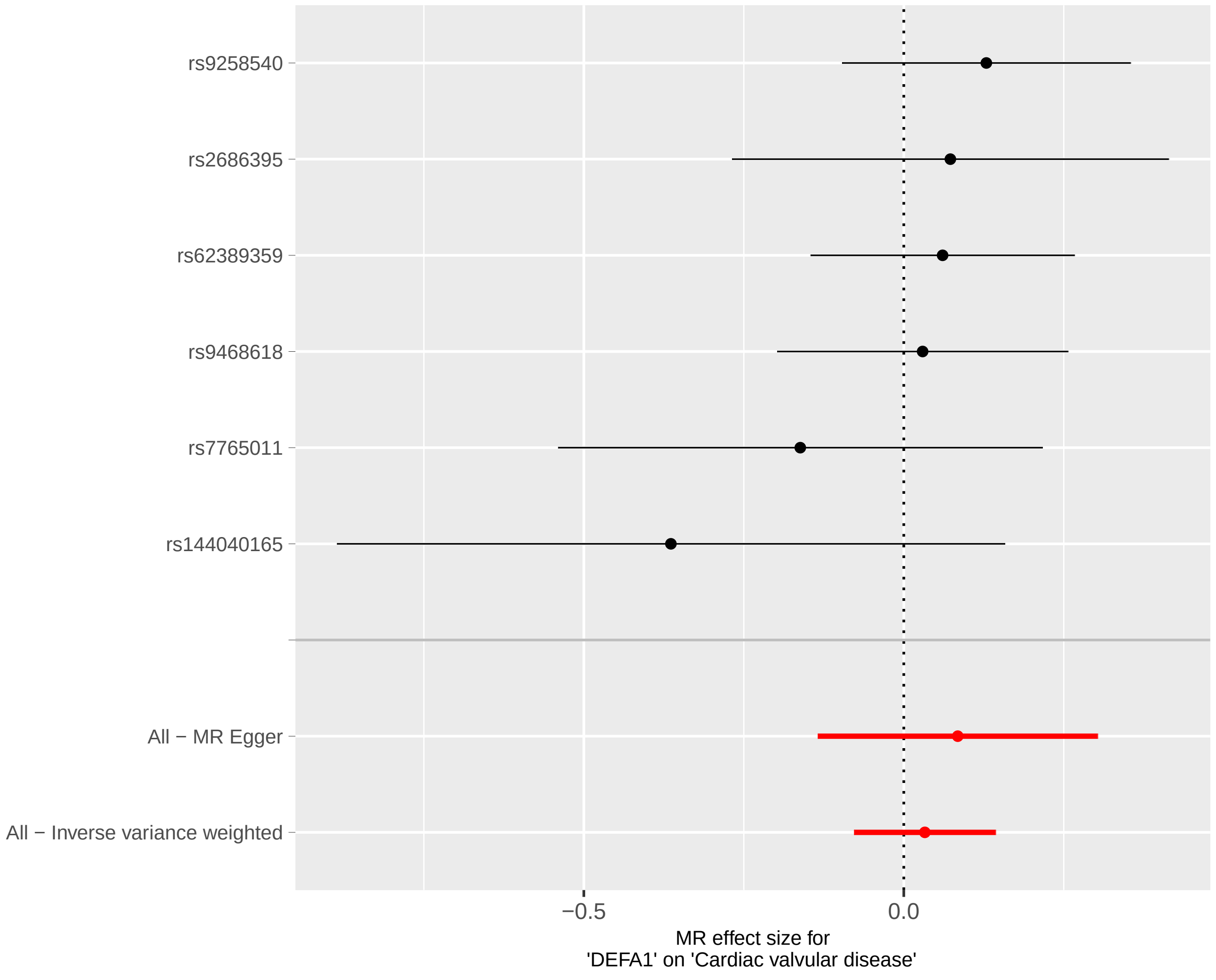
rs9258540
rs2686395
rs62389359
rs9468618
rs7765011
rs144040165
All − MR Egger
All − Inverse variance weighted
−0.5
0.0
MR effect size for
'DEFA1' on 'Cardiac valvular disease'

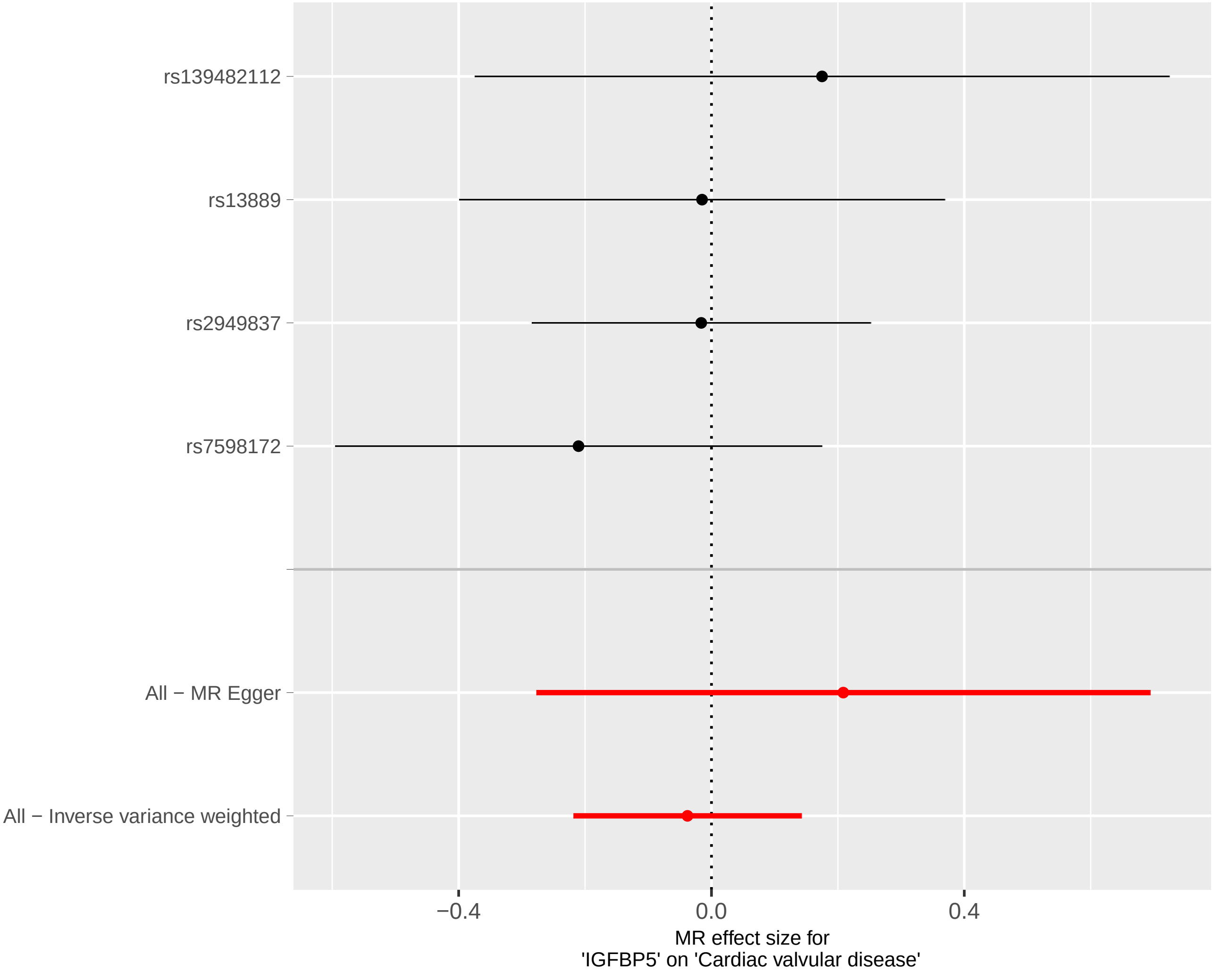

rs139482112
rs13889
rs2949837
rs7598172
All − MR Egger
All − Inverse variance weighted
−0.4
0.0
0.4
MR effect size for
'IGFBP5' on 'Cardiac valvular disease'

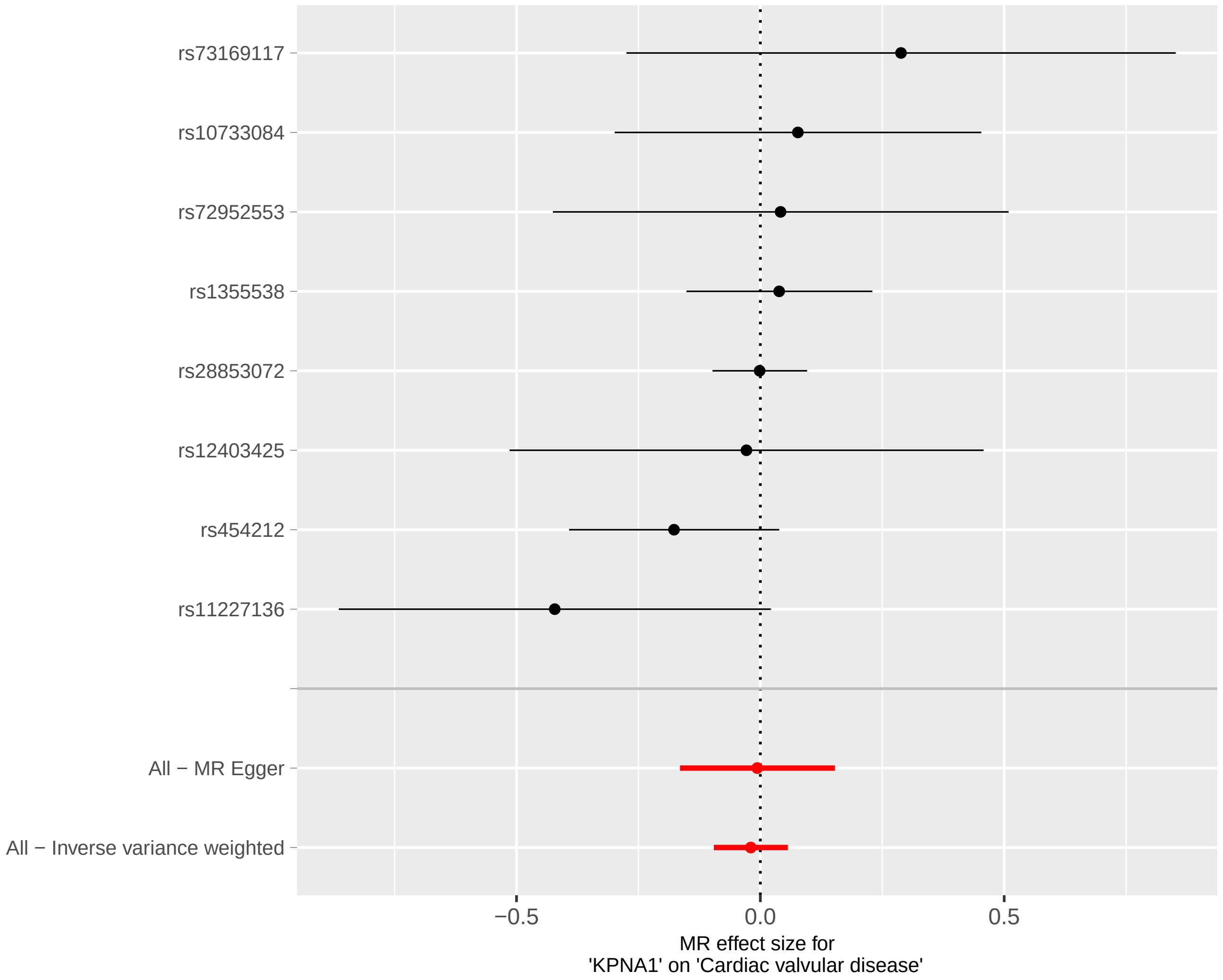
rs73169117
rs10733084
rs72952553
rs1355538
rs28853072
rs12403425
rs454212
rs11227136
All − MR Egger
All − Inverse variance weighted
−0.5
0.0
0.5
MR effect size for
'KPNA1' on 'Cardiac valvular disease'

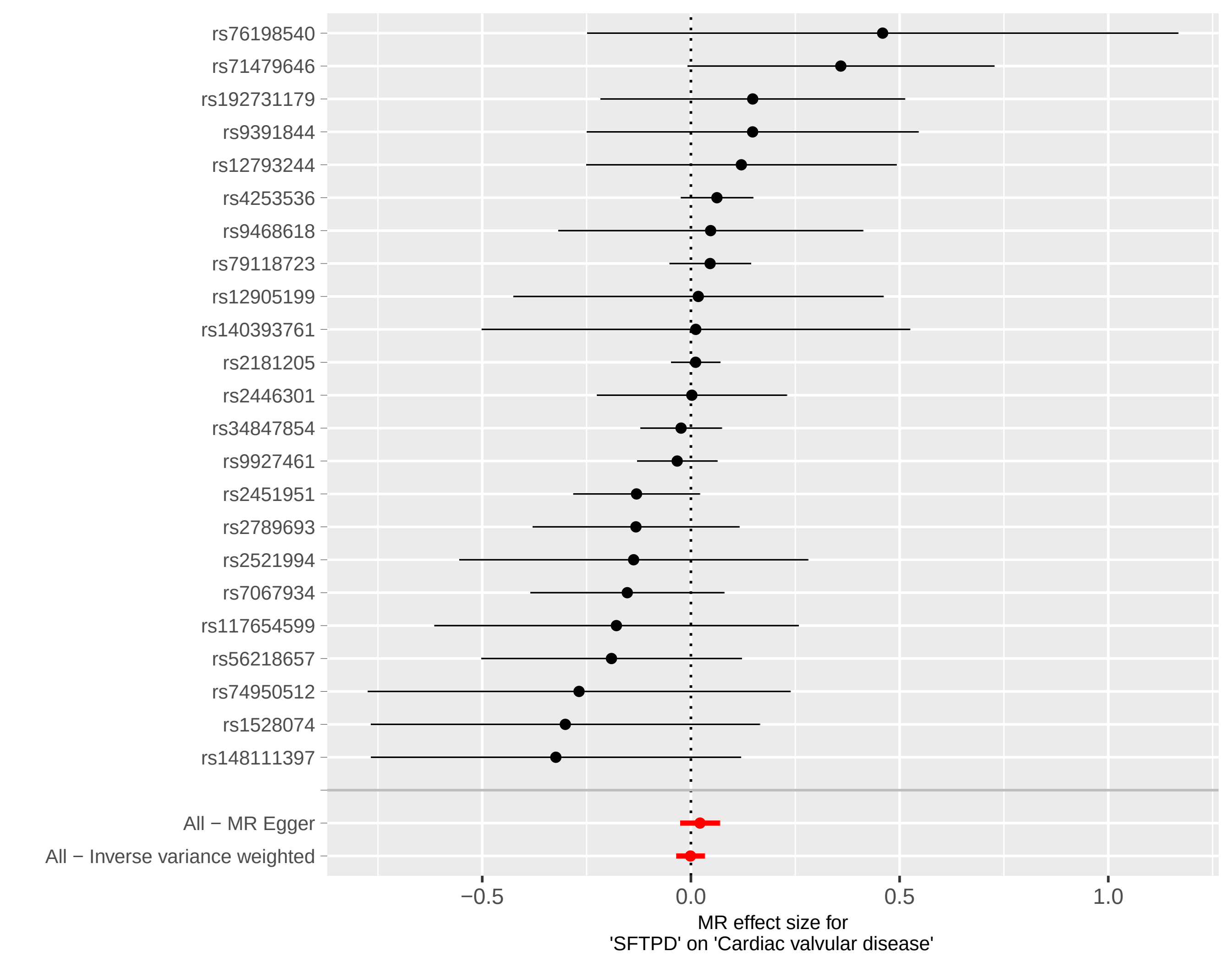

rs76198540
rs71479646
rs192731179
rs9391844
rs12793244
rs4253536
rs9468618
rs79118723
rs12905199
rs140393761
rs2181205
rs2446301
rs34847854
rs9927461
rs2451951
rs2789693
rs2521994
rs7067934
rs117654599
rs56218657
rs74950512
rs1528074
rs148111397
All − MR Egger
All − Inverse variance weighted
−0.5
0.0
0.5
1.0
MR effect size for
'SFTPD' on 'Cardiac valvular disease'

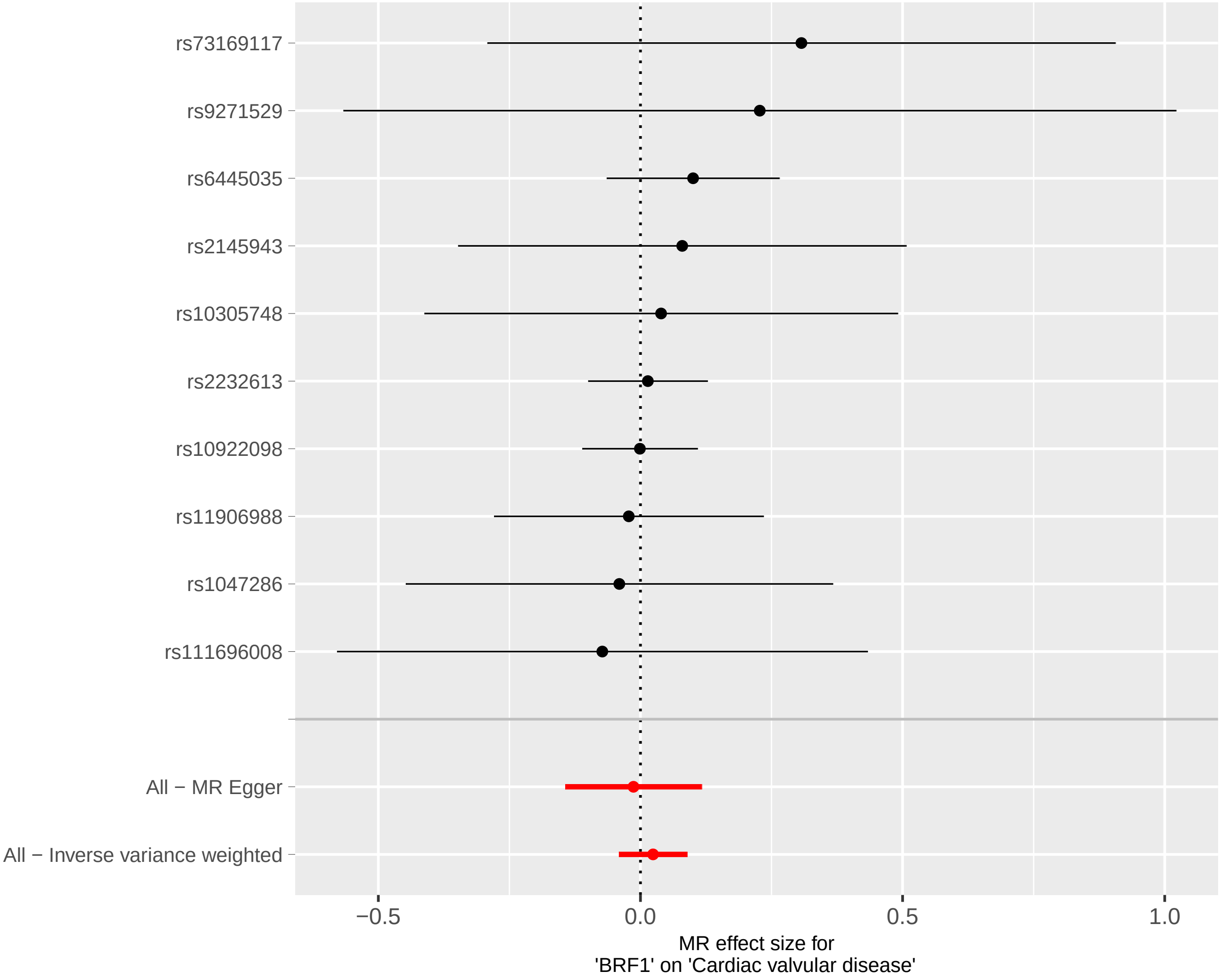

rs73169117
rs9271529
rs6445035
rs2145943
rs10305748
rs2232613
rs10922098
rs11906988
rs1047286
rs111696008
All − MR Egger
All − Inverse variance weighted
−0.5
0.0
0.5
1.0
MR effect size for
'BRF1' on 'Cardiac valvular disease'

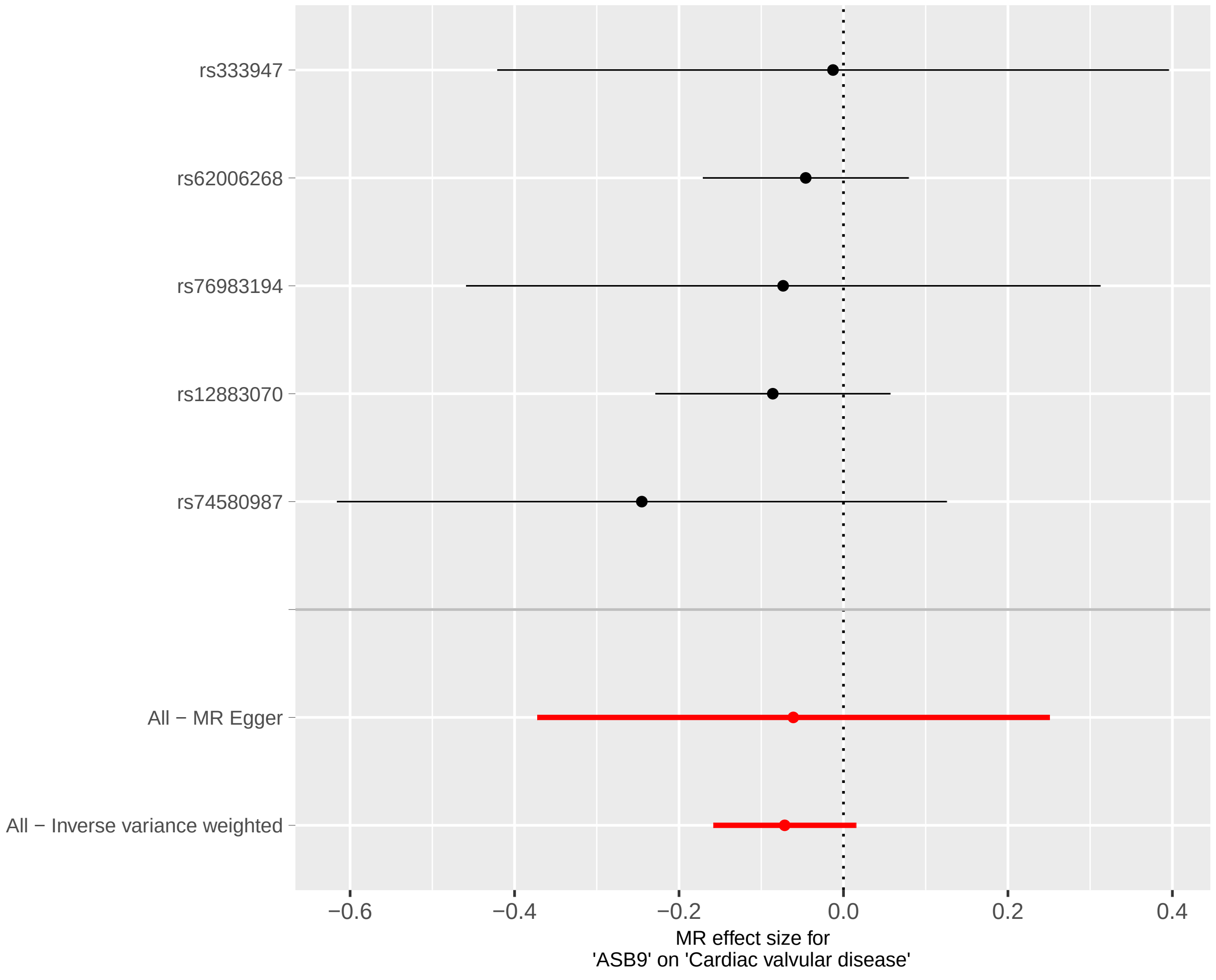
rs333947
rs62006268
rs76983194
rs12883070
rs74580987
All − MR Egger
All − Inverse variance weighted
−0.6
−0.4
−0.2
0.0
0.2
0.4
MR effect size for
'ASB9' on 'Cardiac valvular disease'

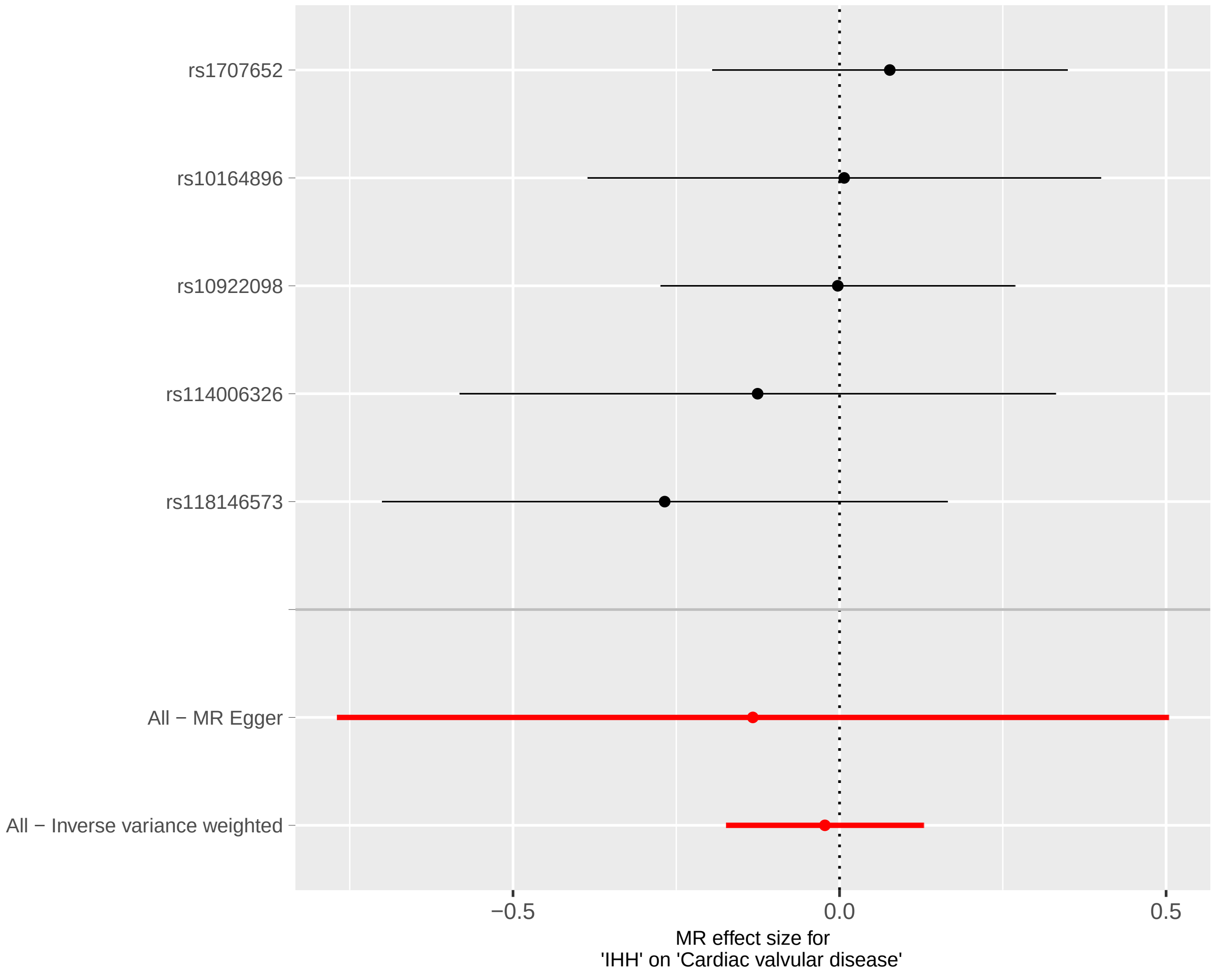
rs1707652
rs10164896
rs10922098
rs114006326
rs118146573
All − MR Egger
All − Inverse variance weighted
−0.5
0.0
0.5
MR effect size for
'IHH' on 'Cardiac valvular disease'

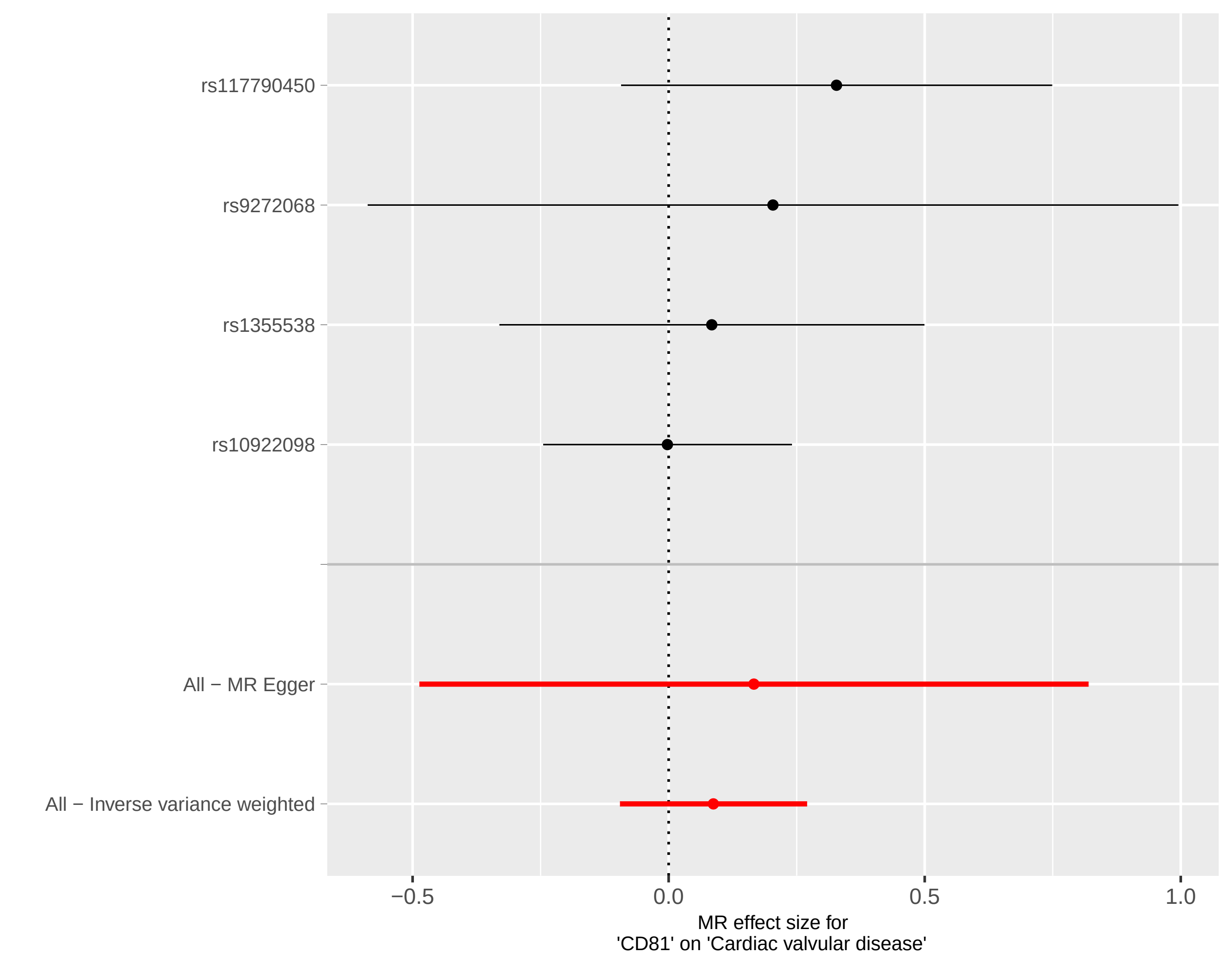
rs117790450
rs9272068
rs1355538
rs10922098
All − MR Egger
All − Inverse variance weighted
−0.5
0.0
0.5
1.0
MR effect size for
'CD81' on 'Cardiac valvular disease'

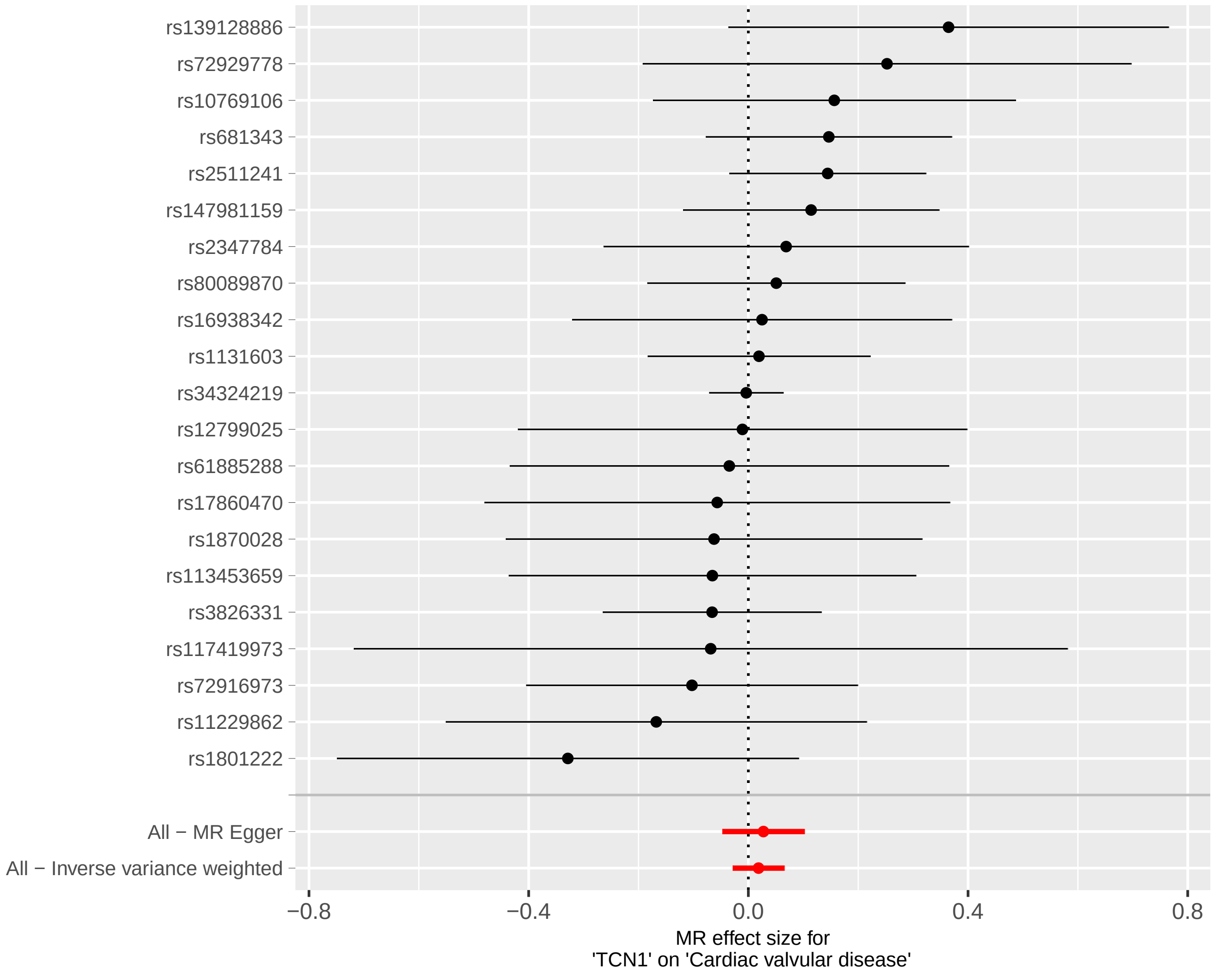

rs139128886
rs72929778
rs10769106
rs681343
rs2511241
rs147981159
rs2347784
rs80089870
rs16938342
rs1131603
rs34324219
rs12799025
rs61885288
rs17860470
rs1870028
rs113453659
rs3826331
rs117419973
rs72916973
rs11229862
rs1801222
All − MR Egger
All − Inverse variance weighted
−0.8
−0.4
0.0
0.4
0.8
MR effect size for
'TCN1' on 'Cardiac valvular disease'

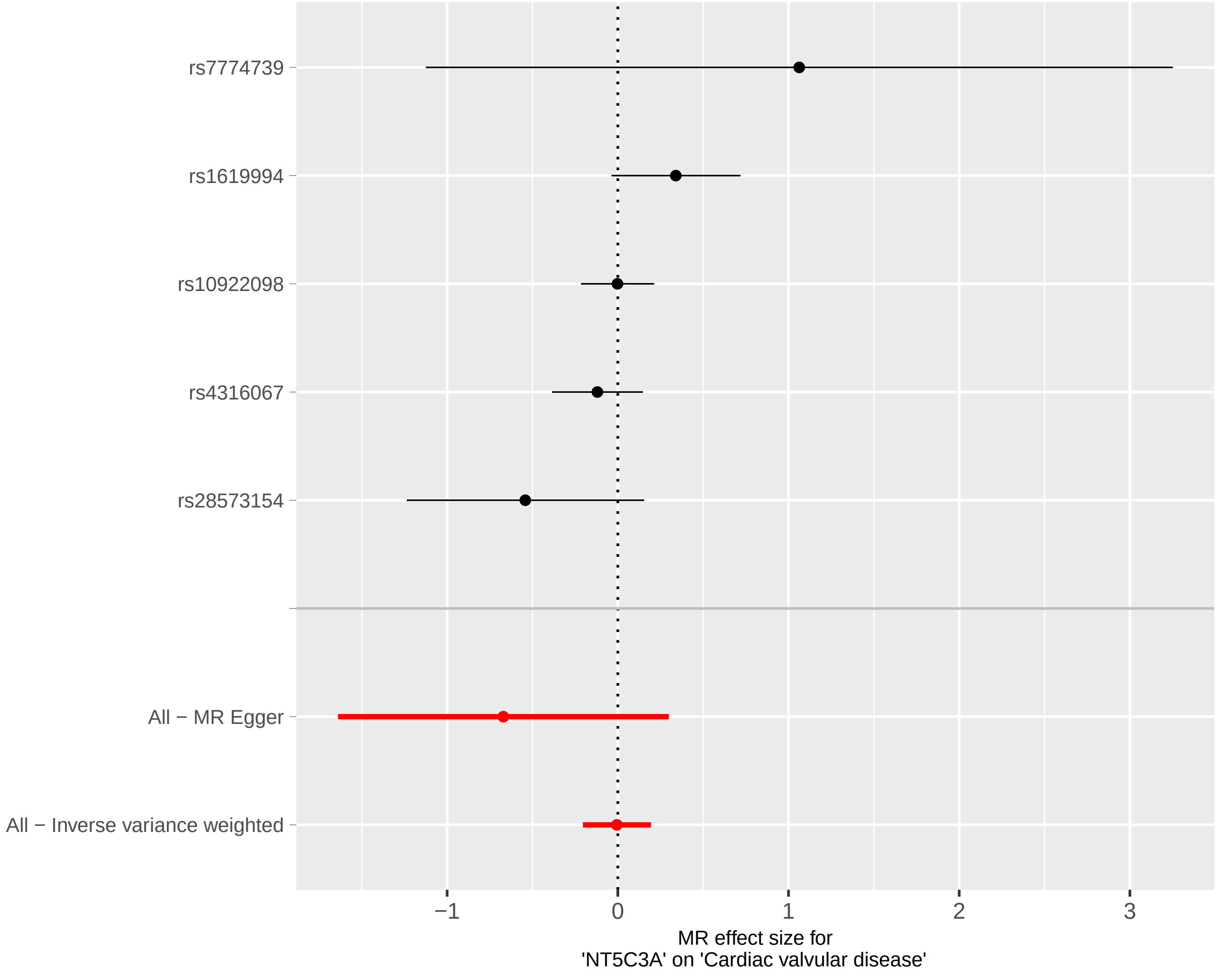

rs7774739
rs1619994
rs10922098
rs4316067
rs28573154
All − MR Egger
All − Inverse variance weighted
−1
0
1
2
3
MR effect size for
'NT5C3A' on 'Cardiac valvular disease'

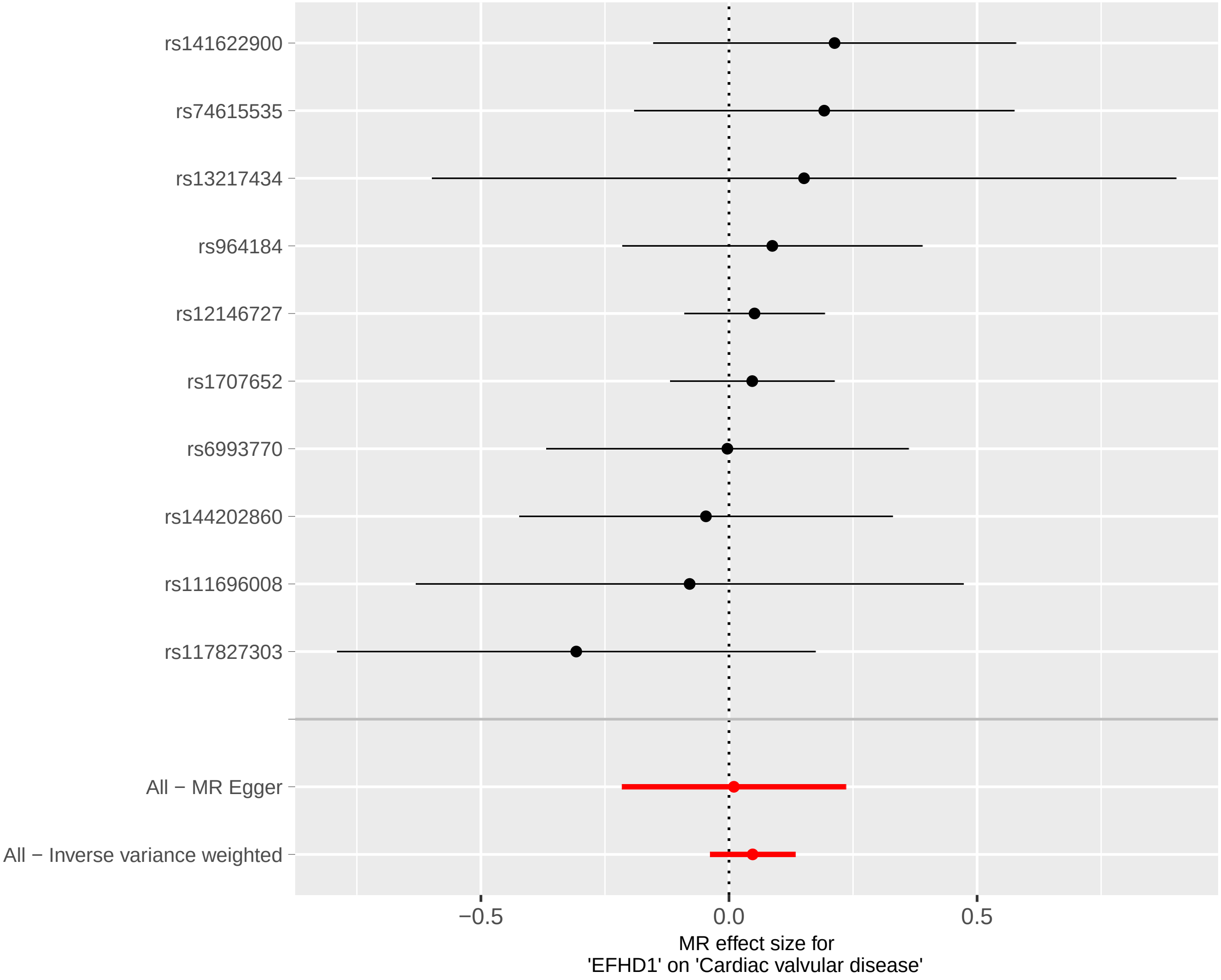

rs141622900
rs74615535
rs13217434
rs964184
rs12146727
rs1707652
rs6993770
rs144202860
rs111696008
rs117827303
All − MR Egger
All − Inverse variance weighted
−0.5
0.0
0.5
MR effect size for
'EFHD1' on 'Cardiac valvular disease'

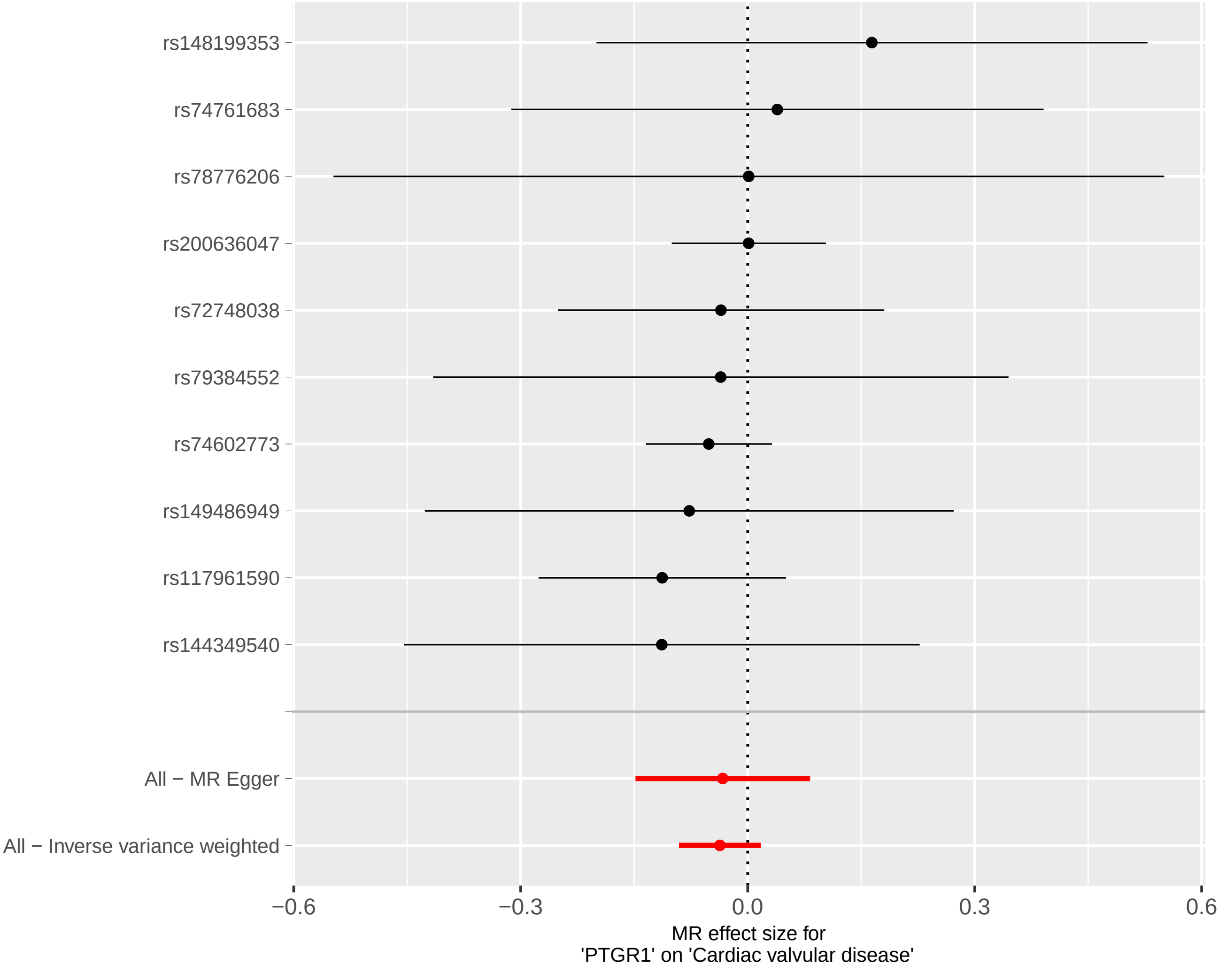
rs148199353
rs74761683
rs78776206
rs200636047
rs72748038
rs79384552
rs74602773
rs149486949
rs117961590
rs144349540
All − MR Egger
All − Inverse variance weighted
−0.6
−0.3
0.0
0.3
0.6
MR effect size for
'PTGR1' on 'Cardiac valvular disease'

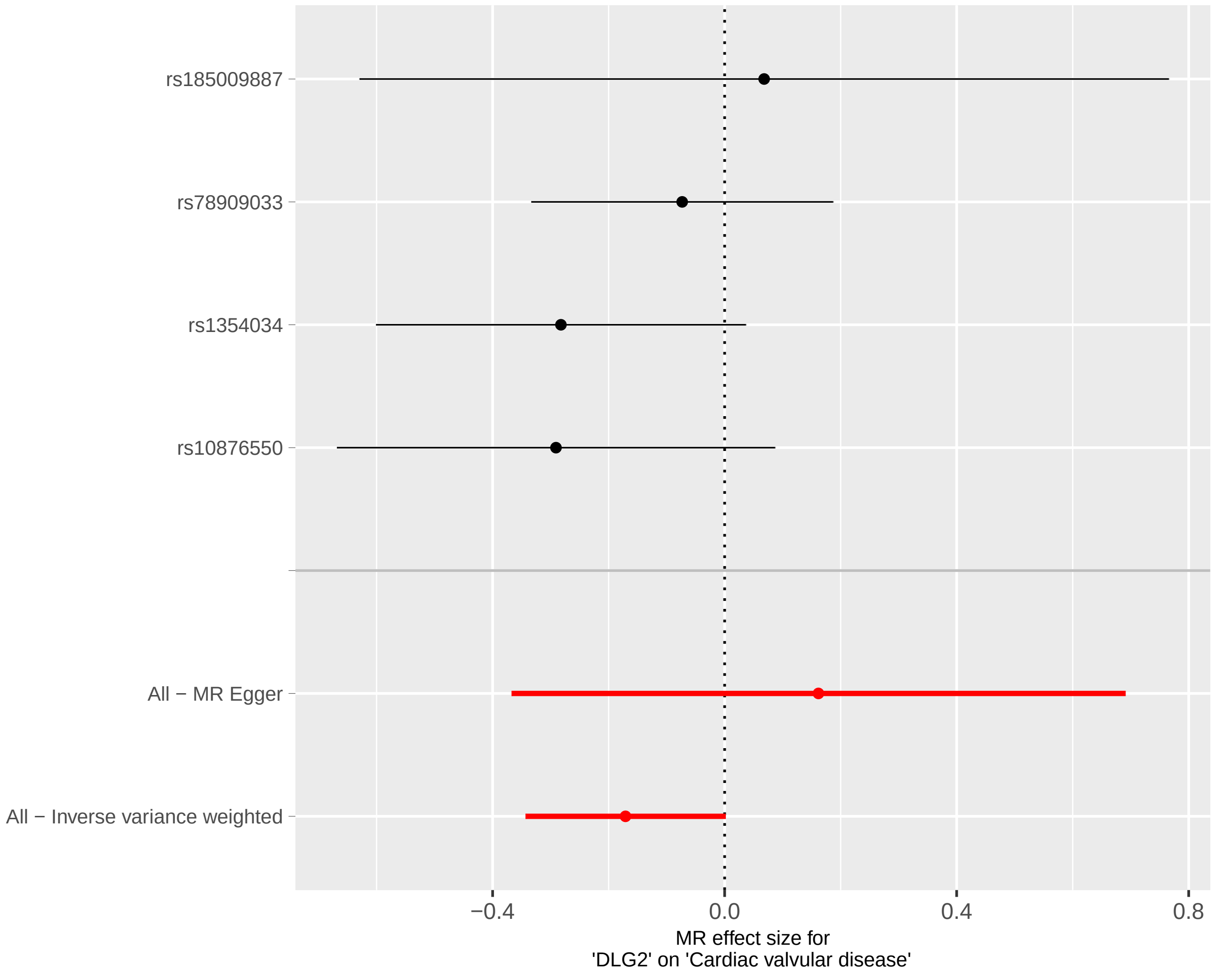
rs185009887
rs78909033
rs1354034
rs10876550
All − MR Egger
All − Inverse variance weighted
−0.4
0.0
0.4
0.8
MR effect size for
'DLG2' on 'Cardiac valvular disease'

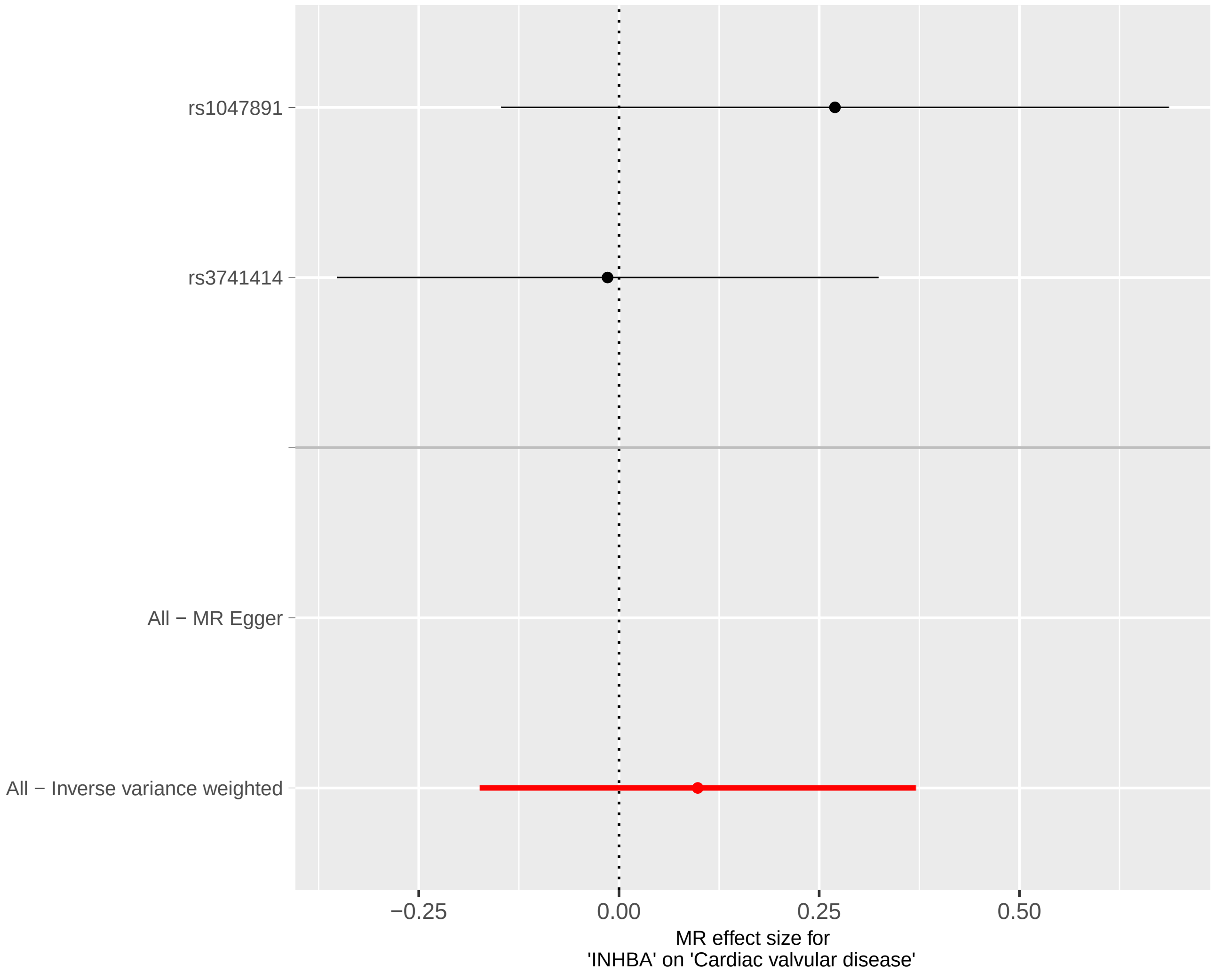
rs1047891
rs3741414
All − MR Egger
All − Inverse variance weighted
−0.25
0.00
0.25
0.50
MR effect size for
'INHBA' on 'Cardiac valvular disease'

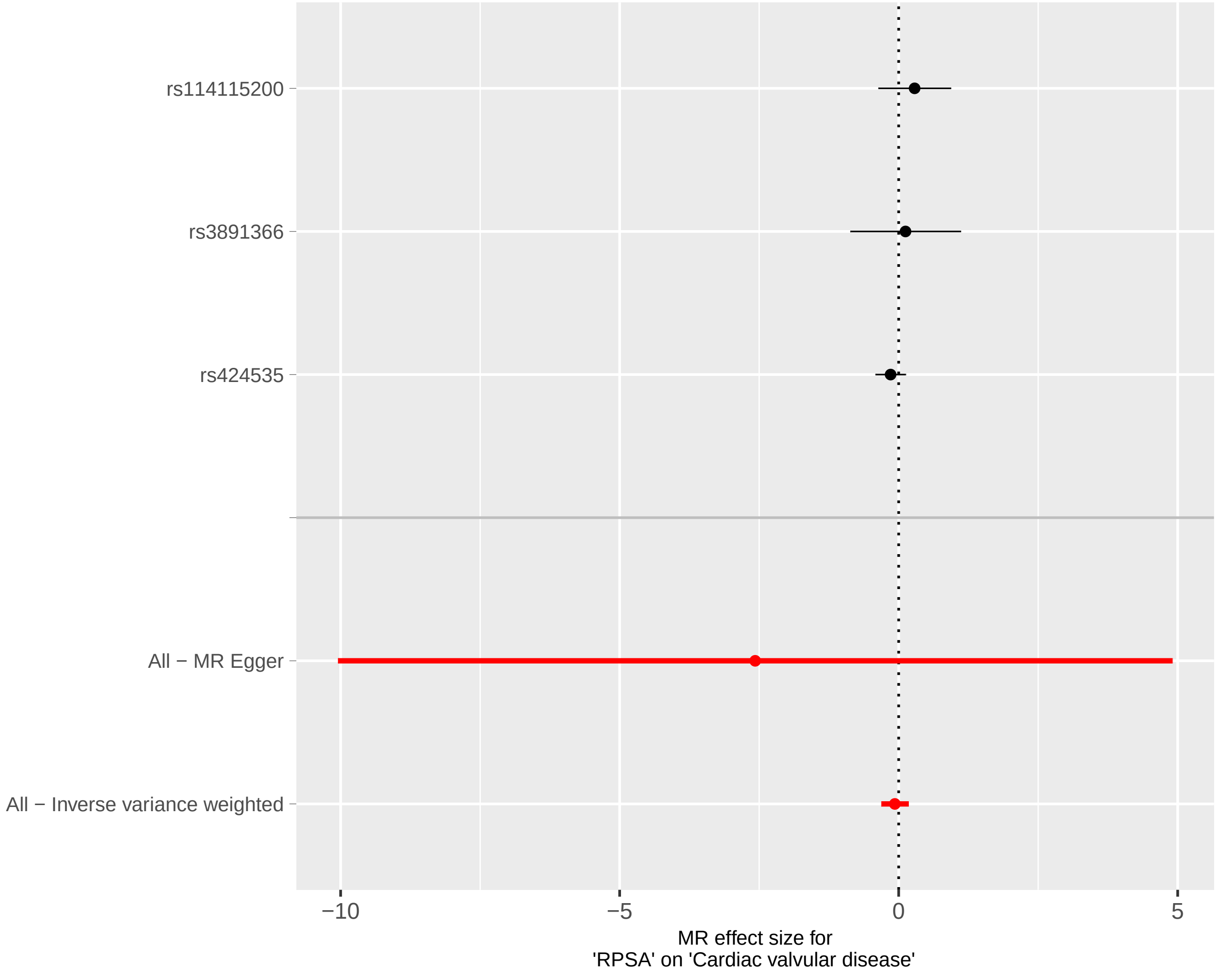
rs114115200
rs3891366
rs424535
All − MR Egger
All − Inverse variance weighted
−10
−5
0
5
MR effect size for
'RPSA' on 'Cardiac valvular disease'

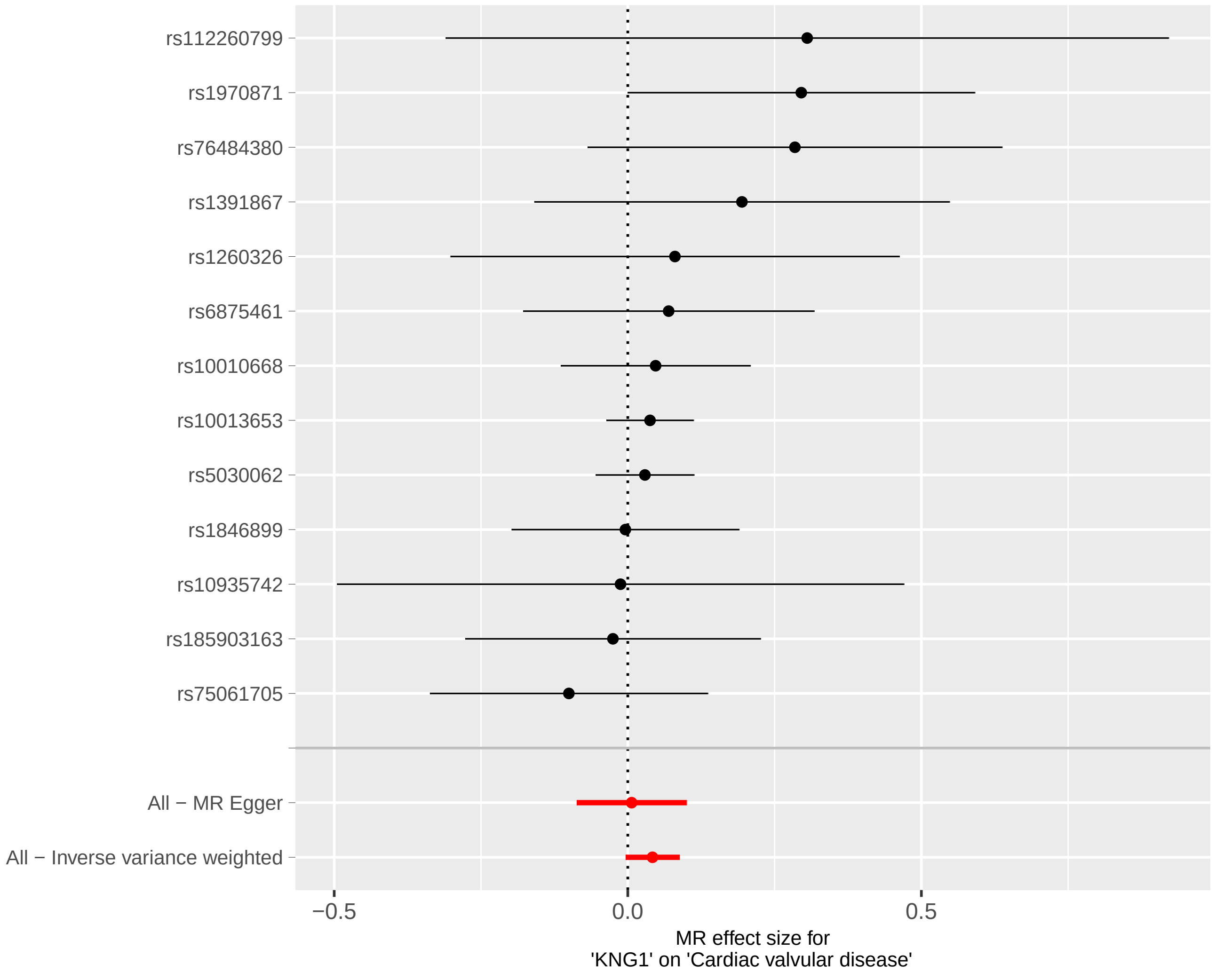
rs112260799
rs1970871
rs76484380
rs1391867
rs1260326
rs6875461
rs10010668
rs10013653
rs5030062
rs1846899
rs10935742
rs185903163
rs75061705
All − MR Egger
All − Inverse variance weighted
−0.5
0.0
0.5
MR effect size for
'KNG1' on 'Cardiac valvular disease'

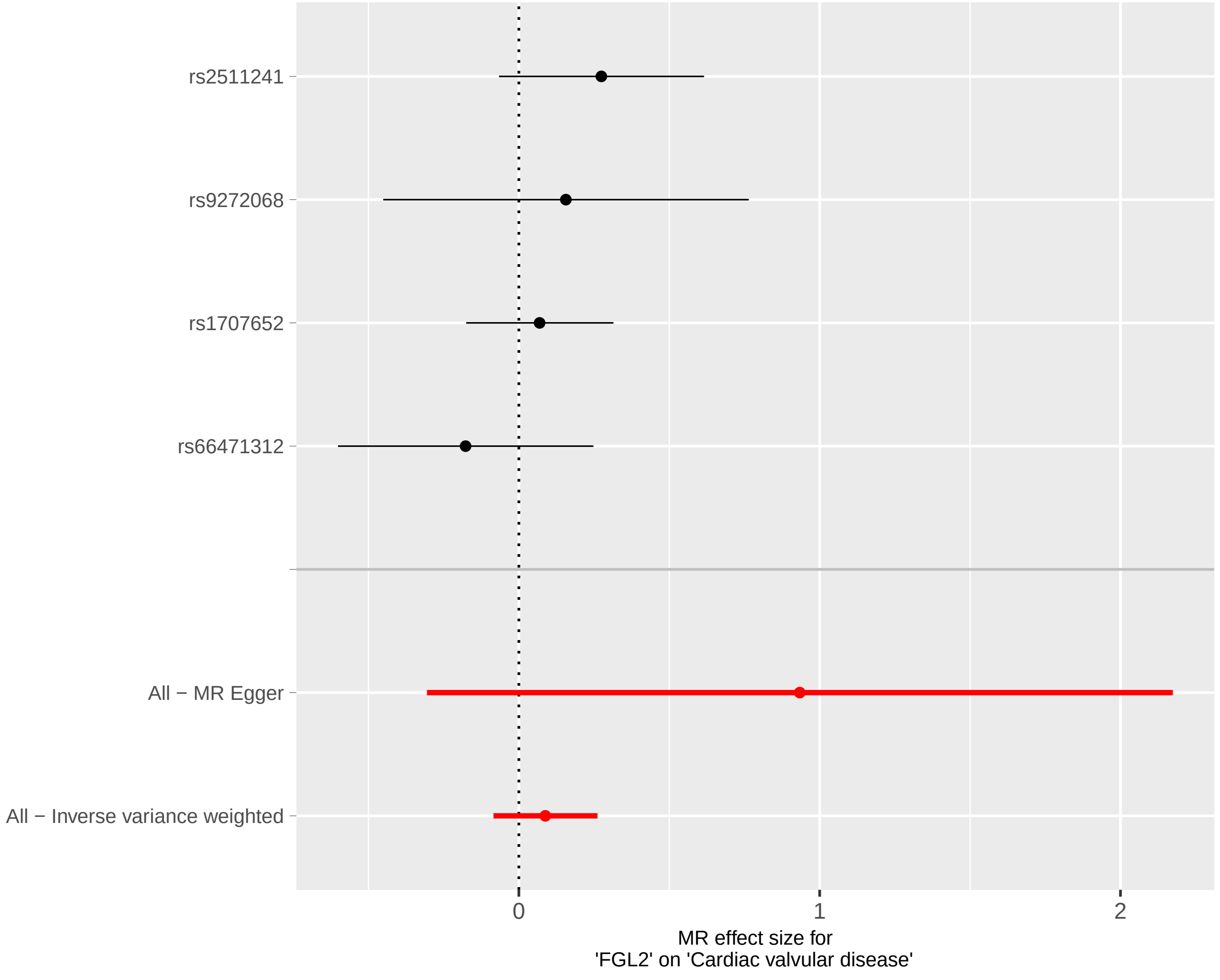
rs2511241
rs9272068
rs1707652
rs66471312
All − MR Egger
All − Inverse variance weighted
0
1
2
MR effect size for
'FGL2' on 'Cardiac valvular disease'

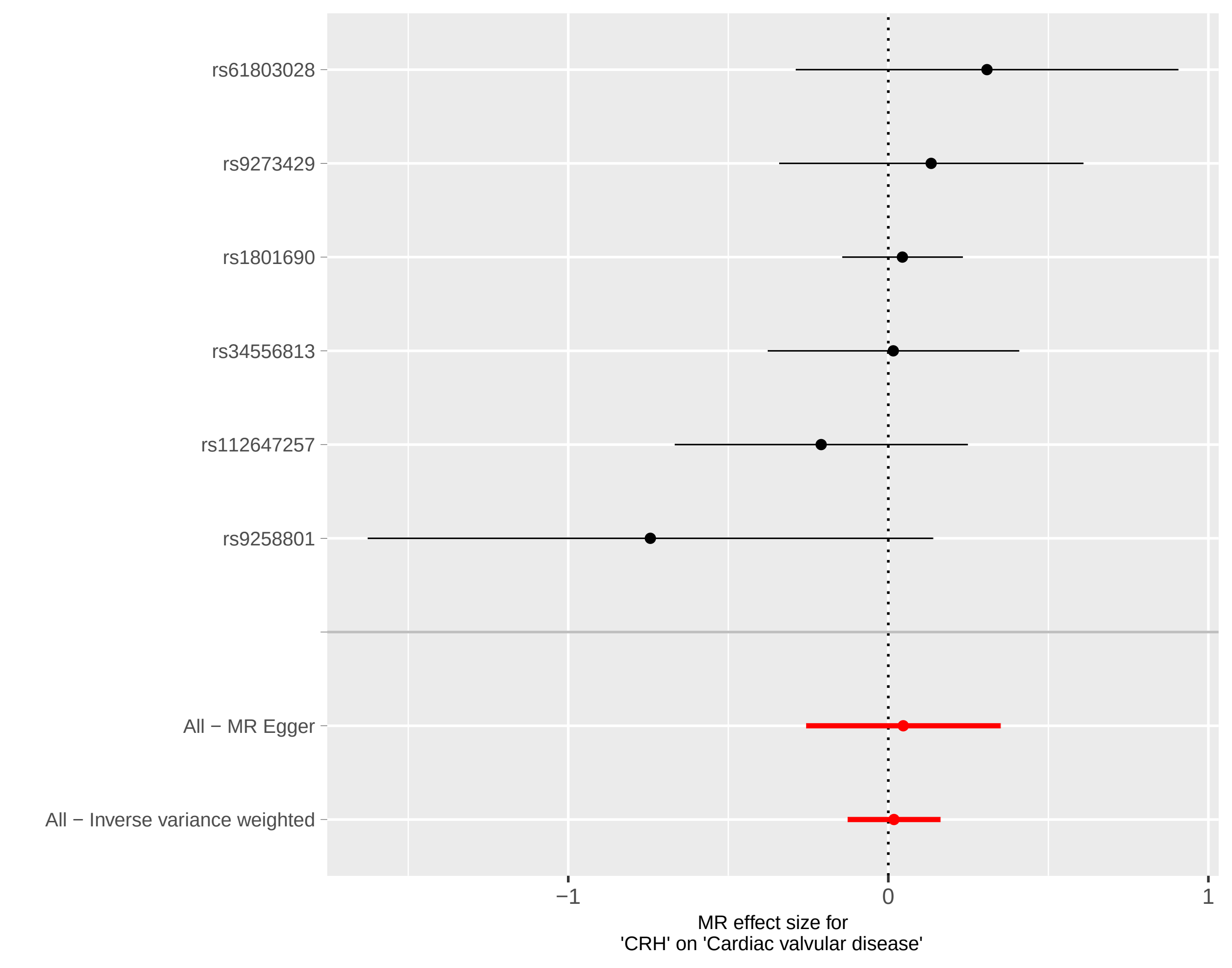
rs61803028
rs9273429
rs1801690
rs34556813
rs112647257
rs9258801
All − MR Egger
All − Inverse variance weighted
−1
0
1
MR effect size for
'CRH' on 'Cardiac valvular disease'

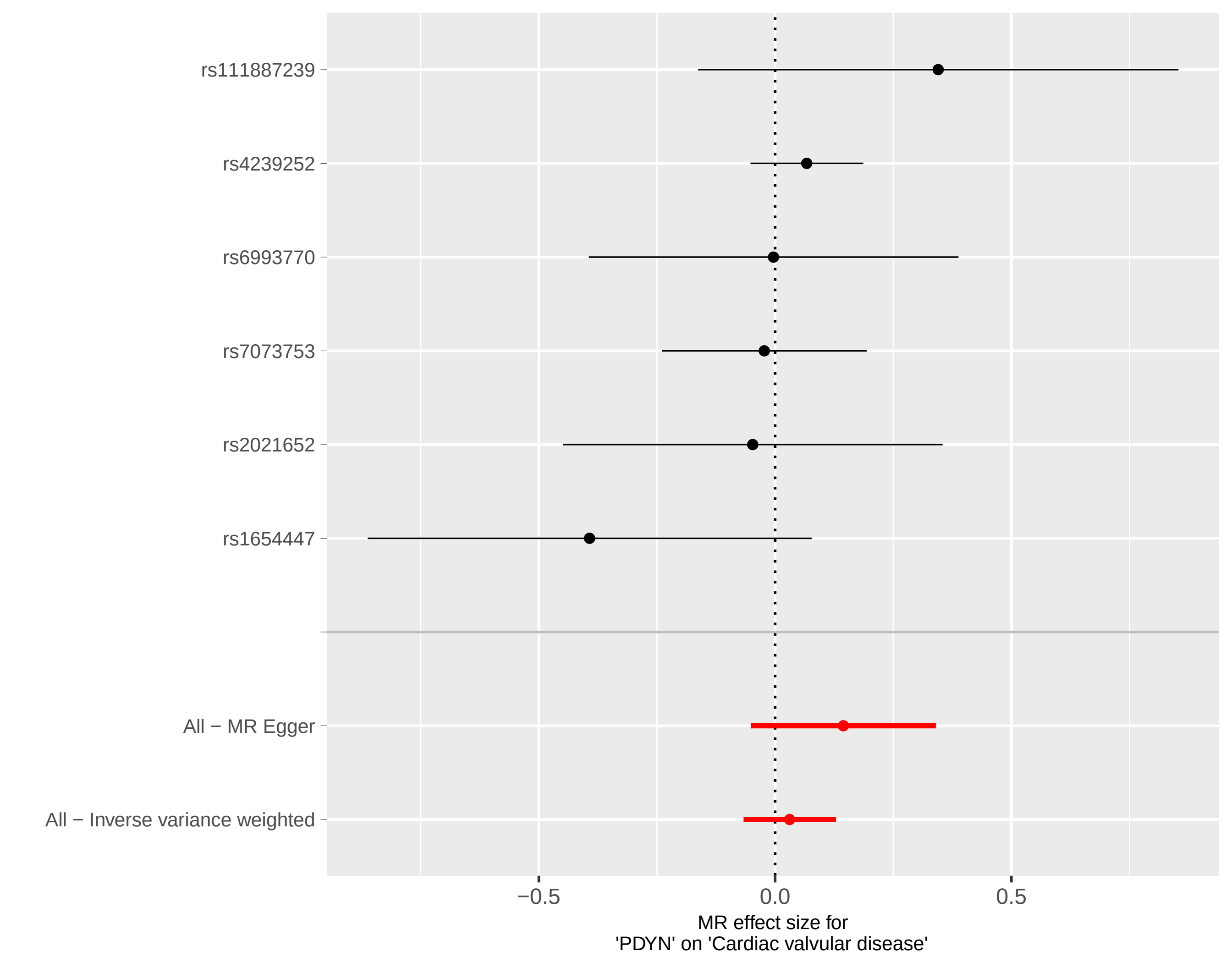
rs111887239
rs4239252
rs6993770
rs7073753
rs2021652
rs1654447
All − MR Egger
All − Inverse variance weighted
−0.5
0.0
0.5
MR effect size for
'PDYN' on 'Cardiac valvular disease'

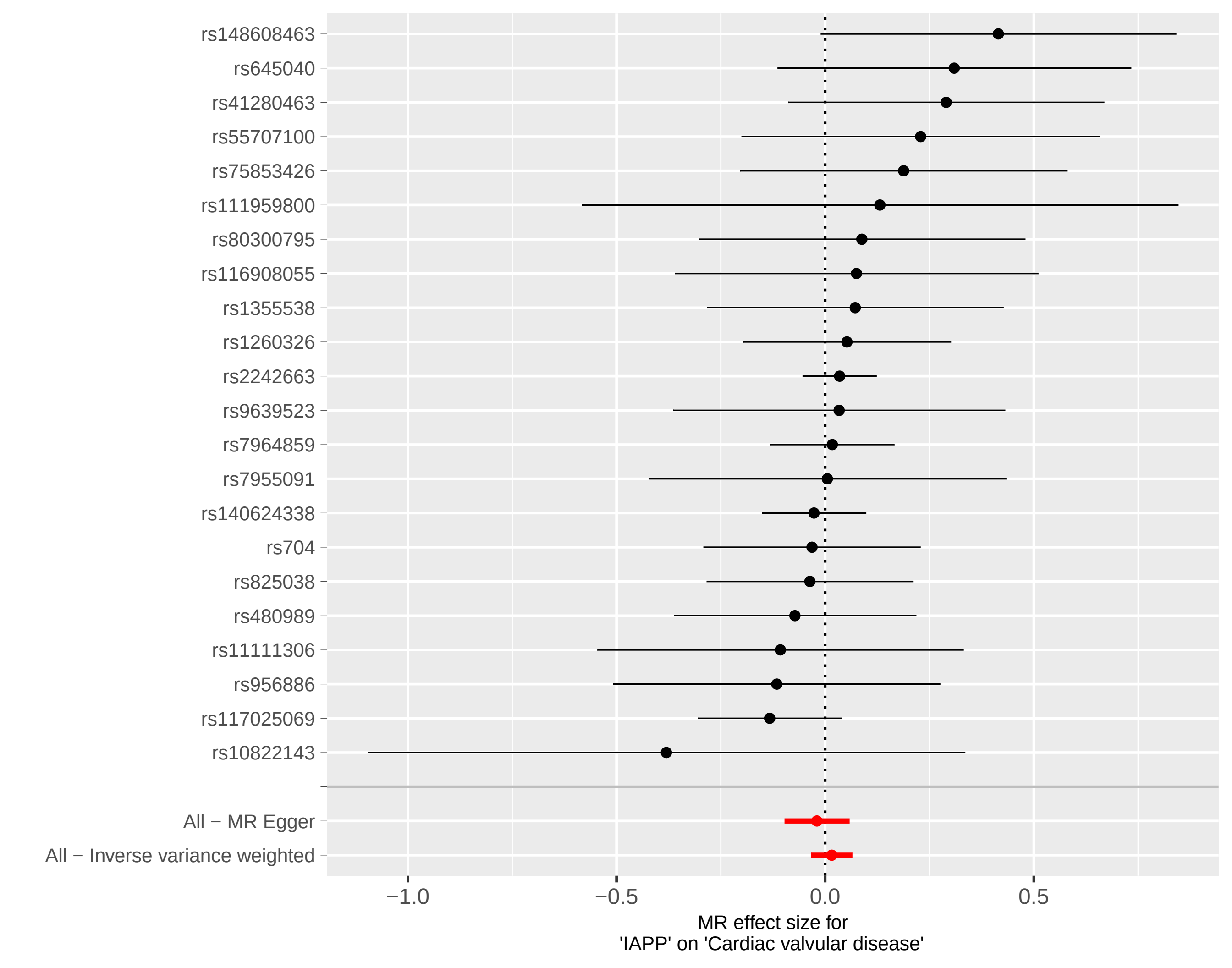
rs148608463
rs645040
rs41280463
rs55707100
rs75853426
rs111959800
rs80300795
rs116908055
rs1355538
rs1260326
rs2242663
rs9639523
rs7964859
rs7955091
rs140624338
rs704
rs825038
rs480989
rs11111306
rs956886
rs117025069
rs10822143
All − MR Egger
All − Inverse variance weighted
−1.0
−0.5
0.0
0.5
MR effect size for
'IAPP' on 'Cardiac valvular disease'

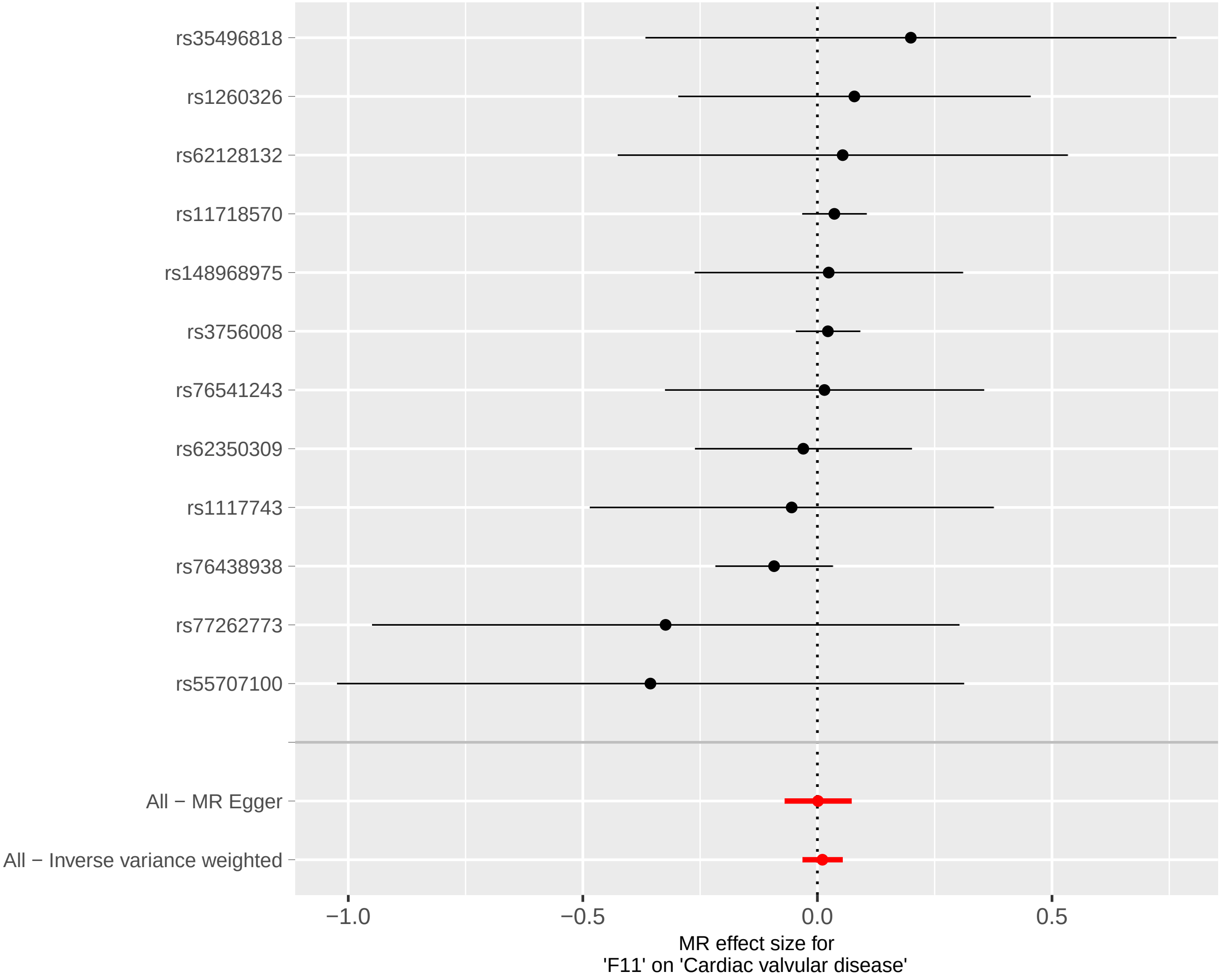

rs35496818
rs1260326
rs62128132
rs11718570
rs148968975
rs3756008
rs76541243
rs62350309
rs1117743
rs76438938
rs77262773
rs55707100
All − MR Egger
All − Inverse variance weighted
−1.0
−0.5
0.0
0.5
MR effect size for
'F11' on 'Cardiac valvular disease'

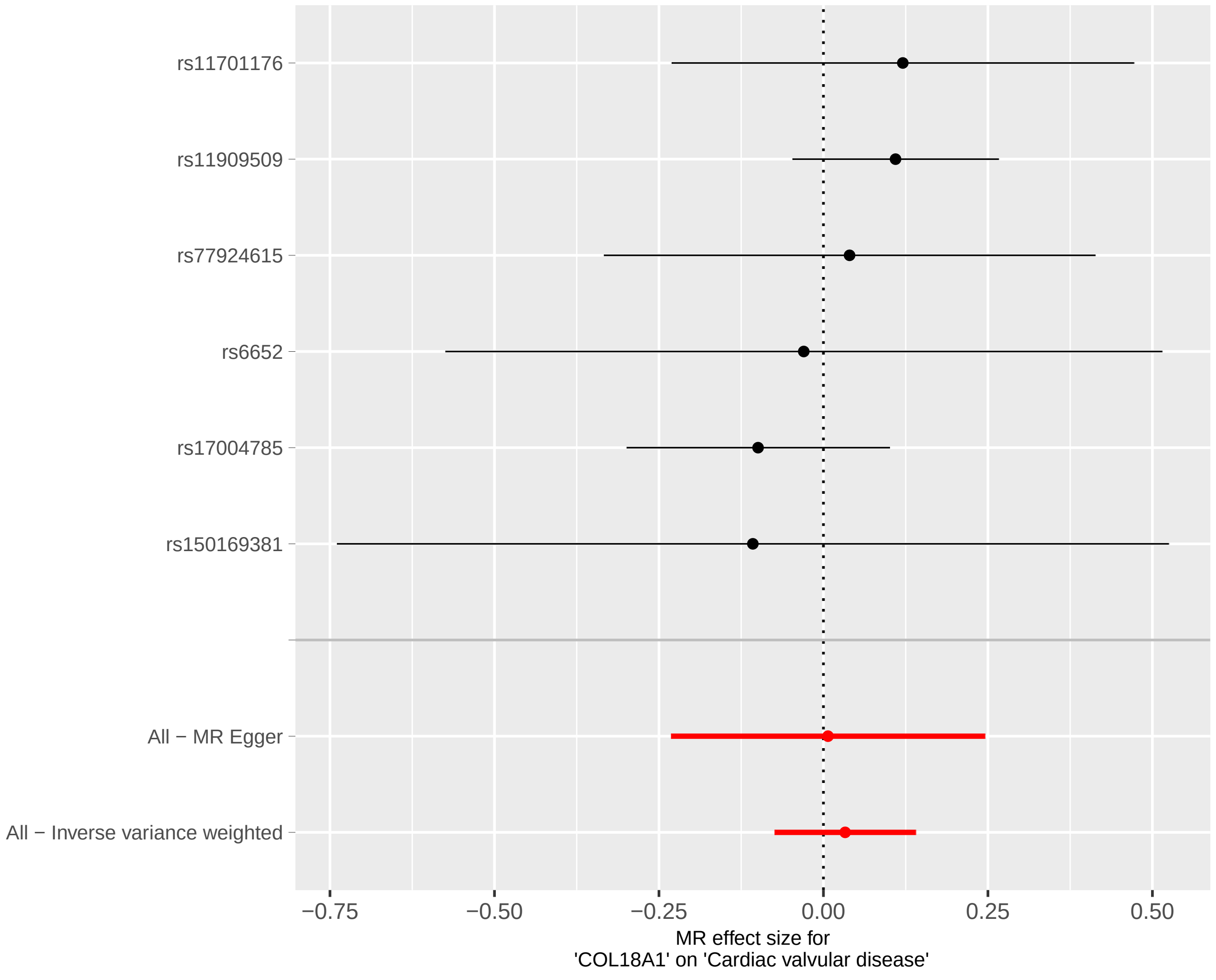

rs11701176
rs11909509
rs77924615
rs6652
rs17004785
rs150169381
All − MR Egger
All − Inverse variance weighted
−0.75
−0.50
−0.25
0.00
0.25
0.50
MR effect size for
'COL18A1' on 'Cardiac valvular disease'

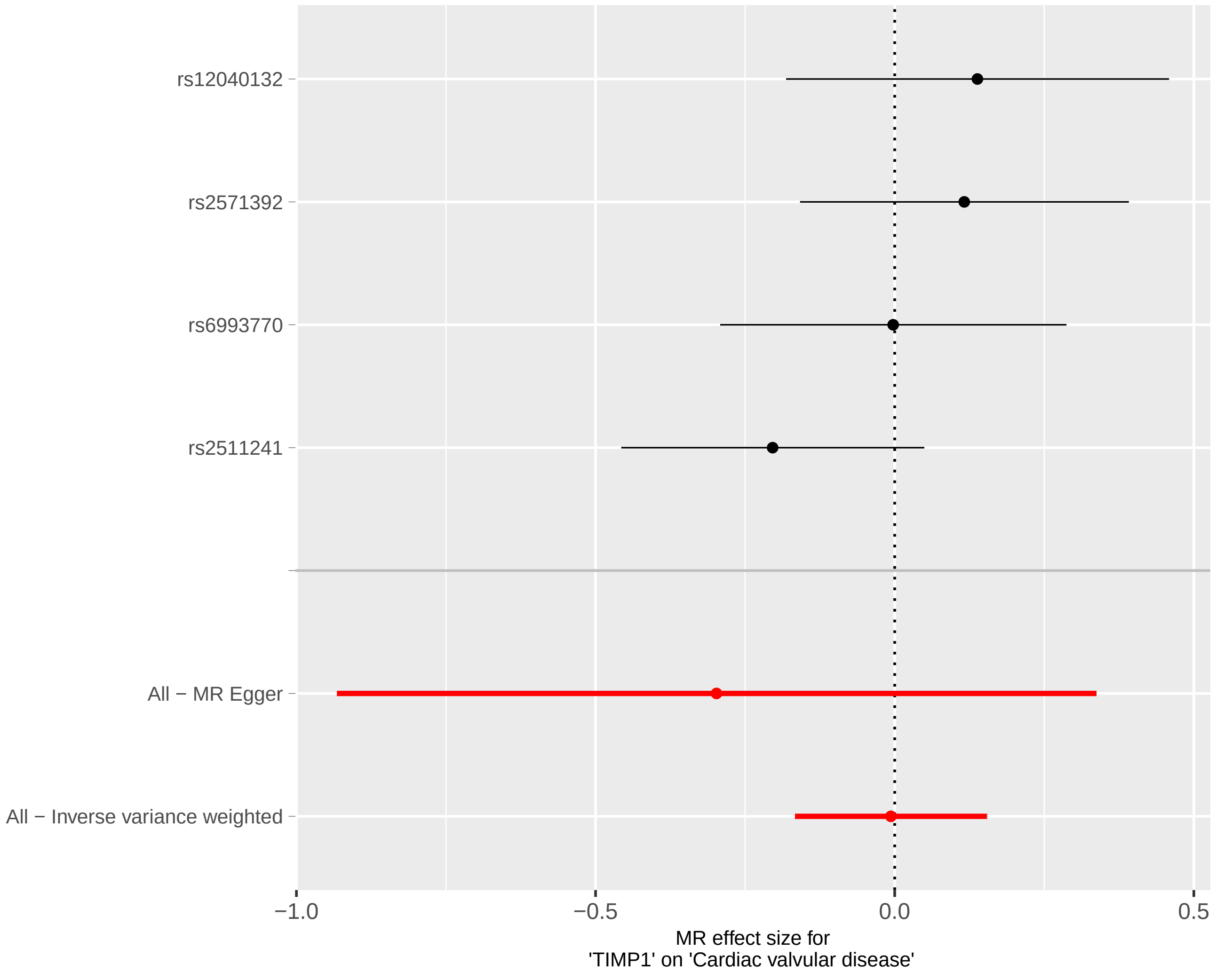

rs12040132
rs2571392
rs6993770
rs2511241
All − MR Egger
All − Inverse variance weighted
−1.0
−0.5
0.0
0.5
MR effect size for
'TIMP1' on 'Cardiac valvular disease'

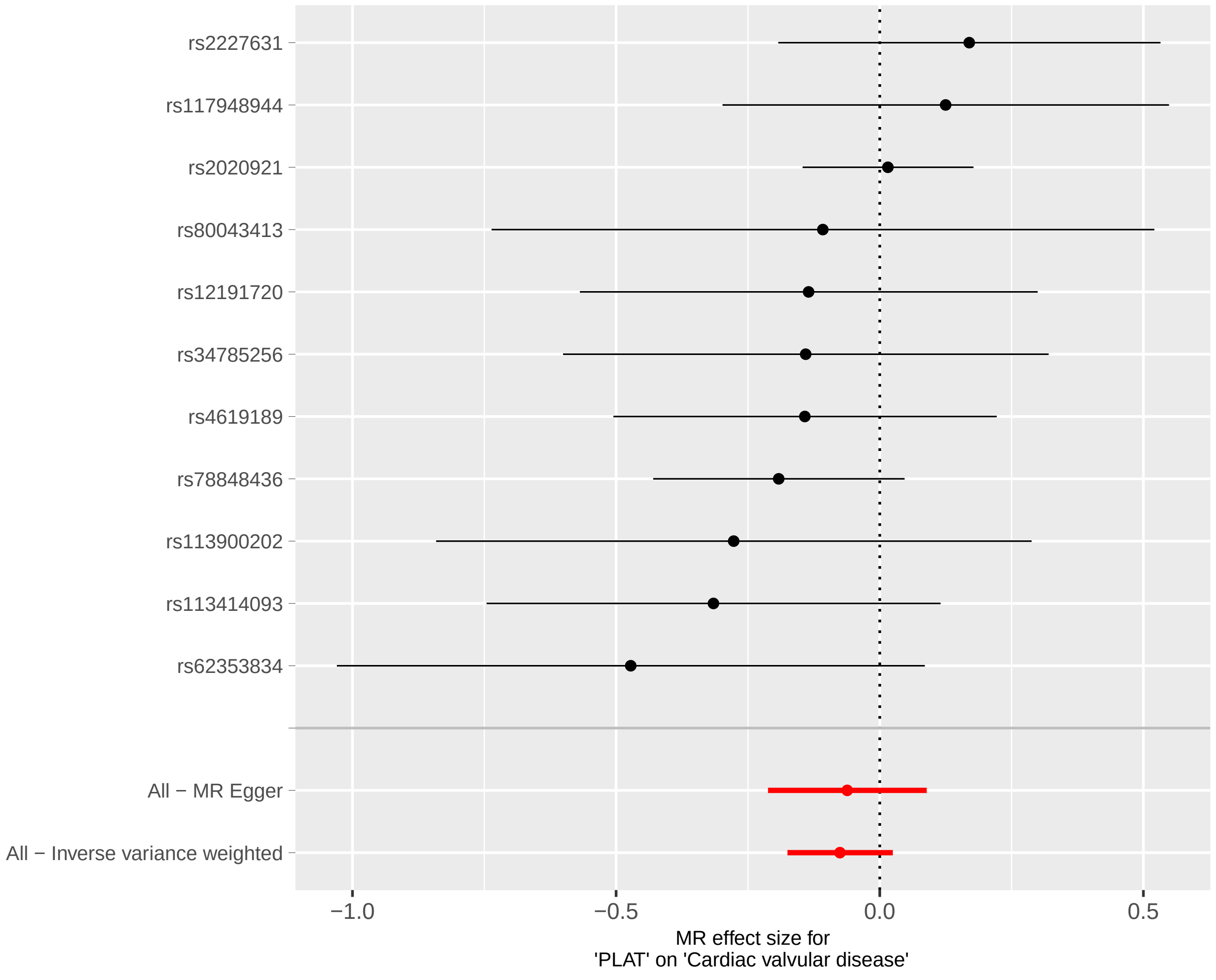

rs2227631
rs117948944
rs2020921
rs80043413
rs12191720
rs34785256
rs4619189
rs78848436
rs113900202
rs113414093
rs62353834
All − MR Egger
All − Inverse variance weighted
−1.0
−0.5
0.0
0.5
MR effect size for
'PLAT' on 'Cardiac valvular disease'

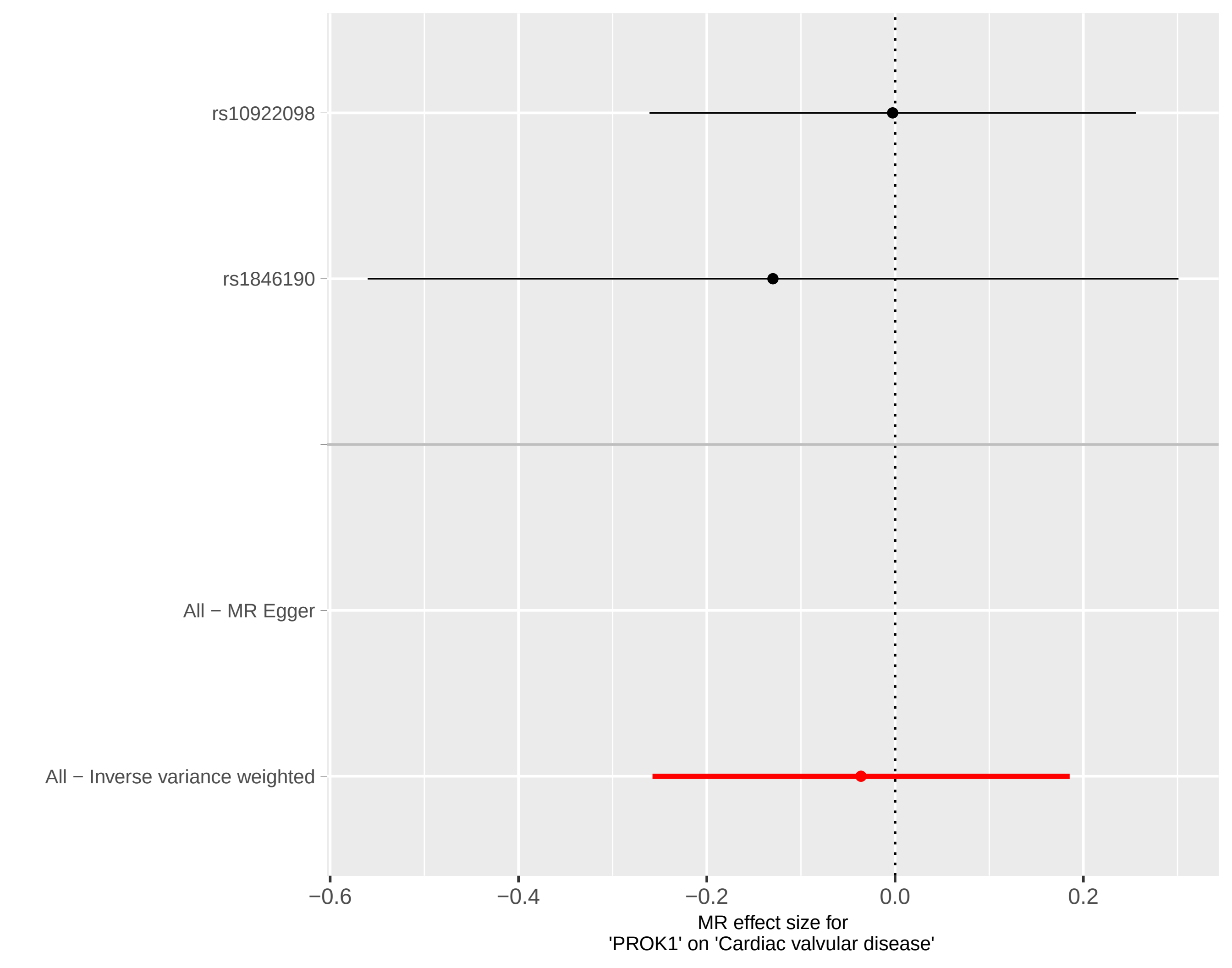
rs10922098
rs1846190
All − MR Egger
All − Inverse variance weighted
−0.6
−0.4
−0.2
0.0
0.2
MR effect size for
'PROK1' on 'Cardiac valvular disease'

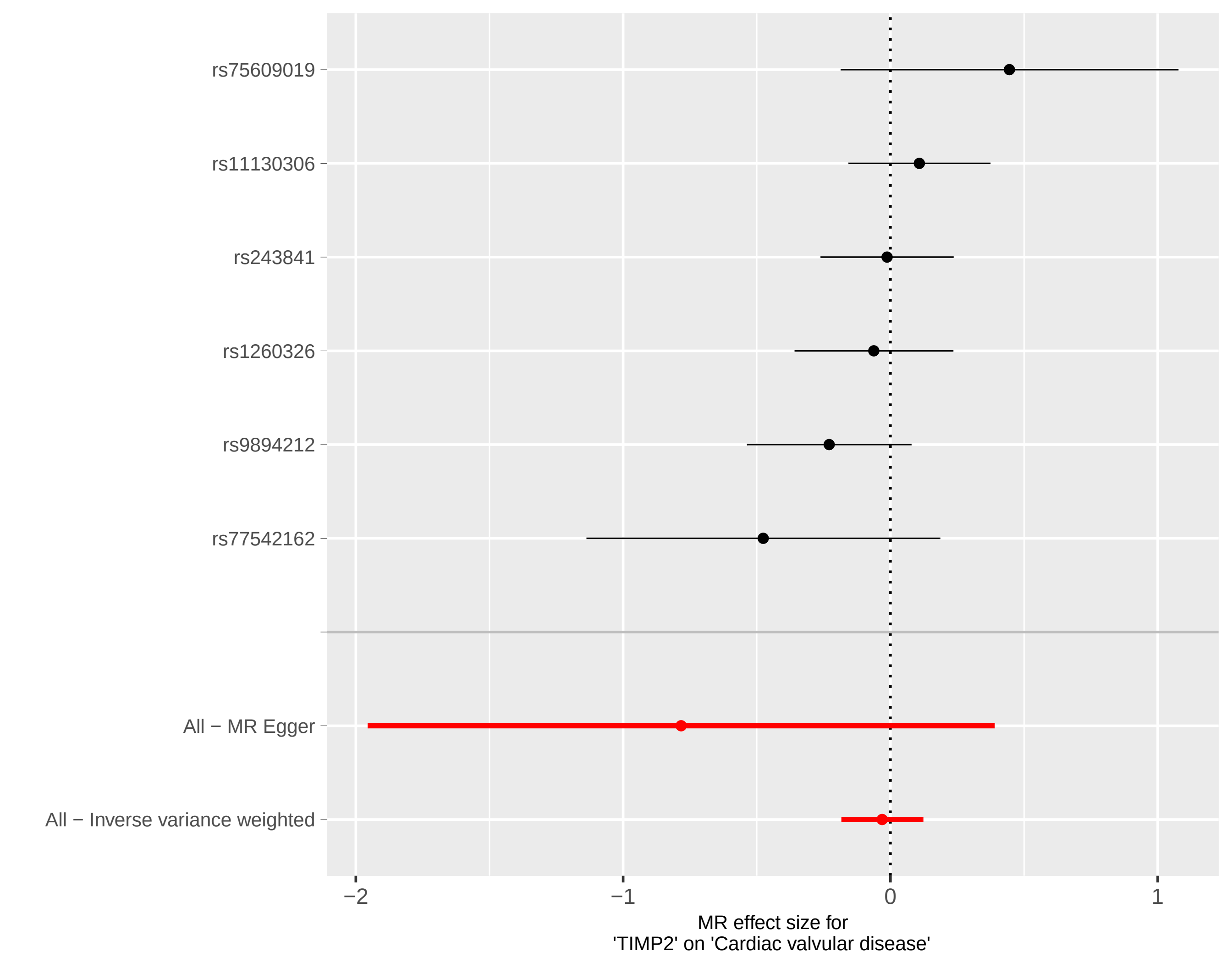
rs75609019
rs11130306
rs243841
rs1260326
rs9894212
rs77542162
All − MR Egger
All − Inverse variance weighted
−2
−1
0
1
MR effect size for
'TIMP2' on 'Cardiac valvular disease'

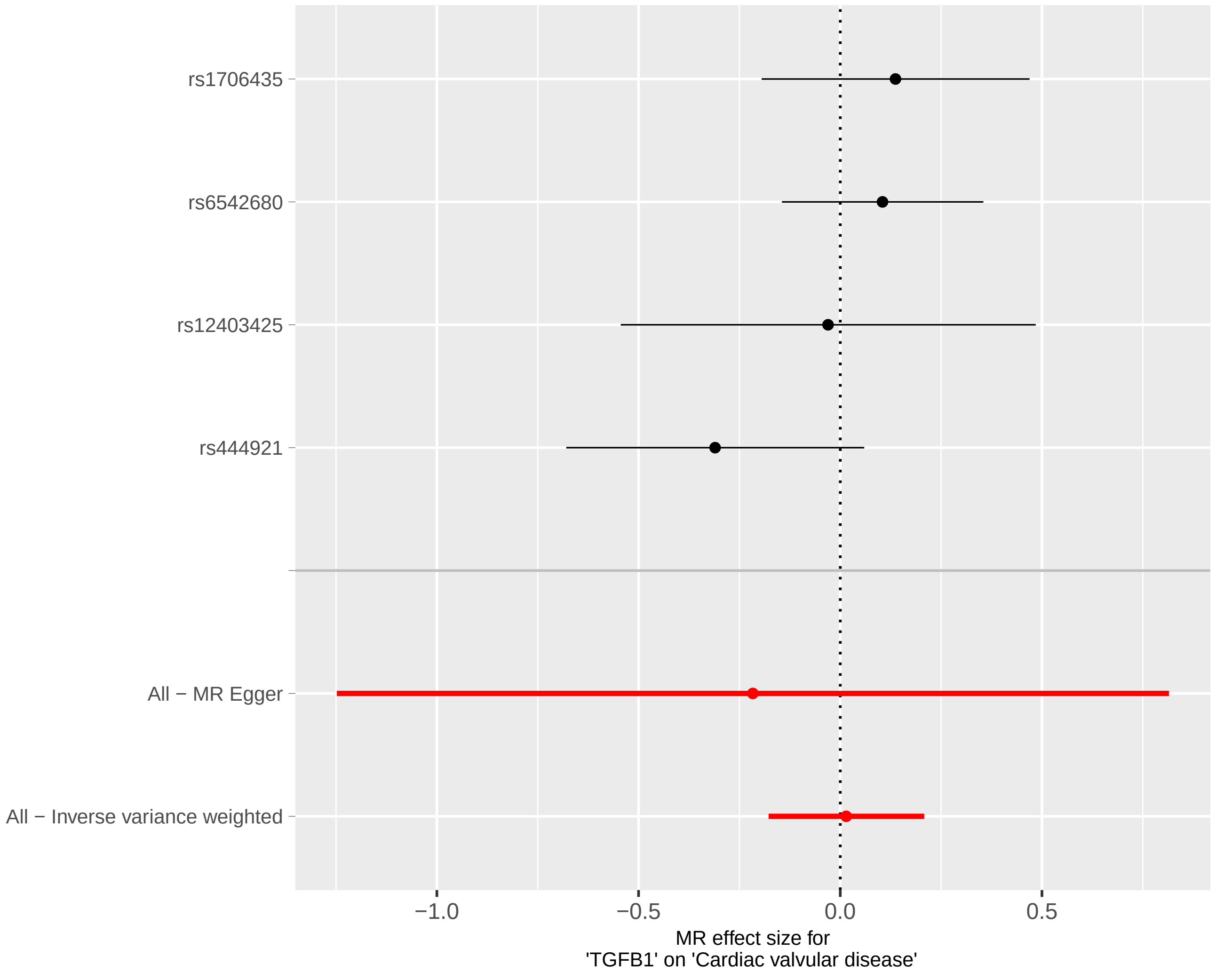
rs1706435
rs6542680
rs12403425
rs444921
All − MR Egger
All − Inverse variance weighted
−1.0
−0.5
0.0
0.5
MR effect size for
'TGFB1' on 'Cardiac valvular disease'

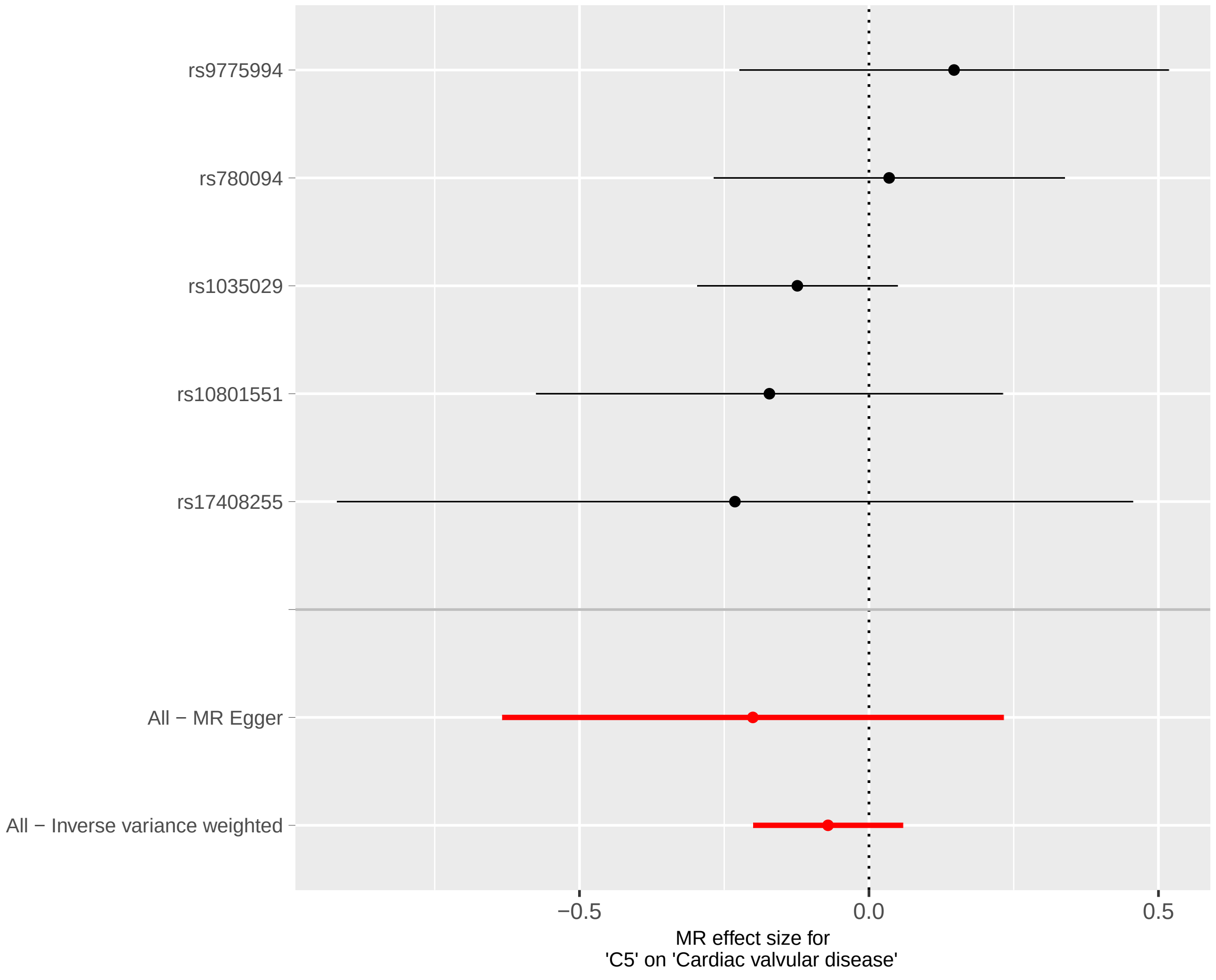

rs9775994
rs780094
rs1035029
rs10801551
rs17408255
All − MR Egger
All − Inverse variance weighted
−0.5
0.0
0.5
MR effect size for
'C5' on 'Cardiac valvular disease'

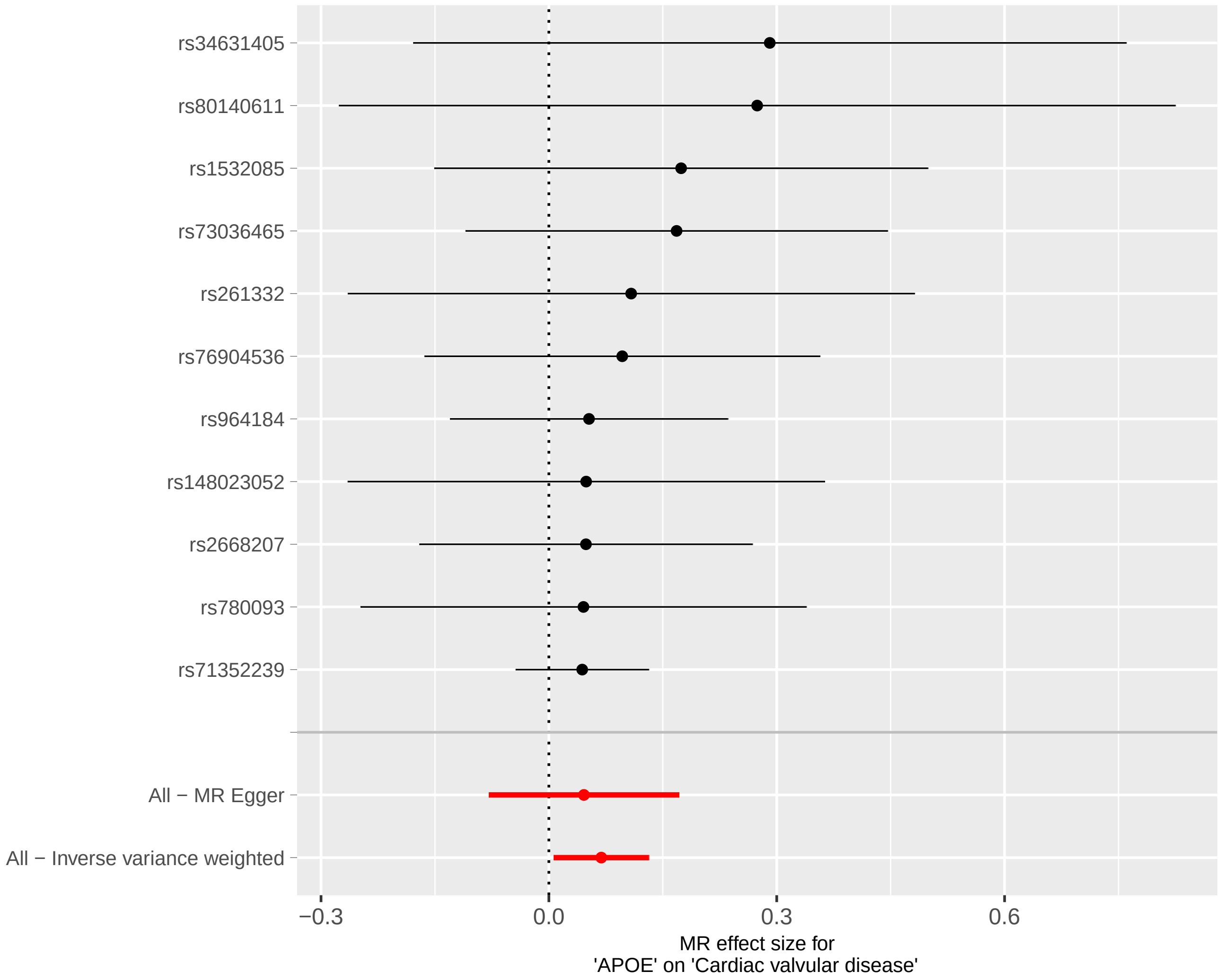
rs34631405
rs80140611
rs1532085
rs73036465
rs261332
rs76904536
rs964184
rs148023052
rs2668207
rs780093
rs71352239
All − MR Egger
All − Inverse variance weighted
−0.3
0.0
0.3
0.6
MR effect size for
'APOE' on 'Cardiac valvular disease'

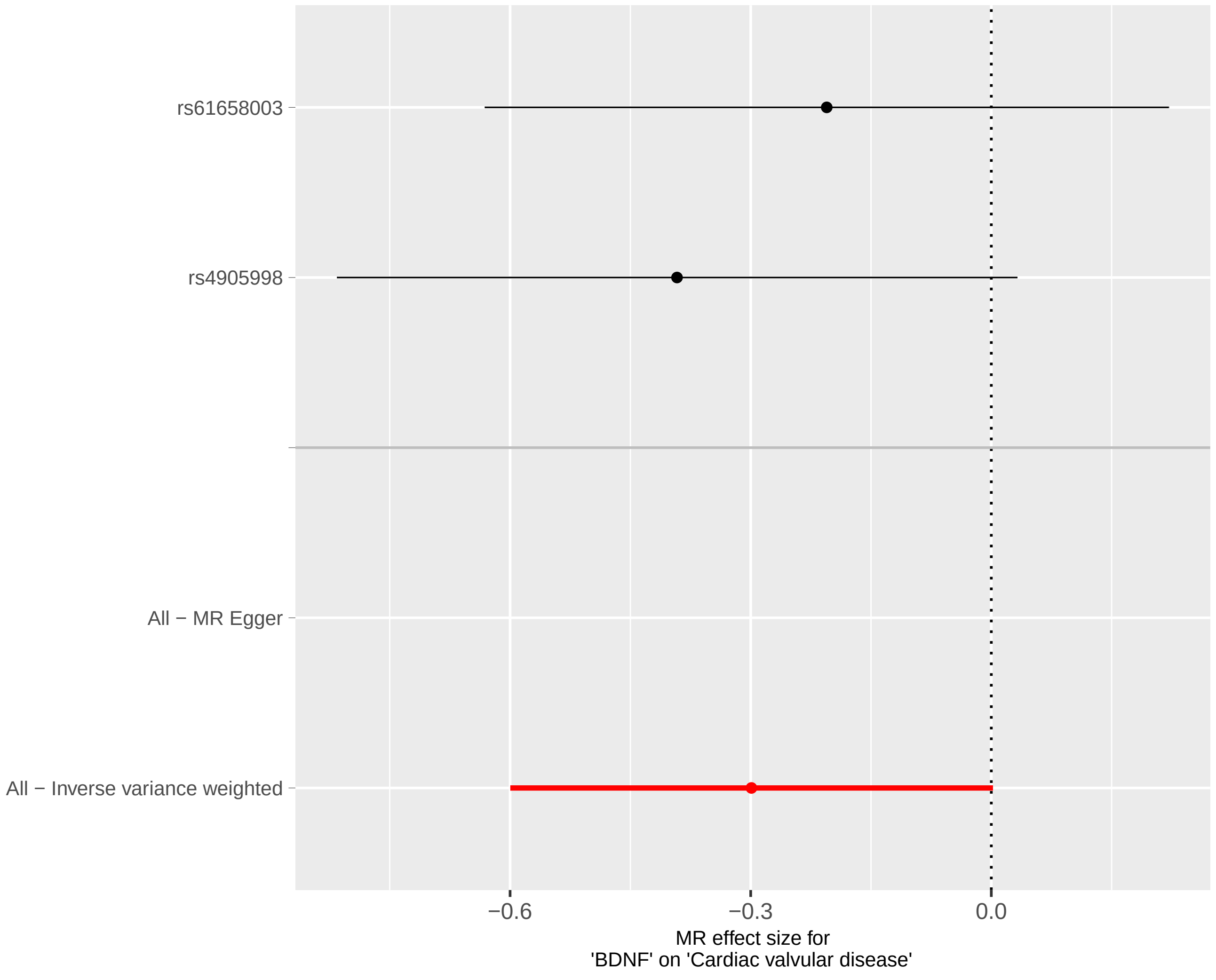
rs61658003
rs4905998
All − MR Egger
All − Inverse variance weighted
−0.6
−0.3
0.0
MR effect size for
'BDNF' on 'Cardiac valvular disease'

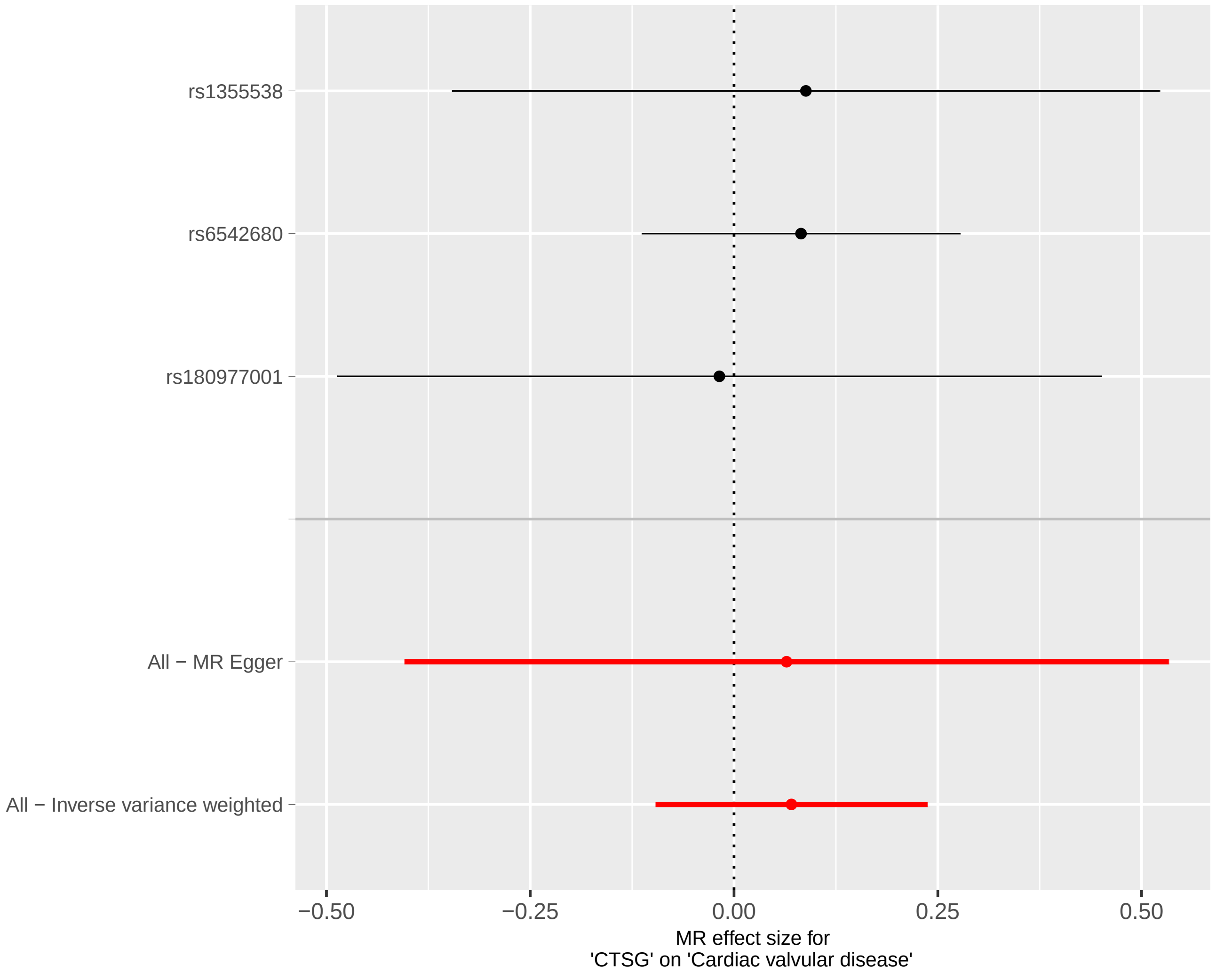
rs1355538
rs6542680
rs180977001
All − MR Egger
All − Inverse variance weighted
−0.50
−0.25
0.00
0.25
0.50
MR effect size for
'CTSG' on 'Cardiac valvular disease'

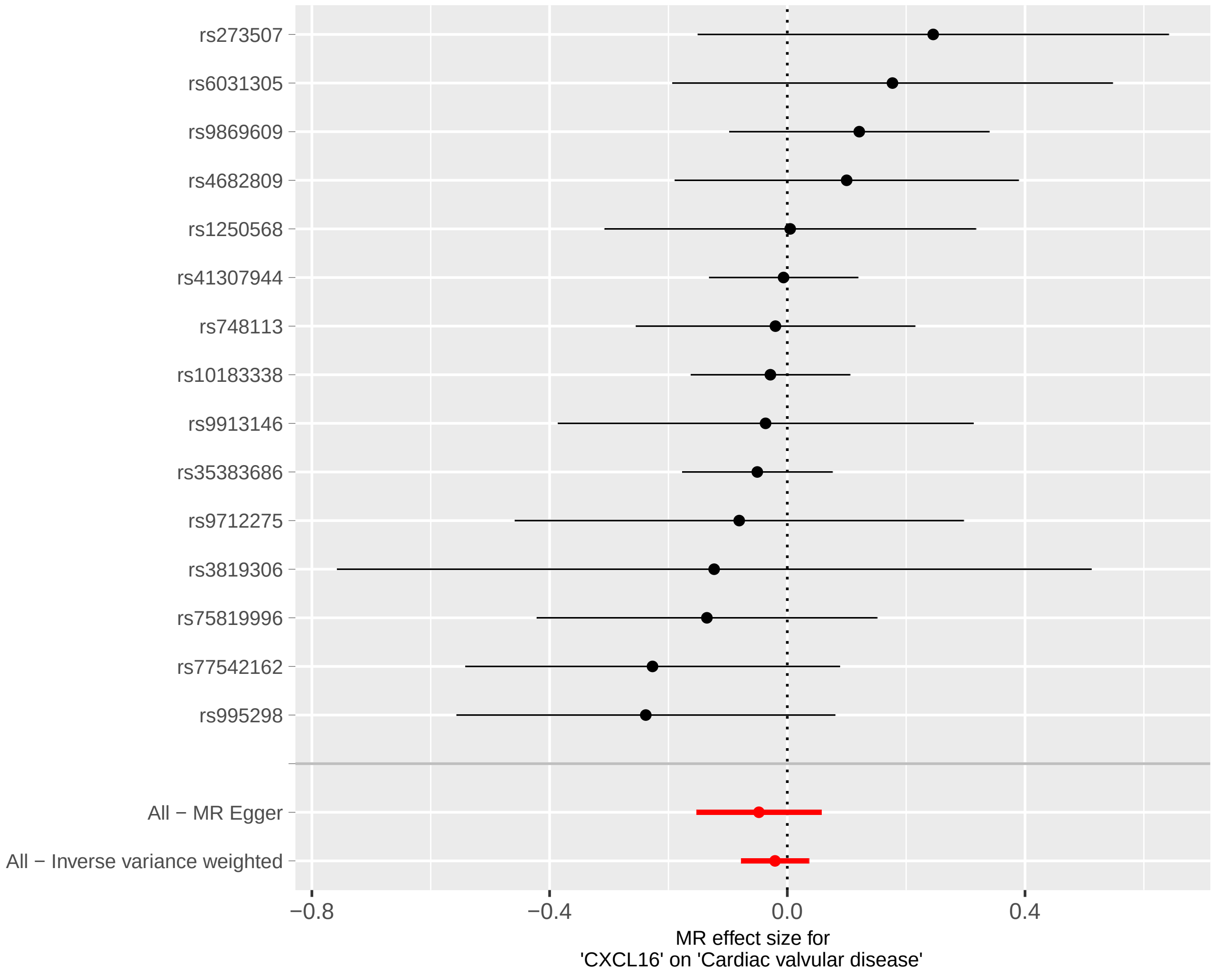

rs273507
rs6031305
rs9869609
rs4682809
rs1250568
rs41307944
rs748113
rs10183338
rs9913146
rs35383686
rs9712275
rs3819306
rs75819996
rs77542162
rs995298
All − MR Egger
All − Inverse variance weighted
−0.8
−0.4
0.0
0.4
MR effect size for
'CXCL16' on 'Cardiac valvular disease'

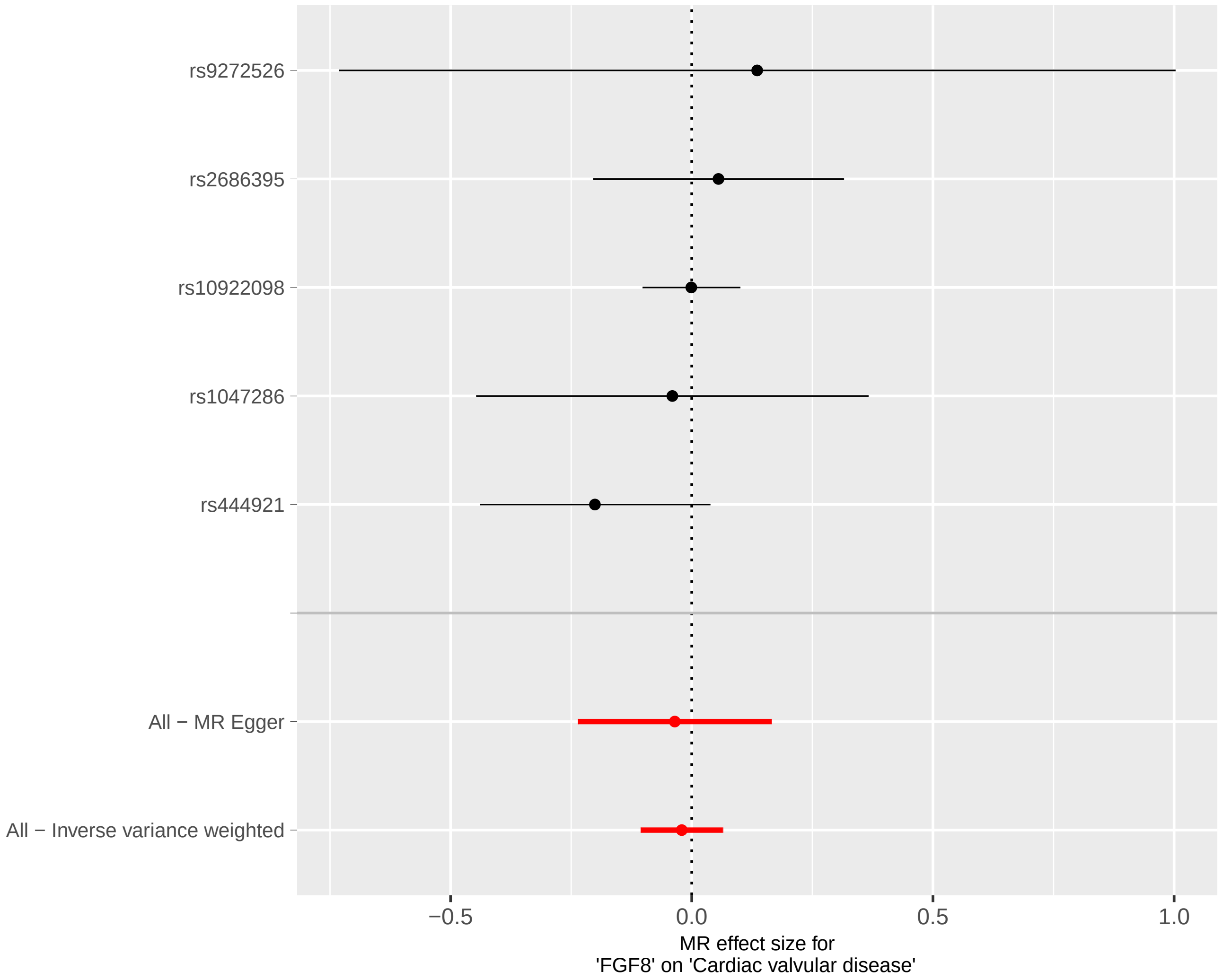
rs9272526
rs2686395
rs10922098
rs1047286
rs444921
All − MR Egger
All − Inverse variance weighted
−0.5
0.0
0.5
1.0
MR effect size for
'FGF8' on 'Cardiac valvular disease'

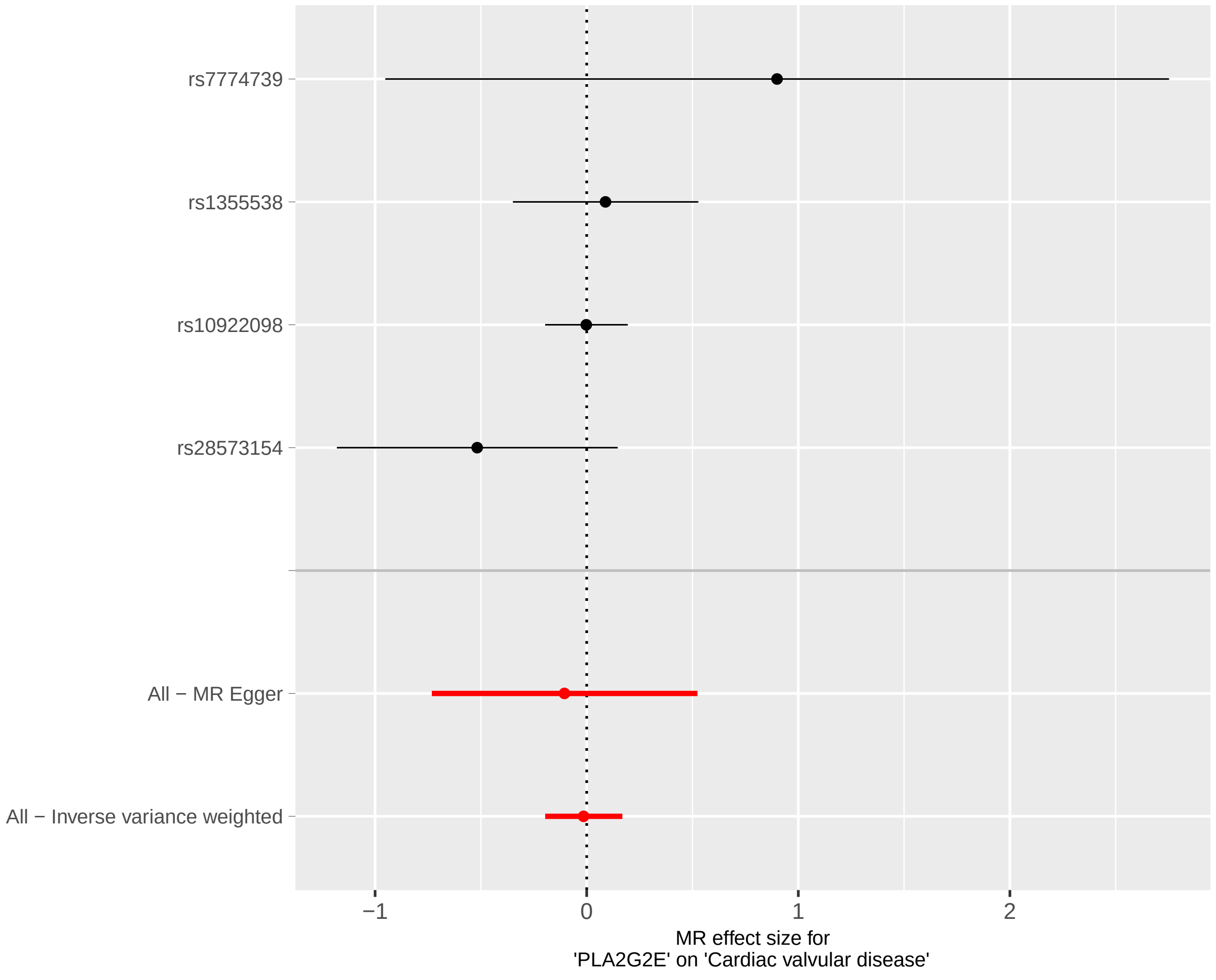

rs7774739
rs1355538
rs10922098
rs28573154
All − MR Egger
All − Inverse variance weighted
−1
0
1
2
MR effect size for
'PLA2G2E' on 'Cardiac valvular disease'

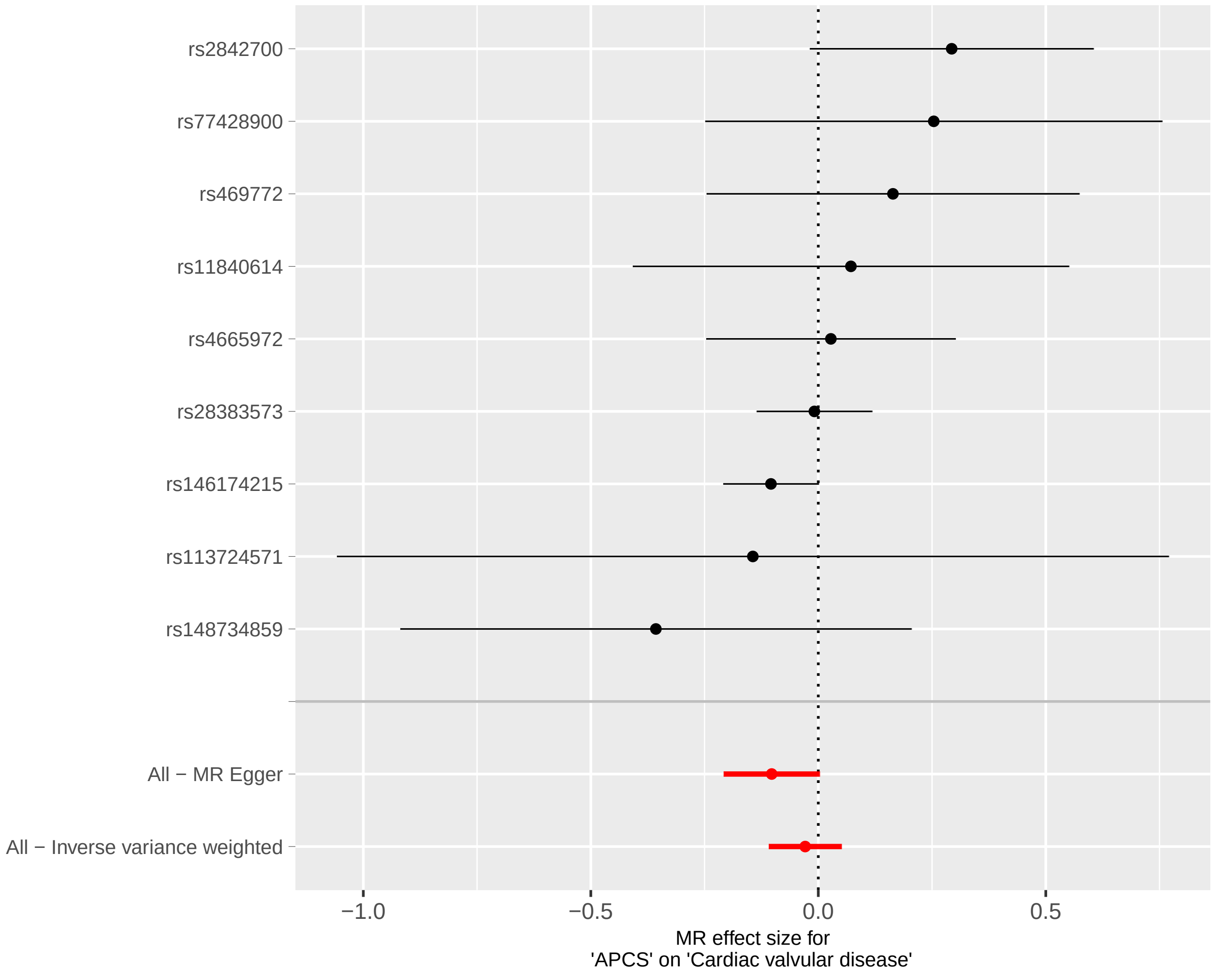
rs2842700
rs77428900
rs469772
rs11840614
rs4665972
rs28383573
rs146174215
rs113724571
rs148734859
All − MR Egger
All − Inverse variance weighted
−1.0
−0.5
0.0
0.5
MR effect size for
'APCS' on 'Cardiac valvular disease'

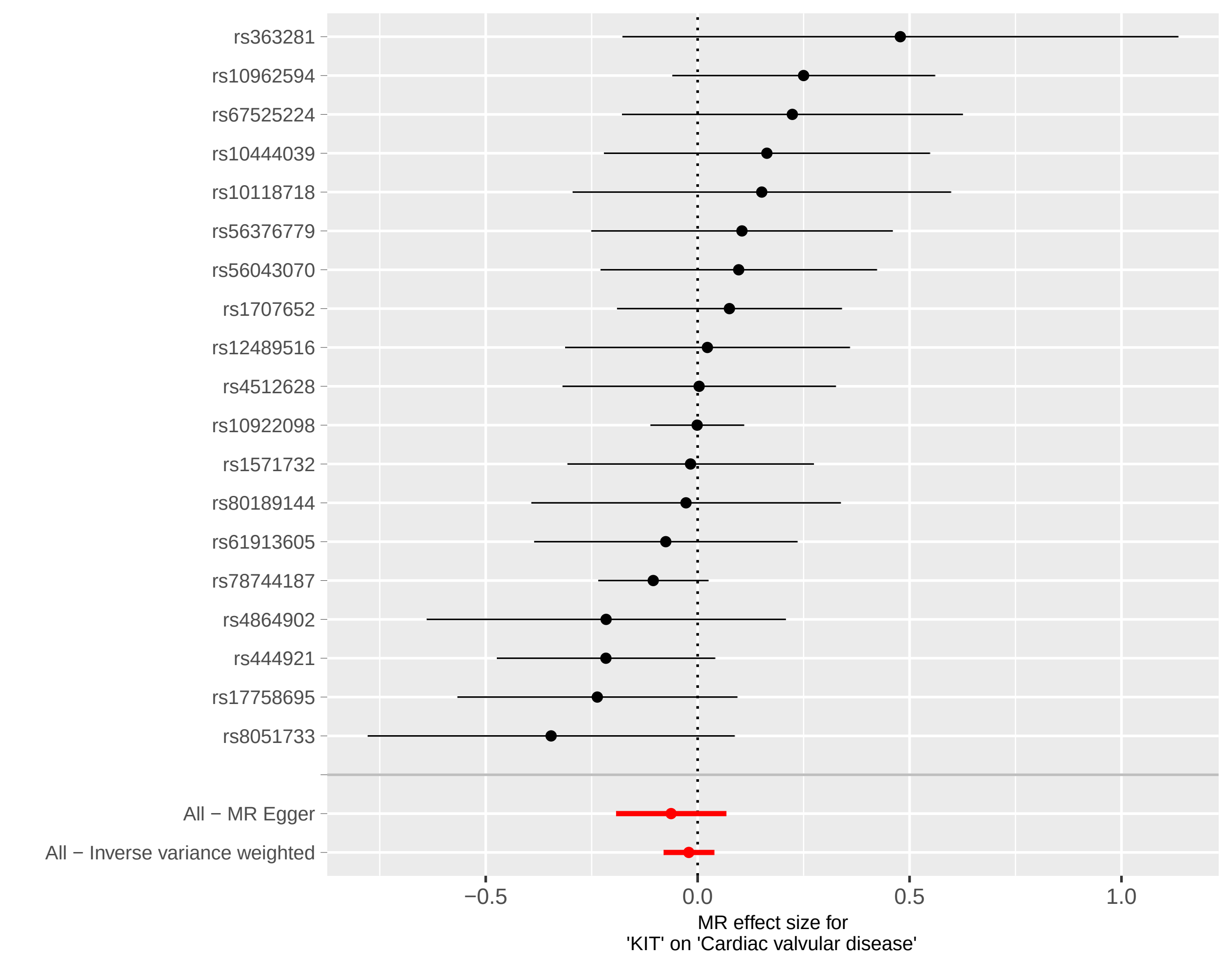
rs363281
rs10962594
rs67525224
rs10444039
rs10118718
rs56376779
rs56043070
rs1707652
rs12489516
rs4512628
rs10922098
rs1571732
rs80189144
rs61913605
rs78744187
rs4864902
rs444921
rs17758695
rs8051733
All − MR Egger
All − Inverse variance weighted
−0.5
0.0
0.5
1.0
MR effect size for
'KIT' on 'Cardiac valvular disease'

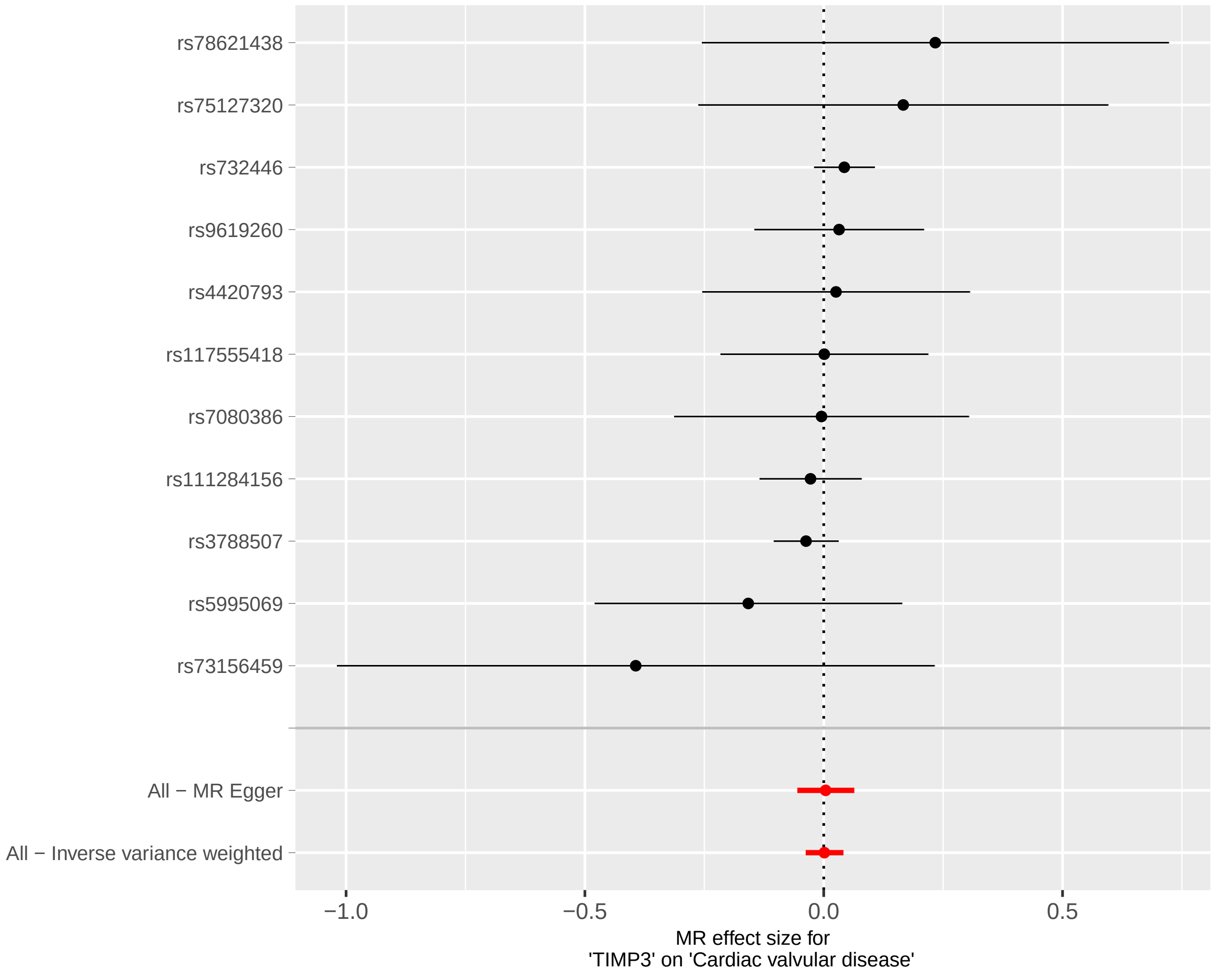

rs78621438
rs75127320
rs732446
rs9619260
rs4420793
rs117555418
rs7080386
rs111284156
rs3788507
rs5995069
rs73156459
All − MR Egger
All − Inverse variance weighted
−1.0
−0.5
0.0
0.5
MR effect size for
'TIMP3' on 'Cardiac valvular disease'

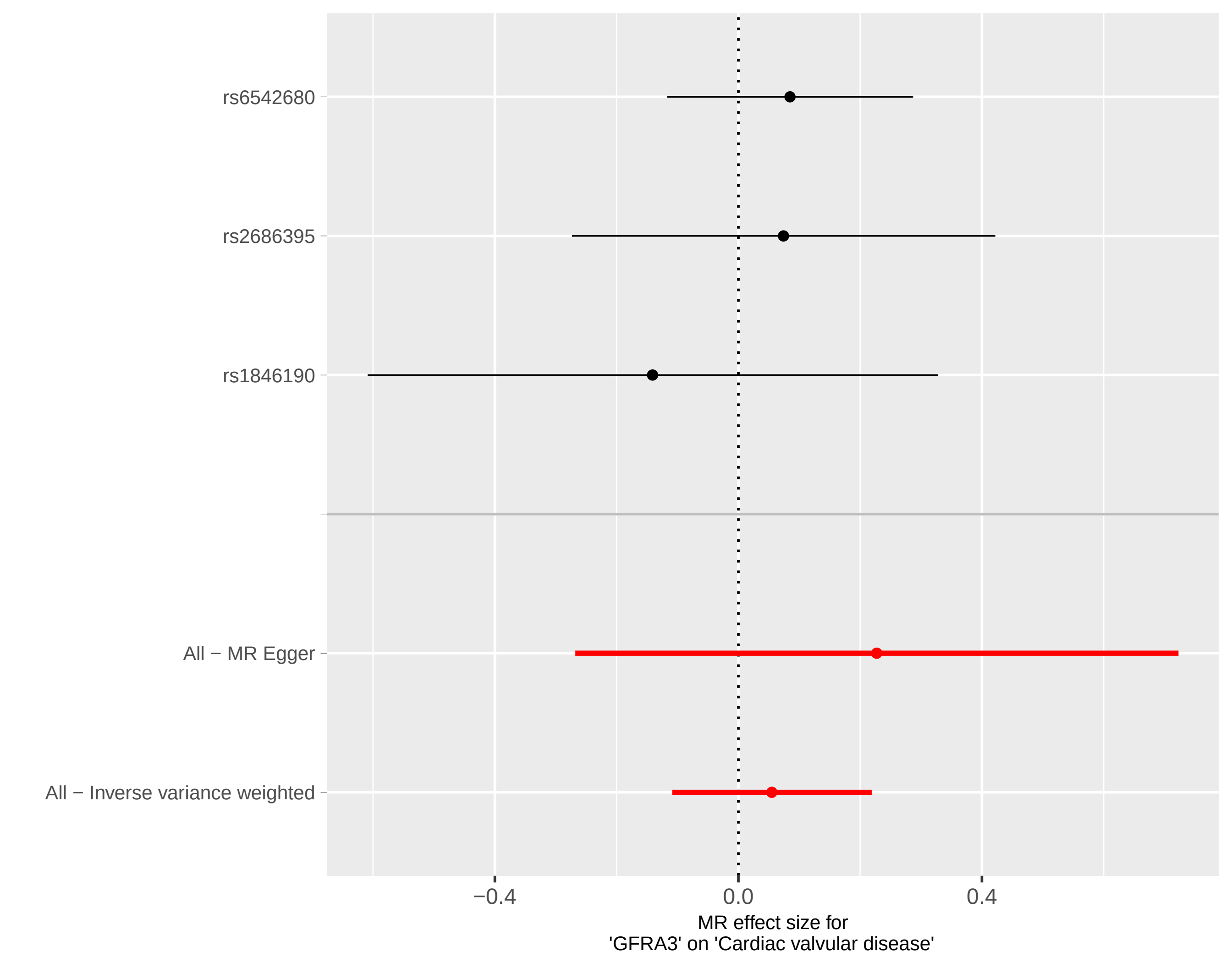
rs6542680
rs2686395
rs1846190
All − MR Egger
All − Inverse variance weighted
−0.4
0.0
0.4
MR effect size for
'GFRA3' on 'Cardiac valvular disease'

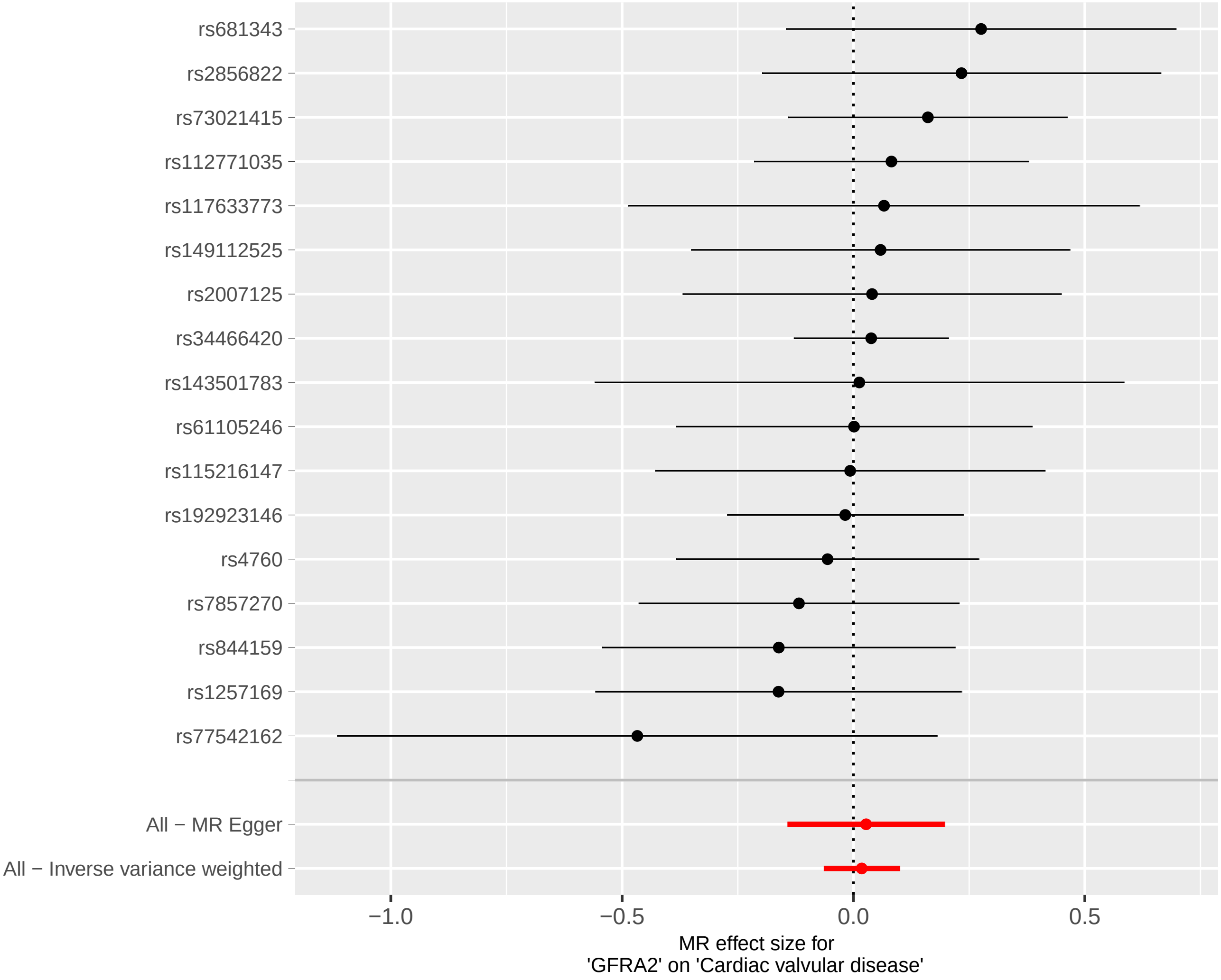

rs681343
rs2856822
rs73021415
rs112771035
rs117633773
rs149112525
rs2007125
rs34466420
rs143501783
rs61105246
rs115216147
rs192923146
rs4760
rs7857270
rs844159
rs1257169
rs77542162
All − MR Egger
All − Inverse variance weighted
−1.0
−0.5
0.0
0.5
MR effect size for
'GFRA2' on 'Cardiac valvular disease'

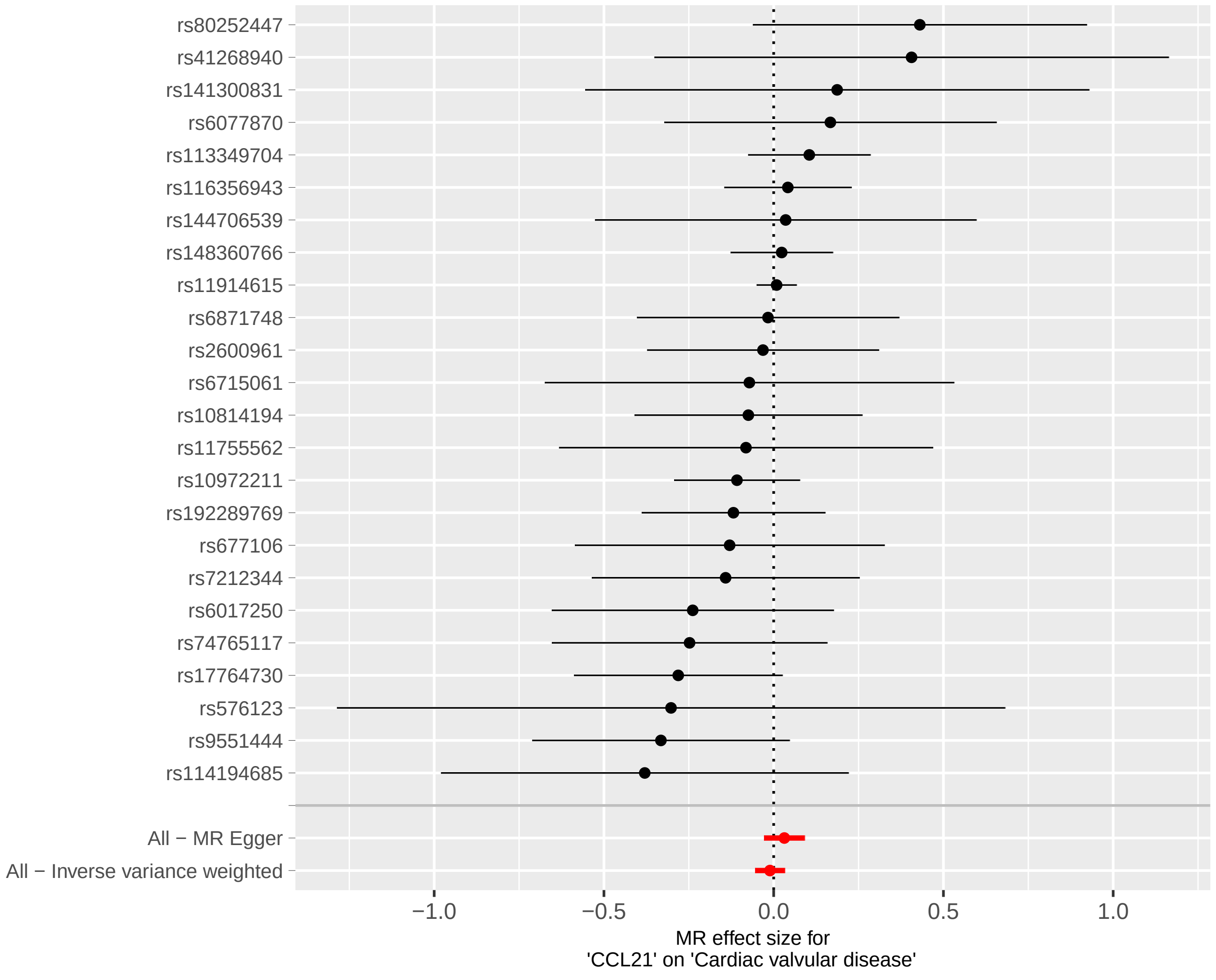

rs80252447
rs41268940
rs141300831
rs6077870
rs113349704
rs116356943
rs144706539
rs148360766
rs11914615
rs6871748
rs2600961
rs6715061
rs10814194
rs11755562
rs10972211
rs192289769
rs677106
rs7212344
rs6017250
rs74765117
rs17764730
rs576123
rs9551444
rs114194685
All − MR Egger
All − Inverse variance weighted
−1.0
−0.5
0.0
0.5
1.0
MR effect size for
'CCL21' on 'Cardiac valvular disease'

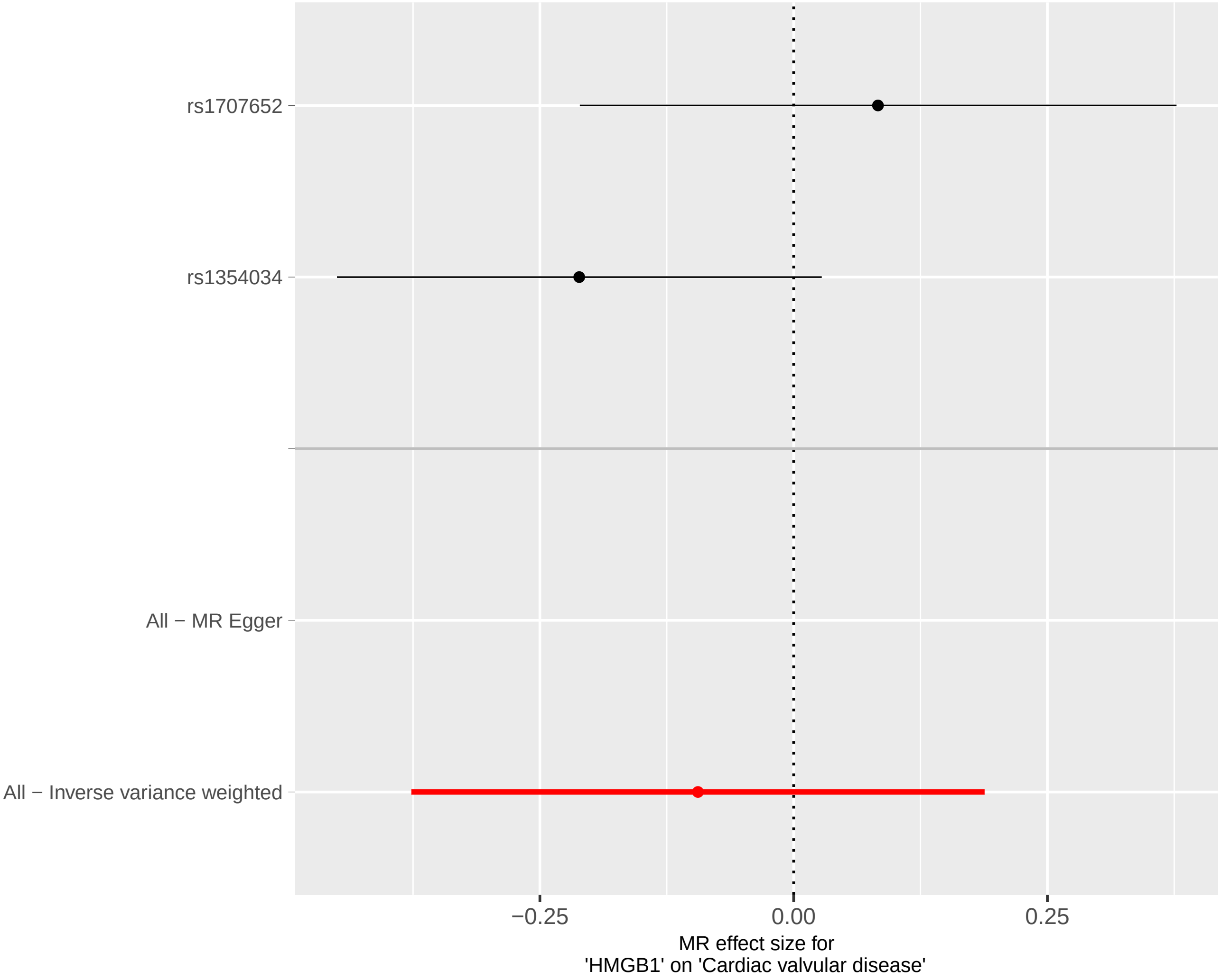

rs1707652
rs1354034
All − MR Egger
All − Inverse variance weighted
−0.25
0.00
0.25
MR effect size for
'HMGB1' on 'Cardiac valvular disease'

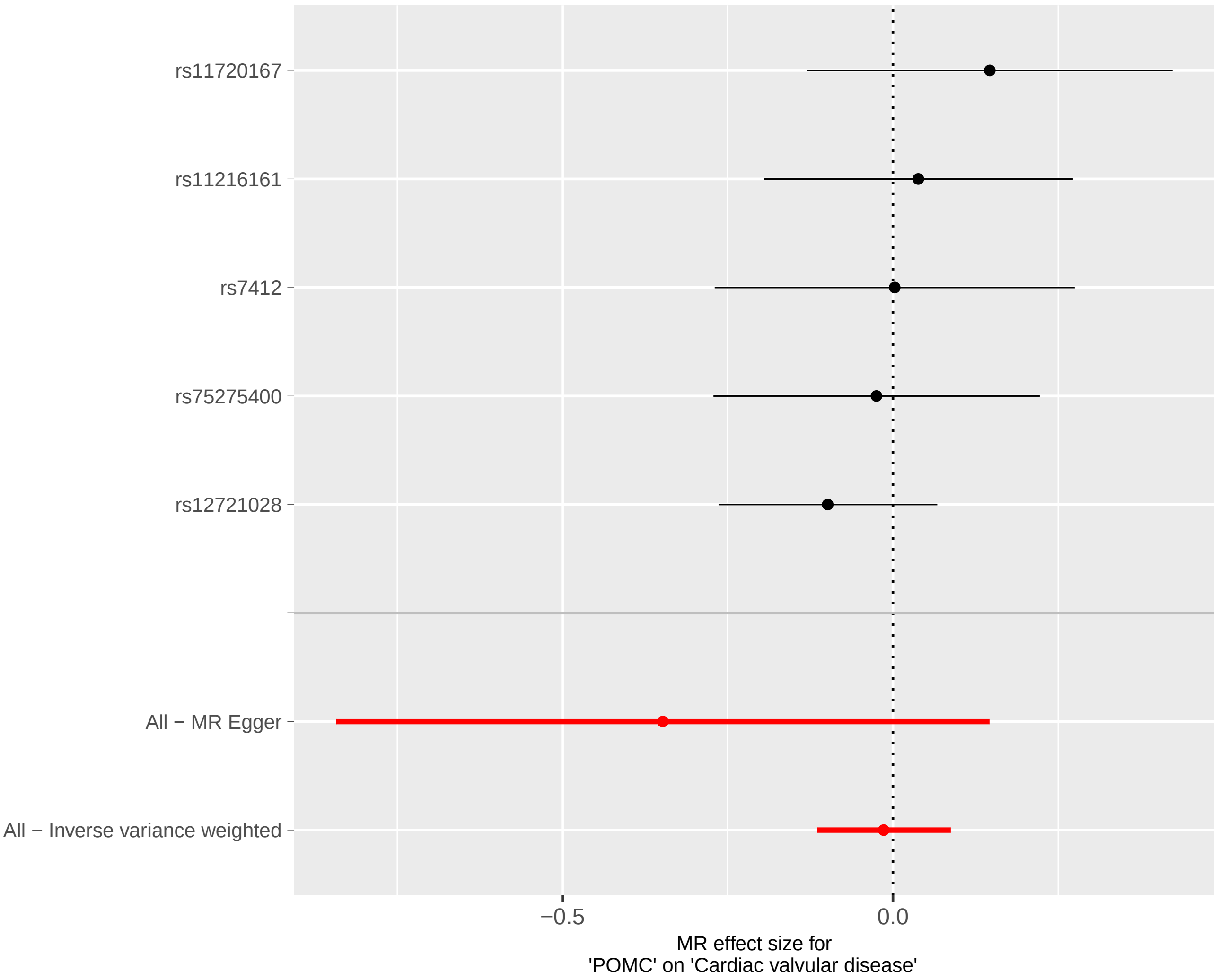
rs11720167
rs11216161
rs7412
rs75275400
rs12721028
All − MR Egger
All − Inverse variance weighted
−0.5
0.0
MR effect size for
'POMC' on 'Cardiac valvular disease'

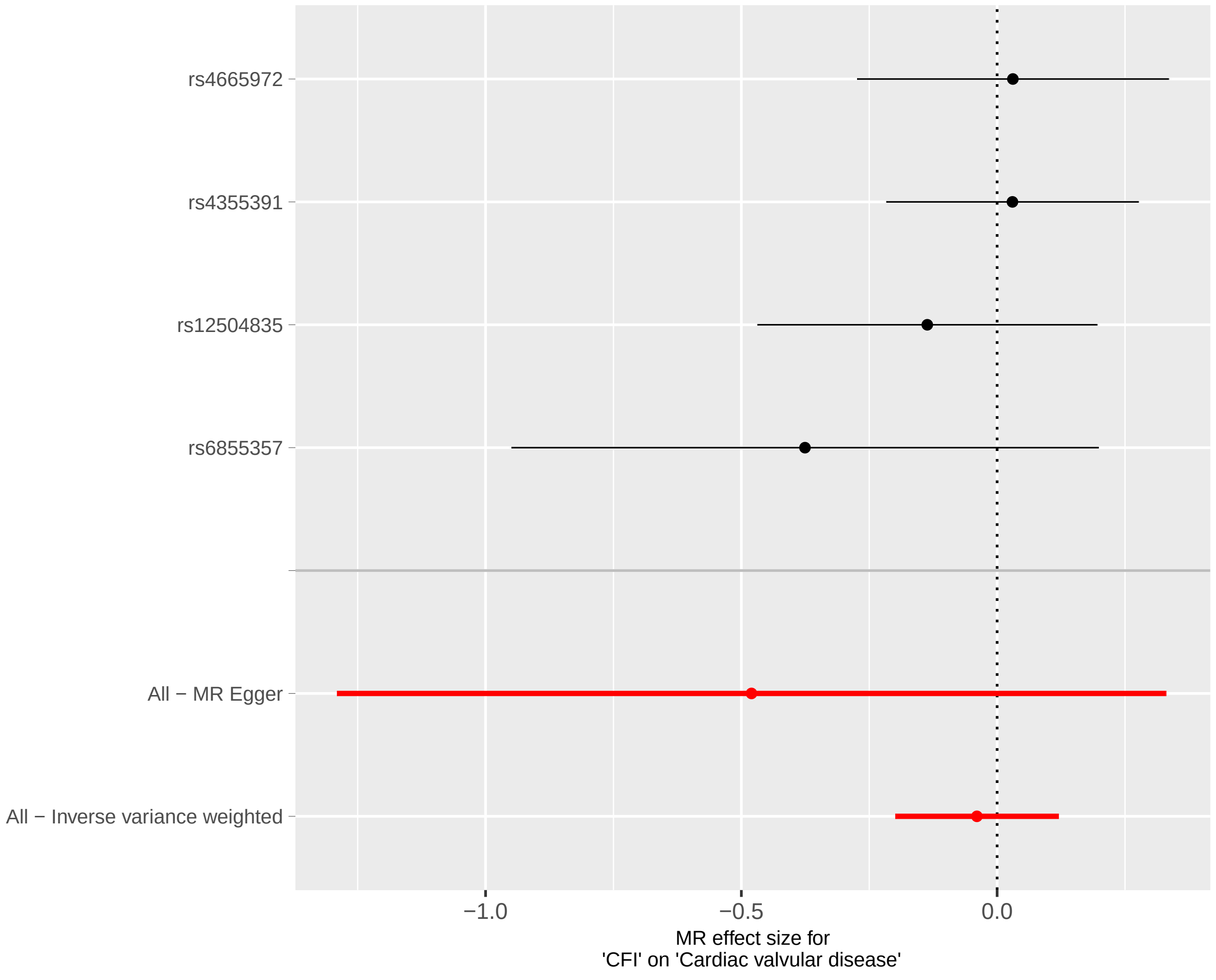
rs4665972
rs4355391
rs12504835
rs6855357
All − MR Egger
All − Inverse variance weighted
−1.0
−0.5
0.0
MR effect size for
'CFI' on 'Cardiac valvular disease'

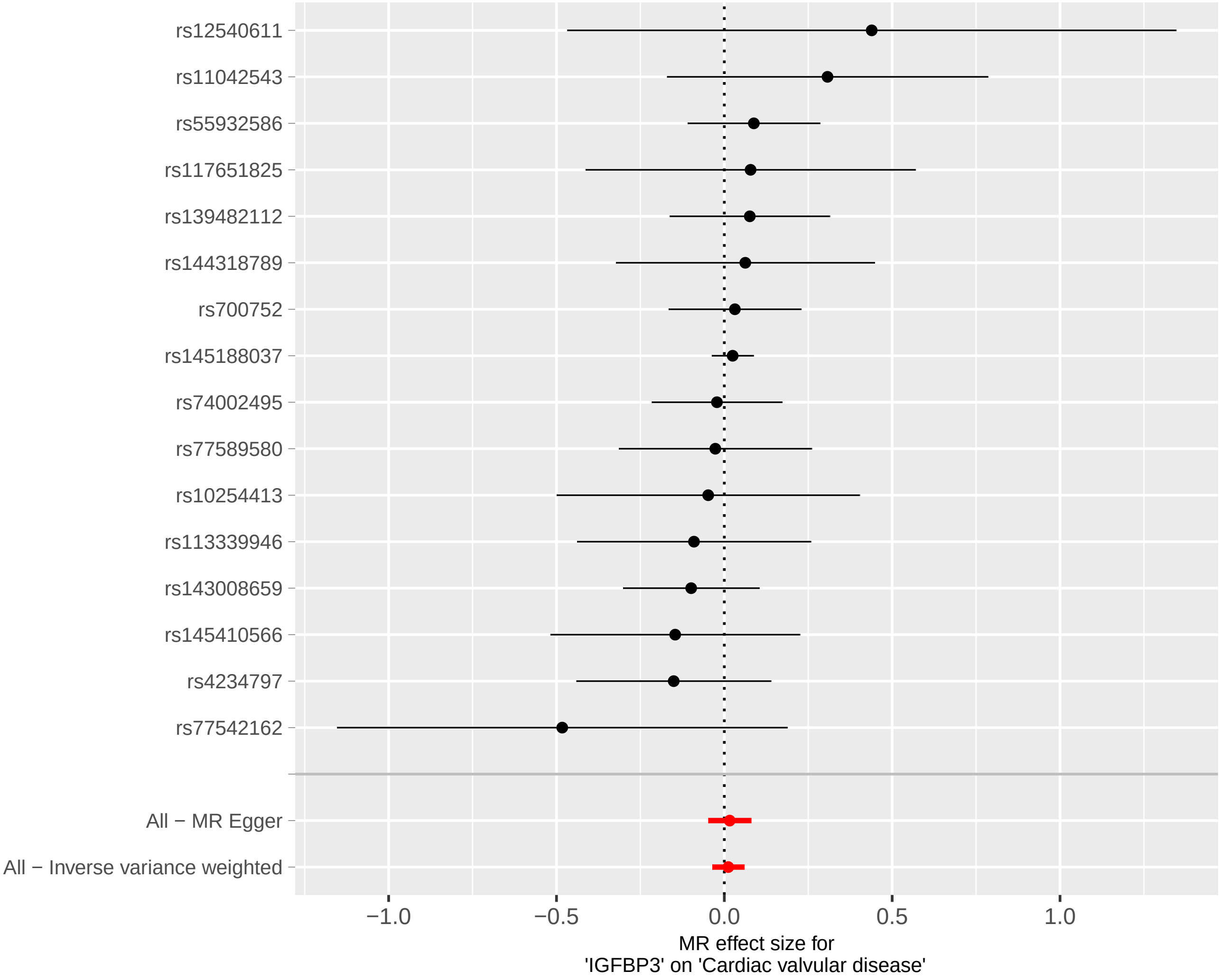
rs12540611
rs11042543
rs55932586
rs117651825
rs139482112
rs144318789
rs700752
rs145188037
rs74002495
rs77589580
rs10254413
rs113339946
rs143008659
rs145410566
rs4234797
rs77542162
All − MR Egger
All − Inverse variance weighted
−1.0
−0.5
0.0
0.5
1.0
MR effect size for
'IGFBP3' on 'Cardiac valvular disease'

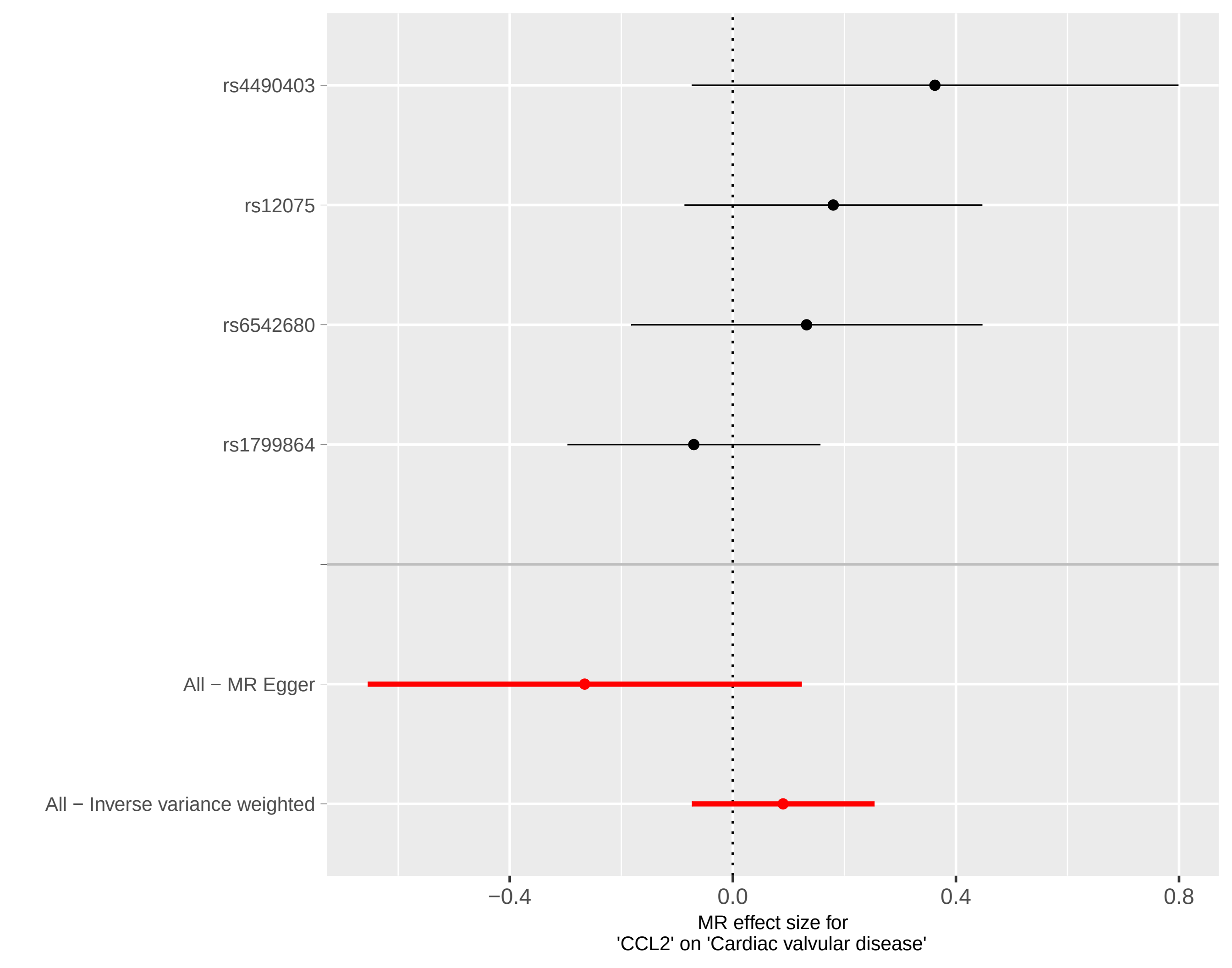
rs4490403
rs12075
rs6542680
rs1799864
All − MR Egger
All − Inverse variance weighted
−0.4
0.0
0.4
0.8
MR effect size for
'CCL2' on 'Cardiac valvular disease'

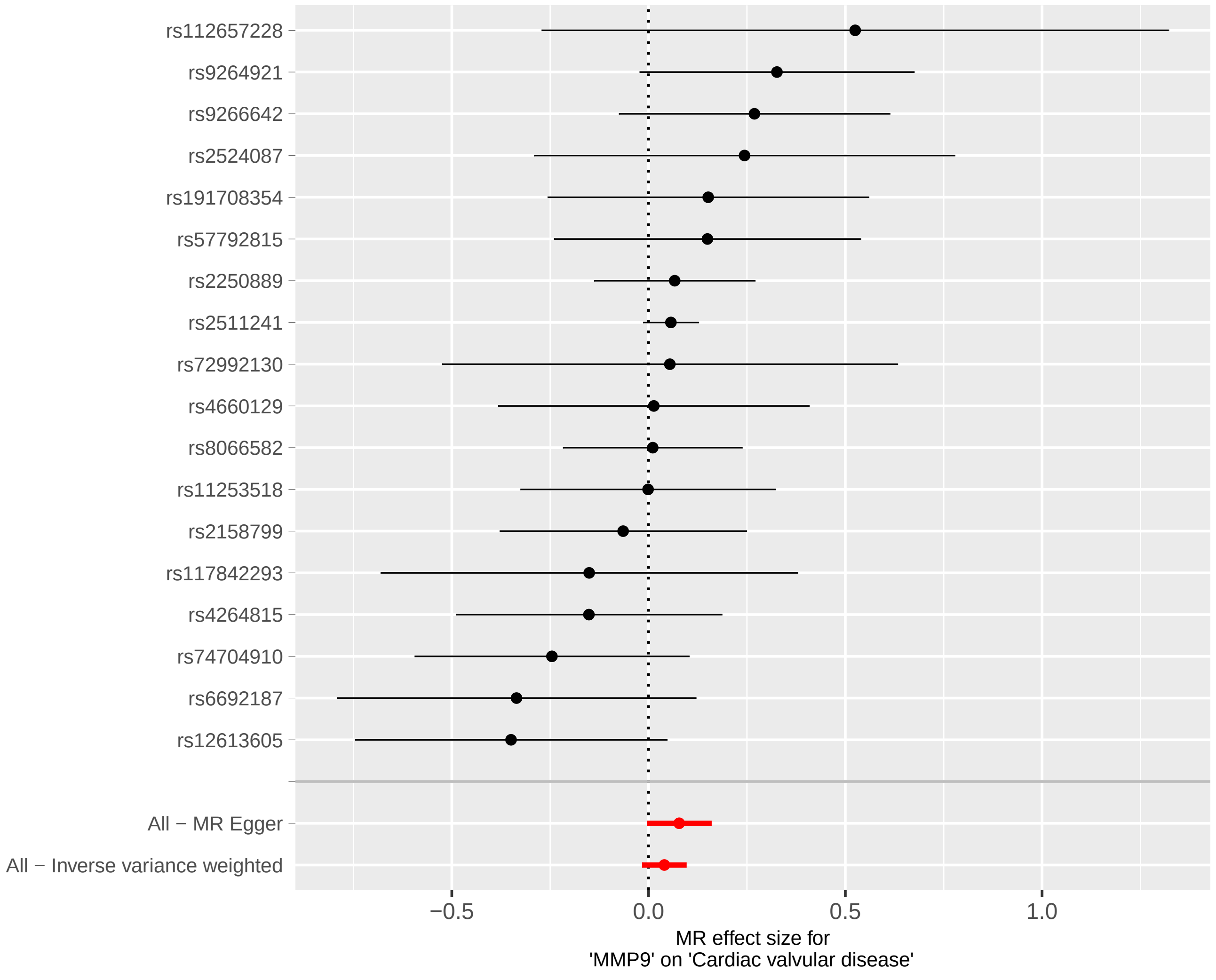

rs112657228
rs9264921
rs9266642
rs2524087
rs191708354
rs57792815
rs2250889
rs2511241
rs72992130
rs4660129
rs8066582
rs11253518
rs2158799
rs117842293
rs4264815
rs74704910
rs6692187
rs12613605
All − MR Egger
All − Inverse variance weighted
−0.5
0.0
0.5
1.0
MR effect size for
'MMP9' on 'Cardiac valvular disease'

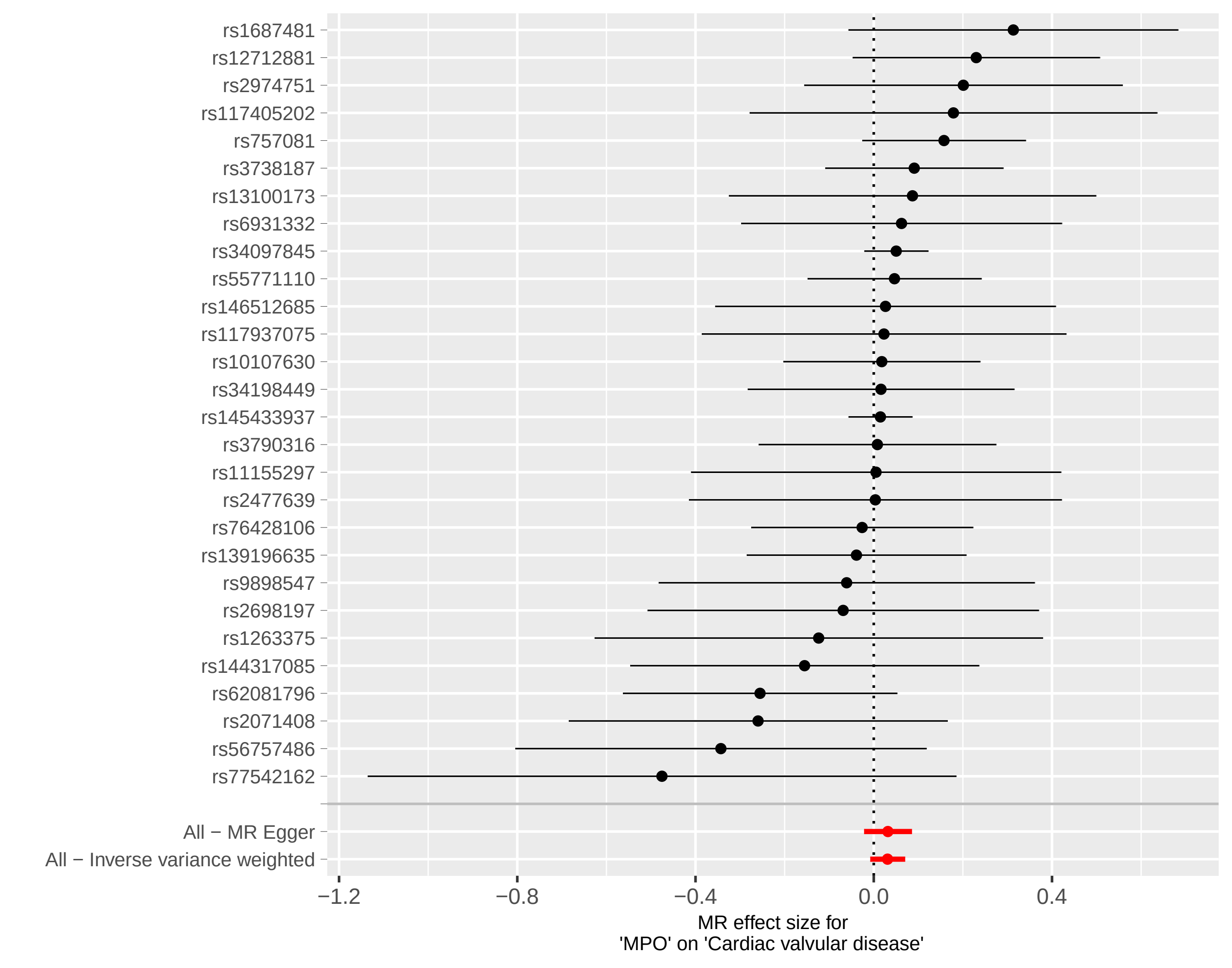
rs1687481
rs12712881
rs2974751
rs117405202
rs757081
rs3738187
rs13100173
rs6931332
rs34097845
rs55771110
rs146512685
rs117937075
rs10107630
rs34198449
rs145433937
rs3790316
rs11155297
rs2477639
rs76428106
rs139196635
rs9898547
rs2698197
rs1263375
rs144317085
rs62081796
rs2071408
rs56757486
rs77542162
All − MR Egger
All − Inverse variance weighted
−1.2
−0.8
−0.4
0.0
0.4
MR effect size for
'MPO' on 'Cardiac valvular disease'

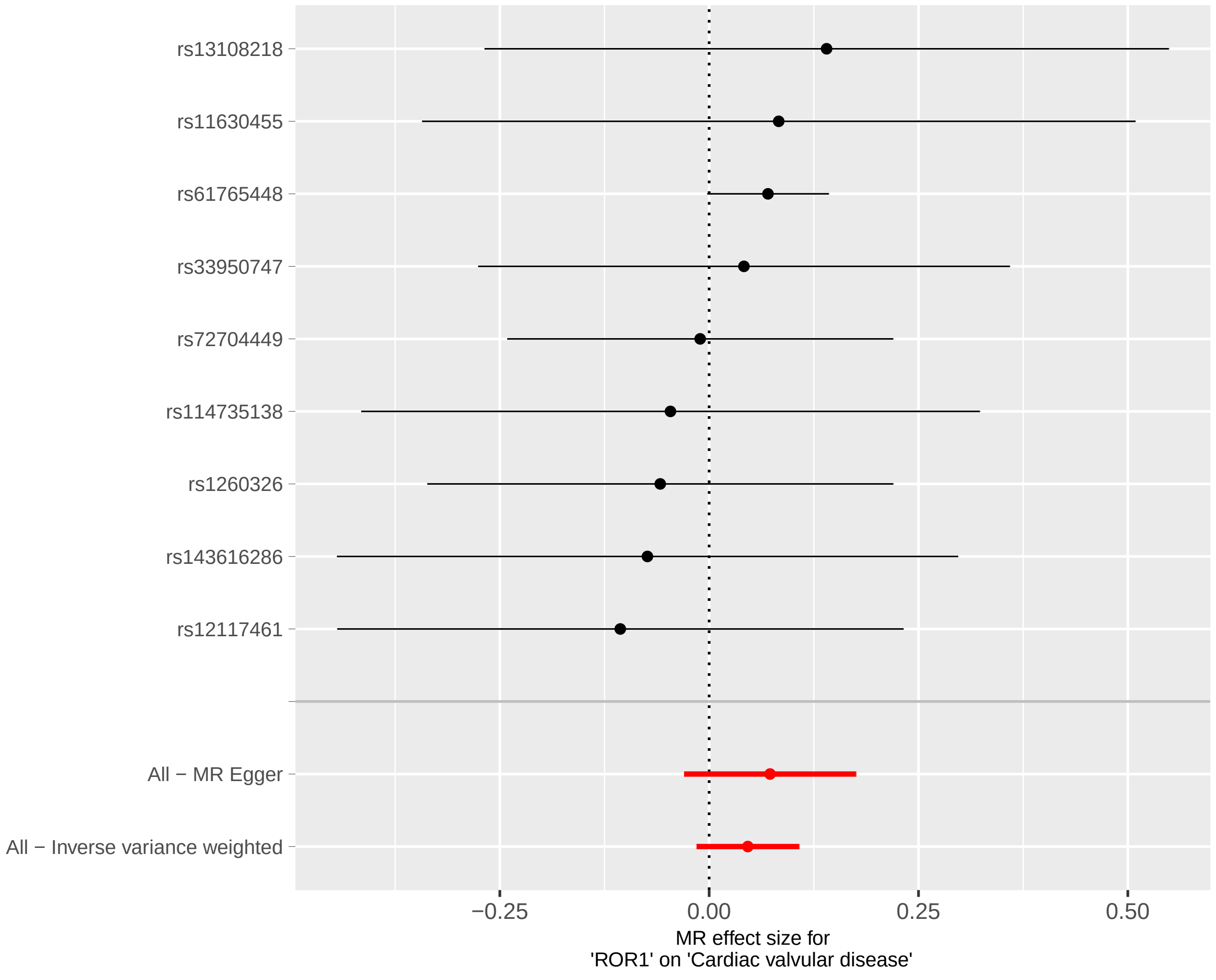

rs13108218
rs11630455
rs61765448
rs33950747
rs72704449
rs114735138
rs1260326
rs143616286
rs12117461
All − MR Egger
All − Inverse variance weighted
−0.25
0.00
0.25
0.50
MR effect size for
'ROR1' on 'Cardiac valvular disease'

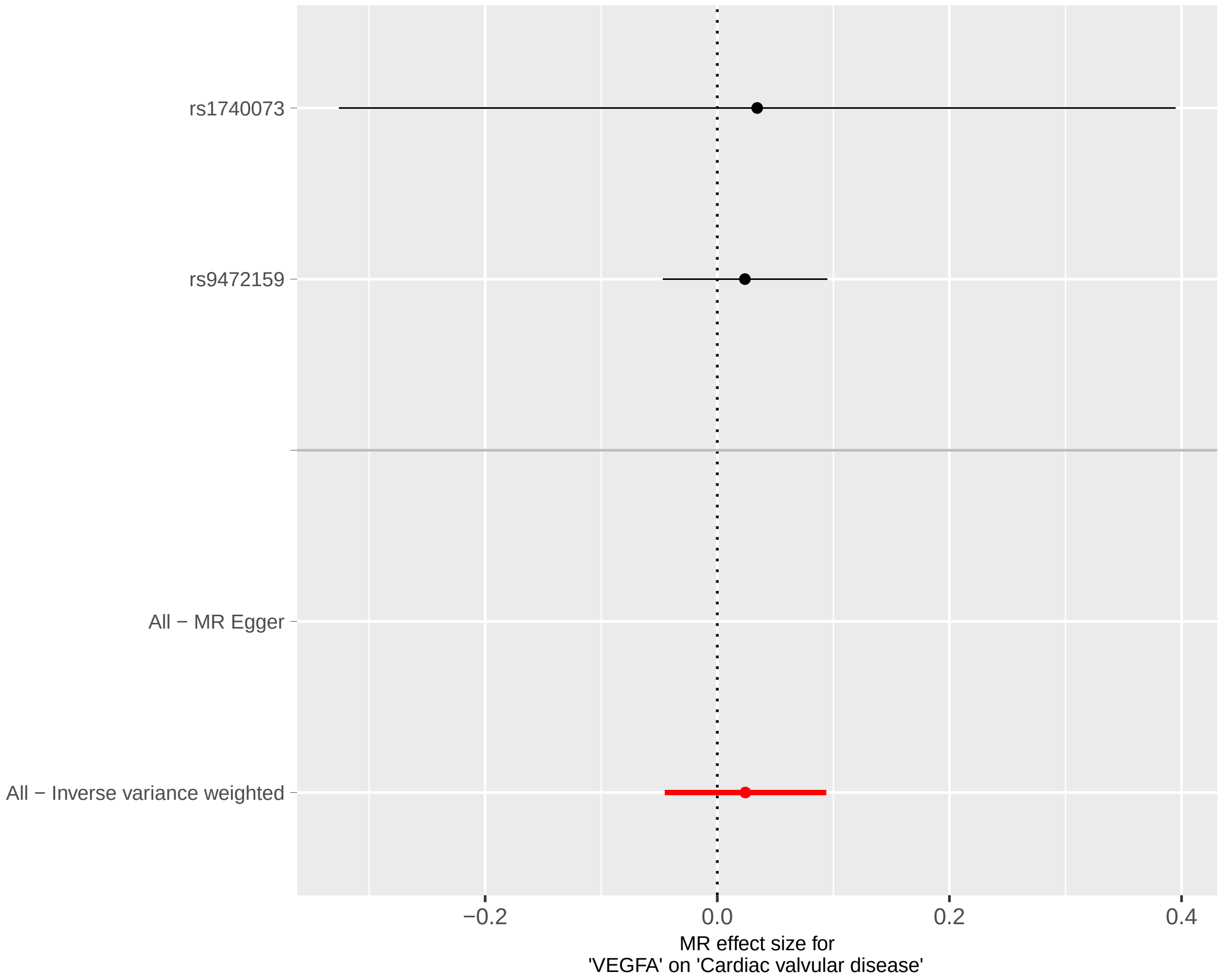
rs1740073
rs9472159
All − MR Egger
All − Inverse variance weighted
−0.2
0.0
0.2
0.4
MR effect size for
'VEGFA' on 'Cardiac valvular disease'

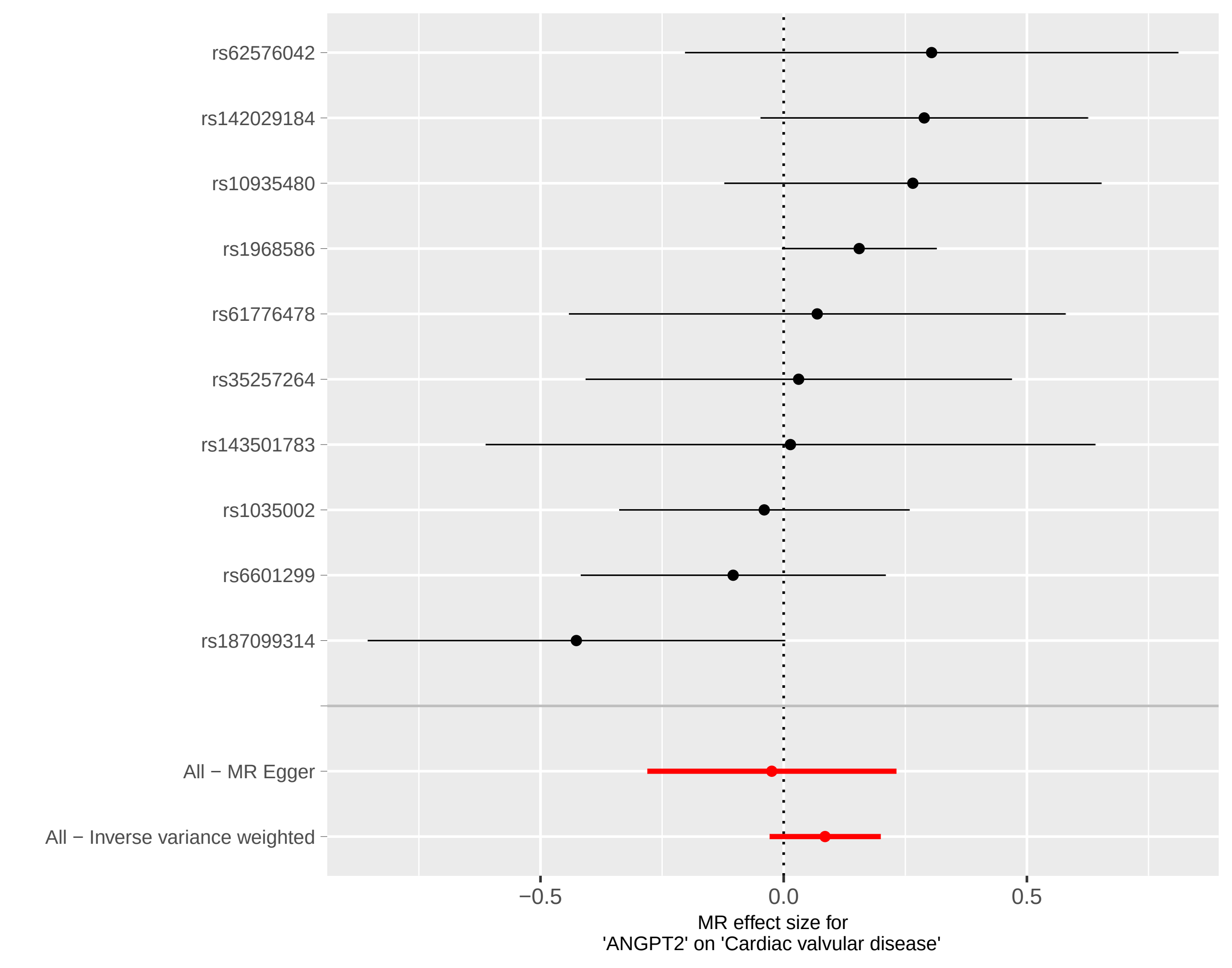
rs62576042
rs142029184
rs10935480
rs1968586
rs61776478
rs35257264
rs143501783
rs1035002
rs6601299
rs187099314
All − MR Egger
All − Inverse variance weighted
−0.5
0.0
0.5
MR effect size for
'ANGPT2' on 'Cardiac valvular disease'

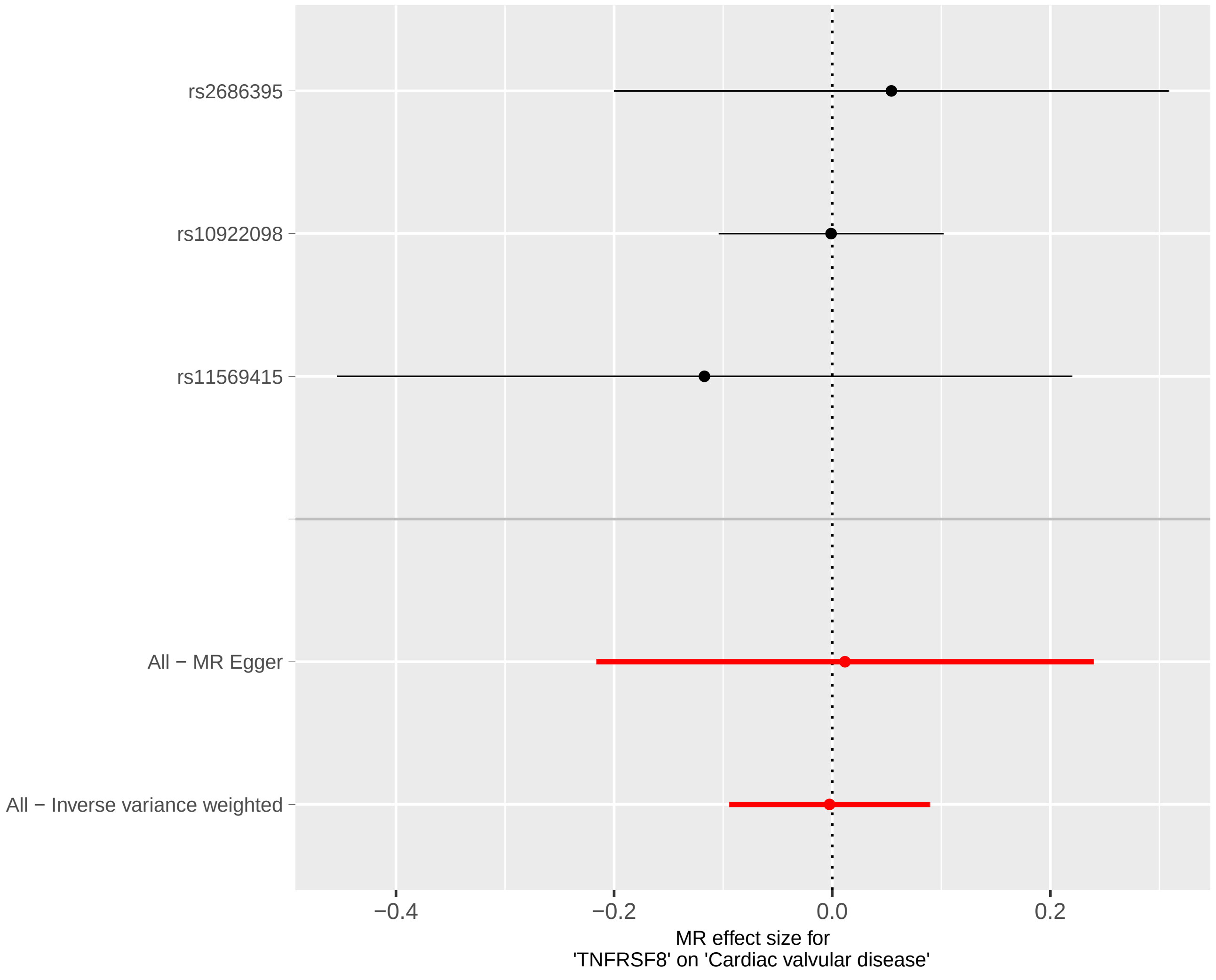

rs2686395
rs10922098
rs11569415
All − MR Egger
All − Inverse variance weighted
−0.4
−0.2
0.0
0.2
MR effect size for
'TNFRSF8' on 'Cardiac valvular disease'

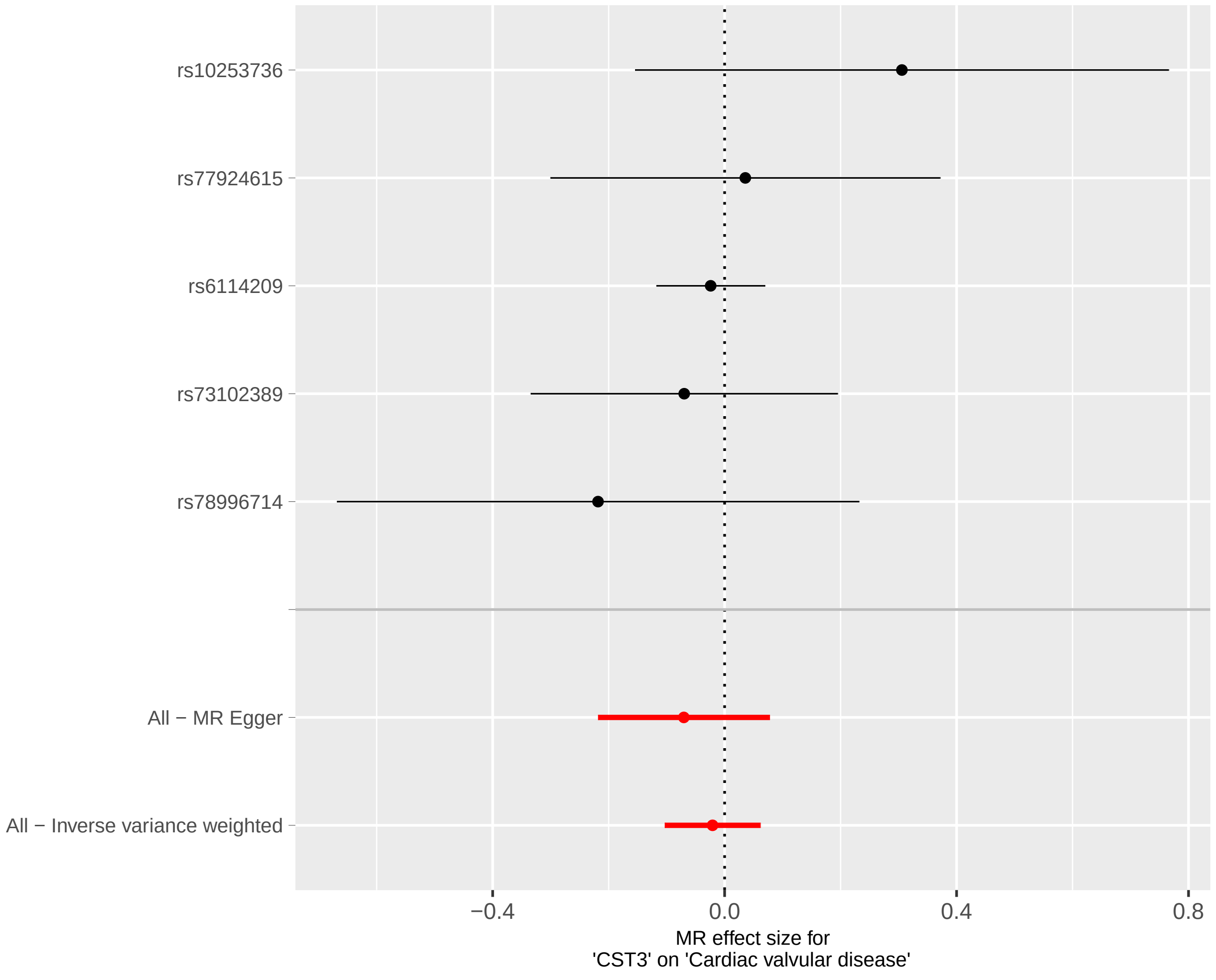
rs10253736
rs77924615
rs6114209
rs73102389
rs78996714
All − MR Egger
All − Inverse variance weighted
−0.4
0.0
0.4
0.8
MR effect size for
'CST3' on 'Cardiac valvular disease'

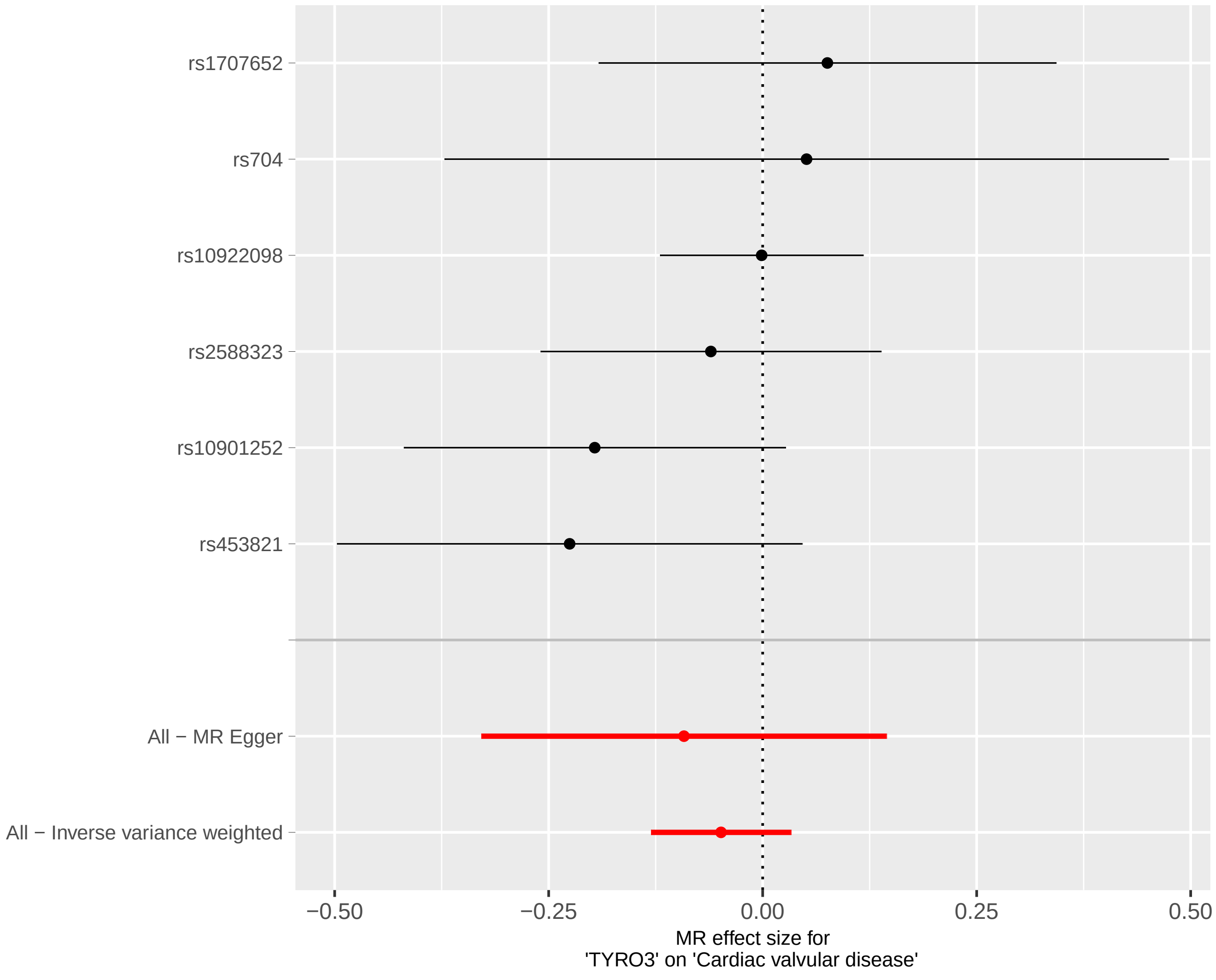
rs1707652
rs704
rs10922098
rs2588323
rs10901252
rs453821
All − MR Egger
All − Inverse variance weighted
−0.50
−0.25
0.00
0.25
0.50
MR effect size for
'TYRO3' on 'Cardiac valvular disease'

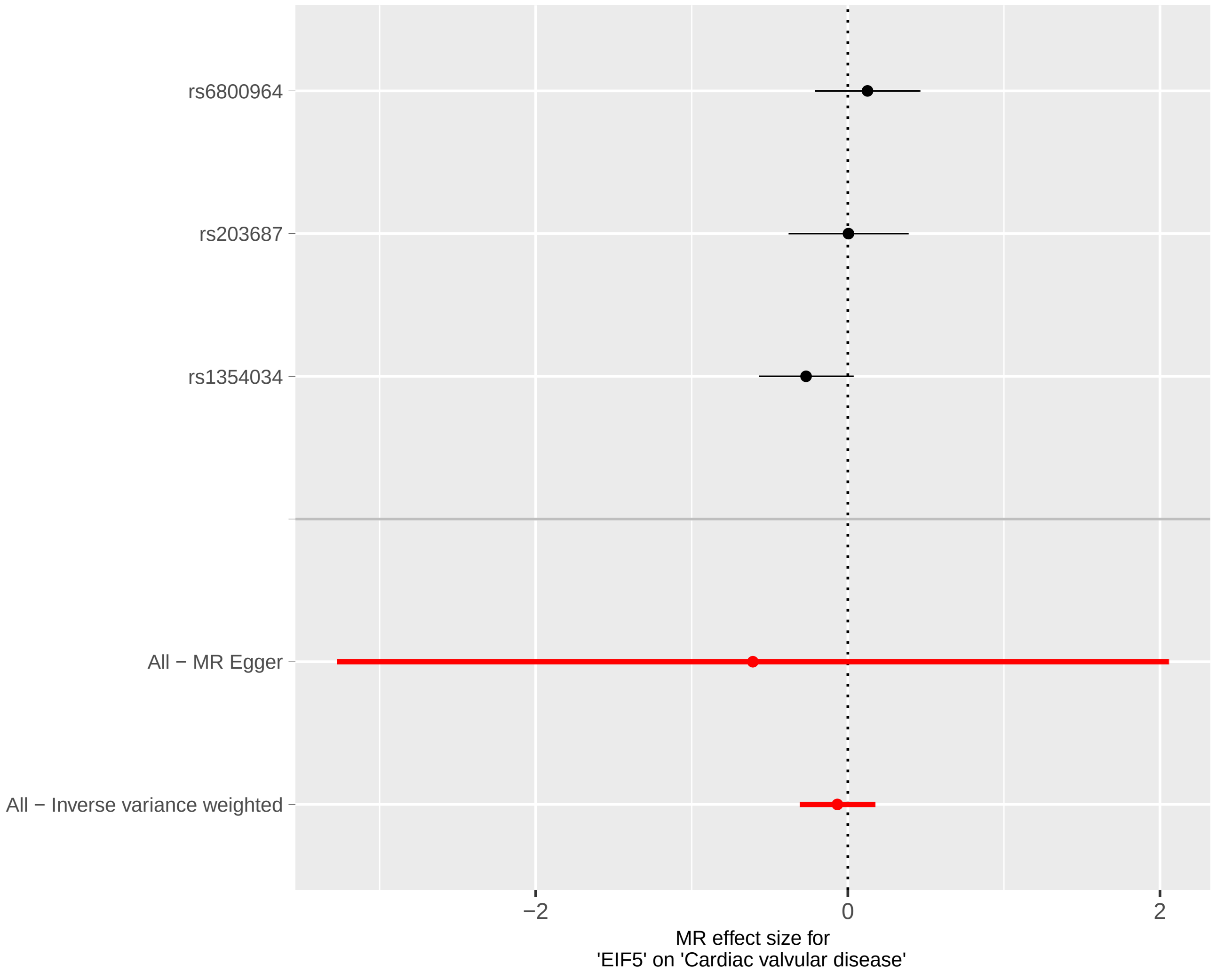
rs6800964
rs203687
rs1354034
All − MR Egger
All − Inverse variance weighted
−2
0
2
MR effect size for
'EIF5' on 'Cardiac valvular disease'

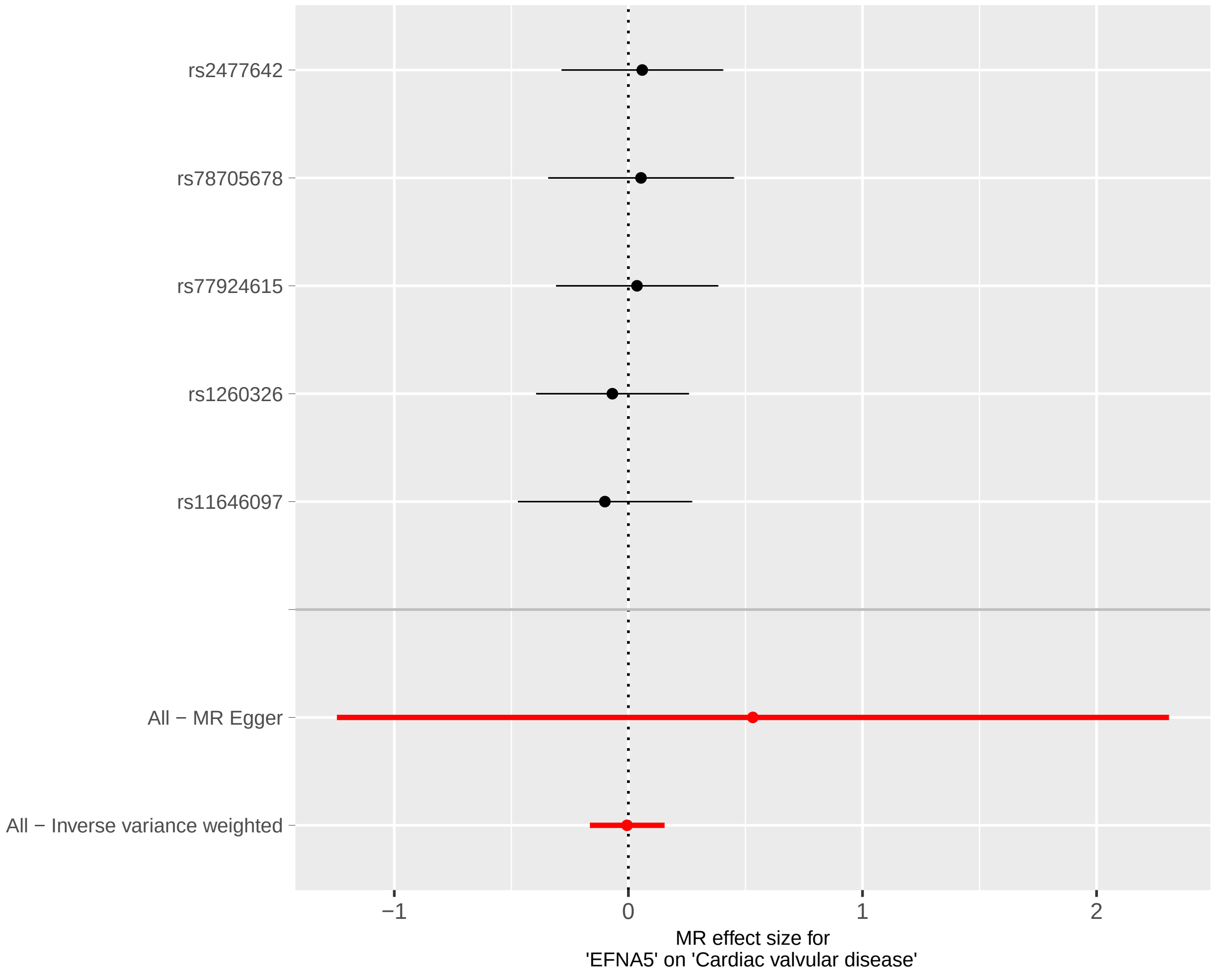

rs2477642
rs78705678
rs77924615
rs1260326
rs11646097
All − MR Egger
All − Inverse variance weighted
−1
0
1
2
MR effect size for
'EFNA5' on 'Cardiac valvular disease'

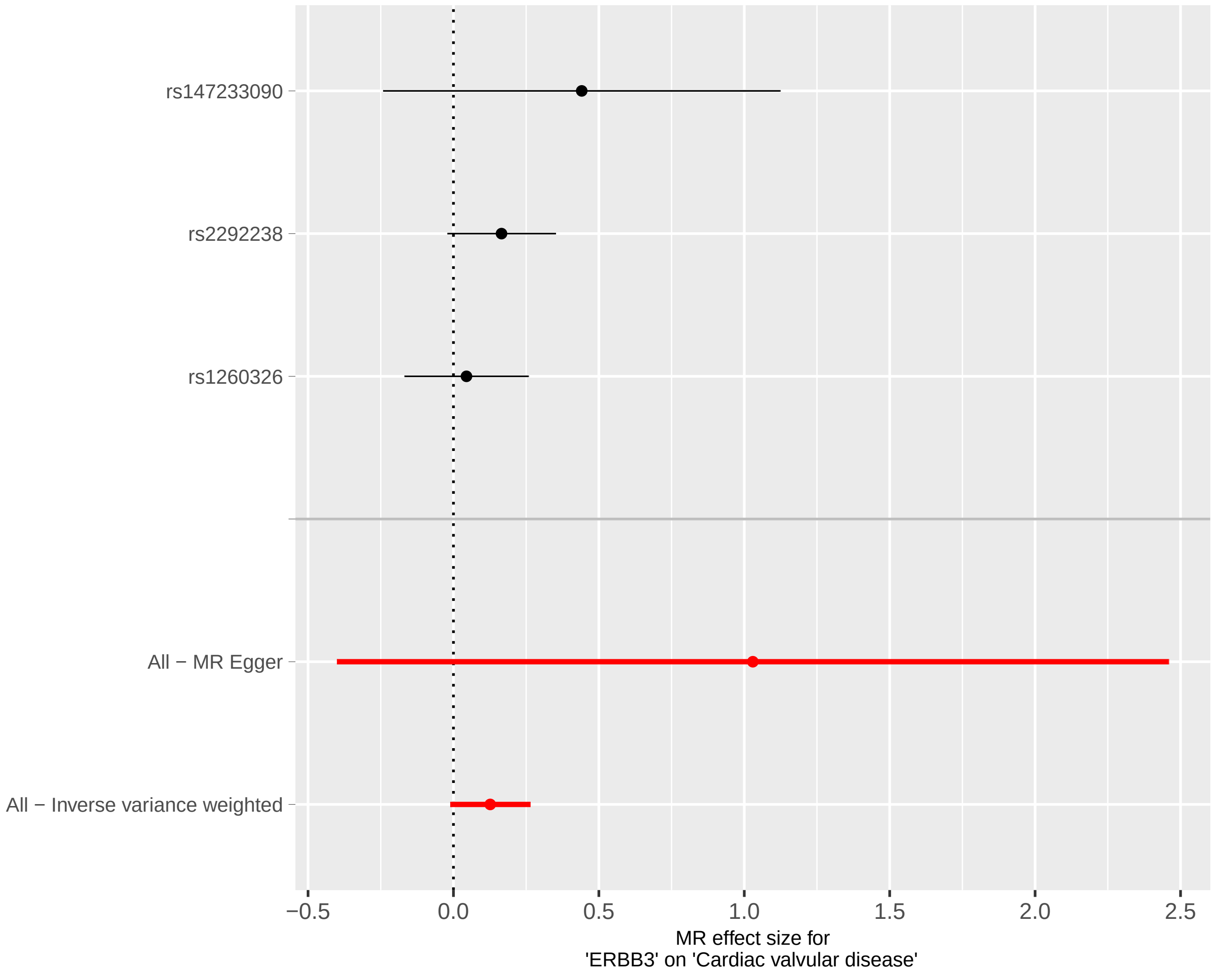
rs147233090
rs2292238
rs1260326
All − MR Egger
All − Inverse variance weighted
−0.5
0.0
0.5
1.0
1.5
2.0
2.5
MR effect size for
'ERBB3' on 'Cardiac valvular disease'

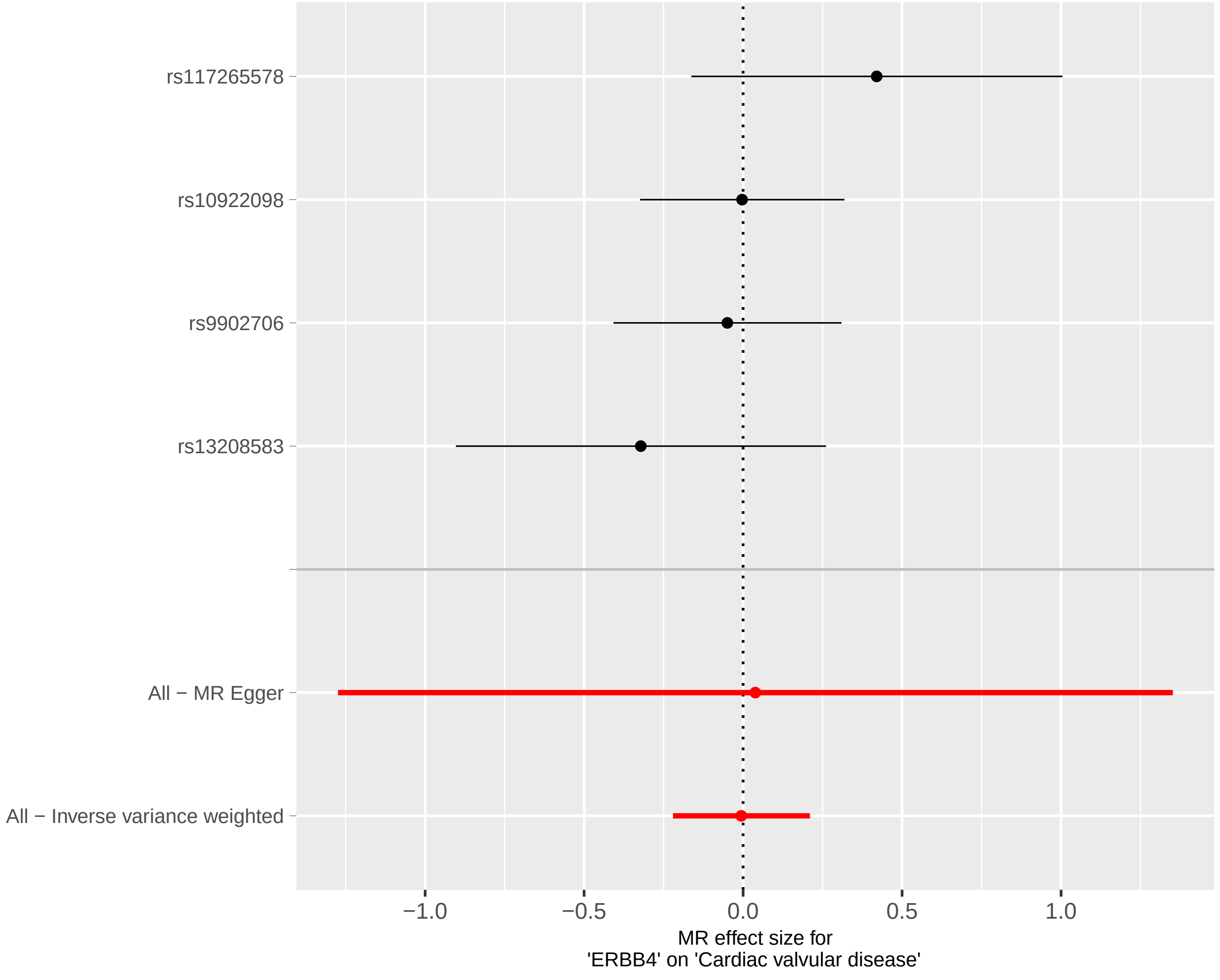
rs117265578
rs10922098
rs9902706
rs13208583
All − MR Egger
All − Inverse variance weighted
−1.0
−0.5
0.0
0.5
1.0
MR effect size for
'ERBB4' on 'Cardiac valvular disease'

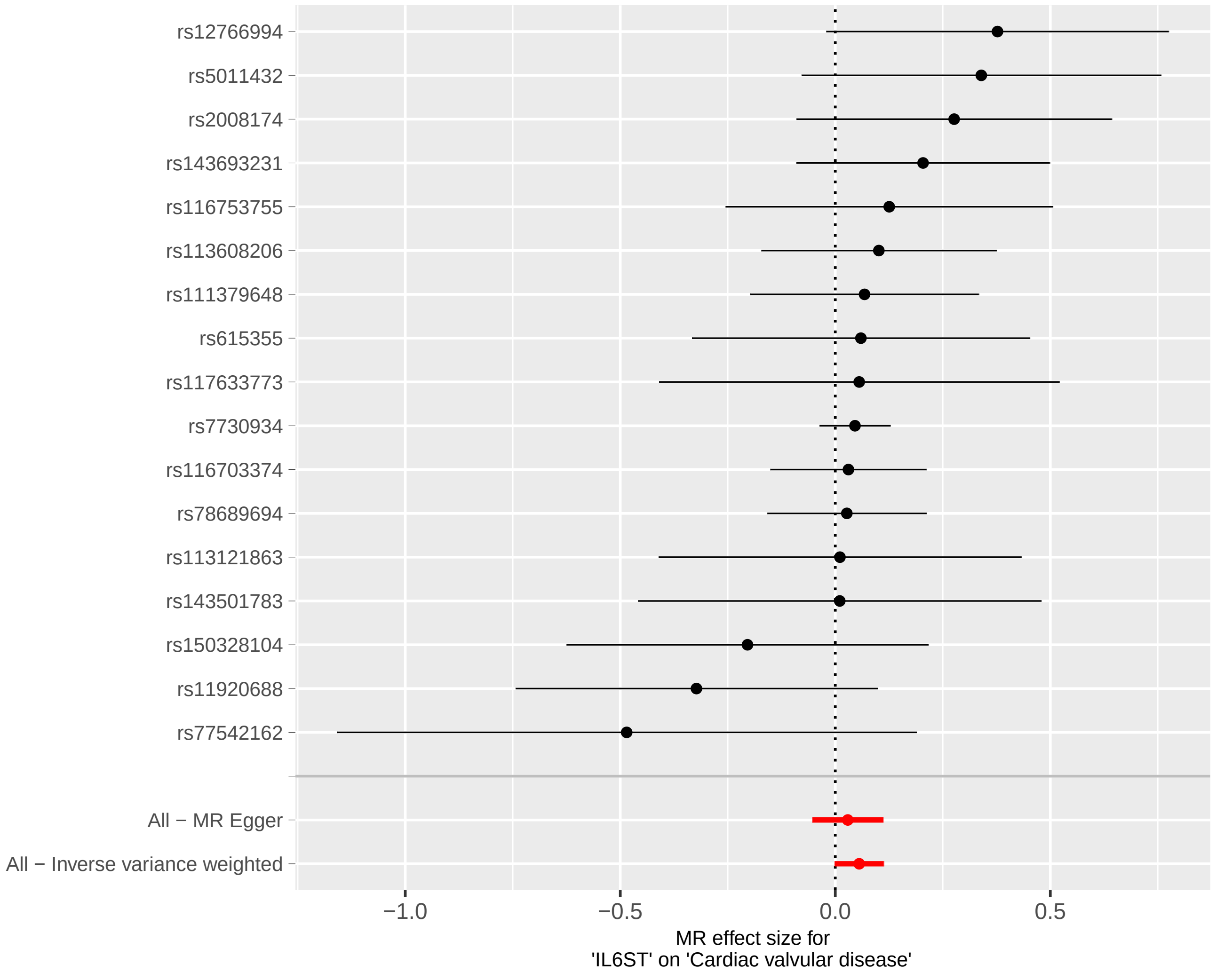

rs12766994
rs5011432
rs2008174
rs143693231
rs116753755
rs113608206
rs111379648
rs615355
rs117633773
rs7730934
rs116703374
rs78689694
rs113121863
rs143501783
rs150328104
rs11920688
rs77542162
All − MR Egger
All − Inverse variance weighted
−1.0
−0.5
0.0
0.5
MR effect size for
'IL6ST' on 'Cardiac valvular disease'

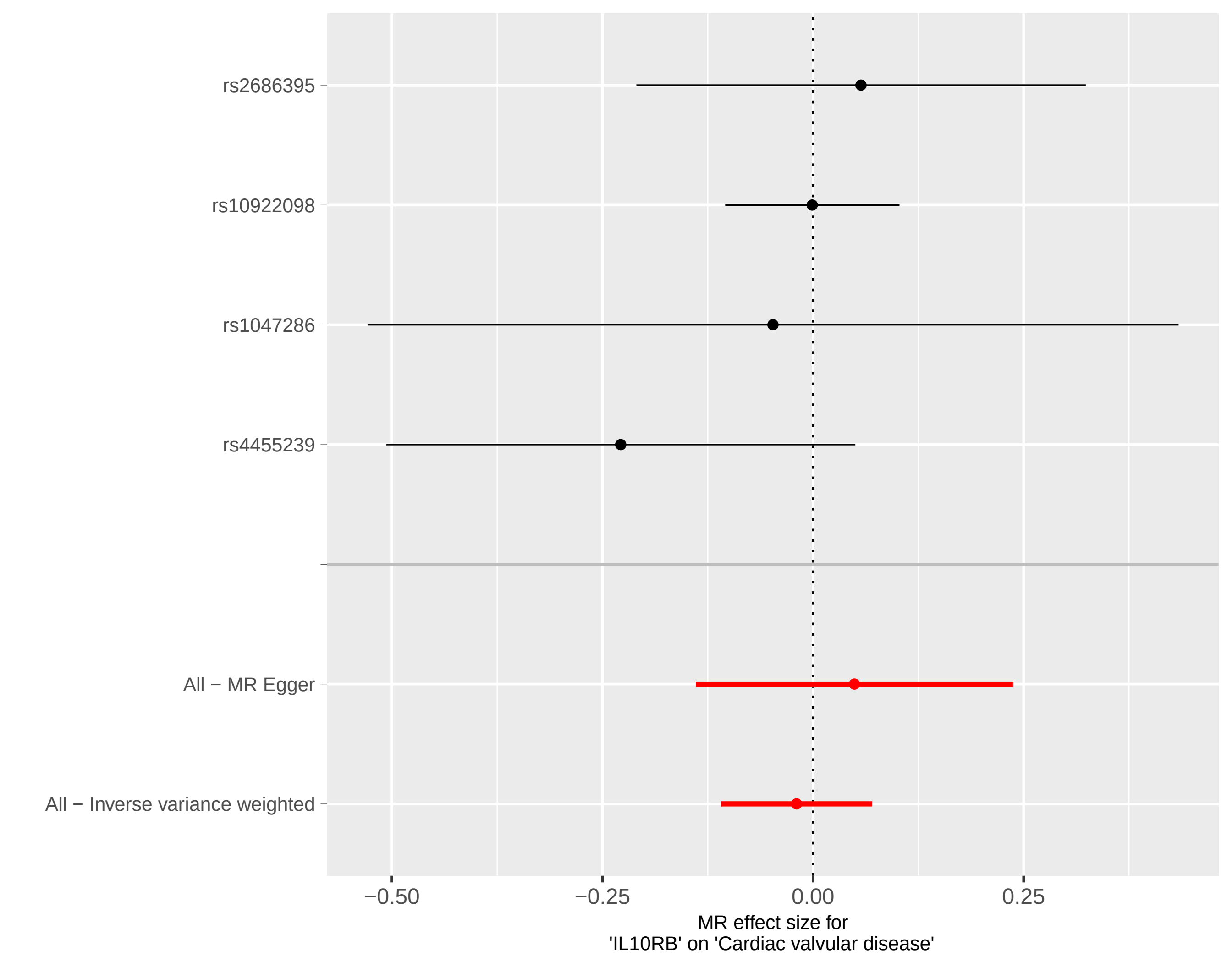

rs2686395
rs10922098
rs1047286
rs4455239
All − MR Egger
All − Inverse variance weighted
−0.50
−0.25
0.00
0.25
MR effect size for
'IL10RB' on 'Cardiac valvular disease'

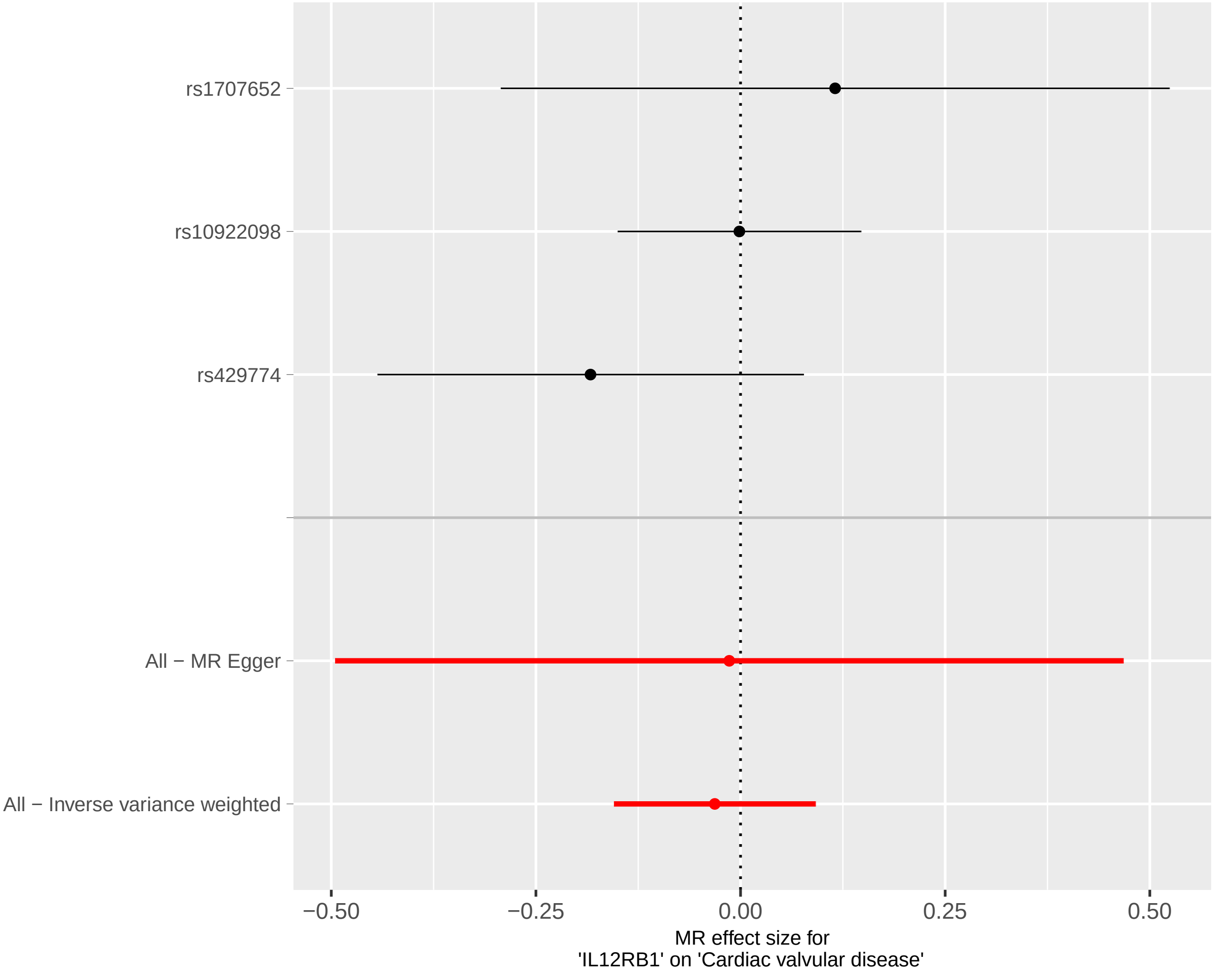
rs1707652
rs10922098
rs429774
All − MR Egger
All − Inverse variance weighted
−0.50
−0.25
0.00
0.25
0.50
MR effect size for
'IL12RB1' on 'Cardiac valvular disease'

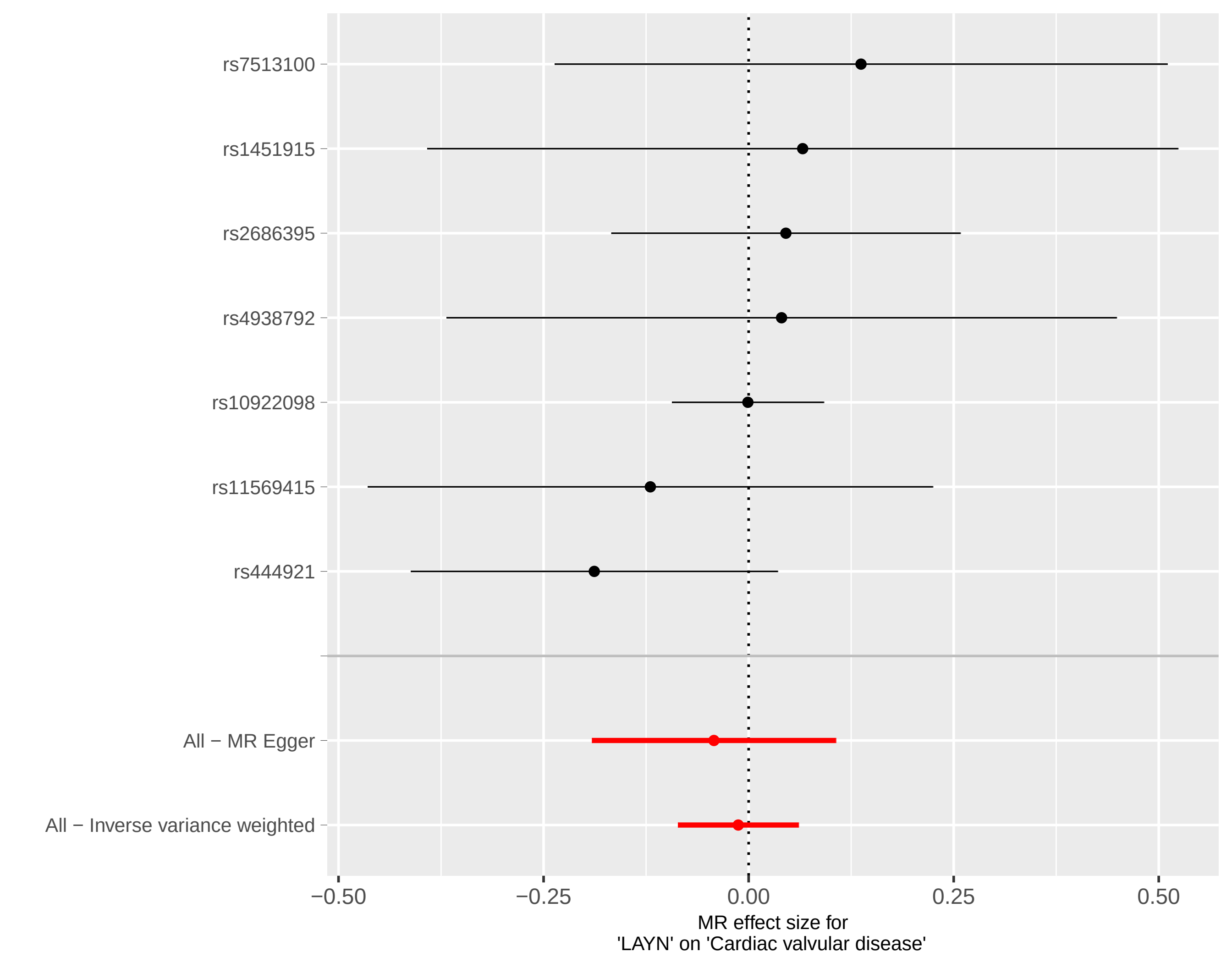
rs7513100
rs1451915
rs2686395
rs4938792
rs10922098
rs11569415
rs444921
All − MR Egger
All − Inverse variance weighted
−0.50
−0.25
0.00
0.25
0.50
MR effect size for
'LAYN' on 'Cardiac valvular disease'

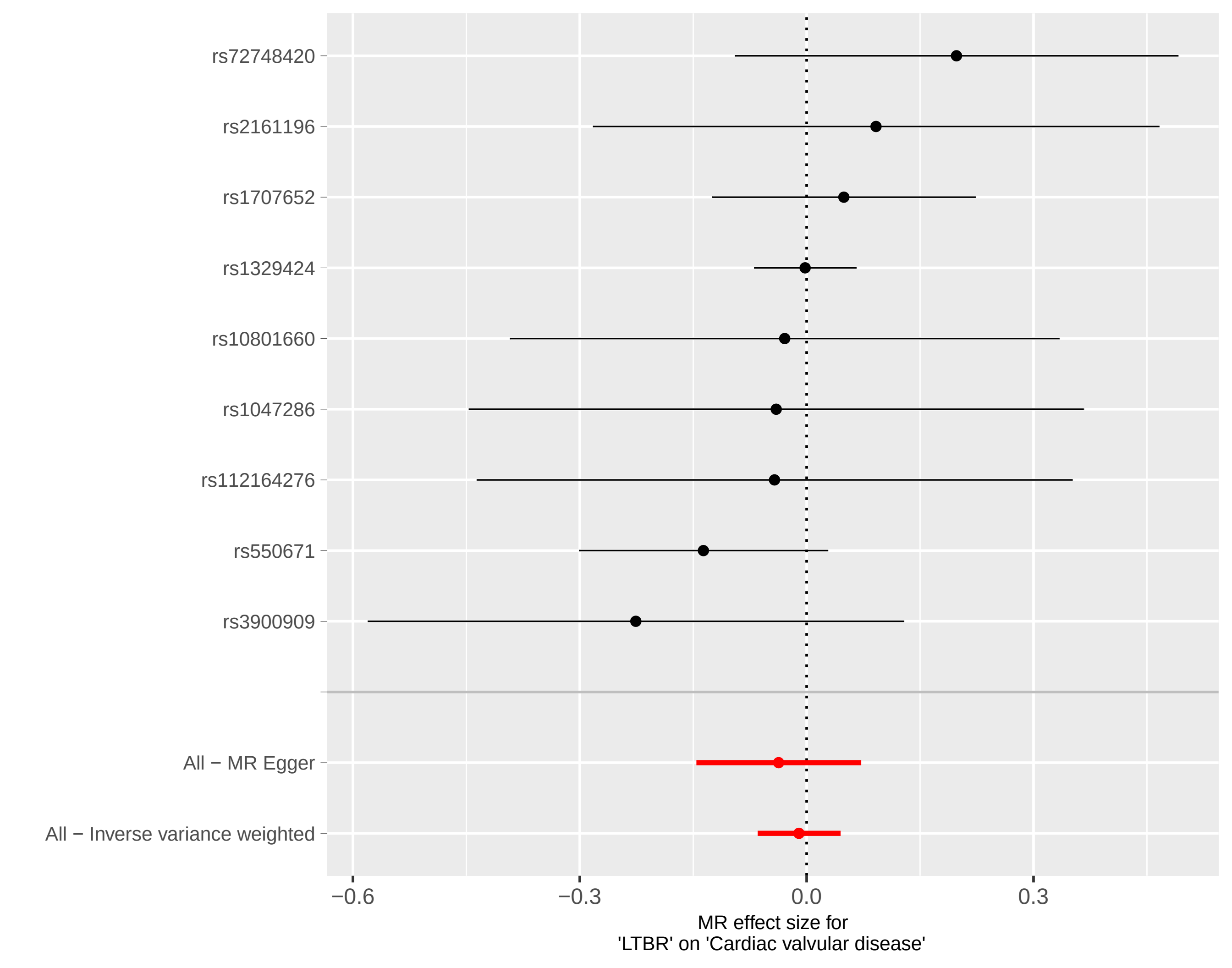

rs72748420
rs2161196
rs1707652
rs1329424
rs10801660
rs1047286
rs112164276
rs550671
rs3900909
All − MR Egger
All − Inverse variance weighted
−0.6
−0.3
0.0
0.3
MR effect size for
'LTBR' on 'Cardiac valvular disease'

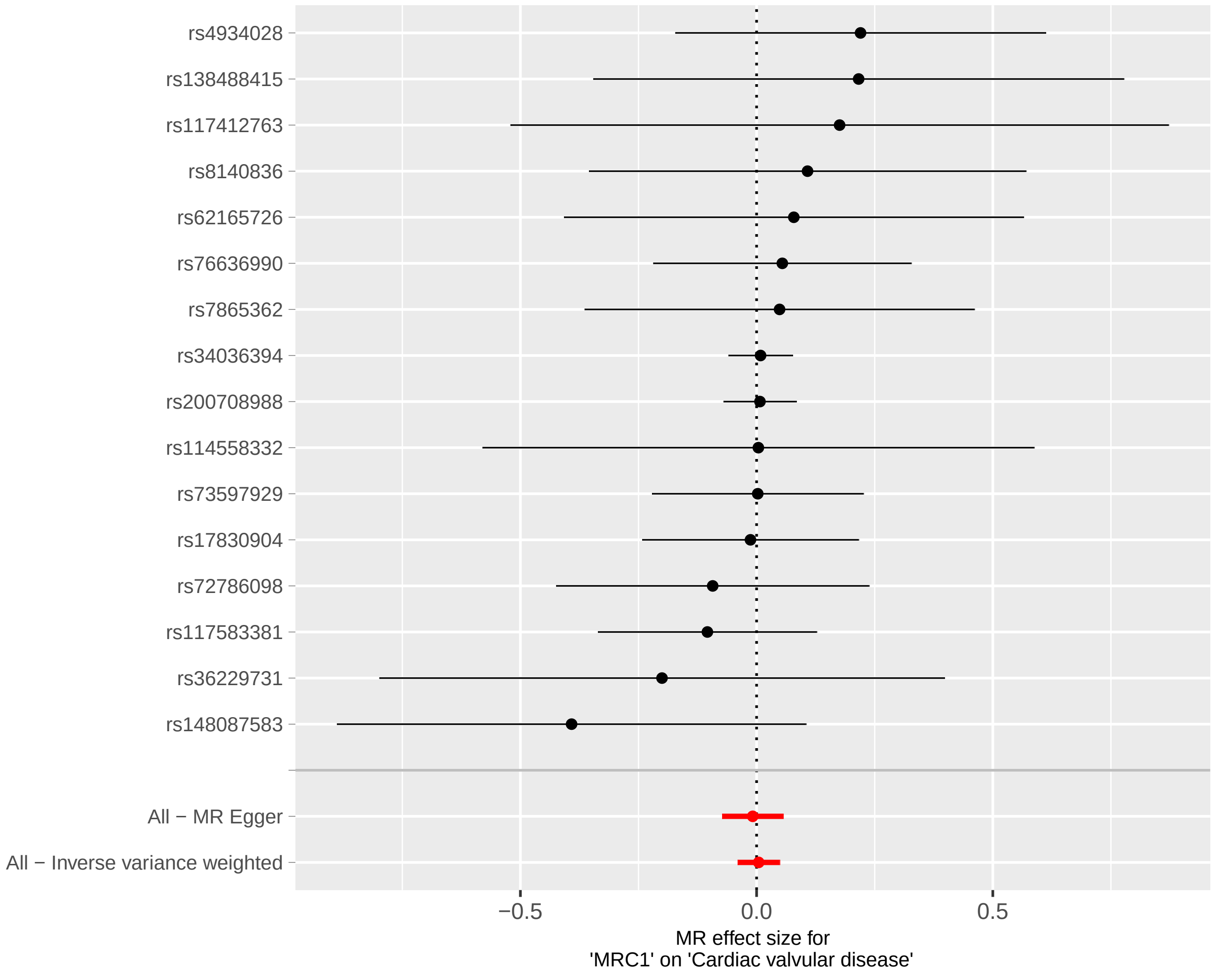
rs4934028
rs138488415
rs117412763
rs8140836
rs62165726
rs76636990
rs7865362
rs34036394
rs200708988
rs114558332
rs73597929
rs17830904
rs72786098
rs117583381
rs36229731
rs148087583
All − MR Egger
All − Inverse variance weighted
−0.5
0.0
0.5
MR effect size for
'MRC1' on 'Cardiac valvular disease'

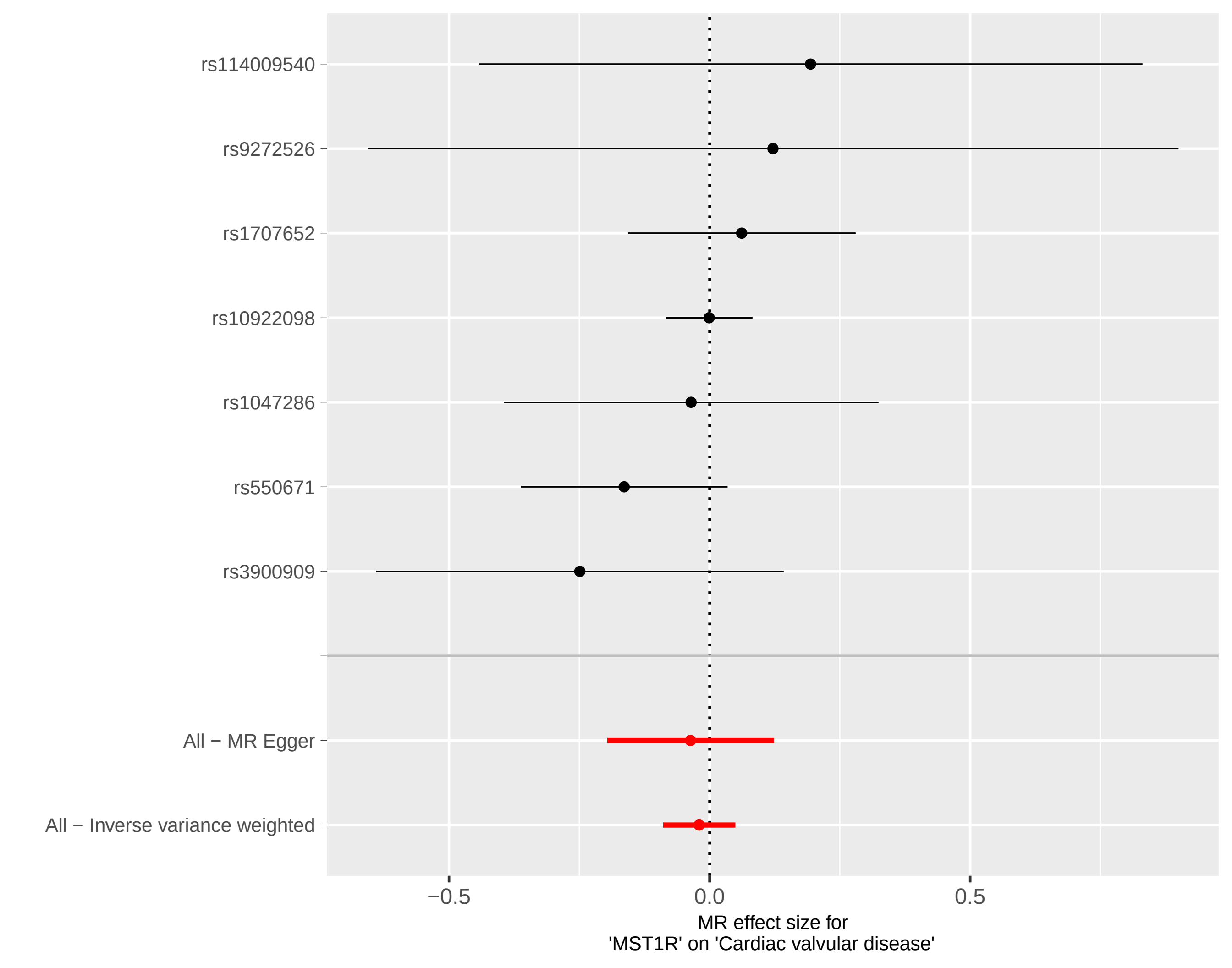
rs114009540
rs9272526
rs1707652
rs10922098
rs1047286
rs550671
rs3900909
All − MR Egger
All − Inverse variance weighted
−0.5
0.0
0.5
MR effect size for
'MST1R' on 'Cardiac valvular disease'

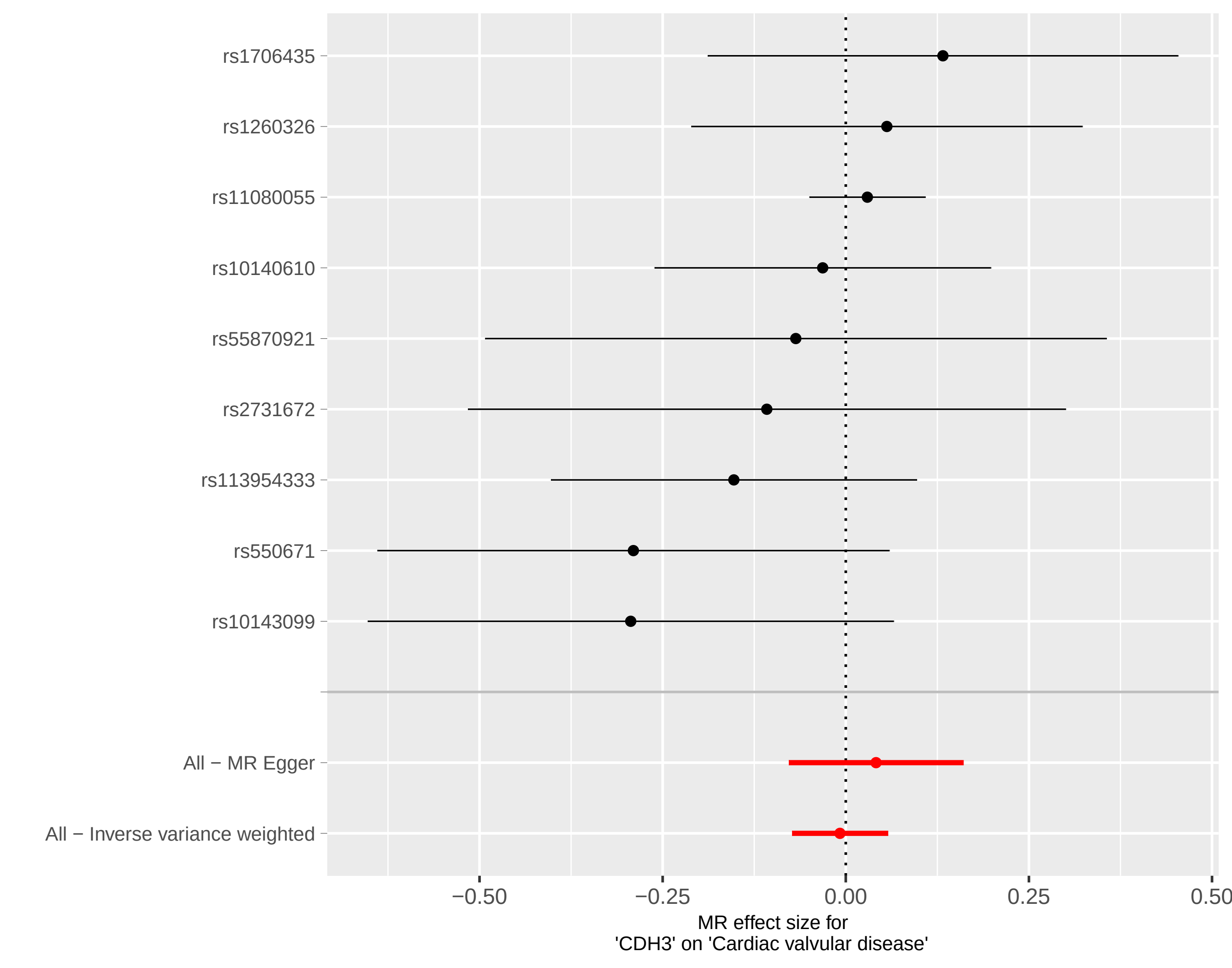
rs1706435
rs1260326
rs11080055
rs10140610
rs55870921
rs2731672
rs113954333
rs550671
rs10143099
All − MR Egger
All − Inverse variance weighted
−0.50
−0.25
0.00
0.25
0.50
MR effect size for
'CDH3' on 'Cardiac valvular disease'

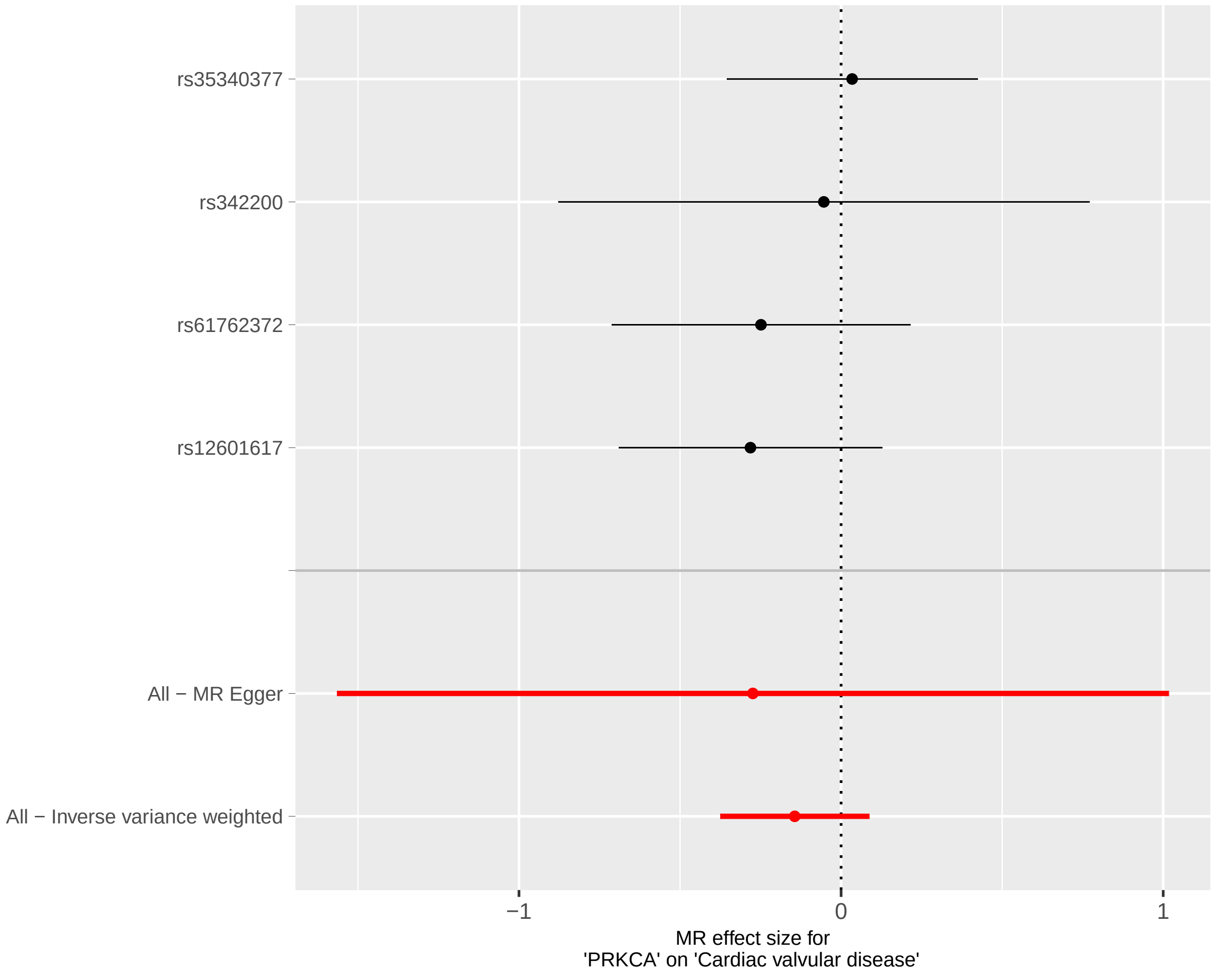
rs35340377
rs342200
rs61762372
rs12601617
All − MR Egger
All − Inverse variance weighted
−1
0
1
MR effect size for
'PRKCA' on 'Cardiac valvular disease'

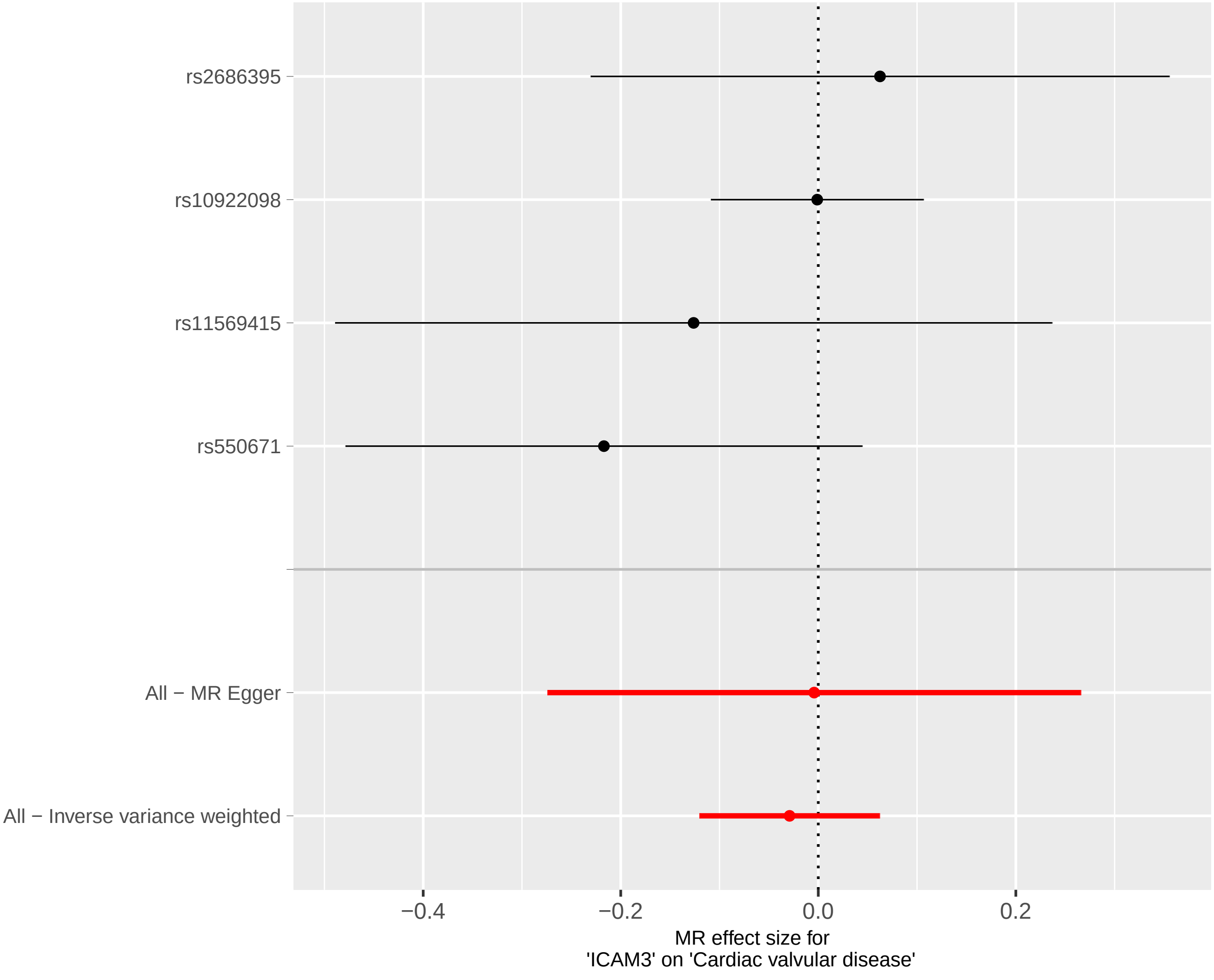
rs2686395
rs10922098
rs11569415
rs550671
All − MR Egger
All − Inverse variance weighted
−0.4
−0.2
0.0
0.2
MR effect size for
'ICAM3' on 'Cardiac valvular disease'

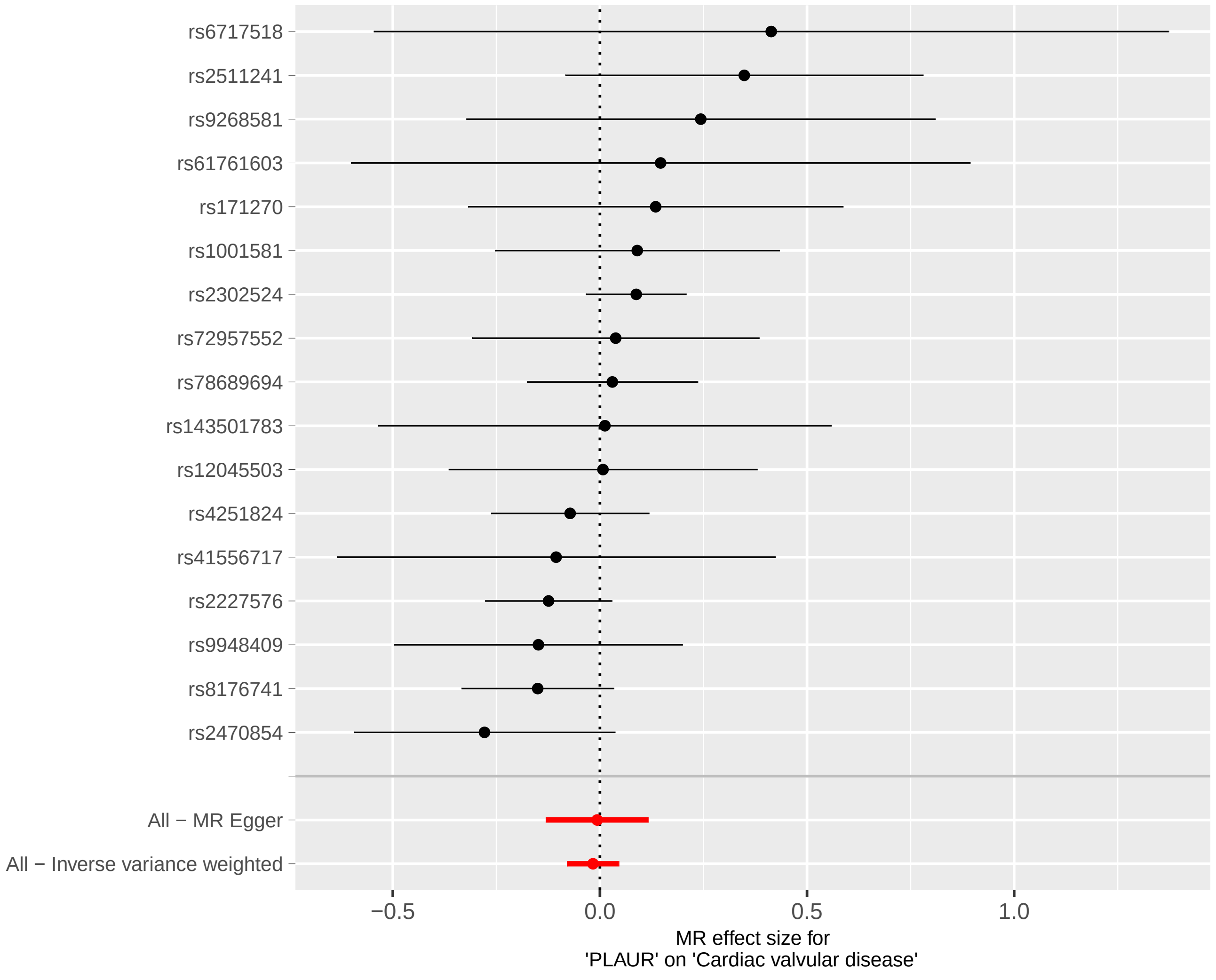
rs6717518
rs2511241
rs9268581
rs61761603
rs171270
rs1001581
rs2302524
rs72957552
rs78689694
rs143501783
rs12045503
rs4251824
rs41556717
rs2227576
rs9948409
rs8176741
rs2470854
All − MR Egger
All − Inverse variance weighted
−0.5
0.0
0.5
1.0
MR effect size for
'PLAUR' on 'Cardiac valvular disease'

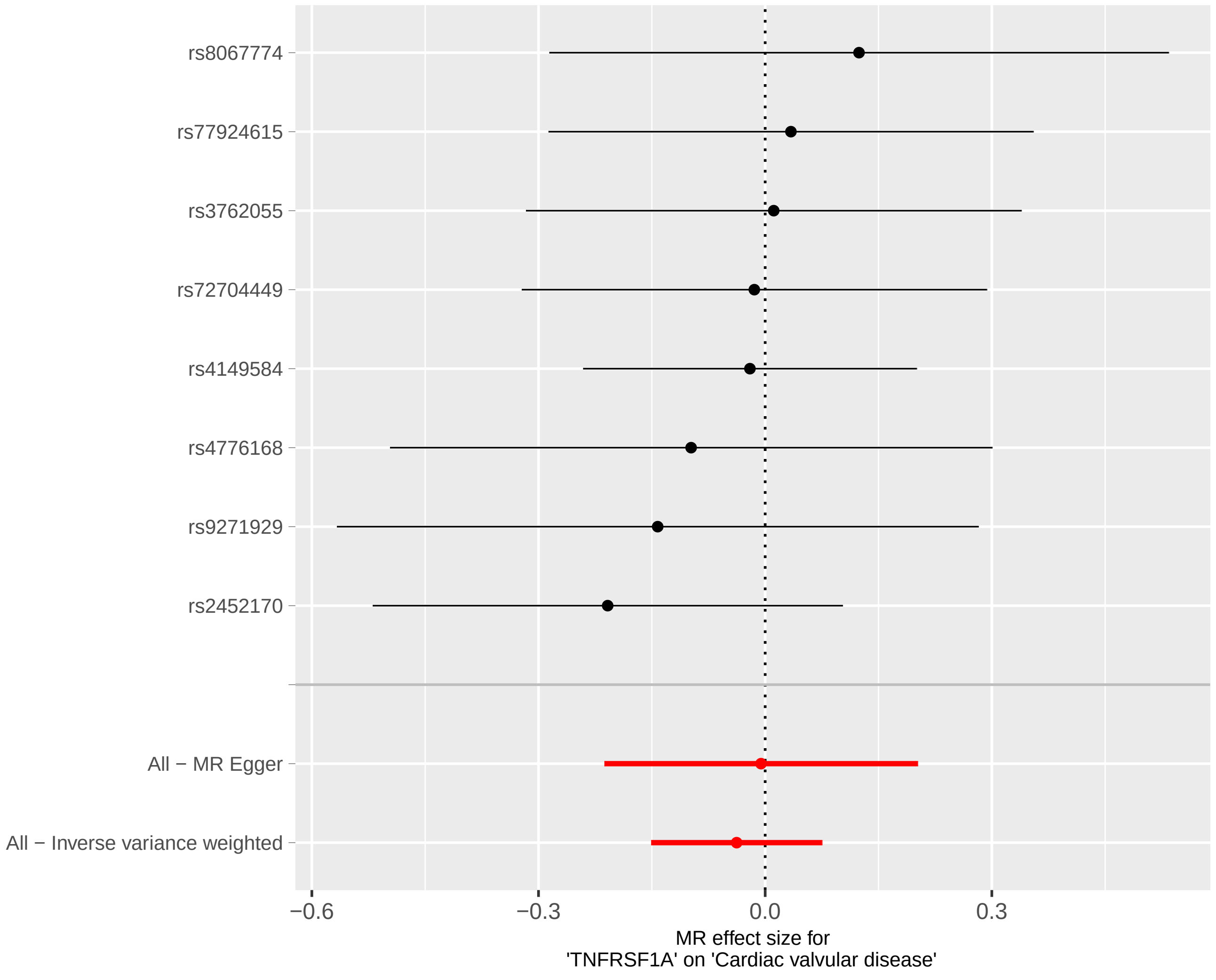

rs8067774
rs77924615
rs3762055
rs72704449
rs4149584
rs4776168
rs9271929
rs2452170
All − MR Egger
All − Inverse variance weighted
−0.6
−0.3
0.0
0.3
MR effect size for
'TNFRSF1A' on 'Cardiac valvular disease'

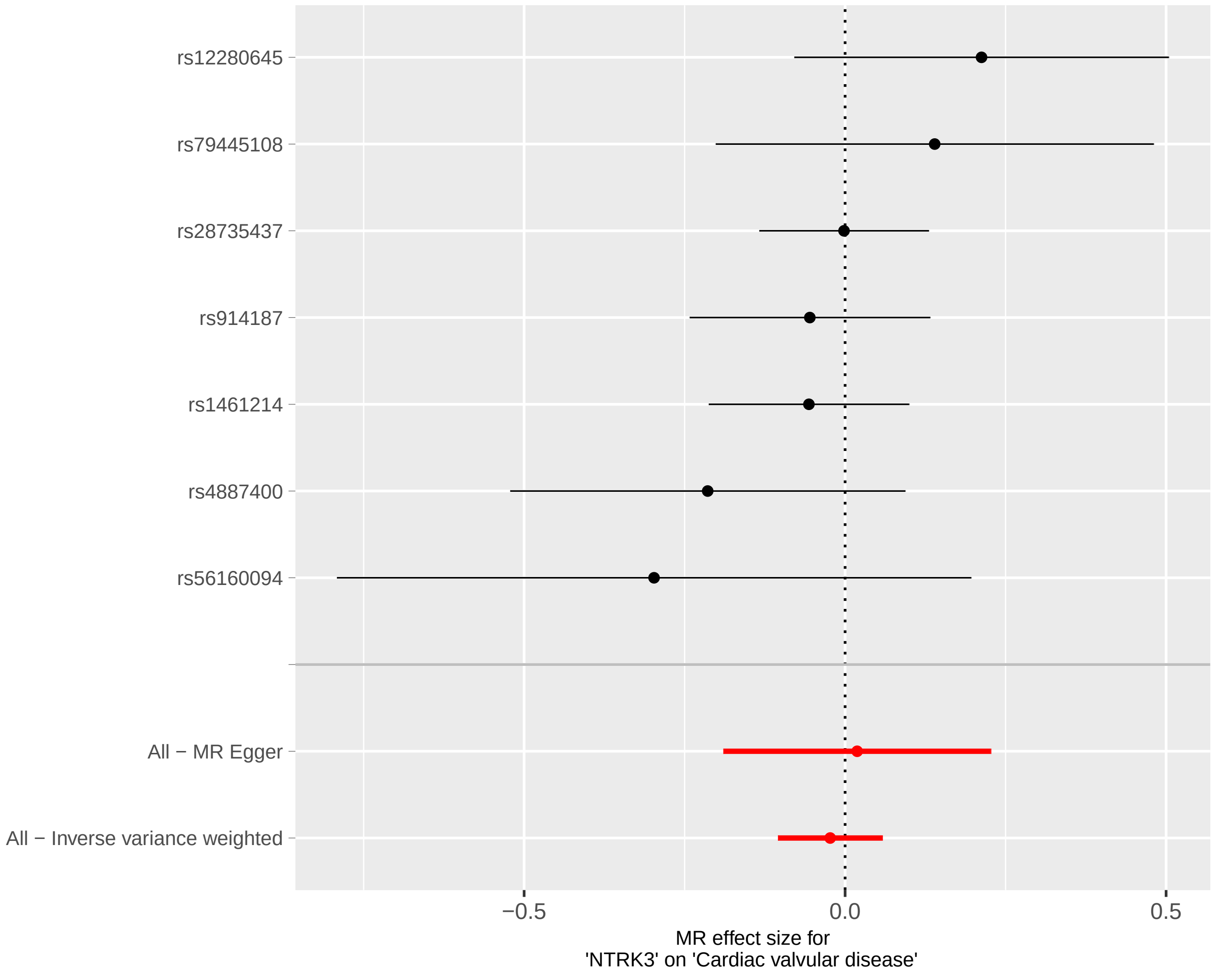
rs12280645
rs79445108
rs28735437
rs914187
rs1461214
rs4887400
rs56160094
All − MR Egger
All − Inverse variance weighted
−0.5
0.0
0.5
MR effect size for
'NTRK3' on 'Cardiac valvular disease'

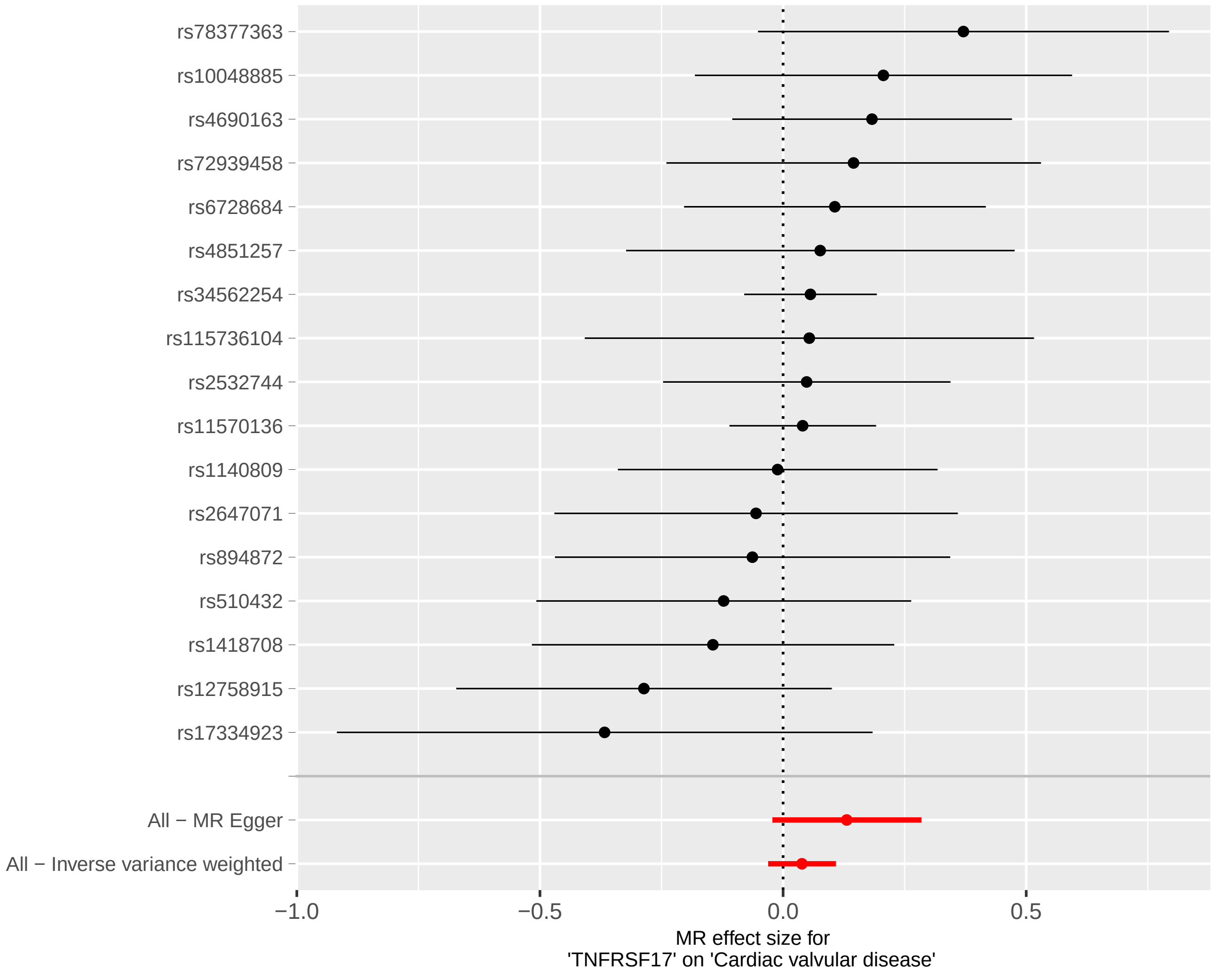
rs78377363
rs10048885
rs4690163
rs72939458
rs6728684
rs4851257
rs34562254
rs115736104
rs2532744
rs11570136
rs1140809
rs2647071
rs894872
rs510432
rs1418708
rs12758915
rs17334923
All − MR Egger
All − Inverse variance weighted
−1.0
−0.5
0.0
0.5
MR effect size for
'TNFRSF17' on 'Cardiac valvular disease'

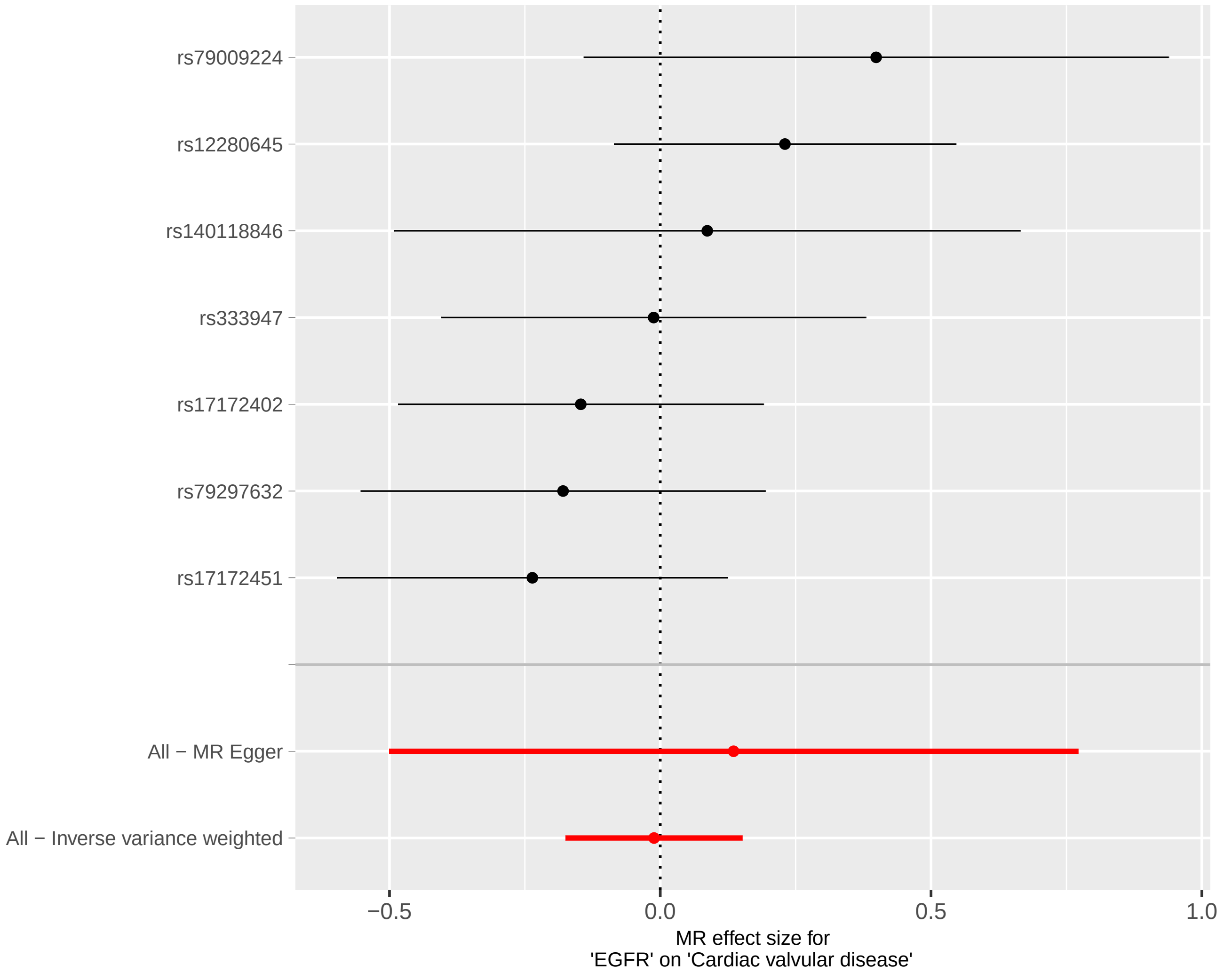
rs79009224
rs12280645
rs140118846
rs333947
rs17172402
rs79297632
rs17172451
All − MR Egger
All − Inverse variance weighted
−0.5
0.0
0.5
1.0
MR effect size for
'EGFR' on 'Cardiac valvular disease'

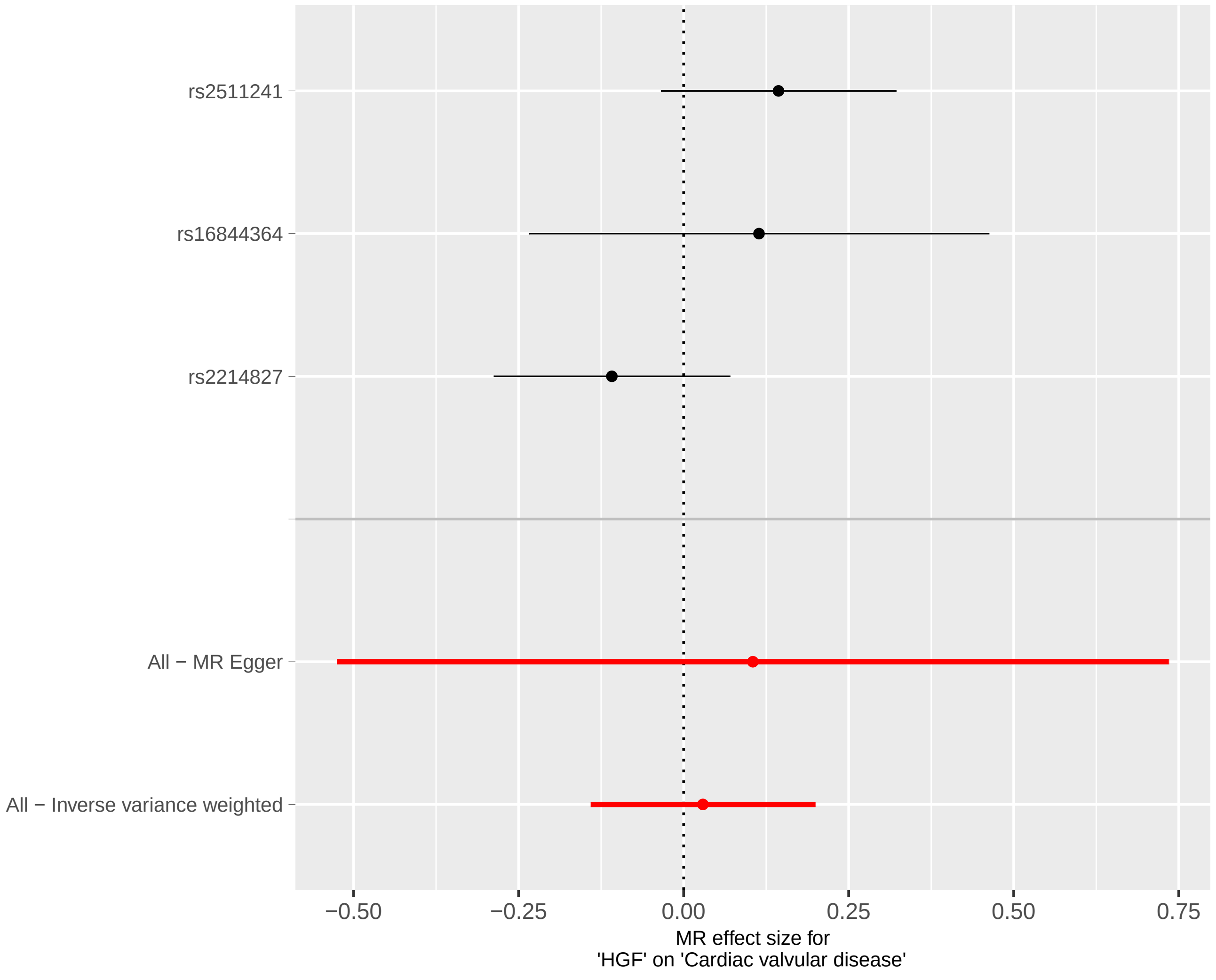
rs2511241
rs16844364
rs2214827
All − MR Egger
All − Inverse variance weighted
−0.50
−0.25
0.00
0.25
0.50
0.75
MR effect size for
'HGF' on 'Cardiac valvular disease'

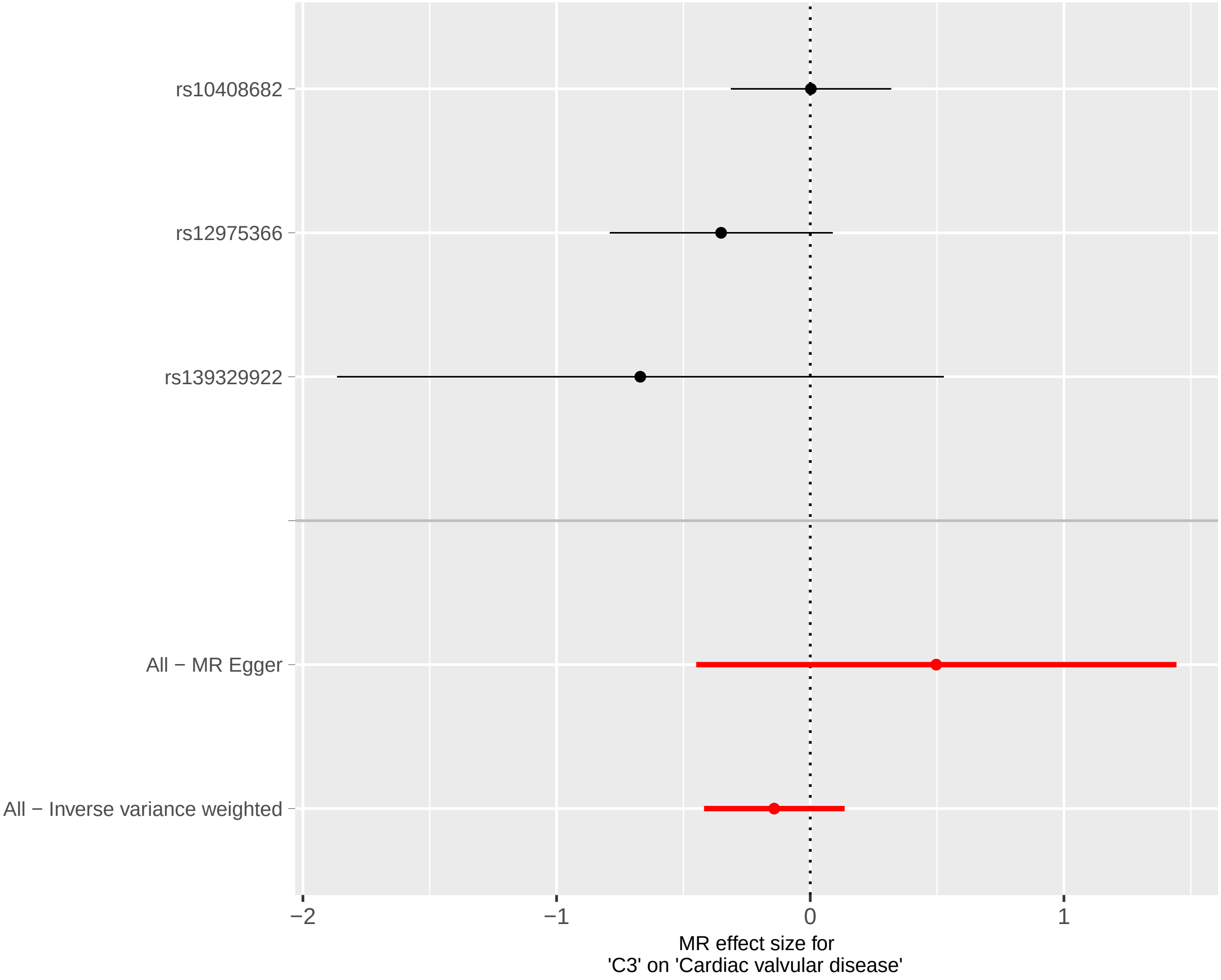

rs10408682
rs12975366
rs139329922
All − MR Egger
All − Inverse variance weighted
−2
−1
0
1
MR effect size for
'C3' on 'Cardiac valvular disease'

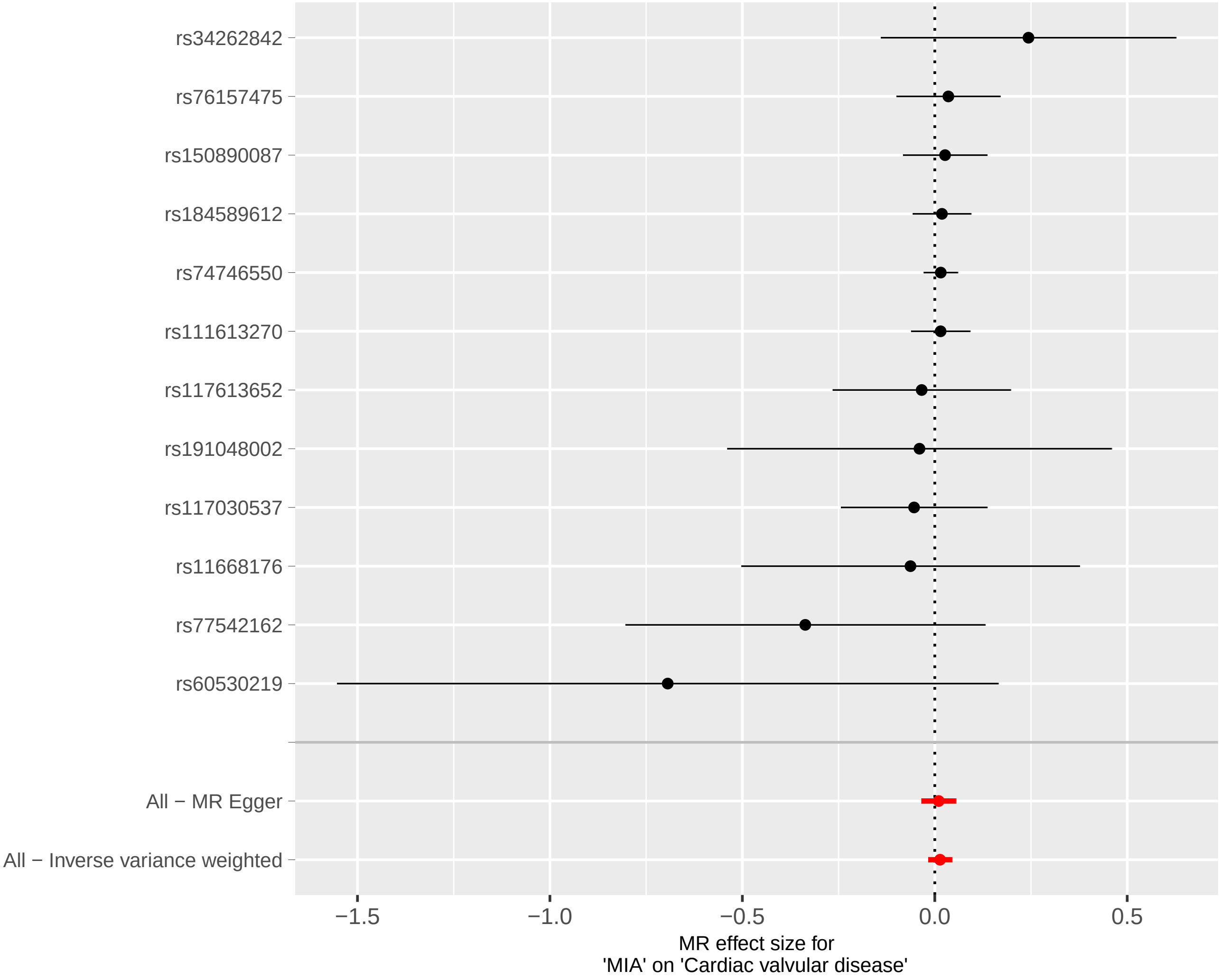
rs34262842
rs76157475
rs150890087
rs184589612
rs74746550
rs111613270
rs117613652
rs191048002
rs117030537
rs11668176
rs77542162
rs60530219
All − MR Egger
All − Inverse variance weighted
−1.5
−1.0
−0.5
0.0
0.5
MR effect size for
'MIA' on 'Cardiac valvular disease'

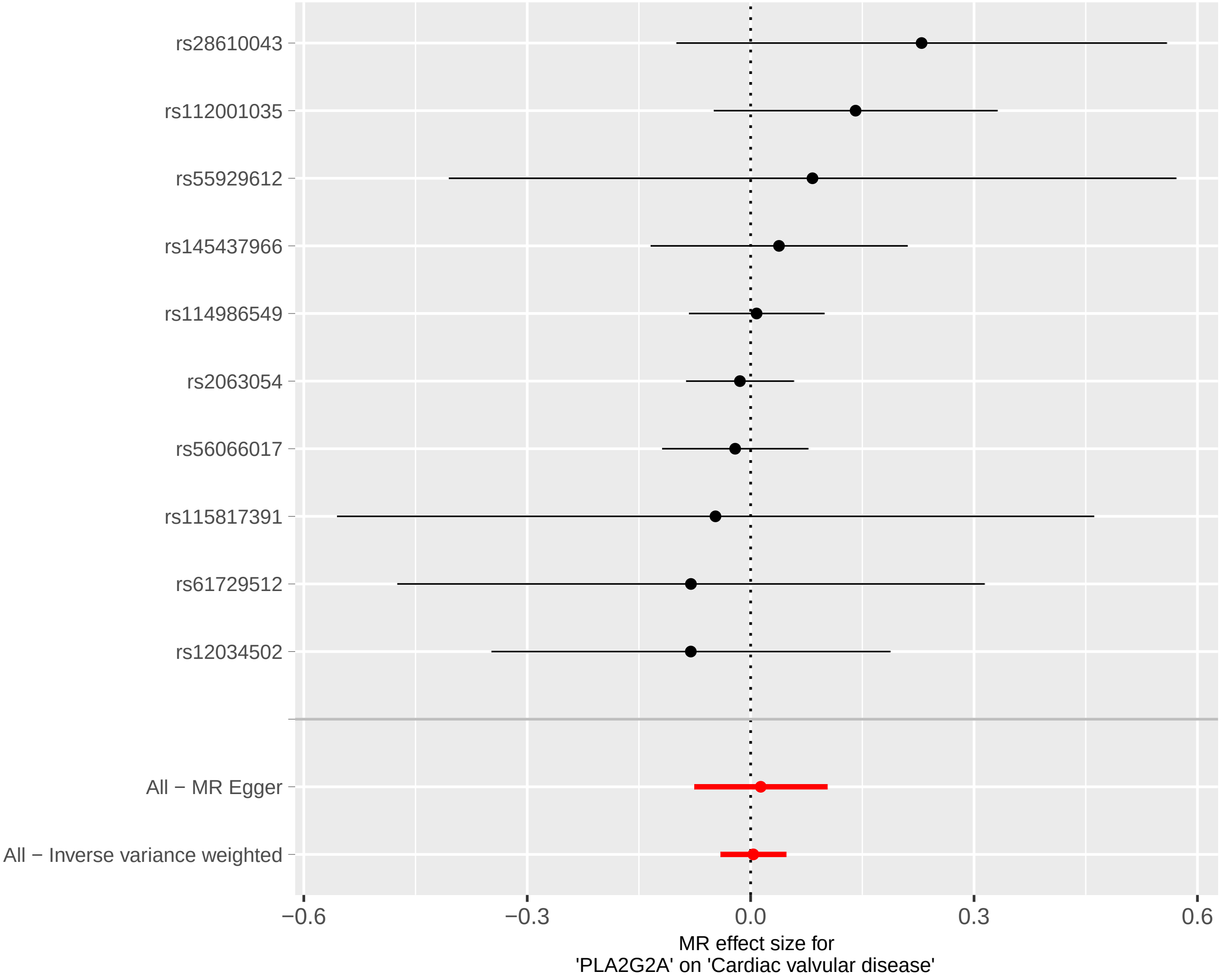
rs28610043
rs112001035
rs55929612
rs145437966
rs114986549
rs2063054
rs56066017
rs115817391
rs61729512
rs12034502
All − MR Egger
All − Inverse variance weighted
−0.6
−0.3
0.0
0.3
0.6
MR effect size for
'PLA2G2A' on 'Cardiac valvular disease'

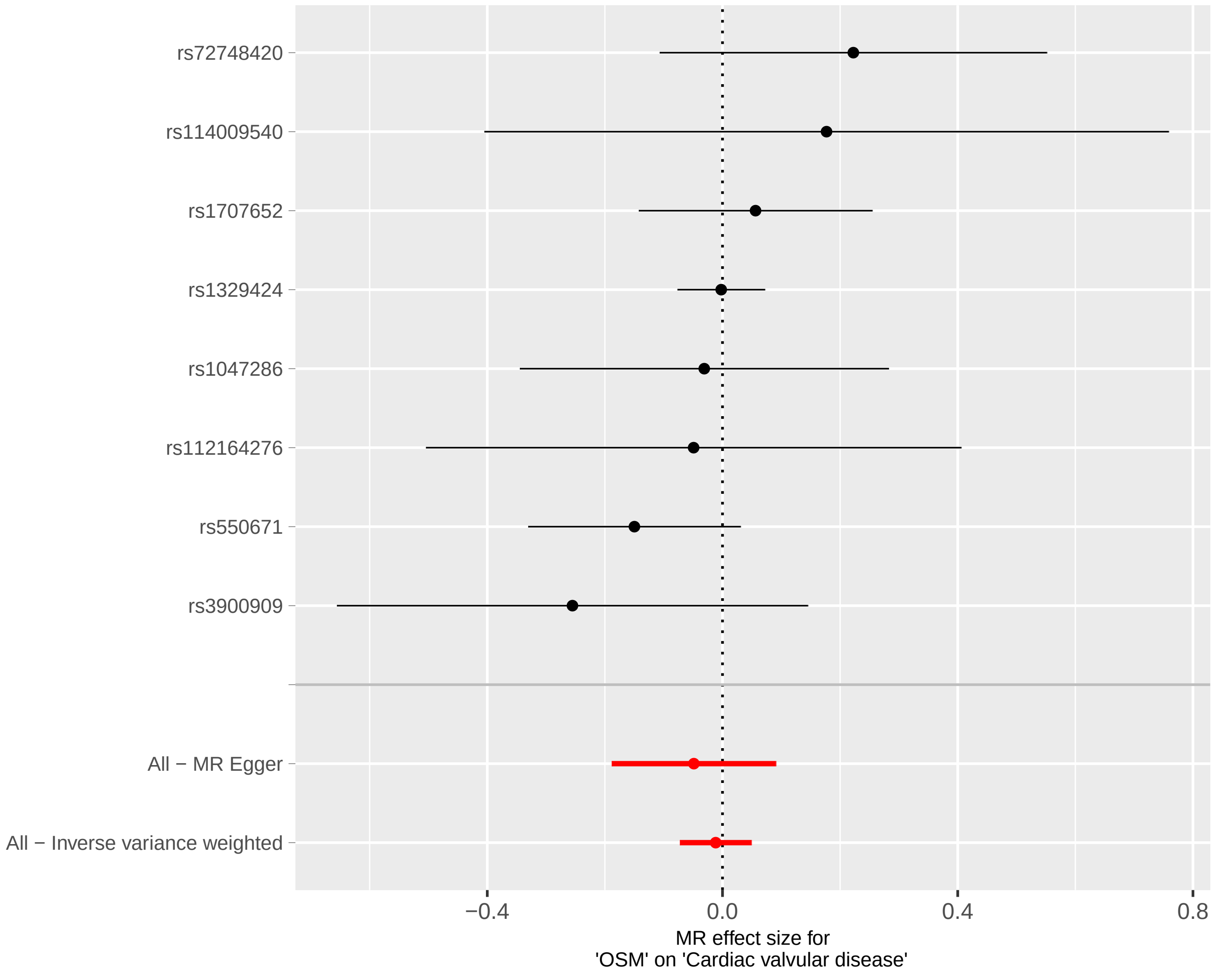
rs72748420
rs114009540
rs1707652
rs1329424
rs1047286
rs112164276
rs550671
rs3900909
All − MR Egger
All − Inverse variance weighted
−0.4
0.0
0.4
0.8
MR effect size for
'OSM' on 'Cardiac valvular disease'

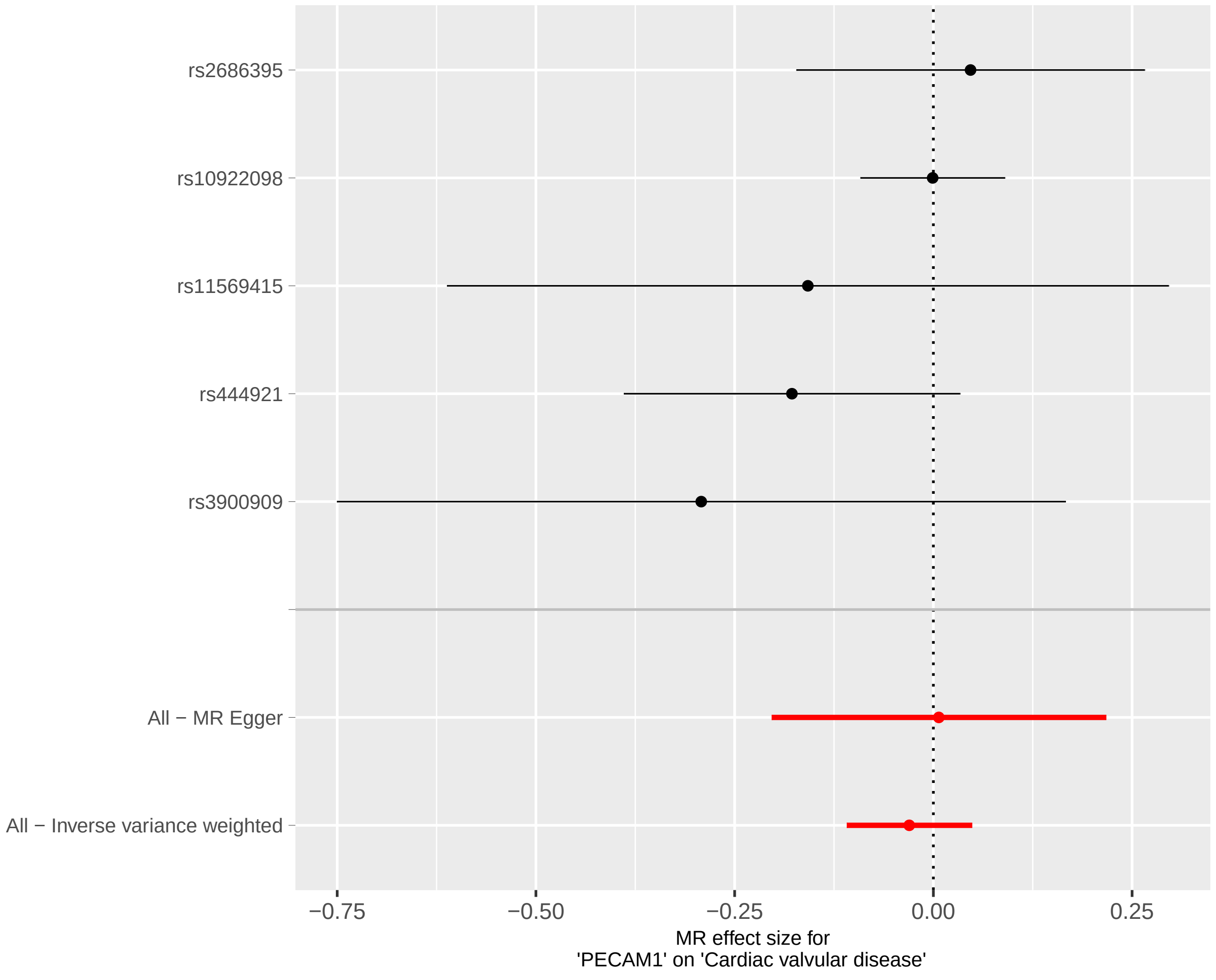
rs2686395
rs10922098
rs11569415
rs444921
rs3900909
All − MR Egger
All − Inverse variance weighted
−0.75
−0.50
−0.25
0.00
0.25
MR effect size for
'PECAM1' on 'Cardiac valvular disease'

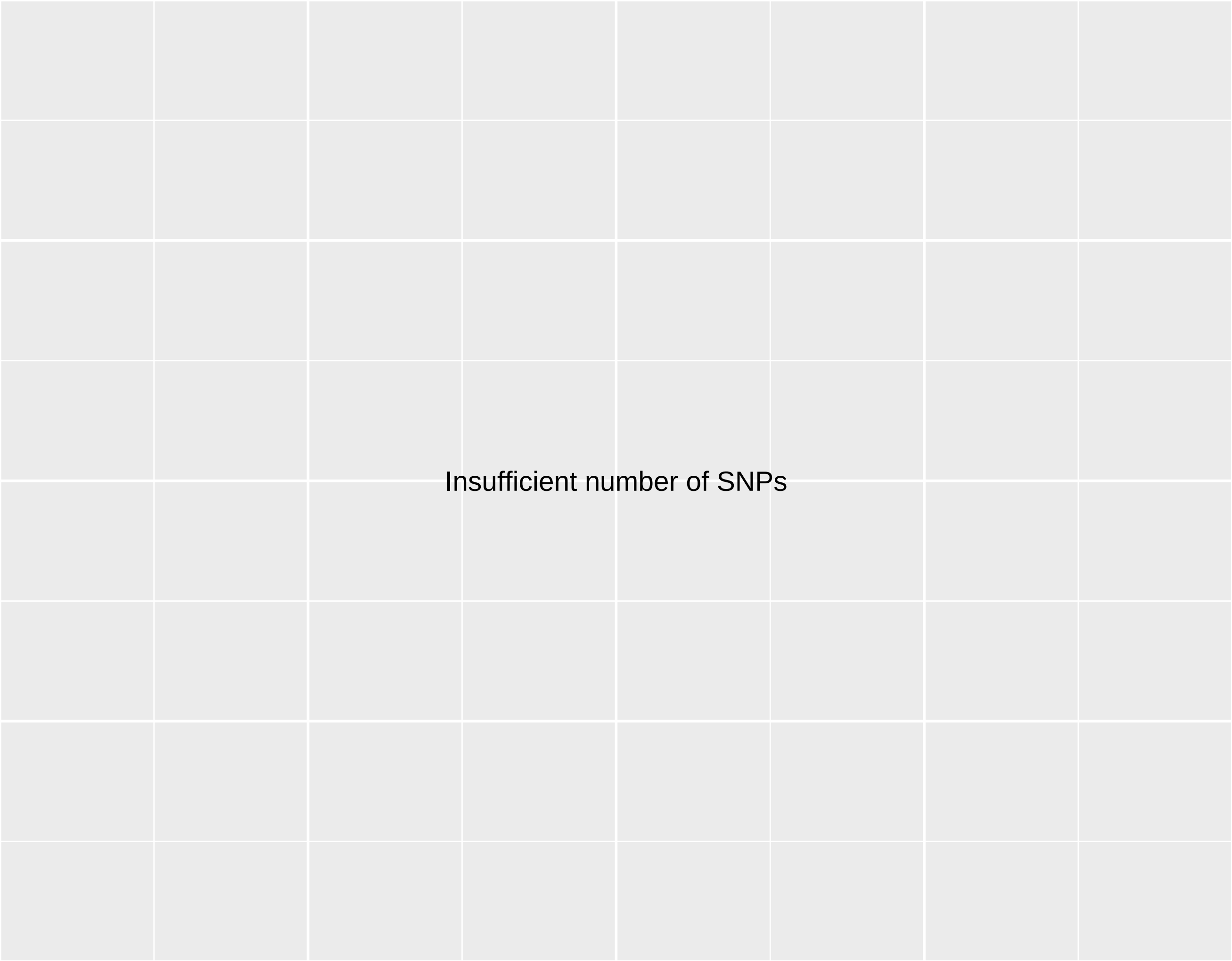
Insufficient number of SNPs

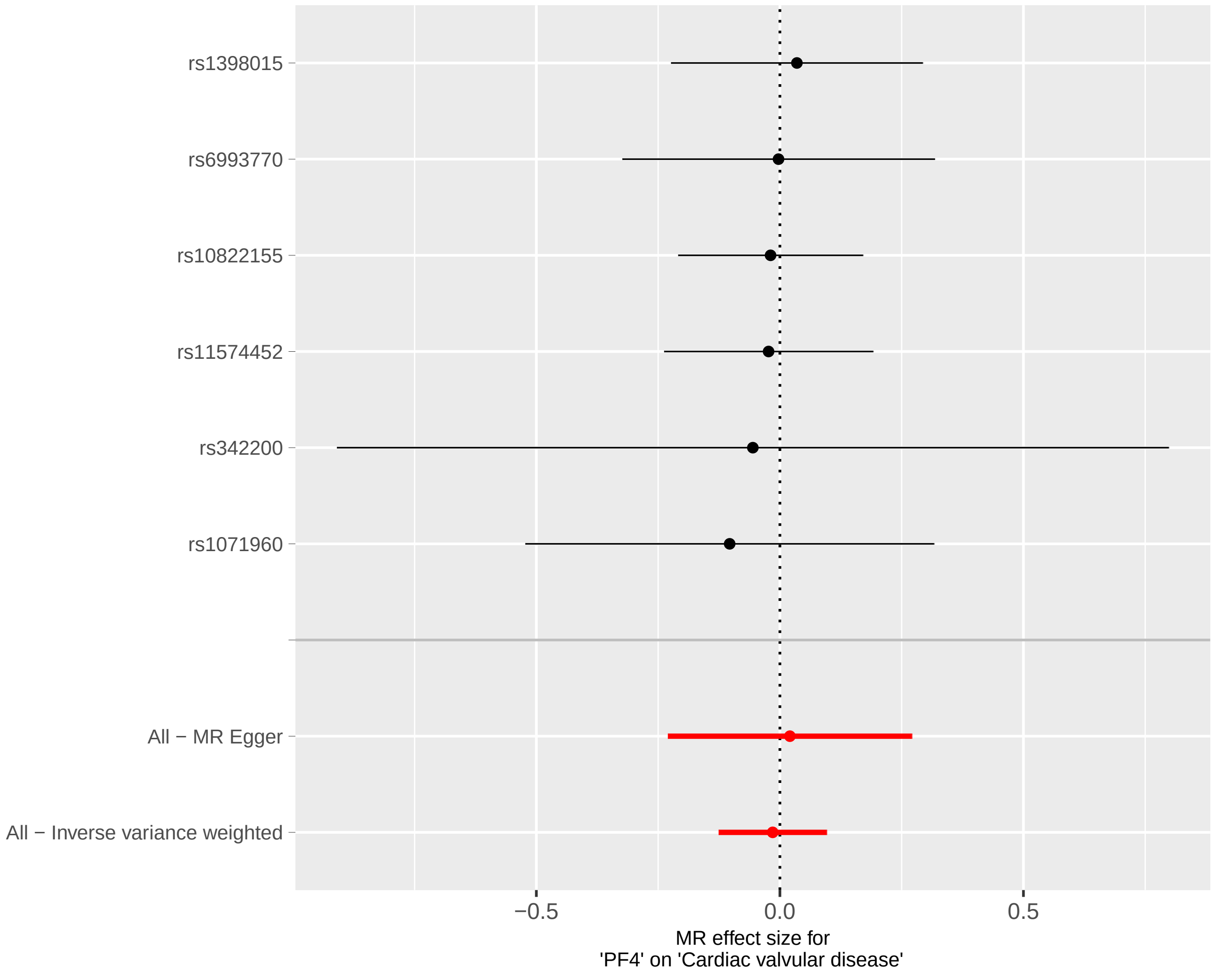
rs1398015
rs6993770
rs10822155
rs11574452
rs342200
rs1071960
All − MR Egger
All − Inverse variance weighted
−0.5
0.0
0.5
MR effect size for
'PF4' on 'Cardiac valvular disease'

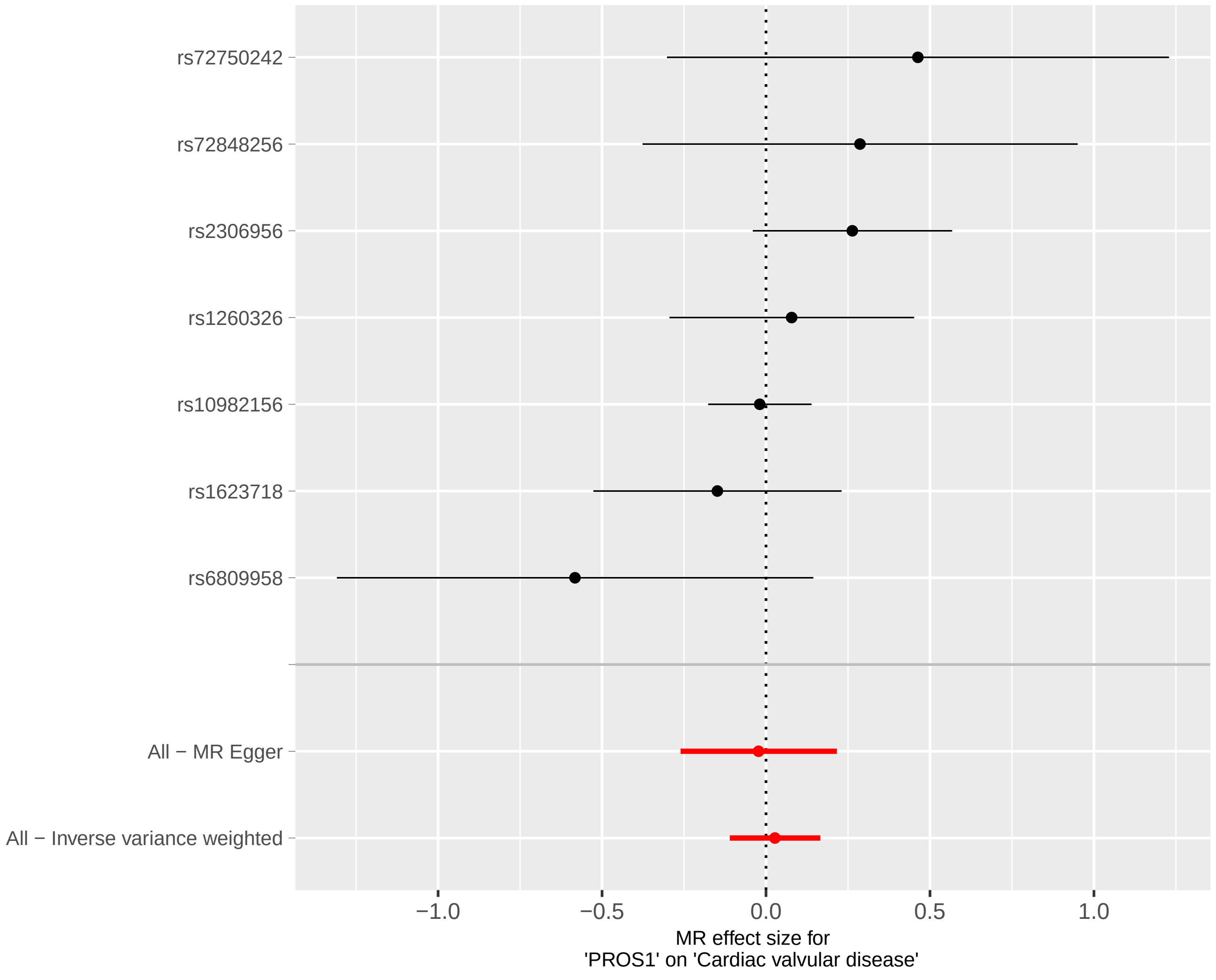
rs72750242
rs72848256
rs2306956
rs1260326
rs10982156
rs1623718
rs6809958
All − MR Egger
All − Inverse variance weighted
−1.0
−0.5
0.0
0.5
1.0
MR effect size for
'PROS1' on 'Cardiac valvular disease'

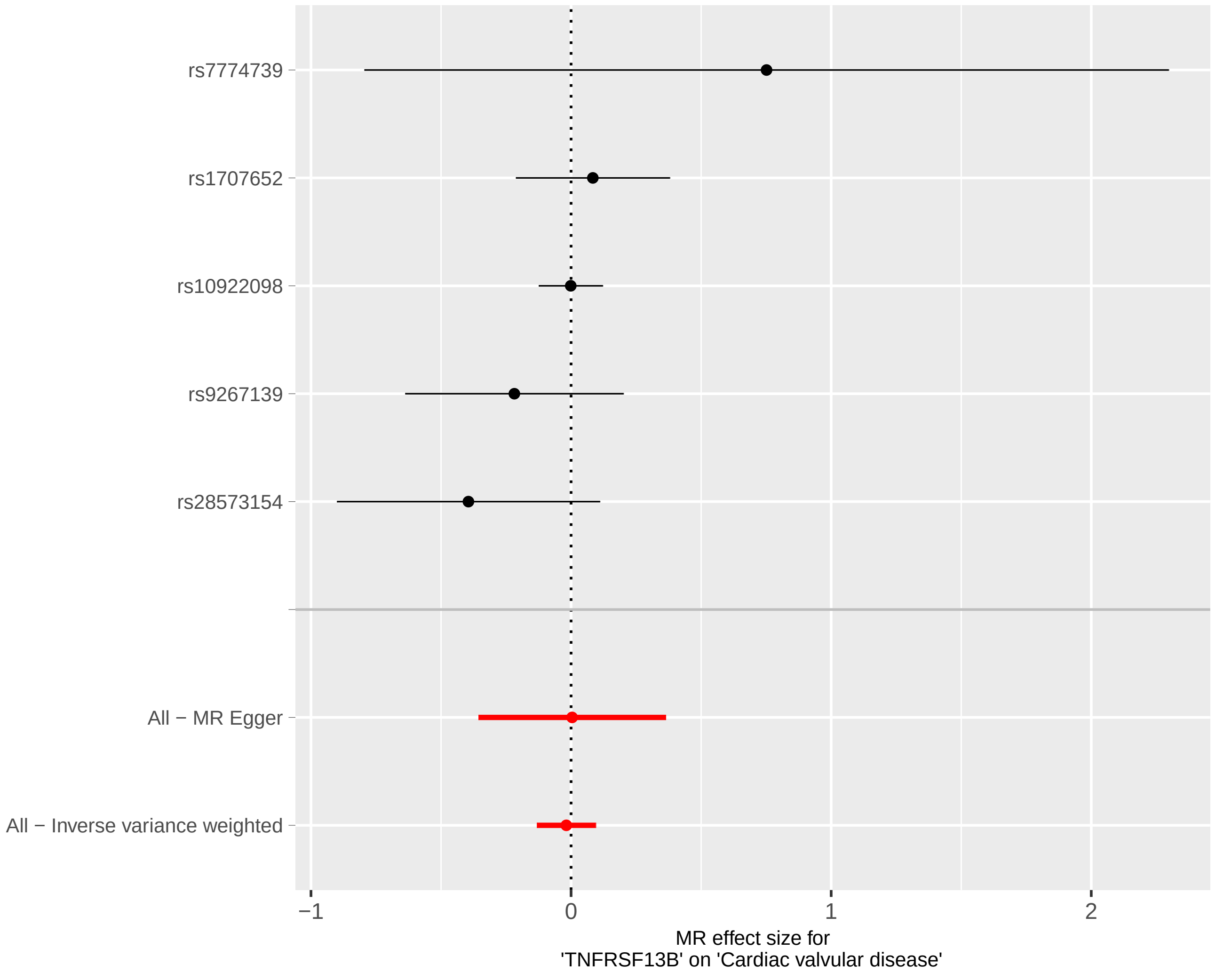
rs7774739
rs1707652
rs10922098
rs9267139
rs28573154
All − MR Egger
All − Inverse variance weighted
−1
0
1
2
MR effect size for
'TNFRSF13B' on 'Cardiac valvular disease'

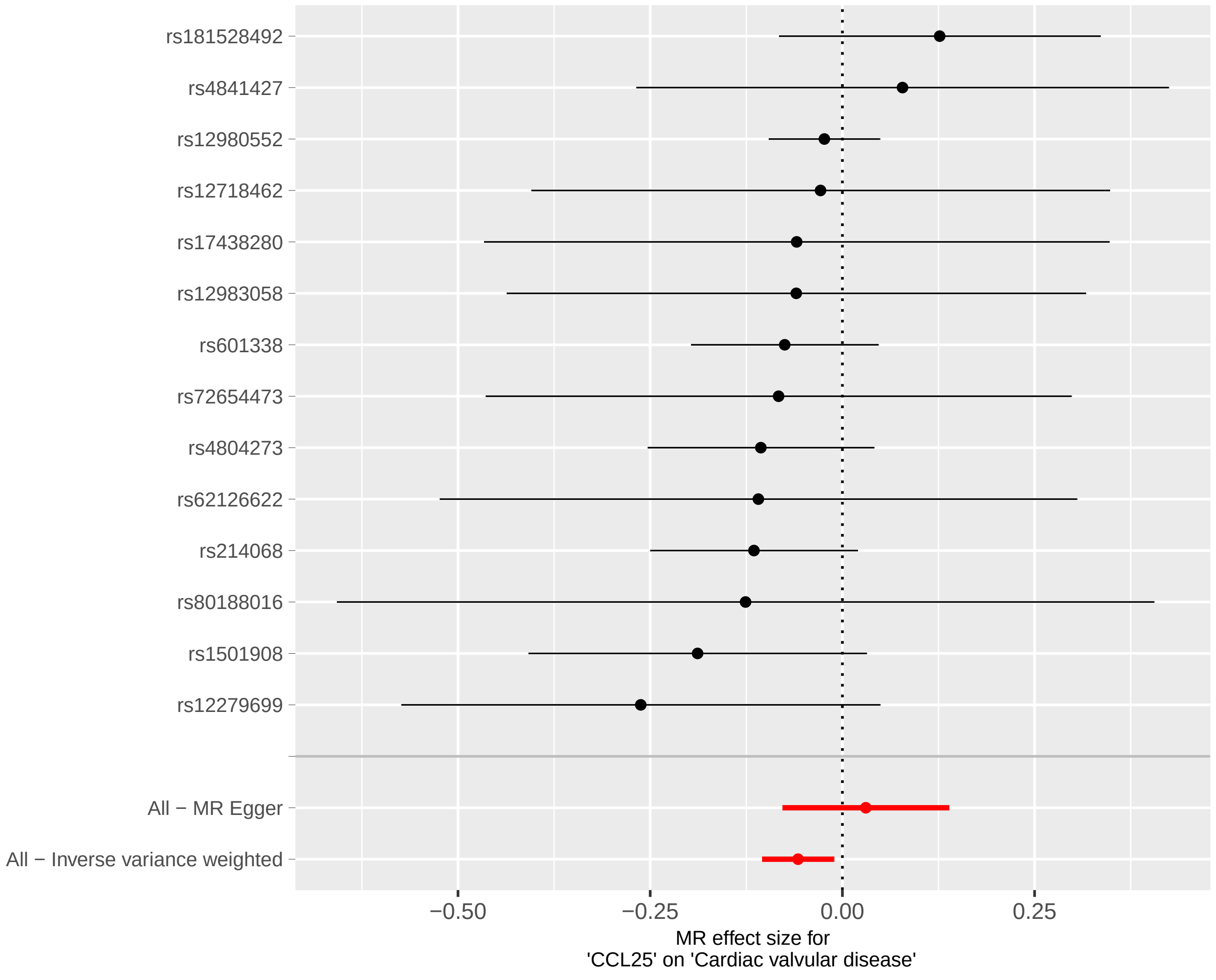

rs181528492
rs4841427
rs12980552
rs12718462
rs17438280
rs12983058
rs601338
rs72654473
rs4804273
rs62126622
rs214068
rs80188016
rs1501908
rs12279699
All − MR Egger
All − Inverse variance weighted
−0.50
−0.25
0.00
0.25
MR effect size for
'CCL25' on 'Cardiac valvular disease'

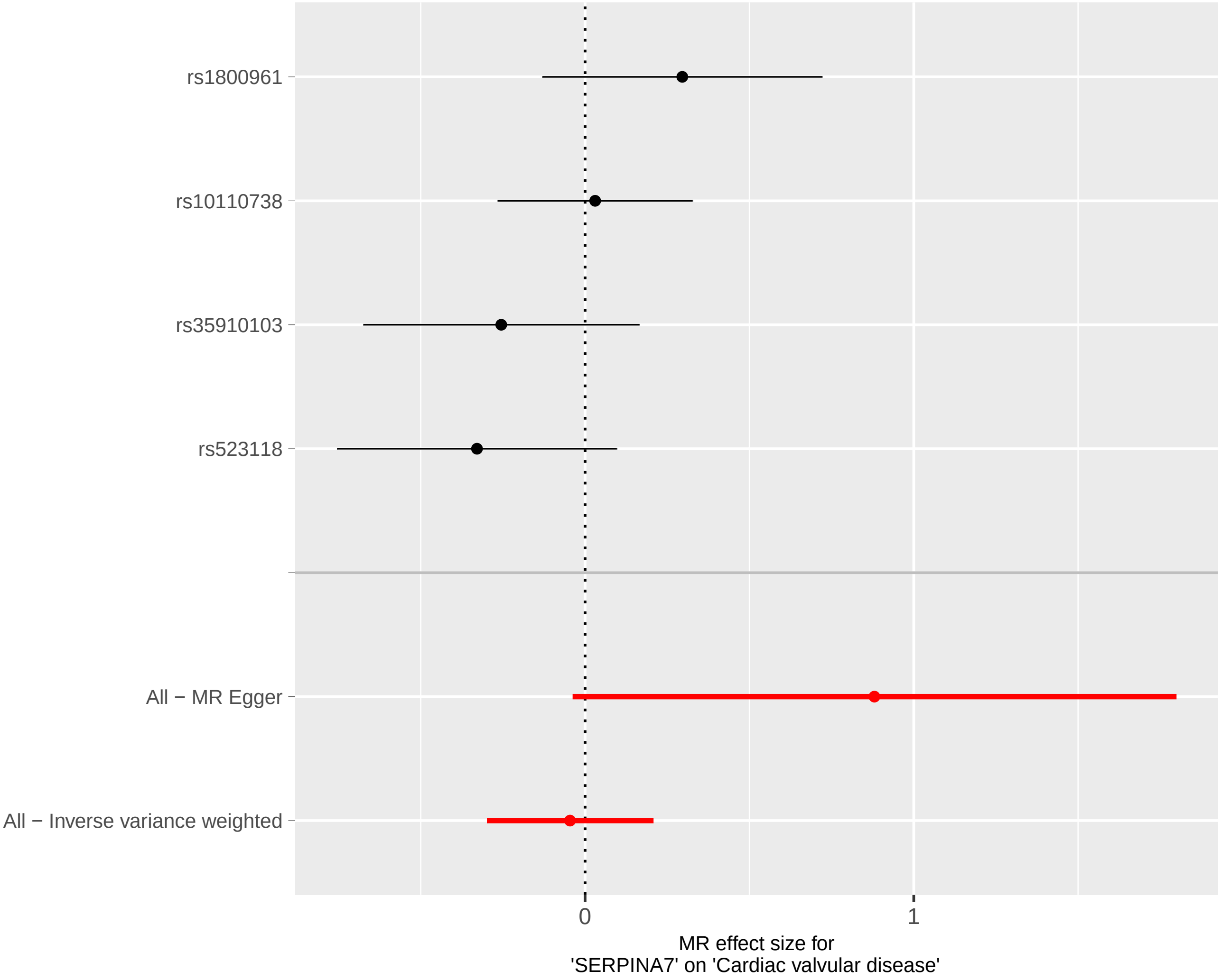

rs1800961
rs10110738
rs35910103
rs523118
All − MR Egger
All − Inverse variance weighted
0
1
MR effect size for
'SERPINA7' on 'Cardiac valvular disease'

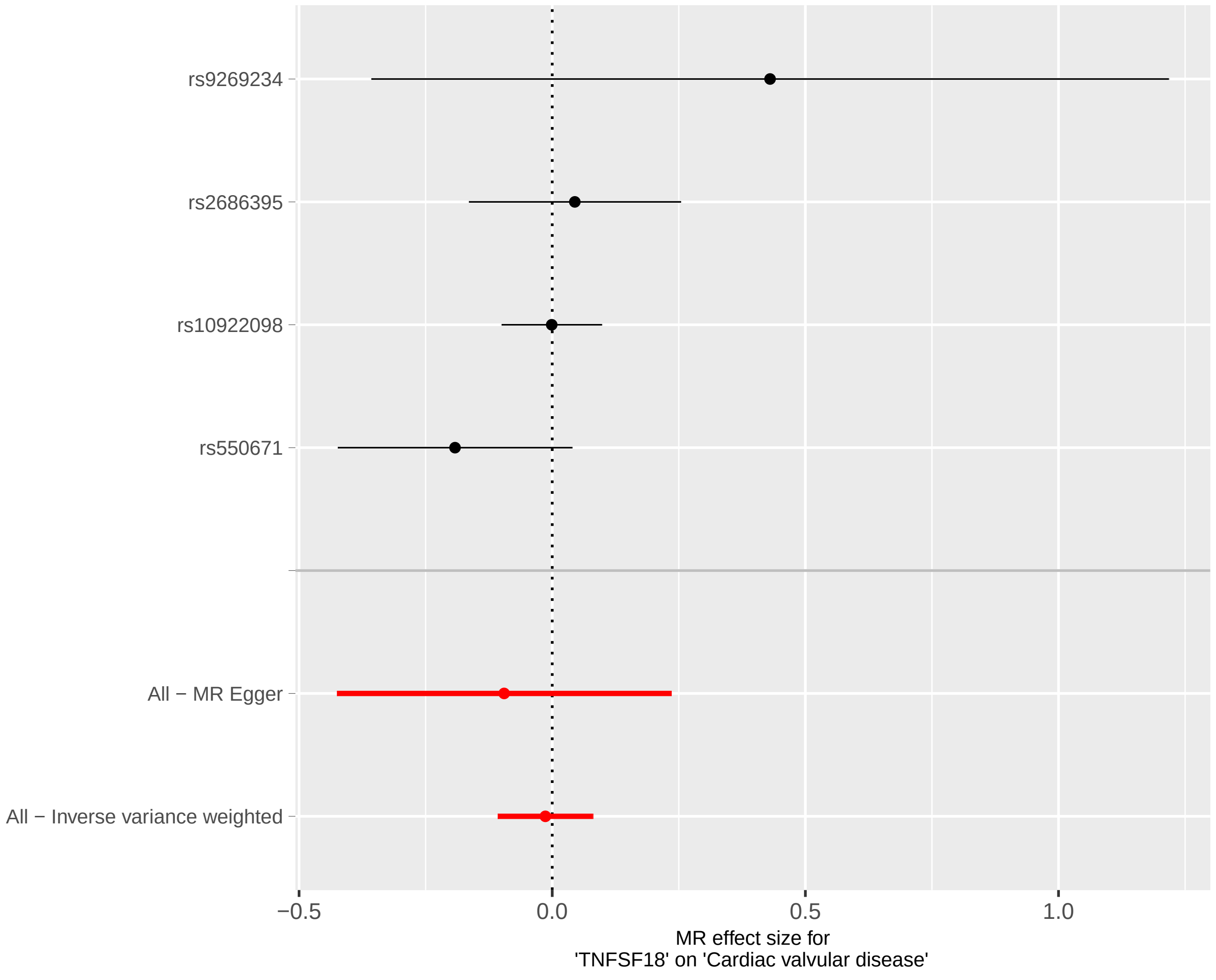
rs9269234
rs2686395
rs10922098
rs550671
All − MR Egger
All − Inverse variance weighted
−0.5
0.0
0.5
1.0
MR effect size for
'TNFSF18' on 'Cardiac valvular disease'

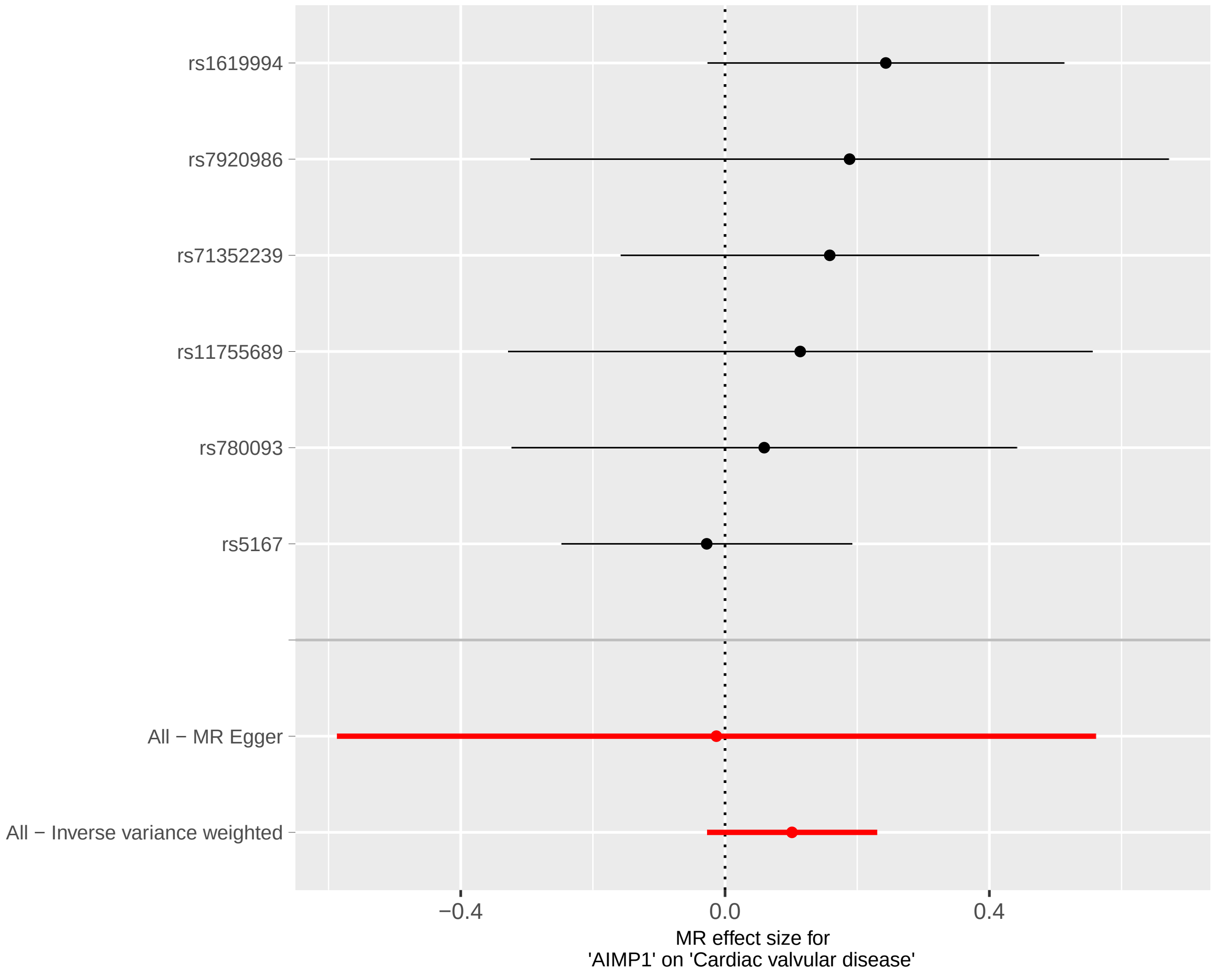
rs1619994
rs7920986
rs71352239
rs11755689
rs780093
rs5167
All − MR Egger
All − Inverse variance weighted
−0.4
0.0
0.4
MR effect size for
'AIMP1' on 'Cardiac valvular disease'

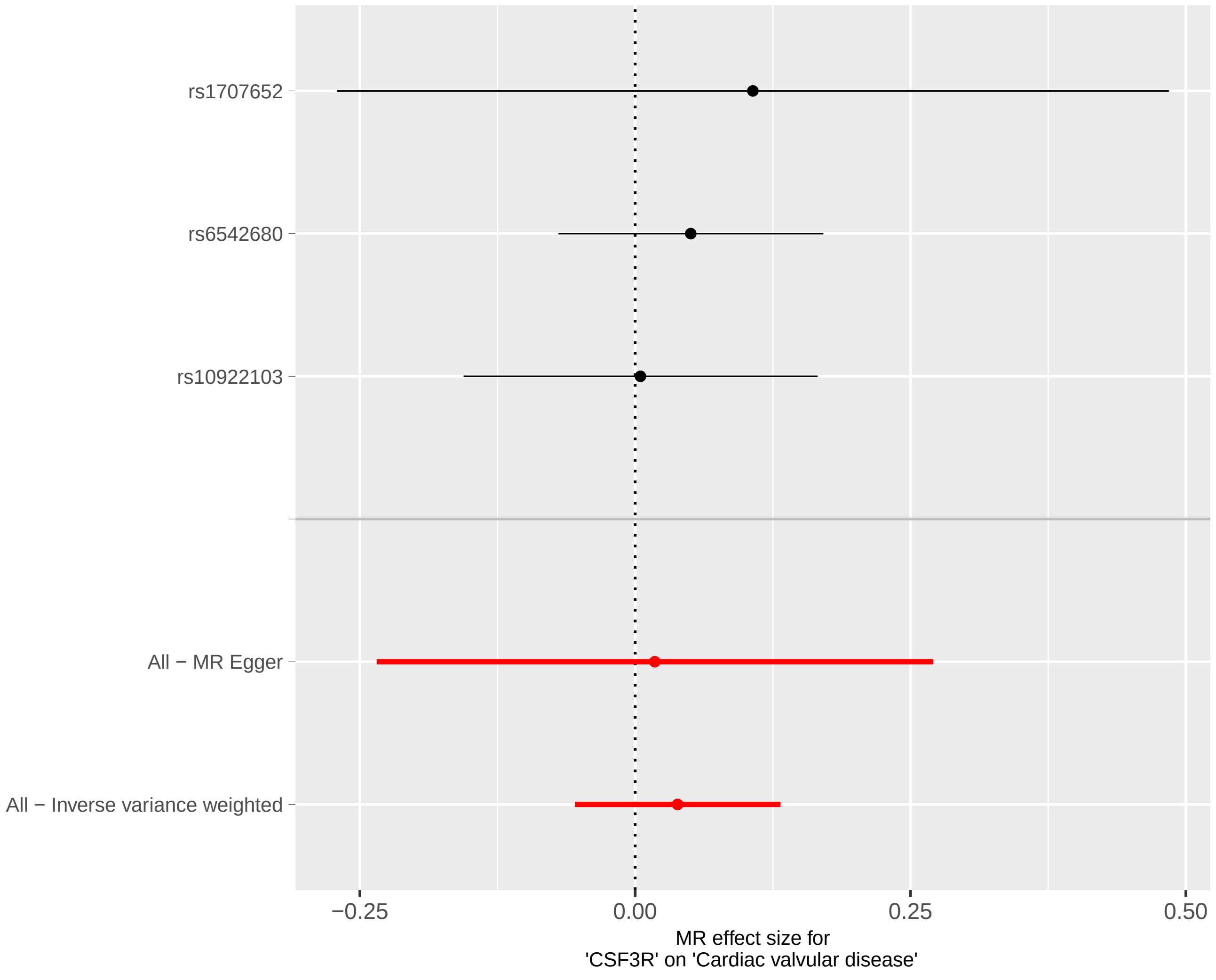
rs1707652
rs6542680
rs10922103
All − MR Egger
All − Inverse variance weighted
−0.25
0.00
0.25
0.50
MR effect size for
'CSF3R' on 'Cardiac valvular disease'

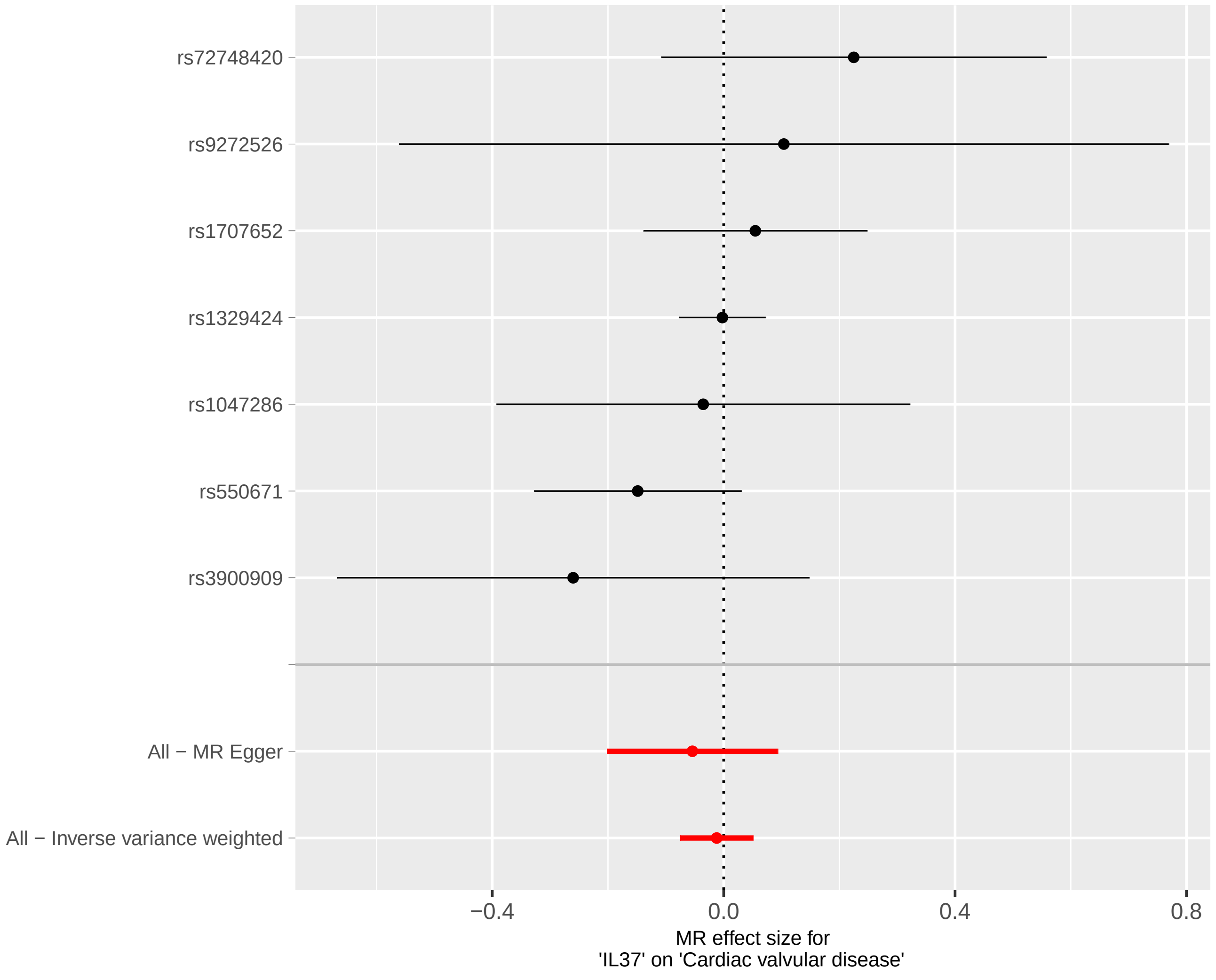

rs72748420
rs9272526
rs1707652
rs1329424
rs1047286
rs550671
rs3900909
All − MR Egger
All − Inverse variance weighted
−0.4
0.0
0.4
0.8
MR effect size for
'IL37' on 'Cardiac valvular disease'

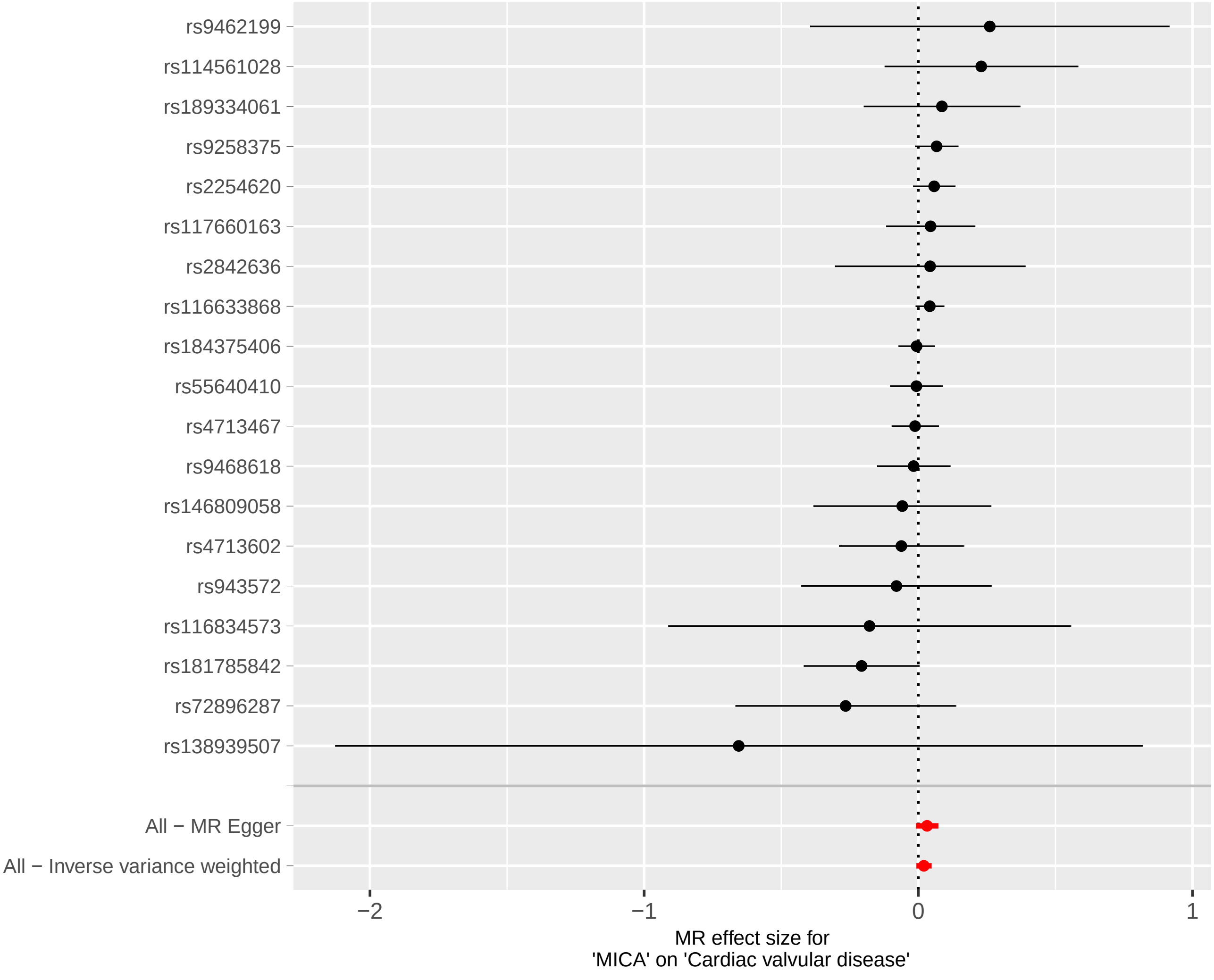
rs9462199
rs114561028
rs189334061
rs9258375
rs2254620
rs117660163
rs2842636
rs116633868
rs184375406
rs55640410
rs4713467
rs9468618
rs146809058
rs4713602
rs943572
rs116834573
rs181785842
rs72896287
rs138939507
All − MR Egger
All − Inverse variance weighted
−2
−1
0
1
MR effect size for
'MICA' on 'Cardiac valvular disease'

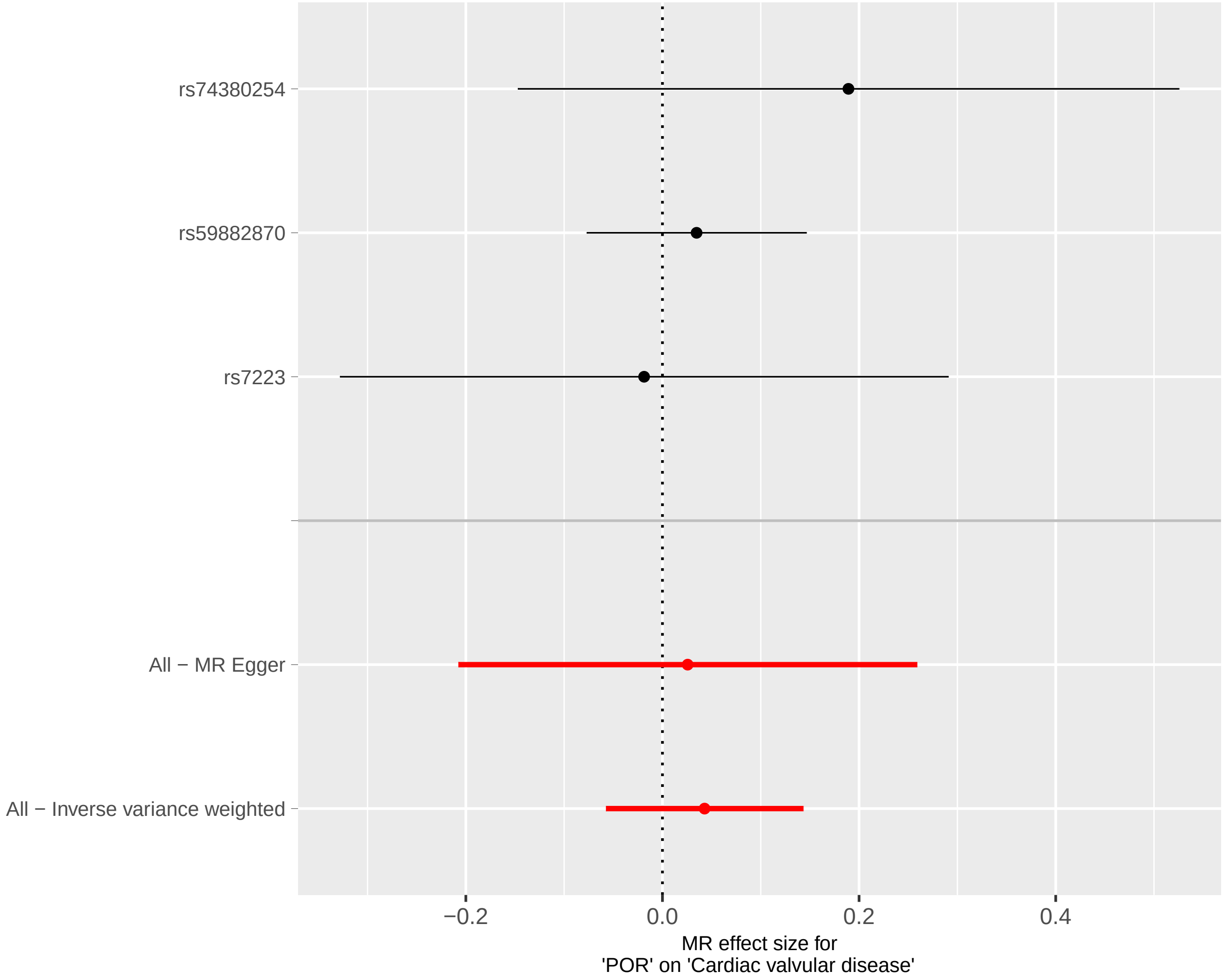

rs74380254
rs59882870
rs7223
All − MR Egger
All − Inverse variance weighted
−0.2
0.0
0.2
0.4
MR effect size for
'POR' on 'Cardiac valvular disease'

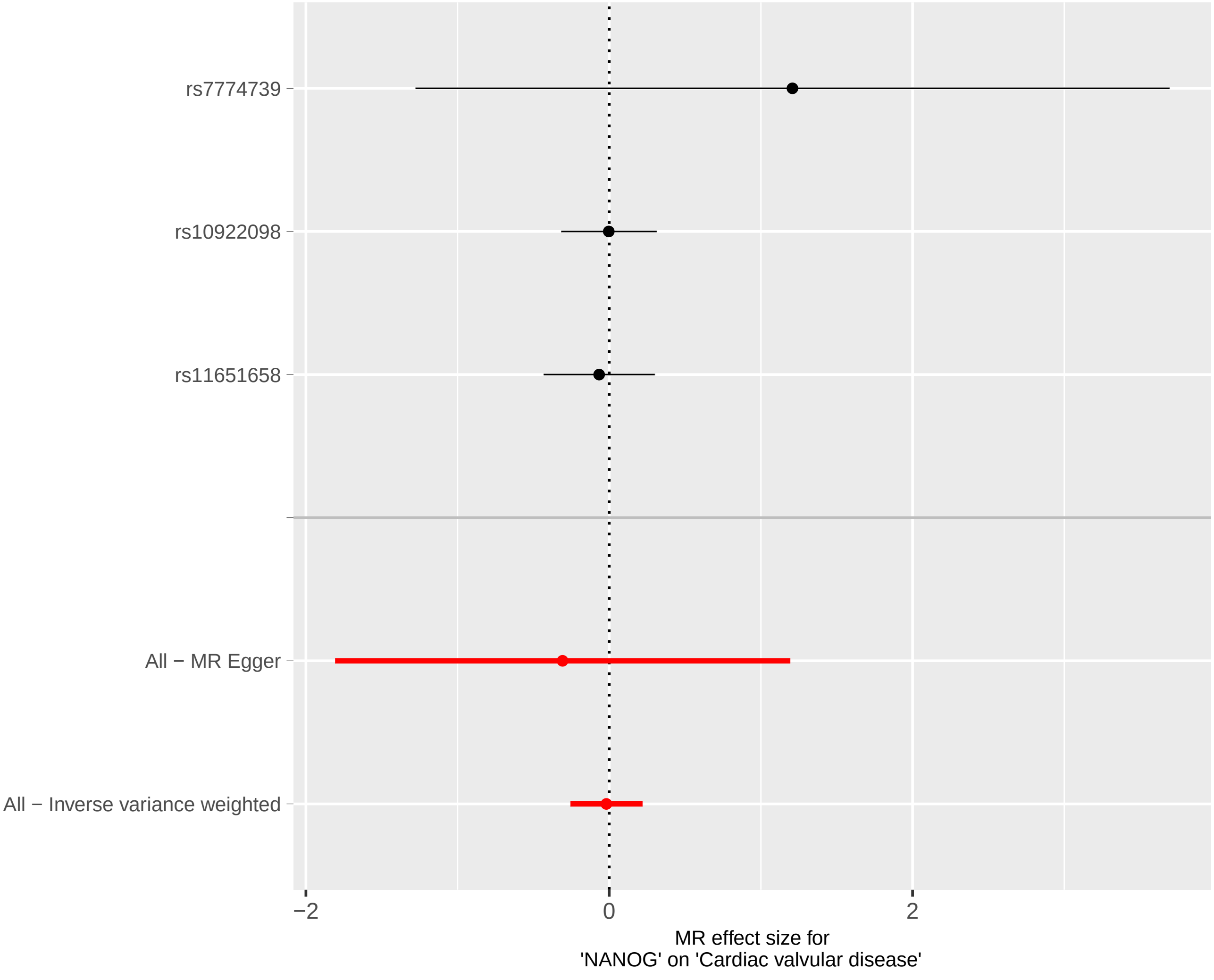
rs7774739
rs10922098
rs11651658
All − MR Egger
All − Inverse variance weighted
−2
0
2
MR effect size for
'NANOG' on 'Cardiac valvular disease'

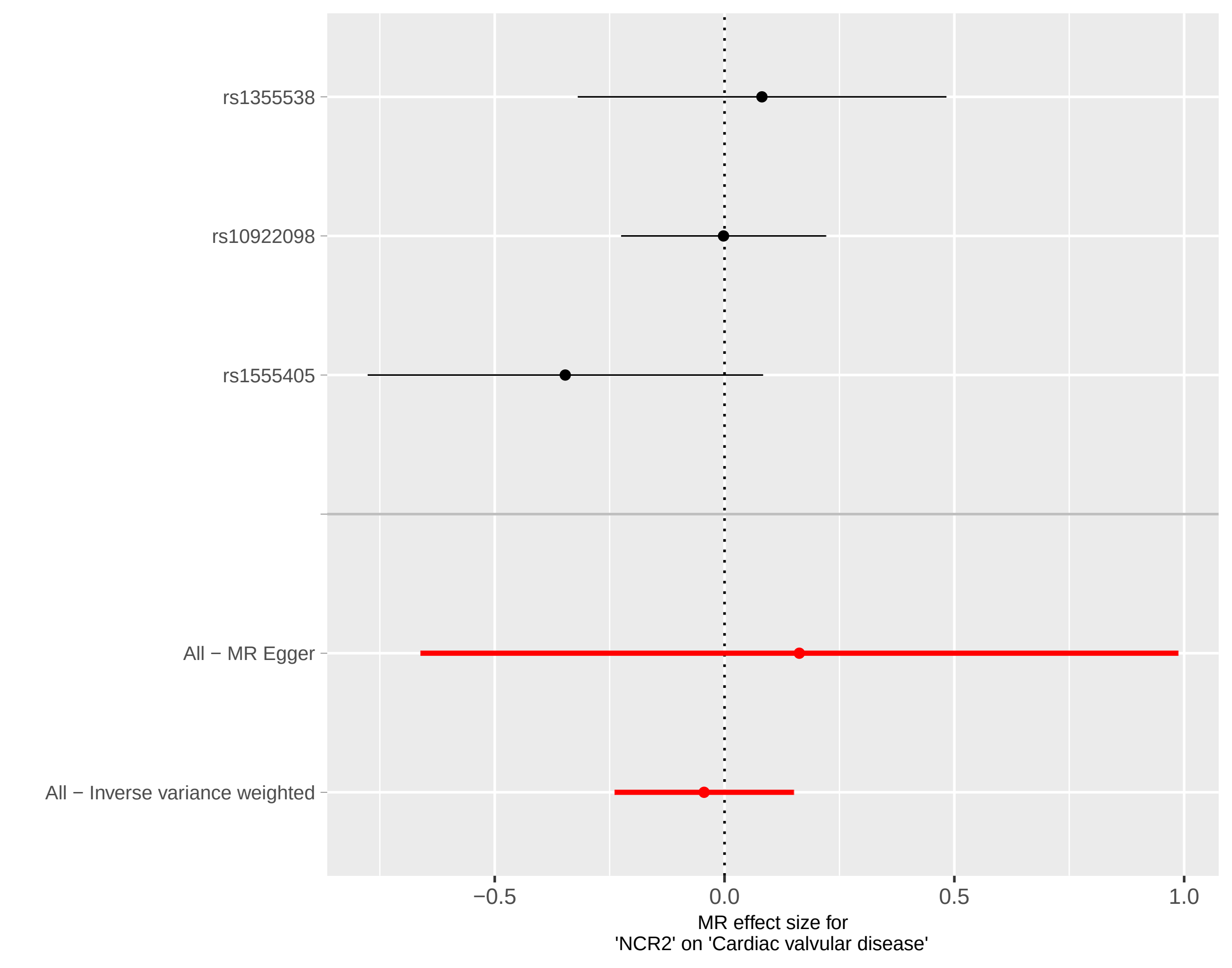

rs1355538
rs10922098
rs1555405
All − MR Egger
All − Inverse variance weighted
−0.5
0.0
0.5
1.0
MR effect size for
'NCR2' on 'Cardiac valvular disease'

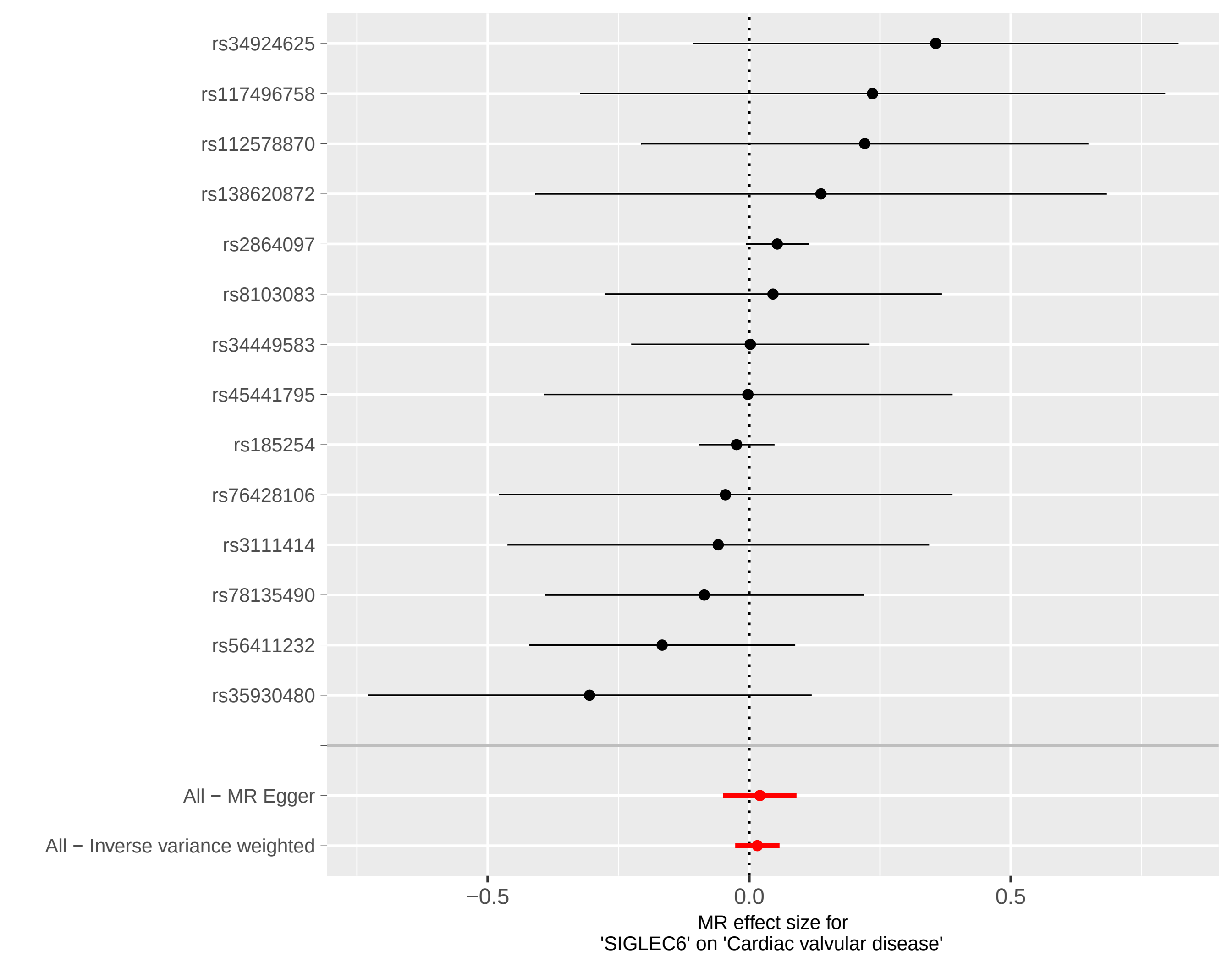
rs34924625
rs117496758
rs112578870
rs138620872
rs2864097
rs8103083
rs34449583
rs45441795
rs185254
rs76428106
rs3111414
rs78135490
rs56411232
rs35930480
All − MR Egger
All − Inverse variance weighted
−0.5
0.0
0.5
MR effect size for
'SIGLEC6' on 'Cardiac valvular disease'

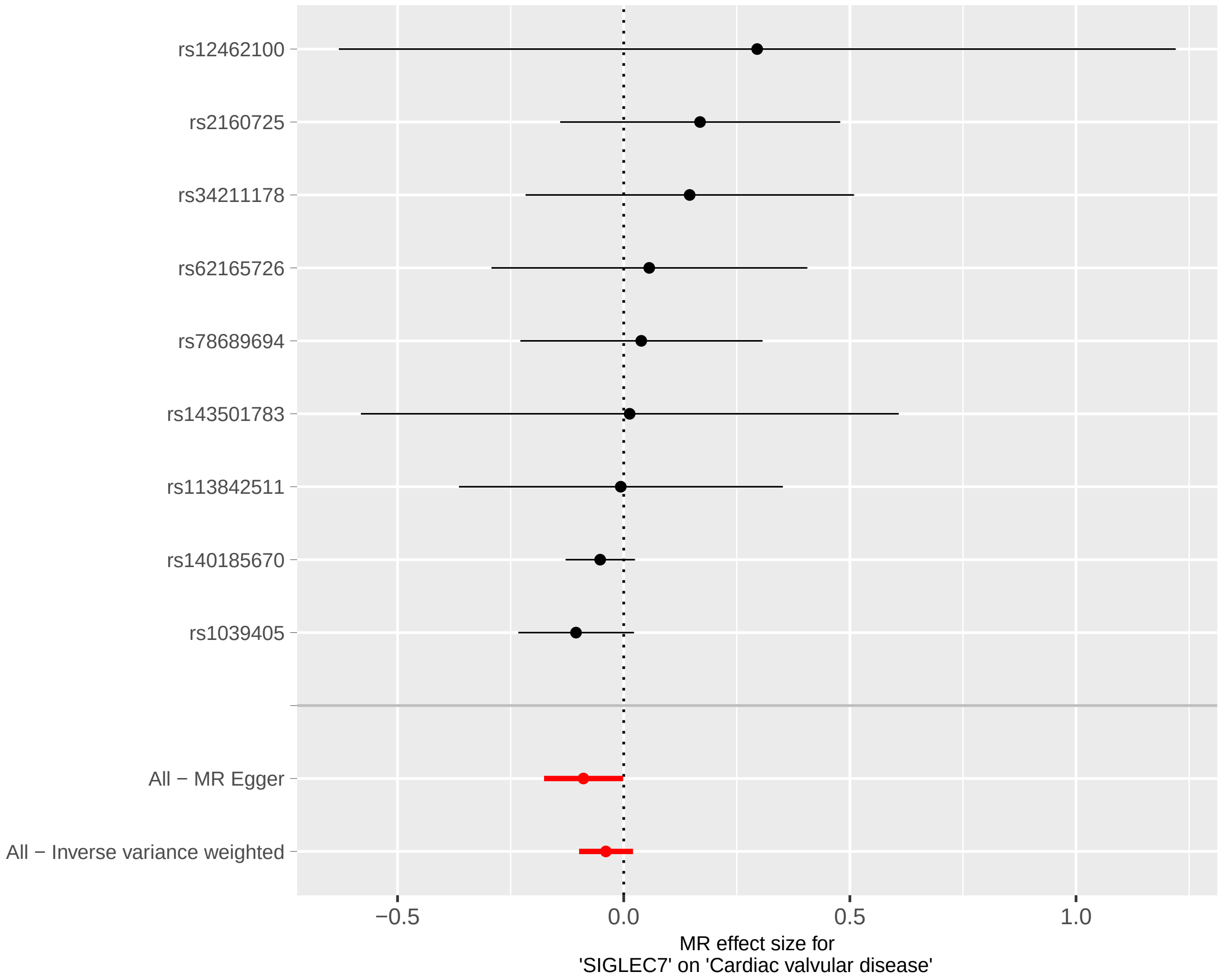
rs12462100
rs2160725
rs34211178
rs62165726
rs78689694
rs143501783
rs113842511
rs140185670
rs1039405
All − MR Egger
All − Inverse variance weighted
−0.5
0.0
0.5
1.0
MR effect size for
'SIGLEC7' on 'Cardiac valvular disease'

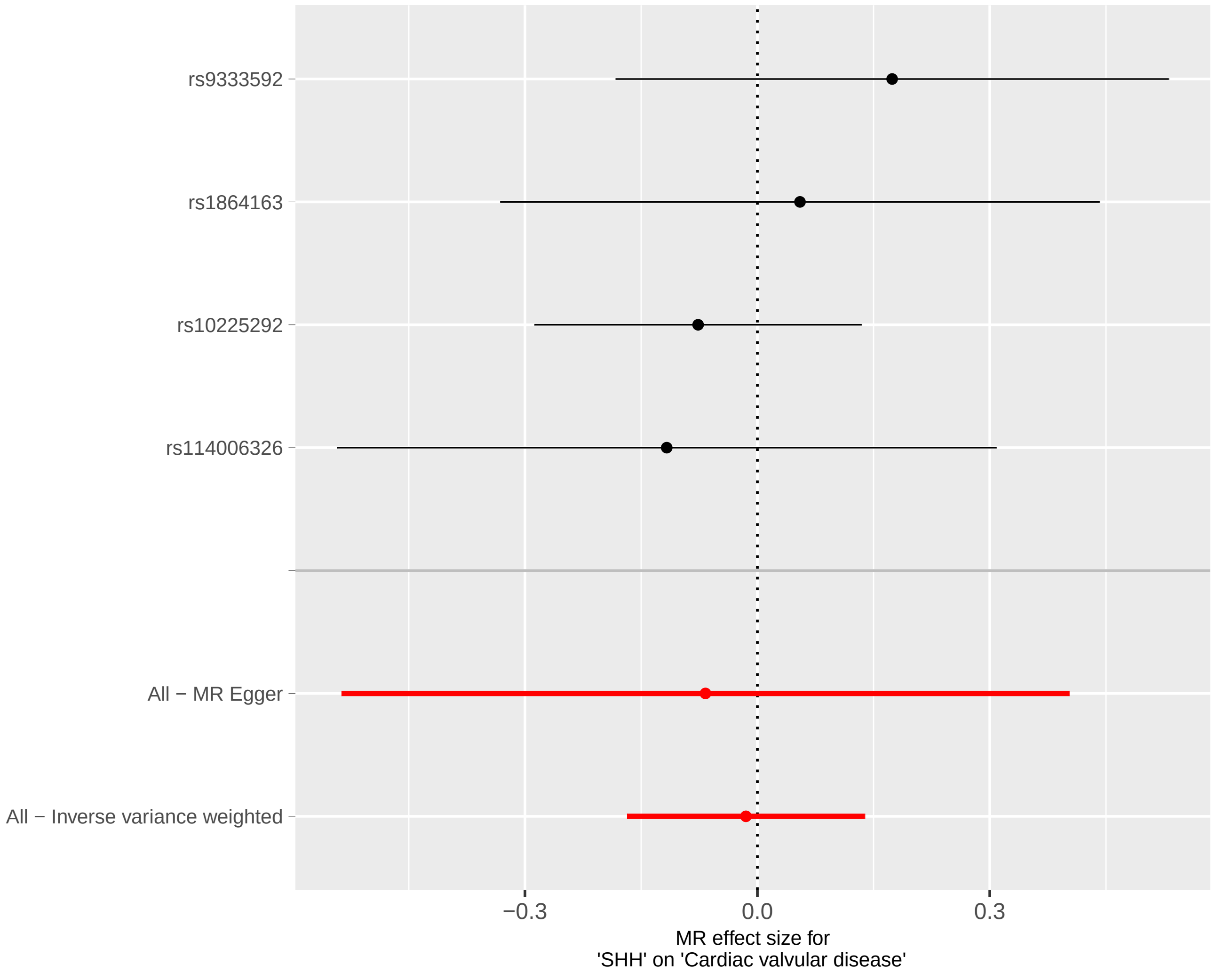
rs9333592
rs1864163
rs10225292
rs114006326
All − MR Egger
All − Inverse variance weighted
−0.3
0.0
0.3
MR effect size for
'SHH' on 'Cardiac valvular disease'

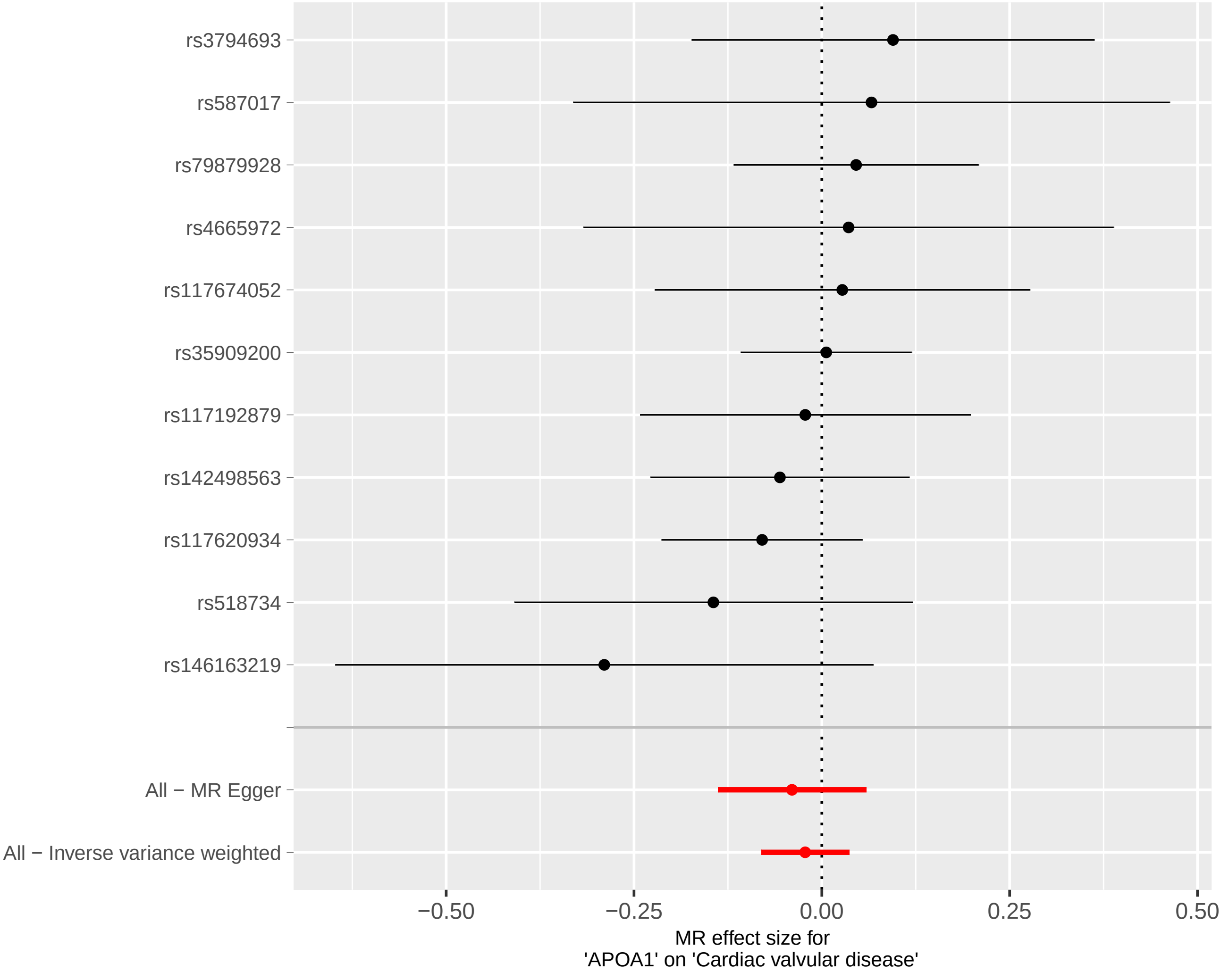
rs3794693
rs587017
rs79879928
rs4665972
rs117674052
rs35909200
rs117192879
rs142498563
rs117620934
rs518734
rs146163219
All − MR Egger
All − Inverse variance weighted
−0.50
−0.25
0.00
0.25
0.50
MR effect size for
'APOA1' on 'Cardiac valvular disease'

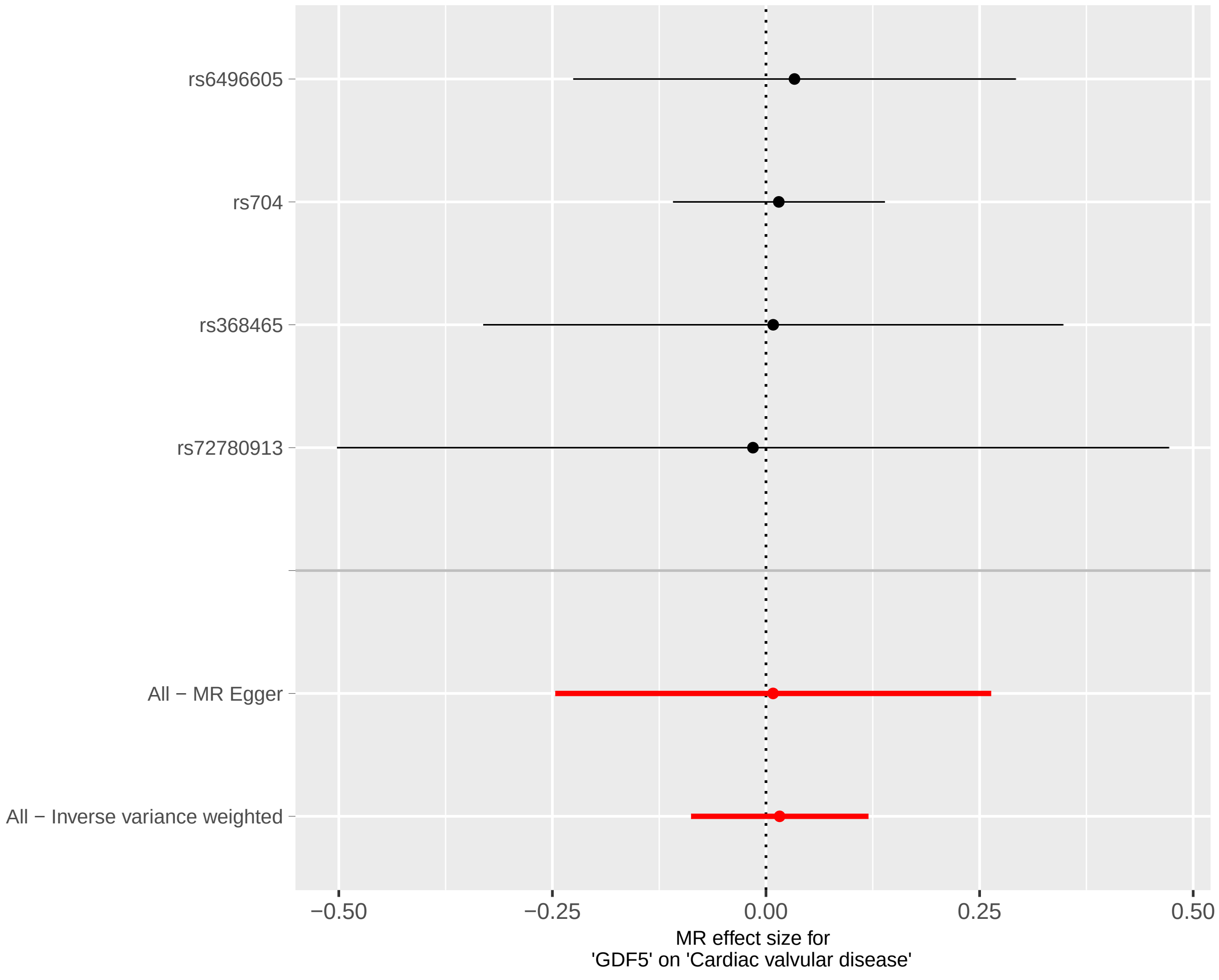

rs6496605
rs704
rs368465
rs72780913
All − MR Egger
All − Inverse variance weighted
−0.50
−0.25
0.00
0.25
0.50
MR effect size for
'GDF5' on 'Cardiac valvular disease'

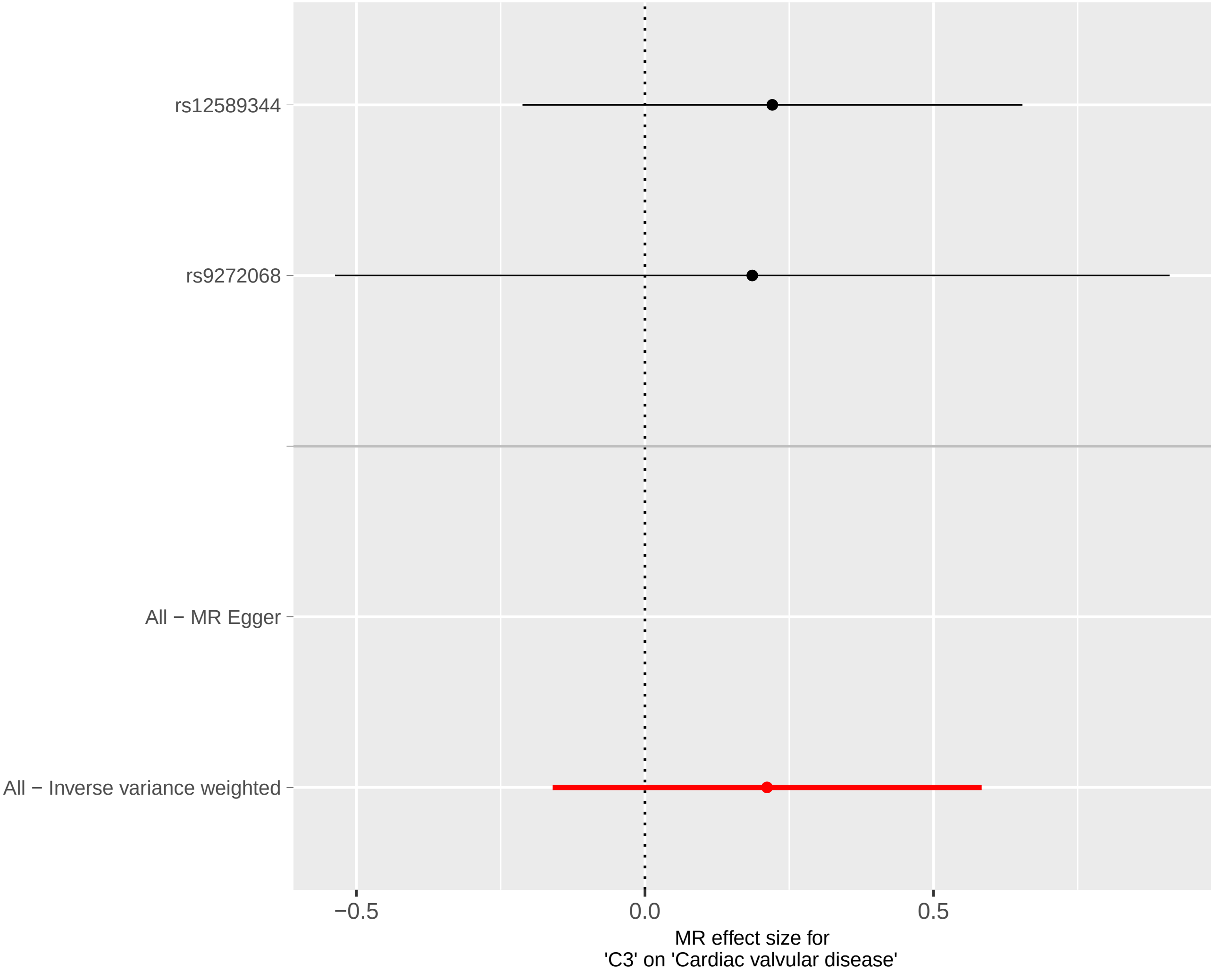
rs12589344
rs9272068
All − MR Egger
All − Inverse variance weighted
−0.5
0.0
0.5
MR effect size for
'C3' on 'Cardiac valvular disease'

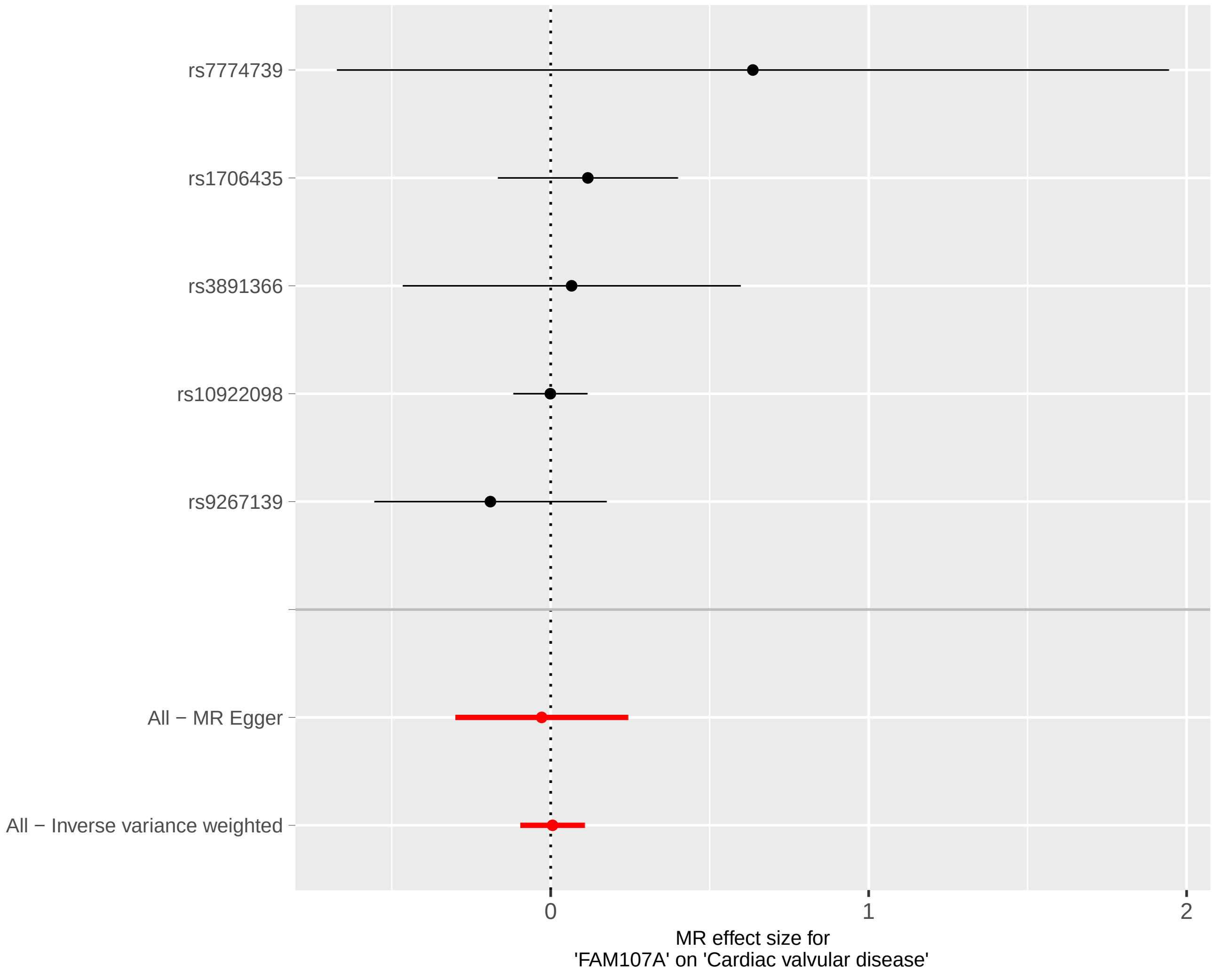
rs7774739
rs1706435
rs3891366
rs10922098
rs9267139
All − MR Egger
All − Inverse variance weighted
0
1
2
MR effect size for
'FAM107A' on 'Cardiac valvular disease'

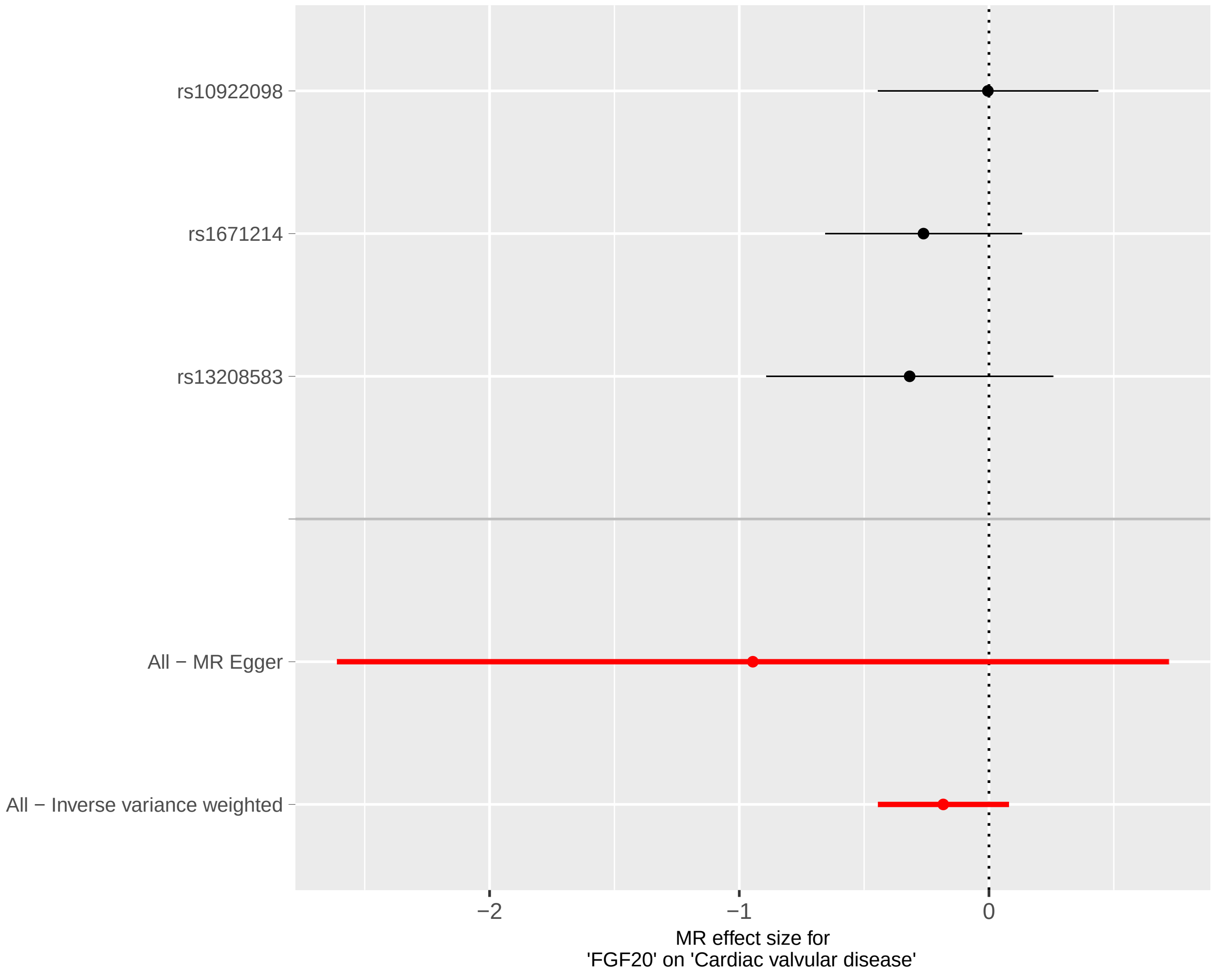
rs10922098
rs1671214
rs13208583
All − MR Egger
All − Inverse variance weighted
−2
−1
0
MR effect size for
'FGF20' on 'Cardiac valvular disease'

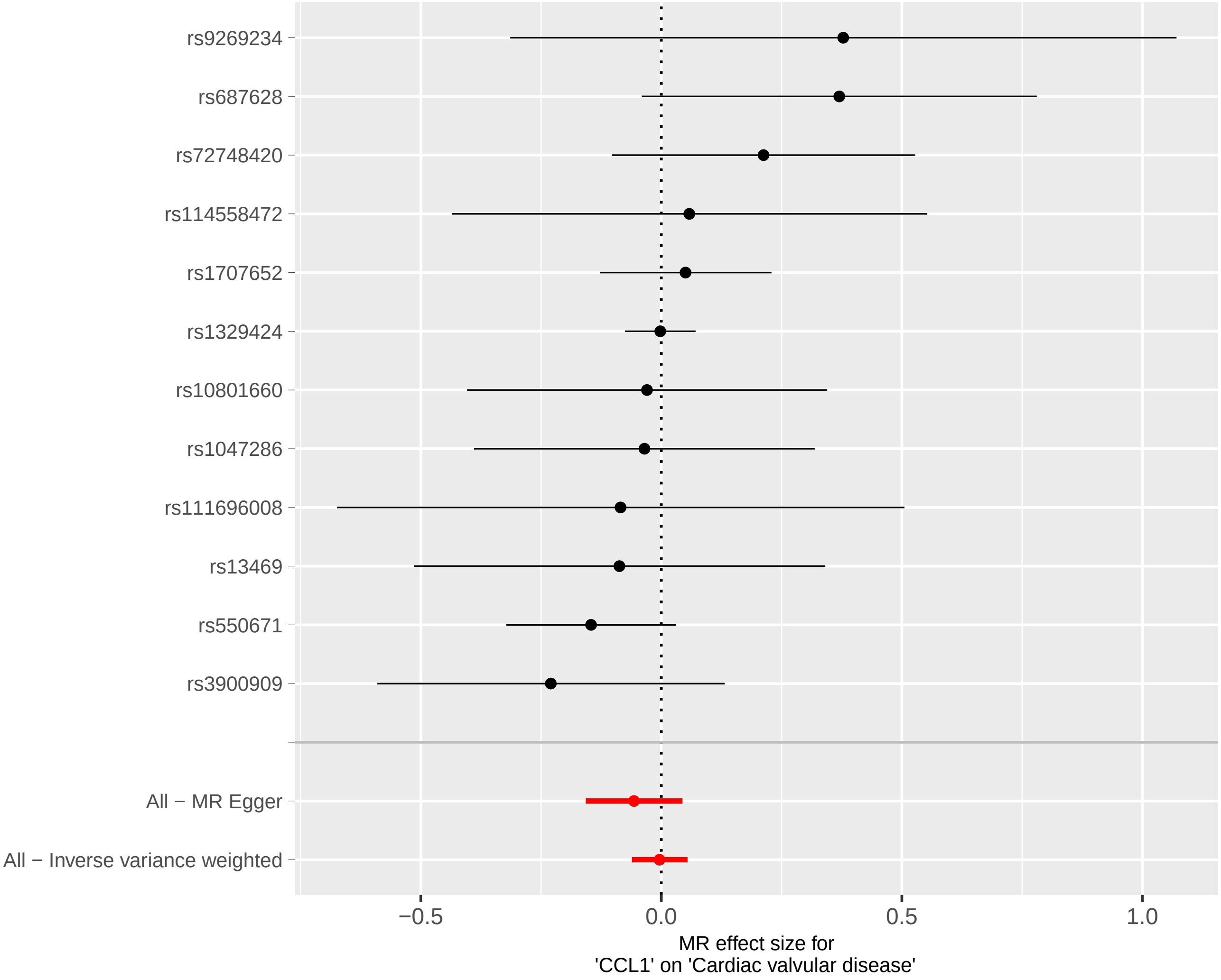
rs9269234
rs687628
rs72748420
rs114558472
rs1707652
rs1329424
rs10801660
rs1047286
rs111696008
rs13469
rs550671
rs3900909
All − MR Egger
All − Inverse variance weighted
−0.5
0.0
0.5
1.0
MR effect size for
'CCL1' on 'Cardiac valvular disease'

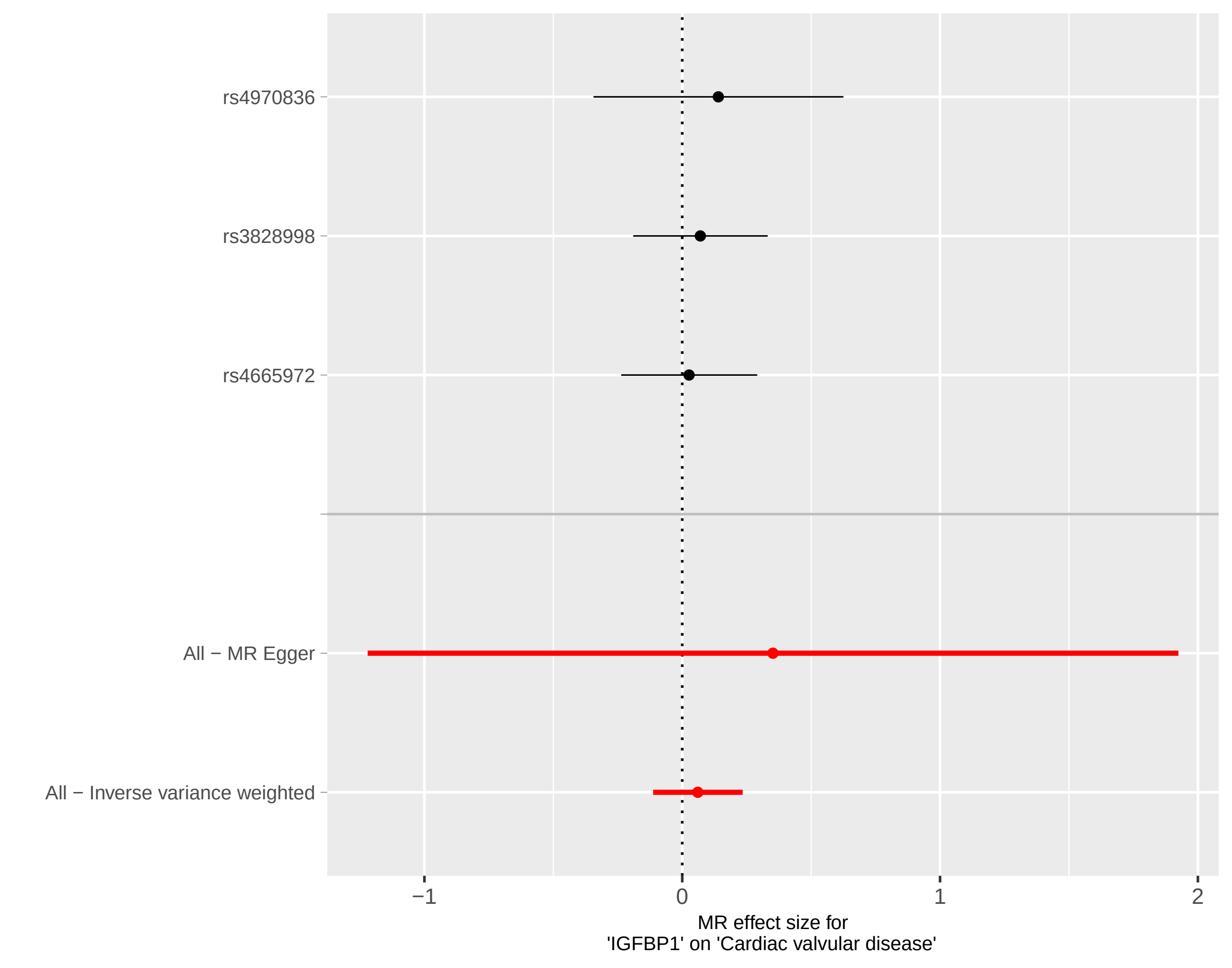
rs4970836
rs3828998
rs4665972
All − MR Egger
All − Inverse variance weighted
−1
0
1
2
MR effect size for
'IGFBP1' on 'Cardiac valvular disease'

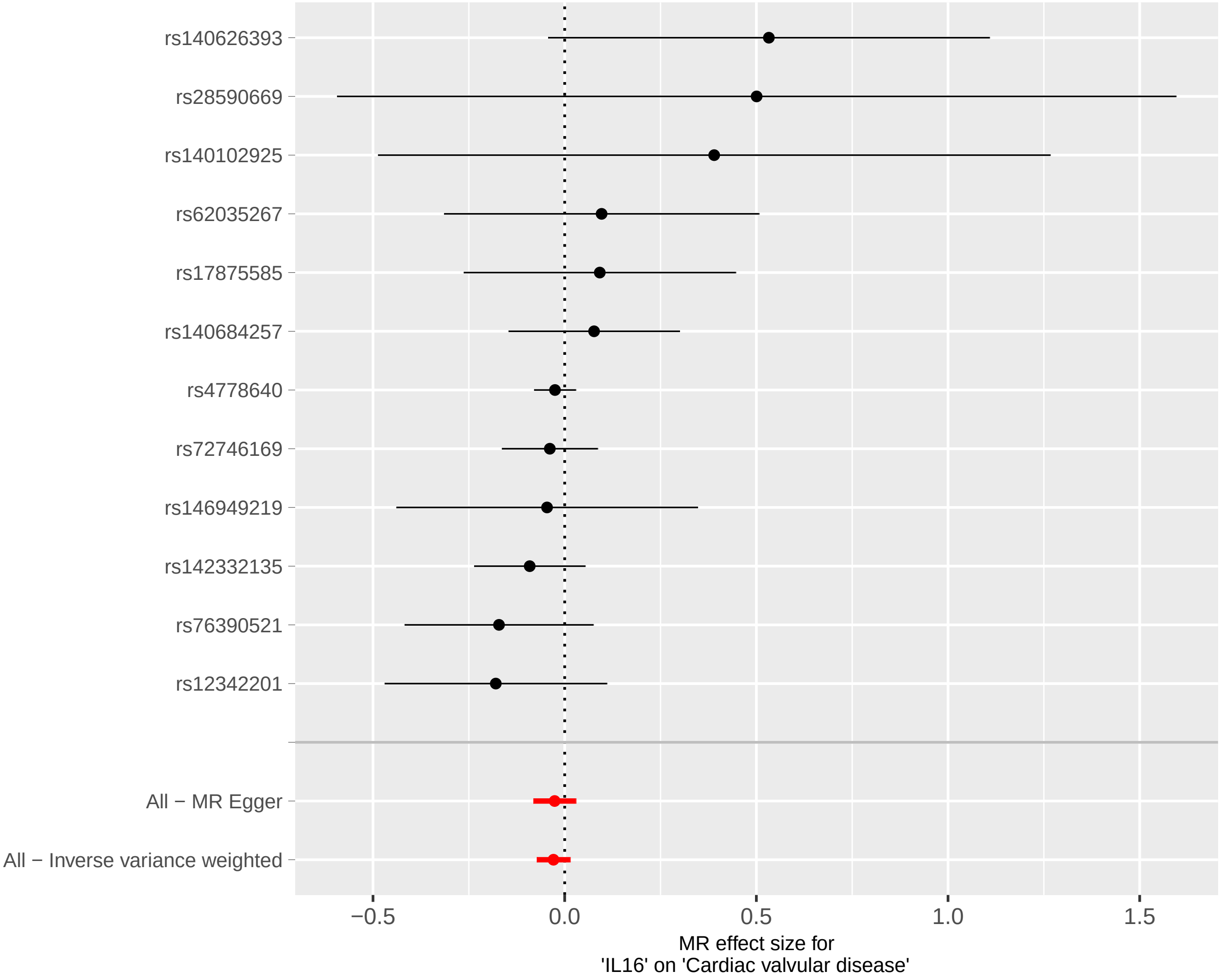
rs140626393
rs28590669
rs140102925
rs62035267
rs17875585
rs140684257
rs4778640
rs72746169
rs146949219
rs142332135
rs76390521
rs12342201
All − MR Egger
All − Inverse variance weighted
−0.5
0.0
0.5
1.0
1.5
MR effect size for
'IL16' on 'Cardiac valvular disease'

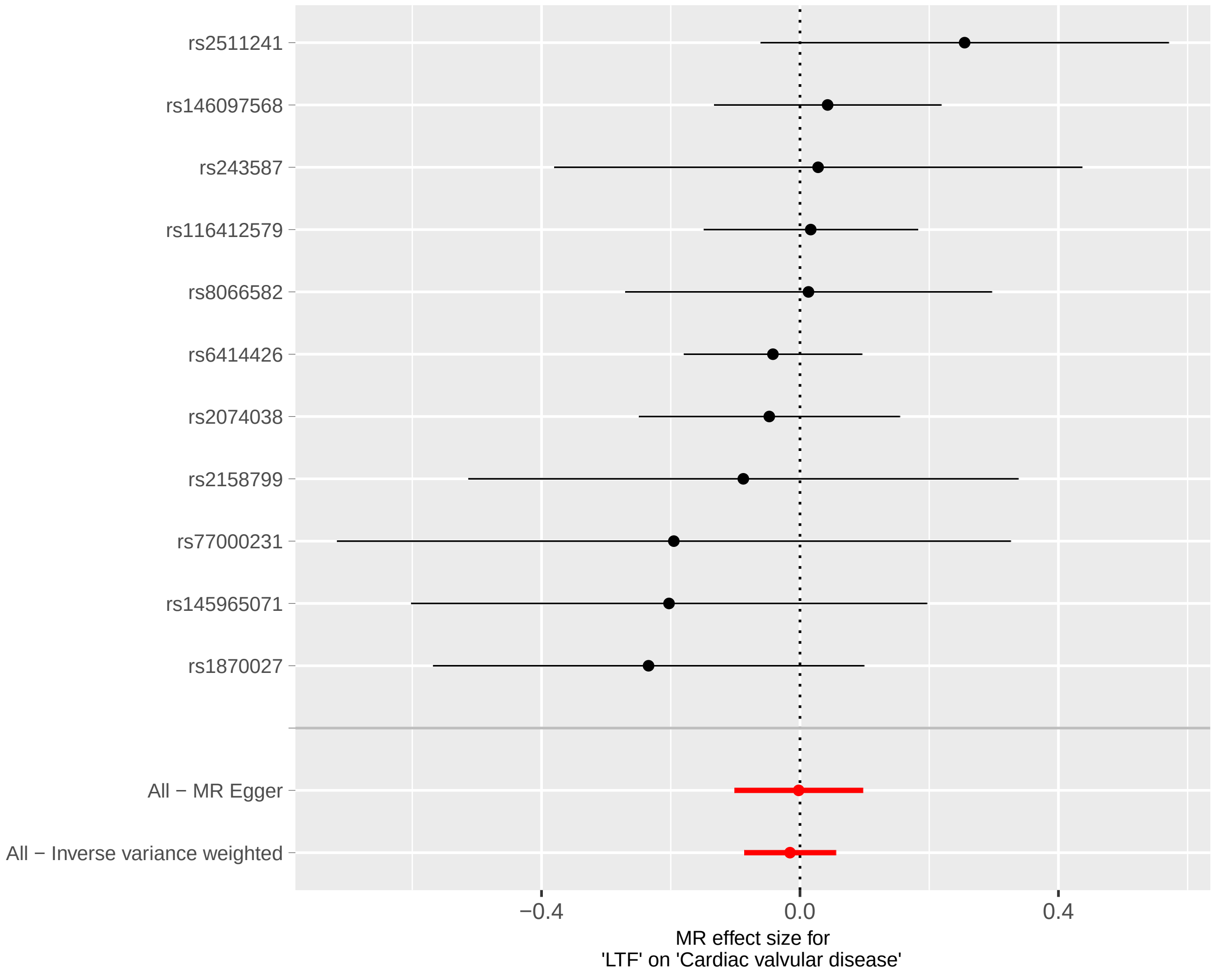
rs2511241
rs146097568
rs243587
rs116412579
rs8066582
rs6414426
rs2074038
rs2158799
rs77000231
rs145965071
rs1870027
All − MR Egger
All − Inverse variance weighted
−0.4
0.0
0.4
MR effect size for
'LTF' on 'Cardiac valvular disease'

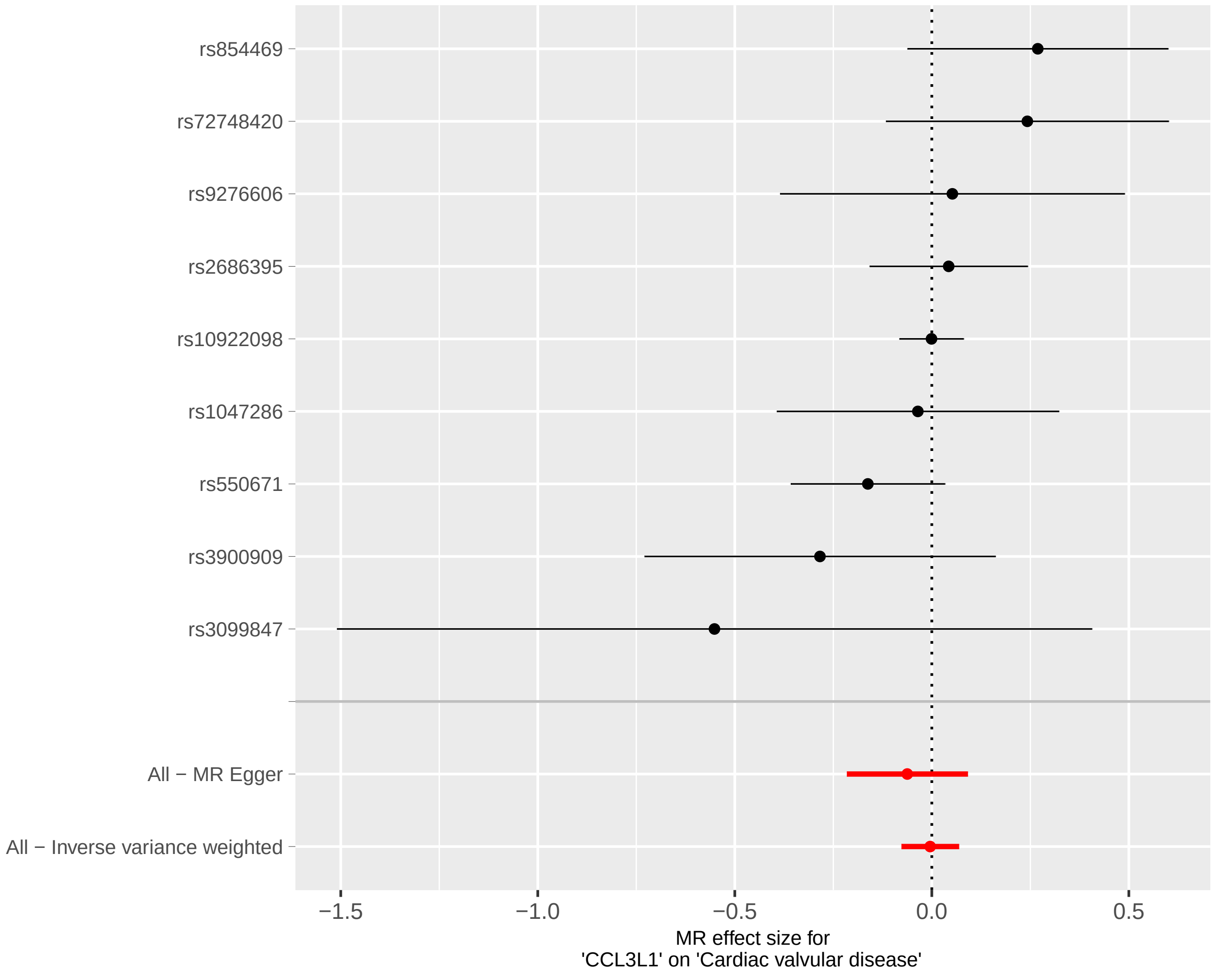
rs854469
rs72748420
rs9276606
rs2686395
rs10922098
rs1047286
rs550671
rs3900909
rs3099847
All − MR Egger
All − Inverse variance weighted
−1.5
−1.0
−0.5
0.0
0.5
MR effect size for
'CCL3L1' on 'Cardiac valvular disease'

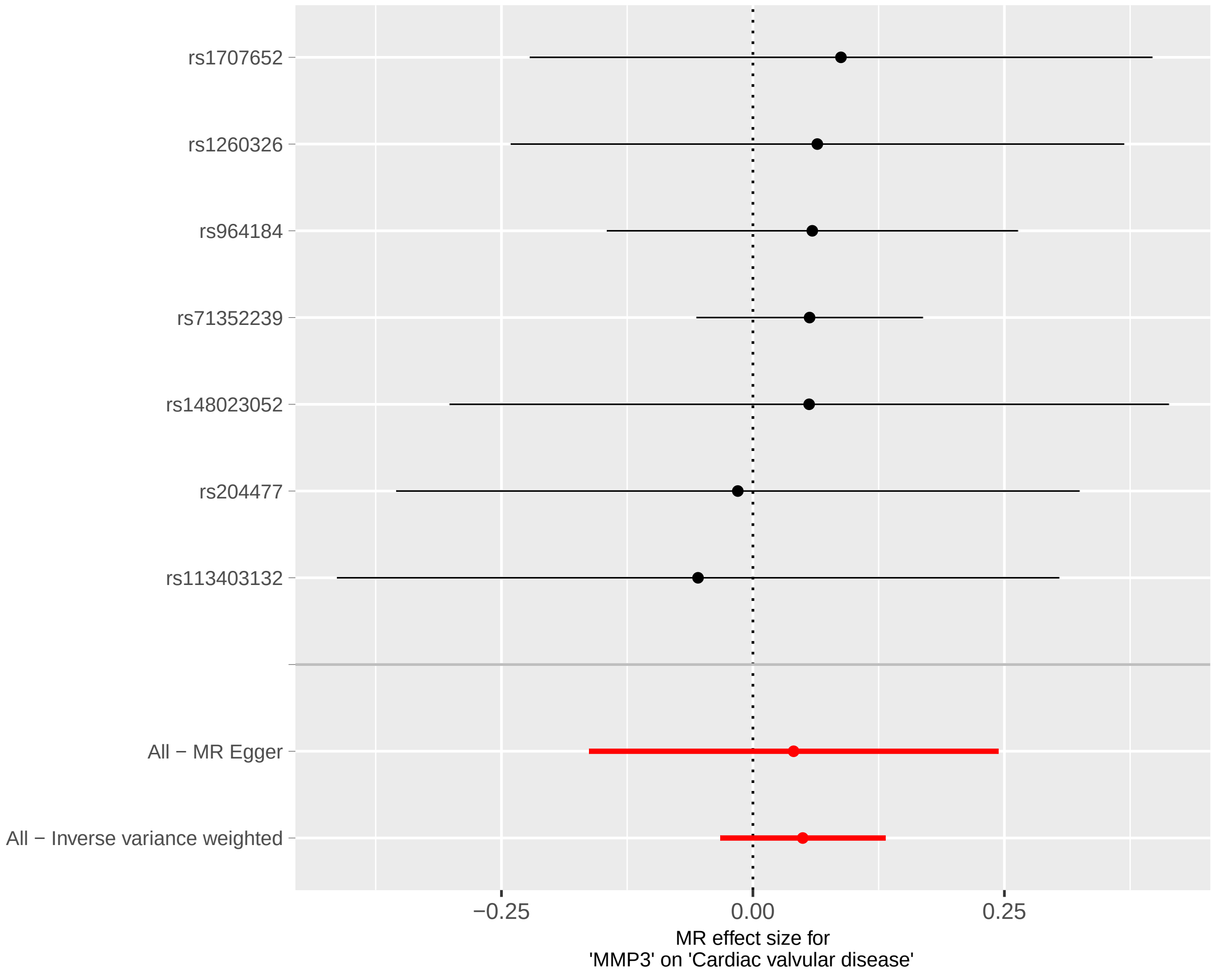
rs1707652
rs1260326
rs964184
rs71352239
rs148023052
rs204477
rs113403132
All − MR Egger
All − Inverse variance weighted
−0.25
0.00
0.25
MR effect size for
'MMP3' on 'Cardiac valvular disease'

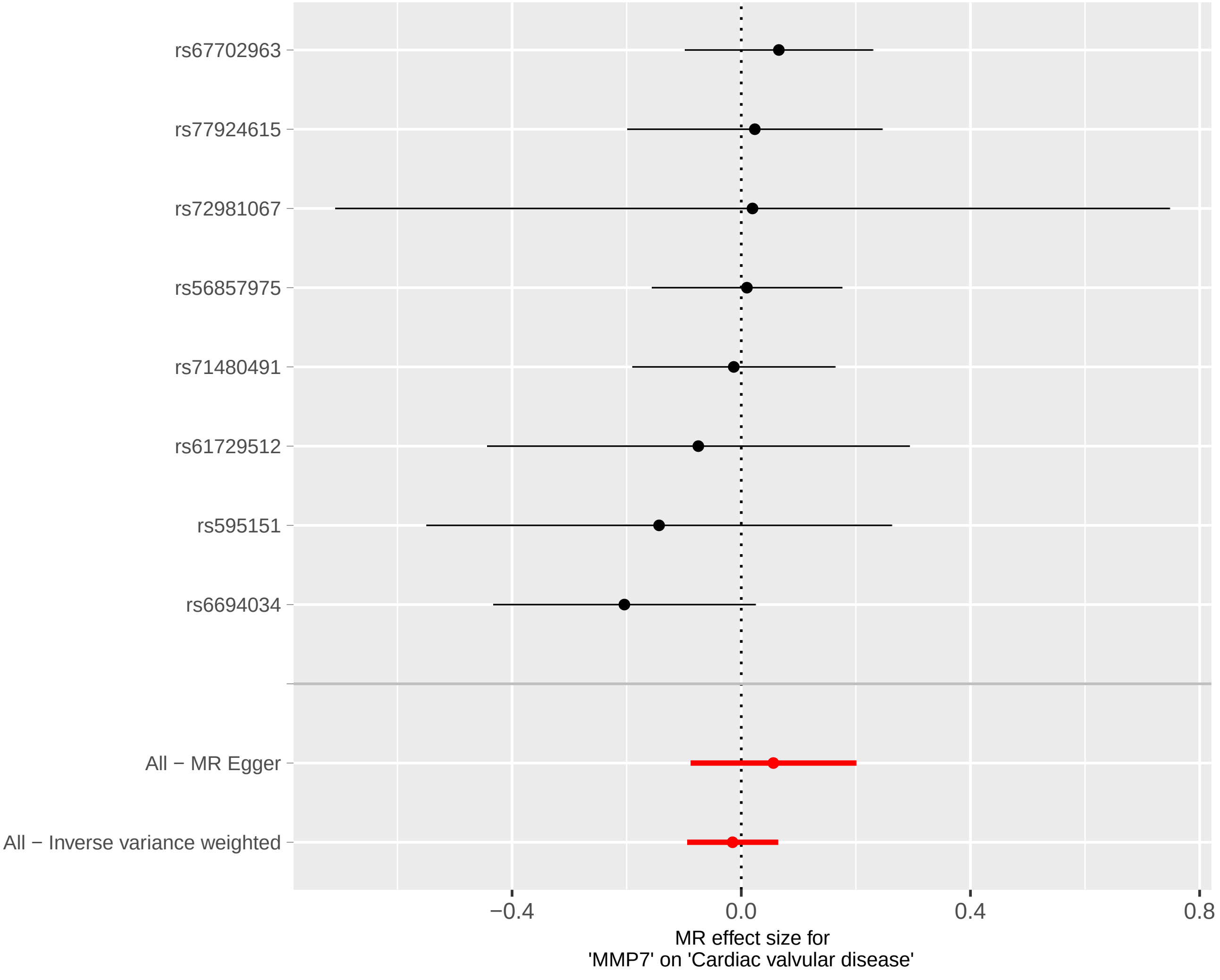
rs67702963
rs77924615
rs72981067
rs56857975
rs71480491
rs61729512
rs595151
rs6694034
All − MR Egger
All − Inverse variance weighted
−0.4
0.0
0.4
0.8
MR effect size for
'MMP7' on 'Cardiac valvular disease'

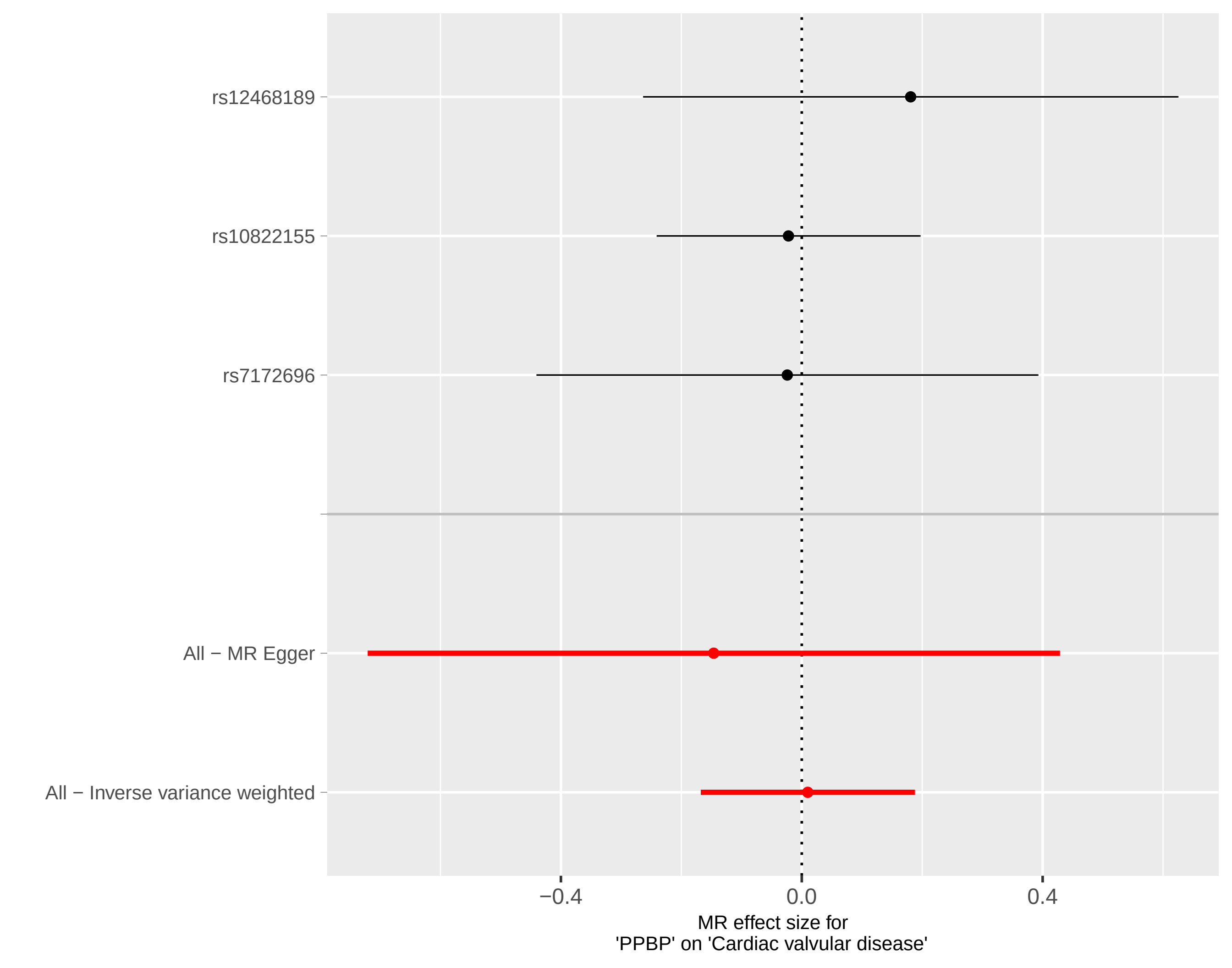
rs12468189
rs10822155
rs7172696
All − MR Egger
All − Inverse variance weighted
−0.4
0.0
0.4
MR effect size for
'PPBP' on 'Cardiac valvular disease'

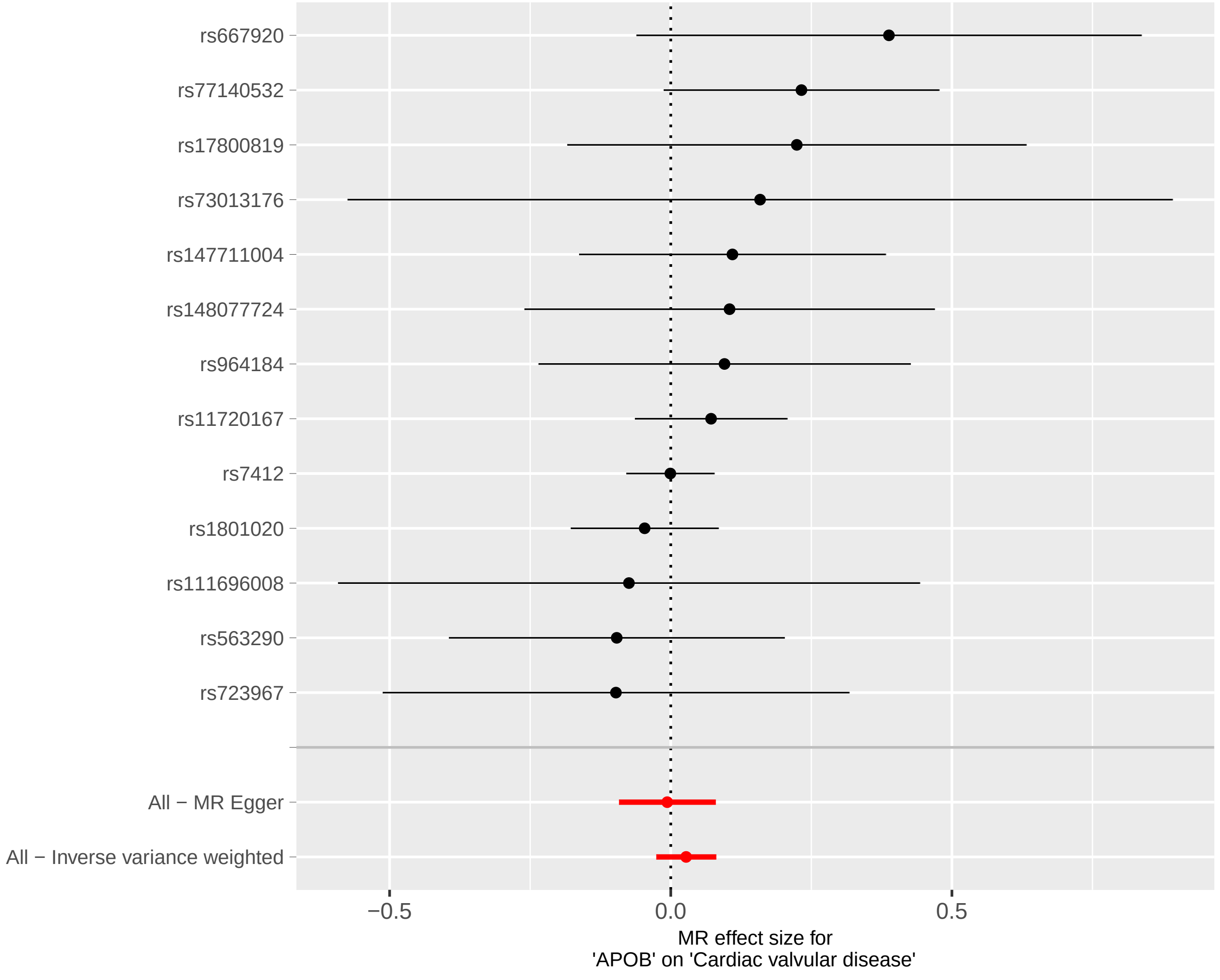

rs667920
rs77140532
rs17800819
rs73013176
rs147711004
rs148077724
rs964184
rs11720167
rs7412
rs1801020
rs111696008
rs563290
rs723967
All − MR Egger
All − Inverse variance weighted
−0.5
0.0
0.5
MR effect size for
'APOB' on 'Cardiac valvular disease'

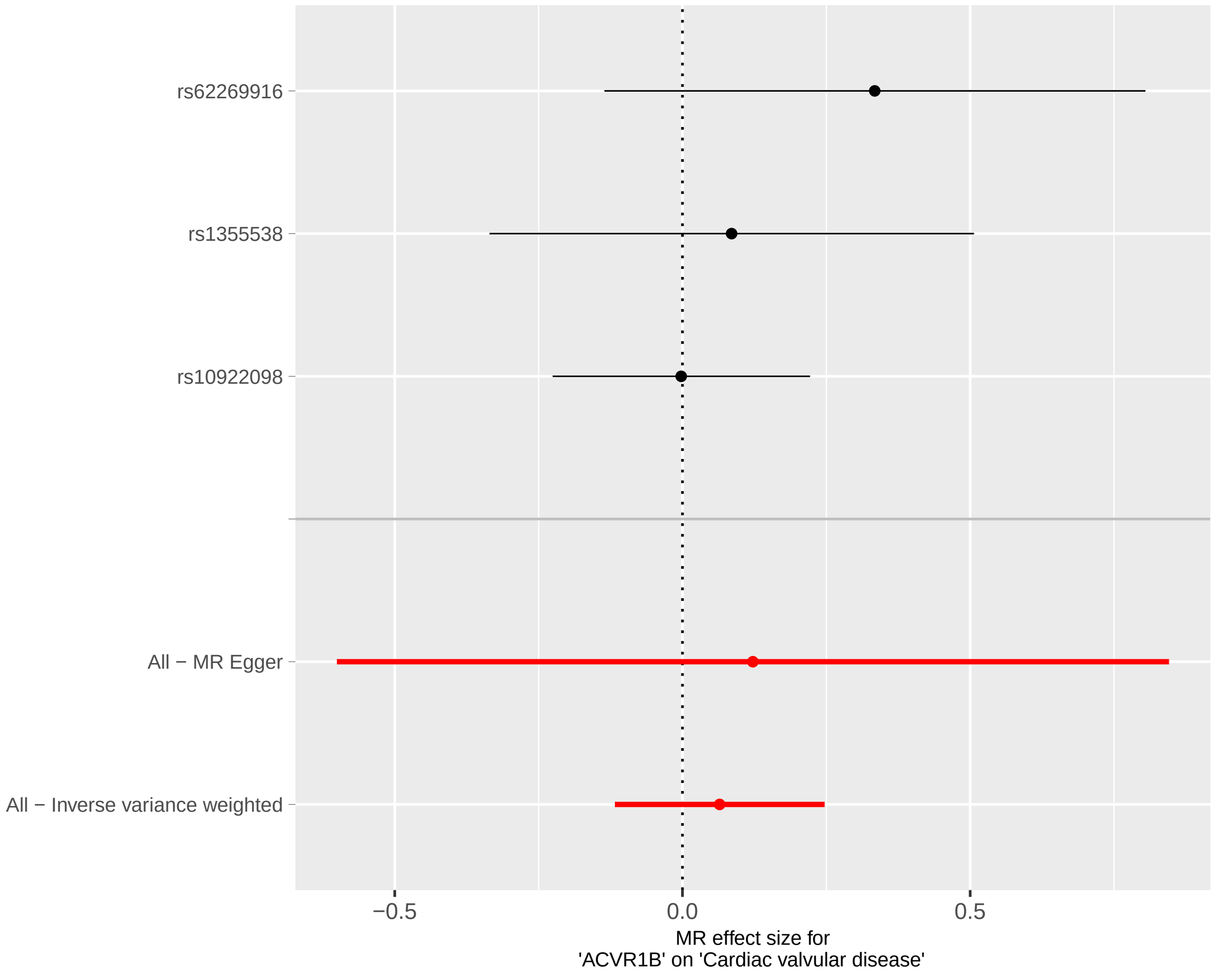
rs62269916
rs1355538
rs10922098
All − MR Egger
All − Inverse variance weighted
−0.5
0.0
0.5
MR effect size for
'ACVR1B' on 'Cardiac valvular disease'

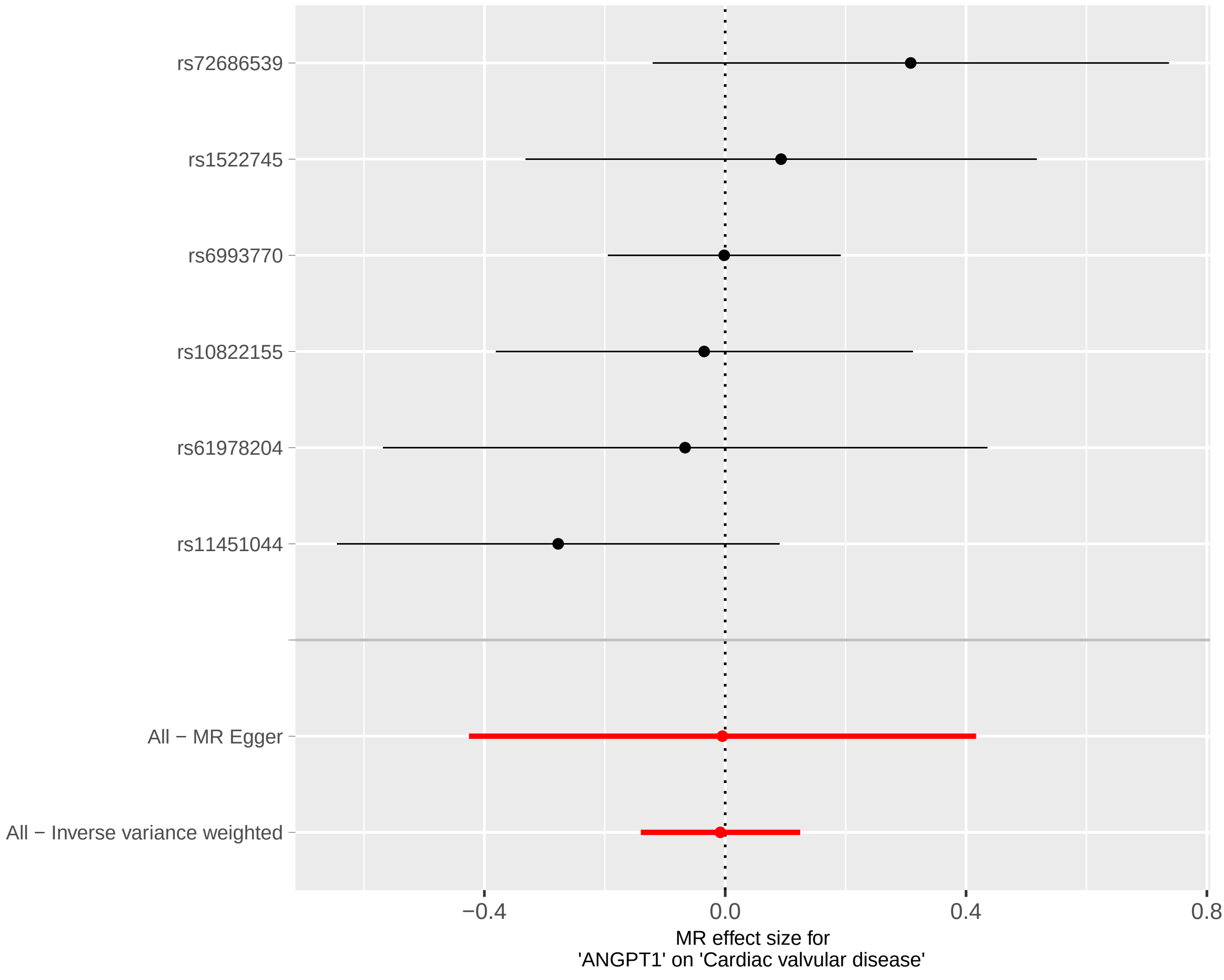
rs72686539
rs1522745
rs6993770
rs10822155
rs61978204
rs11451044
All − MR Egger
All − Inverse variance weighted
−0.4
0.0
0.4
0.8
MR effect size for
'ANGPT1' on 'Cardiac valvular disease'

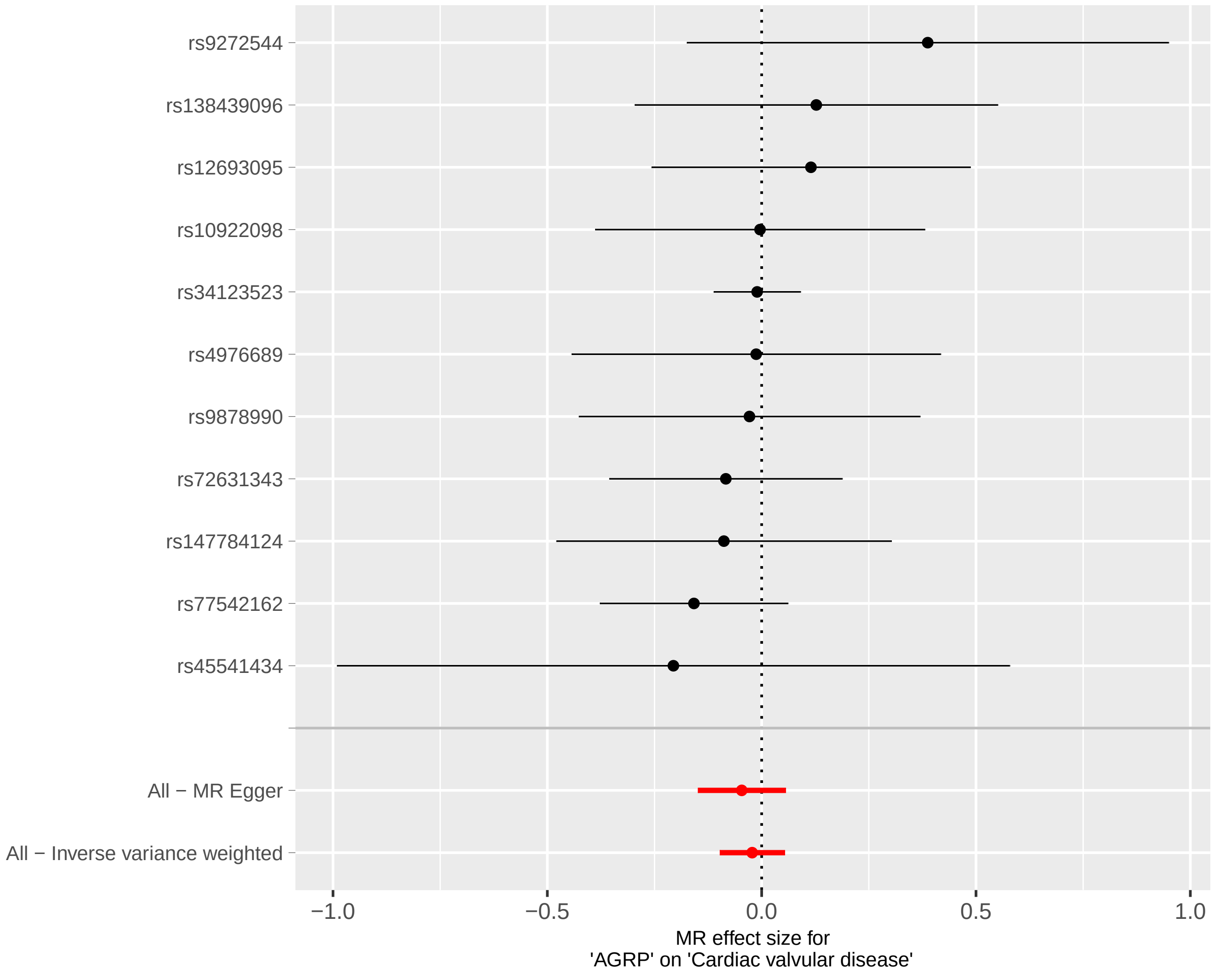
rs9272544
rs138439096
rs12693095
rs10922098
rs34123523
rs4976689
rs9878990
rs72631343
rs147784124
rs77542162
rs45541434
All − MR Egger
All − Inverse variance weighted
−1.0
−0.5
0.0
0.5
1.0
MR effect size for
'AGRP' on 'Cardiac valvular disease'

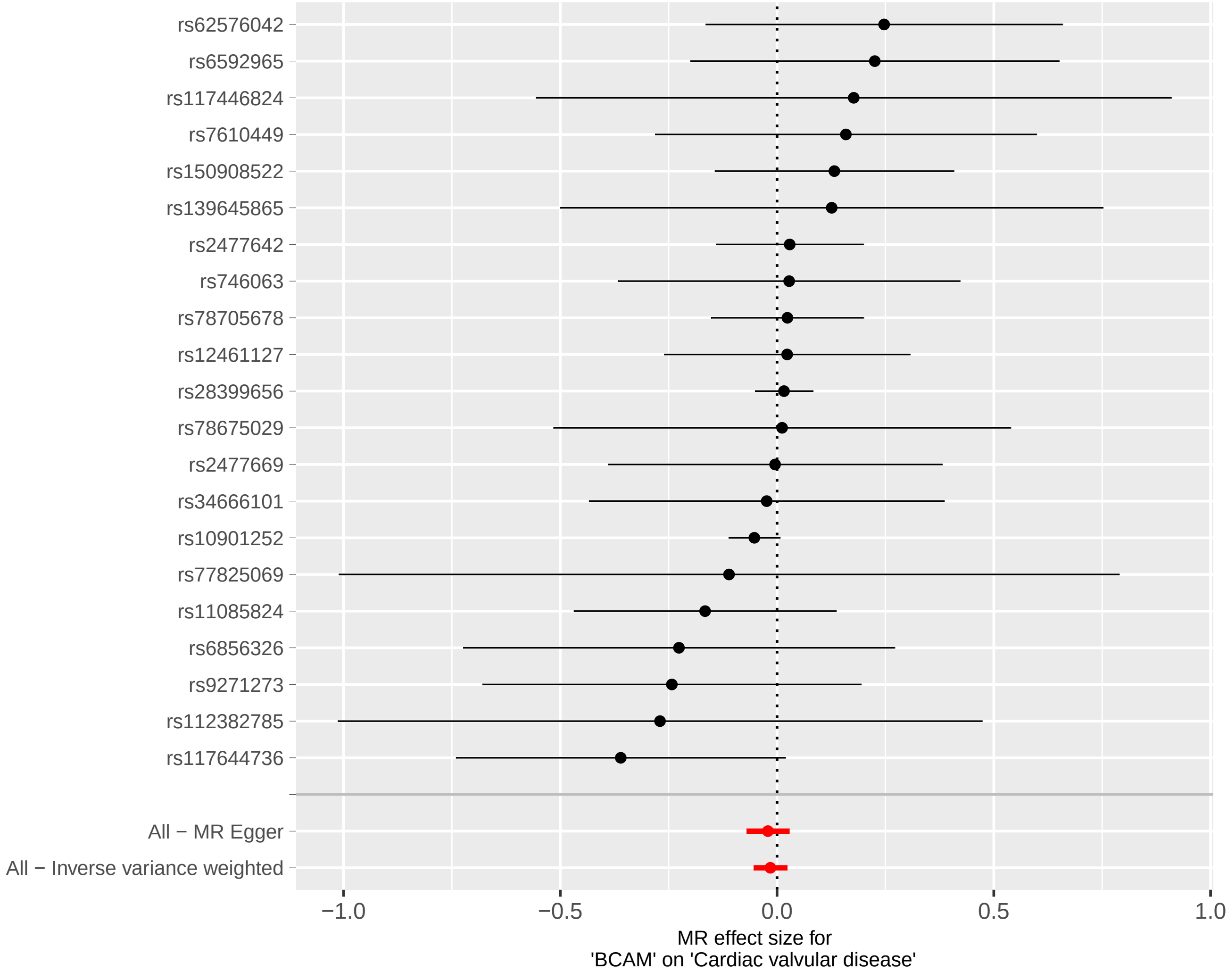

rs62576042
rs6592965
rs117446824
rs7610449
rs150908522
rs139645865
rs2477642
rs746063
rs78705678
rs12461127
rs28399656
rs78675029
rs2477669
rs34666101
rs10901252
rs77825069
rs11085824
rs6856326
rs9271273
rs112382785
rs117644736
All − MR Egger
All − Inverse variance weighted
−1.0
−0.5
0.0
0.5
1.0
MR effect size for
'BCAM' on 'Cardiac valvular disease'

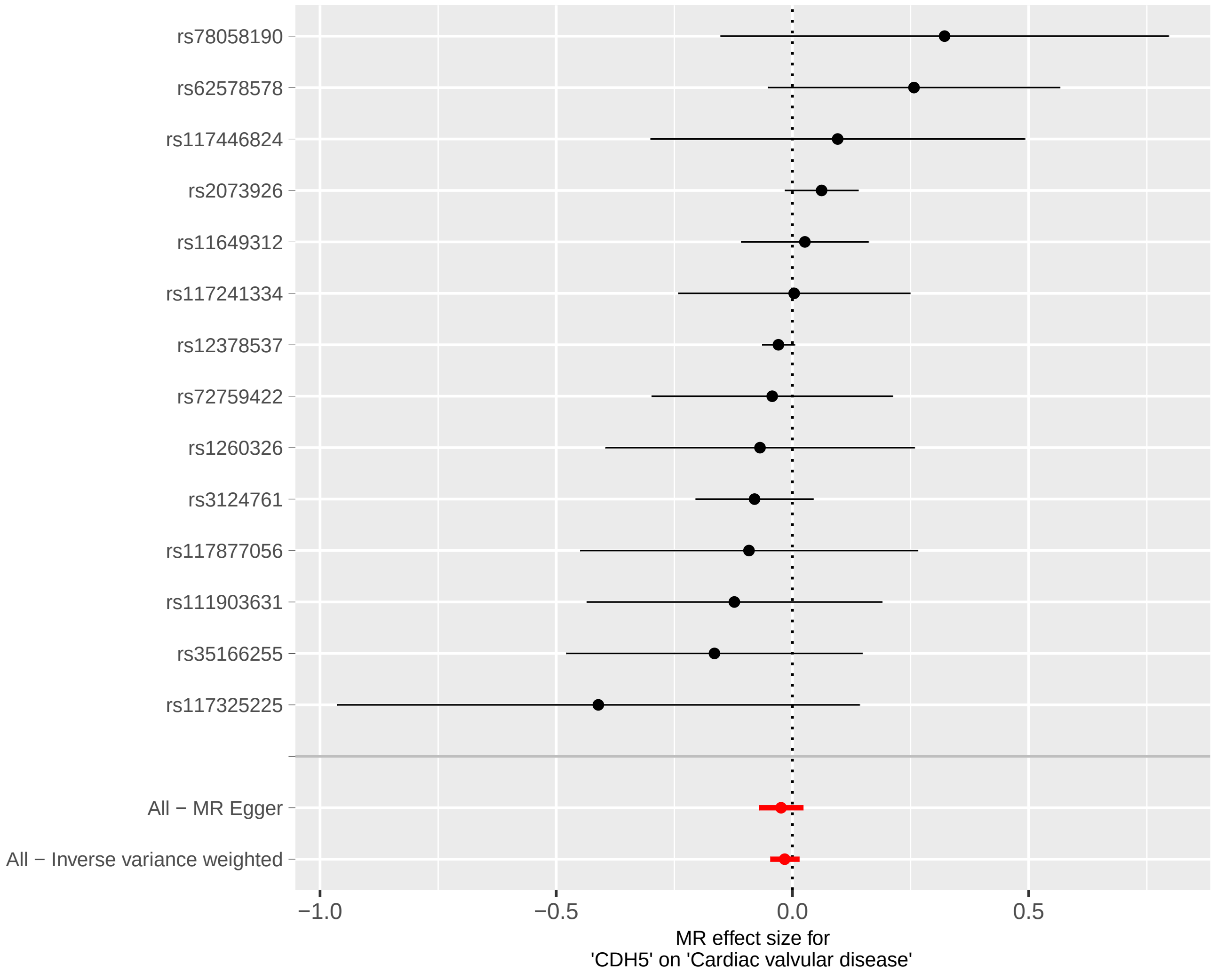
rs78058190
rs62578578
rs117446824
rs2073926
rs11649312
rs117241334
rs12378537
rs72759422
rs1260326
rs3124761
rs117877056
rs111903631
rs35166255
rs117325225
All − MR Egger
All − Inverse variance weighted
−1.0
−0.5
0.0
0.5
MR effect size for
'CDH5' on 'Cardiac valvular disease'

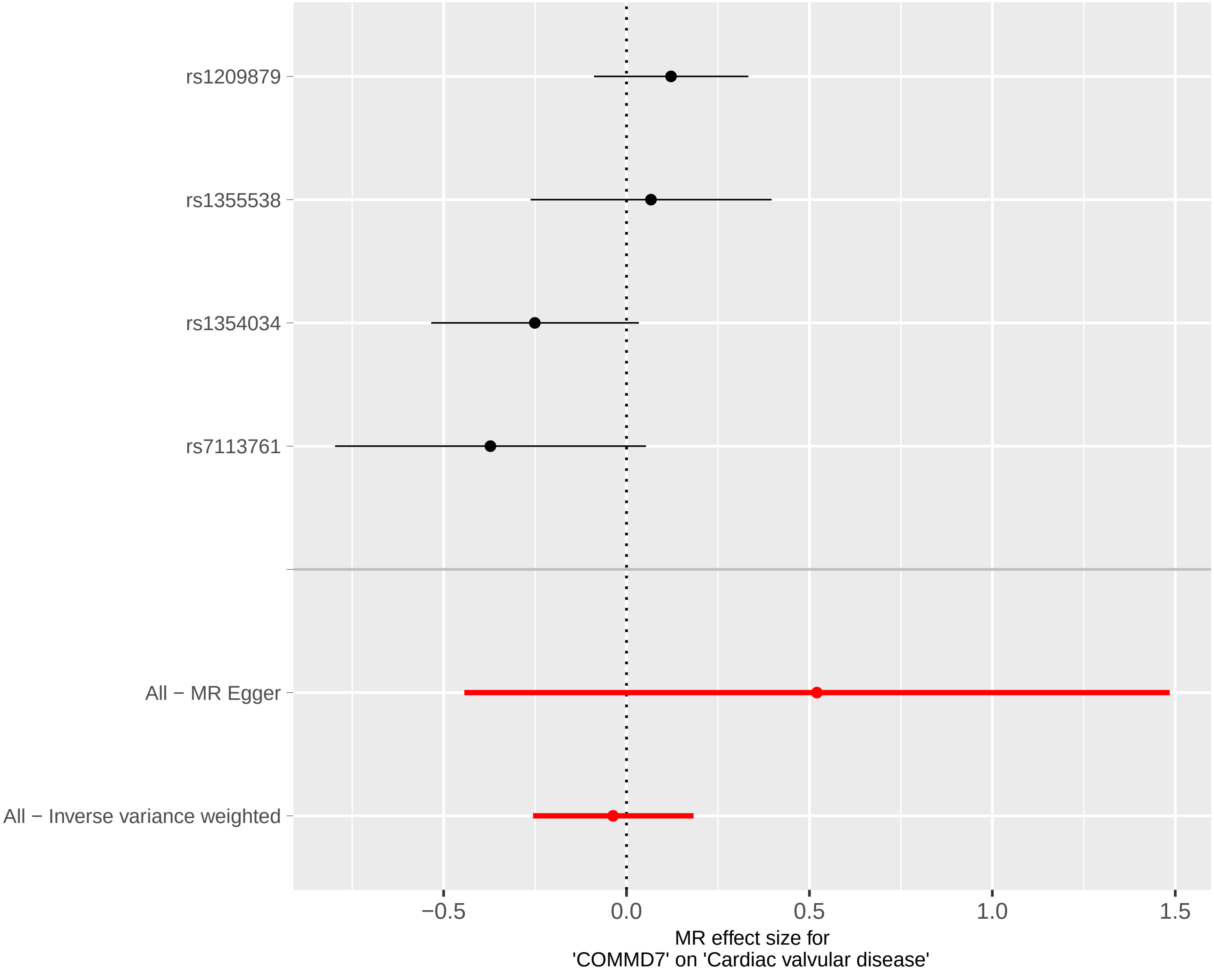

rs1209879
rs1355538
rs1354034
rs7113761
All − MR Egger
All − Inverse variance weighted
−0.5
0.0
0.5
1.0
1.5
MR effect size for
'COMMD7' on 'Cardiac valvular disease'

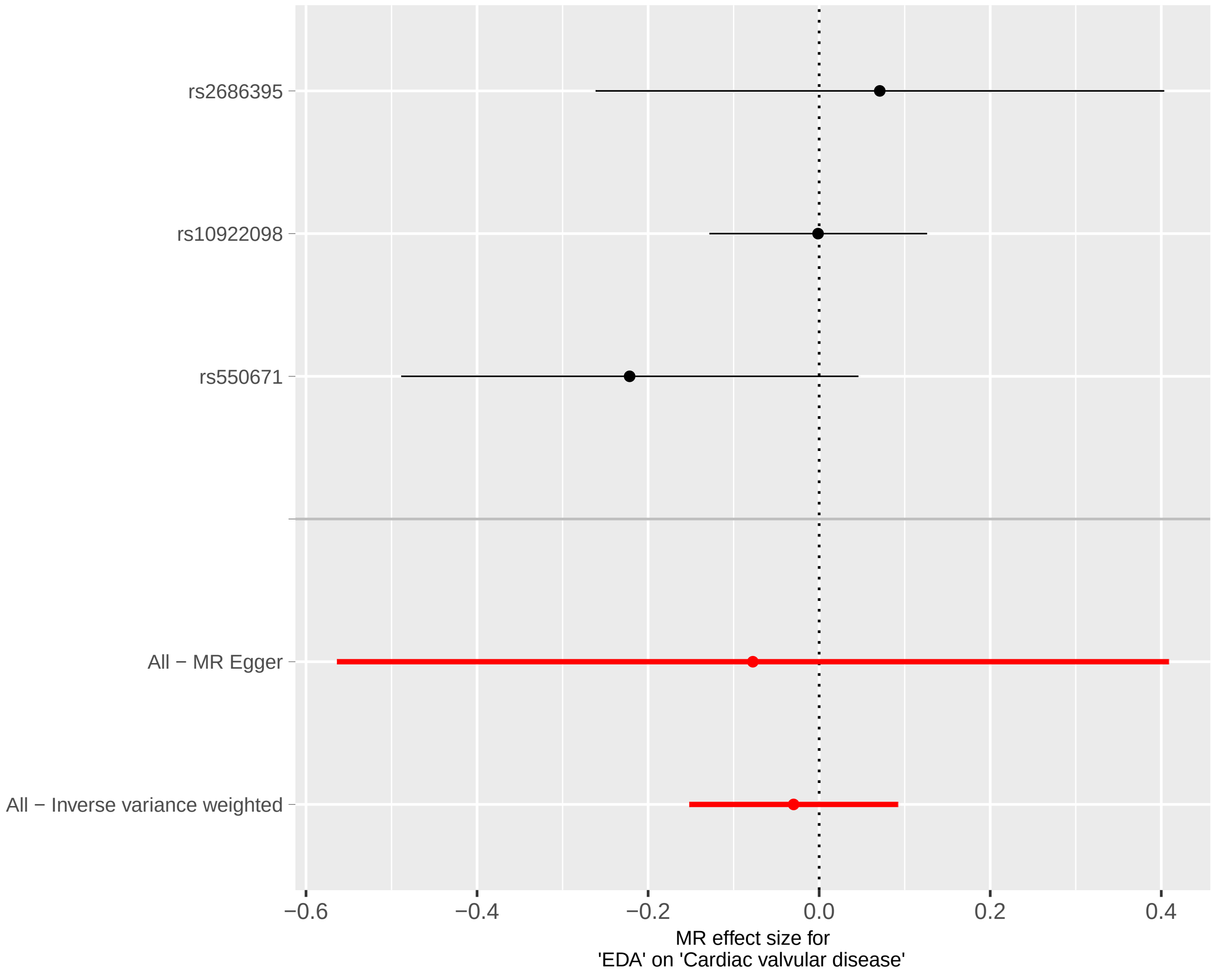
rs2686395
rs10922098
rs550671
All − MR Egger
All − Inverse variance weighted
−0.6
−0.4
−0.2
0.0
0.2
0.4
MR effect size for
'EDA' on 'Cardiac valvular disease'

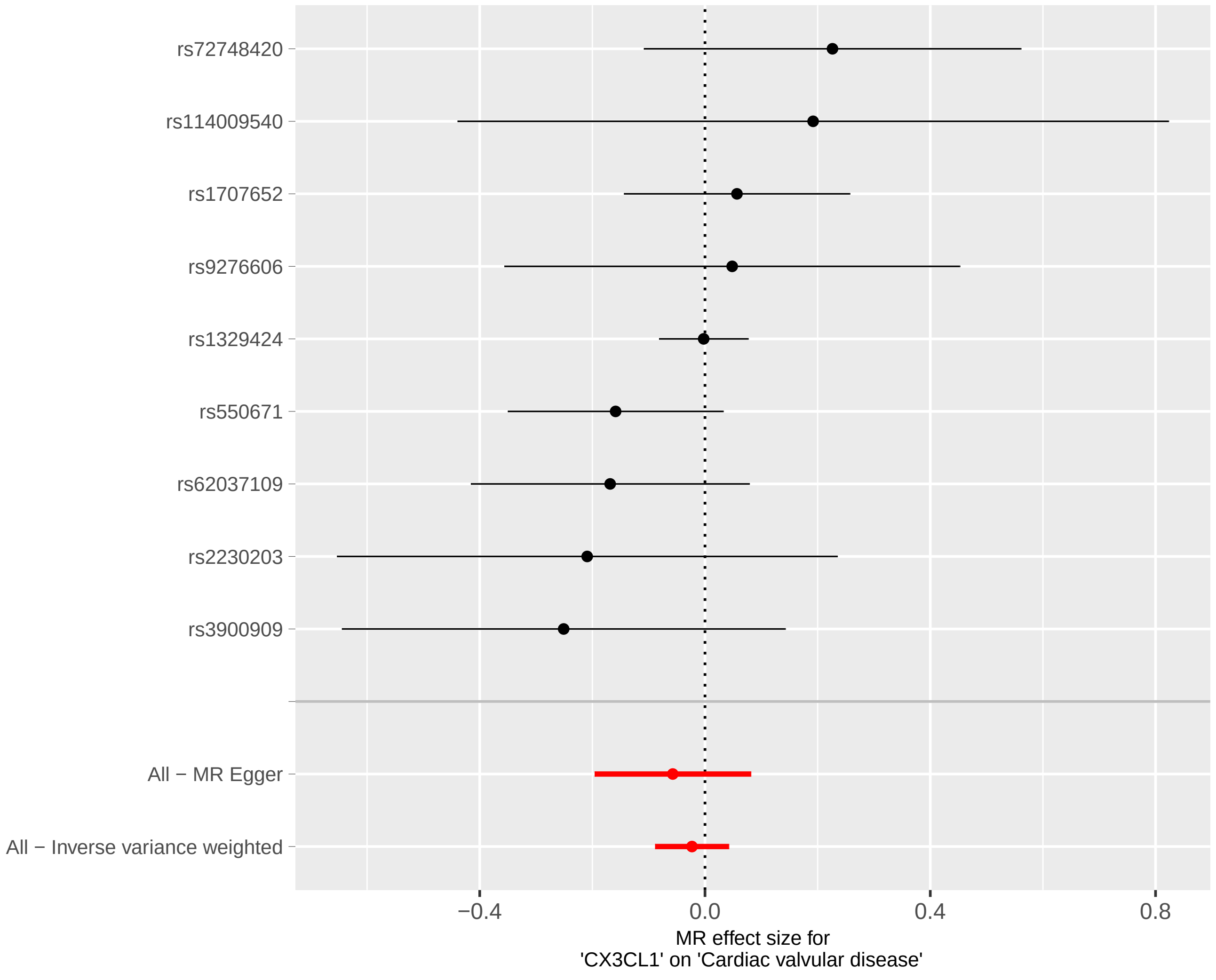
rs72748420
rs114009540
rs1707652
rs9276606
rs1329424
rs550671
rs62037109
rs2230203
rs3900909
All − MR Egger
All − Inverse variance weighted
−0.4
0.0
0.4
0.8
MR effect size for
'CX3CL1' on 'Cardiac valvular disease'

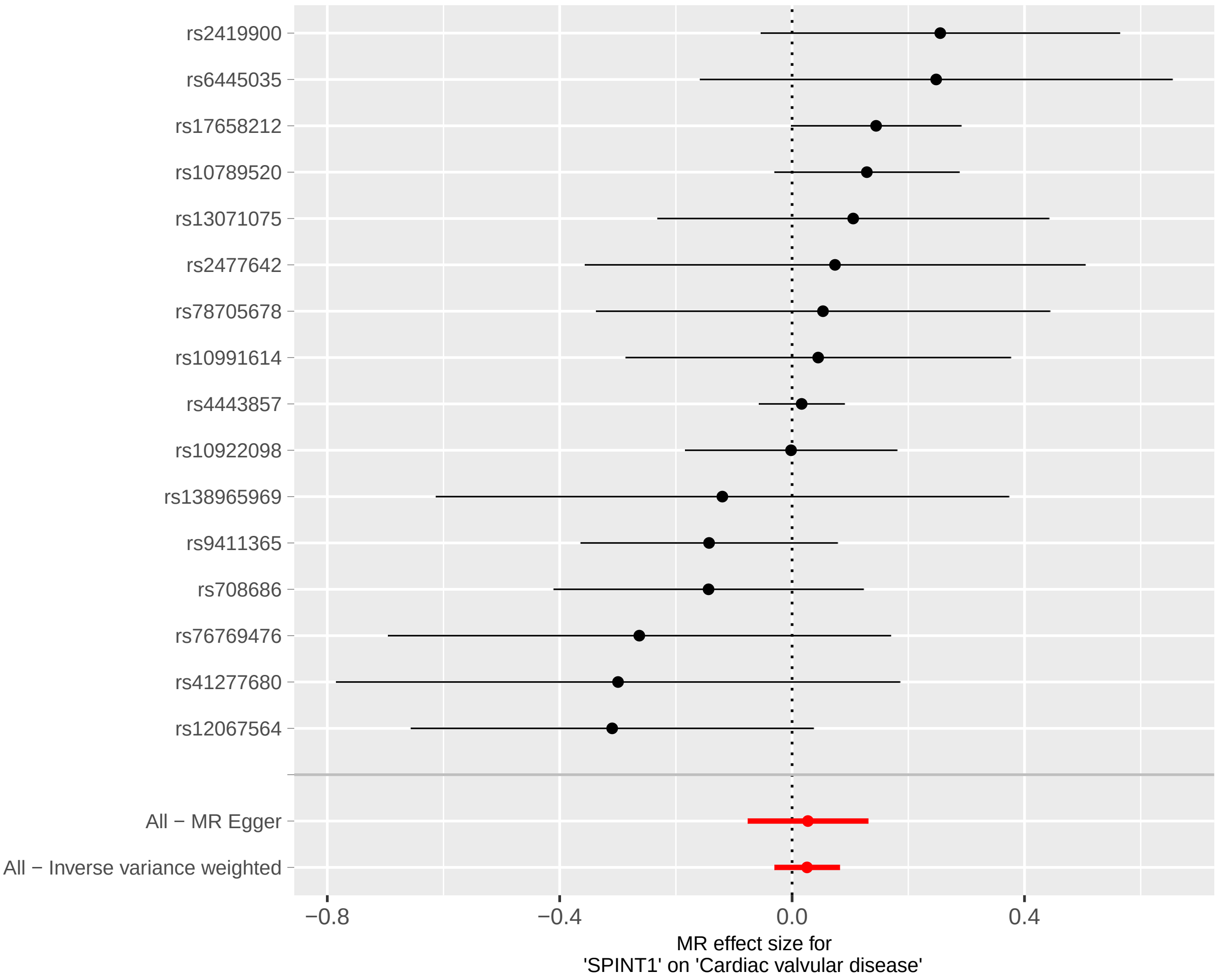
rs2419900
rs6445035
rs17658212
rs10789520
rs13071075
rs2477642
rs78705678
rs10991614
rs4443857
rs10922098
rs138965969
rs9411365
rs708686
rs76769476
rs41277680
rs12067564
All − MR Egger
All − Inverse variance weighted
−0.8
−0.4
0.0
0.4
MR effect size for
'SPINT1' on 'Cardiac valvular disease'

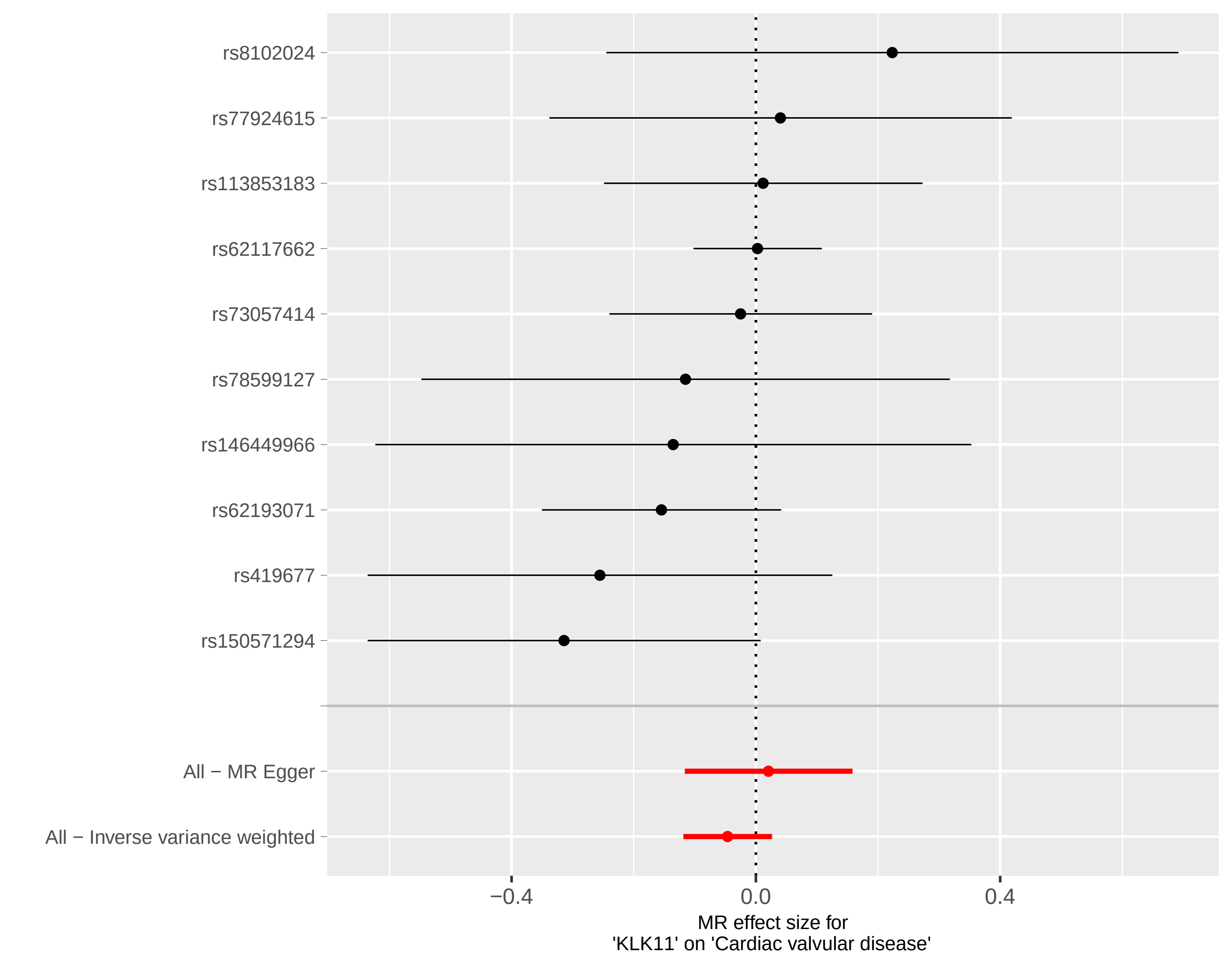

rs8102024
rs77924615
rs113853183
rs62117662
rs73057414
rs78599127
rs146449966
rs62193071
rs419677
rs150571294
All − MR Egger
All − Inverse variance weighted
−0.4
0.0
0.4
MR effect size for
'KLK11' on 'Cardiac valvular disease'

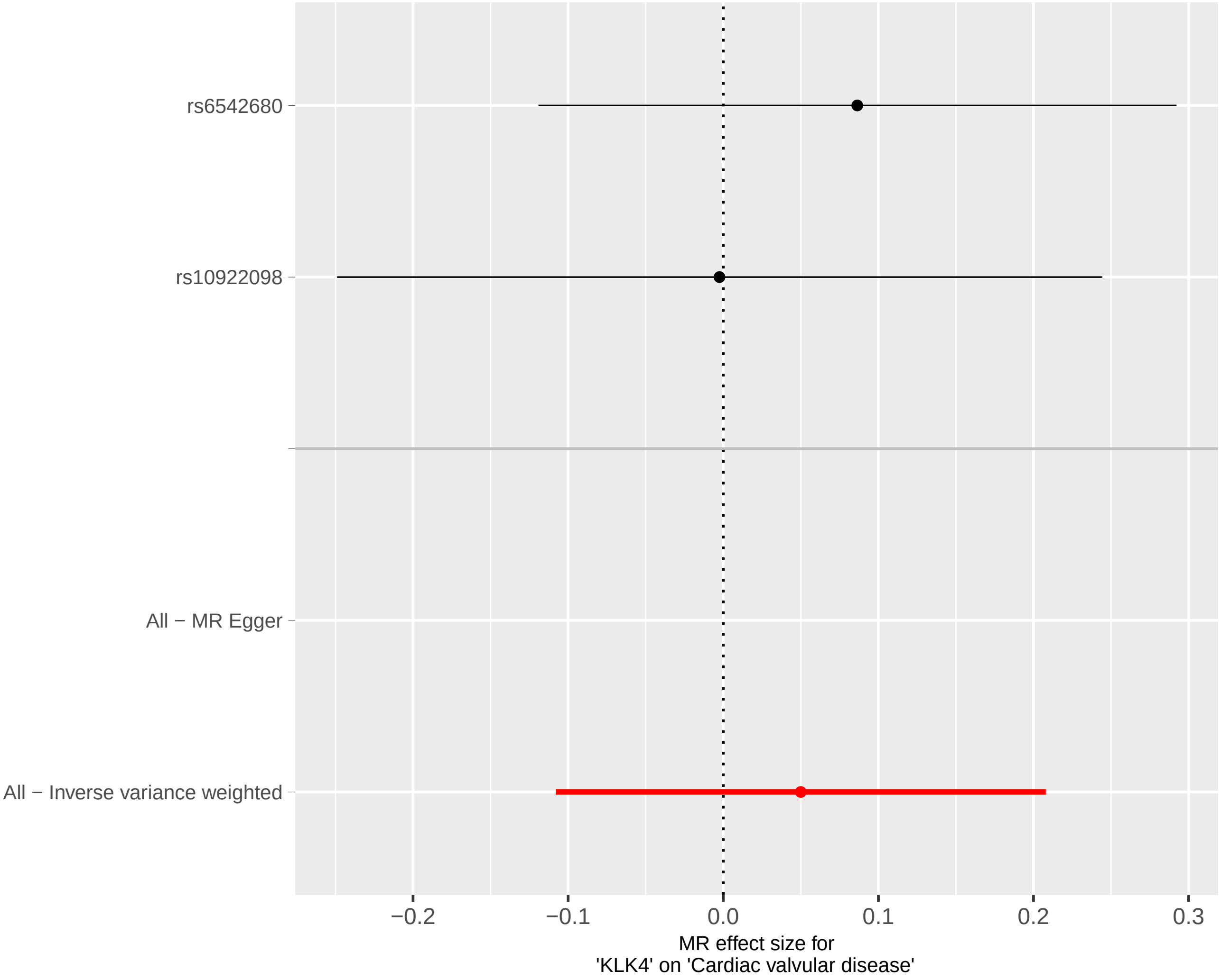

rs6542680
rs10922098
All − MR Egger
All − Inverse variance weighted
−0.2
−0.1
0.0
0.1
0.2
0.3
MR effect size for
'KLK4' on 'Cardiac valvular disease'

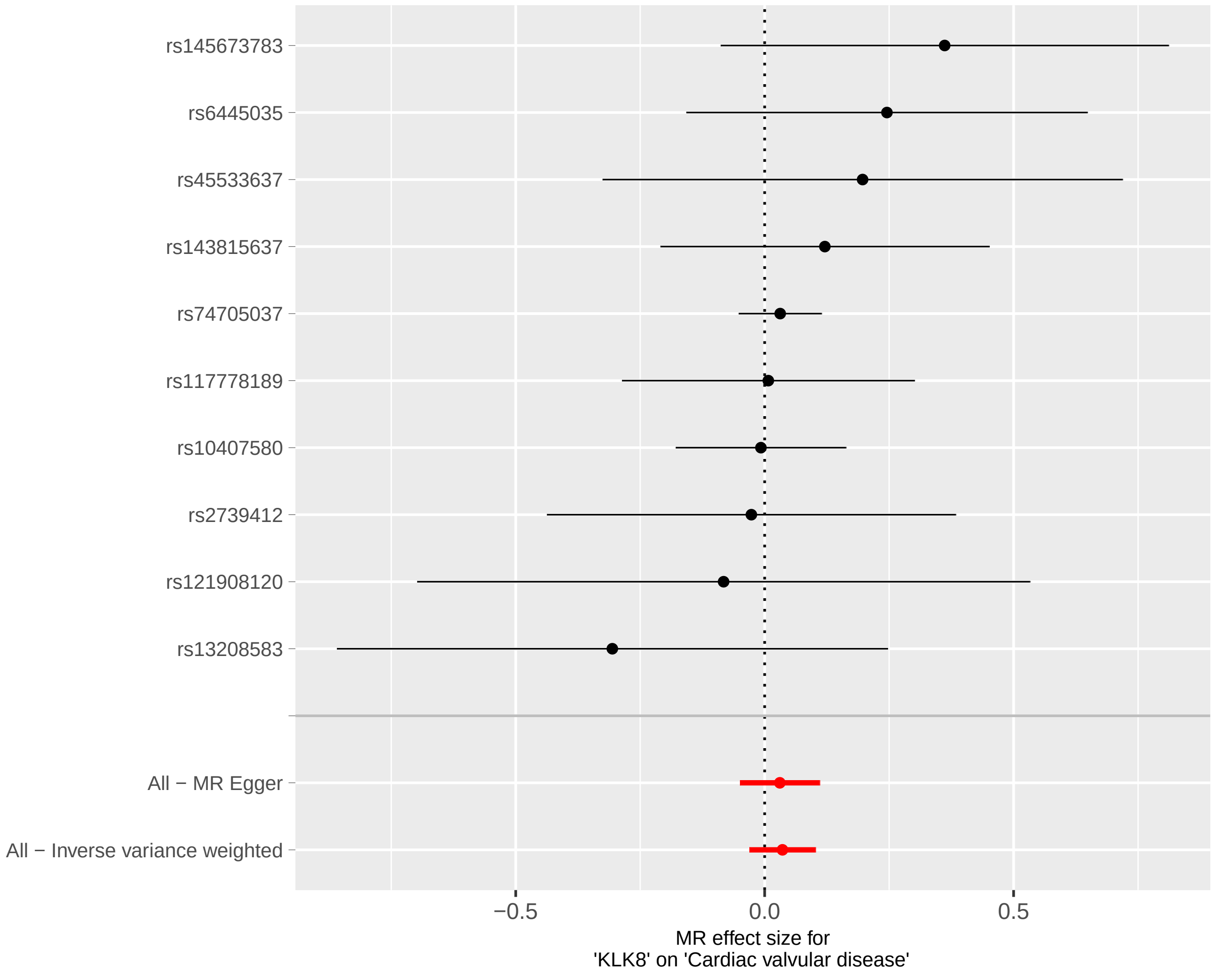

rs145673783
rs6445035
rs45533637
rs143815637
rs74705037
rs117778189
rs10407580
rs2739412
rs121908120
rs13208583
All − MR Egger
All − Inverse variance weighted
−0.5
0.0
0.5
MR effect size for
'KLK8' on 'Cardiac valvular disease'

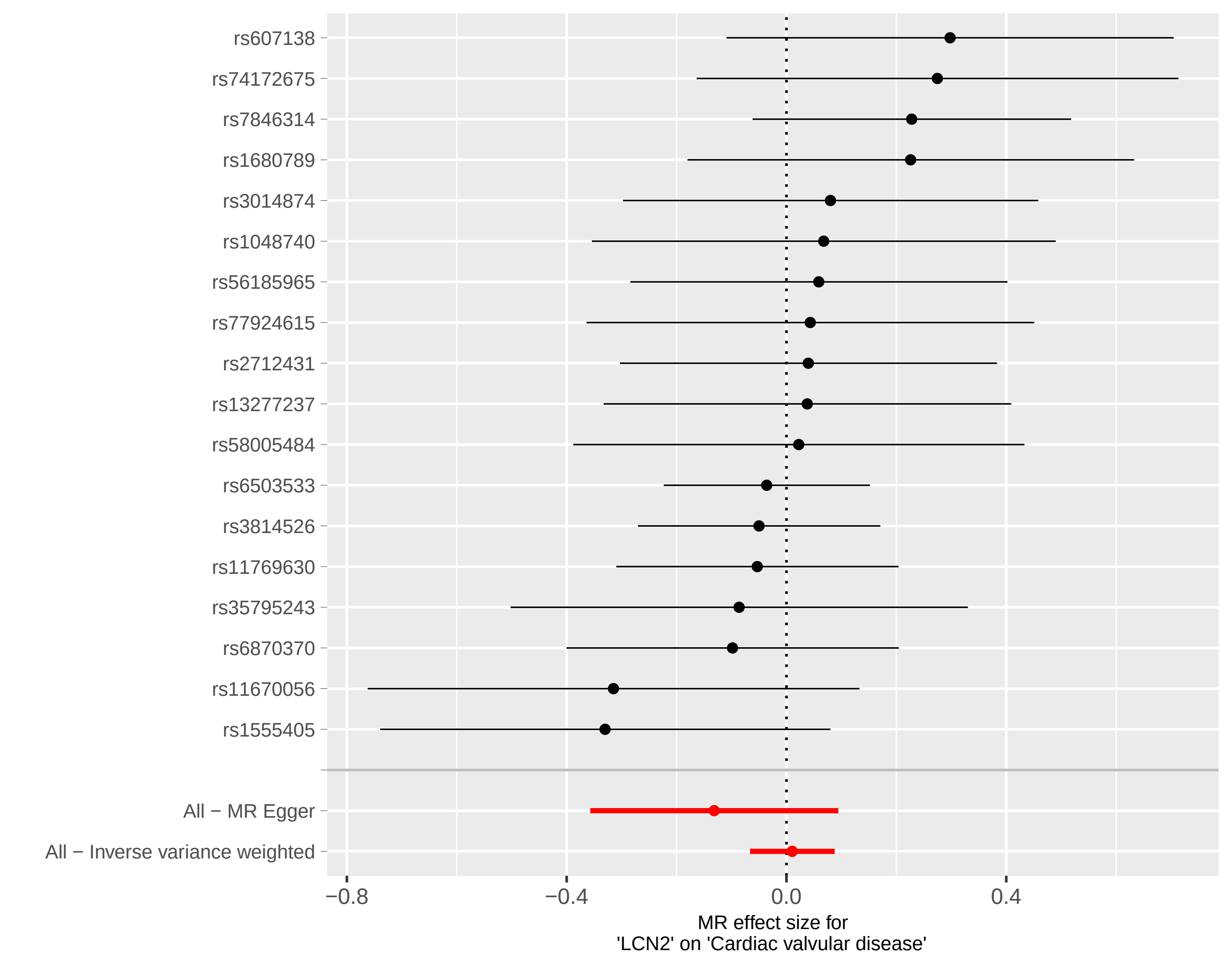
rs607138
rs74172675
rs7846314
rs1680789
rs3014874
rs1048740
rs56185965
rs77924615
rs2712431
rs13277237
rs58005484
rs6503533
rs3814526
rs11769630
rs35795243
rs6870370
rs11670056
rs1555405
All − MR Egger
All − Inverse variance weighted
−0.8
−0.4
0.0
0.4
MR effect size for
'LCN2' on 'Cardiac valvular disease'

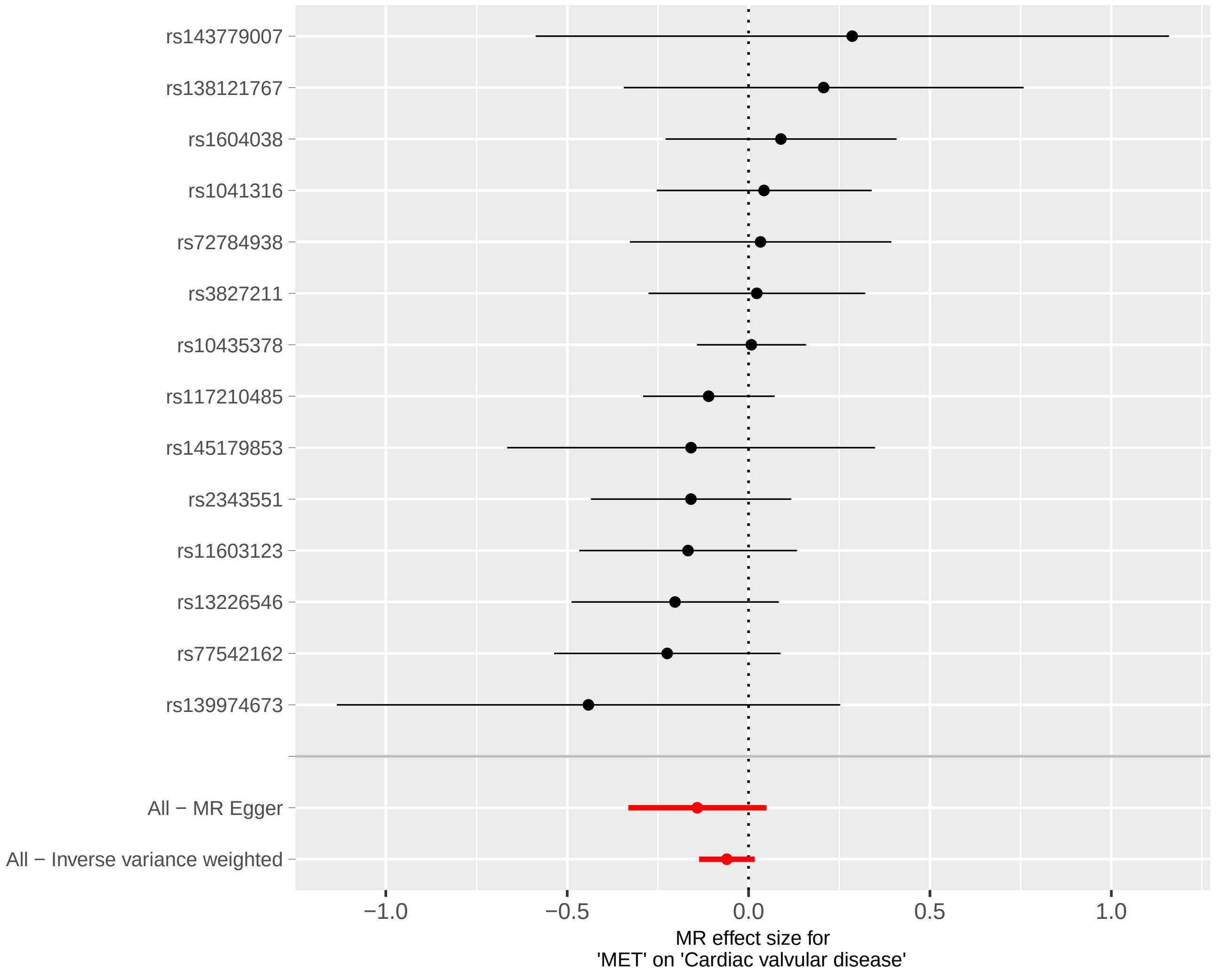
rs143779007
rs138121767
rs1604038
rs1041316
rs72784938
rs3827211
rs10435378
rs117210485
rs145179853
rs2343551
rs11603123
rs13226546
rs77542162
rs139974673
All − MR Egger
All − Inverse variance weighted
−1.0
−0.5
0.0
0.5
1.0
MR effect size for
'MET' on 'Cardiac valvular disease'

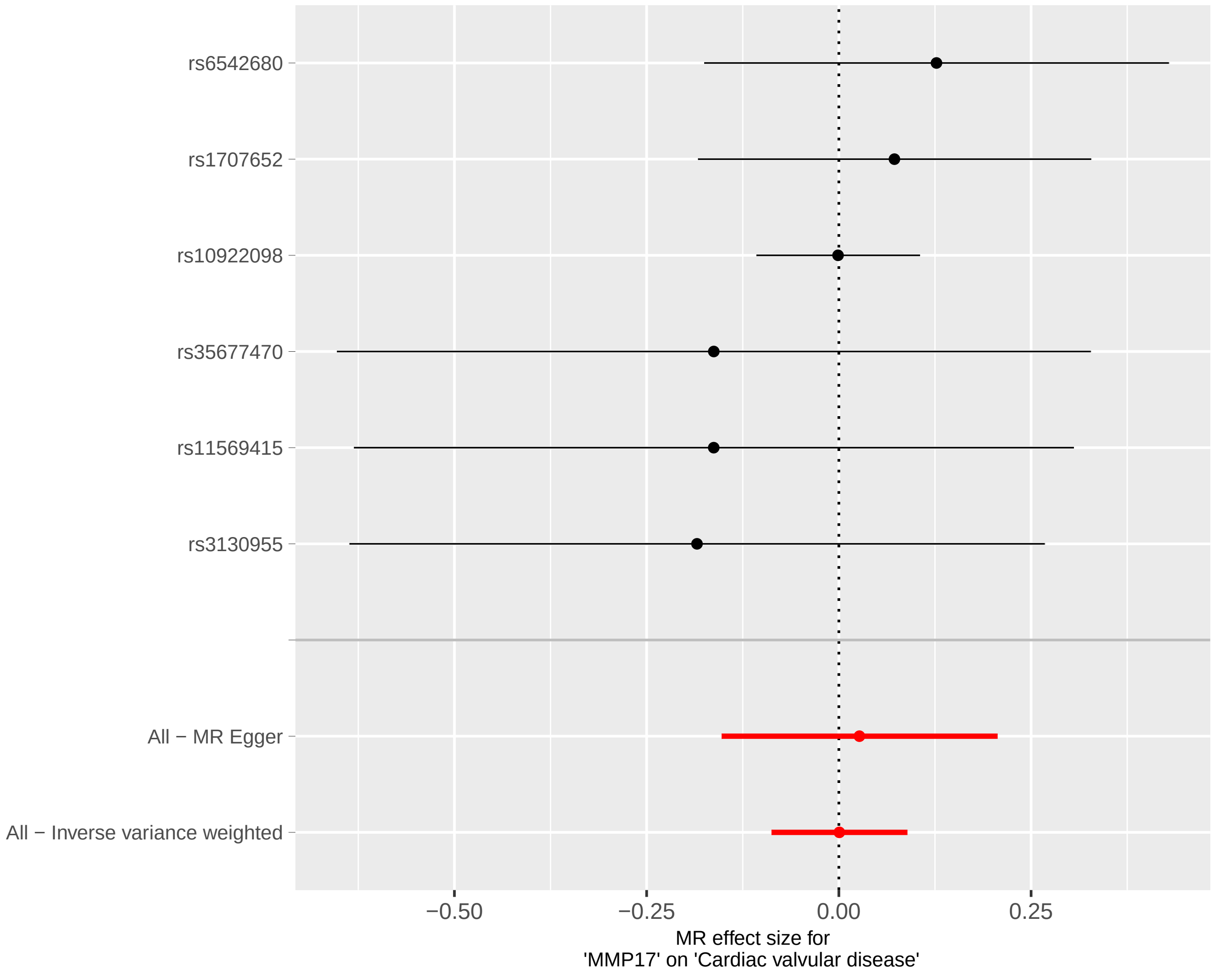
rs6542680
rs1707652
rs10922098
rs35677470
rs11569415
rs3130955
All − MR Egger
All − Inverse variance weighted
−0.50
−0.25
0.00
0.25
MR effect size for
'MMP17' on 'Cardiac valvular disease'

Insufficient number of SNPs

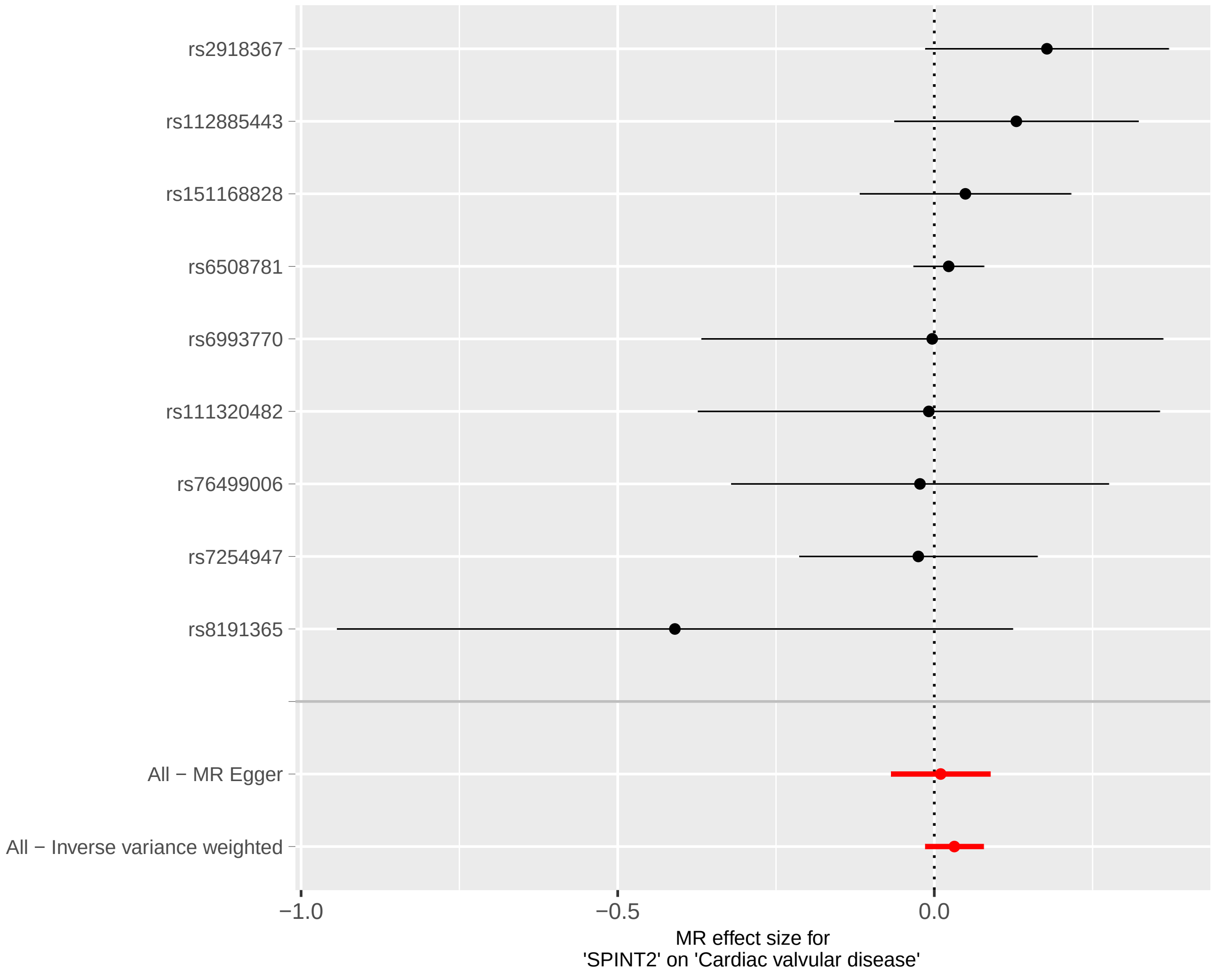
rs2918367
rs112885443
rs151168828
rs6508781
rs6993770
rs111320482
rs76499006
rs7254947
rs8191365
All − MR Egger
All − Inverse variance weighted
−1.0
−0.5
0.0
MR effect size for
'SPINT2' on 'Cardiac valvular disease'

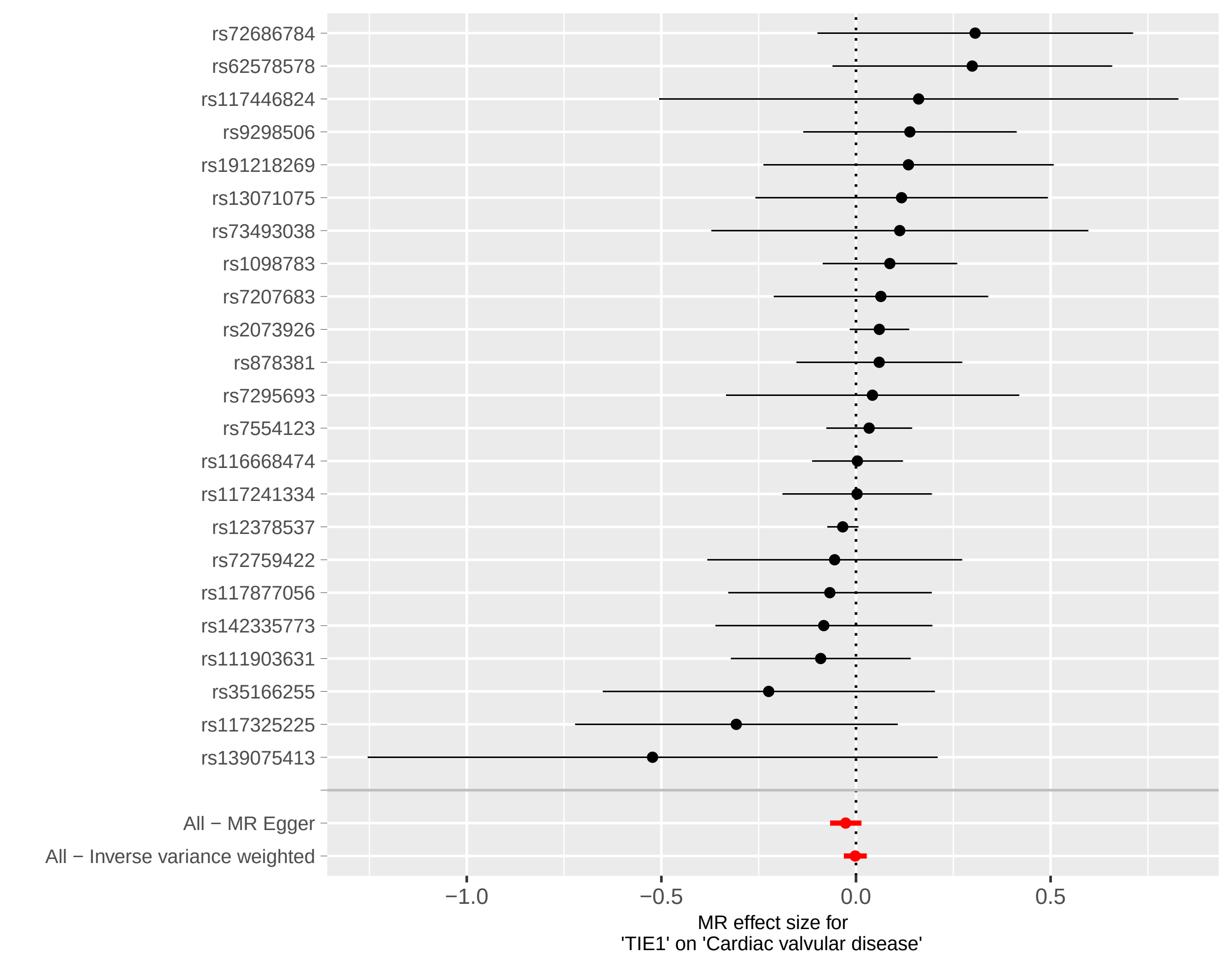
rs72686784
rs62578578
rs117446824
rs9298506
rs191218269
rs13071075
rs73493038
rs1098783
rs7207683
rs2073926
rs878381
rs7295693
rs7554123
rs116668474
rs117241334
rs12378537
rs72759422
rs117877056
rs142335773
rs111903631
rs35166255
rs117325225
rs139075413
All − MR Egger
All − Inverse variance weighted
−1.0
−0.5
0.0
0.5
MR effect size for
'TIE1' on 'Cardiac valvular disease'

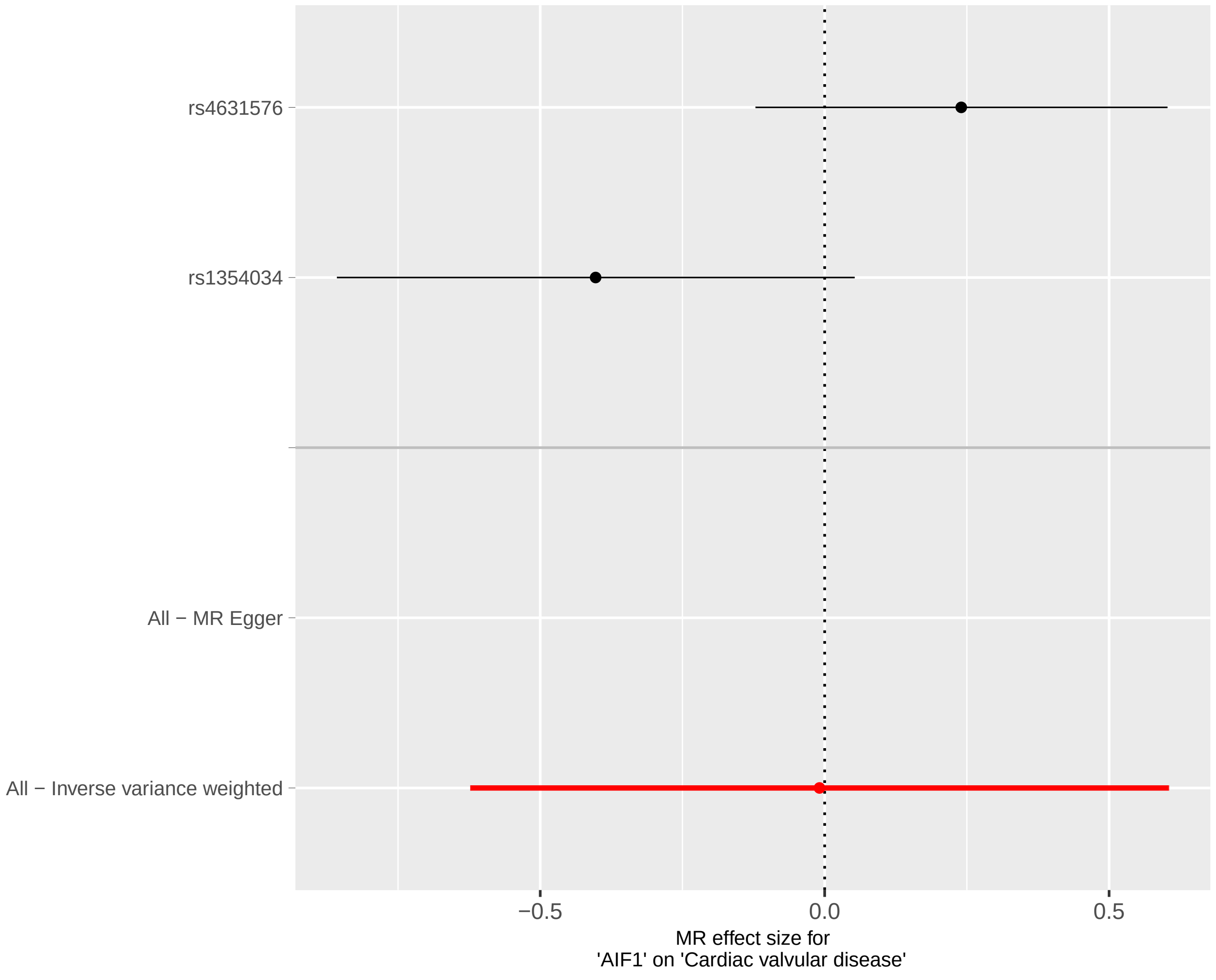
rs4631576
rs1354034
All − MR Egger
All − Inverse variance weighted
−0.5
0.0
0.5
MR effect size for
'AIF1' on 'Cardiac valvular disease'

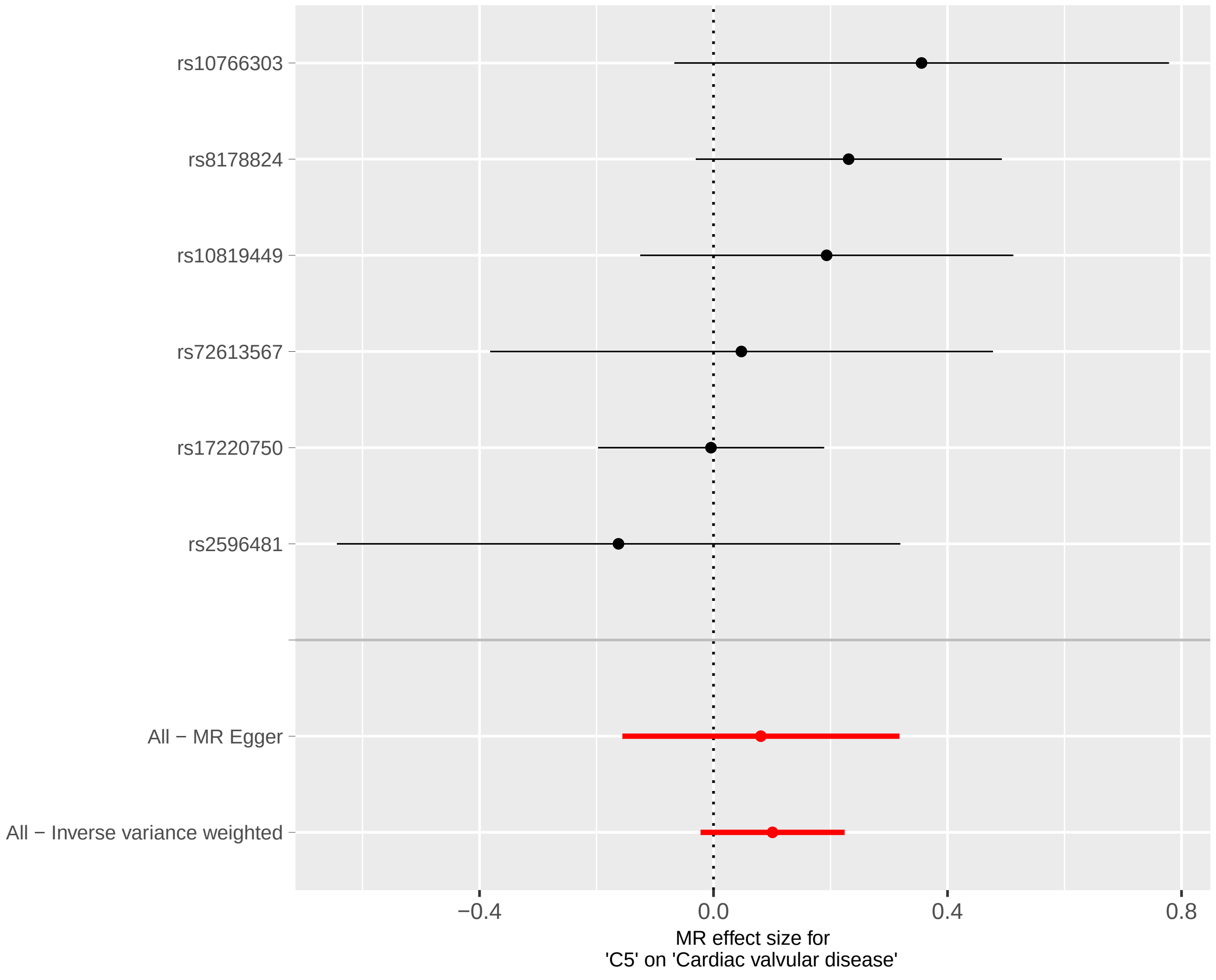

rs10766303
rs8178824
rs10819449
rs72613567
rs17220750
rs2596481
All − MR Egger
All − Inverse variance weighted
−0.4
0.0
0.4
0.8
MR effect size for
'C5' on 'Cardiac valvular disease'

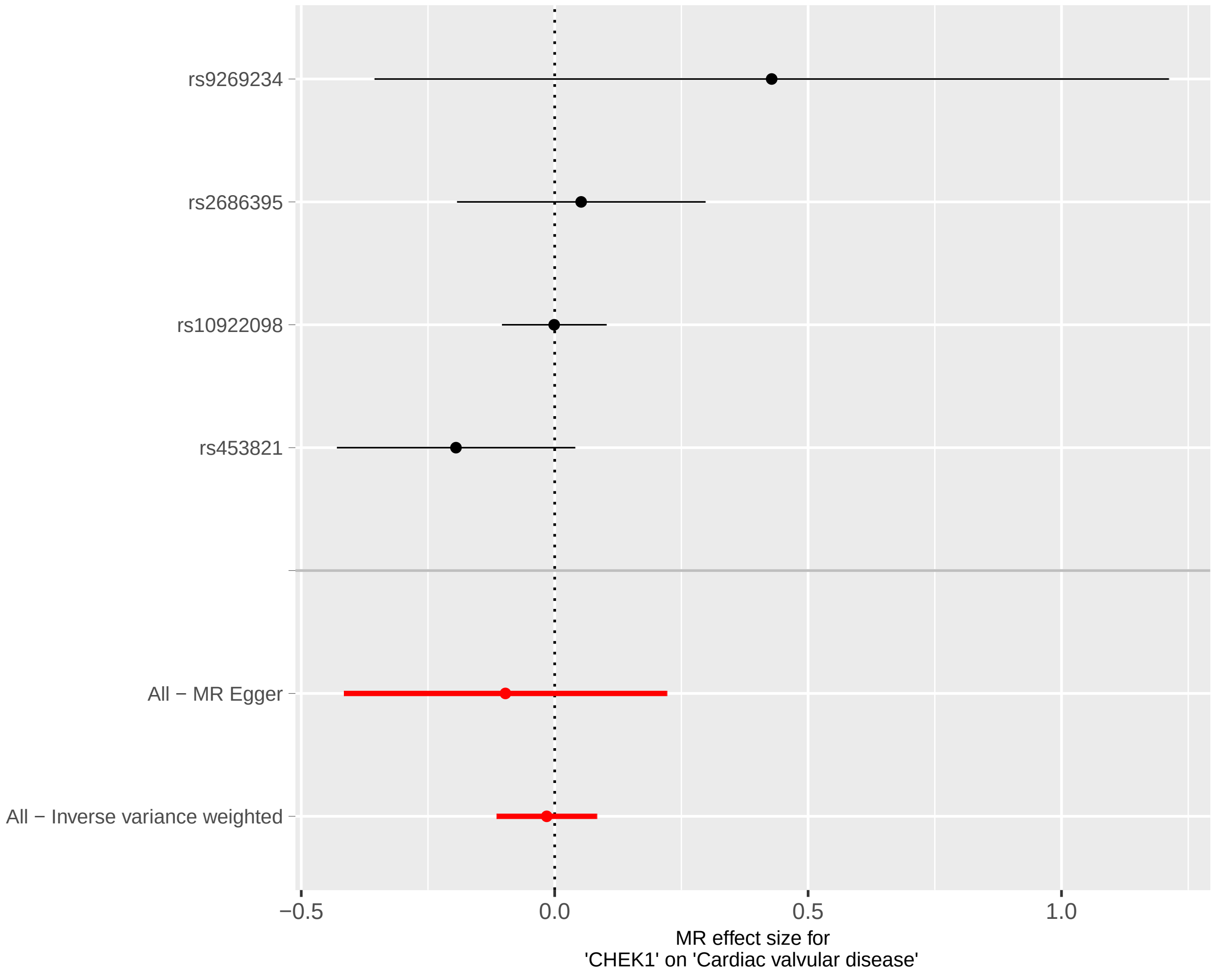
rs9269234
rs2686395
rs10922098
rs453821
All − MR Egger
All − Inverse variance weighted
−0.5
0.0
0.5
1.0
MR effect size for
'CHEK1' on 'Cardiac valvular disease'

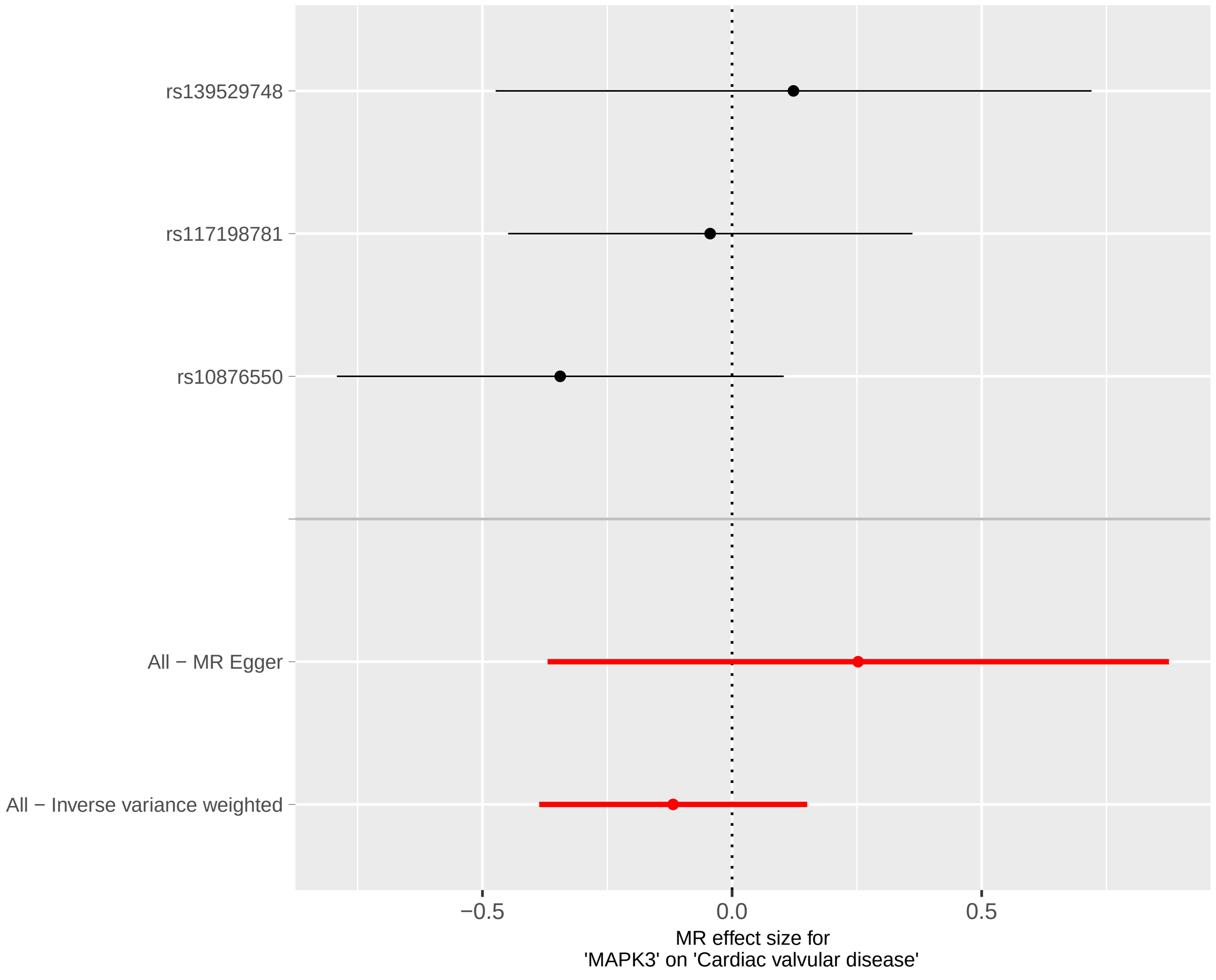
rs139529748
rs117198781
rs10876550
All − MR Egger
All − Inverse variance weighted
−0.5
0.0
0.5
MR effect size for
'MAPK3' on 'Cardiac valvular disease'

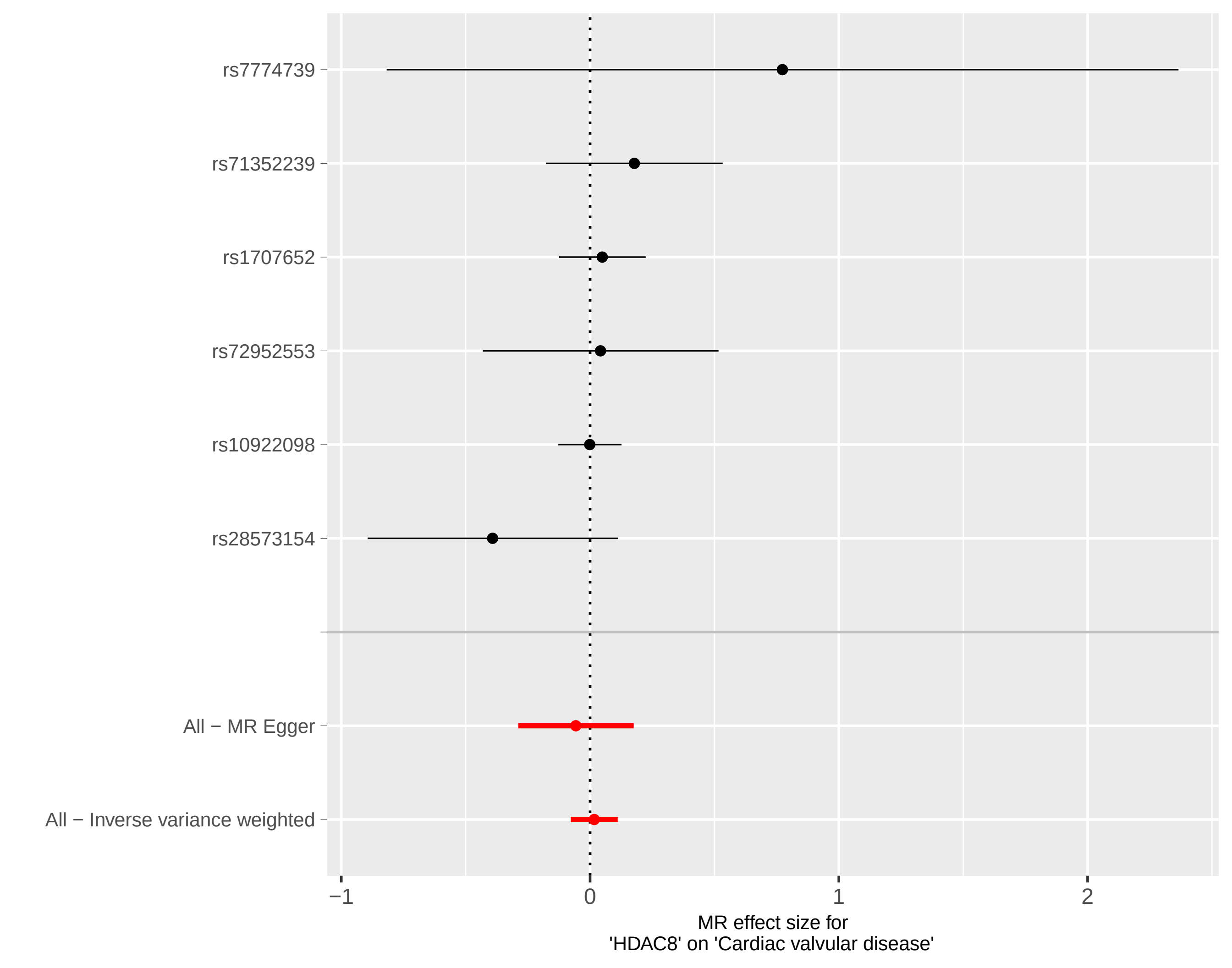
rs7774739
rs71352239
rs1707652
rs72952553
rs10922098
rs28573154
All − MR Egger
All − Inverse variance weighted
−1
0
1
2
MR effect size for
'HDAC8' on 'Cardiac valvular disease'

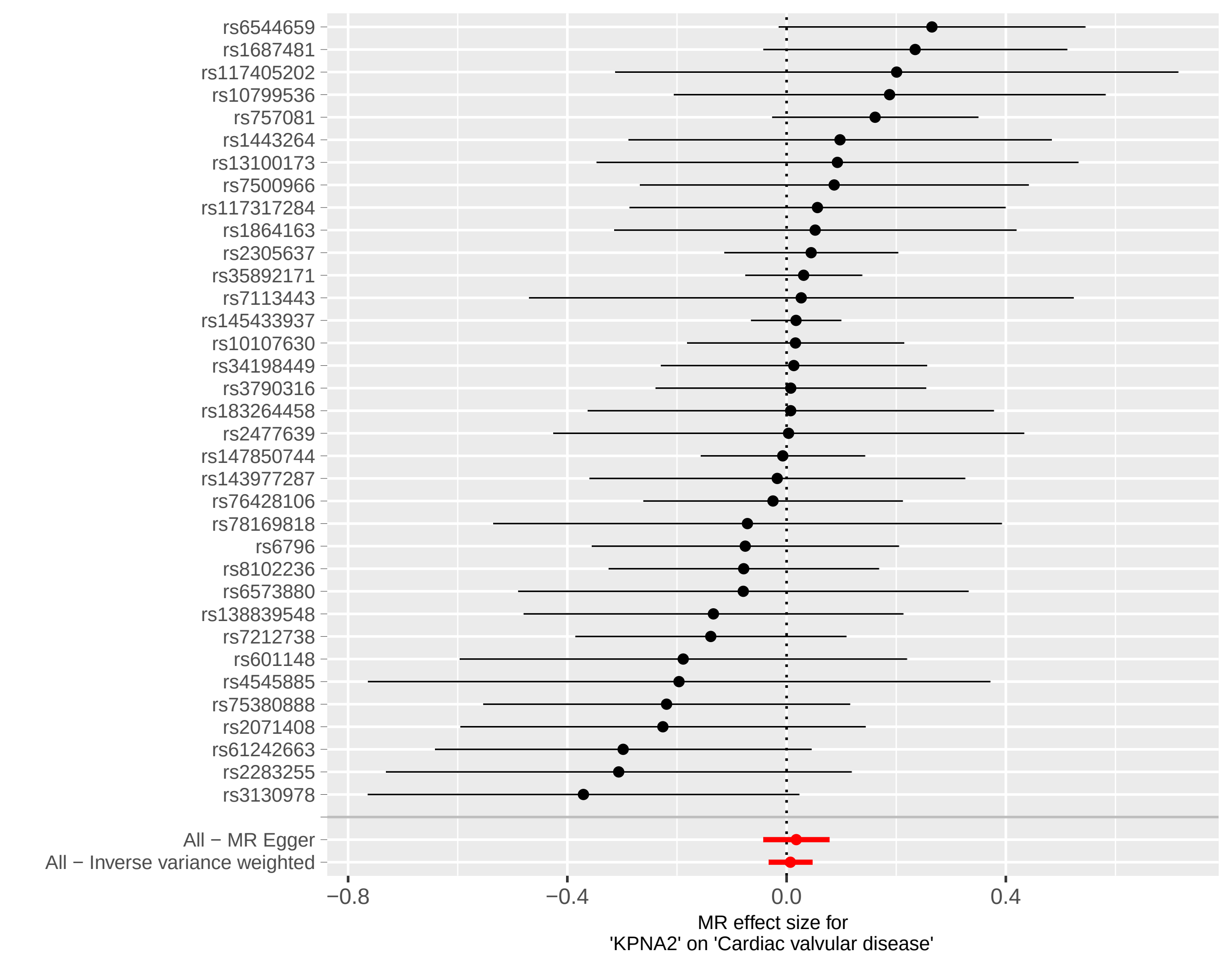
rs6544659
rs1687481
rs117405202
rs10799536
rs757081
rs1443264
rs13100173
rs7500966
rs117317284
rs1864163
rs2305637
rs35892171
rs7113443
rs145433937
rs10107630
rs34198449
rs3790316
rs183264458
rs2477639
rs147850744
rs143977287
rs76428106
rs78169818
rs6796
rs8102236
rs6573880
rs138839548
rs7212738
rs601148
rs4545885
rs75380888
rs2071408
rs61242663
rs2283255
rs3130978
All − MR Egger
All − Inverse variance weighted
−0.8
−0.4
0.0
0.4
MR effect size for
'KPNA2' on 'Cardiac valvular disease'

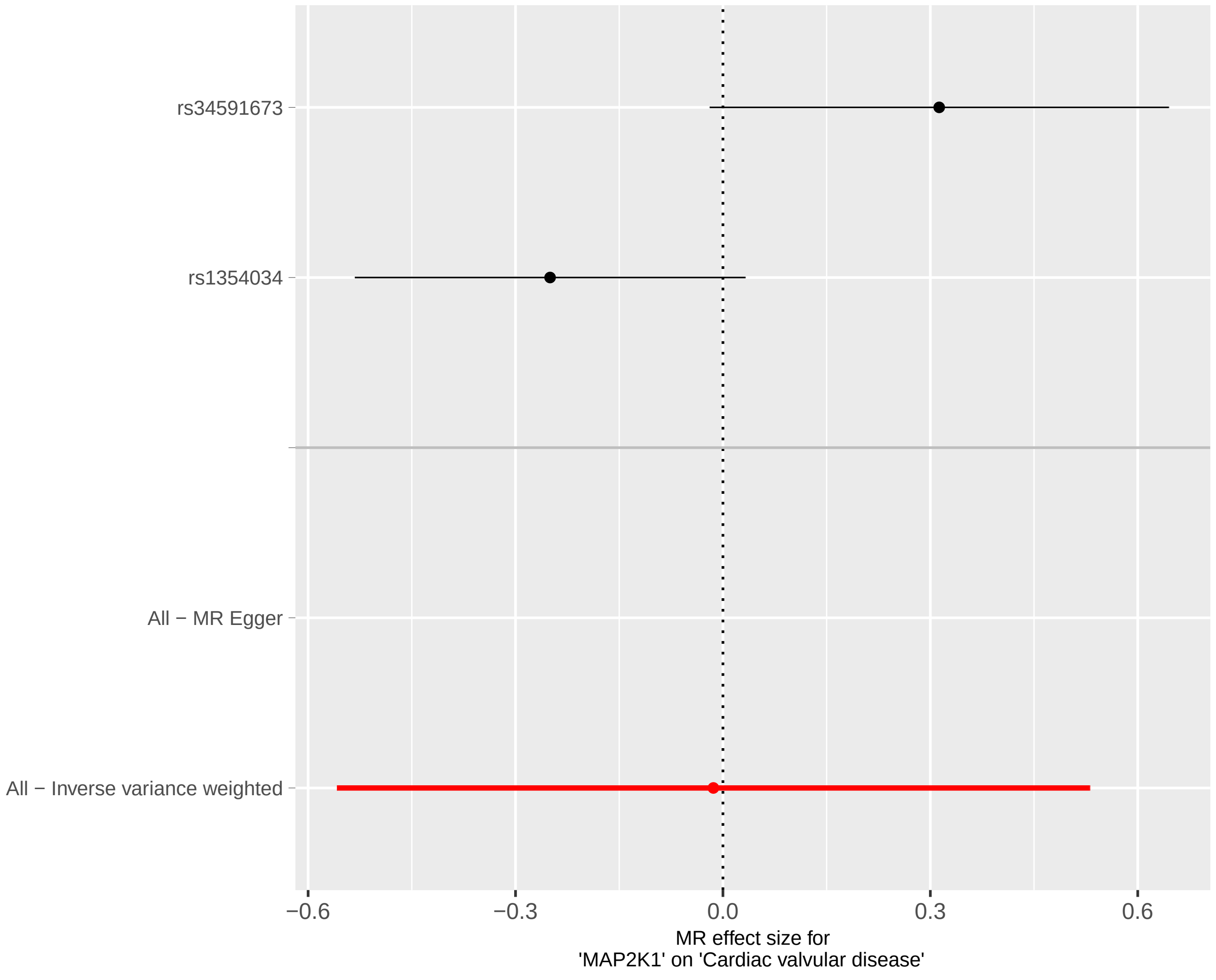
rs34591673
rs1354034
All − MR Egger
All − Inverse variance weighted
−0.6
−0.3
0.0
0.3
0.6
MR effect size for
'MAP2K1' on 'Cardiac valvular disease'

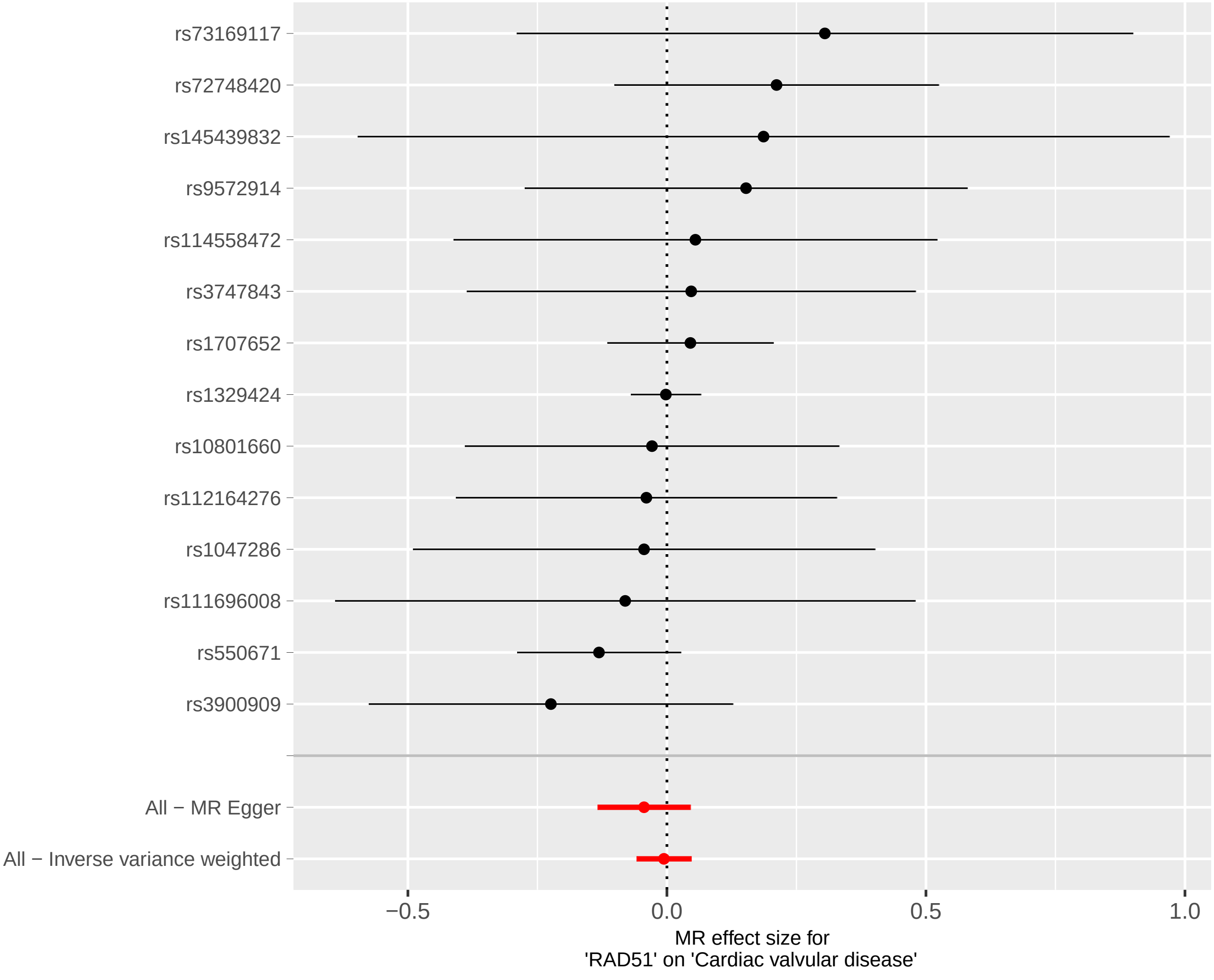
rs73169117
rs72748420
rs145439832
rs9572914
rs114558472
rs3747843
rs1707652
rs1329424
rs10801660
rs112164276
rs1047286
rs111696008
rs550671
rs3900909
All − MR Egger
All − Inverse variance weighted
−0.5
0.0
0.5
1.0
MR effect size for
'RAD51' on 'Cardiac valvular disease'

Insufficient number of SNPs

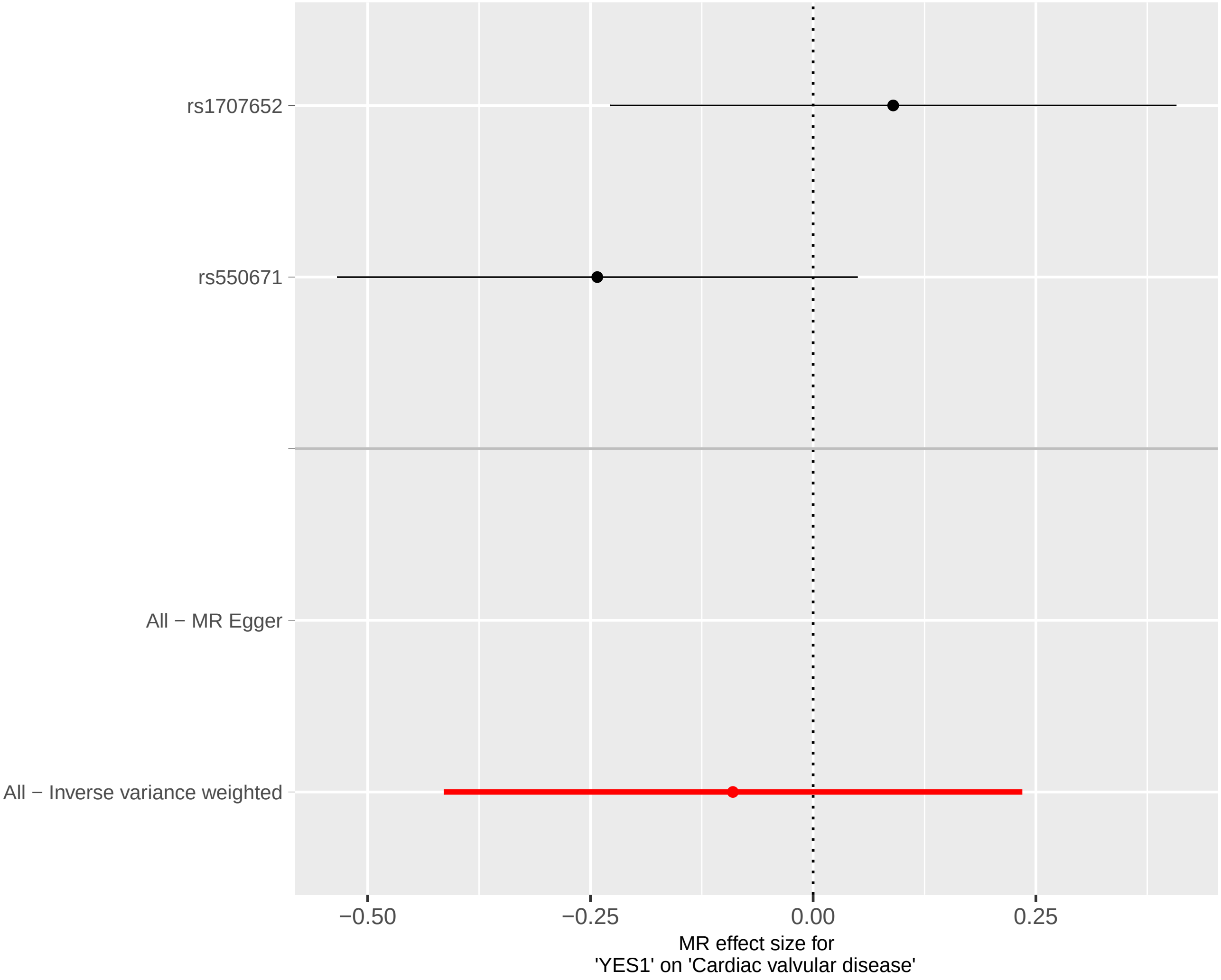
rs1707652
rs550671
All − MR Egger
All − Inverse variance weighted
−0.50
−0.25
0.00
0.25
MR effect size for
'YES1' on 'Cardiac valvular disease'

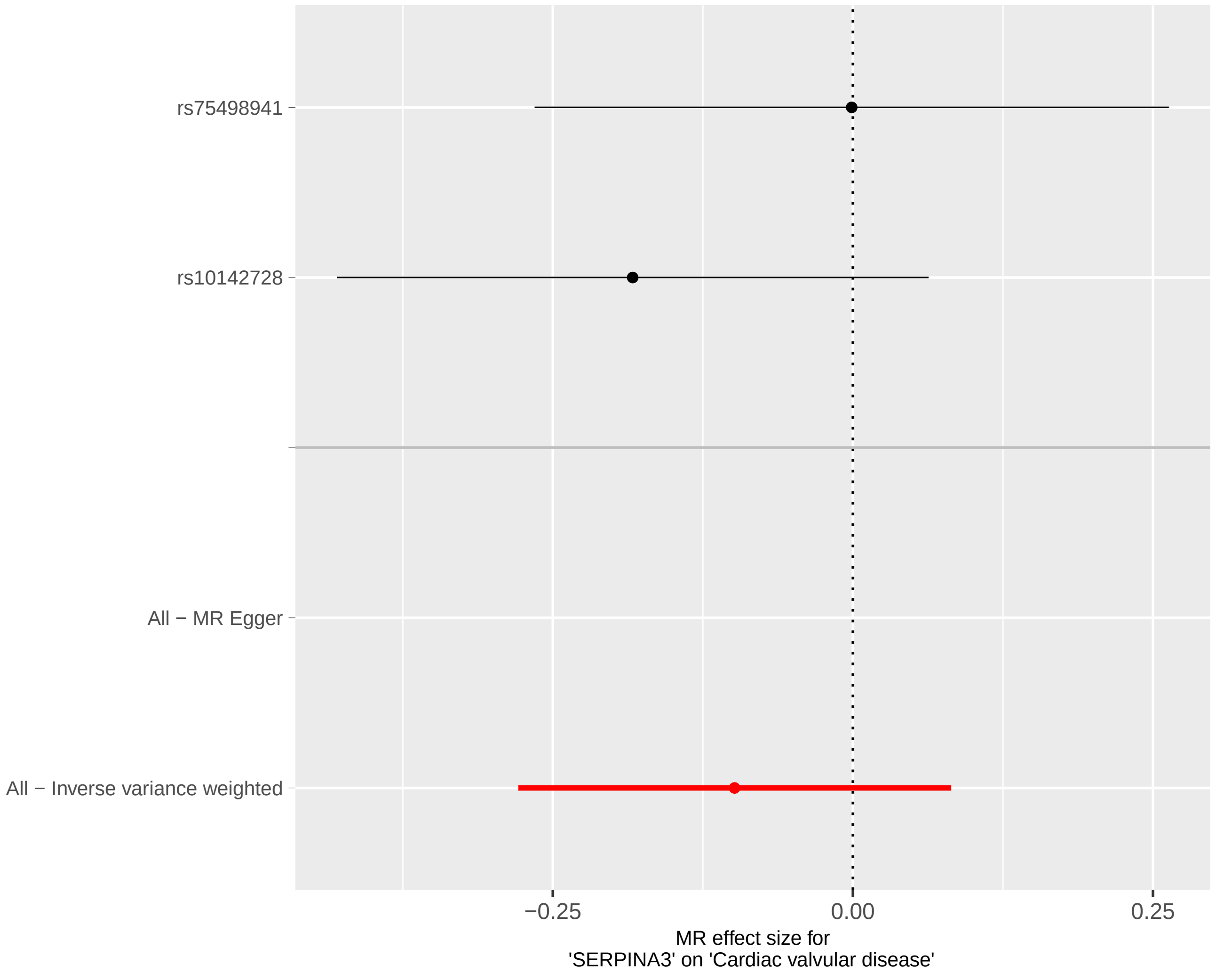
rs75498941
rs10142728
All − MR Egger
All − Inverse variance weighted
−0.25
0.00
0.25
MR effect size for
'SERPINA3' on 'Cardiac valvular disease'

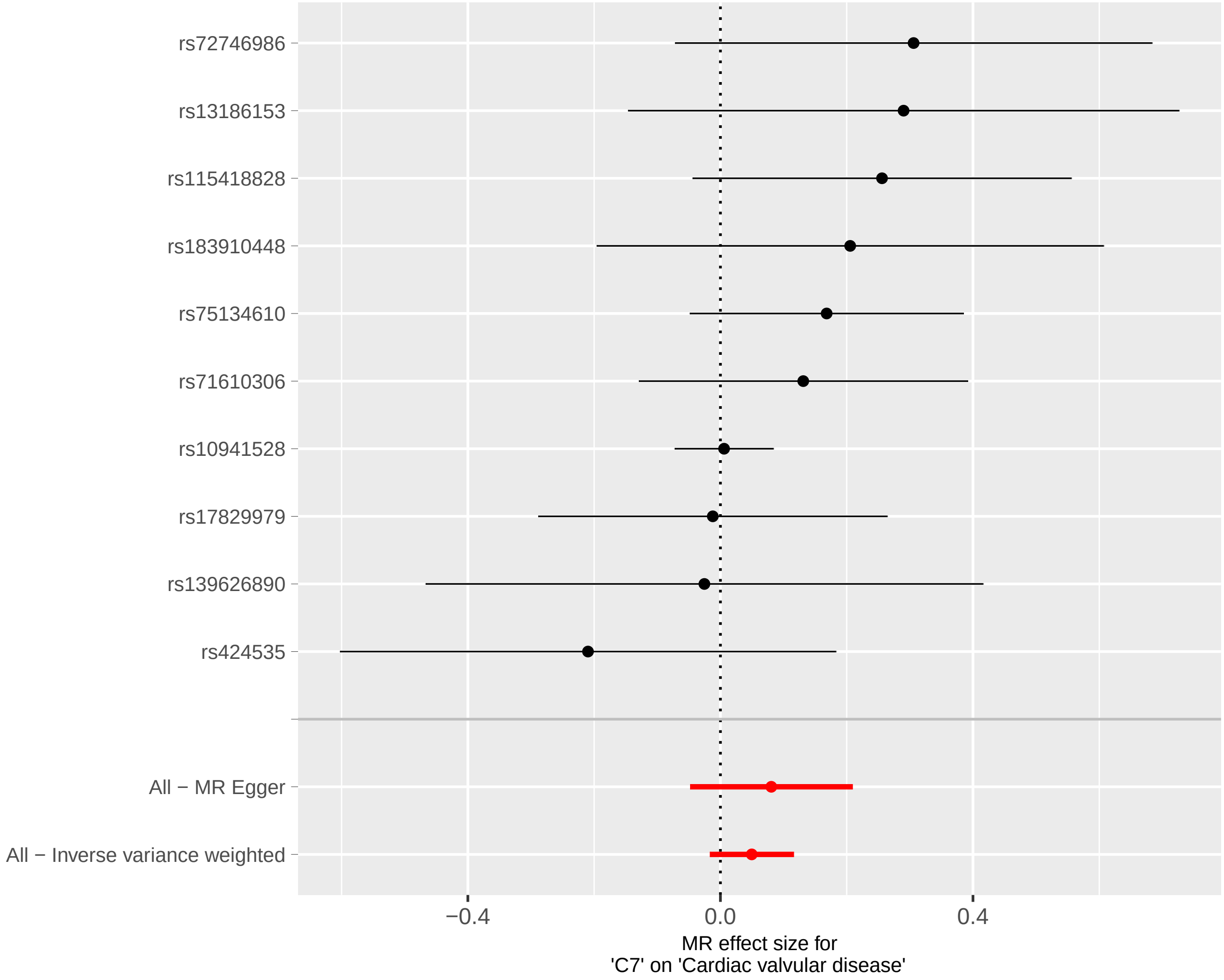
rs72746986
rs13186153
rs115418828
rs183910448
rs75134610
rs71610306
rs10941528
rs17829979
rs139626890
rs424535
All − MR Egger
All − Inverse variance weighted
−0.4
0.0
0.4
MR effect size for
'C7' on 'Cardiac valvular disease'

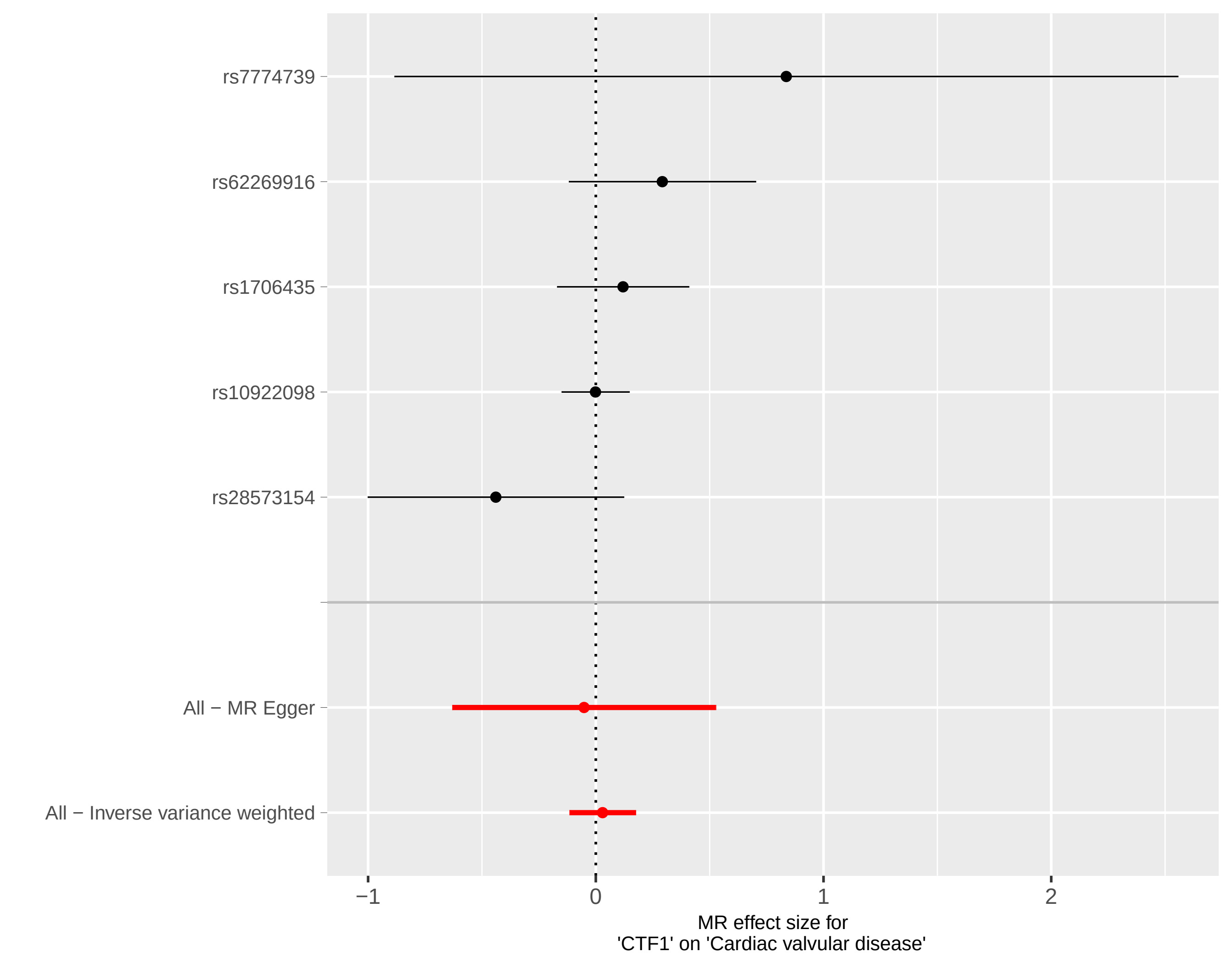
rs7774739
rs62269916
rs1706435
rs10922098
rs28573154
All − MR Egger
All − Inverse variance weighted
−1
0
1
2
MR effect size for
'CTF1' on 'Cardiac valvular disease'

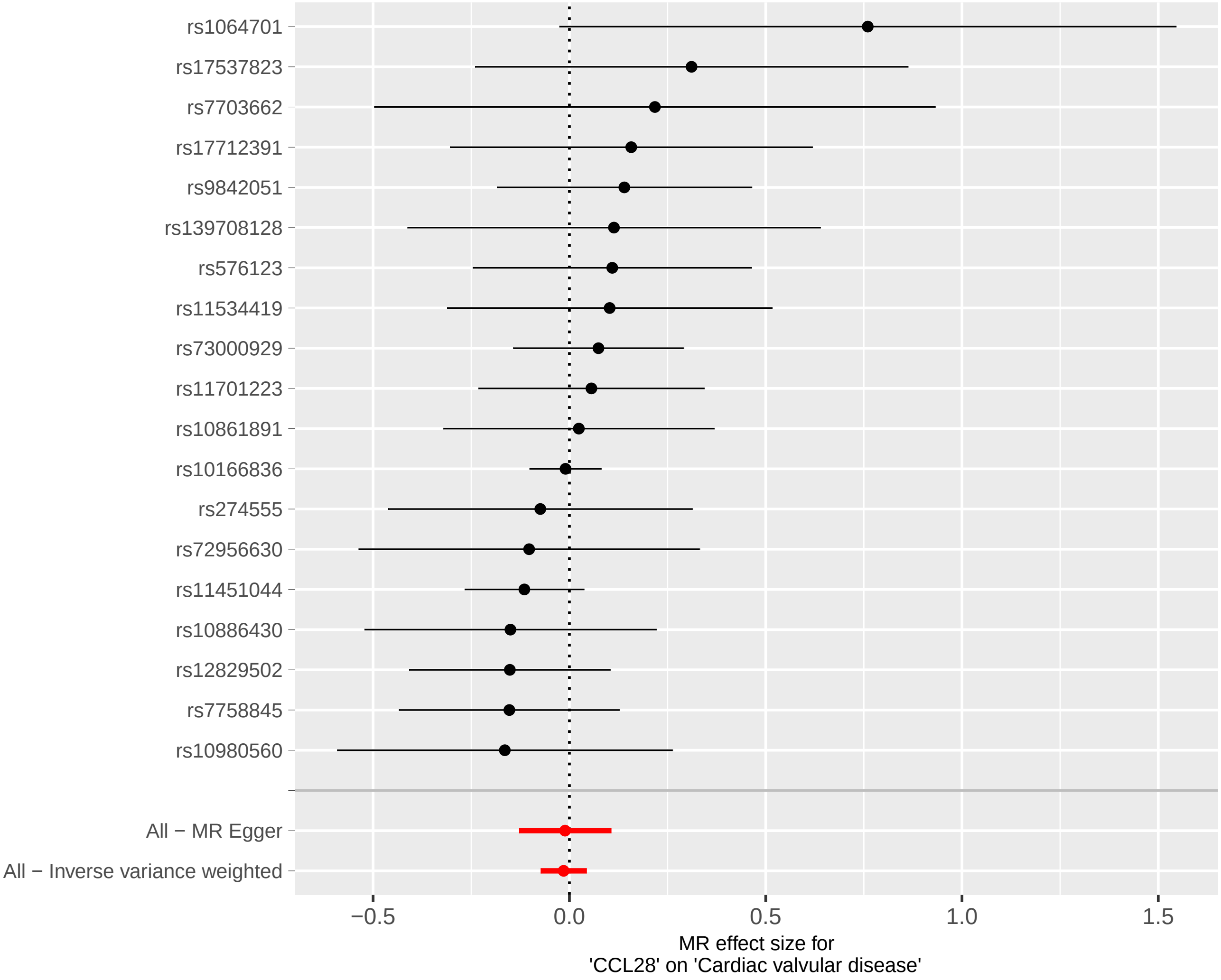
rs1064701
rs17537823
rs7703662
rs17712391
rs9842051
rs139708128
rs576123
rs11534419
rs73000929
rs11701223
rs10861891
rs10166836
rs274555
rs72956630
rs11451044
rs10886430
rs12829502
rs7758845
rs10980560
All − MR Egger
All − Inverse variance weighted
−0.5
0.0
0.5
1.0
1.5
MR effect size for
'CCL28' on 'Cardiac valvular disease'

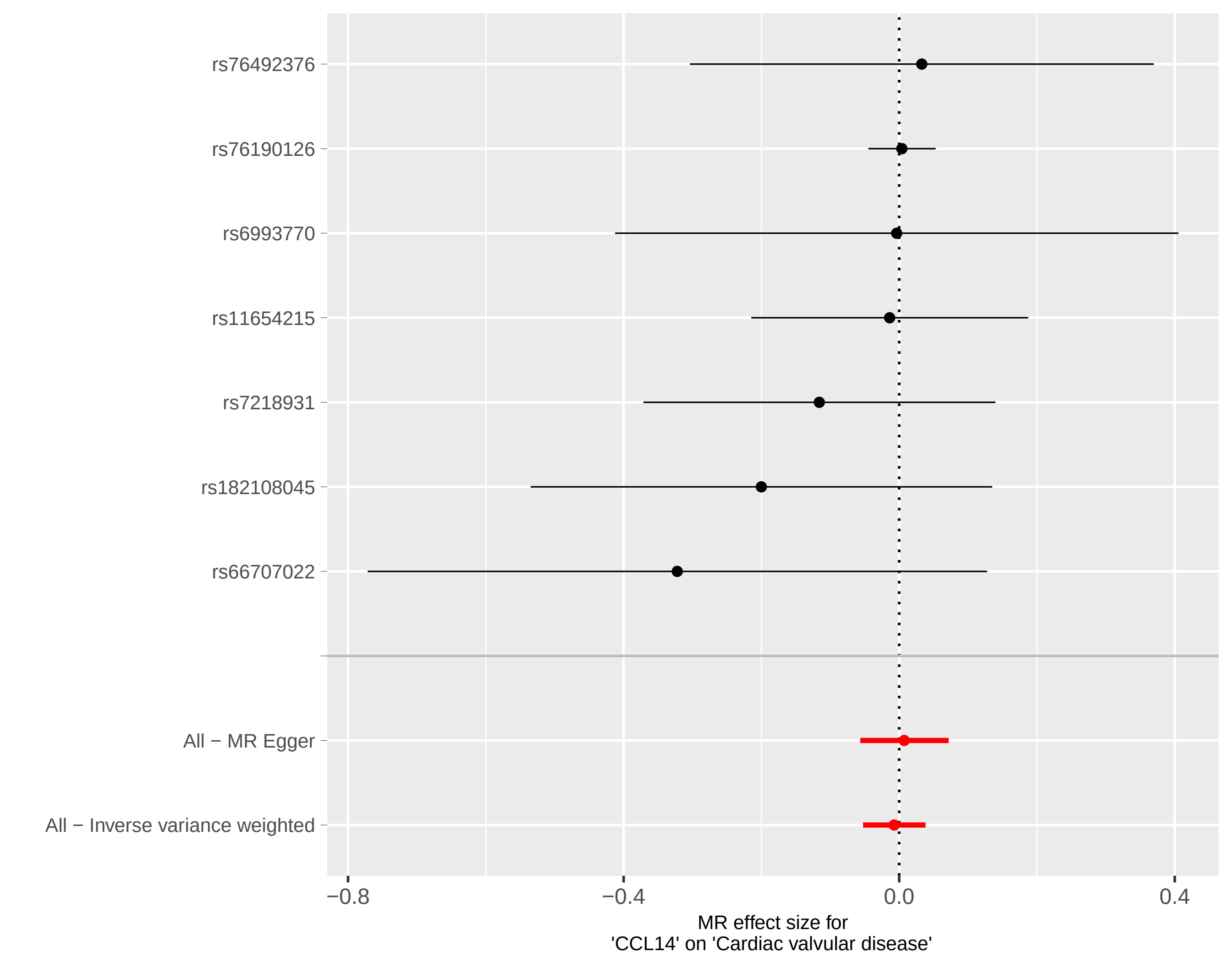
rs76492376
rs76190126
rs6993770
rs11654215
rs7218931
rs182108045
rs66707022
All − MR Egger
All − Inverse variance weighted
−0.8
−0.4
0.0
0.4
MR effect size for
'CCL14' on 'Cardiac valvular disease'

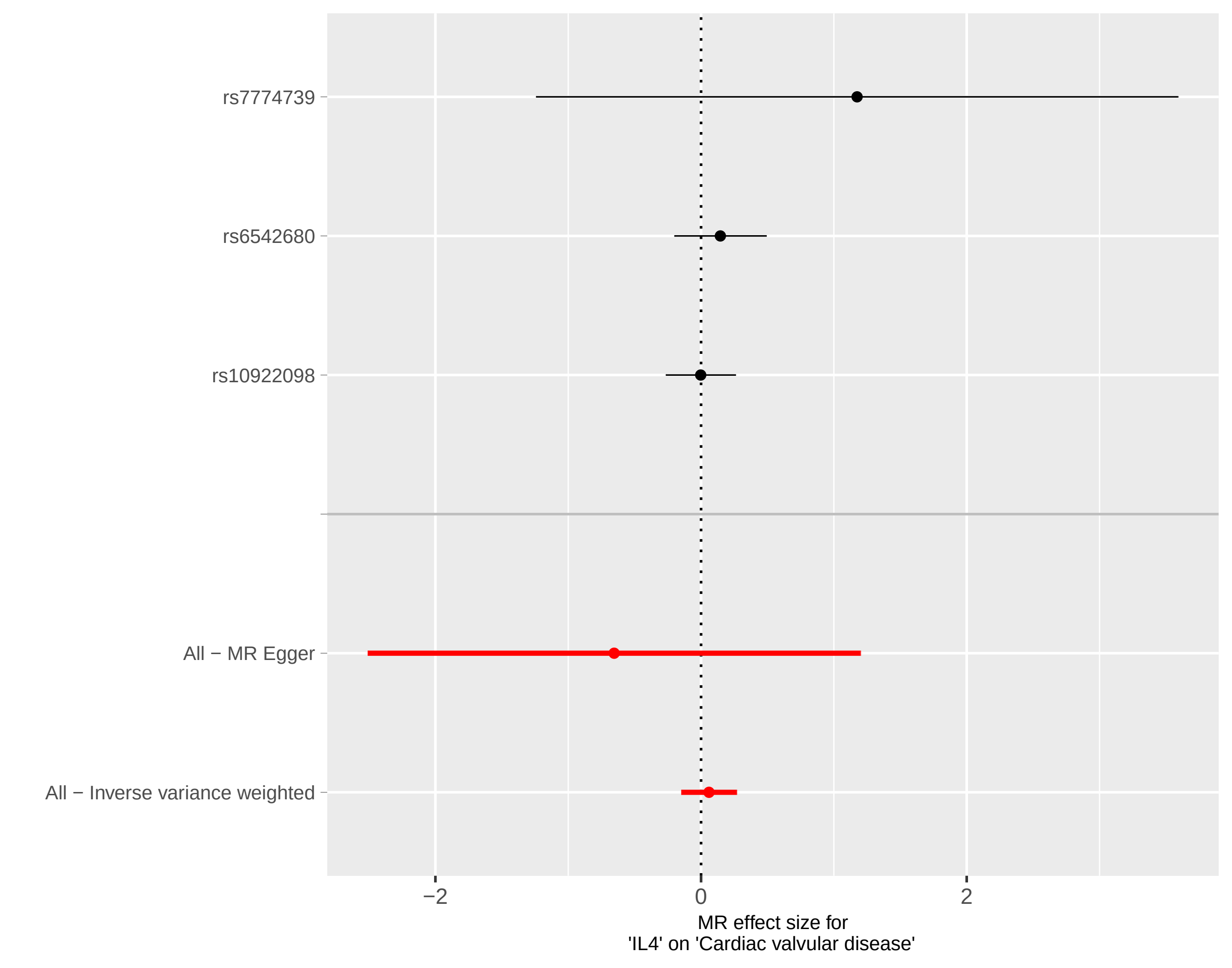
rs7774739
rs6542680
rs10922098
All − MR Egger
All − Inverse variance weighted
−2
0
2
MR effect size for
'IL4' on 'Cardiac valvular disease'

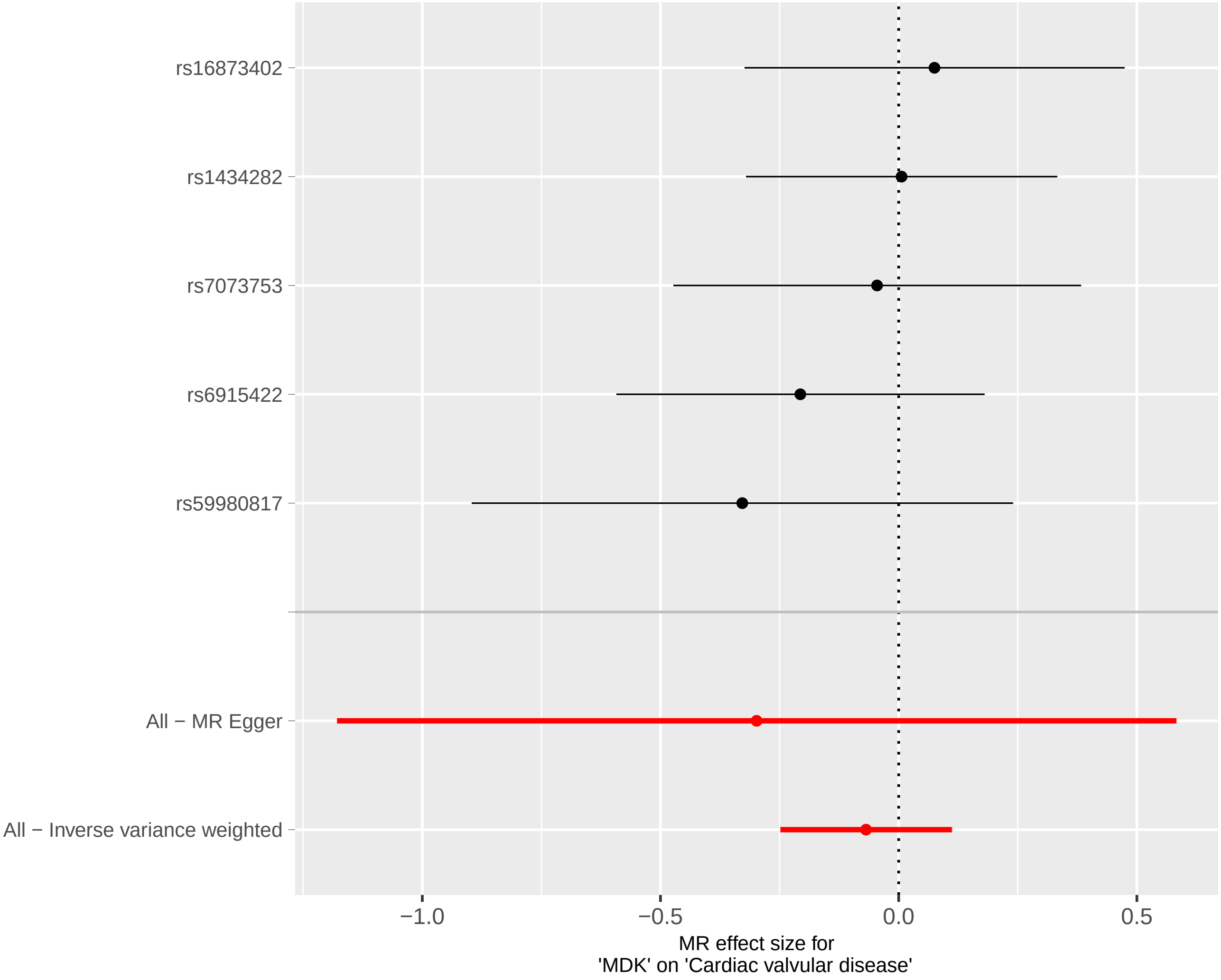
rs16873402
rs1434282
rs7073753
rs6915422
rs59980817
All − MR Egger
All − Inverse variance weighted
−1.0
−0.5
0.0
0.5
MR effect size for
'MDK' on 'Cardiac valvular disease'

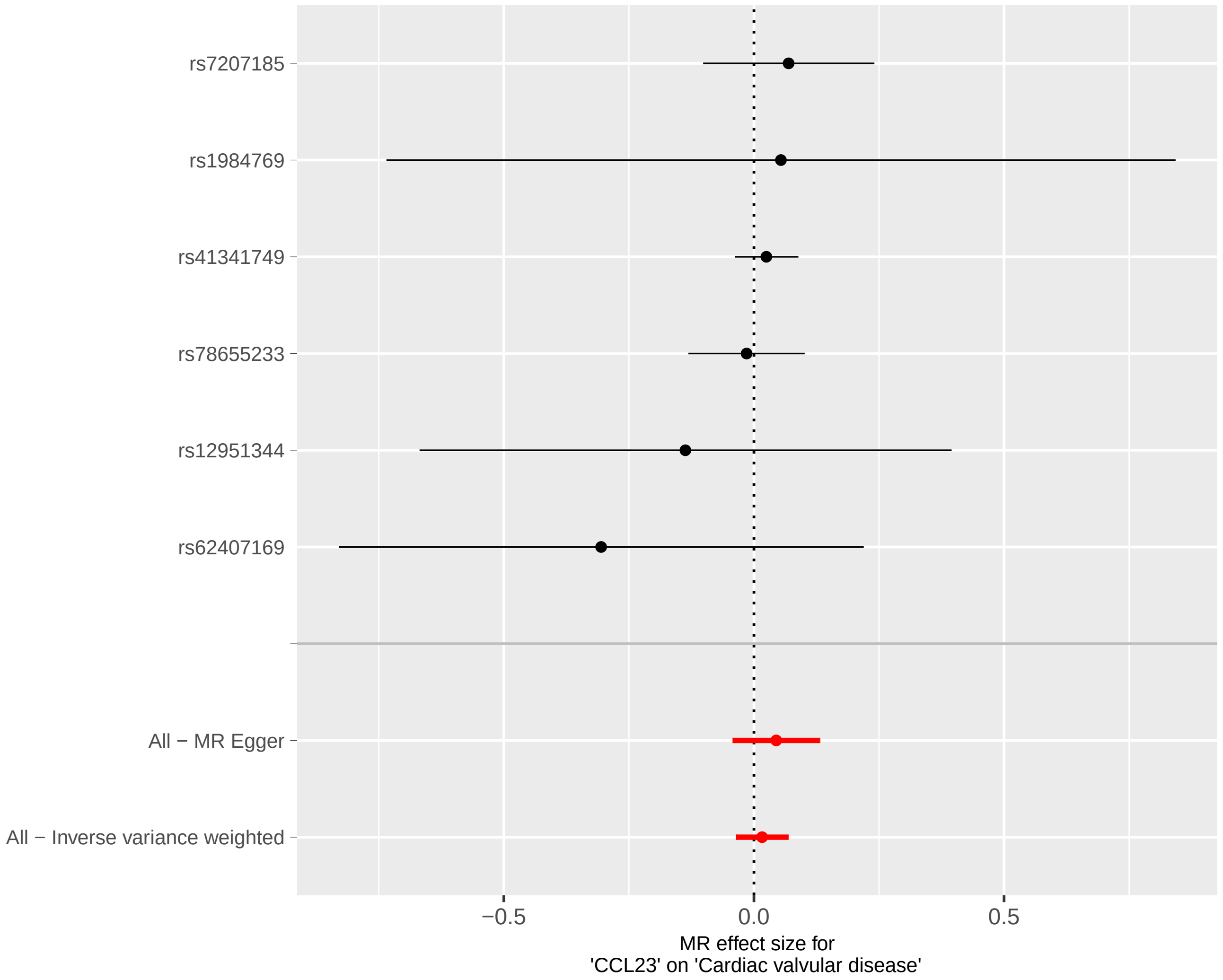

rs7207185
rs1984769
rs41341749
rs78655233
rs12951344
rs62407169
All − MR Egger
All − Inverse variance weighted
−0.5
0.0
0.5
MR effect size for
'CCL23' on 'Cardiac valvular disease'

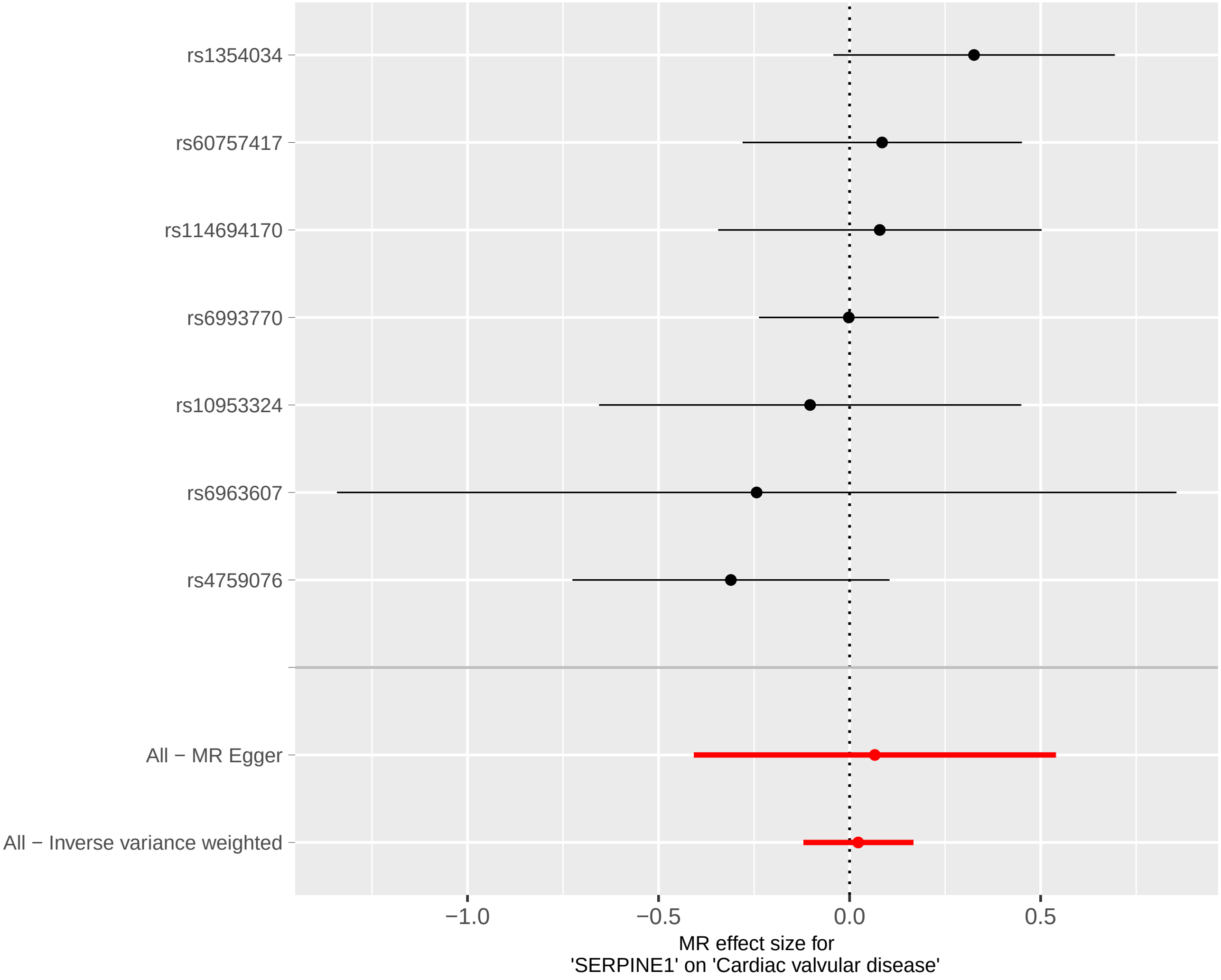

rs1354034
rs60757417
rs114694170
rs6993770
rs10953324
rs6963607
rs4759076
All − MR Egger
All − Inverse variance weighted
−1.0
−0.5
0.0
0.5
MR effect size for
'SERPINE1' on 'Cardiac valvular disease'

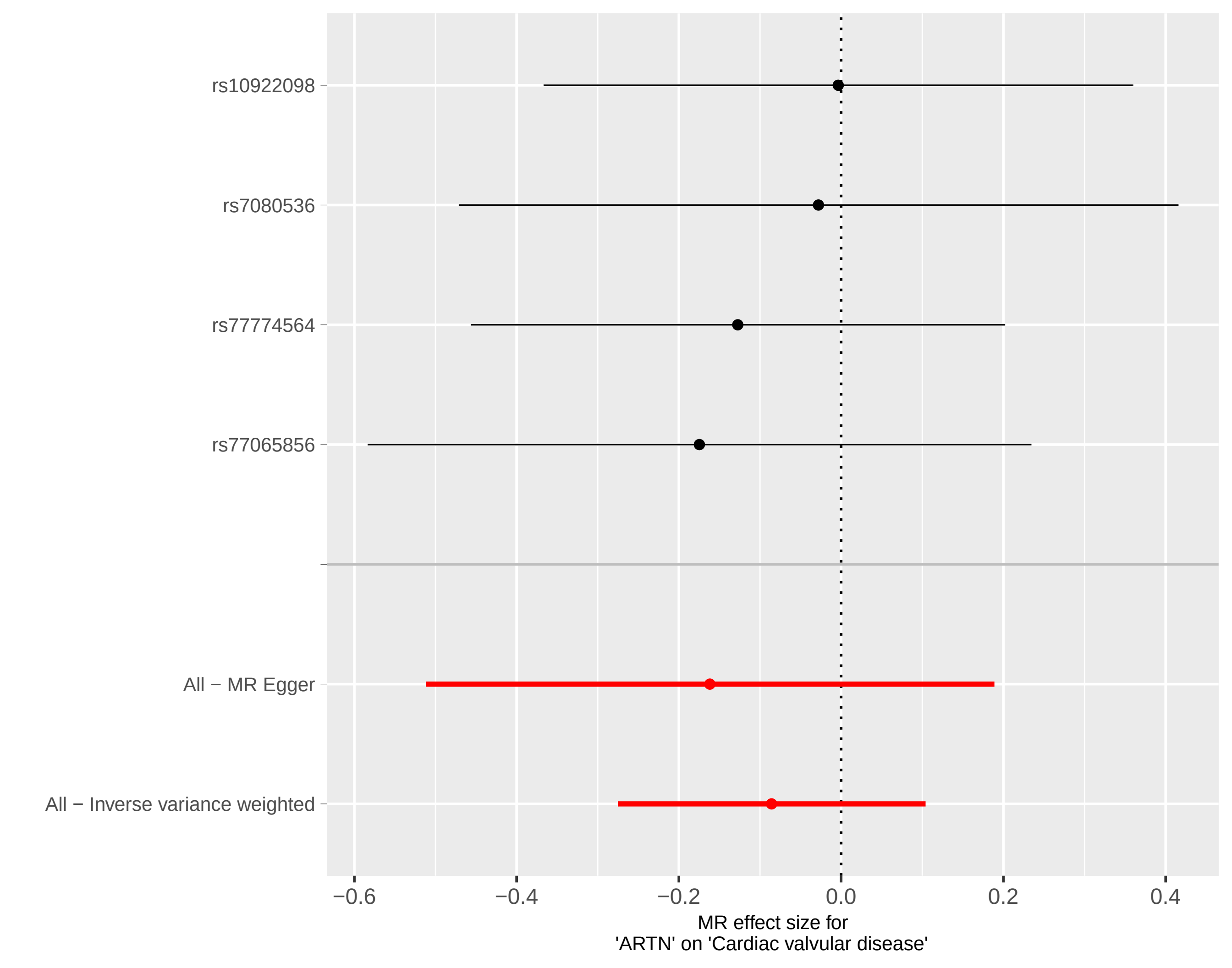
rs10922098
rs7080536
rs77774564
rs77065856
All − MR Egger
All − Inverse variance weighted
−0.6
−0.4
−0.2
0.0
0.2
0.4
MR effect size for
'ARTN' on 'Cardiac valvular disease'

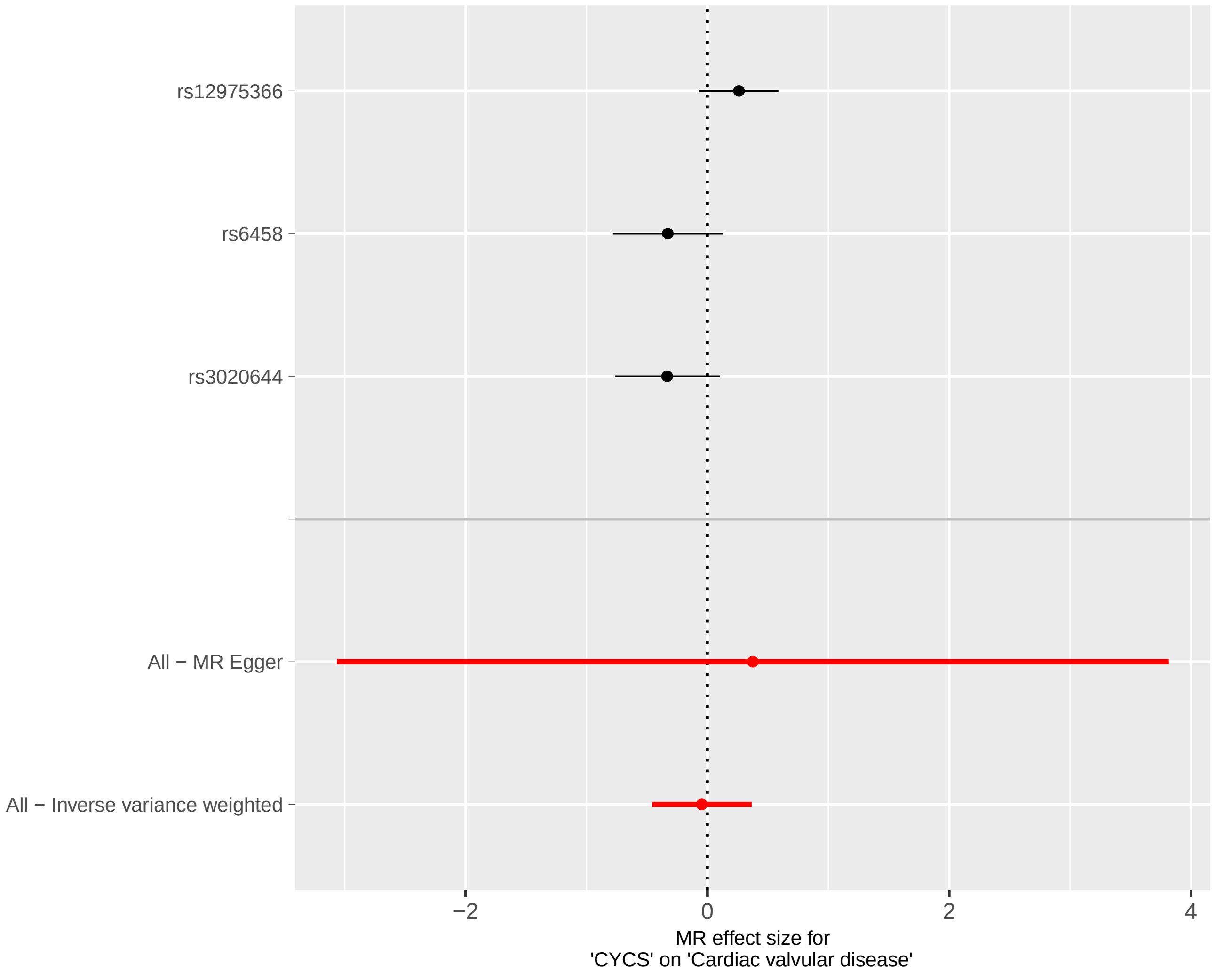
rs12975366
rs6458
rs3020644
All − MR Egger
All − Inverse variance weighted
−2
0
2
4
MR effect size for
'CYCS' on 'Cardiac valvular disease'

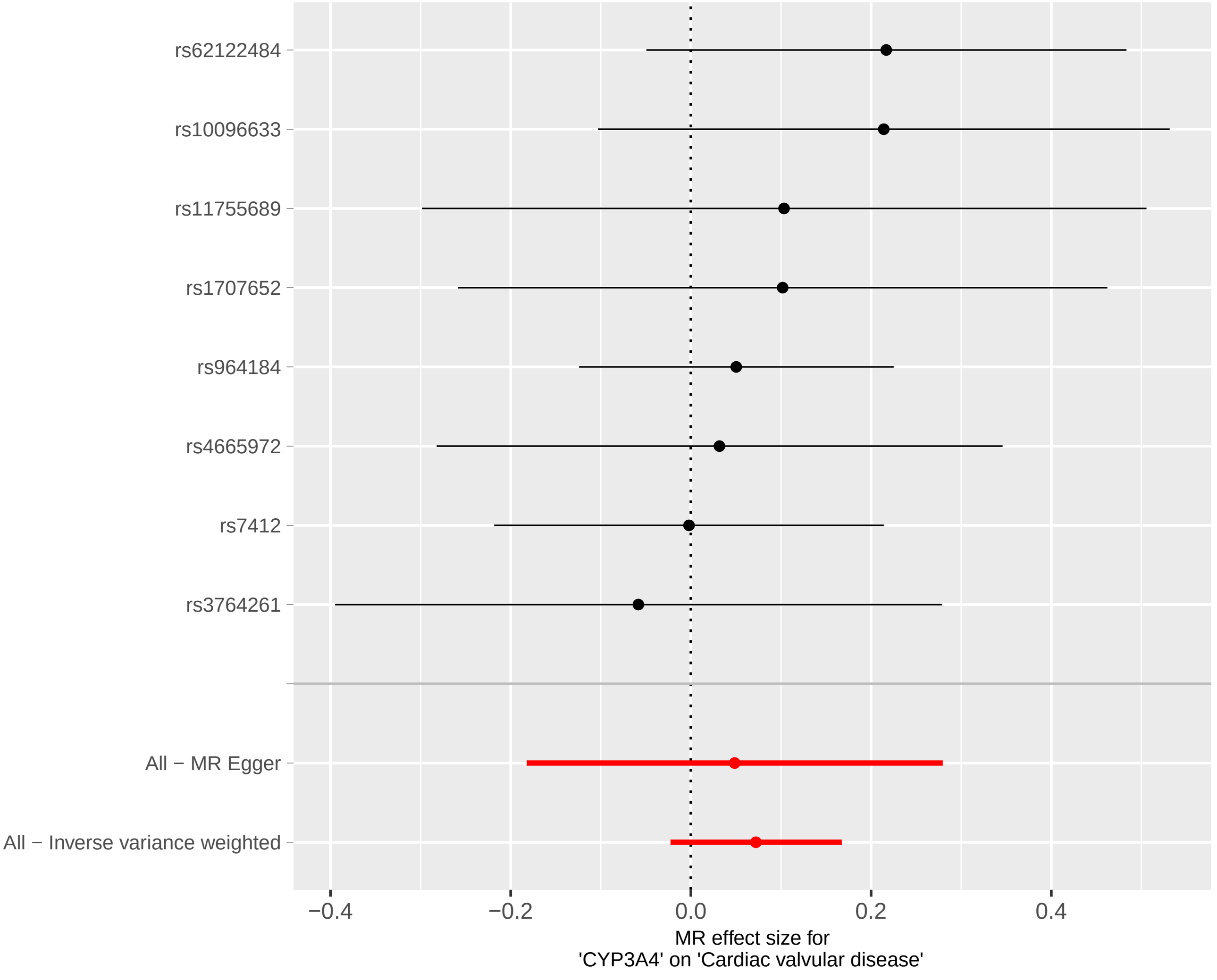
rs62122484
rs10096633
rs11755689
rs1707652
rs964184
rs4665972
rs7412
rs3764261
All − MR Egger
All − Inverse variance weighted
−0.4
−0.2
0.0
0.2
0.4
MR effect size for
'CYP3A4' on 'Cardiac valvular disease'

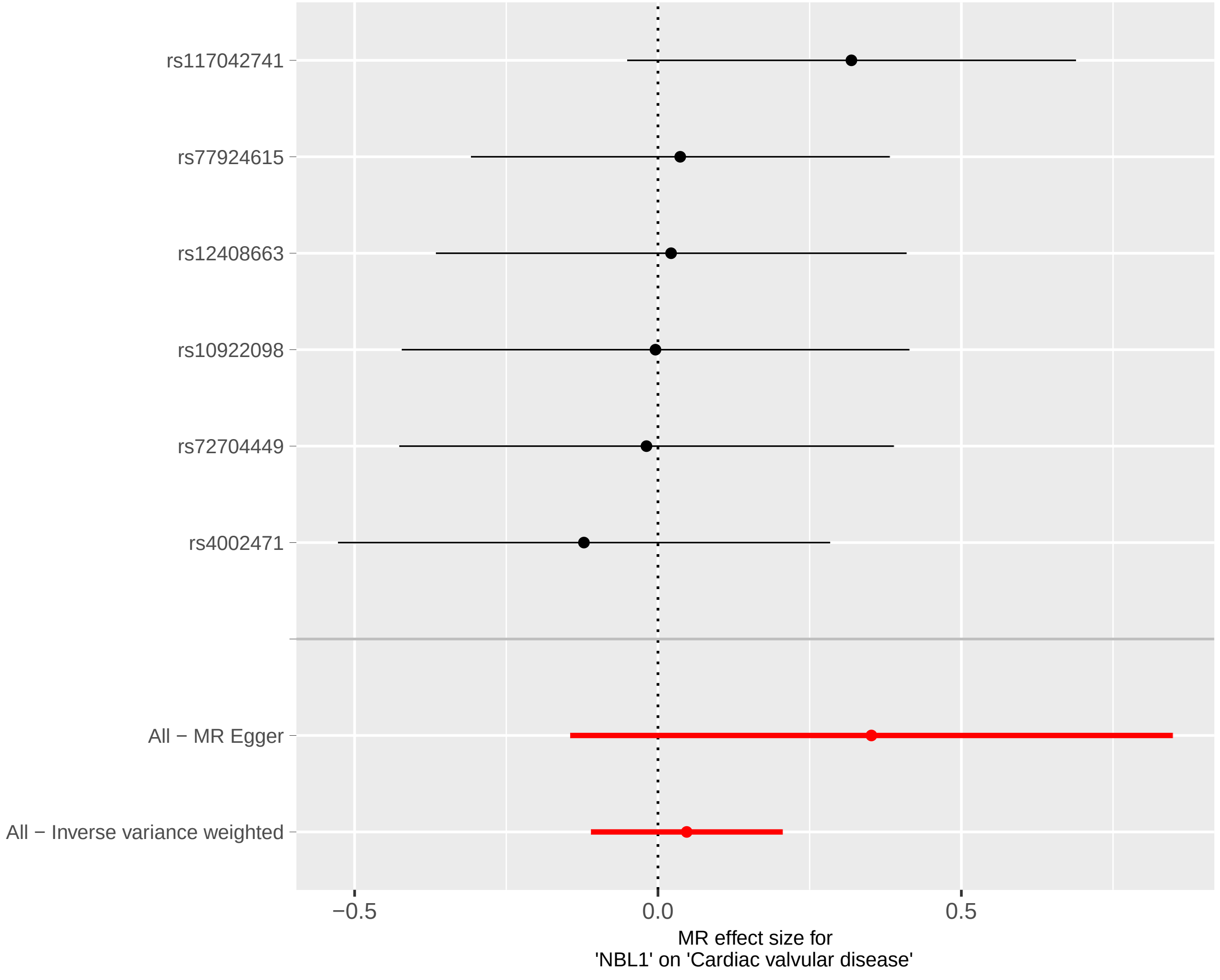
rs117042741
rs77924615
rs12408663
rs10922098
rs72704449
rs4002471
All − MR Egger
All − Inverse variance weighted
−0.5
0.0
0.5
MR effect size for
'NBL1' on 'Cardiac valvular disease'

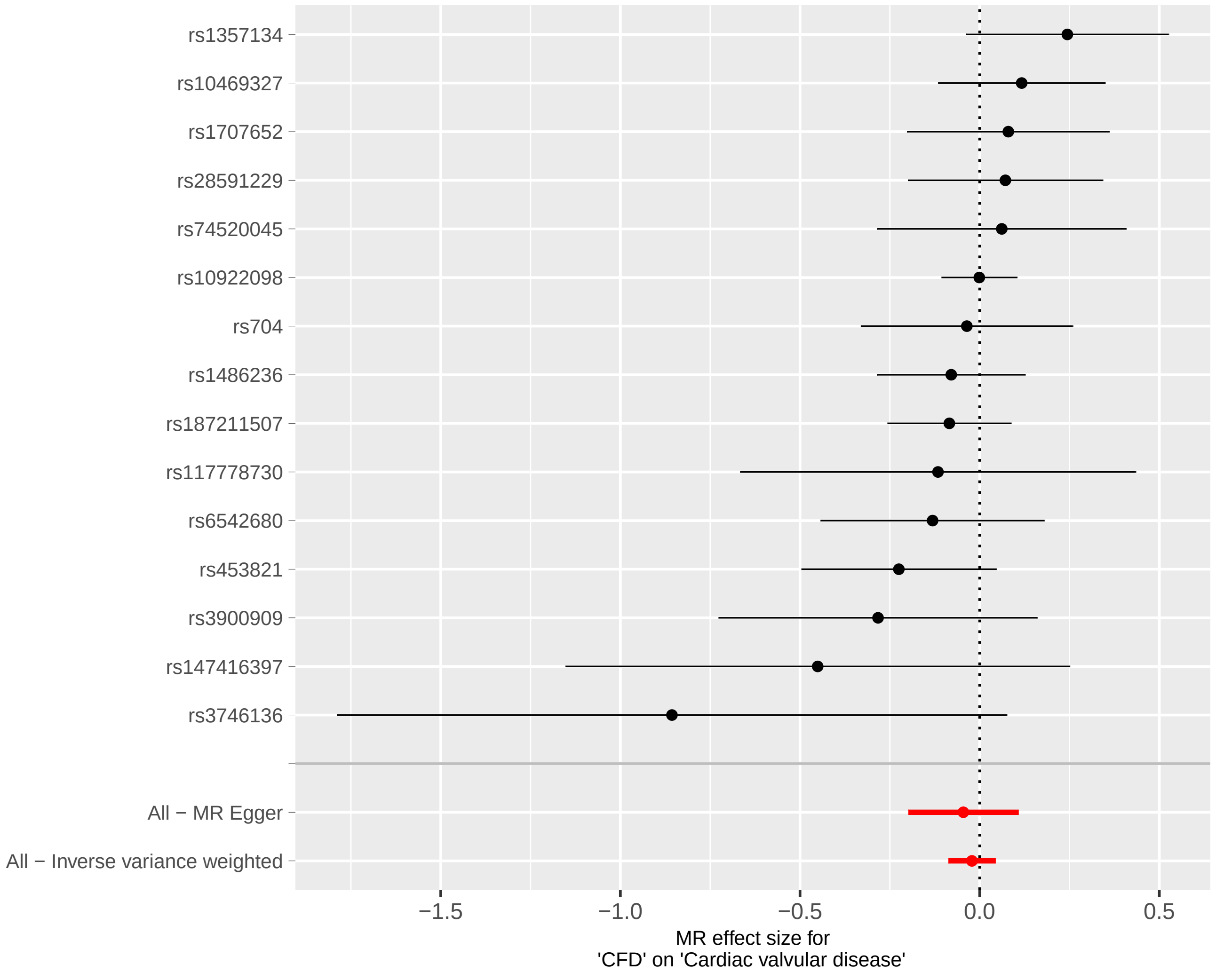

rs1357134
rs10469327
rs1707652
rs28591229
rs74520045
rs10922098
rs704
rs1486236
rs187211507
rs117778730
rs6542680
rs453821
rs3900909
rs147416397
rs3746136
All − MR Egger
All − Inverse variance weighted
−1.5
−1.0
−0.5
0.0
0.5
MR effect size for
'CFD' on 'Cardiac valvular disease'

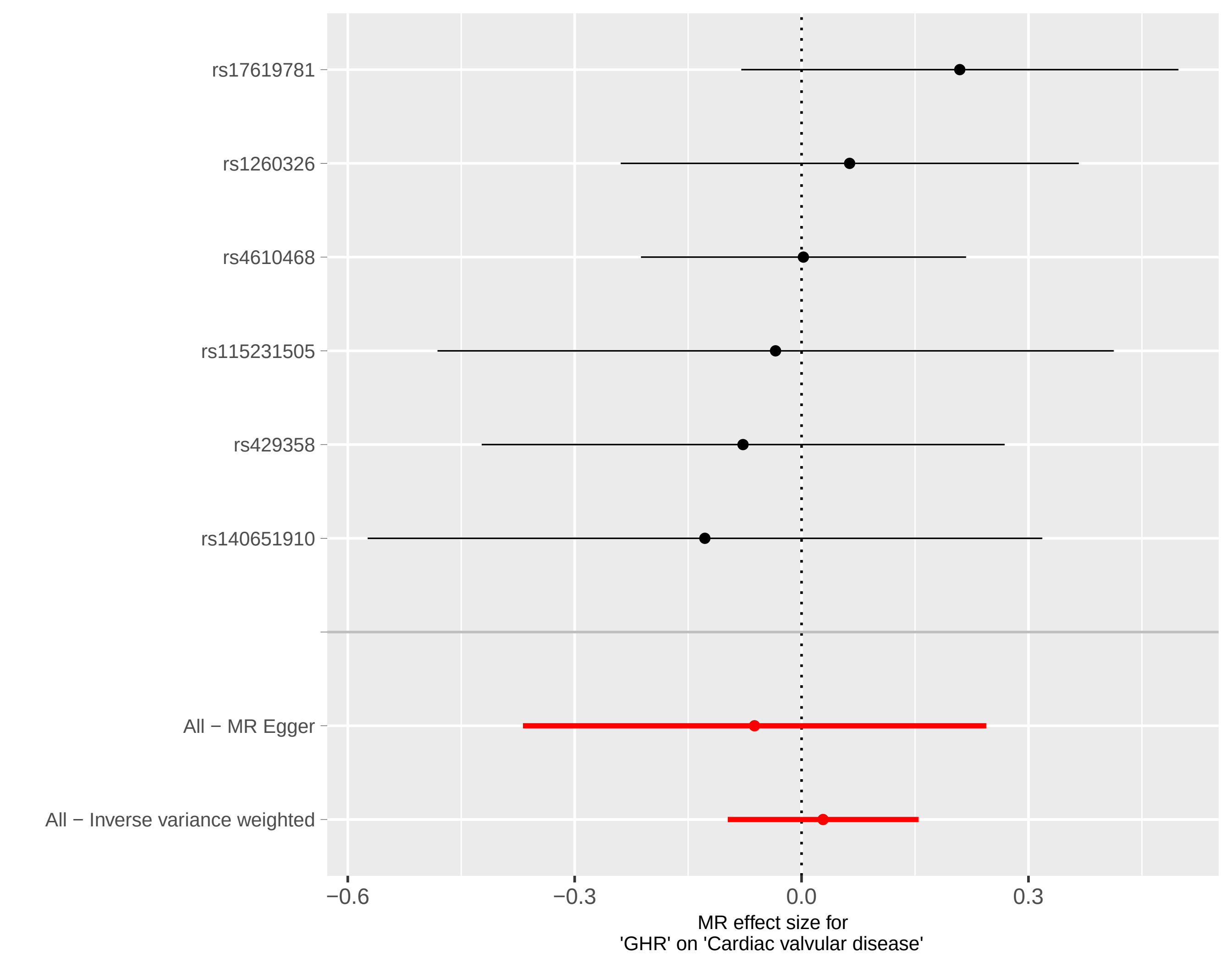
rs17619781
rs1260326
rs4610468
rs115231505
rs429358
rs140651910
All − MR Egger
All − Inverse variance weighted
−0.6
−0.3
0.0
0.3
MR effect size for
'GHR' on 'Cardiac valvular disease'

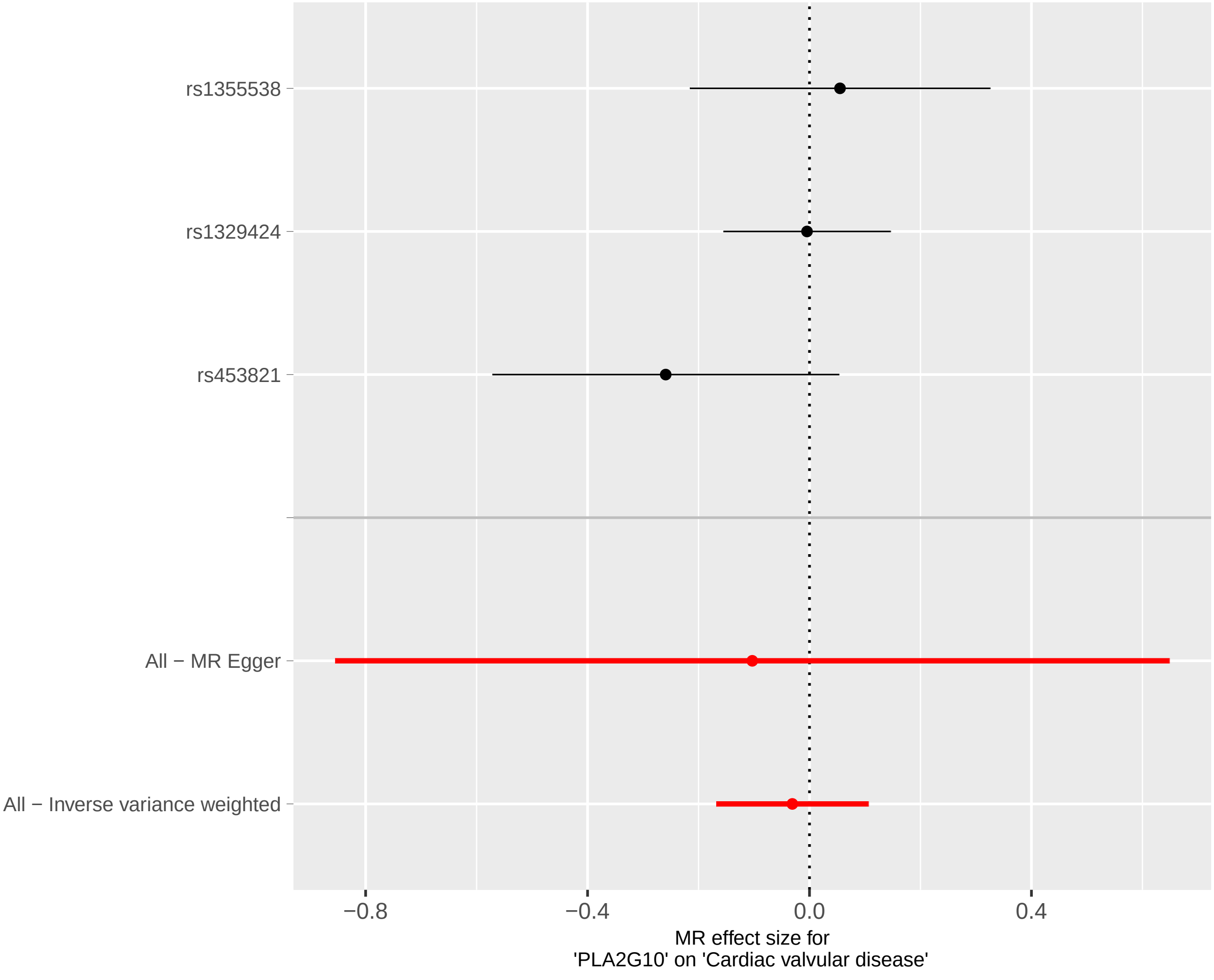
rs1355538
rs1329424
rs453821
All − MR Egger
All − Inverse variance weighted
−0.8
−0.4
0.0
0.4
MR effect size for
'PLA2G10' on 'Cardiac valvular disease'

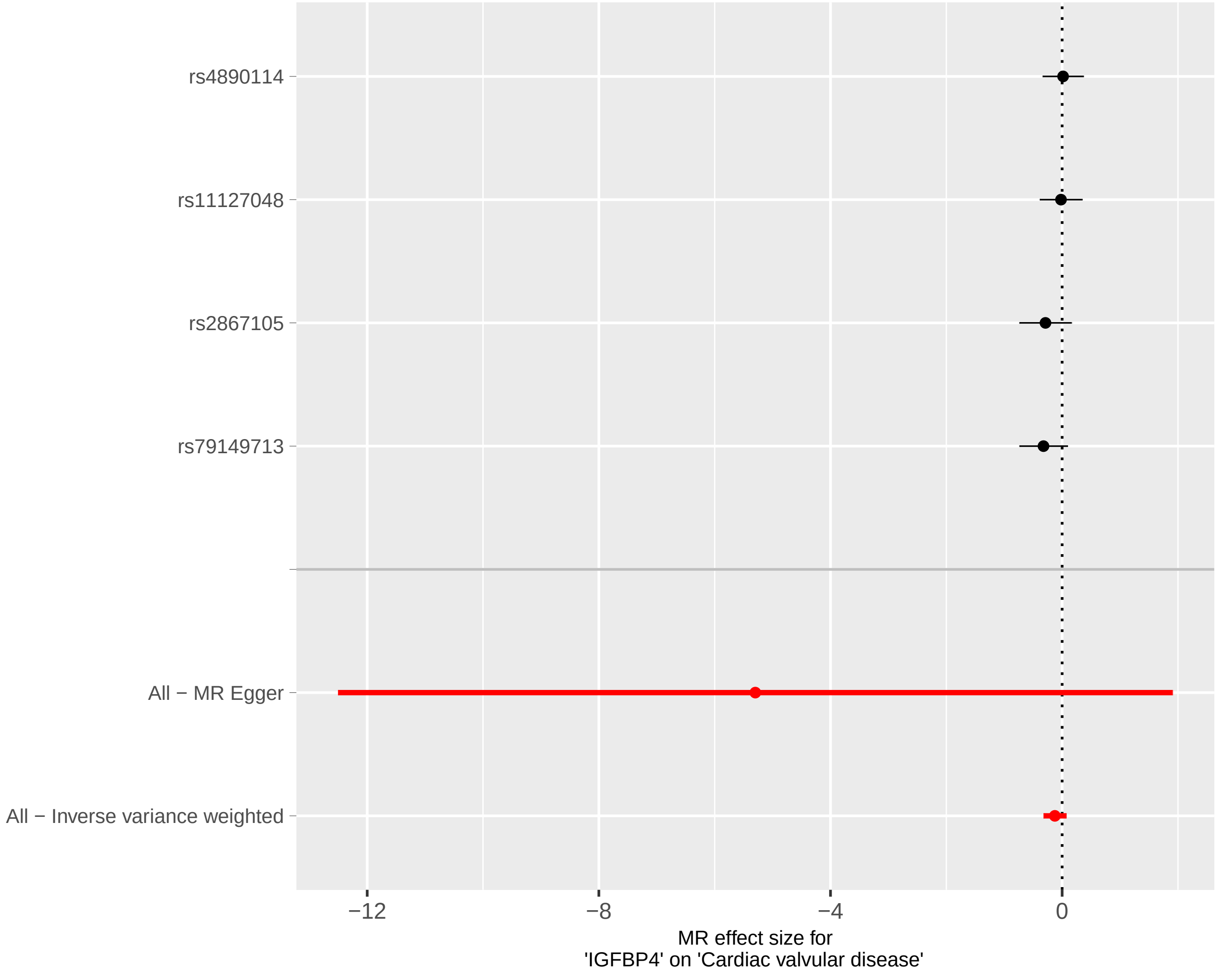
rs4890114
rs11127048
rs2867105
rs79149713
All − MR Egger
All − Inverse variance weighted
−12
−8
−4
0
MR effect size for
'IGFBP4' on 'Cardiac valvular disease'

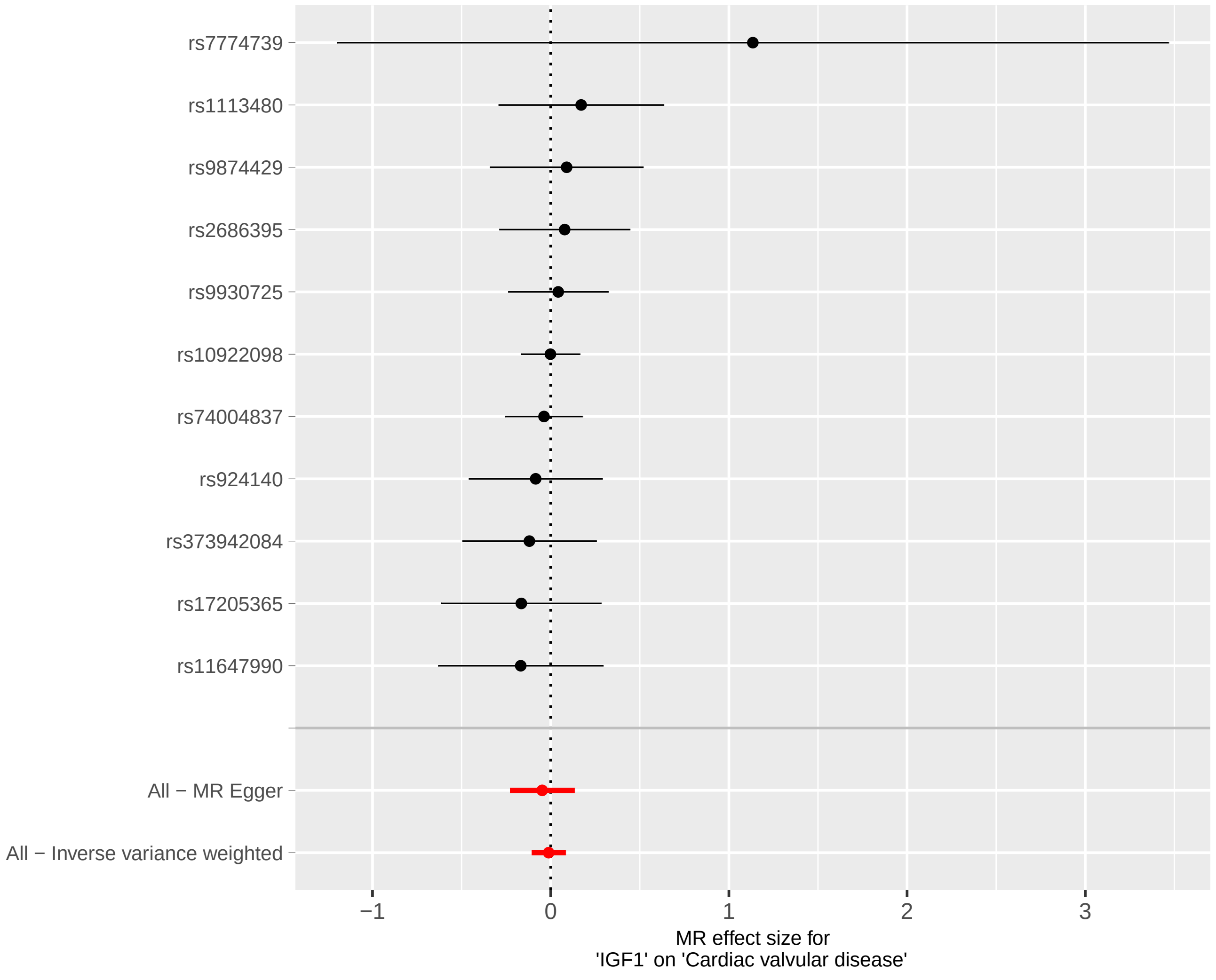
rs7774739
rs1113480
rs9874429
rs2686395
rs9930725
rs10922098
rs74004837
rs924140
rs373942084
rs17205365
rs11647990
All − MR Egger
All − Inverse variance weighted
−1
0
1
2
3
MR effect size for
'IGF1' on 'Cardiac valvular disease'

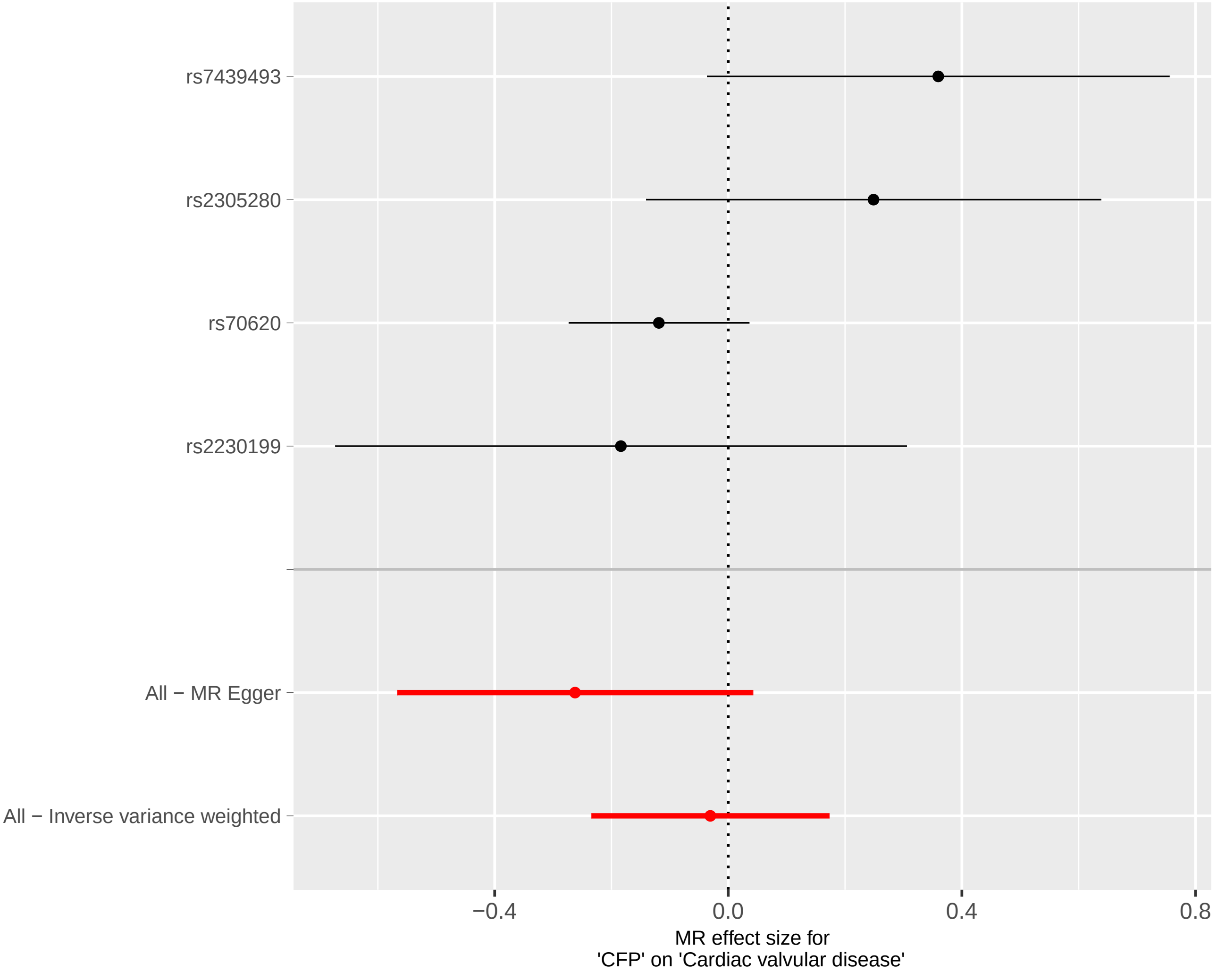

rs7439493
rs2305280
rs70620
rs2230199
All − MR Egger
All − Inverse variance weighted
−0.4
0.0
0.4
0.8
MR effect size for
'CFP' on 'Cardiac valvular disease'

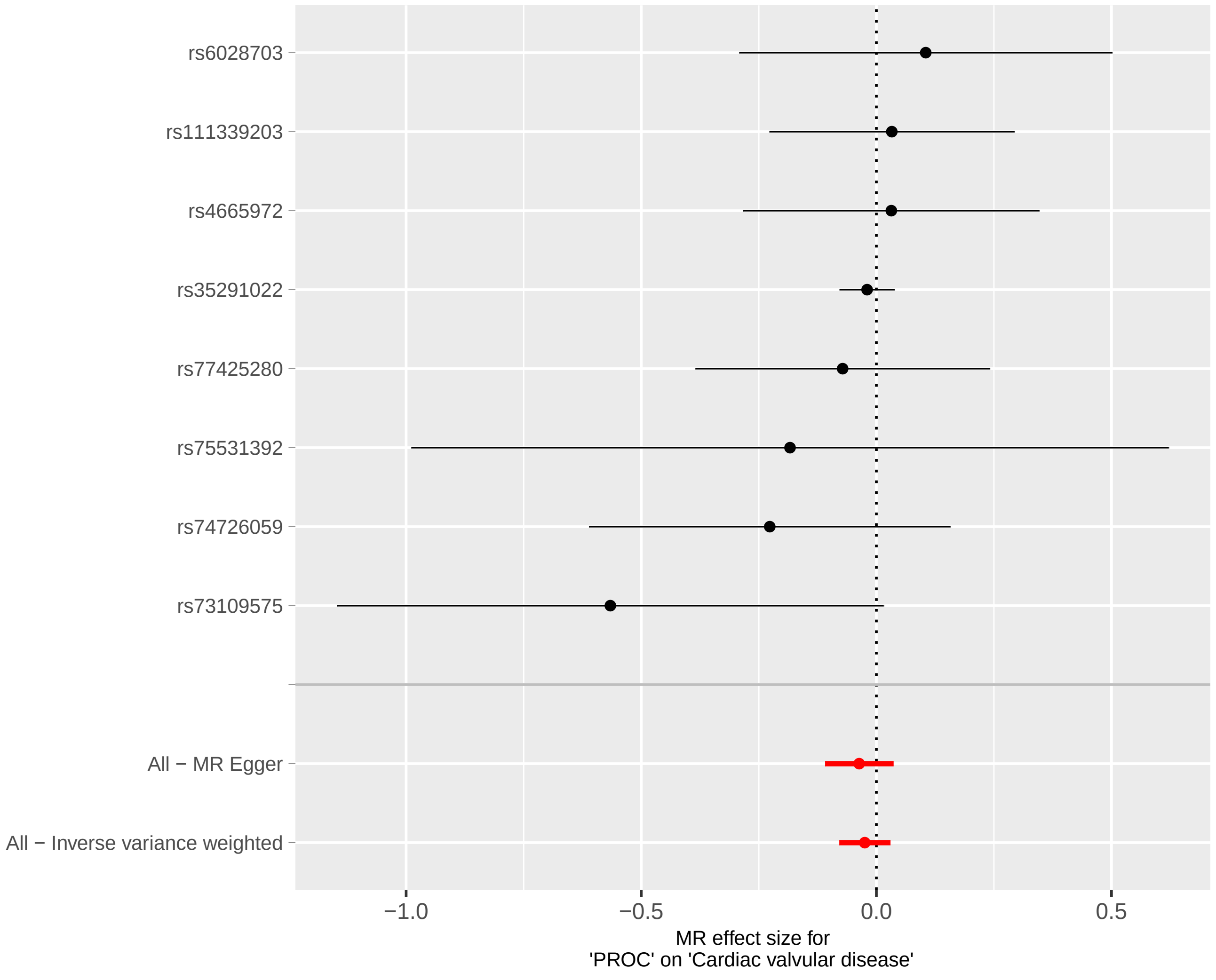
rs6028703
rs111339203
rs4665972
rs35291022
rs77425280
rs75531392
rs74726059
rs73109575
All − MR Egger
All − Inverse variance weighted
−1.0
−0.5
0.0
0.5
MR effect size for
'PROC' on 'Cardiac valvular disease'

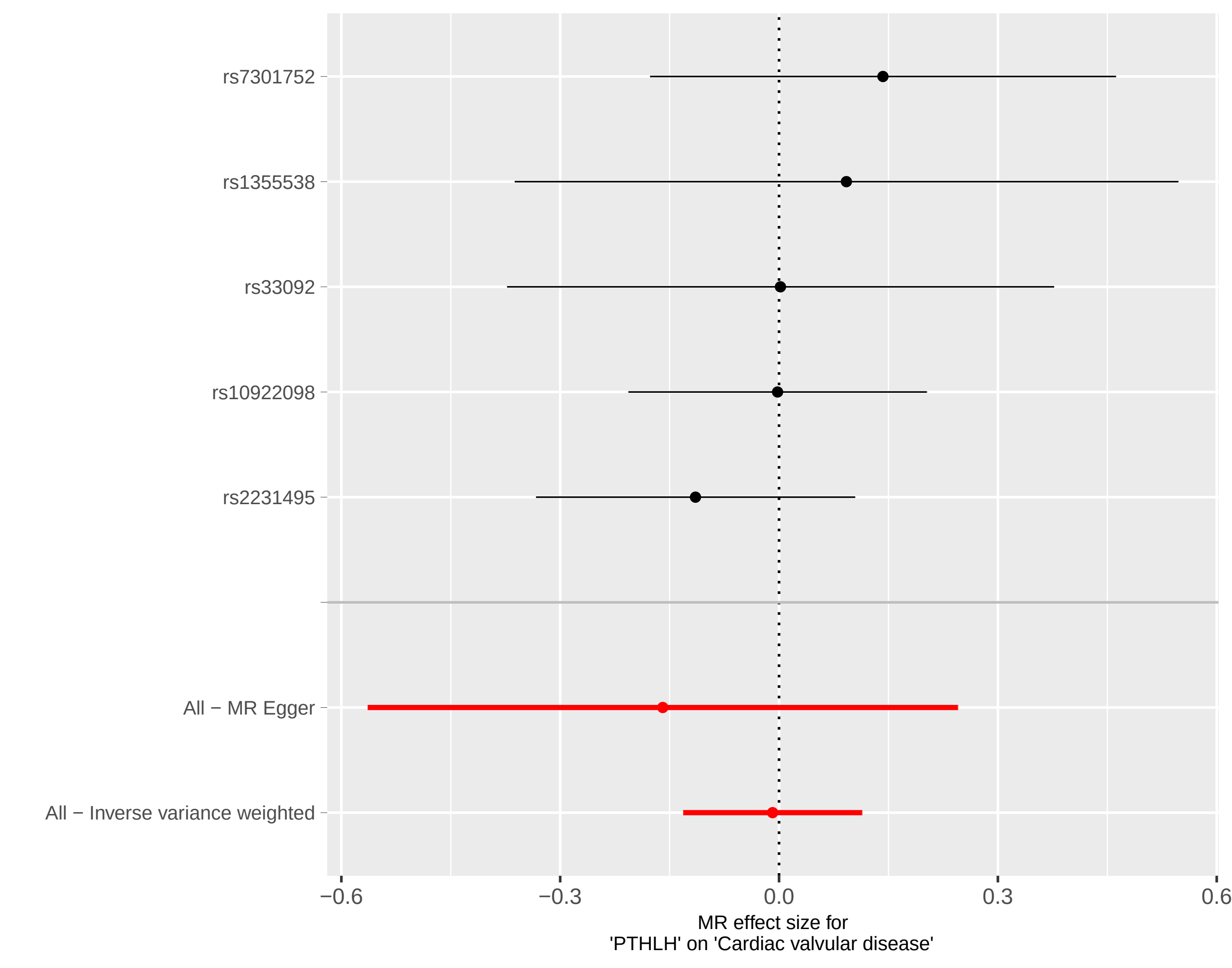

rs7301752
rs1355538
rs33092
rs10922098
rs2231495
All − MR Egger
All − Inverse variance weighted
−0.6
−0.3
0.0
0.3
0.6
MR effect size for
'PTHLH' on 'Cardiac valvular disease'

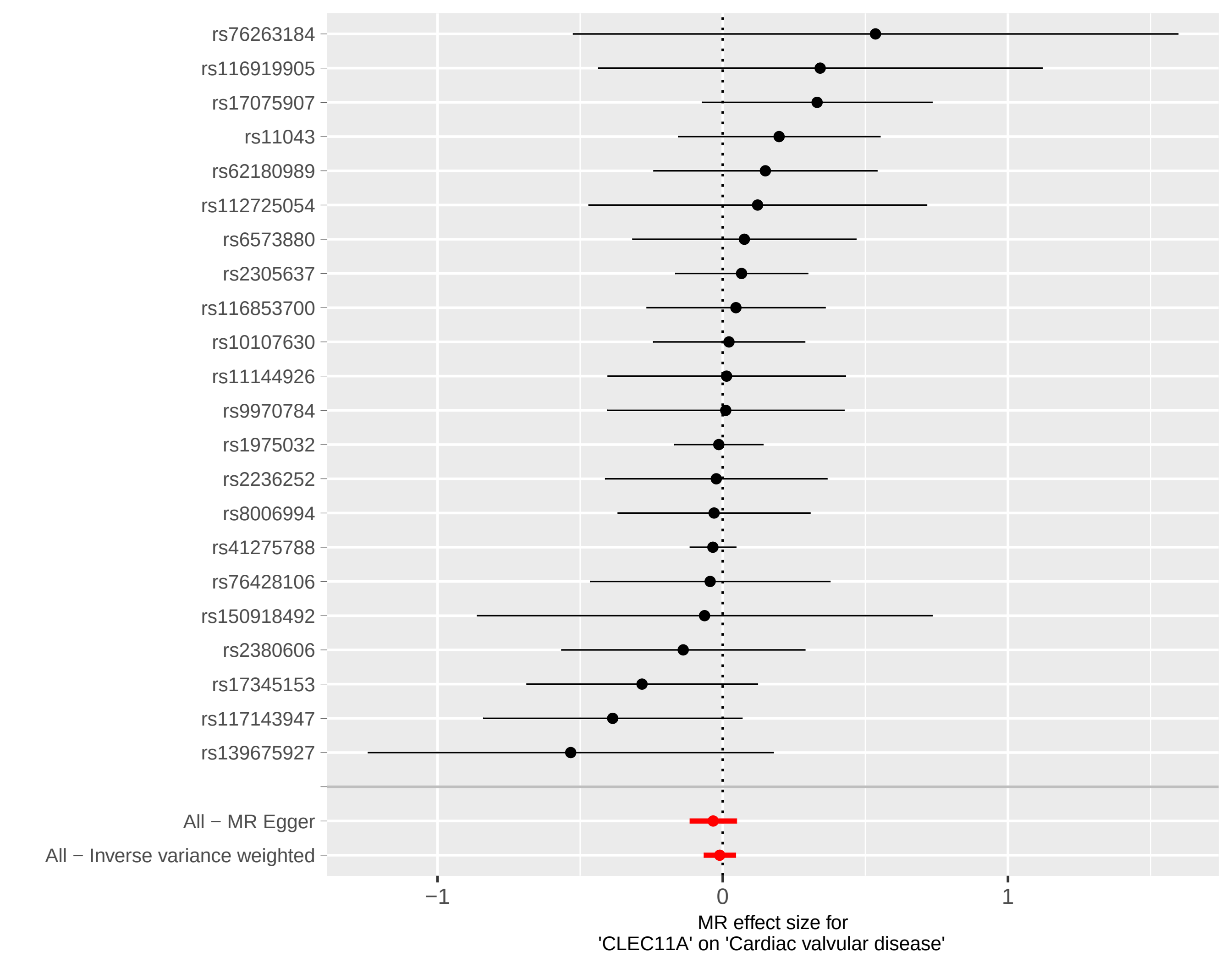
rs76263184
rs116919905
rs17075907
rs11043
rs62180989
rs112725054
rs6573880
rs2305637
rs116853700
rs10107630
rs11144926
rs9970784
rs1975032
rs2236252
rs8006994
rs41275788
rs76428106
rs150918492
rs2380606
rs17345153
rs117143947
rs139675927
All − MR Egger
All − Inverse variance weighted
−1
0
1
MR effect size for
'CLEC11A' on 'Cardiac valvular disease'

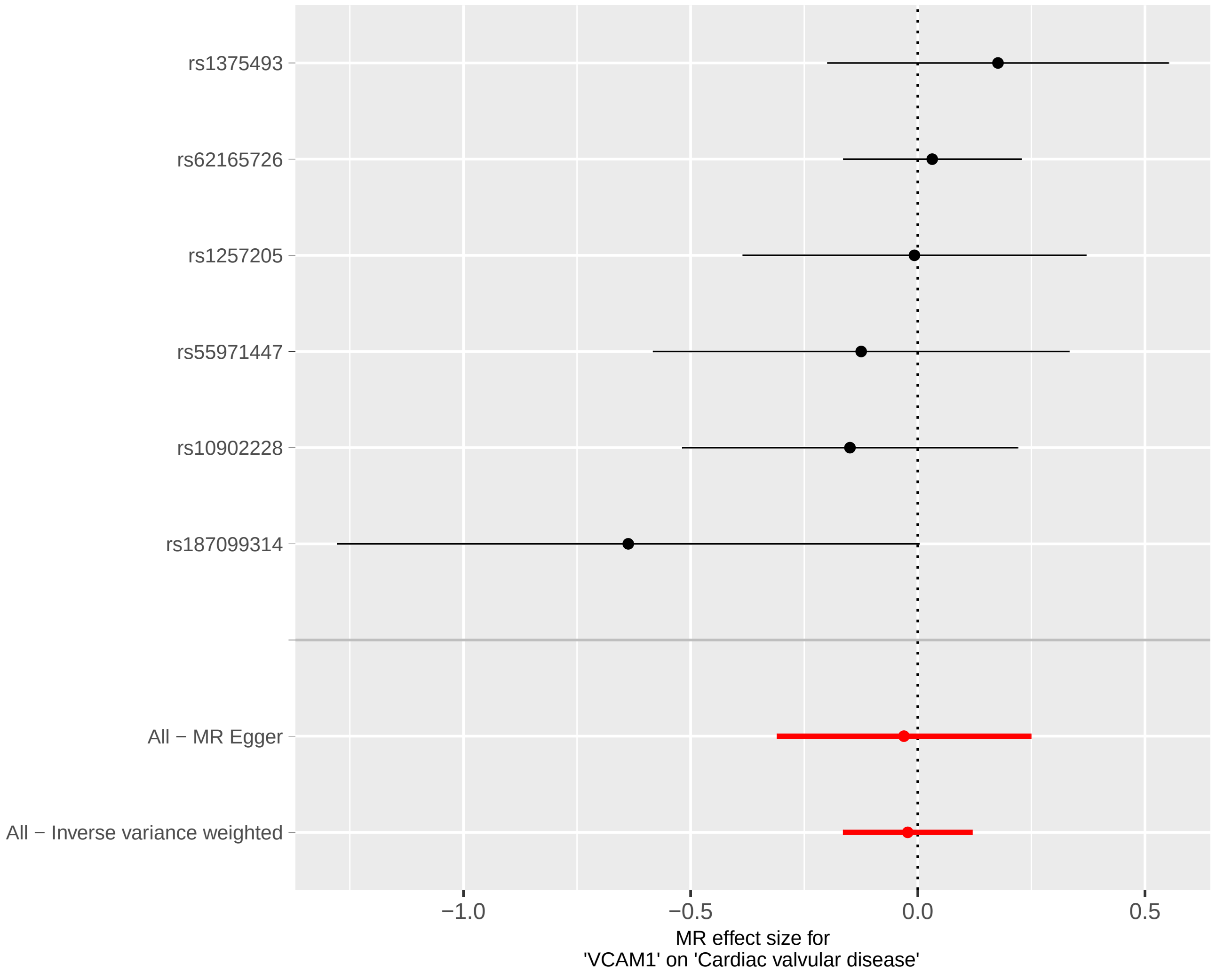
rs1375493
rs62165726
rs1257205
rs55971447
rs10902228
rs187099314
All − MR Egger
All − Inverse variance weighted
−1.0
−0.5
0.0
0.5
MR effect size for
'VCAM1' on 'Cardiac valvular disease'

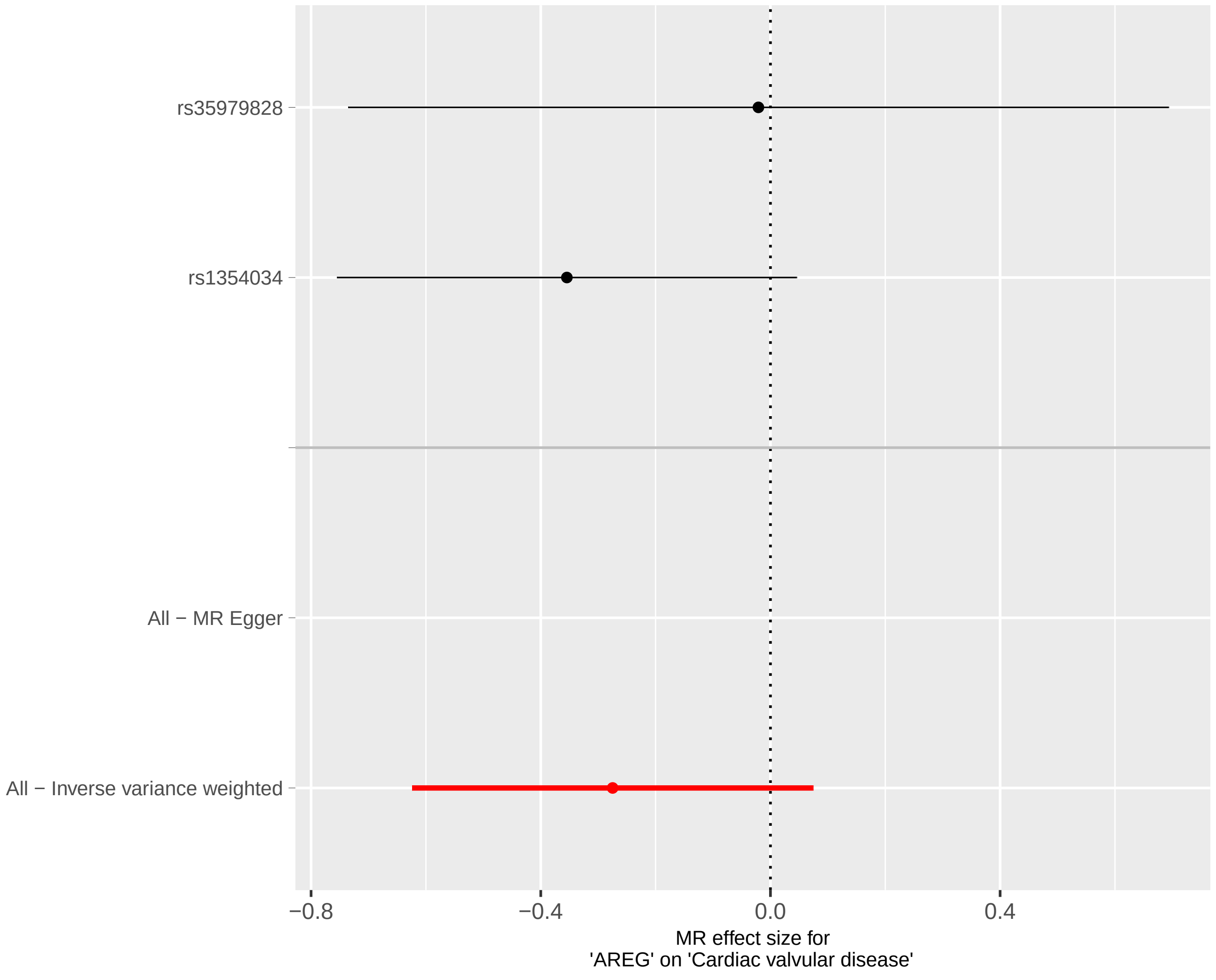
rs35979828
rs1354034
All − MR Egger
All − Inverse variance weighted
−0.8
−0.4
0.0
0.4
MR effect size for
'AREG' on 'Cardiac valvular disease'

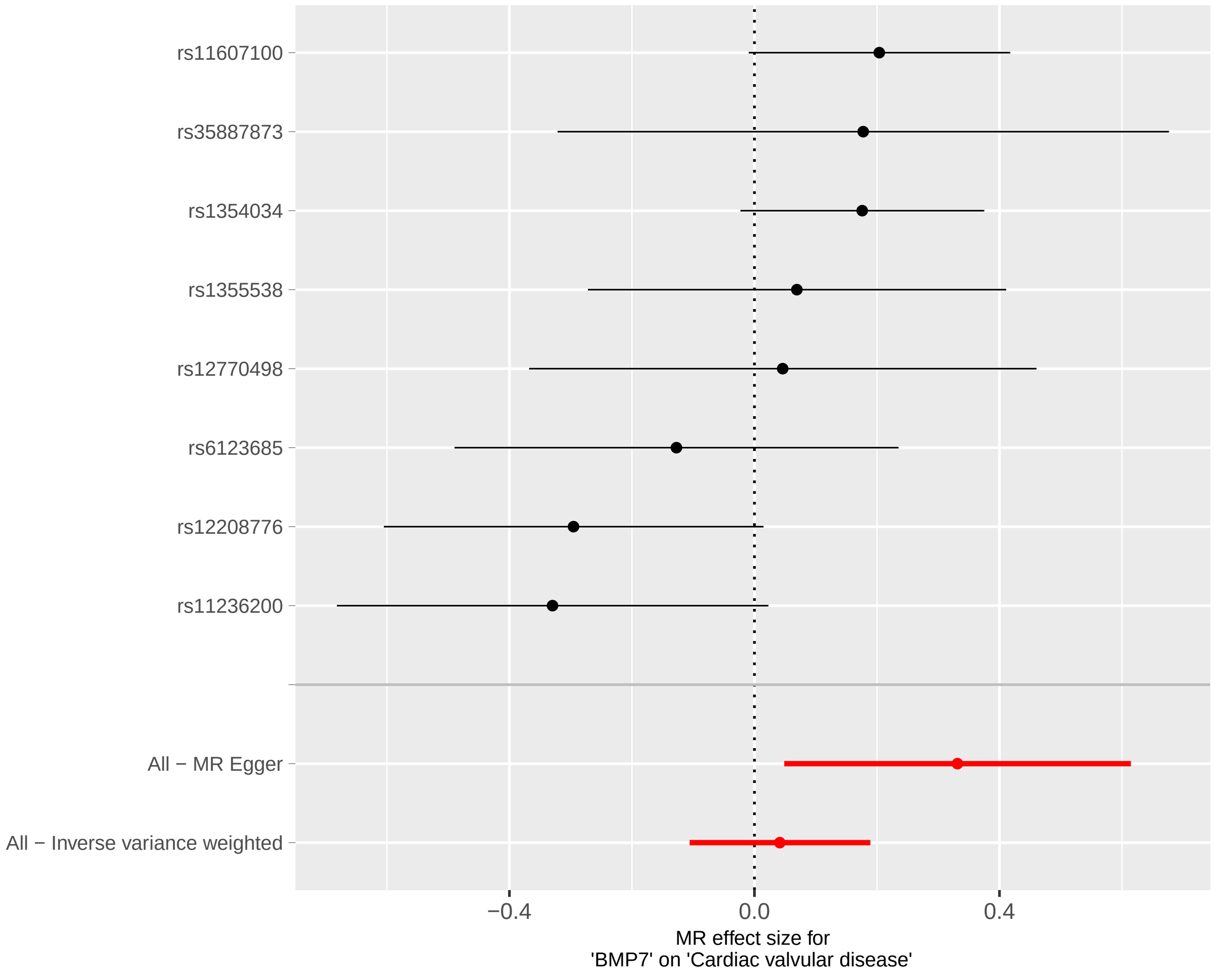
rs11607100
rs35887873
rs1354034
rs1355538
rs12770498
rs6123685
rs12208776
rs11236200
All − MR Egger
All − Inverse variance weighted
−0.4
0.0
0.4
MR effect size for
'BMP7' on 'Cardiac valvular disease'

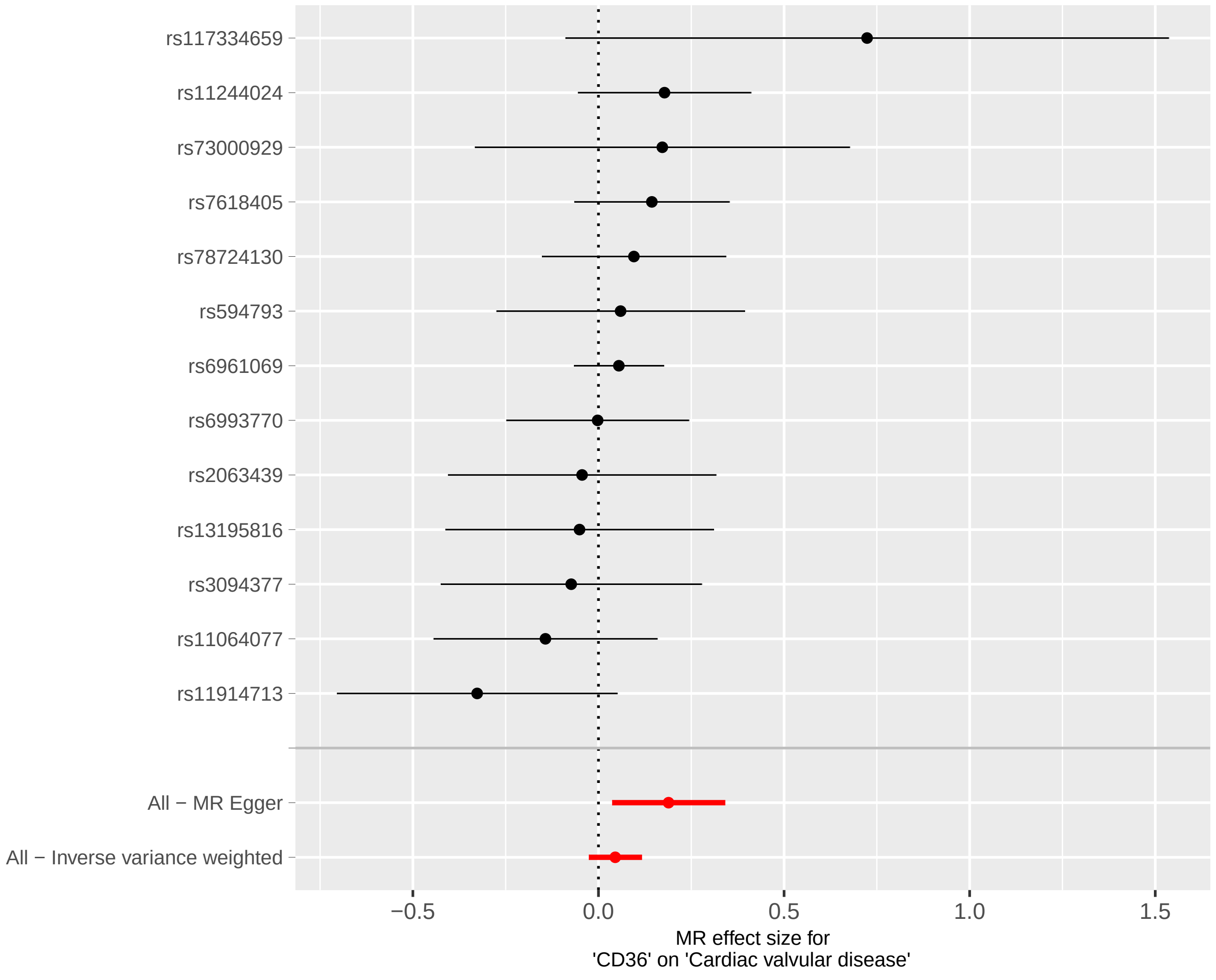

rs117334659
rs11244024
rs73000929
rs7618405
rs78724130
rs594793
rs6961069
rs6993770
rs2063439
rs13195816
rs3094377
rs11064077
rs11914713
All − MR Egger
All − Inverse variance weighted
−0.5
0.0
0.5
1.0
1.5
MR effect size for
'CD36' on 'Cardiac valvular disease'

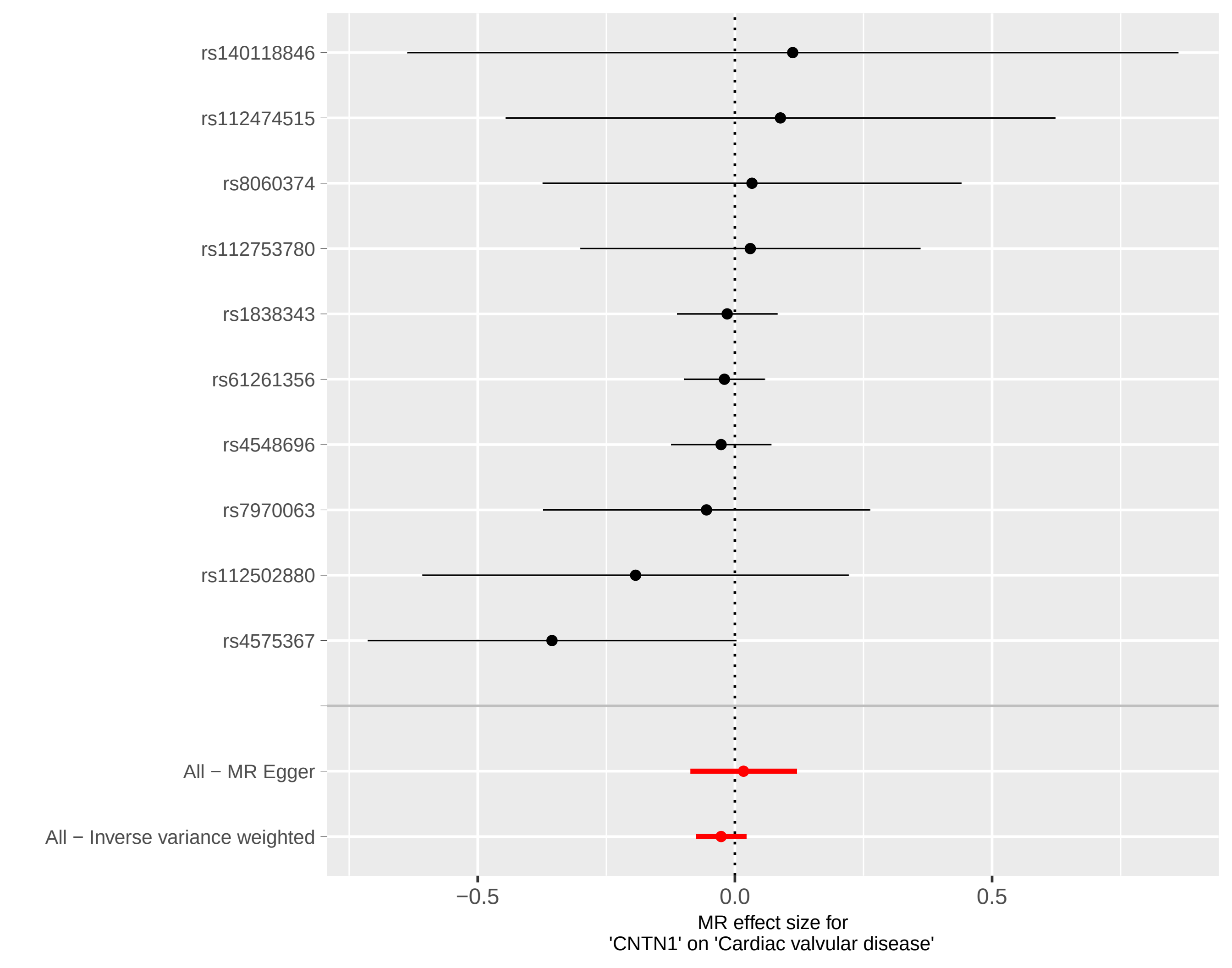

rs140118846
rs112474515
rs8060374
rs112753780
rs1838343
rs61261356
rs4548696
rs7970063
rs112502880
rs4575367
All − MR Egger
All − Inverse variance weighted
−0.5
0.0
0.5
MR effect size for
'CNTN1' on 'Cardiac valvular disease'

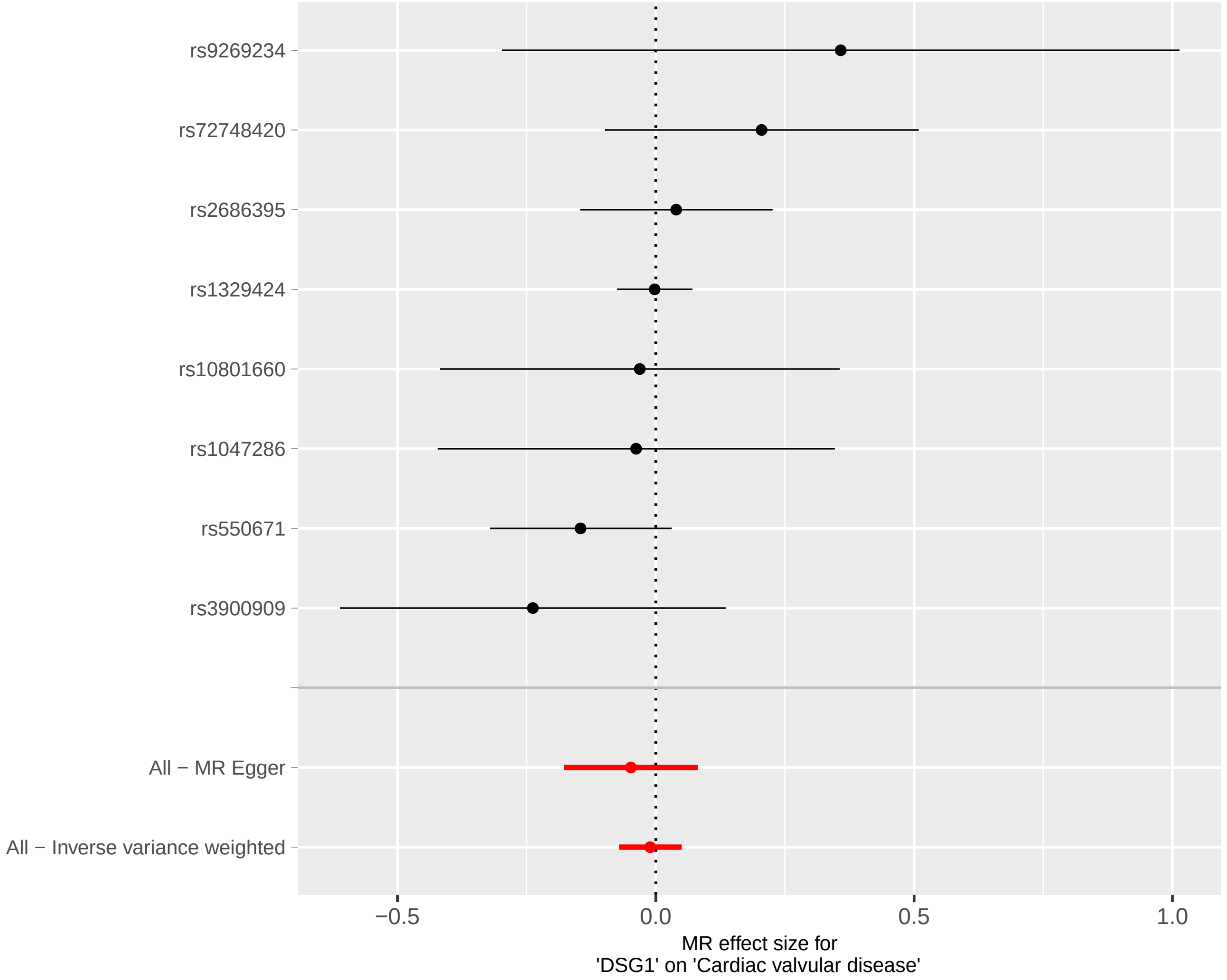
rs9269234
rs72748420
rs2686395
rs1329424
rs10801660
rs1047286
rs550671
rs3900909
All − MR Egger
All − Inverse variance weighted
−0.5
0.0
0.5
1.0
MR effect size for
'DSG1' on 'Cardiac valvular disease'

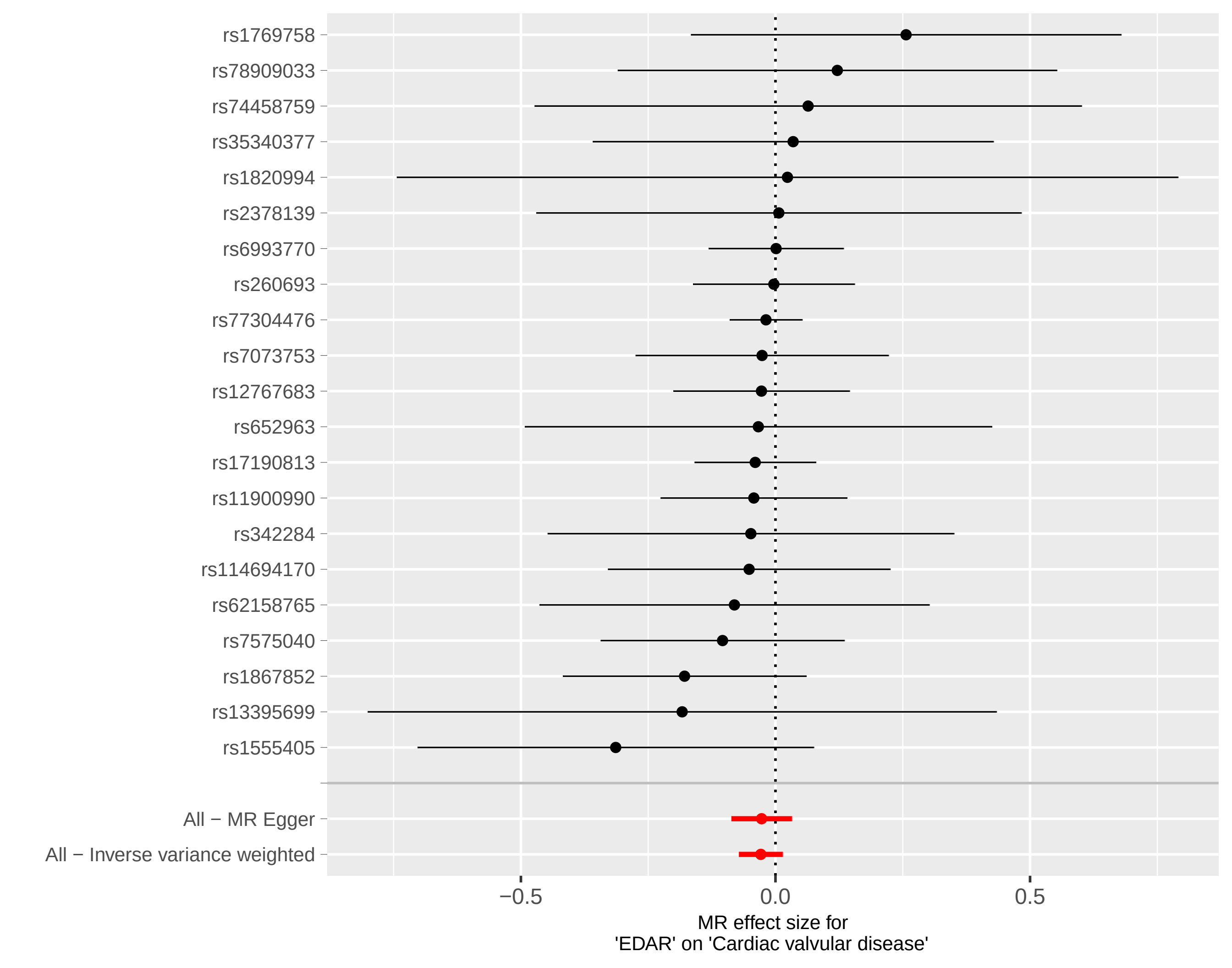

rs1769758
rs78909033
rs74458759
rs35340377
rs1820994
rs2378139
rs6993770
rs260693
rs77304476
rs7073753
rs12767683
rs652963
rs17190813
rs11900990
rs342284
rs114694170
rs62158765
rs7575040
rs1867852
rs13395699
rs1555405
All − MR Egger
All − Inverse variance weighted
−0.5
0.0
0.5
MR effect size for
'EDAR' on 'Cardiac valvular disease'

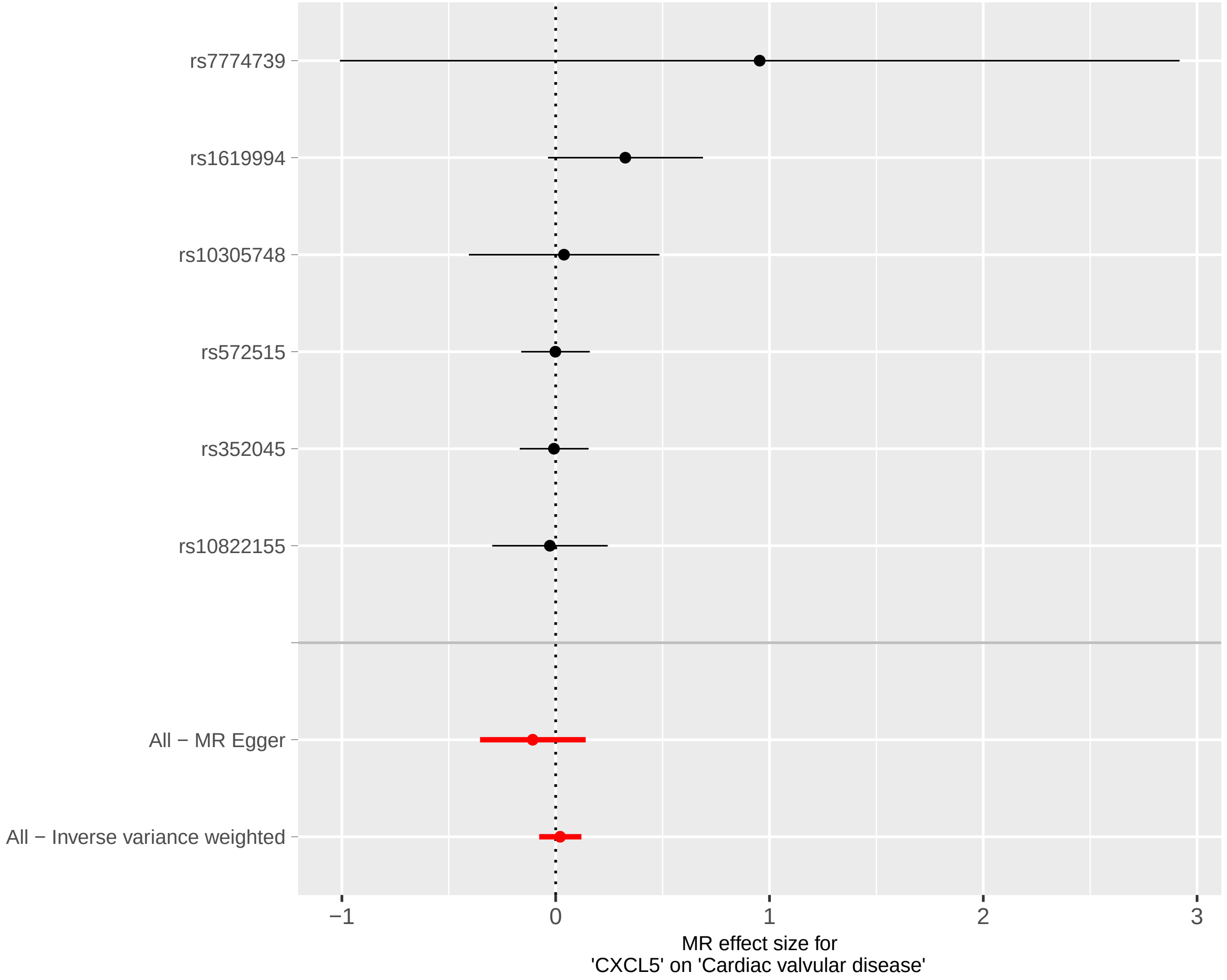
rs7774739
rs1619994
rs10305748
rs572515
rs352045
rs10822155
All − MR Egger
All − Inverse variance weighted
−1
0
1
2
3
MR effect size for
'CXCL5' on 'Cardiac valvular disease'

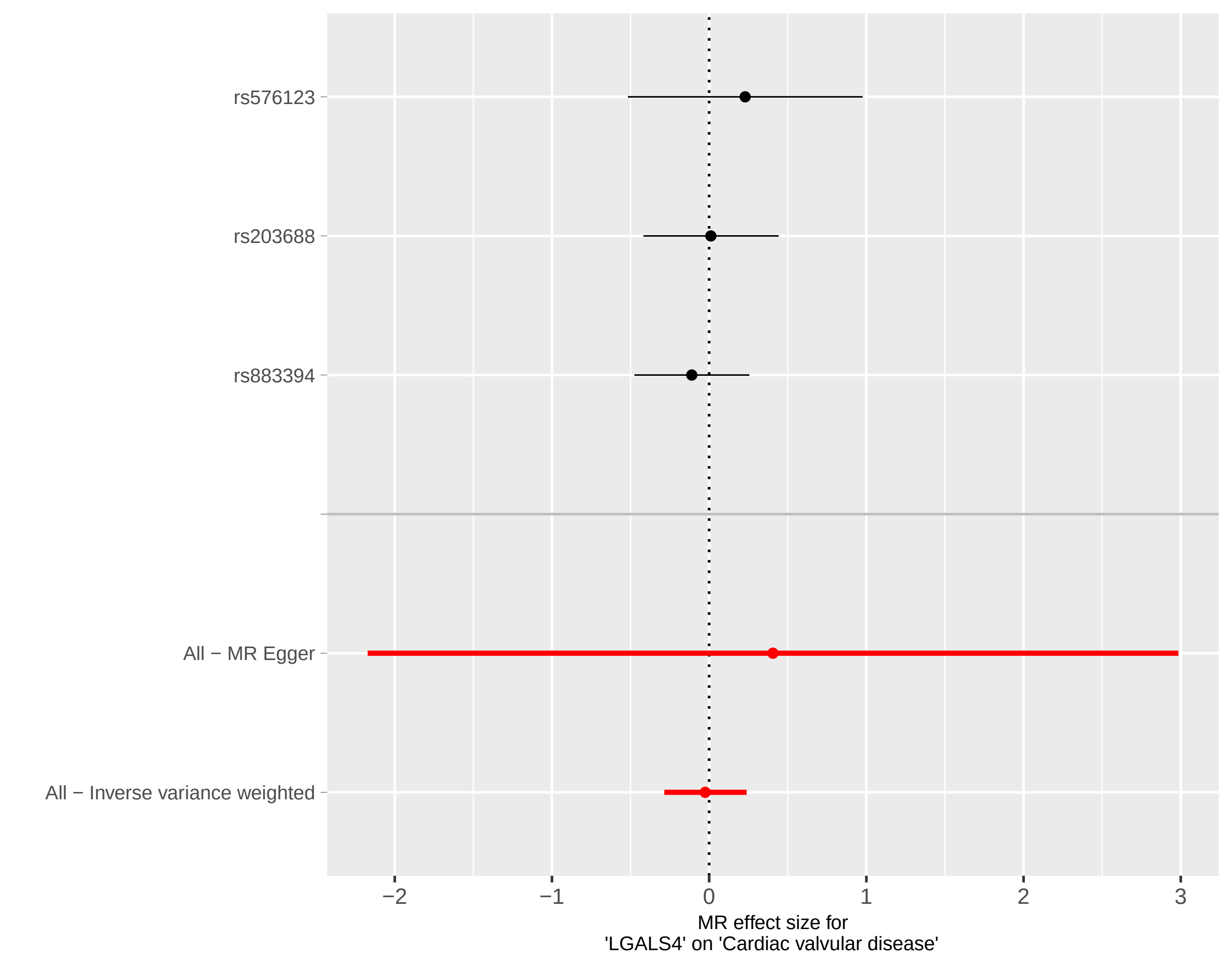
rs576123
rs203688
rs883394
All − MR Egger
All − Inverse variance weighted
−2
−1
0
1
2
3
MR effect size for
'LGALS4' on 'Cardiac valvular disease'

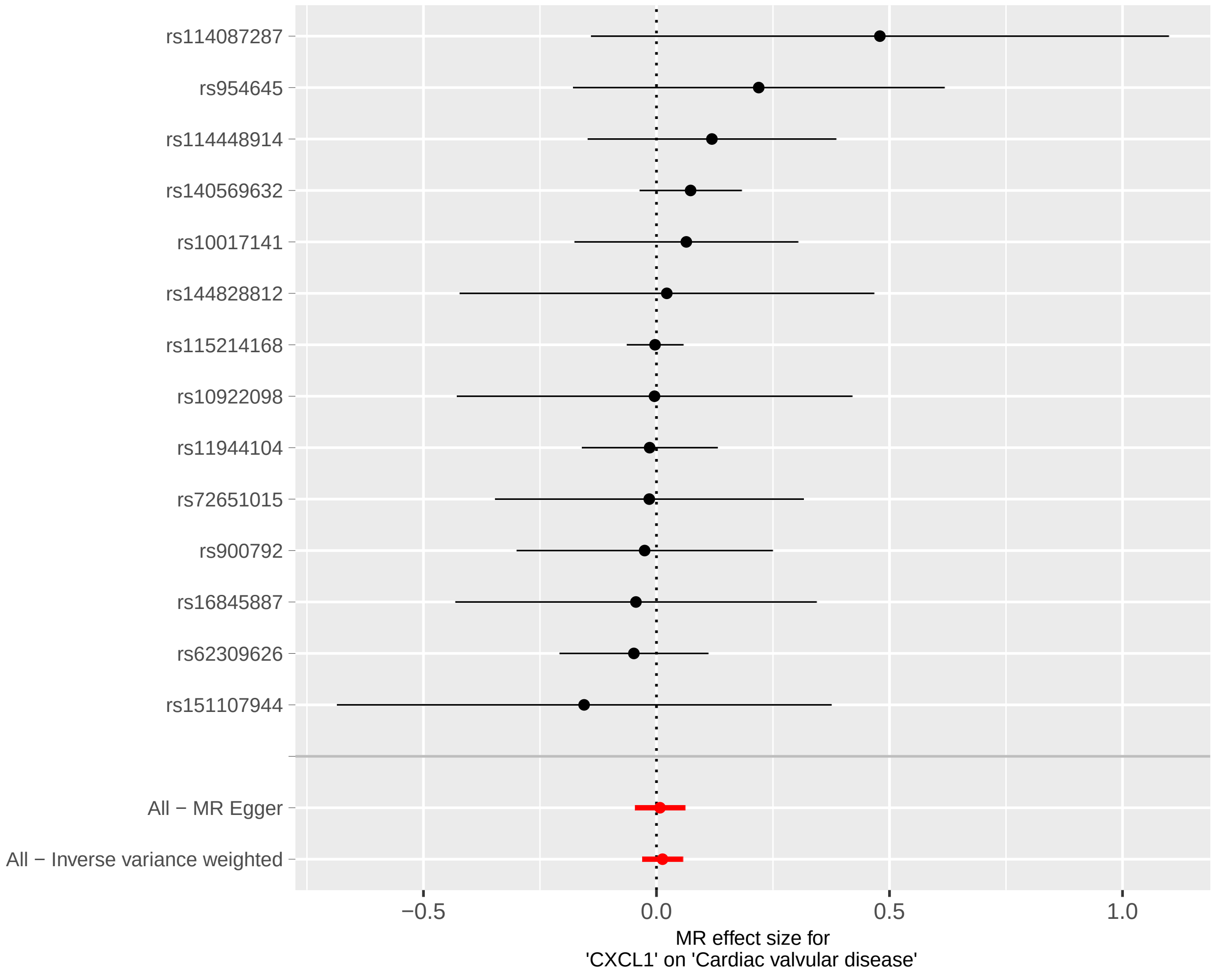
rs114087287
rs954645
rs114448914
rs140569632
rs10017141
rs144828812
rs115214168
rs10922098
rs11944104
rs72651015
rs900792
rs16845887
rs62309626
rs151107944
All − MR Egger
All − Inverse variance weighted
−0.5
0.0
0.5
1.0
MR effect size for
'CXCL1' on 'Cardiac valvular disease'

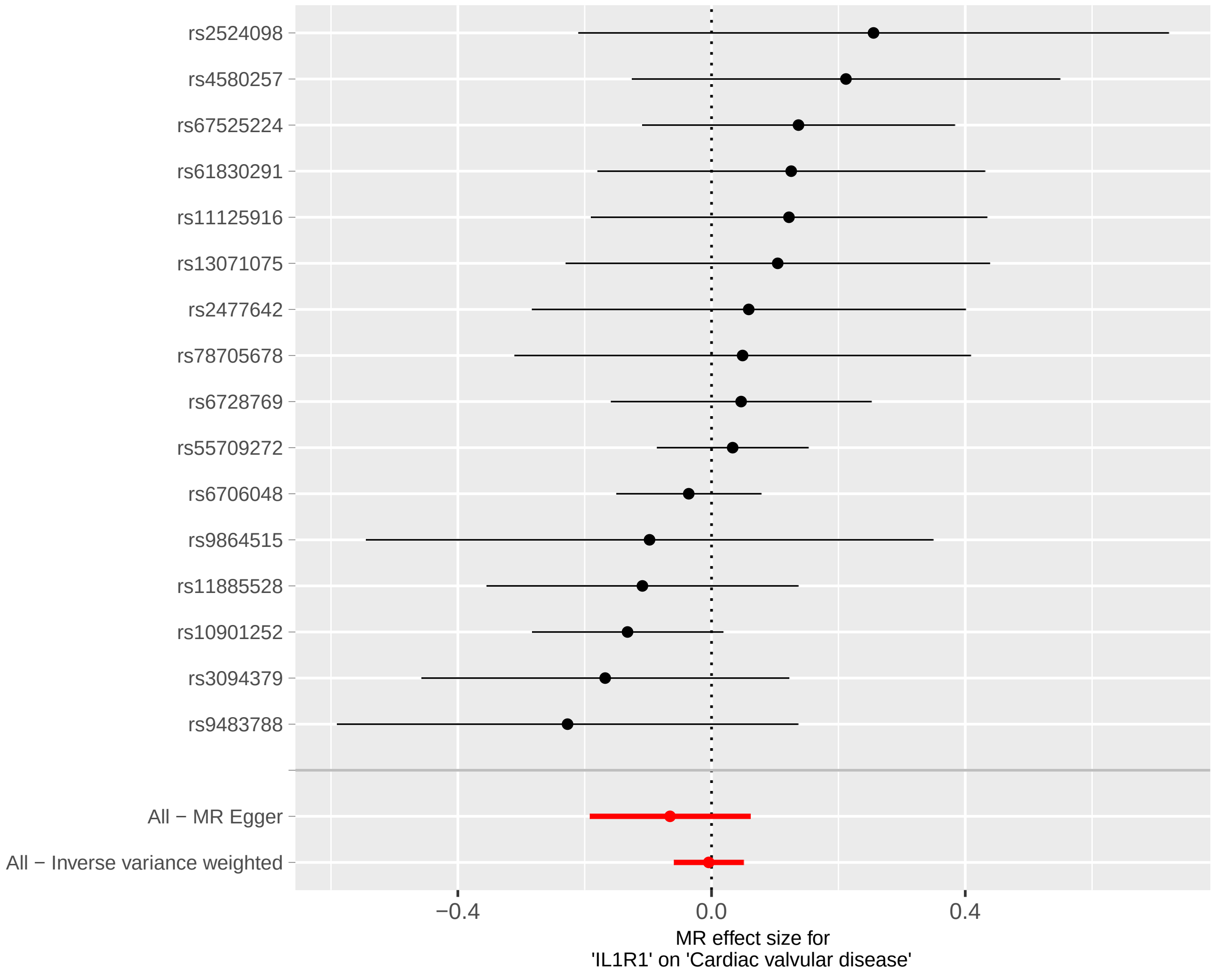

rs2524098
rs4580257
rs67525224
rs61830291
rs11125916
rs13071075
rs2477642
rs78705678
rs6728769
rs55709272
rs6706048
rs9864515
rs11885528
rs10901252
rs3094379
rs9483788
All − MR Egger
All − Inverse variance weighted
−0.4
0.0
0.4
MR effect size for
'IL1R1' on 'Cardiac valvular disease'

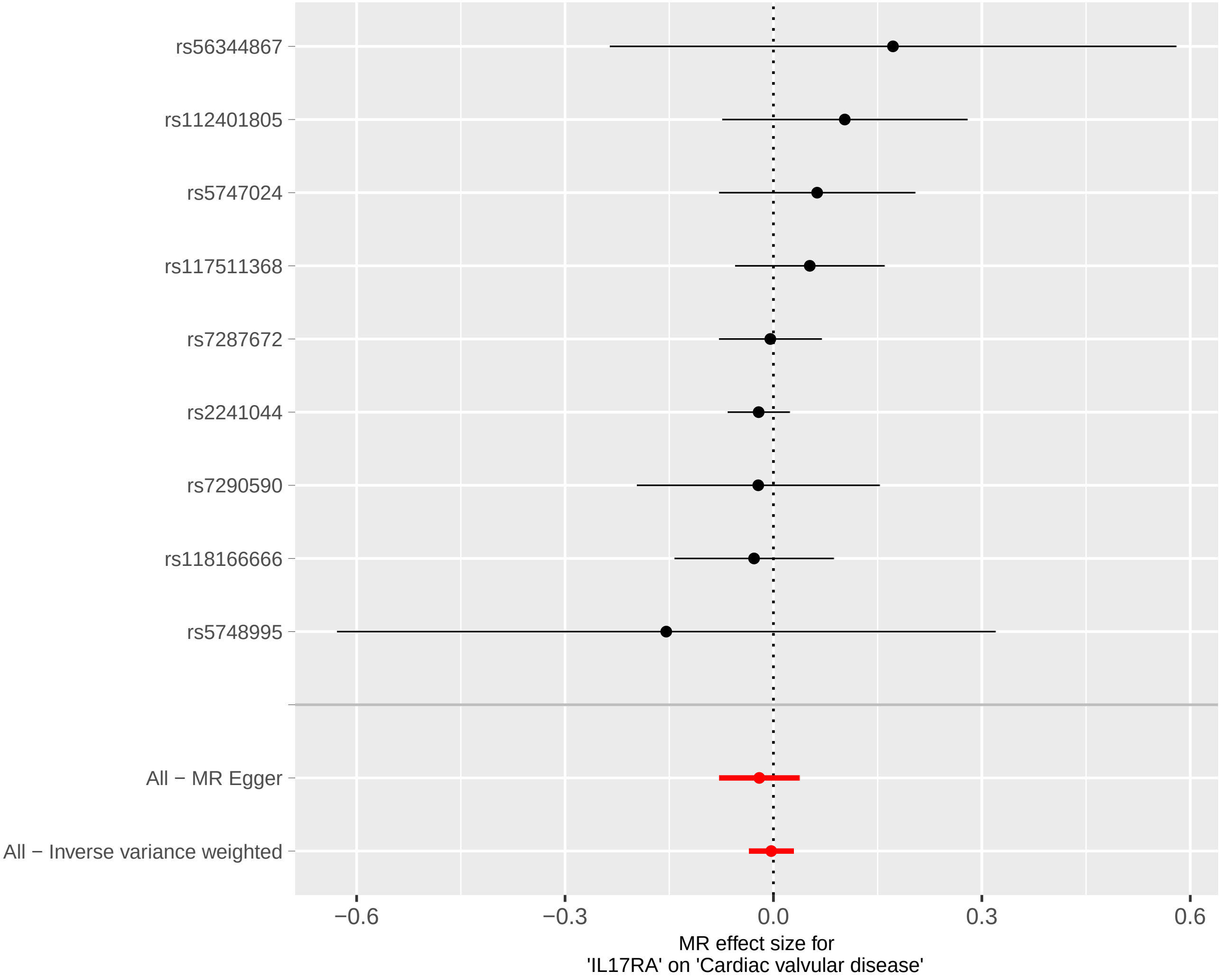
rs56344867
rs112401805
rs5747024
rs117511368
rs7287672
rs2241044
rs7290590
rs118166666
rs5748995
All − MR Egger
All − Inverse variance weighted
−0.6
−0.3
0.0
0.3
0.6
MR effect size for
'IL17RA' on 'Cardiac valvular disease'

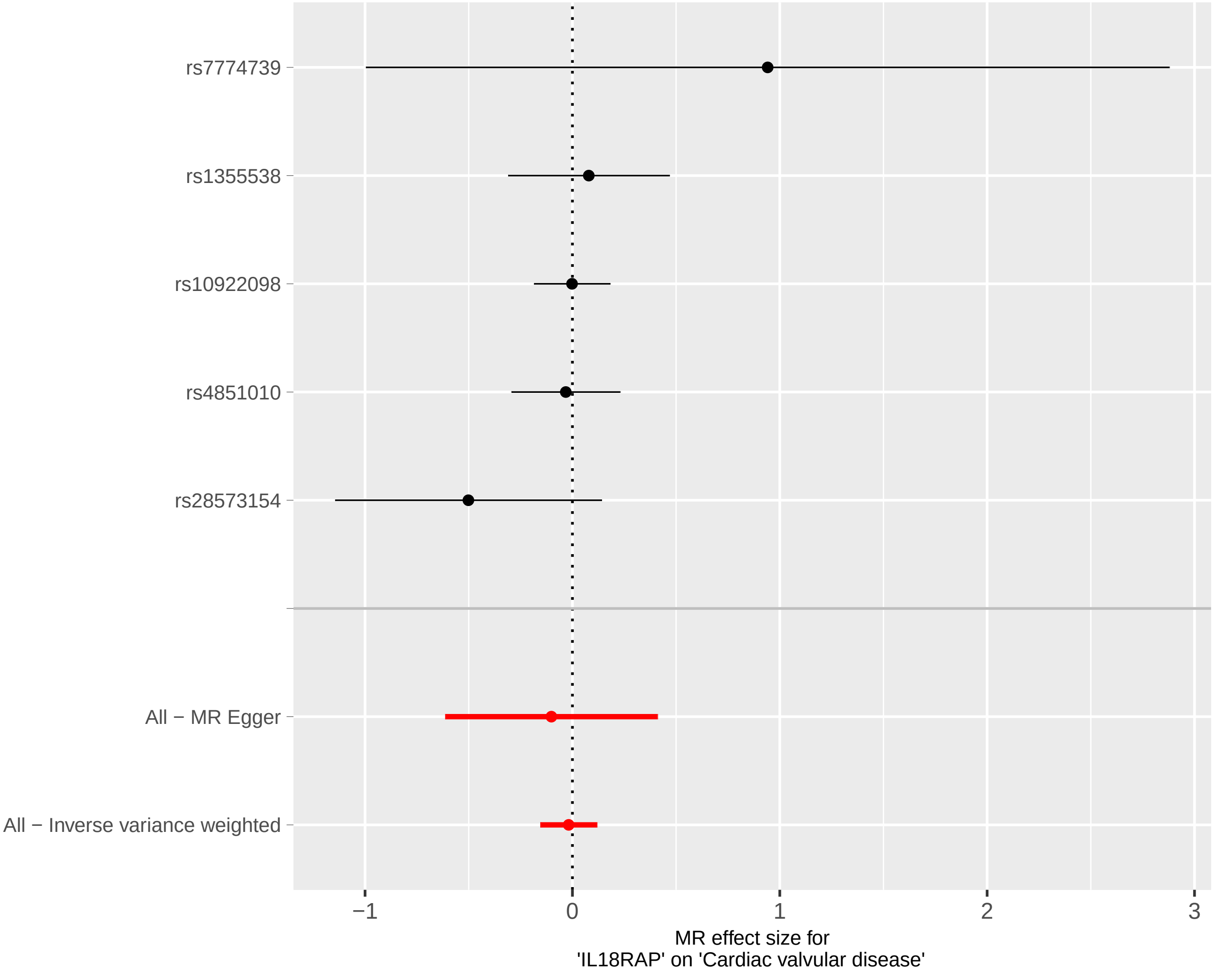
rs7774739
rs1355538
rs10922098
rs4851010
rs28573154
All − MR Egger
All − Inverse variance weighted
−1
0
1
2
3
MR effect size for
'IL18RAP' on 'Cardiac valvular disease'

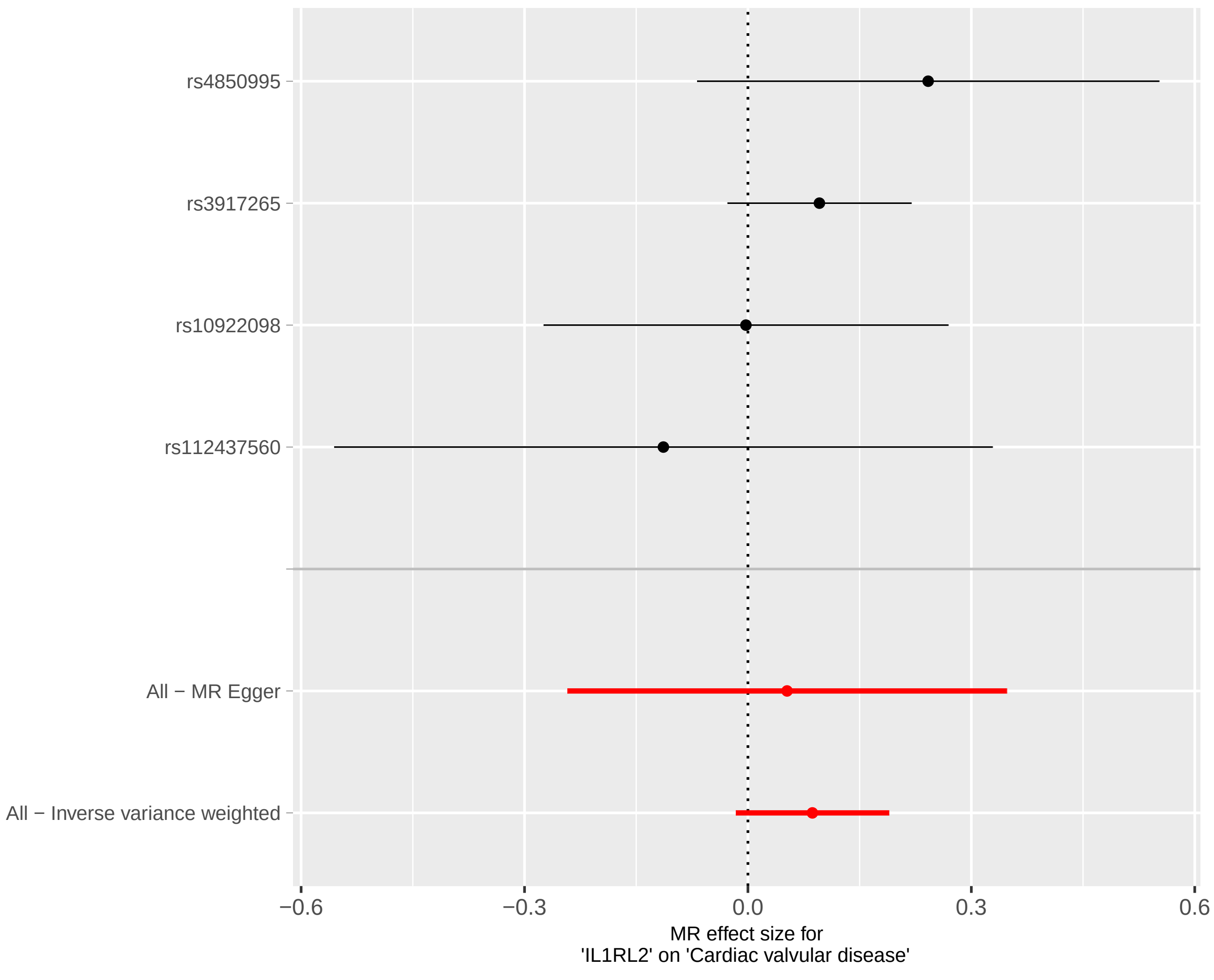
rs4850995
rs3917265
rs10922098
rs112437560
All − MR Egger
All − Inverse variance weighted
−0.6
−0.3
0.0
0.3
0.6
MR effect size for
'IL1RL2' on 'Cardiac valvular disease'

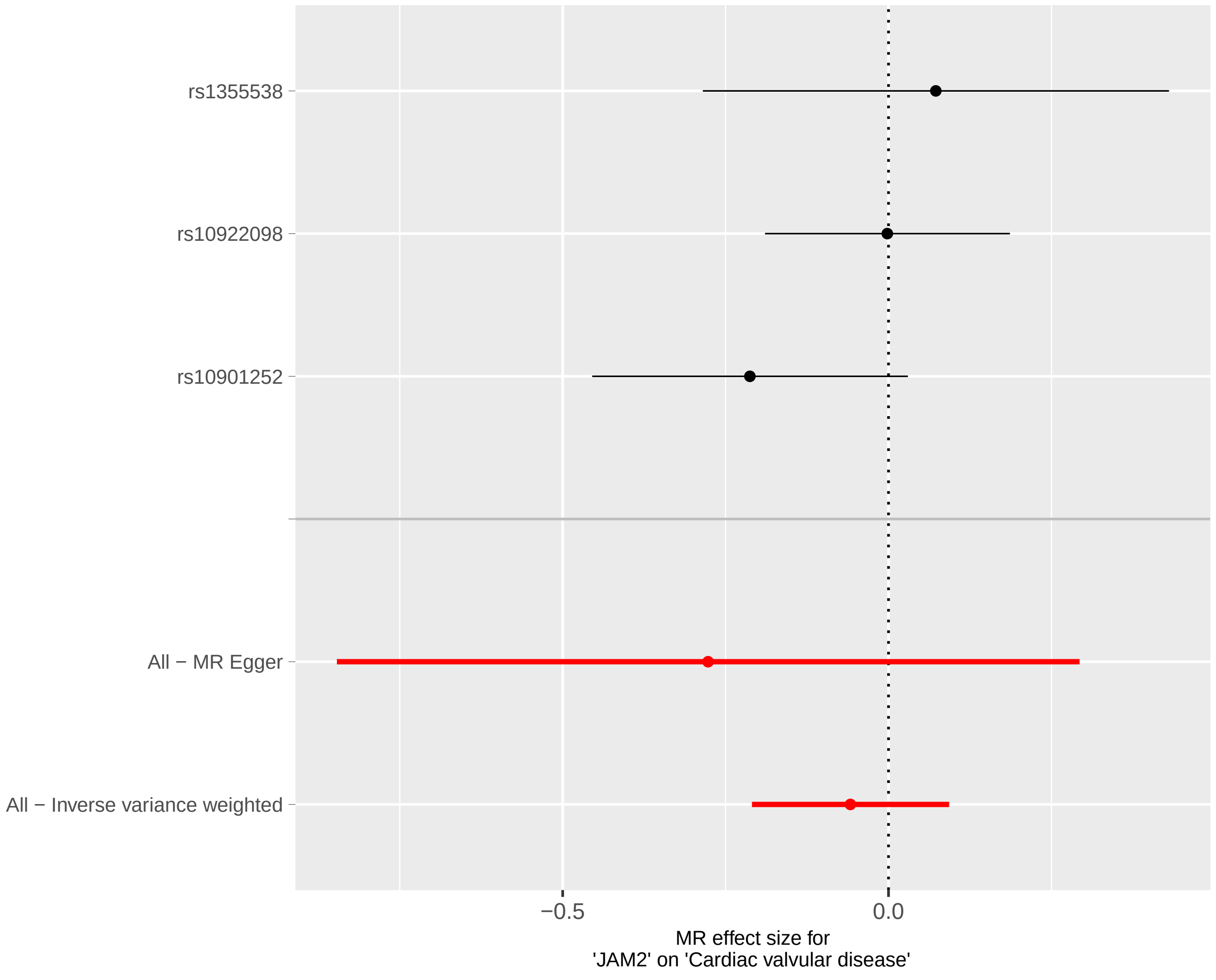
rs1355538
rs10922098
rs10901252
All − MR Egger
All − Inverse variance weighted
−0.5
0.0
MR effect size for
'JAM2' on 'Cardiac valvular disease'

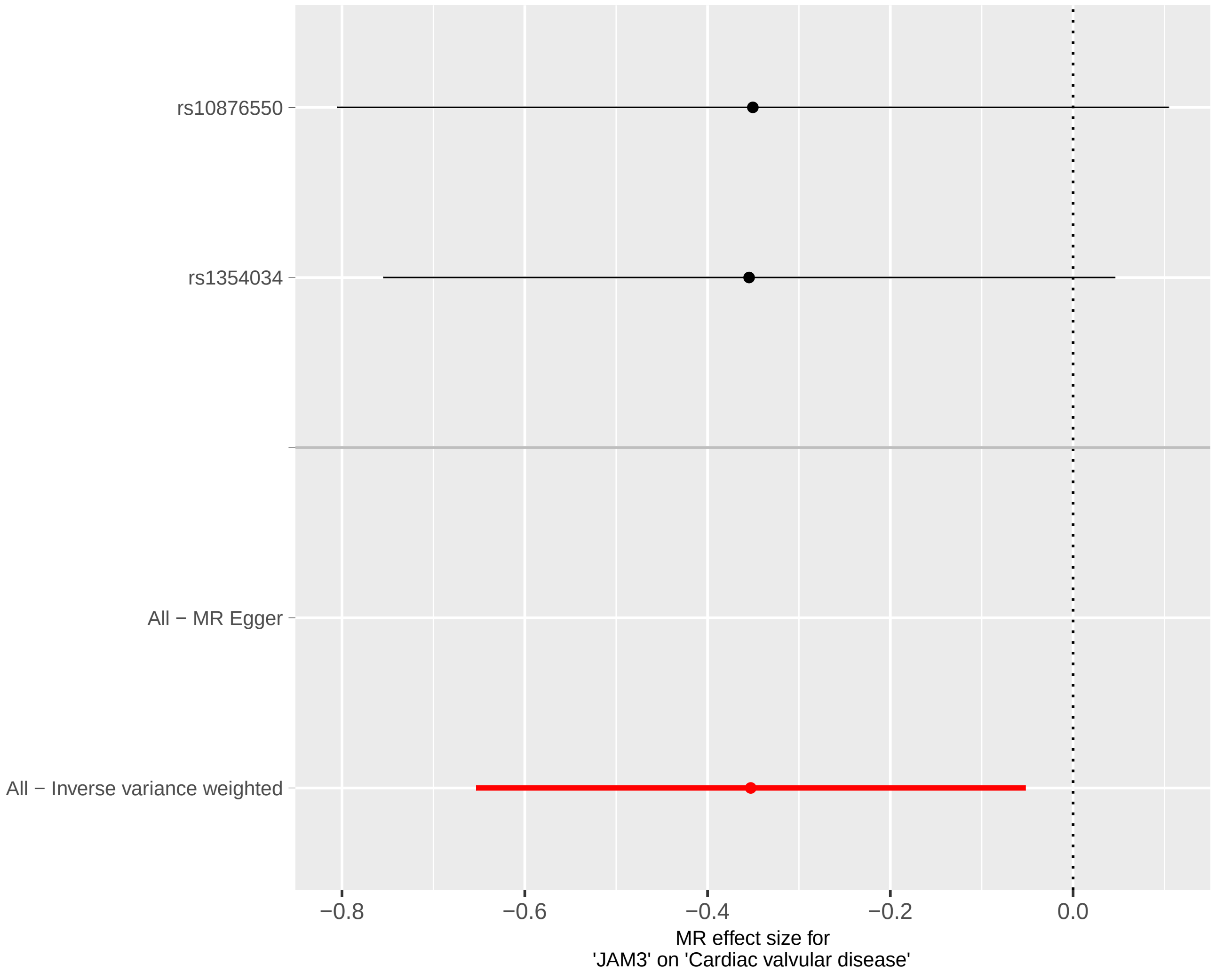
rs10876550
rs1354034
All − MR Egger
All − Inverse variance weighted
−0.8
−0.6
−0.4
−0.2
0.0
MR effect size for
'JAM3' on 'Cardiac valvular disease'

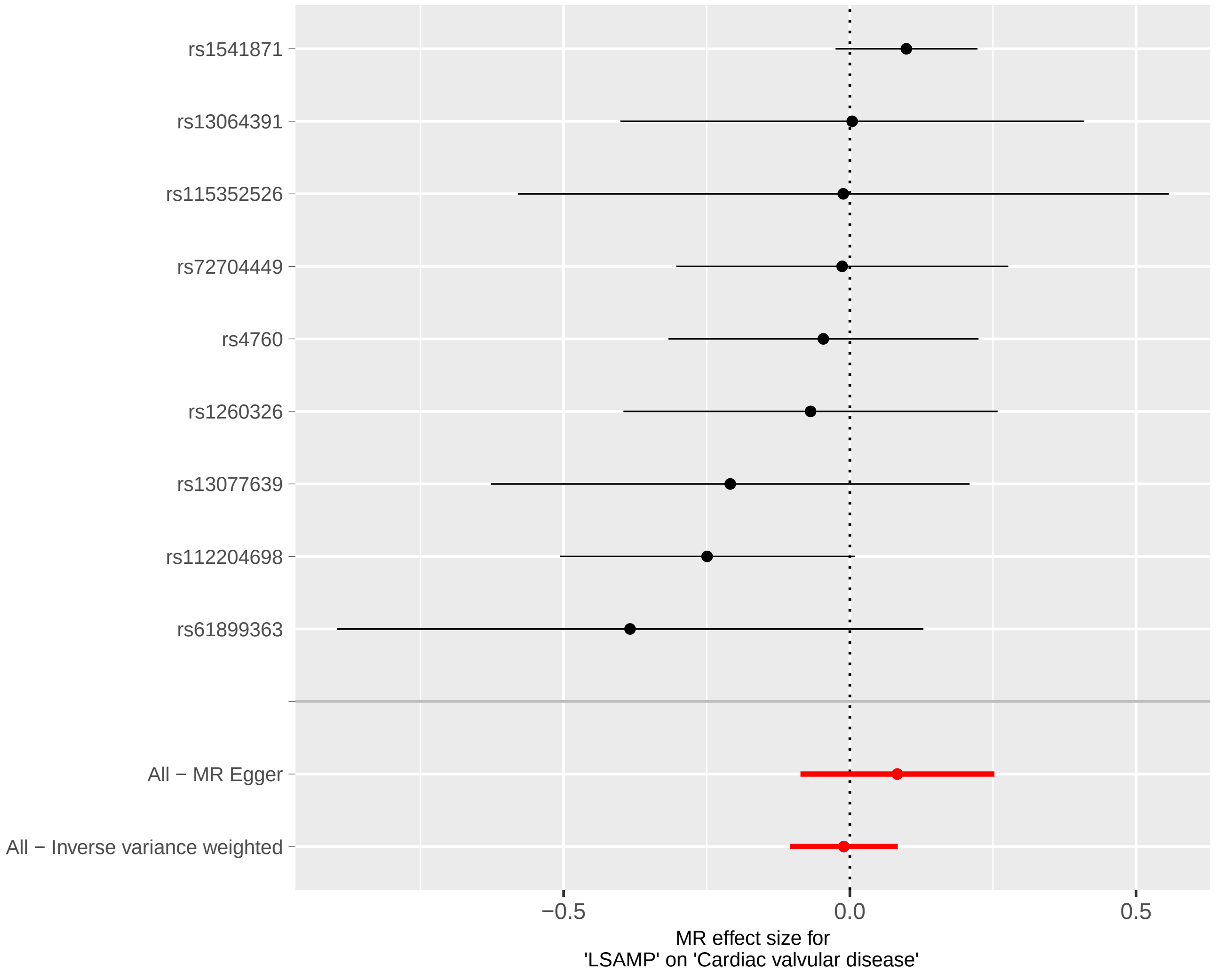
rs1541871
rs13064391
rs115352526
rs72704449
rs4760
rs1260326
rs13077639
rs112204698
rs61899363
All − MR Egger
All − Inverse variance weighted
−0.5
0.0
0.5
MR effect size for
'LSAMP' on 'Cardiac valvular disease'

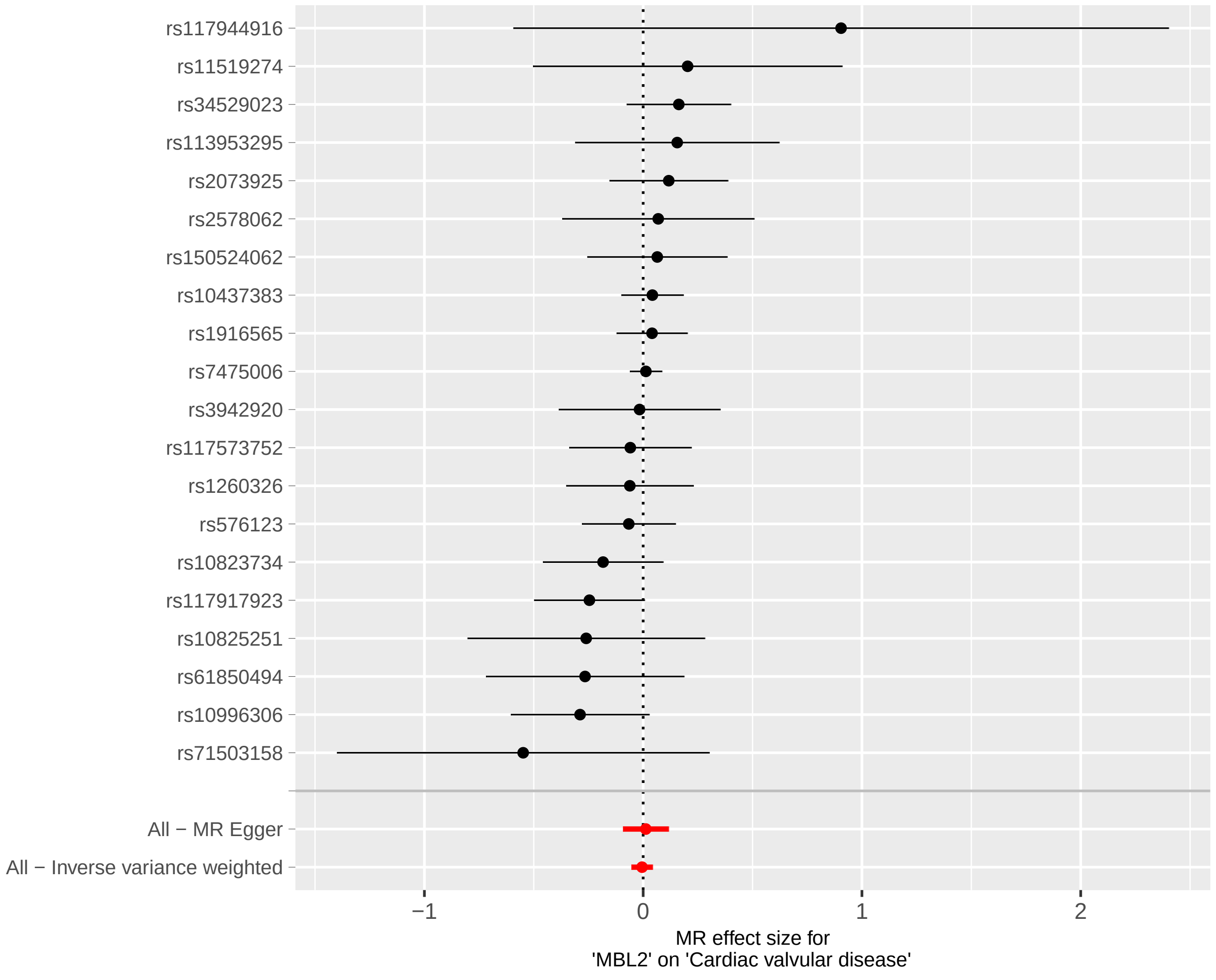

rs117944916
rs11519274
rs34529023
rs113953295
rs2073925
rs2578062
rs150524062
rs10437383
rs1916565
rs7475006
rs3942920
rs117573752
rs1260326
rs576123
rs10823734
rs117917923
rs10825251
rs61850494
rs10996306
rs71503158
All − MR Egger
All − Inverse variance weighted
−1
0
1
2
MR effect size for
'MBL2' on 'Cardiac valvular disease'

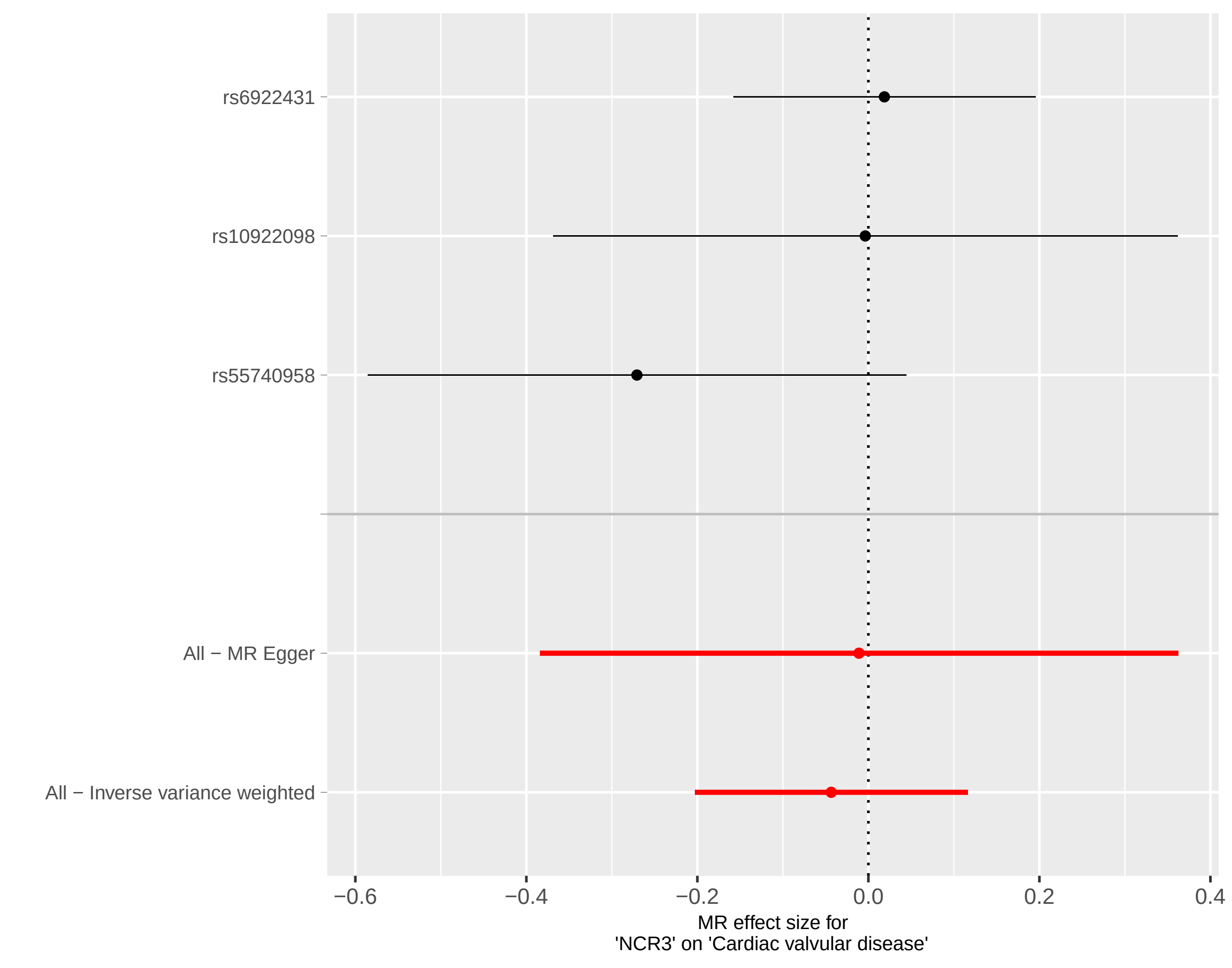
rs6922431
rs10922098
rs55740958
All − MR Egger
All − Inverse variance weighted
−0.6
−0.4
−0.2
0.0
0.2
0.4
MR effect size for
'NCR3' on 'Cardiac valvular disease'

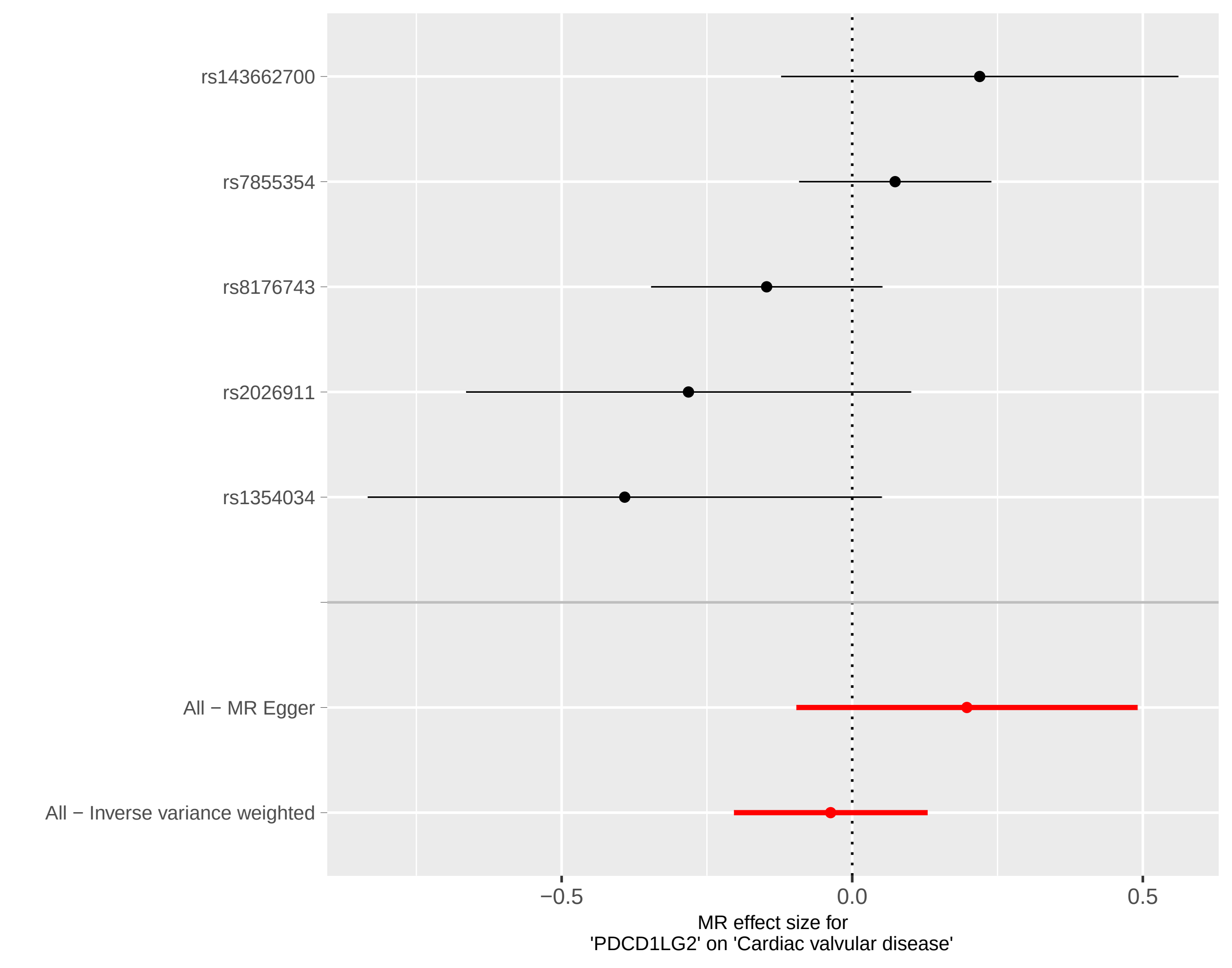
rs143662700
rs7855354
rs8176743
rs2026911
rs1354034
All − MR Egger
All − Inverse variance weighted
−0.5
0.0
0.5
MR effect size for
'PDCD1LG2' on 'Cardiac valvular disease'

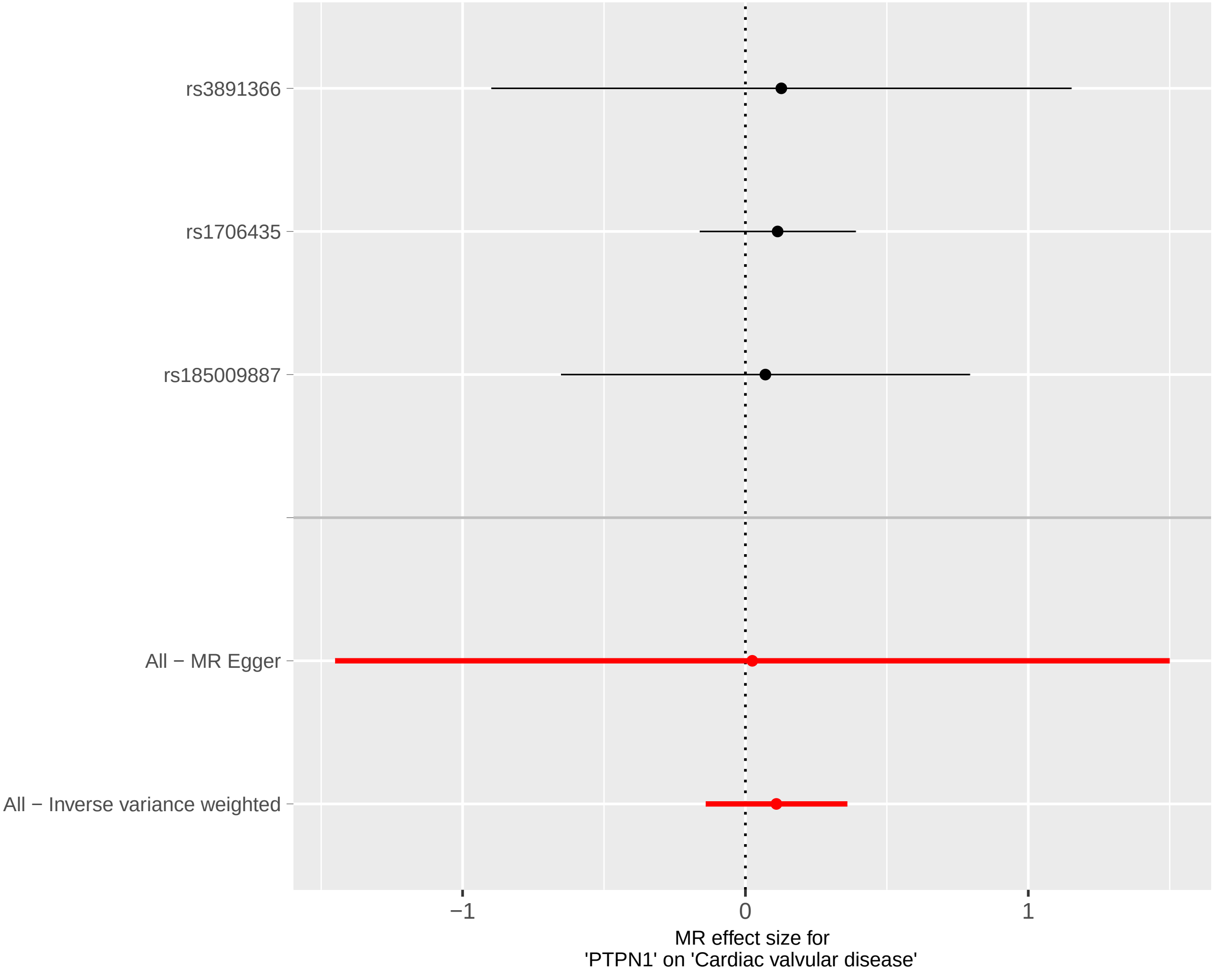
rs3891366
rs1706435
rs185009887
All − MR Egger
All − Inverse variance weighted
−1
0
1
MR effect size for
'PTPN1' on 'Cardiac valvular disease'

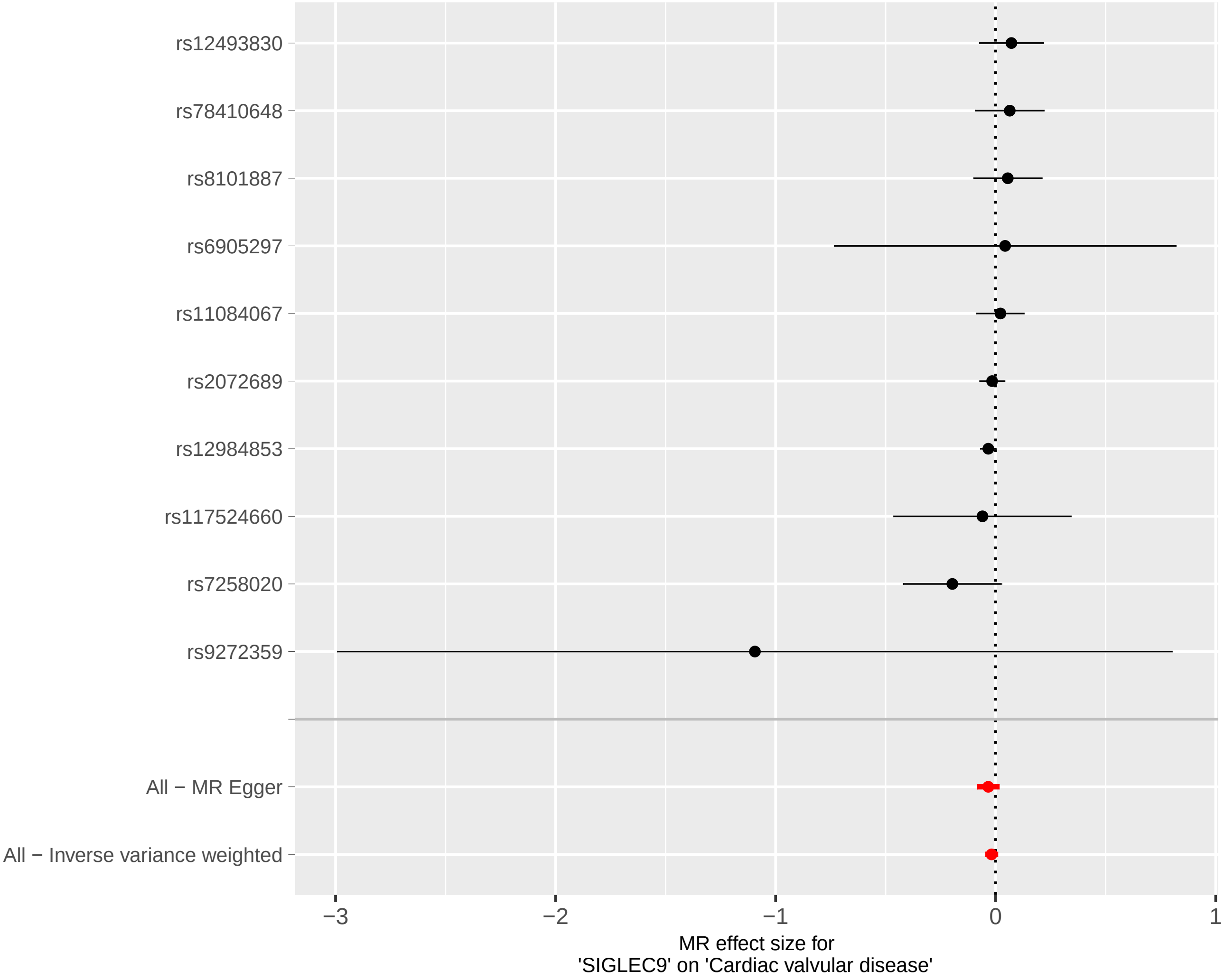
rs12493830
rs78410648
rs8101887
rs6905297
rs11084067
rs2072689
rs12984853
rs117524660
rs7258020
rs9272359
All − MR Egger
All − Inverse variance weighted
−3
−2
−1
0
1
MR effect size for
'SIGLEC9' on 'Cardiac valvular disease'

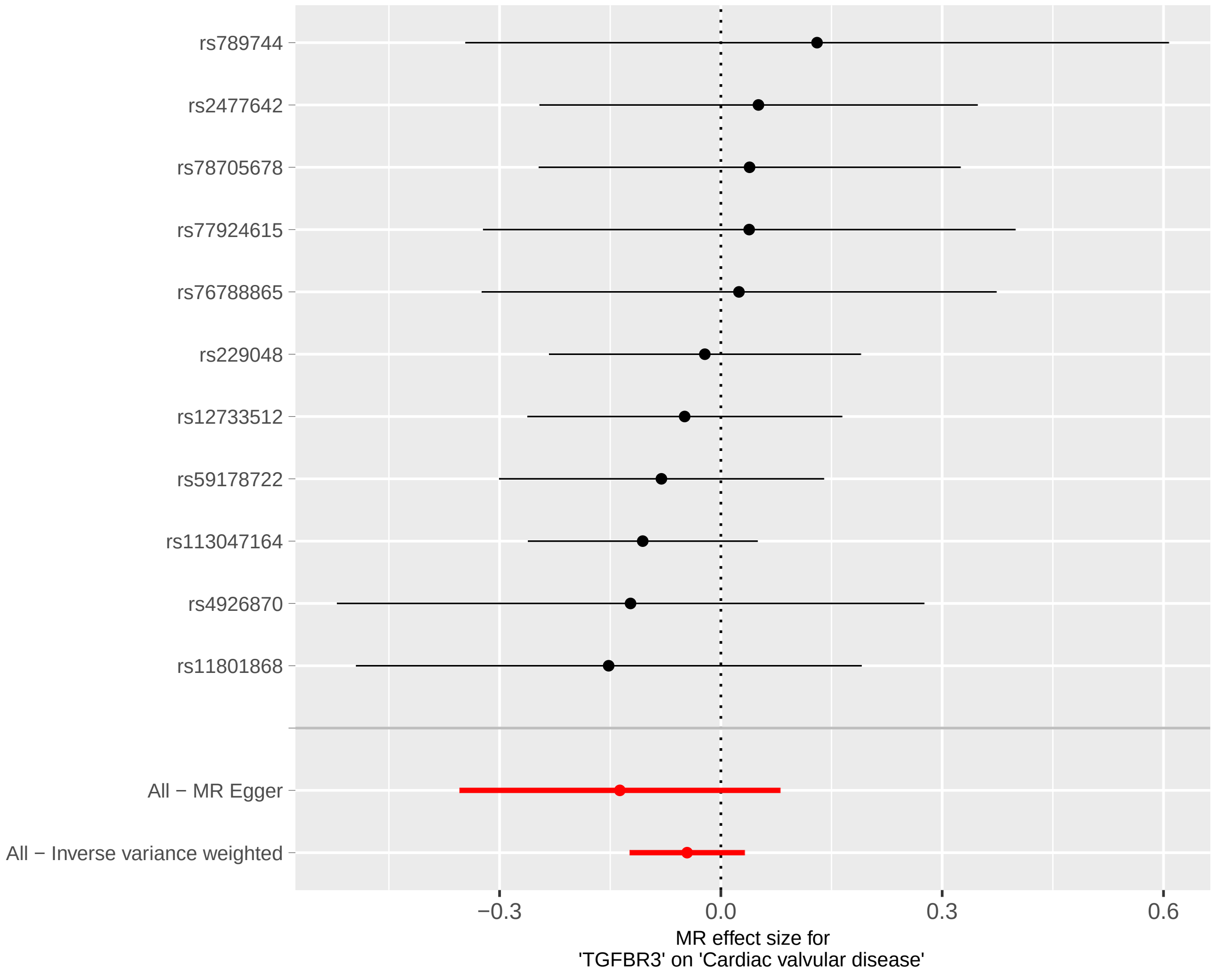
rs789744
rs2477642
rs78705678
rs77924615
rs76788865
rs229048
rs12733512
rs59178722
rs113047164
rs4926870
rs11801868
All − MR Egger
All − Inverse variance weighted
−0.3
0.0
0.3
0.6
MR effect size for
'TGFBR3' on 'Cardiac valvular disease'

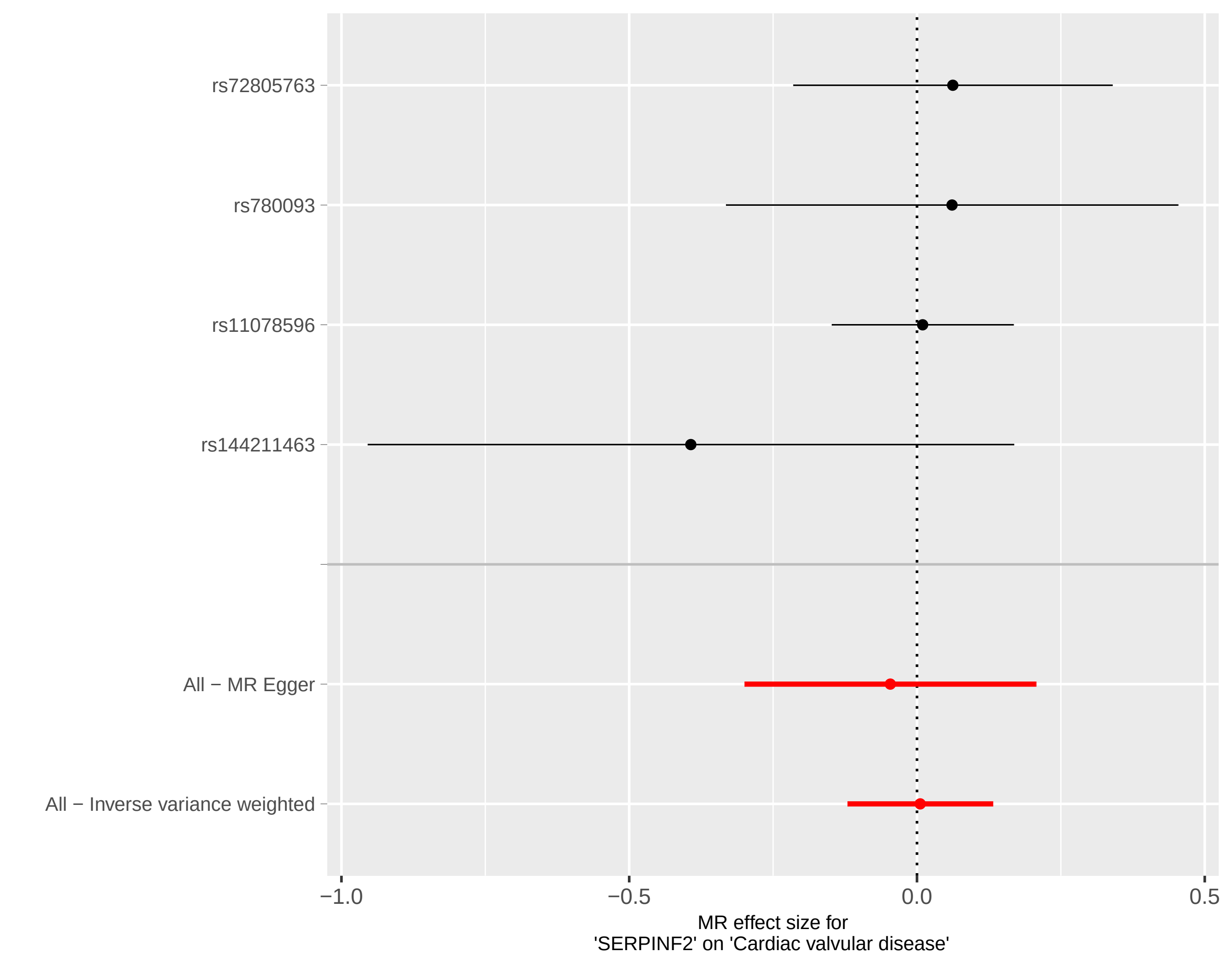
rs72805763
rs780093
rs11078596
rs144211463
All − MR Egger
All − Inverse variance weighted
−1.0
−0.5
0.0
0.5
MR effect size for
'SERPINF2' on 'Cardiac valvular disease'

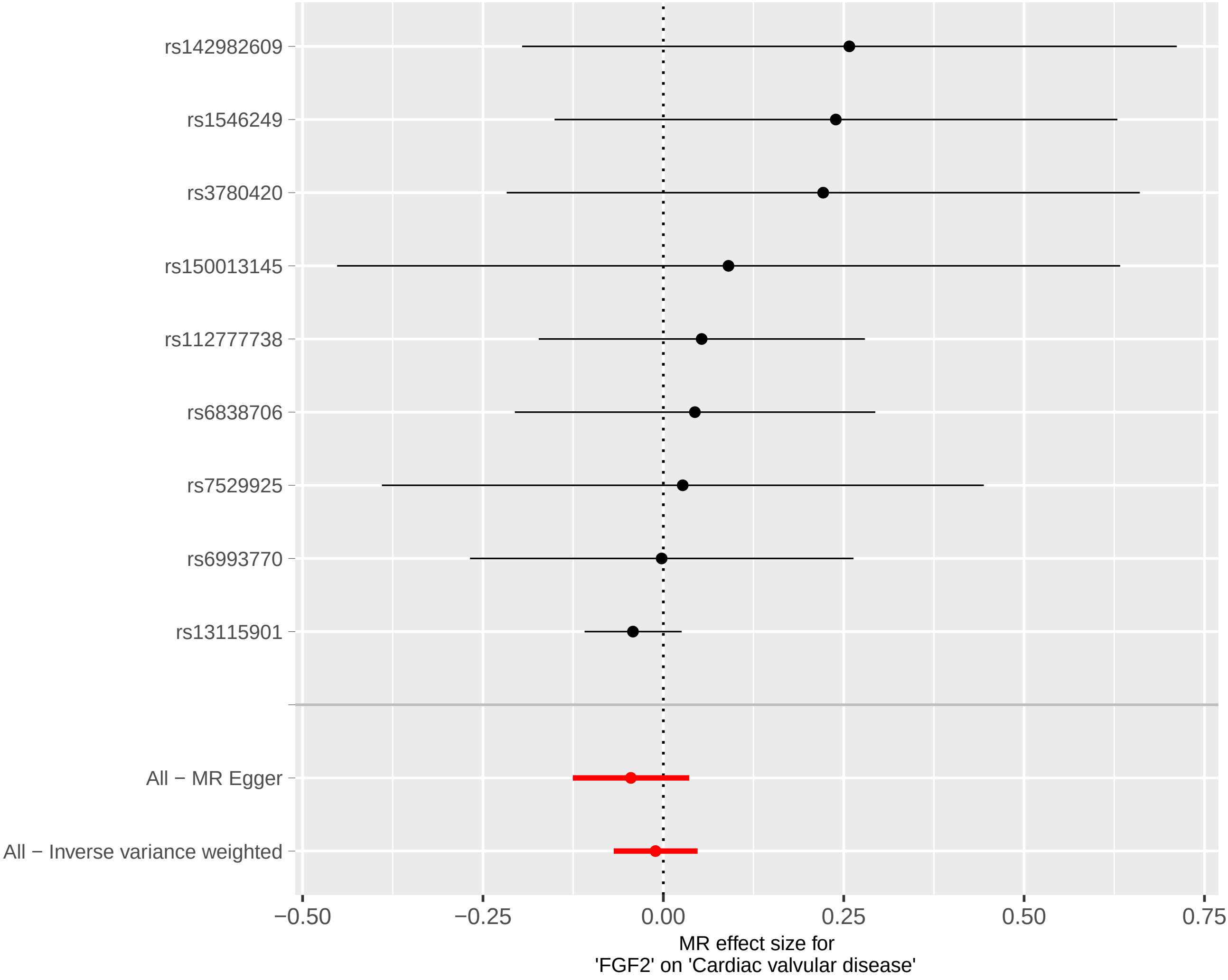
rs142982609
rs1546249
rs3780420
rs150013145
rs112777738
rs6838706
rs7529925
rs6993770
rs13115901
All − MR Egger
All − Inverse variance weighted
−0.50
−0.25
0.00
0.25
0.50
0.75
MR effect size for
'FGF2' on 'Cardiac valvular disease'

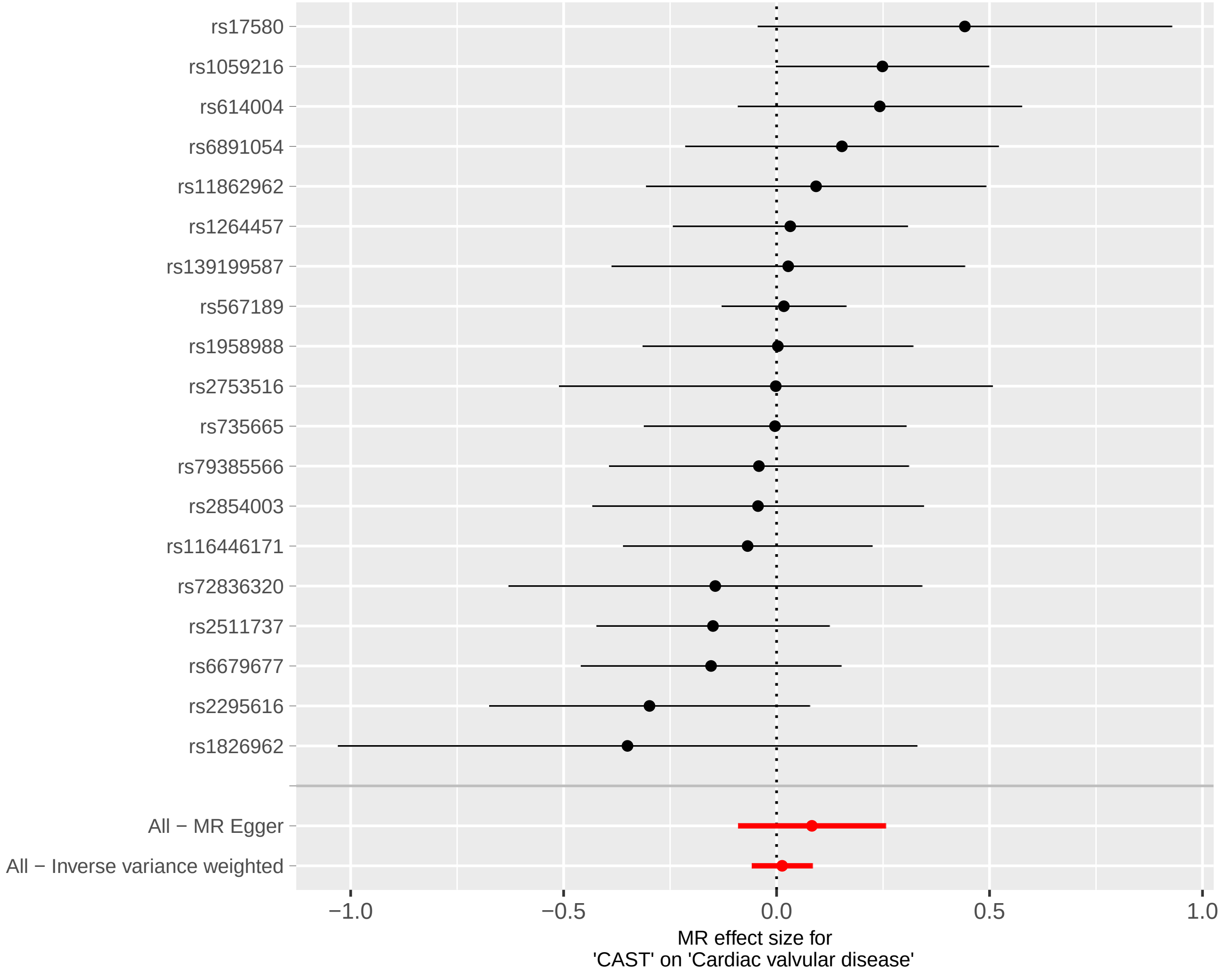
rs17580
rs1059216
rs614004
rs6891054
rs11862962
rs1264457
rs139199587
rs567189
rs1958988
rs2753516
rs735665
rs79385566
rs2854003
rs116446171
rs72836320
rs2511737
rs6679677
rs2295616
rs1826962
All − MR Egger
All − Inverse variance weighted
−1.0
−0.5
0.0
0.5
1.0
MR effect size for
'CAST' on 'Cardiac valvular disease'

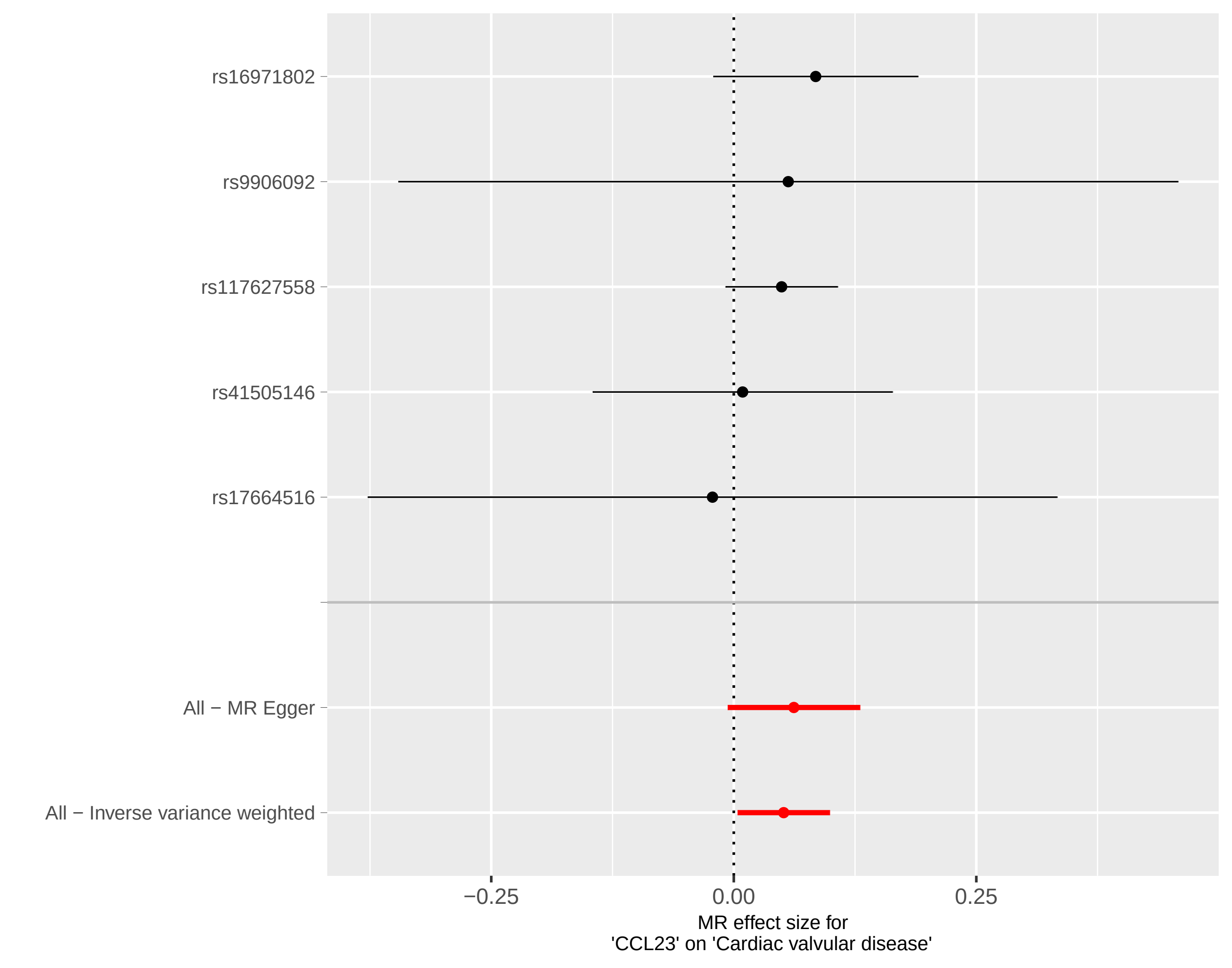
rs16971802
rs9906092
rs117627558
rs41505146
rs17664516
All − MR Egger
All − Inverse variance weighted
−0.25
0.00
0.25
MR effect size for
'CCL23' on 'Cardiac valvular disease'

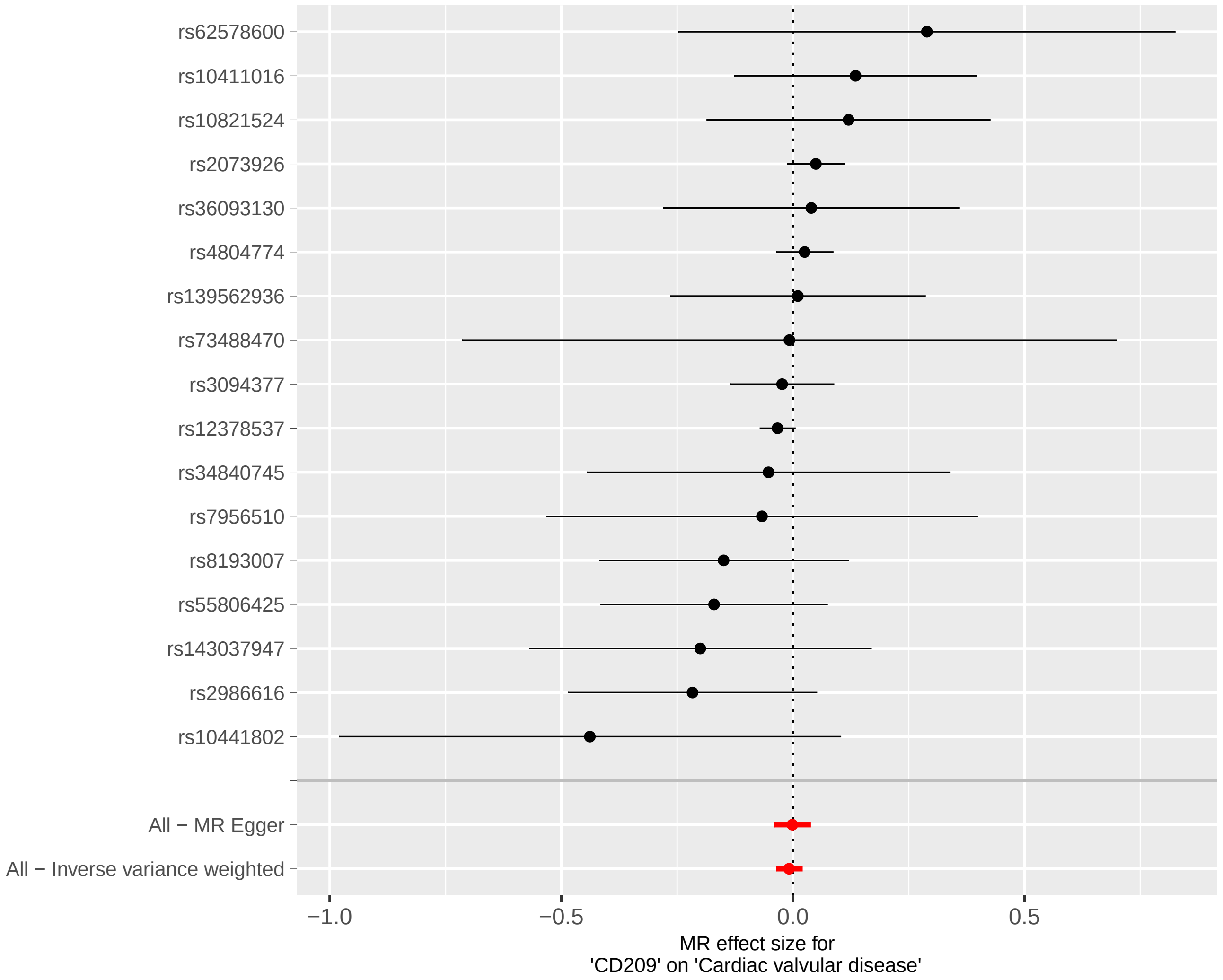
rs62578600
rs10411016
rs10821524
rs2073926
rs36093130
rs4804774
rs139562936
rs73488470
rs3094377
rs12378537
rs34840745
rs7956510
rs8193007
rs55806425
rs143037947
rs2986616
rs10441802
All − MR Egger
All − Inverse variance weighted
−1.0
−0.5
0.0
0.5
MR effect size for
'CD209' on 'Cardiac valvular disease'

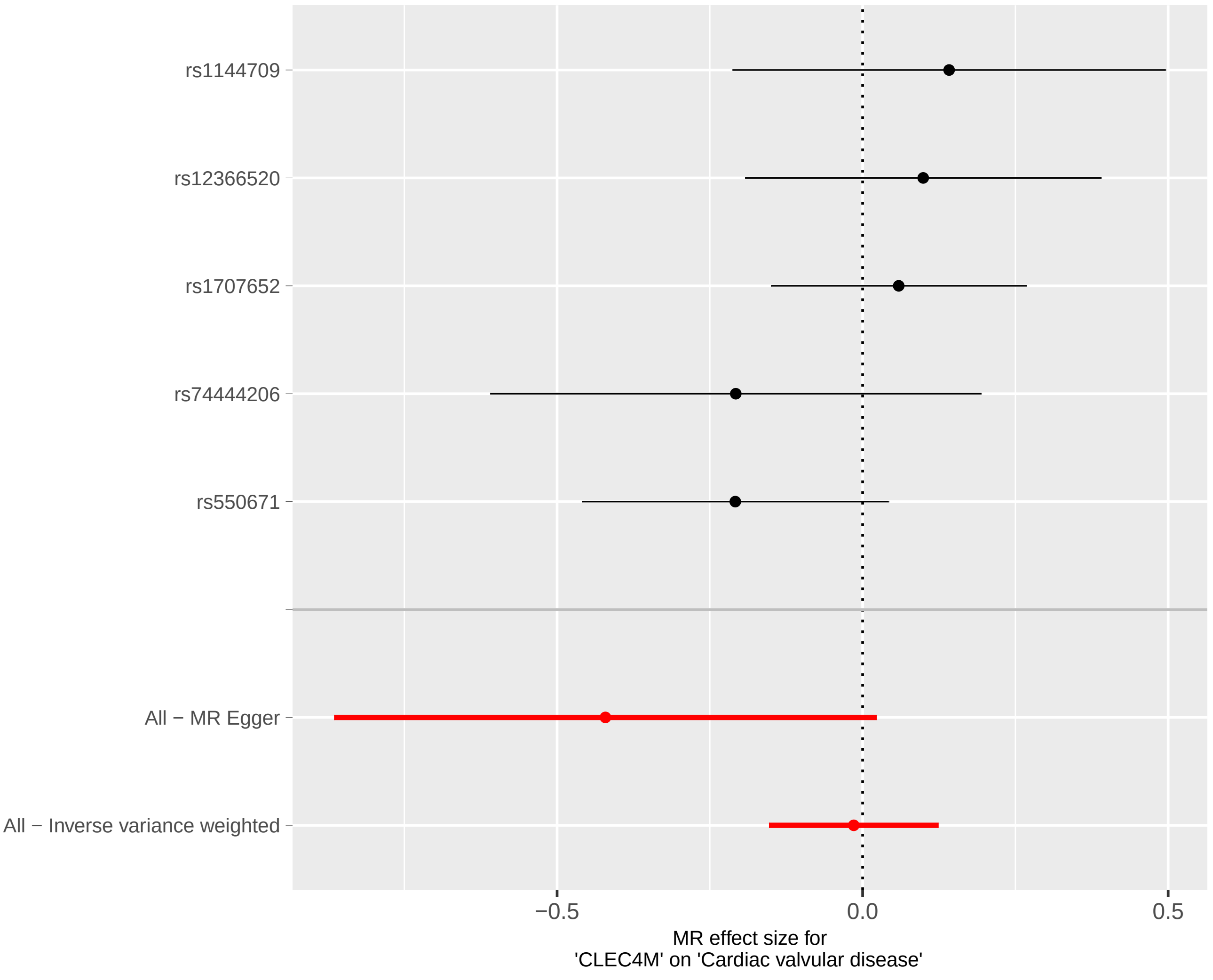
rs1144709
rs12366520
rs1707652
rs74444206
rs550671
All − MR Egger
All − Inverse variance weighted
−0.5
0.0
0.5
MR effect size for
'CLEC4M' on 'Cardiac valvular disease'

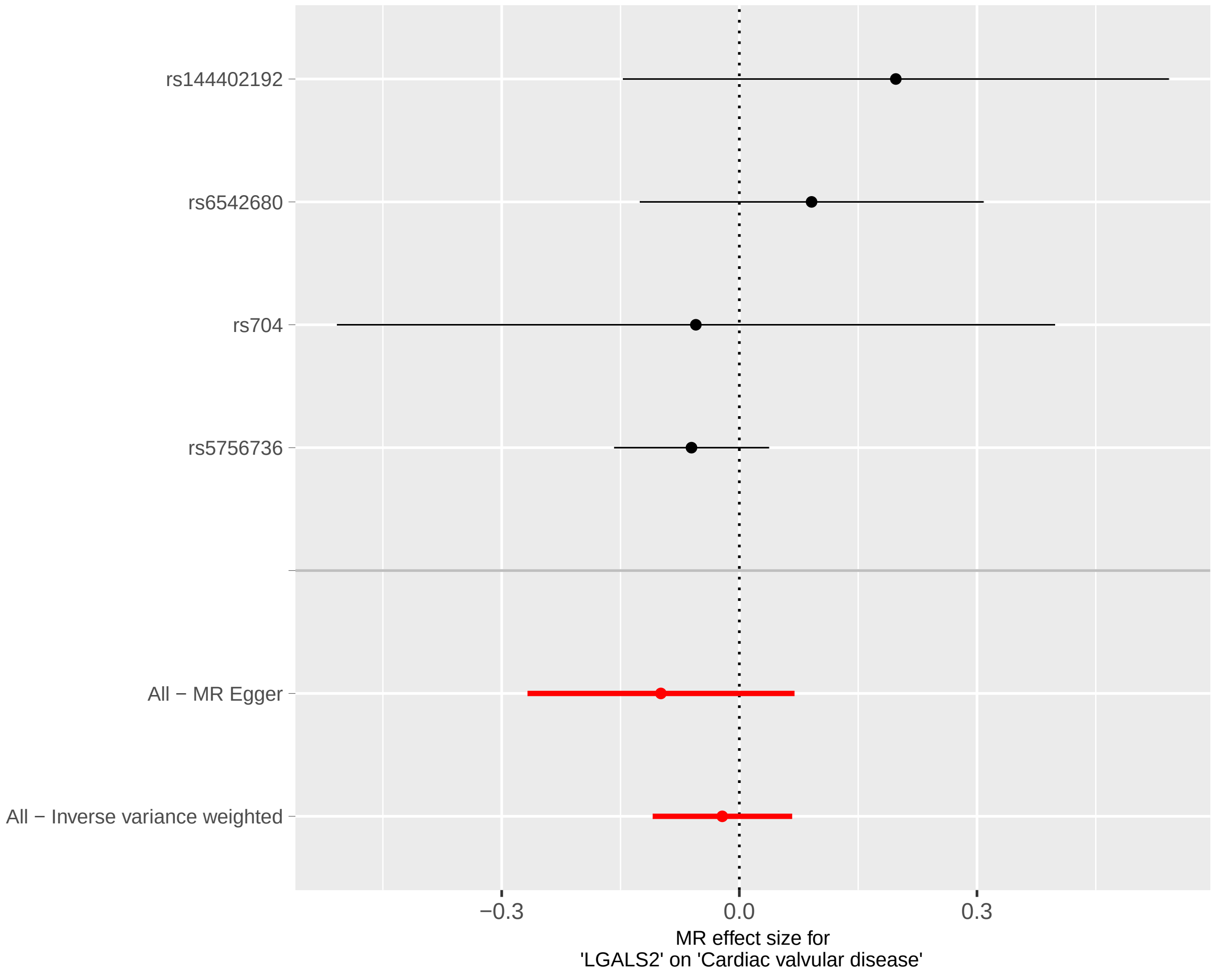
rs144402192
rs6542680
rs704
rs5756736
All − MR Egger
All − Inverse variance weighted
−0.3
0.0
0.3
MR effect size for
'LGALS2' on 'Cardiac valvular disease'

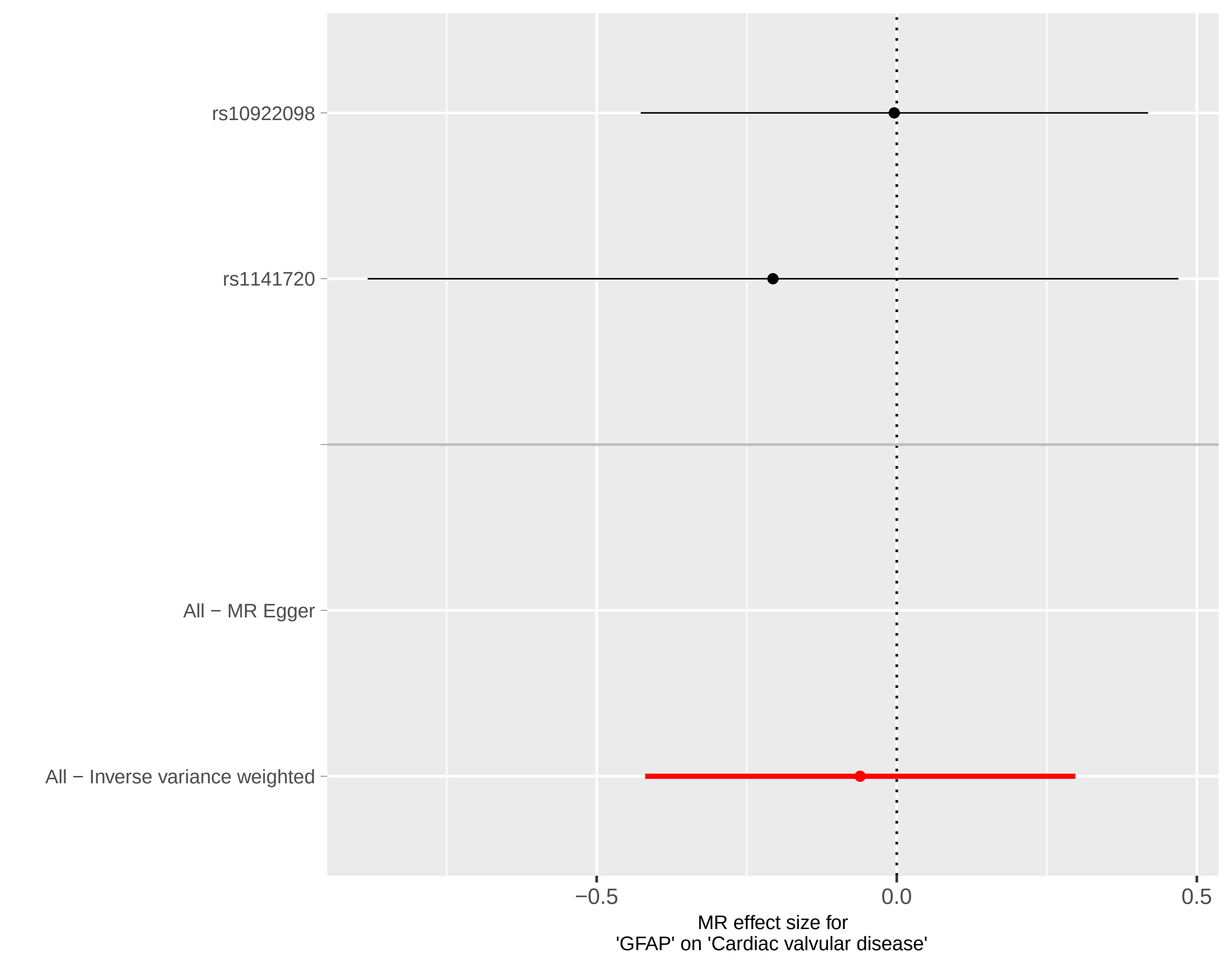
rs10922098
rs1141720
All − MR Egger
All − Inverse variance weighted
−0.5
0.0
0.5
MR effect size for
'GFAP' on 'Cardiac valvular disease'

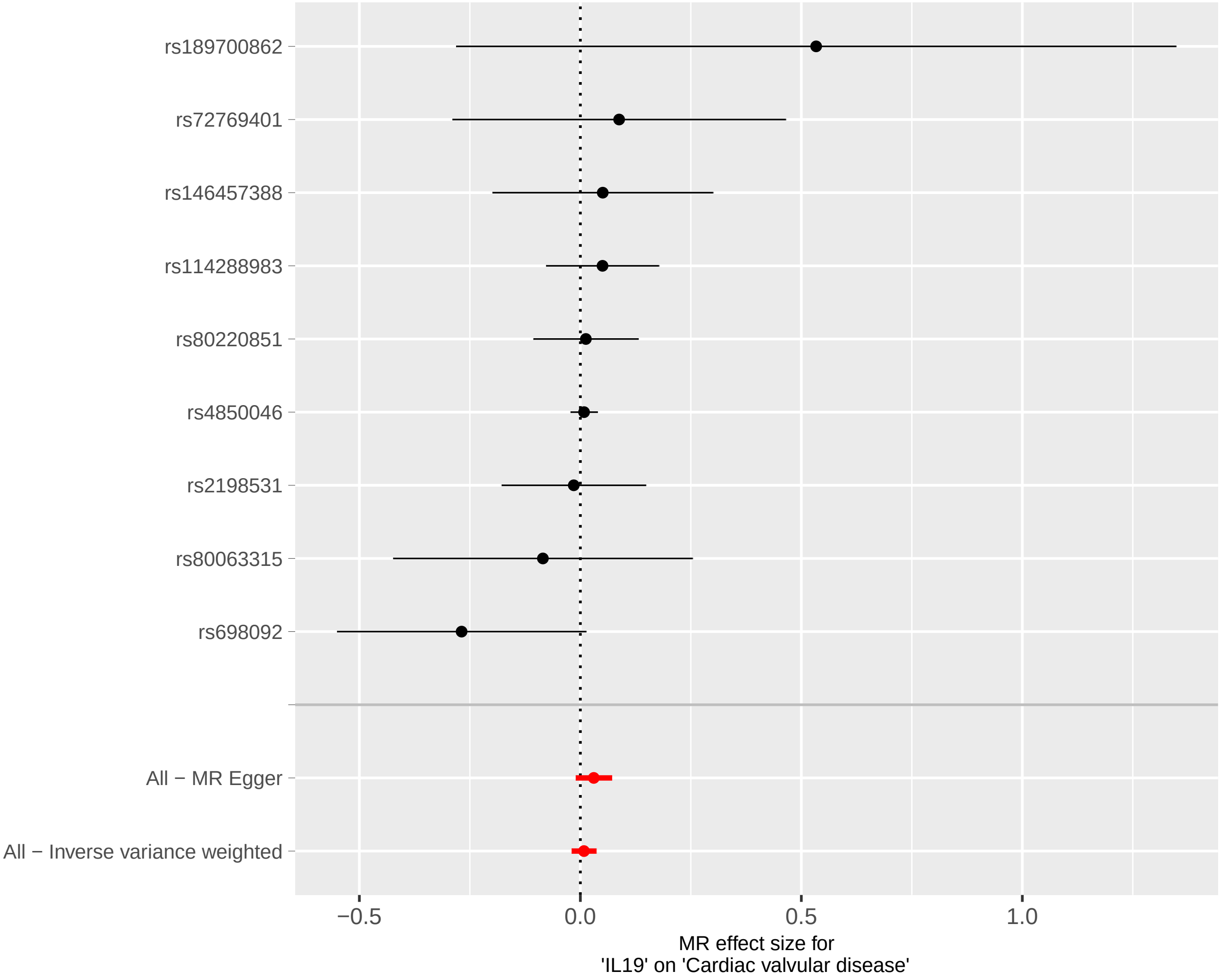

rs189700862
rs72769401
rs146457388
rs114288983
rs80220851
rs4850046
rs2198531
rs80063315
rs698092
All − MR Egger
All − Inverse variance weighted
−0.5
0.0
0.5
1.0
MR effect size for
'IL19' on 'Cardiac valvular disease'

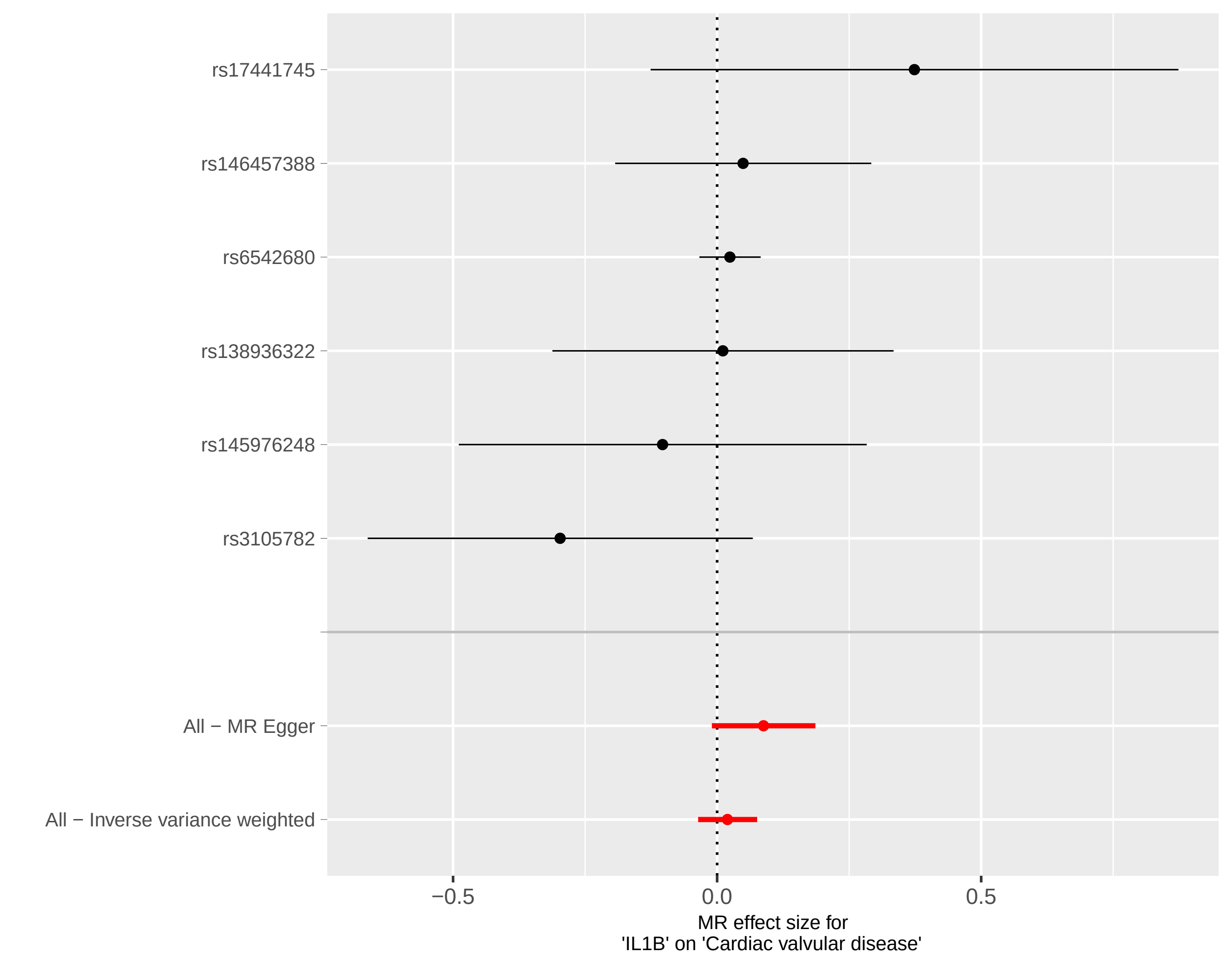

rs17441745
rs146457388
rs6542680
rs138936322
rs145976248
rs3105782
All − MR Egger
All − Inverse variance weighted
−0.5
0.0
0.5
MR effect size for
'IL1B' on 'Cardiac valvular disease'

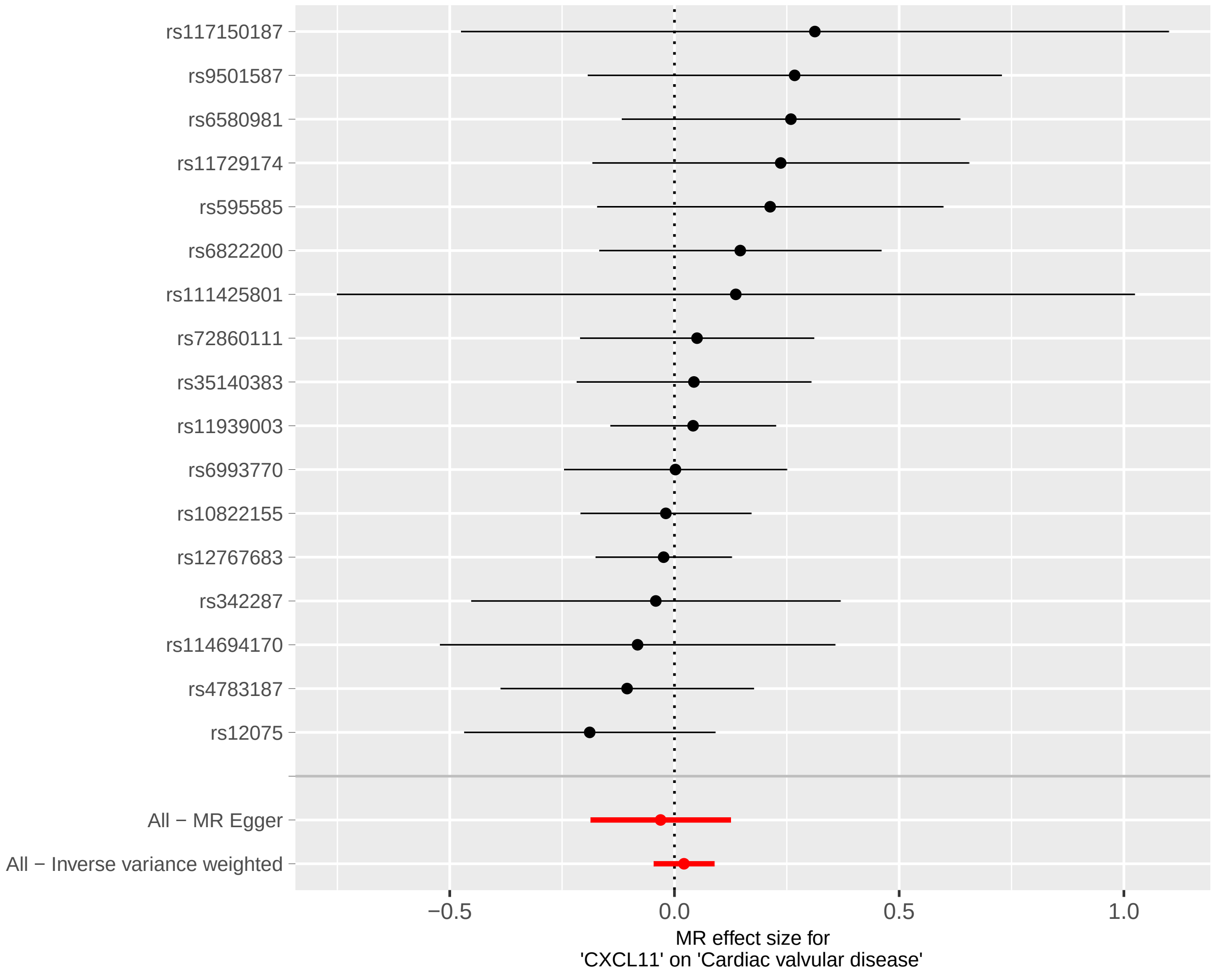

rs117150187
rs9501587
rs6580981
rs11729174
rs595585
rs6822200
rs111425801
rs72860111
rs35140383
rs11939003
rs6993770
rs10822155
rs12767683
rs342287
rs114694170
rs4783187
rs12075
All − MR Egger
All − Inverse variance weighted
−0.5
0.0
0.5
1.0
MR effect size for
'CXCL11' on 'Cardiac valvular disease'

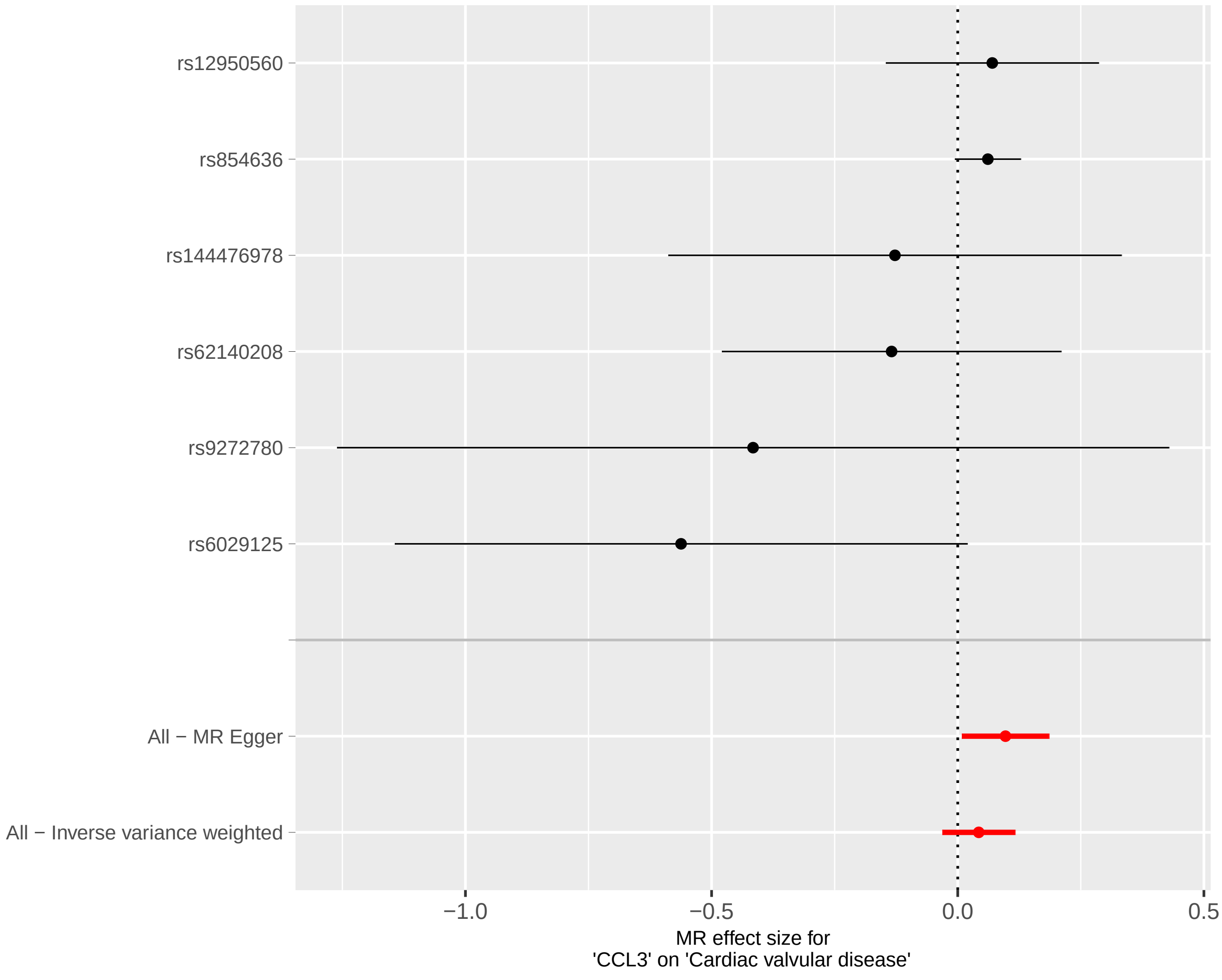
rs12950560
rs854636
rs144476978
rs62140208
rs9272780
rs6029125
All − MR Egger
All − Inverse variance weighted
−1.0
−0.5
0.0
0.5
MR effect size for
'CCL3' on 'Cardiac valvular disease'

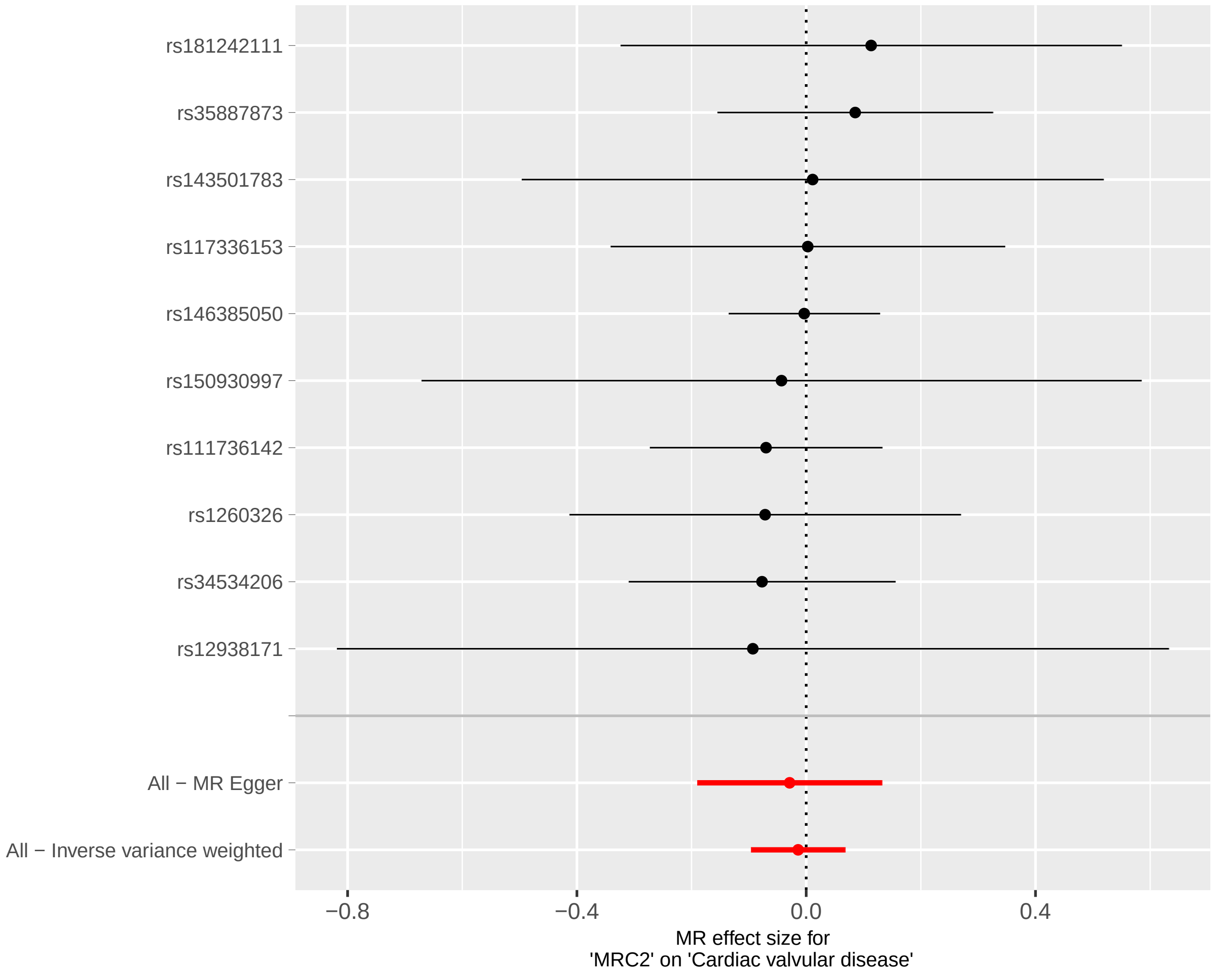
rs181242111
rs35887873
rs143501783
rs117336153
rs146385050
rs150930997
rs111736142
rs1260326
rs34534206
rs12938171
All − MR Egger
All − Inverse variance weighted
−0.8
−0.4
0.0
0.4
MR effect size for
'MRC2' on 'Cardiac valvular disease'

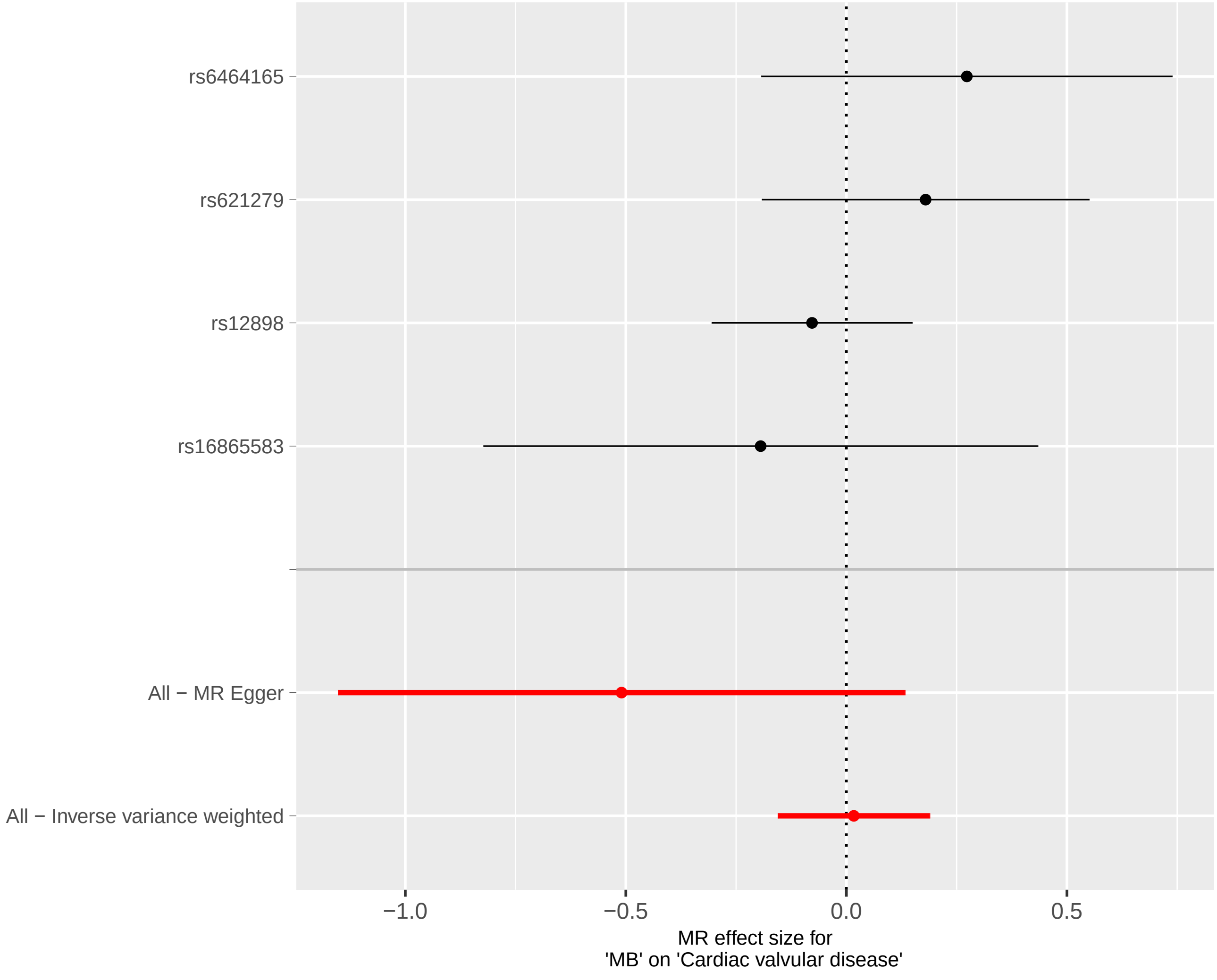
rs6464165
rs621279
rs12898
rs16865583
All − MR Egger
All − Inverse variance weighted
−1.0
−0.5
0.0
0.5
MR effect size for
'MB' on 'Cardiac valvular disease'

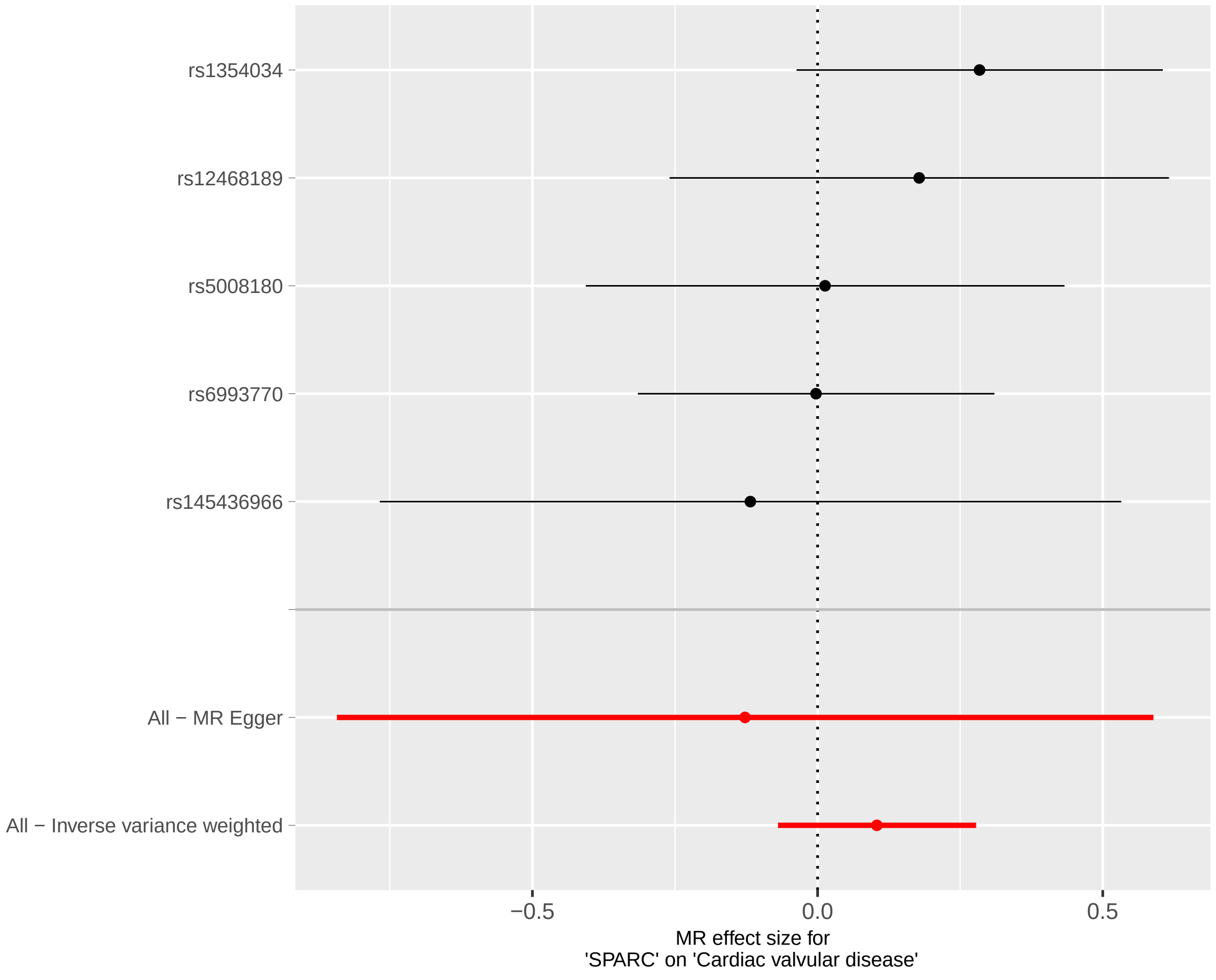
rs1354034
rs12468189
rs5008180
rs6993770
rs145436966
All − MR Egger
All − Inverse variance weighted
−0.5
0.0
0.5
MR effect size for
'SPARC' on 'Cardiac valvular disease'

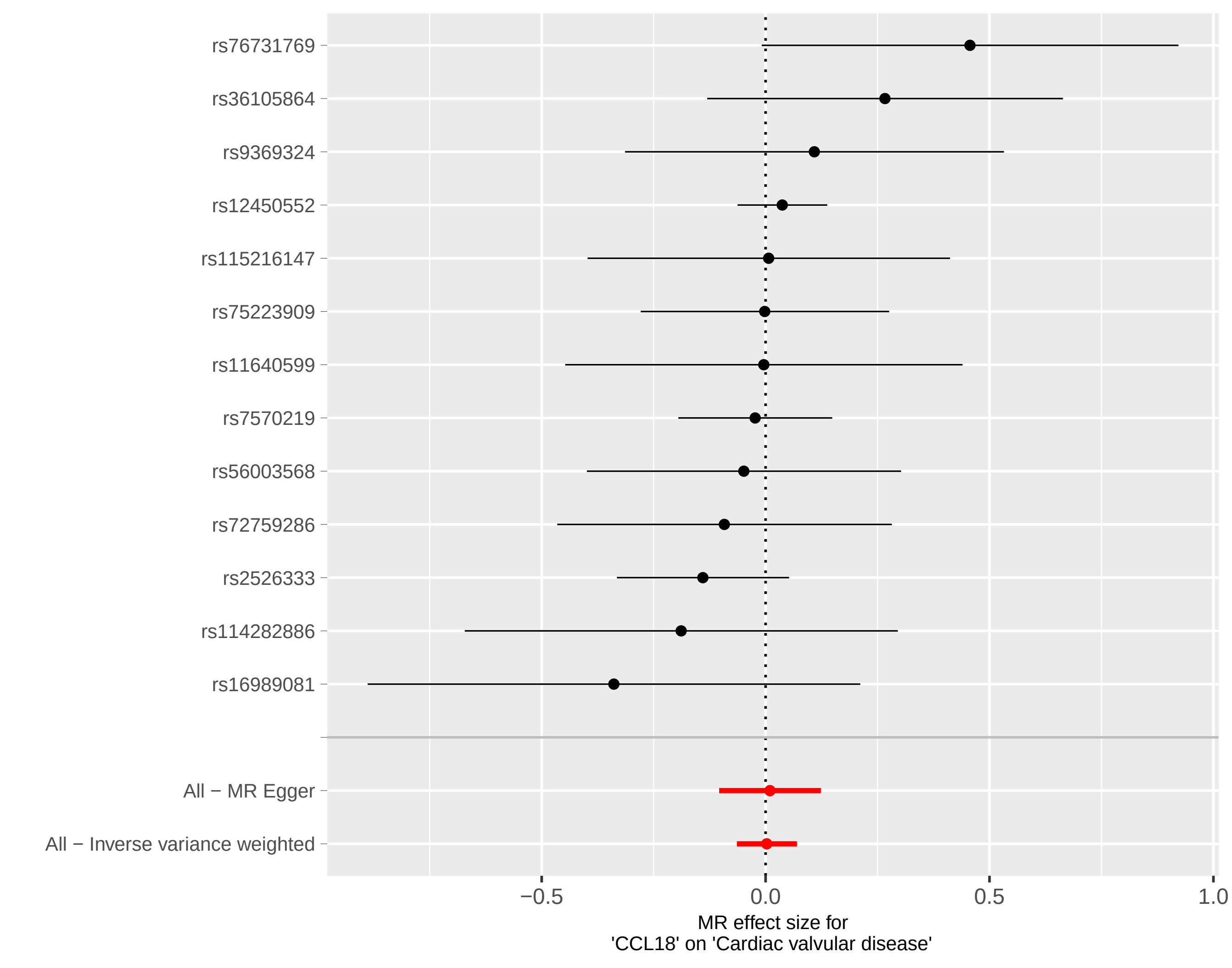
rs76731769
rs36105864
rs9369324
rs12450552
rs115216147
rs75223909
rs11640599
rs7570219
rs56003568
rs72759286
rs2526333
rs114282886
rs16989081
All − MR Egger
All − Inverse variance weighted
−0.5
0.0
0.5
1.0
MR effect size for
'CCL18' on 'Cardiac valvular disease'

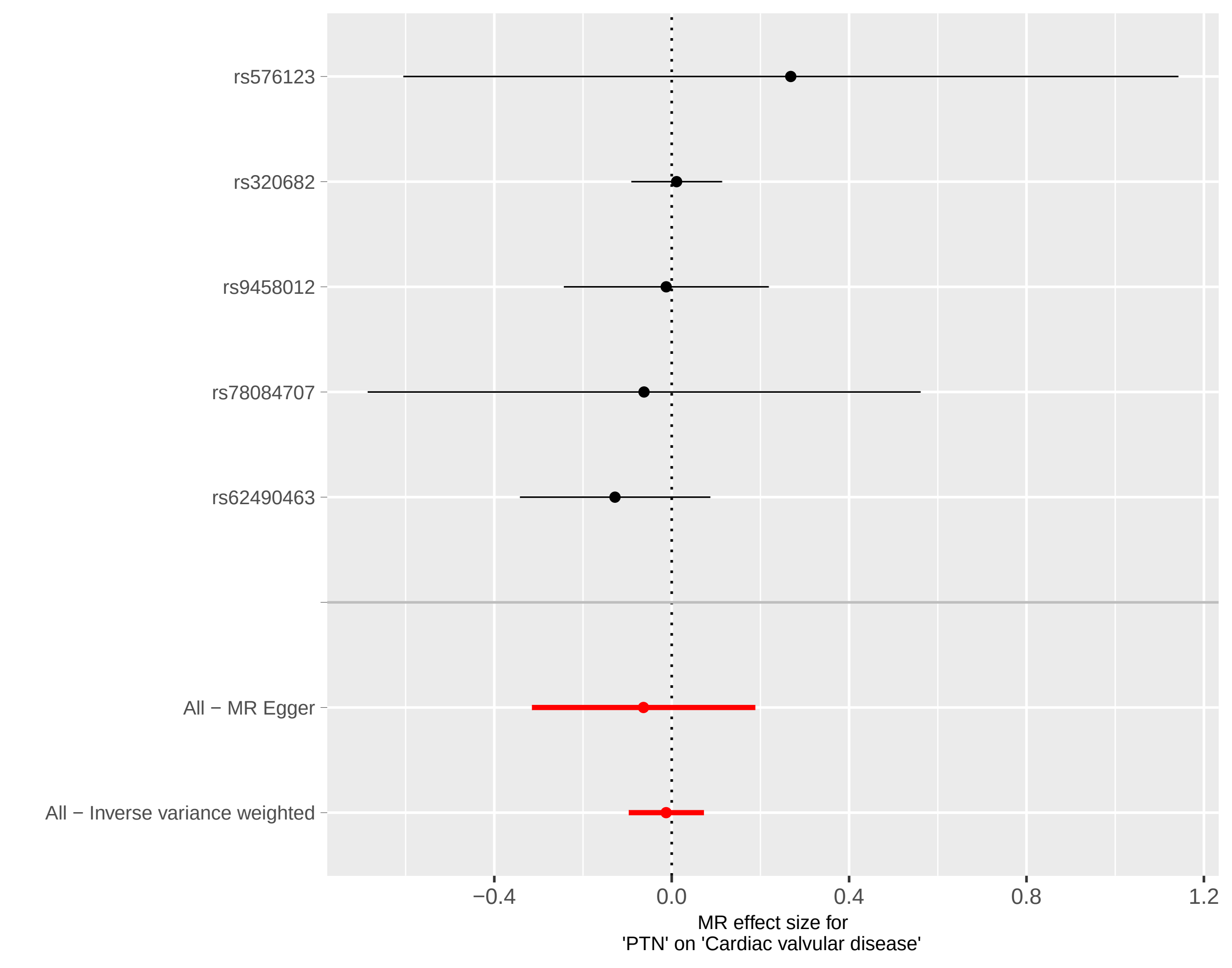
rs576123
rs320682
rs9458012
rs78084707
rs62490463
All − MR Egger
All − Inverse variance weighted
−0.4
0.0
0.4
0.8
1.2
MR effect size for
'PTN' on 'Cardiac valvular disease'

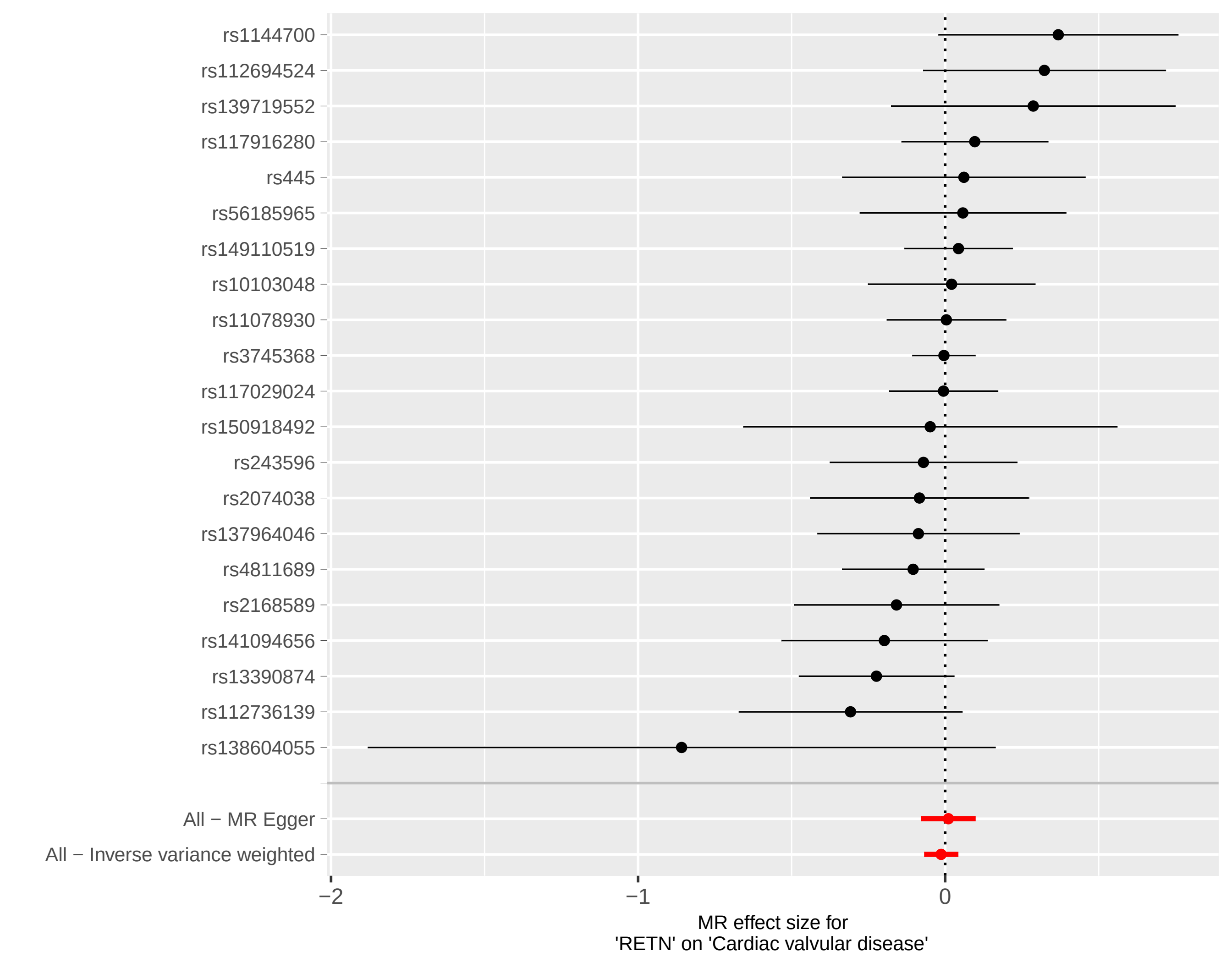
rs1144700
rs112694524
rs139719552
rs117916280
rs445
rs56185965
rs149110519
rs10103048
rs11078930
rs3745368
rs117029024
rs150918492
rs243596
rs2074038
rs137964046
rs4811689
rs2168589
rs141094656
rs13390874
rs112736139
rs138604055
All − MR Egger
All − Inverse variance weighted
−2
−1
0
MR effect size for
'RETN' on 'Cardiac valvular disease'

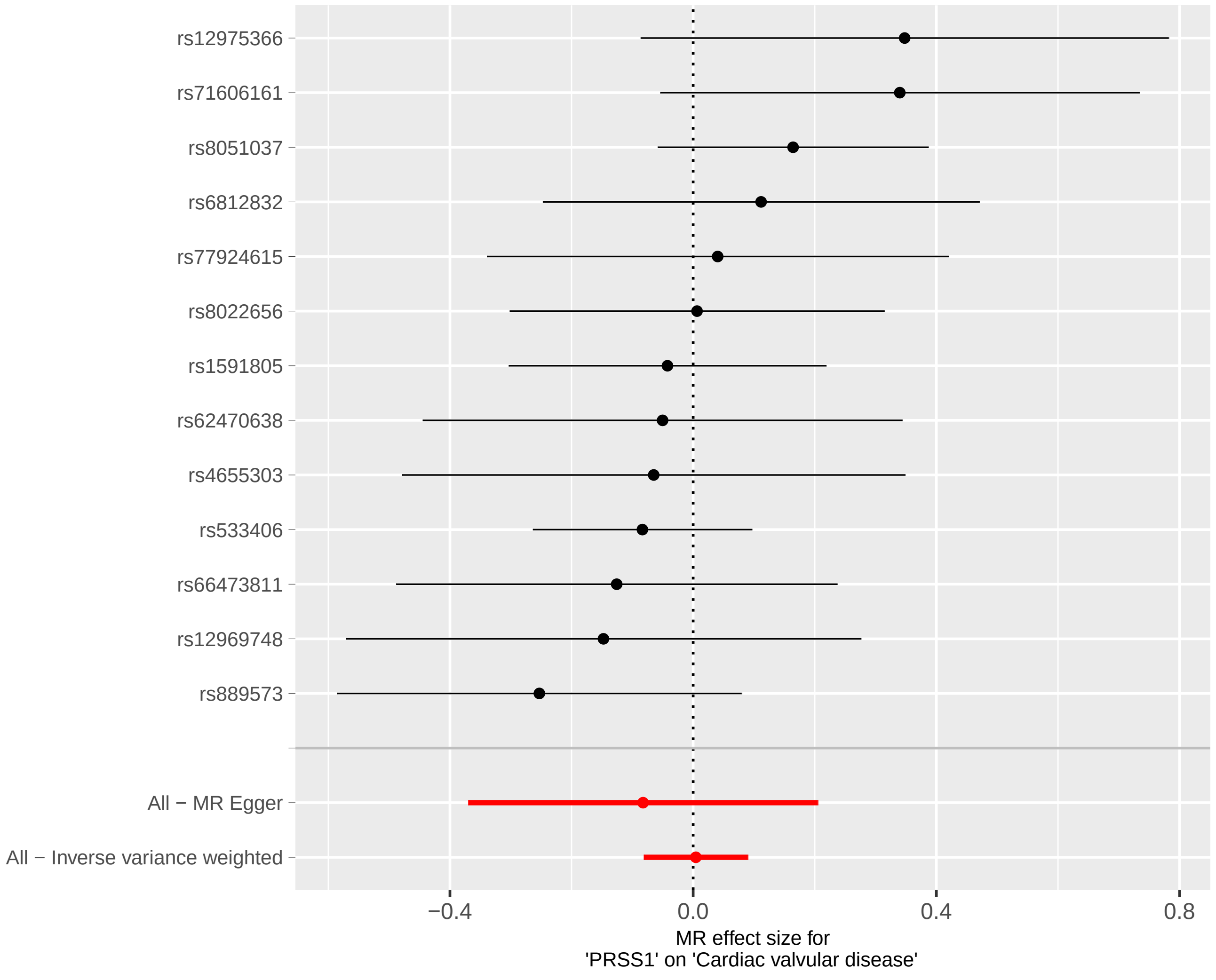

rs12975366
rs71606161
rs8051037
rs6812832
rs77924615
rs8022656
rs1591805
rs62470638
rs4655303
rs533406
rs66473811
rs12969748
rs889573
All − MR Egger
All − Inverse variance weighted
−0.4
0.0
0.4
0.8
MR effect size for
'PRSS1' on 'Cardiac valvular disease'

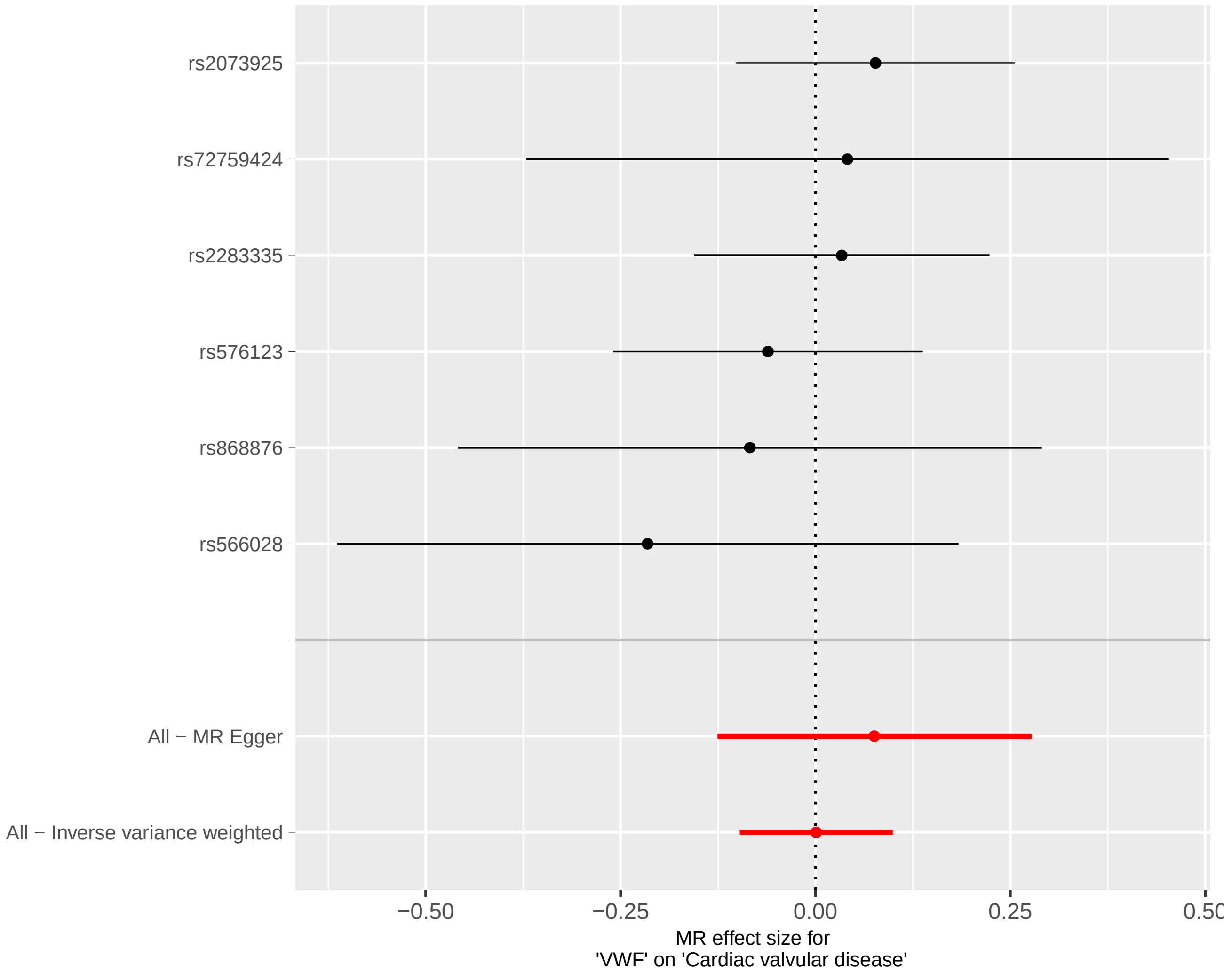
rs2073925
rs72759424
rs2283335
rs576123
rs868876
rs566028
All − MR Egger
All − Inverse variance weighted
−0.50
−0.25
0.00
0.25
0.50
MR effect size for
'VWF' on 'Cardiac valvular disease'

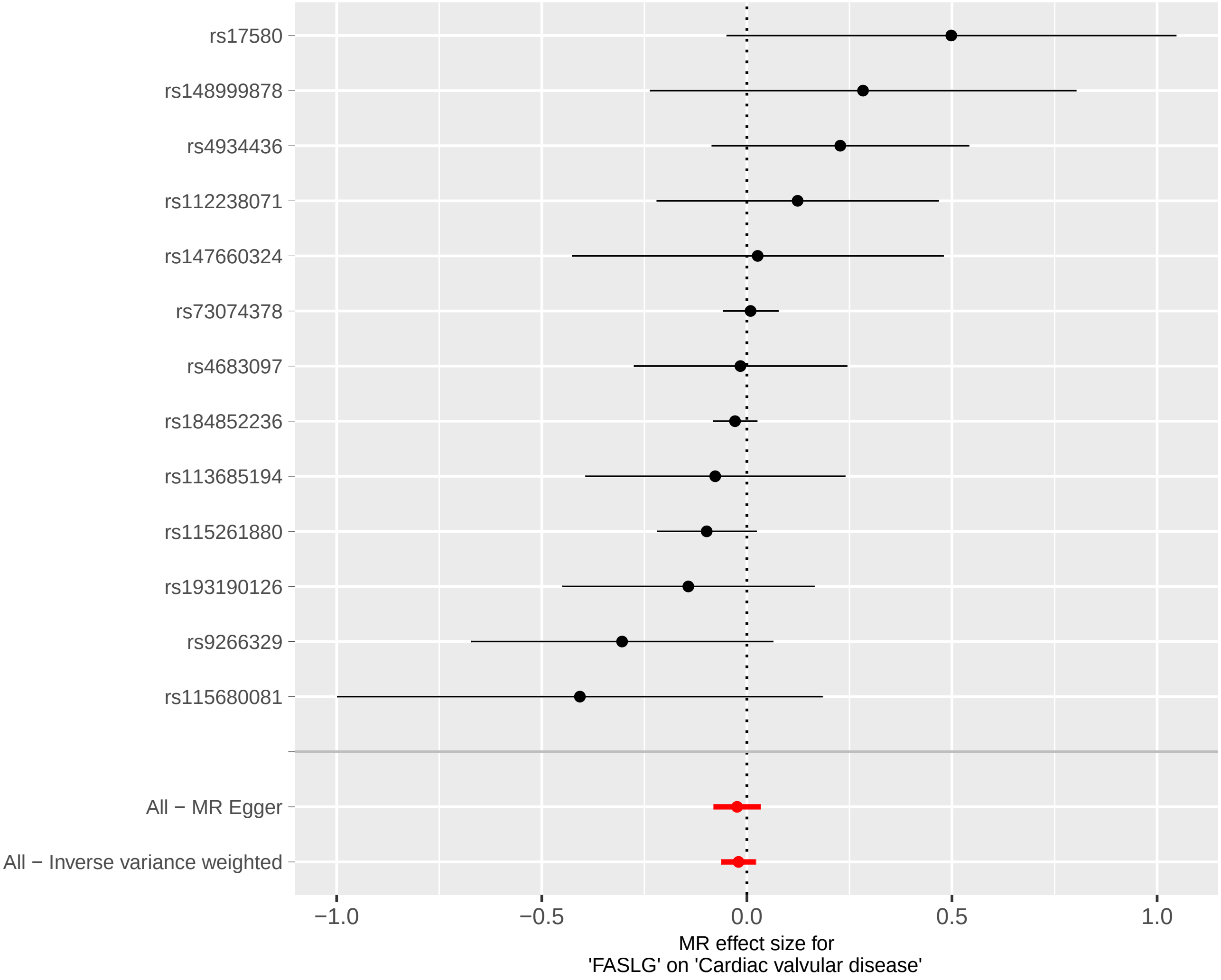

rs17580
rs148999878
rs4934436
rs112238071
rs147660324
rs73074378
rs4683097
rs184852236
rs113685194
rs115261880
rs193190126
rs9266329
rs115680081
All − MR Egger
All − Inverse variance weighted
−1.0
−0.5
0.0
0.5
1.0
MR effect size for
'FASLG' on 'Cardiac valvular disease'

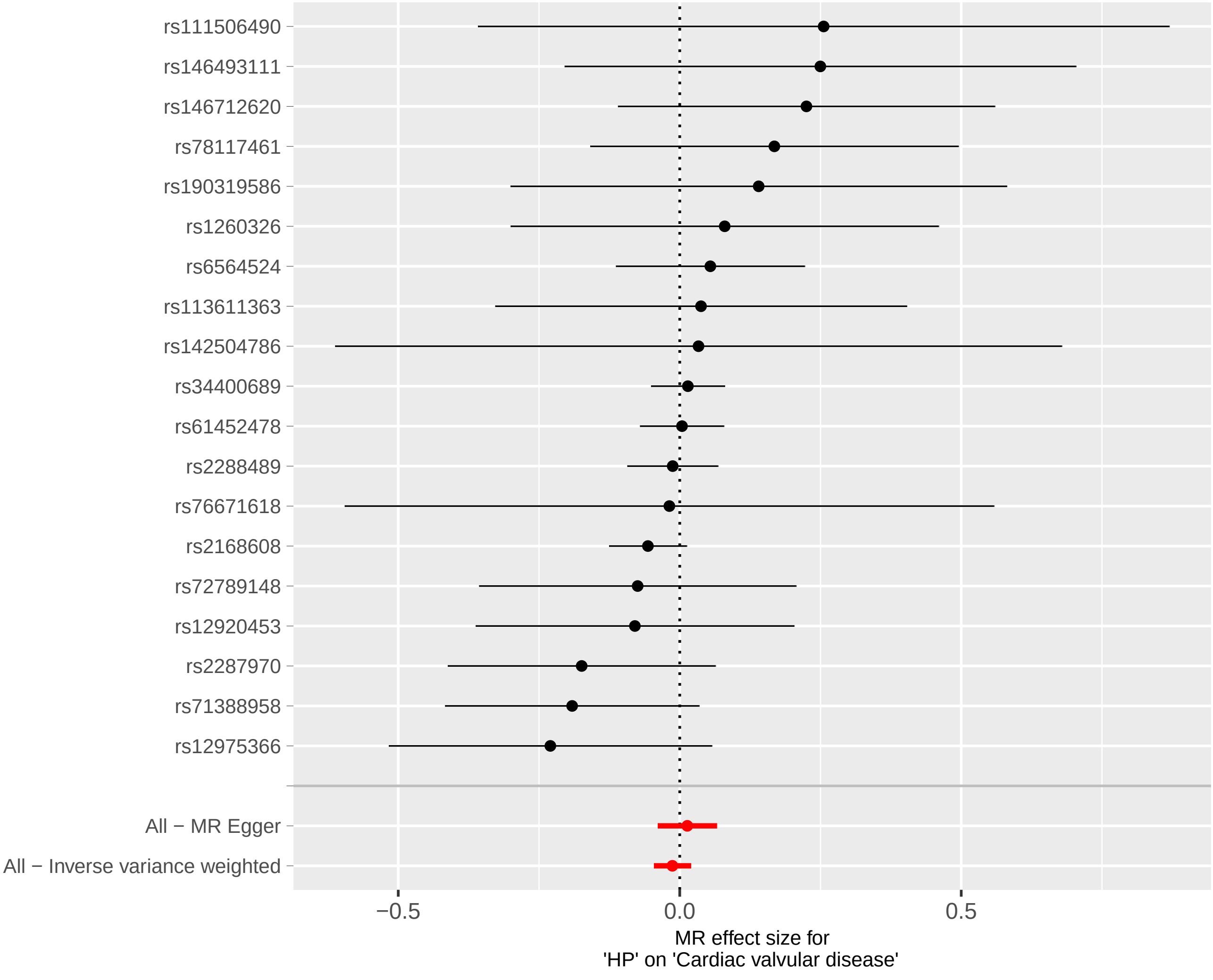
rs111506490
rs146493111
rs146712620
rs78117461
rs190319586
rs1260326
rs6564524
rs113611363
rs142504786
rs34400689
rs61452478
rs2288489
rs76671618
rs2168608
rs72789148
rs12920453
rs2287970
rs71388958
rs12975366
All − MR Egger
All − Inverse variance weighted
−0.5
0.0
0.5
MR effect size for
'HP' on 'Cardiac valvular disease'

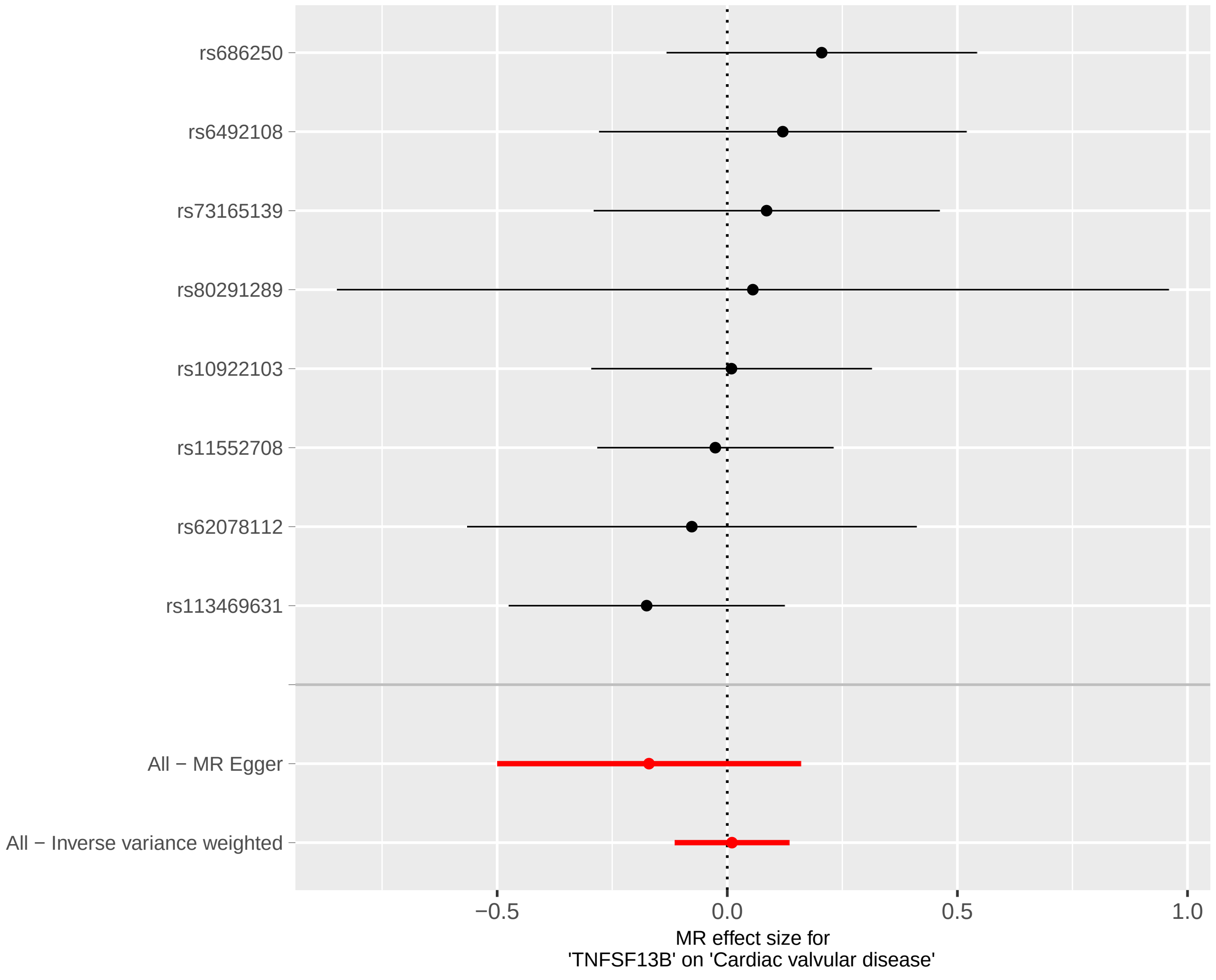

rs686250
rs6492108
rs73165139
rs80291289
rs10922103
rs11552708
rs62078112
rs113469631
All − MR Egger
All − Inverse variance weighted
−0.5
0.0
0.5
1.0
MR effect size for
'TNFSF13B' on 'Cardiac valvular disease'

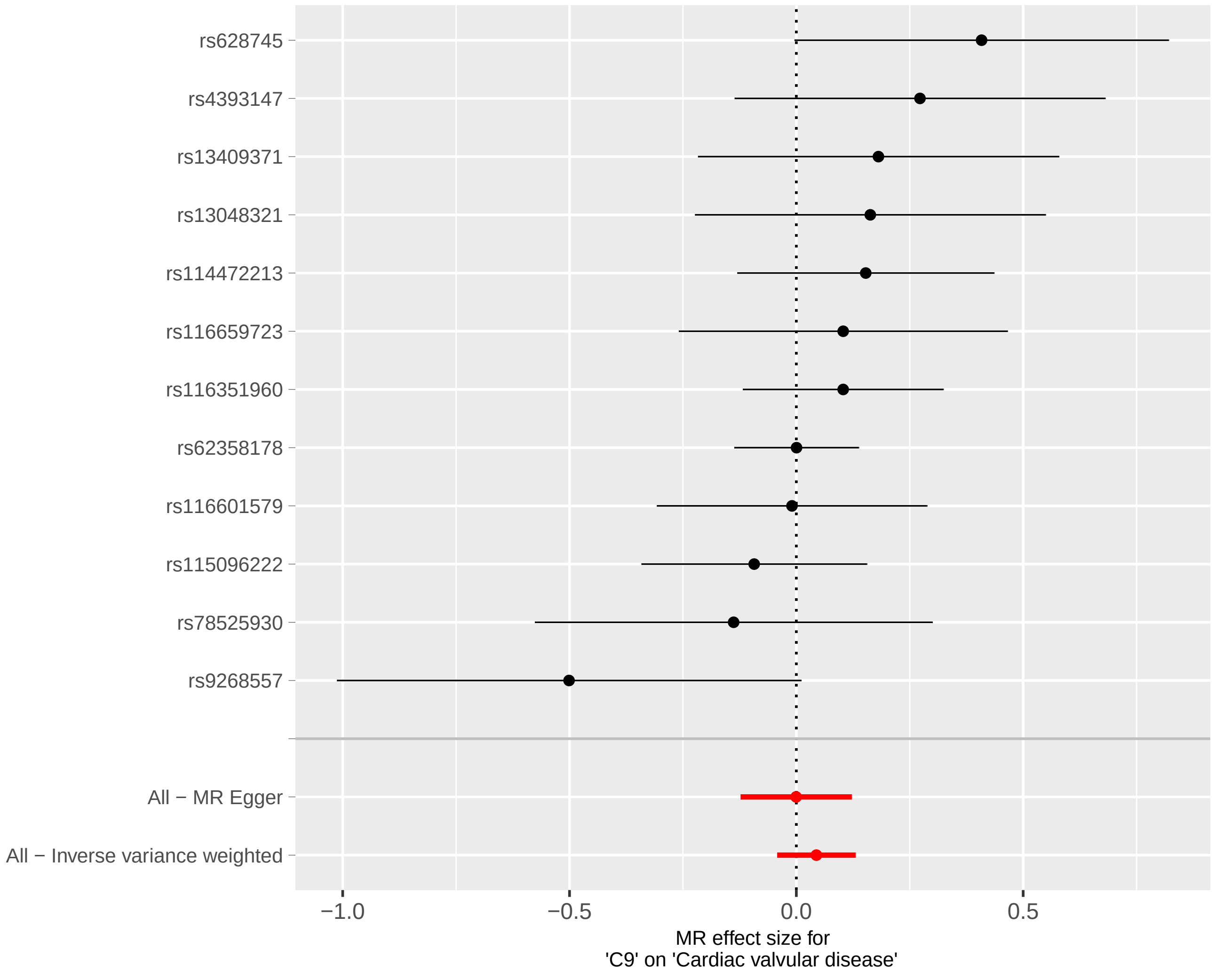
rs628745
rs4393147
rs13409371
rs13048321
rs114472213
rs116659723
rs116351960
rs62358178
rs116601579
rs115096222
rs78525930
rs9268557
All − MR Egger
All − Inverse variance weighted
−1.0
−0.5
0.0
0.5
MR effect size for
'C9' on 'Cardiac valvular disease'

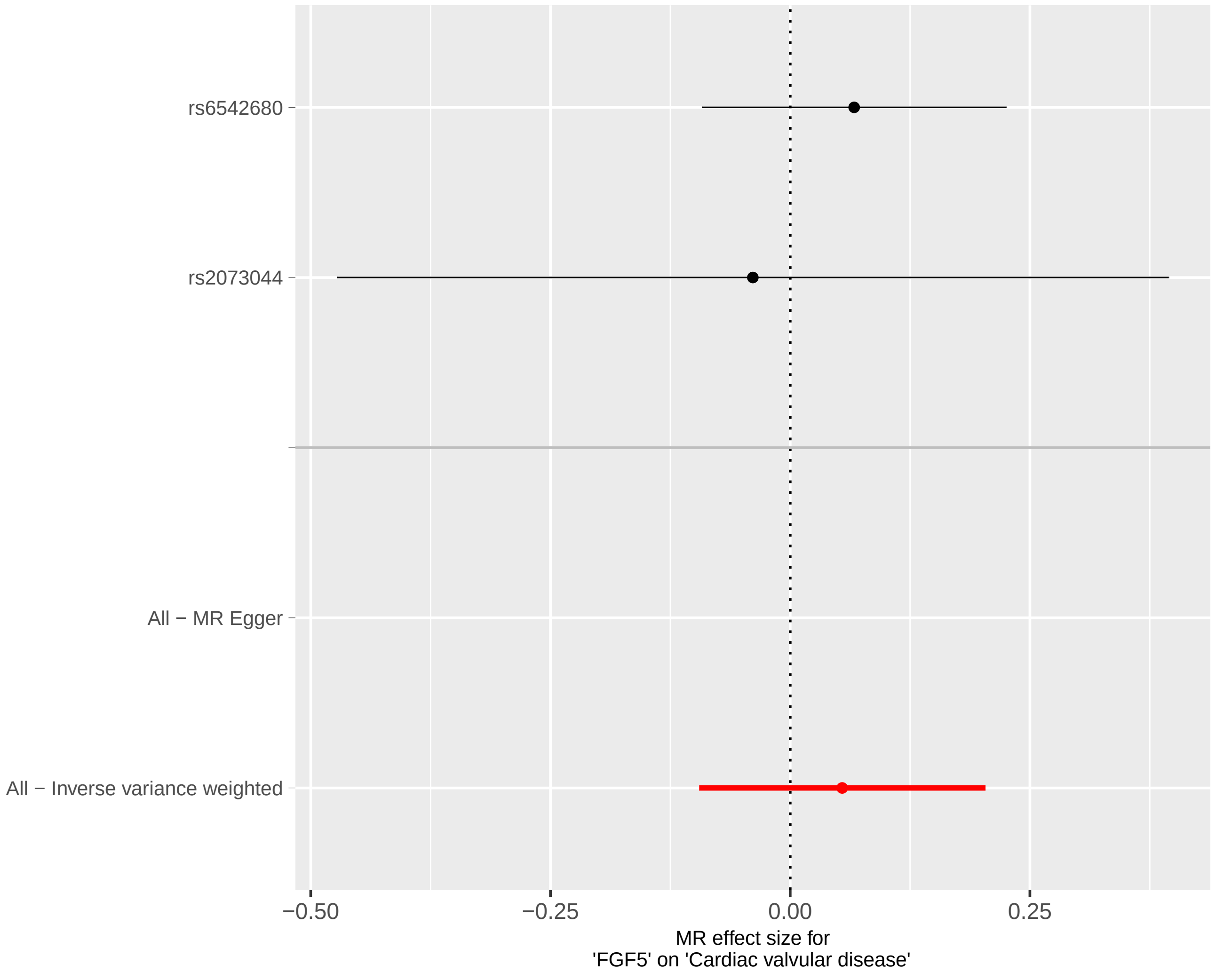
rs6542680
rs2073044
All − MR Egger
All − Inverse variance weighted
−0.50
−0.25
0.00
0.25
MR effect size for
'FGF5' on 'Cardiac valvular disease'

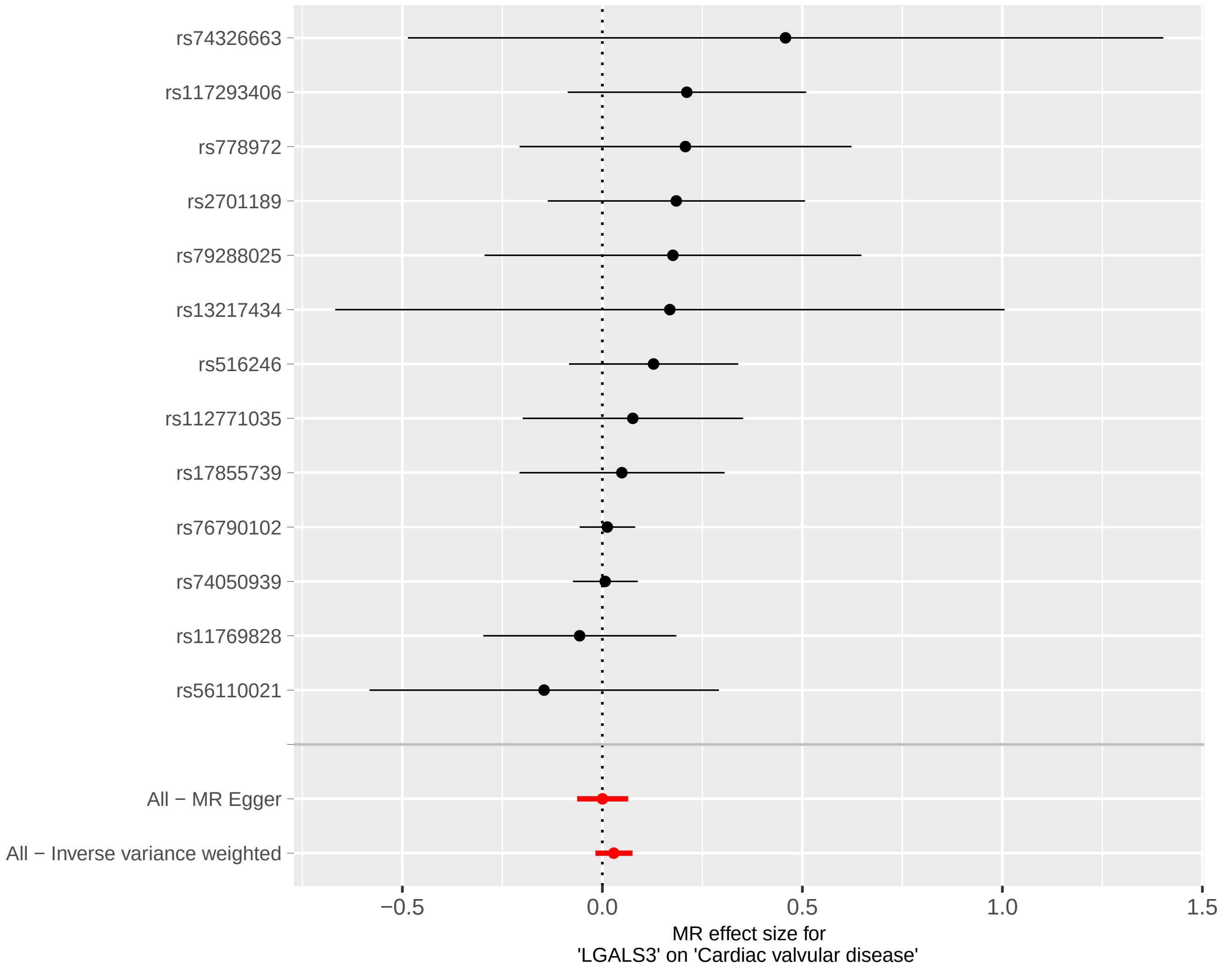
rs74326663
rs117293406
rs778972
rs2701189
rs79288025
rs13217434
rs516246
rs112771035
rs17855739
rs76790102
rs74050939
rs11769828
rs56110021
All − MR Egger
All − Inverse variance weighted
−0.5
0.0
0.5
1.0
1.5
MR effect size for
'LGALS3' on 'Cardiac valvular disease'

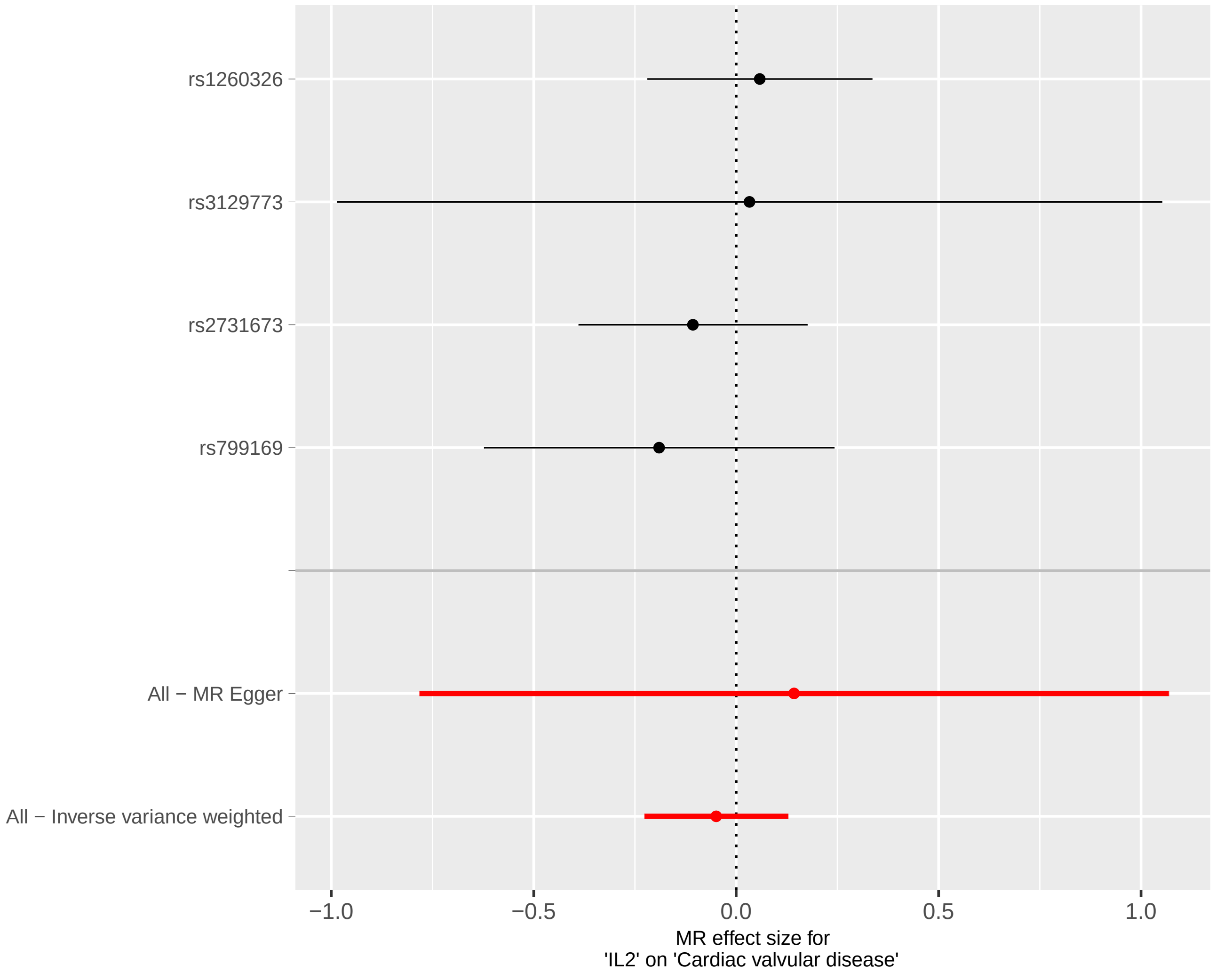

rs1260326
rs3129773
rs2731673
rs799169
All − MR Egger
All − Inverse variance weighted
−1.0
−0.5
0.0
0.5
1.0
MR effect size for
'IL2' on 'Cardiac valvular disease'

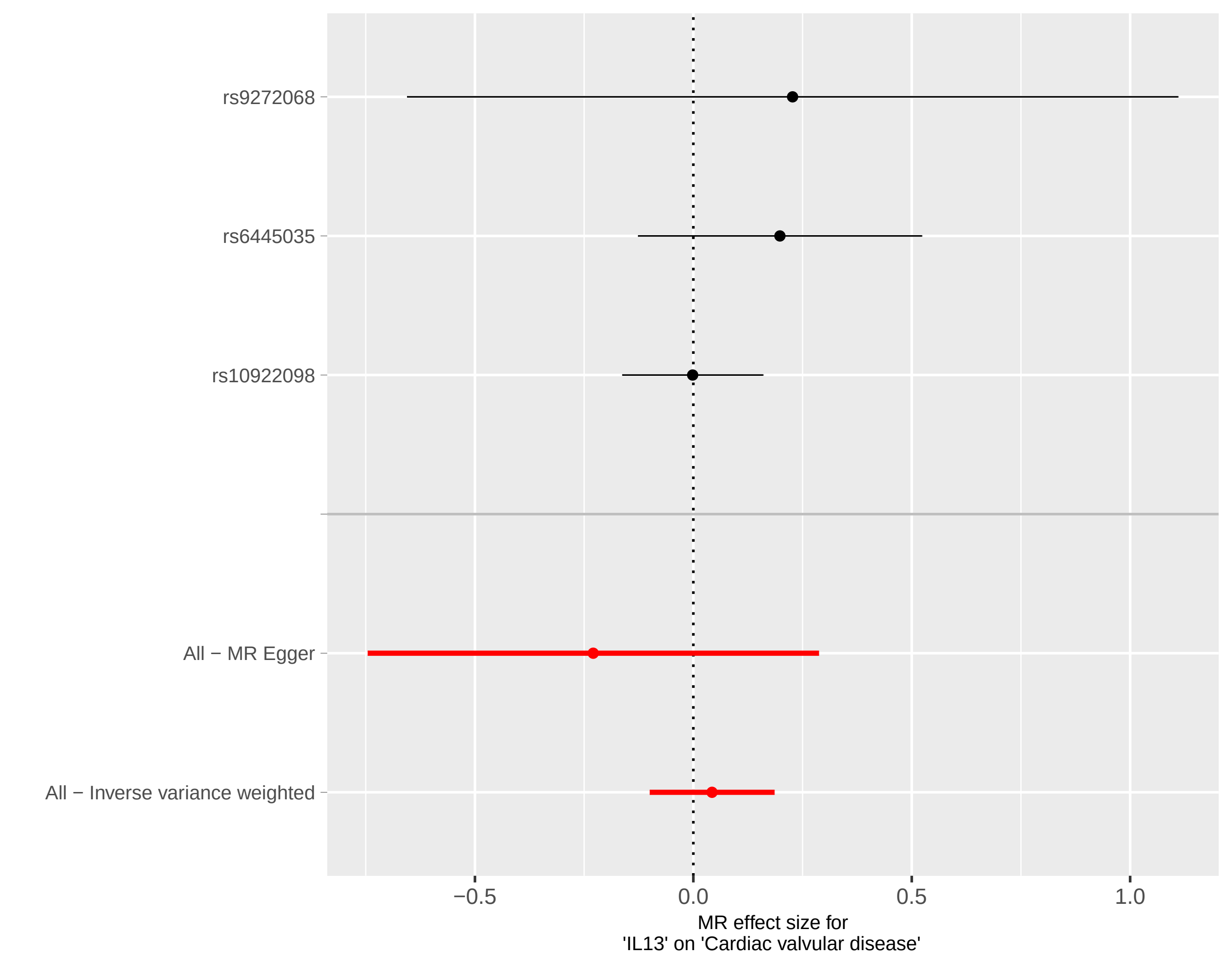
rs9272068
rs6445035
rs10922098
All − MR Egger
All − Inverse variance weighted
−0.5
0.0
0.5
1.0
MR effect size for
'IL13' on 'Cardiac valvular disease'

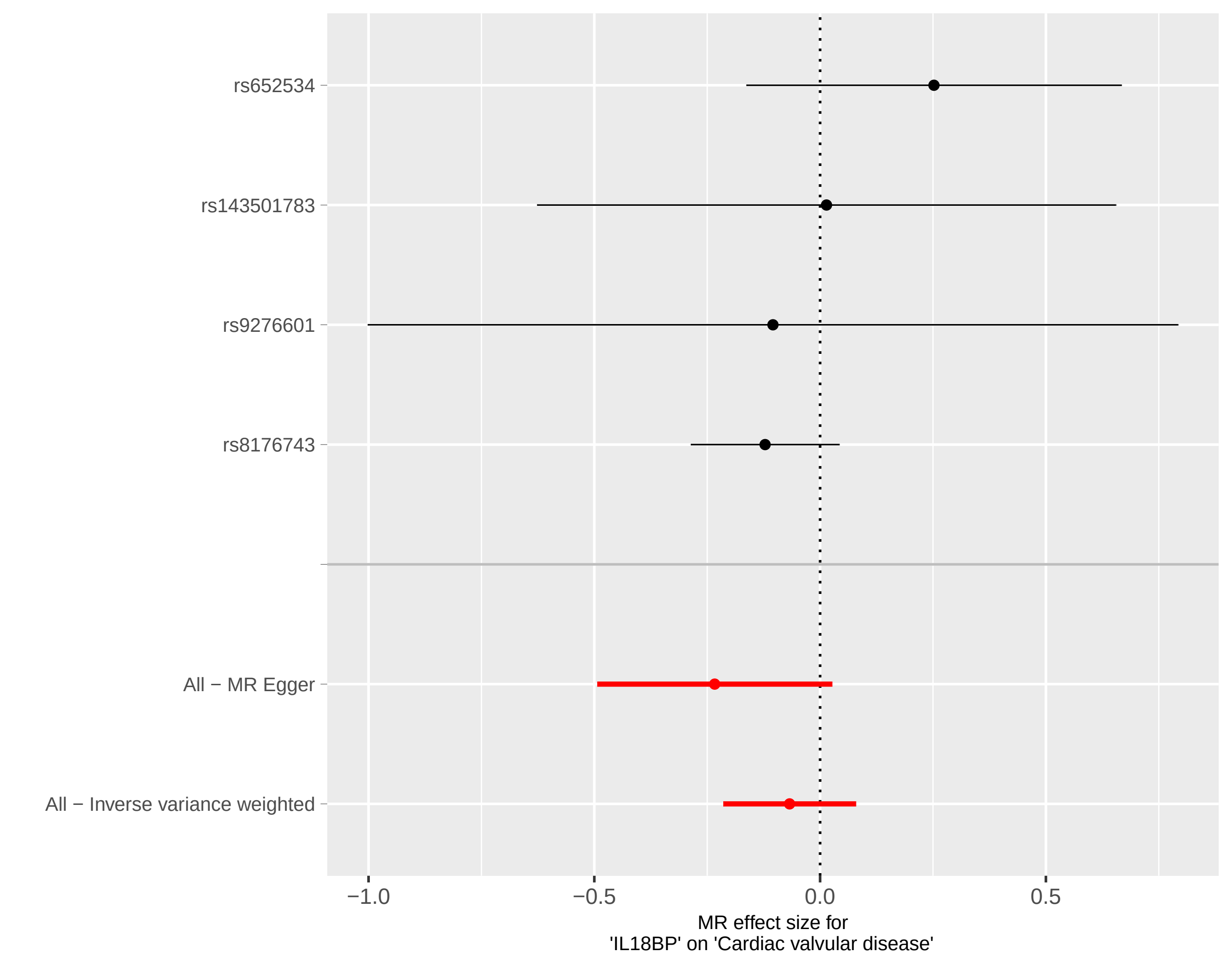
rs652534
rs143501783
rs9276601
rs8176743
All − MR Egger
All − Inverse variance weighted
−1.0
−0.5
0.0
0.5
MR effect size for
'IL18BP' on 'Cardiac valvular disease'

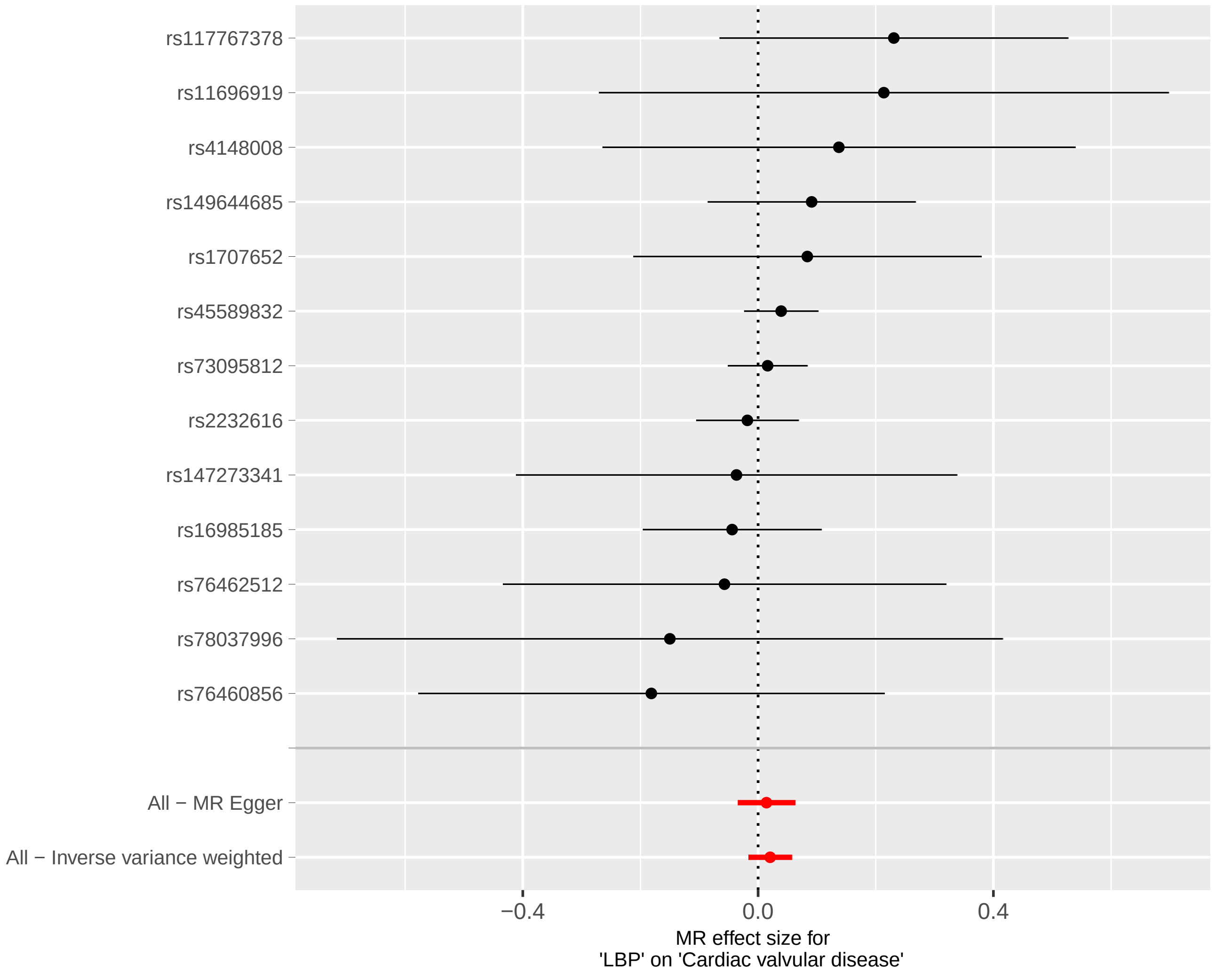
rs117767378
rs11696919
rs4148008
rs149644685
rs1707652
rs45589832
rs73095812
rs2232616
rs147273341
rs16985185
rs76462512
rs78037996
rs76460856
All − MR Egger
All − Inverse variance weighted
−0.4
0.0
0.4
MR effect size for
'LBP' on 'Cardiac valvular disease'

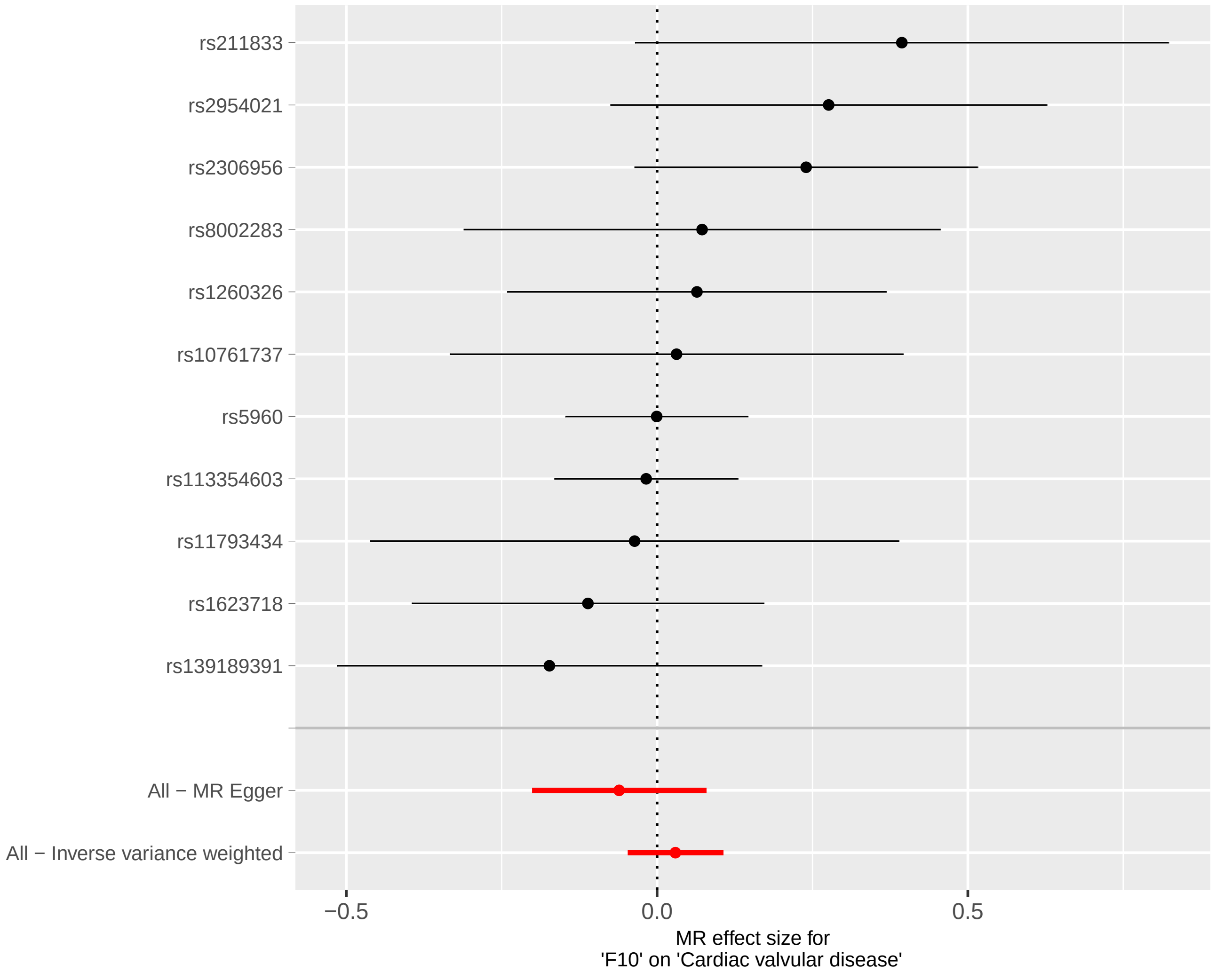
rs211833
rs2954021
rs2306956
rs8002283
rs1260326
rs10761737
rs5960
rs113354603
rs11793434
rs1623718
rs139189391
All − MR Egger
All − Inverse variance weighted
−0.5
0.0
0.5
MR effect size for
'F10' on 'Cardiac valvular disease'

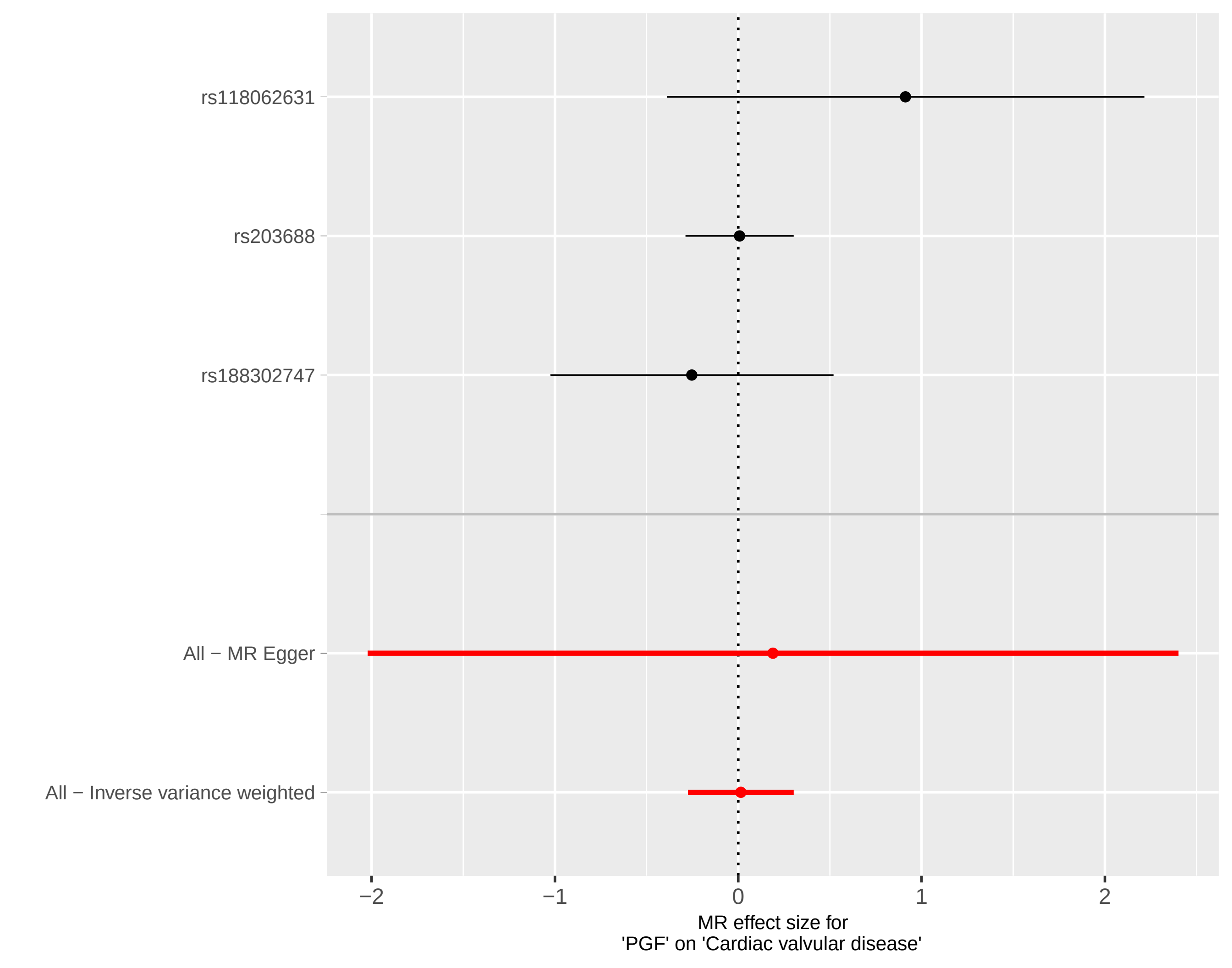
rs118062631
rs203688
rs188302747
All − MR Egger
All − Inverse variance weighted
−2
−1
0
1
2
MR effect size for
'PGF' on 'Cardiac valvular disease'

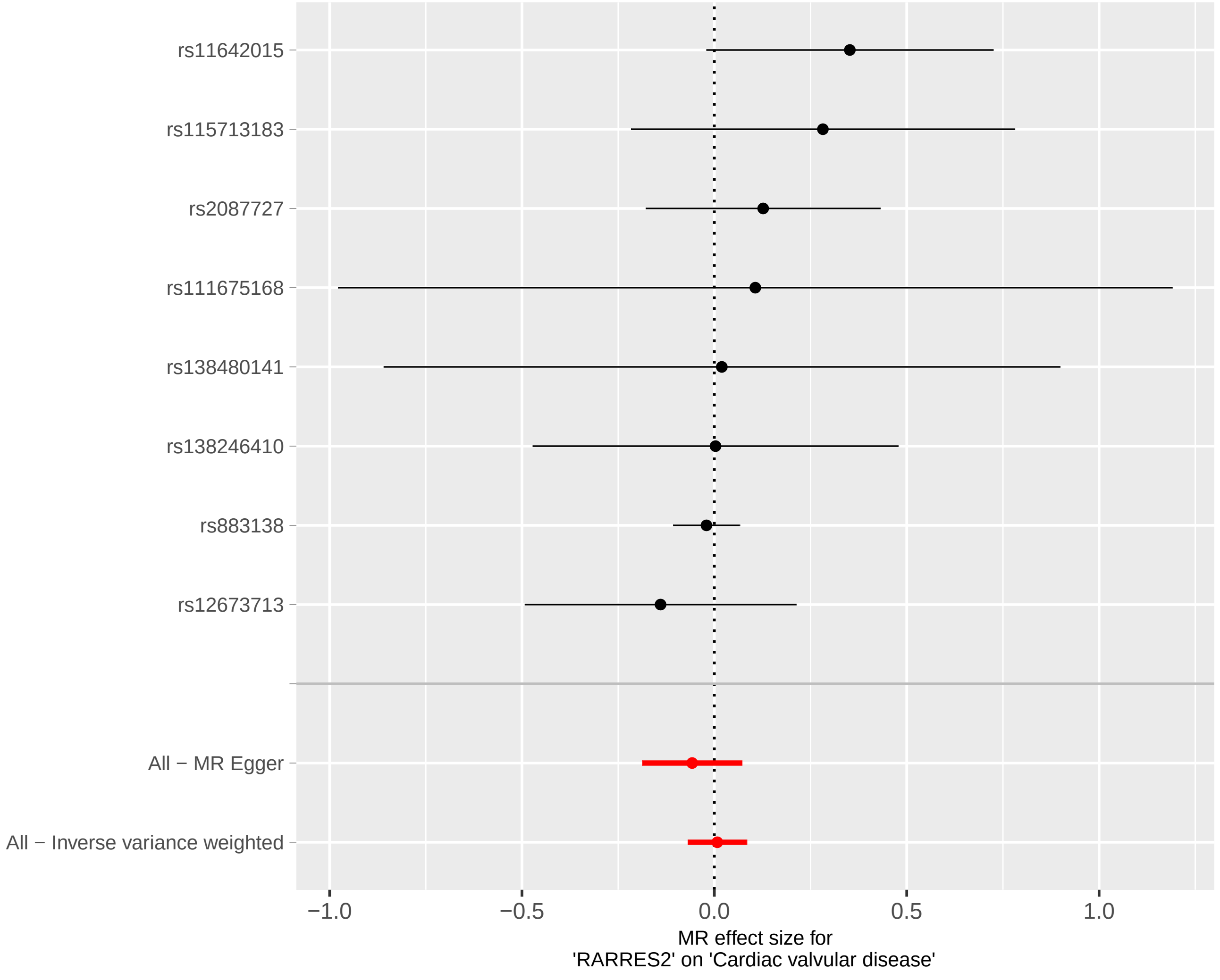

rs11642015
rs115713183
rs2087727
rs111675168
rs138480141
rs138246410
rs883138
rs12673713
All − MR Egger
All − Inverse variance weighted
−1.0
−0.5
0.0
0.5
1.0
MR effect size for
'RARRES2' on 'Cardiac valvular disease'

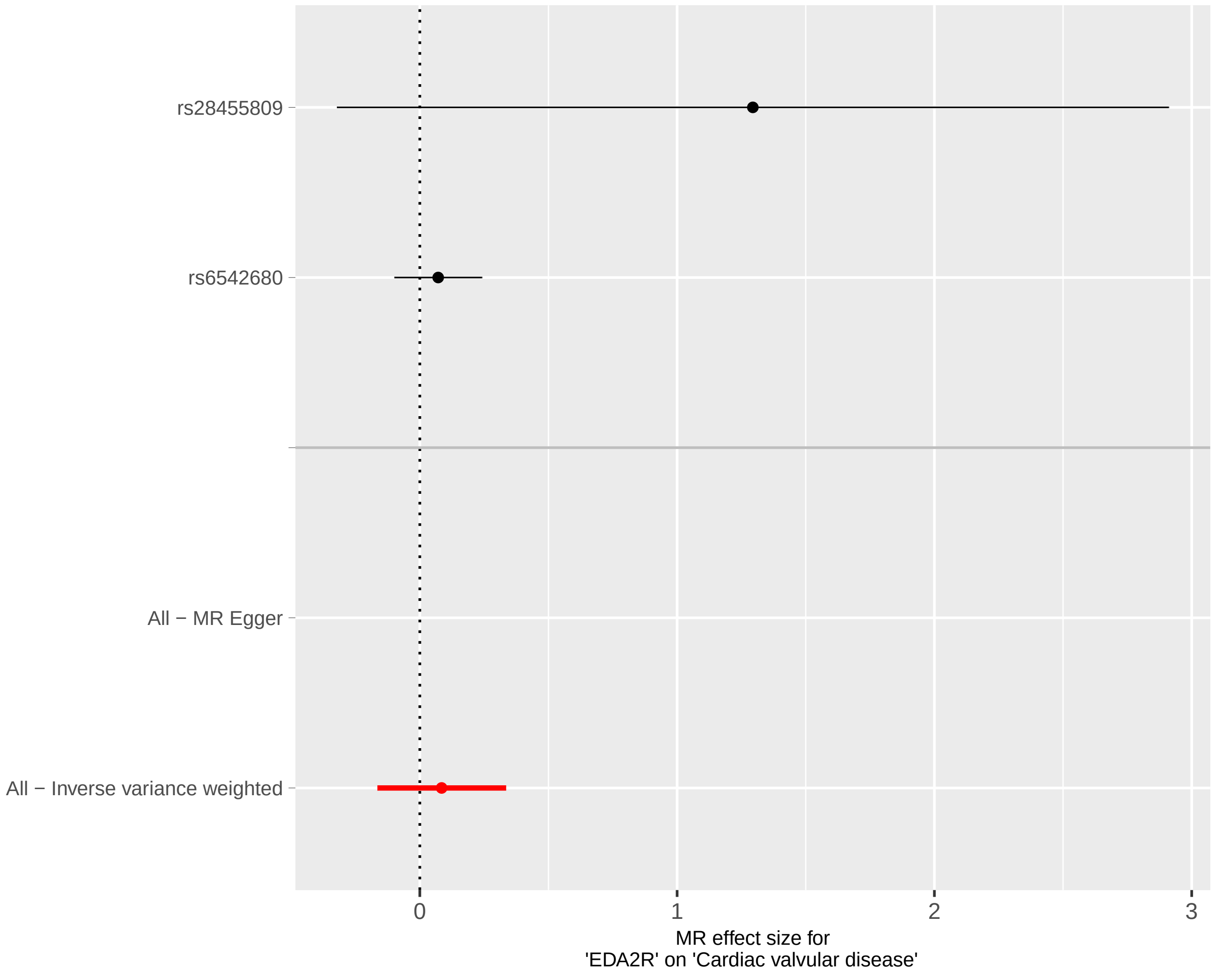

rs28455809
rs6542680
All − MR Egger
All − Inverse variance weighted
0
1
2
3
MR effect size for
'EDA2R' on 'Cardiac valvular disease'

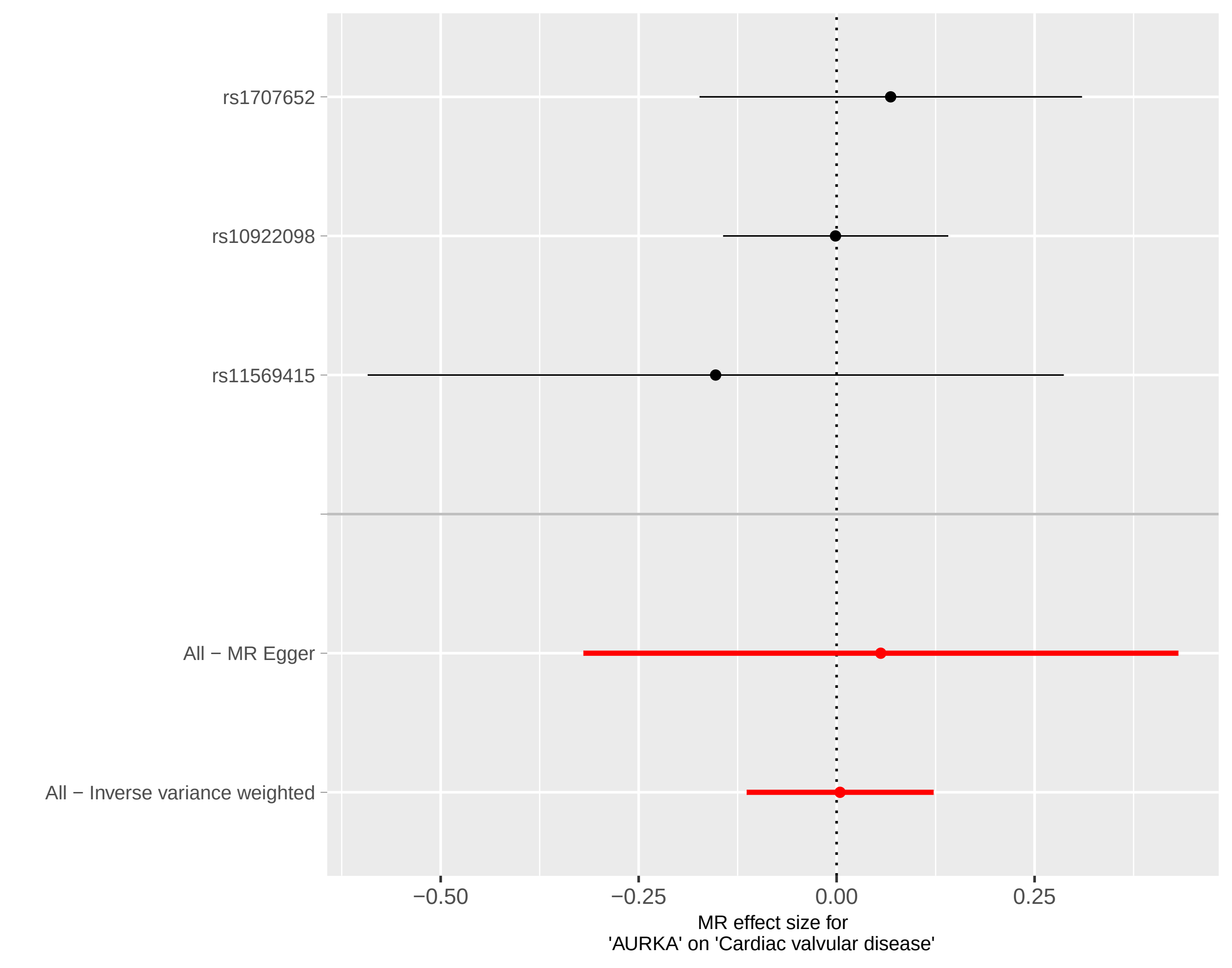
rs1707652
rs10922098
rs11569415
All − MR Egger
All − Inverse variance weighted
−0.50
−0.25
0.00
0.25
MR effect size for
'AURKA' on 'Cardiac valvular disease'

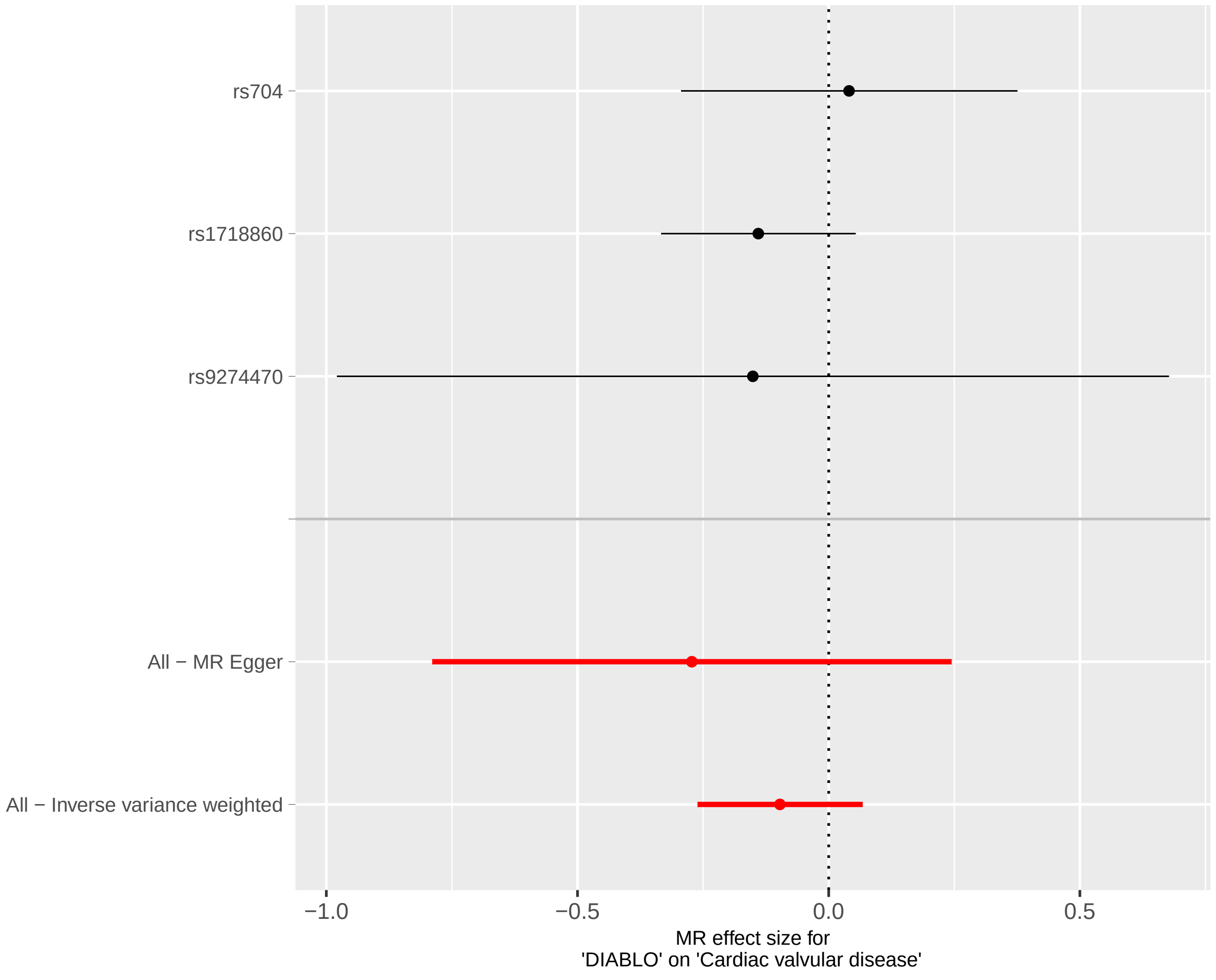
rs704
rs1718860
rs9274470
All − MR Egger
All − Inverse variance weighted
−1.0
−0.5
0.0
0.5
MR effect size for
'DIABLO' on 'Cardiac valvular disease'

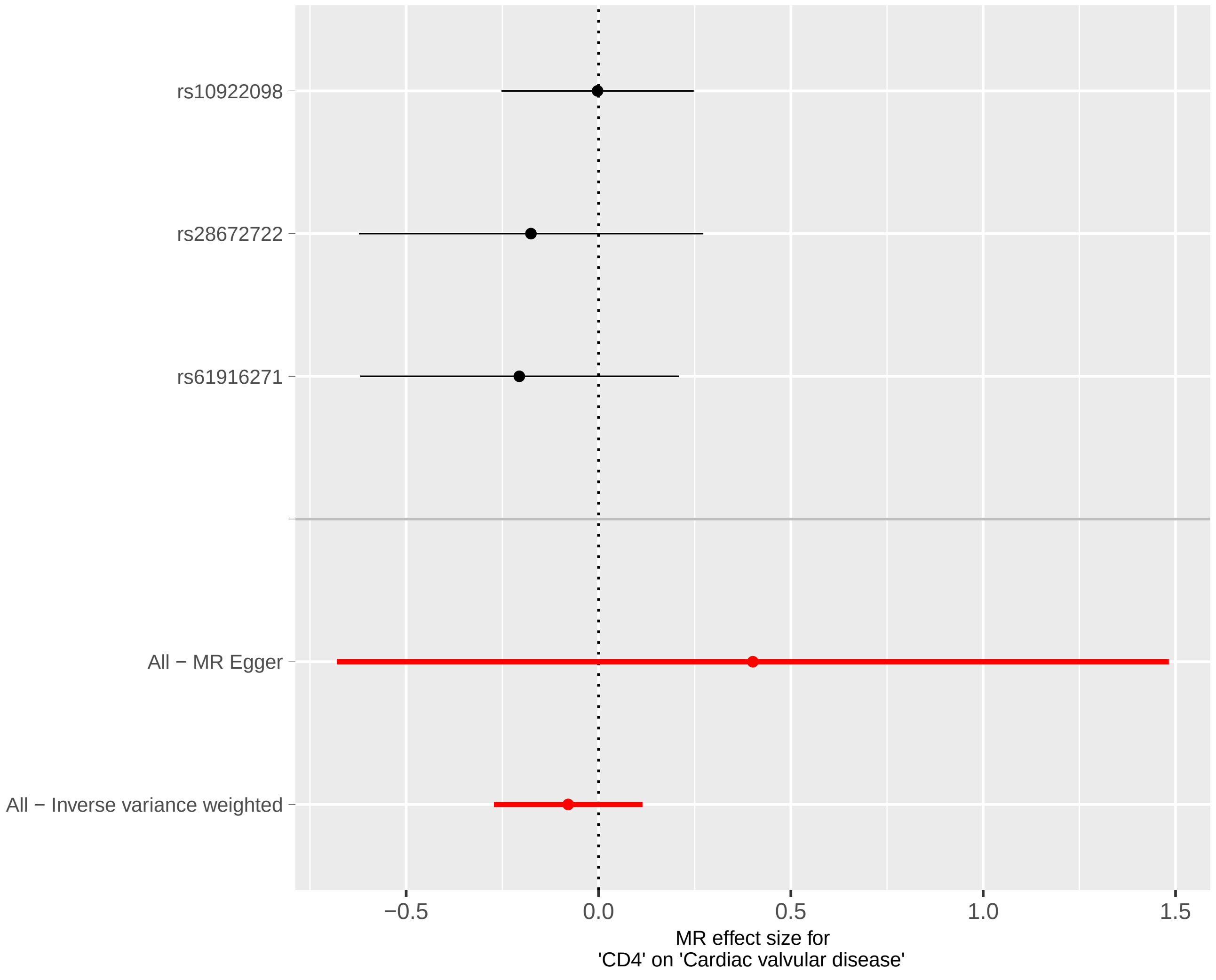
rs10922098
rs28672722
rs61916271
All − MR Egger
All − Inverse variance weighted
−0.5
0.0
0.5
1.0
1.5
MR effect size for
'CD4' on 'Cardiac valvular disease'

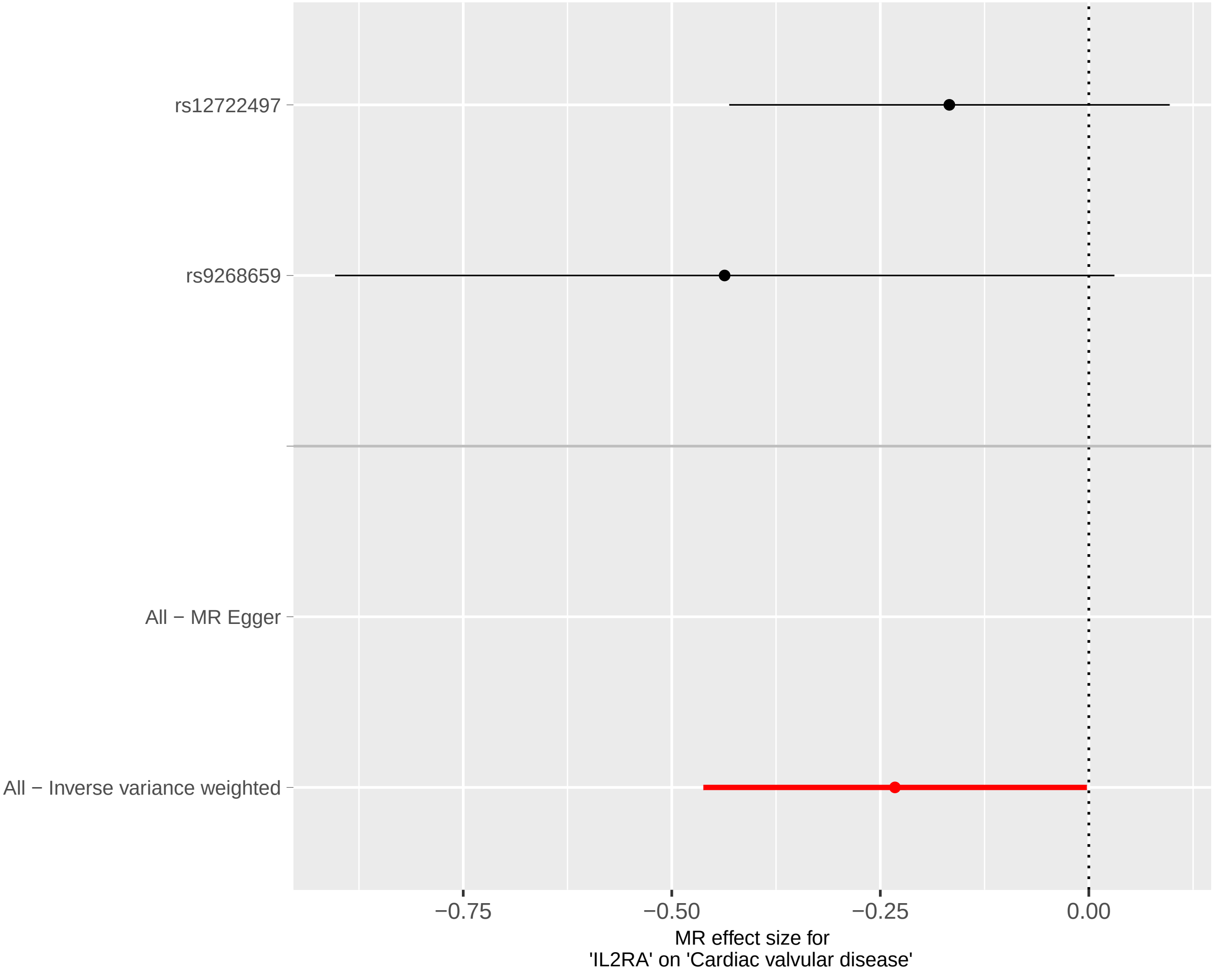
rs12722497
rs9268659
All − MR Egger
All − Inverse variance weighted
−0.75
−0.50
−0.25
0.00
MR effect size for
'IL2RA' on 'Cardiac valvular disease'

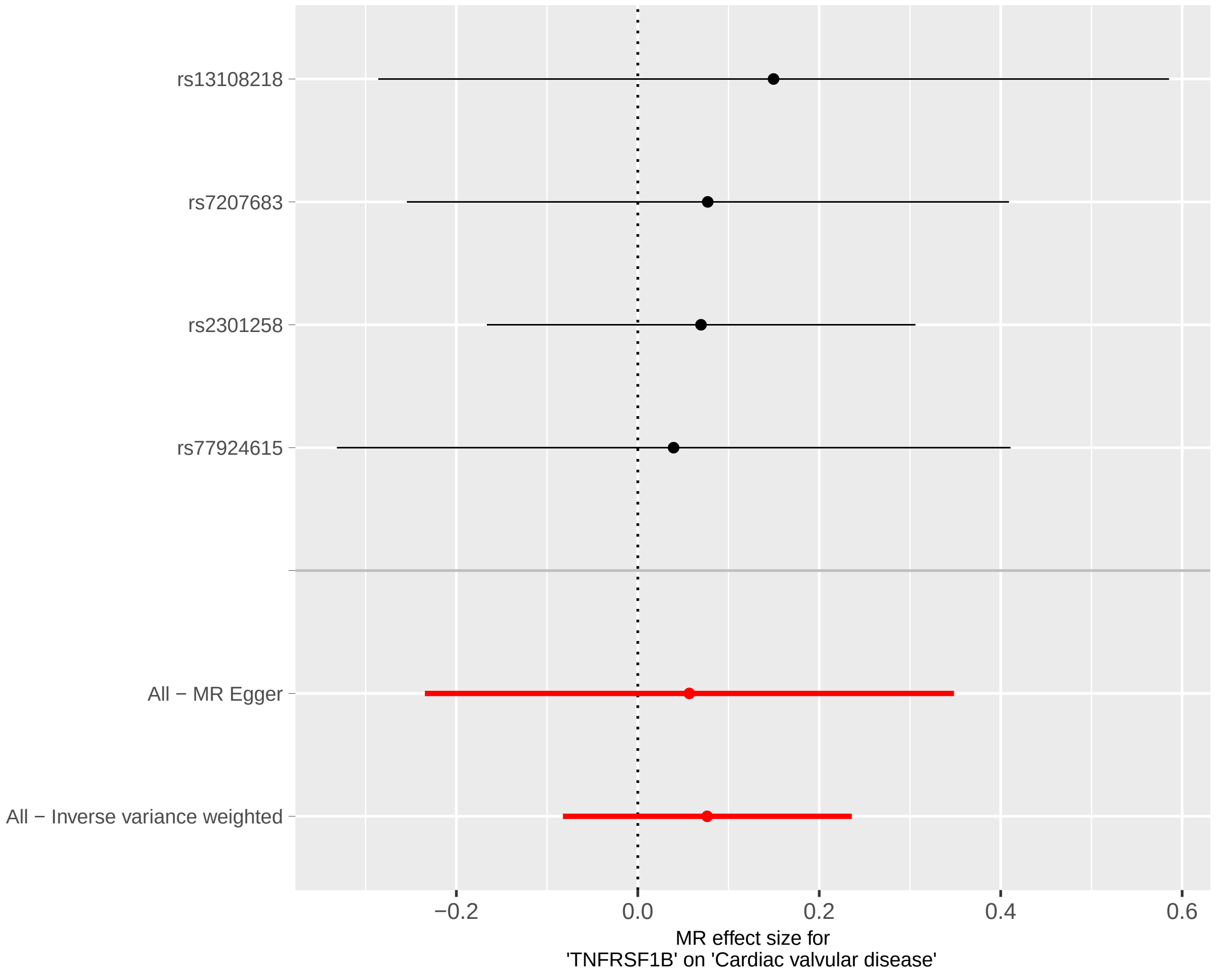

rs13108218
rs7207683
rs2301258
rs77924615
All − MR Egger
All − Inverse variance weighted
−0.2
0.0
0.2
0.4
0.6
MR effect size for
'TNFRSF1B' on 'Cardiac valvular disease'

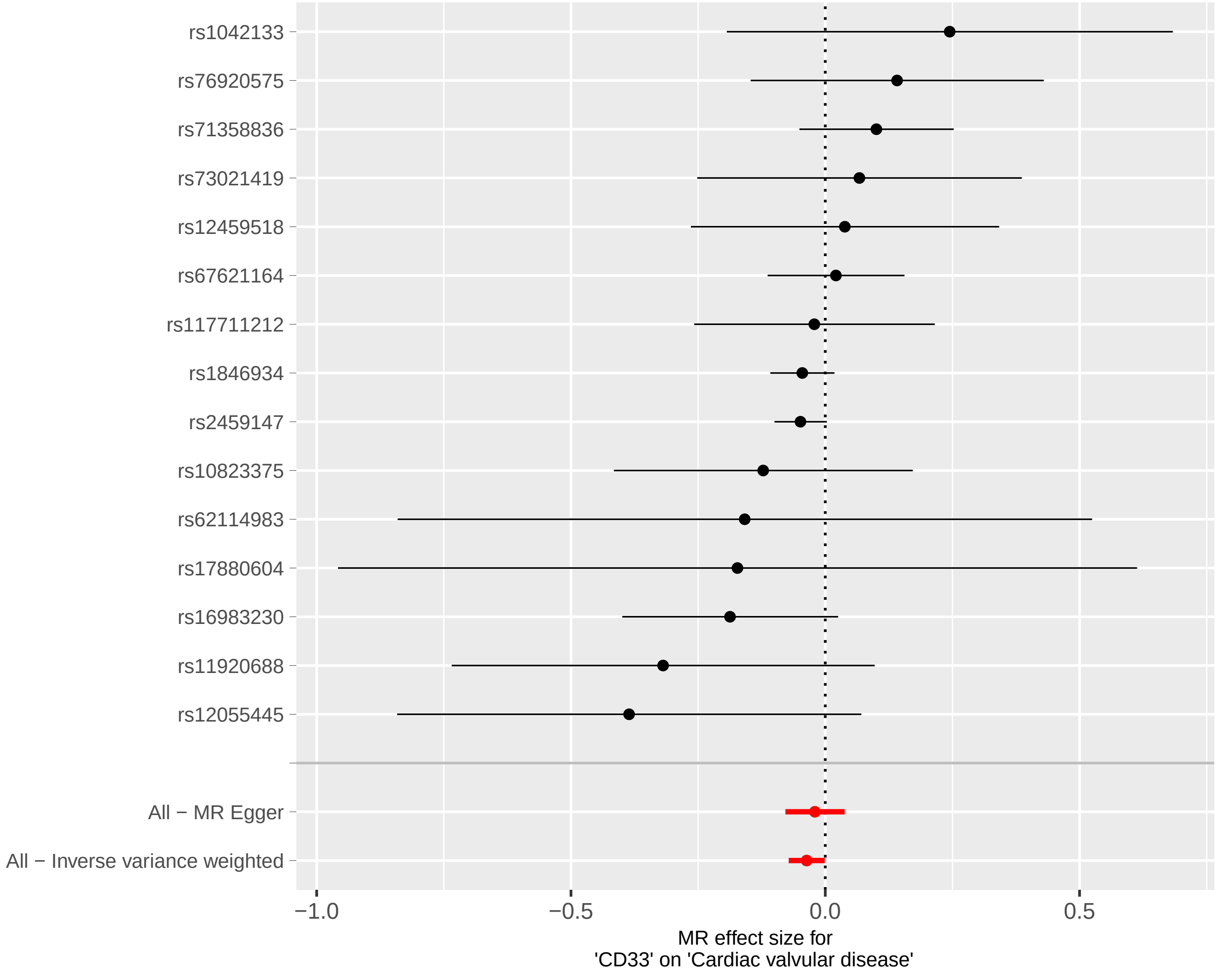
rs1042133
rs76920575
rs71358836
rs73021419
rs12459518
rs67621164
rs117711212
rs1846934
rs2459147
rs10823375
rs62114983
rs17880604
rs16983230
rs11920688
rs12055445
All − MR Egger
All − Inverse variance weighted
−1.0
−0.5
0.0
0.5
MR effect size for
'CD33' on 'Cardiac valvular disease'

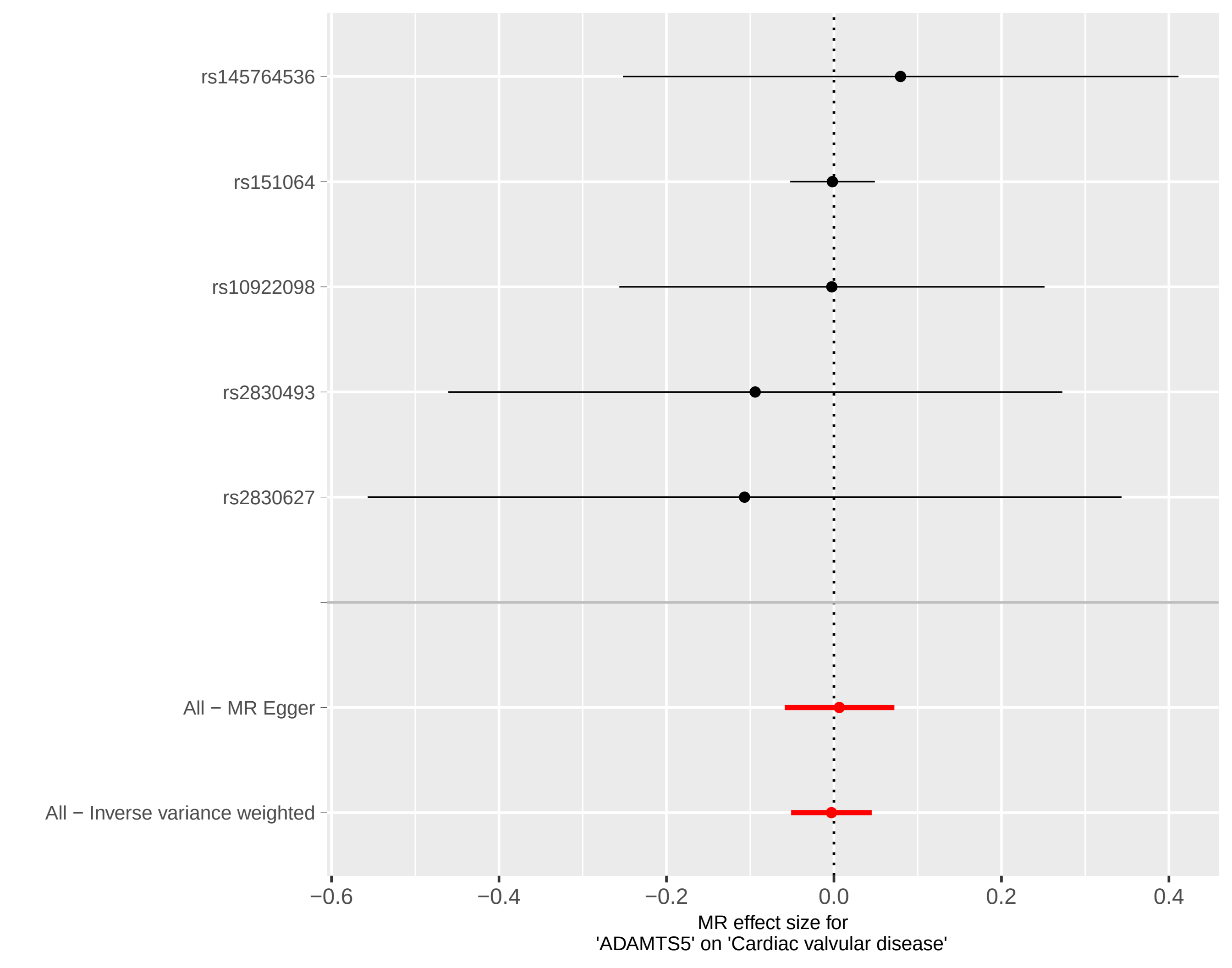
rs145764536
rs151064
rs10922098
rs2830493
rs2830627
All − MR Egger
All − Inverse variance weighted
−0.6
−0.4
−0.2
0.0
0.2
0.4
MR effect size for
'ADAMTS5' on 'Cardiac valvular disease'

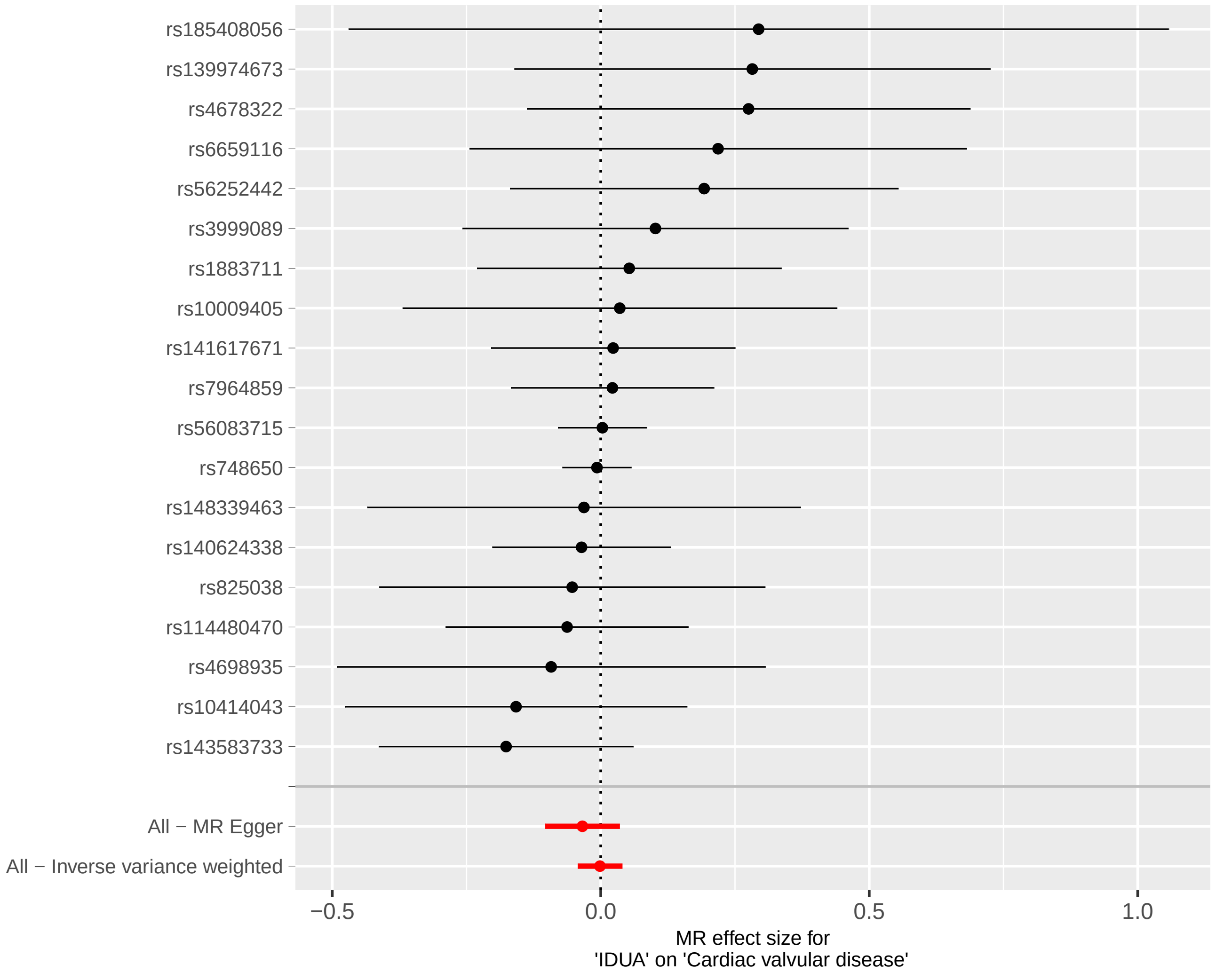

rs185408056
rs139974673
rs4678322
rs6659116
rs56252442
rs3999089
rs1883711
rs10009405
rs141617671
rs7964859
rs56083715
rs748650
rs148339463
rs140624338
rs825038
rs114480470
rs4698935
rs10414043
rs143583733
All − MR Egger
All − Inverse variance weighted
−0.5
0.0
0.5
1.0
MR effect size for
'IDUA' on 'Cardiac valvular disease'

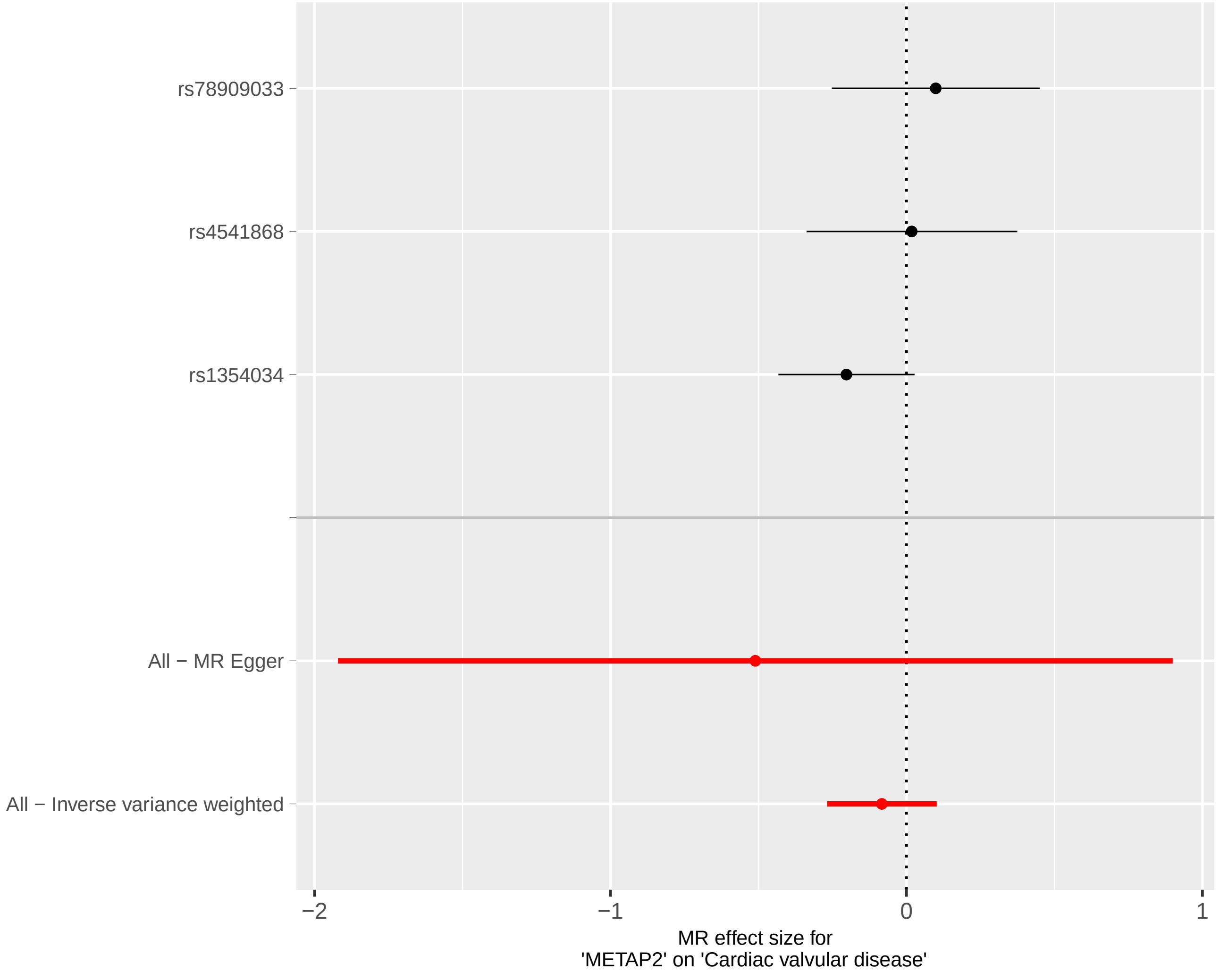
rs78909033
rs4541868
rs1354034
All − MR Egger
All − Inverse variance weighted
−2
−1
0
1
MR effect size for
'METAP2' on 'Cardiac valvular disease'

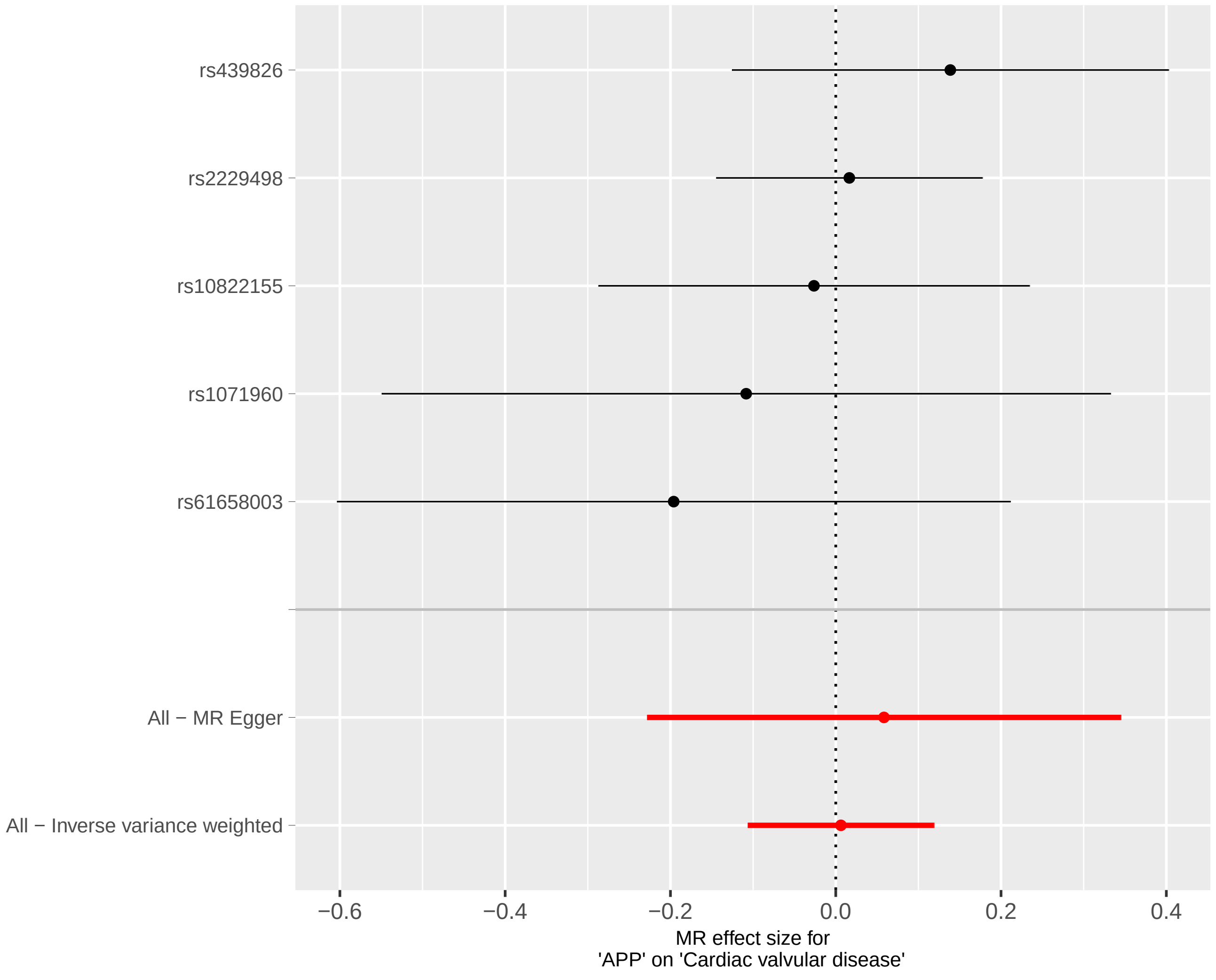

rs439826
rs2229498
rs10822155
rs1071960
rs61658003
All − MR Egger
All − Inverse variance weighted
−0.6
−0.4
−0.2
0.0
0.2
0.4
MR effect size for
'APP' on 'Cardiac valvular disease'

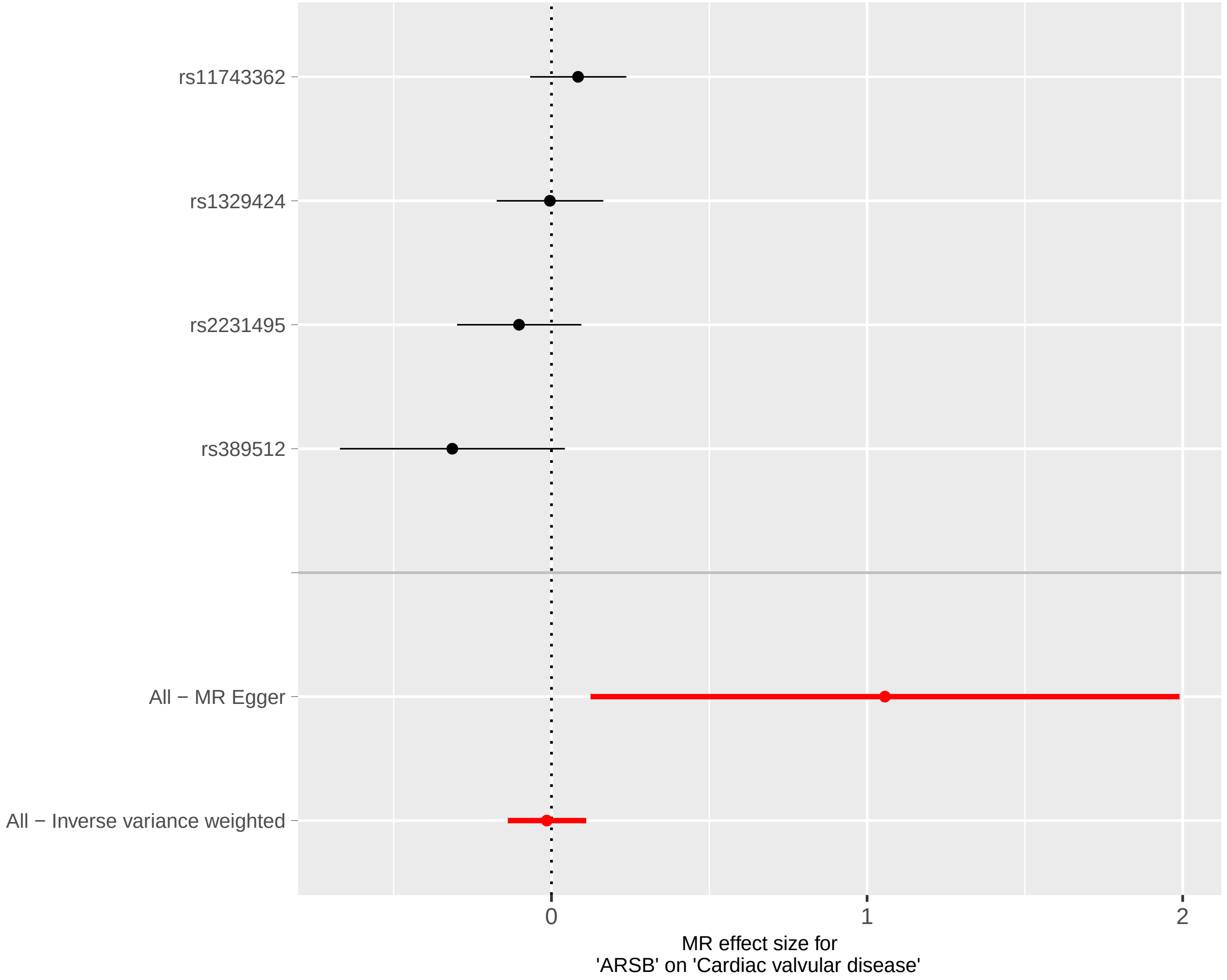

rs11743362
rs1329424
rs2231495
rs389512
All − MR Egger
All − Inverse variance weighted
0
1
2
MR effect size for
'ARSB' on 'Cardiac valvular disease'

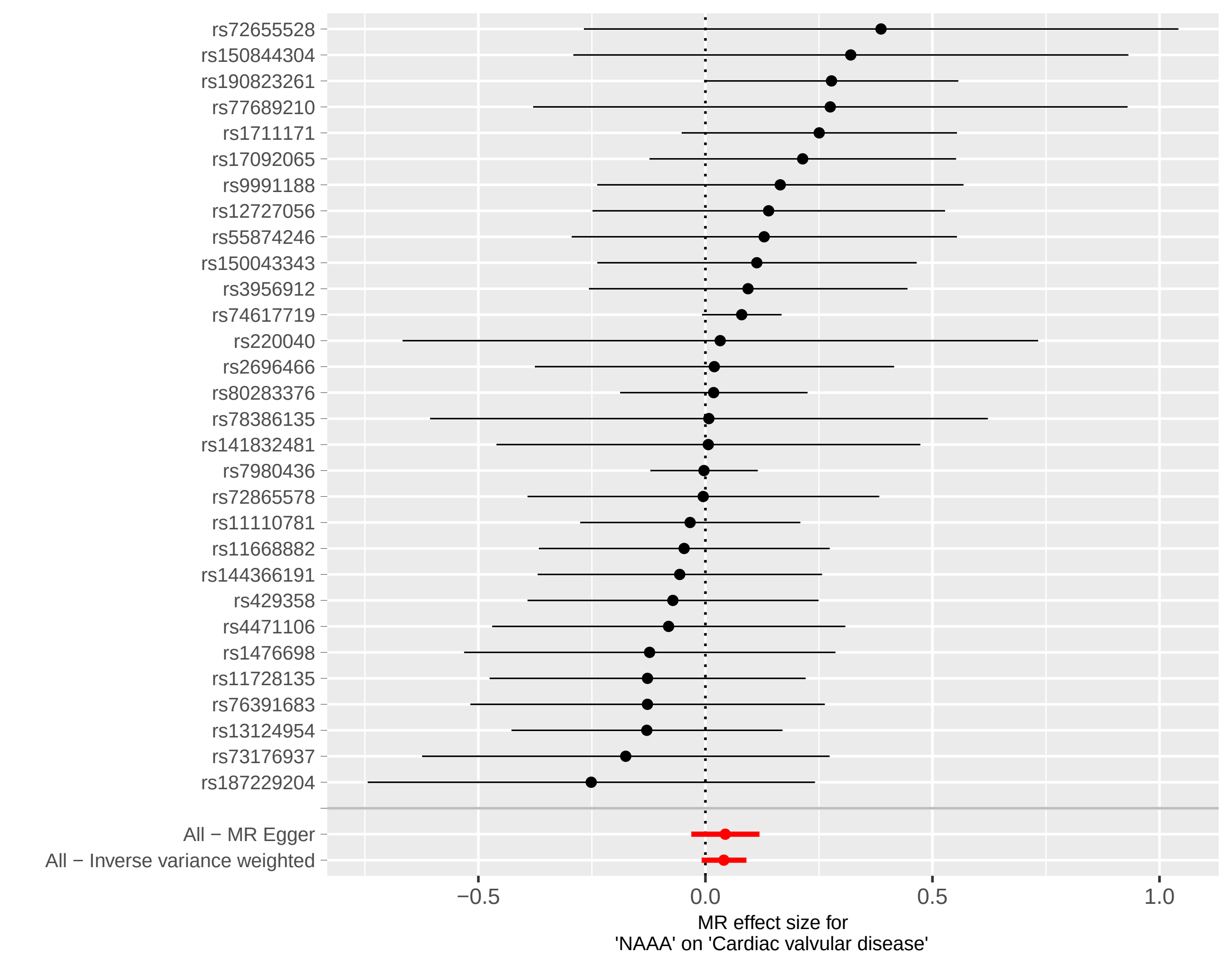
rs72655528
rs150844304
rs190823261
rs77689210
rs1711171
rs17092065
rs9991188
rs12727056
rs55874246
rs150043343
rs3956912
rs74617719
rs220040
rs2696466
rs80283376
rs78386135
rs141832481
rs7980436
rs72865578
rs11110781
rs11668882
rs144366191
rs429358
rs4471106
rs1476698
rs11728135
rs76391683
rs13124954
rs73176937
rs187229204
All − MR Egger
All − Inverse variance weighted
−0.5
0.0
0.5
1.0
MR effect size for
'NAAA' on 'Cardiac valvular disease'

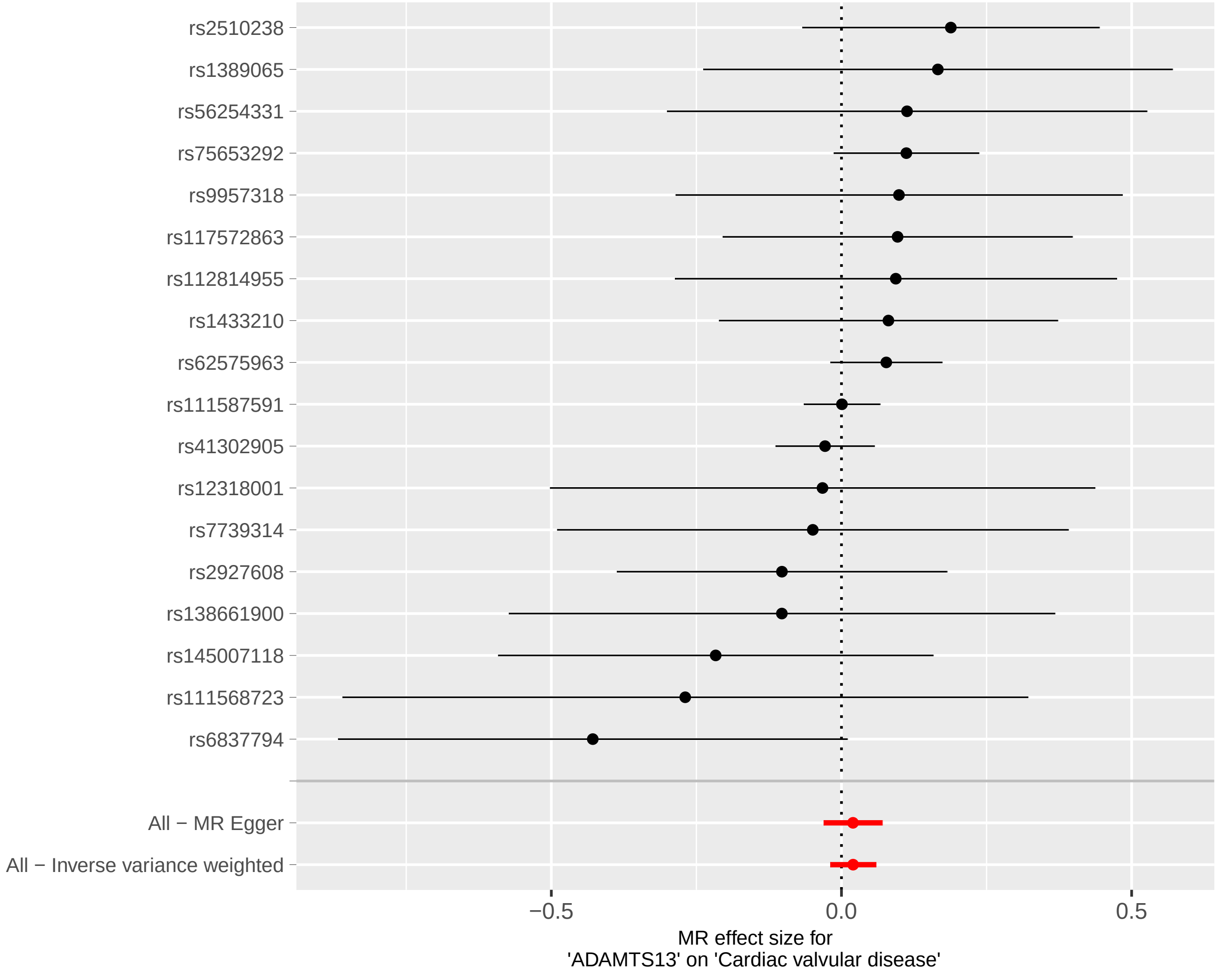

rs2510238
rs1389065
rs56254331
rs75653292
rs9957318
rs117572863
rs112814955
rs1433210
rs62575963
rs111587591
rs41302905
rs12318001
rs7739314
rs2927608
rs138661900
rs145007118
rs111568723
rs6837794
All − MR Egger
All − Inverse variance weighted
−0.5
0.0
0.5
MR effect size for
'ADAMTS13' on 'Cardiac valvular disease'

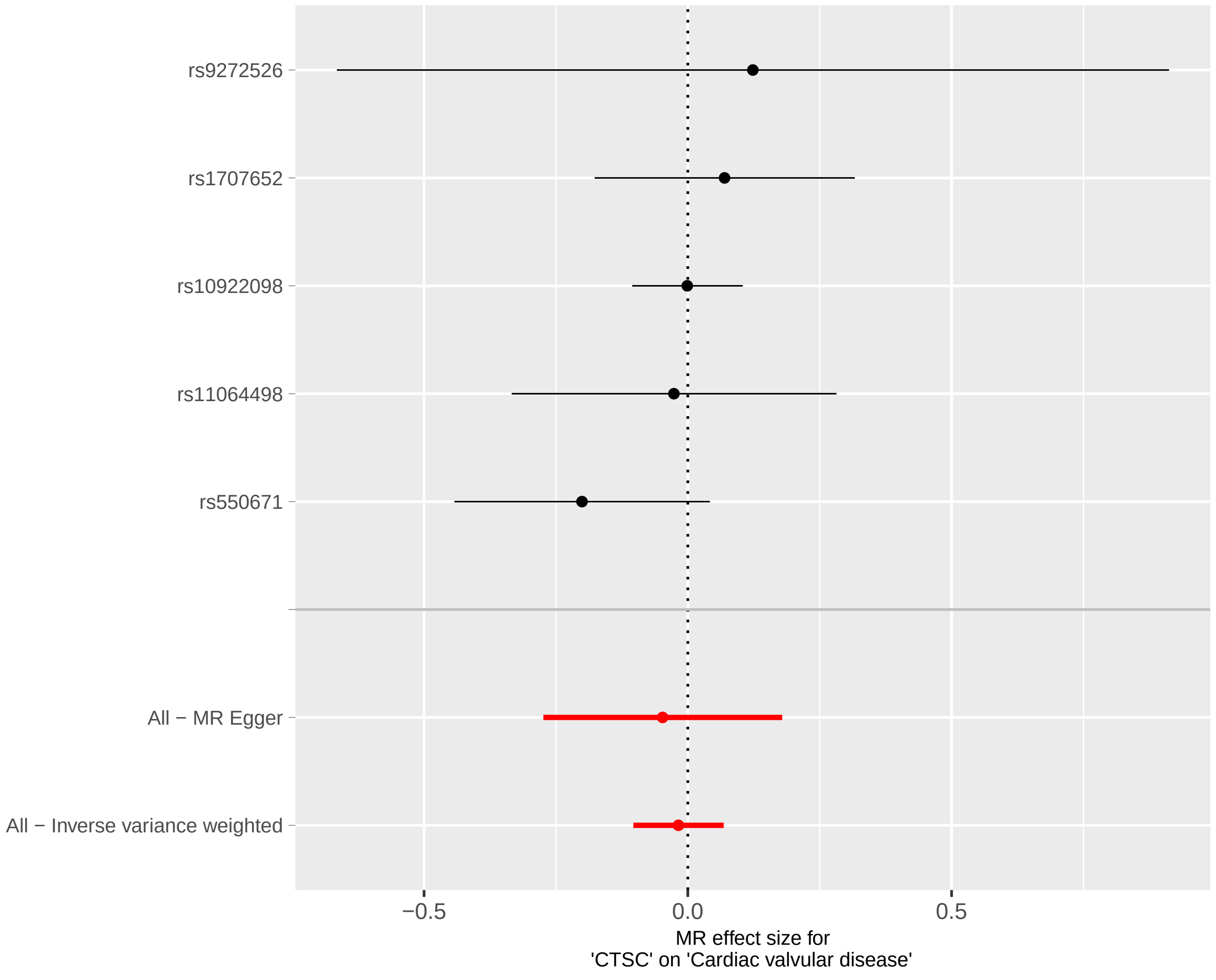
rs9272526
rs1707652
rs10922098
rs11064498
rs550671
All − MR Egger
All − Inverse variance weighted
−0.5
0.0
0.5
MR effect size for
'CTSC' on 'Cardiac valvular disease'

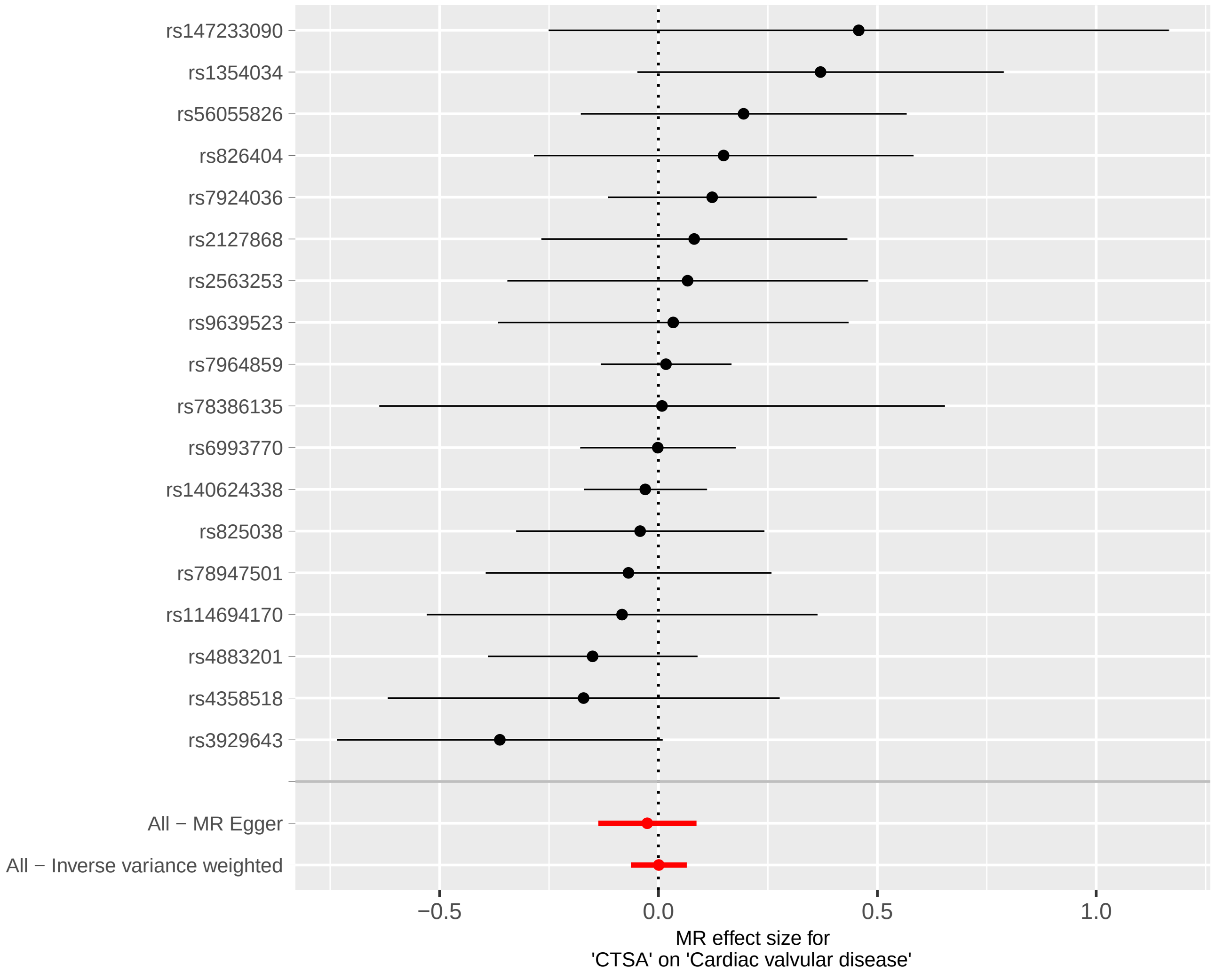
rs147233090
rs1354034
rs56055826
rs826404
rs7924036
rs2127868
rs2563253
rs9639523
rs7964859
rs78386135
rs6993770
rs140624338
rs825038
rs78947501
rs114694170
rs4883201
rs4358518
rs3929643
All − MR Egger
All − Inverse variance weighted
−0.5
0.0
0.5
1.0
MR effect size for
'CTSA' on 'Cardiac valvular disease'

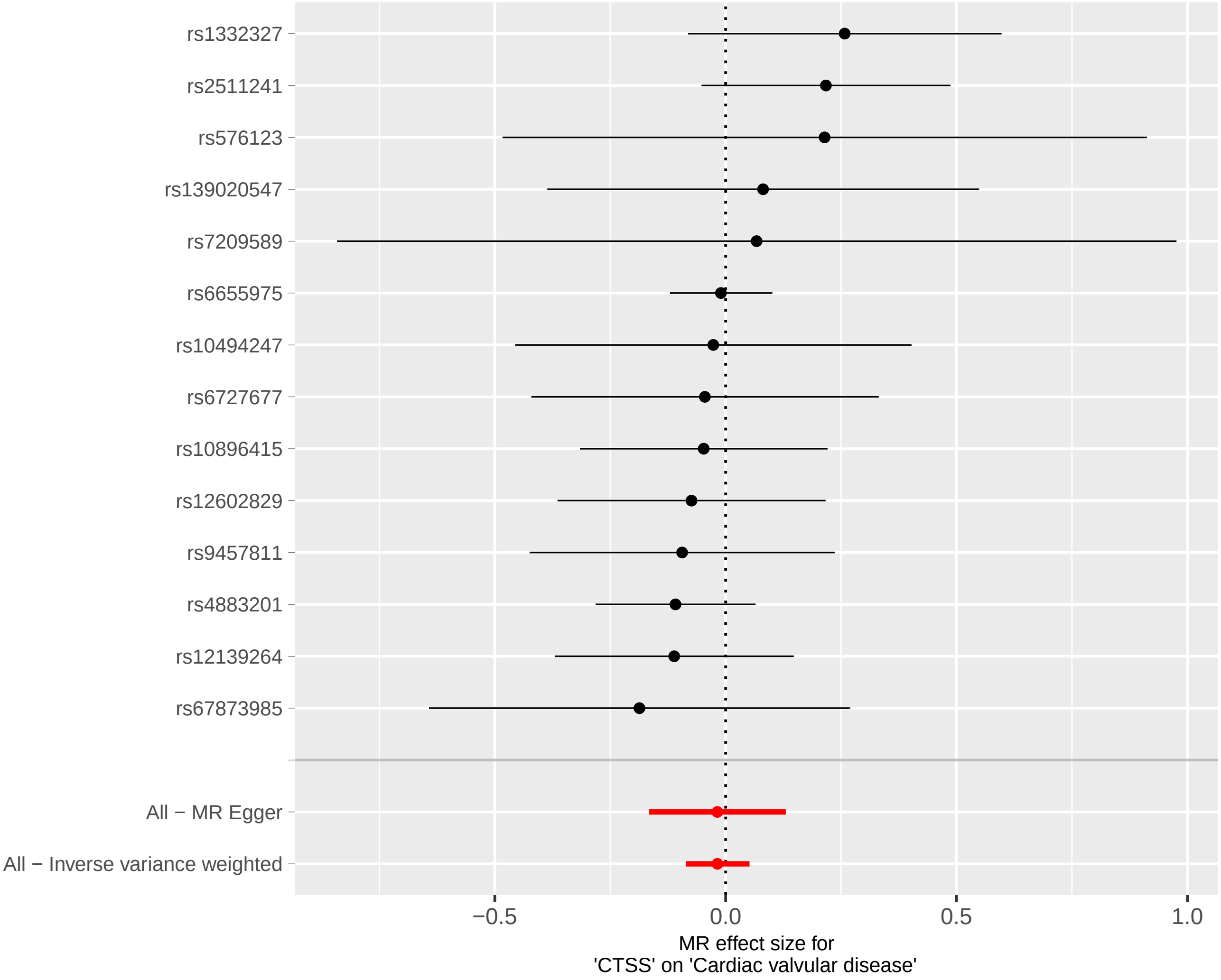
rs1332327
rs2511241
rs576123
rs139020547
rs7209589
rs6655975
rs10494247
rs6727677
rs10896415
rs12602829
rs9457811
rs4883201
rs12139264
rs67873985
All − MR Egger
All − Inverse variance weighted
−0.5
0.0
0.5
1.0
MR effect size for
'CTSS' on 'Cardiac valvular disease'

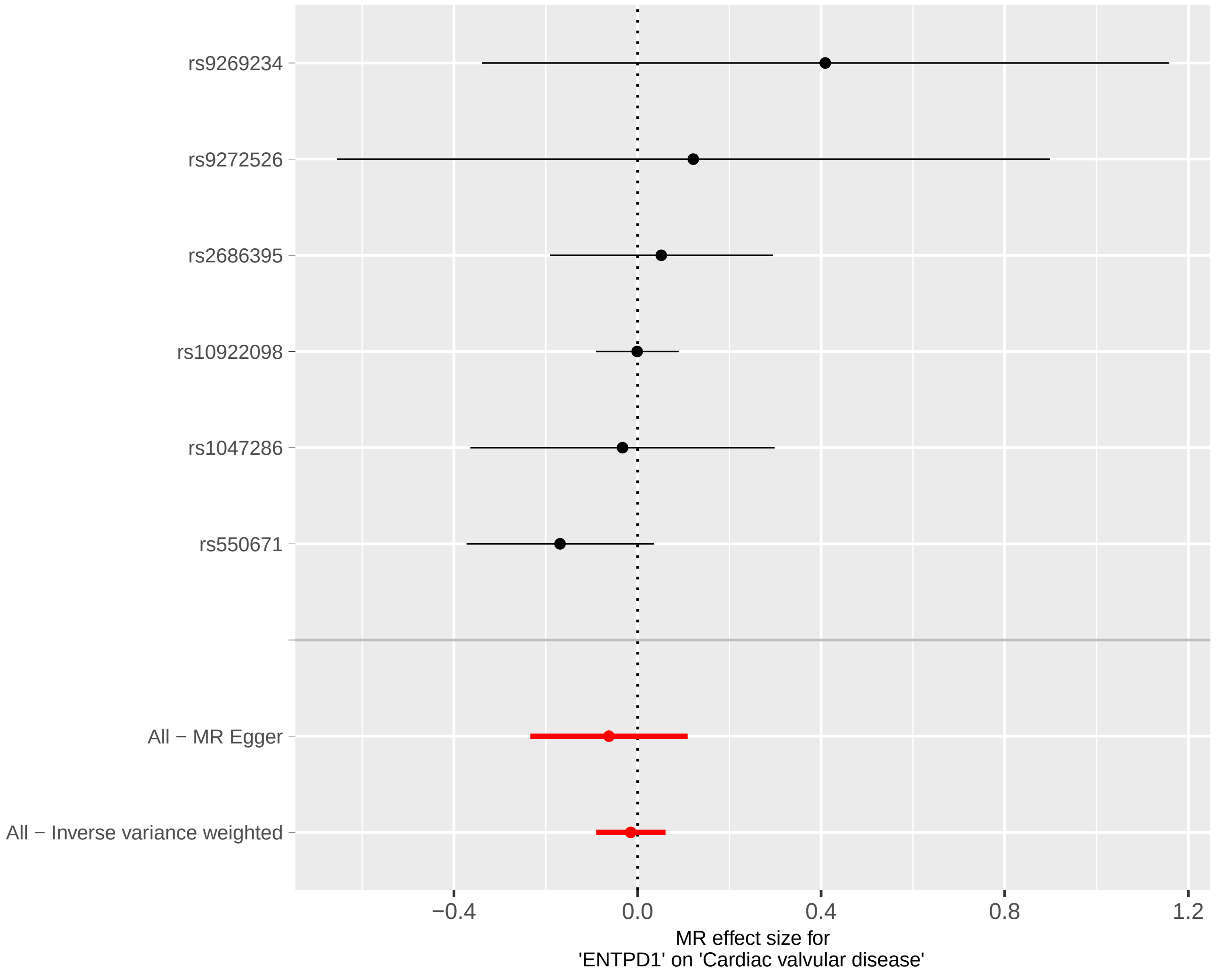
rs9269234
rs9272526
rs2686395
rs10922098
rs1047286
rs550671
All − MR Egger
All − Inverse variance weighted
−0.4
0.0
0.4
0.8
1.2
MR effect size for
'ENTPD1' on 'Cardiac valvular disease'

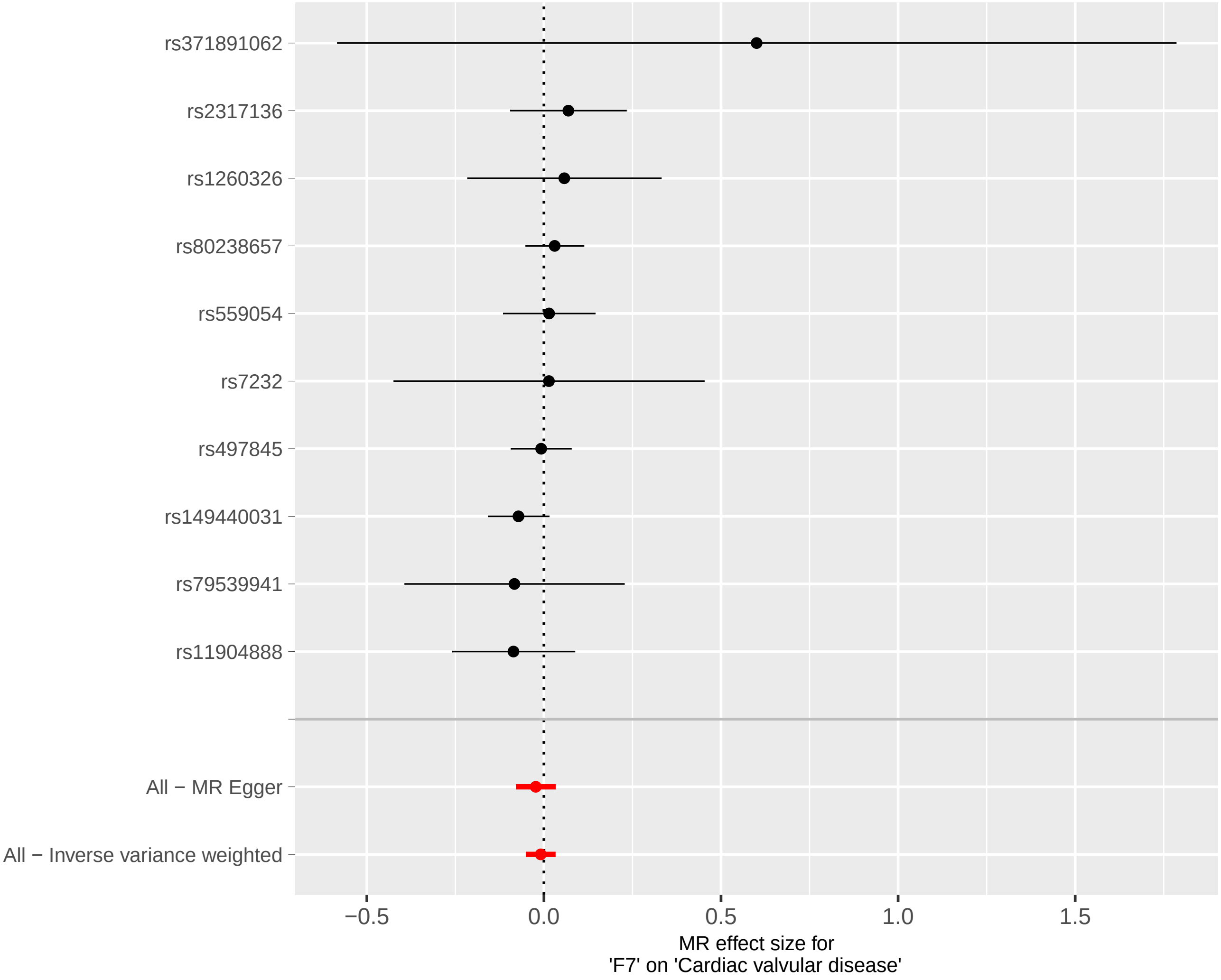

rs371891062
rs2317136
rs1260326
rs80238657
rs559054
rs7232
rs497845
rs149440031
rs79539941
rs11904888
All − MR Egger
All − Inverse variance weighted
−0.5
0.0
0.5
1.0
1.5
MR effect size for
'F7' on 'Cardiac valvular disease'

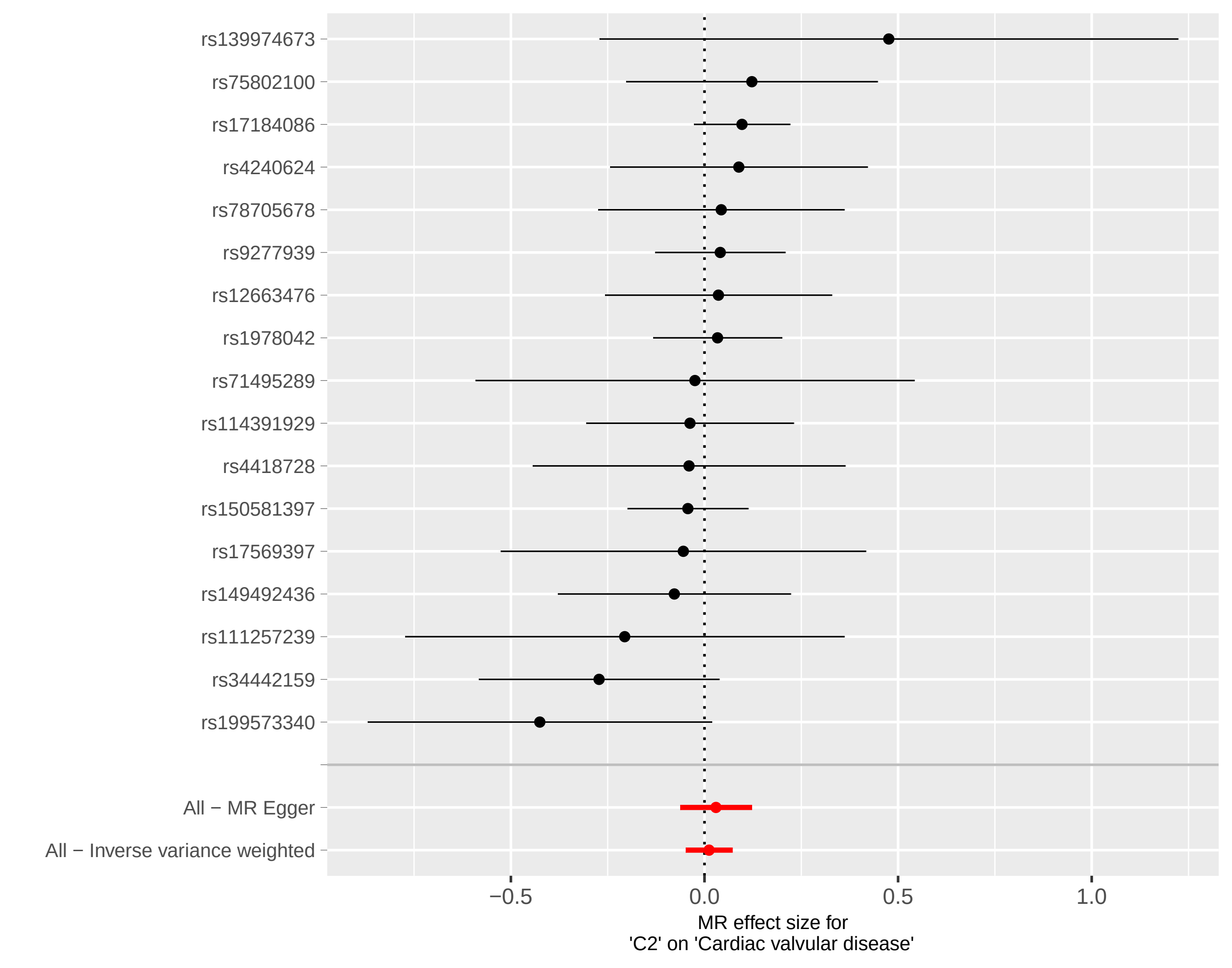
rs139974673
rs75802100
rs17184086
rs4240624
rs78705678
rs9277939
rs12663476
rs1978042
rs71495289
rs114391929
rs4418728
rs150581397
rs17569397
rs149492436
rs111257239
rs34442159
rs199573340
All − MR Egger
All − Inverse variance weighted
−0.5
0.0
0.5
1.0
MR effect size for
'C2' on 'Cardiac valvular disease'

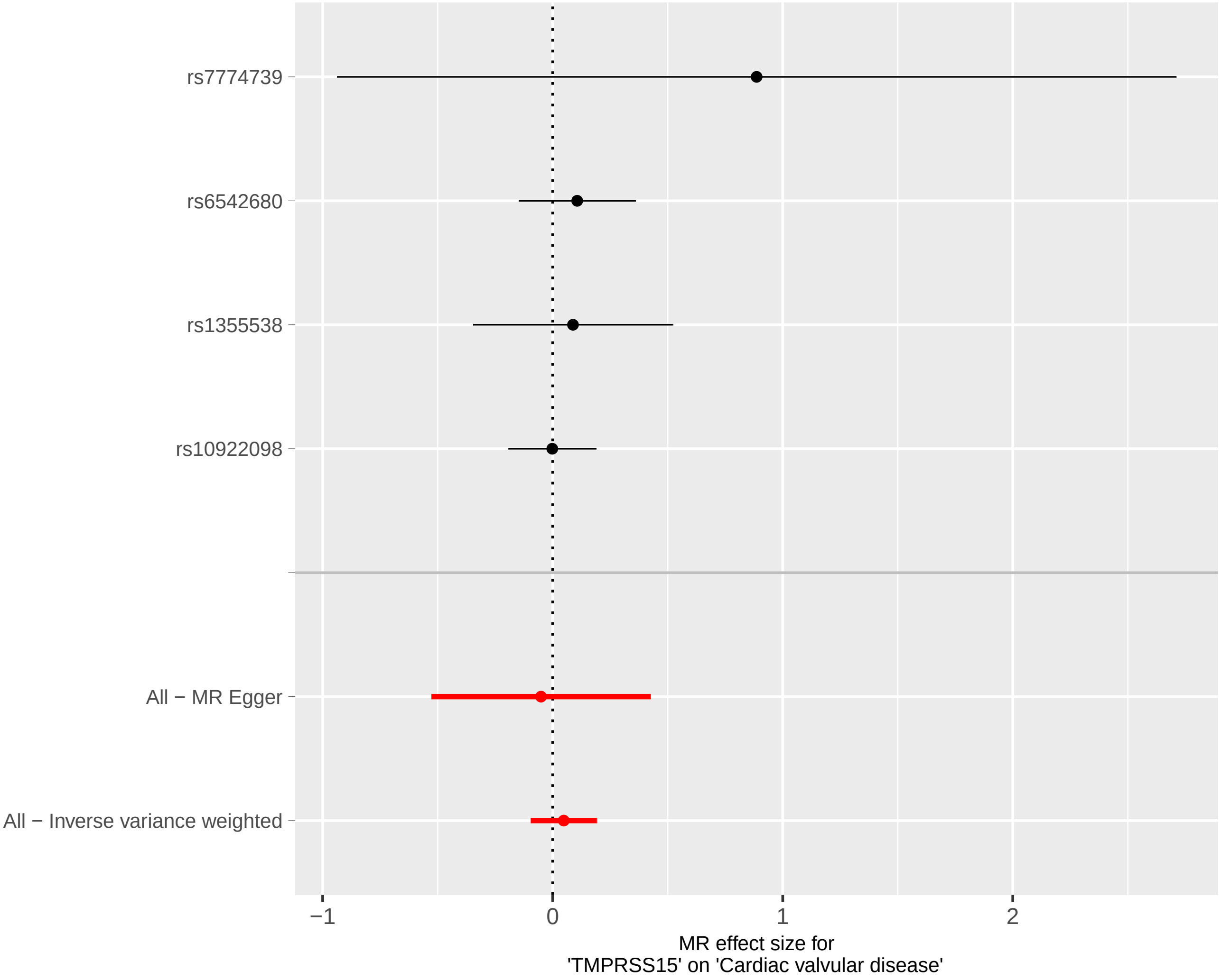
rs7774739
rs6542680
rs1355538
rs10922098
All − MR Egger
All − Inverse variance weighted
−1
0
1
2
MR effect size for
'TMPRSS15' on 'Cardiac valvular disease'

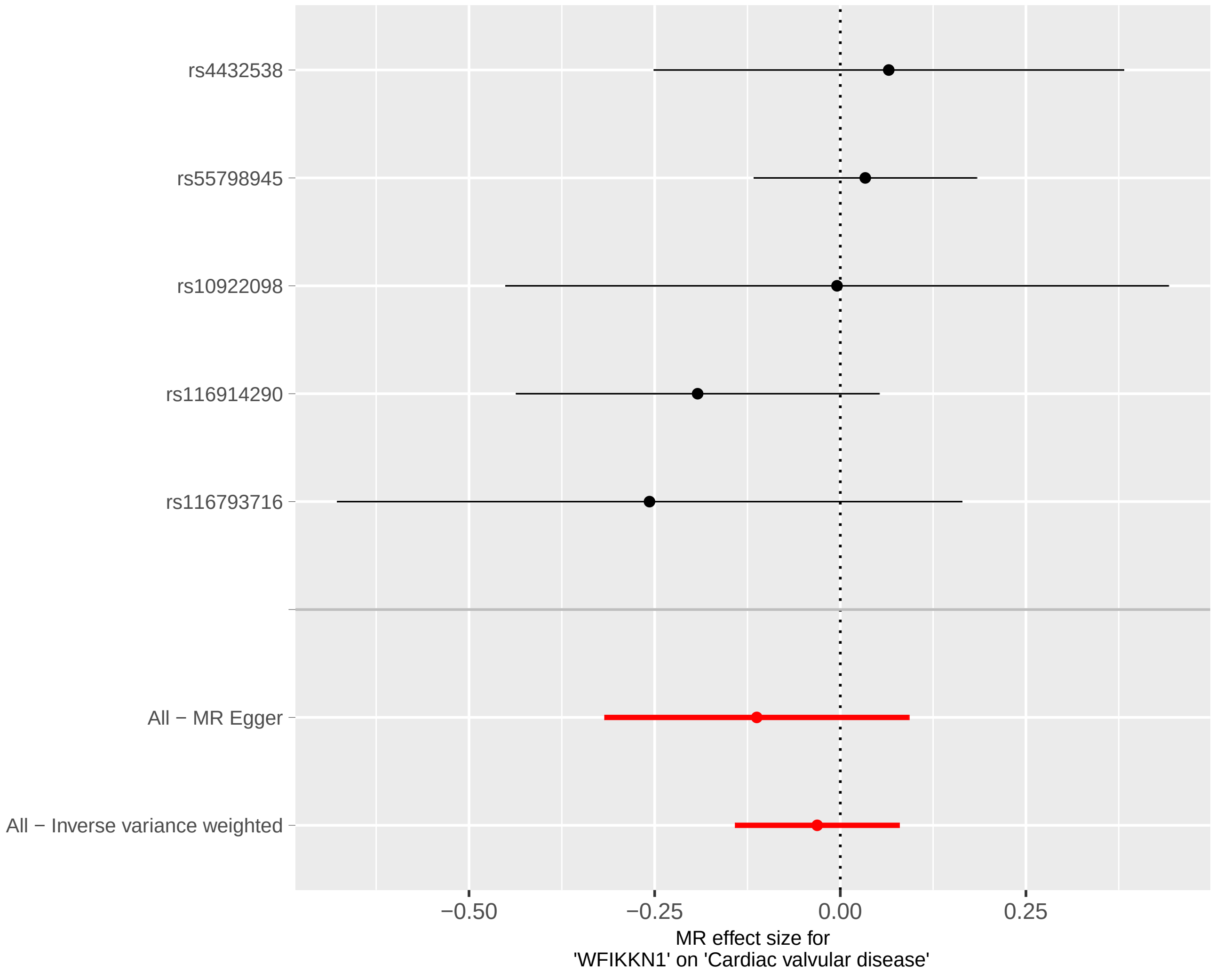
rs4432538
rs55798945
rs10922098
rs116914290
rs116793716
All − MR Egger
All − Inverse variance weighted
−0.50
−0.25
0.00
0.25
MR effect size for
'WFIKKN1' on 'Cardiac valvular disease'

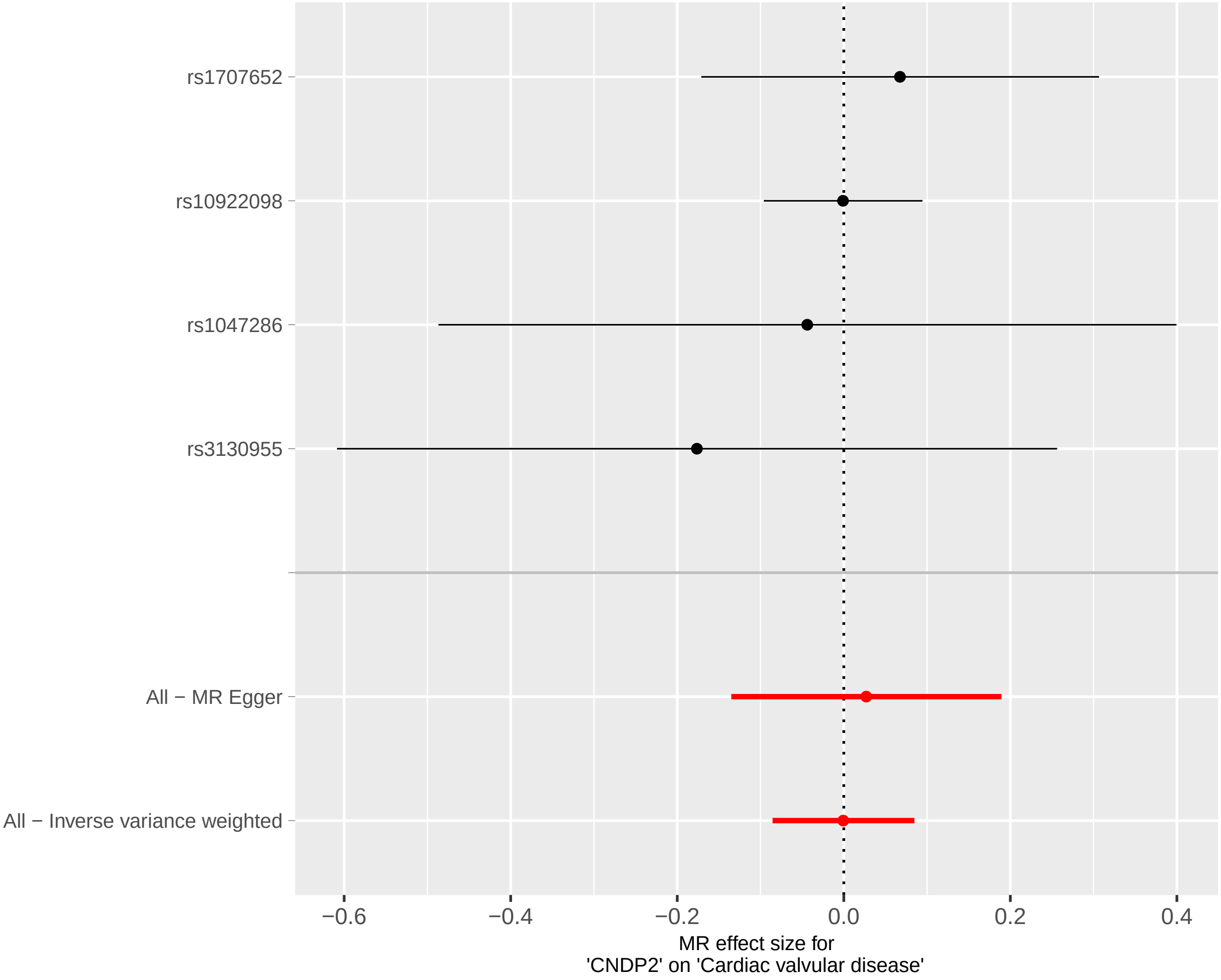
rs1707652
rs10922098
rs1047286
rs3130955
All − MR Egger
All − Inverse variance weighted
−0.6
−0.4
−0.2
0.0
0.2
0.4
MR effect size for
'CNDP2' on 'Cardiac valvular disease'

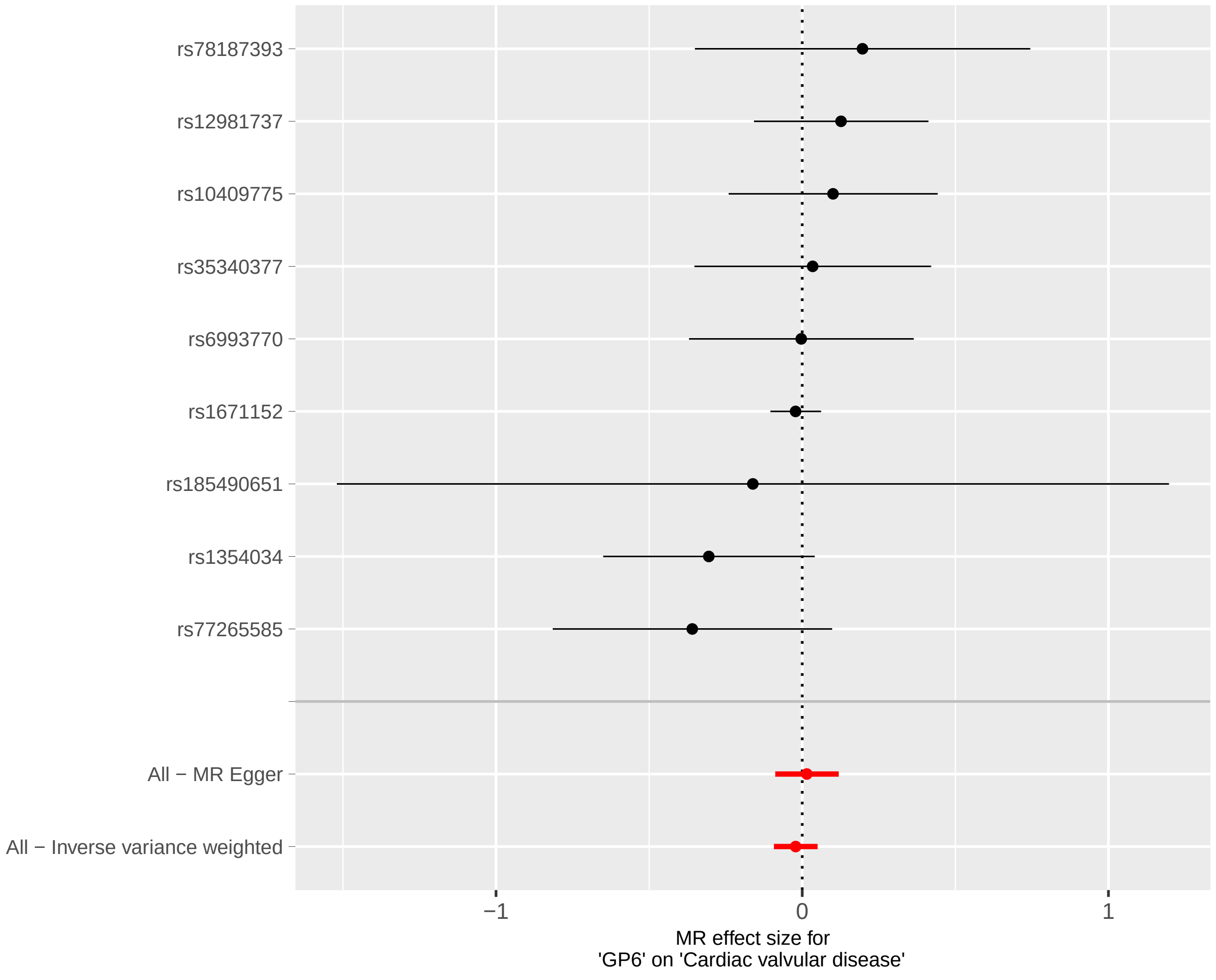
rs78187393
rs12981737
rs10409775
rs35340377
rs6993770
rs1671152
rs185490651
rs1354034
rs77265585
All − MR Egger
All − Inverse variance weighted
−1
0
1
MR effect size for
'GP6' on 'Cardiac valvular disease'

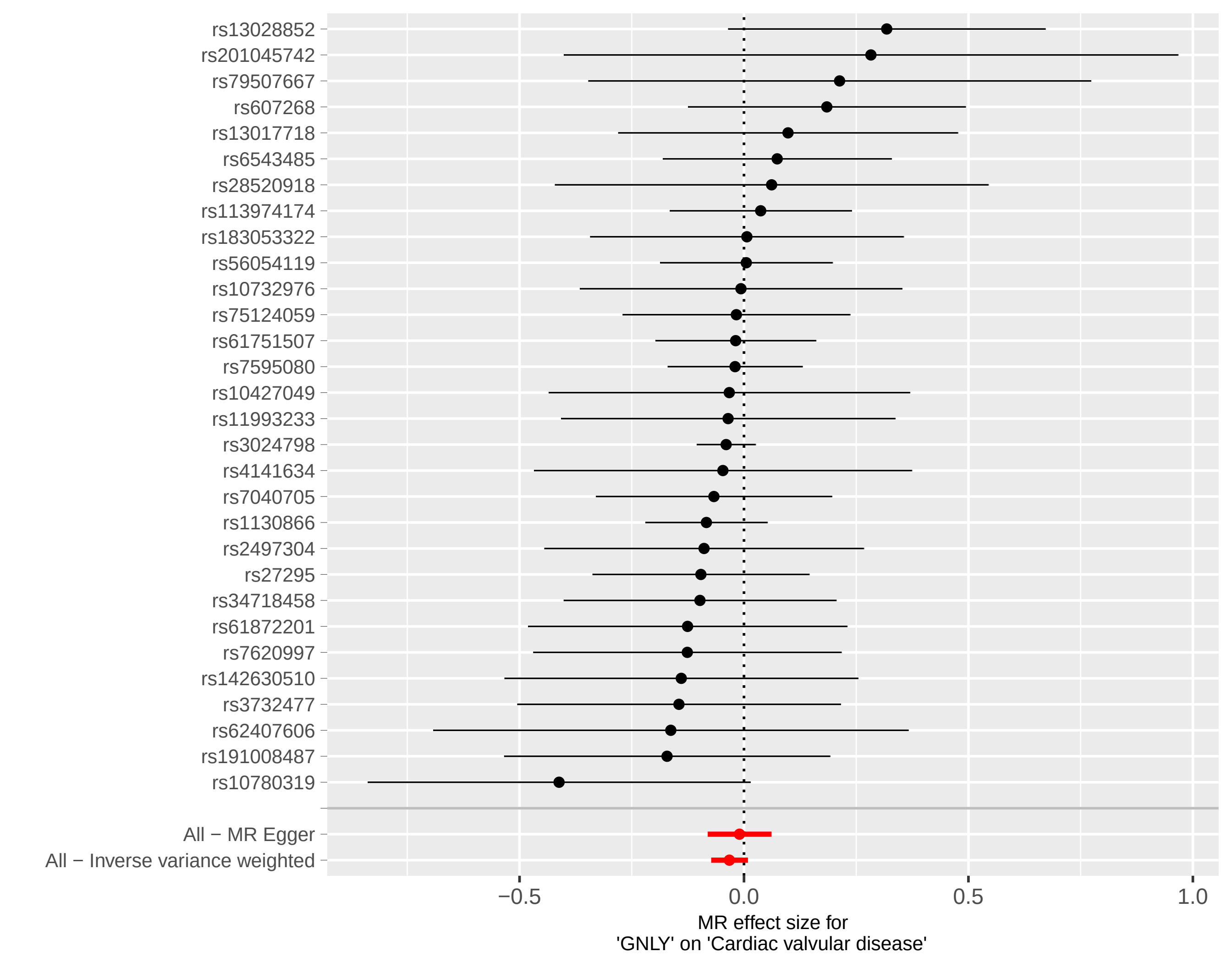
rs13028852
rs201045742
rs79507667
rs607268
rs13017718
rs6543485
rs28520918
rs113974174
rs183053322
rs56054119
rs10732976
rs75124059
rs61751507
rs7595080
rs10427049
rs11993233
rs3024798
rs4141634
rs7040705
rs1130866
rs2497304
rs27295
rs34718458
rs61872201
rs7620997
rs142630510
rs3732477
rs62407606
rs191008487
rs10780319
All − MR Egger
All − Inverse variance weighted
−0.5
0.0
0.5
1.0
MR effect size for
'GNLY' on 'Cardiac valvular disease'

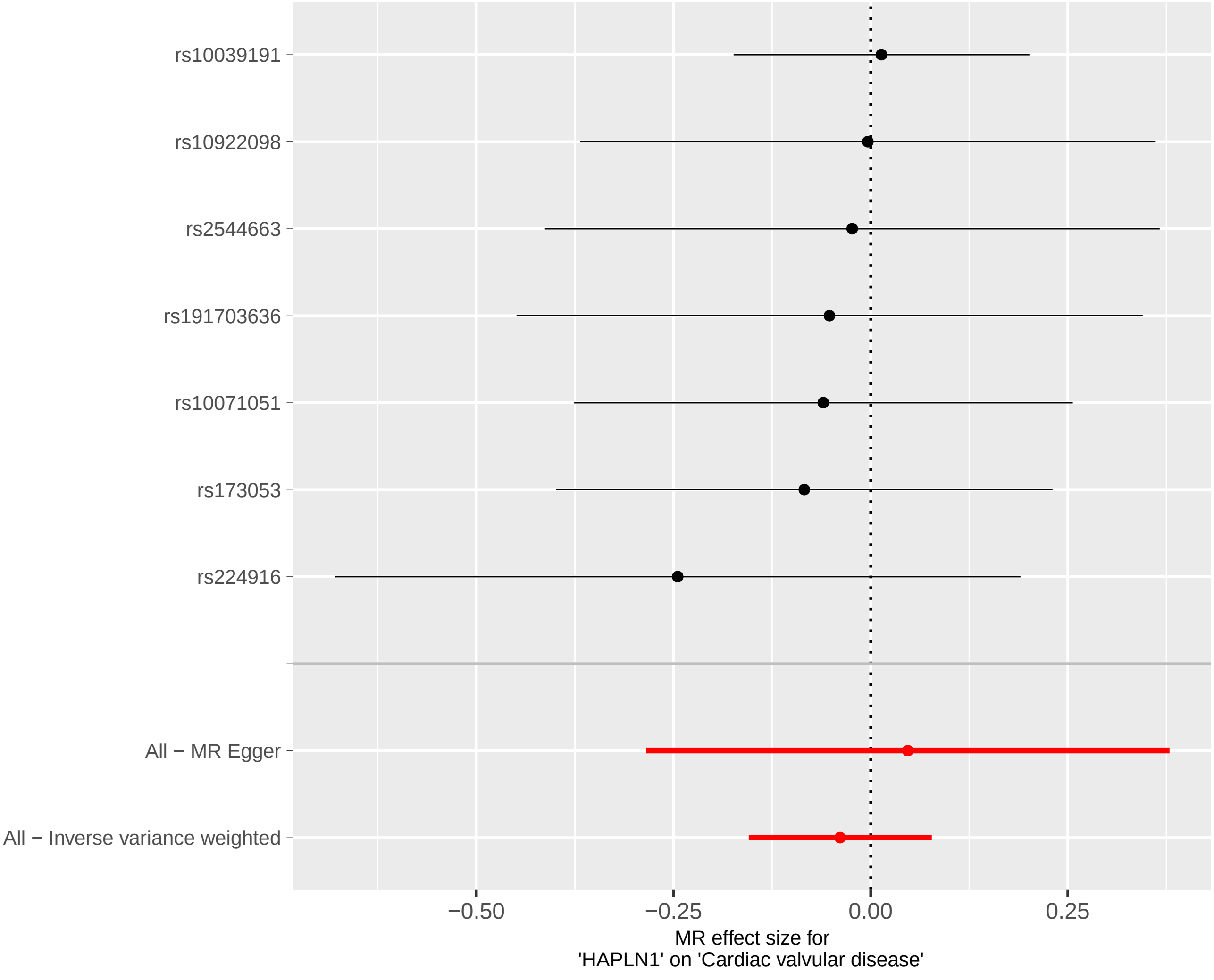
rs10039191
rs10922098
rs2544663
rs191703636
rs10071051
rs173053
rs224916
All − MR Egger
All − Inverse variance weighted
−0.50
−0.25
0.00
0.25
MR effect size for
'HAPLN1' on 'Cardiac valvular disease'

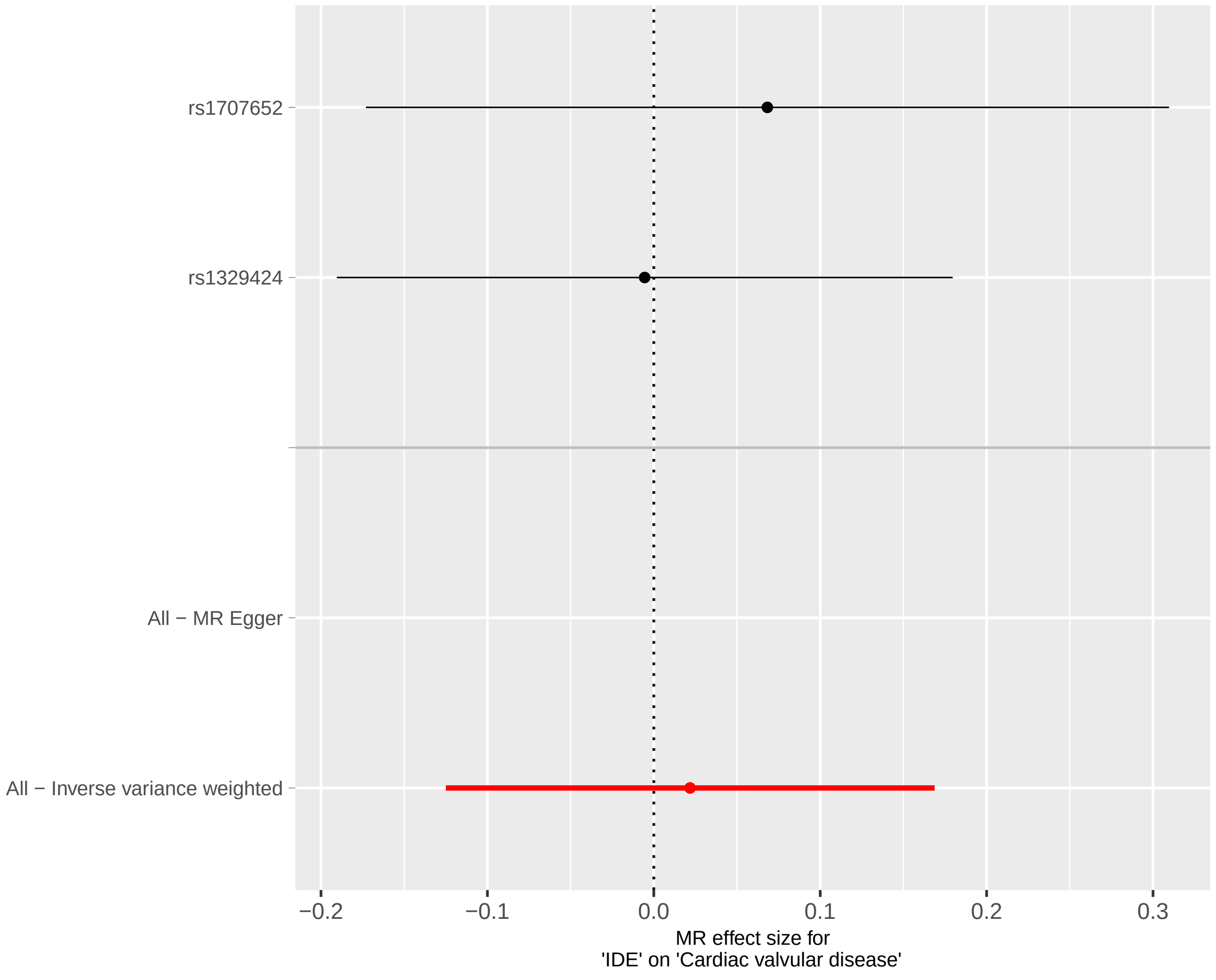
rs1707652
rs1329424
All − MR Egger
All − Inverse variance weighted
−0.2
−0.1
0.0
0.1
0.2
0.3
MR effect size for
'IDE' on 'Cardiac valvular disease'

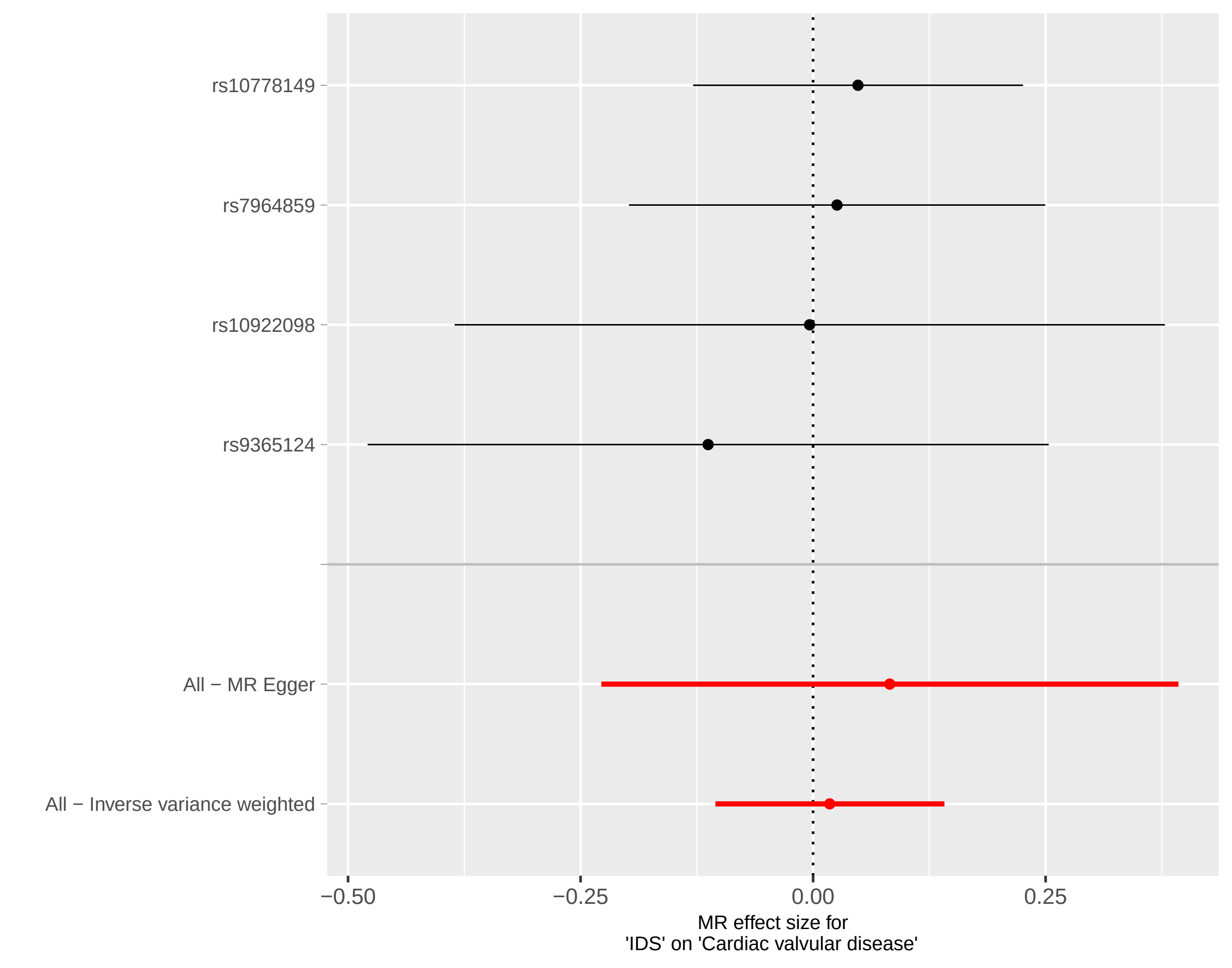
rs10778149
rs7964859
rs10922098
rs9365124
All − MR Egger
All − Inverse variance weighted
−0.50
−0.25
0.00
0.25
MR effect size for
'IDS' on 'Cardiac valvular disease'

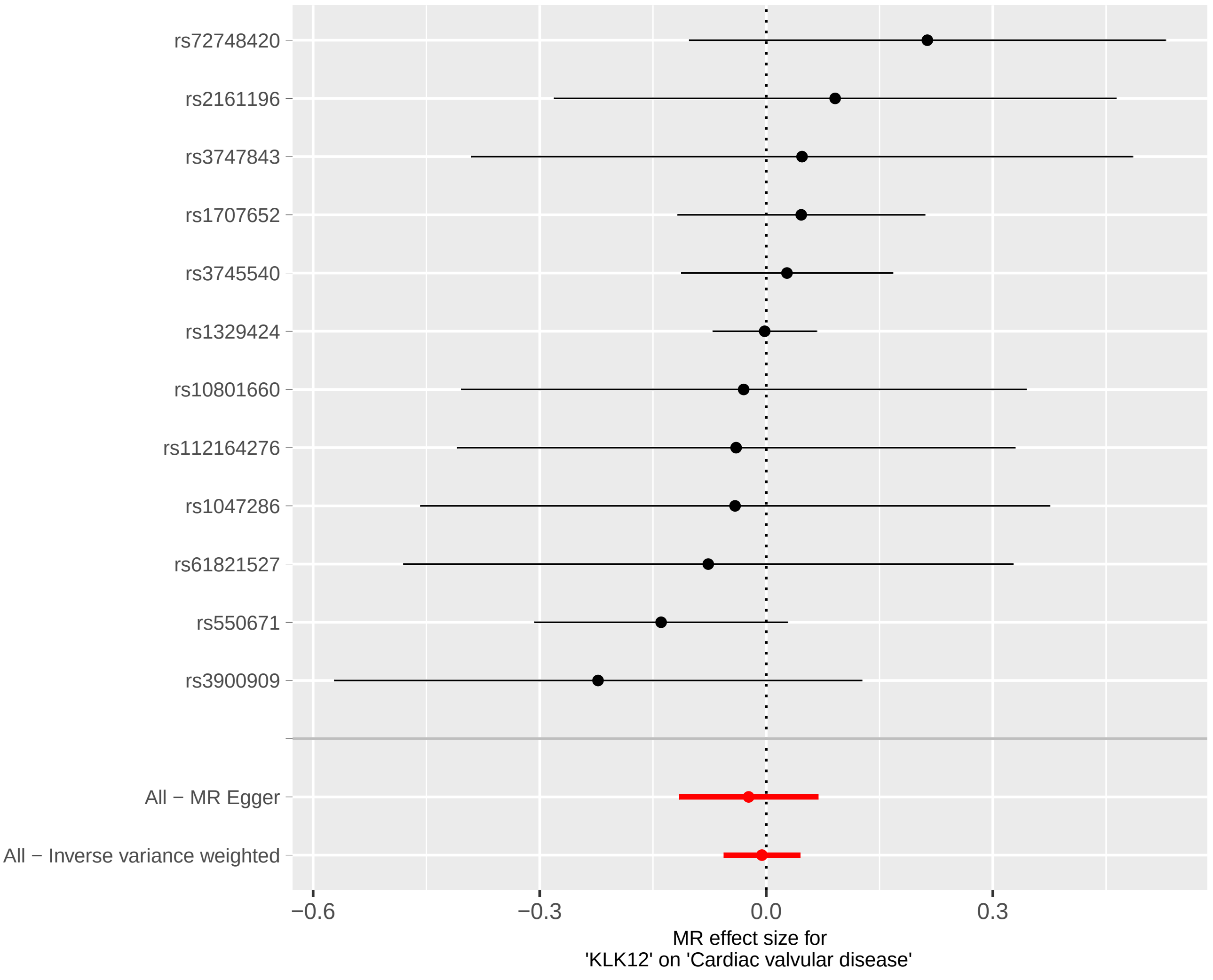

rs72748420
rs2161196
rs3747843
rs1707652
rs3745540
rs1329424
rs10801660
rs112164276
rs1047286
rs61821527
rs550671
rs3900909
All − MR Egger
All − Inverse variance weighted
−0.6
−0.3
0.0
0.3
MR effect size for
'KLK12' on 'Cardiac valvular disease'

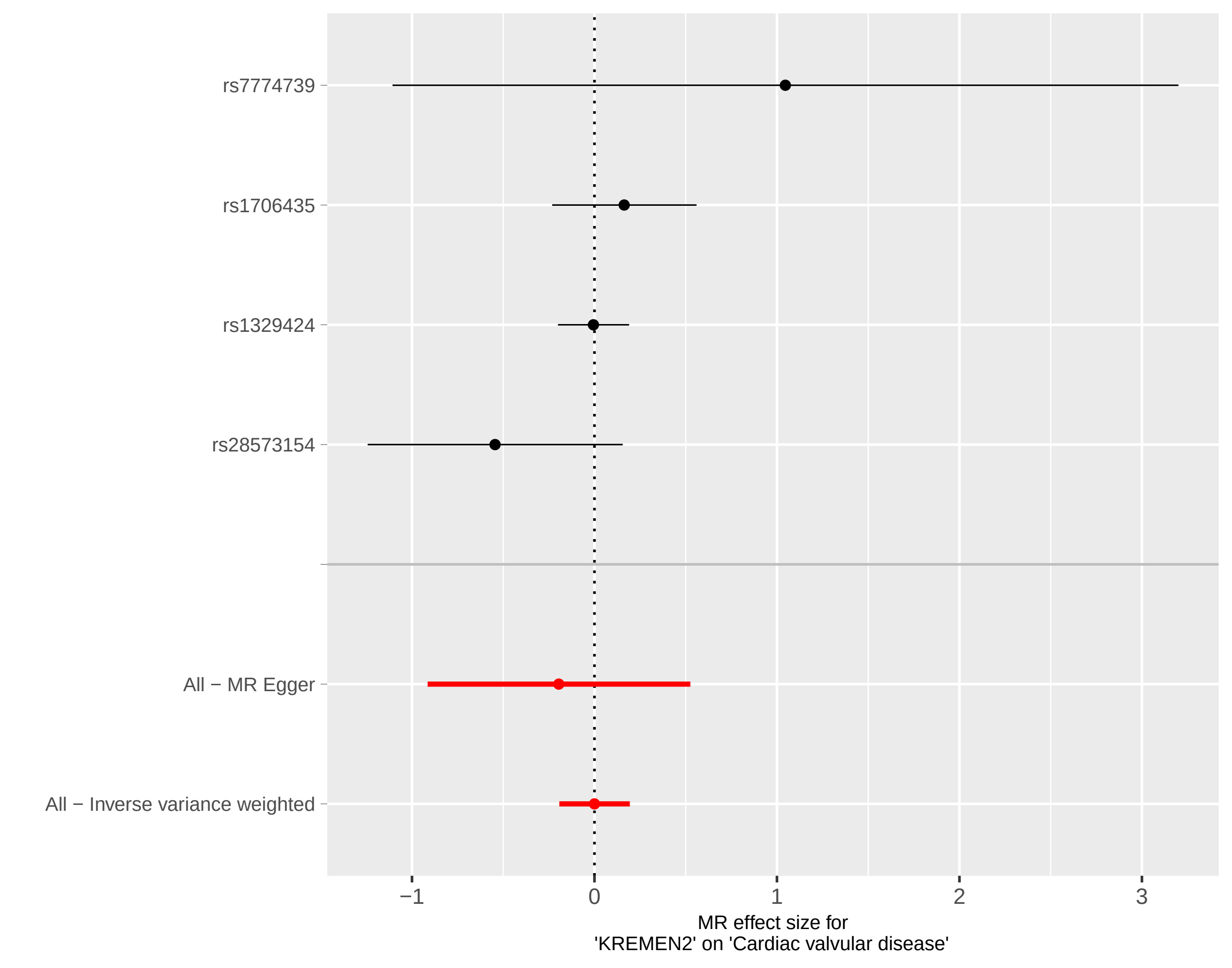
rs7774739
rs1706435
rs1329424
rs28573154
All − MR Egger
All − Inverse variance weighted
−1
0
1
2
3
MR effect size for
'KREMEN2' on 'Cardiac valvular disease'

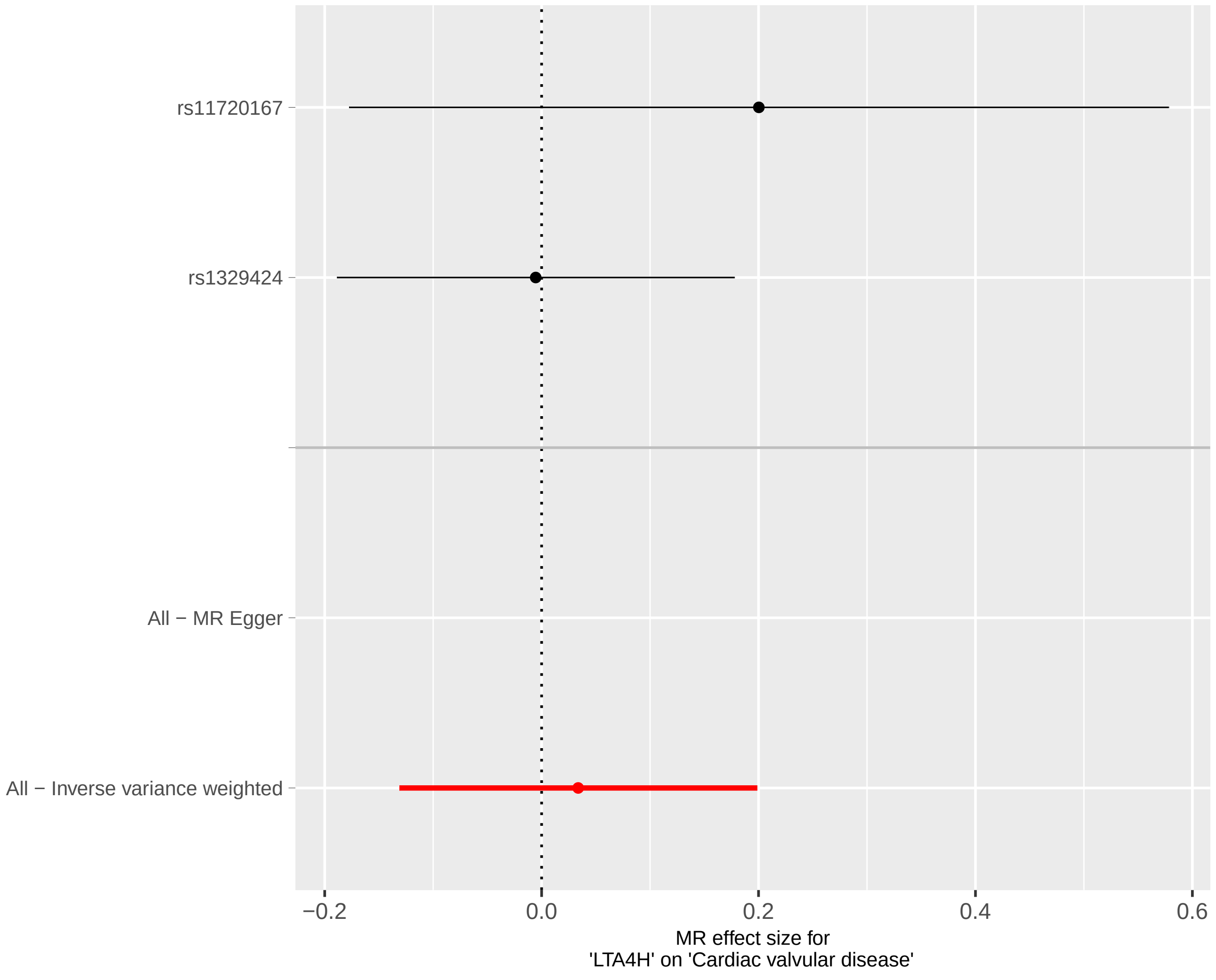

rs11720167
rs1329424
All − MR Egger
All − Inverse variance weighted
−0.2
0.0
0.2
0.4
0.6
MR effect size for
'LTA4H' on 'Cardiac valvular disease'

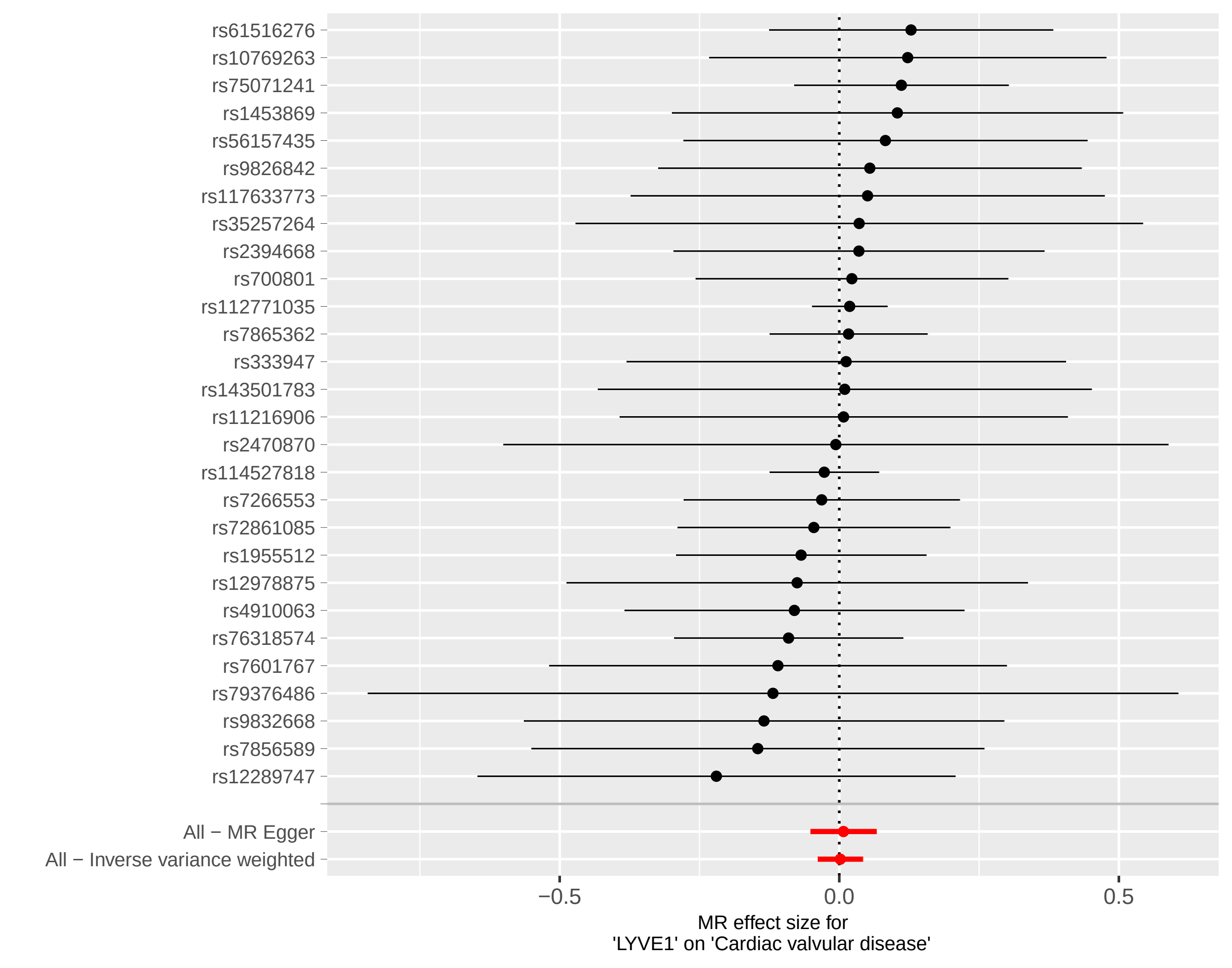
rs61516276
rs10769263
rs75071241
rs1453869
rs56157435
rs9826842
rs117633773
rs35257264
rs2394668
rs700801
rs112771035
rs7865362
rs333947
rs143501783
rs11216906
rs2470870
rs114527818
rs7266553
rs72861085
rs1955512
rs12978875
rs4910063
rs76318574
rs7601767
rs79376486
rs9832668
rs7856589
rs12289747
All − MR Egger
All − Inverse variance weighted
−0.5
0.0
0.5
MR effect size for
'LYVE1' on 'Cardiac valvular disease'

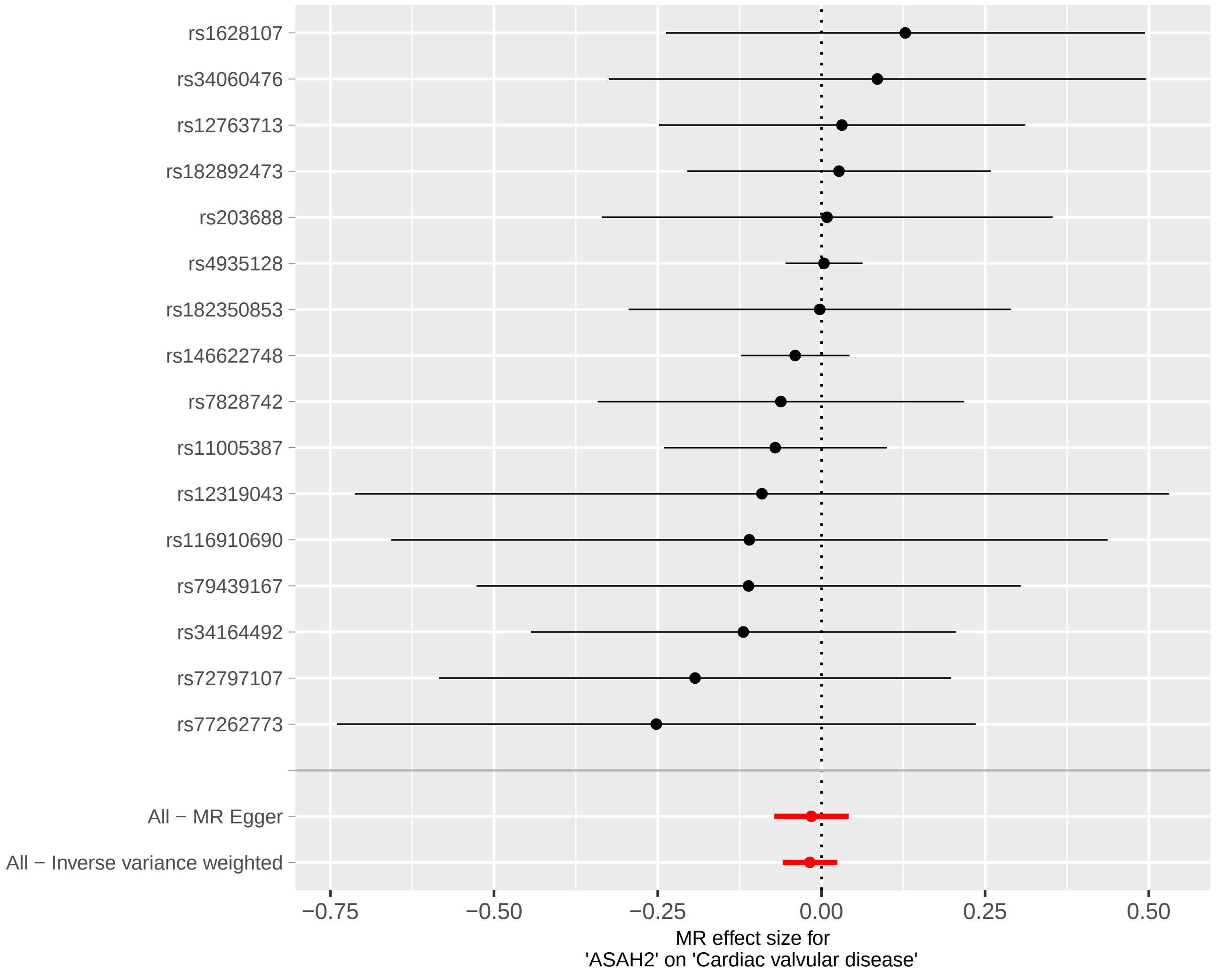
rs1628107
rs34060476
rs12763713
rs182892473
rs203688
rs4935128
rs182350853
rs146622748
rs7828742
rs11005387
rs12319043
rs116910690
rs79439167
rs34164492
rs72797107
rs77262773
All − MR Egger
All − Inverse variance weighted
−0.75
−0.50
−0.25
0.00
0.25
0.50
MR effect size for
'ASAH2' on 'Cardiac valvular disease'

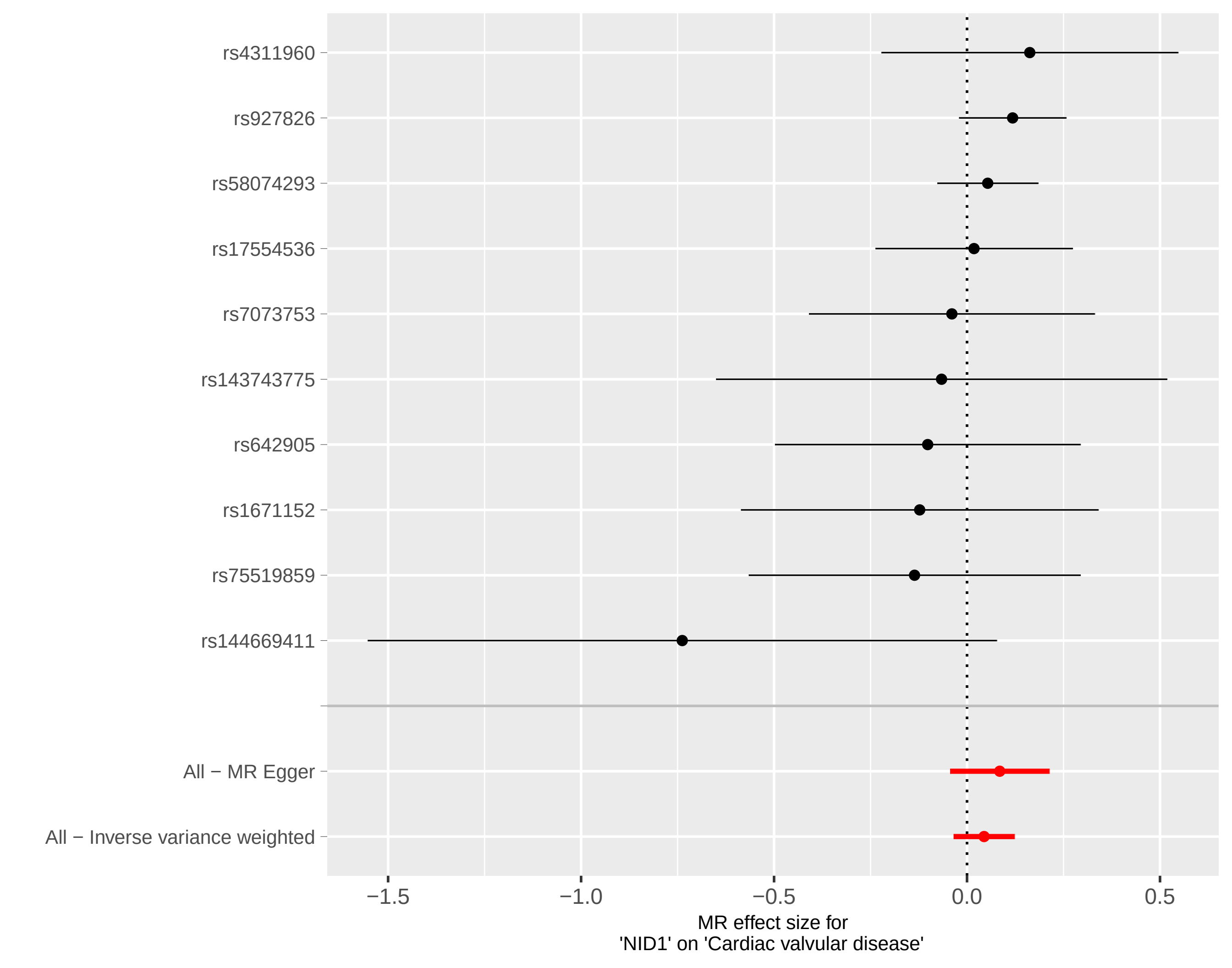
rs4311960
rs927826
rs58074293
rs17554536
rs7073753
rs143743775
rs642905
rs1671152
rs75519859
rs144669411
All − MR Egger
All − Inverse variance weighted
−1.5
−1.0
−0.5
0.0
0.5
MR effect size for
'NID1' on 'Cardiac valvular disease'

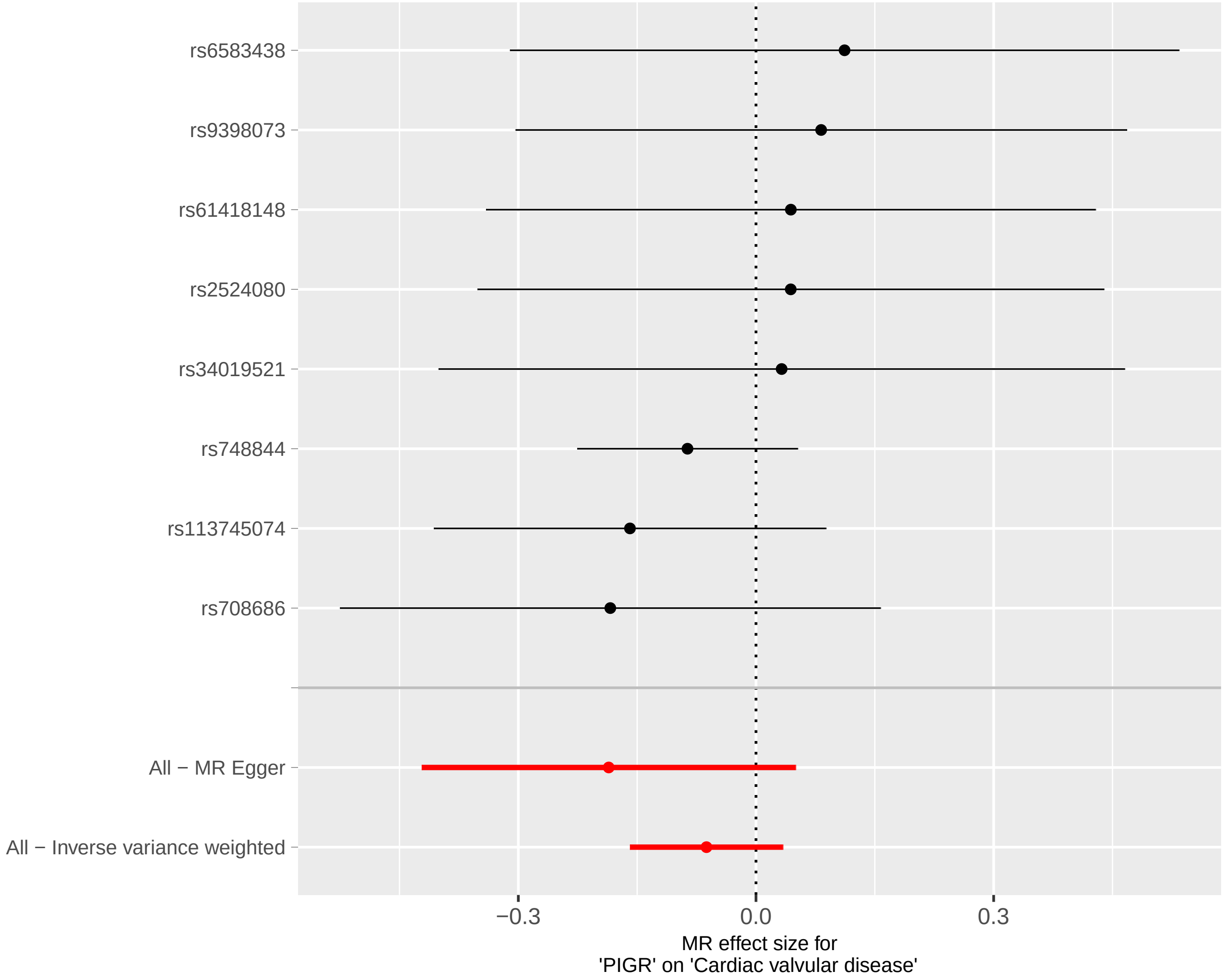
rs6583438
rs9398073
rs61418148
rs2524080
rs34019521
rs748844
rs113745074
rs708686
All − MR Egger
All − Inverse variance weighted
−0.3
0.0
0.3
MR effect size for
'PIGR' on 'Cardiac valvular disease'

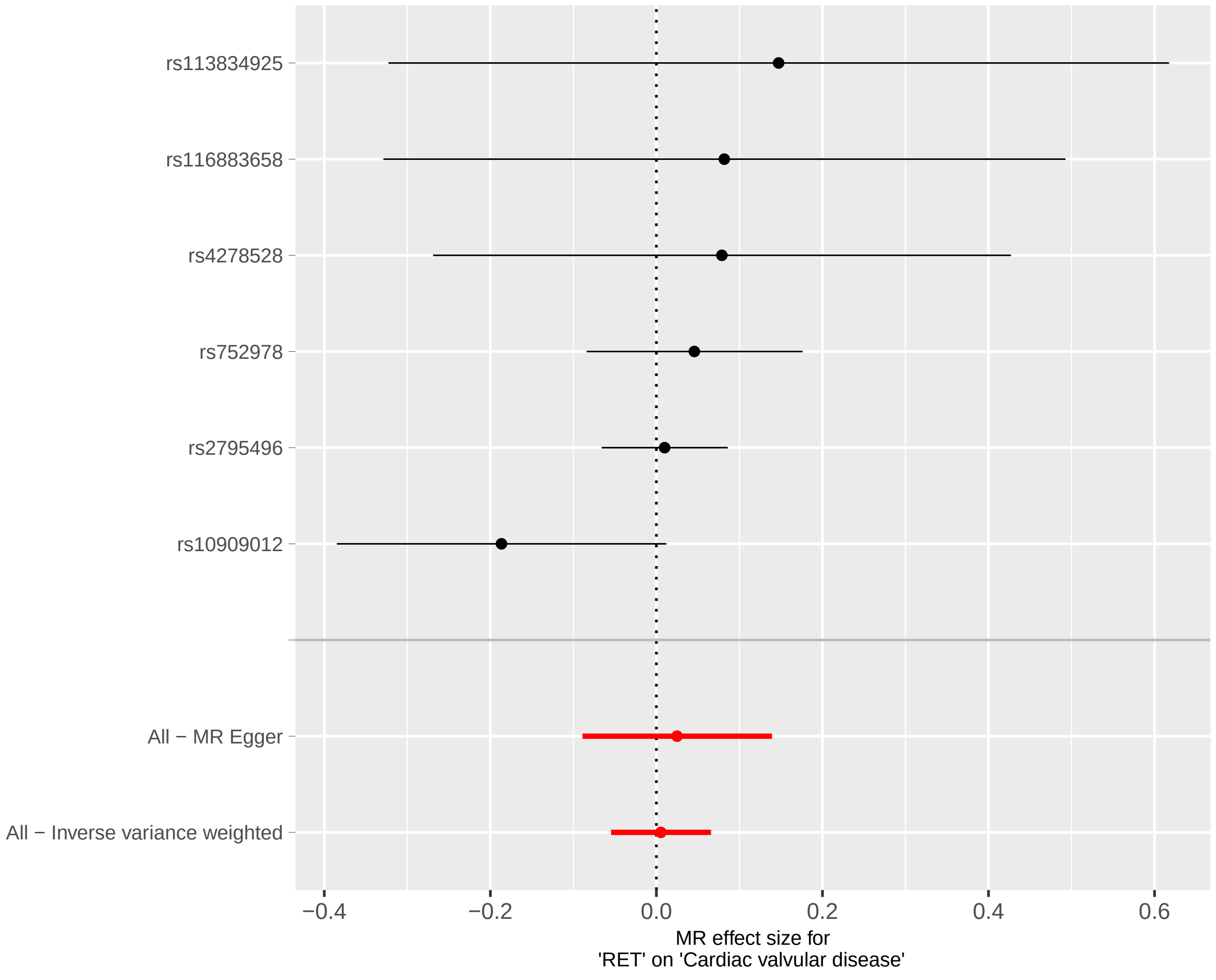
rs113834925
rs116883658
rs4278528
rs752978
rs2795496
rs10909012
All − MR Egger
All − Inverse variance weighted
−0.4
−0.2
0.0
0.2
0.4
0.6
MR effect size for
'RET' on 'Cardiac valvular disease'

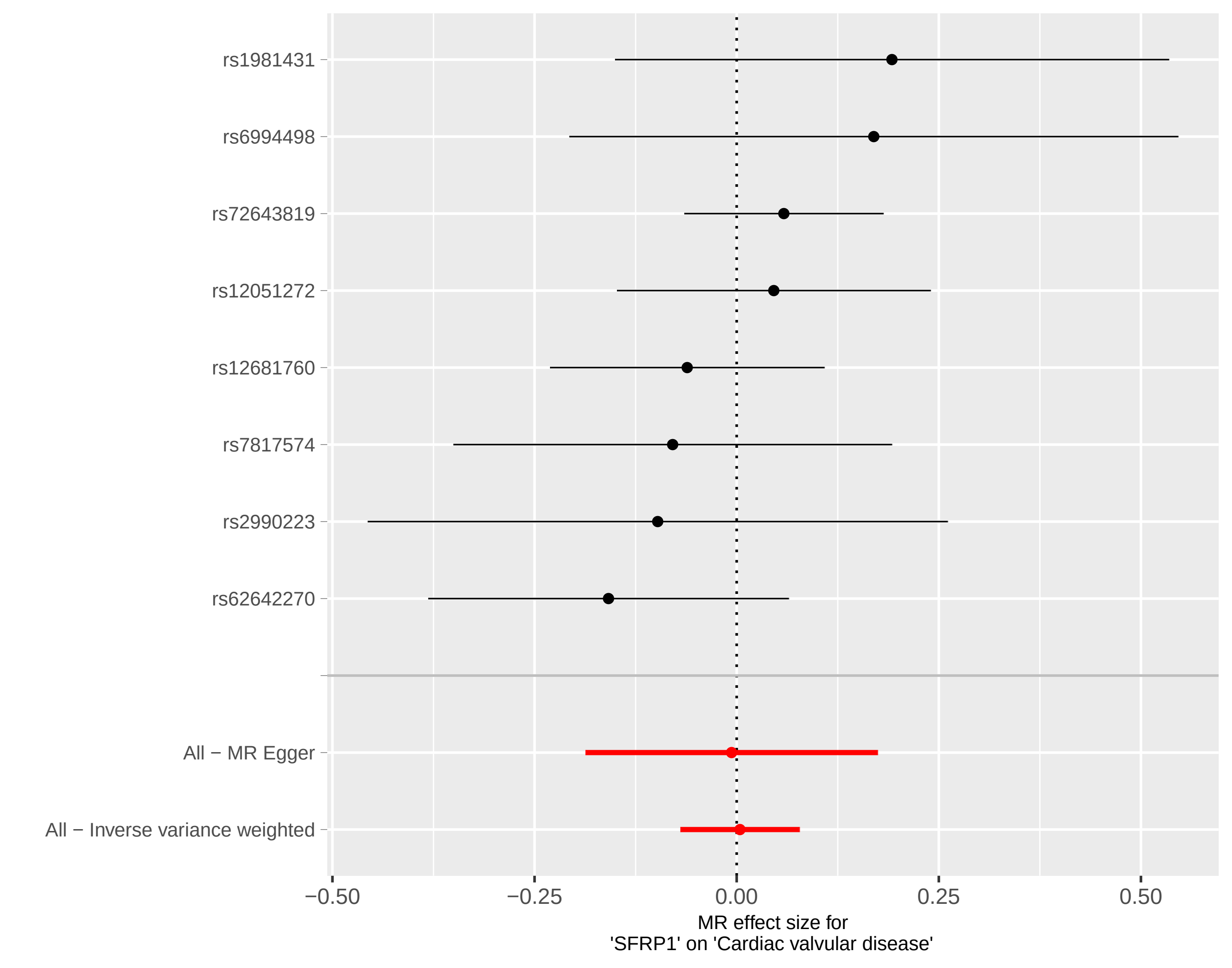

rs1981431
rs6994498
rs72643819
rs12051272
rs12681760
rs7817574
rs2990223
rs62642270
All − MR Egger
All − Inverse variance weighted
−0.50
−0.25
0.00
0.25
0.50
MR effect size for
'SFRP1' on 'Cardiac valvular disease'

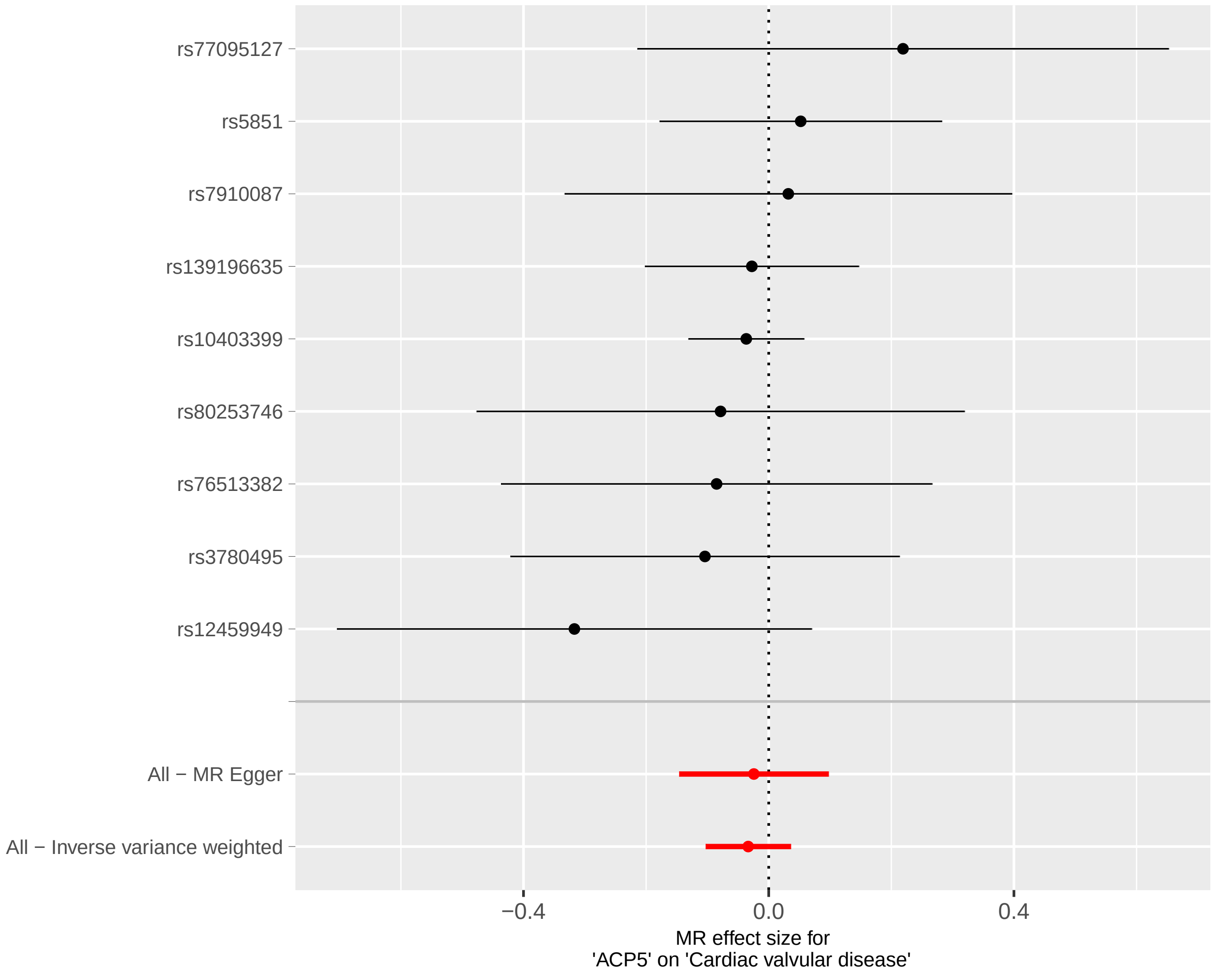
rs77095127
rs5851
rs7910087
rs139196635
rs10403399
rs80253746
rs76513382
rs3780495
rs12459949
All − MR Egger
All − Inverse variance weighted
−0.4
0.0
0.4
MR effect size for
'ACP5' on 'Cardiac valvular disease'

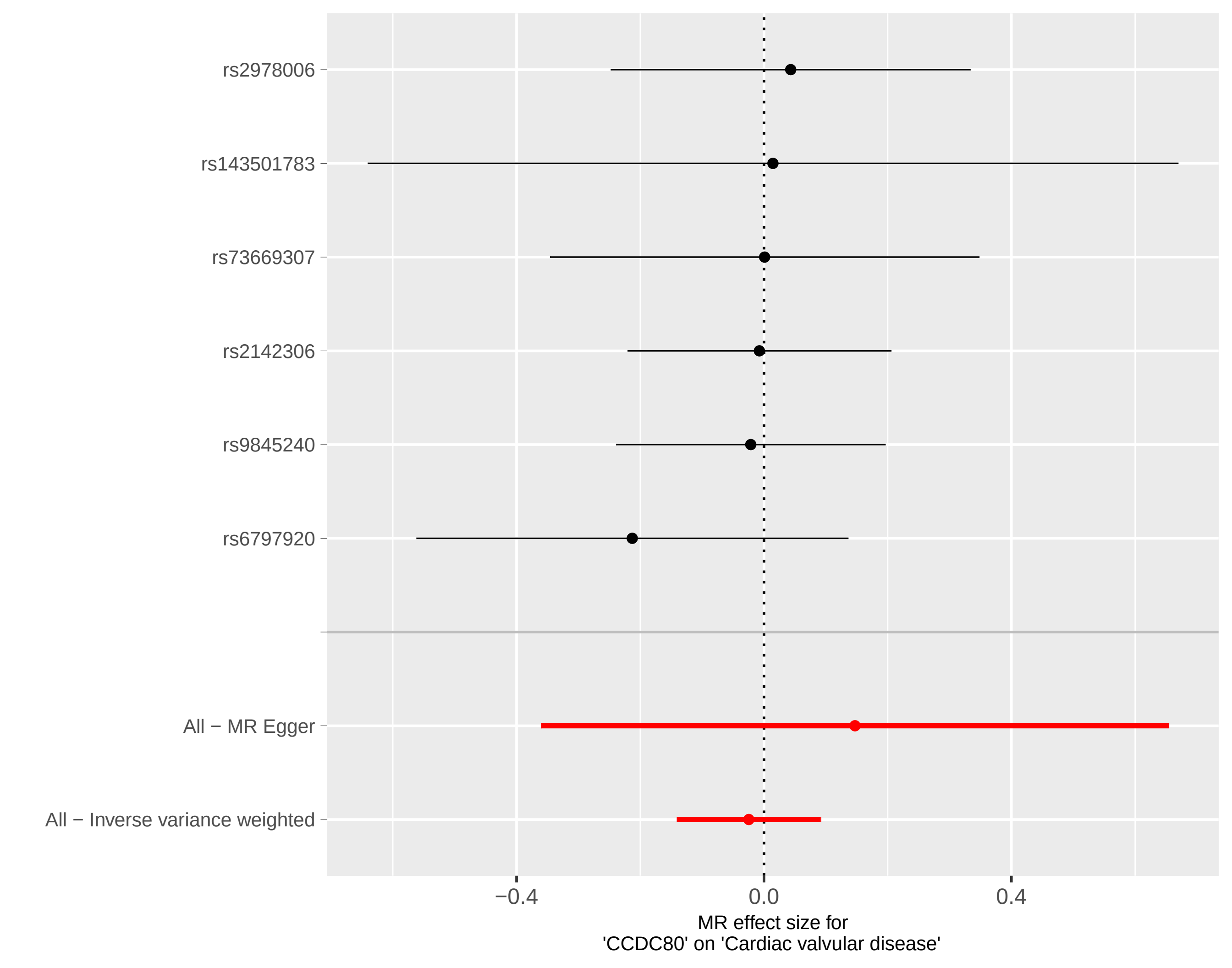
rs2978006
rs143501783
rs73669307
rs2142306
rs9845240
rs6797920
All − MR Egger
All − Inverse variance weighted
−0.4
0.0
0.4
MR effect size for
'CCDC80' on 'Cardiac valvular disease'

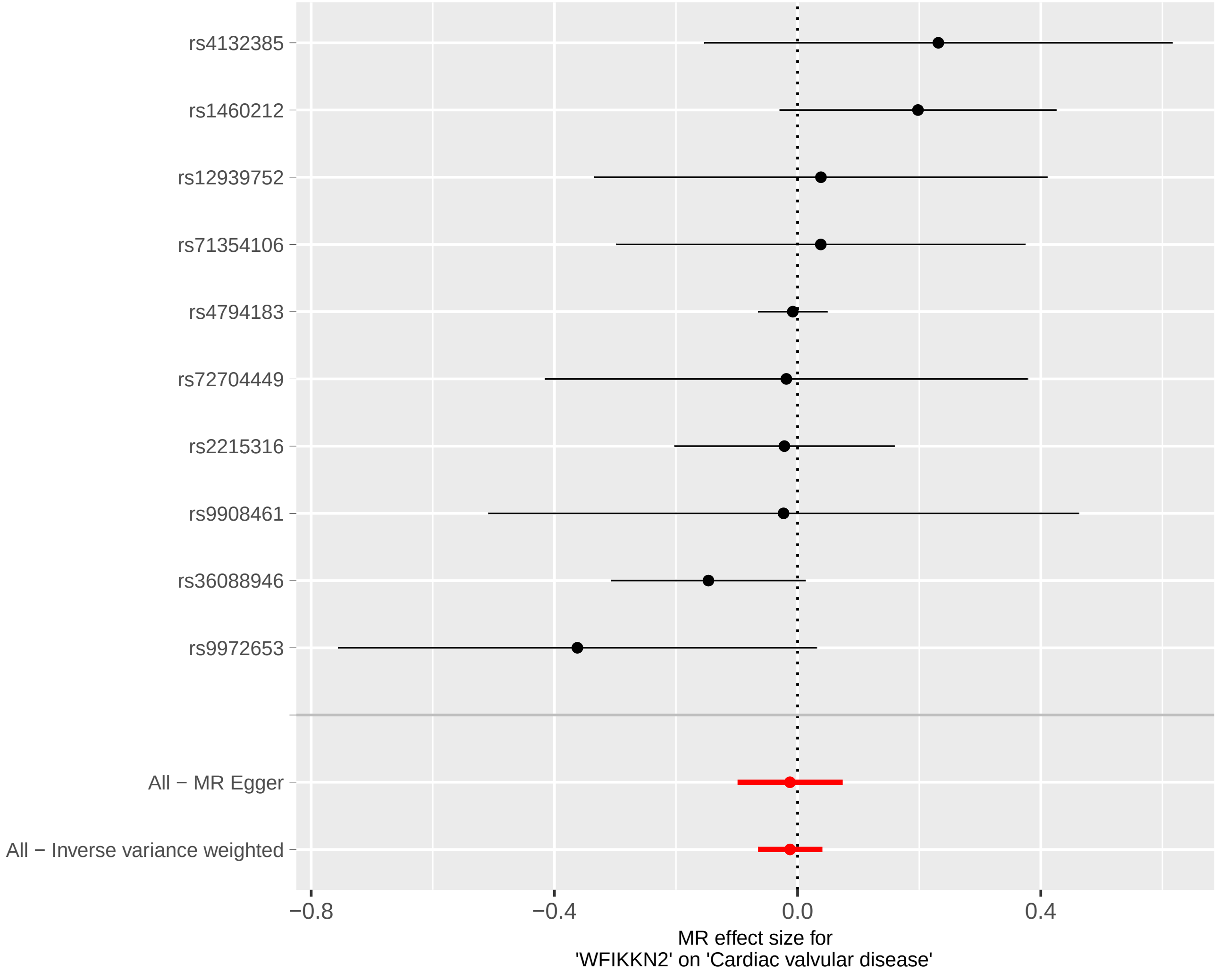
rs4132385
rs1460212
rs12939752
rs71354106
rs4794183
rs72704449
rs2215316
rs9908461
rs36088946
rs9972653
All − MR Egger
All − Inverse variance weighted
−0.8
−0.4
0.0
0.4
MR effect size for
'WFIKKN2' on 'Cardiac valvular disease'

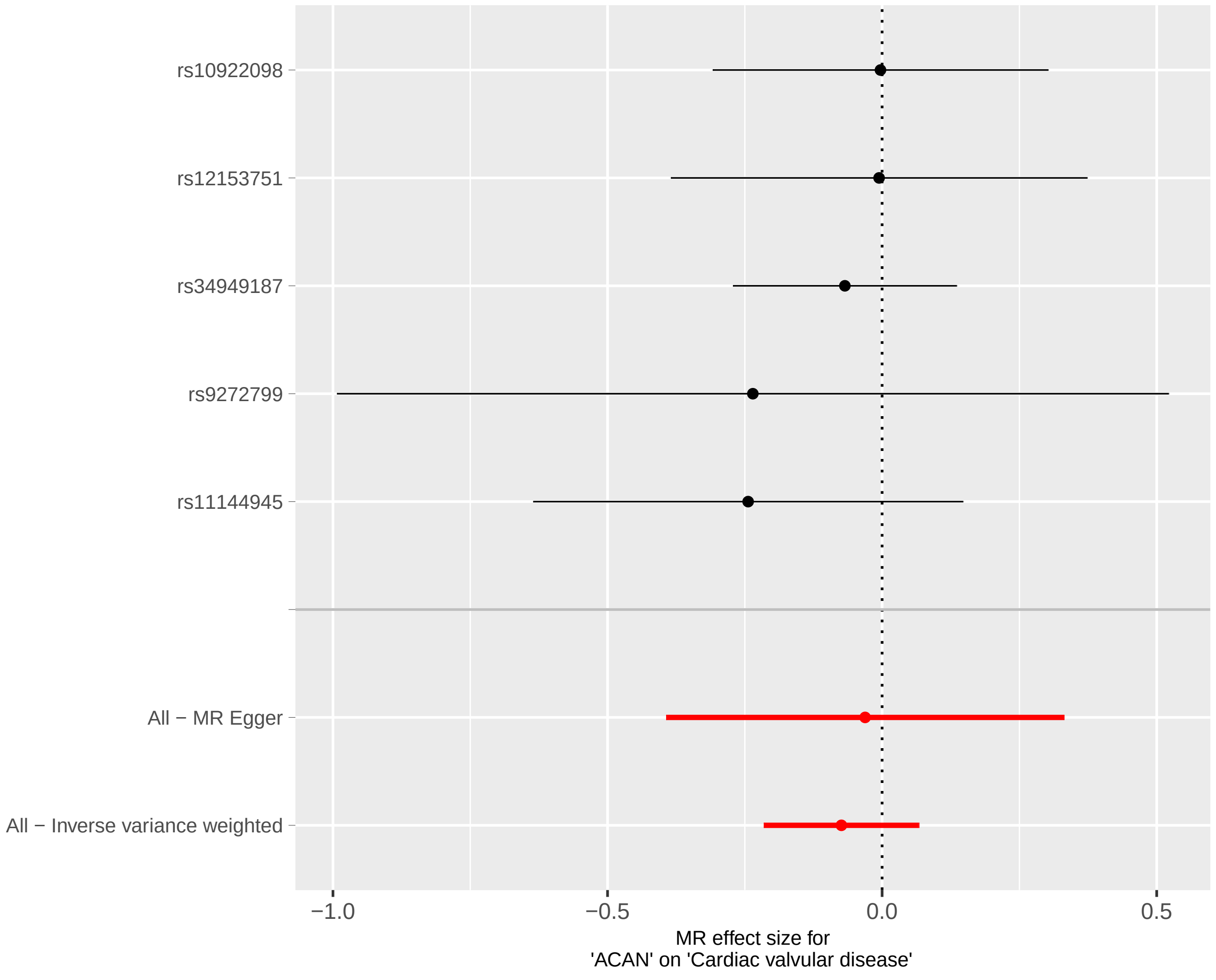

rs10922098
rs12153751
rs34949187
rs9272799
rs11144945
All − MR Egger
All − Inverse variance weighted
−1.0
−0.5
0.0
0.5
MR effect size for
'ACAN' on 'Cardiac valvular disease'

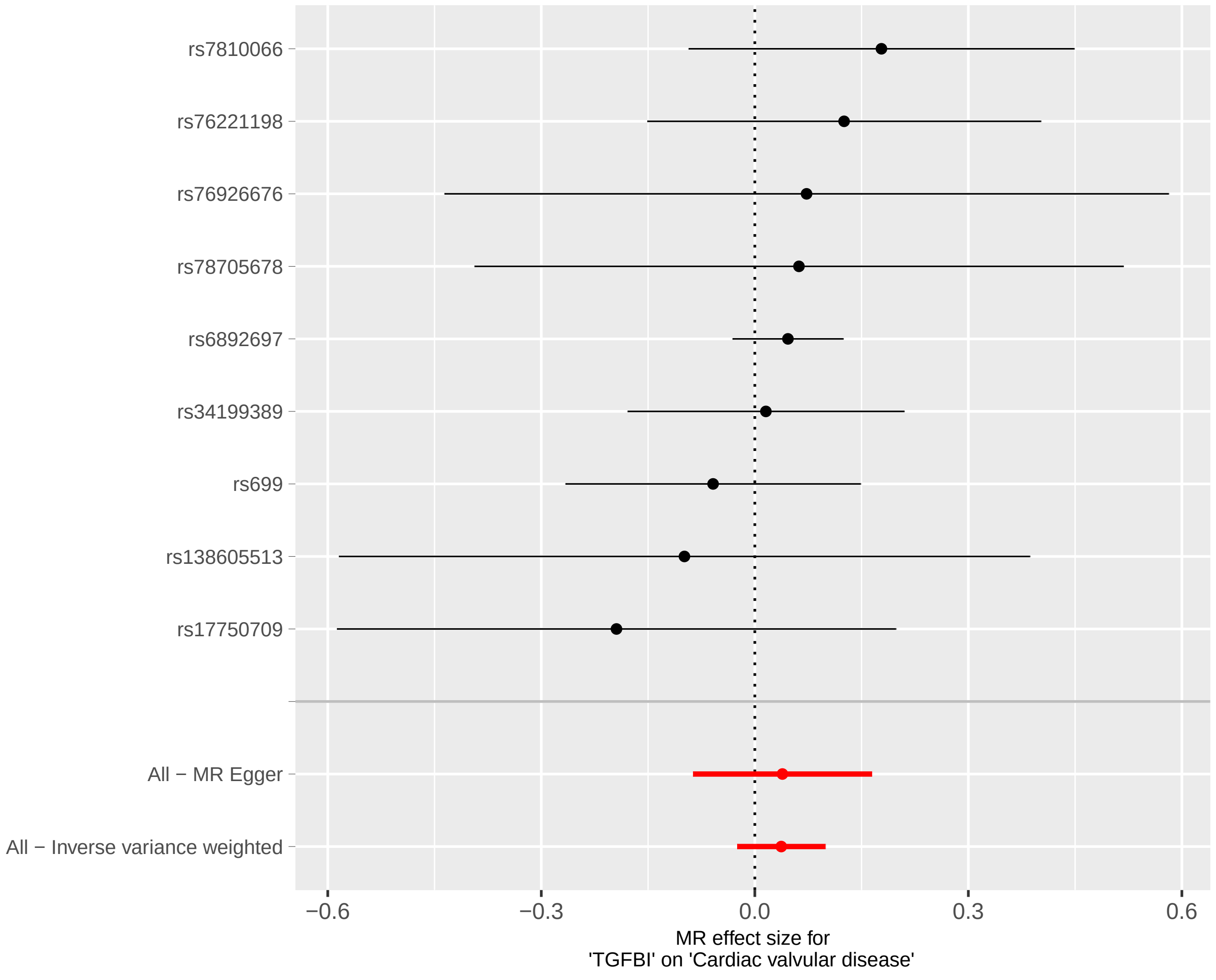
rs7810066
rs76221198
rs76926676
rs78705678
rs6892697
rs34199389
rs699
rs138605513
rs17750709
All − MR Egger
All − Inverse variance weighted
−0.6
−0.3
0.0
0.3
0.6
MR effect size for
'TGFBI' on 'Cardiac valvular disease'

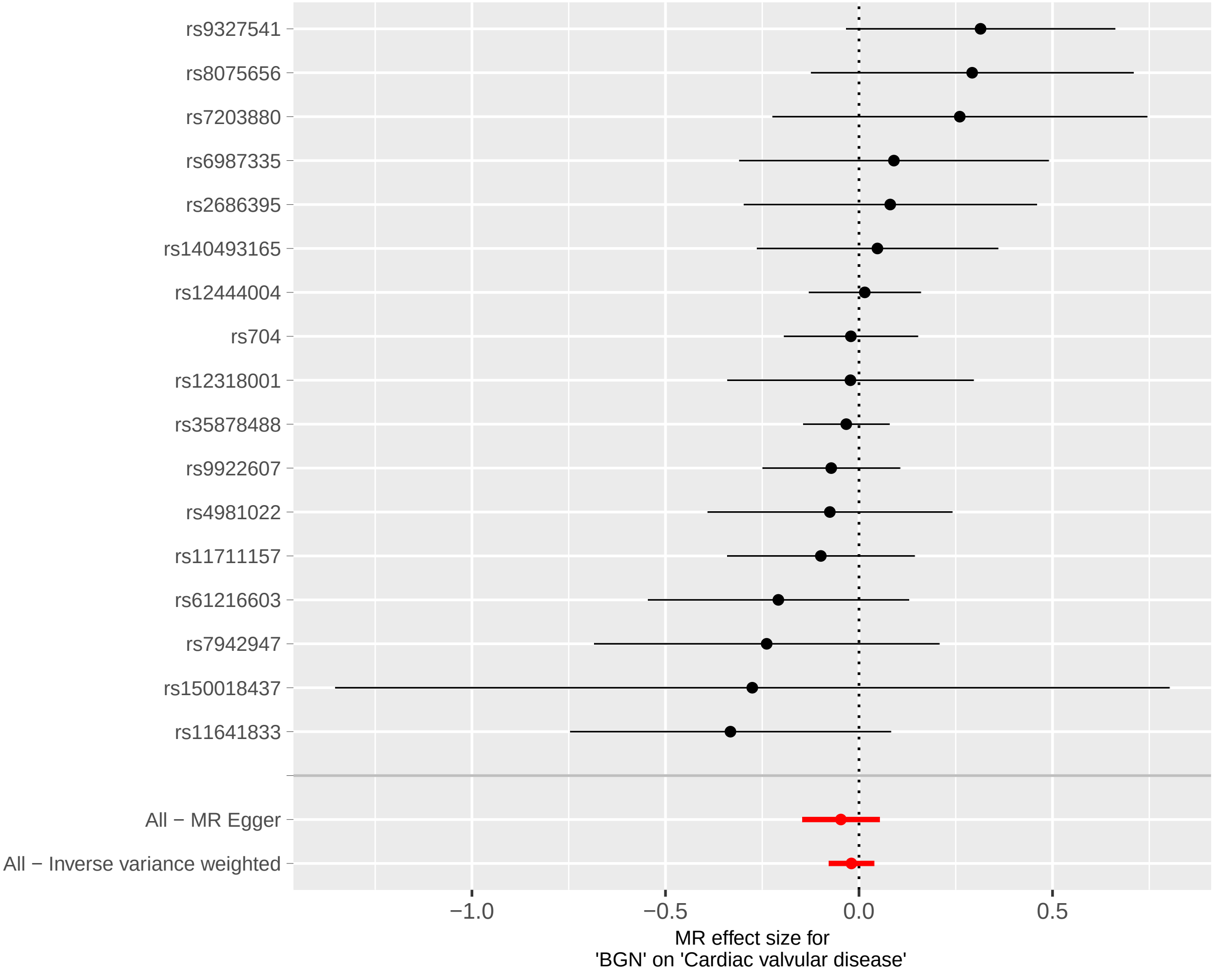
rs9327541
rs8075656
rs7203880
rs6987335
rs2686395
rs140493165
rs12444004
rs704
rs12318001
rs35878488
rs9922607
rs4981022
rs11711157
rs61216603
rs7942947
rs150018437
rs11641833
All − MR Egger
All − Inverse variance weighted
−1.0
−0.5
0.0
0.5
MR effect size for
'BGN' on 'Cardiac valvular disease'

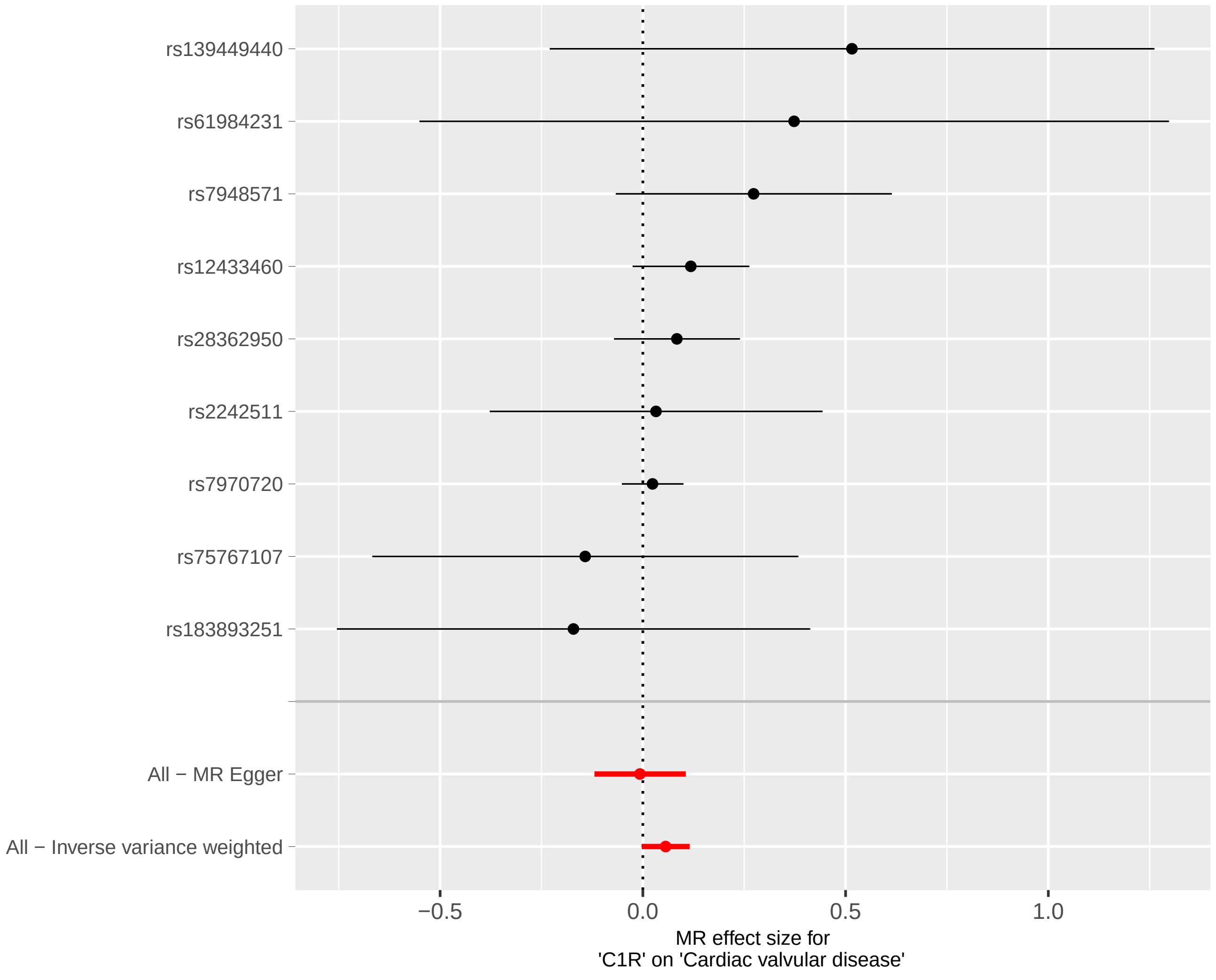
rs139449440
rs61984231
rs7948571
rs12433460
rs28362950
rs2242511
rs7970720
rs75767107
rs183893251
All − MR Egger
All − Inverse variance weighted
−0.5
0.0
0.5
1.0
MR effect size for
'C1R' on 'Cardiac valvular disease'

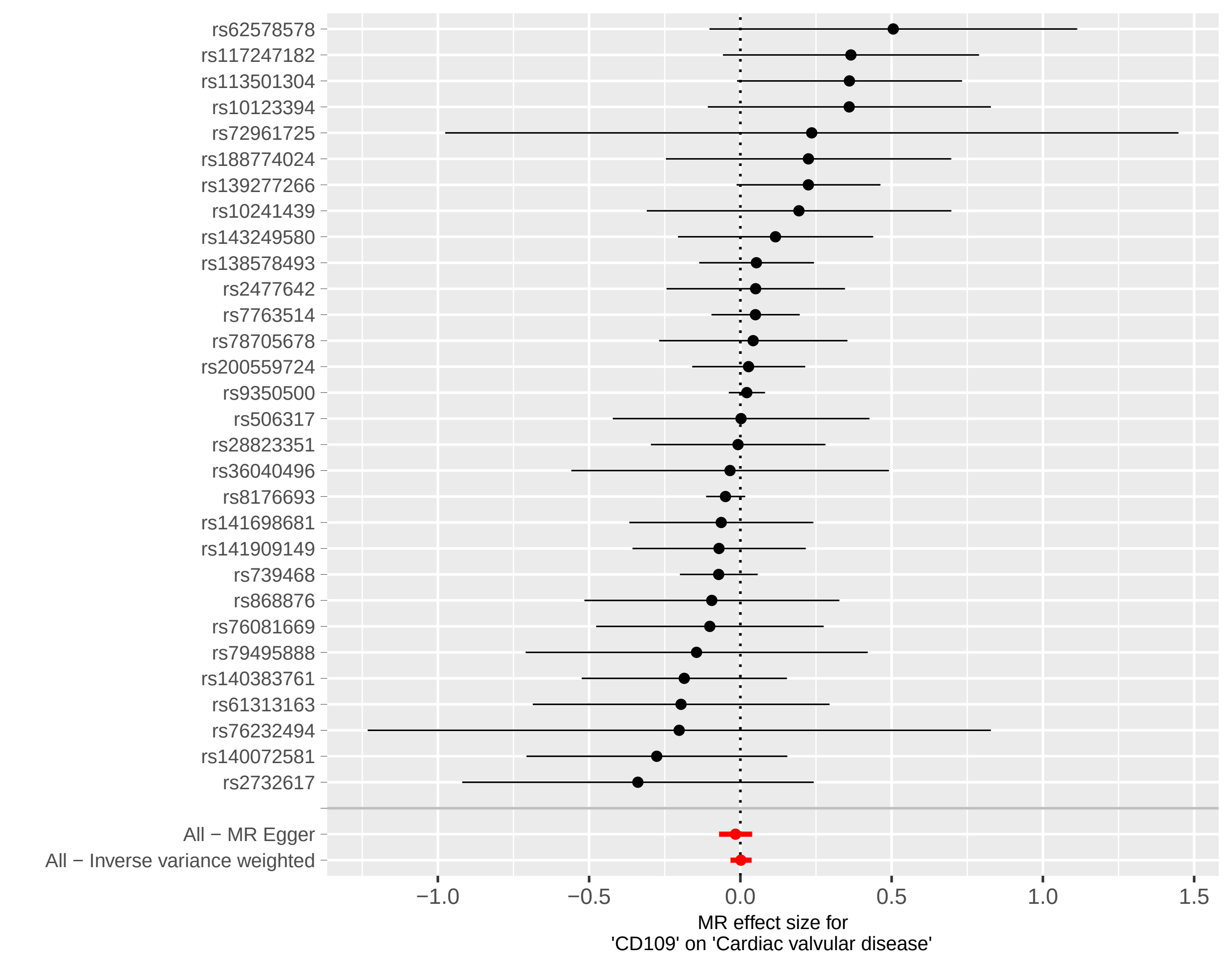
rs62578578
rs117247182
rs113501304
rs10123394
rs72961725
rs188774024
rs139277266
rs10241439
rs143249580
rs138578493
rs2477642
rs7763514
rs78705678
rs200559724
rs9350500
rs506317
rs28823351
rs36040496
rs8176693
rs141698681
rs141909149
rs739468
rs868876
rs76081669
rs79495888
rs140383761
rs61313163
rs76232494
rs140072581
rs2732617
All − MR Egger
All − Inverse variance weighted
−1.0
−0.5
0.0
0.5
1.0
1.5
MR effect size for
'CD109' on 'Cardiac valvular disease'

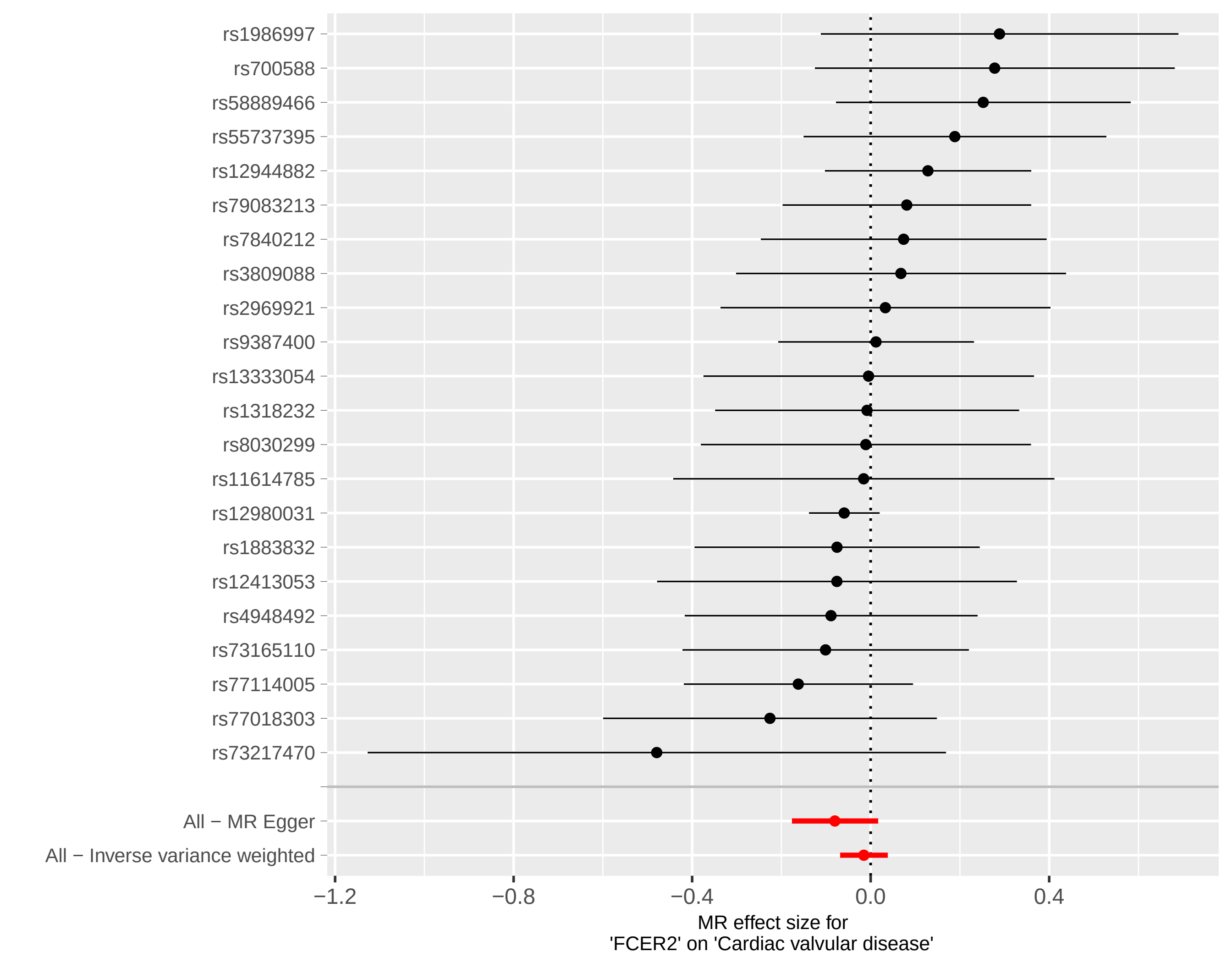

rs1986997
rs700588
rs58889466
rs55737395
rs12944882
rs79083213
rs7840212
rs3809088
rs2969921
rs9387400
rs13333054
rs1318232
rs8030299
rs11614785
rs12980031
rs1883832
rs12413053
rs4948492
rs73165110
rs77114005
rs77018303
rs73217470
All − MR Egger
All − Inverse variance weighted
−1.2
−0.8
−0.4
0.0
0.4
MR effect size for
'FCER2' on 'Cardiac valvular disease'

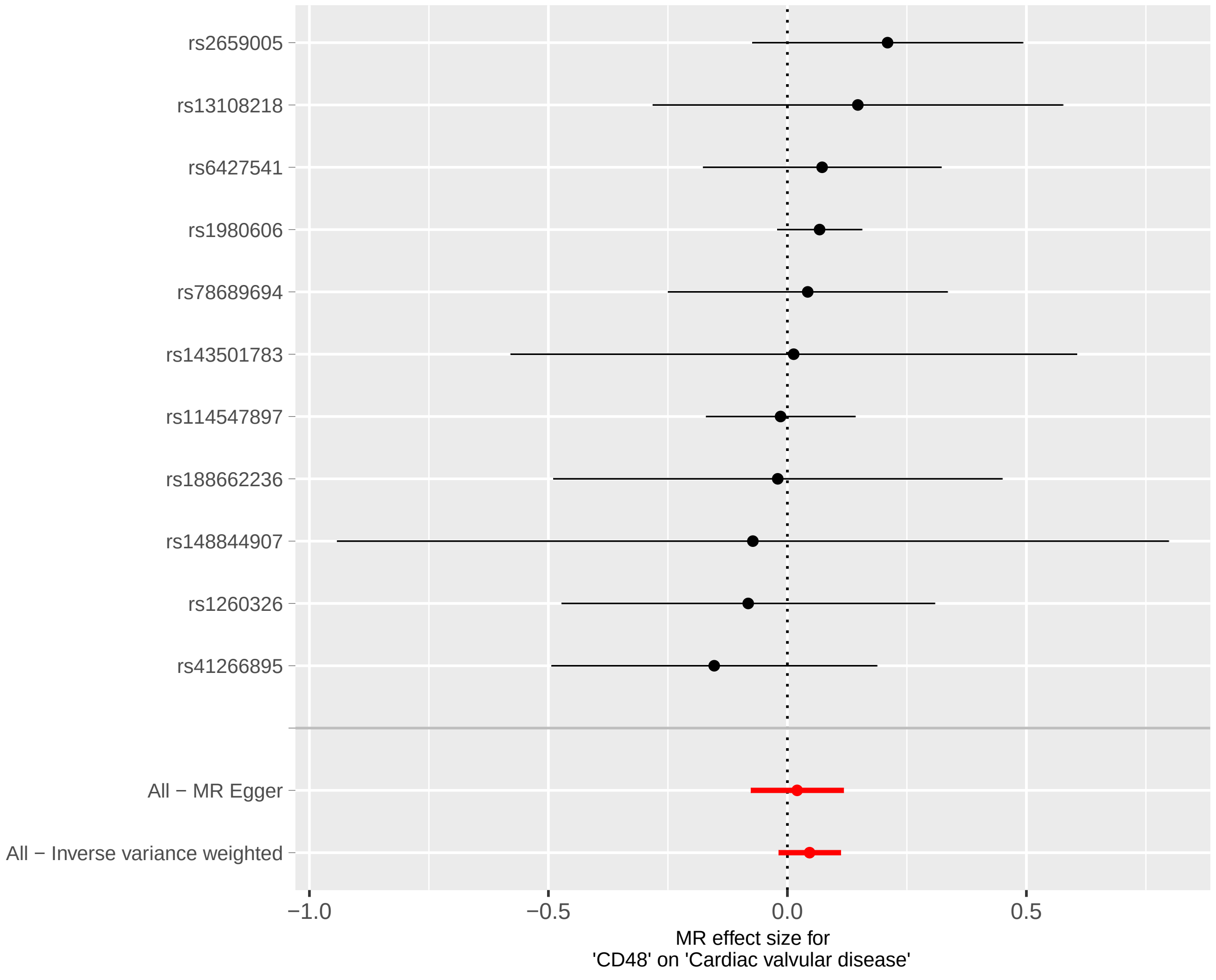
rs2659005
rs13108218
rs6427541
rs1980606
rs78689694
rs143501783
rs114547897
rs188662236
rs148844907
rs1260326
rs41266895
All − MR Egger
All − Inverse variance weighted
−1.0
−0.5
0.0
0.5
MR effect size for
'CD48' on 'Cardiac valvular disease'

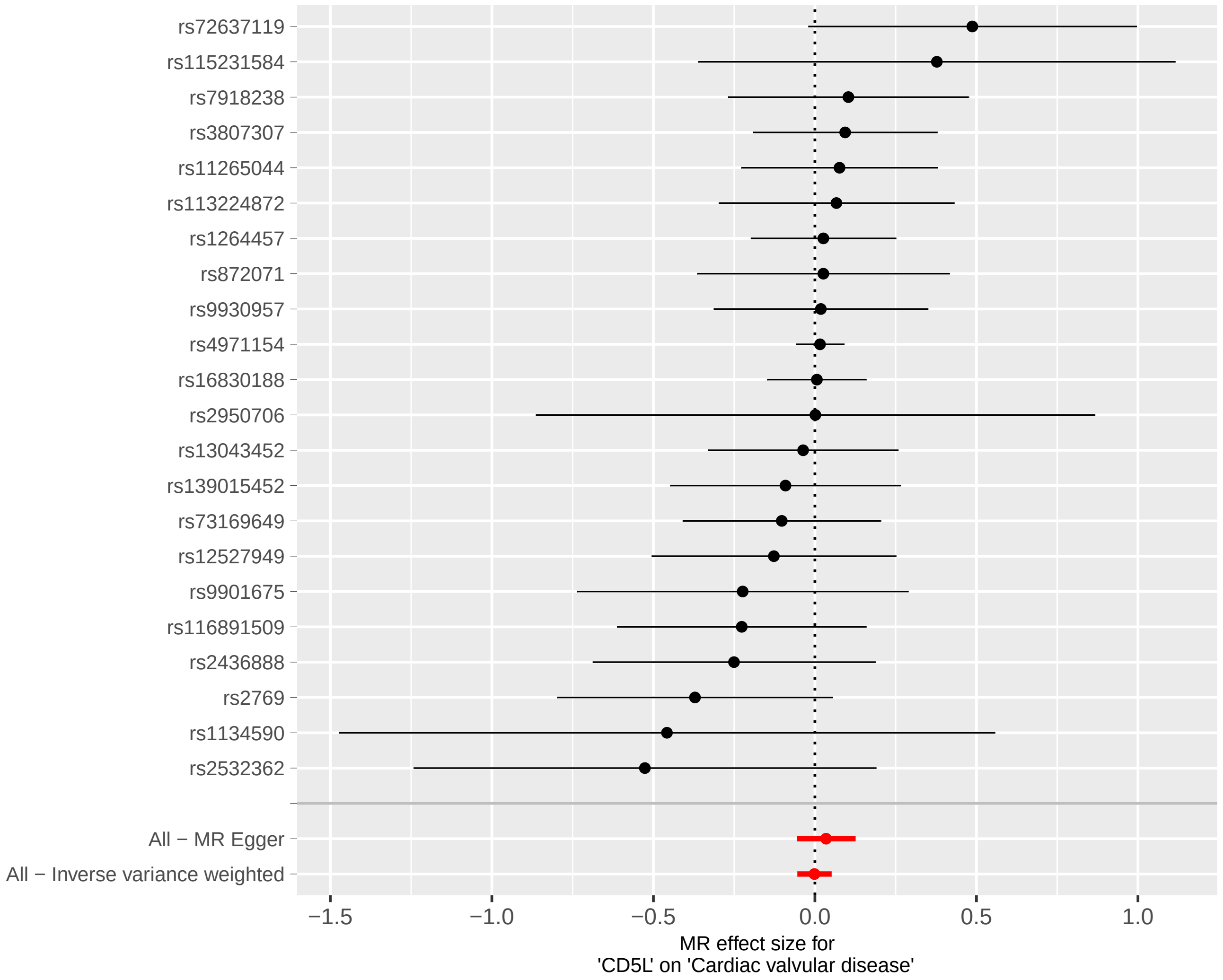
rs72637119
rs115231584
rs7918238
rs3807307
rs11265044
rs113224872
rs1264457
rs872071
rs9930957
rs4971154
rs16830188
rs2950706
rs13043452
rs139015452
rs73169649
rs12527949
rs9901675
rs116891509
rs2436888
rs2769
rs1134590
rs2532362
All − MR Egger
All − Inverse variance weighted
−1.5
−1.0
−0.5
0.0
0.5
1.0
MR effect size for
'CD5L' on 'Cardiac valvular disease'

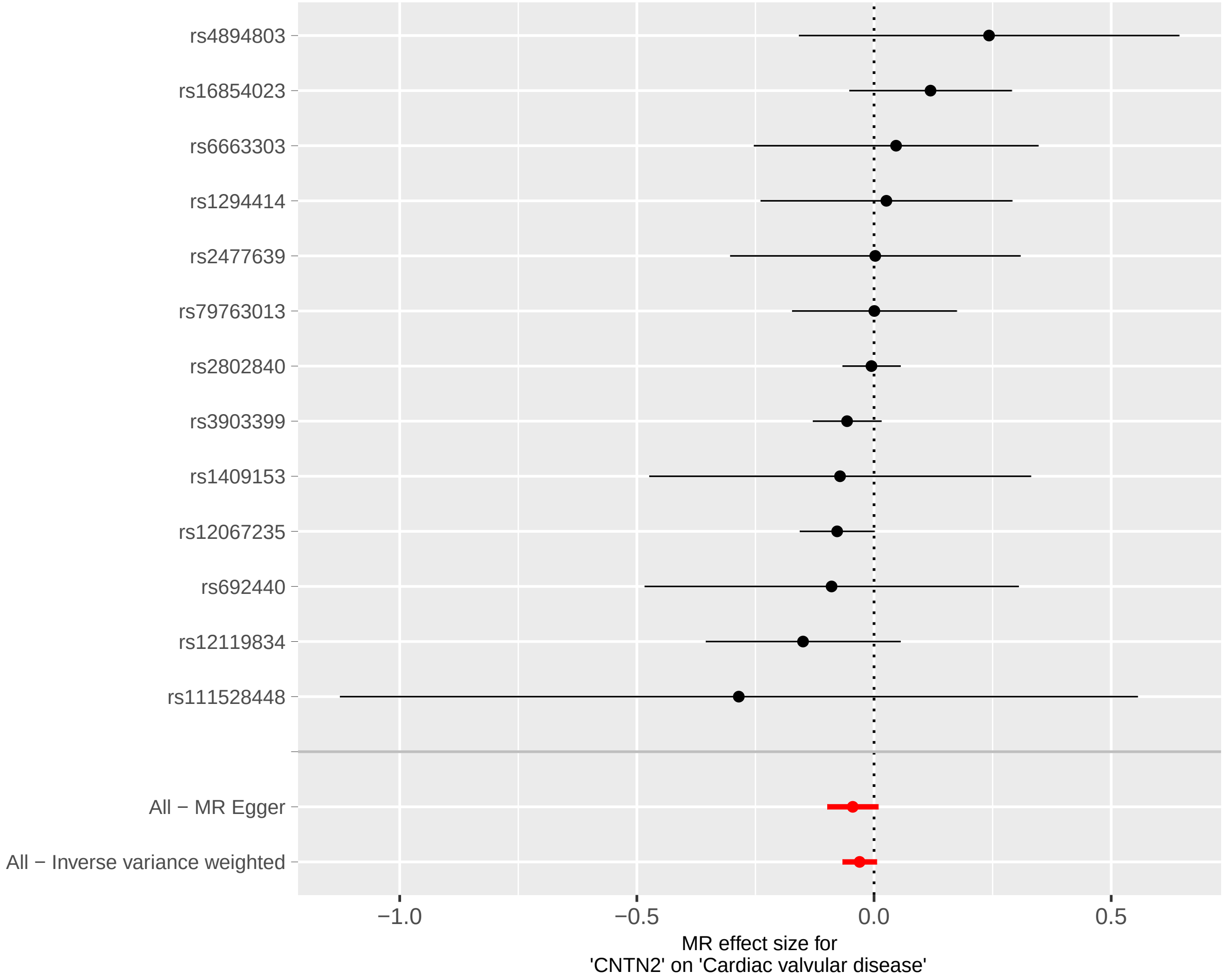
rs4894803
rs16854023
rs6663303
rs1294414
rs2477639
rs79763013
rs2802840
rs3903399
rs1409153
rs12067235
rs692440
rs12119834
rs111528448
All − MR Egger
All − Inverse variance weighted
−1.0
−0.5
0.0
0.5
MR effect size for
'CNTN2' on 'Cardiac valvular disease'

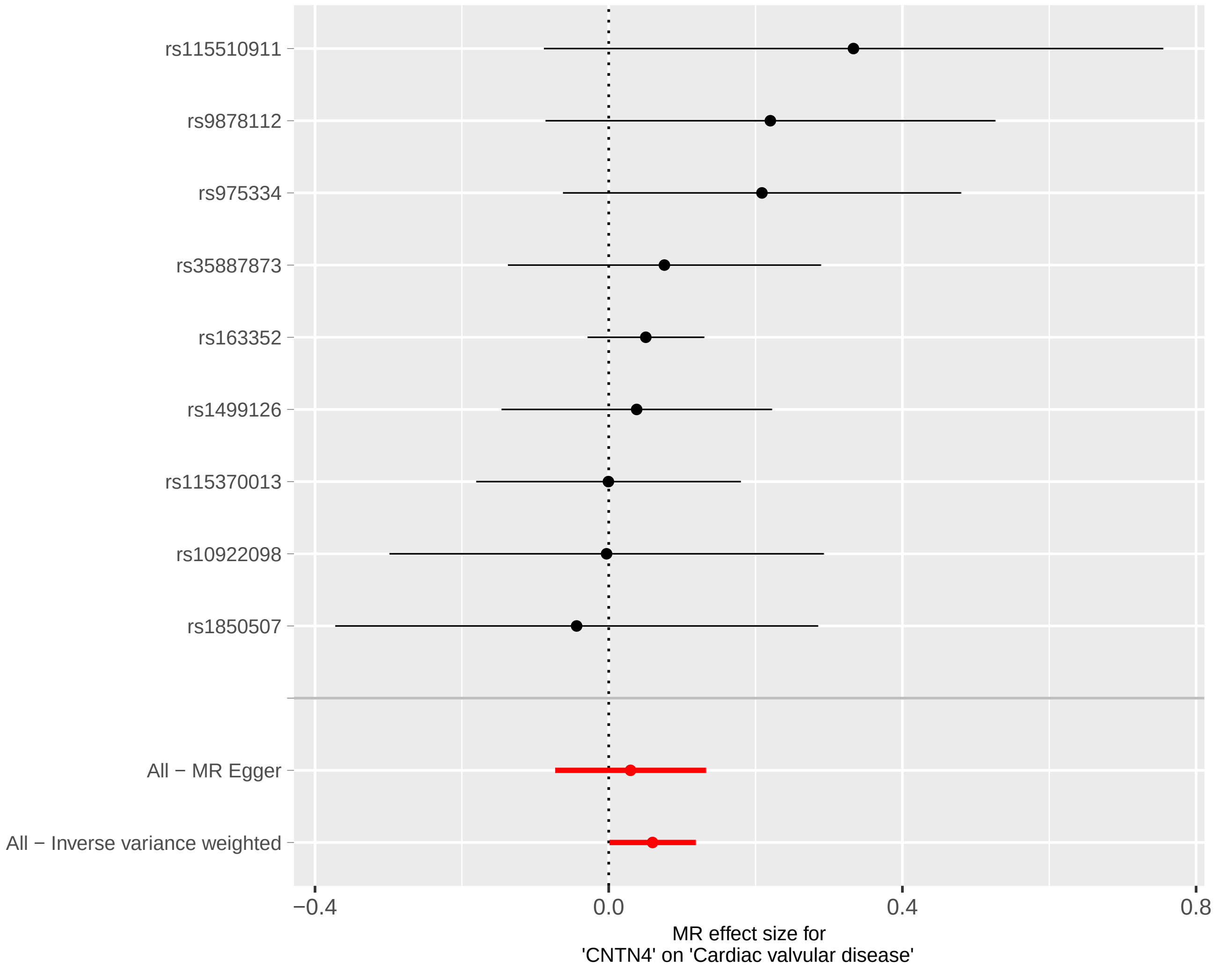
rs115510911
rs9878112
rs975334
rs35887873
rs163352
rs1499126
rs115370013
rs10922098
rs1850507
All − MR Egger
All − Inverse variance weighted
−0.4
0.0
0.4
0.8
MR effect size for
'CNTN4' on 'Cardiac valvular disease'

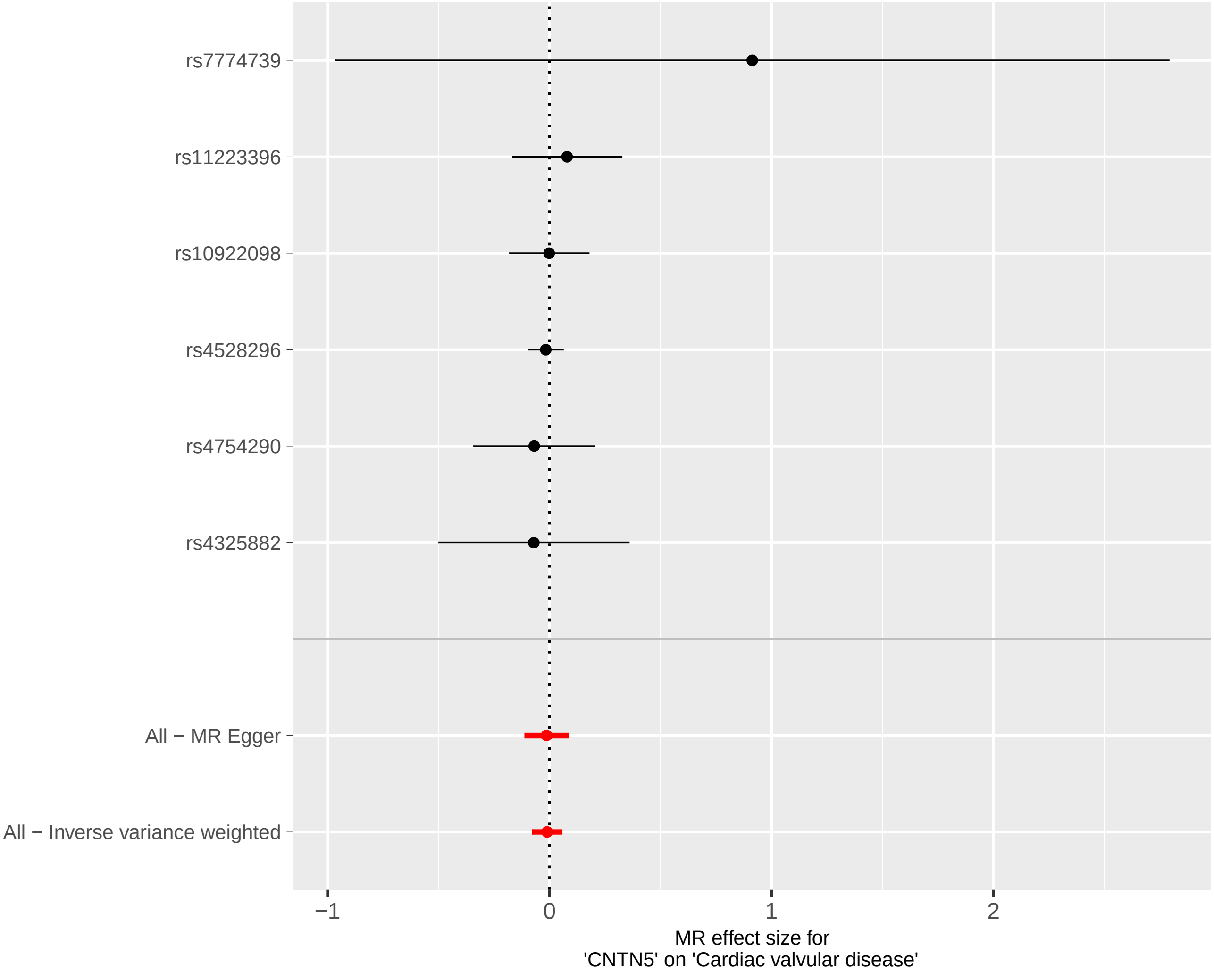

rs7774739
rs11223396
rs10922098
rs4528296
rs4754290
rs4325882
All − MR Egger
All − Inverse variance weighted
−1
0
1
2
MR effect size for
'CNTN5' on 'Cardiac valvular disease'

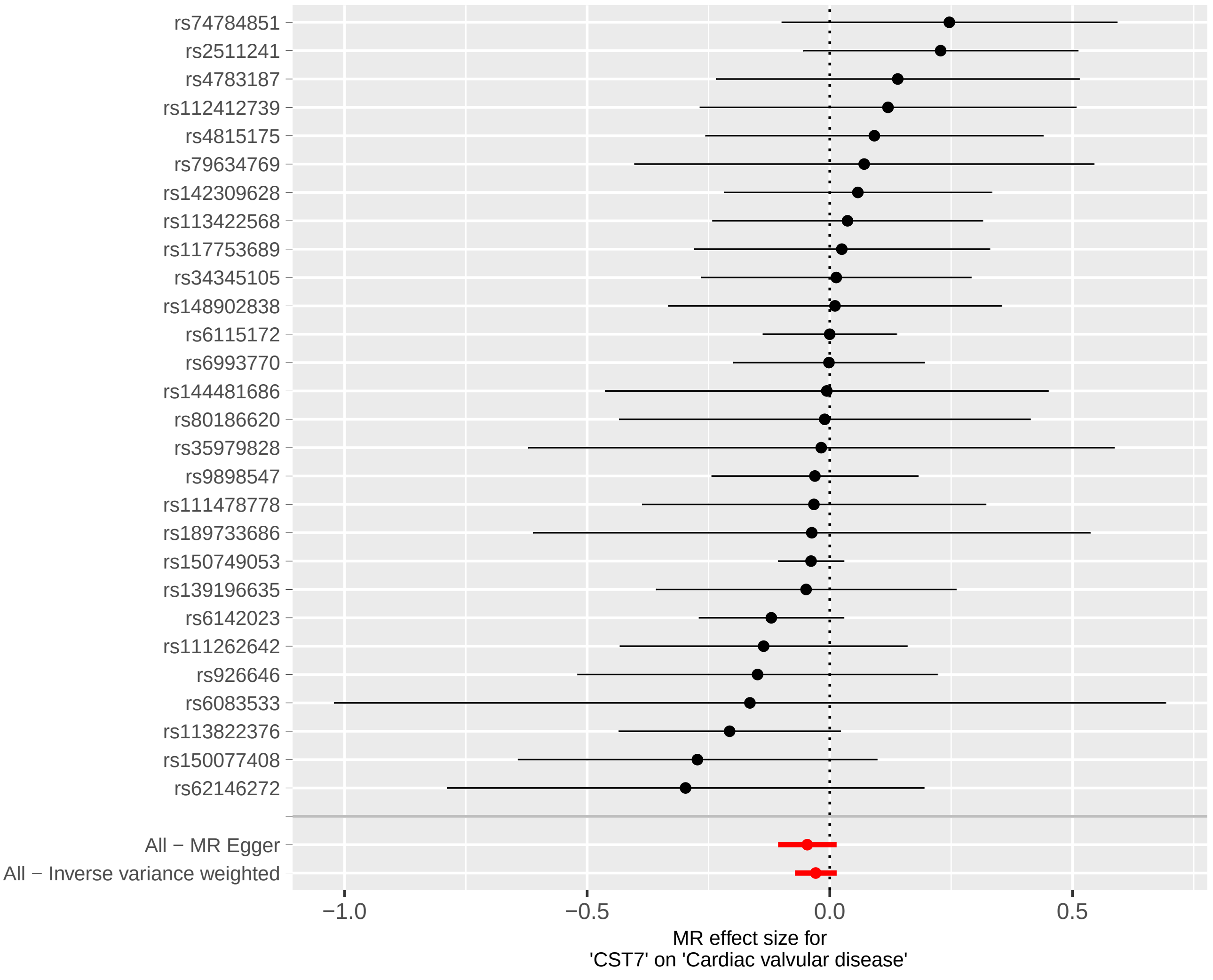

rs74784851
rs2511241
rs4783187
rs112412739
rs4815175
rs79634769
rs142309628
rs113422568
rs117753689
rs34345105
rs148902838
rs6115172
rs6993770
rs144481686
rs80186620
rs35979828
rs9898547
rs111478778
rs189733686
rs150749053
rs139196635
rs6142023
rs111262642
rs926646
rs6083533
rs113822376
rs150077408
rs62146272
All − MR Egger
All − Inverse variance weighted
−1.0
−0.5
0.0
0.5
MR effect size for
'CST7' on 'Cardiac valvular disease'

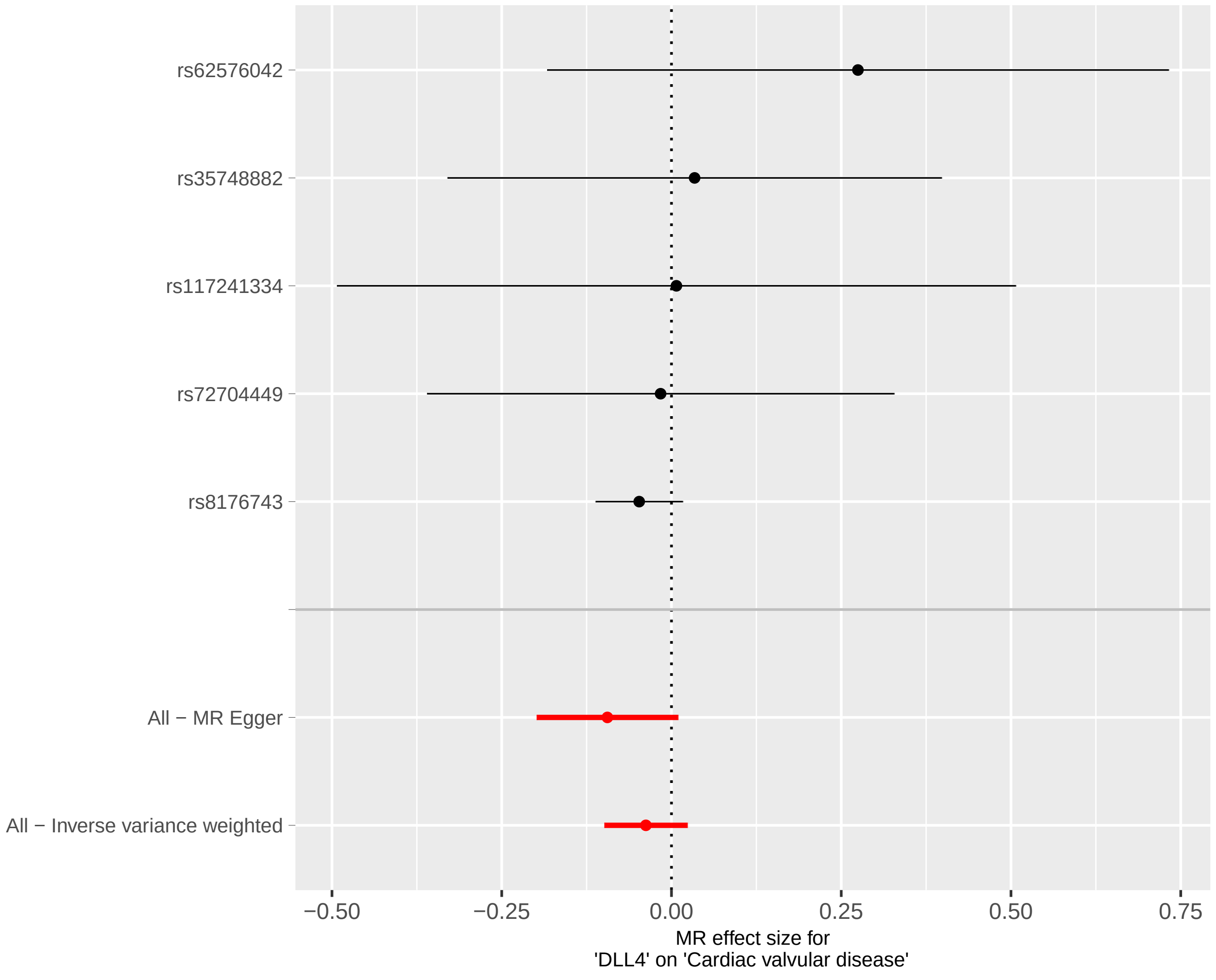

rs62576042
rs35748882
rs117241334
rs72704449
rs8176743
All − MR Egger
All − Inverse variance weighted
−0.50
−0.25
0.00
0.25
0.50
0.75
MR effect size for
'DLL4' on 'Cardiac valvular disease'

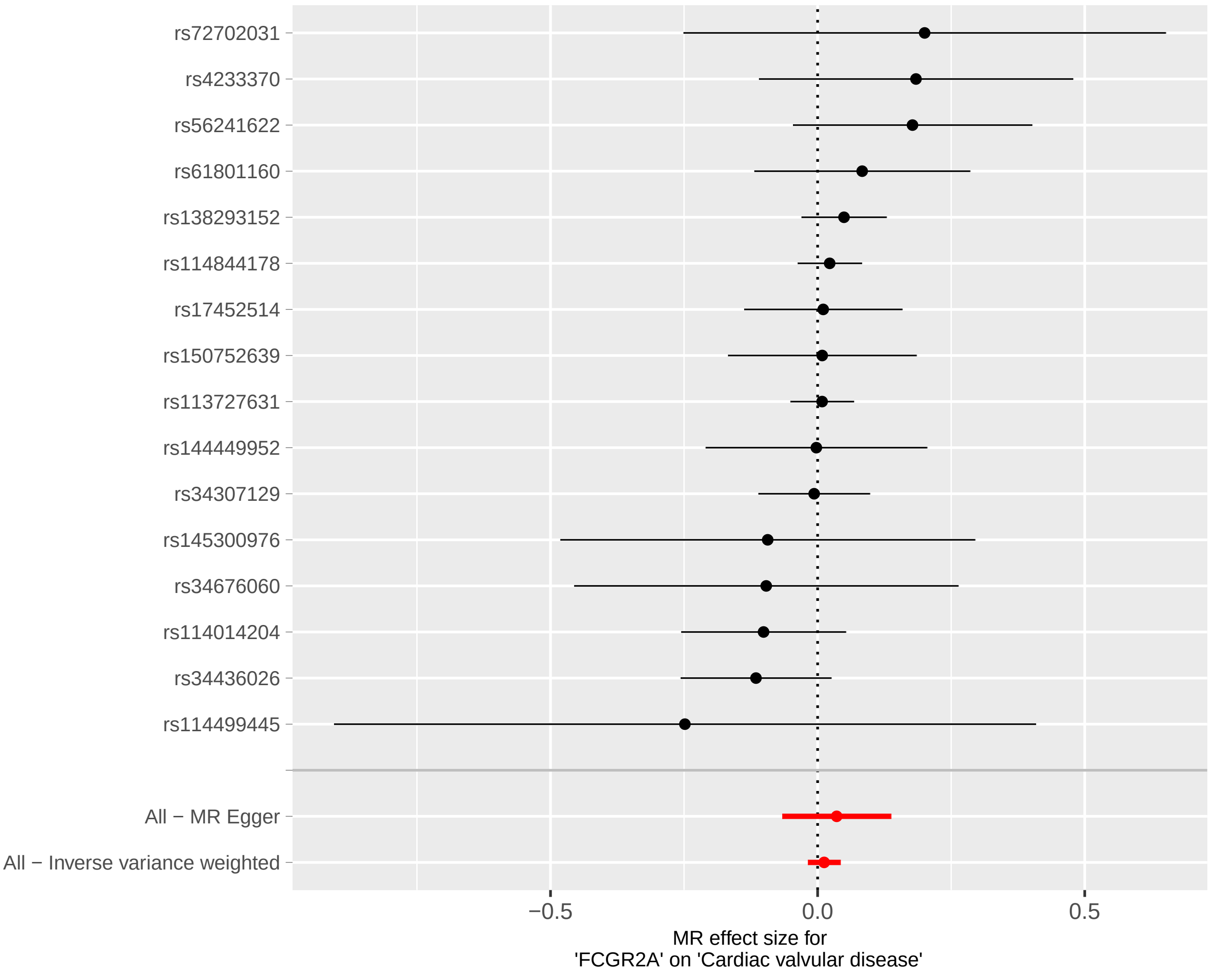
rs72702031
rs4233370
rs56241622
rs61801160
rs138293152
rs114844178
rs17452514
rs150752639
rs113727631
rs144449952
rs34307129
rs145300976
rs34676060
rs114014204
rs34436026
rs114499445
All − MR Egger
All − Inverse variance weighted
−0.5
0.0
0.5
MR effect size for
'FCGR2A' on 'Cardiac valvular disease'

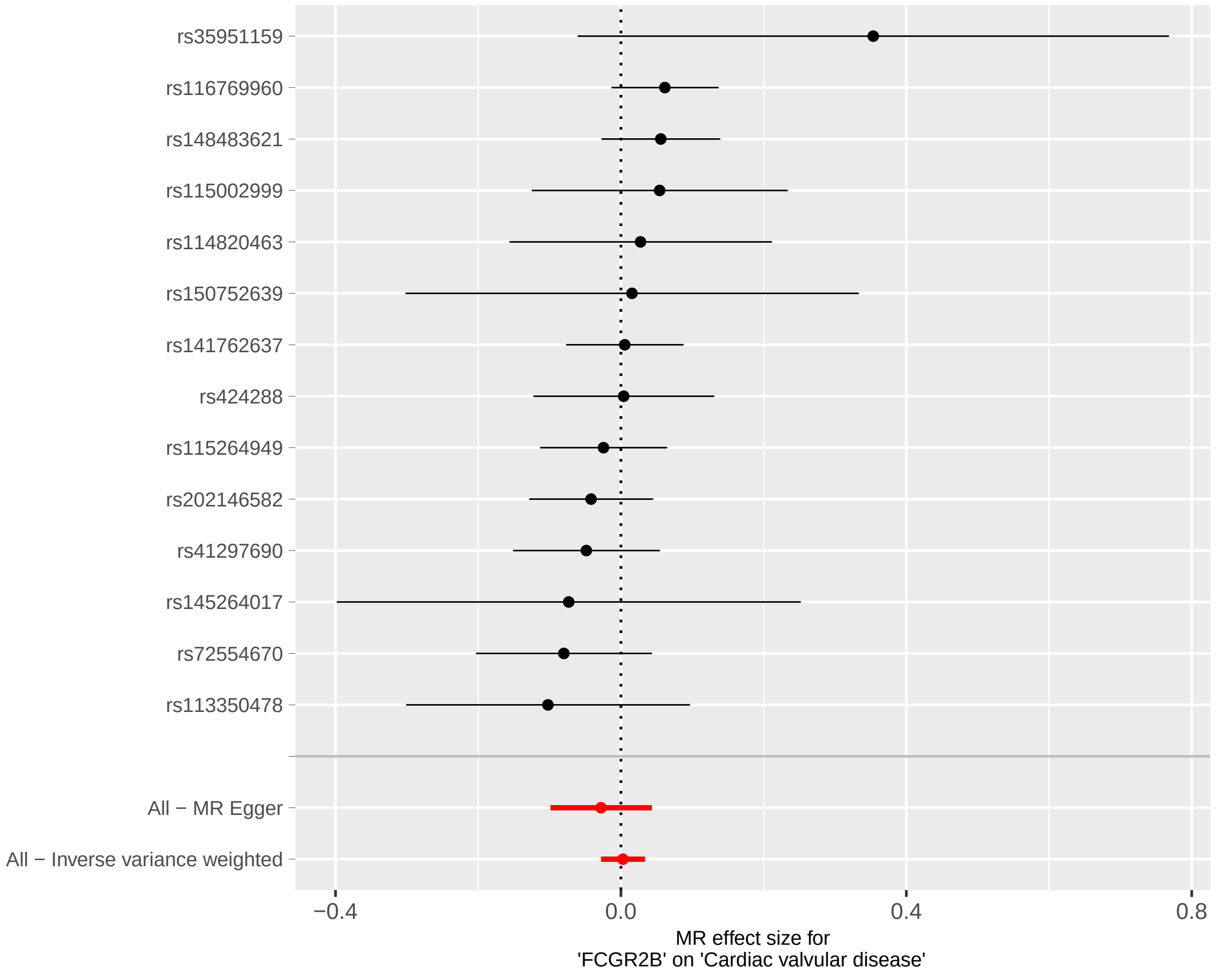
rs35951159
rs116769960
rs148483621
rs115002999
rs114820463
rs150752639
rs141762637
rs424288
rs115264949
rs202146582
rs41297690
rs145264017
rs72554670
rs113350478
All − MR Egger
All − Inverse variance weighted
−0.4
0.0
0.4
0.8
MR effect size for
'FCGR2B' on 'Cardiac valvular disease'

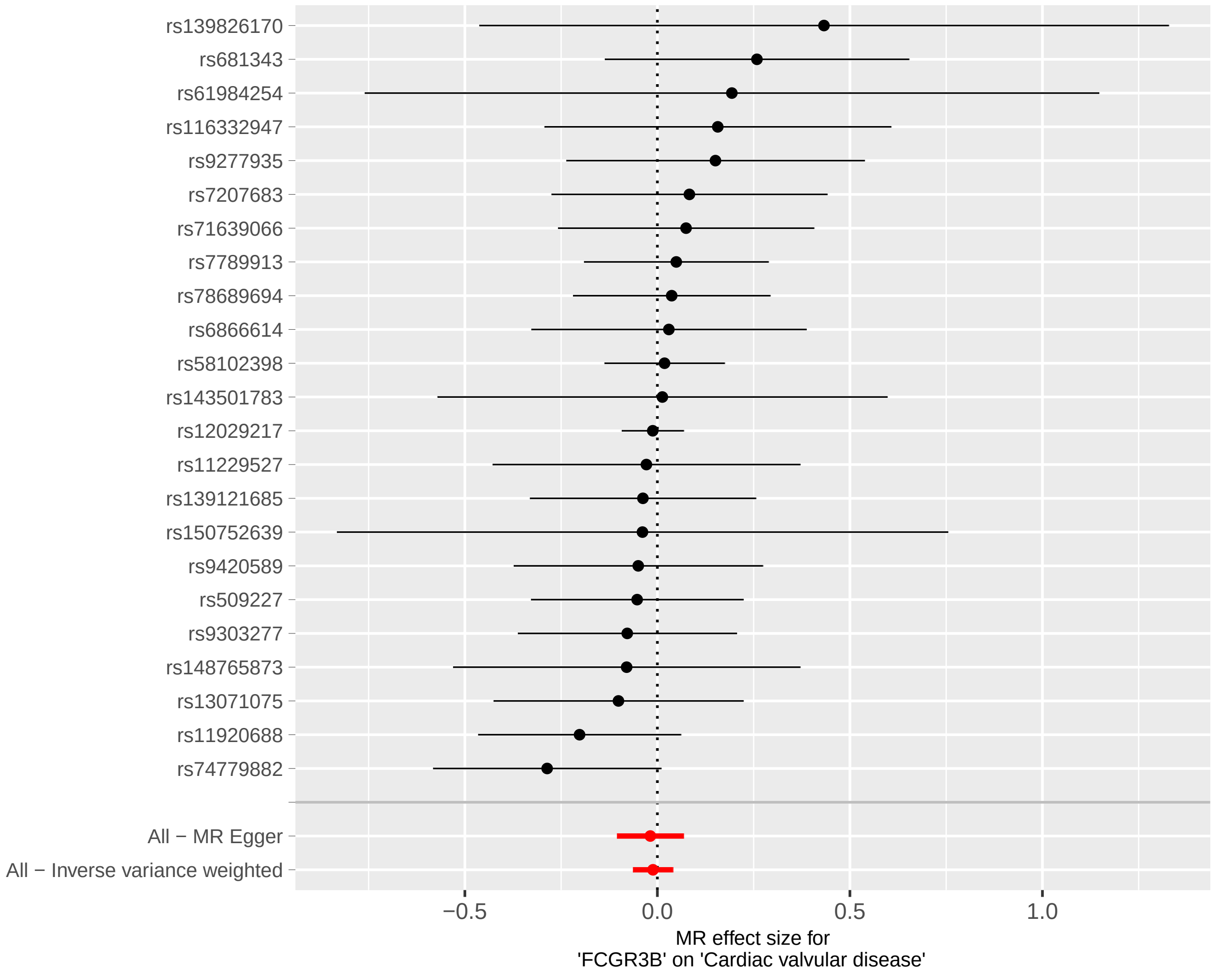

rs139826170
rs681343
rs61984254
rs116332947
rs9277935
rs7207683
rs71639066
rs7789913
rs78689694
rs6866614
rs58102398
rs143501783
rs12029217
rs11229527
rs139121685
rs150752639
rs9420589
rs509227
rs9303277
rs148765873
rs13071075
rs11920688
rs74779882
All − MR Egger
All − Inverse variance weighted
−0.5
0.0
0.5
1.0
MR effect size for
'FCGR3B' on 'Cardiac valvular disease'

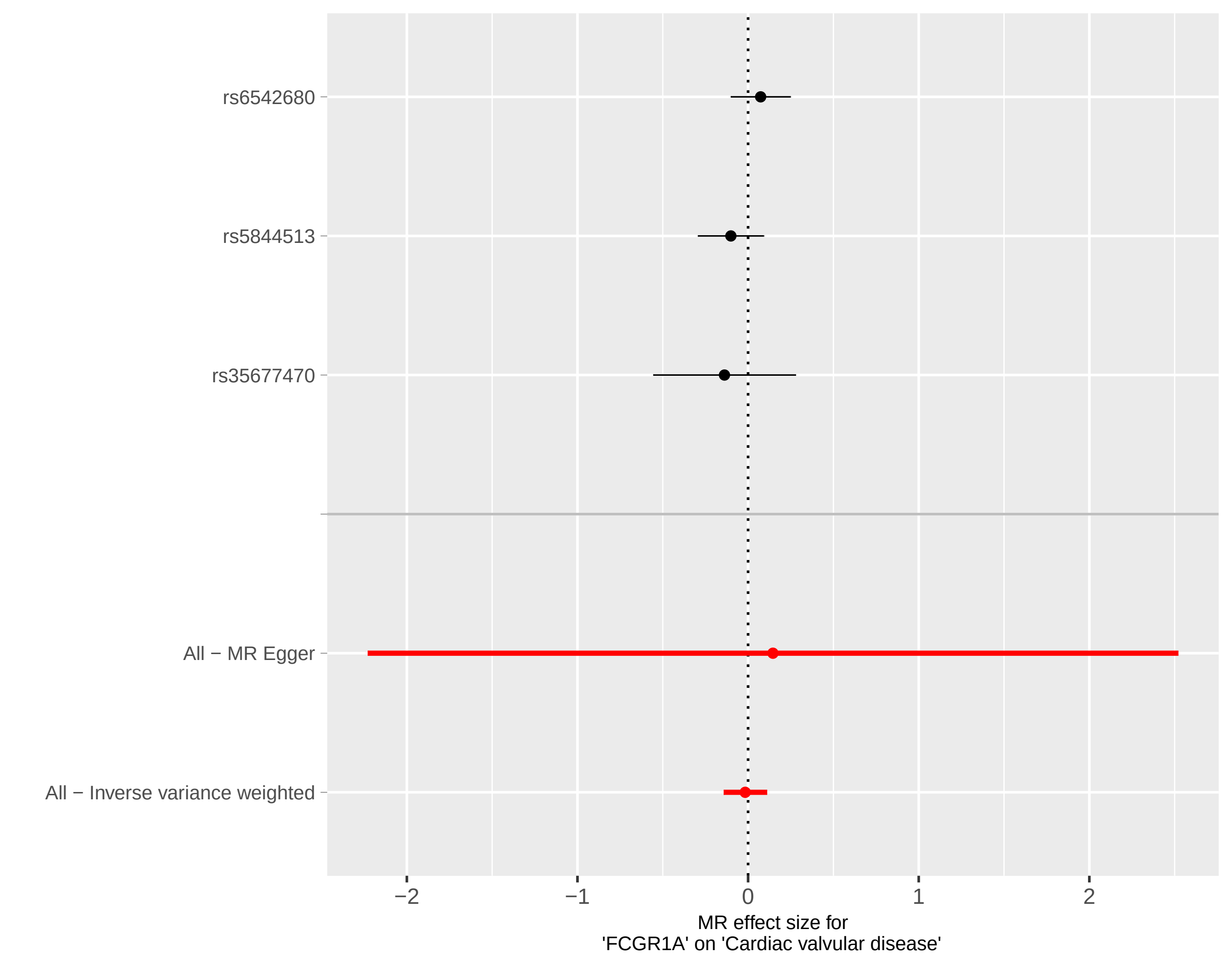

rs6542680
rs5844513
rs35677470
All − MR Egger
All − Inverse variance weighted
−2
−1
0
1
2
MR effect size for
'FCGR1A' on 'Cardiac valvular disease'

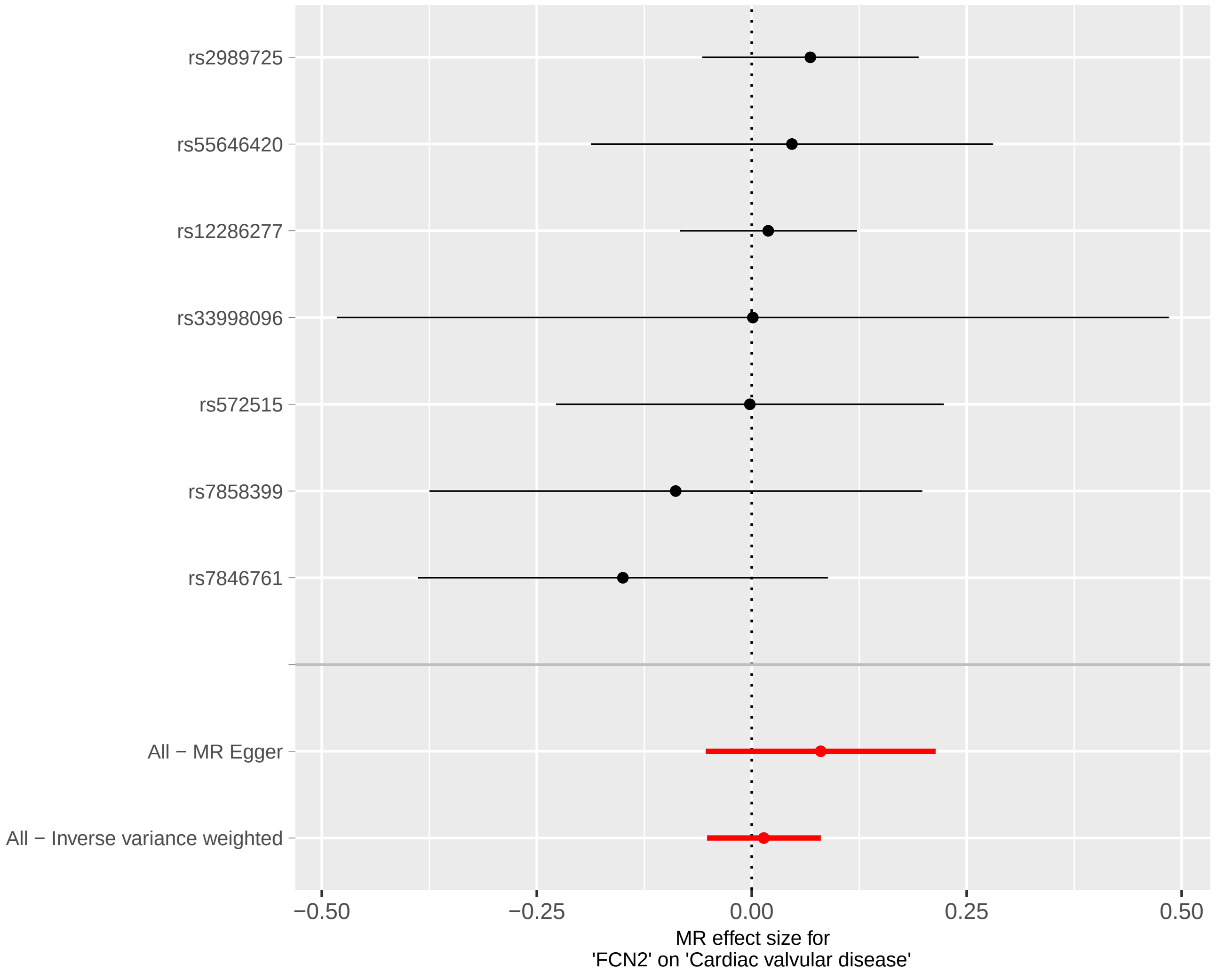
rs2989725
rs55646420
rs12286277
rs33998096
rs572515
rs7858399
rs7846761
All − MR Egger
All − Inverse variance weighted
−0.50
−0.25
0.00
0.25
0.50
MR effect size for
'FCN2' on 'Cardiac valvular disease'

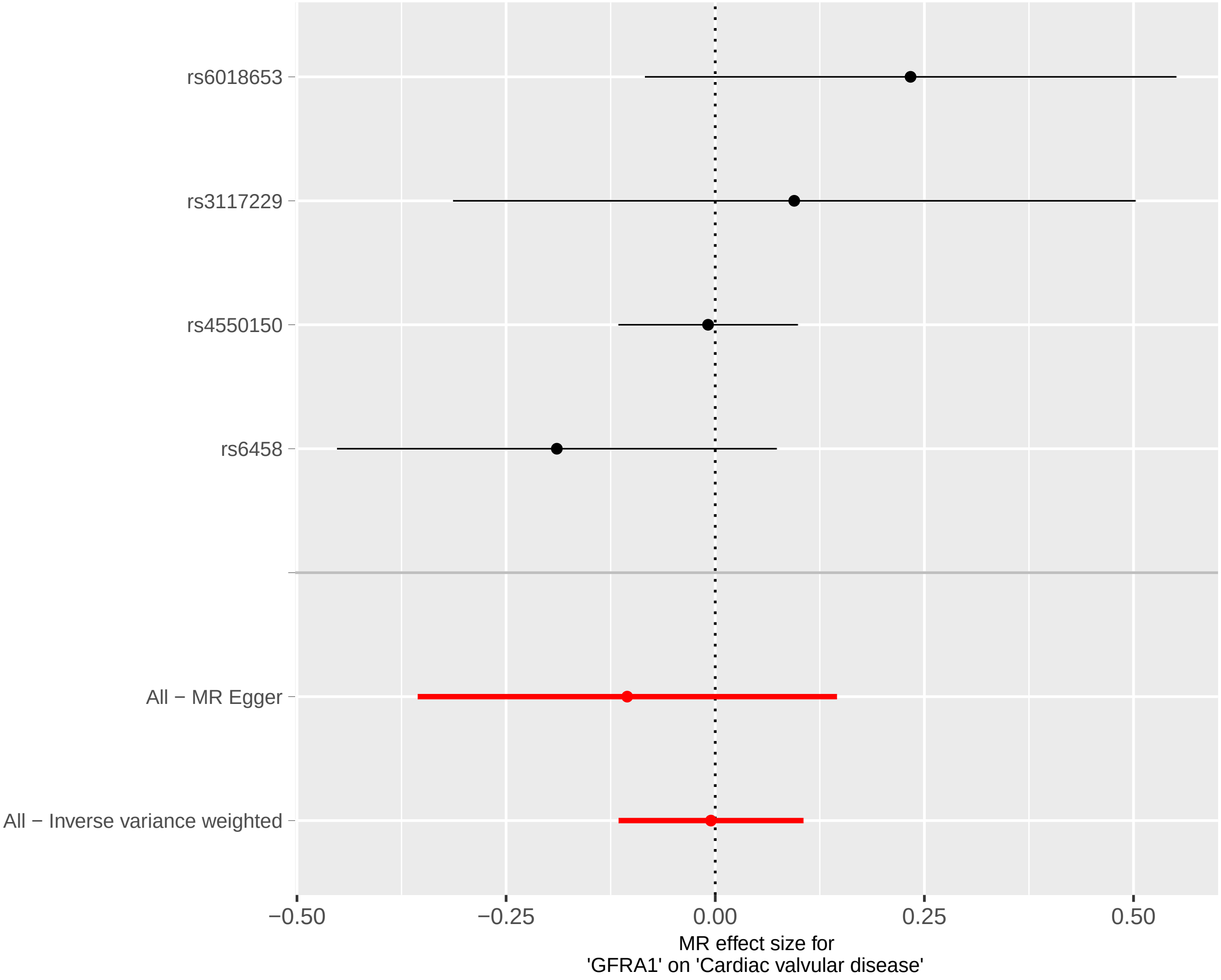
rs6018653
rs3117229
rs4550150
rs6458
All − MR Egger
All − Inverse variance weighted
−0.50
−0.25
0.00
0.25
0.50
MR effect size for
'GFRA1' on 'Cardiac valvular disease'

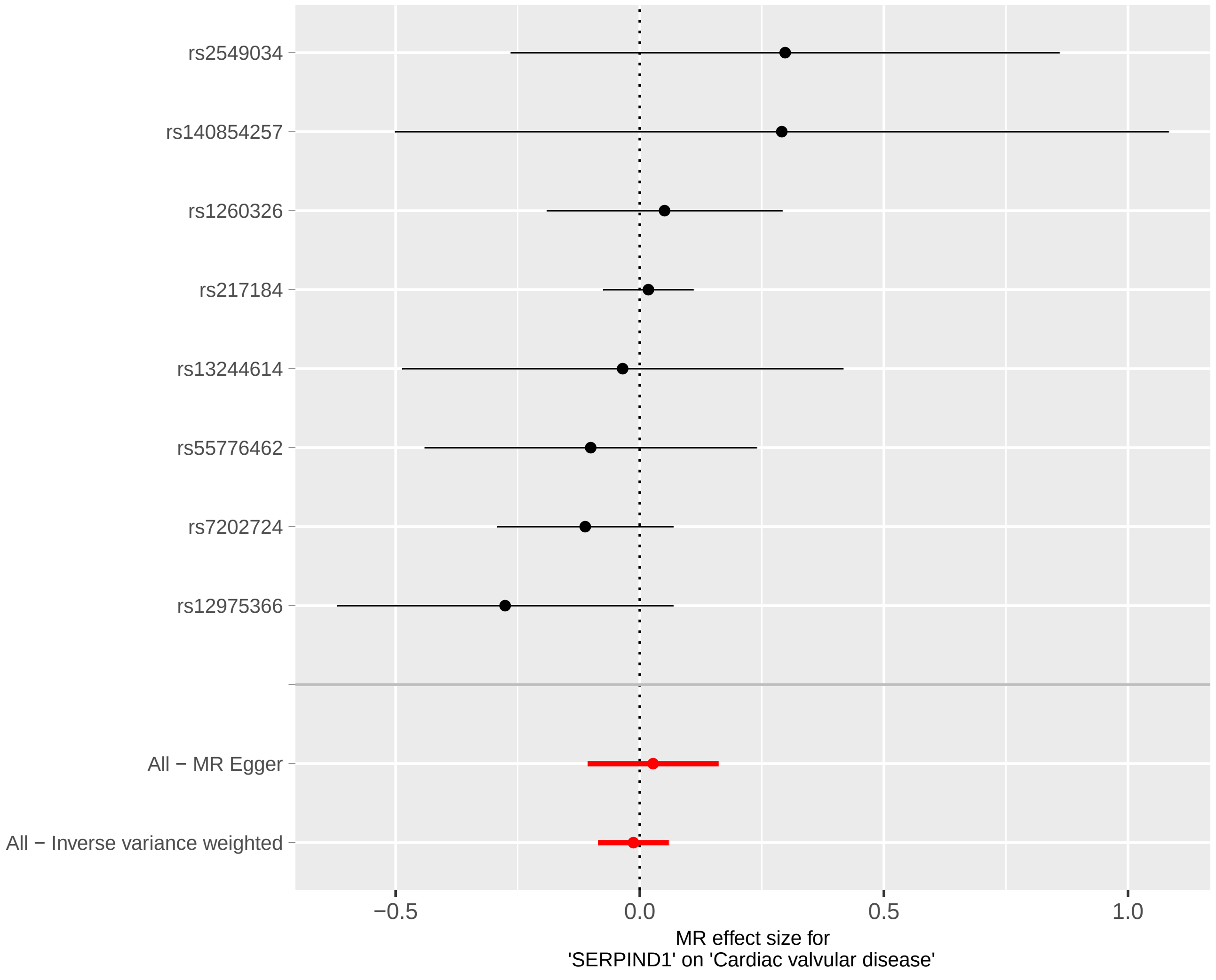
rs2549034
rs140854257
rs1260326
rs217184
rs13244614
rs55776462
rs7202724
rs12975366
All − MR Egger
All − Inverse variance weighted
−0.5
0.0
0.5
1.0
MR effect size for
'SERPIND1' on 'Cardiac valvular disease'

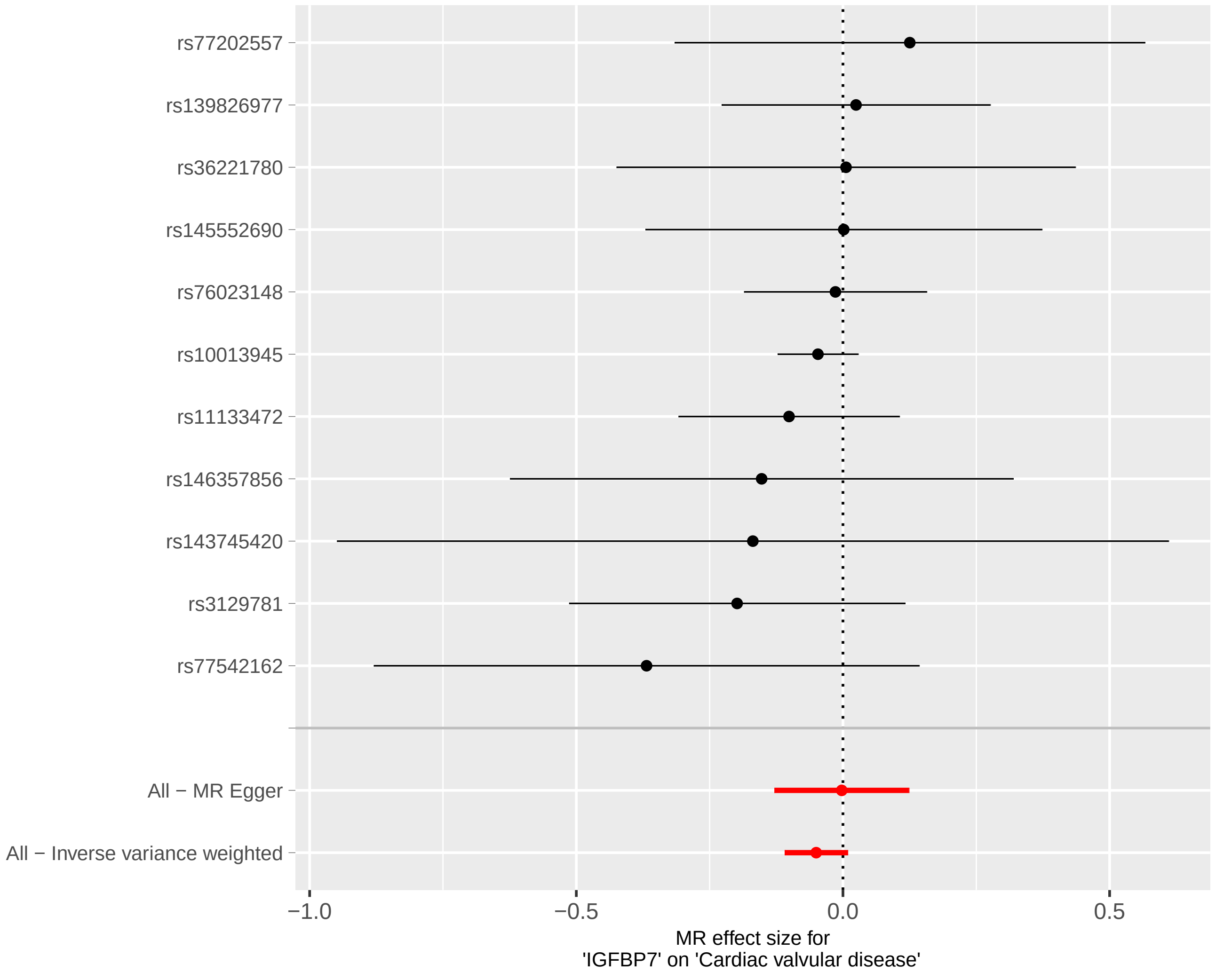
rs77202557
rs139826977
rs36221780
rs145552690
rs76023148
rs10013945
rs11133472
rs146357856
rs143745420
rs3129781
rs77542162
All − MR Egger
All − Inverse variance weighted
−1.0
−0.5
0.0
0.5
MR effect size for
'IGFBP7' on 'Cardiac valvular disease'

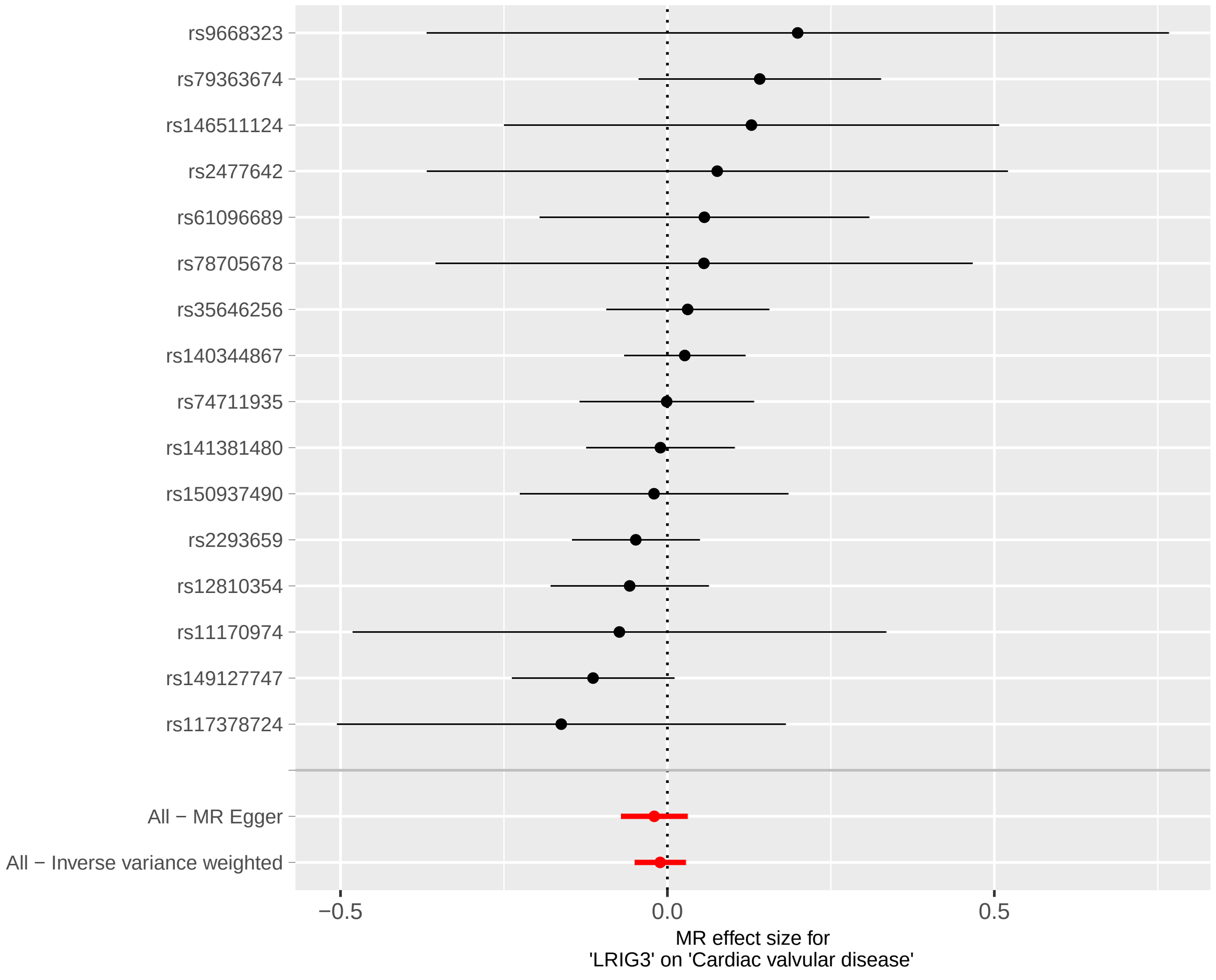
rs9668323
rs79363674
rs146511124
rs2477642
rs61096689
rs78705678
rs35646256
rs140344867
rs74711935
rs141381480
rs150937490
rs2293659
rs12810354
rs11170974
rs149127747
rs117378724
All − MR Egger
All − Inverse variance weighted
−0.5
0.0
0.5
MR effect size for
'LRIG3' on 'Cardiac valvular disease'

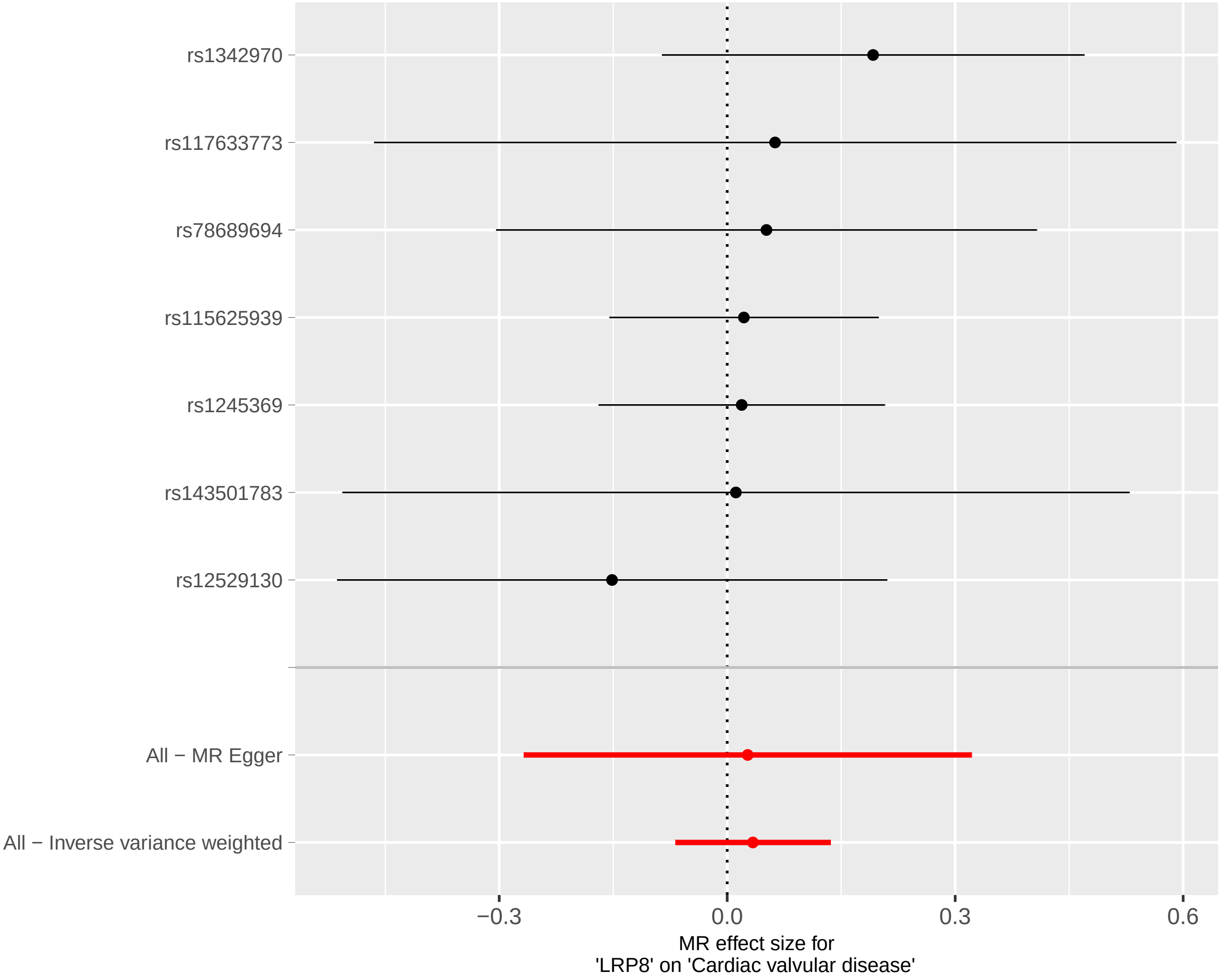

rs1342970
rs117633773
rs78689694
rs115625939
rs1245369
rs143501783
rs12529130
All − MR Egger
All − Inverse variance weighted
−0.3
0.0
0.3
0.6
MR effect size for
'LRP8' on 'Cardiac valvular disease'

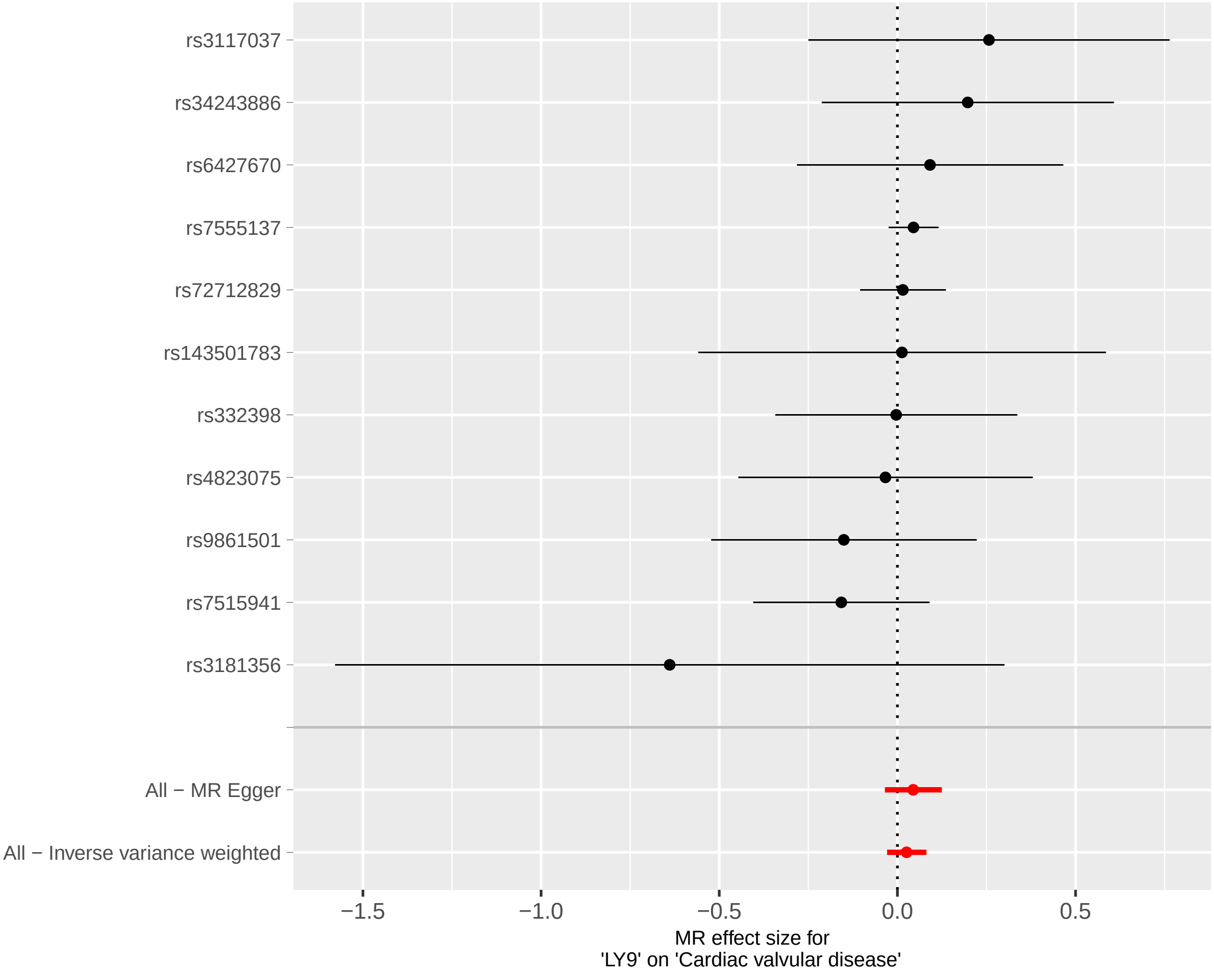
rs3117037
rs34243886
rs6427670
rs7555137
rs72712829
rs143501783
rs332398
rs4823075
rs9861501
rs7515941
rs3181356
All − MR Egger
All − Inverse variance weighted
−1.5
−1.0
−0.5
0.0
0.5
MR effect size for
'LY9' on 'Cardiac valvular disease'

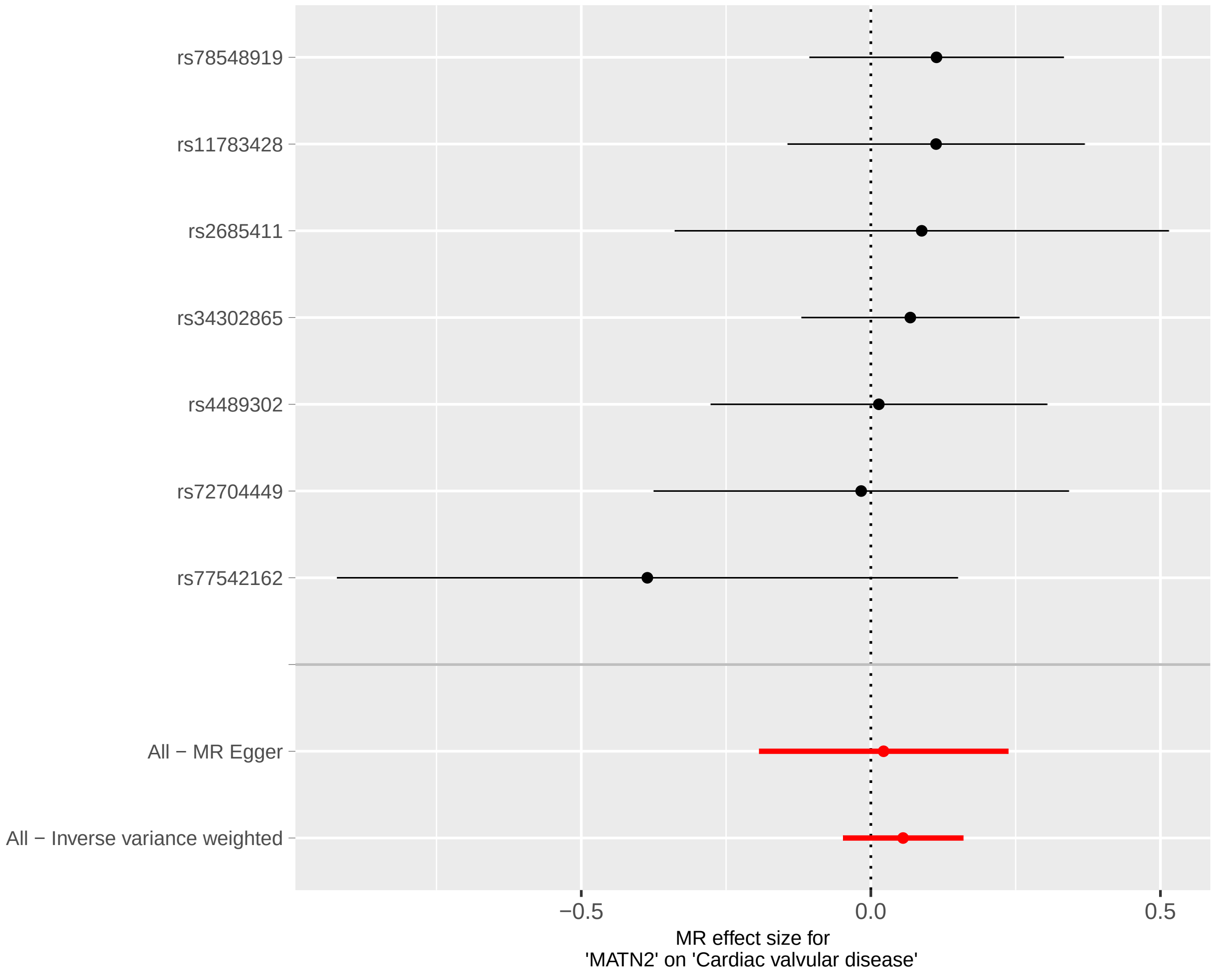
rs78548919
rs11783428
rs2685411
rs34302865
rs4489302
rs72704449
rs77542162
All − MR Egger
All − Inverse variance weighted
−0.5
0.0
0.5
MR effect size for
'MATN2' on 'Cardiac valvular disease'

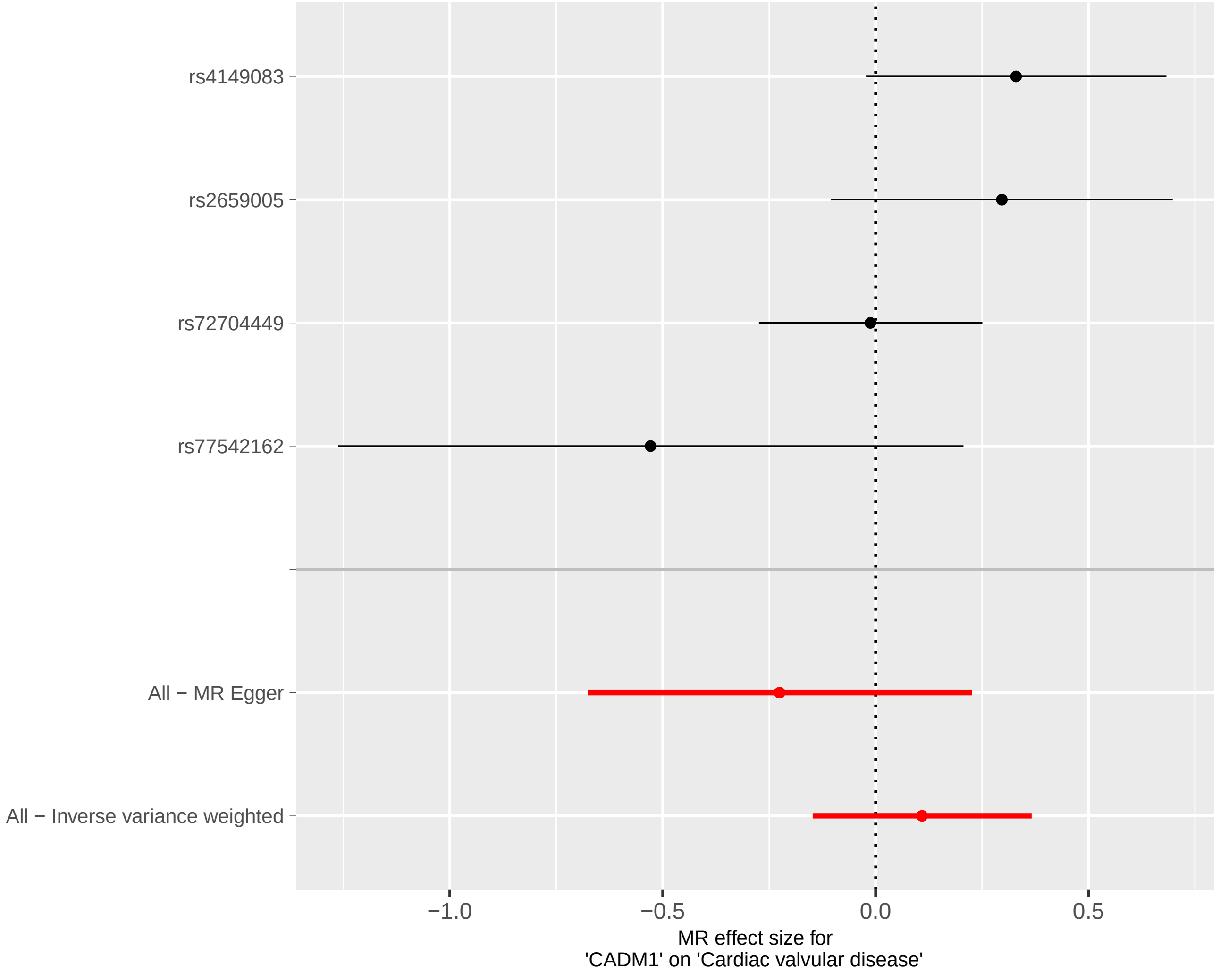
rs4149083
rs2659005
rs72704449
rs77542162
All − MR Egger
All − Inverse variance weighted
−1.0
−0.5
0.0
0.5
MR effect size for
'CADM1' on 'Cardiac valvular disease'

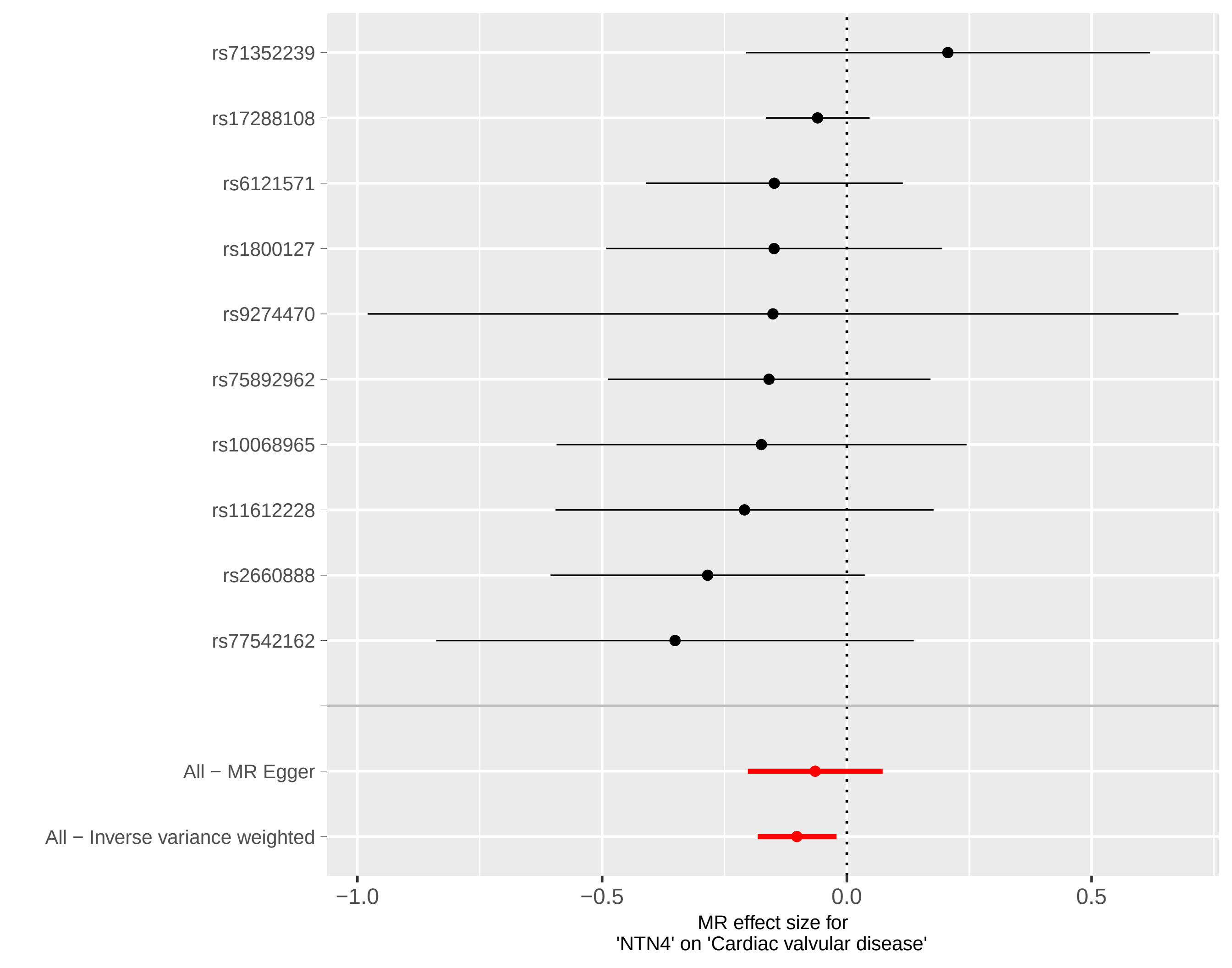
rs71352239
rs17288108
rs6121571
rs1800127
rs9274470
rs75892962
rs10068965
rs11612228
rs2660888
rs77542162
All − MR Egger
All − Inverse variance weighted
−1.0
−0.5
0.0
0.5
MR effect size for
'NTN4' on 'Cardiac valvular disease'

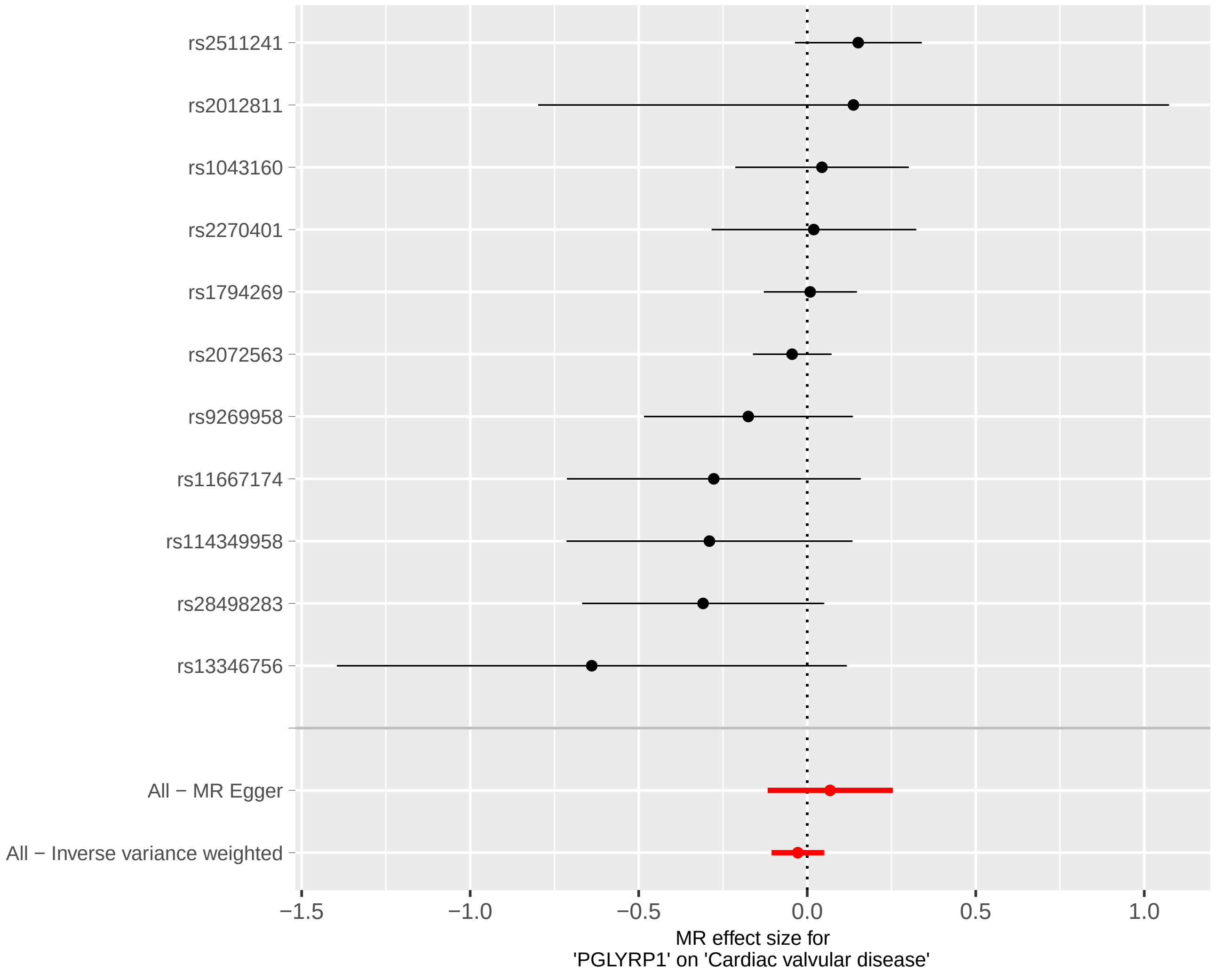
rs2511241
rs2012811
rs1043160
rs2270401
rs1794269
rs2072563
rs9269958
rs11667174
rs114349958
rs28498283
rs13346756
All − MR Egger
All − Inverse variance weighted
−1.5
−1.0
−0.5
0.0
0.5
1.0
MR effect size for
'PGLYRP1' on 'Cardiac valvular disease'

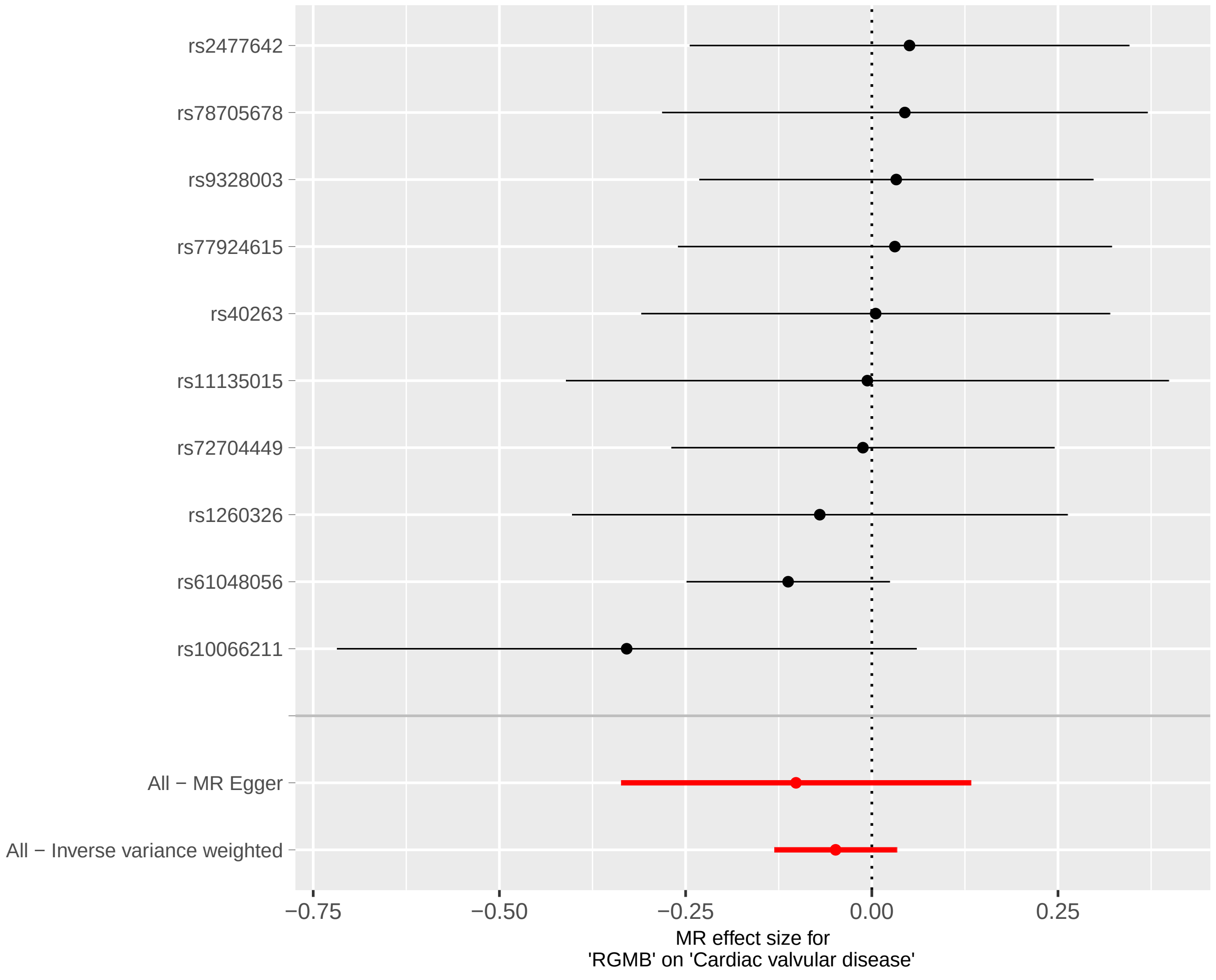
rs2477642
rs78705678
rs9328003
rs77924615
rs40263
rs11135015
rs72704449
rs1260326
rs61048056
rs10066211
All − MR Egger
All − Inverse variance weighted
−0.75
−0.50
−0.25
0.00
0.25
MR effect size for
'RGMB' on 'Cardiac valvular disease'

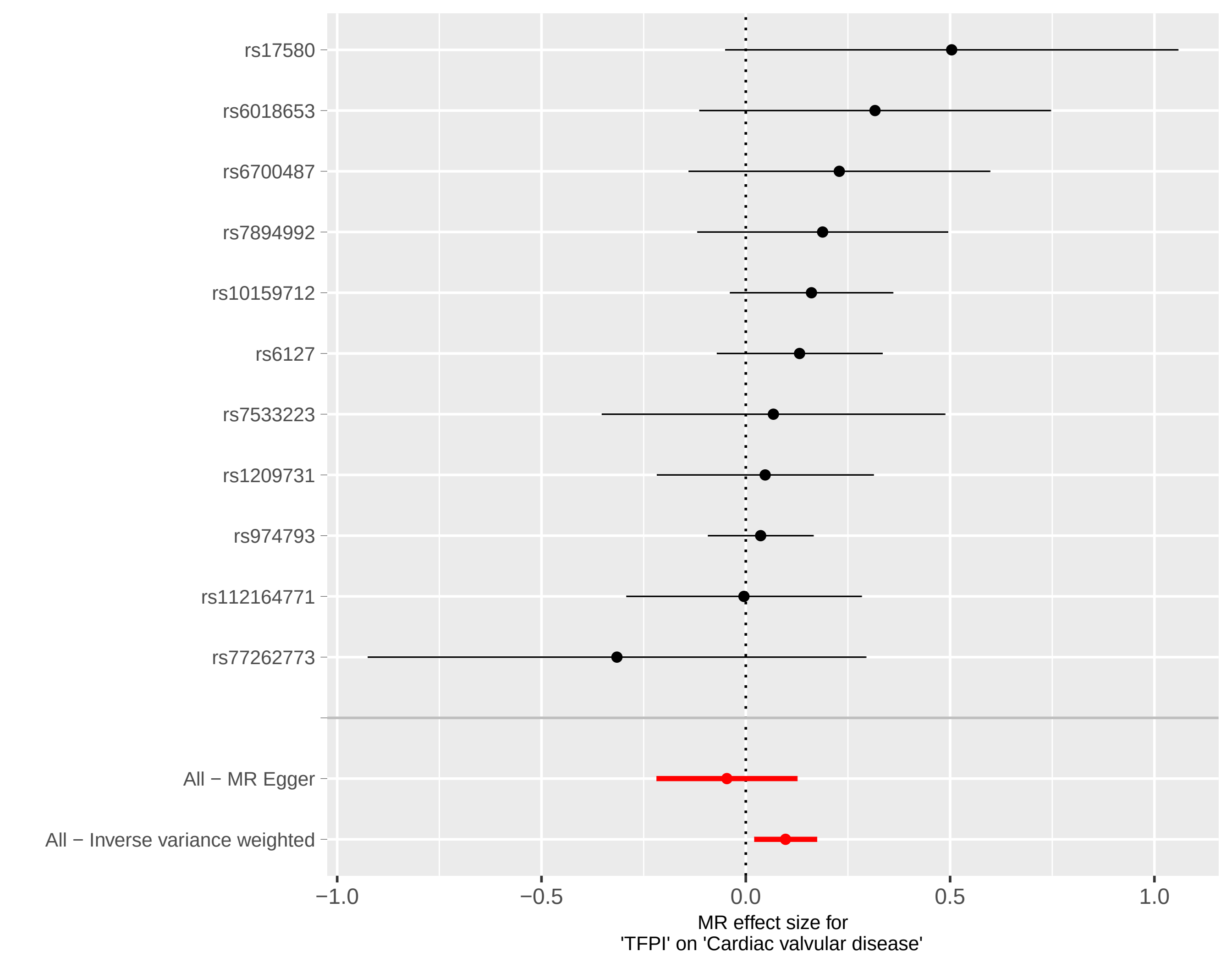
rs17580
rs6018653
rs6700487
rs7894992
rs10159712
rs6127
rs7533223
rs1209731
rs974793
rs112164771
rs77262773
All − MR Egger
All − Inverse variance weighted
−1.0
−0.5
0.0
0.5
1.0
MR effect size for
'TFPI' on 'Cardiac valvular disease'

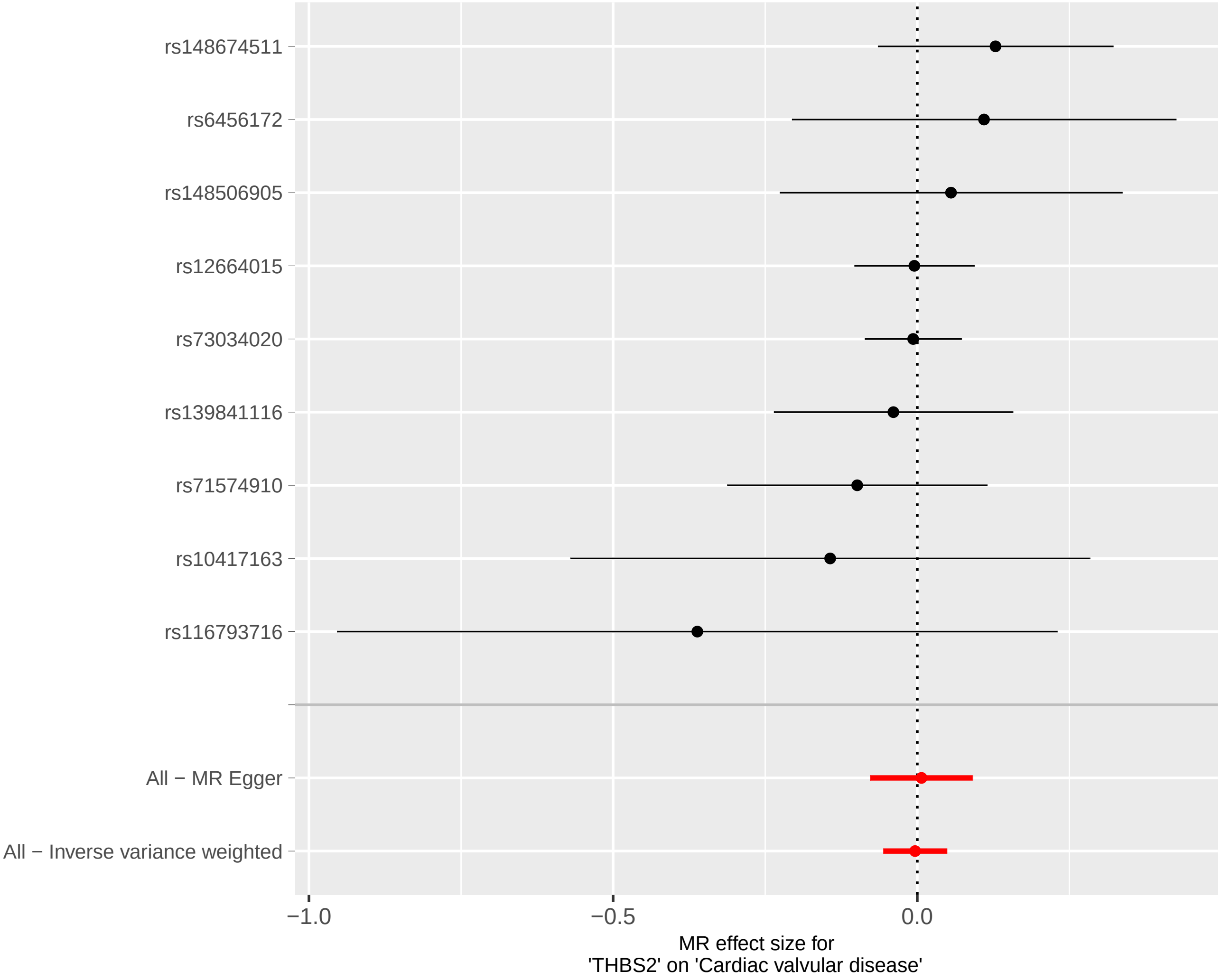
rs148674511
rs6456172
rs148506905
rs12664015
rs73034020
rs139841116
rs71574910
rs10417163
rs116793716
All − MR Egger
All − Inverse variance weighted
−1.0
−0.5
0.0
MR effect size for
'THBS2' on 'Cardiac valvular disease'

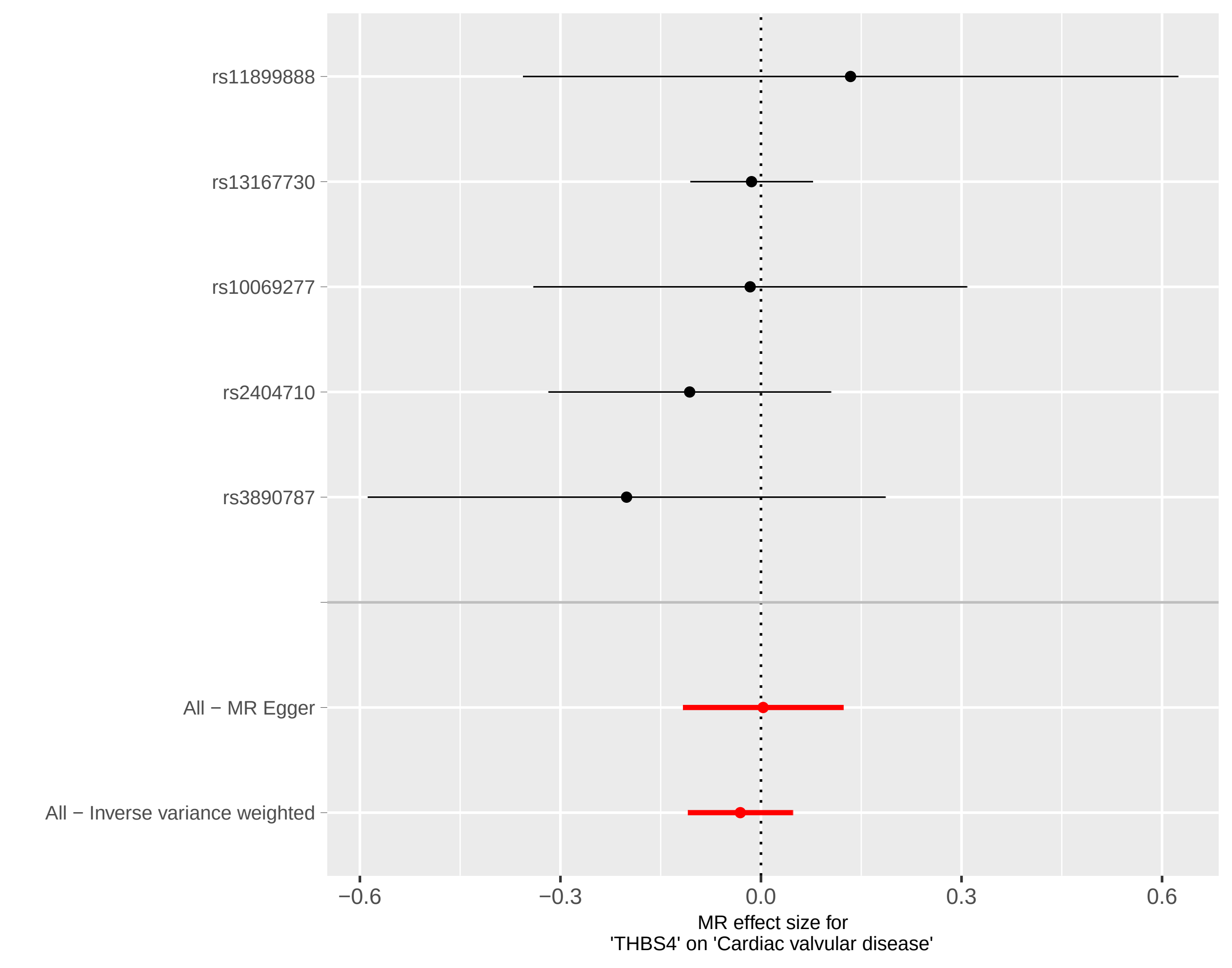
rs11899888
rs13167730
rs10069277
rs2404710
rs3890787
All − MR Egger
All − Inverse variance weighted
−0.6
−0.3
0.0
0.3
0.6
MR effect size for
'THBS4' on 'Cardiac valvular disease'

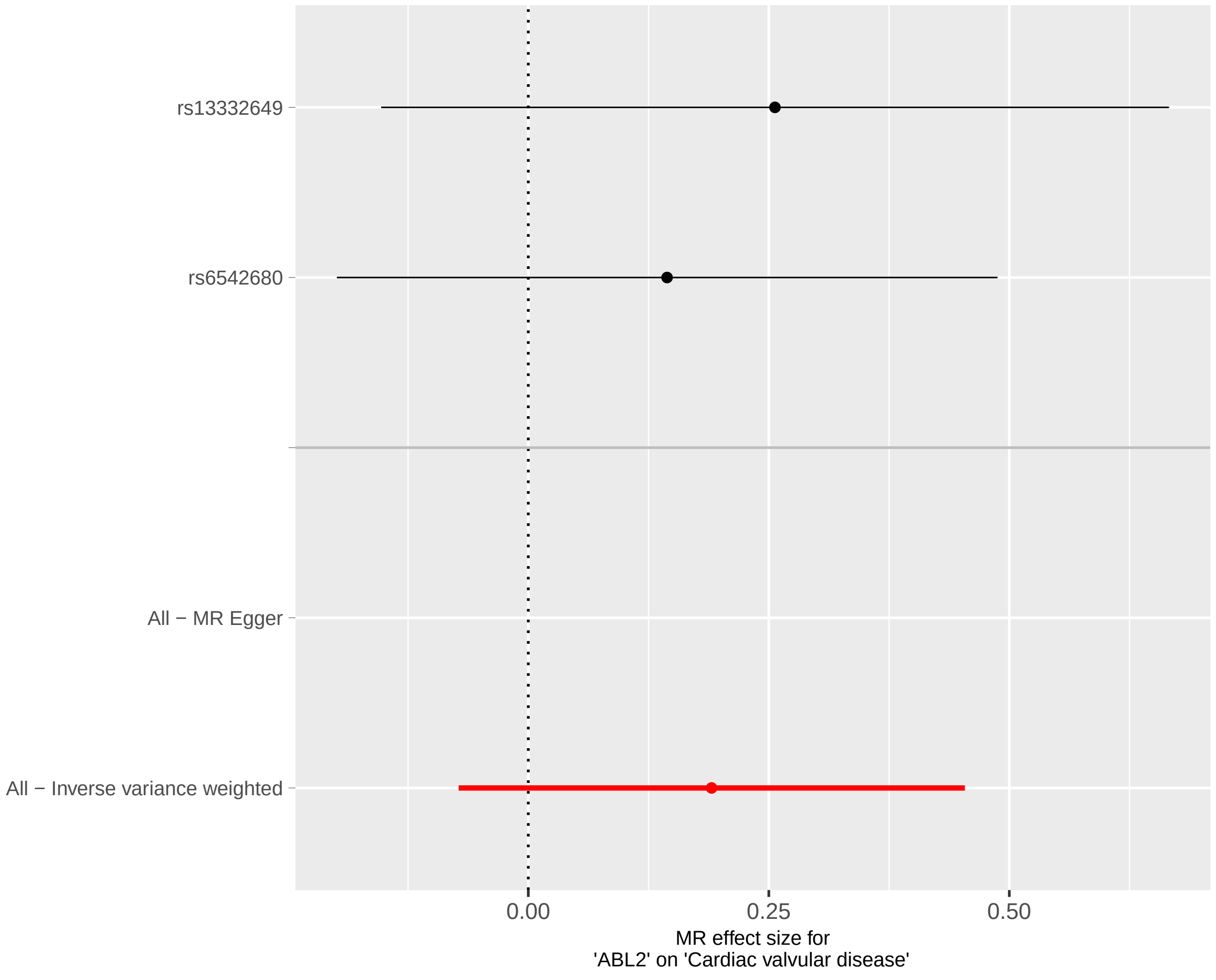
rs13332649
rs6542680
All − MR Egger
All − Inverse variance weighted
0.00
0.25
0.50
MR effect size for
'ABL2' on 'Cardiac valvular disease'

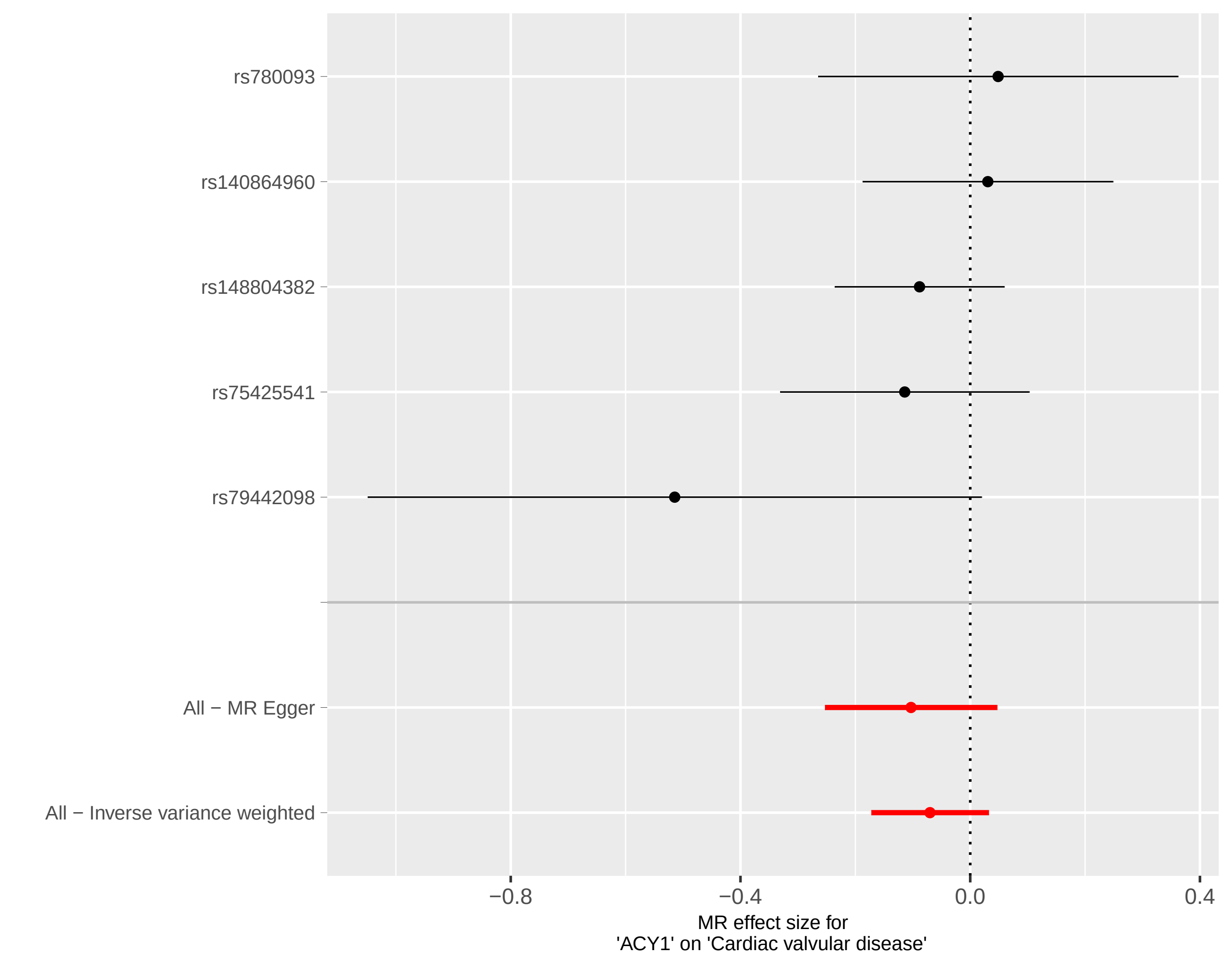
rs780093
rs140864960
rs148804382
rs75425541
rs79442098
All − MR Egger
All − Inverse variance weighted
−0.8
−0.4
0.0
0.4
MR effect size for
'ACY1' on 'Cardiac valvular disease'

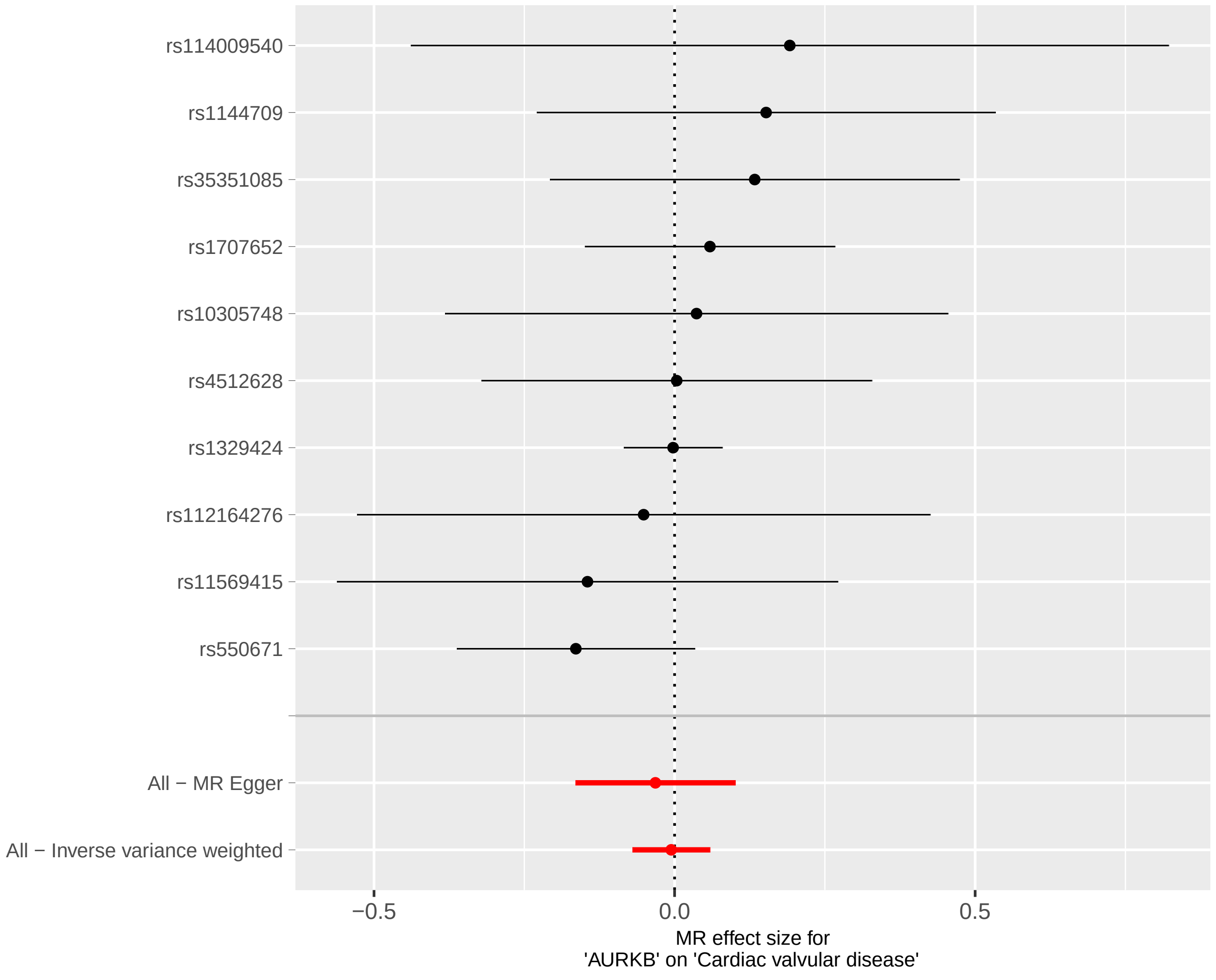
rs114009540
rs1144709
rs35351085
rs1707652
rs10305748
rs4512628
rs1329424
rs112164276
rs11569415
rs550671
All − MR Egger
All − Inverse variance weighted
−0.5
0.0
0.5
MR effect size for
'AURKB' on 'Cardiac valvular disease'

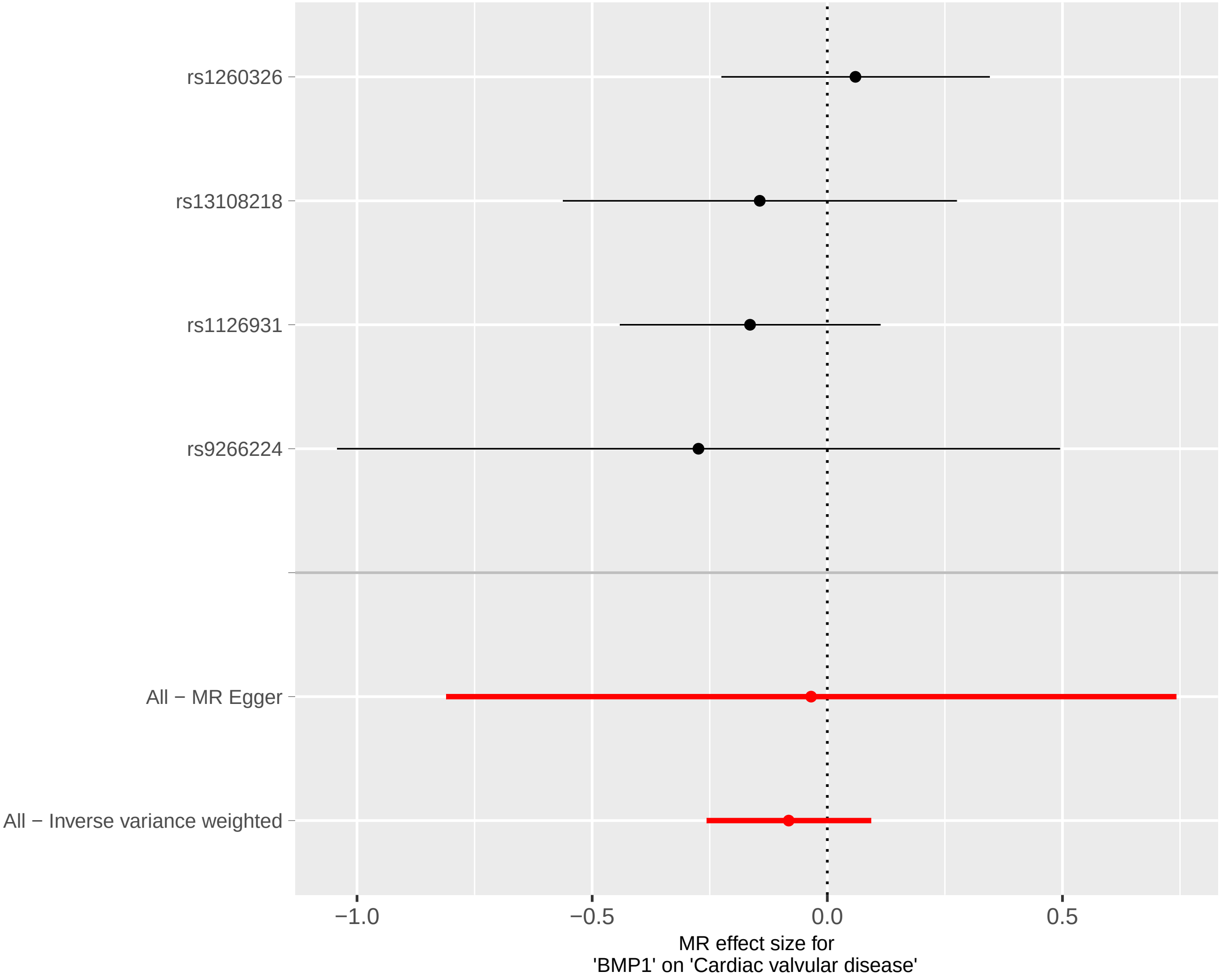
rs1260326
rs13108218
rs1126931
rs9266224
All − MR Egger
All − Inverse variance weighted
−1.0
−0.5
0.0
0.5
MR effect size for
'BMP1' on 'Cardiac valvular disease'

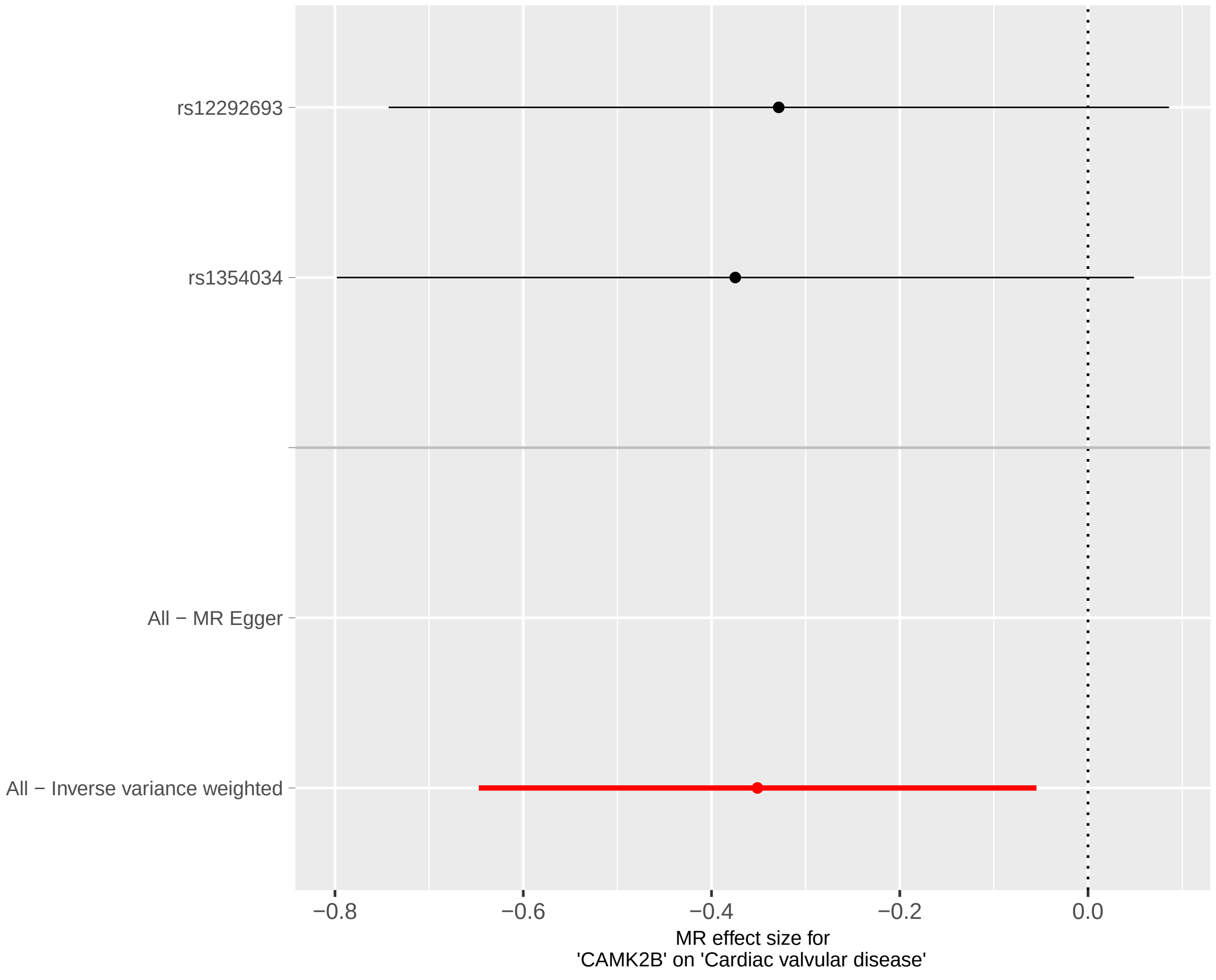
rs12292693
rs1354034
All − MR Egger
All − Inverse variance weighted
−0.8
−0.6
−0.4
−0.2
0.0
MR effect size for
'CAMK2B' on 'Cardiac valvular disease'

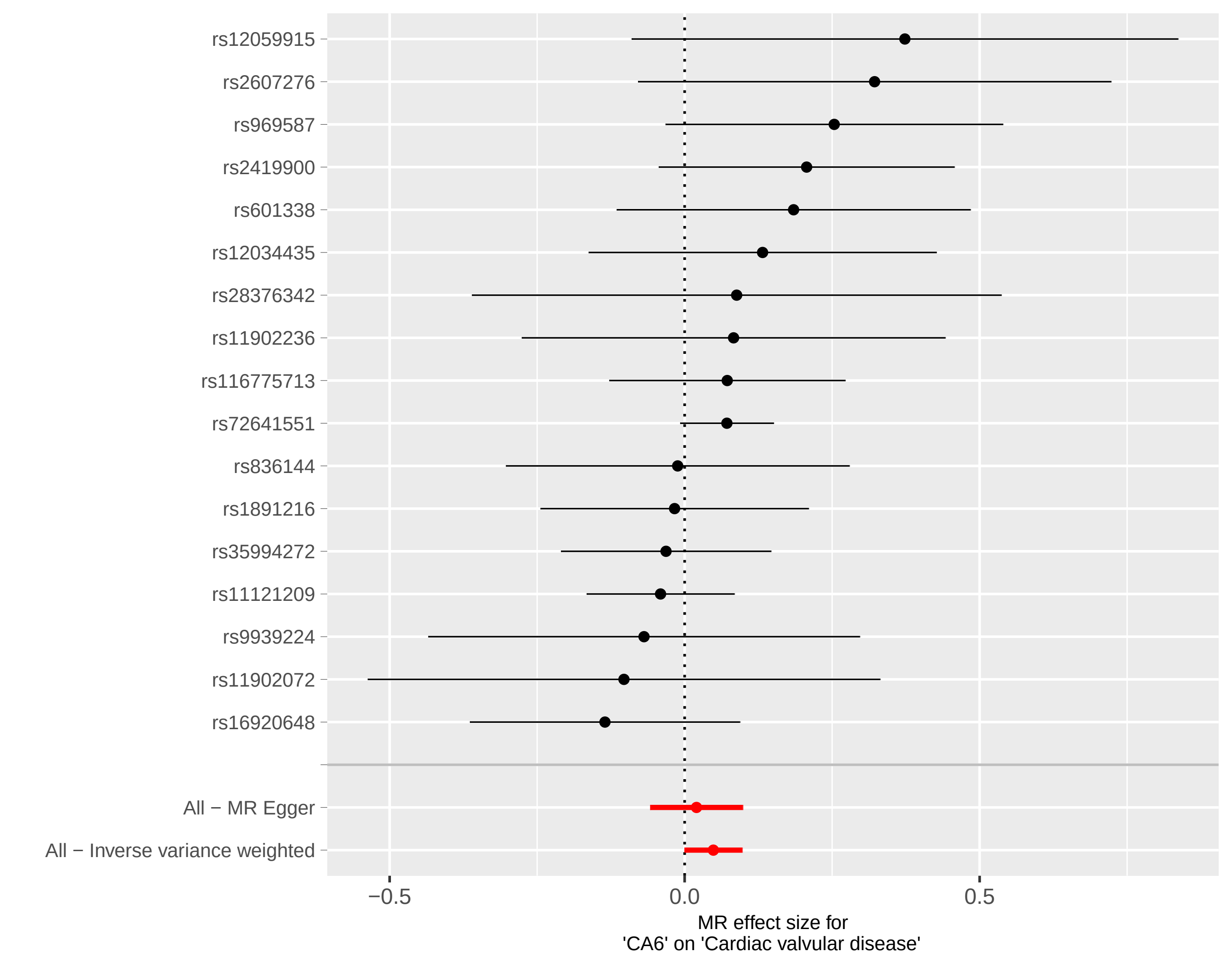
rs12059915
rs2607276
rs969587
rs2419900
rs601338
rs12034435
rs28376342
rs11902236
rs116775713
rs72641551
rs836144
rs1891216
rs35994272
rs11121209
rs9939224
rs11902072
rs16920648
All − MR Egger
All − Inverse variance weighted
−0.5
0.0
0.5
MR effect size for
'CA6' on 'Cardiac valvular disease'

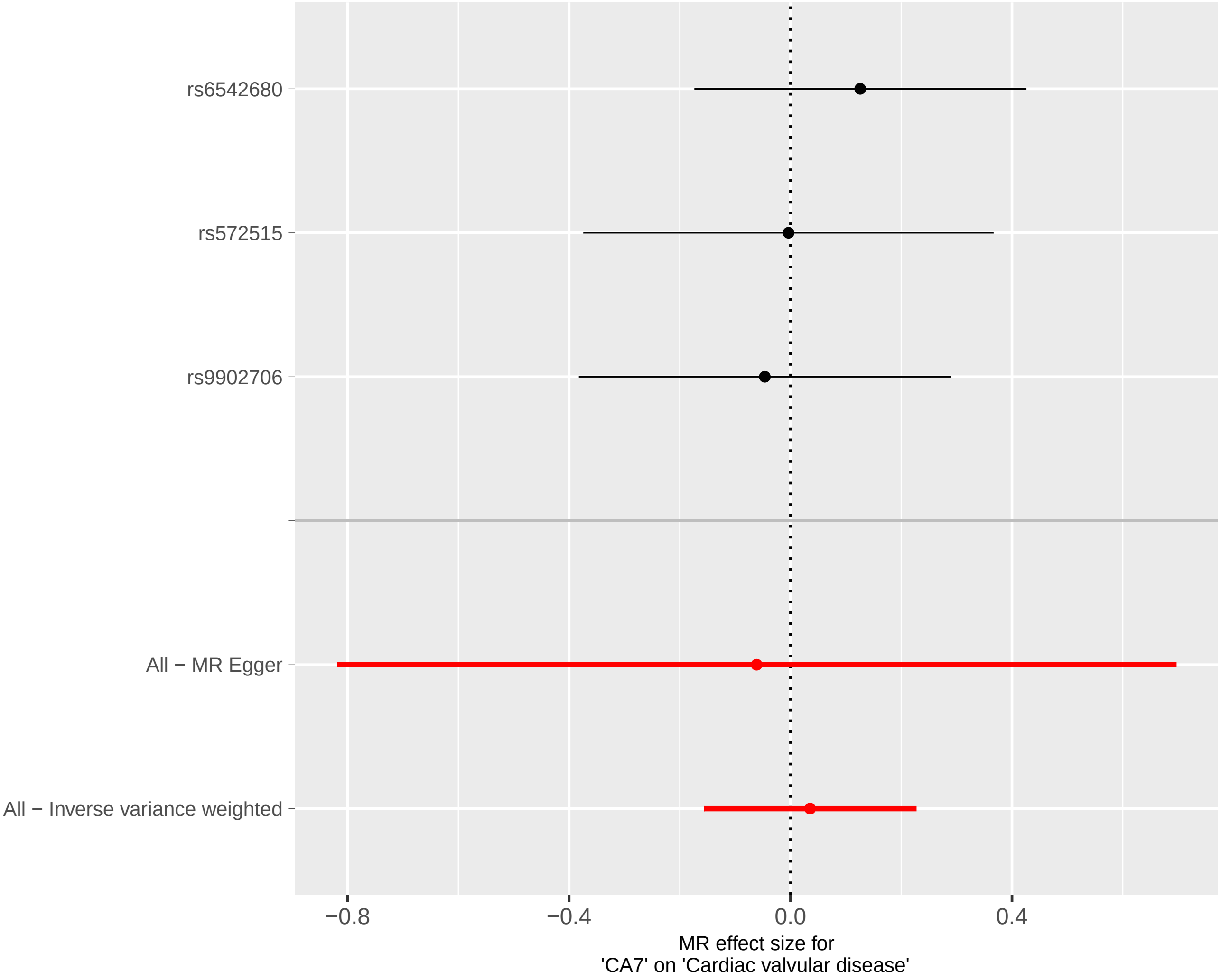
rs6542680
rs572515
rs9902706
All − MR Egger
All − Inverse variance weighted
−0.8
−0.4
0.0
0.4
MR effect size for
'CA7' on 'Cardiac valvular disease'

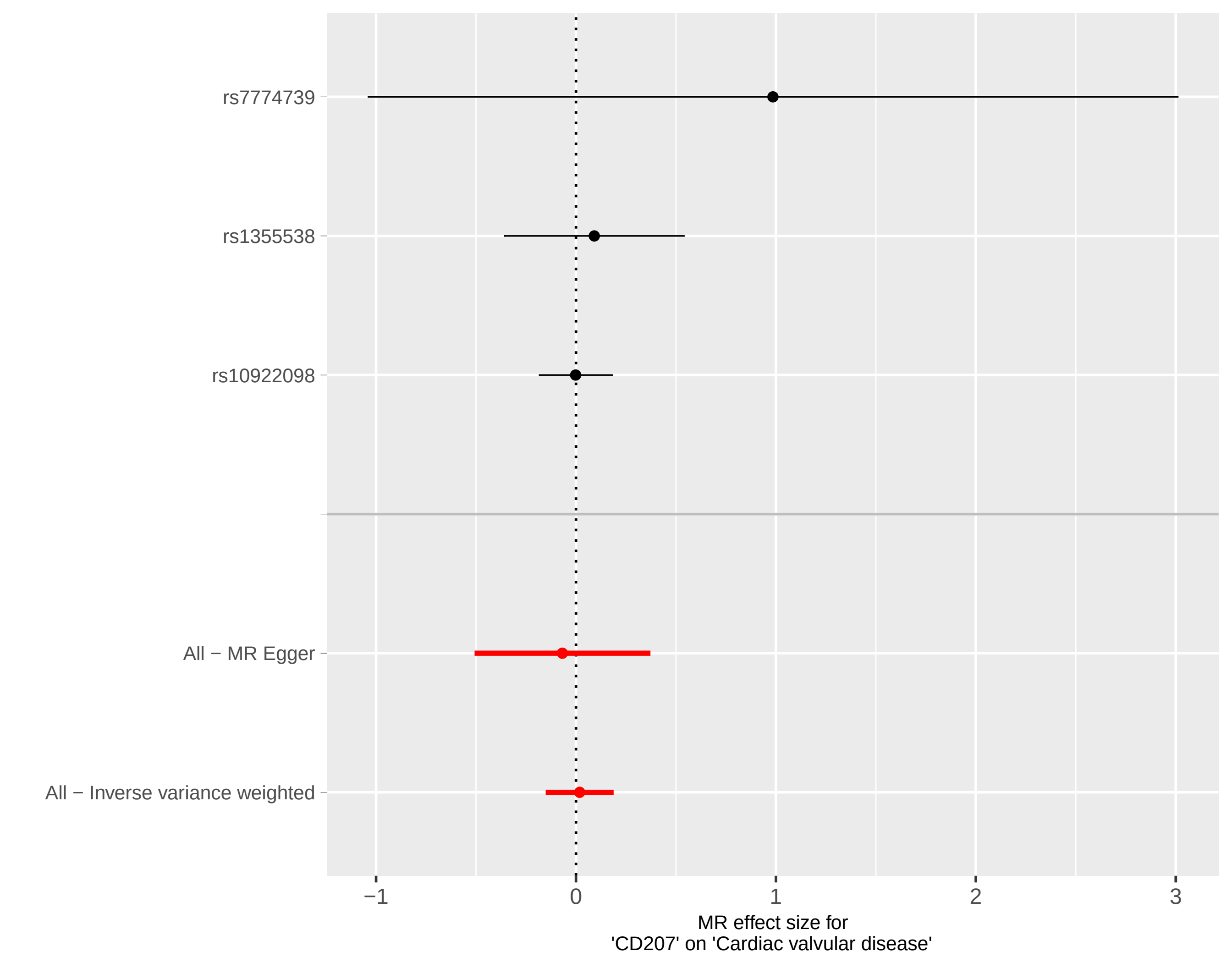
rs7774739
rs1355538
rs10922098
All − MR Egger
All − Inverse variance weighted
−1
0
1
2
3
MR effect size for
'CD207' on 'Cardiac valvular disease'

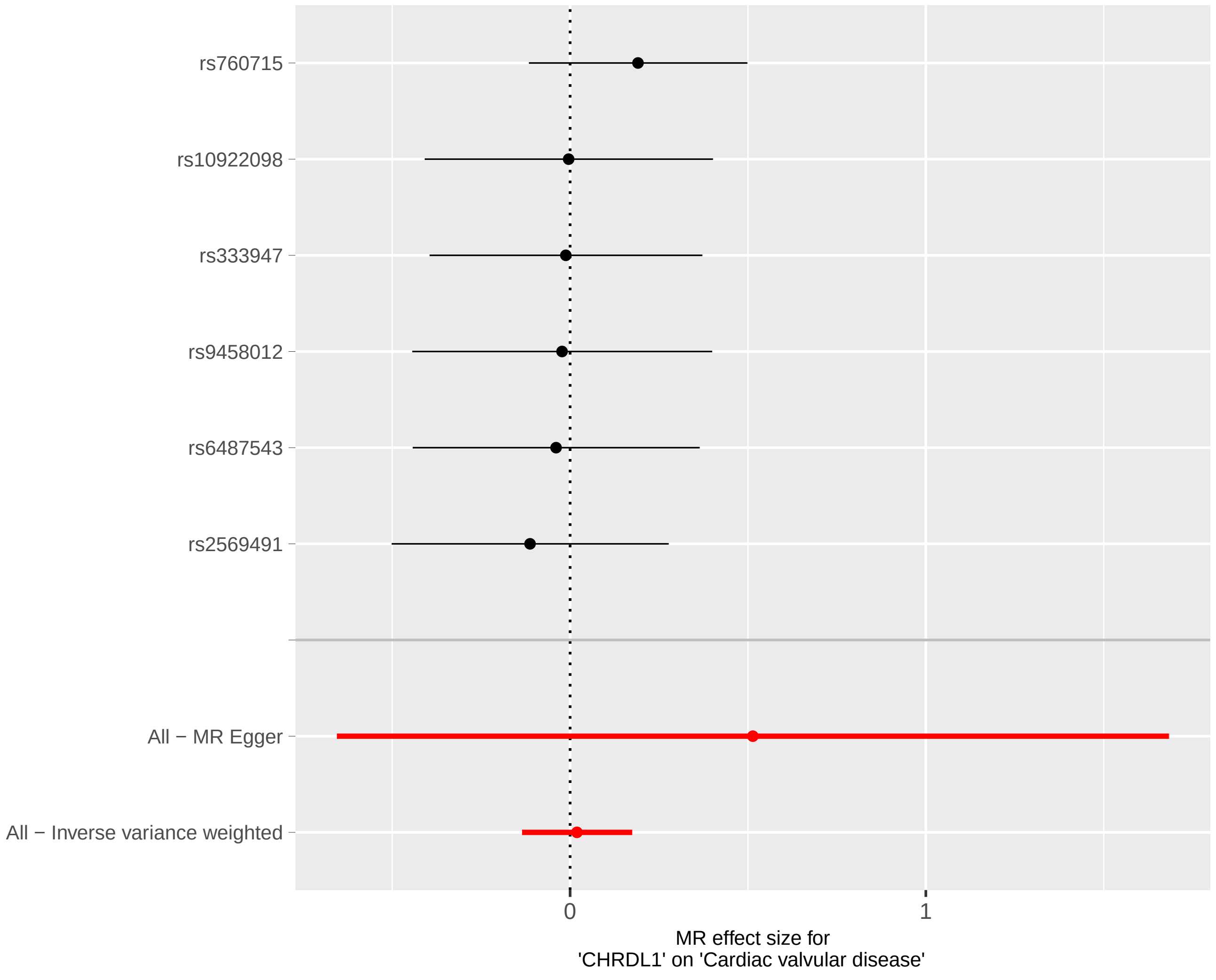
rs760715
rs10922098
rs333947
rs9458012
rs6487543
rs2569491
All − MR Egger
All − Inverse variance weighted
0
1
MR effect size for
'CHRDL1' on 'Cardiac valvular disease'

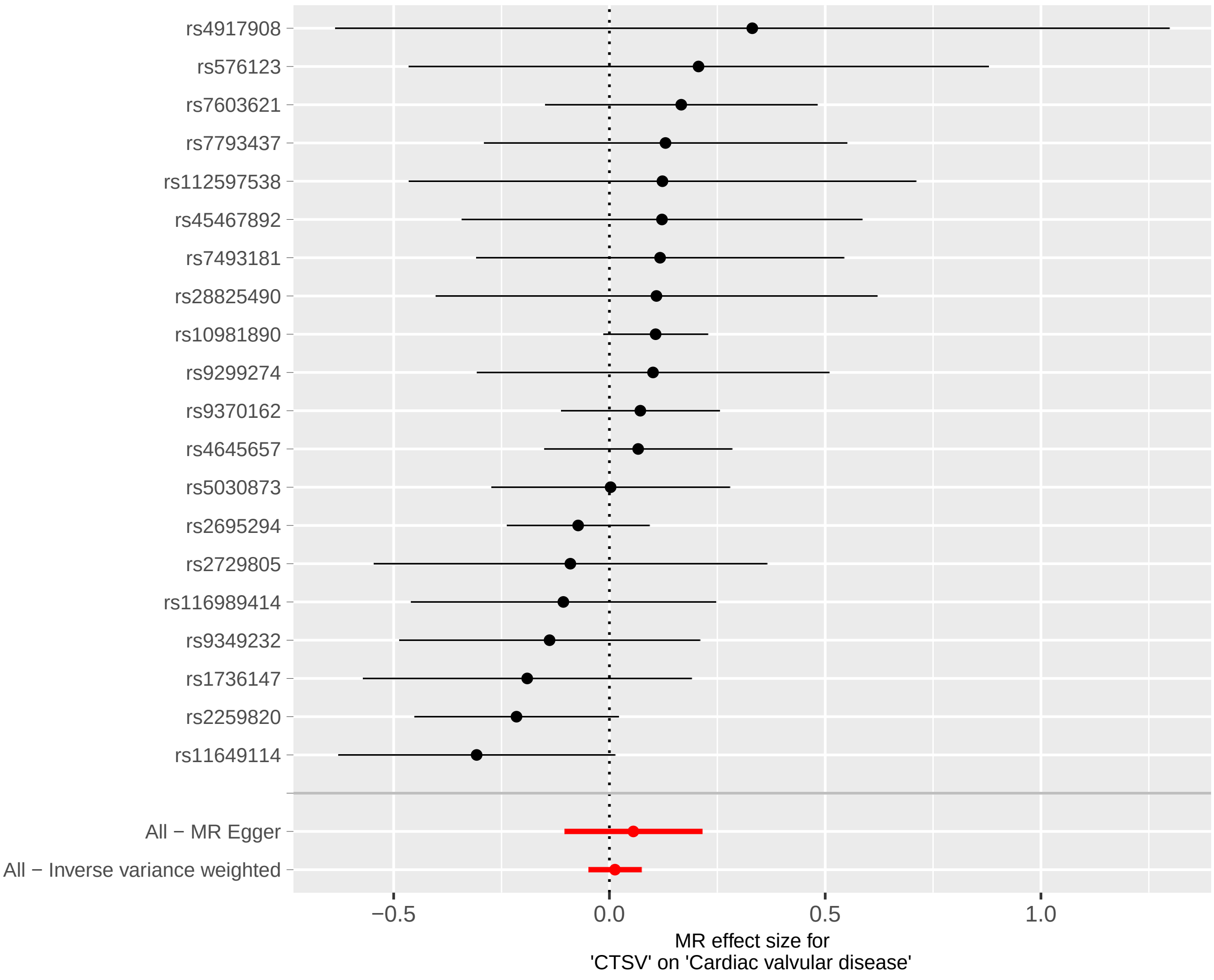

rs4917908
rs576123
rs7603621
rs7793437
rs112597538
rs45467892
rs7493181
rs28825490
rs10981890
rs9299274
rs9370162
rs4645657
rs5030873
rs2695294
rs2729805
rs116989414
rs9349232
rs1736147
rs2259820
rs11649114
All − MR Egger
All − Inverse variance weighted
−0.5
0.0
0.5
1.0
MR effect size for
'CTSV' on 'Cardiac valvular disease'

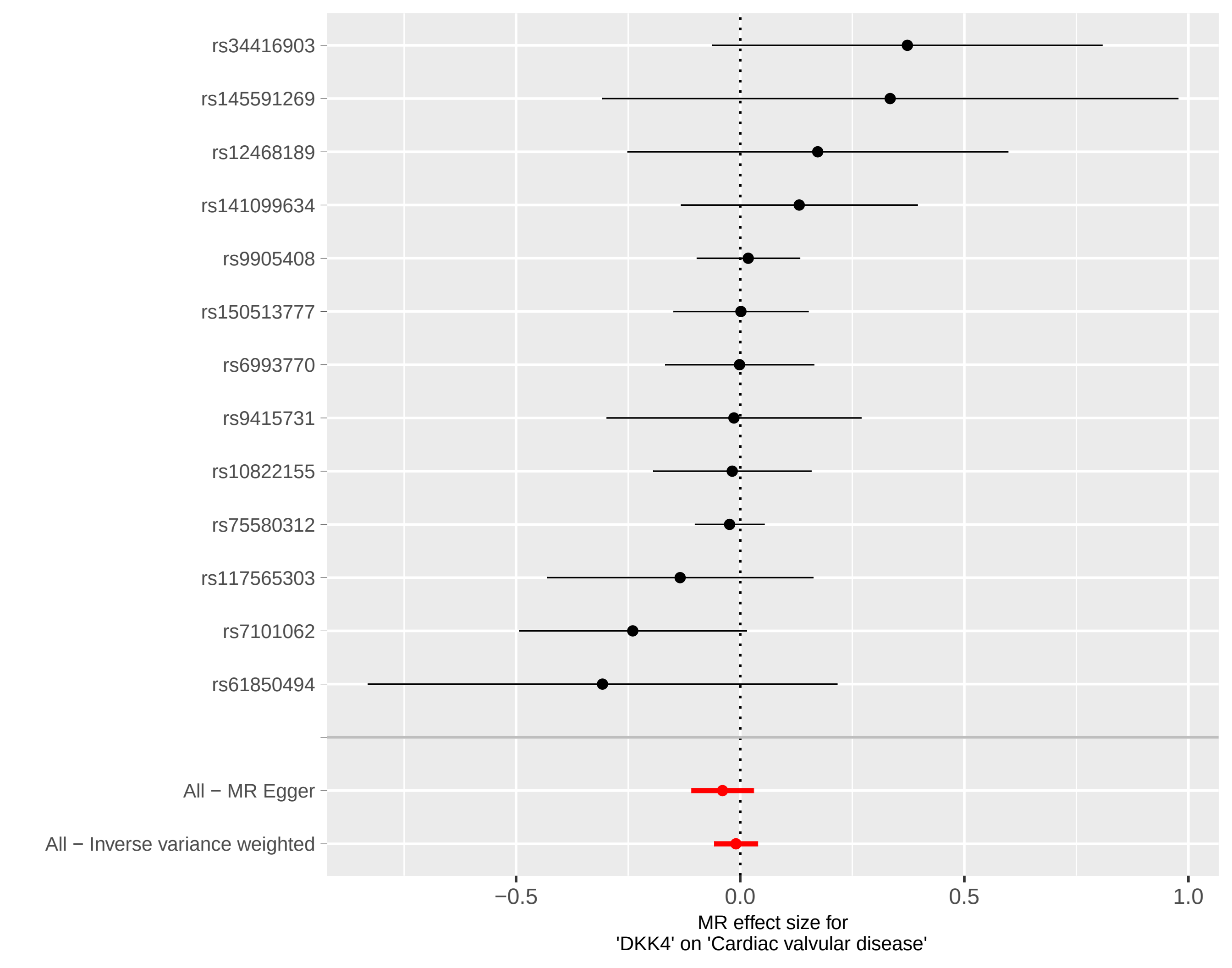

rs34416903
rs145591269
rs12468189
rs141099634
rs9905408
rs150513777
rs6993770
rs9415731
rs10822155
rs75580312
rs117565303
rs7101062
rs61850494
All − MR Egger
All − Inverse variance weighted
−0.5
0.0
0.5
1.0
MR effect size for
'DKK4' on 'Cardiac valvular disease'

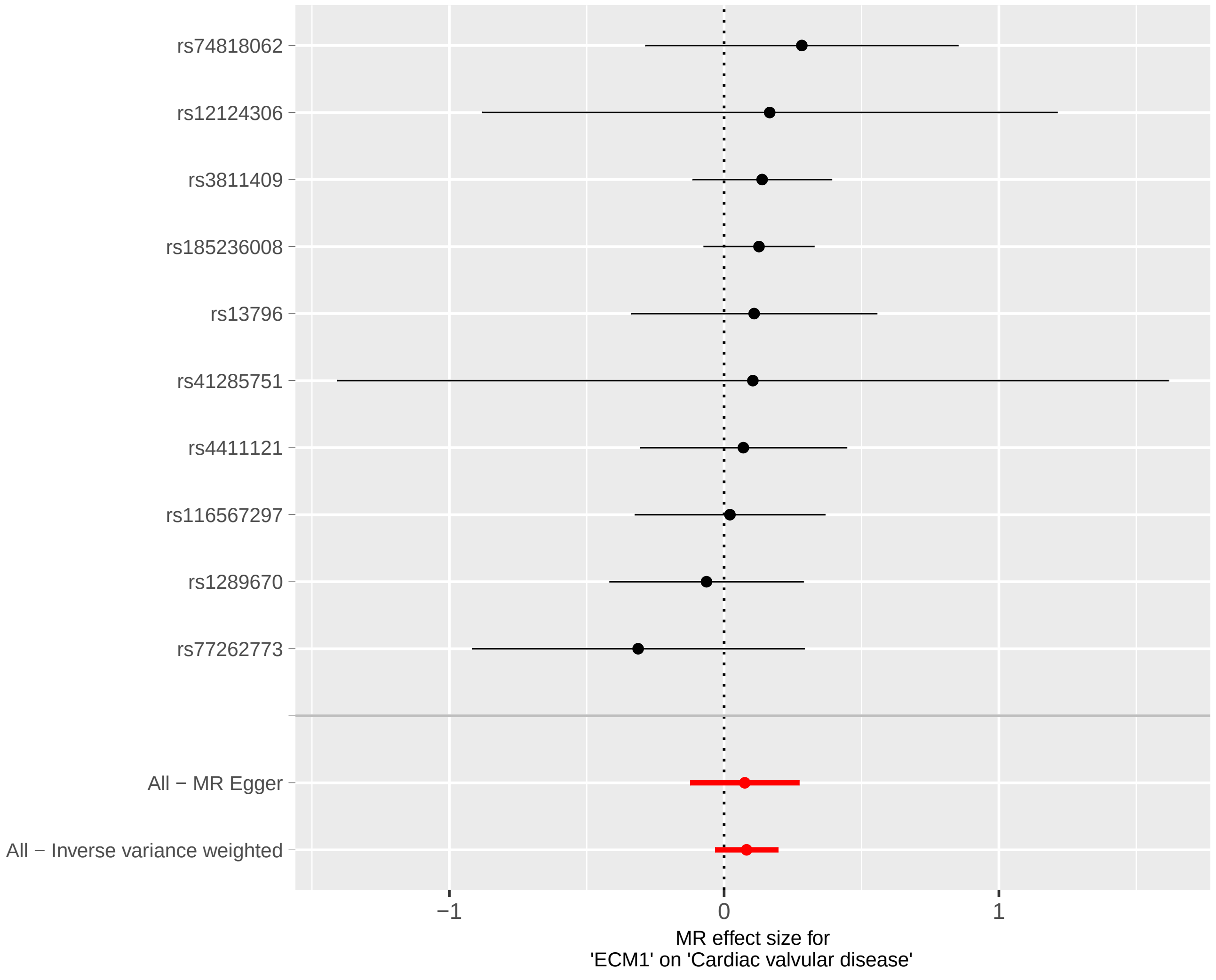
rs74818062
rs12124306
rs3811409
rs185236008
rs13796
rs41285751
rs4411121
rs116567297
rs1289670
rs77262773
All − MR Egger
All − Inverse variance weighted
−1
0
1
MR effect size for
'ECM1' on 'Cardiac valvular disease'

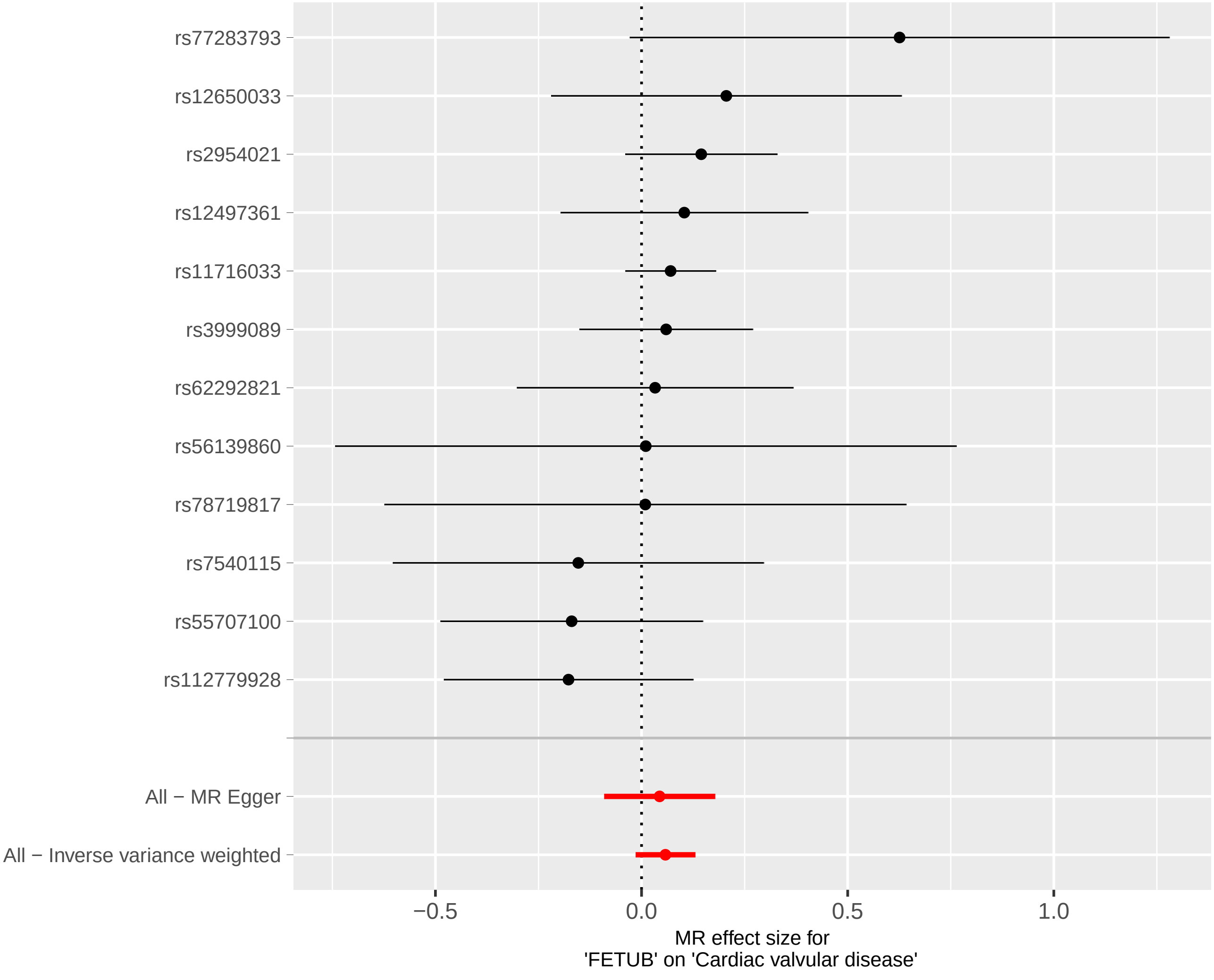

rs77283793
rs12650033
rs2954021
rs12497361
rs11716033
rs3999089
rs62292821
rs56139860
rs78719817
rs7540115
rs55707100
rs112779928
All − MR Egger
All − Inverse variance weighted
−0.5
0.0
0.5
1.0
MR effect size for
'FETUB' on 'Cardiac valvular disease'

Insufficient number of SNPs

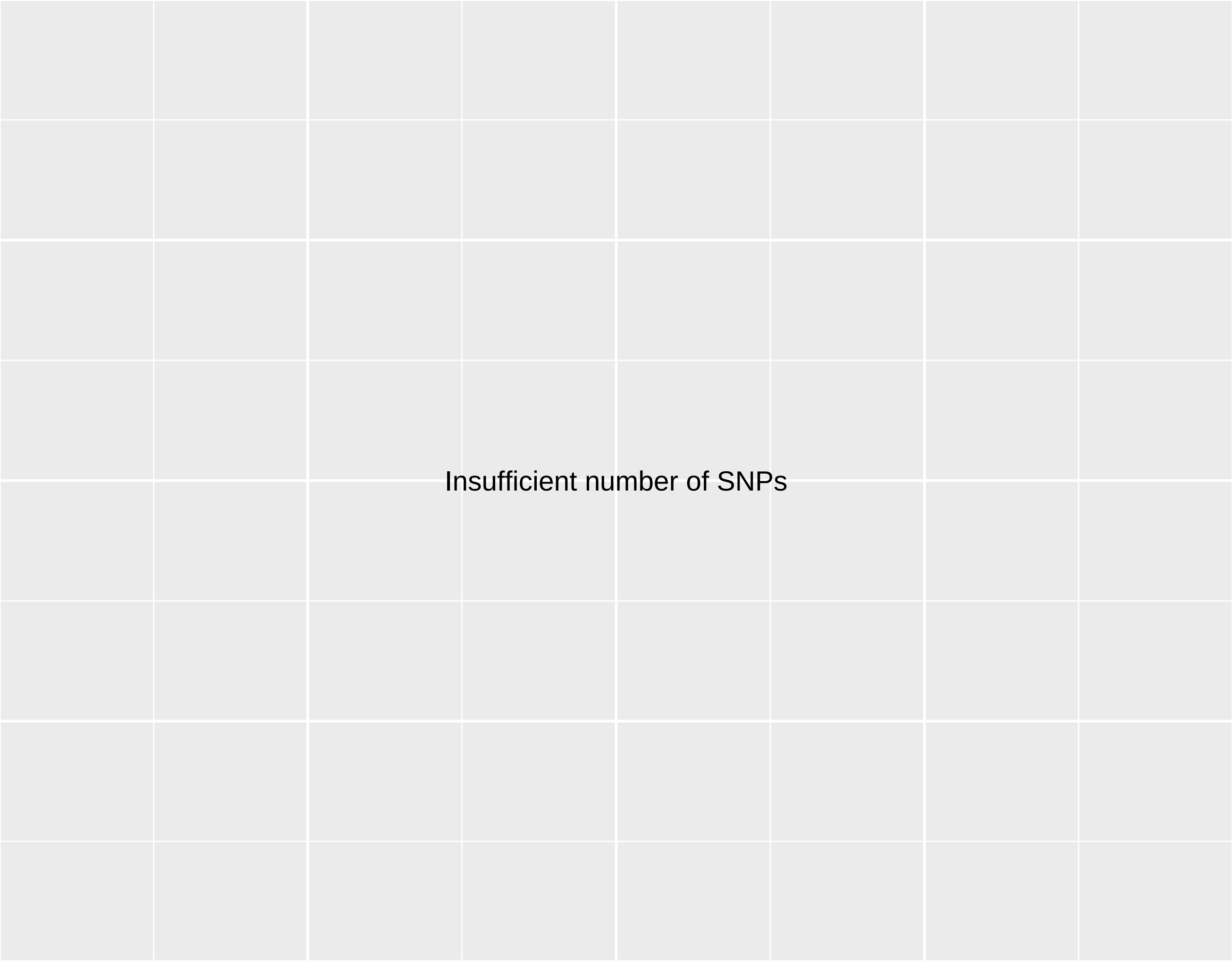
Insufficient number of SNPs

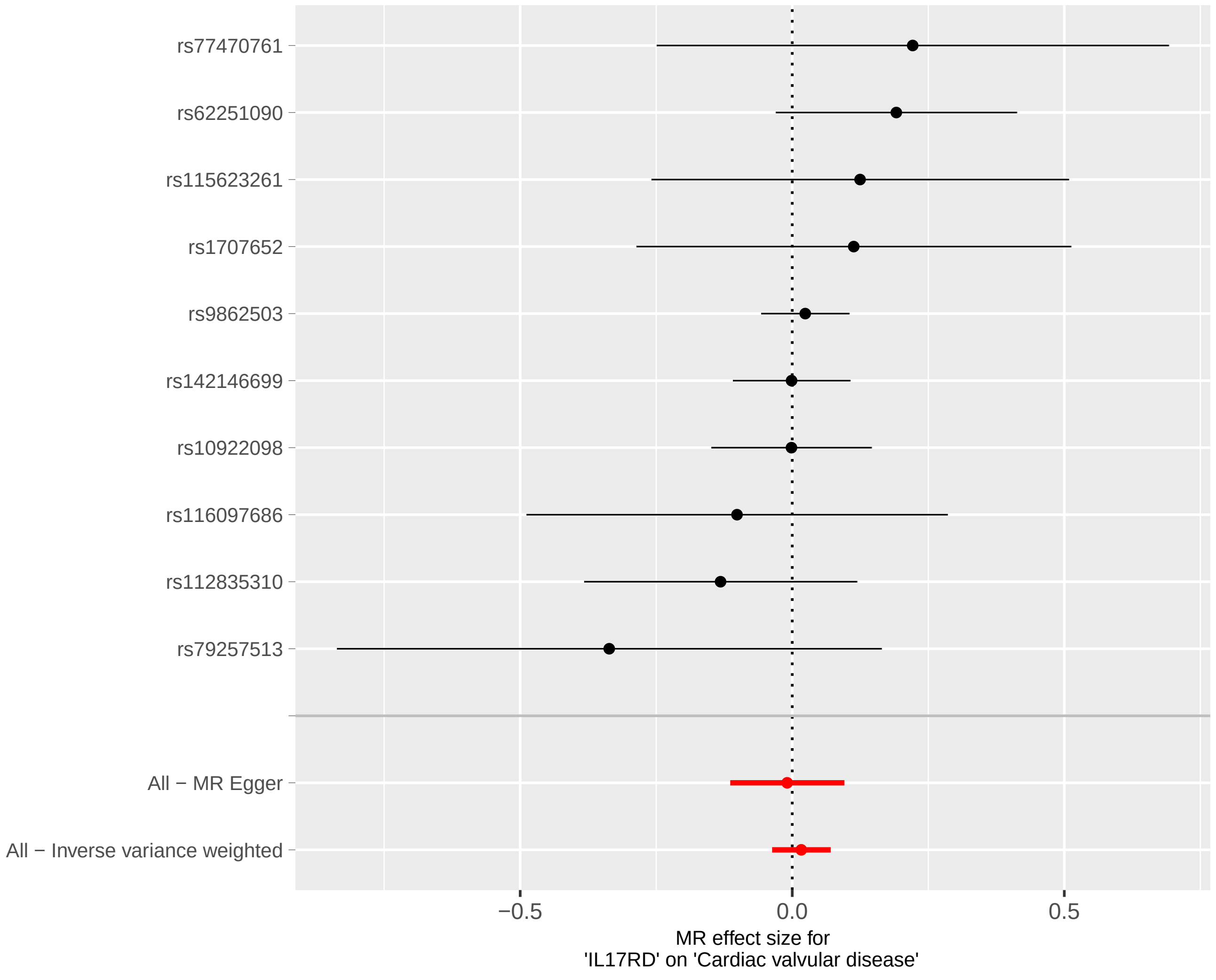
rs77470761
rs62251090
rs115623261
rs1707652
rs9862503
rs142146699
rs10922098
rs116097686
rs112835310
rs79257513
All − MR Egger
All − Inverse variance weighted
−0.5
0.0
0.5
MR effect size for
'IL17RD' on 'Cardiac valvular disease'

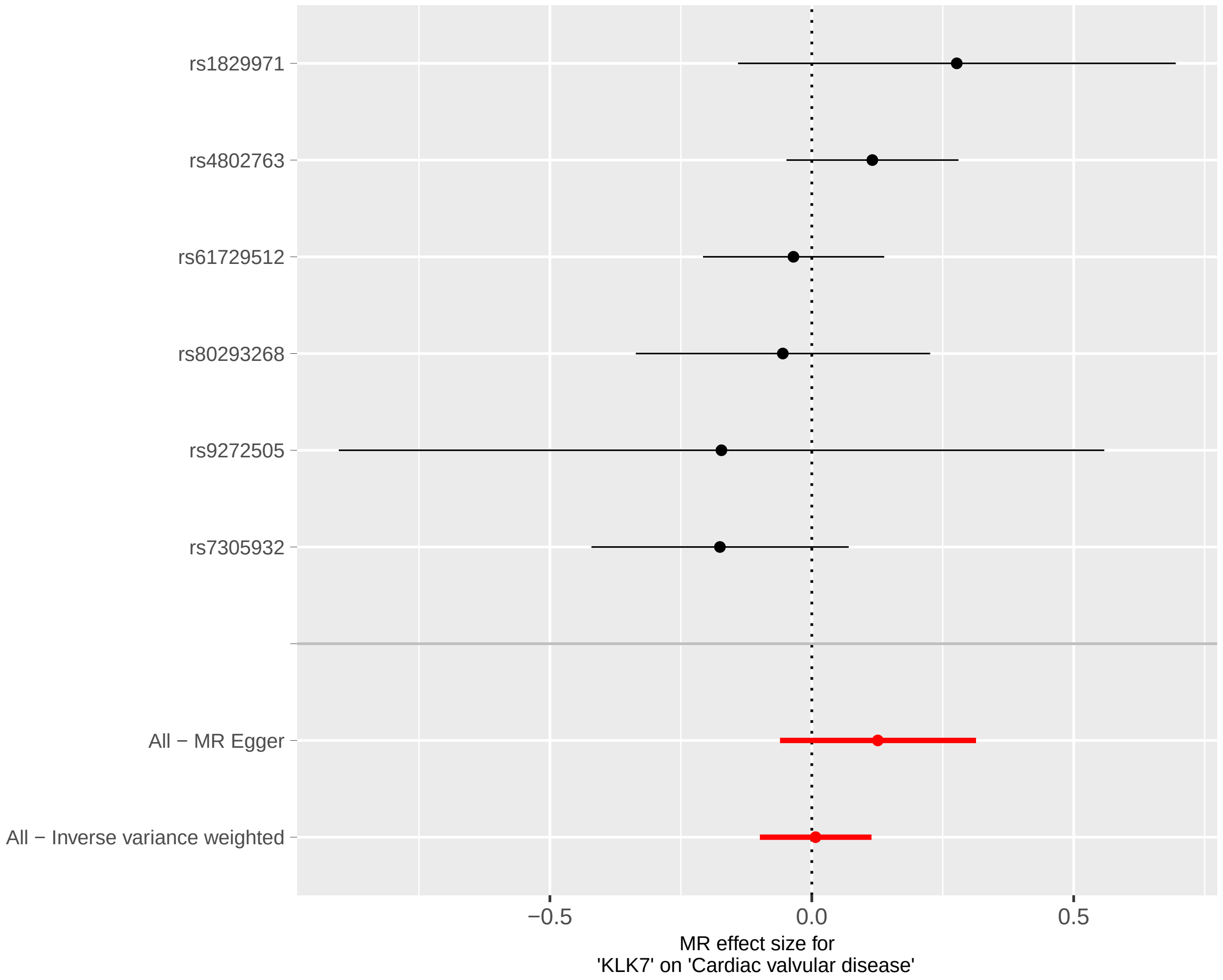
rs1829971
rs4802763
rs61729512
rs80293268
rs9272505
rs7305932
All − MR Egger
All − Inverse variance weighted
−0.5
0.0
0.5
MR effect size for
'KLK7' on 'Cardiac valvular disease'

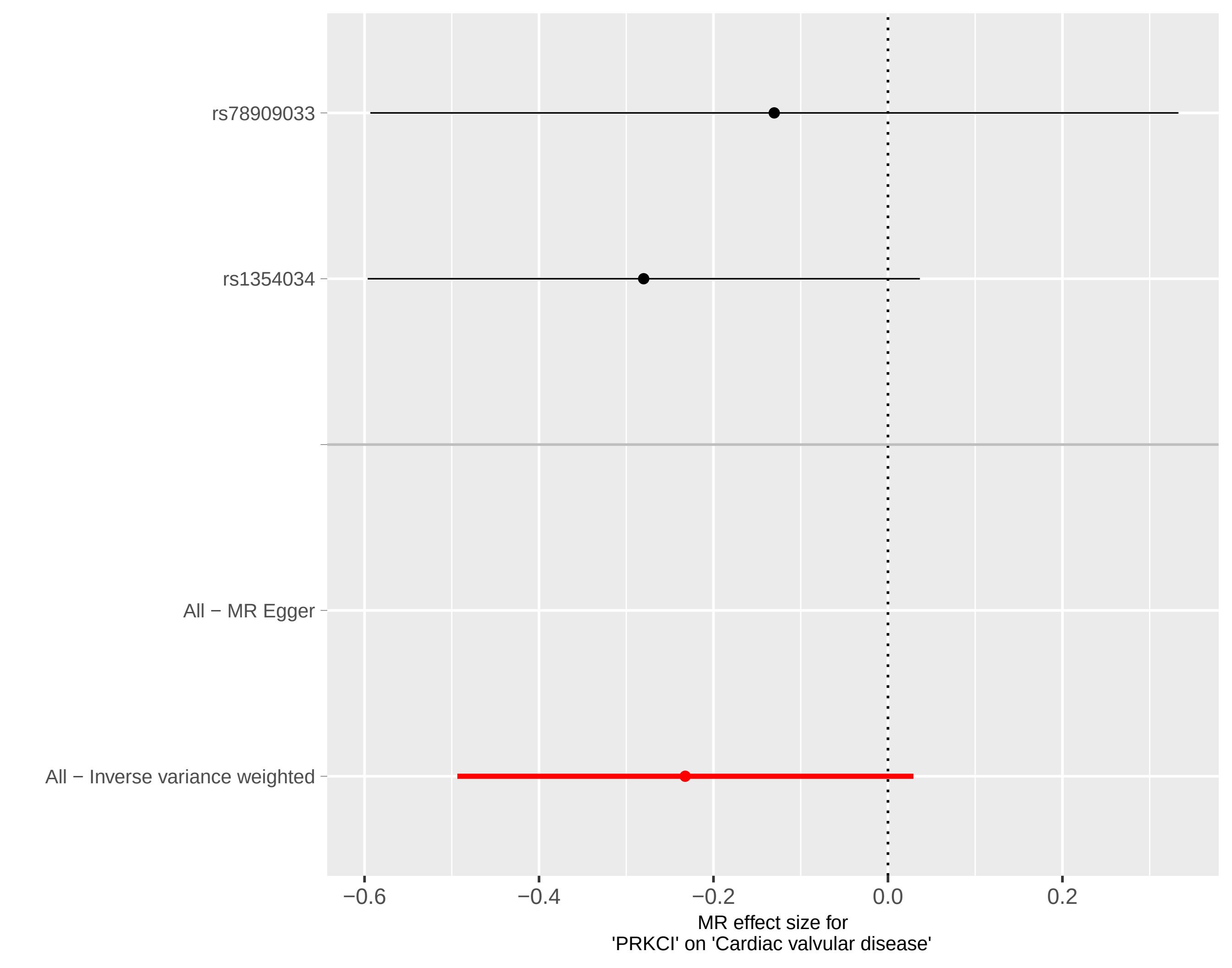
rs78909033
rs1354034
All − MR Egger
All − Inverse variance weighted
−0.6
−0.4
−0.2
0.0
0.2
MR effect size for
'PRKCI' on 'Cardiac valvular disease'

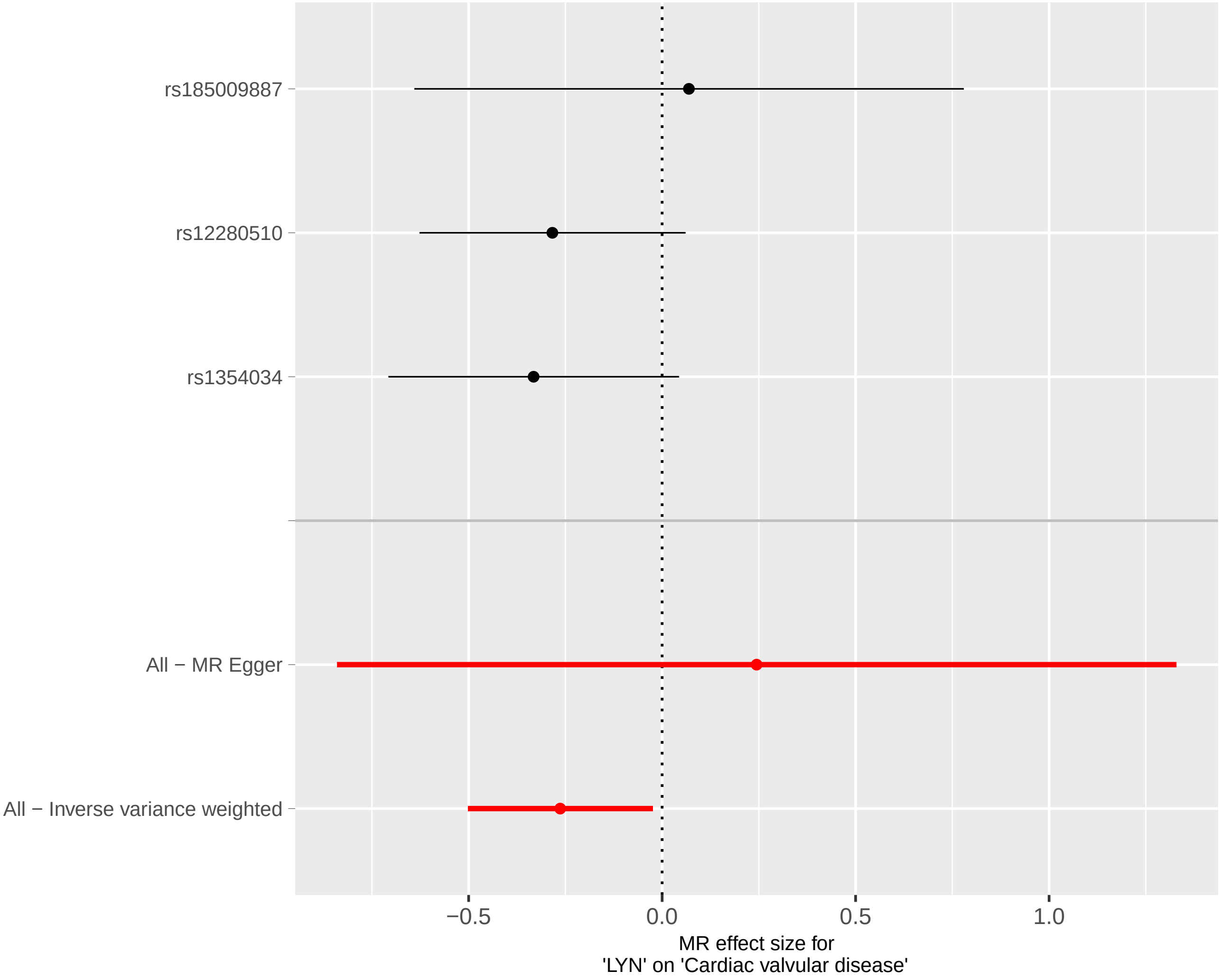
rs185009887
rs12280510
rs1354034
All − MR Egger
All − Inverse variance weighted
−0.5
0.0
0.5
1.0
MR effect size for
'LYN' on 'Cardiac valvular disease'

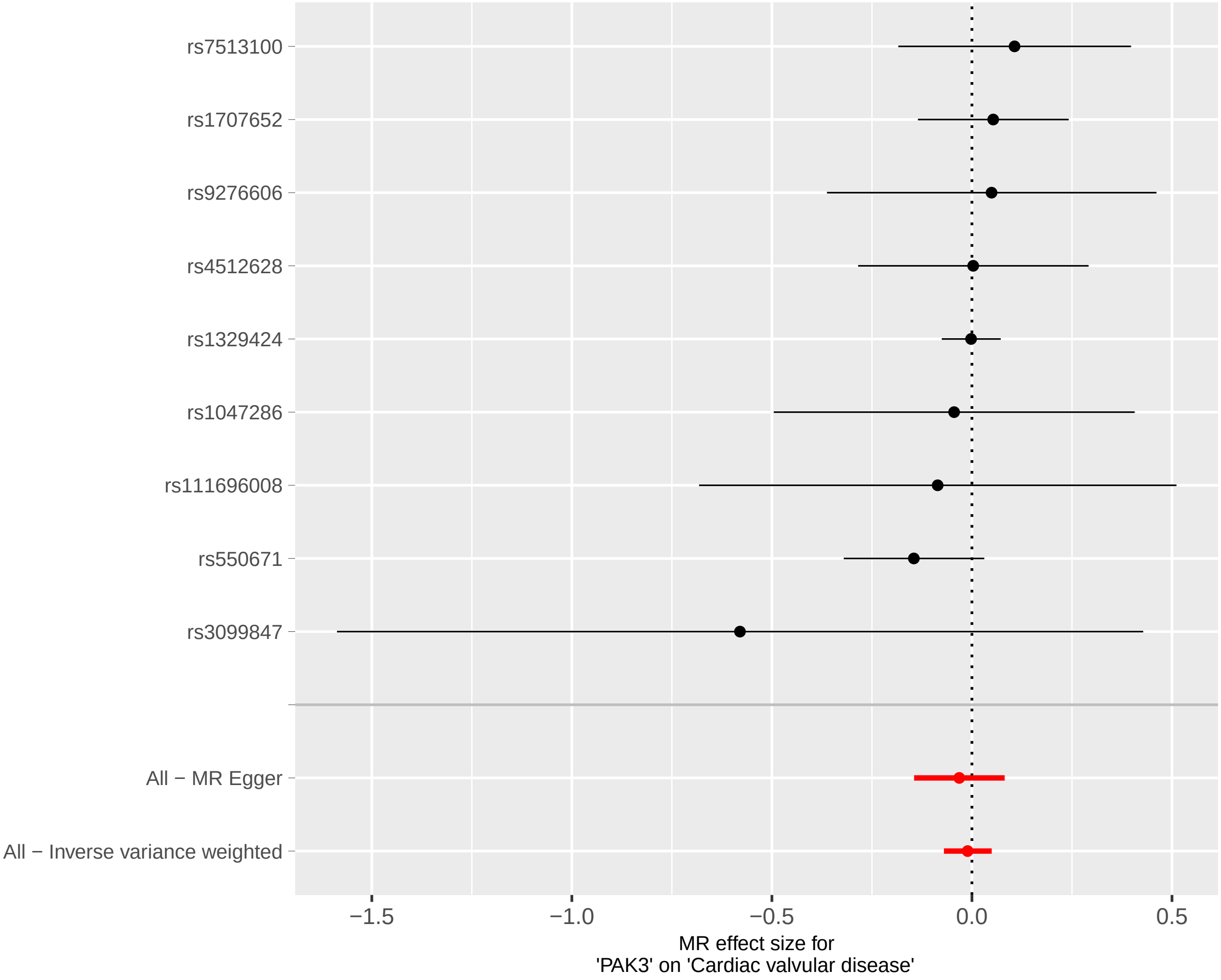
rs7513100
rs1707652
rs9276606
rs4512628
rs1329424
rs1047286
rs111696008
rs550671
rs3099847
All − MR Egger
All − Inverse variance weighted
−1.5
−1.0
−0.5
0.0
0.5
MR effect size for
'PAK3' on 'Cardiac valvular disease'

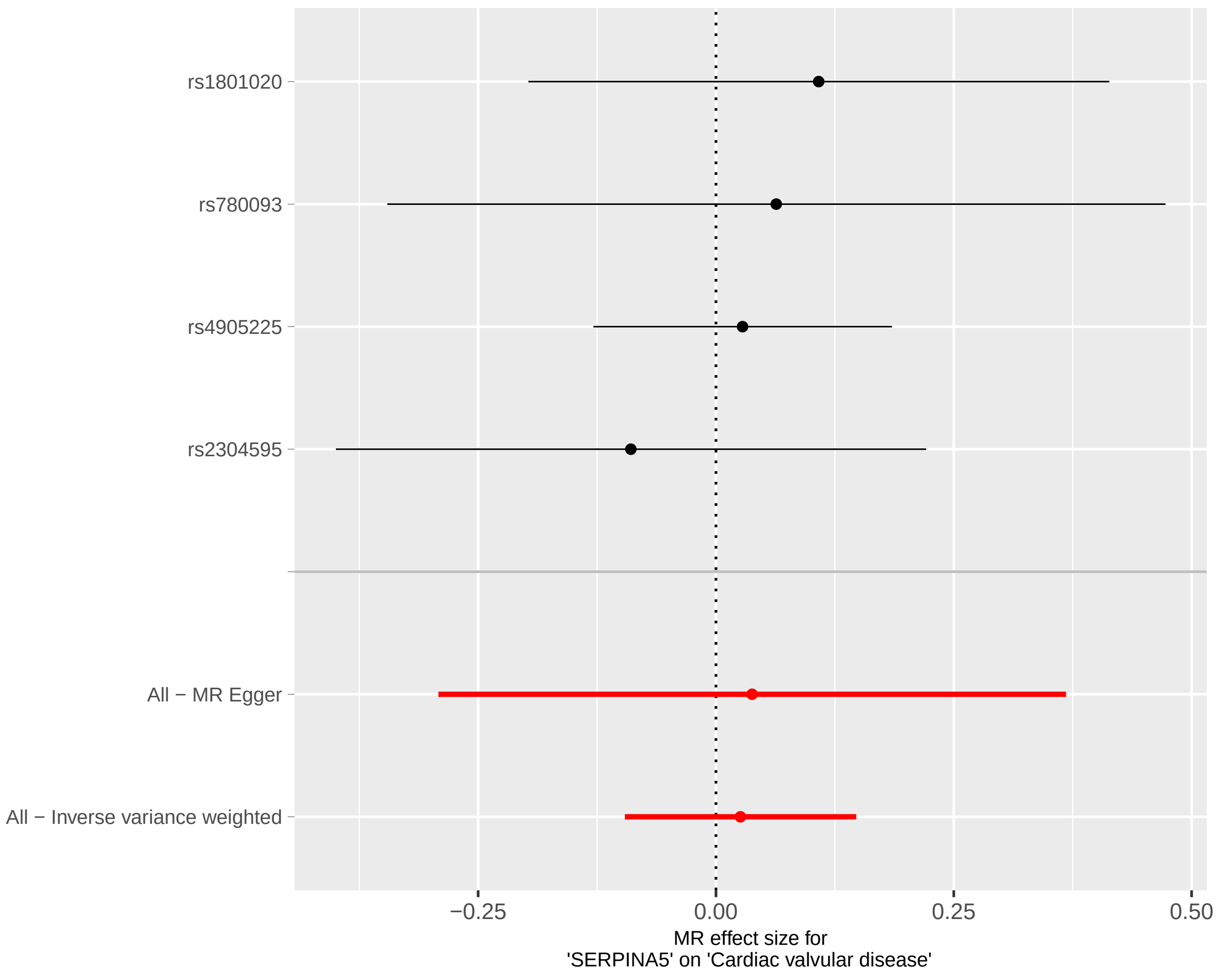
rs1801020
rs780093
rs4905225
rs2304595
All − MR Egger
All − Inverse variance weighted
−0.25
0.00
0.25
0.50
MR effect size for
'SERPINA5' on 'Cardiac valvular disease'

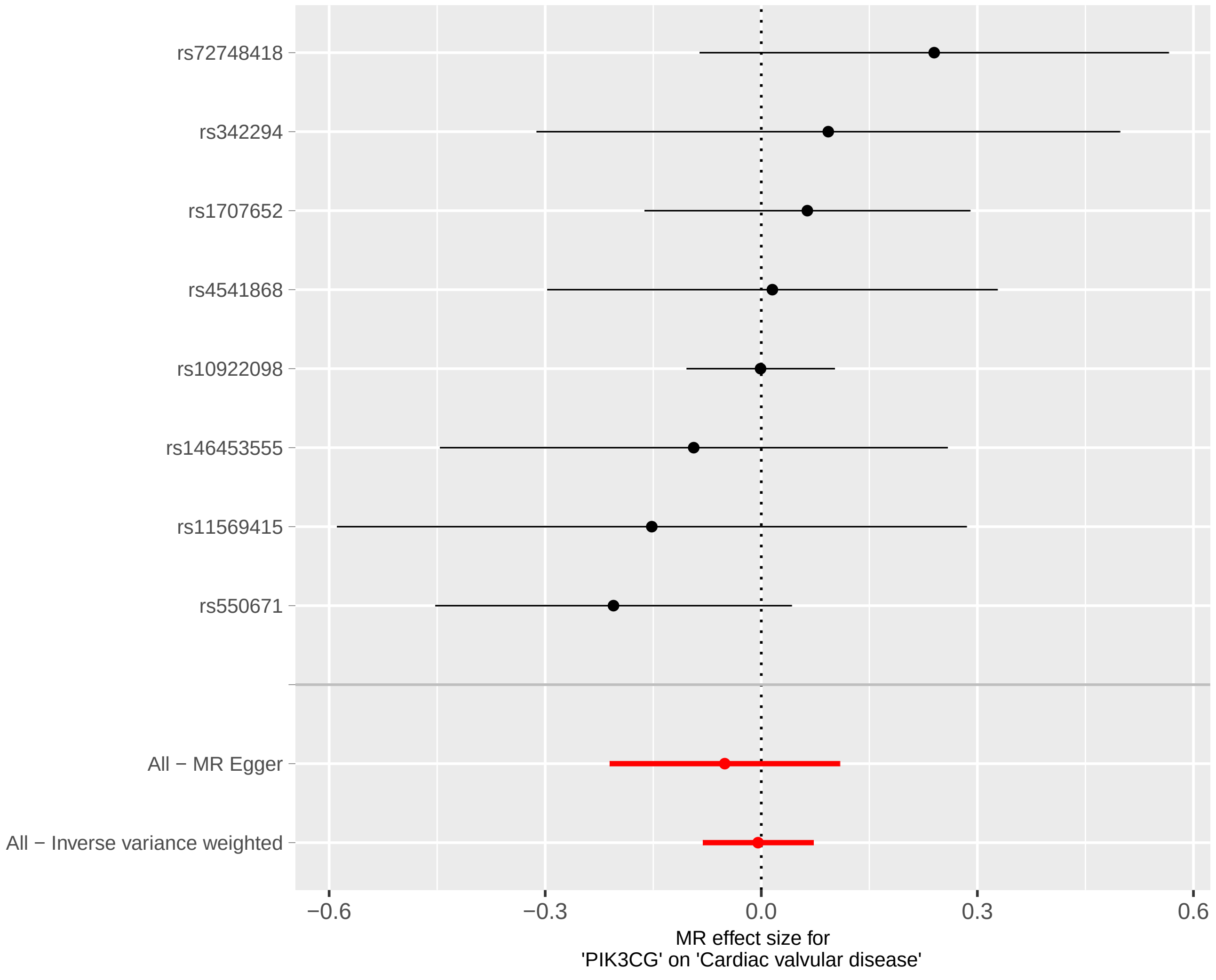
rs72748418
rs342294
rs1707652
rs4541868
rs10922098
rs146453555
rs11569415
rs550671
All − MR Egger
All − Inverse variance weighted
−0.6
−0.3
0.0
0.3
0.6
MR effect size for
'PIK3CG' on 'Cardiac valvular disease'

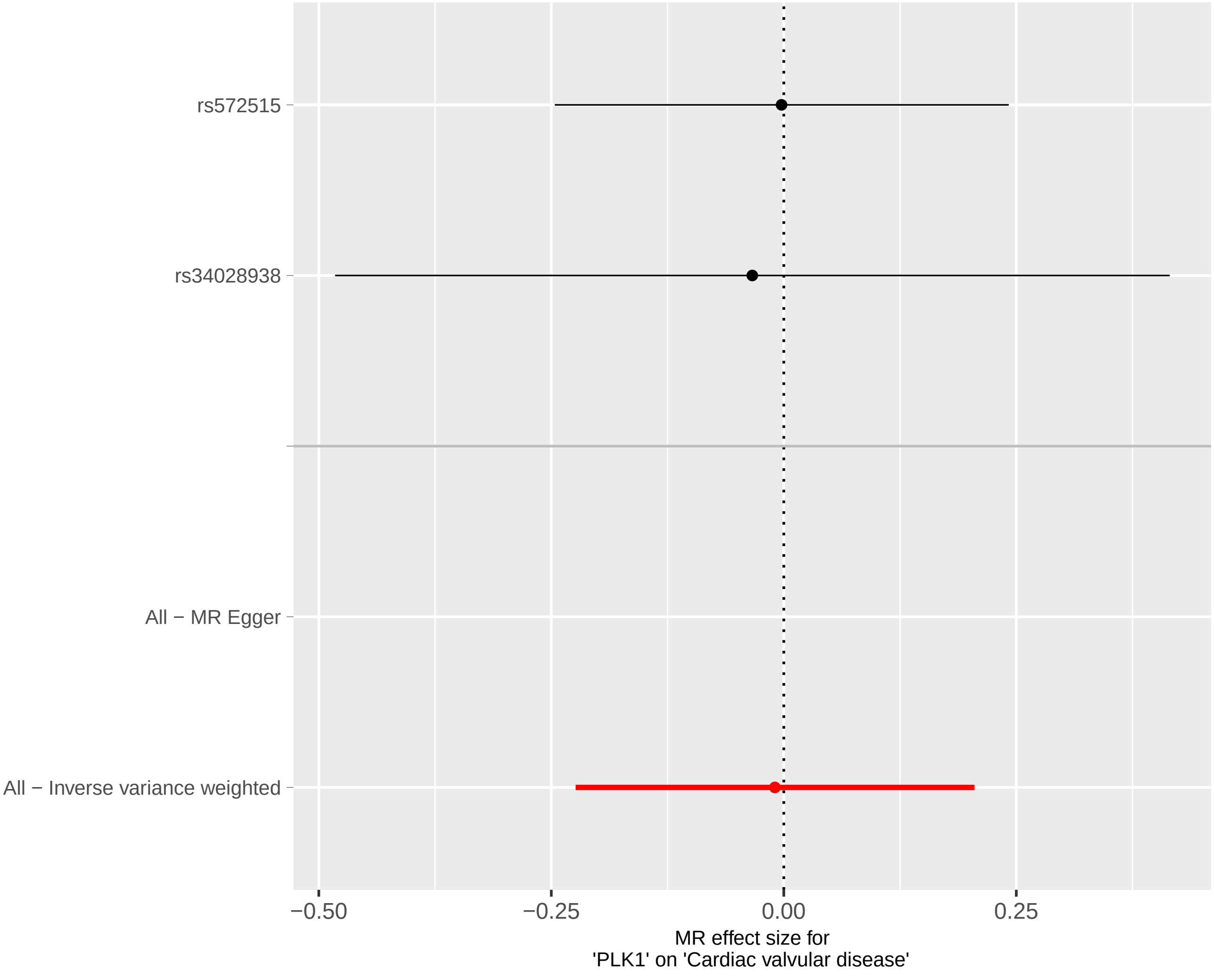
rs572515
rs34028938
All − MR Egger
All − Inverse variance weighted
−0.50
−0.25
0.00
0.25
MR effect size for
'PLK1' on 'Cardiac valvular disease'

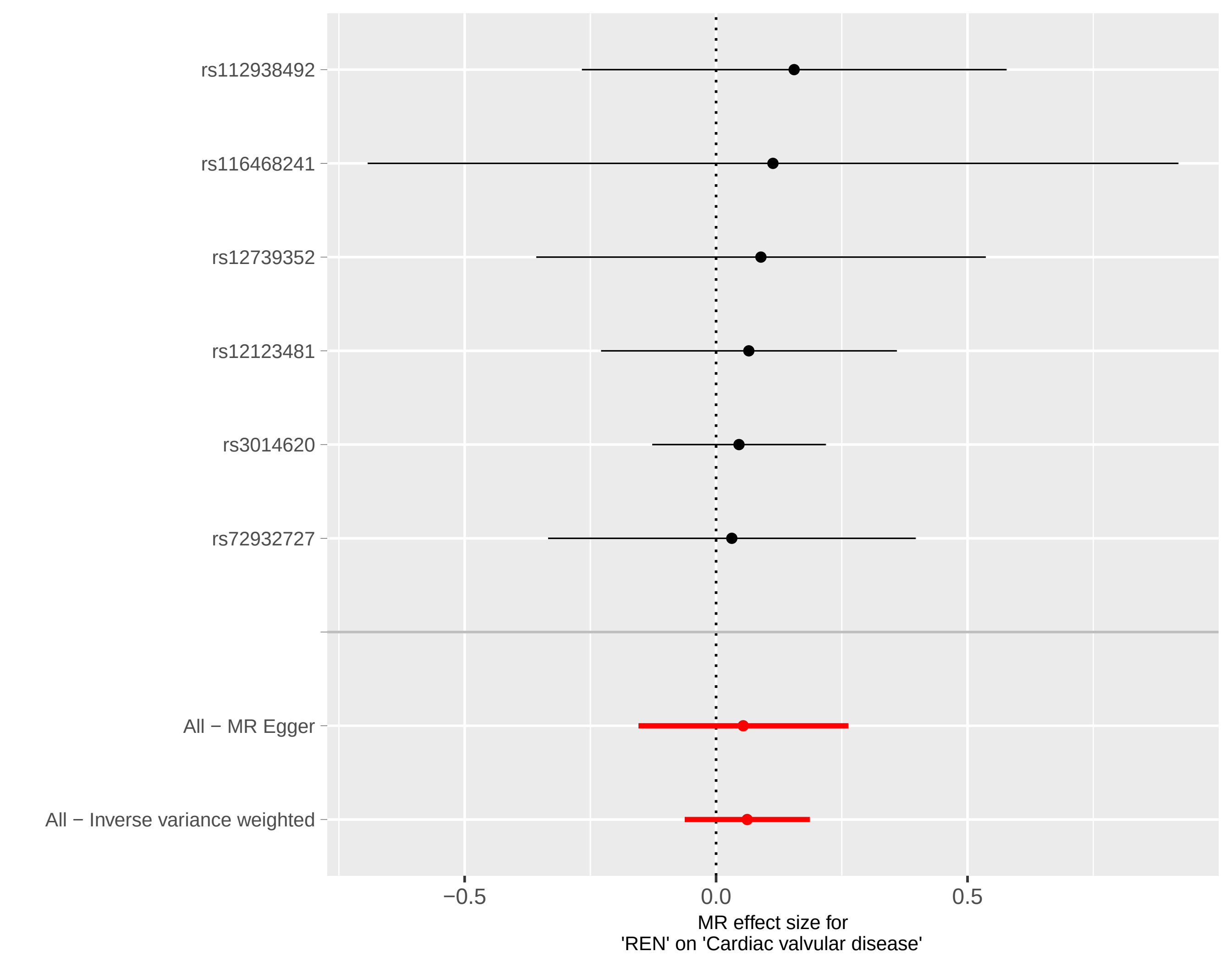
rs112938492
rs116468241
rs12739352
rs12123481
rs3014620
rs72932727
All − MR Egger
All − Inverse variance weighted
−0.5
0.0
0.5
MR effect size for
'REN' on 'Cardiac valvular disease'

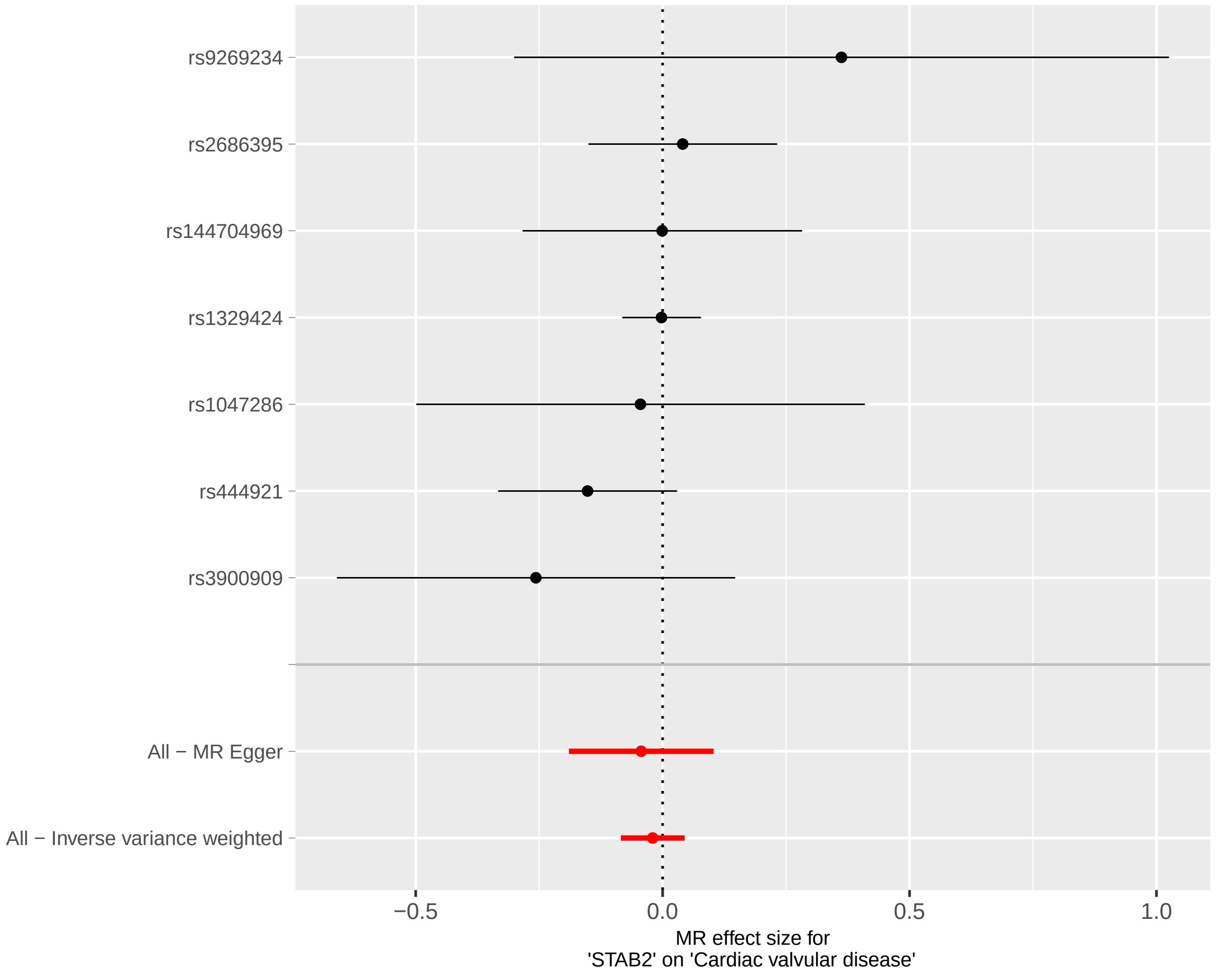

rs9269234
rs2686395
rs144704969
rs1329424
rs1047286
rs444921
rs3900909
All − MR Egger
All − Inverse variance weighted
−0.5
0.0
0.5
1.0
MR effect size for
'STAB2' on 'Cardiac valvular disease'

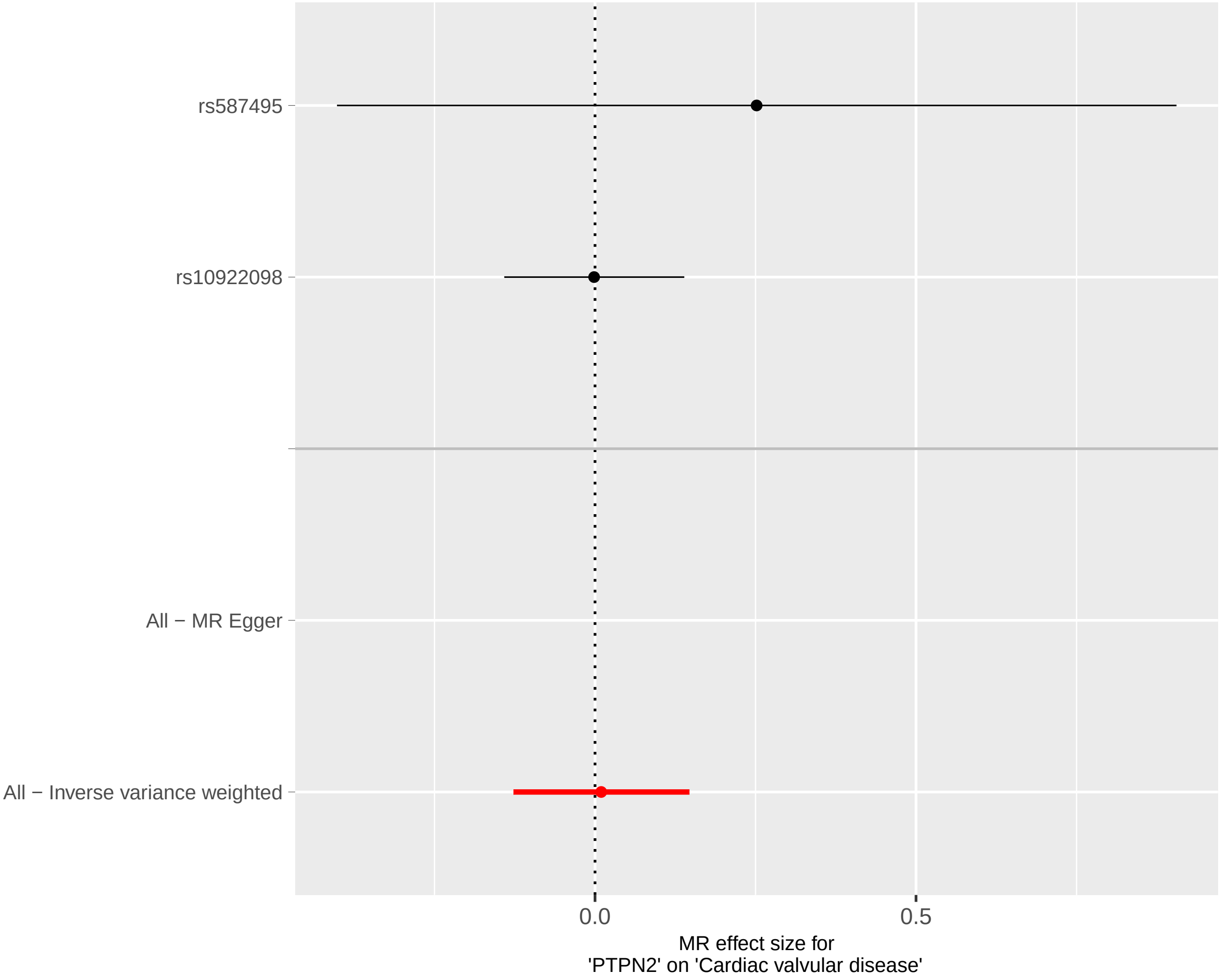

rs587495
rs10922098
All − MR Egger
All − Inverse variance weighted
0.0
0.5
MR effect size for
'PTPN2' on 'Cardiac valvular disease'

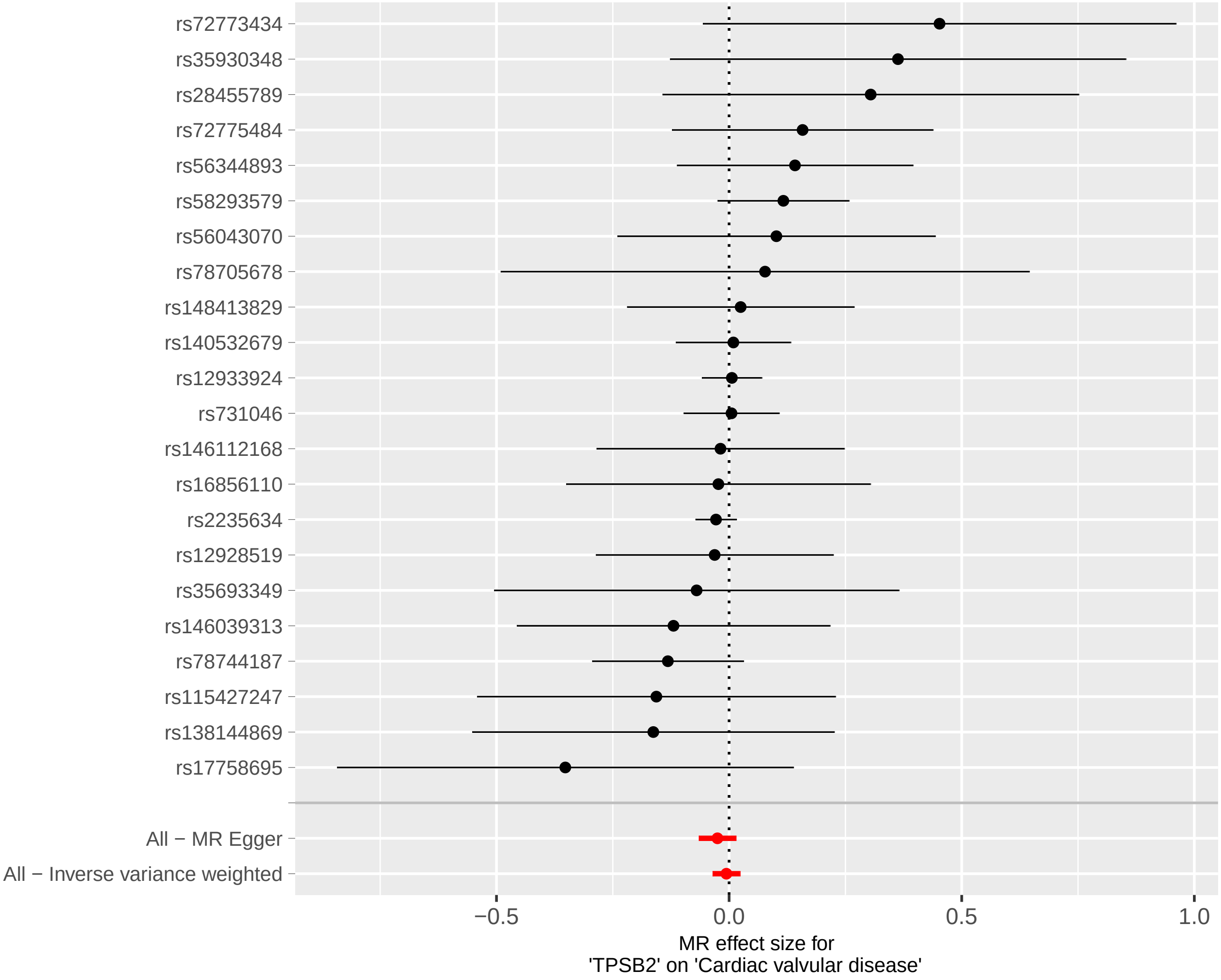
rs72773434
rs35930348
rs28455789
rs72775484
rs56344893
rs58293579
rs56043070
rs78705678
rs148413829
rs140532679
rs12933924
rs731046
rs146112168
rs16856110
rs2235634
rs12928519
rs35693349
rs146039313
rs78744187
rs115427247
rs138144869
rs17758695
All − MR Egger
All − Inverse variance weighted
−0.5
0.0
0.5
1.0
MR effect size for
'TPSB2' on 'Cardiac valvular disease'

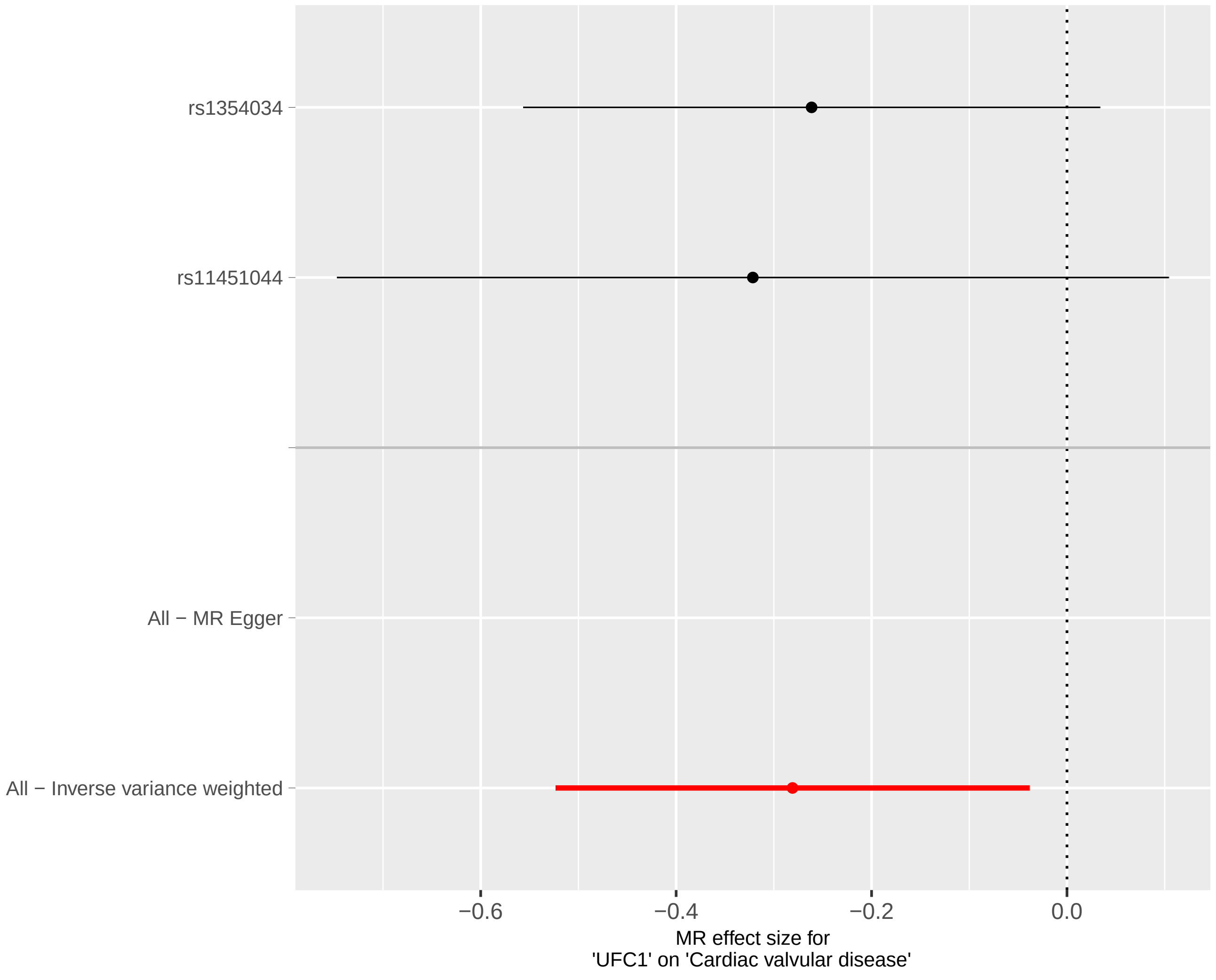
rs1354034
rs11451044
All − MR Egger
All − Inverse variance weighted
−0.6
−0.4
−0.2
0.0
MR effect size for
'UFC1' on 'Cardiac valvular disease'

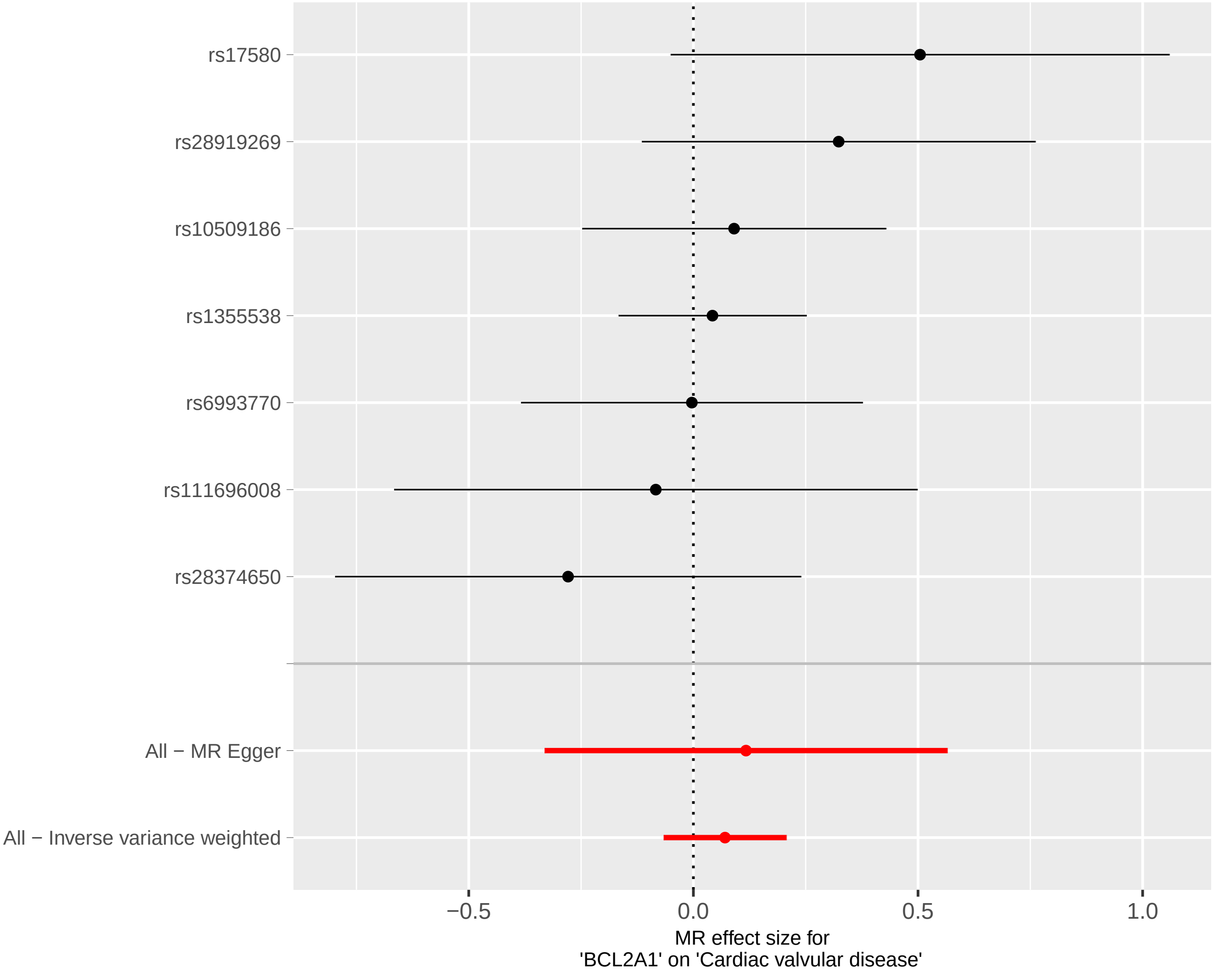
rs17580
rs28919269
rs10509186
rs1355538
rs6993770
rs111696008
rs28374650
All − MR Egger
All − Inverse variance weighted
−0.5
0.0
0.5
1.0
MR effect size for
'BCL2A1' on 'Cardiac valvular disease'

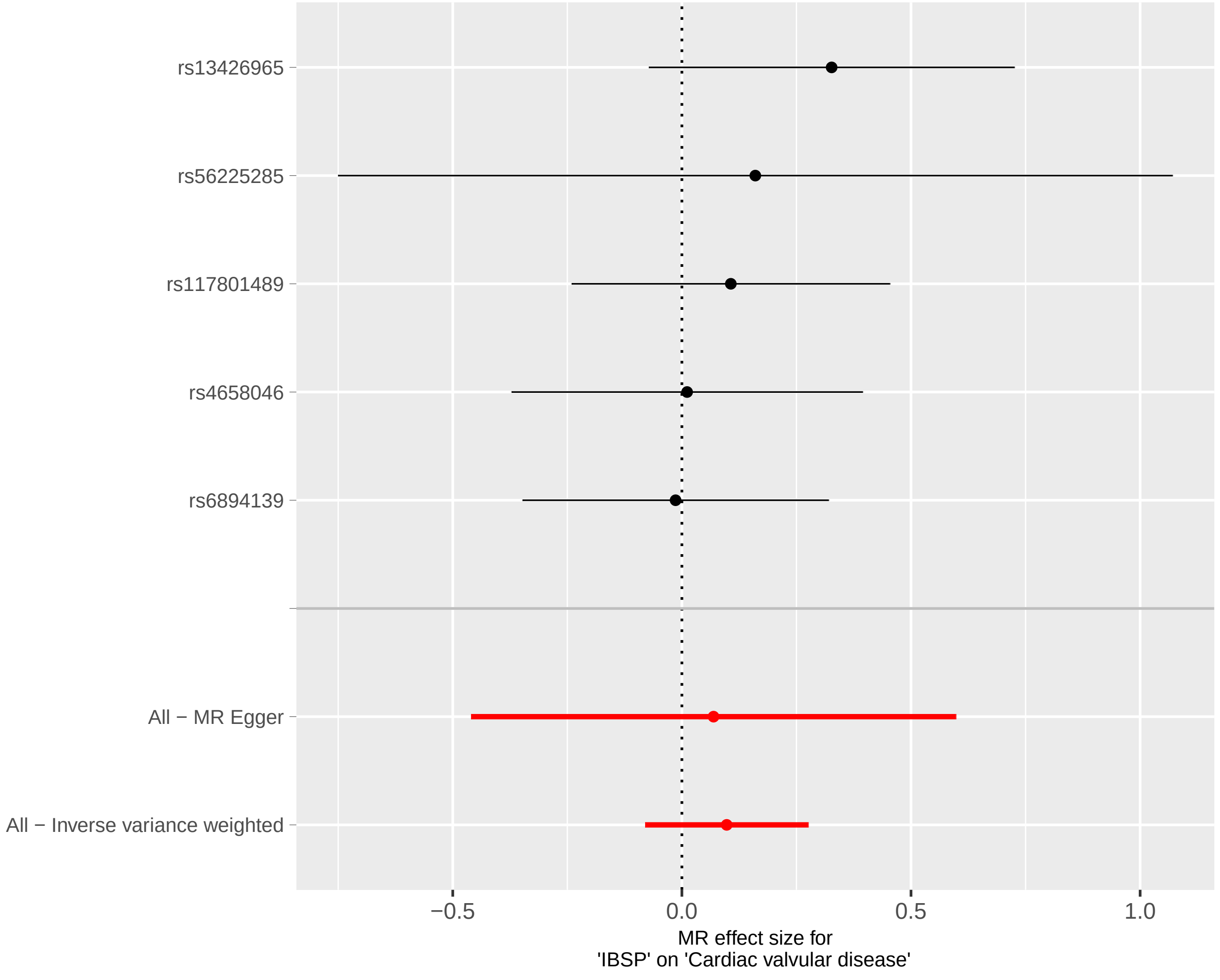
rs13426965
rs56225285
rs117801489
rs4658046
rs6894139
All − MR Egger
All − Inverse variance weighted
−0.5
0.0
0.5
1.0
MR effect size for
'IBSP' on 'Cardiac valvular disease'

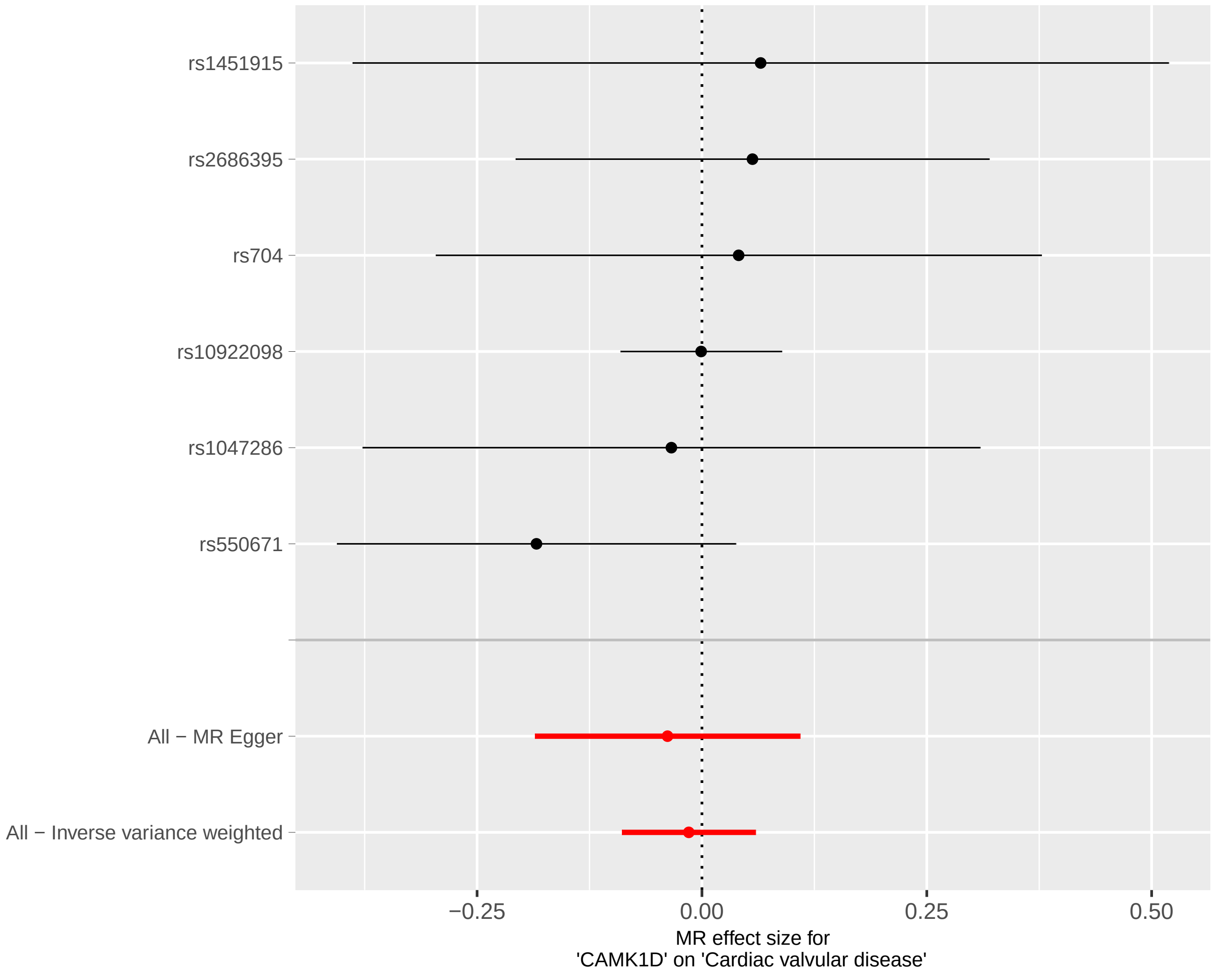

rs1451915
rs2686395
rs704
rs10922098
rs1047286
rs550671
All − MR Egger
All − Inverse variance weighted
−0.25
0.00
0.25
0.50
MR effect size for
'CAMK1D' on 'Cardiac valvular disease'

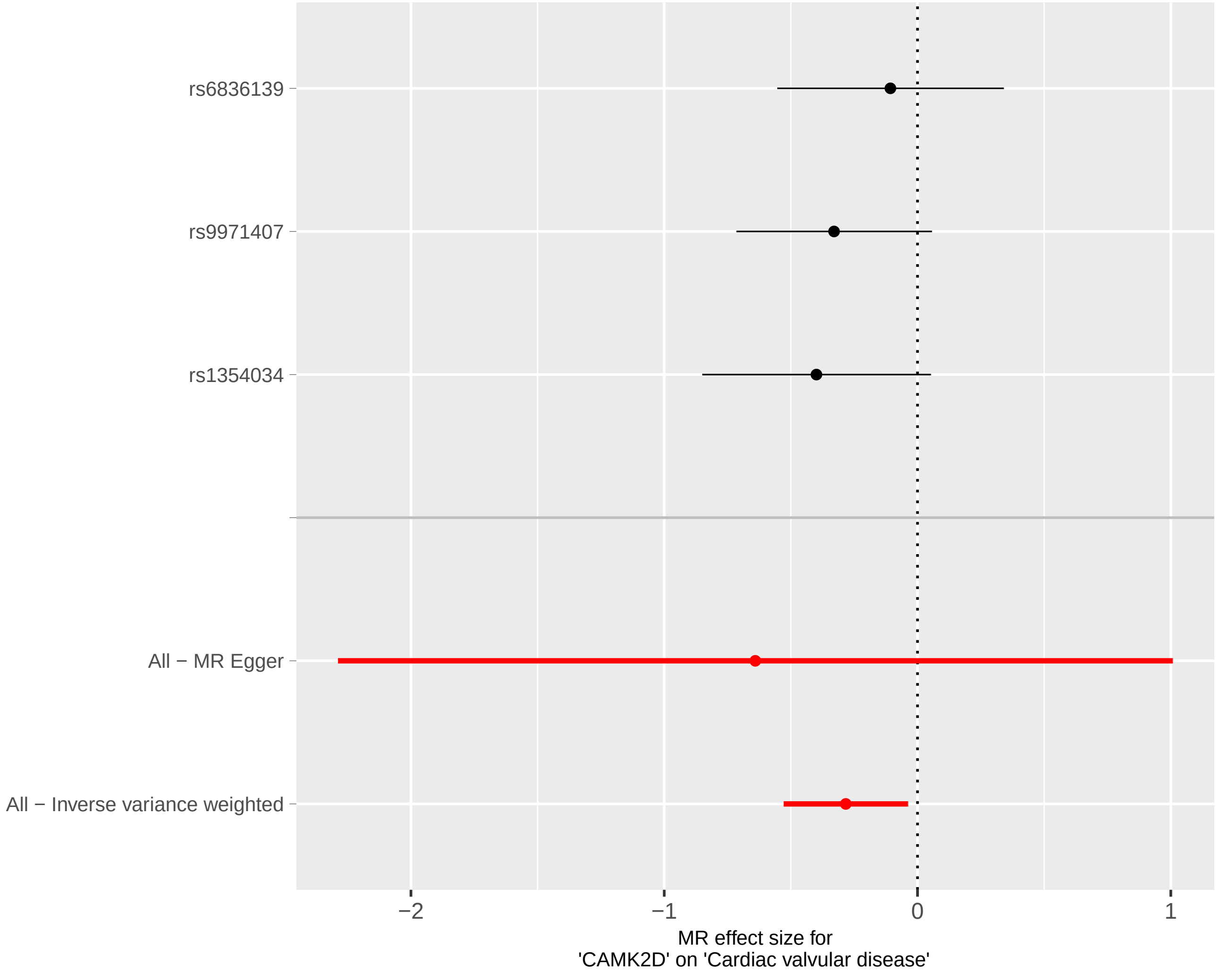
rs6836139
rs9971407
rs1354034
All − MR Egger
All − Inverse variance weighted
−2
−1
0
1
MR effect size for
'CAMK2D' on 'Cardiac valvular disease'

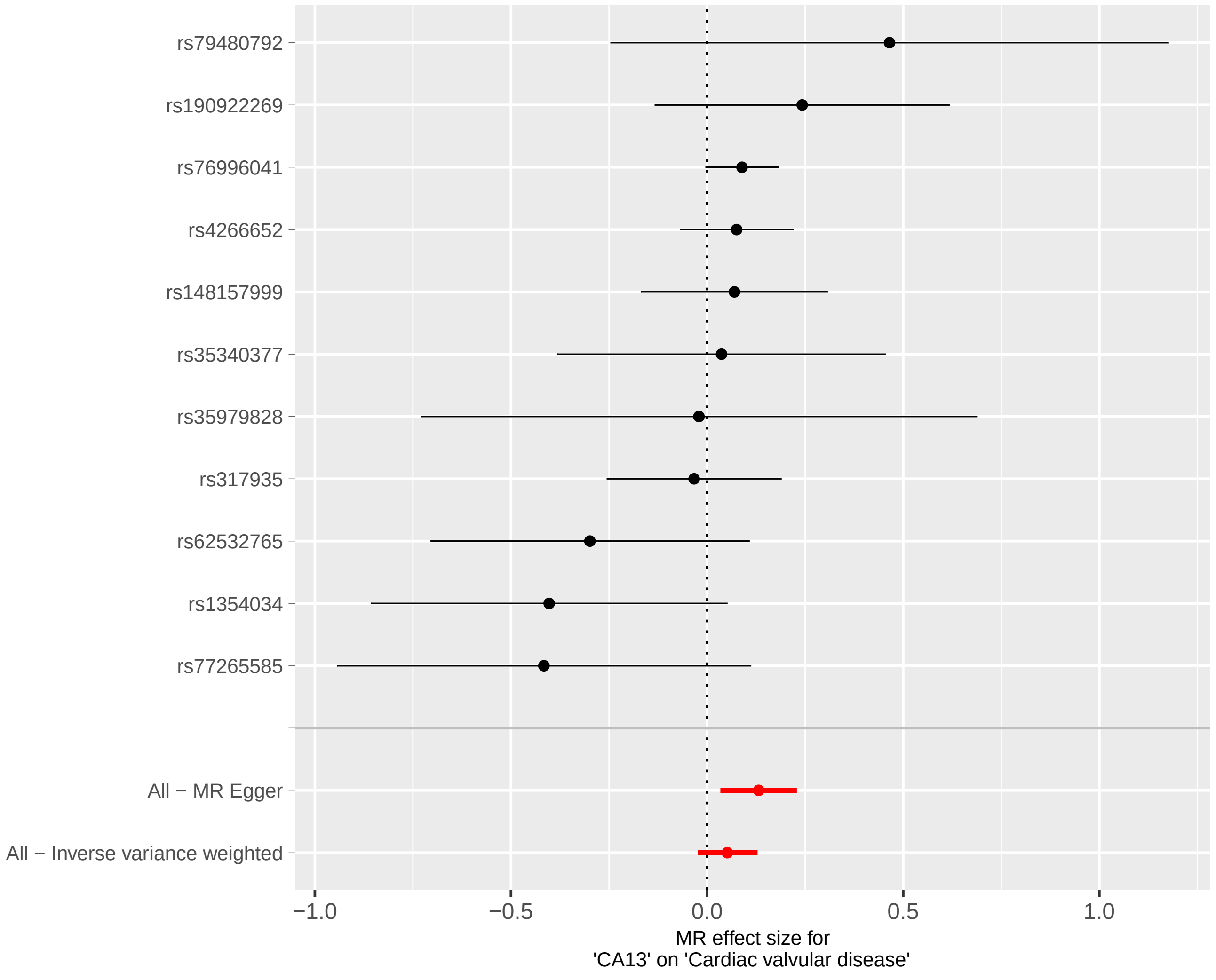
rs79480792
rs190922269
rs76996041
rs4266652
rs148157999
rs35340377
rs35979828
rs317935
rs62532765
rs1354034
rs77265585
All − MR Egger
All − Inverse variance weighted
−1.0
−0.5
0.0
0.5
1.0
MR effect size for
'CA13' on 'Cardiac valvular disease'

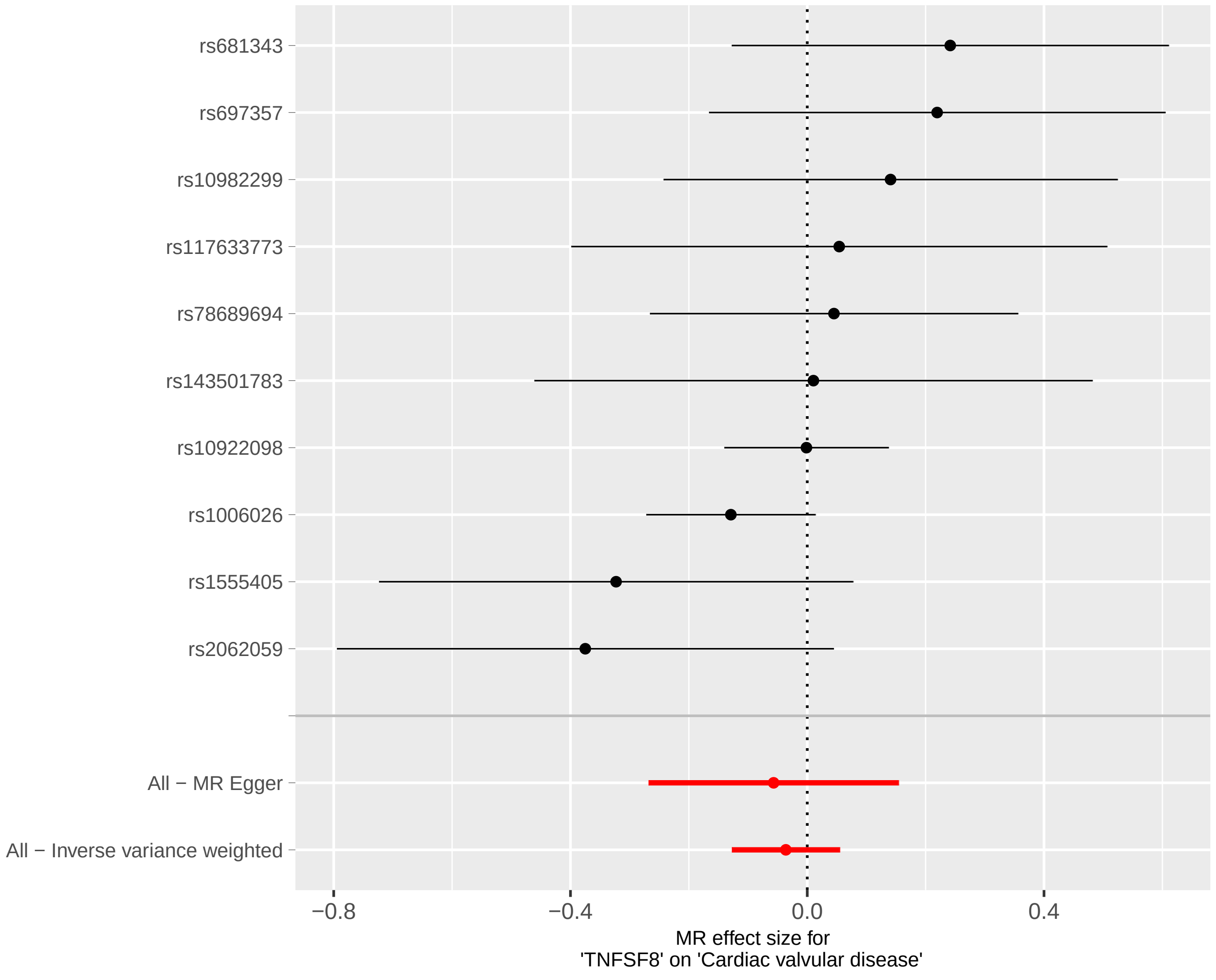
rs681343
rs697357
rs10982299
rs117633773
rs78689694
rs143501783
rs10922098
rs1006026
rs1555405
rs2062059
All − MR Egger
All − Inverse variance weighted
−0.8
−0.4
0.0
0.4
MR effect size for
'TNFSF8' on 'Cardiac valvular disease'

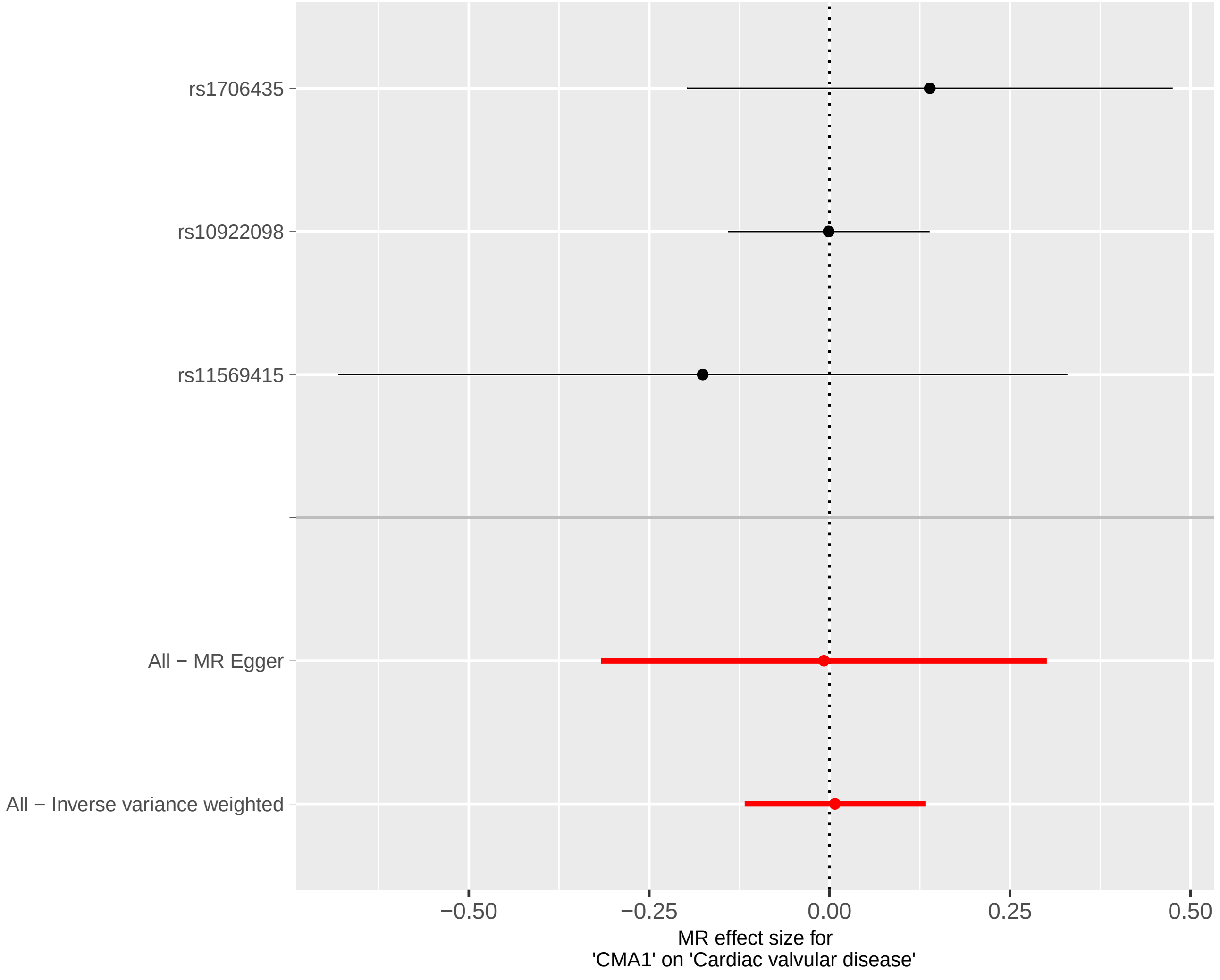

rs1706435
rs10922098
rs11569415
All − MR Egger
All − Inverse variance weighted
−0.50
−0.25
0.00
0.25
0.50
MR effect size for
'CMA1' on 'Cardiac valvular disease'

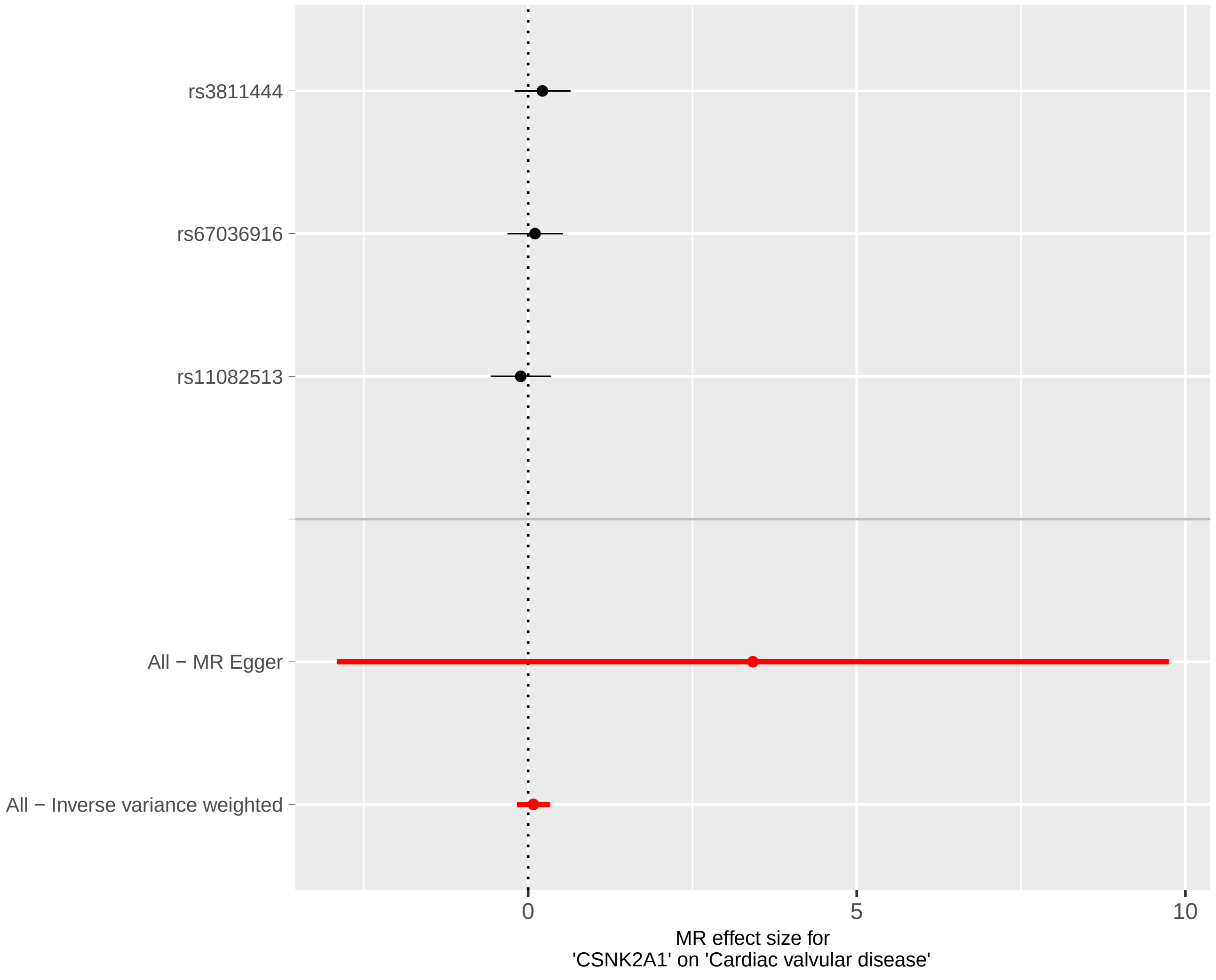
rs3811444
rs67036916
rs11082513
All − MR Egger
All − Inverse variance weighted
0
5
10
MR effect size for
'CSNK2A1' on 'Cardiac valvular disease'

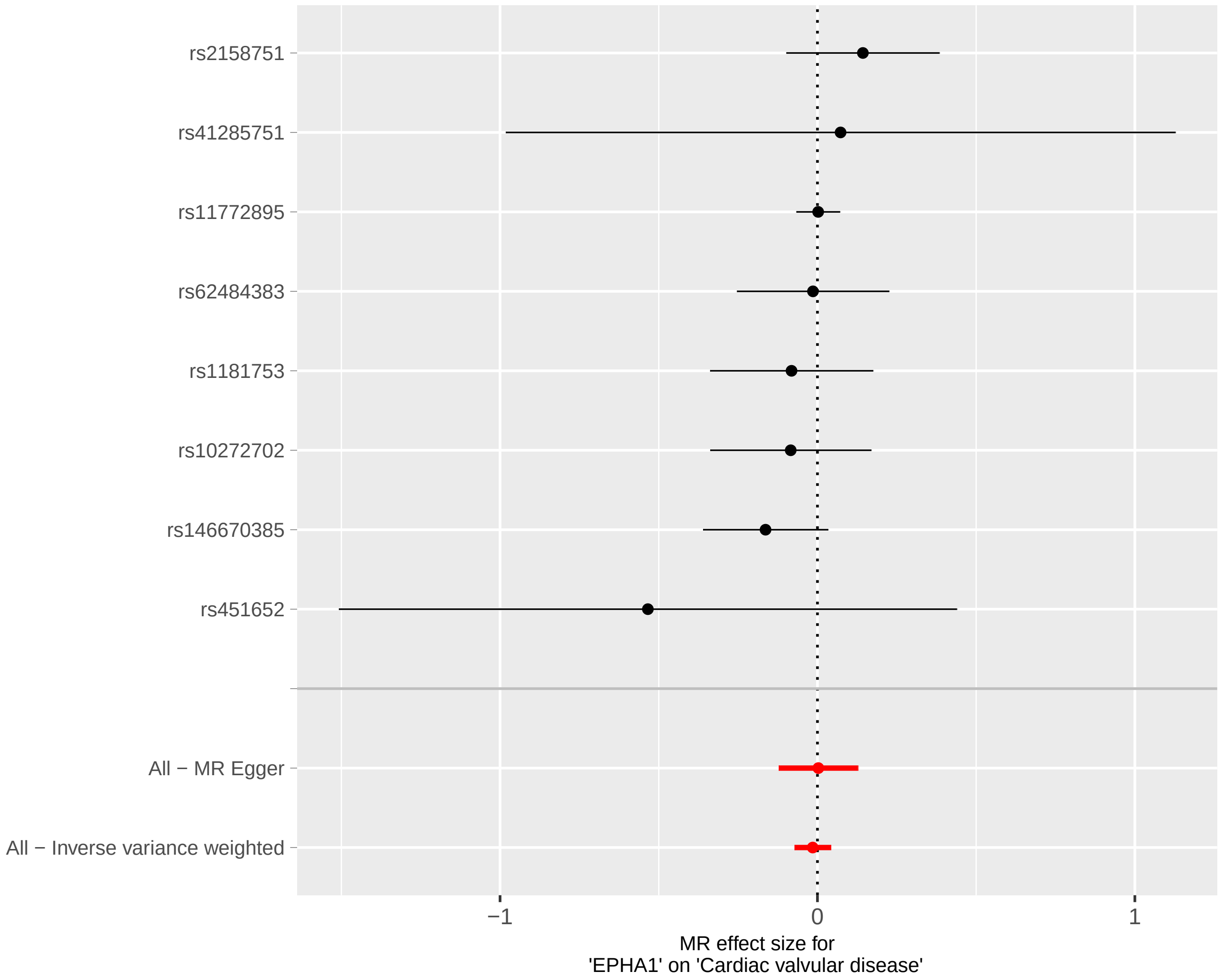
rs2158751
rs41285751
rs11772895
rs62484383
rs1181753
rs10272702
rs146670385
rs451652
All − MR Egger
All − Inverse variance weighted
−1
0
1
MR effect size for
'EPHA1' on 'Cardiac valvular disease'

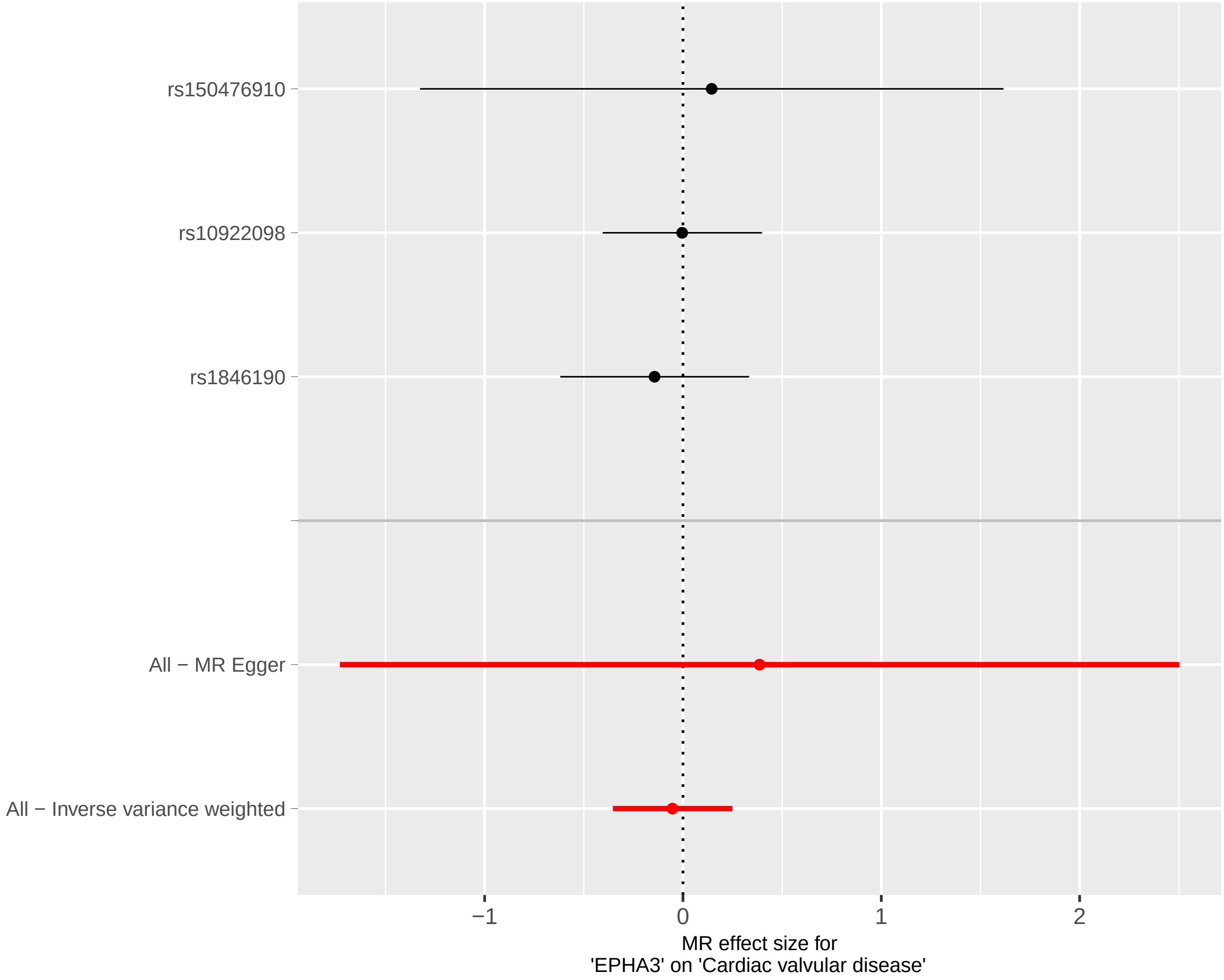
rs150476910
rs10922098
rs1846190
All − MR Egger
All − Inverse variance weighted
−1
0
1
2
MR effect size for
'EPHA3' on 'Cardiac valvular disease'

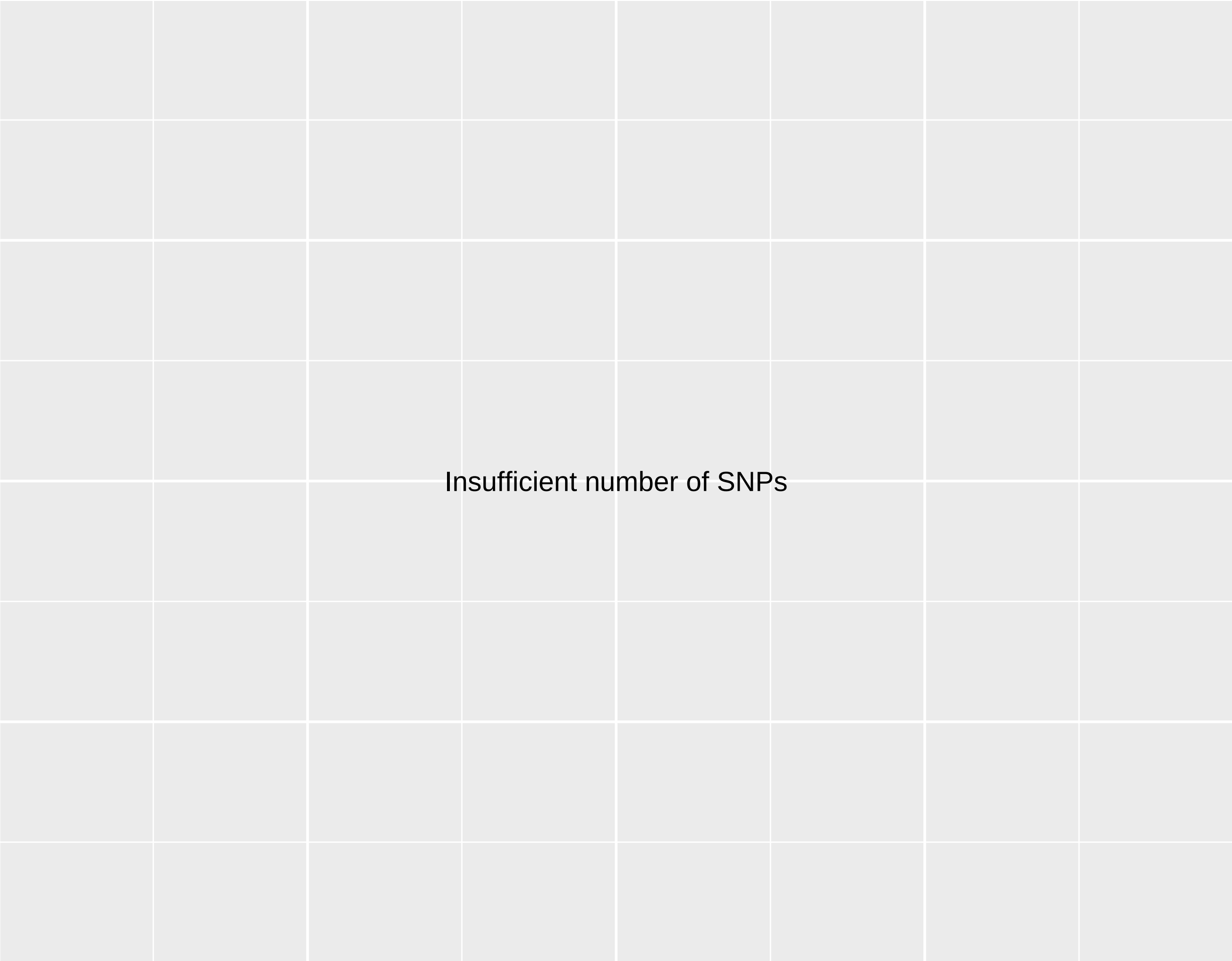
Insufficient number of SNPs

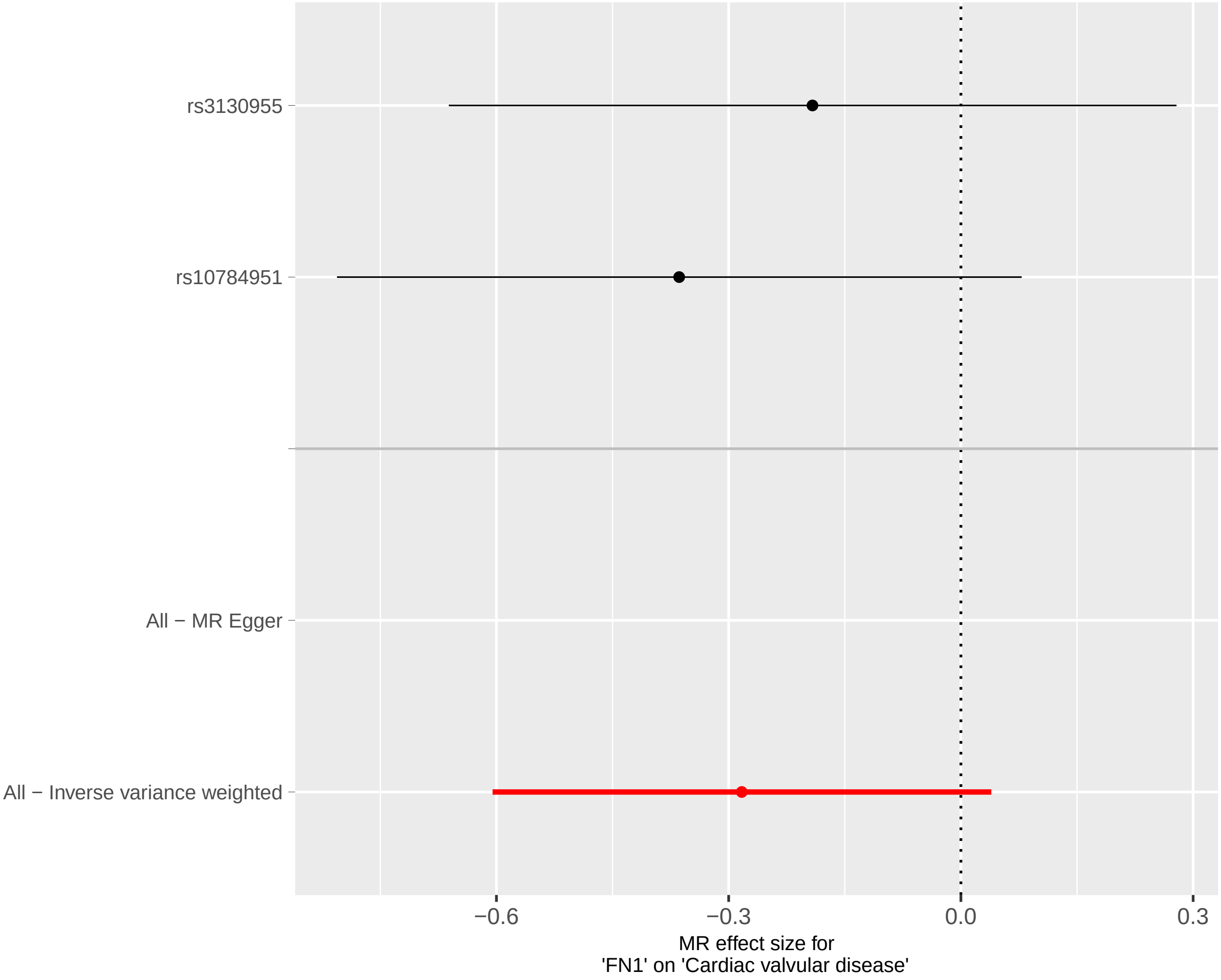
rs3130955
rs10784951
All − MR Egger
All − Inverse variance weighted
−0.6
−0.3
0.0
0.3
MR effect size for
'FN1' on 'Cardiac valvular disease'

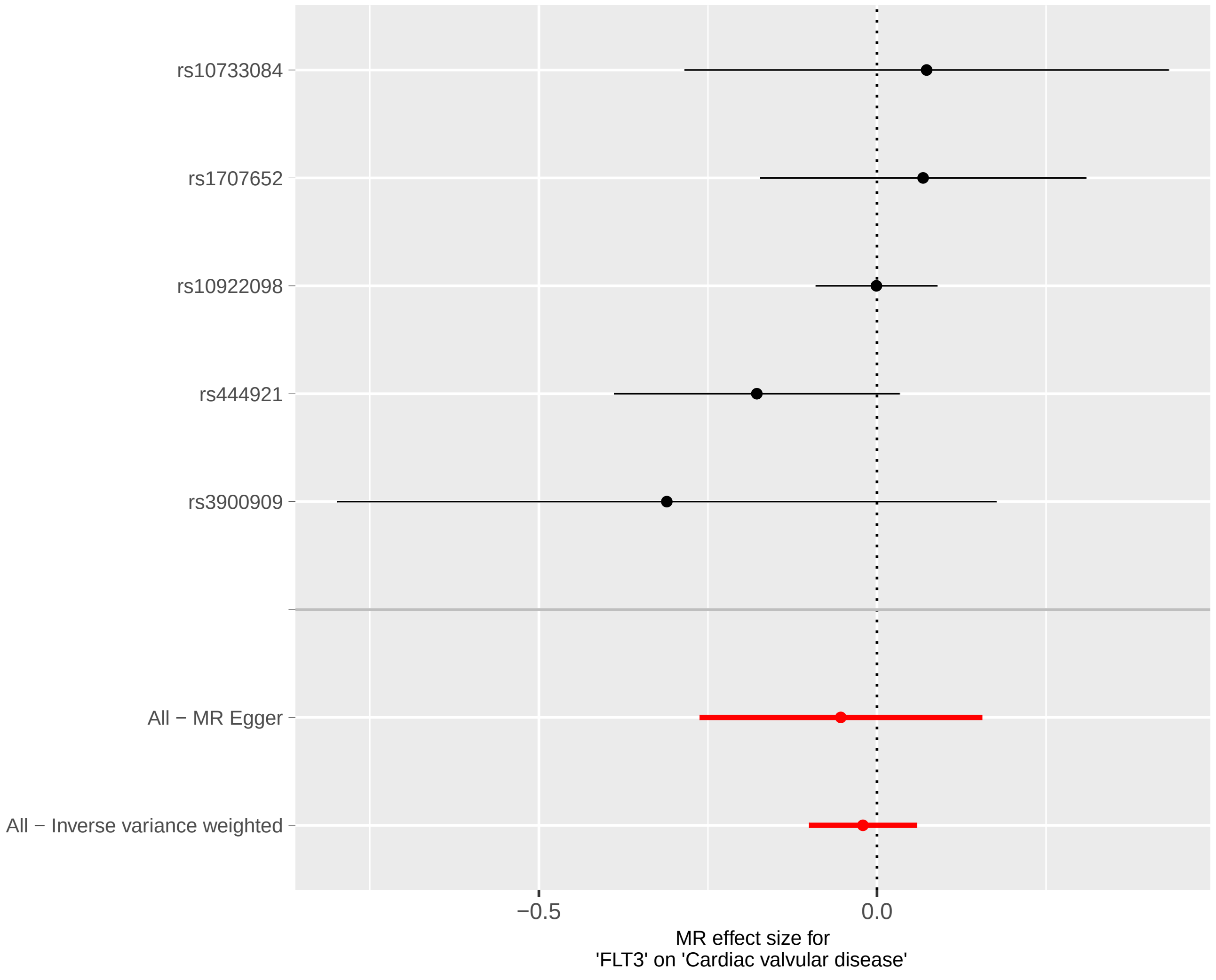
rs10733084
rs1707652
rs10922098
rs444921
rs3900909
All − MR Egger
All − Inverse variance weighted
−0.5
0.0
MR effect size for
'FLT3' on 'Cardiac valvular disease'

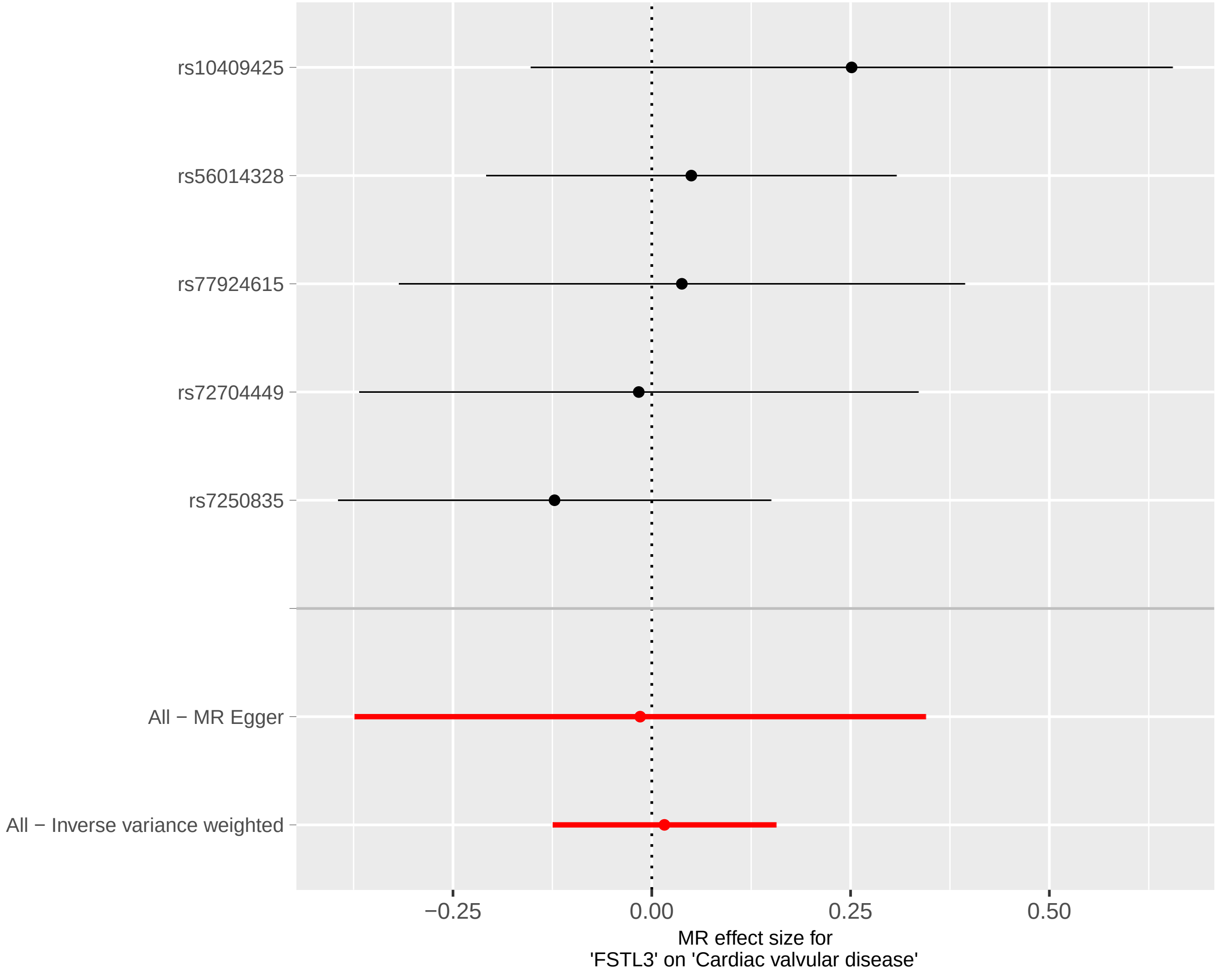
rs10409425
rs56014328
rs77924615
rs72704449
rs7250835
All − MR Egger
All − Inverse variance weighted
−0.25
0.00
0.25
0.50
MR effect size for
'FSTL3' on 'Cardiac valvular disease'

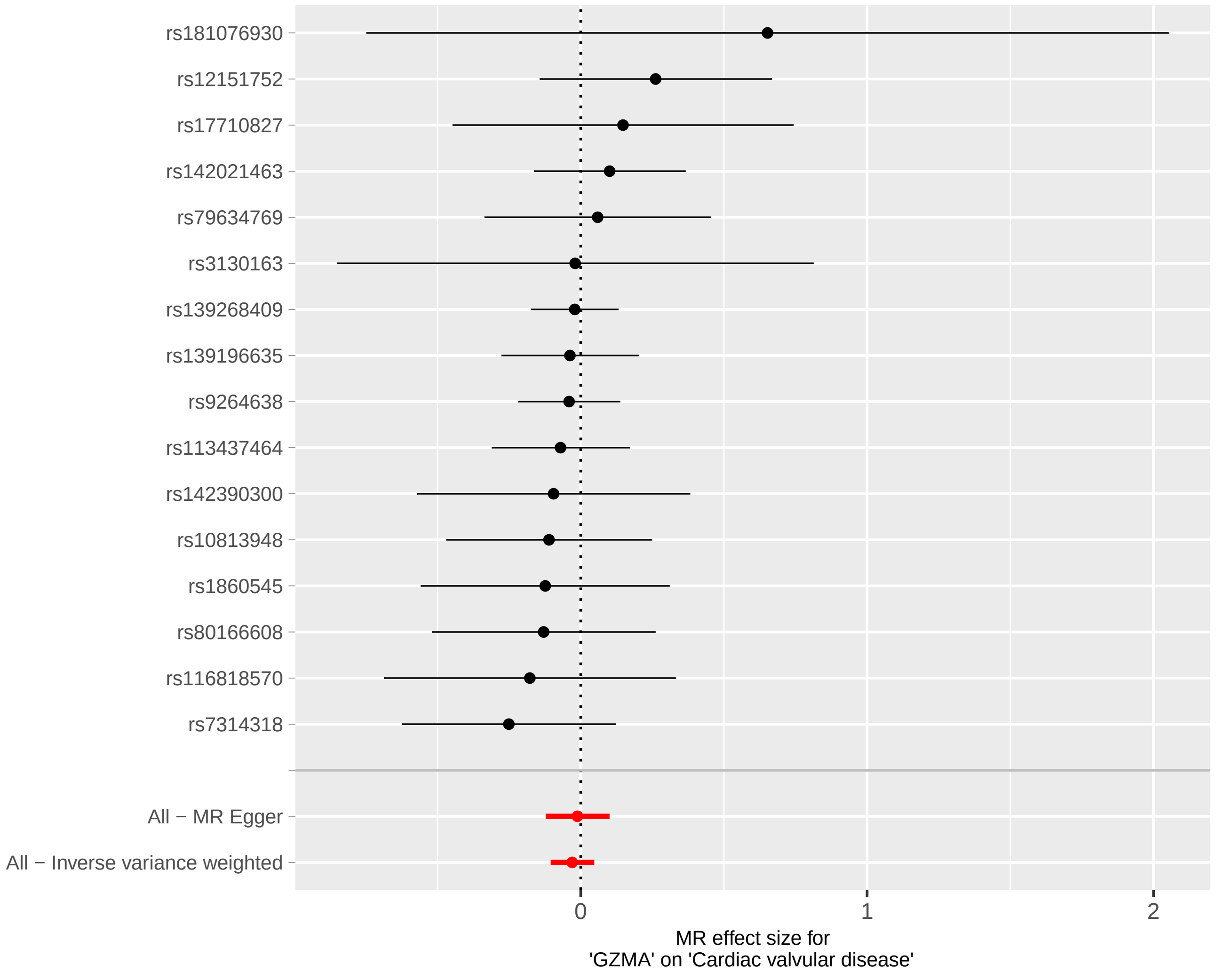
rs181076930
rs12151752
rs17710827
rs142021463
rs79634769
rs3130163
rs139268409
rs139196635
rs9264638
rs113437464
rs142390300
rs10813948
rs1860545
rs80166608
rs116818570
rs7314318
All − MR Egger
All − Inverse variance weighted
0
1
2
MR effect size for
'GZMA' on 'Cardiac valvular disease'

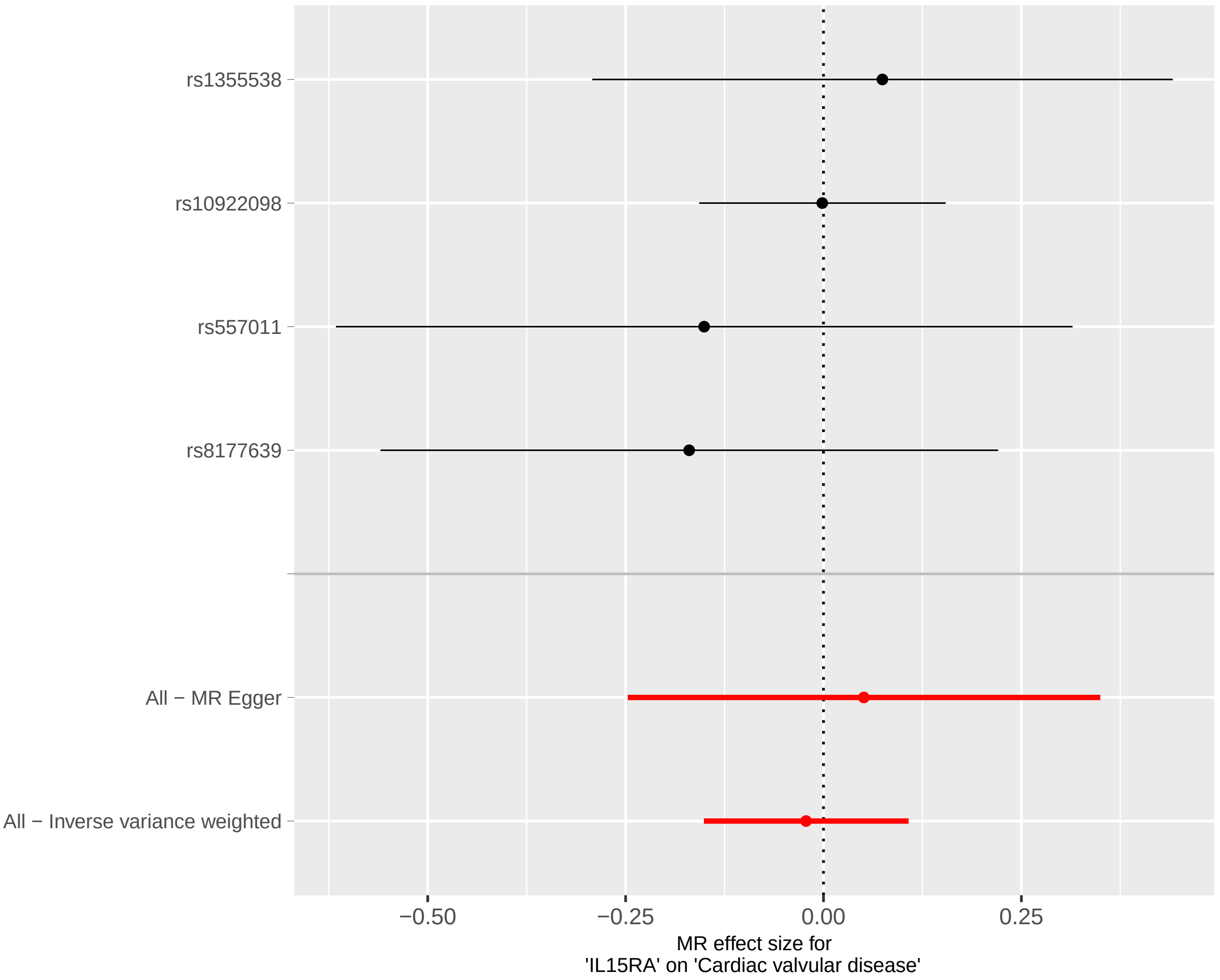
rs1355538
rs10922098
rs557011
rs8177639
All − MR Egger
All − Inverse variance weighted
−0.50
−0.25
0.00
0.25
MR effect size for
'IL15RA' on 'Cardiac valvular disease'

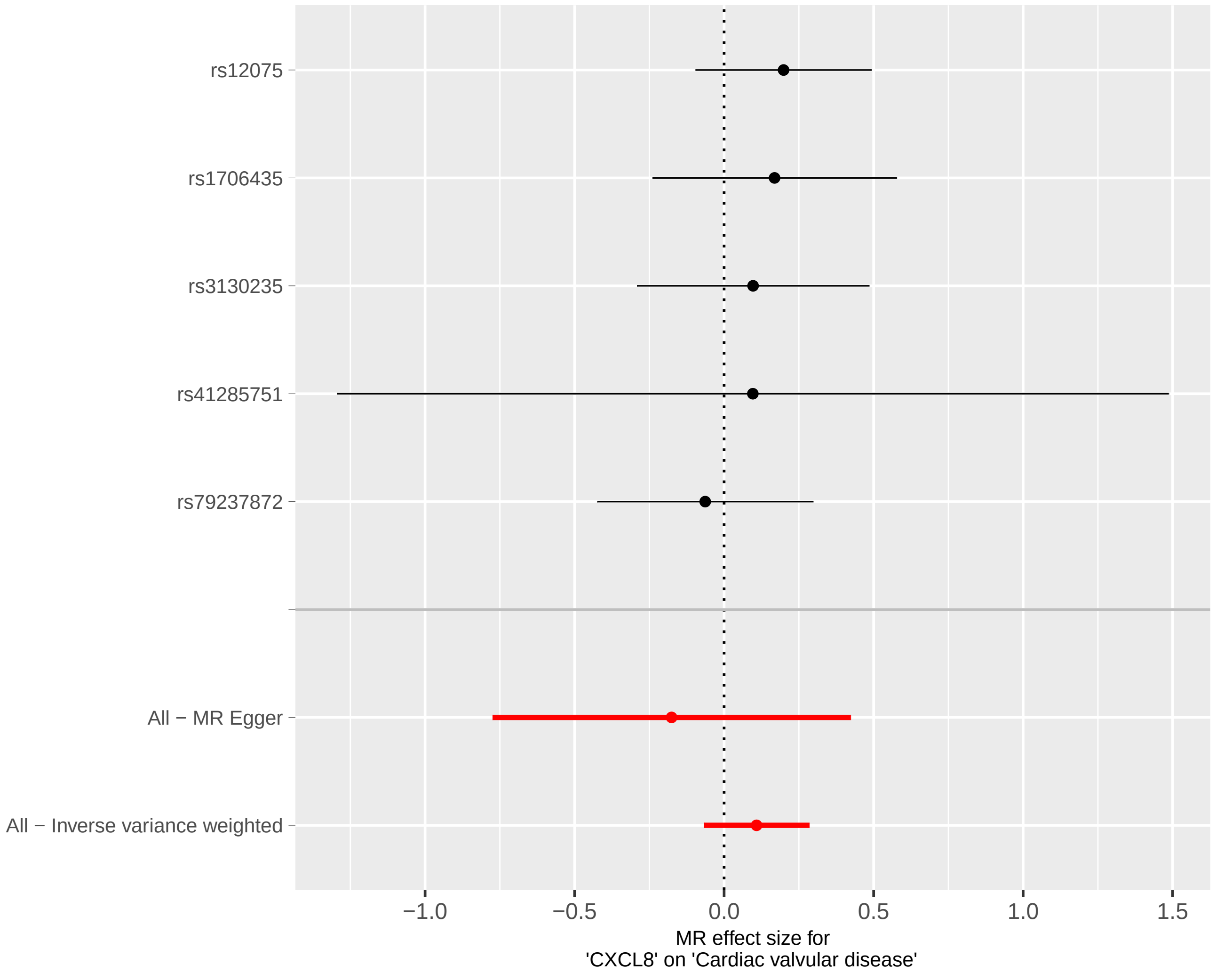

rs12075
rs1706435
rs3130235
rs41285751
rs79237872
All − MR Egger
All − Inverse variance weighted
−1.0
−0.5
0.0
0.5
1.0
1.5
MR effect size for
'CXCL8' on 'Cardiac valvular disease'

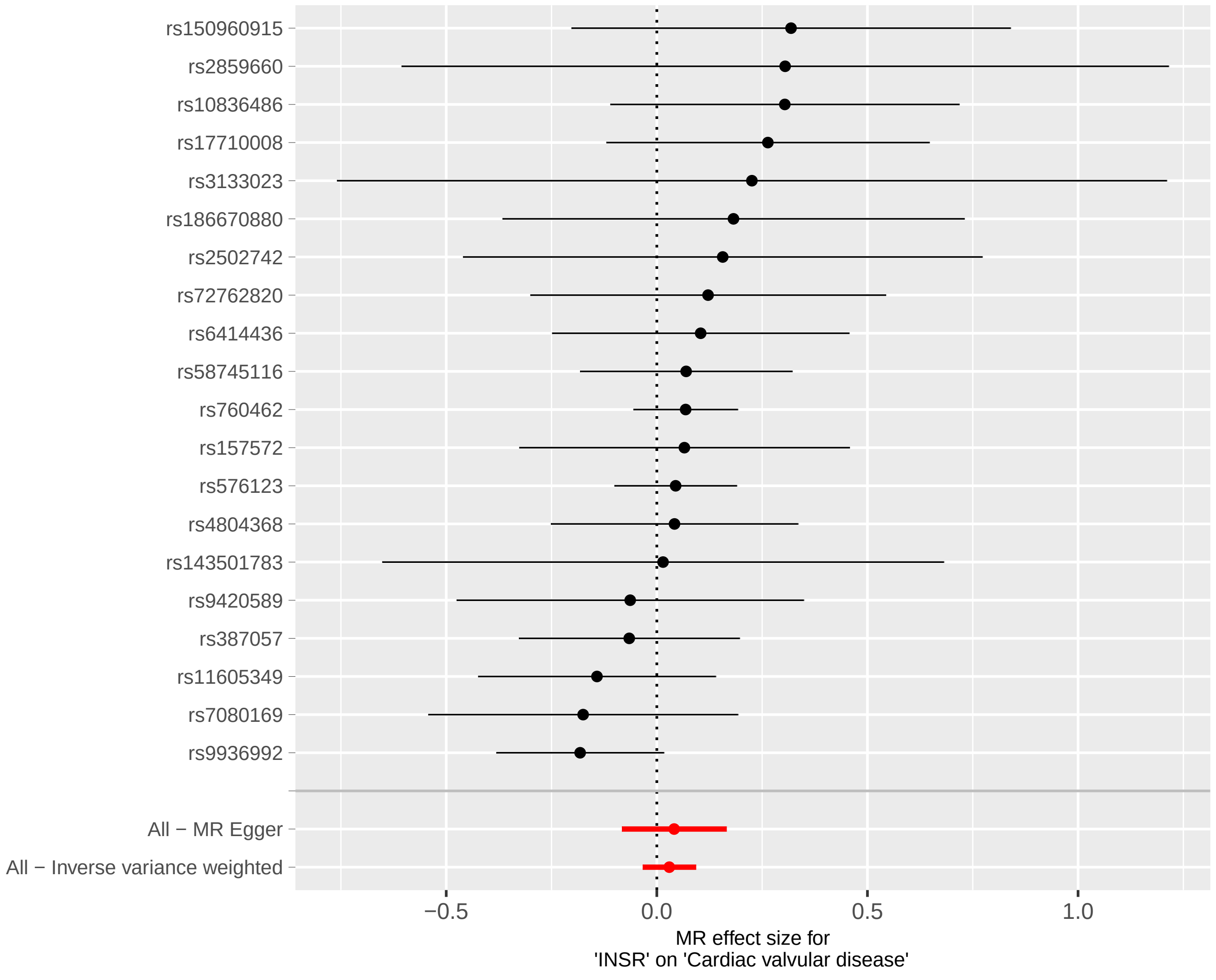
rs150960915
rs2859660
rs10836486
rs17710008
rs3133023
rs186670880
rs2502742
rs72762820
rs6414436
rs58745116
rs760462
rs157572
rs576123
rs4804368
rs143501783
rs9420589
rs387057
rs11605349
rs7080169
rs9936992
All − MR Egger
All − Inverse variance weighted
−0.5
0.0
0.5
1.0
MR effect size for
'INSR' on 'Cardiac valvular disease'

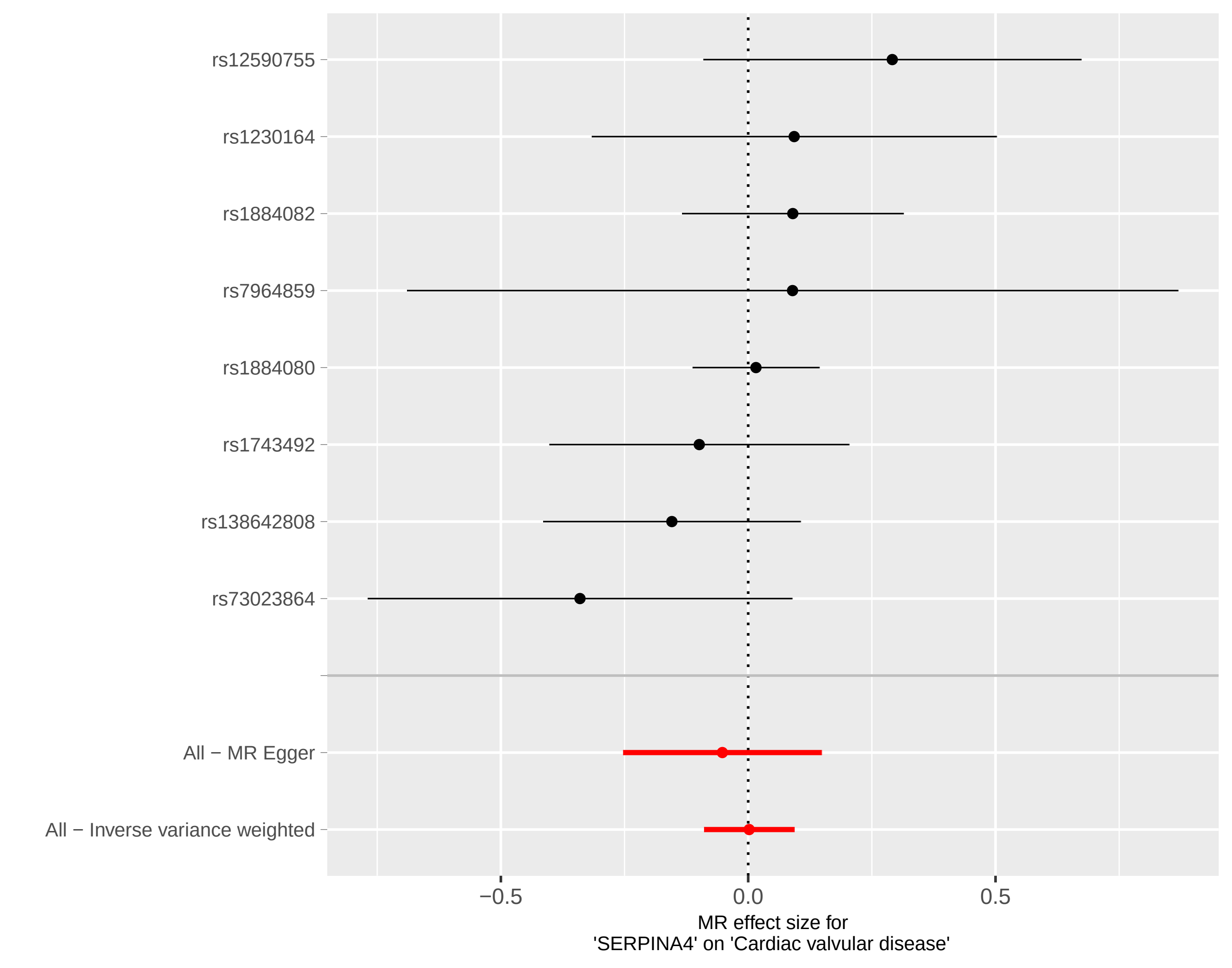

rs12590755
rs1230164
rs1884082
rs7964859
rs1884080
rs1743492
rs138642808
rs73023864
All − MR Egger
All − Inverse variance weighted
−0.5
0.0
0.5
MR effect size for
'SERPINA4' on 'Cardiac valvular disease'

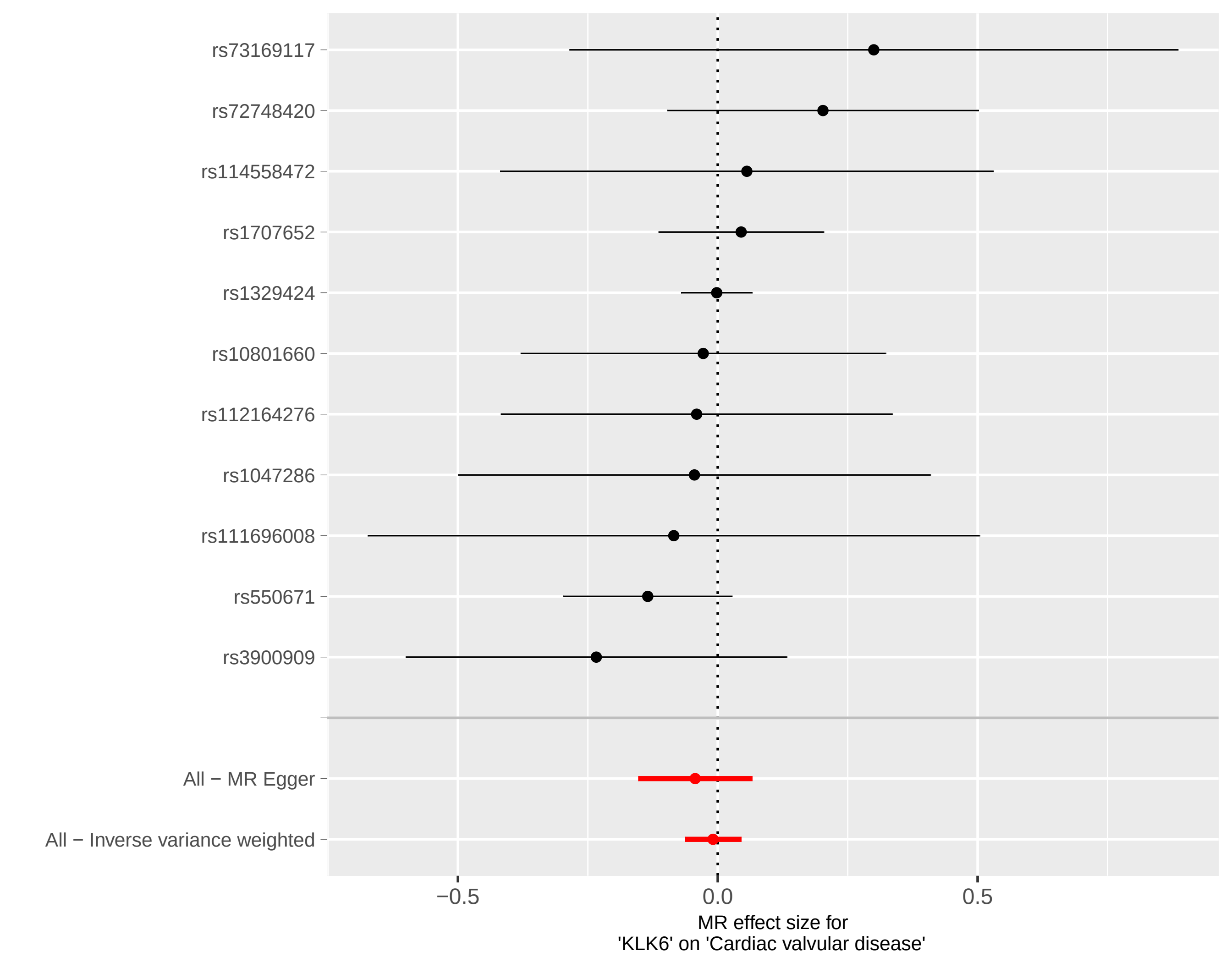
rs73169117
rs72748420
rs114558472
rs1707652
rs1329424
rs10801660
rs112164276
rs1047286
rs111696008
rs550671
rs3900909
All − MR Egger
All − Inverse variance weighted
−0.5
0.0
0.5
MR effect size for
'KLK6' on 'Cardiac valvular disease'

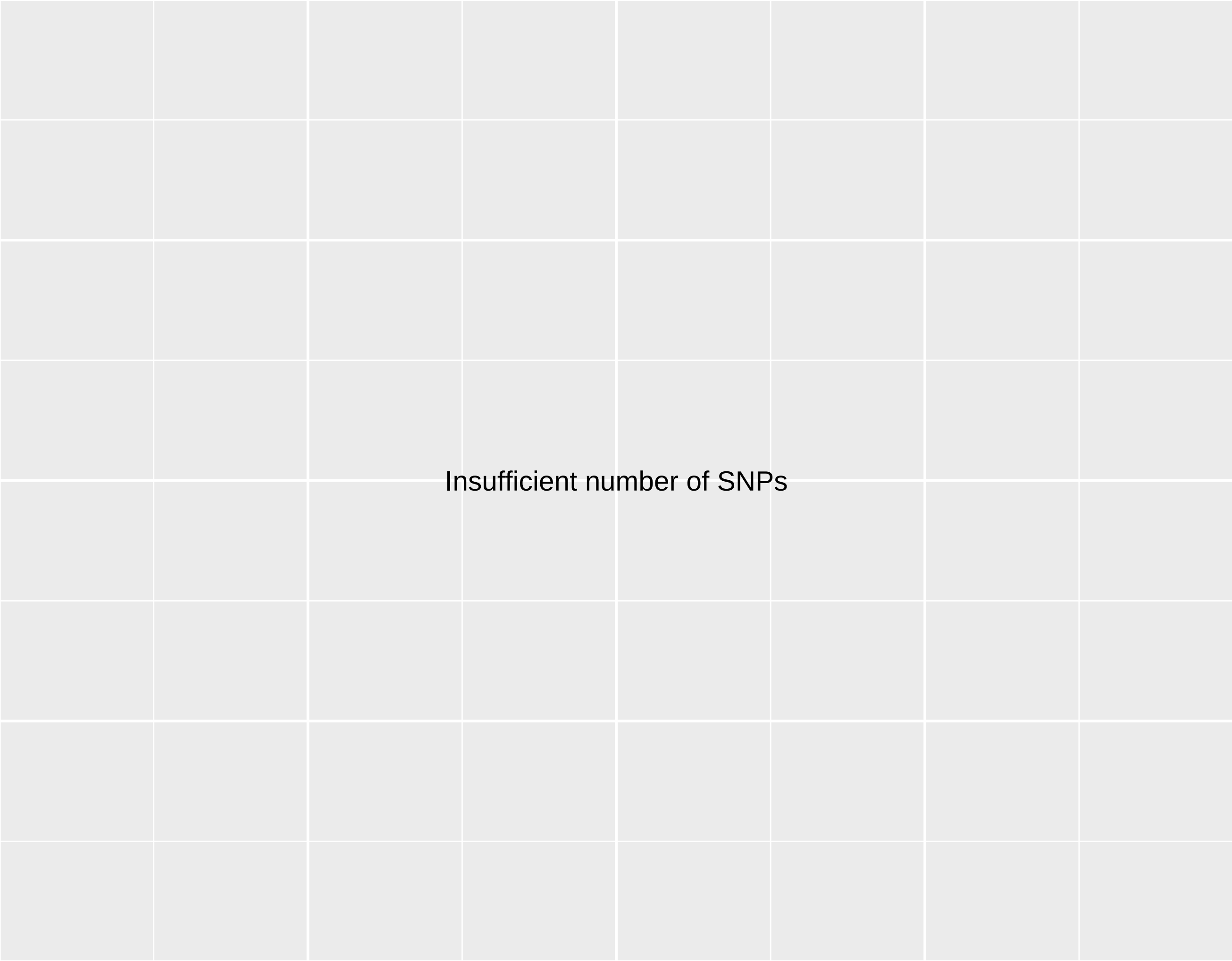
Insufficient number of SNPs

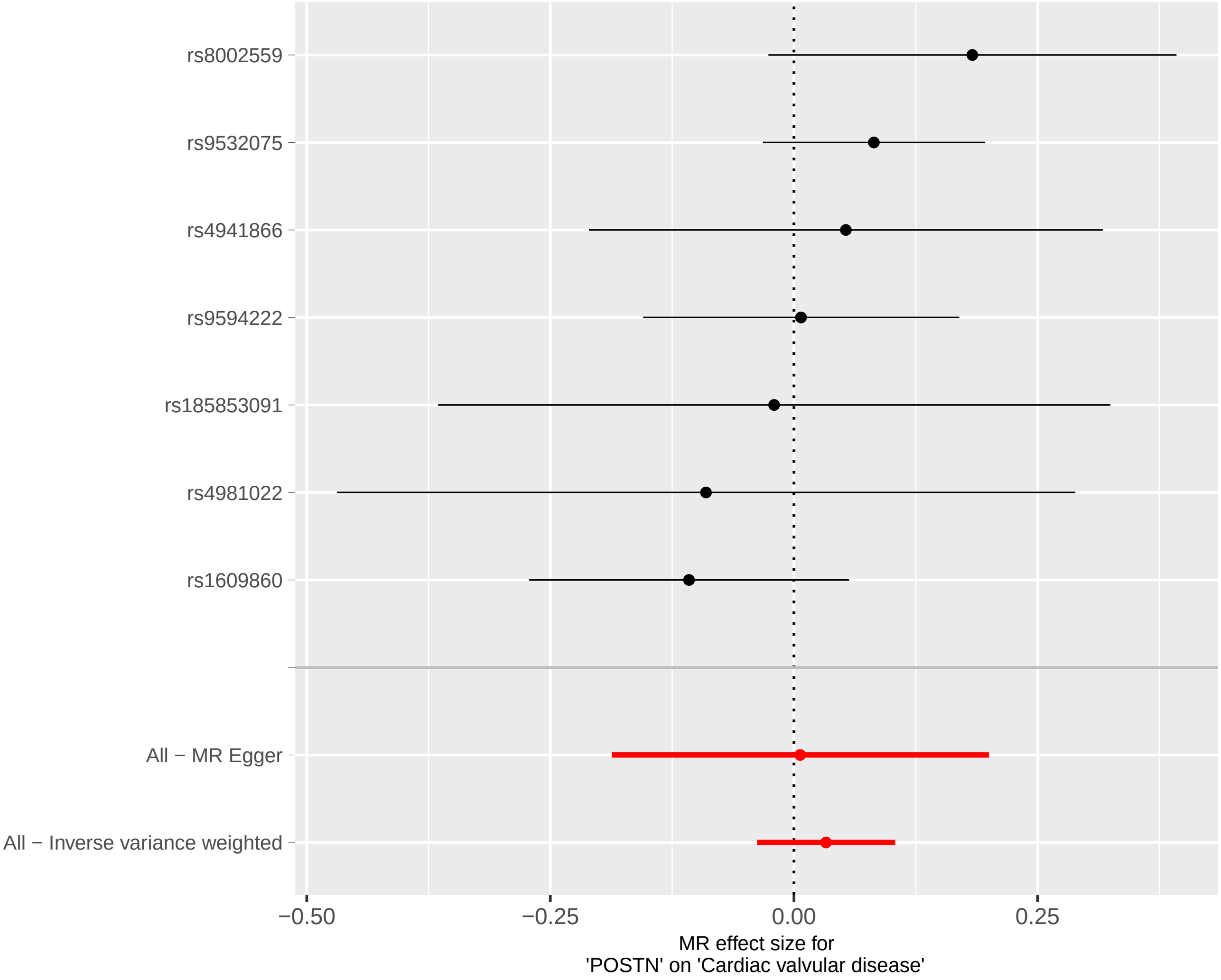
rs8002559
rs9532075
rs4941866
rs9594222
rs185853091
rs4981022
rs1609860
All − MR Egger
All − Inverse variance weighted
−0.50
−0.25
0.00
0.25
MR effect size for
'POSTN' on 'Cardiac valvular disease'

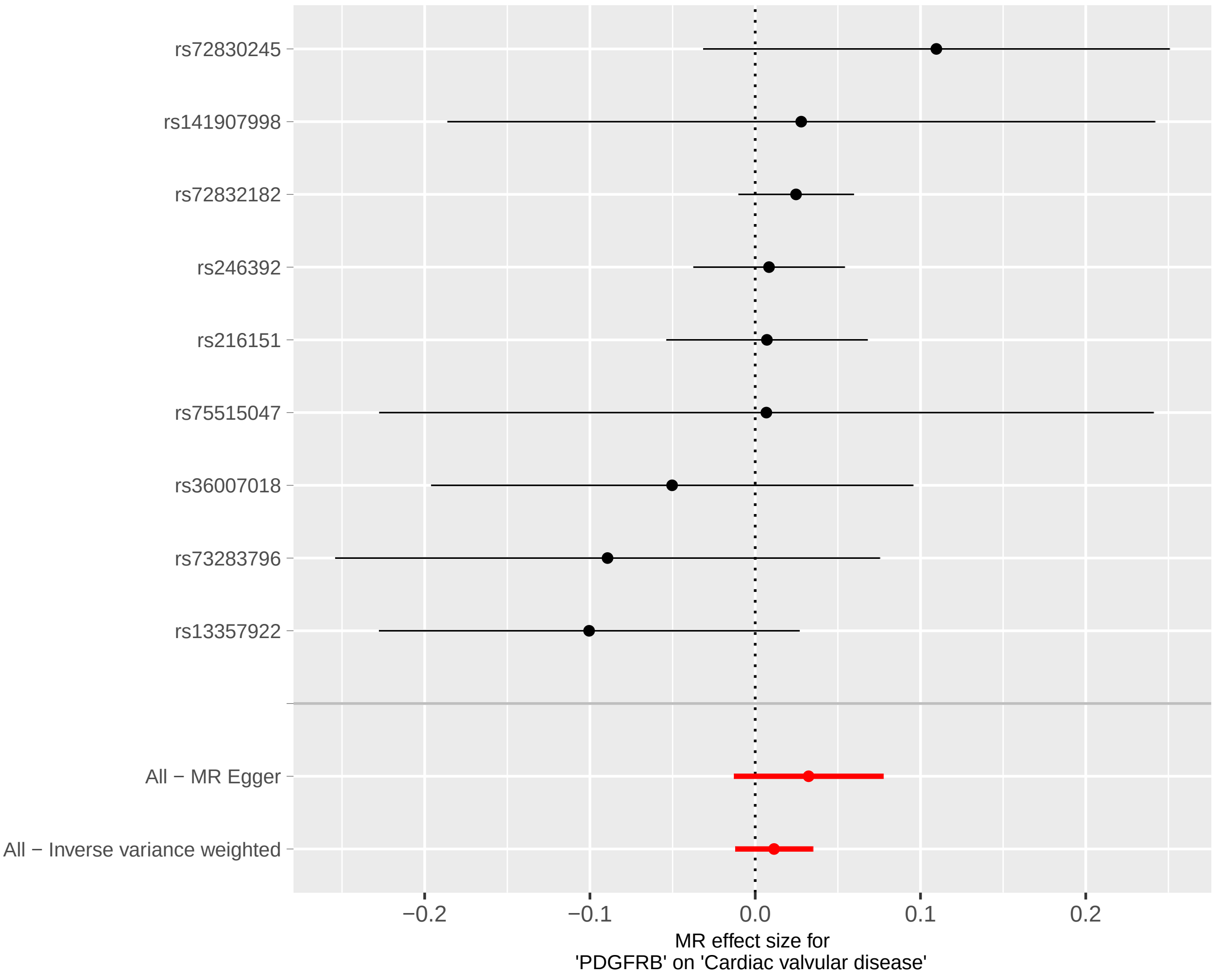

rs72830245
rs141907998
rs72832182
rs246392
rs216151
rs75515047
rs36007018
rs73283796
rs13357922
All − MR Egger
All − Inverse variance weighted
−0.2
−0.1
0.0
0.1
0.2
MR effect size for
'PDGFRB' on 'Cardiac valvular disease'

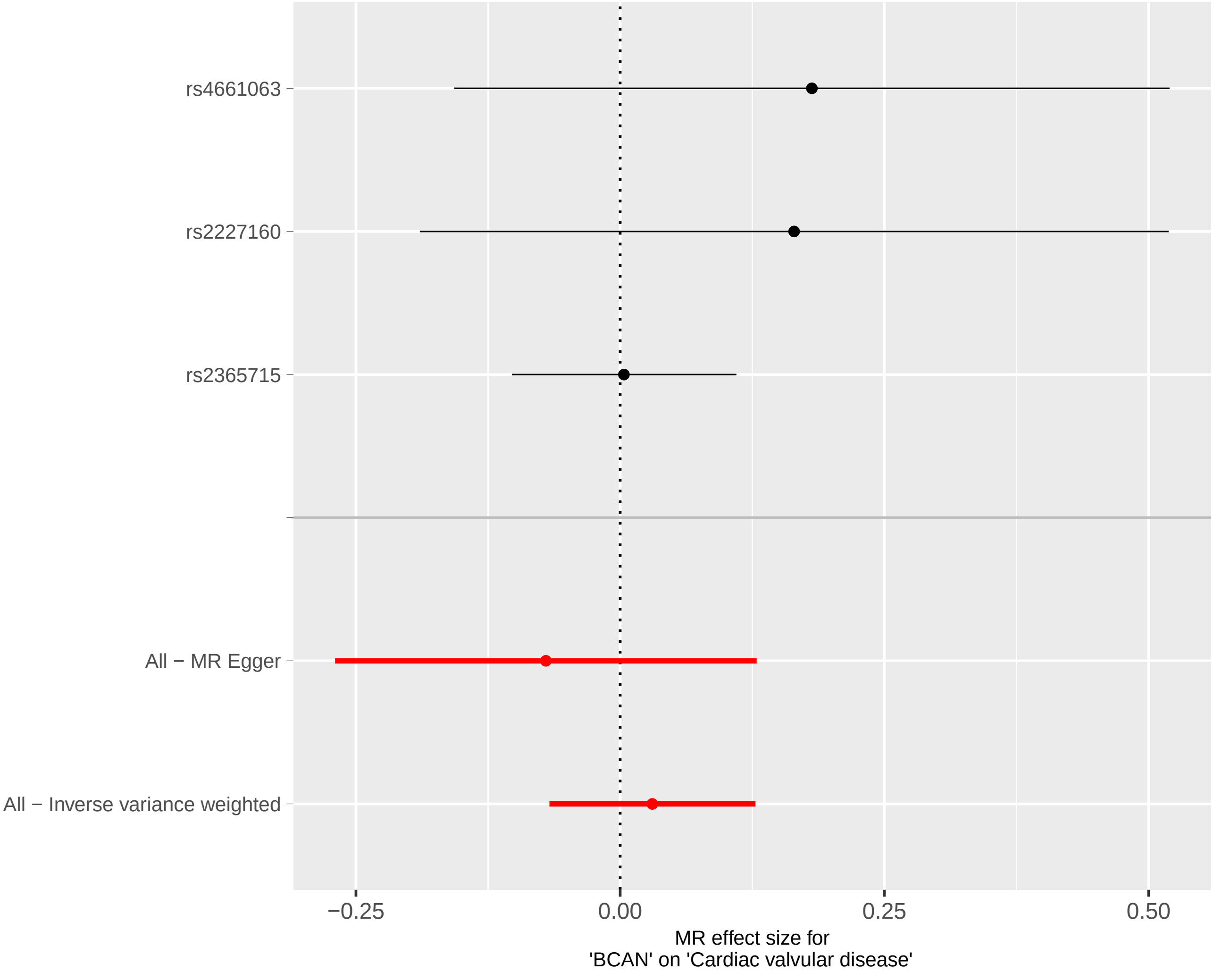

rs4661063
rs2227160
rs2365715
All − MR Egger
All − Inverse variance weighted
−0.25
0.00
0.25
0.50
MR effect size for
'BCAN' on 'Cardiac valvular disease'

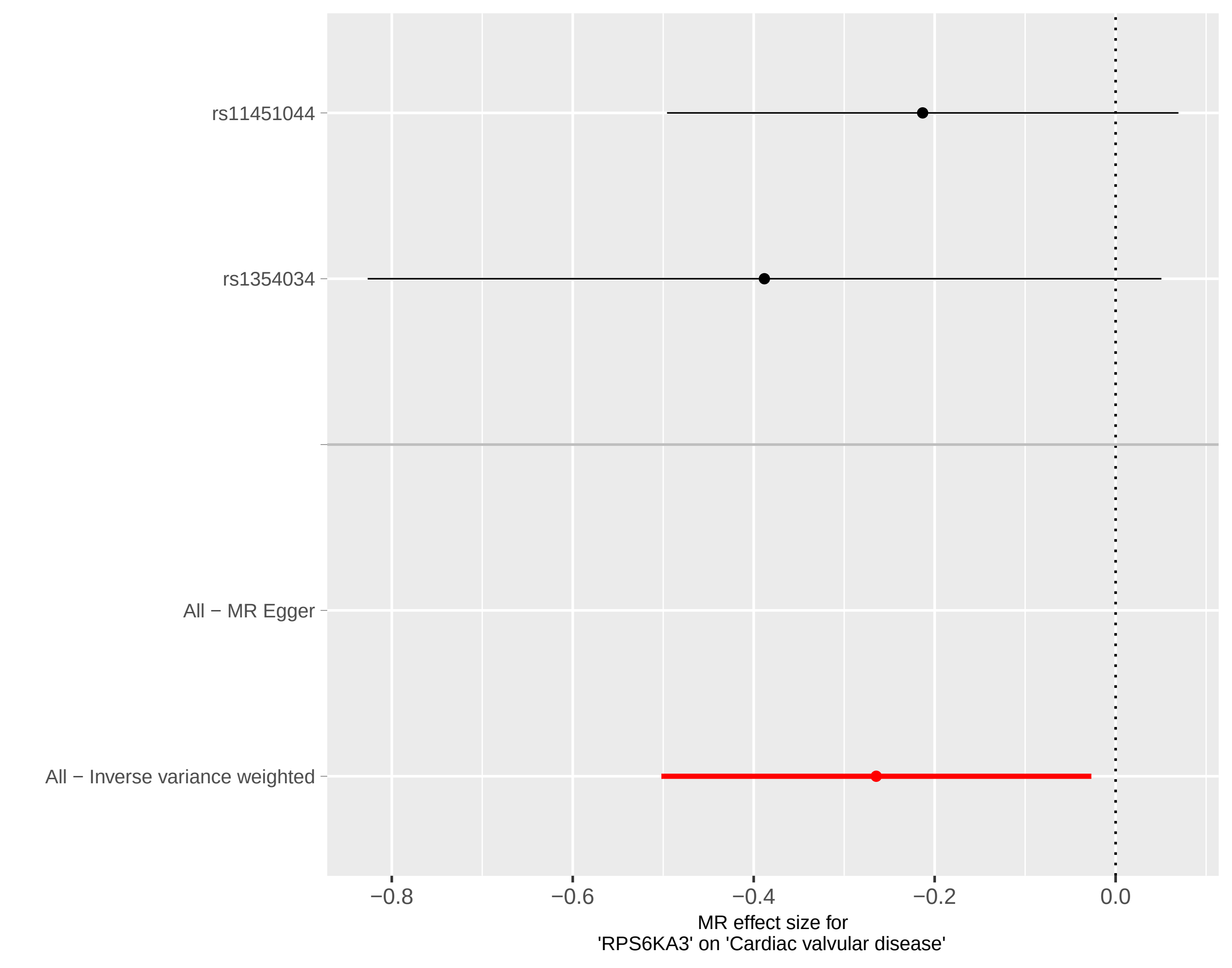
rs11451044
rs1354034
All − MR Egger
All − Inverse variance weighted
−0.8
−0.6
−0.4
−0.2
0.0
MR effect size for
'RPS6KA3' on 'Cardiac valvular disease'

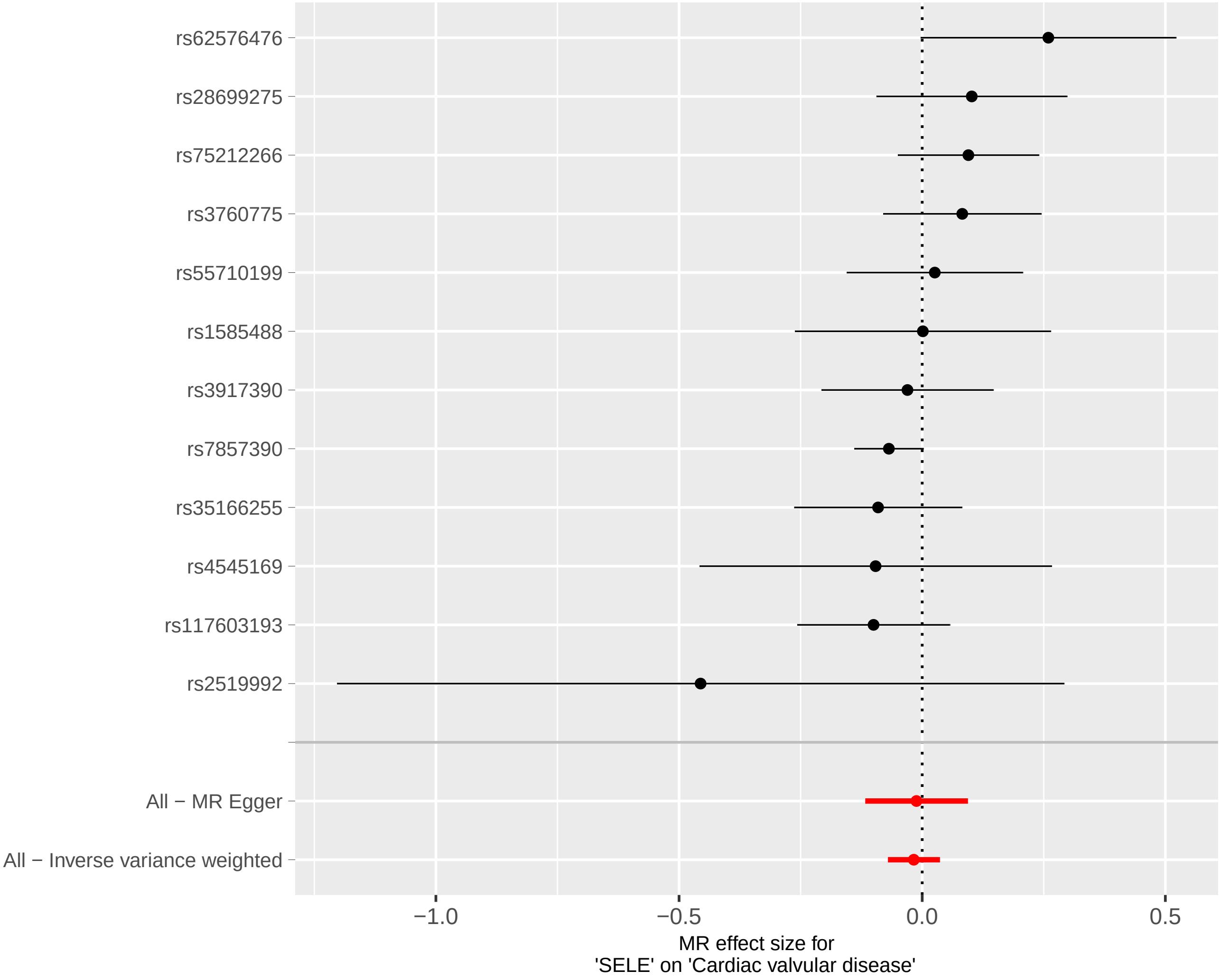
rs62576476
rs28699275
rs75212266
rs3760775
rs55710199
rs1585488
rs3917390
rs7857390
rs35166255
rs4545169
rs117603193
rs2519992
All − MR Egger
All − Inverse variance weighted
−1.0
−0.5
0.0
0.5
MR effect size for
'SELE' on 'Cardiac valvular disease'

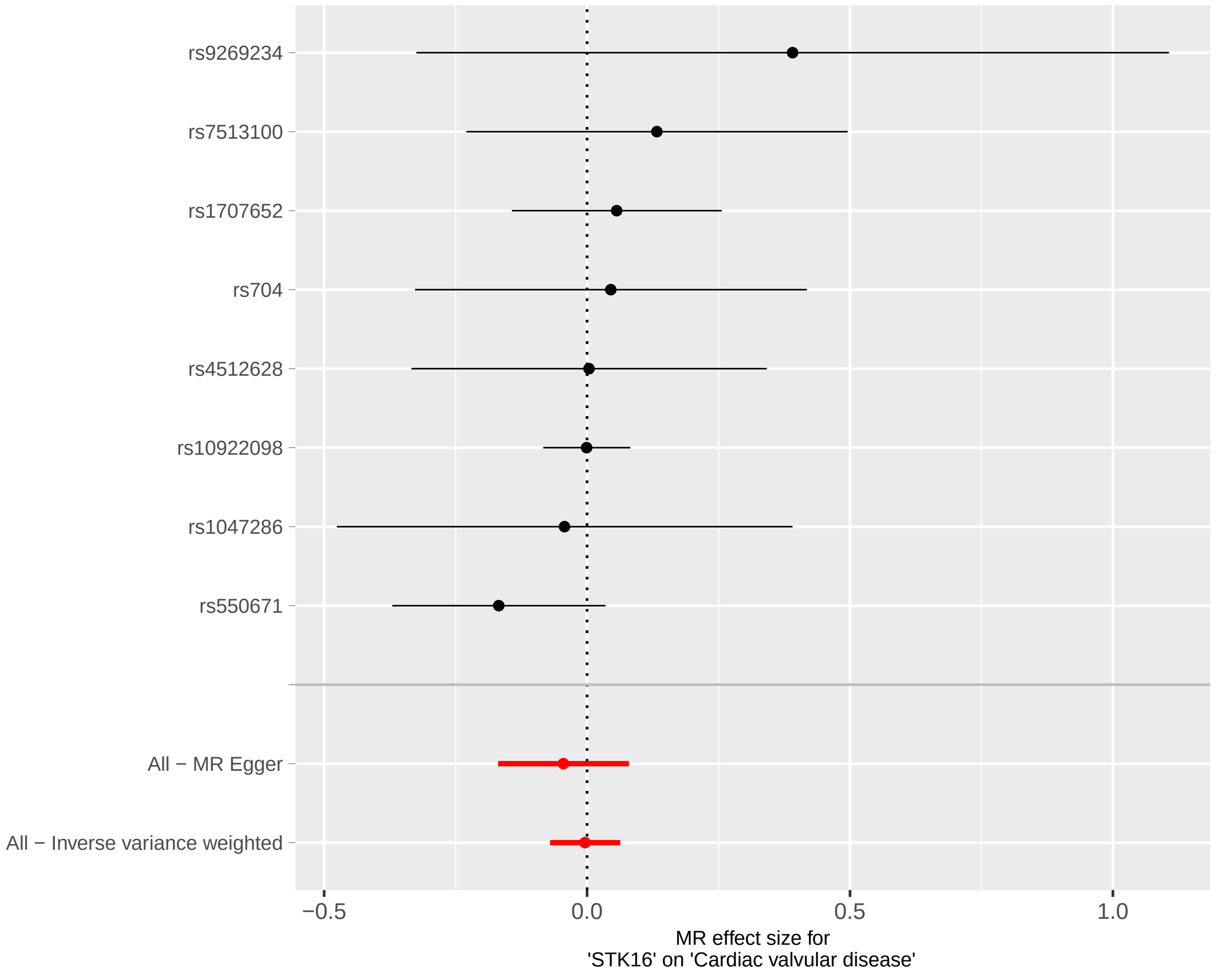
rs9269234
rs7513100
rs1707652
rs704
rs4512628
rs10922098
rs1047286
rs550671
All − MR Egger
All − Inverse variance weighted
−0.5
0.0
0.5
1.0
MR effect size for
'STK16' on 'Cardiac valvular disease'

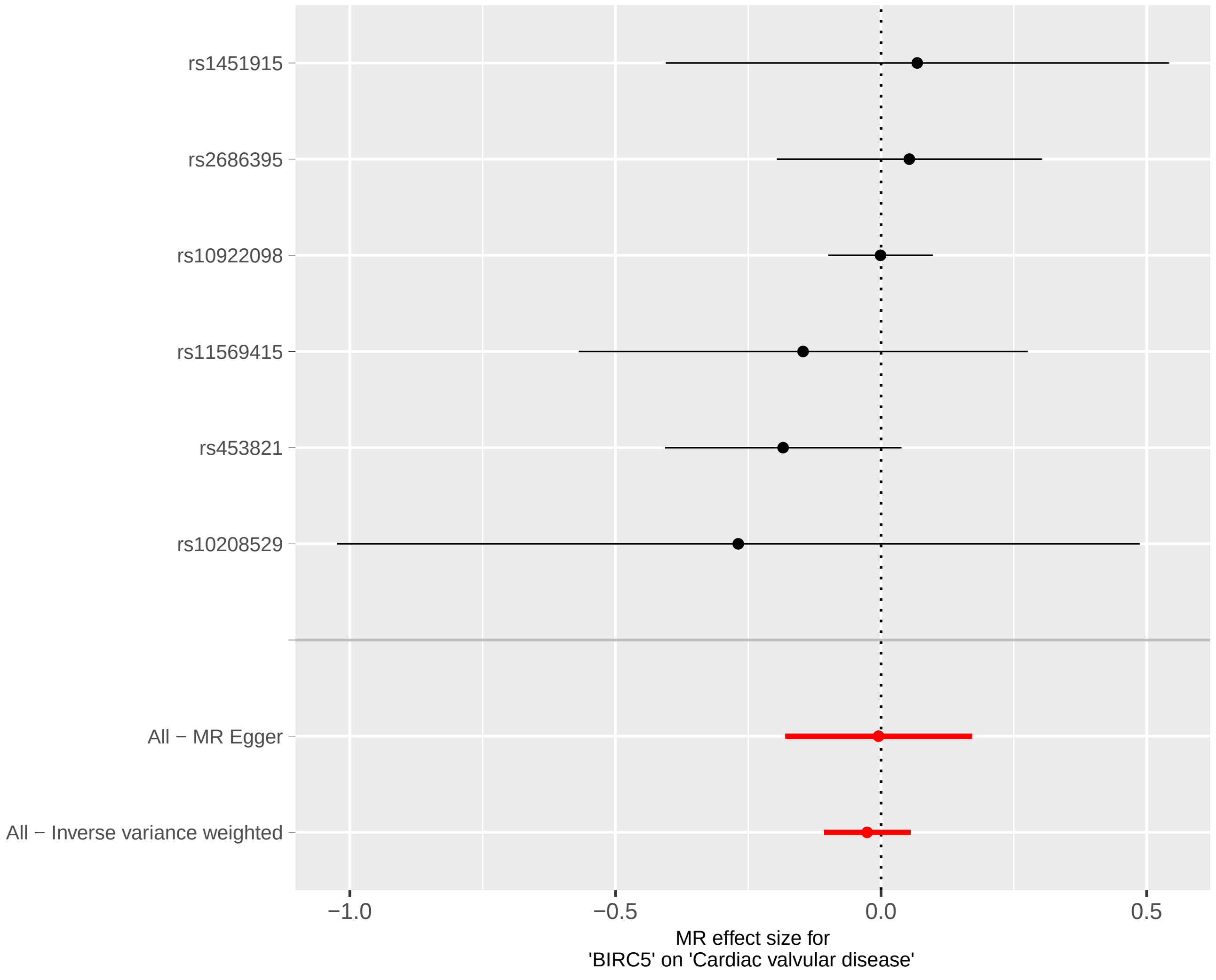
rs1451915
rs2686395
rs10922098
rs11569415
rs453821
rs10208529
All − MR Egger
All − Inverse variance weighted
−1.0
−0.5
0.0
0.5
MR effect size for
'BIRC5' on 'Cardiac valvular disease'

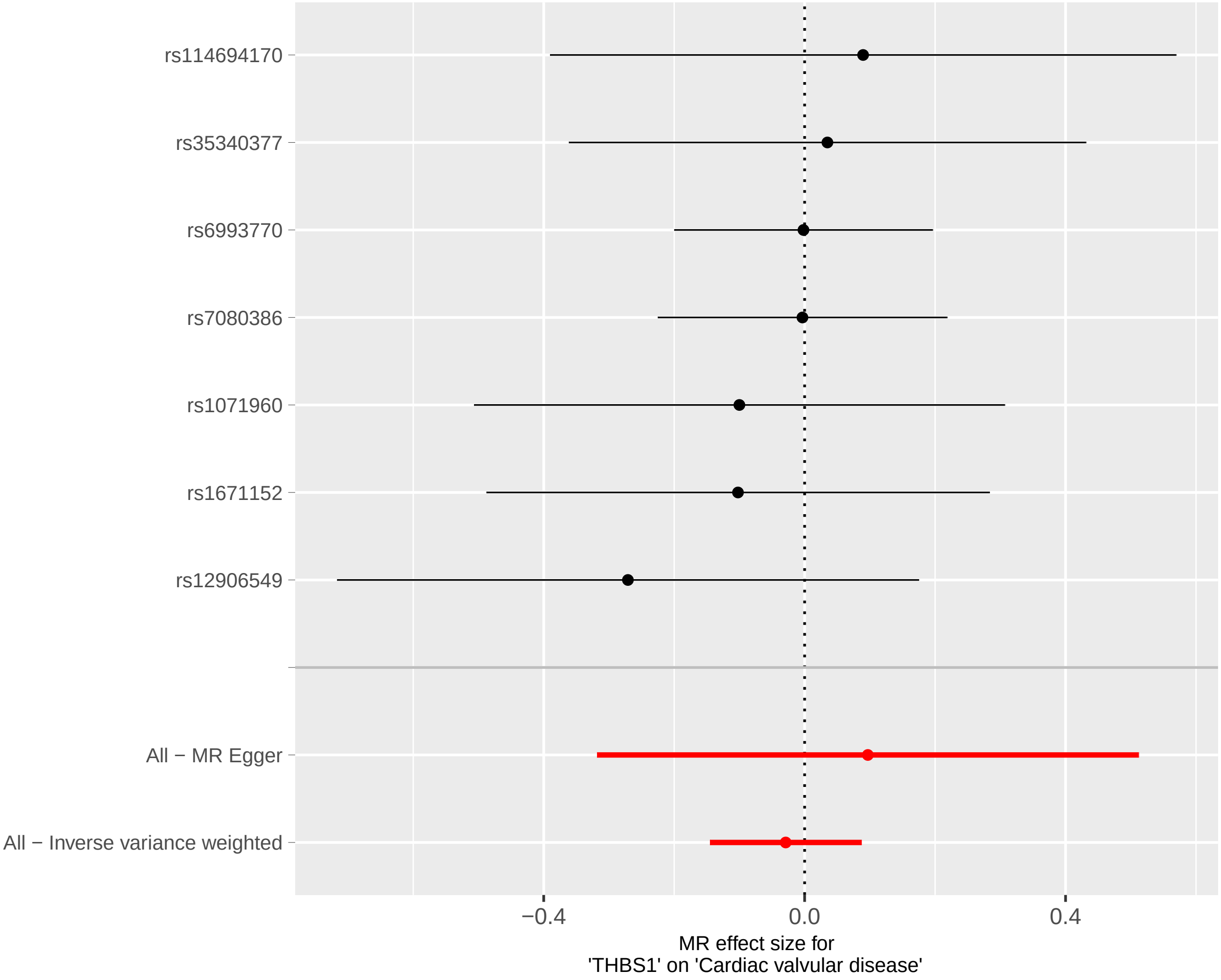
rs114694170
rs35340377
rs6993770
rs7080386
rs1071960
rs1671152
rs12906549
All − MR Egger
All − Inverse variance weighted
−0.4
0.0
0.4
MR effect size for
'THBS1' on 'Cardiac valvular disease'

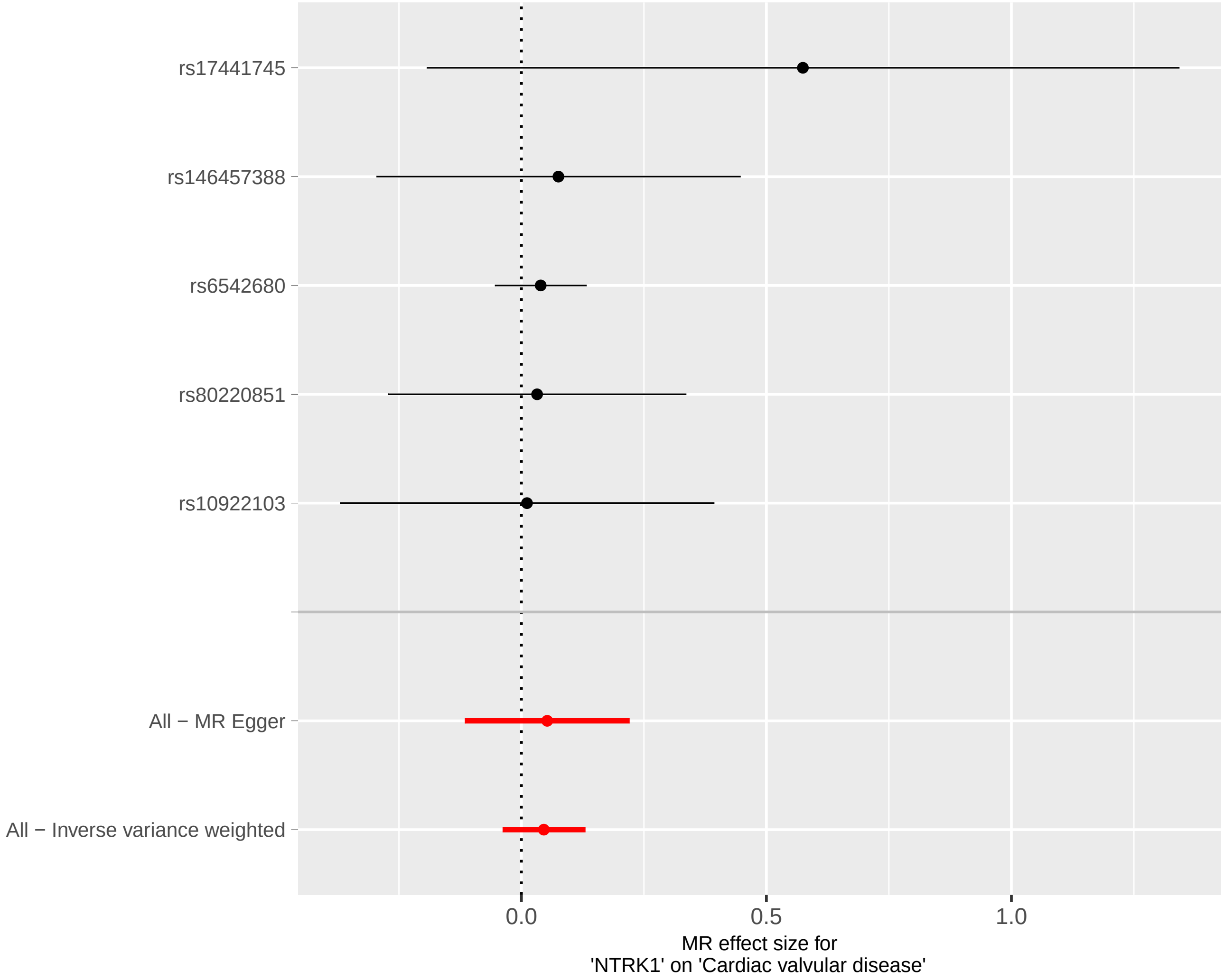

rs17441745
rs146457388
rs6542680
rs80220851
rs10922103
All − MR Egger
All − Inverse variance weighted
0.0
0.5
1.0
MR effect size for
'NTRK1' on 'Cardiac valvular disease'

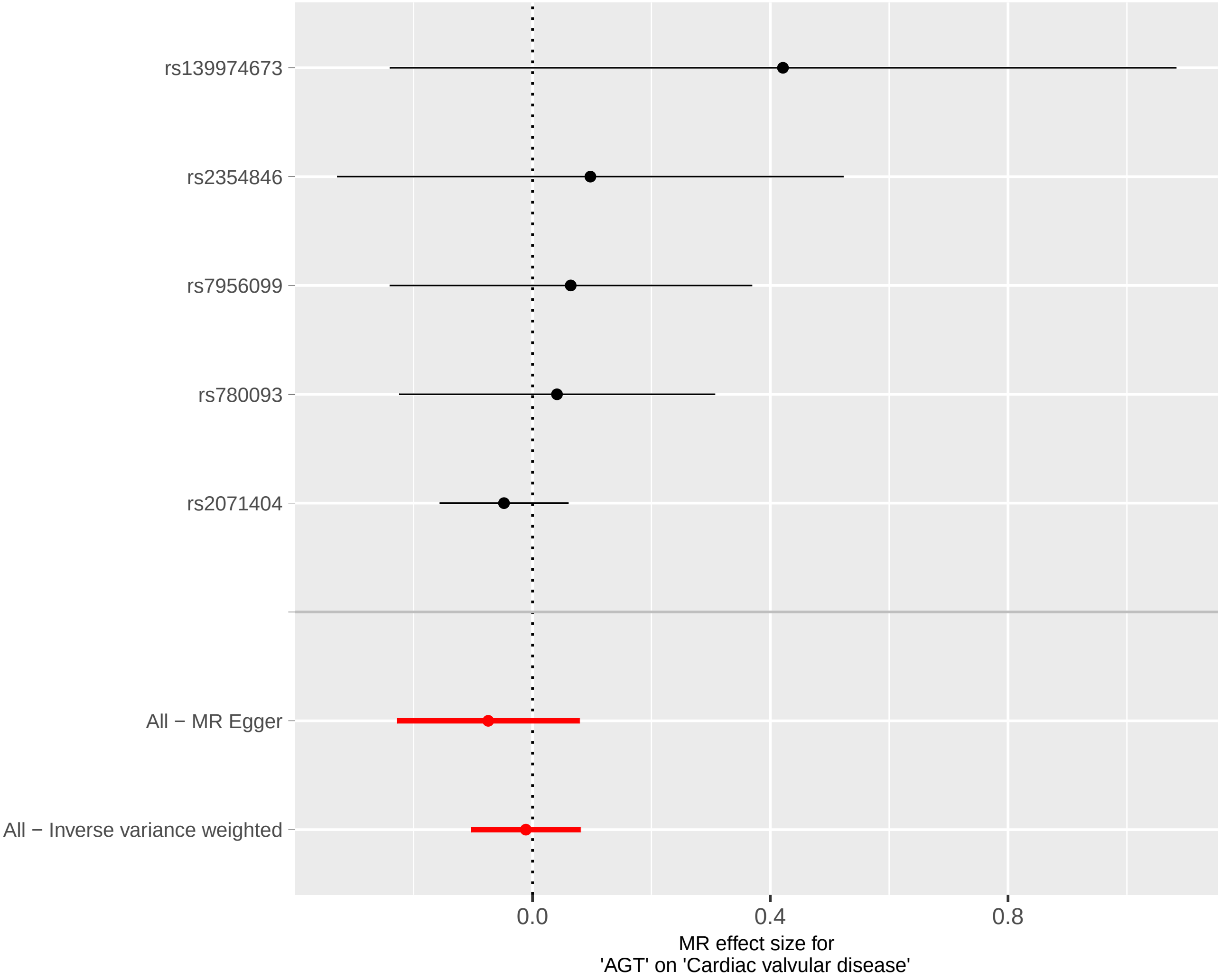
rs139974673
rs2354846
rs7956099
rs780093
rs2071404
All − MR Egger
All − Inverse variance weighted
0.0
0.4
0.8
MR effect size for
'AGT' on 'Cardiac valvular disease'

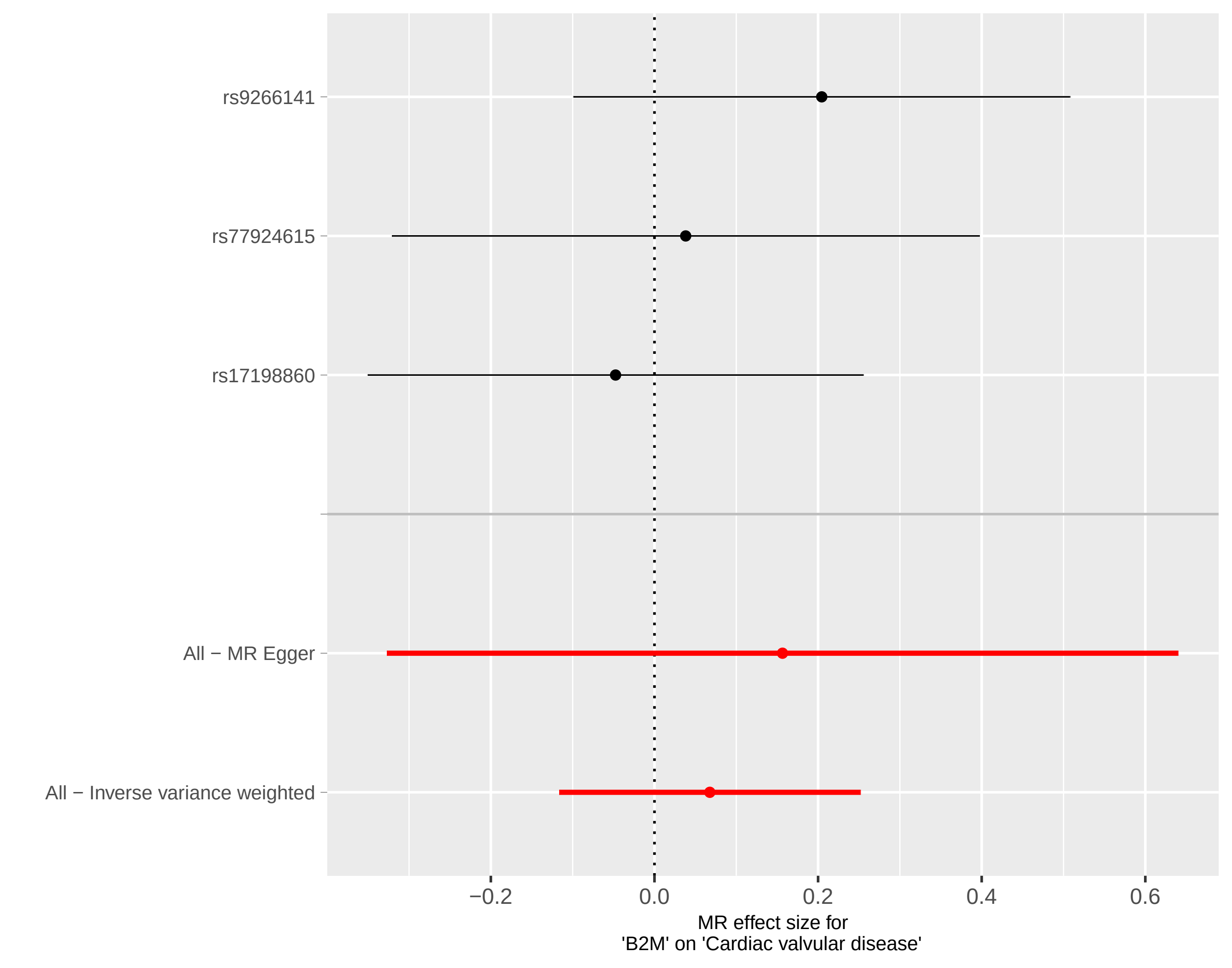
rs9266141
rs77924615
rs17198860
All − MR Egger
All − Inverse variance weighted
−0.2
0.0
0.2
0.4
0.6
MR effect size for
'B2M' on 'Cardiac valvular disease'

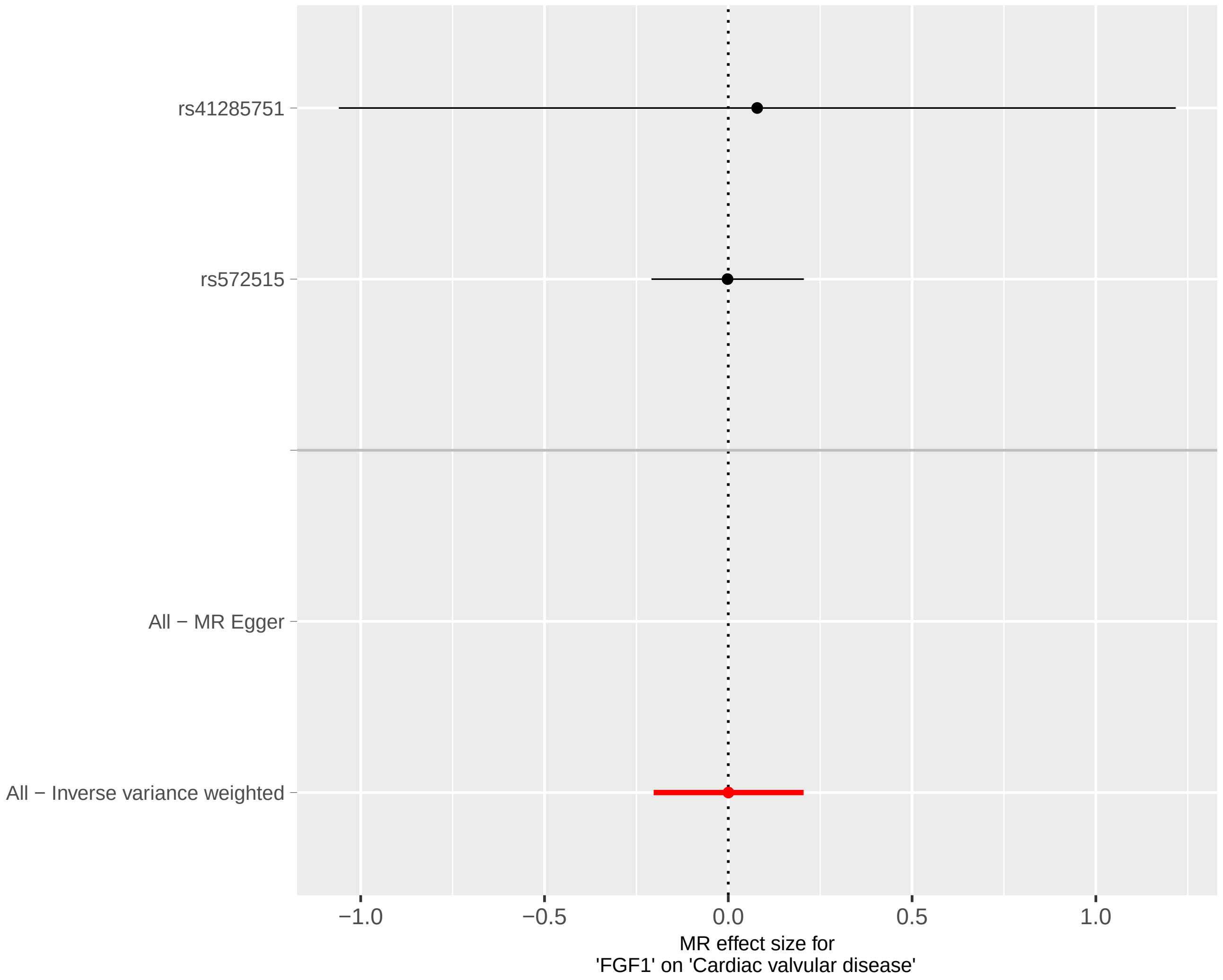
rs41285751
rs572515
All − MR Egger
All − Inverse variance weighted
−1.0
−0.5
0.0
0.5
1.0
MR effect size for
'FGF1' on 'Cardiac valvular disease'

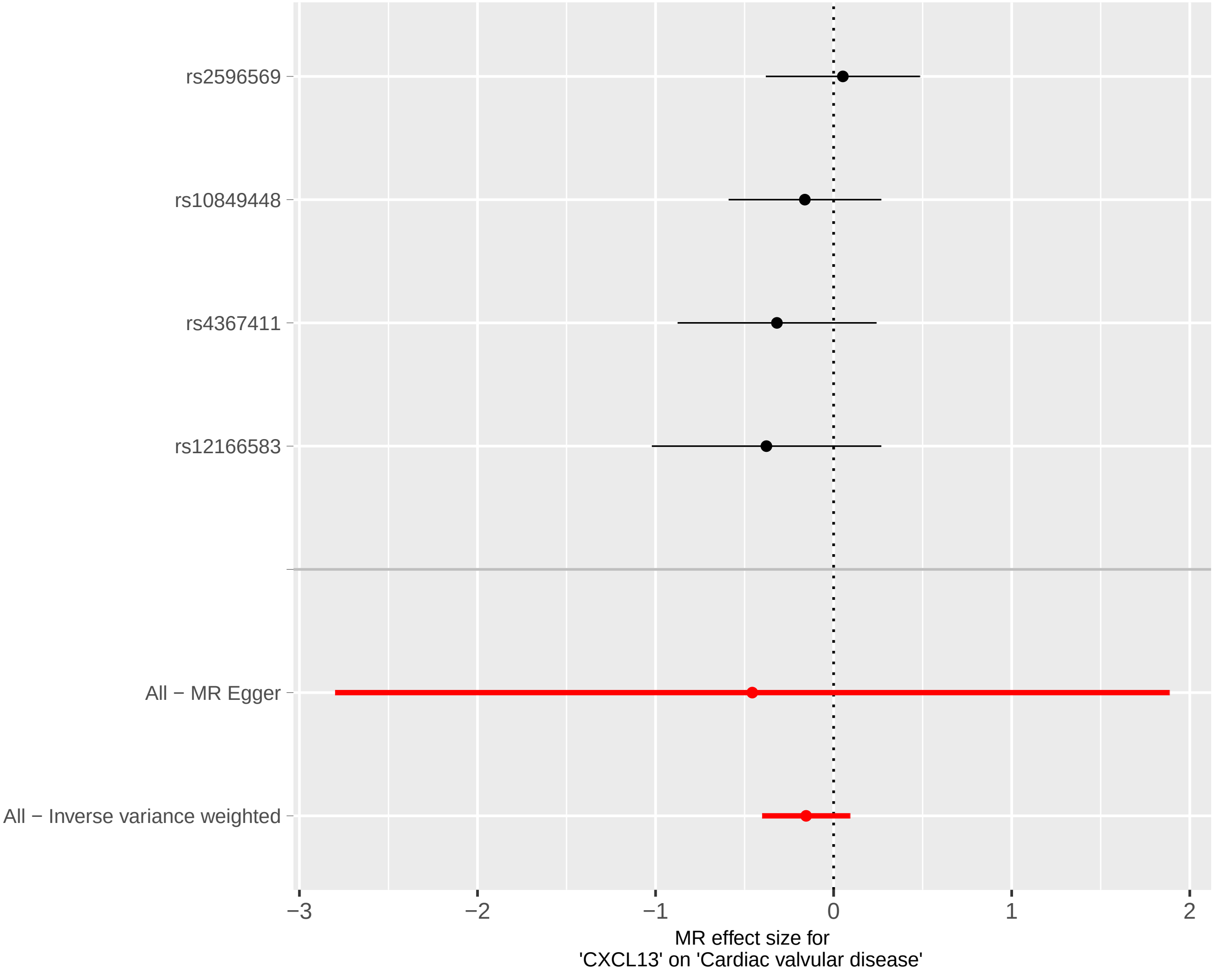

rs2596569
rs10849448
rs4367411
rs12166583
All − MR Egger
All − Inverse variance weighted
−3
−2
−1
0
1
2
MR effect size for
'CXCL13' on 'Cardiac valvular disease'

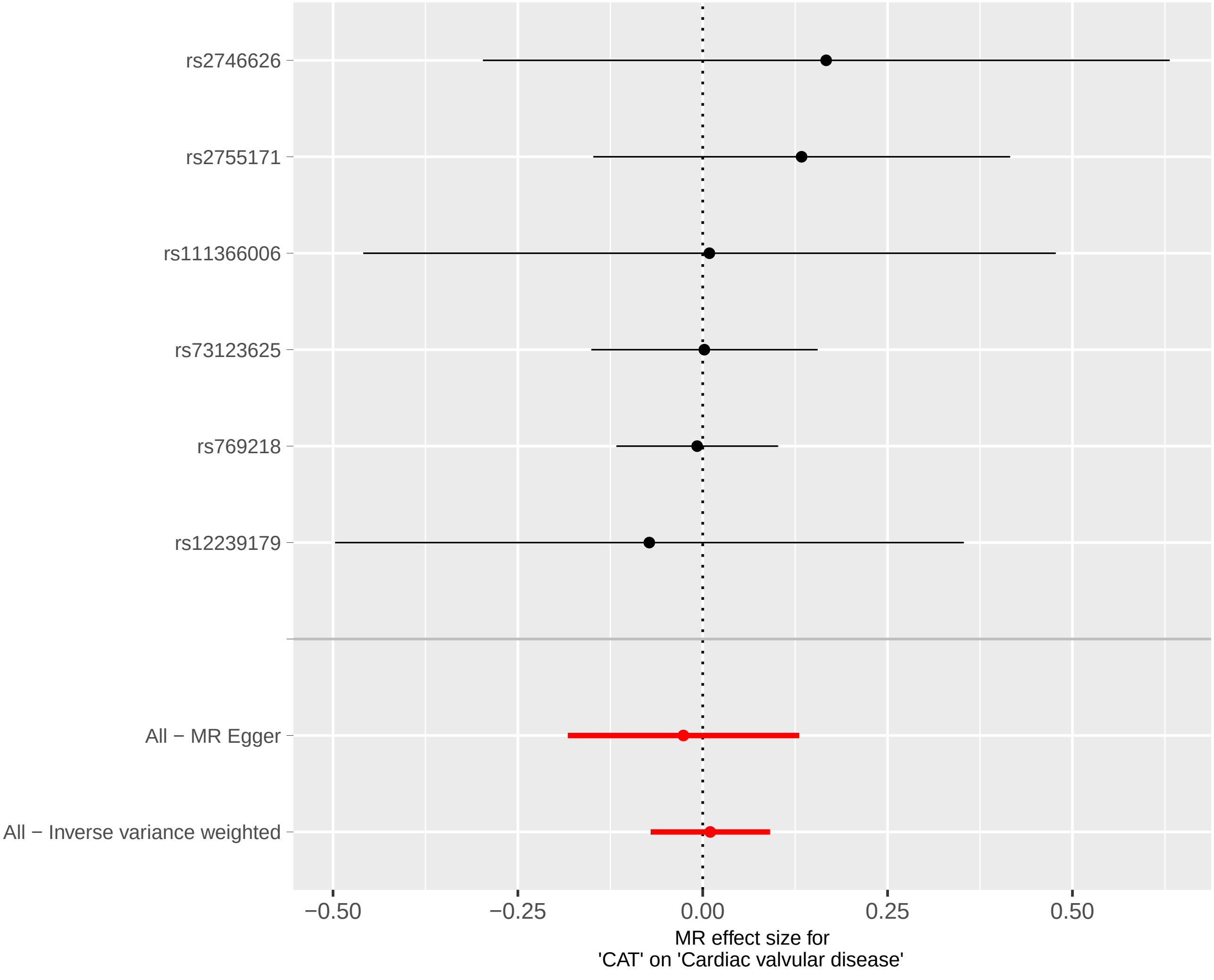
rs2746626
rs2755171
rs111366006
rs73123625
rs769218
rs12239179
All − MR Egger
All − Inverse variance weighted
−0.50
−0.25
0.00
0.25
0.50
MR effect size for
'CAT' on 'Cardiac valvular disease'

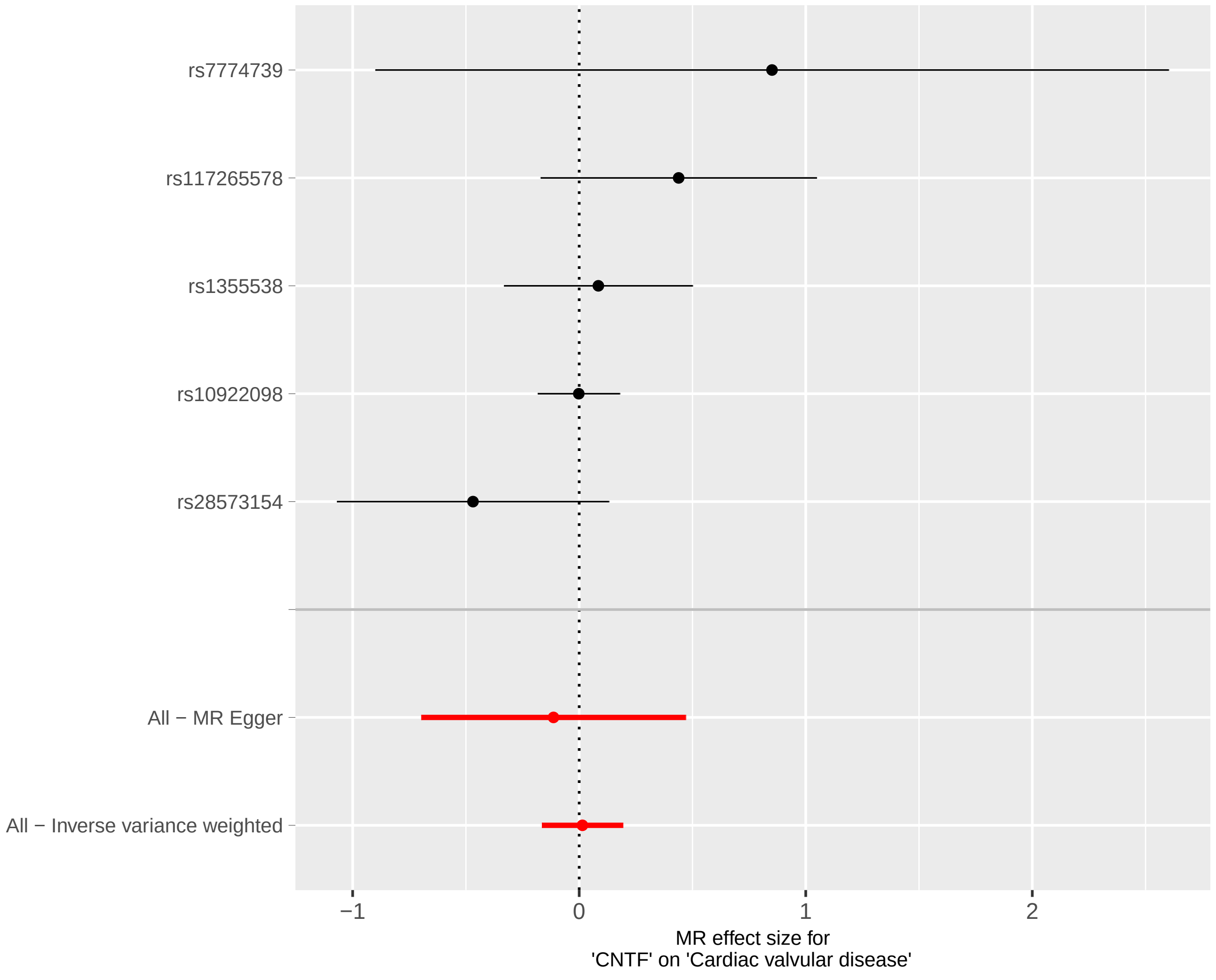
rs7774739
rs117265578
rs1355538
rs10922098
rs28573154
All − MR Egger
All − Inverse variance weighted
−1
0
1
2
MR effect size for
'CNTF' on 'Cardiac valvular disease'

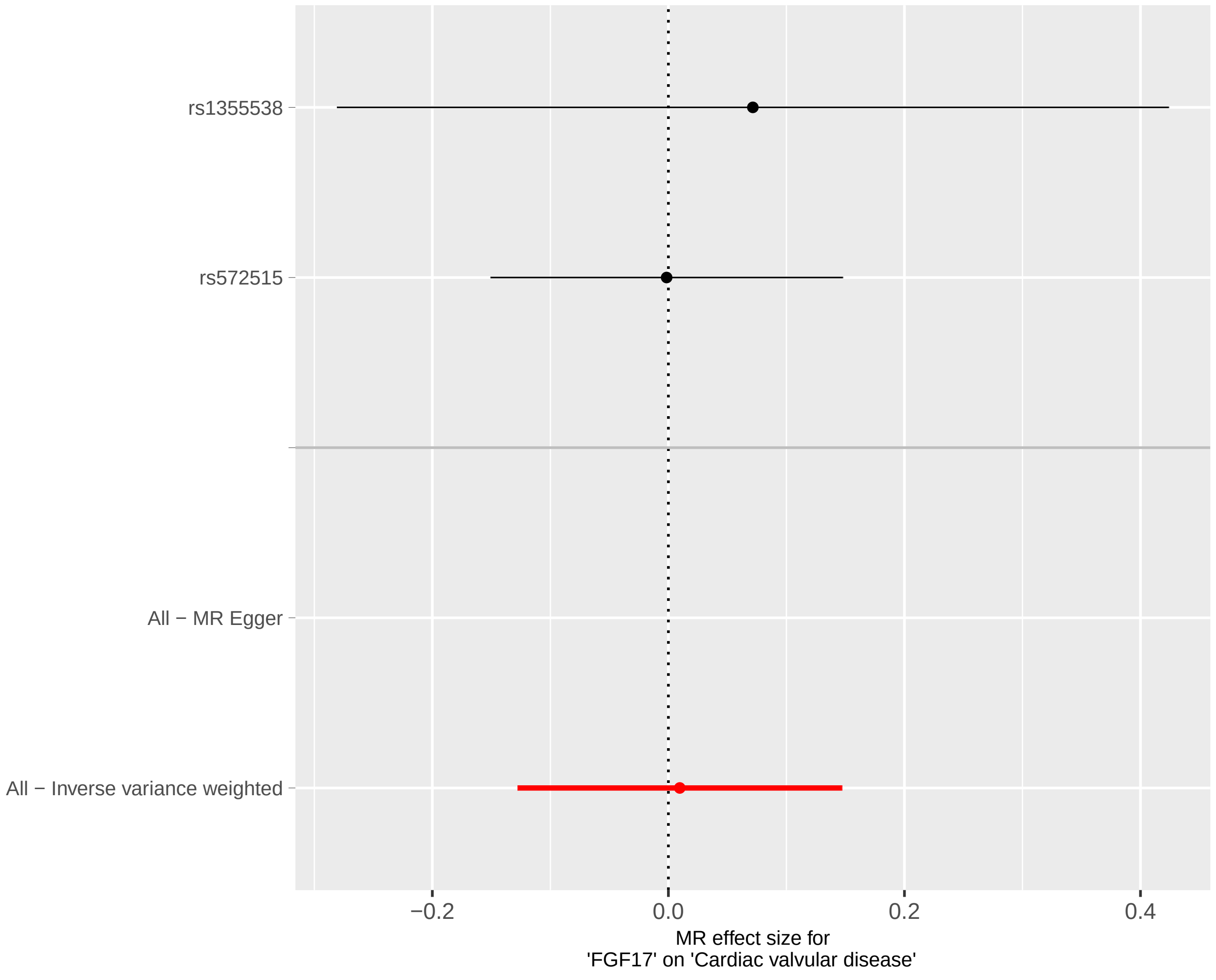
rs1355538
rs572515
All − MR Egger
All − Inverse variance weighted
−0.2
0.0
0.2
0.4
MR effect size for
'FGF17' on 'Cardiac valvular disease'

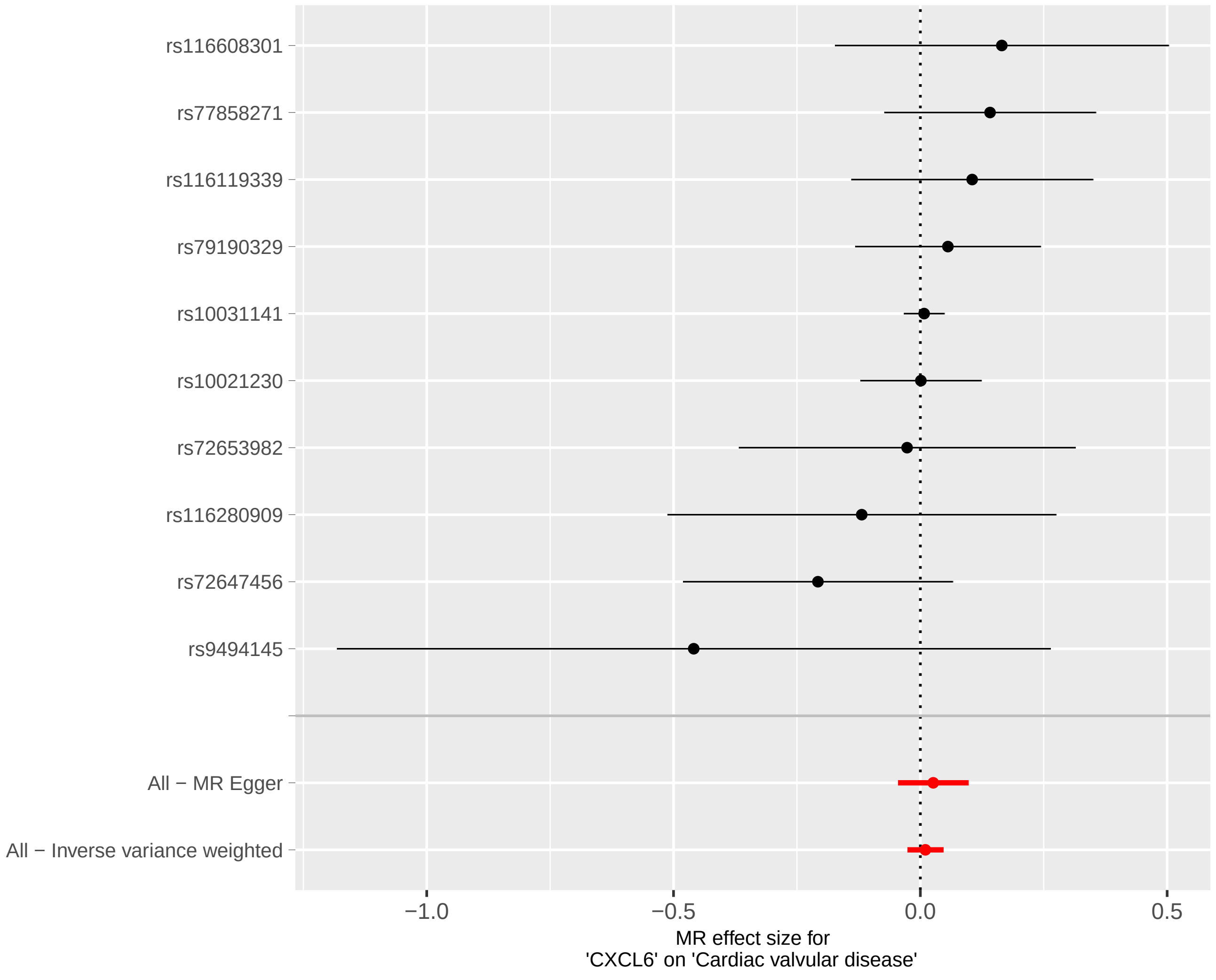

rs116608301
rs77858271
rs116119339
rs79190329
rs10031141
rs10021230
rs72653982
rs116280909
rs72647456
rs9494145
All − MR Egger
All − Inverse variance weighted
−1.0
−0.5
0.0
0.5
MR effect size for
'CXCL6' on 'Cardiac valvular disease'

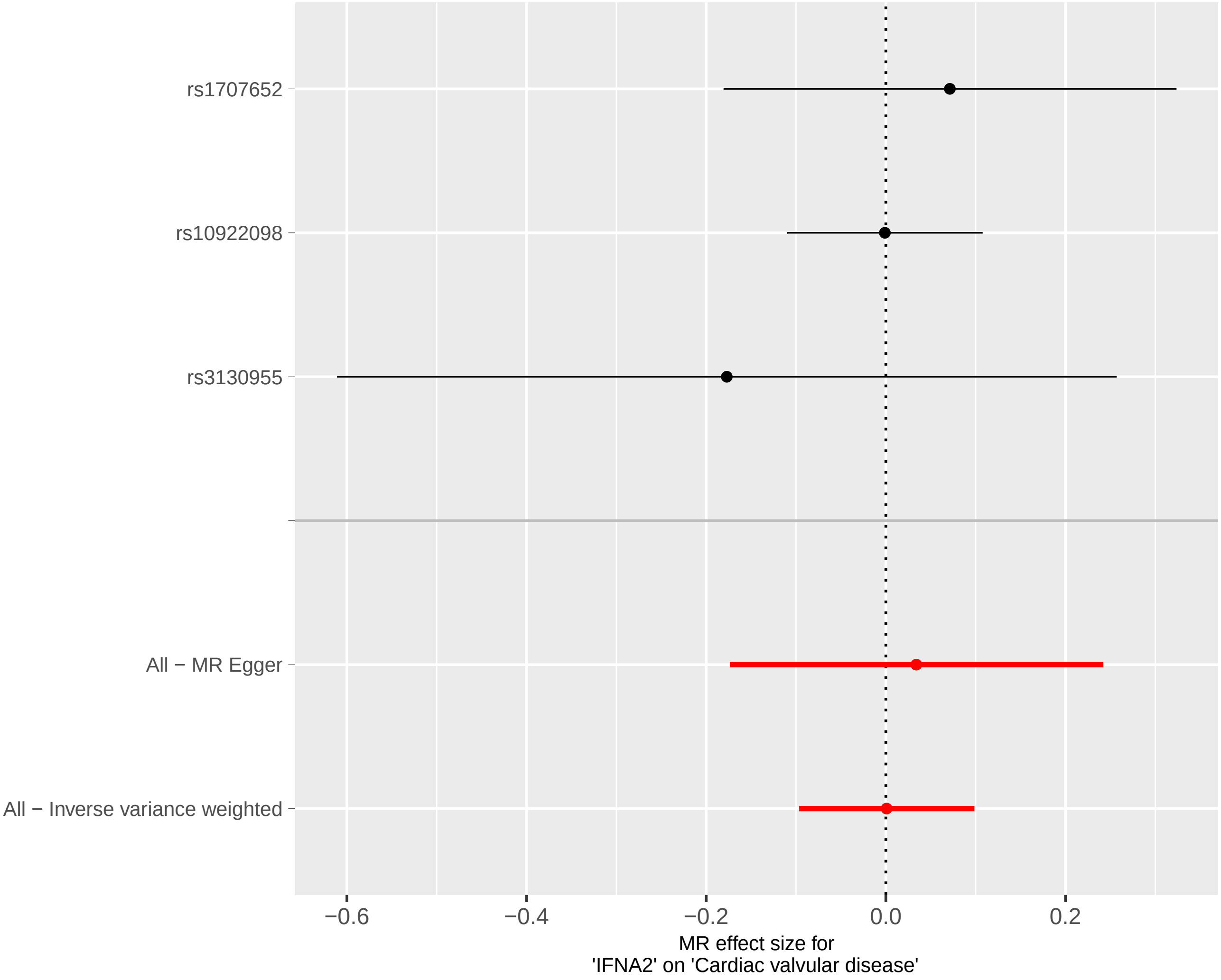
rs1707652
rs10922098
rs3130955
All − MR Egger
All − Inverse variance weighted
−0.6
−0.4
−0.2
0.0
0.2
MR effect size for
'IFNA2' on 'Cardiac valvular disease'

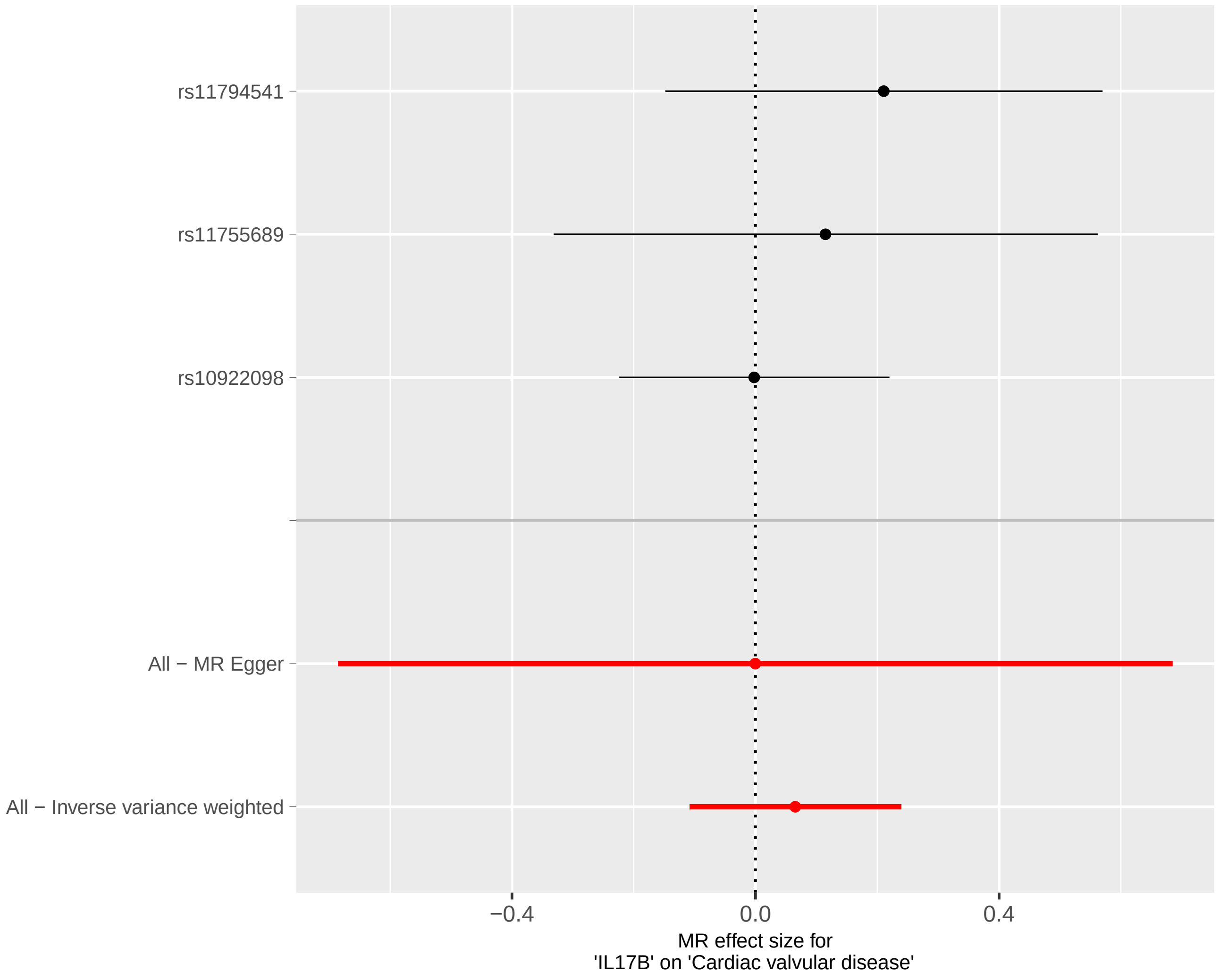
rs11794541
rs11755689
rs10922098
All − MR Egger
All − Inverse variance weighted
−0.4
0.0
0.4
MR effect size for
'IL17B' on 'Cardiac valvular disease'

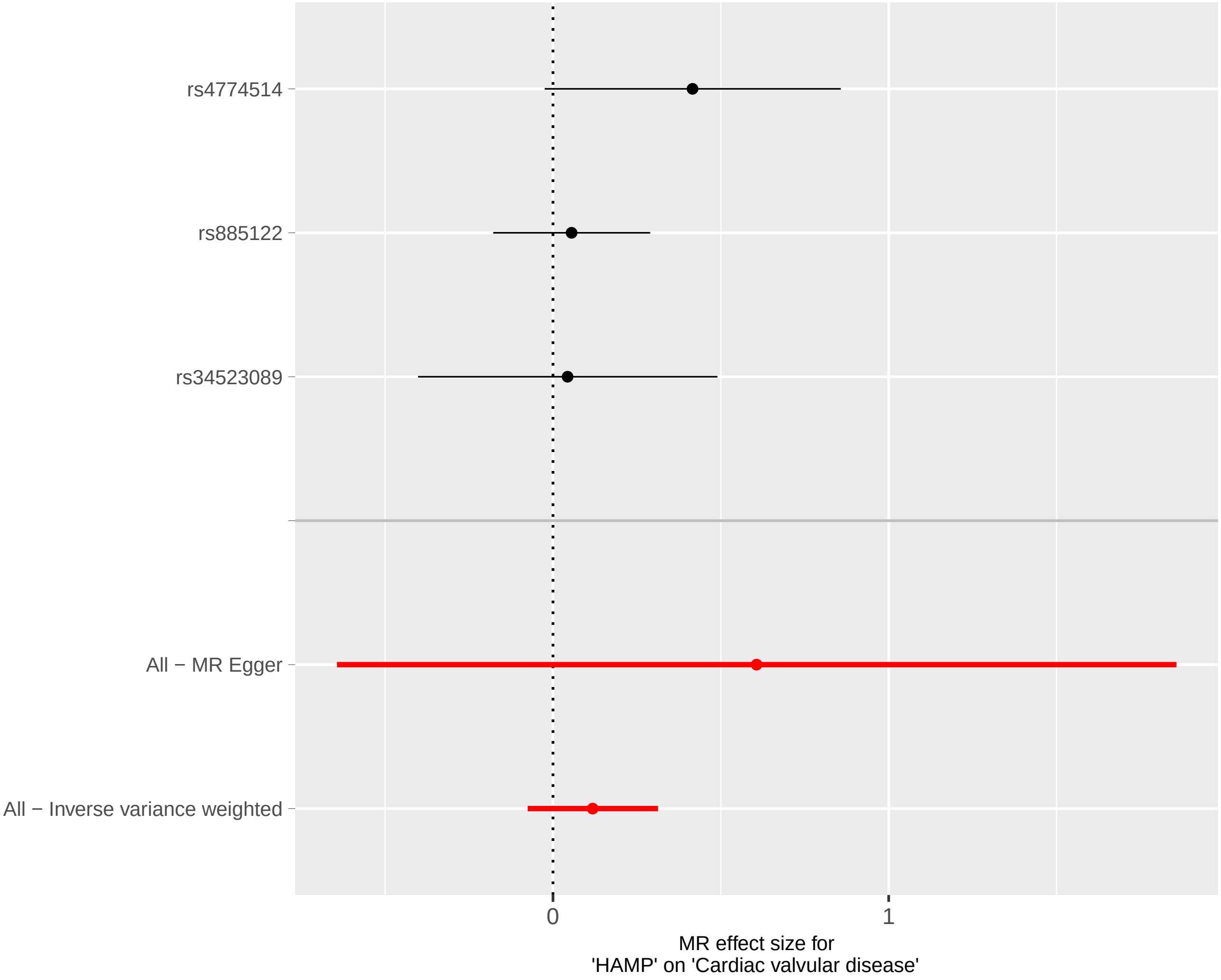
rs4774514
rs885122
rs34523089
All − MR Egger
All − Inverse variance weighted
0
1
MR effect size for
'HAMP' on 'Cardiac valvular disease'

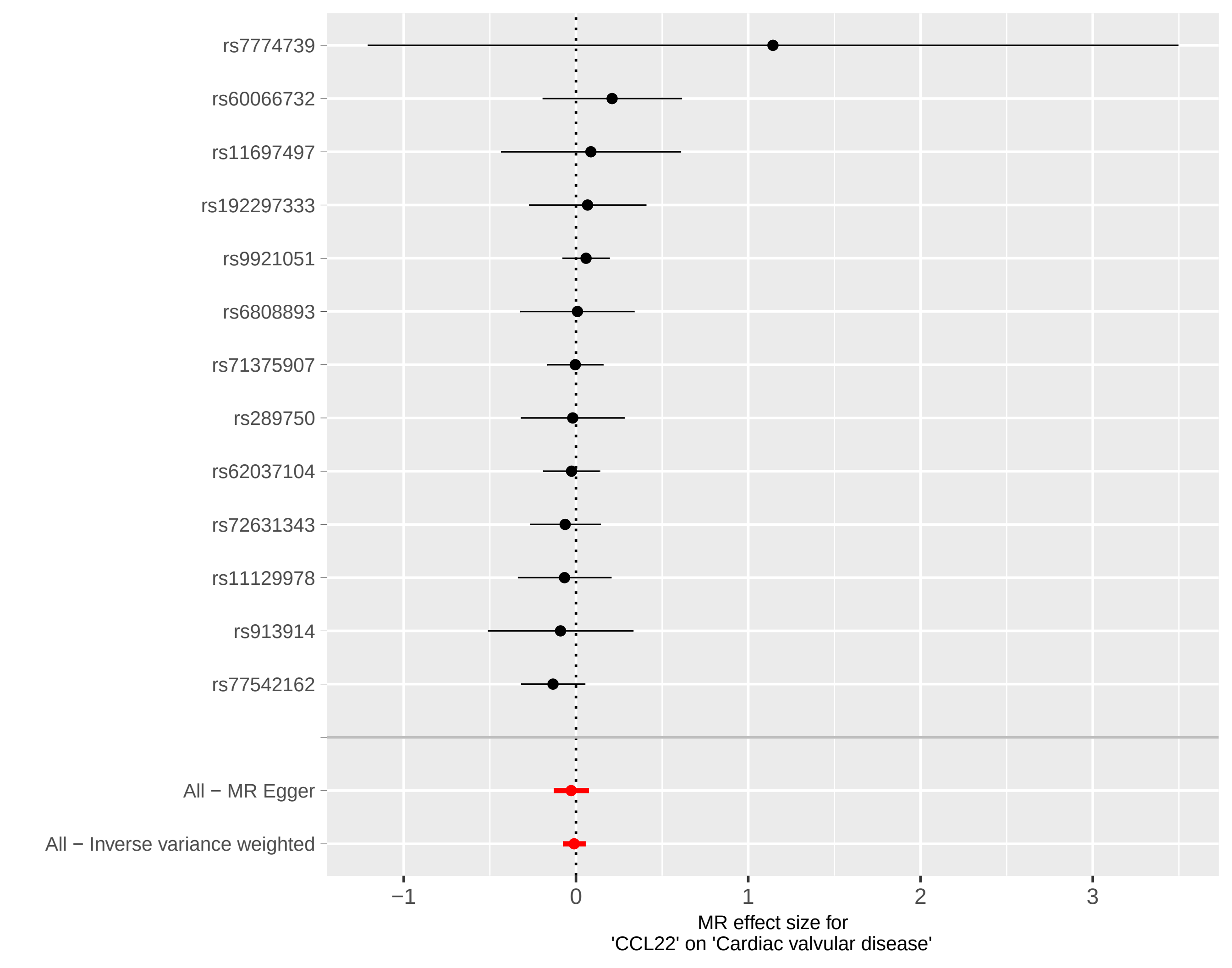
rs7774739
rs60066732
rs11697497
rs192297333
rs9921051
rs6808893
rs71375907
rs289750
rs62037104
rs72631343
rs11129978
rs913914
rs77542162
All − MR Egger
All − Inverse variance weighted
−1
0
1
2
3
MR effect size for
'CCL22' on 'Cardiac valvular disease'

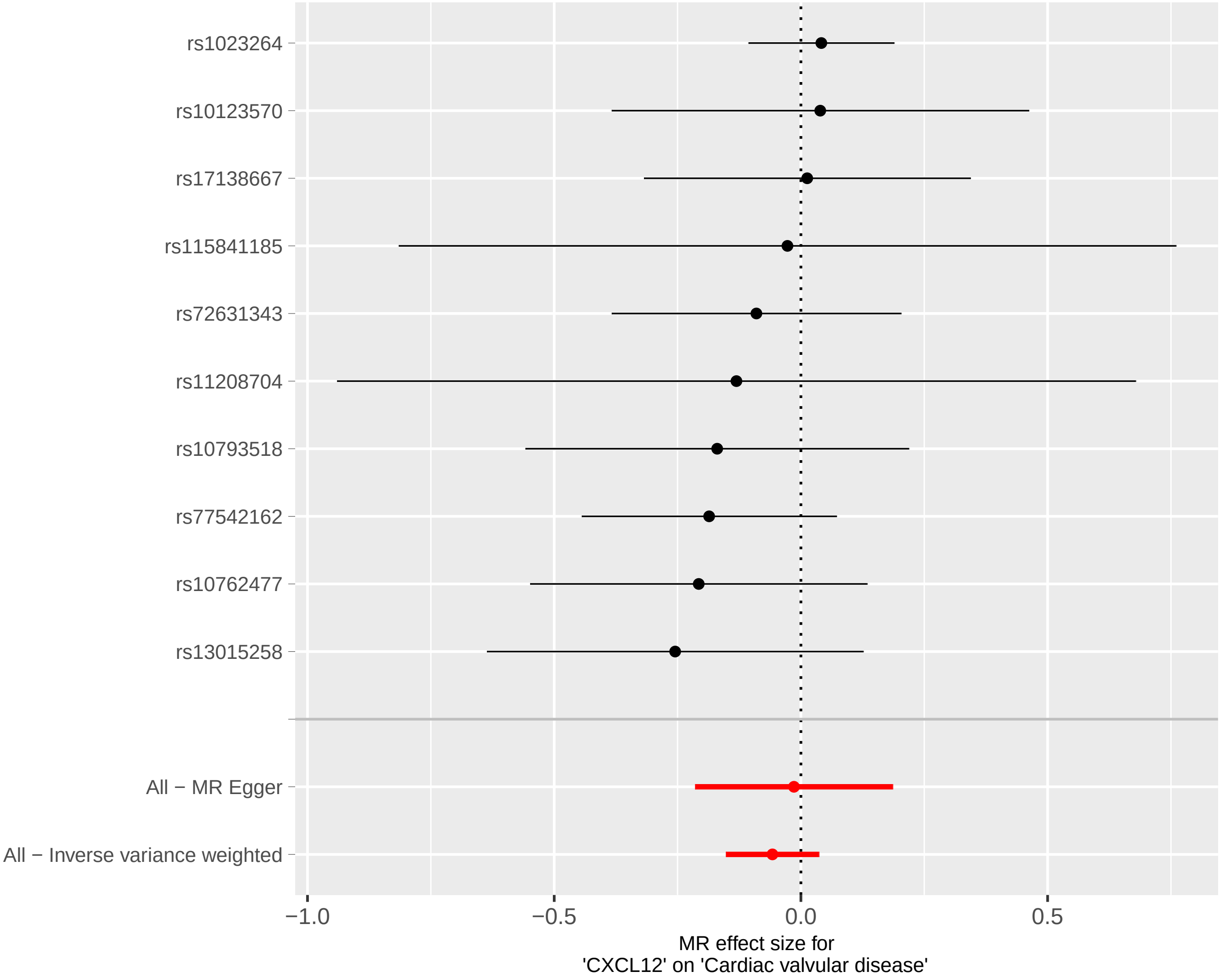
rs1023264
rs10123570
rs17138667
rs115841185
rs72631343
rs11208704
rs10793518
rs77542162
rs10762477
rs13015258
All − MR Egger
All − Inverse variance weighted
−1.0
−0.5
0.0
0.5
MR effect size for
'CXCL12' on 'Cardiac valvular disease'

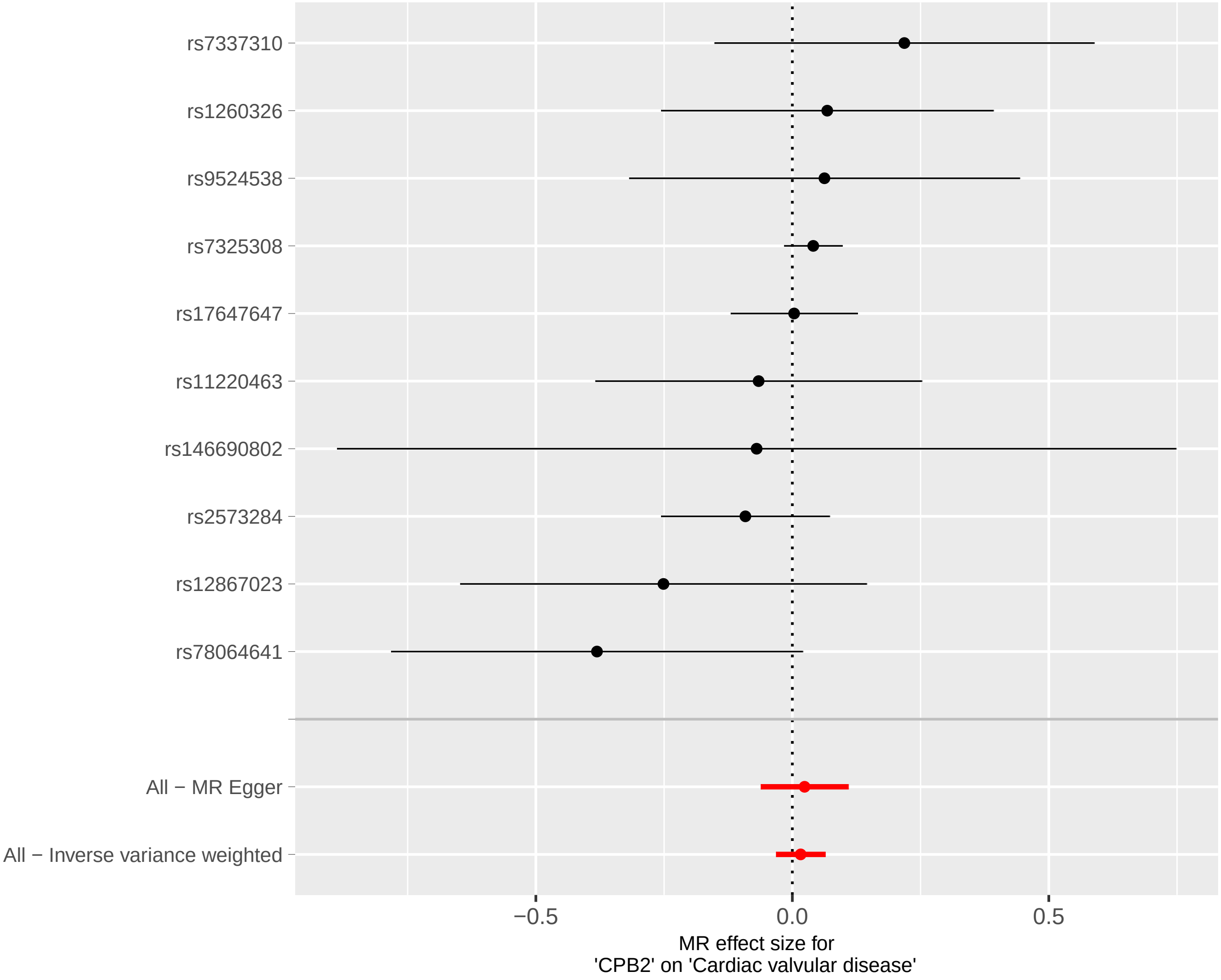
rs7337310
rs1260326
rs9524538
rs7325308
rs17647647
rs11220463
rs146690802
rs2573284
rs12867023
rs78064641
All − MR Egger
All − Inverse variance weighted
−0.5
0.0
0.5
MR effect size for
'CPB2' on 'Cardiac valvular disease'

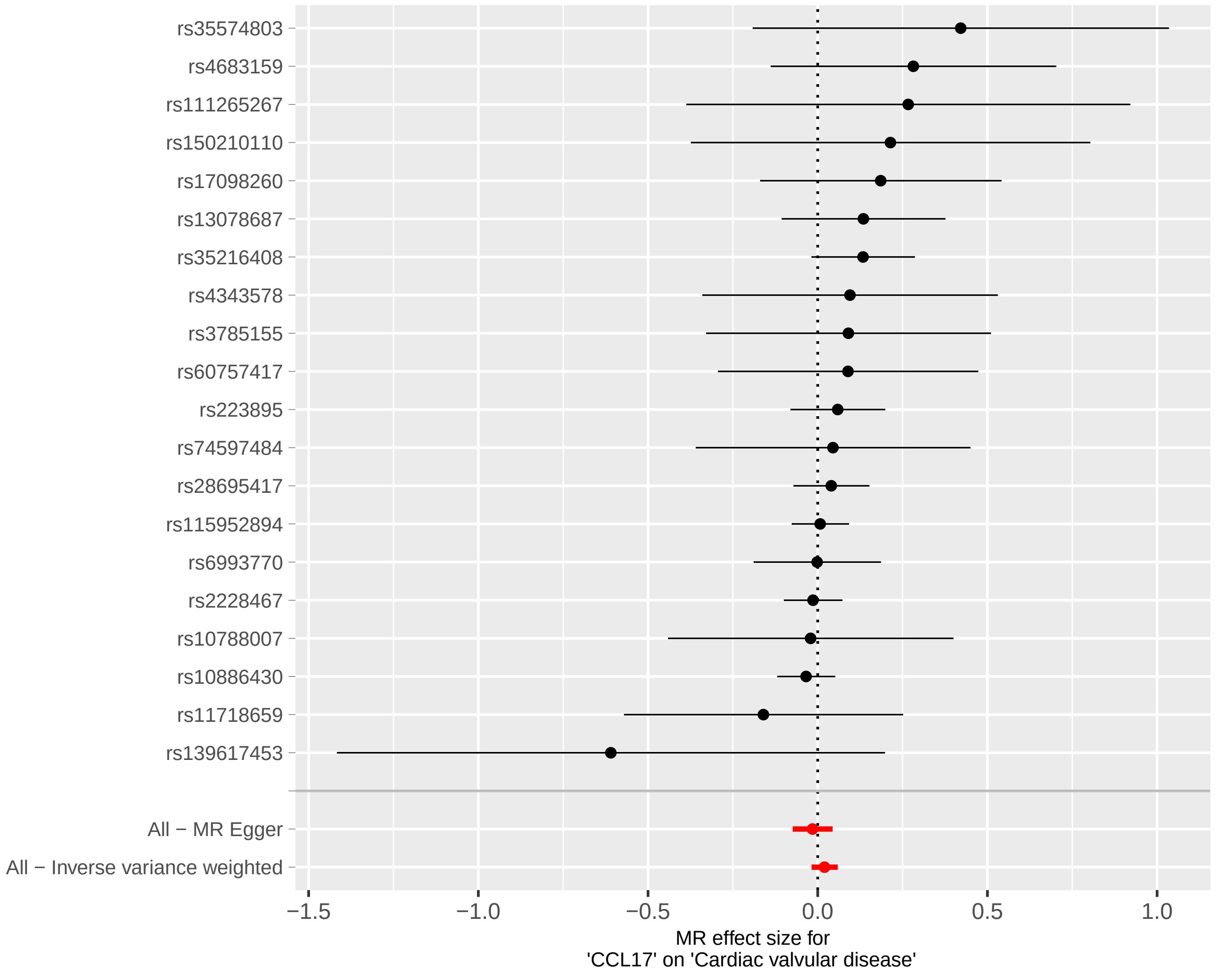
rs35574803
rs4683159
rs111265267
rs150210110
rs17098260
rs13078687
rs35216408
rs4343578
rs3785155
rs60757417
rs223895
rs74597484
rs28695417
rs115952894
rs6993770
rs2228467
rs10788007
rs10886430
rs11718659
rs139617453
All − MR Egger
All − Inverse variance weighted
−1.5
−1.0
−0.5
0.0
0.5
1.0
MR effect size for
'CCL17' on 'Cardiac valvular disease'

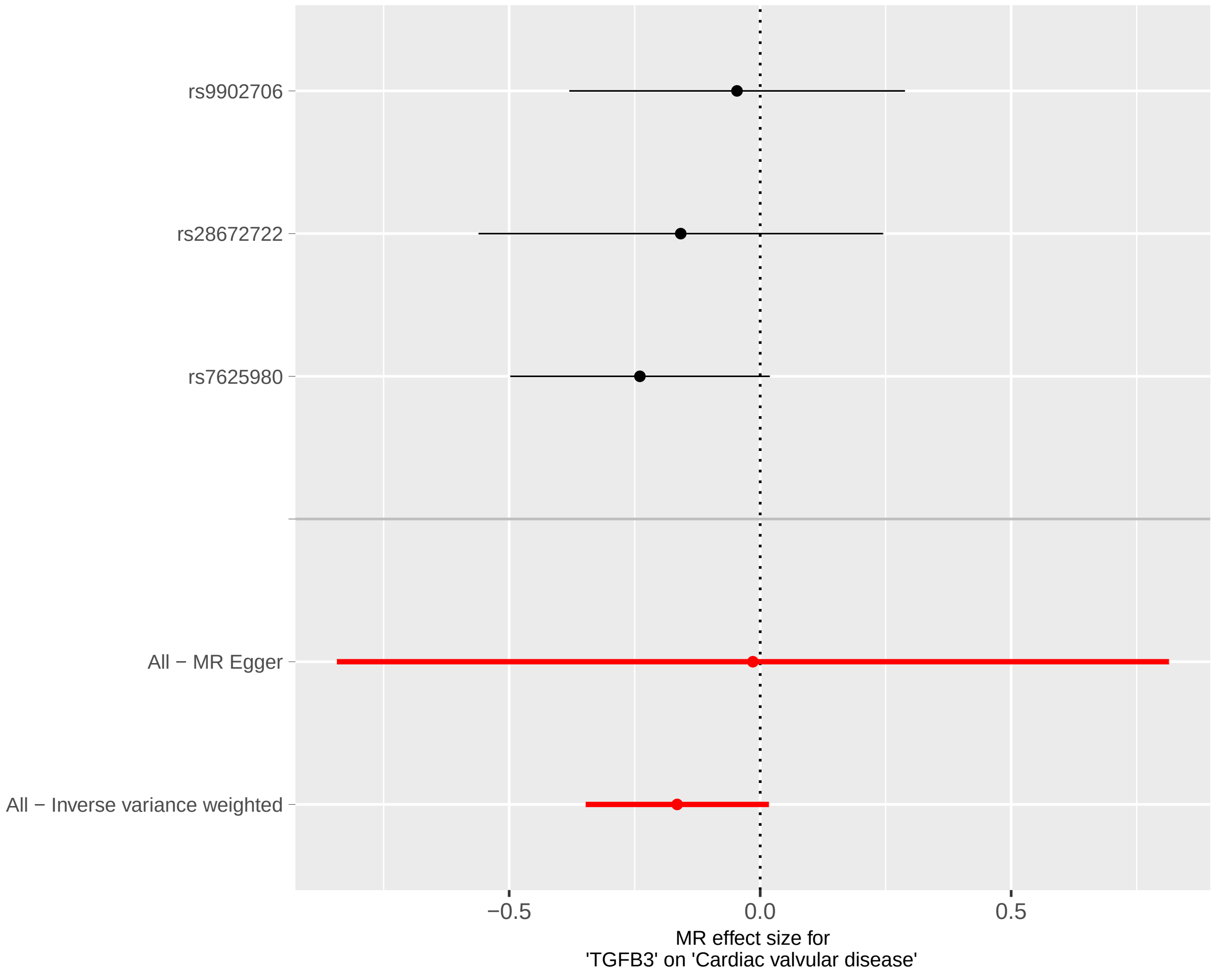
rs9902706
rs28672722
rs7625980
All − MR Egger
All − Inverse variance weighted
−0.5
0.0
0.5
MR effect size for
'TGFB3' on 'Cardiac valvular disease'

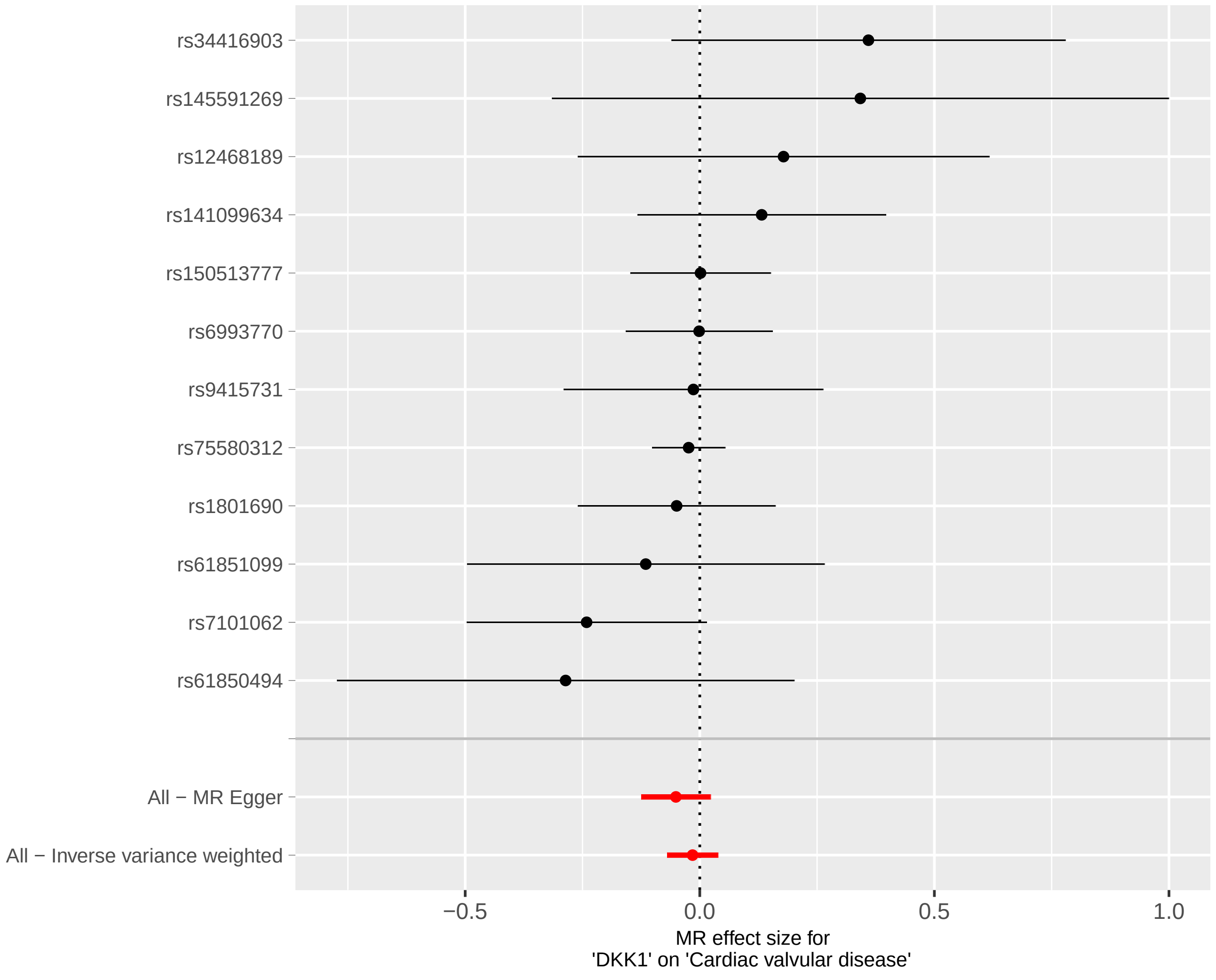
rs34416903
rs145591269
rs12468189
rs141099634
rs150513777
rs6993770
rs9415731
rs75580312
rs1801690
rs61851099
rs7101062
rs61850494
All − MR Egger
All − Inverse variance weighted
−0.5
0.0
0.5
1.0
MR effect size for
'DKK1' on 'Cardiac valvular disease'

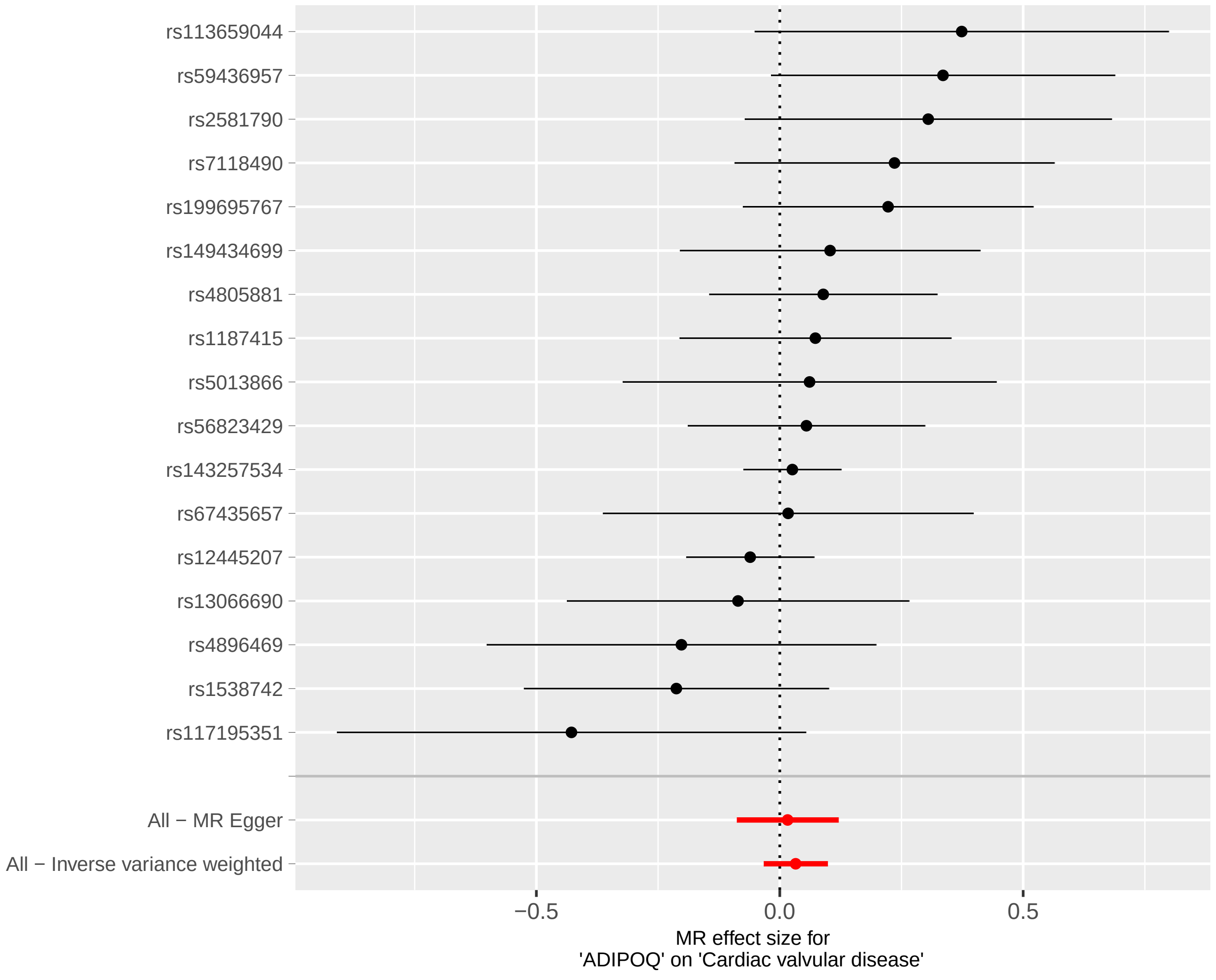
rs113659044
rs59436957
rs2581790
rs7118490
rs199695767
rs149434699
rs4805881
rs1187415
rs5013866
rs56823429
rs143257534
rs67435657
rs12445207
rs13066690
rs4896469
rs1538742
rs117195351
All − MR Egger
All − Inverse variance weighted
−0.5
0.0
0.5
MR effect size for
'ADIPOQ' on 'Cardiac valvular disease'

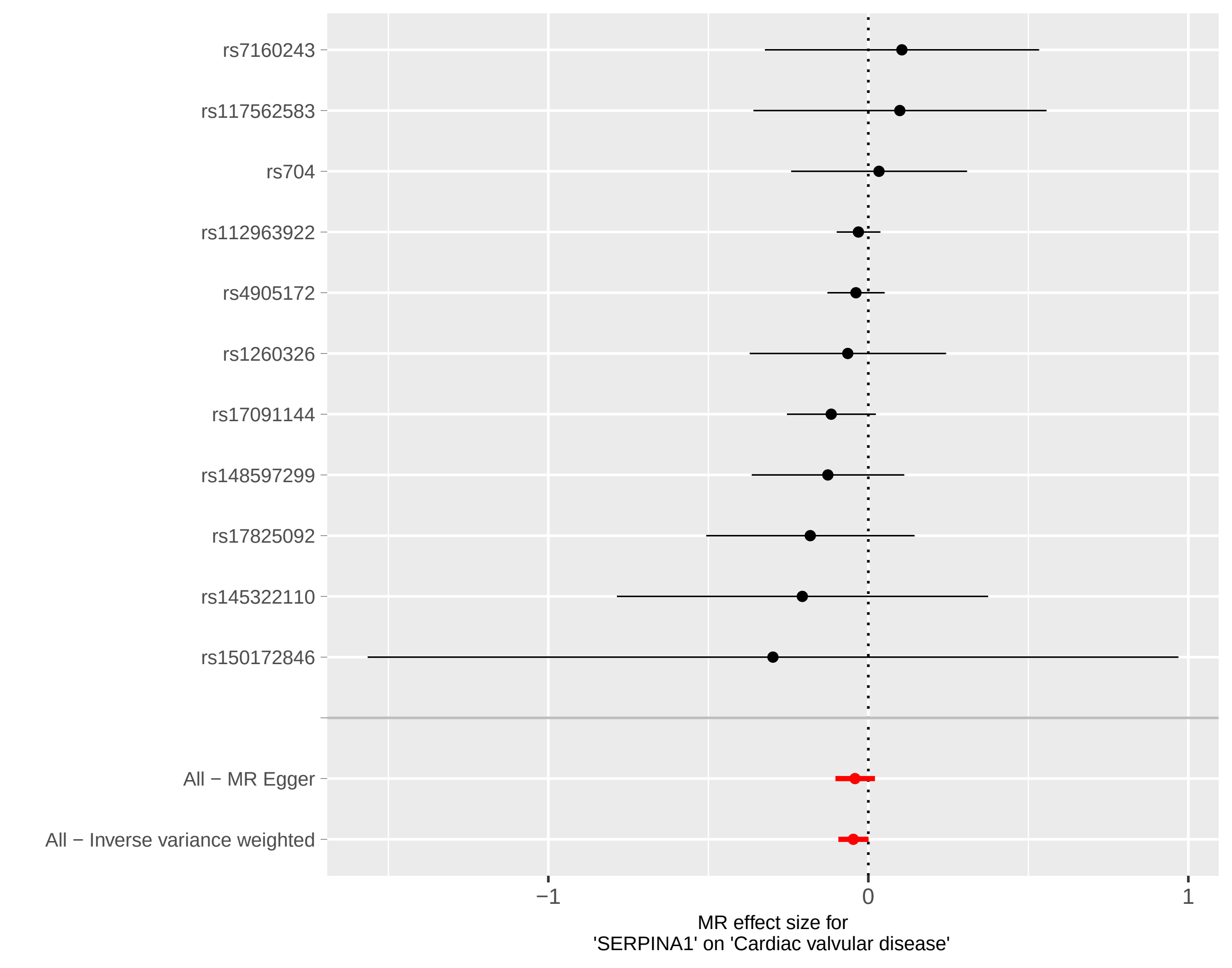

rs7160243
rs117562583
rs704
rs112963922
rs4905172
rs1260326
rs17091144
rs148597299
rs17825092
rs145322110
rs150172846
All − MR Egger
All − Inverse variance weighted
−1
0
1
MR effect size for
'SERPINA1' on 'Cardiac valvular disease'

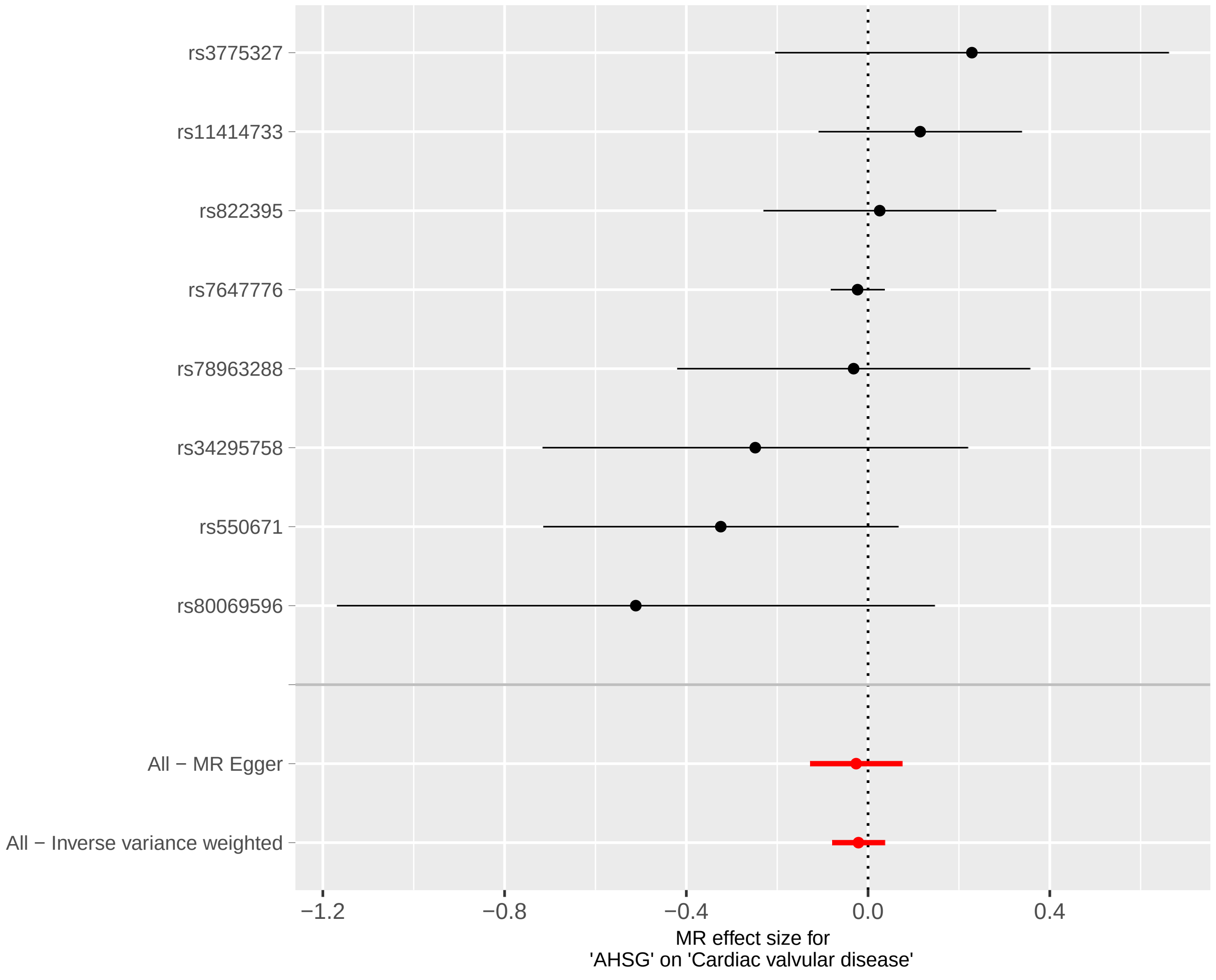
rs3775327
rs11414733
rs822395
rs7647776
rs78963288
rs34295758
rs550671
rs80069596
All − MR Egger
All − Inverse variance weighted
−1.2
−0.8
−0.4
0.0
0.4
MR effect size for
'AHSG' on 'Cardiac valvular disease'

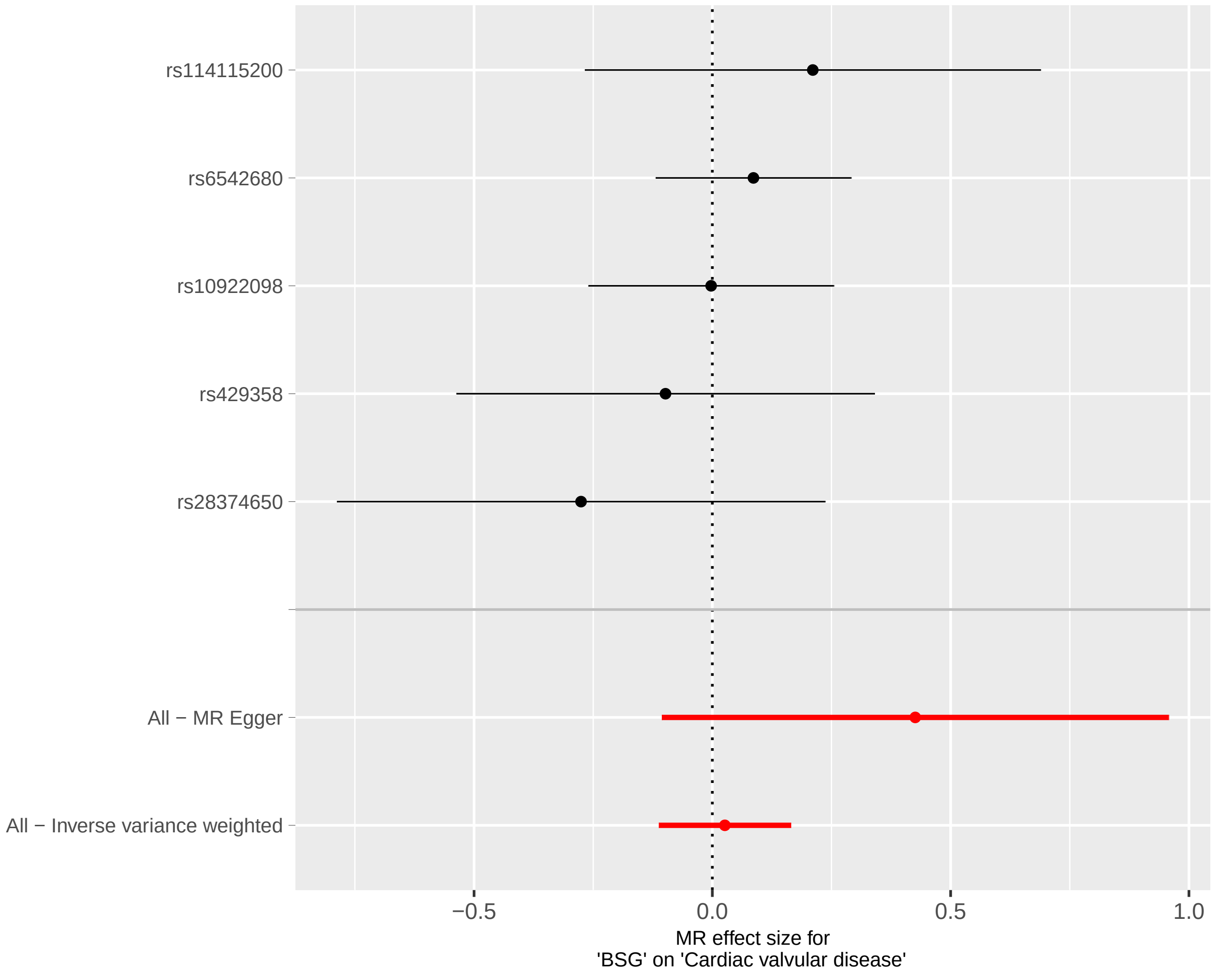
rs114115200
rs6542680
rs10922098
rs429358
rs28374650
All − MR Egger
All − Inverse variance weighted
−0.5
0.0
0.5
1.0
MR effect size for
'BSG' on 'Cardiac valvular disease'

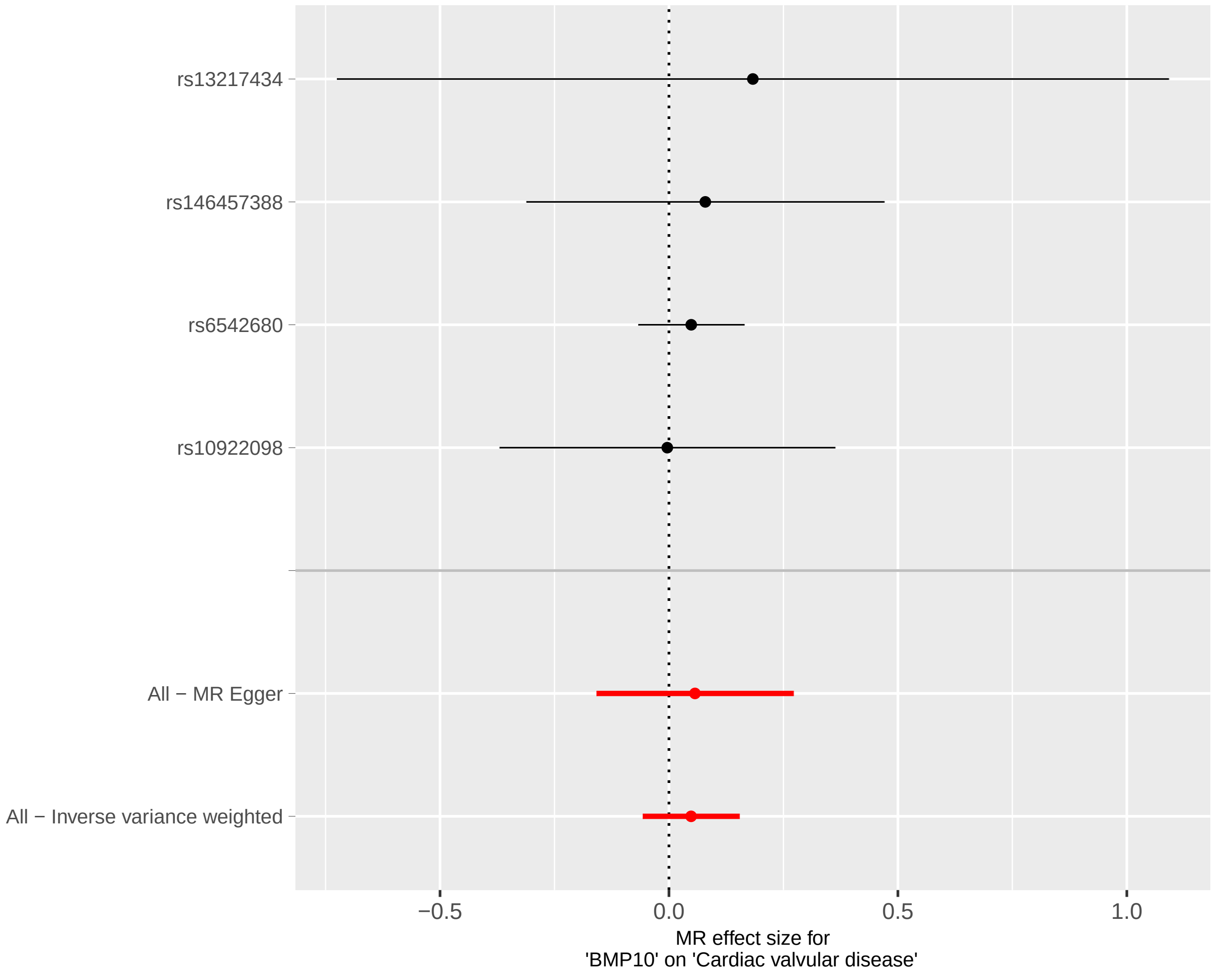
rs13217434
rs146457388
rs6542680
rs10922098
All − MR Egger
All − Inverse variance weighted
−0.5
0.0
0.5
1.0
MR effect size for
'BMP10' on 'Cardiac valvular disease'

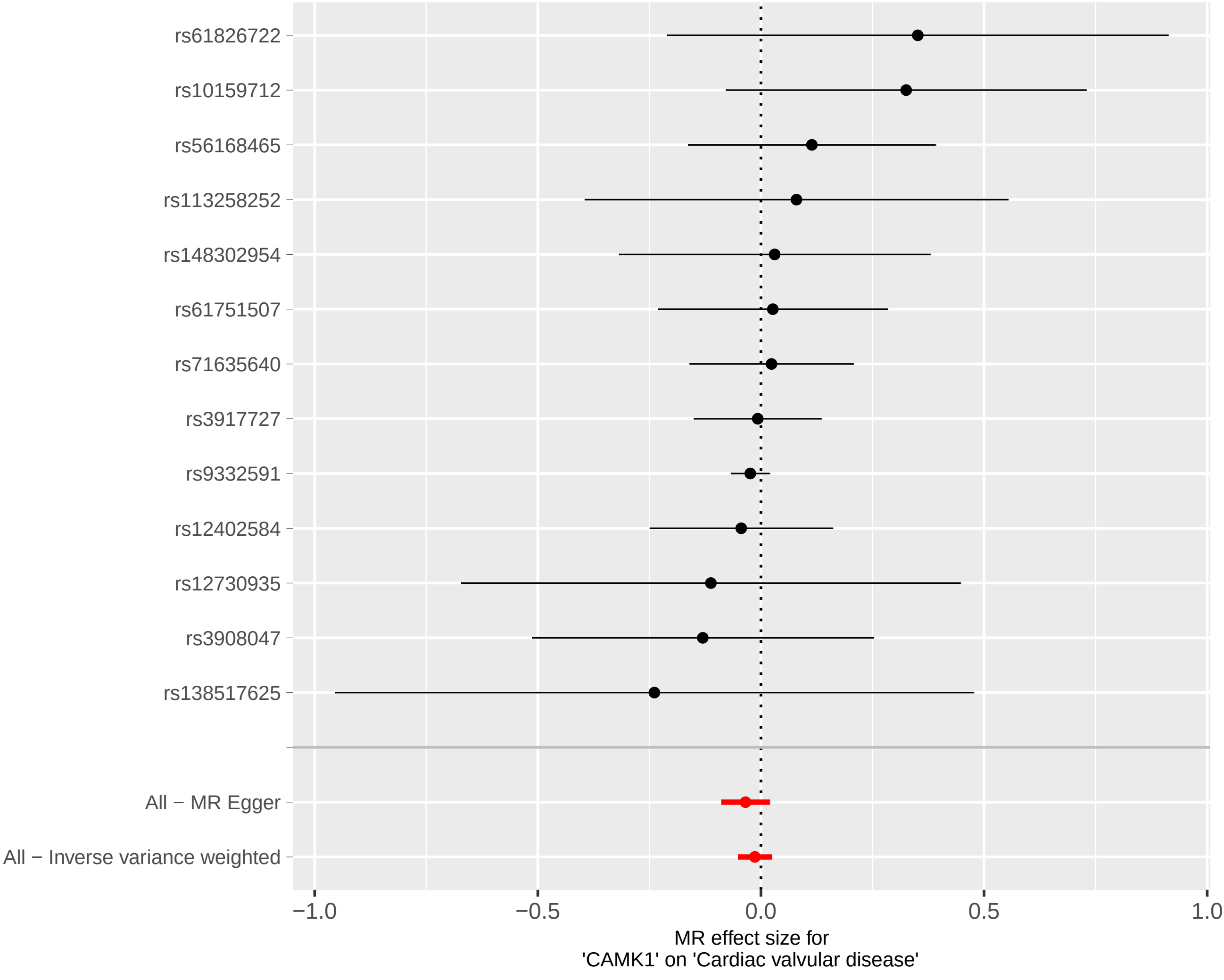
rs61826722
rs10159712
rs56168465
rs113258252
rs148302954
rs61751507
rs71635640
rs3917727
rs9332591
rs12402584
rs12730935
rs3908047
rs138517625
All − MR Egger
All − Inverse variance weighted
−1.0
−0.5
0.0
0.5
1.0
MR effect size for
'CAMK1' on 'Cardiac valvular disease'

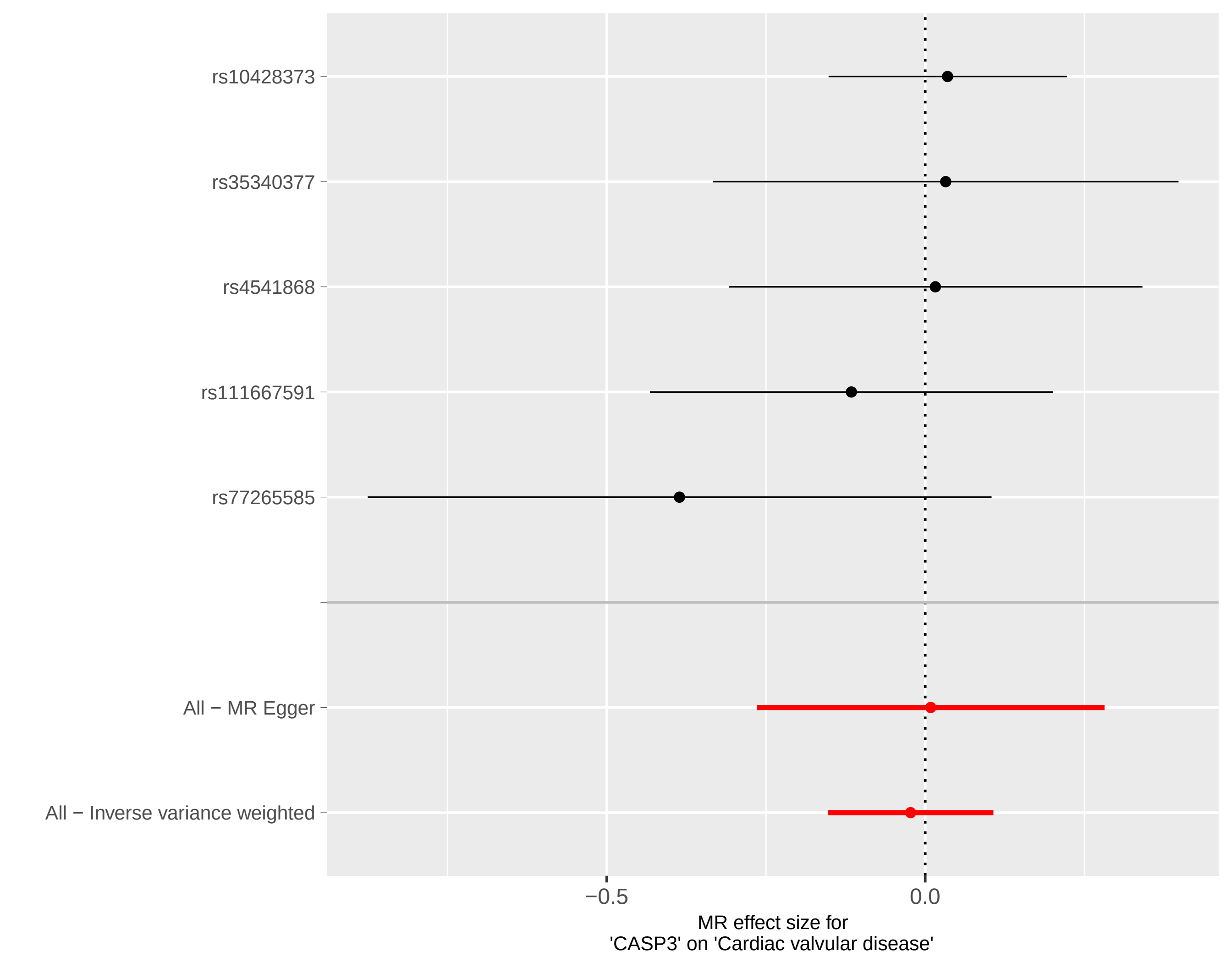
rs10428373
rs35340377
rs4541868
rs111667591
rs77265585
All − MR Egger
All − Inverse variance weighted
−0.5
0.0
MR effect size for
'CASP3' on 'Cardiac valvular disease'

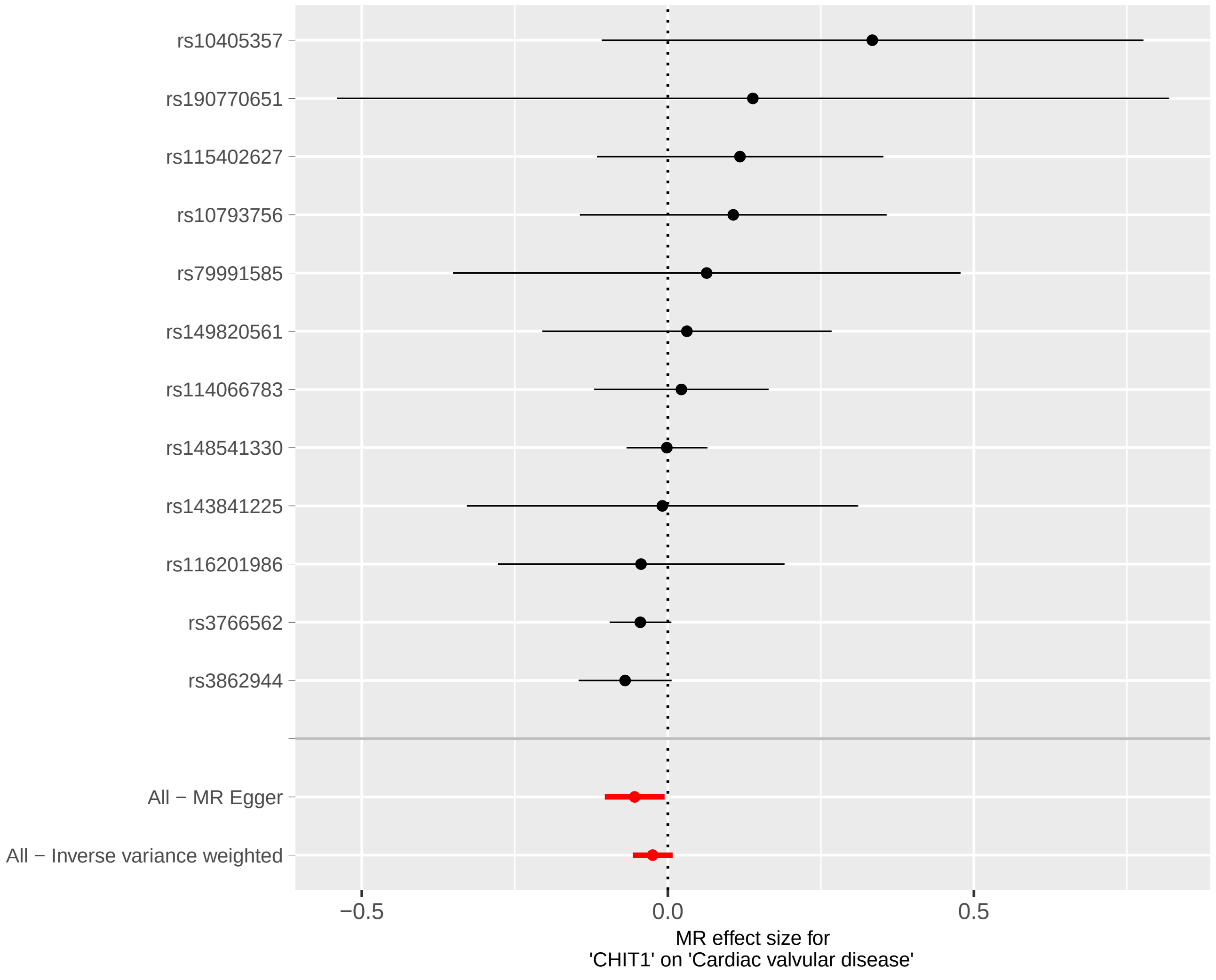
rs10405357
rs190770651
rs115402627
rs10793756
rs79991585
rs149820561
rs114066783
rs148541330
rs143841225
rs116201986
rs3766562
rs3862944
All − MR Egger
All − Inverse variance weighted
−0.5
0.0
0.5
MR effect size for
'CHIT1' on 'Cardiac valvular disease'

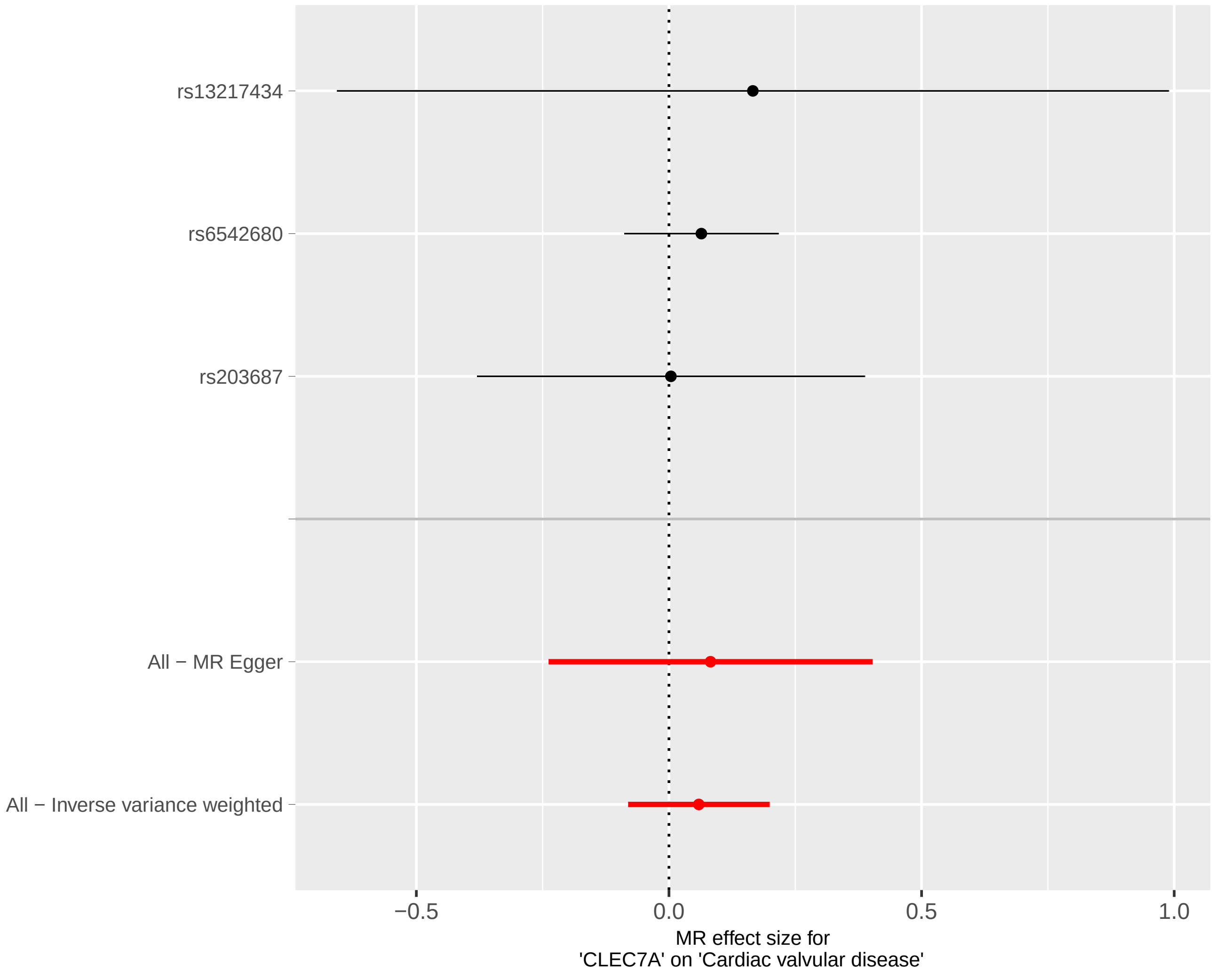
rs13217434
rs6542680
rs203687
All − MR Egger
All − Inverse variance weighted
−0.5
0.0
0.5
1.0
MR effect size for
'CLEC7A' on 'Cardiac valvular disease'

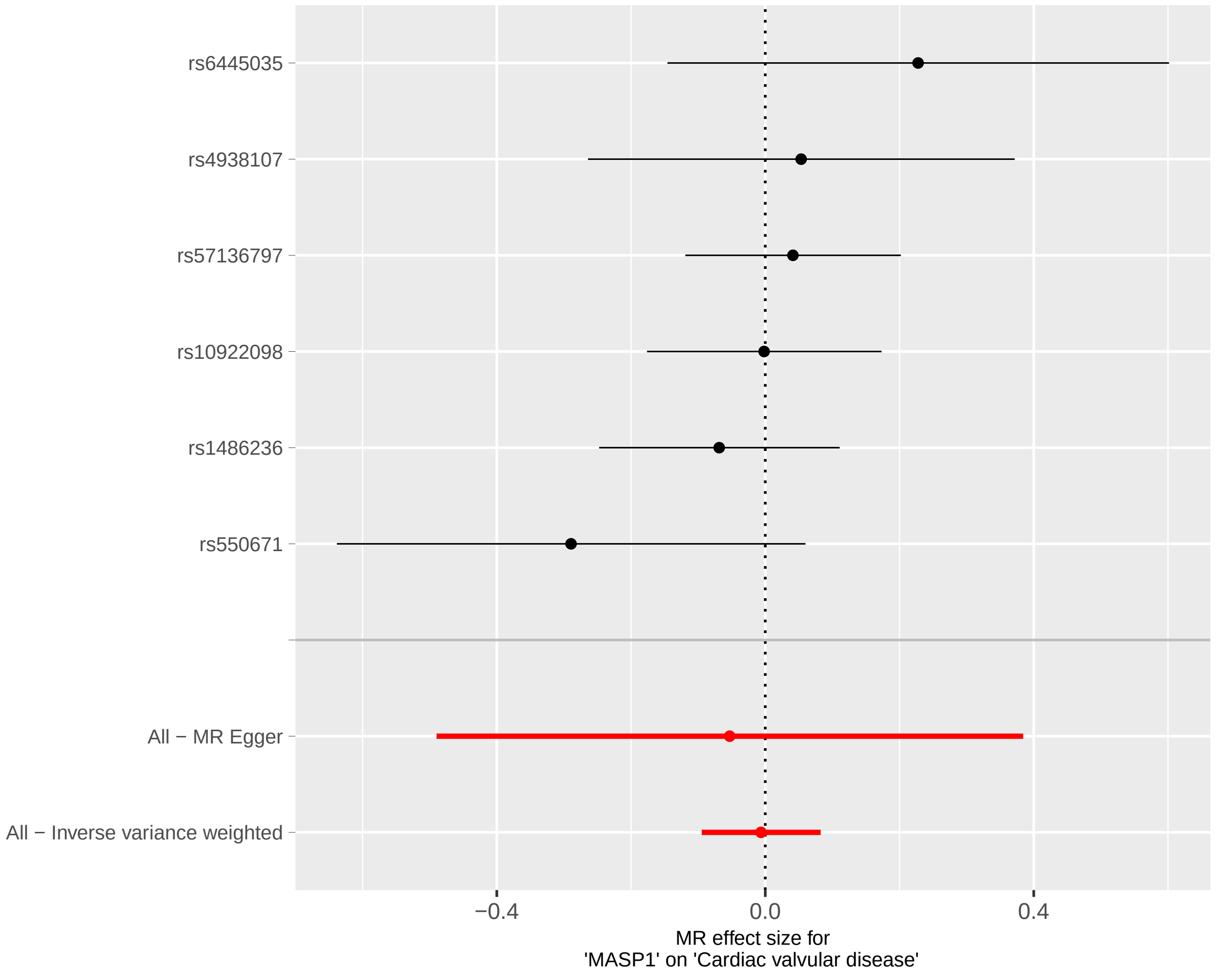
rs6445035
rs4938107
rs57136797
rs10922098
rs1486236
rs550671
All − MR Egger
All − Inverse variance weighted
−0.4
0.0
0.4
MR effect size for
'MASP1' on 'Cardiac valvular disease'

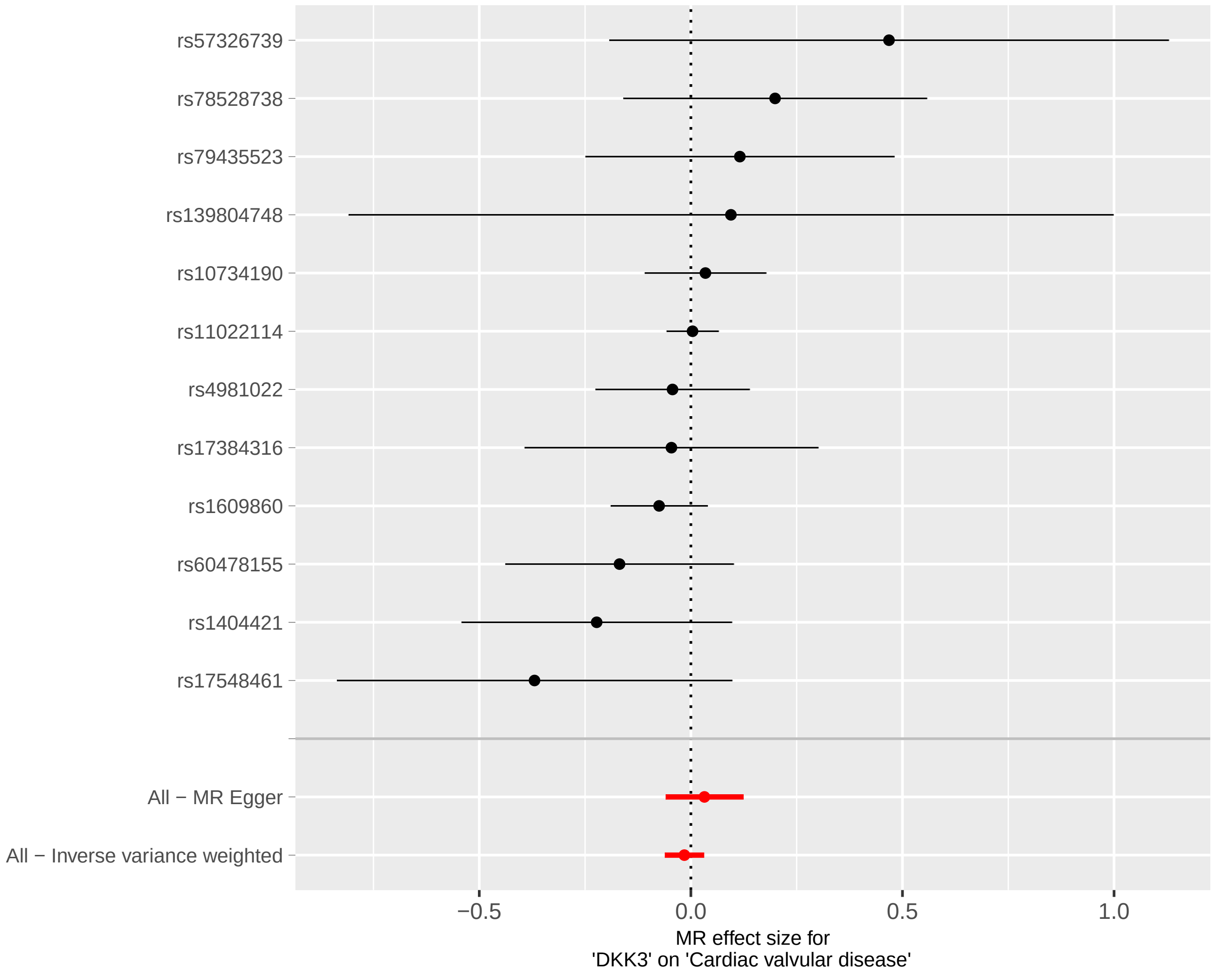
rs57326739
rs78528738
rs79435523
rs139804748
rs10734190
rs11022114
rs4981022
rs17384316
rs1609860
rs60478155
rs1404421
rs17548461
All − MR Egger
All − Inverse variance weighted
−0.5
0.0
0.5
1.0
MR effect size for
'DKK3' on 'Cardiac valvular disease'

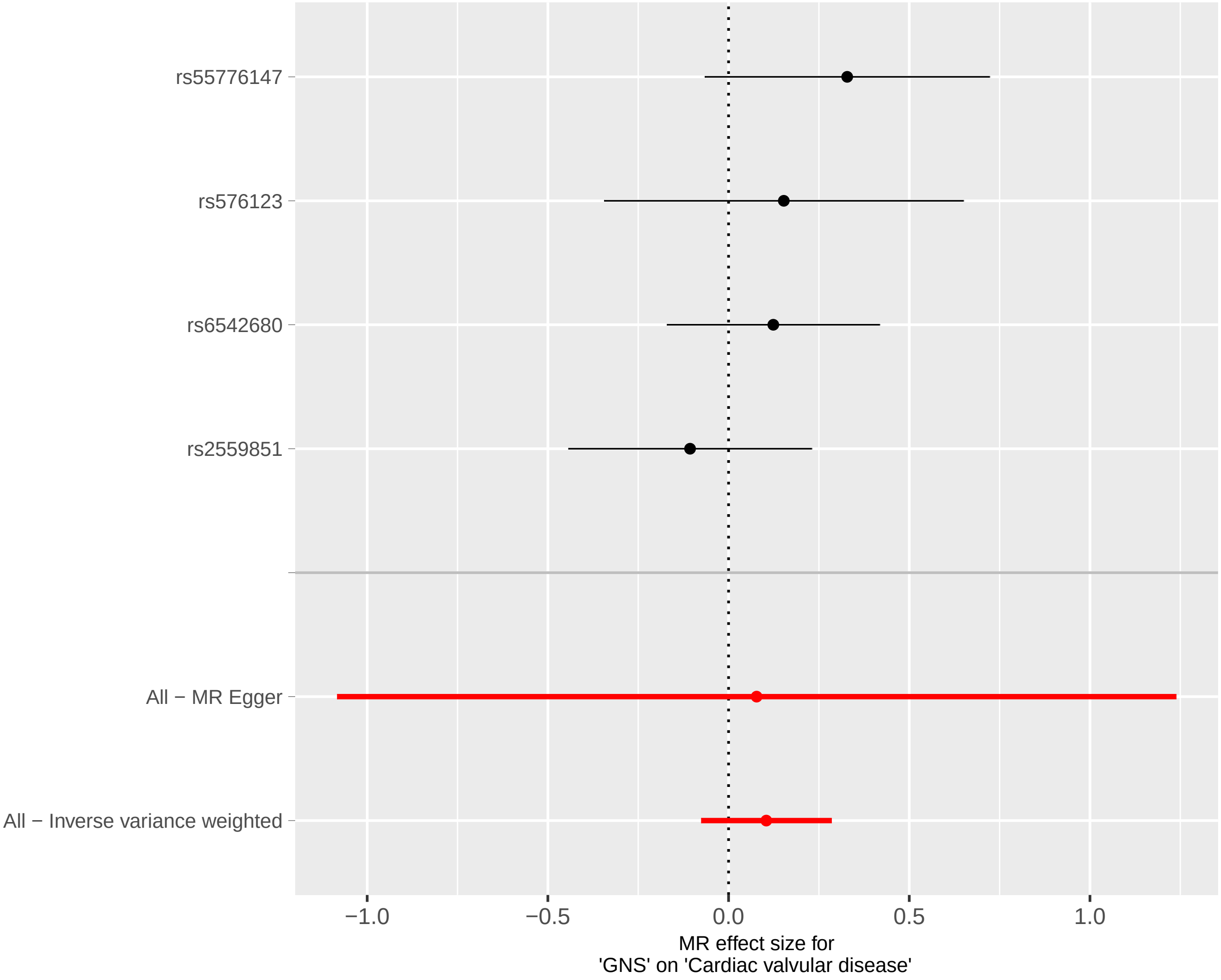

rs55776147
rs576123
rs6542680
rs2559851
All − MR Egger
All − Inverse variance weighted
−1.0
−0.5
0.0
0.5
1.0
MR effect size for
'GNS' on 'Cardiac valvular disease'

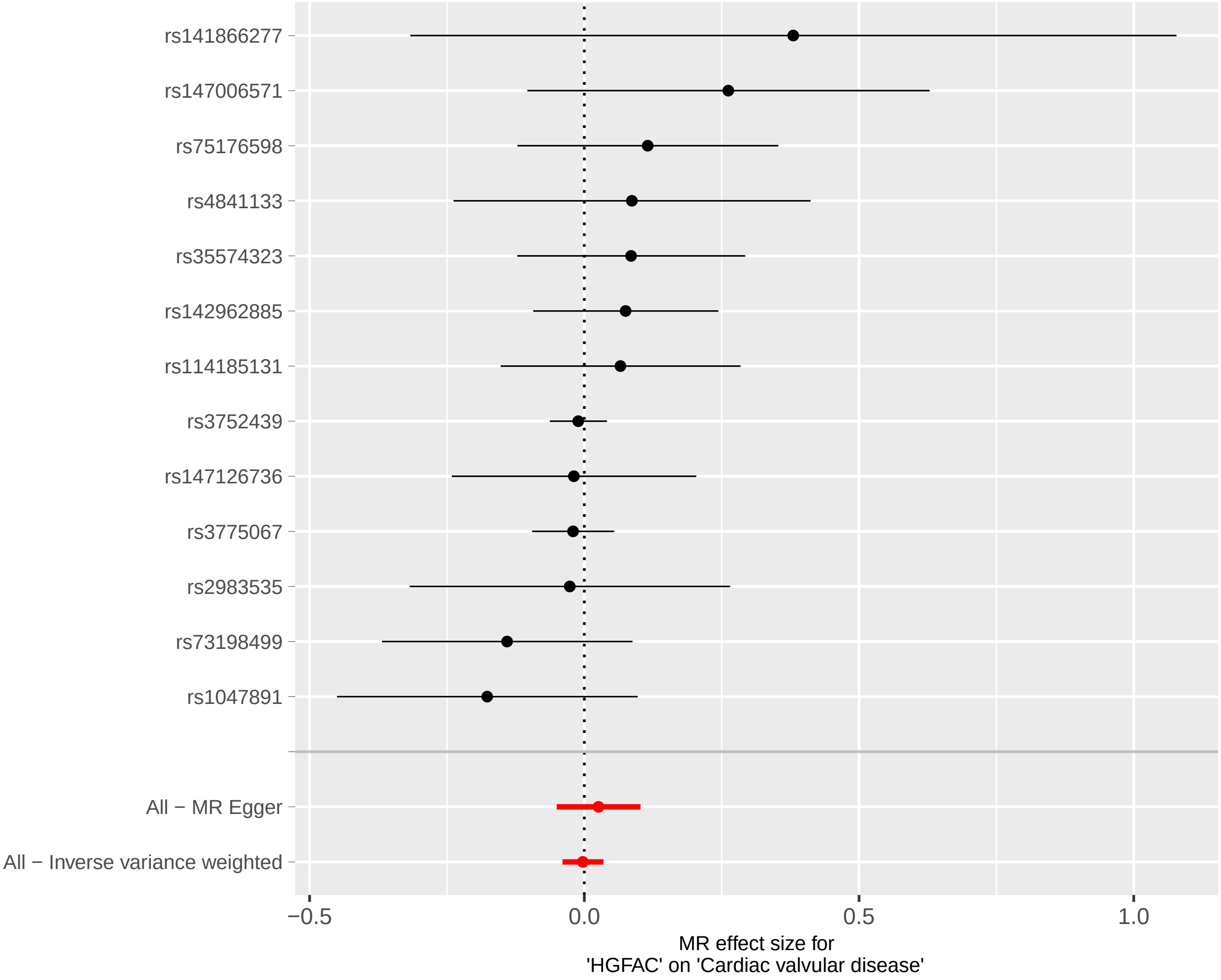
rs141866277
rs147006571
rs75176598
rs4841133
rs35574323
rs142962885
rs114185131
rs3752439
rs147126736
rs3775067
rs2983535
rs73198499
rs1047891
All − MR Egger
All − Inverse variance weighted
−0.5
0.0
0.5
1.0
MR effect size for
'HGFAC' on 'Cardiac valvular disease'

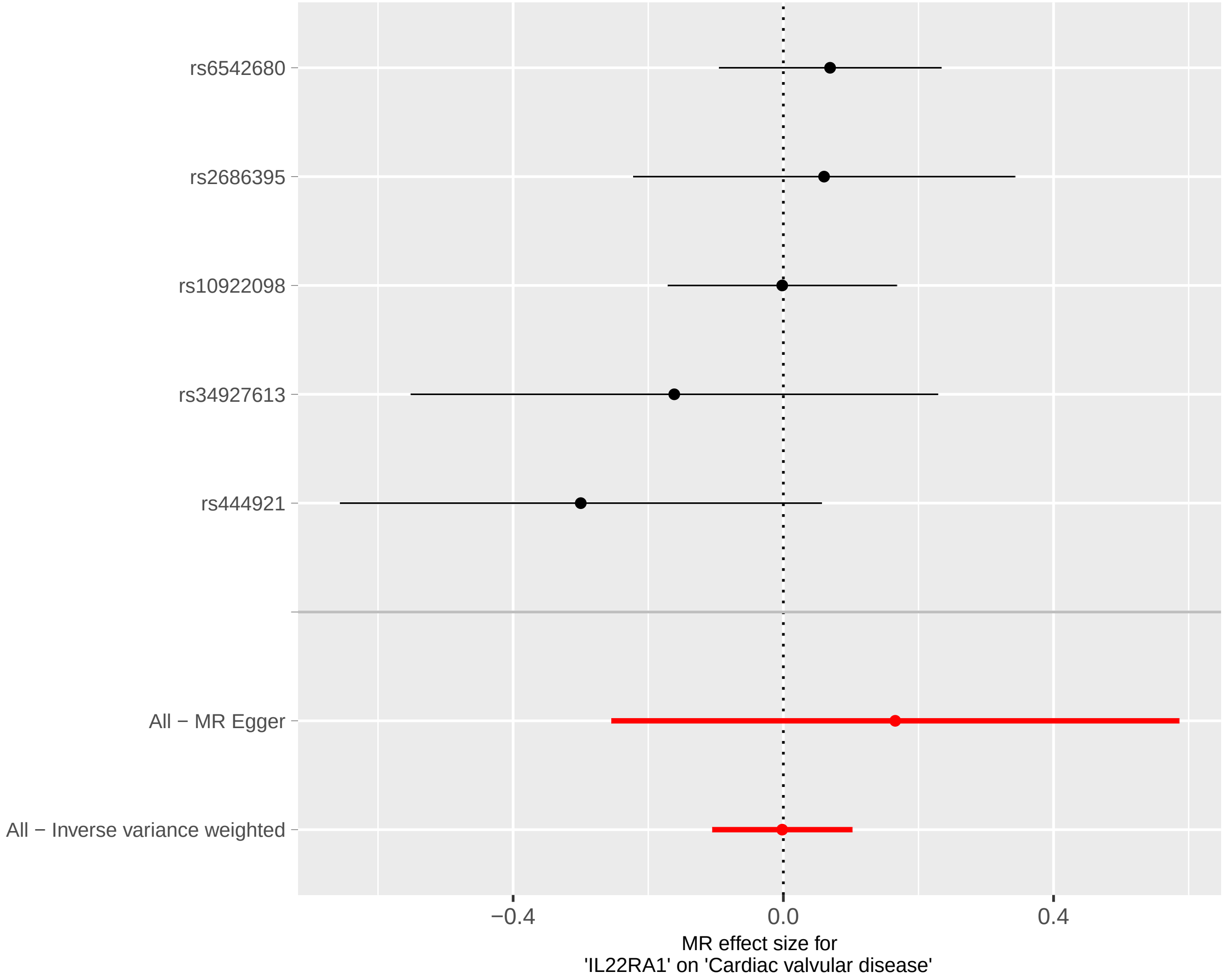
rs6542680
rs2686395
rs10922098
rs34927613
rs444921
All − MR Egger
All − Inverse variance weighted
−0.4
0.0
0.4
MR effect size for
'IL22RA1' on 'Cardiac valvular disease'

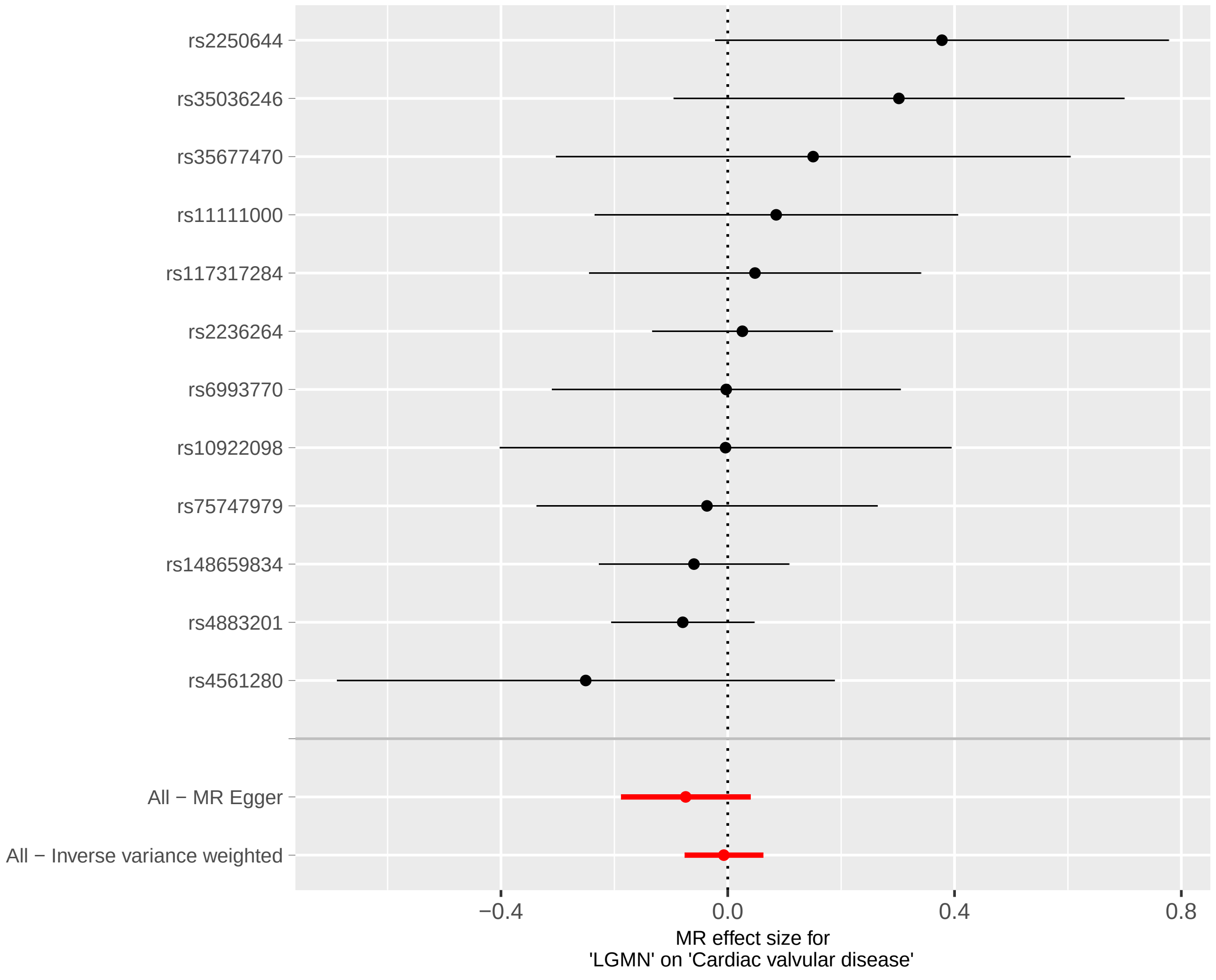

rs2250644
rs35036246
rs35677470
rs11111000
rs117317284
rs2236264
rs6993770
rs10922098
rs75747979
rs148659834
rs4883201
rs4561280
All − MR Egger
All − Inverse variance weighted
−0.4
0.0
0.4
0.8
MR effect size for
'LGMN' on 'Cardiac valvular disease'

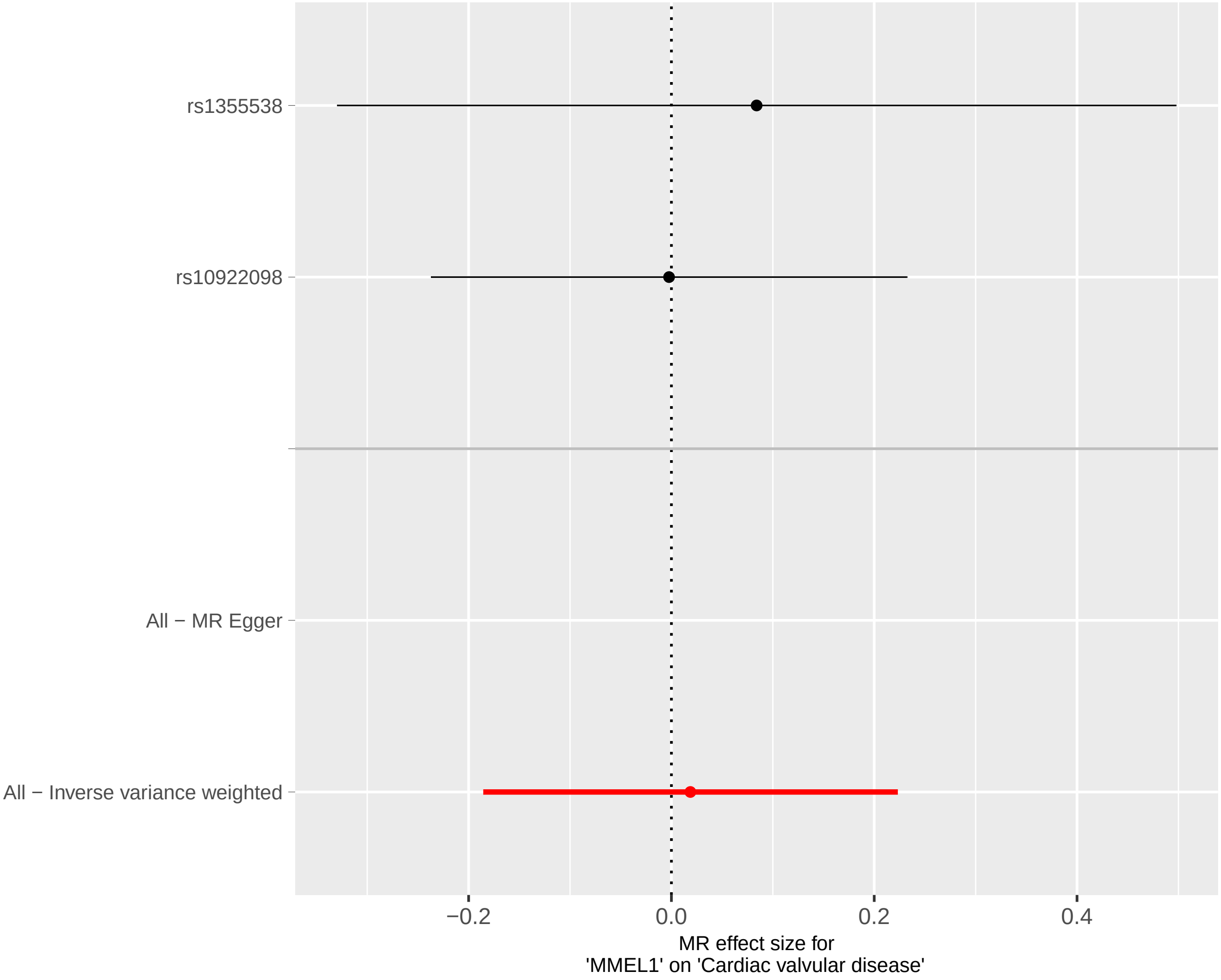
rs1355538
rs10922098
All − MR Egger
All − Inverse variance weighted
−0.2
0.0
0.2
0.4
MR effect size for
'MMEL1' on 'Cardiac valvular disease'

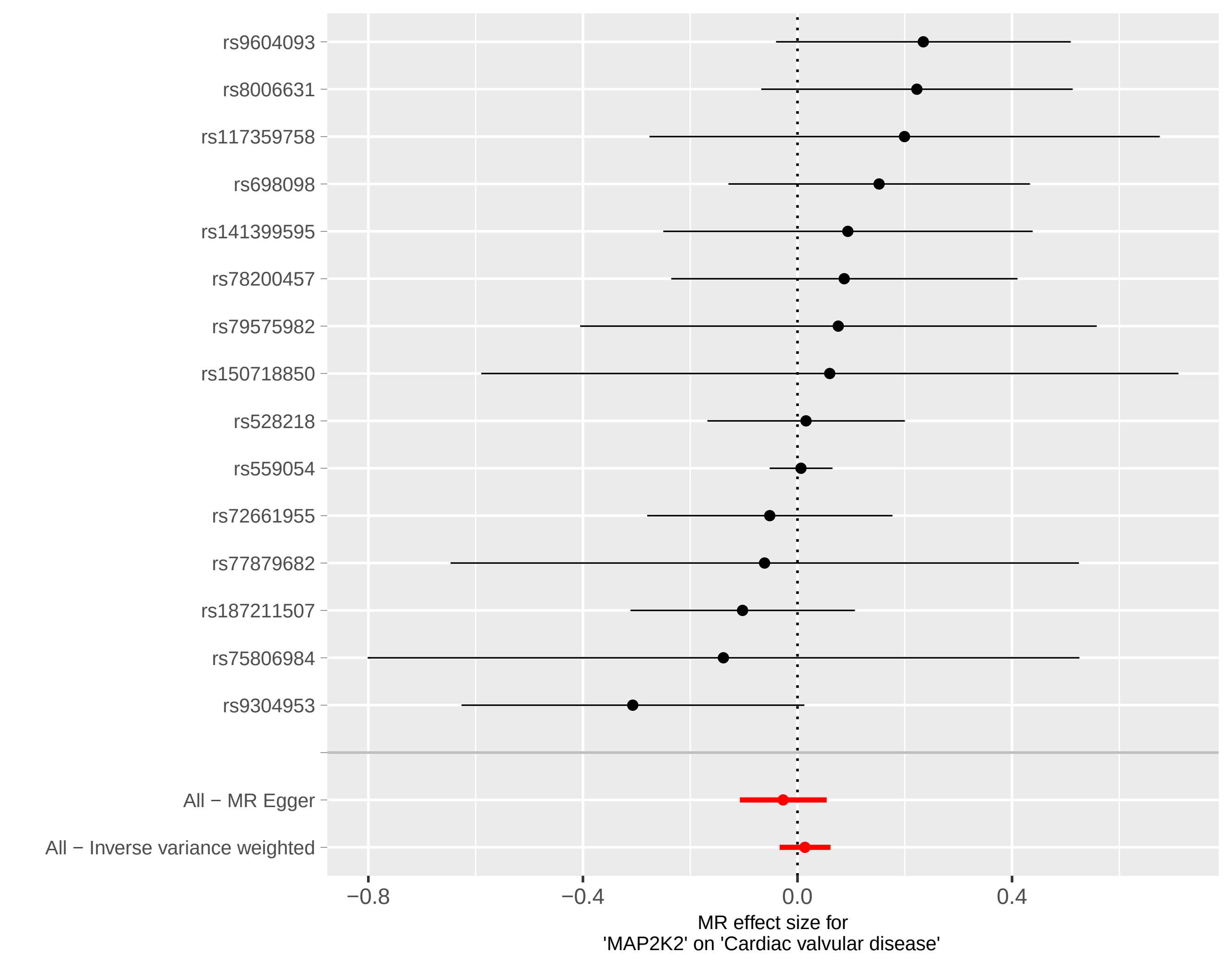
rs9604093
rs8006631
rs117359758
rs698098
rs141399595
rs78200457
rs79575982
rs150718850
rs528218
rs559054
rs72661955
rs77879682
rs187211507
rs75806984
rs9304953
All − MR Egger
All − Inverse variance weighted
−0.8
−0.4
0.0
0.4
MR effect size for
'MAP2K2' on 'Cardiac valvular disease'

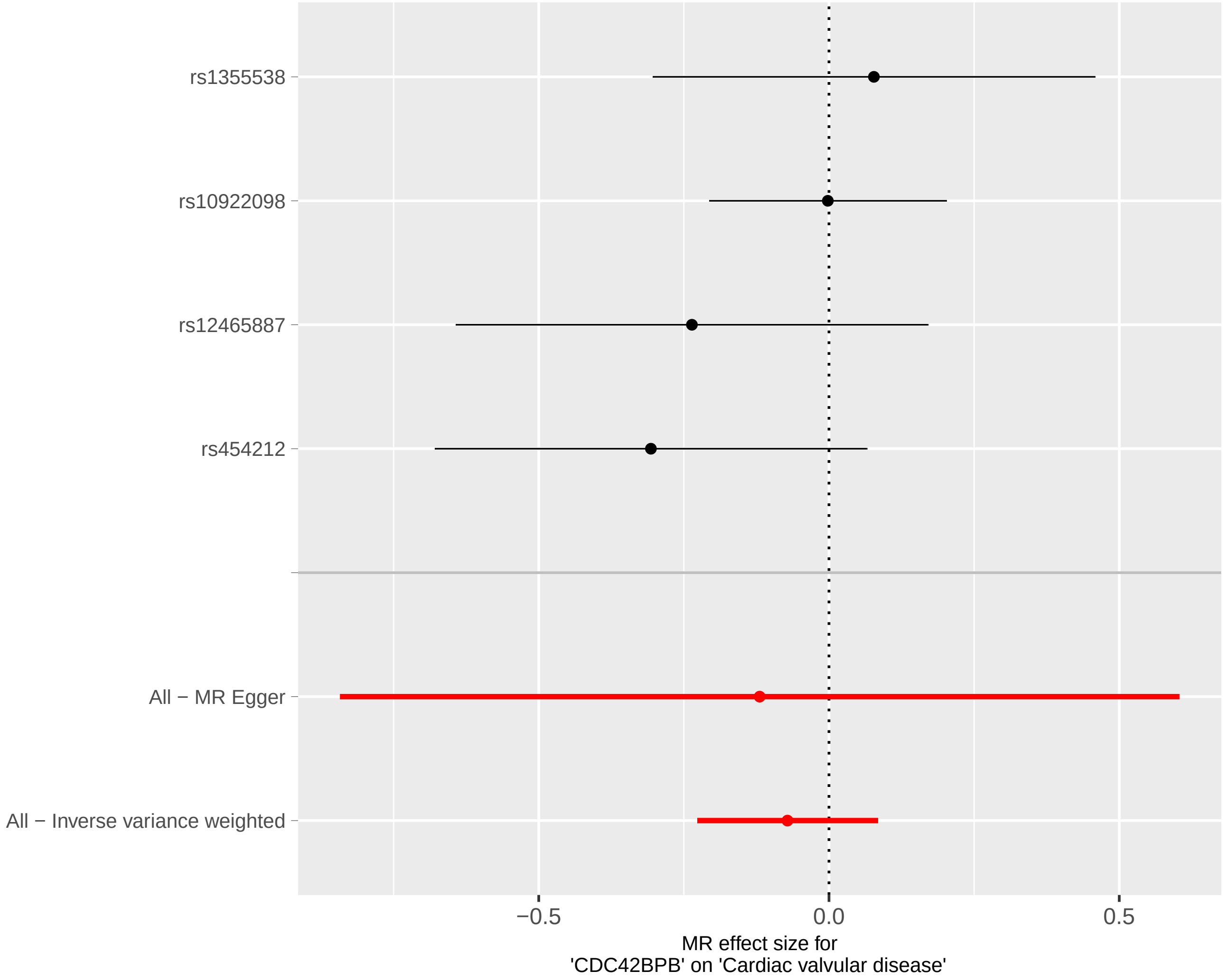
rs1355538
rs10922098
rs12465887
rs454212
All − MR Egger
All − Inverse variance weighted
−0.5
0.0
0.5
MR effect size for
'CDC42BPB' on 'Cardiac valvular disease'

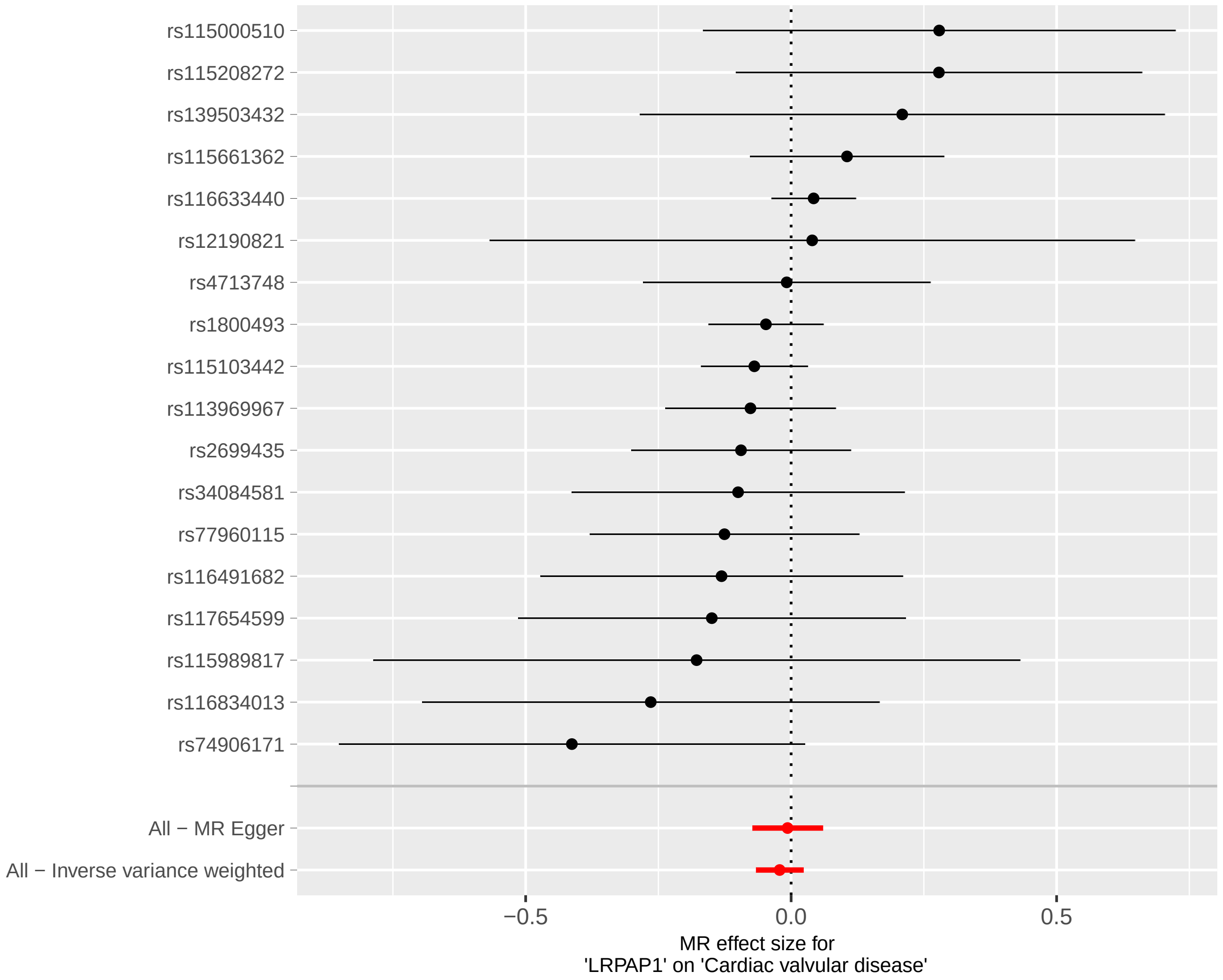
rs115000510
rs115208272
rs139503432
rs115661362
rs116633440
rs12190821
rs4713748
rs1800493
rs115103442
rs113969967
rs2699435
rs34084581
rs77960115
rs116491682
rs117654599
rs115989817
rs116834013
rs74906171
All − MR Egger
All − Inverse variance weighted
−0.5
0.0
0.5
MR effect size for
'LRPAP1' on 'Cardiac valvular disease'

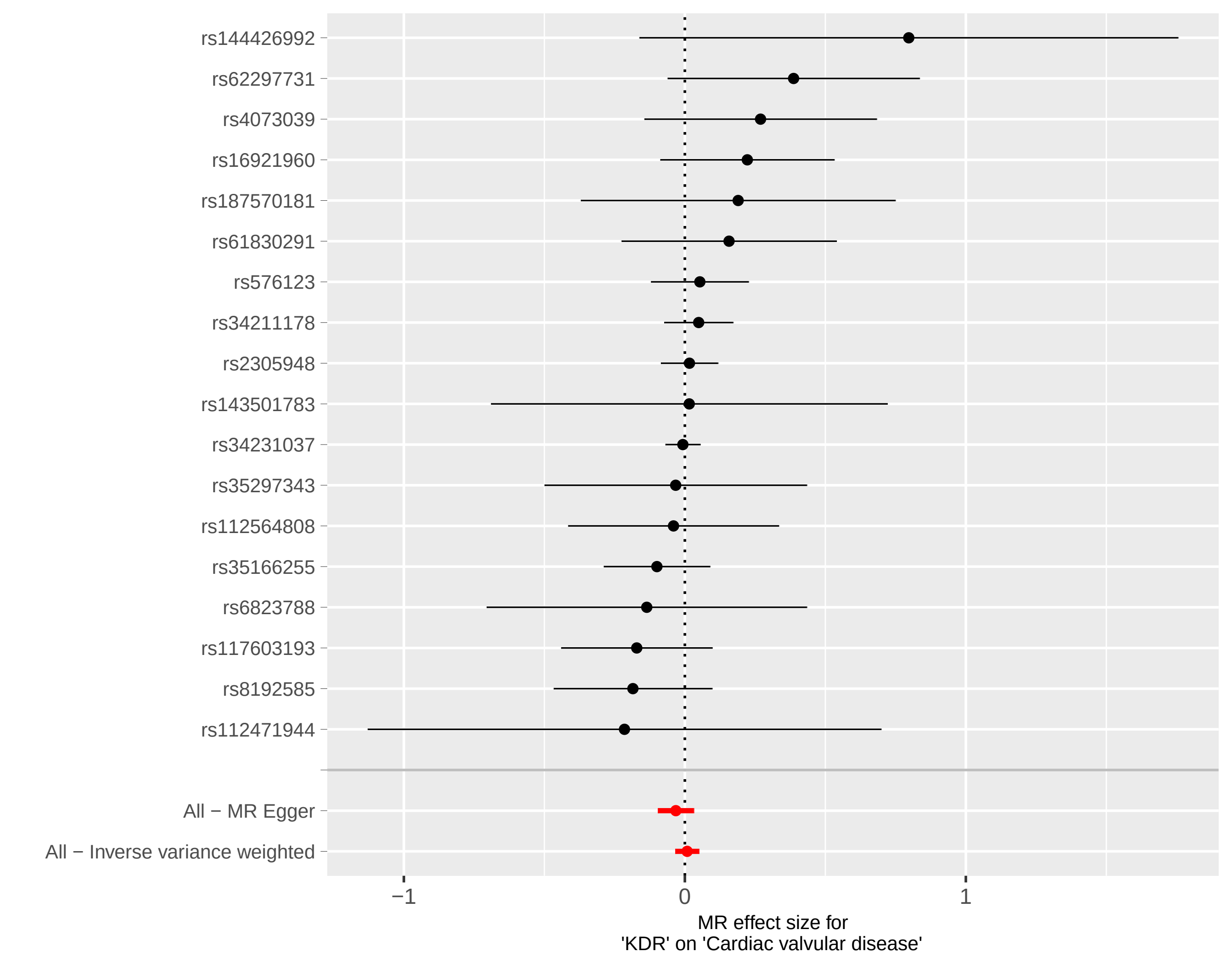
rs144426992
rs62297731
rs4073039
rs16921960
rs187570181
rs61830291
rs576123
rs34211178
rs2305948
rs143501783
rs34231037
rs35297343
rs112564808
rs35166255
rs6823788
rs117603193
rs8192585
rs112471944
All − MR Egger
All − Inverse variance weighted
−1
0
1
MR effect size for
'KDR' on 'Cardiac valvular disease'

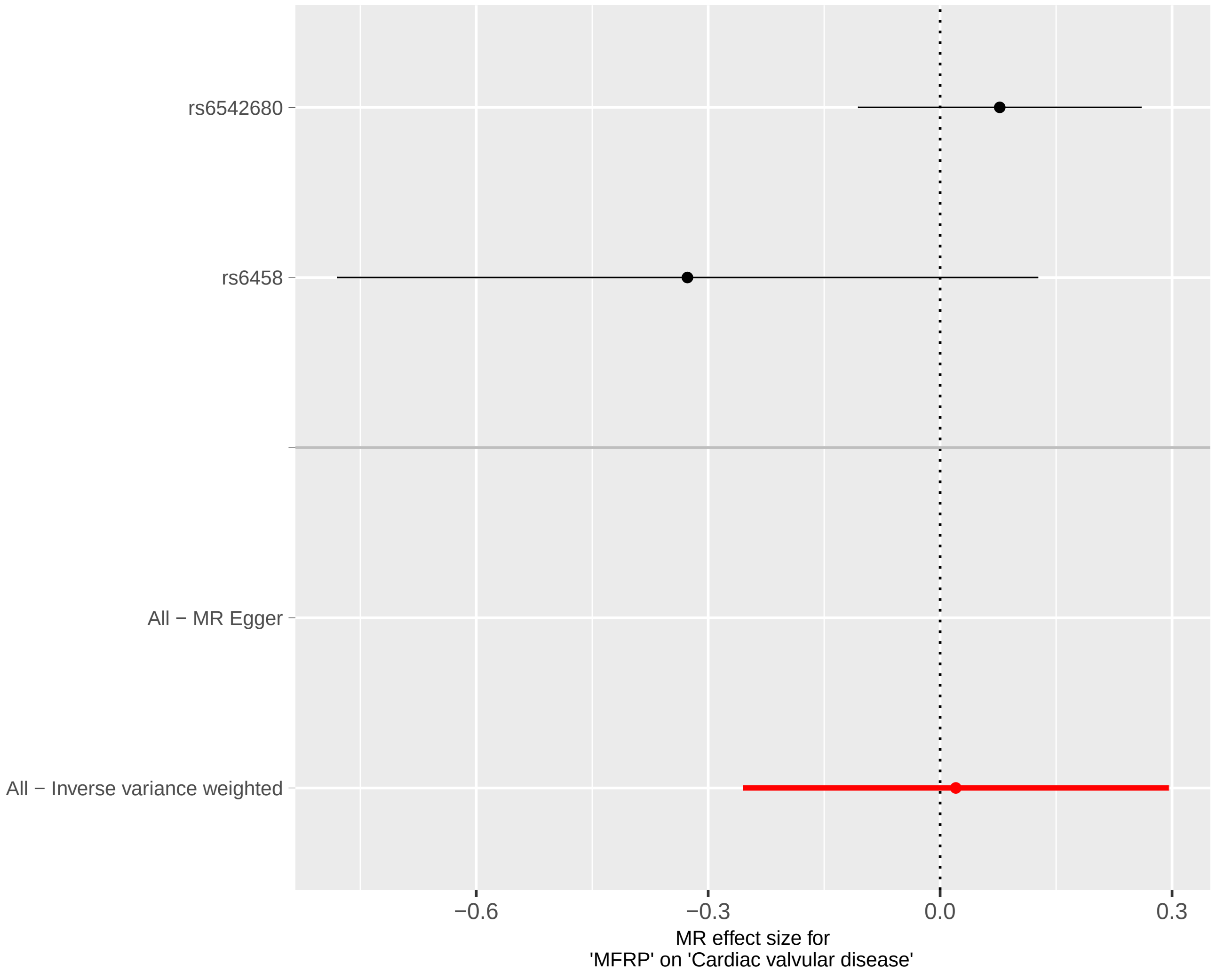

rs6542680
rs6458
All − MR Egger
All − Inverse variance weighted
−0.6
−0.3
0.0
0.3
MR effect size for
'MFRP' on 'Cardiac valvular disease'

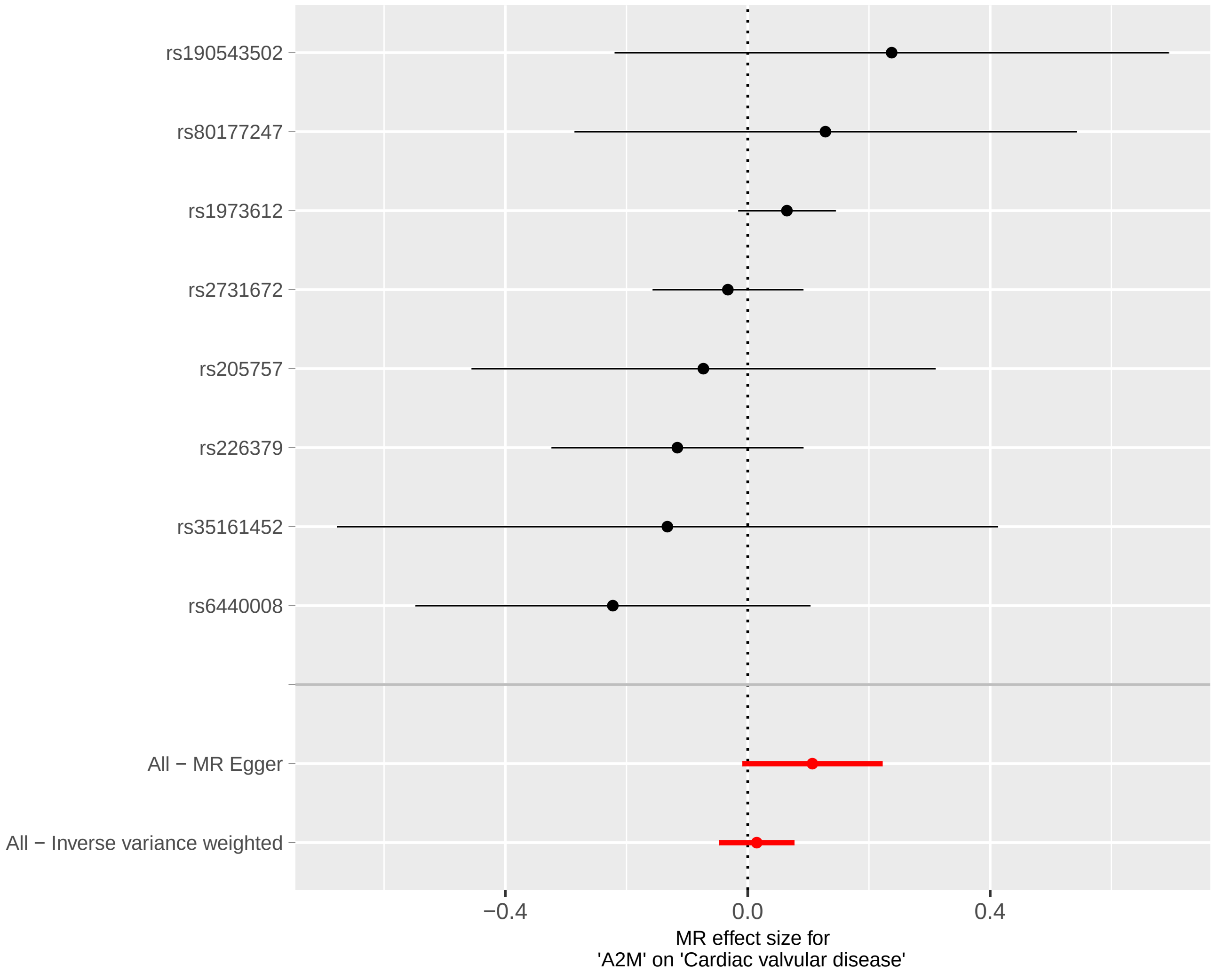
rs190543502
rs80177247
rs1973612
rs2731672
rs205757
rs226379
rs35161452
rs6440008
All − MR Egger
All − Inverse variance weighted
−0.4
0.0
0.4
MR effect size for
'A2M' on 'Cardiac valvular disease'

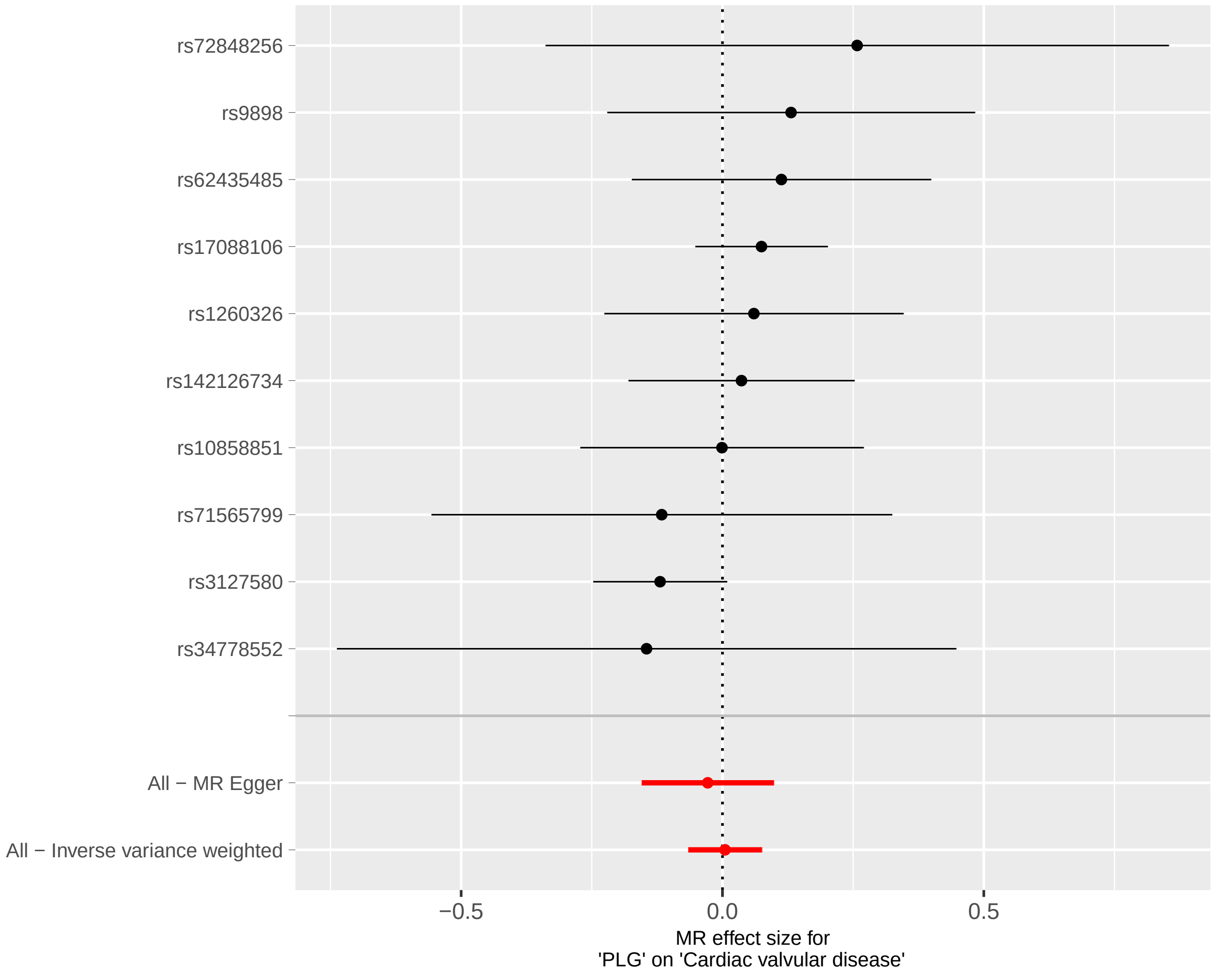
rs72848256
rs9898
rs62435485
rs17088106
rs1260326
rs142126734
rs10858851
rs71565799
rs3127580
rs34778552
All − MR Egger
All − Inverse variance weighted
−0.5
0.0
0.5
MR effect size for
'PLG' on 'Cardiac valvular disease'

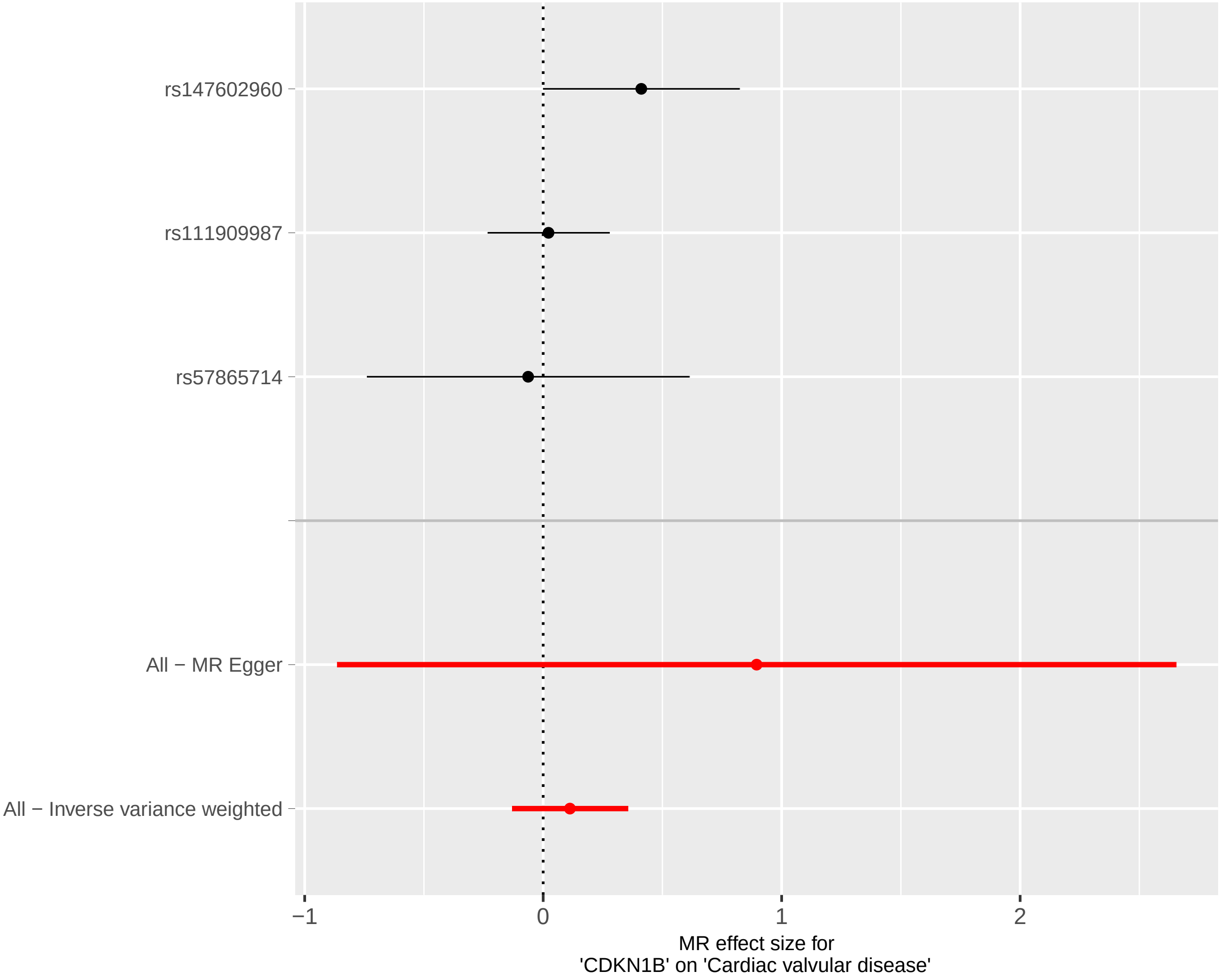
rs147602960
rs111909987
rs57865714
All − MR Egger
All − Inverse variance weighted
−1
0
1
2
MR effect size for
'CDKN1B' on 'Cardiac valvular disease'

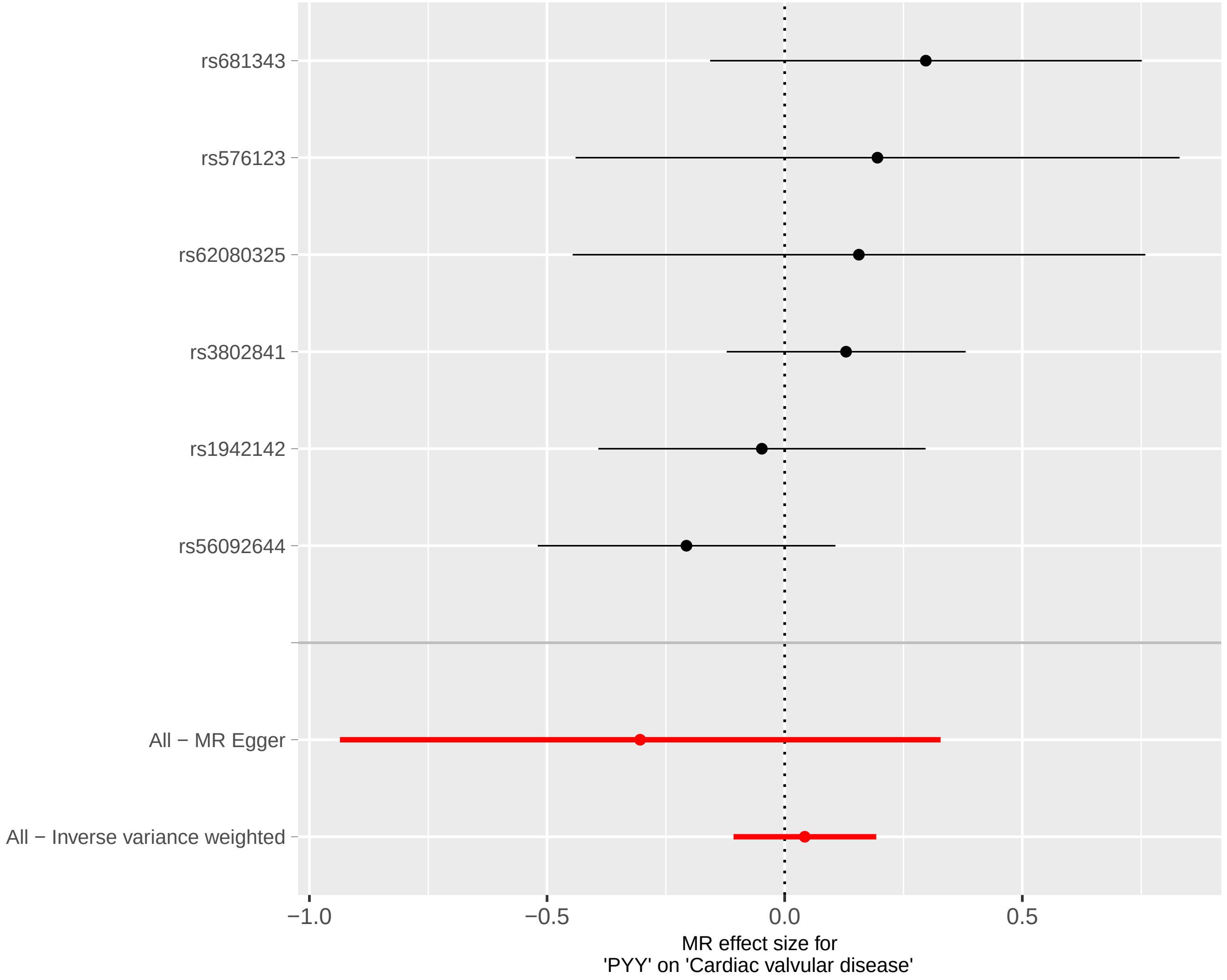
rs681343
rs576123
rs62080325
rs3802841
rs1942142
rs56092644
All − MR Egger
All − Inverse variance weighted
−1.0
−0.5
0.0
0.5
MR effect size for
'PYY' on 'Cardiac valvular disease'

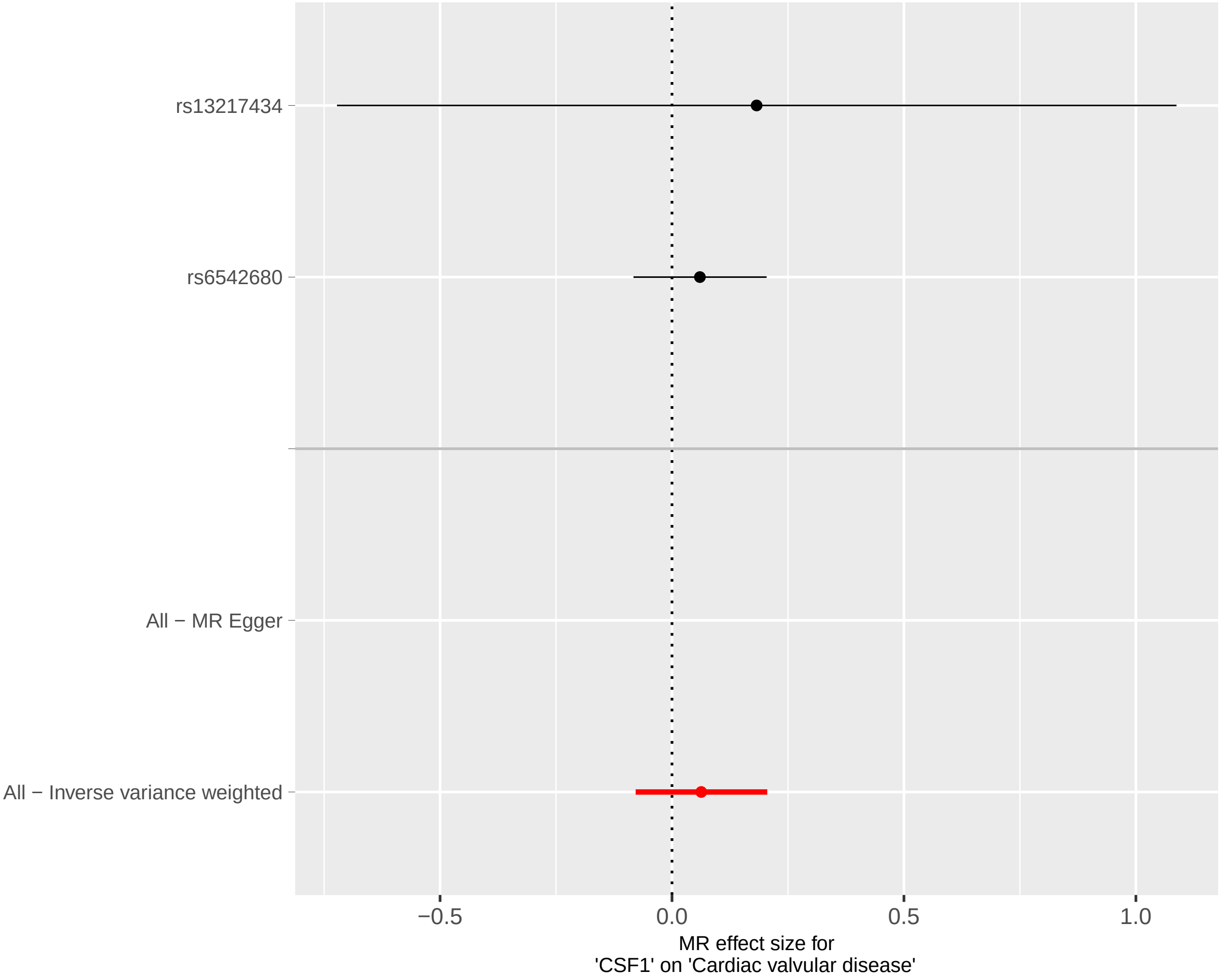
rs13217434
rs6542680
All − MR Egger
All − Inverse variance weighted
−0.5
0.0
0.5
1.0
MR effect size for
'CSF1' on 'Cardiac valvular disease'

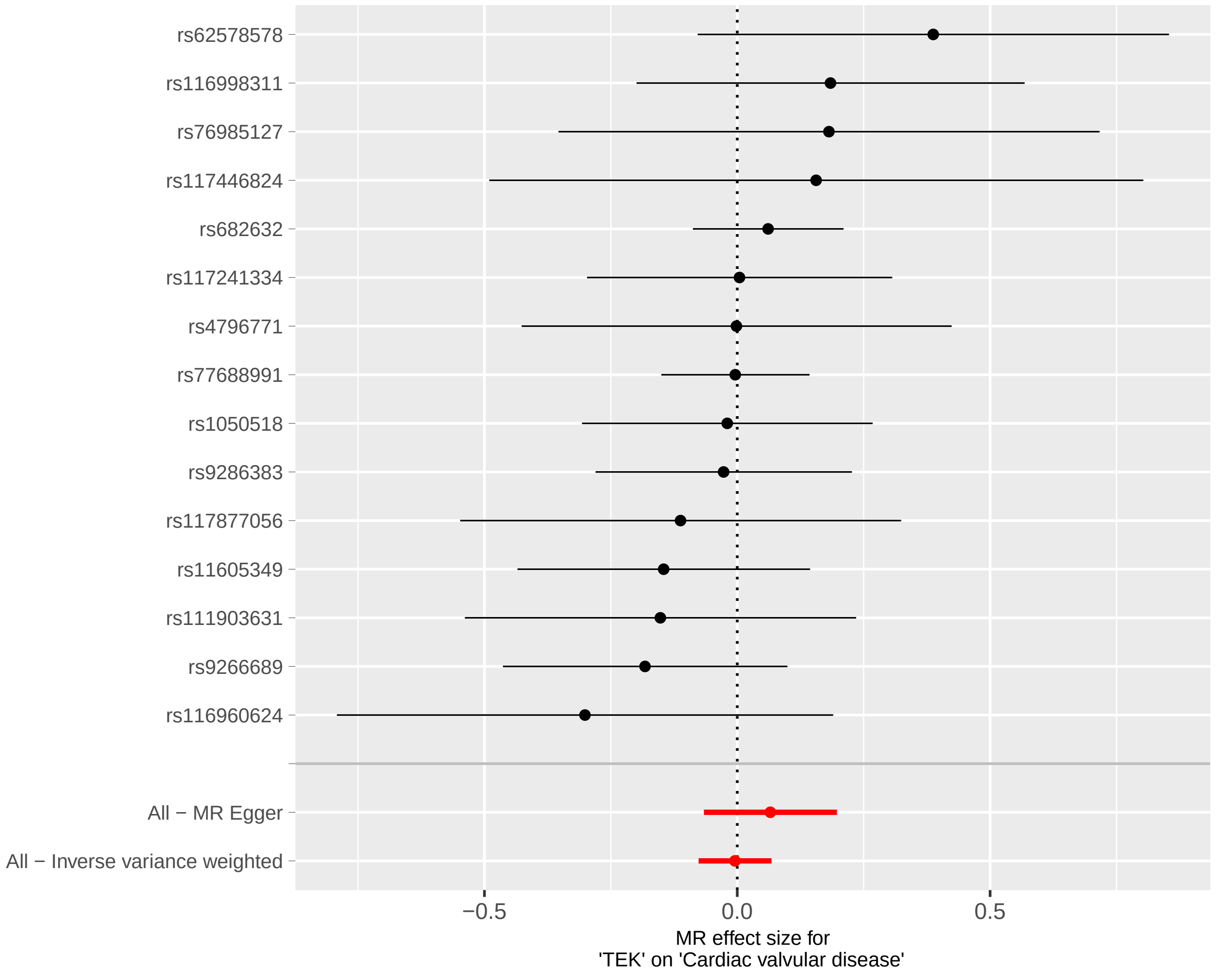
rs62578578
rs116998311
rs76985127
rs117446824
rs682632
rs117241334
rs4796771
rs77688991
rs1050518
rs9286383
rs117877056
rs11605349
rs111903631
rs9266689
rs116960624
All − MR Egger
All − Inverse variance weighted
−0.5
0.0
0.5
MR effect size for
'TEK' on 'Cardiac valvular disease'

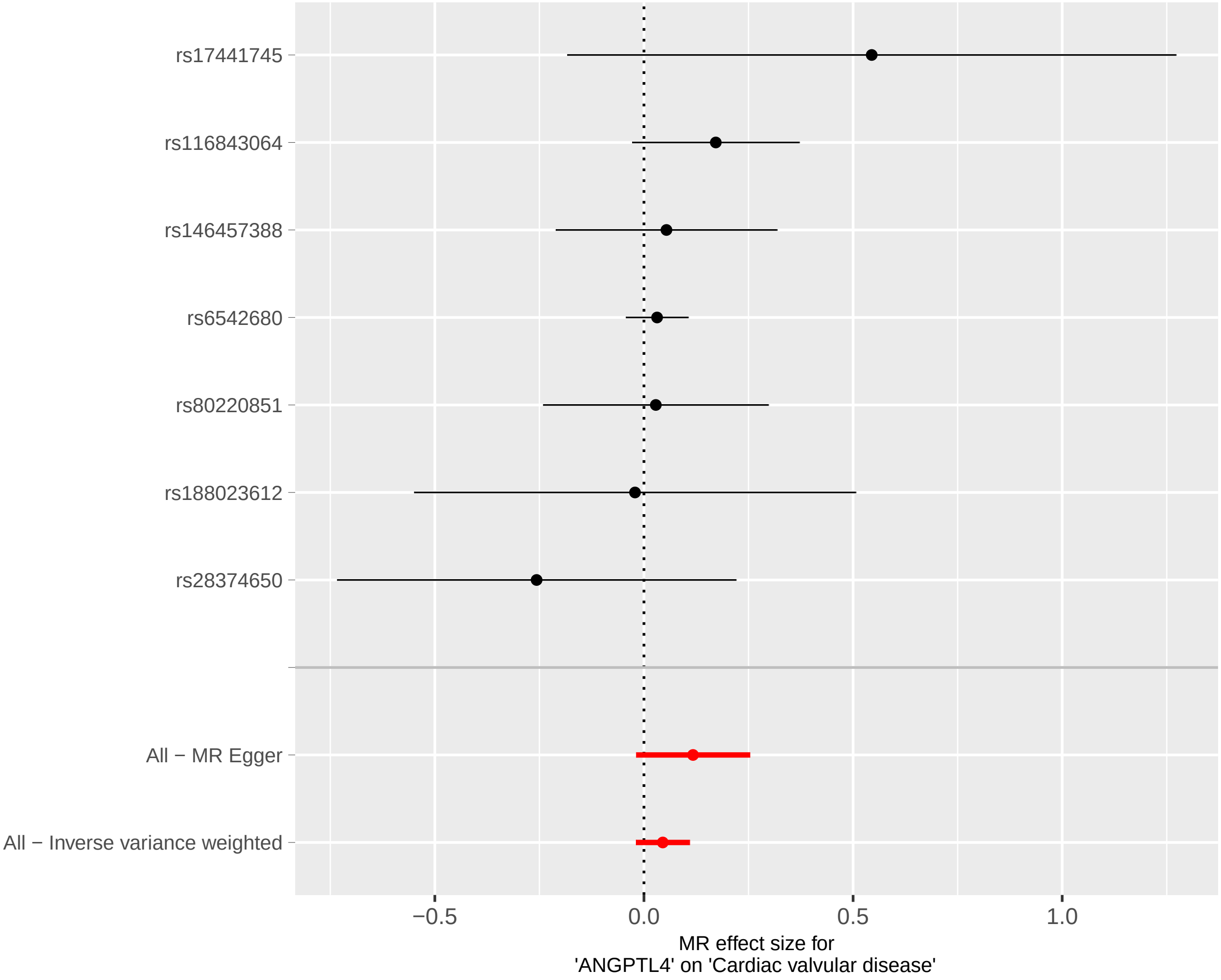
rs17441745
rs116843064
rs146457388
rs6542680
rs80220851
rs188023612
rs28374650
All − MR Egger
All − Inverse variance weighted
−0.5
0.0
0.5
1.0
MR effect size for
'ANGPTL4' on 'Cardiac valvular disease'

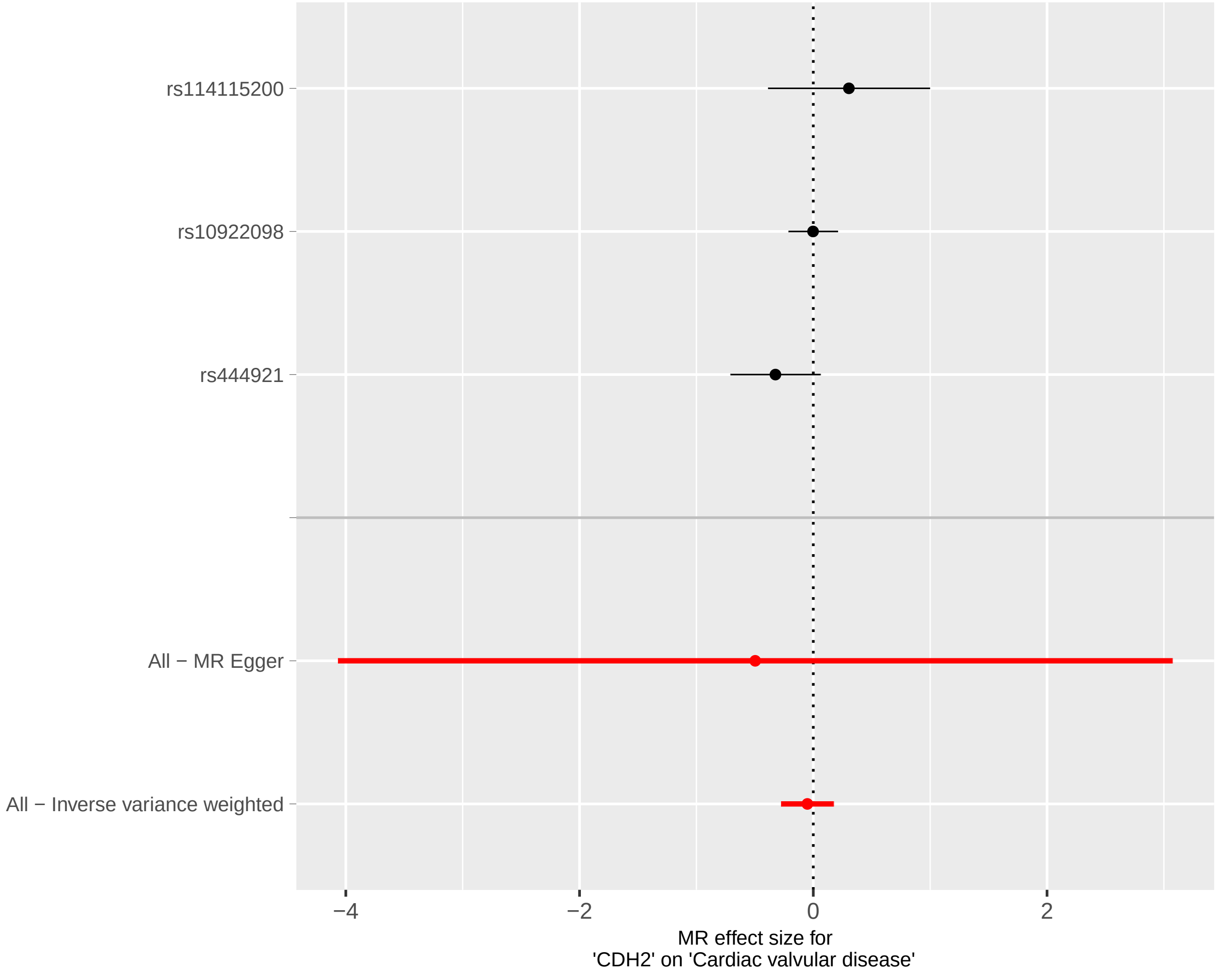

rs114115200
rs10922098
rs444921
All − MR Egger
All − Inverse variance weighted
−4
−2
0
2
MR effect size for
'CDH2' on 'Cardiac valvular disease'

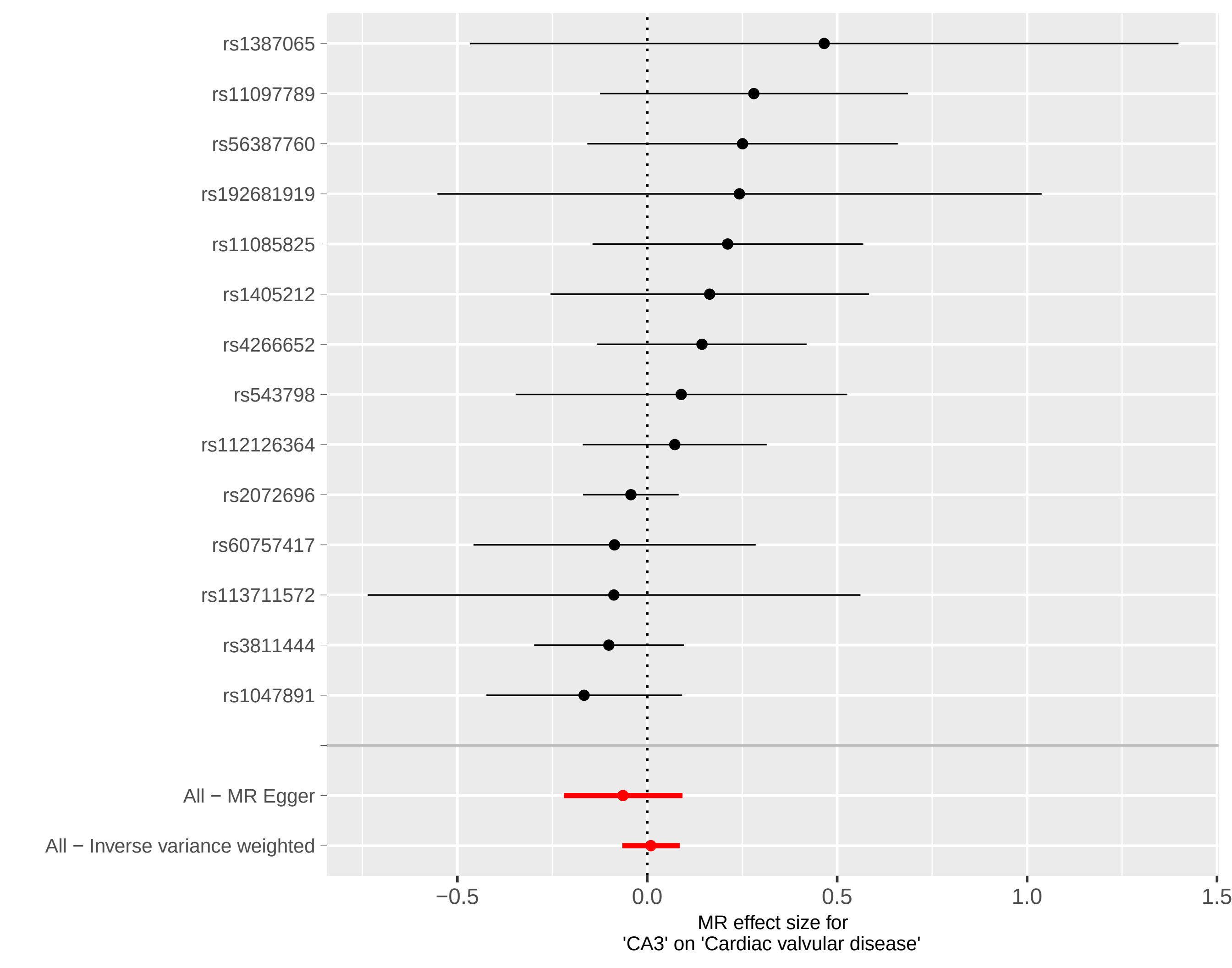
rs1387065
rs11097789
rs56387760
rs192681919
rs11085825
rs1405212
rs4266652
rs543798
rs112126364
rs2072696
rs60757417
rs113711572
rs3811444
rs1047891
All − MR Egger
All − Inverse variance weighted
−0.5
0.0
0.5
1.0
1.5
MR effect size for
'CA3' on 'Cardiac valvular disease'

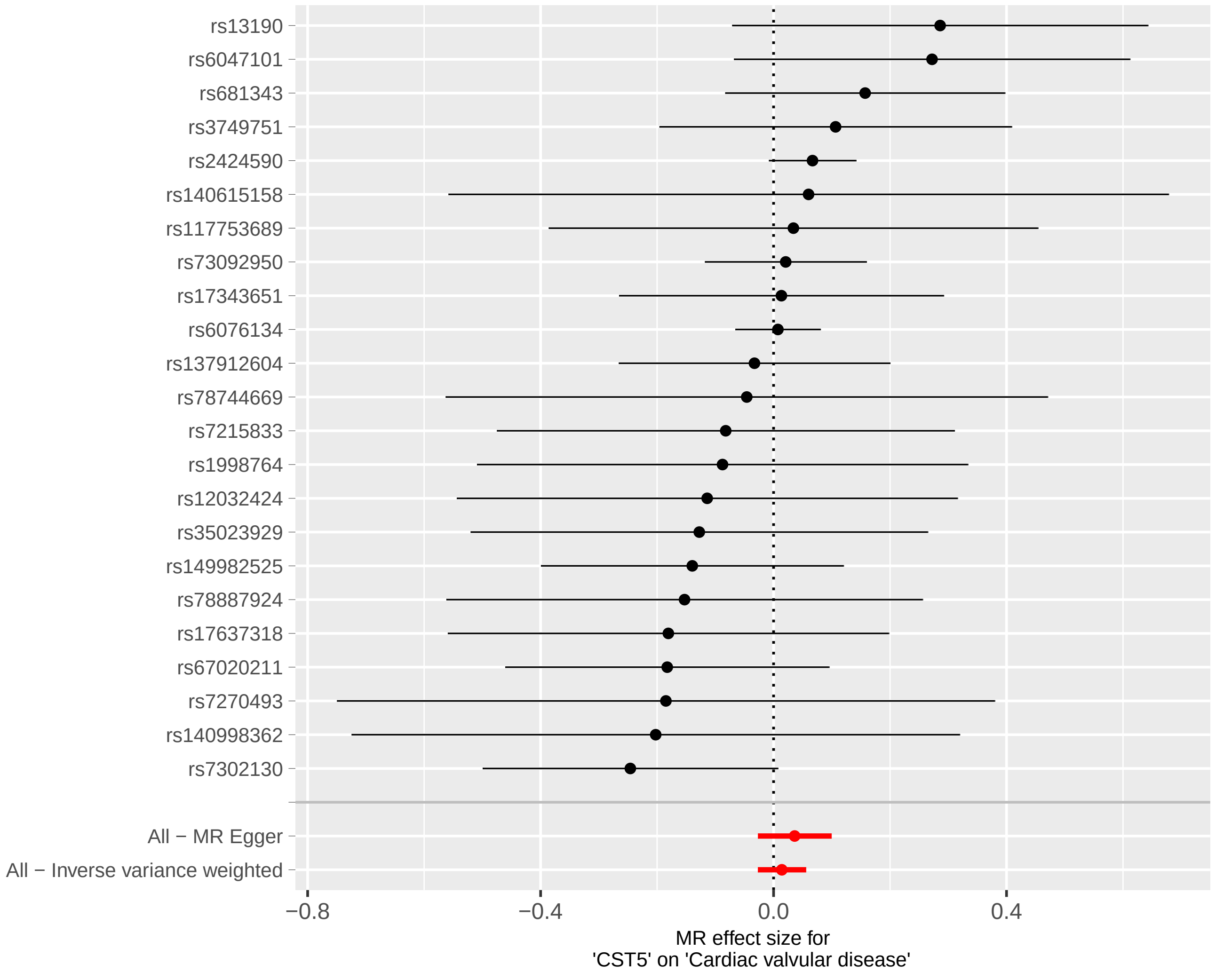

rs13190
rs6047101
rs681343
rs3749751
rs2424590
rs140615158
rs117753689
rs73092950
rs17343651
rs6076134
rs137912604
rs78744669
rs7215833
rs1998764
rs12032424
rs35023929
rs149982525
rs78887924
rs17637318
rs67020211
rs7270493
rs140998362
rs7302130
All − MR Egger
All − Inverse variance weighted
−0.8
−0.4
0.0
0.4
MR effect size for
'CST5' on 'Cardiac valvular disease'

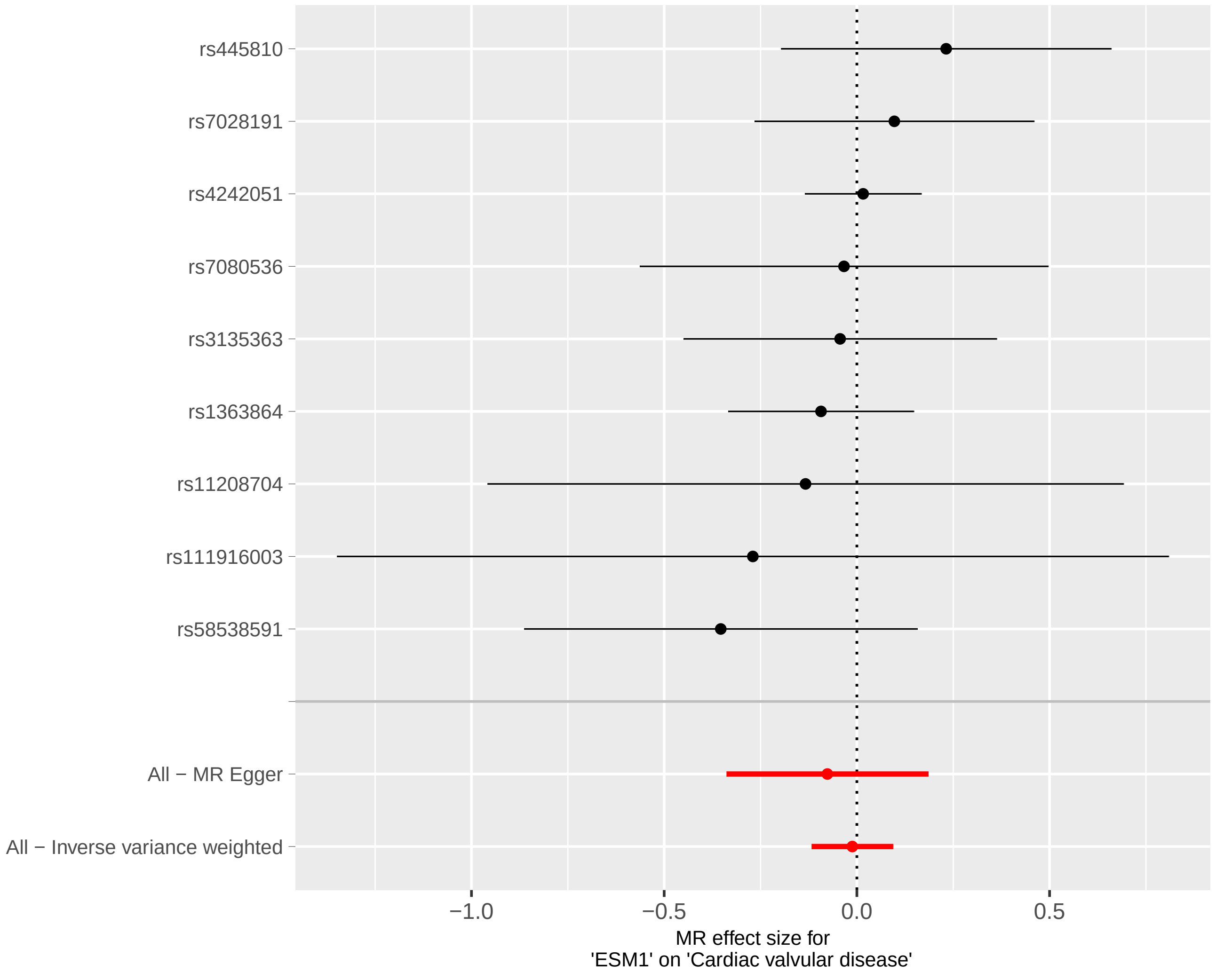
rs445810
rs7028191
rs4242051
rs7080536
rs3135363
rs1363864
rs11208704
rs111916003
rs58538591
All − MR Egger
All − Inverse variance weighted
−1.0
−0.5
0.0
0.5
MR effect size for
'ESM1' on 'Cardiac valvular disease'

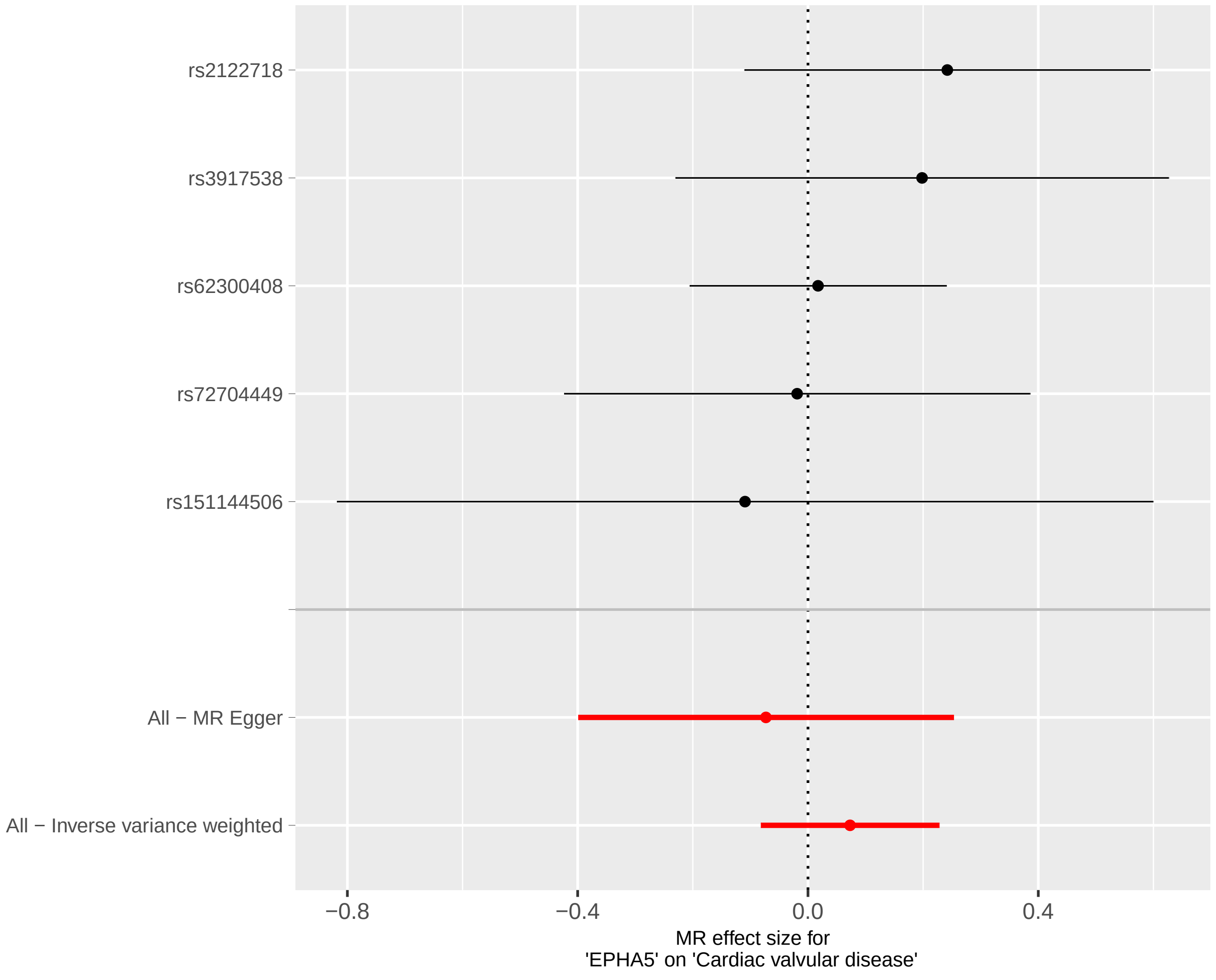
rs2122718
rs3917538
rs62300408
rs72704449
rs151144506
All − MR Egger
All − Inverse variance weighted
−0.8
−0.4
0.0
0.4
MR effect size for
'EPHA5' on 'Cardiac valvular disease'

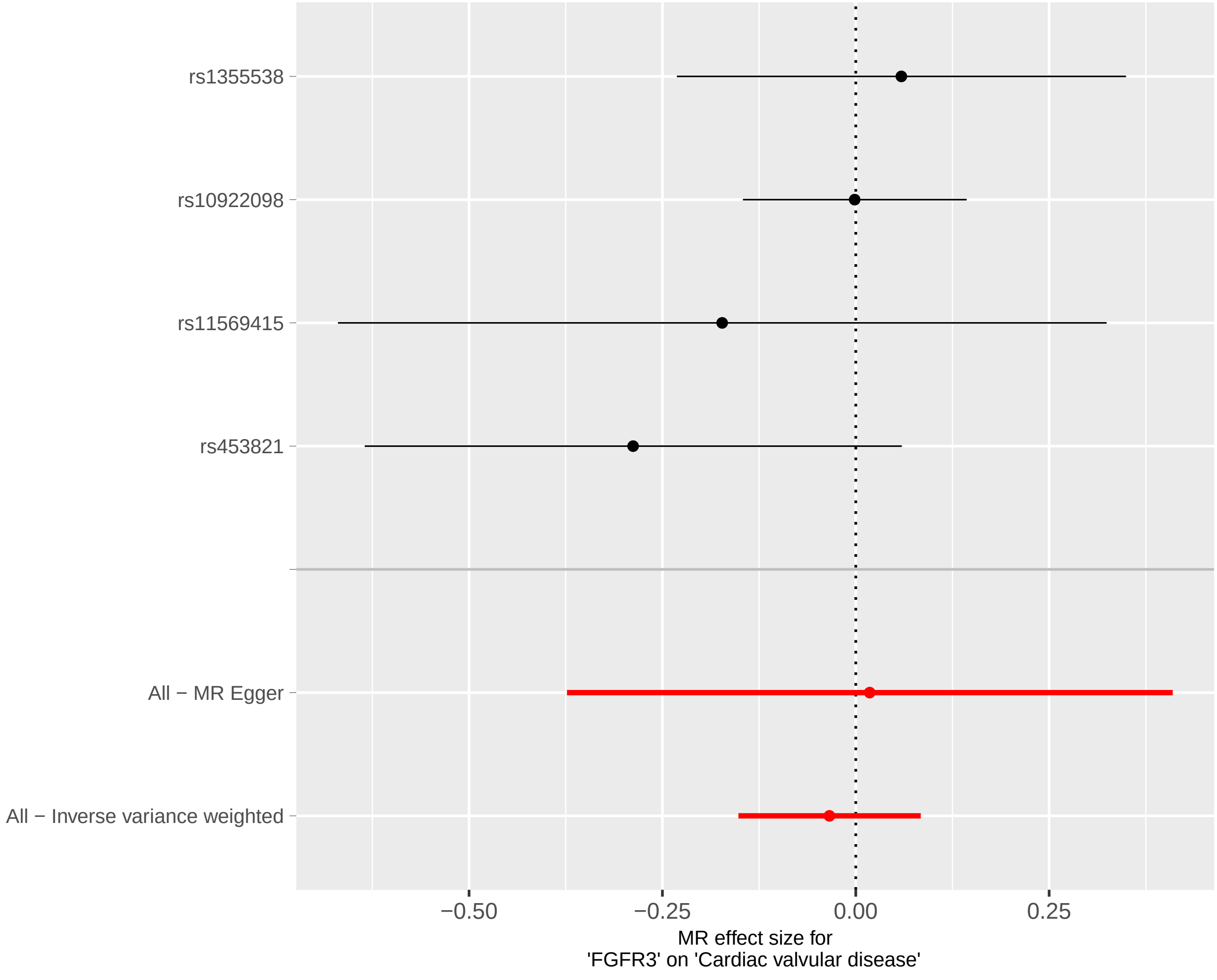
rs1355538
rs10922098
rs11569415
rs453821
All − MR Egger
All − Inverse variance weighted
−0.50
−0.25
0.00
0.25
MR effect size for
'FGFR3' on 'Cardiac valvular disease'

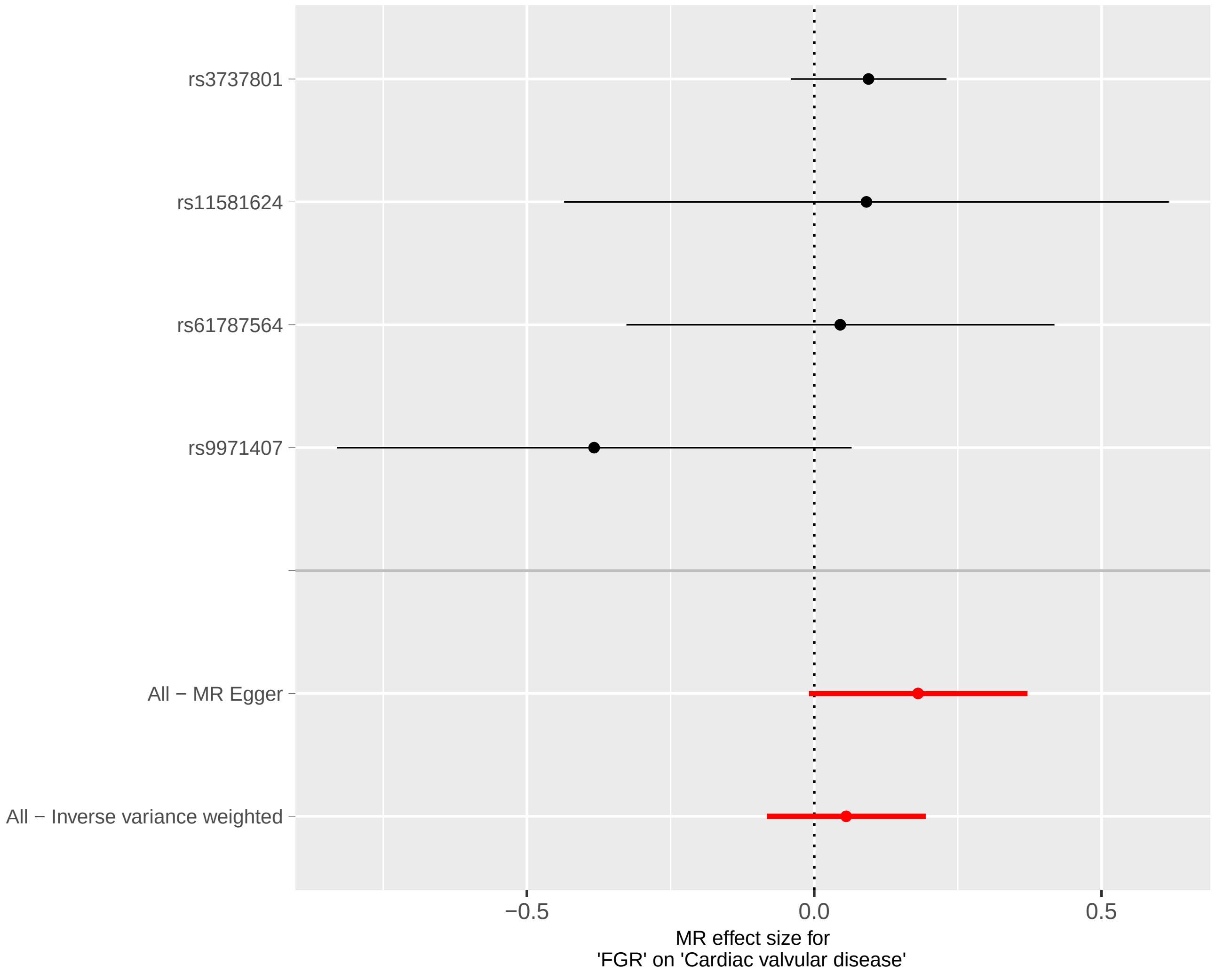
rs3737801
rs11581624
rs61787564
rs9971407
All − MR Egger
All − Inverse variance weighted
−0.5
0.0
0.5
MR effect size for
'FGR' on 'Cardiac valvular disease'

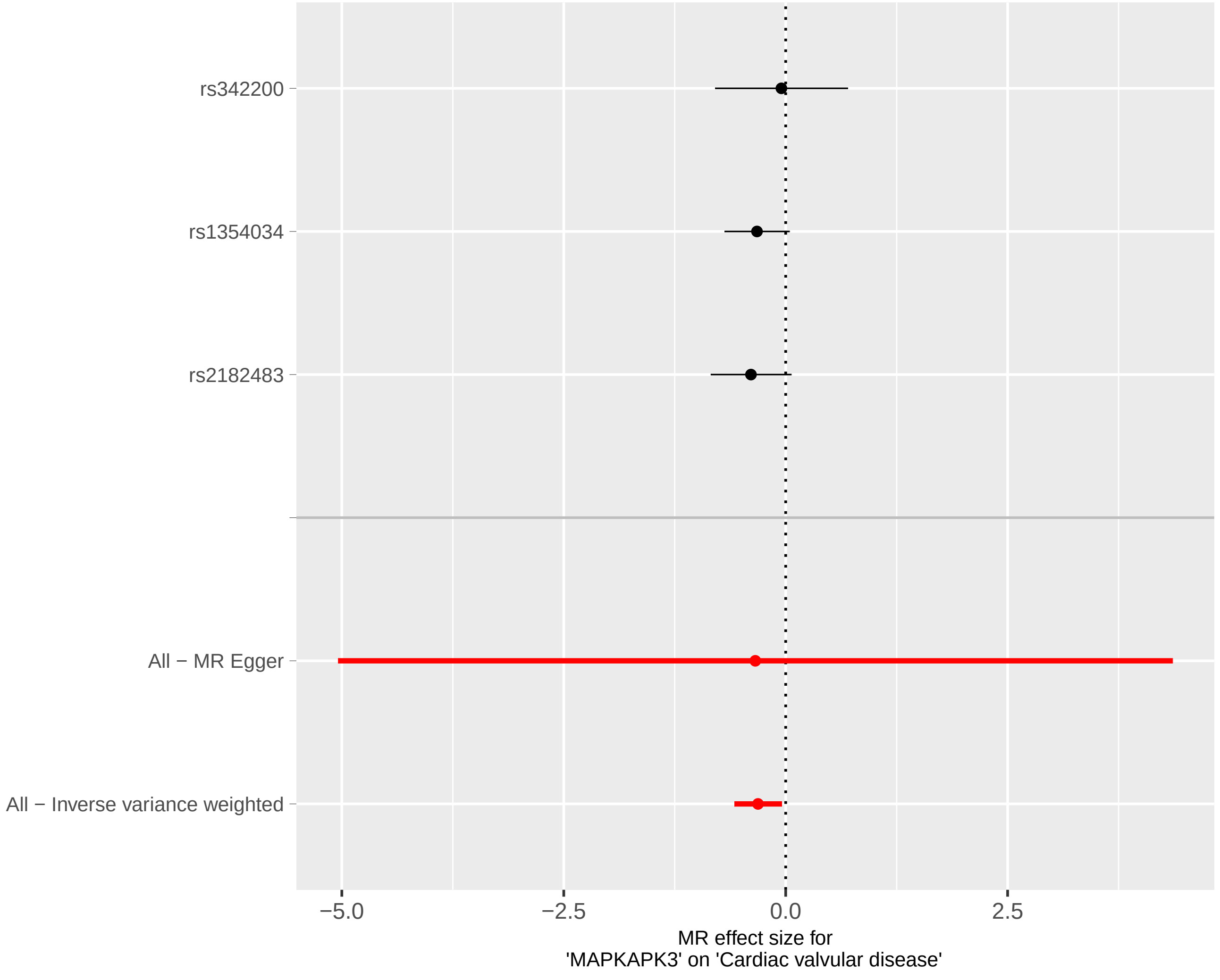
rs342200
rs1354034
rs2182483
All − MR Egger
All − Inverse variance weighted
−5.0
−2.5
0.0
2.5
MR effect size for
'MAPKAPK3' on 'Cardiac valvular disease'

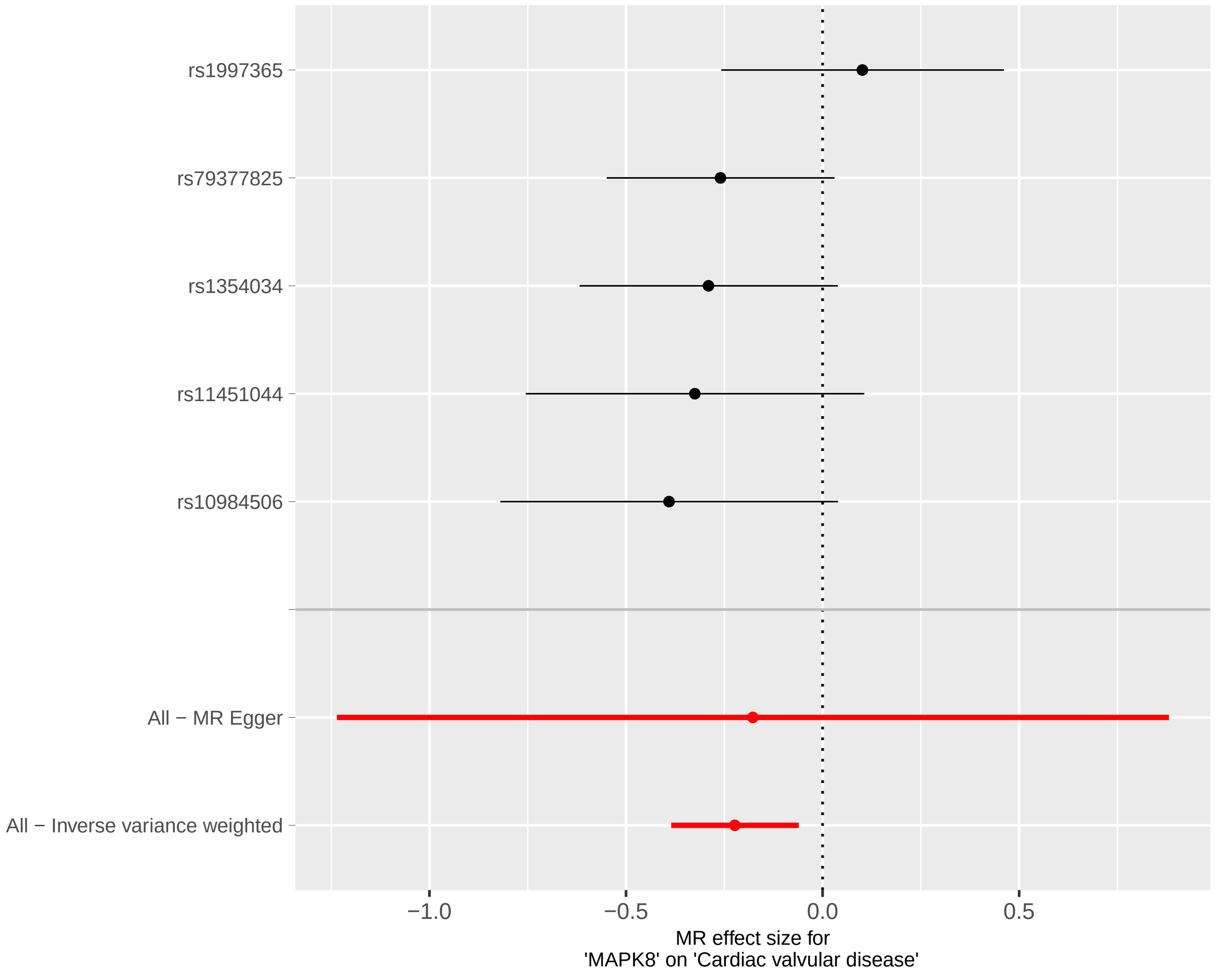
rs1997365
rs79377825
rs1354034
rs11451044
rs10984506
All − MR Egger
All − Inverse variance weighted
−1.0
−0.5
0.0
0.5
MR effect size for
'MAPK8' on 'Cardiac valvular disease'

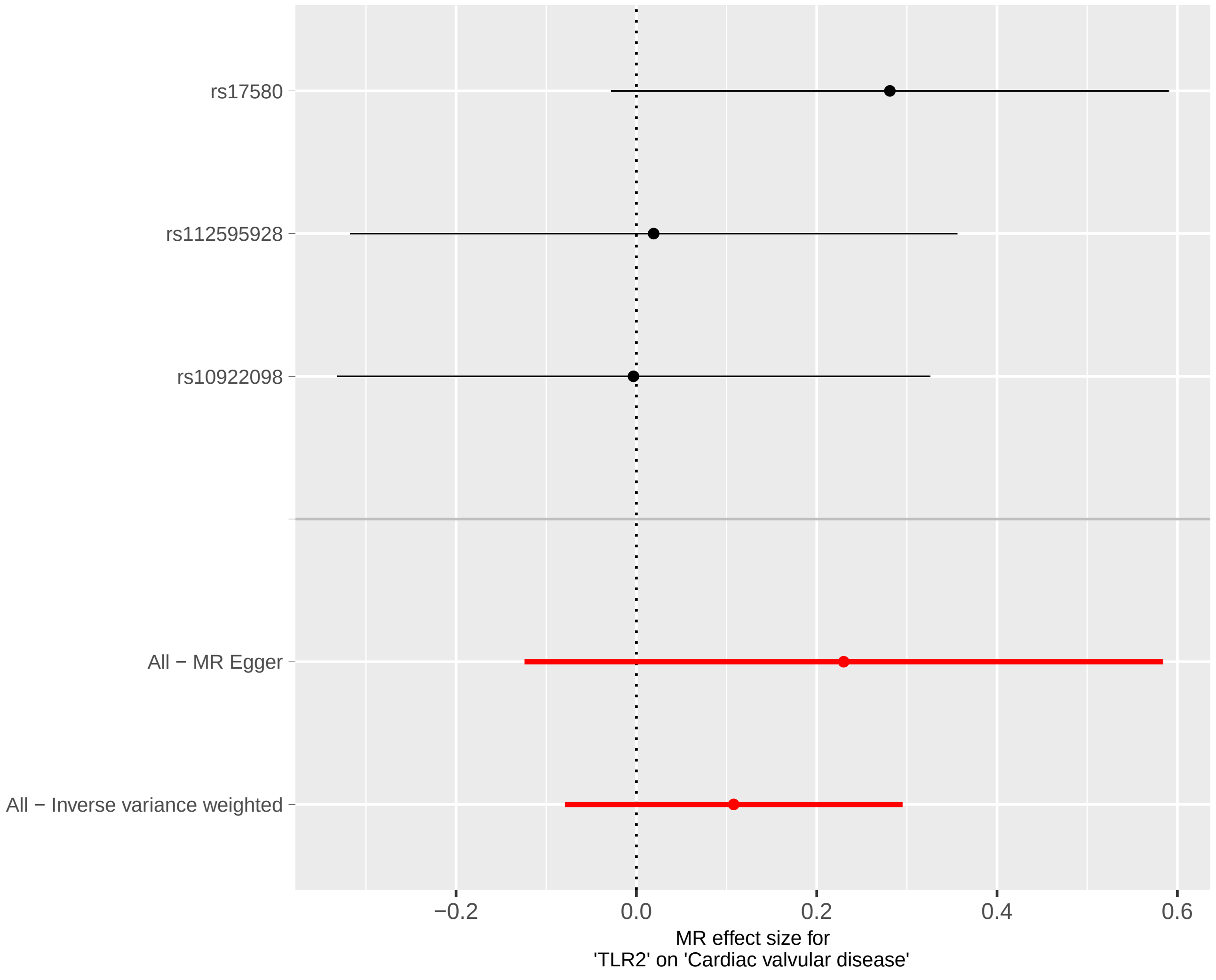
rs17580
rs112595928
rs10922098
All − MR Egger
All − Inverse variance weighted
−0.2
0.0
0.2
0.4
0.6
MR effect size for
'TLR2' on 'Cardiac valvular disease'

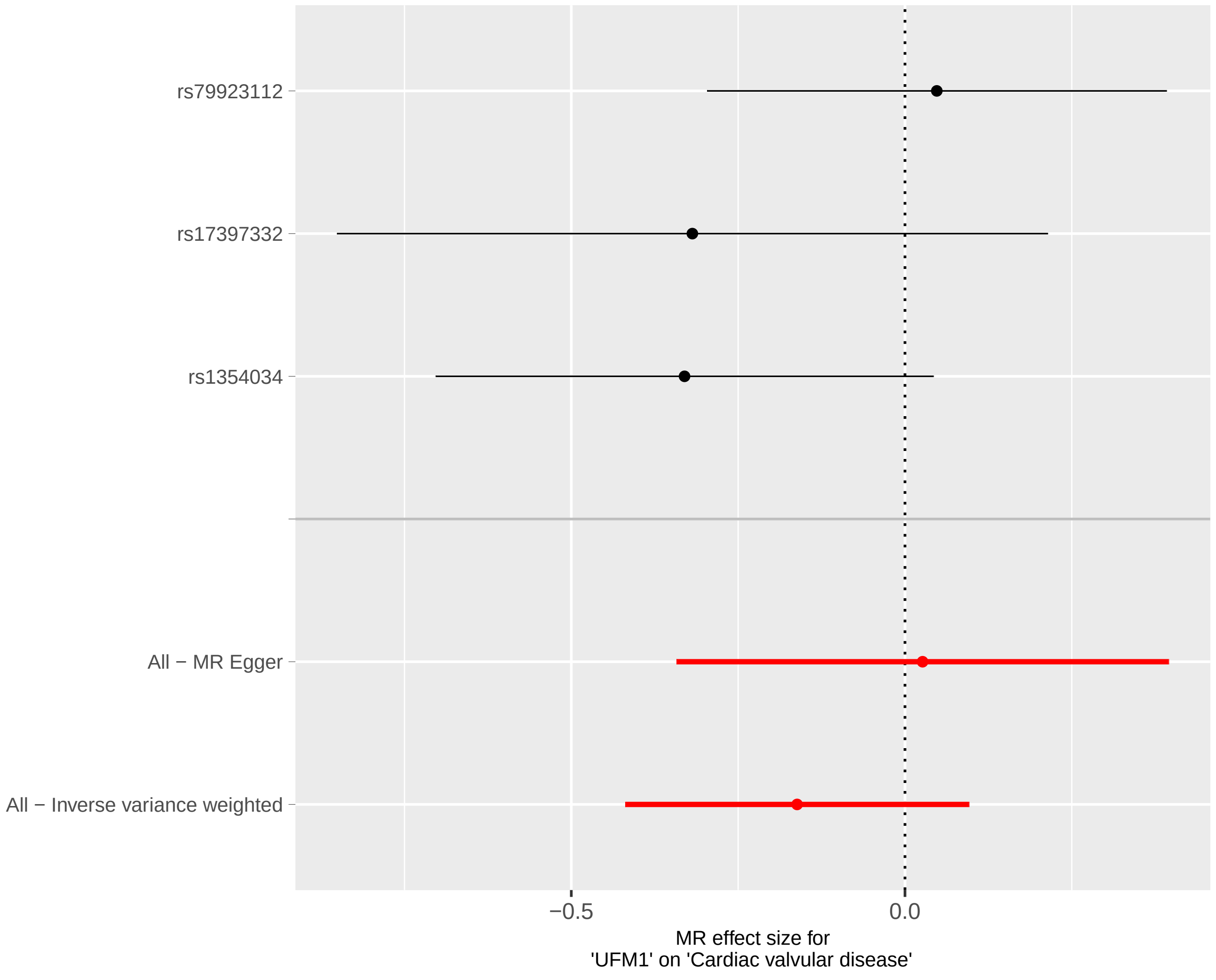
rs79923112
rs17397332
rs1354034
All − MR Egger
All − Inverse variance weighted
−0.5
0.0
MR effect size for
'UFM1' on 'Cardiac valvular disease'

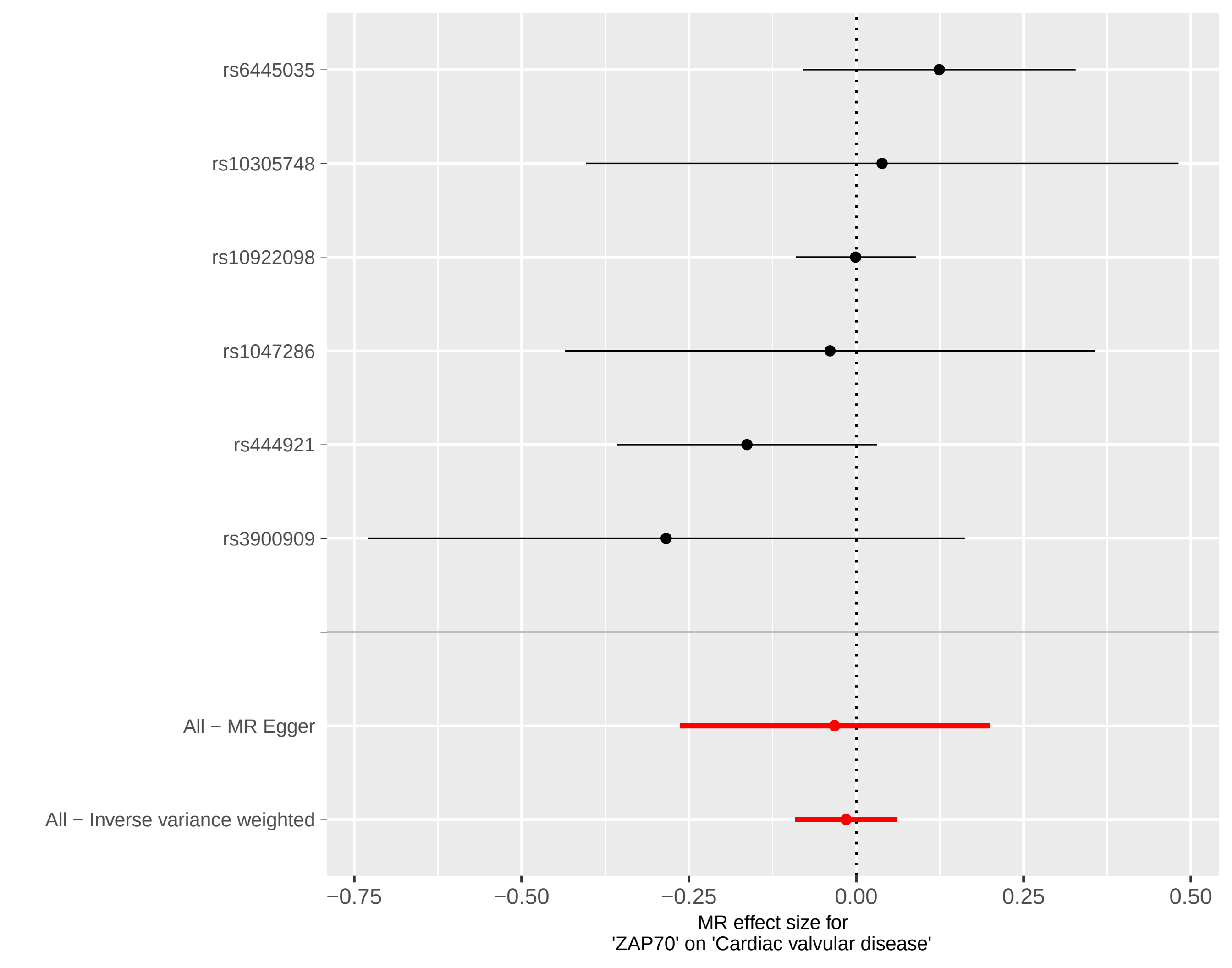
rs6445035
rs10305748
rs10922098
rs1047286
rs444921
rs3900909
All − MR Egger
All − Inverse variance weighted
−0.75
−0.50
−0.25
0.00
0.25
0.50
MR effect size for
'ZAP70' on 'Cardiac valvular disease'

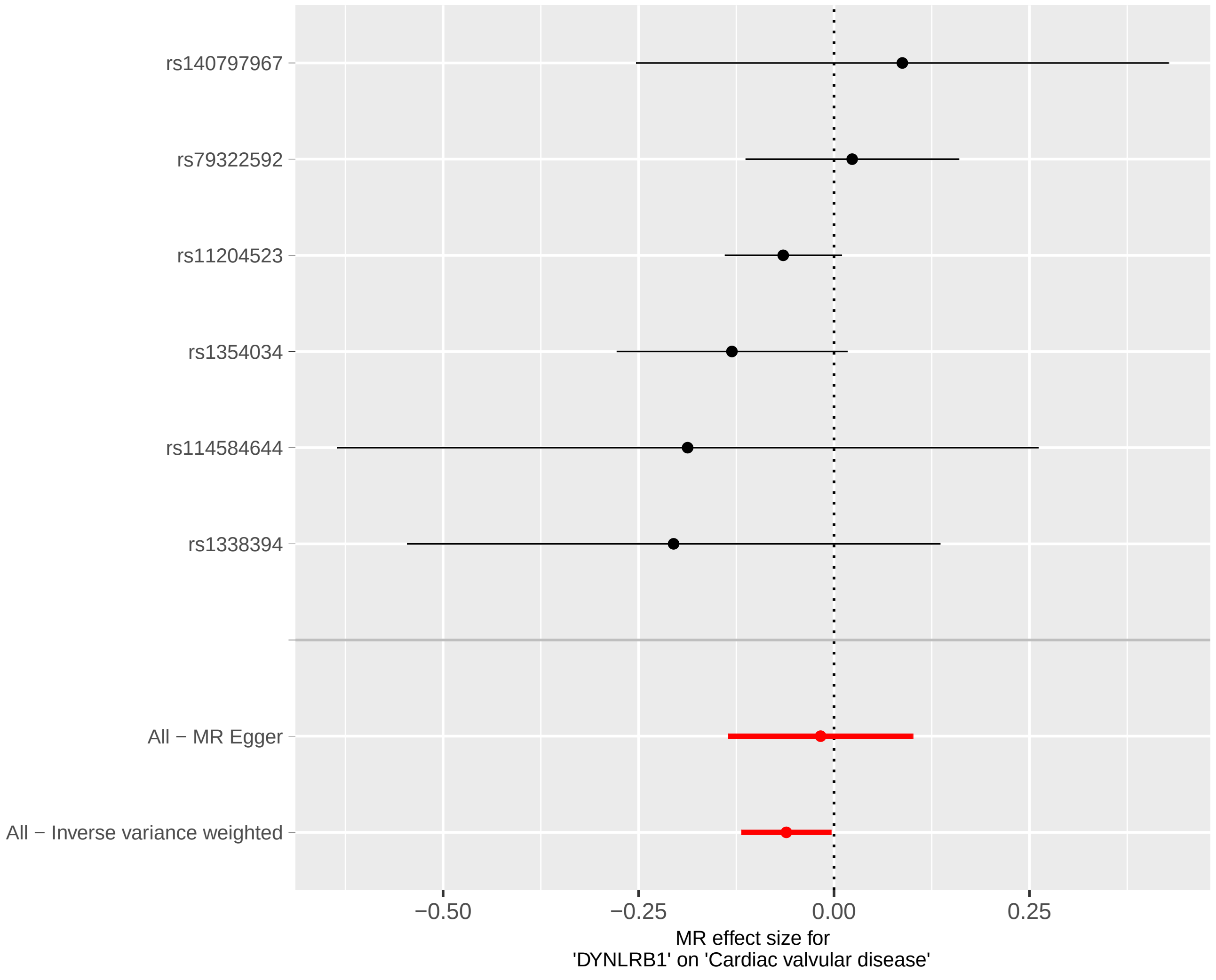
rs140797967
rs79322592
rs11204523
rs1354034
rs114584644
rs1338394
All − MR Egger
All − Inverse variance weighted
−0.50
−0.25
0.00
0.25
MR effect size for
'DYNLRB1' on 'Cardiac valvular disease'

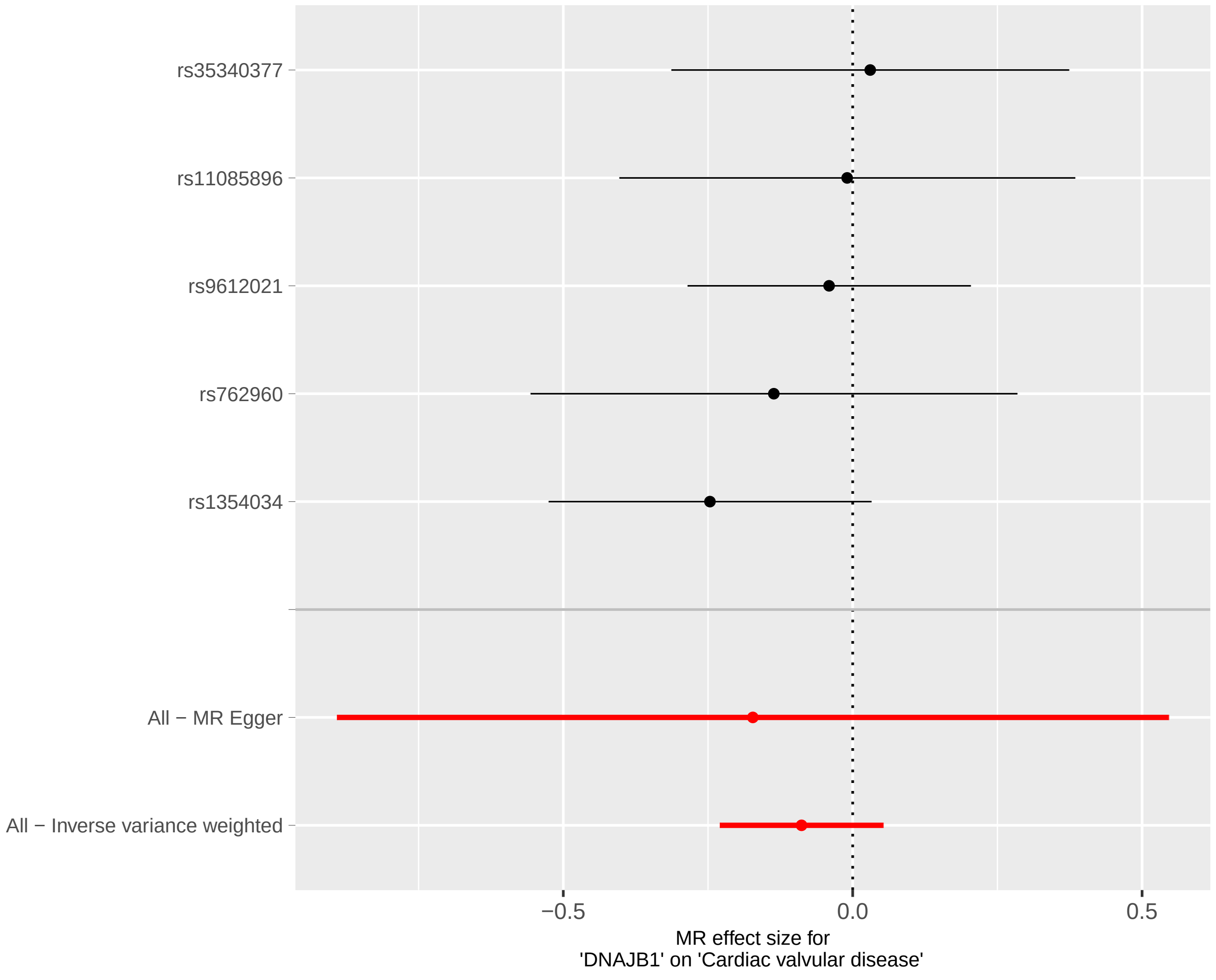
rs35340377
rs11085896
rs9612021
rs762960
rs1354034
All − MR Egger
All − Inverse variance weighted
−0.5
0.0
0.5
MR effect size for
'DNAJB1' on 'Cardiac valvular disease'

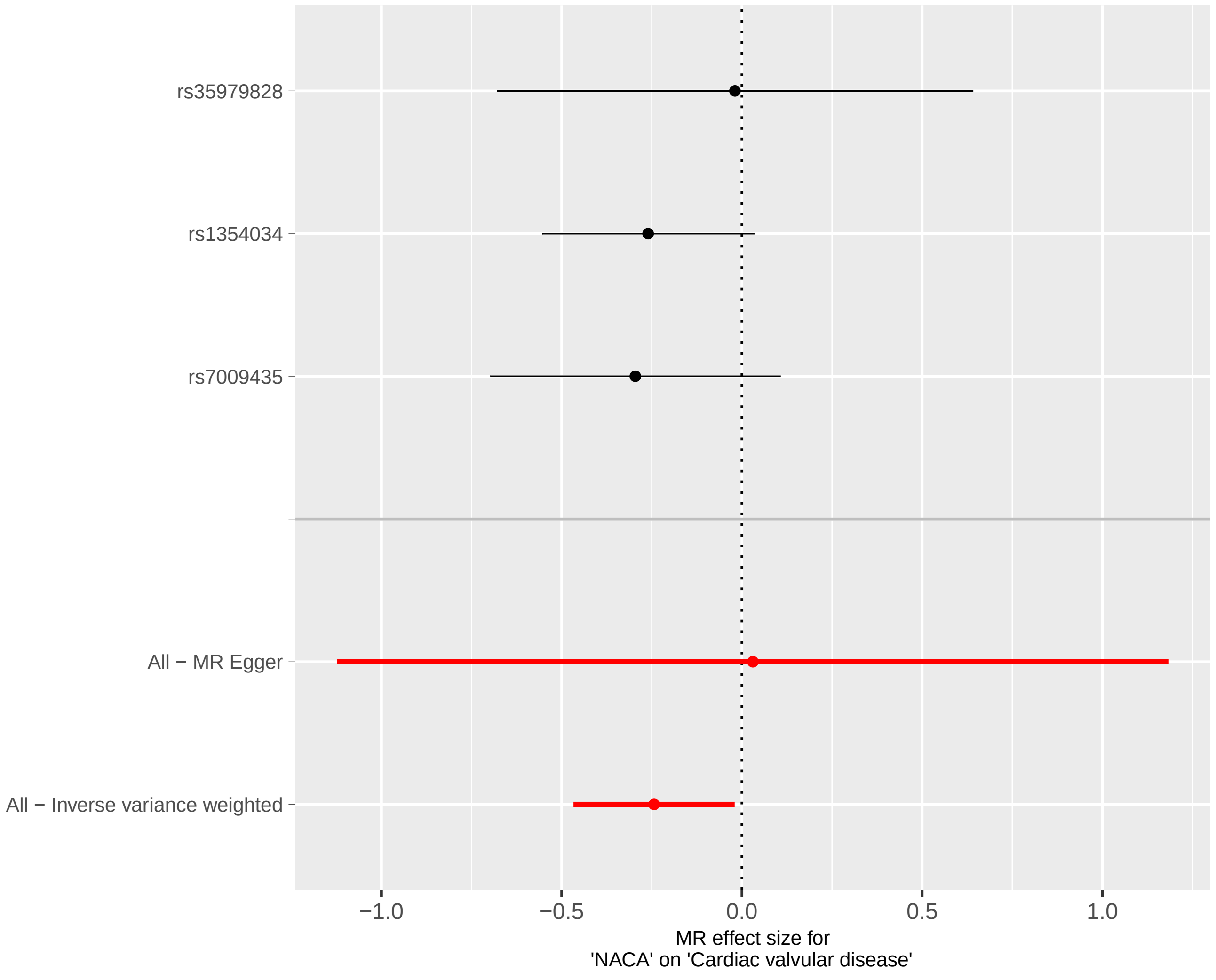
rs35979828
rs1354034
rs7009435
All − MR Egger
All − Inverse variance weighted
−1.0
−0.5
0.0
0.5
1.0
MR effect size for
'NACA' on 'Cardiac valvular disease'

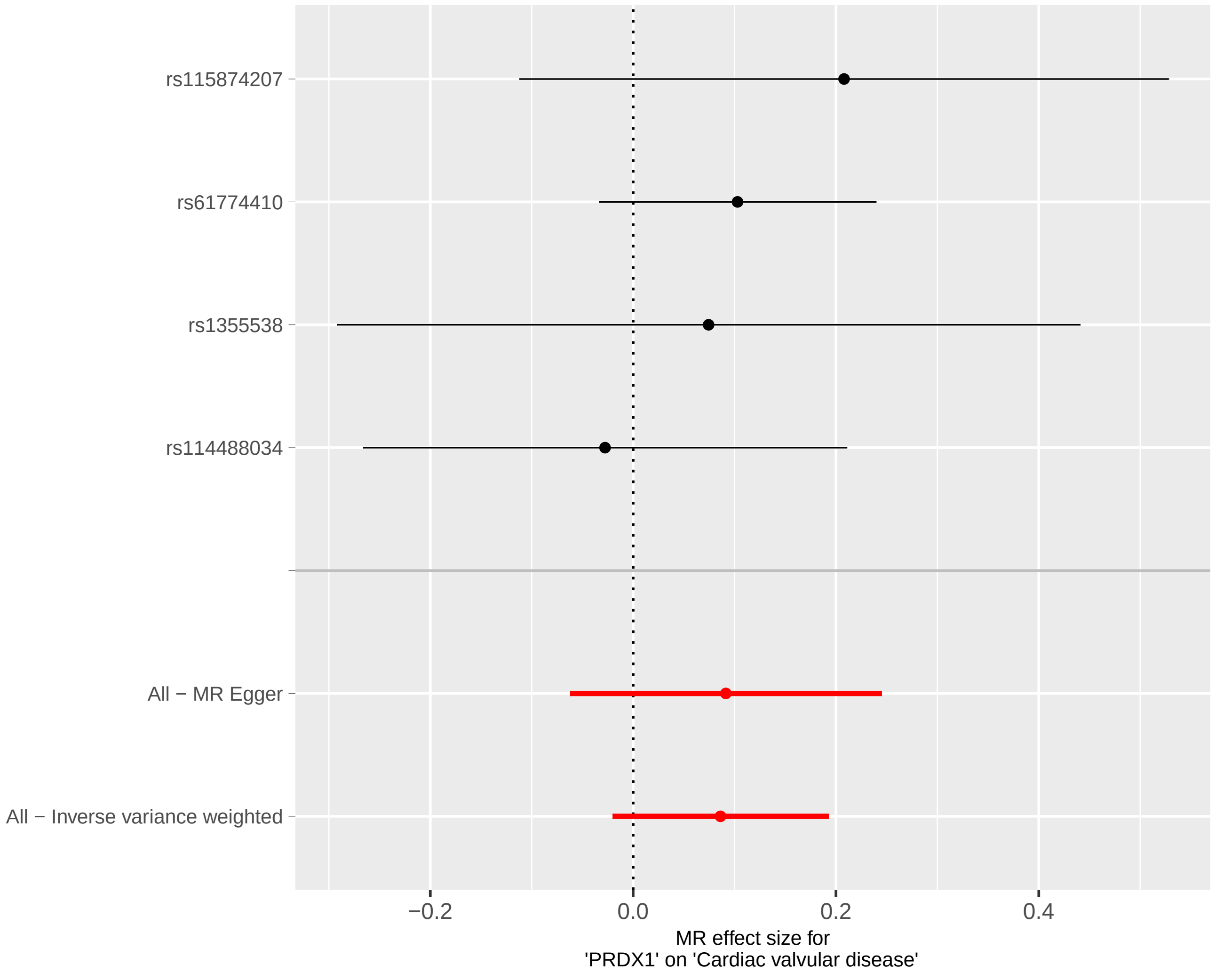
rs115874207
rs61774410
rs1355538
rs114488034
All − MR Egger
All − Inverse variance weighted
−0.2
0.0
0.2
0.4
MR effect size for
'PRDX1' on 'Cardiac valvular disease'

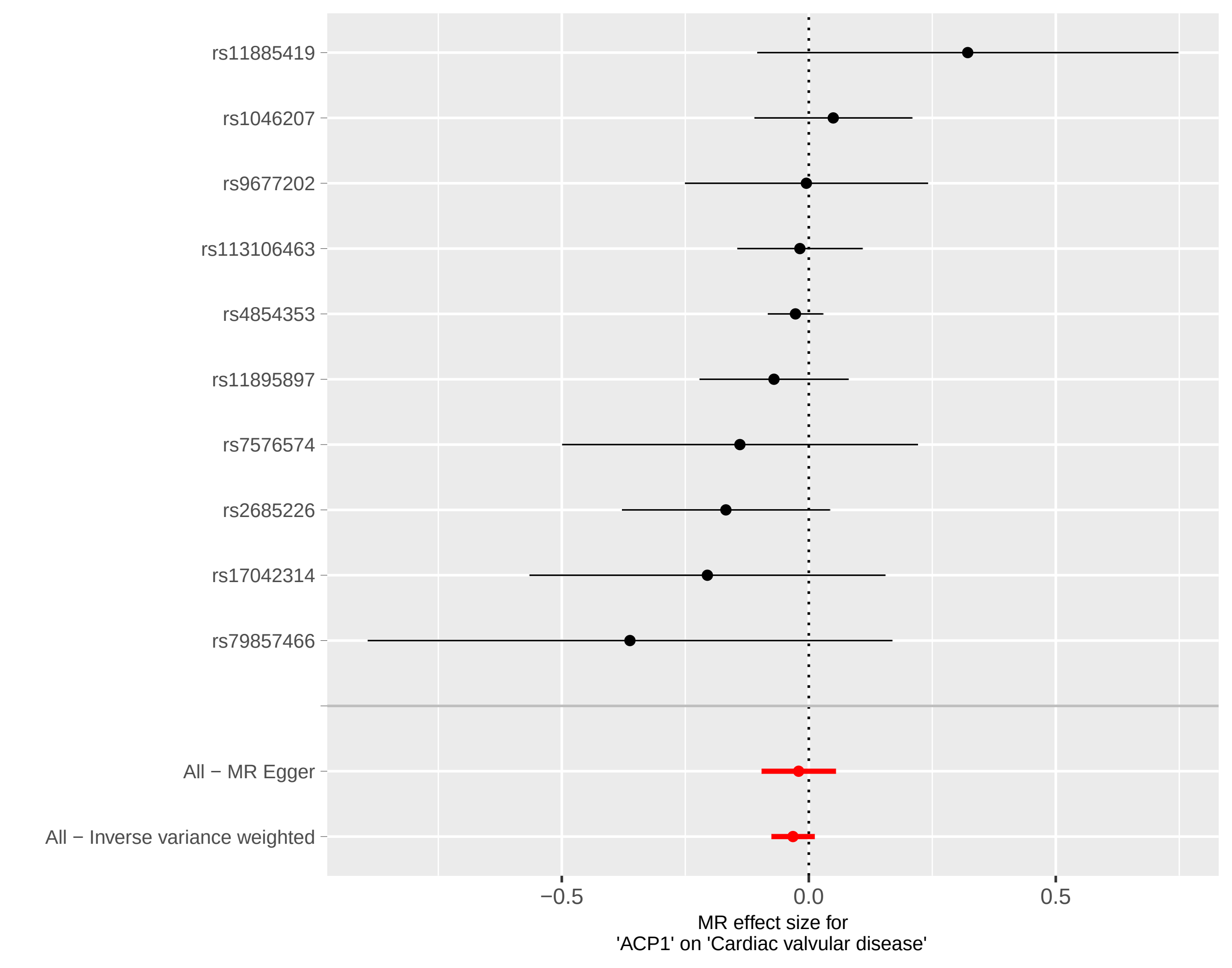
rs11885419
rs1046207
rs9677202
rs113106463
rs4854353
rs11895897
rs7576574
rs2685226
rs17042314
rs79857466
All − MR Egger
All − Inverse variance weighted
−0.5
0.0
0.5
MR effect size for
'ACP1' on 'Cardiac valvular disease'

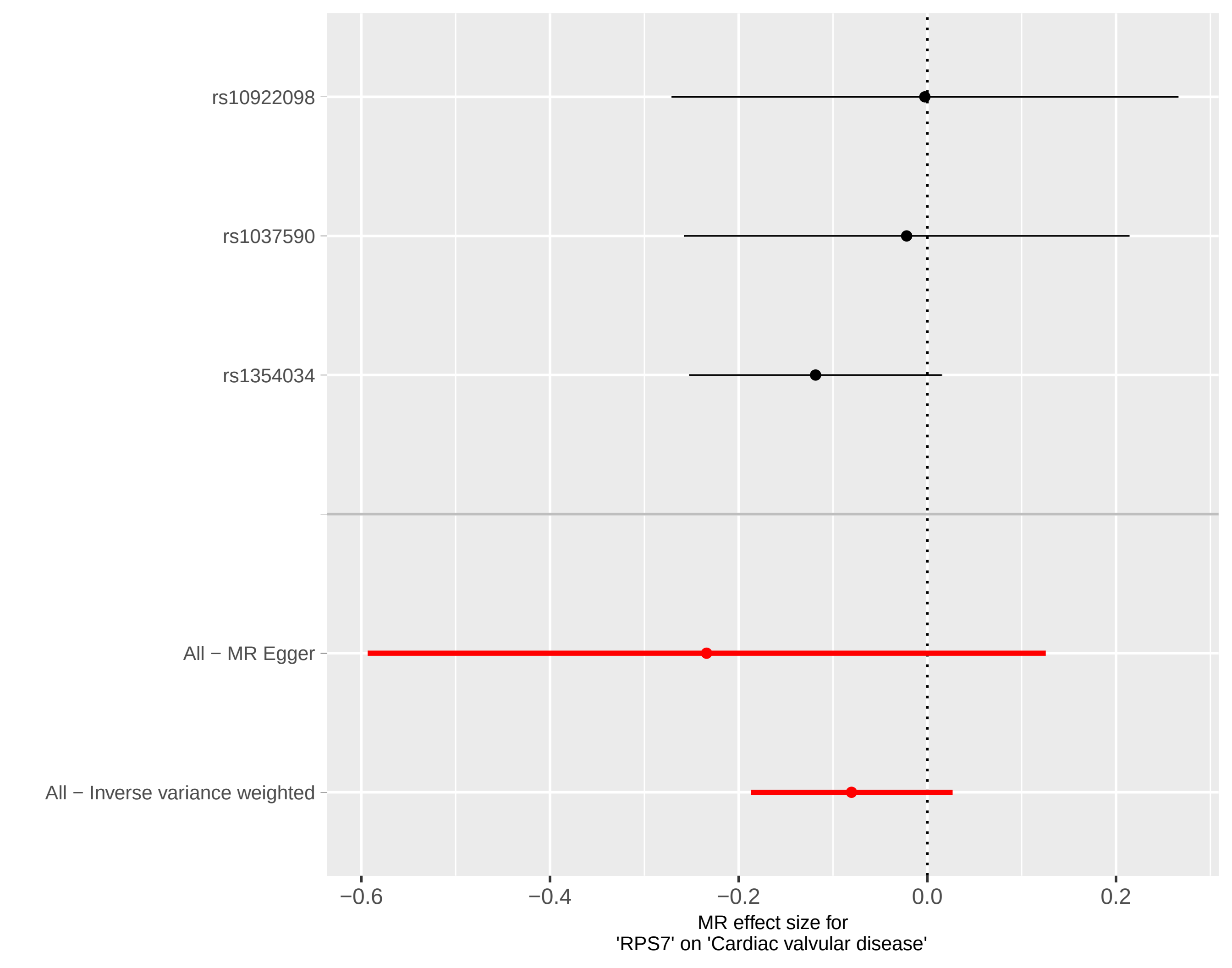
rs10922098
rs1037590
rs1354034
All − MR Egger
All − Inverse variance weighted
−0.6
−0.4
−0.2
0.0
0.2
MR effect size for
'RPS7' on 'Cardiac valvular disease'

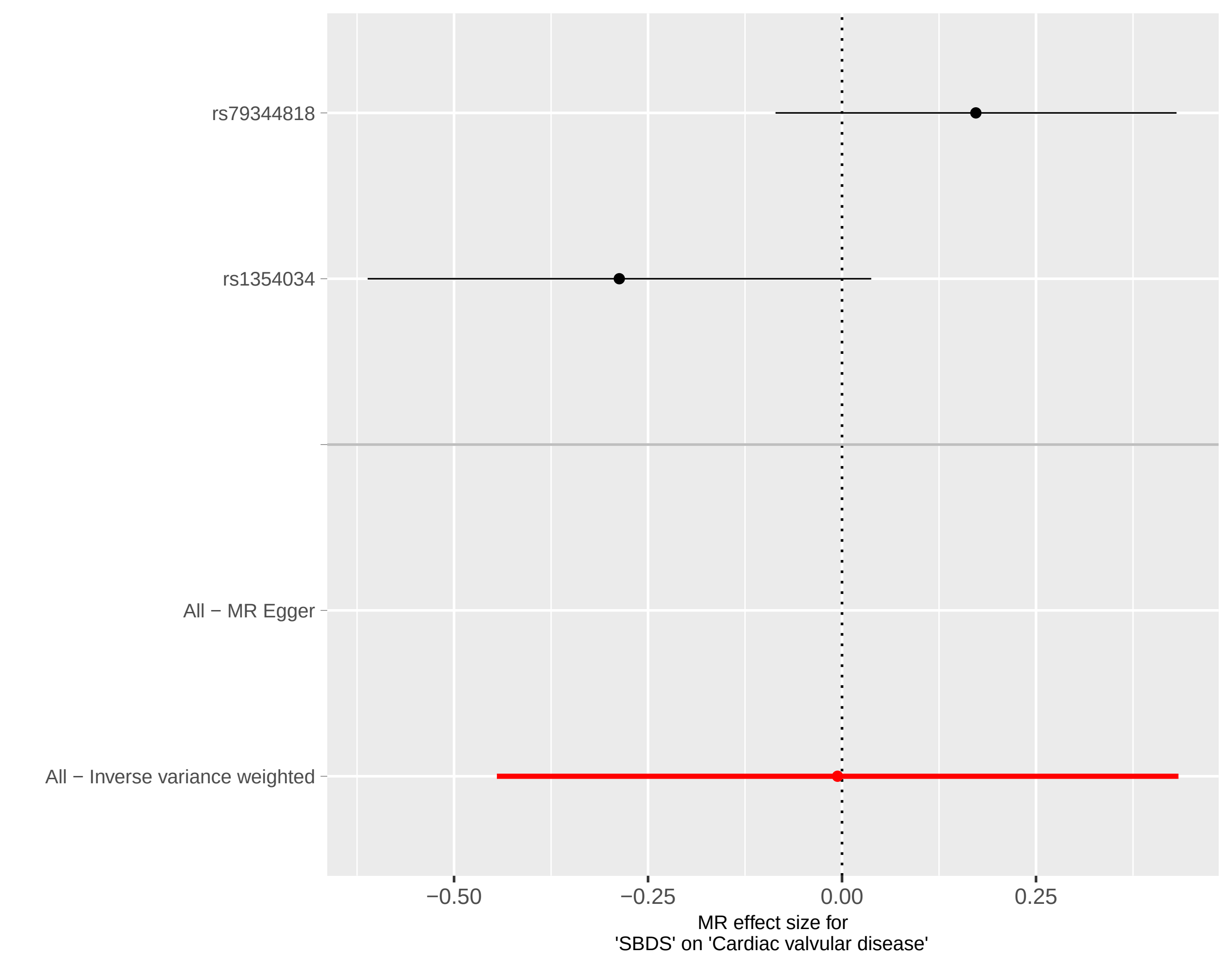
rs79344818
rs1354034
All − MR Egger
All − Inverse variance weighted
−0.50
−0.25
0.00
0.25
MR effect size for
'SBDS' on 'Cardiac valvular disease'

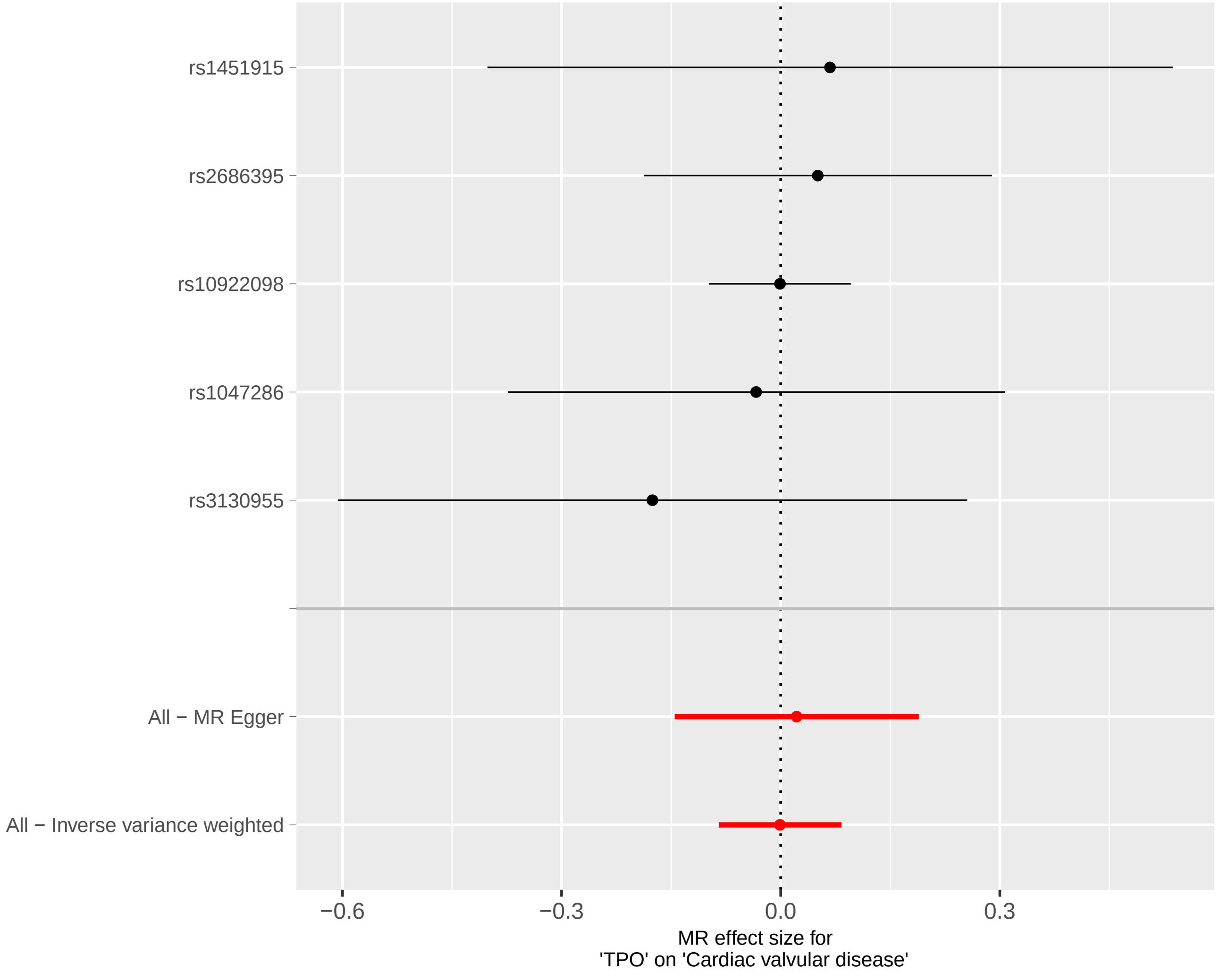
rs1451915
rs2686395
rs10922098
rs1047286
rs3130955
All − MR Egger
All − Inverse variance weighted
−0.6
−0.3
0.0
0.3
MR effect size for
'TPO' on 'Cardiac valvular disease'

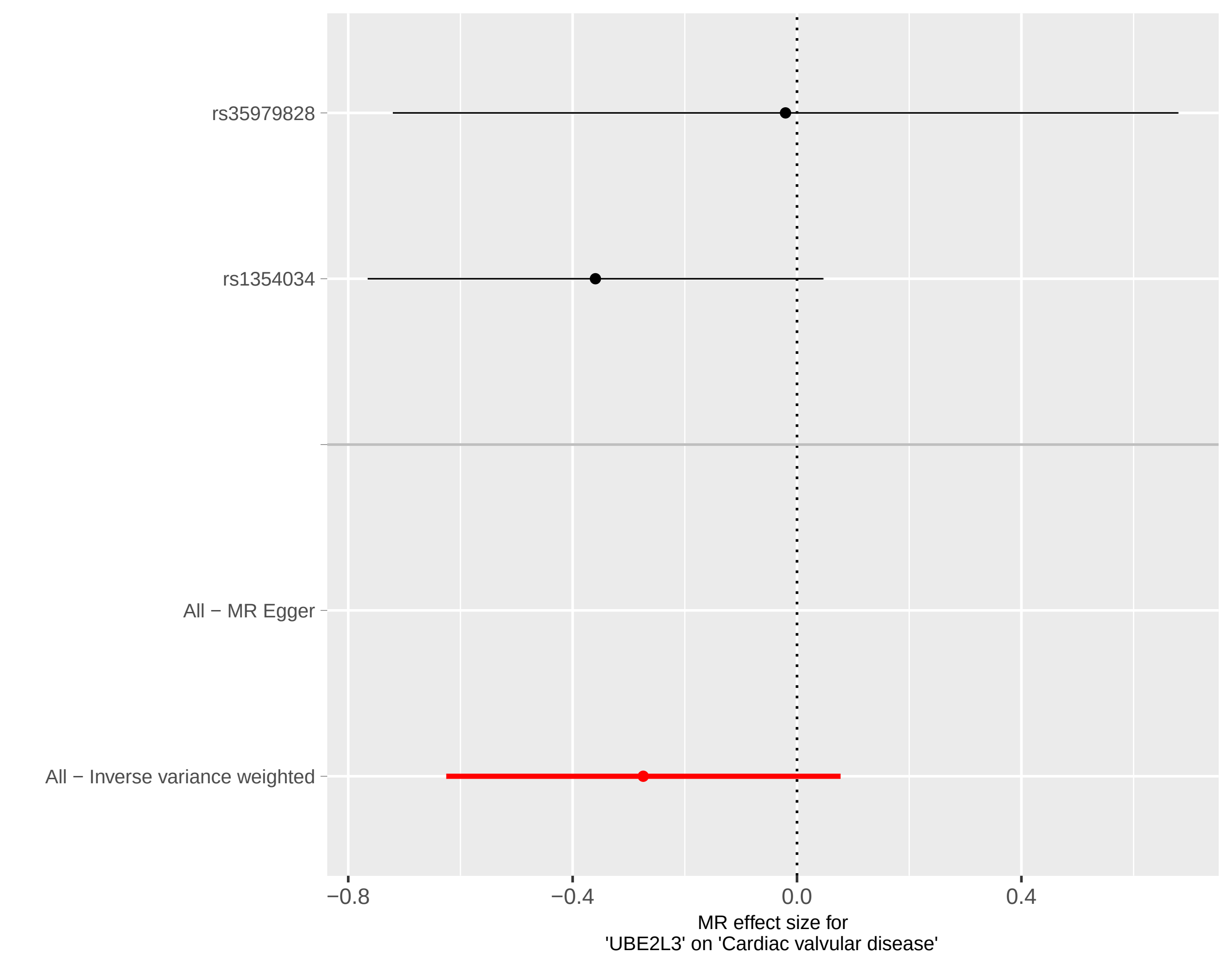
rs35979828
rs1354034
All − MR Egger
All − Inverse variance weighted
−0.8
−0.4
0.0
0.4
MR effect size for
'UBE2L3' on 'Cardiac valvular disease'

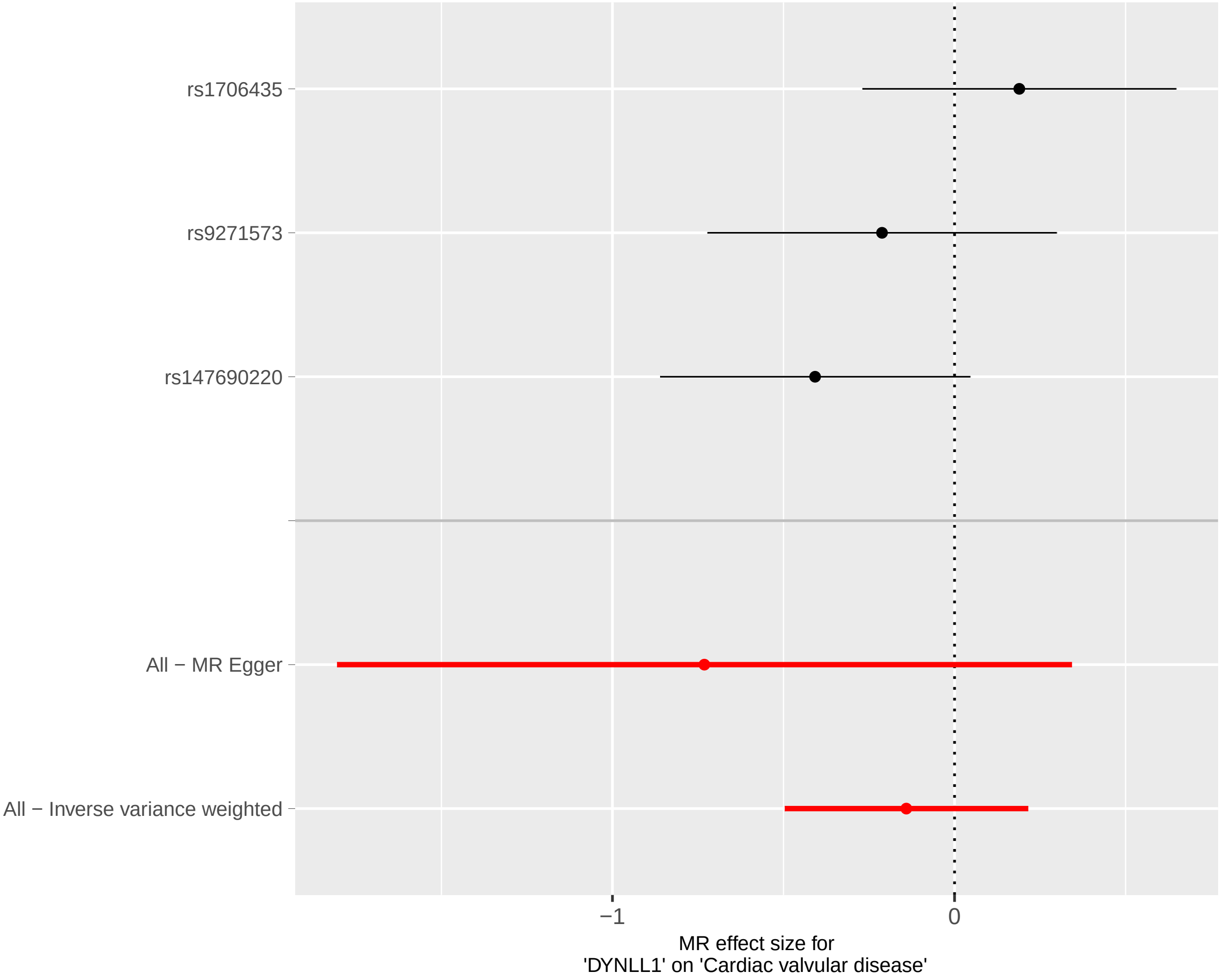

rs1706435
rs9271573
rs147690220
All − MR Egger
All − Inverse variance weighted
−1
0
MR effect size for
'DYNLL1' on 'Cardiac valvular disease'

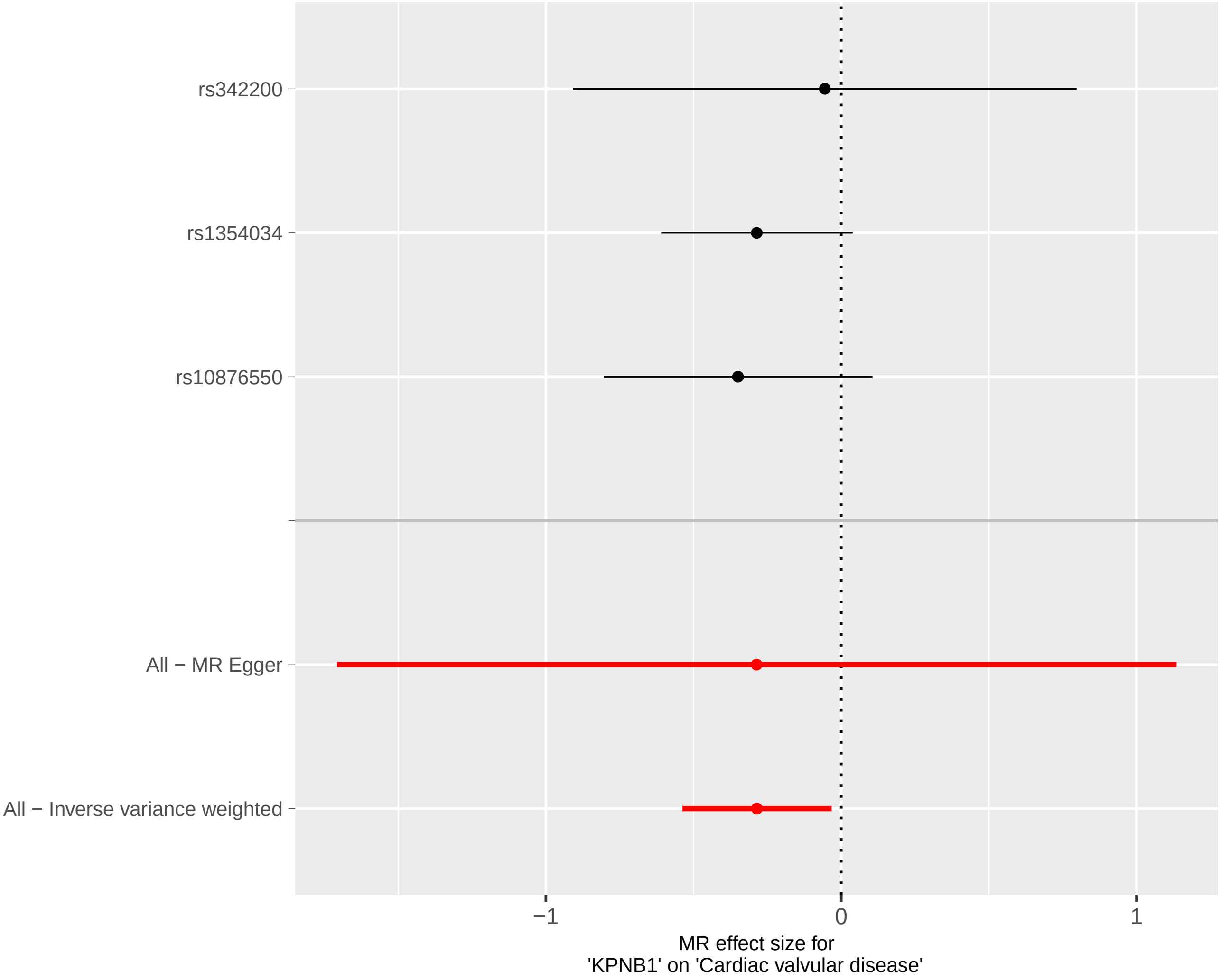
rs342200
rs1354034
rs10876550
All − MR Egger
All − Inverse variance weighted
−1
0
1
MR effect size for
'KPNB1' on 'Cardiac valvular disease'

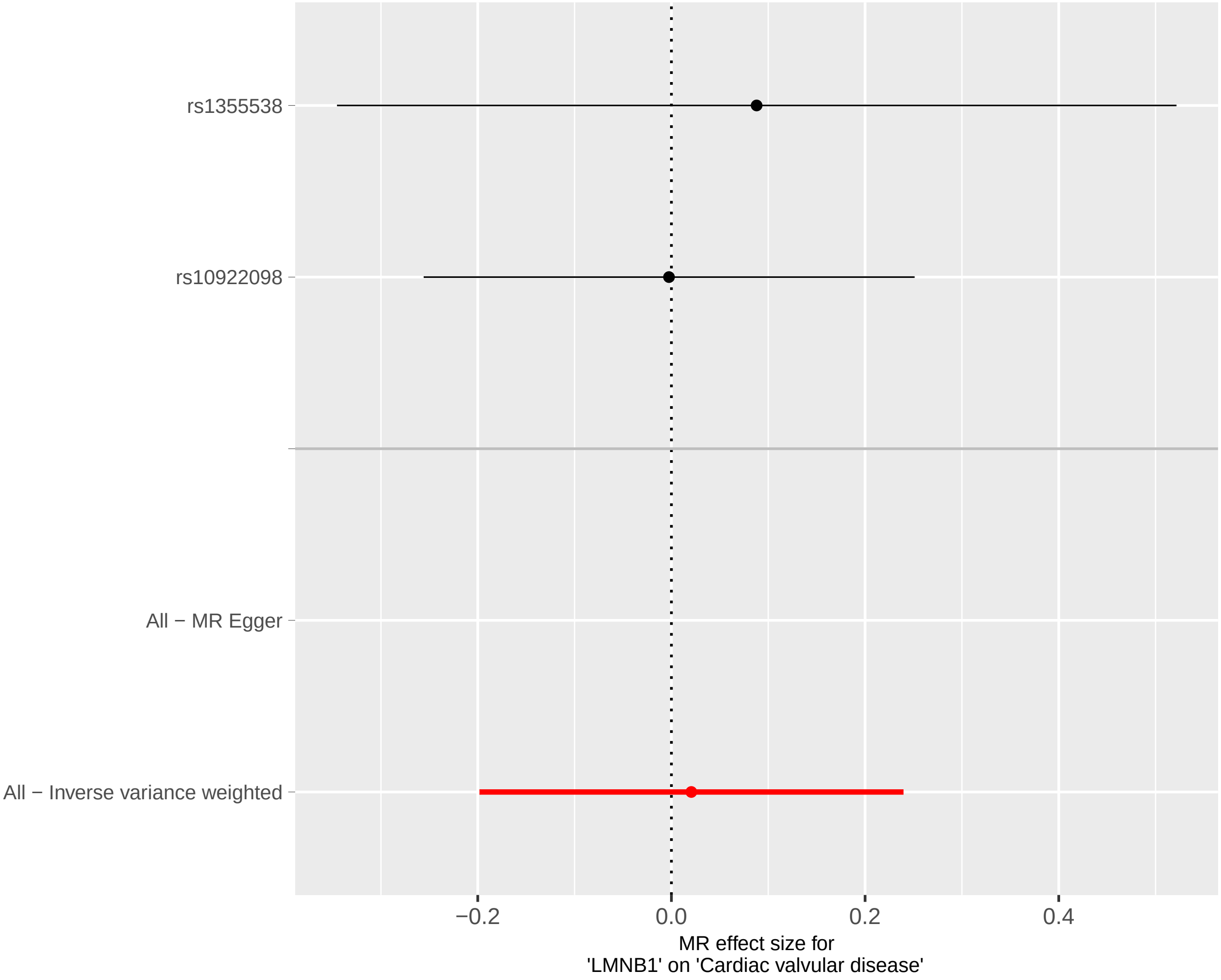

rs1355538
rs10922098
All − MR Egger
All − Inverse variance weighted
−0.2
0.0
0.2
0.4
MR effect size for
'LMNB1' on 'Cardiac valvular disease'

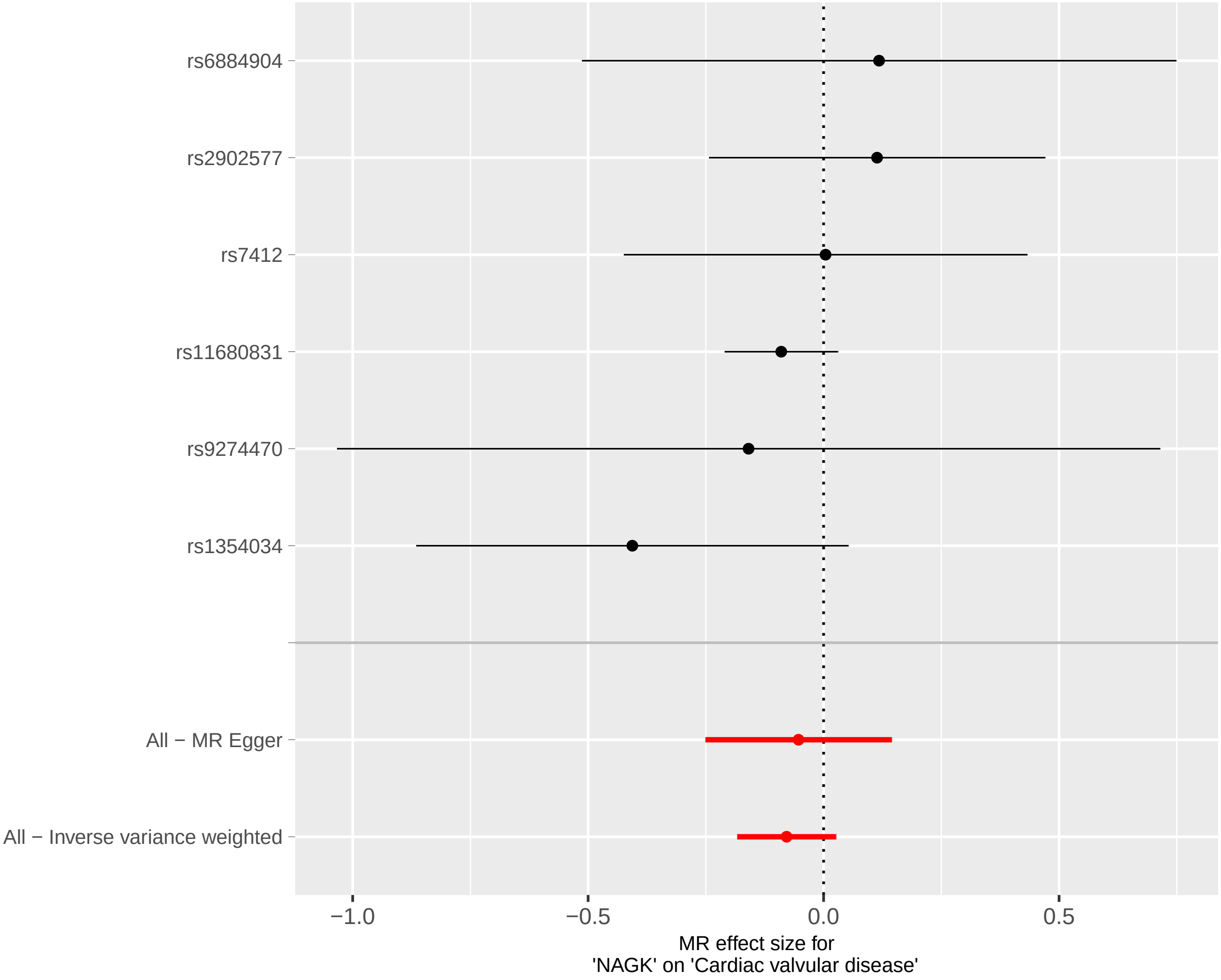
rs6884904
rs2902577
rs7412
rs11680831
rs9274470
rs1354034
All − MR Egger
All − Inverse variance weighted
−1.0
−0.5
0.0
0.5
MR effect size for
'NAGK' on 'Cardiac valvular disease'

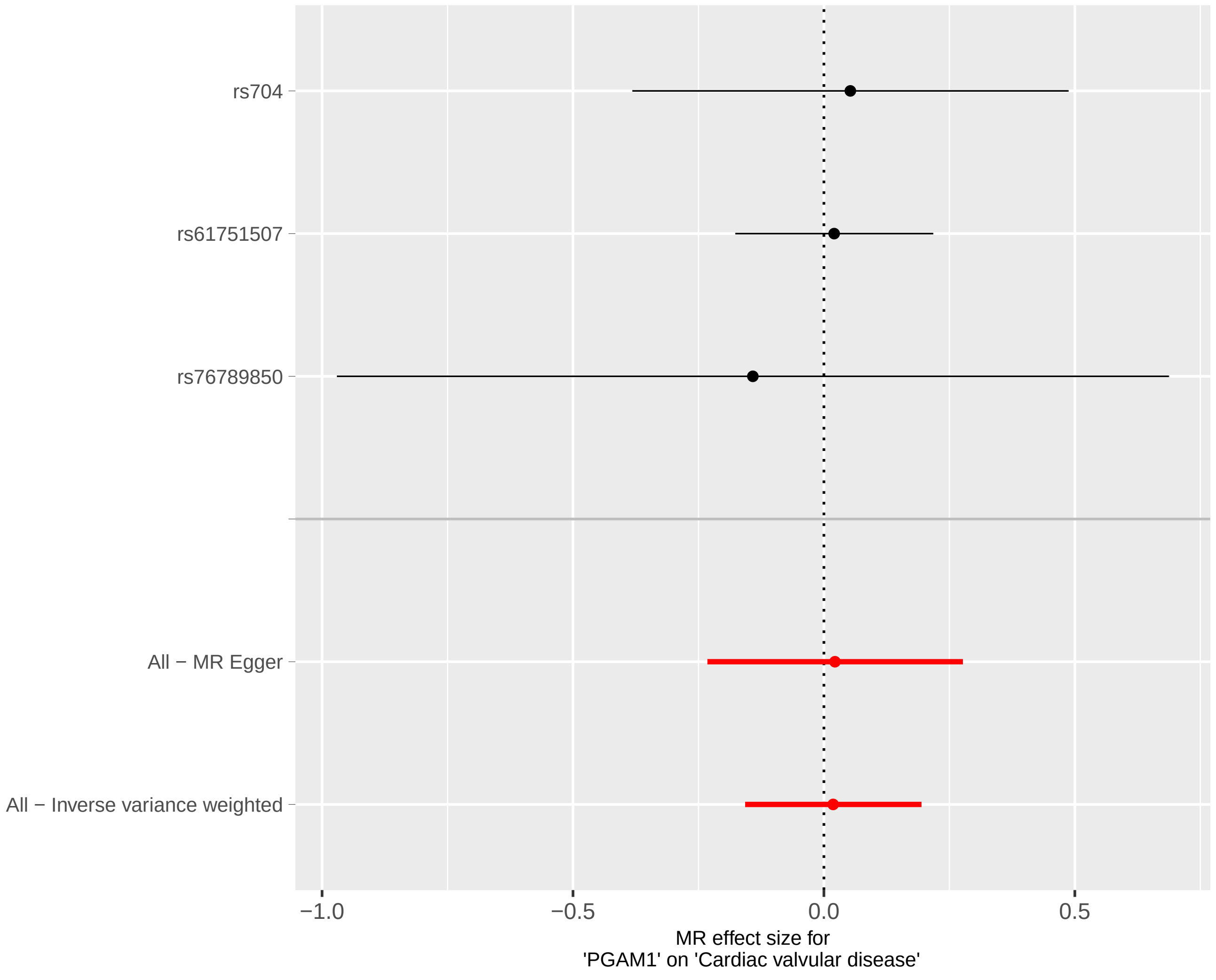
rs704
rs61751507
rs76789850
All − MR Egger
All − Inverse variance weighted
−1.0
−0.5
0.0
0.5
MR effect size for
'PGAM1' on 'Cardiac valvular disease'

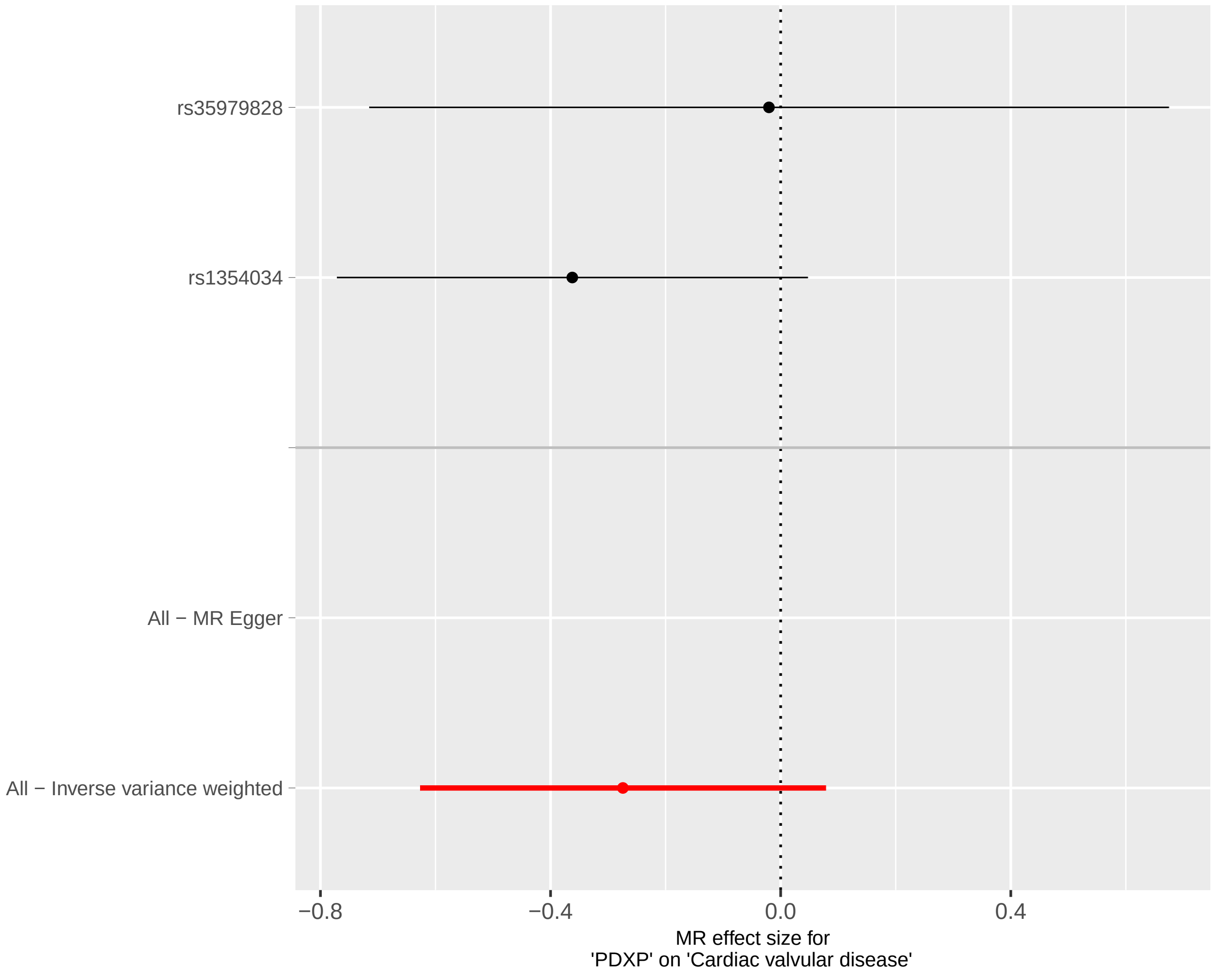
rs35979828
rs1354034
All − MR Egger
All − Inverse variance weighted
−0.8
−0.4
0.0
0.4
MR effect size for
'PDXP' on 'Cardiac valvular disease'

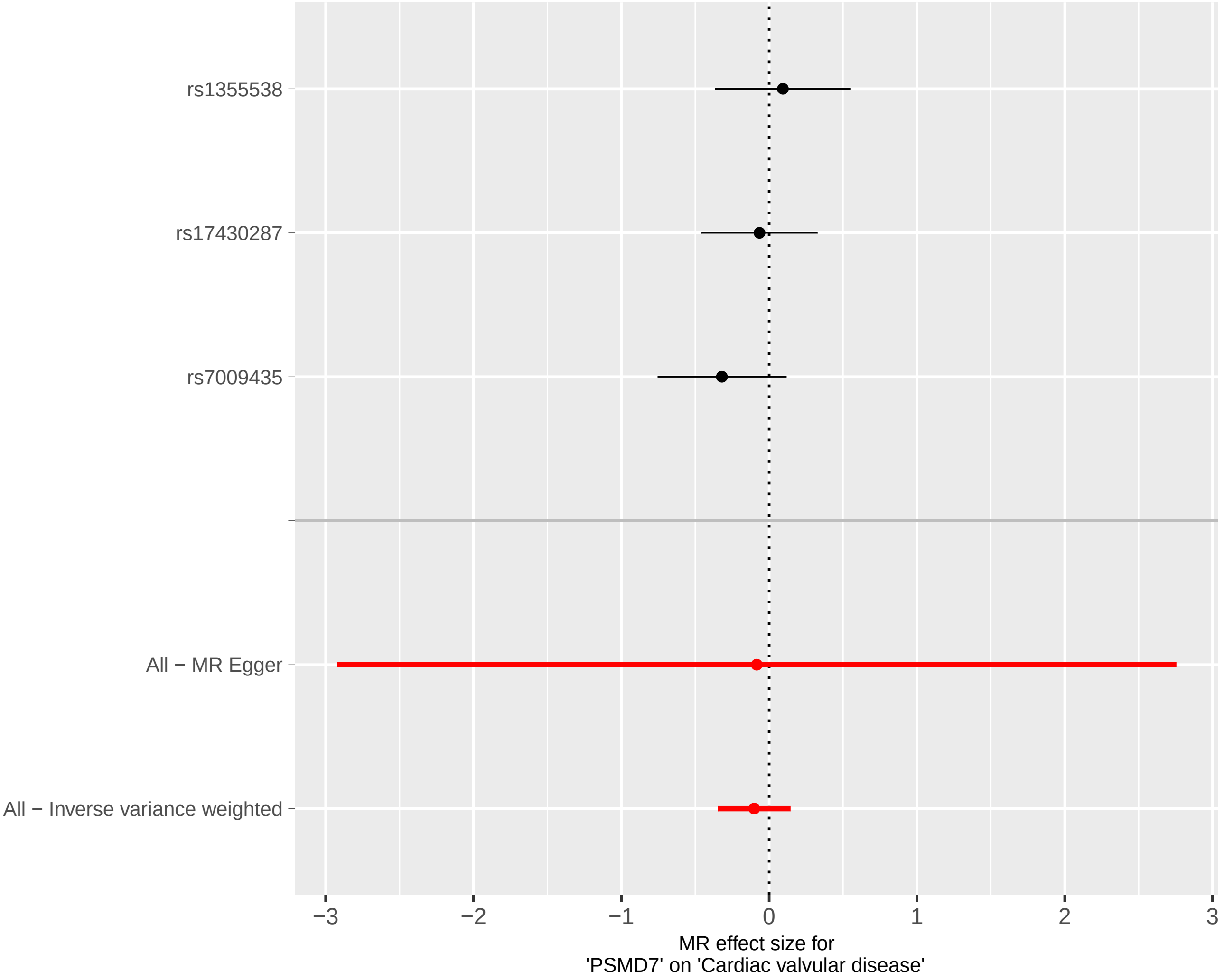
rs1355538
rs17430287
rs7009435
All − MR Egger
All − Inverse variance weighted
−3
−2
−1
0
1
2
3
MR effect size for
'PSMD7' on 'Cardiac valvular disease'

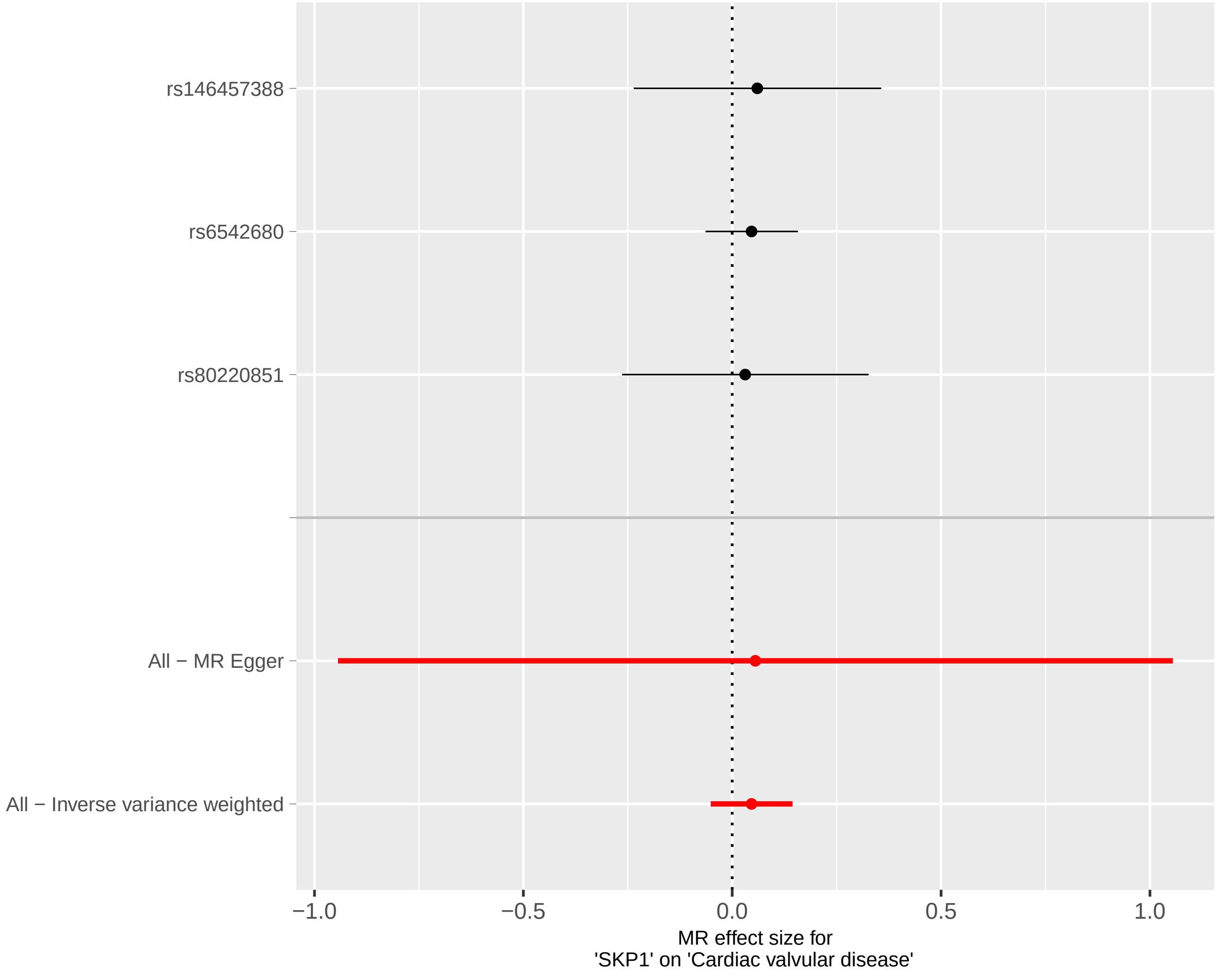
rs146457388
rs6542680
rs80220851
All − MR Egger
All − Inverse variance weighted
−1.0
−0.5
0.0
0.5
1.0
MR effect size for
'SKP1' on 'Cardiac valvular disease'

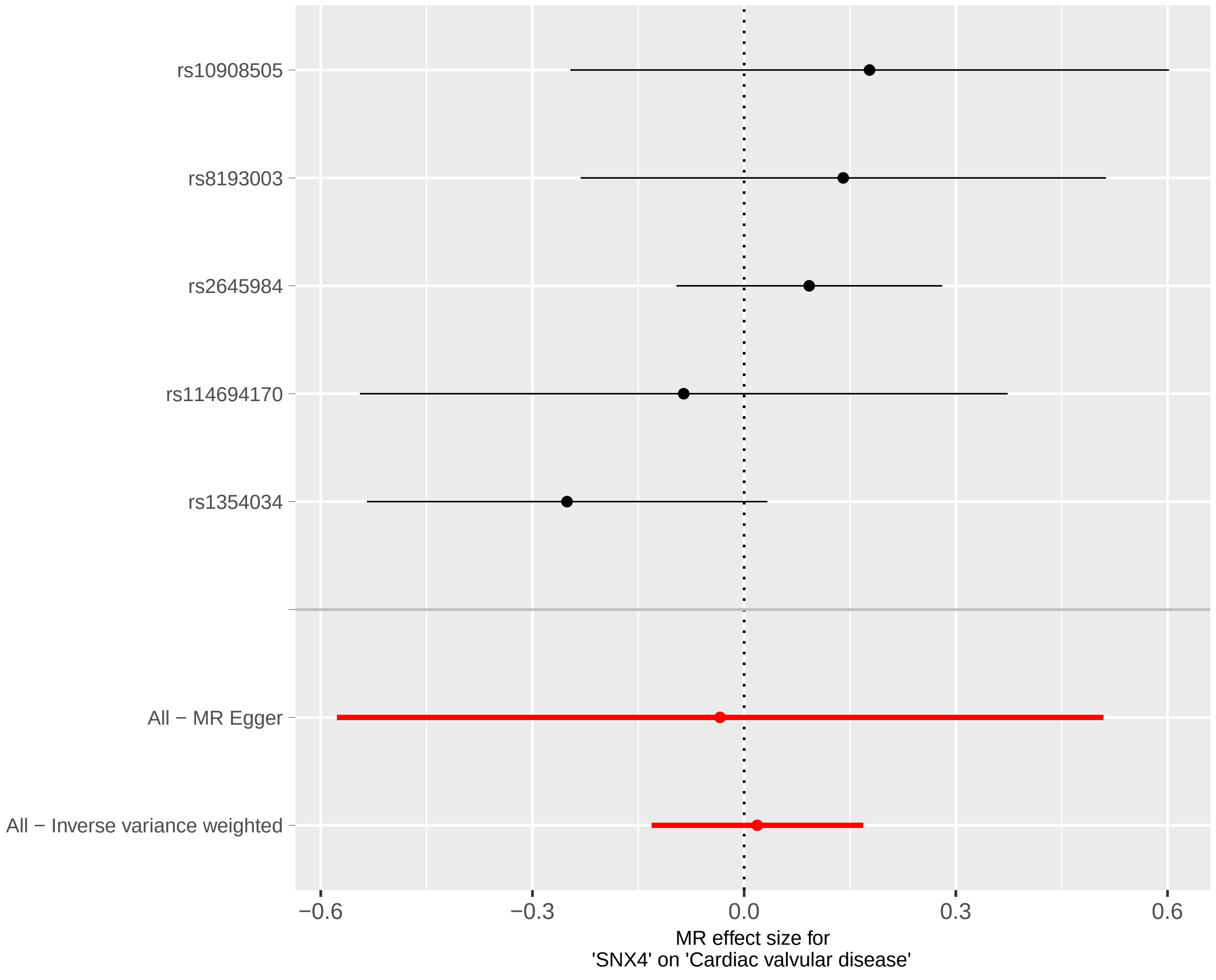
rs10908505
rs8193003
rs2645984
rs114694170
rs1354034
All − MR Egger
All − Inverse variance weighted
−0.6
−0.3
0.0
0.3
0.6
MR effect size for
'SNX4' on 'Cardiac valvular disease'

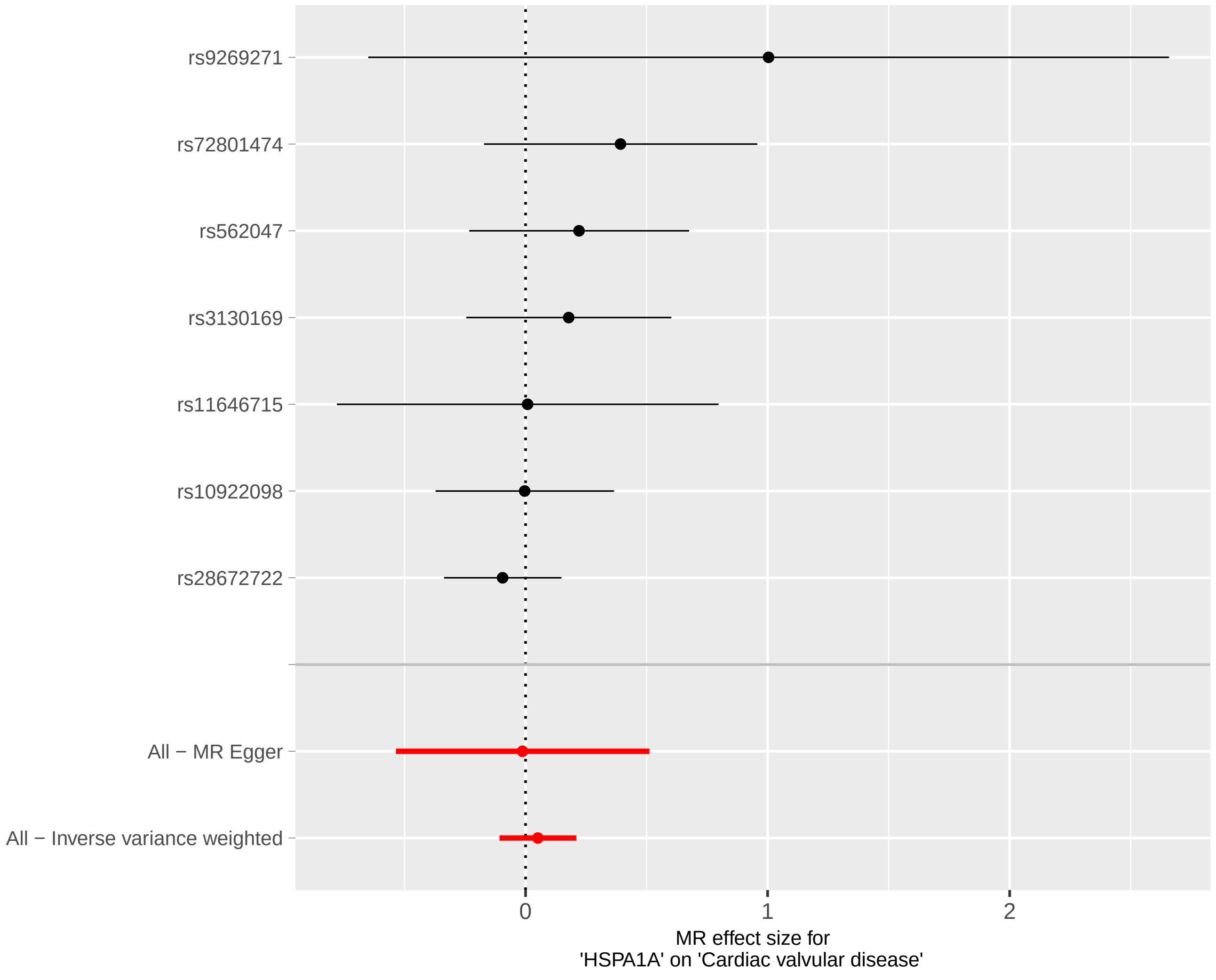
rs9269271
rs72801474
rs562047
rs3130169
rs11646715
rs10922098
rs28672722
All − MR Egger
All − Inverse variance weighted
0
1
2
MR effect size for
'HSPA1A' on 'Cardiac valvular disease'

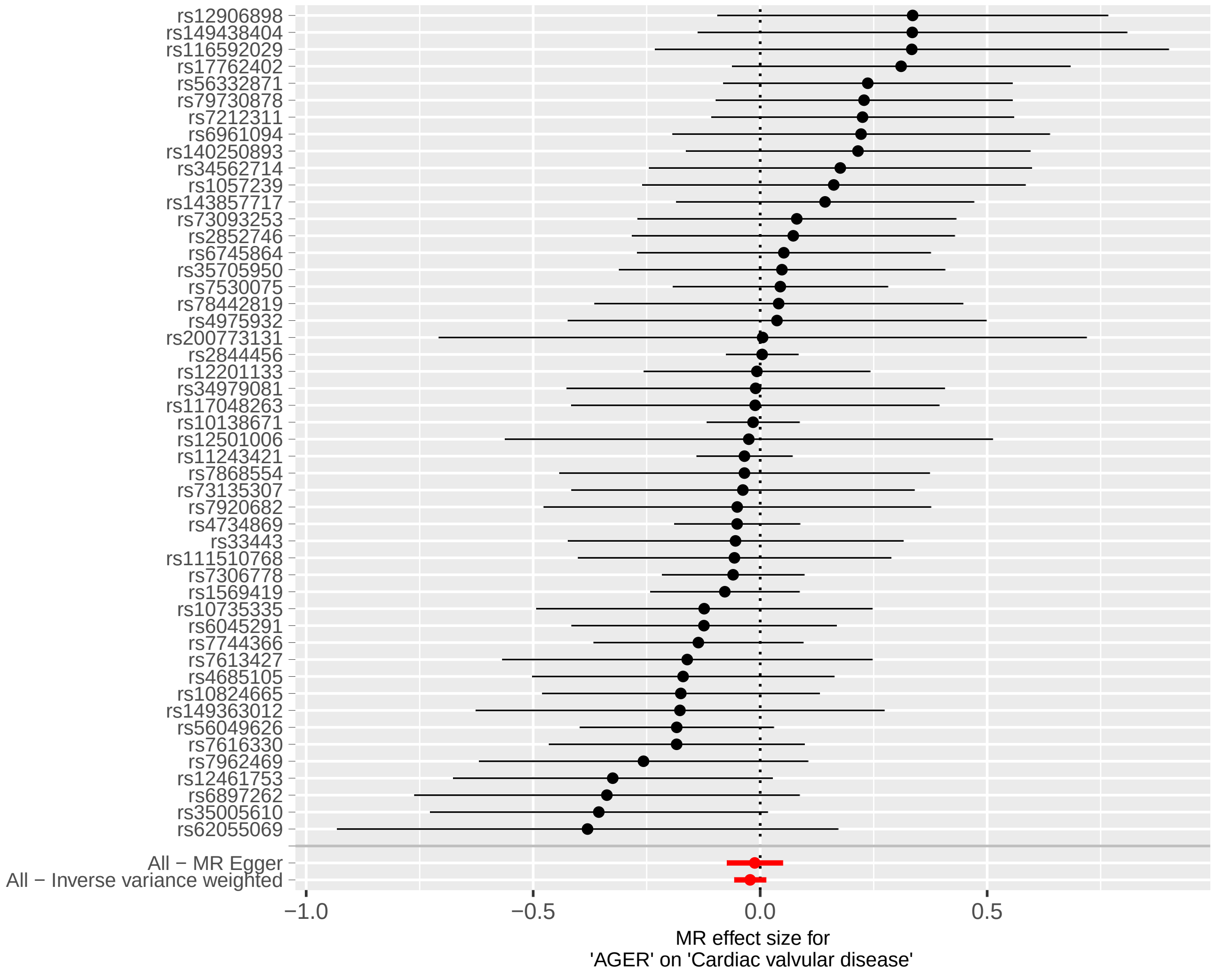
rs12906898
rs149438404
rs116592029
rs17762402
rs56332871
rs79730878
rs7212311
rs6961094
rs140250893
rs34562714
rs1057239
rs143857717
rs73093253
rs2852746
rs6745864
rs35705950
rs7530075
rs78442819
rs4975932
rs200773131
rs2844456
rs12201133
rs34979081
rs117048263
rs10138671
rs12501006
rs11243421
rs7868554
rs73135307
rs7920682
rs4734869
rs33443
rs111510768
rs7306778
rs1569419
rs10735335
rs6045291
rs7744366
rs7613427
rs4685105
rs10824665
rs149363012
rs56049626
rs7616330
rs7962469
rs12461753
rs6897262
rs35005610
rs62055069
All − MR Egger
All − Inverse variance weighted
−1.0
−0.5
0.0
0.5
MR effect size for
'AGER' on 'Cardiac valvular disease'

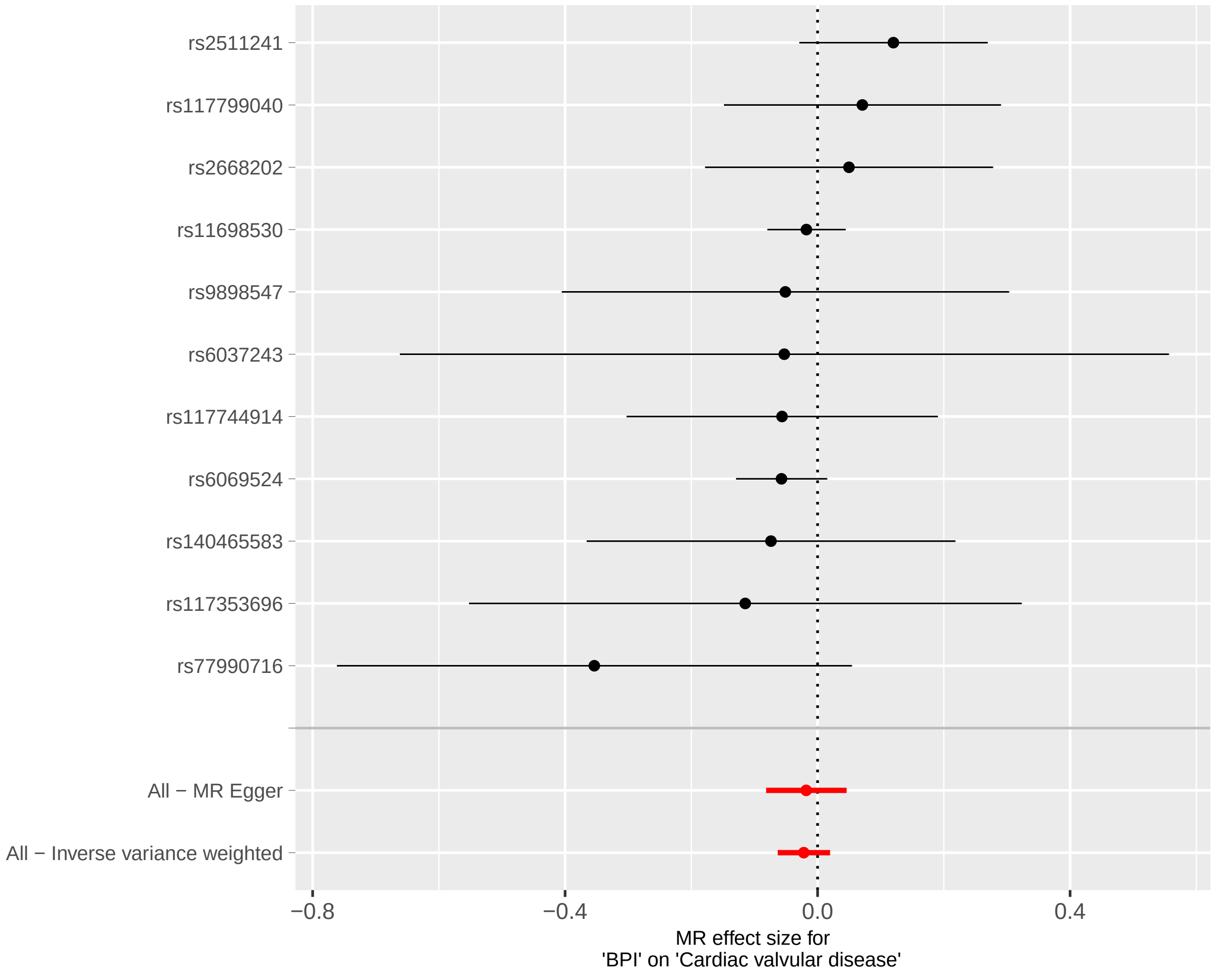
rs2511241
rs117799040
rs2668202
rs11698530
rs9898547
rs6037243
rs117744914
rs6069524
rs140465583
rs117353696
rs77990716
All − MR Egger
All − Inverse variance weighted
−0.8
−0.4
0.0
0.4
MR effect size for
'BPI' on 'Cardiac valvular disease'

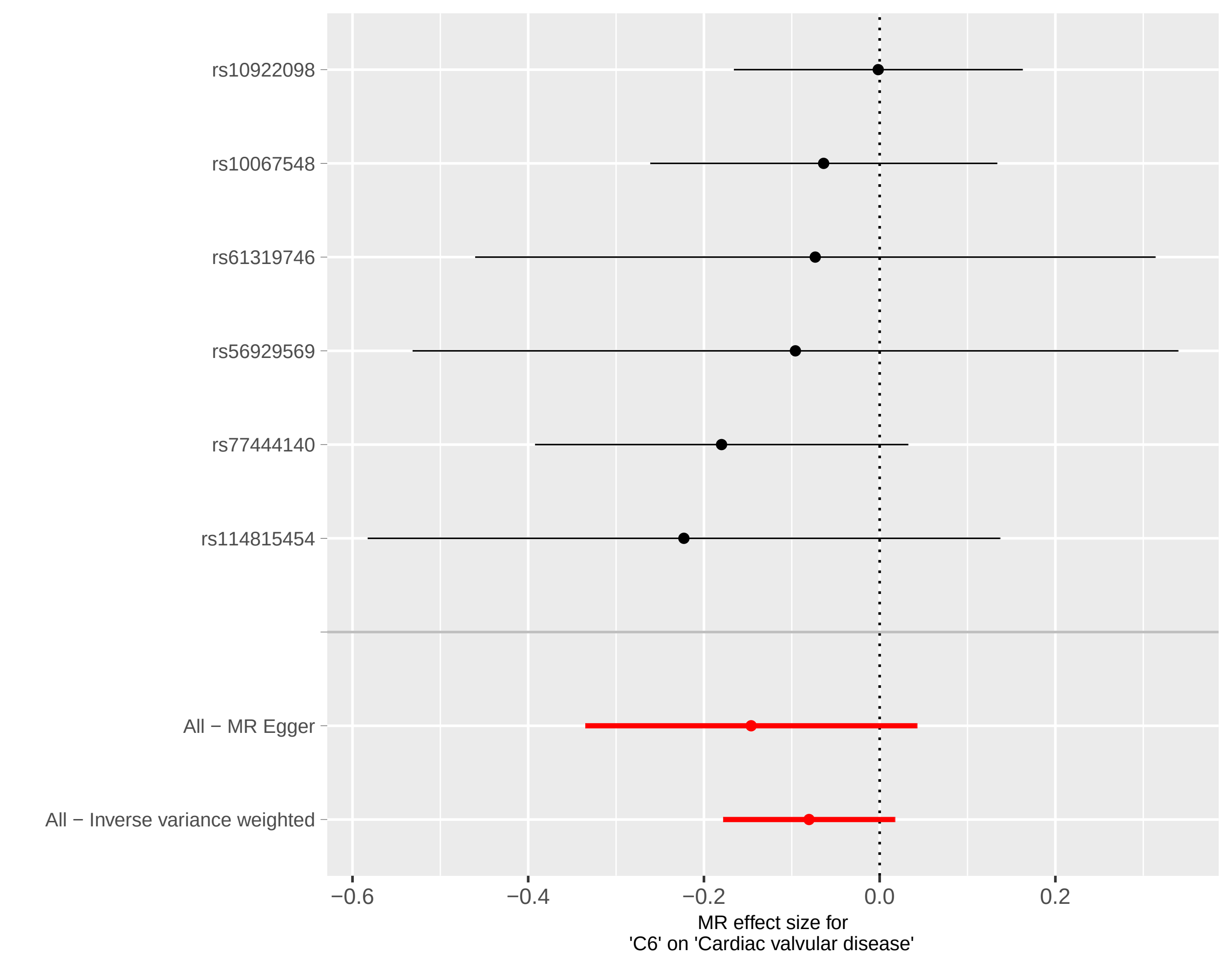
rs10922098
rs10067548
rs61319746
rs56929569
rs77444140
rs114815454
All − MR Egger
All − Inverse variance weighted
−0.6
−0.4
−0.2
0.0
0.2
MR effect size for
'C6' on 'Cardiac valvular disease'

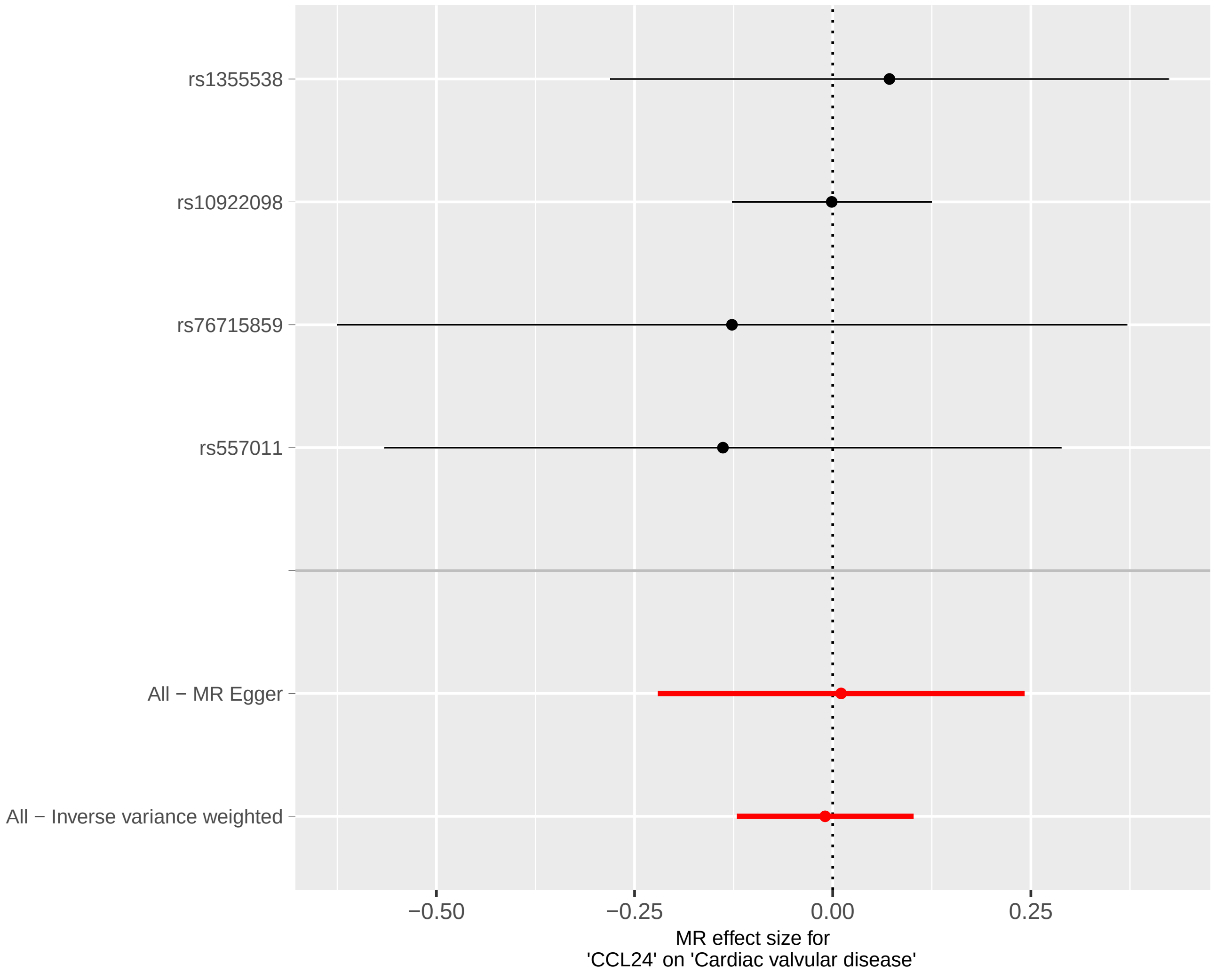

rs1355538
rs10922098
rs76715859
rs557011
All − MR Egger
All − Inverse variance weighted
−0.50
−0.25
0.00
0.25
MR effect size for
'CCL24' on 'Cardiac valvular disease'

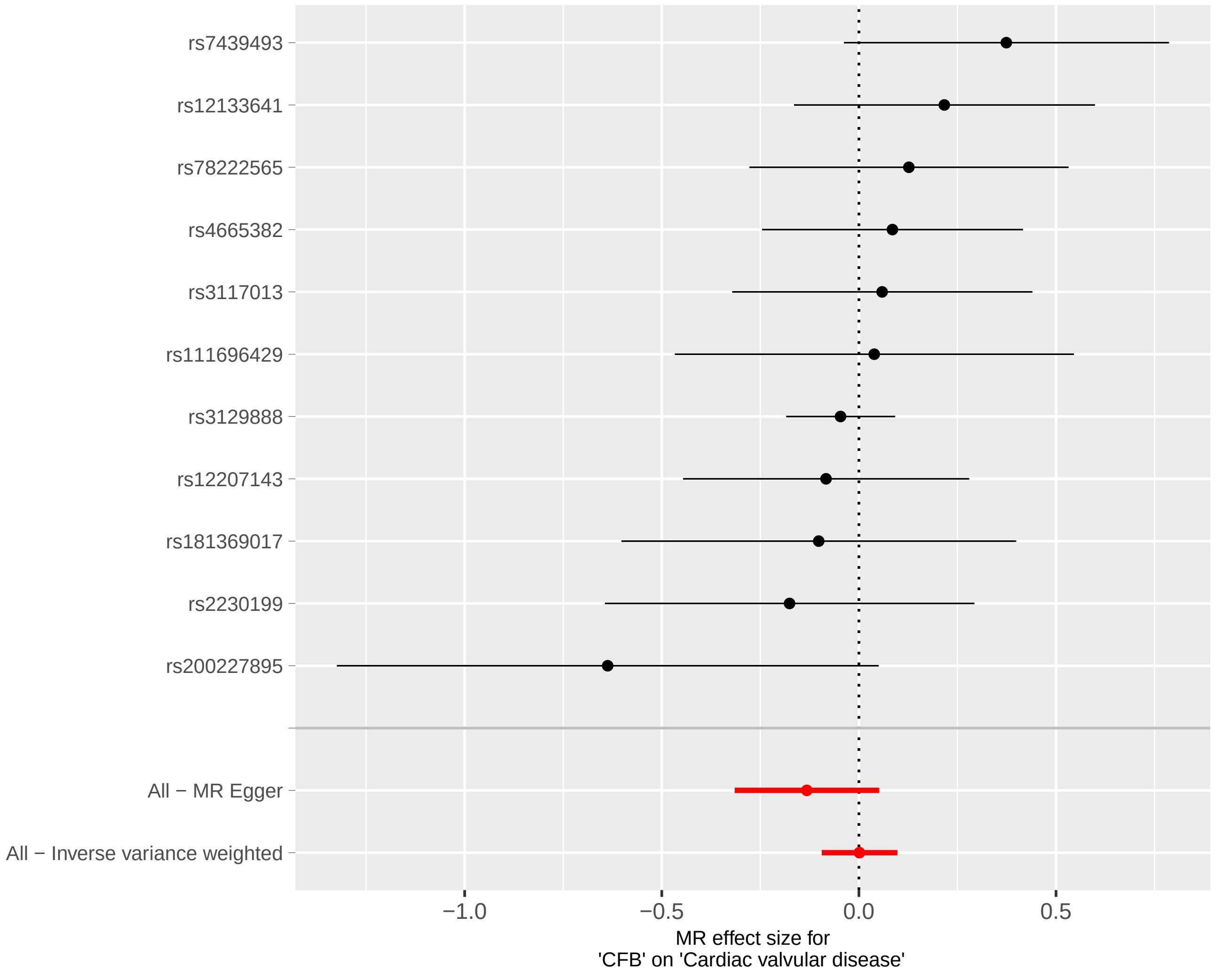

rs7439493
rs12133641
rs78222565
rs4665382
rs3117013
rs111696429
rs3129888
rs12207143
rs181369017
rs2230199
rs200227895
All − MR Egger
All − Inverse variance weighted
−1.0
−0.5
0.0
0.5
MR effect size for
'CFB' on 'Cardiac valvular disease'

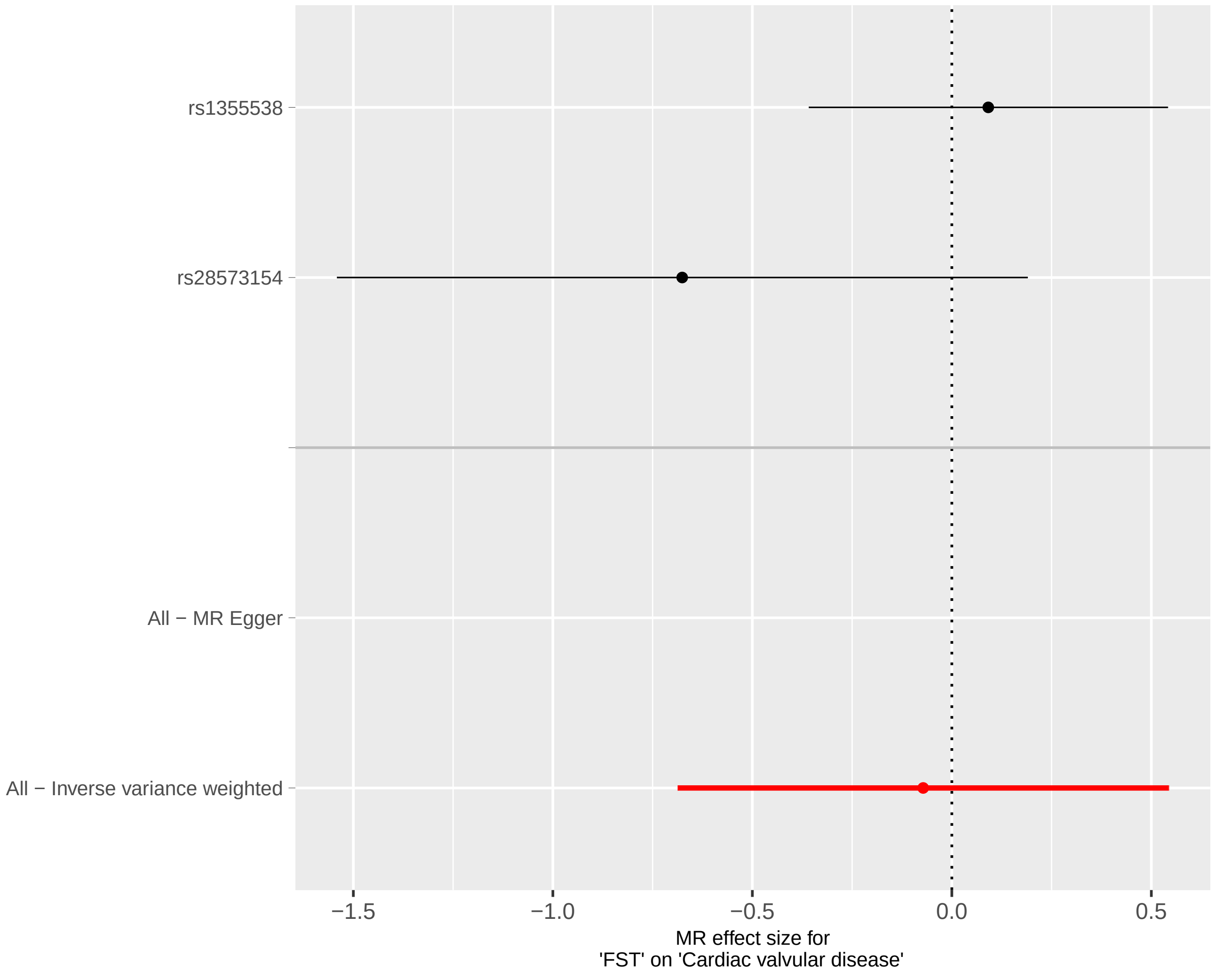

rs1355538
rs28573154
All − MR Egger
All − Inverse variance weighted
−1.5
−1.0
−0.5
0.0
0.5
MR effect size for
'FST' on 'Cardiac valvular disease'

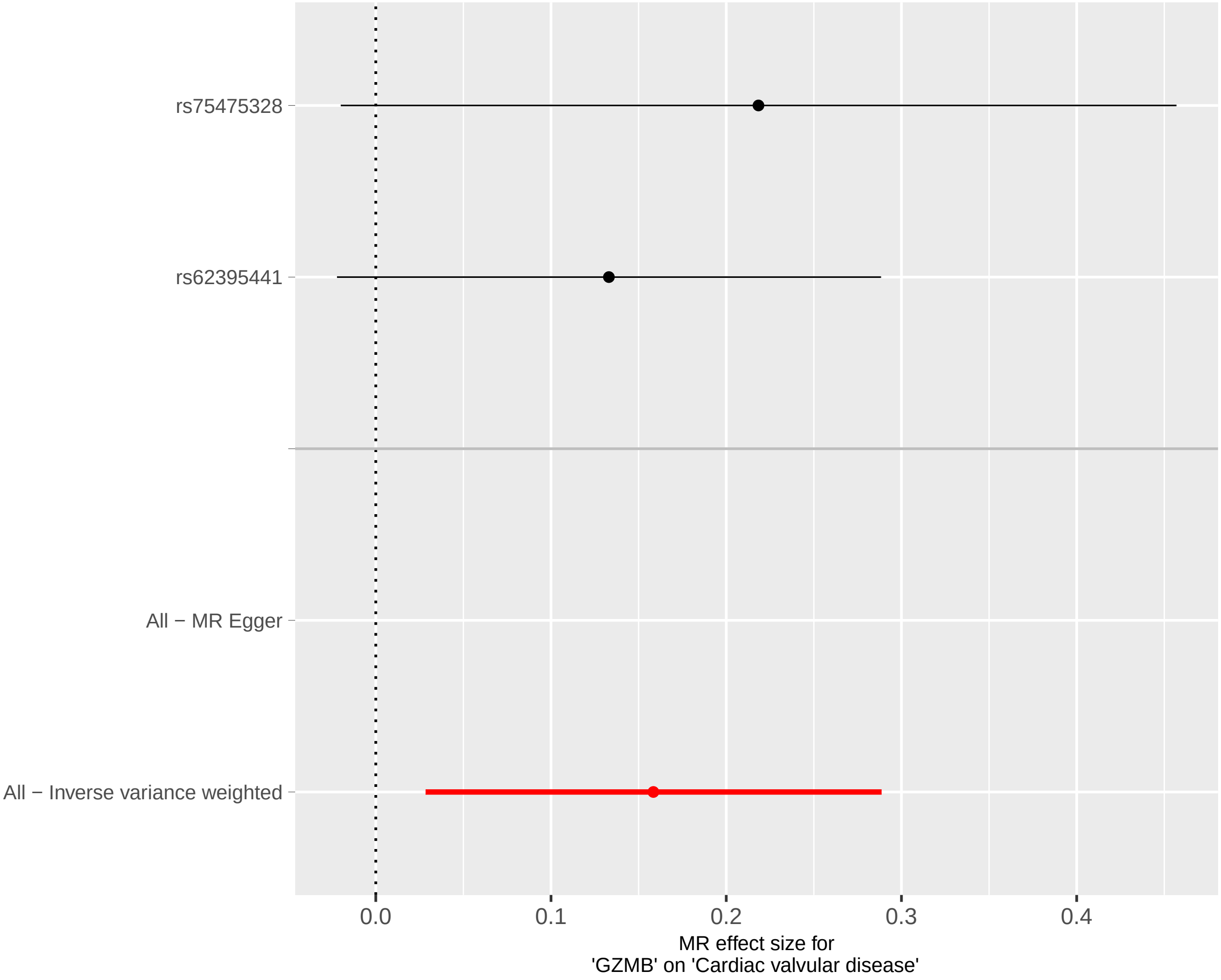

rs75475328
rs62395441
All − MR Egger
All − Inverse variance weighted
0.0
0.1
0.2
0.3
0.4
MR effect size for
'GZMB' on 'Cardiac valvular disease'

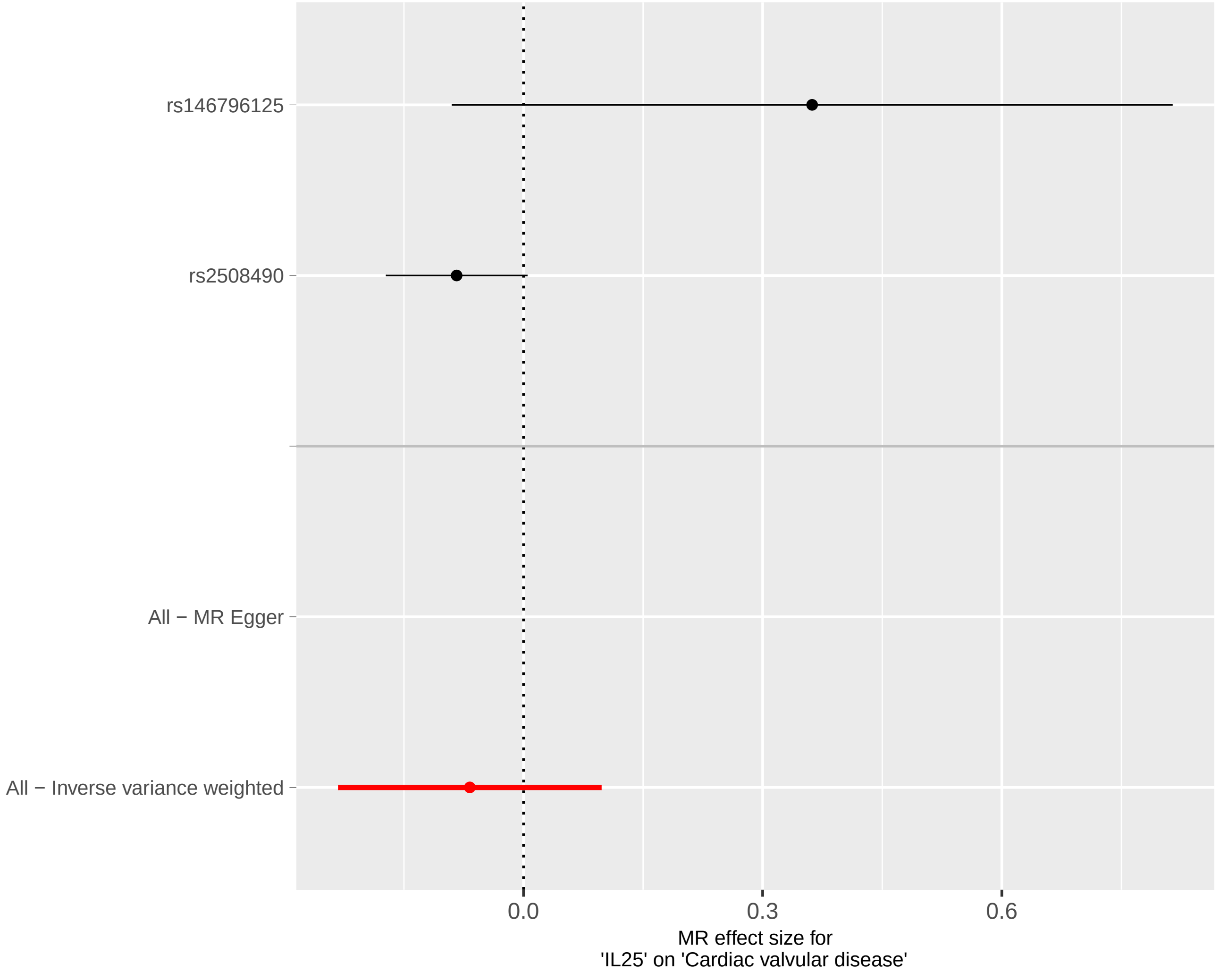

rs146796125
rs2508490
All − MR Egger
All − Inverse variance weighted
0.0
0.3
0.6
MR effect size for
'IL25' on 'Cardiac valvular disease'

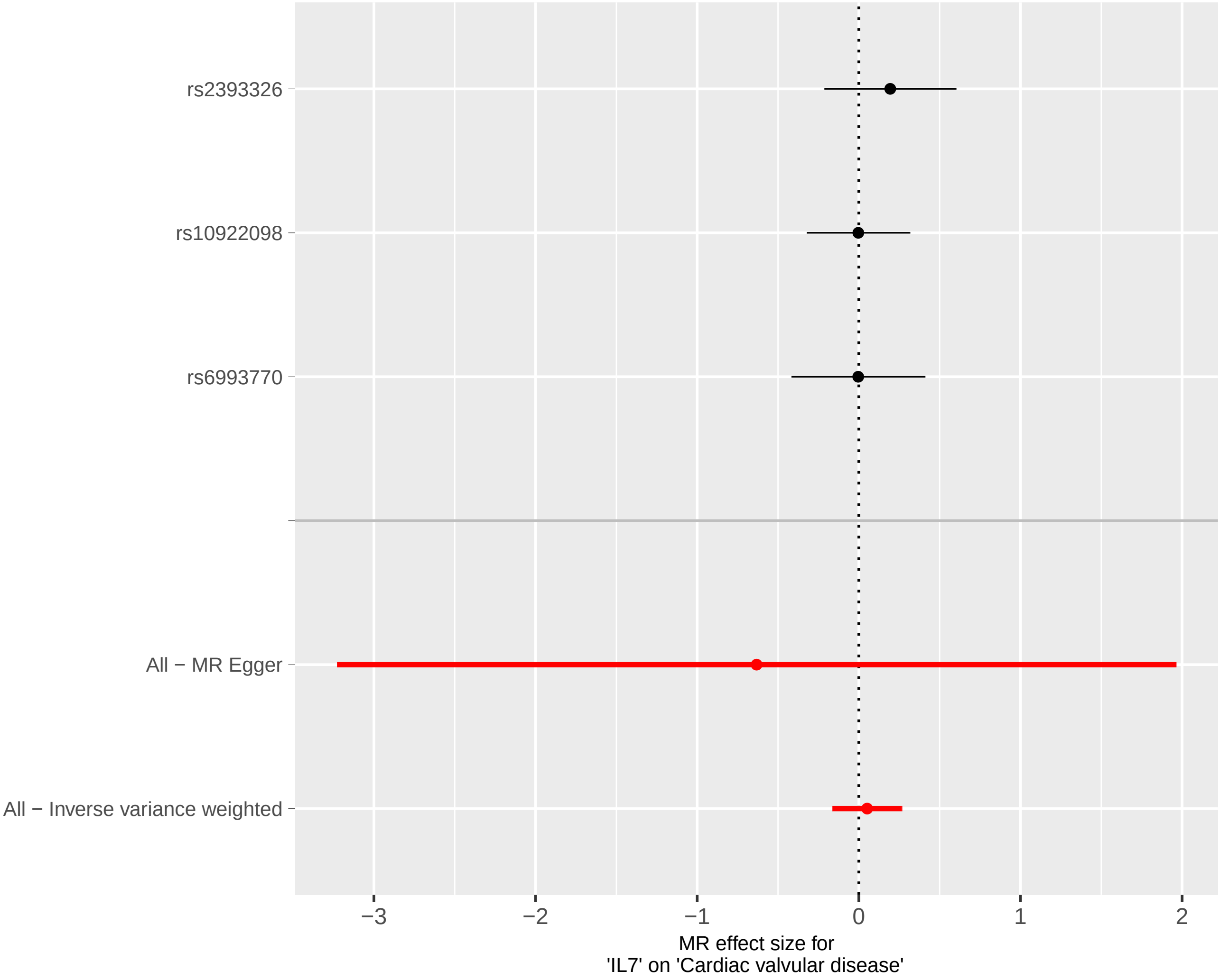
rs2393326
rs10922098
rs6993770
All − MR Egger
All − Inverse variance weighted
−3
−2
−1
0
1
2
MR effect size for
'IL7' on 'Cardiac valvular disease'

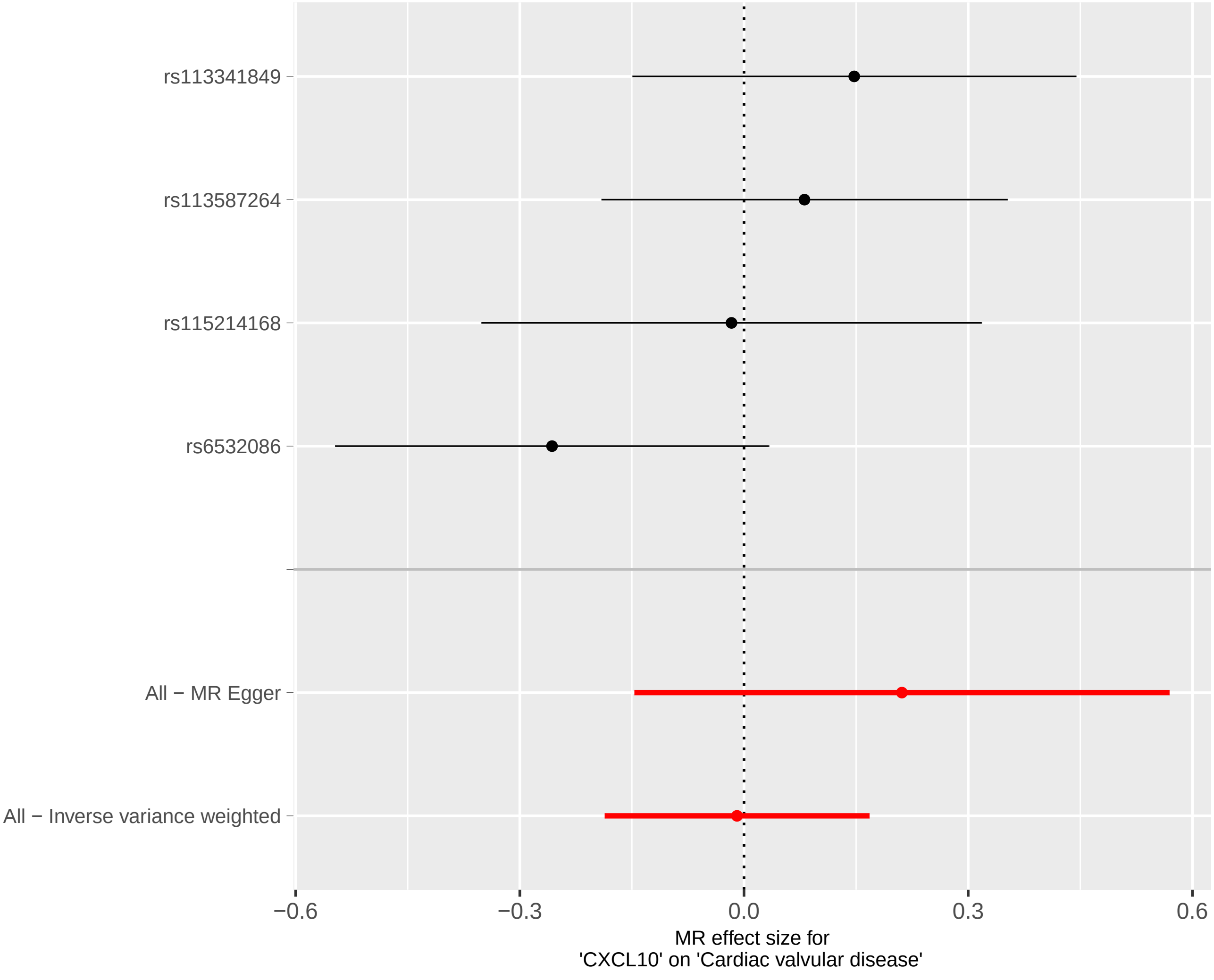
rs113341849
rs113587264
rs115214168
rs6532086
All − MR Egger
All − Inverse variance weighted
−0.6
−0.3
0.0
0.3
0.6
MR effect size for
'CXCL10' on 'Cardiac valvular disease'

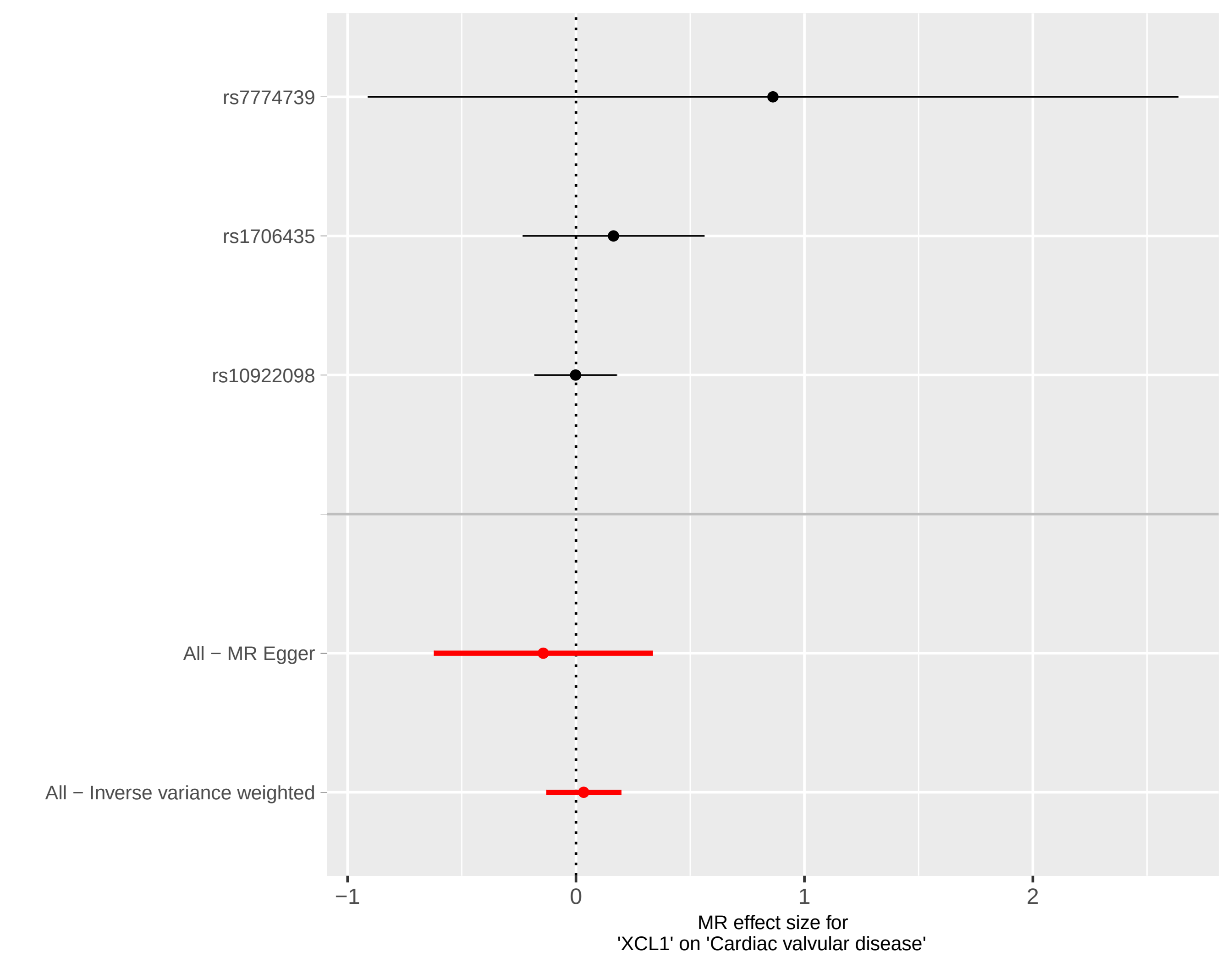
rs7774739
rs1706435
rs10922098
All − MR Egger
All − Inverse variance weighted
−1
0
1
2
MR effect size for
'XCL1' on 'Cardiac valvular disease'

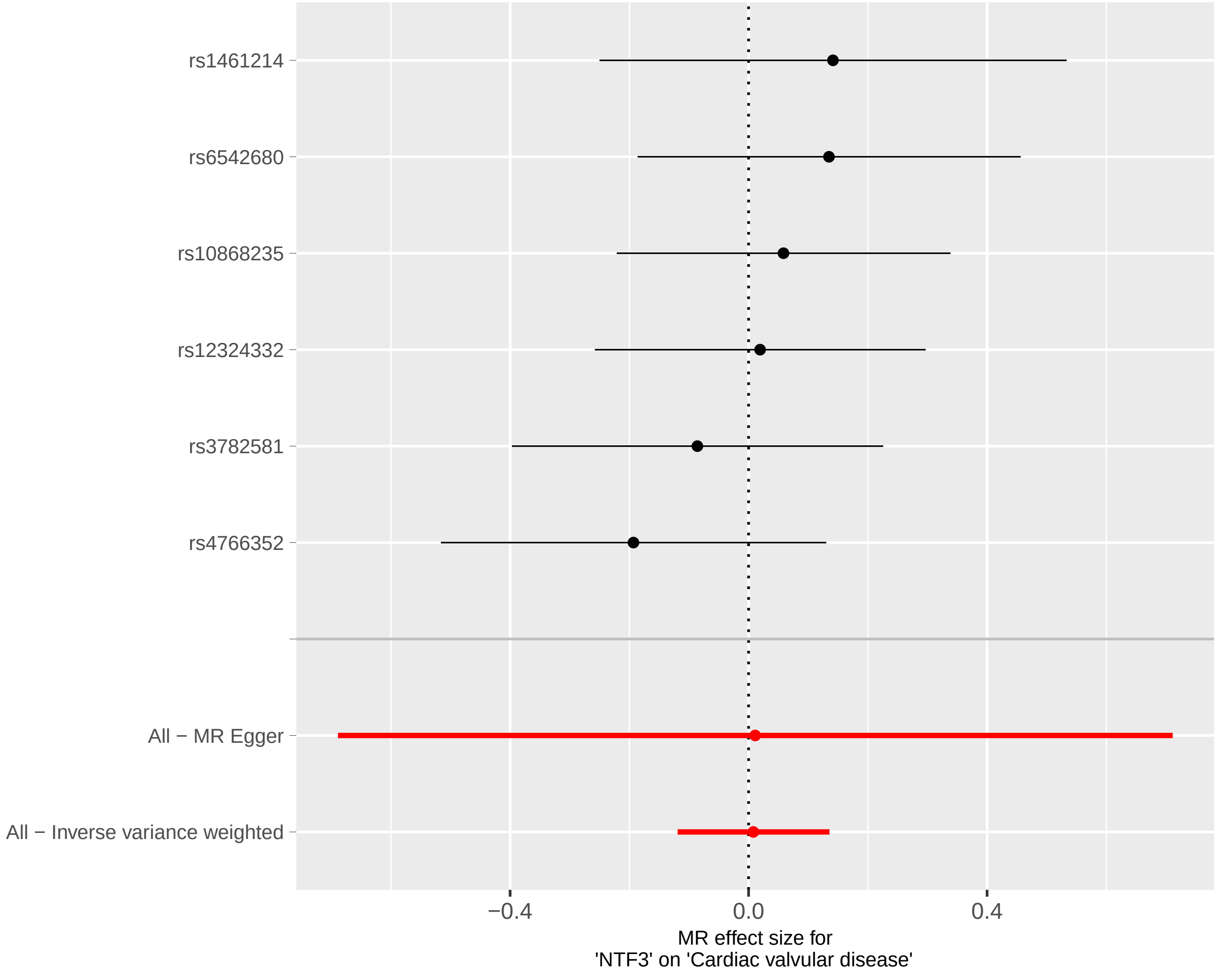
rs1461214
rs6542680
rs10868235
rs12324332
rs3782581
rs4766352
All − MR Egger
All − Inverse variance weighted
−0.4
0.0
0.4
MR effect size for
'NTF3' on 'Cardiac valvular disease'

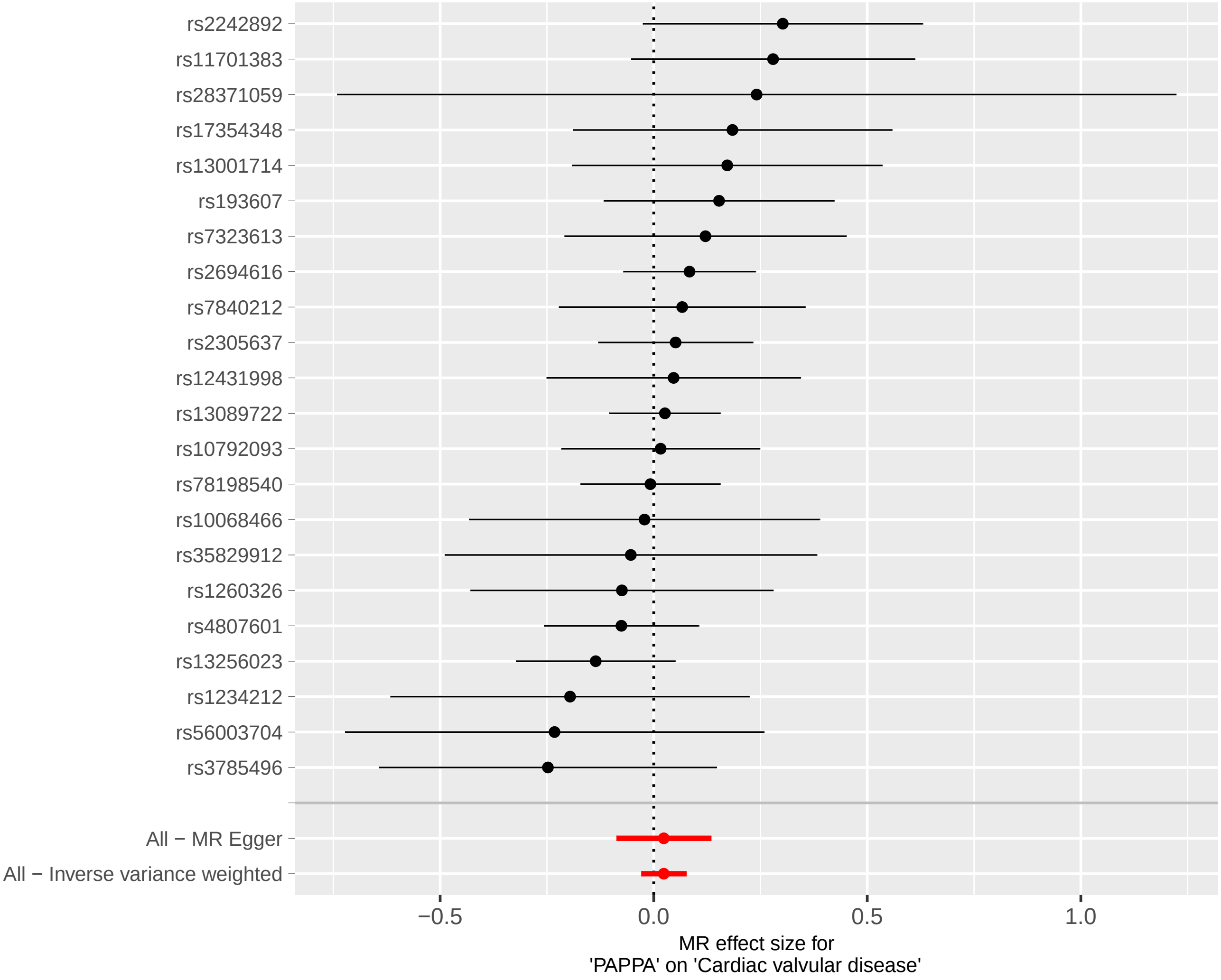

rs2242892
rs11701383
rs28371059
rs17354348
rs13001714
rs193607
rs7323613
rs2694616
rs7840212
rs2305637
rs12431998
rs13089722
rs10792093
rs78198540
rs10068466
rs35829912
rs1260326
rs4807601
rs13256023
rs1234212
rs56003704
rs3785496
All − MR Egger
All − Inverse variance weighted
−0.5
0.0
0.5
1.0
MR effect size for
'PAPPA' on 'Cardiac valvular disease'

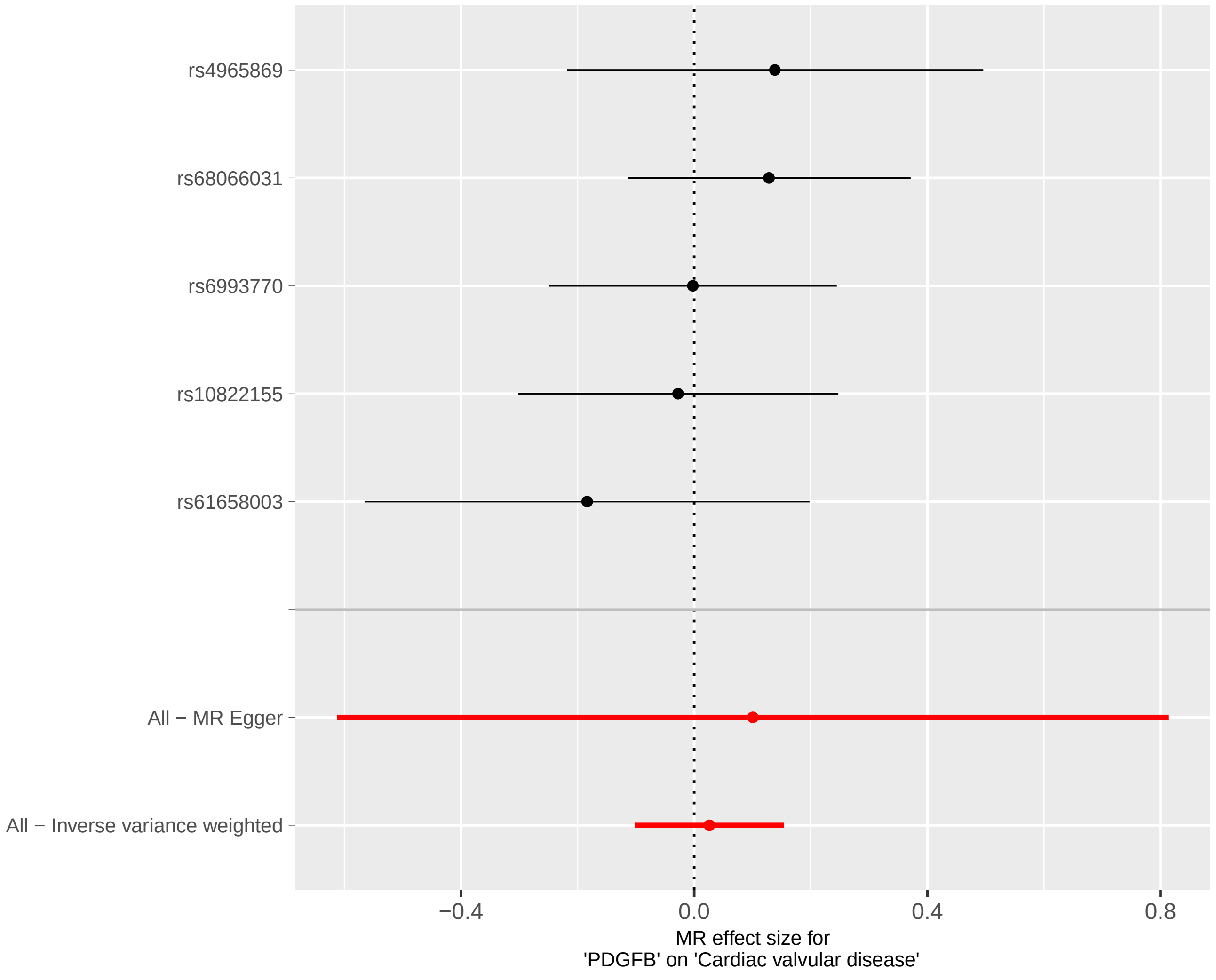
rs4965869
rs68066031
rs6993770
rs10822155
rs61658003
All − MR Egger
All − Inverse variance weighted
−0.4
0.0
0.4
0.8
MR effect size for
'PDGFB' on 'Cardiac valvular disease'

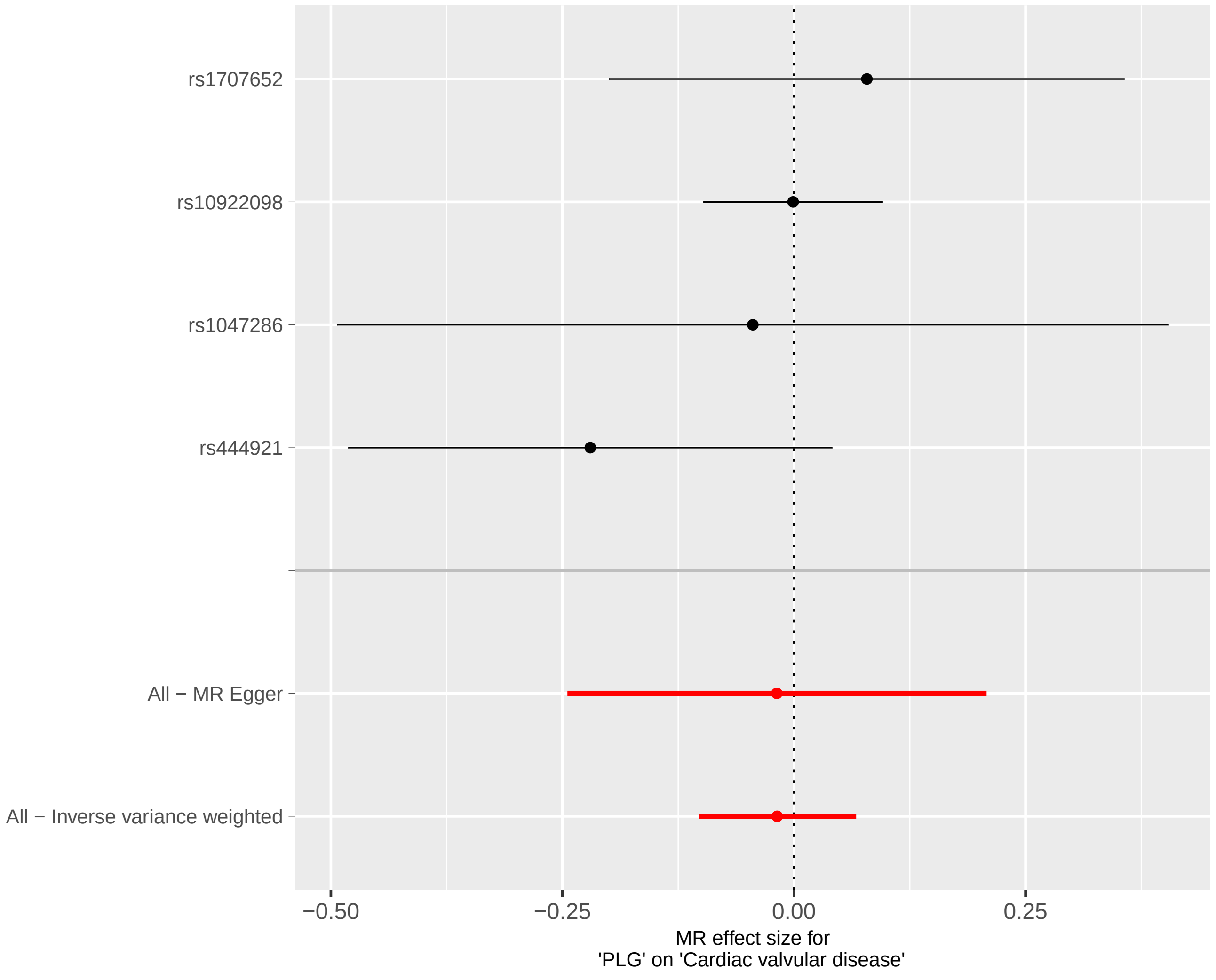

rs1707652
rs10922098
rs1047286
rs444921
All − MR Egger
All − Inverse variance weighted
−0.50
−0.25
0.00
0.25
MR effect size for
'PLG' on 'Cardiac valvular disease'

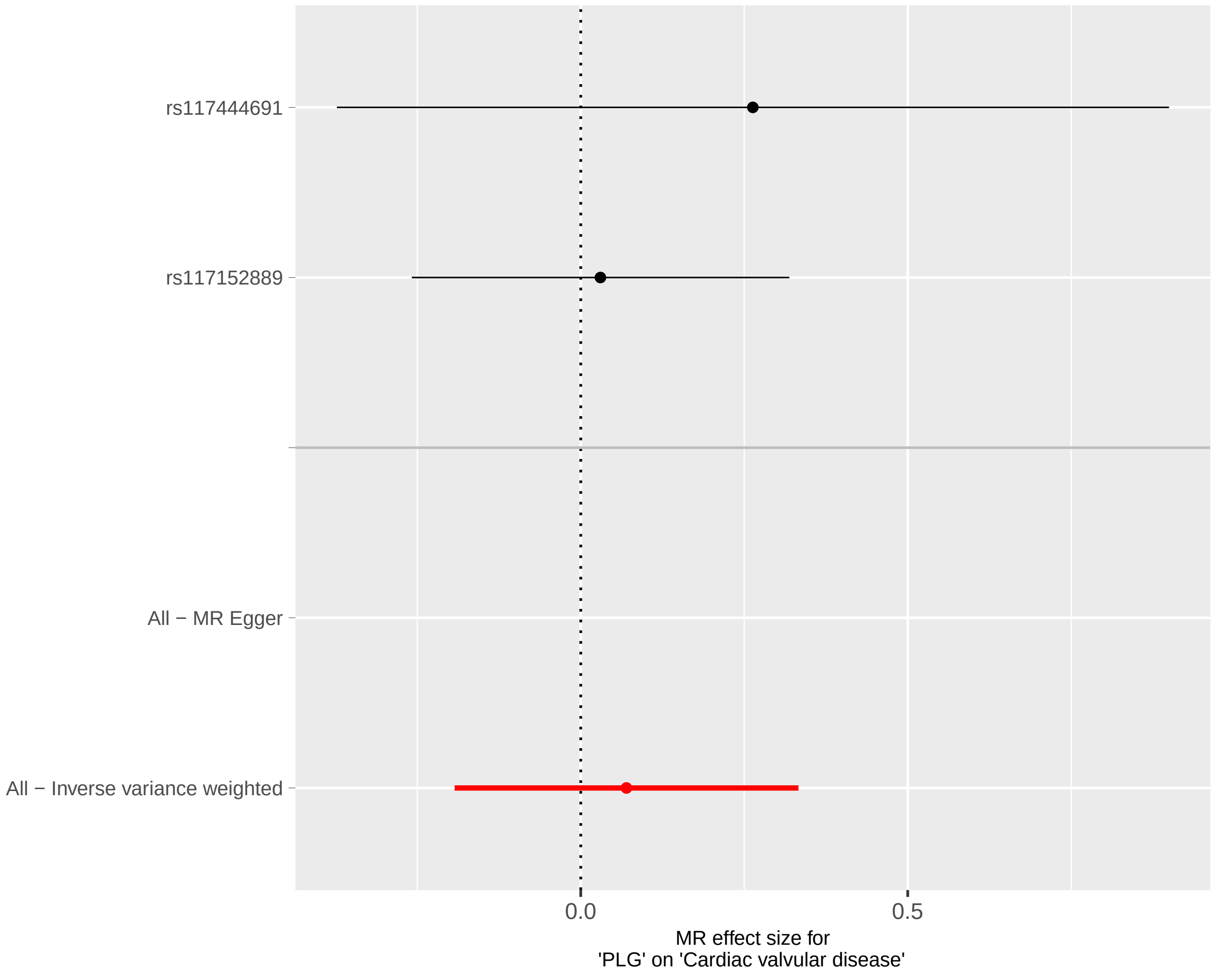
rs117444691
rs117152889
All − MR Egger
All − Inverse variance weighted
0.0
0.5
MR effect size for
'PLG' on 'Cardiac valvular disease'

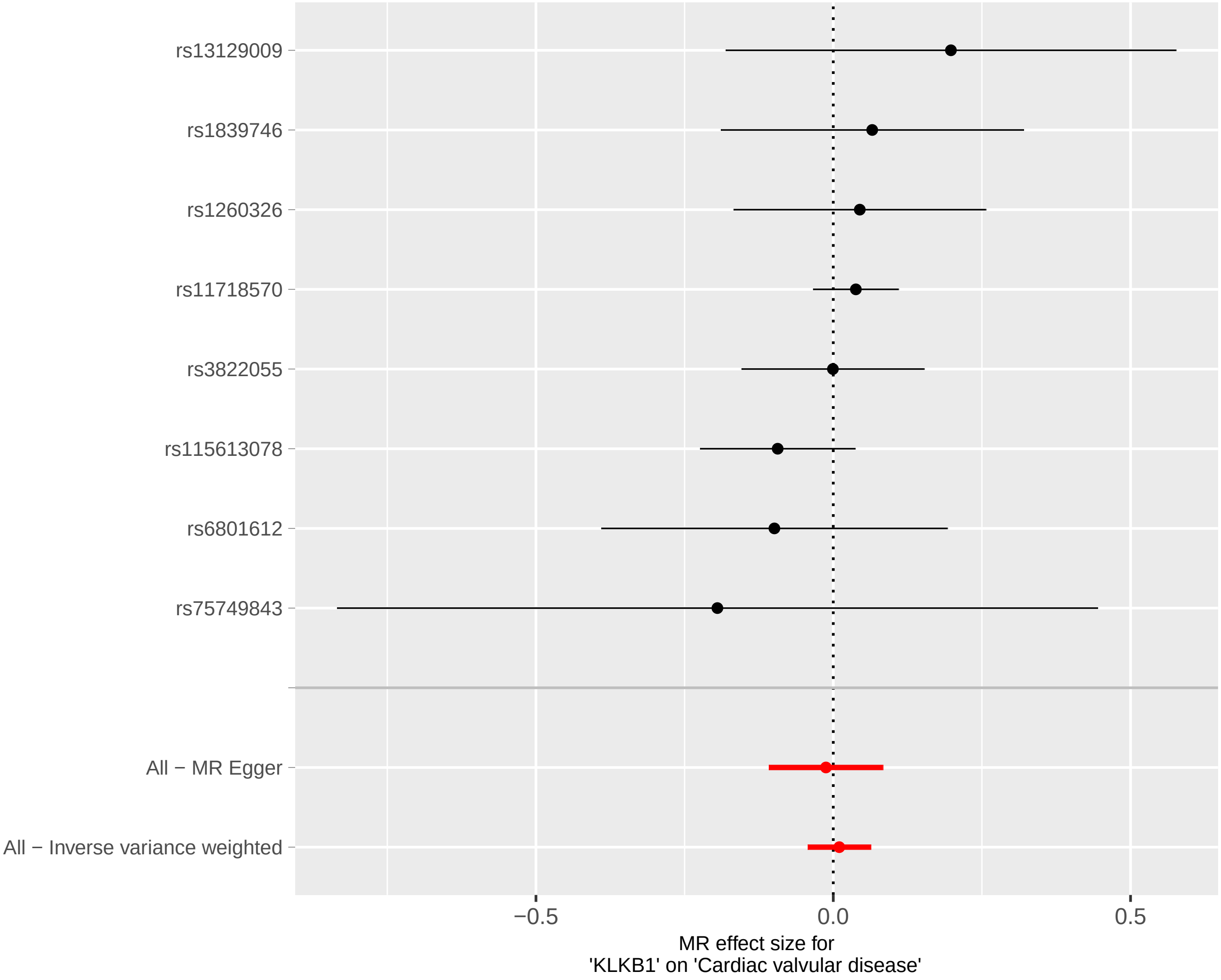
rs13129009
rs1839746
rs1260326
rs11718570
rs3822055
rs115613078
rs6801612
rs75749843
All − MR Egger
All − Inverse variance weighted
−0.5
0.0
0.5
MR effect size for
'KLKB1' on 'Cardiac valvular disease'

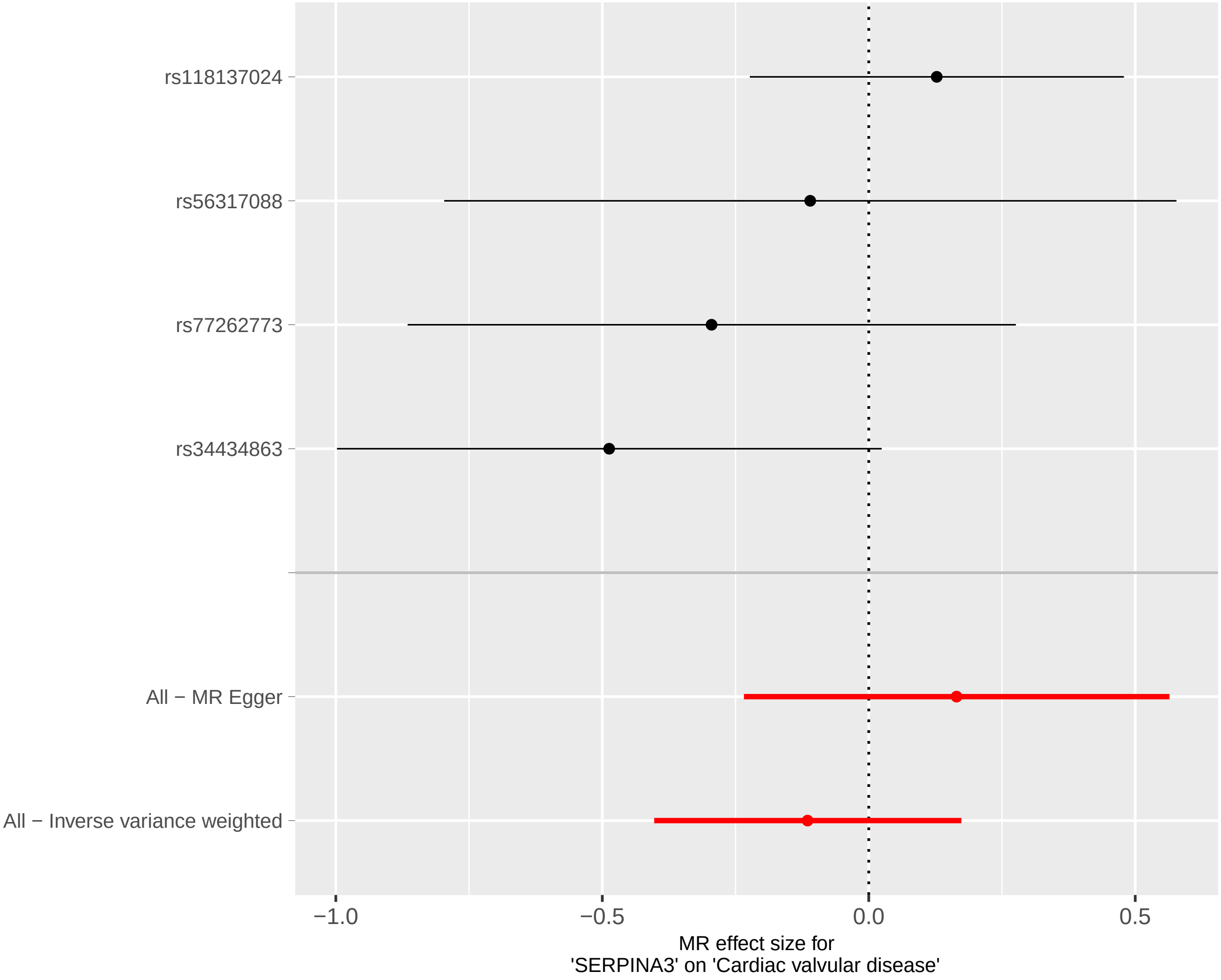

rs118137024
rs56317088
rs77262773
rs34434863
All − MR Egger
All − Inverse variance weighted
−1.0
−0.5
0.0
0.5
MR effect size for
'SERPINA3' on 'Cardiac valvular disease'

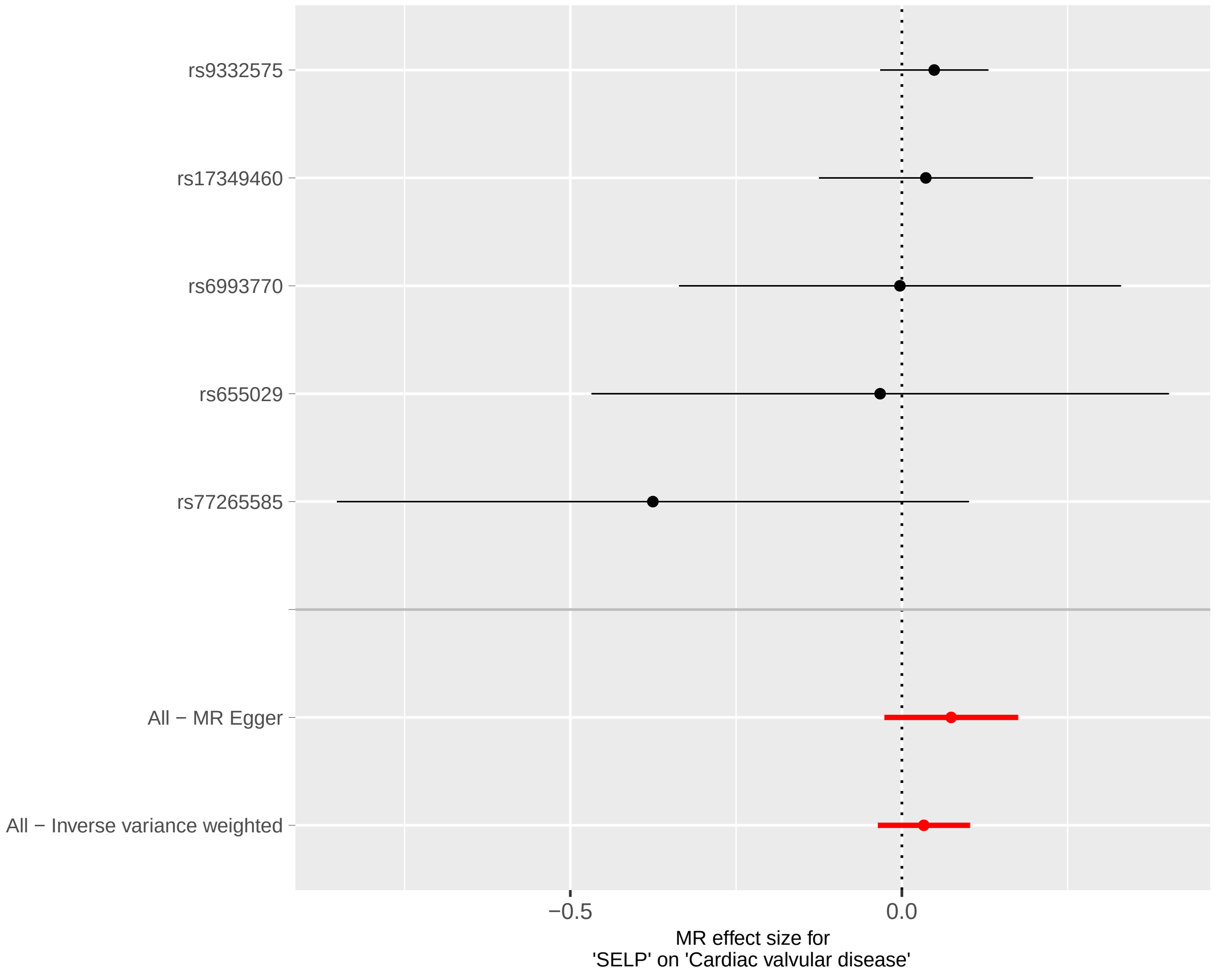

rs9332575
rs17349460
rs6993770
rs655029
rs77265585
All − MR Egger
All − Inverse variance weighted
−0.5
0.0
MR effect size for
'SELP' on 'Cardiac valvular disease'

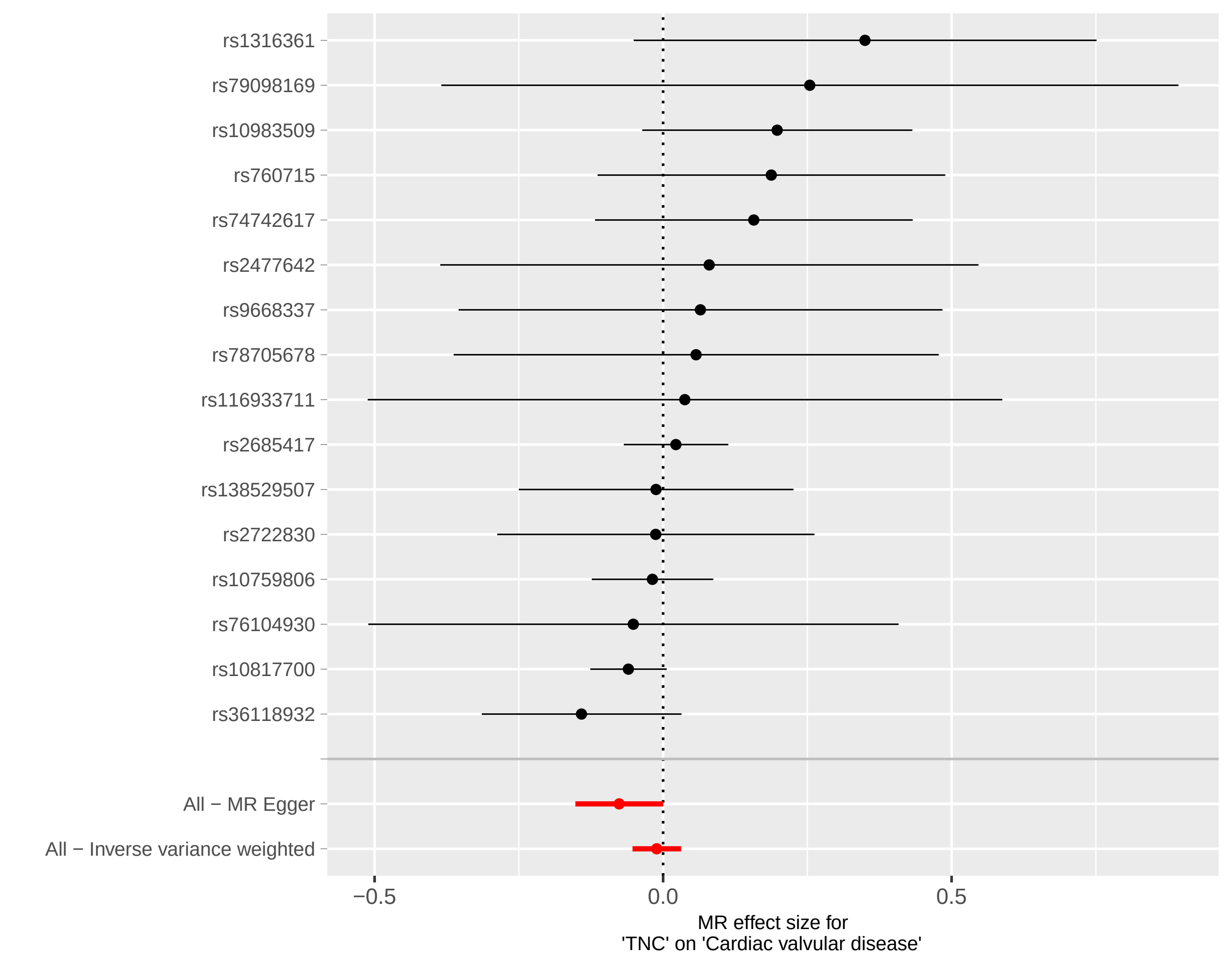
rs1316361
rs79098169
rs10983509
rs760715
rs74742617
rs2477642
rs9668337
rs78705678
rs116933711
rs2685417
rs138529507
rs2722830
rs10759806
rs76104930
rs10817700
rs36118932
All − MR Egger
All − Inverse variance weighted
−0.5
0.0
0.5
MR effect size for
'TNC' on 'Cardiac valvular disease'

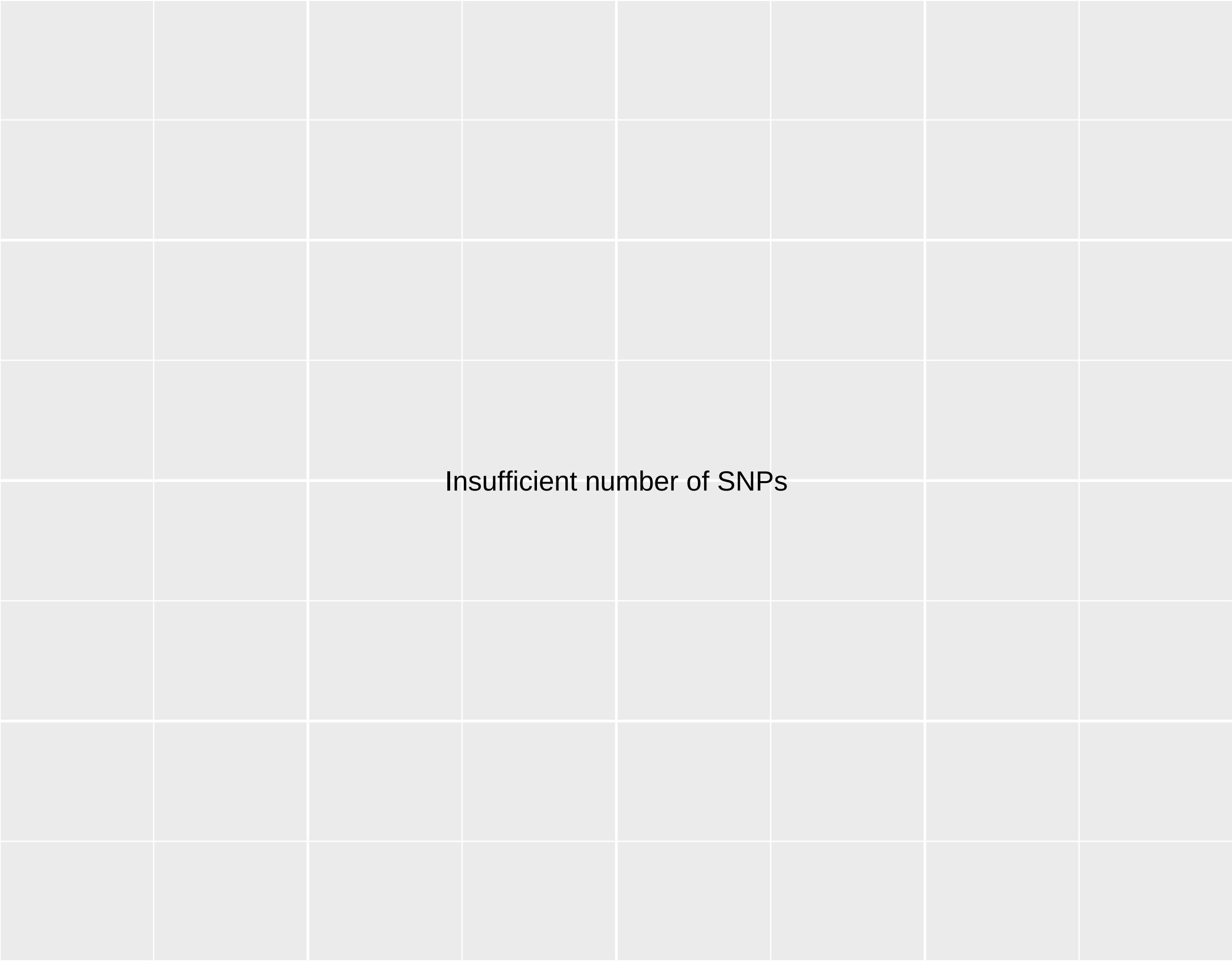
Insufficient number of SNPs

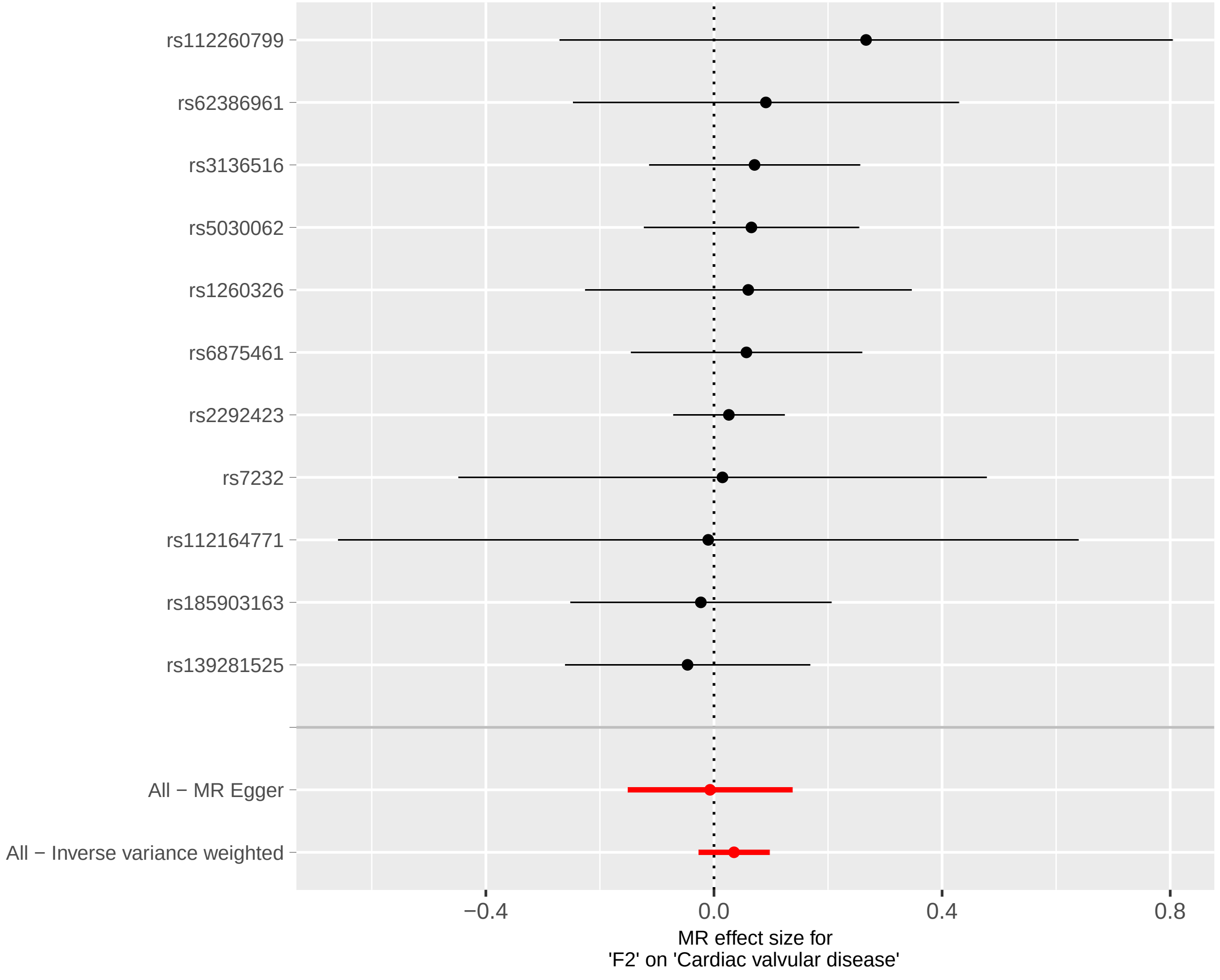
rs112260799
rs62386961
rs3136516
rs5030062
rs1260326
rs6875461
rs2292423
rs7232
rs112164771
rs185903163
rs139281525
All − MR Egger
All − Inverse variance weighted
−0.4
0.0
0.4
0.8
MR effect size for
'F2' on 'Cardiac valvular disease'

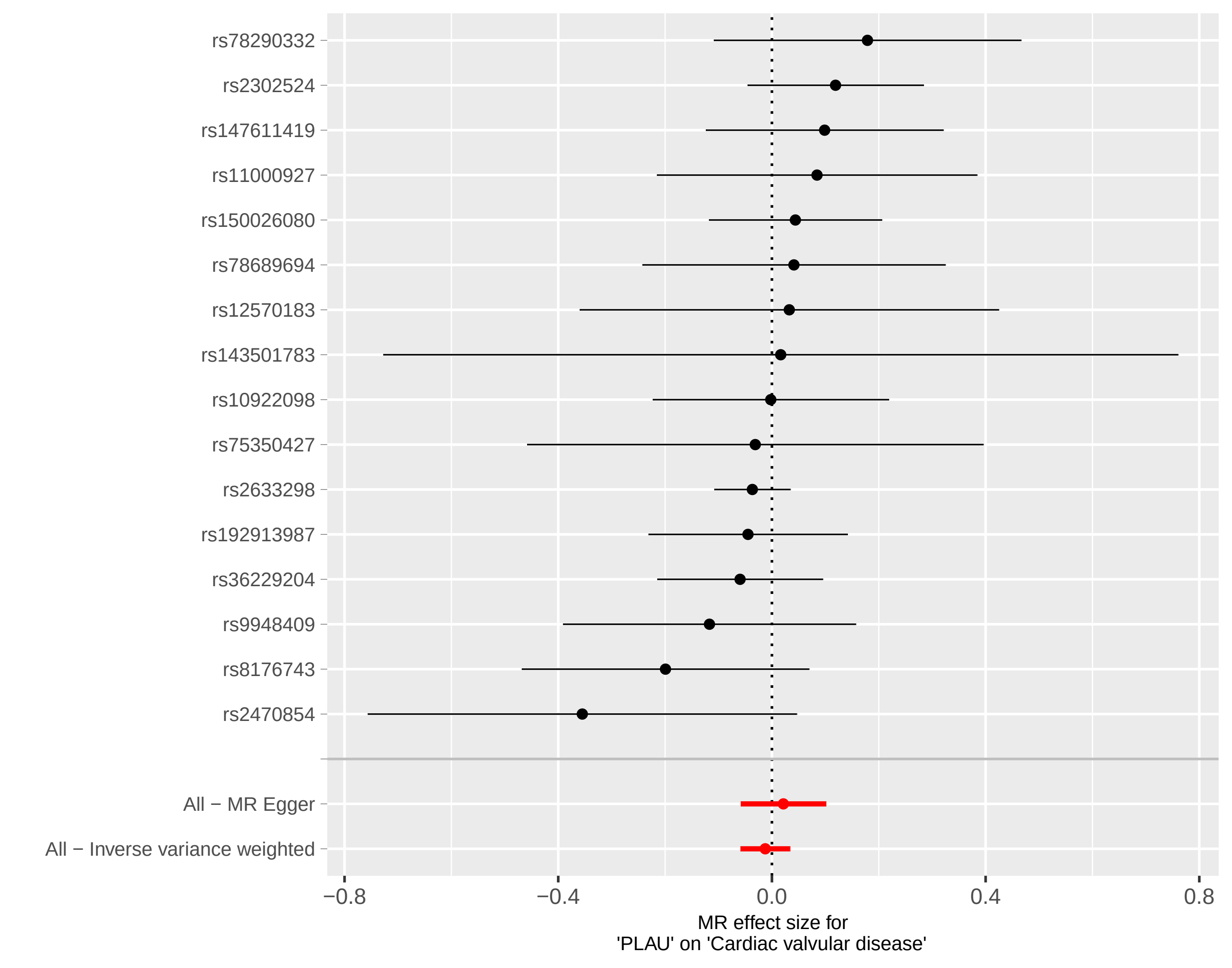

rs78290332
rs2302524
rs147611419
rs11000927
rs150026080
rs78689694
rs12570183
rs143501783
rs10922098
rs75350427
rs2633298
rs192913987
rs36229204
rs9948409
rs8176743
rs2470854
All − MR Egger
All − Inverse variance weighted
−0.8
−0.4
0.0
0.4
0.8
MR effect size for
'PLAU' on 'Cardiac valvular disease'

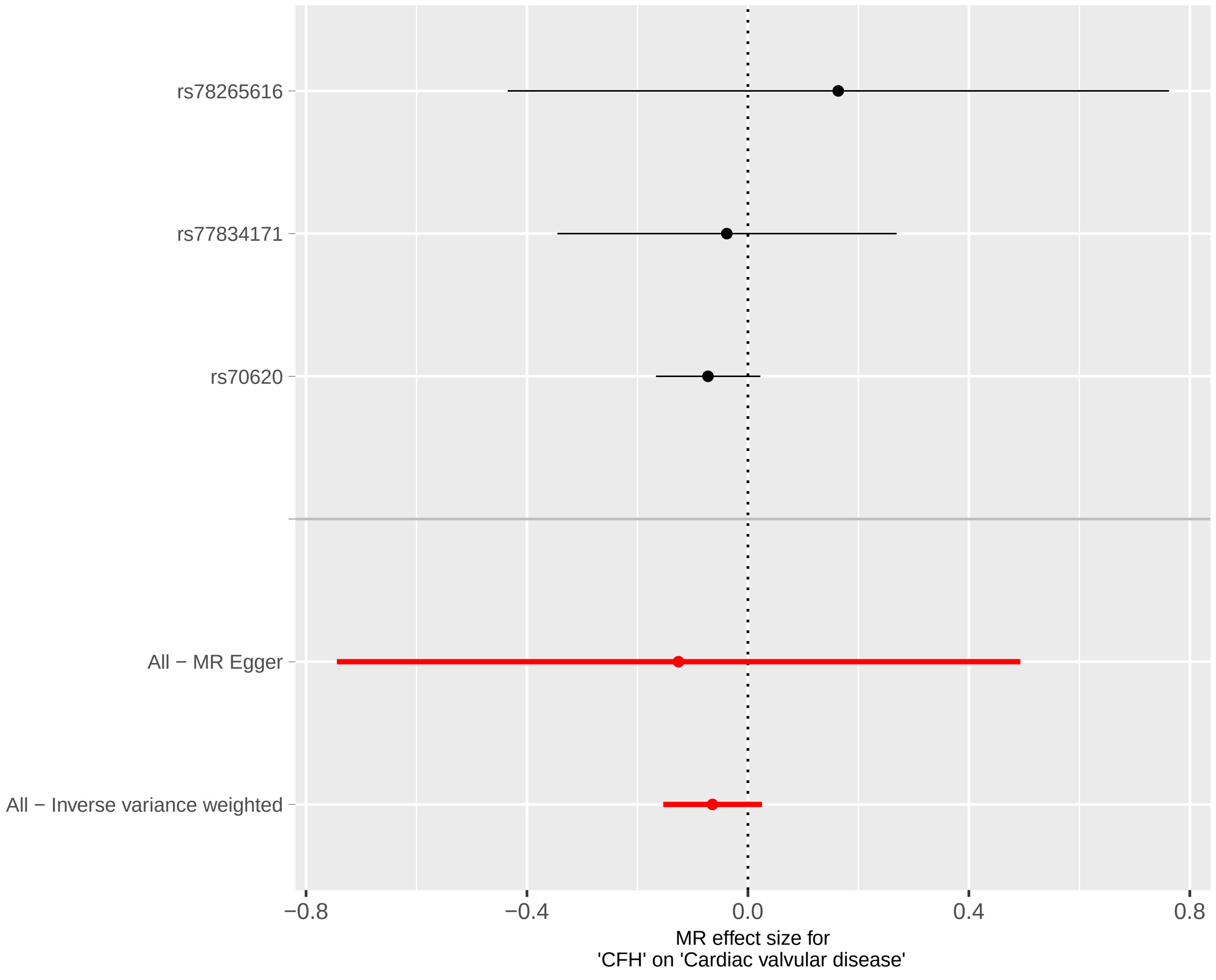

rs78265616
rs77834171
rs70620
All − MR Egger
All − Inverse variance weighted
−0.8
−0.4
0.0
0.4
0.8
MR effect size for
'CFH' on 'Cardiac valvular disease'

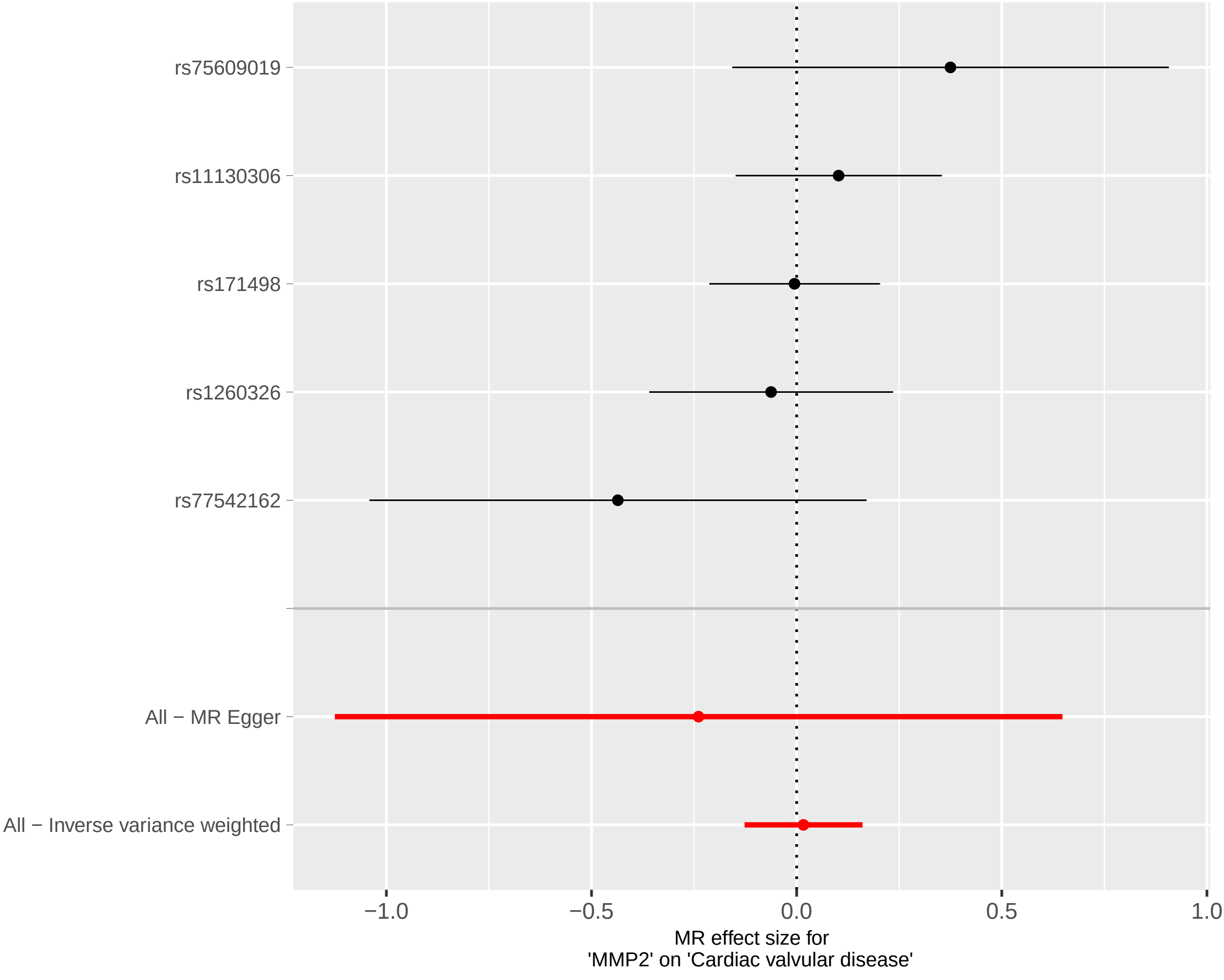
rs75609019
rs11130306
rs171498
rs1260326
rs77542162
All − MR Egger
All − Inverse variance weighted
−1.0
−0.5
0.0
0.5
1.0
MR effect size for
'MMP2' on 'Cardiac valvular disease'

Insufficient number of SNPs

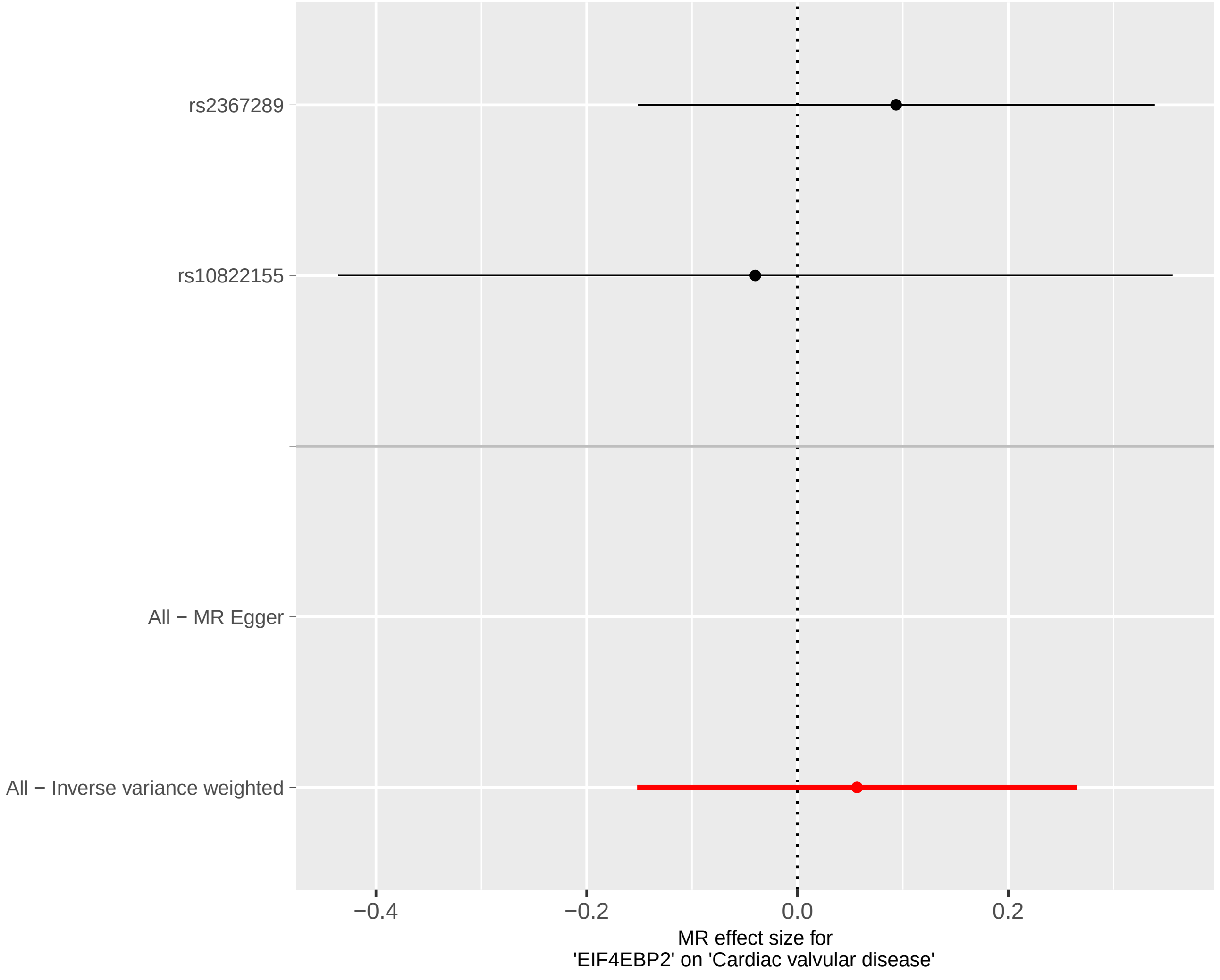
rs2367289
rs10822155
All − MR Egger
All − Inverse variance weighted
−0.4
−0.2
0.0
0.2
MR effect size for
'EIF4EBP2' on 'Cardiac valvular disease'

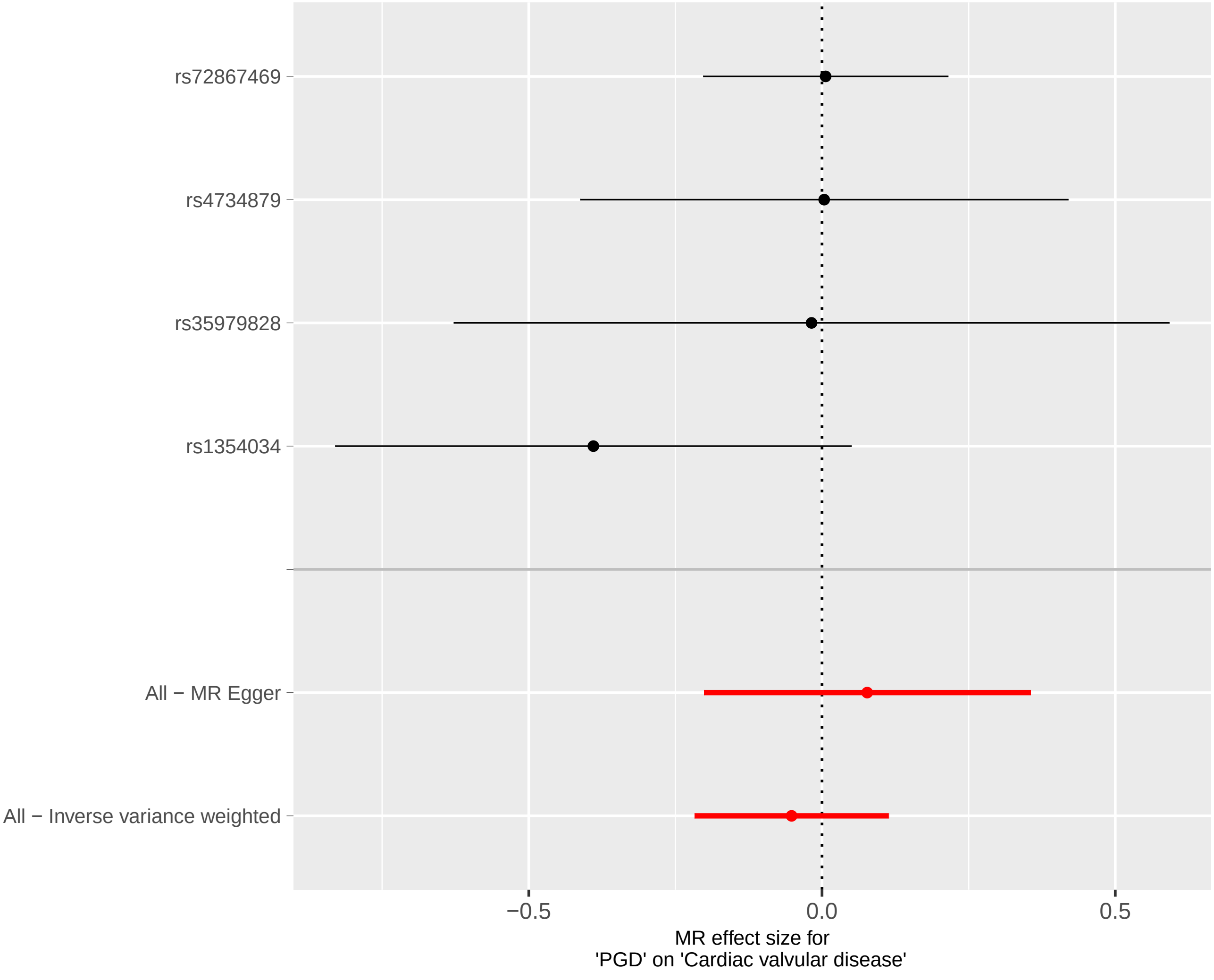
rs72867469
rs4734879
rs35979828
rs1354034
All − MR Egger
All − Inverse variance weighted
−0.5
0.0
0.5
MR effect size for
'PGD' on 'Cardiac valvular disease'

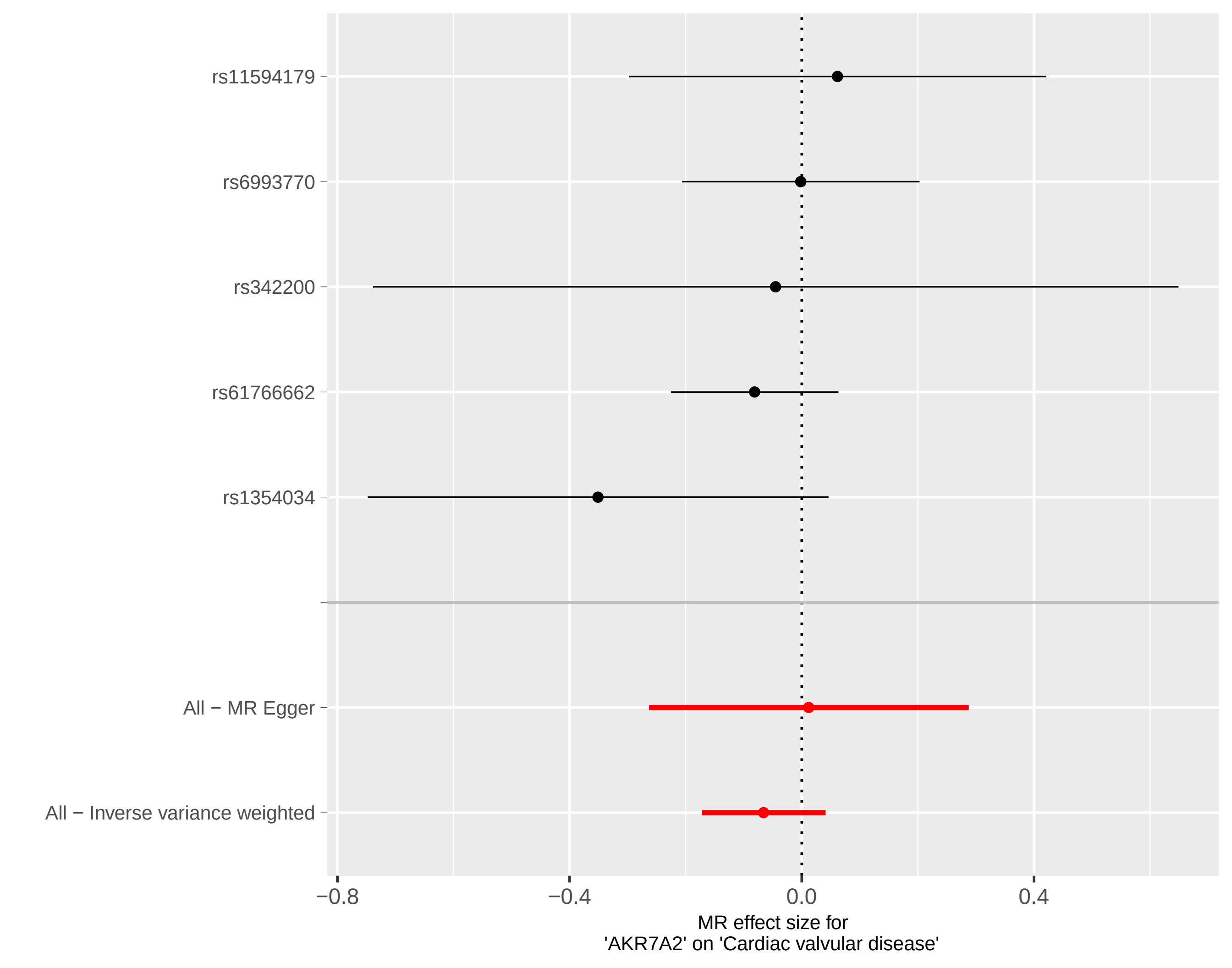

rs11594179
rs6993770
rs342200
rs61766662
rs1354034
All − MR Egger
All − Inverse variance weighted
−0.8
−0.4
0.0
0.4
MR effect size for
'AKR7A2' on 'Cardiac valvular disease'

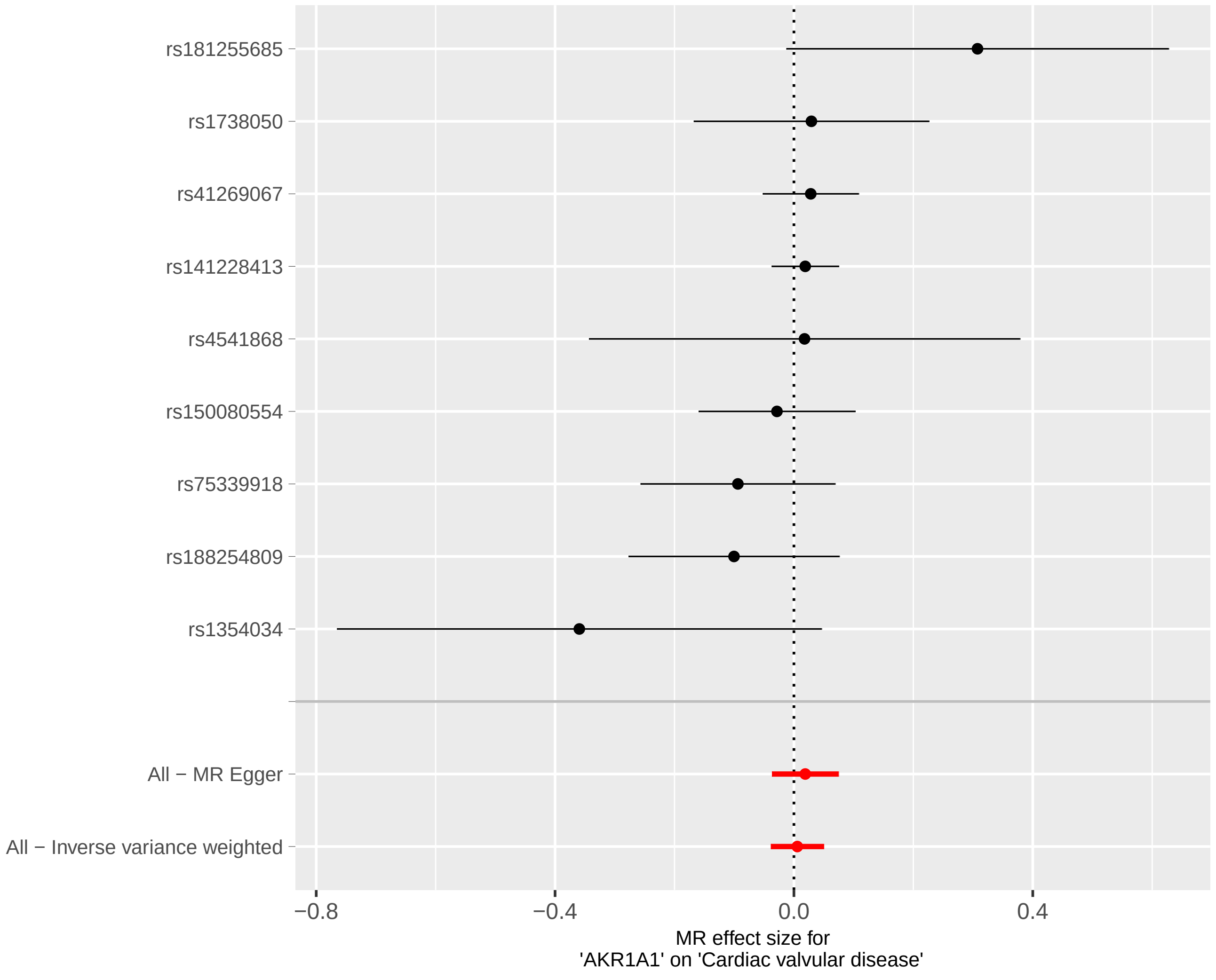
rs181255685
rs1738050
rs41269067
rs141228413
rs4541868
rs150080554
rs75339918
rs188254809
rs1354034
All − MR Egger
All − Inverse variance weighted
−0.8
−0.4
0.0
0.4
MR effect size for
'AKR1A1' on 'Cardiac valvular disease'

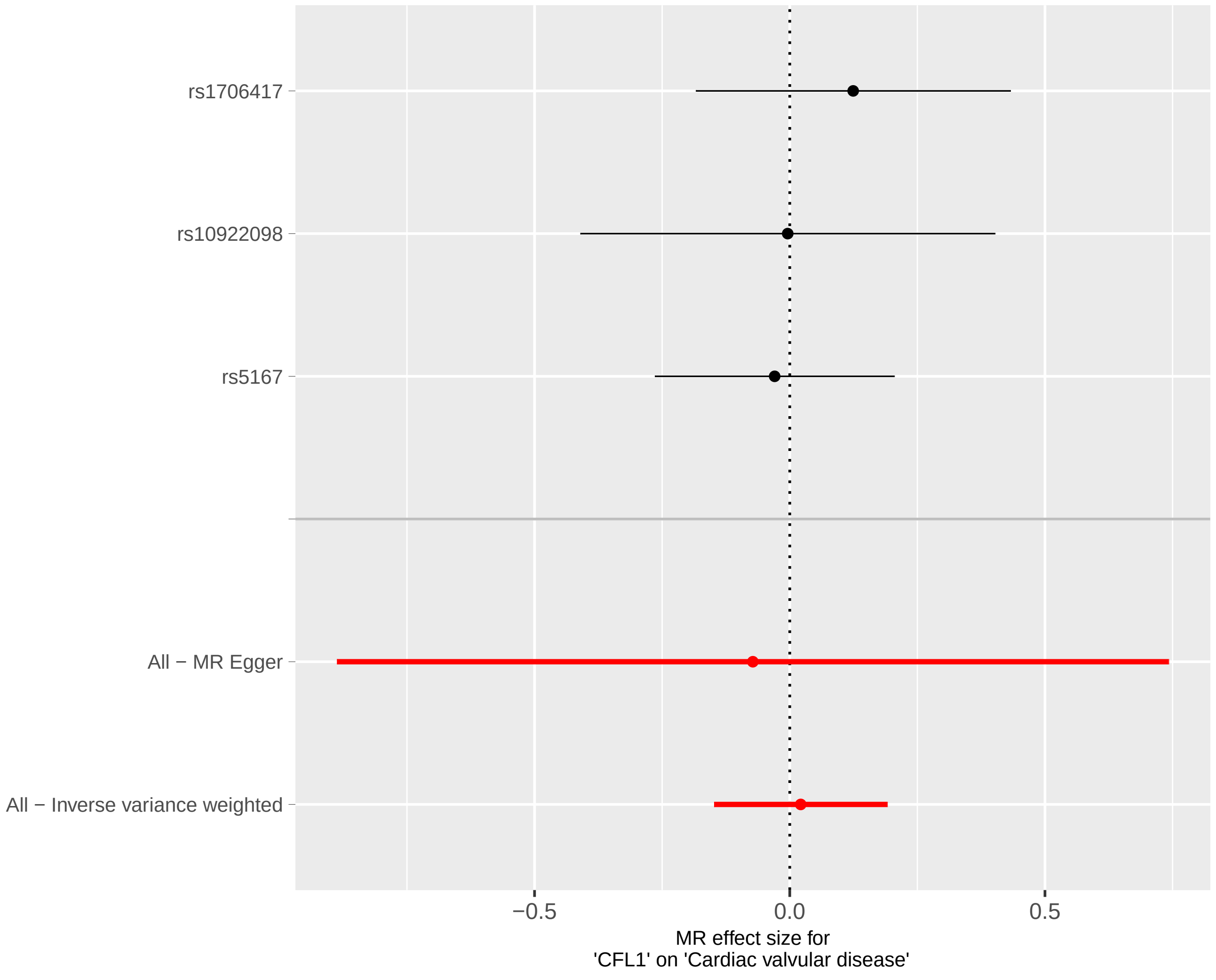
rs1706417
rs10922098
rs5167
All − MR Egger
All − Inverse variance weighted
−0.5
0.0
0.5
MR effect size for
'CFL1' on 'Cardiac valvular disease'

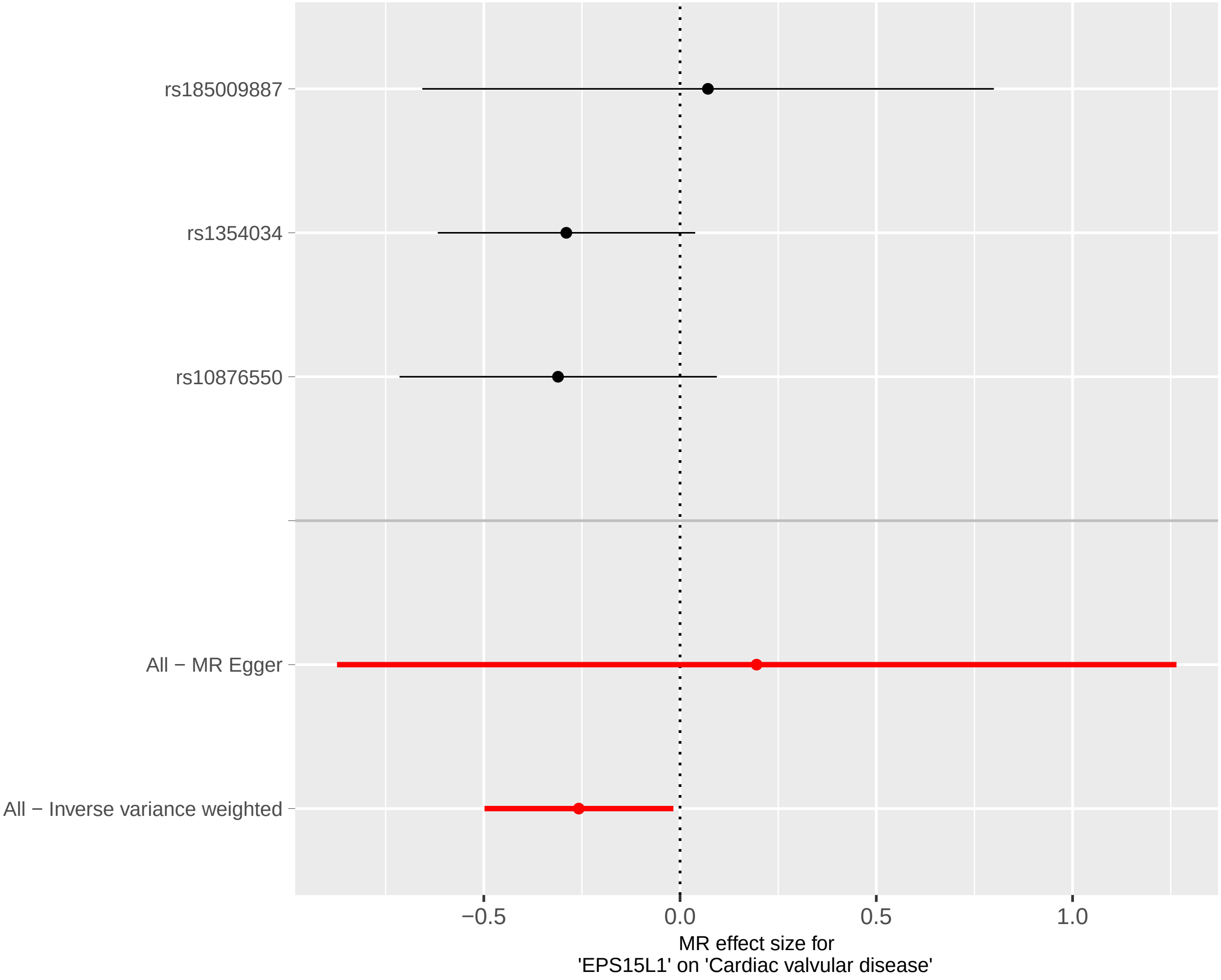
rs185009887
rs1354034
rs10876550
All − MR Egger
All − Inverse variance weighted
−0.5
0.0
0.5
1.0
MR effect size for
'EPS15L1' on 'Cardiac valvular disease'

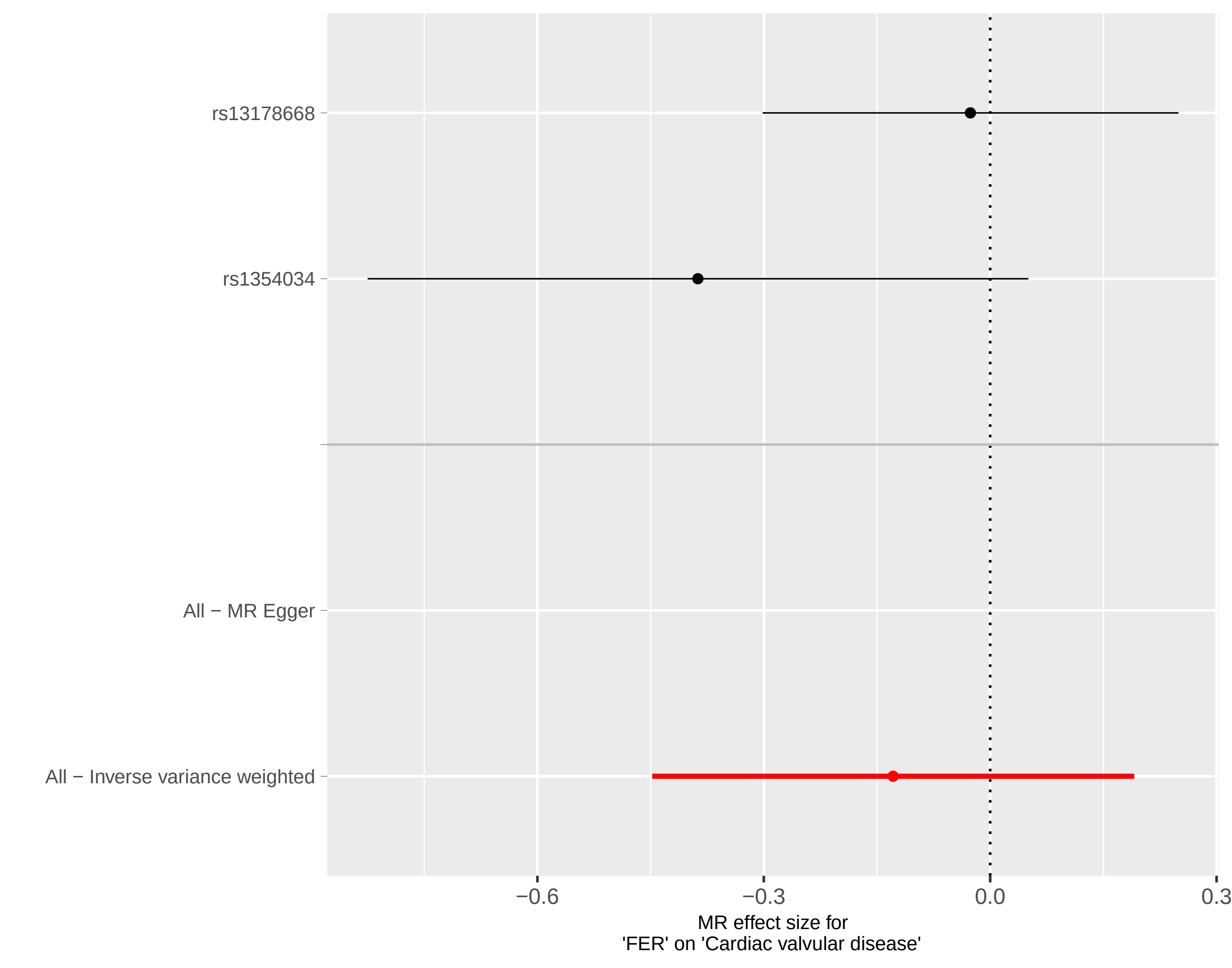
rs13178668
rs1354034
All − MR Egger
All − Inverse variance weighted
−0.6
−0.3
0.0
0.3
MR effect size for
'FER' on 'Cardiac valvular disease'

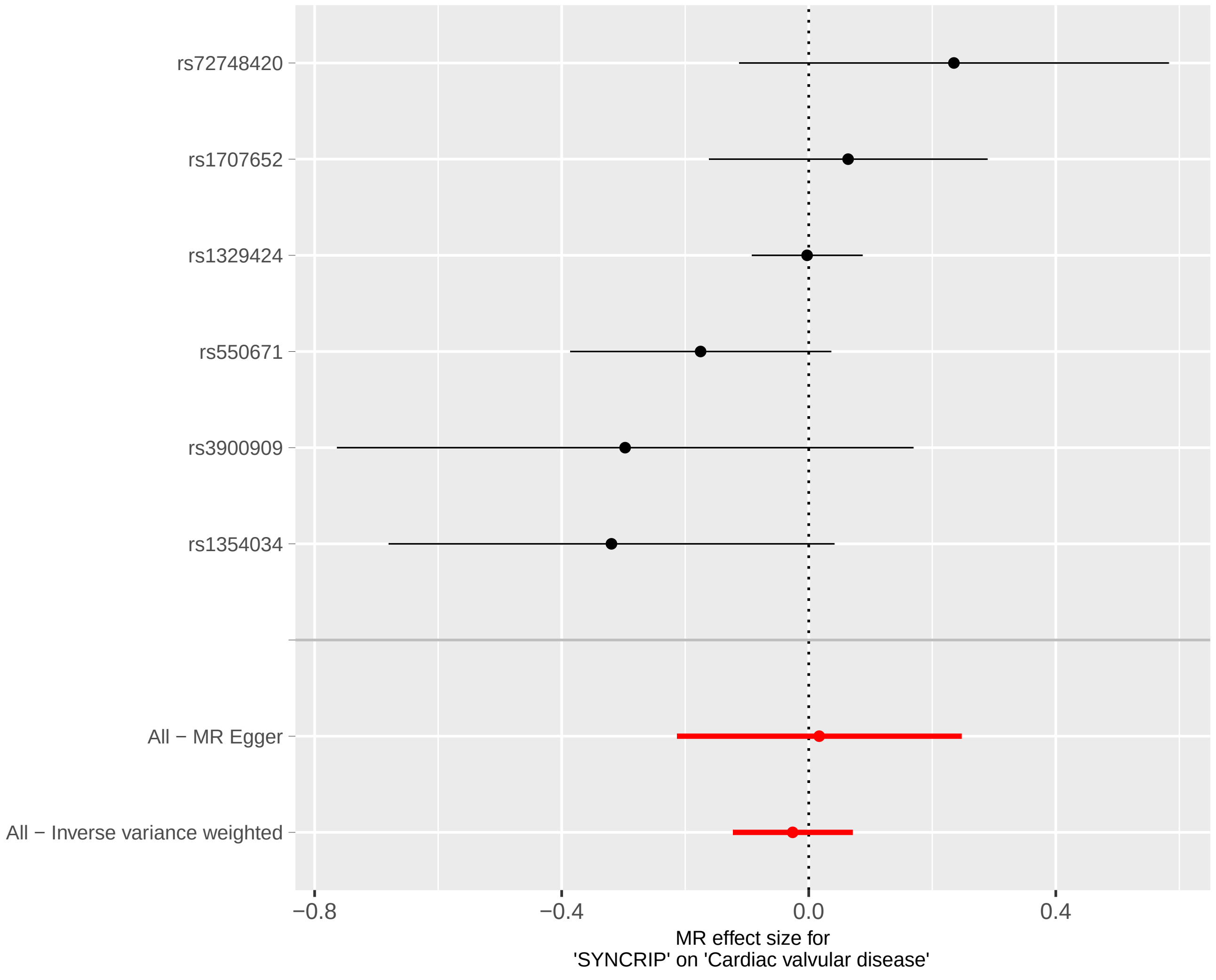
rs72748420
rs1707652
rs1329424
rs550671
rs3900909
rs1354034
All − MR Egger
All − Inverse variance weighted
−0.8
−0.4
0.0
0.4
MR effect size for
'SYNCRIP' on 'Cardiac valvular disease'

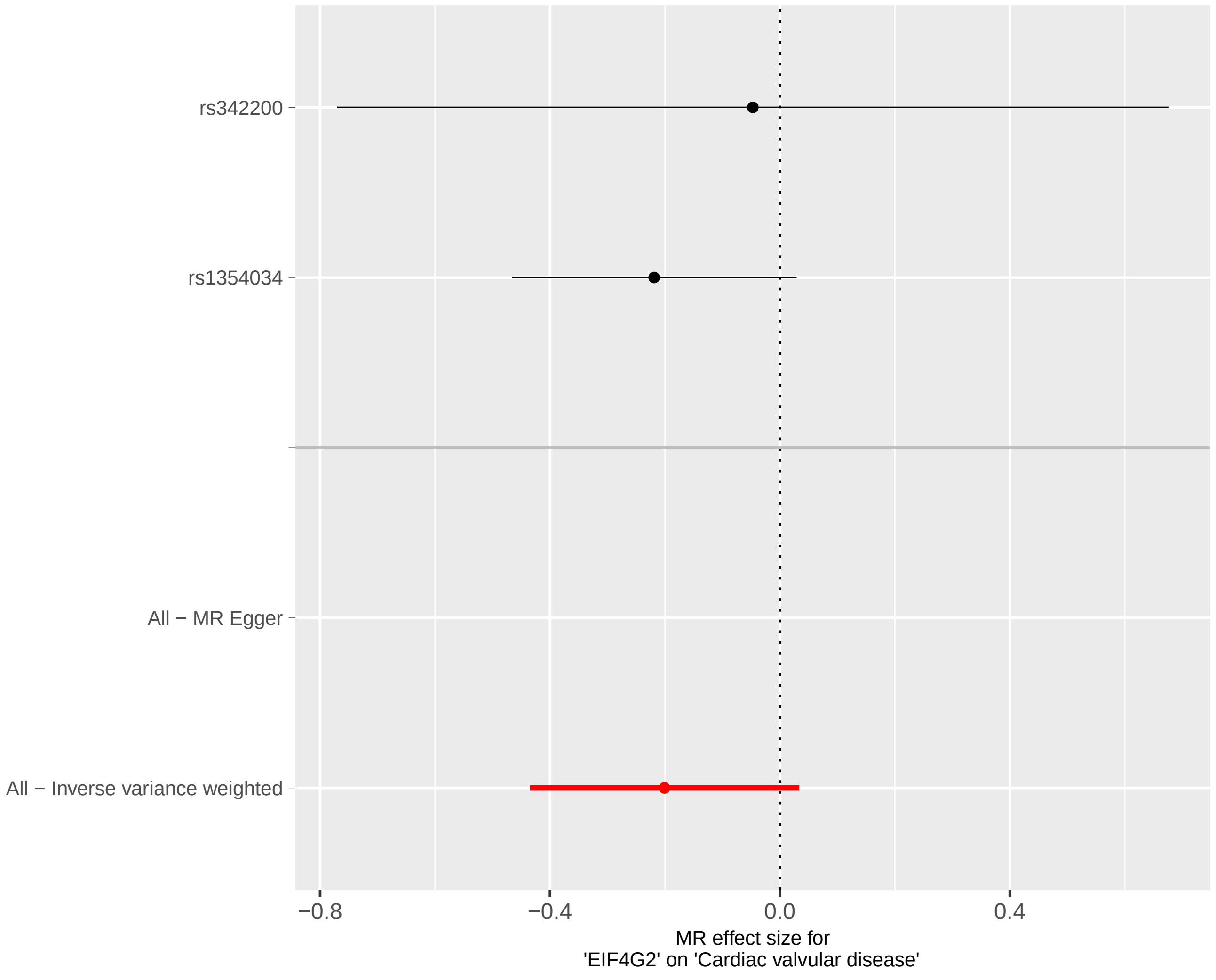
rs342200
rs1354034
All − MR Egger
All − Inverse variance weighted
−0.8
−0.4
0.0
0.4
MR effect size for
'EIF4G2' on 'Cardiac valvular disease'

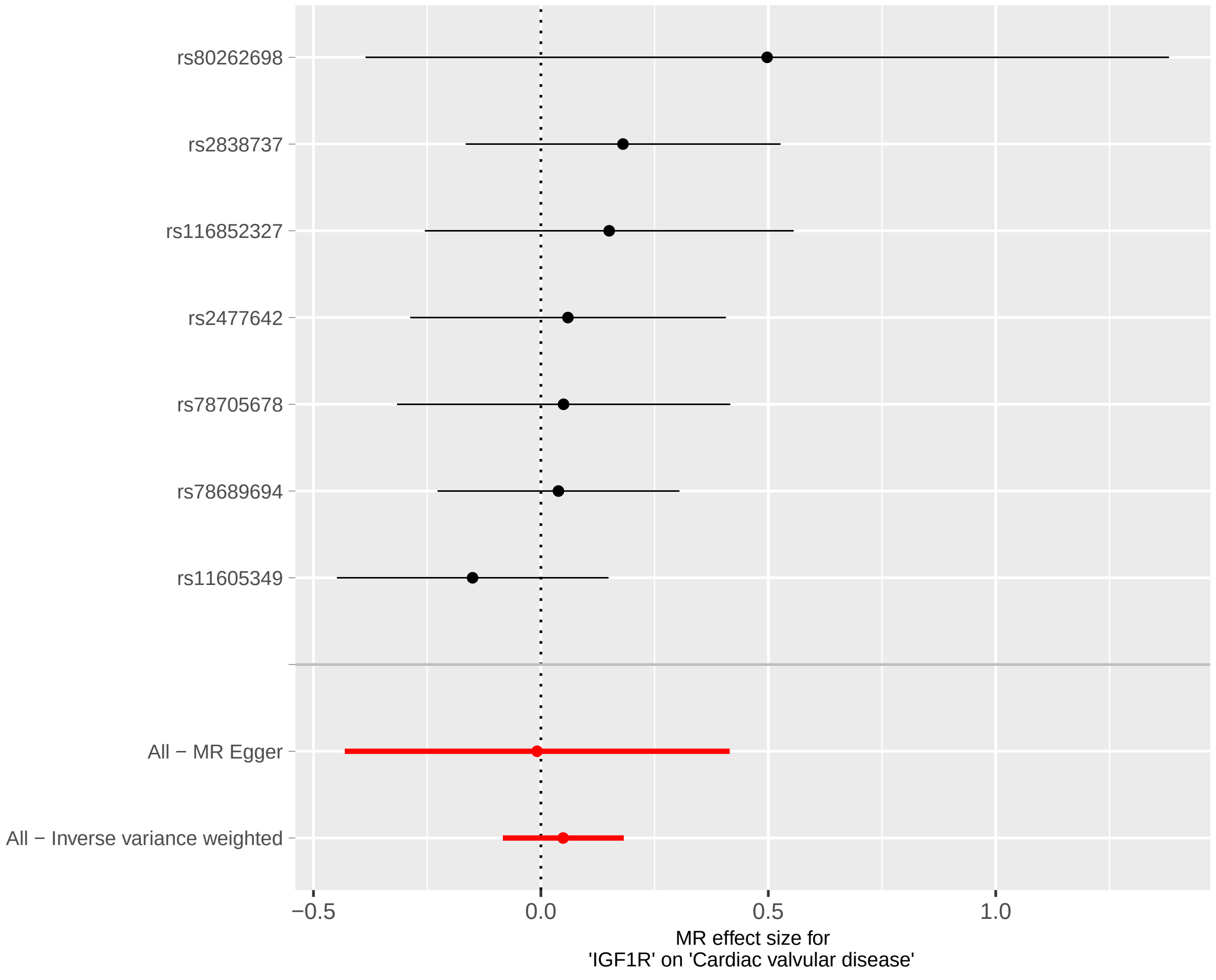
rs80262698
rs2838737
rs116852327
rs2477642
rs78705678
rs78689694
rs11605349
All − MR Egger
All − Inverse variance weighted
−0.5
0.0
0.5
1.0
MR effect size for
'IGF1R' on 'Cardiac valvular disease'

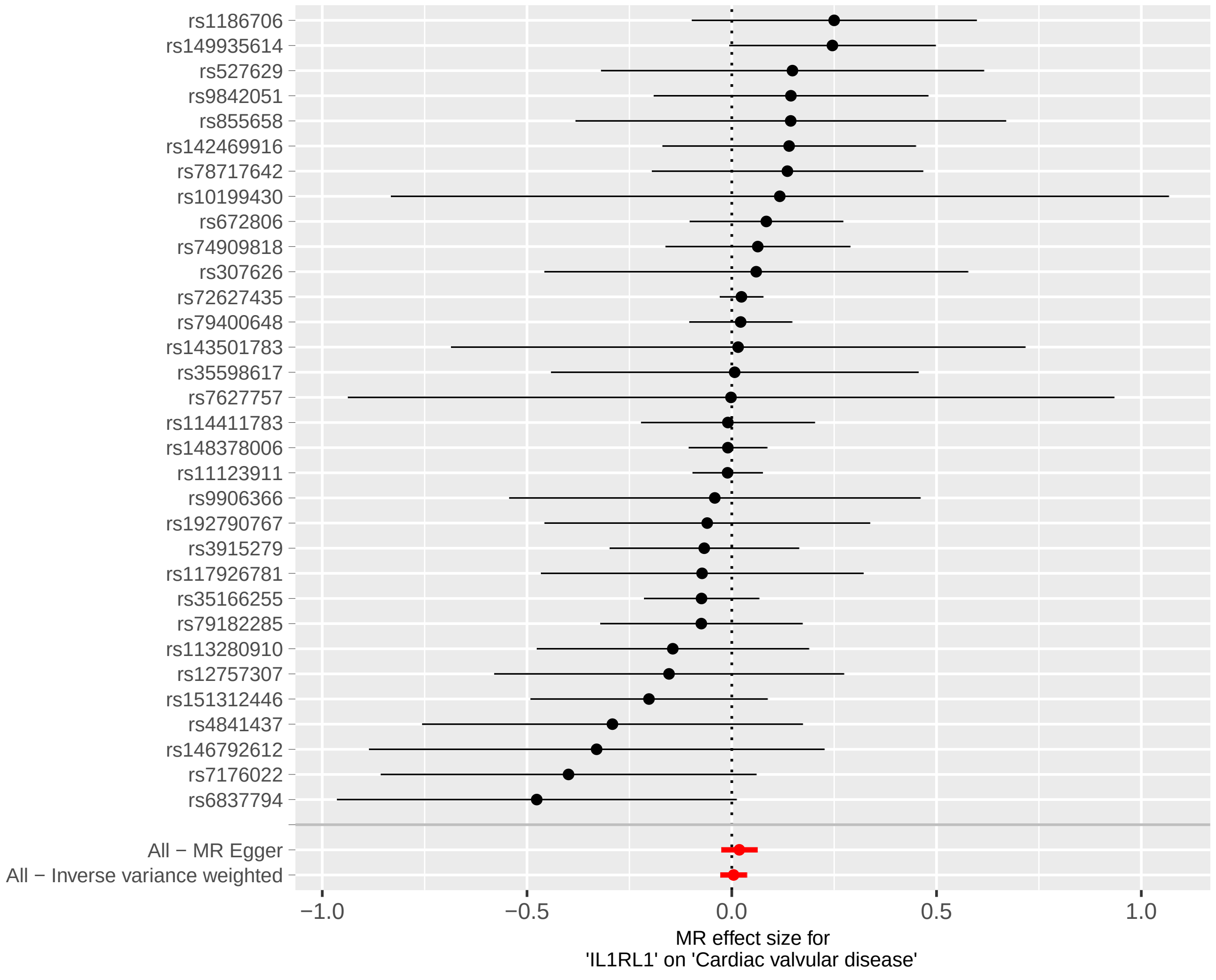

rs1186706
rs149935614
rs527629
rs9842051
rs855658
rs142469916
rs78717642
rs10199430
rs672806
rs74909818
rs307626
rs72627435
rs79400648
rs143501783
rs35598617
rs7627757
rs114411783
rs148378006
rs11123911
rs9906366
rs192790767
rs3915279
rs117926781
rs35166255
rs79182285
rs113280910
rs12757307
rs151312446
rs4841437
rs146792612
rs7176022
rs6837794
All − MR Egger
All − Inverse variance weighted
−1.0
−0.5
0.0
0.5
1.0
MR effect size for
'IL1RL1' on 'Cardiac valvular disease'

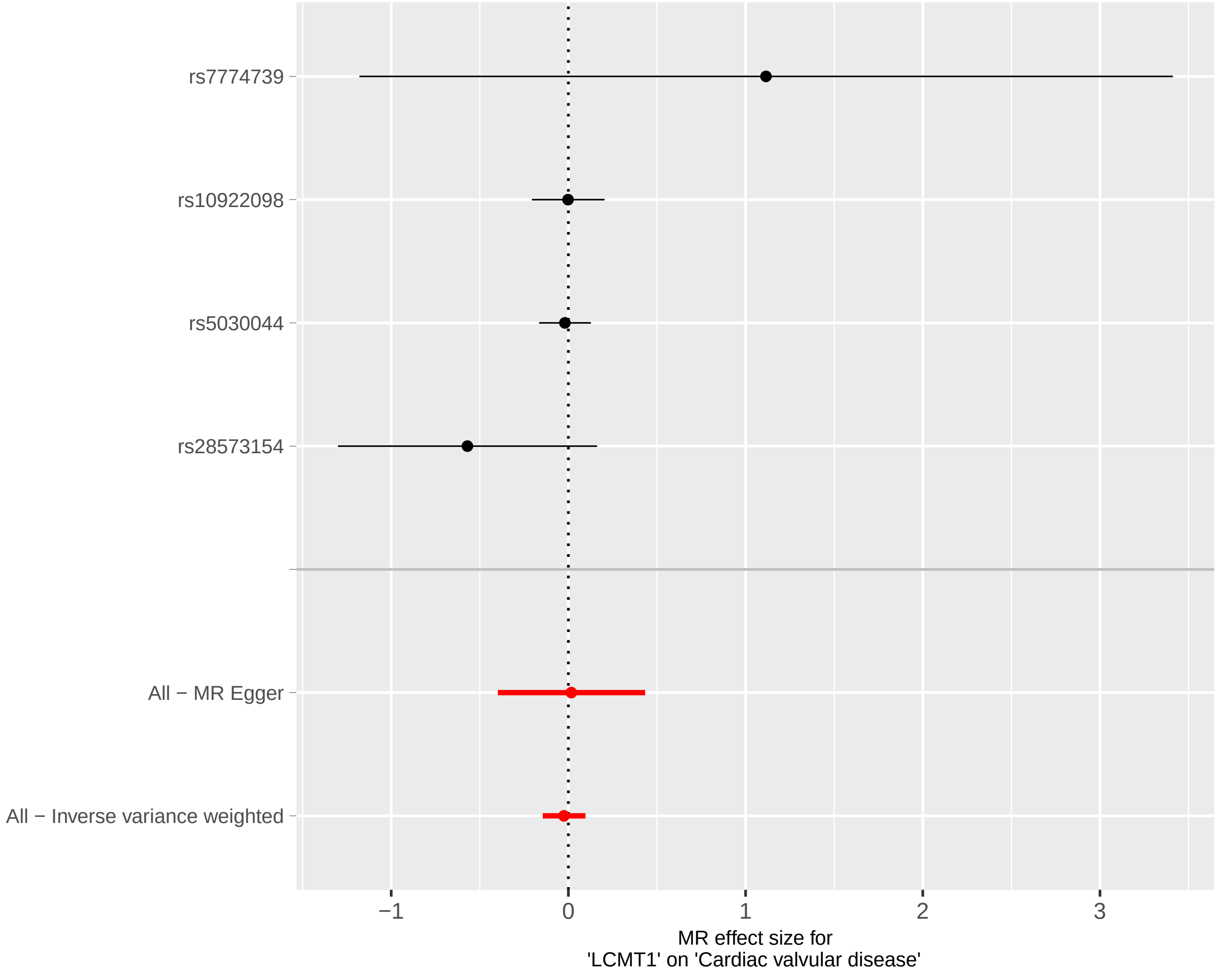
rs7774739
rs10922098
rs5030044
rs28573154
All − MR Egger
All − Inverse variance weighted
−1
0
1
2
3
MR effect size for
'LCMT1' on 'Cardiac valvular disease'

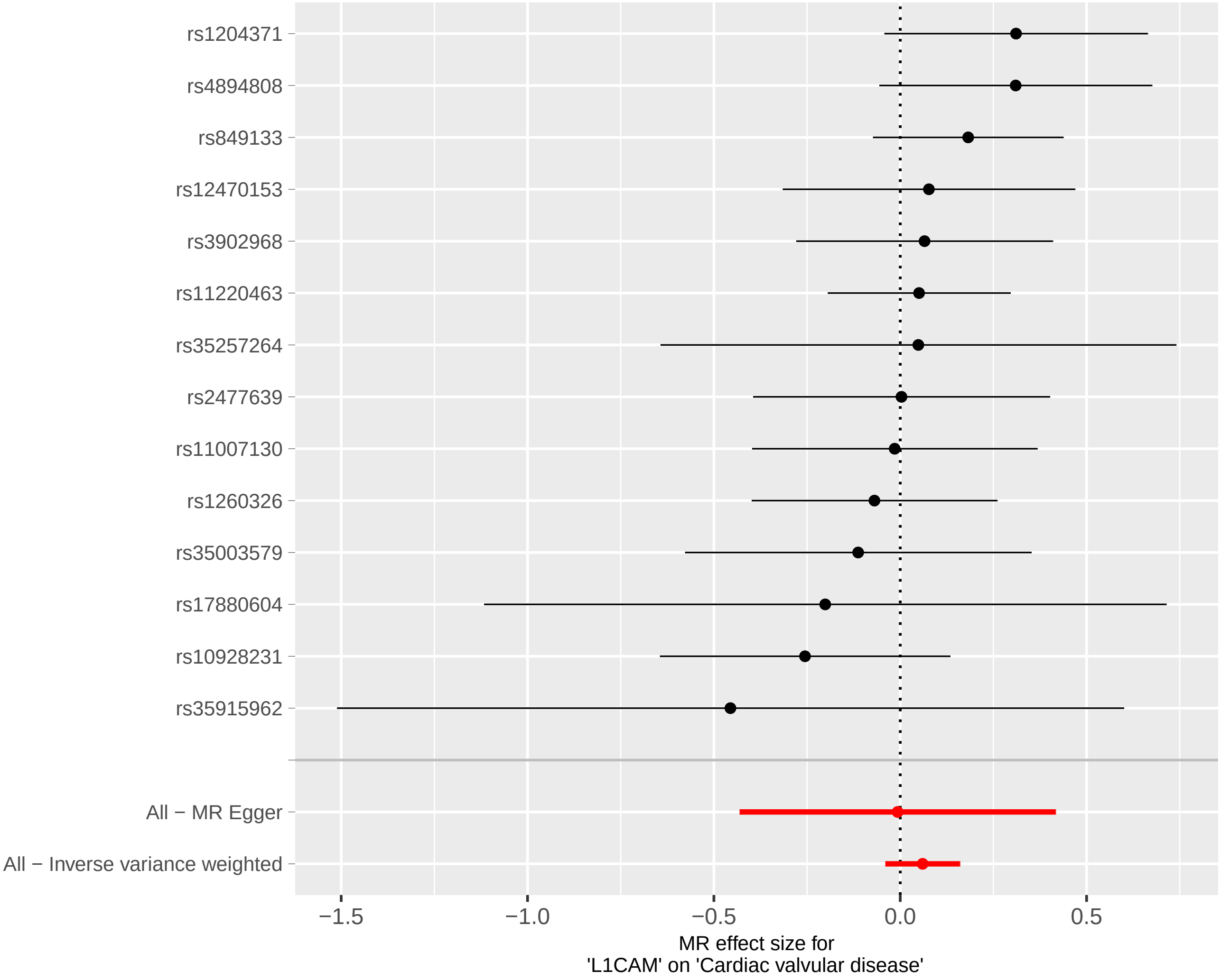
rs1204371
rs4894808
rs849133
rs12470153
rs3902968
rs11220463
rs35257264
rs2477639
rs11007130
rs1260326
rs35003579
rs17880604
rs10928231
rs35915962
All − MR Egger
All − Inverse variance weighted
−1.5
−1.0
−0.5
0.0
0.5
MR effect size for
'L1CAM' on 'Cardiac valvular disease'

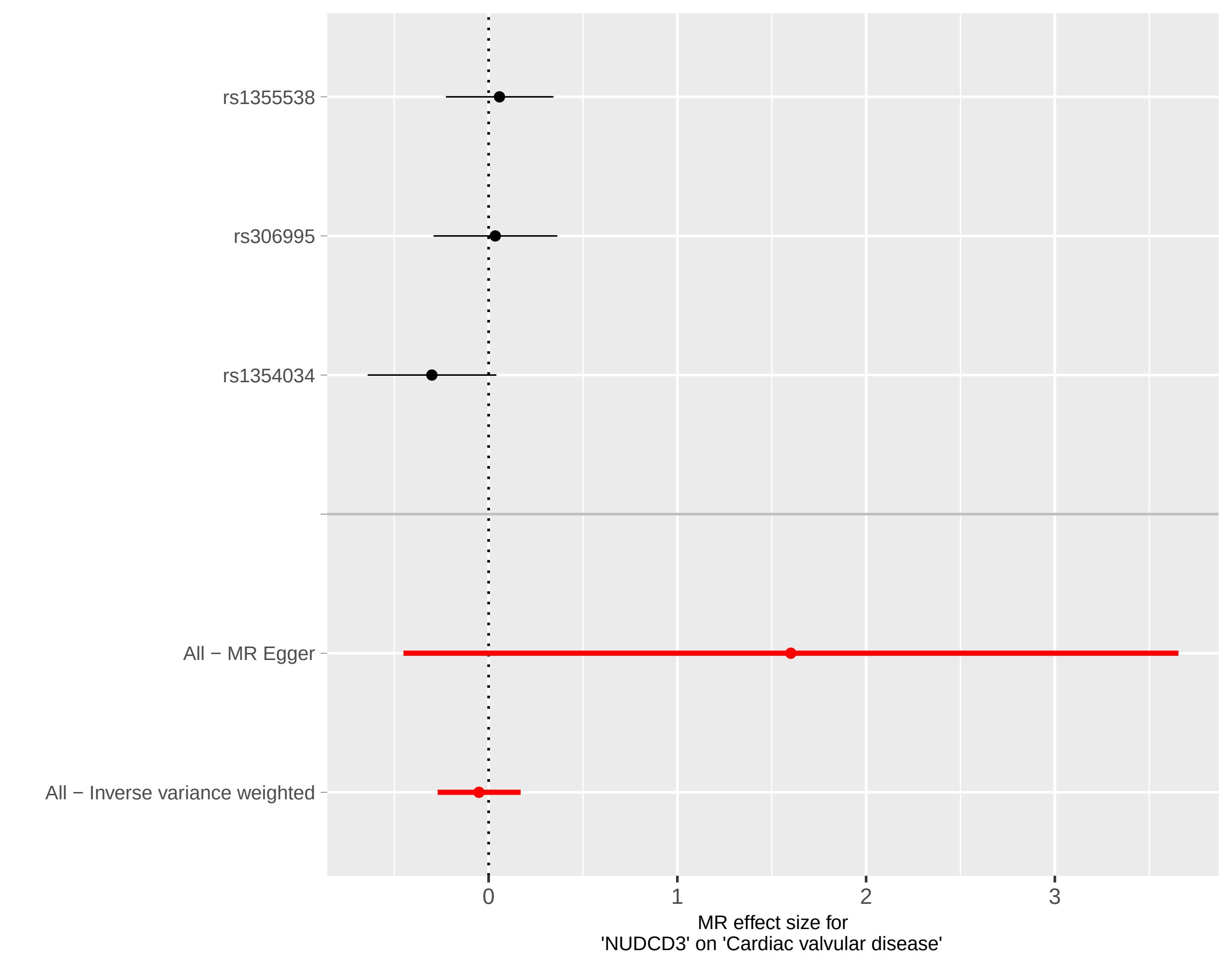

rs1355538
rs306995
rs1354034
All − MR Egger
All − Inverse variance weighted
0
1
2
3
MR effect size for
'NUDCD3' on 'Cardiac valvular disease'

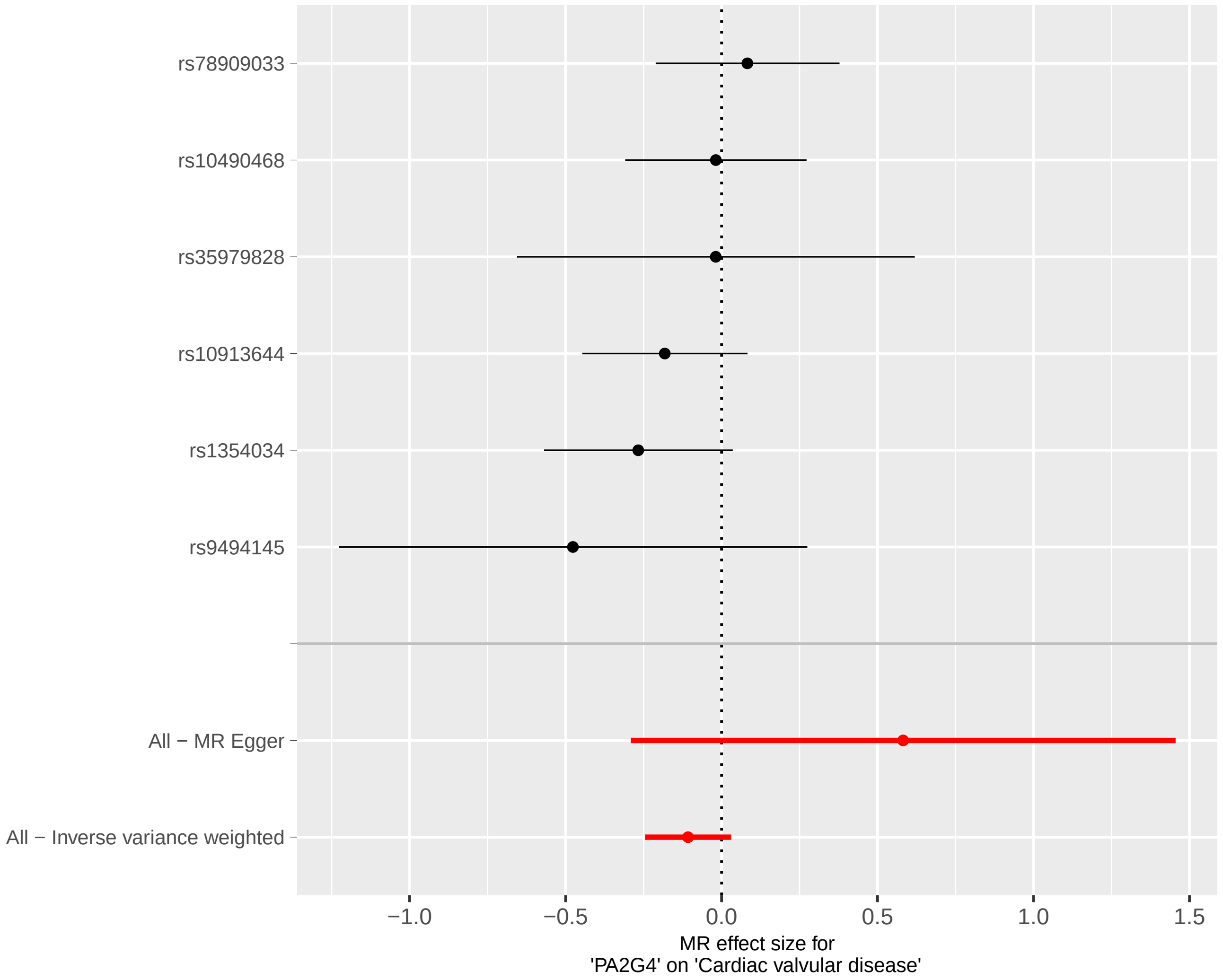
rs78909033
rs10490468
rs35979828
rs10913644
rs1354034
rs9494145
All − MR Egger
All − Inverse variance weighted
−1.0
−0.5
0.0
0.5
1.0
1.5
MR effect size for
'PA2G4' on 'Cardiac valvular disease'

Insufficient number of SNPs

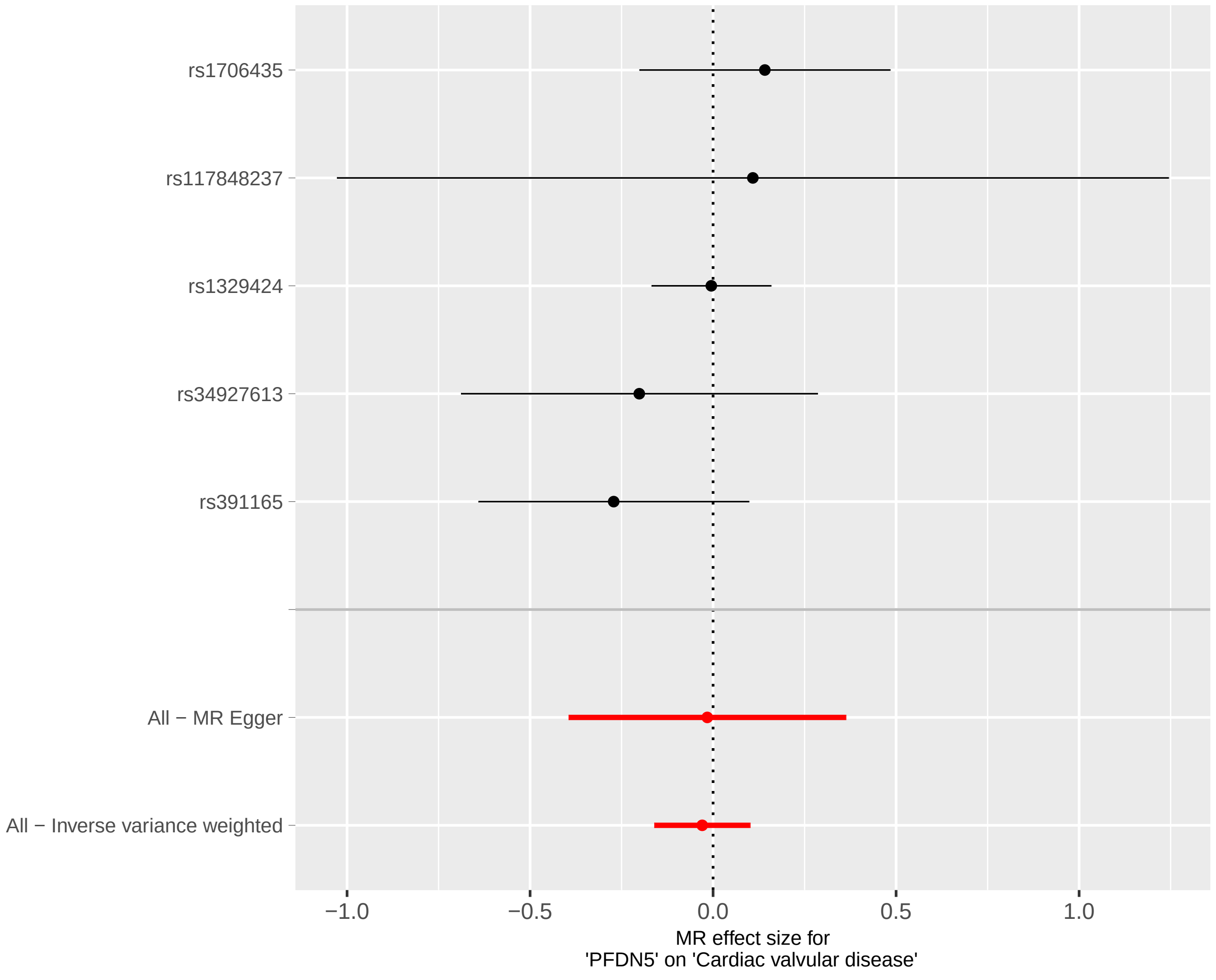
rs1706435
rs117848237
rs1329424
rs34927613
rs391165
All − MR Egger
All − Inverse variance weighted
−1.0
−0.5
0.0
0.5
1.0
MR effect size for
'PFDN5' on 'Cardiac valvular disease'

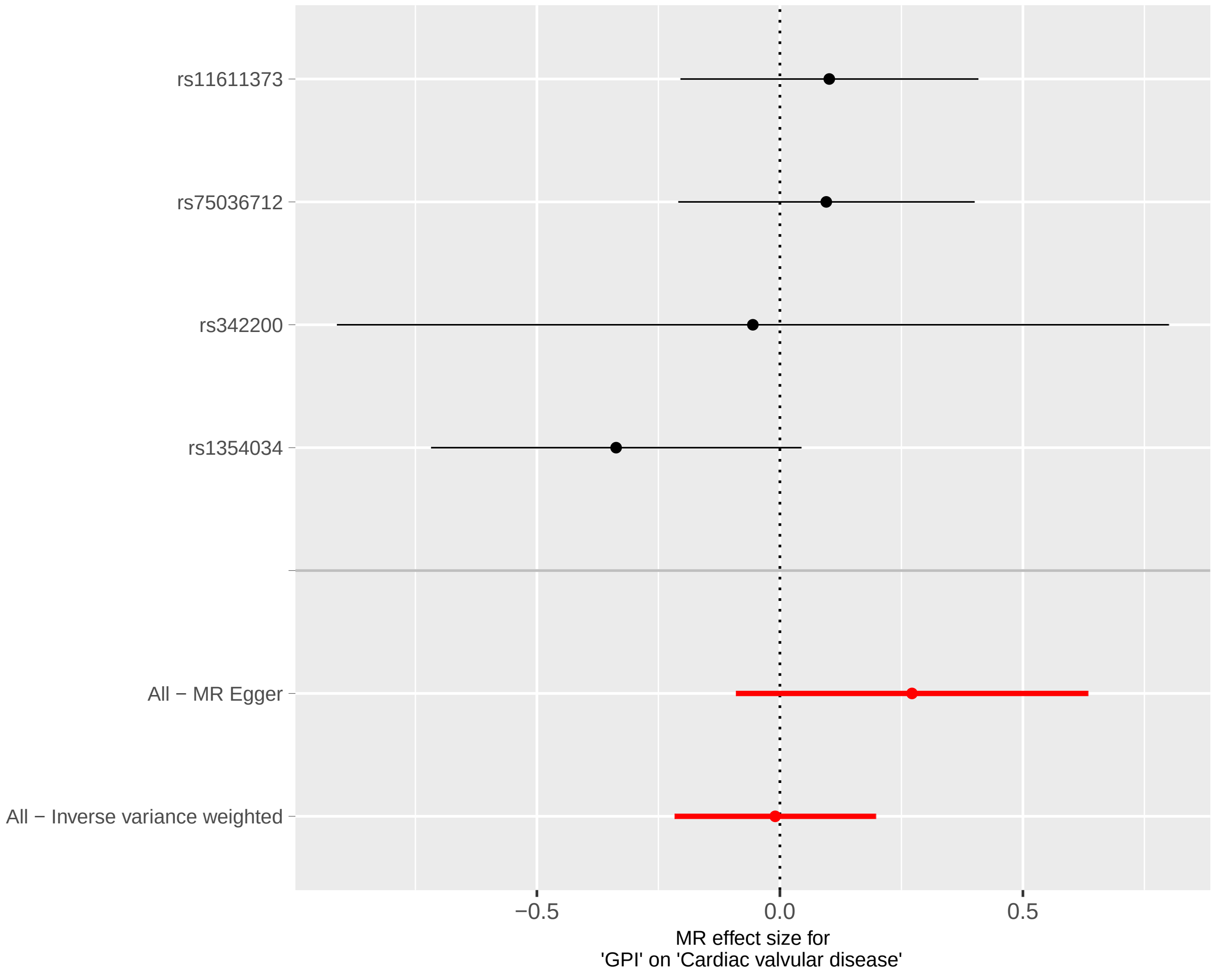
rs11611373
rs75036712
rs342200
rs1354034
All − MR Egger
All − Inverse variance weighted
−0.5
0.0
0.5
MR effect size for
'GPI' on 'Cardiac valvular disease'

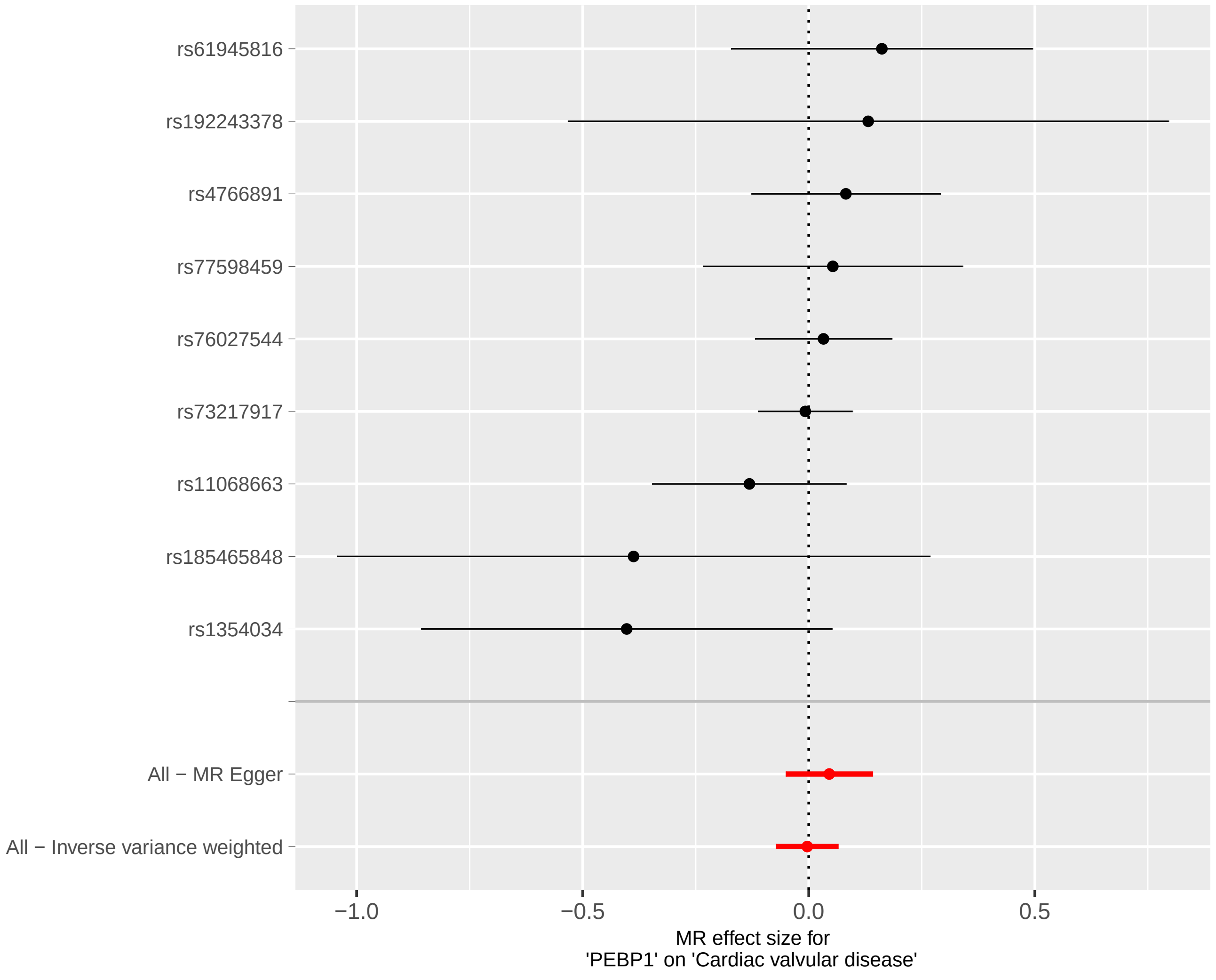

rs61945816
rs192243378
rs4766891
rs77598459
rs76027544
rs73217917
rs11068663
rs185465848
rs1354034
All − MR Egger
All − Inverse variance weighted
−1.0
−0.5
0.0
0.5
MR effect size for
'PEBP1' on 'Cardiac valvular disease'

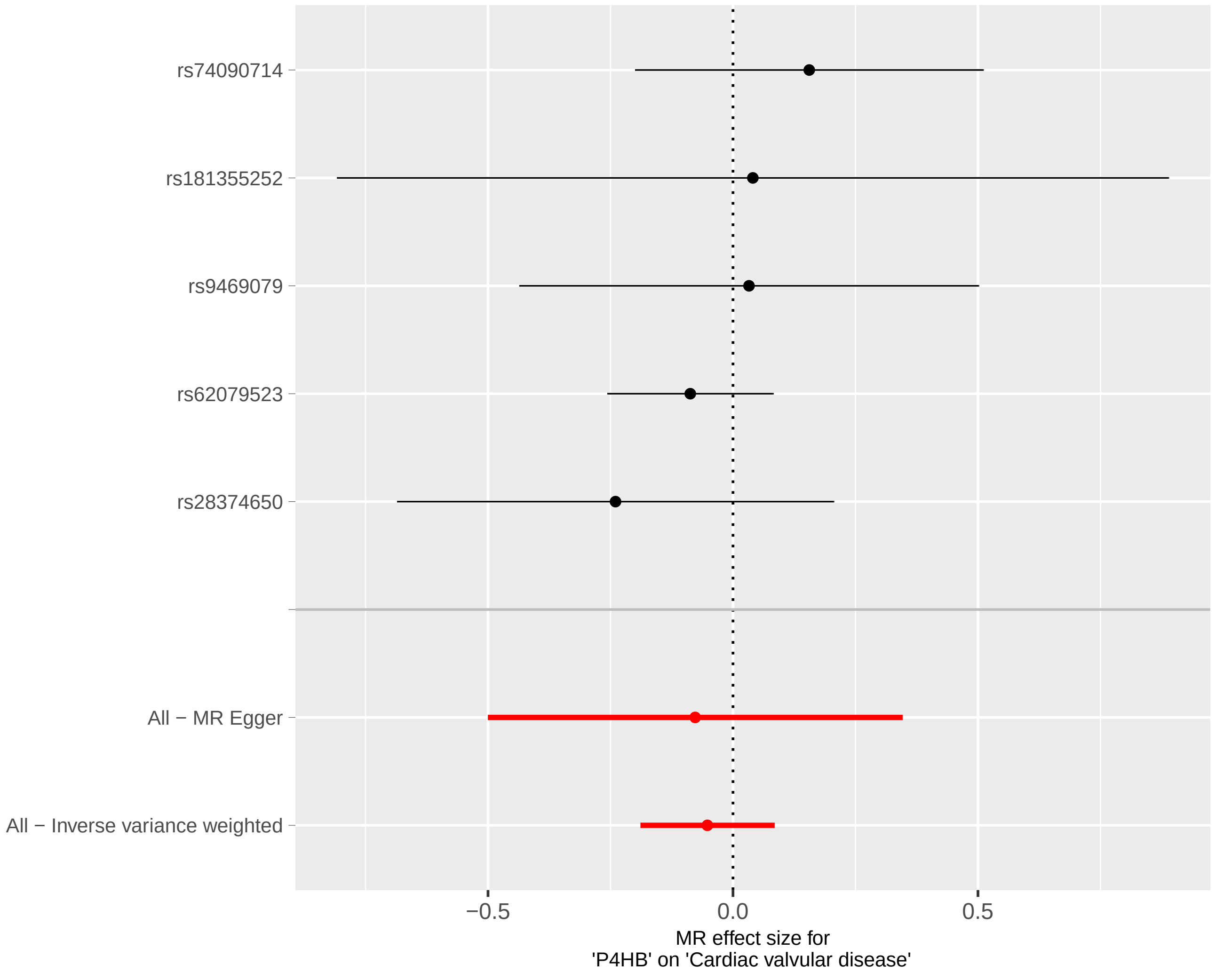

rs74090714
rs181355252
rs9469079
rs62079523
rs28374650
All − MR Egger
All − Inverse variance weighted
−0.5
0.0
0.5
MR effect size for
'P4HB' on 'Cardiac valvular disease'

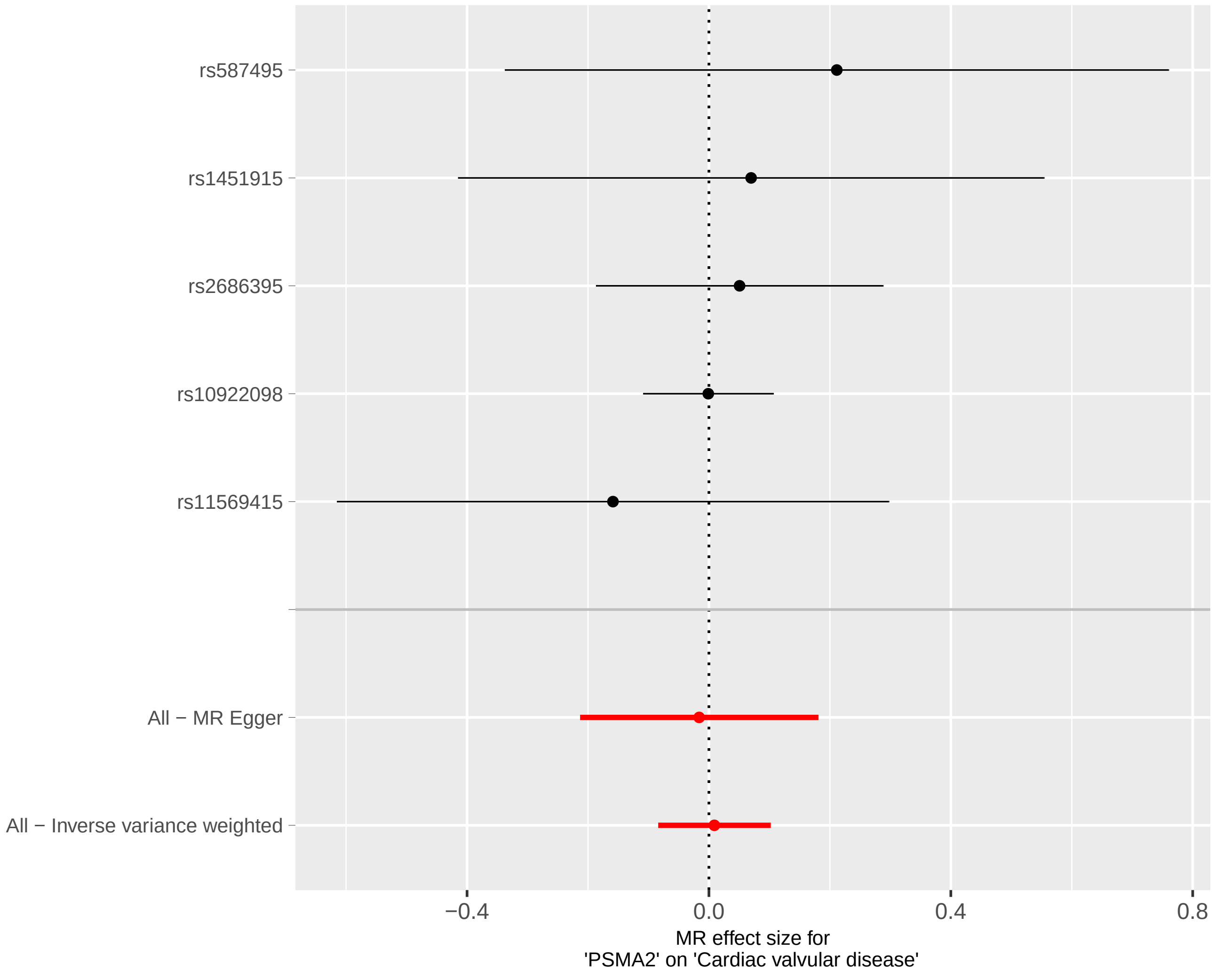

rs587495
rs1451915
rs2686395
rs10922098
rs11569415
All − MR Egger
All − Inverse variance weighted
−0.4
0.0
0.4
0.8
MR effect size for
'PSMA2' on 'Cardiac valvular disease'

Insufficient number of SNPs

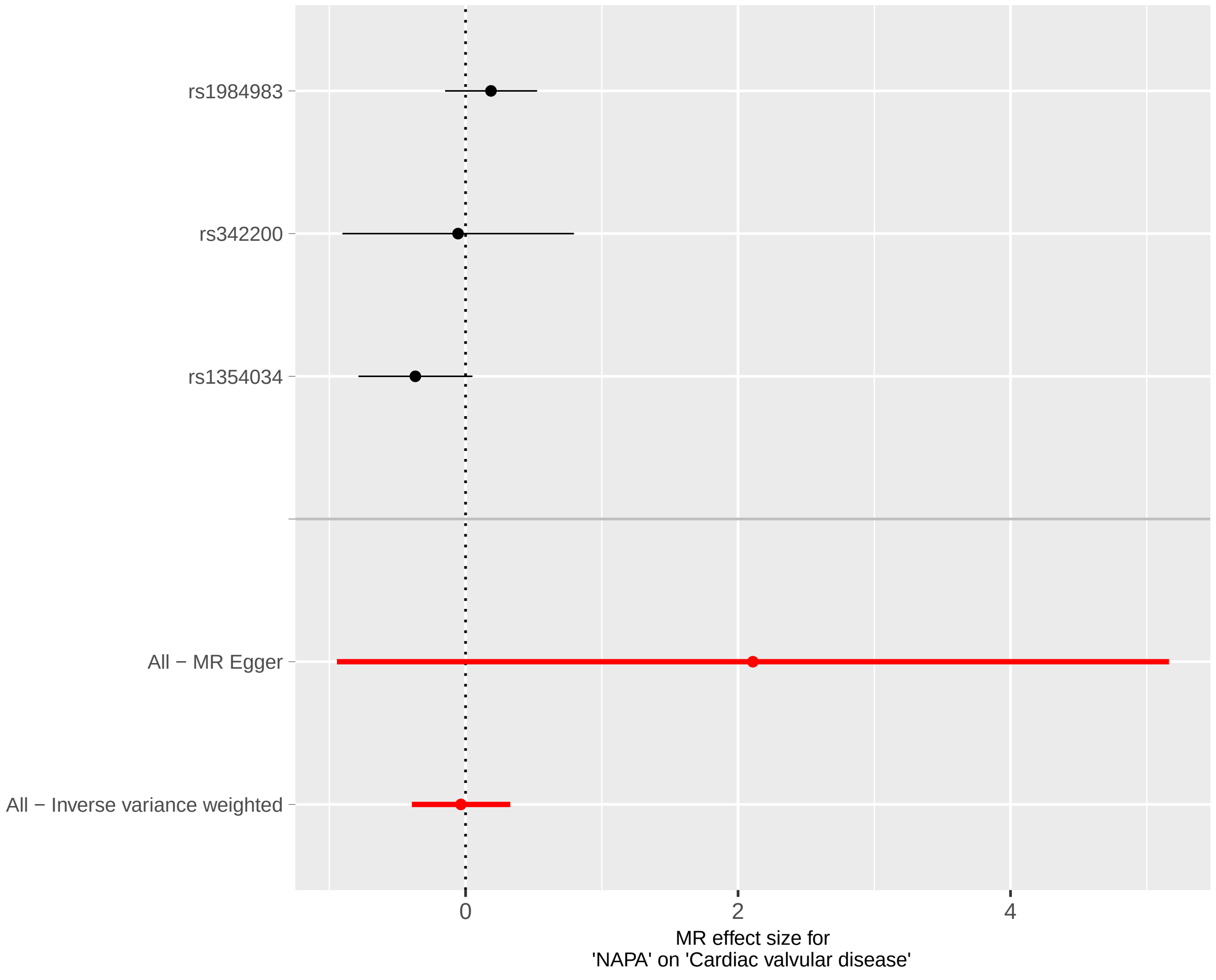

rs1984983
rs342200
rs1354034
All − MR Egger
All − Inverse variance weighted
0
2
4
MR effect size for
'NAPA' on 'Cardiac valvular disease'

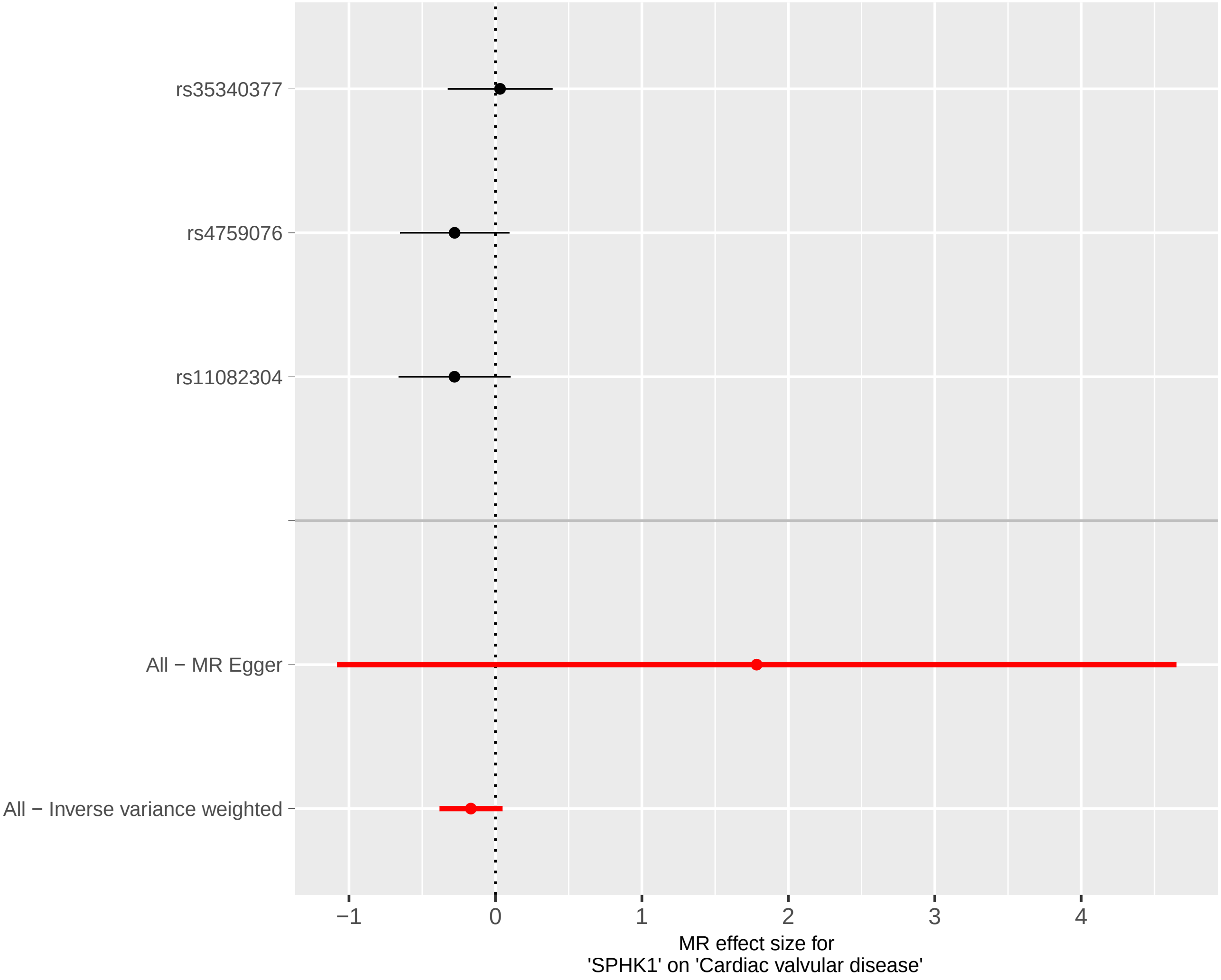

rs35340377
rs4759076
rs11082304
All − MR Egger
All − Inverse variance weighted
−1
0
1
2
3
4
MR effect size for
'SPHK1' on 'Cardiac valvular disease'

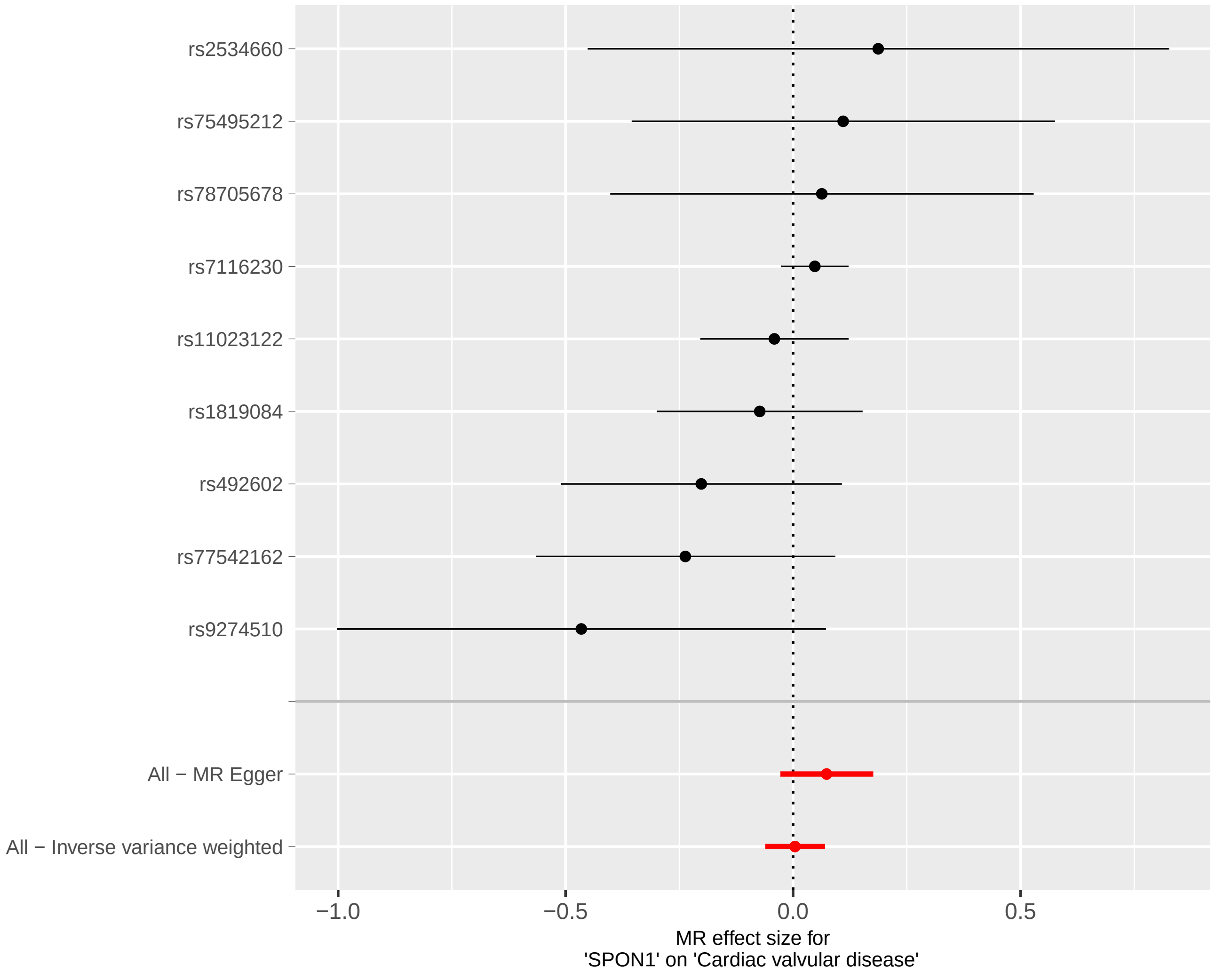
rs2534660
rs75495212
rs78705678
rs7116230
rs11023122
rs1819084
rs492602
rs77542162
rs9274510
All − MR Egger
All − Inverse variance weighted
−1.0
−0.5
0.0
0.5
MR effect size for
'SPON1' on 'Cardiac valvular disease'

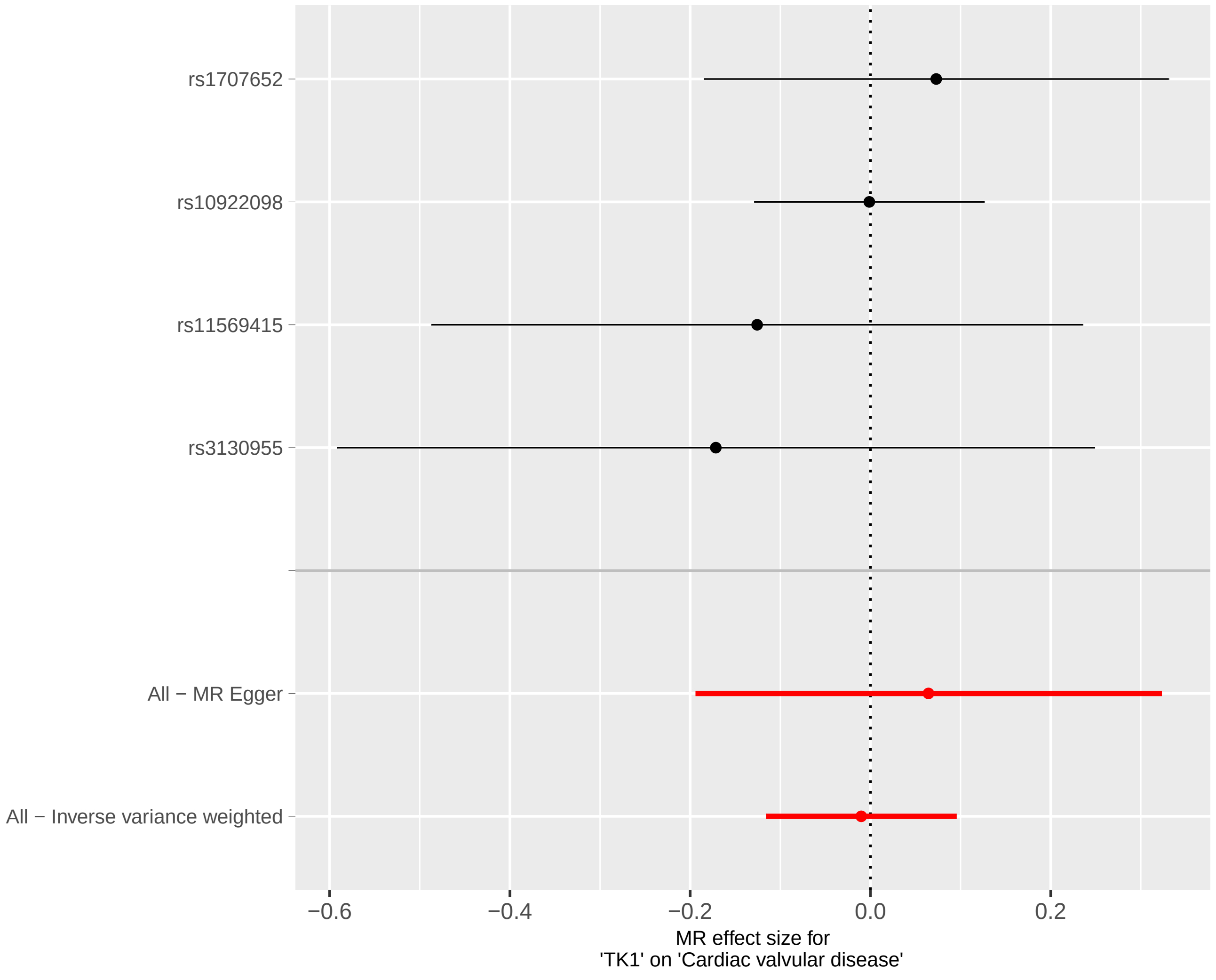
rs1707652
rs10922098
rs11569415
rs3130955
All − MR Egger
All − Inverse variance weighted
−0.6
−0.4
−0.2
0.0
0.2
MR effect size for
'TK1' on 'Cardiac valvular disease'

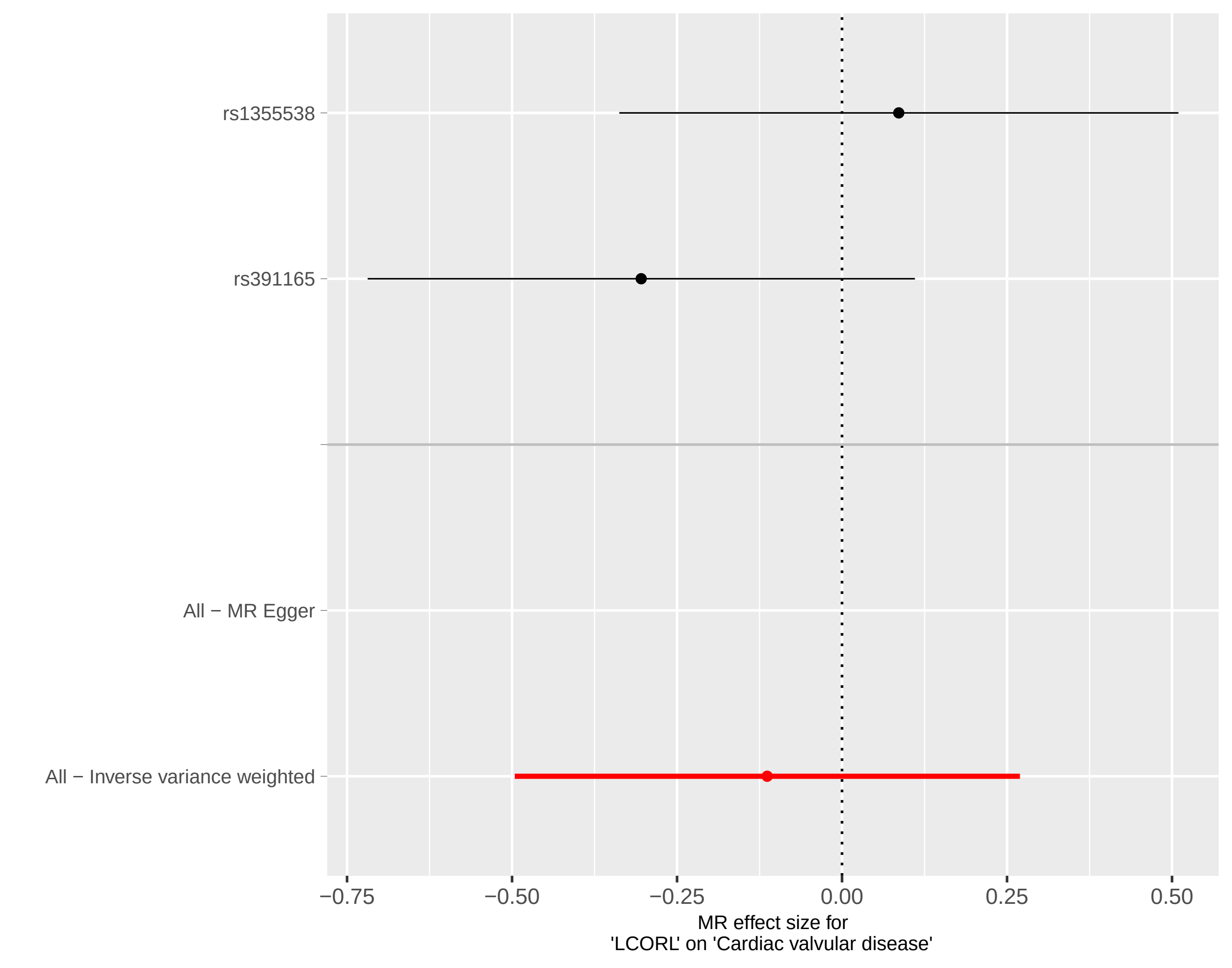

rs1355538
rs391165
All − MR Egger
All − Inverse variance weighted
−0.75
−0.50
−0.25
0.00
0.25
0.50
MR effect size for
'LCORL' on 'Cardiac valvular disease'

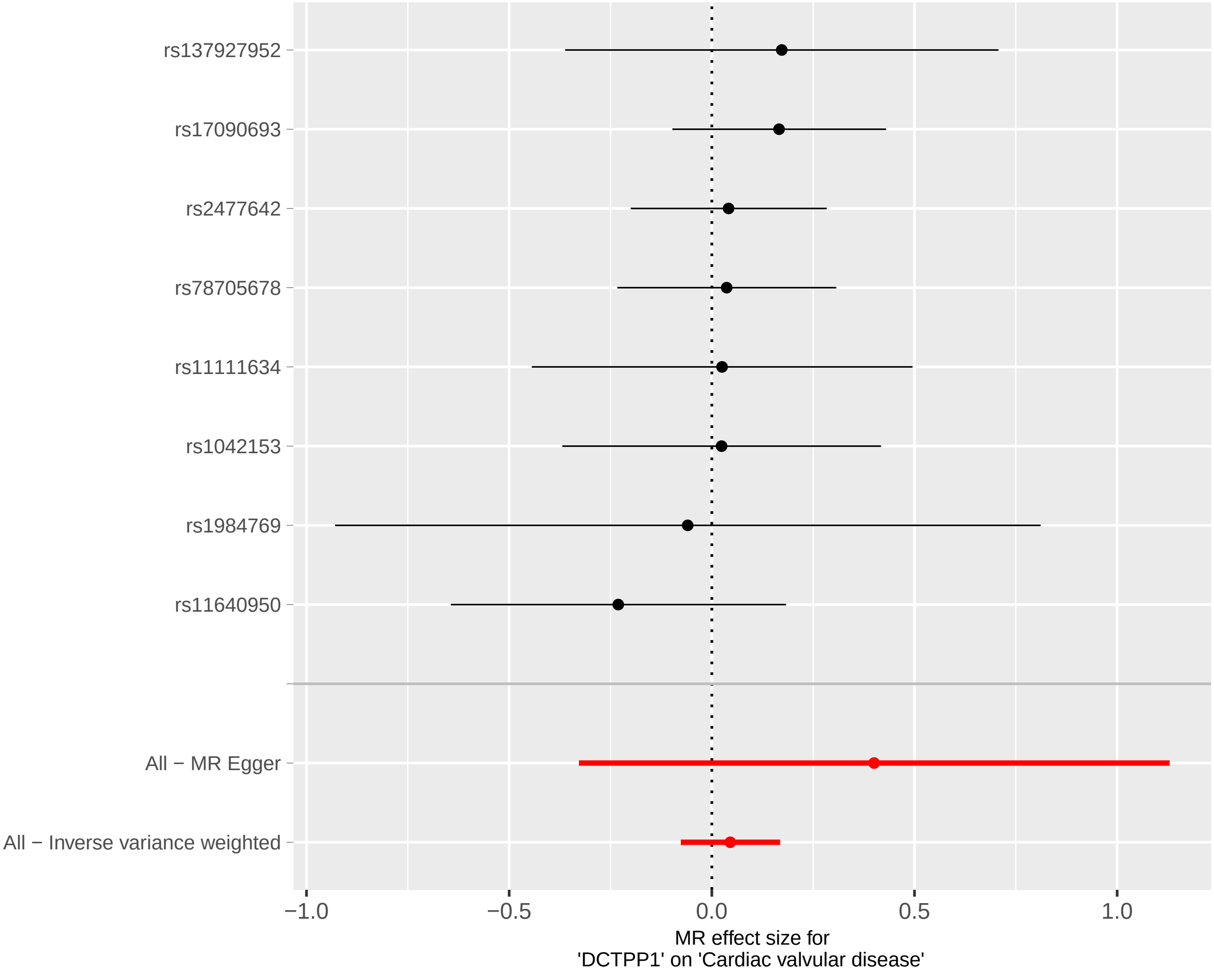
rs137927952
rs17090693
rs2477642
rs78705678
rs11111634
rs1042153
rs1984769
rs11640950
All − MR Egger
All − Inverse variance weighted
−1.0
−0.5
0.0
0.5
1.0
MR effect size for
'DCTPP1' on 'Cardiac valvular disease'

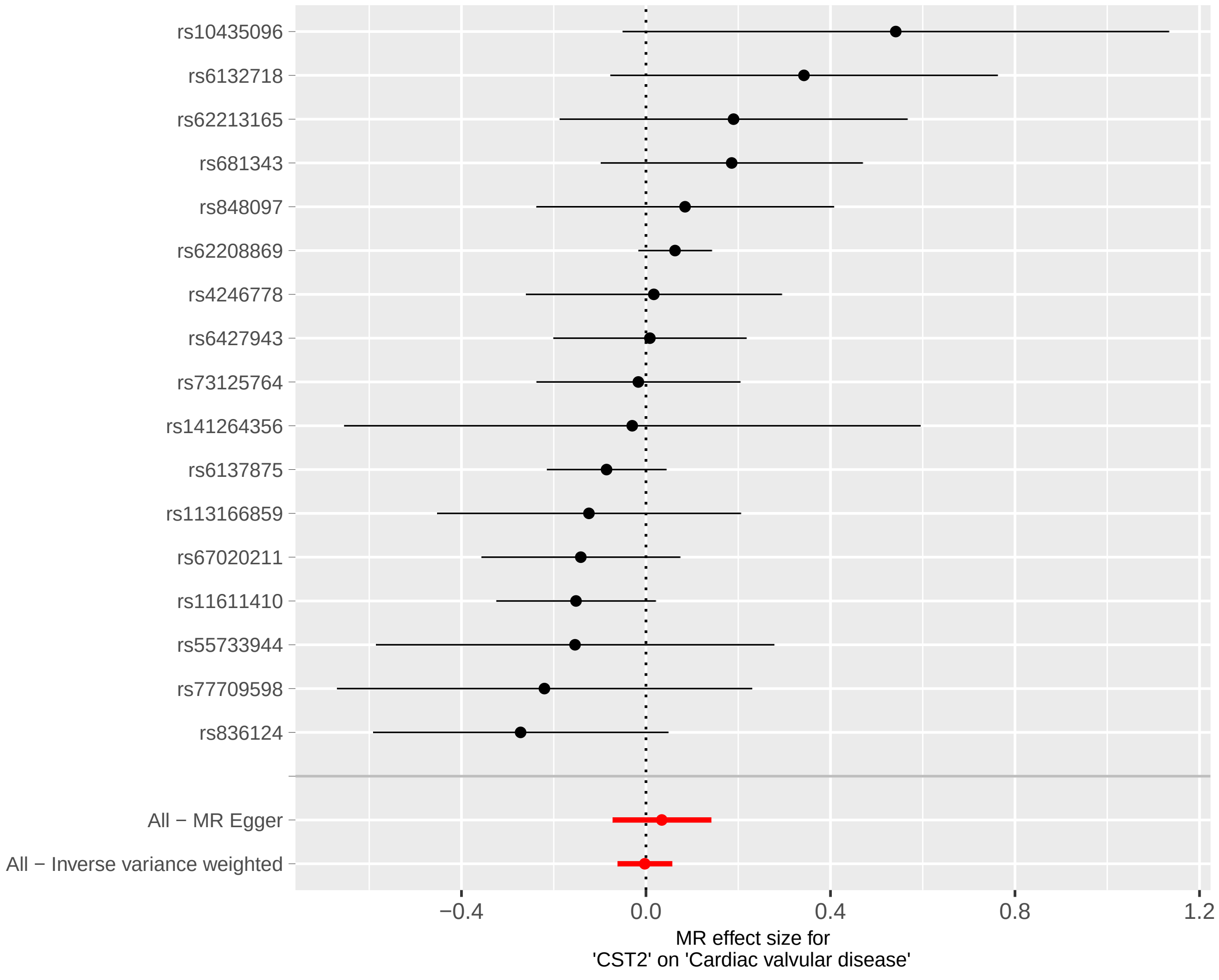
rs10435096
rs6132718
rs62213165
rs681343
rs848097
rs62208869
rs4246778
rs6427943
rs73125764
rs141264356
rs6137875
rs113166859
rs67020211
rs11611410
rs55733944
rs77709598
rs836124
All − MR Egger
All − Inverse variance weighted
−0.4
0.0
0.4
0.8
1.2
MR effect size for
'CST2' on 'Cardiac valvular disease'

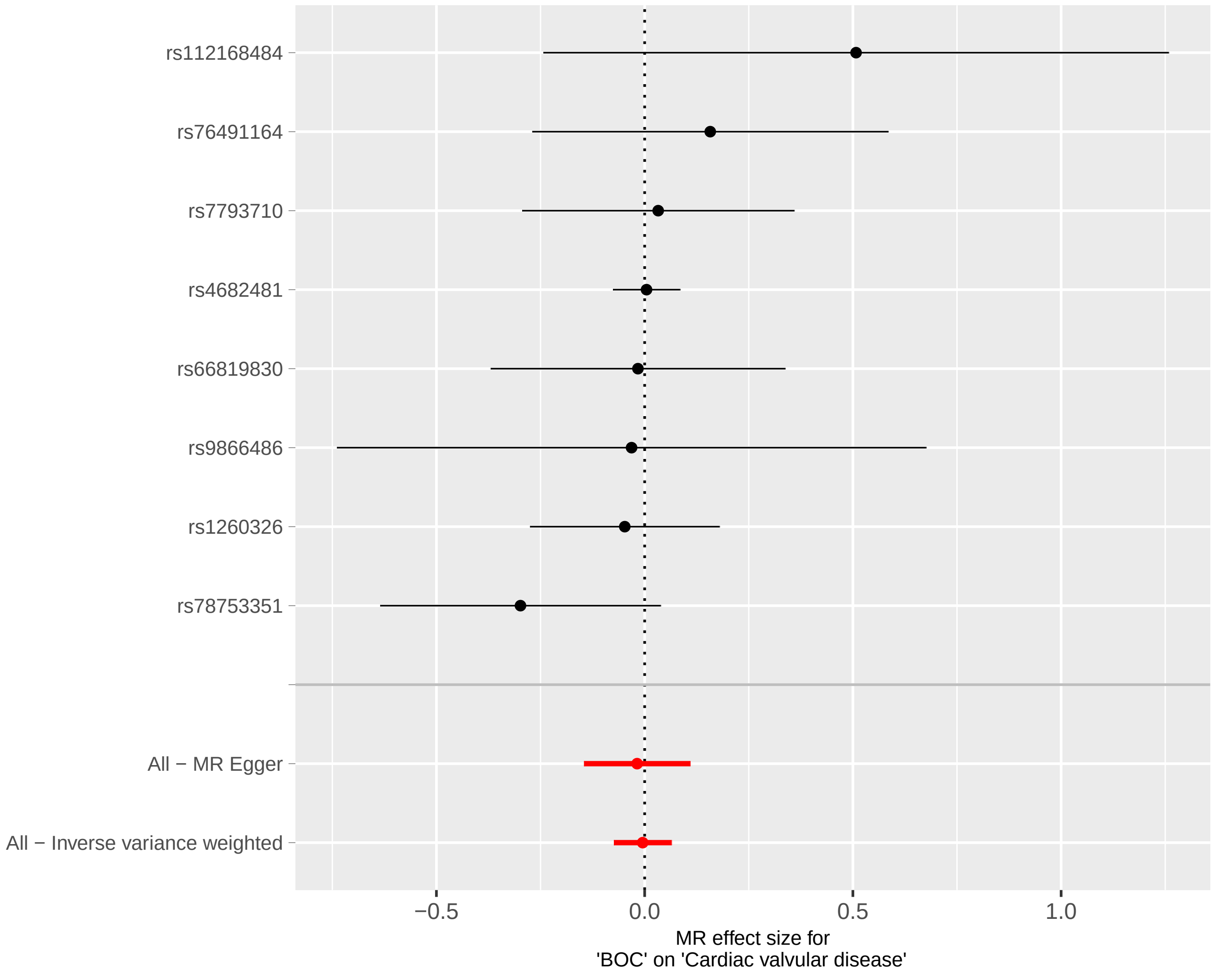
rs112168484
rs76491164
rs7793710
rs4682481
rs66819830
rs9866486
rs1260326
rs78753351
All − MR Egger
All − Inverse variance weighted
−0.5
0.0
0.5
1.0
MR effect size for
'BOC' on 'Cardiac valvular disease'

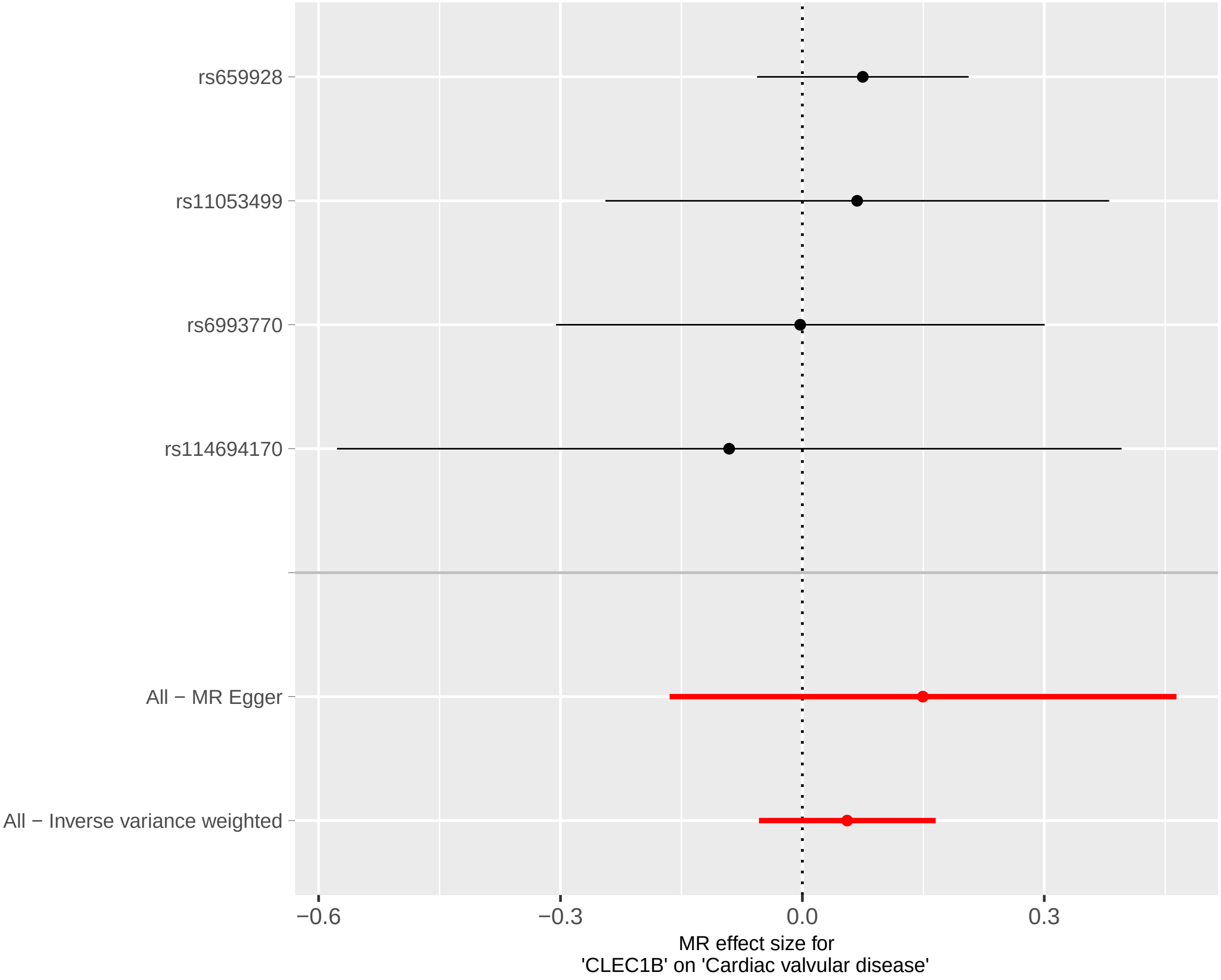
rs659928
rs11053499
rs6993770
rs114694170
All − MR Egger
All − Inverse variance weighted
−0.6
−0.3
0.0
0.3
MR effect size for
'CLEC1B' on 'Cardiac valvular disease'

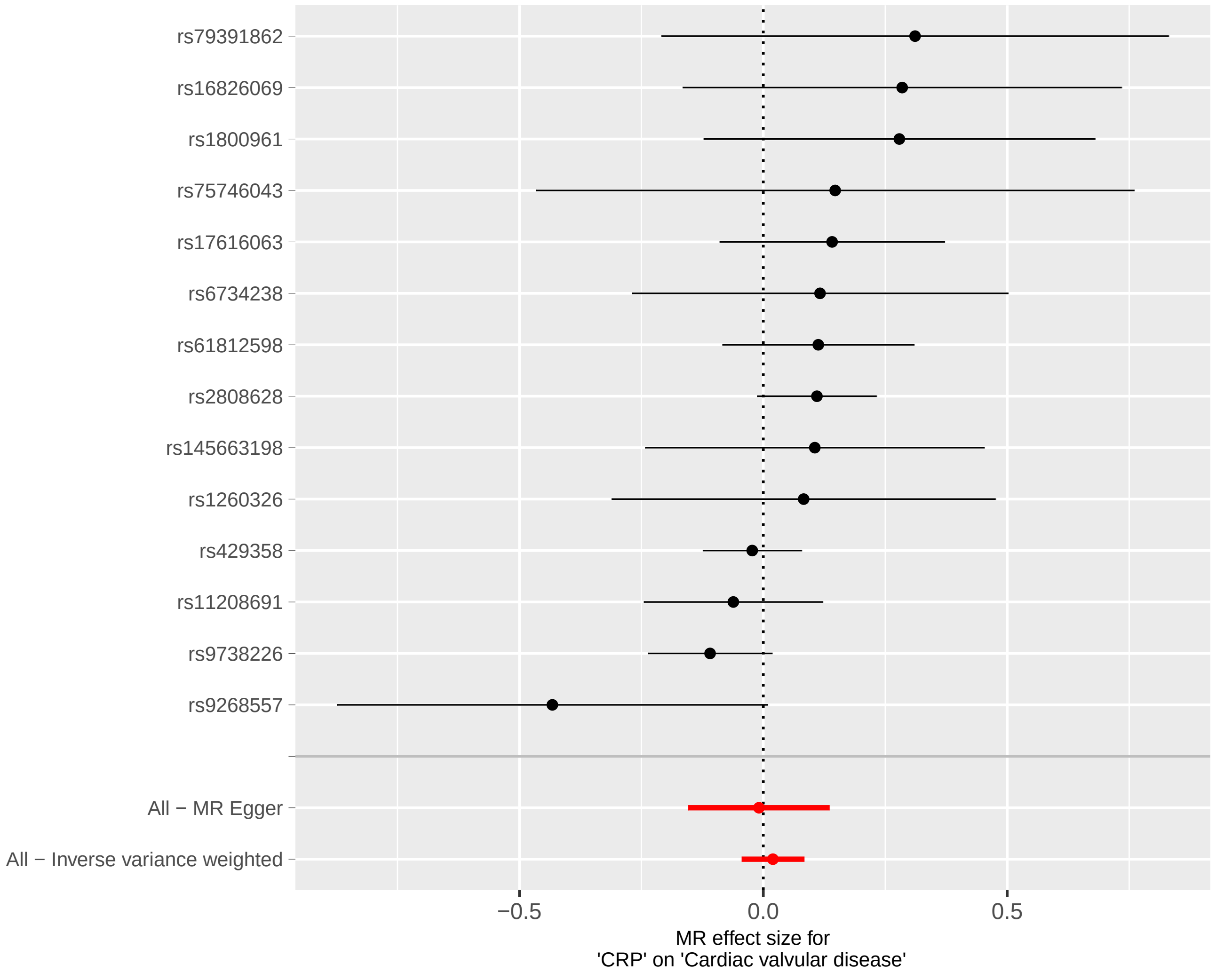
rs79391862
rs16826069
rs1800961
rs75746043
rs17616063
rs6734238
rs61812598
rs2808628
rs145663198
rs1260326
rs429358
rs11208691
rs9738226
rs9268557
All − MR Egger
All − Inverse variance weighted
−0.5
0.0
0.5
MR effect size for
'CRP' on 'Cardiac valvular disease'

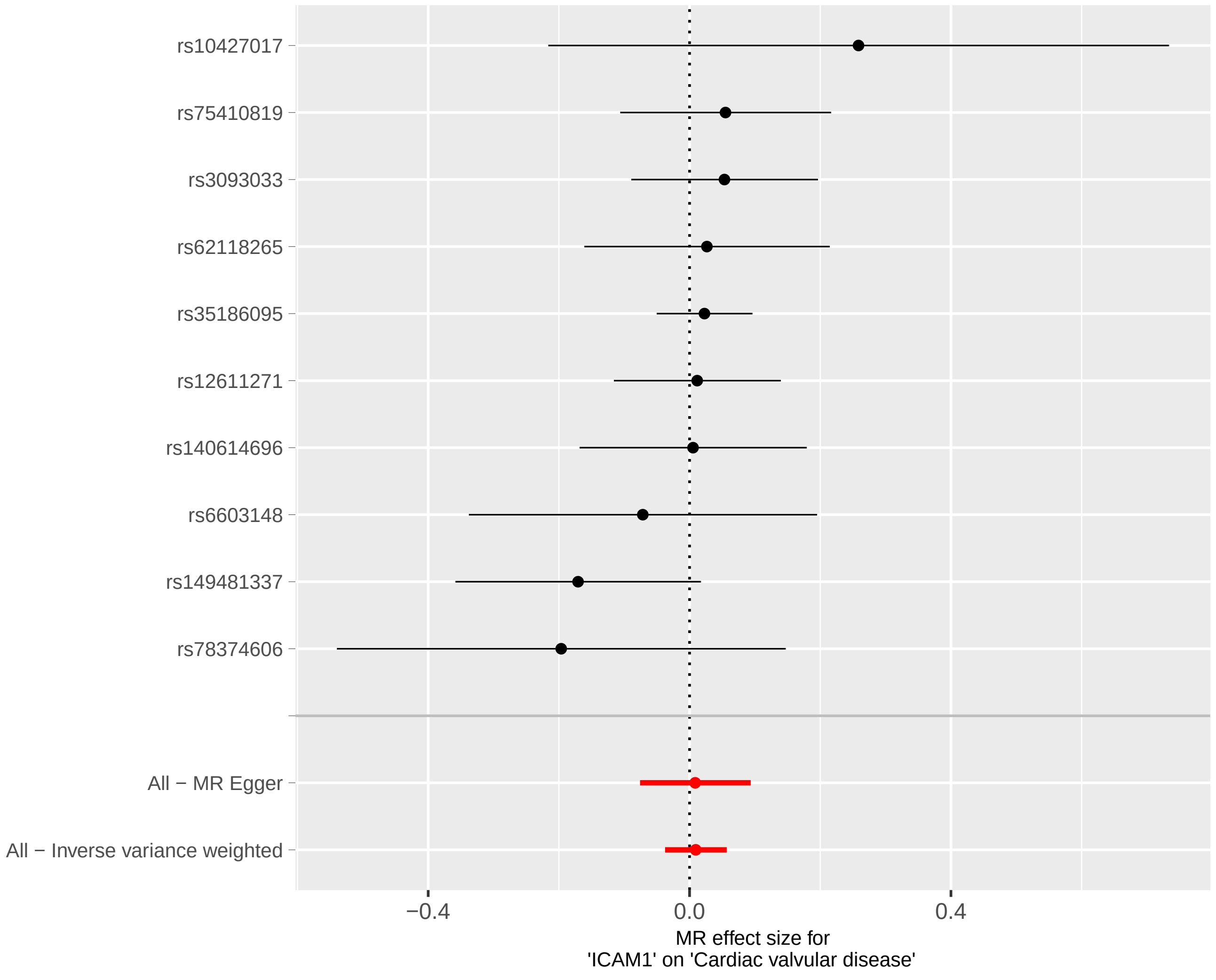
rs10427017
rs75410819
rs3093033
rs62118265
rs35186095
rs12611271
rs140614696
rs6603148
rs149481337
rs78374606
All − MR Egger
All − Inverse variance weighted
−0.4
0.0
0.4
MR effect size for
'ICAM1' on 'Cardiac valvular disease'

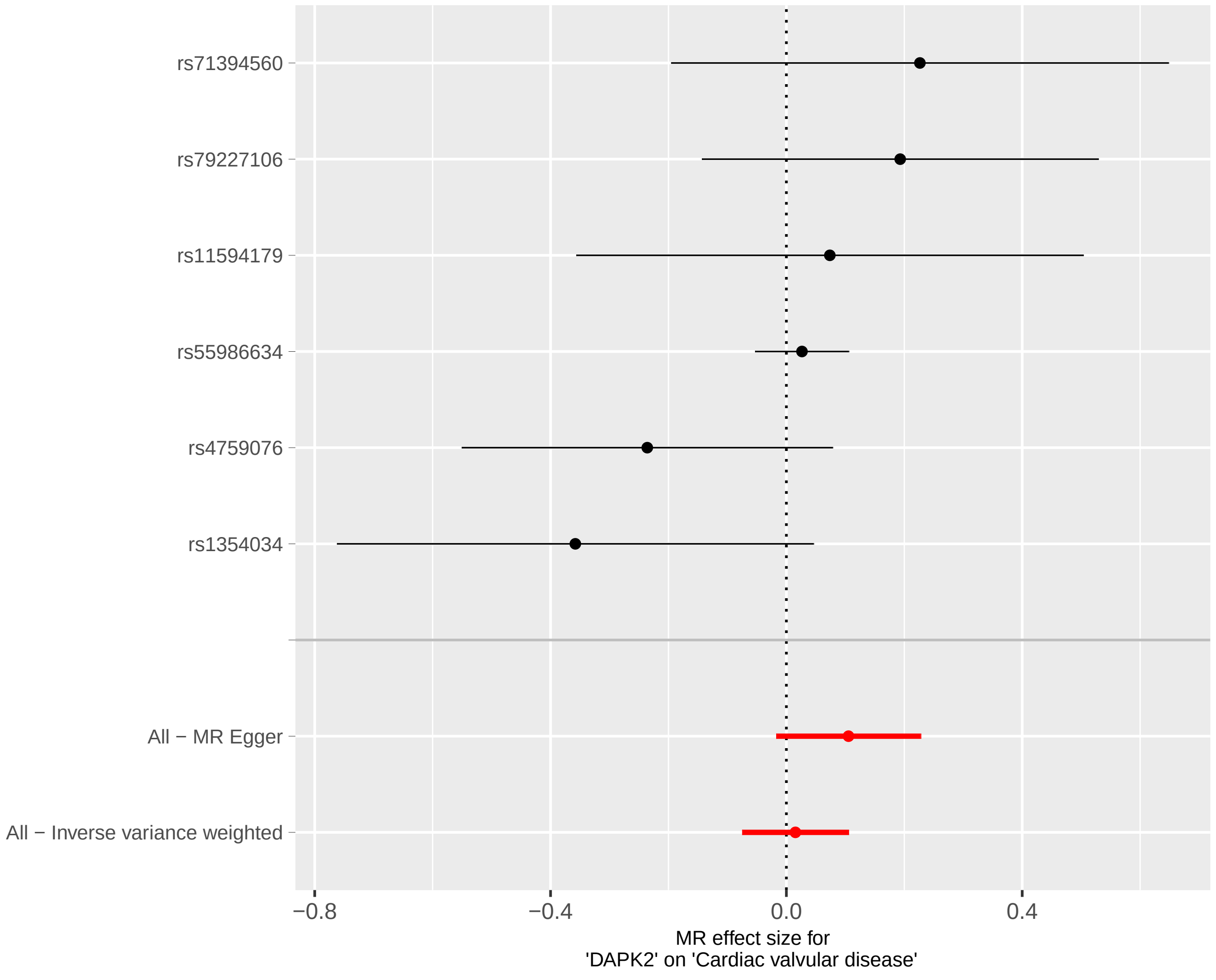
rs71394560
rs79227106
rs11594179
rs55986634
rs4759076
rs1354034
All − MR Egger
All − Inverse variance weighted
−0.8
−0.4
0.0
0.4
MR effect size for
'DAPK2' on 'Cardiac valvular disease'

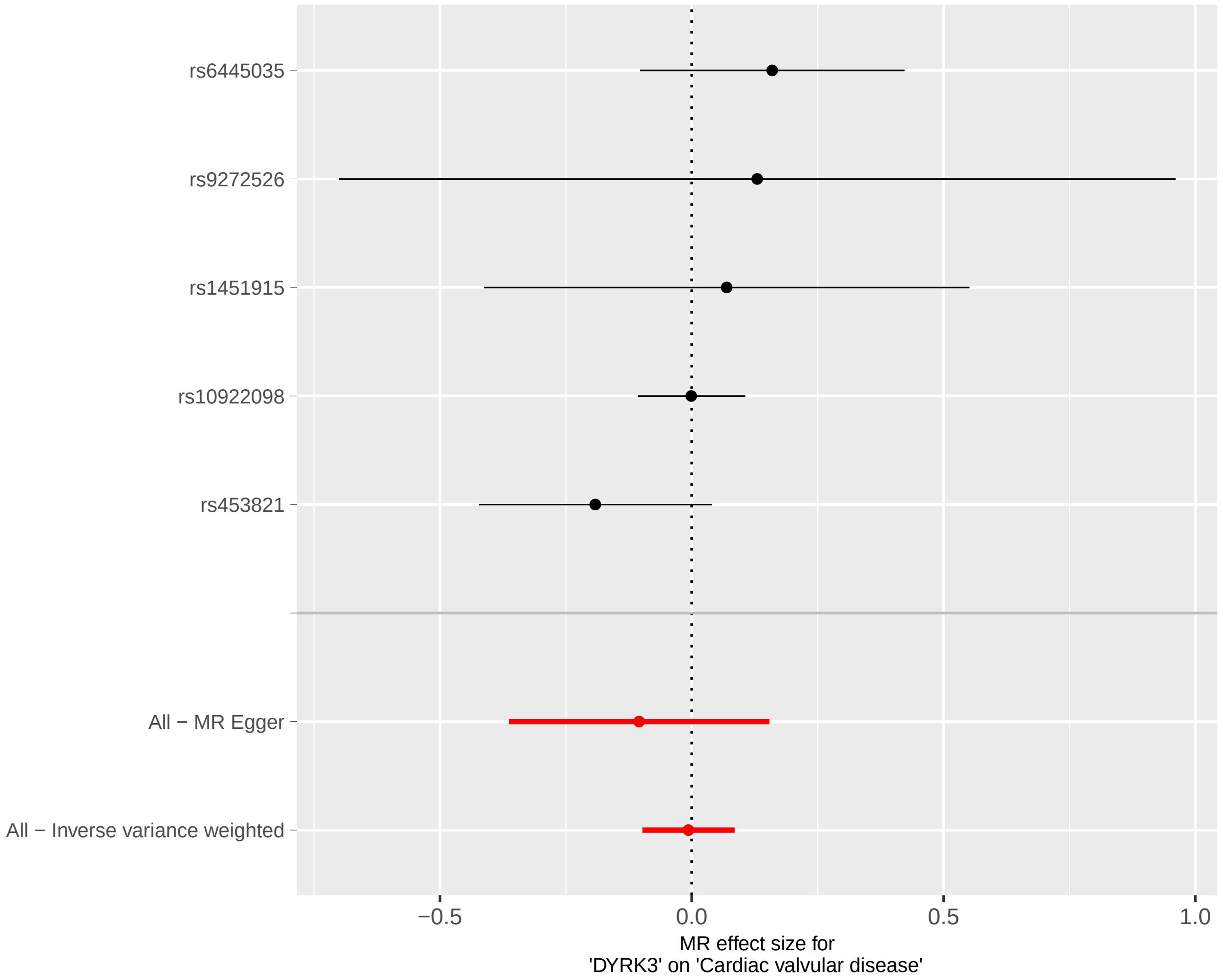
rs6445035
rs9272526
rs1451915
rs10922098
rs453821
All − MR Egger
All − Inverse variance weighted
−0.5
0.0
0.5
1.0
MR effect size for
'DYRK3' on 'Cardiac valvular disease'

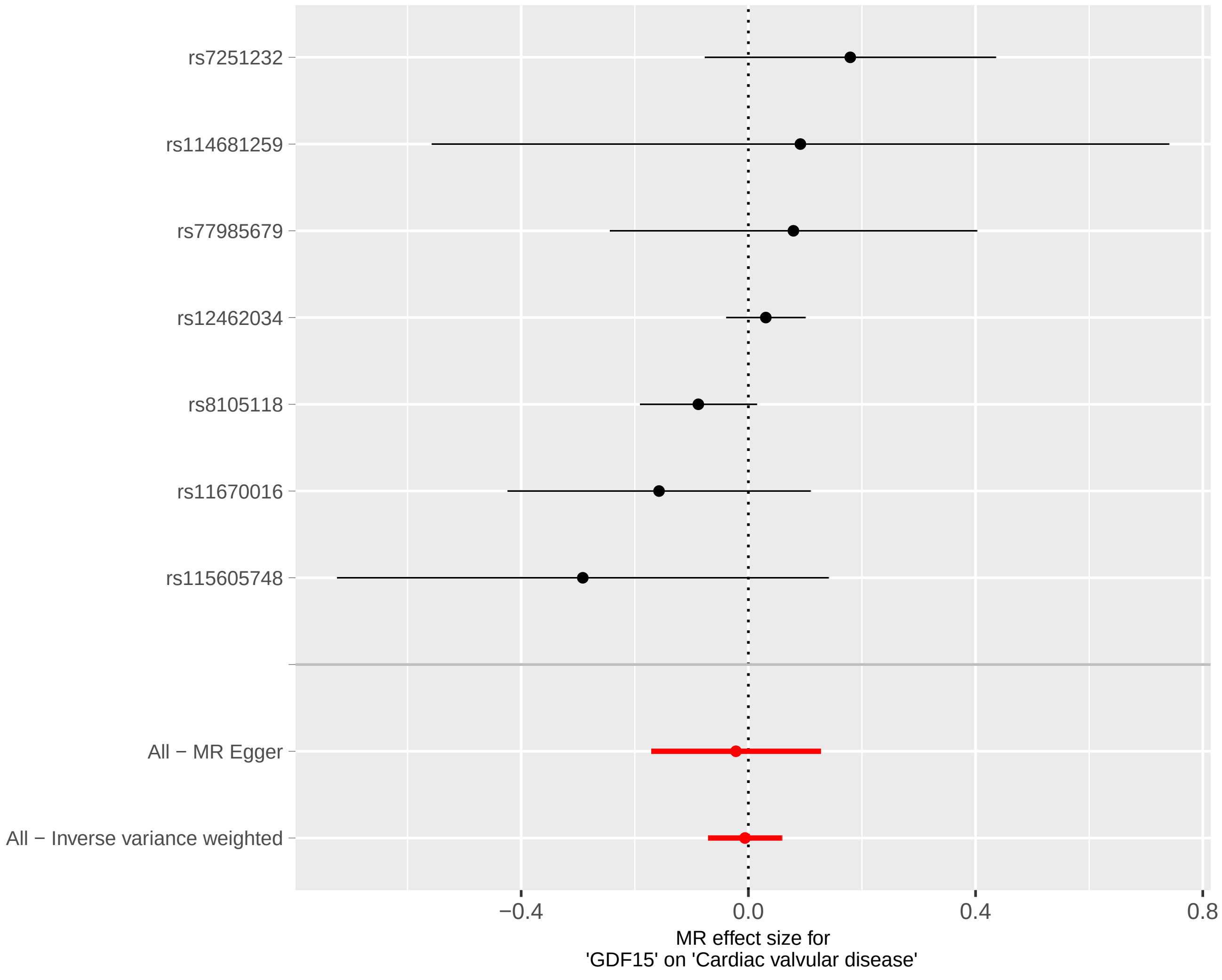
rs7251232
rs114681259
rs77985679
rs12462034
rs8105118
rs11670016
rs115605748
All − MR Egger
All − Inverse variance weighted
−0.4
0.0
0.4
0.8
MR effect size for
'GDF15' on 'Cardiac valvular disease'

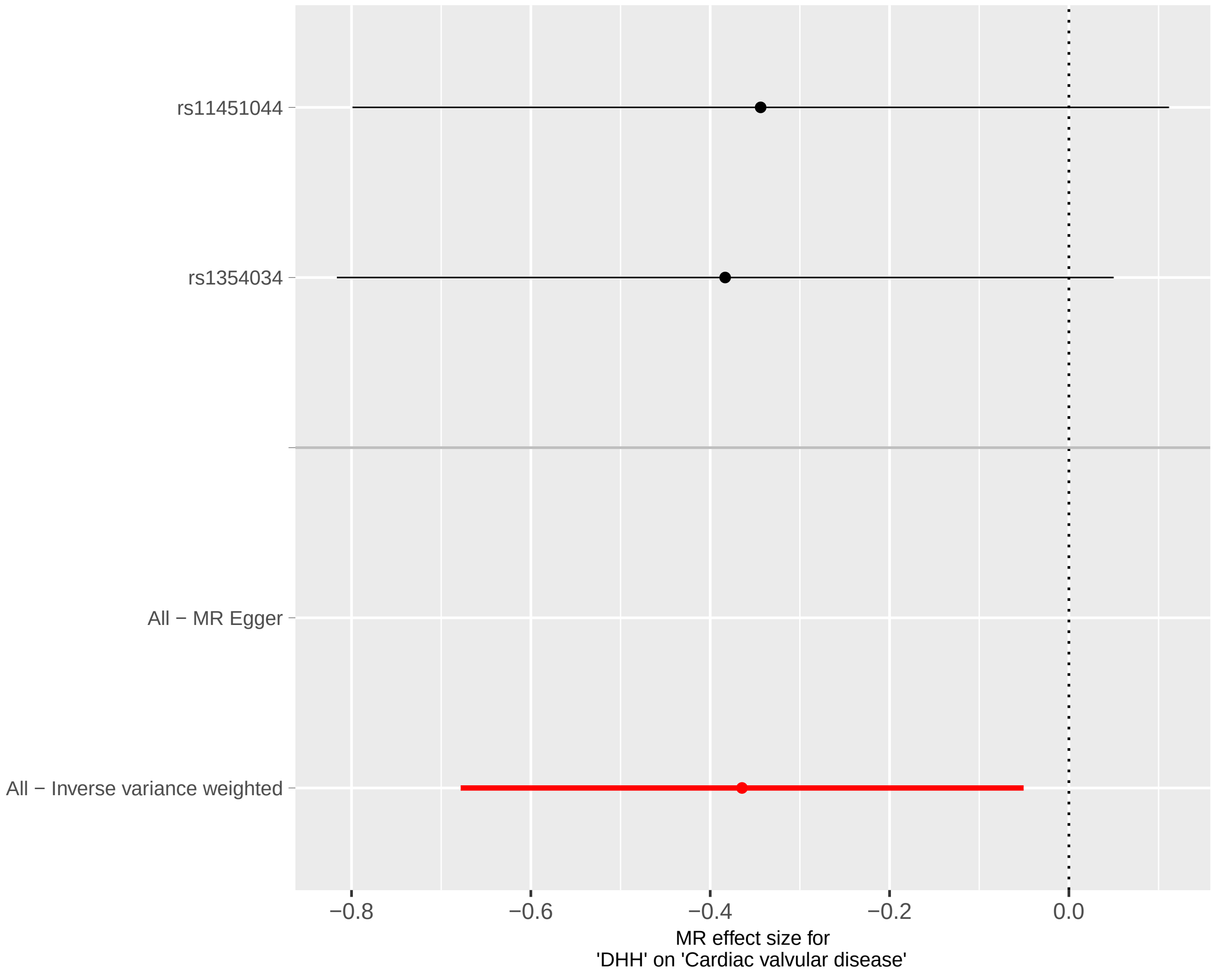
rs11451044
rs1354034
All − MR Egger
All − Inverse variance weighted
−0.8
−0.6
−0.4
−0.2
0.0
MR effect size for
'DHH' on 'Cardiac valvular disease'

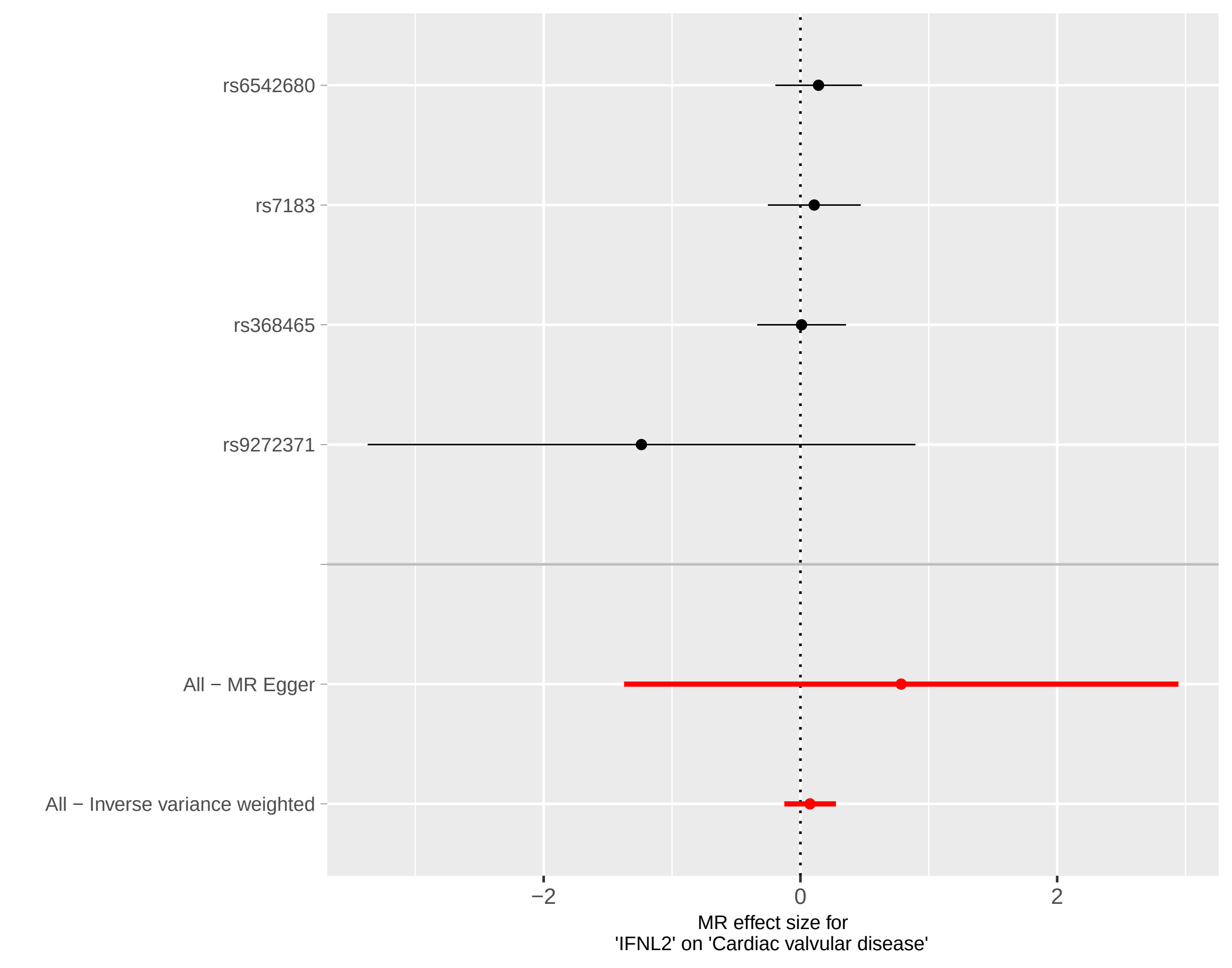
rs6542680
rs7183
rs368465
rs9272371
All − MR Egger
All − Inverse variance weighted
−2
0
2
MR effect size for
'IFNL2' on 'Cardiac valvular disease'

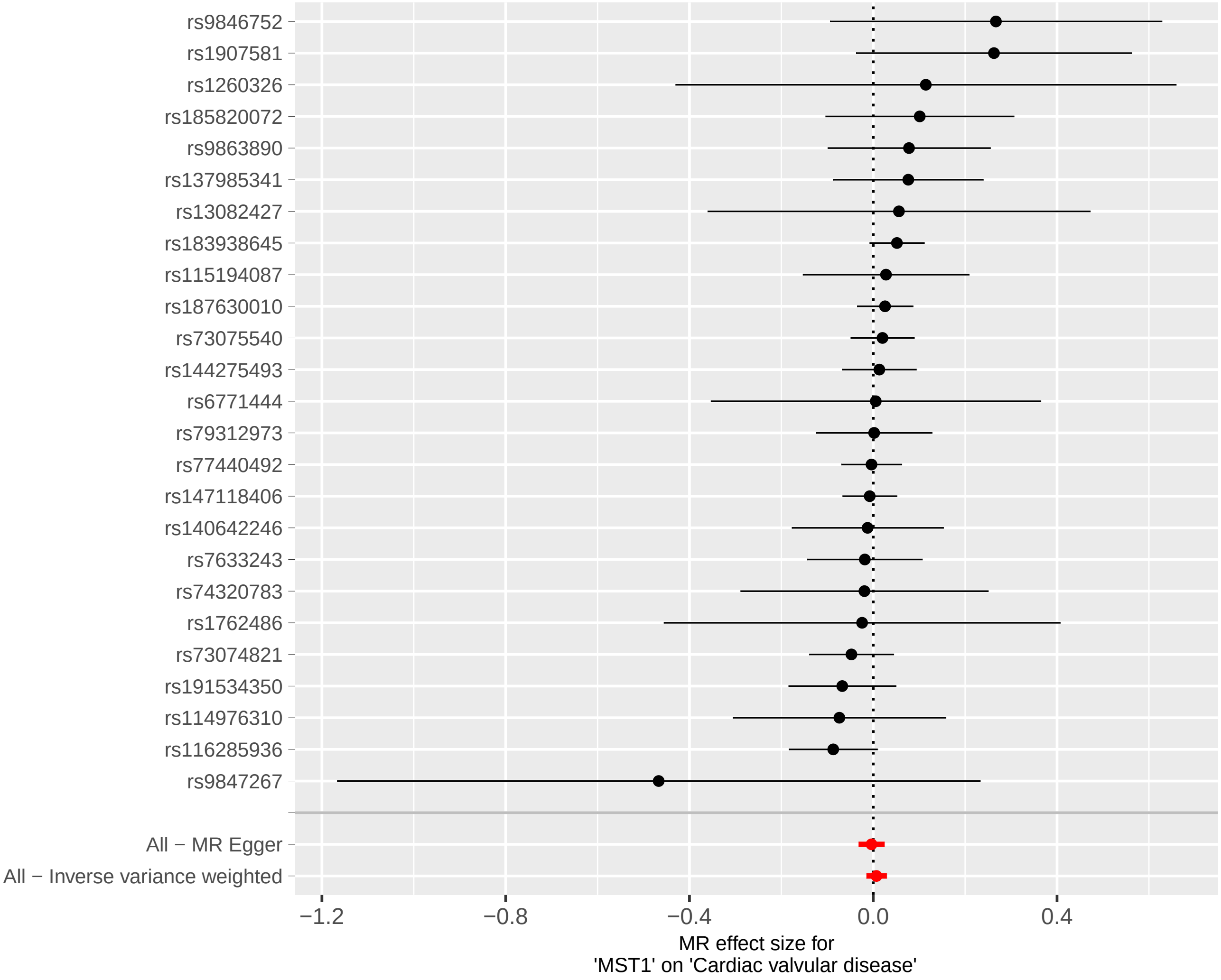
rs9846752
rs1907581
rs1260326
rs185820072
rs9863890
rs137985341
rs13082427
rs183938645
rs115194087
rs187630010
rs73075540
rs144275493
rs6771444
rs79312973
rs77440492
rs147118406
rs140642246
rs7633243
rs74320783
rs1762486
rs73074821
rs191534350
rs114976310
rs116285936
rs9847267
All − MR Egger
All − Inverse variance weighted
−1.2
−0.8
−0.4
0.0
0.4
MR effect size for
'MST1' on 'Cardiac valvular disease'

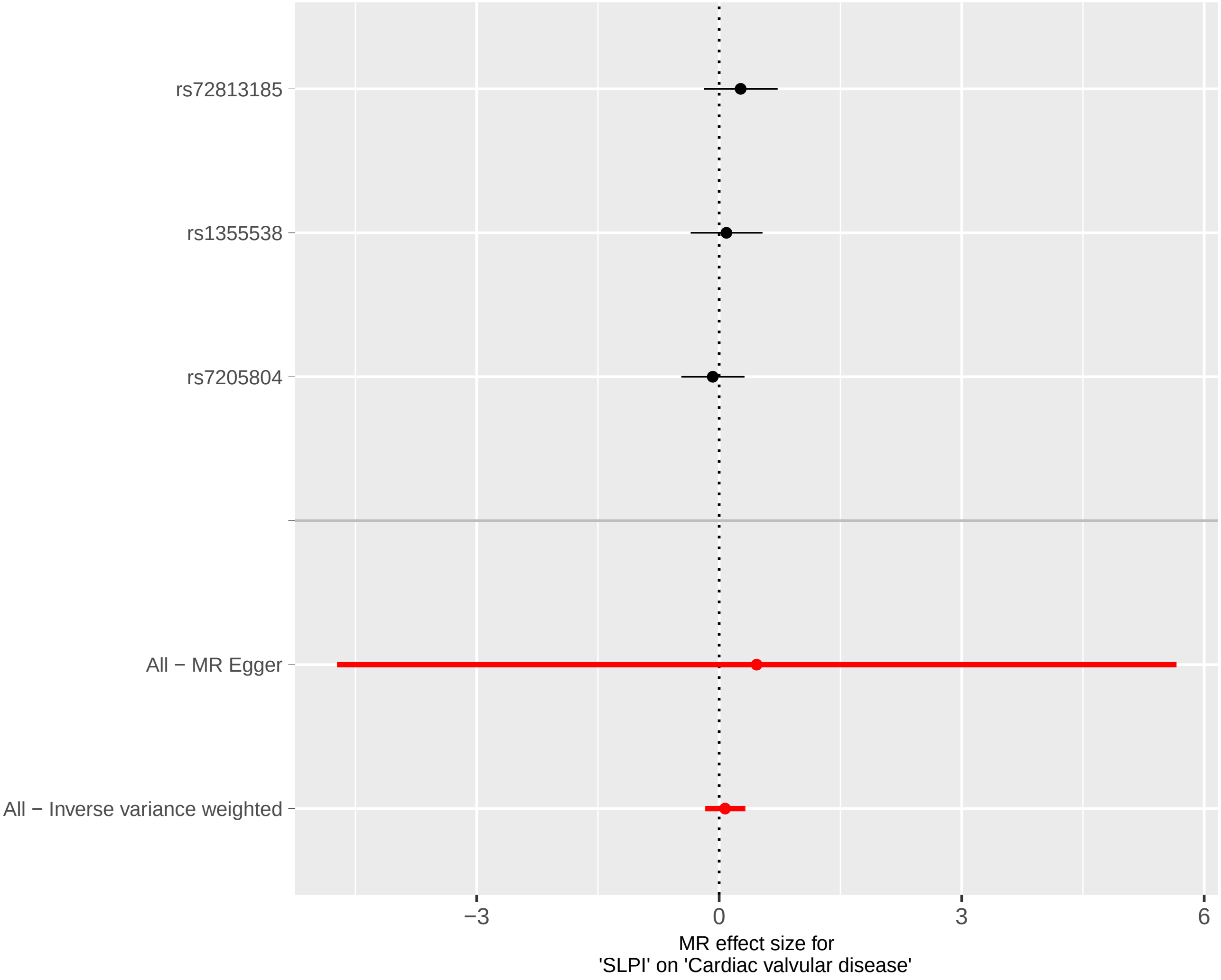

rs72813185
rs1355538
rs7205804
All − MR Egger
All − Inverse variance weighted
−3
0
3
6
MR effect size for
'SLPI' on 'Cardiac valvular disease'

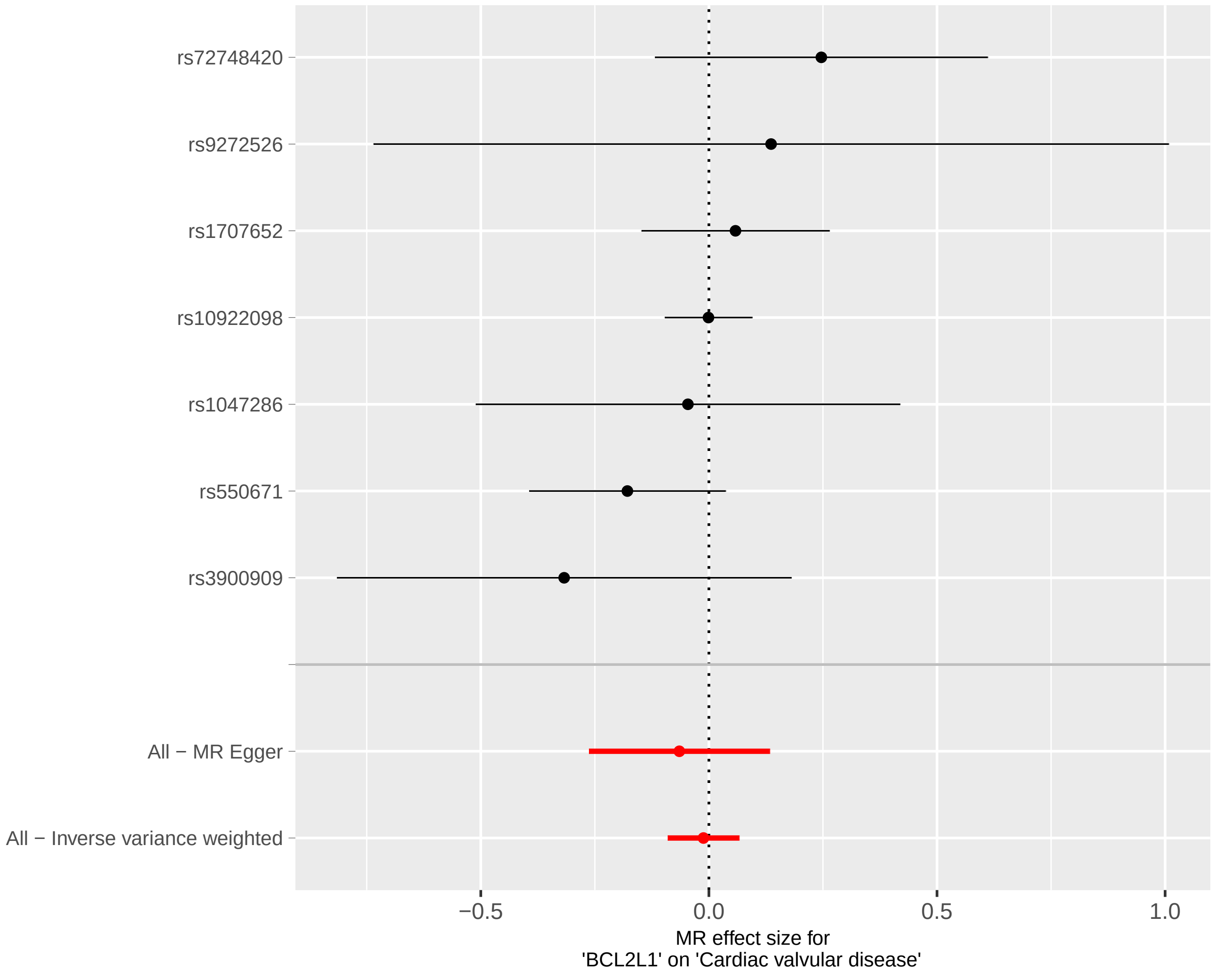
rs72748420
rs9272526
rs1707652
rs10922098
rs1047286
rs550671
rs3900909
All − MR Egger
All − Inverse variance weighted
−0.5
0.0
0.5
1.0
MR effect size for
'BCL2L1' on 'Cardiac valvular disease'

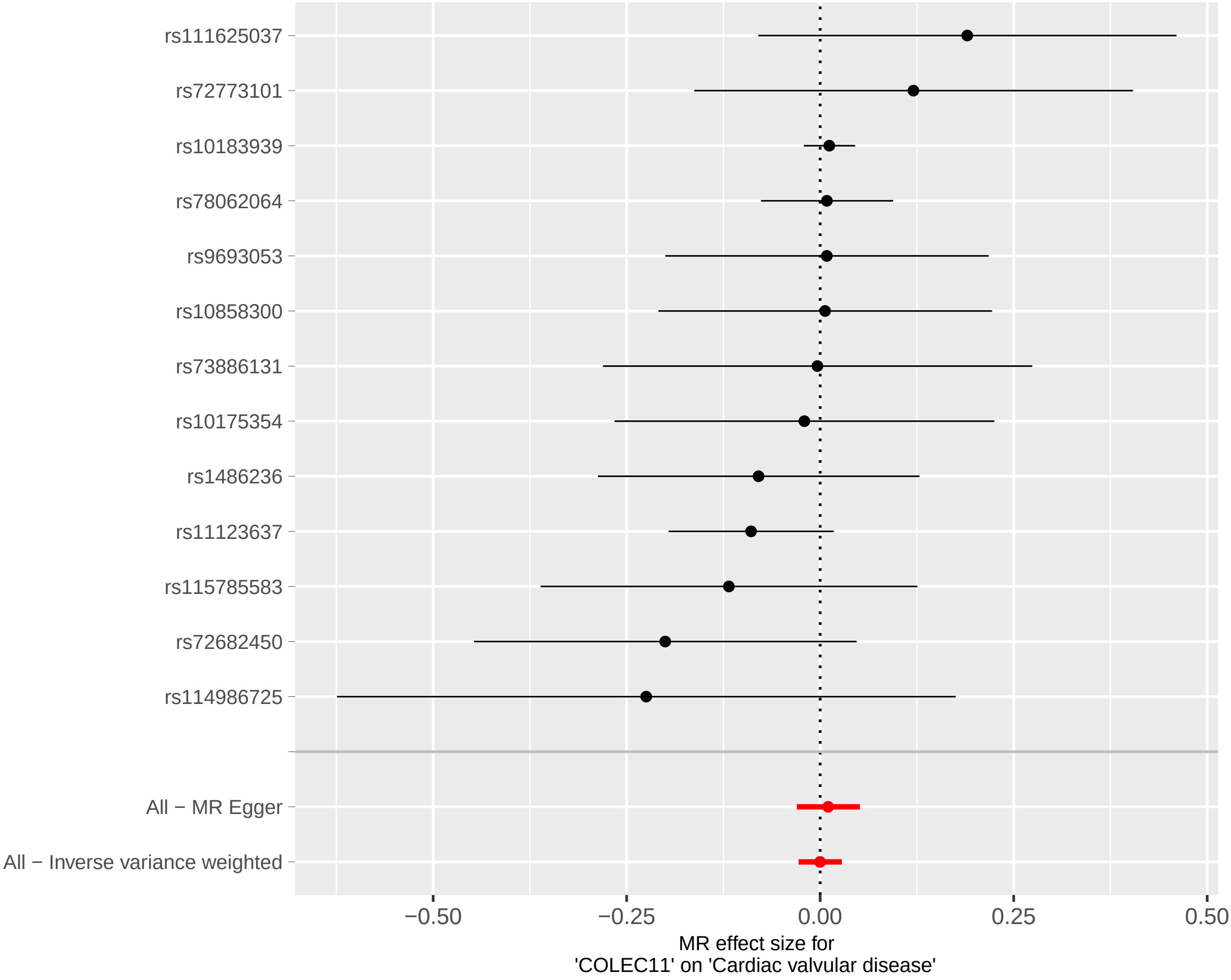
rs111625037
rs72773101
rs10183939
rs78062064
rs9693053
rs10858300
rs73886131
rs10175354
rs1486236
rs11123637
rs115785583
rs72682450
rs114986725
All − MR Egger
All − Inverse variance weighted
−0.50
−0.25
0.00
0.25
0.50
MR effect size for
'COLEC11' on 'Cardiac valvular disease'

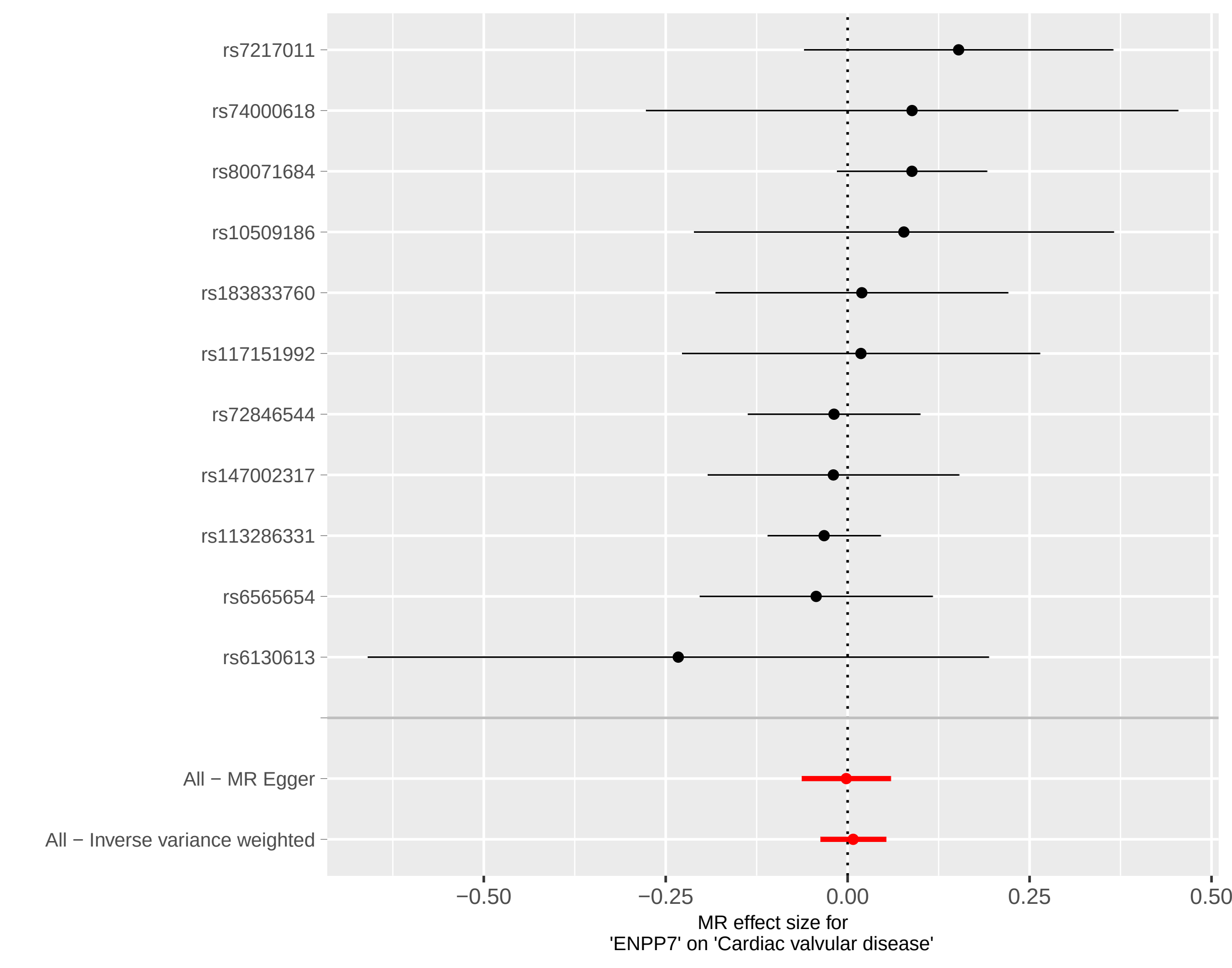
rs7217011
rs74000618
rs80071684
rs10509186
rs183833760
rs117151992
rs72846544
rs147002317
rs113286331
rs6565654
rs6130613
All − MR Egger
All − Inverse variance weighted
−0.50
−0.25
0.00
0.25
0.50
MR effect size for
'ENPP7' on 'Cardiac valvular disease'

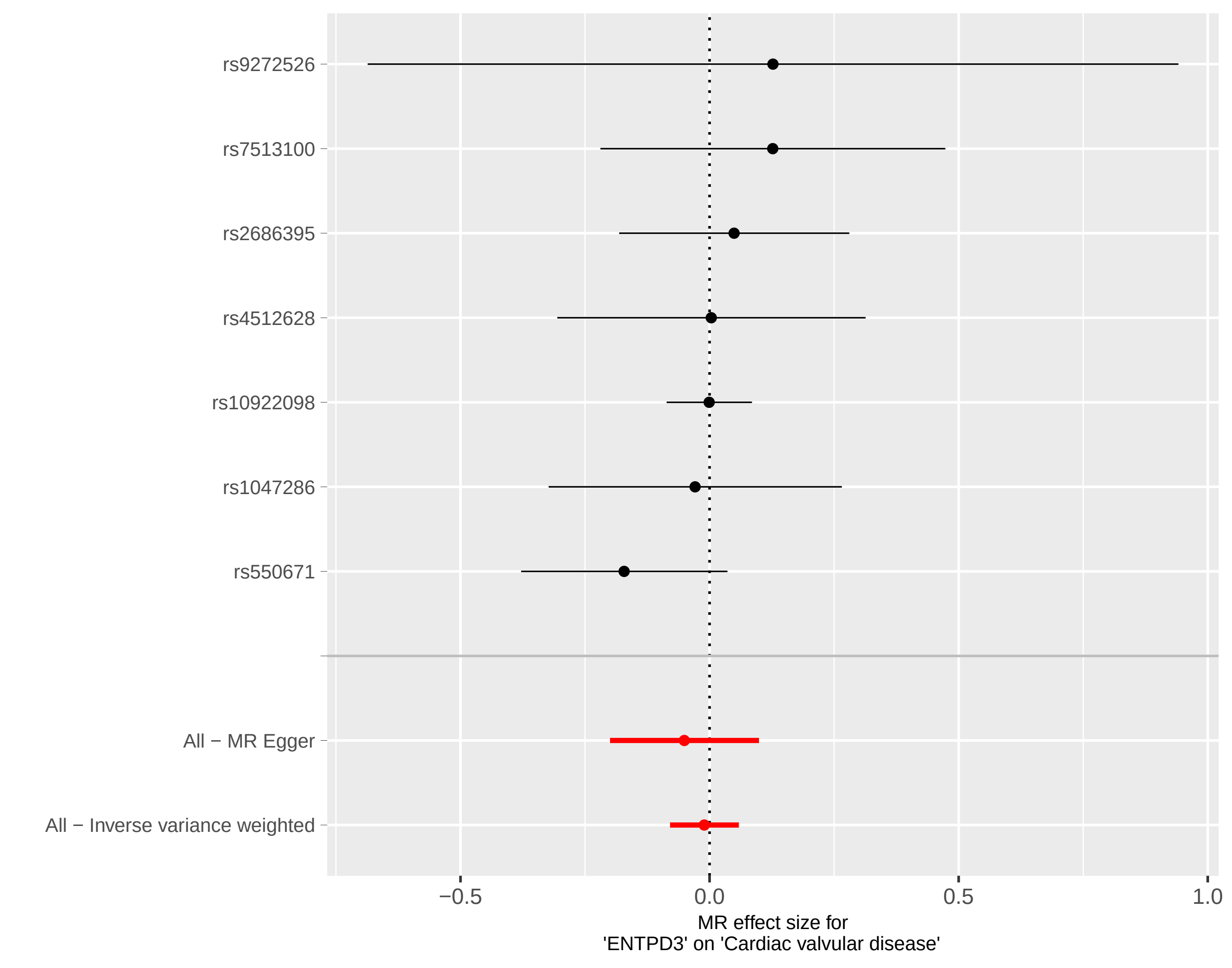
rs9272526
rs7513100
rs2686395
rs4512628
rs10922098
rs1047286
rs550671
All − MR Egger
All − Inverse variance weighted
−0.5
0.0
0.5
1.0
MR effect size for
'ENTPD3' on 'Cardiac valvular disease'

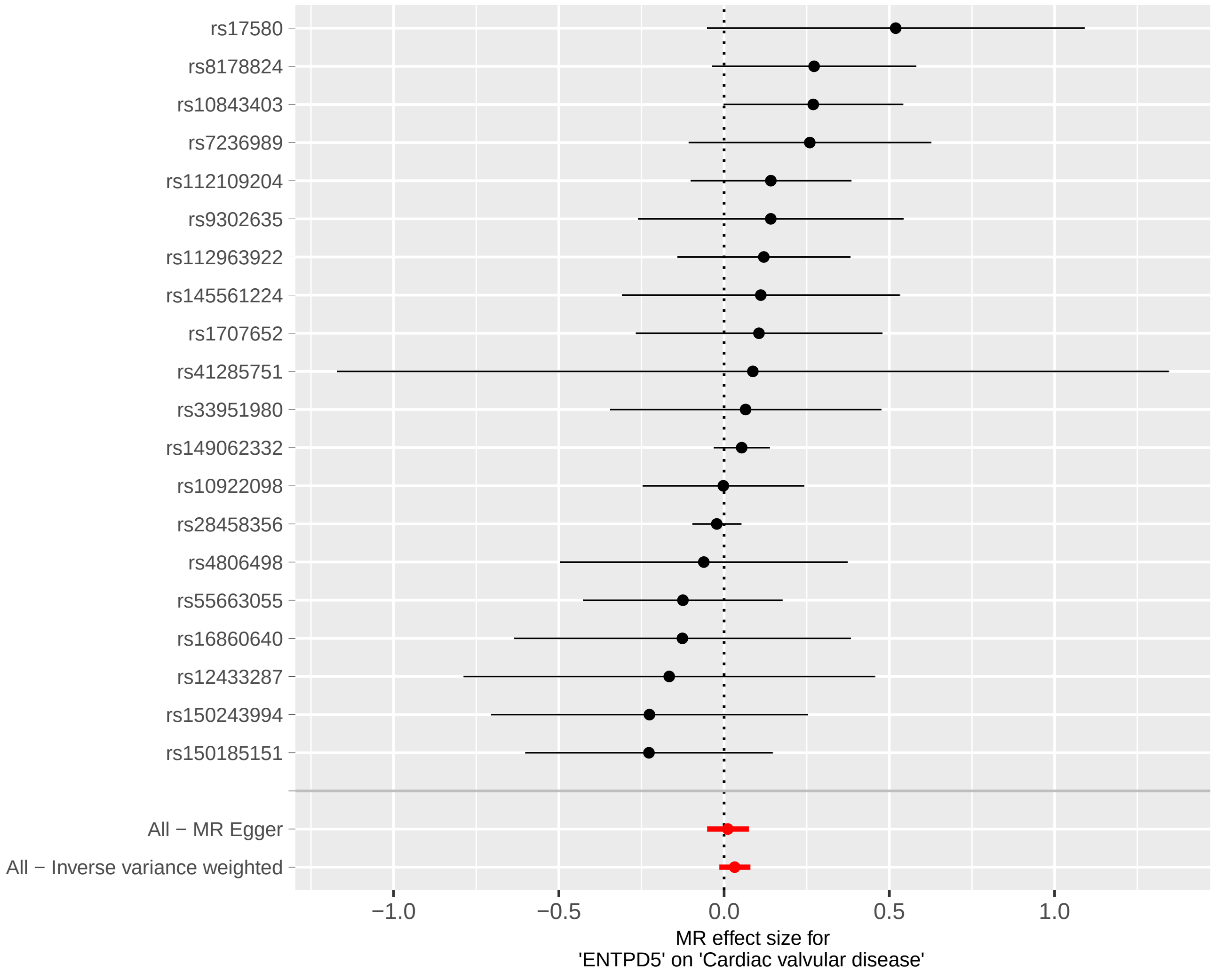
rs17580
rs8178824
rs10843403
rs7236989
rs112109204
rs9302635
rs112963922
rs145561224
rs1707652
rs41285751
rs33951980
rs149062332
rs10922098
rs28458356
rs4806498
rs55663055
rs16860640
rs12433287
rs150243994
rs150185151
All − MR Egger
All − Inverse variance weighted
−1.0
−0.5
0.0
0.5
1.0
MR effect size for
'ENTPD5' on 'Cardiac valvular disease'

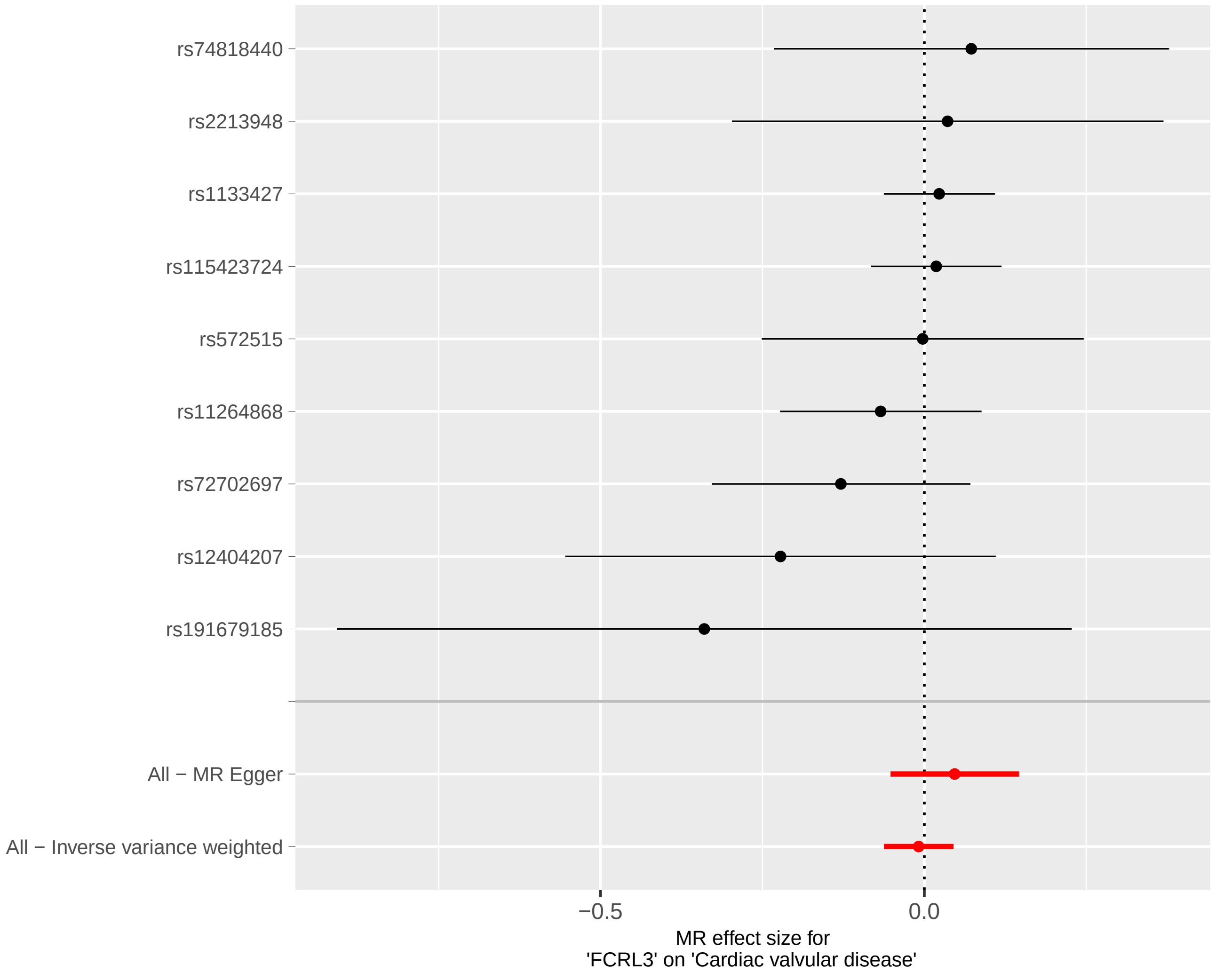
rs74818440
rs2213948
rs1133427
rs115423724
rs572515
rs11264868
rs72702697
rs12404207
rs191679185
All − MR Egger
All − Inverse variance weighted
−0.5
0.0
MR effect size for
'FCRL3' on 'Cardiac valvular disease'

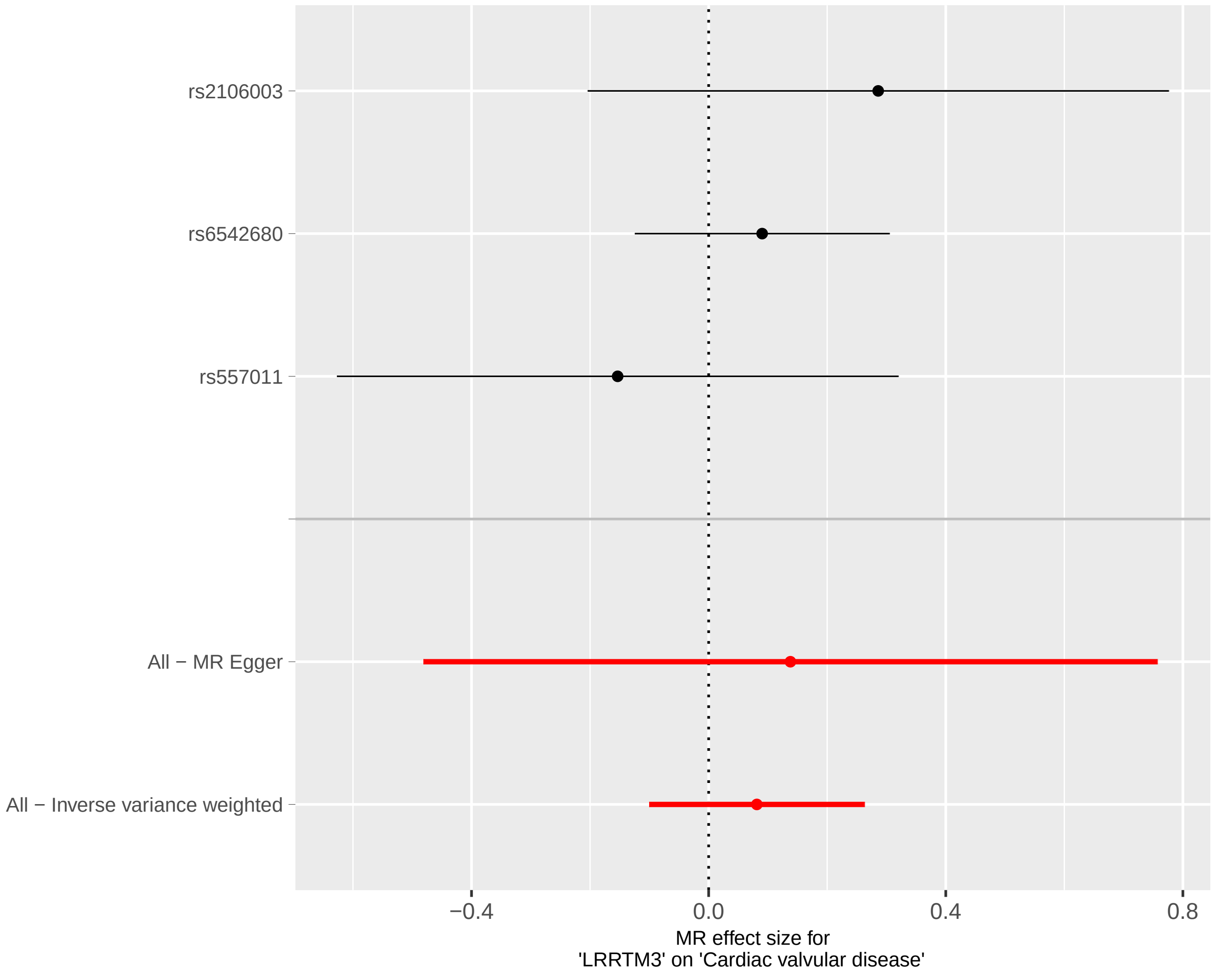
rs2106003
rs6542680
rs557011
All − MR Egger
All − Inverse variance weighted
−0.4
0.0
0.4
0.8
MR effect size for
'LRRTM3' on 'Cardiac valvular disease'

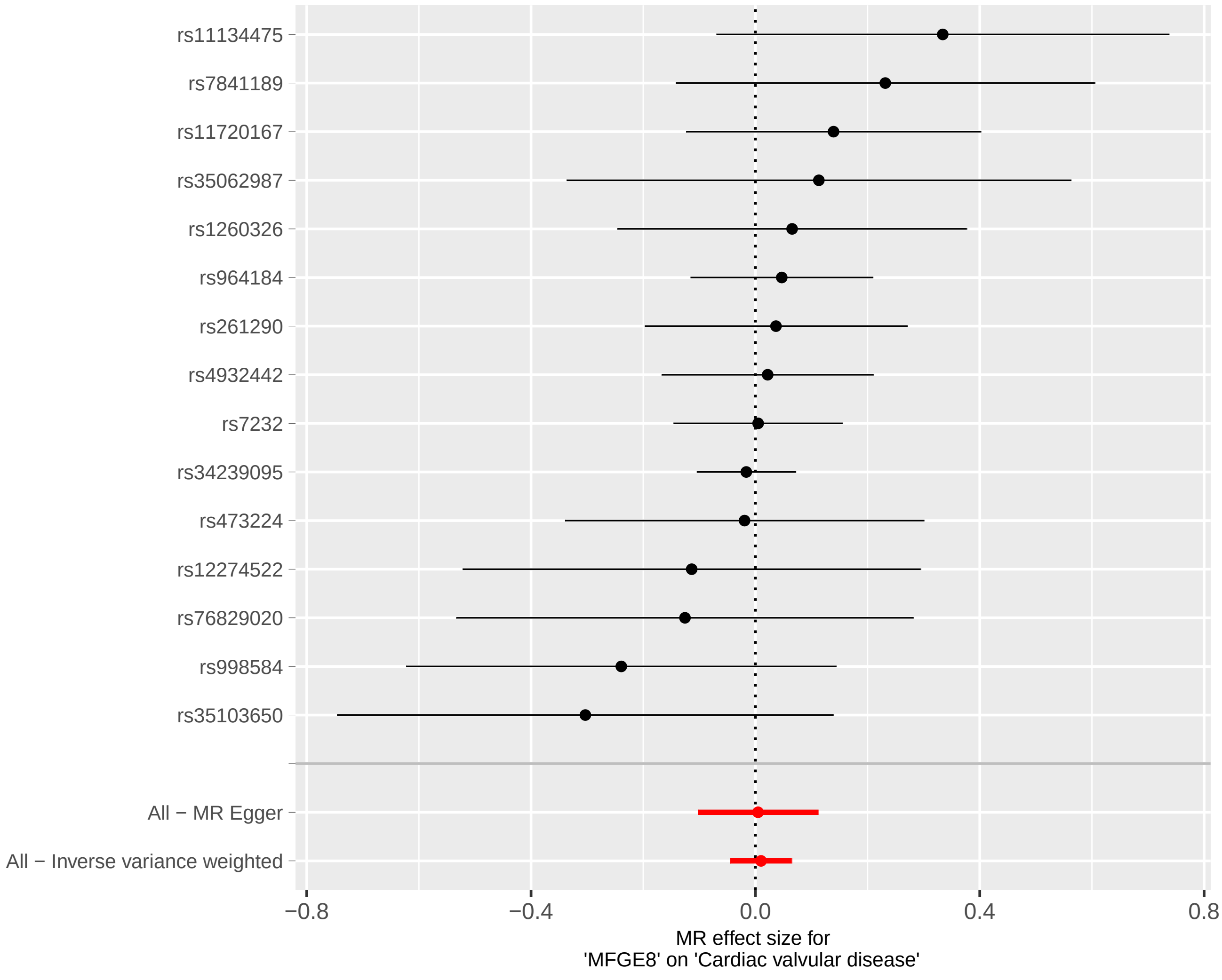
rs11134475
rs7841189
rs11720167
rs35062987
rs1260326
rs964184
rs261290
rs4932442
rs7232
rs34239095
rs473224
rs12274522
rs76829020
rs998584
rs35103650
All − MR Egger
All − Inverse variance weighted
−0.8
−0.4
0.0
0.4
0.8
MR effect size for
'MFGE8' on 'Cardiac valvular disease'

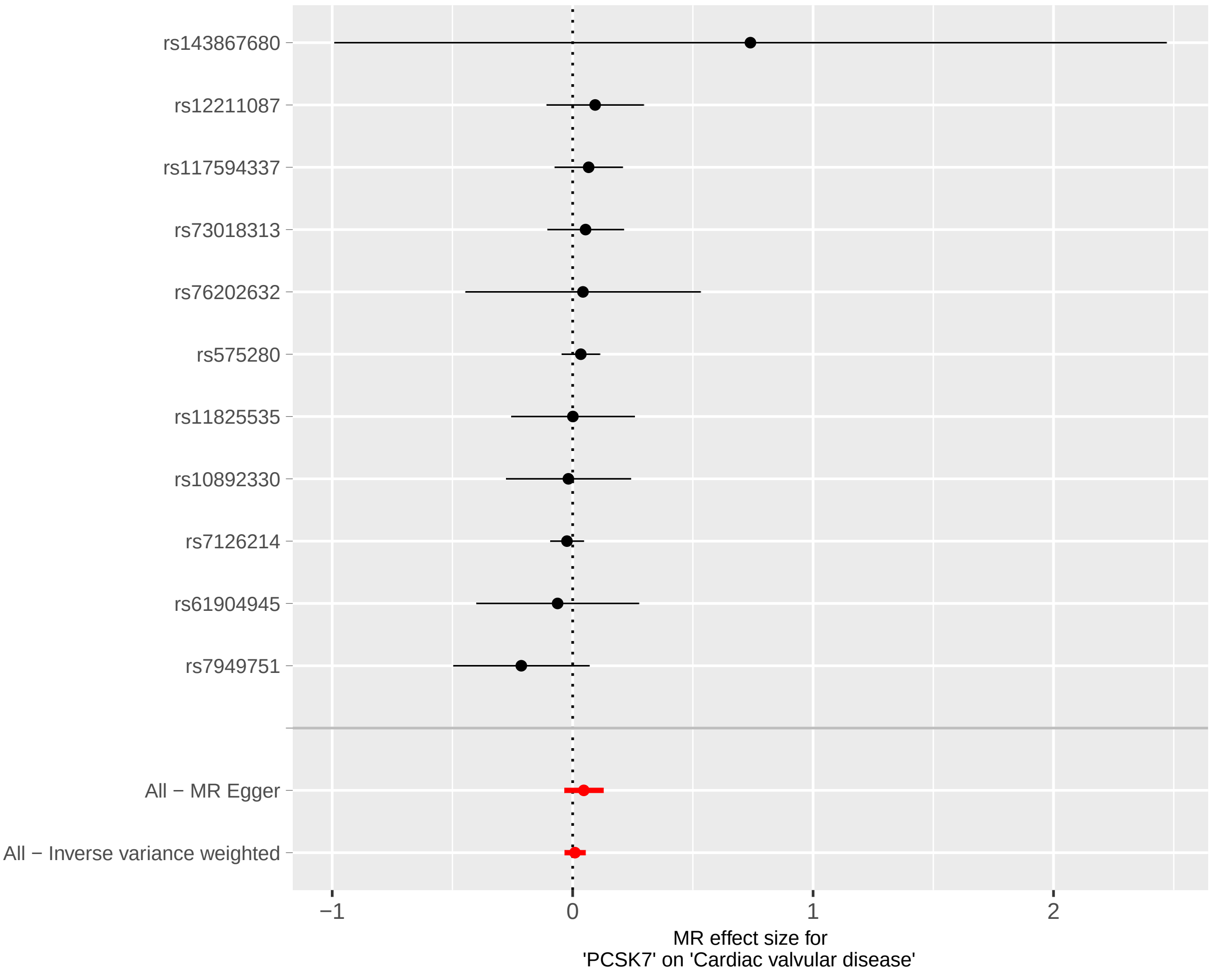
rs143867680
rs12211087
rs117594337
rs73018313
rs76202632
rs575280
rs11825535
rs10892330
rs7126214
rs61904945
rs7949751
All − MR Egger
All − Inverse variance weighted
−1
0
1
2
MR effect size for
'PCSK7' on 'Cardiac valvular disease'

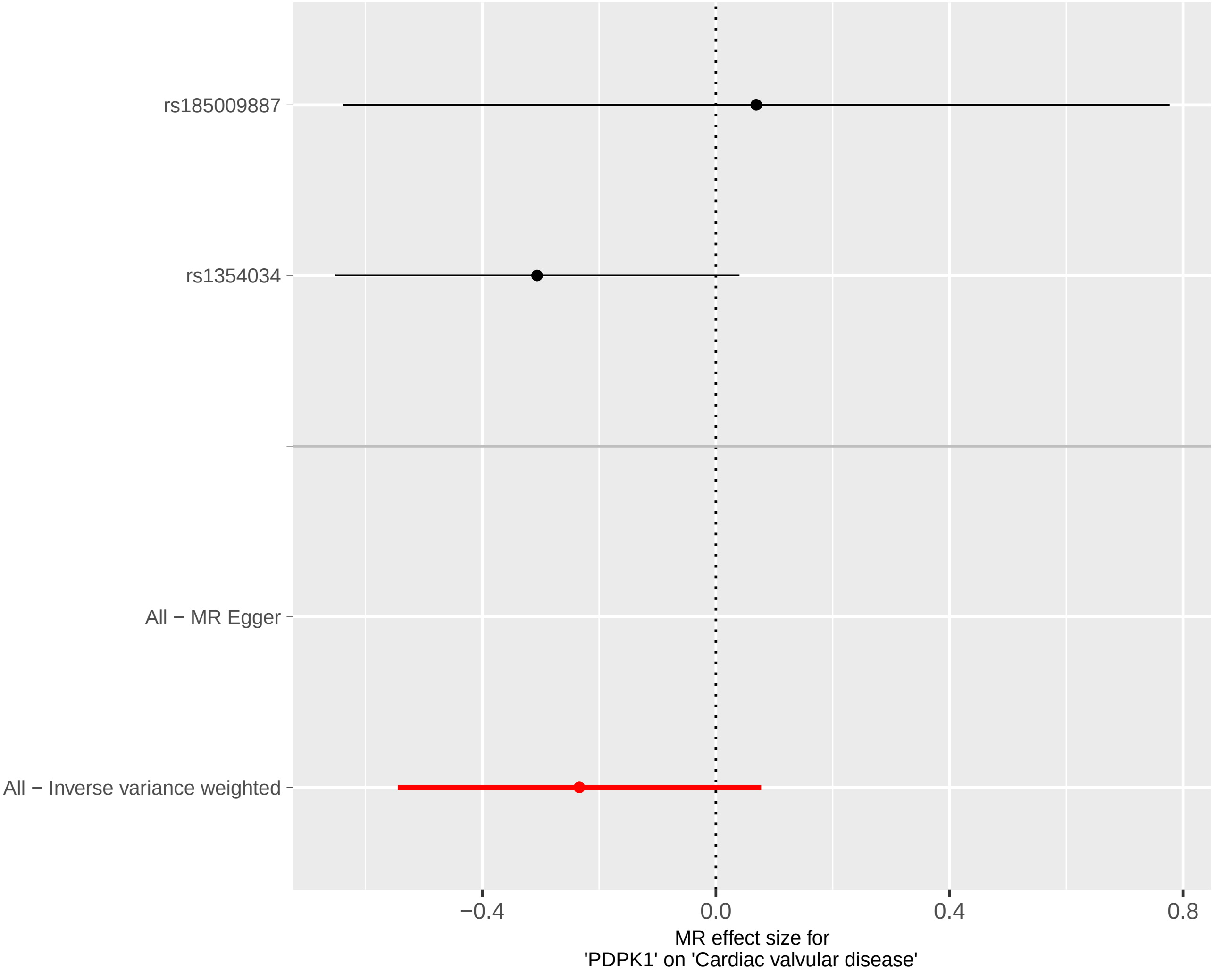
rs185009887
rs1354034
All − MR Egger
All − Inverse variance weighted
−0.4
0.0
0.4
0.8
MR effect size for
'PDPK1' on 'Cardiac valvular disease'

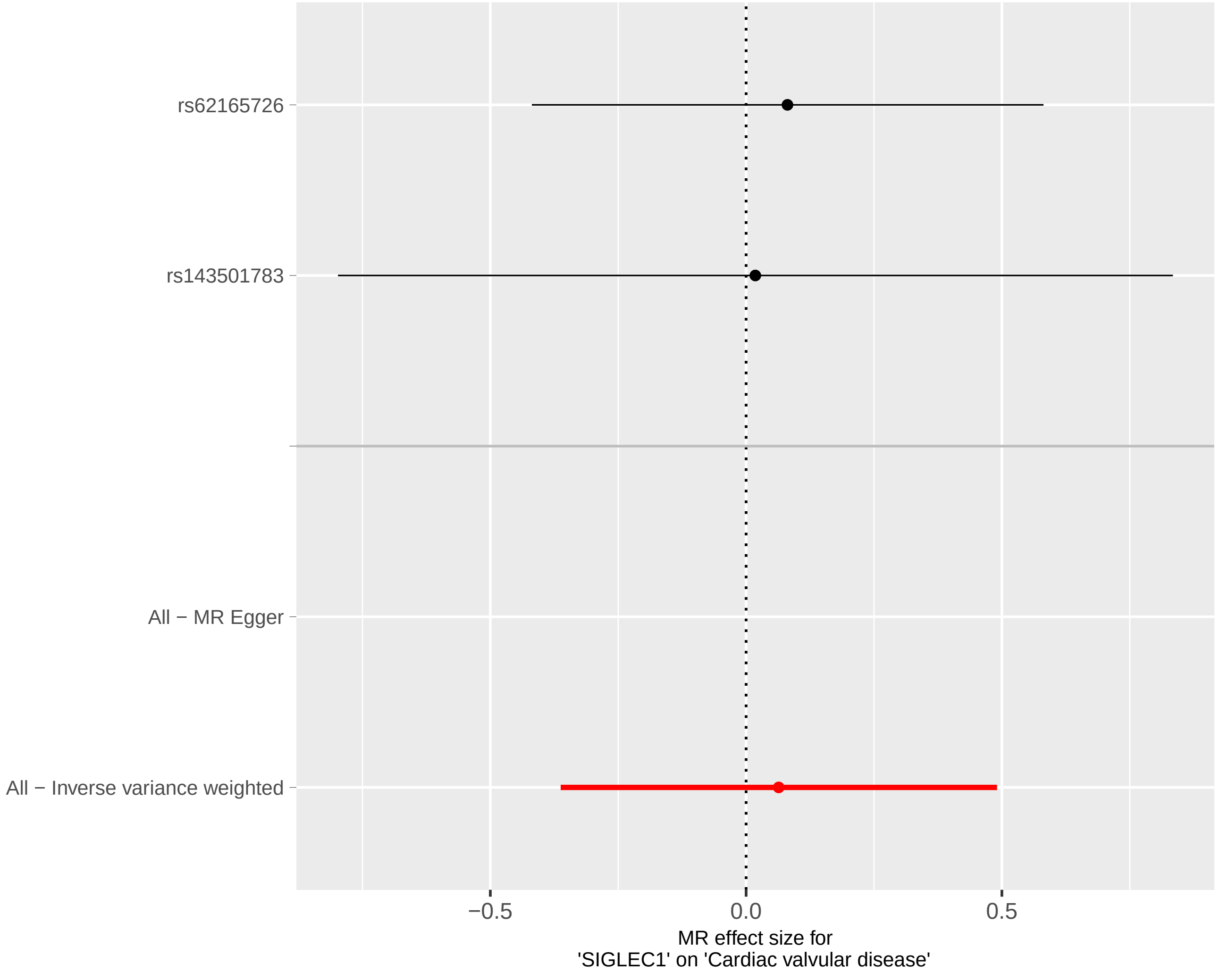

rs62165726
rs143501783
All − MR Egger
All − Inverse variance weighted
−0.5
0.0
0.5
MR effect size for
'SIGLEC1' on 'Cardiac valvular disease'

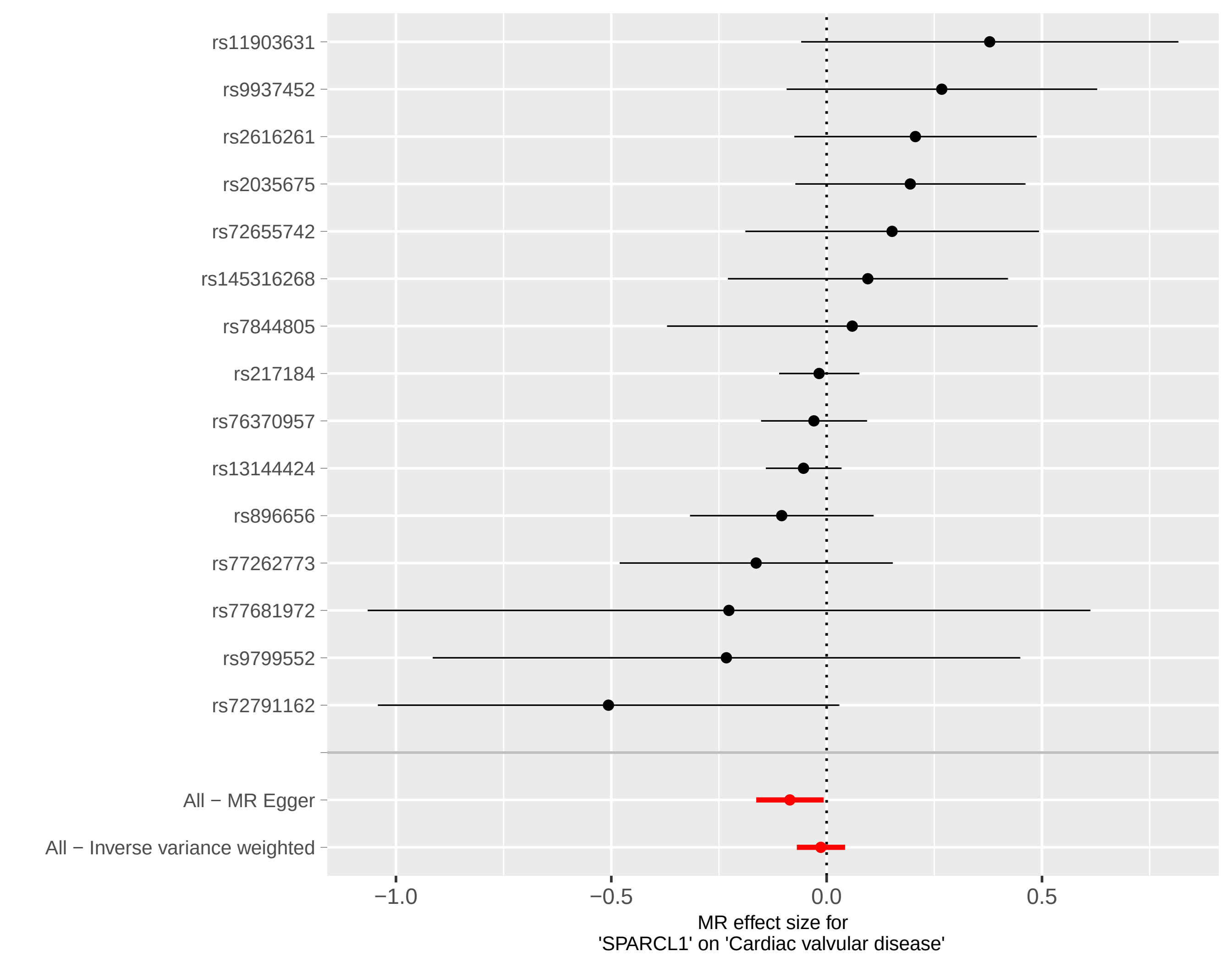

rs11903631
rs9937452
rs2616261
rs2035675
rs72655742
rs145316268
rs7844805
rs217184
rs76370957
rs13144424
rs896656
rs77262773
rs77681972
rs9799552
rs72791162
All − MR Egger
All − Inverse variance weighted
−1.0
−0.5
0.0
0.5
MR effect size for
'SPARCL1' on 'Cardiac valvular disease'

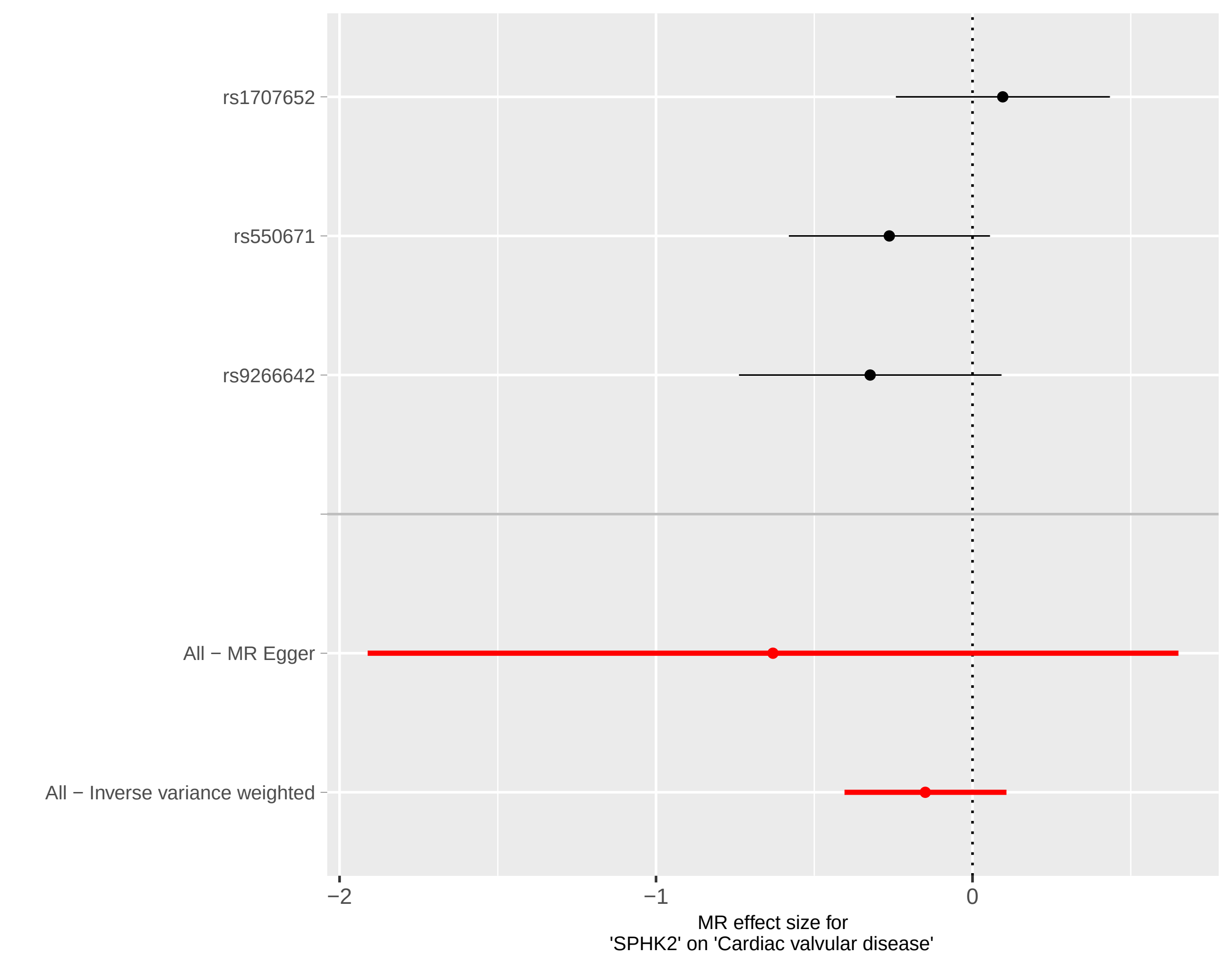
rs1707652
rs550671
rs9266642
All − MR Egger
All − Inverse variance weighted
−2
−1
0
MR effect size for
'SPHK2' on 'Cardiac valvular disease'

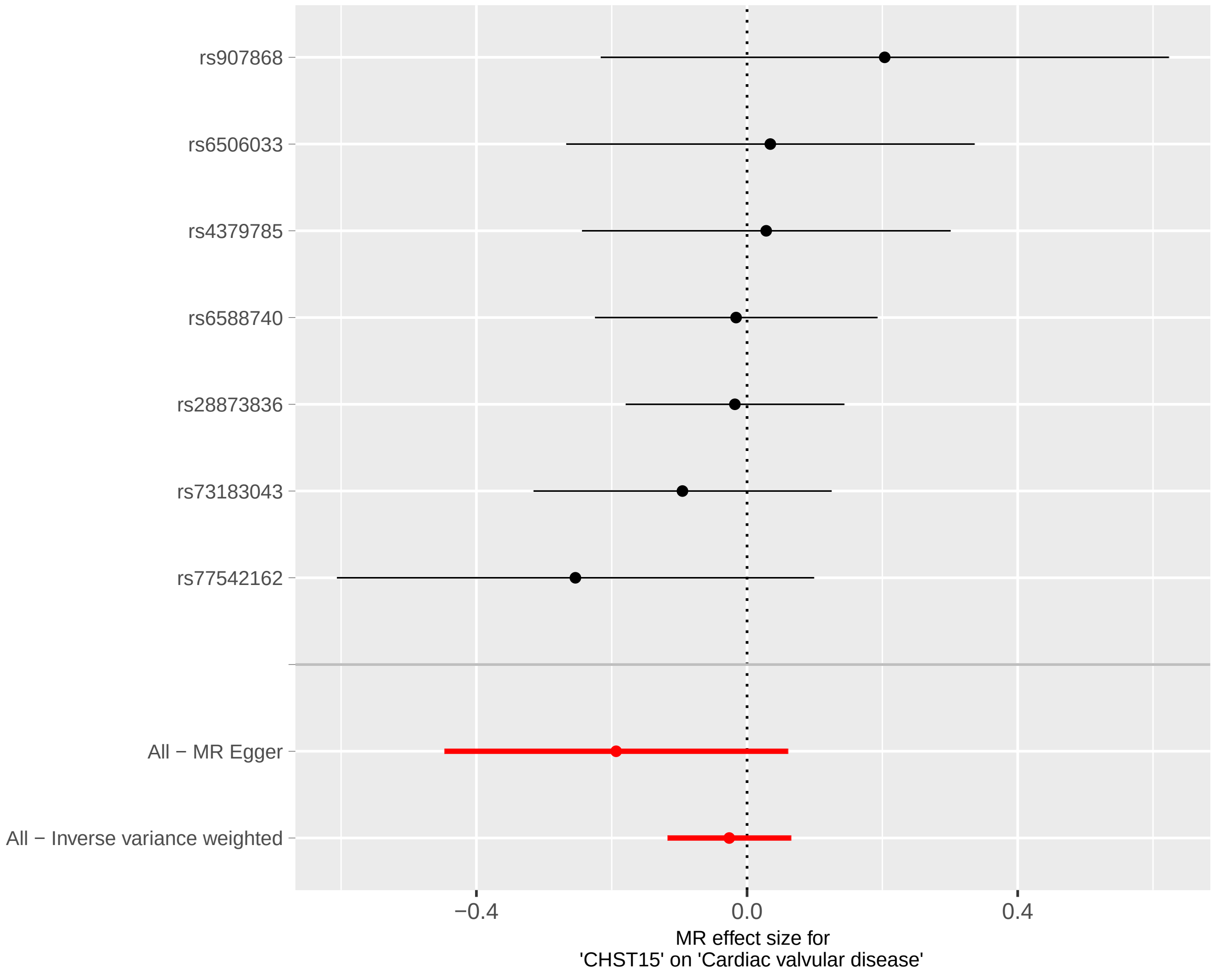

rs907868
rs6506033
rs4379785
rs6588740
rs28873836
rs73183043
rs77542162
All − MR Egger
All − Inverse variance weighted
−0.4
0.0
0.4
MR effect size for
'CHST15' on 'Cardiac valvular disease'

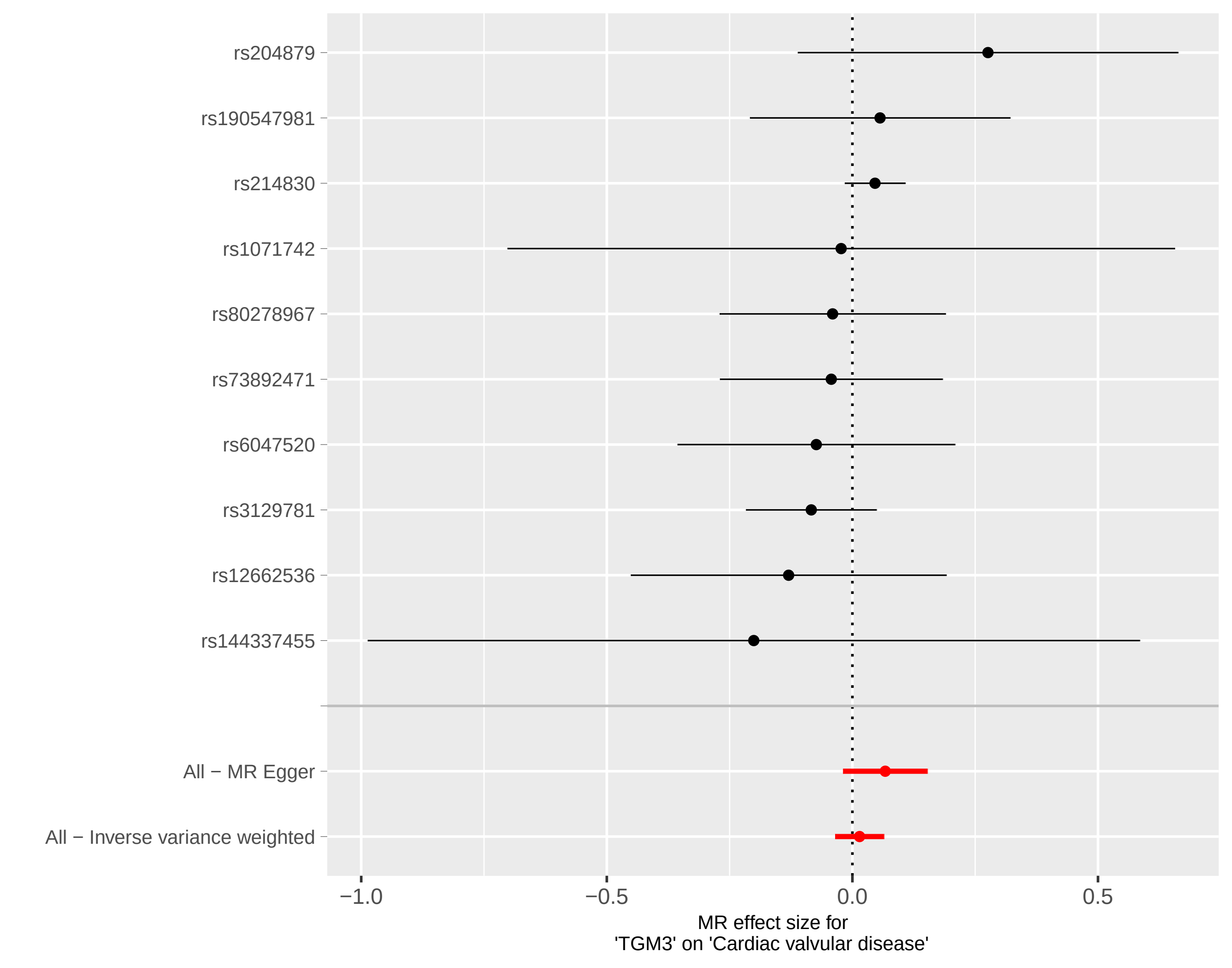
rs204879
rs190547981
rs214830
rs1071742
rs80278967
rs73892471
rs6047520
rs3129781
rs12662536
rs144337455
All − MR Egger
All − Inverse variance weighted
−1.0
−0.5
0.0
0.5
MR effect size for
'TGM3' on 'Cardiac valvular disease'

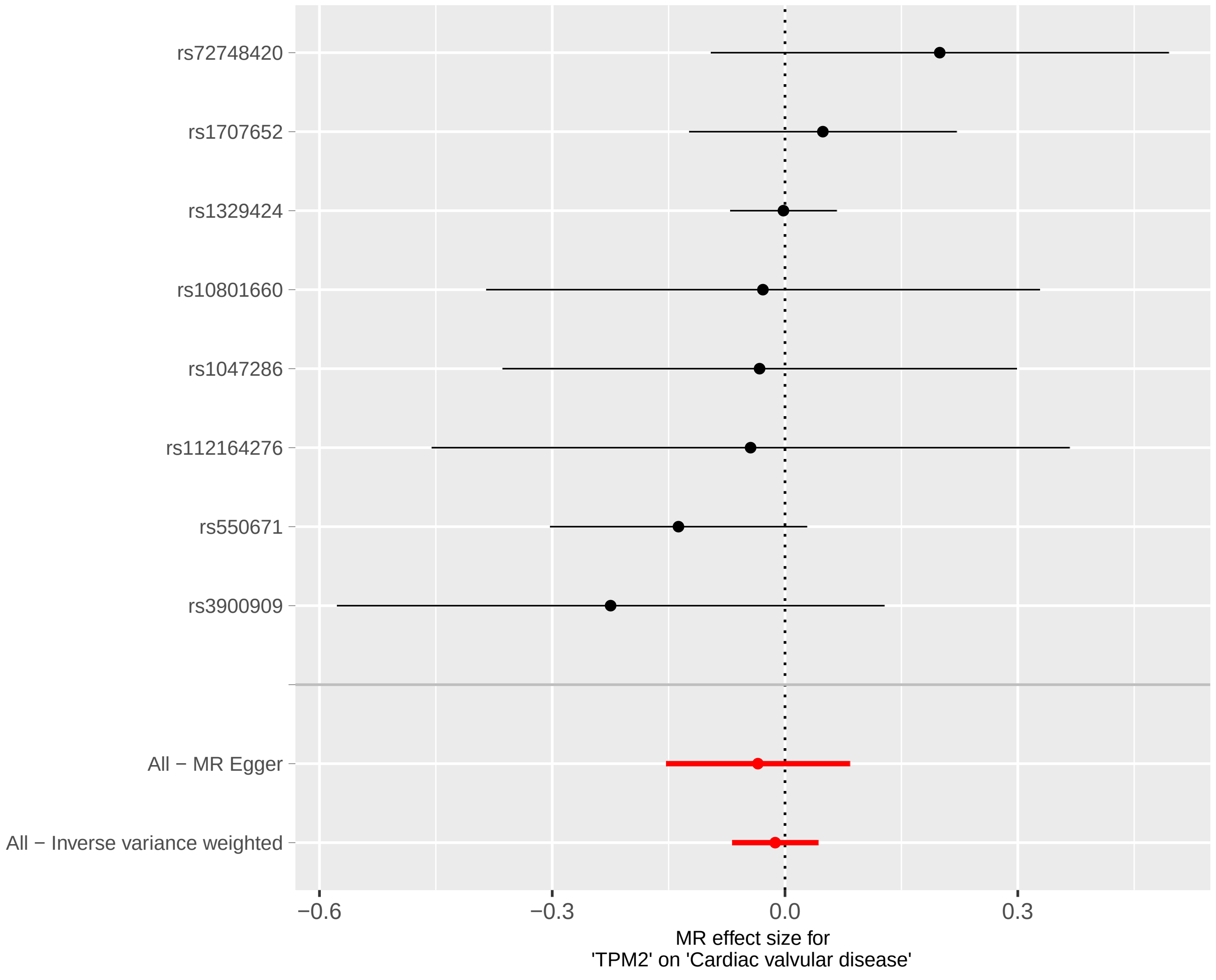
rs72748420
rs1707652
rs1329424
rs10801660
rs1047286
rs112164276
rs550671
rs3900909
All − MR Egger
All − Inverse variance weighted
−0.6
−0.3
0.0
0.3
MR effect size for
'TPM2' on 'Cardiac valvular disease'

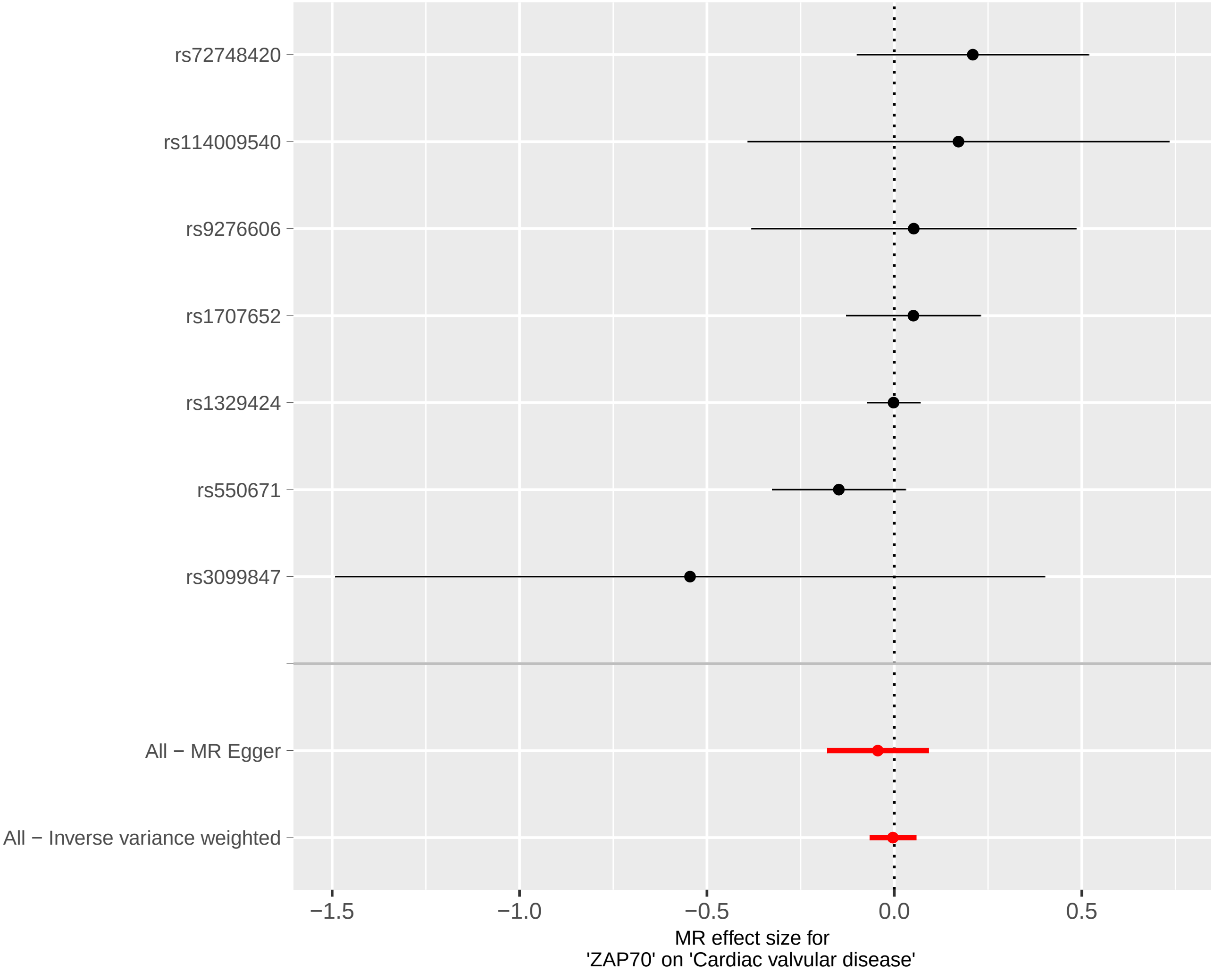

rs72748420
rs114009540
rs9276606
rs1707652
rs1329424
rs550671
rs3099847
All − MR Egger
All − Inverse variance weighted
−1.5
−1.0
−0.5
0.0
0.5
MR effect size for
'ZAP70' on 'Cardiac valvular disease'

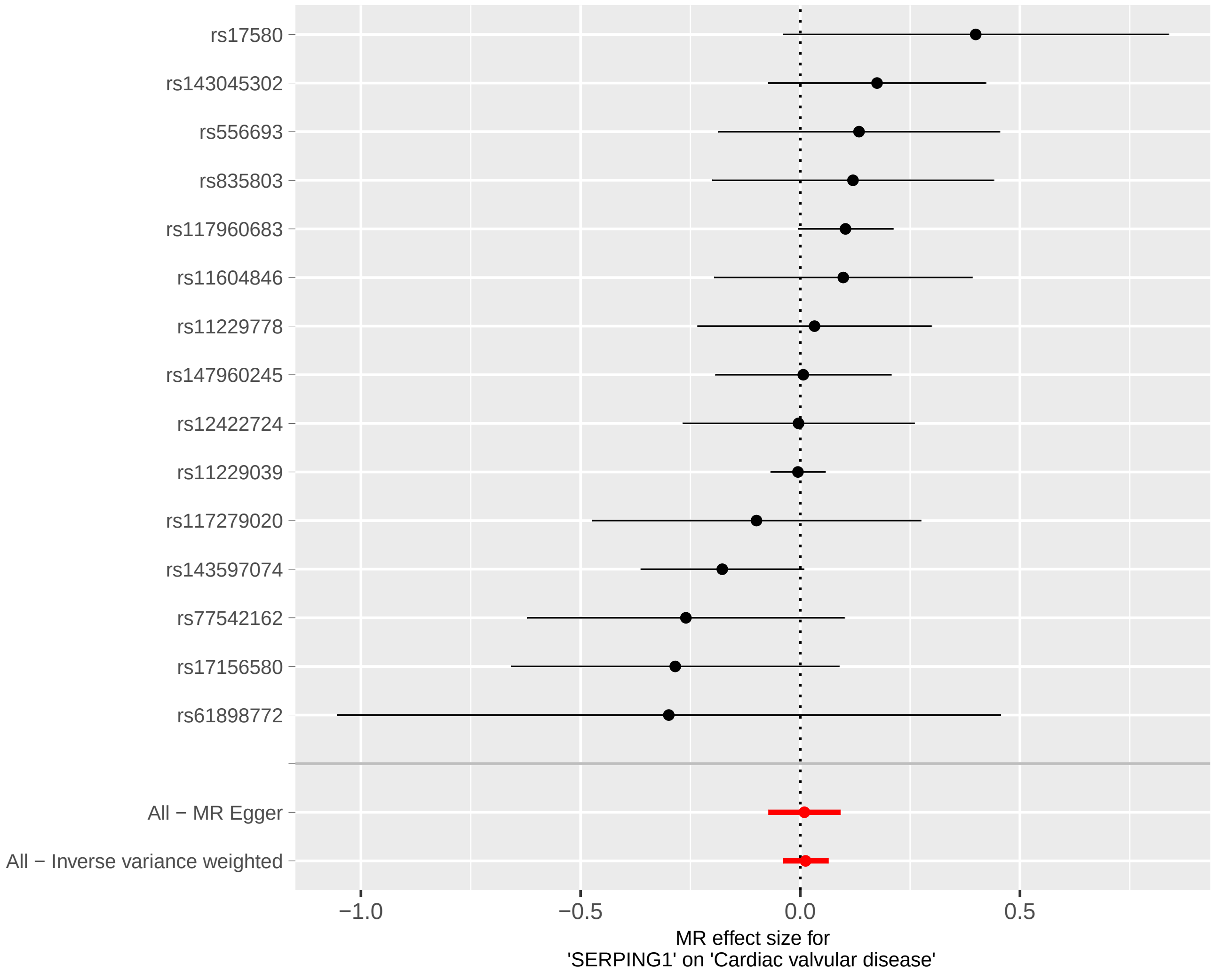
rs17580
rs143045302
rs556693
rs835803
rs117960683
rs11604846
rs11229778
rs147960245
rs12422724
rs11229039
rs117279020
rs143597074
rs77542162
rs17156580
rs61898772
All − MR Egger
All − Inverse variance weighted
−1.0
−0.5
0.0
0.5
MR effect size for
'SERPING1' on 'Cardiac valvular disease'

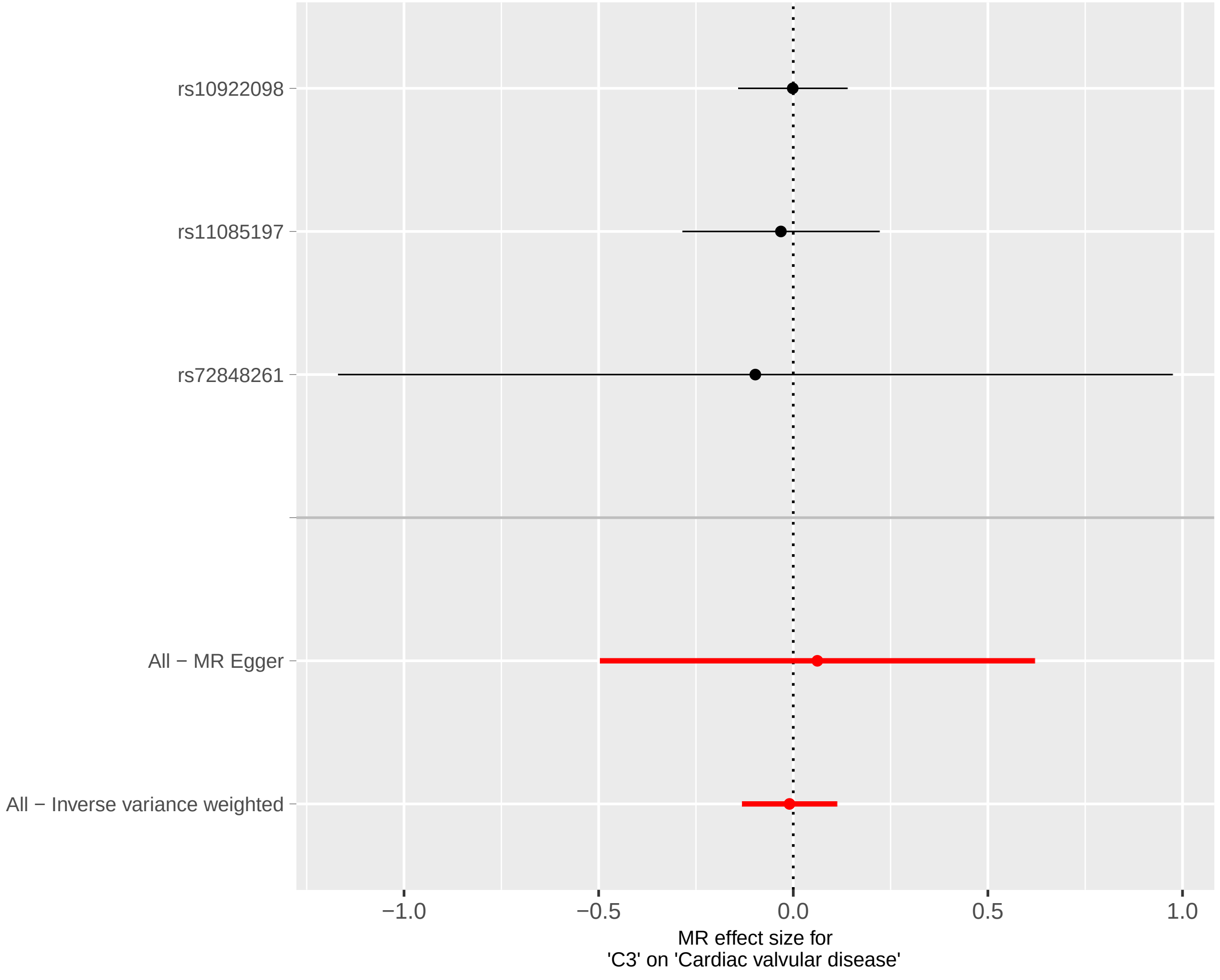
rs10922098
rs11085197
rs72848261
All − MR Egger
All − Inverse variance weighted
−1.0
−0.5
0.0
0.5
1.0
MR effect size for
'C3' on 'Cardiac valvular disease'

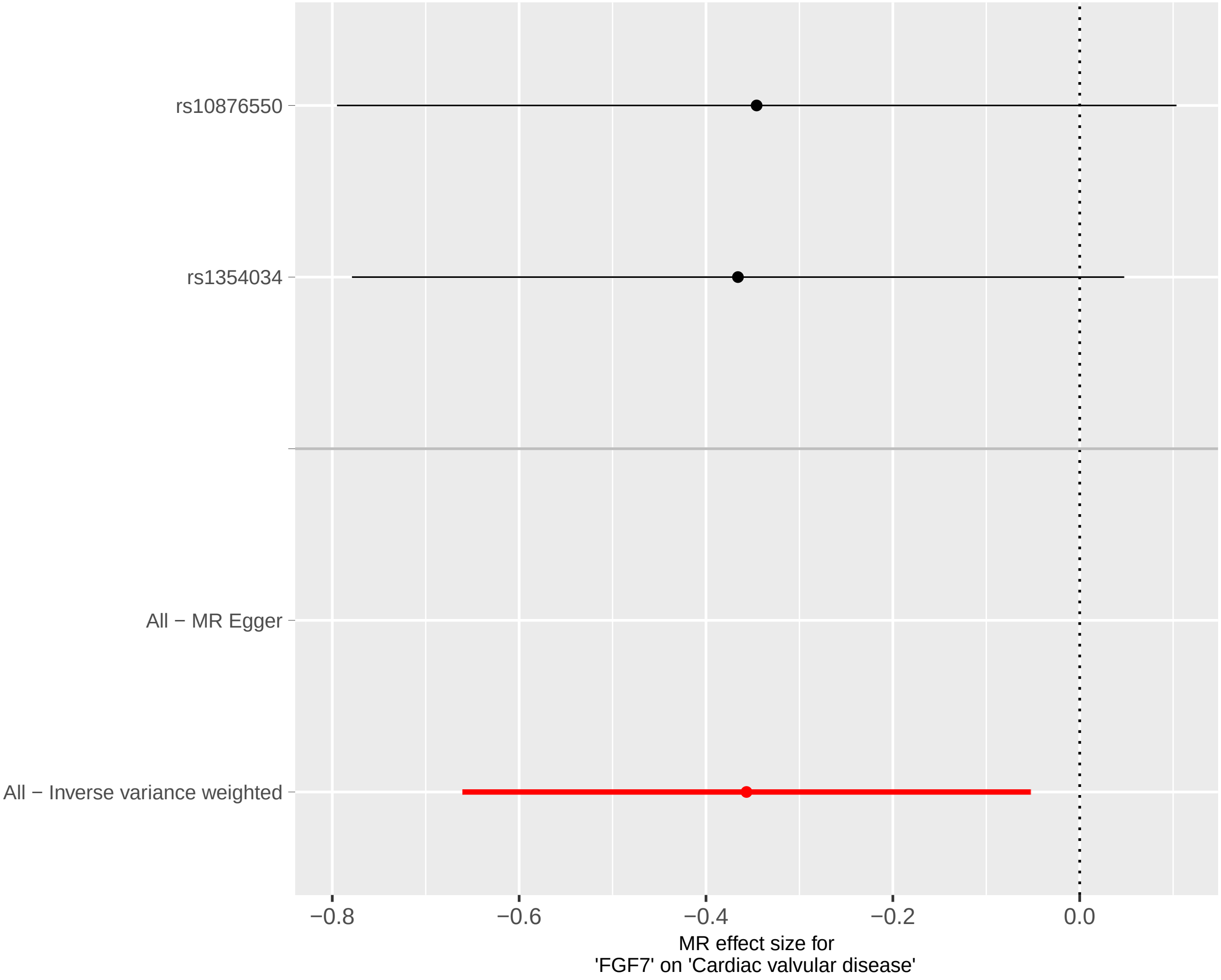
rs10876550
rs1354034
All − MR Egger
All − Inverse variance weighted
−0.8
−0.6
−0.4
−0.2
0.0
MR effect size for
'FGF7' on 'Cardiac valvular disease'

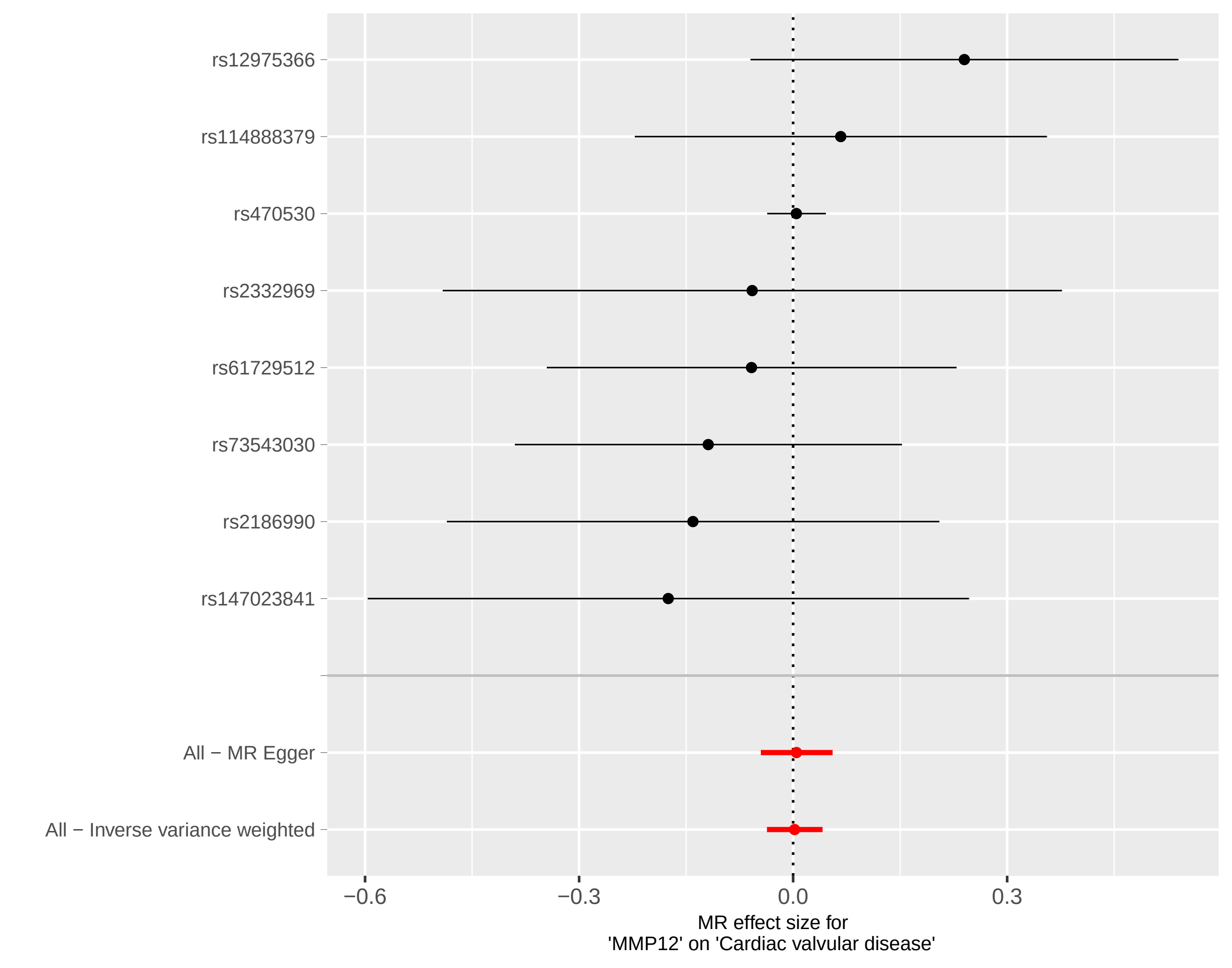

rs12975366
rs114888379
rs470530
rs2332969
rs61729512
rs73543030
rs2186990
rs147023841
All − MR Egger
All − Inverse variance weighted
−0.6
−0.3
0.0
0.3
MR effect size for
'MMP12' on 'Cardiac valvular disease'

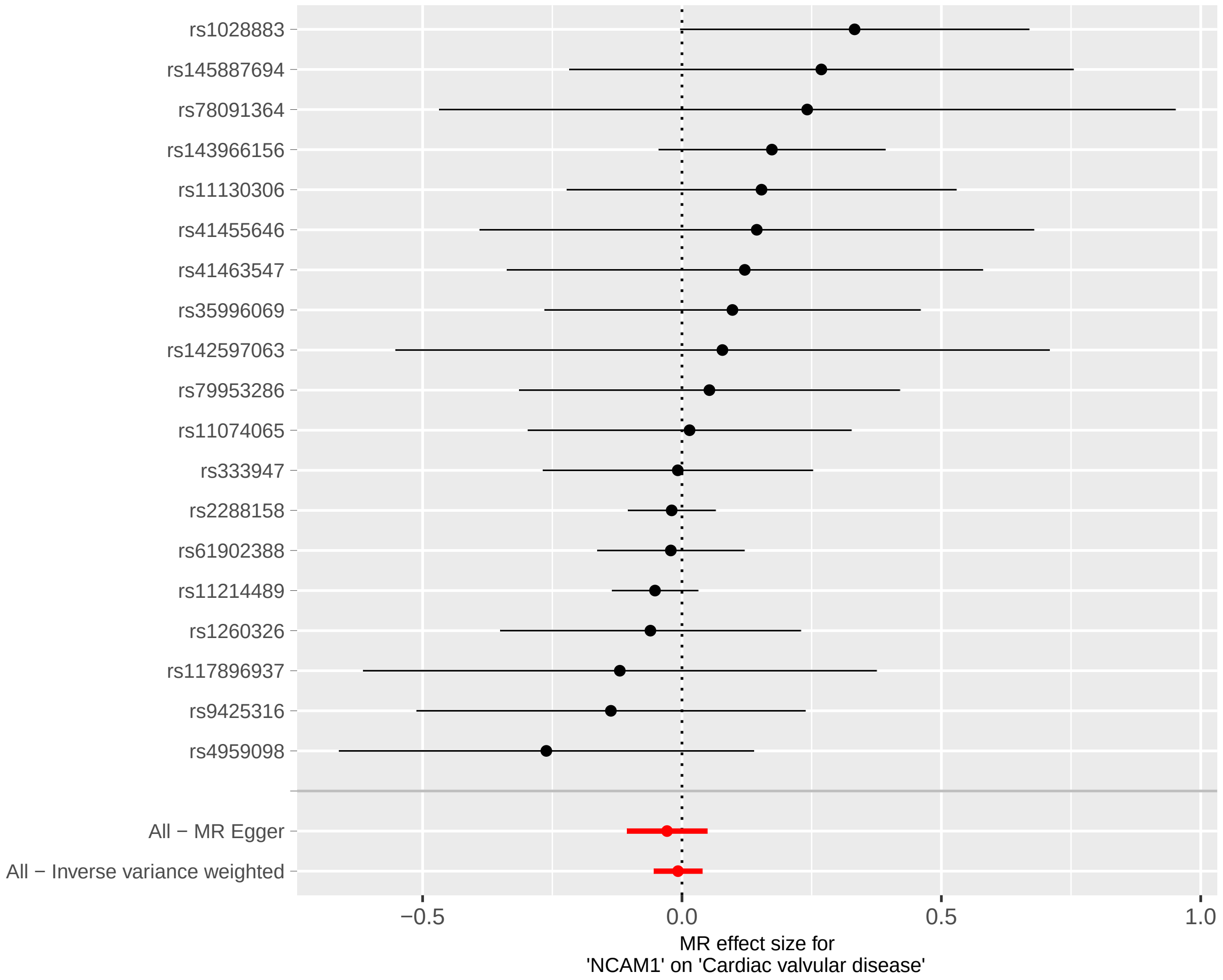
rs1028883
rs145887694
rs78091364
rs143966156
rs11130306
rs41455646
rs41463547
rs35996069
rs142597063
rs79953286
rs11074065
rs333947
rs2288158
rs61902388
rs11214489
rs1260326
rs117896937
rs9425316
rs4959098
All − MR Egger
All − Inverse variance weighted
−0.5
0.0
0.5
1.0
MR effect size for
'NCAM1' on 'Cardiac valvular disease'

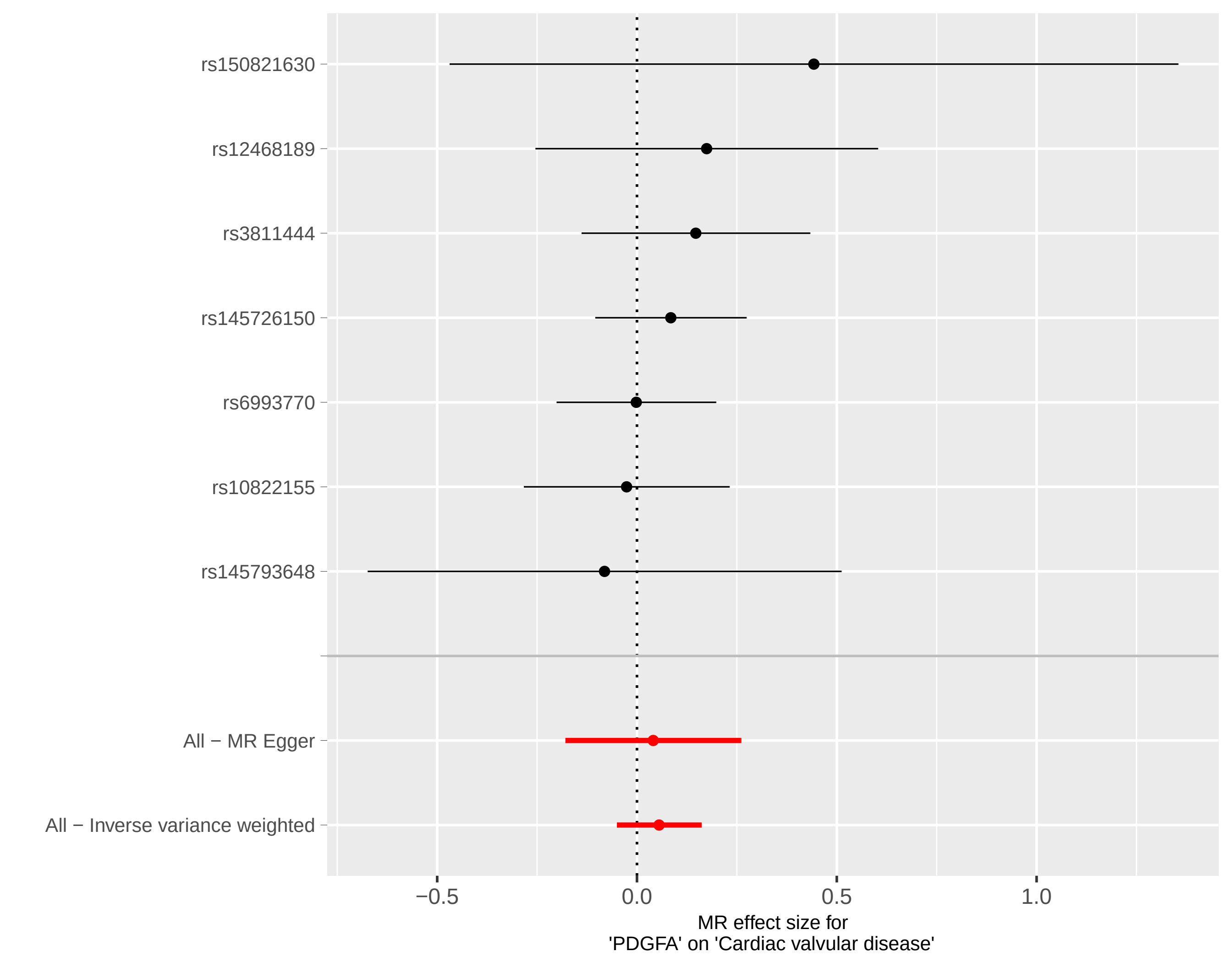
rs150821630
rs12468189
rs3811444
rs145726150
rs6993770
rs10822155
rs145793648
All − MR Egger
All − Inverse variance weighted
−0.5
0.0
0.5
1.0
MR effect size for
'PDGFA' on 'Cardiac valvular disease'

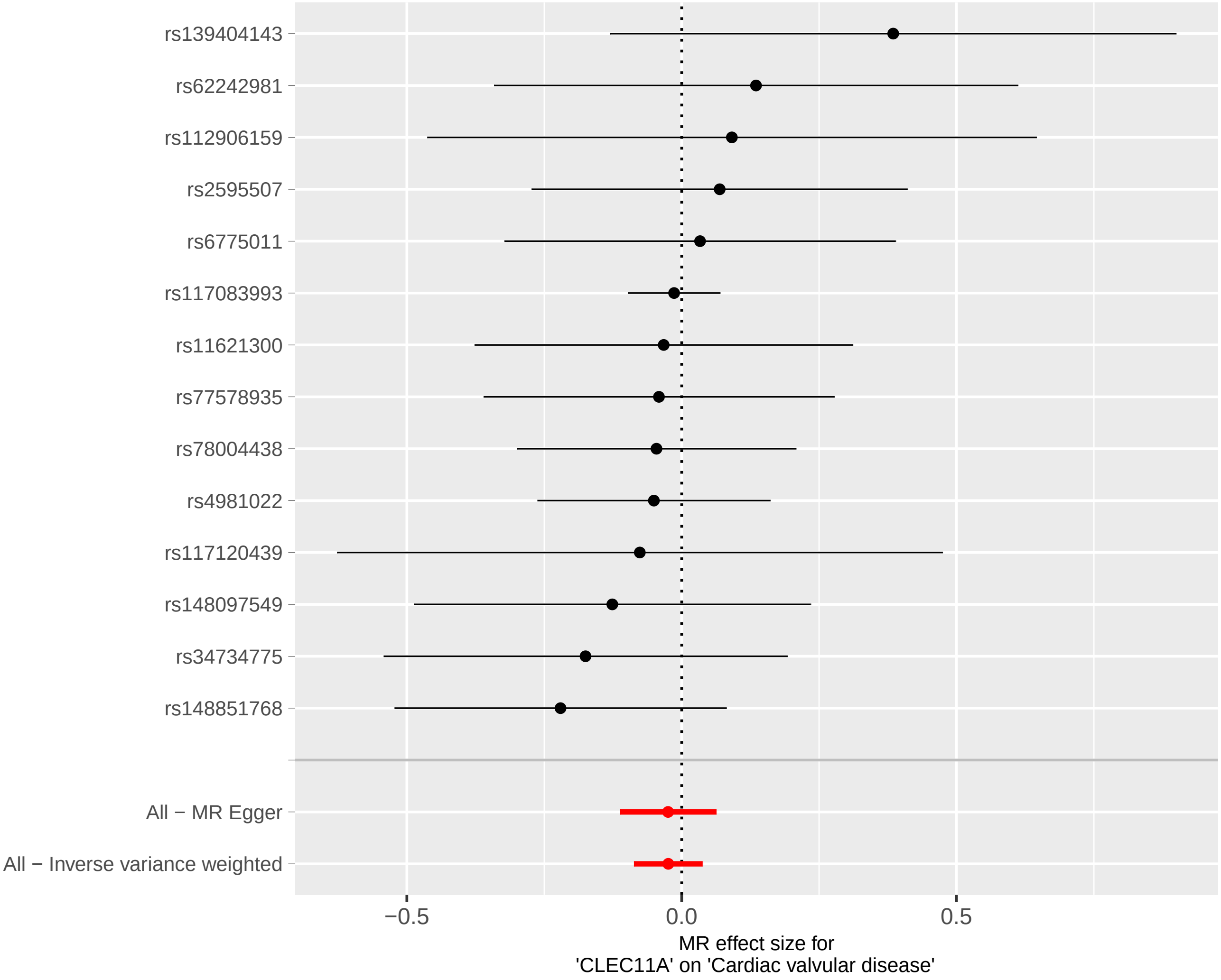
rs139404143
rs62242981
rs112906159
rs2595507
rs6775011
rs117083993
rs11621300
rs77578935
rs78004438
rs4981022
rs117120439
rs148097549
rs34734775
rs148851768
All − MR Egger
All − Inverse variance weighted
−0.5
0.0
0.5
MR effect size for
'CLEC11A' on 'Cardiac valvular disease'

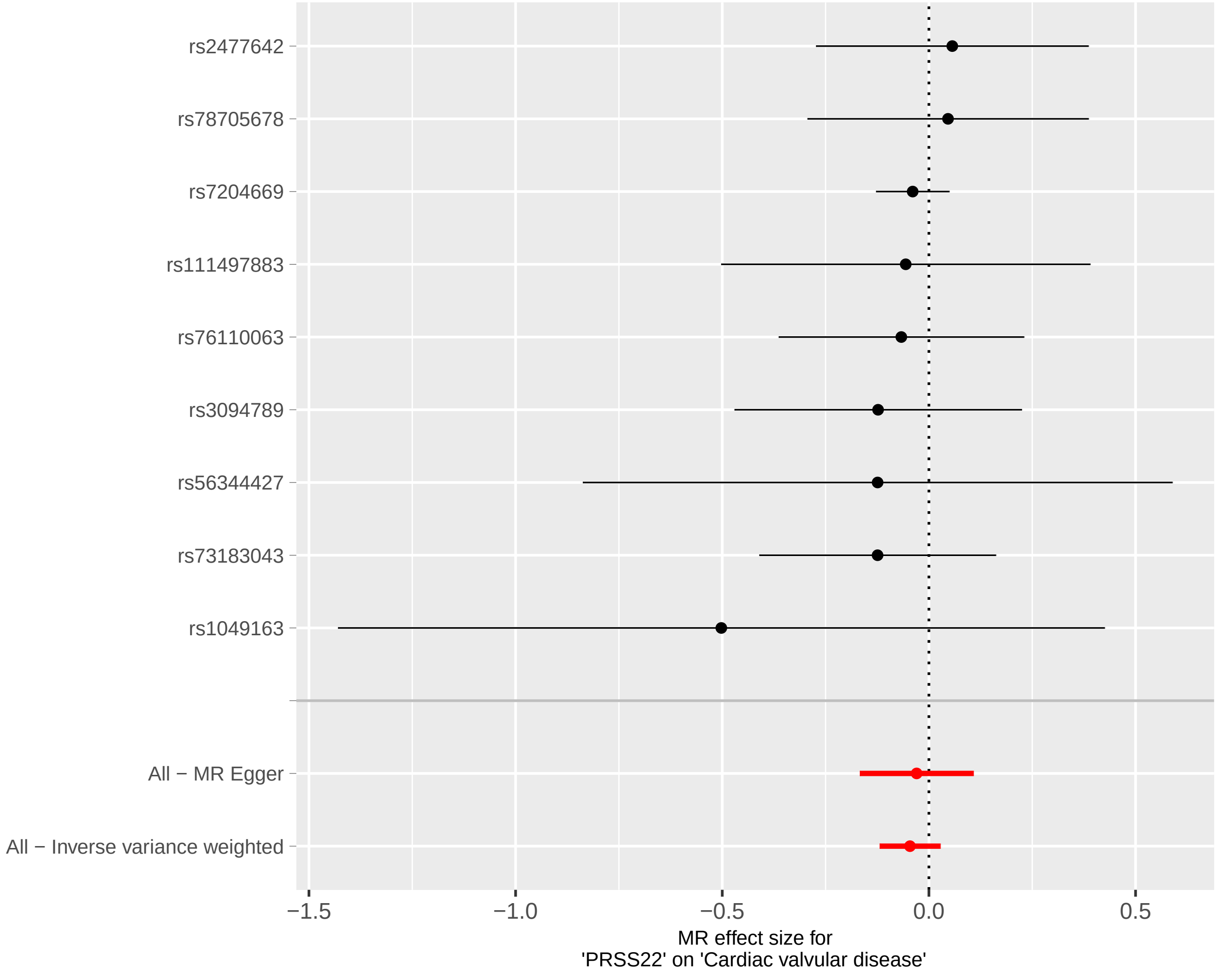
rs2477642
rs78705678
rs7204669
rs111497883
rs76110063
rs3094789
rs56344427
rs73183043
rs1049163
All − MR Egger
All − Inverse variance weighted
−1.5
−1.0
−0.5
0.0
0.5
MR effect size for
'PRSS22' on 'Cardiac valvular disease'

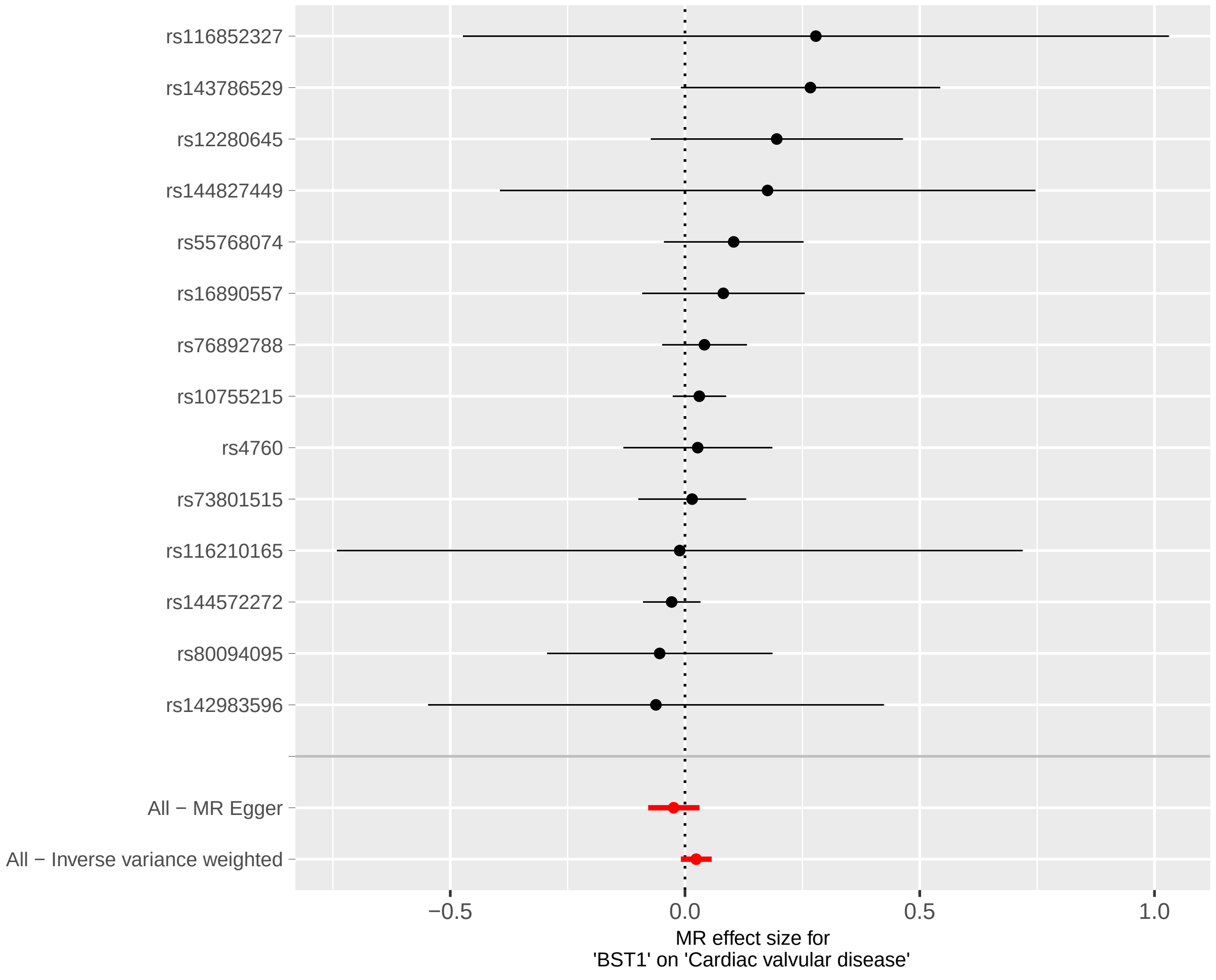
rs116852327
rs143786529
rs12280645
rs144827449
rs55768074
rs16890557
rs76892788
rs10755215
rs4760
rs73801515
rs116210165
rs144572272
rs80094095
rs142983596
All − MR Egger
All − Inverse variance weighted
−0.5
0.0
0.5
1.0
MR effect size for
'BST1' on 'Cardiac valvular disease'

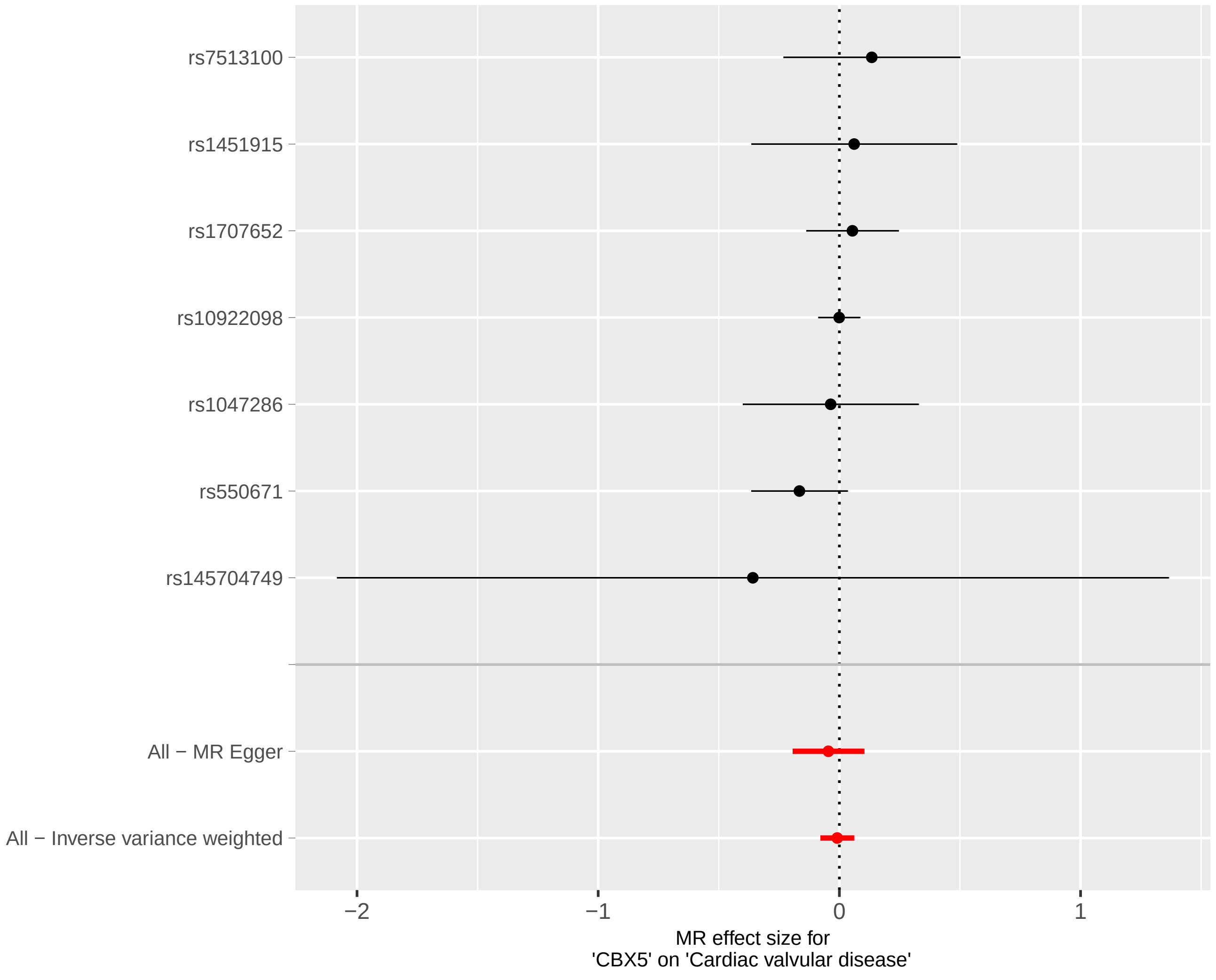
rs7513100
rs1451915
rs1707652
rs10922098
rs1047286
rs550671
rs145704749
All − MR Egger
All − Inverse variance weighted
−2
−1
0
1
MR effect size for
'CBX5' on 'Cardiac valvular disease'

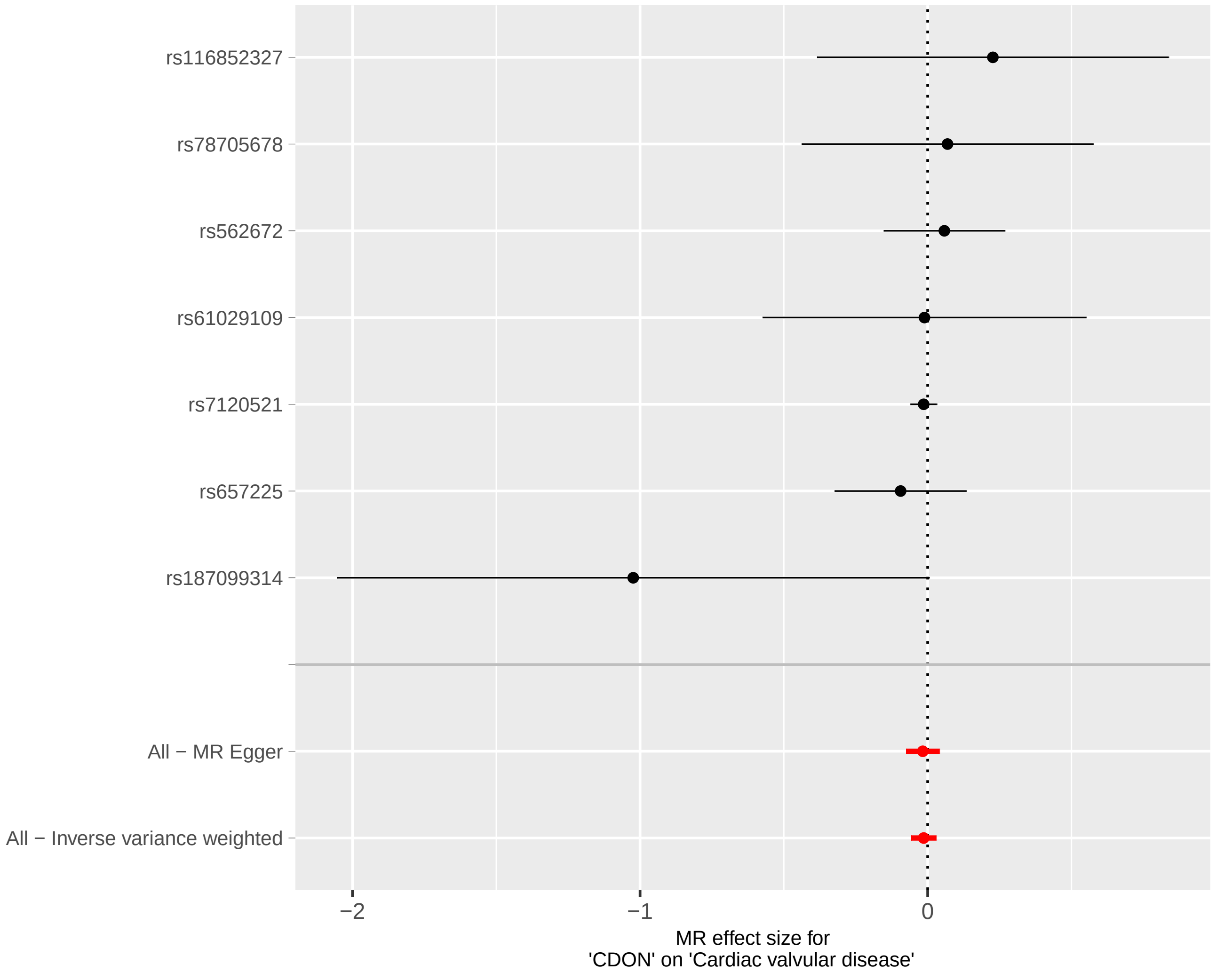
rs116852327
rs78705678
rs562672
rs61029109
rs7120521
rs657225
rs187099314
All − MR Egger
All − Inverse variance weighted
−2
−1
0
MR effect size for
'CDON' on 'Cardiac valvular disease'

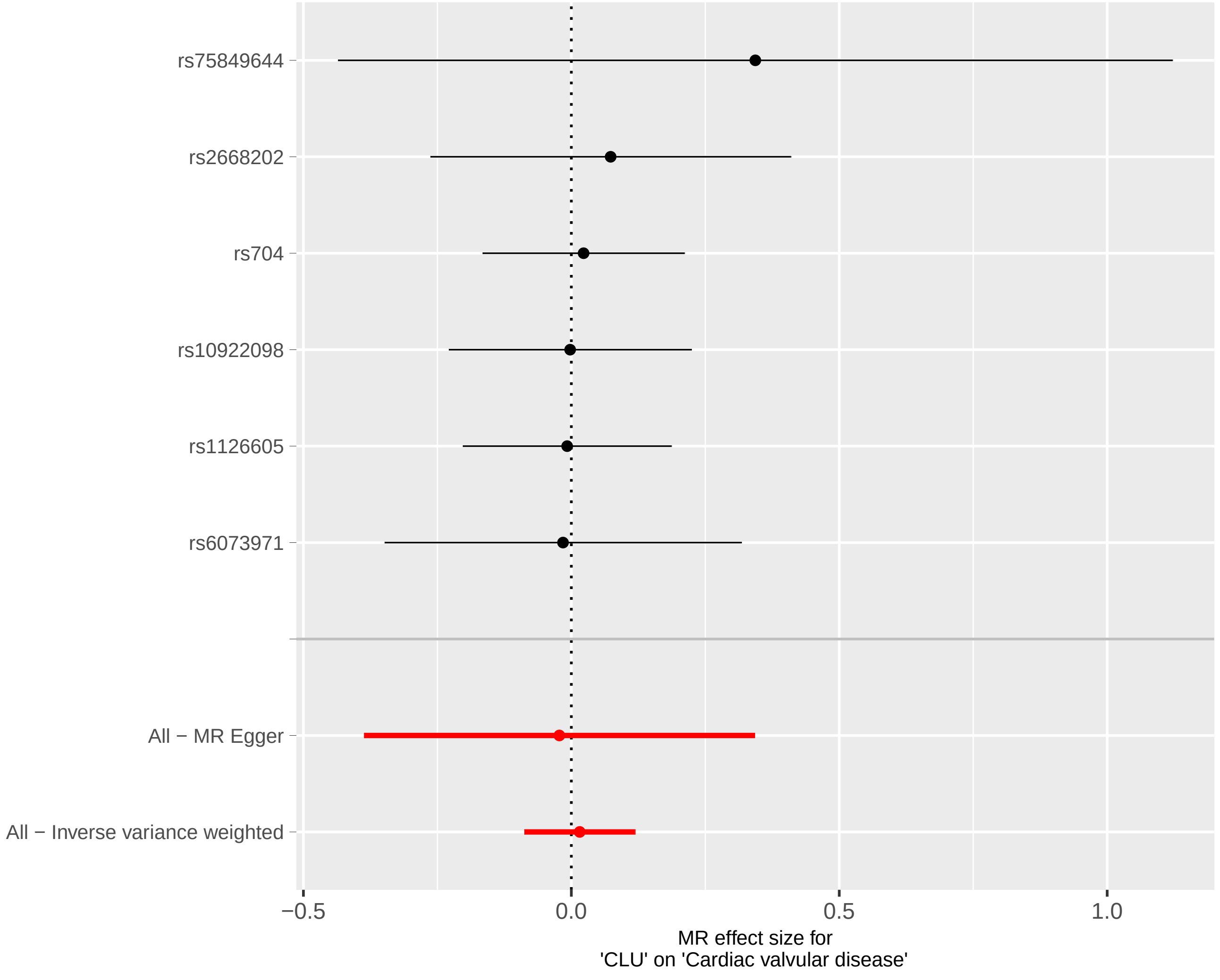
rs75849644
rs2668202
rs704
rs10922098
rs1126605
rs6073971
All − MR Egger
All − Inverse variance weighted
−0.5
0.0
0.5
1.0
MR effect size for
'CLU' on 'Cardiac valvular disease'

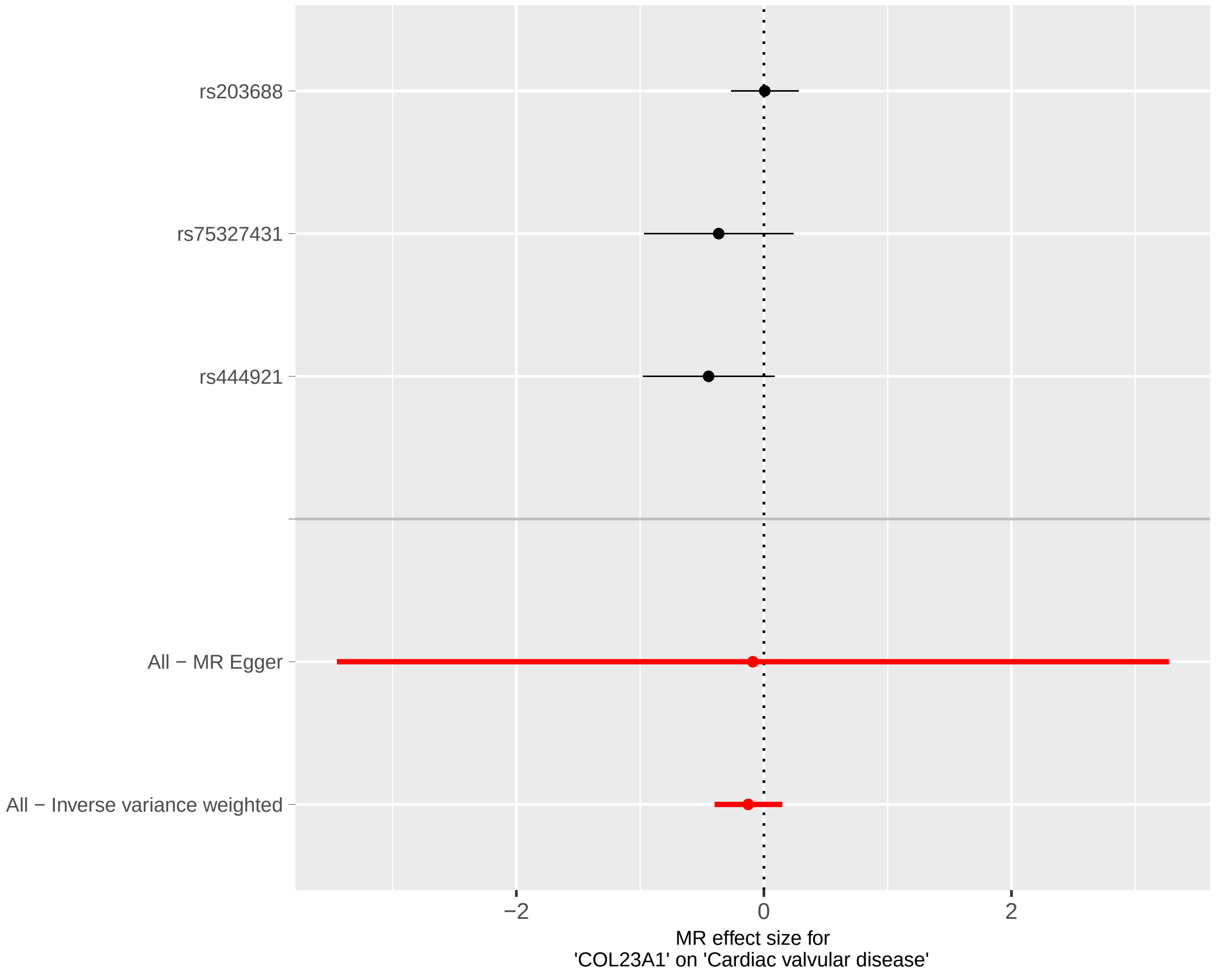
rs203688
rs75327431
rs444921
All − MR Egger
All − Inverse variance weighted
−2
0
2
MR effect size for
'COL23A1' on 'Cardiac valvular disease'

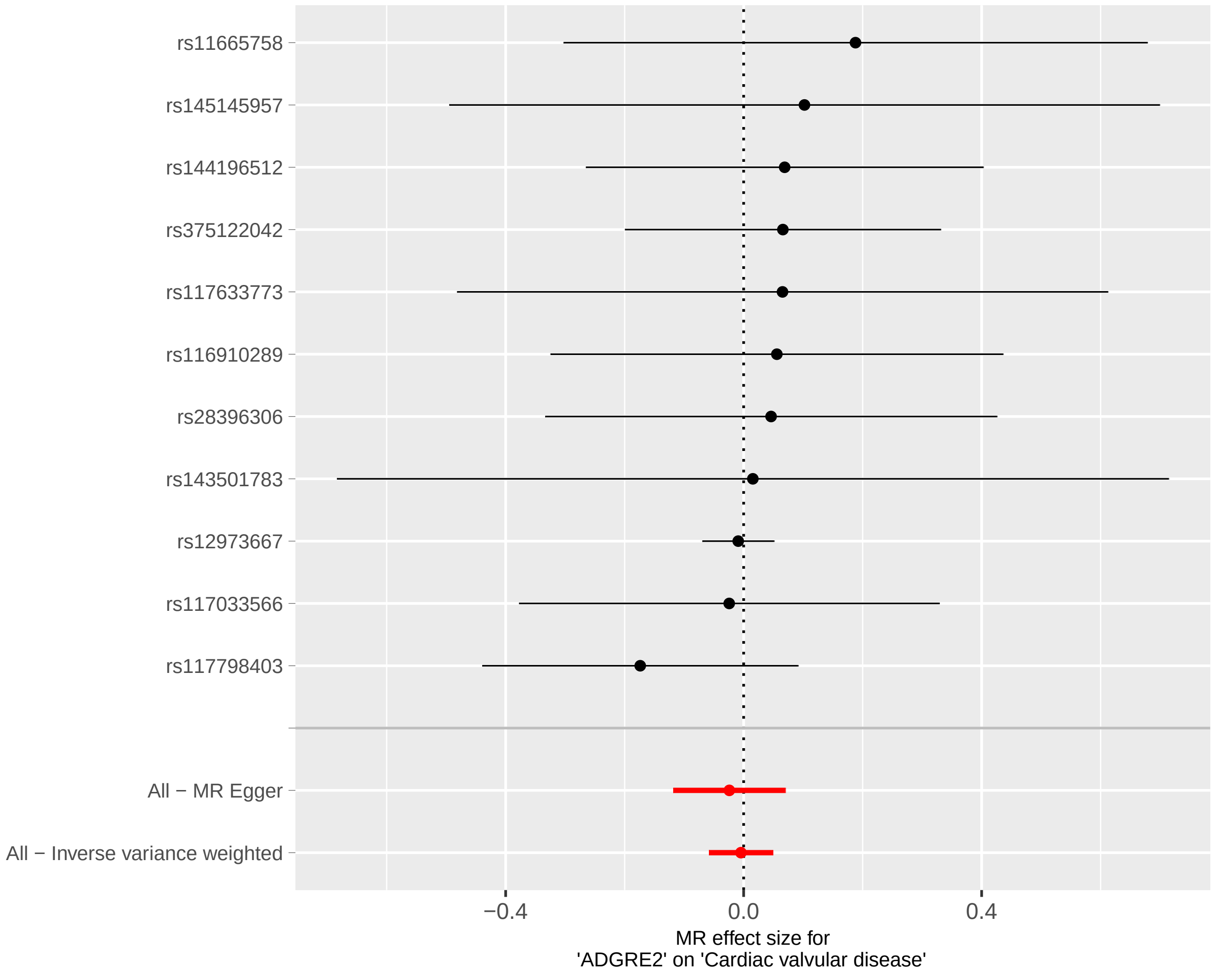
rs11665758
rs145145957
rs144196512
rs375122042
rs117633773
rs116910289
rs28396306
rs143501783
rs12973667
rs117033566
rs117798403
All − MR Egger
All − Inverse variance weighted
−0.4
0.0
0.4
MR effect size for
'ADGRE2' on 'Cardiac valvular disease'

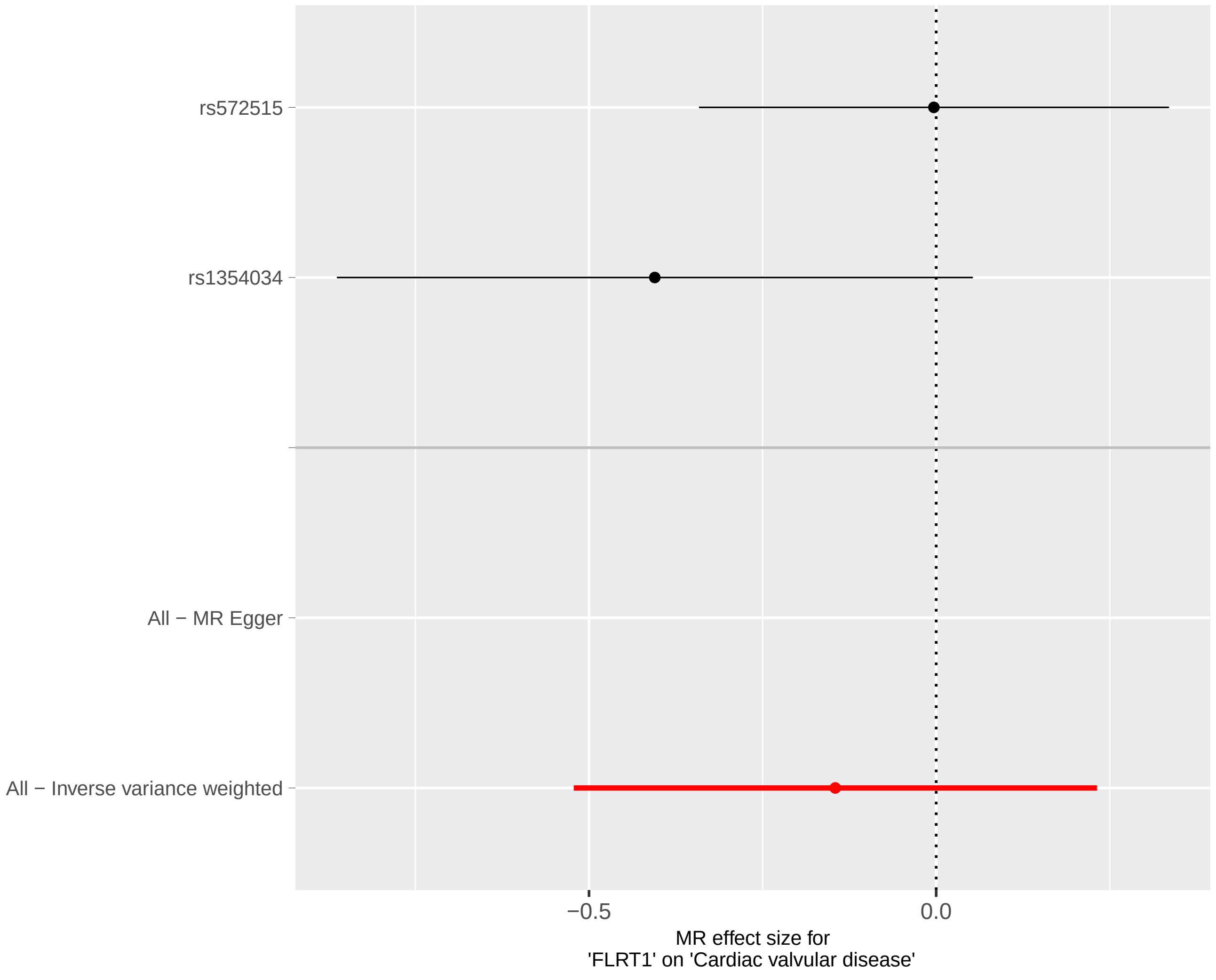

rs572515
rs1354034
All − MR Egger
All − Inverse variance weighted
−0.5
0.0
MR effect size for
'FLRT1' on 'Cardiac valvular disease'

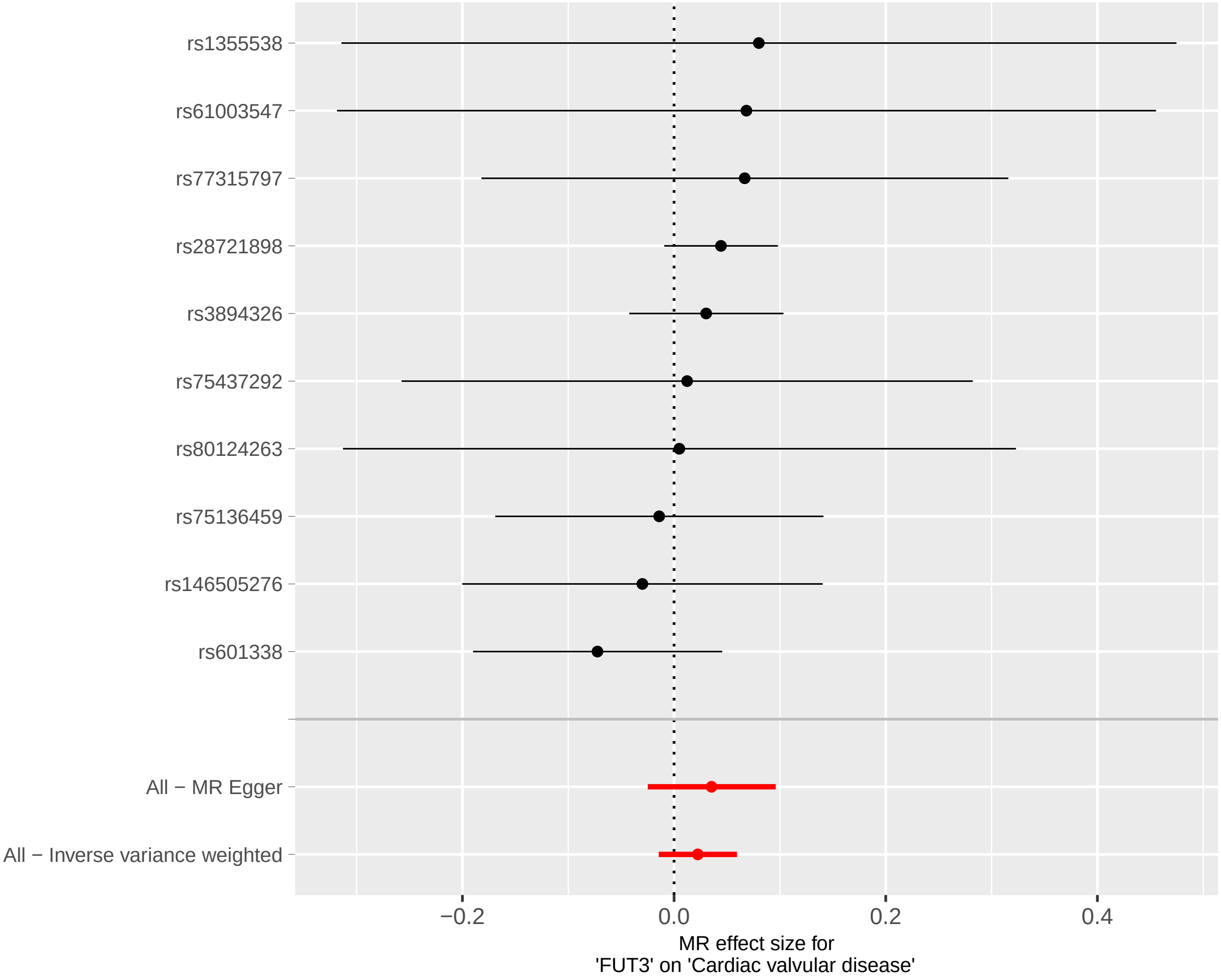

rs1355538
rs61003547
rs77315797
rs28721898
rs3894326
rs75437292
rs80124263
rs75136459
rs146505276
rs601338
All − MR Egger
All − Inverse variance weighted
−0.2
0.0
0.2
0.4
MR effect size for
'FUT3' on 'Cardiac valvular disease'

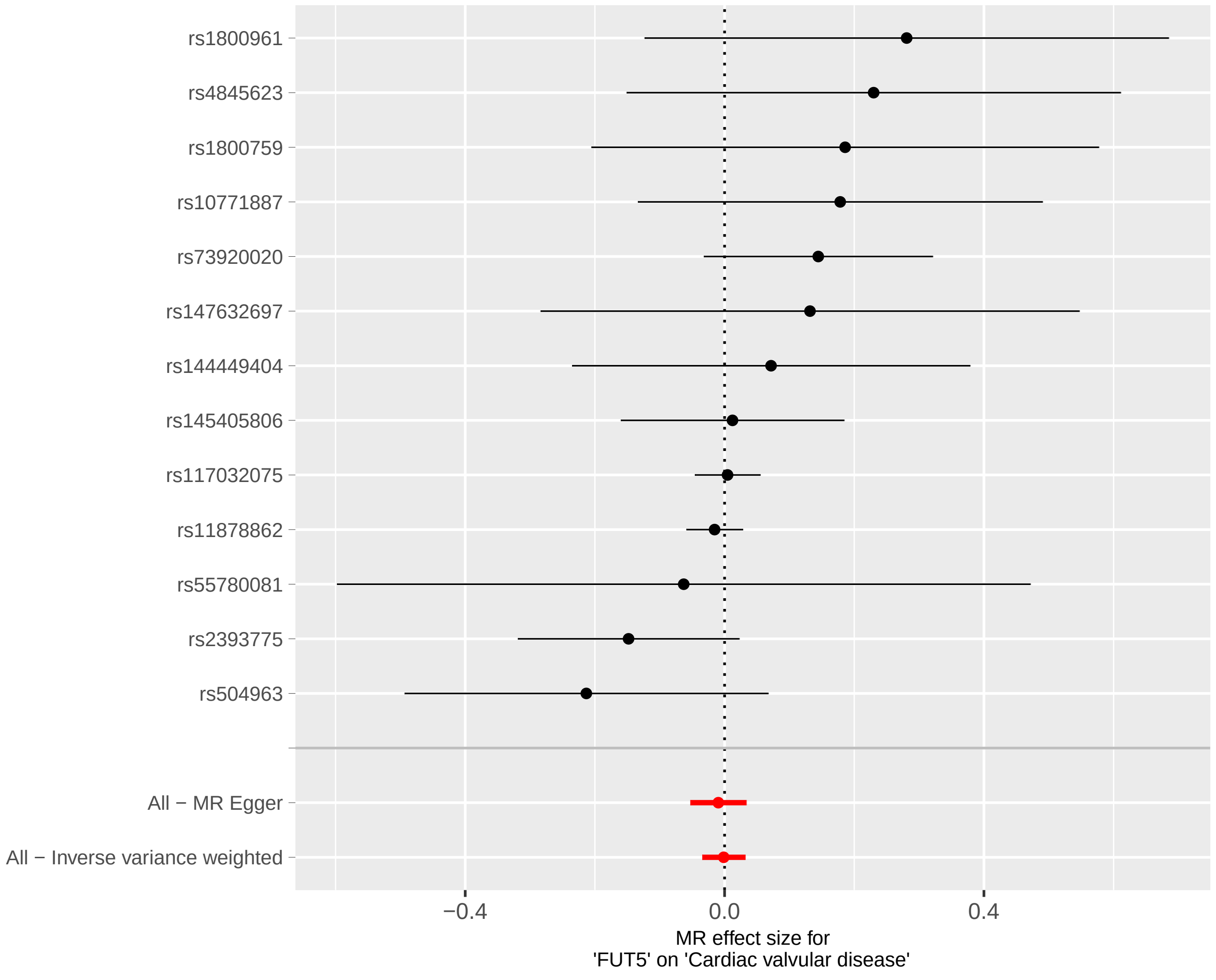
rs1800961
rs4845623
rs1800759
rs10771887
rs73920020
rs147632697
rs144449404
rs145405806
rs117032075
rs11878862
rs55780081
rs2393775
rs504963
All − MR Egger
All − Inverse variance weighted
−0.4
0.0
0.4
MR effect size for
'FUT5' on 'Cardiac valvular disease'

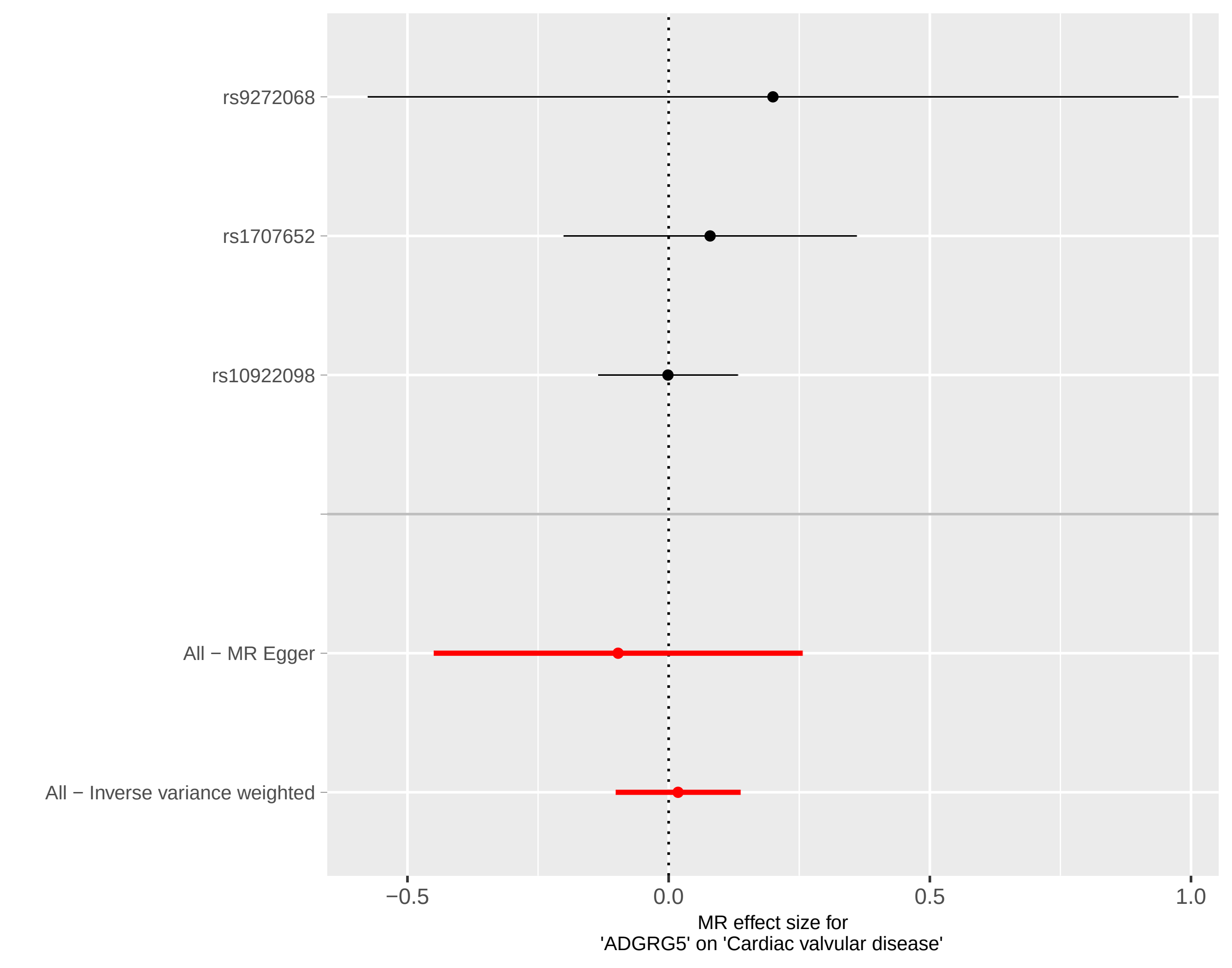

rs9272068
rs1707652
rs10922098
All − MR Egger
All − Inverse variance weighted
−0.5
0.0
0.5
1.0
MR effect size for
'ADGRG5' on 'Cardiac valvular disease'

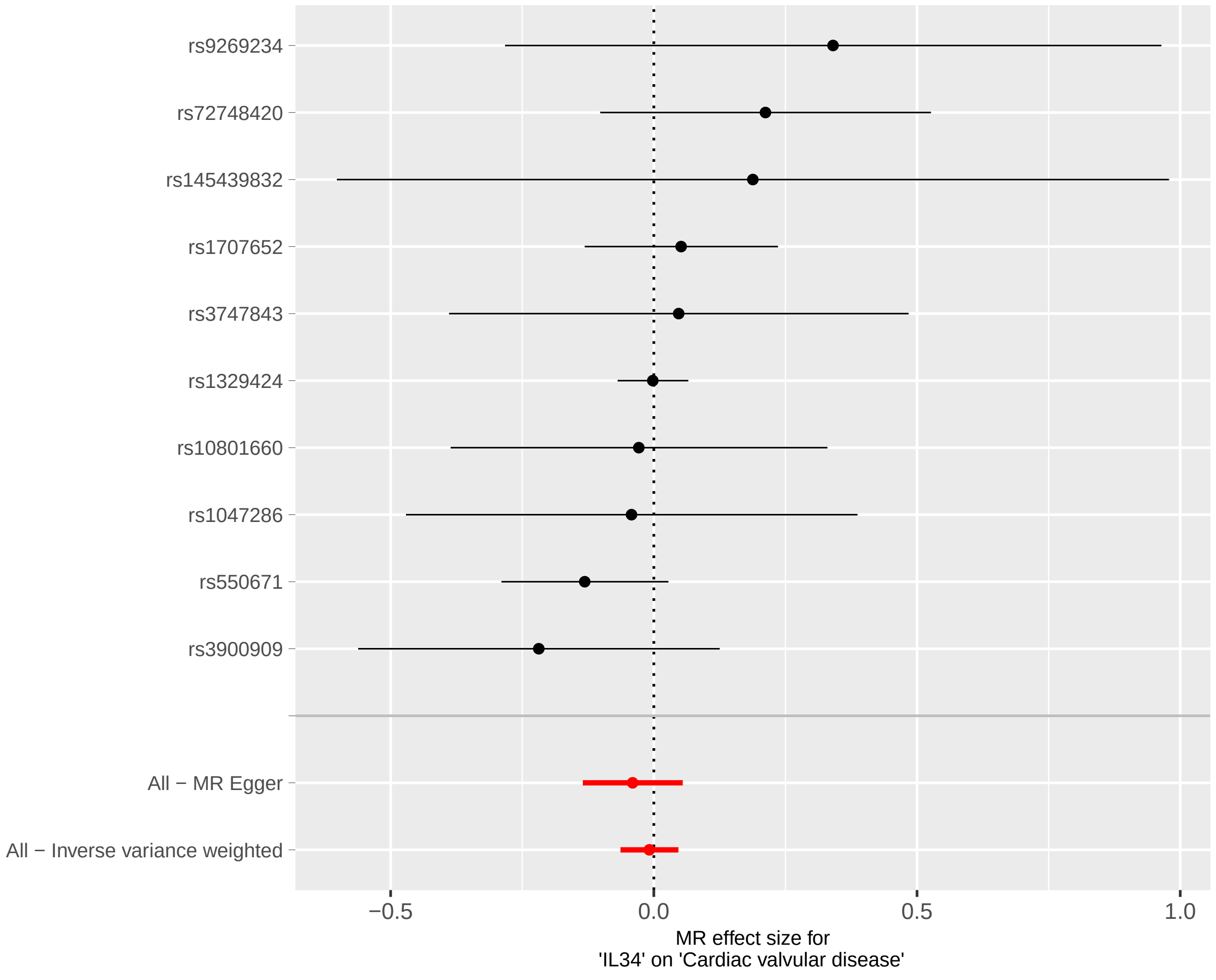

rs9269234
rs72748420
rs145439832
rs1707652
rs3747843
rs1329424
rs10801660
rs1047286
rs550671
rs3900909
All − MR Egger
All − Inverse variance weighted
−0.5
0.0
0.5
1.0
MR effect size for
'IL34' on 'Cardiac valvular disease'

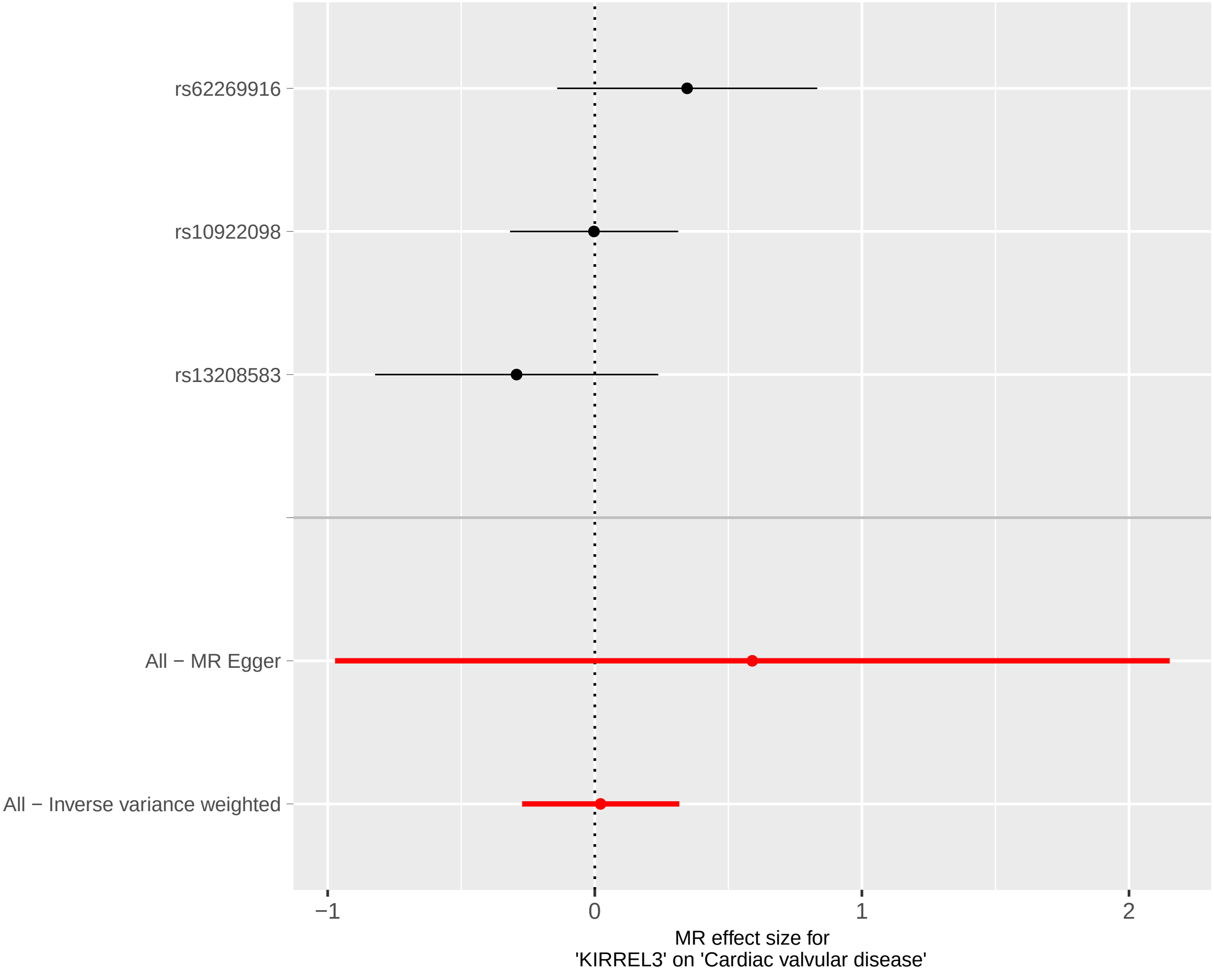
rs62269916
rs10922098
rs13208583
All − MR Egger
All − Inverse variance weighted
−1
0
1
2
MR effect size for
'KIRREL3' on 'Cardiac valvular disease'

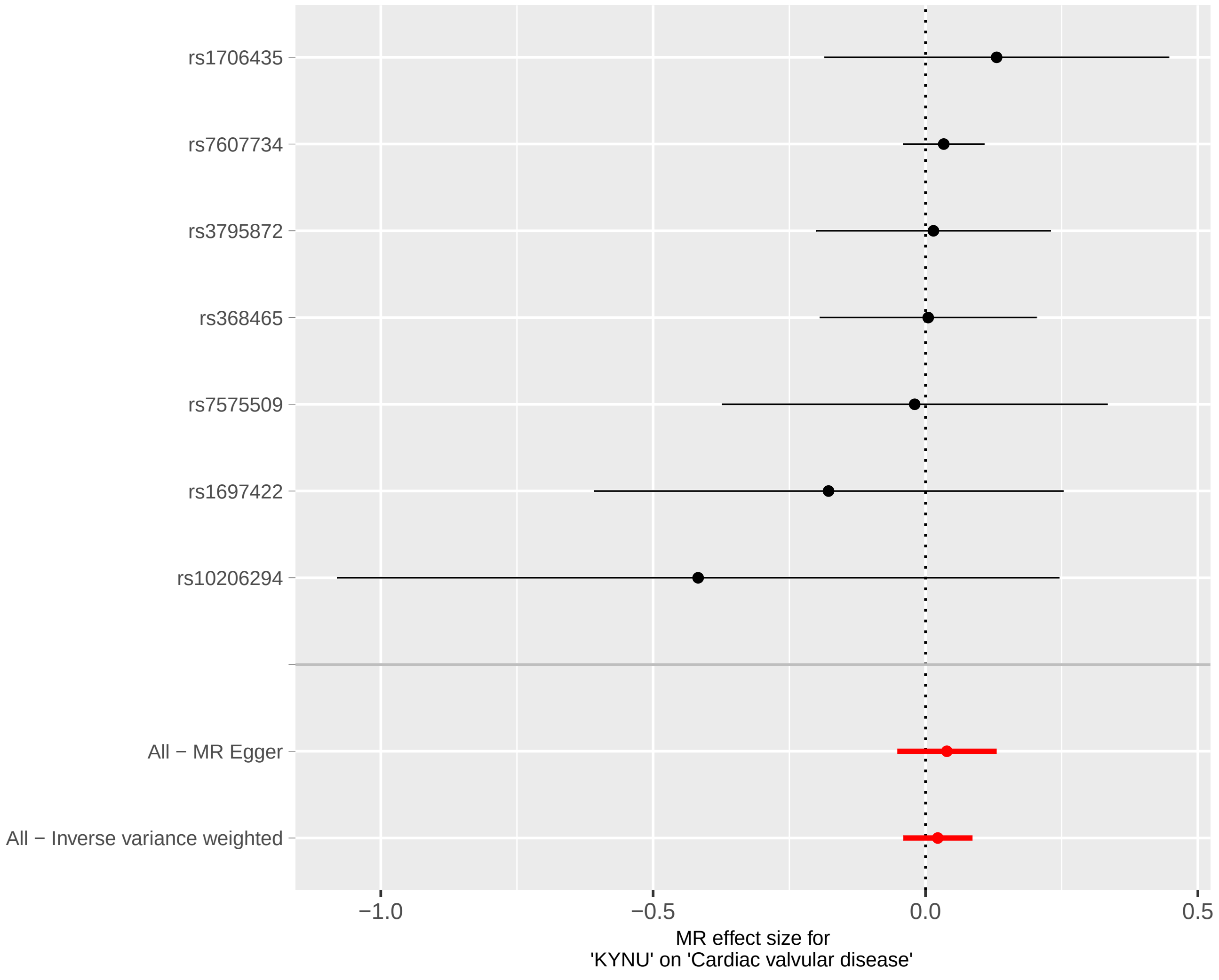

rs1706435
rs7607734
rs3795872
rs368465
rs7575509
rs1697422
rs10206294
All − MR Egger
All − Inverse variance weighted
−1.0
−0.5
0.0
0.5
MR effect size for
'KYNU' on 'Cardiac valvular disease'

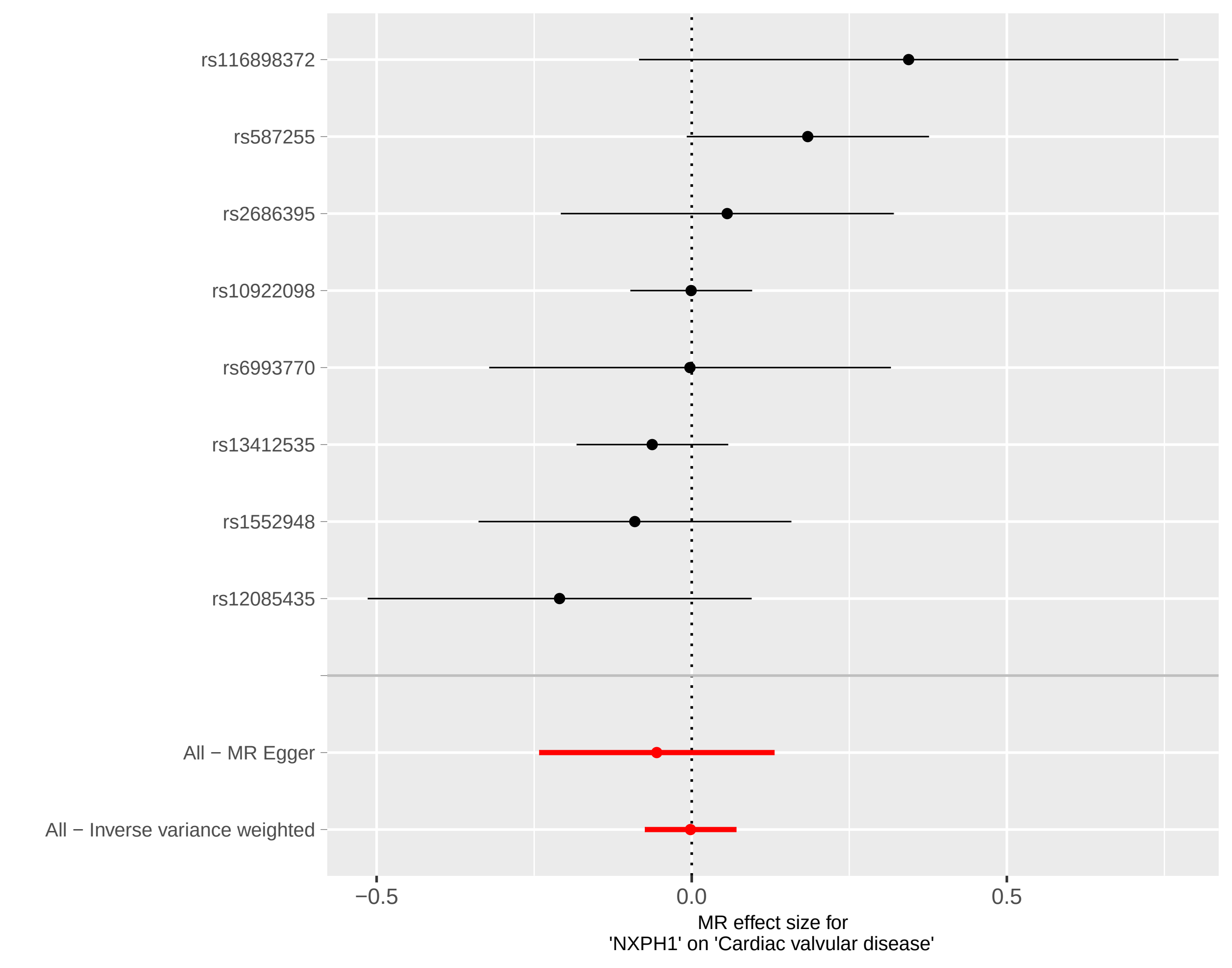
rs116898372
rs587255
rs2686395
rs10922098
rs6993770
rs13412535
rs1552948
rs12085435
All − MR Egger
All − Inverse variance weighted
−0.5
0.0
0.5
MR effect size for
'NXPH1' on 'Cardiac valvular disease'

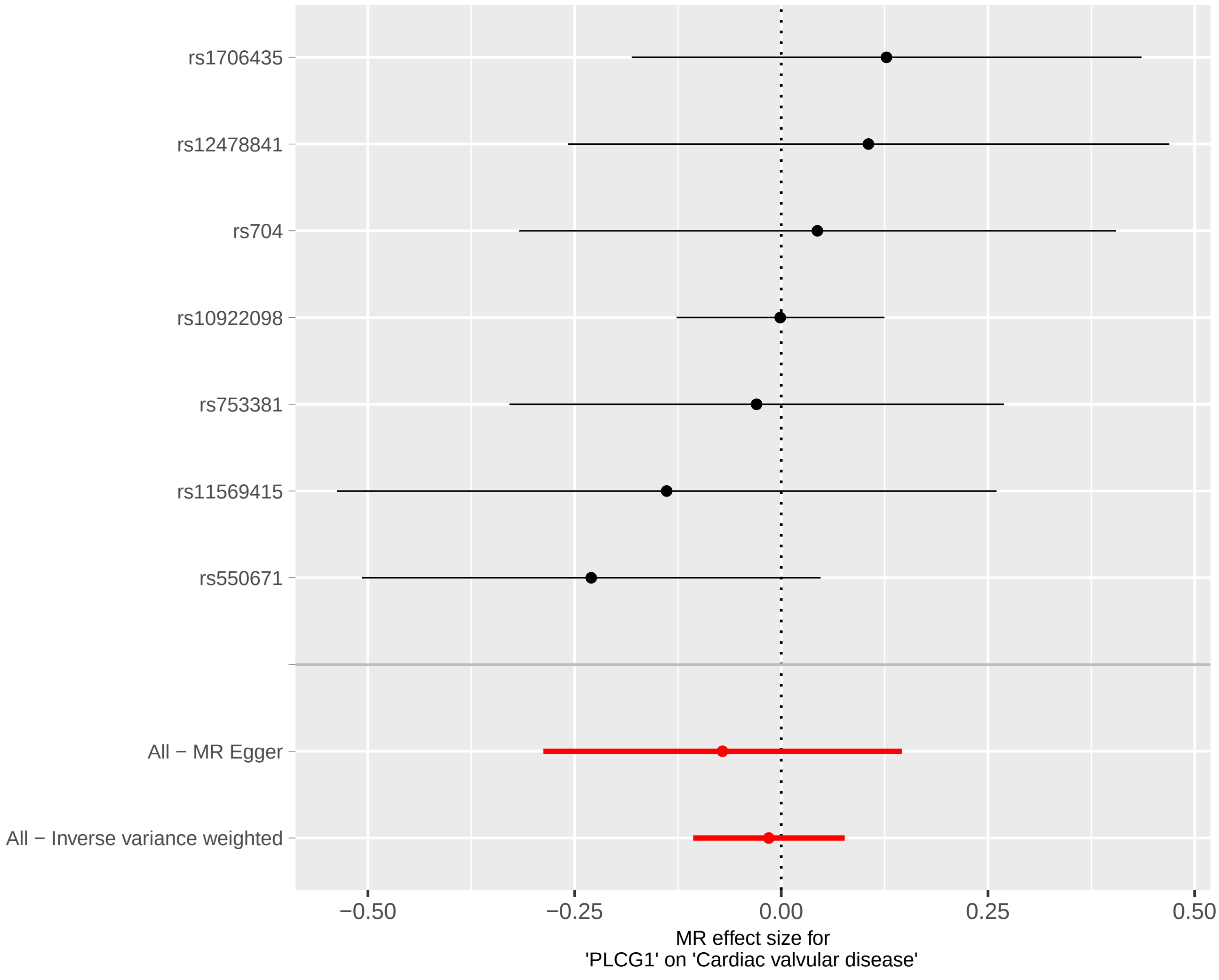
rs1706435
rs12478841
rs704
rs10922098
rs753381
rs11569415
rs550671
All − MR Egger
All − Inverse variance weighted
−0.50
−0.25
0.00
0.25
0.50
MR effect size for
'PLCG1' on 'Cardiac valvular disease'

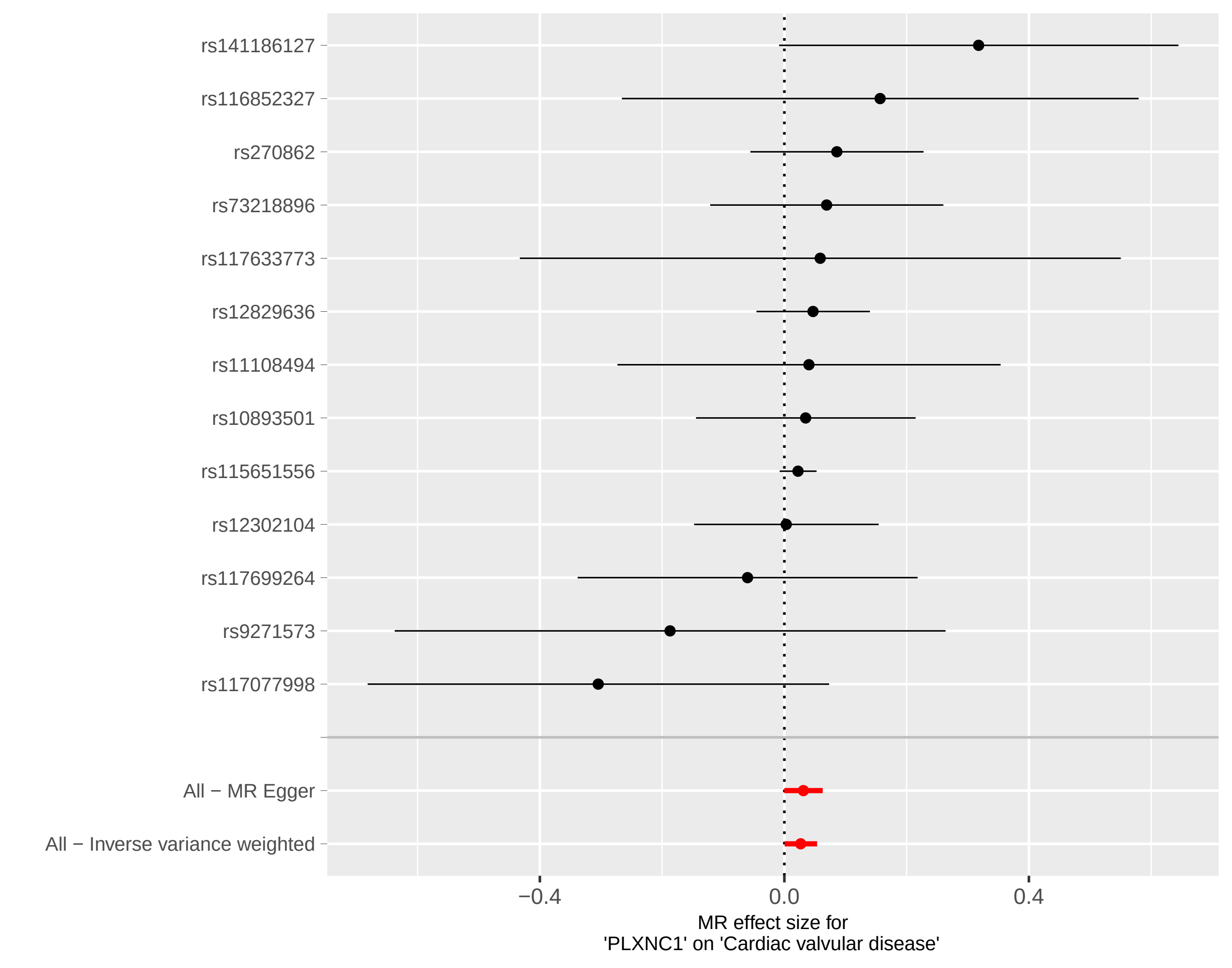
rs141186127
rs116852327
rs270862
rs73218896
rs117633773
rs12829636
rs11108494
rs10893501
rs115651556
rs12302104
rs117699264
rs9271573
rs117077998
All − MR Egger
All − Inverse variance weighted
−0.4
0.0
0.4
MR effect size for
'PLXNC1' on 'Cardiac valvular disease'

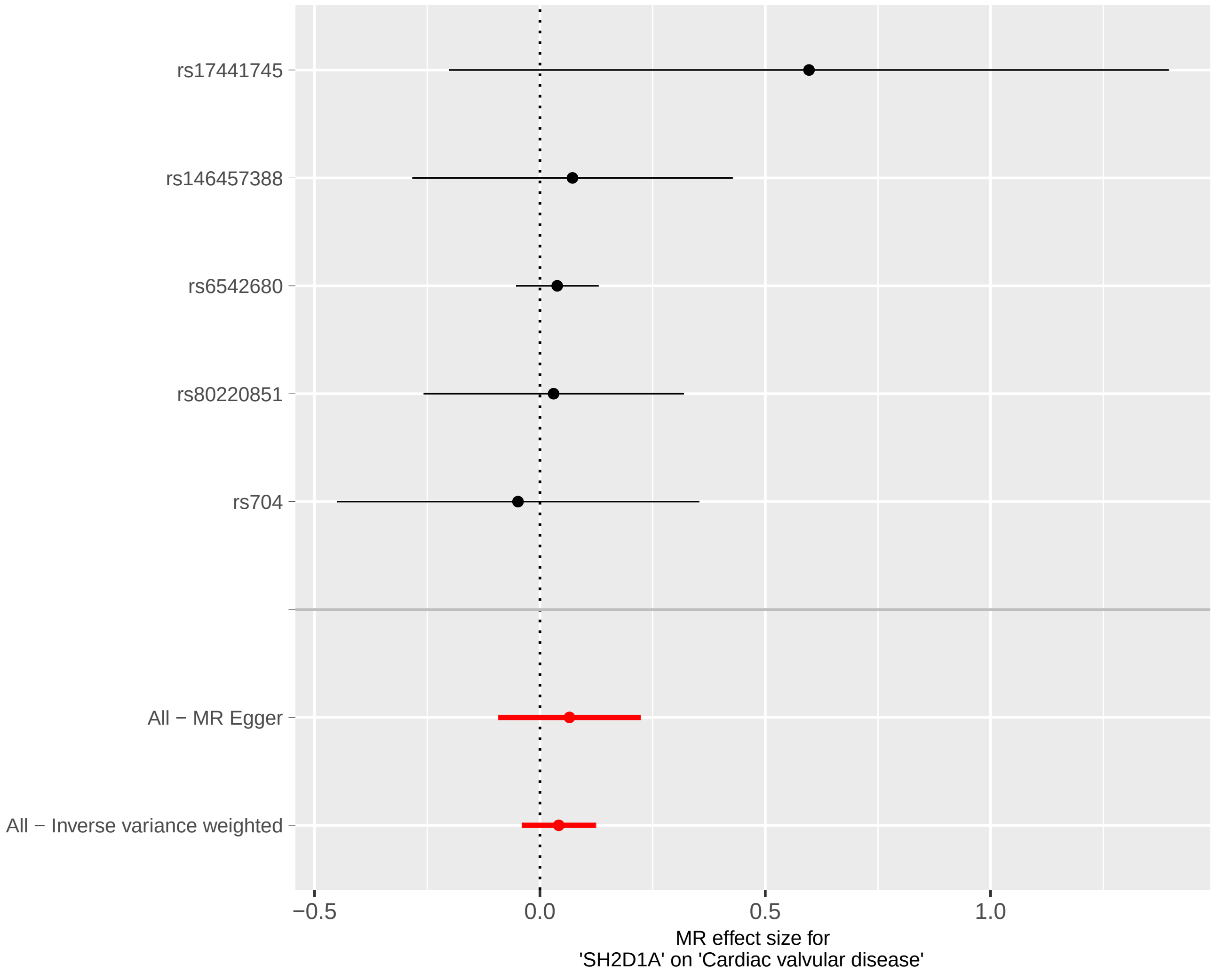
rs17441745
rs146457388
rs6542680
rs80220851
rs704
All − MR Egger
All − Inverse variance weighted
−0.5
0.0
0.5
1.0
MR effect size for
'SH2D1A' on 'Cardiac valvular disease'

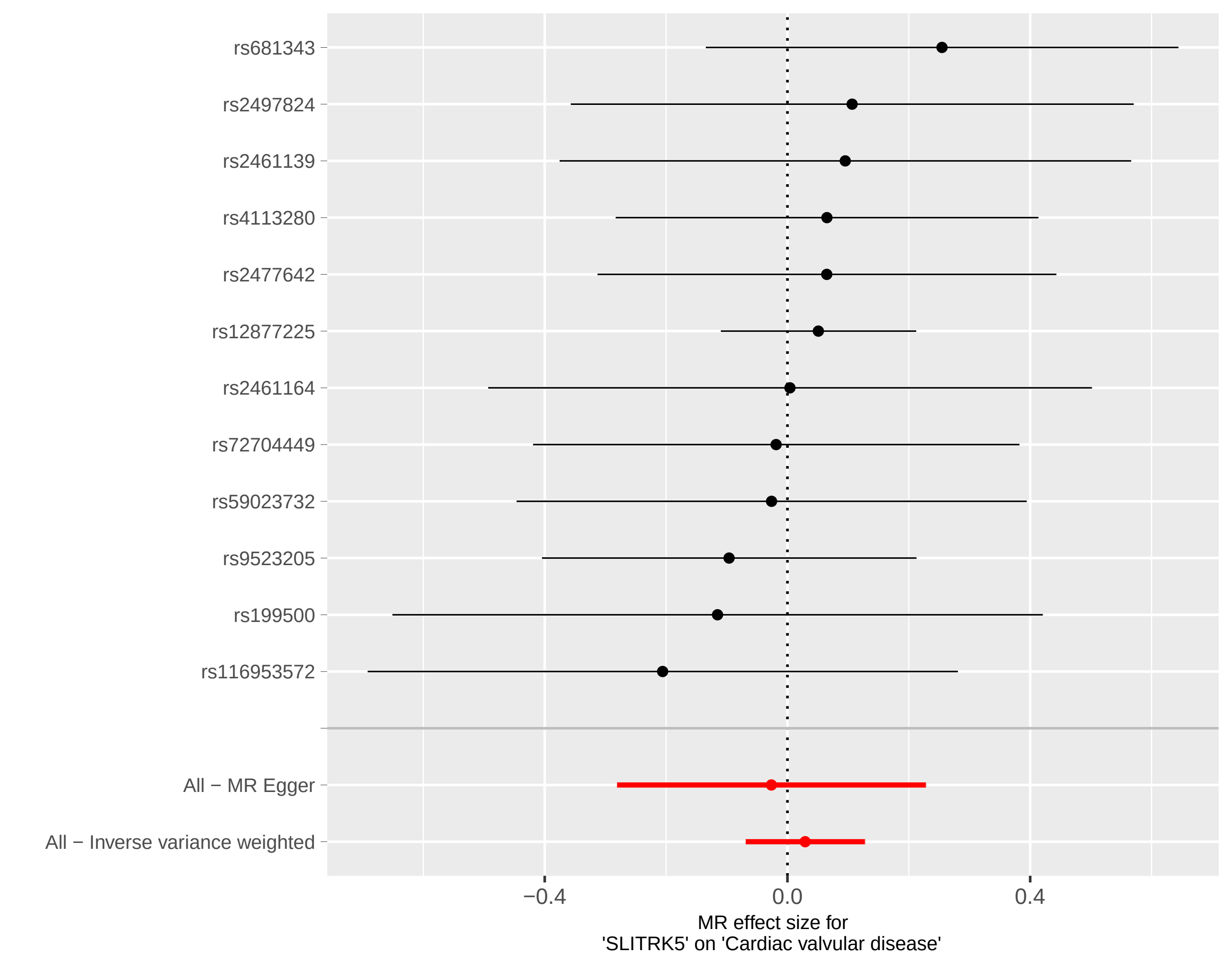
rs681343
rs2497824
rs2461139
rs4113280
rs2477642
rs12877225
rs2461164
rs72704449
rs59023732
rs9523205
rs199500
rs116953572
All − MR Egger
All − Inverse variance weighted
−0.4
0.0
0.4
MR effect size for
'SLITRK5' on 'Cardiac valvular disease'

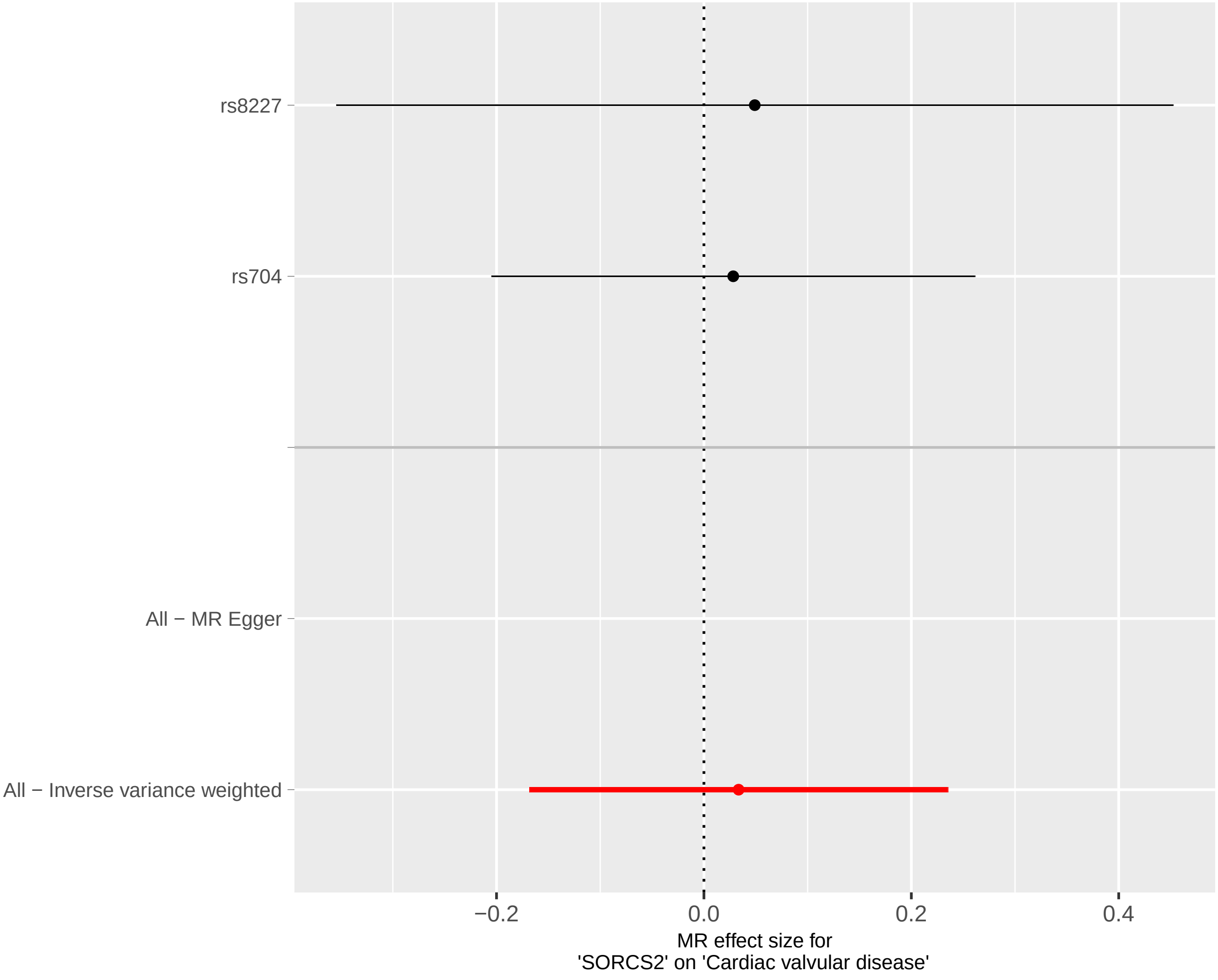

rs8227
rs704
All − MR Egger
All − Inverse variance weighted
−0.2
0.0
0.2
0.4
MR effect size for
'SORCS2' on 'Cardiac valvular disease'

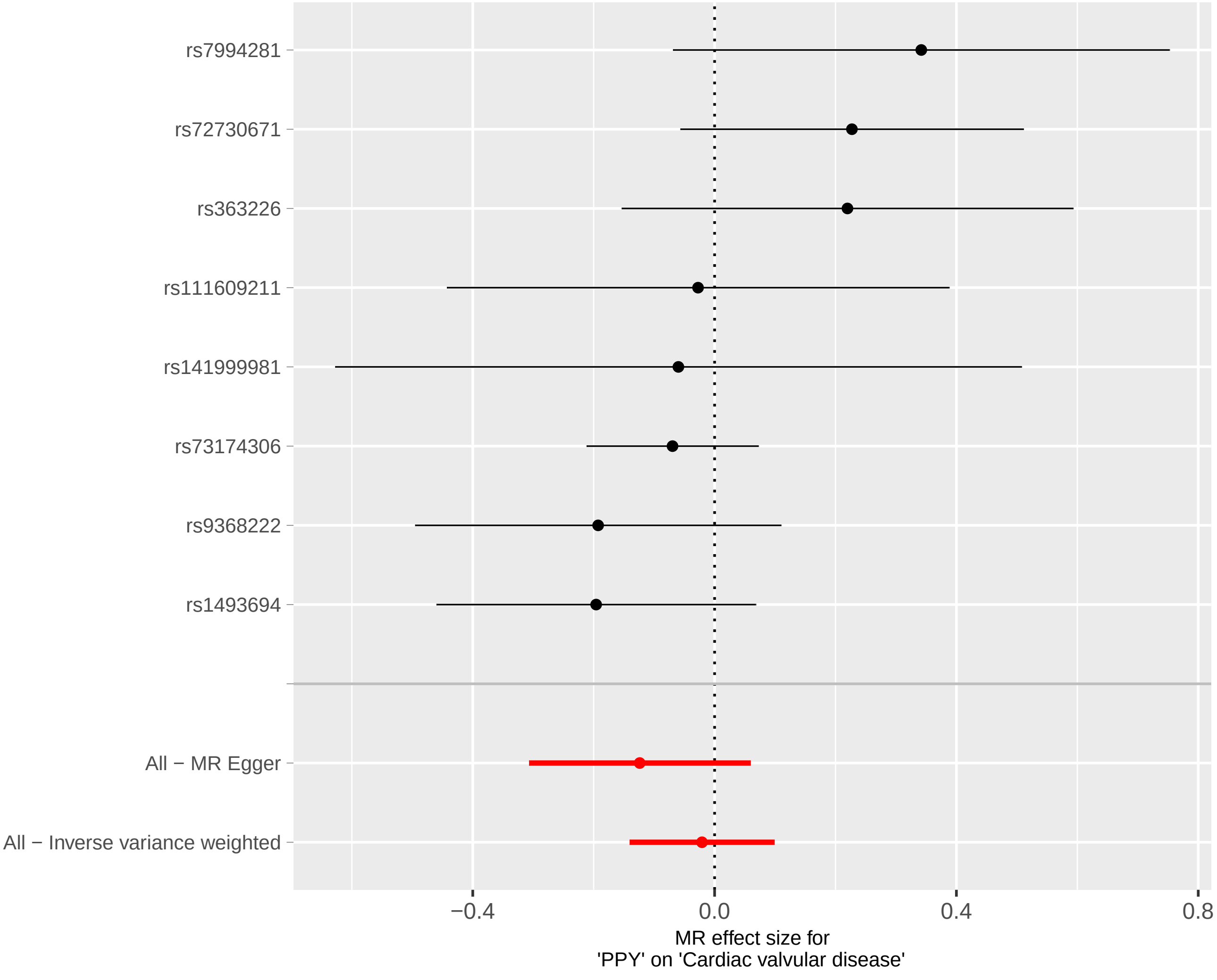
rs7994281
rs72730671
rs363226
rs111609211
rs141999981
rs73174306
rs9368222
rs1493694
All − MR Egger
All − Inverse variance weighted
−0.4
0.0
0.4
0.8
MR effect size for
'PPY' on 'Cardiac valvular disease'

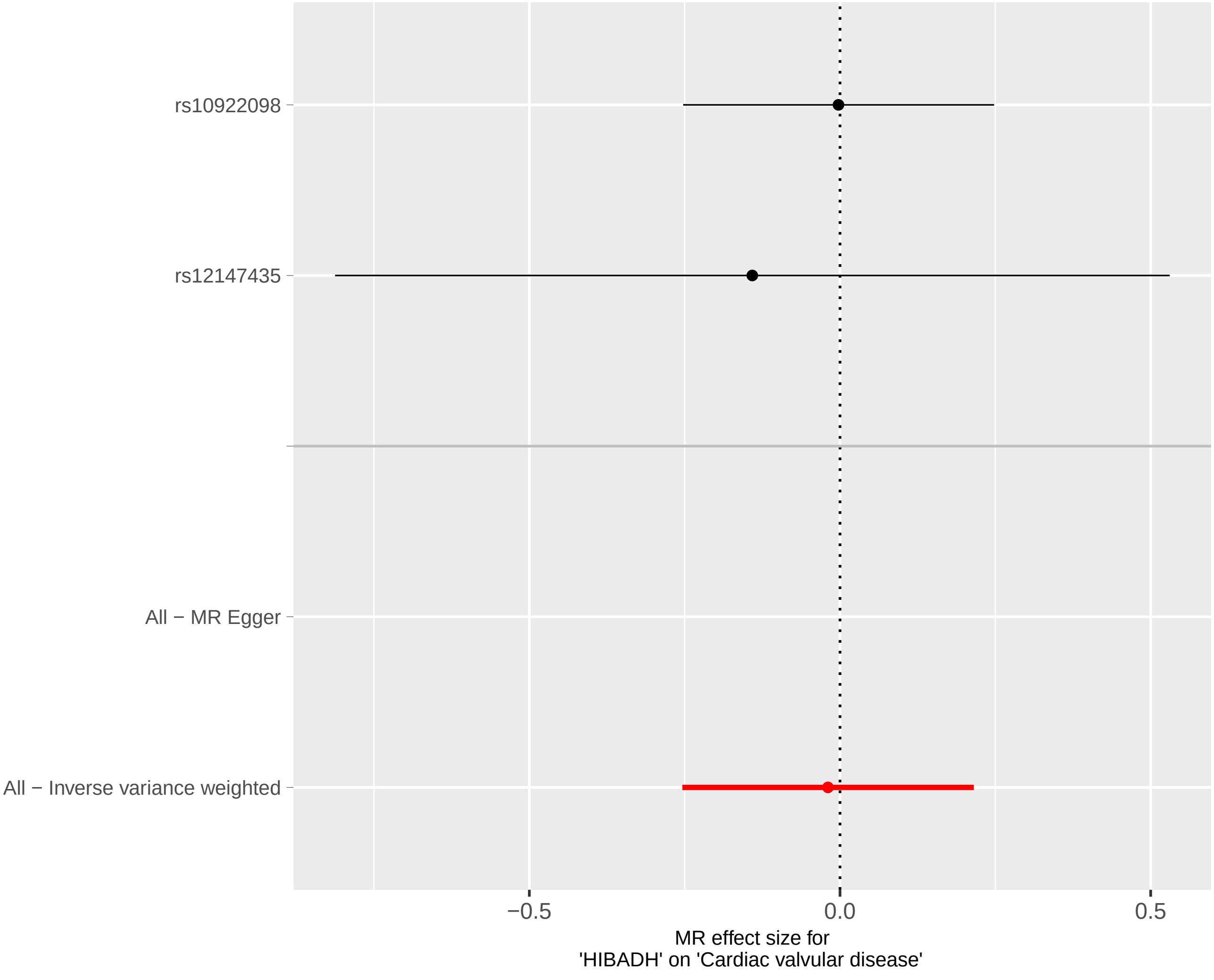
rs10922098
rs12147435
All − MR Egger
All − Inverse variance weighted
−0.5
0.0
0.5
MR effect size for
'HIBADH' on 'Cardiac valvular disease'

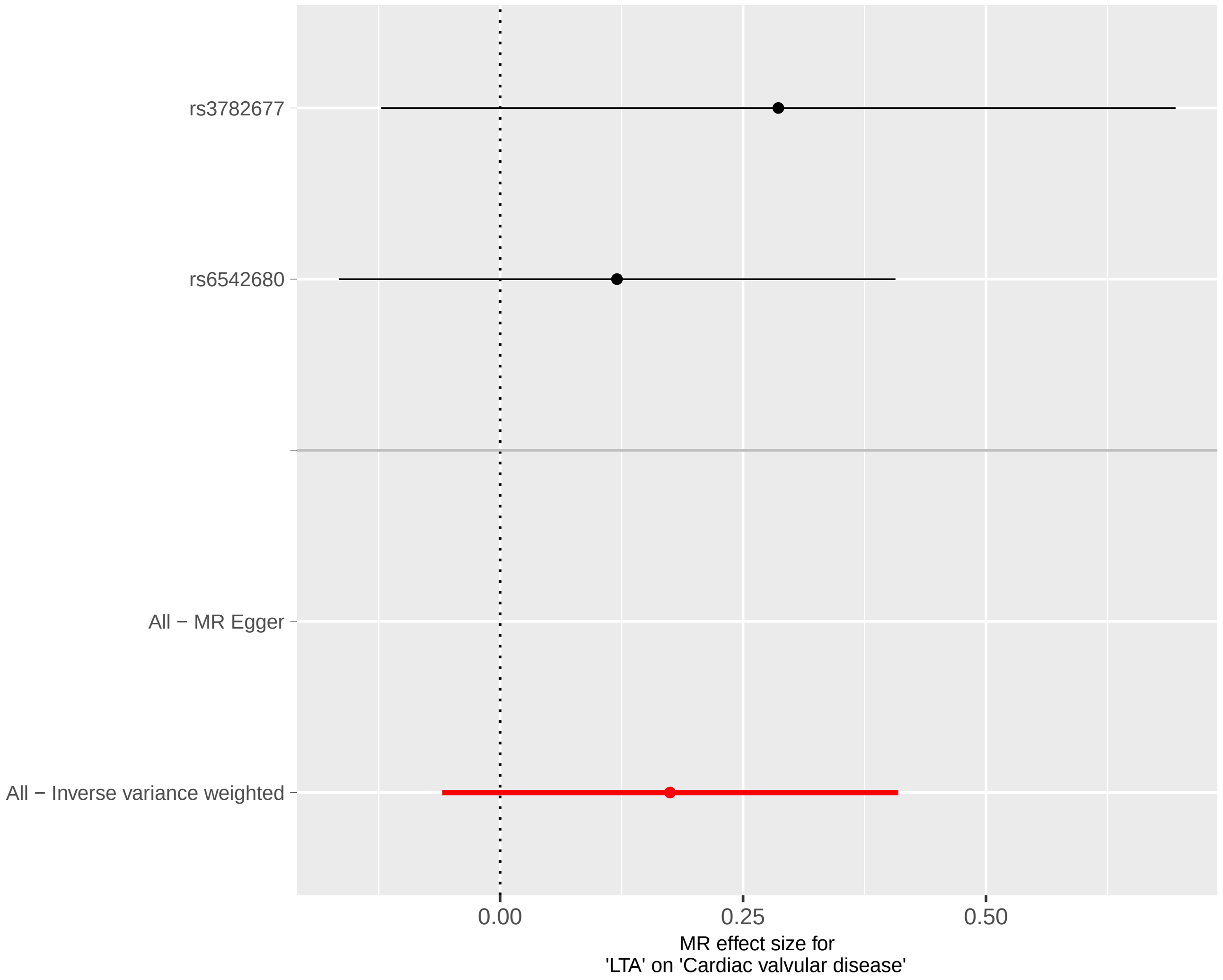

rs3782677
rs6542680
All − MR Egger
All − Inverse variance weighted
0.00
0.25
0.50
MR effect size for
'LTA' on 'Cardiac valvular disease'

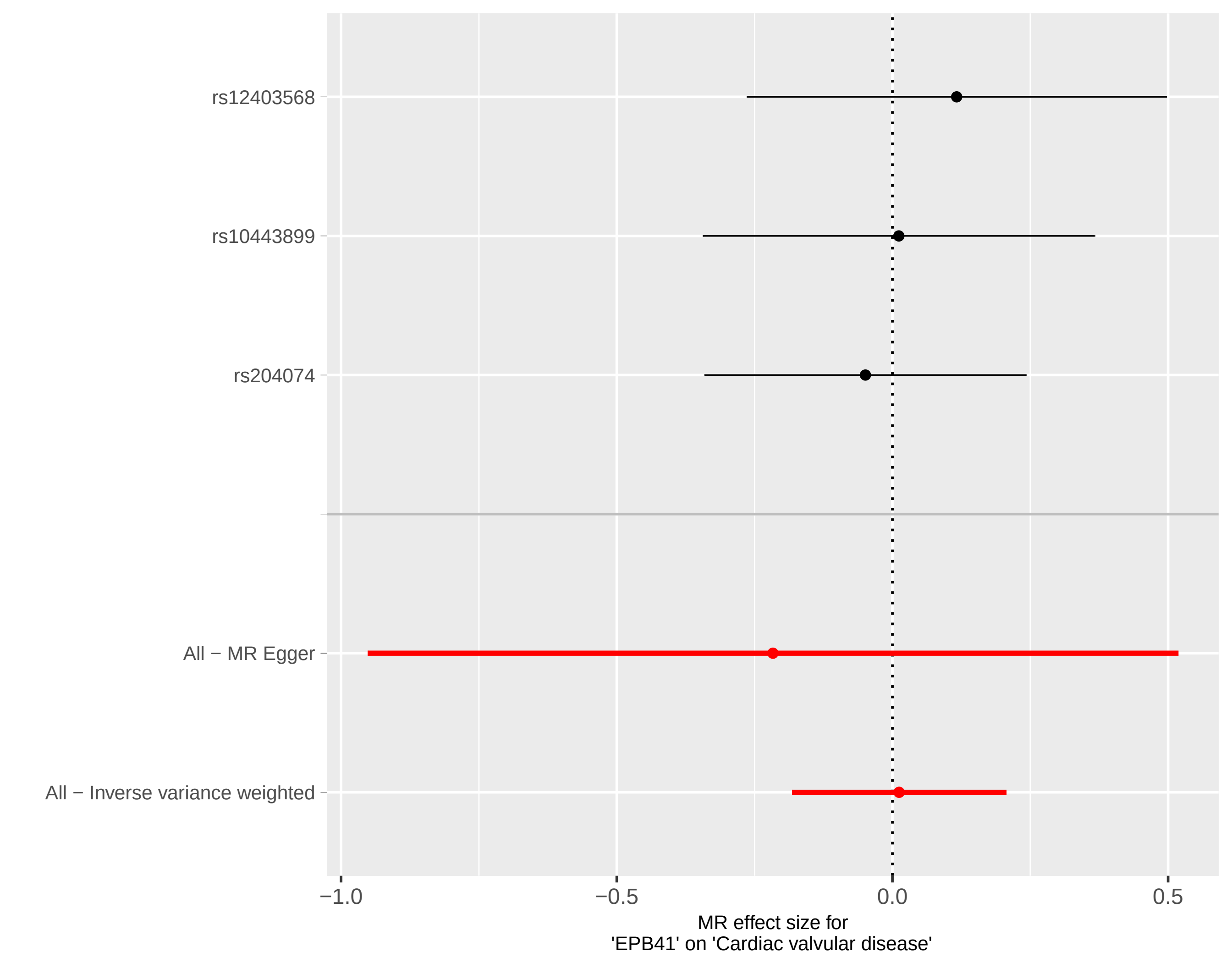
rs12403568
rs10443899
rs204074
All − MR Egger
All − Inverse variance weighted
−1.0
−0.5
0.0
0.5
MR effect size for
'EPB41' on 'Cardiac valvular disease'

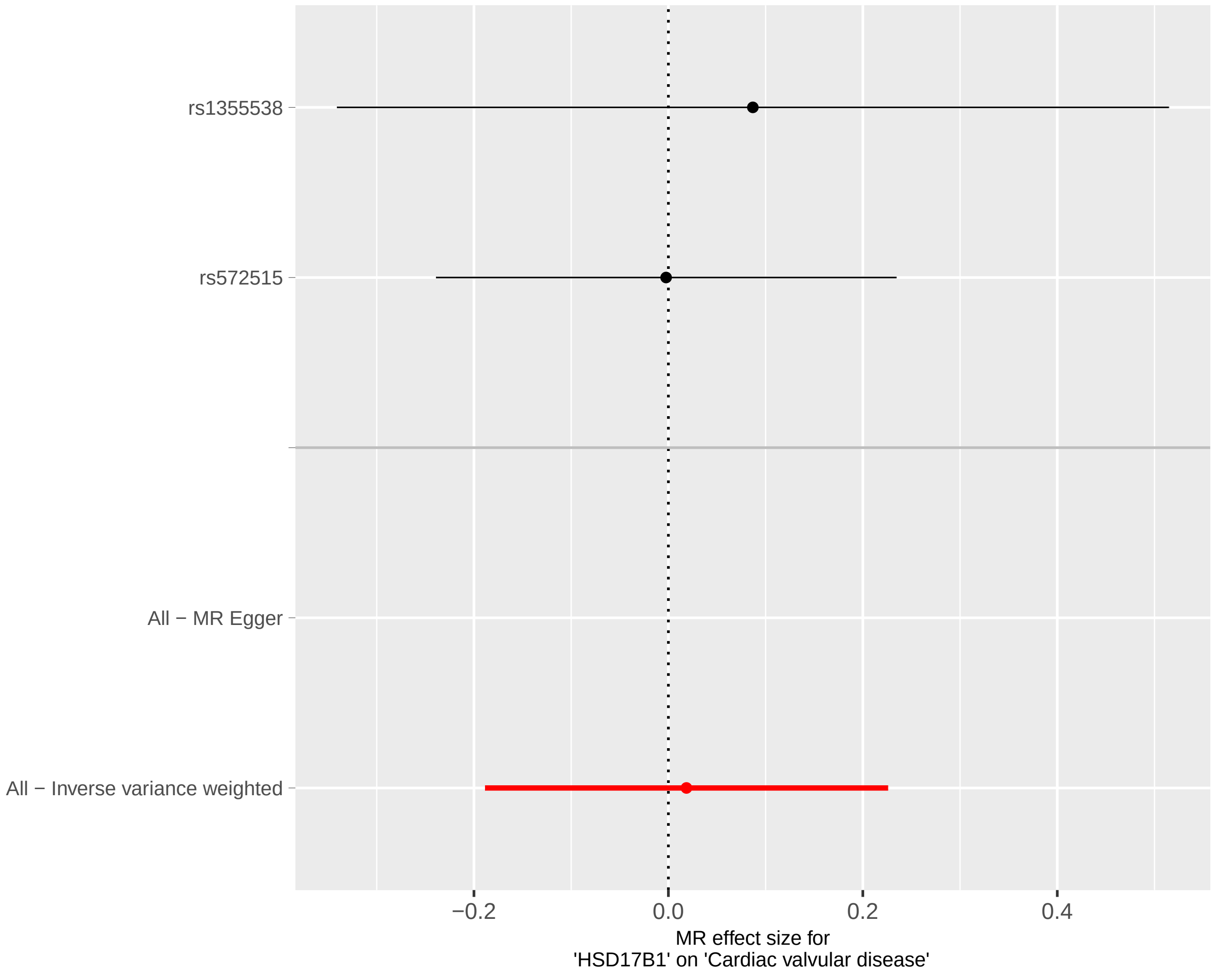
rs1355538
rs572515
All − MR Egger
All − Inverse variance weighted
−0.2
0.0
0.2
0.4
MR effect size for
'HSD17B1' on 'Cardiac valvular disease'

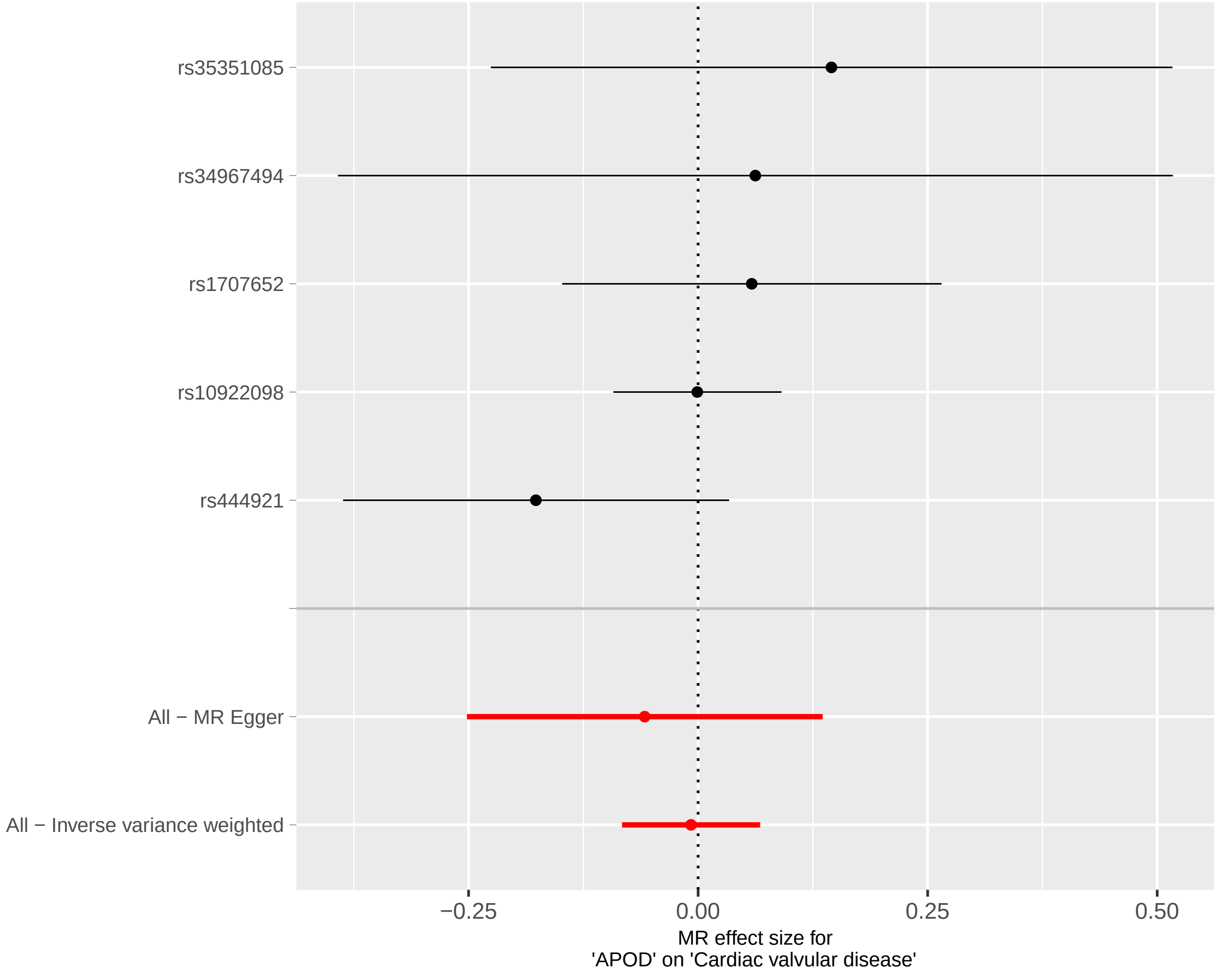

rs35351085
rs34967494
rs1707652
rs10922098
rs444921
All − MR Egger
All − Inverse variance weighted
−0.25
0.00
0.25
0.50
MR effect size for
'APOD' on 'Cardiac valvular disease'

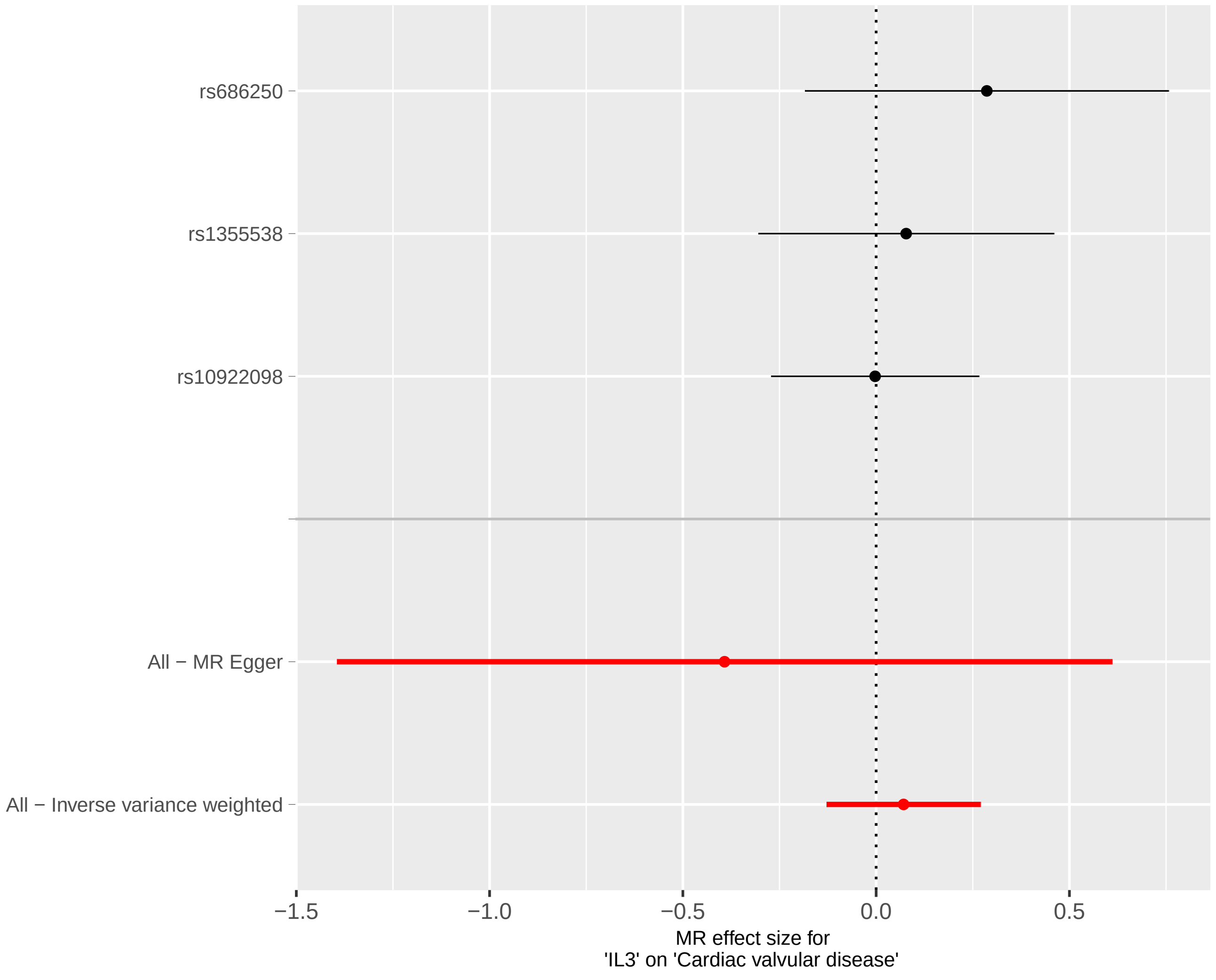
rs686250
rs1355538
rs10922098
All − MR Egger
All − Inverse variance weighted
−1.5
−1.0
−0.5
0.0
0.5
MR effect size for
'IL3' on 'Cardiac valvular disease'

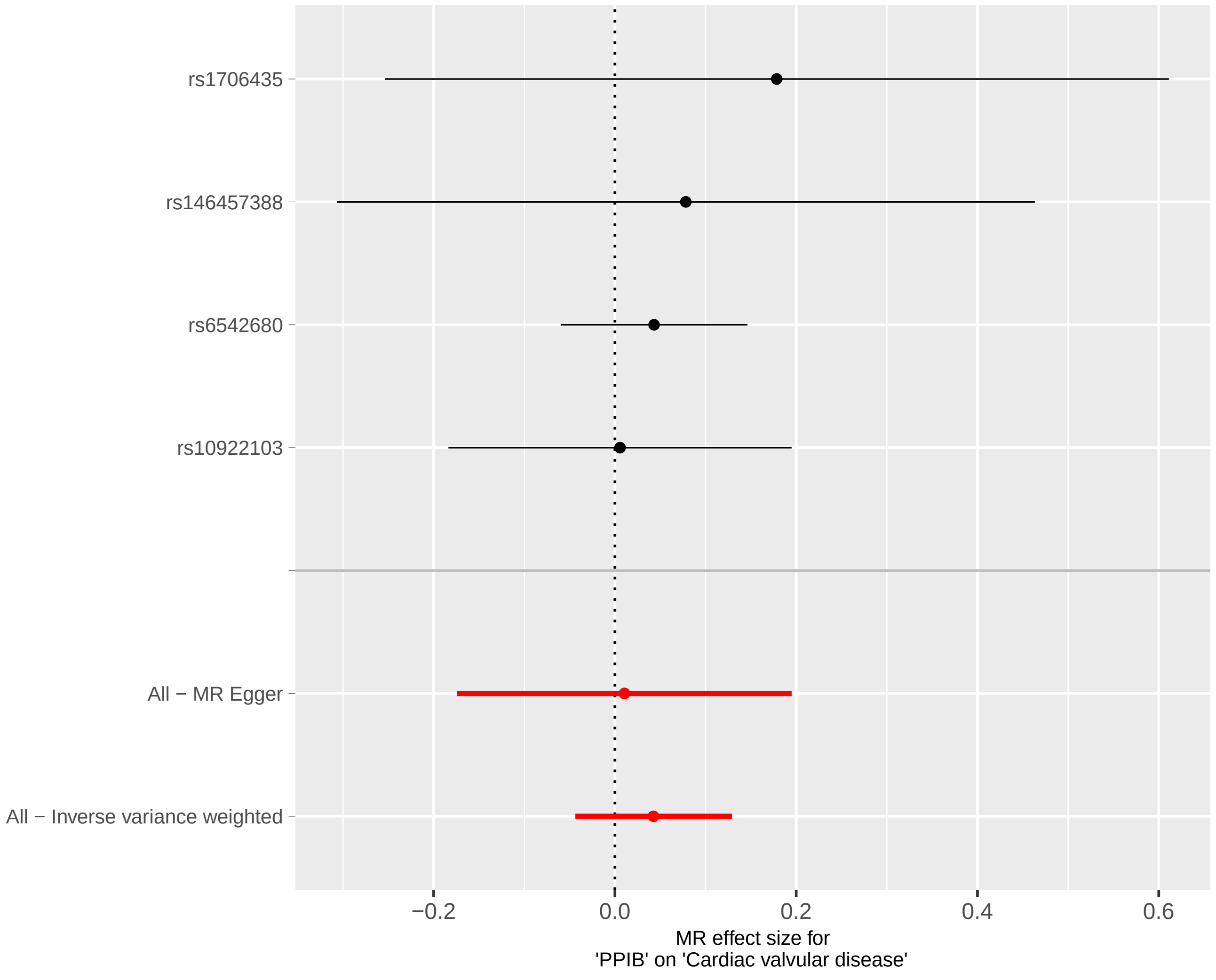
rs1706435
rs146457388
rs6542680
rs10922103
All − MR Egger
All − Inverse variance weighted
−0.2
0.0
0.2
0.4
0.6
MR effect size for
'PPIB' on 'Cardiac valvular disease'

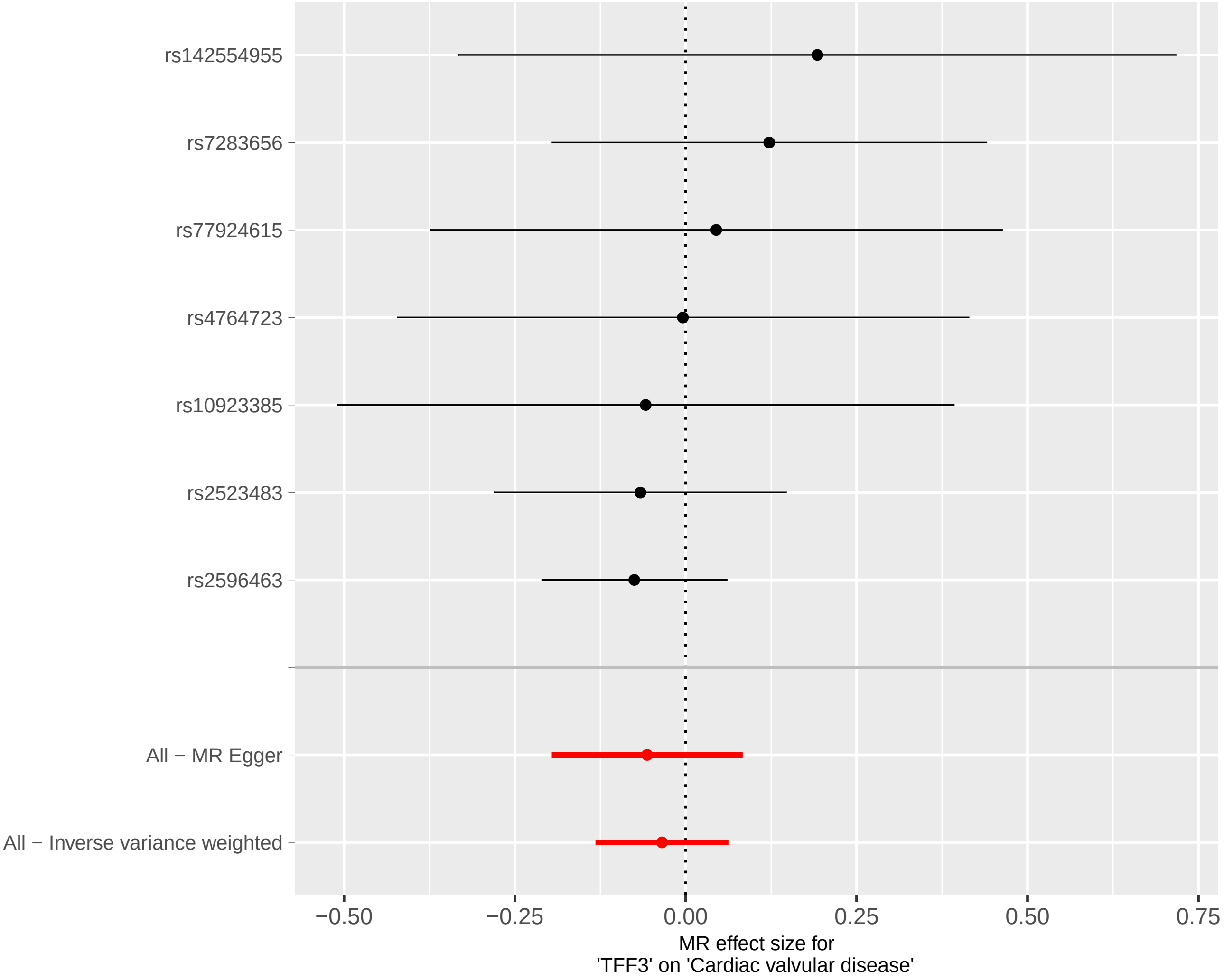
rs142554955
rs7283656
rs77924615
rs4764723
rs10923385
rs2523483
rs2596463
All − MR Egger
All − Inverse variance weighted
−0.50
−0.25
0.00
0.25
0.50
0.75
MR effect size for
'TFF3' on 'Cardiac valvular disease'

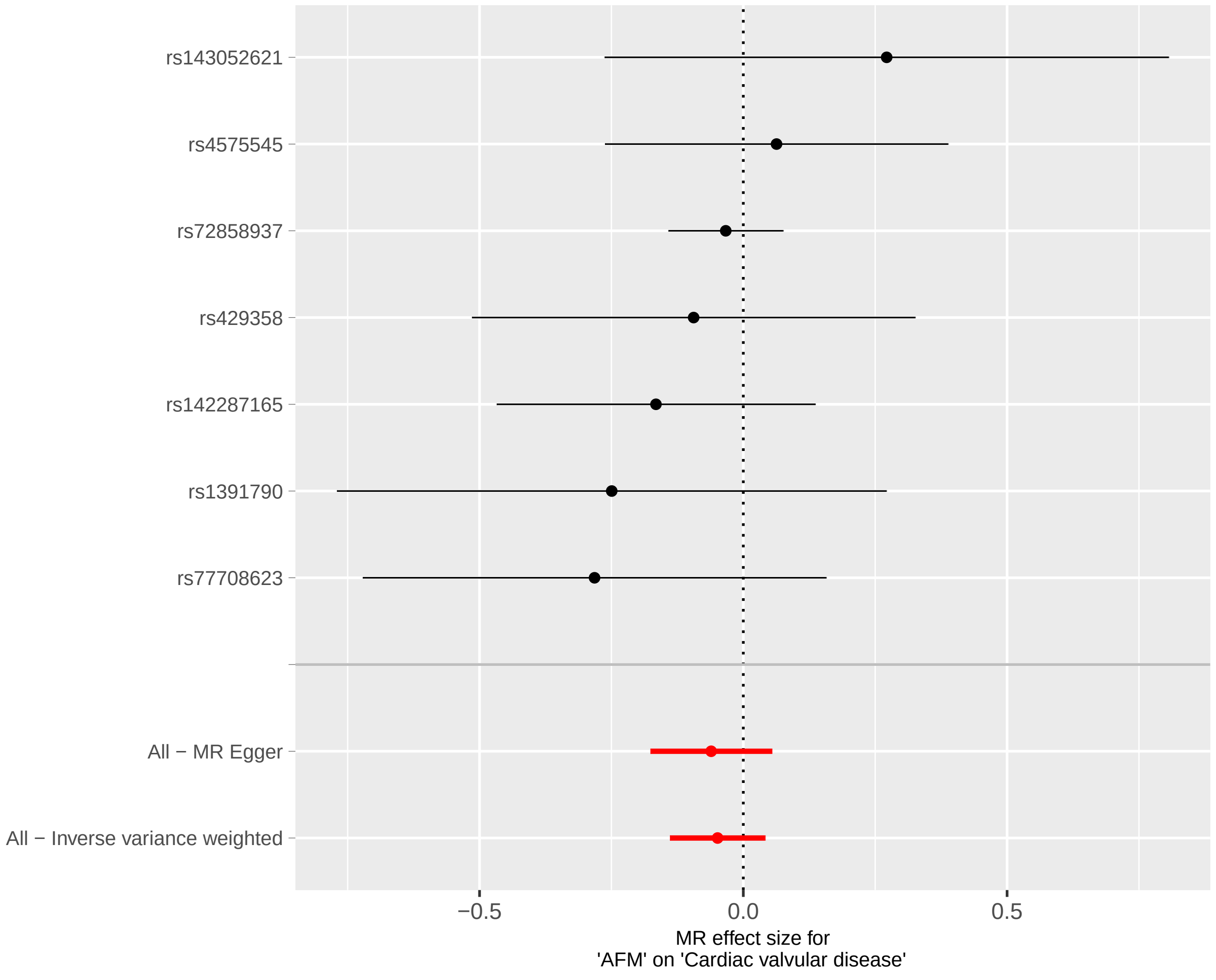
rs143052621
rs4575545
rs72858937
rs429358
rs142287165
rs1391790
rs77708623
All − MR Egger
All − Inverse variance weighted
−0.5
0.0
0.5
MR effect size for
'AFM' on 'Cardiac valvular disease'

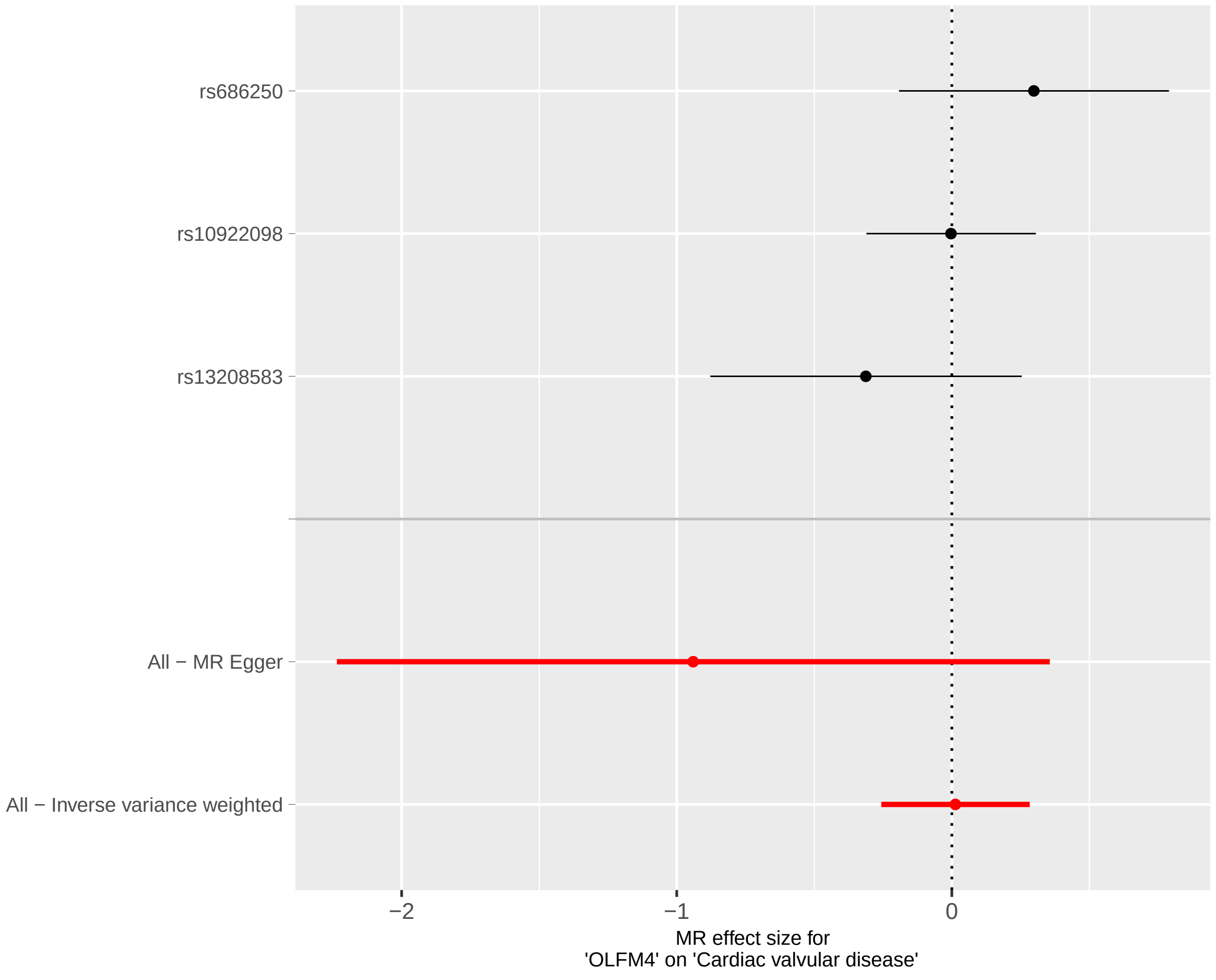
rs686250
rs10922098
rs13208583
All − MR Egger
All − Inverse variance weighted
−2
−1
0
MR effect size for
'OLFM4' on 'Cardiac valvular disease'

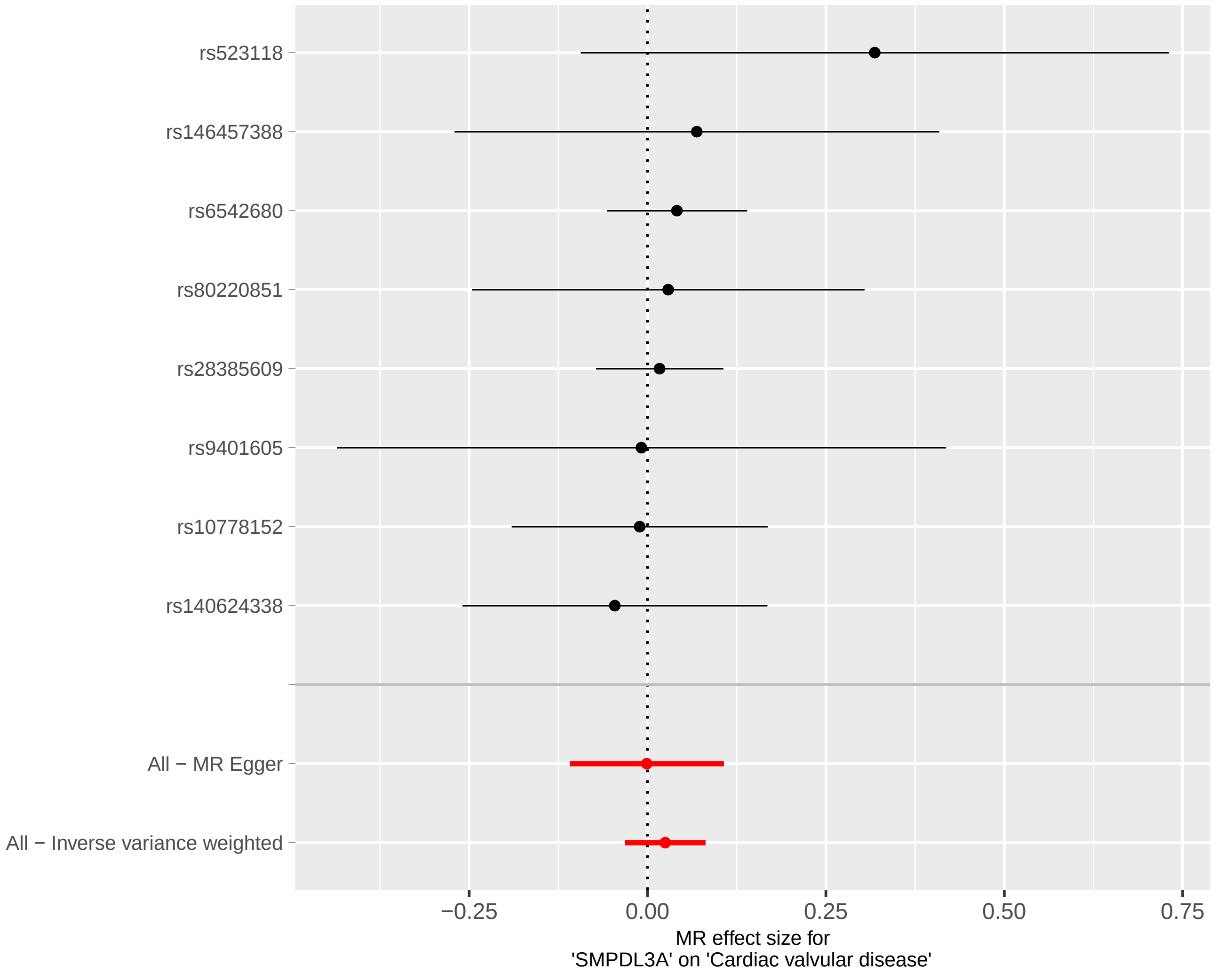
rs523118
rs146457388
rs6542680
rs80220851
rs28385609
rs9401605
rs10778152
rs140624338
All − MR Egger
All − Inverse variance weighted
−0.25
0.00
0.25
0.50
0.75
MR effect size for
'SMPDL3A' on 'Cardiac valvular disease'

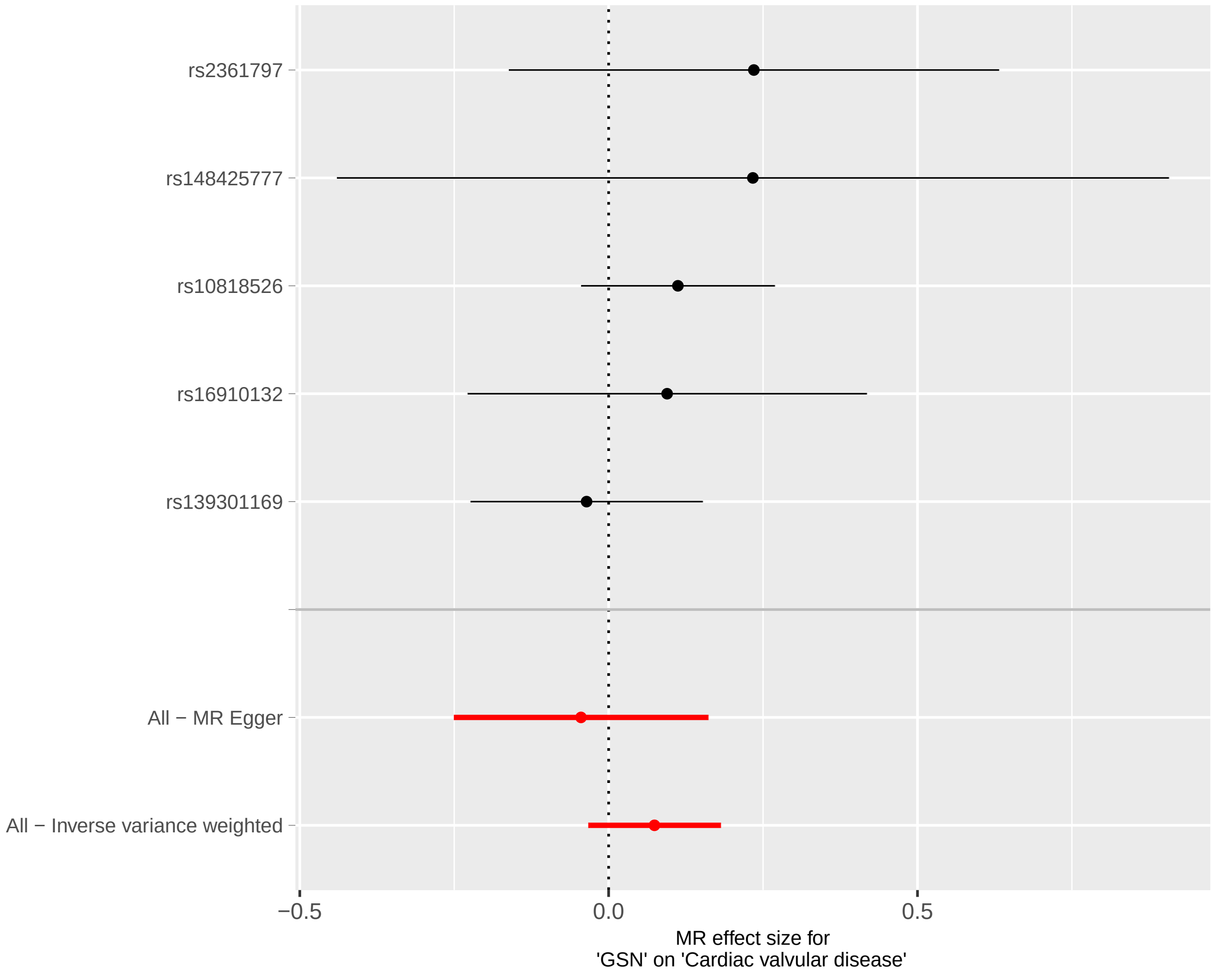
rs2361797
rs148425777
rs10818526
rs16910132
rs139301169
All − MR Egger
All − Inverse variance weighted
−0.5
0.0
0.5
MR effect size for
'GSN' on 'Cardiac valvular disease'

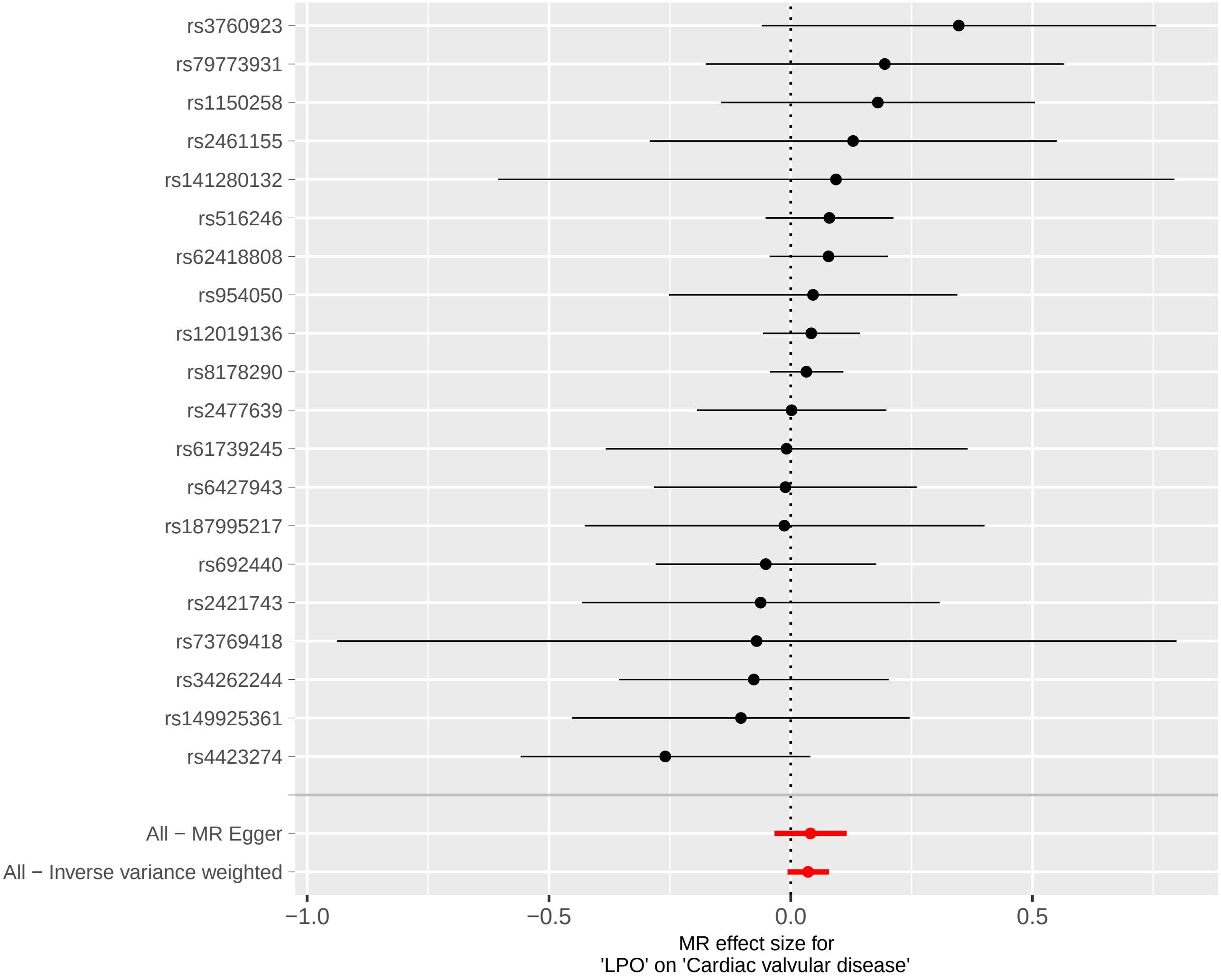
rs3760923
rs79773931
rs1150258
rs2461155
rs141280132
rs516246
rs62418808
rs954050
rs12019136
rs8178290
rs2477639
rs61739245
rs6427943
rs187995217
rs692440
rs2421743
rs73769418
rs34262244
rs149925361
rs4423274
All − MR Egger
All − Inverse variance weighted
−1.0
−0.5
0.0
0.5
MR effect size for
'LPO' on 'Cardiac valvular disease'

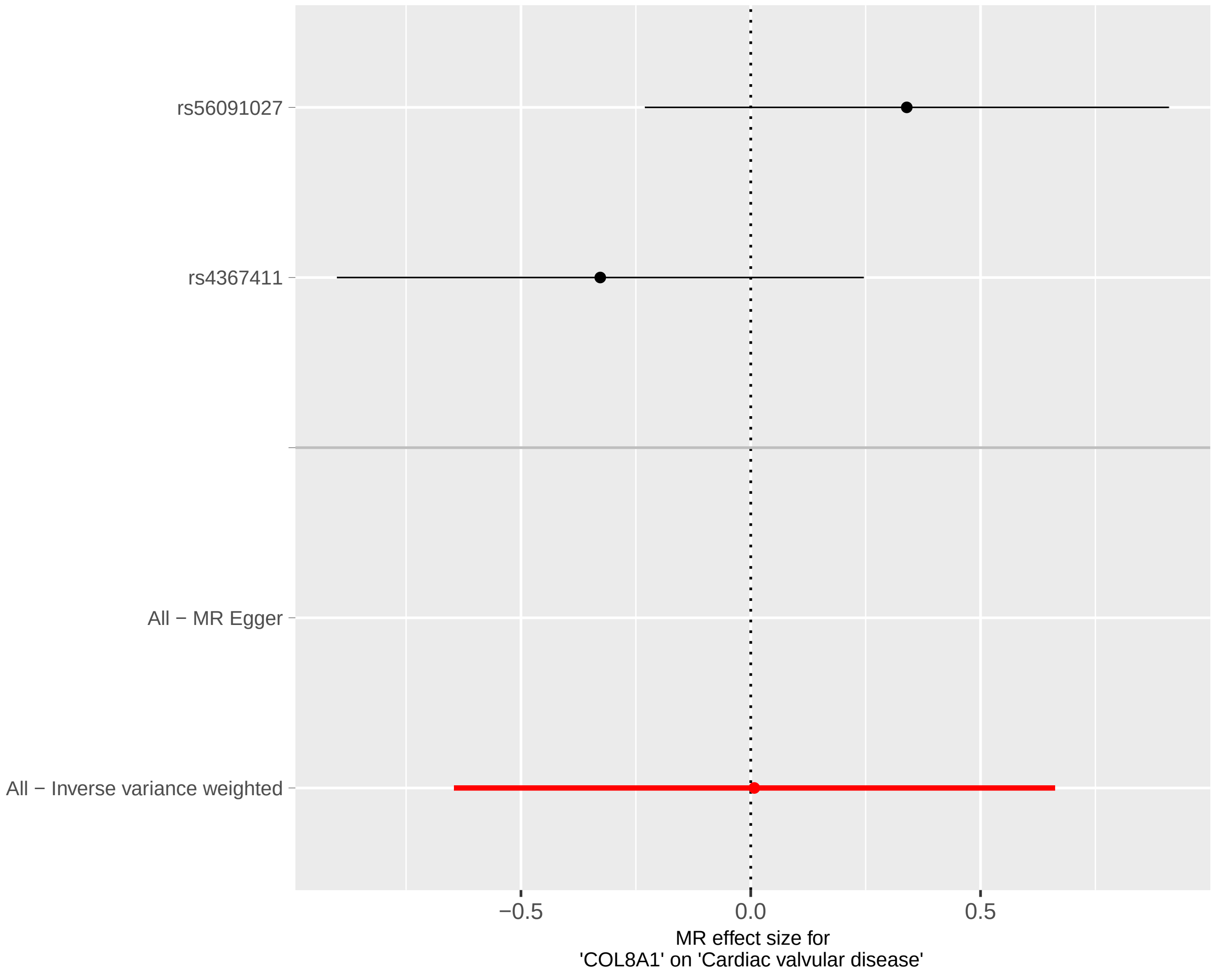
rs56091027
rs4367411
All − MR Egger
All − Inverse variance weighted
−0.5
0.0
0.5
MR effect size for
'COL8A1' on 'Cardiac valvular disease'

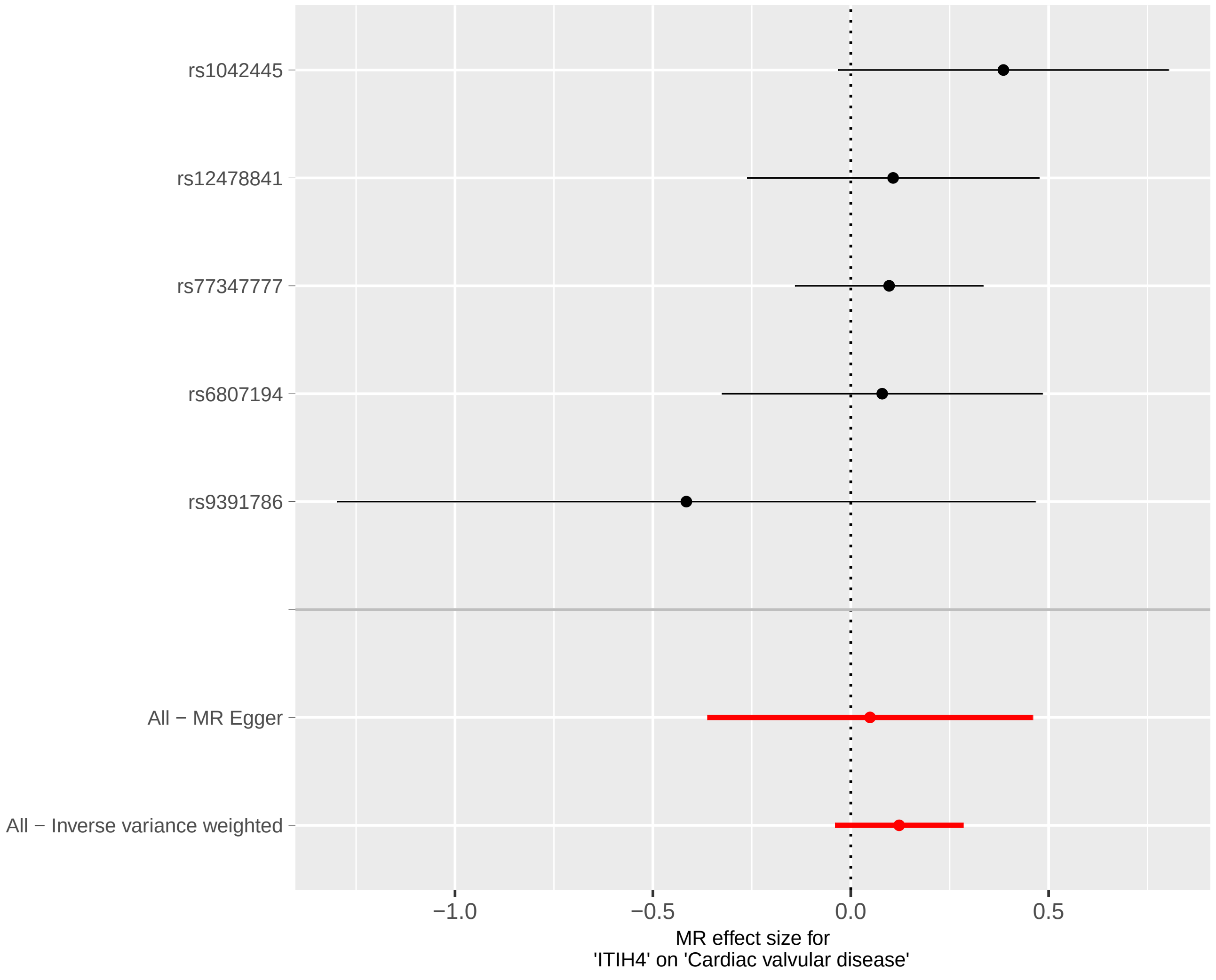
rs1042445
rs12478841
rs77347777
rs6807194
rs9391786
All − MR Egger
All − Inverse variance weighted
−1.0
−0.5
0.0
0.5
MR effect size for
'ITIH4' on 'Cardiac valvular disease'

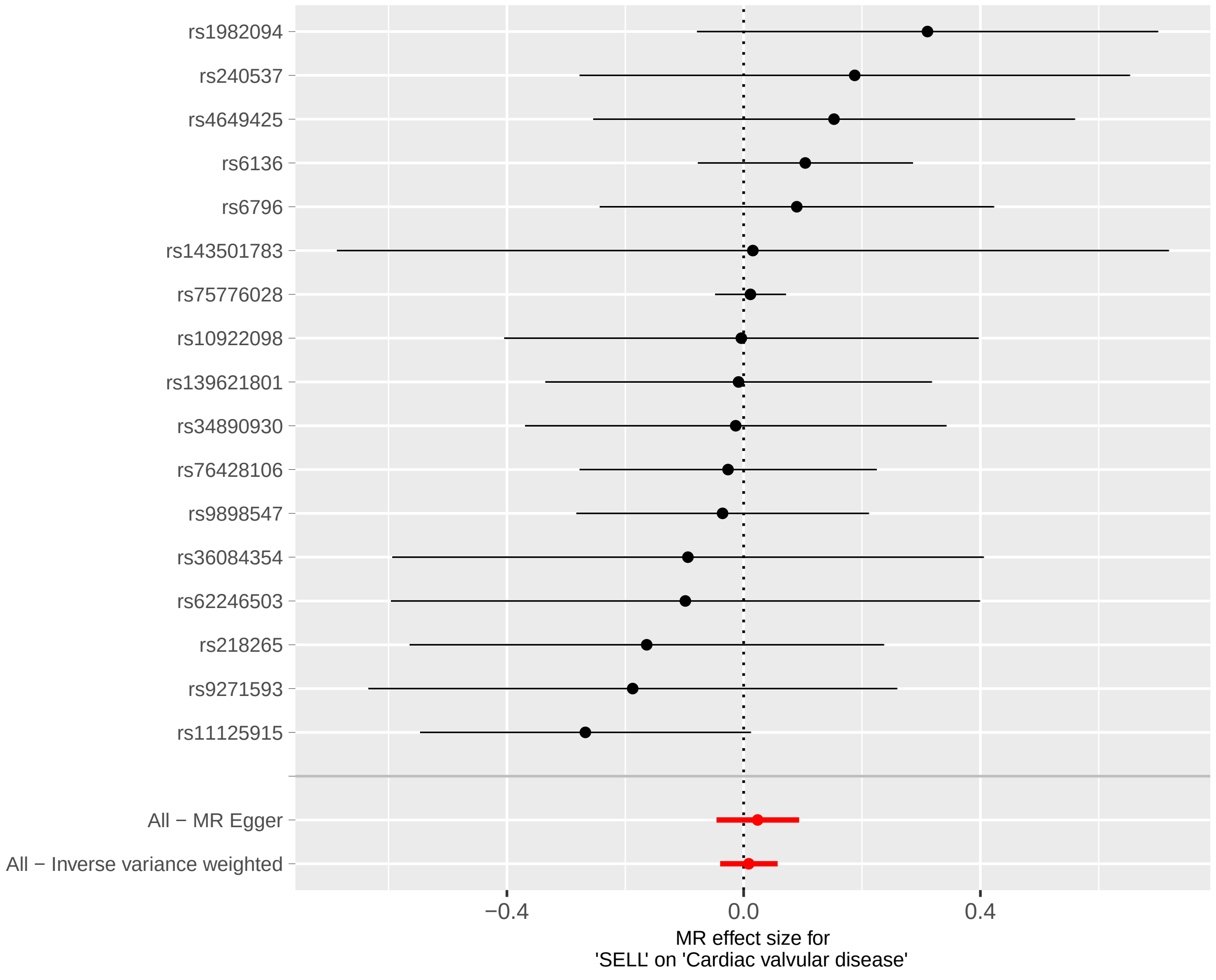
rs1982094
rs240537
rs4649425
rs6136
rs6796
rs143501783
rs75776028
rs10922098
rs139621801
rs34890930
rs76428106
rs9898547
rs36084354
rs62246503
rs218265
rs9271593
rs11125915
All − MR Egger
All − Inverse variance weighted
−0.4
0.0
0.4
MR effect size for
'SELL' on 'Cardiac valvular disease'

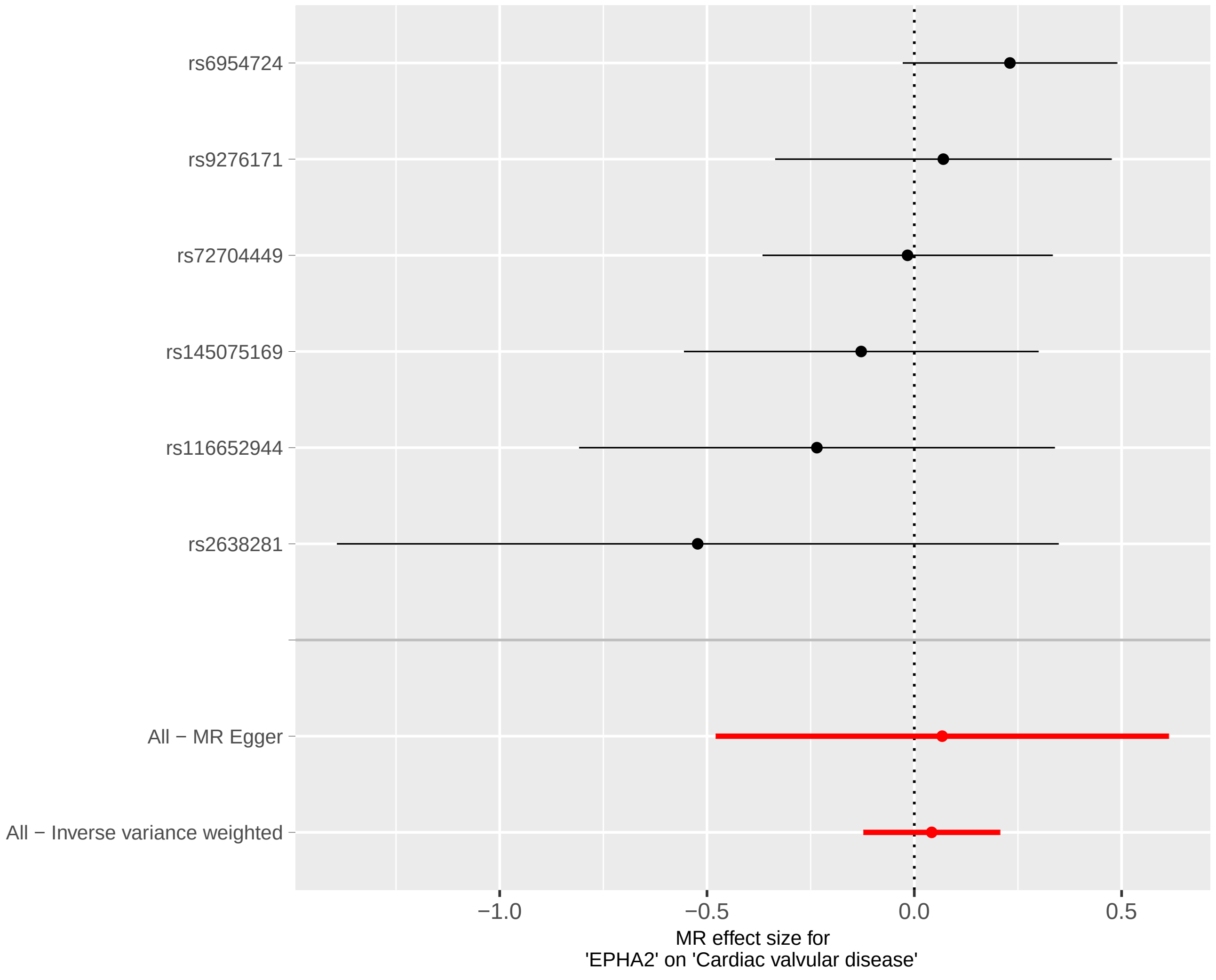
rs6954724
rs9276171
rs72704449
rs145075169
rs116652944
rs2638281
All − MR Egger
All − Inverse variance weighted
−1.0
−0.5
0.0
0.5
MR effect size for
'EPHA2' on 'Cardiac valvular disease'

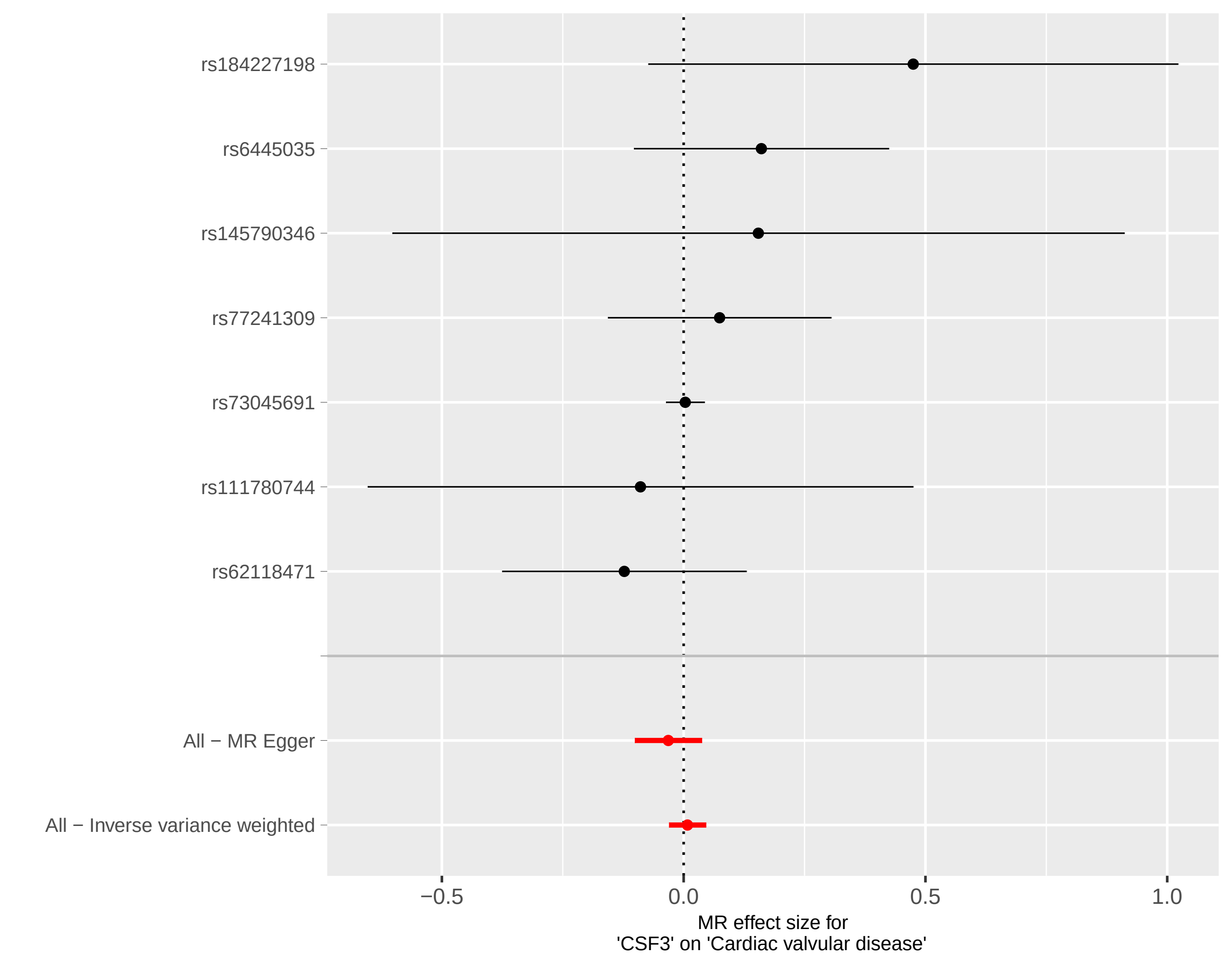
rs184227198
rs6445035
rs145790346
rs77241309
rs73045691
rs111780744
rs62118471
All − MR Egger
All − Inverse variance weighted
−0.5
0.0
0.5
1.0
MR effect size for
'CSF3' on 'Cardiac valvular disease'

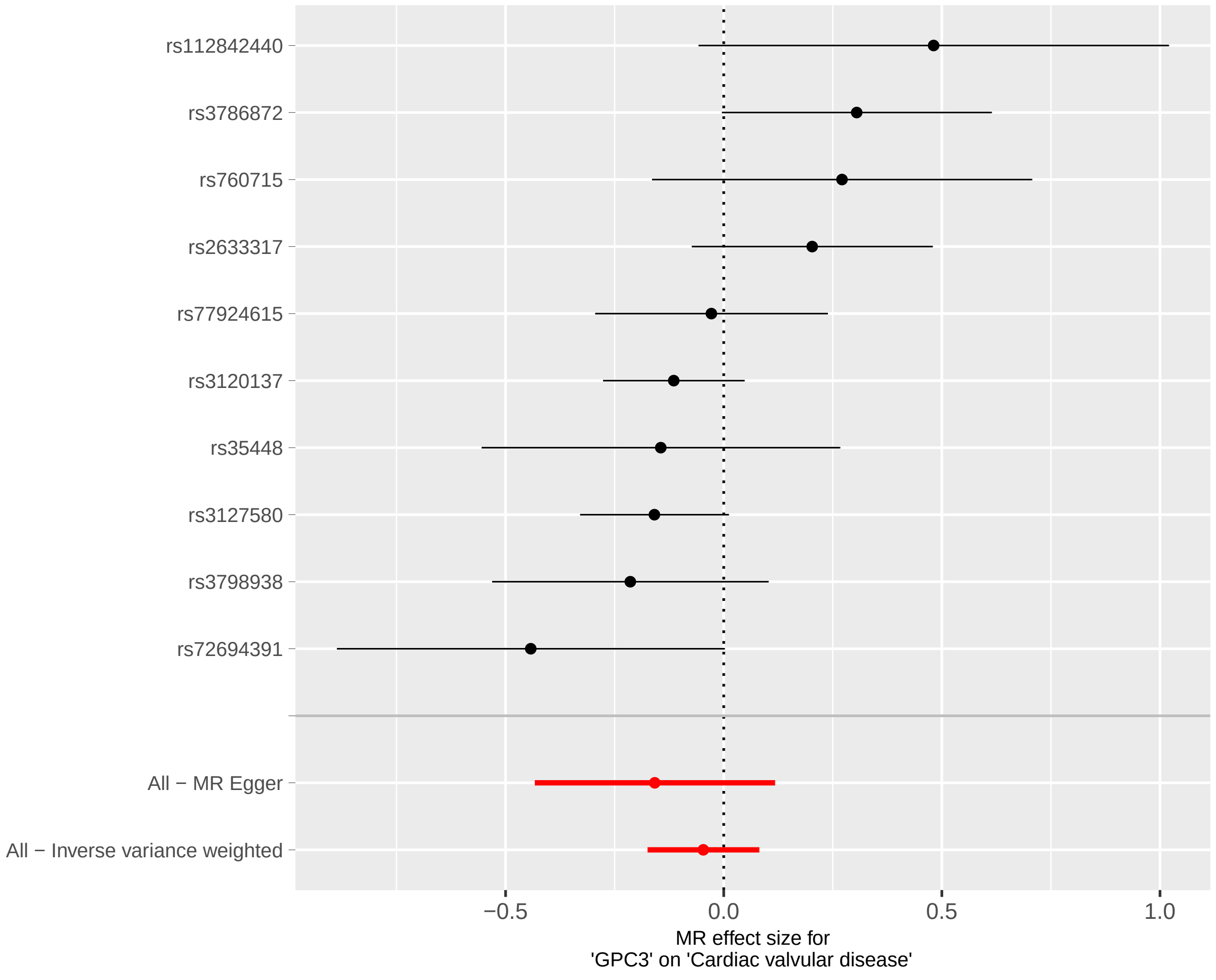

rs112842440
rs3786872
rs760715
rs2633317
rs77924615
rs3120137
rs35448
rs3127580
rs3798938
rs72694391
All − MR Egger
All − Inverse variance weighted
−0.5
0.0
0.5
1.0
MR effect size for
'GPC3' on 'Cardiac valvular disease'

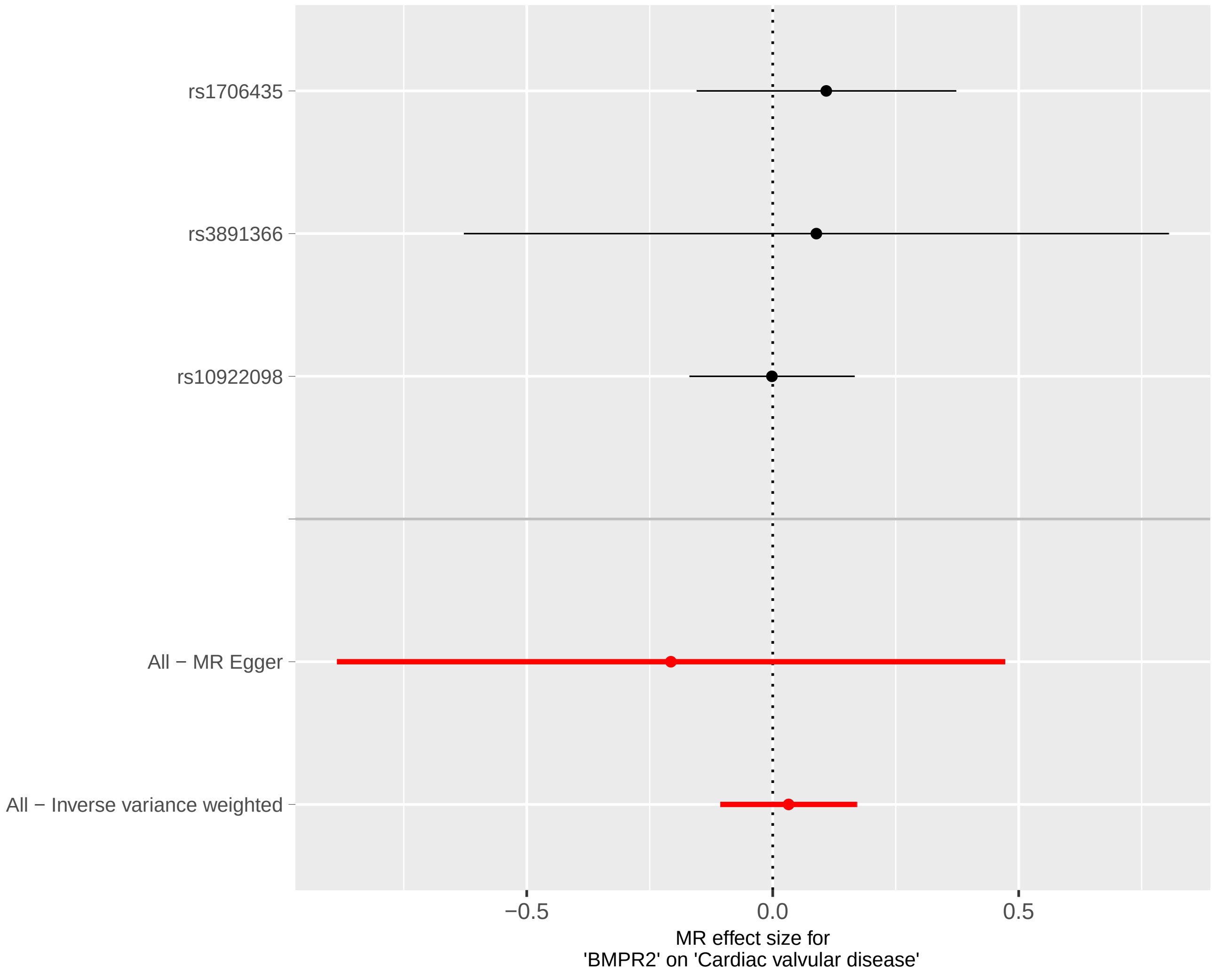
rs1706435
rs3891366
rs10922098
All − MR Egger
All − Inverse variance weighted
−0.5
0.0
0.5
MR effect size for
'BMPR2' on 'Cardiac valvular disease'

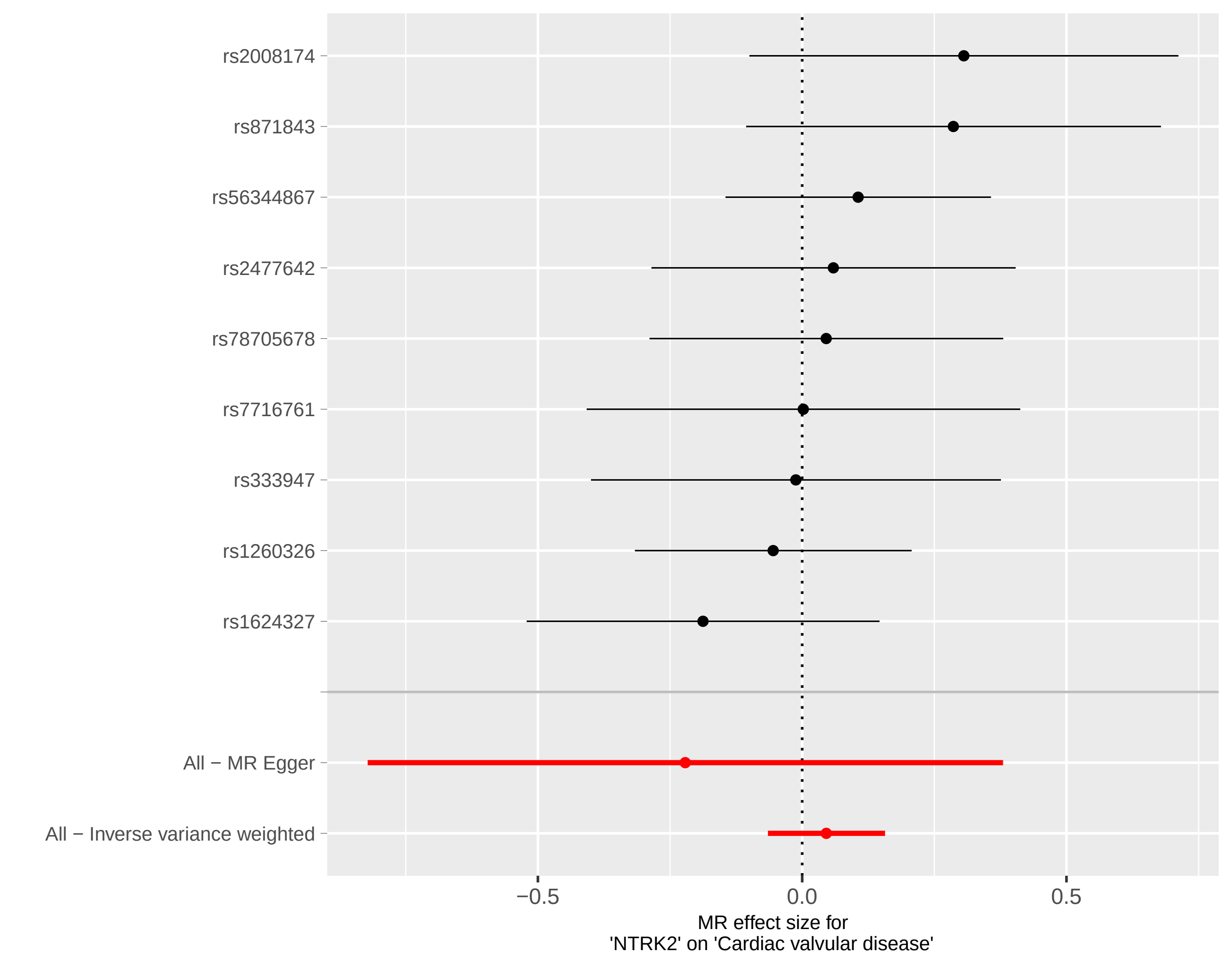
rs2008174
rs871843
rs56344867
rs2477642
rs78705678
rs7716761
rs333947
rs1260326
rs1624327
All − MR Egger
All − Inverse variance weighted
−0.5
0.0
0.5
MR effect size for
'NTRK2' on 'Cardiac valvular disease'

Insufficient number of SNPs

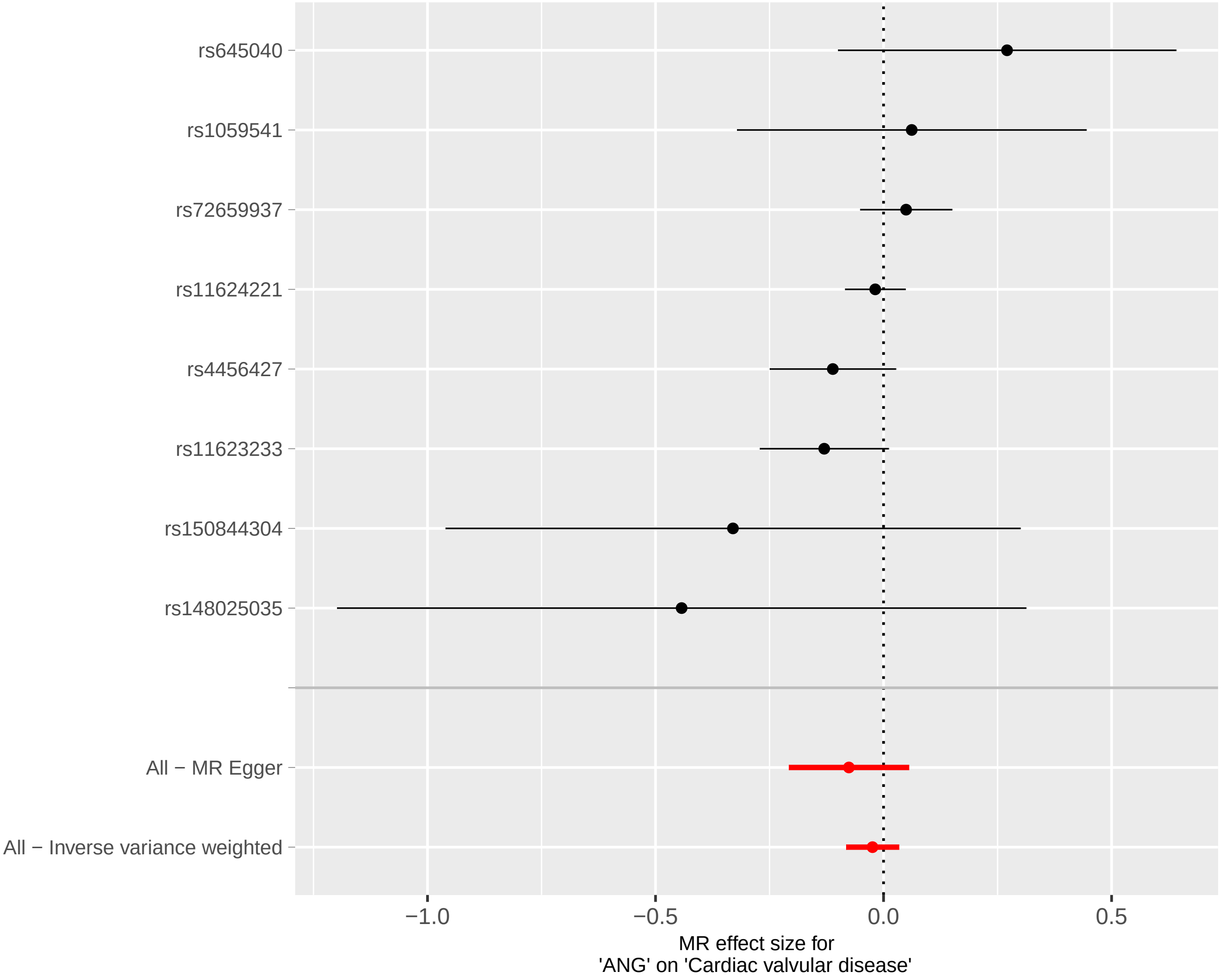
rs645040
rs1059541
rs72659937
rs11624221
rs4456427
rs11623233
rs150844304
rs148025035
All − MR Egger
All − Inverse variance weighted
−1.0
−0.5
0.0
0.5
MR effect size for
'ANG' on 'Cardiac valvular disease'

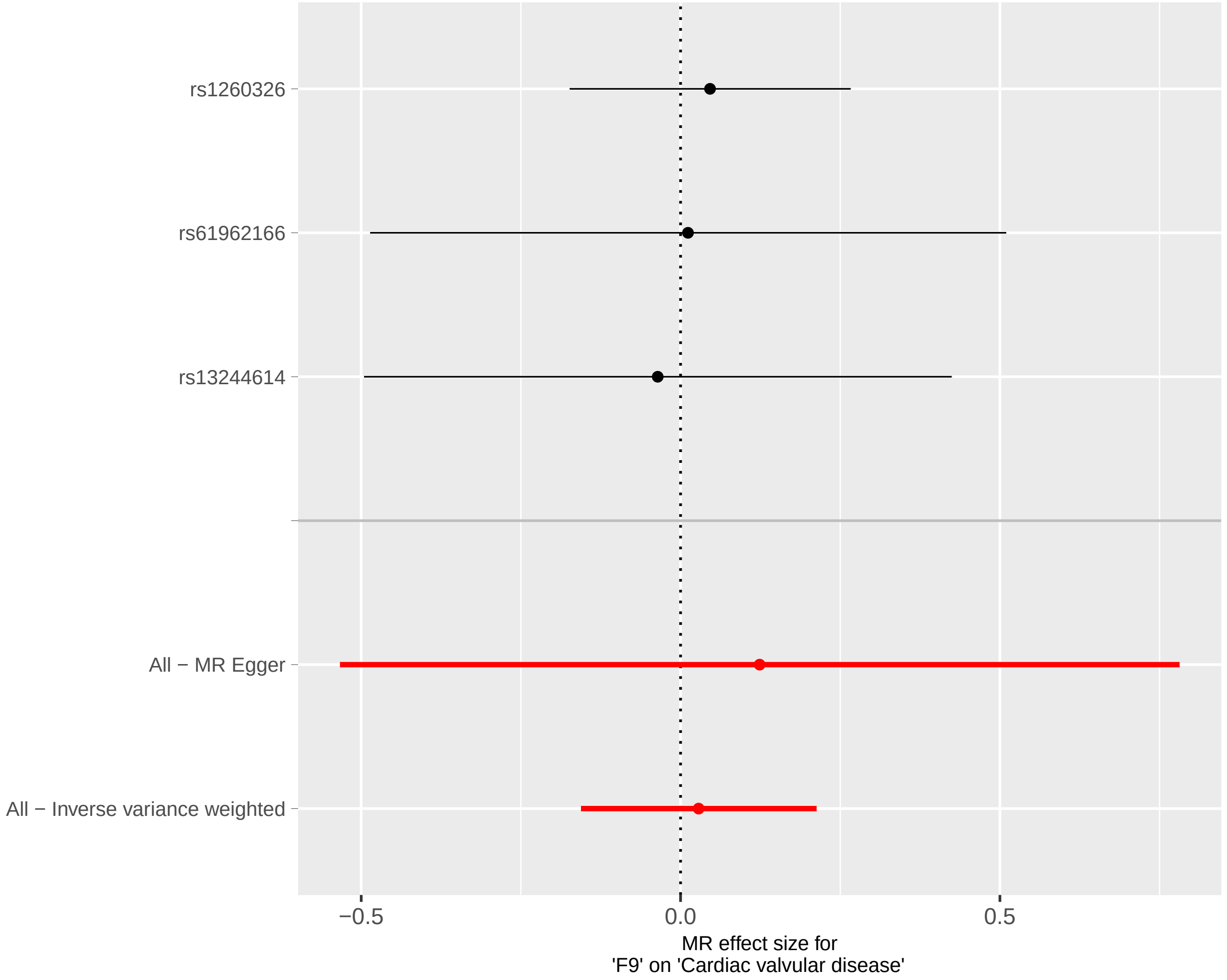

rs1260326
rs61962166
rs13244614
All − MR Egger
All − Inverse variance weighted
−0.5
0.0
0.5
MR effect size for
'F9' on 'Cardiac valvular disease'

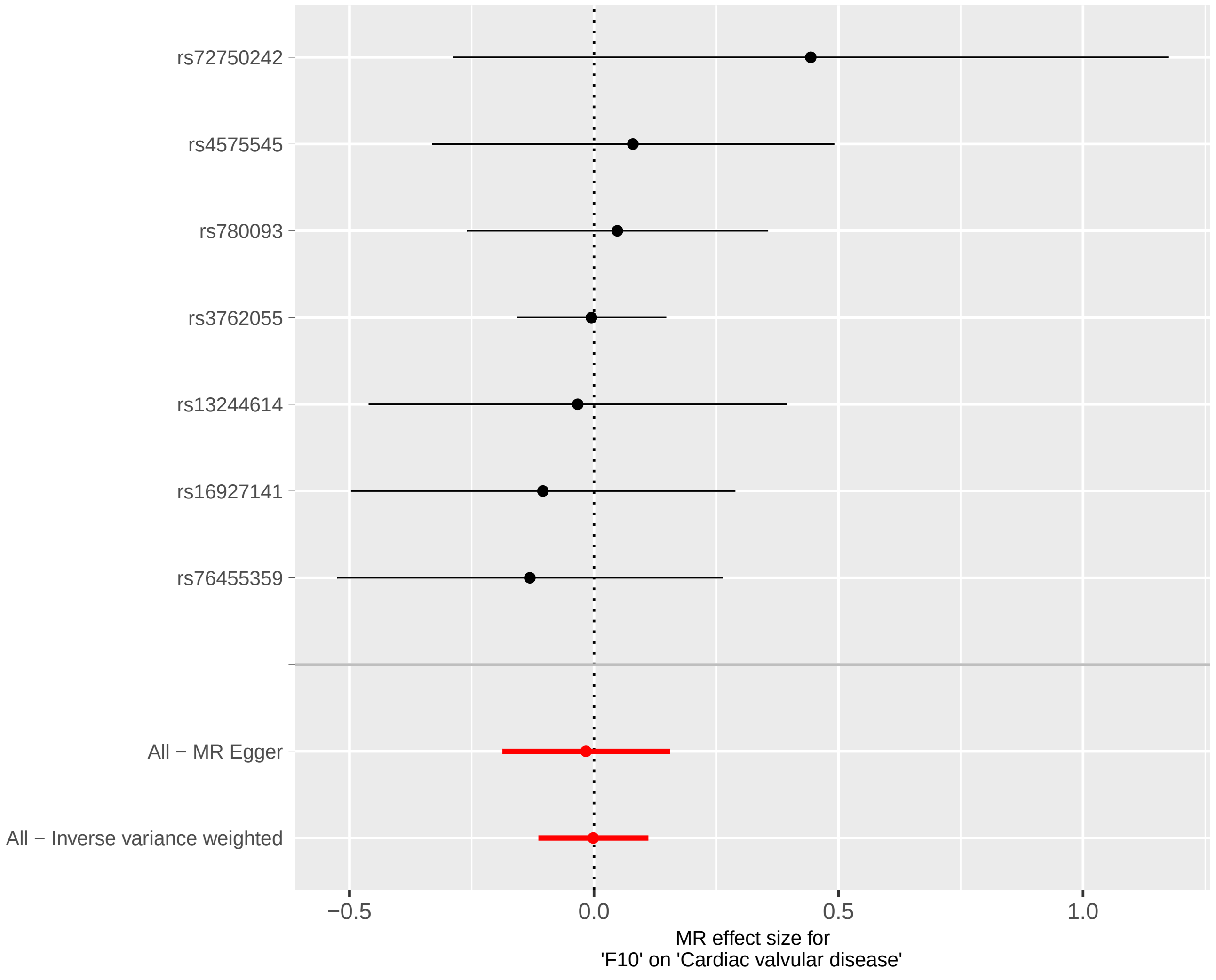
rs72750242
rs4575545
rs780093
rs3762055
rs13244614
rs16927141
rs76455359
All − MR Egger
All − Inverse variance weighted
−0.5
0.0
0.5
1.0
MR effect size for
'F10' on 'Cardiac valvular disease'

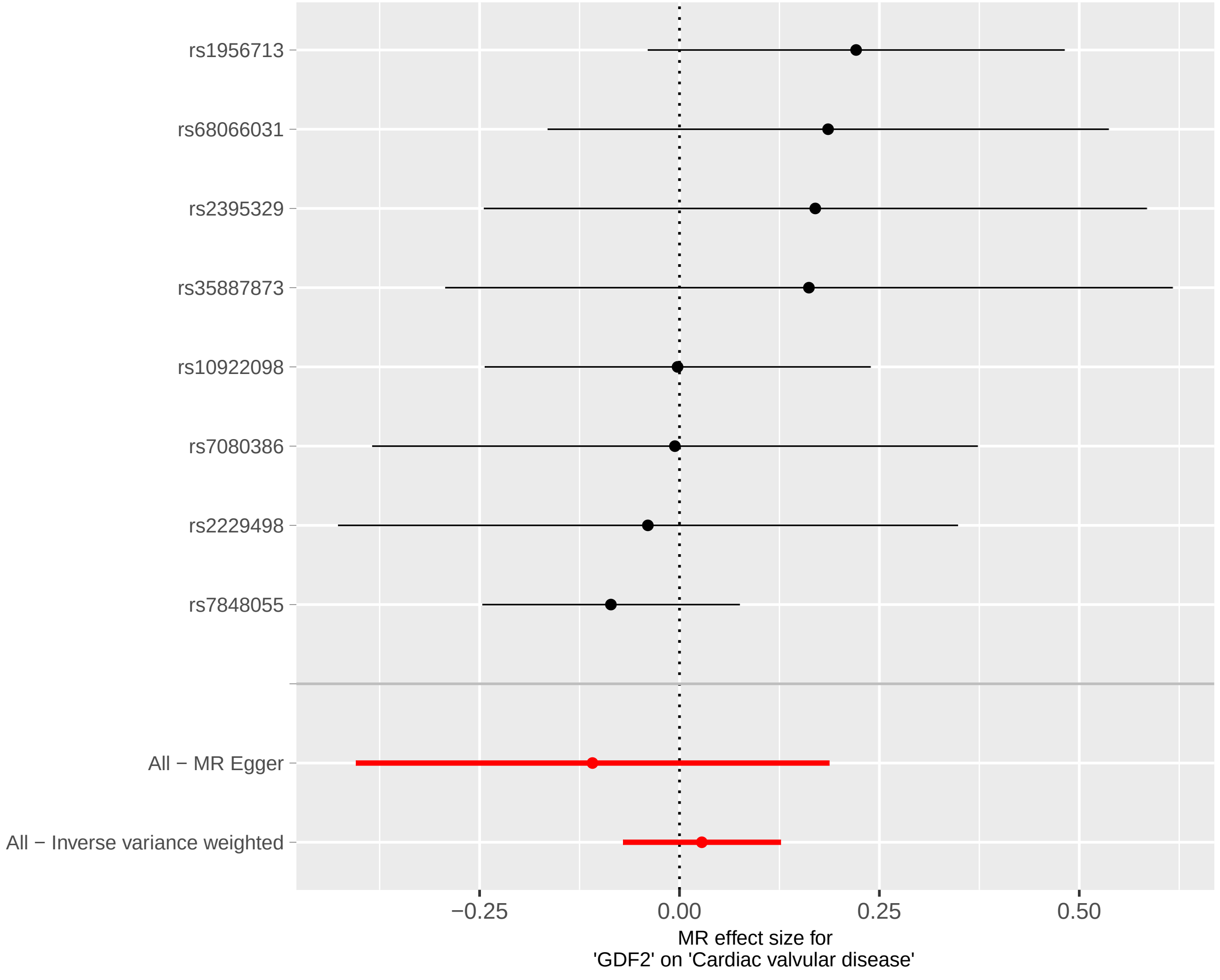
rs1956713
rs68066031
rs2395329
rs35887873
rs10922098
rs7080386
rs2229498
rs7848055
All − MR Egger
All − Inverse variance weighted
−0.25
0.00
0.25
0.50
MR effect size for
'GDF2' on 'Cardiac valvular disease'

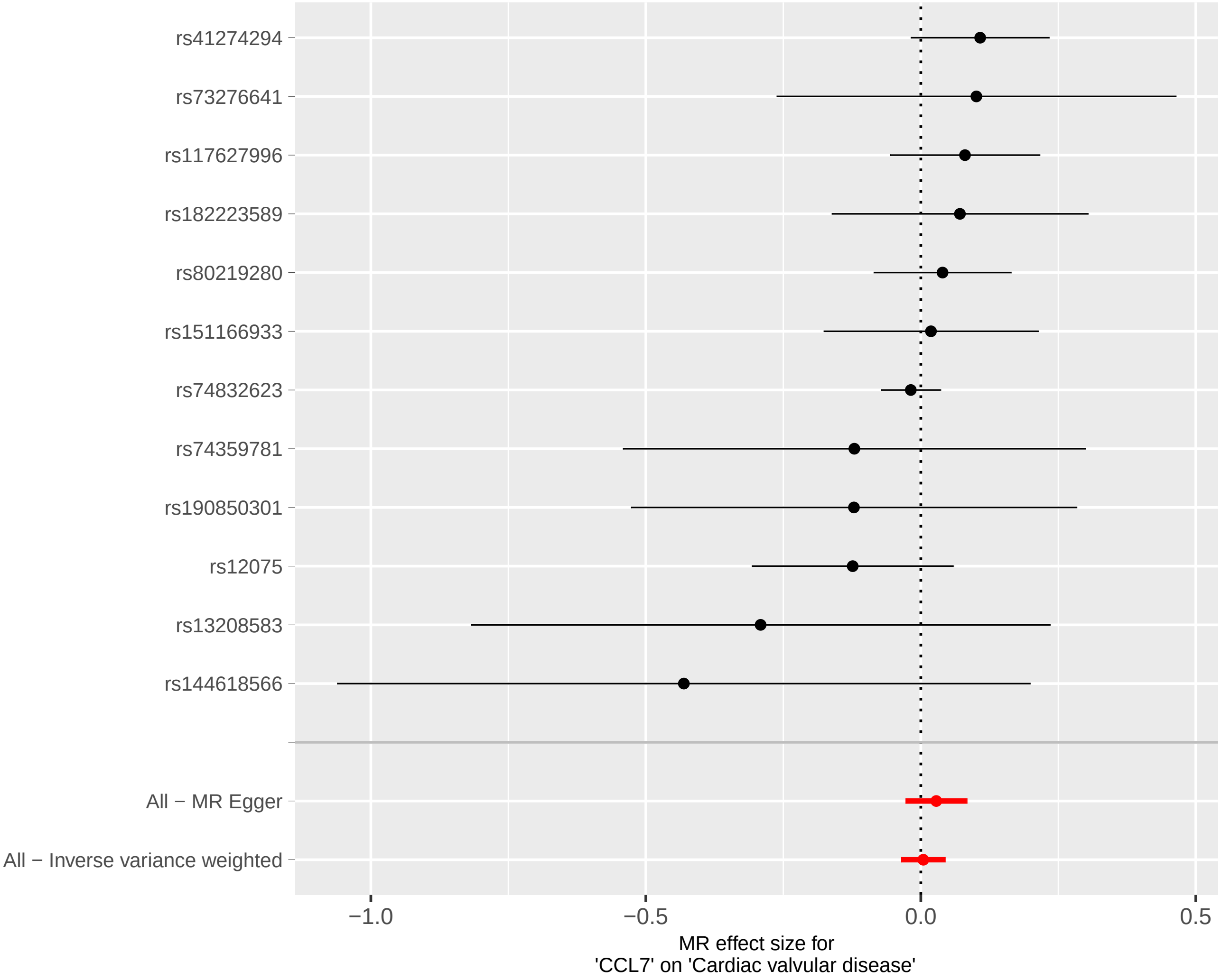
rs41274294
rs73276641
rs117627996
rs182223589
rs80219280
rs151166933
rs74832623
rs74359781
rs190850301
rs12075
rs13208583
rs144618566
All − MR Egger
All − Inverse variance weighted
−1.0
−0.5
0.0
0.5
MR effect size for
'CCL7' on 'Cardiac valvular disease'

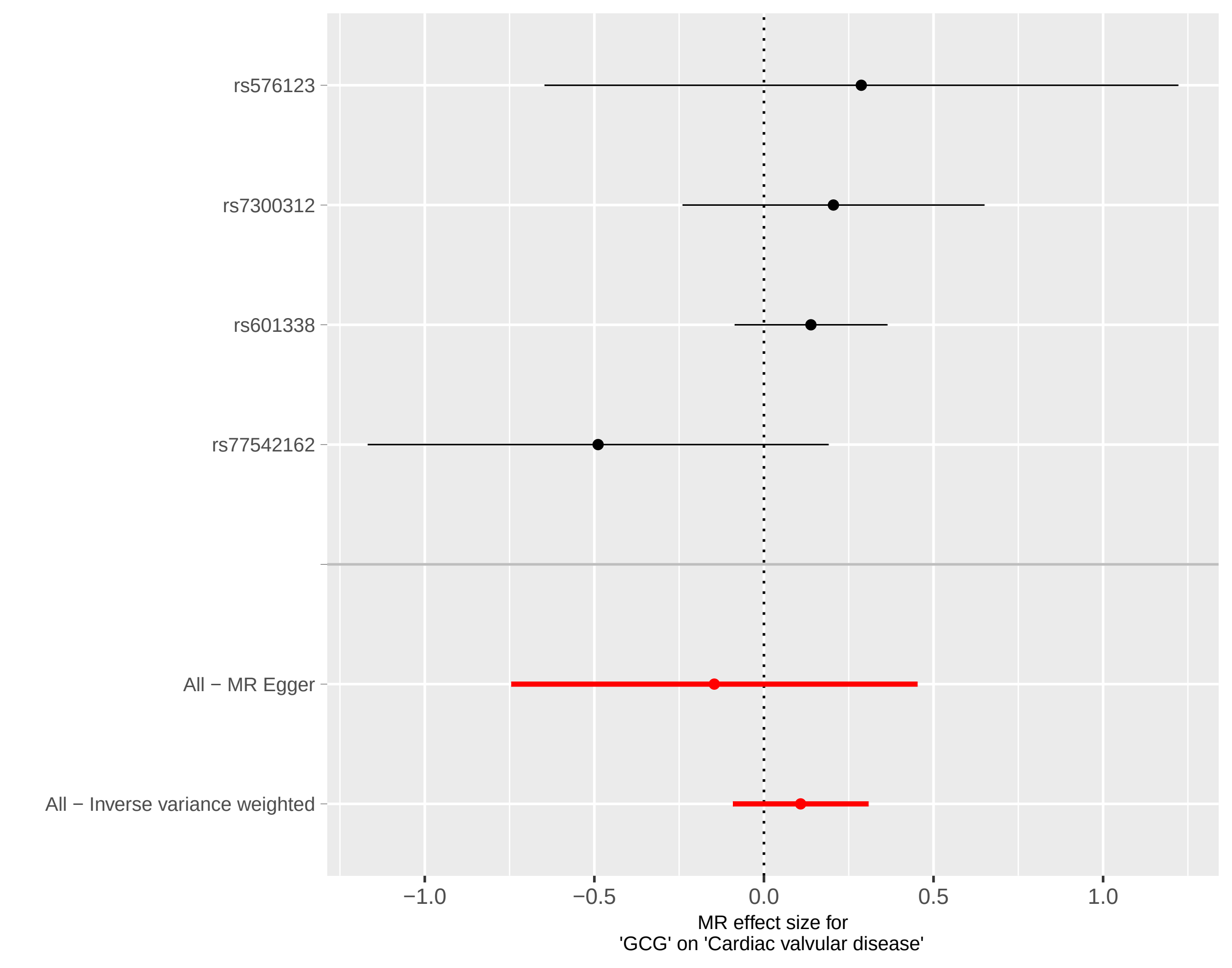
rs576123
rs7300312
rs601338
rs77542162
All − MR Egger
All − Inverse variance weighted
−1.0
−0.5
0.0
0.5
1.0
MR effect size for
'GCG' on 'Cardiac valvular disease'

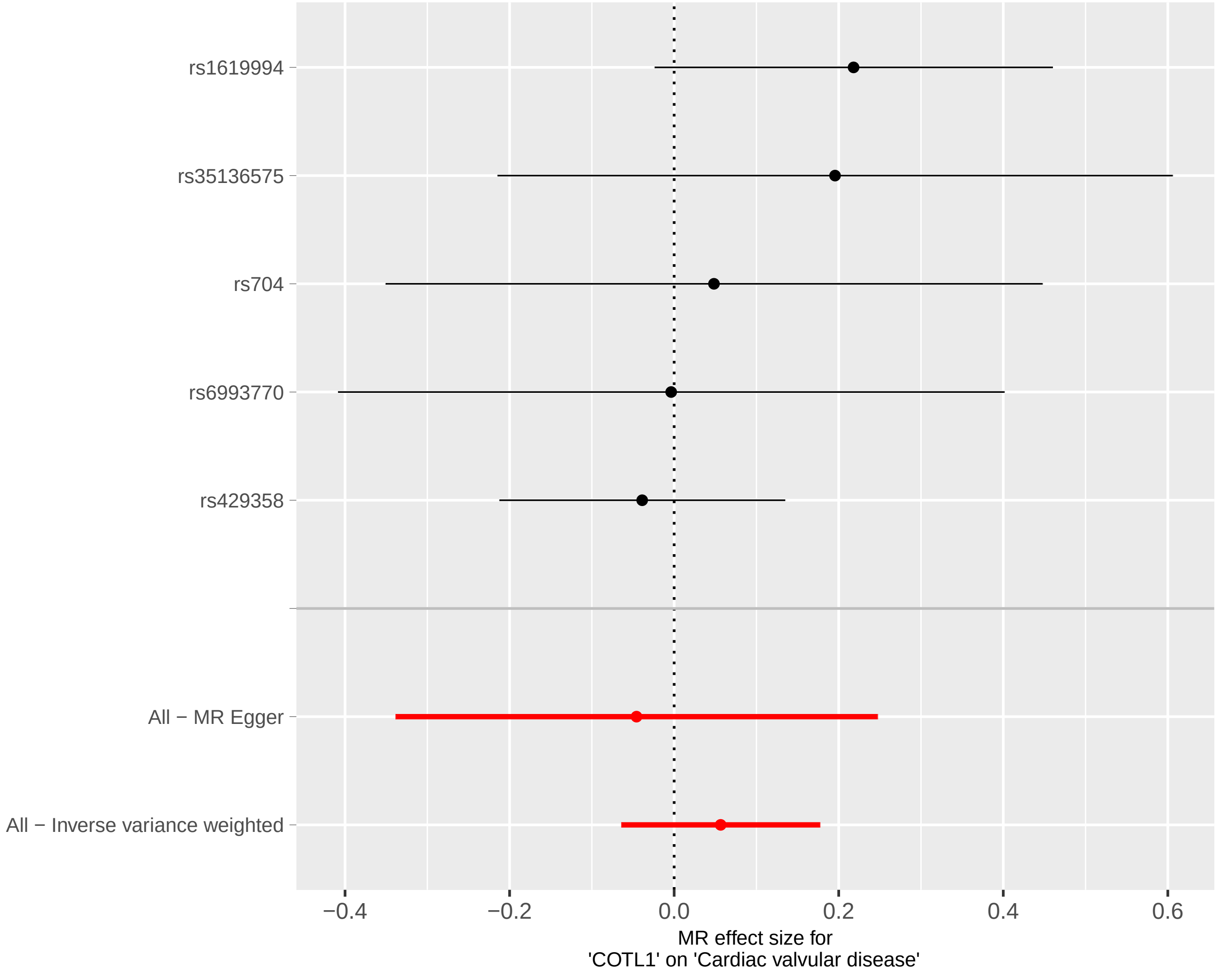
rs1619994
rs35136575
rs704
rs6993770
rs429358
All − MR Egger
All − Inverse variance weighted
−0.4
−0.2
0.0
0.2
0.4
0.6
MR effect size for
'COTL1' on 'Cardiac valvular disease'

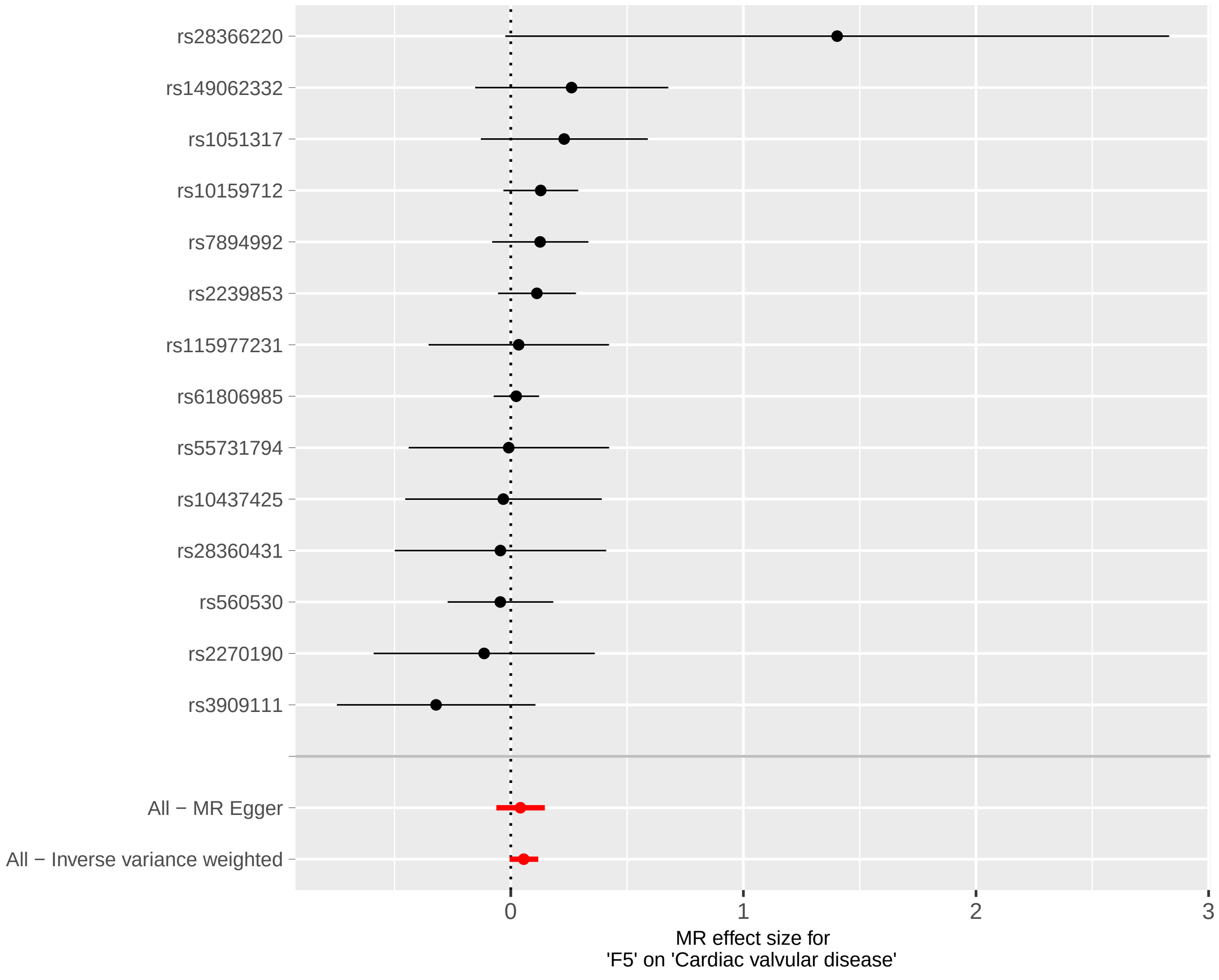

rs28366220
rs149062332
rs1051317
rs10159712
rs7894992
rs2239853
rs115977231
rs61806985
rs55731794
rs10437425
rs28360431
rs560530
rs2270190
rs3909111
All − MR Egger
All − Inverse variance weighted
0
1
2
3
MR effect size for
'F5' on 'Cardiac valvular disease'

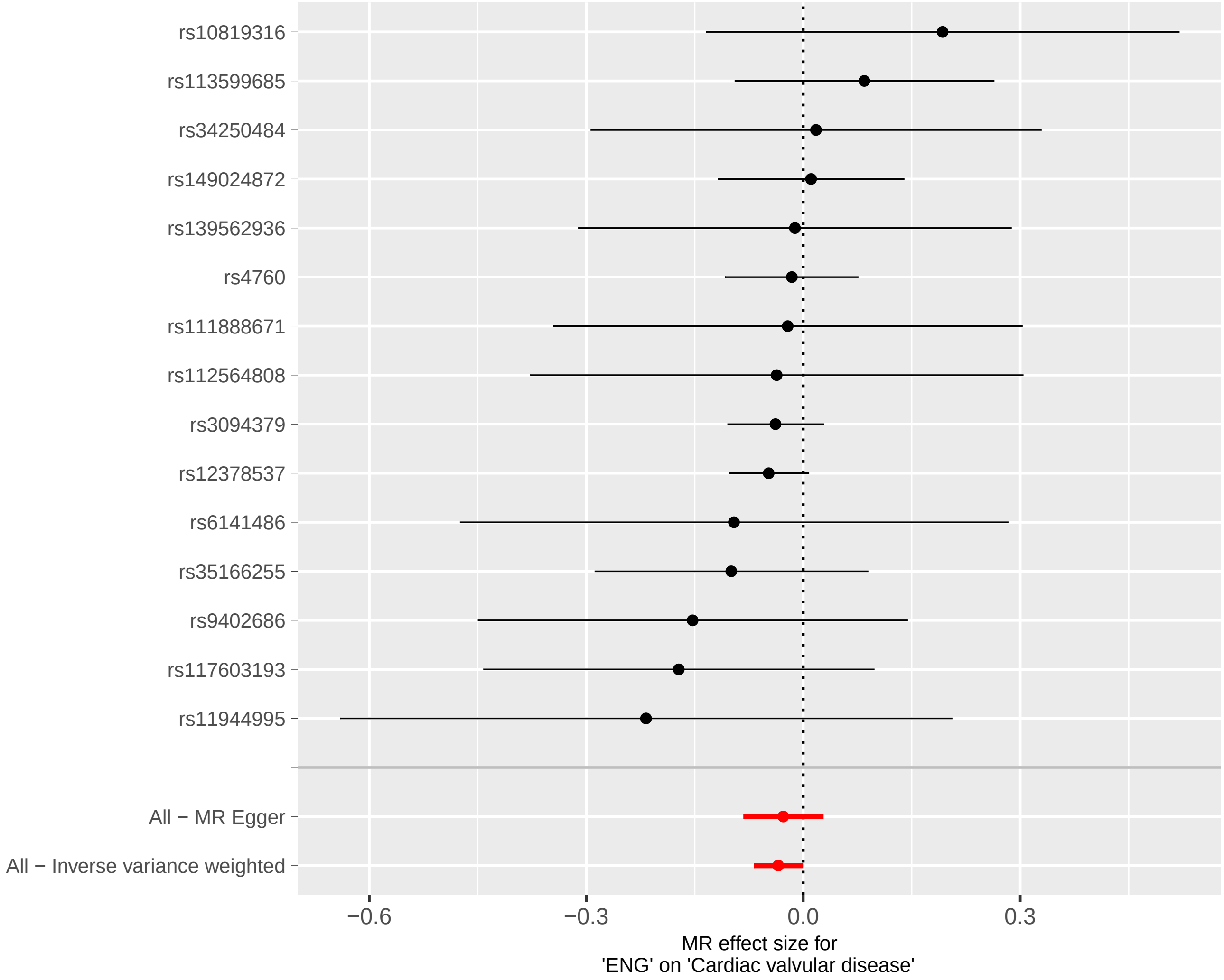
rs10819316
rs113599685
rs34250484
rs149024872
rs139562936
rs4760
rs111888671
rs112564808
rs3094379
rs12378537
rs6141486
rs35166255
rs9402686
rs117603193
rs11944995
All − MR Egger
All − Inverse variance weighted
−0.6
−0.3
0.0
0.3
MR effect size for
'ENG' on 'Cardiac valvular disease'

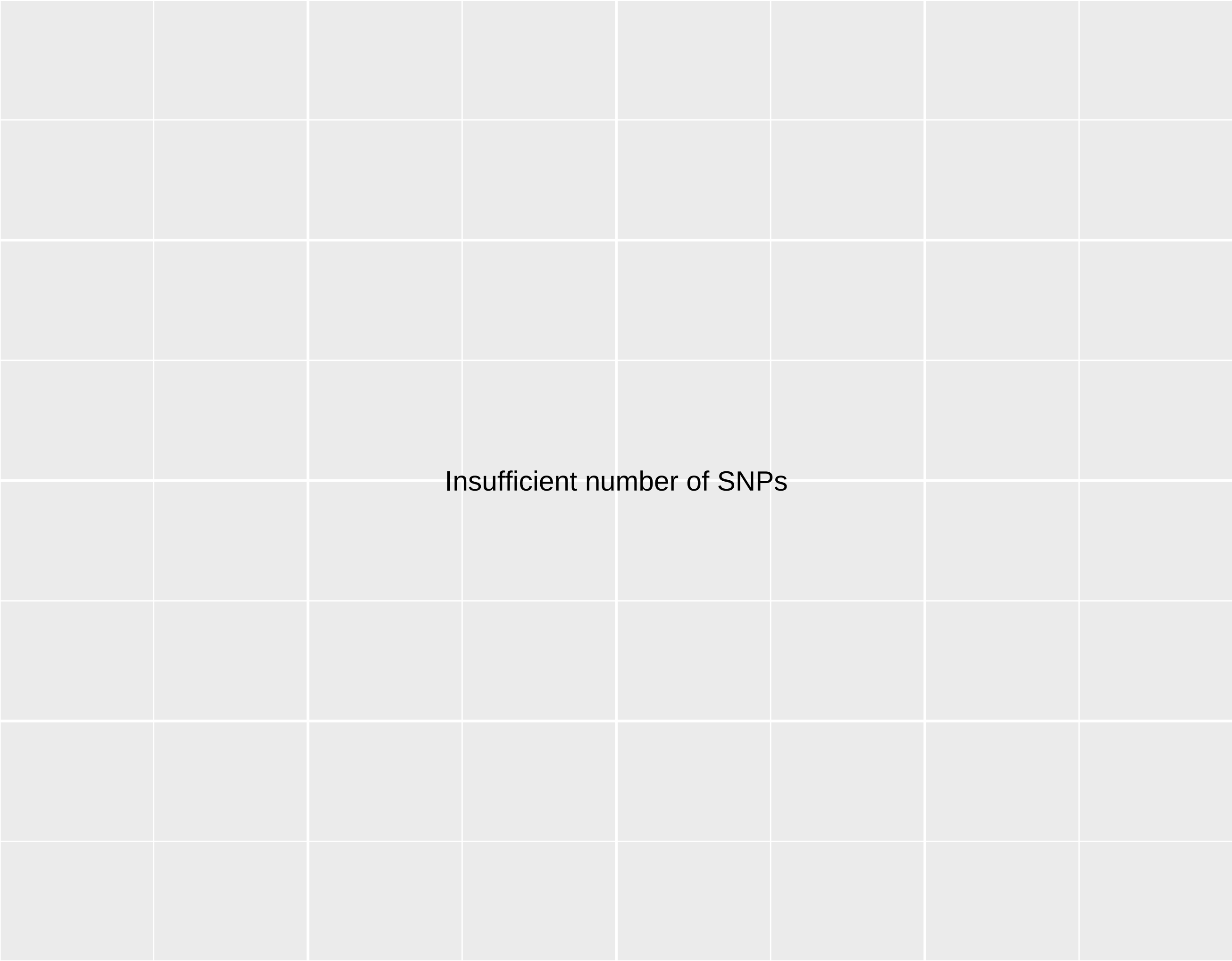
Insufficient number of SNPs

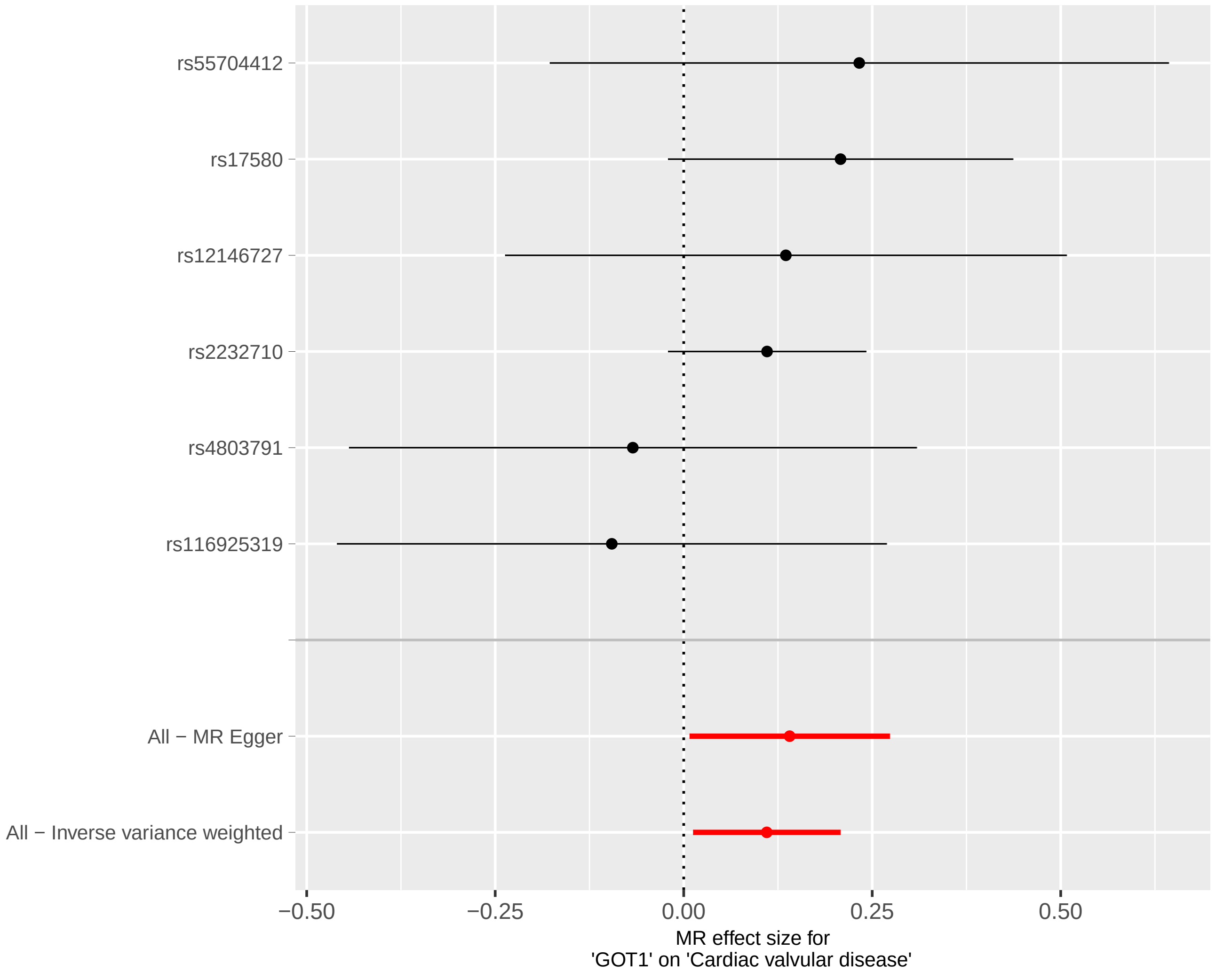

rs55704412
rs17580
rs12146727
rs2232710
rs4803791
rs116925319
All − MR Egger
All − Inverse variance weighted
−0.50
−0.25
0.00
0.25
0.50
MR effect size for
'GOT1' on 'Cardiac valvular disease'

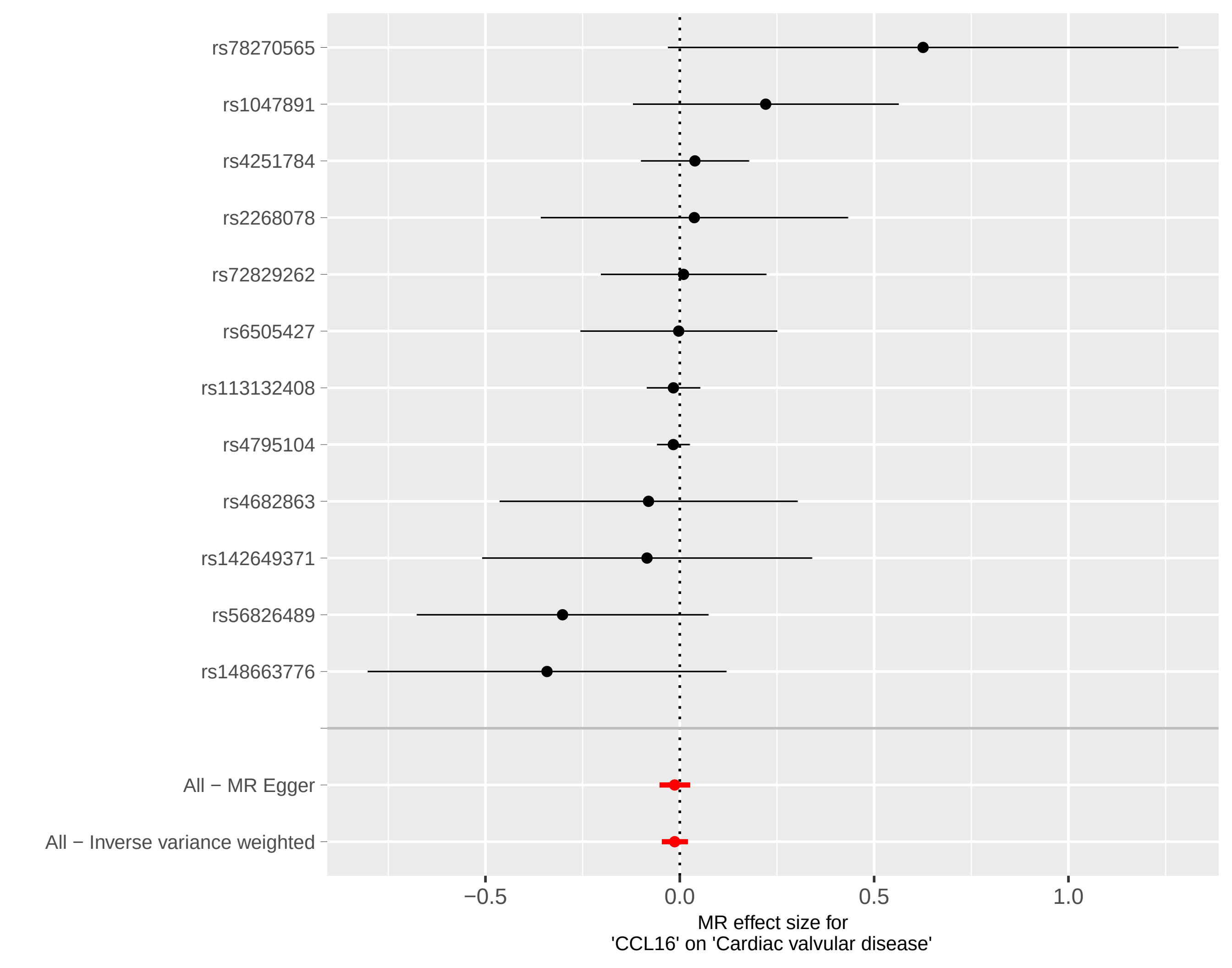

rs78270565
rs1047891
rs4251784
rs2268078
rs72829262
rs6505427
rs113132408
rs4795104
rs4682863
rs142649371
rs56826489
rs148663776
All − MR Egger
All − Inverse variance weighted
−0.5
0.0
0.5
1.0
MR effect size for
'CCL16' on 'Cardiac valvular disease'

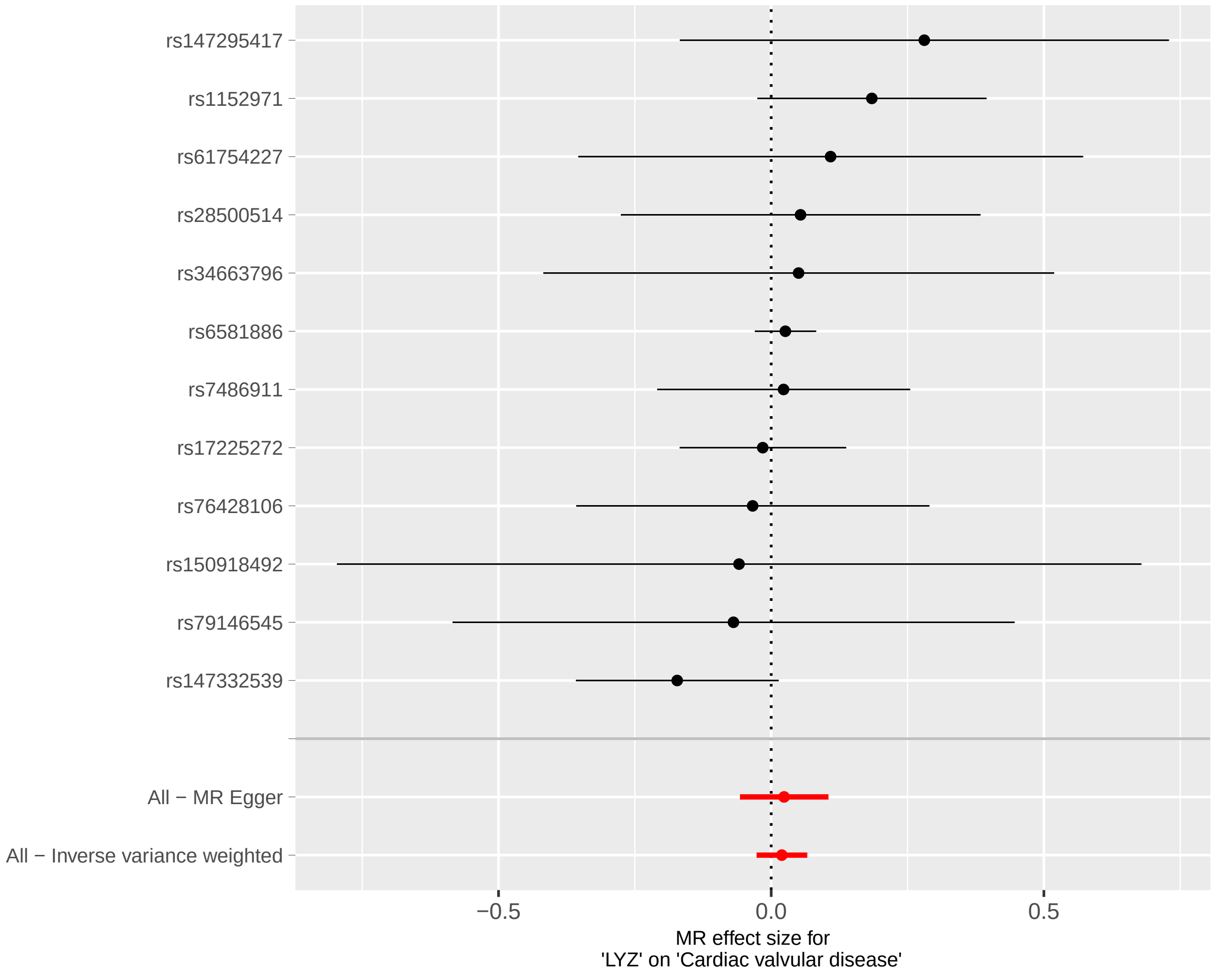

rs147295417
rs1152971
rs61754227
rs28500514
rs34663796
rs6581886
rs7486911
rs17225272
rs76428106
rs150918492
rs79146545
rs147332539
All − MR Egger
All − Inverse variance weighted
−0.5
0.0
0.5
MR effect size for
'LYZ' on 'Cardiac valvular disease'

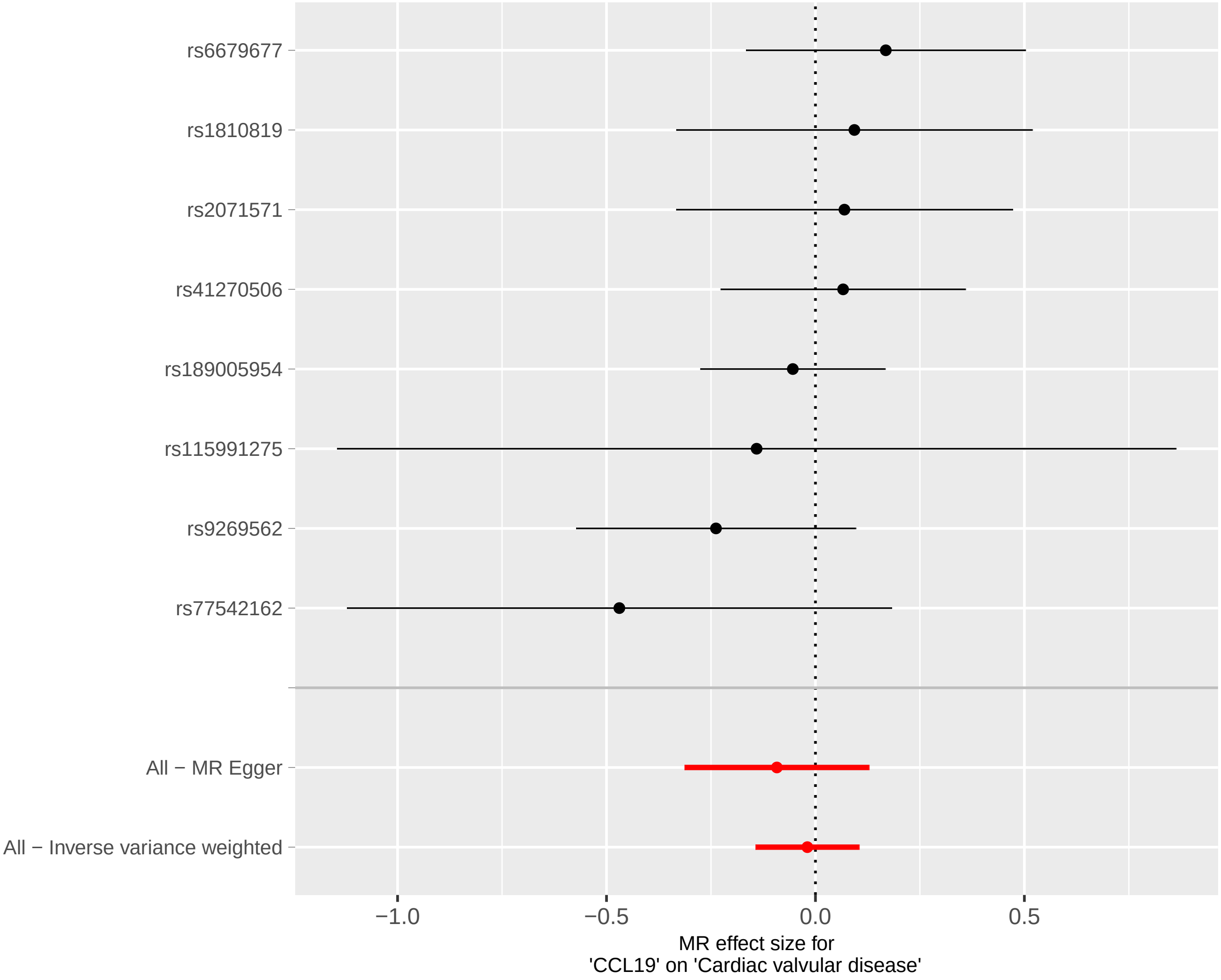

rs6679677
rs1810819
rs2071571
rs41270506
rs189005954
rs115991275
rs9269562
rs77542162
All − MR Egger
All − Inverse variance weighted
−1.0
−0.5
0.0
0.5
MR effect size for
'CCL19' on 'Cardiac valvular disease'

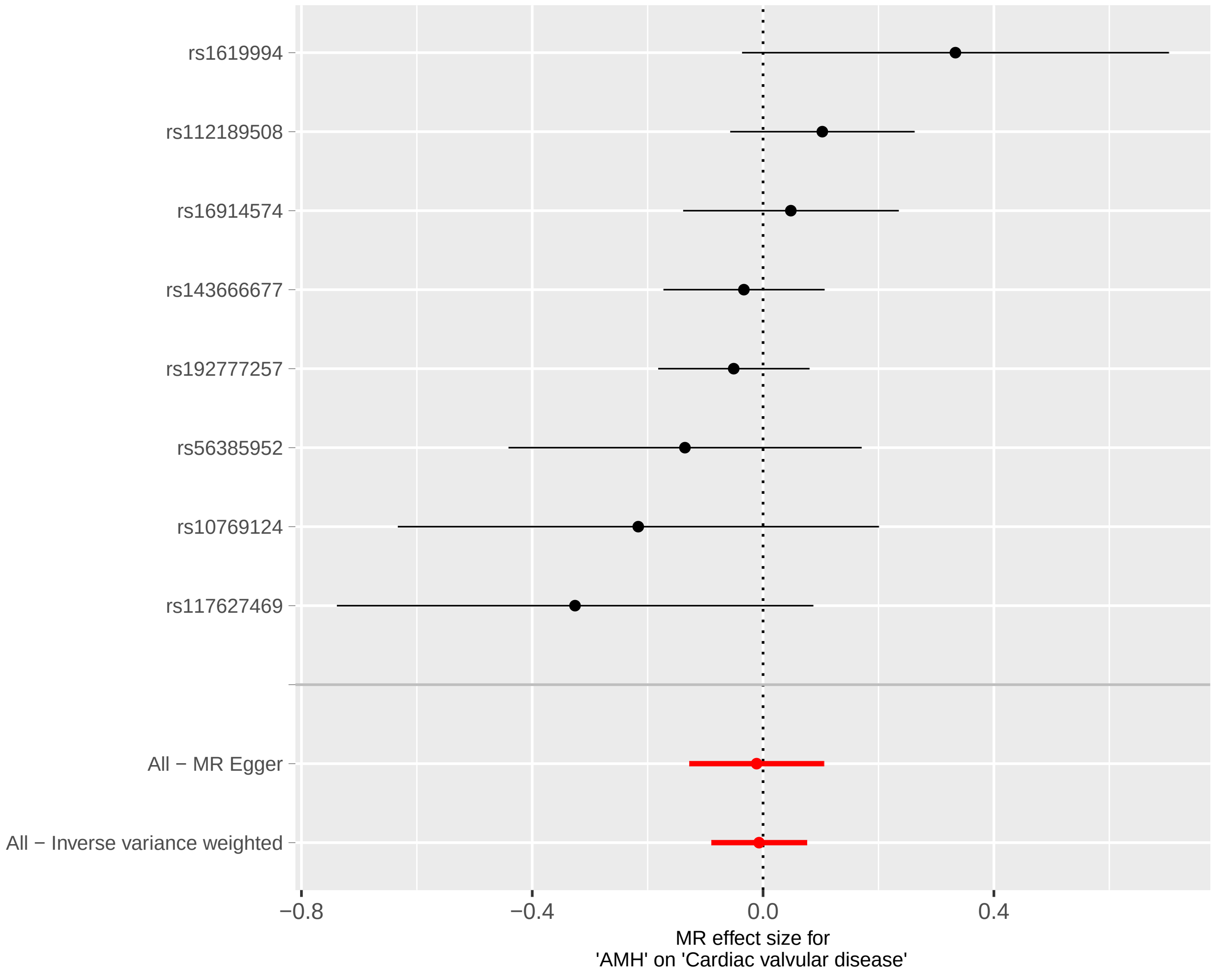

rs1619994
rs112189508
rs16914574
rs143666677
rs192777257
rs56385952
rs10769124
rs117627469
All − MR Egger
All − Inverse variance weighted
−0.8
−0.4
0.0
0.4
MR effect size for
'AMH' on 'Cardiac valvular disease'

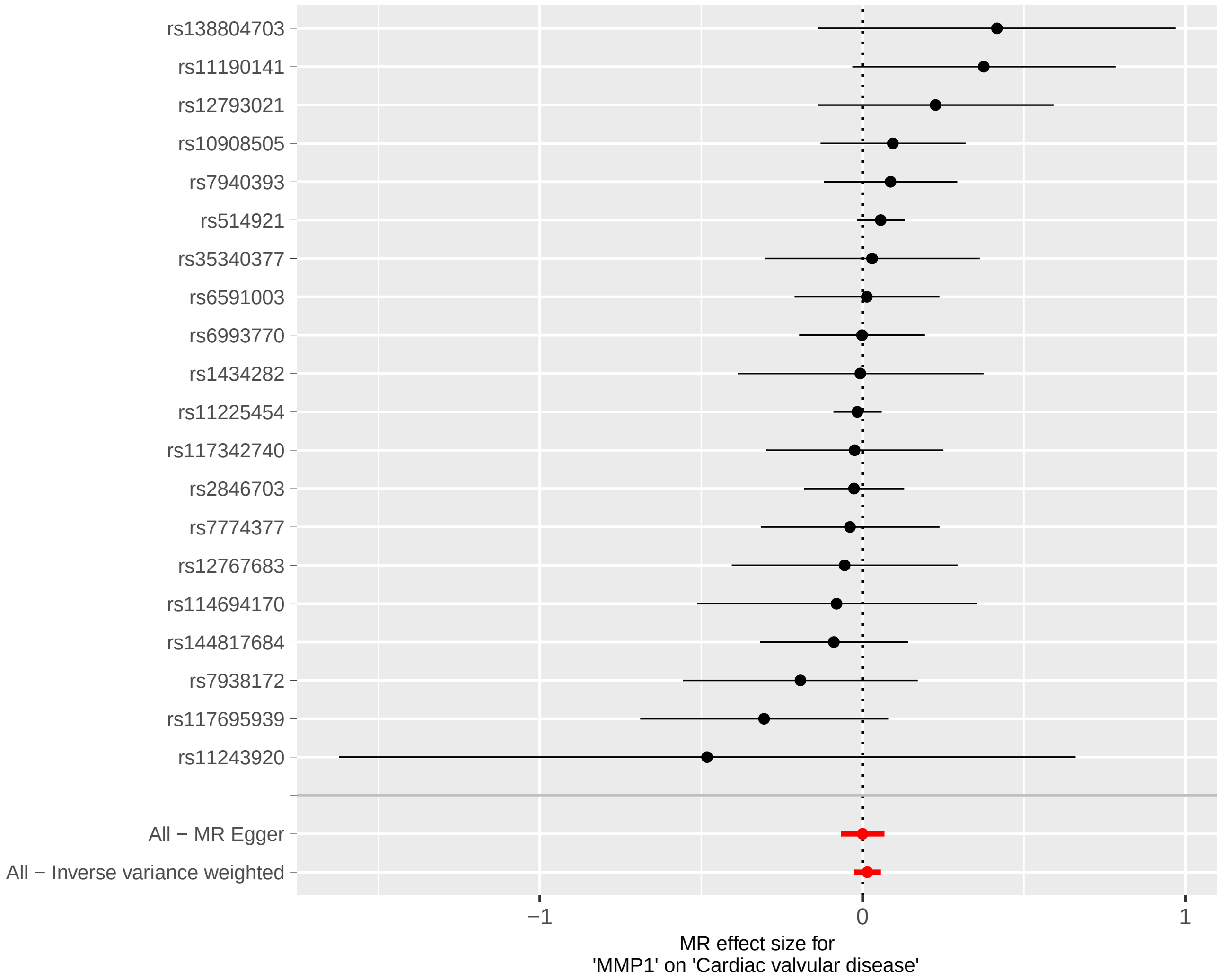
rs138804703
rs11190141
rs12793021
rs10908505
rs7940393
rs514921
rs35340377
rs6591003
rs6993770
rs1434282
rs11225454
rs117342740
rs2846703
rs7774377
rs12767683
rs114694170
rs144817684
rs7938172
rs117695939
rs11243920
All − MR Egger
All − Inverse variance weighted
−1
0
1
MR effect size for
'MMP1' on 'Cardiac valvular disease'

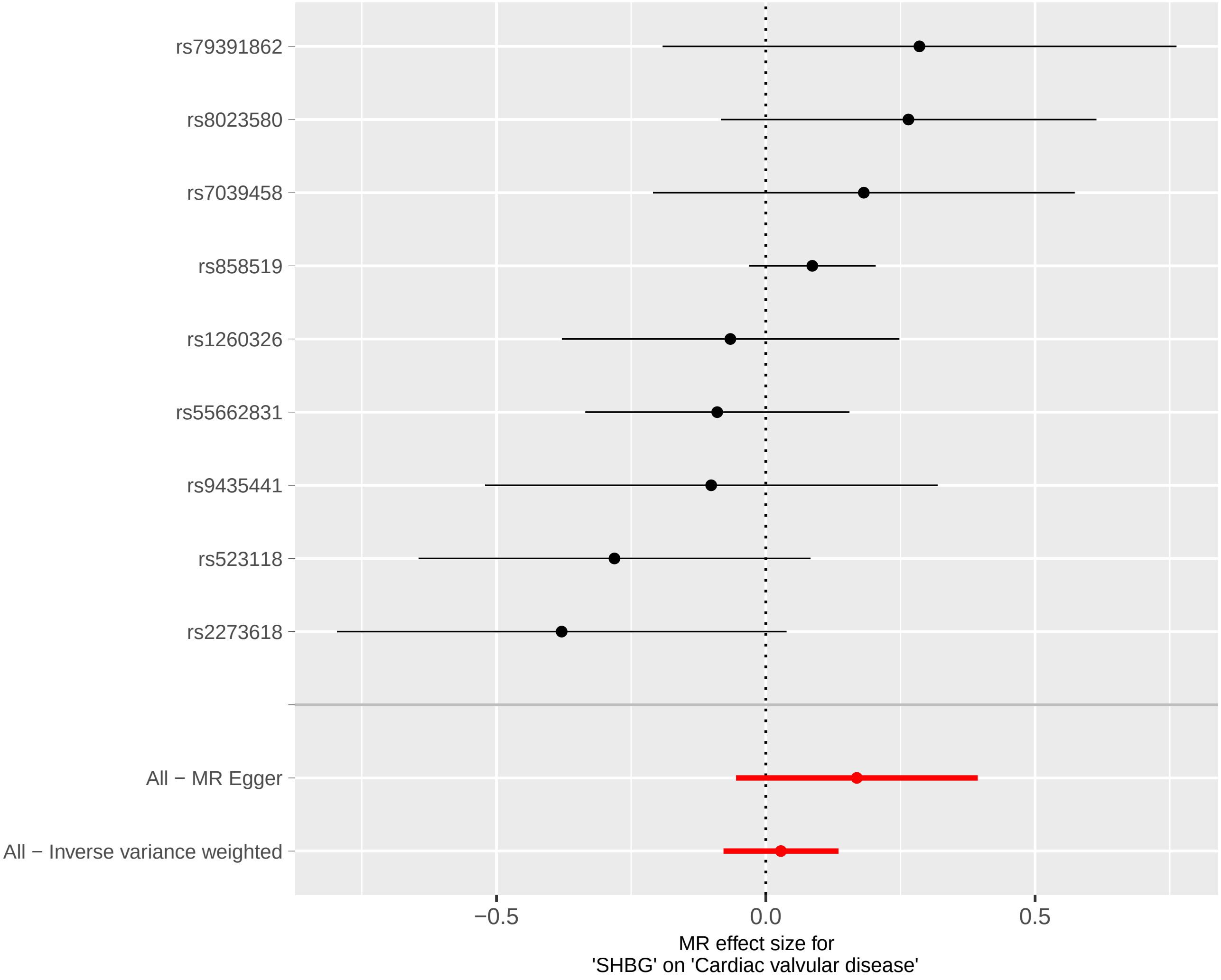

rs79391862
rs8023580
rs7039458
rs858519
rs1260326
rs55662831
rs9435441
rs523118
rs2273618
All − MR Egger
All − Inverse variance weighted
−0.5
0.0
0.5
MR effect size for
'SHBG' on 'Cardiac valvular disease'

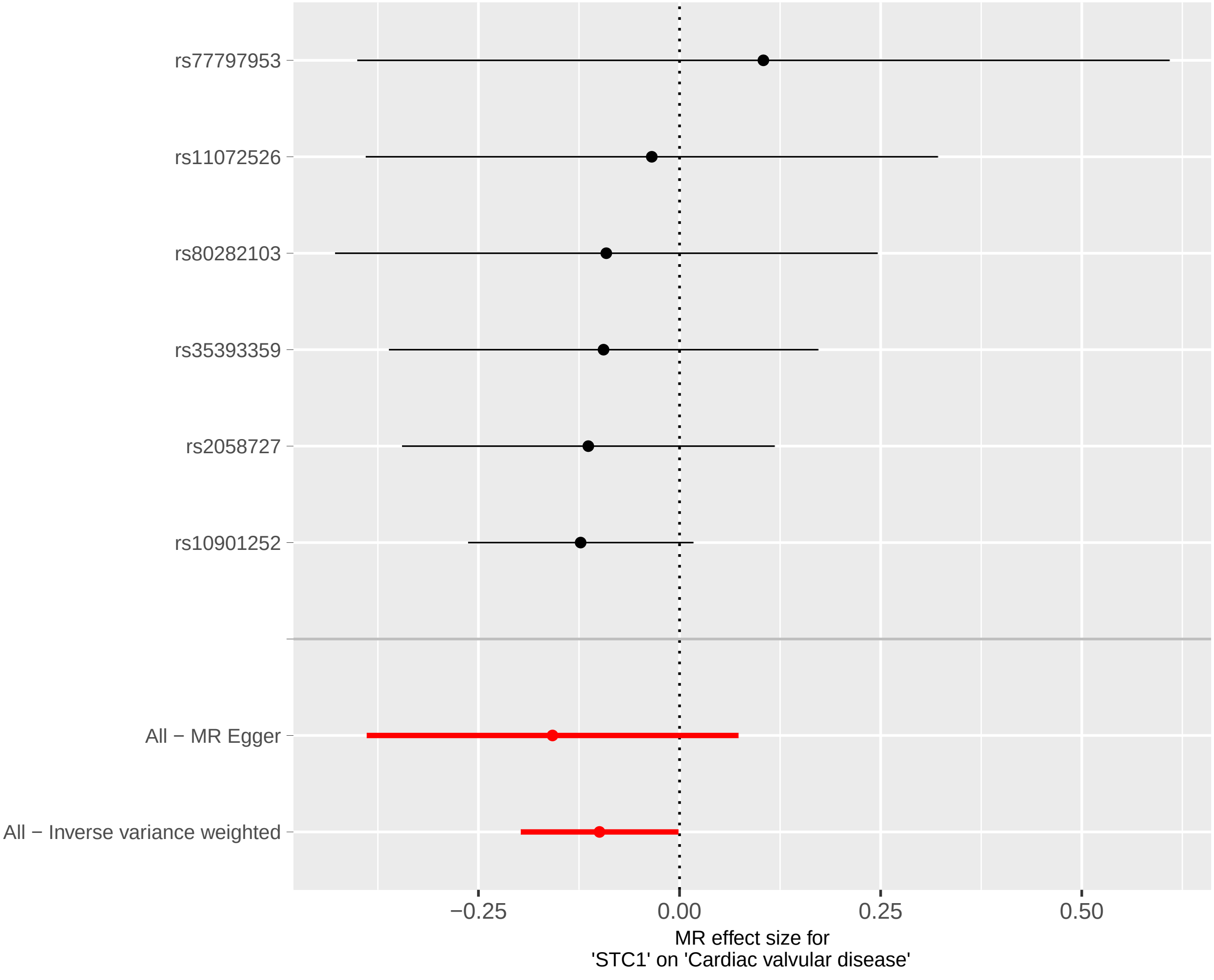
rs77797953
rs11072526
rs80282103
rs35393359
rs2058727
rs10901252
All − MR Egger
All − Inverse variance weighted
−0.25
0.00
0.25
0.50
MR effect size for
'STC1' on 'Cardiac valvular disease'

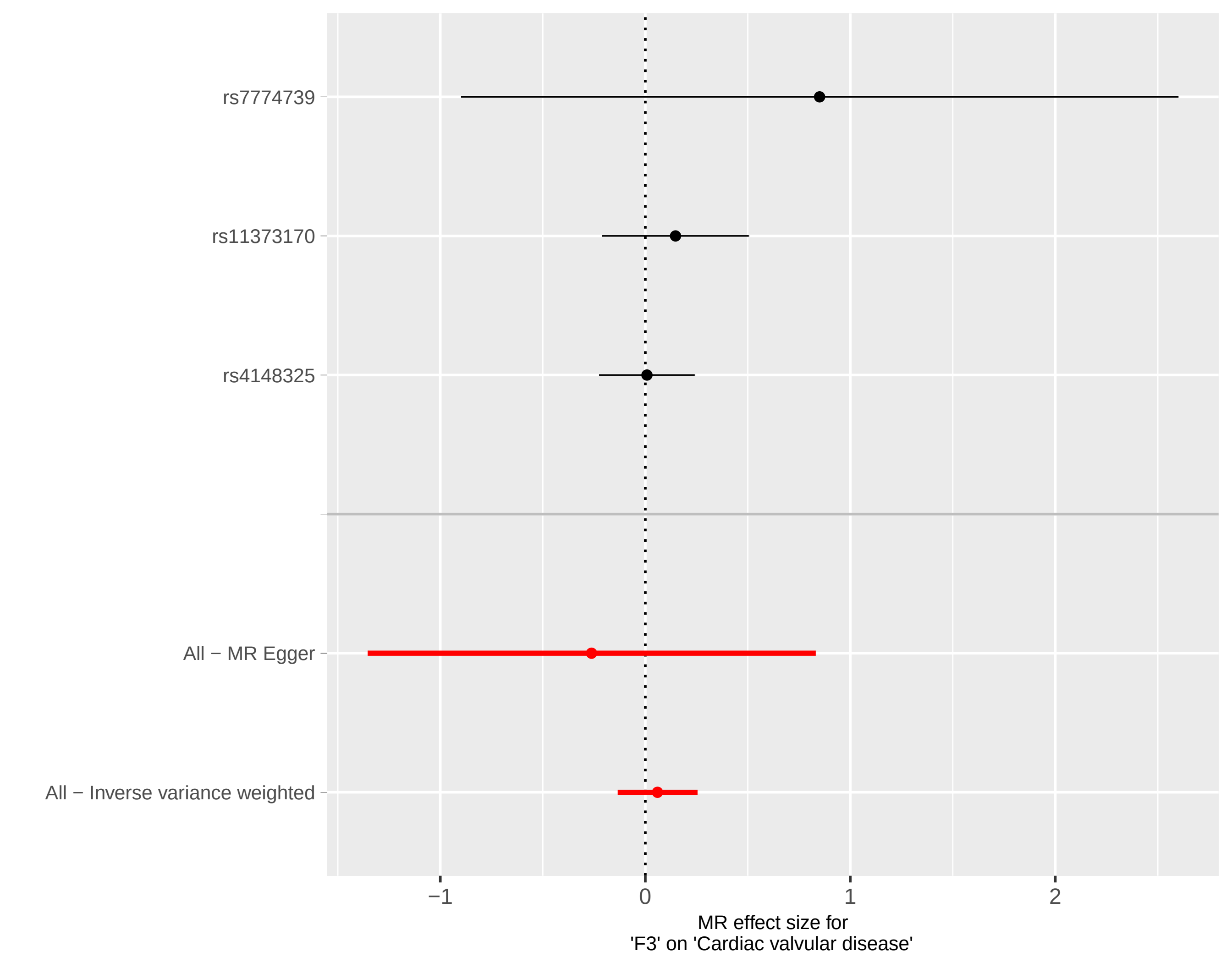

rs7774739
rs11373170
rs4148325
All − MR Egger
All − Inverse variance weighted
−1
0
1
2
MR effect size for
'F3' on 'Cardiac valvular disease'

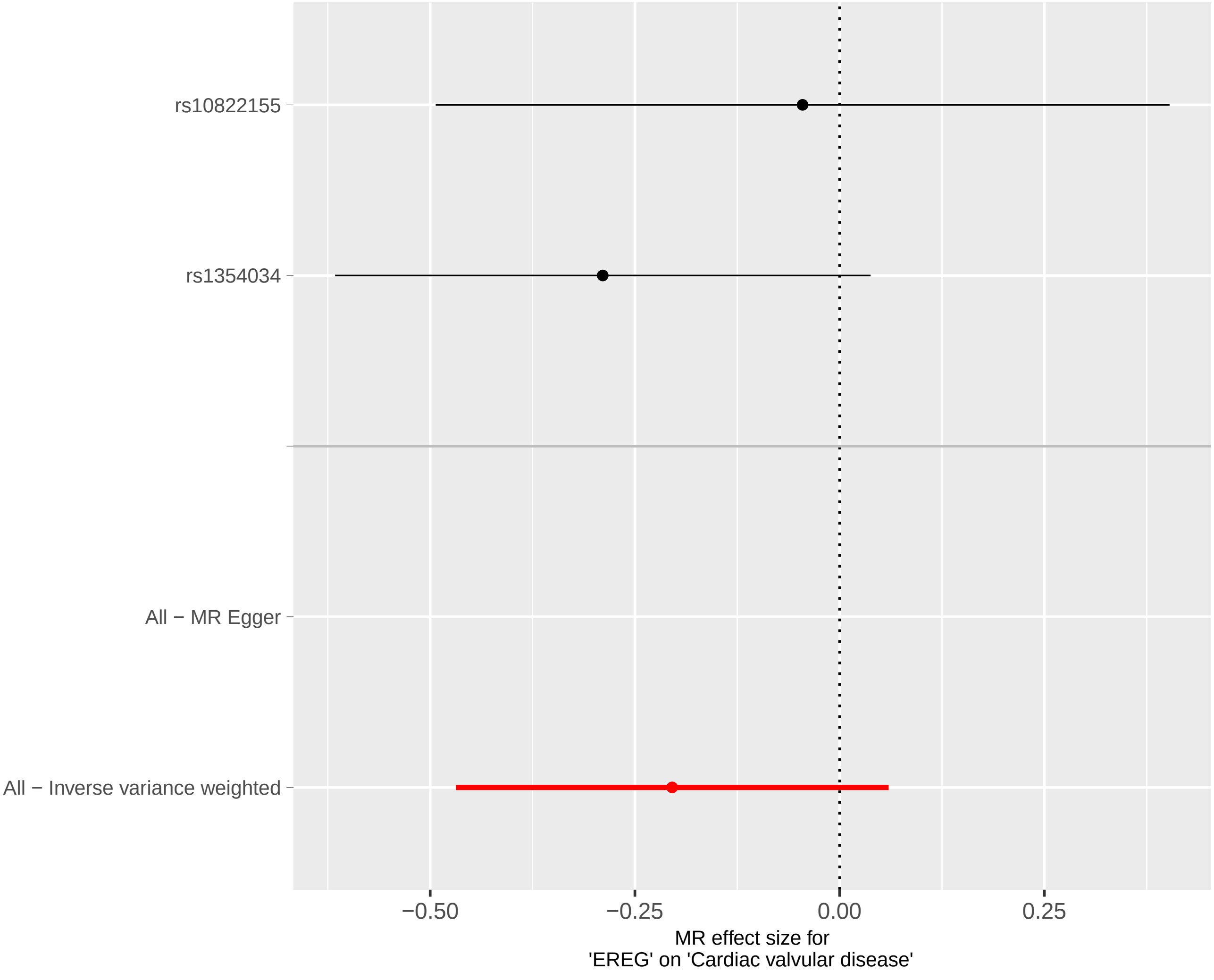

rs10822155
rs1354034
All − MR Egger
All − Inverse variance weighted
−0.50
−0.25
0.00
0.25
MR effect size for
'EREG' on 'Cardiac valvular disease'

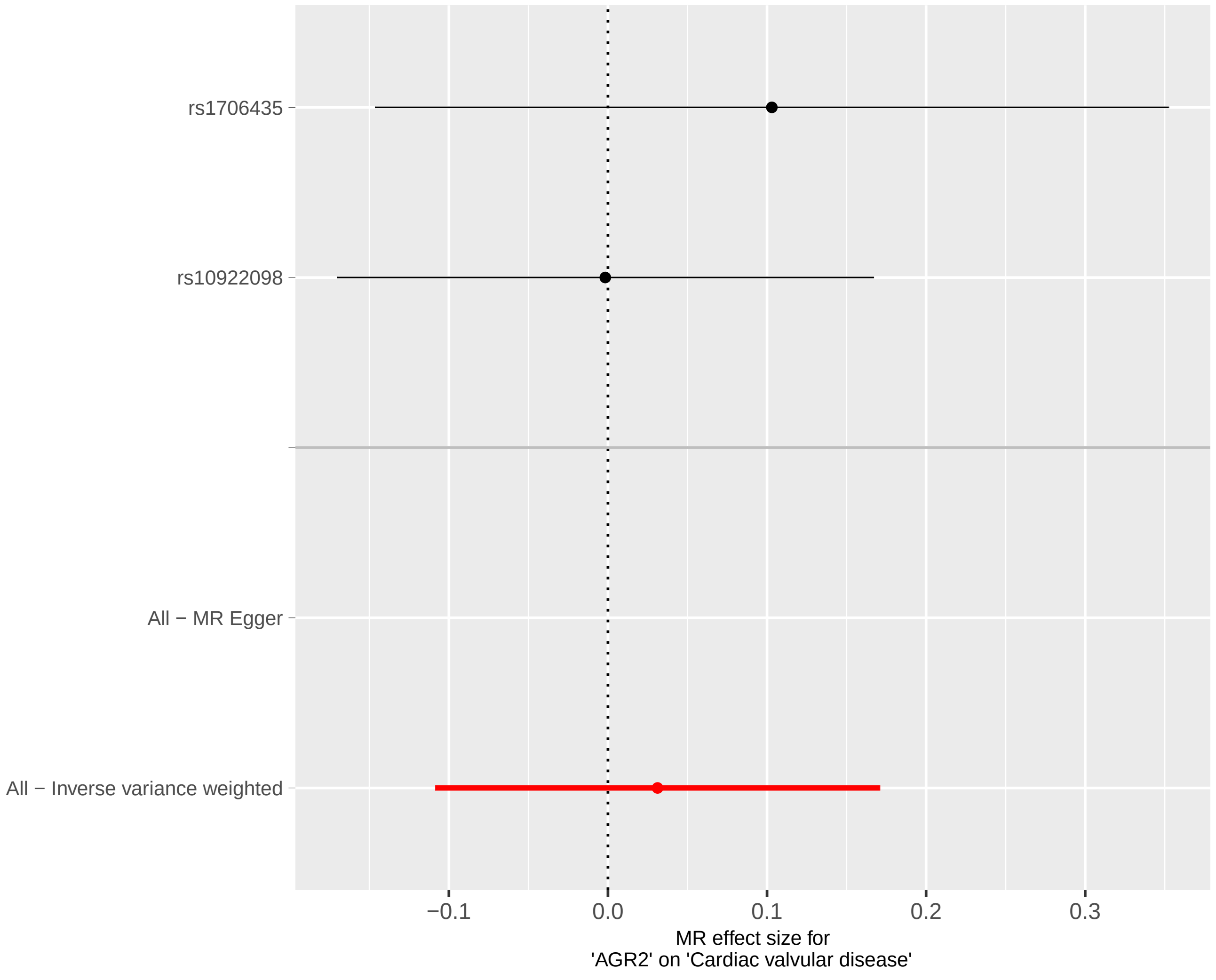
rs1706435
rs10922098
All − MR Egger
All − Inverse variance weighted
−0.1
0.0
0.1
0.2
0.3
MR effect size for
'AGR2' on 'Cardiac valvular disease'

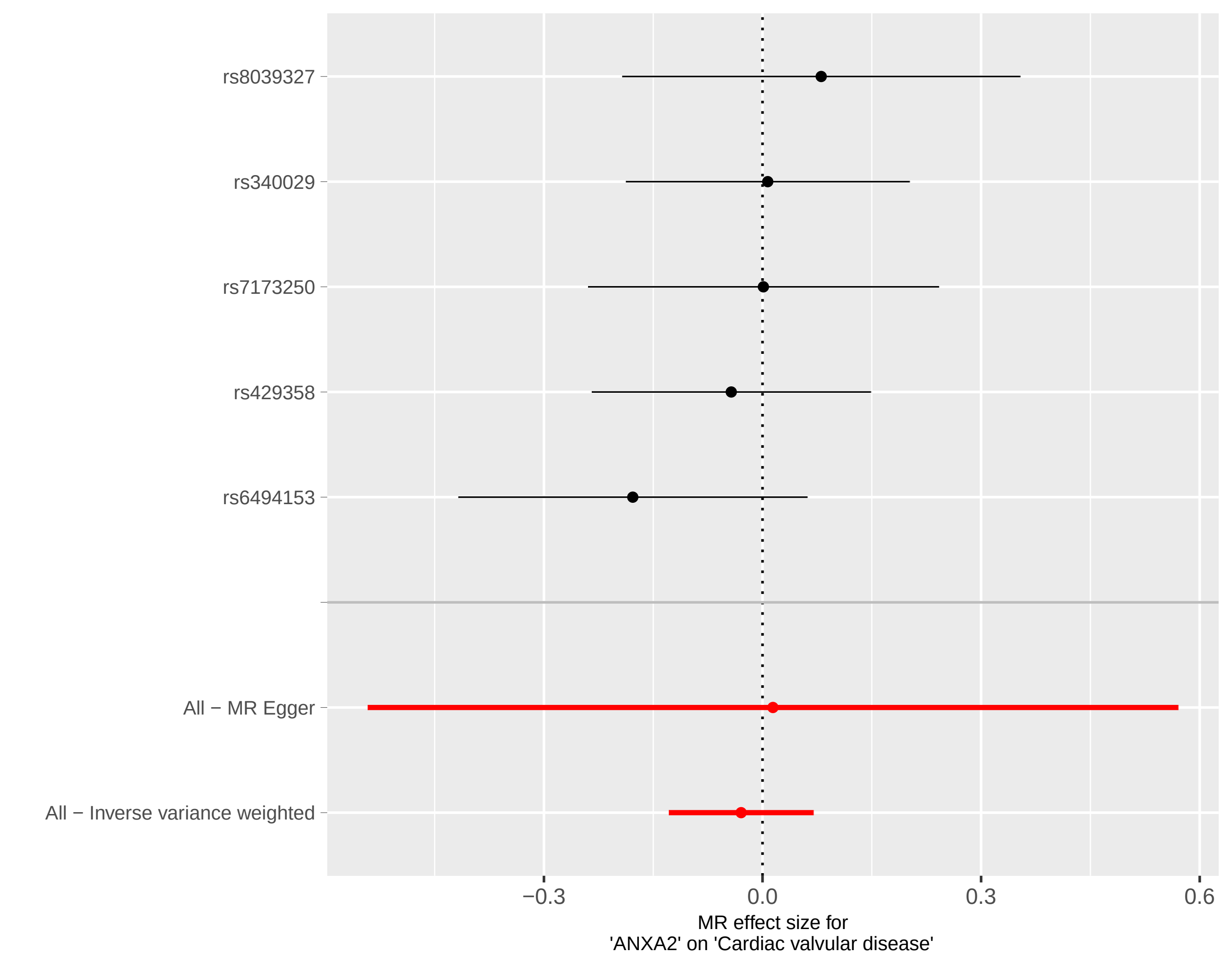
rs8039327
rs340029
rs7173250
rs429358
rs6494153
All − MR Egger
All − Inverse variance weighted
−0.3
0.0
0.3
0.6
MR effect size for
'ANXA2' on 'Cardiac valvular disease'

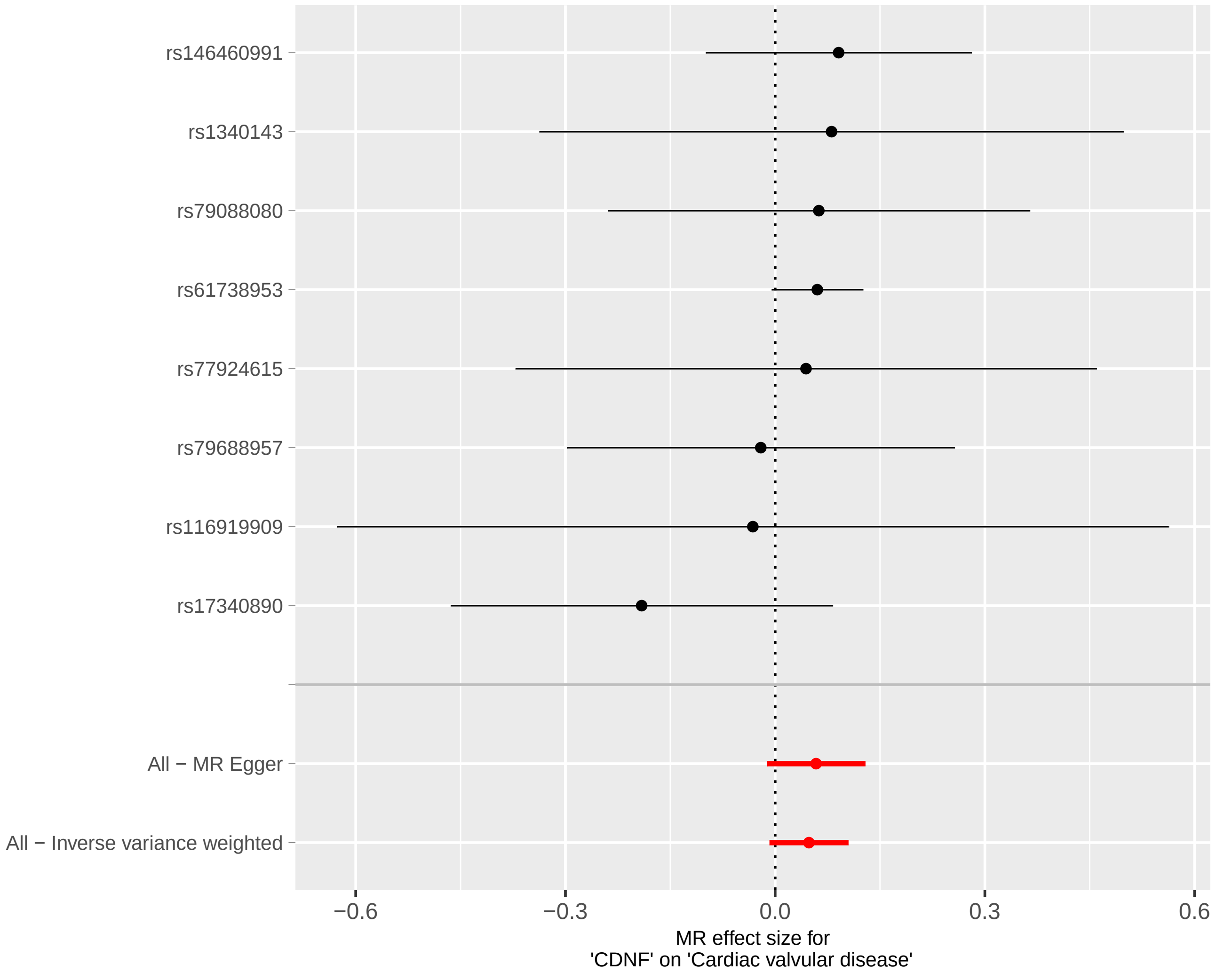
rs146460991
rs1340143
rs79088080
rs61738953
rs77924615
rs79688957
rs116919909
rs17340890
All − MR Egger
All − Inverse variance weighted
−0.6
−0.3
0.0
0.3
0.6
MR effect size for
'CDNF' on 'Cardiac valvular disease'

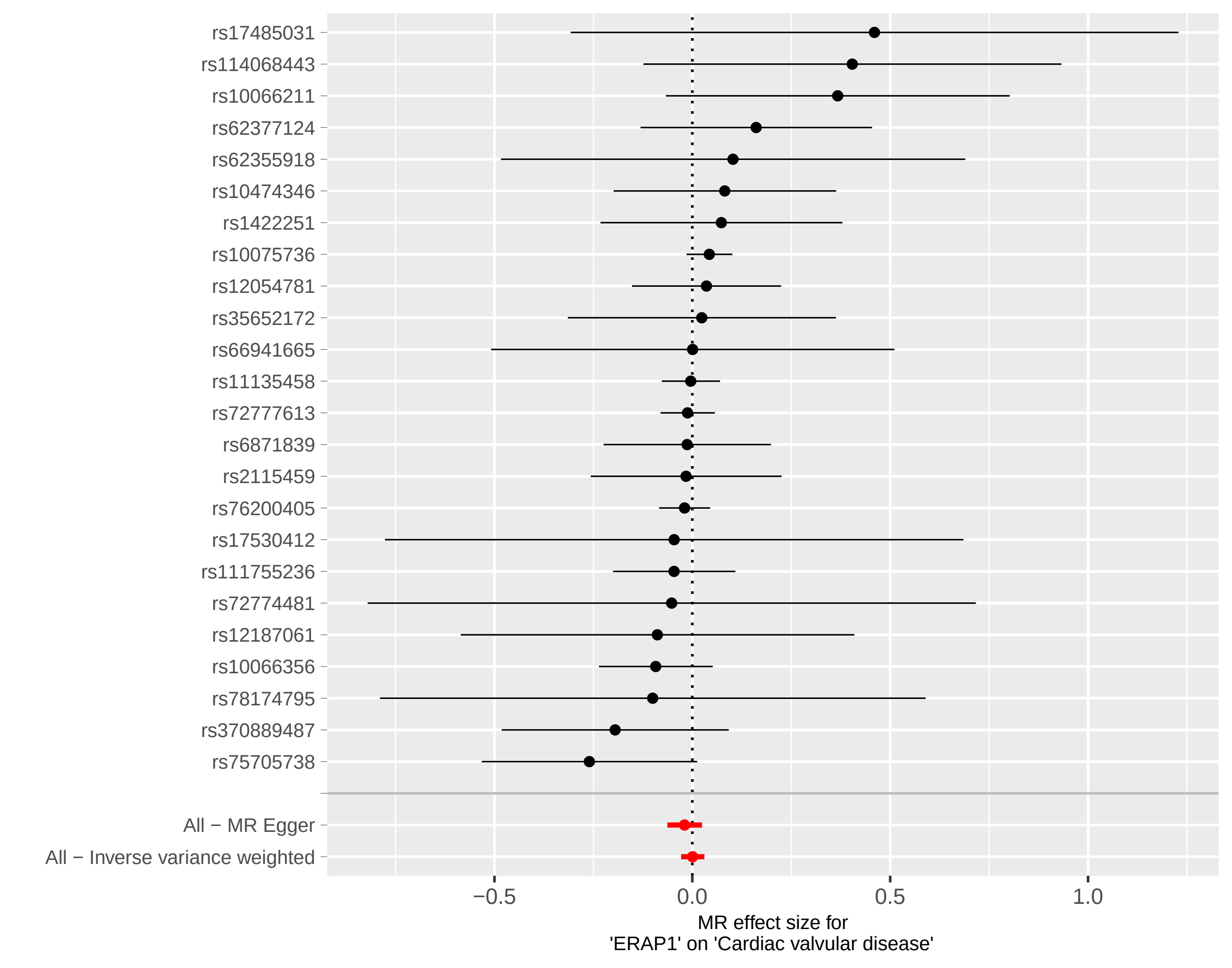
rs17485031
rs114068443
rs10066211
rs62377124
rs62355918
rs10474346
rs1422251
rs10075736
rs12054781
rs35652172
rs66941665
rs11135458
rs72777613
rs6871839
rs2115459
rs76200405
rs17530412
rs111755236
rs72774481
rs12187061
rs10066356
rs78174795
rs370889487
rs75705738
All − MR Egger
All − Inverse variance weighted
−0.5
0.0
0.5
1.0
MR effect size for
'ERAP1' on 'Cardiac valvular disease'

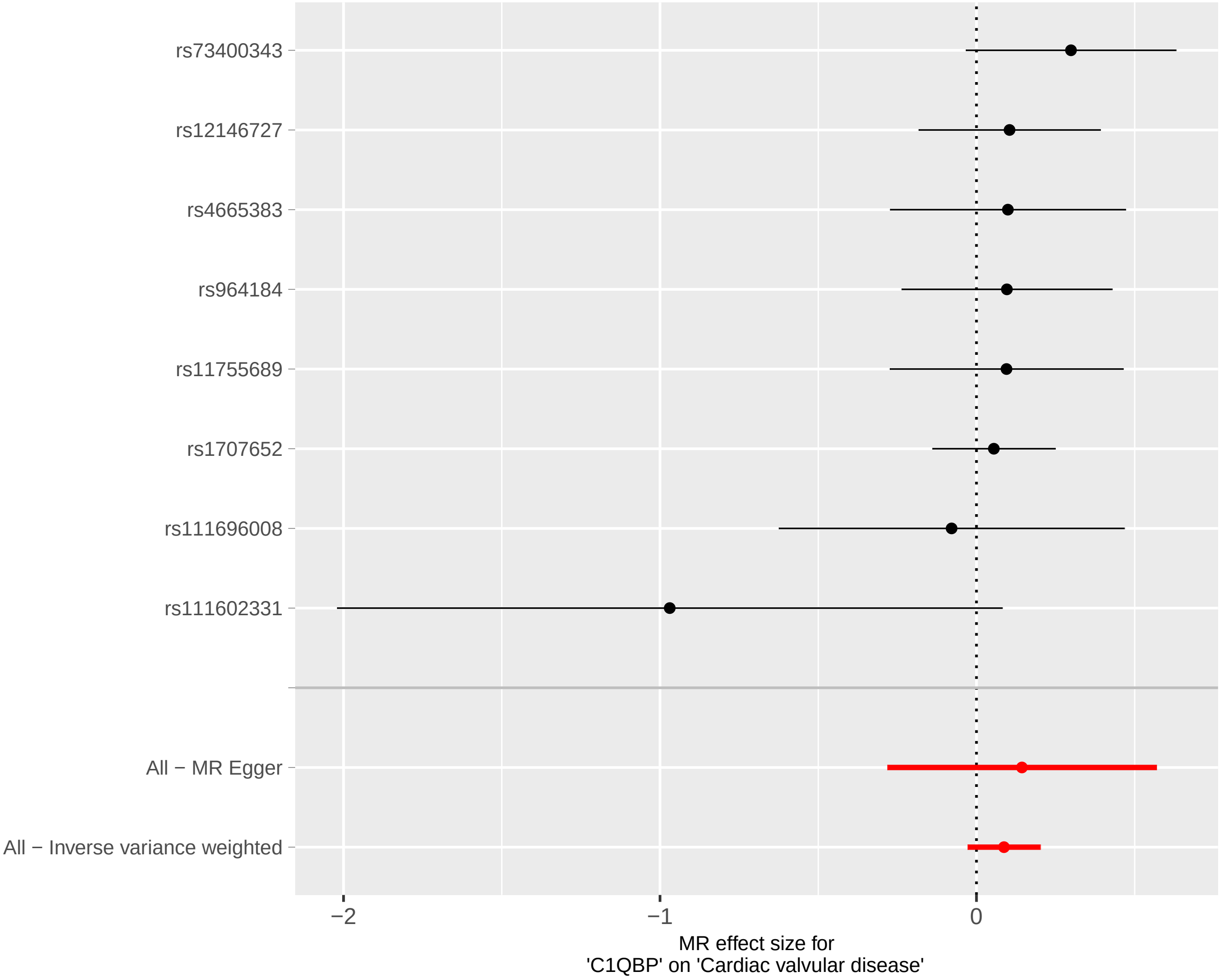
rs73400343
rs12146727
rs4665383
rs964184
rs11755689
rs1707652
rs111696008
rs111602331
All − MR Egger
All − Inverse variance weighted
−2
−1
0
MR effect size for
'C1QBP' on 'Cardiac valvular disease'

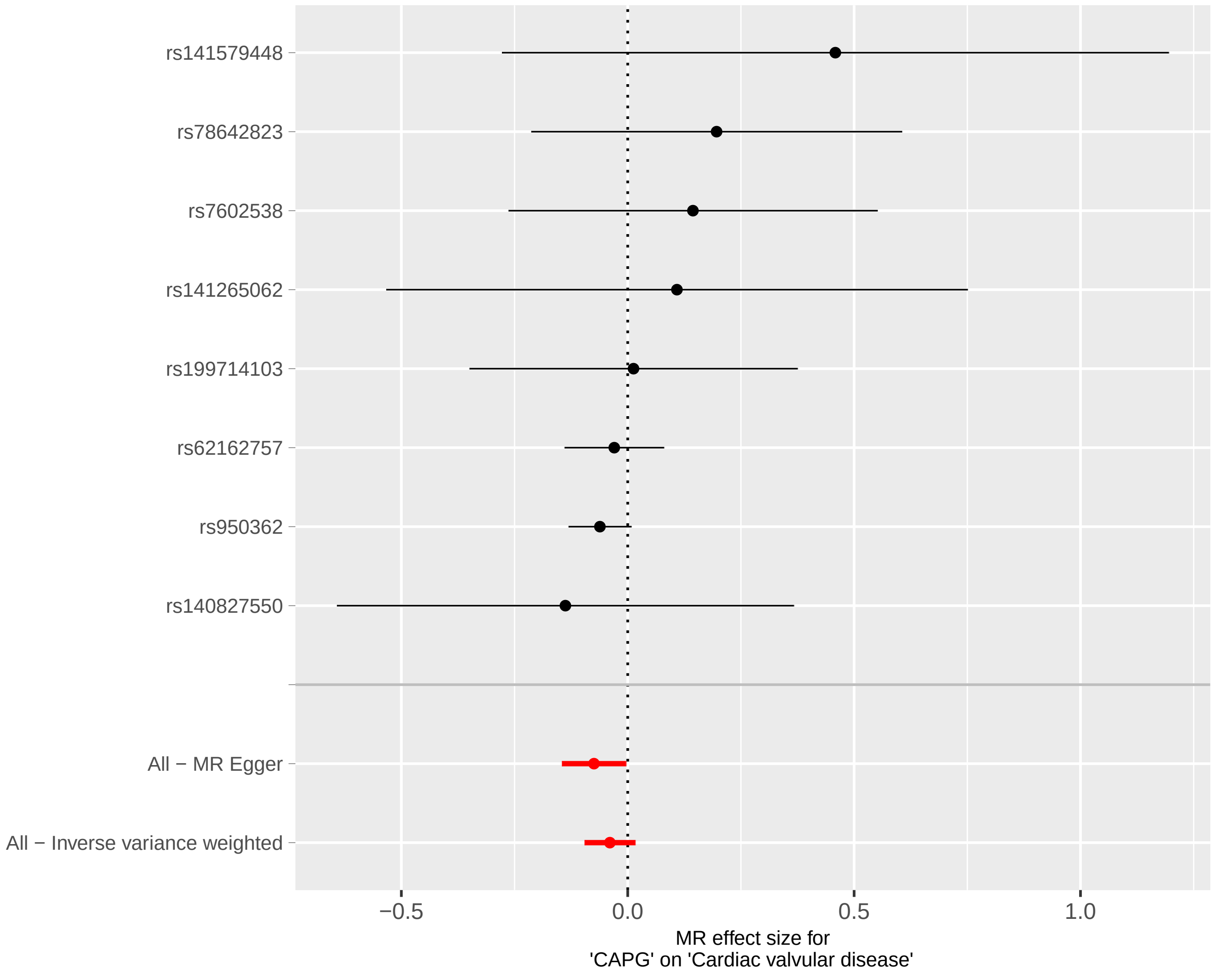
rs141579448
rs78642823
rs7602538
rs141265062
rs199714103
rs62162757
rs950362
rs140827550
All − MR Egger
All − Inverse variance weighted
−0.5
0.0
0.5
1.0
MR effect size for
'CAPG' on 'Cardiac valvular disease'

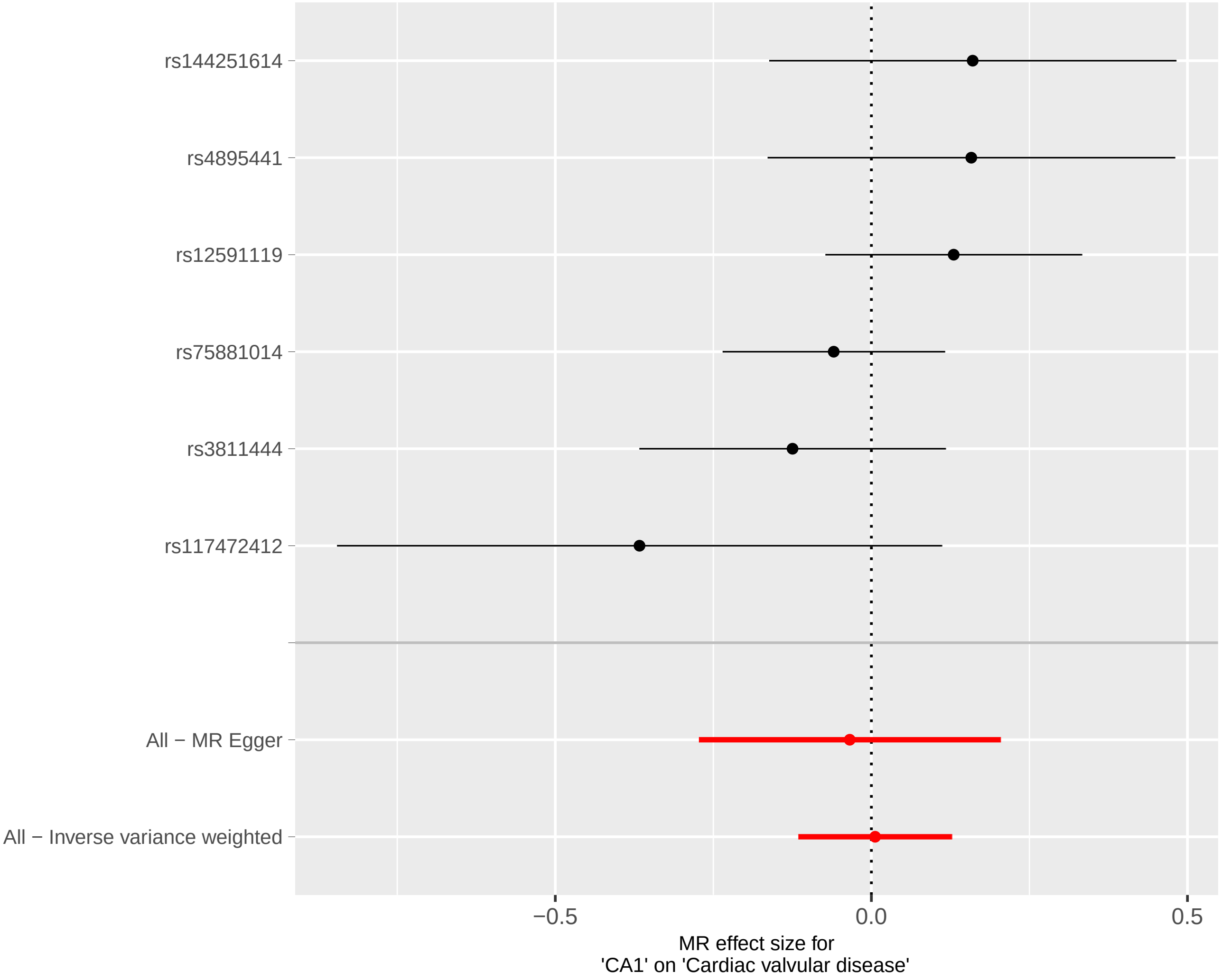
rs144251614
rs4895441
rs12591119
rs75881014
rs3811444
rs117472412
All − MR Egger
All − Inverse variance weighted
−0.5
0.0
0.5
MR effect size for
'CA1' on 'Cardiac valvular disease'

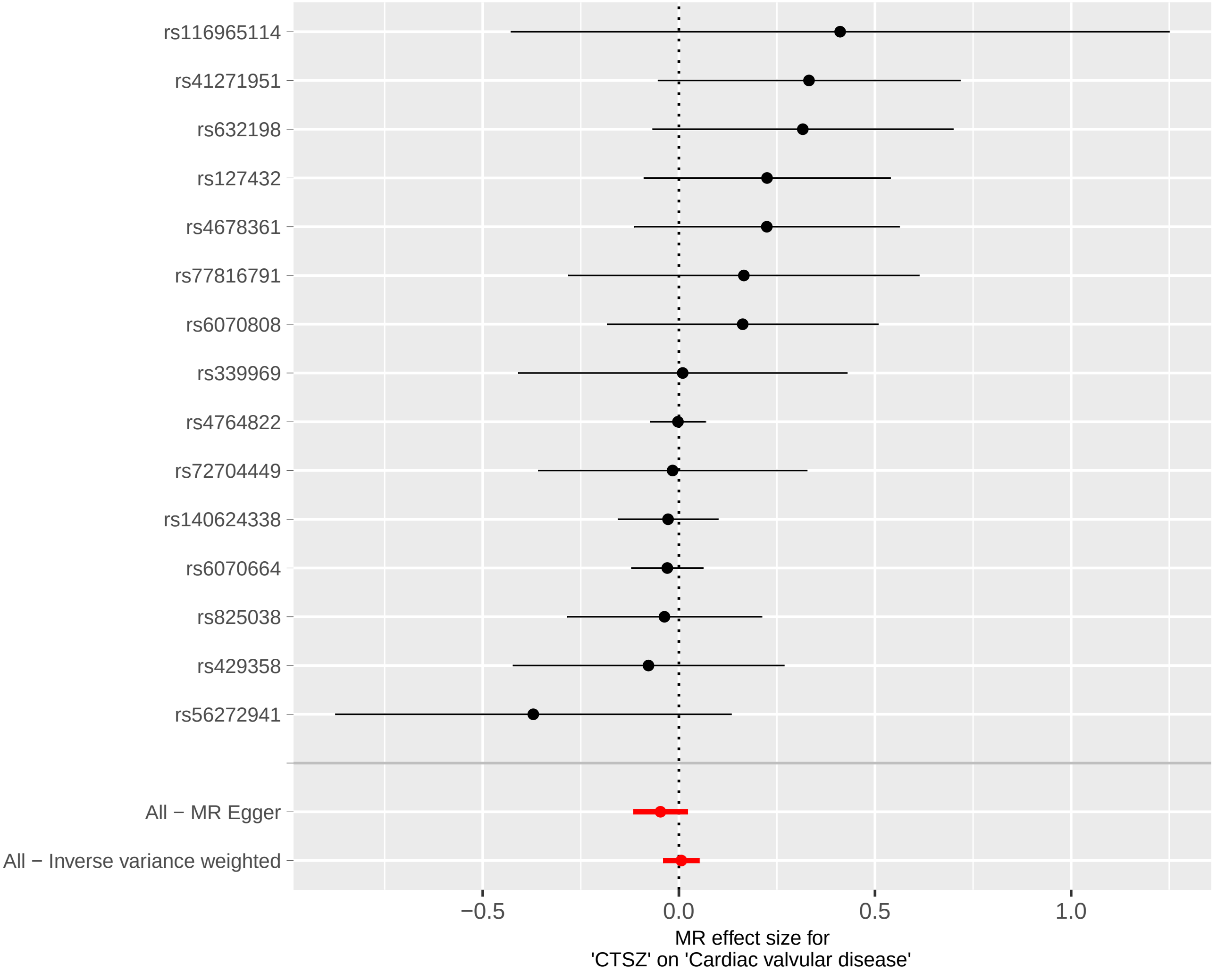
rs116965114
rs41271951
rs632198
rs127432
rs4678361
rs77816791
rs6070808
rs339969
rs4764822
rs72704449
rs140624338
rs6070664
rs825038
rs429358
rs56272941
All − MR Egger
All − Inverse variance weighted
−0.5
0.0
0.5
1.0
MR effect size for
'CTSZ' on 'Cardiac valvular disease'

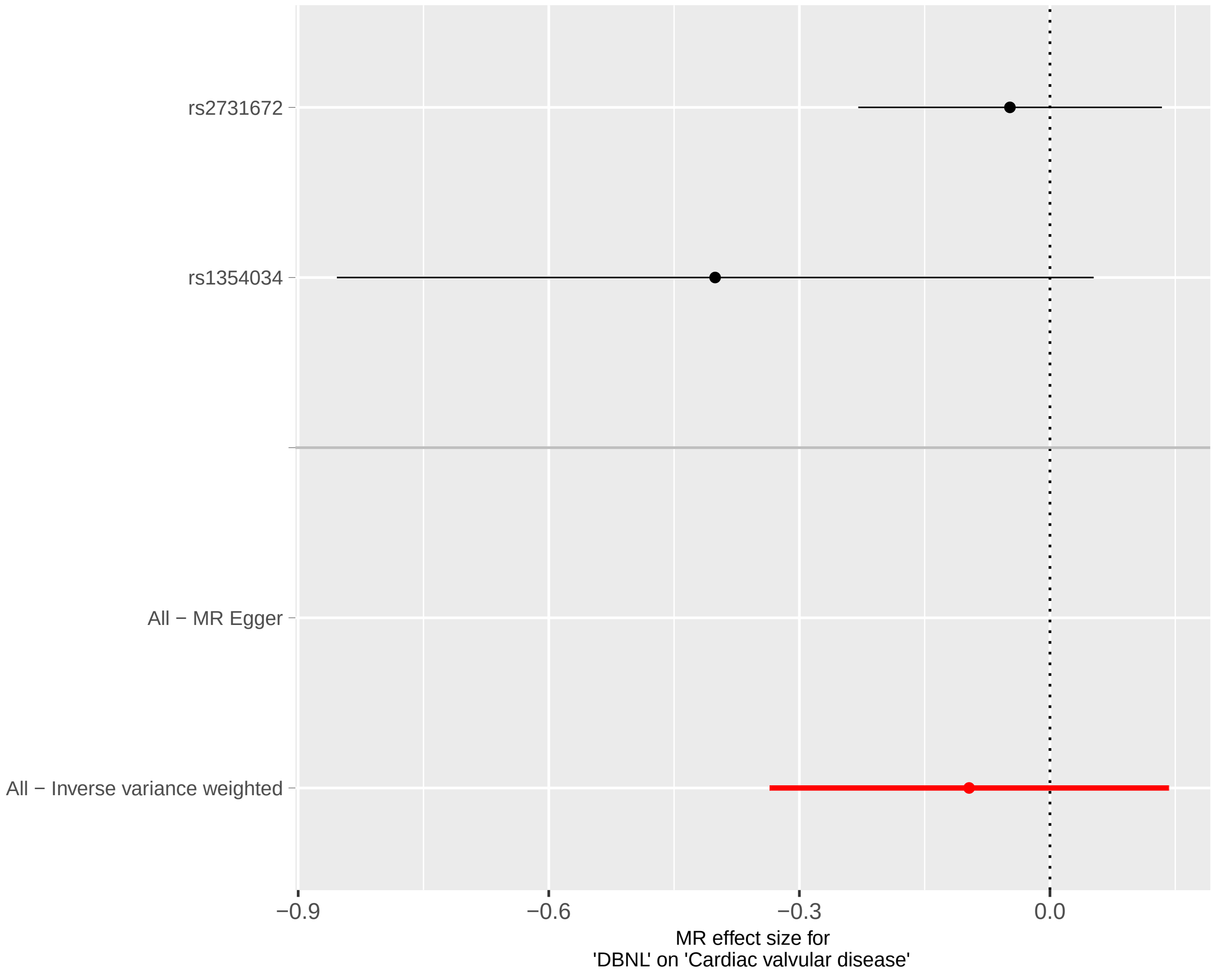

rs2731672
rs1354034
All − MR Egger
All − Inverse variance weighted
−0.9
−0.6
−0.3
0.0
MR effect size for
'DBNL' on 'Cardiac valvular disease'

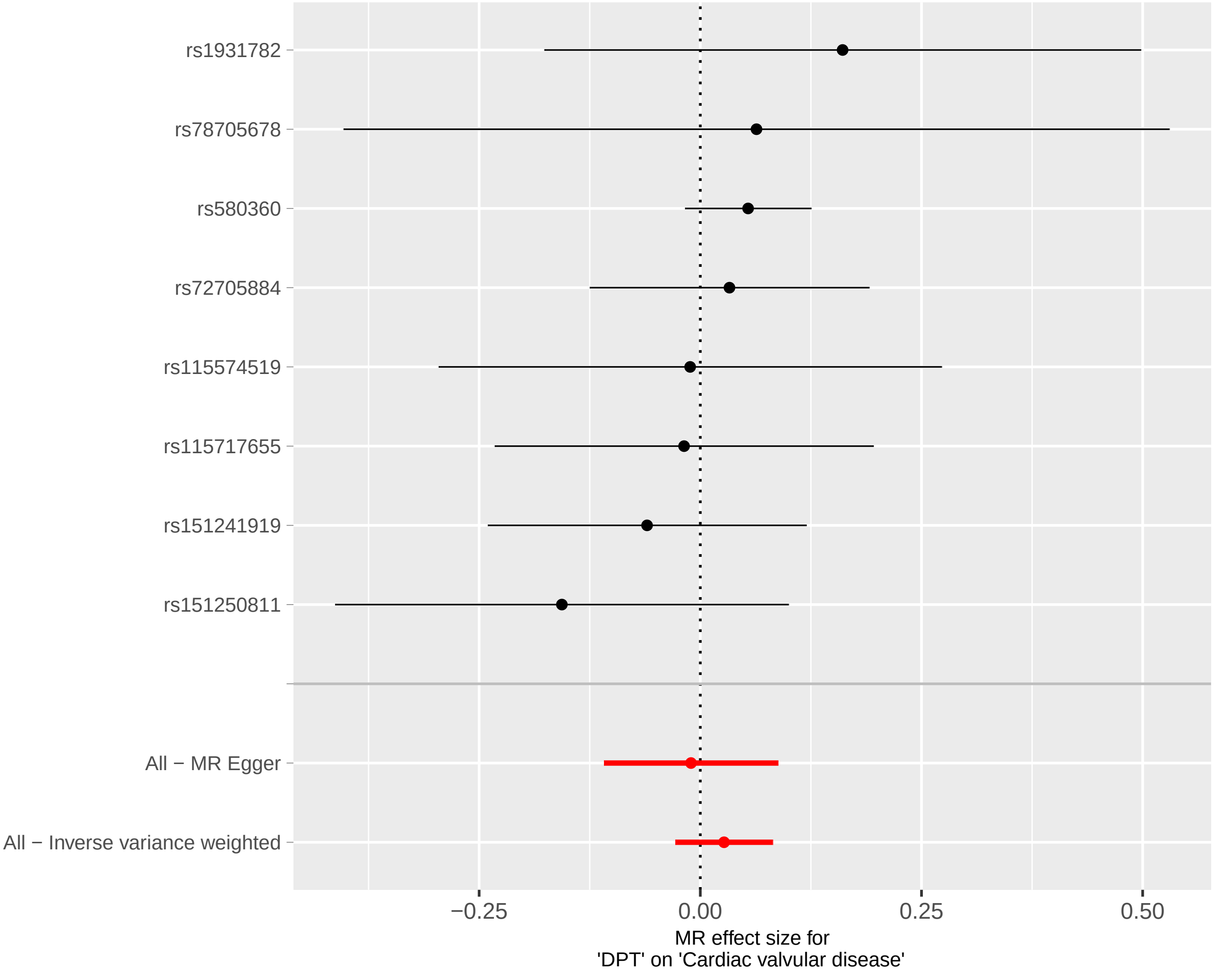

rs1931782
rs78705678
rs580360
rs72705884
rs115574519
rs115717655
rs151241919
rs151250811
All − MR Egger
All − Inverse variance weighted
−0.25
0.00
0.25
0.50
MR effect size for
'DPT' on 'Cardiac valvular disease'

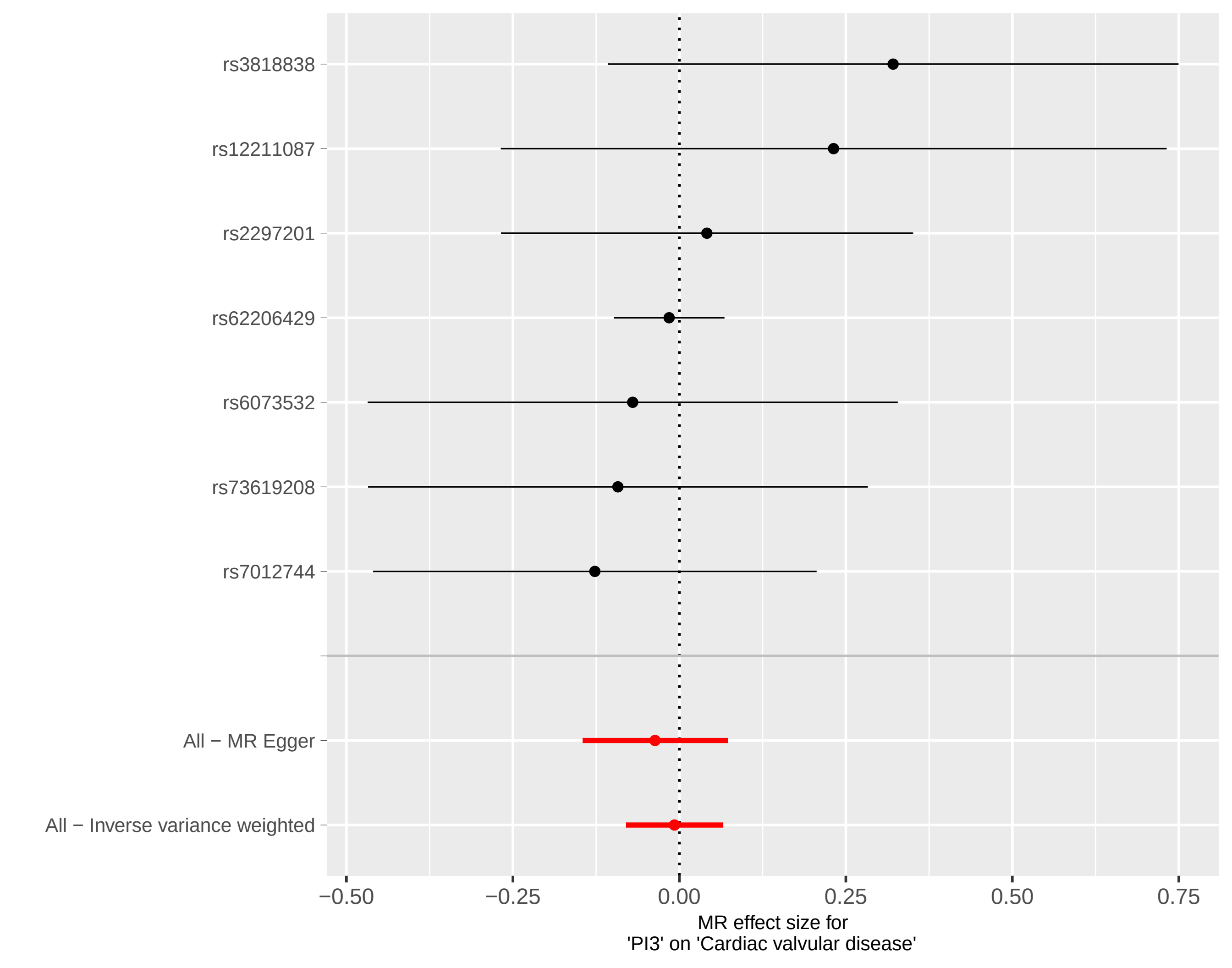
rs3818838
rs12211087
rs2297201
rs62206429
rs6073532
rs73619208
rs7012744
All − MR Egger
All − Inverse variance weighted
−0.50
−0.25
0.00
0.25
0.50
0.75
MR effect size for
'PI3' on 'Cardiac valvular disease'

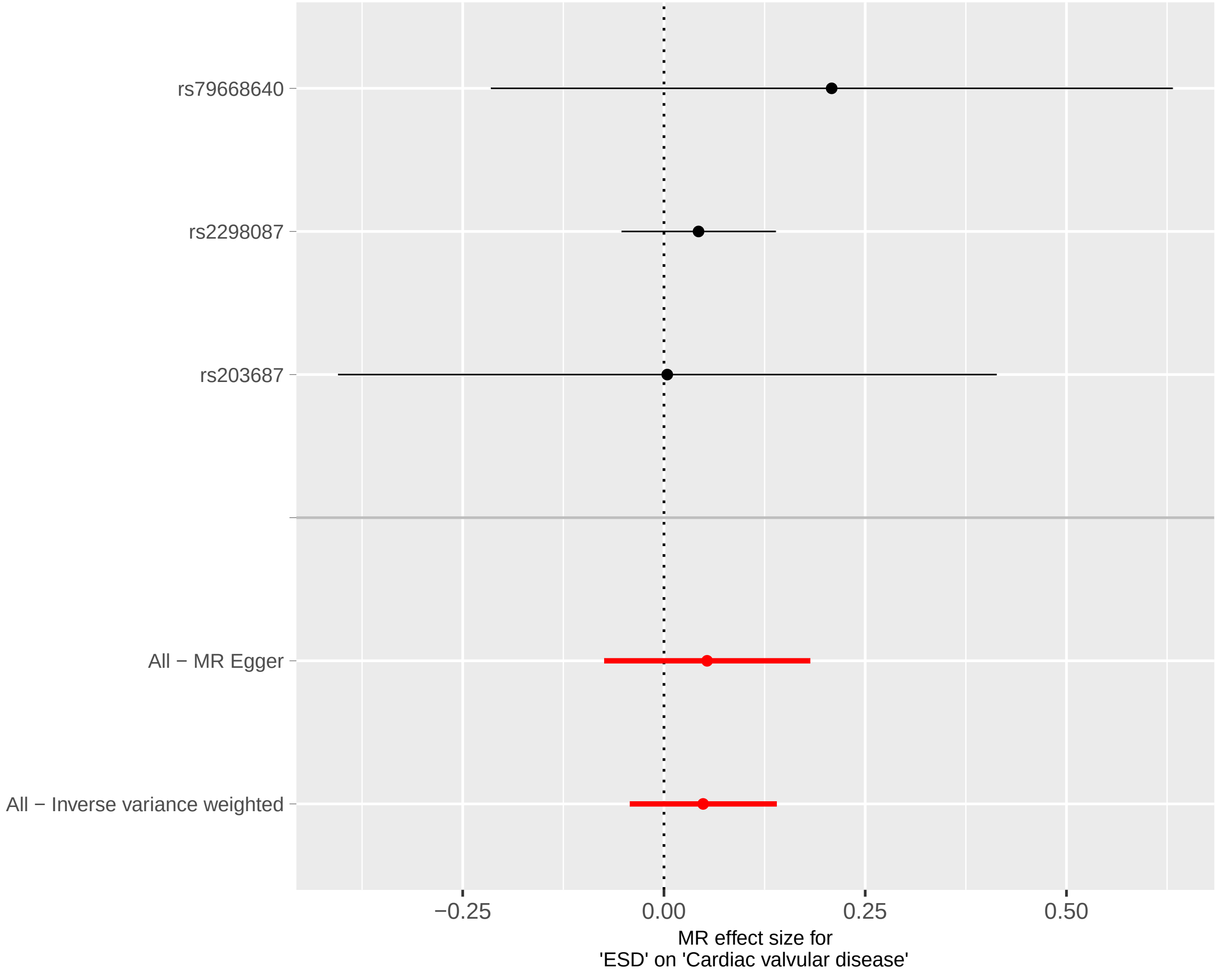
rs79668640
rs2298087
rs203687
All − MR Egger
All − Inverse variance weighted
−0.25
0.00
0.25
0.50
MR effect size for
'ESD' on 'Cardiac valvular disease'

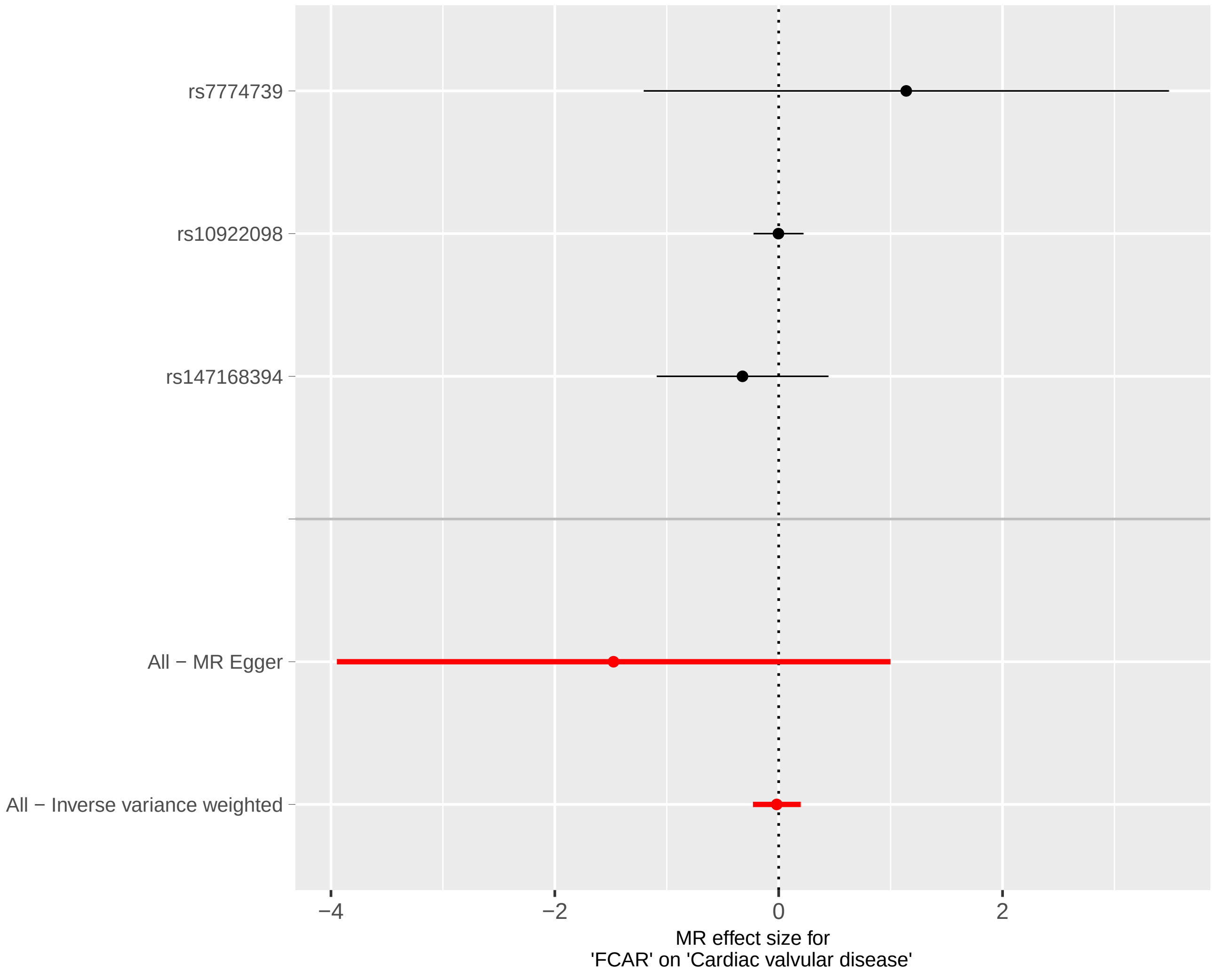
rs7774739
rs10922098
rs147168394
All − MR Egger
All − Inverse variance weighted
−4
−2
0
2
MR effect size for
'FCAR' on 'Cardiac valvular disease'

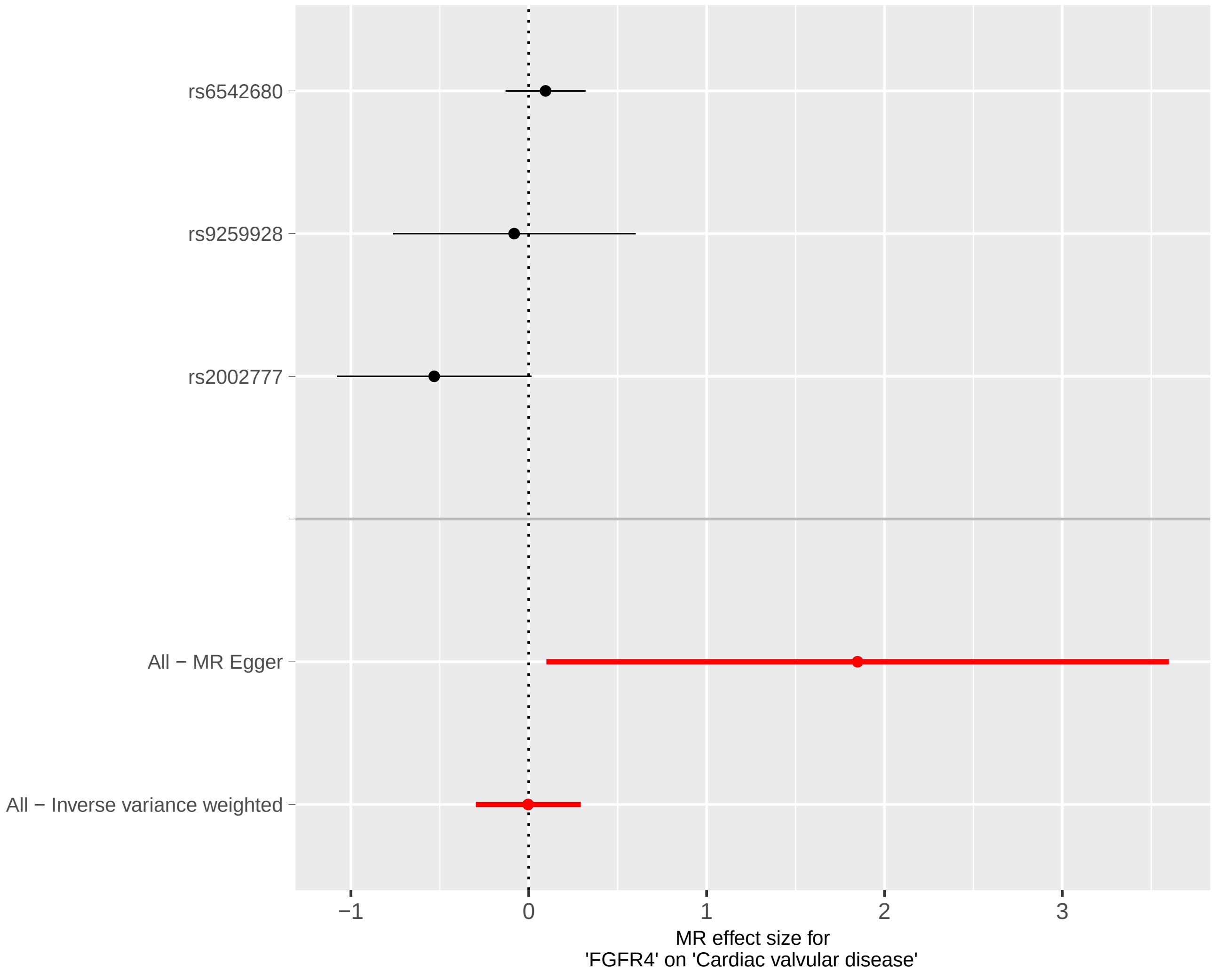
rs6542680
rs9259928
rs2002777
All − MR Egger
All − Inverse variance weighted
−1
0
1
2
3
MR effect size for
'FGFR4' on 'Cardiac valvular disease'

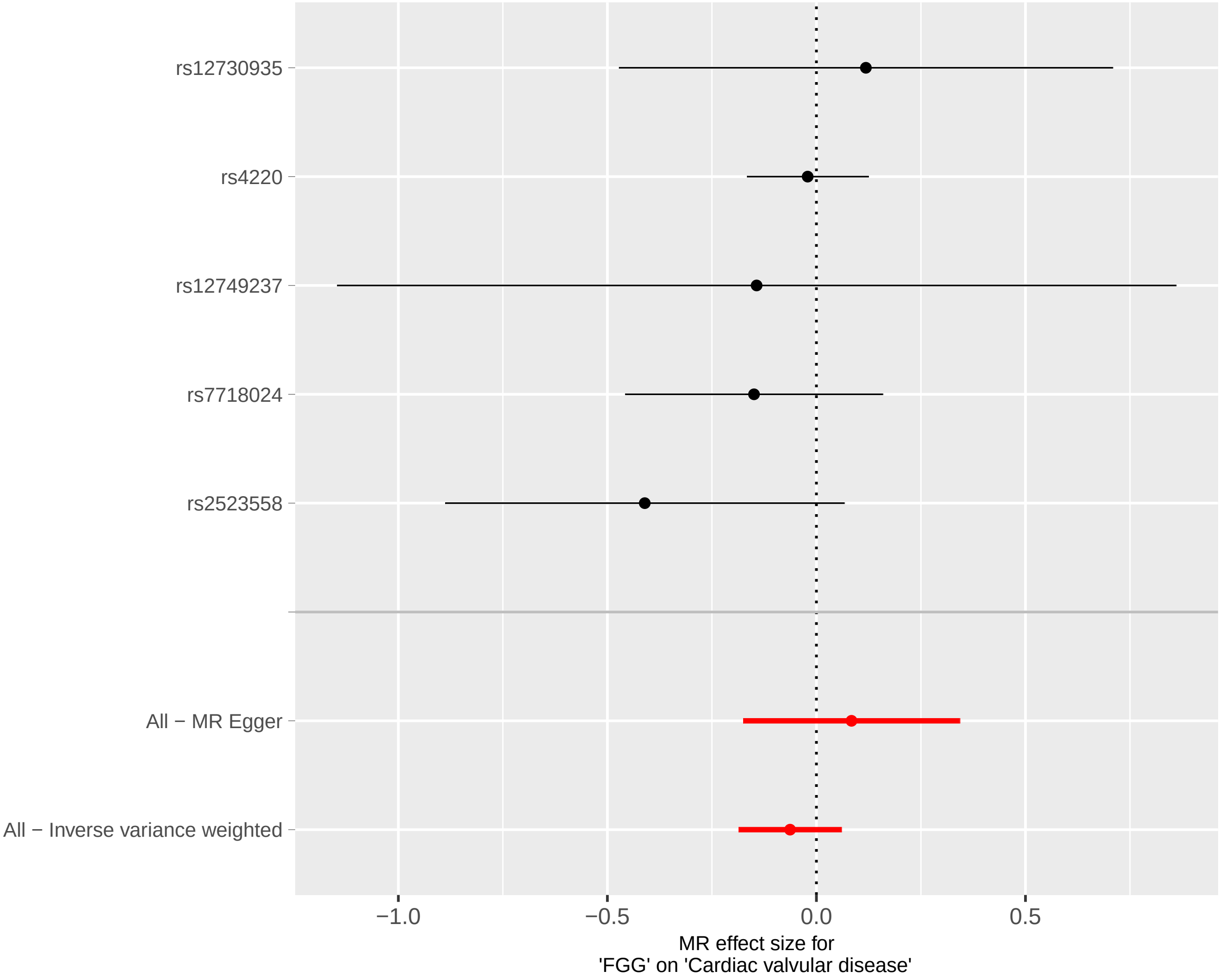
rs12730935
rs4220
rs12749237
rs7718024
rs2523558
All − MR Egger
All − Inverse variance weighted
−1.0
−0.5
0.0
0.5
MR effect size for
'FGG' on 'Cardiac valvular disease'

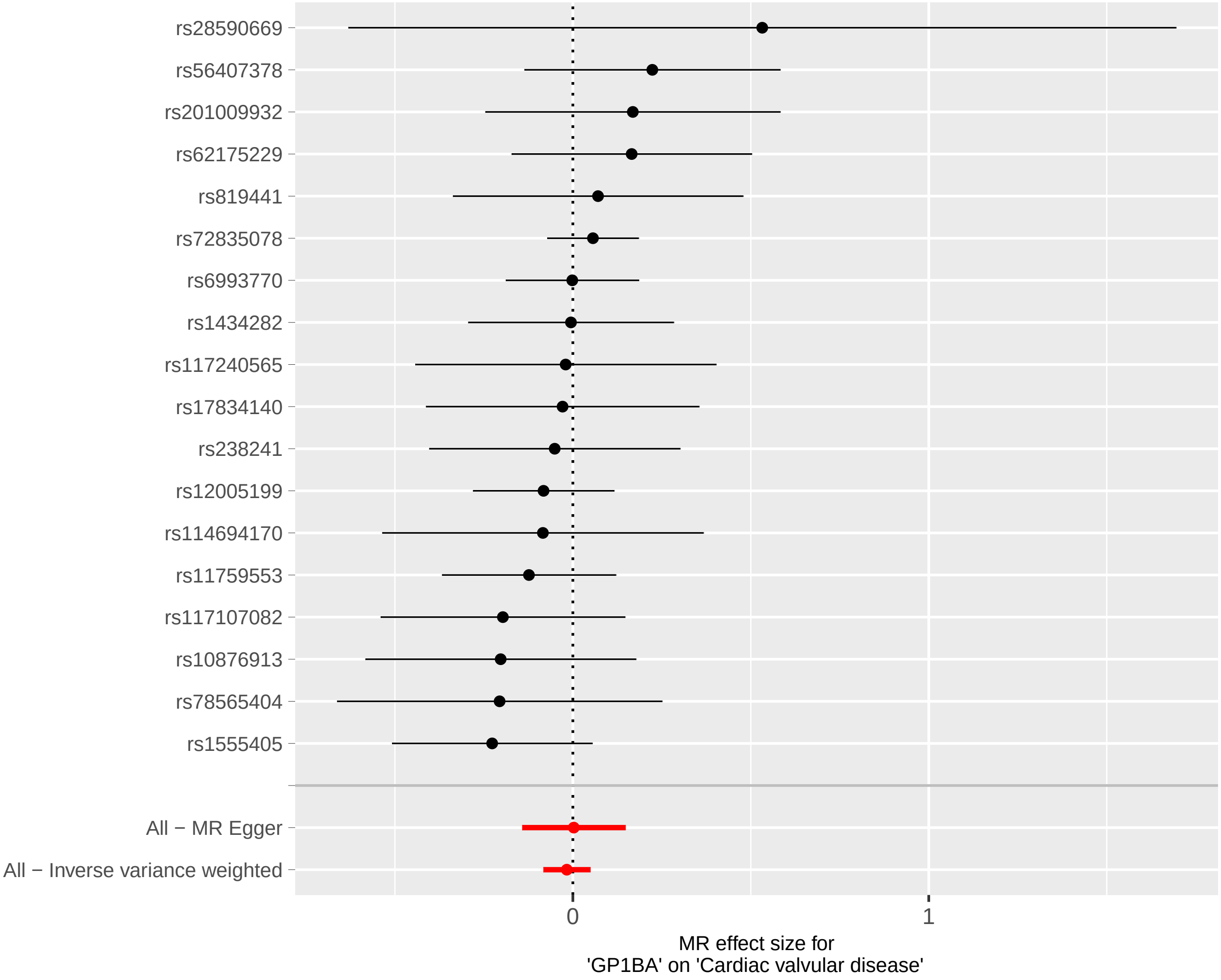
rs28590669
rs56407378
rs201009932
rs62175229
rs819441
rs72835078
rs6993770
rs1434282
rs117240565
rs17834140
rs238241
rs12005199
rs114694170
rs11759553
rs117107082
rs10876913
rs78565404
rs1555405
All − MR Egger
All − Inverse variance weighted
0
1
MR effect size for
'GP1BA' on 'Cardiac valvular disease'

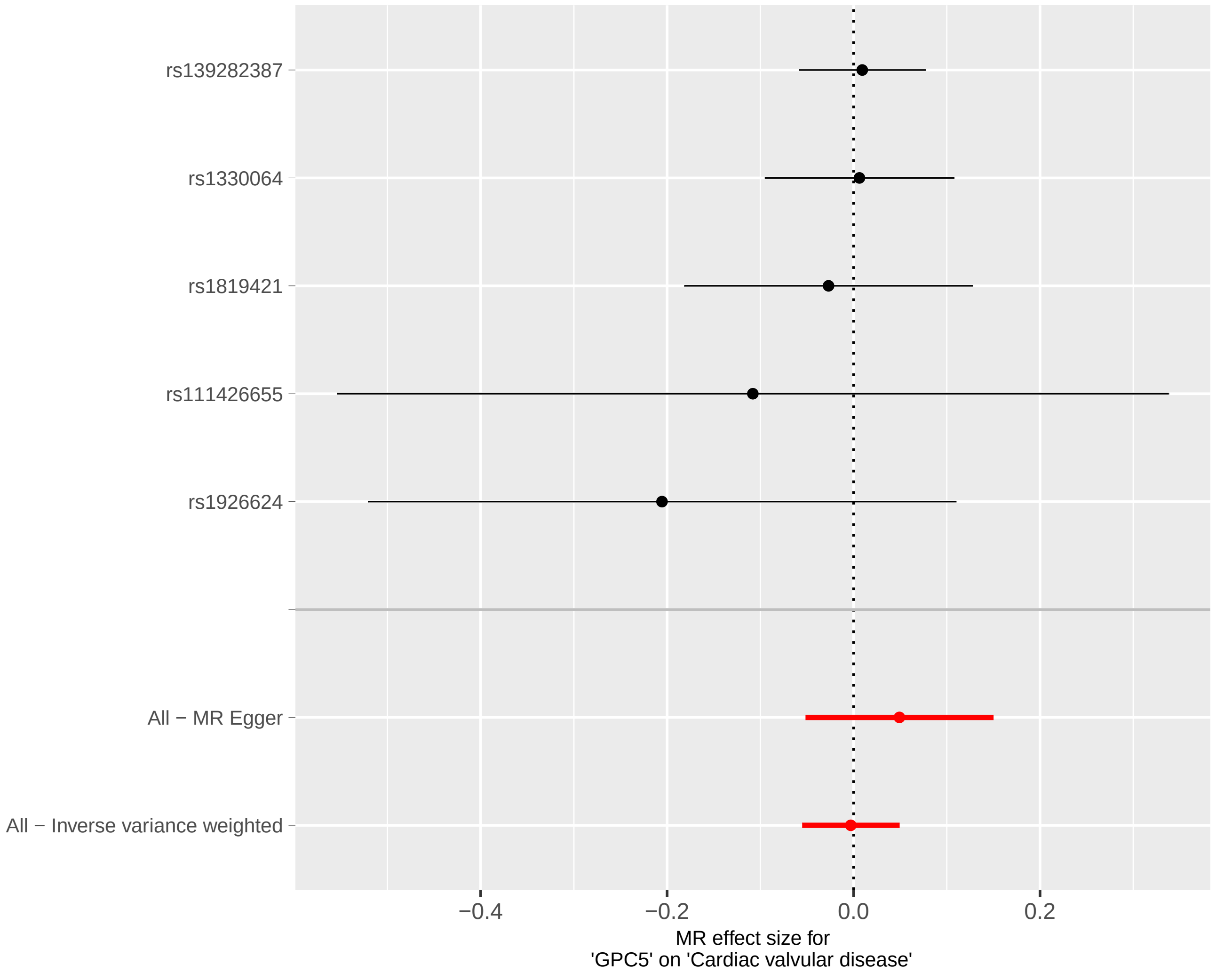

rs139282387
rs1330064
rs1819421
rs111426655
rs1926624
All − MR Egger
All − Inverse variance weighted
−0.4
−0.2
0.0
0.2
MR effect size for
'GPC5' on 'Cardiac valvular disease'

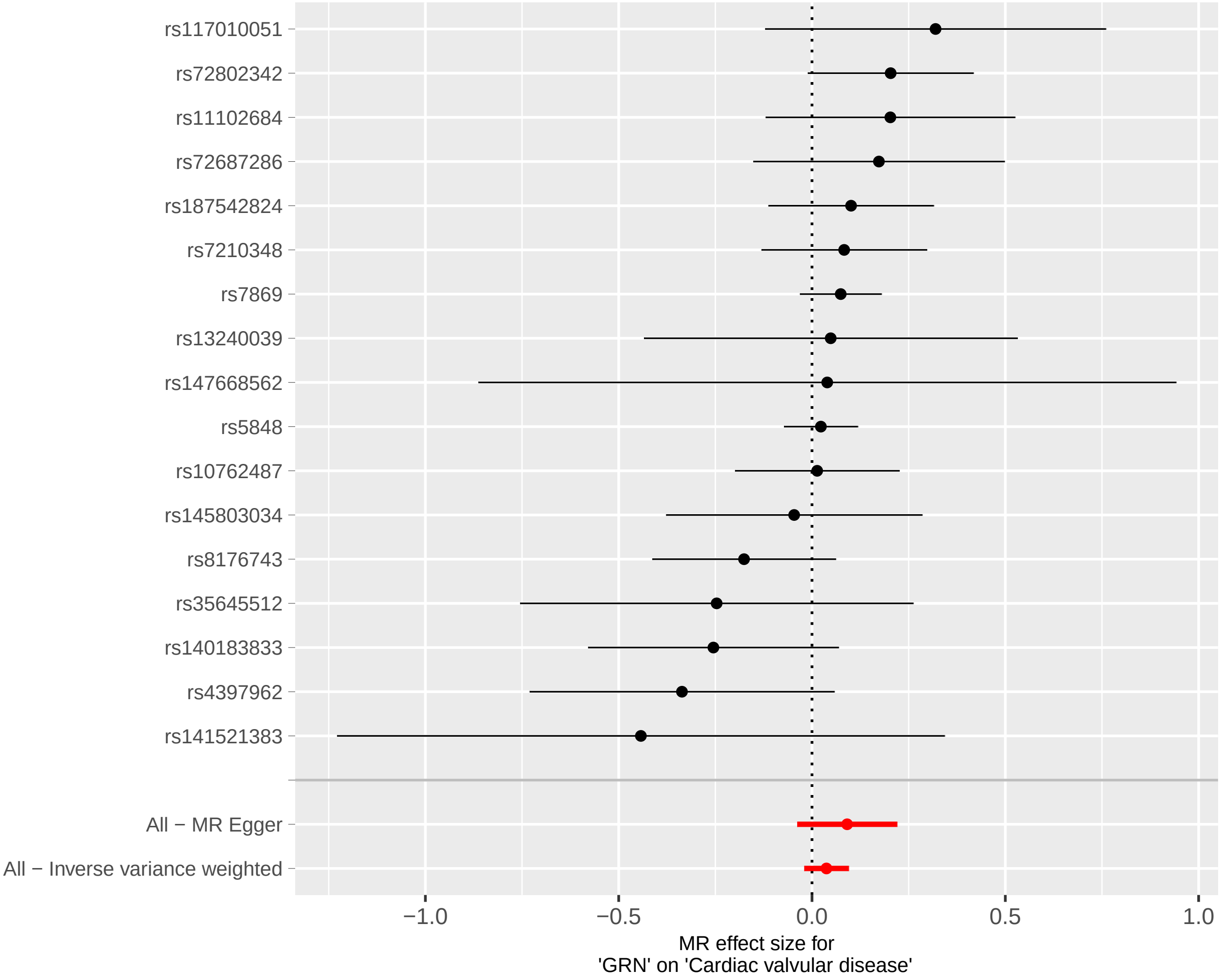
rs117010051
rs72802342
rs11102684
rs72687286
rs187542824
rs7210348
rs7869
rs13240039
rs147668562
rs5848
rs10762487
rs145803034
rs8176743
rs35645512
rs140183833
rs4397962
rs141521383
All − MR Egger
All − Inverse variance weighted
−1.0
−0.5
0.0
0.5
1.0
MR effect size for
'GRN' on 'Cardiac valvular disease'

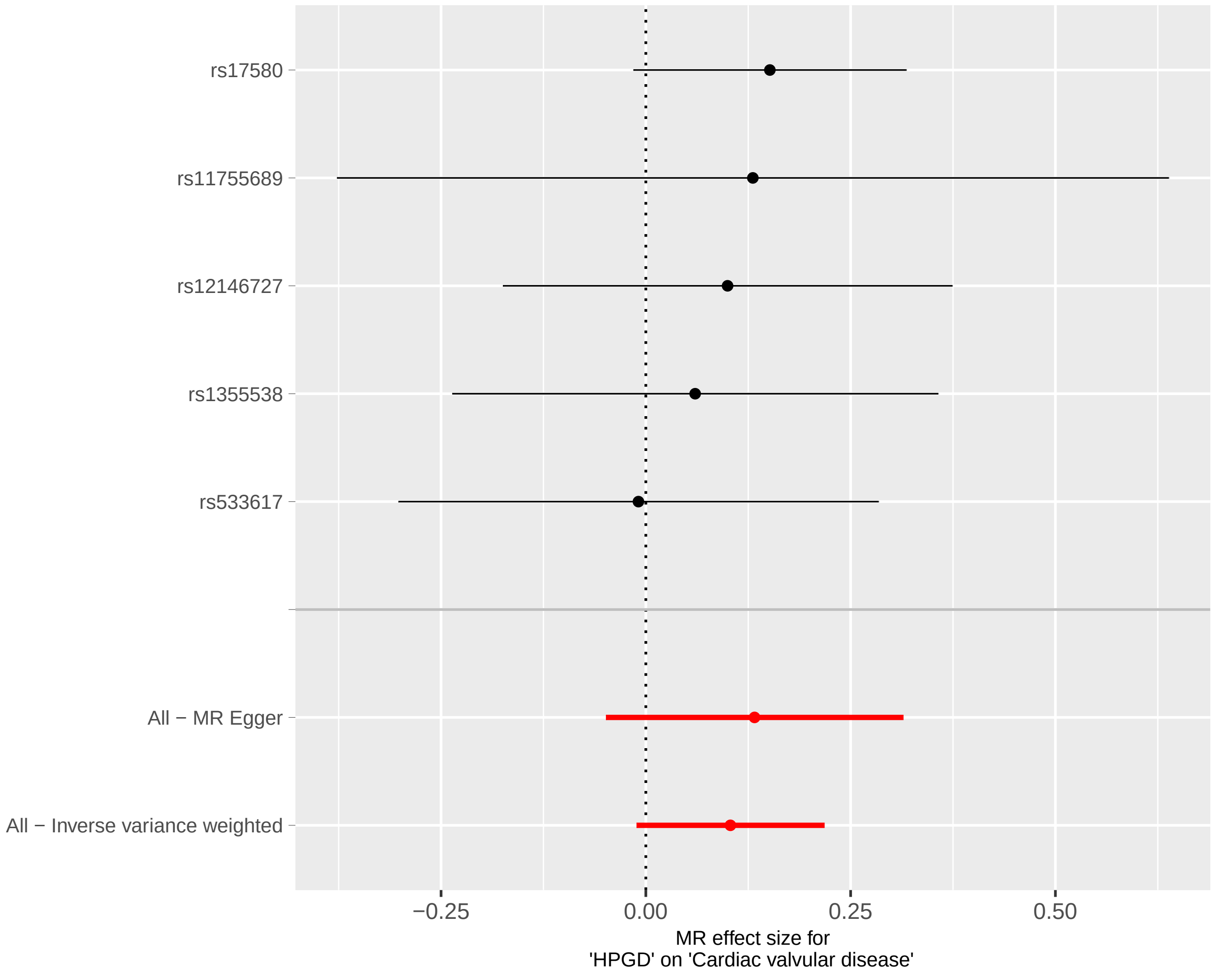

rs17580
rs11755689
rs12146727
rs1355538
rs533617
All − MR Egger
All − Inverse variance weighted
−0.25
0.00
0.25
0.50
MR effect size for
'HPGD' on 'Cardiac valvular disease'

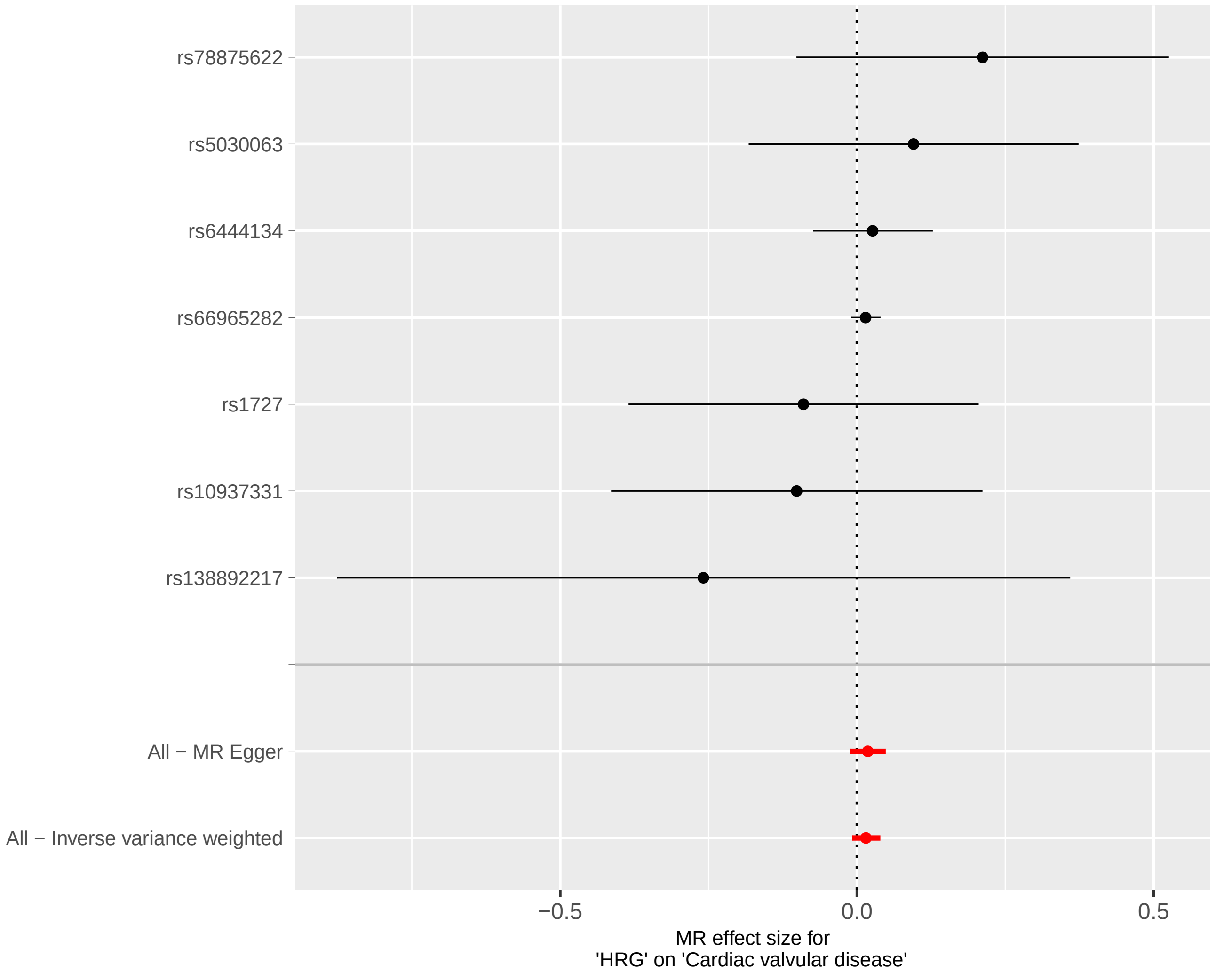

rs78875622
rs5030063
rs6444134
rs66965282
rs1727
rs10937331
rs138892217
All − MR Egger
All − Inverse variance weighted
−0.5
0.0
0.5
MR effect size for
'HRG' on 'Cardiac valvular disease'

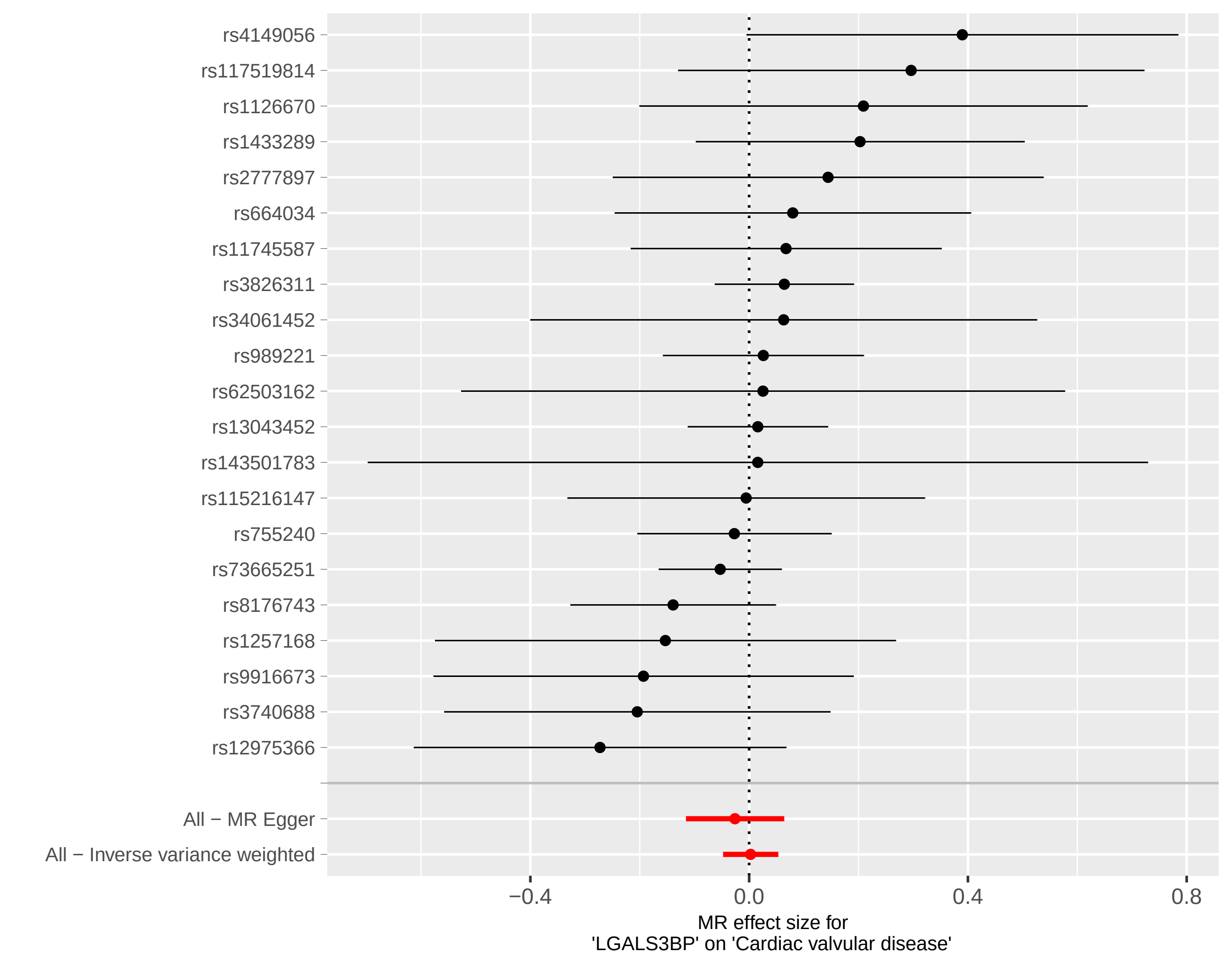
rs4149056
rs117519814
rs1126670
rs1433289
rs2777897
rs664034
rs11745587
rs3826311
rs34061452
rs989221
rs62503162
rs13043452
rs143501783
rs115216147
rs755240
rs73665251
rs8176743
rs1257168
rs9916673
rs3740688
rs12975366
All − MR Egger
All − Inverse variance weighted
−0.4
0.0
0.4
0.8
MR effect size for
'LGALS3BP' on 'Cardiac valvular disease'

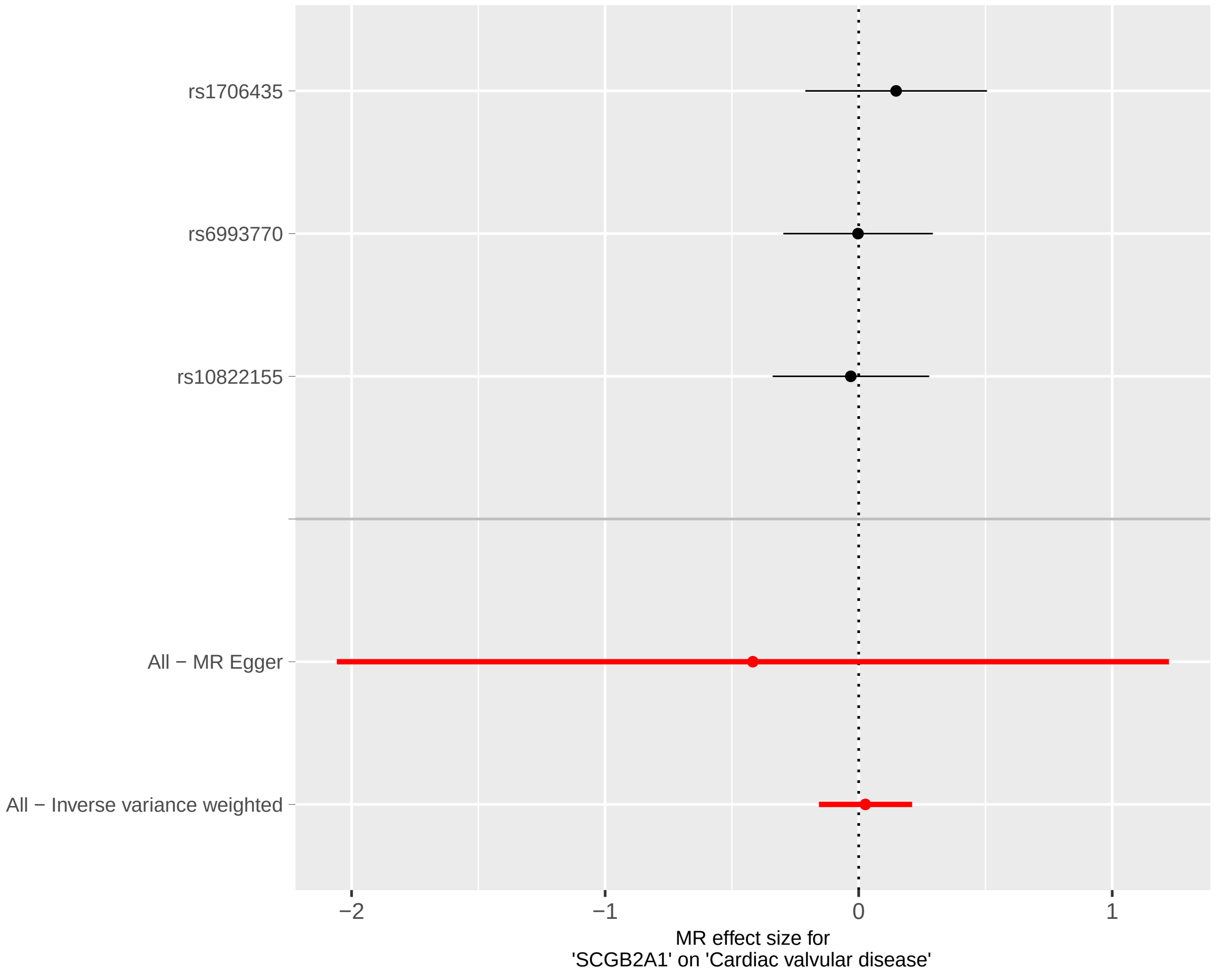
rs1706435
rs6993770
rs10822155
All − MR Egger
All − Inverse variance weighted
−2
−1
0
1
MR effect size for
'SCGB2A1' on 'Cardiac valvular disease'

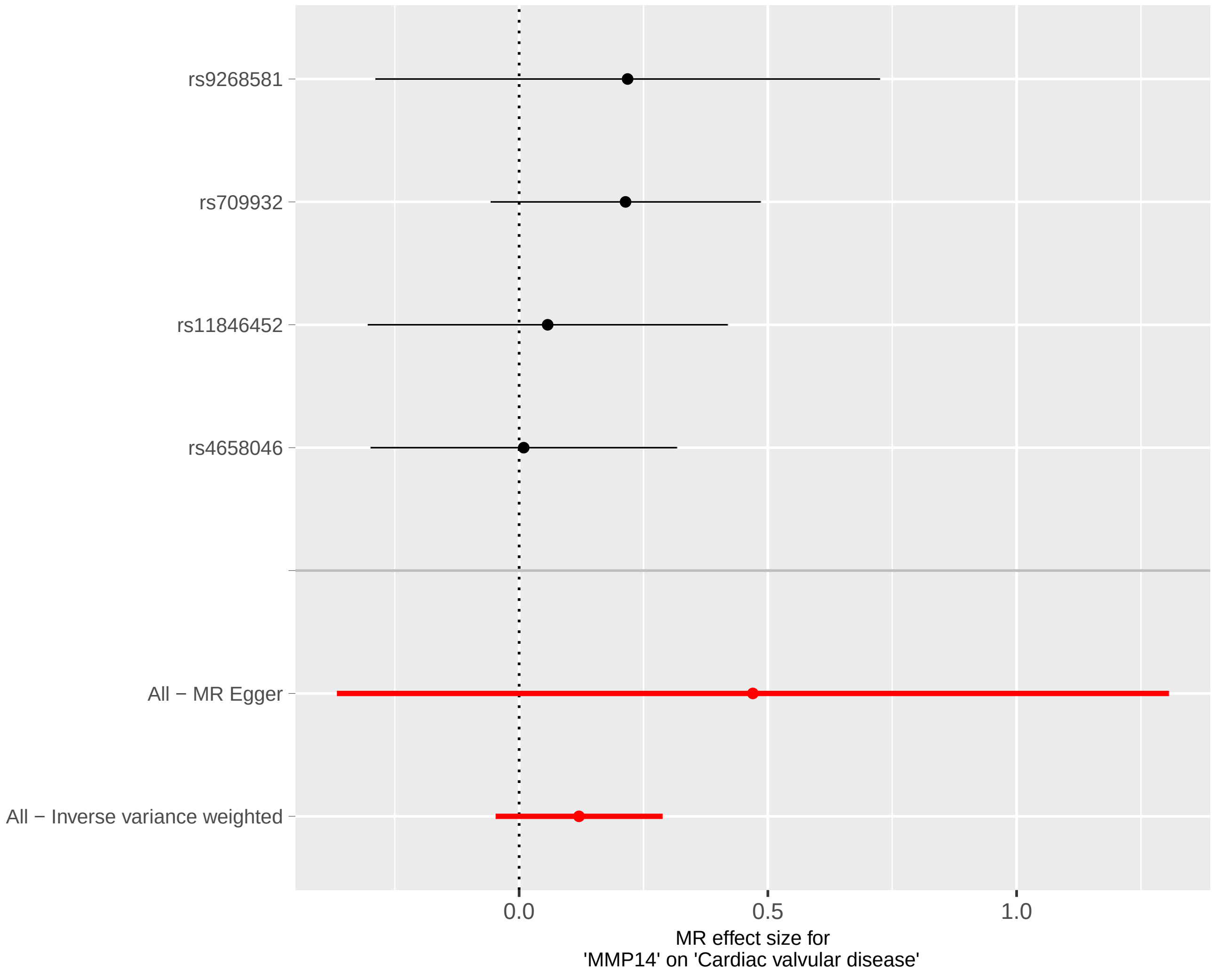
rs9268581
rs709932
rs11846452
rs4658046
All − MR Egger
All − Inverse variance weighted
0.0
0.5
1.0
MR effect size for
'MMP14' on 'Cardiac valvular disease'

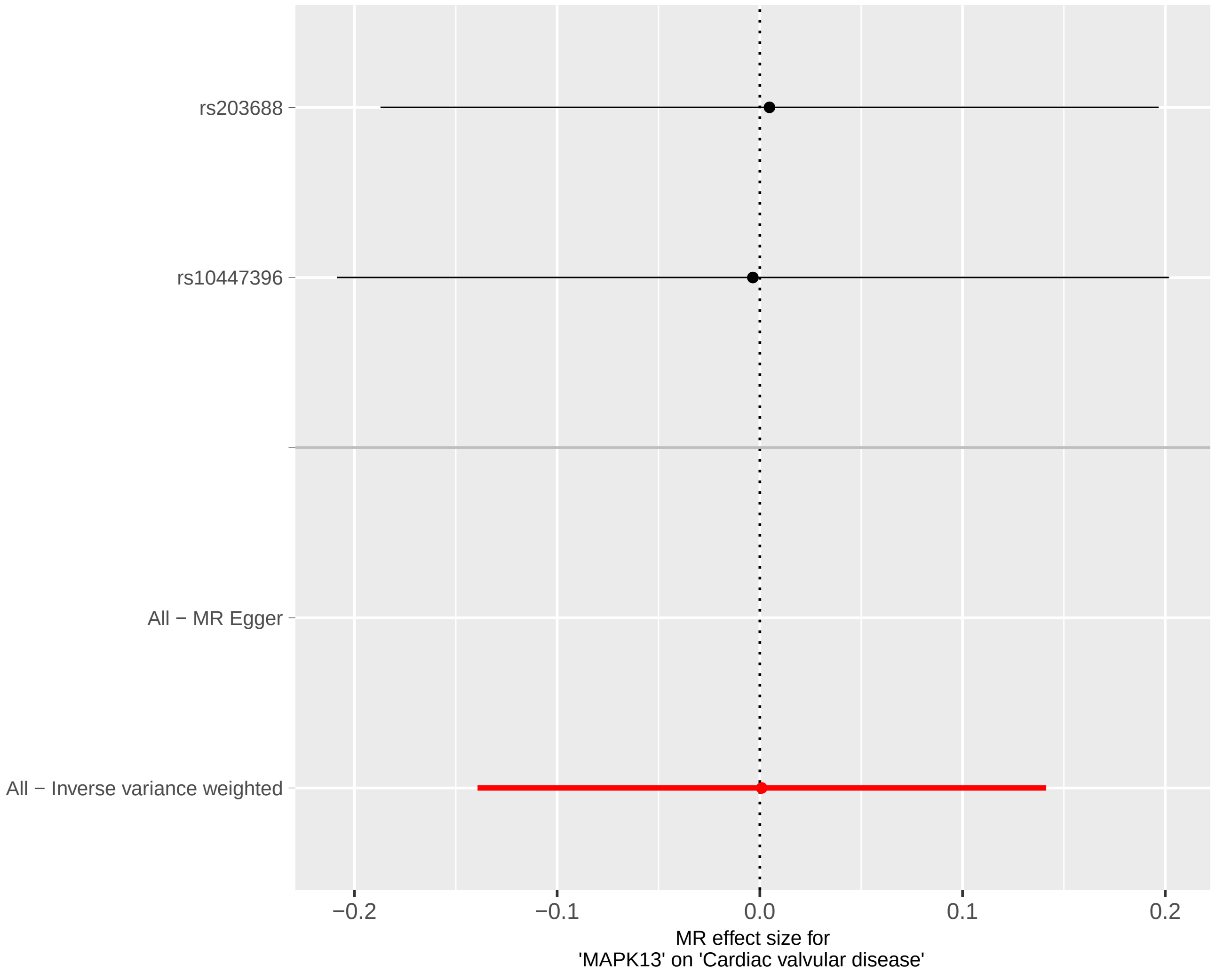
rs203688
rs10447396
All − MR Egger
All − Inverse variance weighted
−0.2
−0.1
0.0
0.1
0.2
MR effect size for
'MAPK13' on 'Cardiac valvular disease'

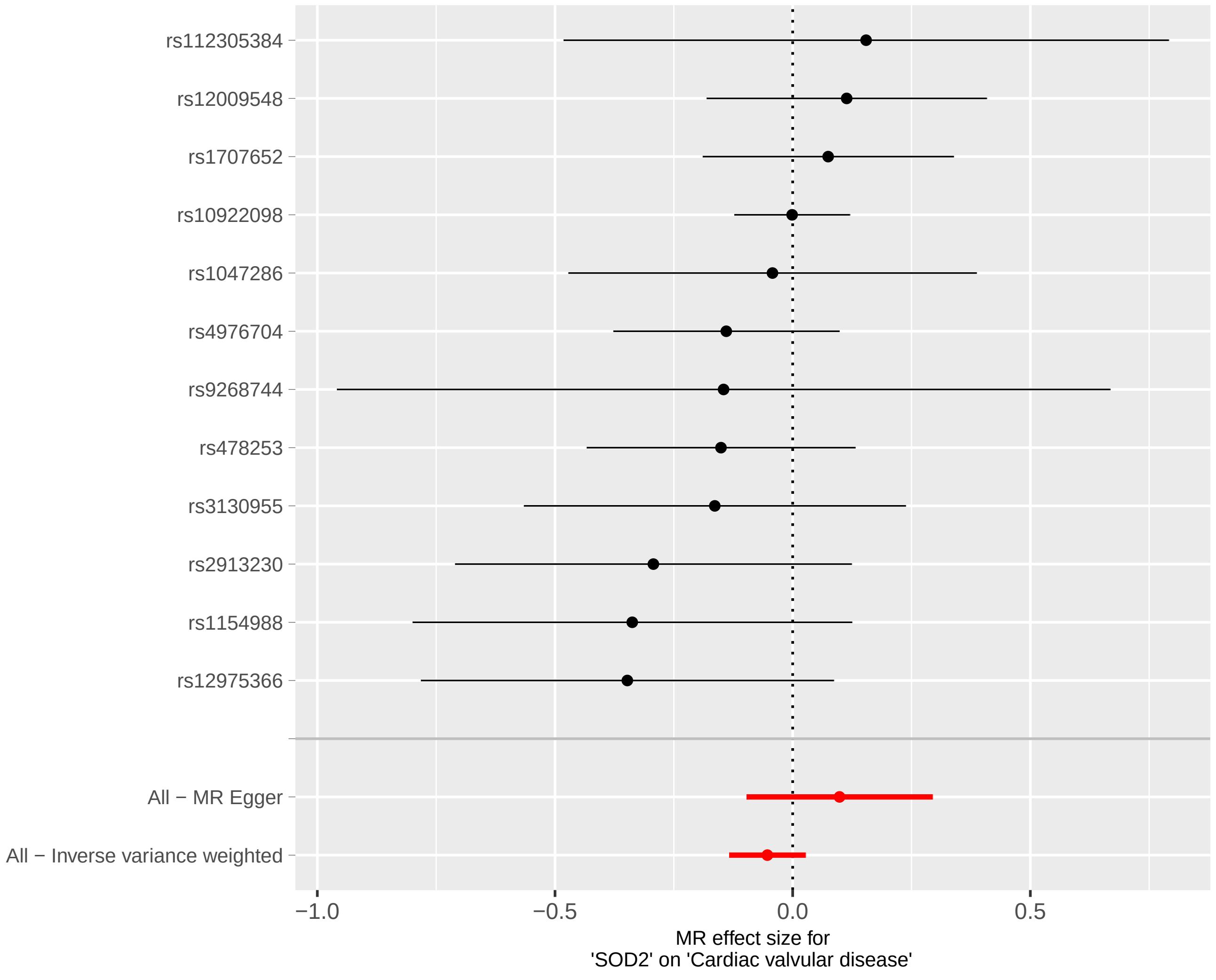

rs112305384
rs12009548
rs1707652
rs10922098
rs1047286
rs4976704
rs9268744
rs478253
rs3130955
rs2913230
rs1154988
rs12975366
All − MR Egger
All − Inverse variance weighted
−1.0
−0.5
0.0
0.5
MR effect size for
'SOD2' on 'Cardiac valvular disease'

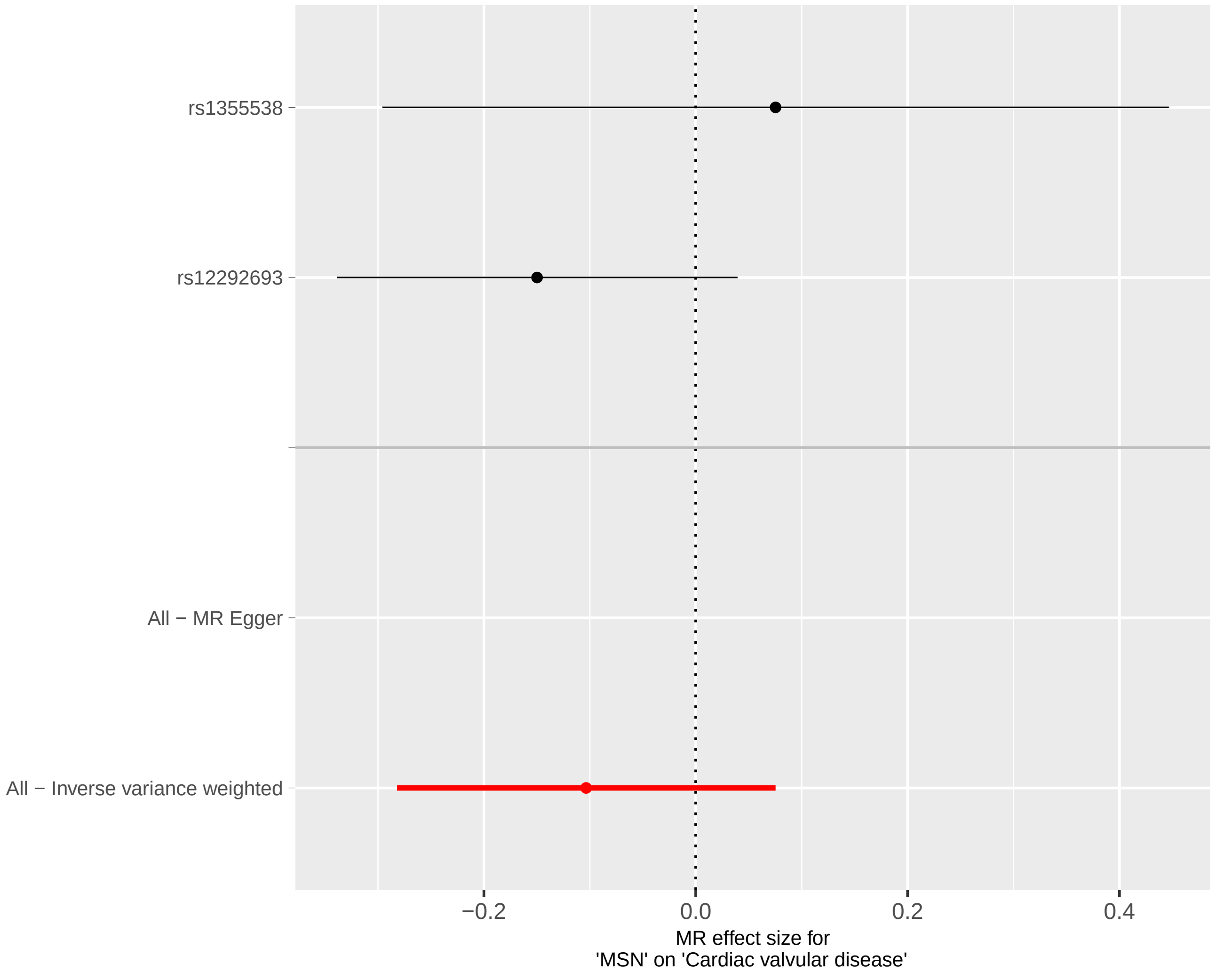
rs1355538
rs12292693
All − MR Egger
All − Inverse variance weighted
−0.2
0.0
0.2
0.4
MR effect size for
'MSN' on 'Cardiac valvular disease'

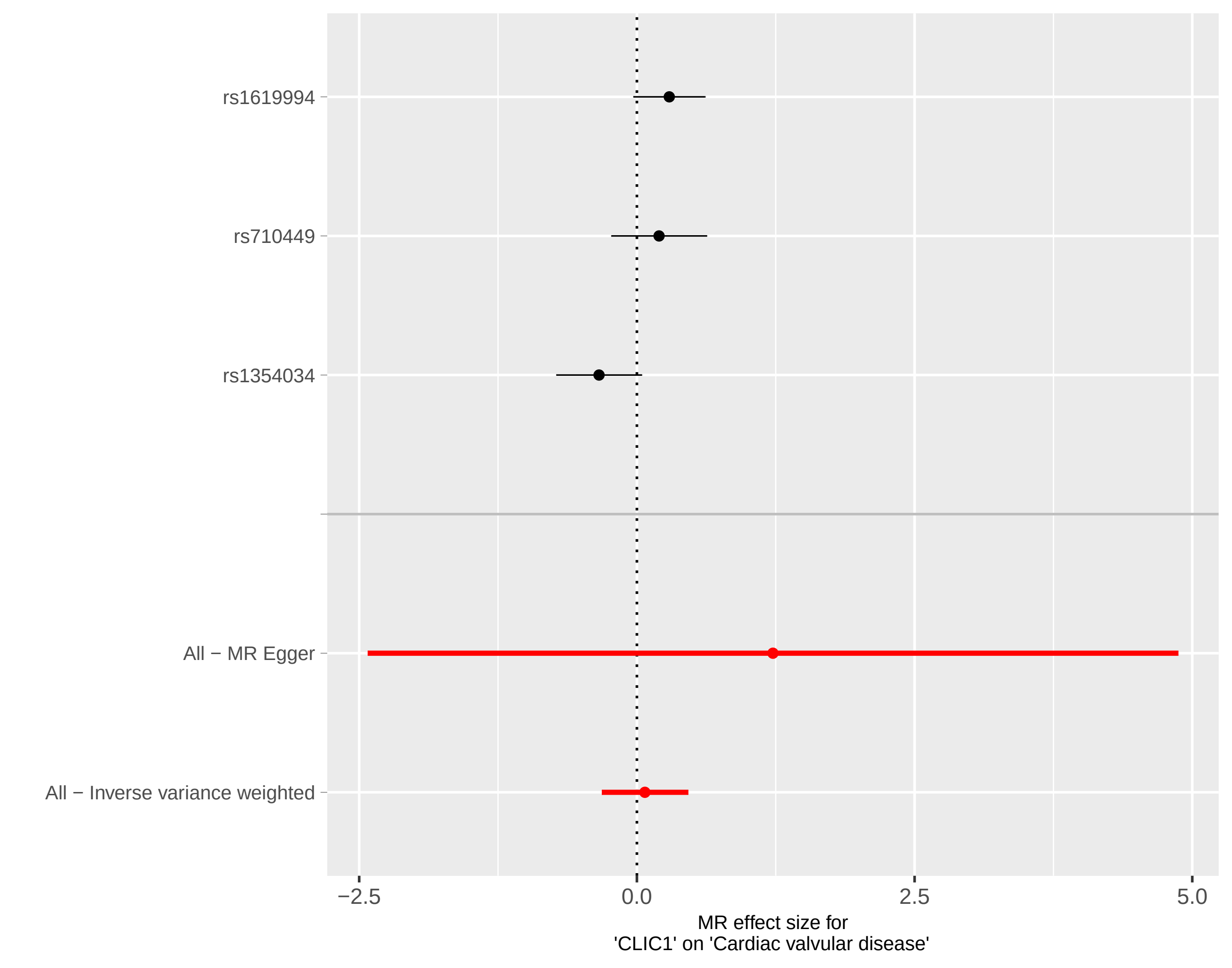
rs1619994
rs710449
rs1354034
All − MR Egger
All − Inverse variance weighted
−2.5
0.0
2.5
5.0
MR effect size for
'CLIC1' on 'Cardiac valvular disease'

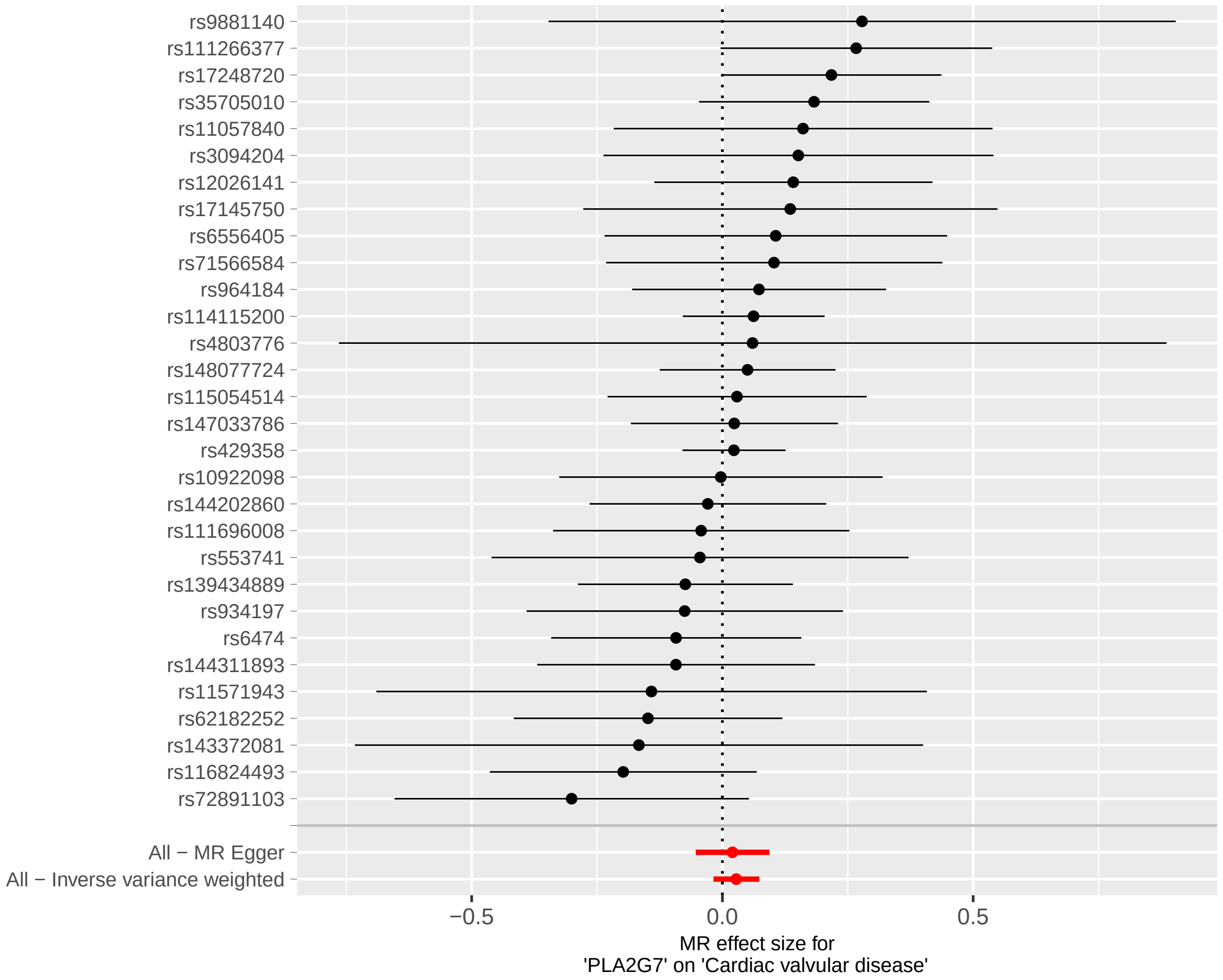

rs9881140
rs111266377
rs17248720
rs35705010
rs11057840
rs3094204
rs12026141
rs17145750
rs6556405
rs71566584
rs964184
rs114115200
rs4803776
rs148077724
rs115054514
rs147033786
rs429358
rs10922098
rs144202860
rs111696008
rs553741
rs139434889
rs934197
rs6474
rs144311893
rs11571943
rs62182252
rs143372081
rs116824493
rs72891103
All − MR Egger
All − Inverse variance weighted
−0.5
0.0
0.5
MR effect size for
'PLA2G7' on 'Cardiac valvular disease'

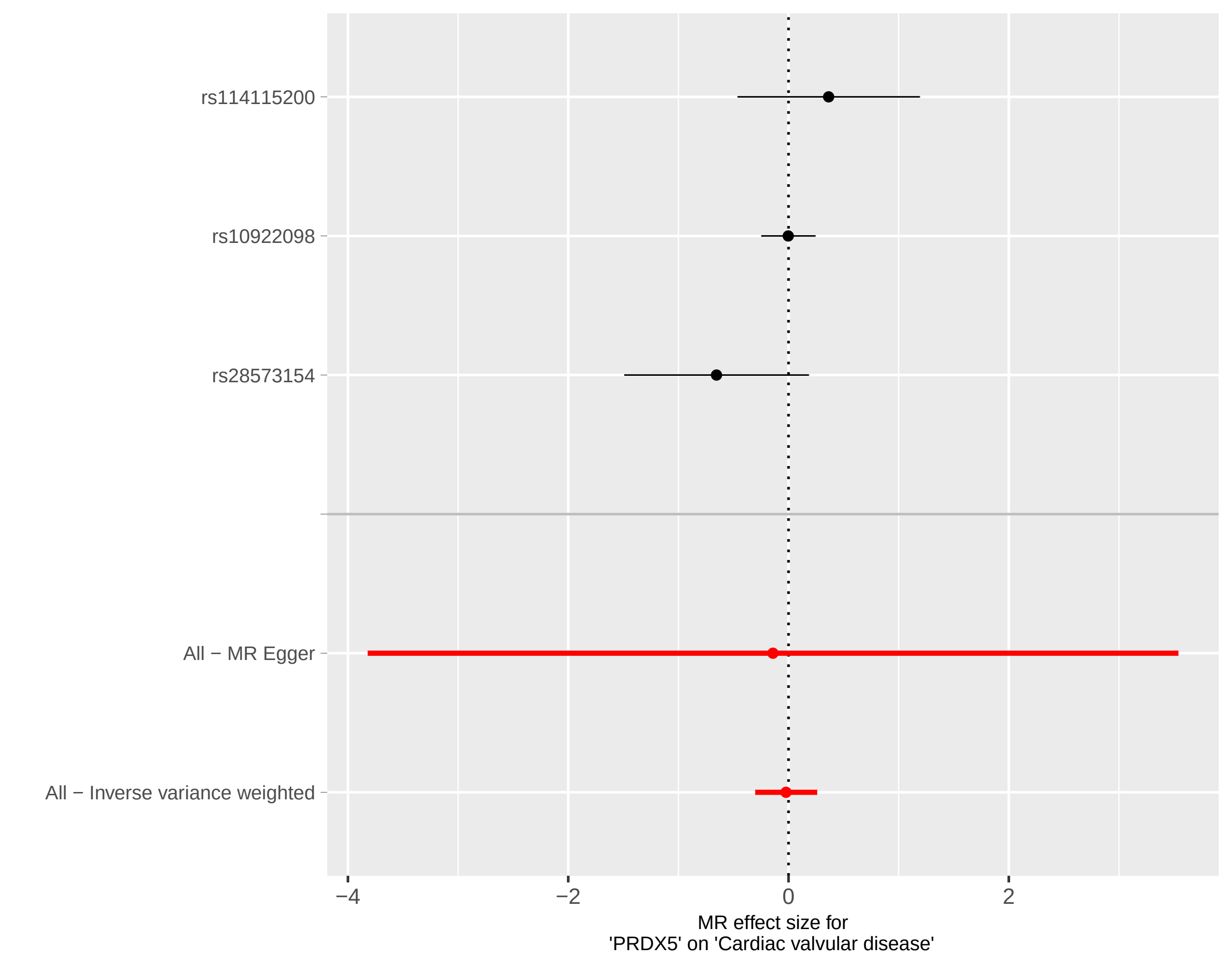

rs114115200
rs10922098
rs28573154
All − MR Egger
All − Inverse variance weighted
−4
−2
0
2
MR effect size for
'PRDX5' on 'Cardiac valvular disease'

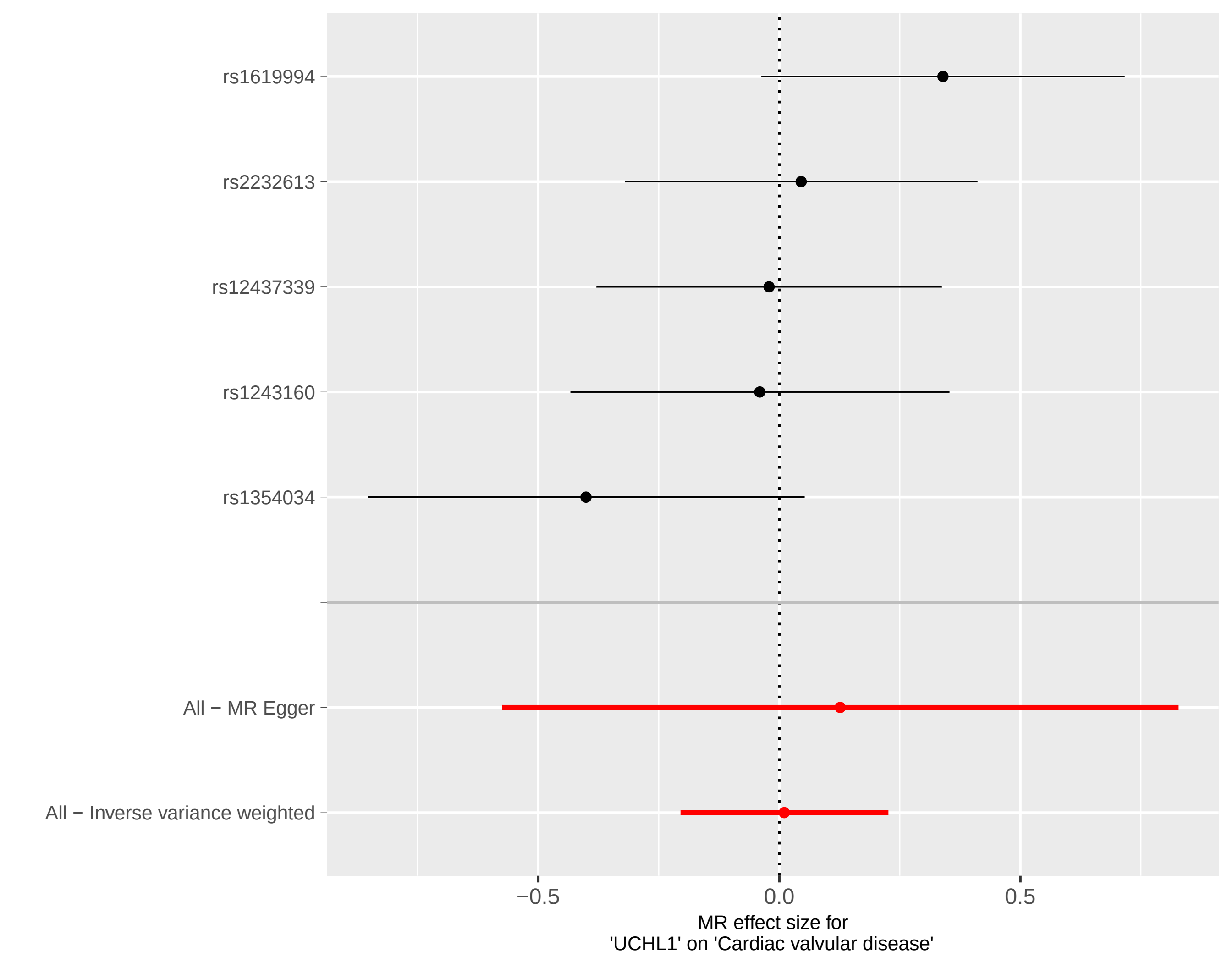
rs1619994
rs2232613
rs12437339
rs1243160
rs1354034
All − MR Egger
All − Inverse variance weighted
−0.5
0.0
0.5
MR effect size for
'UCHL1' on 'Cardiac valvular disease'

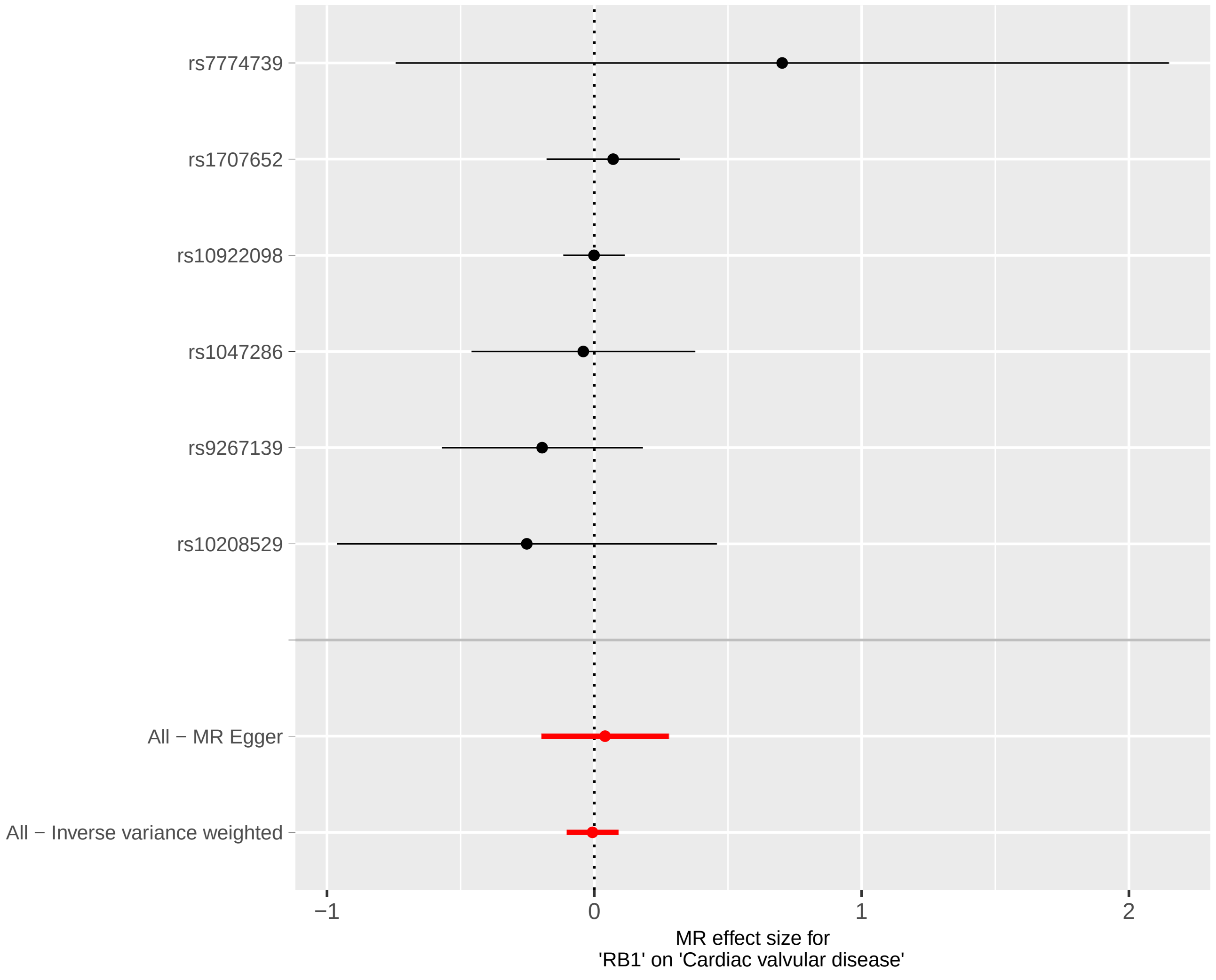

rs7774739
rs1707652
rs10922098
rs1047286
rs9267139
rs10208529
All − MR Egger
All − Inverse variance weighted
−1
0
1
2
MR effect size for
'RB1' on 'Cardiac valvular disease'

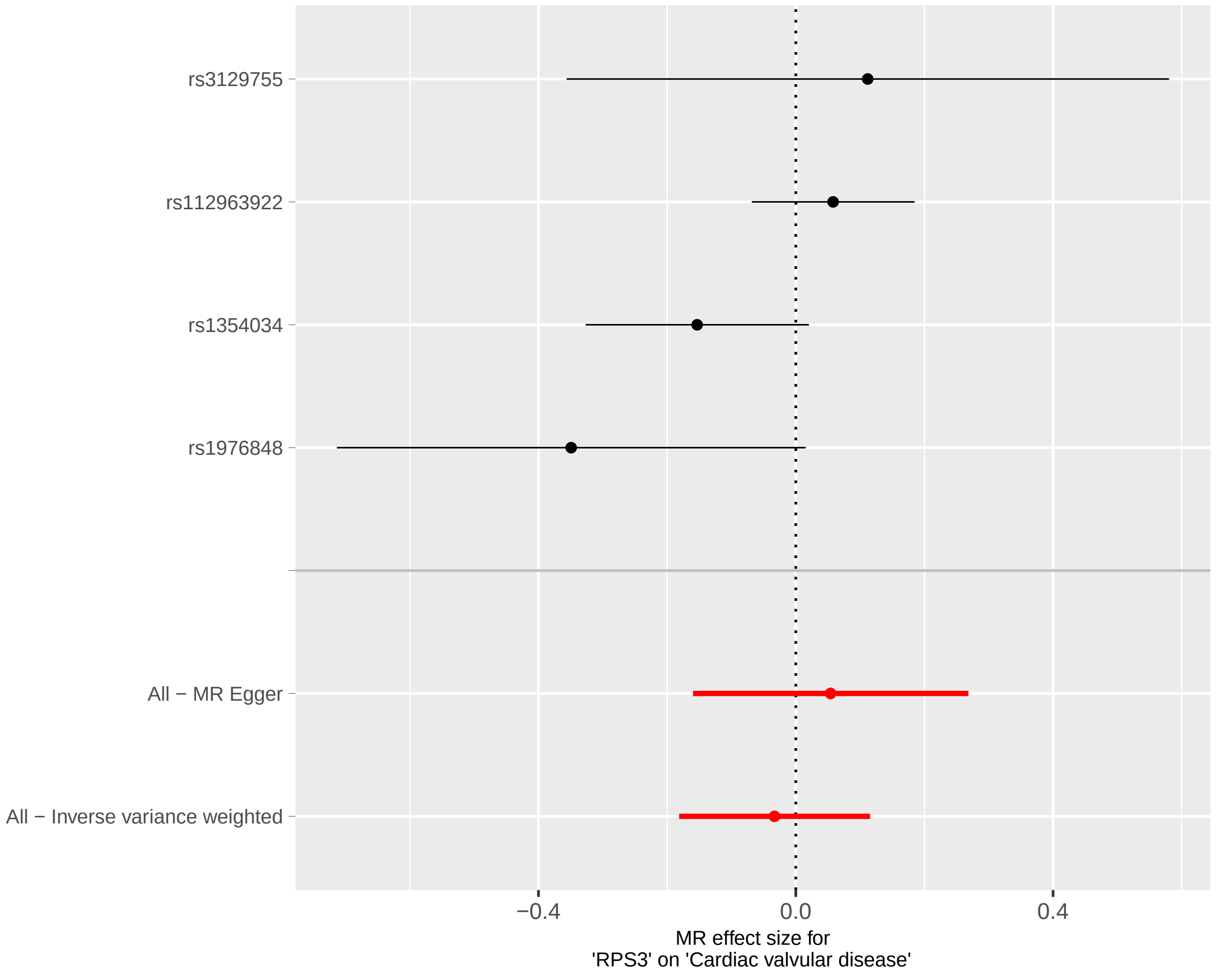
rs3129755
rs112963922
rs1354034
rs1976848
All − MR Egger
All − Inverse variance weighted
−0.4
0.0
0.4
MR effect size for
'RPS3' on 'Cardiac valvular disease'

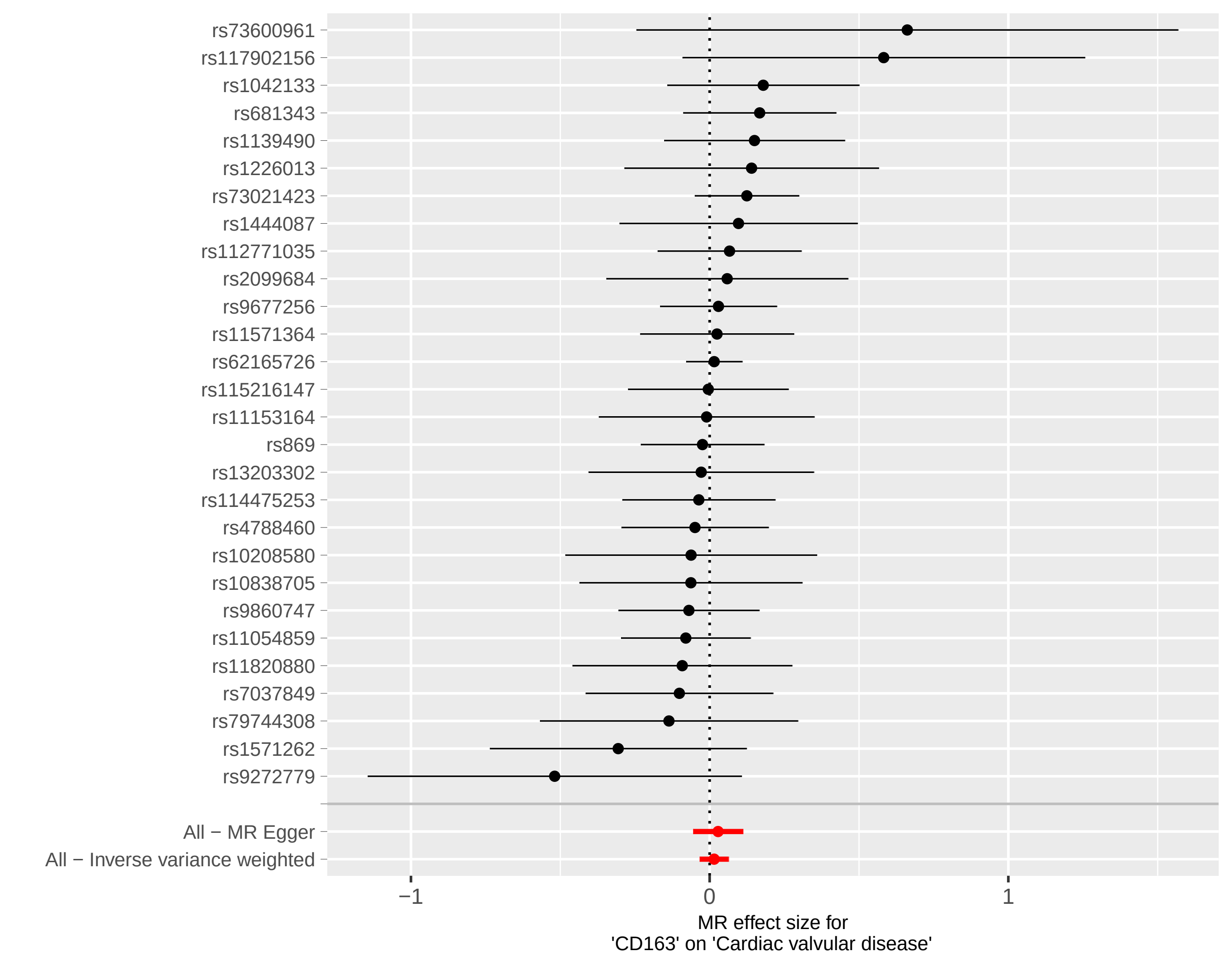
rs73600961
rs117902156
rs1042133
rs681343
rs1139490
rs1226013
rs73021423
rs1444087
rs112771035
rs2099684
rs9677256
rs11571364
rs62165726
rs115216147
rs11153164
rs869
rs13203302
rs114475253
rs4788460
rs10208580
rs10838705
rs9860747
rs11054859
rs11820880
rs7037849
rs79744308
rs1571262
rs9272779
All − MR Egger
All − Inverse variance weighted
−1
0
1
MR effect size for
'CD163' on 'Cardiac valvular disease'

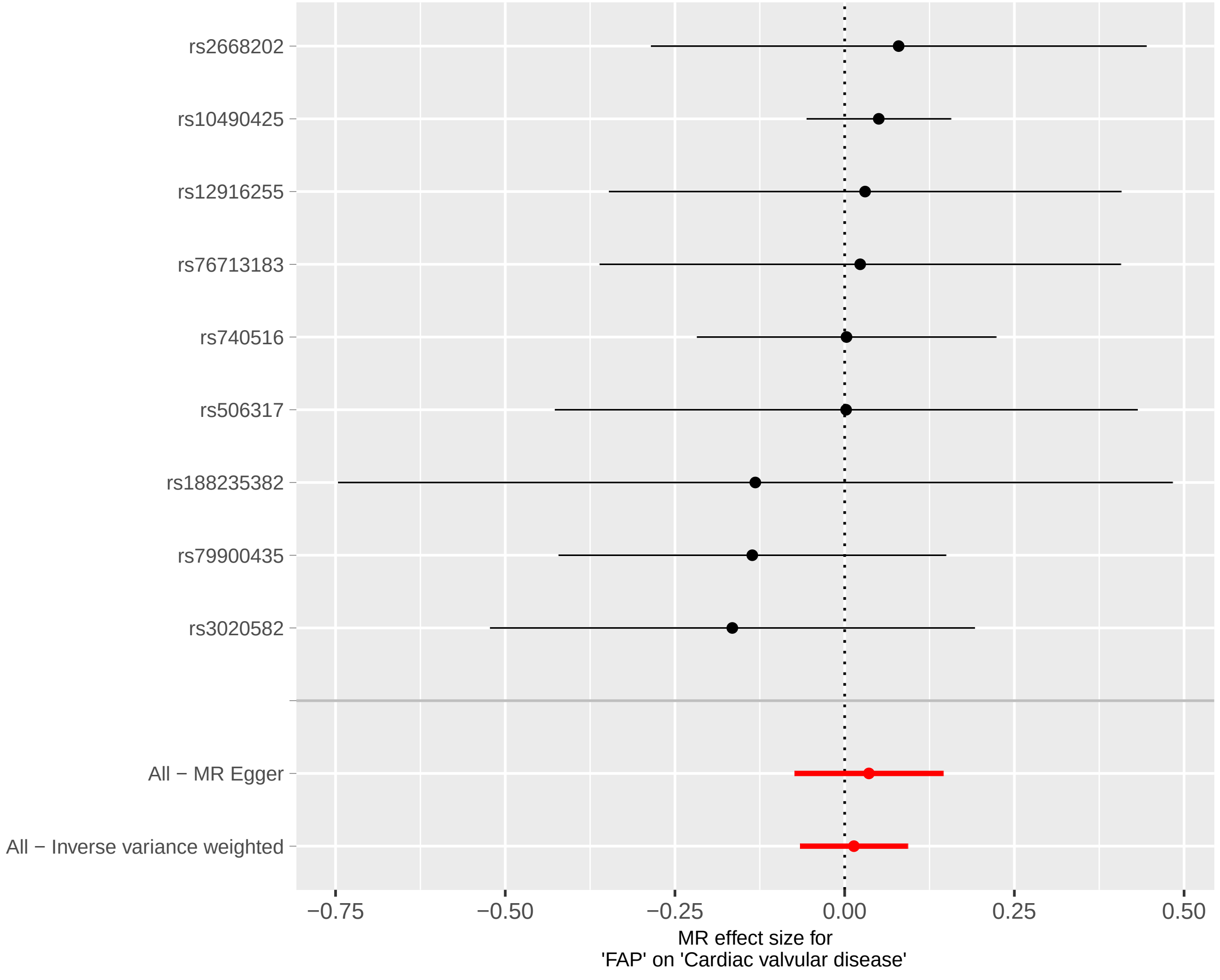

rs2668202
rs10490425
rs12916255
rs76713183
rs740516
rs506317
rs188235382
rs79900435
rs3020582
All − MR Egger
All − Inverse variance weighted
−0.75
−0.50
−0.25
0.00
0.25
0.50
MR effect size for
'FAP' on 'Cardiac valvular disease'

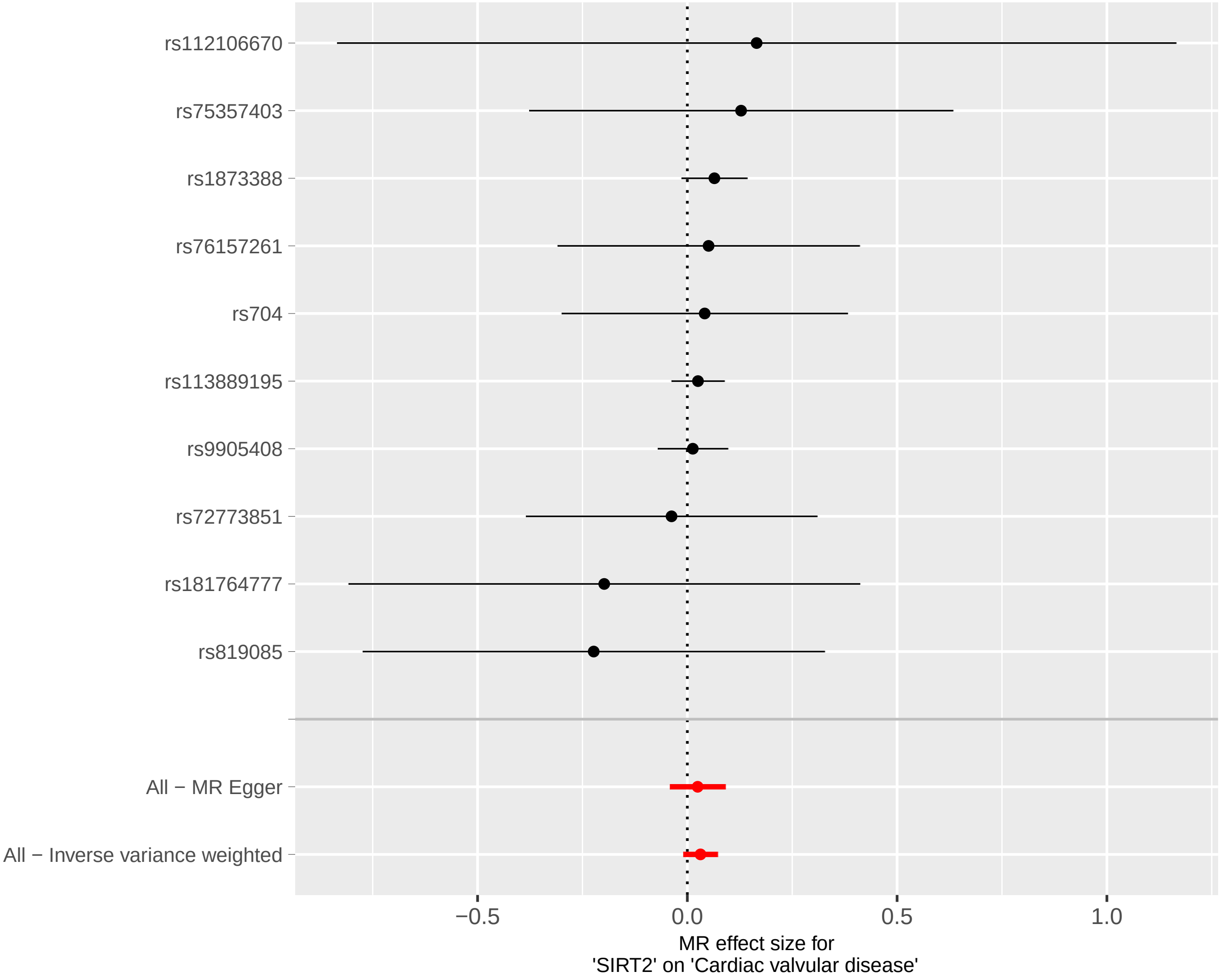
rs112106670
rs75357403
rs1873388
rs76157261
rs704
rs113889195
rs9905408
rs72773851
rs181764777
rs819085
All − MR Egger
All − Inverse variance weighted
−0.5
0.0
0.5
1.0
MR effect size for
'SIRT2' on 'Cardiac valvular disease'

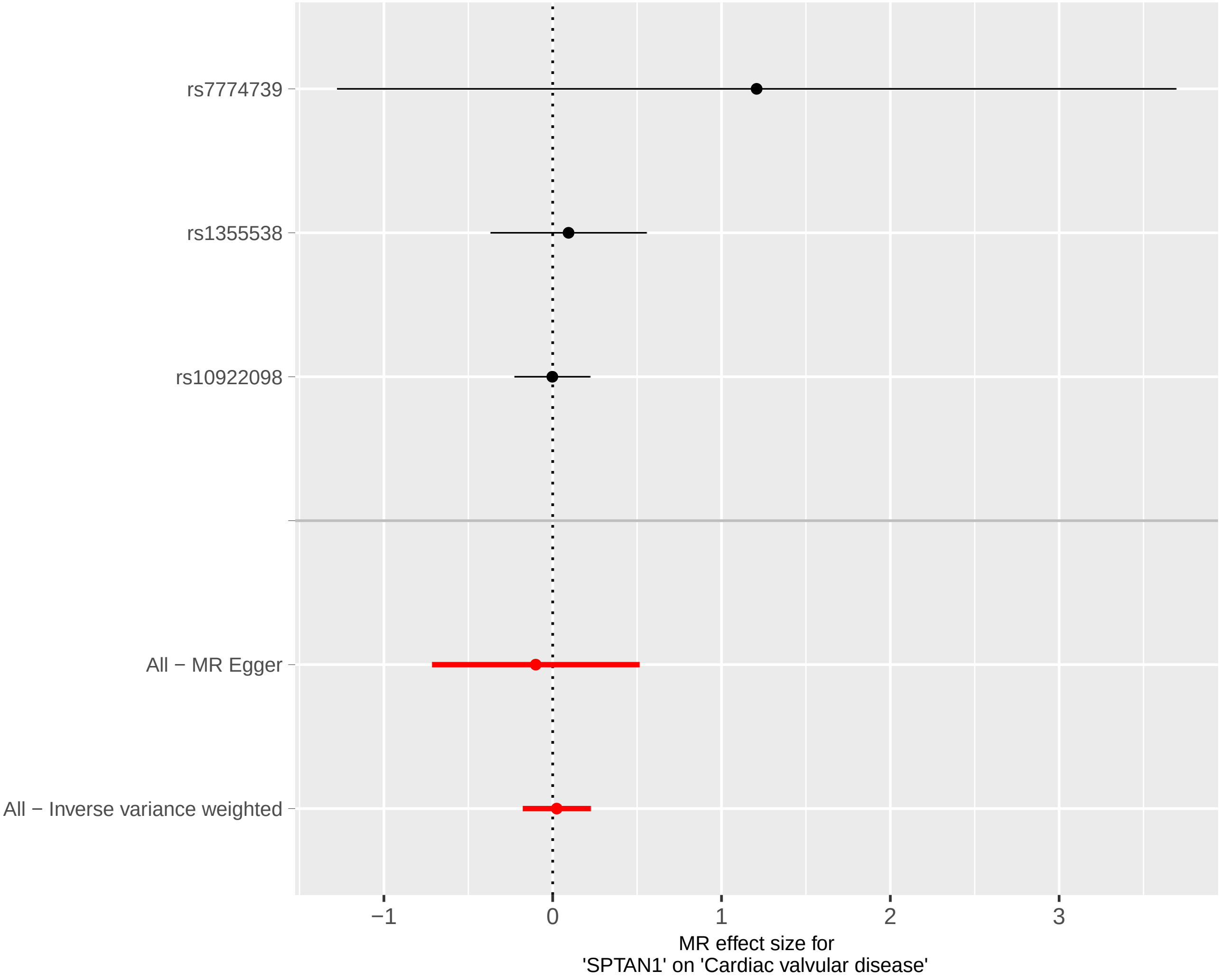
rs7774739
rs1355538
rs10922098
All − MR Egger
All − Inverse variance weighted
−1
0
1
2
3
MR effect size for
'SPTAN1' on 'Cardiac valvular disease'

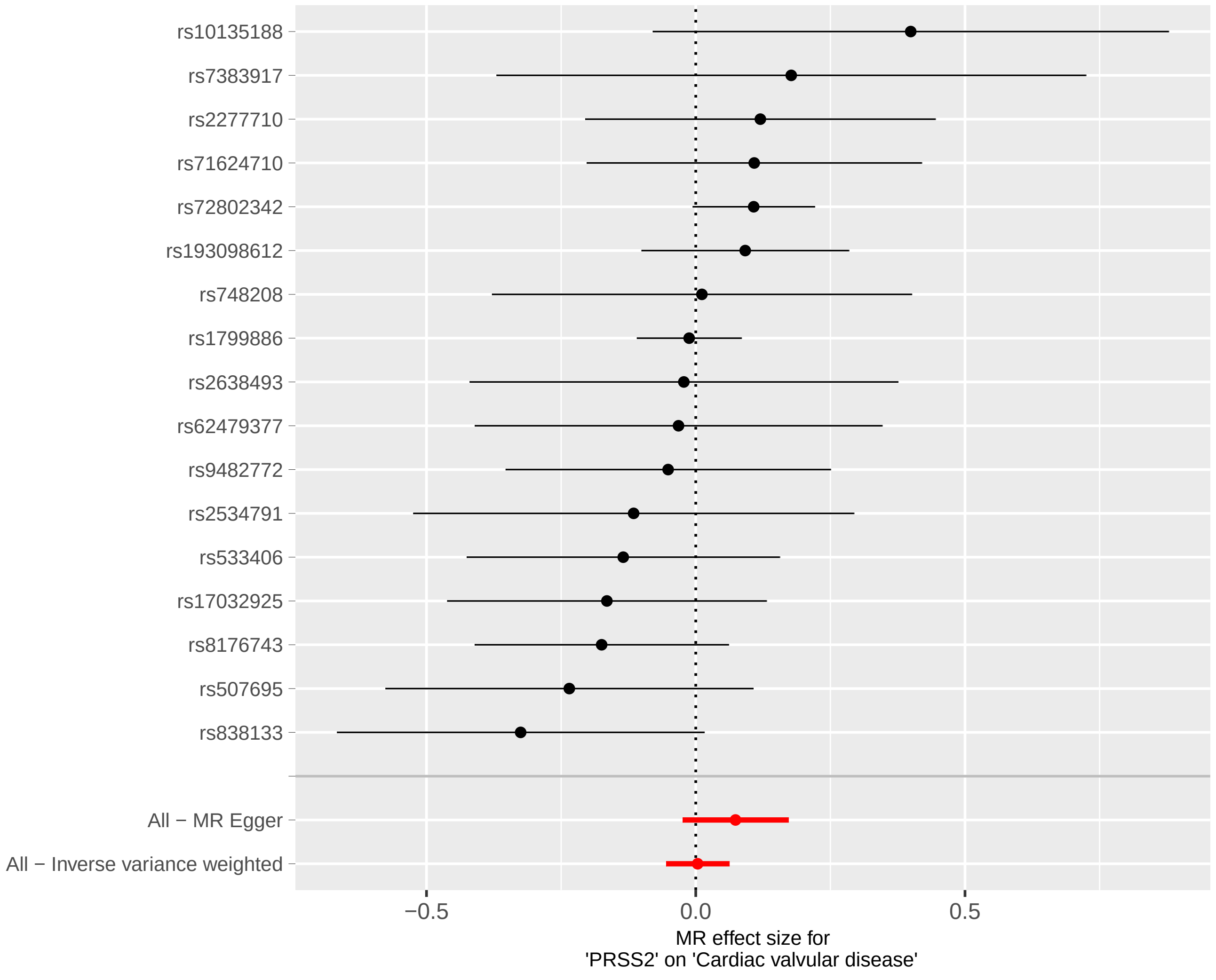
rs10135188
rs7383917
rs2277710
rs71624710
rs72802342
rs193098612
rs748208
rs1799886
rs2638493
rs62479377
rs9482772
rs2534791
rs533406
rs17032925
rs8176743
rs507695
rs838133
All − MR Egger
All − Inverse variance weighted
−0.5
0.0
0.5
MR effect size for
'PRSS2' on 'Cardiac valvular disease'

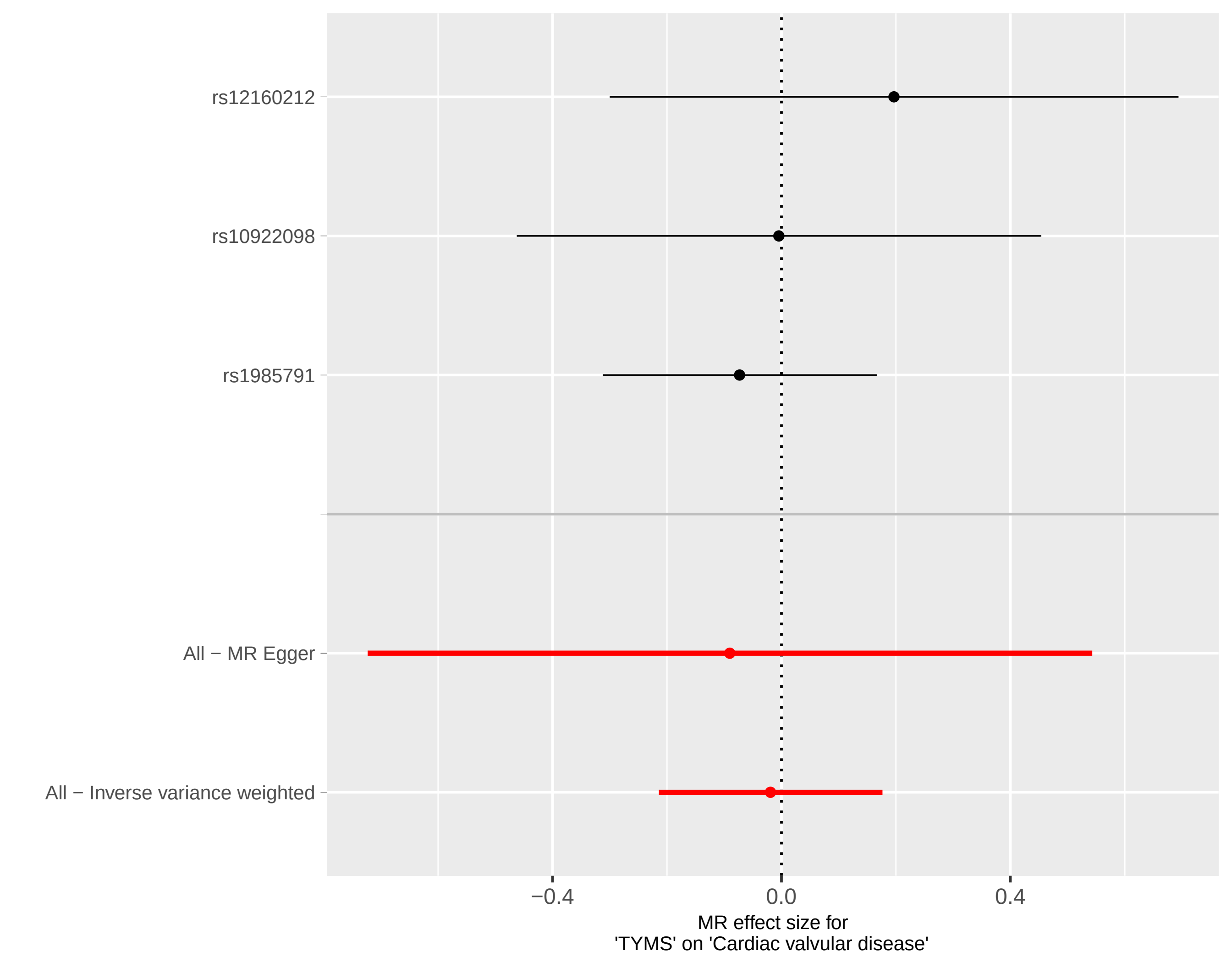
rs12160212
rs10922098
rs1985791
All − MR Egger
All − Inverse variance weighted
−0.4
0.0
0.4
MR effect size for
'TYMS' on 'Cardiac valvular disease'

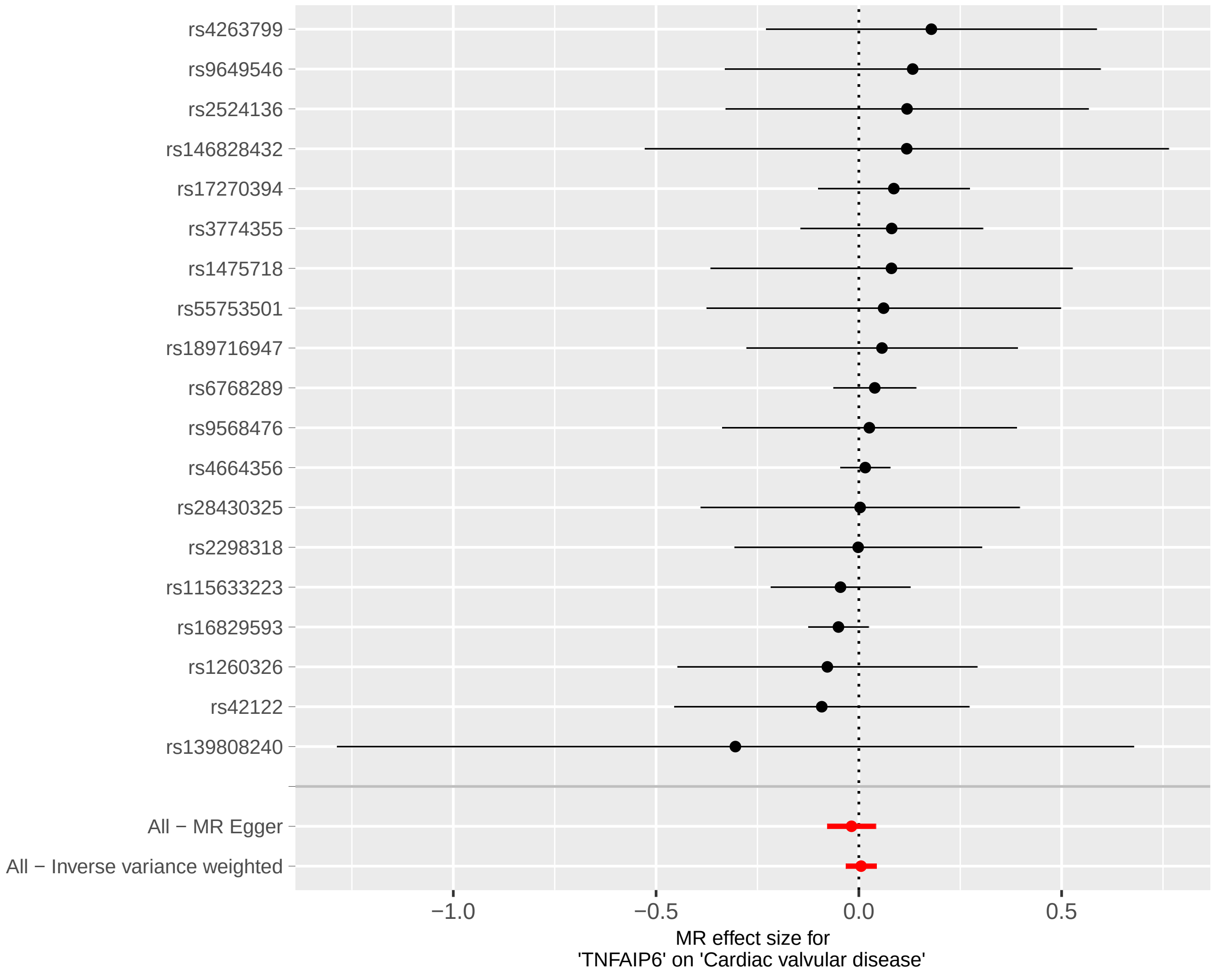
rs4263799
rs9649546
rs2524136
rs146828432
rs17270394
rs3774355
rs1475718
rs55753501
rs189716947
rs6768289
rs9568476
rs4664356
rs28430325
rs2298318
rs115633223
rs16829593
rs1260326
rs42122
rs139808240
All − MR Egger
All − Inverse variance weighted
−1.0
−0.5
0.0
0.5
MR effect size for
'TNFAIP6' on 'Cardiac valvular disease'

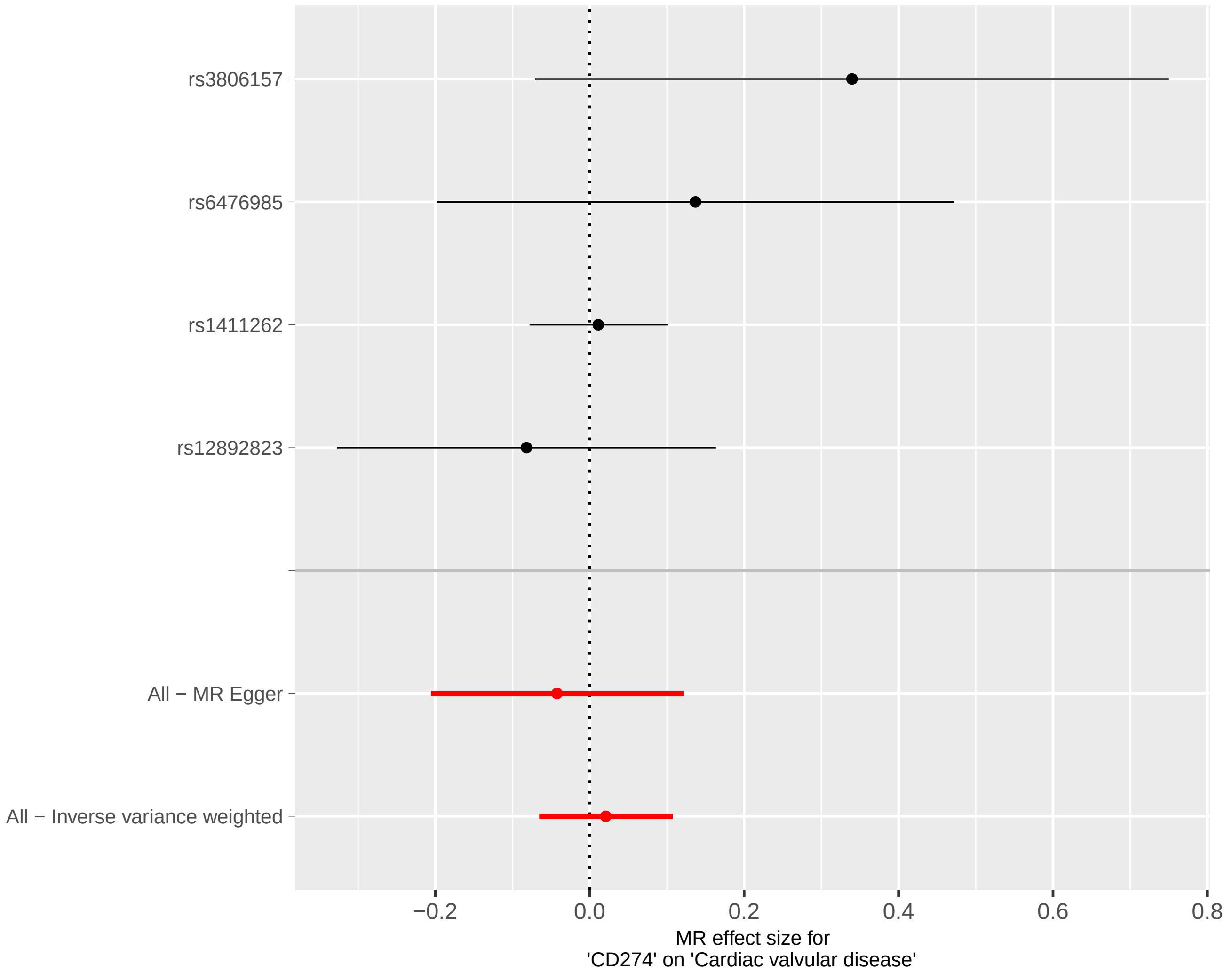
rs3806157
rs6476985
rs1411262
rs12892823
All − MR Egger
All − Inverse variance weighted
−0.2
0.0
0.2
0.4
0.6
0.8
MR effect size for
'CD274' on 'Cardiac valvular disease'

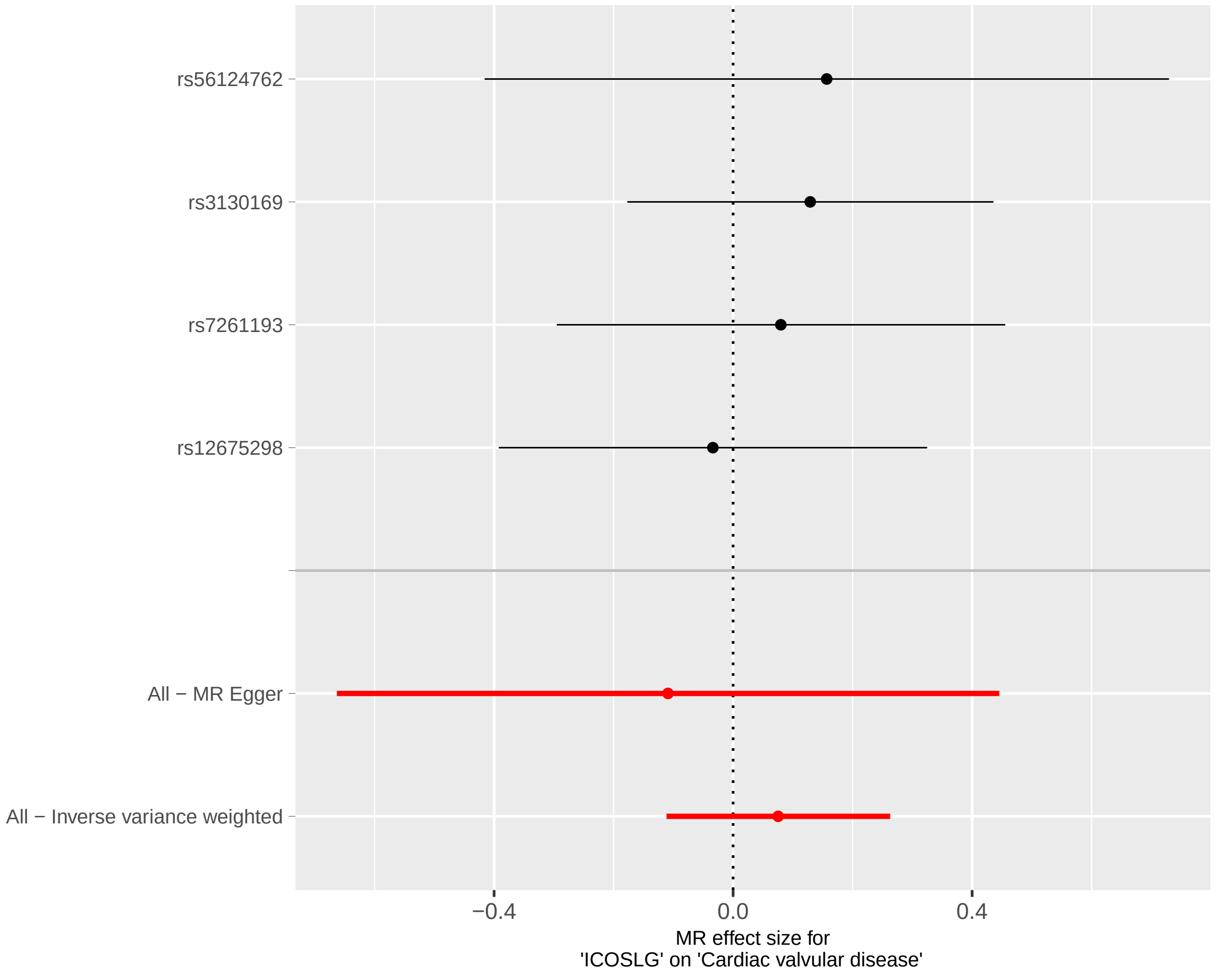

rs56124762
rs3130169
rs7261193
rs12675298
All − MR Egger
All − Inverse variance weighted
−0.4
0.0
0.4
MR effect size for
'ICOSLG' on 'Cardiac valvular disease'

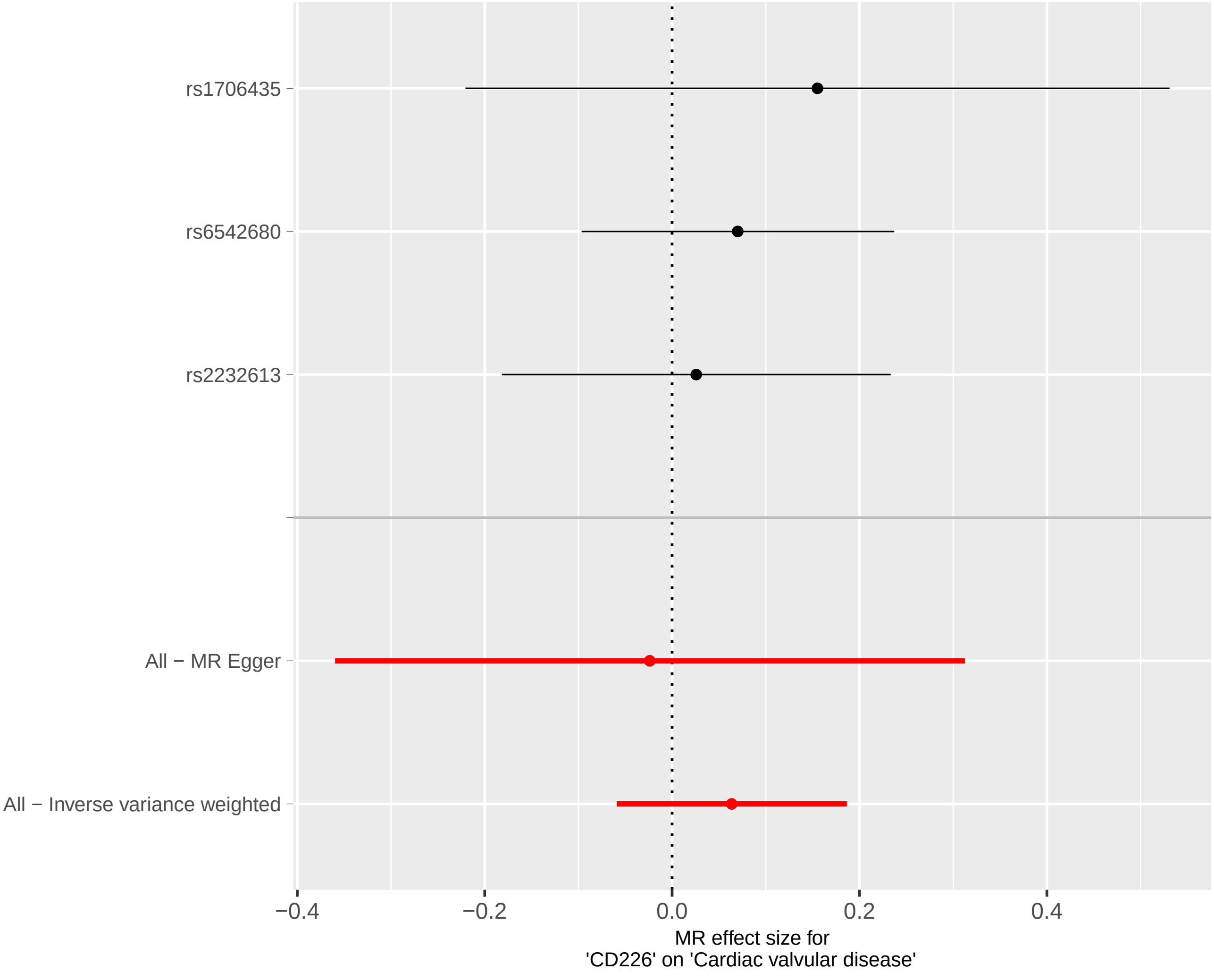
rs1706435
rs6542680
rs2232613
All − MR Egger
All − Inverse variance weighted
−0.4
−0.2
0.0
0.2
0.4
MR effect size for
'CD226' on 'Cardiac valvular disease'

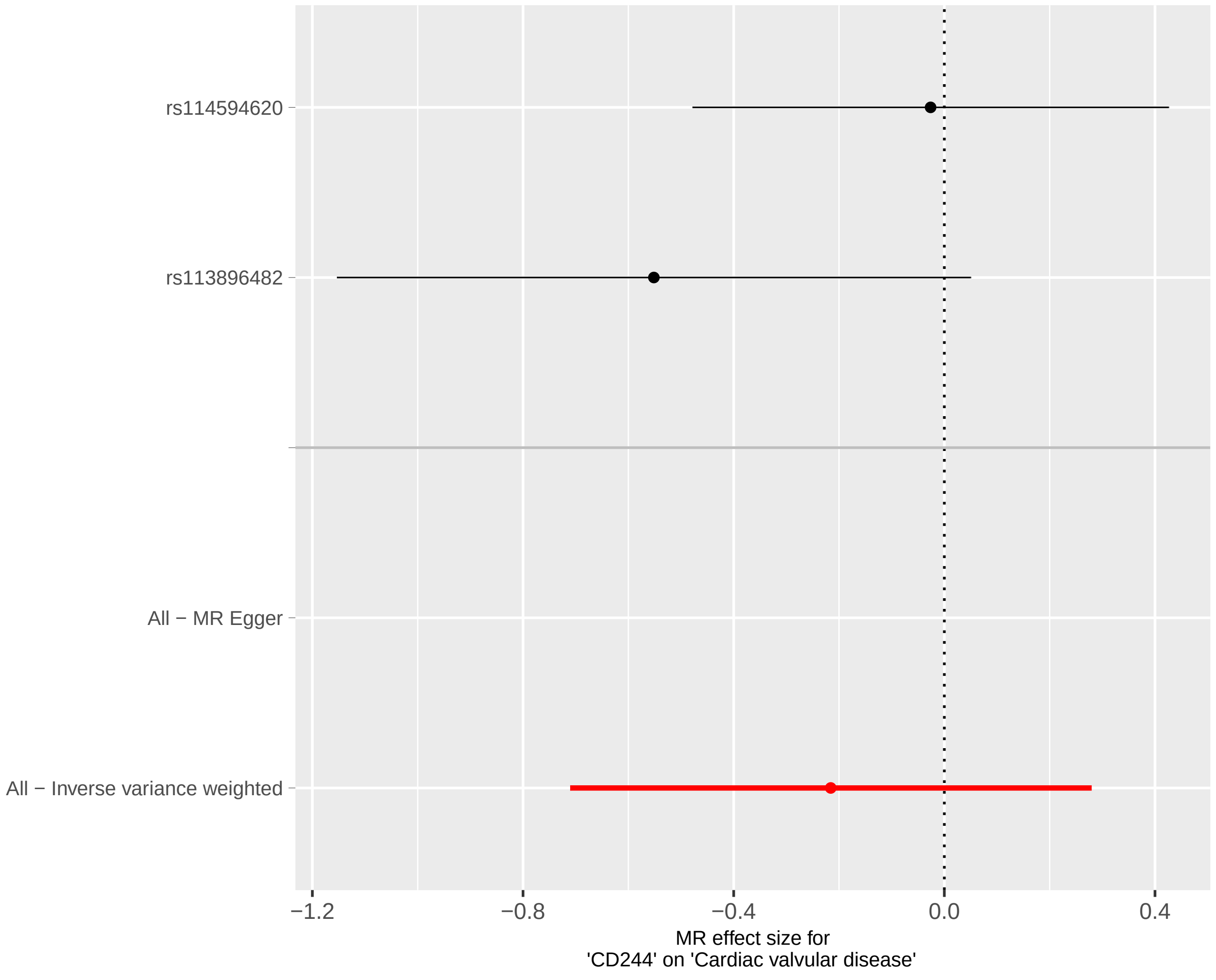
rs114594620
rs113896482
All − MR Egger
All − Inverse variance weighted
−1.2
−0.8
−0.4
0.0
0.4
MR effect size for
'CD244' on 'Cardiac valvular disease'

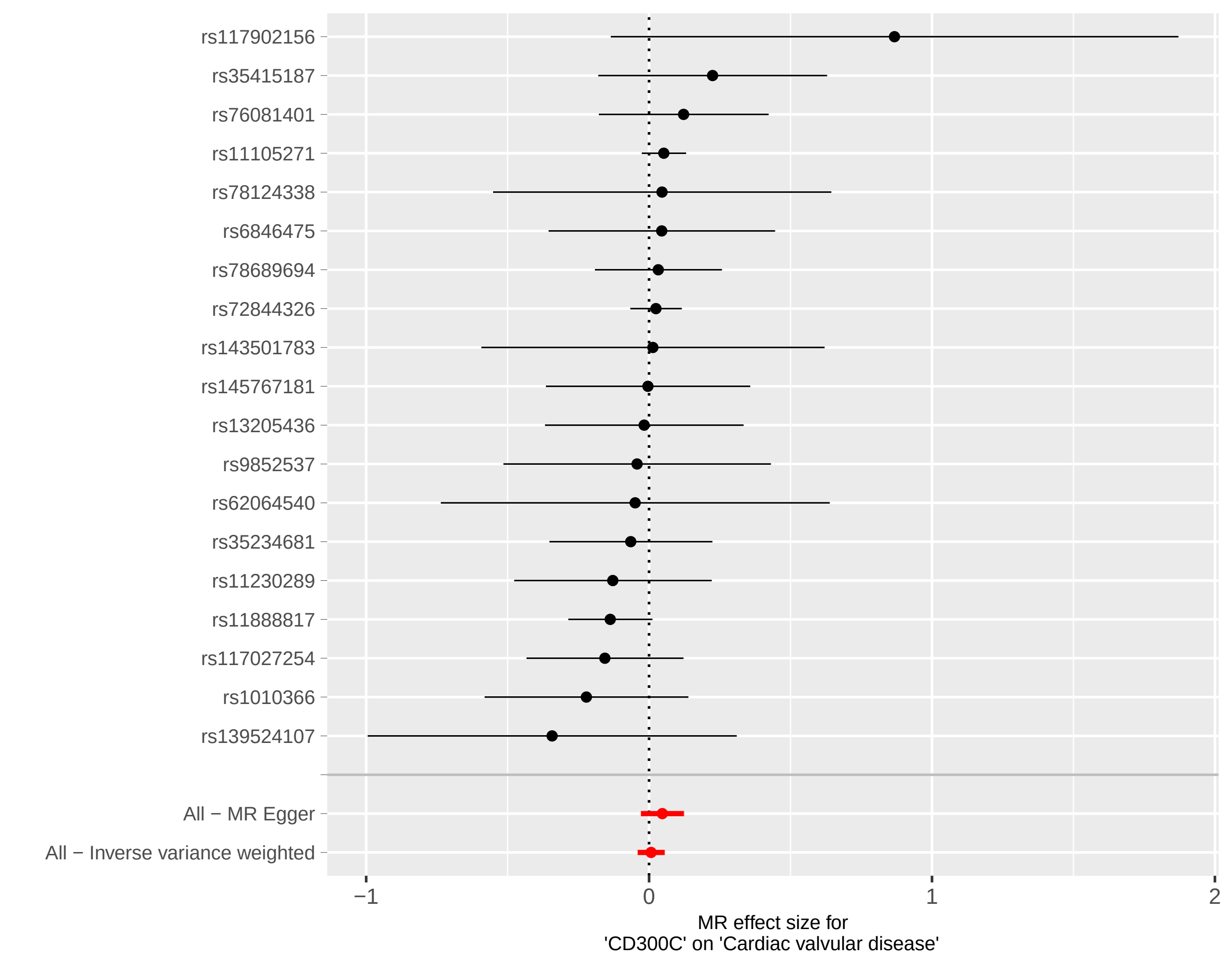
rs117902156
rs35415187
rs76081401
rs11105271
rs78124338
rs6846475
rs78689694
rs72844326
rs143501783
rs145767181
rs13205436
rs9852537
rs62064540
rs35234681
rs11230289
rs11888817
rs117027254
rs1010366
rs139524107
All − MR Egger
All − Inverse variance weighted
−1
0
1
2
MR effect size for
'CD300C' on 'Cardiac valvular disease'

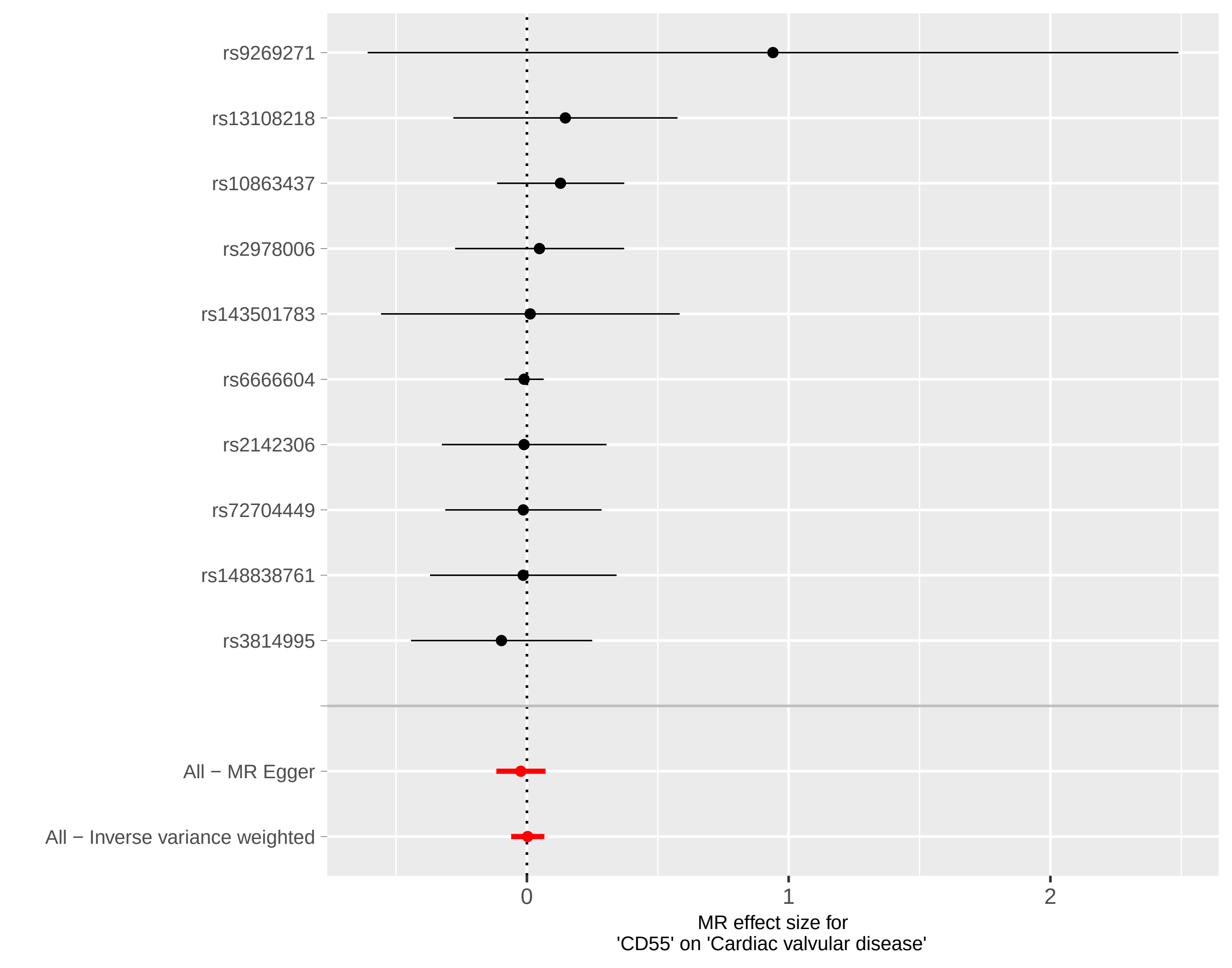
rs9269271
rs13108218
rs10863437
rs2978006
rs143501783
rs6666604
rs2142306
rs72704449
rs148838761
rs3814995
All − MR Egger
All − Inverse variance weighted
0
1
2
MR effect size for
'CD55' on 'Cardiac valvular disease'

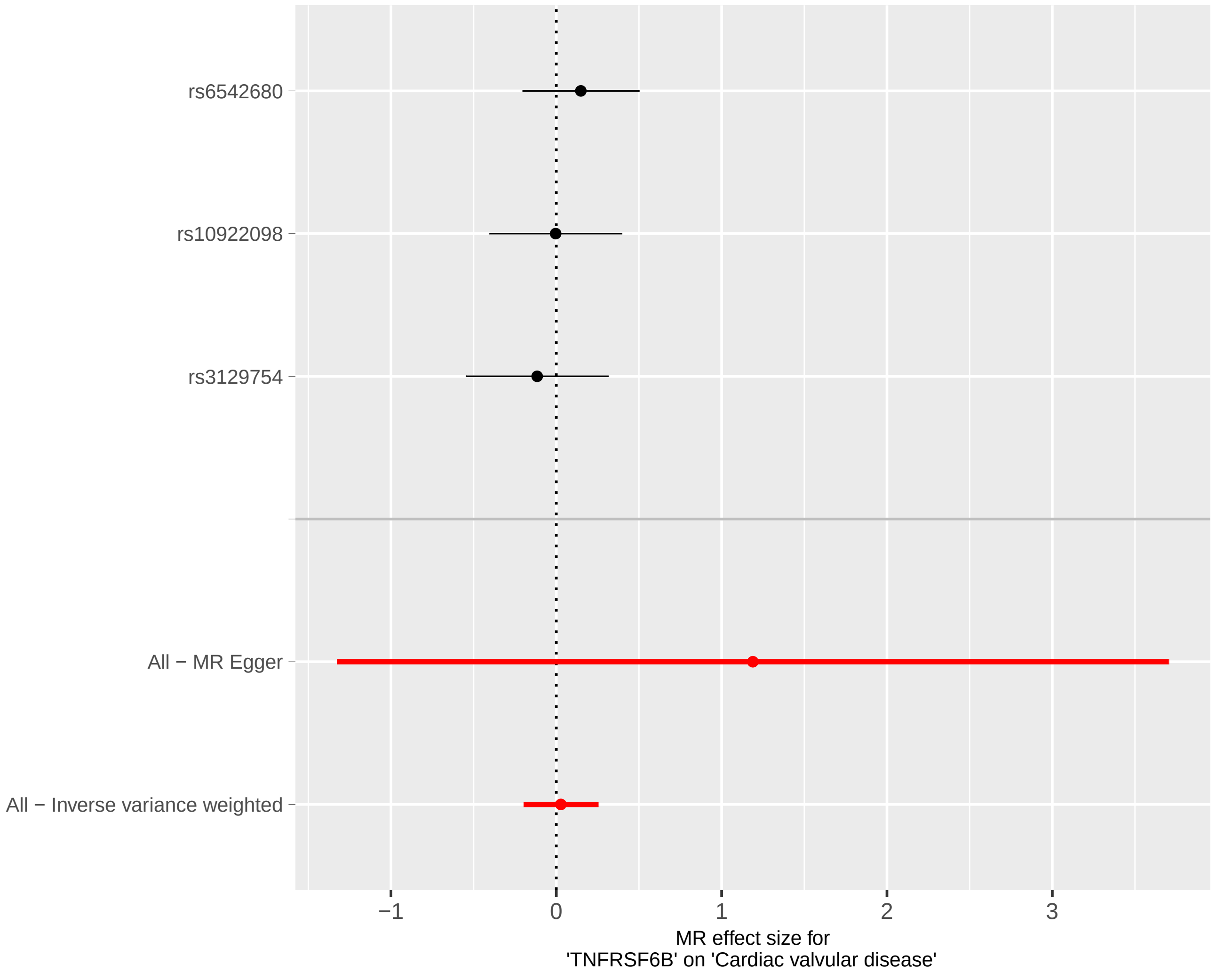
rs6542680
rs10922098
rs3129754
All − MR Egger
All − Inverse variance weighted
−1
0
1
2
3
MR effect size for
'TNFRSF6B' on 'Cardiac valvular disease'

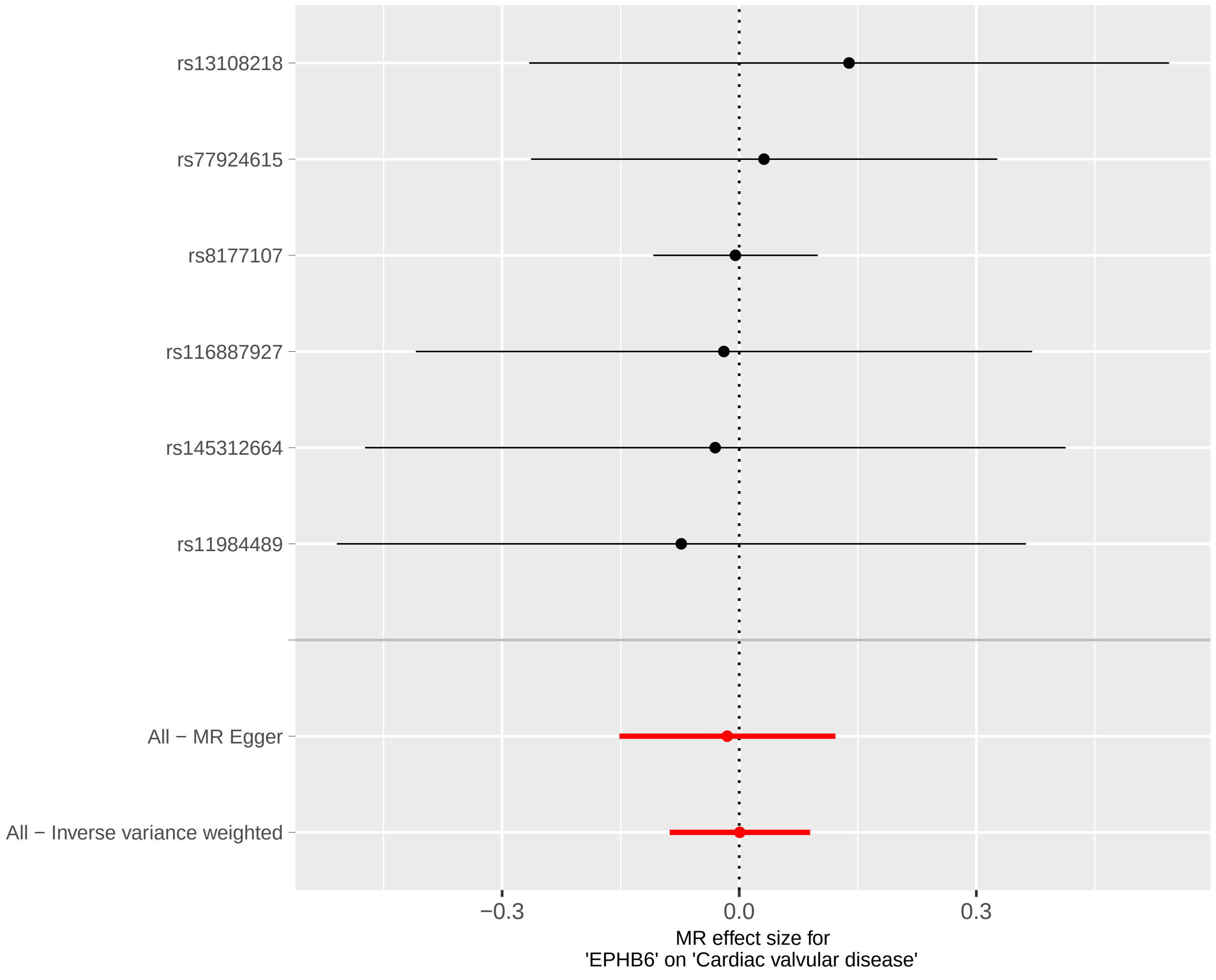
rs13108218
rs77924615
rs8177107
rs116887927
rs145312664
rs11984489
All − MR Egger
All − Inverse variance weighted
−0.3
0.0
0.3
MR effect size for
'EPHB6' on 'Cardiac valvular disease'

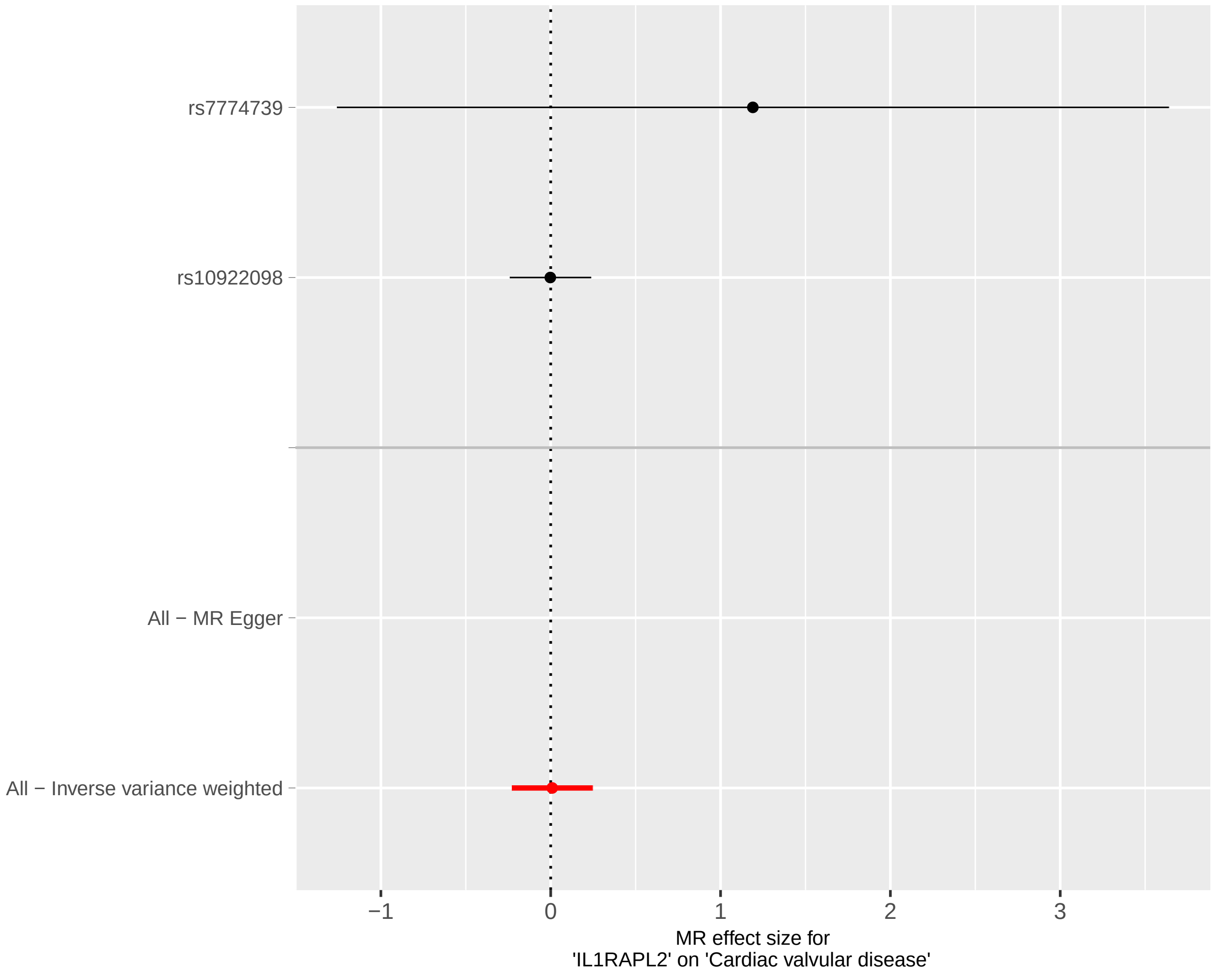
rs7774739
rs10922098
All − MR Egger
All − Inverse variance weighted
−1
0
1
2
3
MR effect size for
'IL1RAPL2' on 'Cardiac valvular disease'

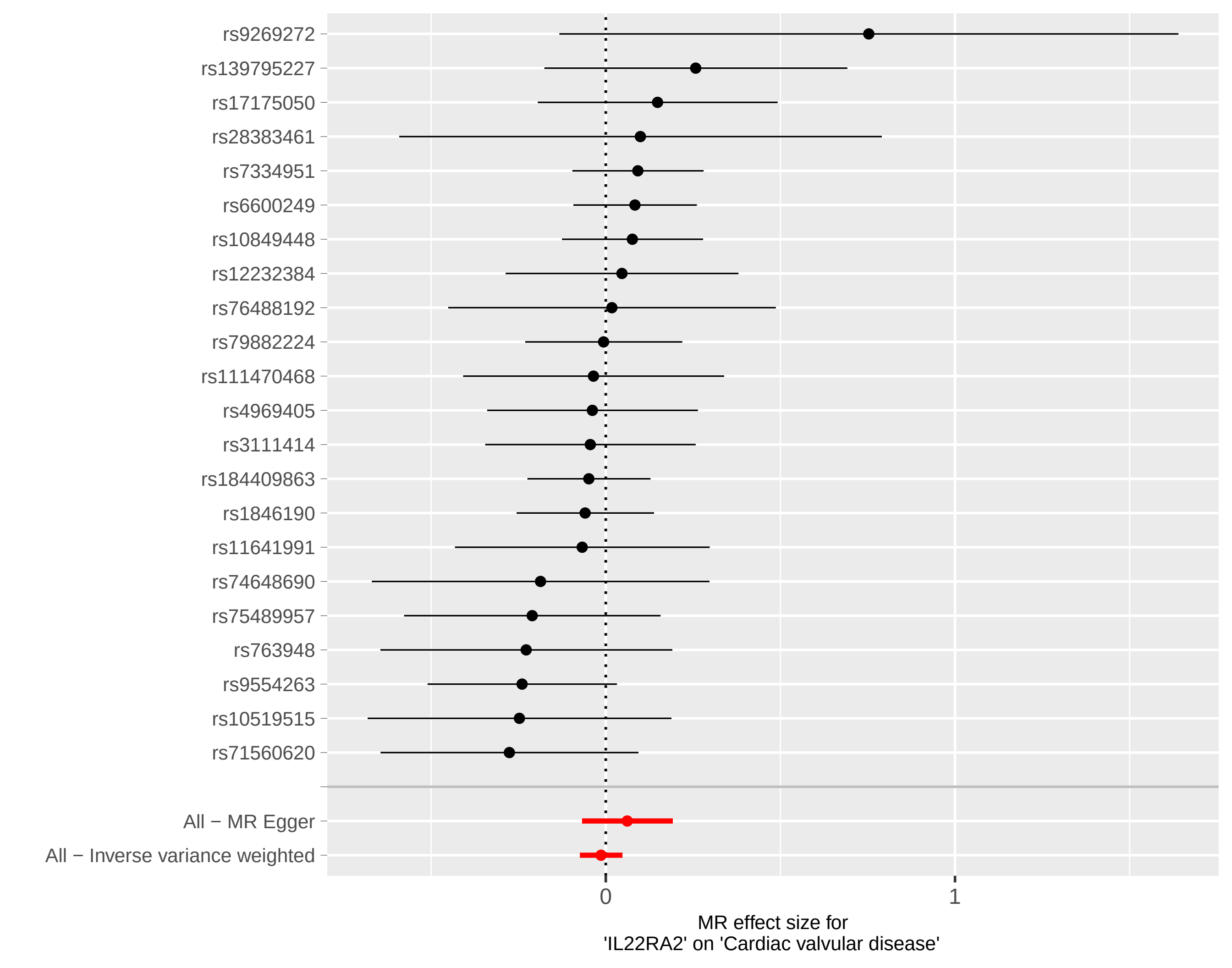
rs9269272
rs139795227
rs17175050
rs28383461
rs7334951
rs6600249
rs10849448
rs12232384
rs76488192
rs79882224
rs111470468
rs4969405
rs3111414
rs184409863
rs1846190
rs11641991
rs74648690
rs75489957
rs763948
rs9554263
rs10519515
rs71560620
All − MR Egger
All − Inverse variance weighted
0
1
MR effect size for
'IL22RA2' on 'Cardiac valvular disease'

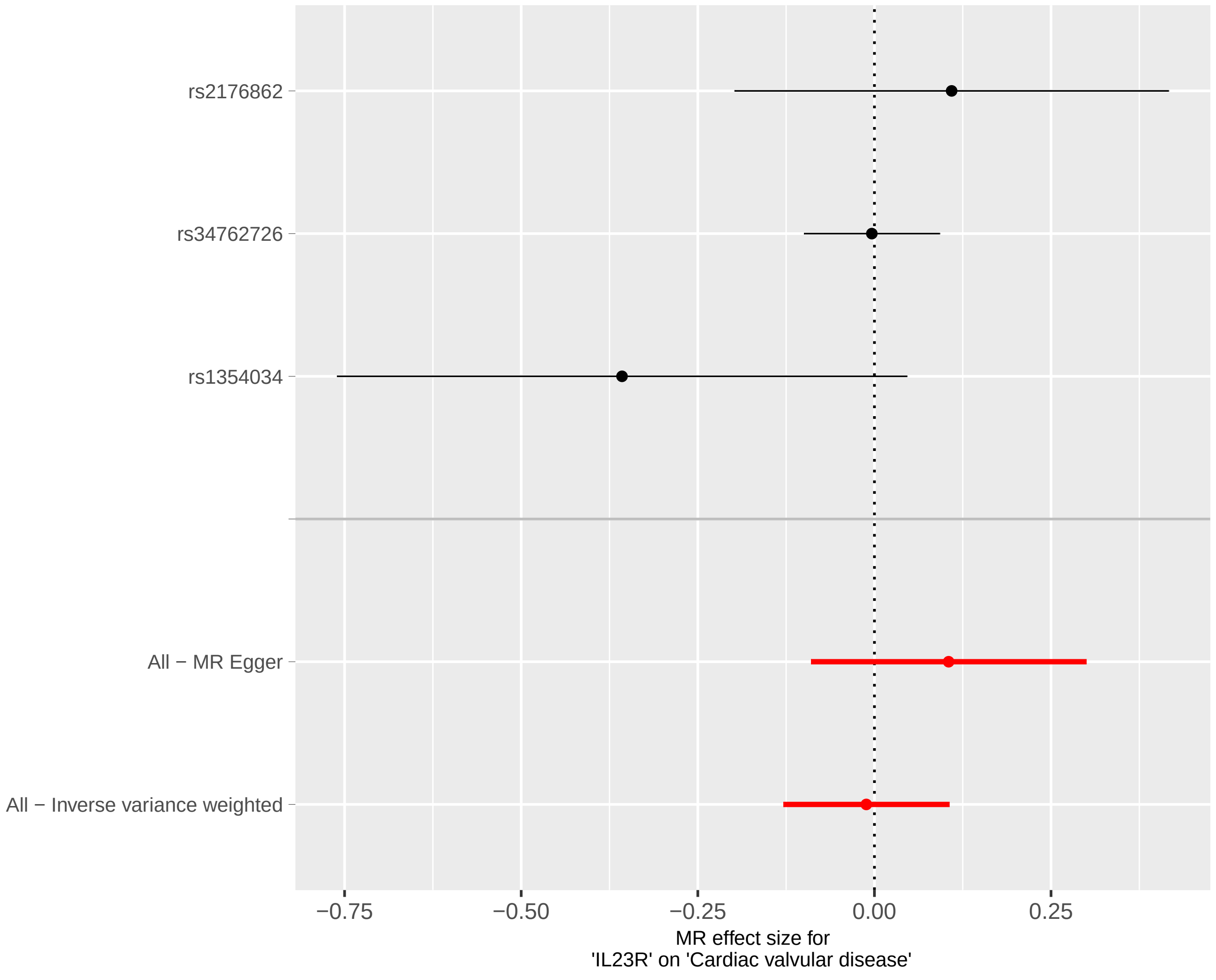
rs2176862
rs34762726
rs1354034
All − MR Egger
All − Inverse variance weighted
−0.75
−0.50
−0.25
0.00
0.25
MR effect size for
'IL23R' on 'Cardiac valvular disease'

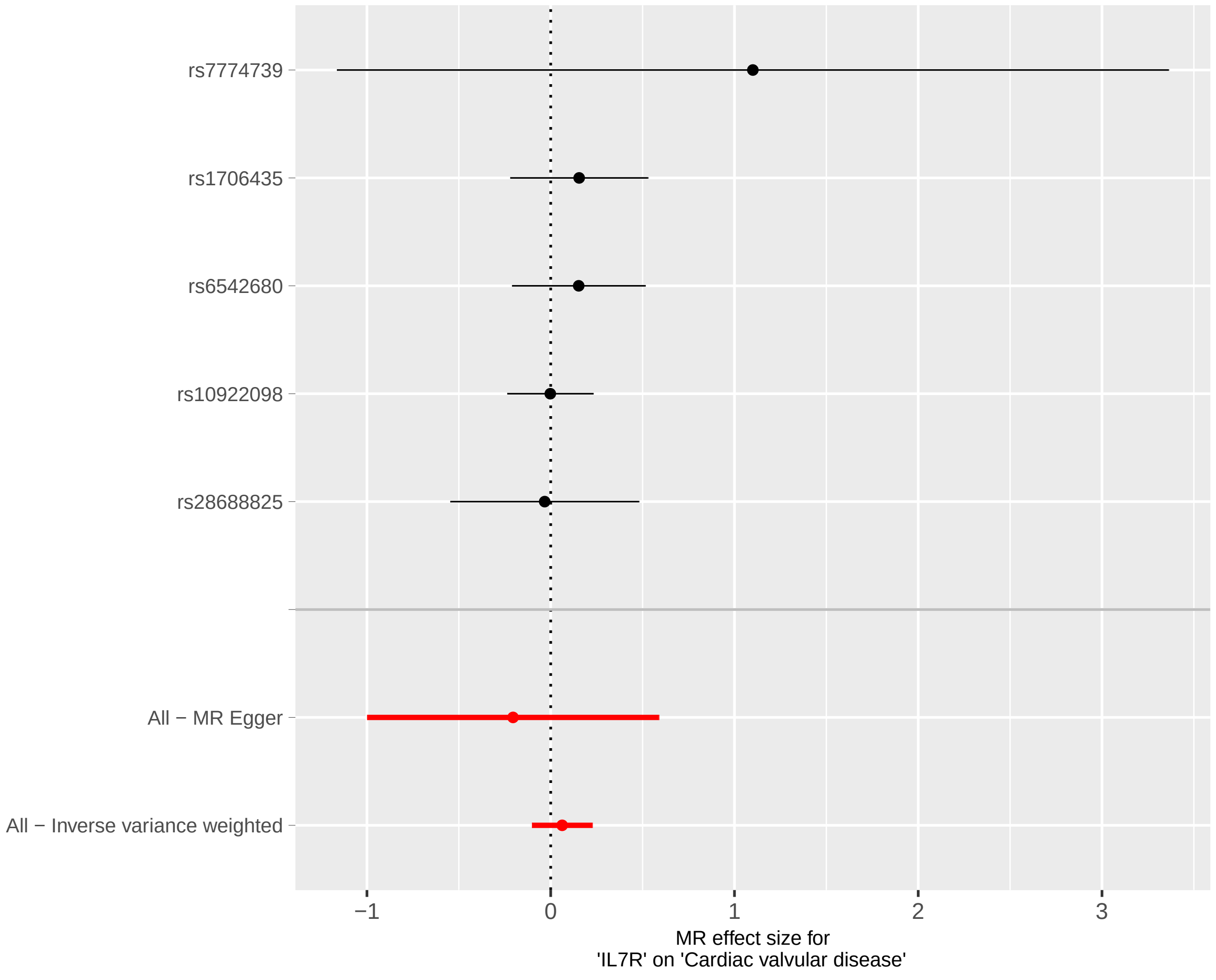
rs7774739
rs1706435
rs6542680
rs10922098
rs28688825
All − MR Egger
All − Inverse variance weighted
−1
0
1
2
3
MR effect size for
'IL7R' on 'Cardiac valvular disease'

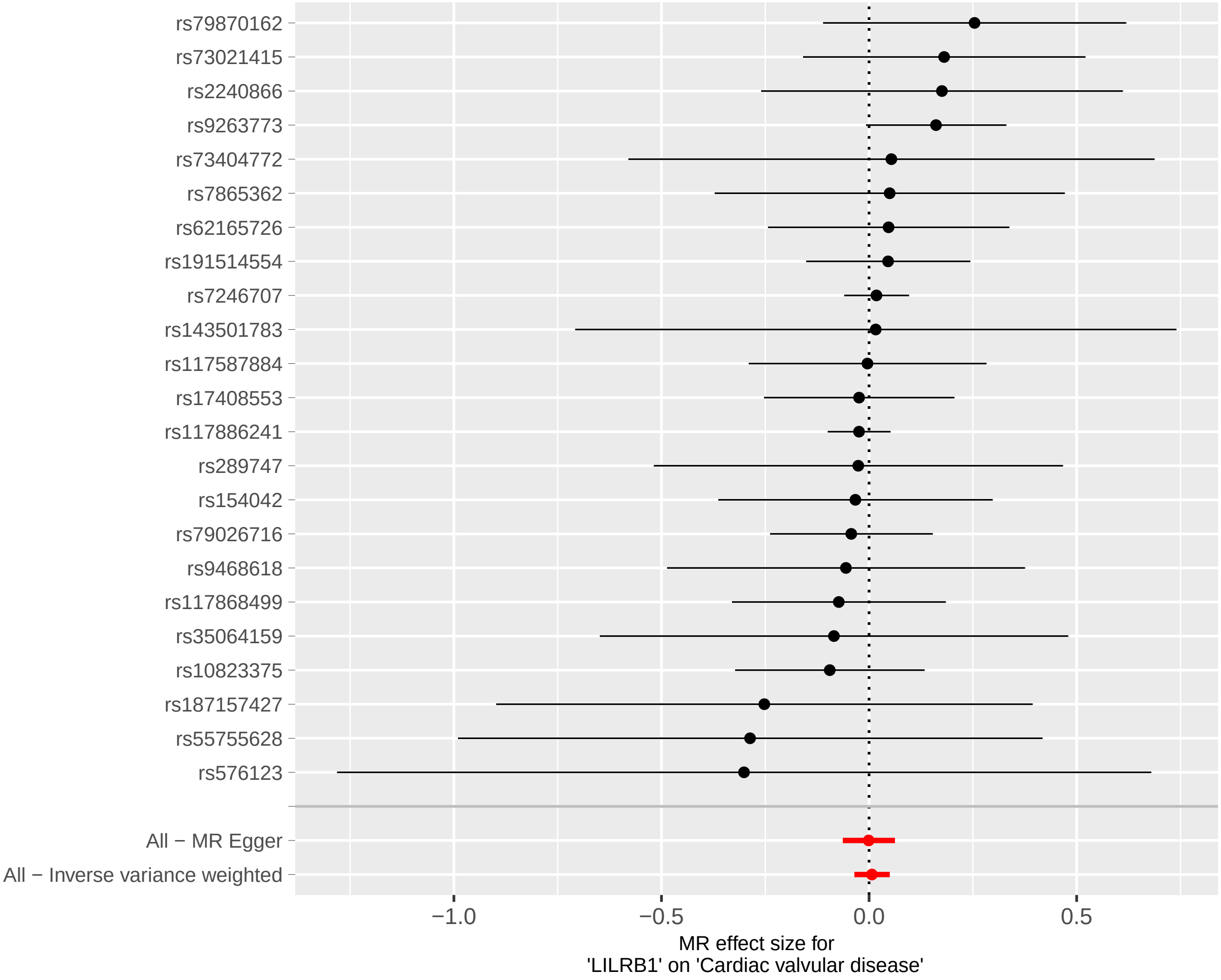
rs79870162
rs73021415
rs2240866
rs9263773
rs73404772
rs7865362
rs62165726
rs191514554
rs7246707
rs143501783
rs117587884
rs17408553
rs117886241
rs289747
rs154042
rs79026716
rs9468618
rs117868499
rs35064159
rs10823375
rs187157427
rs55755628
rs576123
All − MR Egger
All − Inverse variance weighted
−1.0
−0.5
0.0
0.5
MR effect size for
'LILRB1' on 'Cardiac valvular disease'

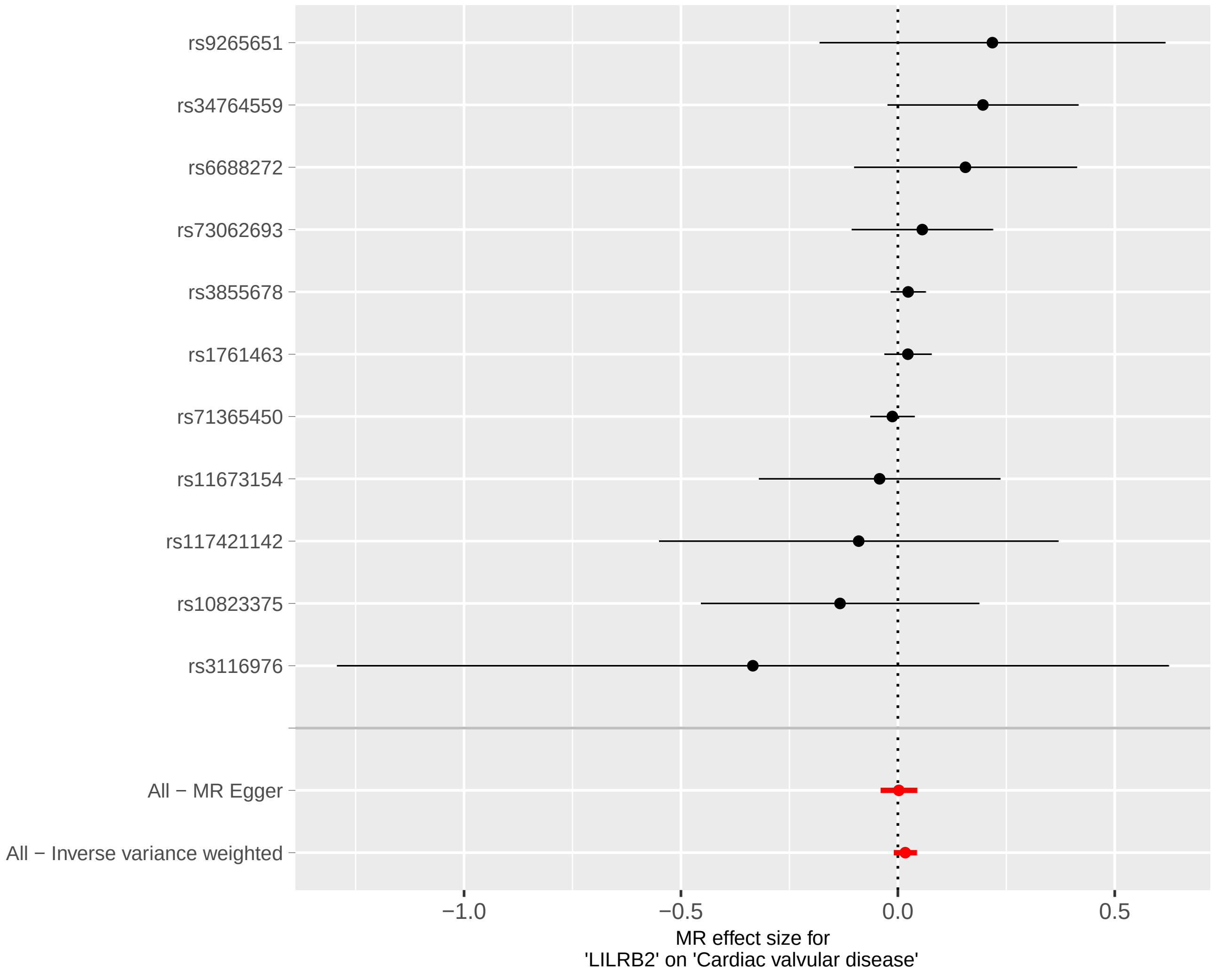

rs9265651
rs34764559
rs6688272
rs73062693
rs3855678
rs1761463
rs71365450
rs11673154
rs117421142
rs10823375
rs3116976
All − MR Egger
All − Inverse variance weighted
−1.0
−0.5
0.0
0.5
MR effect size for
'LILRB2' on 'Cardiac valvular disease'

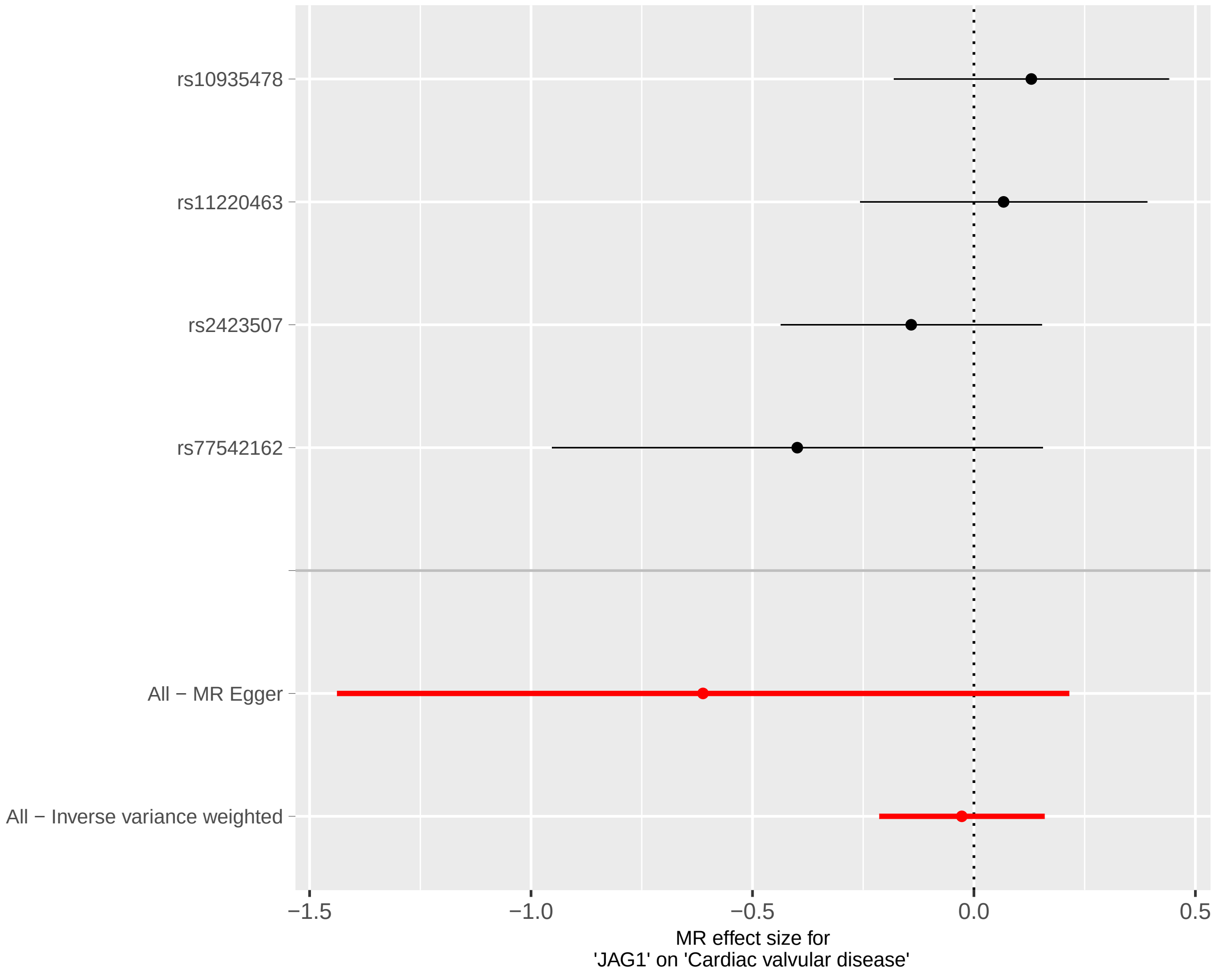
rs10935478
rs11220463
rs2423507
rs77542162
All − MR Egger
All − Inverse variance weighted
−1.5
−1.0
−0.5
0.0
0.5
MR effect size for
'JAG1' on 'Cardiac valvular disease'

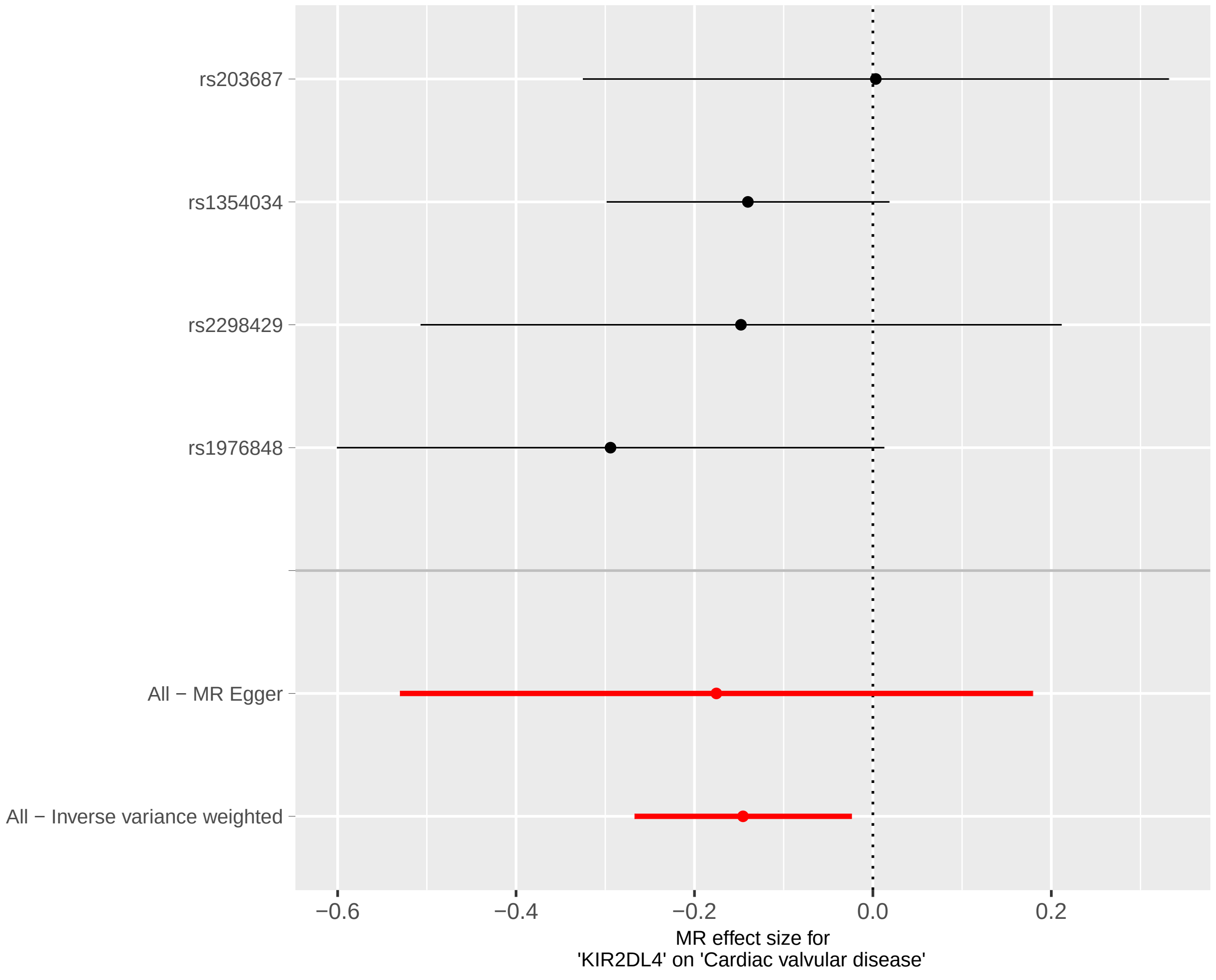
rs203687
rs1354034
rs2298429
rs1976848
All − MR Egger
All − Inverse variance weighted
−0.6
−0.4
−0.2
0.0
0.2
MR effect size for
'KIR2DL4' on 'Cardiac valvular disease'

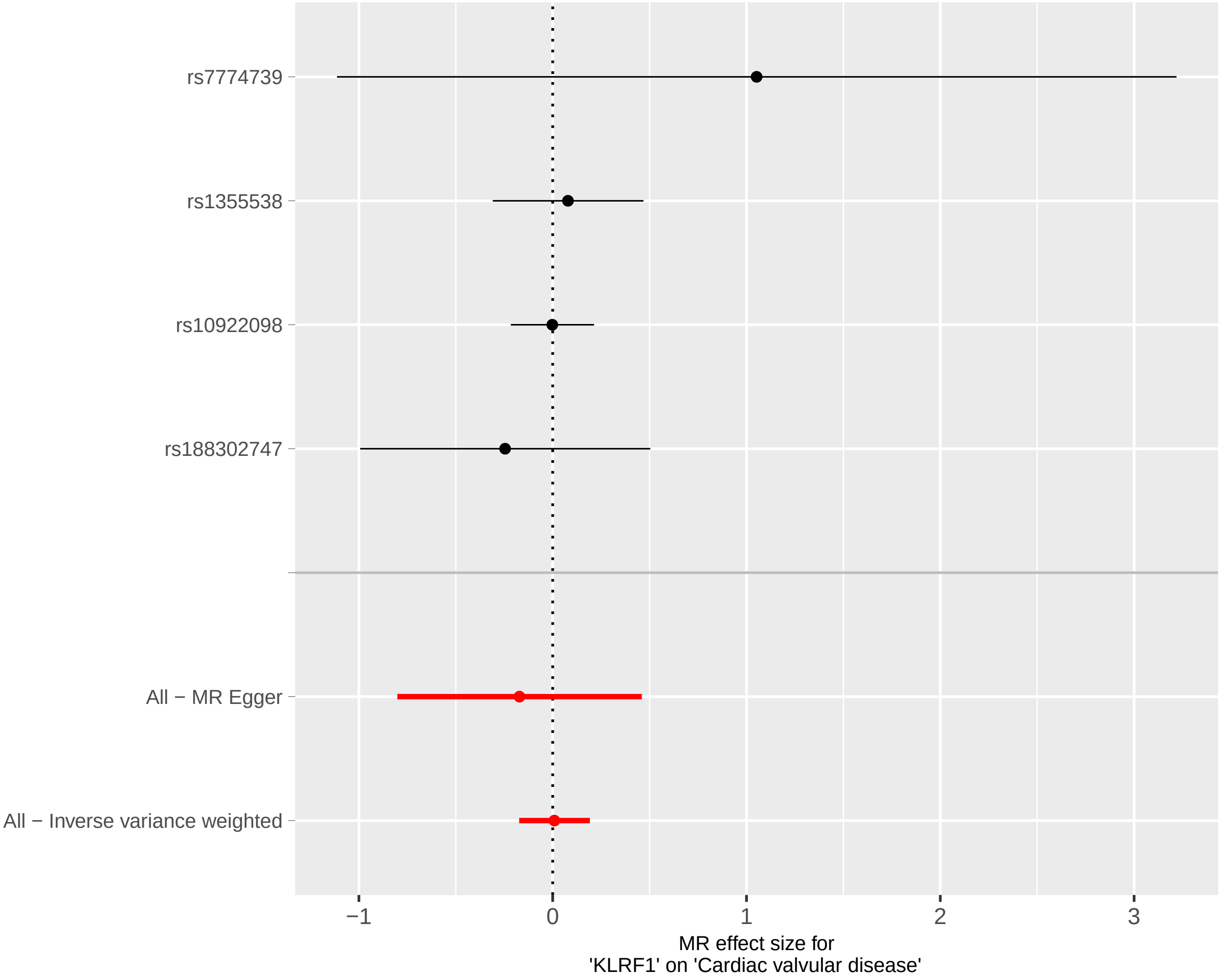

rs7774739
rs1355538
rs10922098
rs188302747
All − MR Egger
All − Inverse variance weighted
−1
0
1
2
3
MR effect size for
'KLRF1' on 'Cardiac valvular disease'

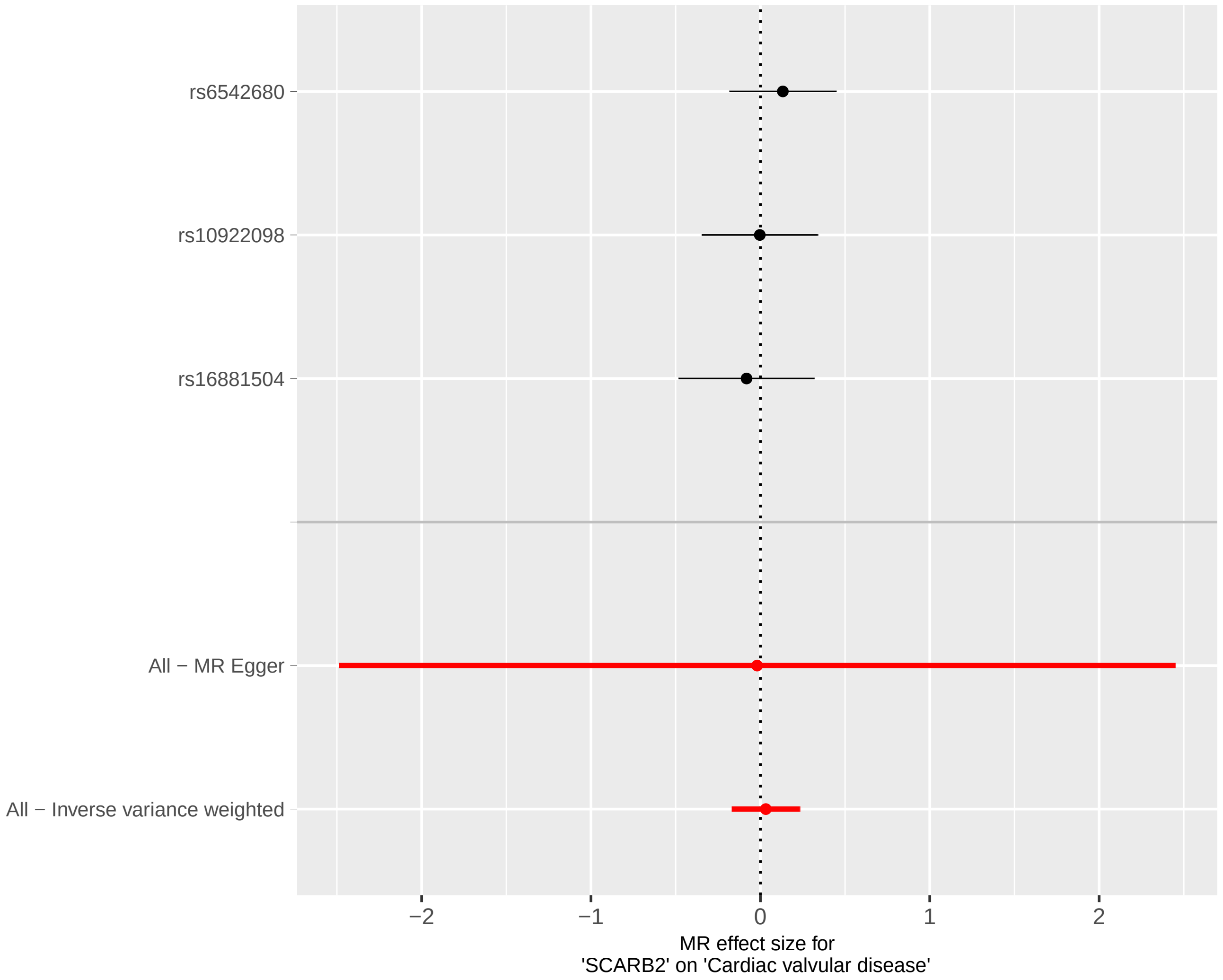
rs6542680
rs10922098
rs16881504
All − MR Egger
All − Inverse variance weighted
−2
−1
0
1
2
MR effect size for
'SCARB2' on 'Cardiac valvular disease'

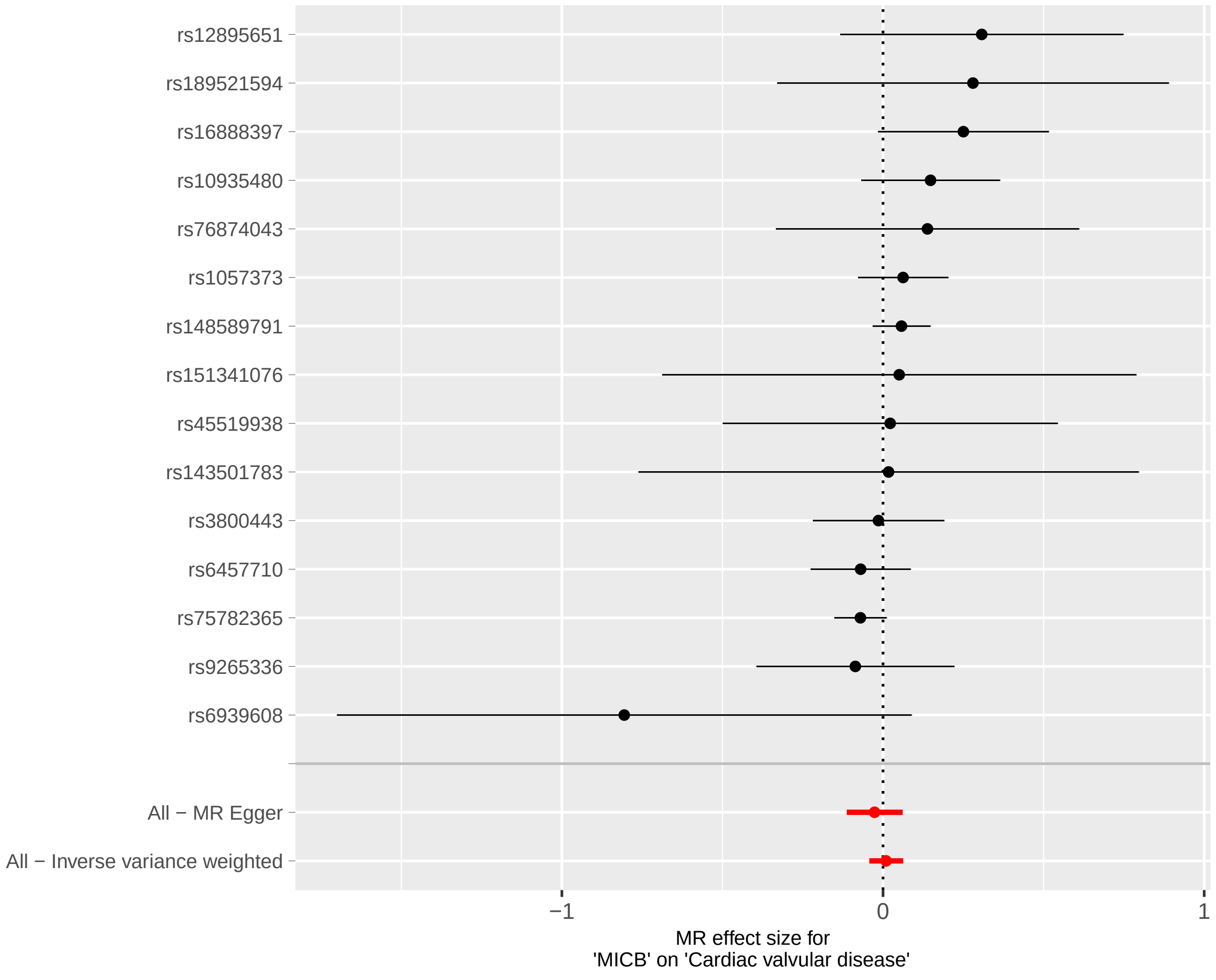
rs12895651
rs189521594
rs16888397
rs10935480
rs76874043
rs1057373
rs148589791
rs151341076
rs45519938
rs143501783
rs3800443
rs6457710
rs75782365
rs9265336
rs6939608
All − MR Egger
All − Inverse variance weighted
−1
0
1
MR effect size for
'MICB' on 'Cardiac valvular disease'

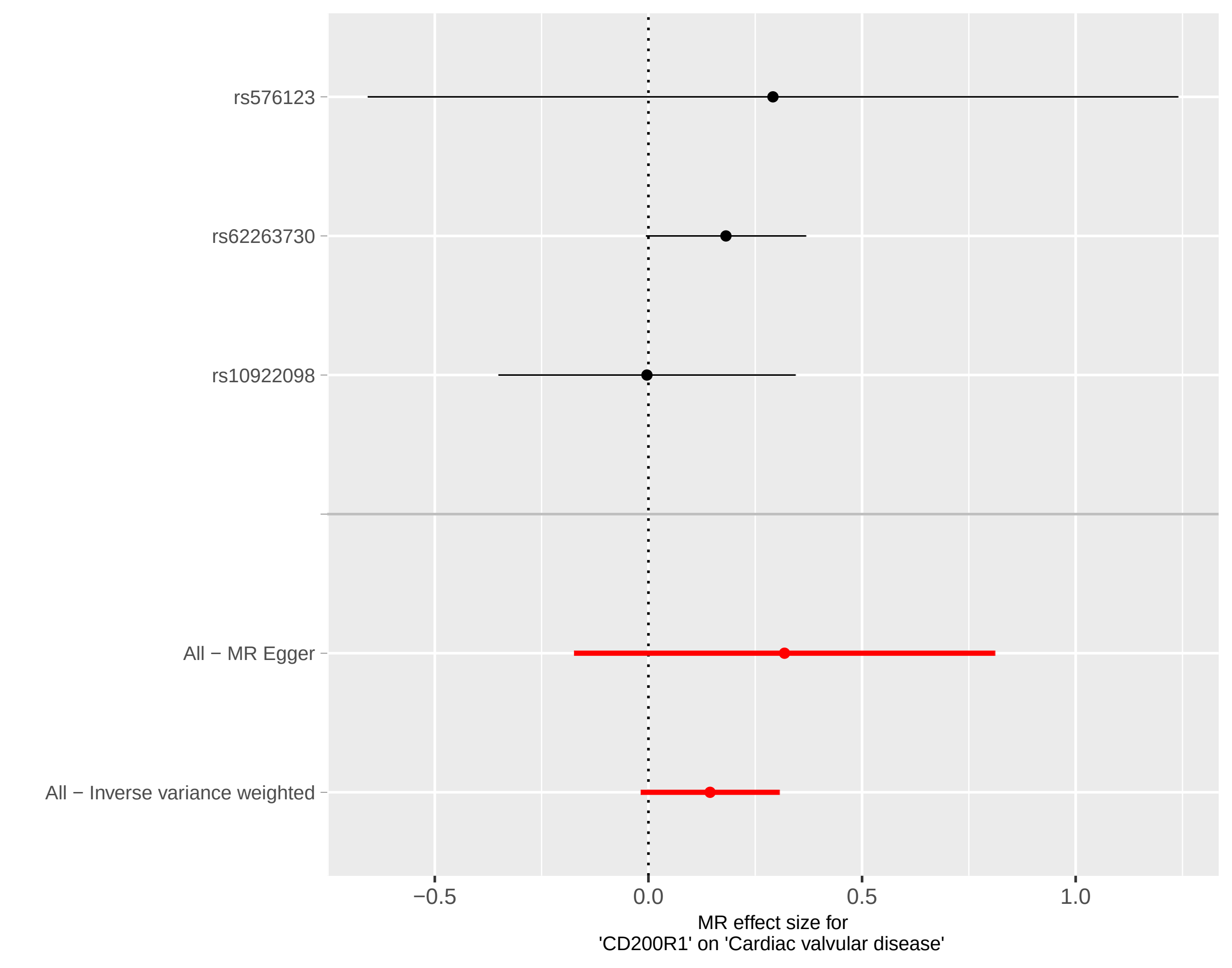
rs576123
rs62263730
rs10922098
All − MR Egger
All − Inverse variance weighted
−0.5
0.0
0.5
1.0
MR effect size for
'CD200R1' on 'Cardiac valvular disease'

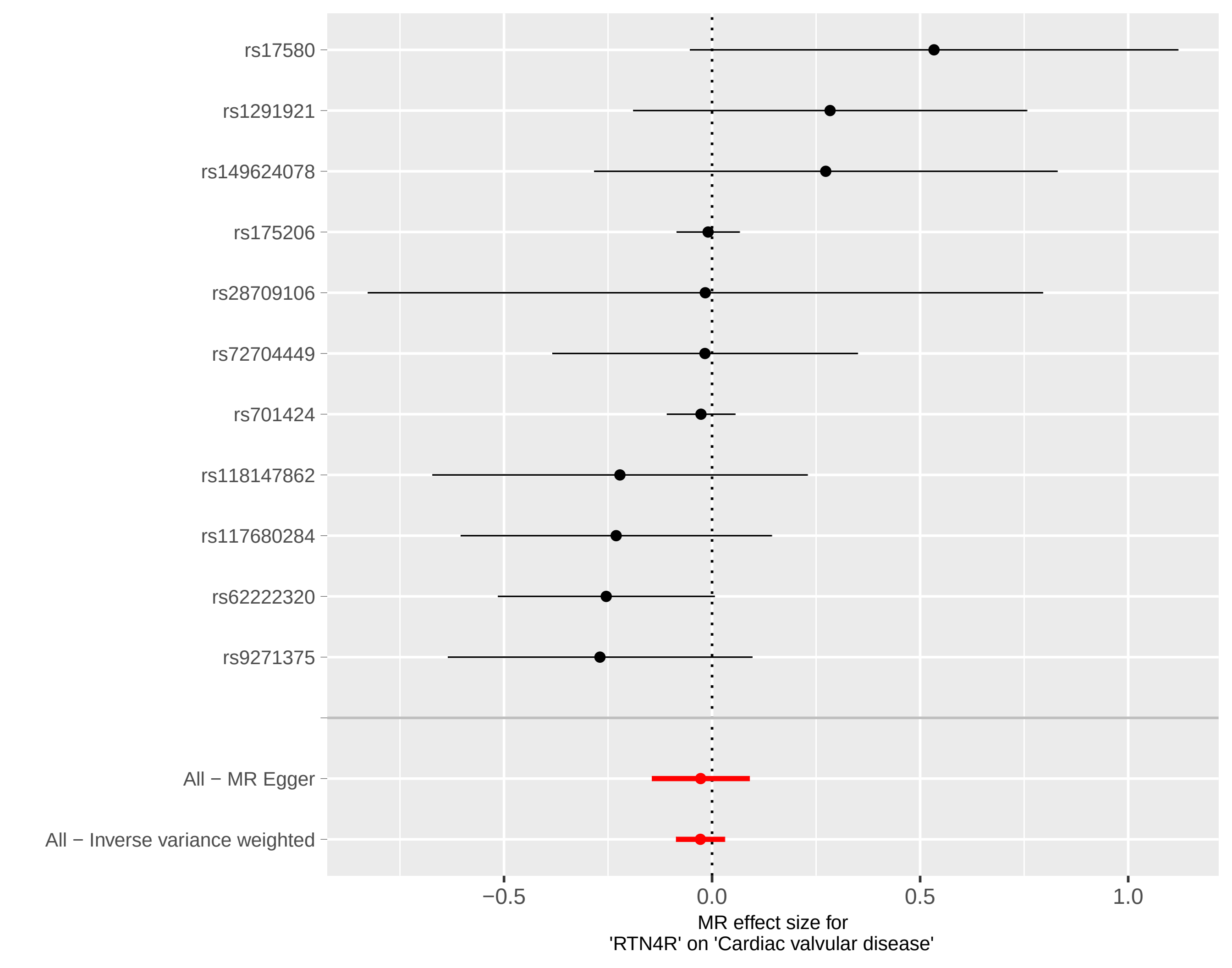

rs17580
rs1291921
rs149624078
rs175206
rs28709106
rs72704449
rs701424
rs118147862
rs117680284
rs62222320
rs9271375
All − MR Egger
All − Inverse variance weighted
−0.5
0.0
0.5
1.0
MR effect size for
'RTN4R' on 'Cardiac valvular disease'

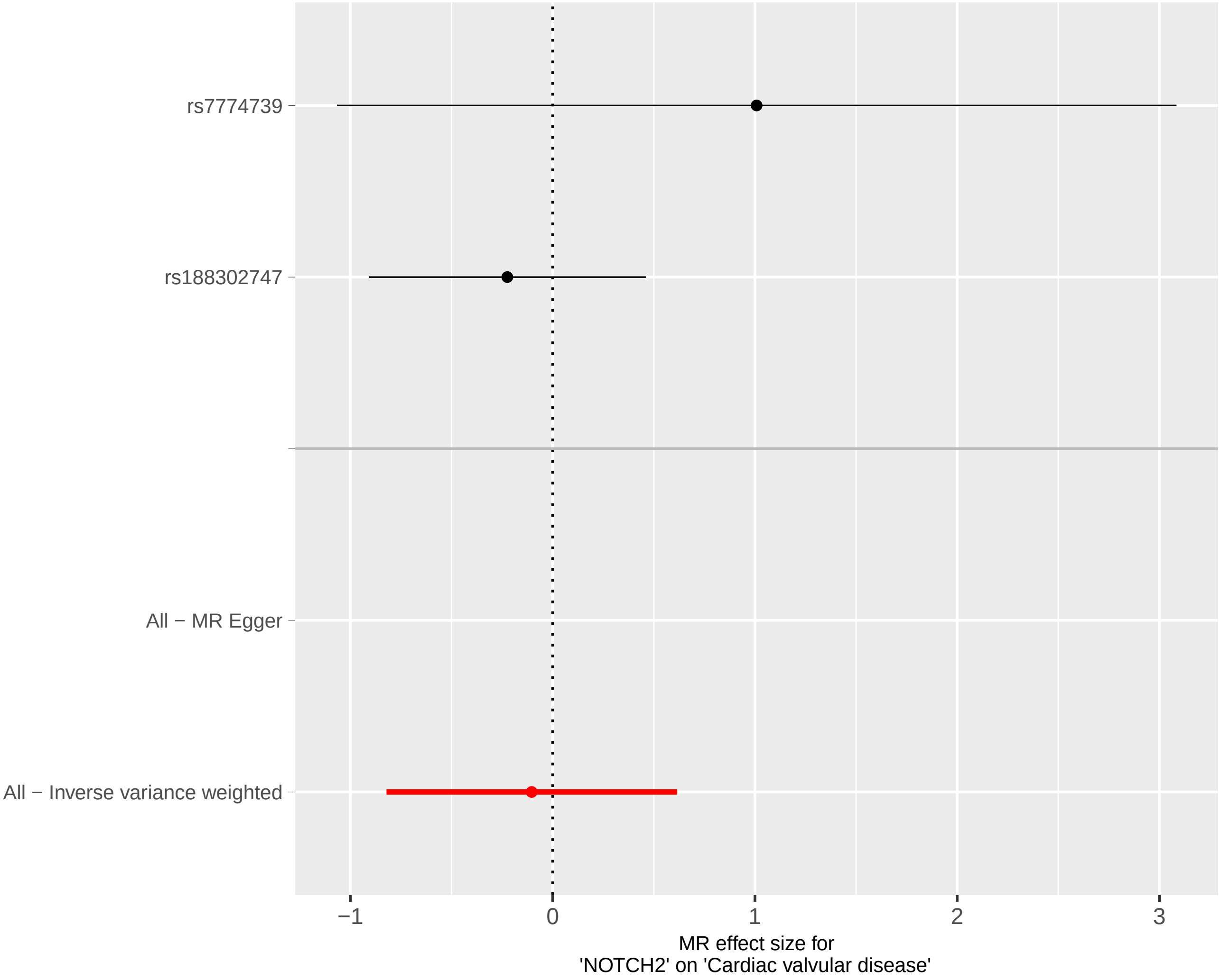

rs7774739
rs188302747
All − MR Egger
All − Inverse variance weighted
−1
0
1
2
3
MR effect size for
'NOTCH2' on 'Cardiac valvular disease'

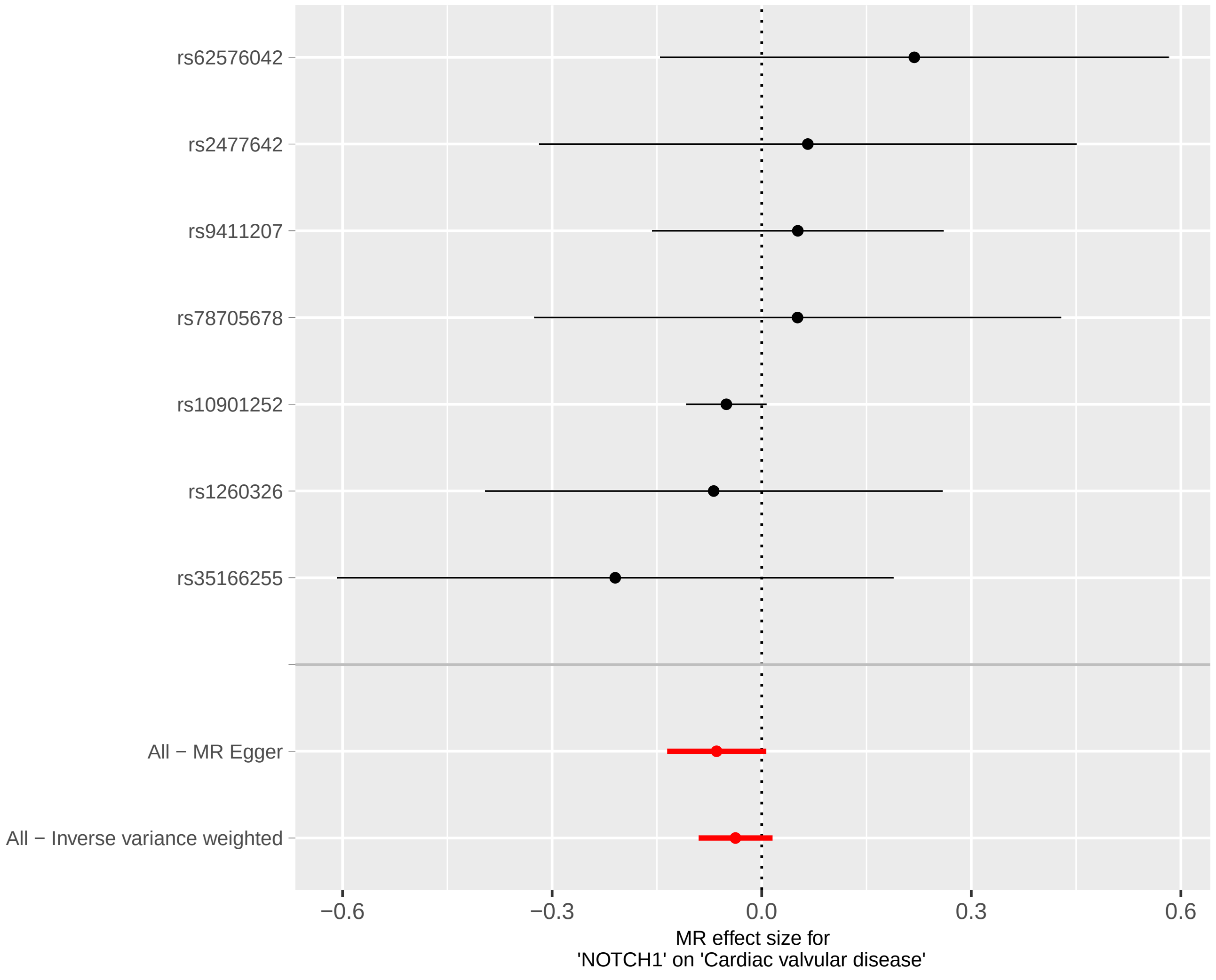

rs62576042
rs2477642
rs9411207
rs78705678
rs10901252
rs1260326
rs35166255
All − MR Egger
All − Inverse variance weighted
−0.6
−0.3
0.0
0.3
0.6
MR effect size for
'NOTCH1' on 'Cardiac valvular disease'

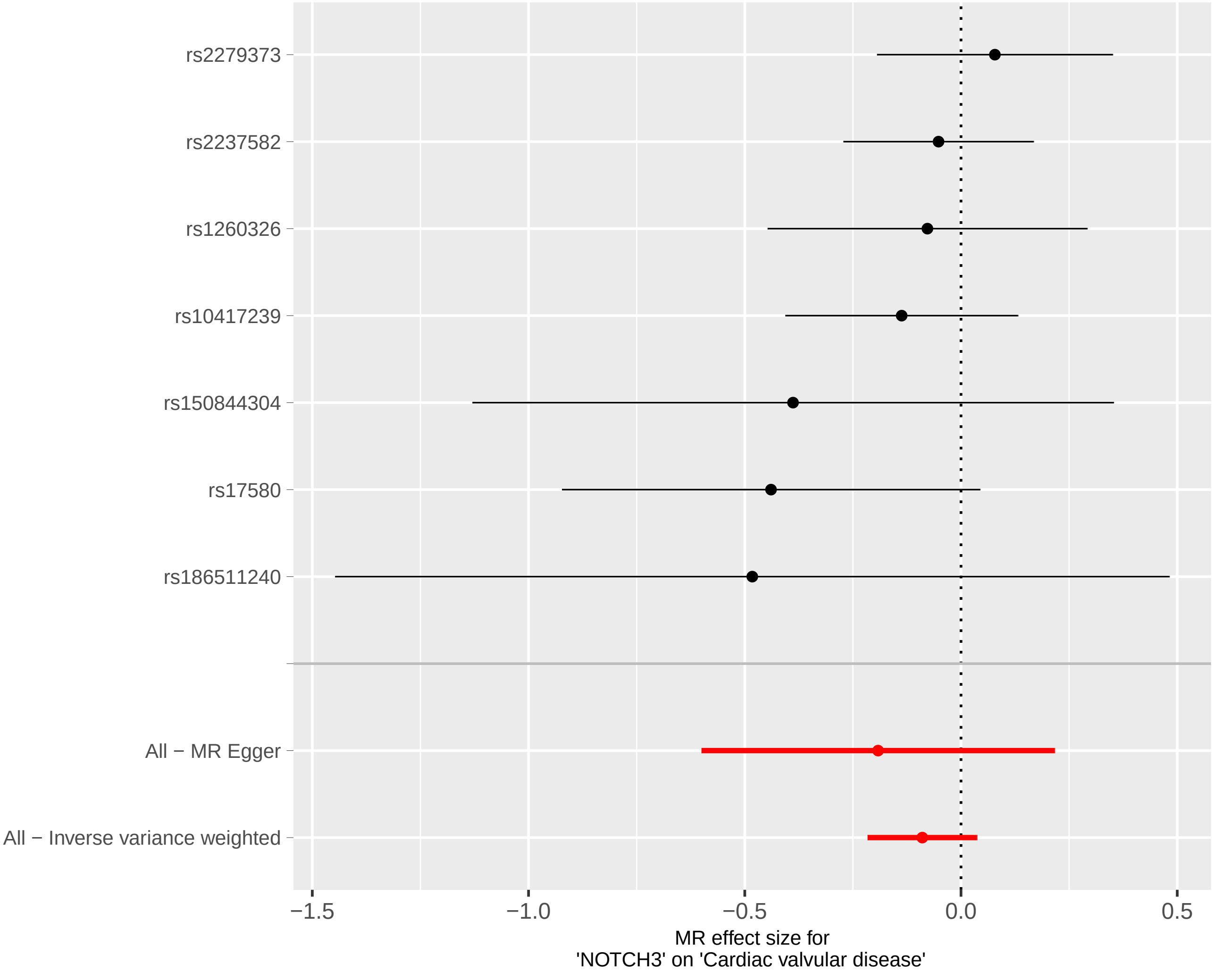
rs2279373
rs2237582
rs1260326
rs10417239
rs150844304
rs17580
rs186511240
All − MR Egger
All − Inverse variance weighted
−1.5
−1.0
−0.5
0.0
0.5
MR effect size for
'NOTCH3' on 'Cardiac valvular disease'

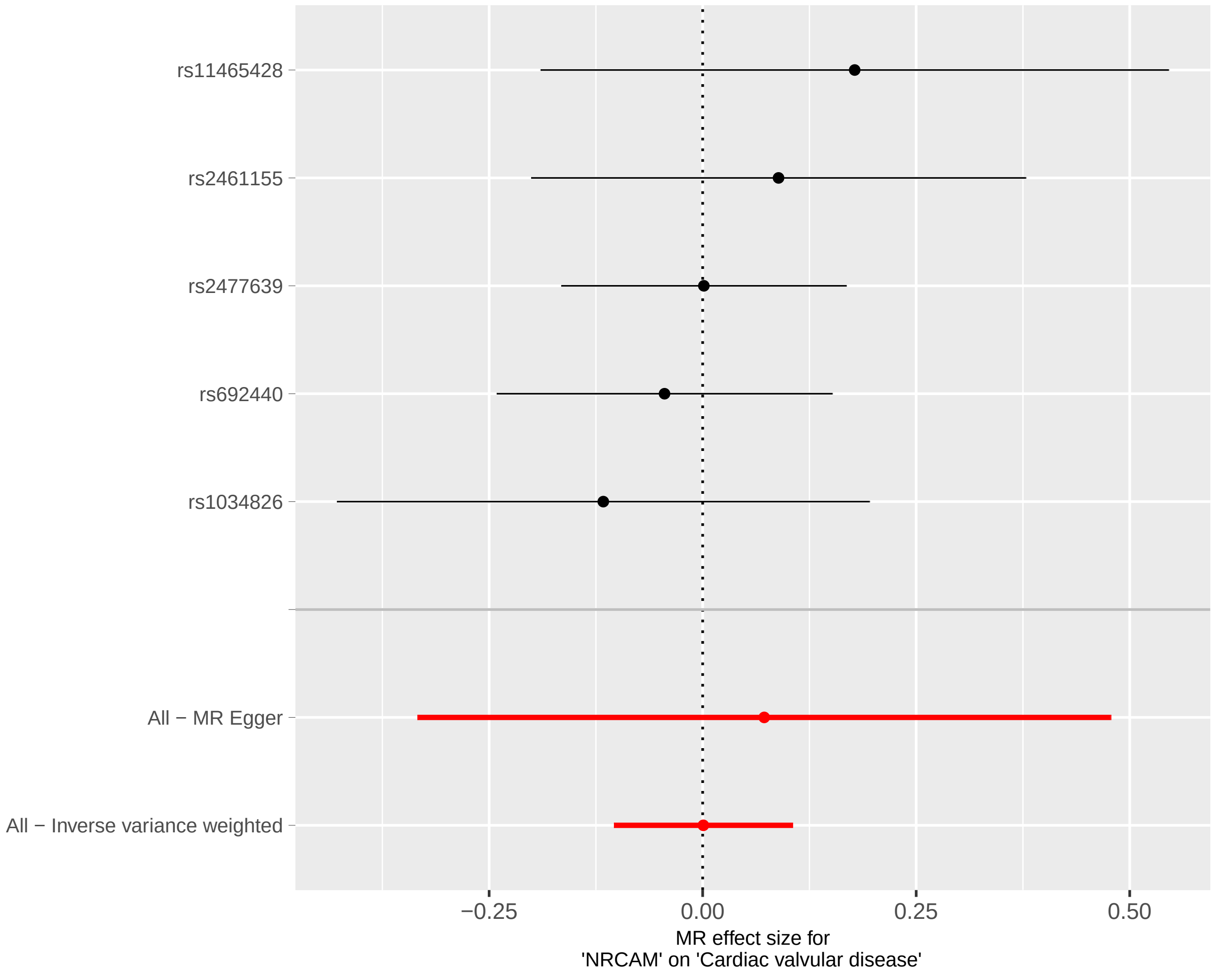

rs11465428
rs2461155
rs2477639
rs692440
rs1034826
All − MR Egger
All − Inverse variance weighted
−0.25
0.00
0.25
0.50
MR effect size for
'NRCAM' on 'Cardiac valvular disease'

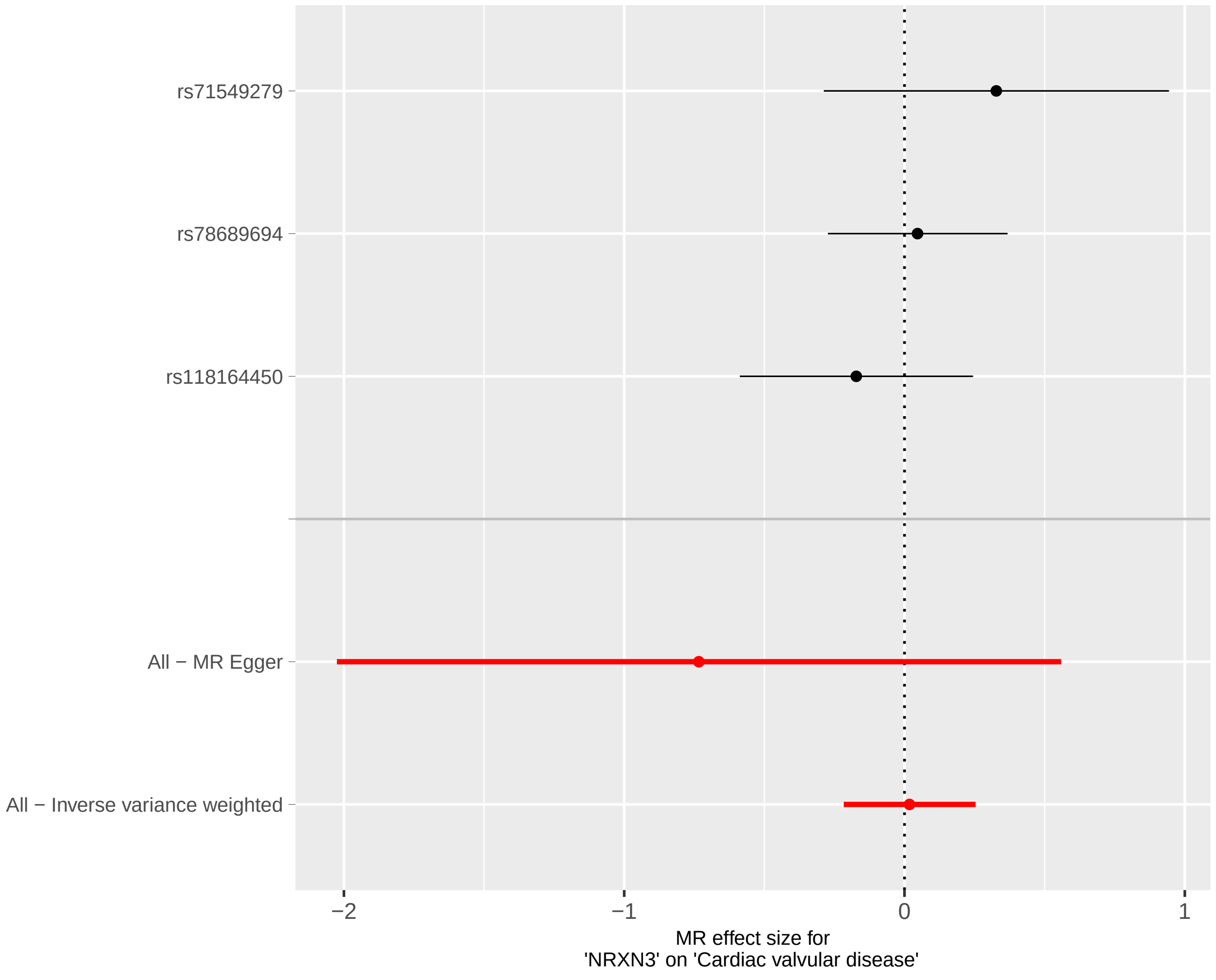
rs71549279
rs78689694
rs118164450
All − MR Egger
All − Inverse variance weighted
−2
−1
0
1
MR effect size for
'NRXN3' on 'Cardiac valvular disease'

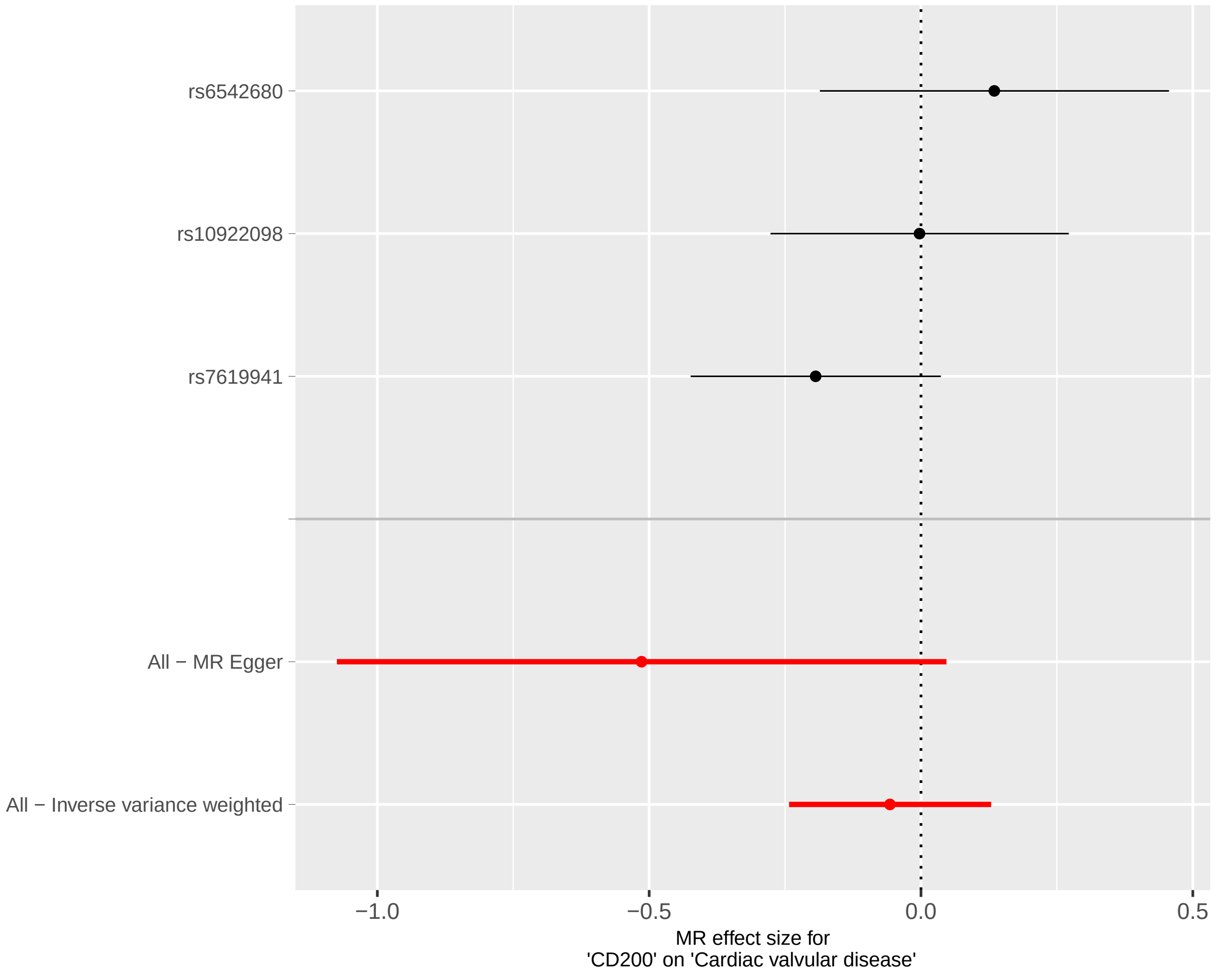

rs6542680
rs10922098
rs7619941
All − MR Egger
All − Inverse variance weighted
−1.0
−0.5
0.0
0.5
MR effect size for
'CD200' on 'Cardiac valvular disease'

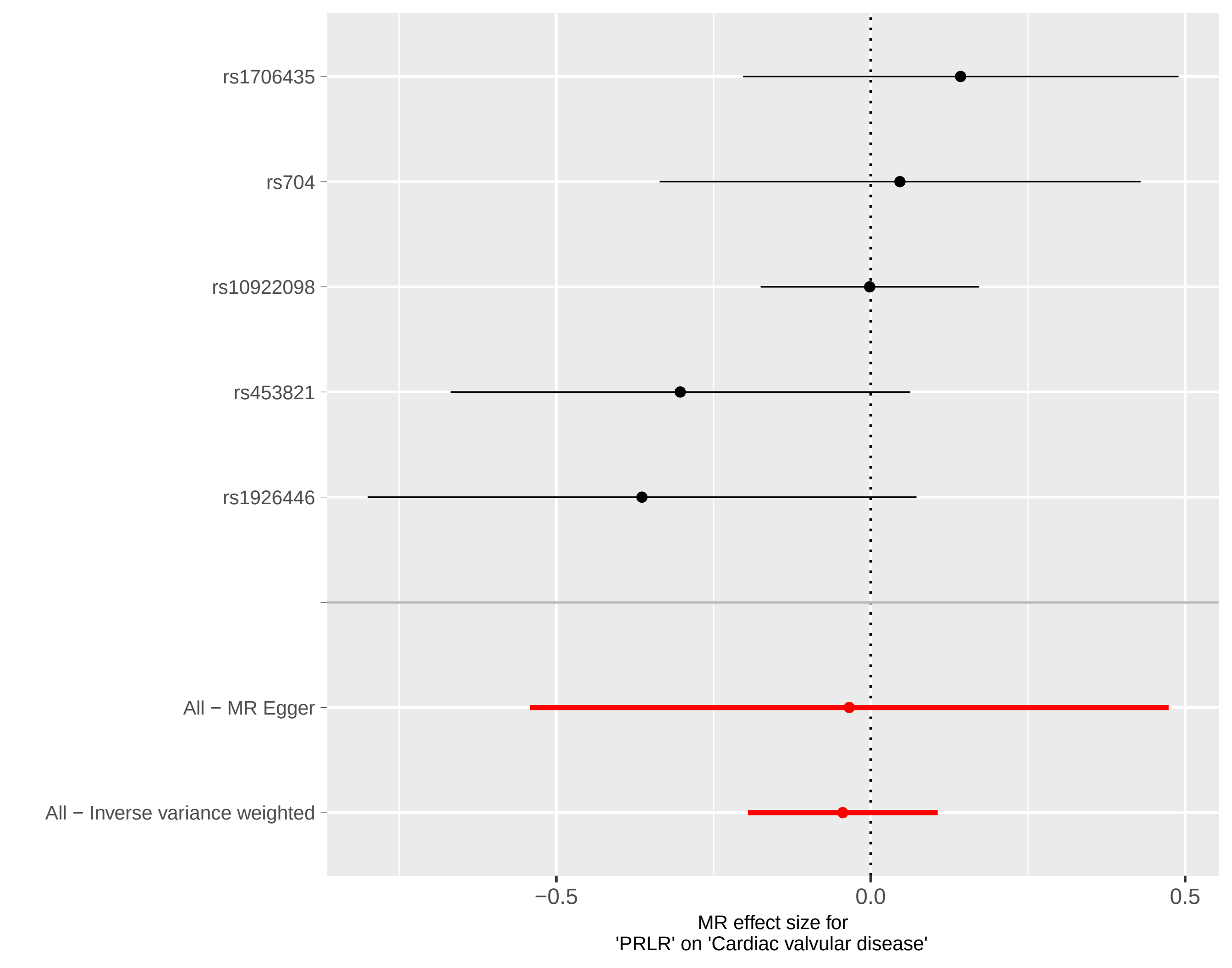
rs1706435
rs704
rs10922098
rs453821
rs1926446
All − MR Egger
All − Inverse variance weighted
−0.5
0.0
0.5
MR effect size for
'PRLR' on 'Cardiac valvular disease'

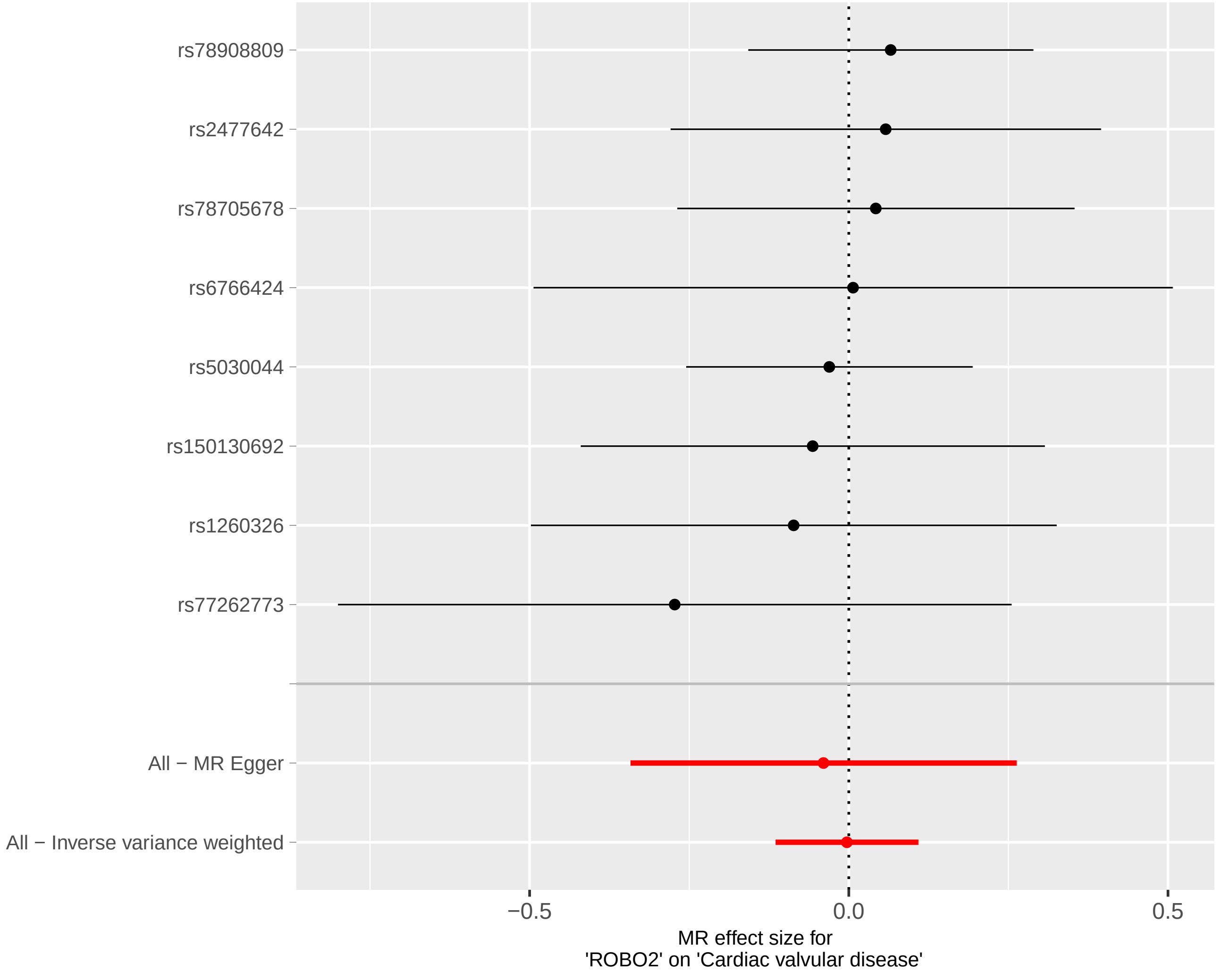
rs78908809
rs2477642
rs78705678
rs6766424
rs5030044
rs150130692
rs1260326
rs77262773
All − MR Egger
All − Inverse variance weighted
−0.5
0.0
0.5
MR effect size for
'ROBO2' on 'Cardiac valvular disease'

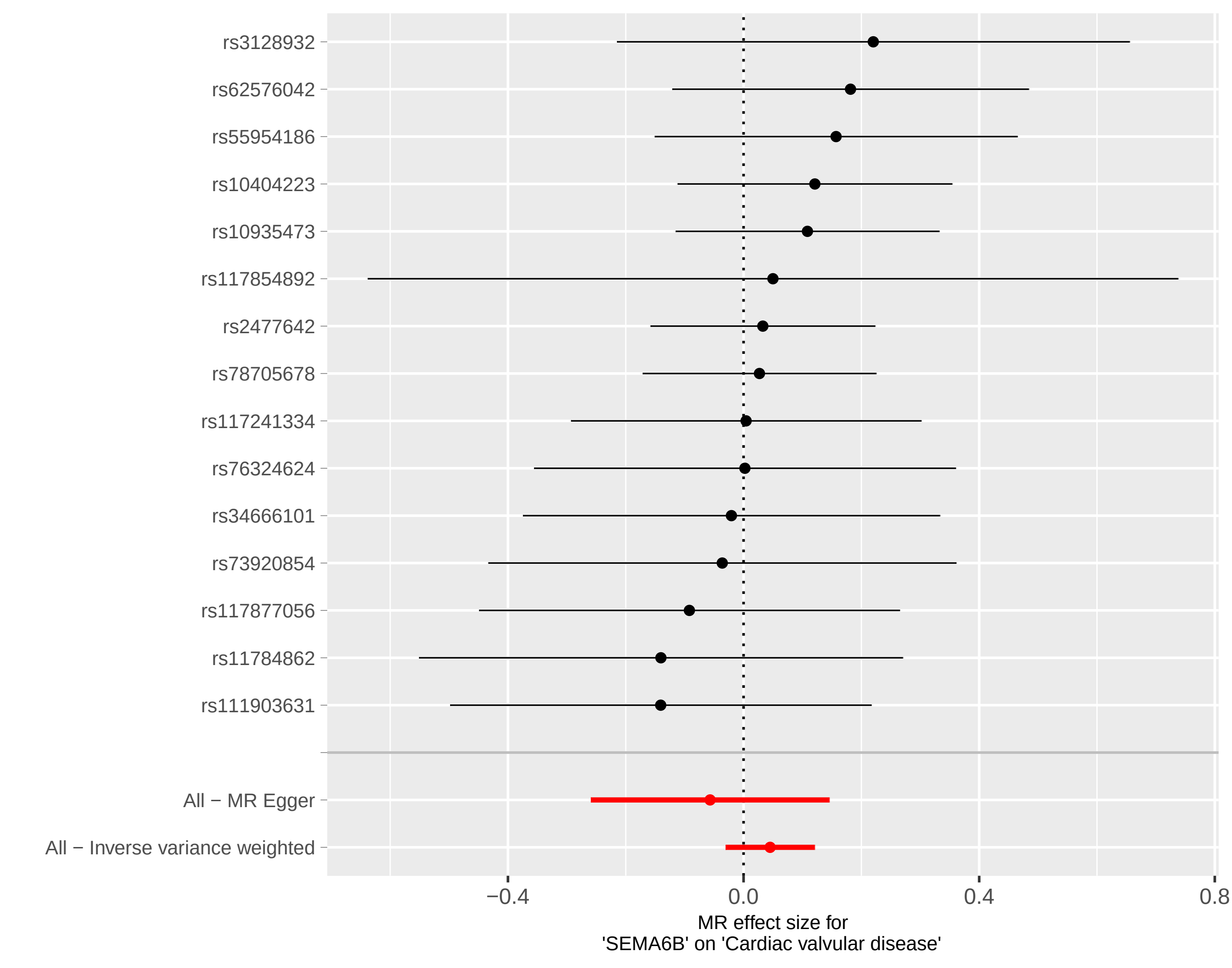
rs3128932
rs62576042
rs55954186
rs10404223
rs10935473
rs117854892
rs2477642
rs78705678
rs117241334
rs76324624
rs34666101
rs73920854
rs117877056
rs11784862
rs111903631
All − MR Egger
All − Inverse variance weighted
−0.4
0.0
0.4
0.8
MR effect size for
'SEMA6B' on 'Cardiac valvular disease'

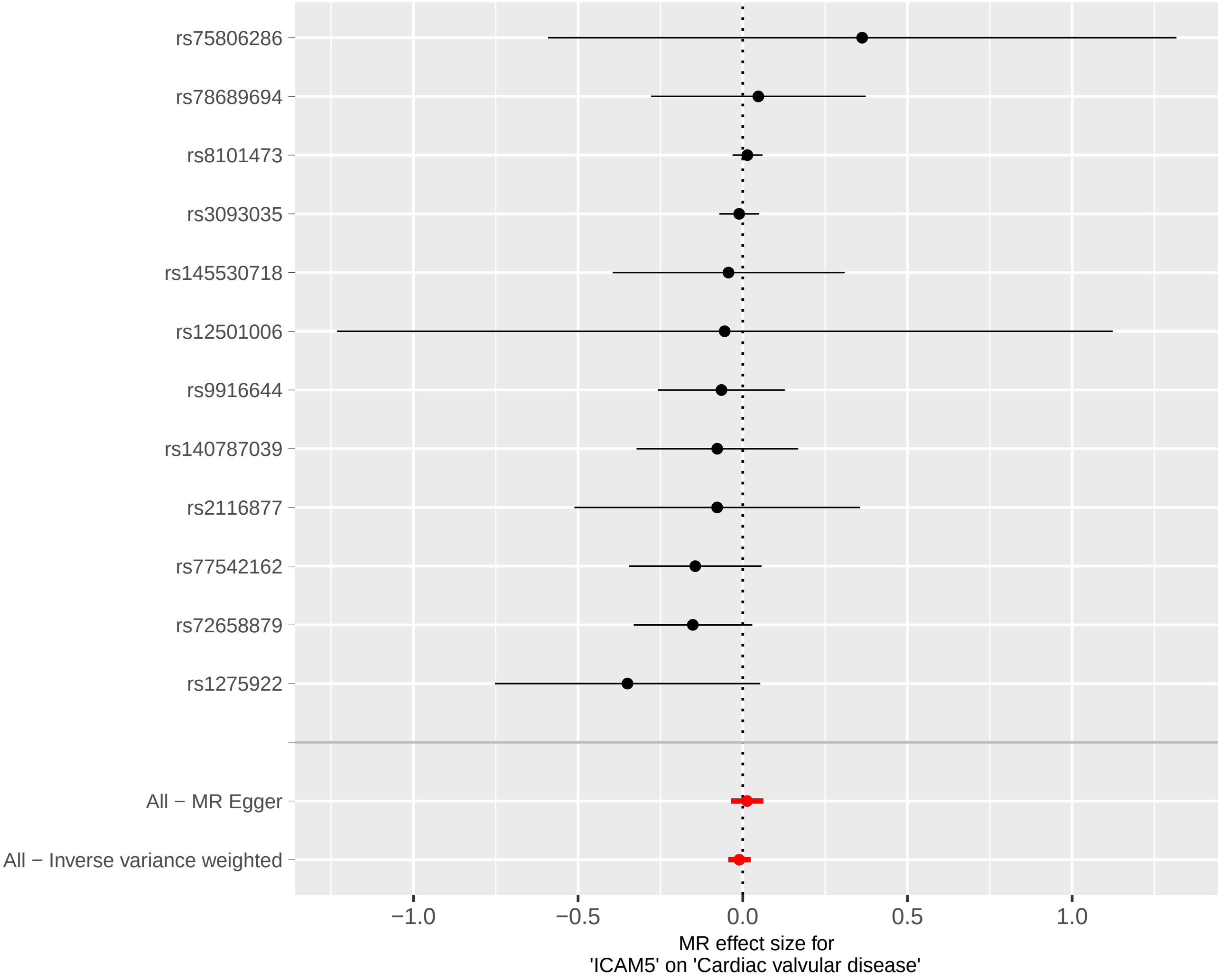
rs75806286
rs78689694
rs8101473
rs3093035
rs145530718
rs12501006
rs9916644
rs140787039
rs2116877
rs77542162
rs72658879
rs1275922
All − MR Egger
All − Inverse variance weighted
−1.0
−0.5
0.0
0.5
1.0
MR effect size for
'ICAM5' on 'Cardiac valvular disease'

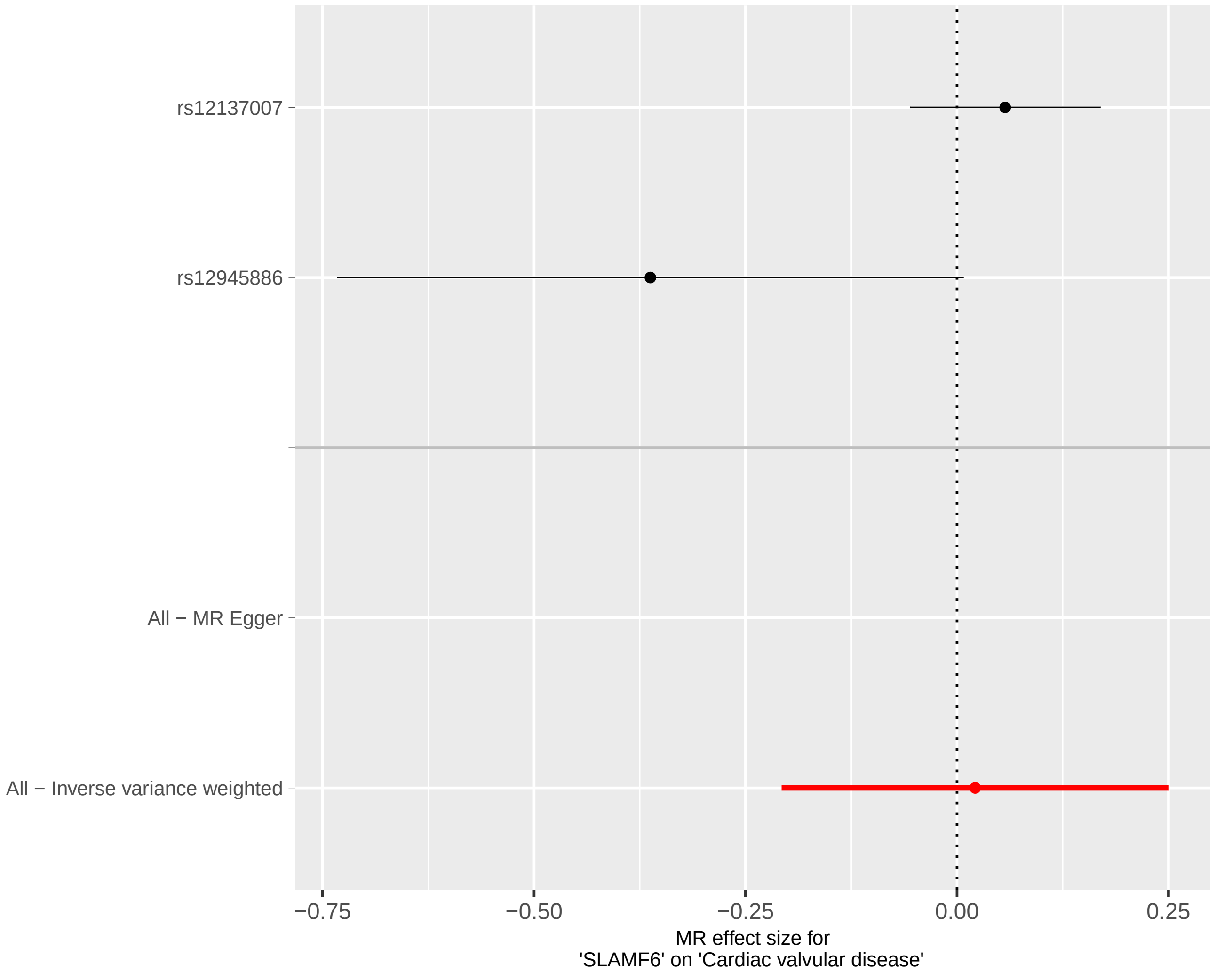
rs12137007
rs12945886
All − MR Egger
All − Inverse variance weighted
−0.75
−0.50
−0.25
0.00
0.25
MR effect size for
'SLAMF6' on 'Cardiac valvular disease'

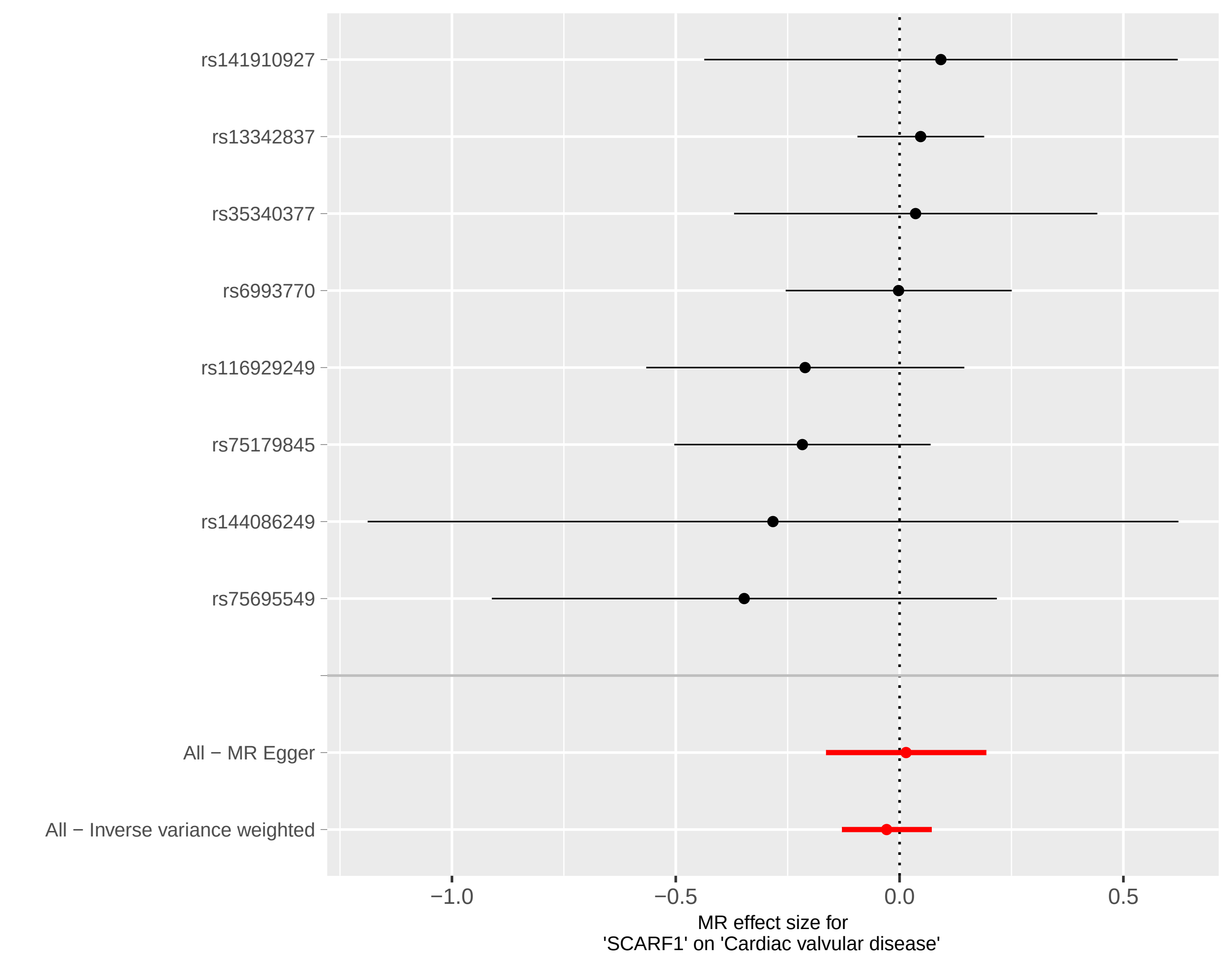
rs141910927
rs13342837
rs35340377
rs6993770
rs116929249
rs75179845
rs144086249
rs75695549
All − MR Egger
All − Inverse variance weighted
−1.0
−0.5
0.0
0.5
MR effect size for
'SCARF1' on 'Cardiac valvular disease'

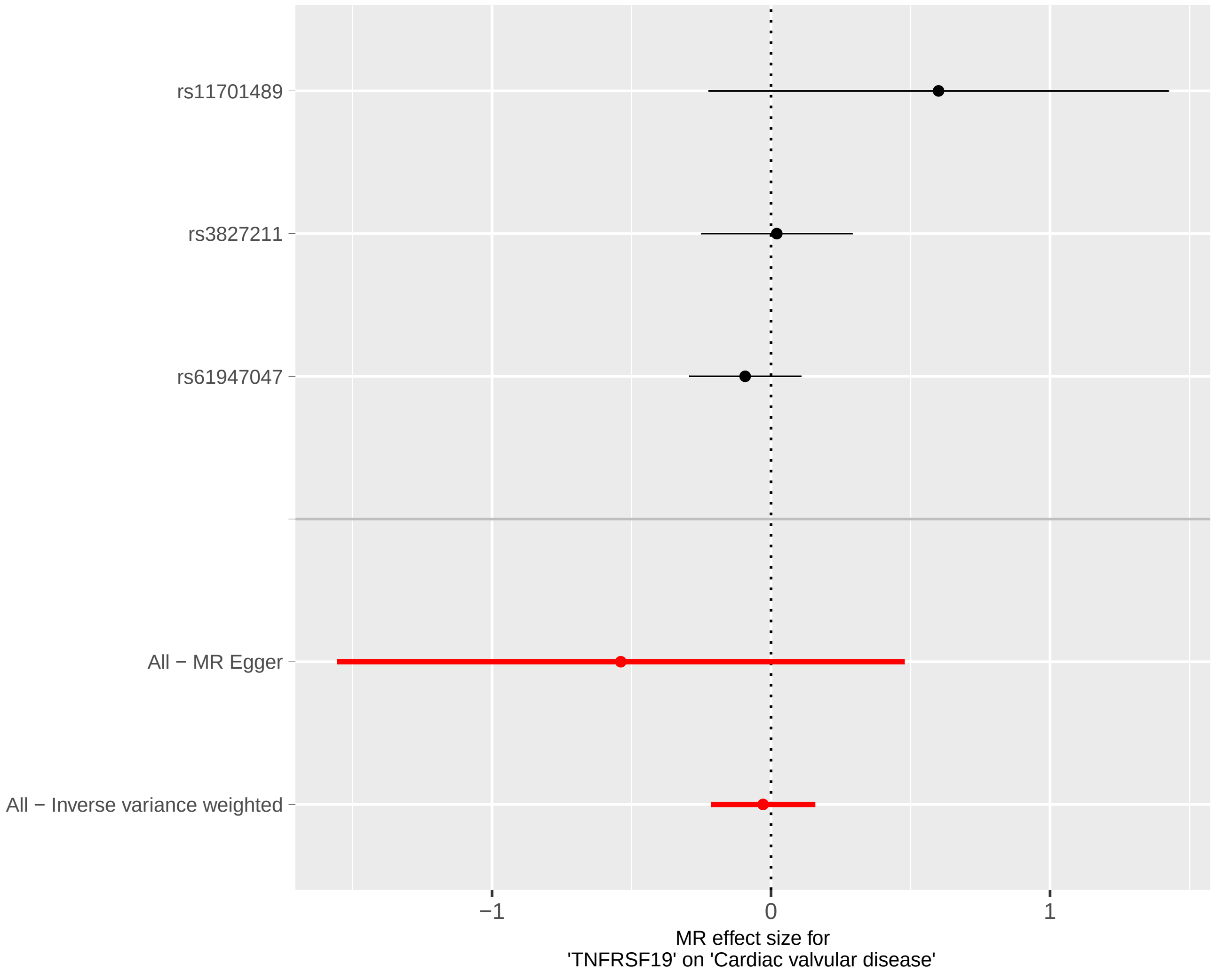

rs11701489
rs3827211
rs61947047
All − MR Egger
All − Inverse variance weighted
−1
0
1
MR effect size for
'TNFRSF19' on 'Cardiac valvular disease'

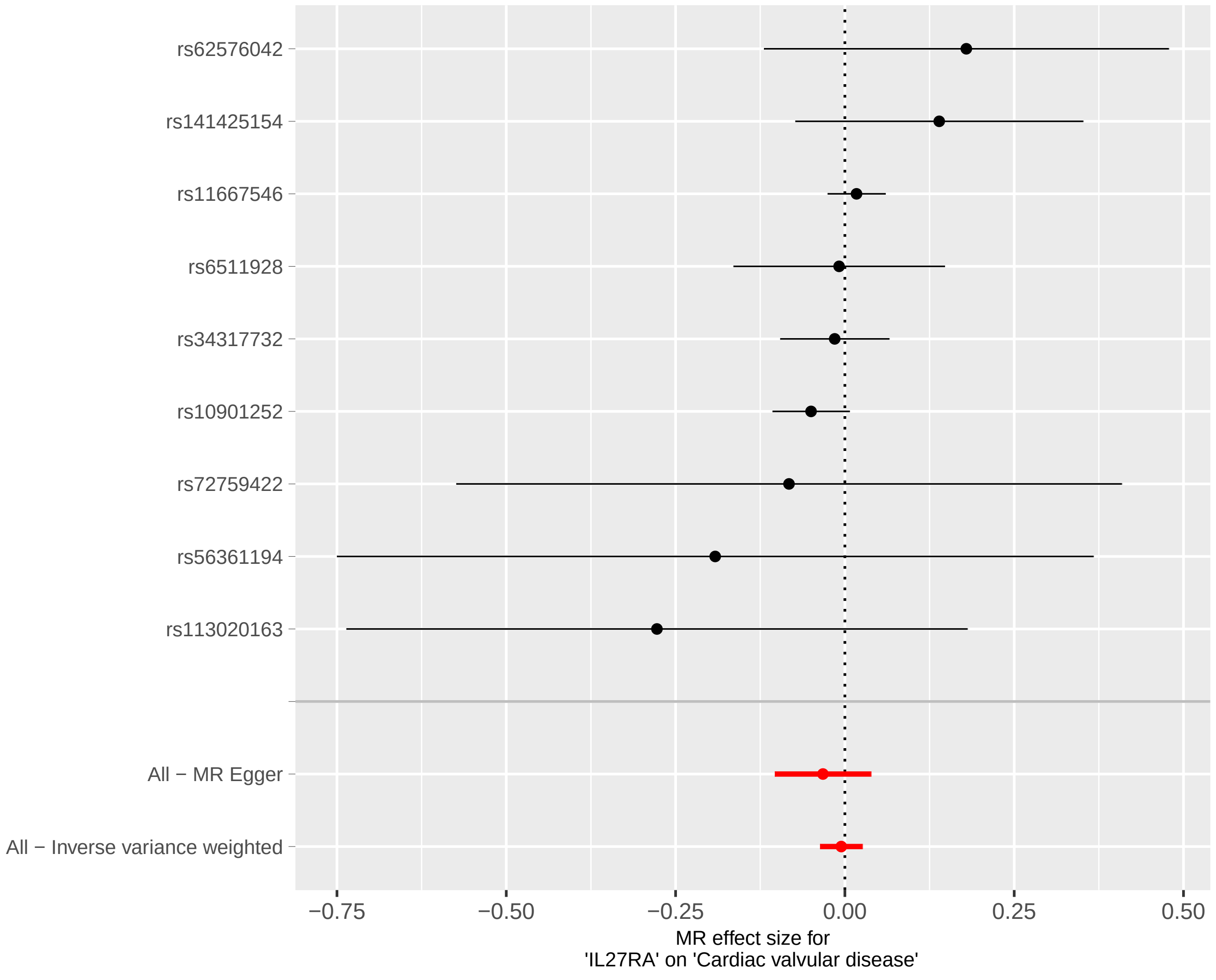

rs62576042
rs141425154
rs11667546
rs6511928
rs34317732
rs10901252
rs72759422
rs56361194
rs113020163
All − MR Egger
All − Inverse variance weighted
−0.75
−0.50
−0.25
0.00
0.25
0.50
MR effect size for
'IL27RA' on 'Cardiac valvular disease'

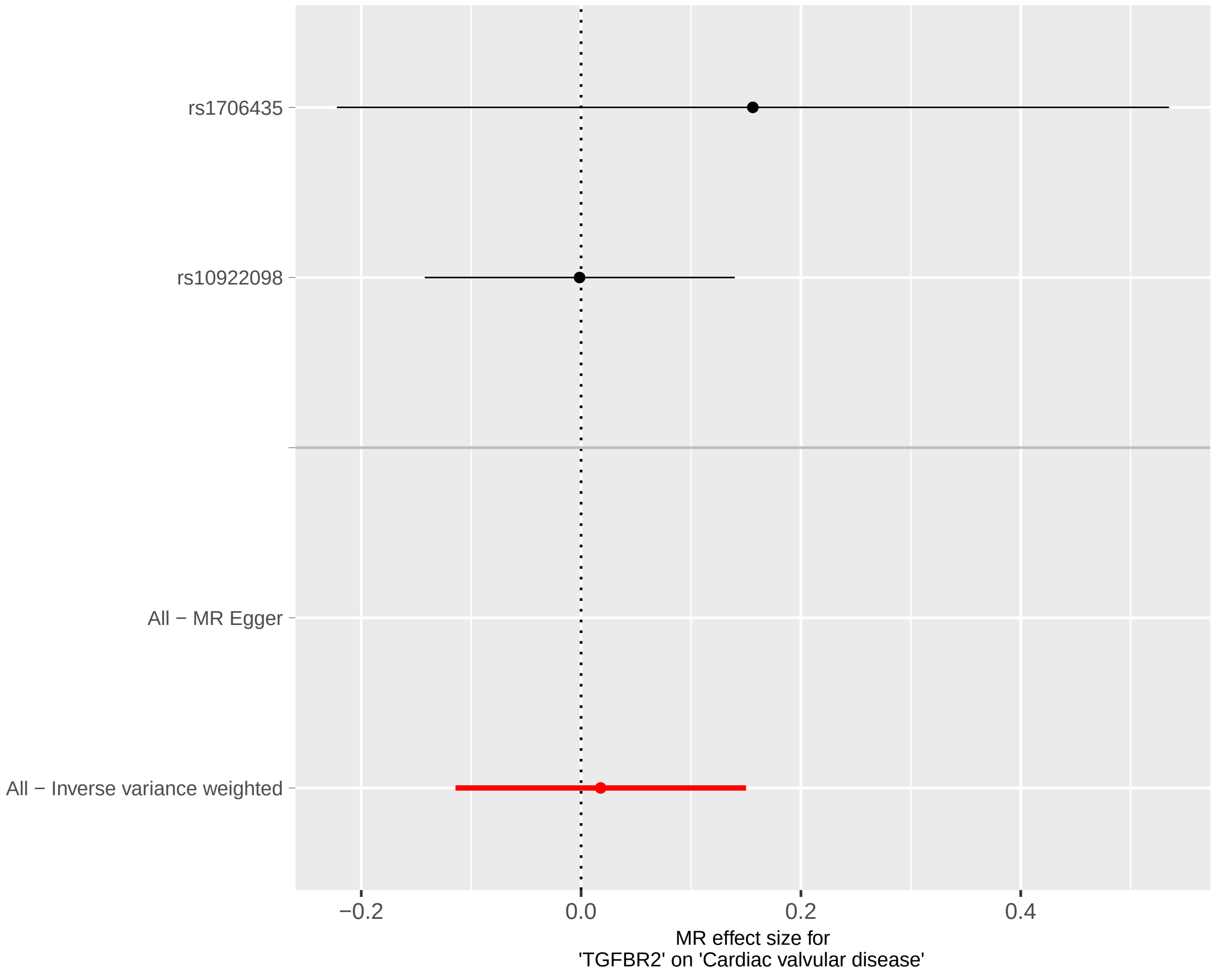
rs1706435
rs10922098
All − MR Egger
All − Inverse variance weighted
−0.2
0.0
0.2
0.4
MR effect size for
'TGFBR2' on 'Cardiac valvular disease'

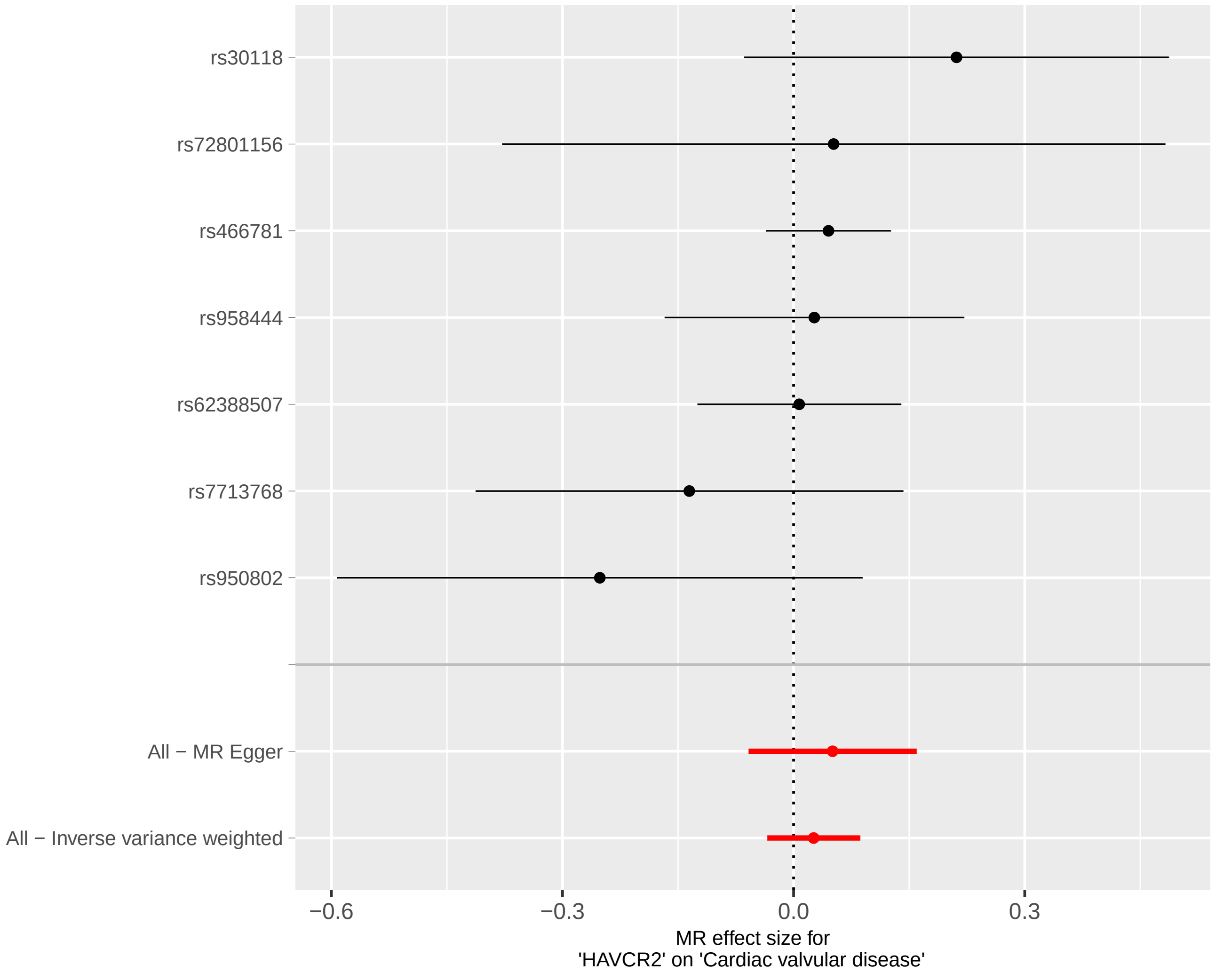
rs30118
rs72801156
rs466781
rs958444
rs62388507
rs7713768
rs950802
All − MR Egger
All − Inverse variance weighted
−0.6
−0.3
0.0
0.3
MR effect size for
'HAVCR2' on 'Cardiac valvular disease'

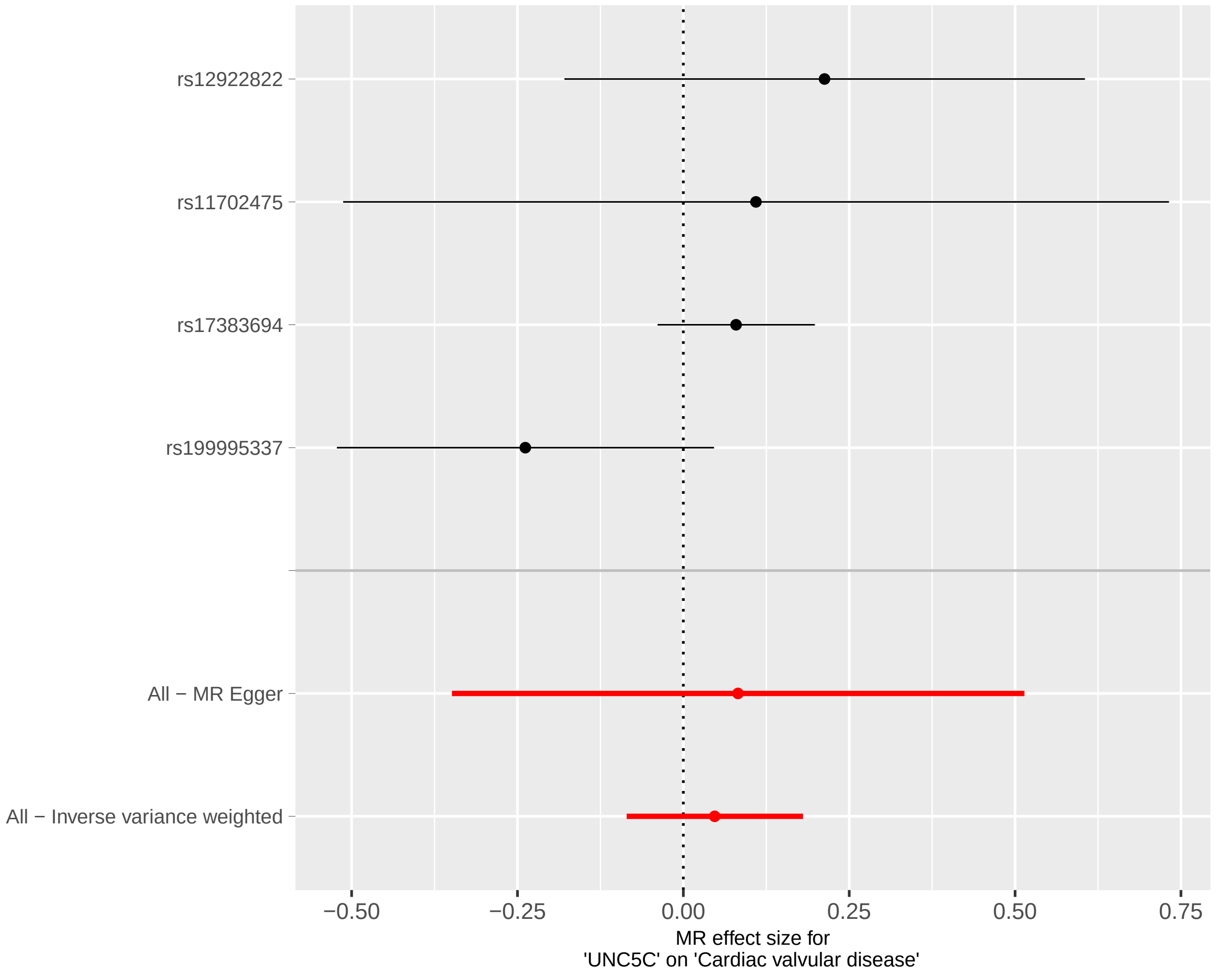

rs12922822
rs11702475
rs17383694
rs199995337
All − MR Egger
All − Inverse variance weighted
−0.50
−0.25
0.00
0.25
0.50
0.75
MR effect size for
'UNC5C' on 'Cardiac valvular disease'

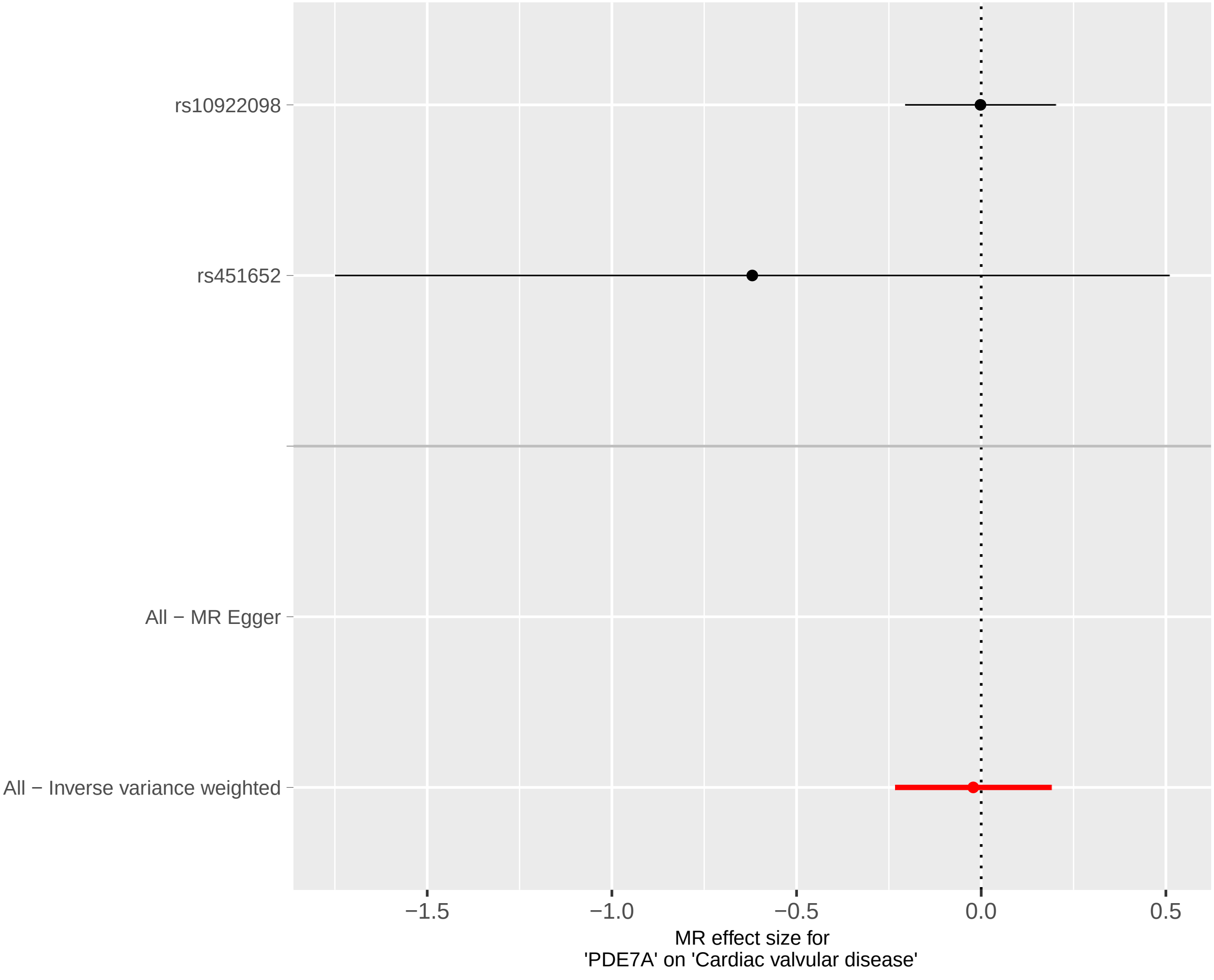

rs10922098
rs451652
All − MR Egger
All − Inverse variance weighted
−1.5
−1.0
−0.5
0.0
0.5
MR effect size for
'PDE7A' on 'Cardiac valvular disease'

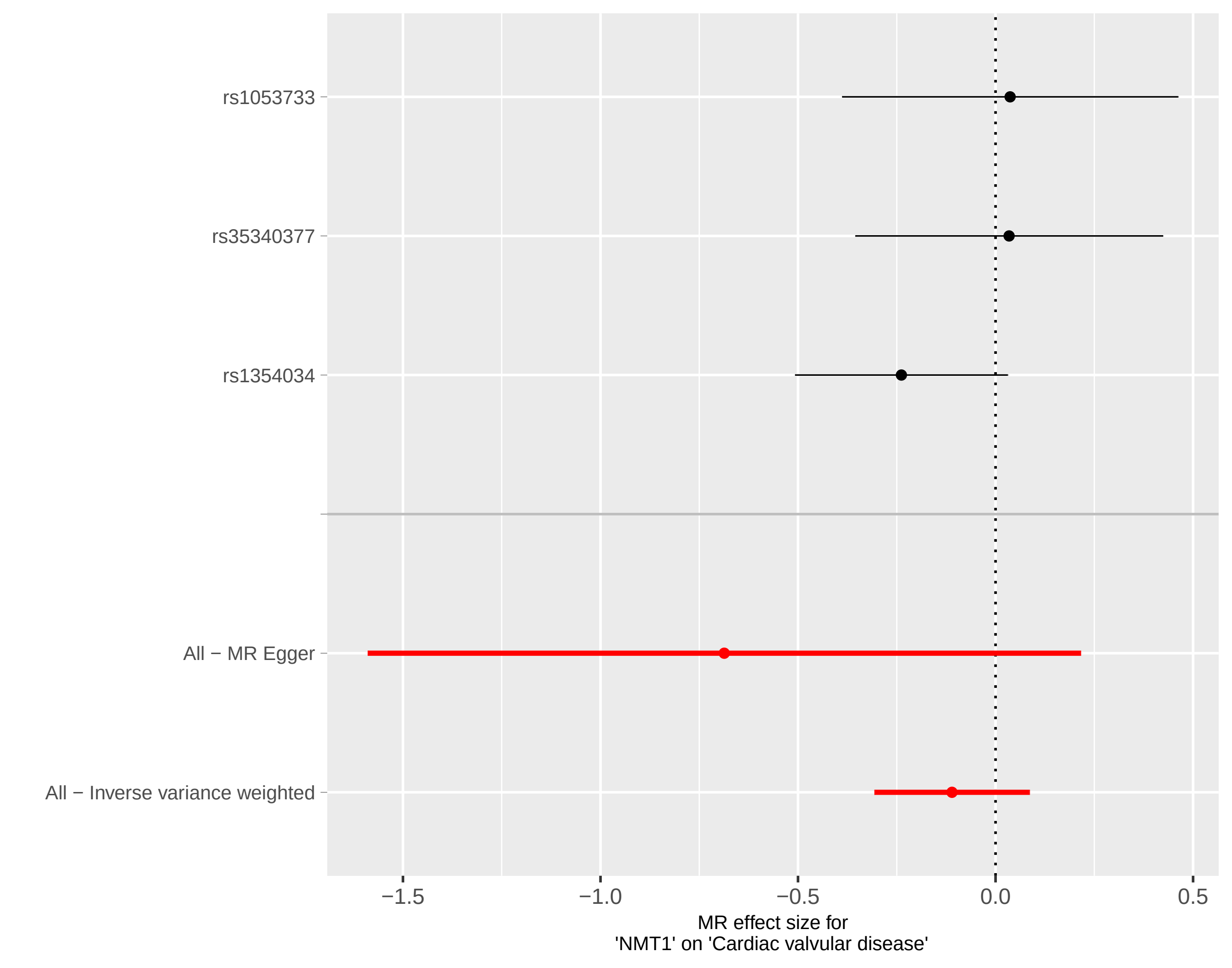

rs1053733
rs35340377
rs1354034
All − MR Egger
All − Inverse variance weighted
−1.5
−1.0
−0.5
0.0
0.5
MR effect size for
'NMT1' on 'Cardiac valvular disease'

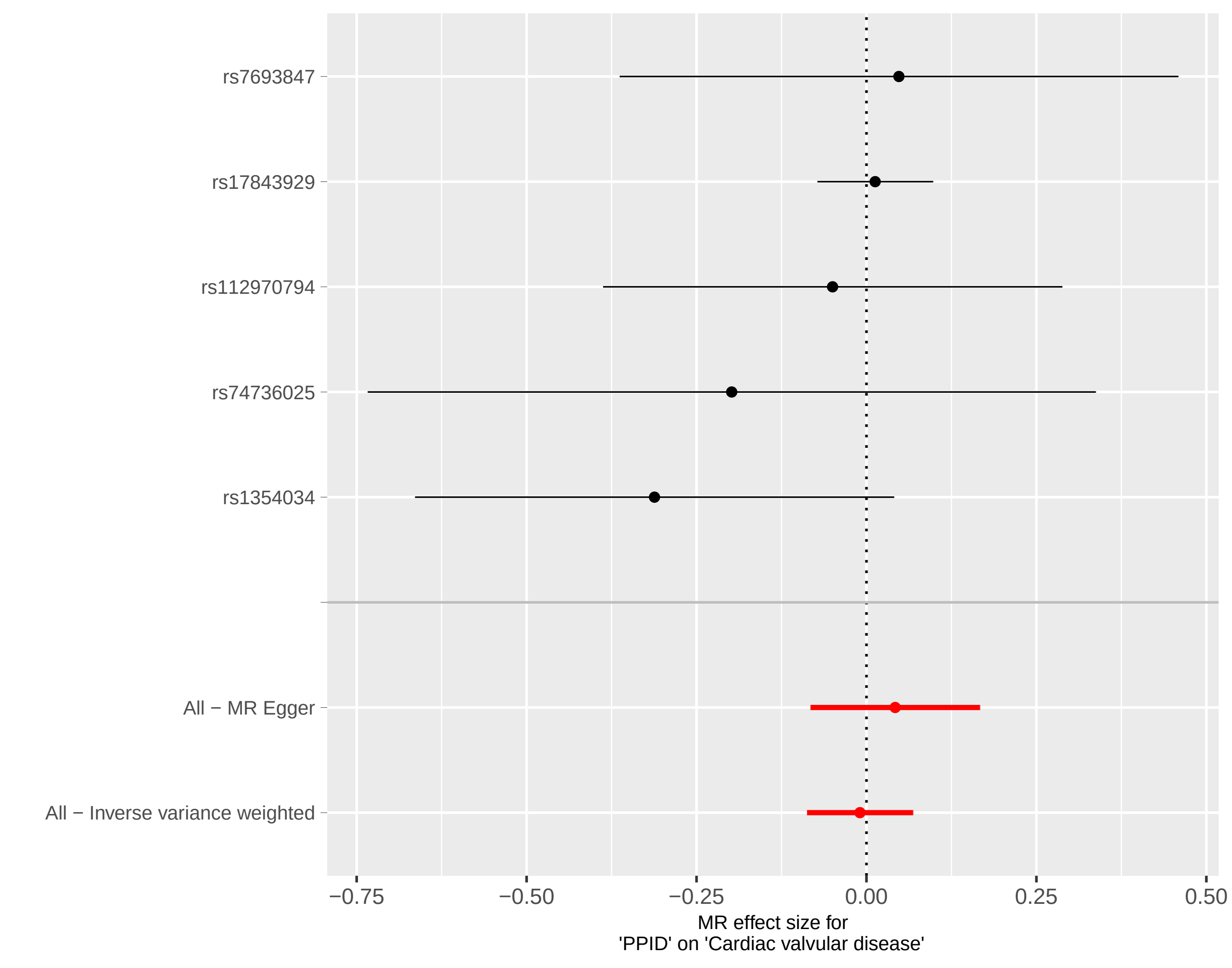
rs7693847
rs17843929
rs112970794
rs74736025
rs1354034
All − MR Egger
All − Inverse variance weighted
−0.75
−0.50
−0.25
0.00
0.25
0.50
MR effect size for
'PPID' on 'Cardiac valvular disease'

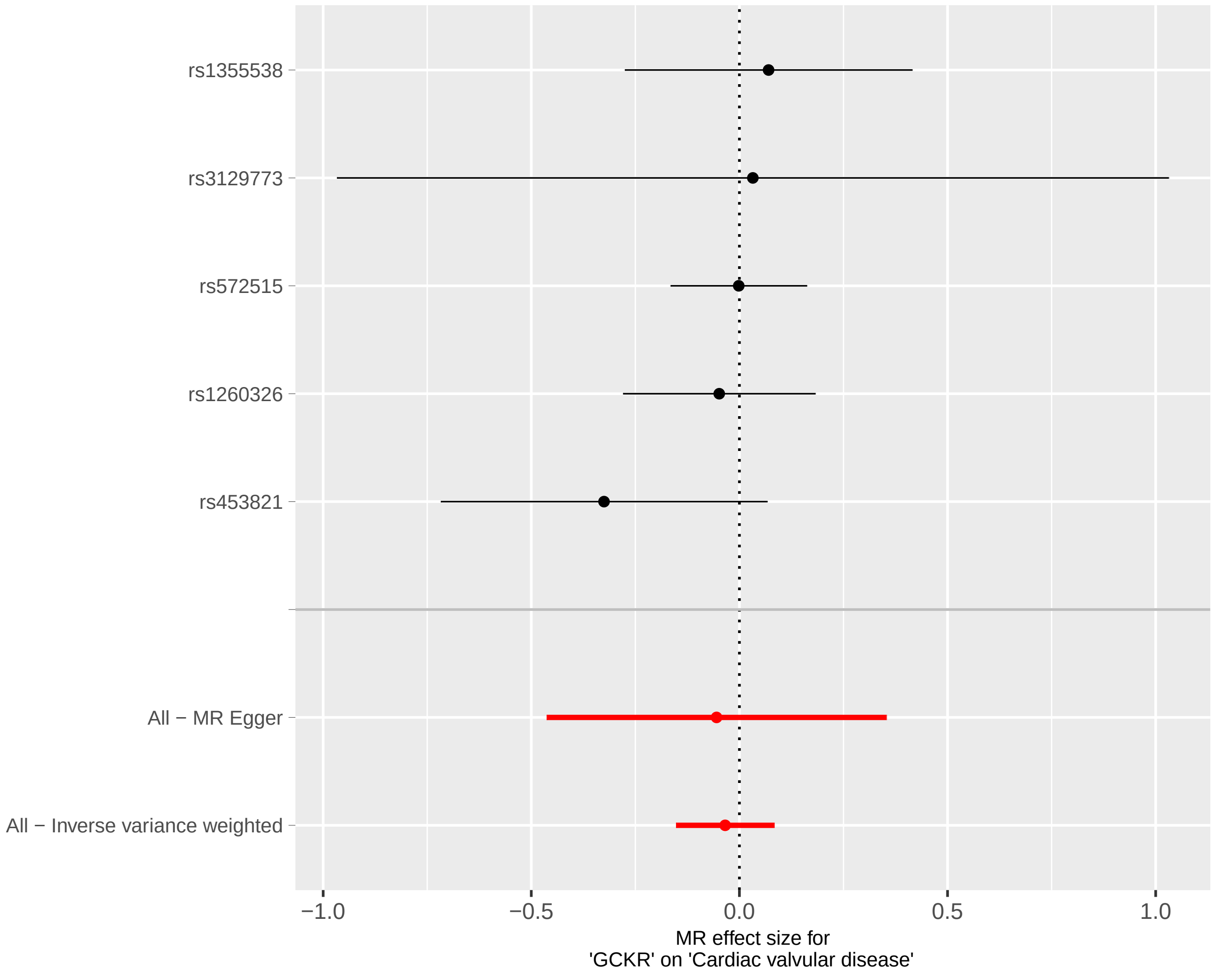
rs1355538
rs3129773
rs572515
rs1260326
rs453821
All − MR Egger
All − Inverse variance weighted
−1.0
−0.5
0.0
0.5
1.0
MR effect size for
'GCKR' on 'Cardiac valvular disease'

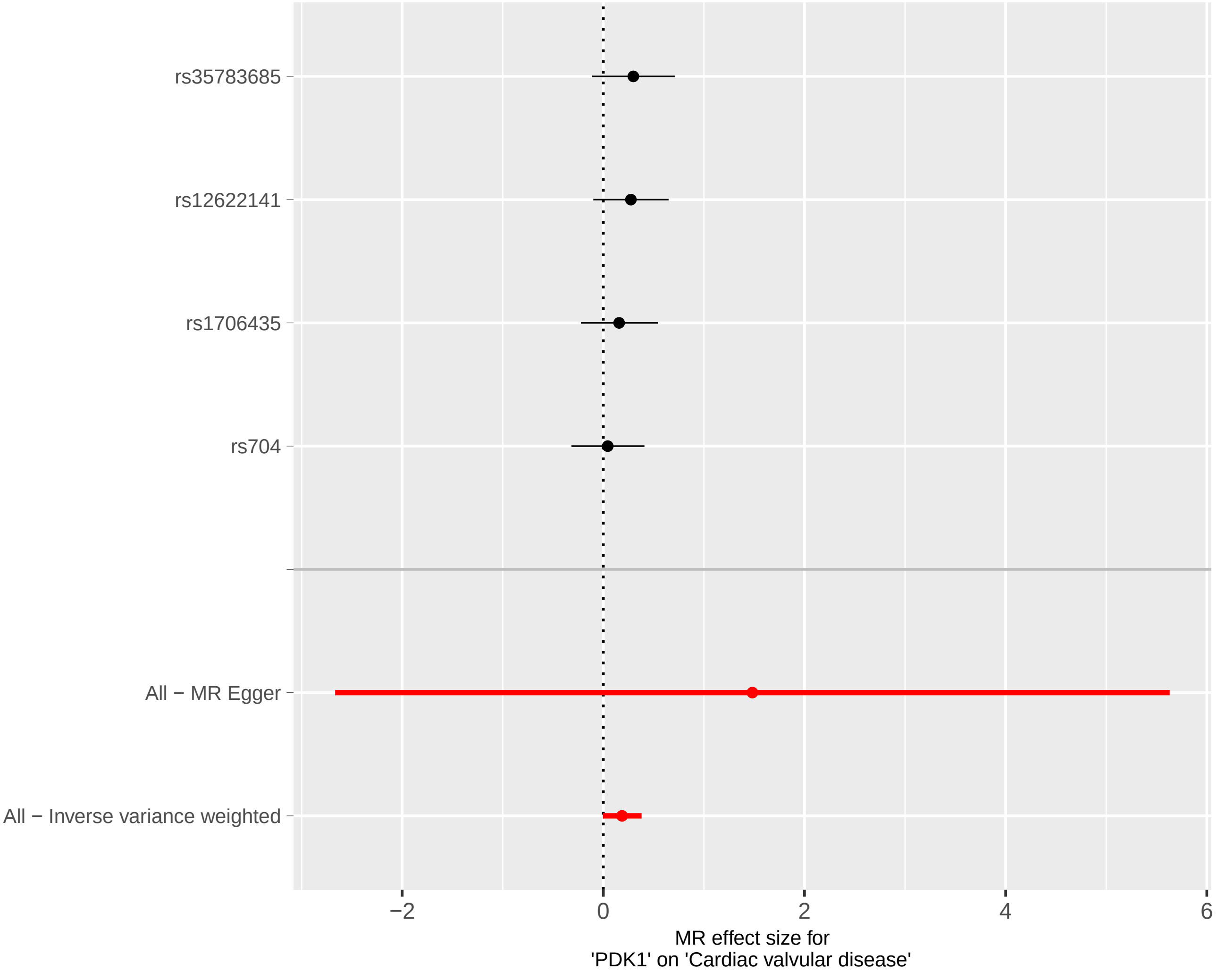
rs35783685
rs12622141
rs1706435
rs704
All − MR Egger
All − Inverse variance weighted
−2
0
2
4
6
MR effect size for
'PDK1' on 'Cardiac valvular disease'

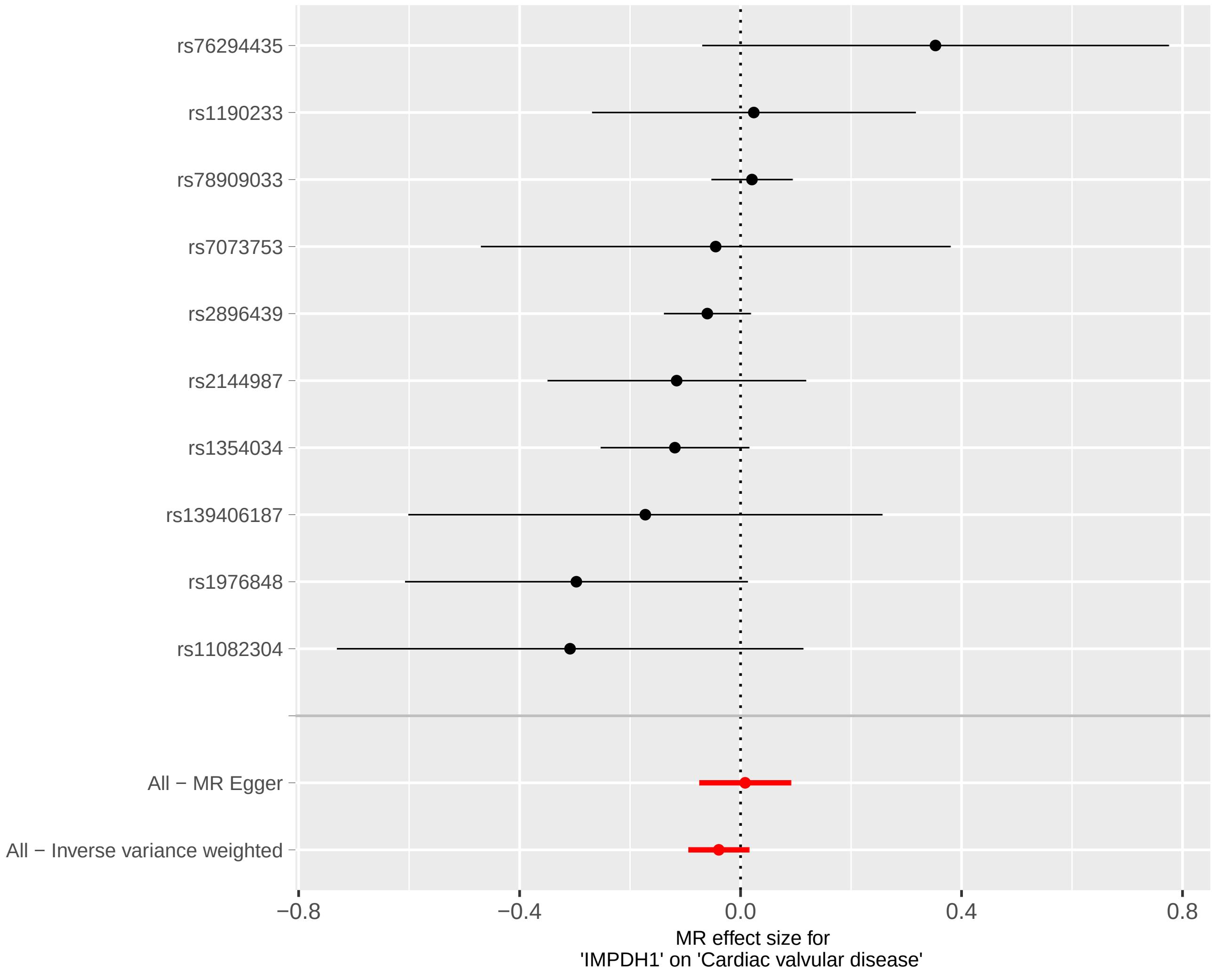
rs76294435
rs1190233
rs78909033
rs7073753
rs2896439
rs2144987
rs1354034
rs139406187
rs1976848
rs11082304
All − MR Egger
All − Inverse variance weighted
−0.8
−0.4
0.0
0.4
0.8
MR effect size for
'IMPDH1' on 'Cardiac valvular disease'

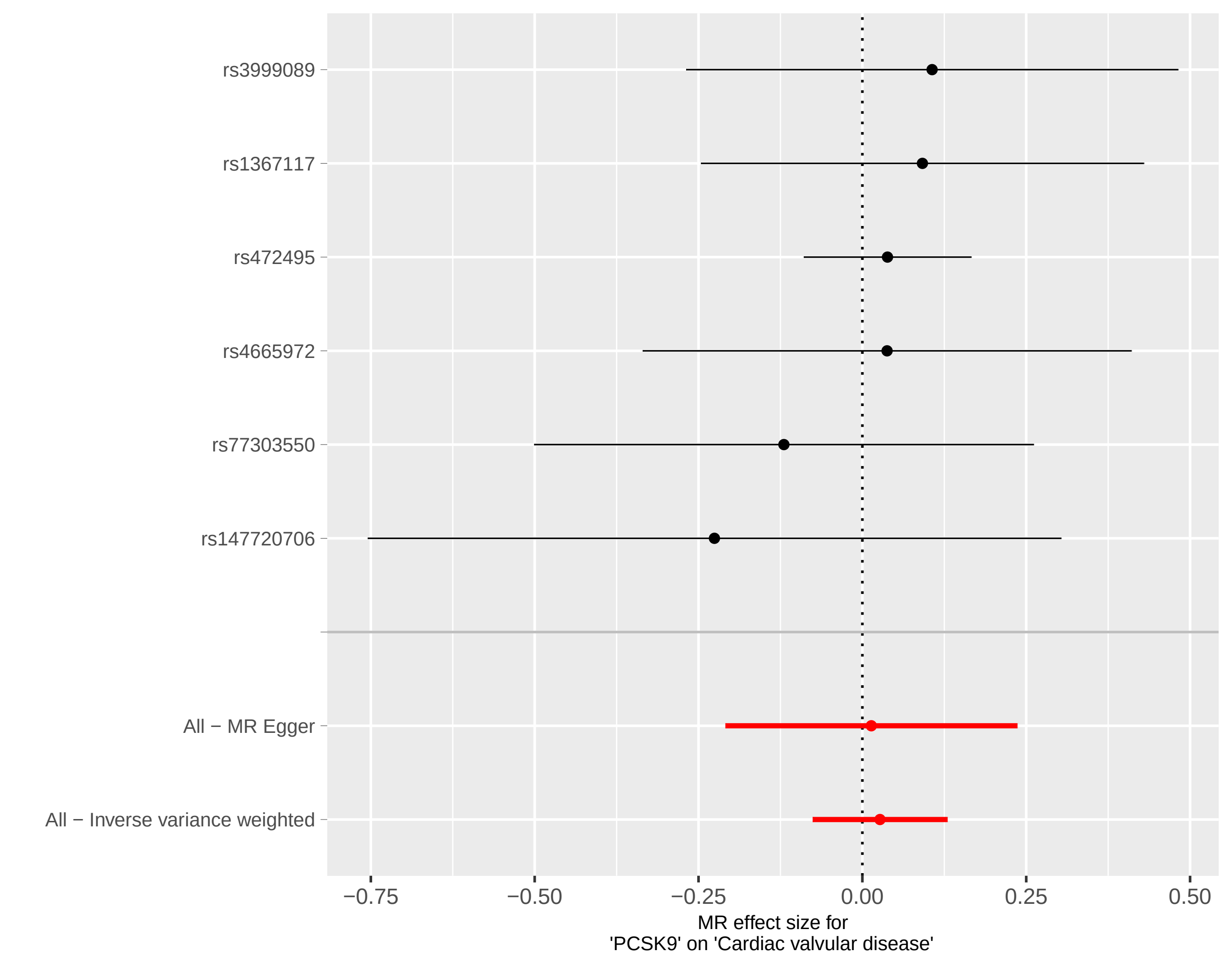
rs3999089
rs1367117
rs472495
rs4665972
rs77303550
rs147720706
All − MR Egger
All − Inverse variance weighted
−0.75
−0.50
−0.25
0.00
0.25
0.50
MR effect size for
'PCSK9' on 'Cardiac valvular disease'

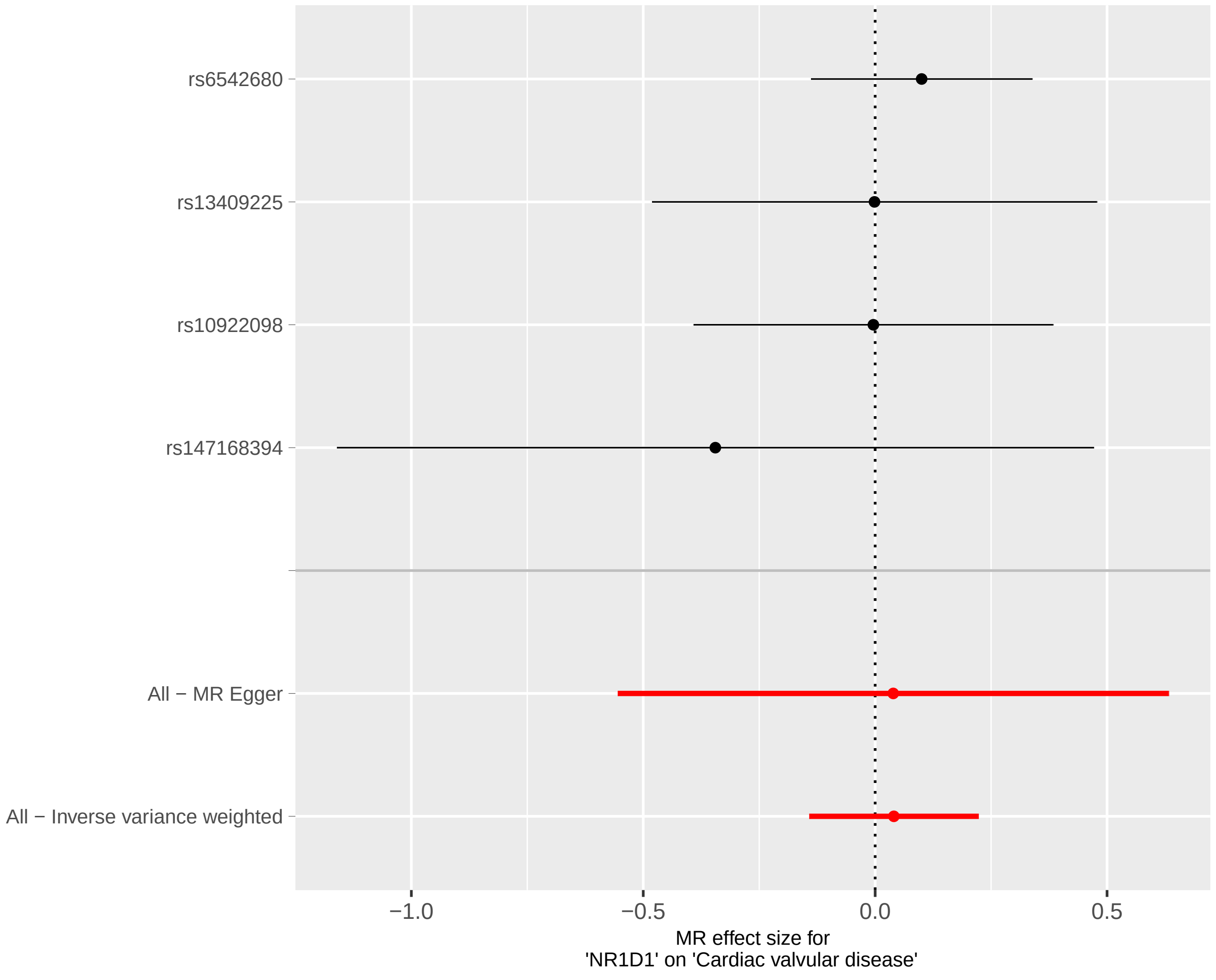
rs6542680
rs13409225
rs10922098
rs147168394
All − MR Egger
All − Inverse variance weighted
−1.0
−0.5
0.0
0.5
MR effect size for
'NR1D1' on 'Cardiac valvular disease'

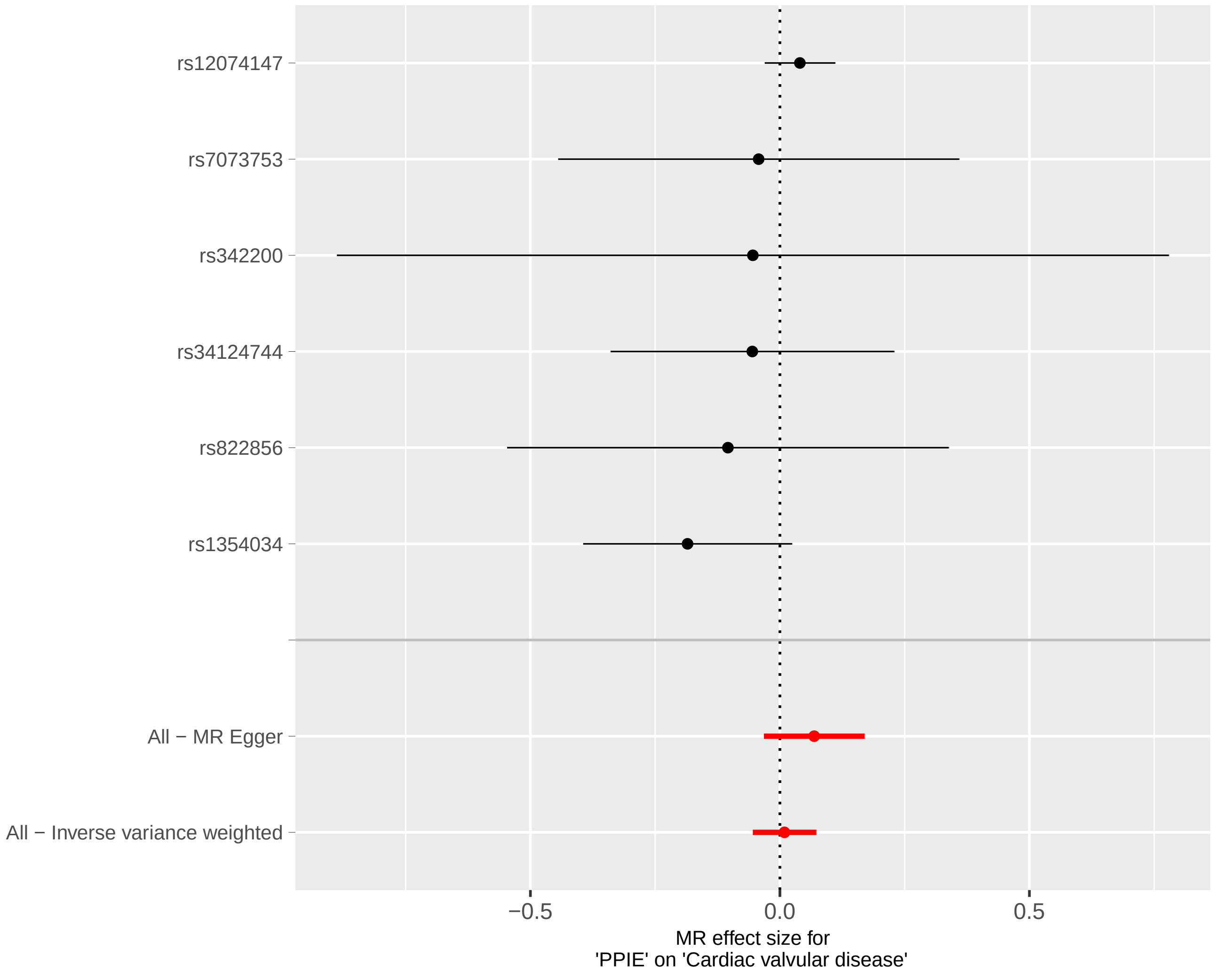
rs12074147
rs7073753
rs342200
rs34124744
rs822856
rs1354034
All − MR Egger
All − Inverse variance weighted
−0.5
0.0
0.5
MR effect size for
'PPIE' on 'Cardiac valvular disease'

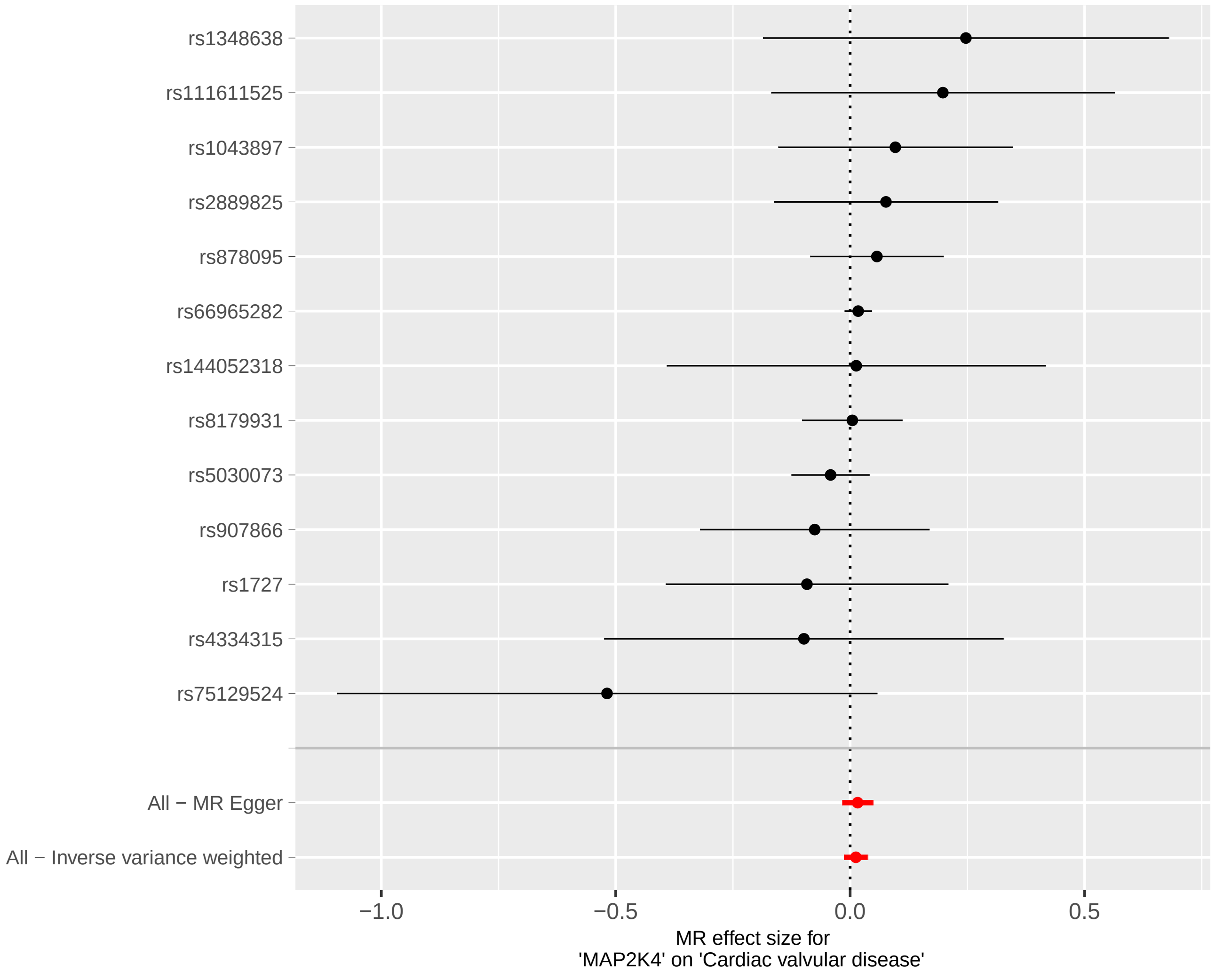
rs1348638
rs111611525
rs1043897
rs2889825
rs878095
rs66965282
rs144052318
rs8179931
rs5030073
rs907866
rs1727
rs4334315
rs75129524
All − MR Egger
All − Inverse variance weighted
−1.0
−0.5
0.0
0.5
MR effect size for
'MAP2K4' on 'Cardiac valvular disease'

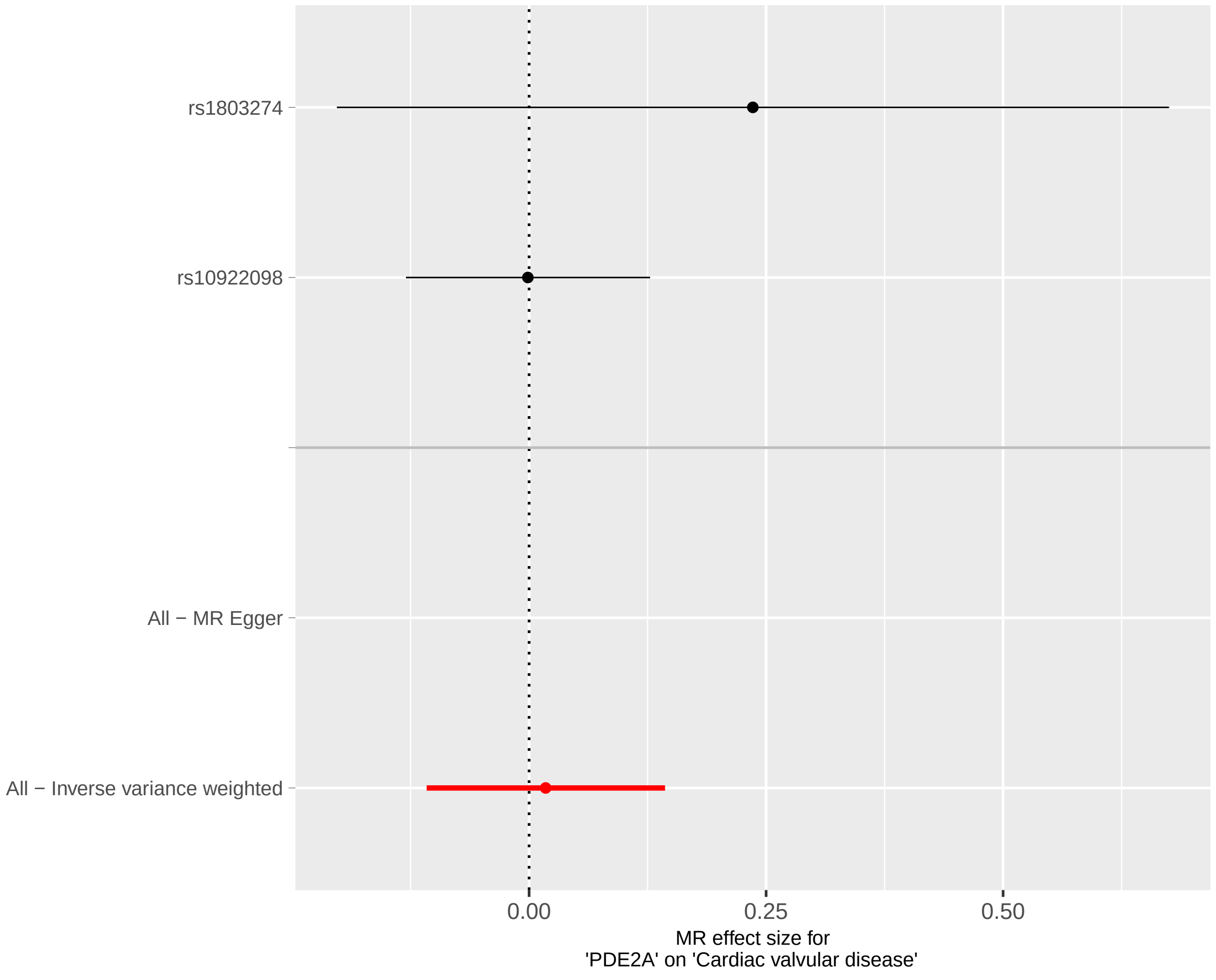

rs1803274
rs10922098
All − MR Egger
All − Inverse variance weighted
0.00
0.25
0.50
MR effect size for
'PDE2A' on 'Cardiac valvular disease'

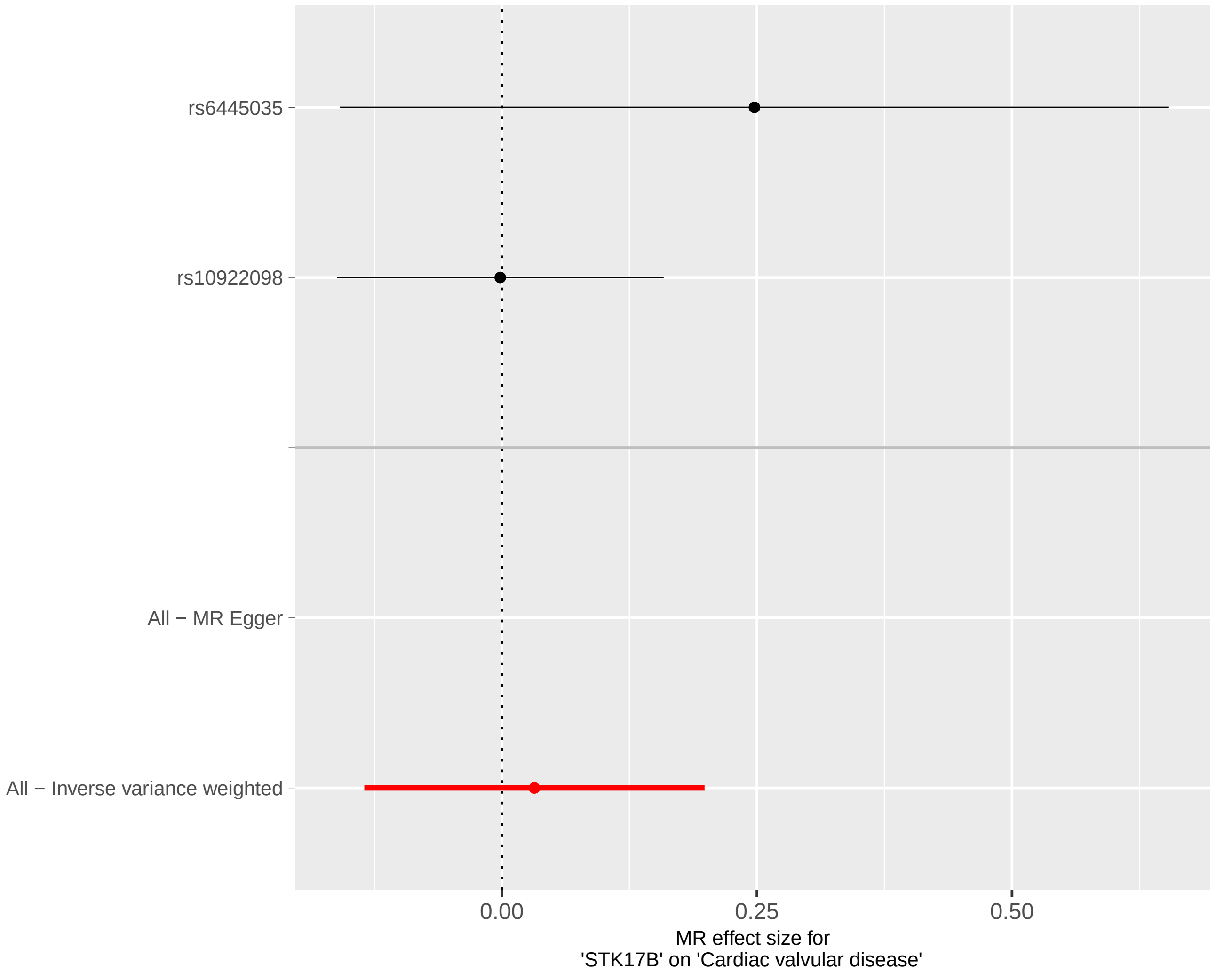

rs6445035
rs10922098
All − MR Egger
All − Inverse variance weighted
0.00
0.25
0.50
MR effect size for
'STK17B' on 'Cardiac valvular disease'

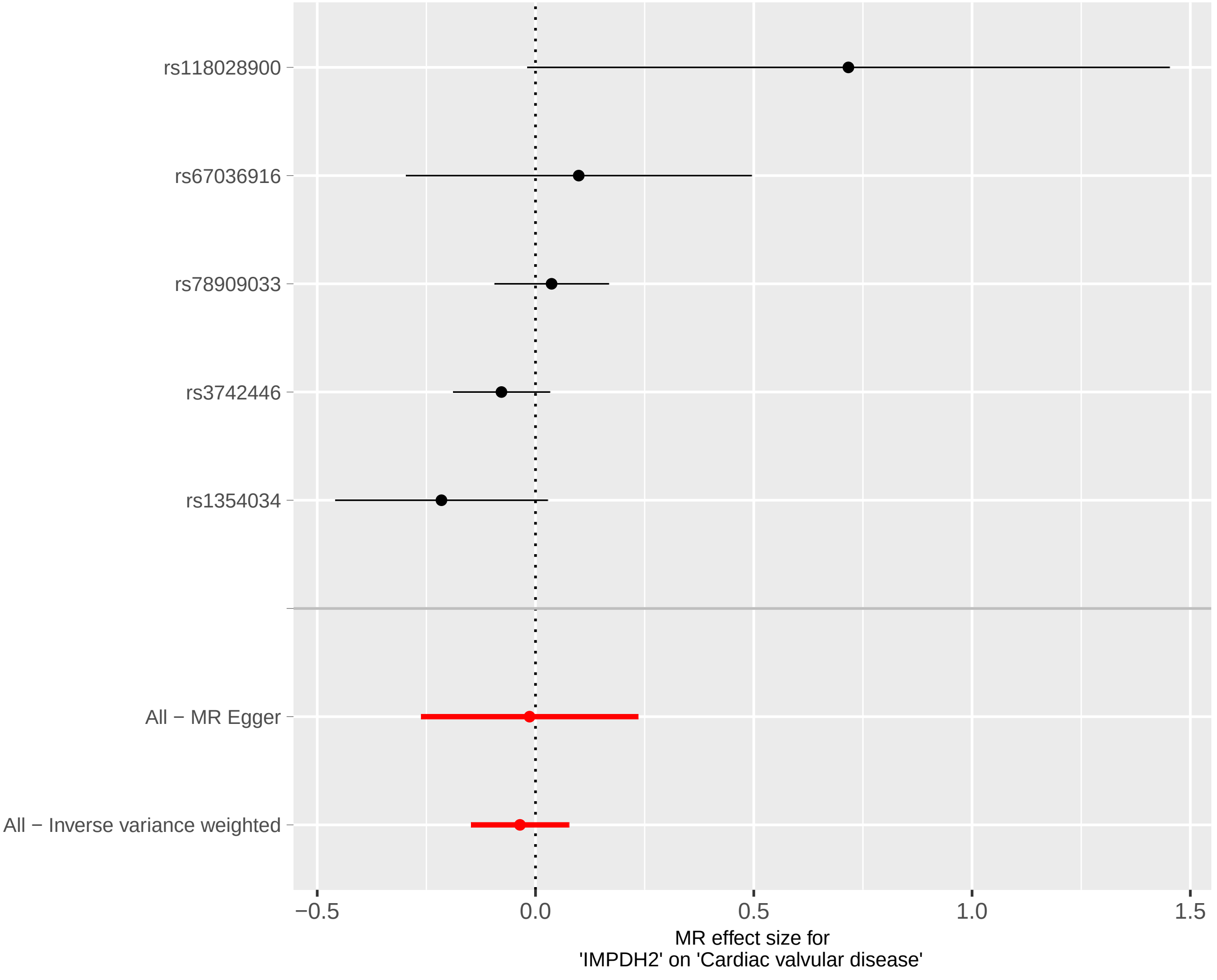
rs118028900
rs67036916
rs78909033
rs3742446
rs1354034
All − MR Egger
All − Inverse variance weighted
−0.5
0.0
0.5
1.0
1.5
MR effect size for
'IMPDH2' on 'Cardiac valvular disease'

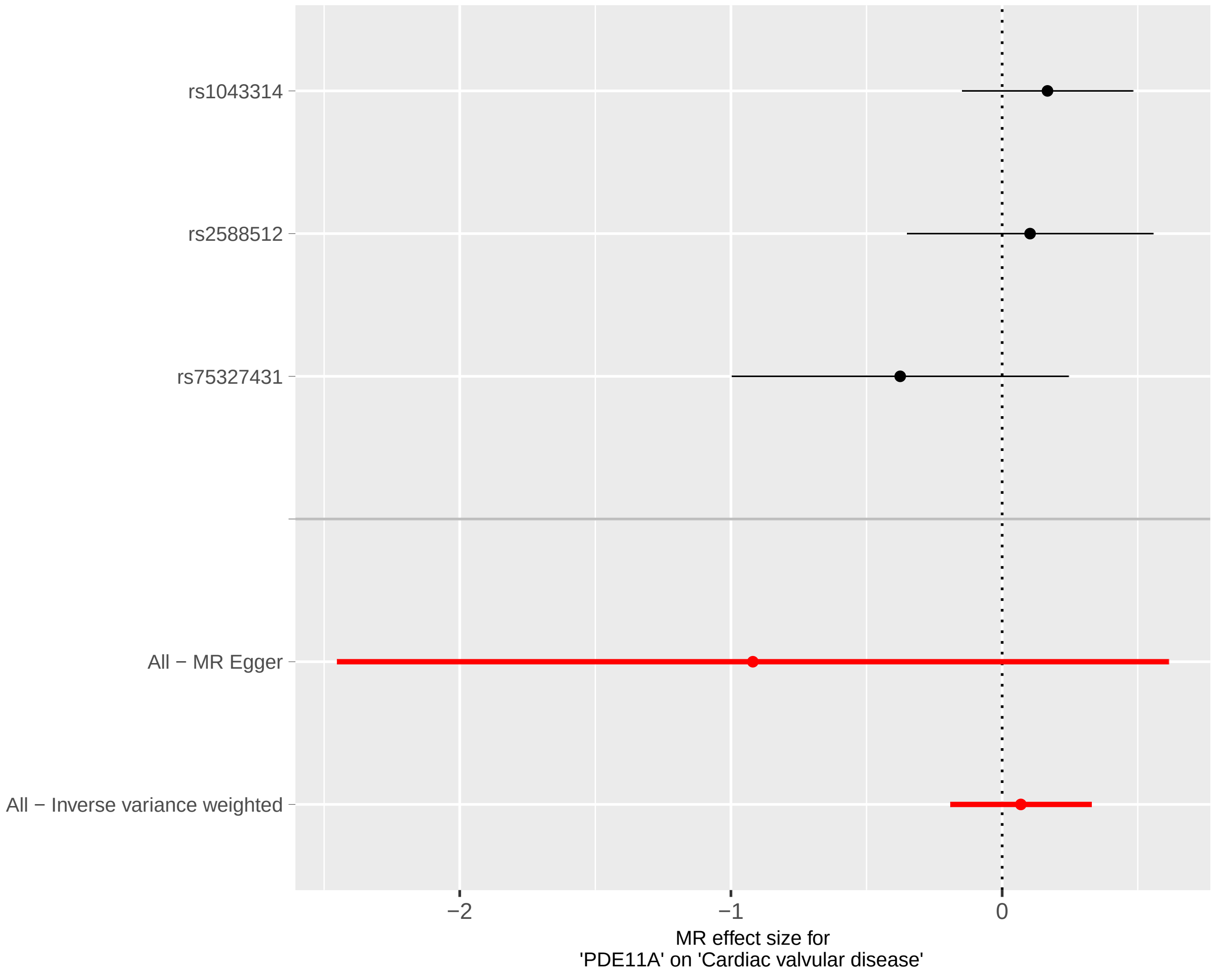
rs1043314
rs2588512
rs75327431
All − MR Egger
All − Inverse variance weighted
−2
−1
0
MR effect size for
'PDE11A' on 'Cardiac valvular disease'

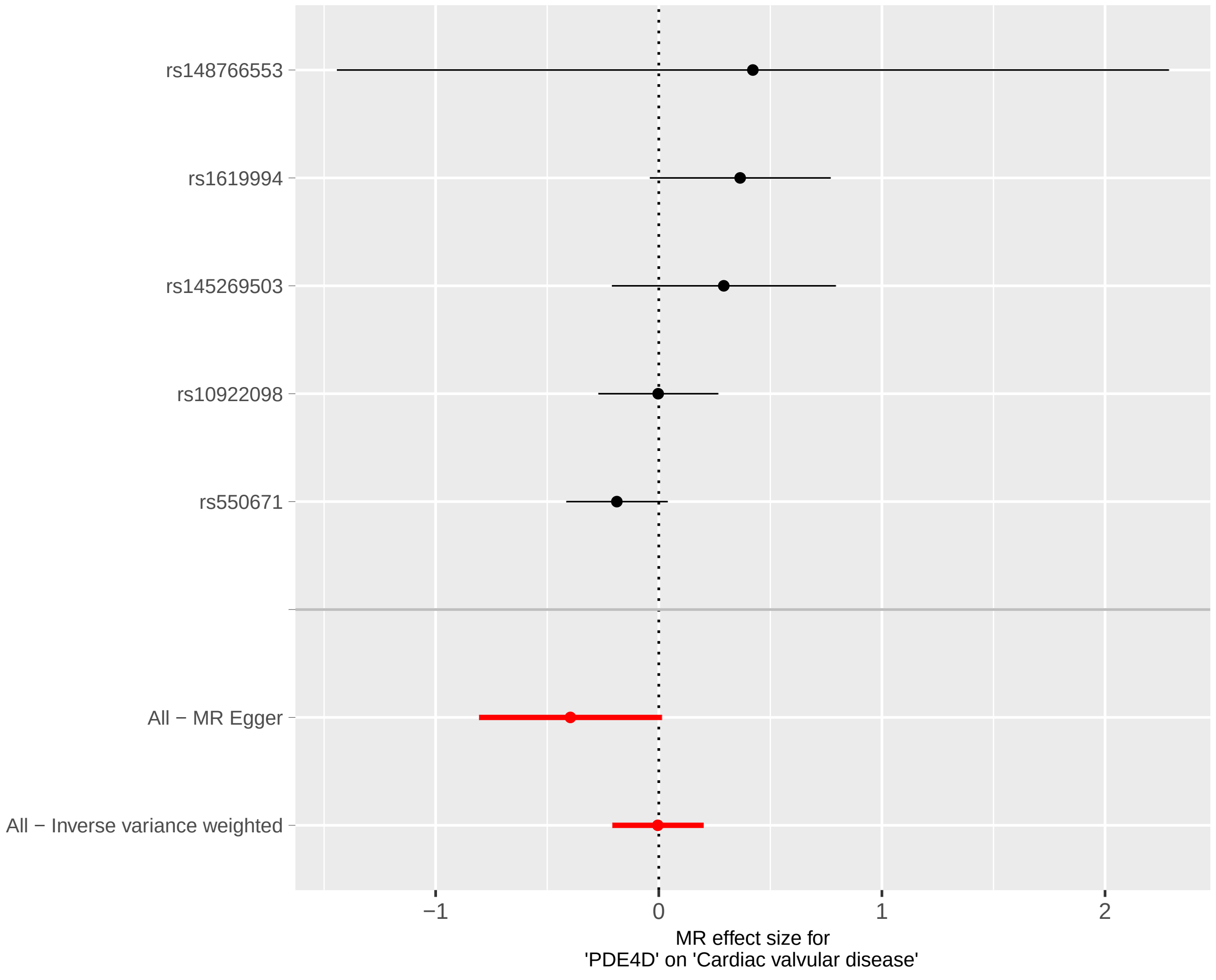

rs148766553
rs1619994
rs145269503
rs10922098
rs550671
All − MR Egger
All − Inverse variance weighted
−1
0
1
2
MR effect size for
'PDE4D' on 'Cardiac valvular disease'

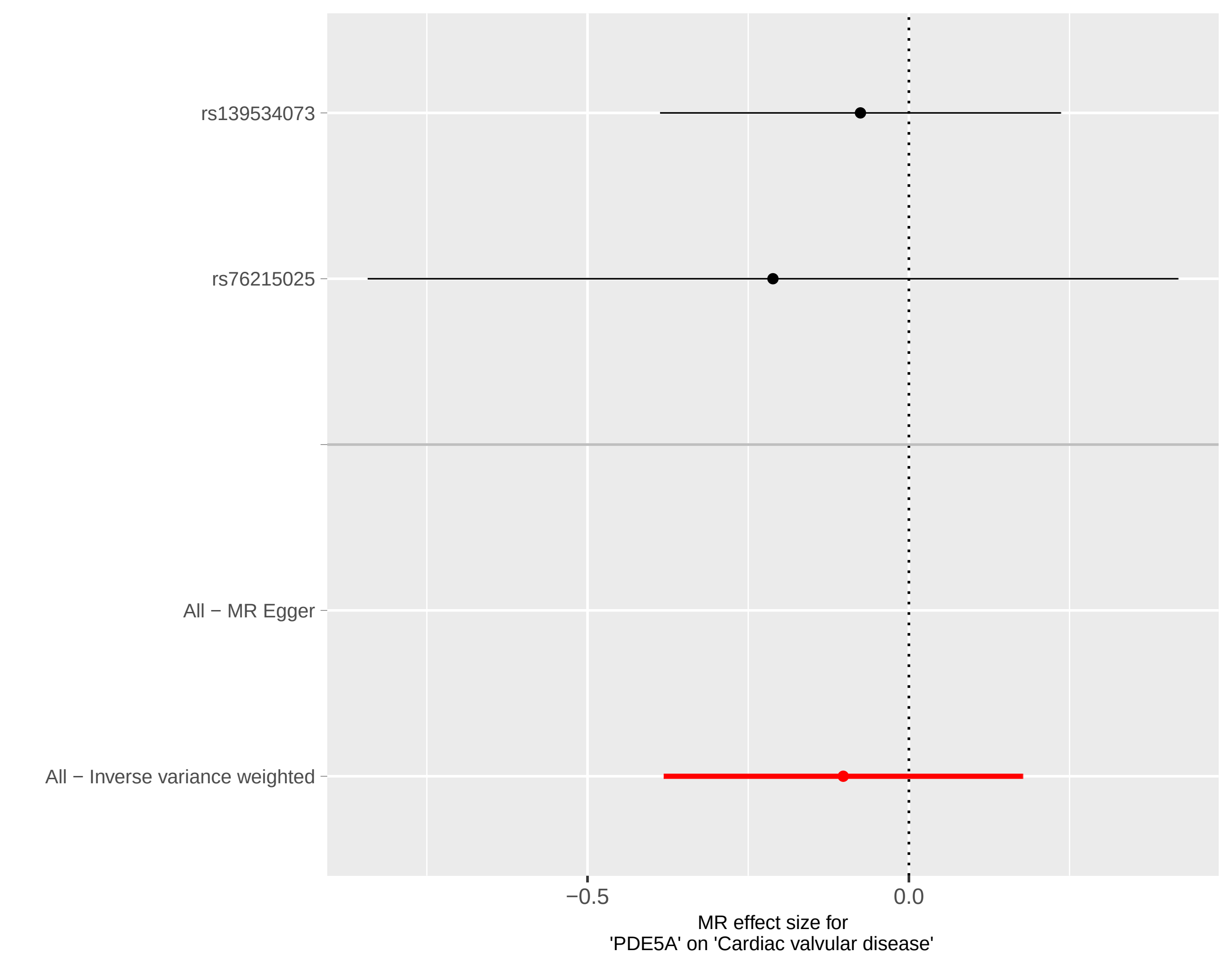
rs139534073
rs76215025
All − MR Egger
All − Inverse variance weighted
−0.5
0.0
MR effect size for
'PDE5A' on 'Cardiac valvular disease'

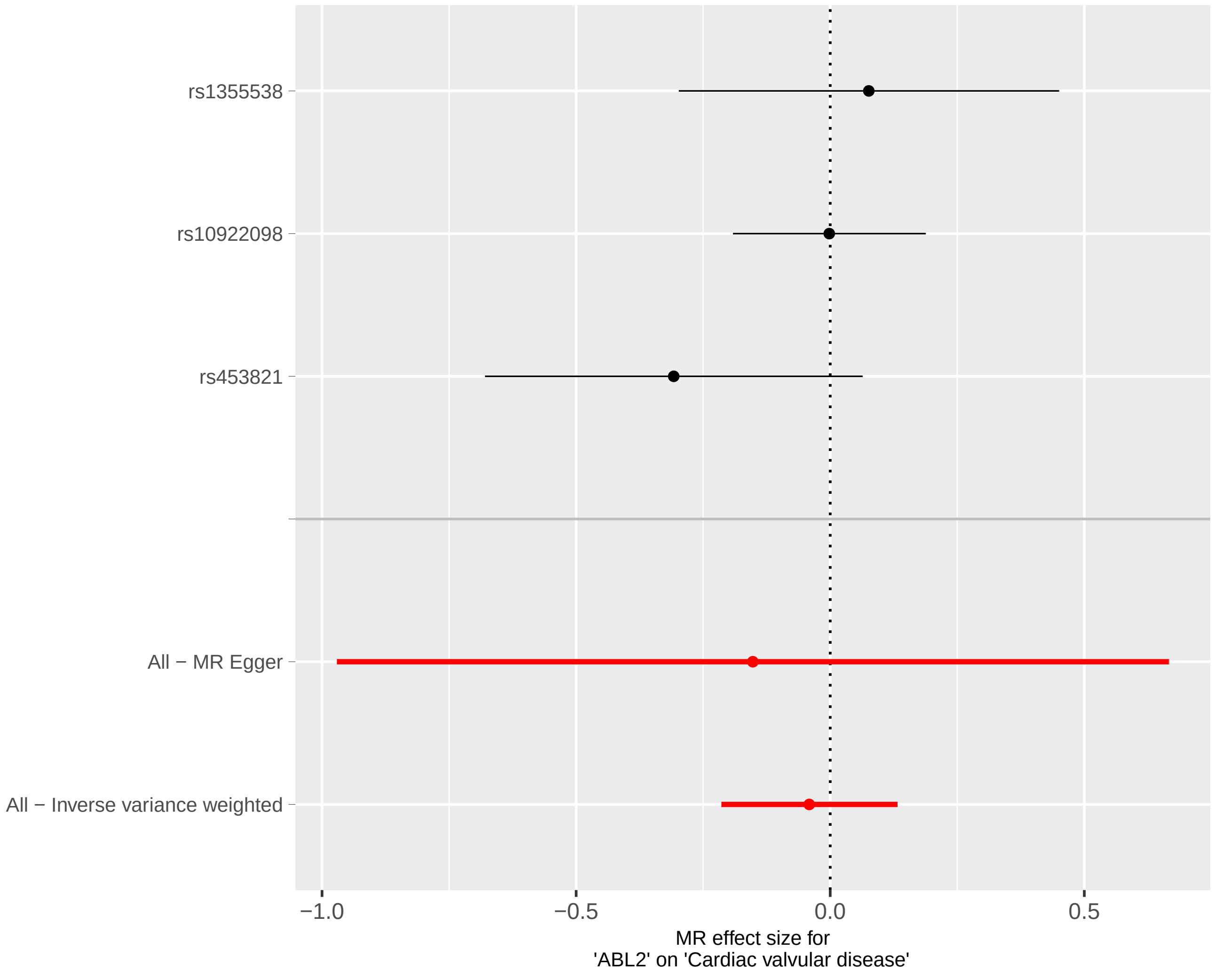
rs1355538
rs10922098
rs453821
All − MR Egger
All − Inverse variance weighted
−1.0
−0.5
0.0
0.5
MR effect size for
'ABL2' on 'Cardiac valvular disease'

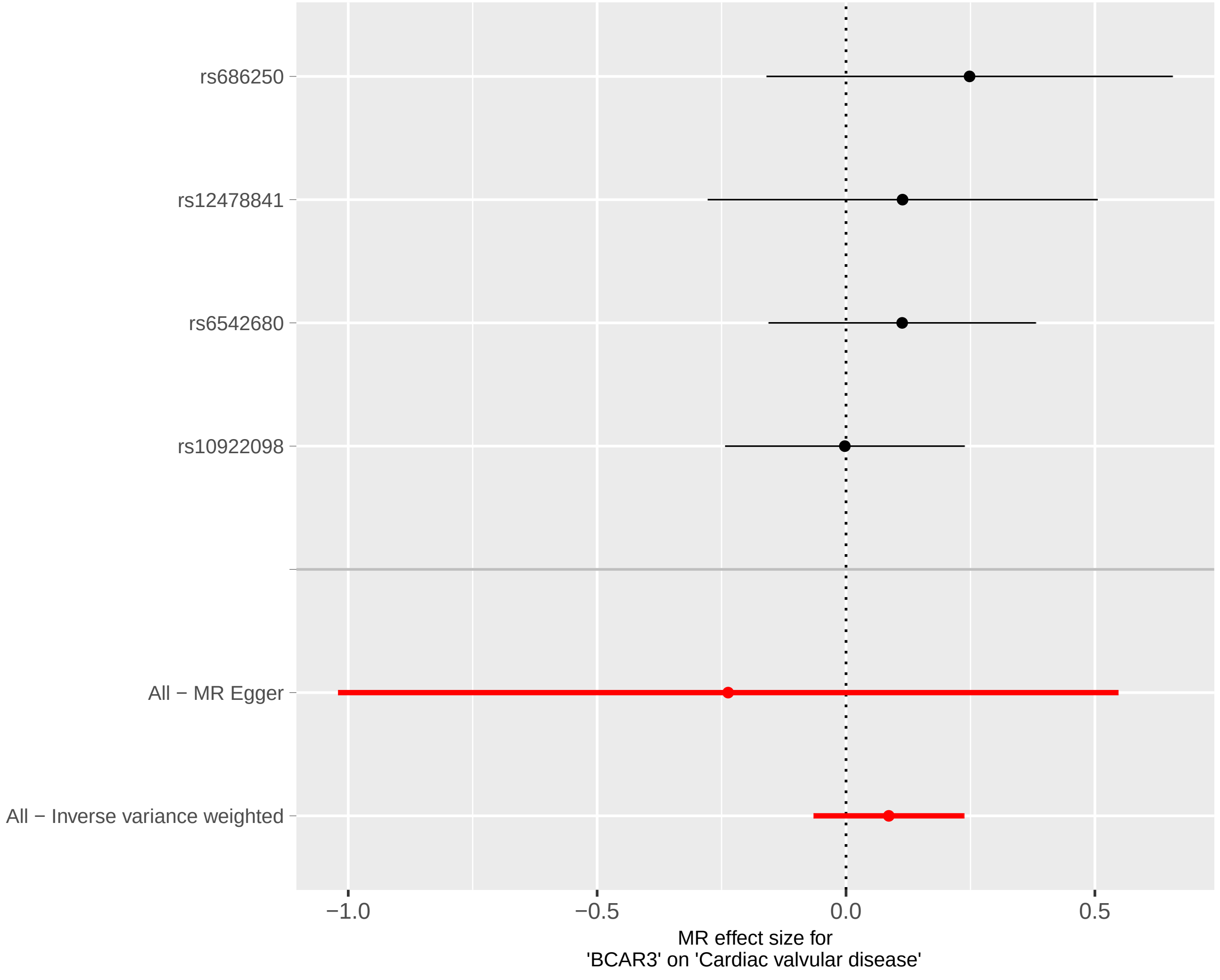
rs686250
rs12478841
rs6542680
rs10922098
All − MR Egger
All − Inverse variance weighted
−1.0
−0.5
0.0
0.5
MR effect size for
'BCAR3' on 'Cardiac valvular disease'

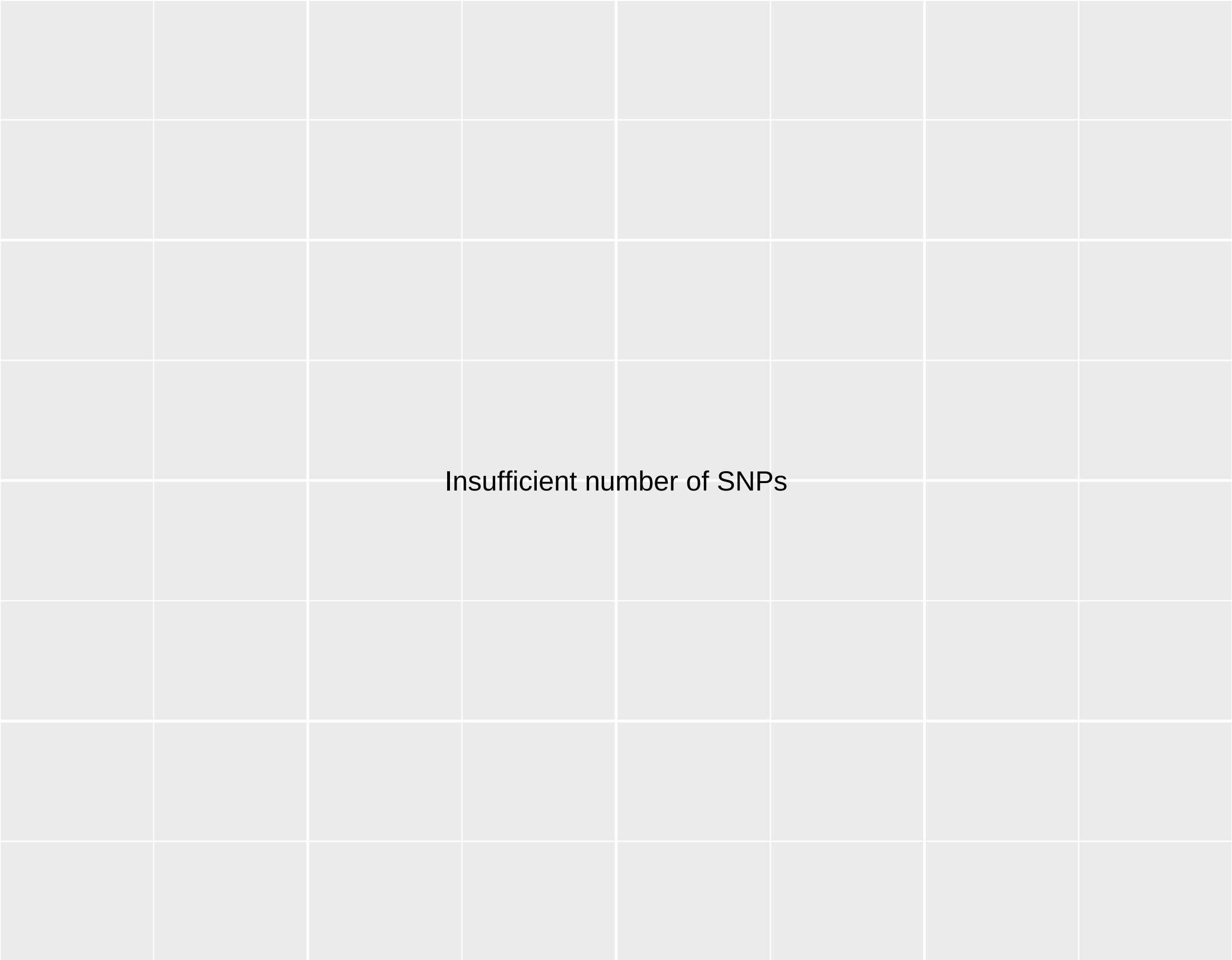
Insufficient number of SNPs

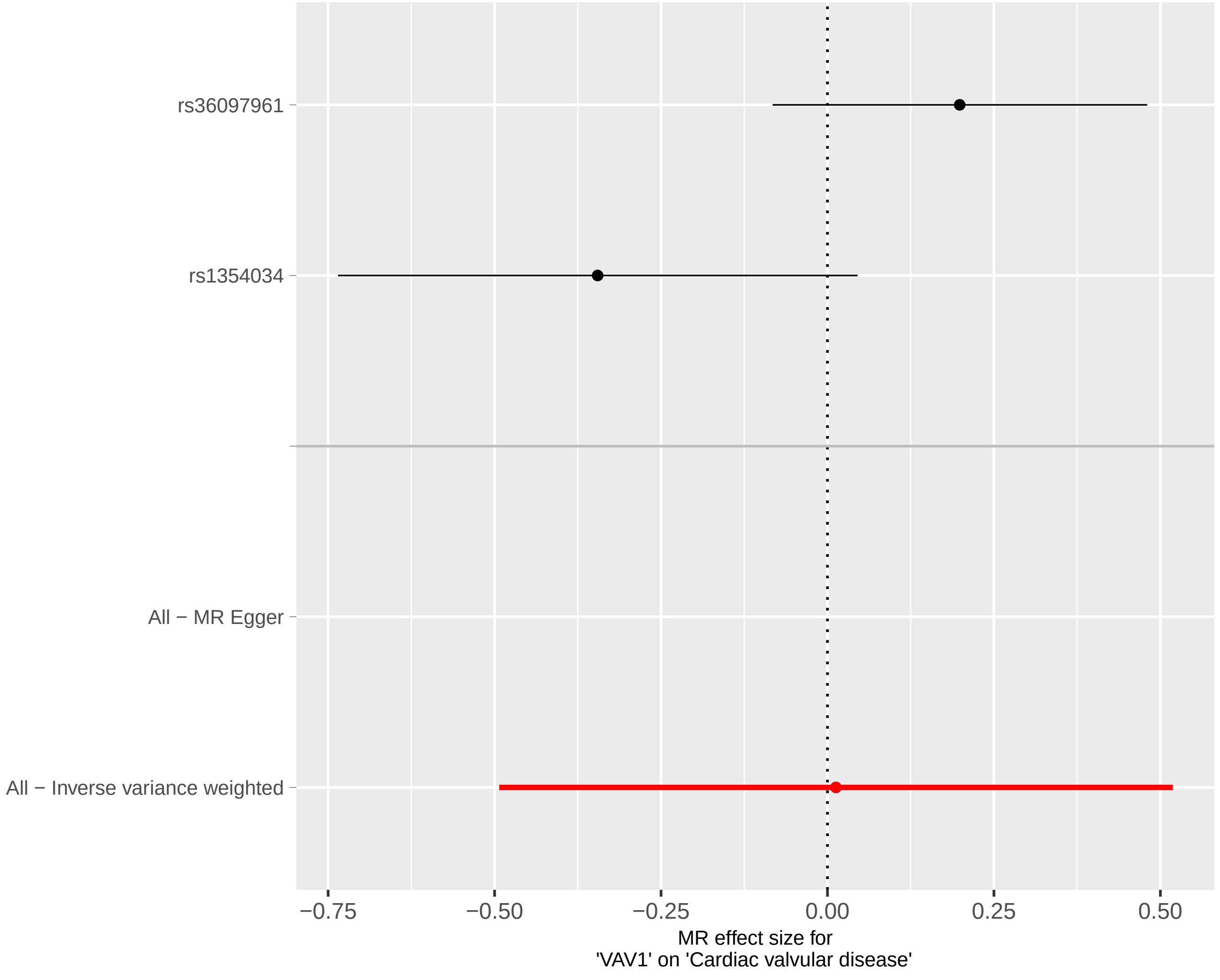
rs36097961
rs1354034
All − MR Egger
All − Inverse variance weighted
−0.75
−0.50
−0.25
0.00
0.25
0.50
MR effect size for
'VAV1' on 'Cardiac valvular disease'

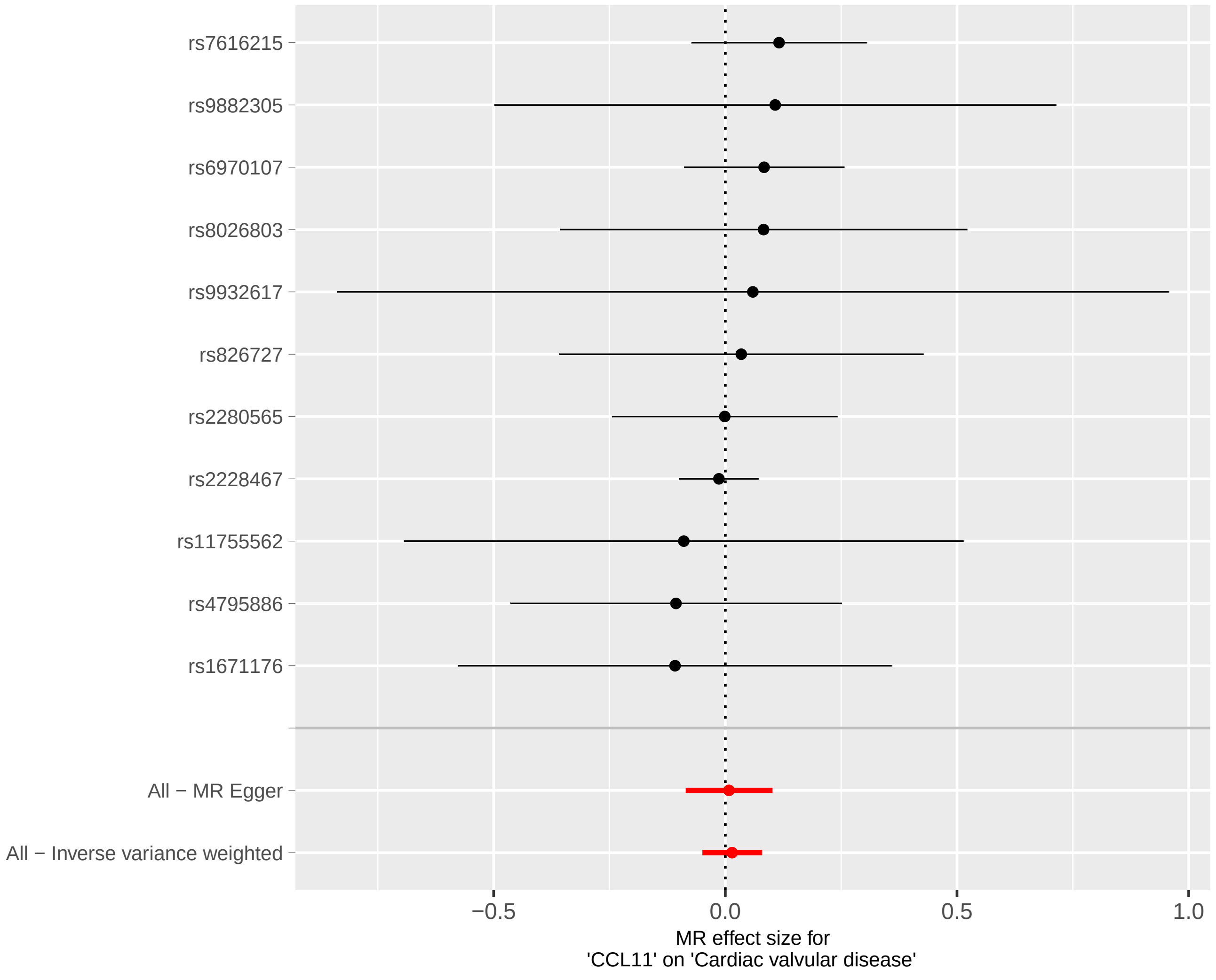
rs7616215
rs9882305
rs6970107
rs8026803
rs9932617
rs826727
rs2280565
rs2228467
rs11755562
rs4795886
rs1671176
All − MR Egger
All − Inverse variance weighted
−0.5
0.0
0.5
1.0
MR effect size for
'CCL11' on 'Cardiac valvular disease'

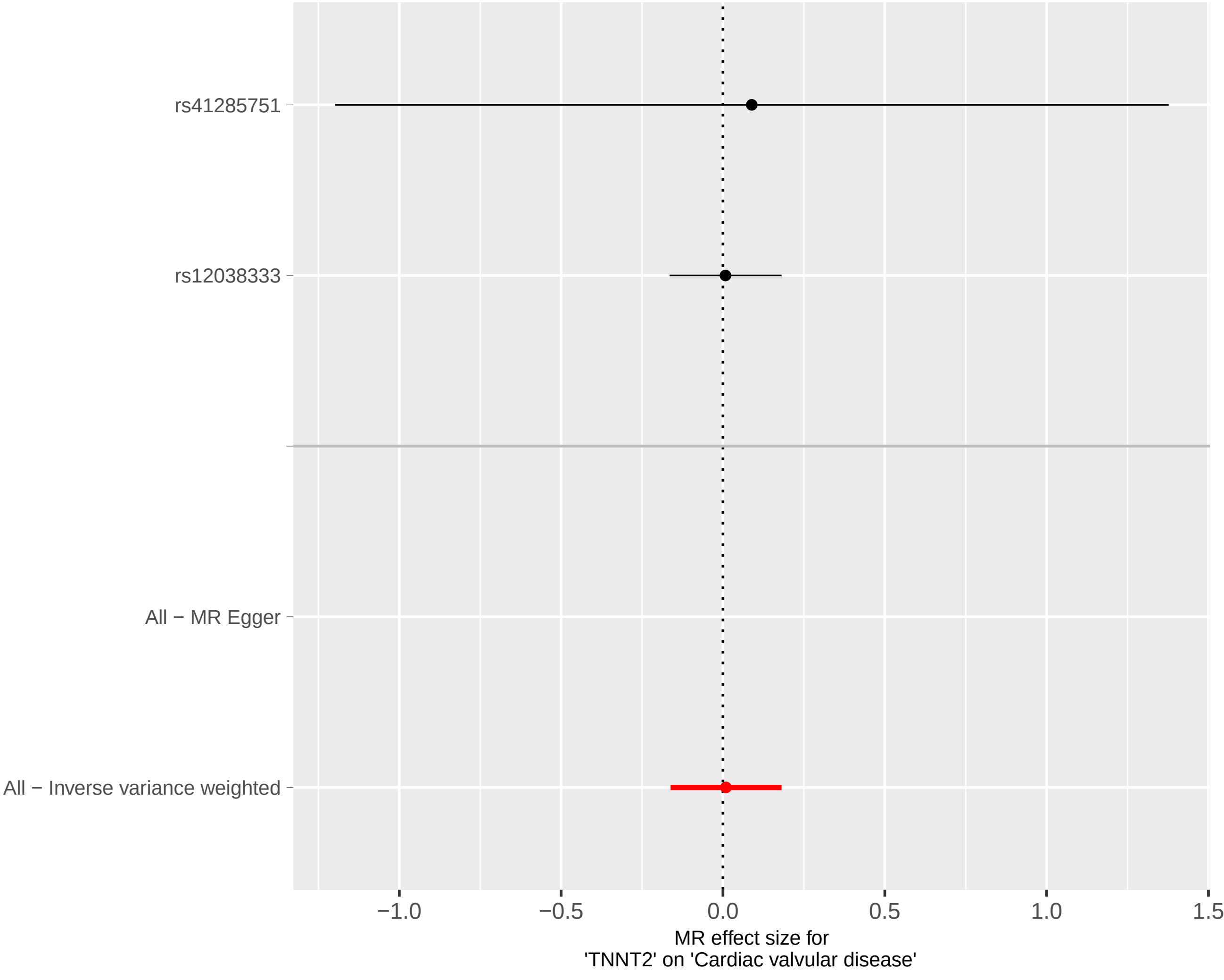
rs41285751
rs12038333
All − MR Egger
All − Inverse variance weighted
−1.0
−0.5
0.0
0.5
1.0
1.5
MR effect size for
'TNNT2' on 'Cardiac valvular disease'

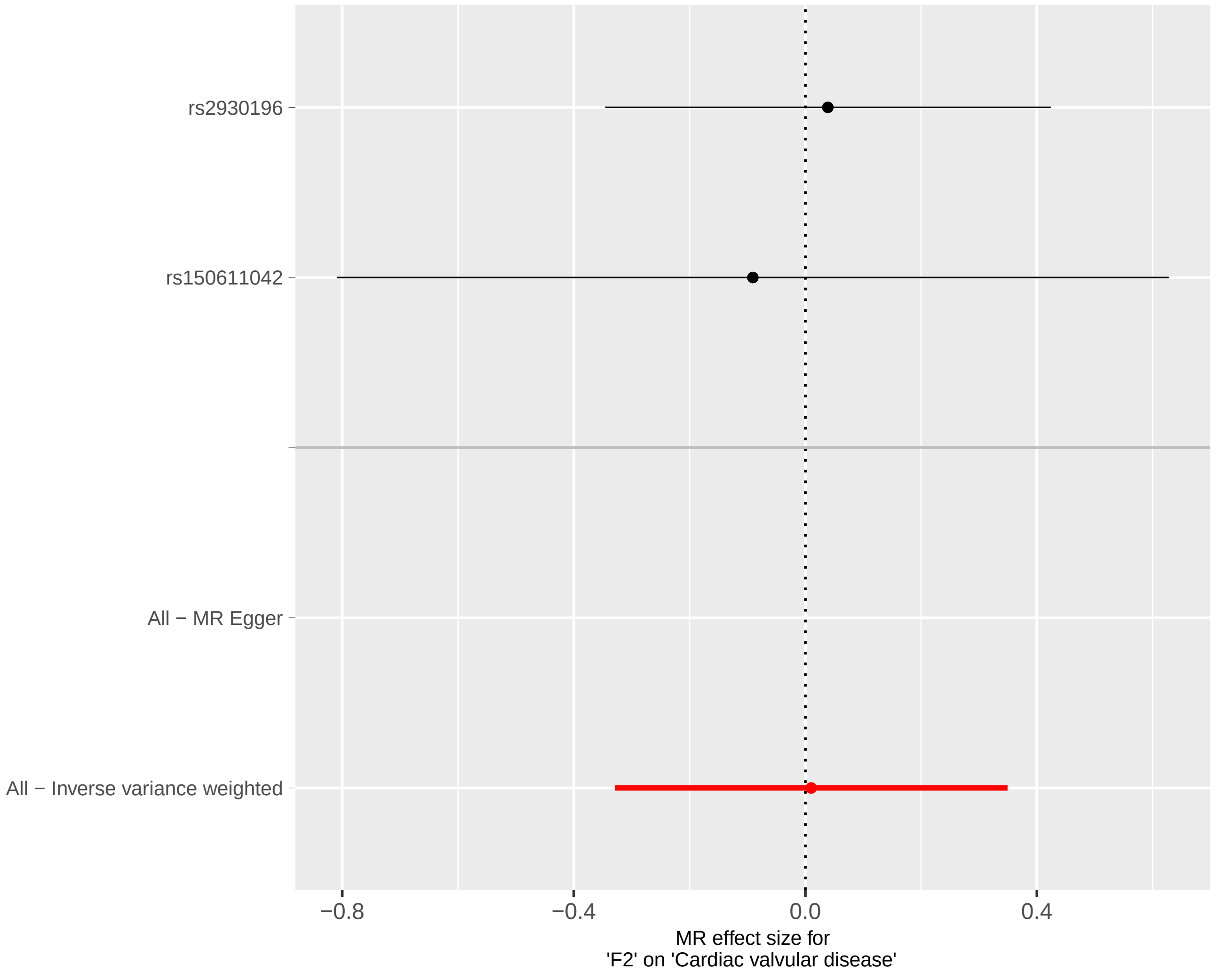
rs2930196
rs150611042
All − MR Egger
All − Inverse variance weighted
−0.8
−0.4
0.0
0.4
MR effect size for
'F2' on 'Cardiac valvular disease'

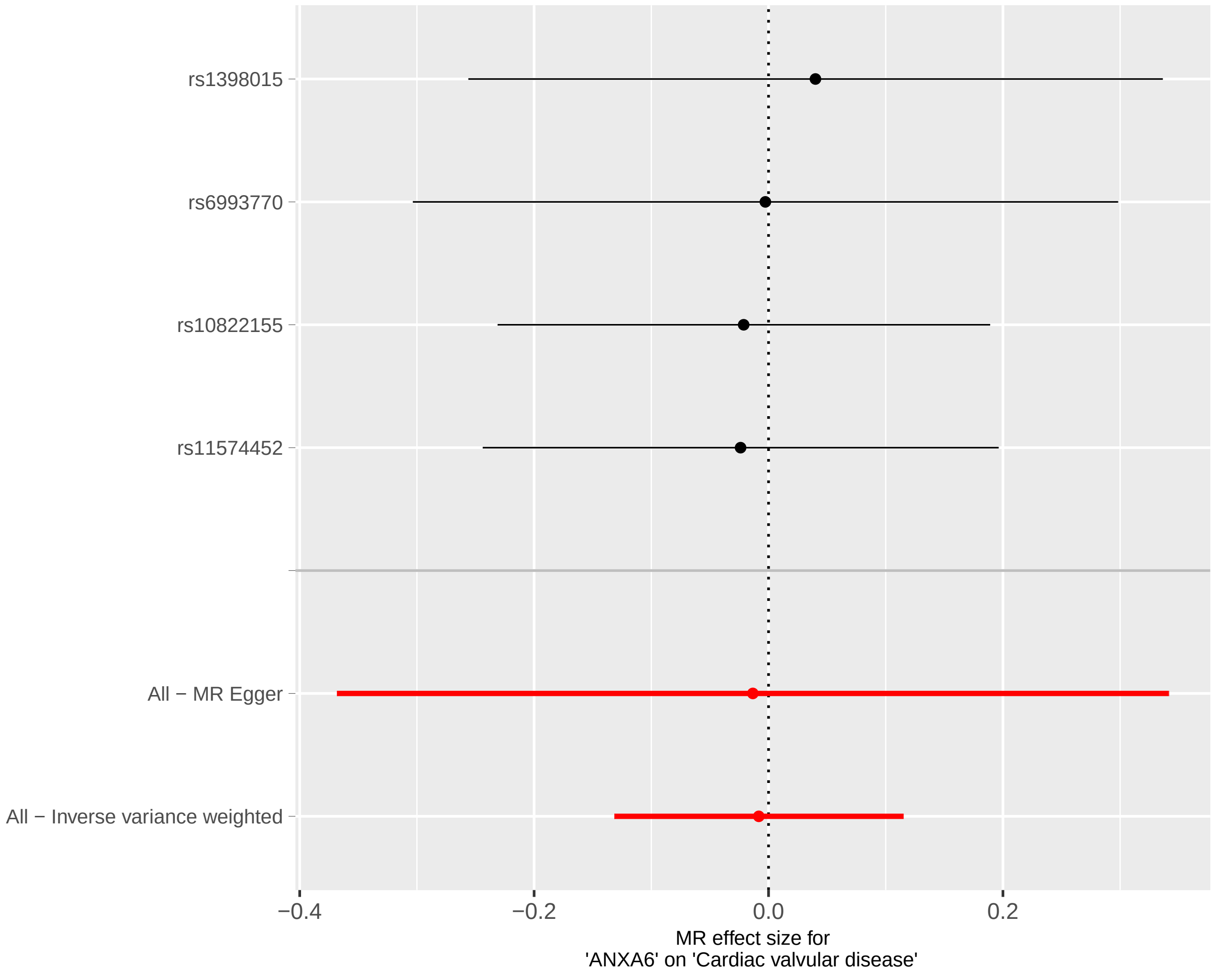
rs1398015
rs6993770
rs10822155
rs11574452
All − MR Egger
All − Inverse variance weighted
−0.4
−0.2
0.0
0.2
MR effect size for
'ANXA6' on 'Cardiac valvular disease'

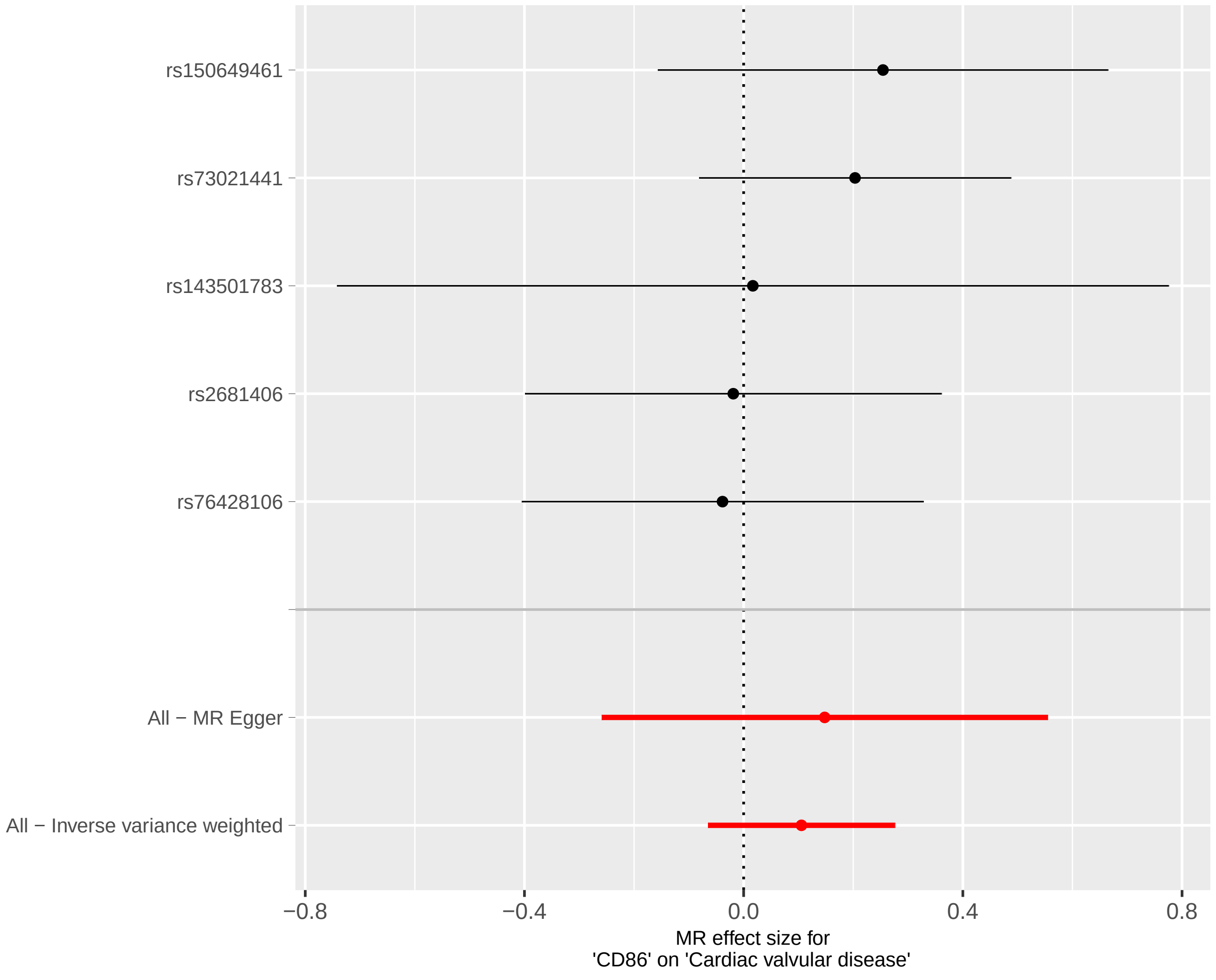
rs150649461
rs73021441
rs143501783
rs2681406
rs76428106
All − MR Egger
All − Inverse variance weighted
−0.8
−0.4
0.0
0.4
0.8
MR effect size for
'CD86' on 'Cardiac valvular disease'

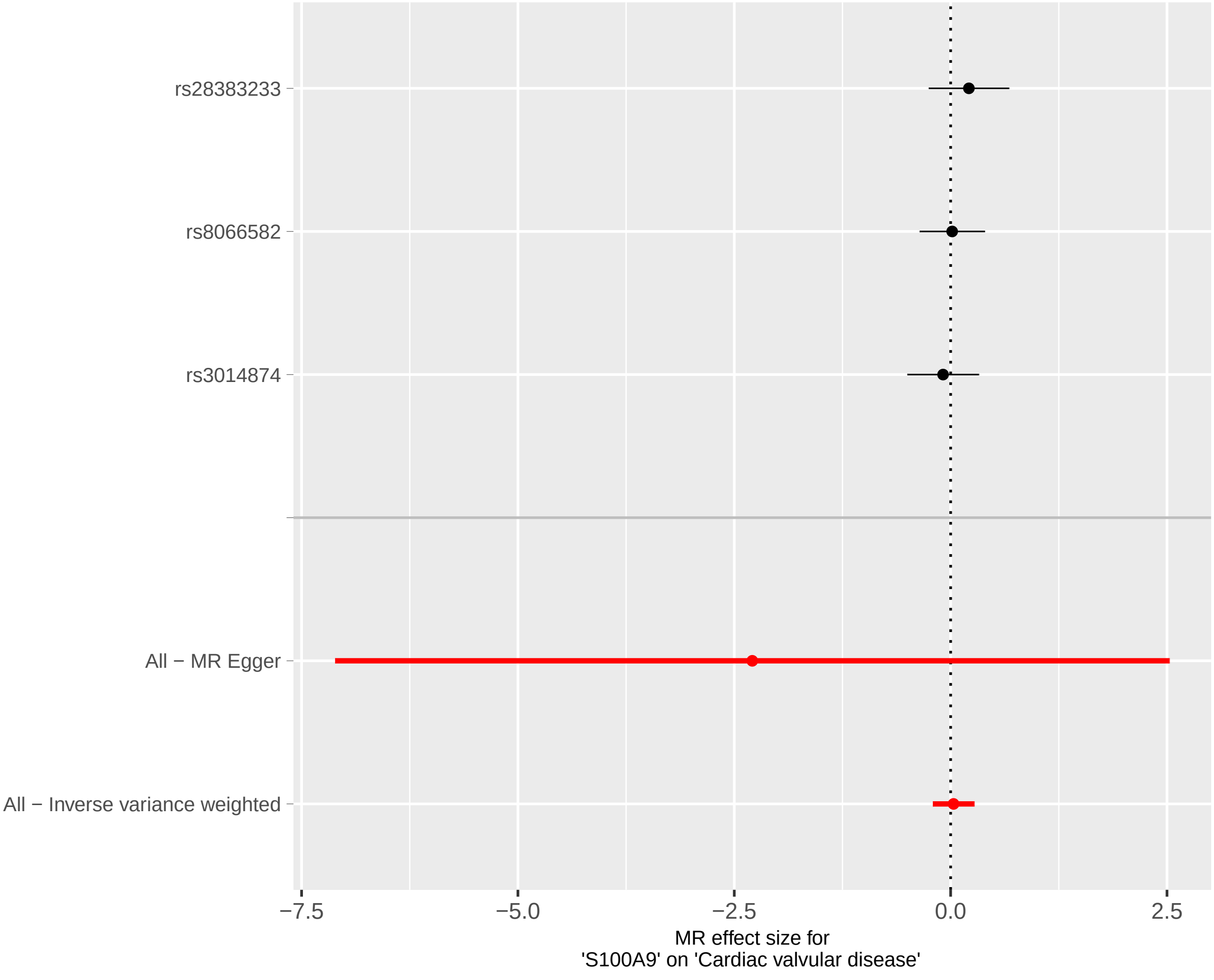

rs28383233
rs8066582
rs3014874
All − MR Egger
All − Inverse variance weighted
−7.5
−5.0
−2.5
0.0
2.5
MR effect size for
'S100A9' on 'Cardiac valvular disease'

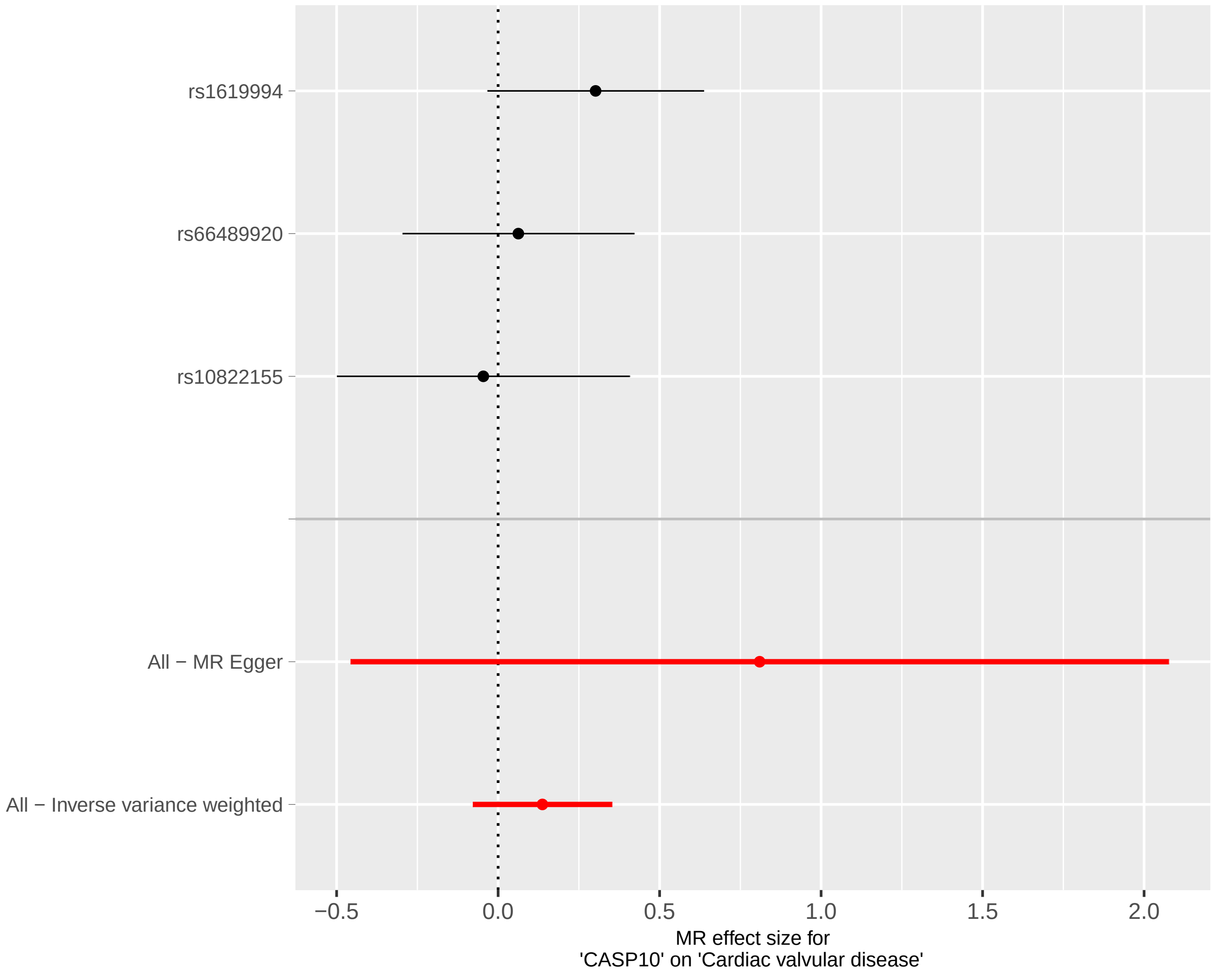
rs1619994
rs66489920
rs10822155
All − MR Egger
All − Inverse variance weighted
−0.5
0.0
0.5
1.0
1.5
2.0
MR effect size for
'CASP10' on 'Cardiac valvular disease'

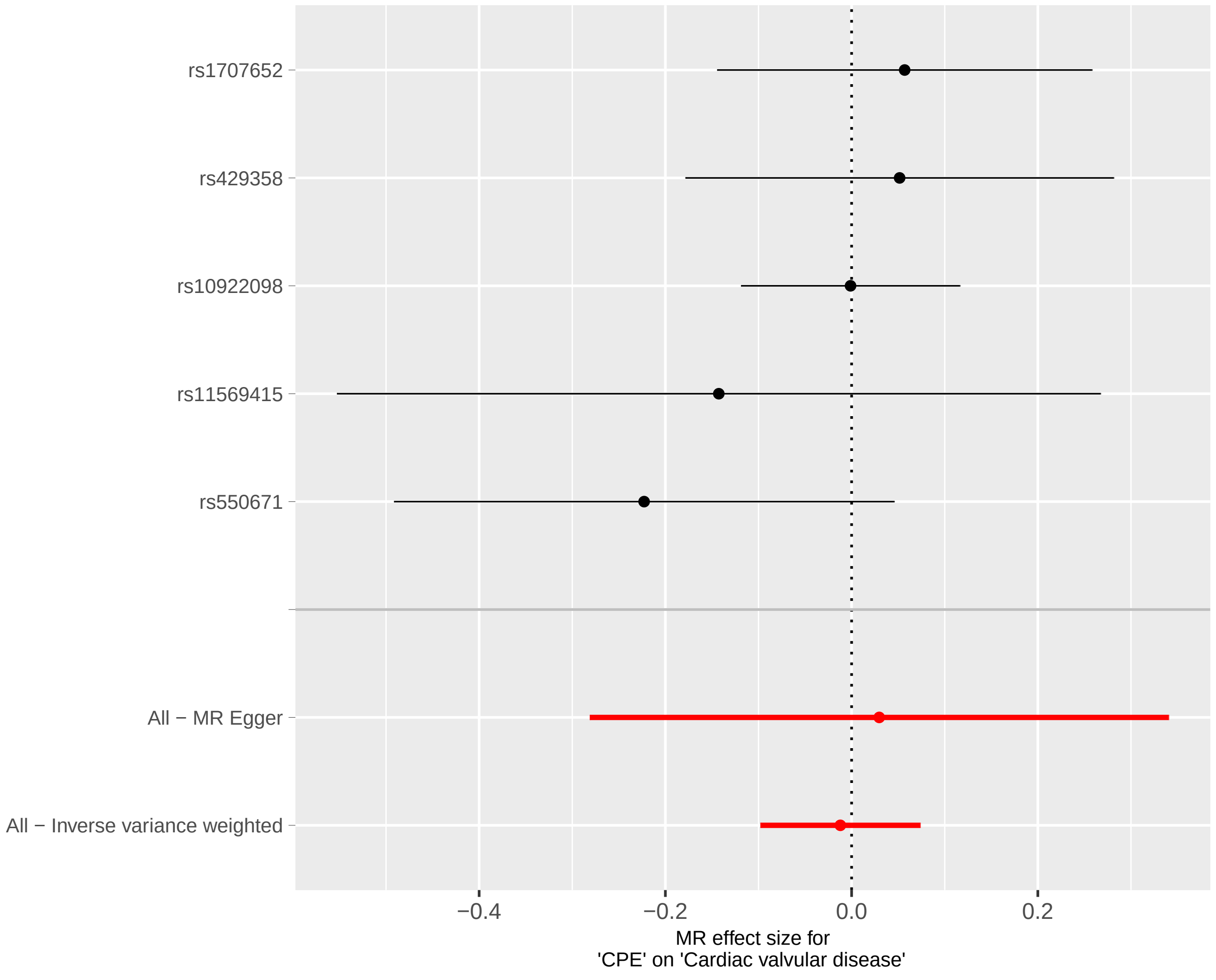
rs1707652
rs429358
rs10922098
rs11569415
rs550671
All − MR Egger
All − Inverse variance weighted
−0.4
−0.2
0.0
0.2
MR effect size for
'CPE' on 'Cardiac valvular disease'

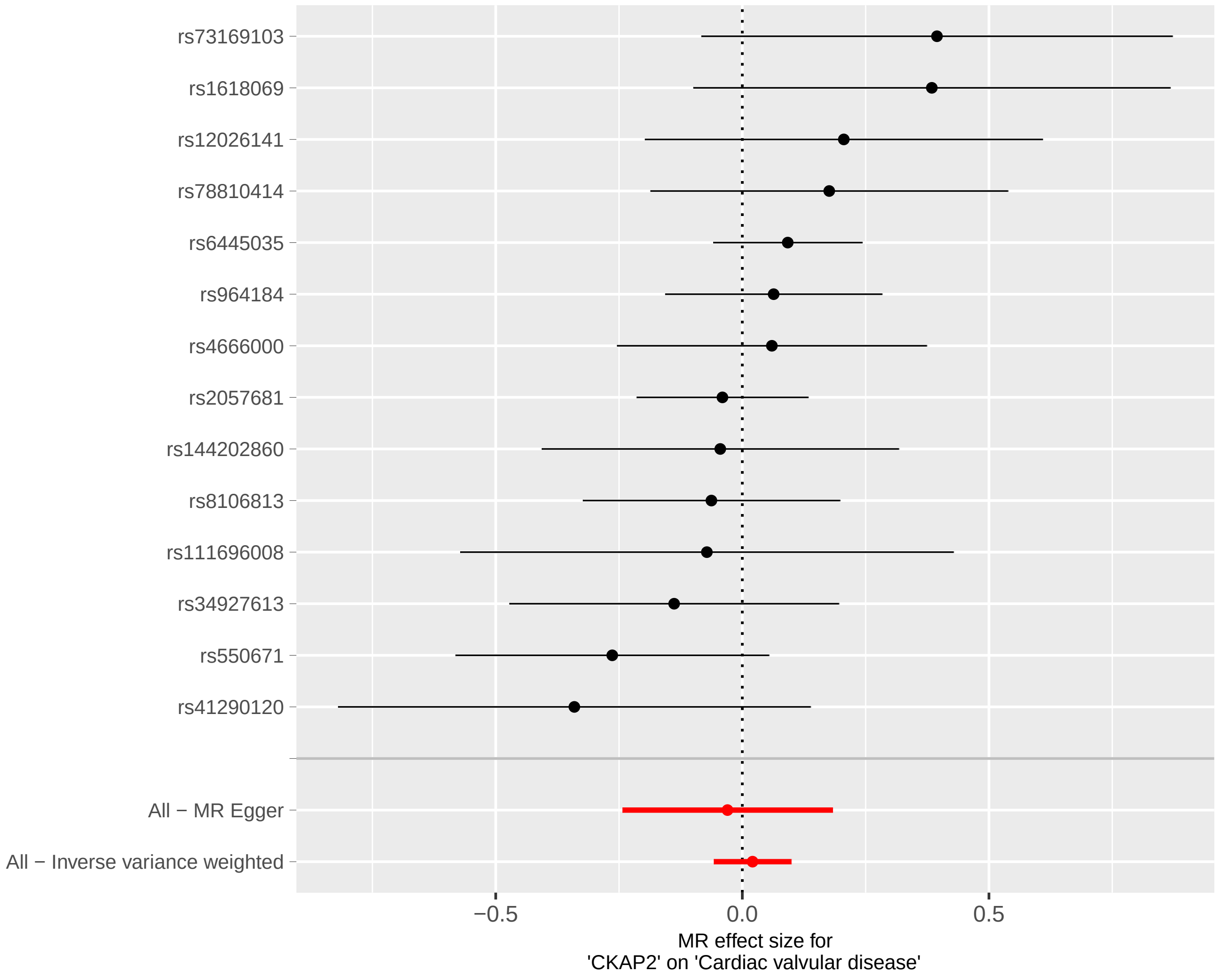
rs73169103
rs1618069
rs12026141
rs78810414
rs6445035
rs964184
rs4666000
rs2057681
rs144202860
rs8106813
rs111696008
rs34927613
rs550671
rs41290120
All − MR Egger
All − Inverse variance weighted
−0.5
0.0
0.5
MR effect size for
'CKAP2' on 'Cardiac valvular disease'

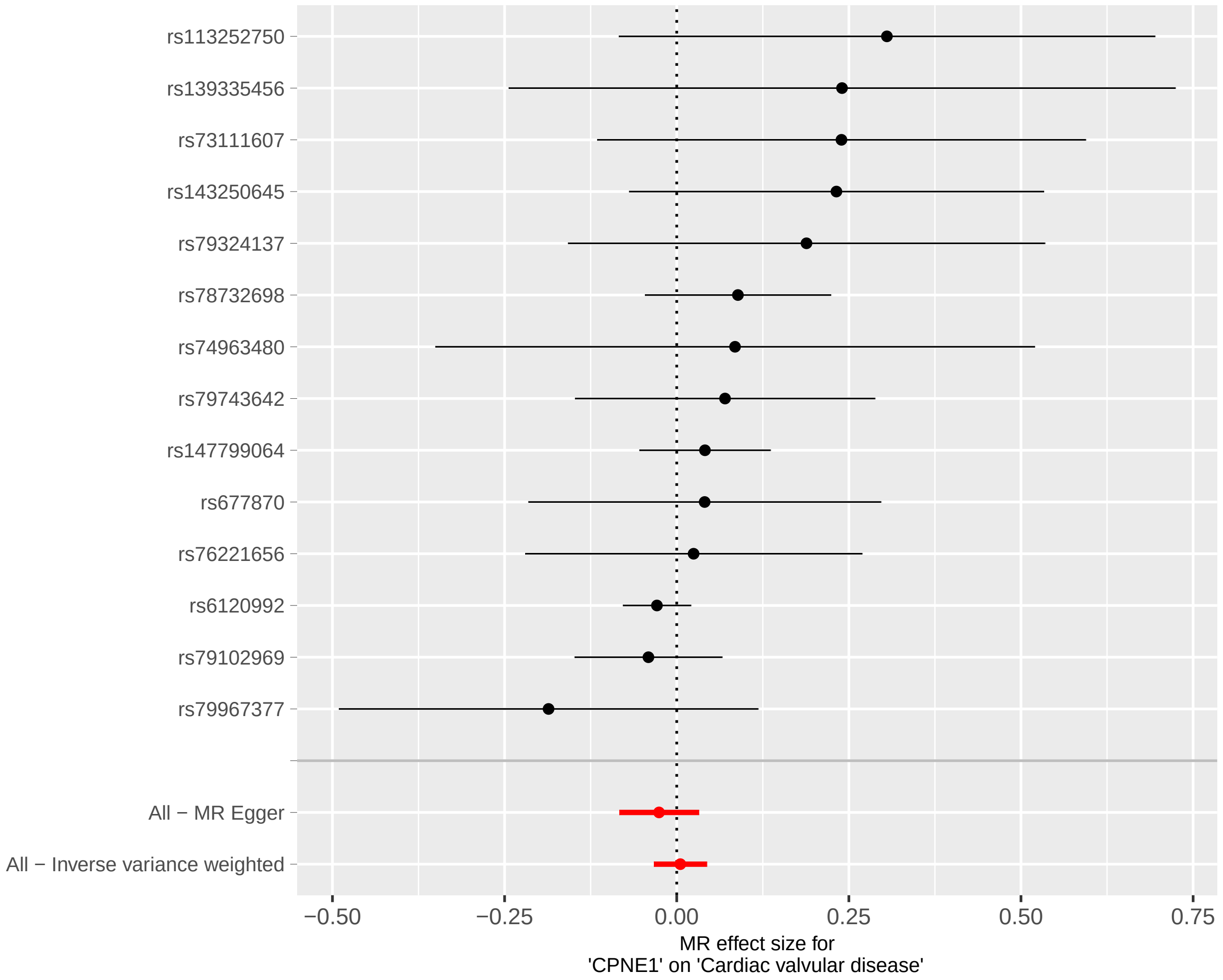
rs113252750
rs139335456
rs73111607
rs143250645
rs79324137
rs78732698
rs74963480
rs79743642
rs147799064
rs677870
rs76221656
rs6120992
rs79102969
rs79967377
All − MR Egger
All − Inverse variance weighted
−0.50
−0.25
0.00
0.25
0.50
0.75
MR effect size for
'CPNE1' on 'Cardiac valvular disease'

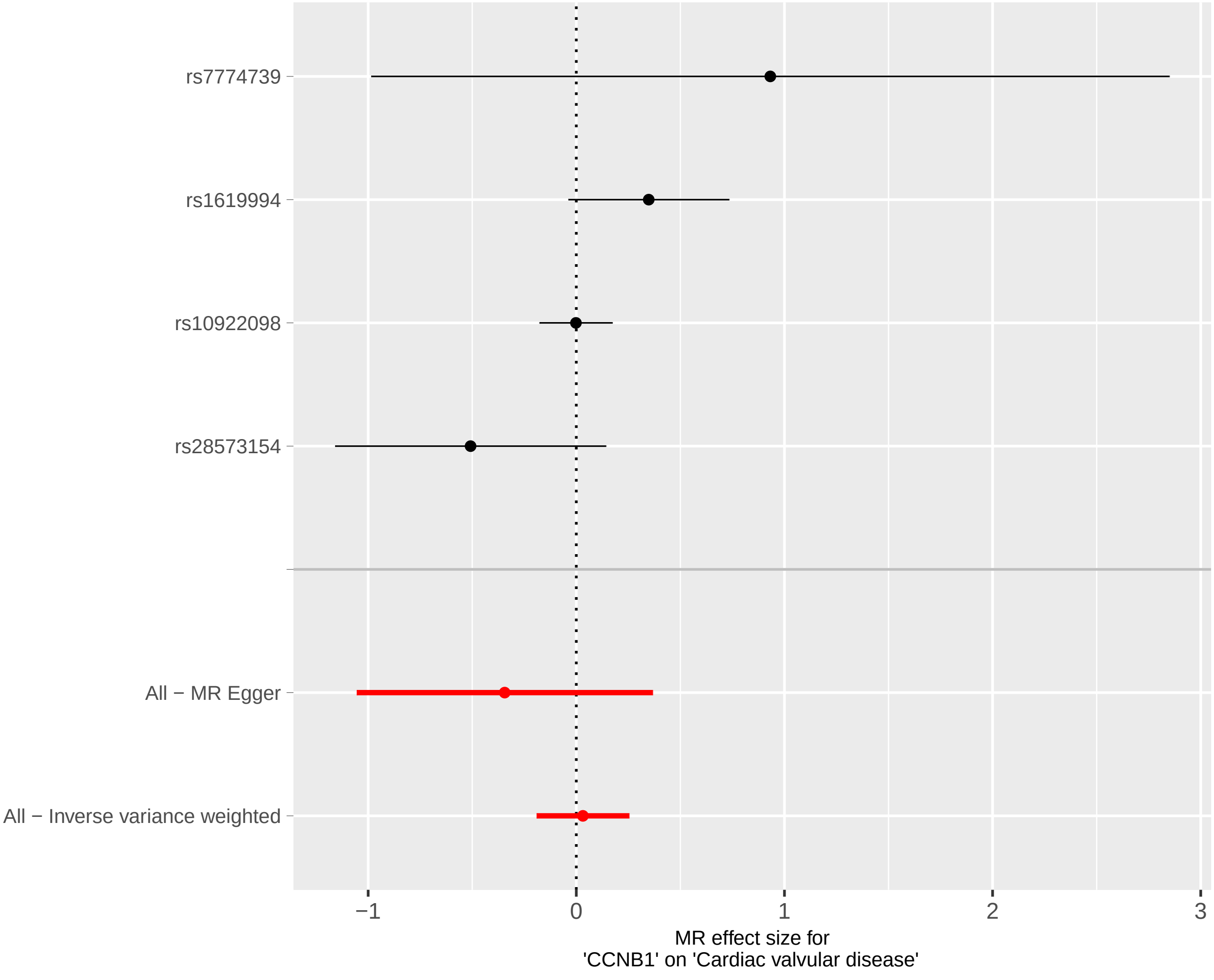
rs7774739
rs1619994
rs10922098
rs28573154
All − MR Egger
All − Inverse variance weighted
−1
0
1
2
3
MR effect size for
'CCNB1' on 'Cardiac valvular disease'

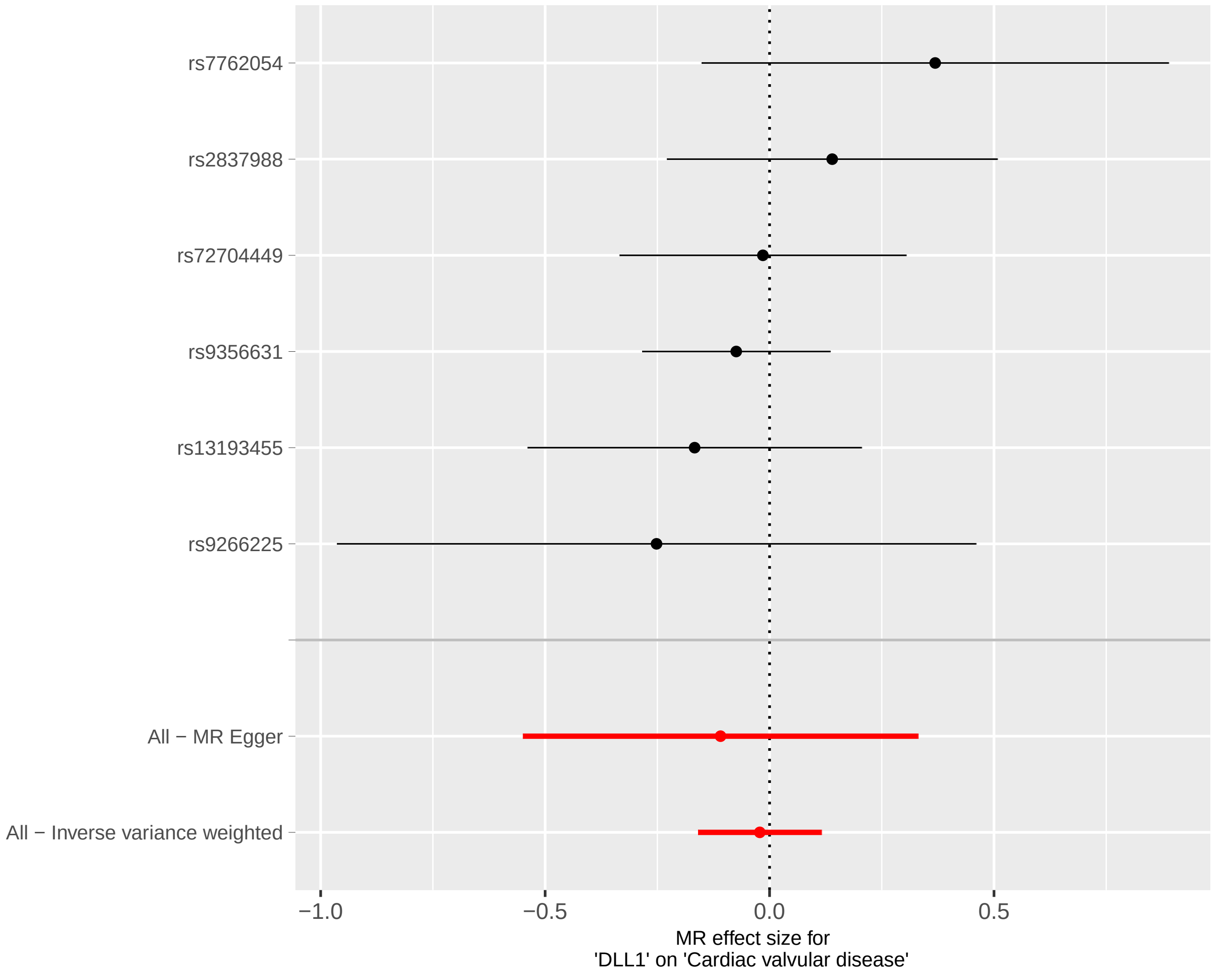
rs7762054
rs2837988
rs72704449
rs9356631
rs13193455
rs9266225
All − MR Egger
All − Inverse variance weighted
−1.0
−0.5
0.0
0.5
MR effect size for
'DLL1' on 'Cardiac valvular disease'

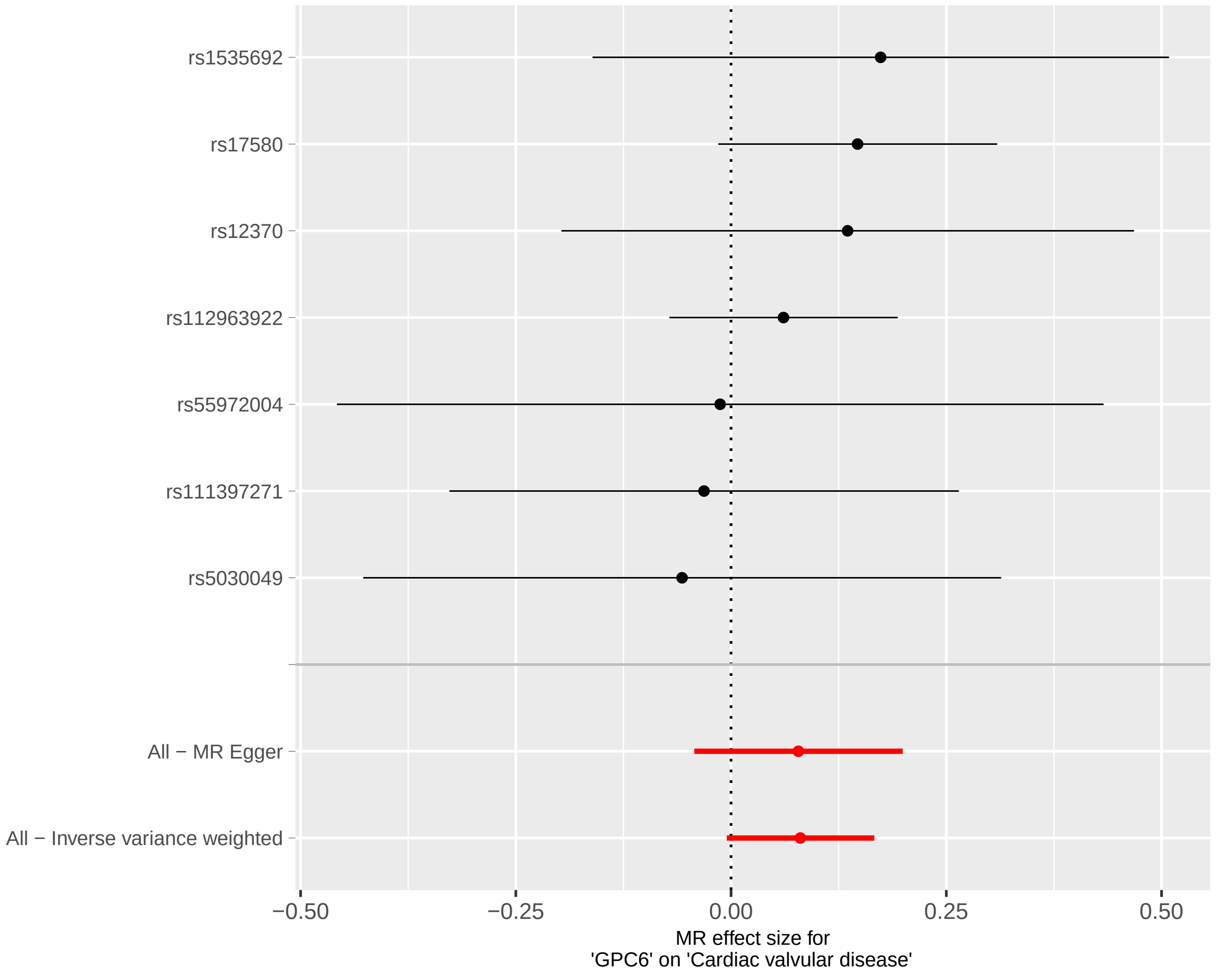
rs1535692
rs17580
rs12370
rs112963922
rs55972004
rs111397271
rs5030049
All − MR Egger
All − Inverse variance weighted
−0.50
−0.25
0.00
0.25
0.50
MR effect size for
'GPC6' on 'Cardiac valvular disease'

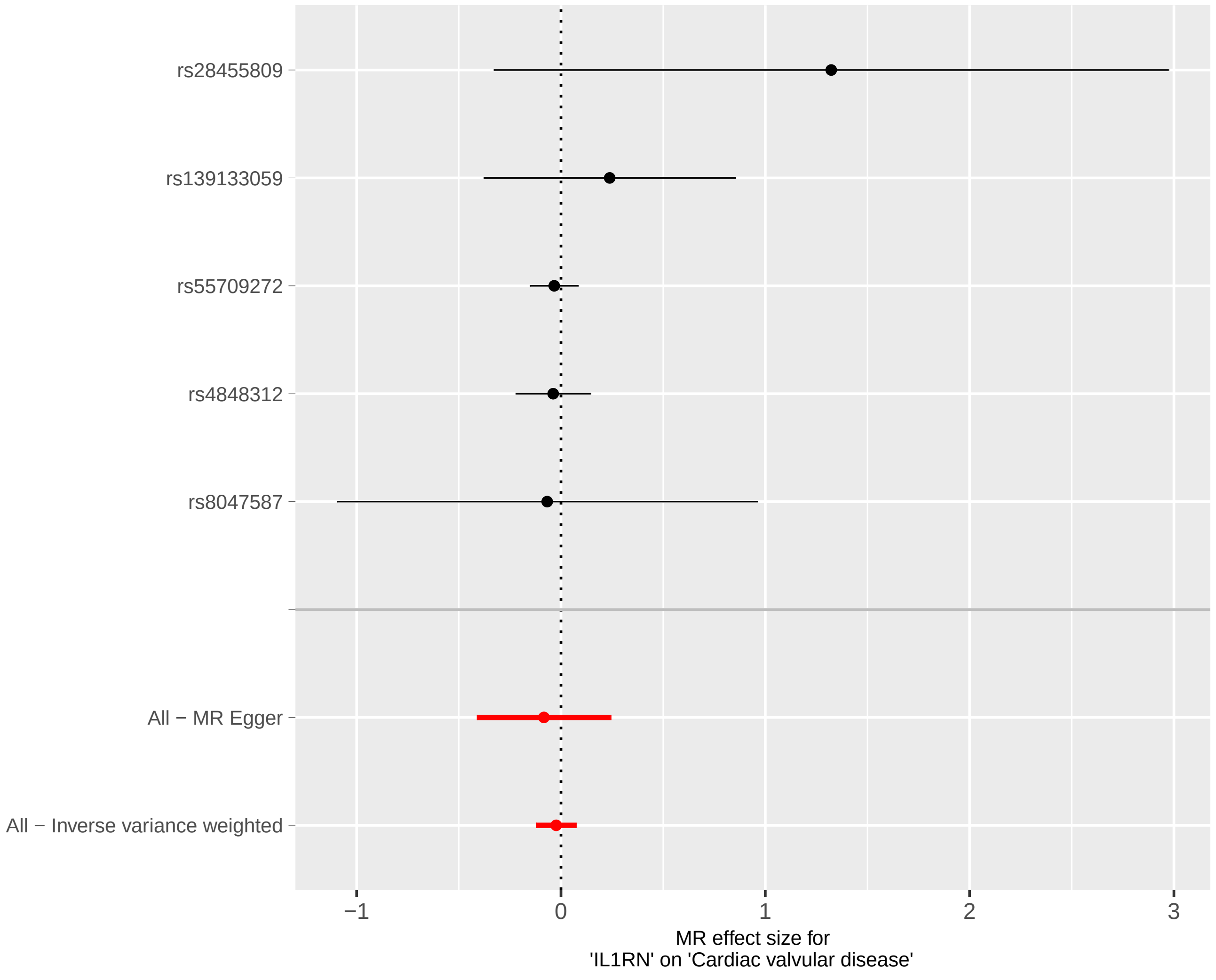

rs28455809
rs139133059
rs55709272
rs4848312
rs8047587
All − MR Egger
All − Inverse variance weighted
−1
0
1
2
3
MR effect size for
'IL1RN' on 'Cardiac valvular disease'

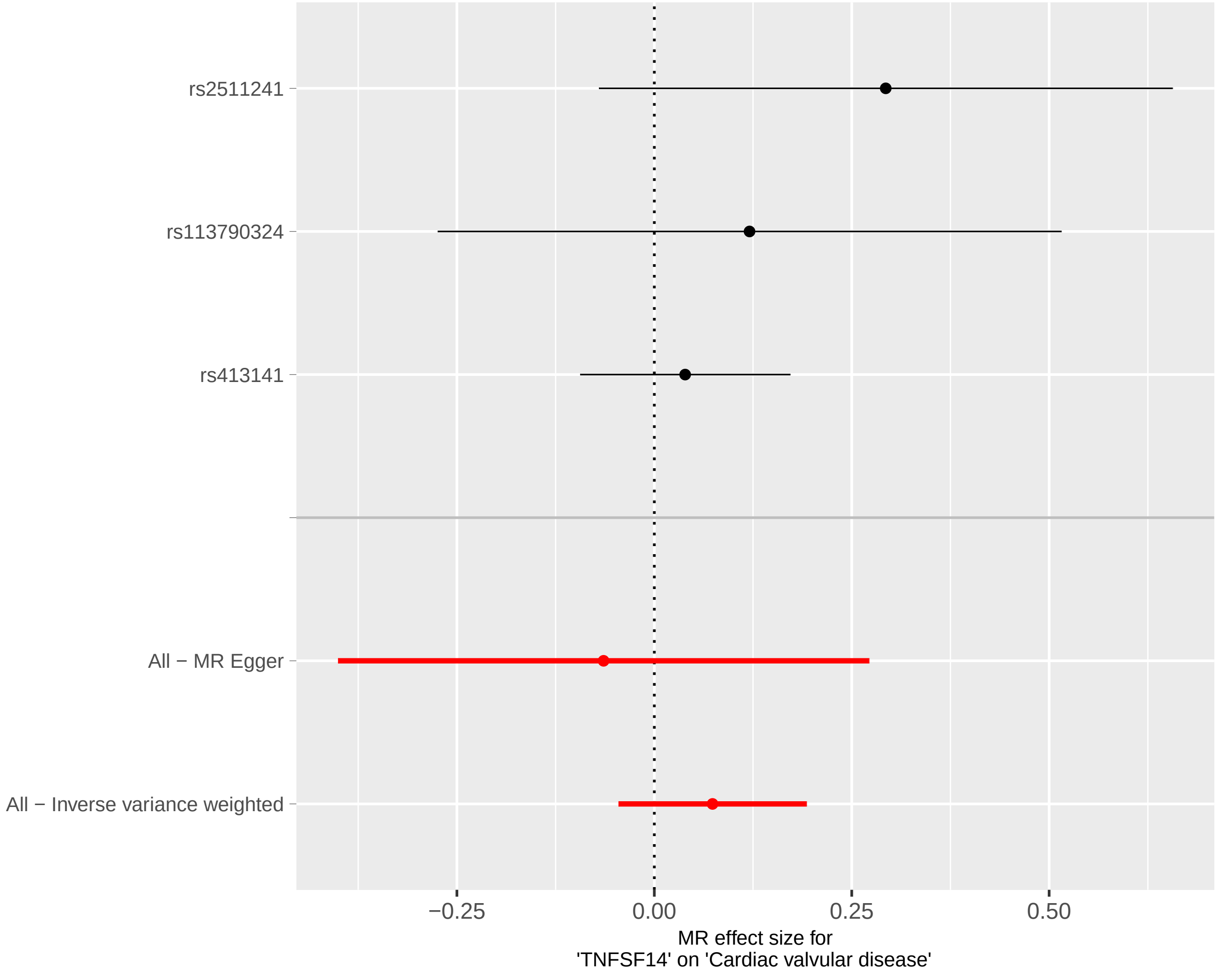
rs2511241
rs113790324
rs413141
All − MR Egger
All − Inverse variance weighted
−0.25
0.00
0.25
0.50
MR effect size for
'TNFSF14' on 'Cardiac valvular disease'

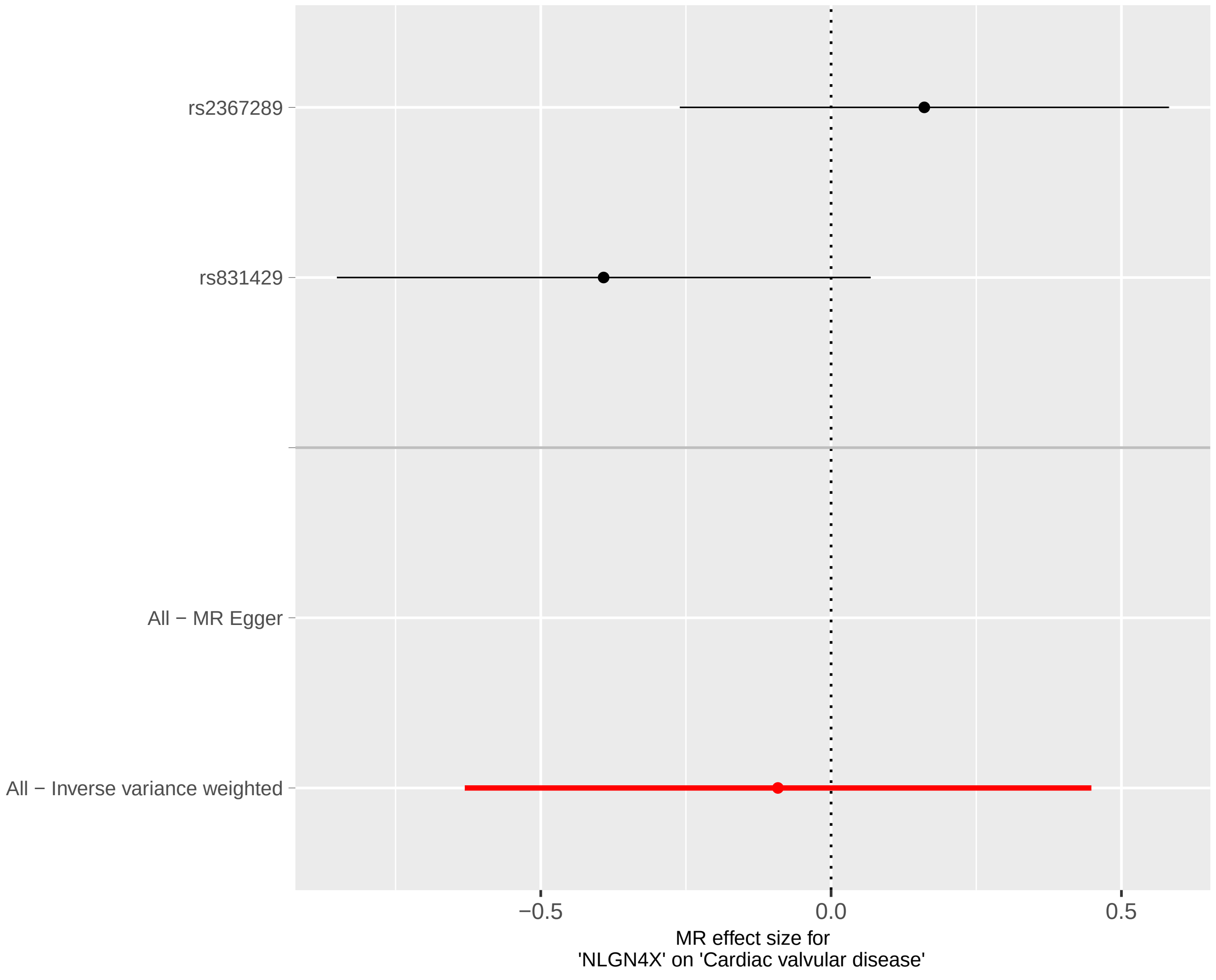
rs2367289
rs831429
All − MR Egger
All − Inverse variance weighted
−0.5
0.0
0.5
MR effect size for
'NLGN4X' on 'Cardiac valvular disease'

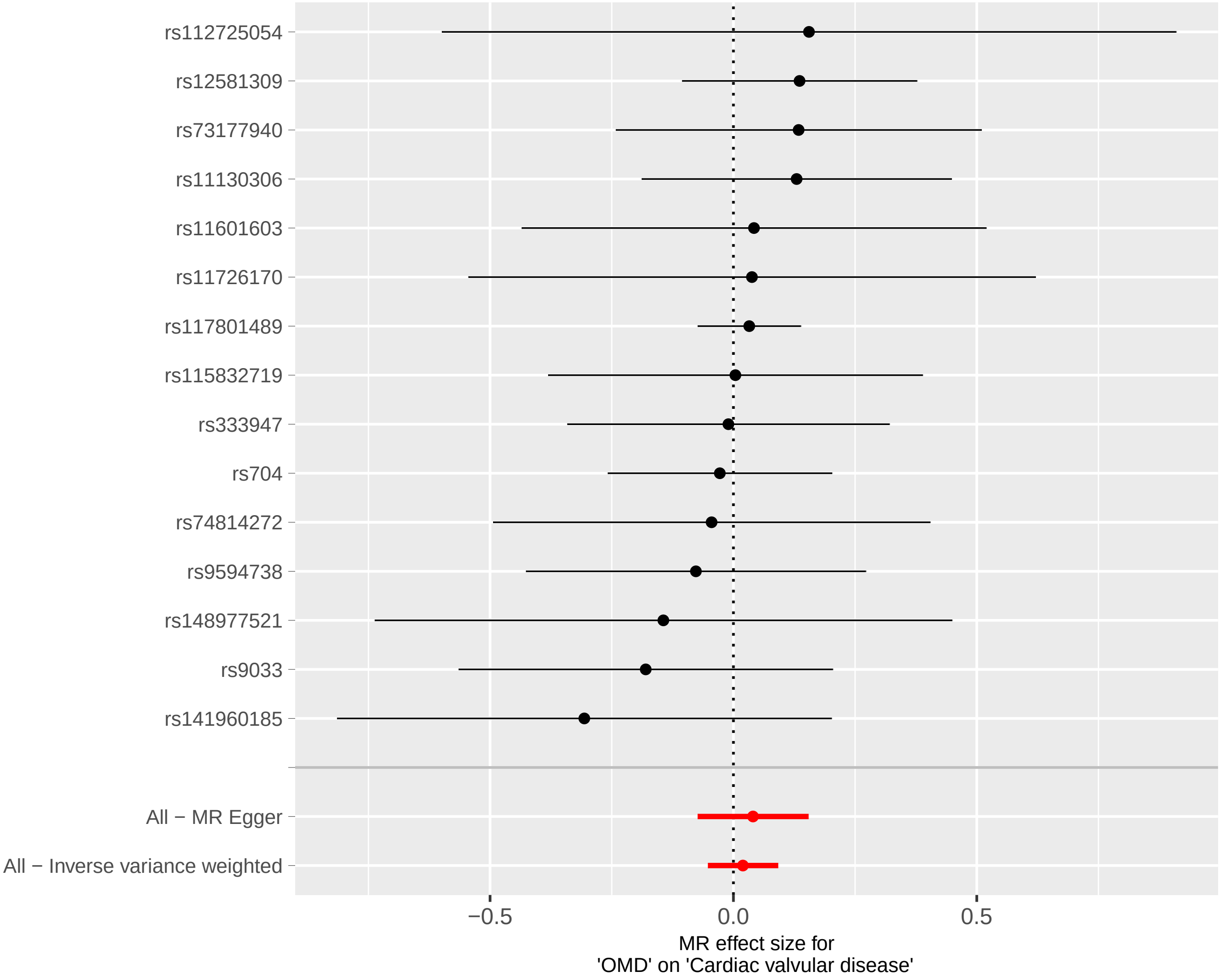

rs112725054
rs12581309
rs73177940
rs11130306
rs11601603
rs11726170
rs117801489
rs115832719
rs333947
rs704
rs74814272
rs9594738
rs148977521
rs9033
rs141960185
All − MR Egger
All − Inverse variance weighted
−0.5
0.0
0.5
MR effect size for
'OMD' on 'Cardiac valvular disease'

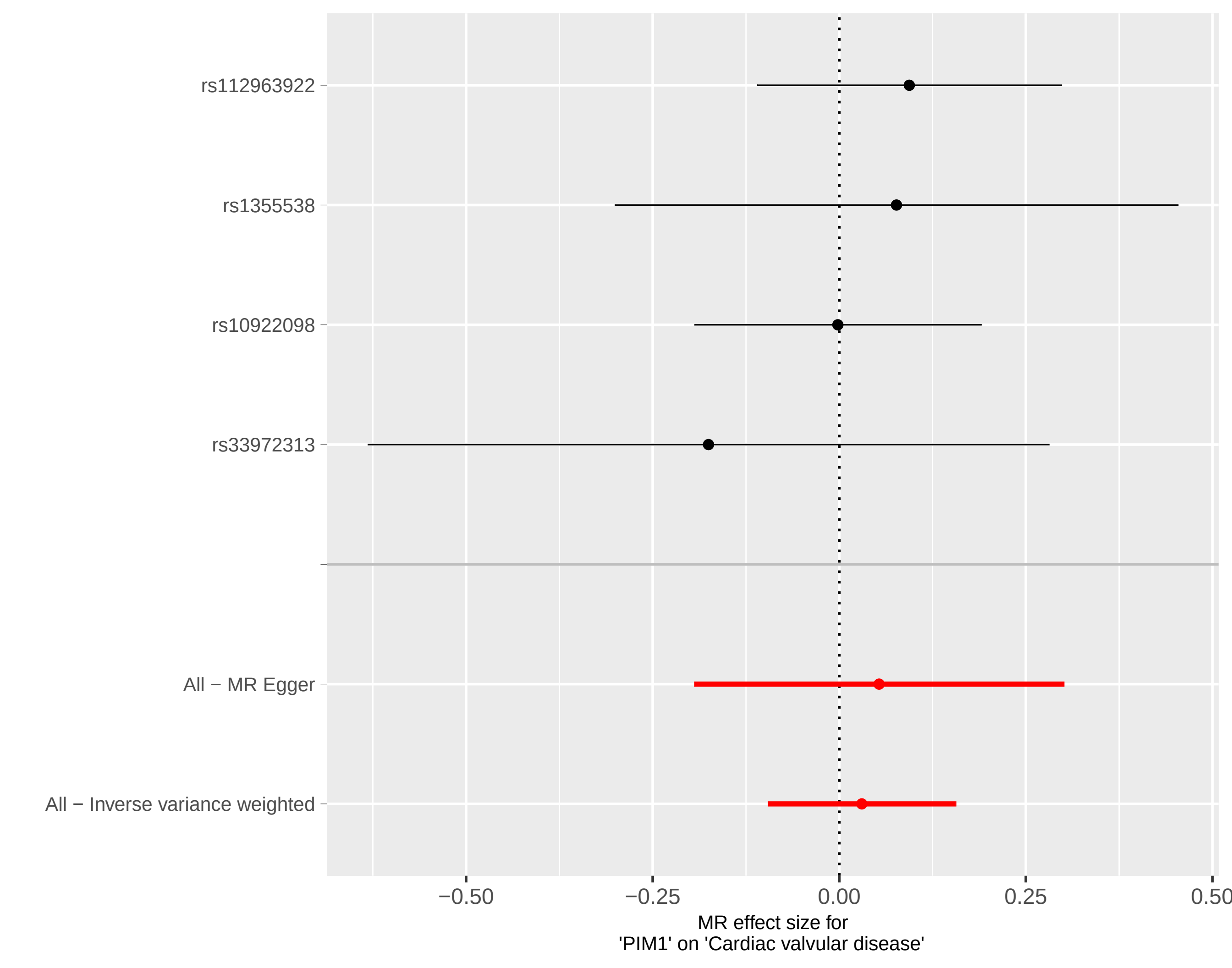
rs112963922
rs1355538
rs10922098
rs33972313
All − MR Egger
All − Inverse variance weighted
−0.50
−0.25
0.00
0.25
0.50
MR effect size for
'PIM1' on 'Cardiac valvular disease'

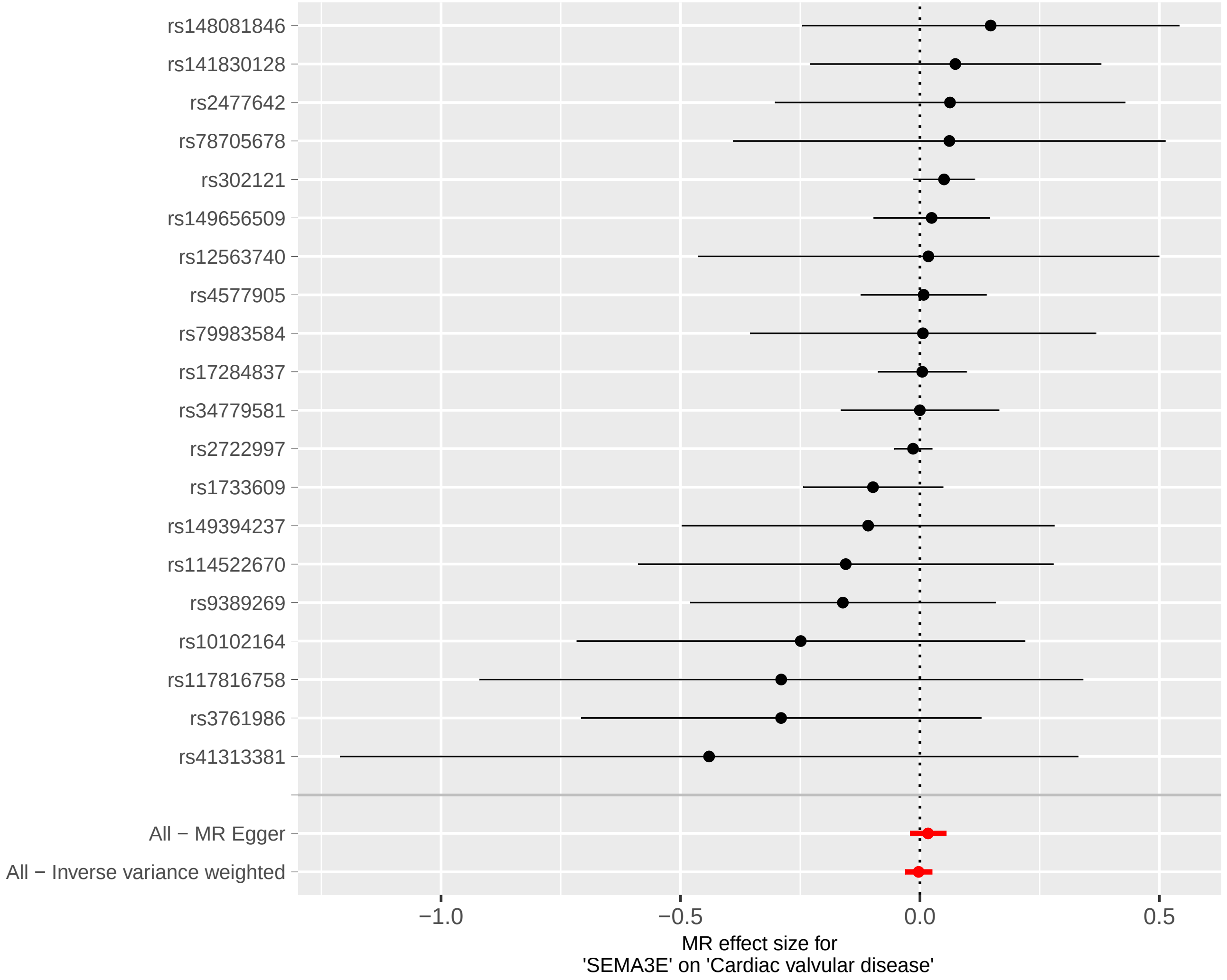
rs148081846
rs141830128
rs2477642
rs78705678
rs302121
rs149656509
rs12563740
rs4577905
rs79983584
rs17284837
rs34779581
rs2722997
rs1733609
rs149394237
rs114522670
rs9389269
rs10102164
rs117816758
rs3761986
rs41313381
All − MR Egger
All − Inverse variance weighted
−1.0
−0.5
0.0
0.5
MR effect size for
'SEMA3E' on 'Cardiac valvular disease'

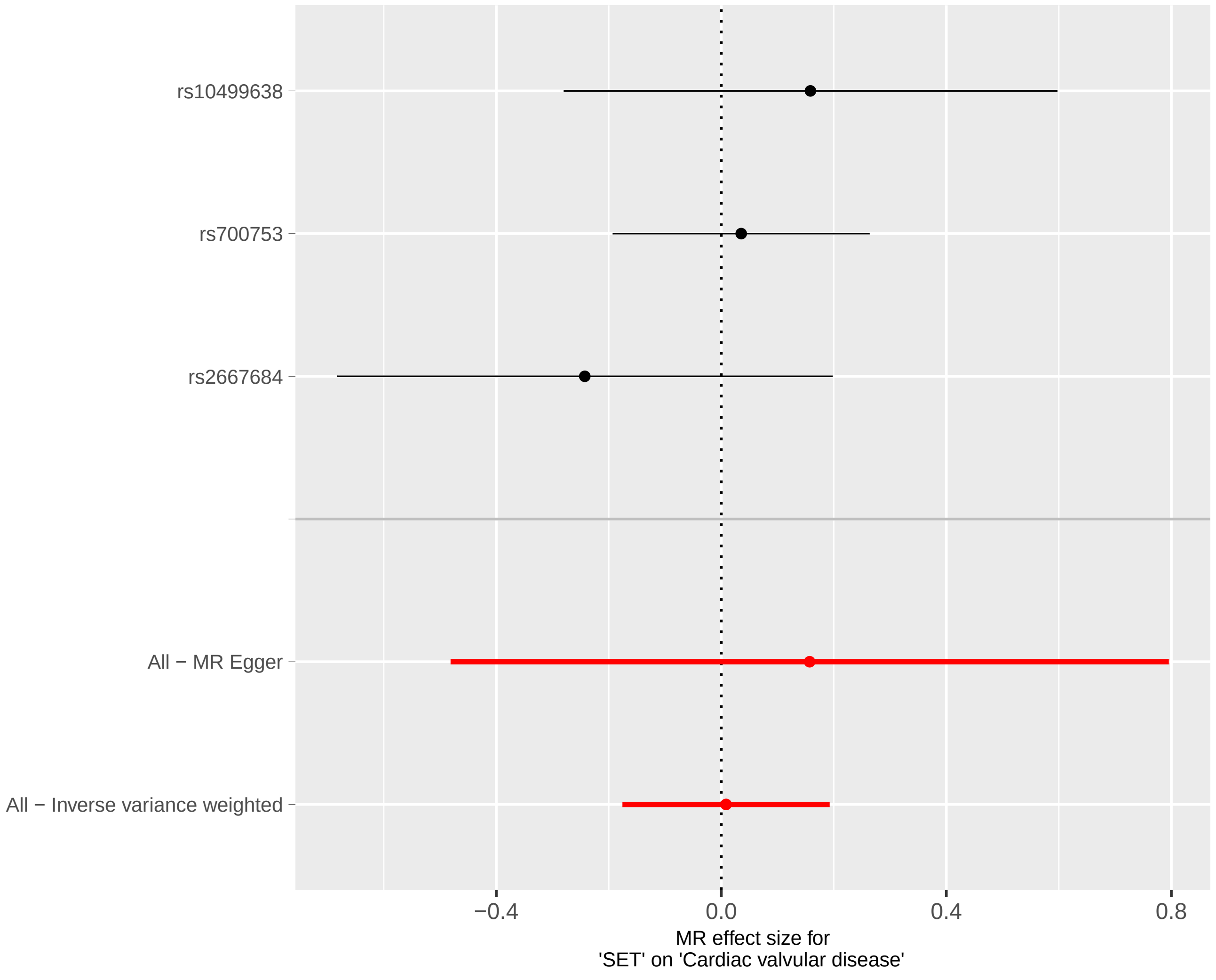

rs10499638
rs700753
rs2667684
All − MR Egger
All − Inverse variance weighted
−0.4
0.0
0.4
0.8
MR effect size for
'SET' on 'Cardiac valvular disease'

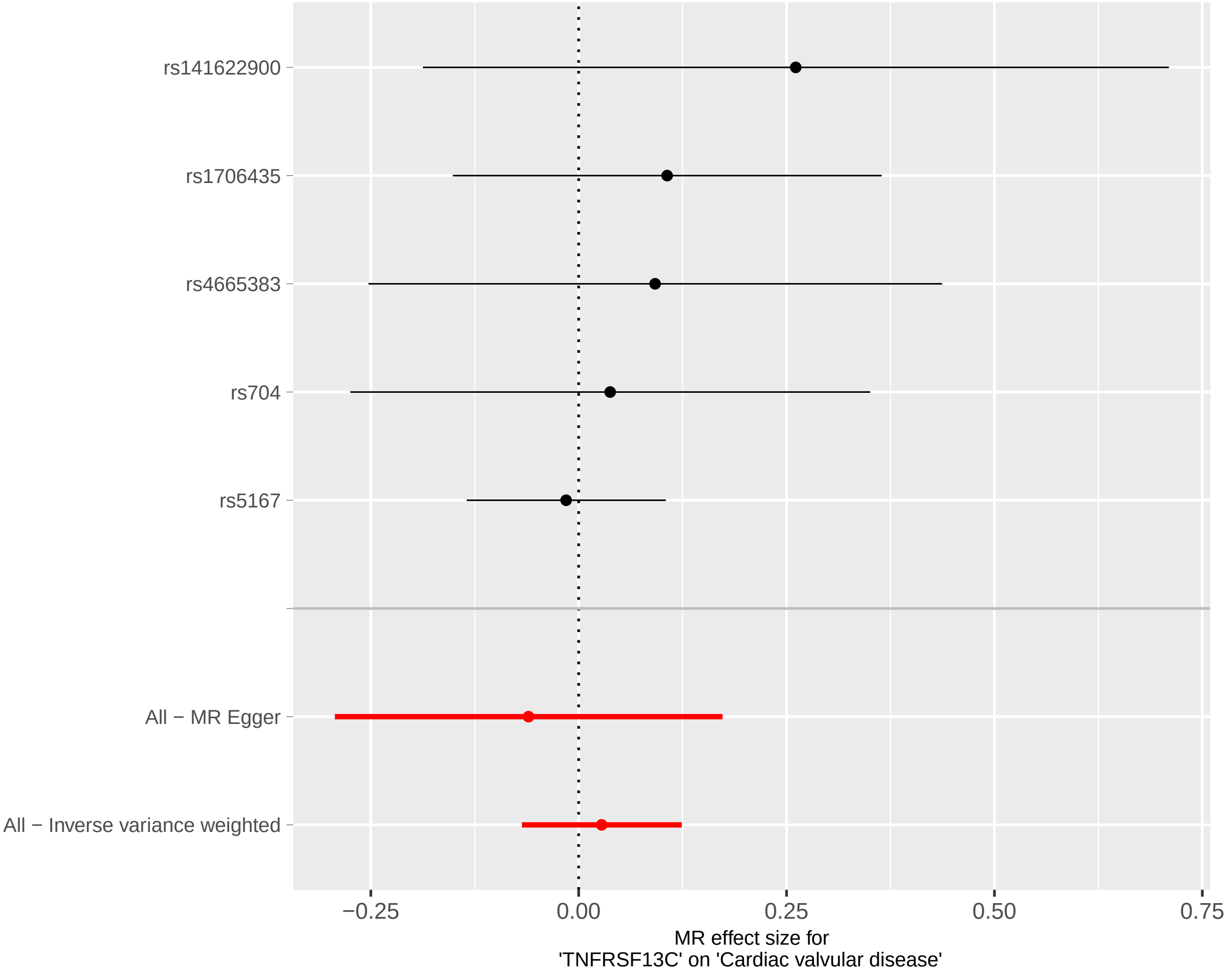

rs141622900
rs1706435
rs4665383
rs704
rs5167
All − MR Egger
All − Inverse variance weighted
−0.25
0.00
0.25
0.50
0.75
MR effect size for
'TNFRSF13C' on 'Cardiac valvular disease'

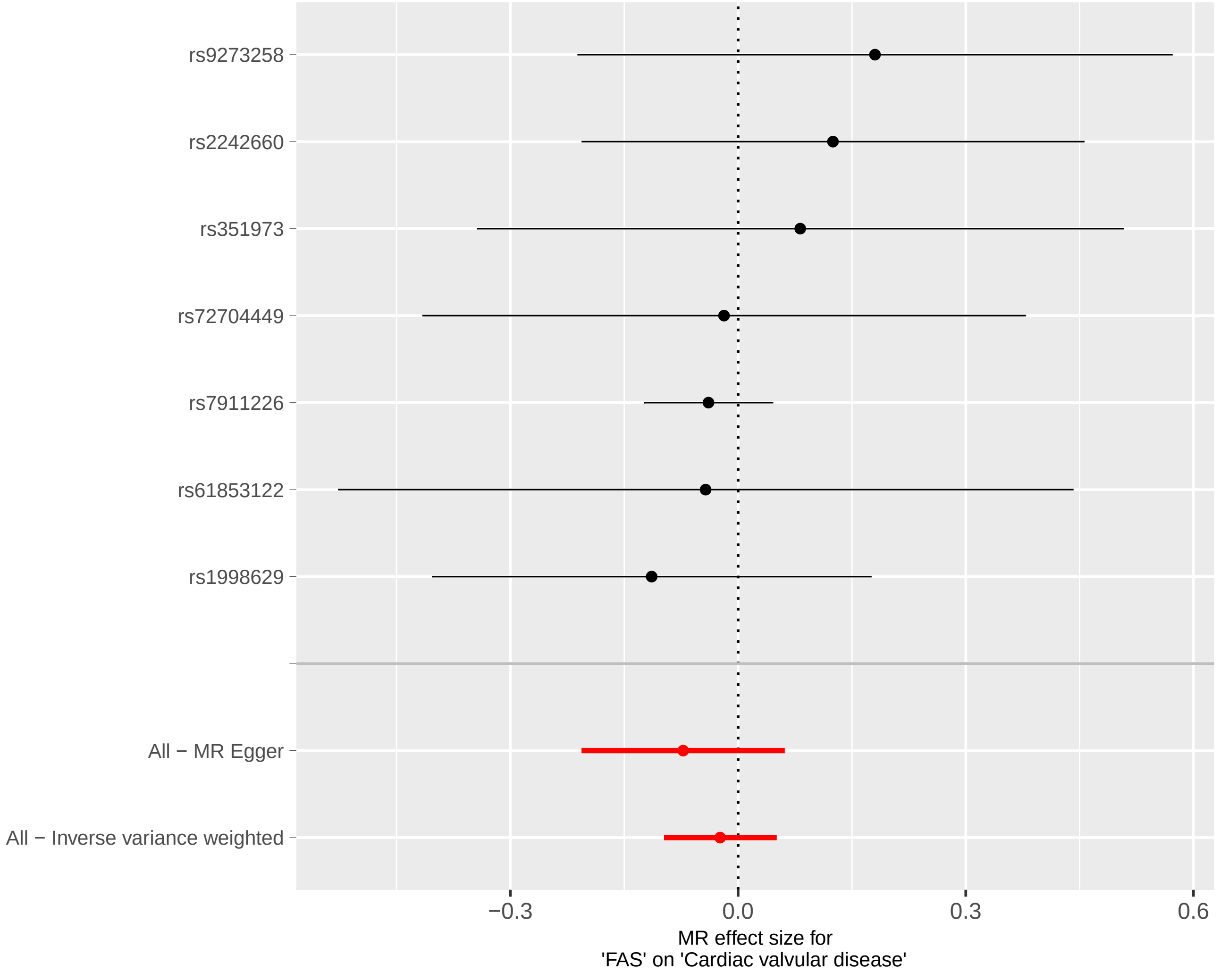
rs9273258
rs2242660
rs351973
rs72704449
rs7911226
rs61853122
rs1998629
All − MR Egger
All − Inverse variance weighted
−0.3
0.0
0.3
0.6
MR effect size for
'FAS' on 'Cardiac valvular disease'

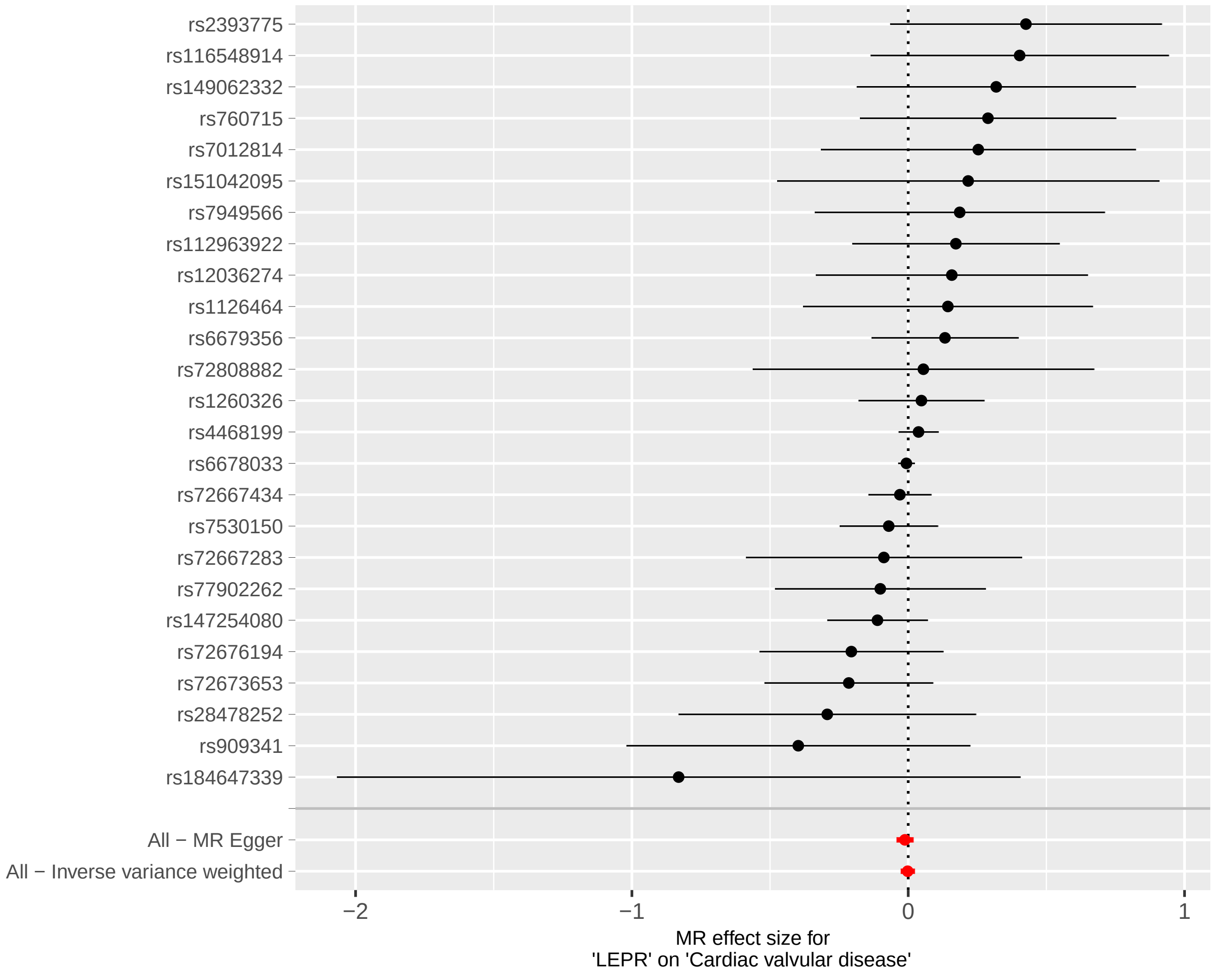

rs2393775
rs116548914
rs149062332
rs760715
rs7012814
rs151042095
rs7949566
rs112963922
rs12036274
rs1126464
rs6679356
rs72808882
rs1260326
rs4468199
rs6678033
rs72667434
rs7530150
rs72667283
rs77902262
rs147254080
rs72676194
rs72673653
rs28478252
rs909341
rs184647339
All − MR Egger
All − Inverse variance weighted
−2
−1
0
1
MR effect size for
'LEPR' on 'Cardiac valvular disease'

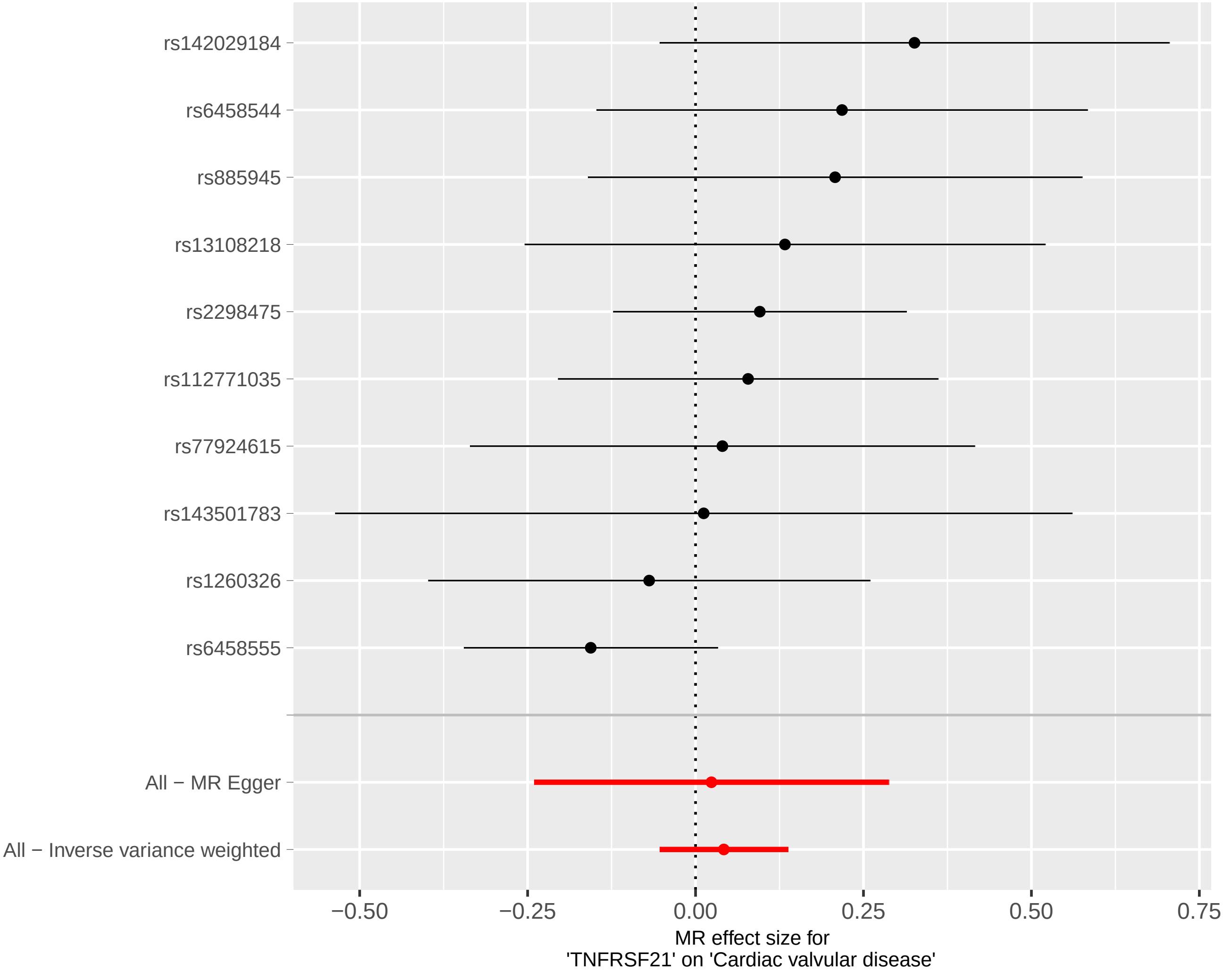
rs142029184
rs6458544
rs885945
rs13108218
rs2298475
rs112771035
rs77924615
rs143501783
rs1260326
rs6458555
All − MR Egger
All − Inverse variance weighted
−0.50
−0.25
0.00
0.25
0.50
0.75
MR effect size for
'TNFRSF21' on 'Cardiac valvular disease'

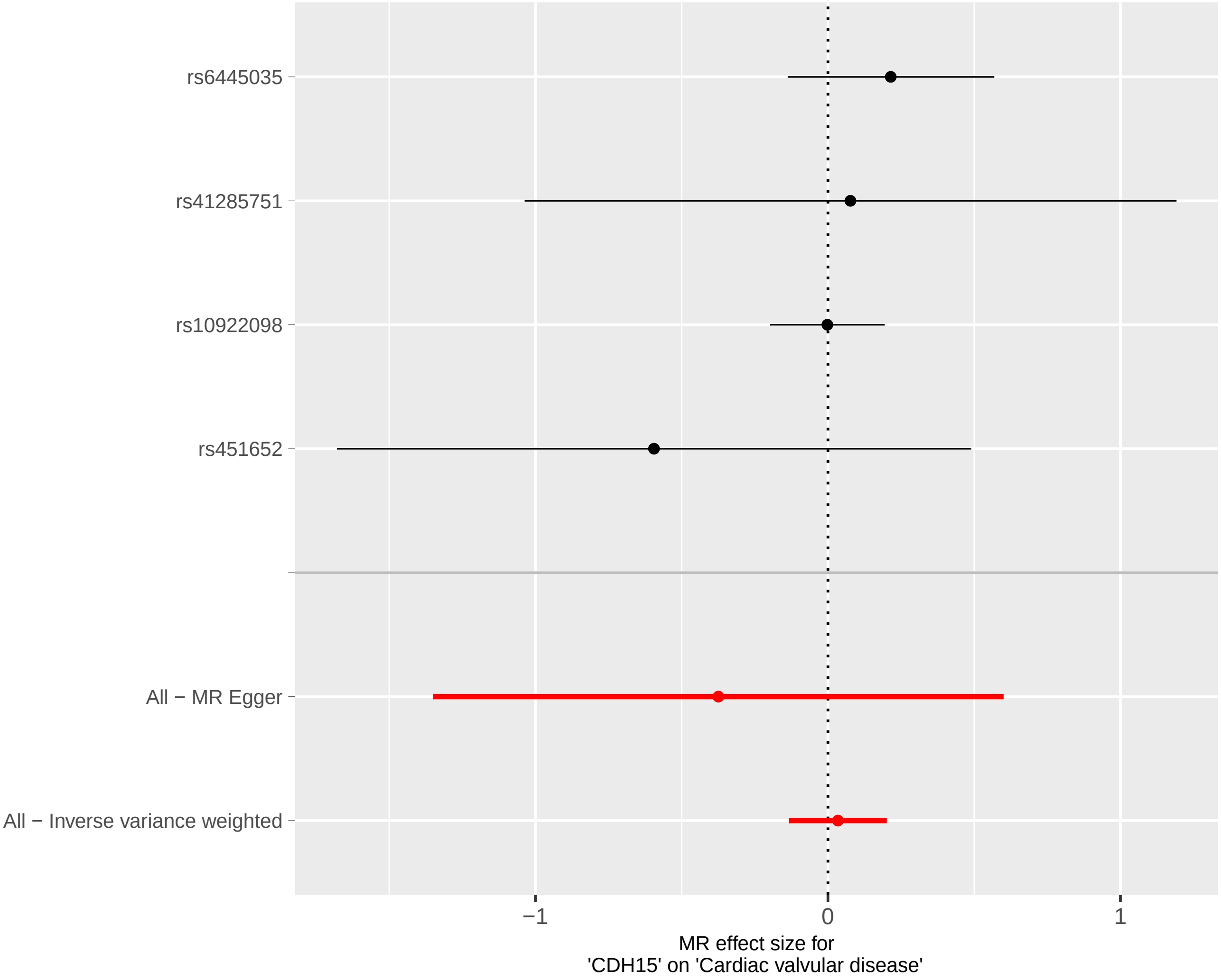
rs6445035
rs41285751
rs10922098
rs451652
All − MR Egger
All − Inverse variance weighted
−1
0
1
MR effect size for
'CDH15' on 'Cardiac valvular disease'

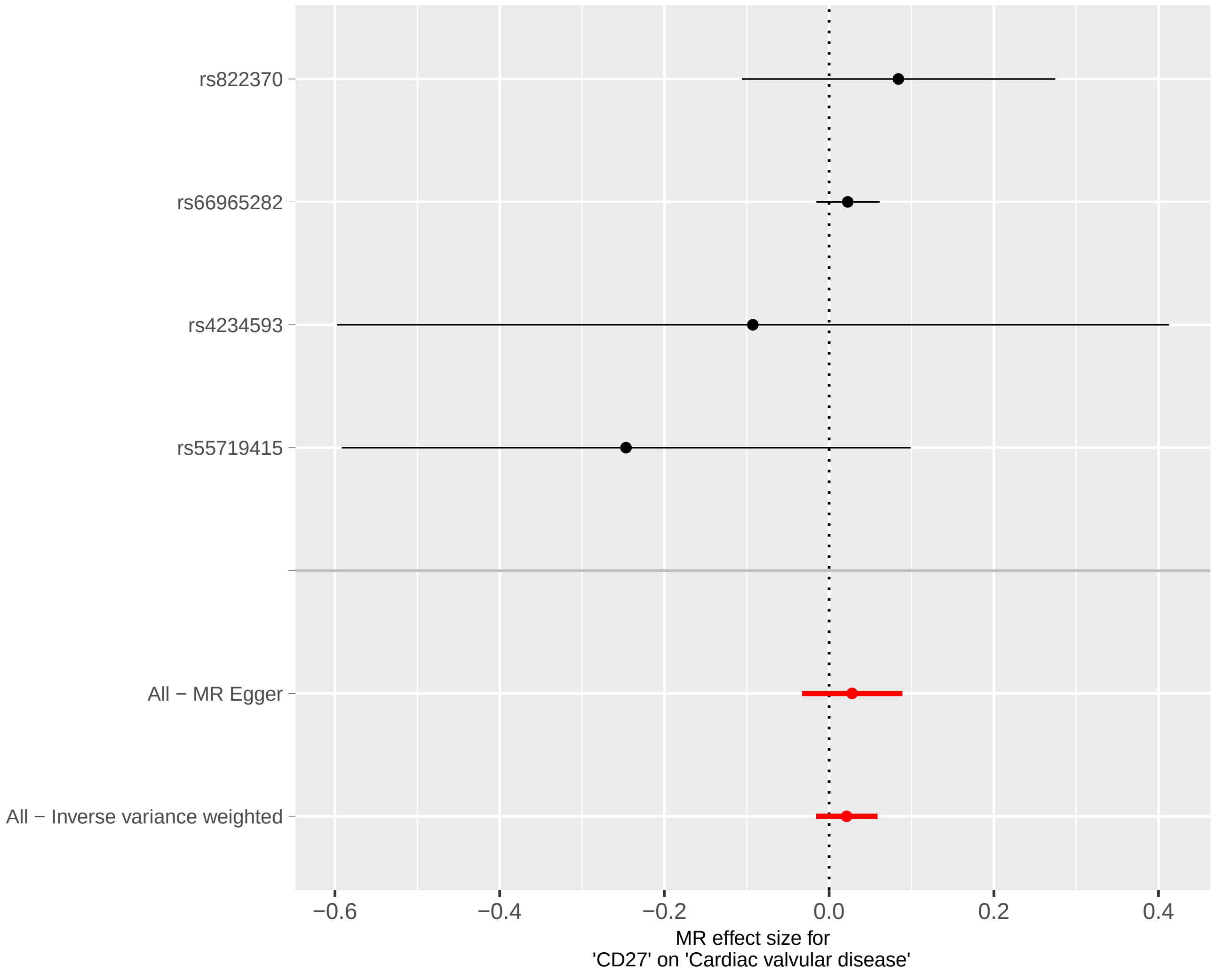
rs822370
rs66965282
rs4234593
rs55719415
All − MR Egger
All − Inverse variance weighted
−0.6
−0.4
−0.2
0.0
0.2
0.4
MR effect size for
'CD27' on 'Cardiac valvular disease'

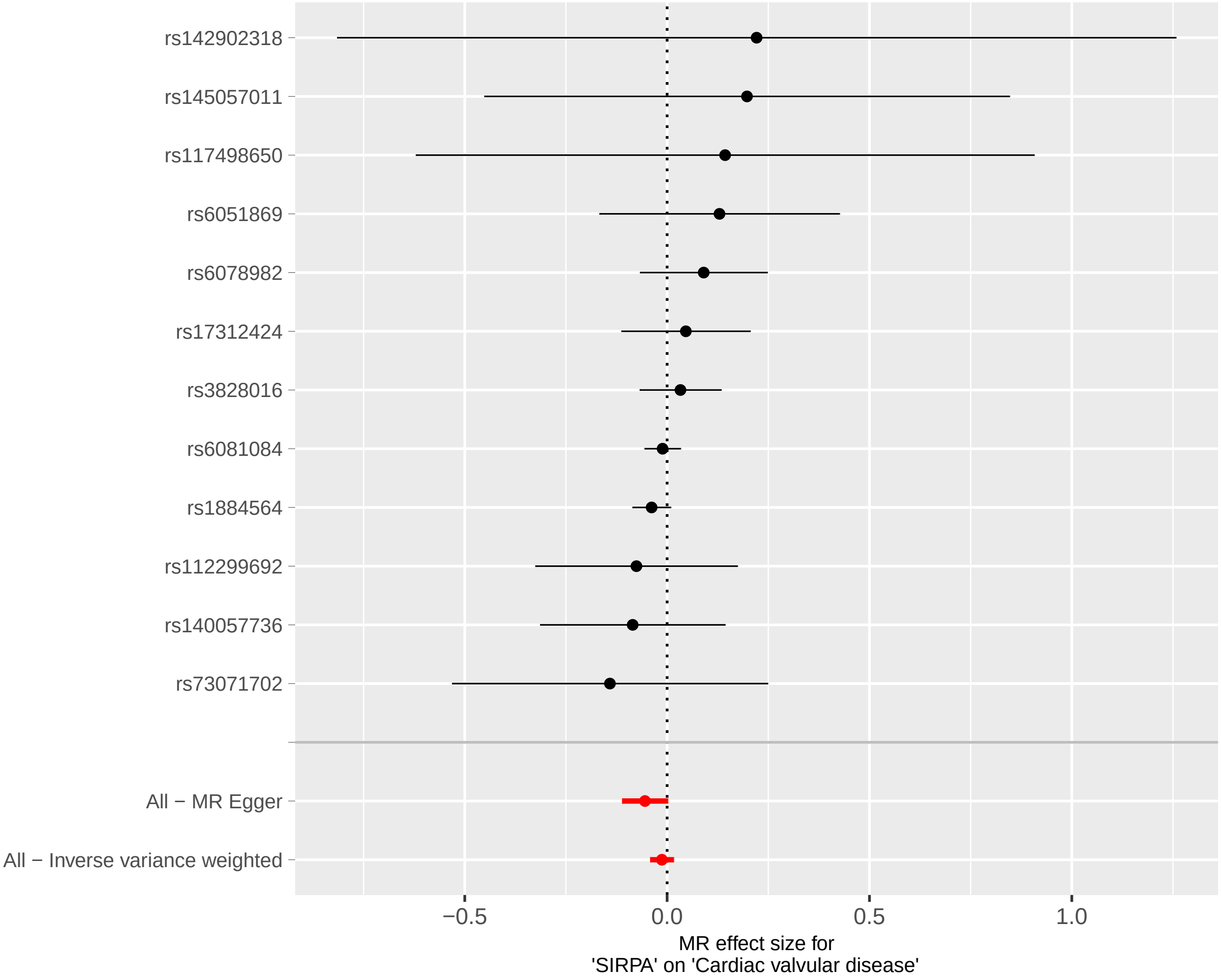
rs142902318
rs145057011
rs117498650
rs6051869
rs6078982
rs17312424
rs3828016
rs6081084
rs1884564
rs112299692
rs140057736
rs73071702
All − MR Egger
All − Inverse variance weighted
−0.5
0.0
0.5
1.0
MR effect size for
'SIRPA' on 'Cardiac valvular disease'

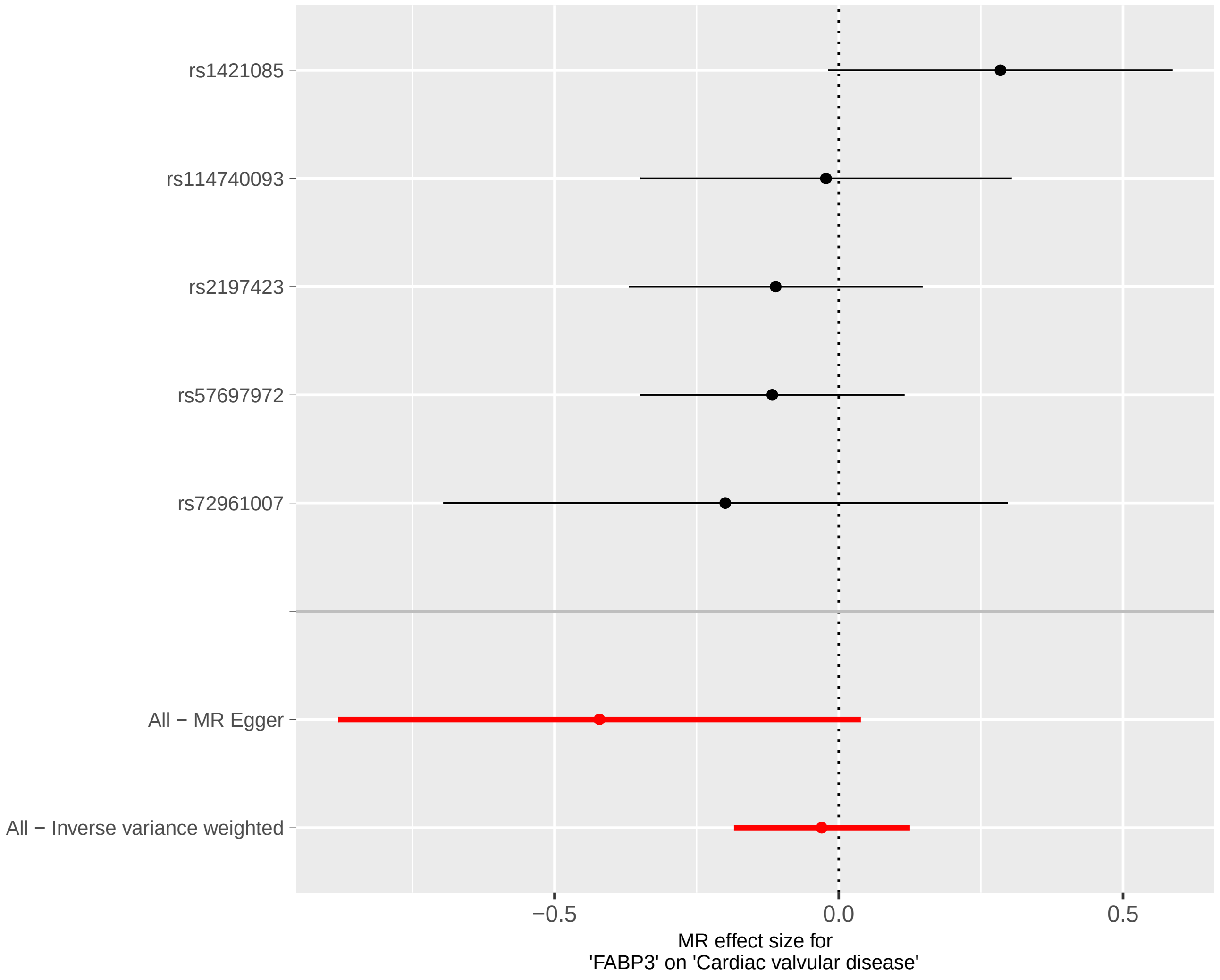

rs1421085
rs114740093
rs2197423
rs57697972
rs72961007
All − MR Egger
All − Inverse variance weighted
−0.5
0.0
0.5
MR effect size for
'FABP3' on 'Cardiac valvular disease'

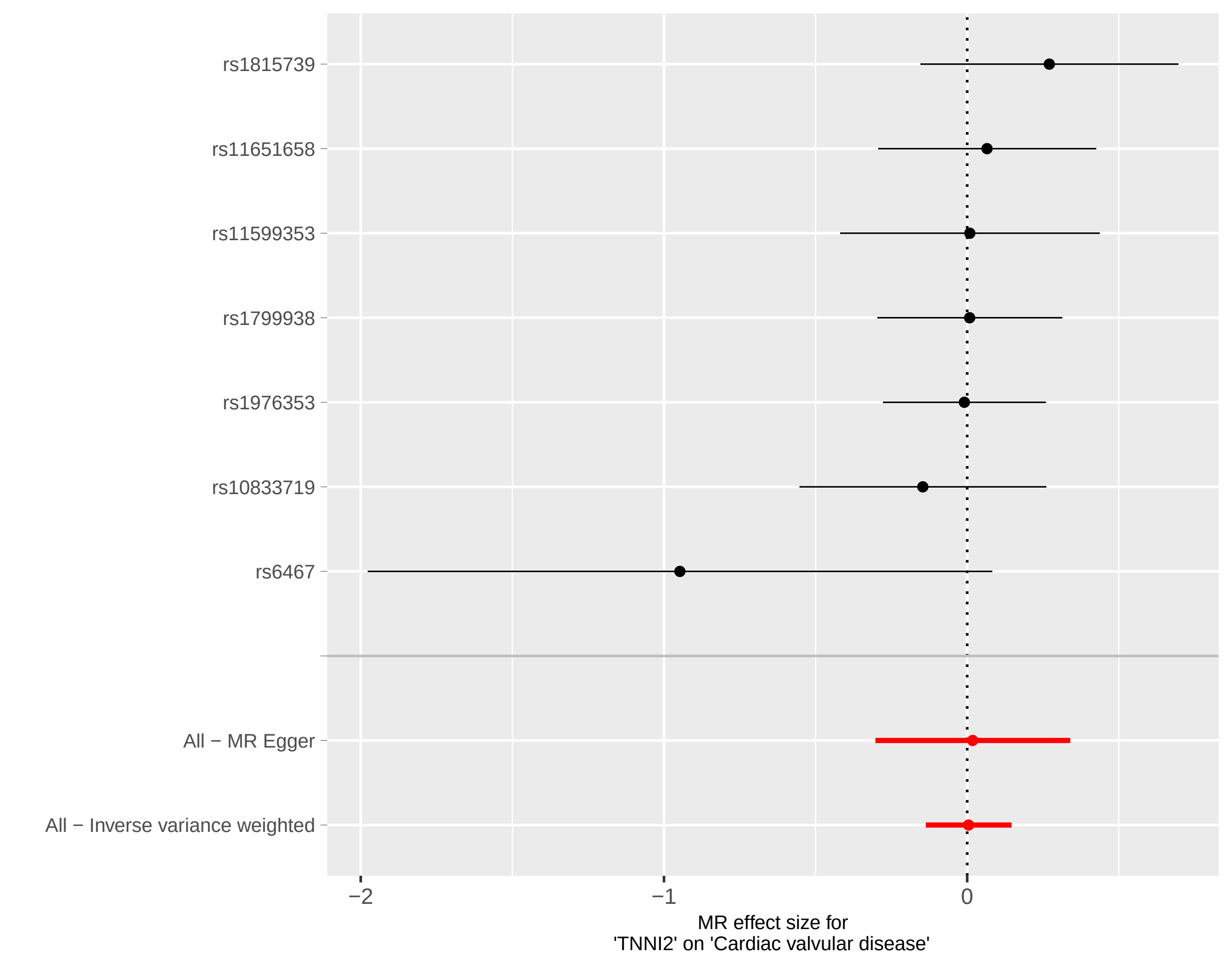

rs1815739
rs11651658
rs11599353
rs1799938
rs1976353
rs10833719
rs6467
All − MR Egger
All − Inverse variance weighted
−2
−1
0
MR effect size for
'TNNI2' on 'Cardiac valvular disease'

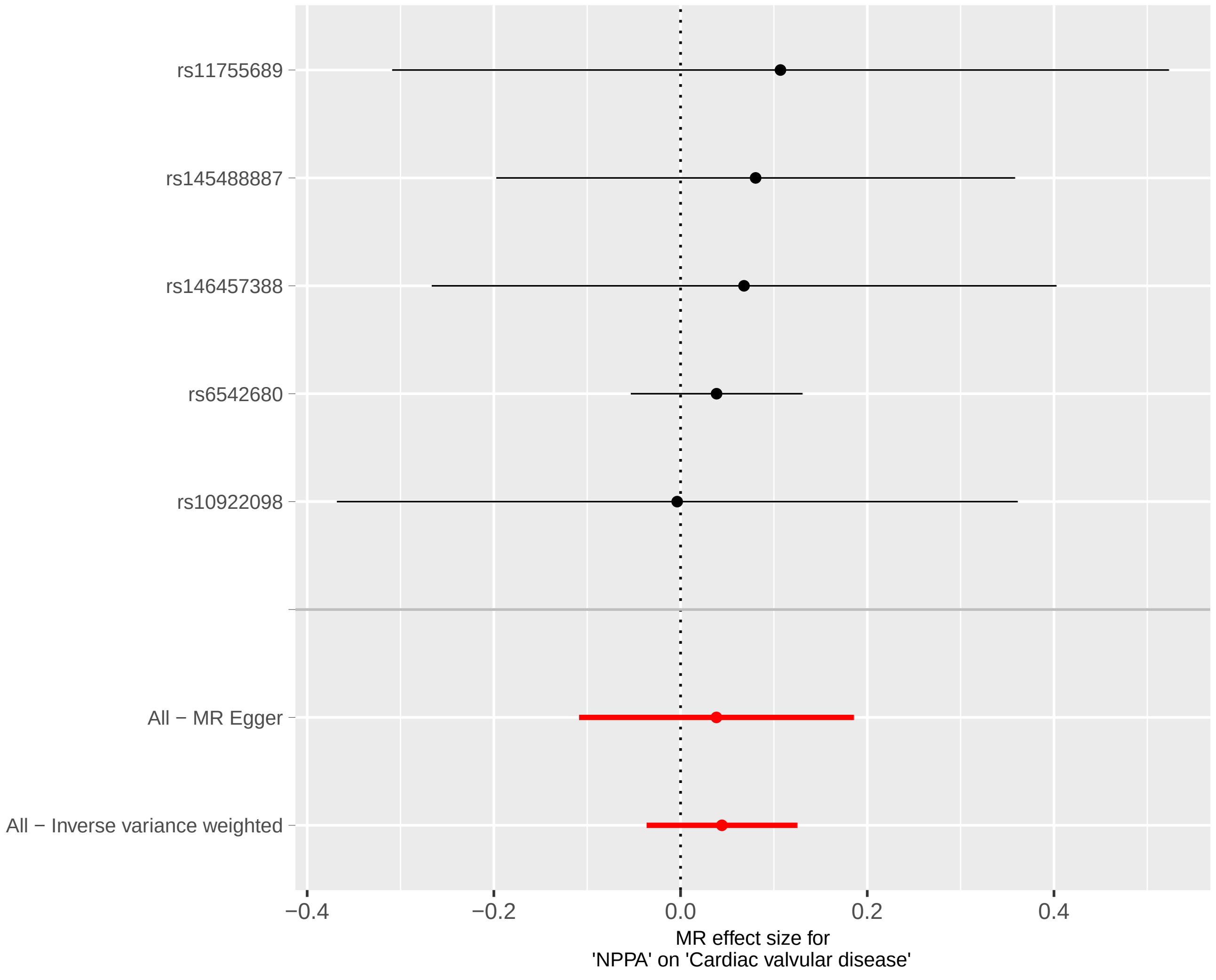
rs11755689
rs145488887
rs146457388
rs6542680
rs10922098
All − MR Egger
All − Inverse variance weighted
−0.4
−0.2
0.0
0.2
0.4
MR effect size for
'NPPA' on 'Cardiac valvular disease'

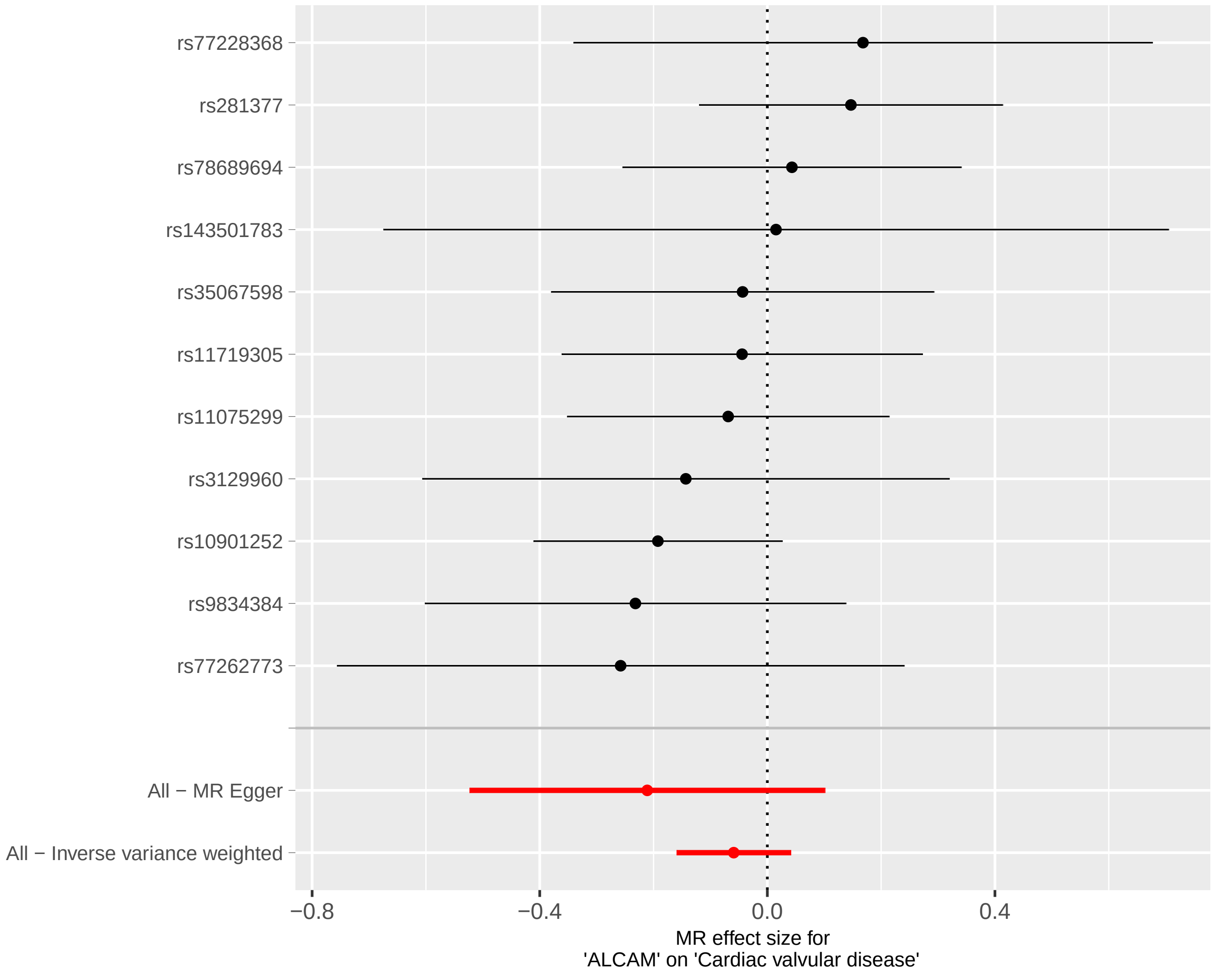
rs77228368
rs281377
rs78689694
rs143501783
rs35067598
rs11719305
rs11075299
rs3129960
rs10901252
rs9834384
rs77262773
All − MR Egger
All − Inverse variance weighted
−0.8
−0.4
0.0
0.4
MR effect size for
'ALCAM' on 'Cardiac valvular disease'

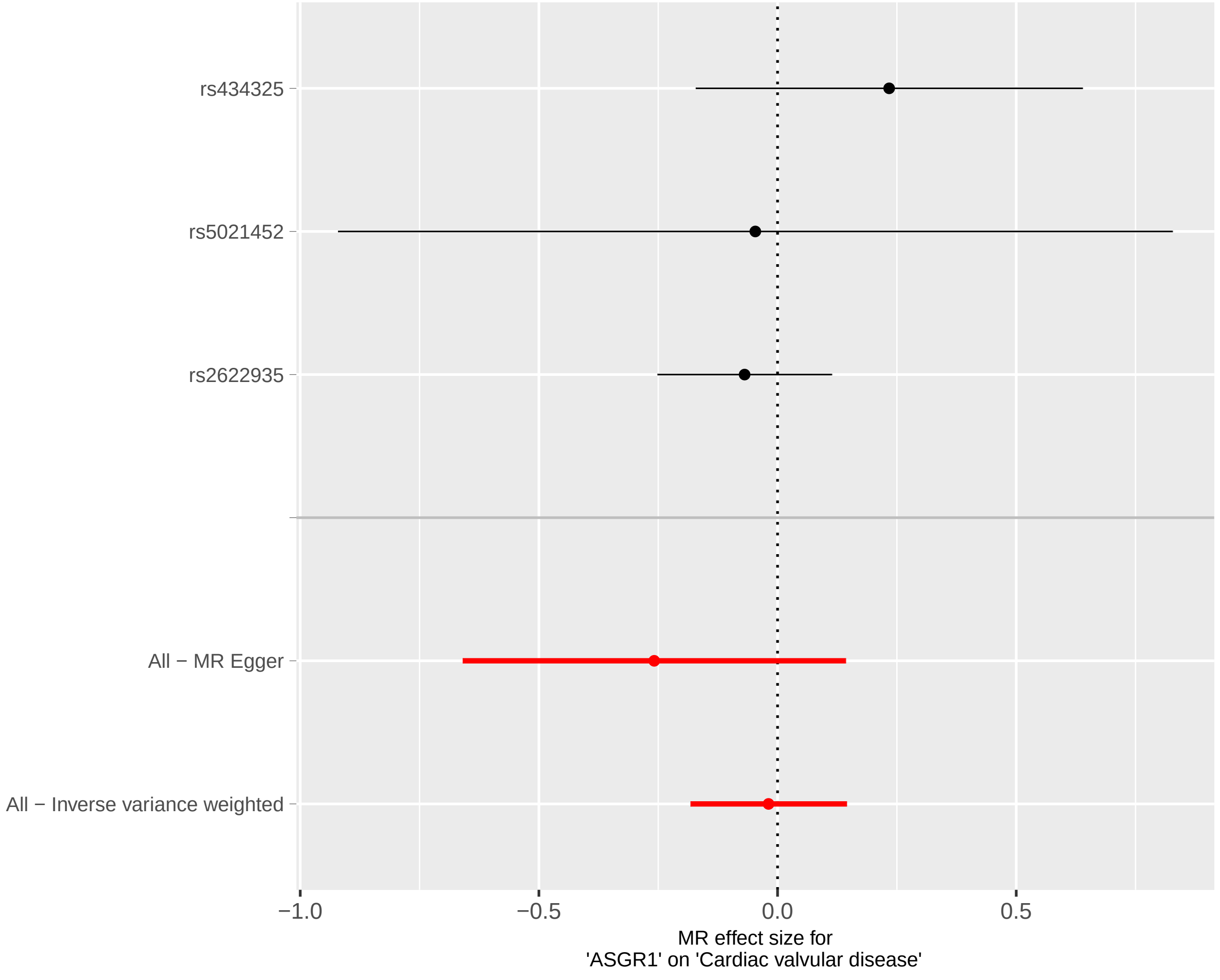
rs434325
rs5021452
rs2622935
All − MR Egger
All − Inverse variance weighted
−1.0
−0.5
0.0
0.5
MR effect size for
'ASGR1' on 'Cardiac valvular disease'

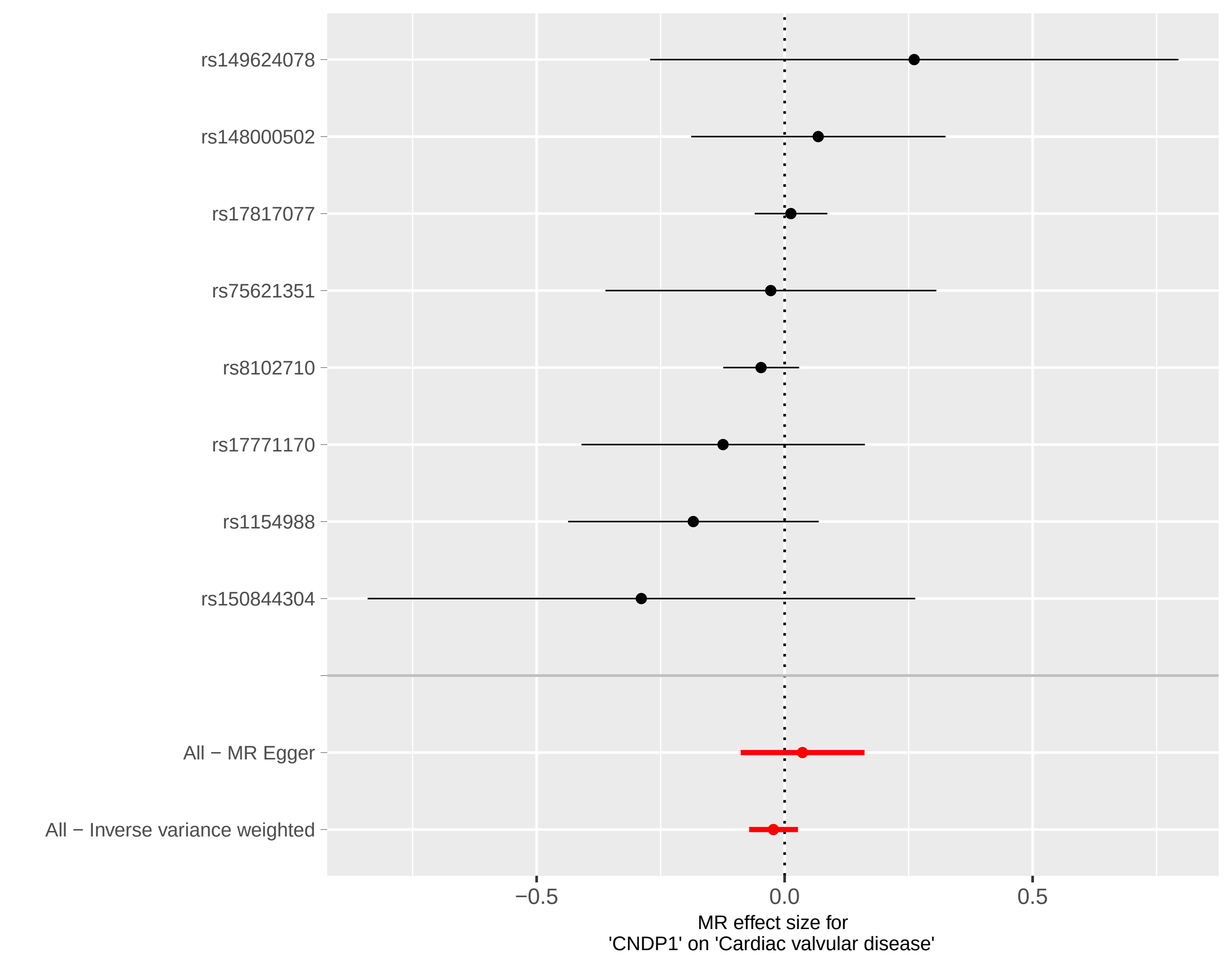

rs149624078
rs148000502
rs17817077
rs75621351
rs8102710
rs17771170
rs1154988
rs150844304
All − MR Egger
All − Inverse variance weighted
−0.5
0.0
0.5
MR effect size for
'CNDP1' on 'Cardiac valvular disease'

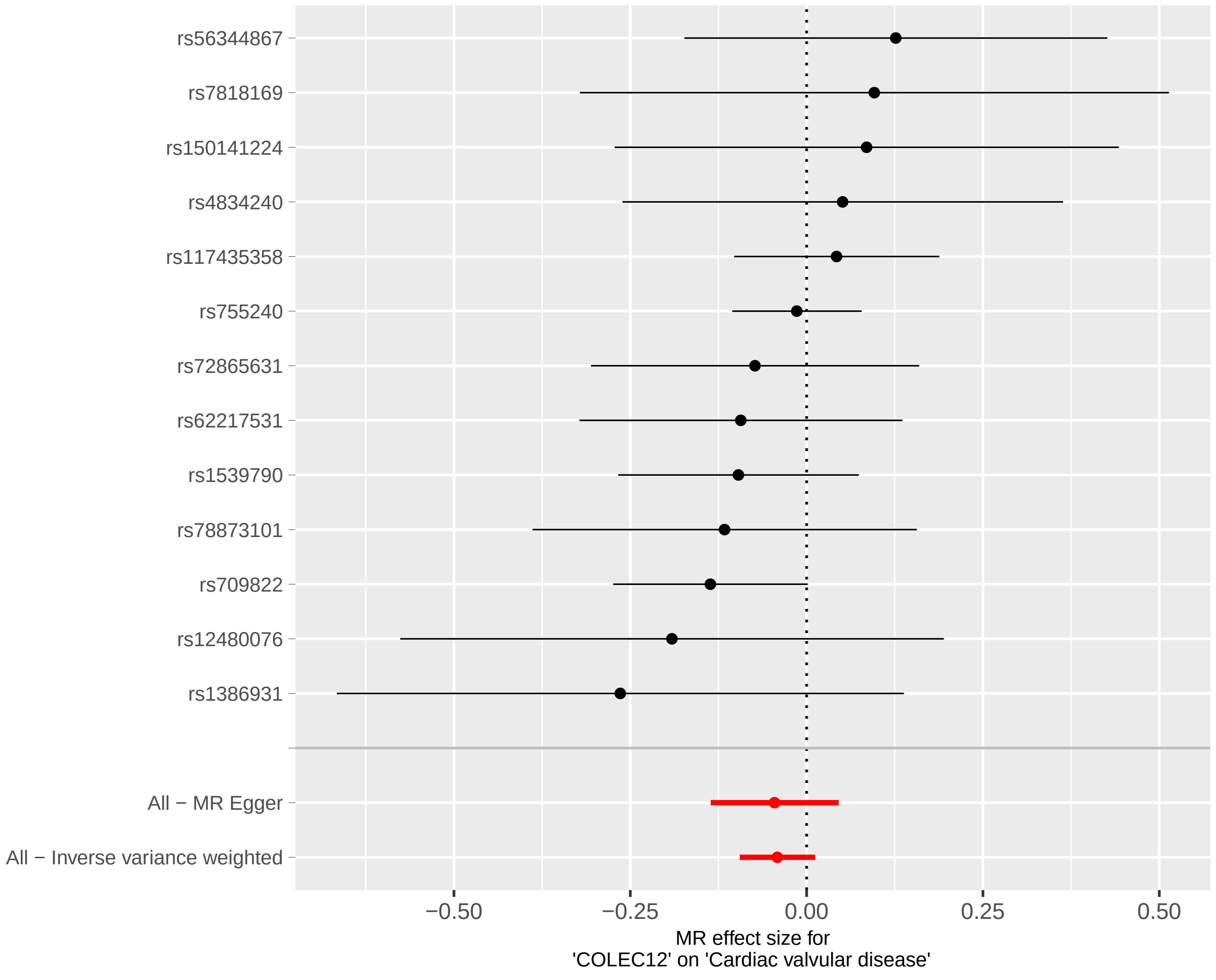
rs56344867
rs7818169
rs150141224
rs4834240
rs117435358
rs755240
rs72865631
rs62217531
rs1539790
rs78873101
rs709822
rs12480076
rs1386931
All − MR Egger
All − Inverse variance weighted
−0.50
−0.25
0.00
0.25
0.50
MR effect size for
'COLEC12' on 'Cardiac valvular disease'

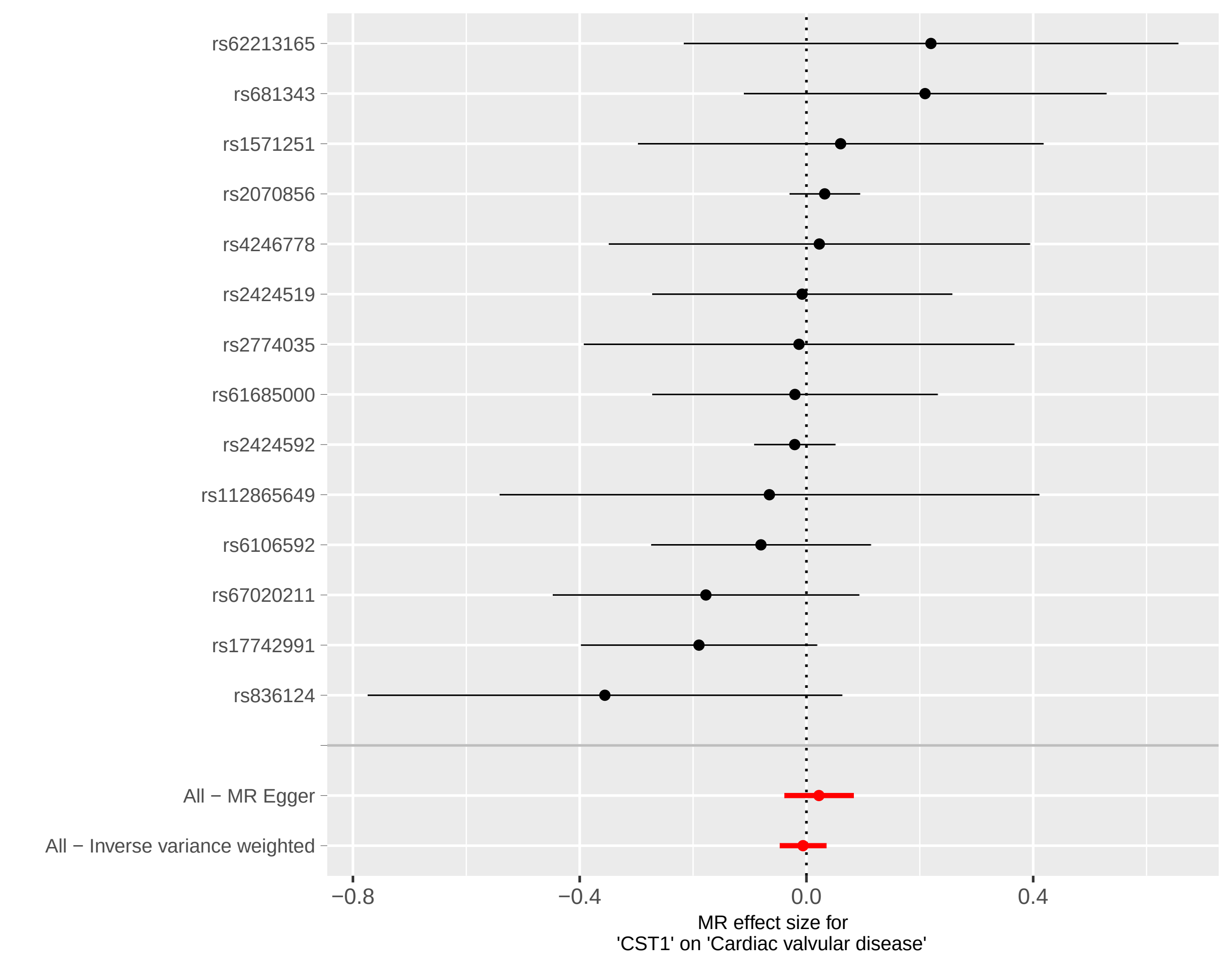
rs62213165
rs681343
rs1571251
rs2070856
rs4246778
rs2424519
rs2774035
rs61685000
rs2424592
rs112865649
rs6106592
rs67020211
rs17742991
rs836124
All − MR Egger
All − Inverse variance weighted
−0.8
−0.4
0.0
0.4
MR effect size for
'CST1' on 'Cardiac valvular disease'

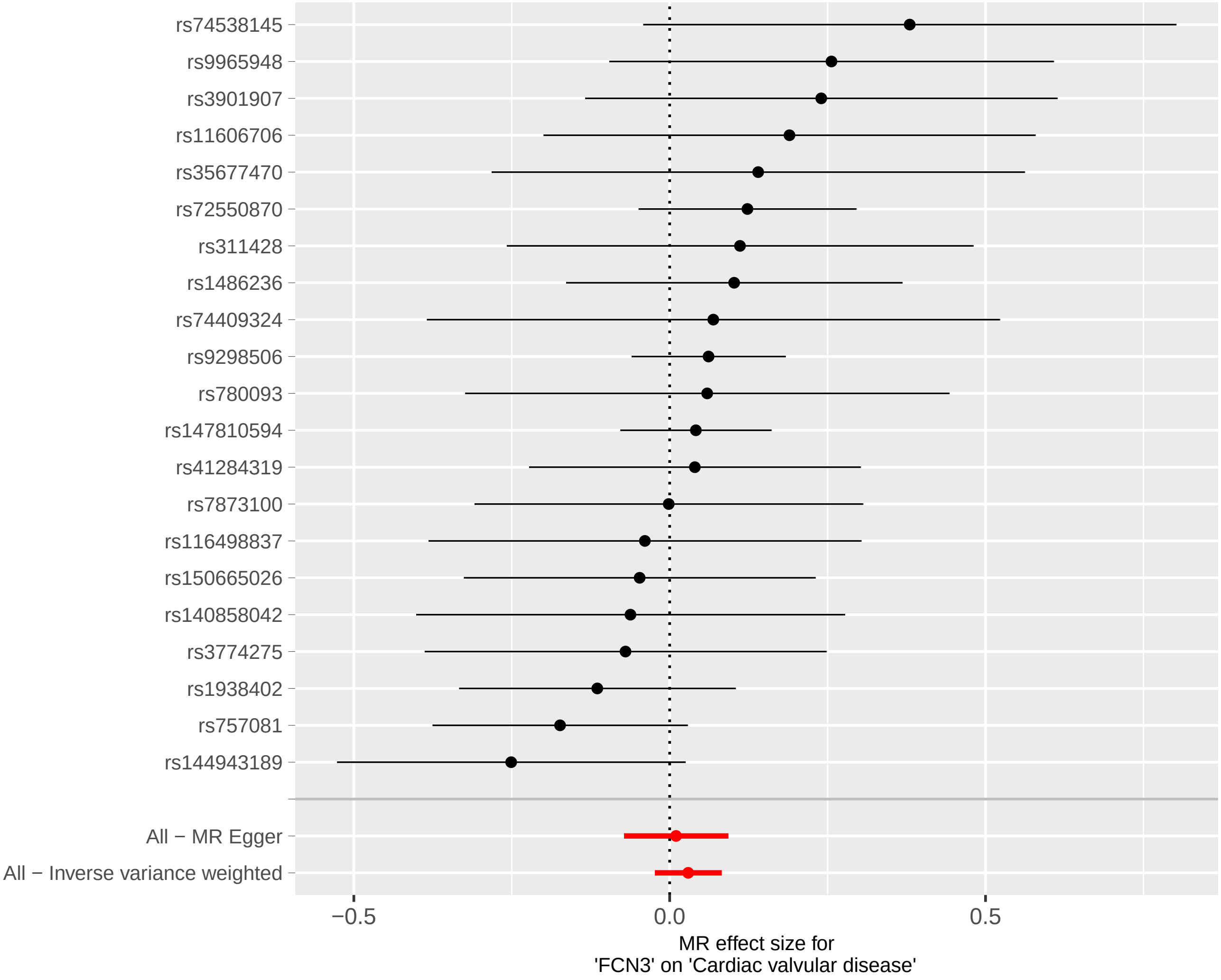
rs74538145
rs9965948
rs3901907
rs11606706
rs35677470
rs72550870
rs311428
rs1486236
rs74409324
rs9298506
rs780093
rs147810594
rs41284319
rs7873100
rs116498837
rs150665026
rs140858042
rs3774275
rs1938402
rs757081
rs144943189
All − MR Egger
All − Inverse variance weighted
−0.5
0.0
0.5
MR effect size for
'FCN3' on 'Cardiac valvular disease'

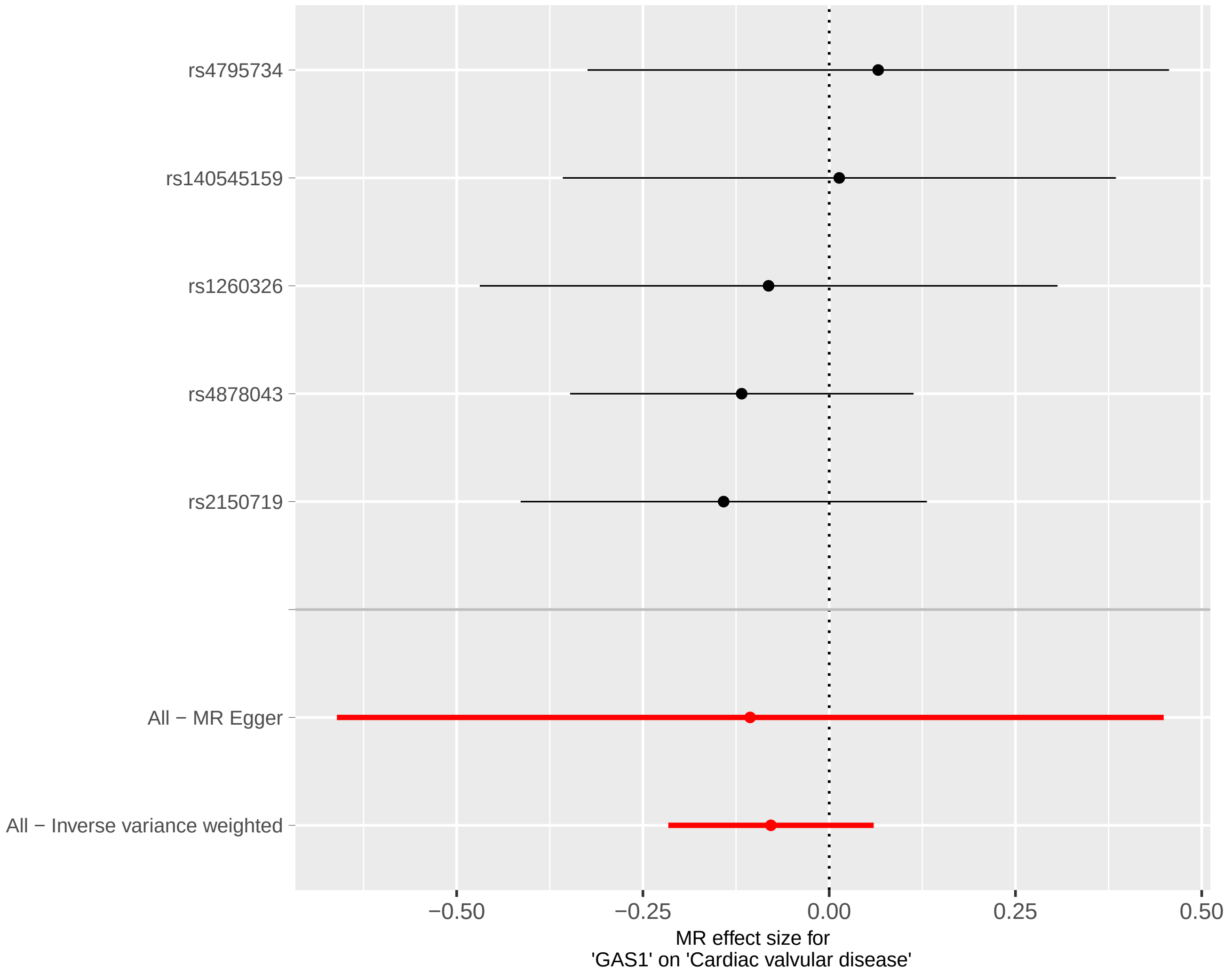

rs4795734
rs140545159
rs1260326
rs4878043
rs2150719
All − MR Egger
All − Inverse variance weighted
−0.50
−0.25
0.00
0.25
0.50
MR effect size for
'GAS1' on 'Cardiac valvular disease'

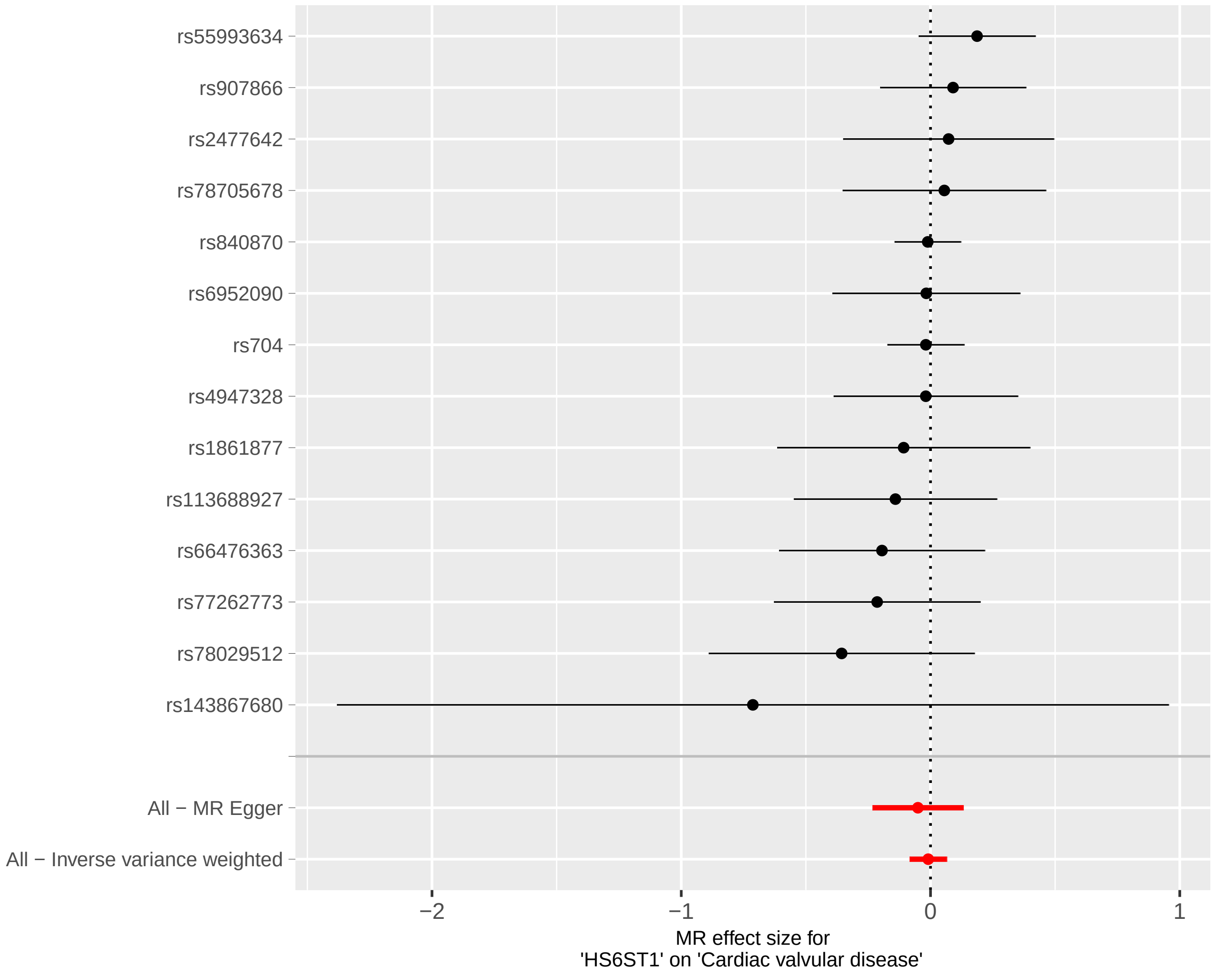
rs55993634
rs907866
rs2477642
rs78705678
rs840870
rs6952090
rs704
rs4947328
rs1861877
rs113688927
rs66476363
rs77262773
rs78029512
rs143867680
All − MR Egger
All − Inverse variance weighted
−2
−1
0
1
MR effect size for
'HS6ST1' on 'Cardiac valvular disease'

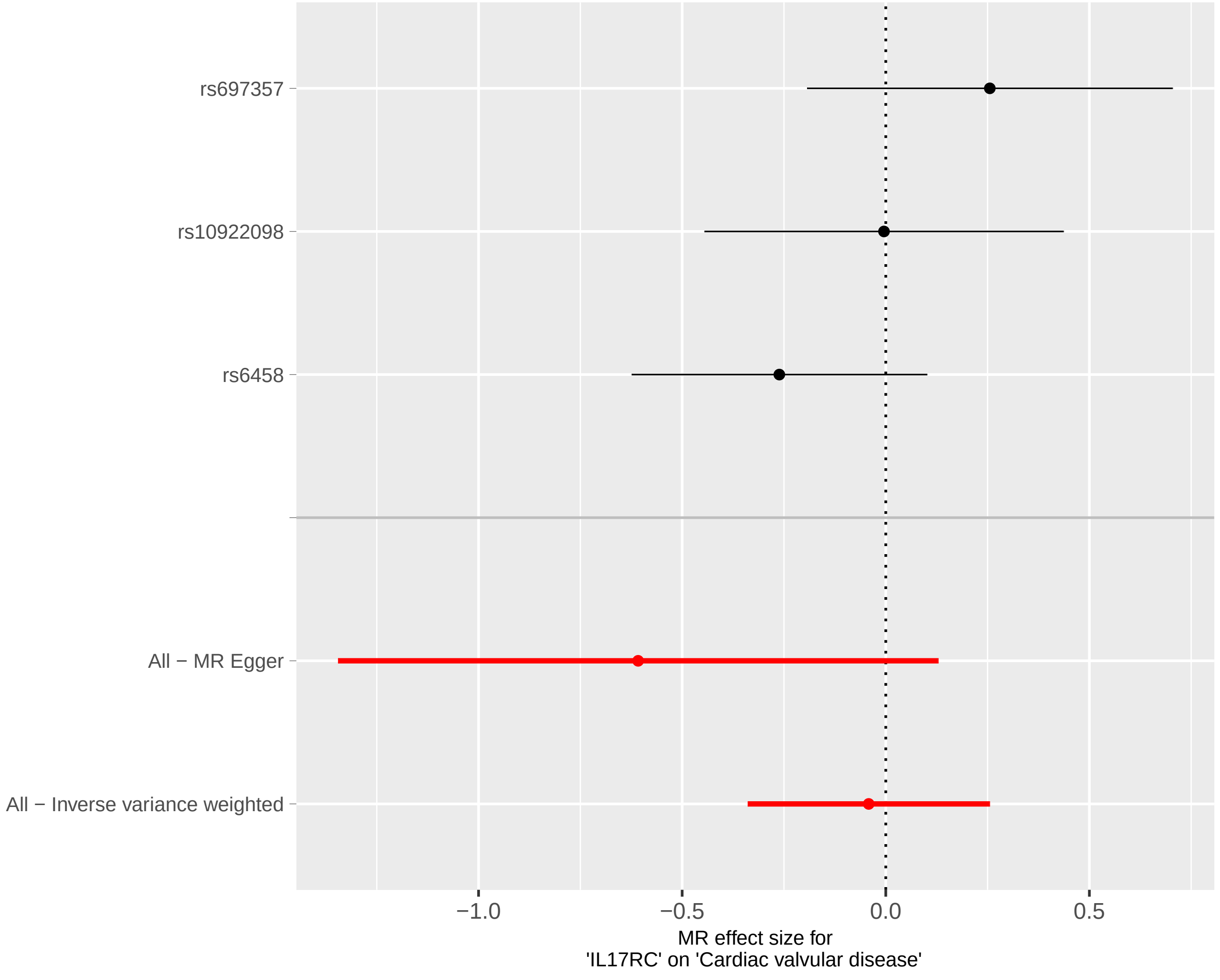
rs697357
rs10922098
rs6458
All − MR Egger
All − Inverse variance weighted
−1.0
−0.5
0.0
0.5
MR effect size for
'IL17RC' on 'Cardiac valvular disease'

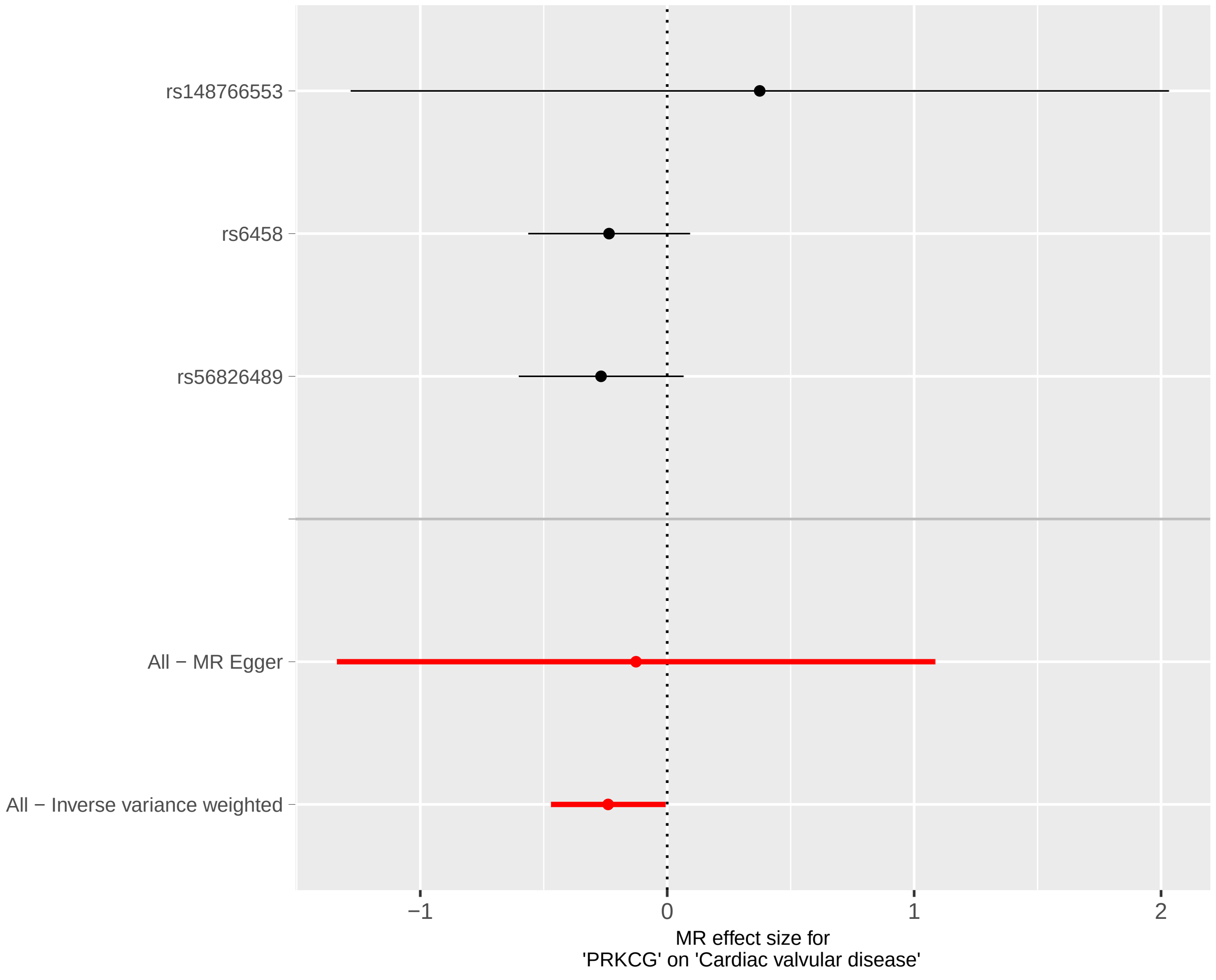
rs148766553
rs6458
rs56826489
All − MR Egger
All − Inverse variance weighted
−1
0
1
2
MR effect size for
'PRKCG' on 'Cardiac valvular disease'

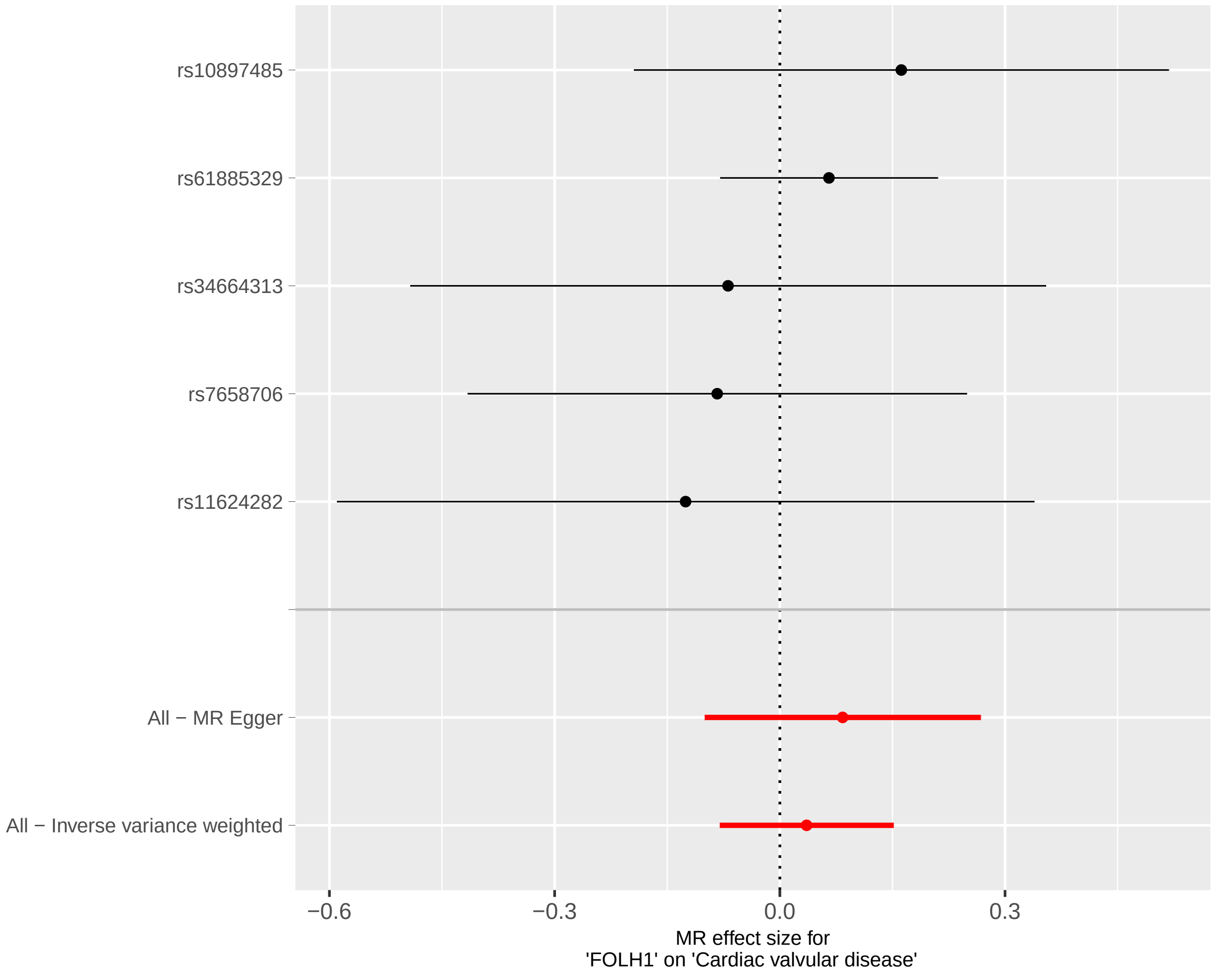

rs10897485
rs61885329
rs34664313
rs7658706
rs11624282
All − MR Egger
All − Inverse variance weighted
−0.6
−0.3
0.0
0.3
MR effect size for
'FOLH1' on 'Cardiac valvular disease'

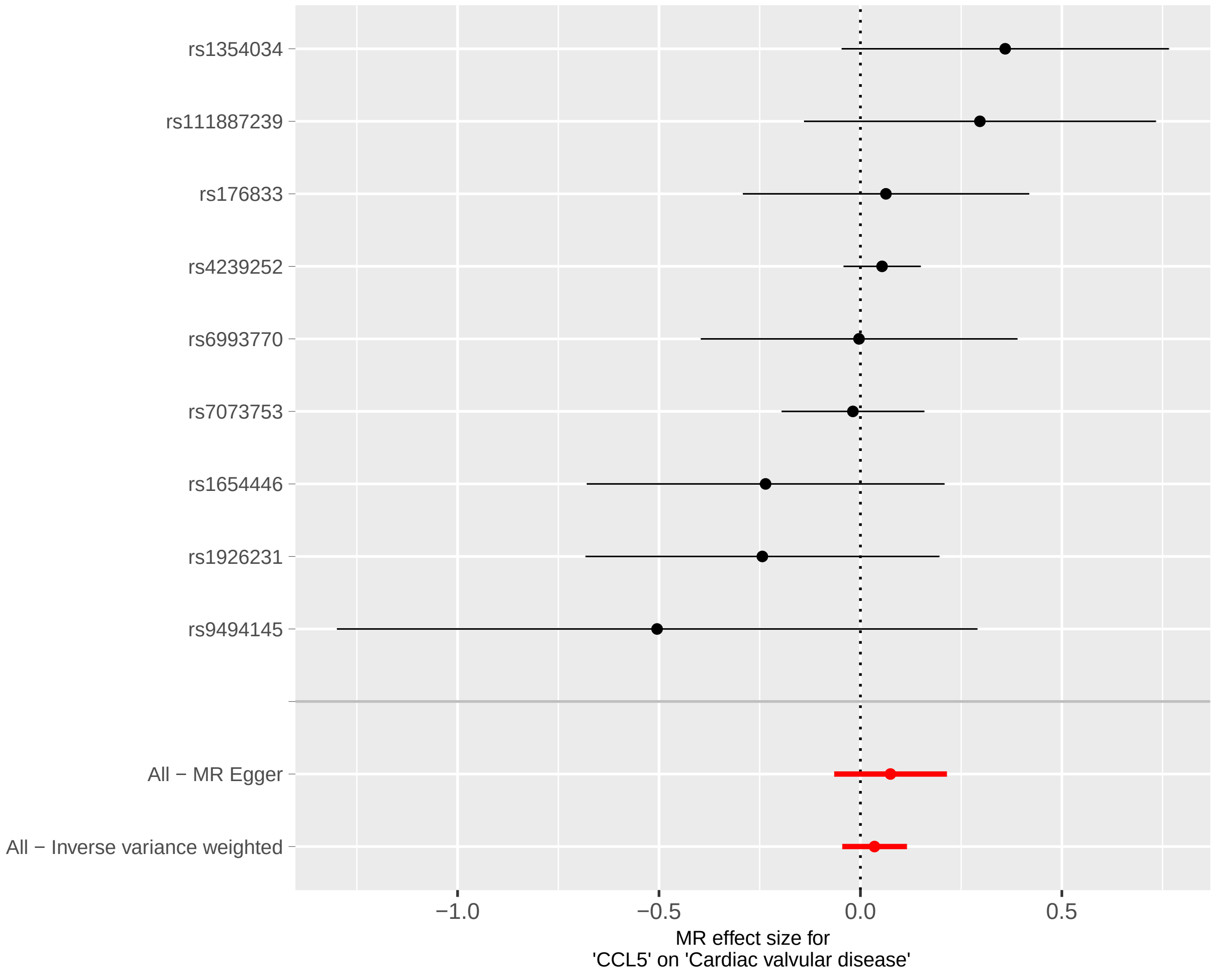
rs1354034
rs111887239
rs176833
rs4239252
rs6993770
rs7073753
rs1654446
rs1926231
rs9494145
All − MR Egger
All − Inverse variance weighted
−1.0
−0.5
0.0
0.5
MR effect size for
'CCL5' on 'Cardiac valvular disease'

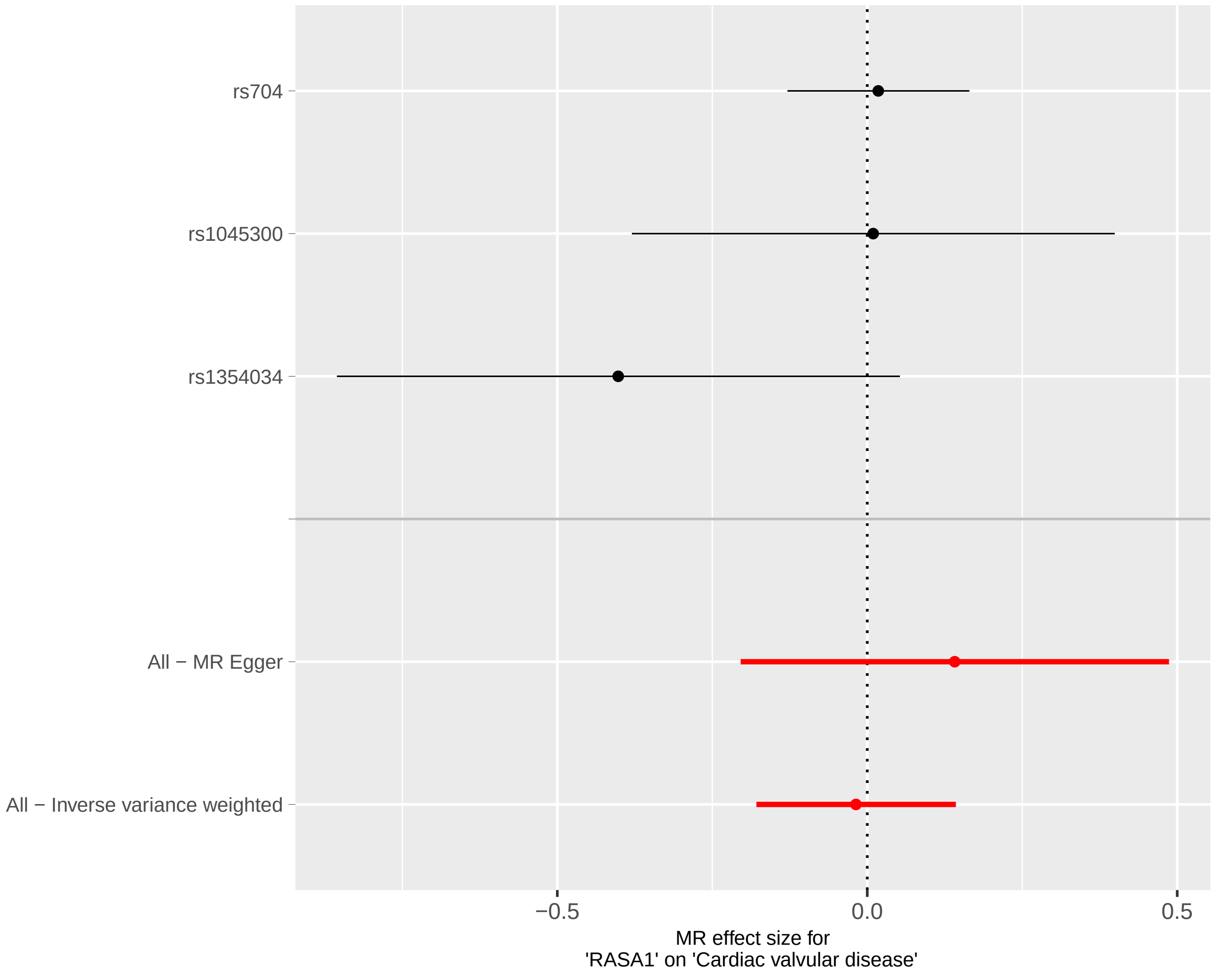
rs704
rs1045300
rs1354034
All − MR Egger
All − Inverse variance weighted
−0.5
0.0
0.5
MR effect size for
'RASA1' on 'Cardiac valvular disease'

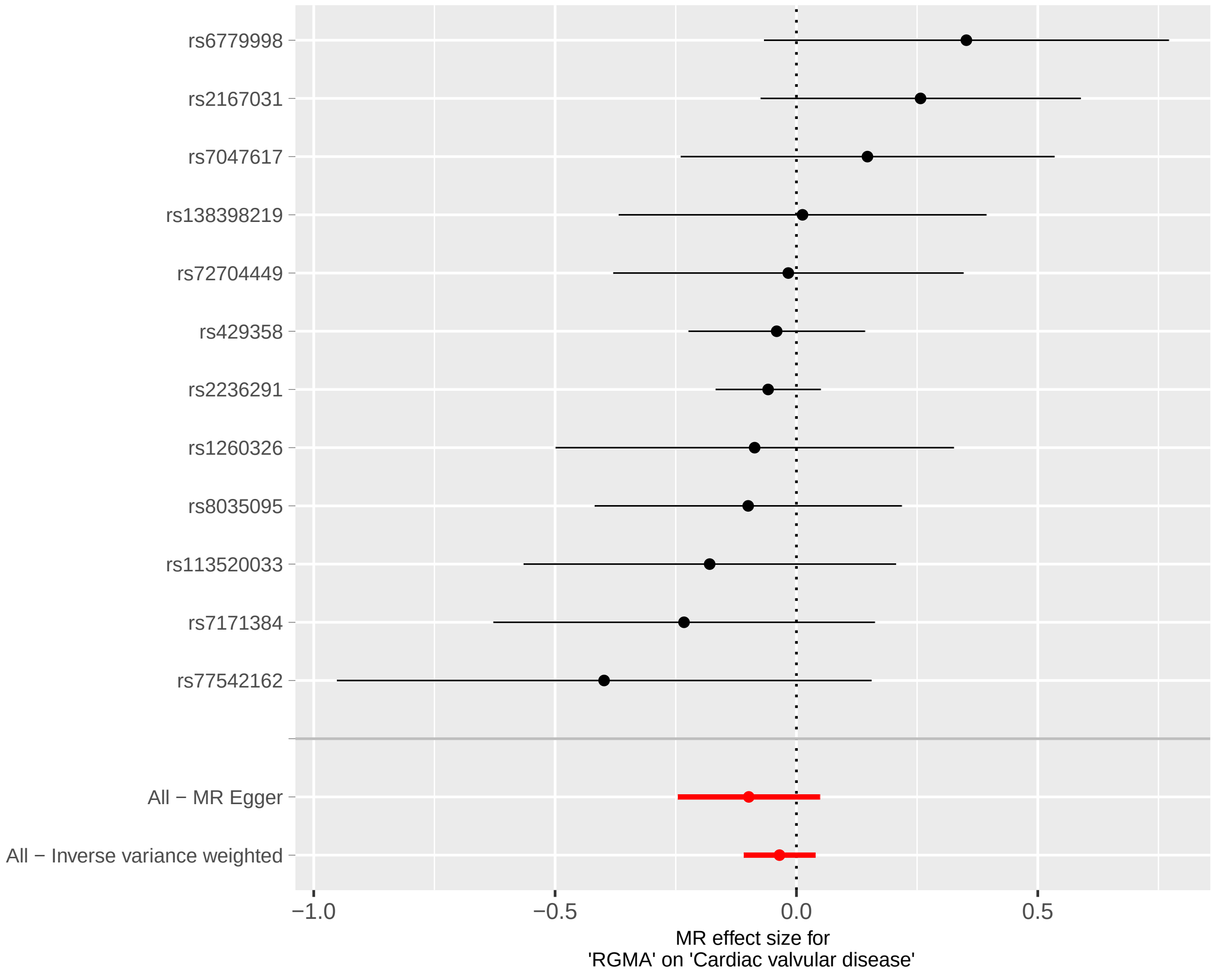

rs6779998
rs2167031
rs7047617
rs138398219
rs72704449
rs429358
rs2236291
rs1260326
rs8035095
rs113520033
rs7171384
rs77542162
All − MR Egger
All − Inverse variance weighted
−1.0
−0.5
0.0
0.5
MR effect size for
'RGMA' on 'Cardiac valvular disease'

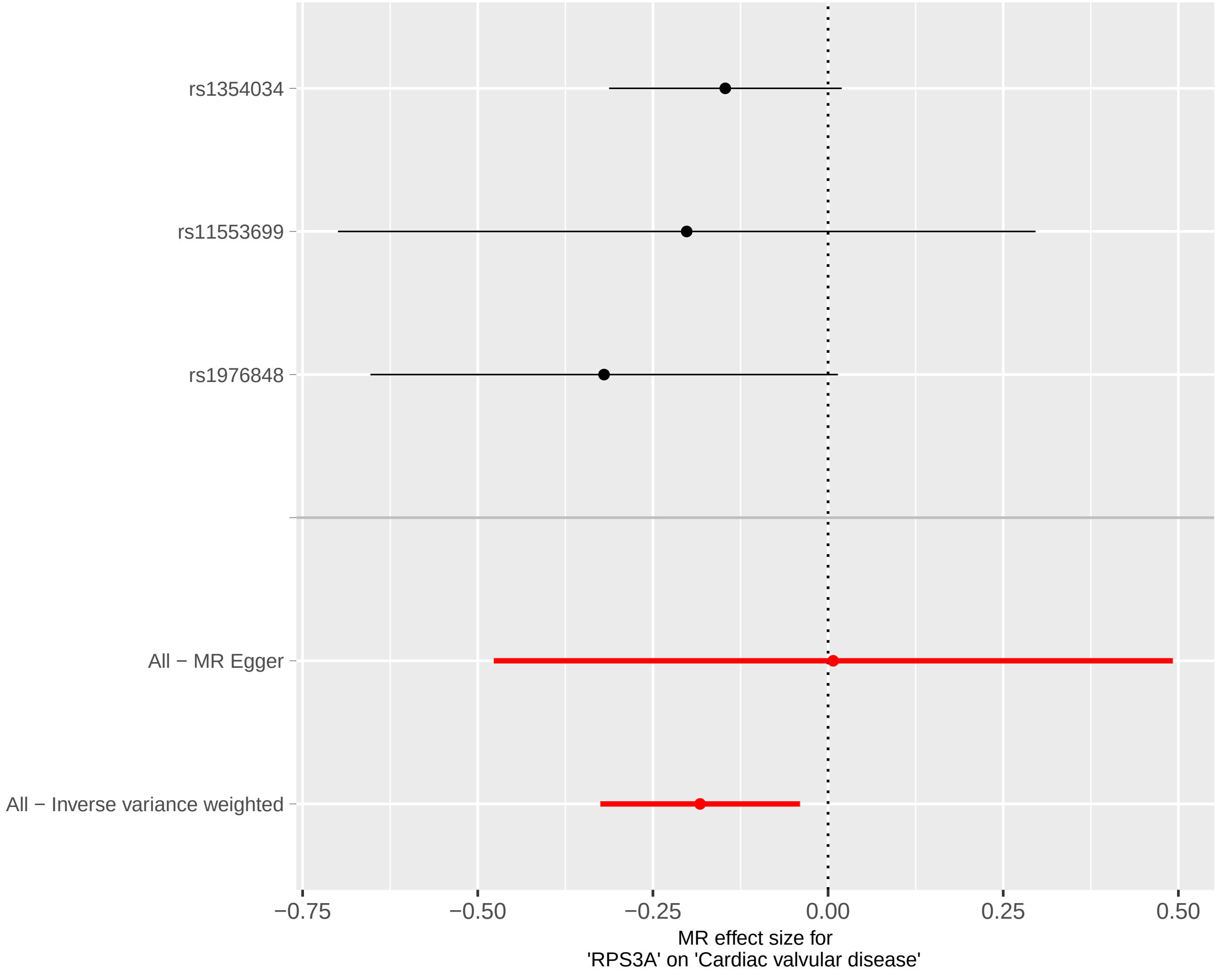
rs1354034
rs11553699
rs1976848
All − MR Egger
All − Inverse variance weighted
−0.75
−0.50
−0.25
0.00
0.25
0.50
MR effect size for
'RPS3A' on 'Cardiac valvular disease'

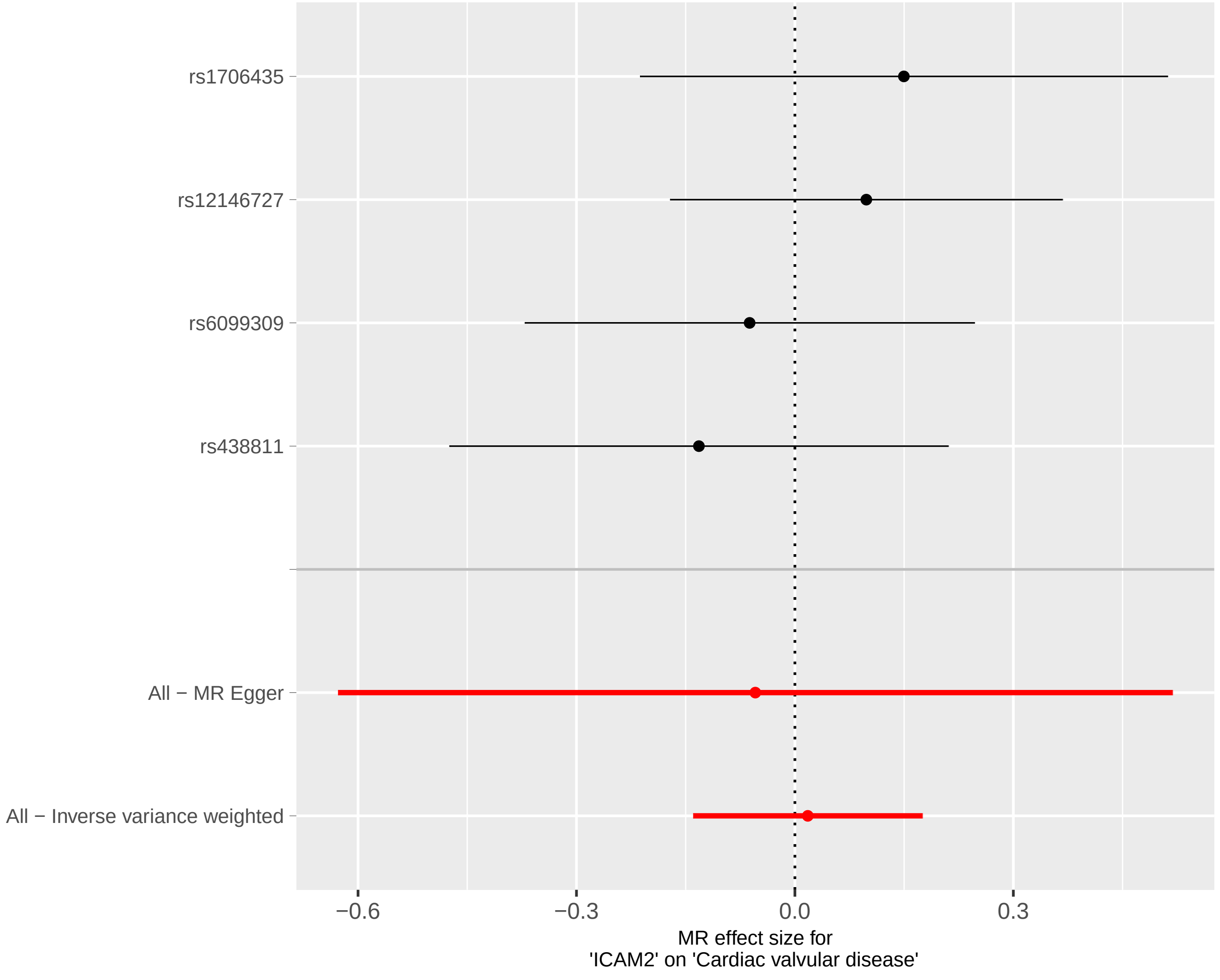
rs1706435
rs12146727
rs6099309
rs438811
All − MR Egger
All − Inverse variance weighted
−0.6
−0.3
0.0
0.3
MR effect size for
'ICAM2' on 'Cardiac valvular disease'

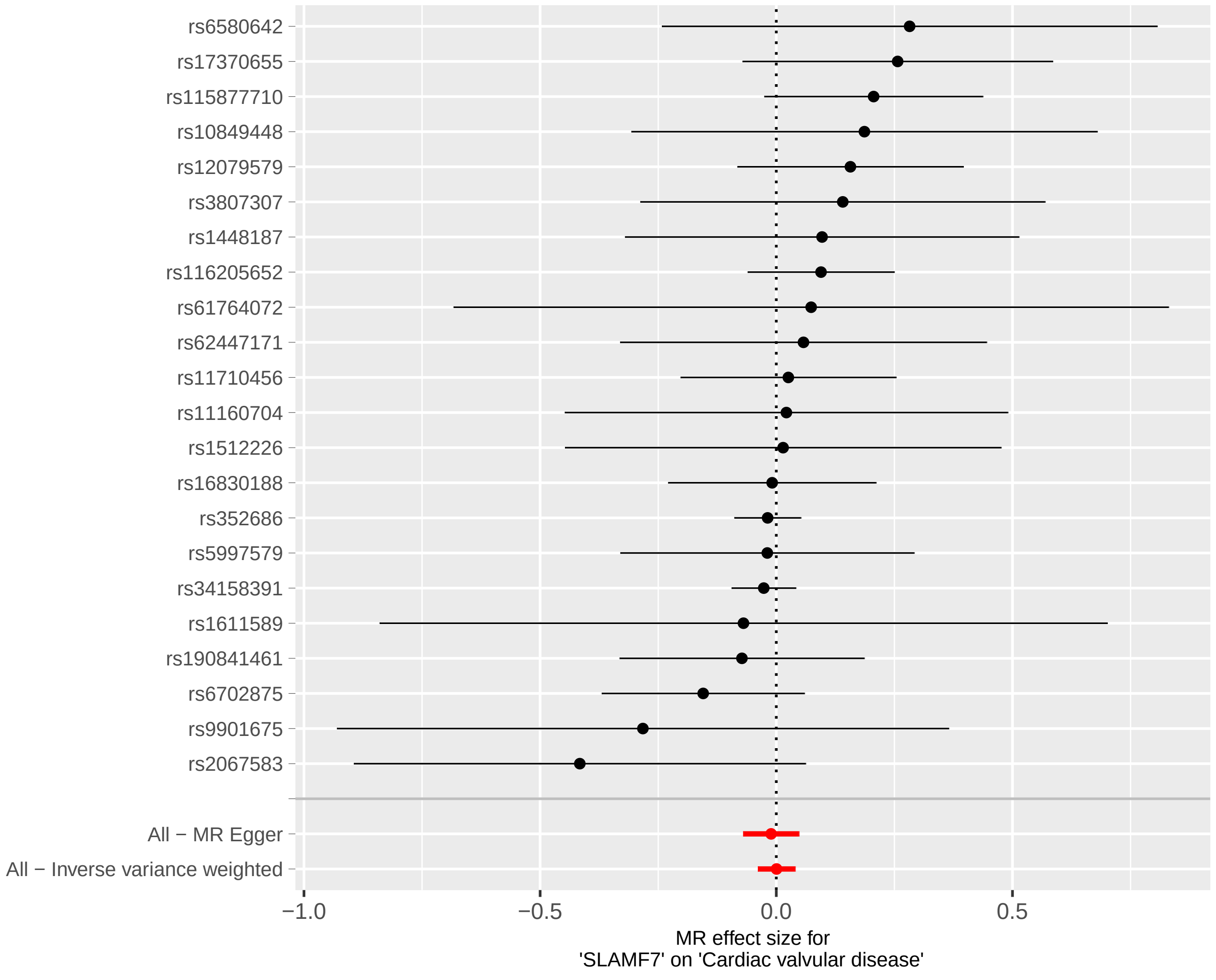

rs6580642
rs17370655
rs115877710
rs10849448
rs12079579
rs3807307
rs1448187
rs116205652
rs61764072
rs62447171
rs11710456
rs11160704
rs1512226
rs16830188
rs352686
rs5997579
rs34158391
rs1611589
rs190841461
rs6702875
rs9901675
rs2067583
All − MR Egger
All − Inverse variance weighted
−1.0
−0.5
0.0
0.5
MR effect size for
'SLAMF7' on 'Cardiac valvular disease'

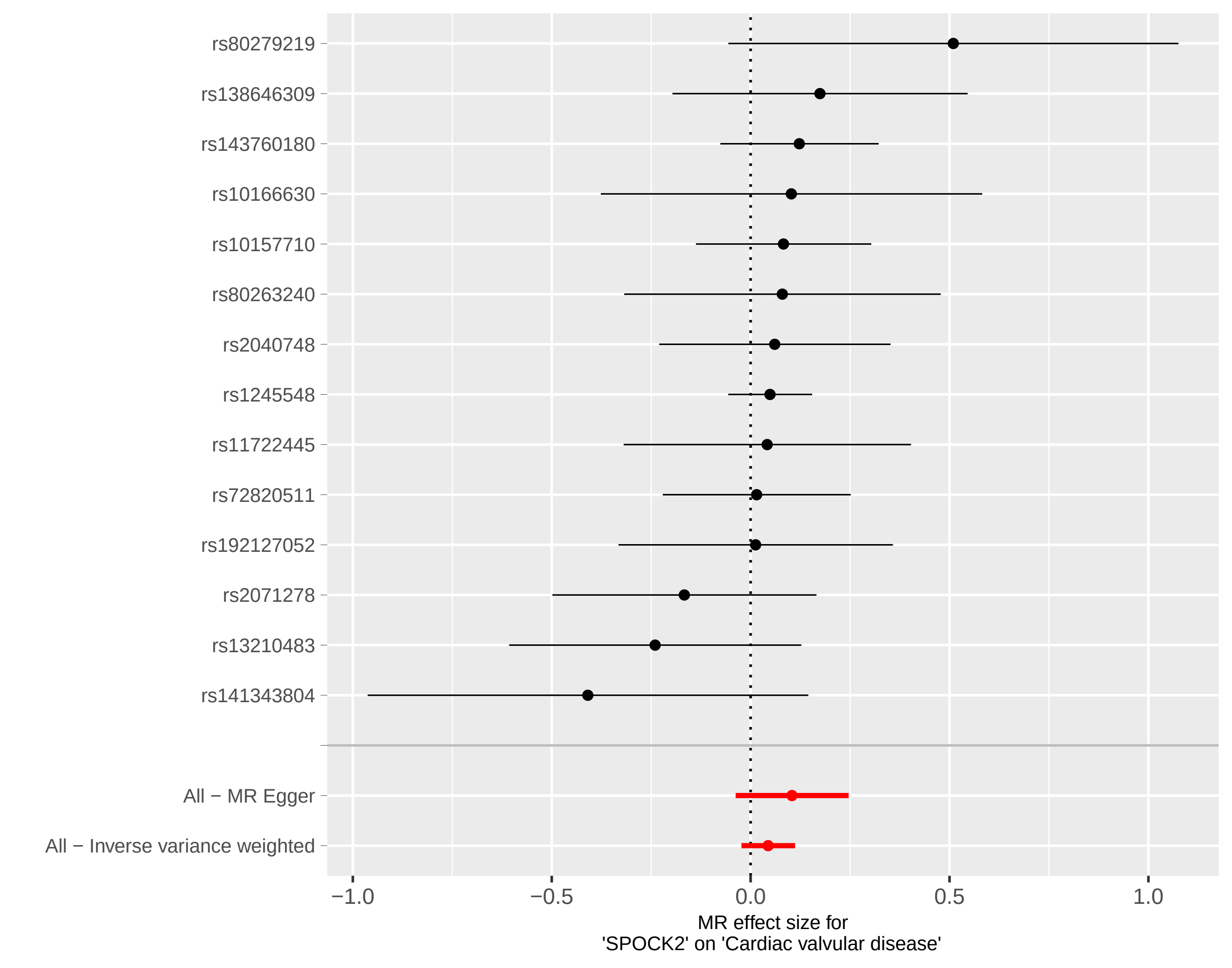
rs80279219
rs138646309
rs143760180
rs10166630
rs10157710
rs80263240
rs2040748
rs1245548
rs11722445
rs72820511
rs192127052
rs2071278
rs13210483
rs141343804
All − MR Egger
All − Inverse variance weighted
−1.0
−0.5
0.0
0.5
1.0
MR effect size for
'SPOCK2' on 'Cardiac valvular disease'

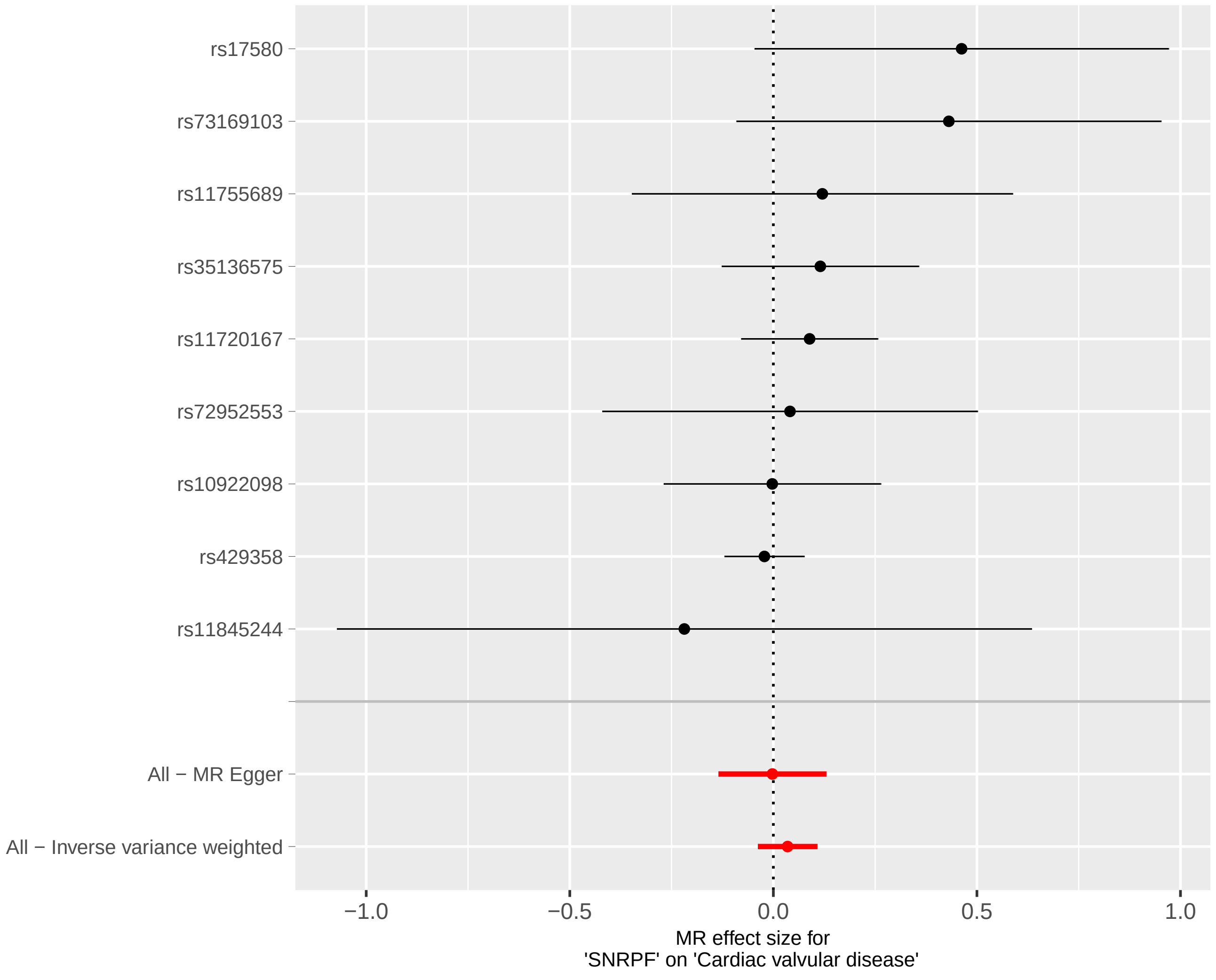
rs17580
rs73169103
rs11755689
rs35136575
rs11720167
rs72952553
rs10922098
rs429358
rs11845244
All − MR Egger
All − Inverse variance weighted
−1.0
−0.5
0.0
0.5
1.0
MR effect size for
'SNRPF' on 'Cardiac valvular disease'

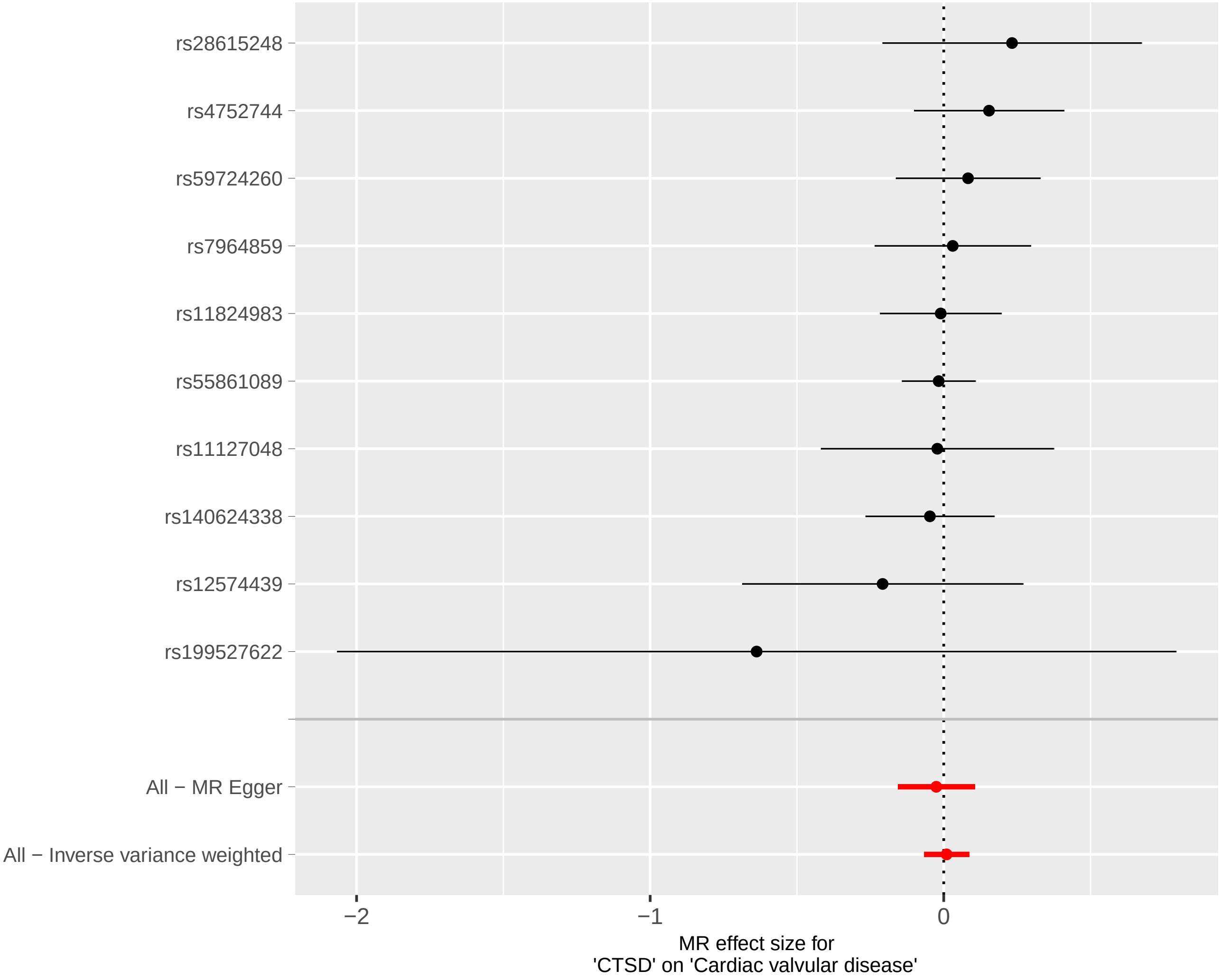
rs28615248
rs4752744
rs59724260
rs7964859
rs11824983
rs55861089
rs11127048
rs140624338
rs12574439
rs199527622
All − MR Egger
All − Inverse variance weighted
−2
−1
0
MR effect size for
'CTSD' on 'Cardiac valvular disease'

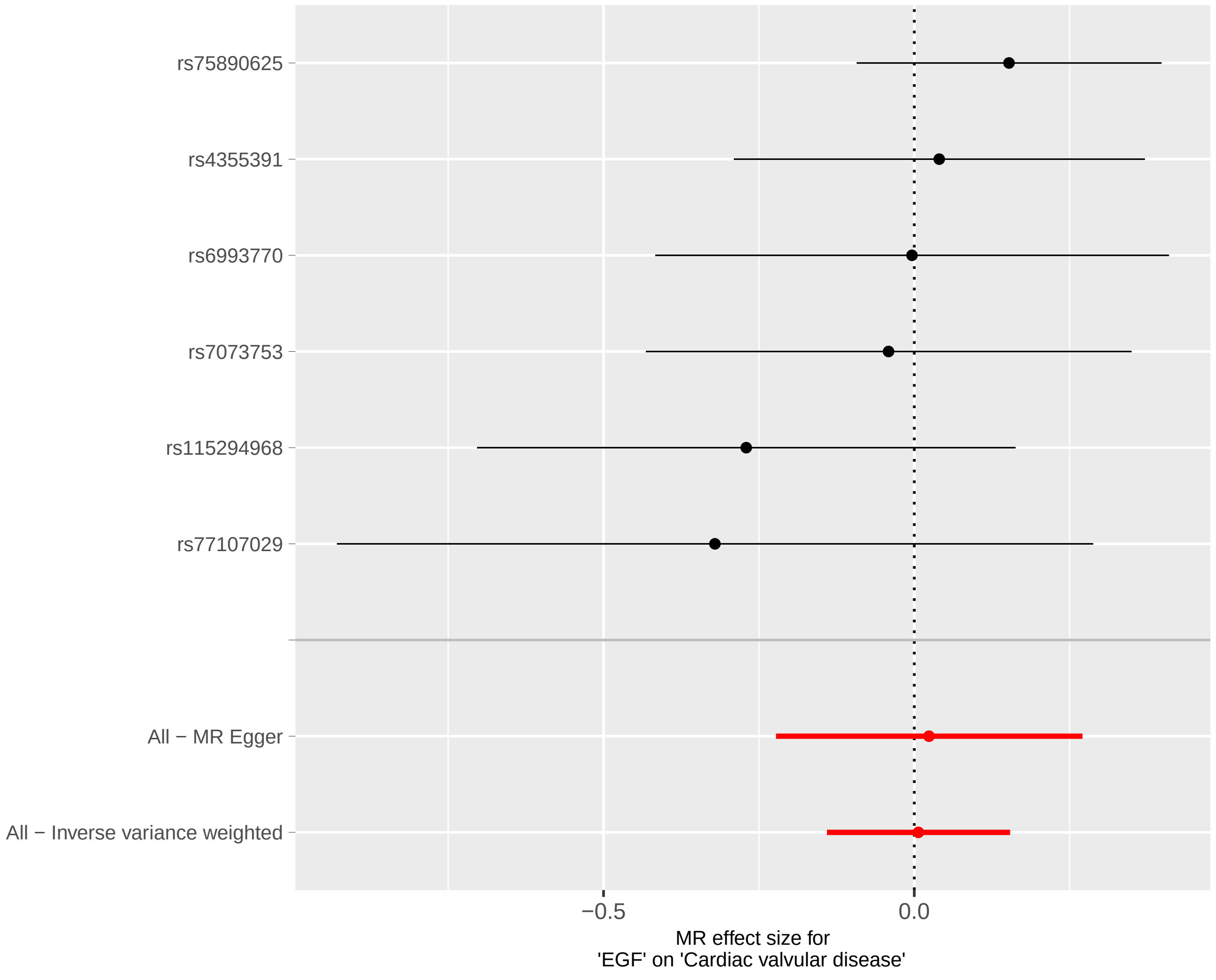
rs75890625
rs4355391
rs6993770
rs7073753
rs115294968
rs77107029
All − MR Egger
All − Inverse variance weighted
−0.5
0.0
MR effect size for
'EGF' on 'Cardiac valvular disease'

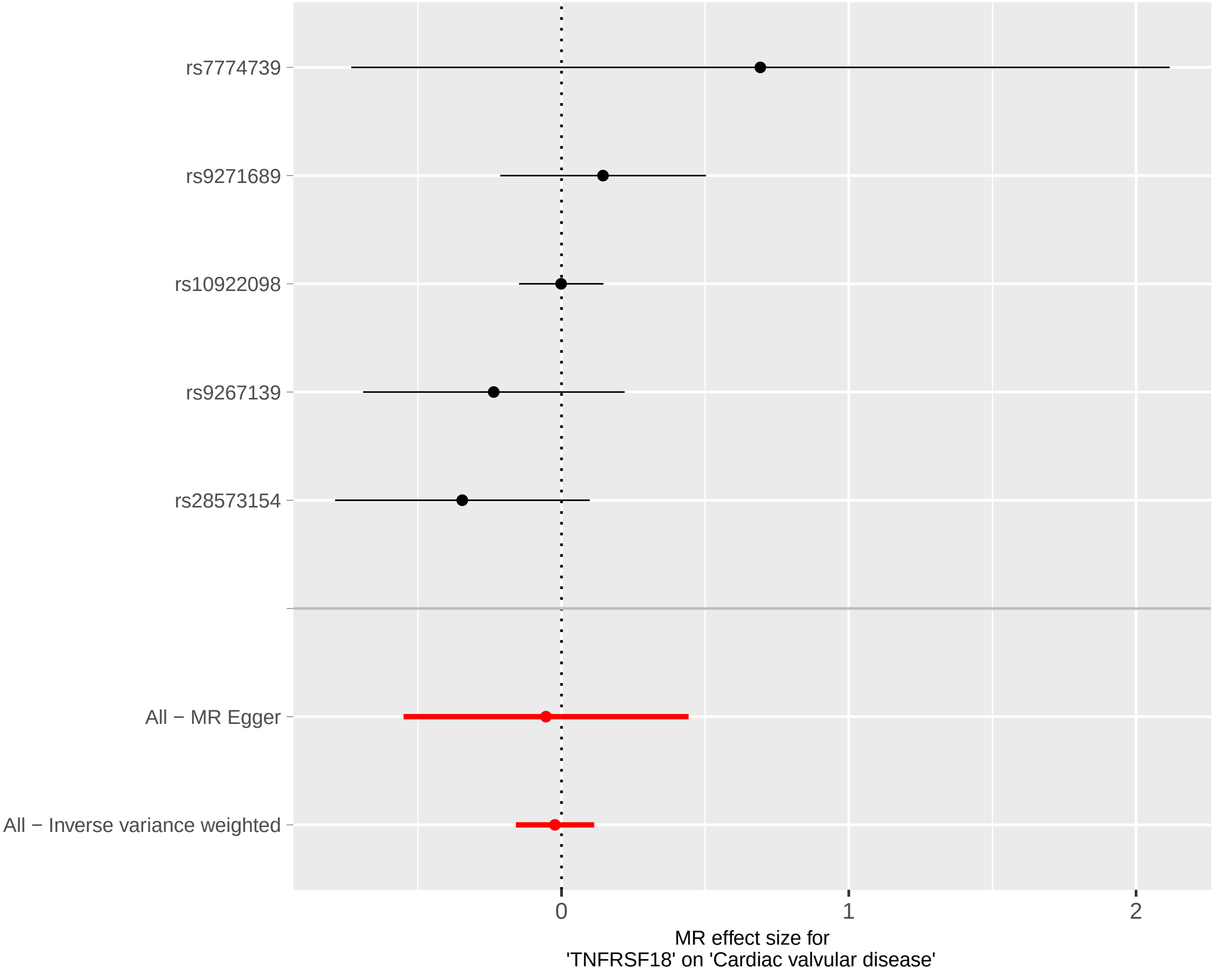
rs7774739
rs9271689
rs10922098
rs9267139
rs28573154
All − MR Egger
All − Inverse variance weighted
0
1
2
MR effect size for
'TNFRSF18' on 'Cardiac valvular disease'

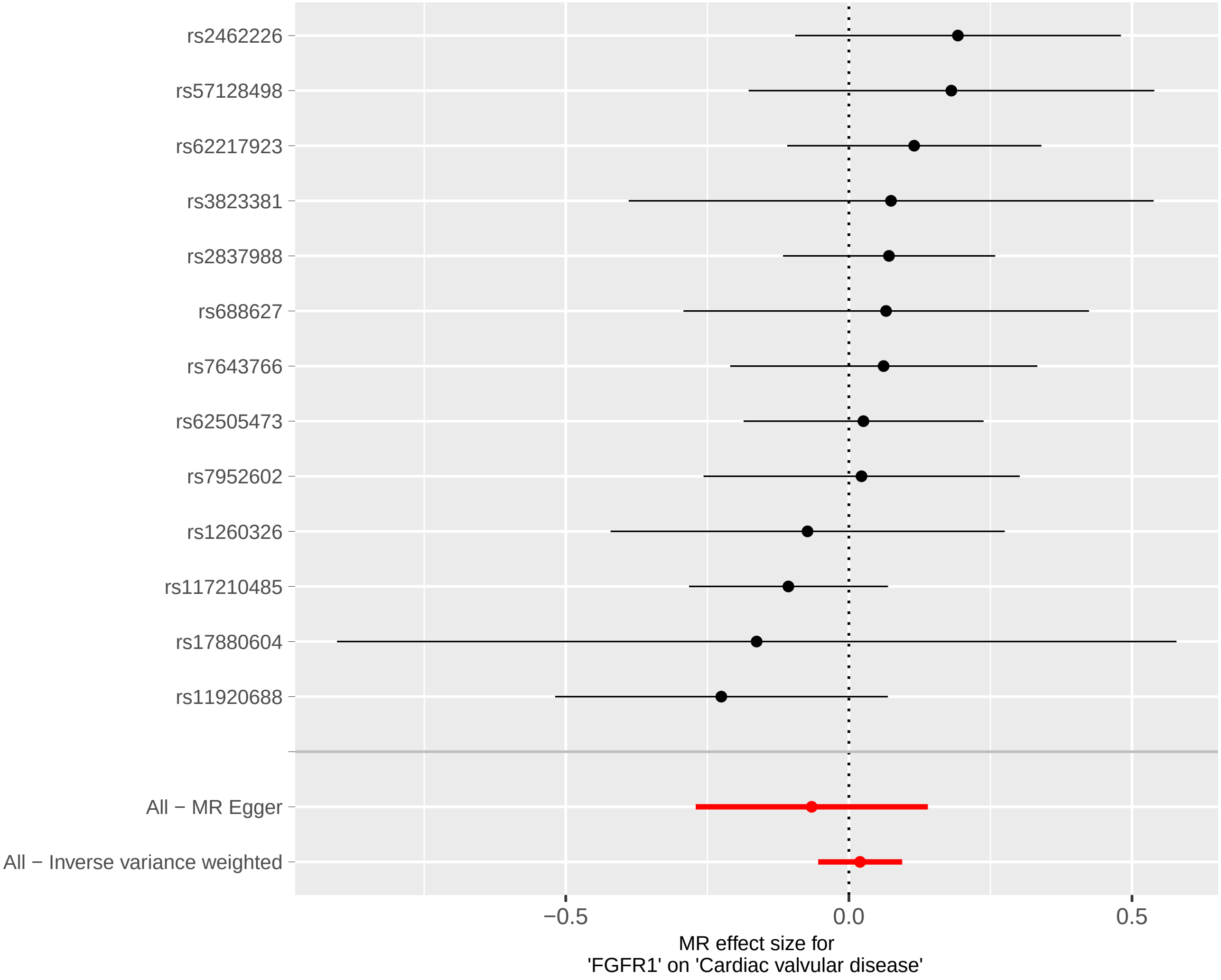
rs2462226
rs57128498
rs62217923
rs3823381
rs2837988
rs688627
rs7643766
rs62505473
rs7952602
rs1260326
rs117210485
rs17880604
rs11920688
All − MR Egger
All − Inverse variance weighted
−0.5
0.0
0.5
MR effect size for
'FGFR1' on 'Cardiac valvular disease'

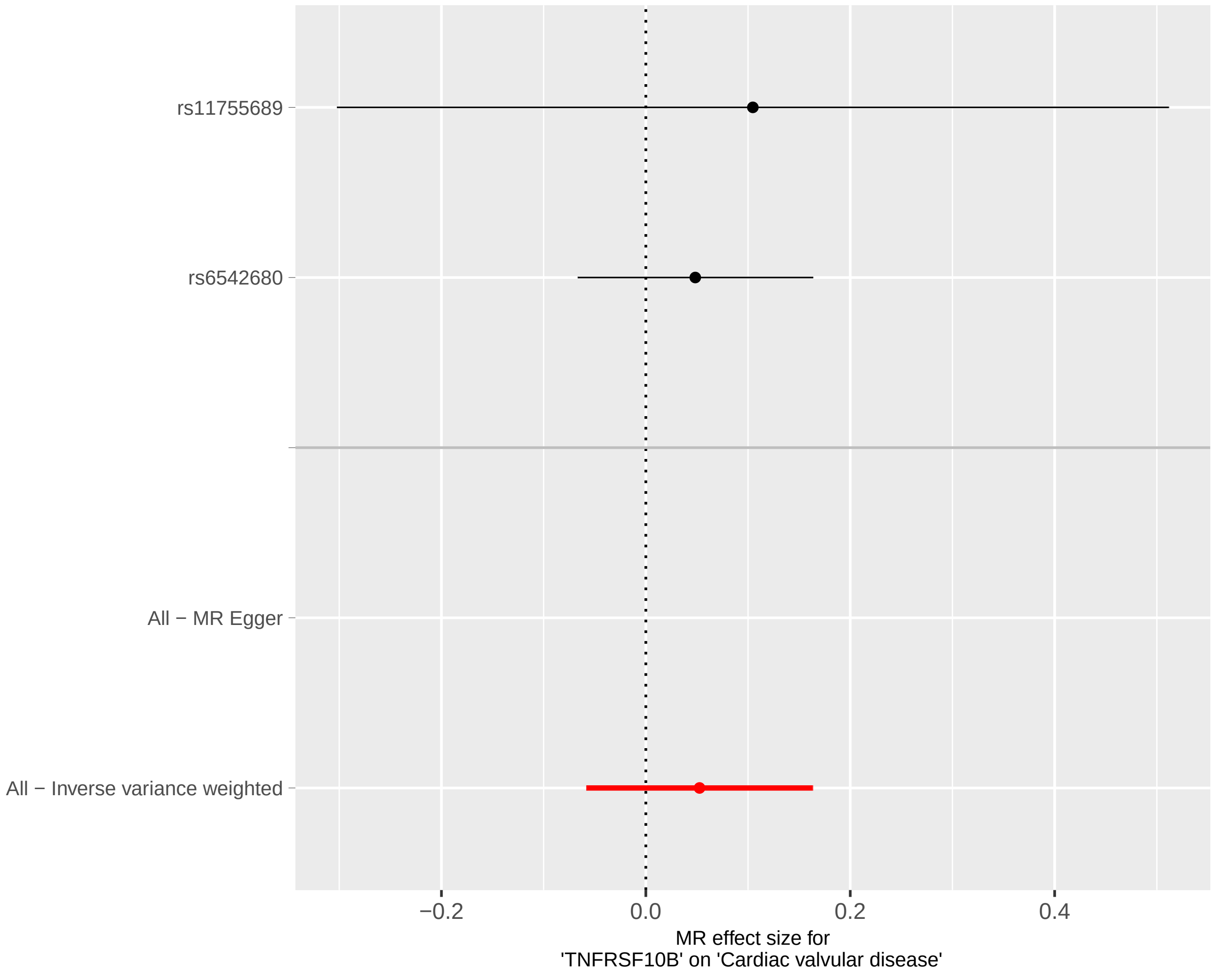

rs11755689
rs6542680
All − MR Egger
All − Inverse variance weighted
−0.2
0.0
0.2
0.4
MR effect size for
'TNFRSF10B' on 'Cardiac valvular disease'

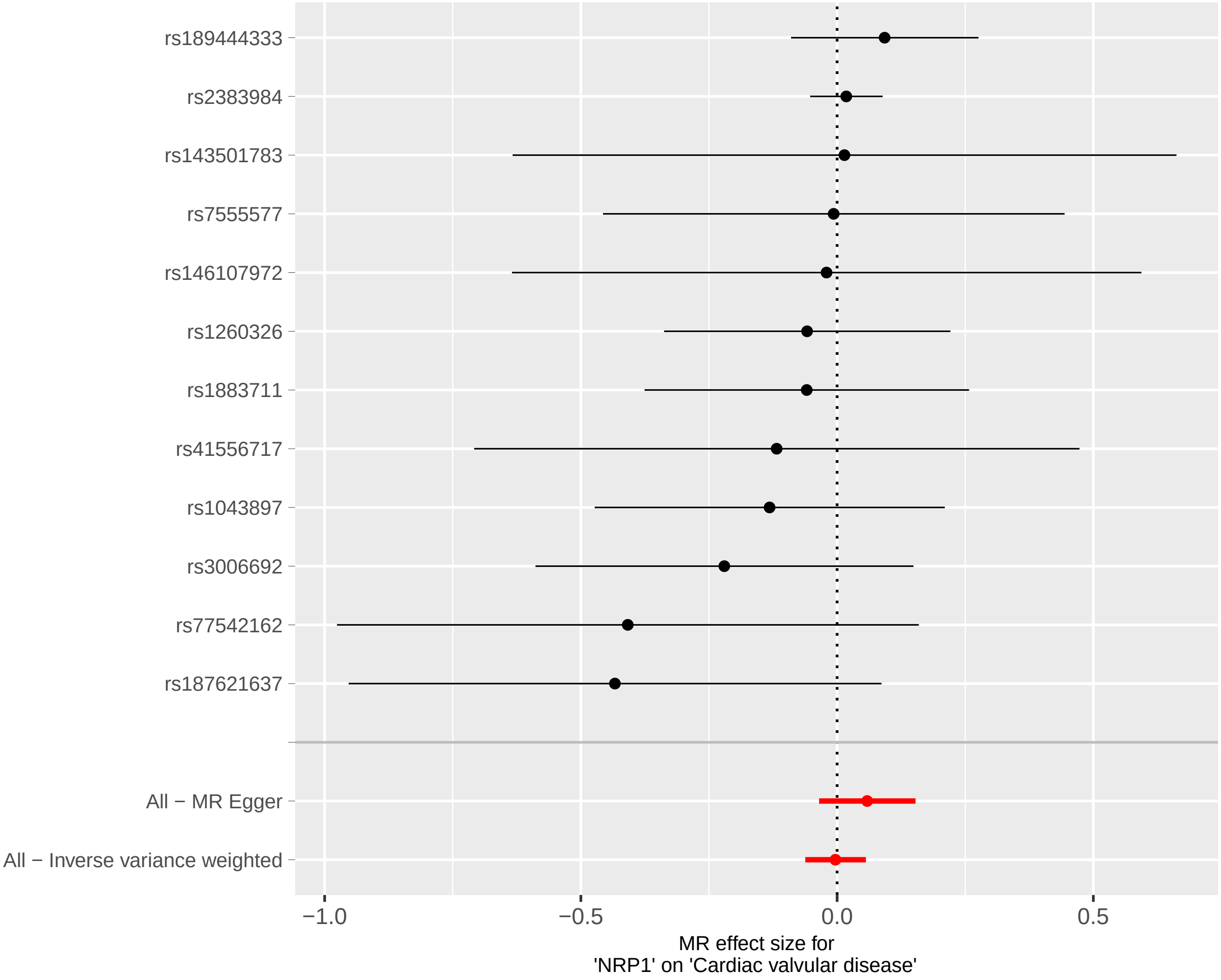
rs189444333
rs2383984
rs143501783
rs7555577
rs146107972
rs1260326
rs1883711
rs41556717
rs1043897
rs3006692
rs77542162
rs187621637
All − MR Egger
All − Inverse variance weighted
−1.0
−0.5
0.0
0.5
MR effect size for
'NRP1' on 'Cardiac valvular disease'

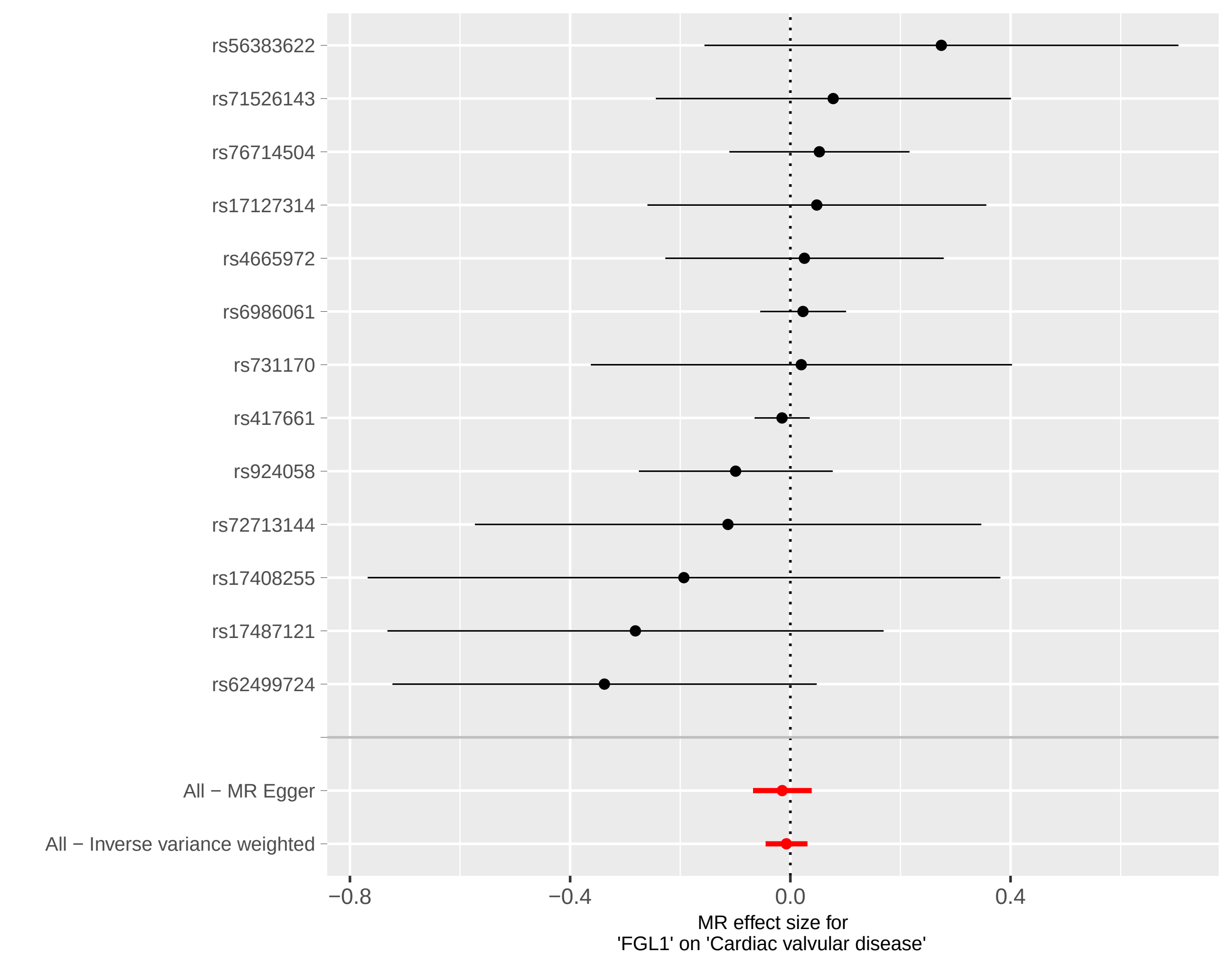

rs56383622
rs71526143
rs76714504
rs17127314
rs4665972
rs6986061
rs731170
rs417661
rs924058
rs72713144
rs17408255
rs17487121
rs62499724
All − MR Egger
All − Inverse variance weighted
−0.8
−0.4
0.0
0.4
MR effect size for
'FGL1' on 'Cardiac valvular disease'

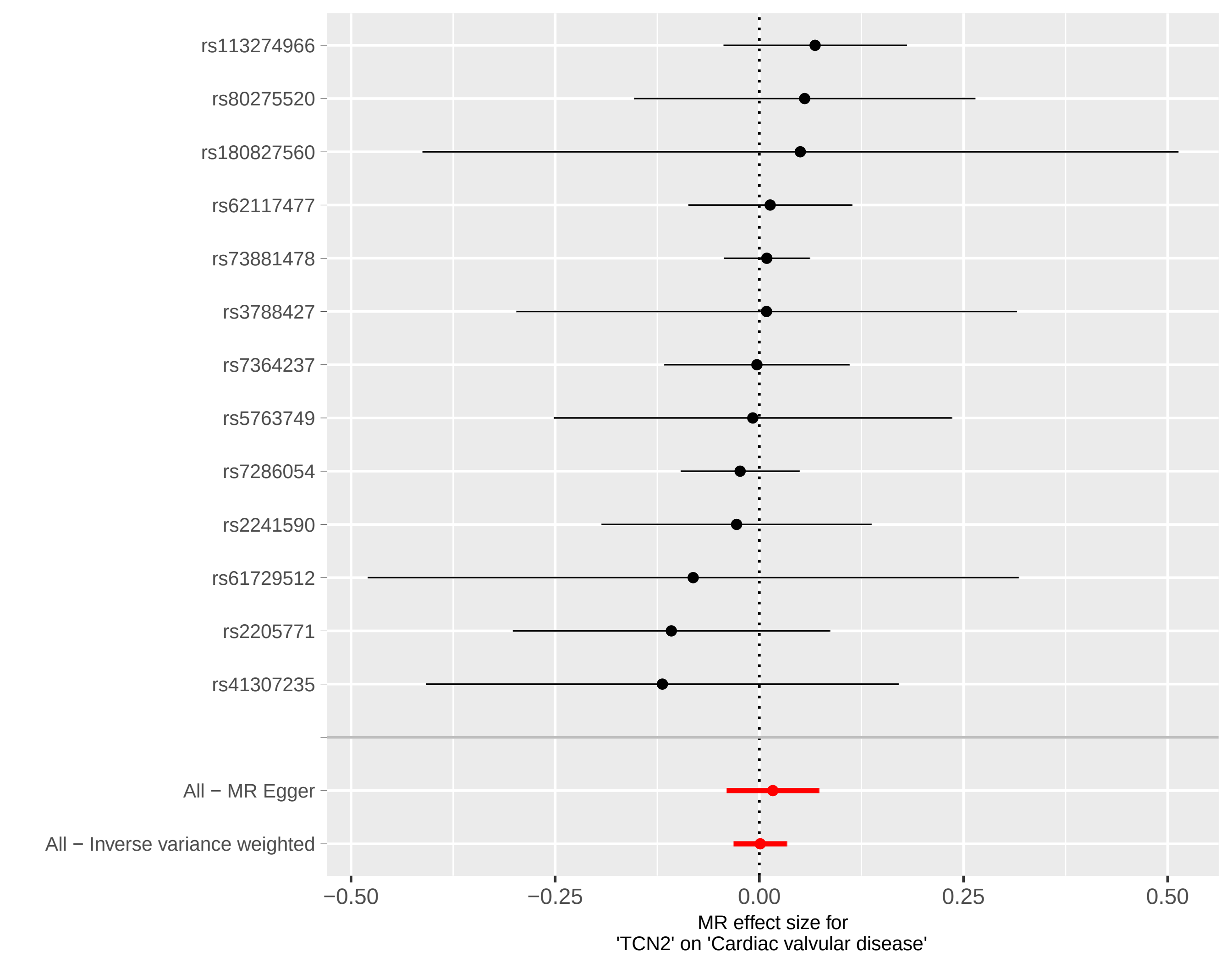

rs113274966
rs80275520
rs180827560
rs62117477
rs73881478
rs3788427
rs7364237
rs5763749
rs7286054
rs2241590
rs61729512
rs2205771
rs41307235
All − MR Egger
All − Inverse variance weighted
−0.50
−0.25
0.00
0.25
0.50
MR effect size for
'TCN2' on 'Cardiac valvular disease'

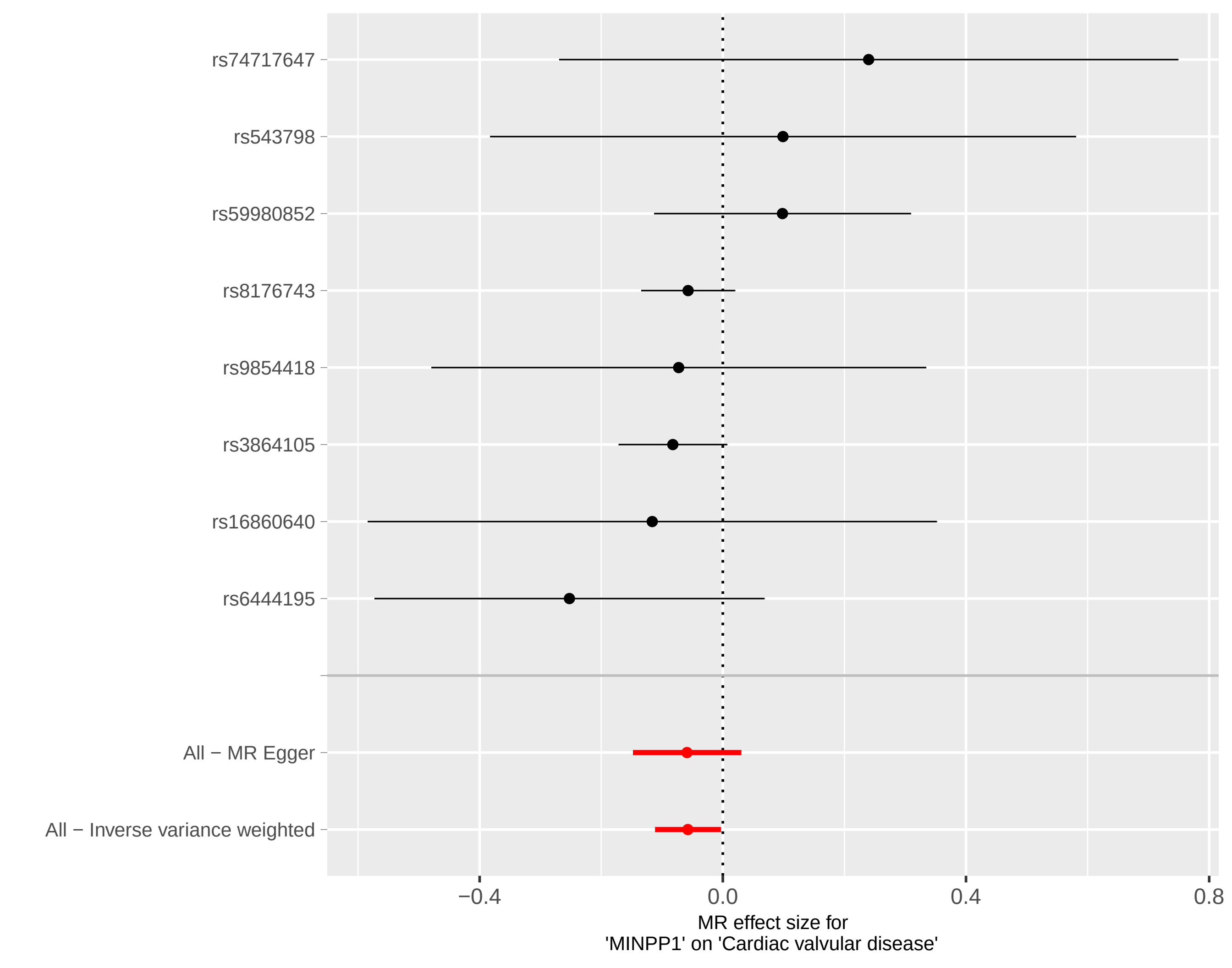
rs74717647
rs543798
rs59980852
rs8176743
rs9854418
rs3864105
rs16860640
rs6444195
All − MR Egger
All − Inverse variance weighted
−0.4
0.0
0.4
0.8
MR effect size for
'MINPP1' on 'Cardiac valvular disease'

Insufficient number of SNPs

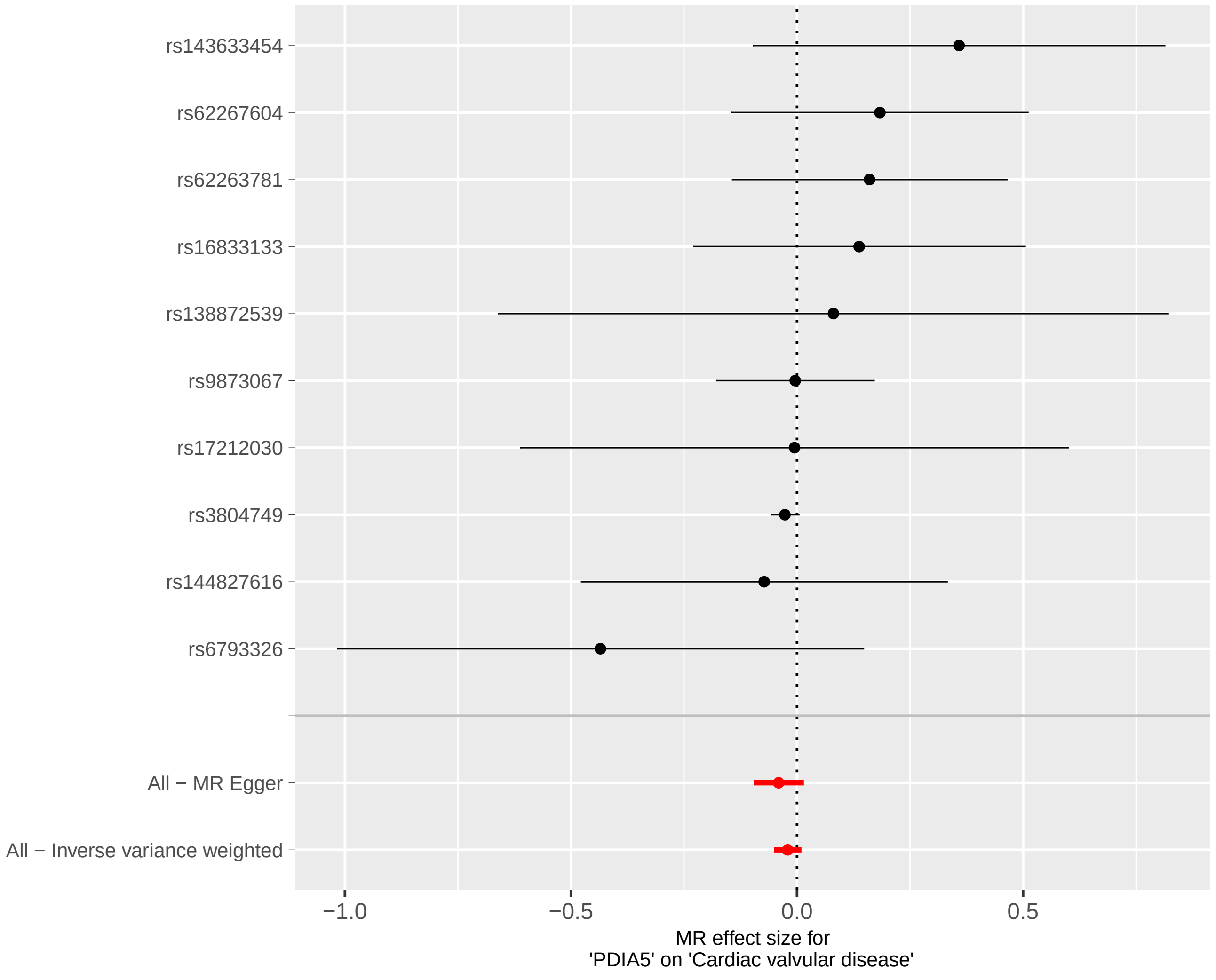

rs143633454
rs62267604
rs62263781
rs16833133
rs138872539
rs9873067
rs17212030
rs3804749
rs144827616
rs6793326
All − MR Egger
All − Inverse variance weighted
−1.0
−0.5
0.0
0.5
MR effect size for
'PDIA5' on 'Cardiac valvular disease'

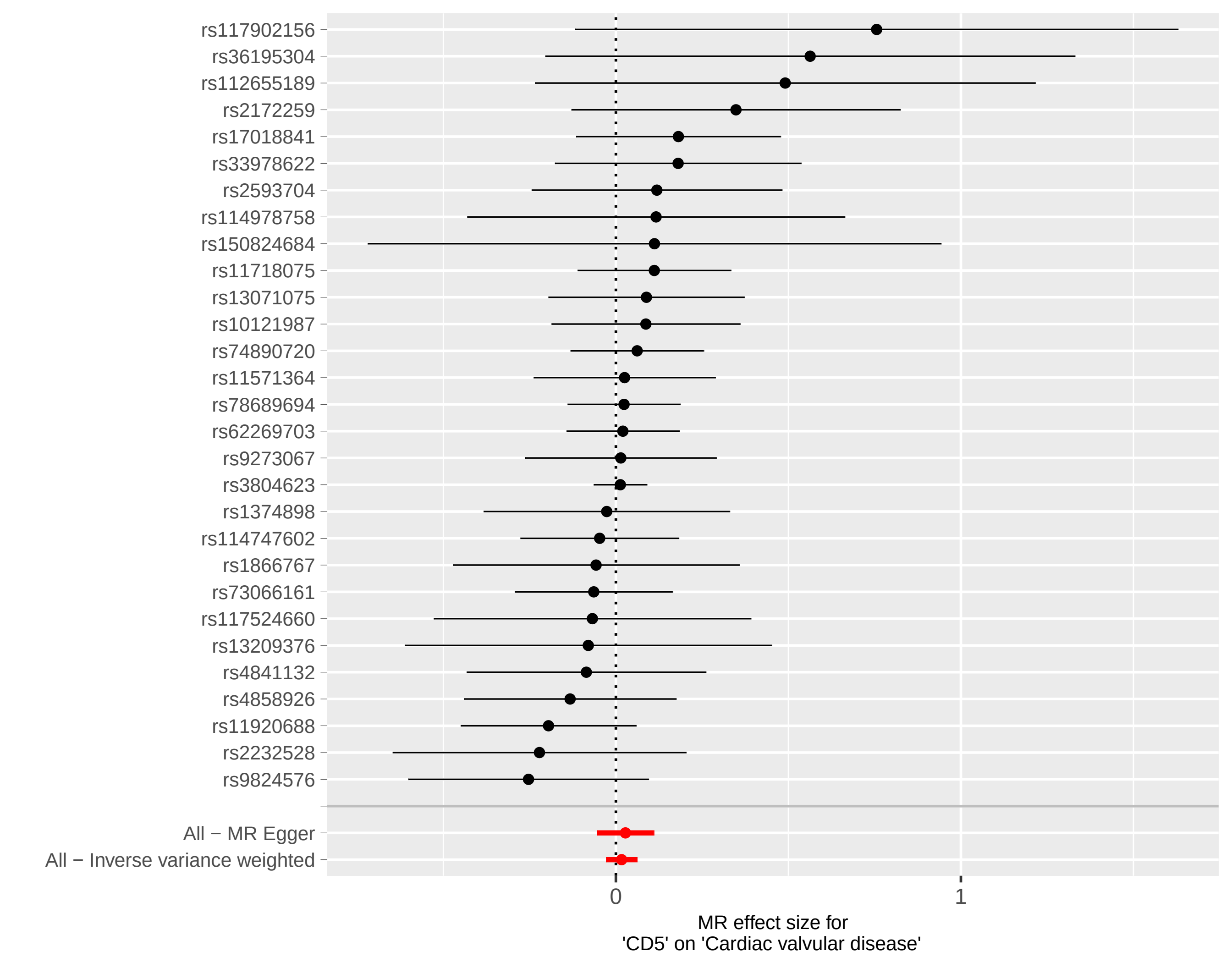
rs117902156
rs36195304
rs112655189
rs2172259
rs17018841
rs33978622
rs2593704
rs114978758
rs150824684
rs11718075
rs13071075
rs10121987
rs74890720
rs11571364
rs78689694
rs62269703
rs9273067
rs3804623
rs1374898
rs114747602
rs1866767
rs73066161
rs117524660
rs13209376
rs4841132
rs4858926
rs11920688
rs2232528
rs9824576
All − MR Egger
All − Inverse variance weighted
0
1
MR effect size for
'CD5' on 'Cardiac valvular disease'

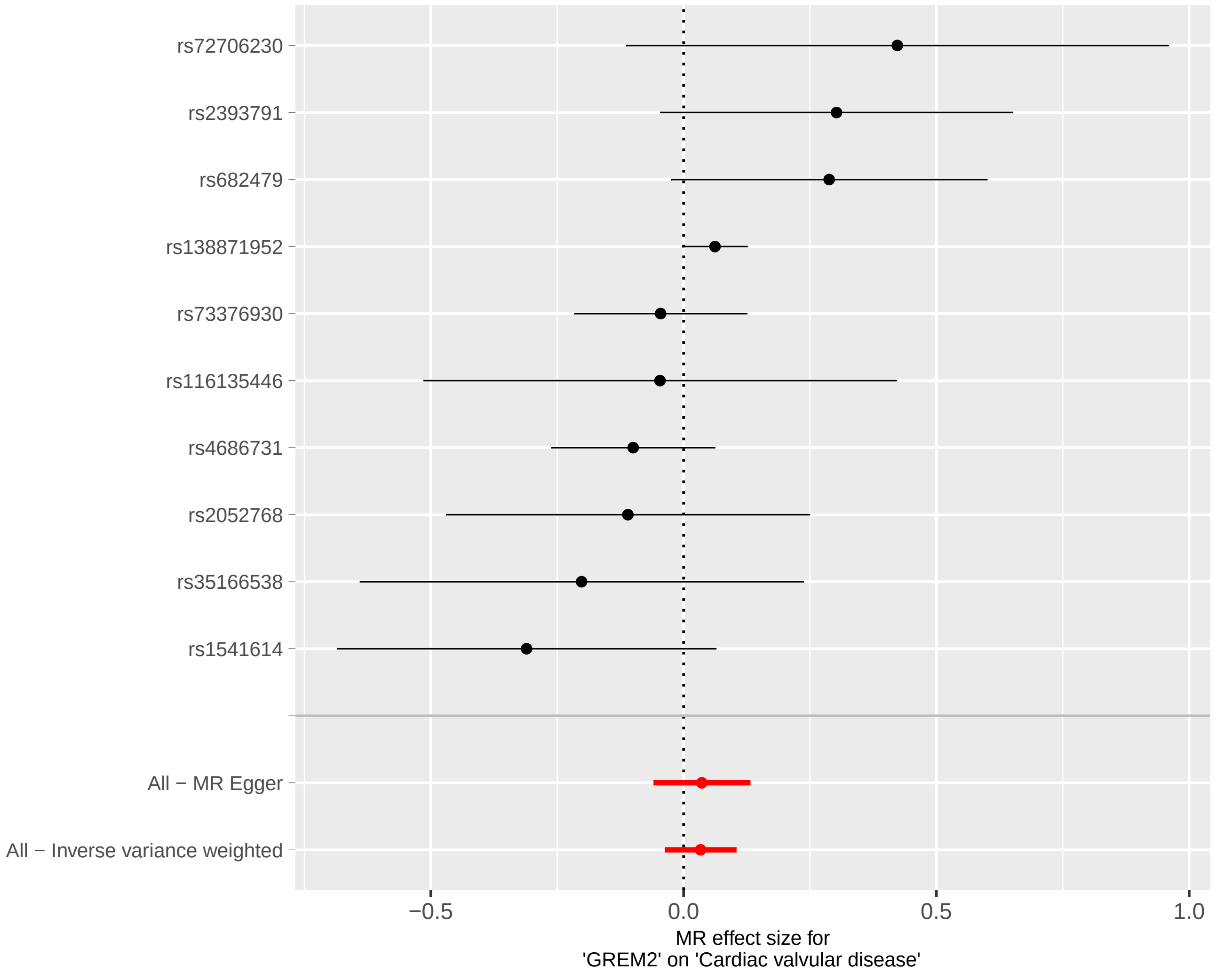
rs72706230
rs2393791
rs682479
rs138871952
rs73376930
rs116135446
rs4686731
rs2052768
rs35166538
rs1541614
All − MR Egger
All − Inverse variance weighted
−0.5
0.0
0.5
1.0
MR effect size for
'GREM2' on 'Cardiac valvular disease'

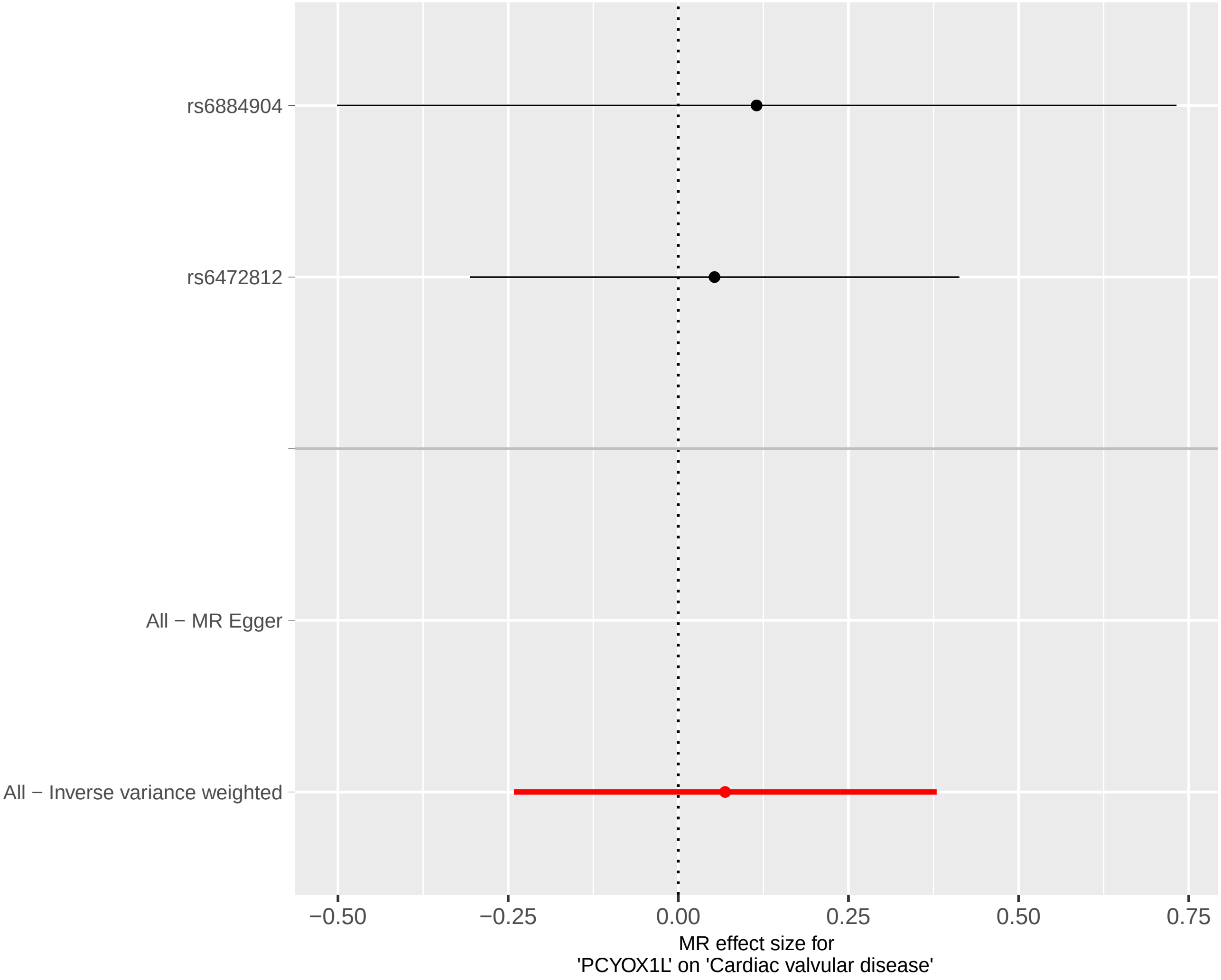
rs6884904
rs6472812
All − MR Egger
All − Inverse variance weighted
−0.50
−0.25
0.00
0.25
0.50
0.75
MR effect size for
'PCYOX1L' on 'Cardiac valvular disease'

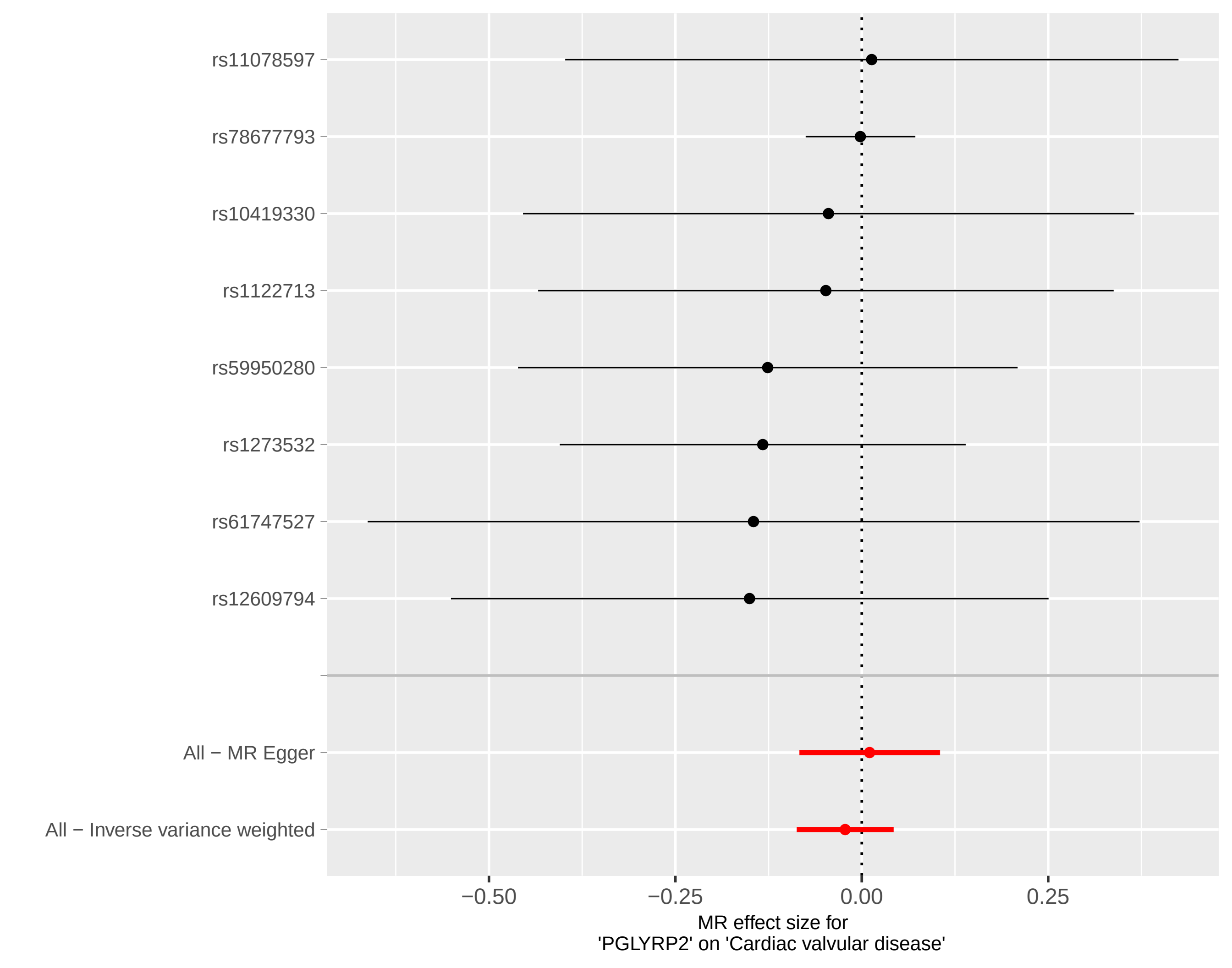
rs11078597
rs78677793
rs10419330
rs1122713
rs59950280
rs1273532
rs61747527
rs12609794
All − MR Egger
All − Inverse variance weighted
−0.50
−0.25
0.00
0.25
MR effect size for
'PGLYRP2' on 'Cardiac valvular disease'

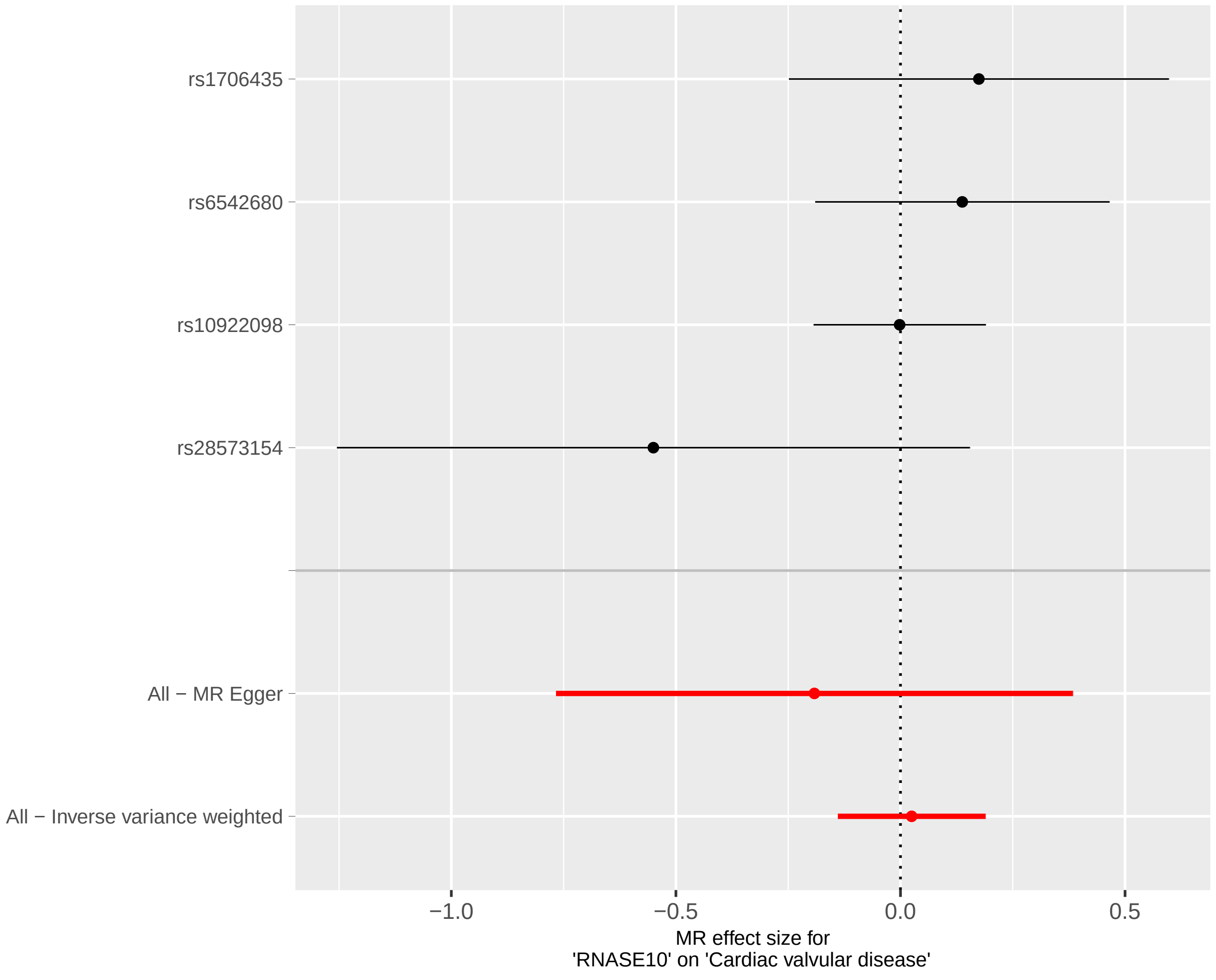
rs1706435
rs6542680
rs10922098
rs28573154
All − MR Egger
All − Inverse variance weighted
−1.0
−0.5
0.0
0.5
MR effect size for
'RNASE10' on 'Cardiac valvular disease'

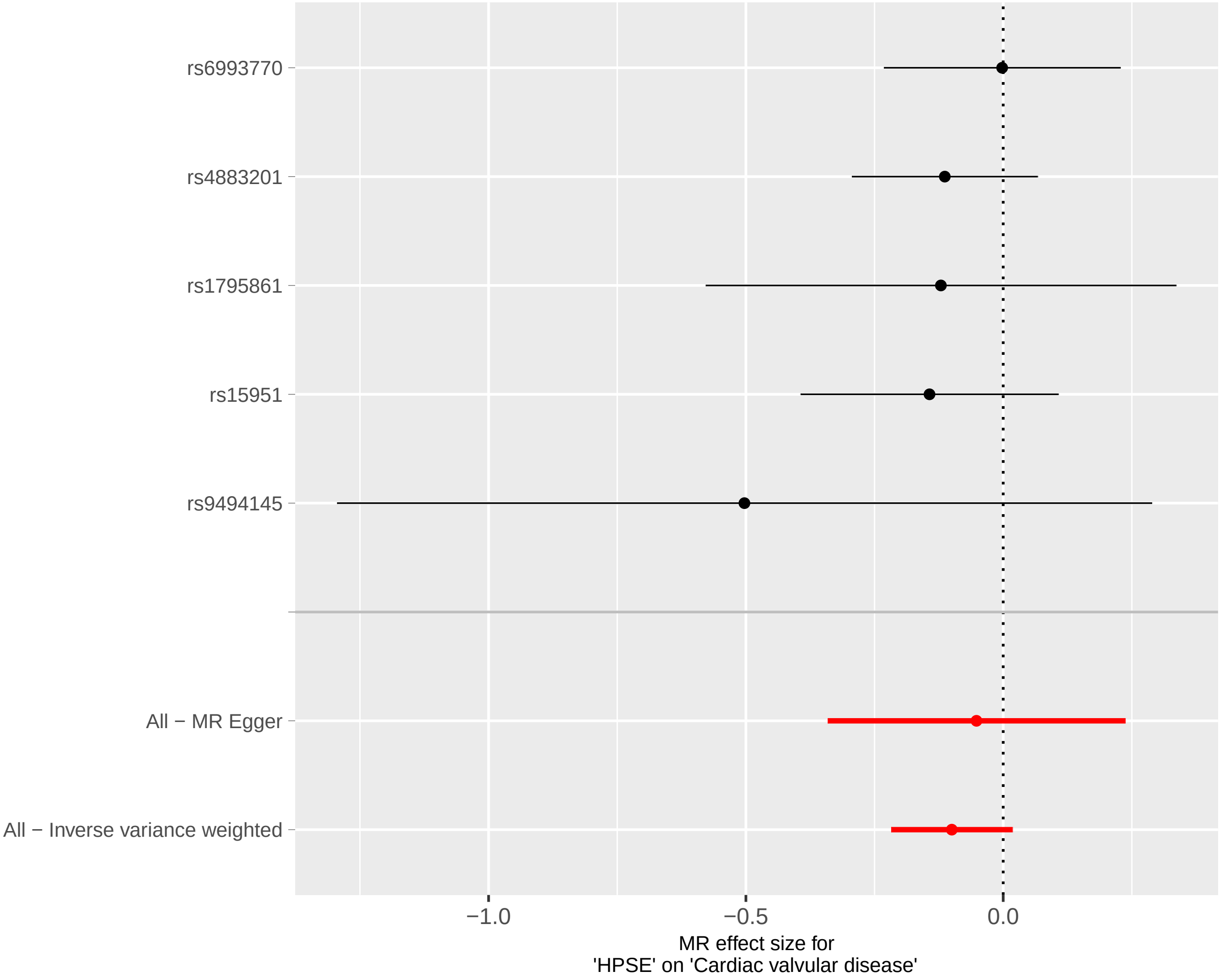
rs6993770
rs4883201
rs1795861
rs15951
rs9494145
All − MR Egger
All − Inverse variance weighted
−1.0
−0.5
0.0
MR effect size for
'HPSE' on 'Cardiac valvular disease'

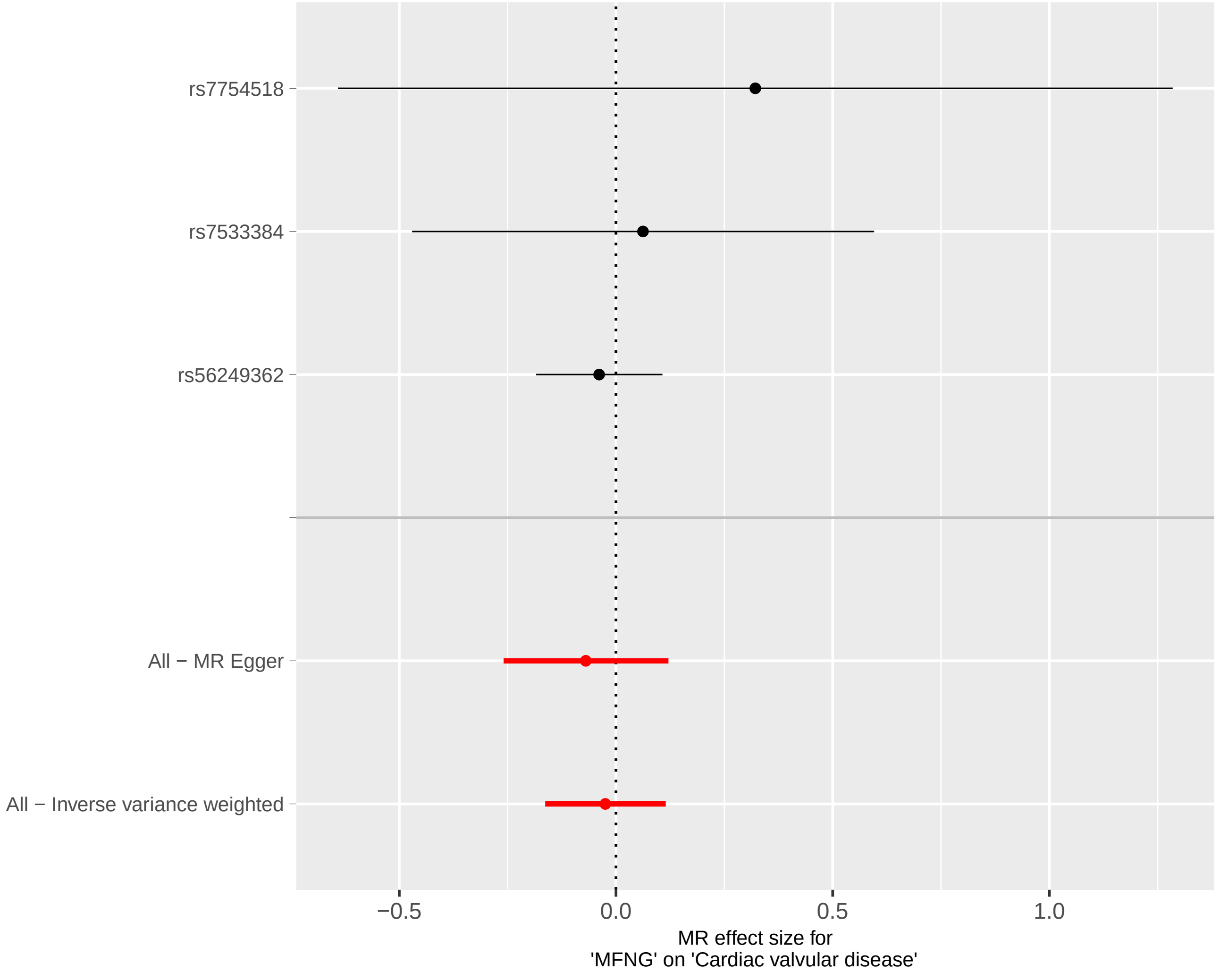
rs7754518
rs7533384
rs56249362
All − MR Egger
All − Inverse variance weighted
−0.5
0.0
0.5
1.0
MR effect size for
'MFNG' on 'Cardiac valvular disease'

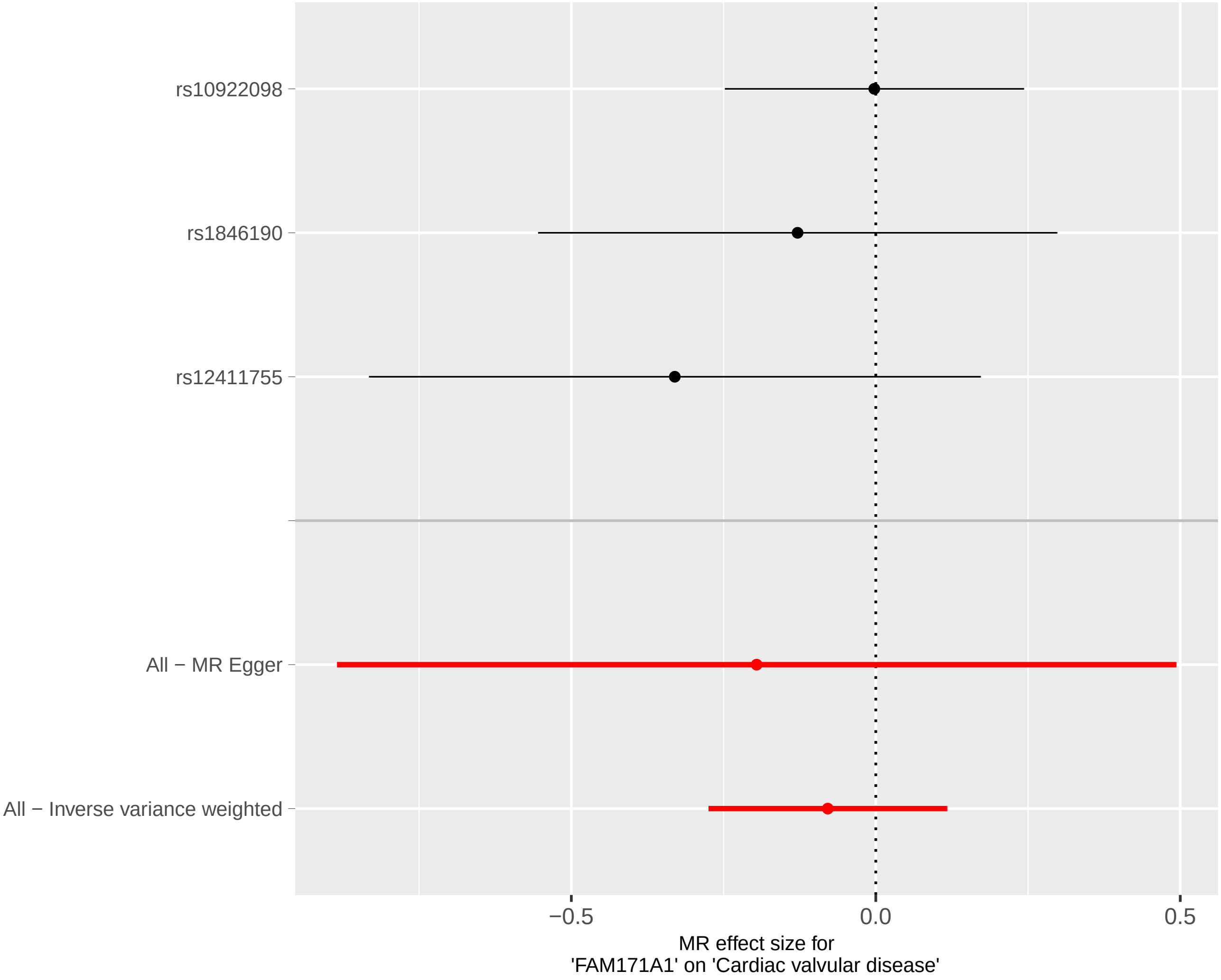
rs10922098
rs1846190
rs12411755
All − MR Egger
All − Inverse variance weighted
−0.5
0.0
0.5
MR effect size for
'FAM171A1' on 'Cardiac valvular disease'

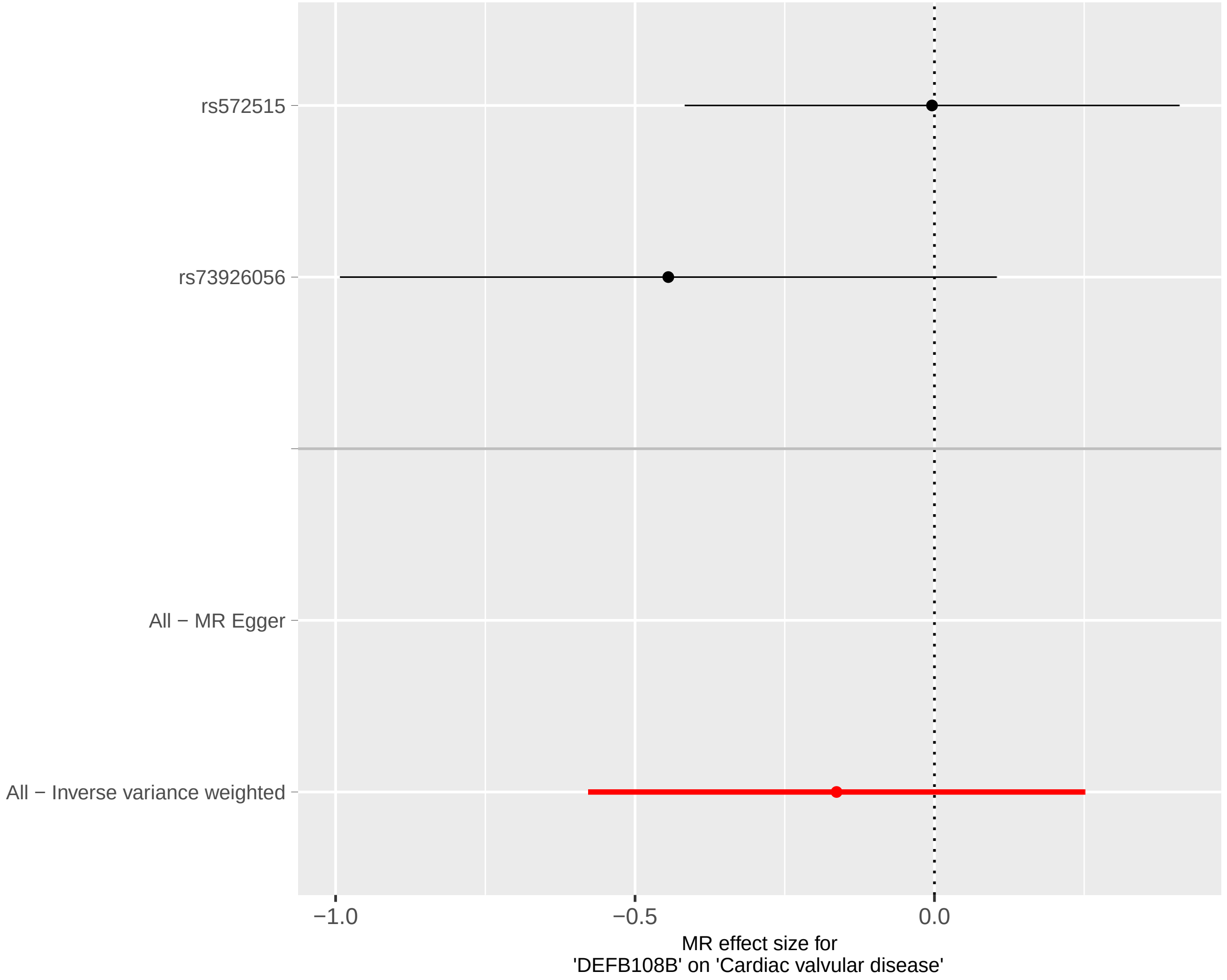
rs572515
rs73926056
All − MR Egger
All − Inverse variance weighted
−1.0
−0.5
0.0
MR effect size for
'DEFB108B' on 'Cardiac valvular disease'

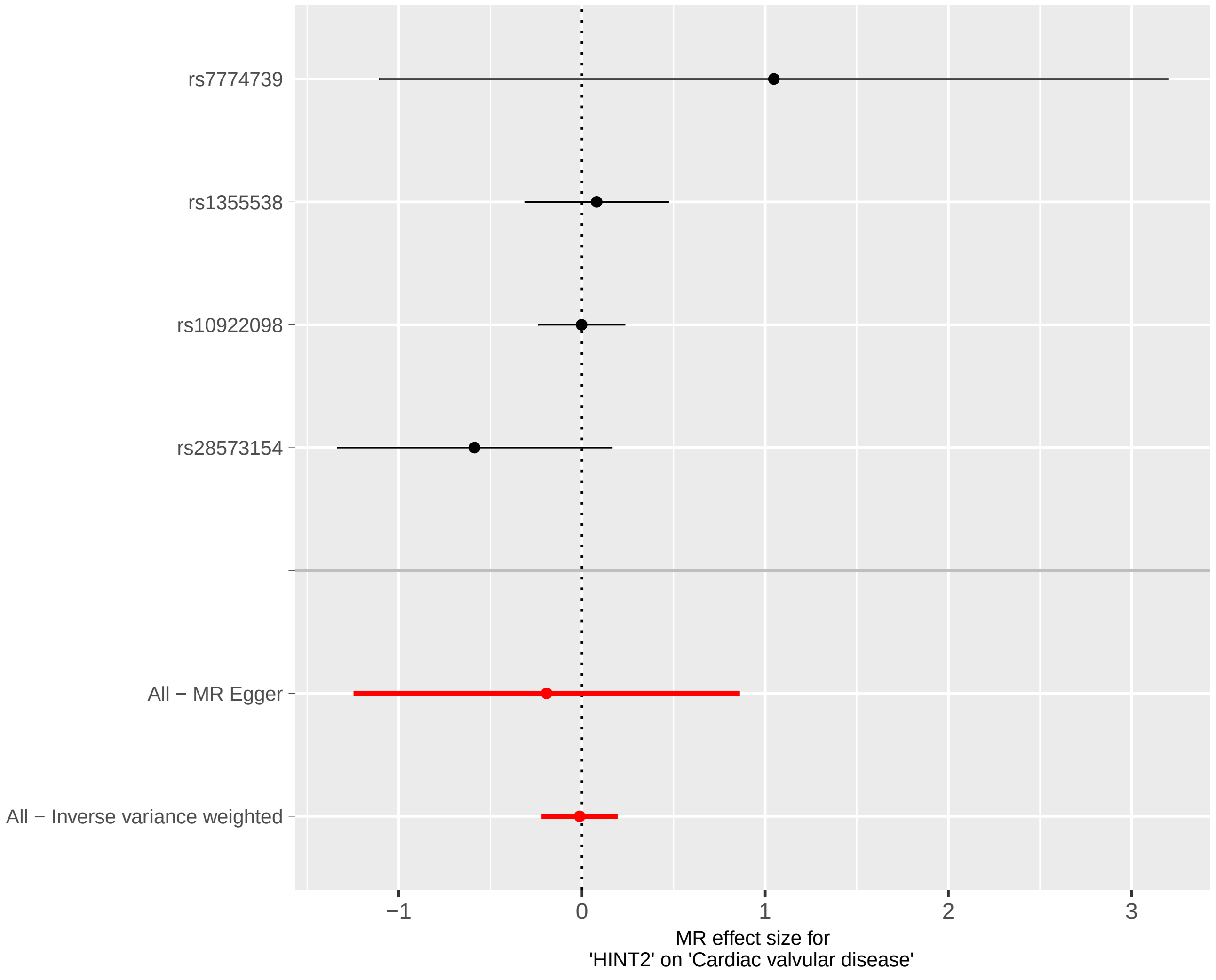
rs7774739
rs1355538
rs10922098
rs28573154
All − MR Egger
All − Inverse variance weighted
−1
0
1
2
3
MR effect size for
'HINT2' on 'Cardiac valvular disease'

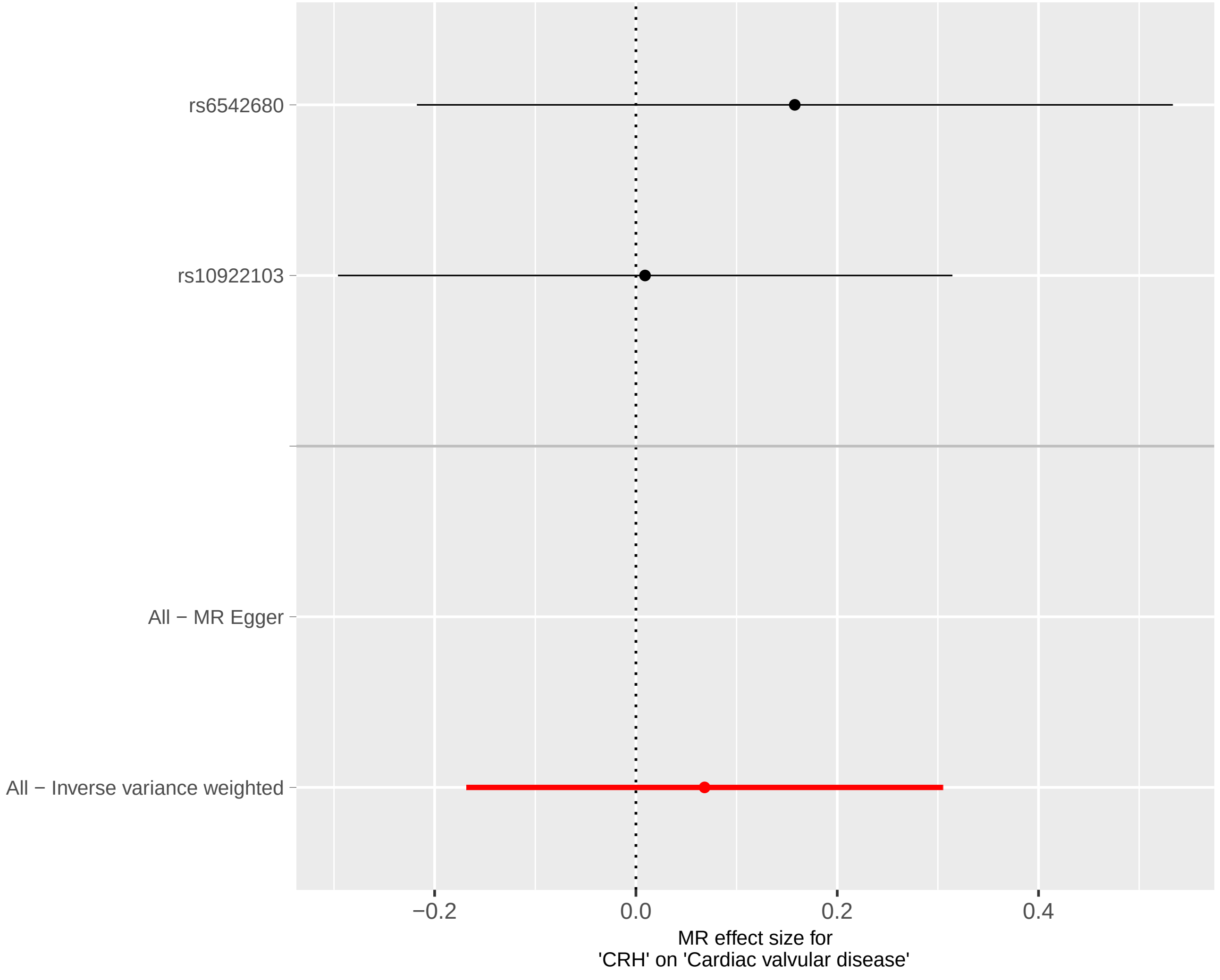

rs6542680
rs10922103
All − MR Egger
All − Inverse variance weighted
−0.2
0.0
0.2
0.4
MR effect size for
'CRH' on 'Cardiac valvular disease'

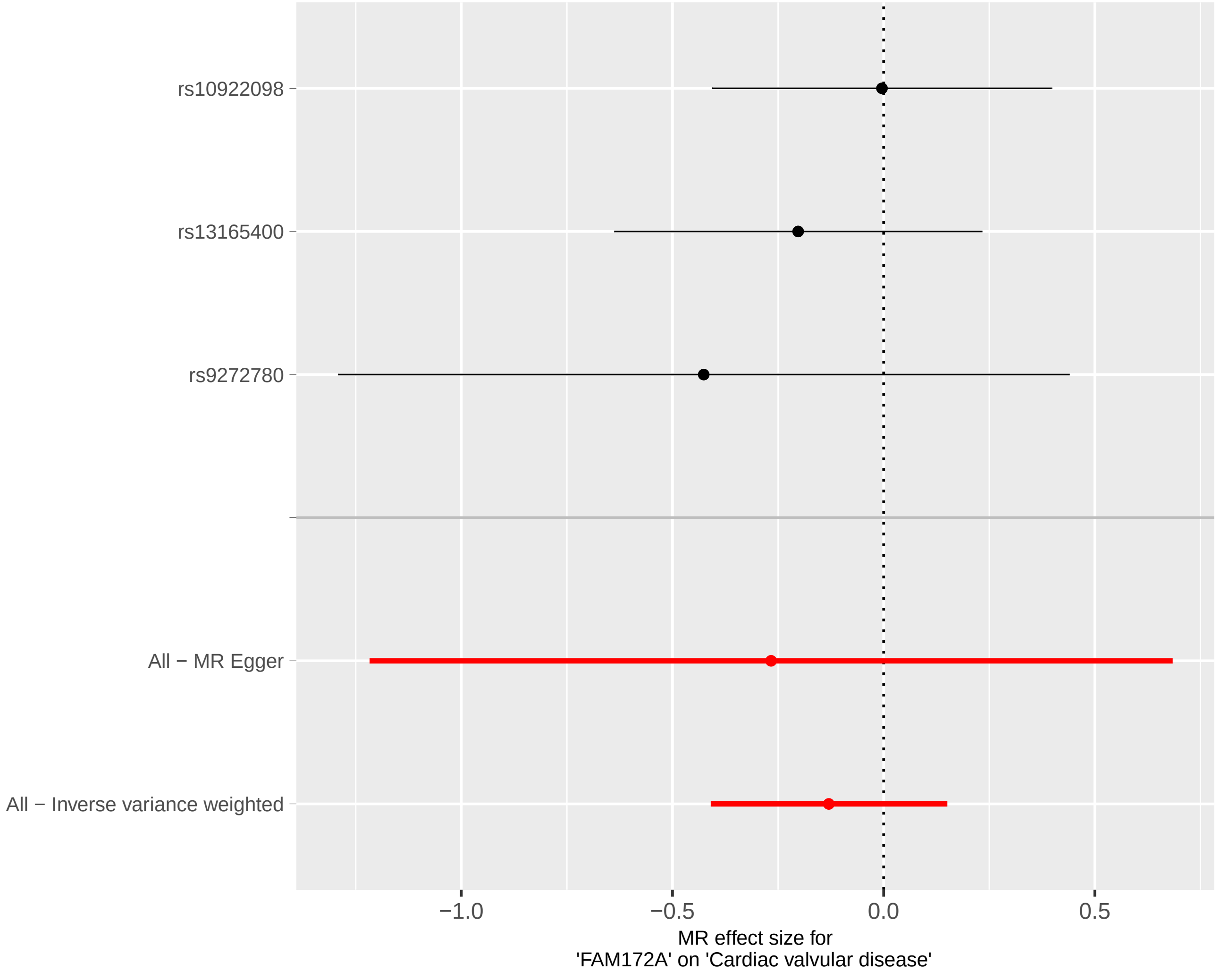
rs10922098
rs13165400
rs9272780
All − MR Egger
All − Inverse variance weighted
−1.0
−0.5
0.0
0.5
MR effect size for
'FAM172A' on 'Cardiac valvular disease'

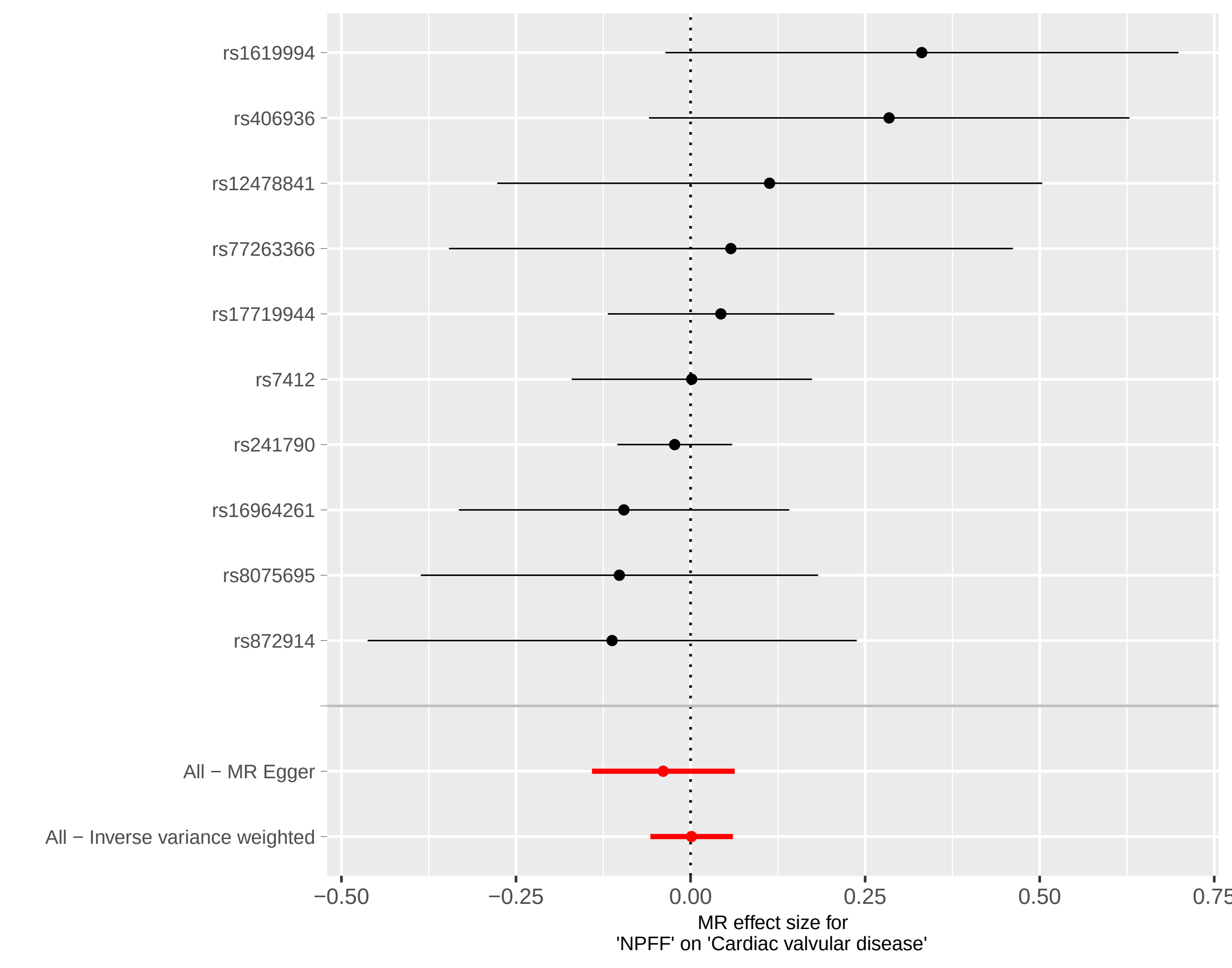
rs1619994
rs406936
rs12478841
rs77263366
rs17719944
rs7412
rs241790
rs16964261
rs8075695
rs872914
All − MR Egger
All − Inverse variance weighted
−0.50
−0.25
0.00
0.25
0.50
0.75
MR effect size for
'NPFF' on 'Cardiac valvular disease'

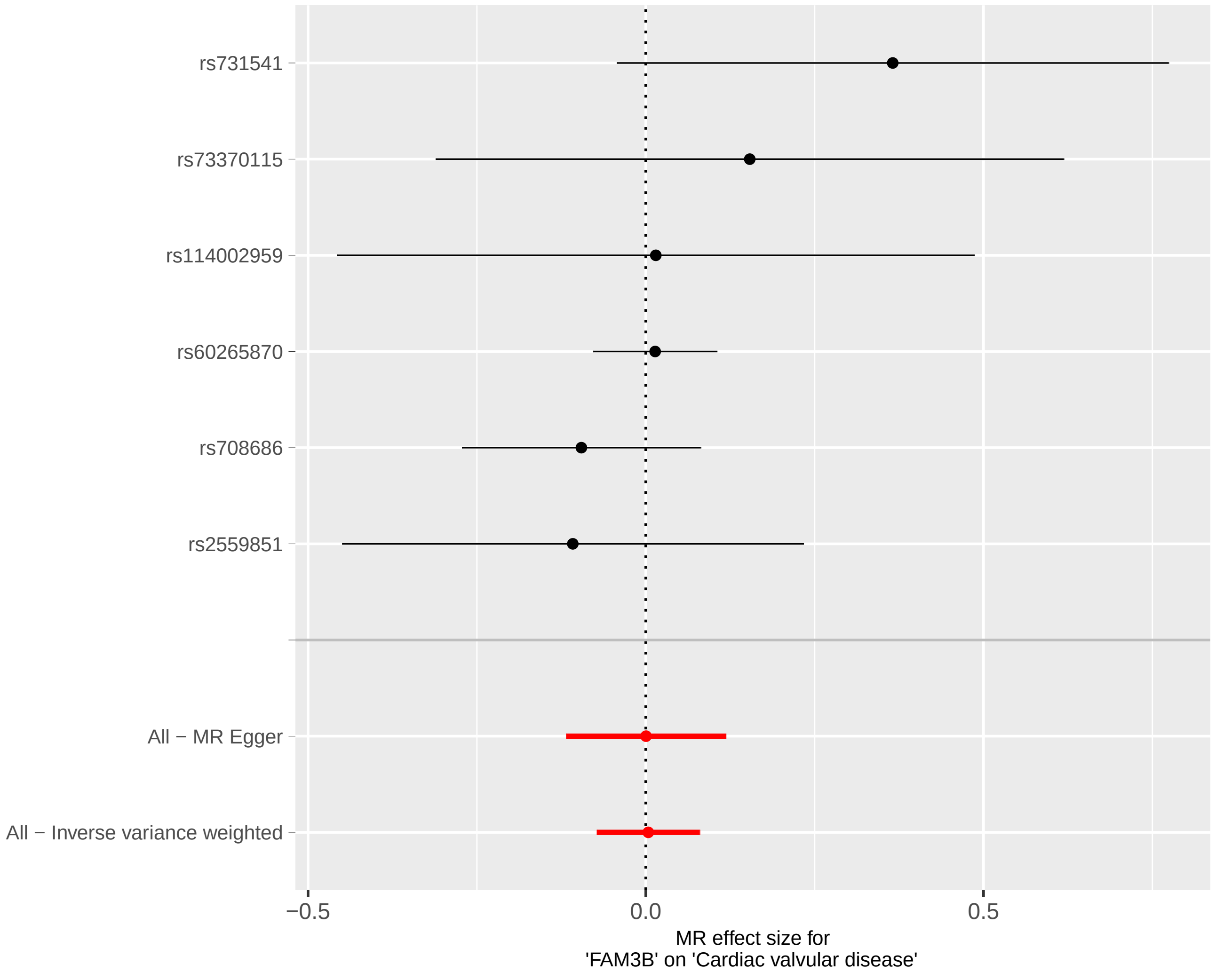
rs731541
rs73370115
rs114002959
rs60265870
rs708686
rs2559851
All − MR Egger
All − Inverse variance weighted
−0.5
0.0
0.5
MR effect size for
'FAM3B' on 'Cardiac valvular disease'

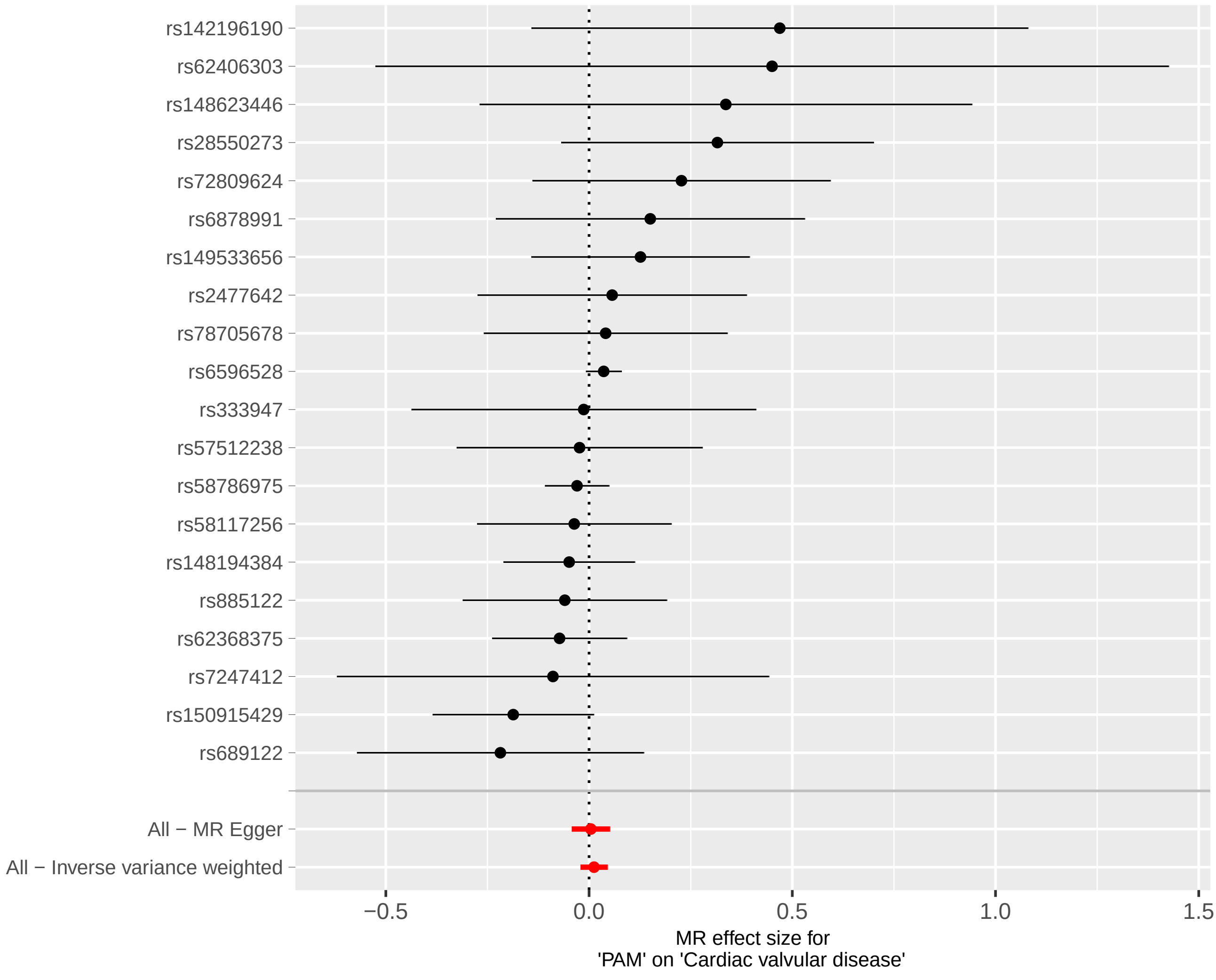
rs142196190
rs62406303
rs148623446
rs28550273
rs72809624
rs6878991
rs149533656
rs2477642
rs78705678
rs6596528
rs333947
rs57512238
rs58786975
rs58117256
rs148194384
rs885122
rs62368375
rs7247412
rs150915429
rs689122
All − MR Egger
All − Inverse variance weighted
−0.5
0.0
0.5
1.0
1.5
MR effect size for
'PAM' on 'Cardiac valvular disease'

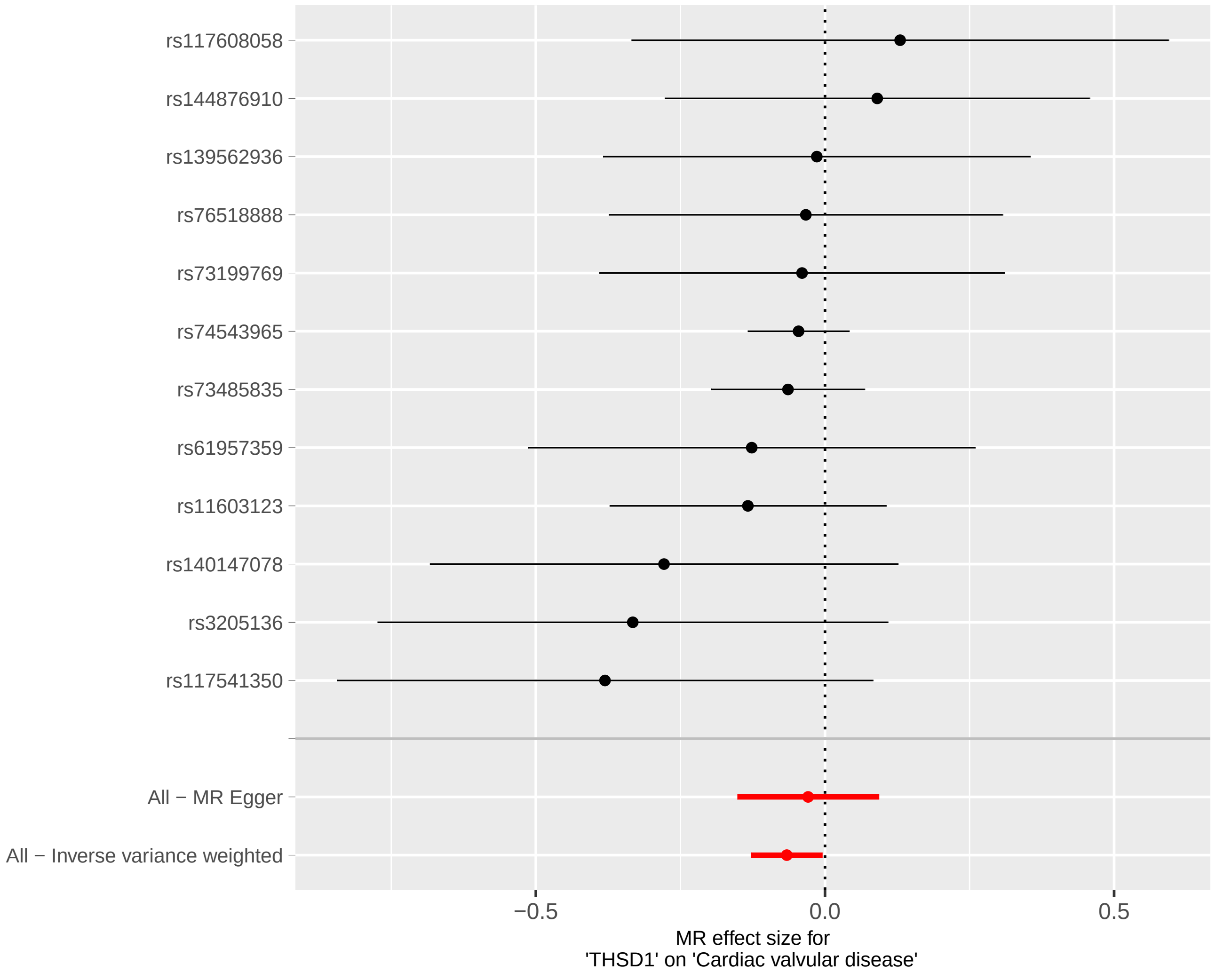
rs117608058
rs144876910
rs139562936
rs76518888
rs73199769
rs74543965
rs73485835
rs61957359
rs11603123
rs140147078
rs3205136
rs117541350
All − MR Egger
All − Inverse variance weighted
−0.5
0.0
0.5
MR effect size for
'THSD1' on 'Cardiac valvular disease'

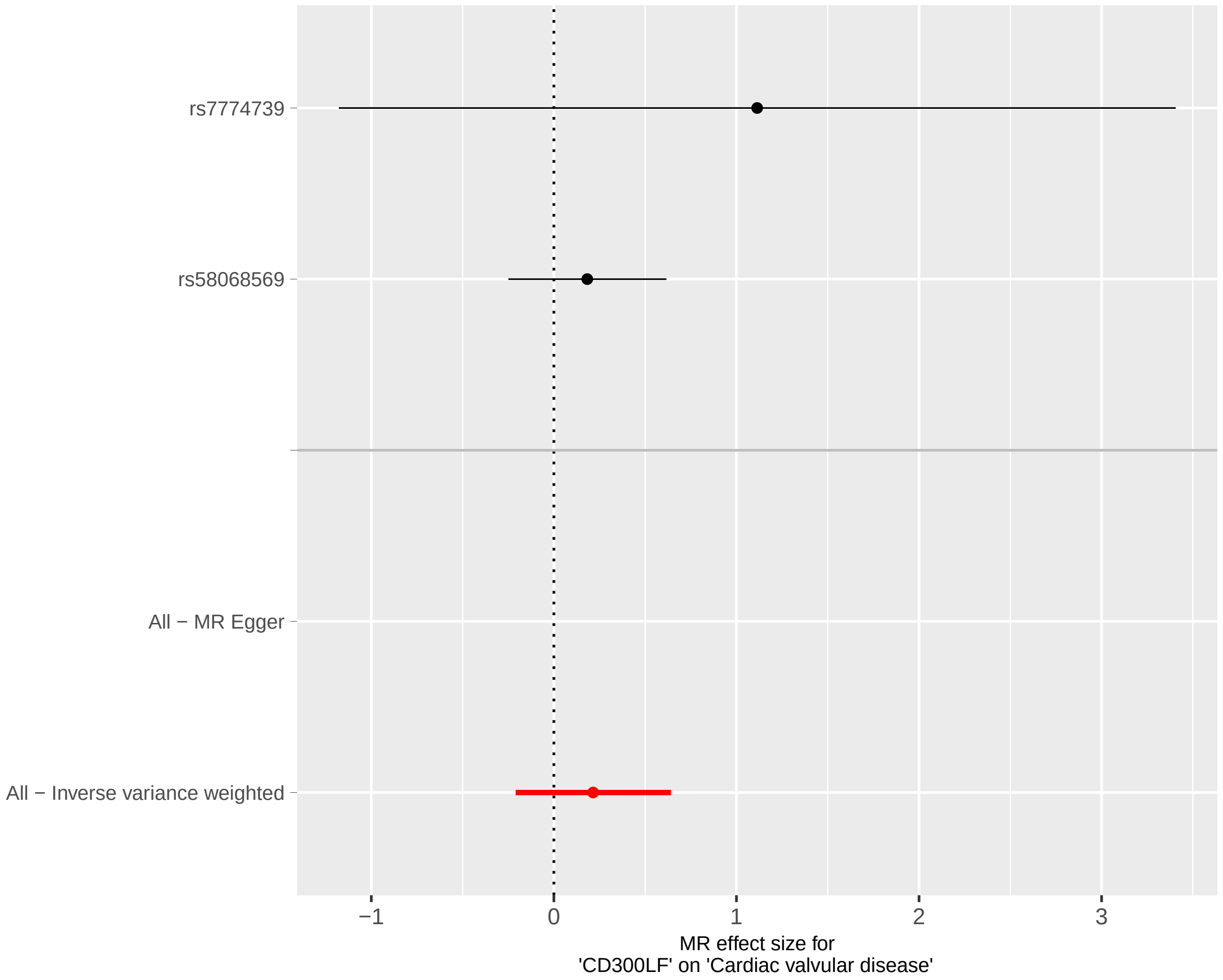
rs7774739
rs58068569
All − MR Egger
All − Inverse variance weighted
−1
0
1
2
3
MR effect size for
'CD300LF' on 'Cardiac valvular disease'

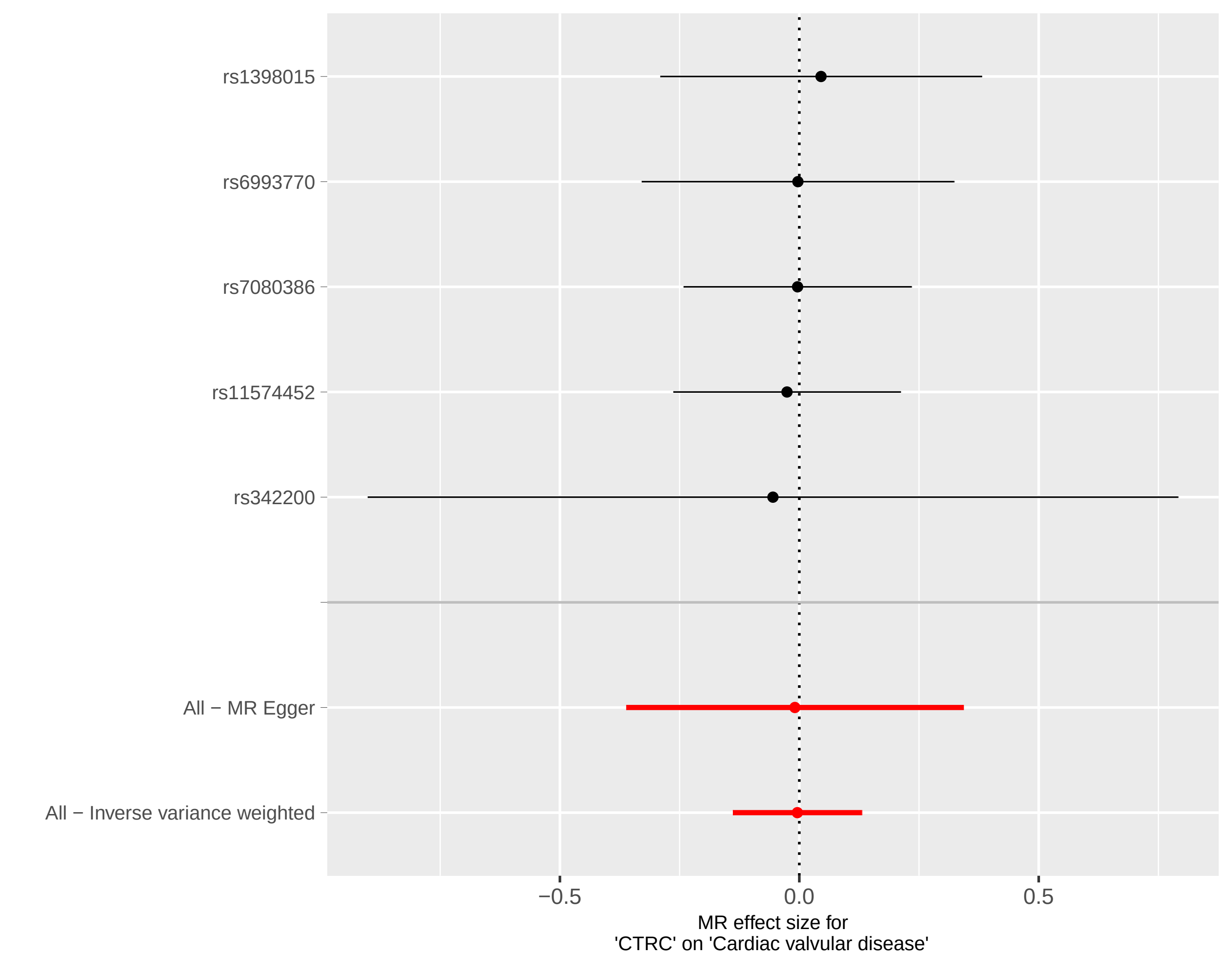
rs1398015
rs6993770
rs7080386
rs11574452
rs342200
All − MR Egger
All − Inverse variance weighted
−0.5
0.0
0.5
MR effect size for
'CTRC' on 'Cardiac valvular disease'

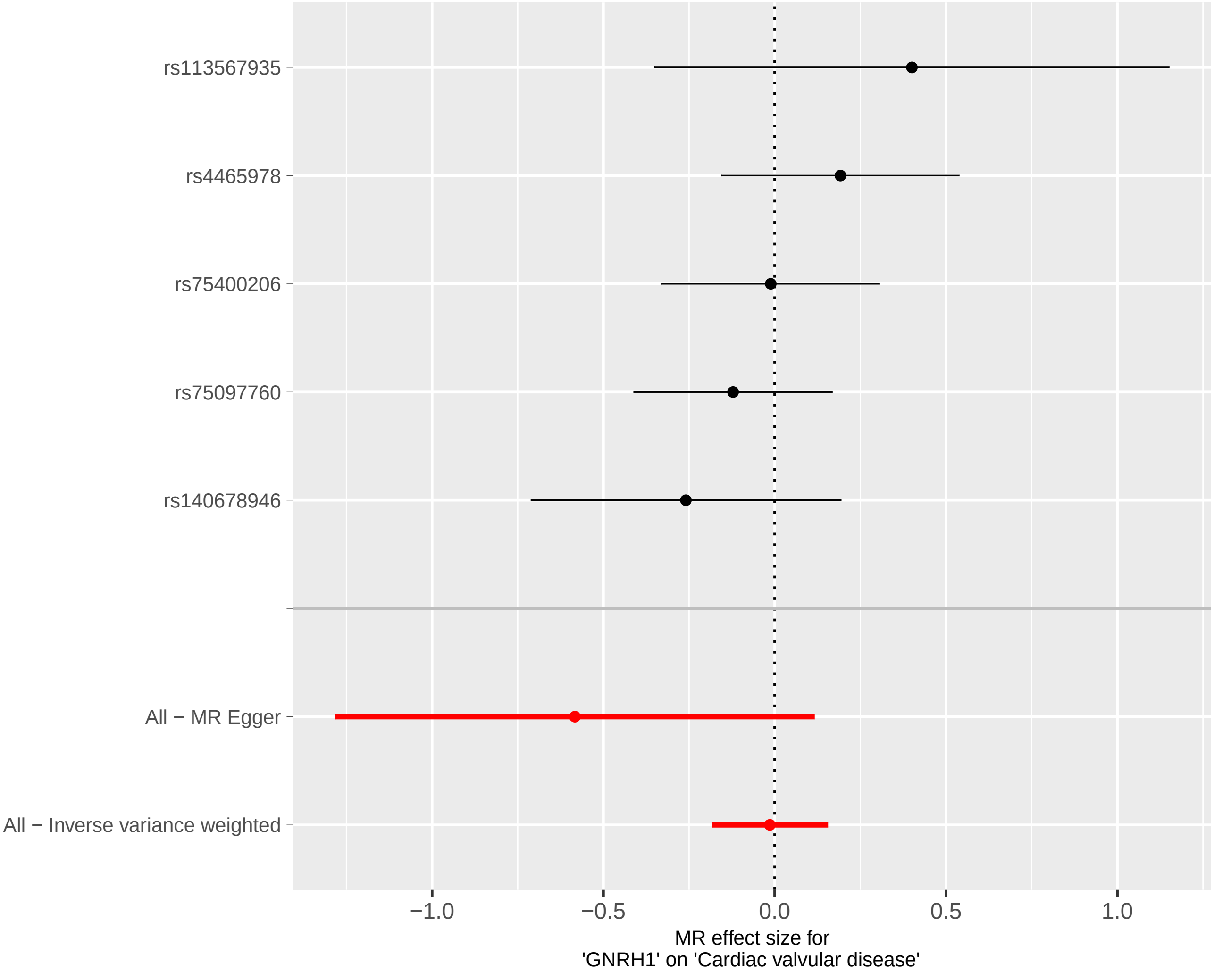

rs113567935
rs4465978
rs75400206
rs75097760
rs140678946
All − MR Egger
All − Inverse variance weighted
−1.0
−0.5
0.0
0.5
1.0
MR effect size for
'GNRH1' on 'Cardiac valvular disease'

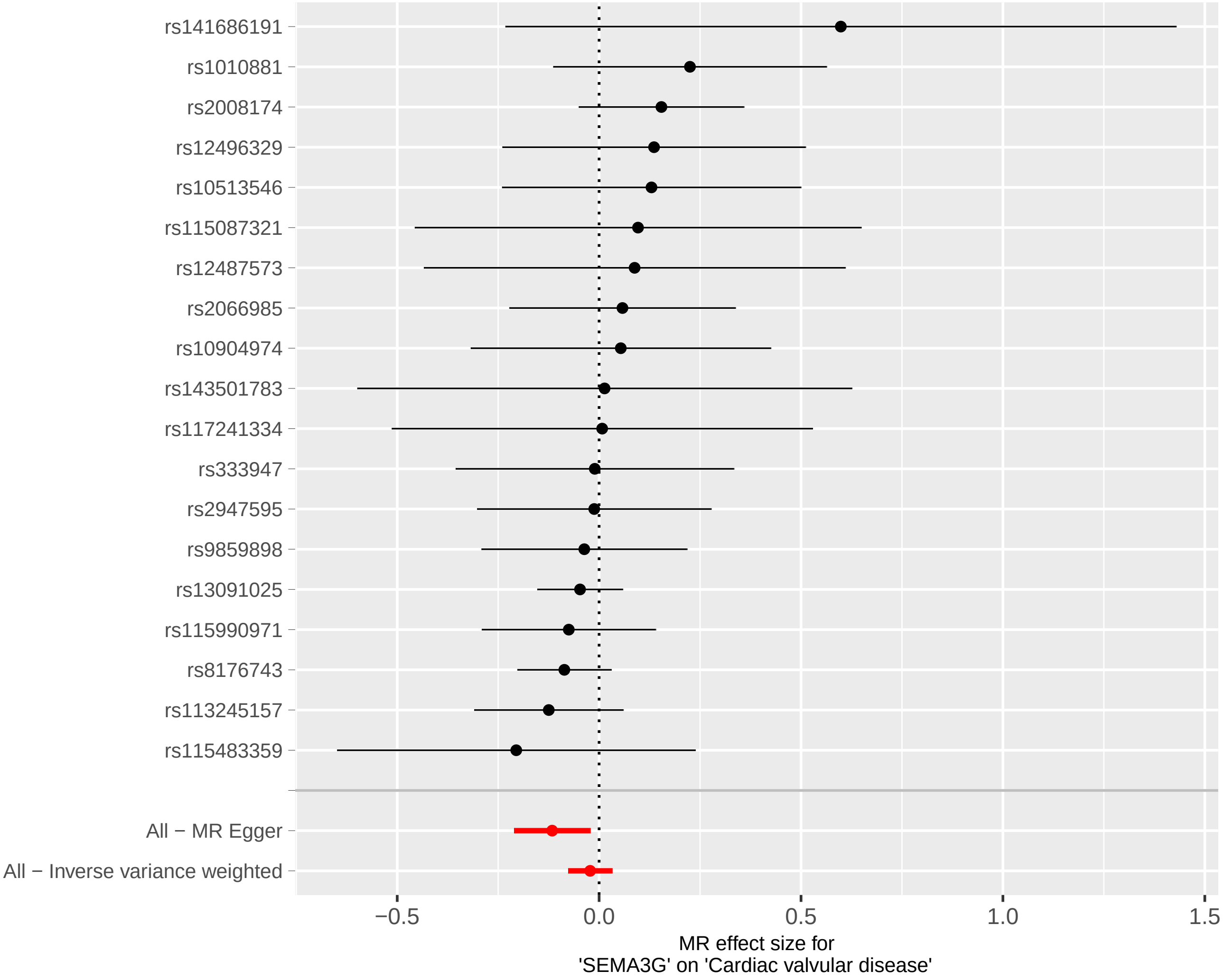
rs141686191
rs1010881
rs2008174
rs12496329
rs10513546
rs115087321
rs12487573
rs2066985
rs10904974
rs143501783
rs117241334
rs333947
rs2947595
rs9859898
rs13091025
rs115990971
rs8176743
rs113245157
rs115483359
All − MR Egger
All − Inverse variance weighted
−0.5
0.0
0.5
1.0
1.5
MR effect size for
'SEMA3G' on 'Cardiac valvular disease'

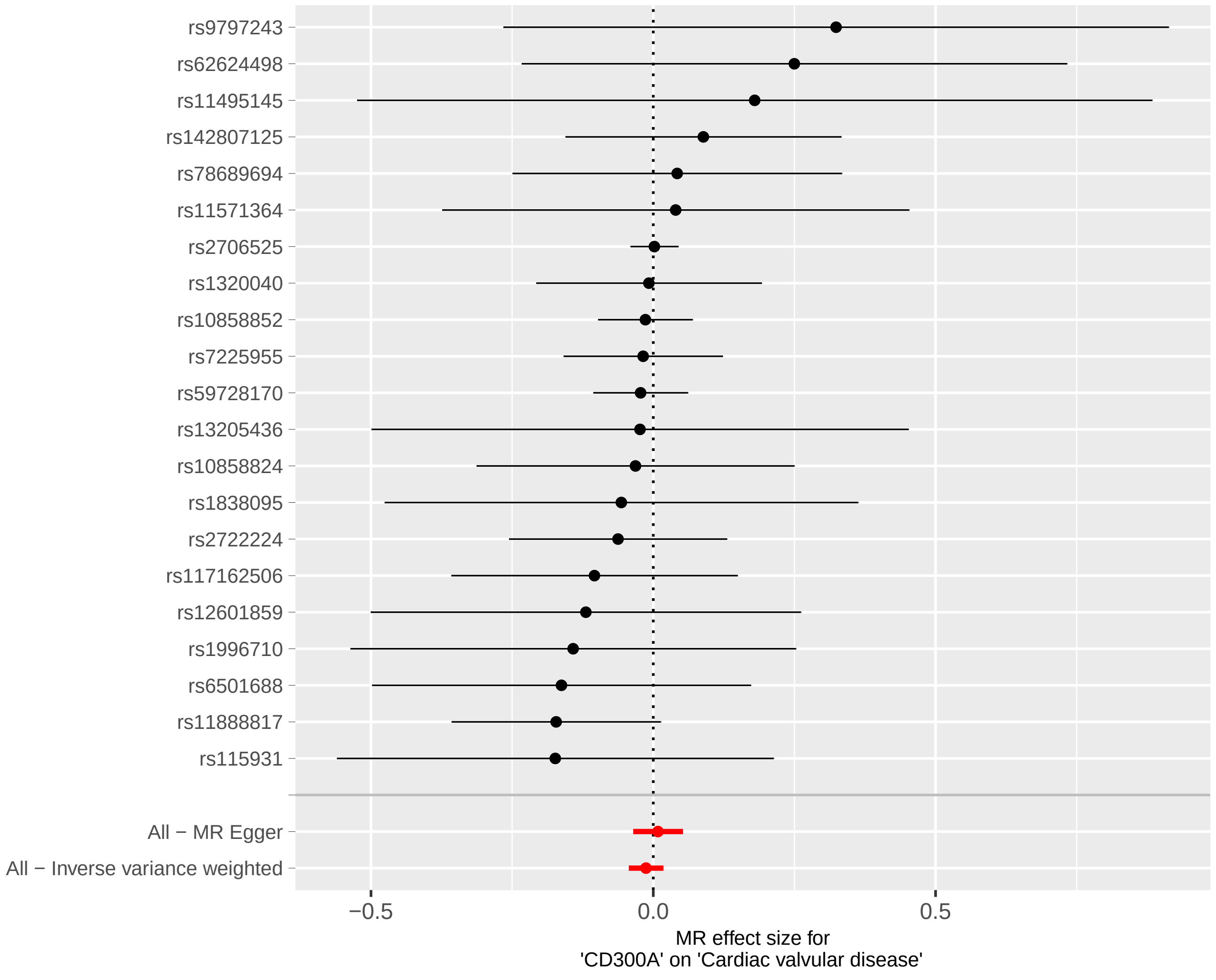
rs9797243
rs62624498
rs11495145
rs142807125
rs78689694
rs11571364
rs2706525
rs1320040
rs10858852
rs7225955
rs59728170
rs13205436
rs10858824
rs1838095
rs2722224
rs117162506
rs12601859
rs1996710
rs6501688
rs11888817
rs115931
All − MR Egger
All − Inverse variance weighted
−0.5
0.0
0.5
MR effect size for
'CD300A' on 'Cardiac valvular disease'

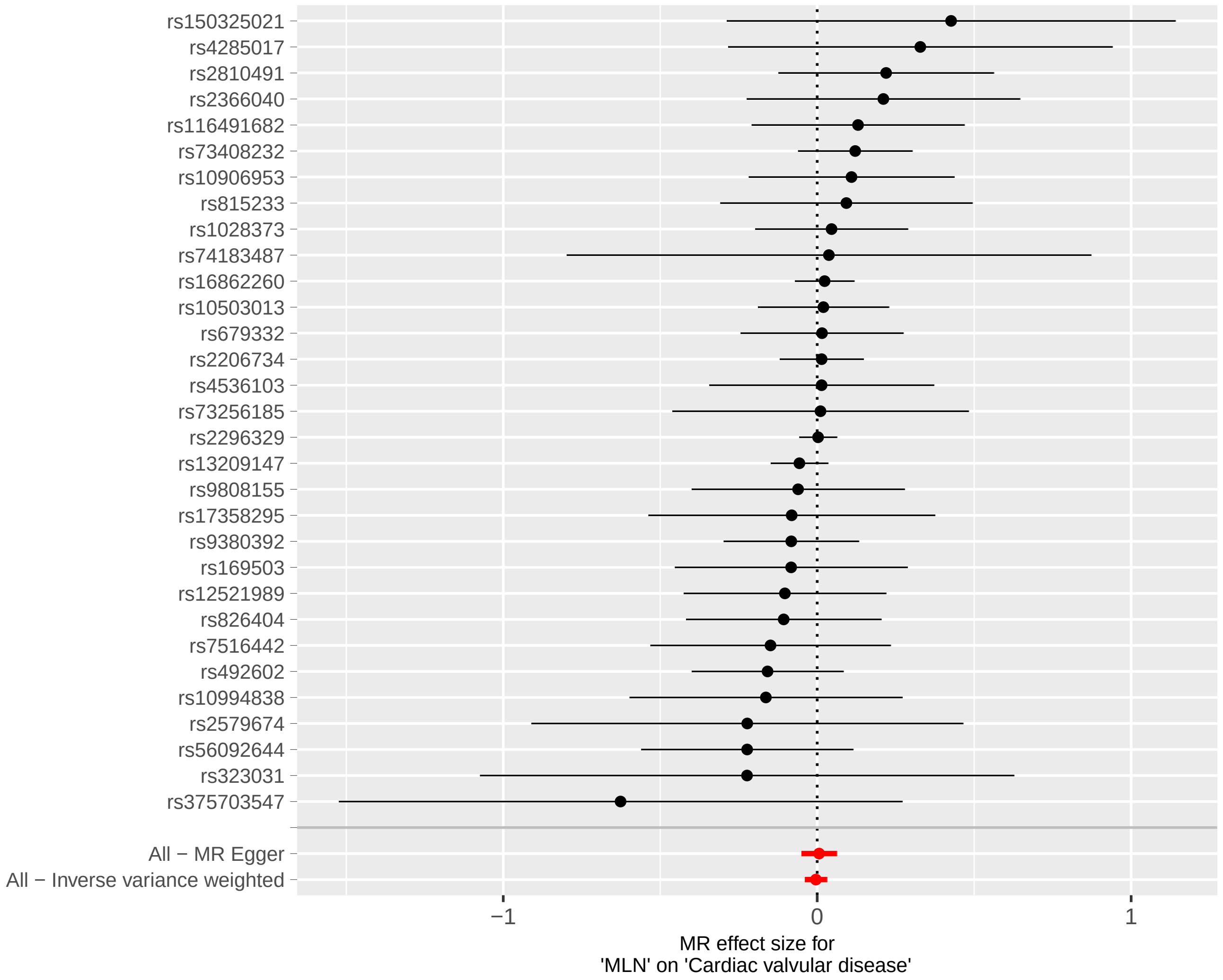
rs150325021
rs4285017
rs2810491
rs2366040
rs116491682
rs73408232
rs10906953
rs815233
rs1028373
rs74183487
rs16862260
rs10503013
rs679332
rs2206734
rs4536103
rs73256185
rs2296329
rs13209147
rs9808155
rs17358295
rs9380392
rs169503
rs12521989
rs826404
rs7516442
rs492602
rs10994838
rs2579674
rs56092644
rs323031
rs375703547
All − MR Egger
All − Inverse variance weighted
−1
0
1
MR effect size for
'MLN' on 'Cardiac valvular disease'

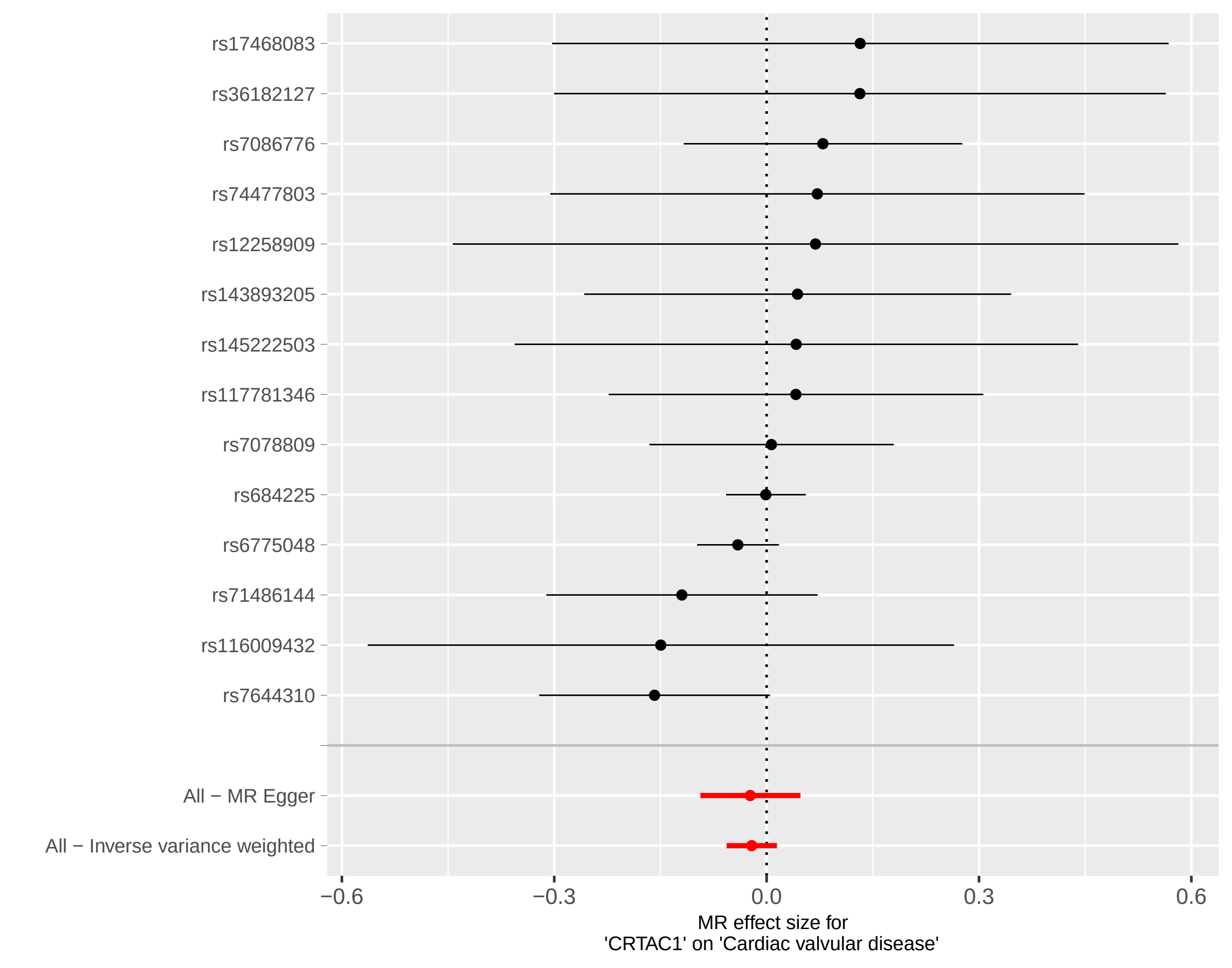

rs17468083
rs36182127
rs7086776
rs74477803
rs12258909
rs143893205
rs145222503
rs117781346
rs7078809
rs684225
rs6775048
rs71486144
rs116009432
rs7644310
All − MR Egger
All − Inverse variance weighted
−0.6
−0.3
0.0
0.3
0.6
MR effect size for
'CRTAC1' on 'Cardiac valvular disease'

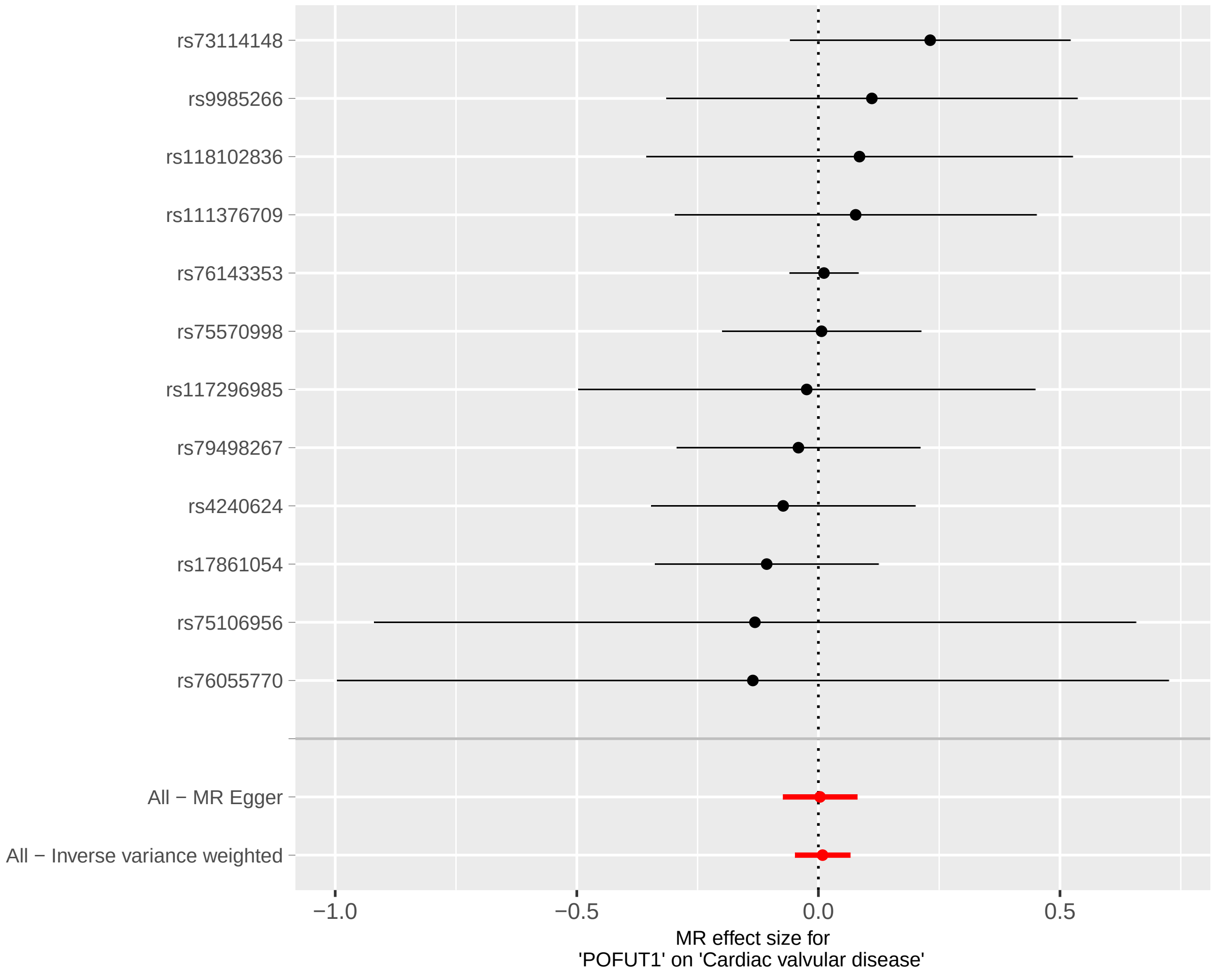
rs73114148
rs9985266
rs118102836
rs111376709
rs76143353
rs75570998
rs117296985
rs79498267
rs4240624
rs17861054
rs75106956
rs76055770
All − MR Egger
All − Inverse variance weighted
−1.0
−0.5
0.0
0.5
MR effect size for
'POFUT1' on 'Cardiac valvular disease'

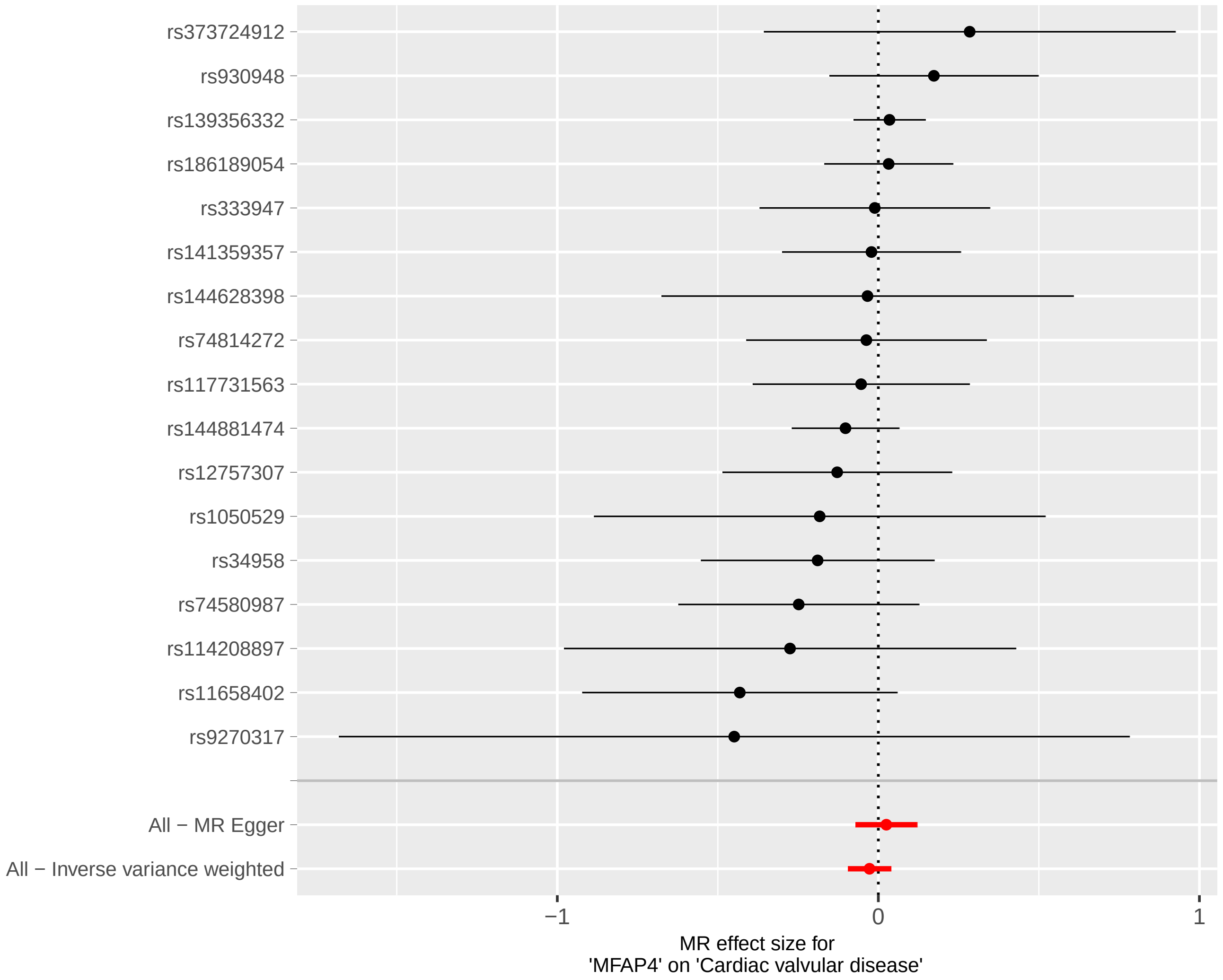
rs373724912
rs930948
rs139356332
rs186189054
rs333947
rs141359357
rs144628398
rs74814272
rs117731563
rs144881474
rs12757307
rs1050529
rs34958
rs74580987
rs114208897
rs11658402
rs9270317
All − MR Egger
All − Inverse variance weighted
−1
0
1
MR effect size for
'MFAP4' on 'Cardiac valvular disease'

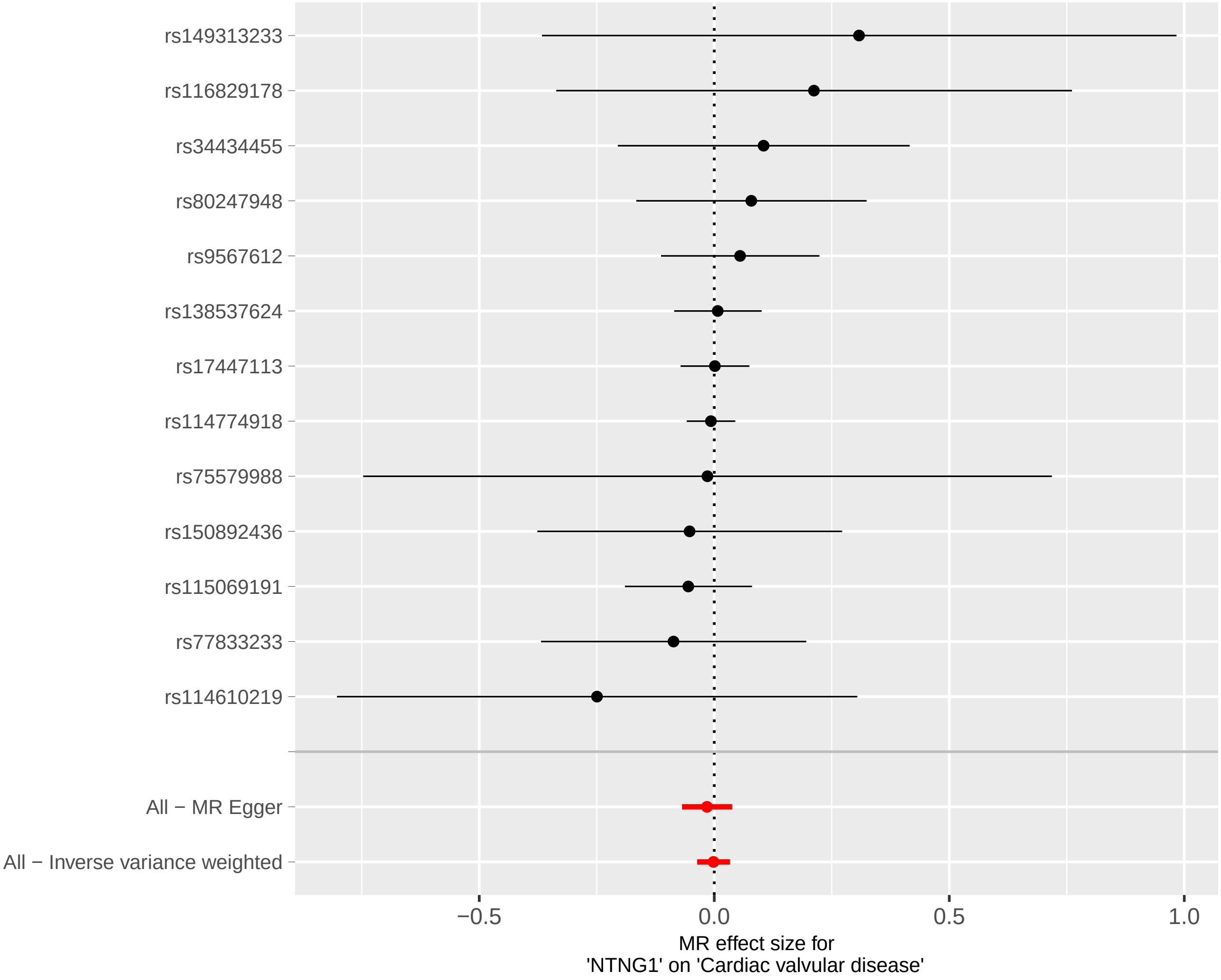
rs149313233
rs116829178
rs34434455
rs80247948
rs9567612
rs138537624
rs17447113
rs114774918
rs75579988
rs150892436
rs115069191
rs77833233
rs114610219
All − MR Egger
All − Inverse variance weighted
−0.5
0.0
0.5
1.0
MR effect size for
'NTNG1' on 'Cardiac valvular disease'

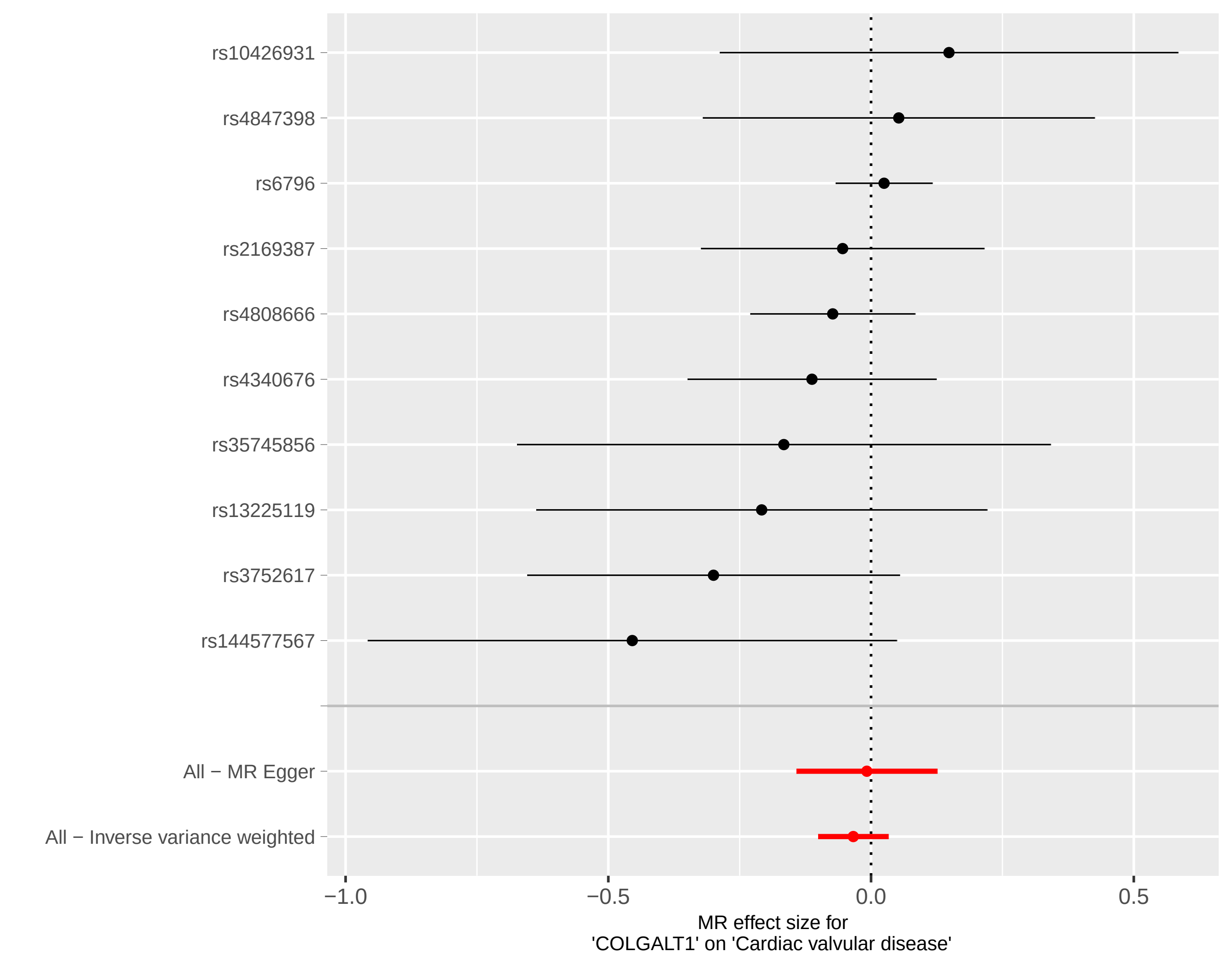
rs10426931
rs4847398
rs6796
rs2169387
rs4808666
rs4340676
rs35745856
rs13225119
rs3752617
rs144577567
All − MR Egger
All − Inverse variance weighted
−1.0
−0.5
0.0
0.5
MR effect size for
'COLGALT1' on 'Cardiac valvular disease'

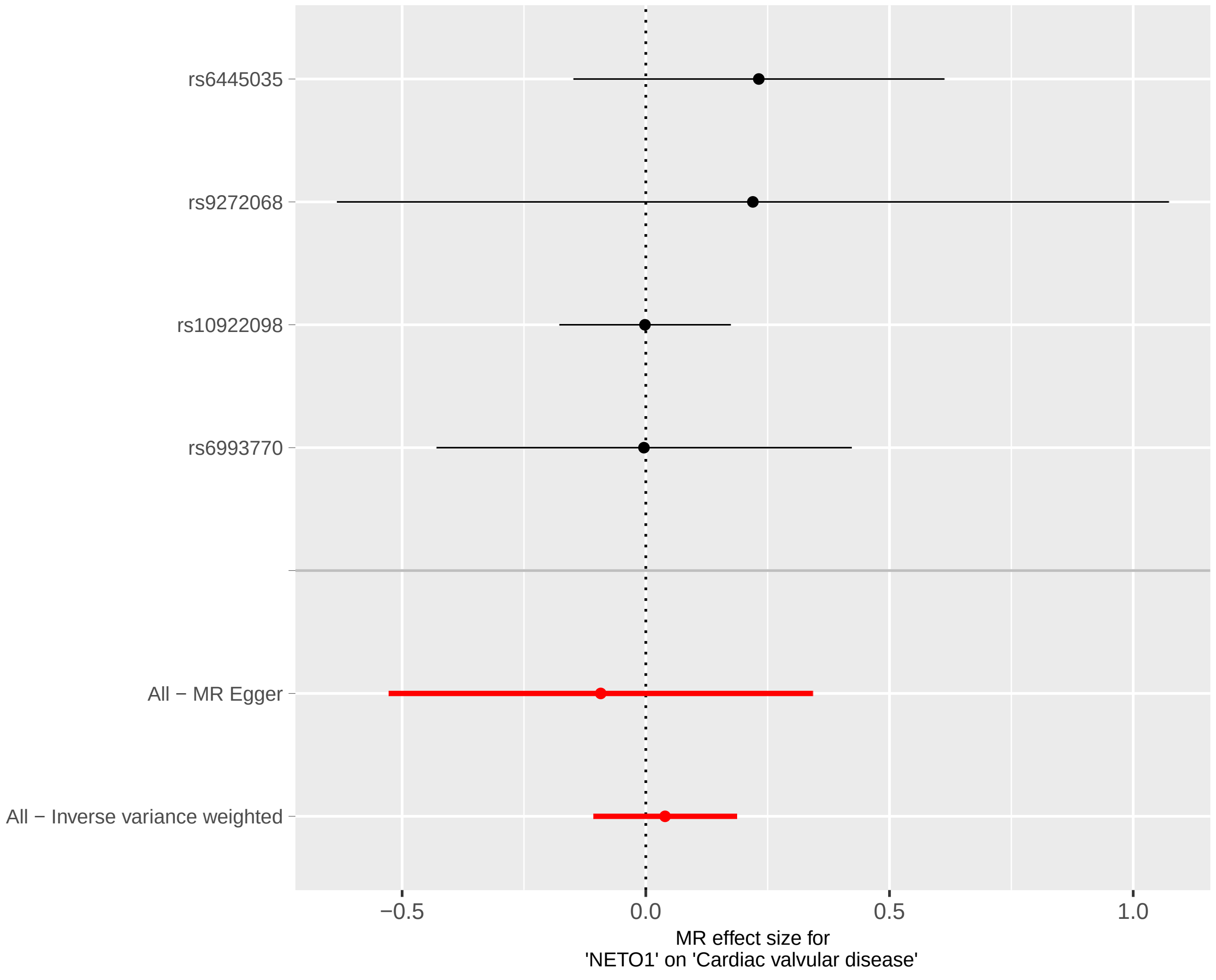
rs6445035
rs9272068
rs10922098
rs6993770
All − MR Egger
All − Inverse variance weighted
−0.5
0.0
0.5
1.0
MR effect size for
'NETO1' on 'Cardiac valvular disease'

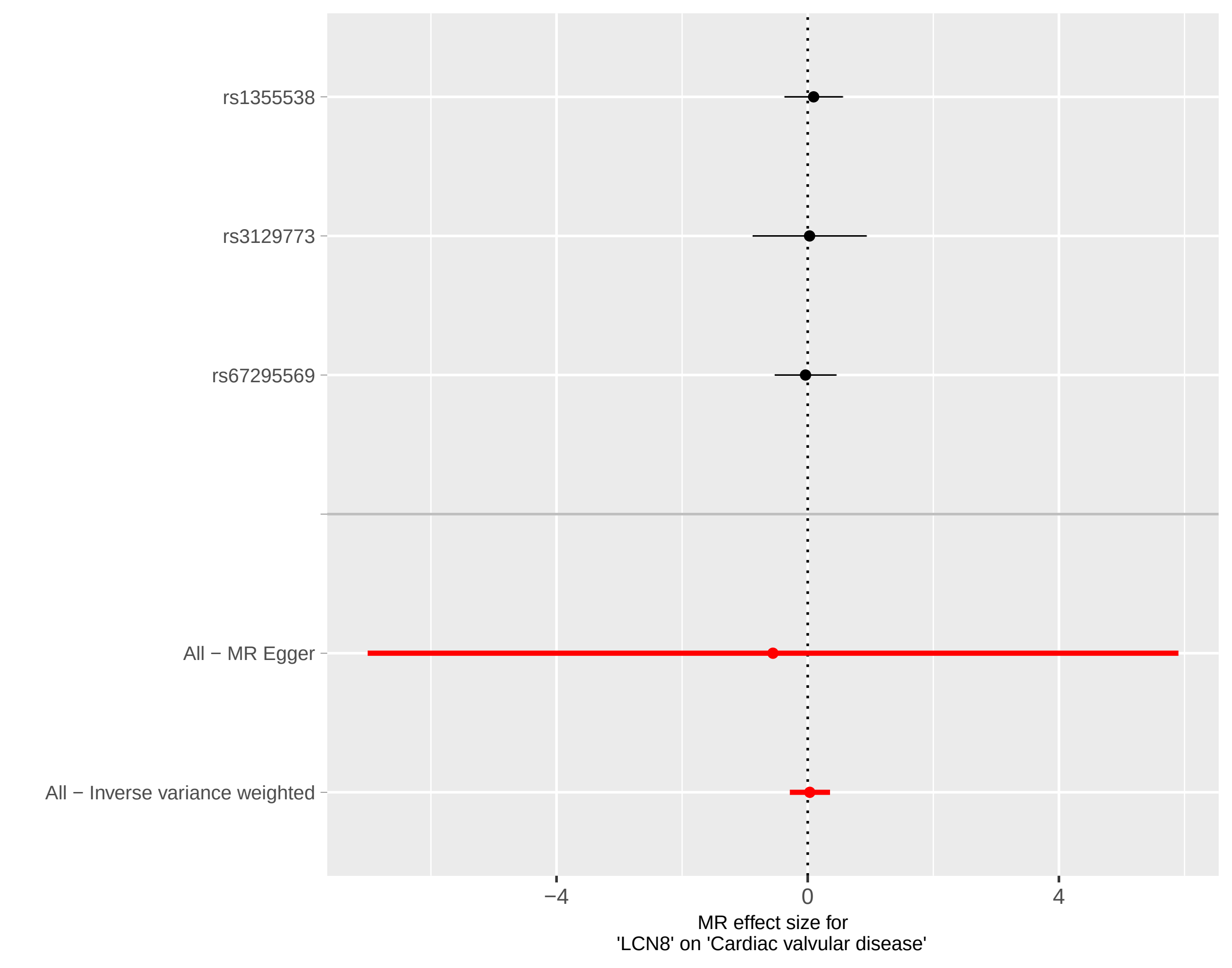
rs1355538
rs3129773
rs67295569
All − MR Egger
All − Inverse variance weighted
−4
0
4
MR effect size for
'LCN8' on 'Cardiac valvular disease'

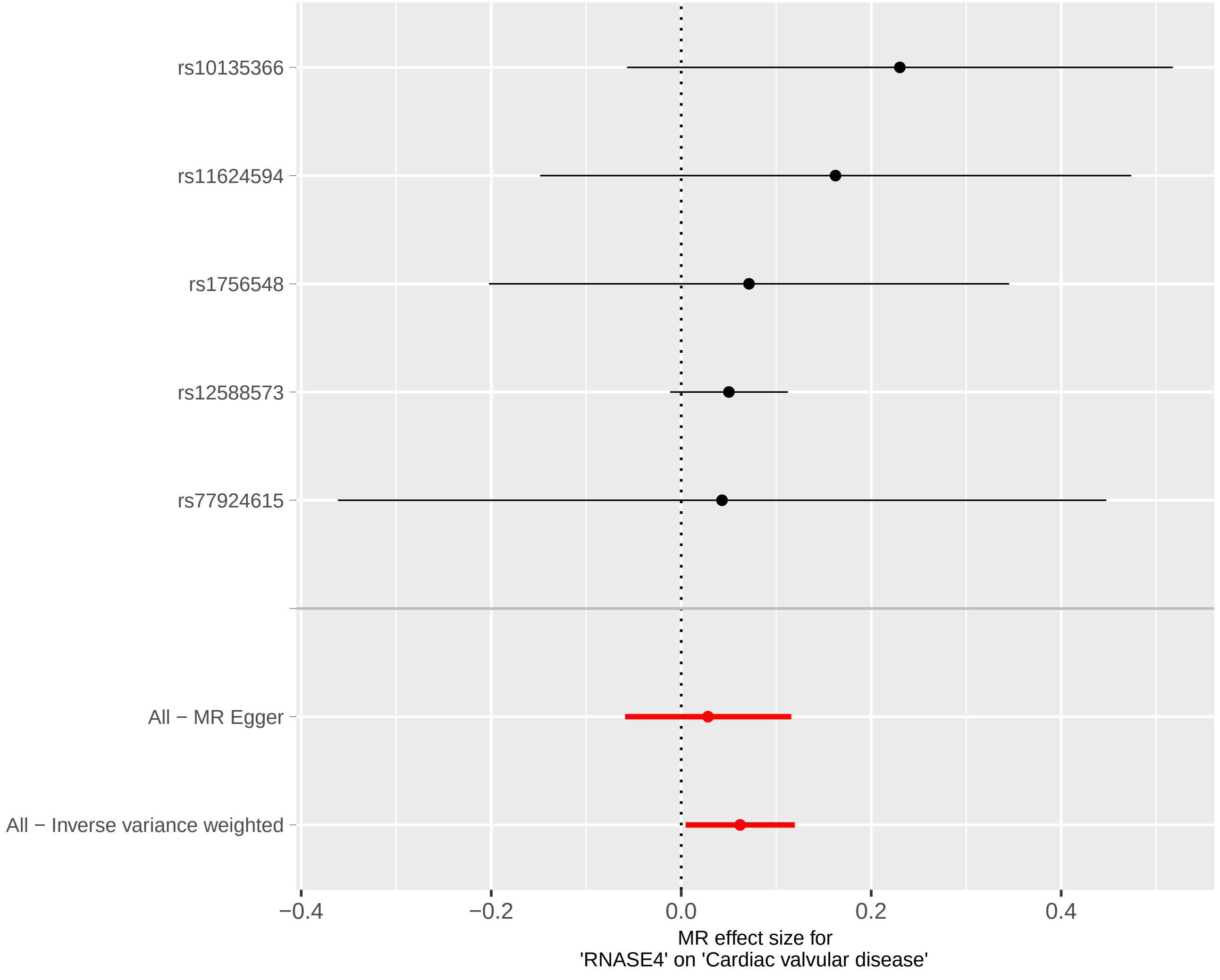
rs10135366
rs11624594
rs1756548
rs12588573
rs77924615
All − MR Egger
All − Inverse variance weighted
−0.4
−0.2
0.0
0.2
0.4
MR effect size for
'RNASE4' on 'Cardiac valvular disease'

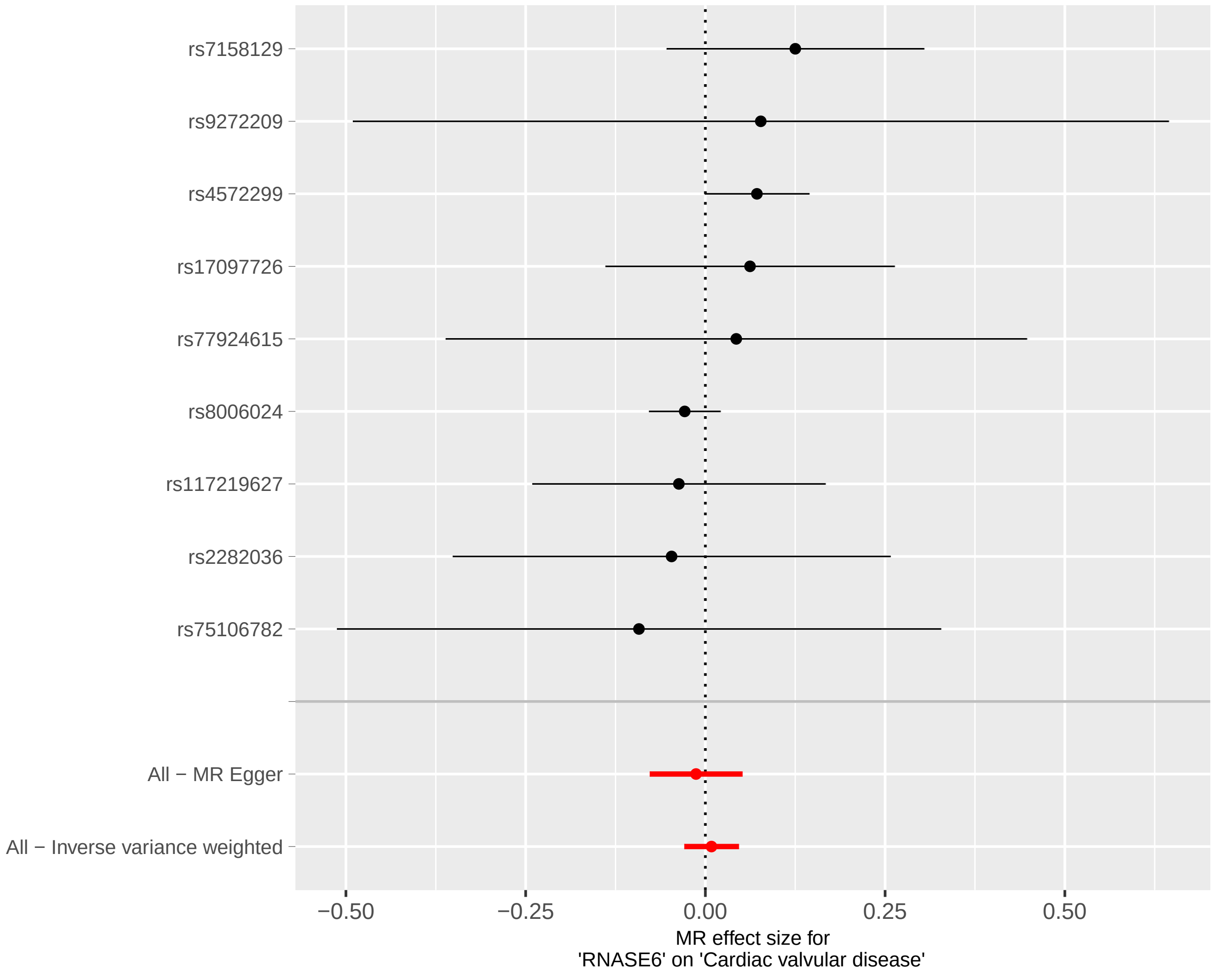
rs7158129
rs9272209
rs4572299
rs17097726
rs77924615
rs8006024
rs117219627
rs2282036
rs75106782
All − MR Egger
All − Inverse variance weighted
−0.50
−0.25
0.00
0.25
0.50
MR effect size for
'RNASE6' on 'Cardiac valvular disease'

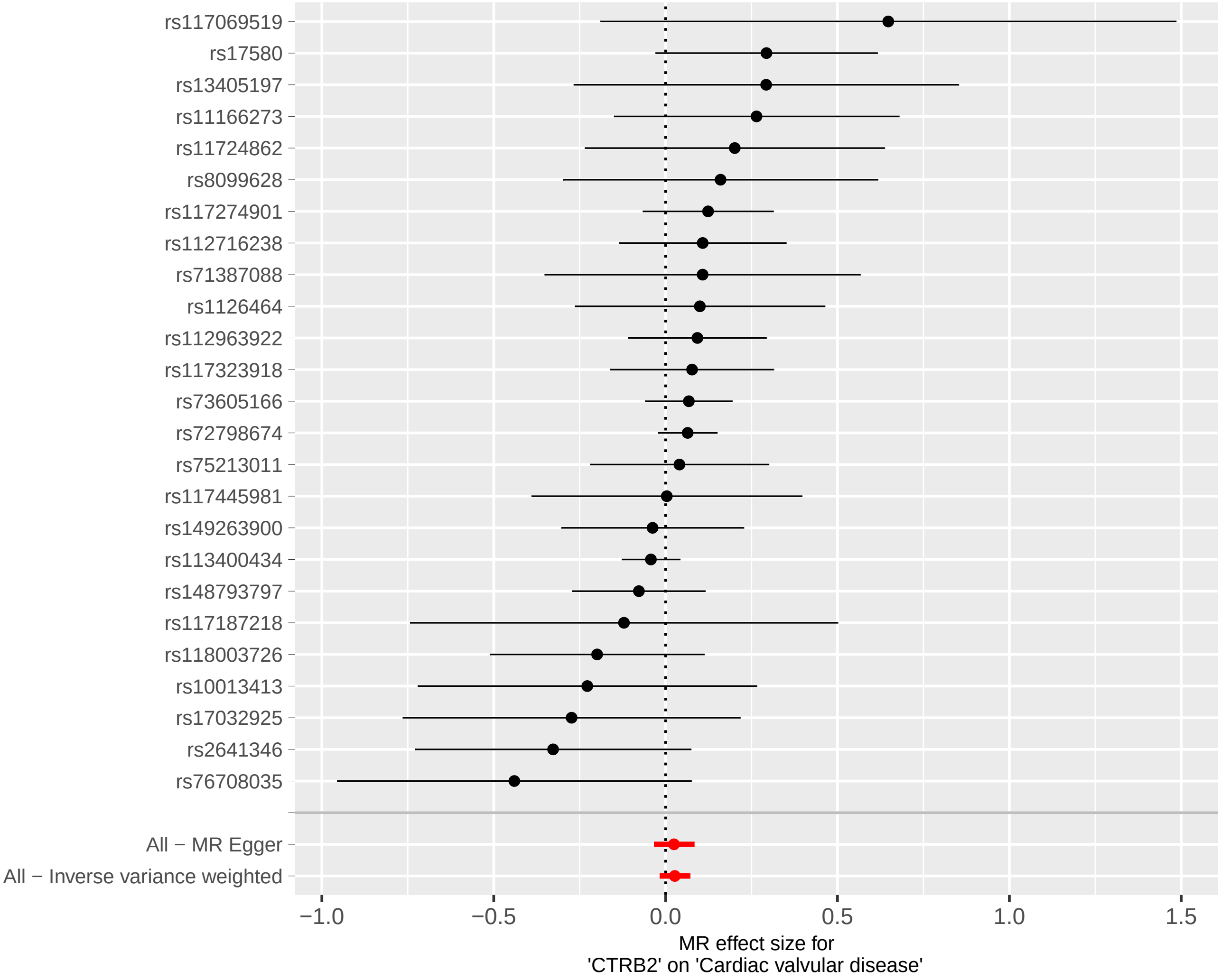
rs117069519
rs17580
rs13405197
rs11166273
rs11724862
rs8099628
rs117274901
rs112716238
rs71387088
rs1126464
rs112963922
rs117323918
rs73605166
rs72798674
rs75213011
rs117445981
rs149263900
rs113400434
rs148793797
rs117187218
rs118003726
rs10013413
rs17032925
rs2641346
rs76708035
All − MR Egger
All − Inverse variance weighted
−1.0
−0.5
0.0
0.5
1.0
1.5
MR effect size for
'CTRB2' on 'Cardiac valvular disease'

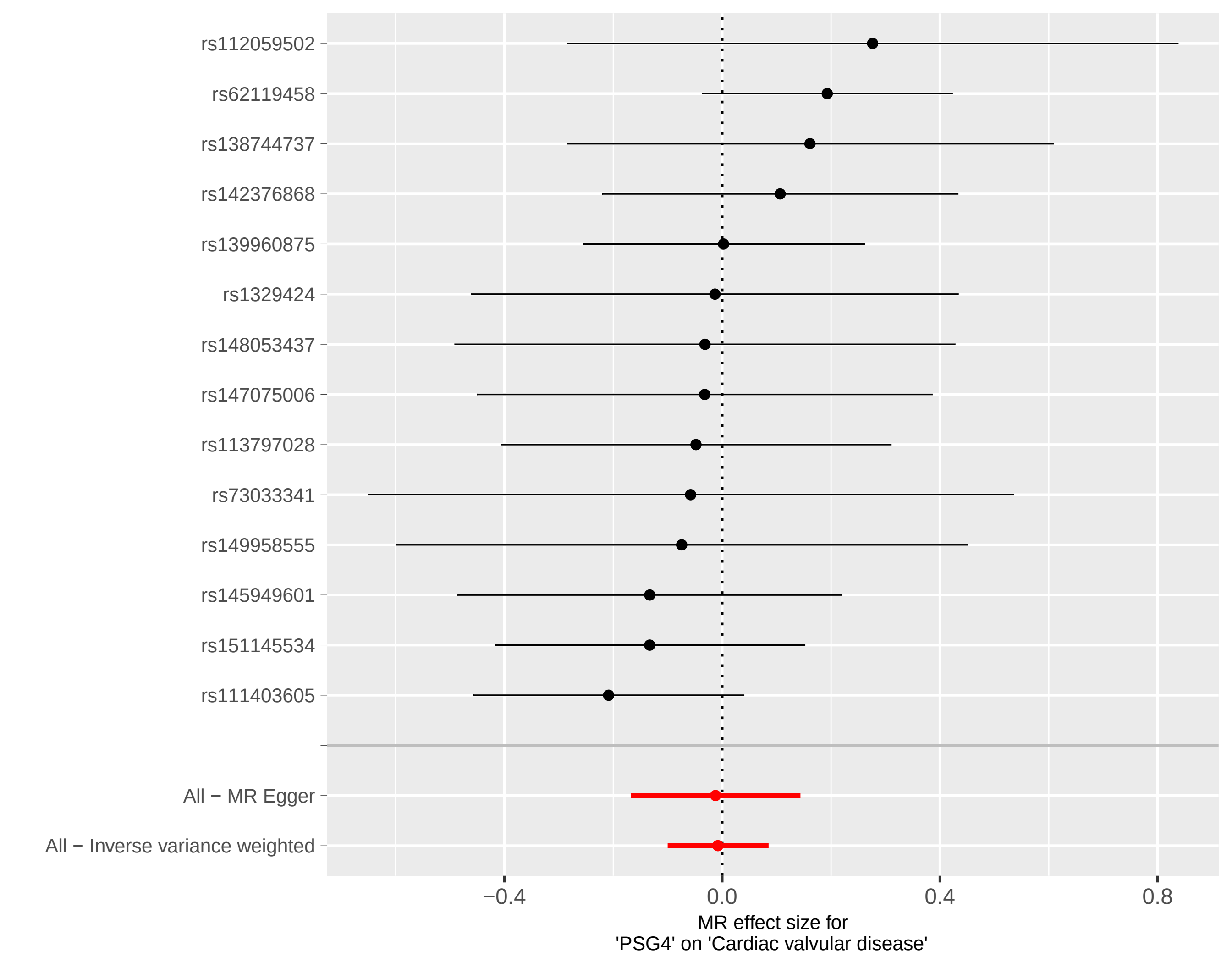
rs112059502
rs62119458
rs138744737
rs142376868
rs139960875
rs1329424
rs148053437
rs147075006
rs113797028
rs73033341
rs149958555
rs145949601
rs151145534
rs111403605
All − MR Egger
All − Inverse variance weighted
−0.4
0.0
0.4
0.8
MR effect size for
'PSG4' on 'Cardiac valvular disease'

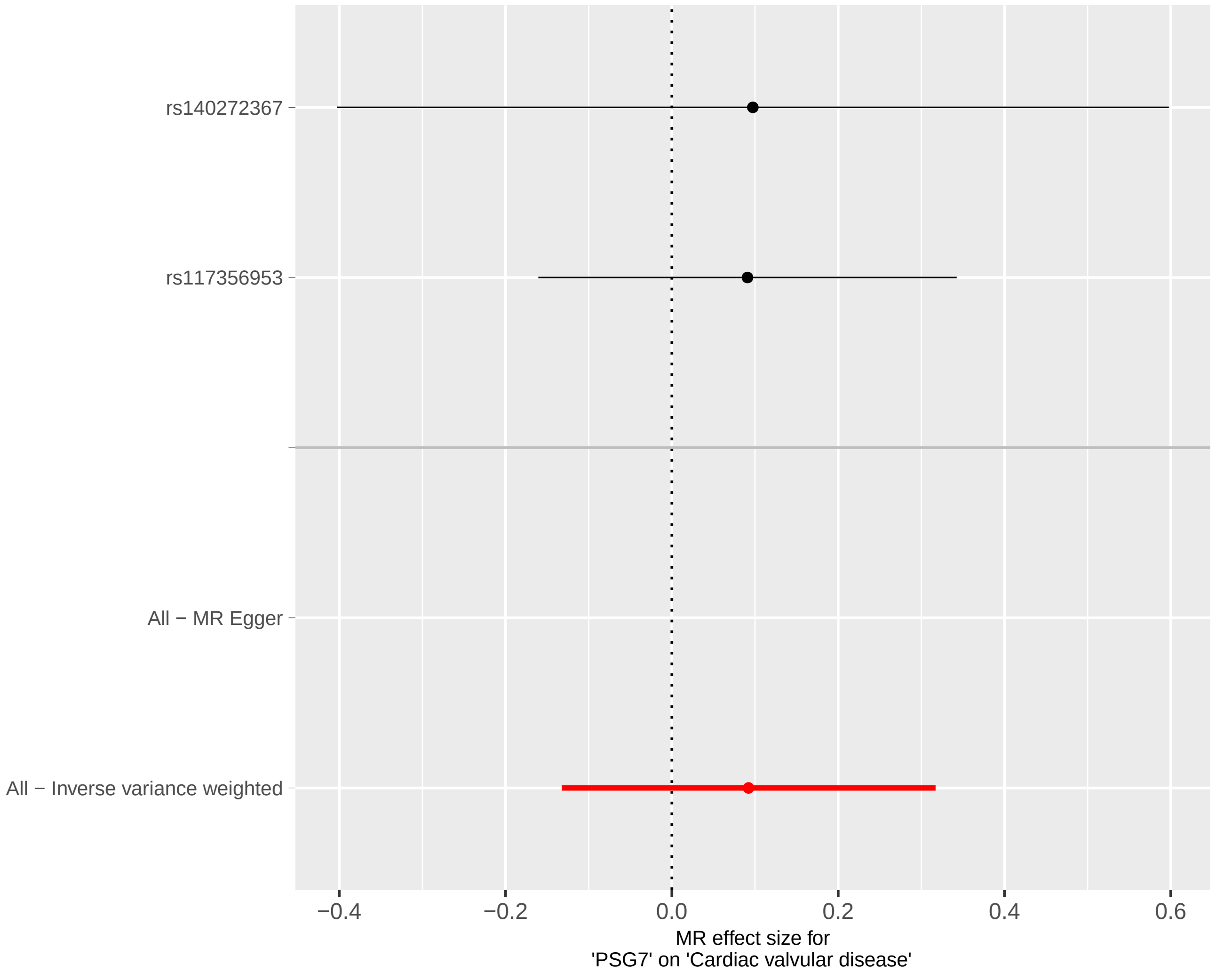
rs140272367
rs117356953
All − MR Egger
All − Inverse variance weighted
−0.4
−0.2
0.0
0.2
0.4
0.6
MR effect size for
'PSG7' on 'Cardiac valvular disease'

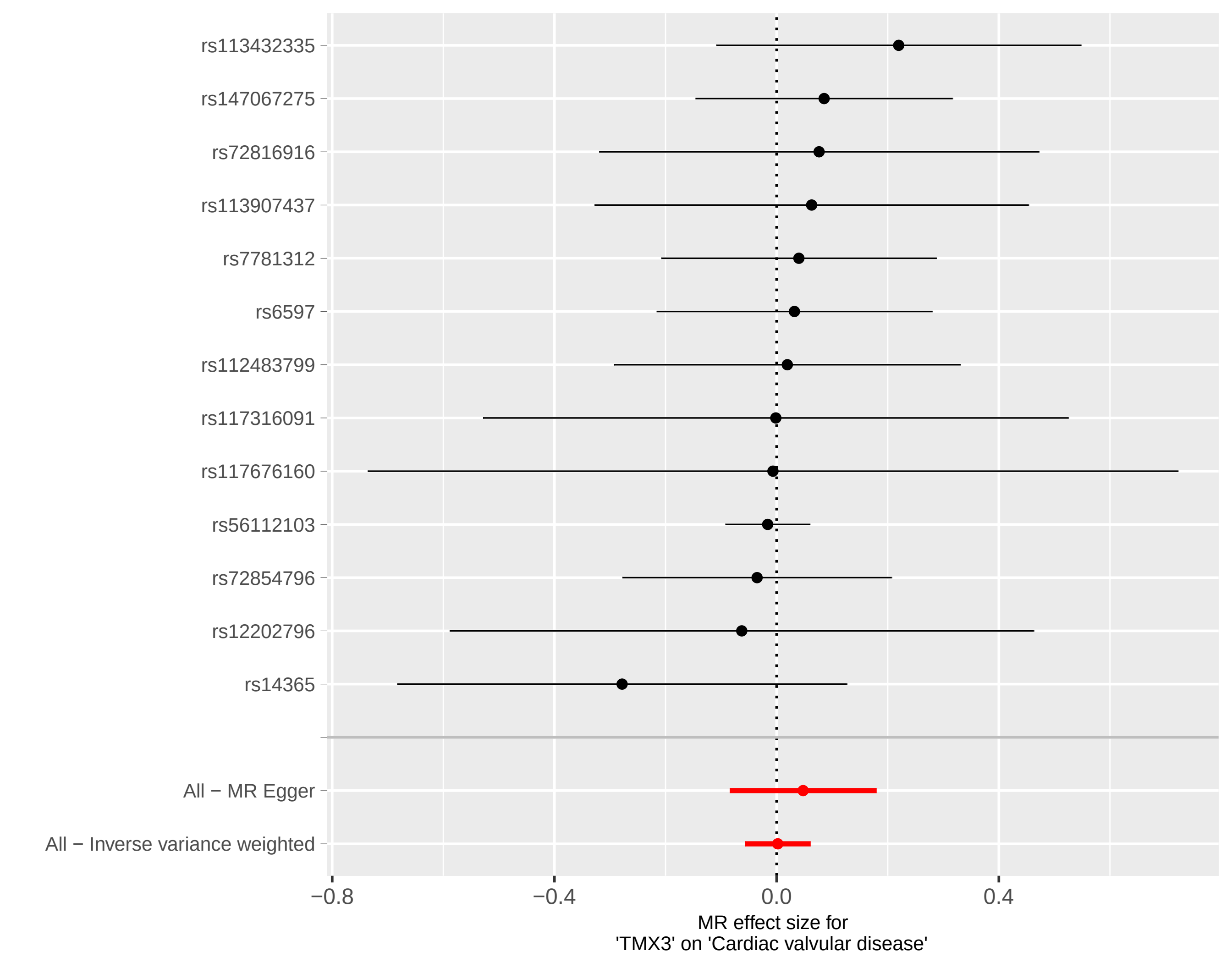
rs113432335
rs147067275
rs72816916
rs113907437
rs7781312
rs6597
rs112483799
rs117316091
rs117676160
rs56112103
rs72854796
rs12202796
rs14365
All − MR Egger
All − Inverse variance weighted
−0.8
−0.4
0.0
0.4
MR effect size for
'TMX3' on 'Cardiac valvular disease'

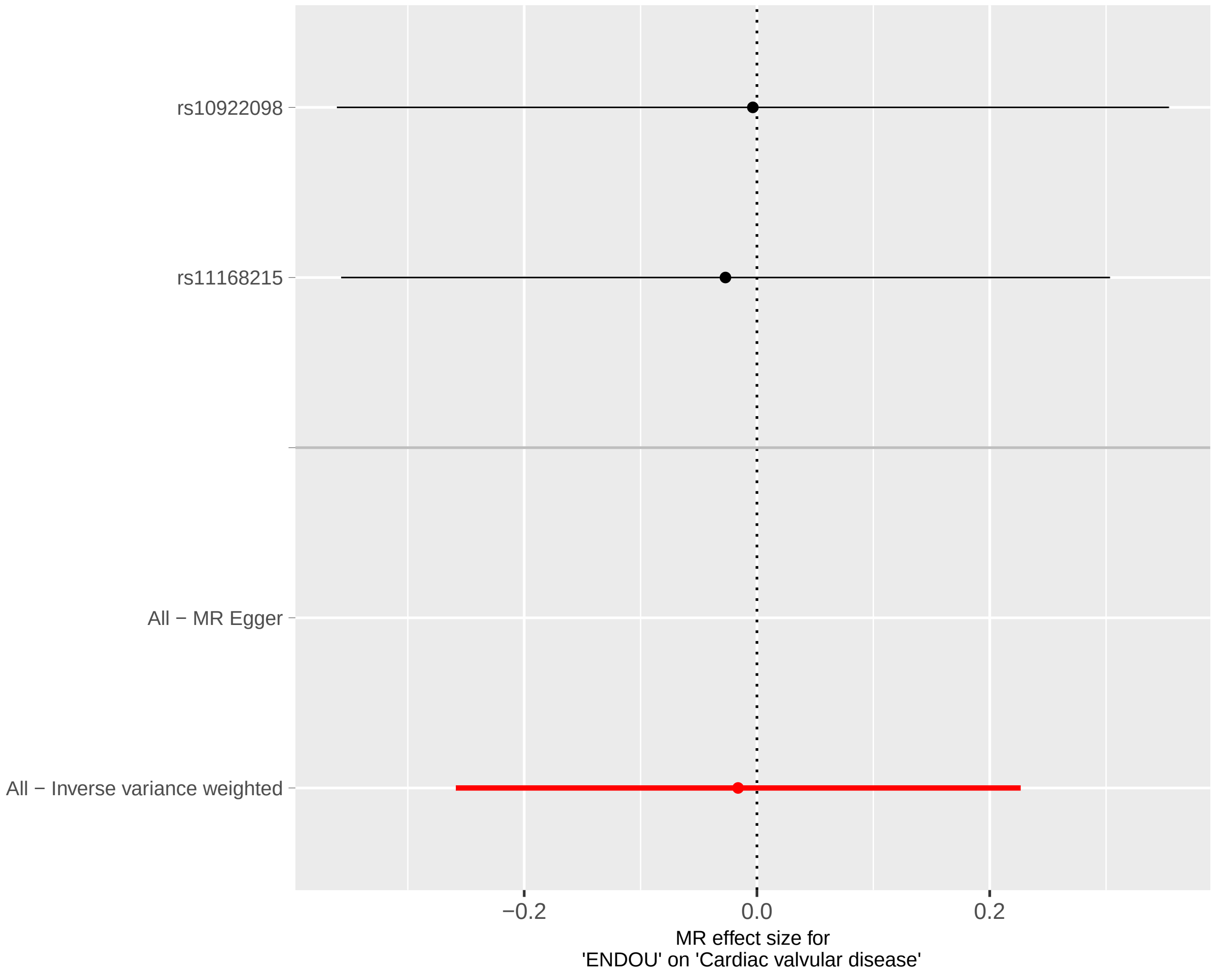

rs10922098
rs11168215
All − MR Egger
All − Inverse variance weighted
−0.2
0.0
0.2
MR effect size for
'ENDOU' on 'Cardiac valvular disease'

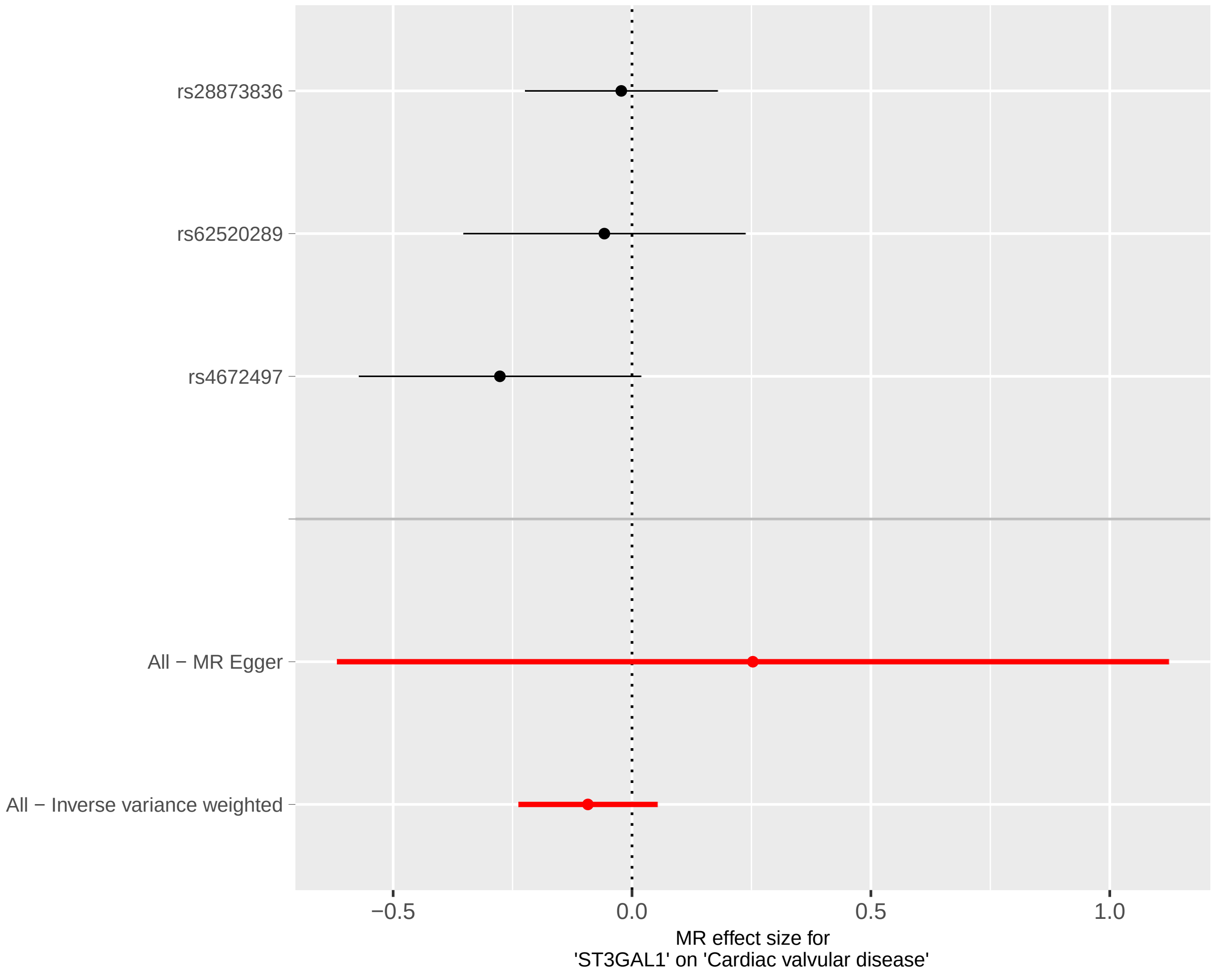

rs28873836
rs62520289
rs4672497
All − MR Egger
All − Inverse variance weighted
−0.5
0.0
0.5
1.0
MR effect size for
'ST3GAL1' on 'Cardiac valvular disease'

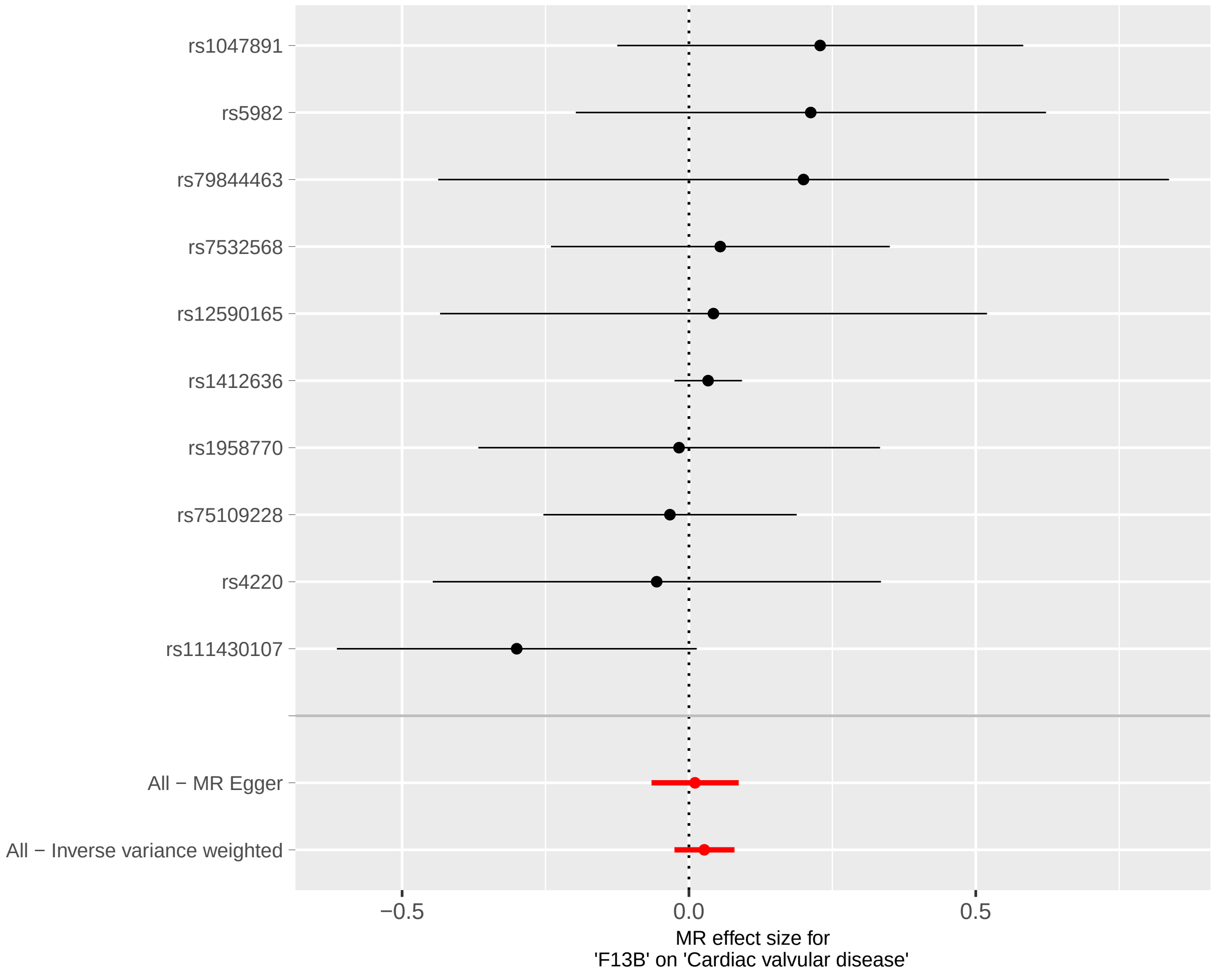
rs1047891
rs5982
rs79844463
rs7532568
rs12590165
rs1412636
rs1958770
rs75109228
rs4220
rs111430107
All − MR Egger
All − Inverse variance weighted
−0.5
0.0
0.5
MR effect size for
'F13B' on 'Cardiac valvular disease'

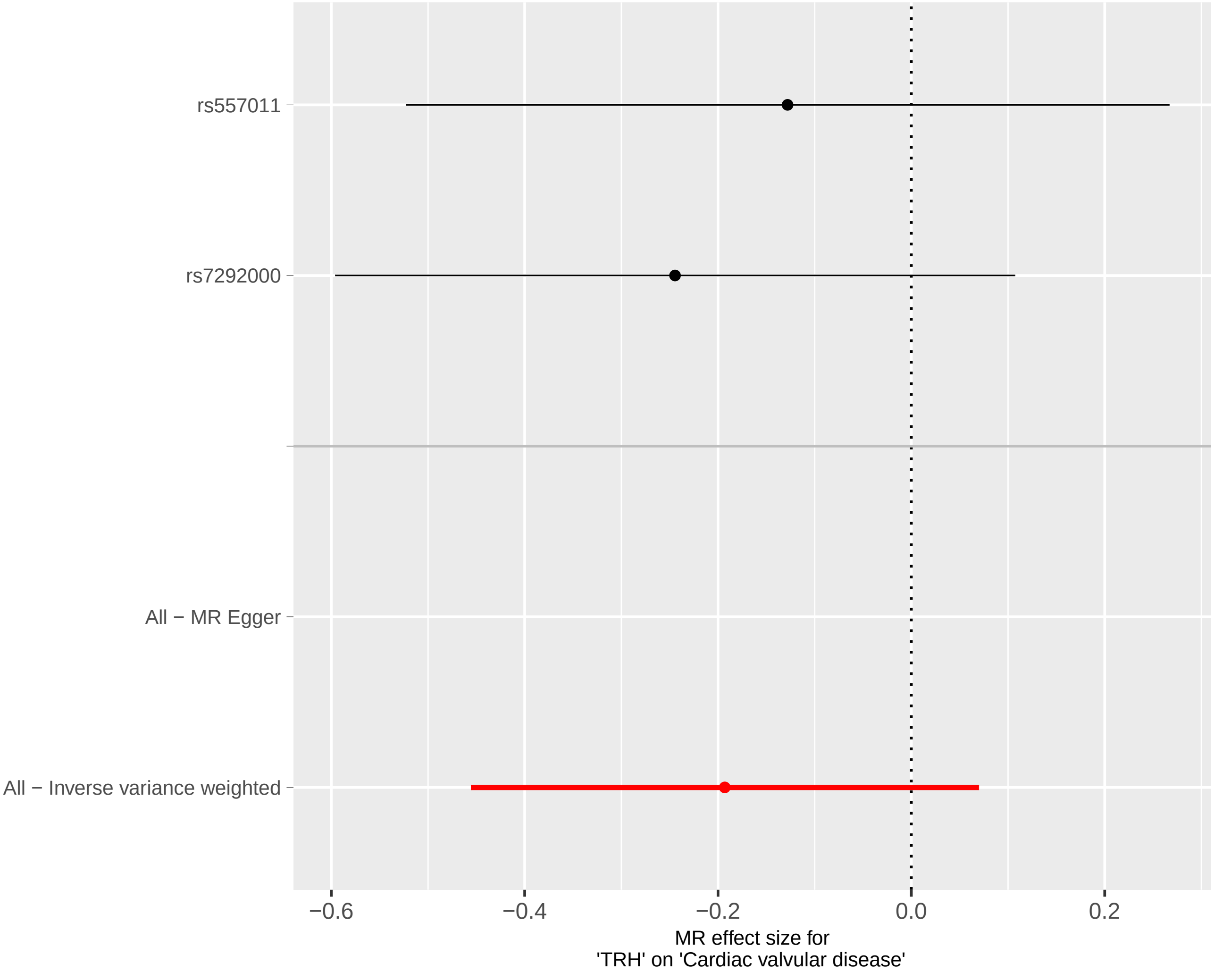
rs557011
rs7292000
All − MR Egger
All − Inverse variance weighted
−0.6
−0.4
−0.2
0.0
0.2
MR effect size for
'TRH' on 'Cardiac valvular disease'

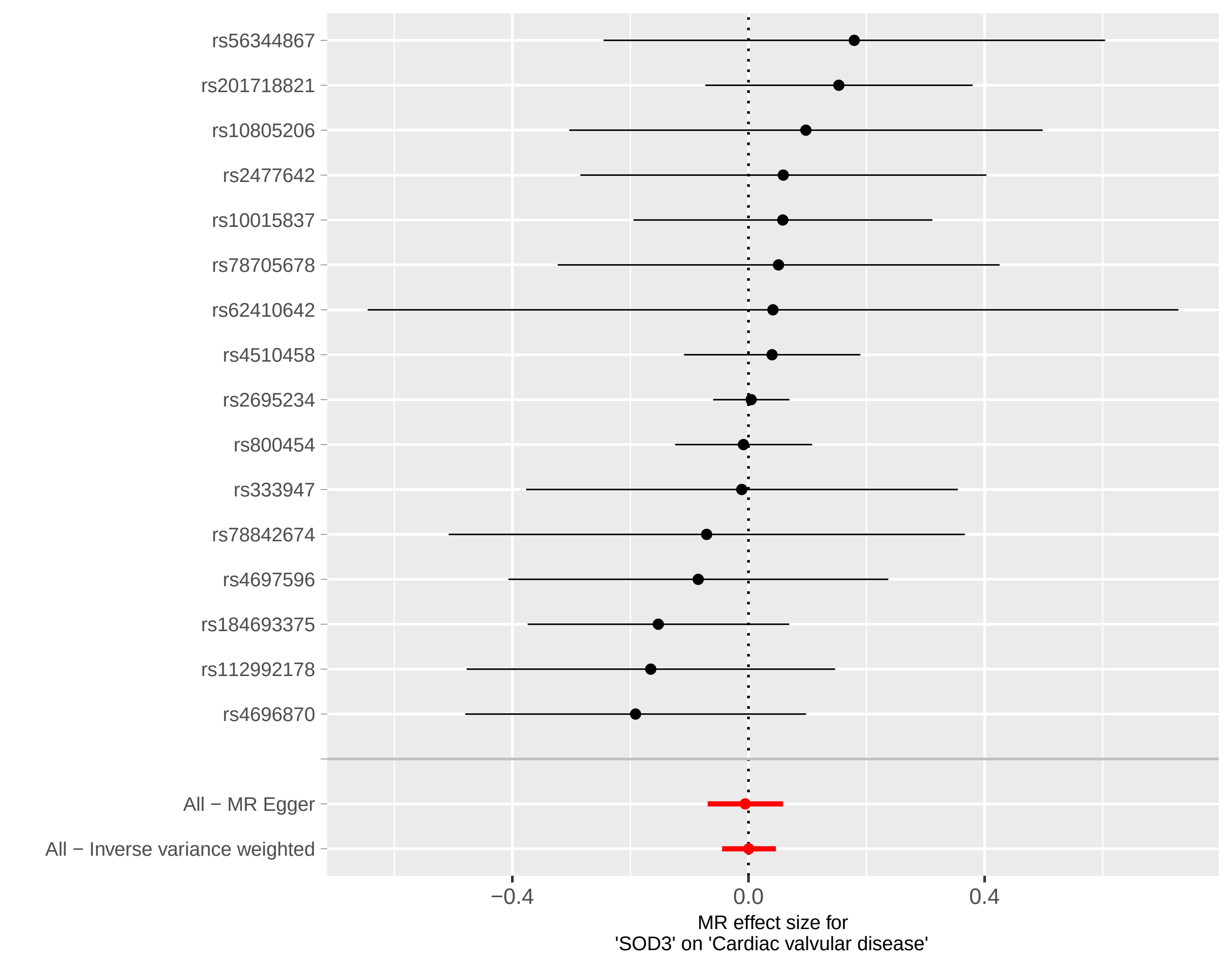
rs56344867
rs201718821
rs10805206
rs2477642
rs10015837
rs78705678
rs62410642
rs4510458
rs2695234
rs800454
rs333947
rs78842674
rs4697596
rs184693375
rs112992178
rs4696870
All − MR Egger
All − Inverse variance weighted
−0.4
0.0
0.4
MR effect size for
'SOD3' on 'Cardiac valvular disease'

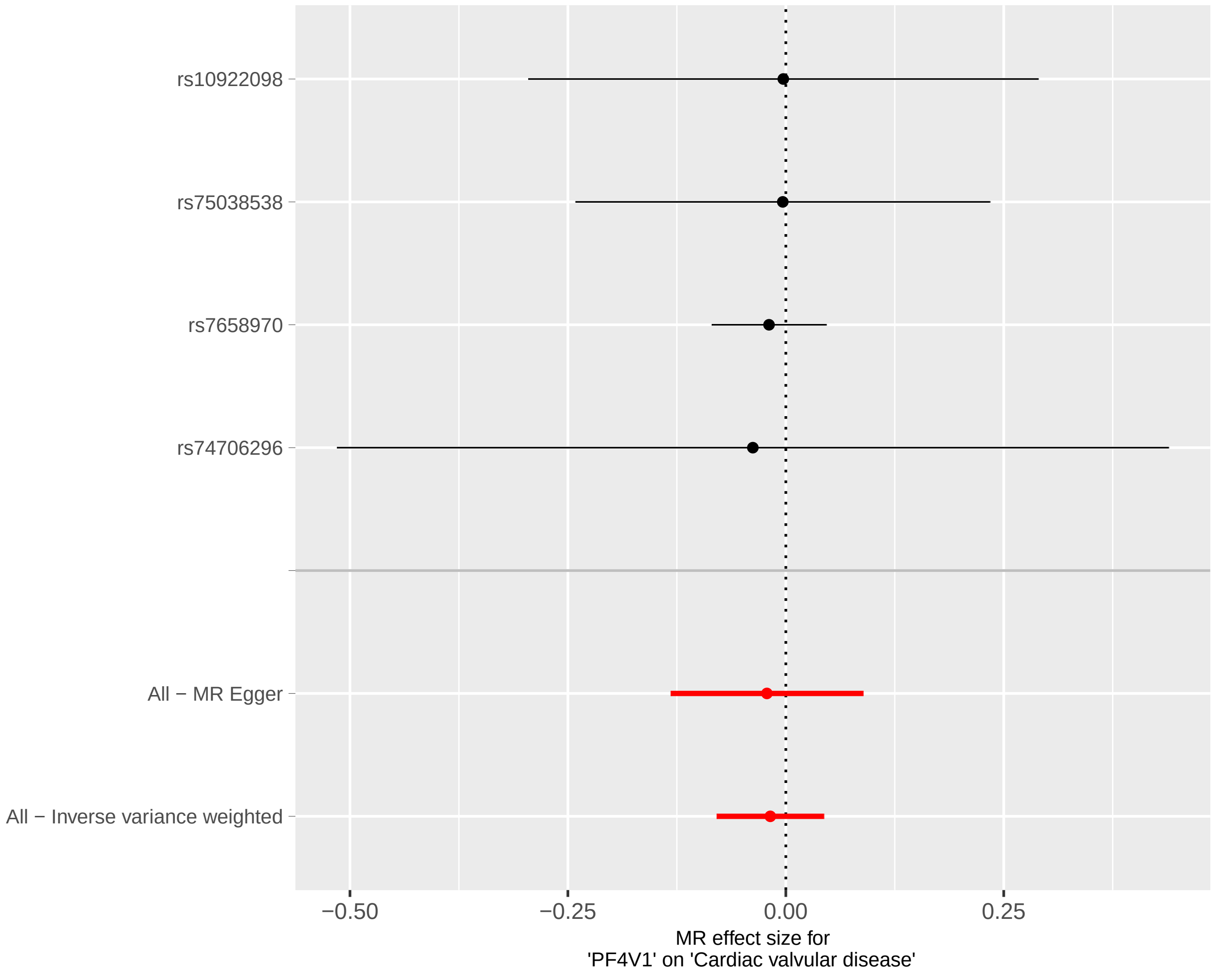
rs10922098
rs75038538
rs7658970
rs74706296
All − MR Egger
All − Inverse variance weighted
−0.50
−0.25
0.00
0.25
MR effect size for
'PF4V1' on 'Cardiac valvular disease'

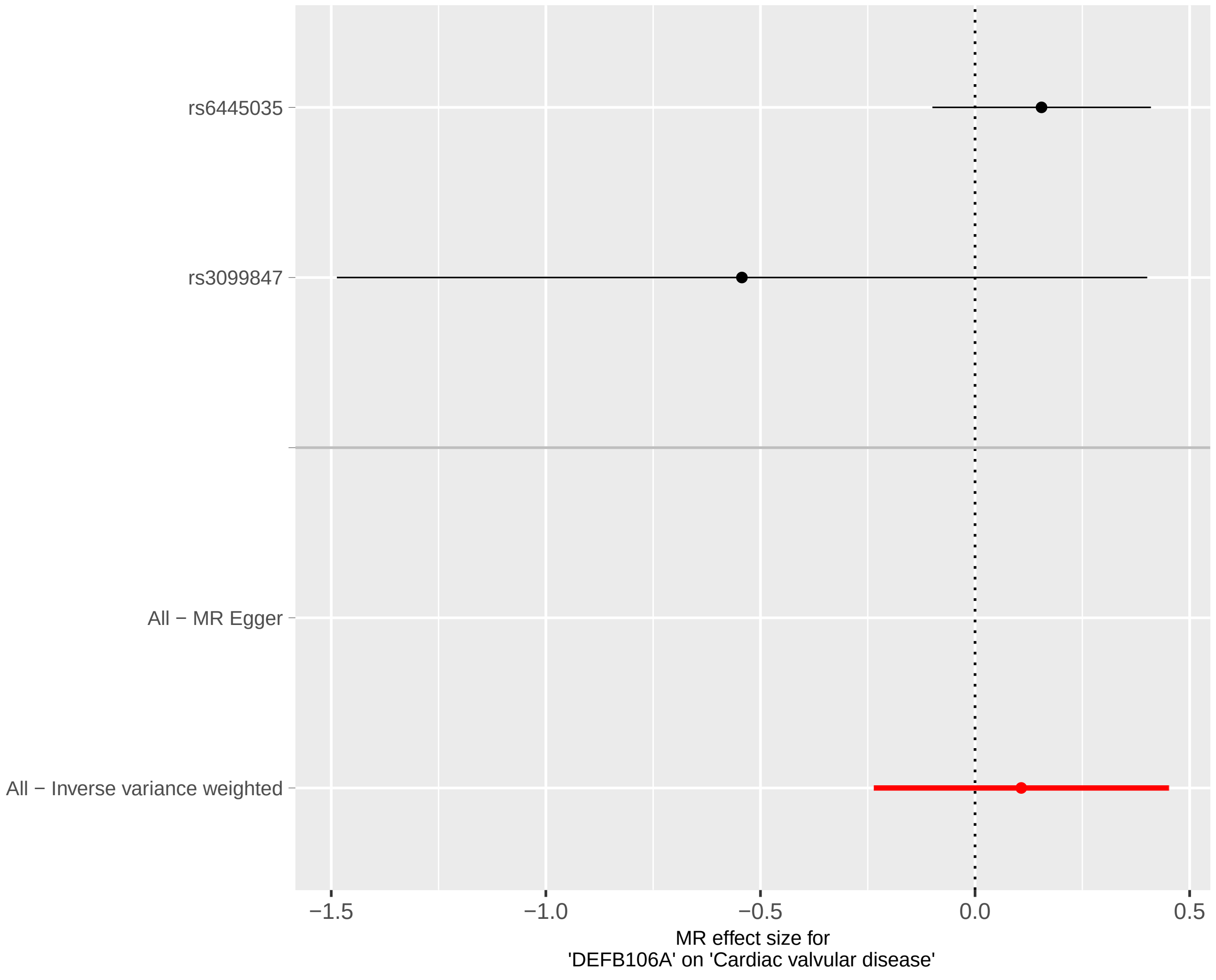
rs6445035
rs3099847
All − MR Egger
All − Inverse variance weighted
−1.5
−1.0
−0.5
0.0
0.5
MR effect size for
'DEFB106A' on 'Cardiac valvular disease'

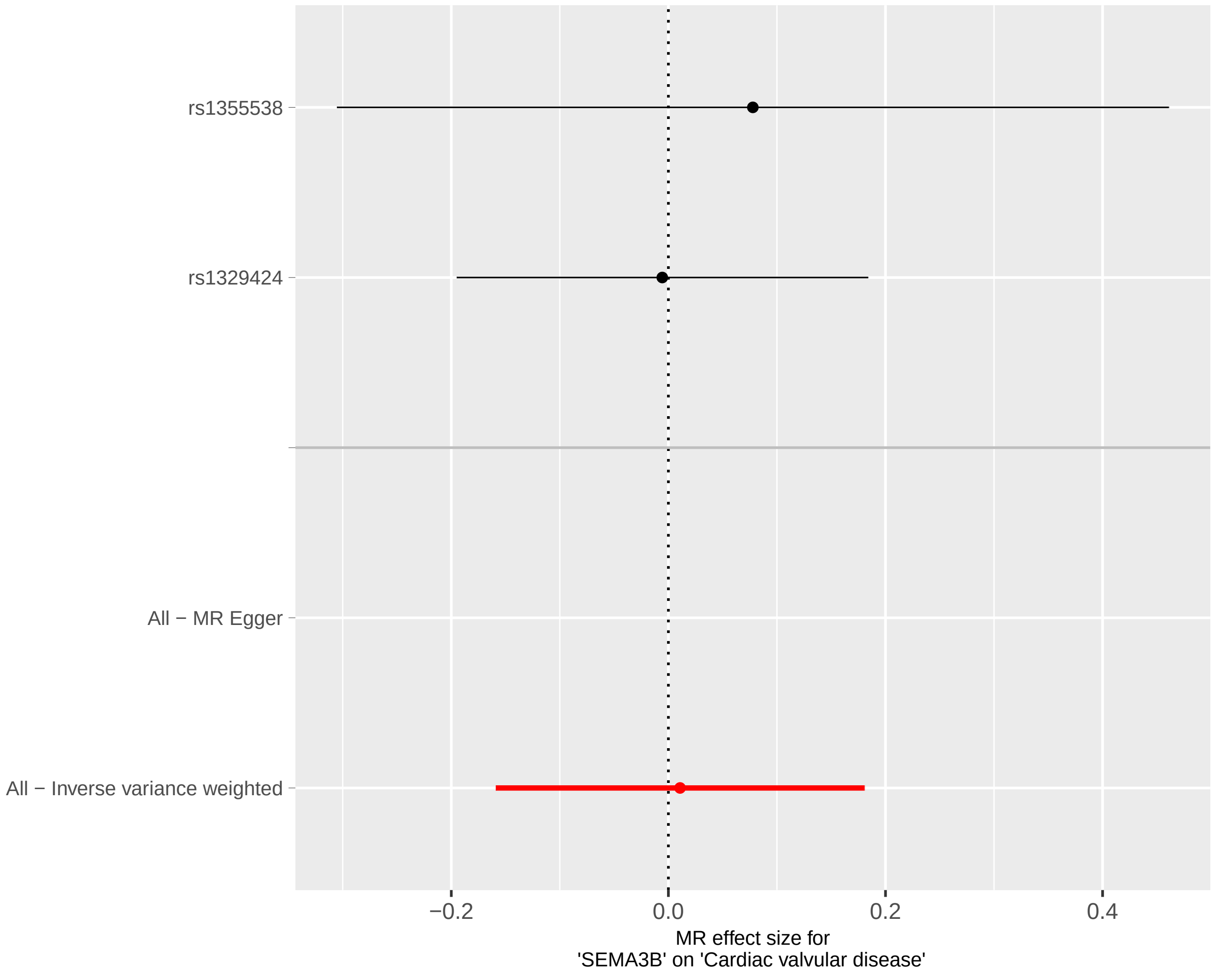
rs1355538
rs1329424
All − MR Egger
All − Inverse variance weighted
−0.2
0.0
0.2
0.4
MR effect size for
'SEMA3B' on 'Cardiac valvular disease'

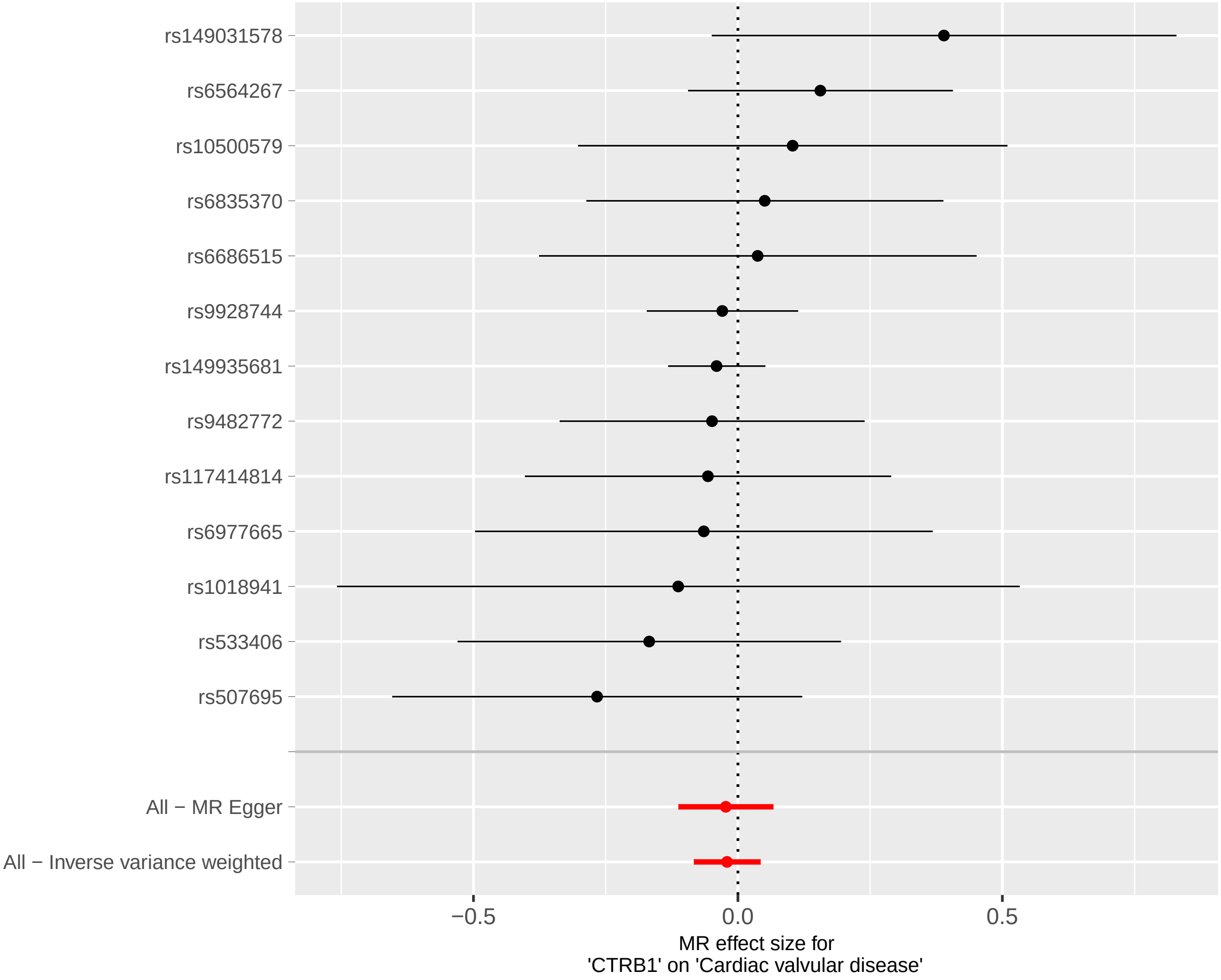
rs149031578
rs6564267
rs10500579
rs6835370
rs6686515
rs9928744
rs149935681
rs9482772
rs117414814
rs6977665
rs1018941
rs533406
rs507695
All − MR Egger
All − Inverse variance weighted
−0.5
0.0
0.5
MR effect size for
'CTRB1' on 'Cardiac valvular disease'

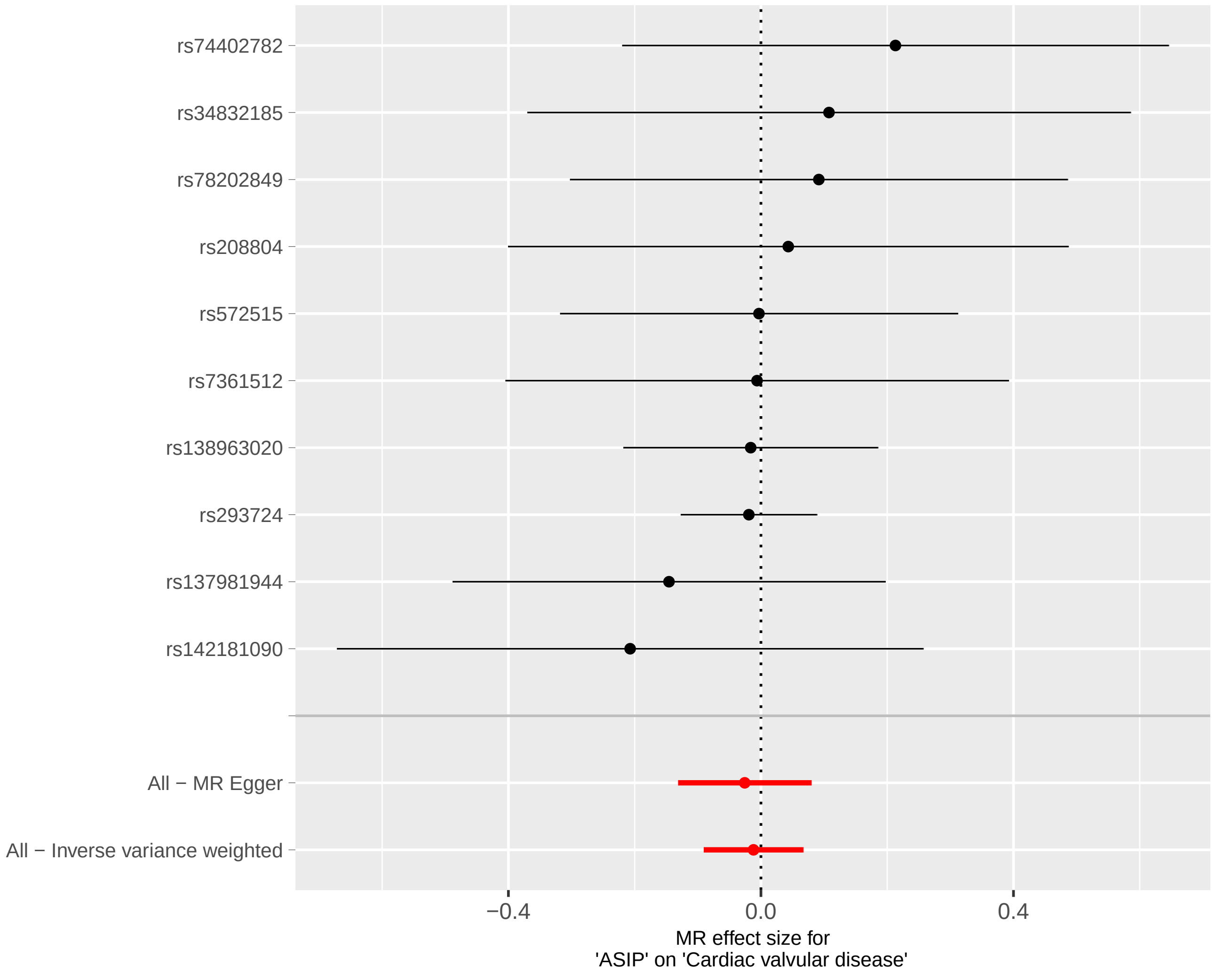
rs74402782
rs34832185
rs78202849
rs208804
rs572515
rs7361512
rs138963020
rs293724
rs137981944
rs142181090
All − MR Egger
All − Inverse variance weighted
−0.4
0.0
0.4
MR effect size for
'ASIP' on 'Cardiac valvular disease'

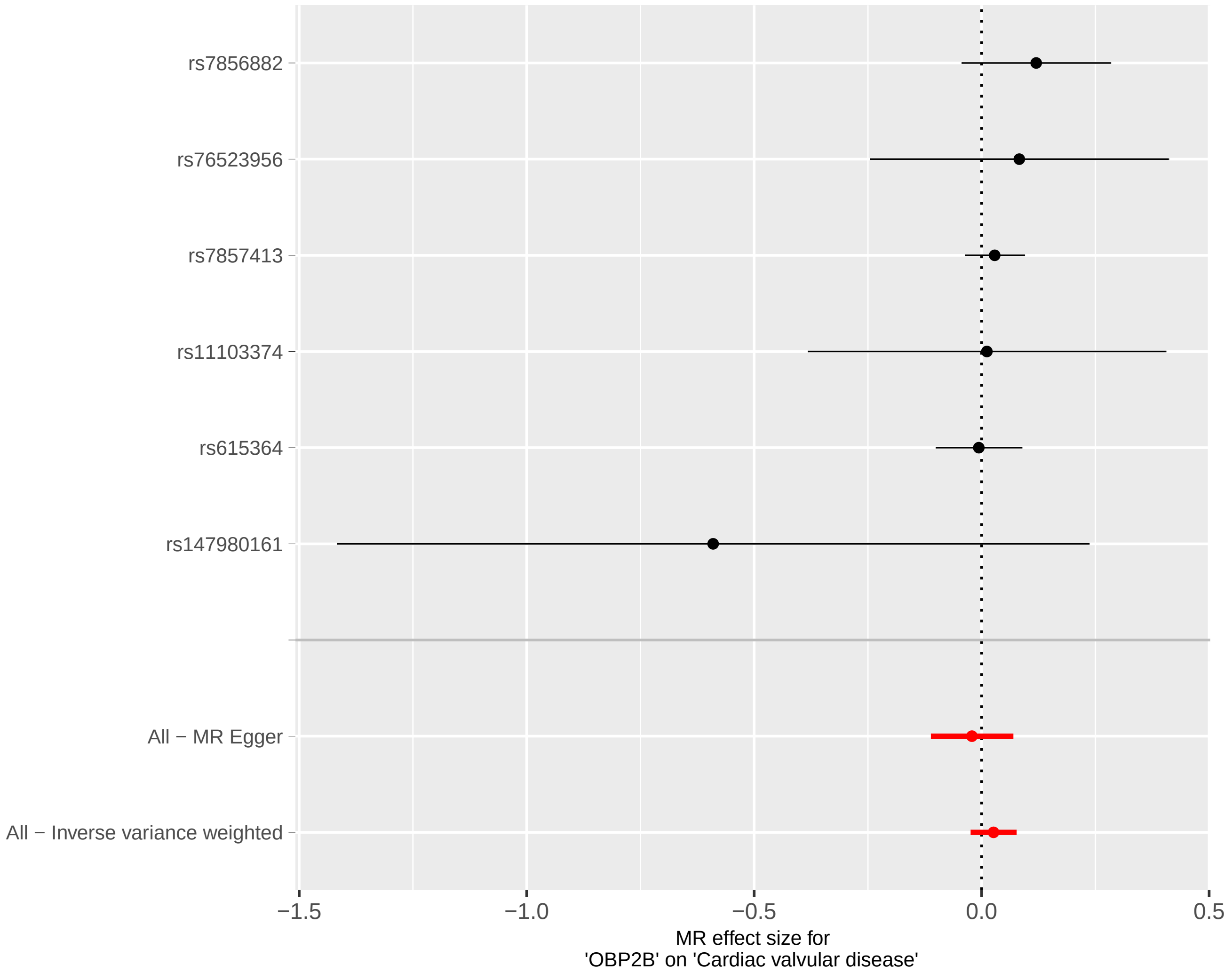

rs7856882
rs76523956
rs7857413
rs11103374
rs615364
rs147980161
All − MR Egger
All − Inverse variance weighted
−1.5
−1.0
−0.5
0.0
0.5
MR effect size for
'OBP2B' on 'Cardiac valvular disease'

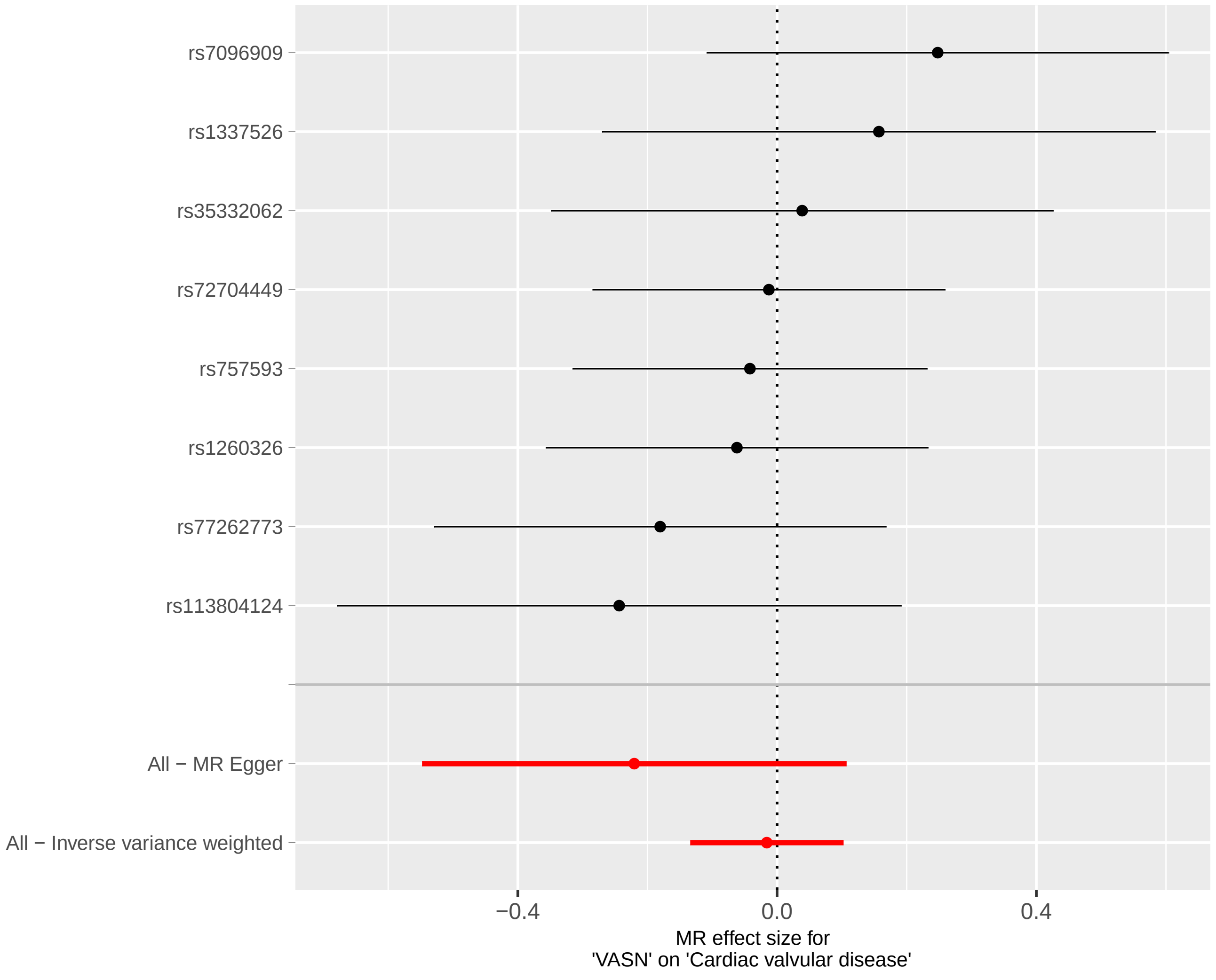

rs7096909
rs1337526
rs35332062
rs72704449
rs757593
rs1260326
rs77262773
rs113804124
All − MR Egger
All − Inverse variance weighted
−0.4
0.0
0.4
MR effect size for
'VASN' on 'Cardiac valvular disease'

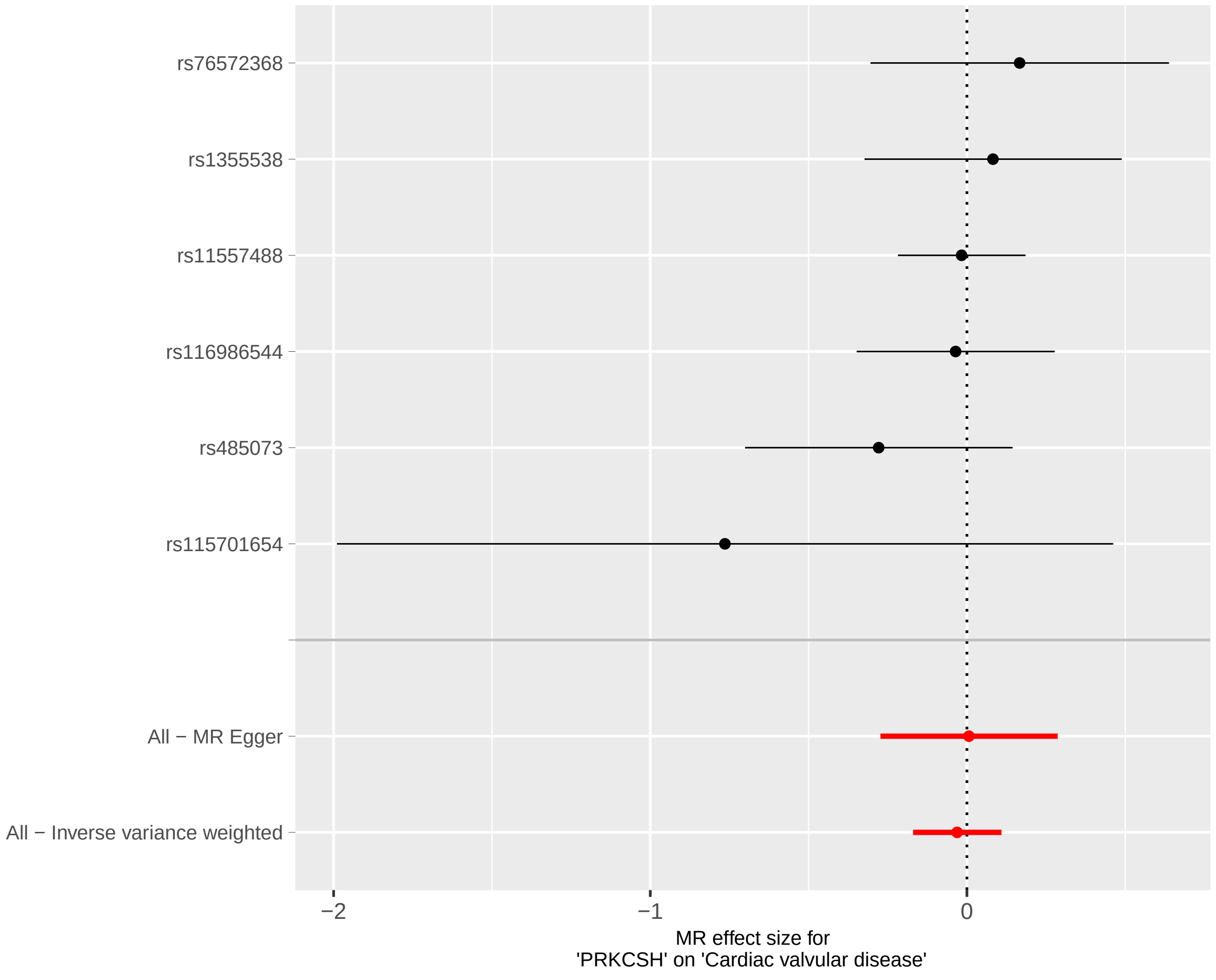
rs76572368
rs1355538
rs11557488
rs116986544
rs485073
rs115701654
All − MR Egger
All − Inverse variance weighted
−2
−1
0
MR effect size for
'PRKCSH' on 'Cardiac valvular disease'

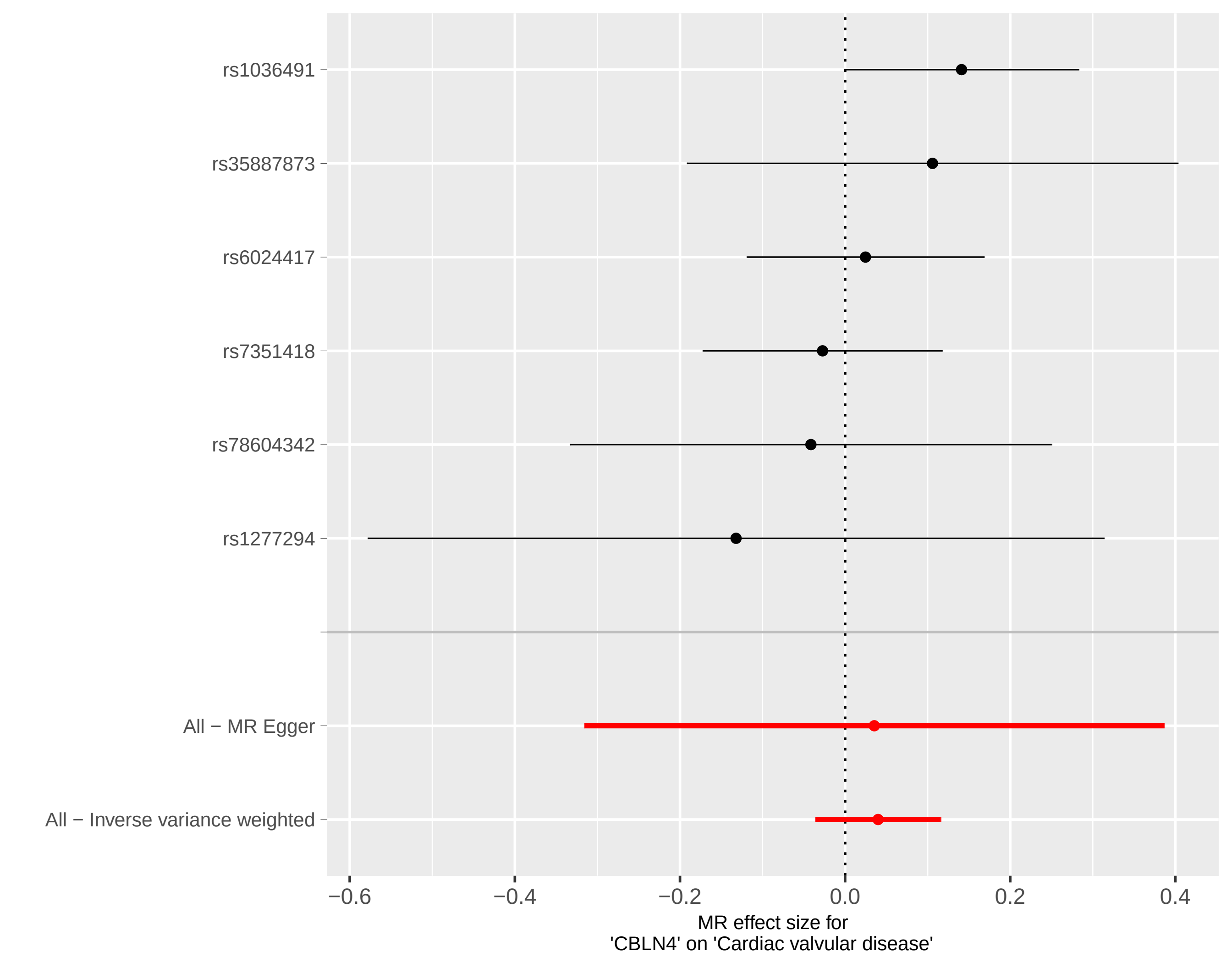
rs1036491
rs35887873
rs6024417
rs7351418
rs78604342
rs1277294
All − MR Egger
All − Inverse variance weighted
−0.6
−0.4
−0.2
0.0
0.2
0.4
MR effect size for
'CBLN4' on 'Cardiac valvular disease'

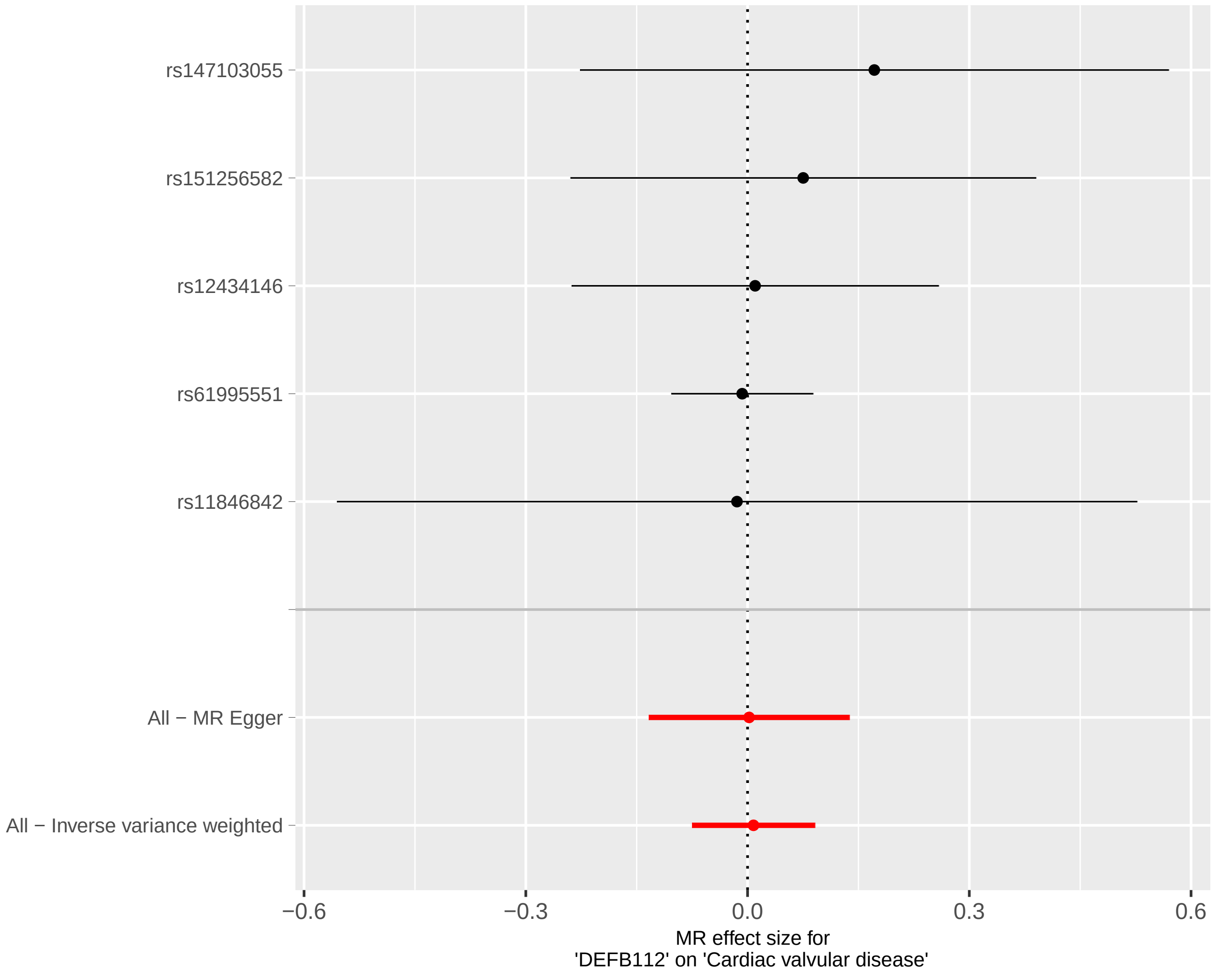
rs147103055
rs151256582
rs12434146
rs61995551
rs11846842
All − MR Egger
All − Inverse variance weighted
−0.6
−0.3
0.0
0.3
0.6
MR effect size for
'DEFB112' on 'Cardiac valvular disease'

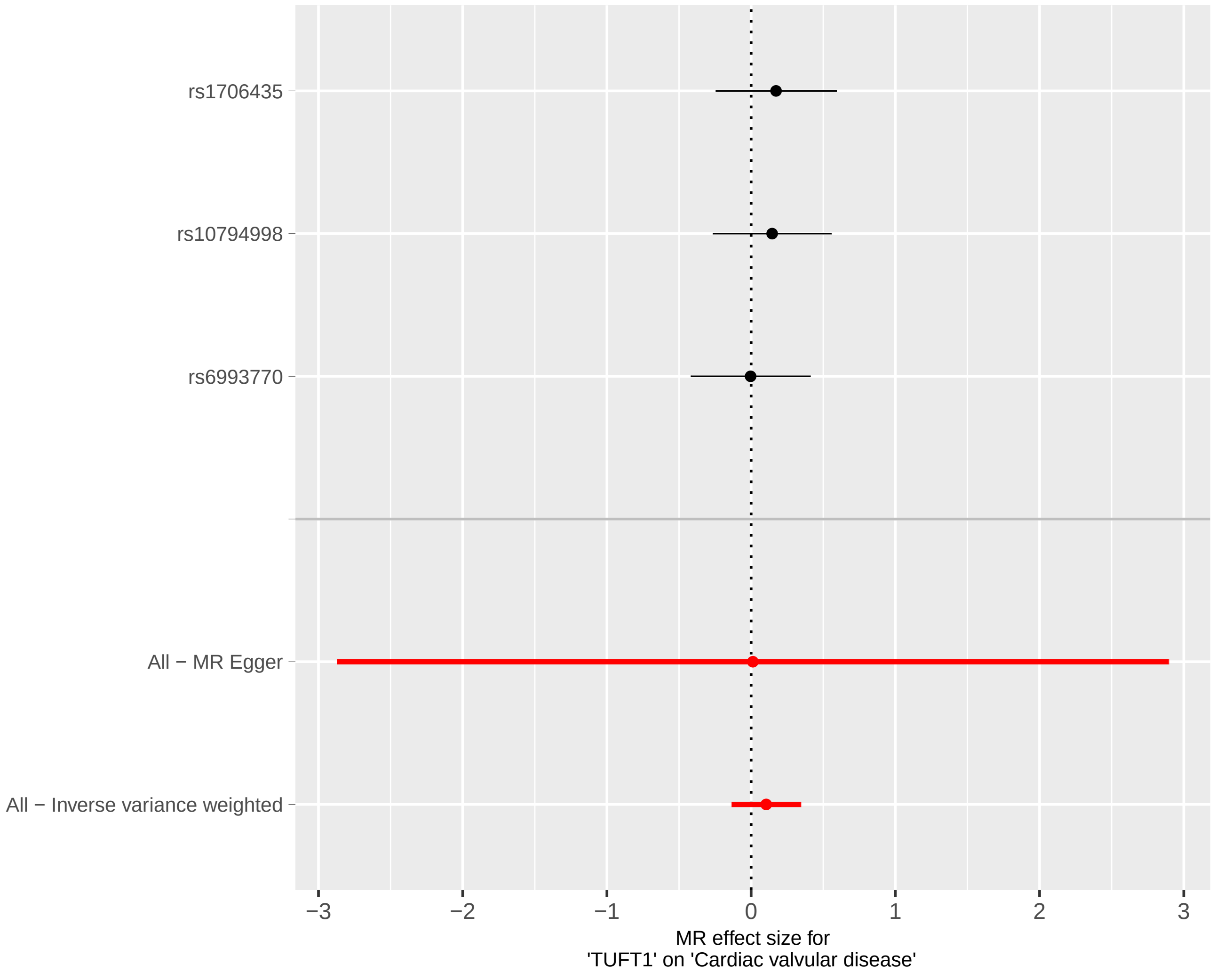
rs1706435
rs10794998
rs6993770
All − MR Egger
All − Inverse variance weighted
−3
−2
−1
0
1
2
3
MR effect size for
'TUFT1' on 'Cardiac valvular disease'

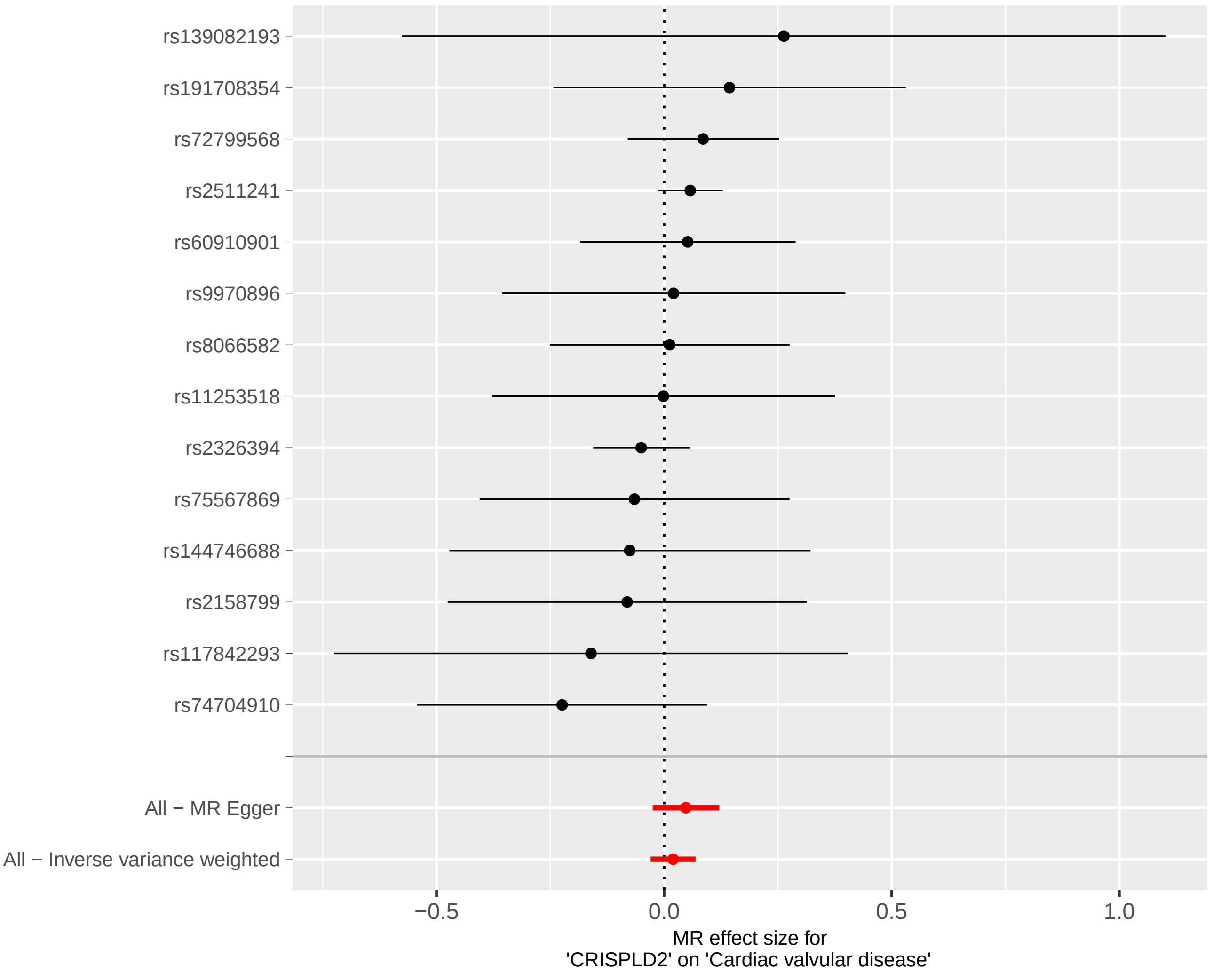
rs139082193
rs191708354
rs72799568
rs2511241
rs60910901
rs9970896
rs8066582
rs11253518
rs2326394
rs75567869
rs144746688
rs2158799
rs117842293
rs74704910
All − MR Egger
All − Inverse variance weighted
−0.5
0.0
0.5
1.0
MR effect size for
'CRISPLD2' on 'Cardiac valvular disease'

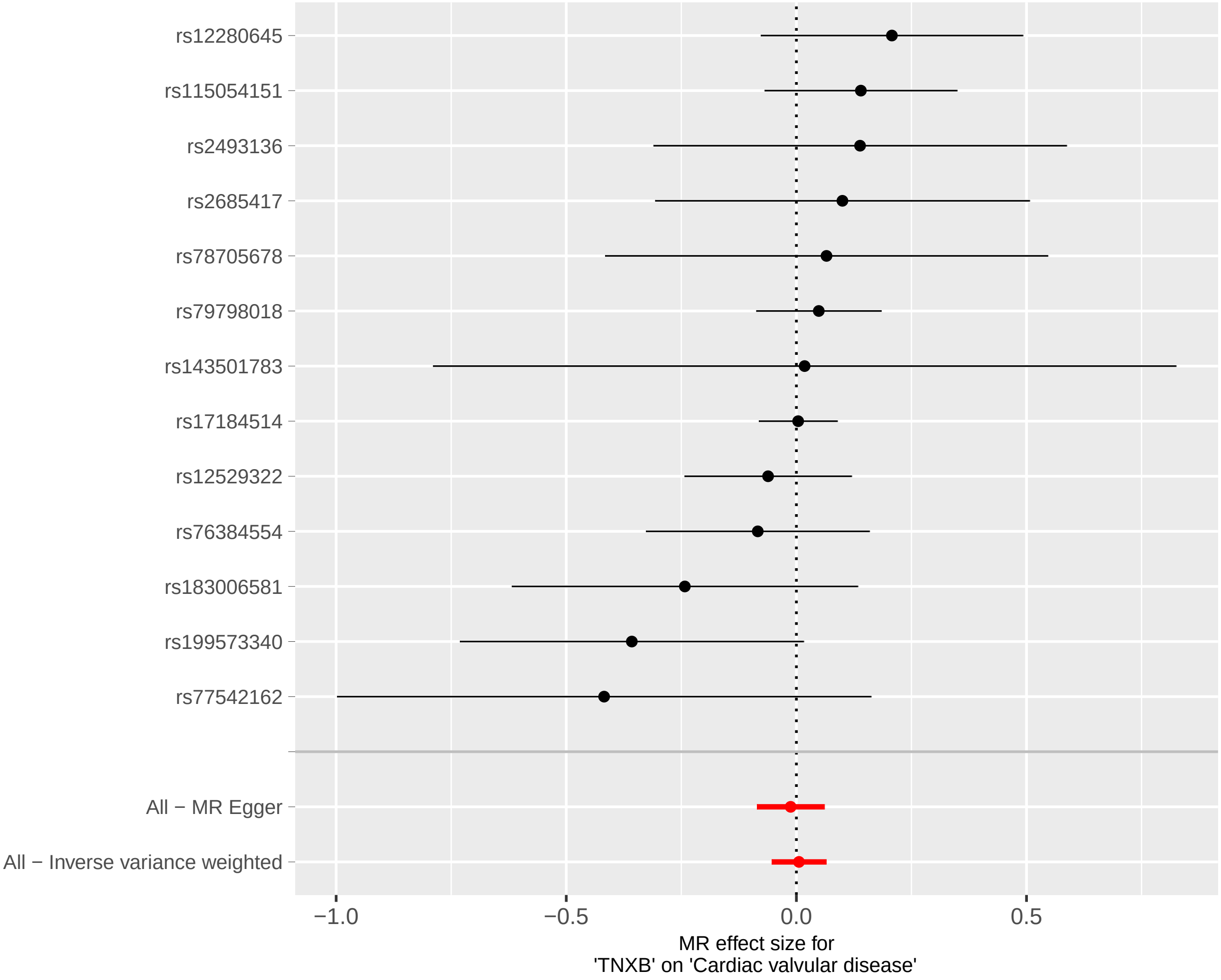
rs12280645
rs115054151
rs2493136
rs2685417
rs78705678
rs79798018
rs143501783
rs17184514
rs12529322
rs76384554
rs183006581
rs199573340
rs77542162
All − MR Egger
All − Inverse variance weighted
−1.0
−0.5
0.0
0.5
MR effect size for
'TNXB' on 'Cardiac valvular disease'

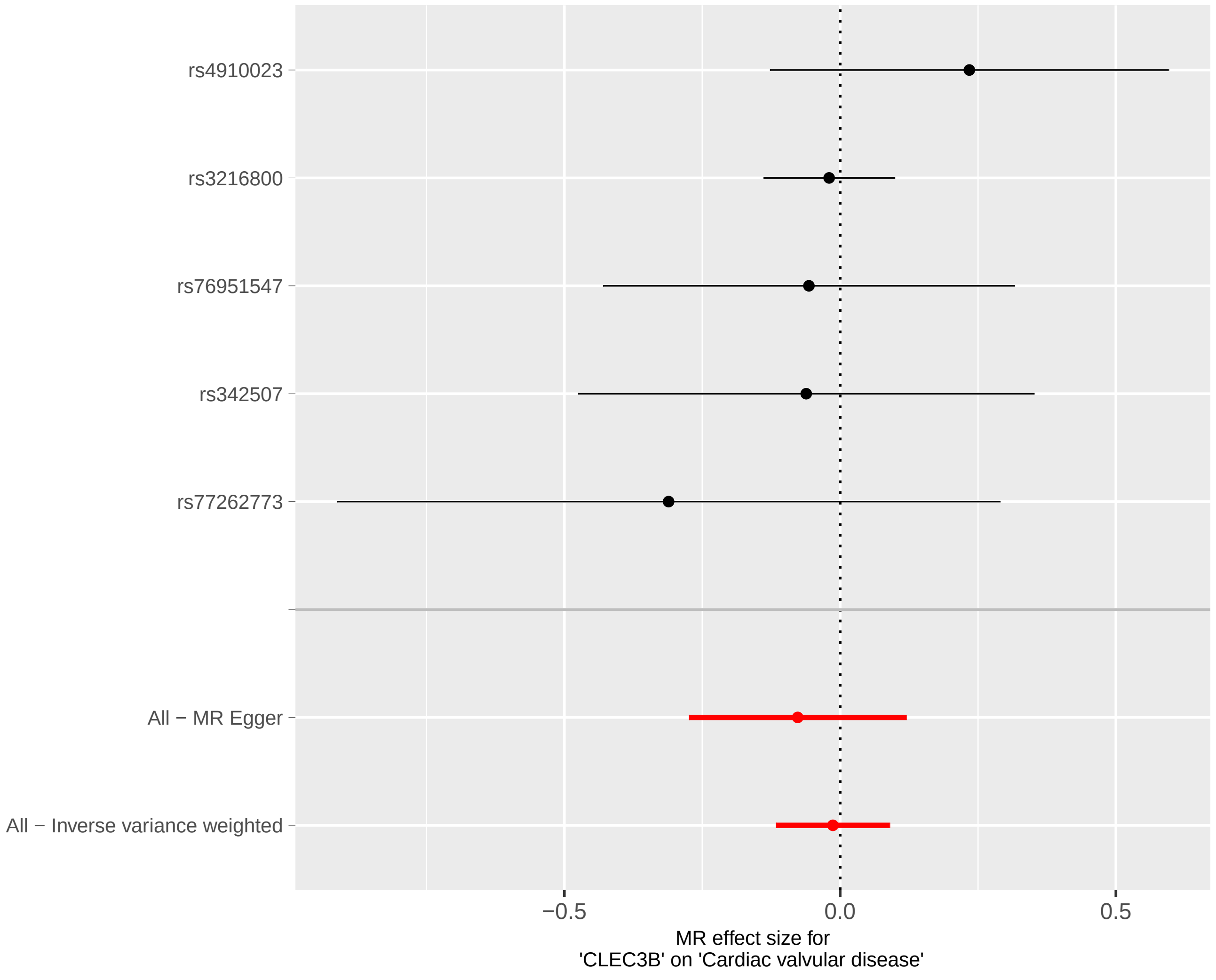
rs4910023
rs3216800
rs76951547
rs342507
rs77262773
All − MR Egger
All − Inverse variance weighted
−0.5
0.0
0.5
MR effect size for
'CLEC3B' on 'Cardiac valvular disease'

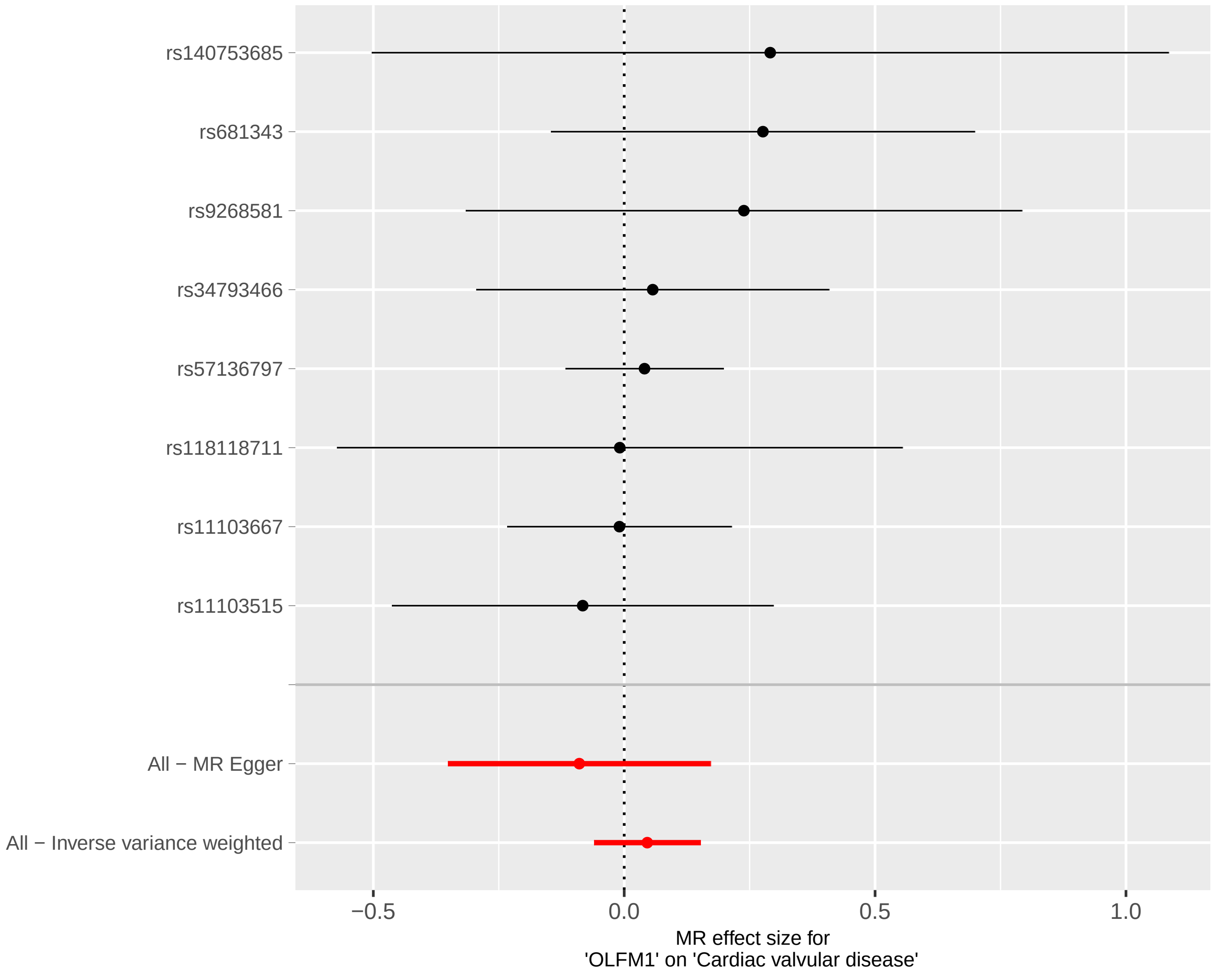
rs140753685
rs681343
rs9268581
rs34793466
rs57136797
rs118118711
rs11103667
rs11103515
All − MR Egger
All − Inverse variance weighted
−0.5
0.0
0.5
1.0
MR effect size for
'OLFM1' on 'Cardiac valvular disease'

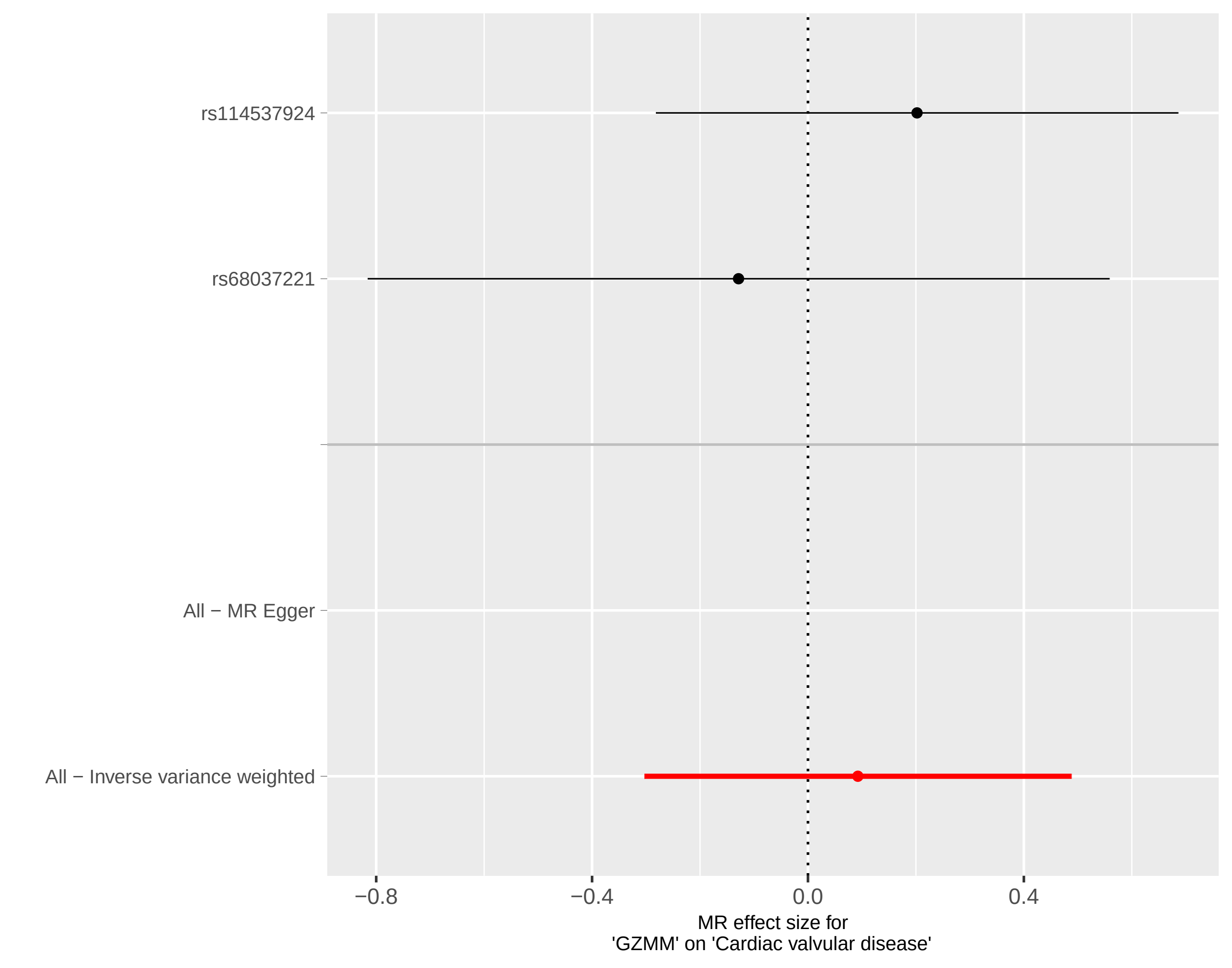

rs114537924
rs68037221
All − MR Egger
All − Inverse variance weighted
−0.8
−0.4
0.0
0.4
MR effect size for
'GZMM' on 'Cardiac valvular disease'

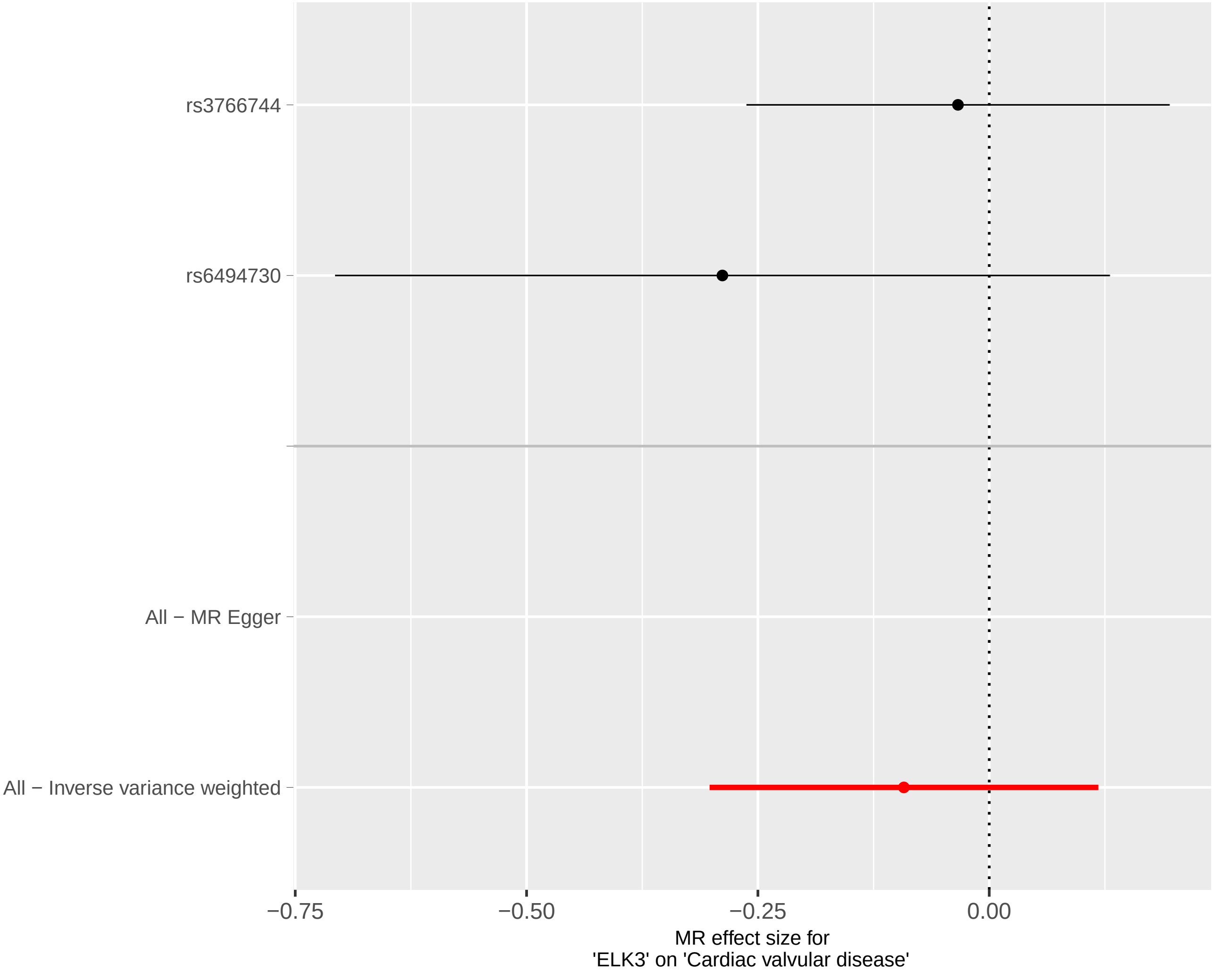
rs3766744
rs6494730
All − MR Egger
All − Inverse variance weighted
−0.75
−0.50
−0.25
0.00
MR effect size for
'ELK3' on 'Cardiac valvular disease'

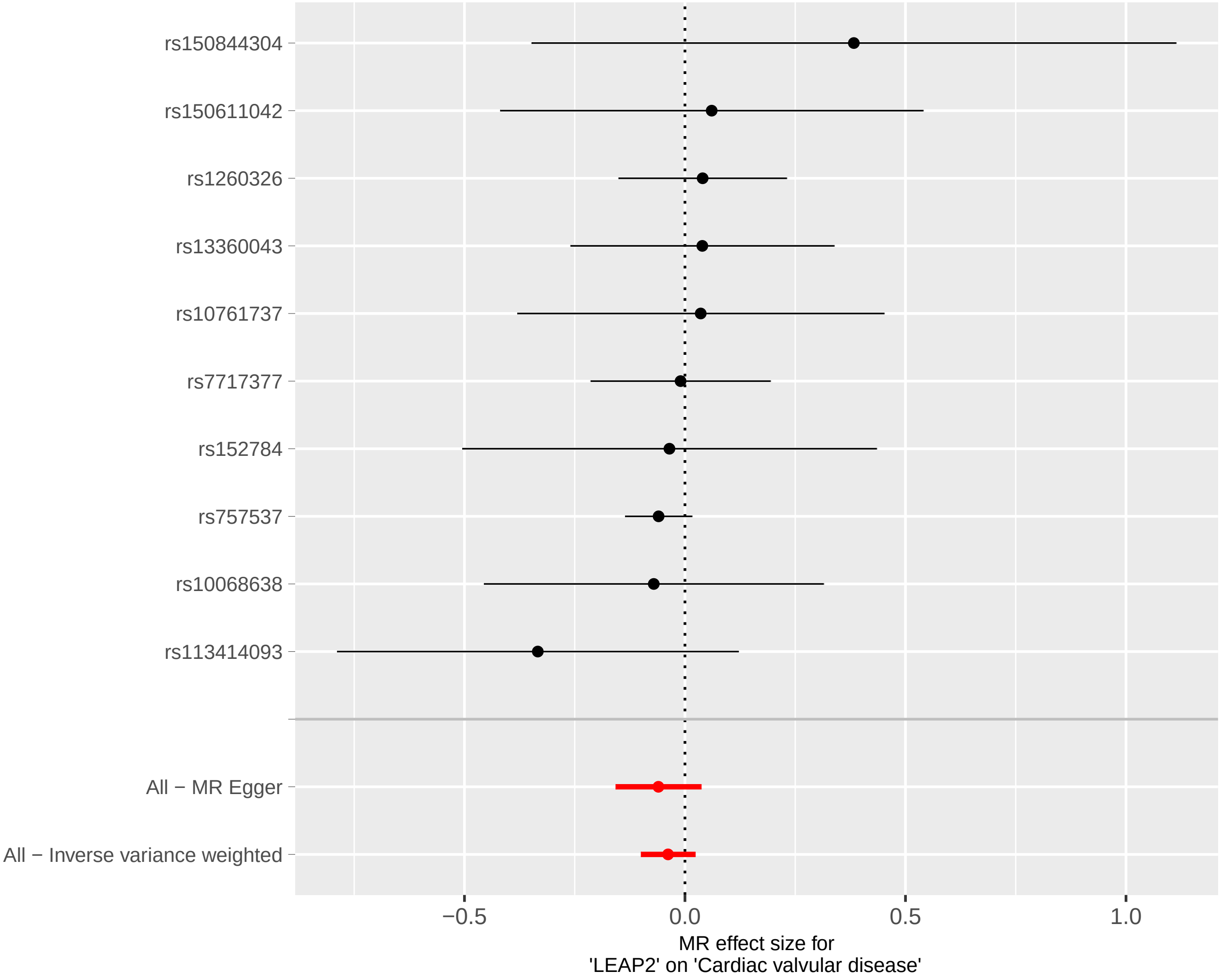
rs150844304
rs150611042
rs1260326
rs13360043
rs10761737
rs7717377
rs152784
rs757537
rs10068638
rs113414093
All − MR Egger
All − Inverse variance weighted
−0.5
0.0
0.5
1.0
MR effect size for
'LEAP2' on 'Cardiac valvular disease'

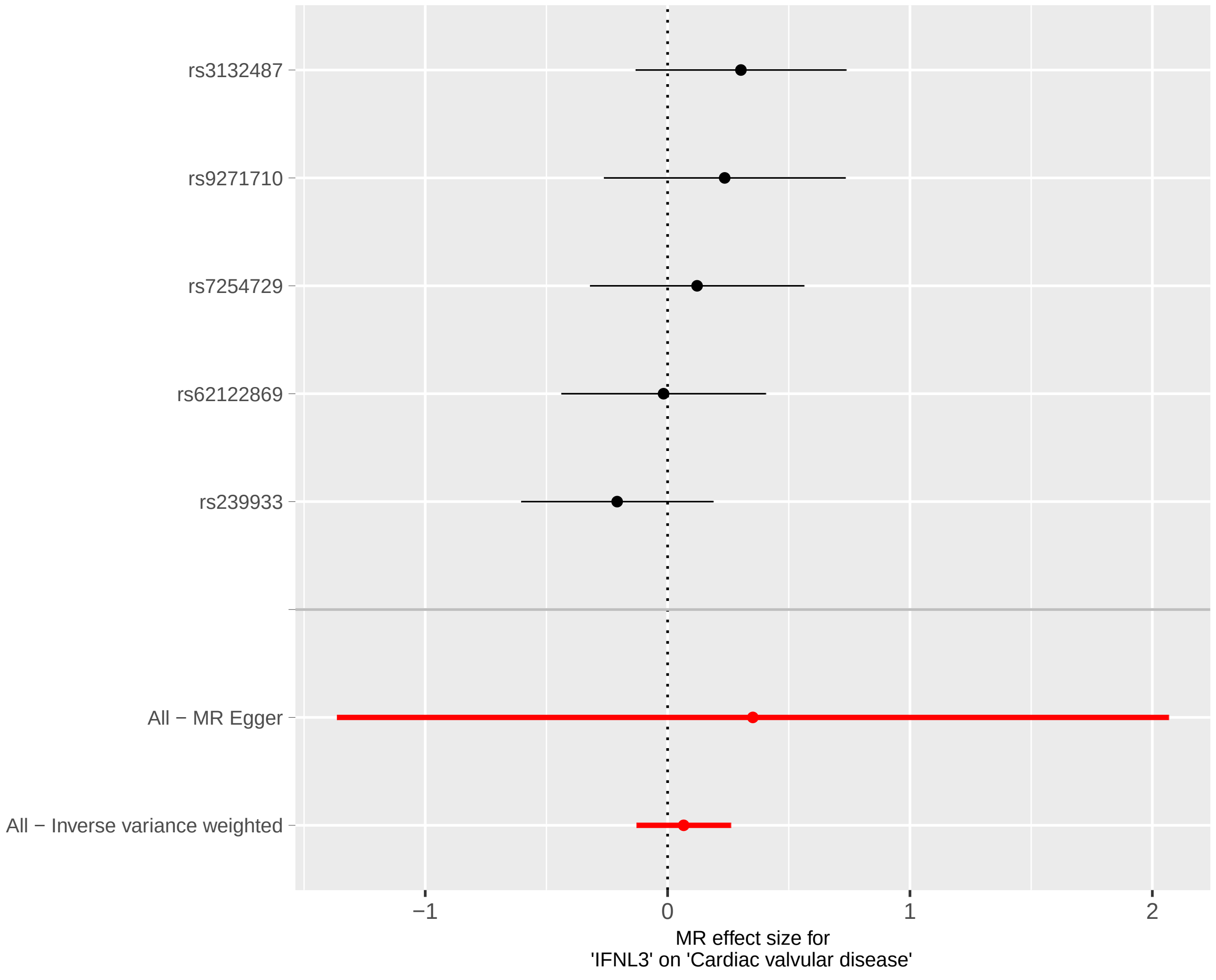
rs3132487
rs9271710
rs7254729
rs62122869
rs239933
All − MR Egger
All − Inverse variance weighted
−1
0
1
2
MR effect size for
'IFNL3' on 'Cardiac valvular disease'

Insufficient number of SNPs

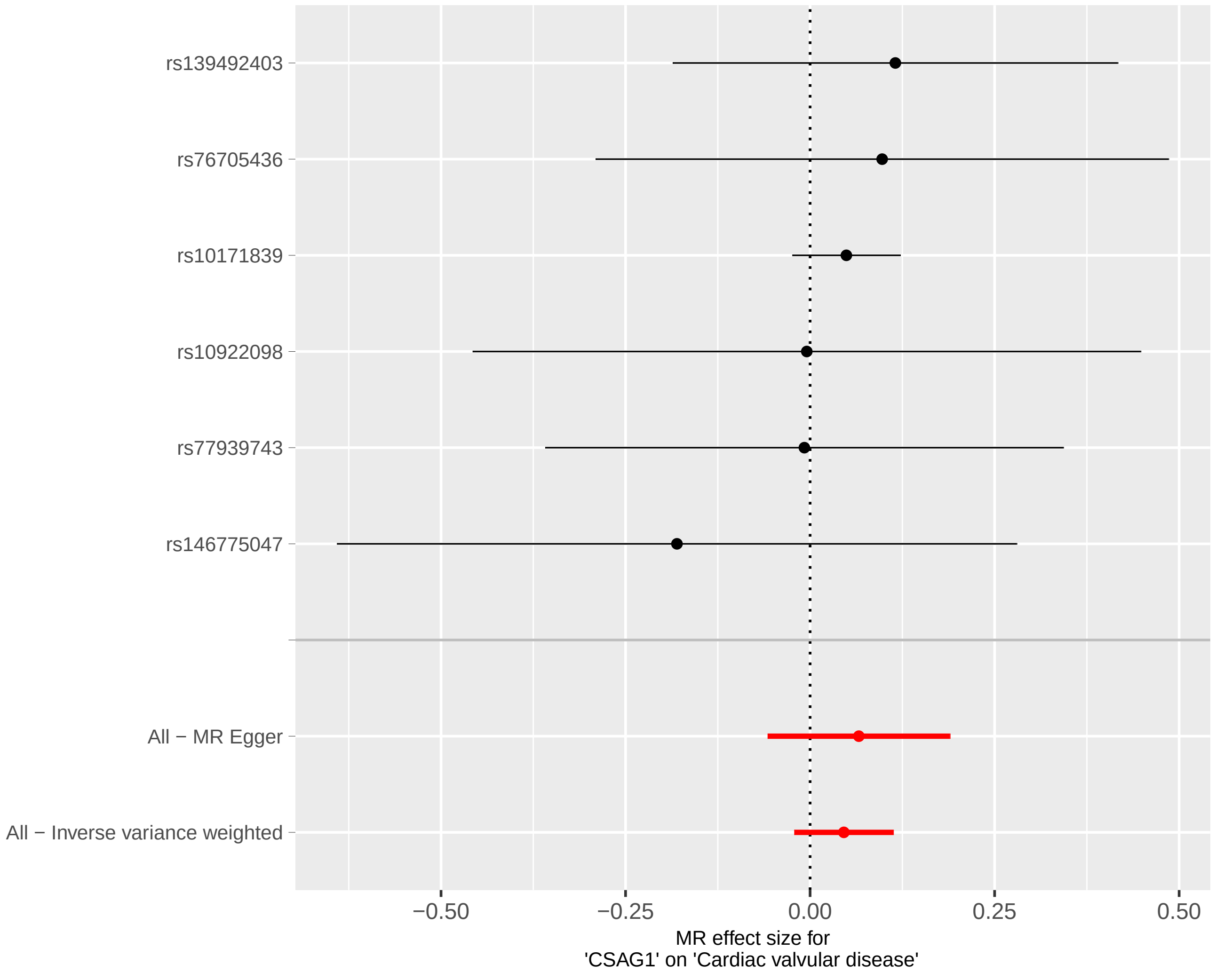
rs139492403
rs76705436
rs10171839
rs10922098
rs77939743
rs146775047
All − MR Egger
All − Inverse variance weighted
−0.50
−0.25
0.00
0.25
0.50
MR effect size for
'CSAG1' on 'Cardiac valvular disease'

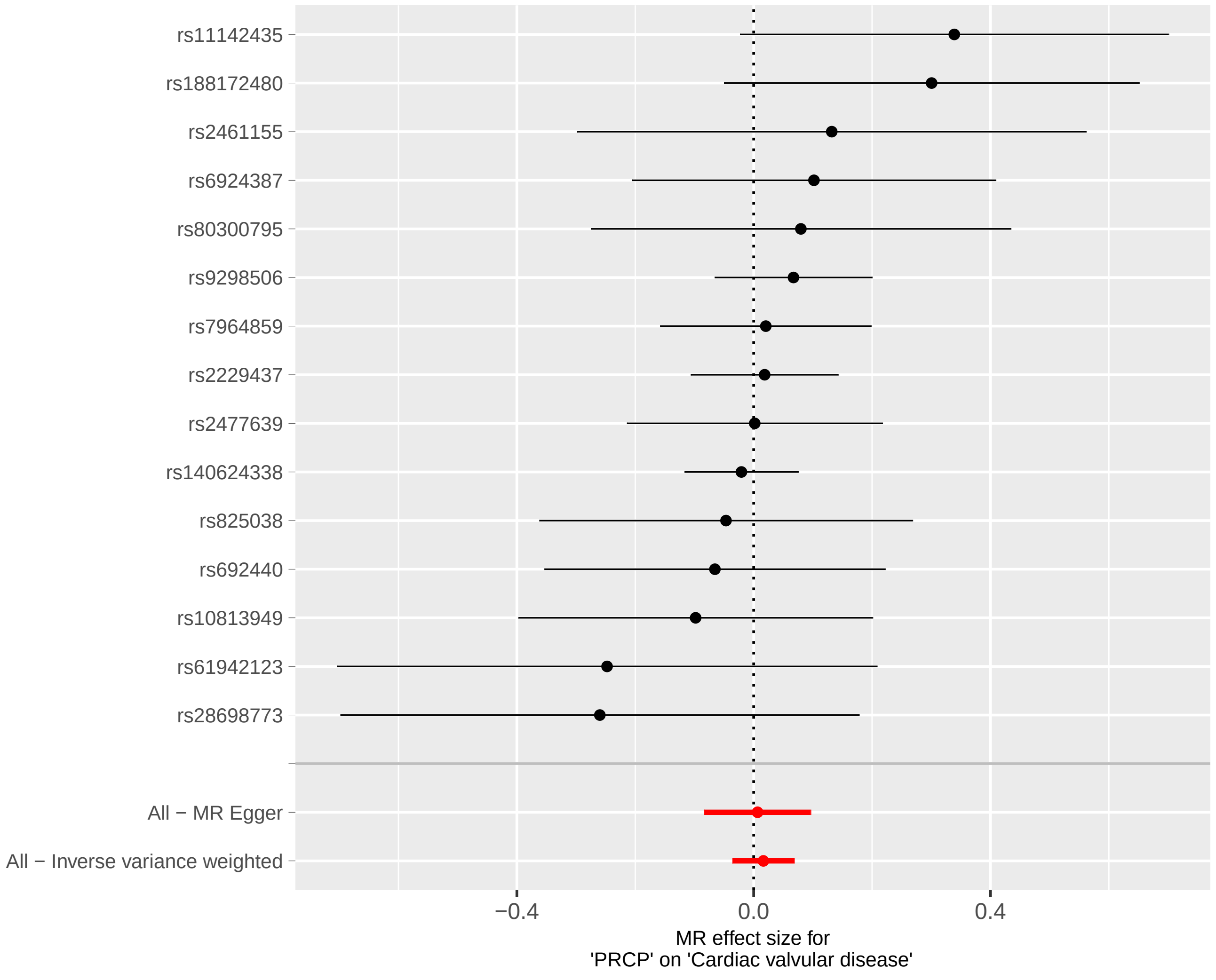

rs11142435
rs188172480
rs2461155
rs6924387
rs80300795
rs9298506
rs7964859
rs2229437
rs2477639
rs140624338
rs825038
rs692440
rs10813949
rs61942123
rs28698773
All − MR Egger
All − Inverse variance weighted
−0.4
0.0
0.4
MR effect size for
'PRCP' on 'Cardiac valvular disease'

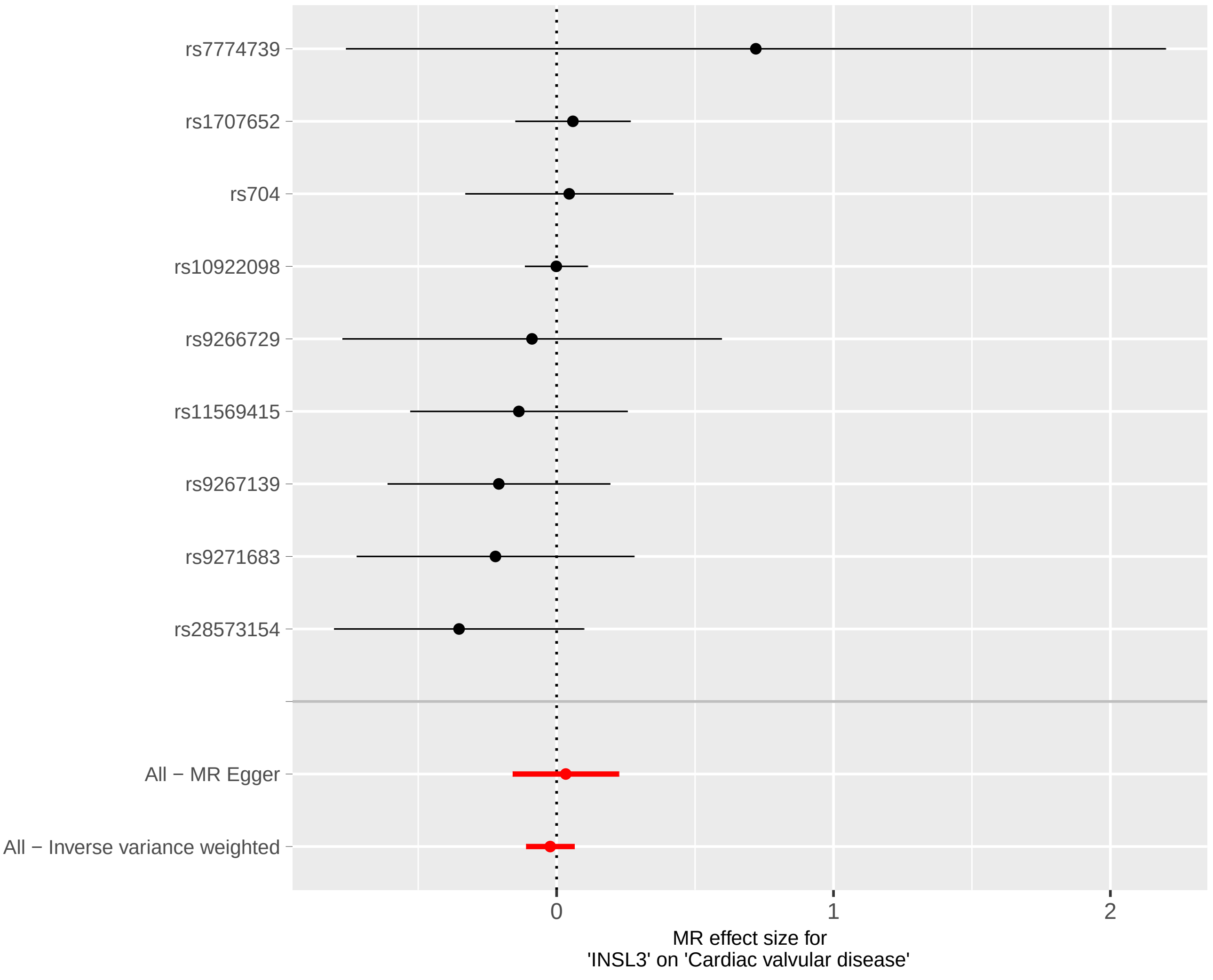
rs7774739
rs1707652
rs704
rs10922098
rs9266729
rs11569415
rs9267139
rs9271683
rs28573154
All − MR Egger
All − Inverse variance weighted
0
1
2
MR effect size for
'INSL3' on 'Cardiac valvular disease'

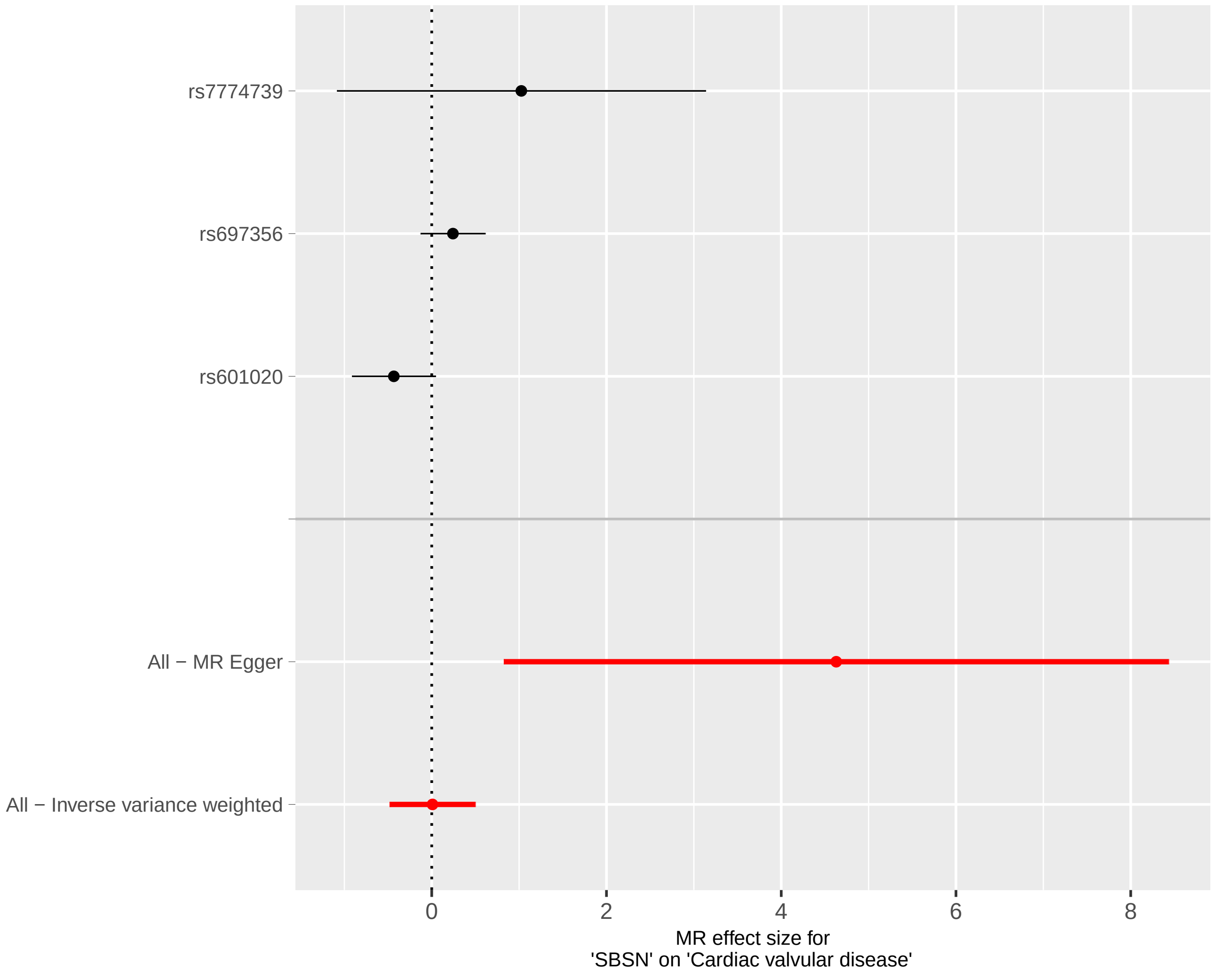
rs7774739
rs697356
rs601020
All − MR Egger
All − Inverse variance weighted
0
2
4
6
8
MR effect size for
'SBSN' on 'Cardiac valvular disease'

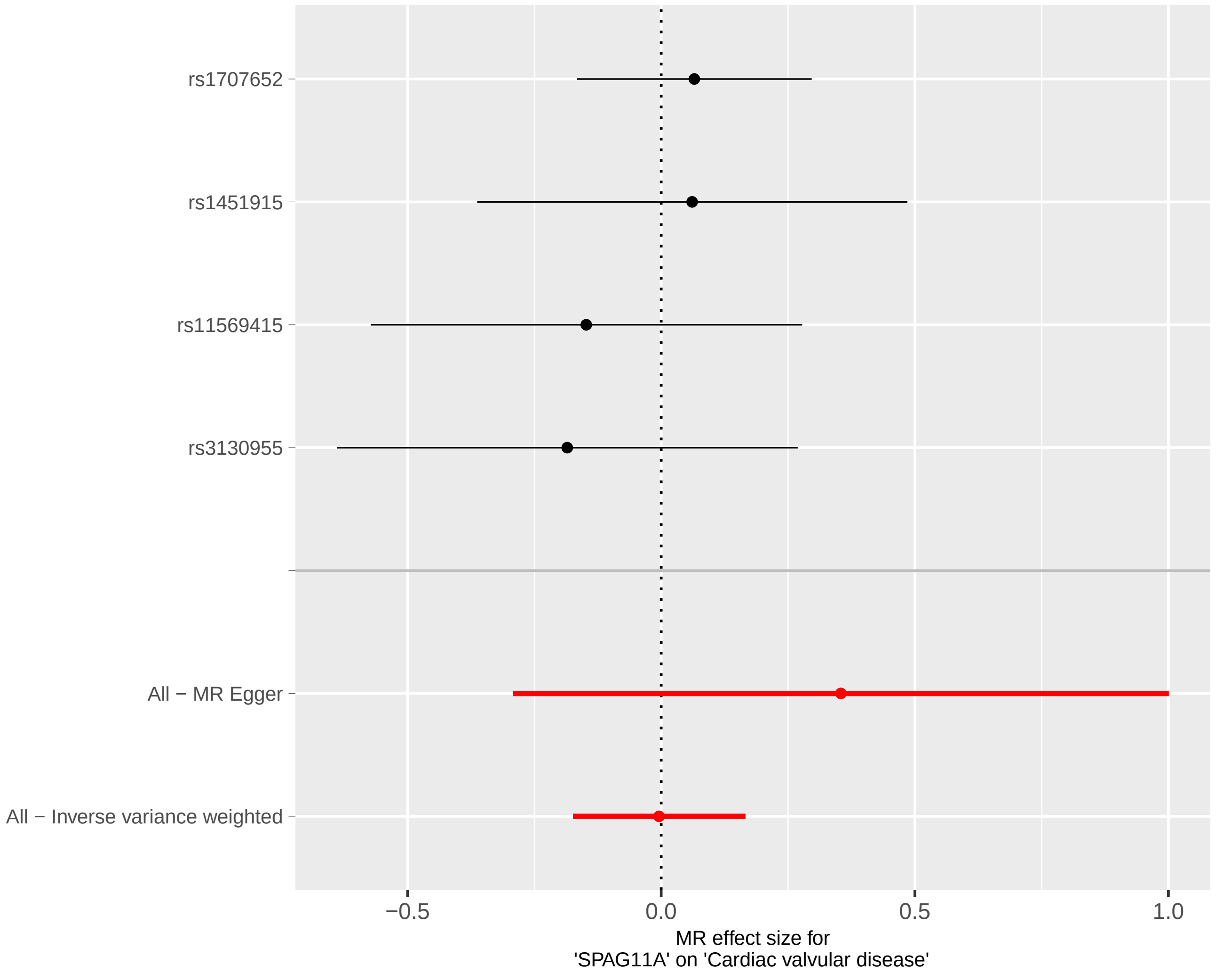
rs1707652
rs1451915
rs11569415
rs3130955
All − MR Egger
All − Inverse variance weighted
−0.5
0.0
0.5
1.0
MR effect size for
'SPAG11A' on 'Cardiac valvular disease'

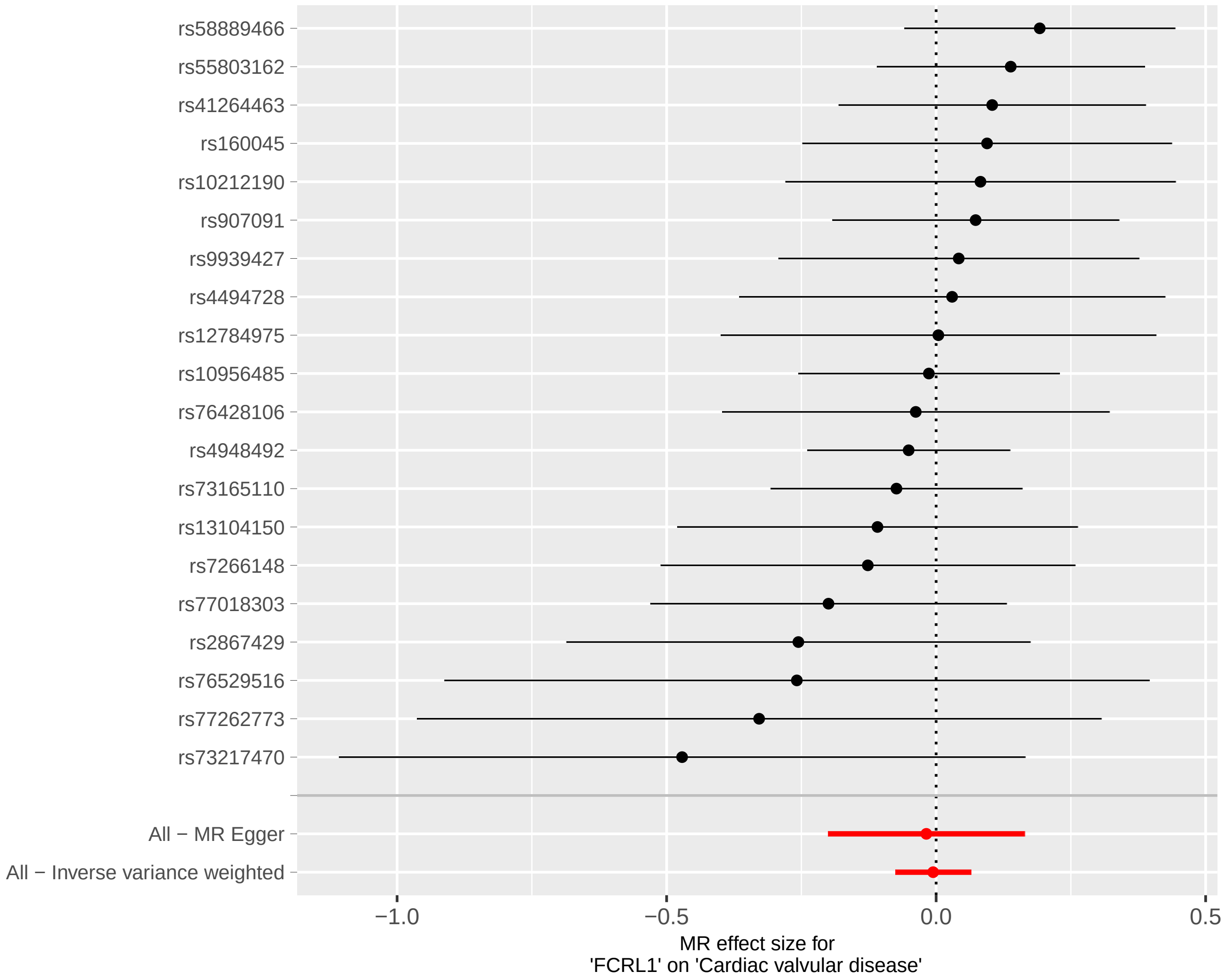

rs58889466
rs55803162
rs41264463
rs160045
rs10212190
rs907091
rs9939427
rs4494728
rs12784975
rs10956485
rs76428106
rs4948492
rs73165110
rs13104150
rs7266148
rs77018303
rs2867429
rs76529516
rs77262773
rs73217470
All − MR Egger
All − Inverse variance weighted
−1.0
−0.5
0.0
0.5
MR effect size for
'FCRL1' on 'Cardiac valvular disease'

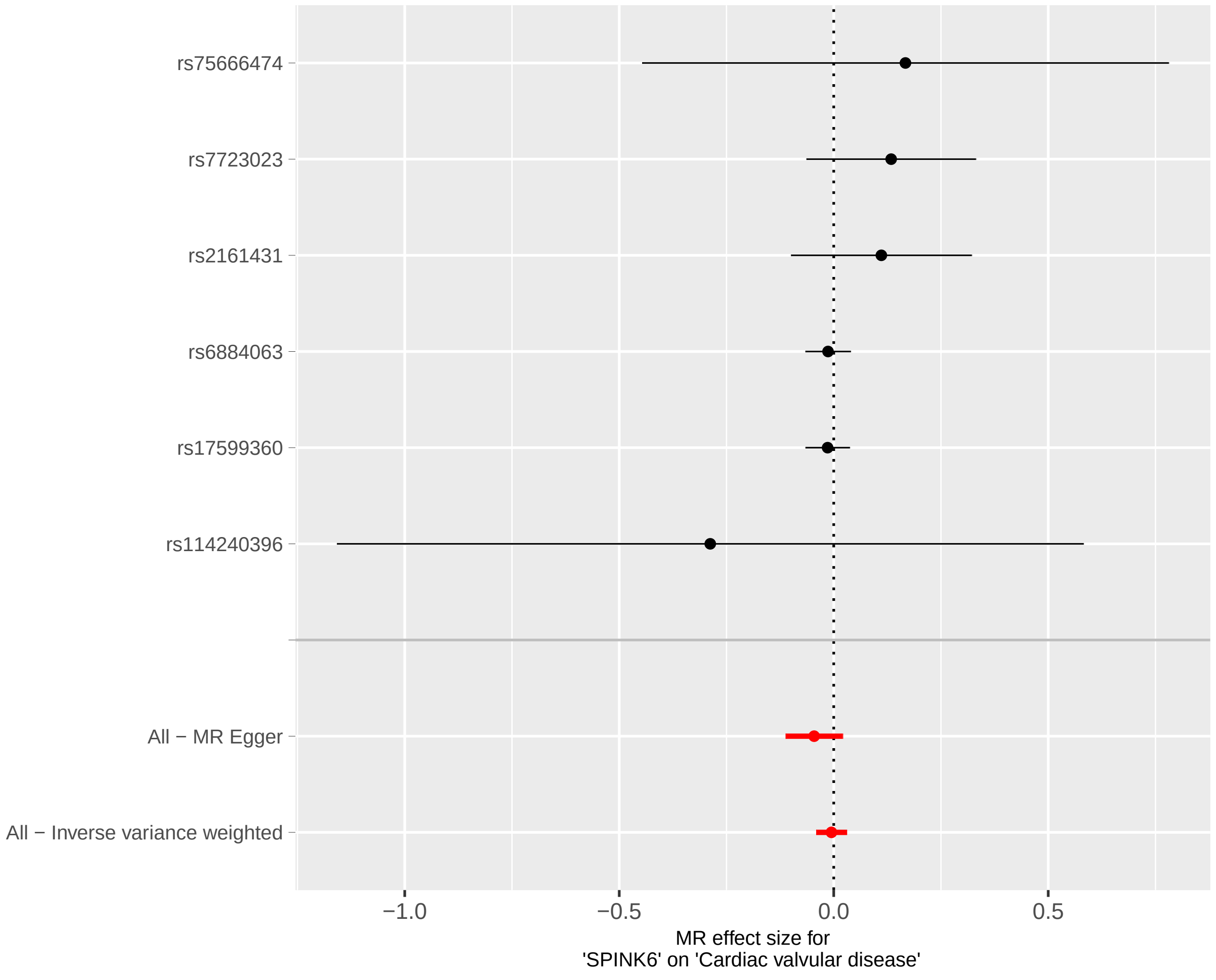

rs75666474
rs7723023
rs2161431
rs6884063
rs17599360
rs114240396
All − MR Egger
All − Inverse variance weighted
−1.0
−0.5
0.0
0.5
MR effect size for
'SPINK6' on 'Cardiac valvular disease'

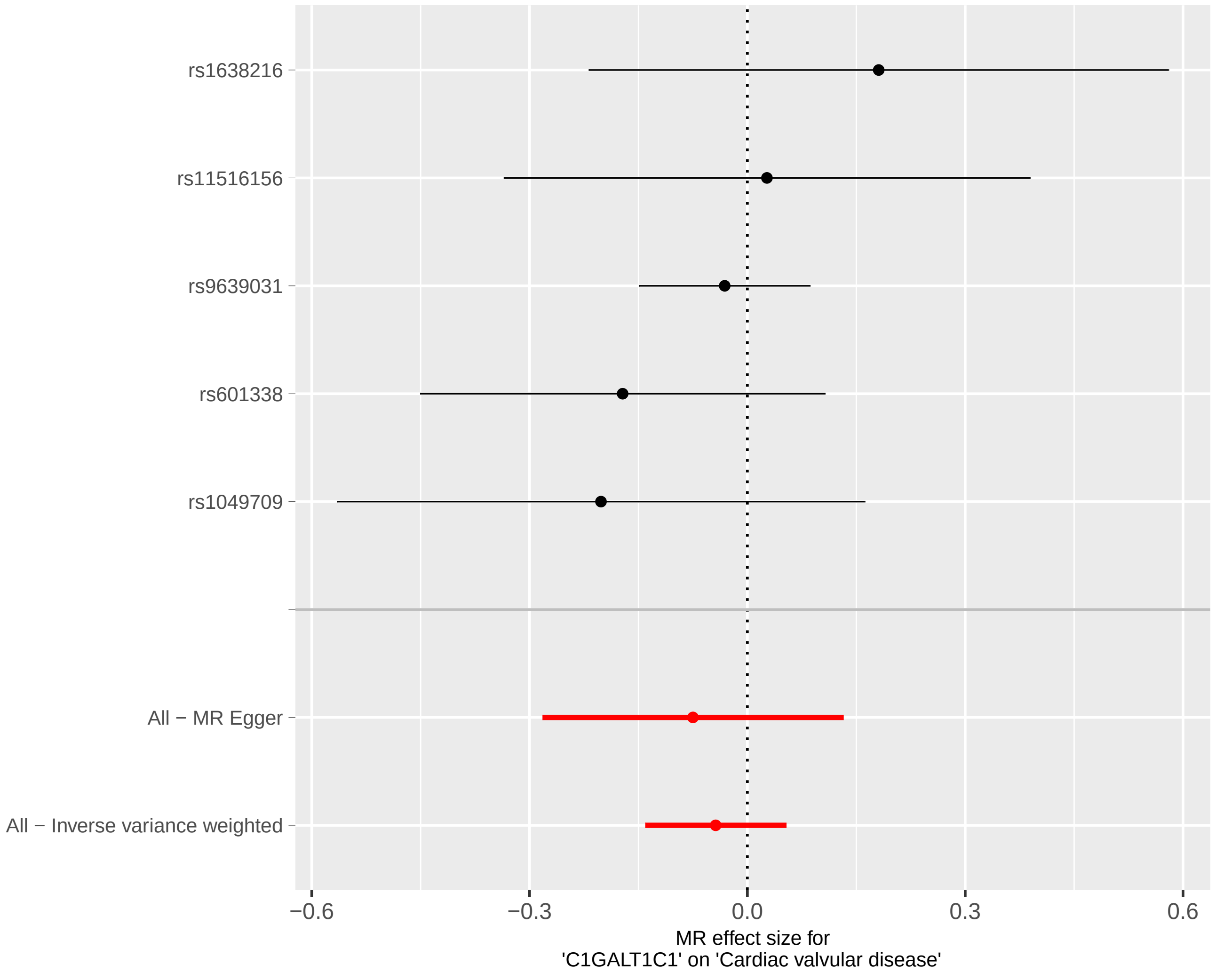
rs1638216
rs11516156
rs9639031
rs601338
rs1049709
All − MR Egger
All − Inverse variance weighted
−0.6
−0.3
0.0
0.3
0.6
MR effect size for
'C1GALT1C1' on 'Cardiac valvular disease'

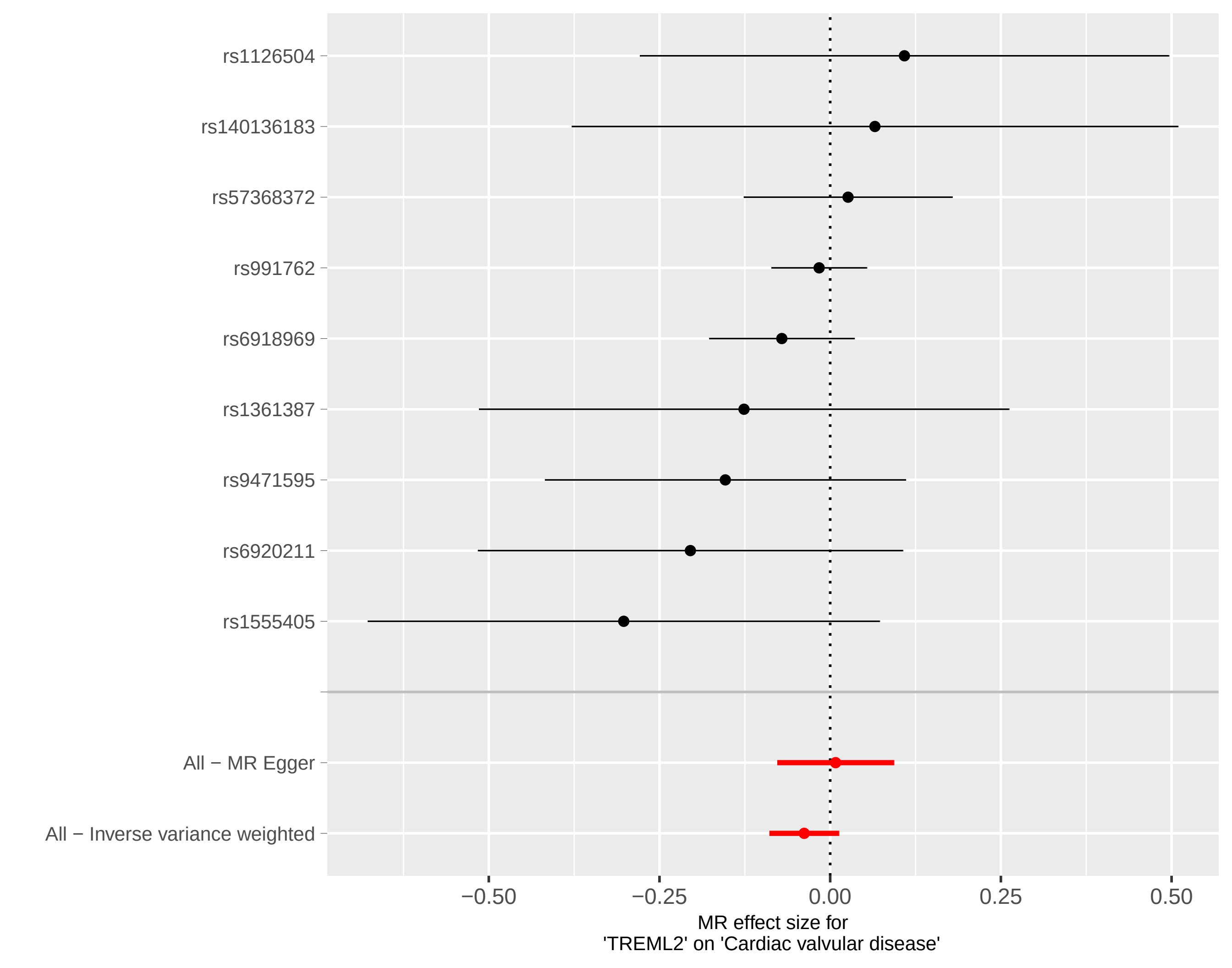
rs1126504
rs140136183
rs57368372
rs991762
rs6918969
rs1361387
rs9471595
rs6920211
rs1555405
All − MR Egger
All − Inverse variance weighted
−0.50
−0.25
0.00
0.25
0.50
MR effect size for
'TREML2' on 'Cardiac valvular disease'

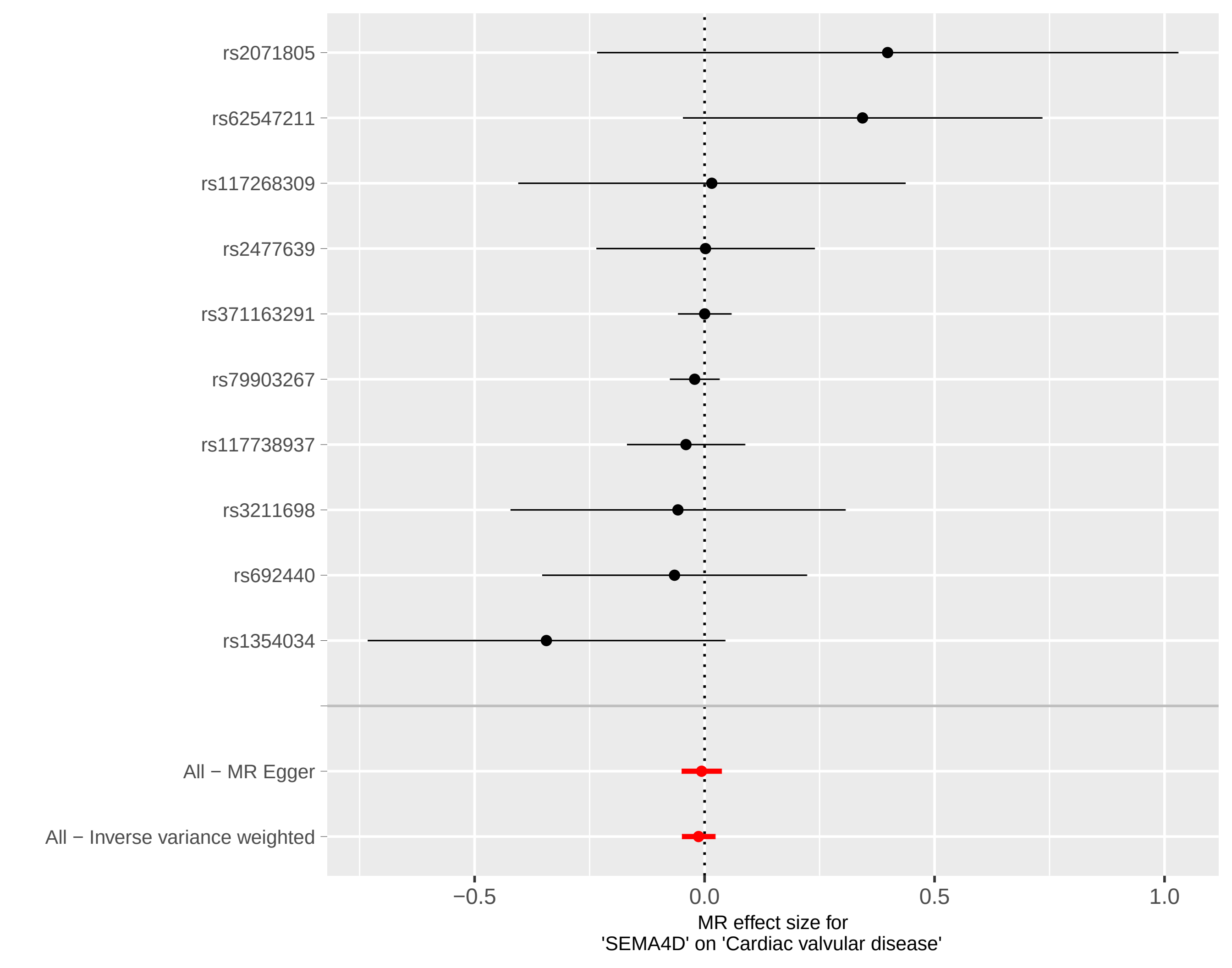
rs2071805
rs62547211
rs117268309
rs2477639
rs371163291
rs79903267
rs117738937
rs3211698
rs692440
rs1354034
All − MR Egger
All − Inverse variance weighted
−0.5
0.0
0.5
1.0
MR effect size for
'SEMA4D' on 'Cardiac valvular disease'

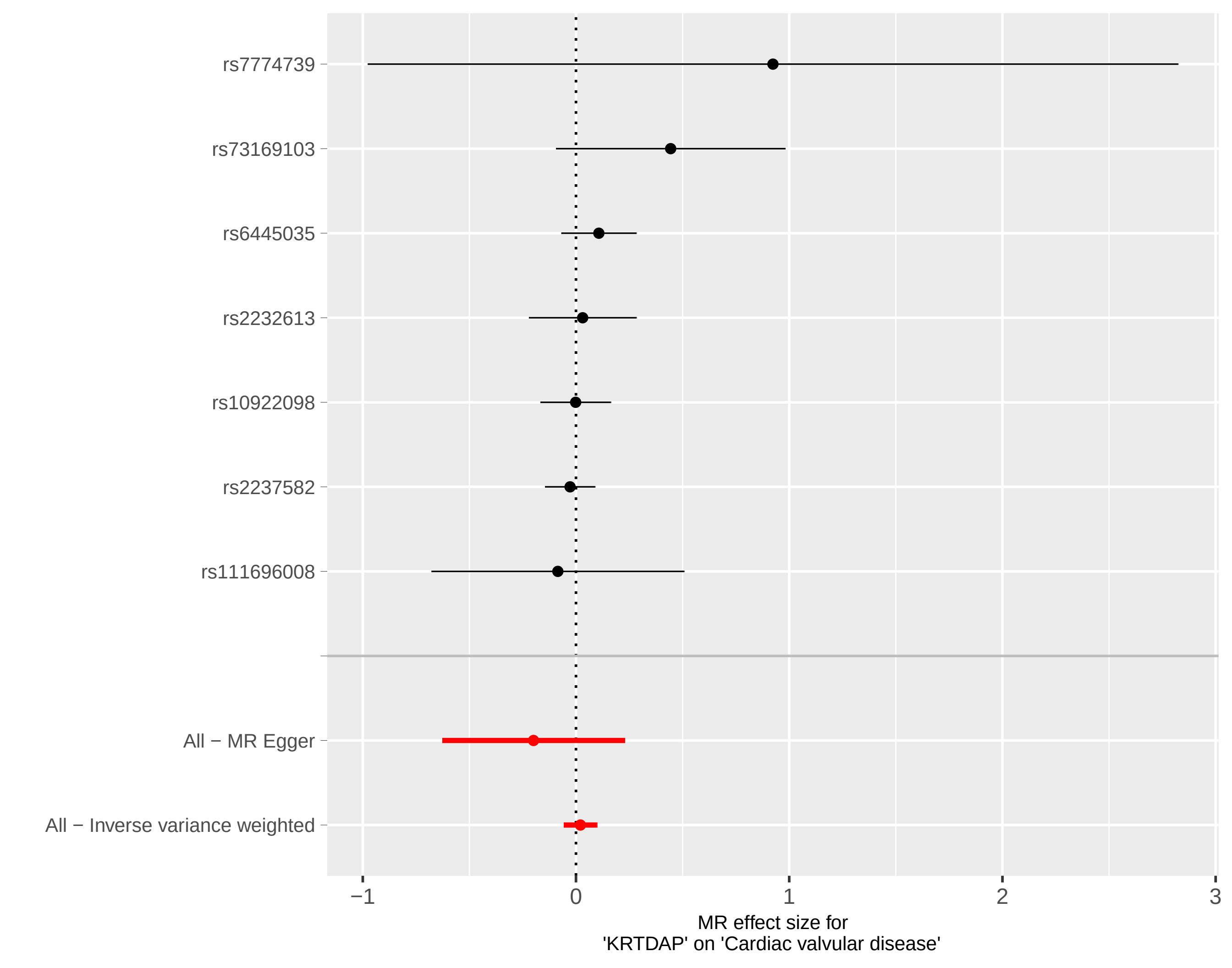
rs7774739
rs73169103
rs6445035
rs2232613
rs10922098
rs2237582
rs111696008
All − MR Egger
All − Inverse variance weighted
−1
0
1
2
3
MR effect size for
'KRTDAP' on 'Cardiac valvular disease'

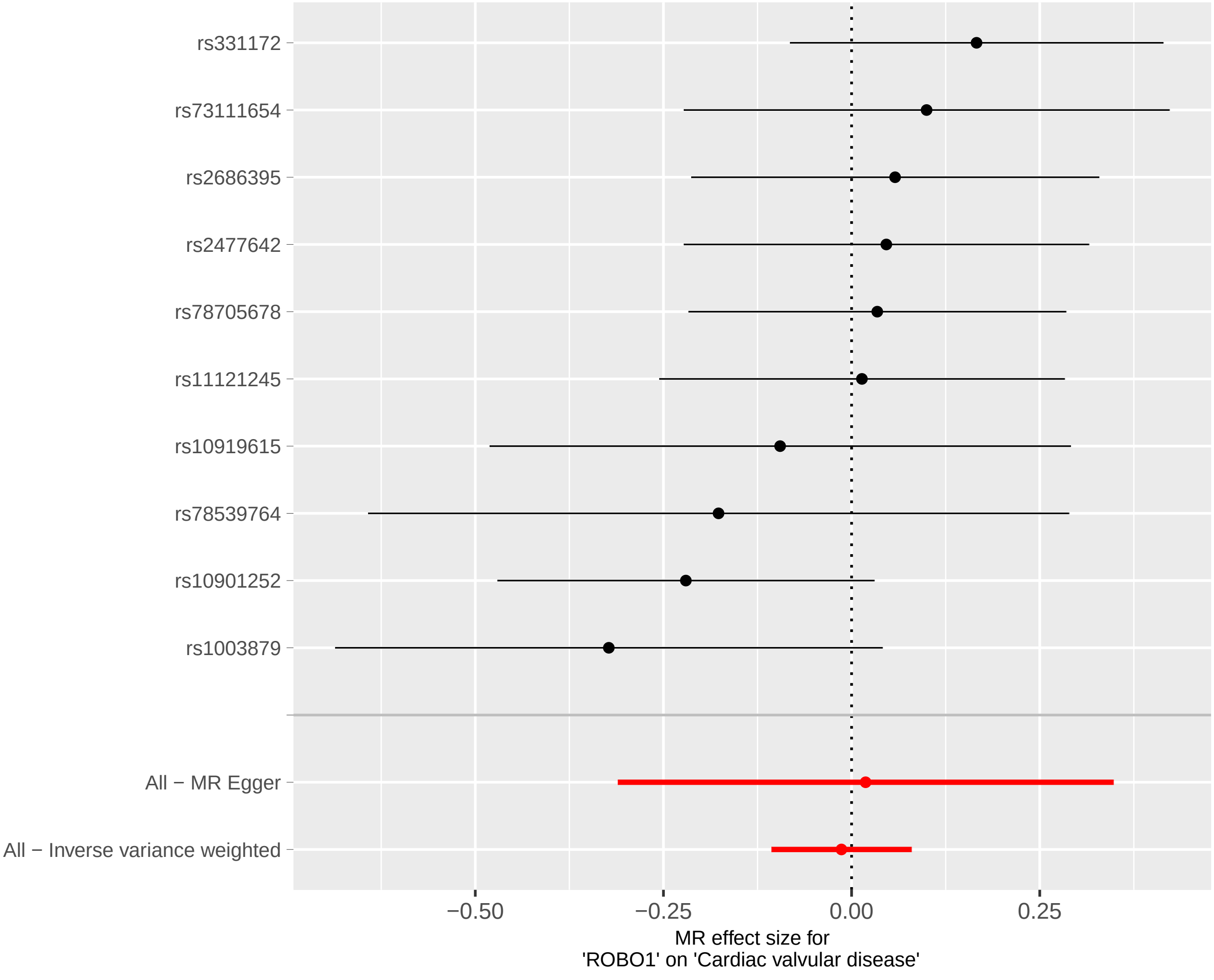
rs331172
rs73111654
rs2686395
rs2477642
rs78705678
rs11121245
rs10919615
rs78539764
rs10901252
rs1003879
All − MR Egger
All − Inverse variance weighted
−0.50
−0.25
0.00
0.25
MR effect size for
'ROBO1' on 'Cardiac valvular disease'

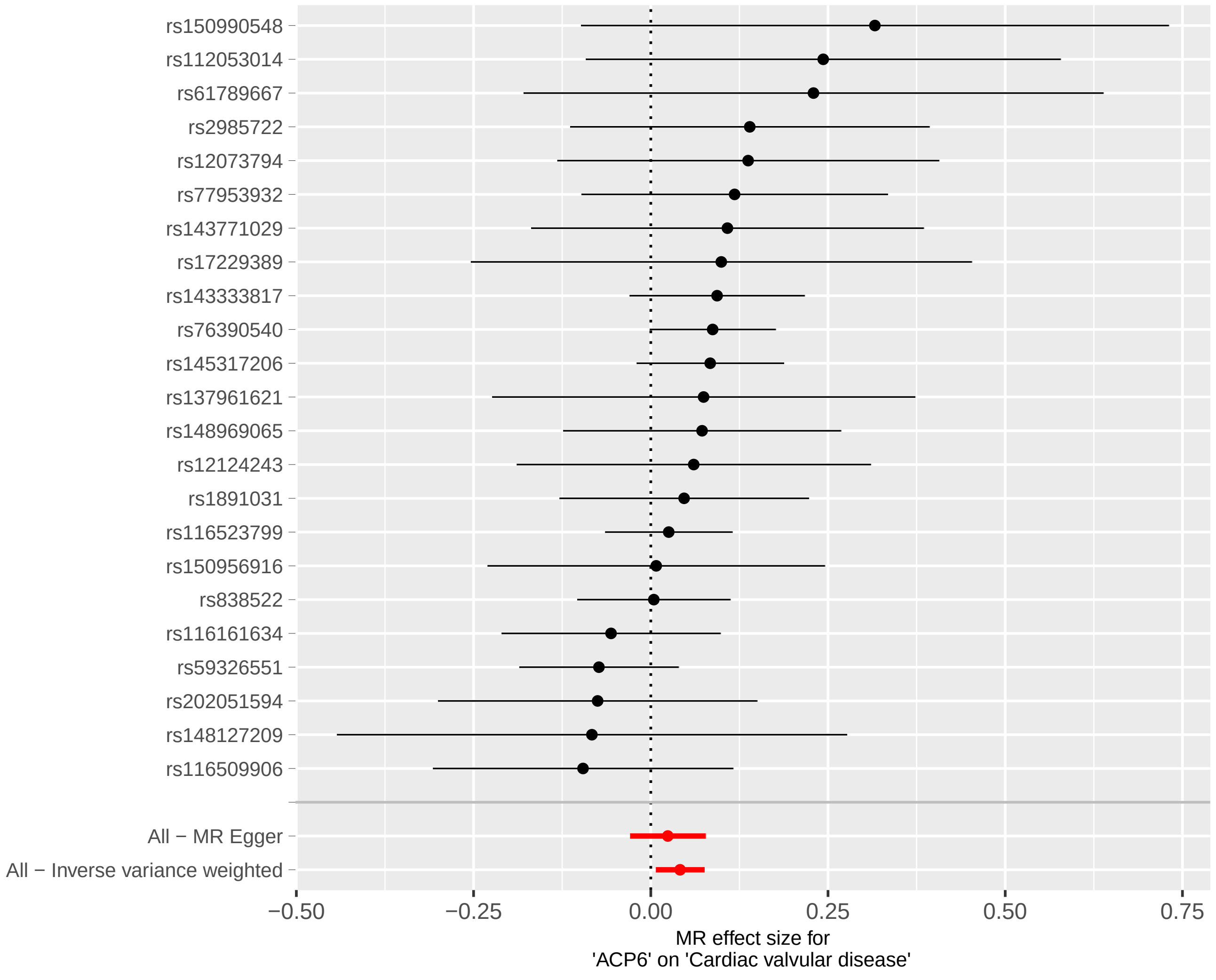
rs150990548
rs112053014
rs61789667
rs2985722
rs12073794
rs77953932
rs143771029
rs17229389
rs143333817
rs76390540
rs145317206
rs137961621
rs148969065
rs12124243
rs1891031
rs116523799
rs150956916
rs838522
rs116161634
rs59326551
rs202051594
rs148127209
rs116509906
All − MR Egger
All − Inverse variance weighted
−0.50
−0.25
0.00
0.25
0.50
0.75
MR effect size for
'ACP6' on 'Cardiac valvular disease'

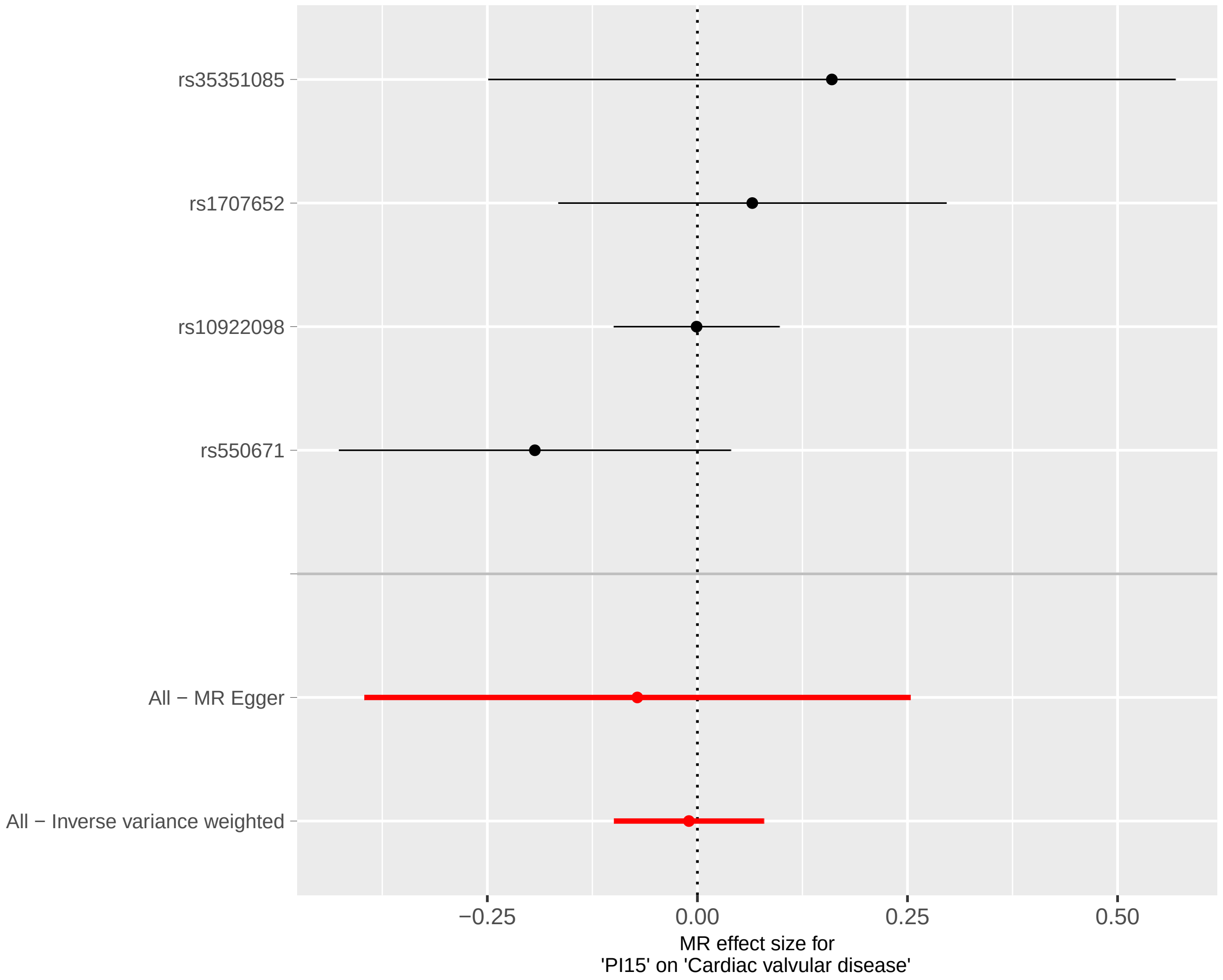
rs35351085
rs1707652
rs10922098
rs550671
All − MR Egger
All − Inverse variance weighted
−0.25
0.00
0.25
0.50
MR effect size for
'PI15' on 'Cardiac valvular disease'

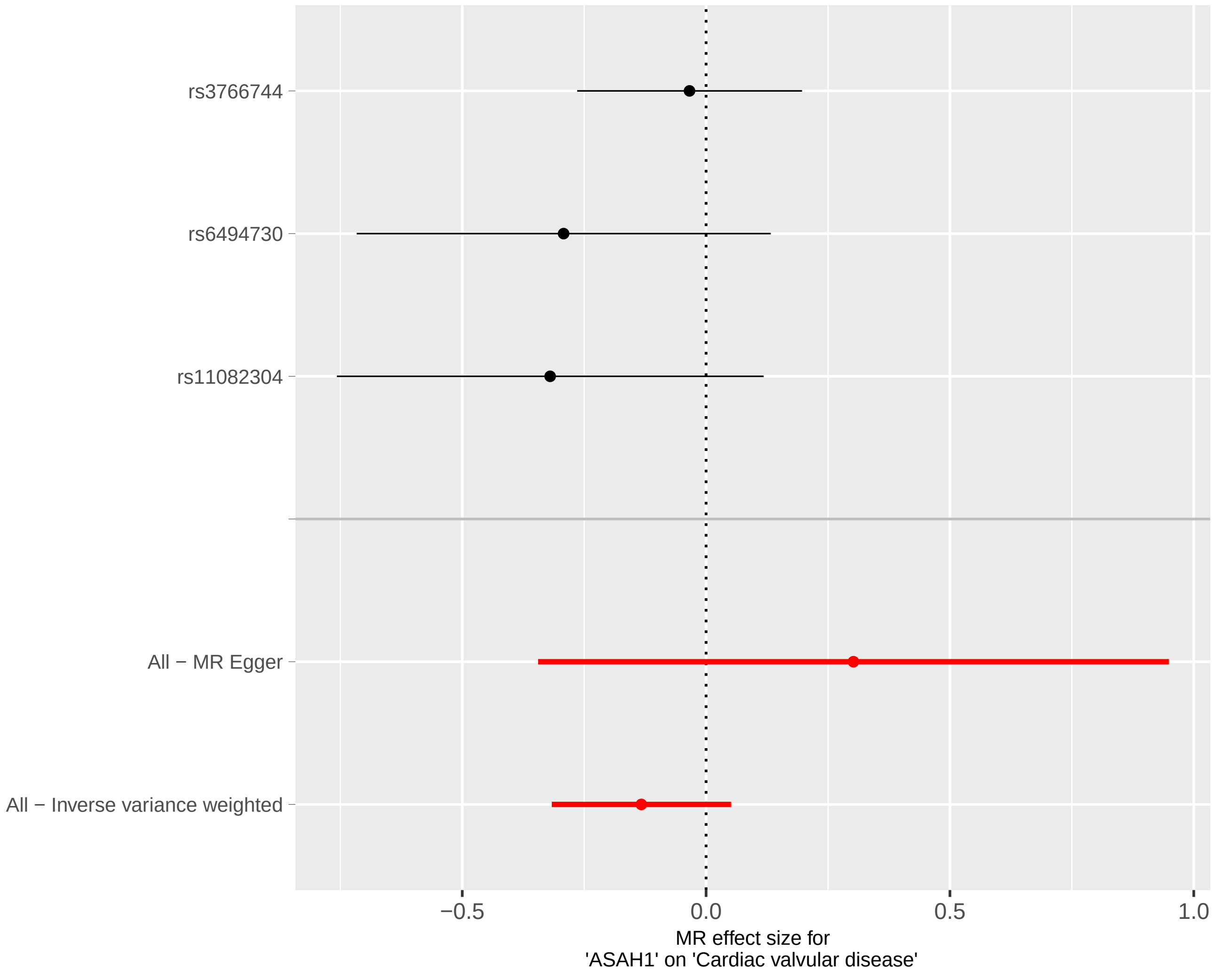
rs3766744
rs6494730
rs11082304
All − MR Egger
All − Inverse variance weighted
−0.5
0.0
0.5
1.0
MR effect size for
'ASAH1' on 'Cardiac valvular disease'

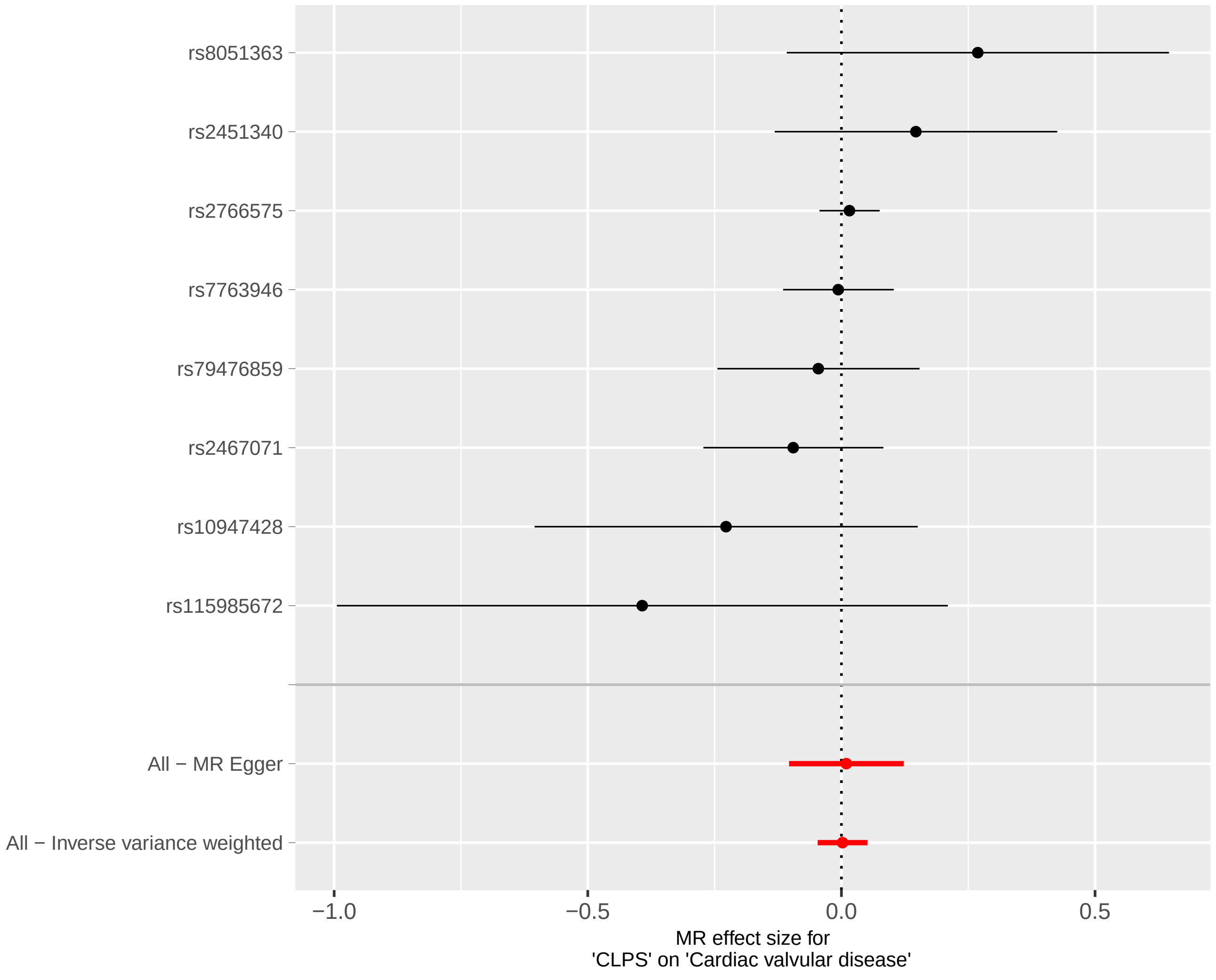

rs8051363
rs2451340
rs2766575
rs7763946
rs79476859
rs2467071
rs10947428
rs115985672
All − MR Egger
All − Inverse variance weighted
−1.0
−0.5
0.0
0.5
MR effect size for
'CLPS' on 'Cardiac valvular disease'

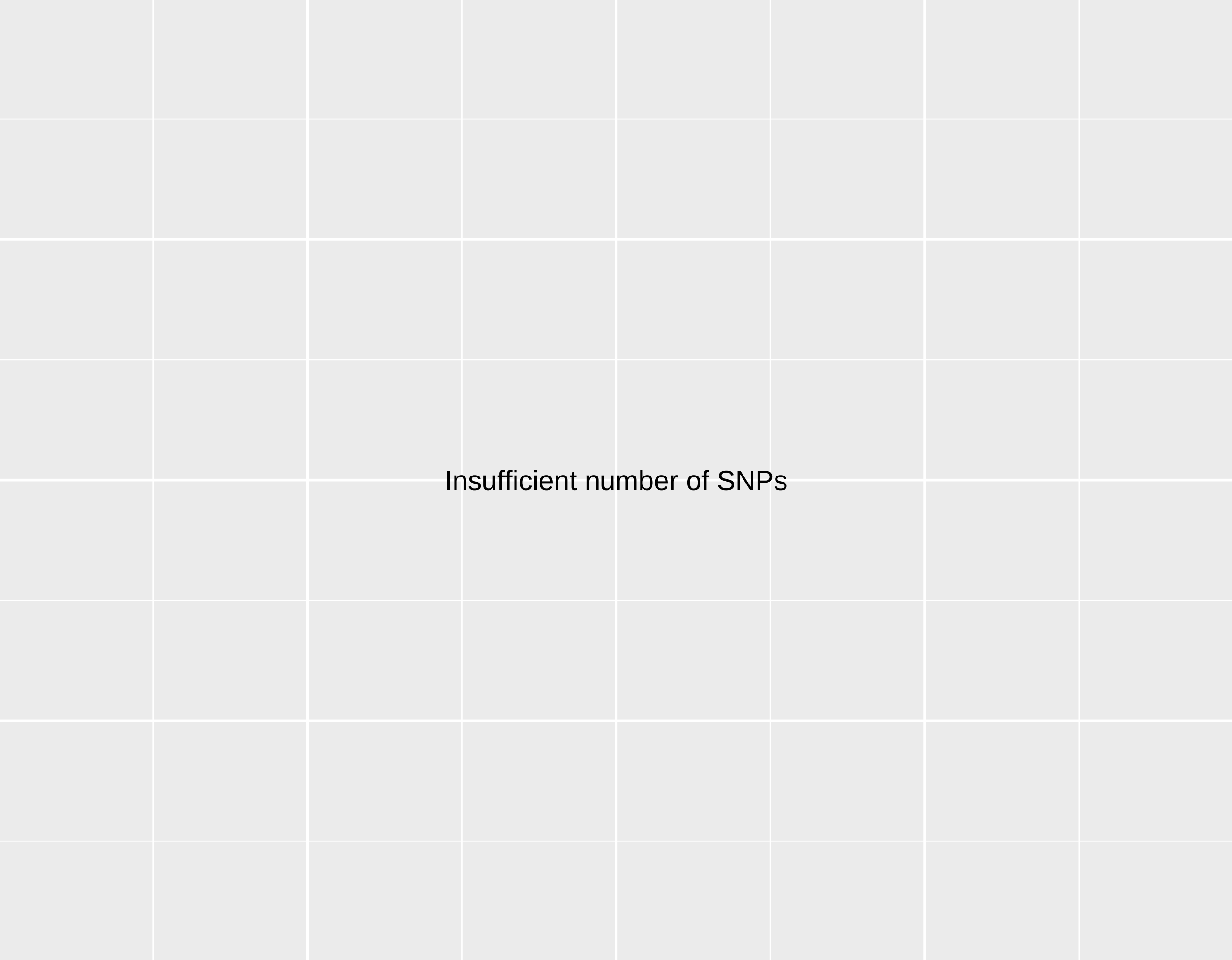
Insufficient number of SNPs

Insufficient number of SNPs

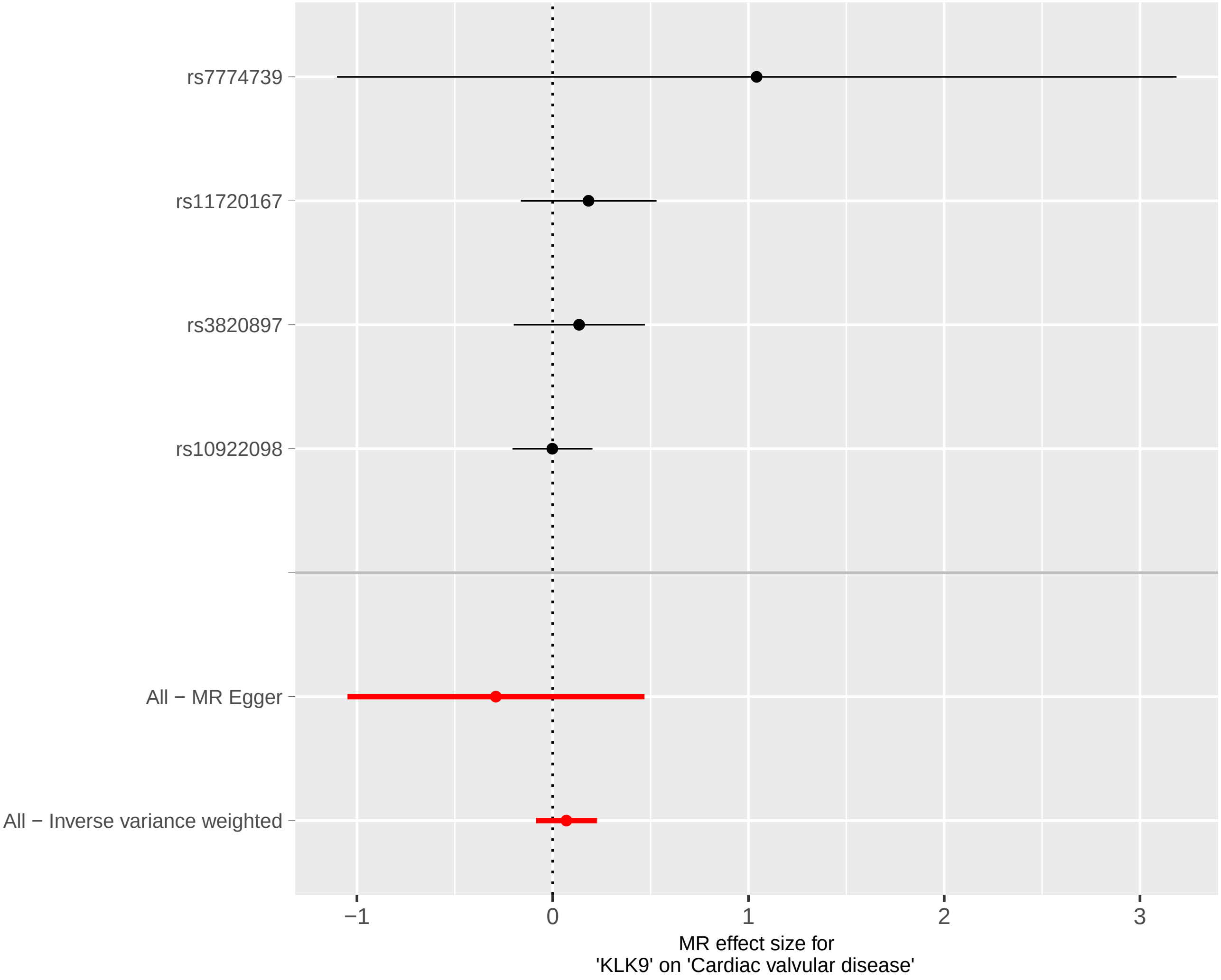
rs7774739
rs11720167
rs3820897
rs10922098
All − MR Egger
All − Inverse variance weighted
−1
0
1
2
3
MR effect size for
'KLK9' on 'Cardiac valvular disease'

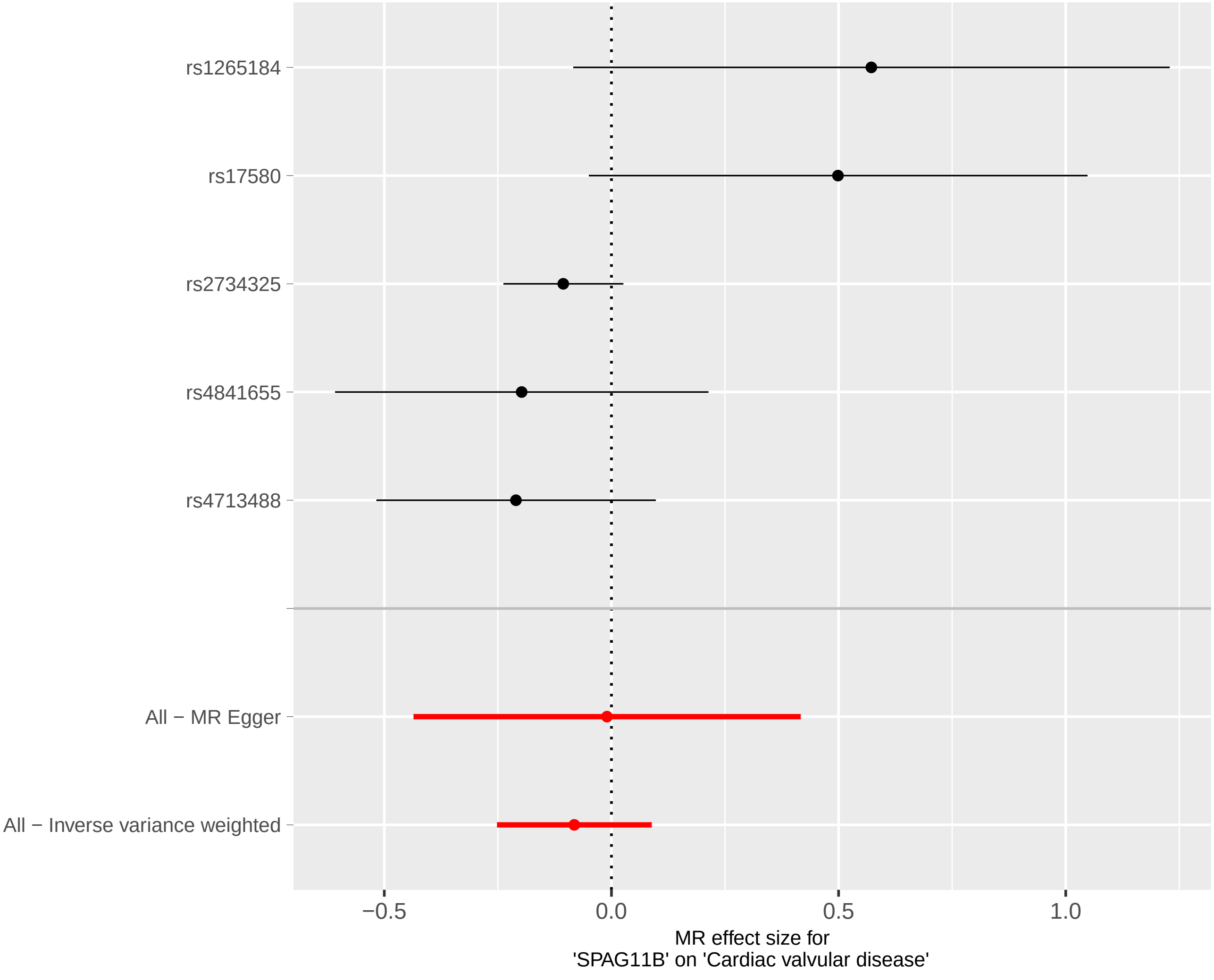
rs1265184
rs17580
rs2734325
rs4841655
rs4713488
All − MR Egger
All − Inverse variance weighted
−0.5
0.0
0.5
1.0
MR effect size for
'SPAG11B' on 'Cardiac valvular disease'

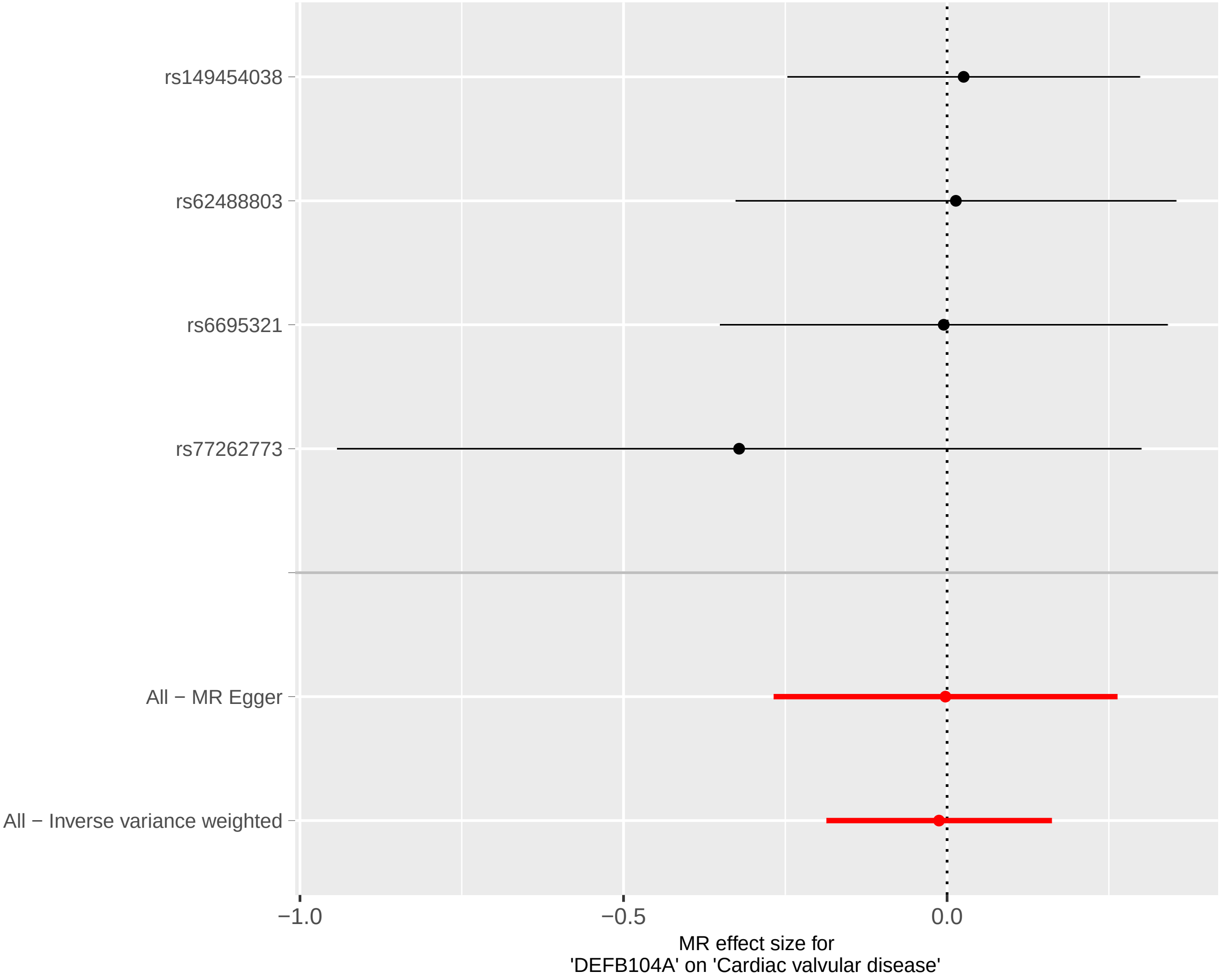
rs149454038
rs62488803
rs6695321
rs77262773
All − MR Egger
All − Inverse variance weighted
−1.0
−0.5
0.0
MR effect size for
'DEFB104A' on 'Cardiac valvular disease'

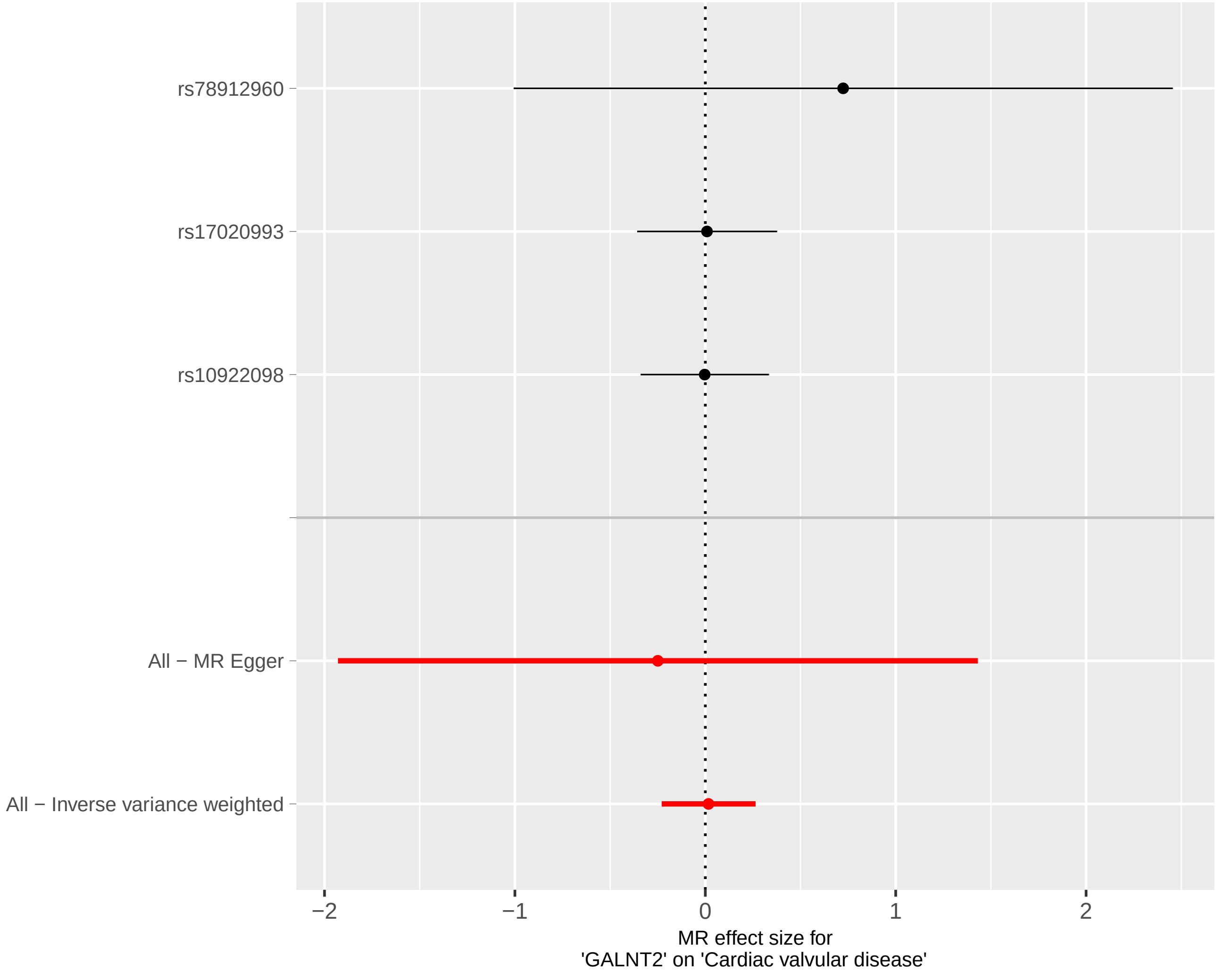
rs78912960
rs17020993
rs10922098
All − MR Egger
All − Inverse variance weighted
−2
−1
0
1
2
MR effect size for
'GALNT2' on 'Cardiac valvular disease'

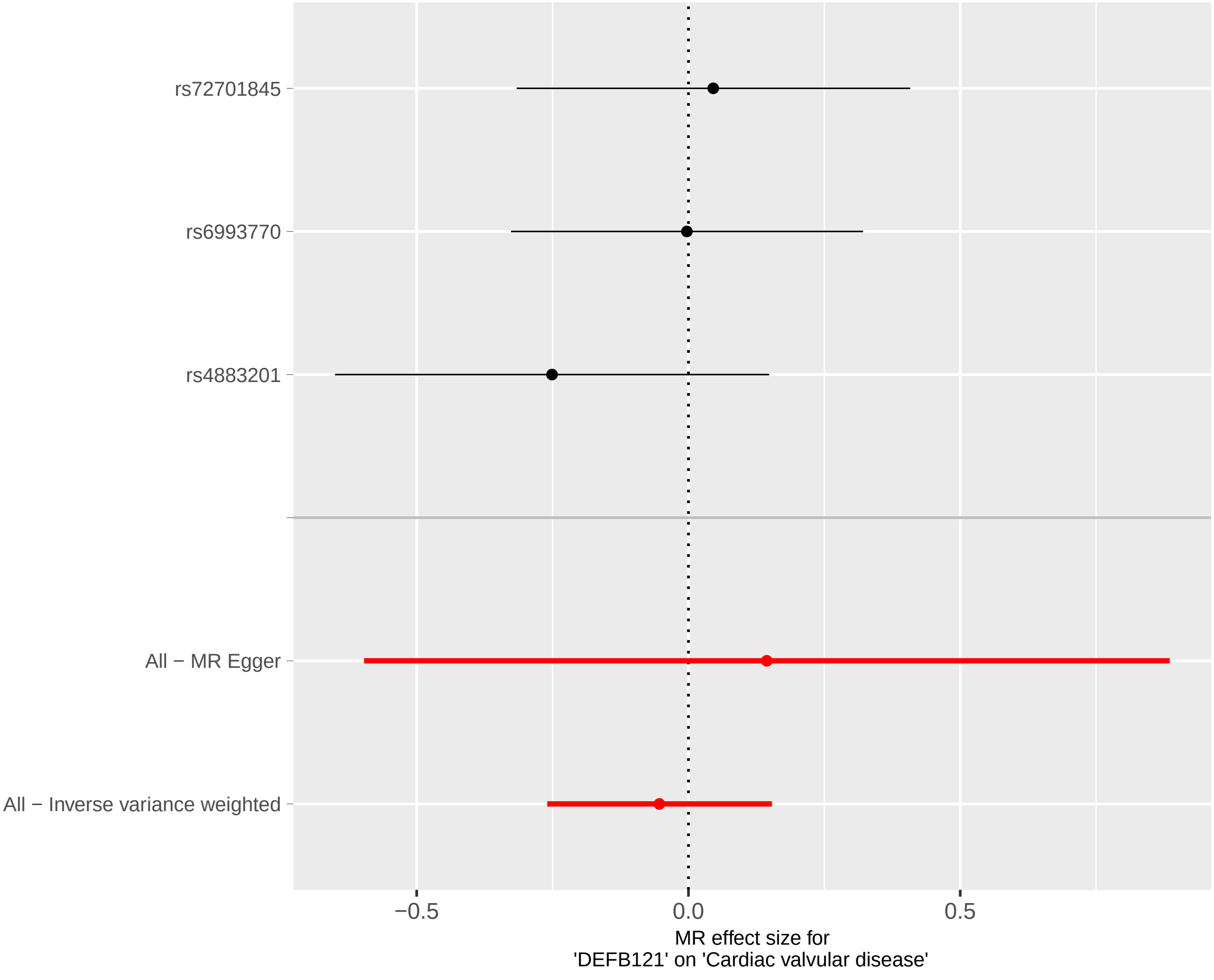
rs72701845
rs6993770
rs4883201
All − MR Egger
All − Inverse variance weighted
−0.5
0.0
0.5
MR effect size for
'DEFB121' on 'Cardiac valvular disease'

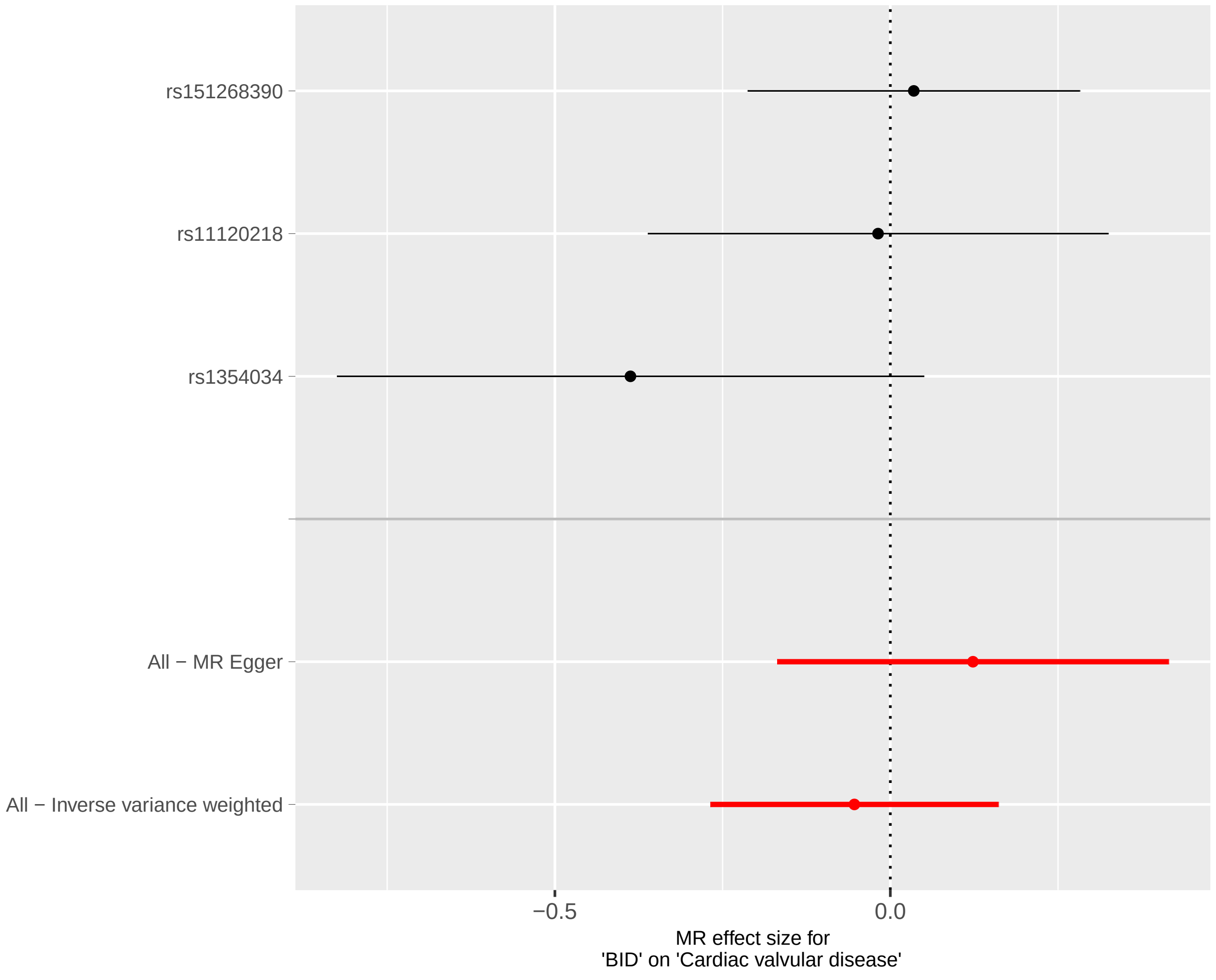
rs151268390
rs11120218
rs1354034
All − MR Egger
All − Inverse variance weighted
−0.5
0.0
MR effect size for
'BID' on 'Cardiac valvular disease'

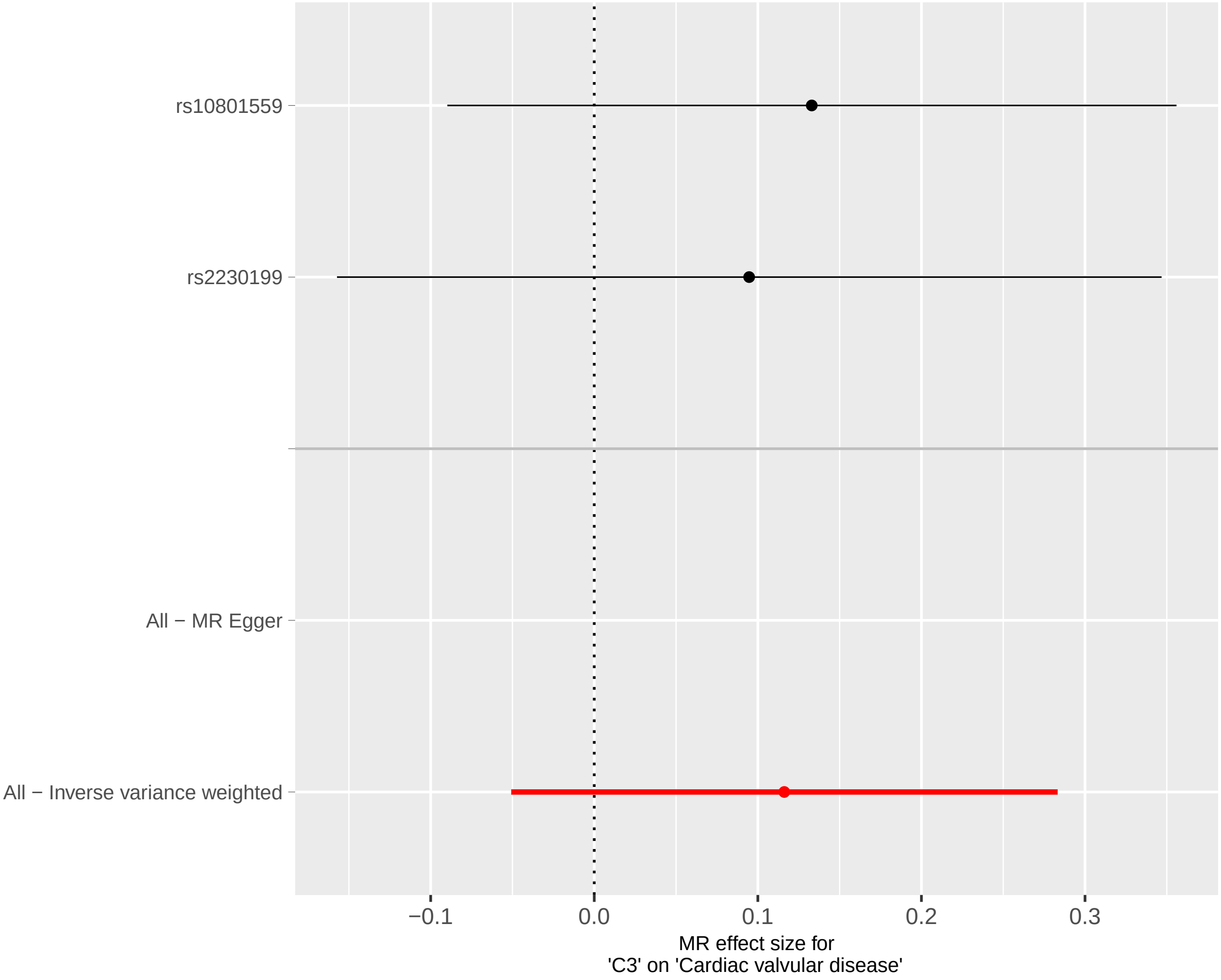

rs10801559
rs2230199
All − MR Egger
All − Inverse variance weighted
−0.1
0.0
0.1
0.2
0.3
MR effect size for
'C3' on 'Cardiac valvular disease'

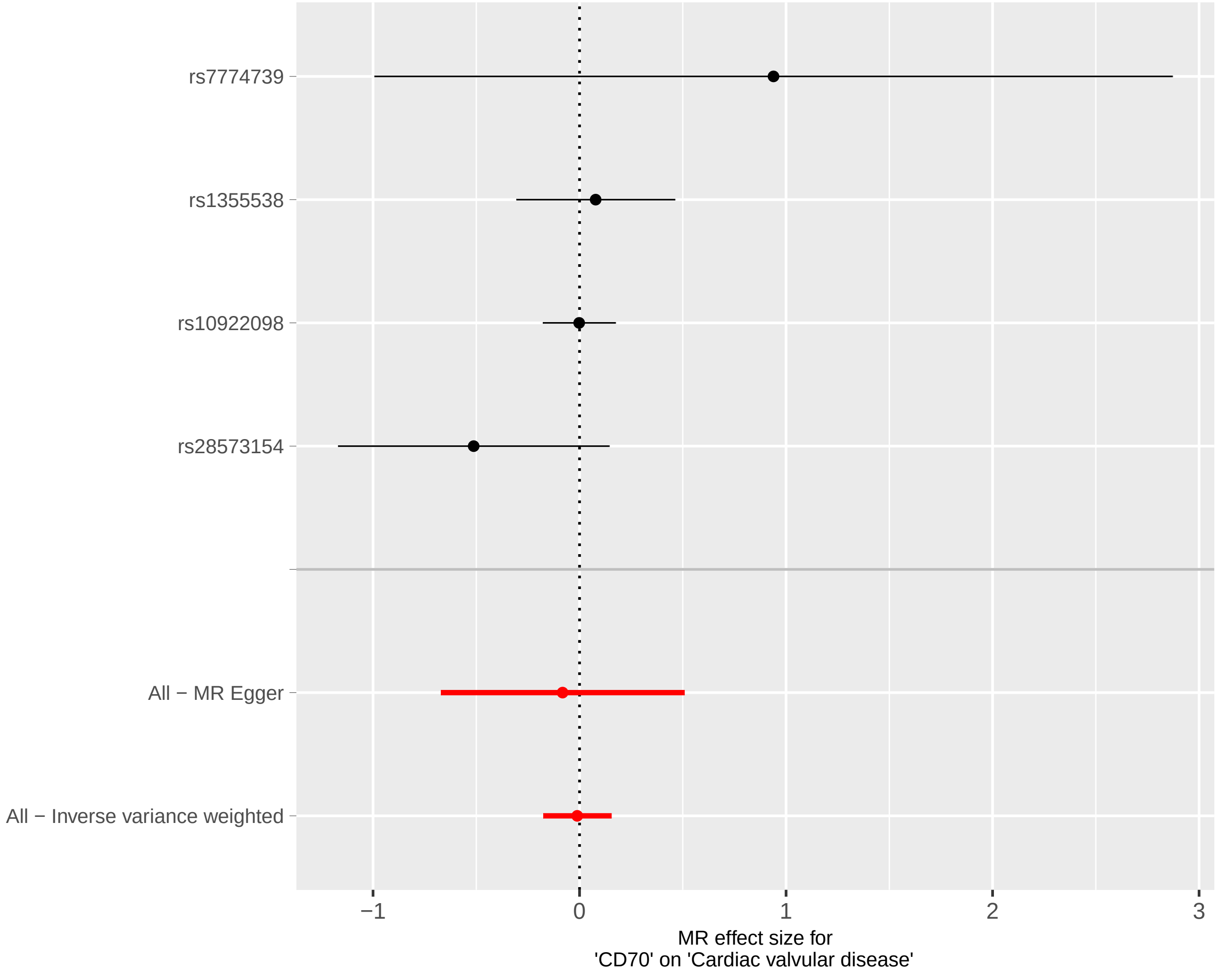

rs7774739
rs1355538
rs10922098
rs28573154
All − MR Egger
All − Inverse variance weighted
−1
0
1
2
3
MR effect size for
'CD70' on 'Cardiac valvular disease'

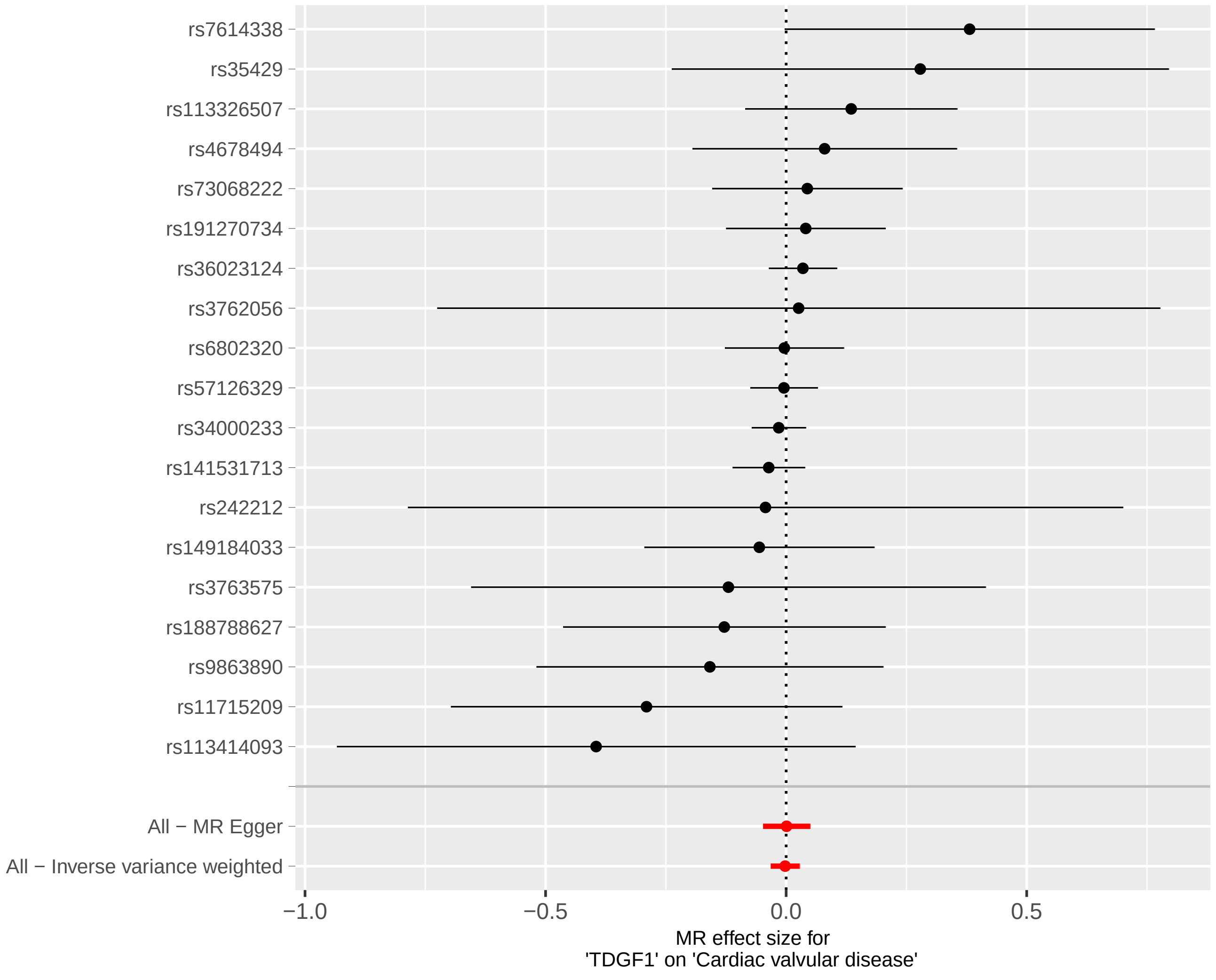
rs7614338
rs35429
rs113326507
rs4678494
rs73068222
rs191270734
rs36023124
rs3762056
rs6802320
rs57126329
rs34000233
rs141531713
rs242212
rs149184033
rs3763575
rs188788627
rs9863890
rs11715209
rs113414093
All − MR Egger
All − Inverse variance weighted
−1.0
−0.5
0.0
0.5
MR effect size for
'TDGF1' on 'Cardiac valvular disease'

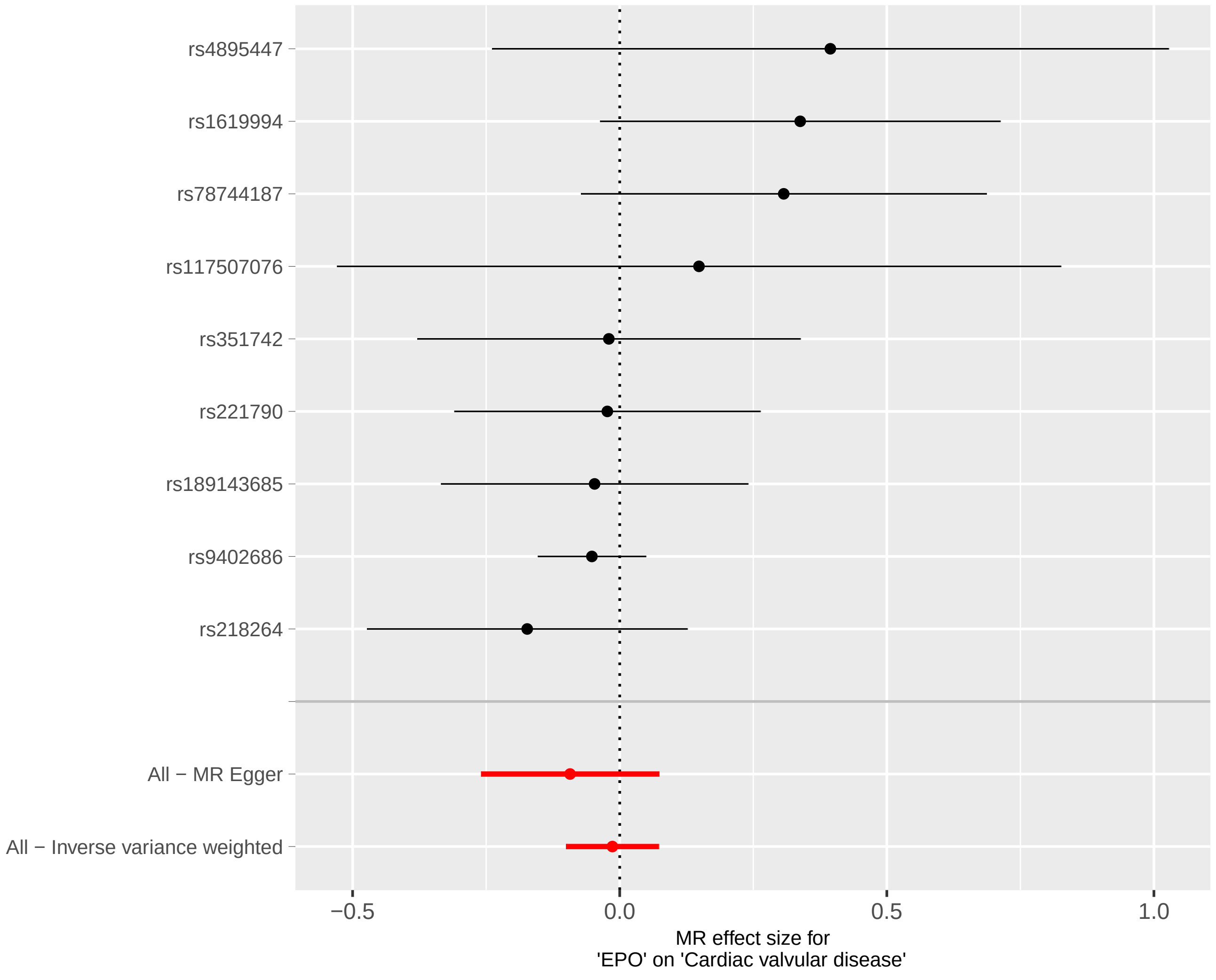
rs4895447
rs1619994
rs78744187
rs117507076
rs351742
rs221790
rs189143685
rs9402686
rs218264
All − MR Egger
All − Inverse variance weighted
−0.5
0.0
0.5
1.0
MR effect size for
'EPO' on 'Cardiac valvular disease'

Insufficient number of SNPs

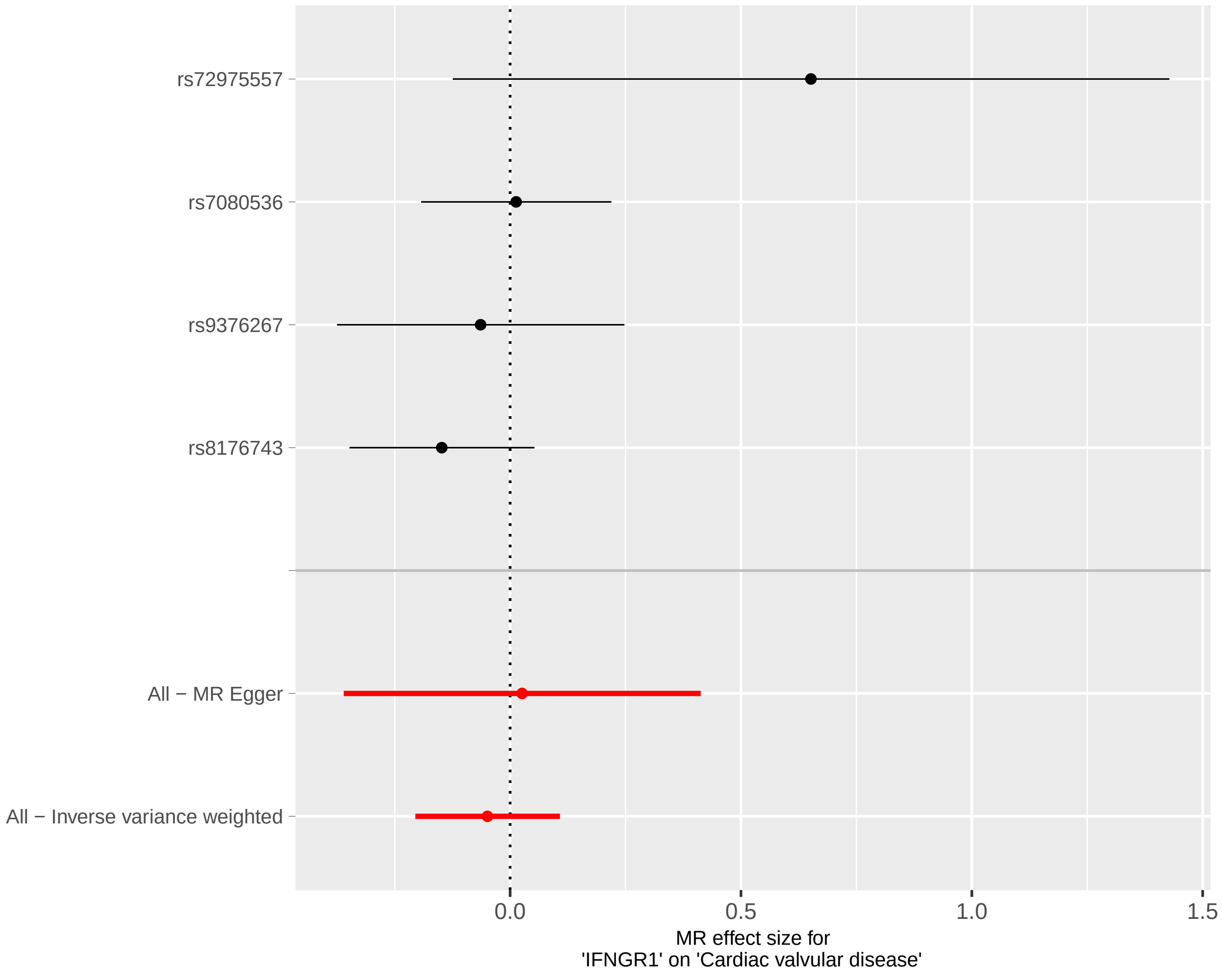

rs72975557
rs7080536
rs9376267
rs8176743
All − MR Egger
All − Inverse variance weighted
0.0
0.5
1.0
1.5
MR effect size for
'IFNGR1' on 'Cardiac valvular disease'

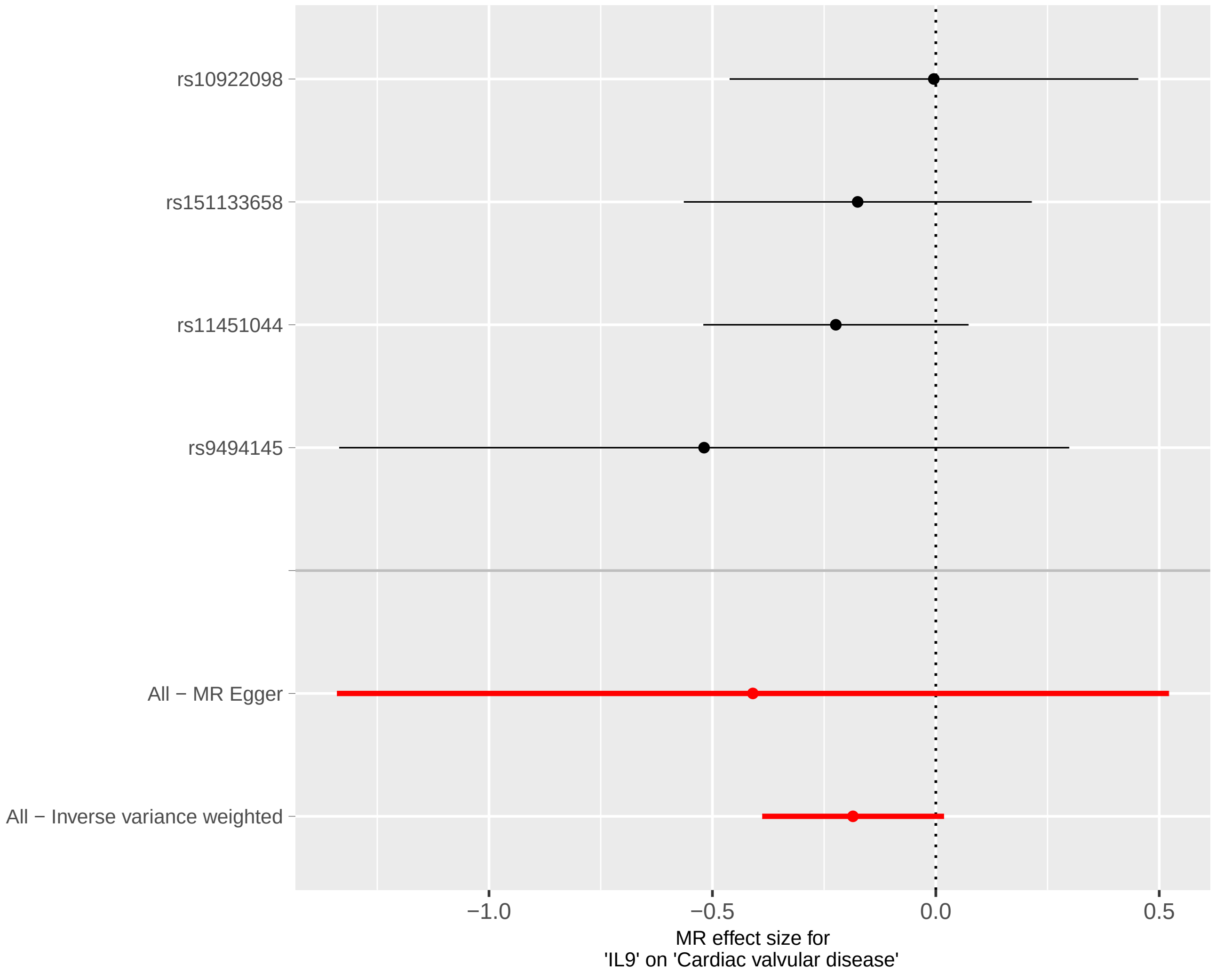
rs10922098
rs151133658
rs11451044
rs9494145
All − MR Egger
All − Inverse variance weighted
−1.0
−0.5
0.0
0.5
MR effect size for
'IL9' on 'Cardiac valvular disease'

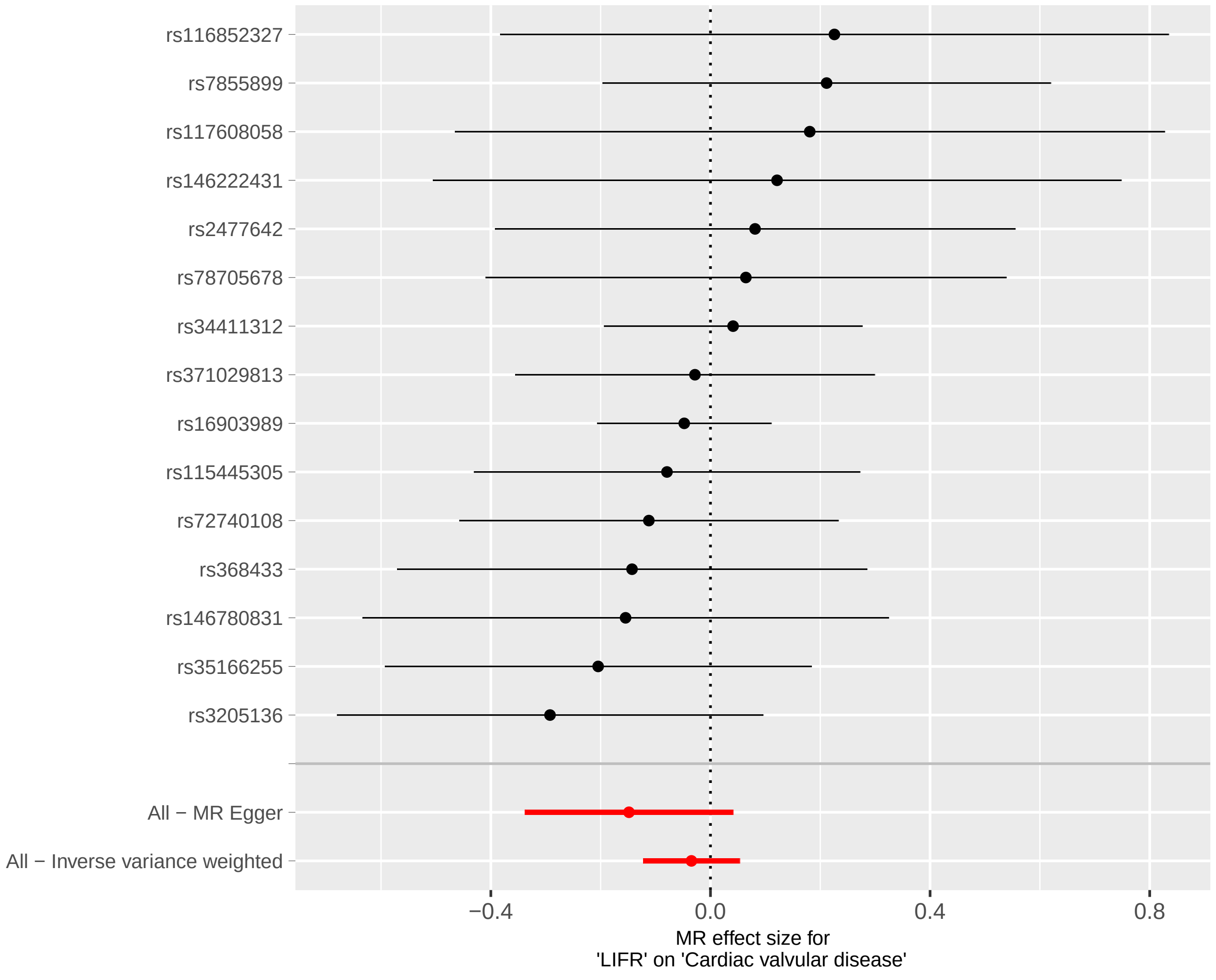

rs116852327
rs7855899
rs117608058
rs146222431
rs2477642
rs78705678
rs34411312
rs371029813
rs16903989
rs115445305
rs72740108
rs368433
rs146780831
rs35166255
rs3205136
All − MR Egger
All − Inverse variance weighted
−0.4
0.0
0.4
0.8
MR effect size for
'LIFR' on 'Cardiac valvular disease'

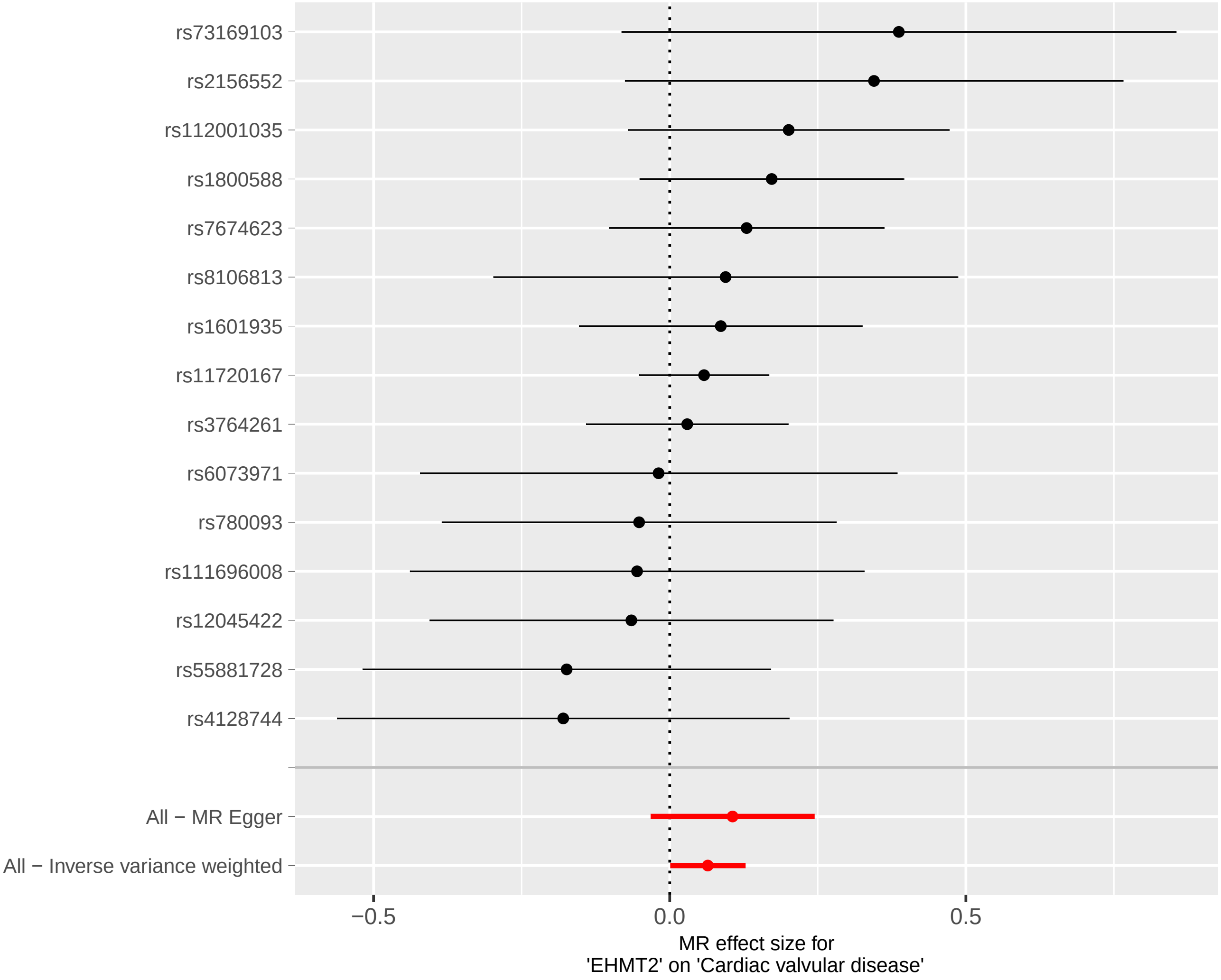
rs73169103
rs2156552
rs112001035
rs1800588
rs7674623
rs8106813
rs1601935
rs11720167
rs3764261
rs6073971
rs780093
rs111696008
rs12045422
rs55881728
rs4128744
All − MR Egger
All − Inverse variance weighted
−0.5
0.0
0.5
MR effect size for
'EHMT2' on 'Cardiac valvular disease'

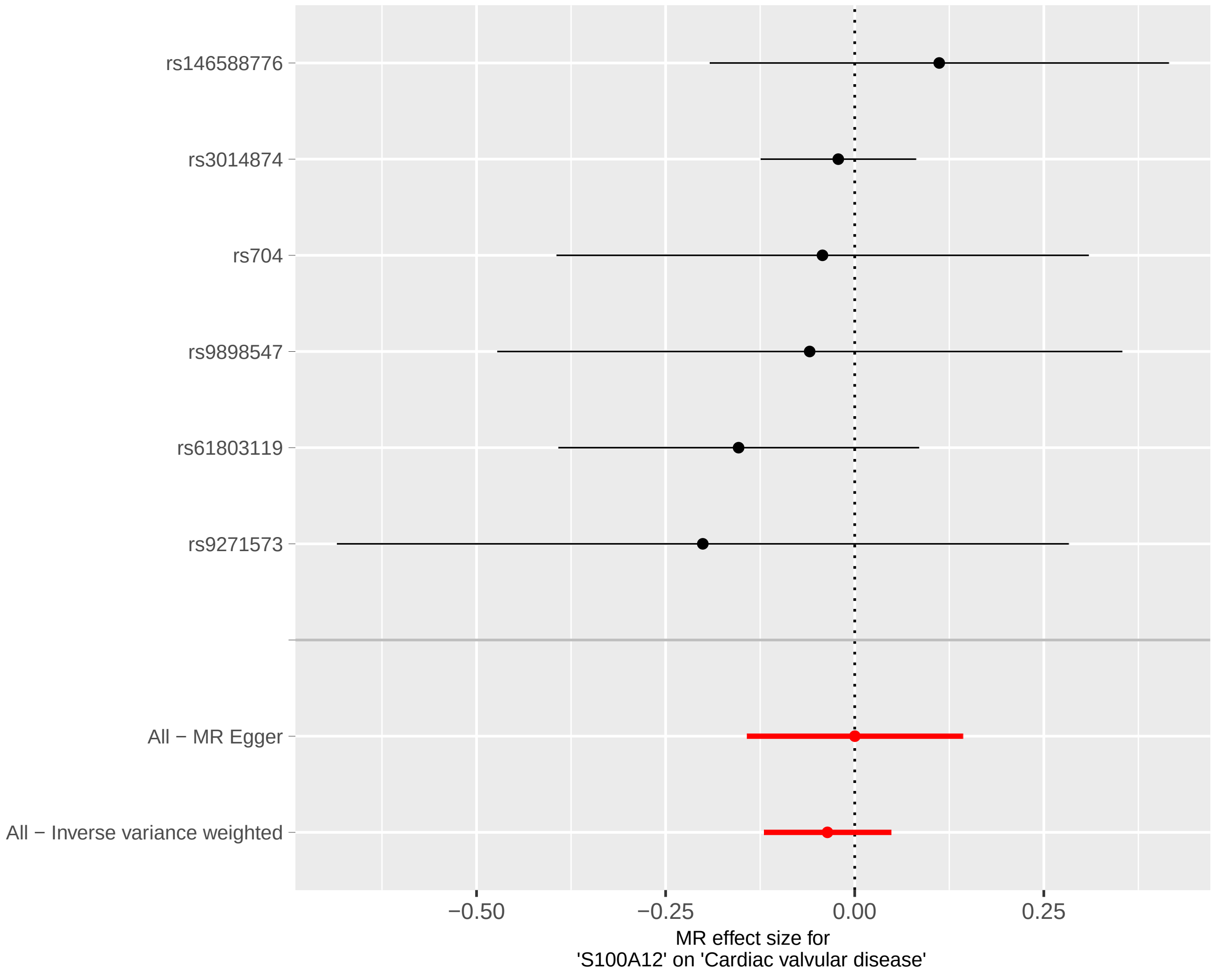

rs146588776
rs3014874
rs704
rs9898547
rs61803119
rs9271573
All − MR Egger
All − Inverse variance weighted
−0.50
−0.25
0.00
0.25
MR effect size for
'S100A12' on 'Cardiac valvular disease'

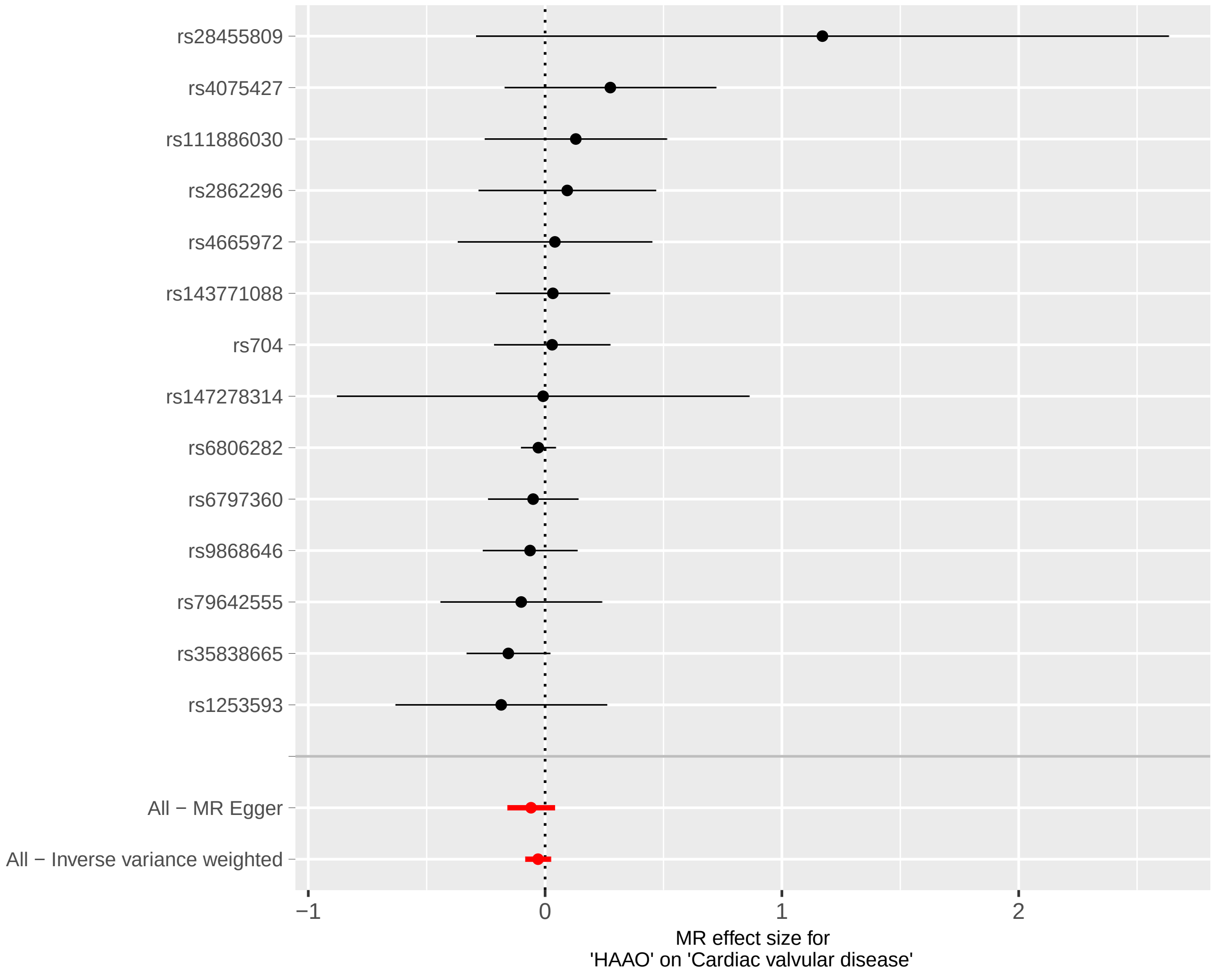

rs28455809
rs4075427
rs111886030
rs2862296
rs4665972
rs143771088
rs704
rs147278314
rs6806282
rs6797360
rs9868646
rs79642555
rs35838665
rs1253593
All − MR Egger
All − Inverse variance weighted
−1
0
1
2
MR effect size for
'HAAO' on 'Cardiac valvular disease'

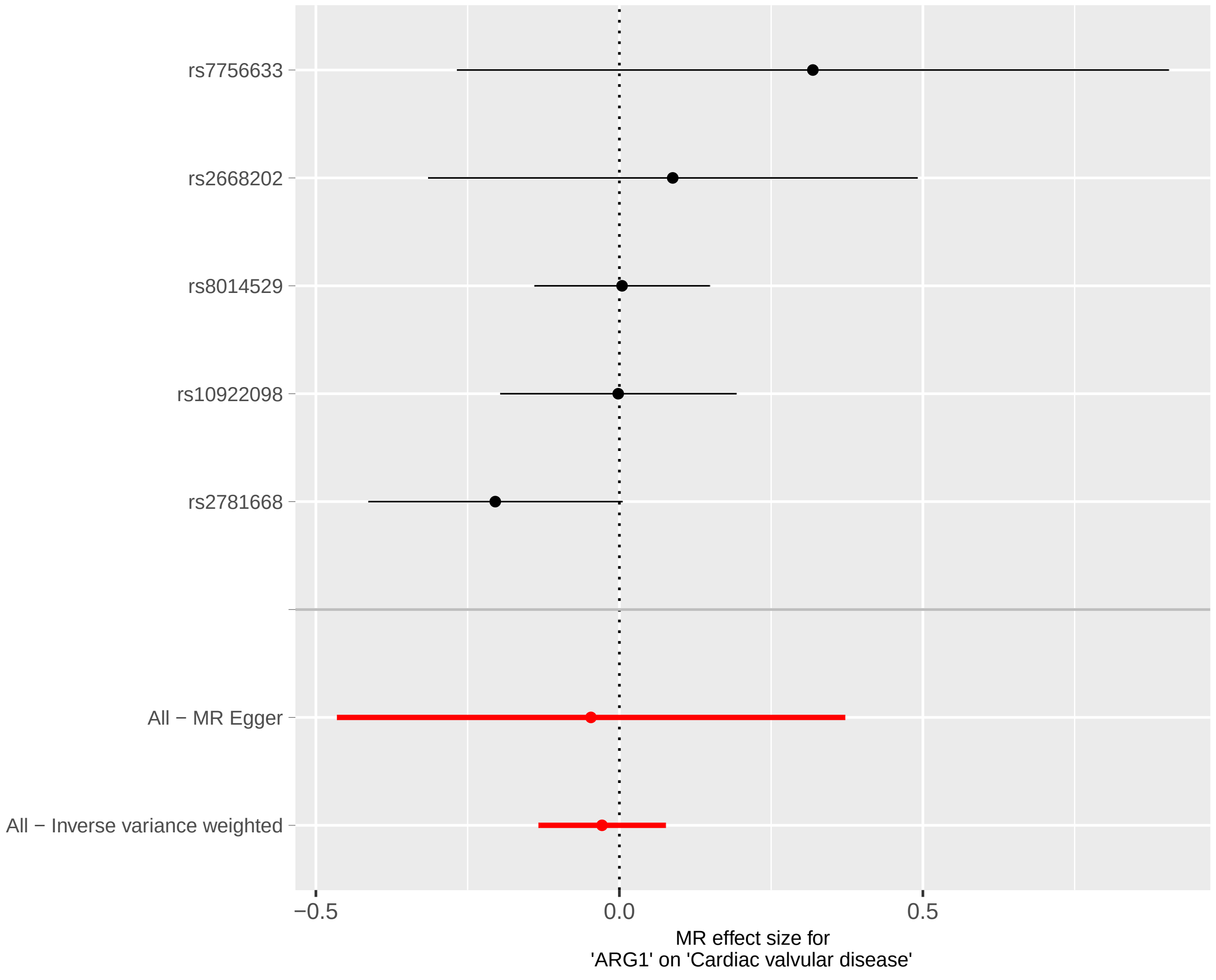
rs7756633
rs2668202
rs8014529
rs10922098
rs2781668
All − MR Egger
All − Inverse variance weighted
−0.5
0.0
0.5
MR effect size for
'ARG1' on 'Cardiac valvular disease'

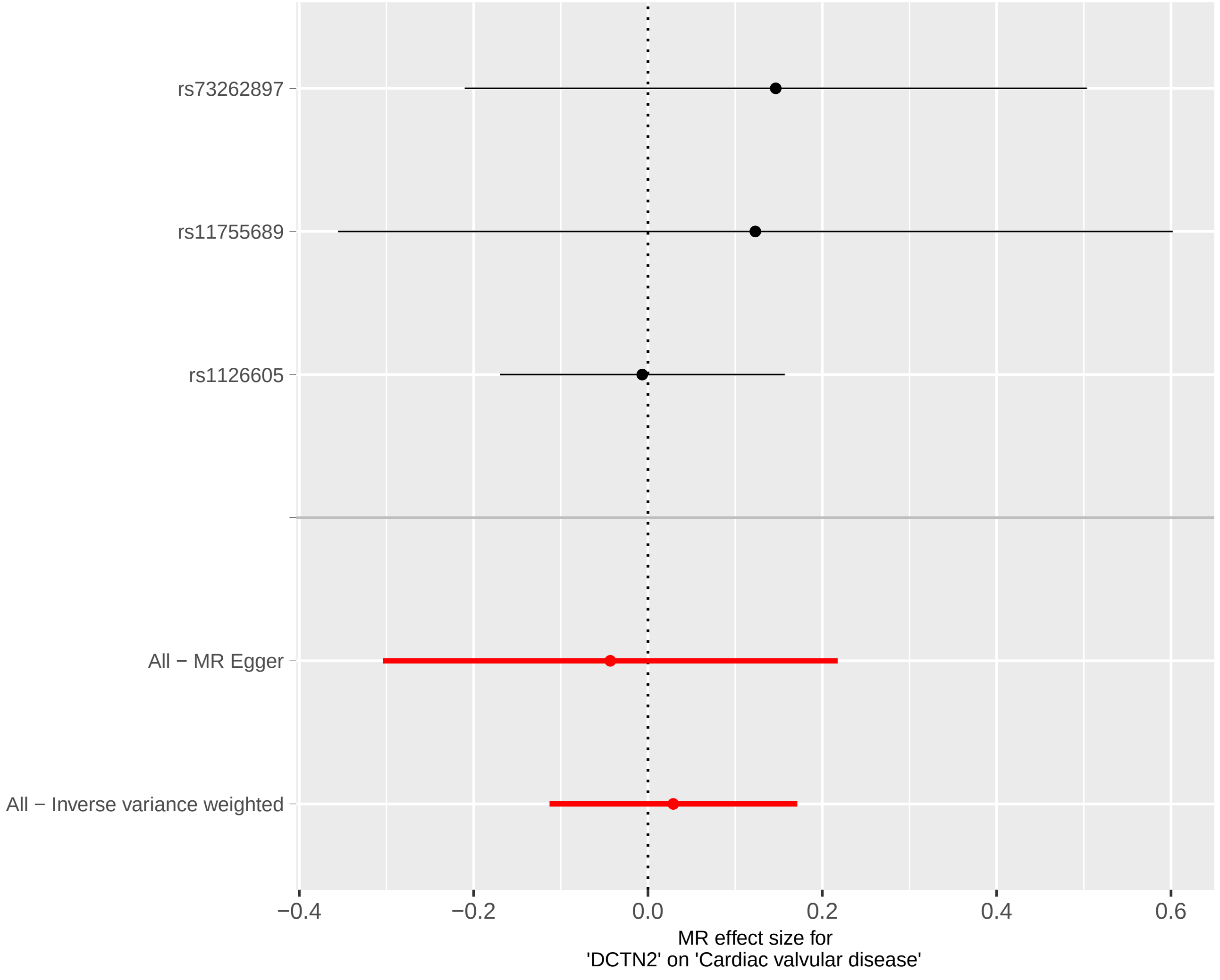
rs73262897
rs11755689
rs1126605
All − MR Egger
All − Inverse variance weighted
−0.4
−0.2
0.0
0.2
0.4
0.6
MR effect size for
'DCTN2' on 'Cardiac valvular disease'

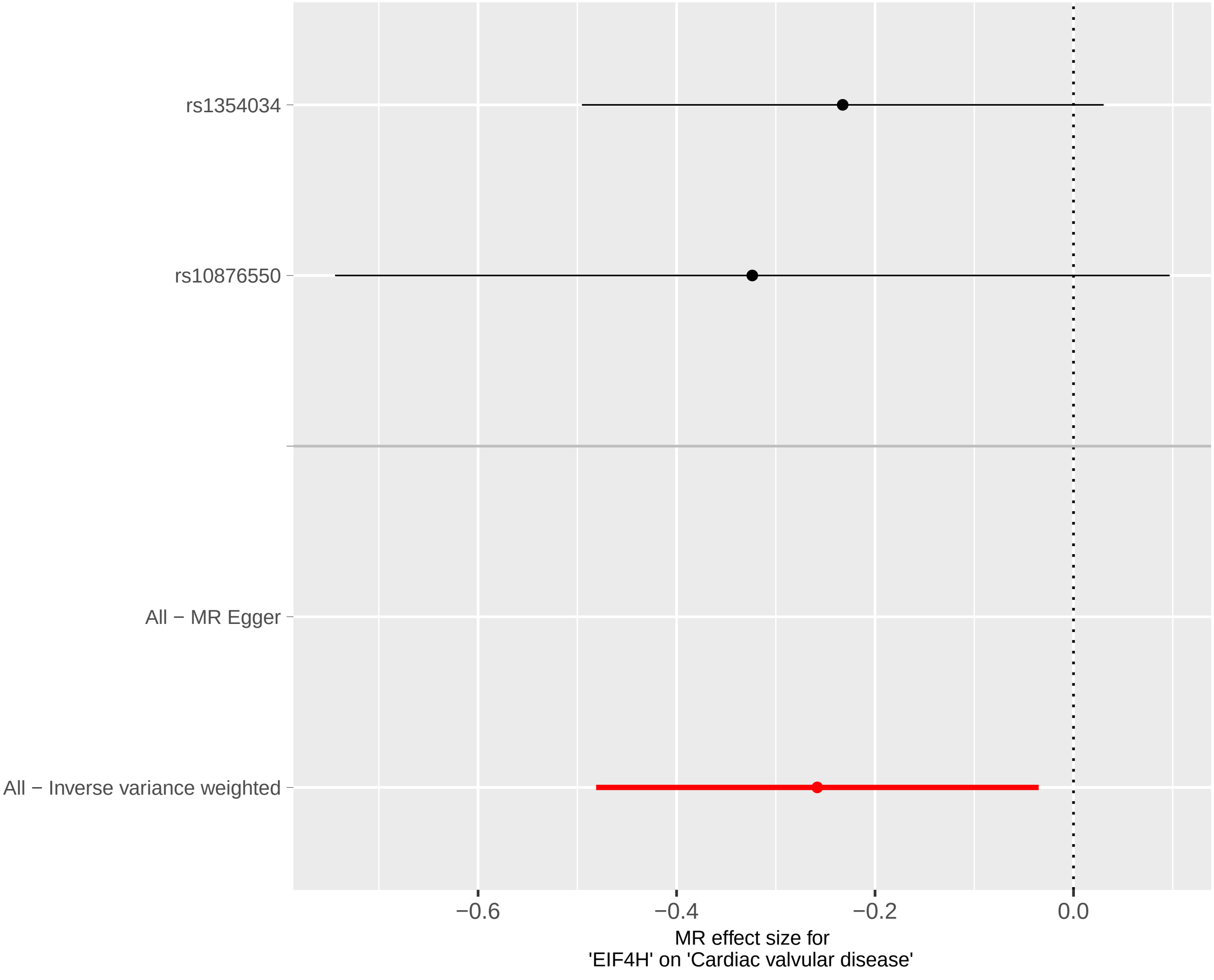
rs1354034
rs10876550
All − MR Egger
All − Inverse variance weighted
−0.6
−0.4
−0.2
0.0
MR effect size for
'EIF4H' on 'Cardiac valvular disease'

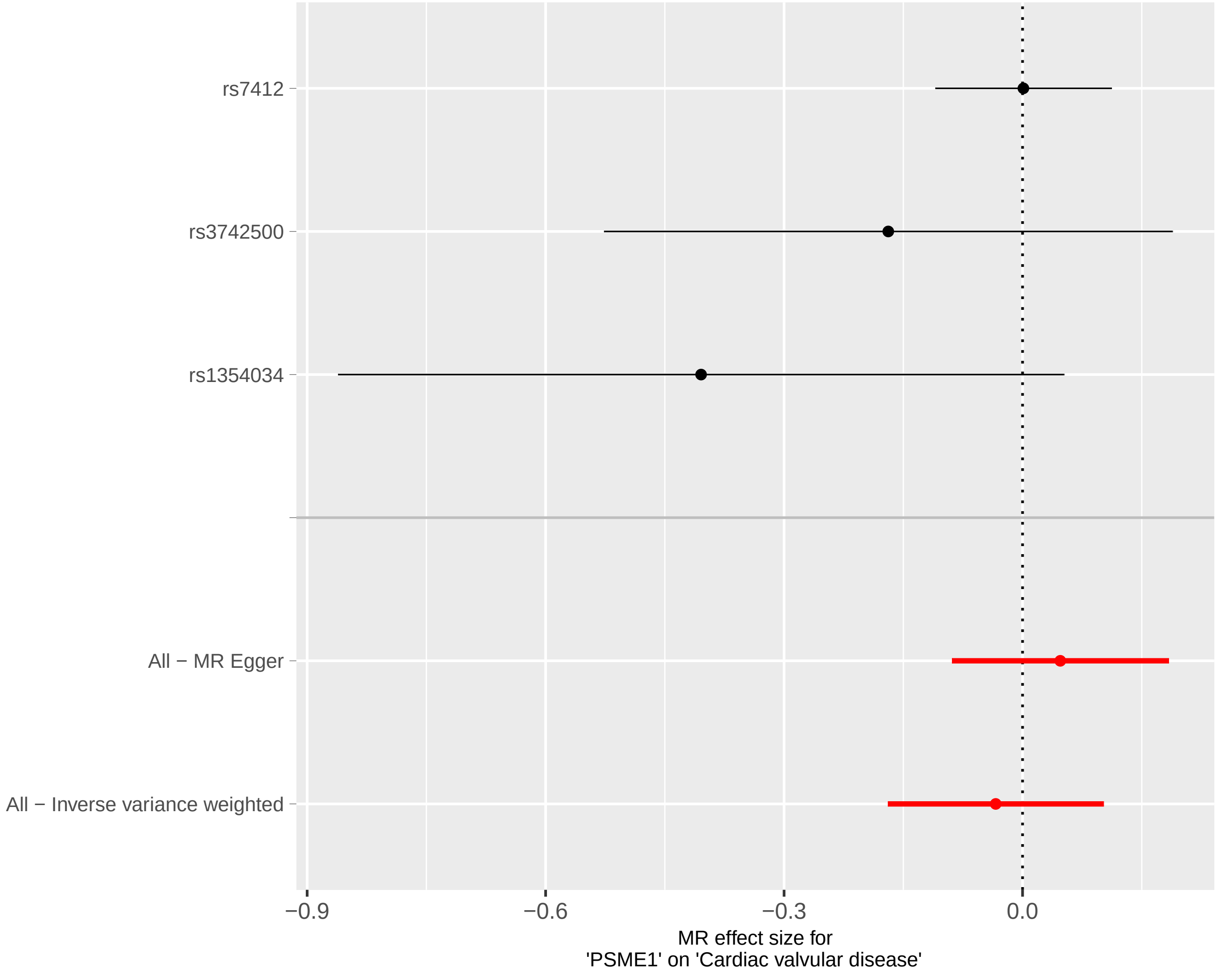
rs7412
rs3742500
rs1354034
All − MR Egger
All − Inverse variance weighted
−0.9
−0.6
−0.3
0.0
MR effect size for
'PSME1' on 'Cardiac valvular disease'

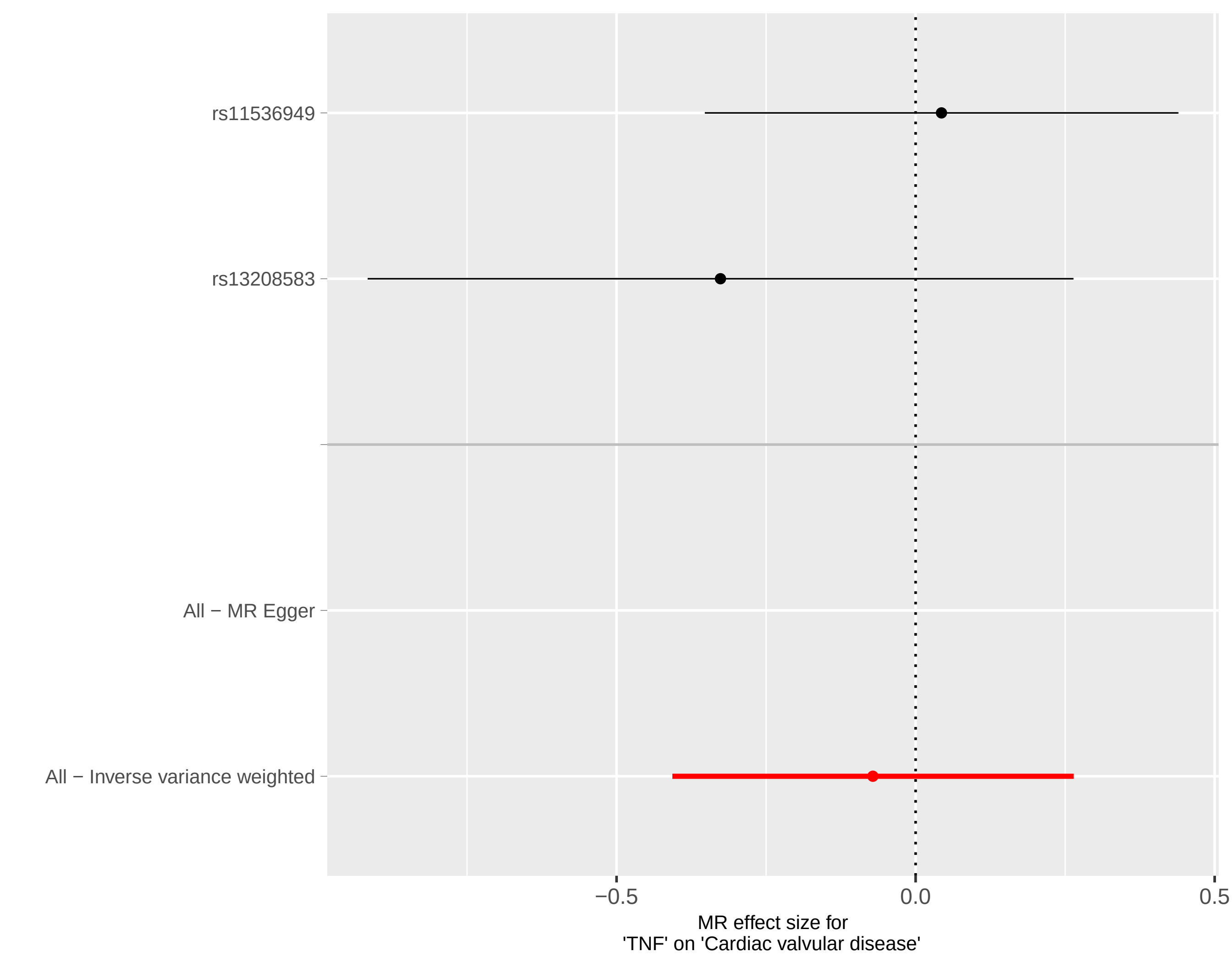
rs11536949
rs13208583
All − MR Egger
All − Inverse variance weighted
−0.5
0.0
0.5
MR effect size for
'TNF' on 'Cardiac valvular disease'

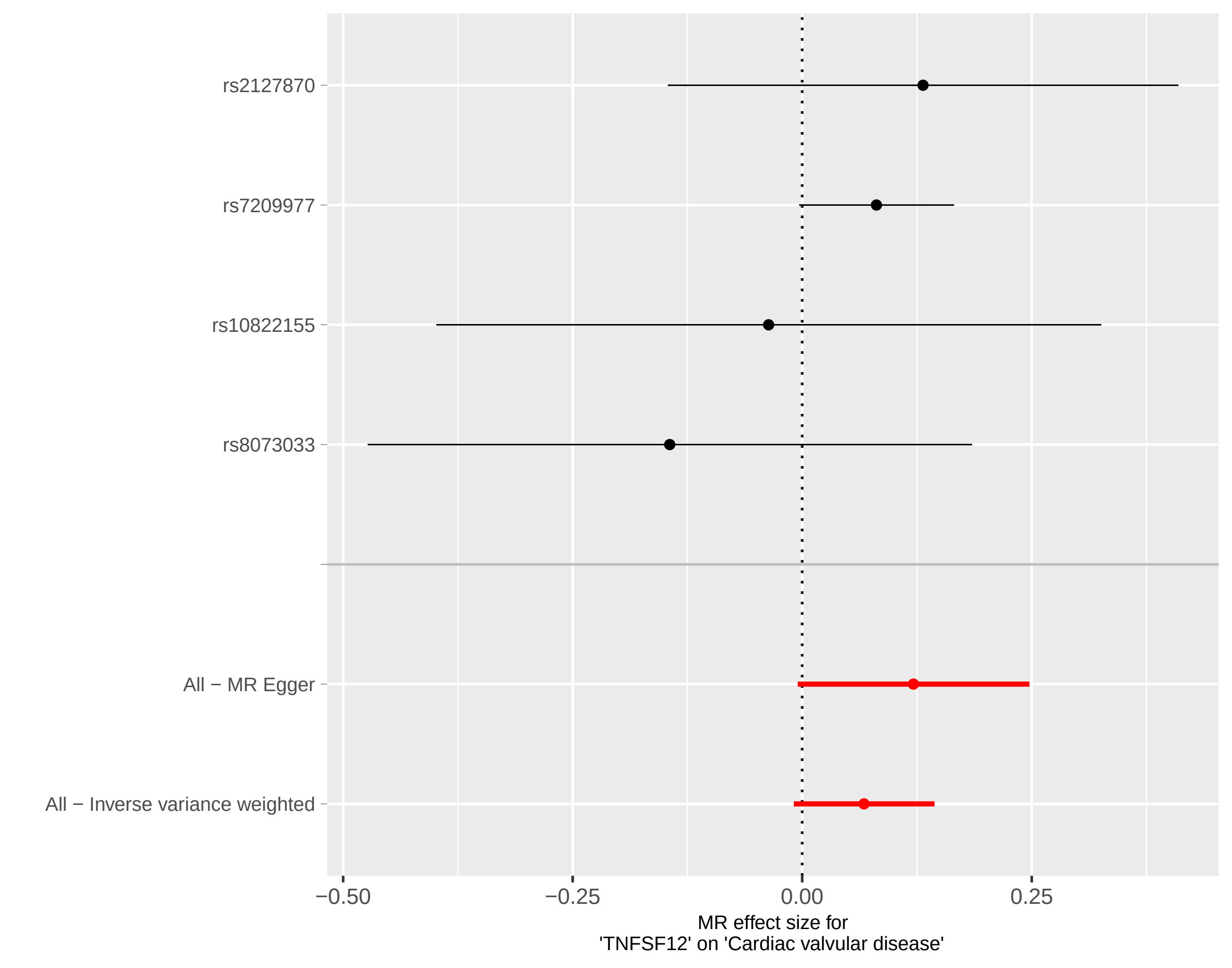
rs2127870
rs7209977
rs10822155
rs8073033
All − MR Egger
All − Inverse variance weighted
−0.50
−0.25
0.00
0.25
MR effect size for
'TNFSF12' on 'Cardiac valvular disease'

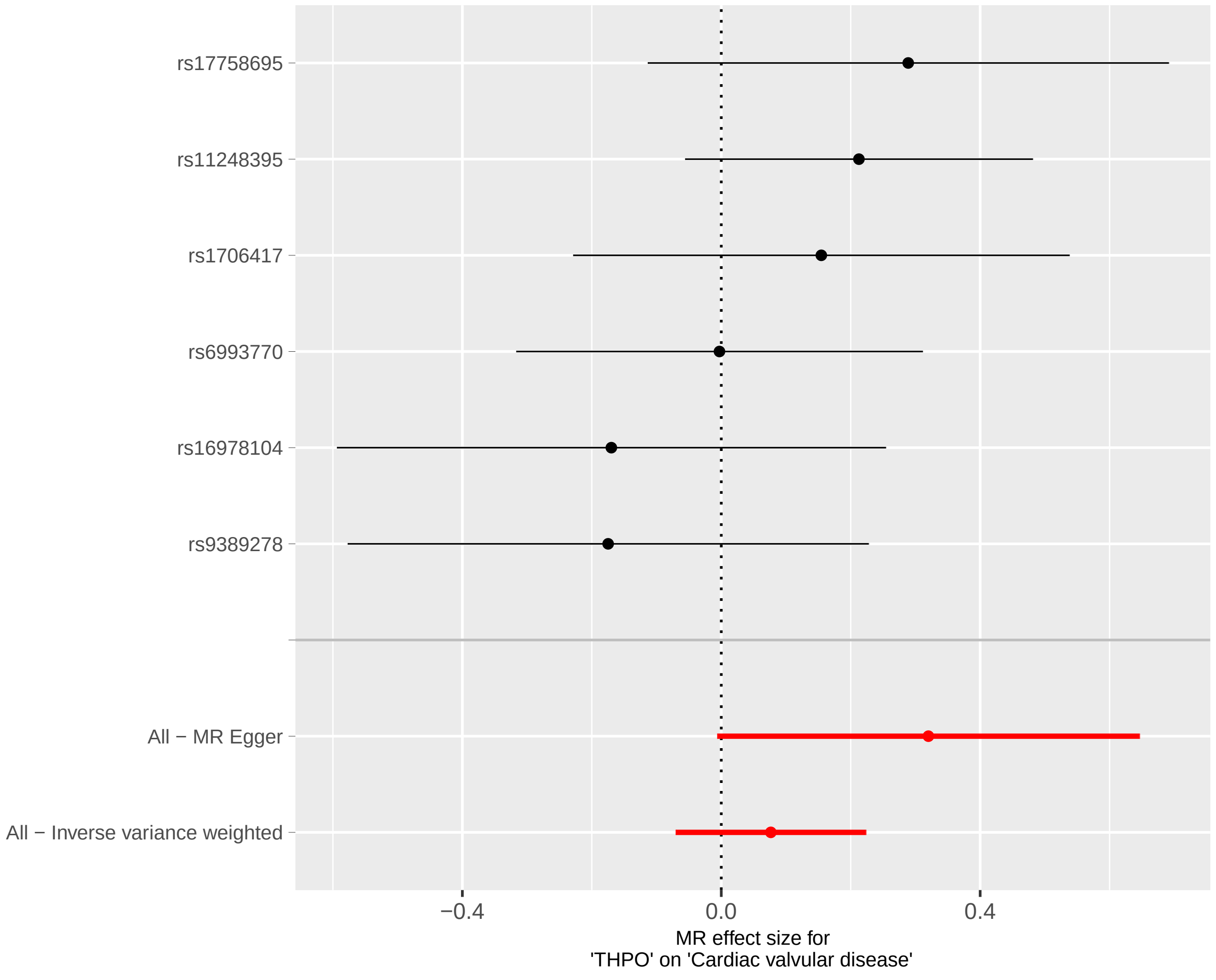
rs17758695
rs11248395
rs1706417
rs6993770
rs16978104
rs9389278
All − MR Egger
All − Inverse variance weighted
−0.4
0.0
0.4
MR effect size for
'THPO' on 'Cardiac valvular disease'

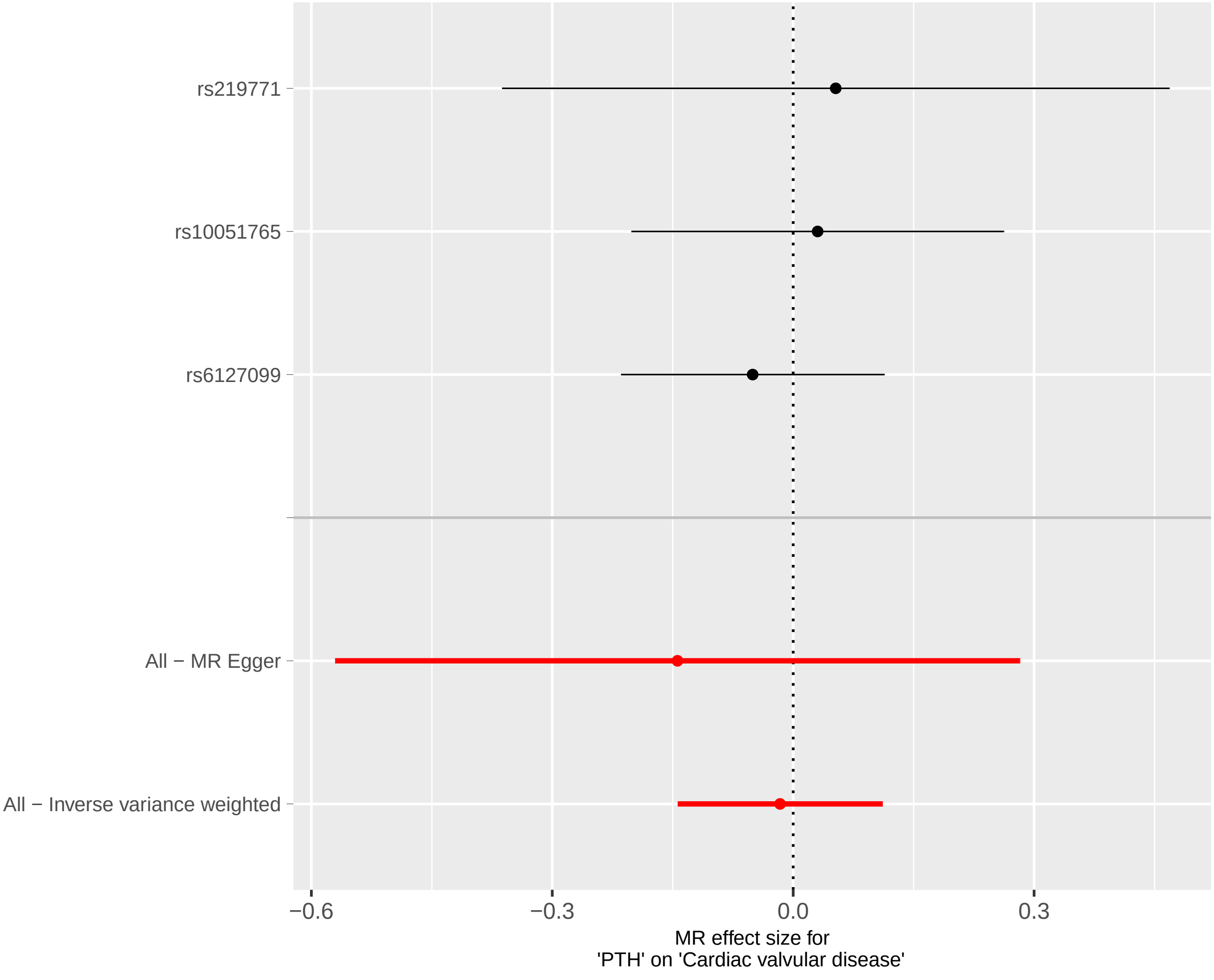
rs219771
rs10051765
rs6127099
All − MR Egger
All − Inverse variance weighted
−0.6
−0.3
0.0
0.3
MR effect size for
'PTH' on 'Cardiac valvular disease'

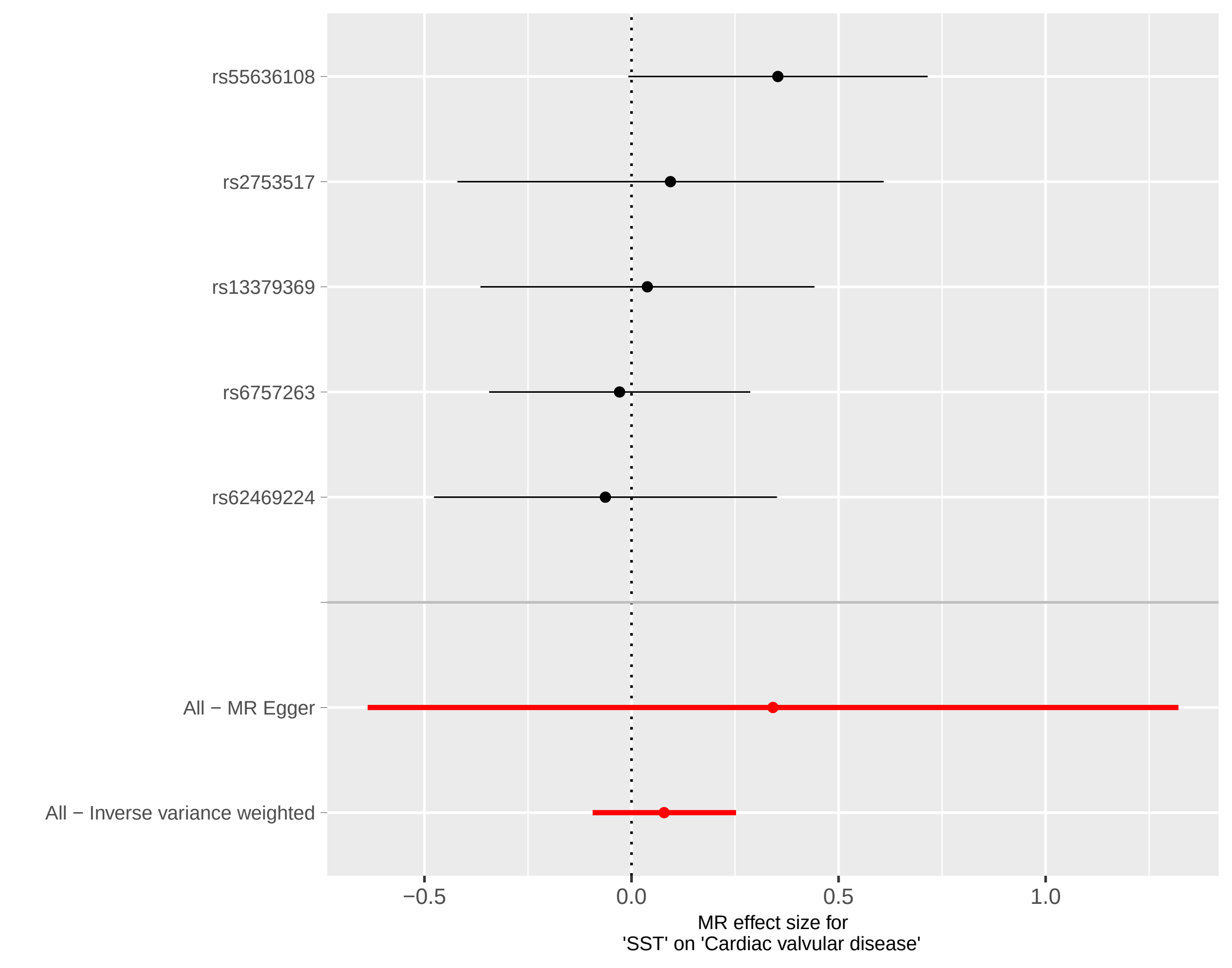

rs55636108
rs2753517
rs13379369
rs6757263
rs62469224
All − MR Egger
All − Inverse variance weighted
−0.5
0.0
0.5
1.0
MR effect size for
'SST' on 'Cardiac valvular disease'

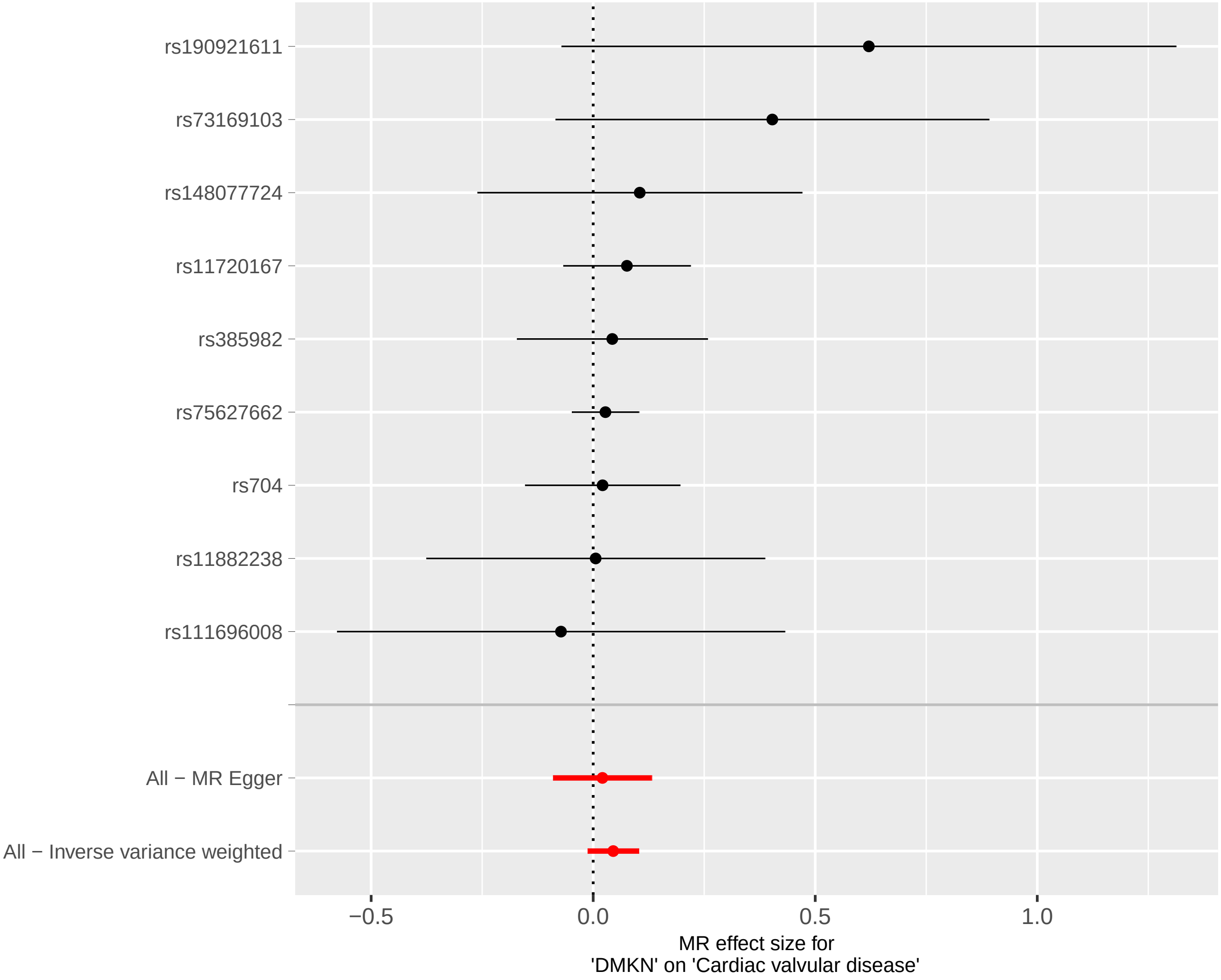
rs190921611
rs73169103
rs148077724
rs11720167
rs385982
rs75627662
rs704
rs11882238
rs111696008
All − MR Egger
All − Inverse variance weighted
−0.5
0.0
0.5
1.0
MR effect size for
'DMKN' on 'Cardiac valvular disease'

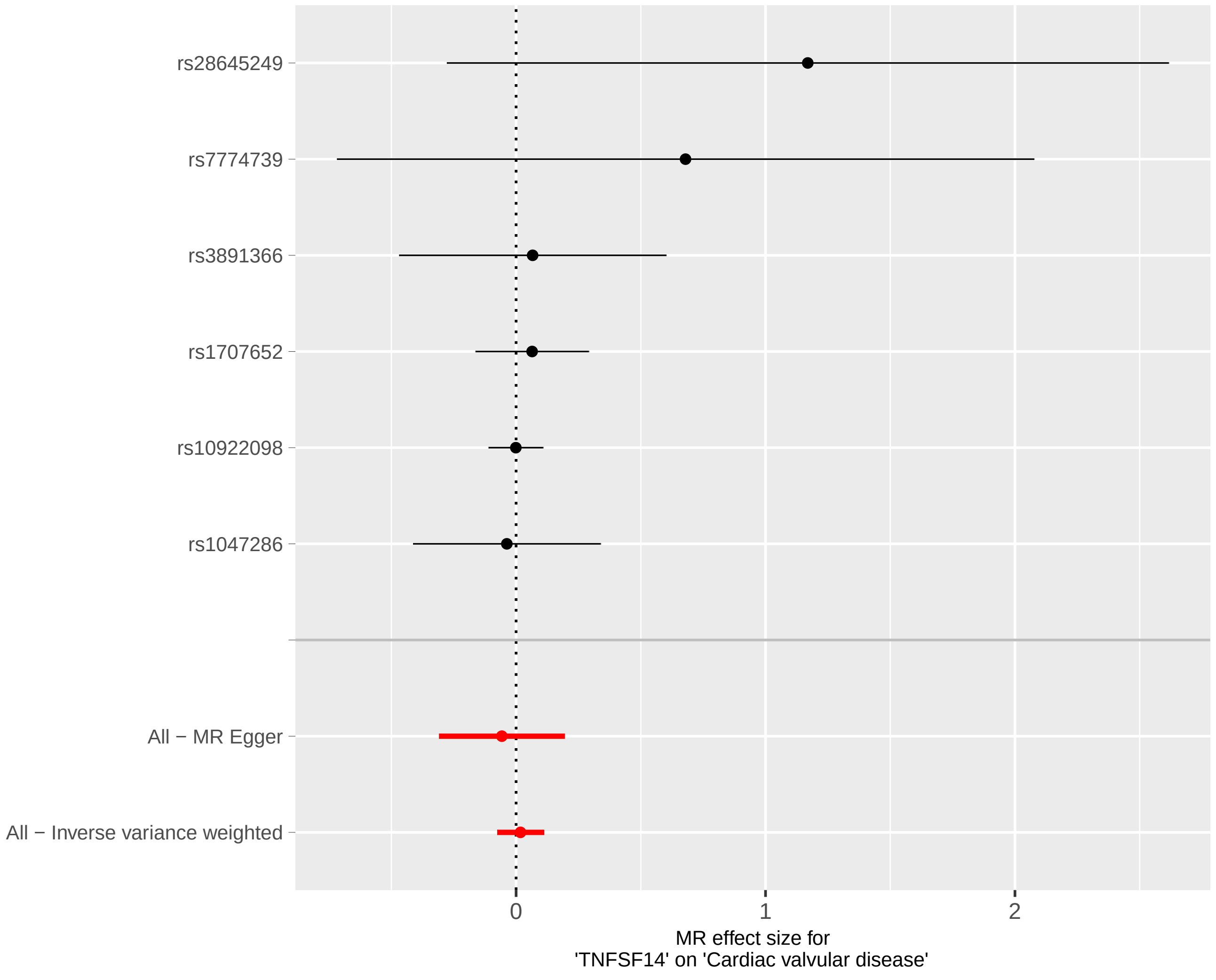
rs28645249
rs7774739
rs3891366
rs1707652
rs10922098
rs1047286
All − MR Egger
All − Inverse variance weighted
0
1
2
MR effect size for
'TNFSF14' on 'Cardiac valvular disease'

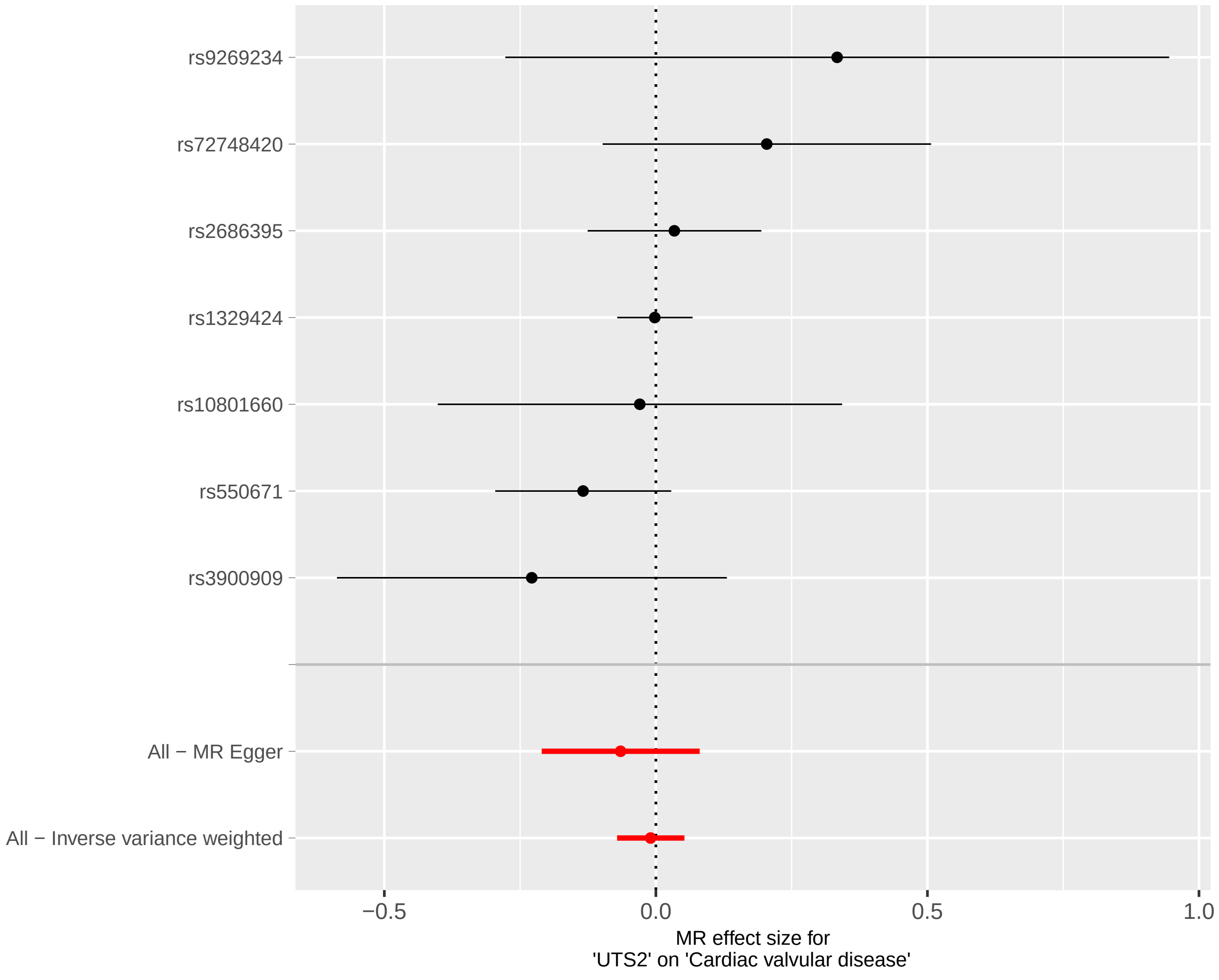

rs9269234
rs72748420
rs2686395
rs1329424
rs10801660
rs550671
rs3900909
All − MR Egger
All − Inverse variance weighted
−0.5
0.0
0.5
1.0
MR effect size for
'UTS2' on 'Cardiac valvular disease'

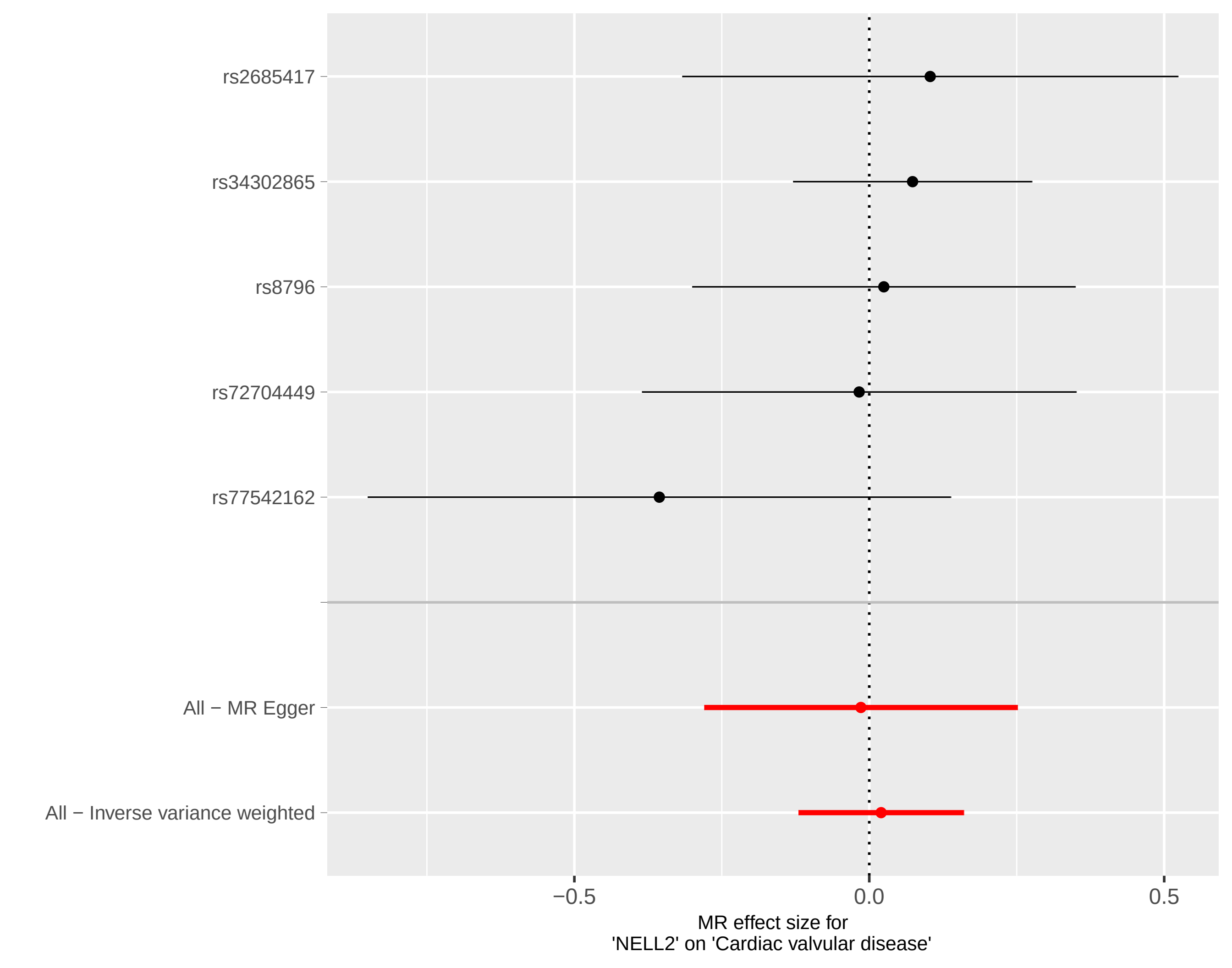
rs2685417
rs34302865
rs8796
rs72704449
rs77542162
All − MR Egger
All − Inverse variance weighted
−0.5
0.0
0.5
MR effect size for
'NELL2' on 'Cardiac valvular disease'

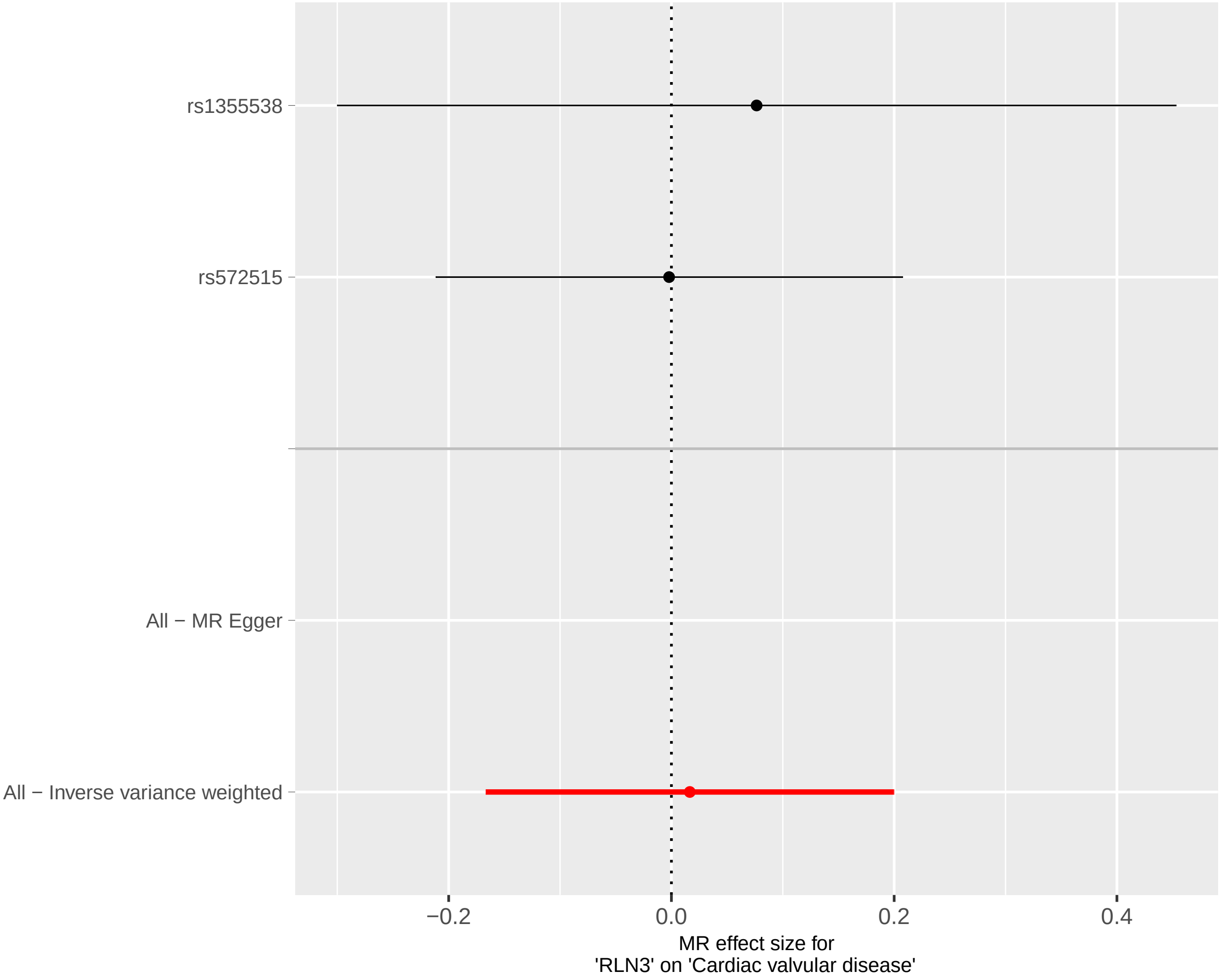
rs1355538
rs572515
All − MR Egger
All − Inverse variance weighted
−0.2
0.0
0.2
0.4
MR effect size for
'RLN3' on 'Cardiac valvular disease'

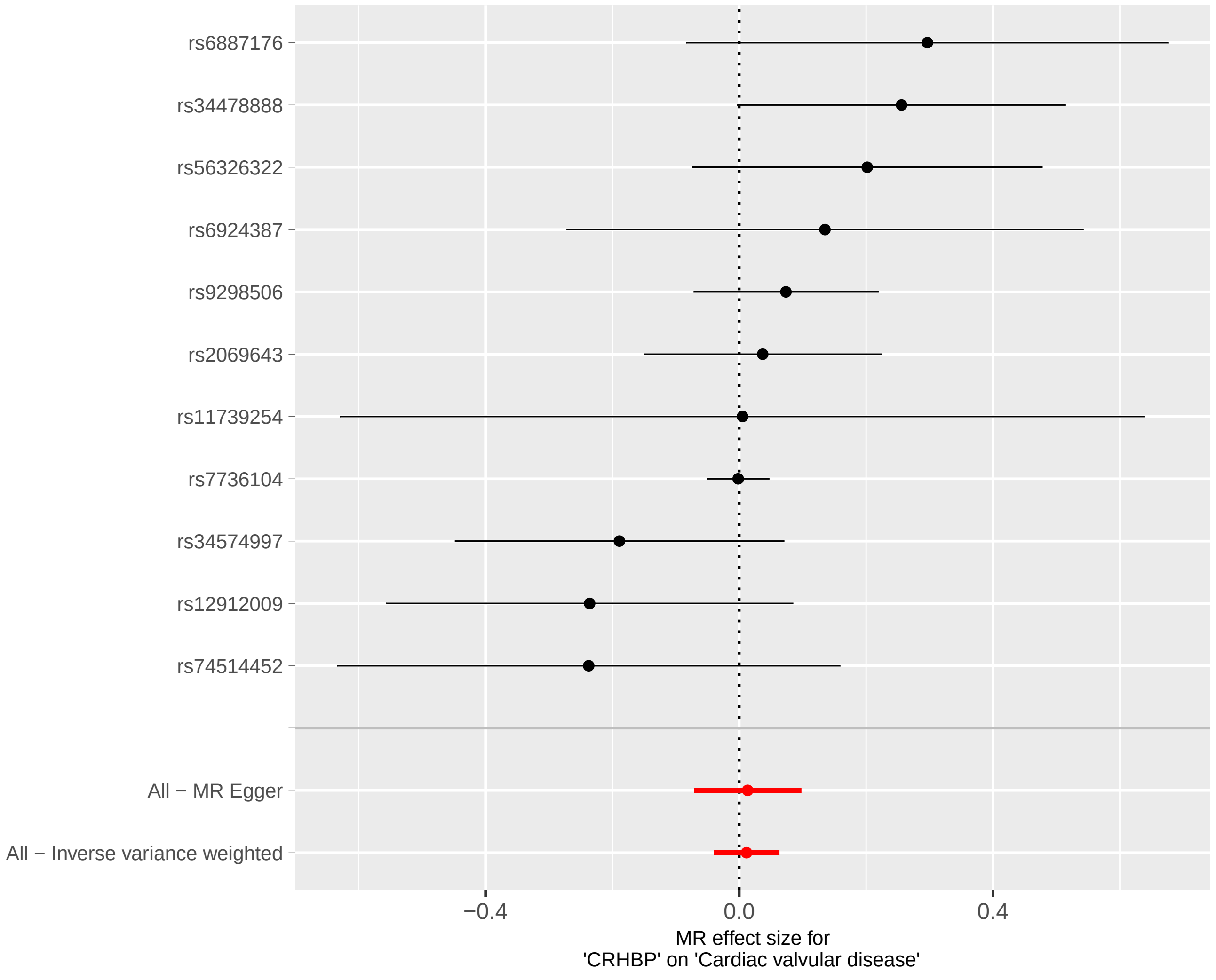
rs6887176
rs34478888
rs56326322
rs6924387
rs9298506
rs2069643
rs11739254
rs7736104
rs34574997
rs12912009
rs74514452
All − MR Egger
All − Inverse variance weighted
−0.4
0.0
0.4
MR effect size for
'CRHBP' on 'Cardiac valvular disease'

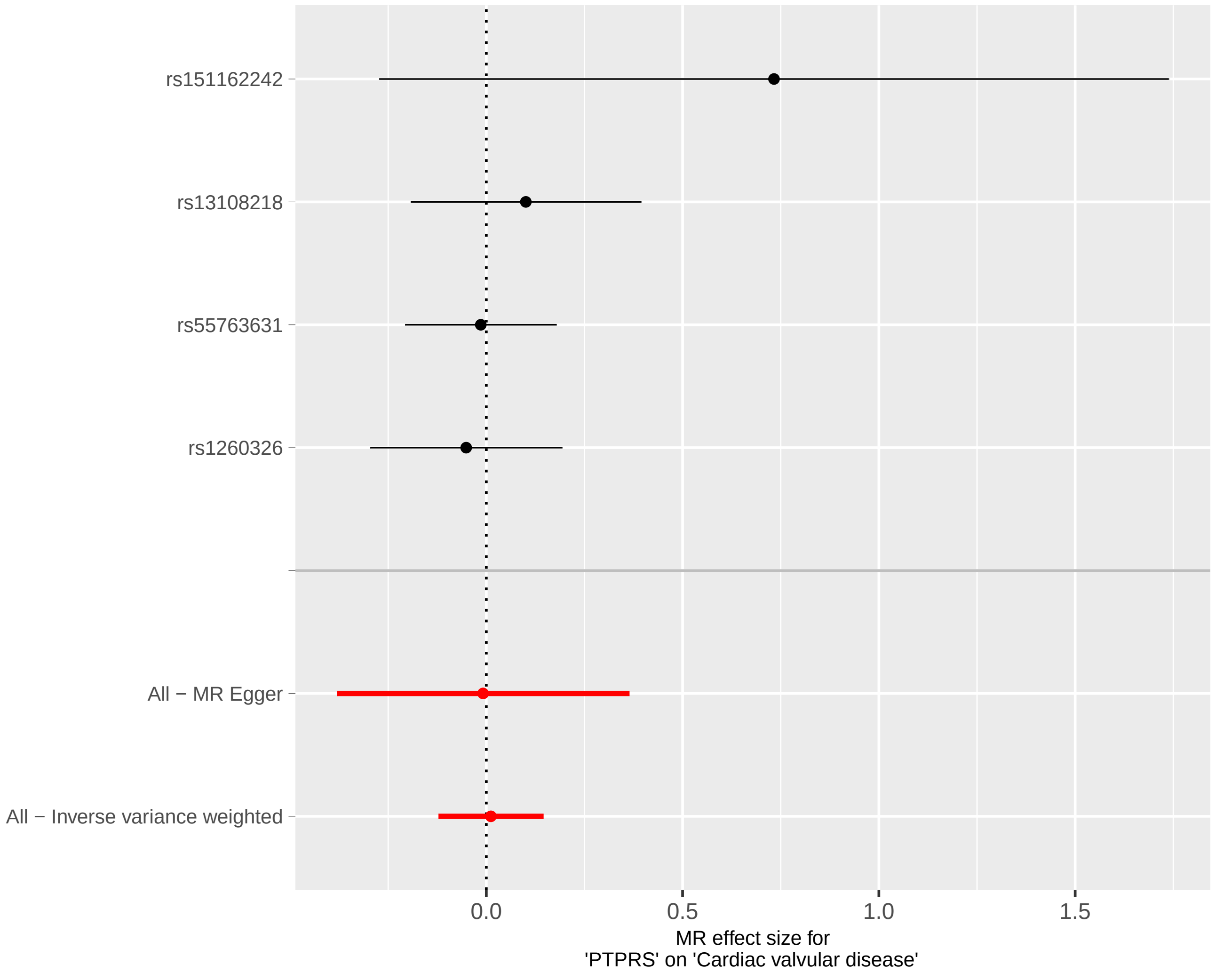
rs151162242
rs13108218
rs55763631
rs1260326
All − MR Egger
All − Inverse variance weighted
0.0
0.5
1.0
1.5
MR effect size for
'PTPRS' on 'Cardiac valvular disease'

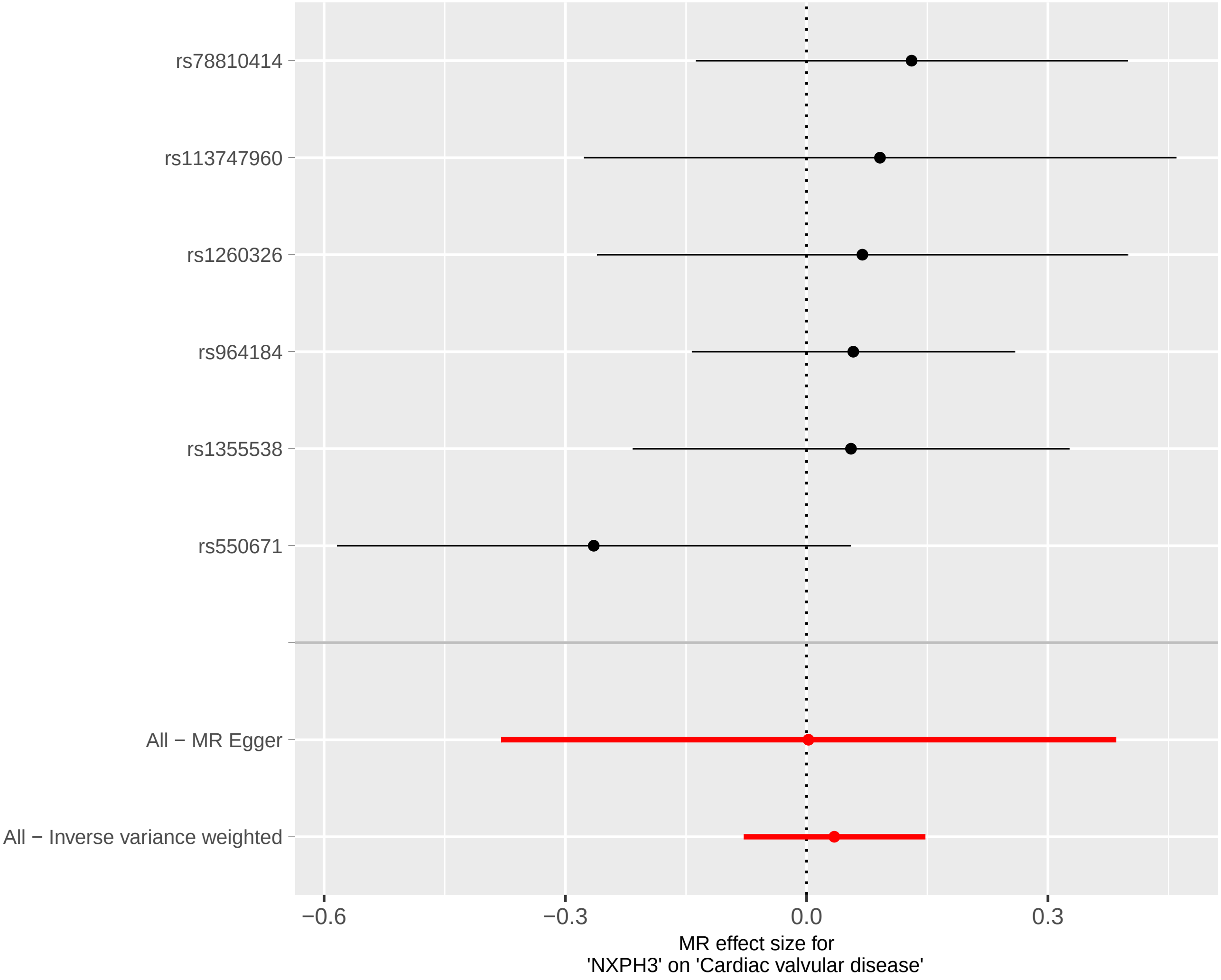
rs78810414
rs113747960
rs1260326
rs964184
rs1355538
rs550671
All − MR Egger
All − Inverse variance weighted
−0.6
−0.3
0.0
0.3
MR effect size for
'NXPH3' on 'Cardiac valvular disease'

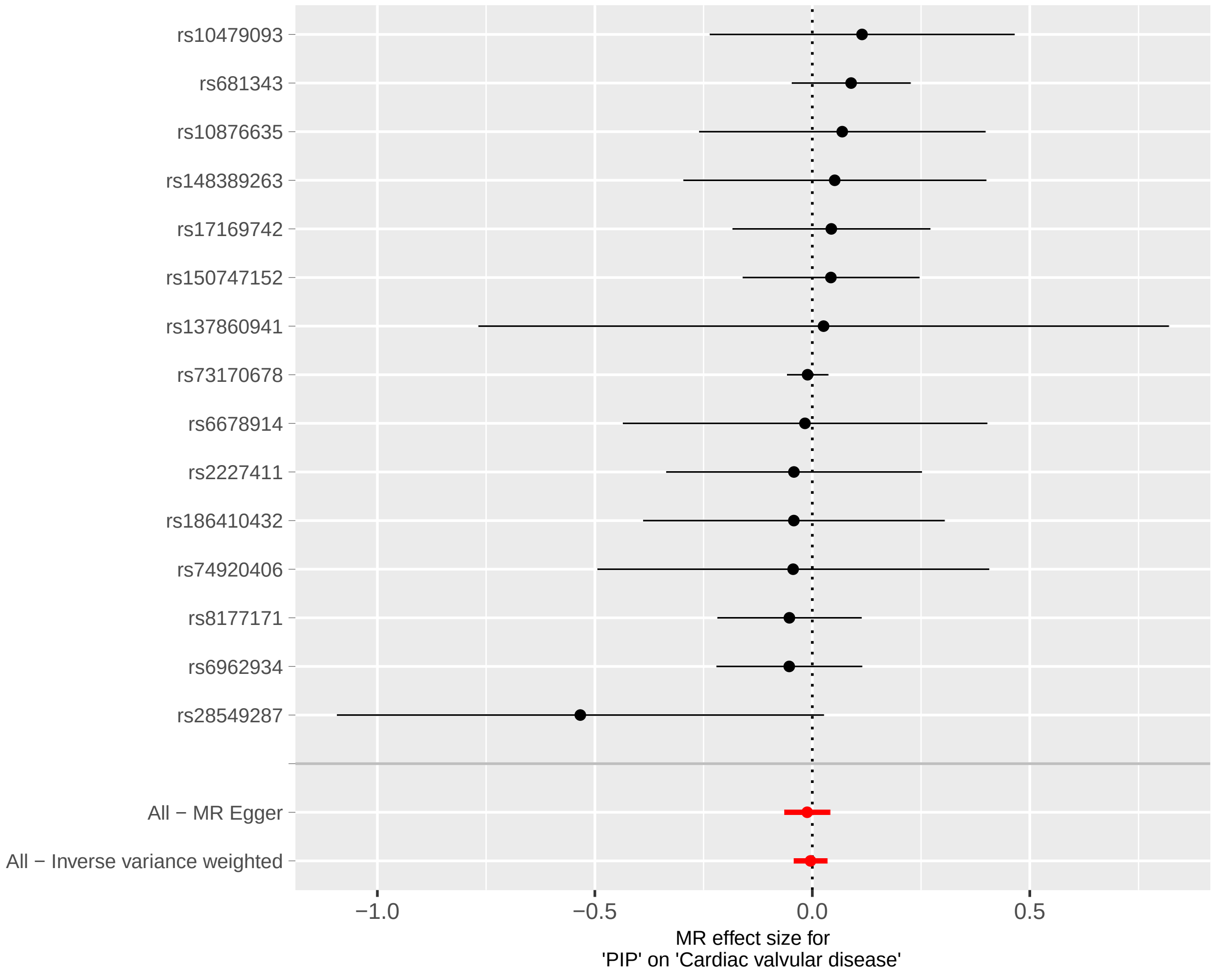

rs10479093
rs681343
rs10876635
rs148389263
rs17169742
rs150747152
rs137860941
rs73170678
rs6678914
rs2227411
rs186410432
rs74920406
rs8177171
rs6962934
rs28549287
All − MR Egger
All − Inverse variance weighted
−1.0
−0.5
0.0
0.5
MR effect size for
'PIP' on 'Cardiac valvular disease'

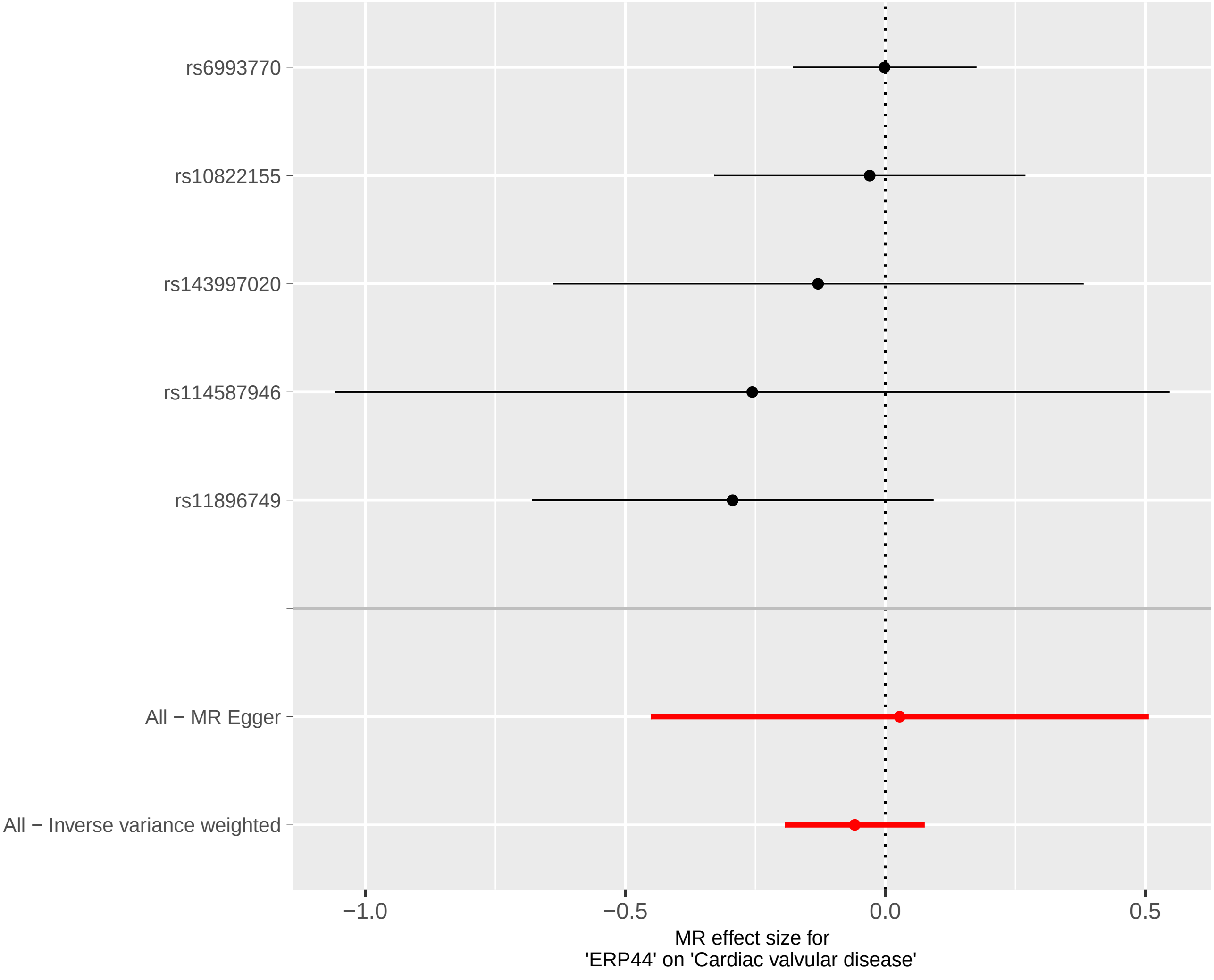

rs6993770
rs10822155
rs143997020
rs114587946
rs11896749
All − MR Egger
All − Inverse variance weighted
−1.0
−0.5
0.0
0.5
MR effect size for
'ERP44' on 'Cardiac valvular disease'

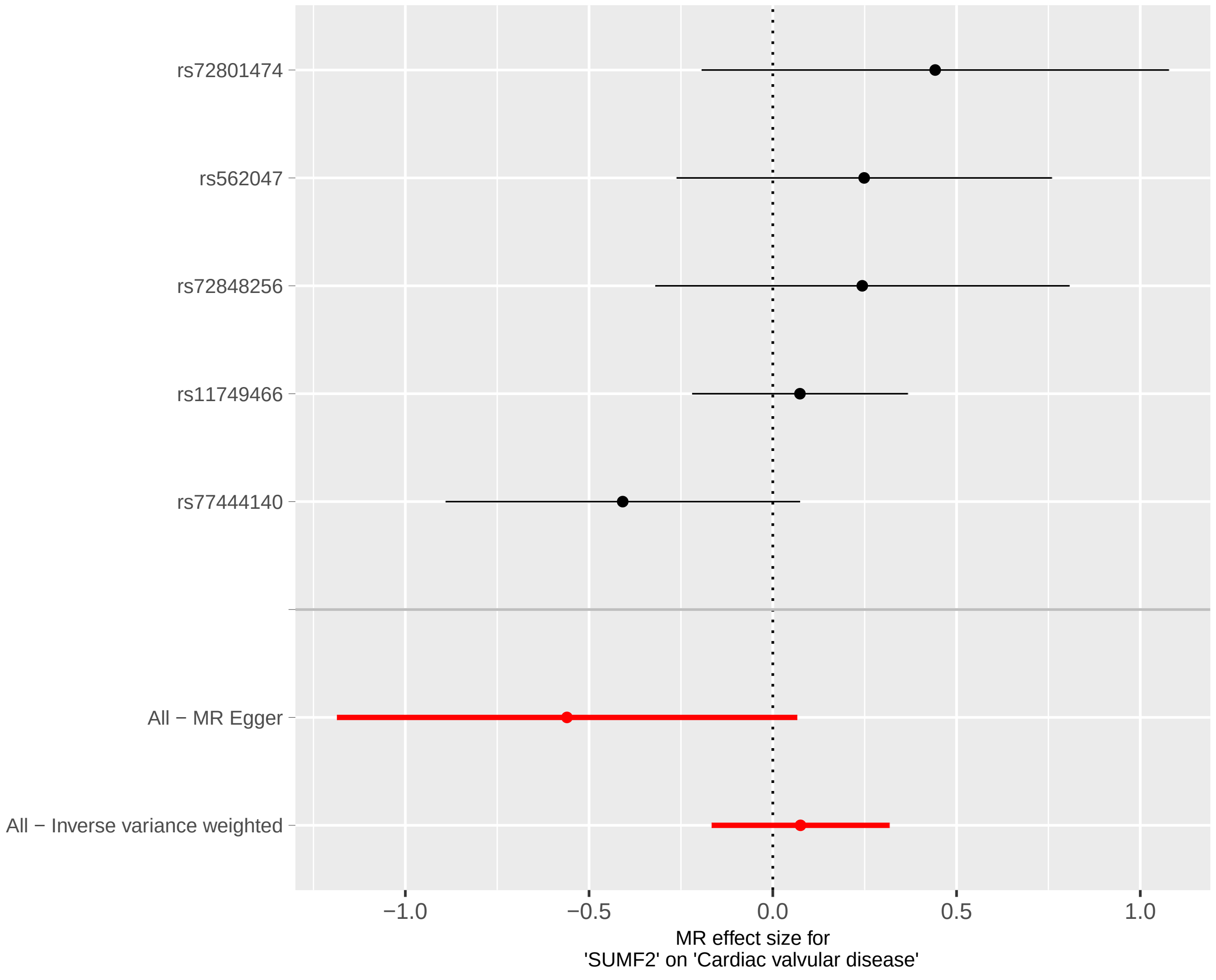
rs72801474
rs562047
rs72848256
rs11749466
rs77444140
All − MR Egger
All − Inverse variance weighted
−1.0
−0.5
0.0
0.5
1.0
MR effect size for
'SUMF2' on 'Cardiac valvular disease'

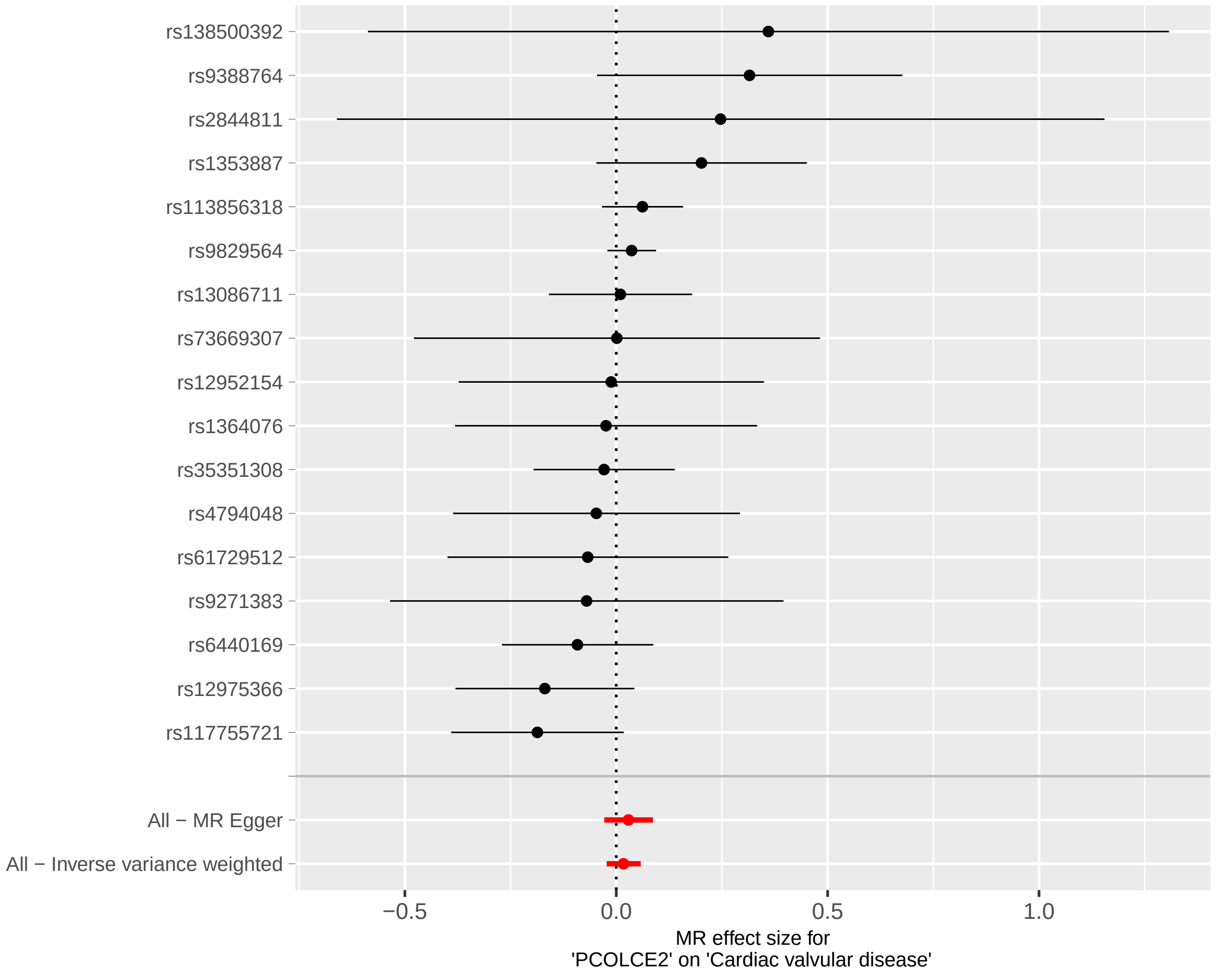

rs138500392
rs9388764
rs2844811
rs1353887
rs113856318
rs9829564
rs13086711
rs73669307
rs12952154
rs1364076
rs35351308
rs4794048
rs61729512
rs9271383
rs6440169
rs12975366
rs117755721
All − MR Egger
All − Inverse variance weighted
−0.5
0.0
0.5
1.0
MR effect size for
'PCOLCE2' on 'Cardiac valvular disease'

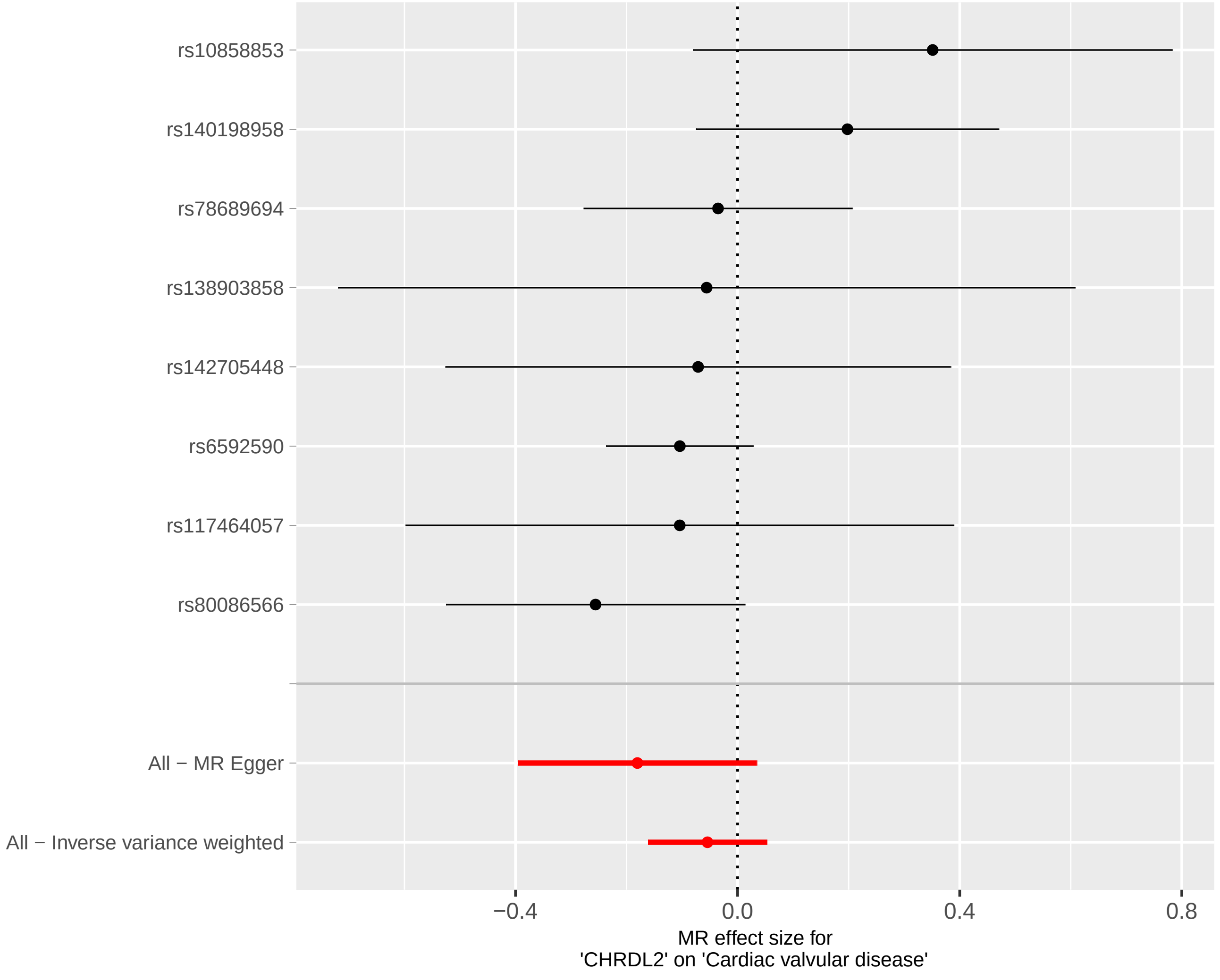
rs10858853
rs140198958
rs78689694
rs138903858
rs142705448
rs6592590
rs117464057
rs80086566
All − MR Egger
All − Inverse variance weighted
−0.4
0.0
0.4
0.8
MR effect size for
'CHRDL2' on 'Cardiac valvular disease'

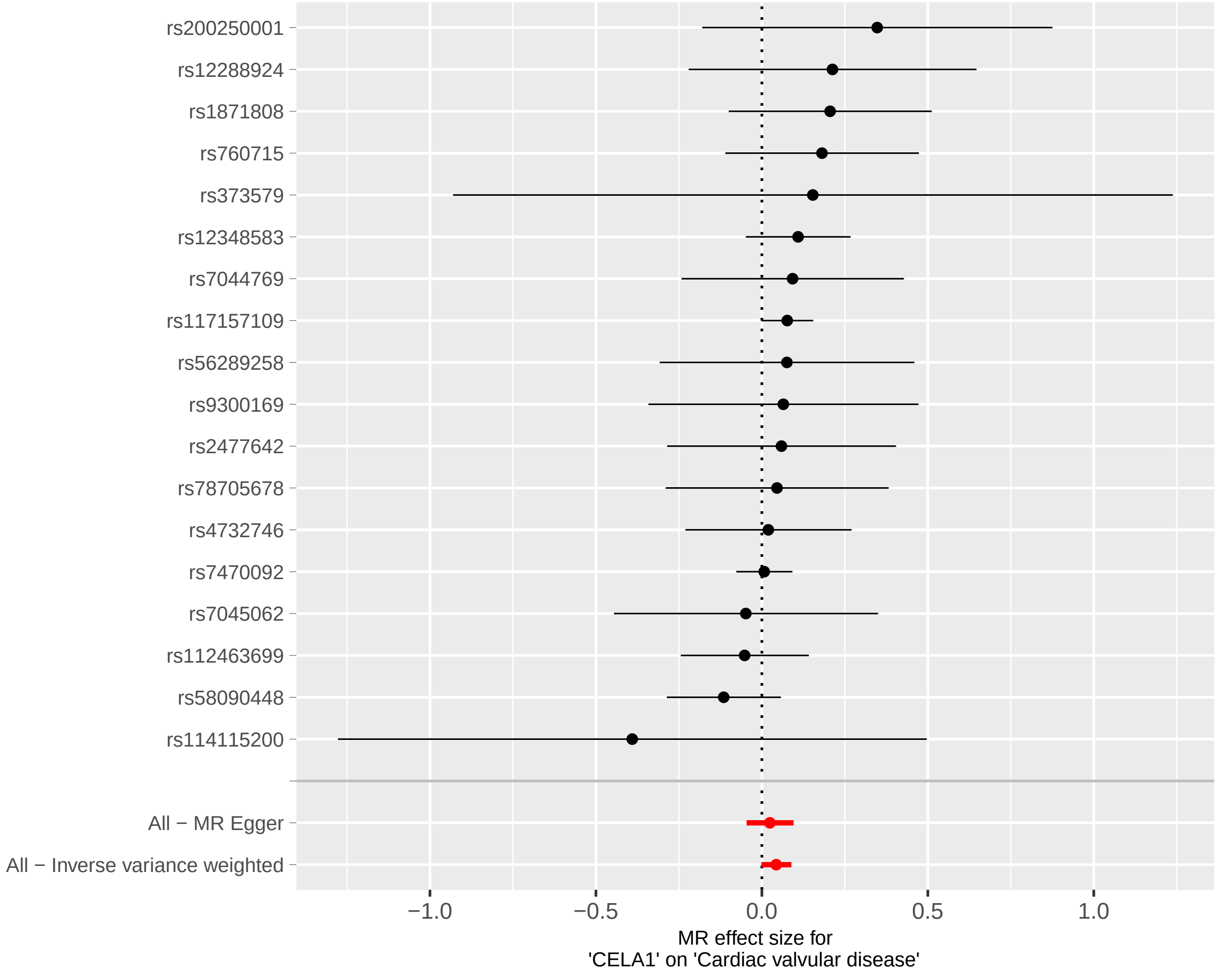

rs200250001
rs12288924
rs1871808
rs760715
rs373579
rs12348583
rs7044769
rs117157109
rs56289258
rs9300169
rs2477642
rs78705678
rs4732746
rs7470092
rs7045062
rs112463699
rs58090448
rs114115200
All − MR Egger
All − Inverse variance weighted
−1.0
−0.5
0.0
0.5
1.0
MR effect size for
'CELA1' on 'Cardiac valvular disease'

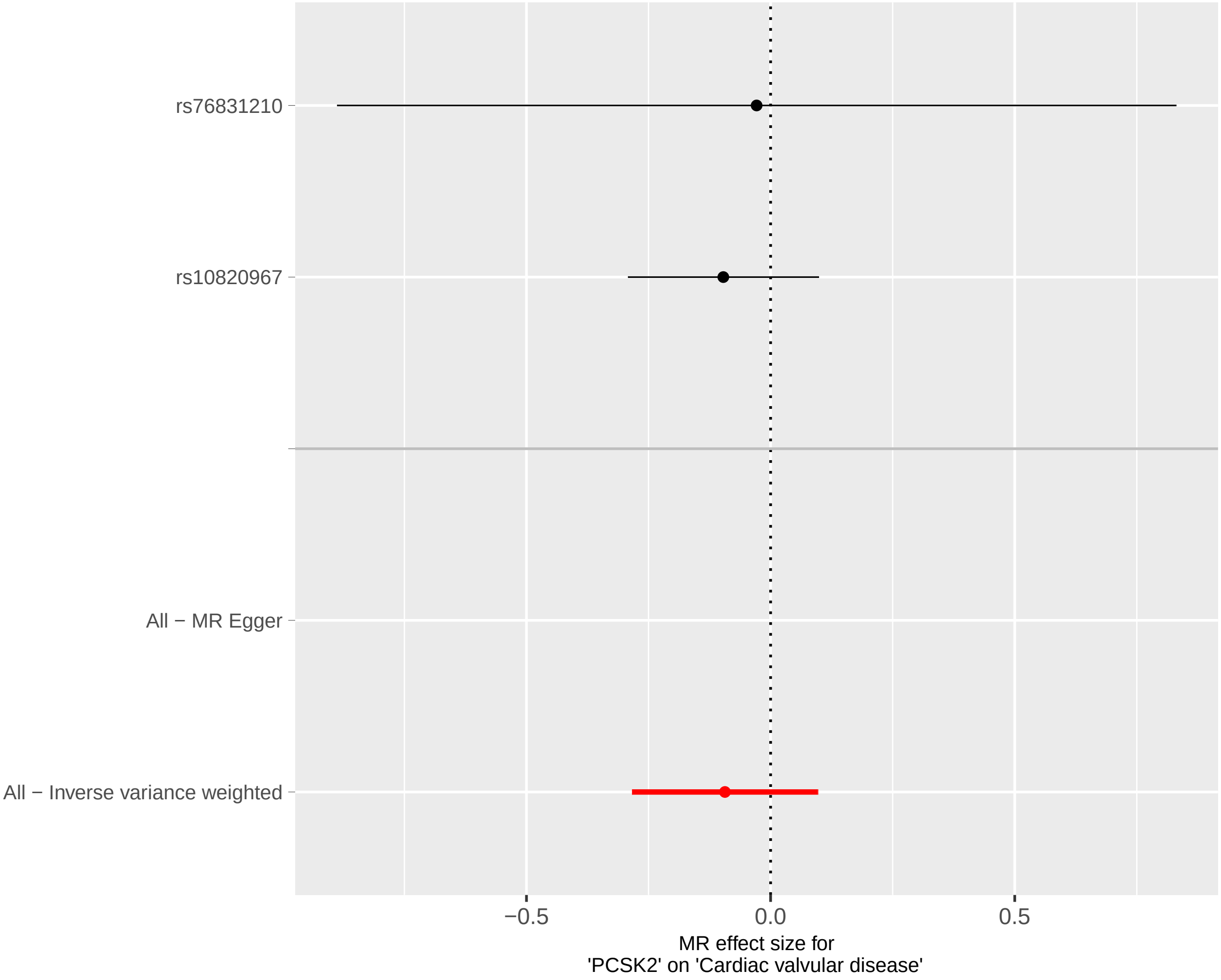
rs76831210
rs10820967
All − MR Egger
All − Inverse variance weighted
−0.5
0.0
0.5
MR effect size for
'PCSK2' on 'Cardiac valvular disease'

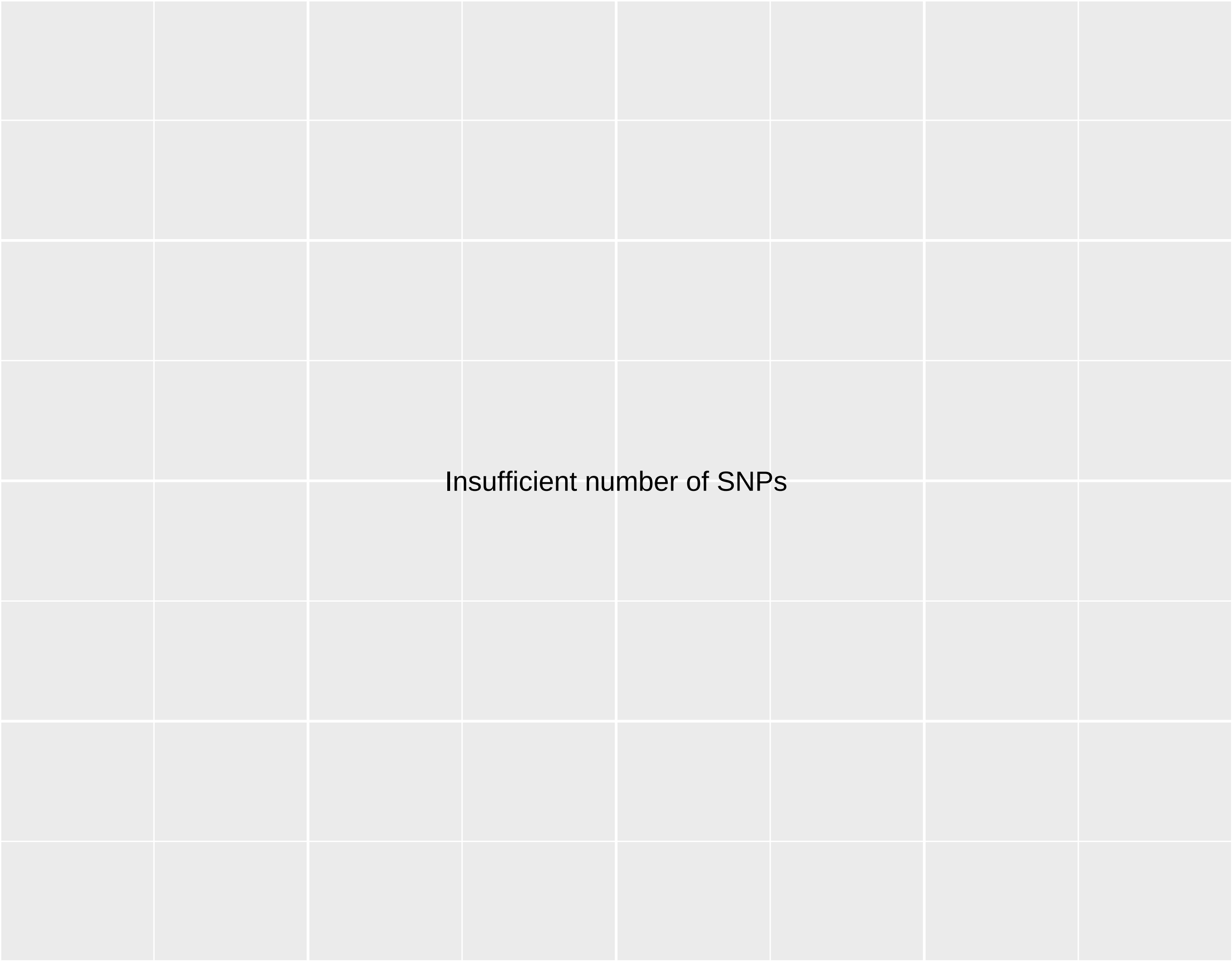
Insufficient number of SNPs

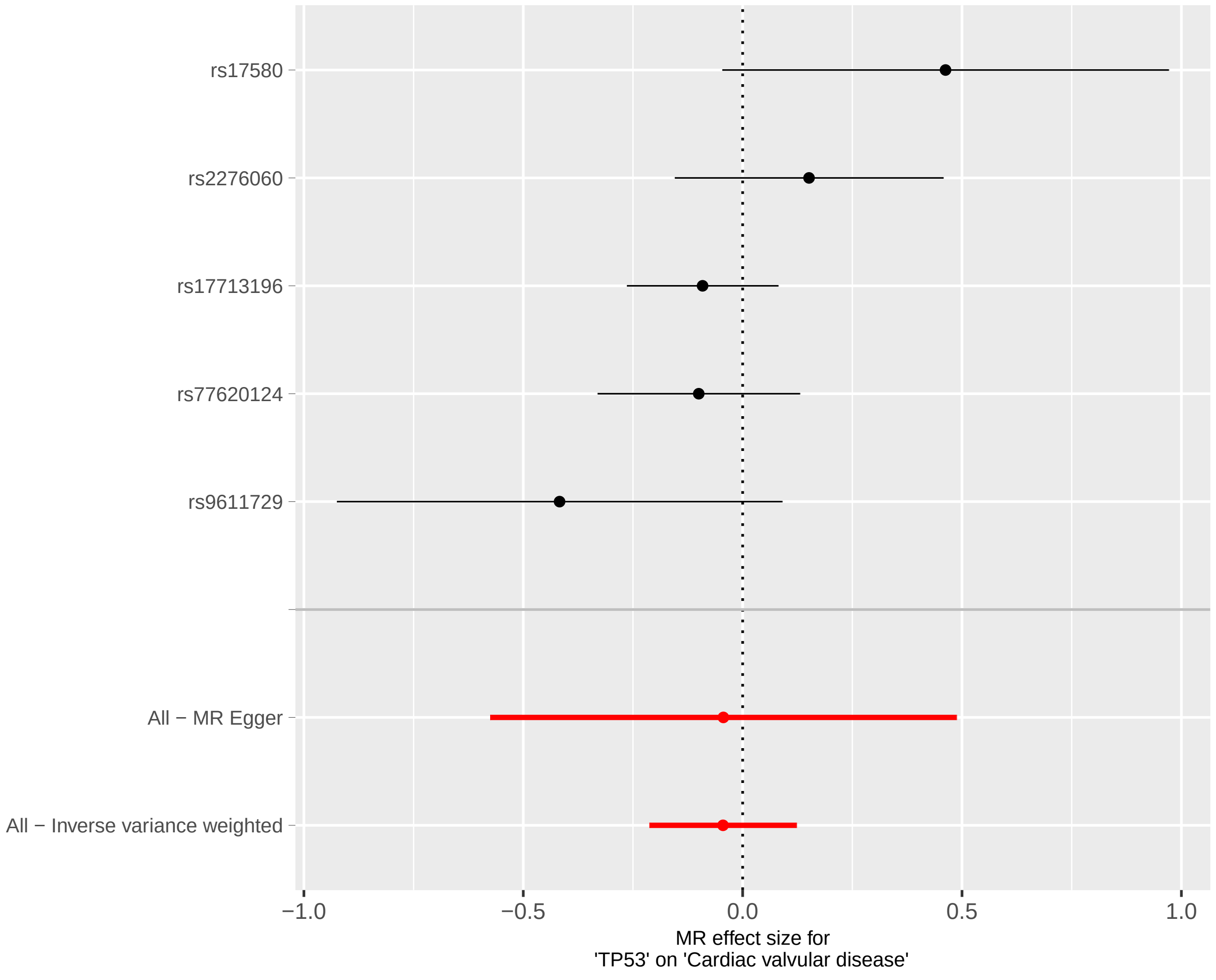
rs17580
rs2276060
rs17713196
rs77620124
rs9611729
All − MR Egger
All − Inverse variance weighted
−1.0
−0.5
0.0
0.5
1.0
MR effect size for
'TP53' on 'Cardiac valvular disease'

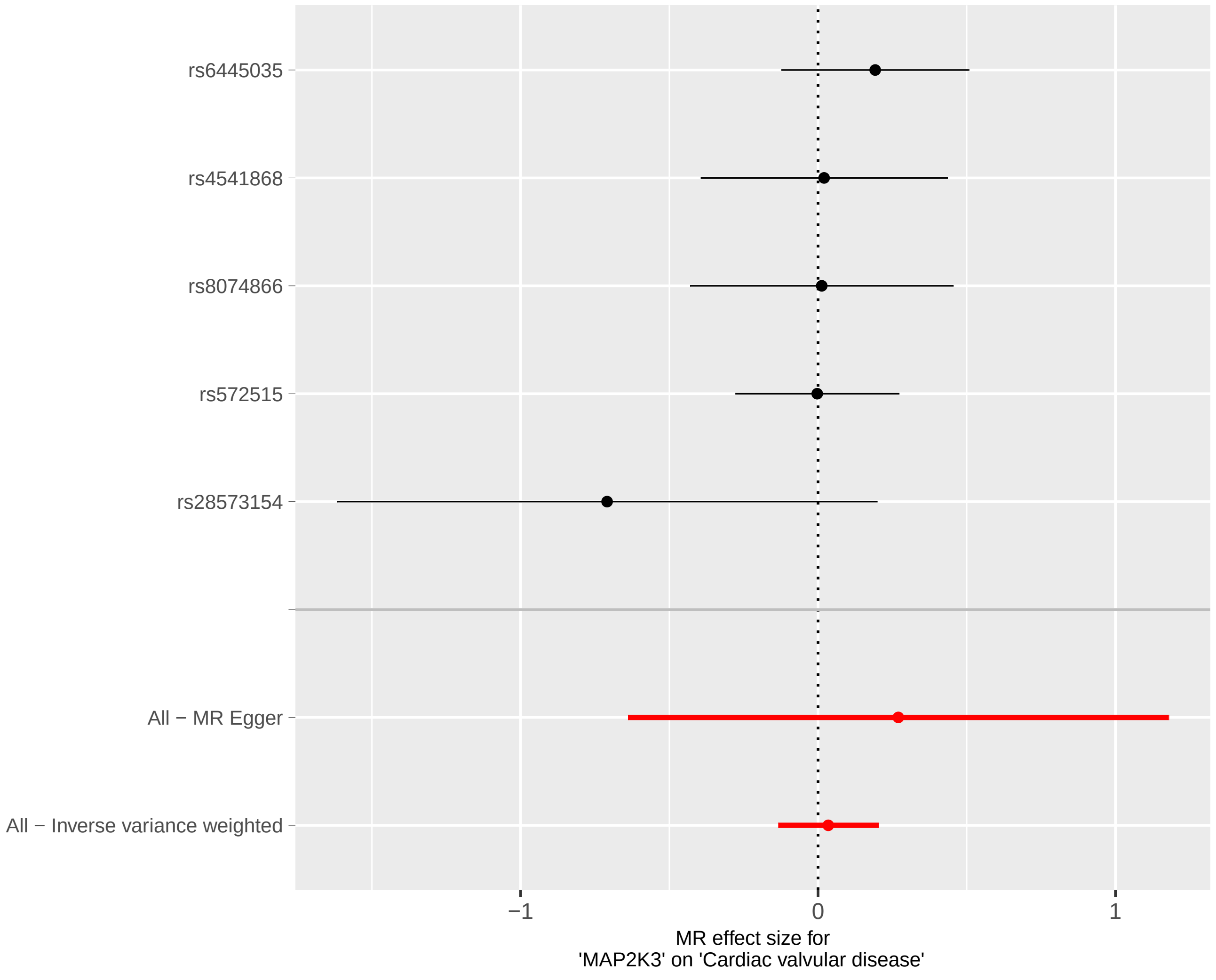
rs6445035
rs4541868
rs8074866
rs572515
rs28573154
All − MR Egger
All − Inverse variance weighted
−1
0
1
MR effect size for
'MAP2K3' on 'Cardiac valvular disease'

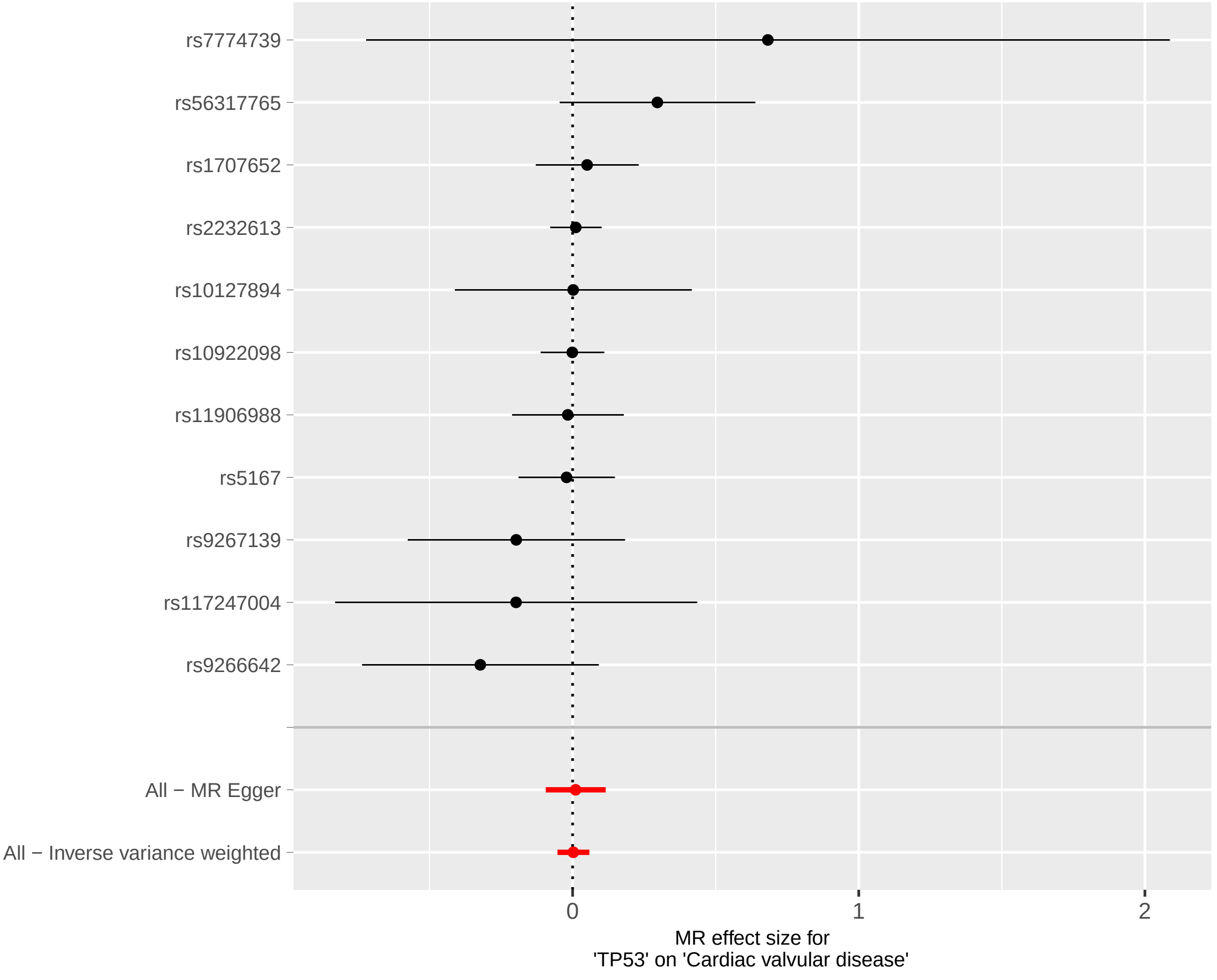

rs7774739
rs56317765
rs1707652
rs2232613
rs10127894
rs10922098
rs11906988
rs5167
rs9267139
rs117247004
rs9266642
All − MR Egger
All − Inverse variance weighted
0
1
2
MR effect size for
'TP53' on 'Cardiac valvular disease'

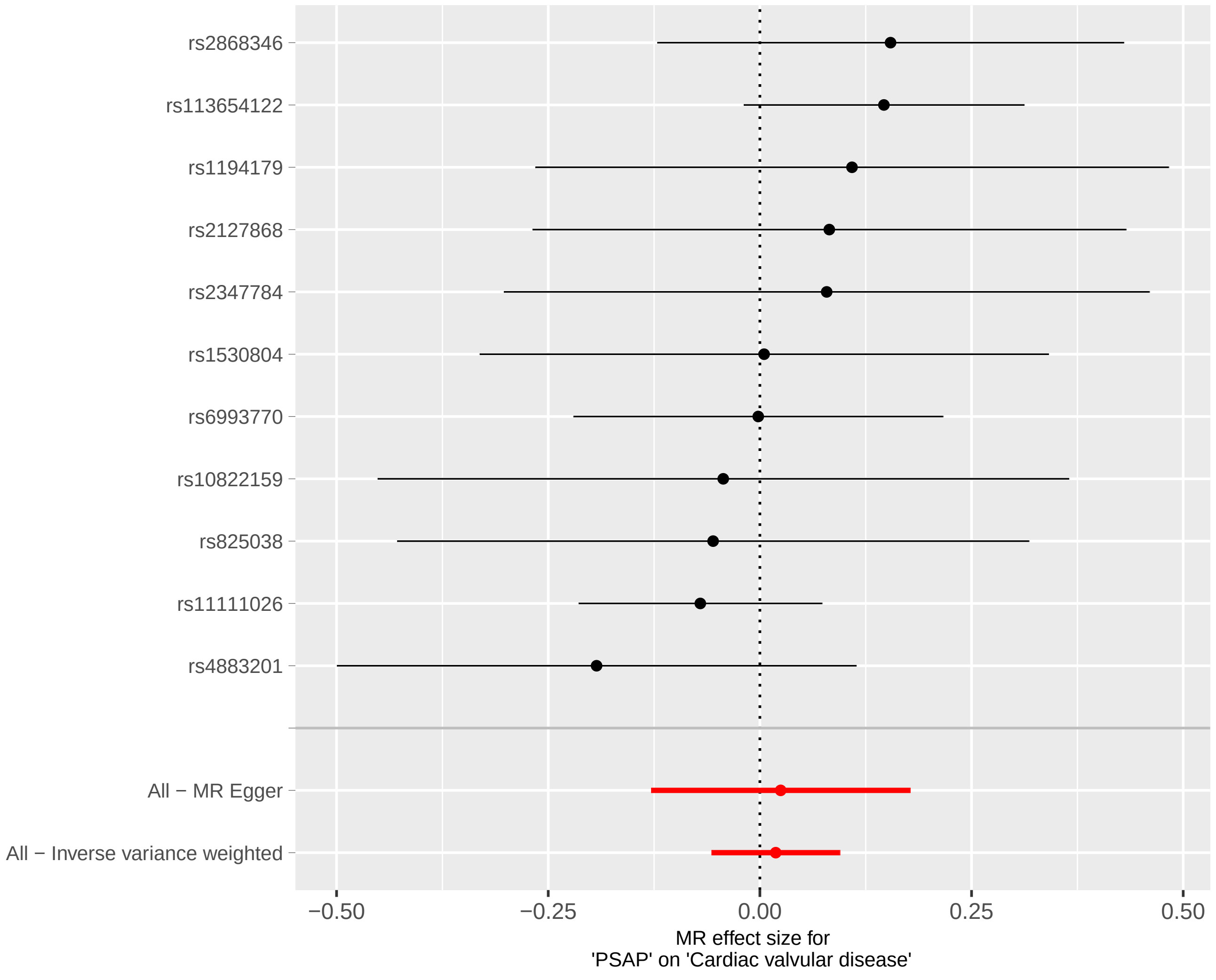

rs2868346
rs113654122
rs1194179
rs2127868
rs2347784
rs1530804
rs6993770
rs10822159
rs825038
rs11111026
rs4883201
All − MR Egger
All − Inverse variance weighted
−0.50
−0.25
0.00
0.25
0.50
MR effect size for
'PSAP' on 'Cardiac valvular disease'

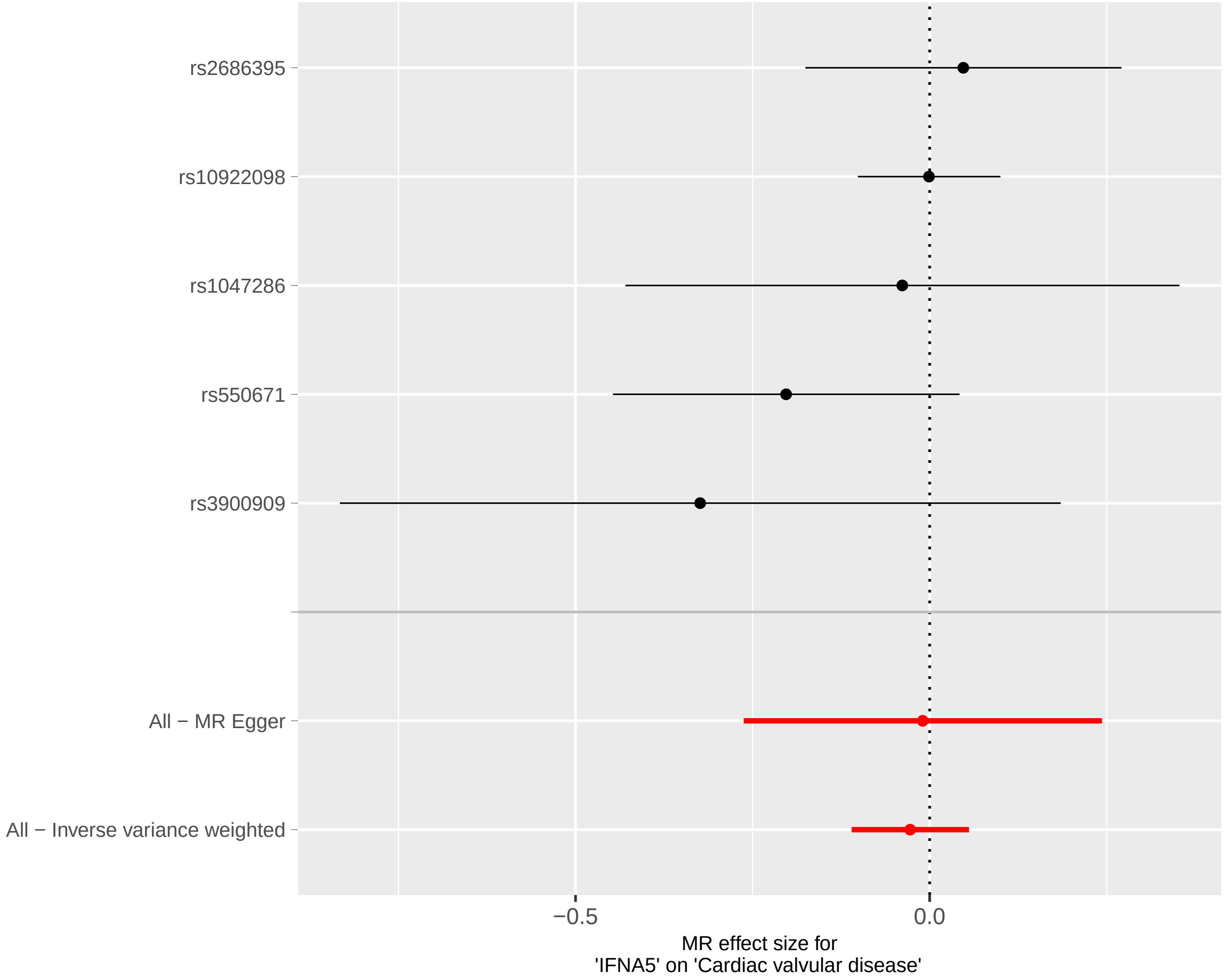
rs2686395
rs10922098
rs1047286
rs550671
rs3900909
All − MR Egger
All − Inverse variance weighted
−0.5
0.0
MR effect size for
'IFNA5' on 'Cardiac valvular disease'

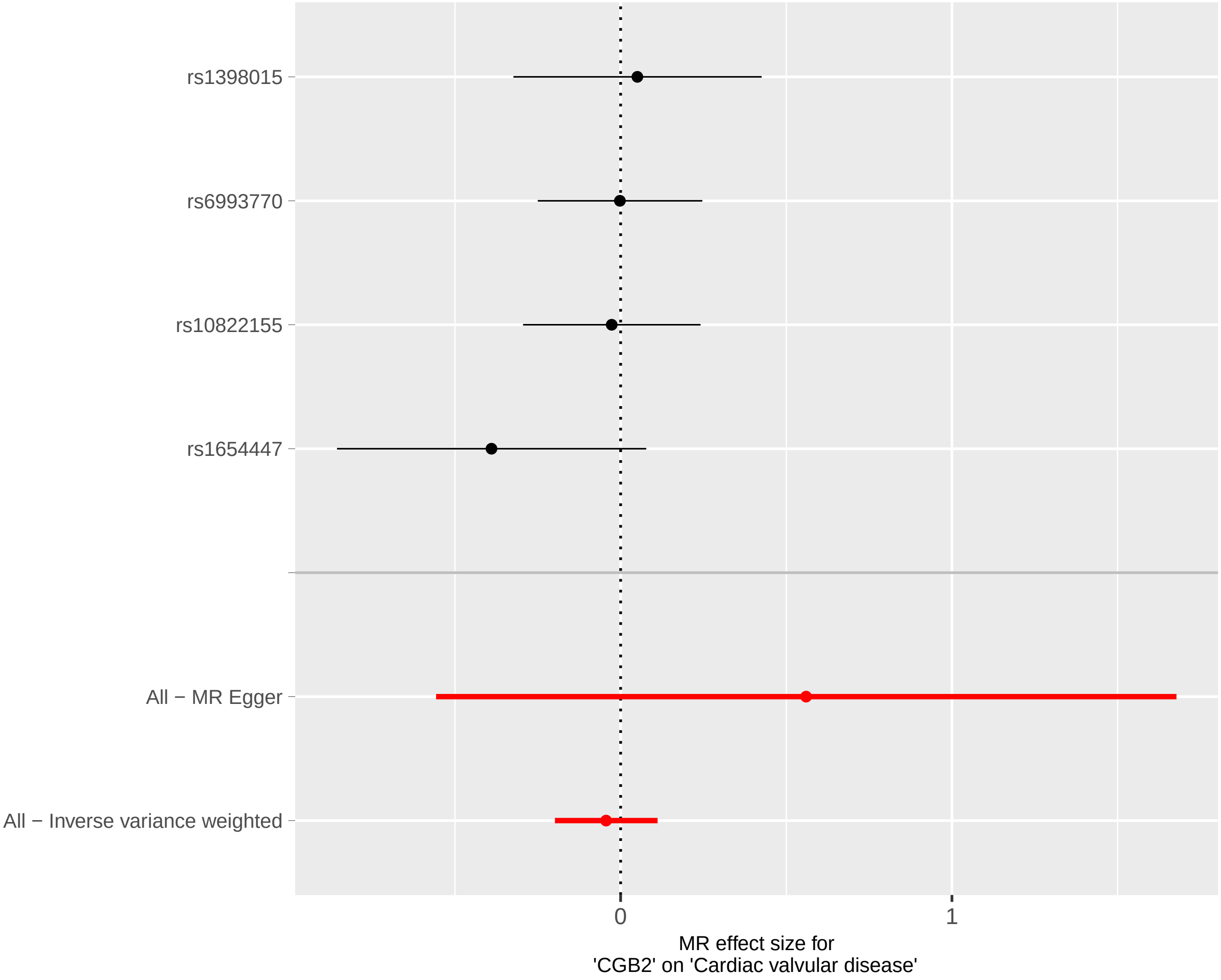
rs1398015
rs6993770
rs10822155
rs1654447
All − MR Egger
All − Inverse variance weighted
0
1
MR effect size for
'CGB2' on 'Cardiac valvular disease'

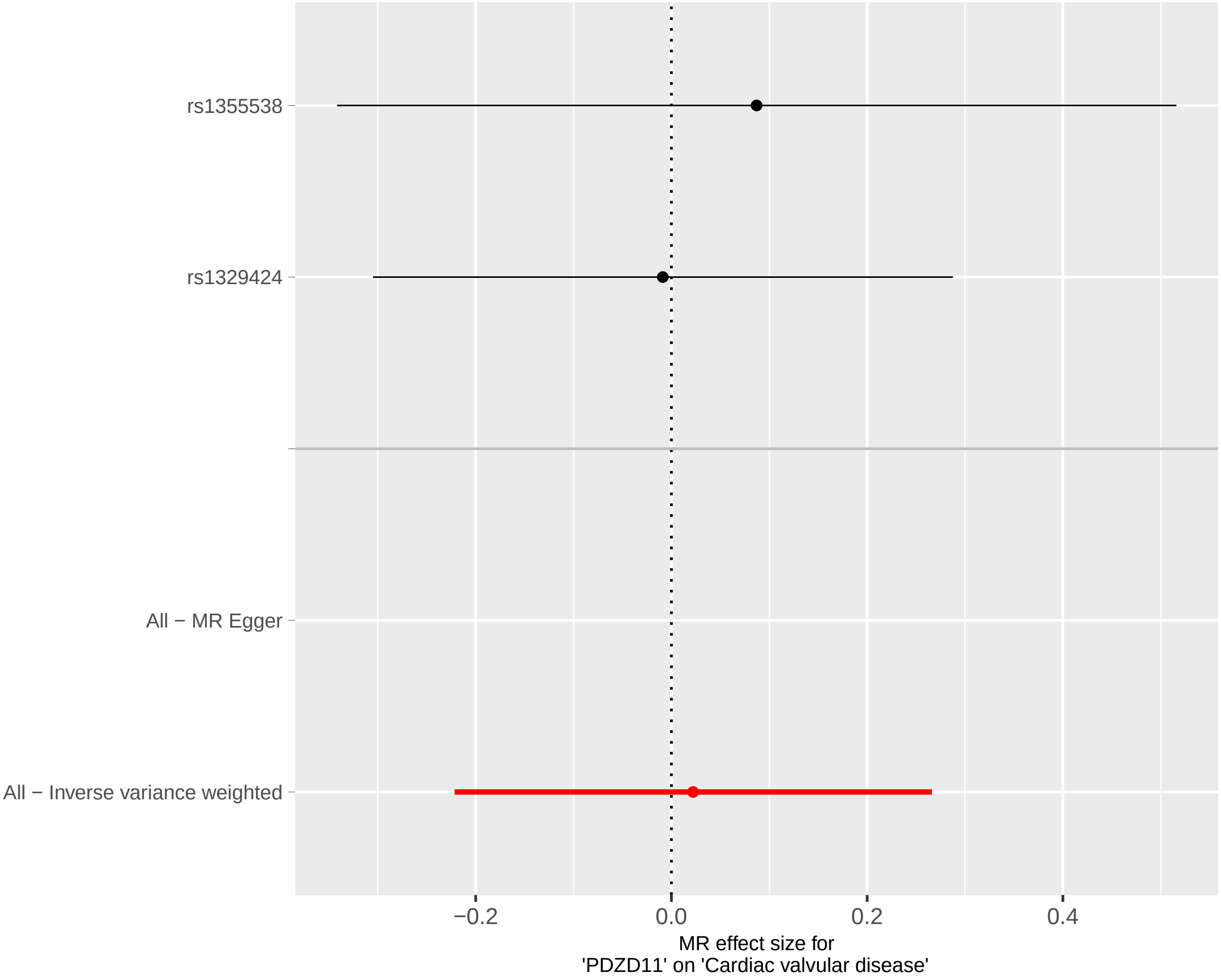
rs1355538
rs1329424
All − MR Egger
All − Inverse variance weighted
−0.2
0.0
0.2
0.4
MR effect size for
'PDZD11' on 'Cardiac valvular disease'

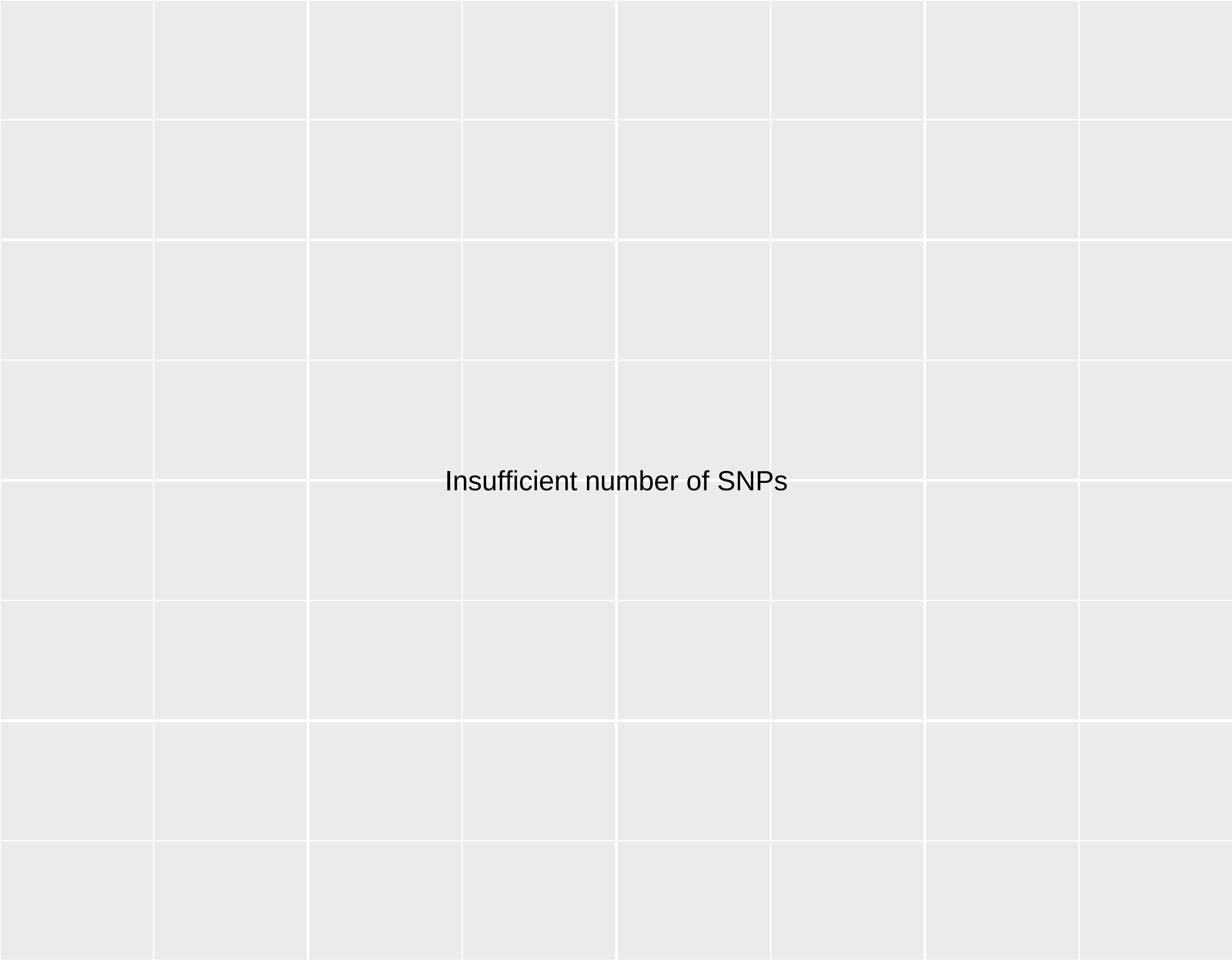
Insufficient number of SNPs

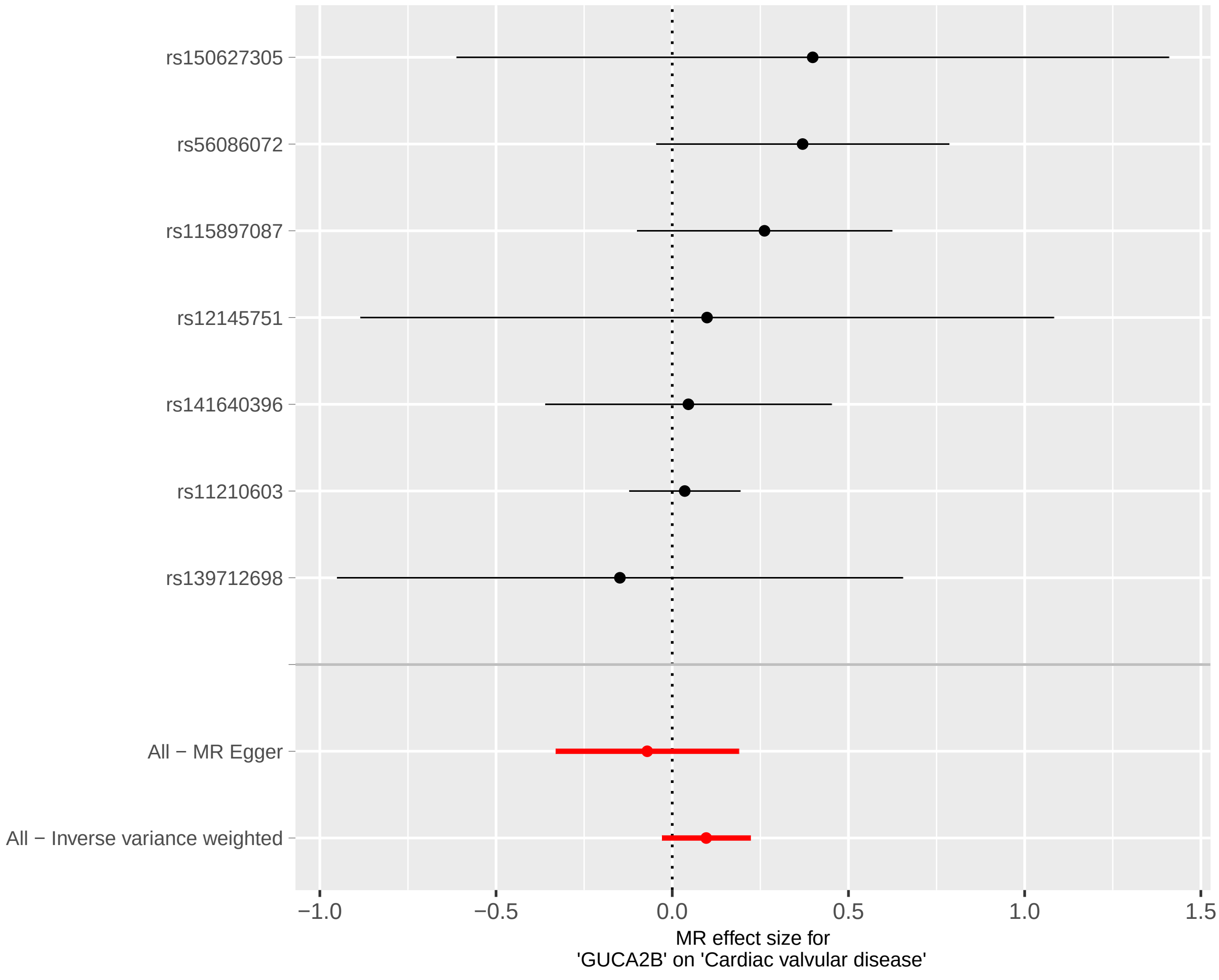
rs150627305
rs56086072
rs115897087
rs12145751
rs141640396
rs11210603
rs139712698
All − MR Egger
All − Inverse variance weighted
−1.0
−0.5
0.0
0.5
1.0
1.5
MR effect size for
'GUCA2B' on 'Cardiac valvular disease'

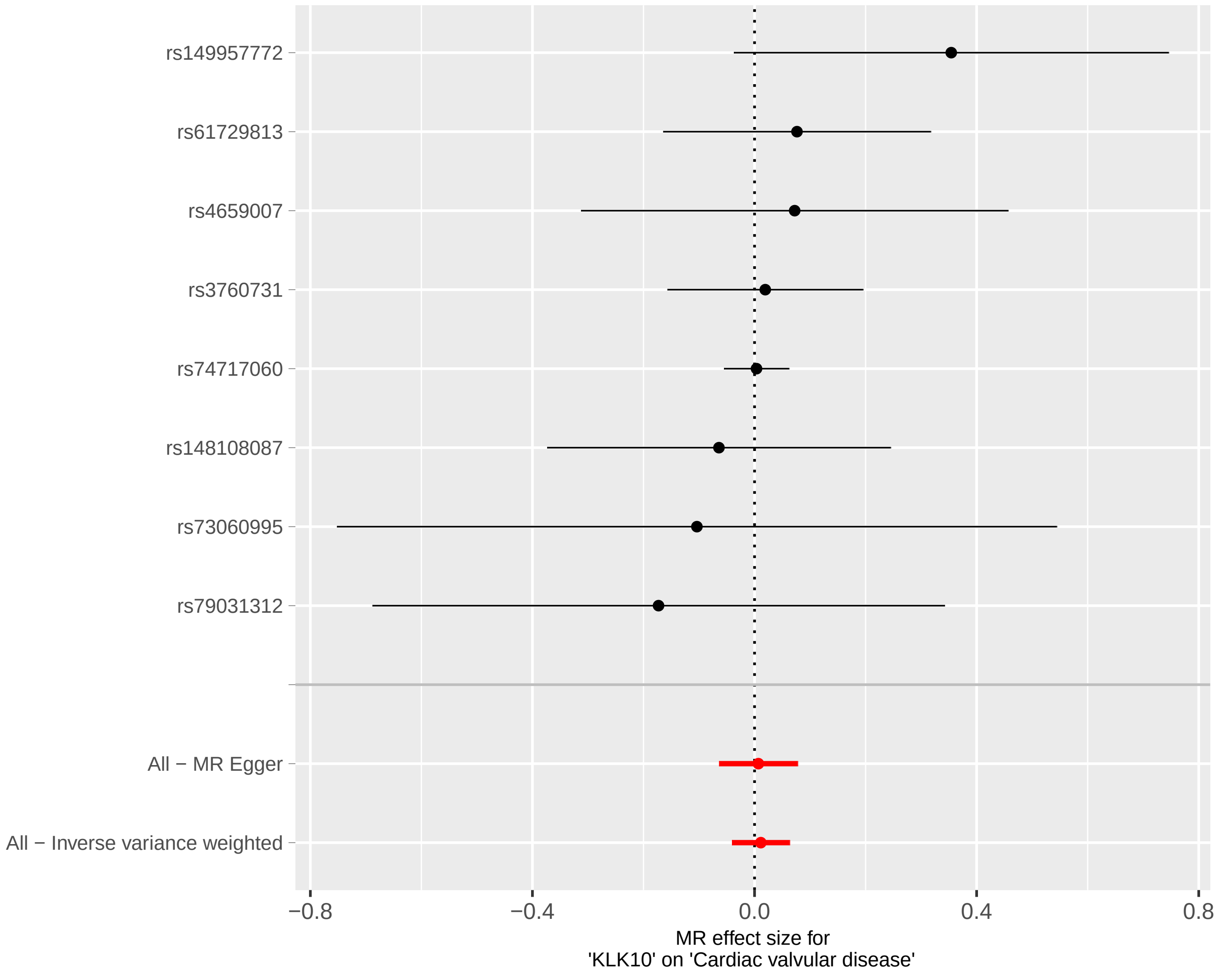

rs149957772
rs61729813
rs4659007
rs3760731
rs74717060
rs148108087
rs73060995
rs79031312
All − MR Egger
All − Inverse variance weighted
−0.8
−0.4
0.0
0.4
0.8
MR effect size for
'KLK10' on 'Cardiac valvular disease'

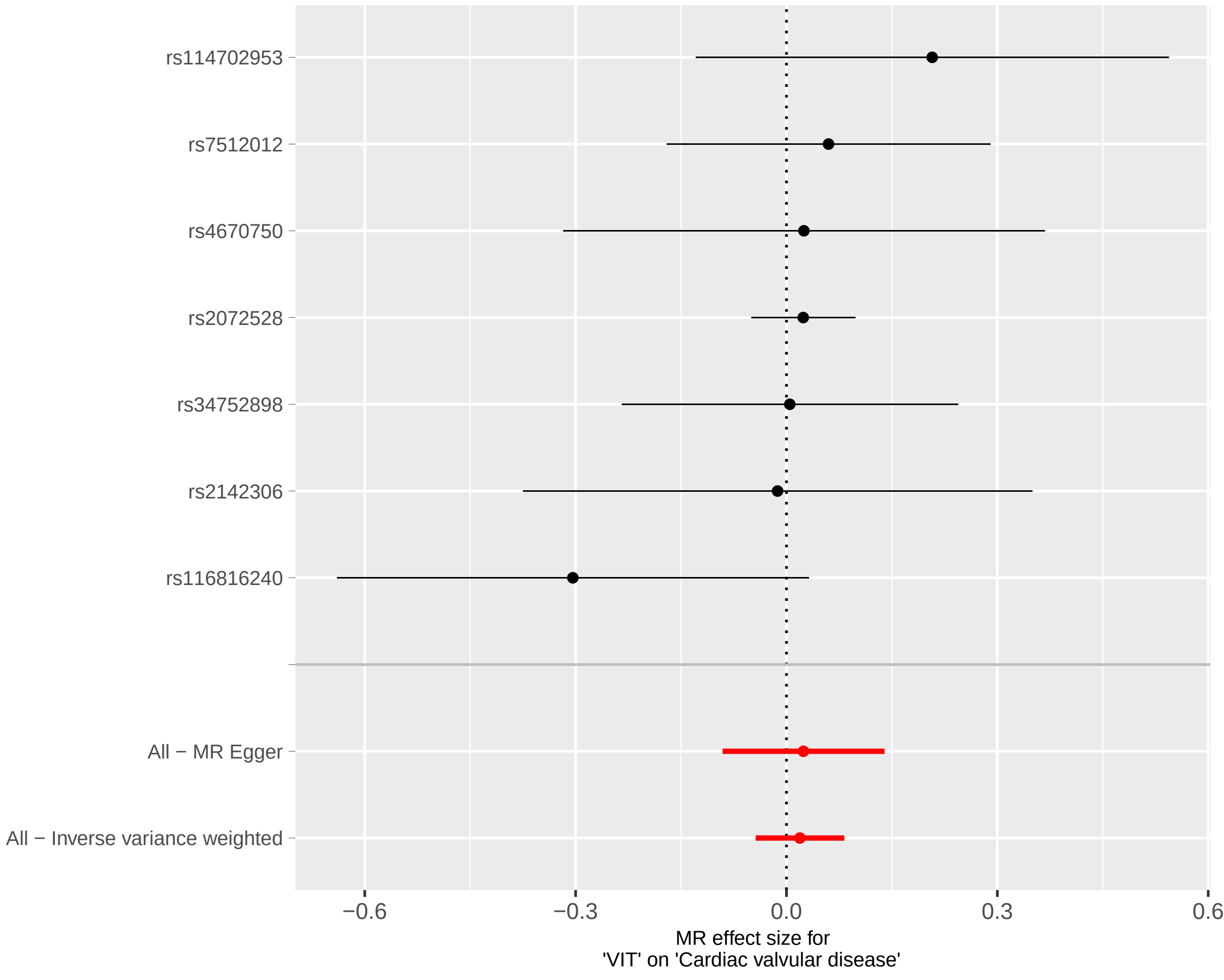
rs114702953
rs7512012
rs4670750
rs2072528
rs34752898
rs2142306
rs116816240
All − MR Egger
All − Inverse variance weighted
−0.6
−0.3
0.0
0.3
0.6
MR effect size for
'VIT' on 'Cardiac valvular disease'

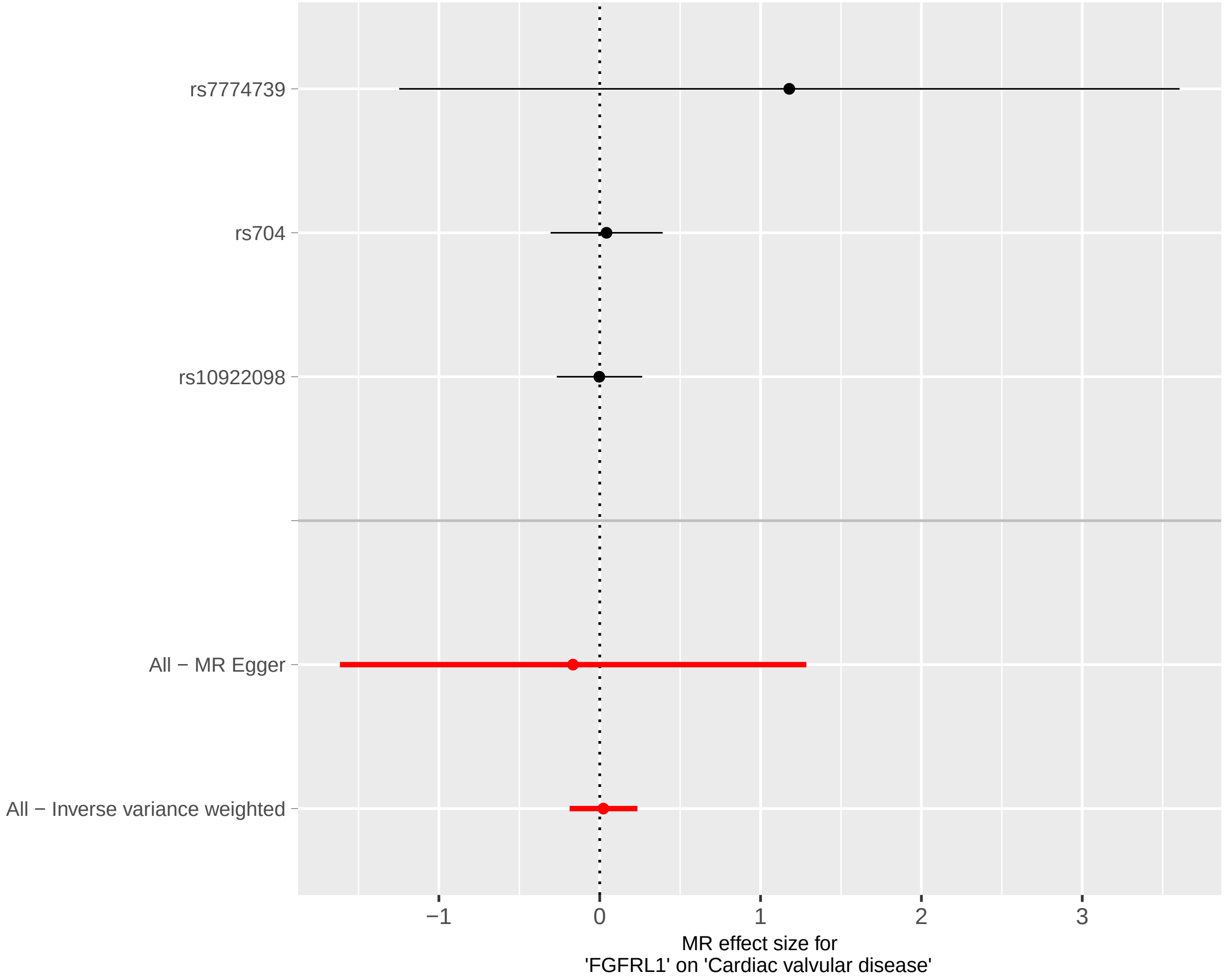
rs7774739
rs704
rs10922098
All − MR Egger
All − Inverse variance weighted
−1
0
1
2
3
MR effect size for
'FGFRL1' on 'Cardiac valvular disease'

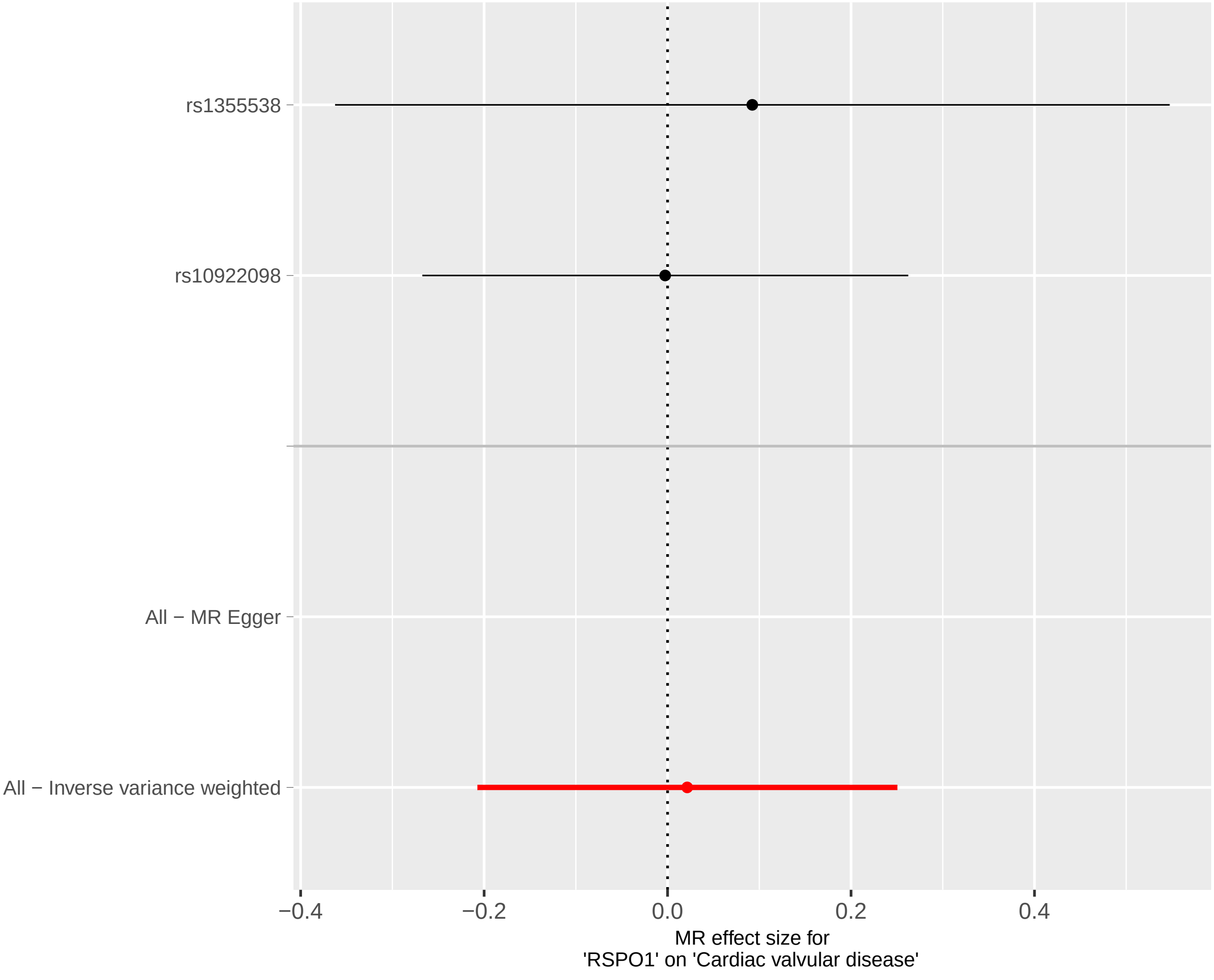
rs1355538
rs10922098
All − MR Egger
All − Inverse variance weighted
−0.4
−0.2
0.0
0.2
0.4
MR effect size for
'RSPO1' on 'Cardiac valvular disease'

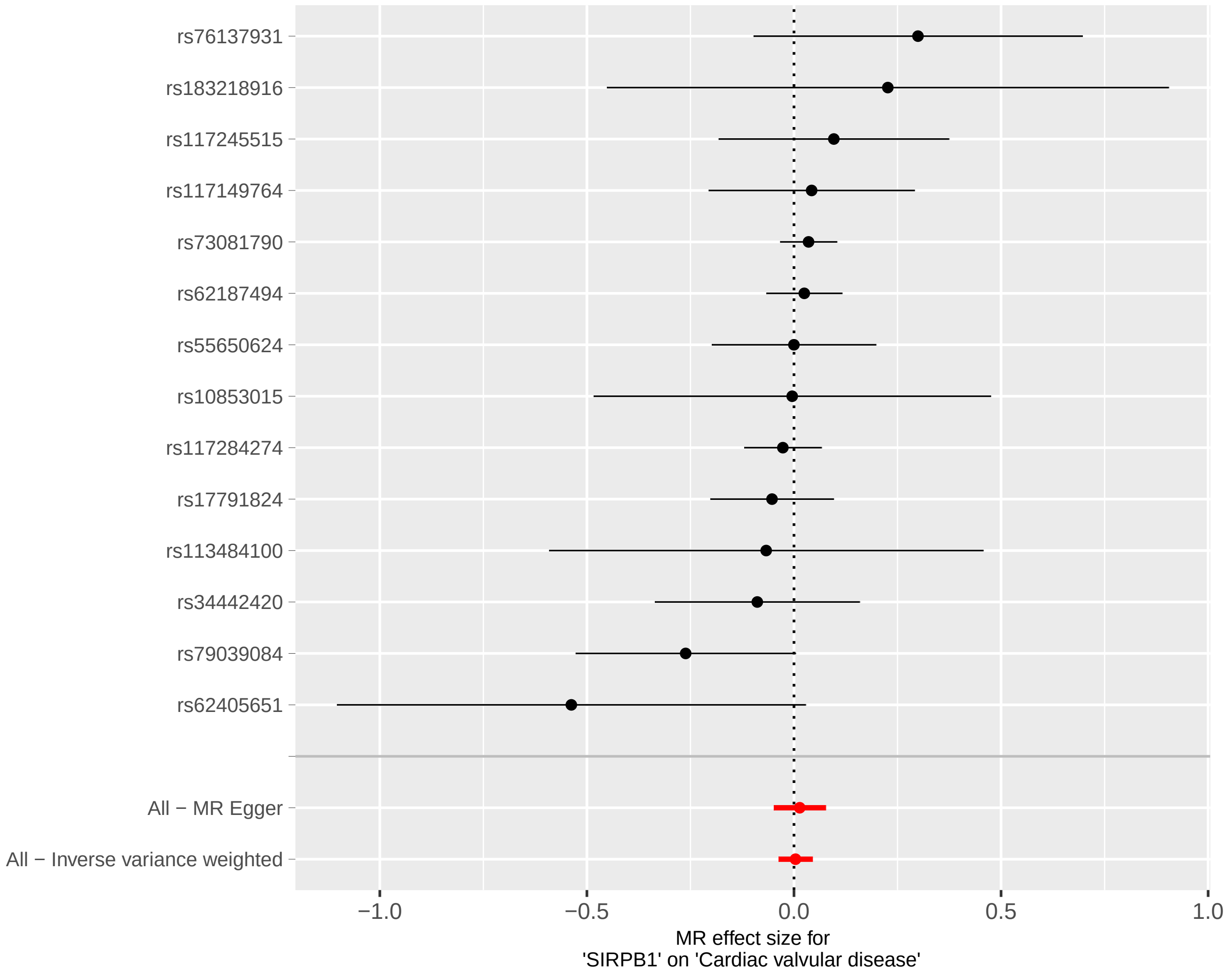
rs76137931
rs183218916
rs117245515
rs117149764
rs73081790
rs62187494
rs55650624
rs10853015
rs117284274
rs17791824
rs113484100
rs34442420
rs79039084
rs62405651
All − MR Egger
All − Inverse variance weighted
−1.0
−0.5
0.0
0.5
1.0
MR effect size for
'SIRPB1' on 'Cardiac valvular disease'

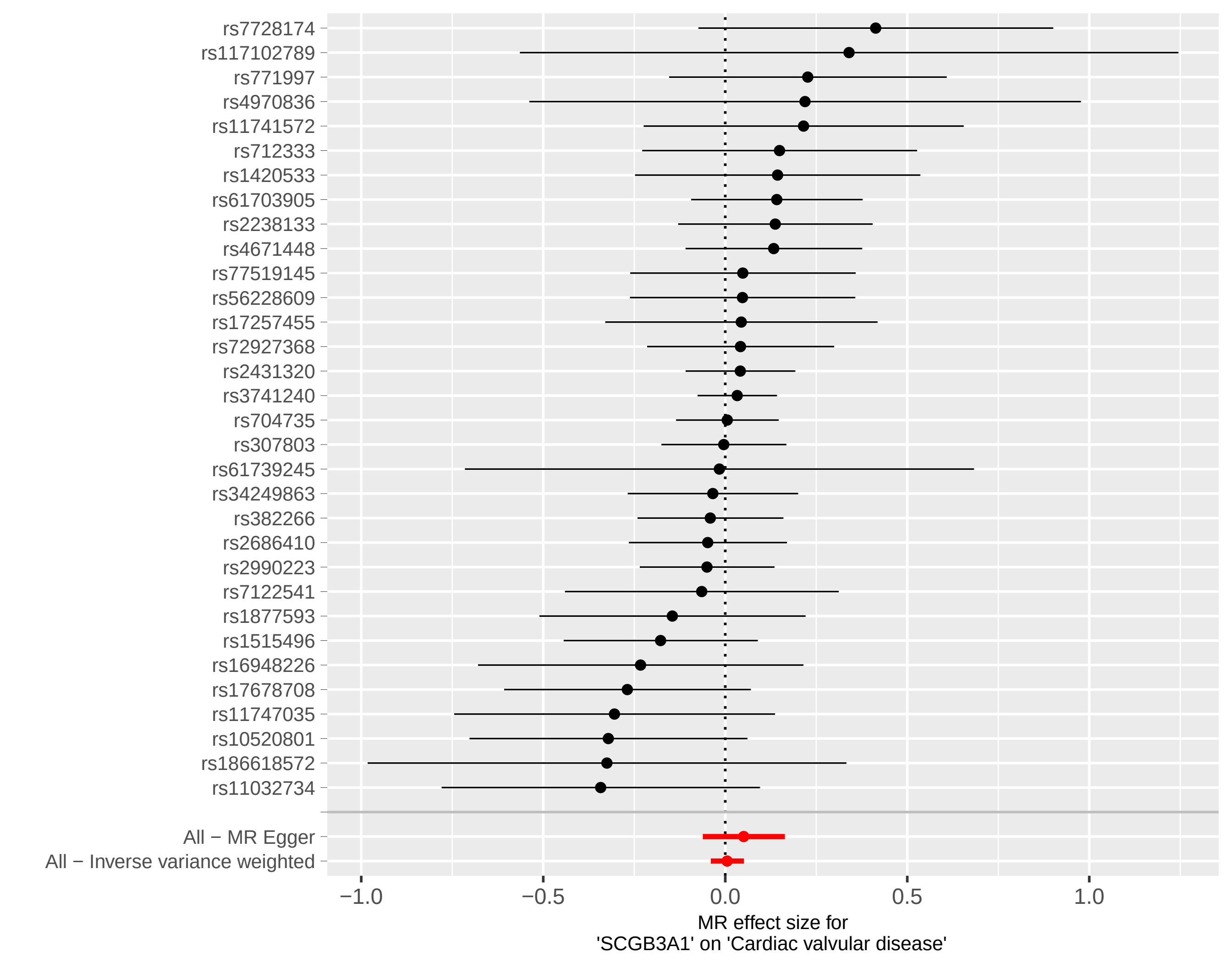
rs7728174
rs117102789
rs771997
rs4970836
rs11741572
rs712333
rs1420533
rs61703905
rs2238133
rs4671448
rs77519145
rs56228609
rs17257455
rs72927368
rs2431320
rs3741240
rs704735
rs307803
rs61739245
rs34249863
rs382266
rs2686410
rs2990223
rs7122541
rs1877593
rs1515496
rs16948226
rs17678708
rs11747035
rs10520801
rs186618572
rs11032734
All − MR Egger
All − Inverse variance weighted
−1.0
−0.5
0.0
0.5
1.0
MR effect size for
'SCGB3A1' on 'Cardiac valvular disease'

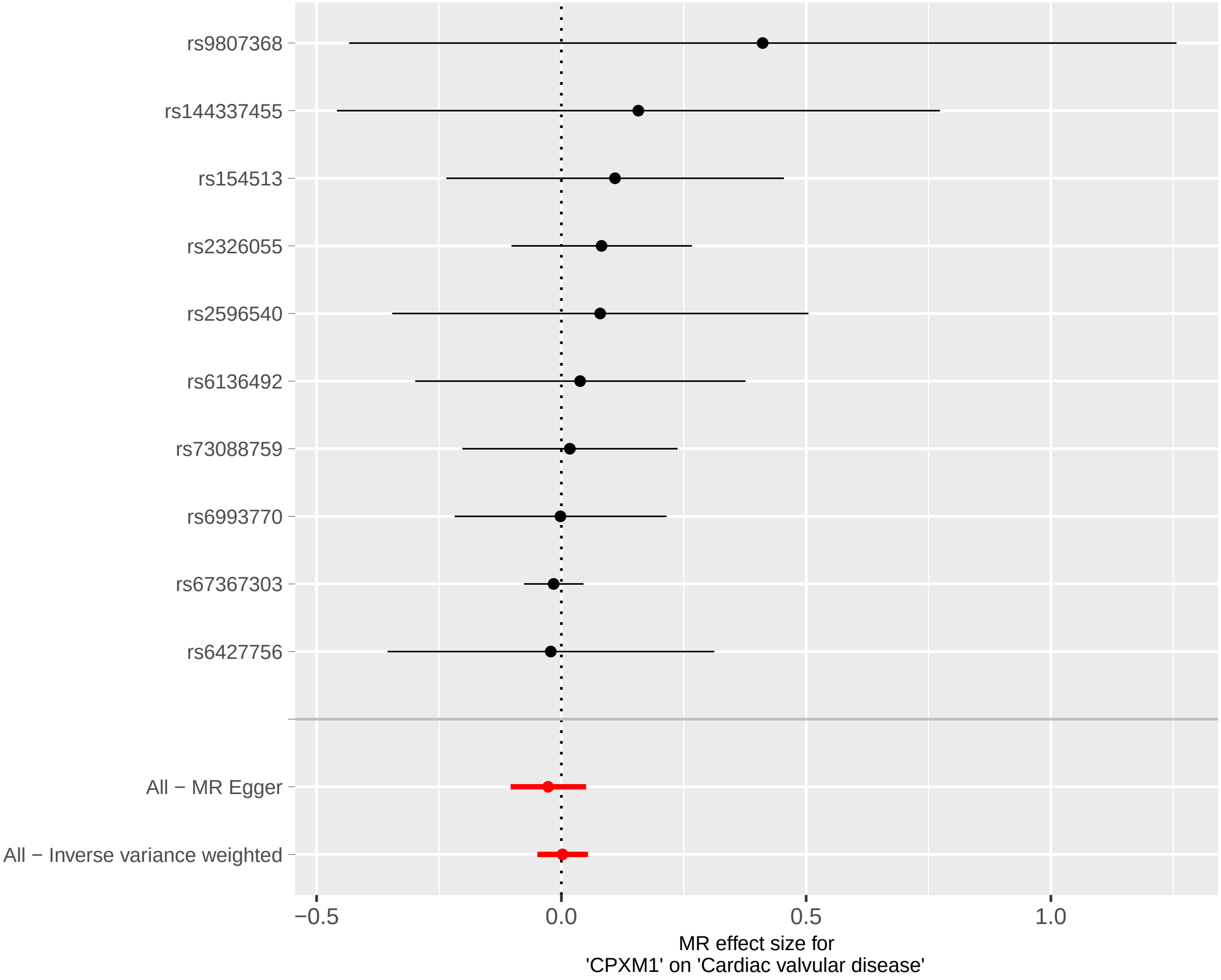

rs9807368
rs144337455
rs154513
rs2326055
rs2596540
rs6136492
rs73088759
rs6993770
rs67367303
rs6427756
All − MR Egger
All − Inverse variance weighted
−0.5
0.0
0.5
1.0
MR effect size for
'CPXM1' on 'Cardiac valvular disease'

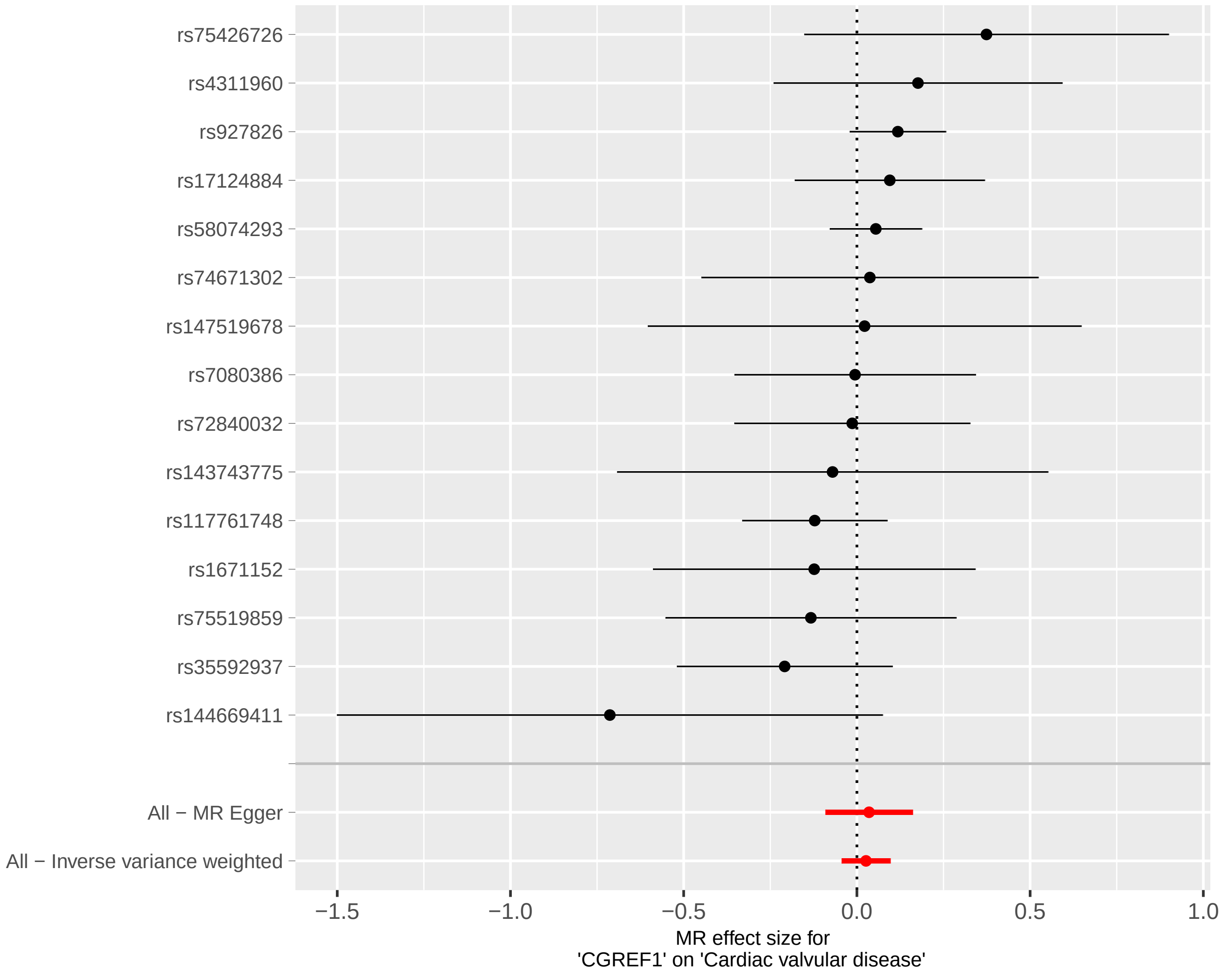
rs75426726
rs4311960
rs927826
rs17124884
rs58074293
rs74671302
rs147519678
rs7080386
rs72840032
rs143743775
rs117761748
rs1671152
rs75519859
rs35592937
rs144669411
All − MR Egger
All − Inverse variance weighted
−1.5
−1.0
−0.5
0.0
0.5
1.0
MR effect size for
'CGREF1' on 'Cardiac valvular disease'

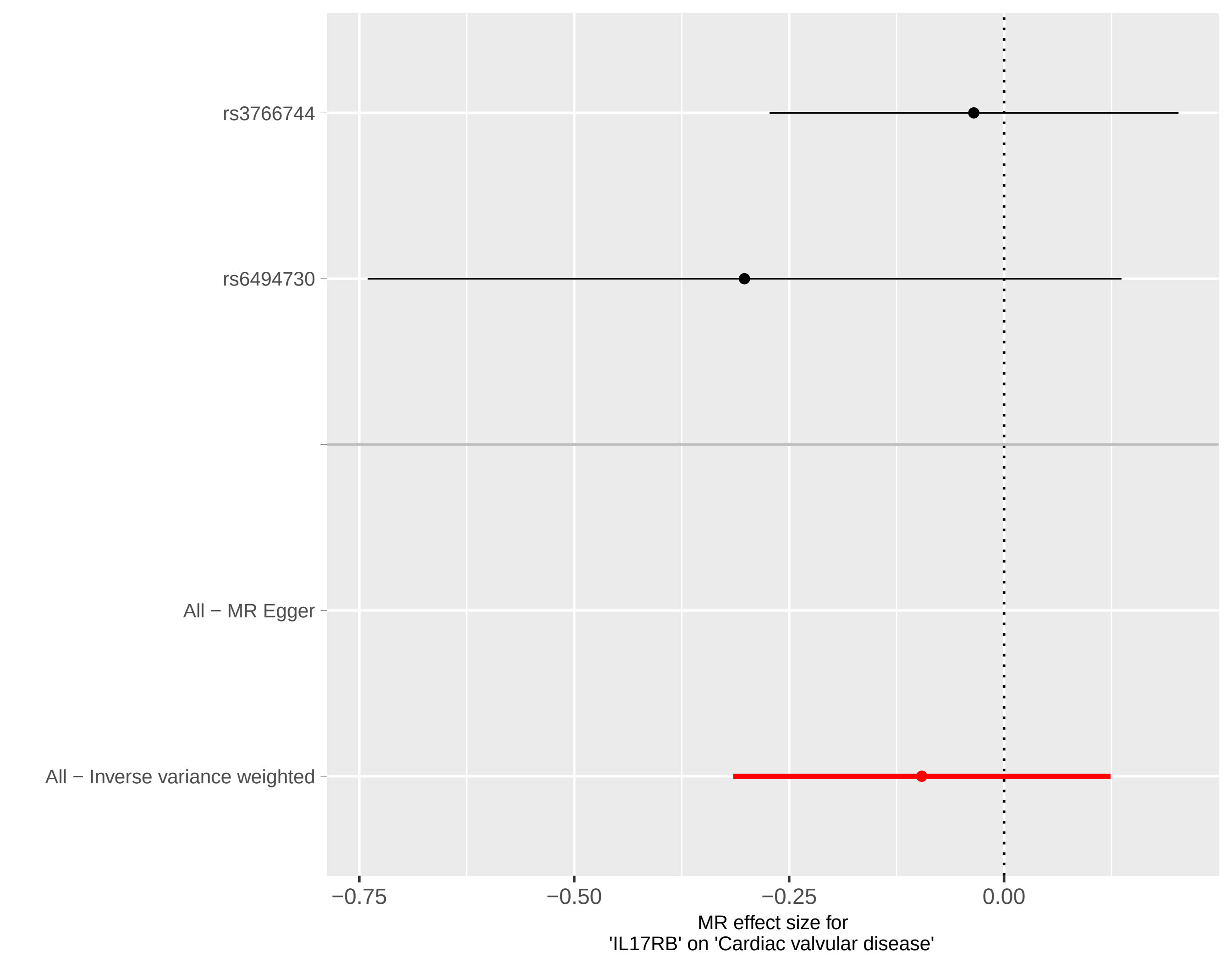

rs3766744
rs6494730
All − MR Egger
All − Inverse variance weighted
−0.75
−0.50
−0.25
0.00
MR effect size for
'IL17RB' on 'Cardiac valvular disease'

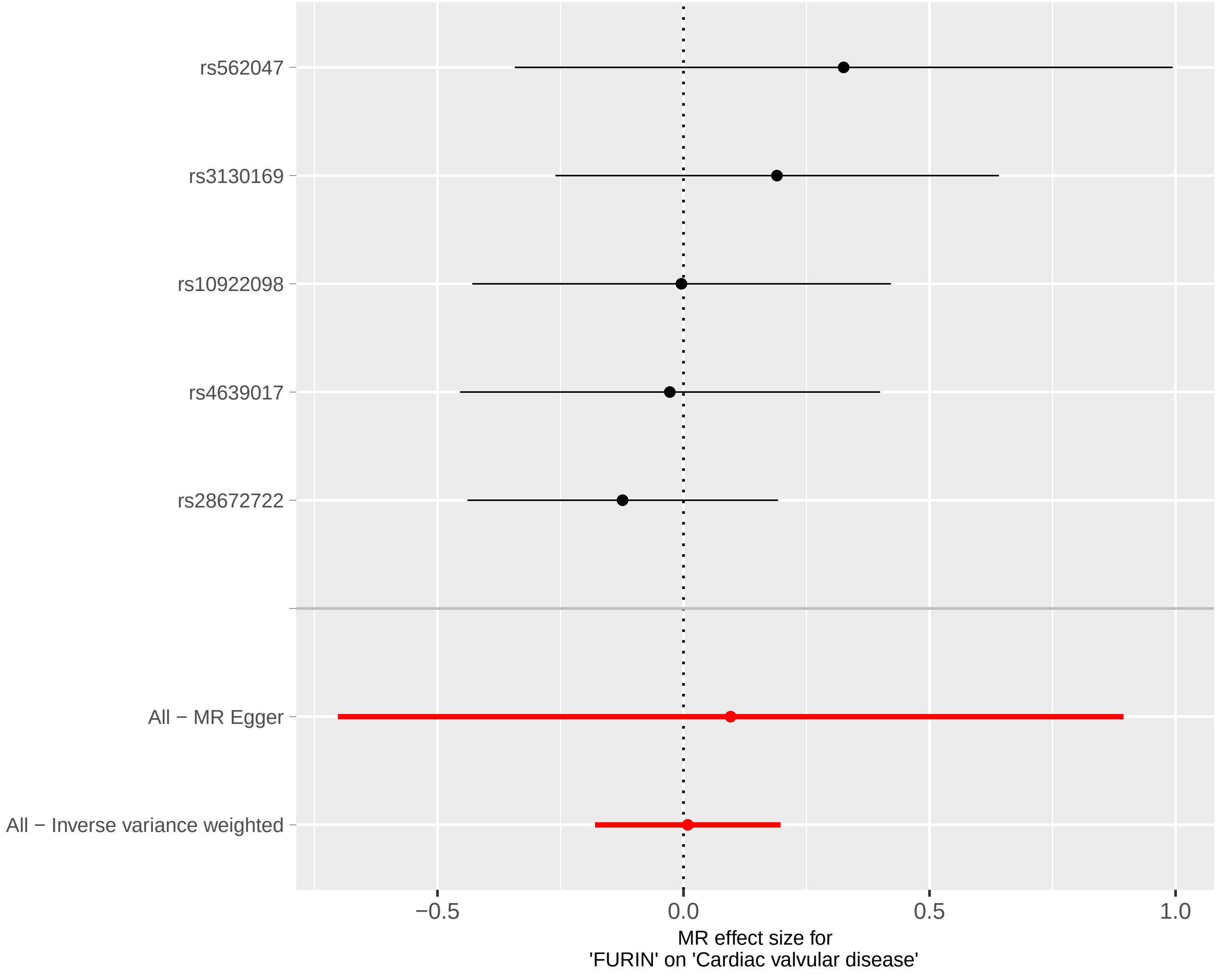

rs562047
rs3130169
rs10922098
rs4639017
rs28672722
All − MR Egger
All − Inverse variance weighted
−0.5
0.0
0.5
1.0
MR effect size for
'FURIN' on 'Cardiac valvular disease'

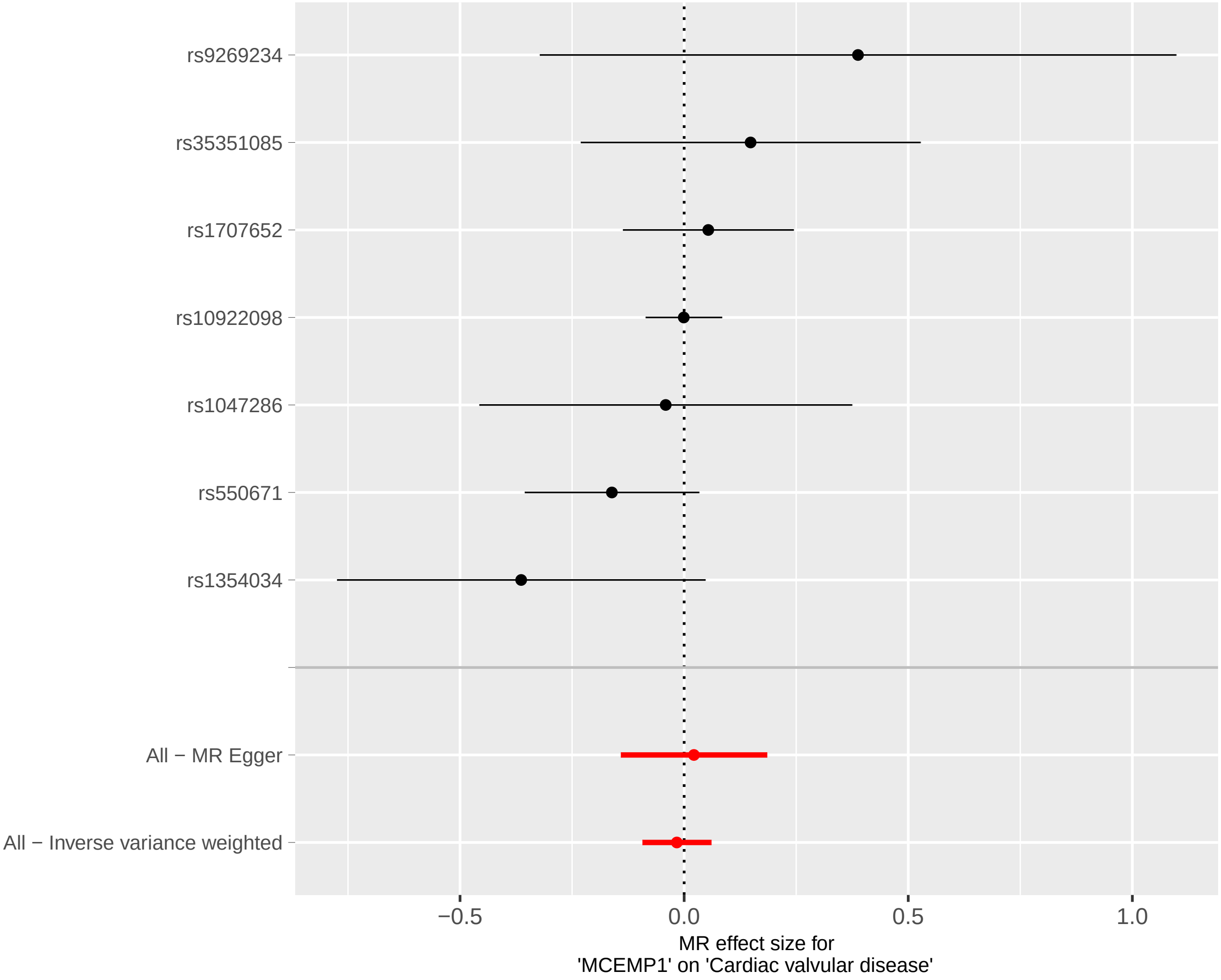

rs9269234
rs35351085
rs1707652
rs10922098
rs1047286
rs550671
rs1354034
All − MR Egger
All − Inverse variance weighted
−0.5
0.0
0.5
1.0
MR effect size for
'MCEMP1' on 'Cardiac valvular disease'

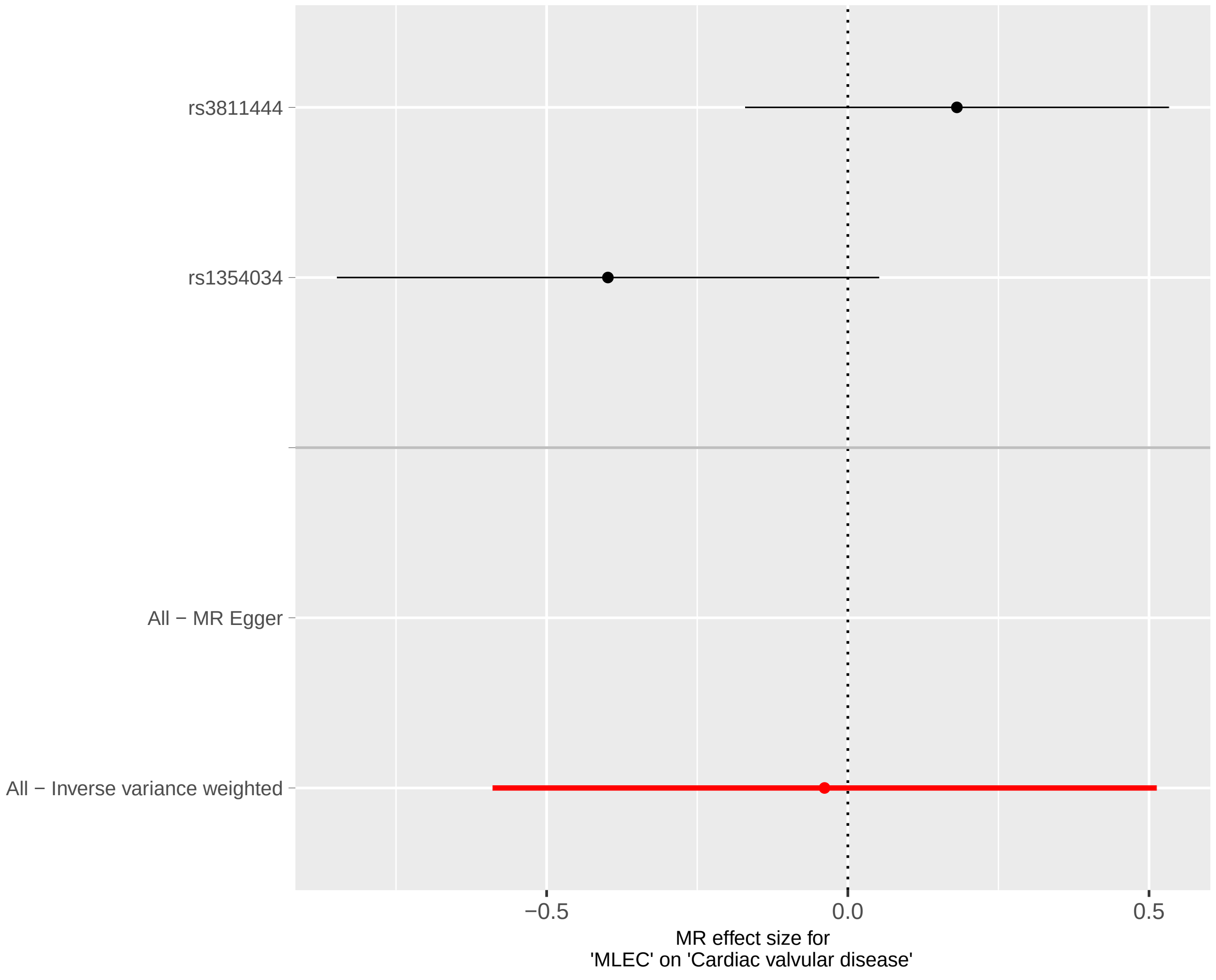
rs3811444
rs1354034
All − MR Egger
All − Inverse variance weighted
−0.5
0.0
0.5
MR effect size for
'MLEC' on 'Cardiac valvular disease'

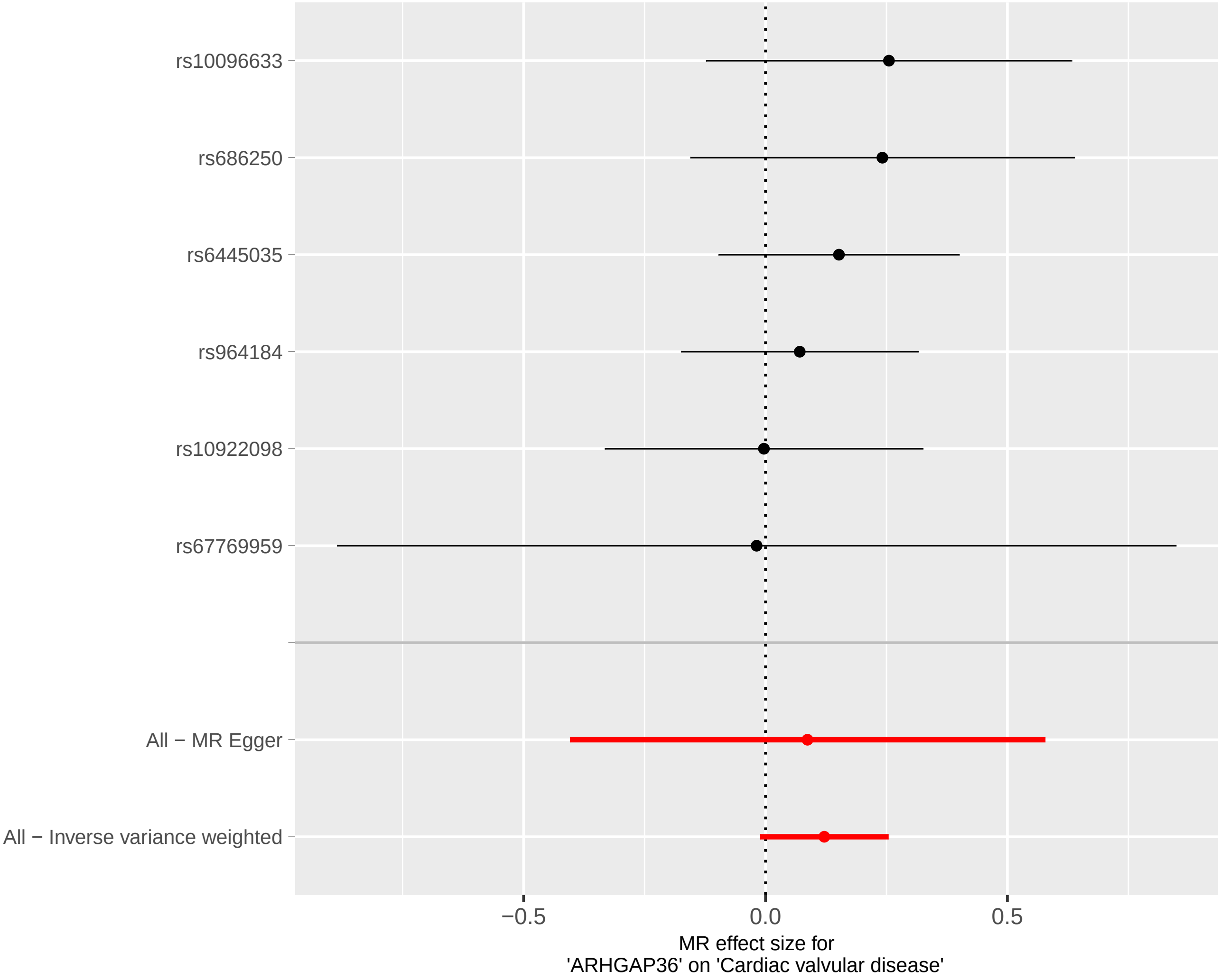

rs10096633
rs686250
rs6445035
rs964184
rs10922098
rs67769959
All − MR Egger
All − Inverse variance weighted
−0.5
0.0
0.5
MR effect size for
'ARHGAP36' on 'Cardiac valvular disease'

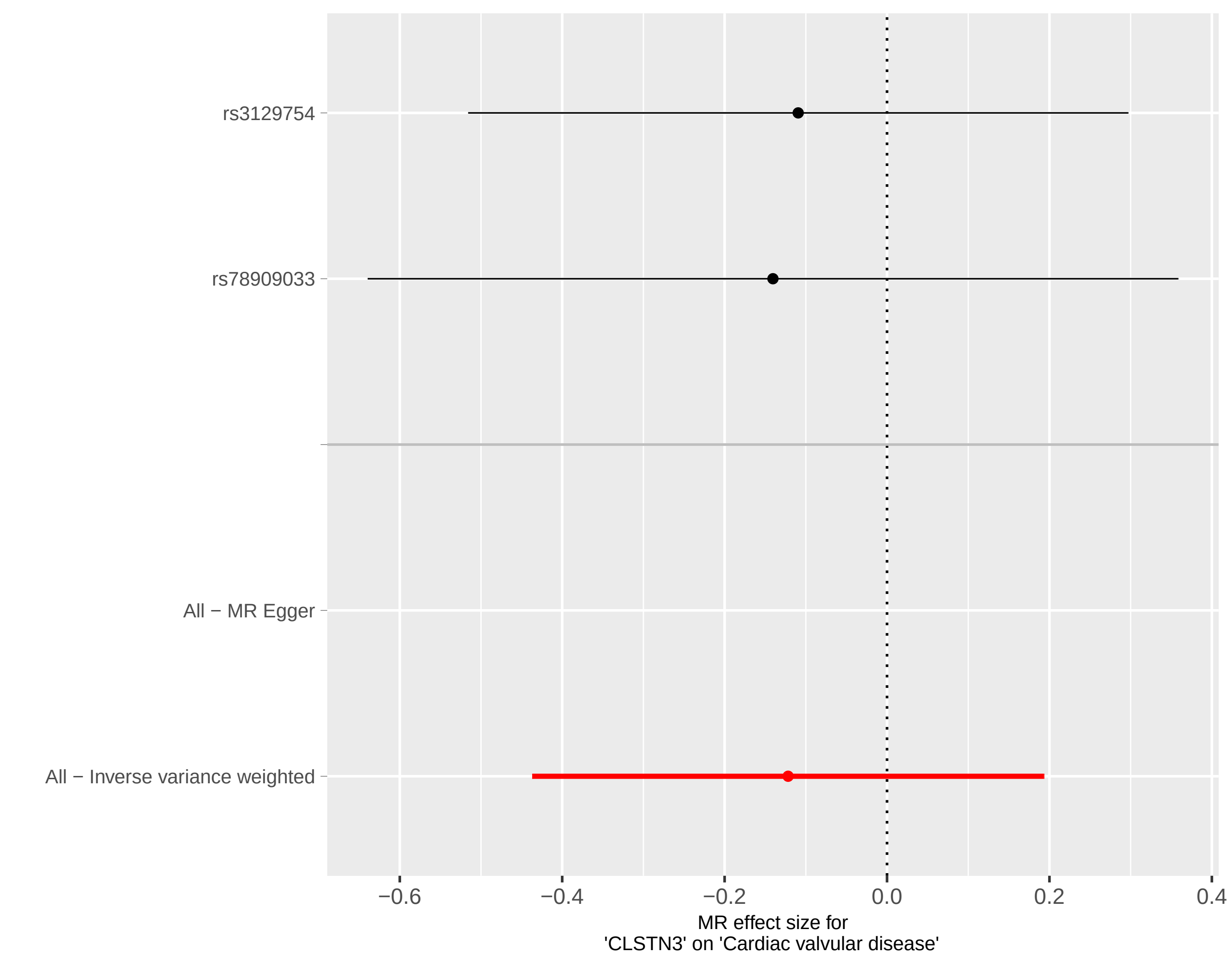
rs3129754
rs78909033
All − MR Egger
All − Inverse variance weighted
−0.6
−0.4
−0.2
0.0
0.2
0.4
MR effect size for
'CLSTN3' on 'Cardiac valvular disease'

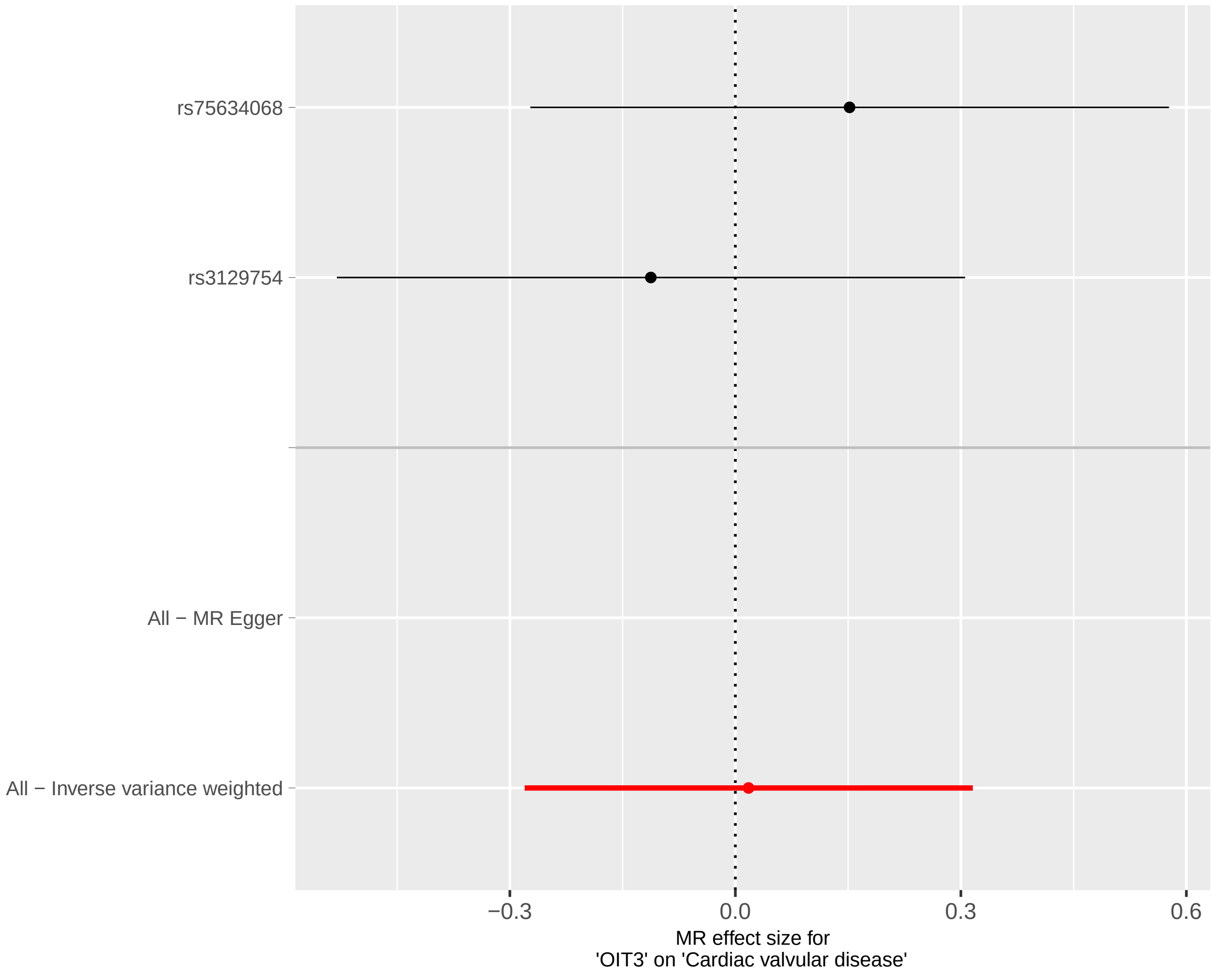
rs75634068
rs3129754
All − MR Egger
All − Inverse variance weighted
−0.3
0.0
0.3
0.6
MR effect size for
'OIT3' on 'Cardiac valvular disease'

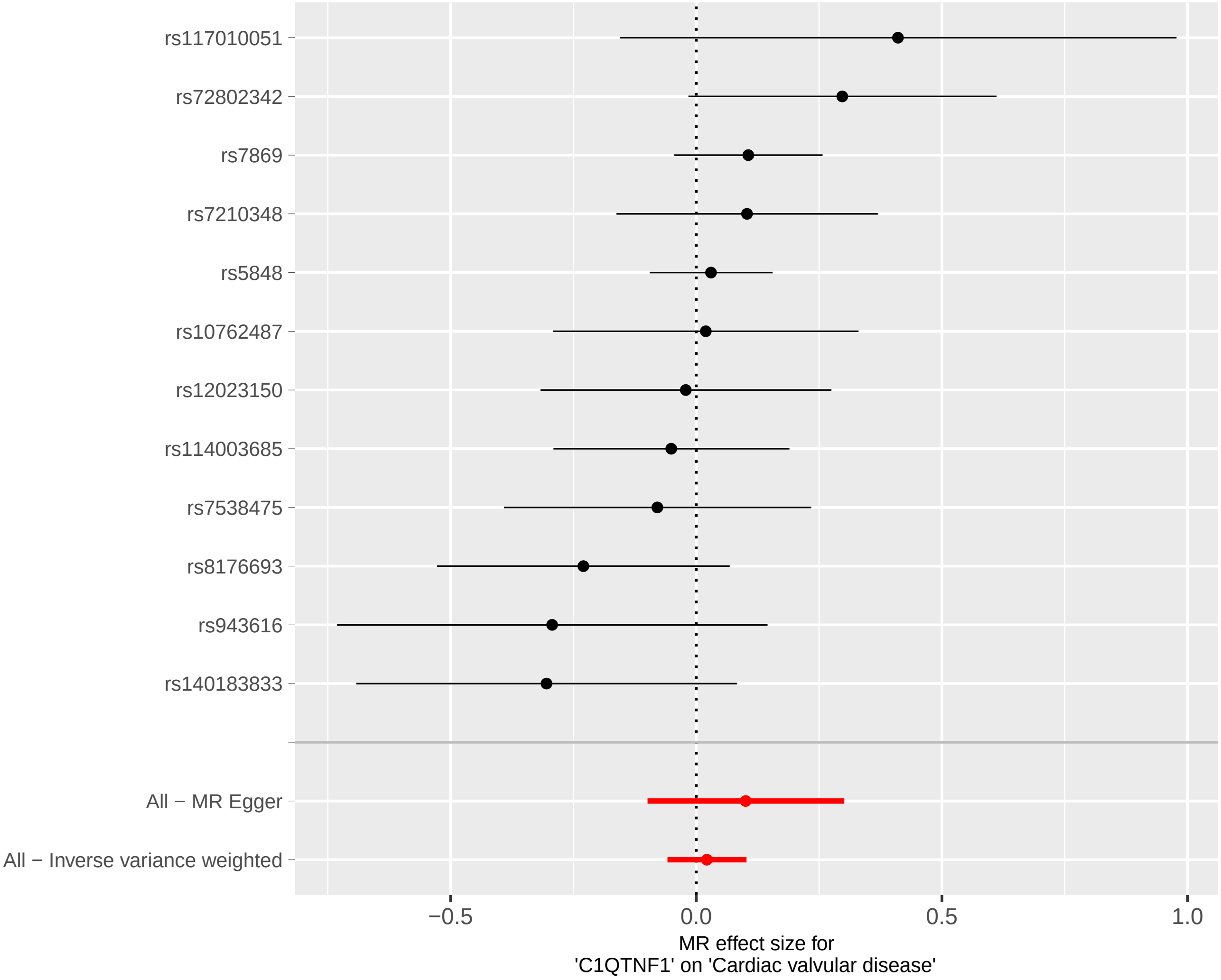
rs117010051
rs72802342
rs7869
rs7210348
rs5848
rs10762487
rs12023150
rs114003685
rs7538475
rs8176693
rs943616
rs140183833
All − MR Egger
All − Inverse variance weighted
−0.5
0.0
0.5
1.0
MR effect size for
'C1QTNF1' on 'Cardiac valvular disease'

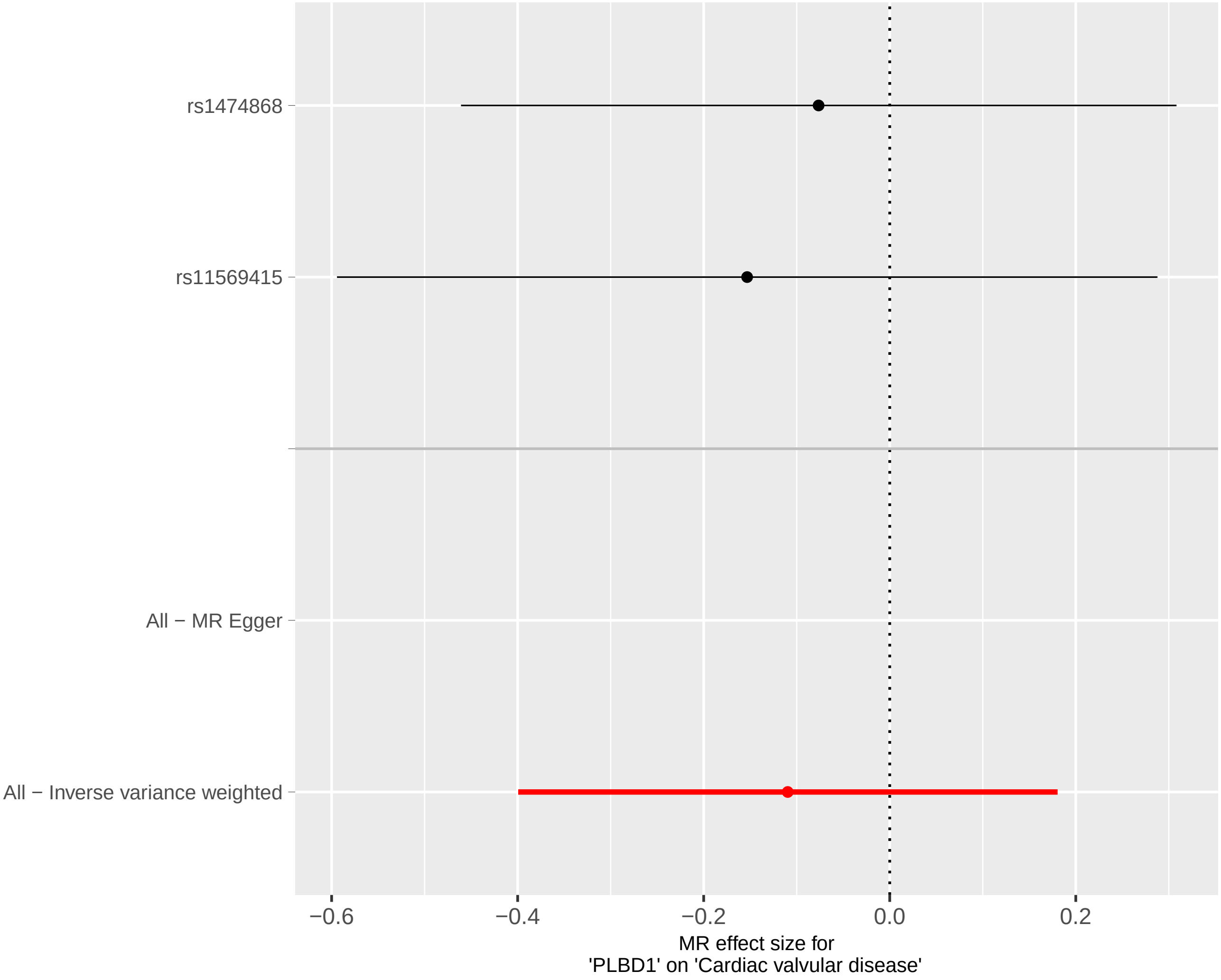

rs1474868
rs11569415
All − MR Egger
All − Inverse variance weighted
−0.6
−0.4
−0.2
0.0
0.2
MR effect size for
'PLBD1' on 'Cardiac valvular disease'

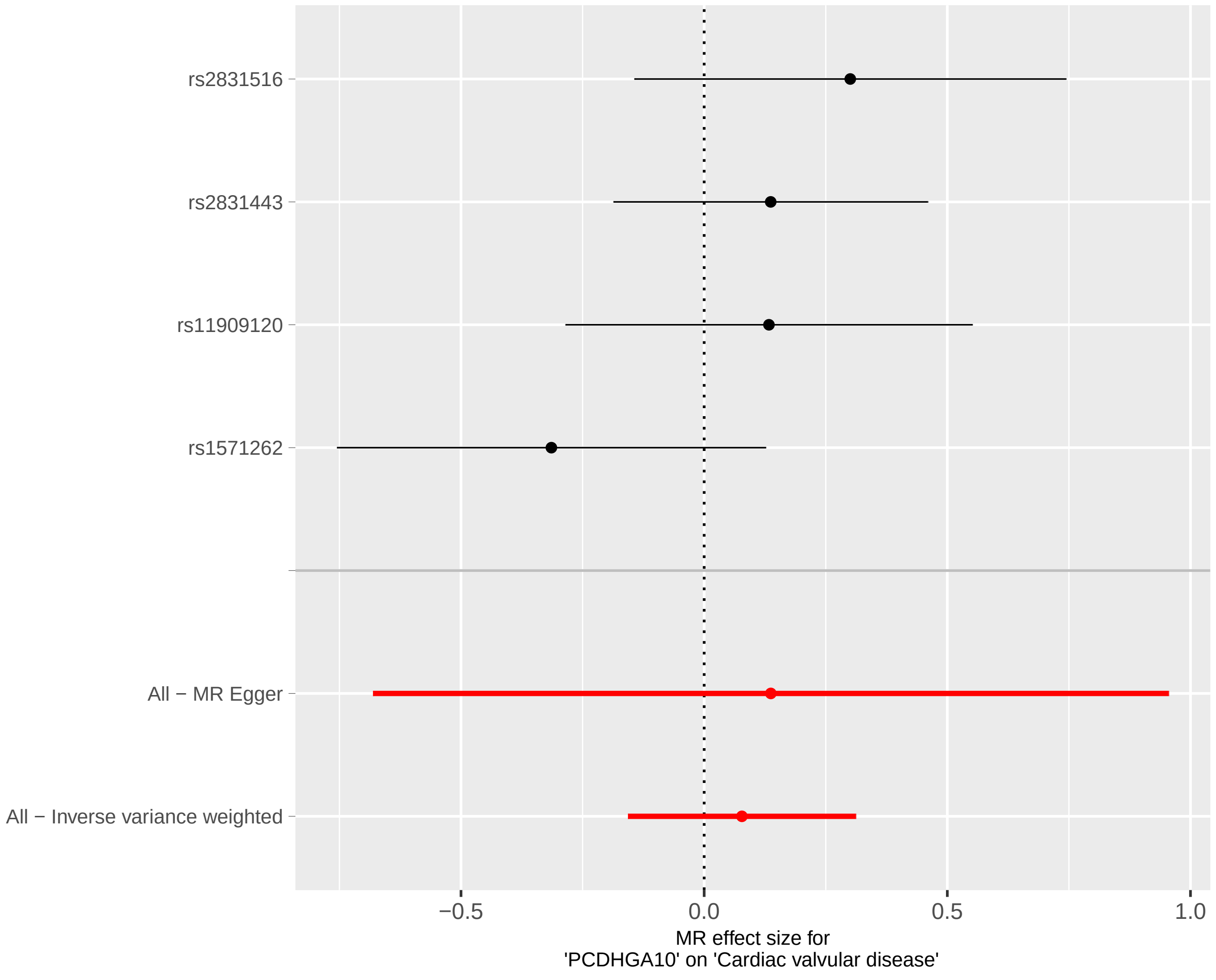
rs2831516
rs2831443
rs11909120
rs1571262
All − MR Egger
All − Inverse variance weighted
−0.5
0.0
0.5
1.0
MR effect size for
'PCDHGA10' on 'Cardiac valvular disease'

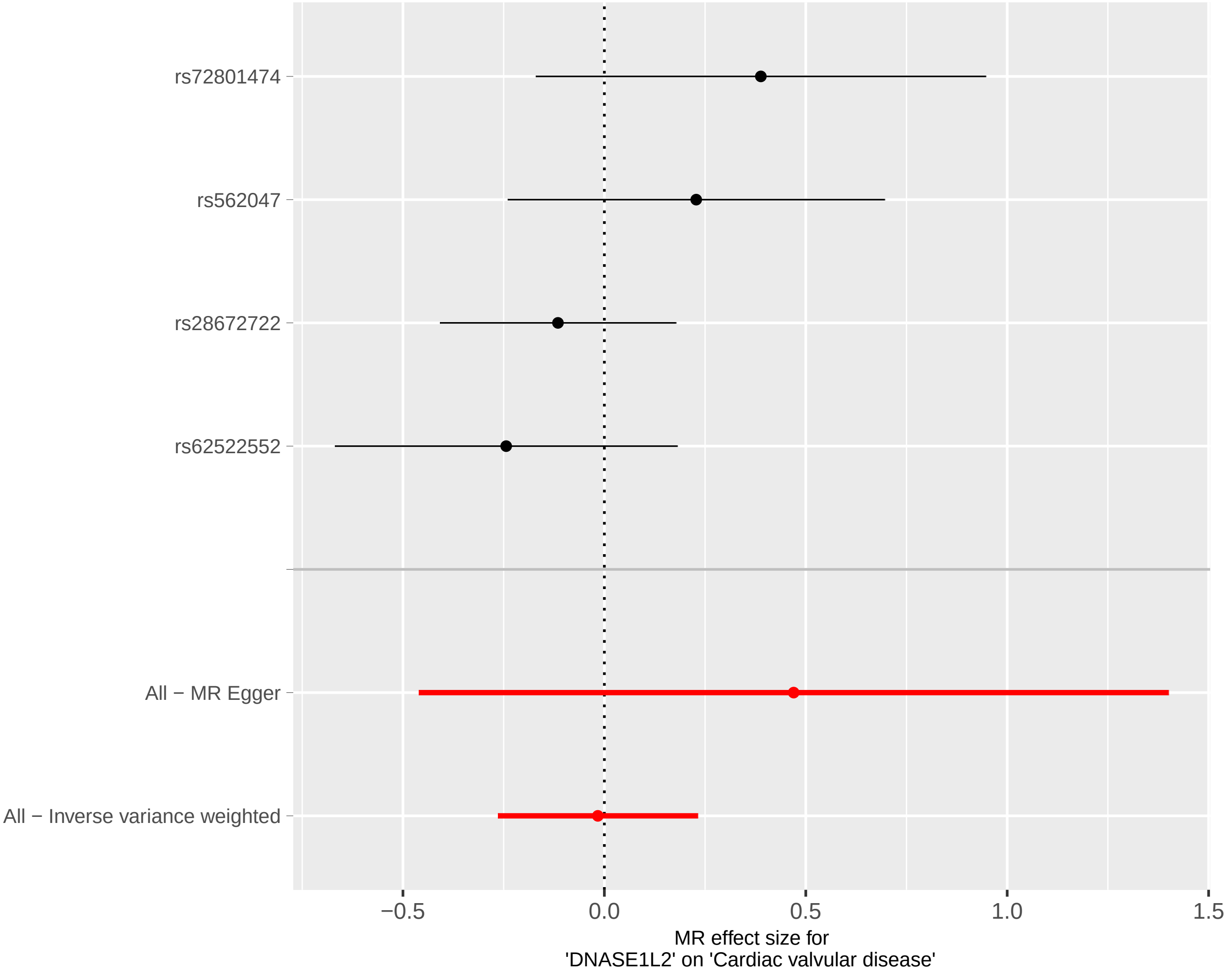
rs72801474
rs562047
rs28672722
rs62522552
All − MR Egger
All − Inverse variance weighted
−0.5
0.0
0.5
1.0
1.5
MR effect size for
'DNASE1L2' on 'Cardiac valvular disease'

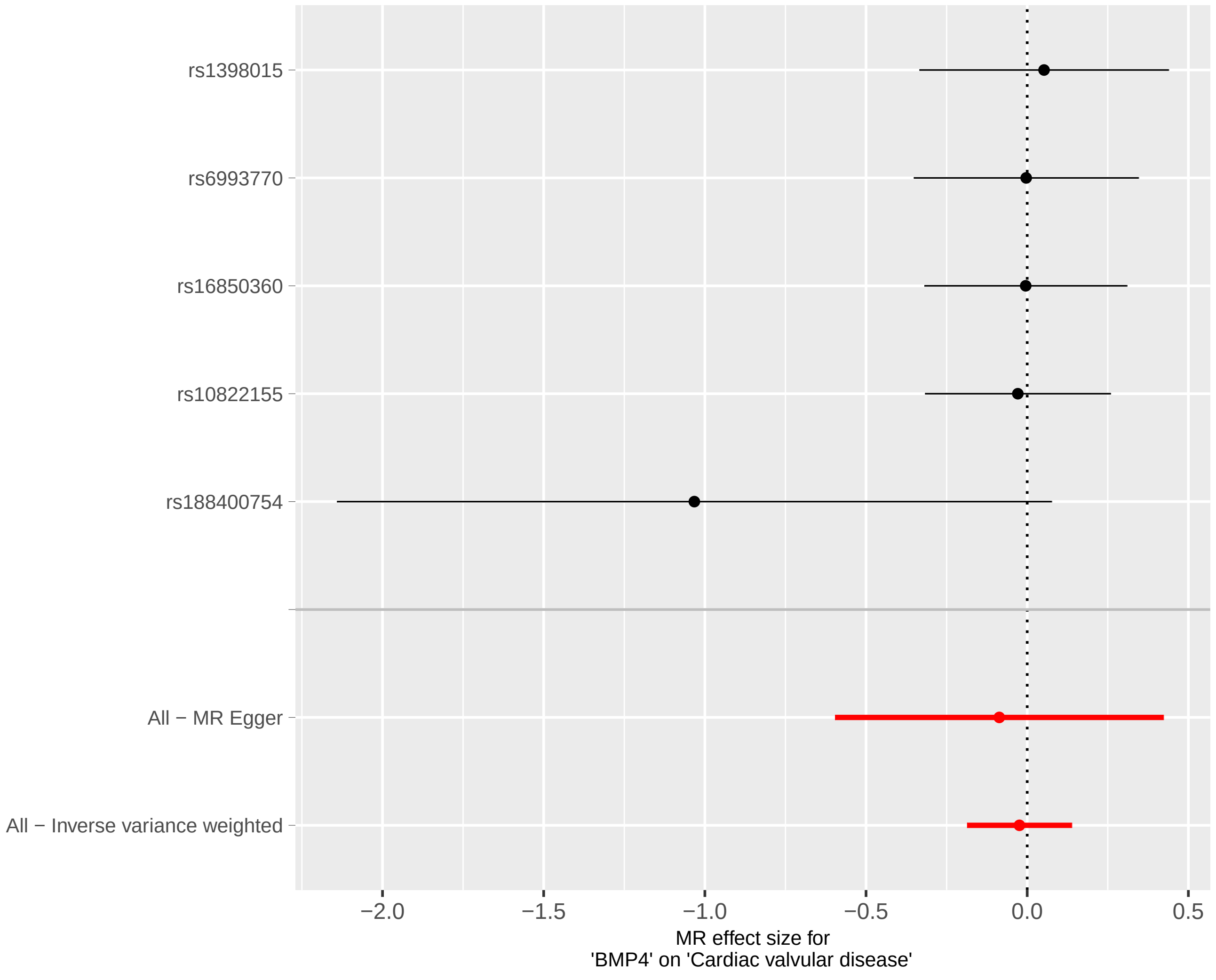
rs1398015
rs6993770
rs16850360
rs10822155
rs188400754
All − MR Egger
All − Inverse variance weighted
−2.0
−1.5
−1.0
−0.5
0.0
0.5
MR effect size for
'BMP4' on 'Cardiac valvular disease'

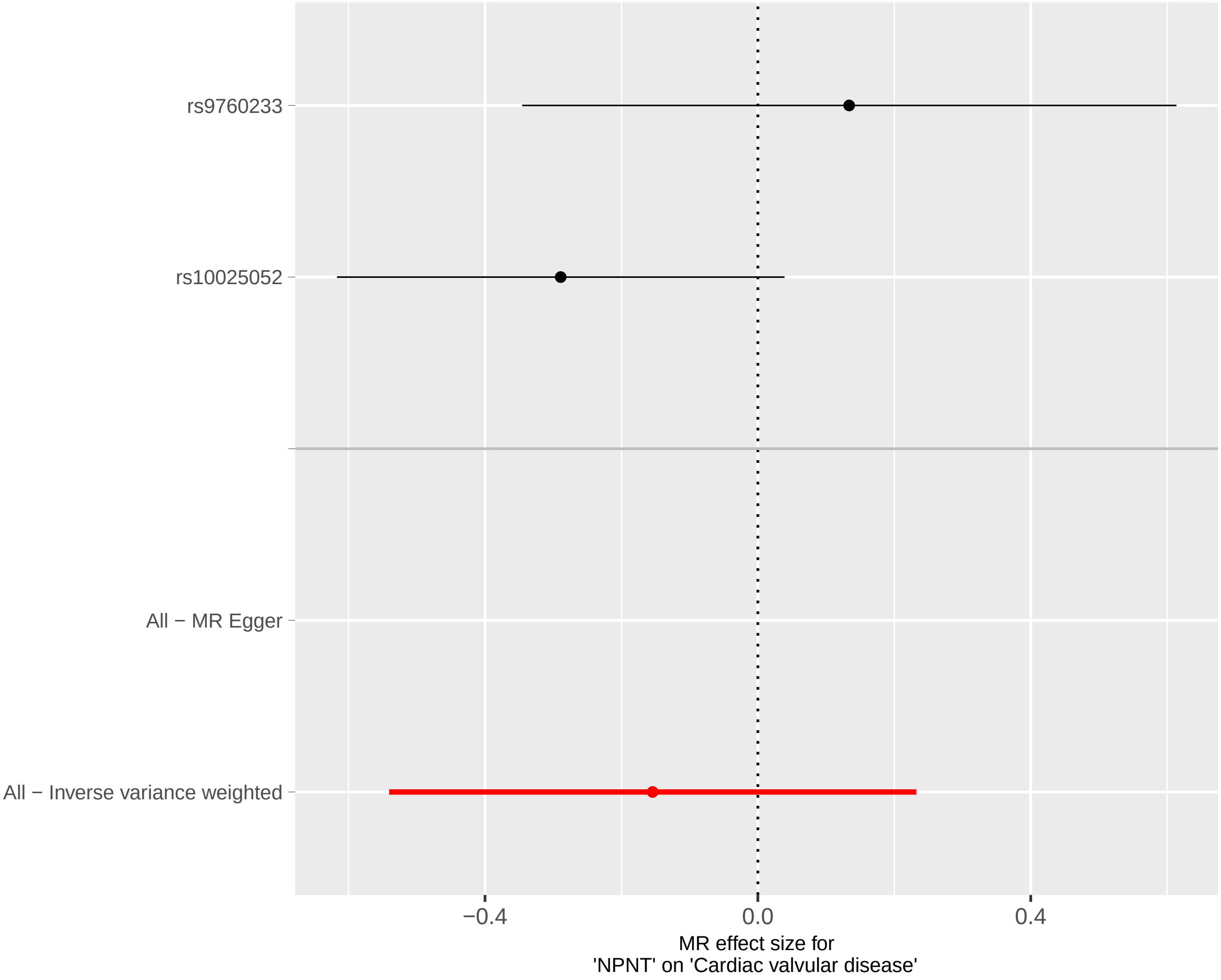

rs9760233
rs10025052
All − MR Egger
All − Inverse variance weighted
−0.4
0.0
0.4
MR effect size for
'NPNT' on 'Cardiac valvular disease'

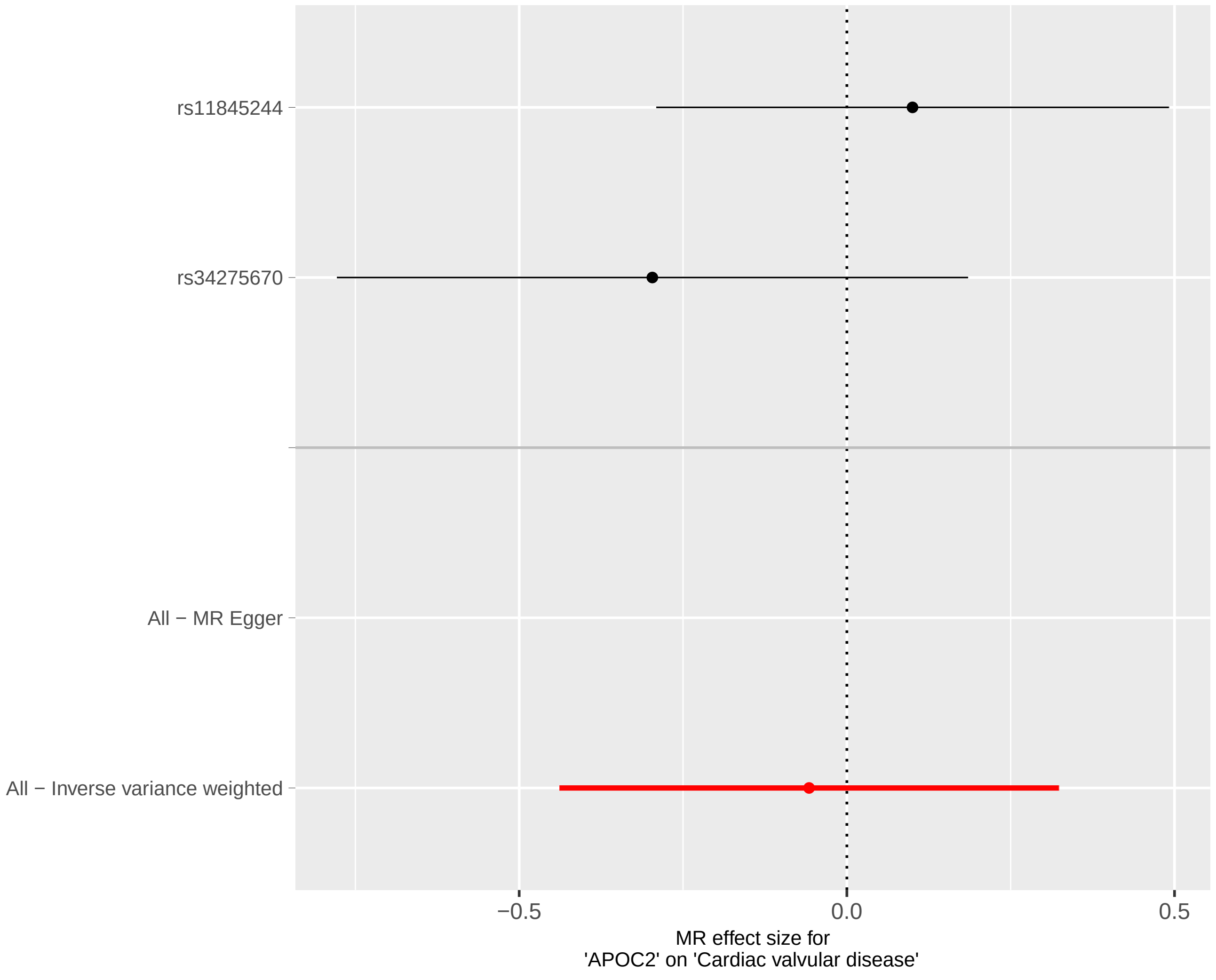

rs11845244
rs34275670
All − MR Egger
All − Inverse variance weighted
−0.5
0.0
0.5
MR effect size for
'APOC2' on 'Cardiac valvular disease'

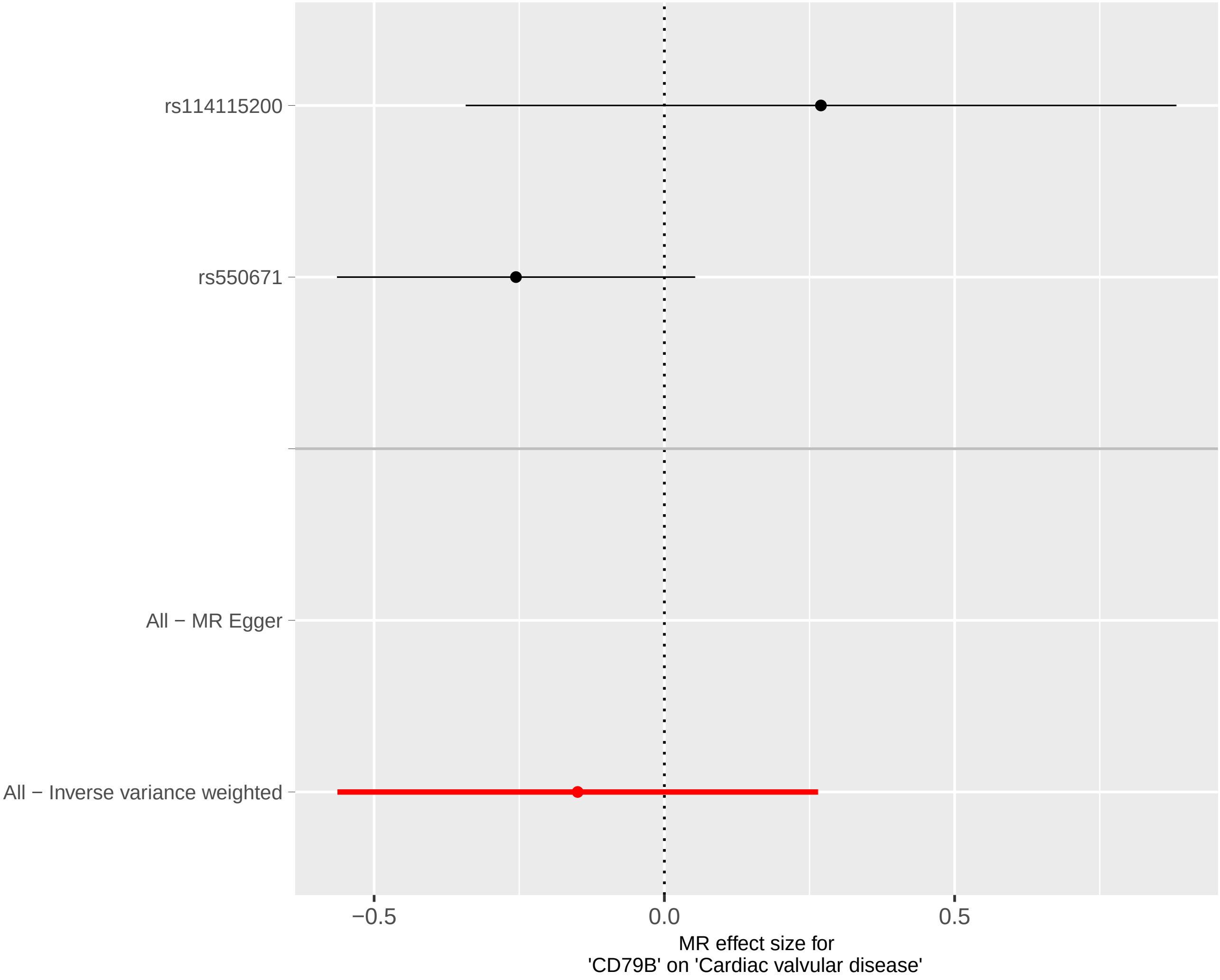
rs114115200
rs550671
All − MR Egger
All − Inverse variance weighted
−0.5
0.0
0.5
MR effect size for
'CD79B' on 'Cardiac valvular disease'

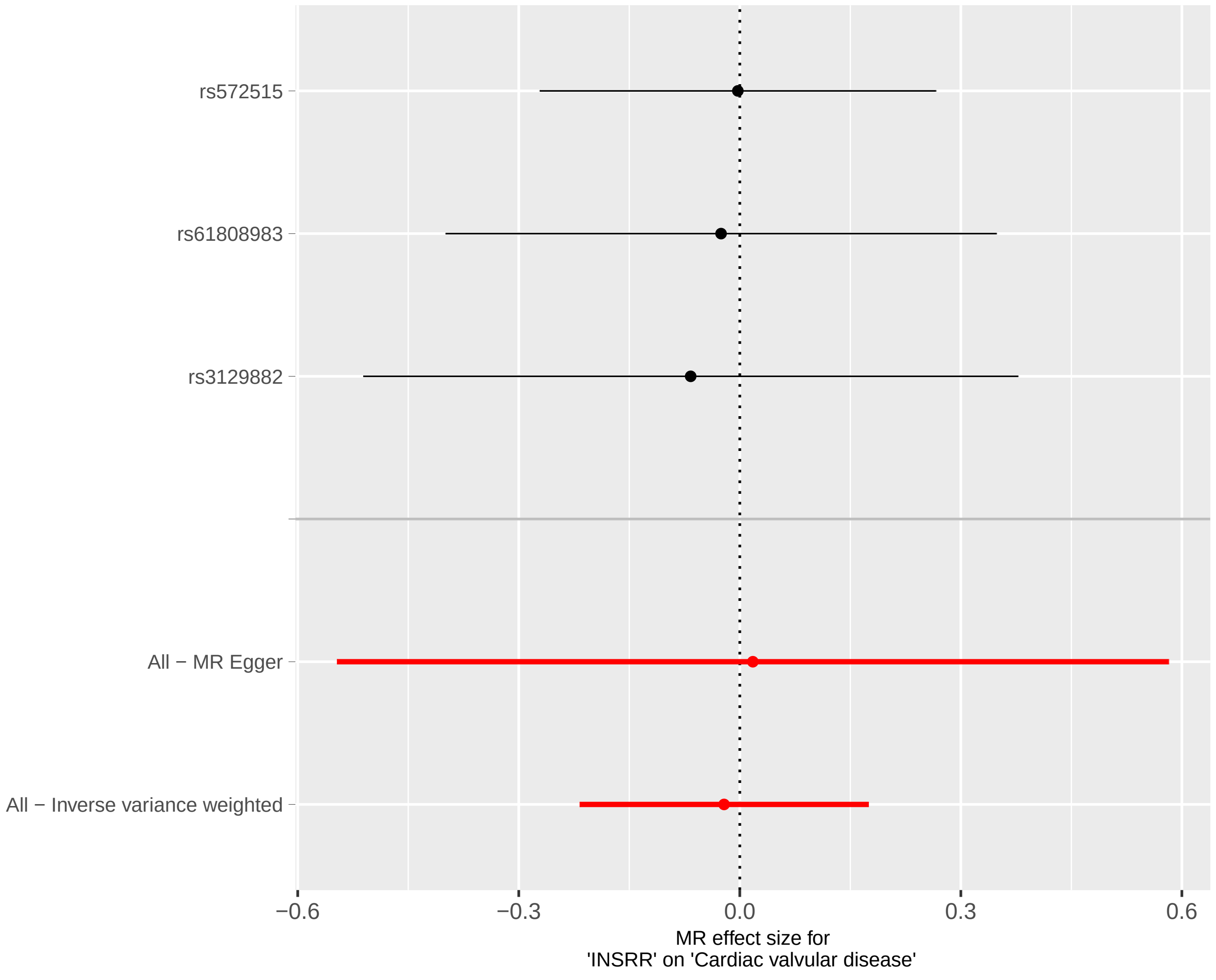
rs572515
rs61808983
rs3129882
All − MR Egger
All − Inverse variance weighted
−0.6
−0.3
0.0
0.3
0.6
MR effect size for
'INSRR' on 'Cardiac valvular disease'

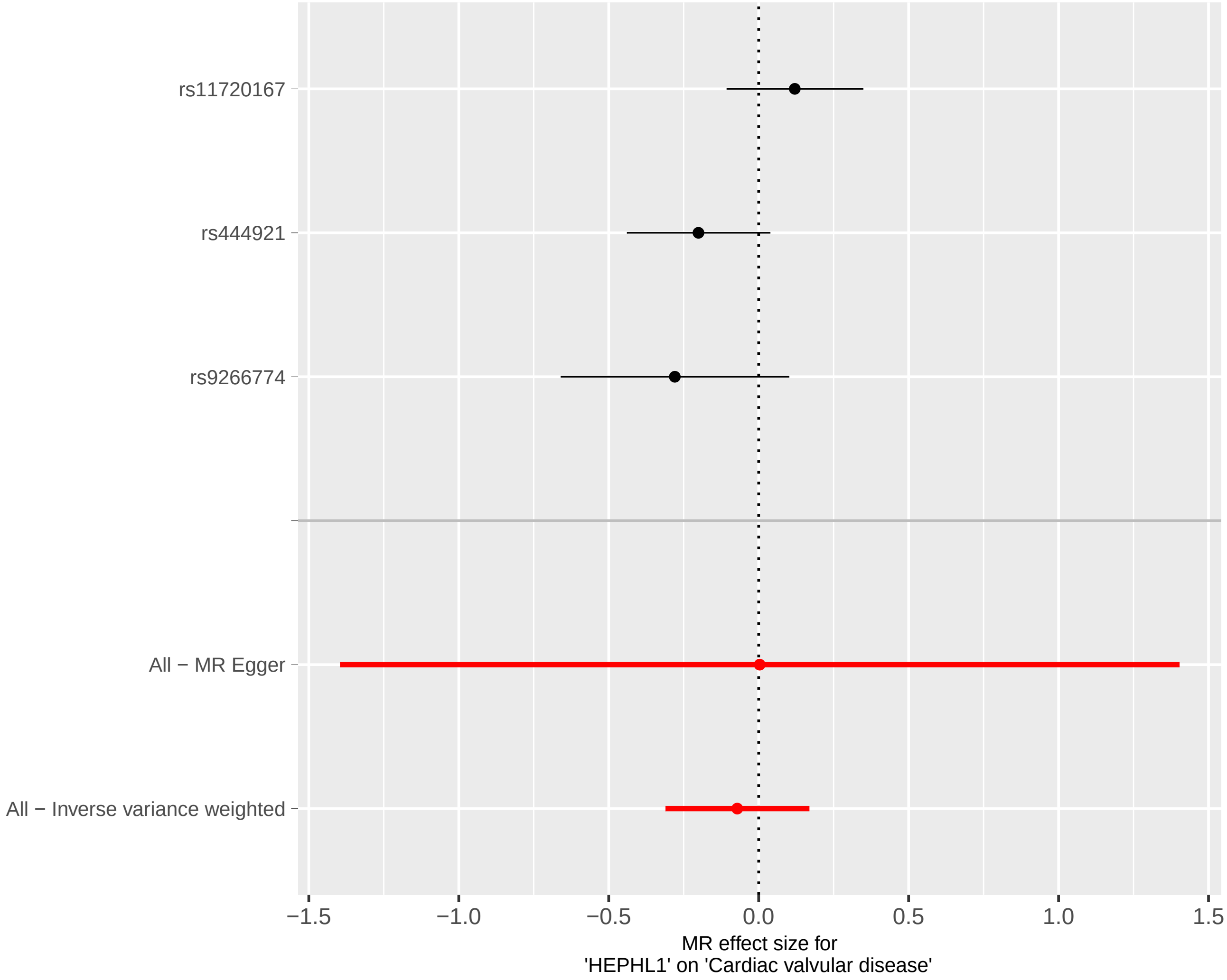
rs11720167
rs444921
rs9266774
All − MR Egger
All − Inverse variance weighted
−1.5
−1.0
−0.5
0.0
0.5
1.0
1.5
MR effect size for
'HEPHL1' on 'Cardiac valvular disease'

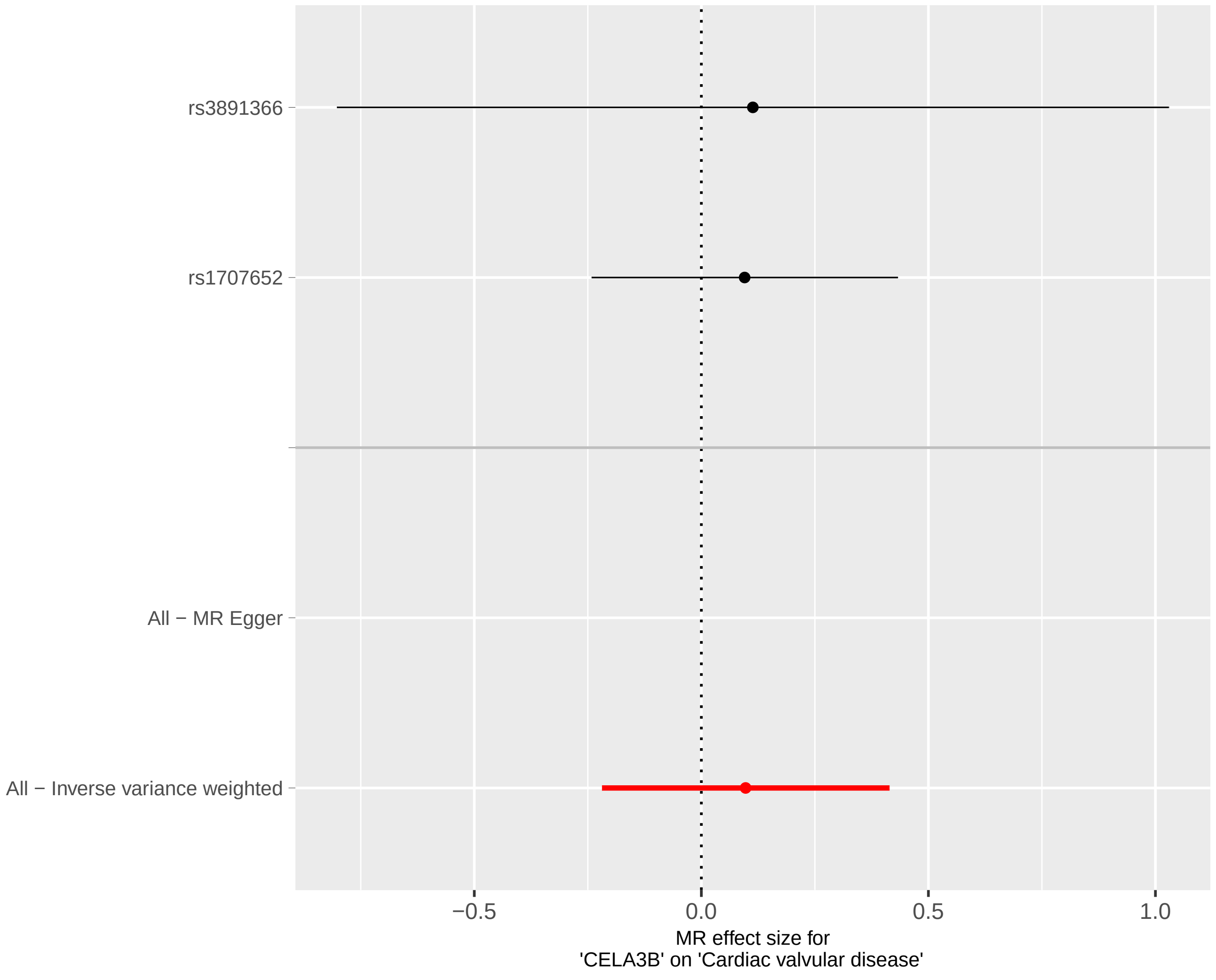
rs3891366
rs1707652
All − MR Egger
All − Inverse variance weighted
−0.5
0.0
0.5
1.0
MR effect size for
'CELA3B' on 'Cardiac valvular disease'

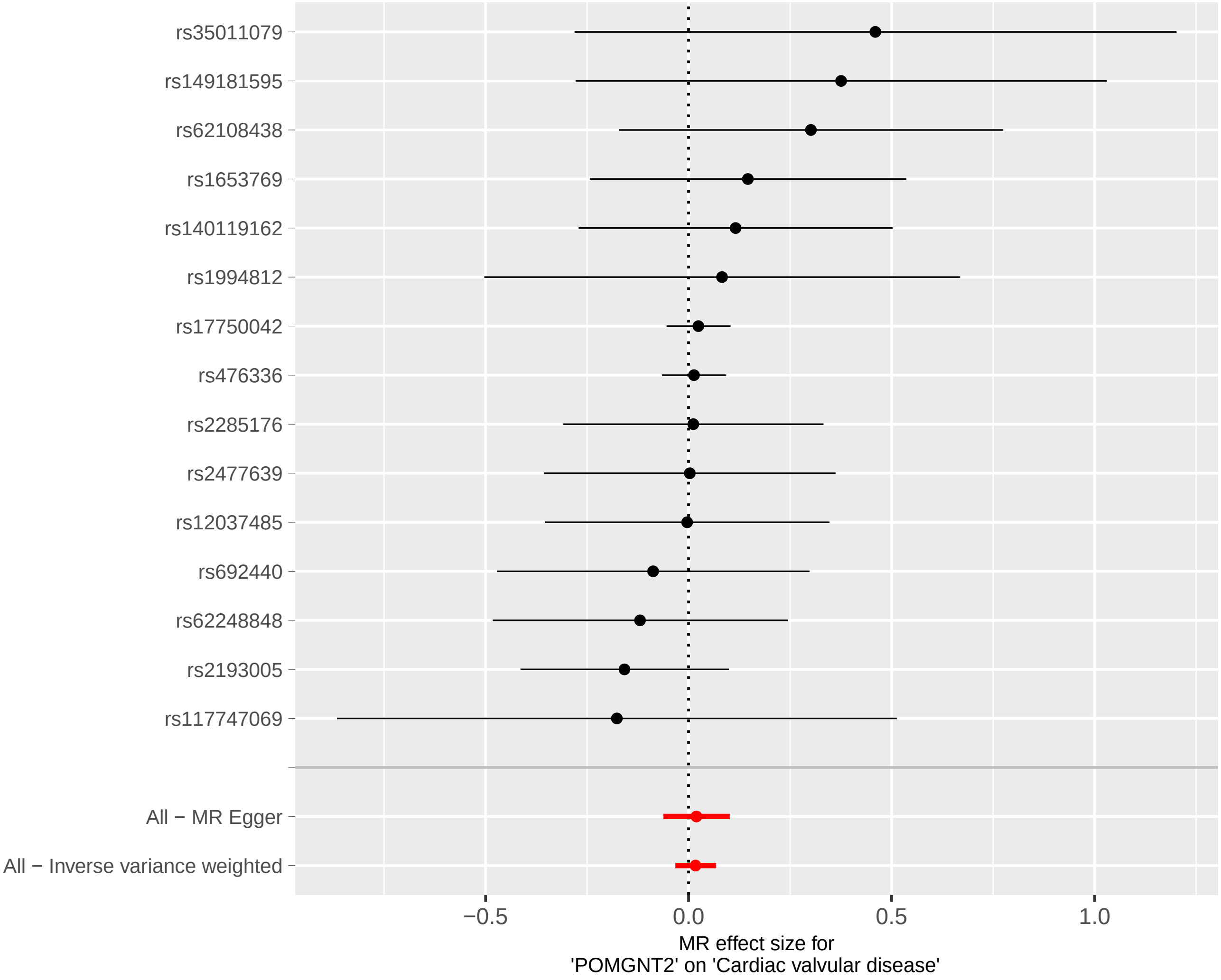
rs35011079
rs149181595
rs62108438
rs1653769
rs140119162
rs1994812
rs17750042
rs476336
rs2285176
rs2477639
rs12037485
rs692440
rs62248848
rs2193005
rs117747069
All − MR Egger
All − Inverse variance weighted
−0.5
0.0
0.5
1.0
MR effect size for
'POMGNT2' on 'Cardiac valvular disease'

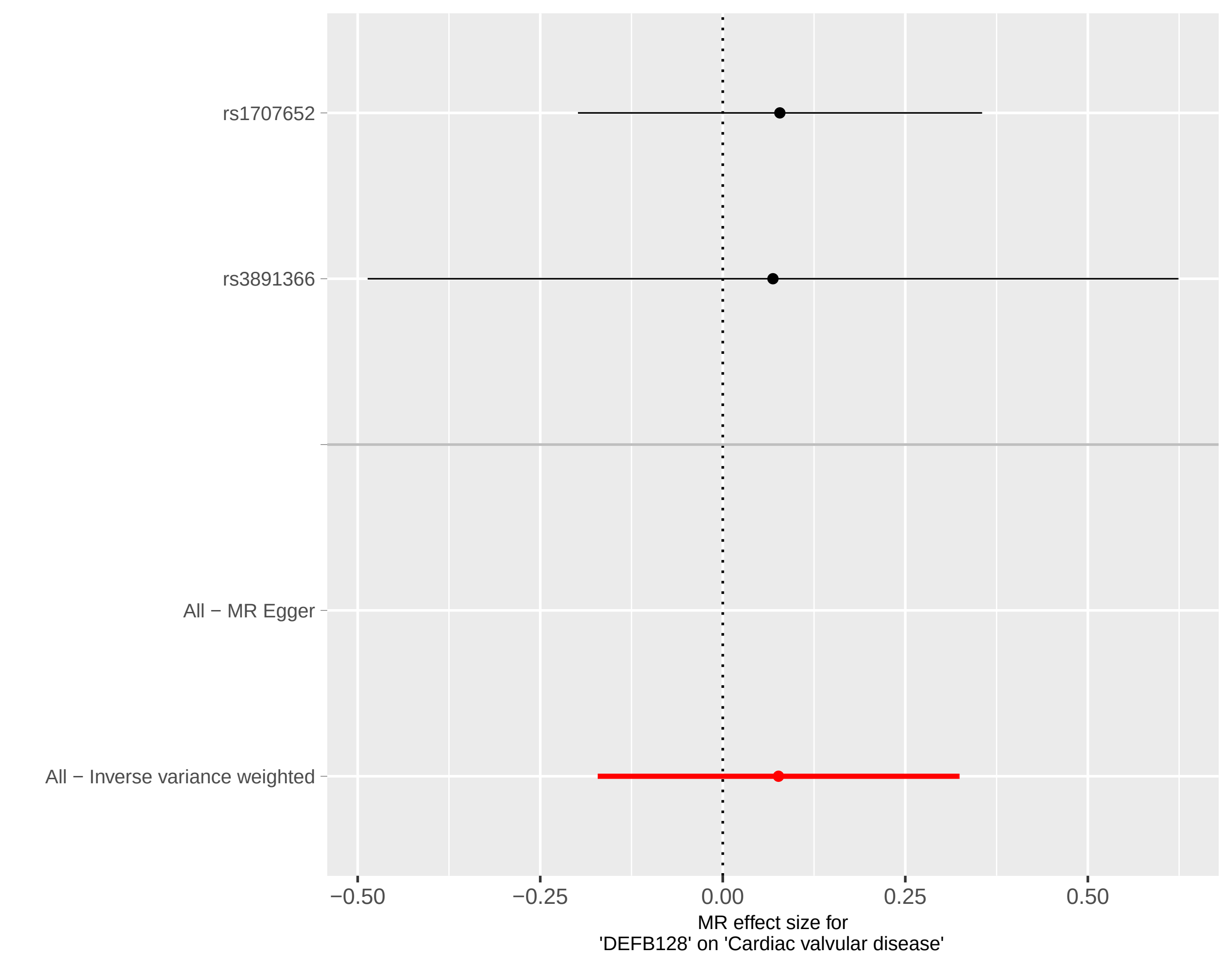
rs1707652
rs3891366
All − MR Egger
All − Inverse variance weighted
−0.50
−0.25
0.00
0.25
0.50
MR effect size for
'DEFB128' on 'Cardiac valvular disease'

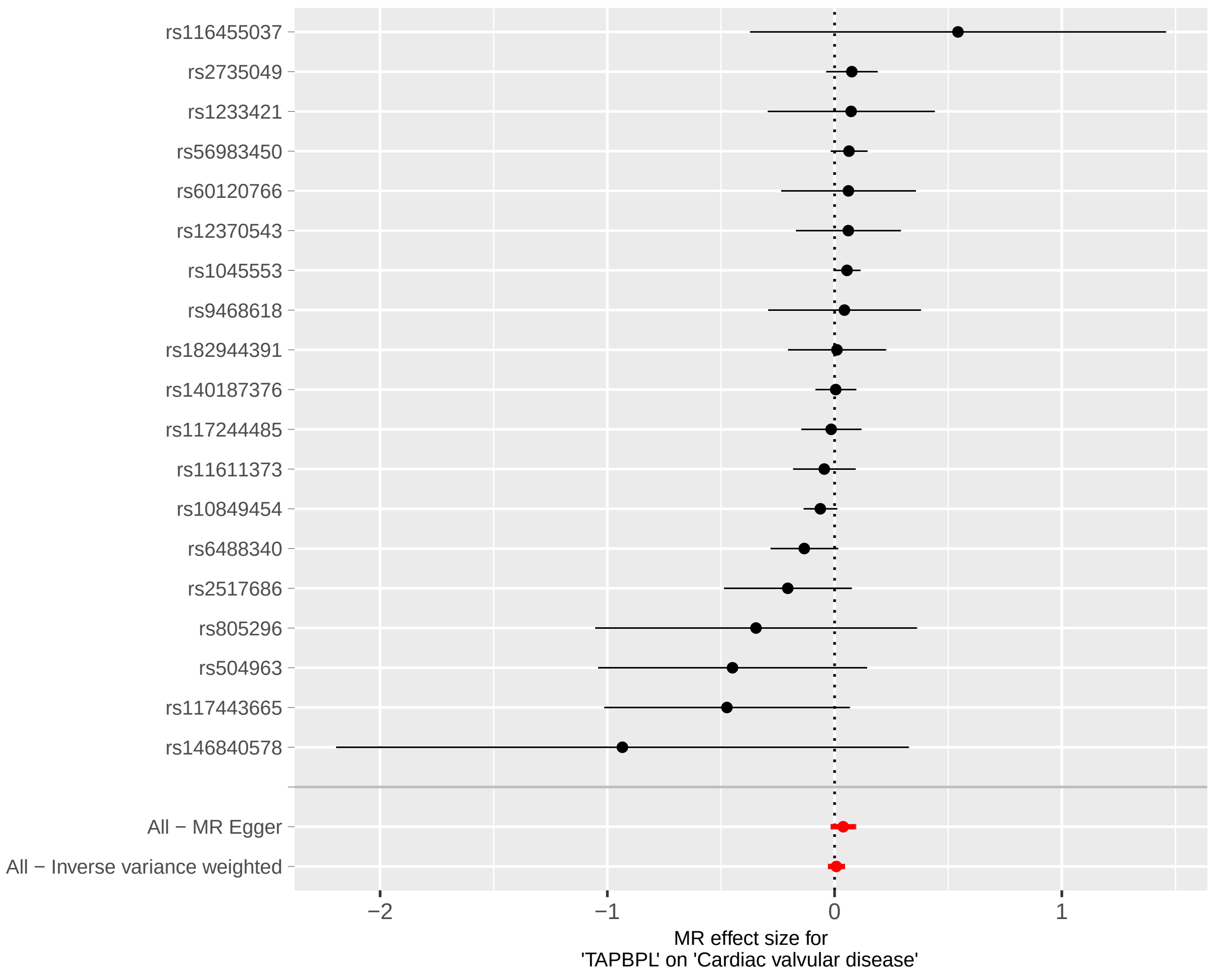
rs116455037
rs2735049
rs1233421
rs56983450
rs60120766
rs12370543
rs1045553
rs9468618
rs182944391
rs140187376
rs117244485
rs11611373
rs10849454
rs6488340
rs2517686
rs805296
rs504963
rs117443665
rs146840578
All − MR Egger
All − Inverse variance weighted
−2
−1
0
1
MR effect size for
'TAPBPL' on 'Cardiac valvular disease'

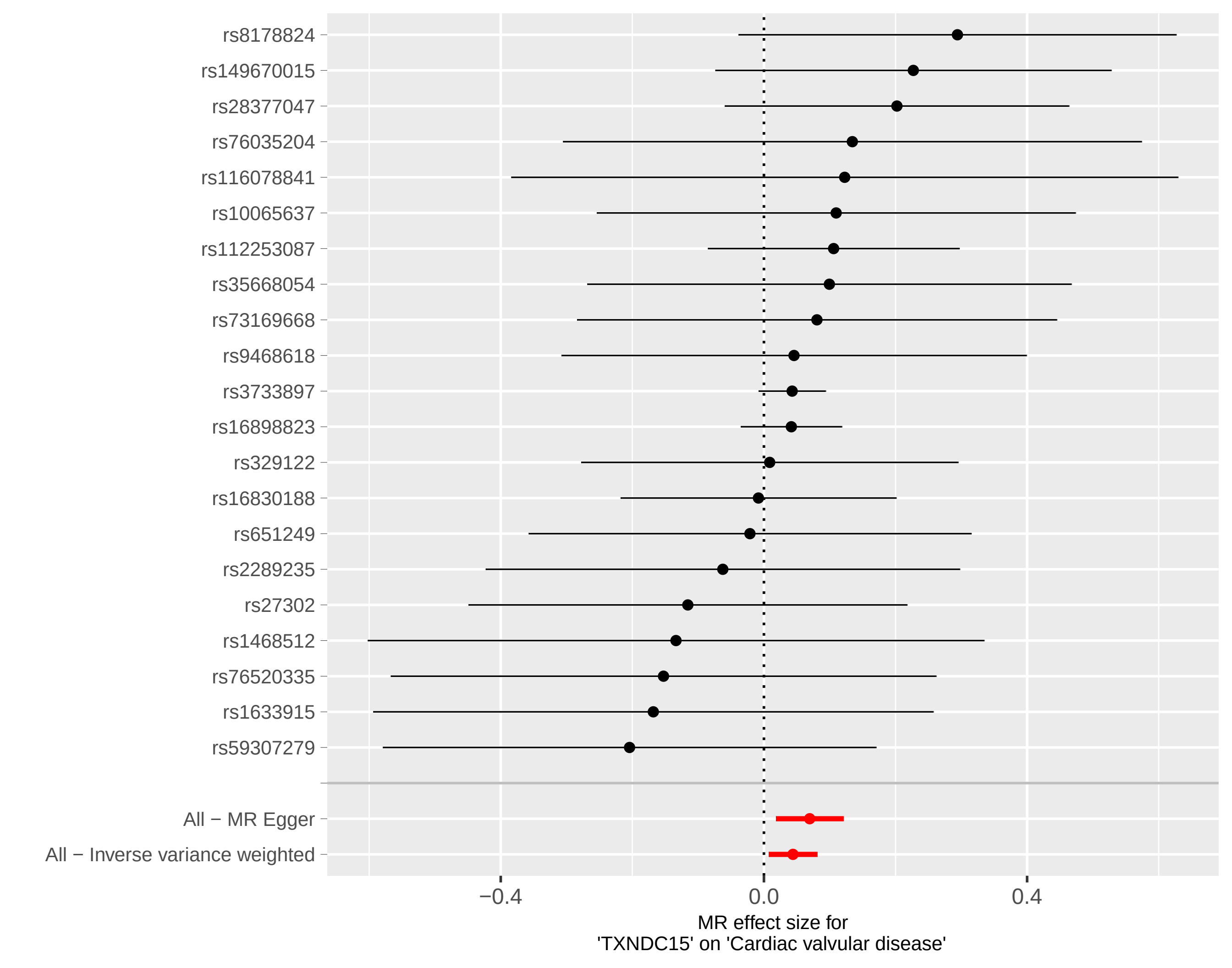

rs8178824
rs149670015
rs28377047
rs76035204
rs116078841
rs10065637
rs112253087
rs35668054
rs73169668
rs9468618
rs3733897
rs16898823
rs329122
rs16830188
rs651249
rs2289235
rs27302
rs1468512
rs76520335
rs1633915
rs59307279
All − MR Egger
All − Inverse variance weighted
−0.4
0.0
0.4
MR effect size for
'TXNDC15' on 'Cardiac valvular disease'

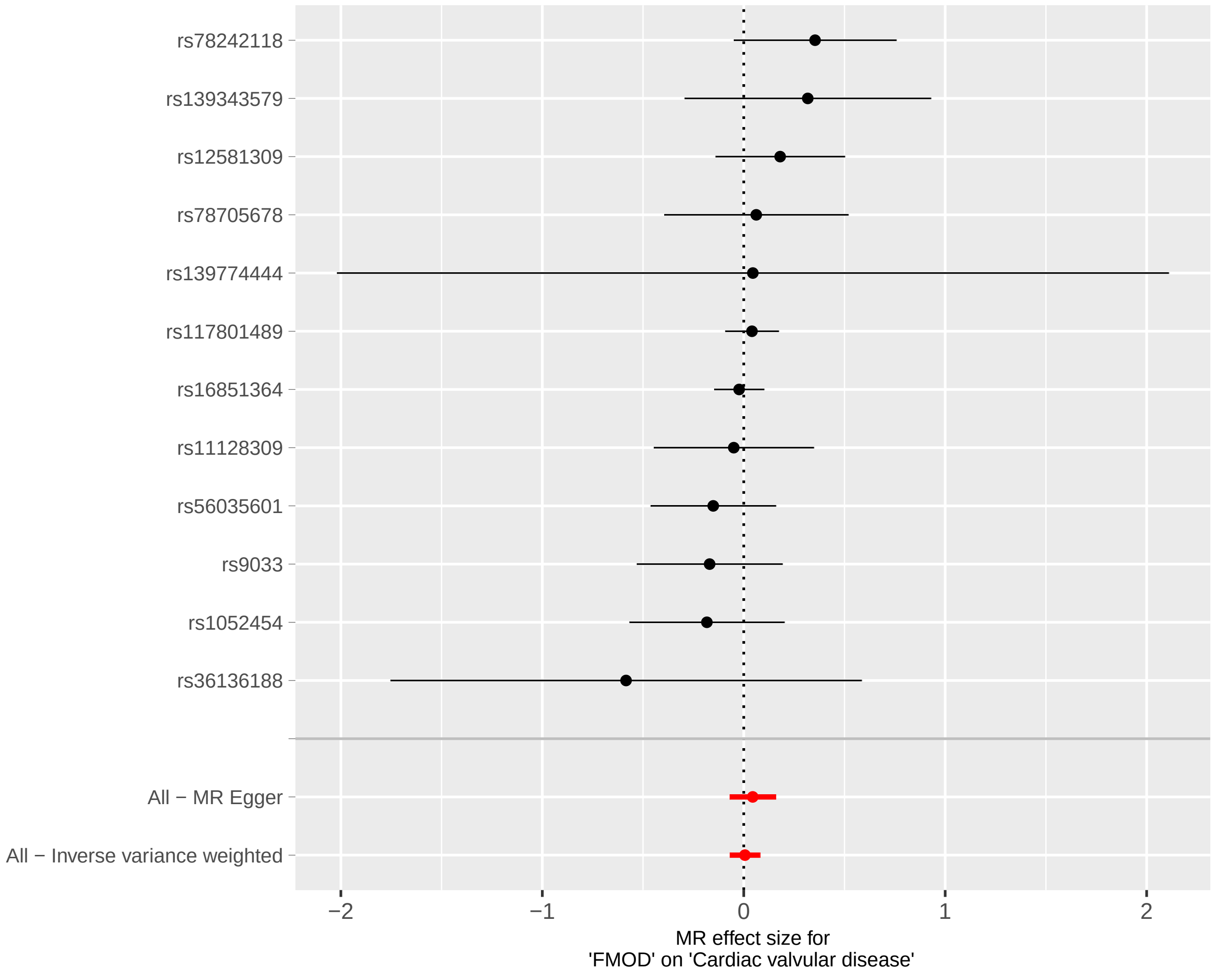
rs78242118
rs139343579
rs12581309
rs78705678
rs139774444
rs117801489
rs16851364
rs11128309
rs56035601
rs9033
rs1052454
rs36136188
All − MR Egger
All − Inverse variance weighted
−2
−1
0
1
2
MR effect size for
'FMOD' on 'Cardiac valvular disease'

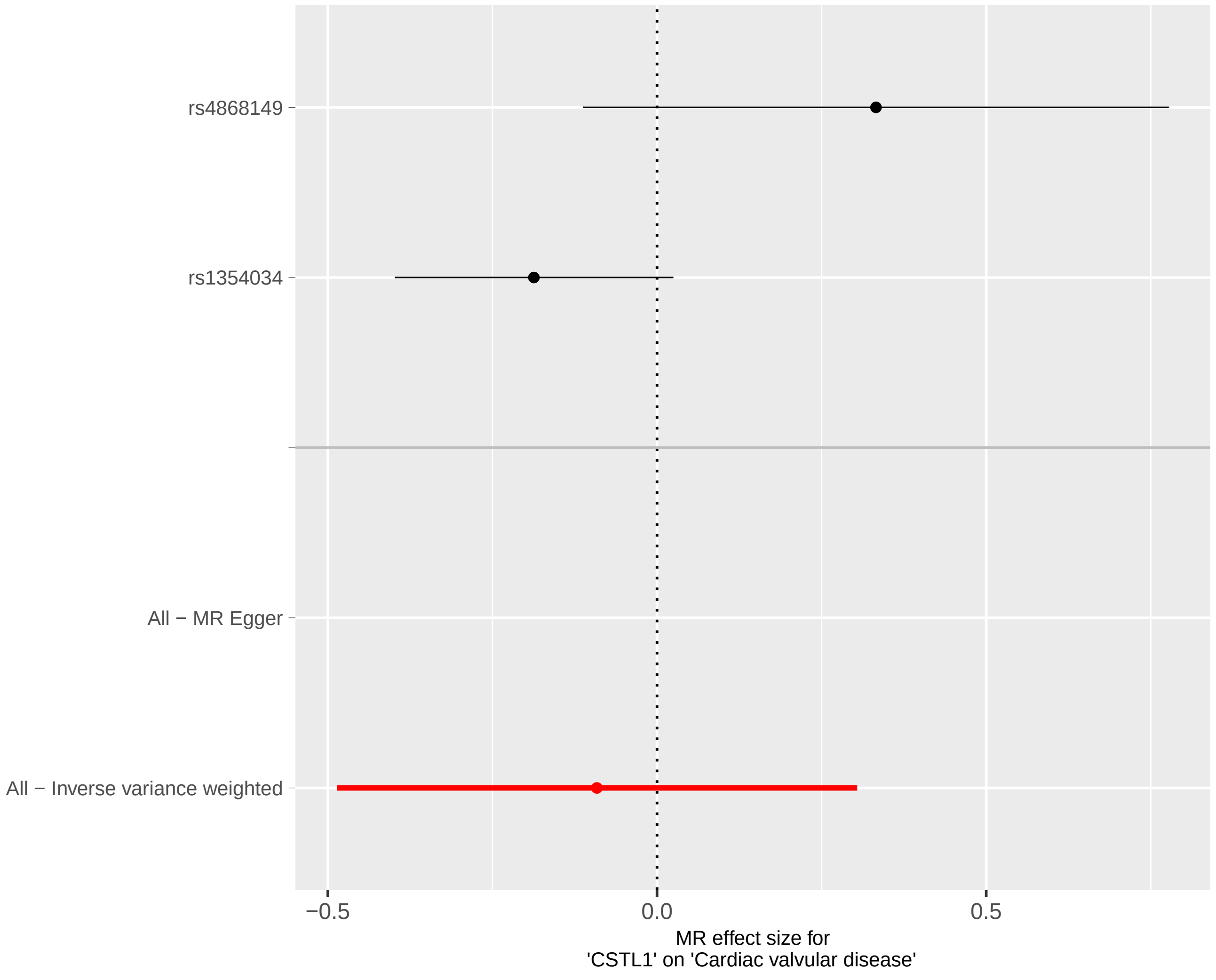
rs4868149
rs1354034
All − MR Egger
All − Inverse variance weighted
−0.5
0.0
0.5
MR effect size for
'CSTL1' on 'Cardiac valvular disease'

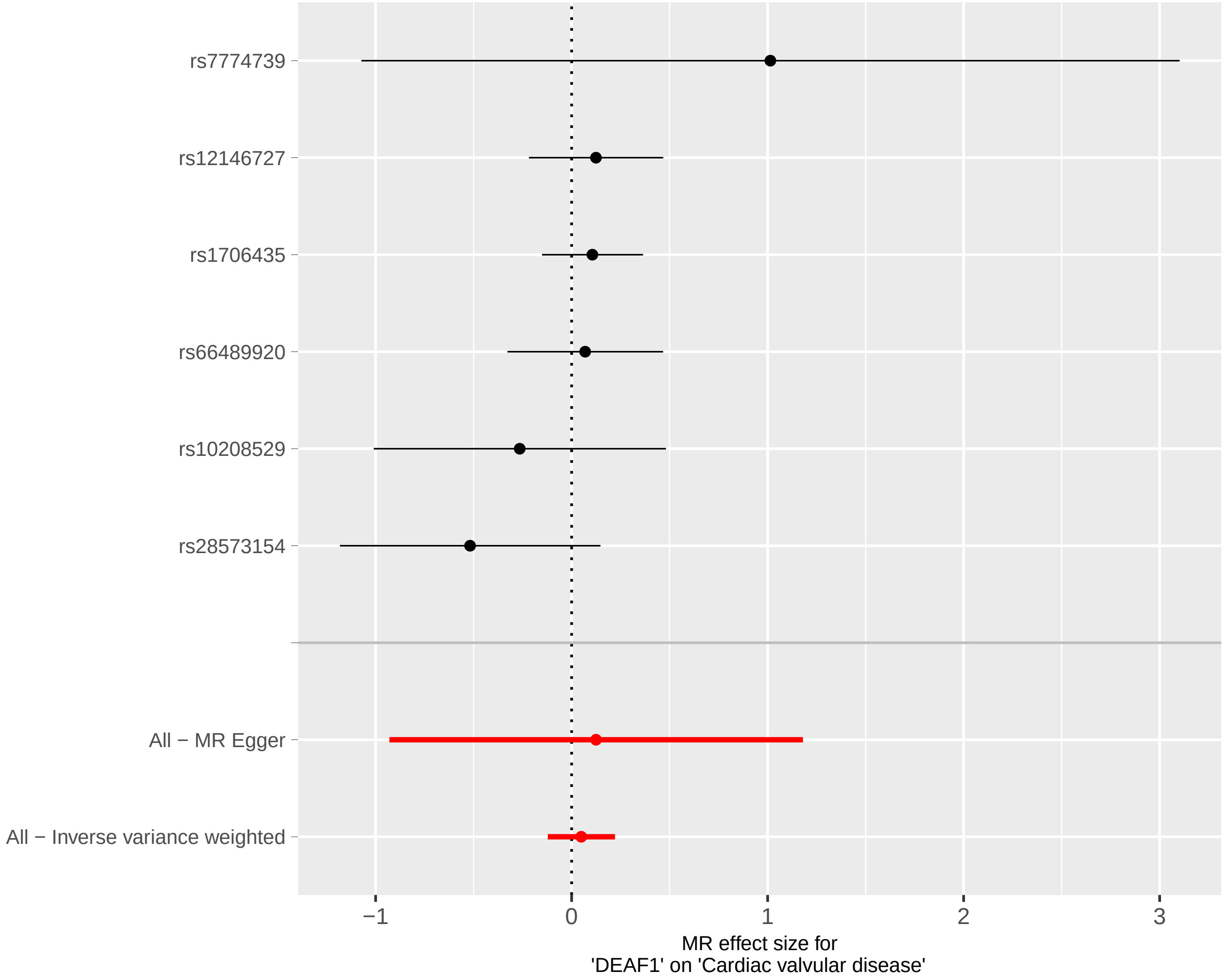
rs7774739
rs12146727
rs1706435
rs66489920
rs10208529
rs28573154
All − MR Egger
All − Inverse variance weighted
−1
0
1
2
3
MR effect size for
'DEAF1' on 'Cardiac valvular disease'

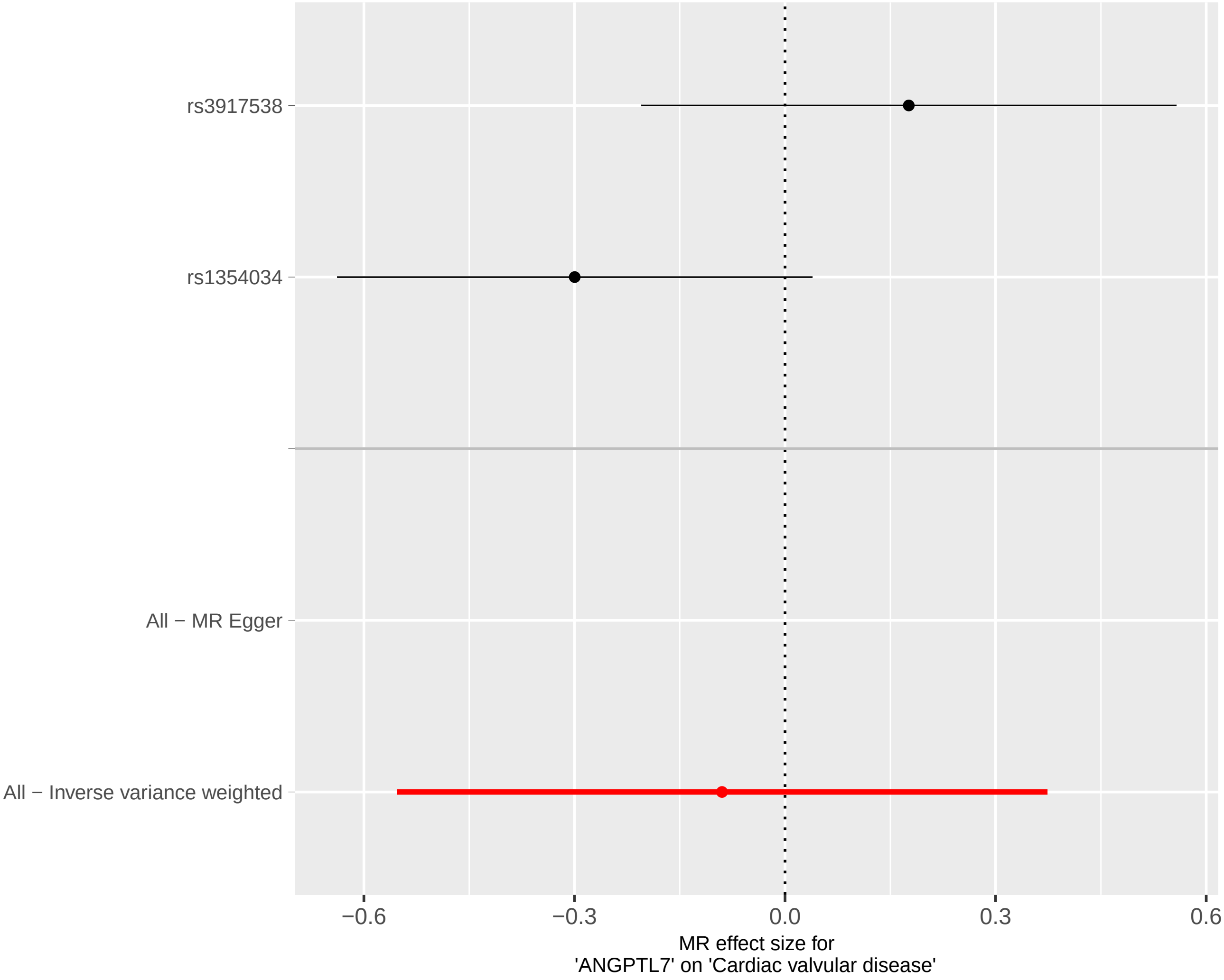
rs3917538
rs1354034
All − MR Egger
All − Inverse variance weighted
−0.6
−0.3
0.0
0.3
0.6
MR effect size for
'ANGPTL7' on 'Cardiac valvular disease'

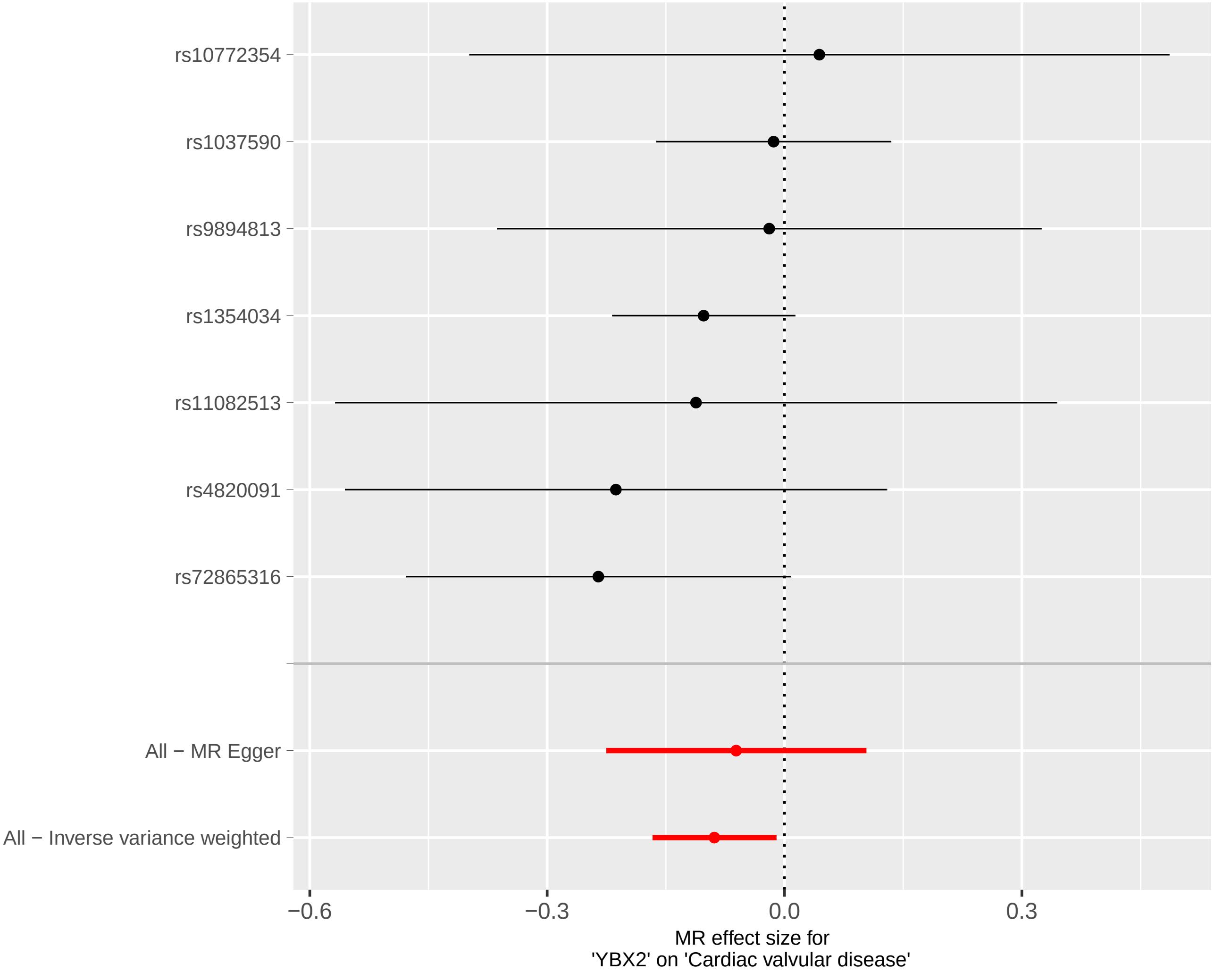

rs10772354
rs1037590
rs9894813
rs1354034
rs11082513
rs4820091
rs72865316
All − MR Egger
All − Inverse variance weighted
−0.6
−0.3
0.0
0.3
MR effect size for
'YBX2' on 'Cardiac valvular disease'

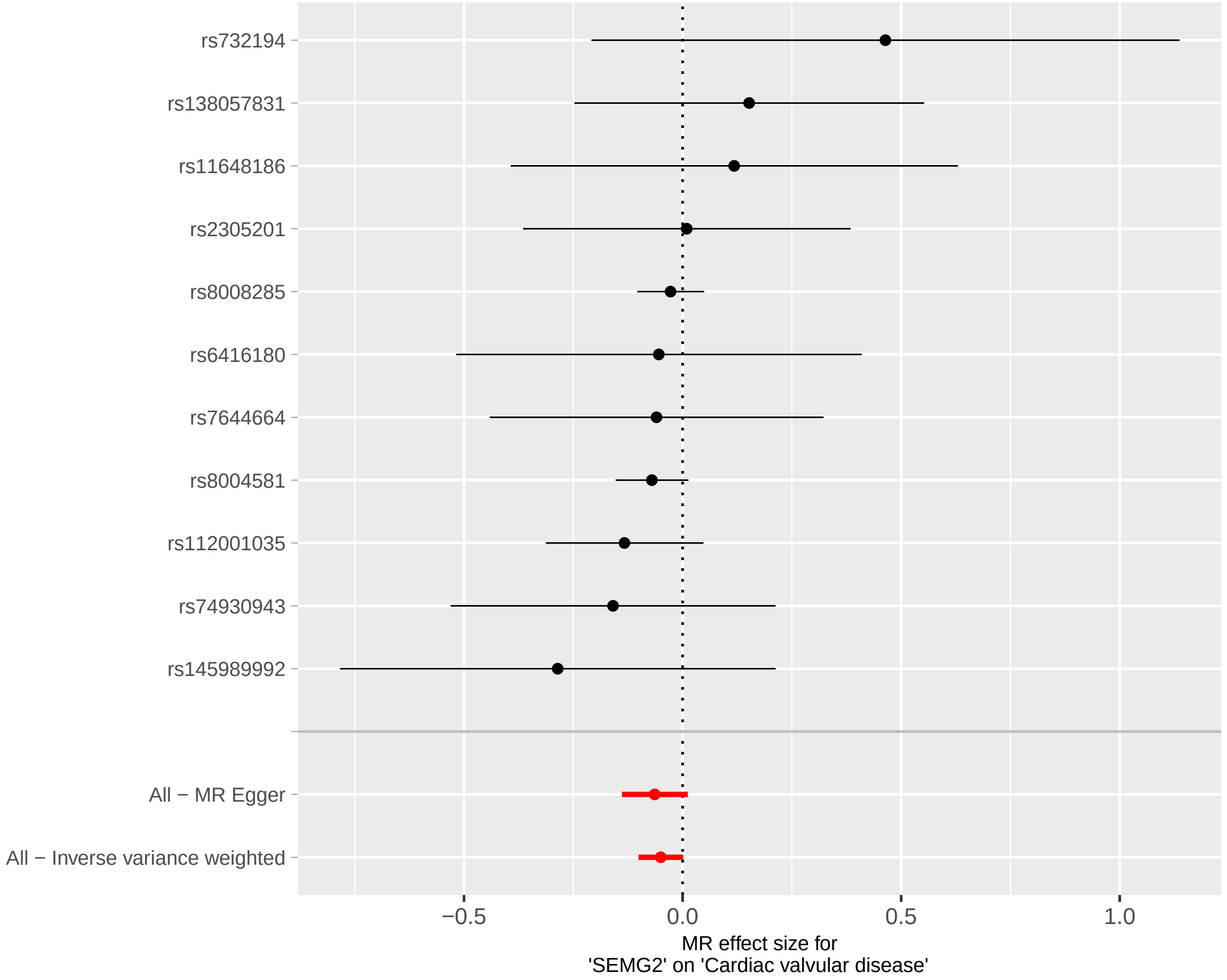
rs732194
rs138057831
rs11648186
rs2305201
rs8008285
rs6416180
rs7644664
rs8004581
rs112001035
rs74930943
rs145989992
All − MR Egger
All − Inverse variance weighted
−0.5
0.0
0.5
1.0
MR effect size for
'SEMG2' on 'Cardiac valvular disease'

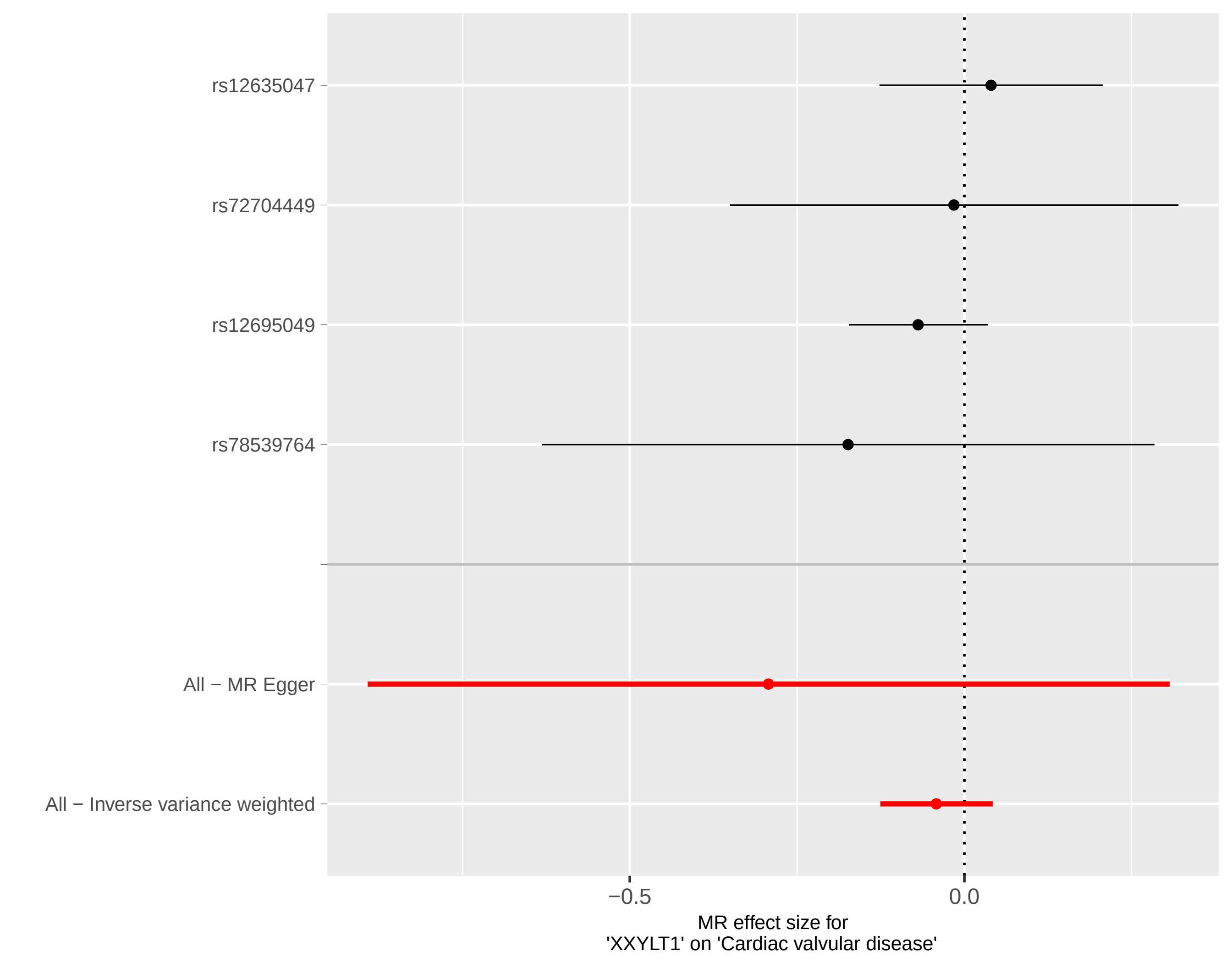
rs12635047
rs72704449
rs12695049
rs78539764
All − MR Egger
All − Inverse variance weighted
−0.5
0.0
MR effect size for
'XXYLT1' on 'Cardiac valvular disease'

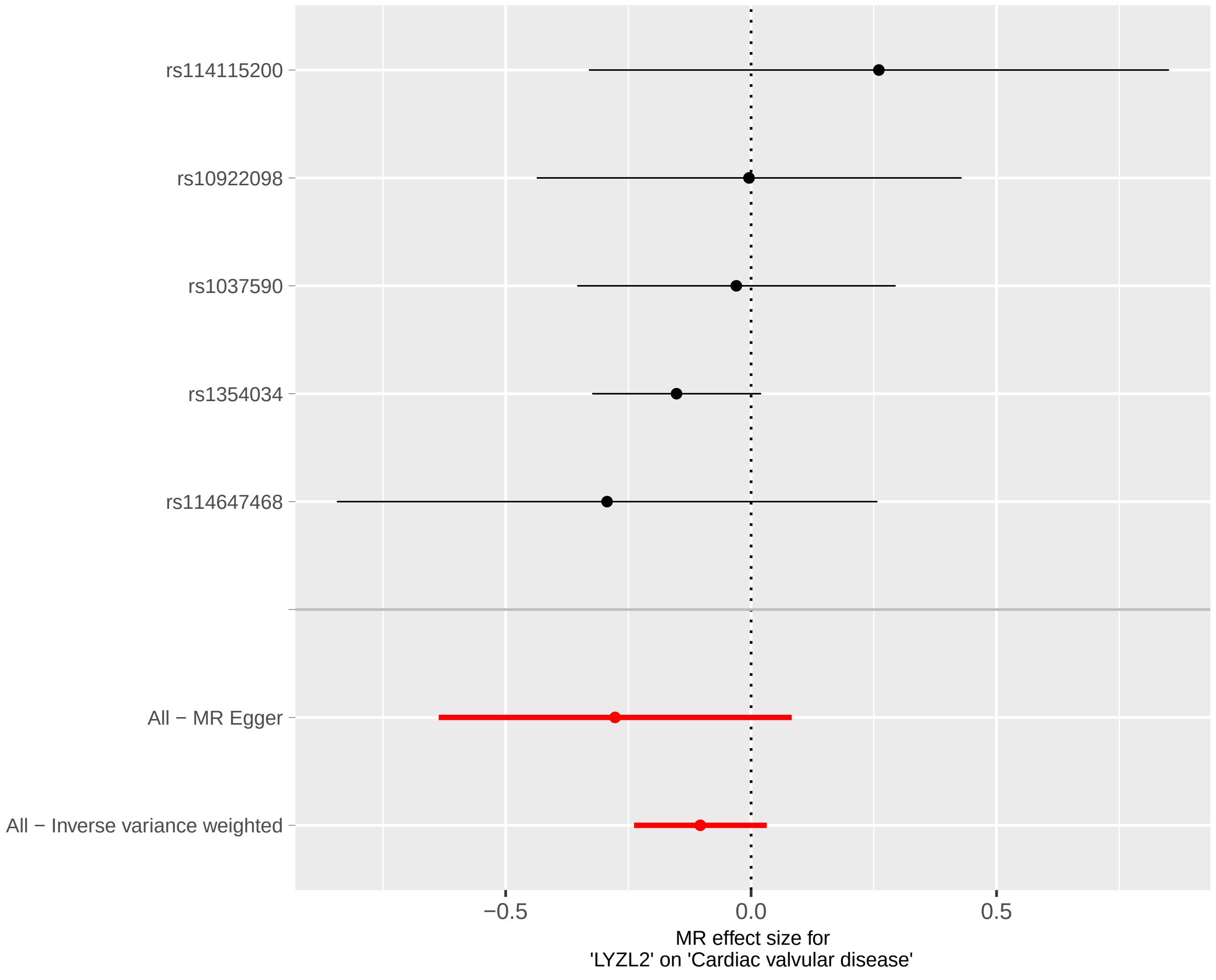
rs114115200
rs10922098
rs1037590
rs1354034
rs114647468
All − MR Egger
All − Inverse variance weighted
−0.5
0.0
0.5
MR effect size for
'LYZL2' on 'Cardiac valvular disease'

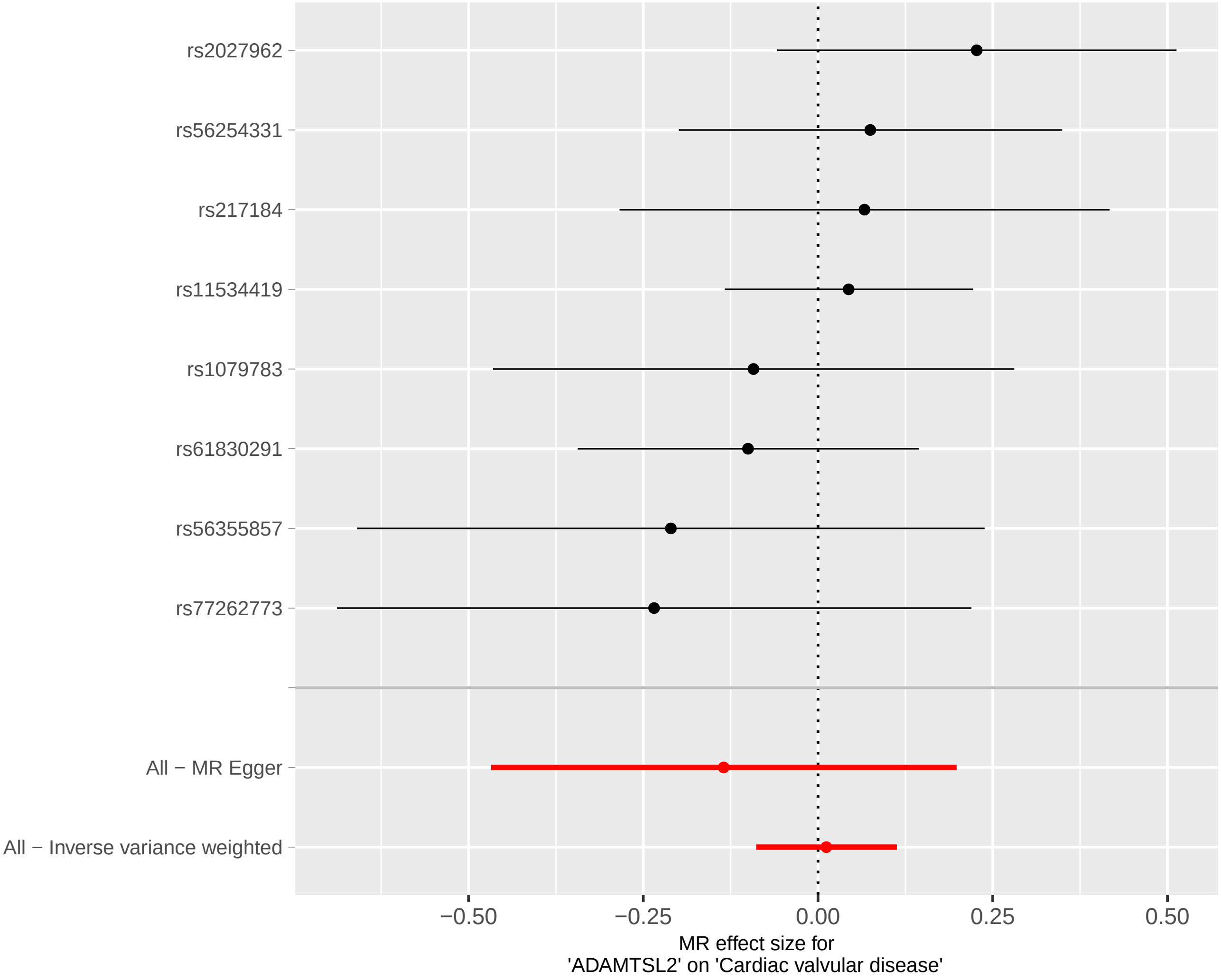
rs2027962
rs56254331
rs217184
rs11534419
rs1079783
rs61830291
rs56355857
rs77262773
All − MR Egger
All − Inverse variance weighted
−0.50
−0.25
0.00
0.25
0.50
MR effect size for
'ADAMTSL2' on 'Cardiac valvular disease'

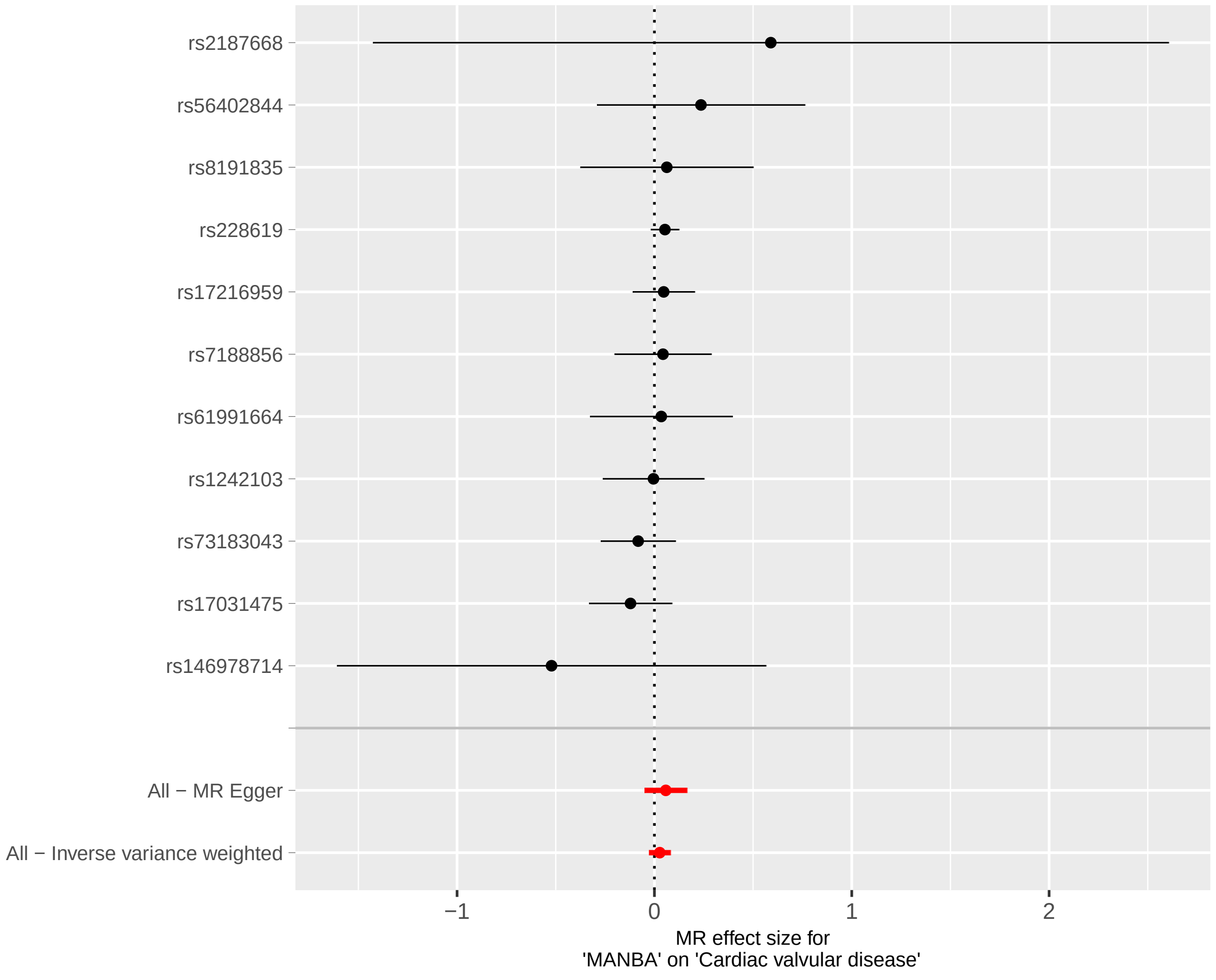
rs2187668
rs56402844
rs8191835
rs228619
rs17216959
rs7188856
rs61991664
rs1242103
rs73183043
rs17031475
rs146978714
All − MR Egger
All − Inverse variance weighted
−1
0
1
2
MR effect size for
'MANBA' on 'Cardiac valvular disease'

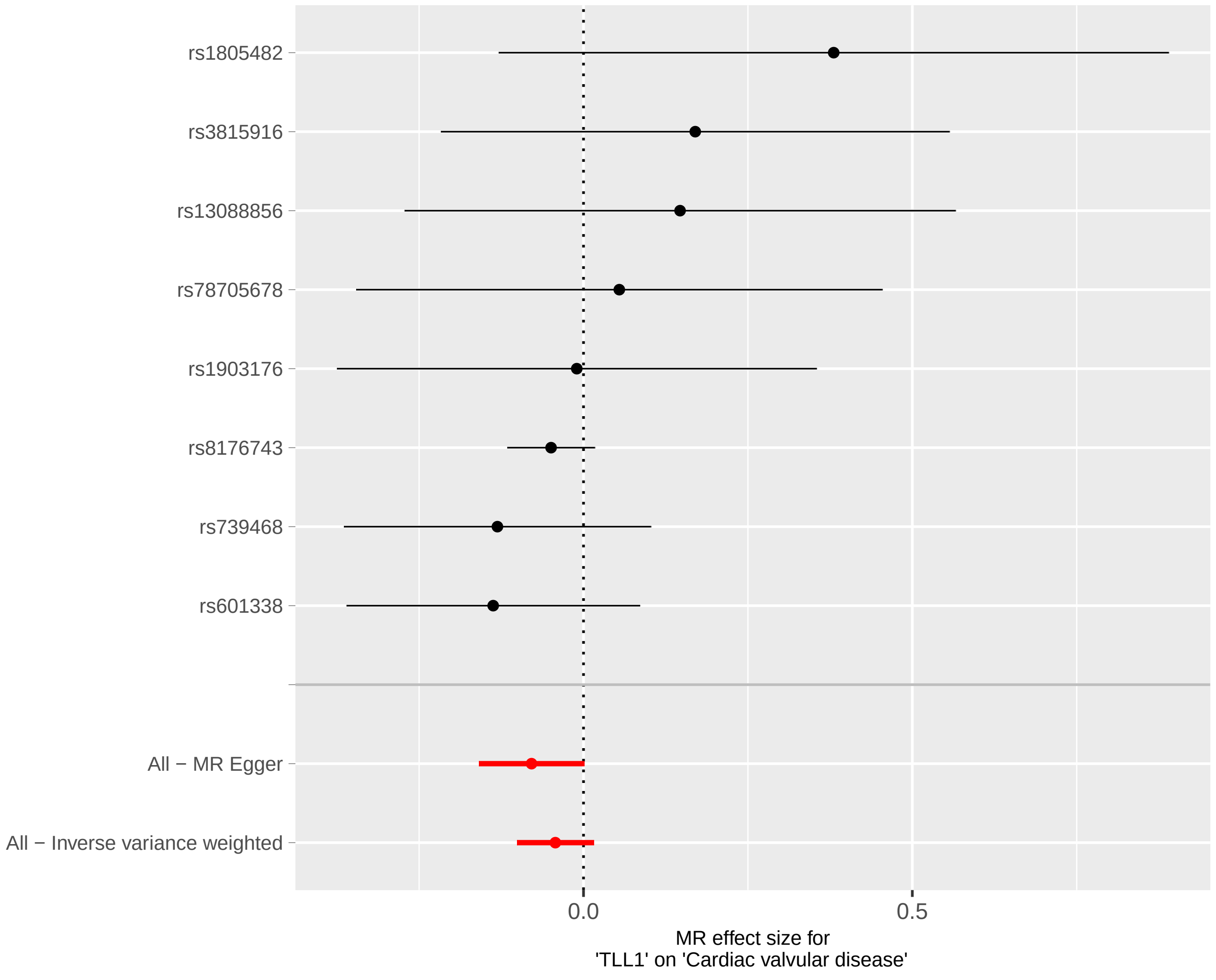
rs1805482
rs3815916
rs13088856
rs78705678
rs1903176
rs8176743
rs739468
rs601338
All − MR Egger
All − Inverse variance weighted
0.0
0.5
MR effect size for
'TLL1' on 'Cardiac valvular disease'

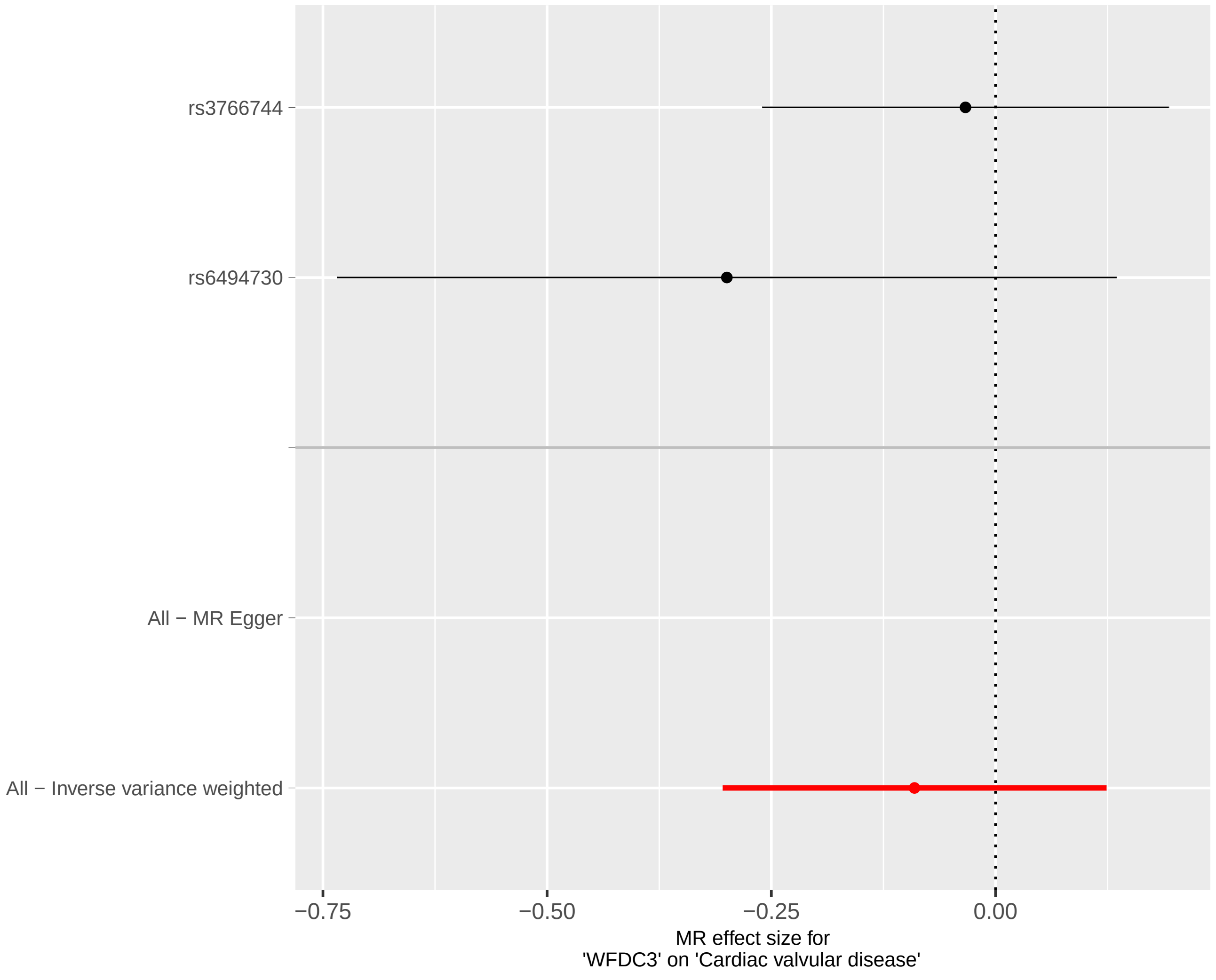

rs3766744
rs6494730
All − MR Egger
All − Inverse variance weighted
−0.75
−0.50
−0.25
0.00
MR effect size for
'WFDC3' on 'Cardiac valvular disease'

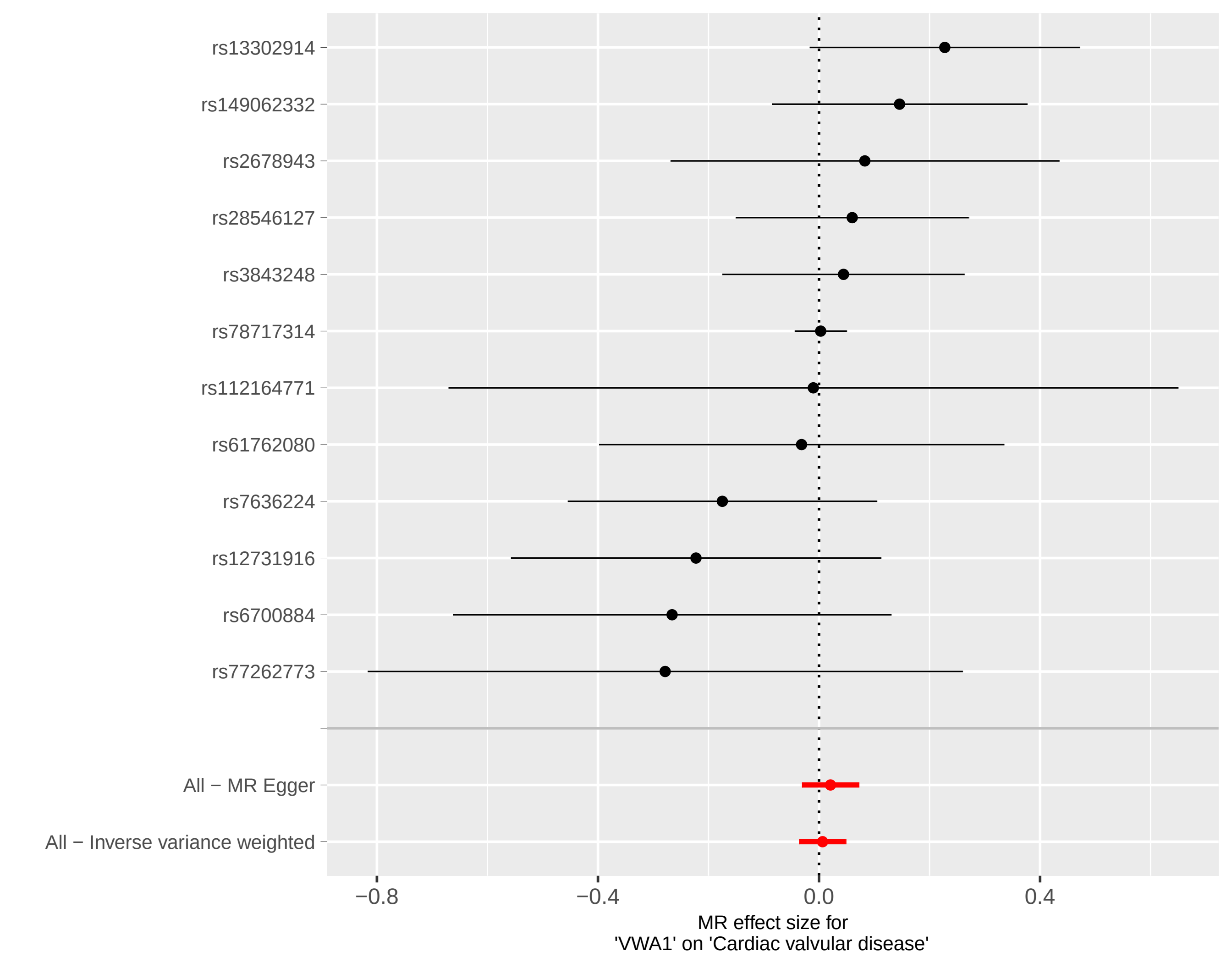
rs13302914
rs149062332
rs2678943
rs28546127
rs3843248
rs78717314
rs112164771
rs61762080
rs7636224
rs12731916
rs6700884
rs77262773
All − MR Egger
All − Inverse variance weighted
−0.8
−0.4
0.0
0.4
MR effect size for
'VWA1' on 'Cardiac valvular disease'

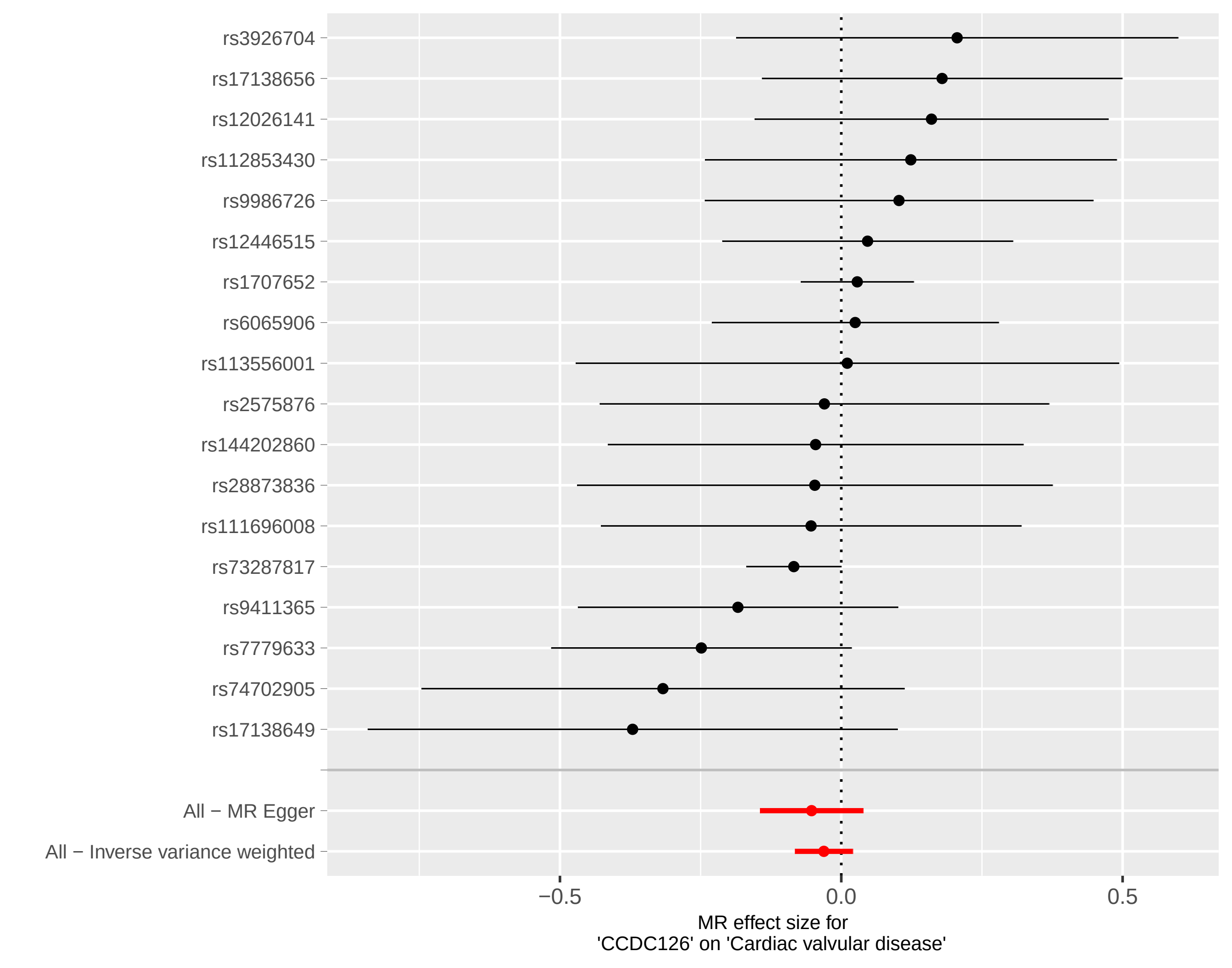

rs3926704
rs17138656
rs12026141
rs112853430
rs9986726
rs12446515
rs1707652
rs6065906
rs113556001
rs2575876
rs144202860
rs28873836
rs111696008
rs73287817
rs9411365
rs7779633
rs74702905
rs17138649
All − MR Egger
All − Inverse variance weighted
−0.5
0.0
0.5
MR effect size for
'CCDC126' on 'Cardiac valvular disease'

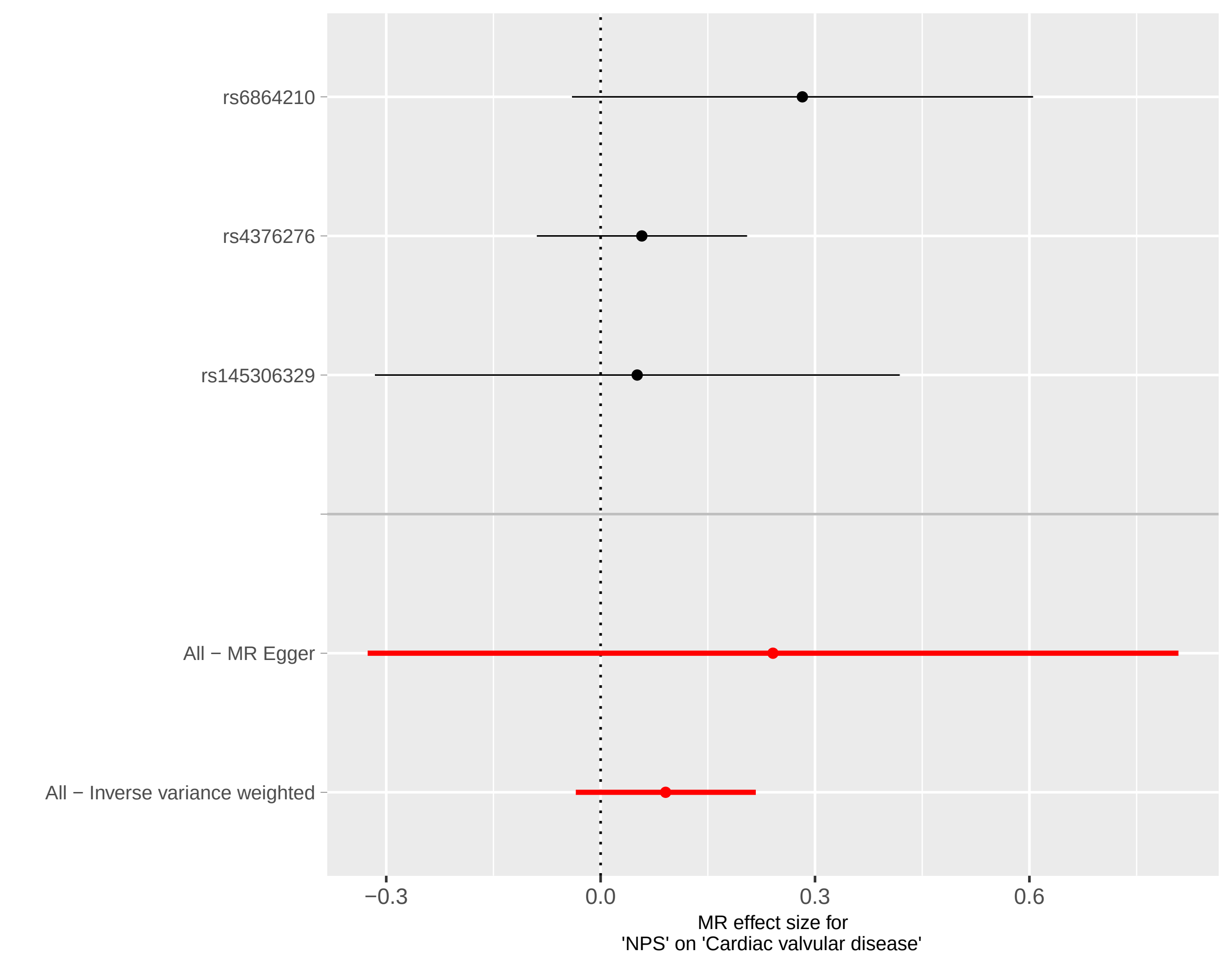
rs6864210
rs4376276
rs145306329
All − MR Egger
All − Inverse variance weighted
−0.3
0.0
0.3
0.6
MR effect size for
'NPS' on 'Cardiac valvular disease'

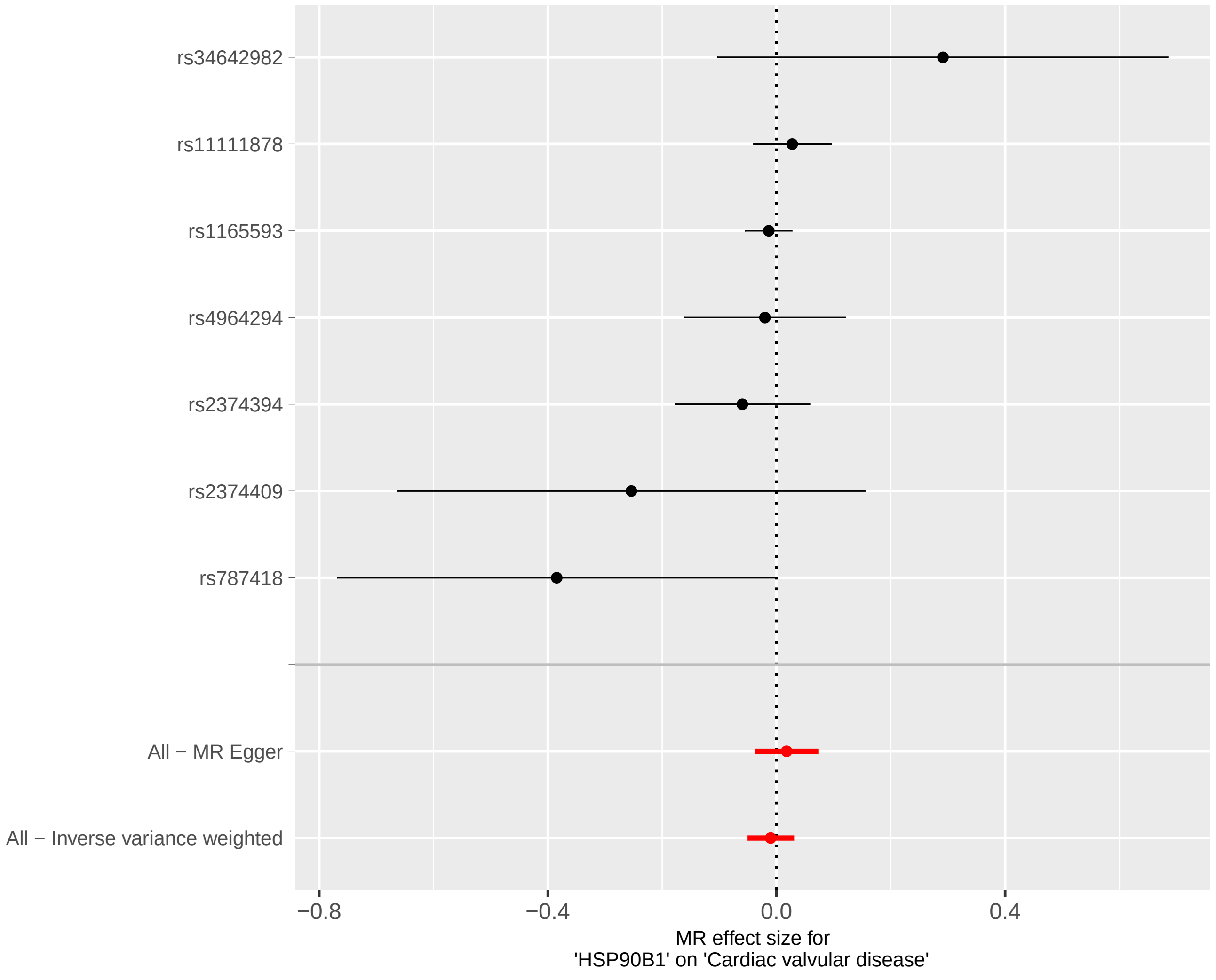
rs34642982
rs11111878
rs1165593
rs4964294
rs2374394
rs2374409
rs787418
All − MR Egger
All − Inverse variance weighted
−0.8
−0.4
0.0
0.4
MR effect size for
'HSP90B1' on 'Cardiac valvular disease'

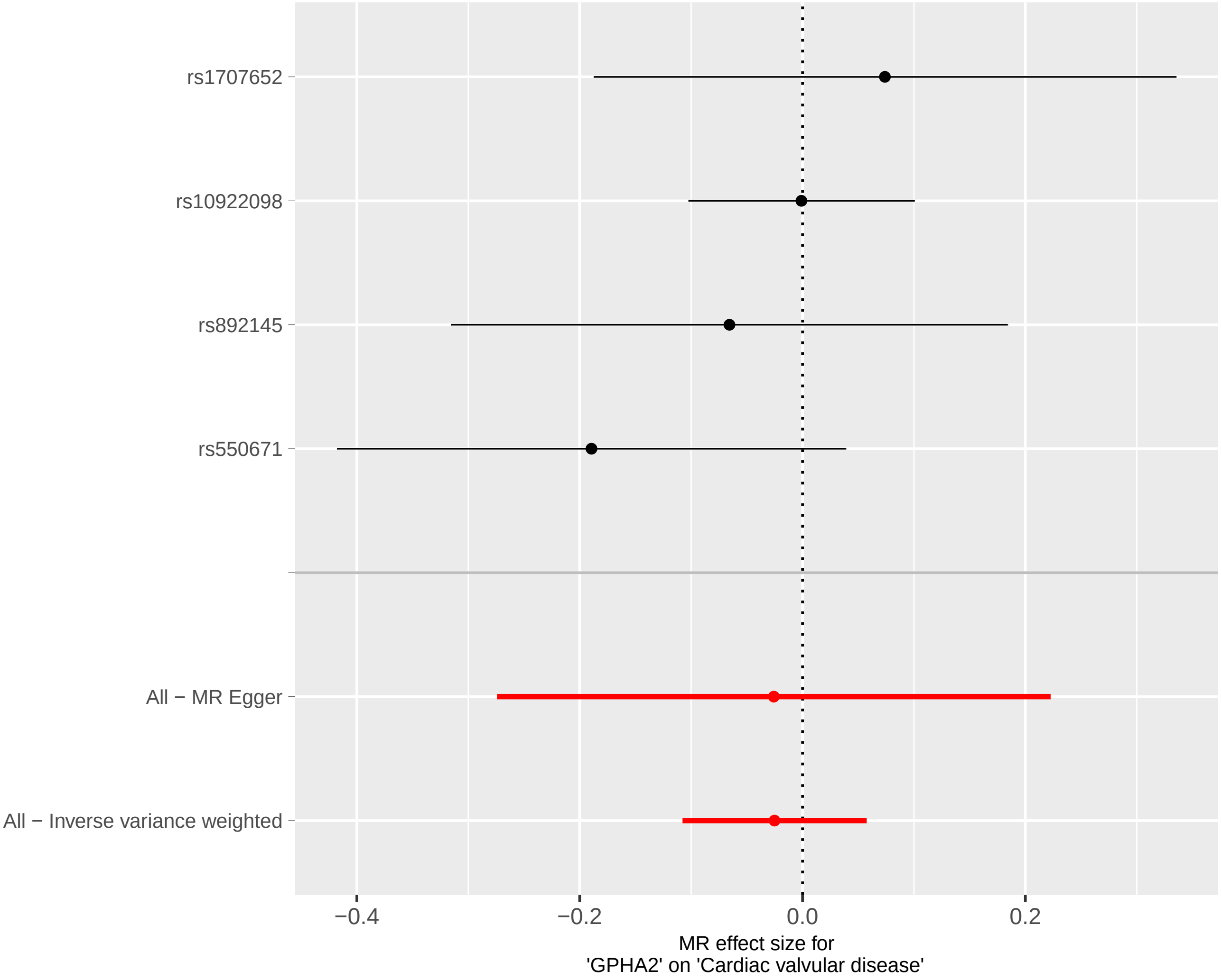
rs1707652
rs10922098
rs892145
rs550671
All − MR Egger
All − Inverse variance weighted
−0.4
−0.2
0.0
0.2
MR effect size for
'GPHA2' on 'Cardiac valvular disease'

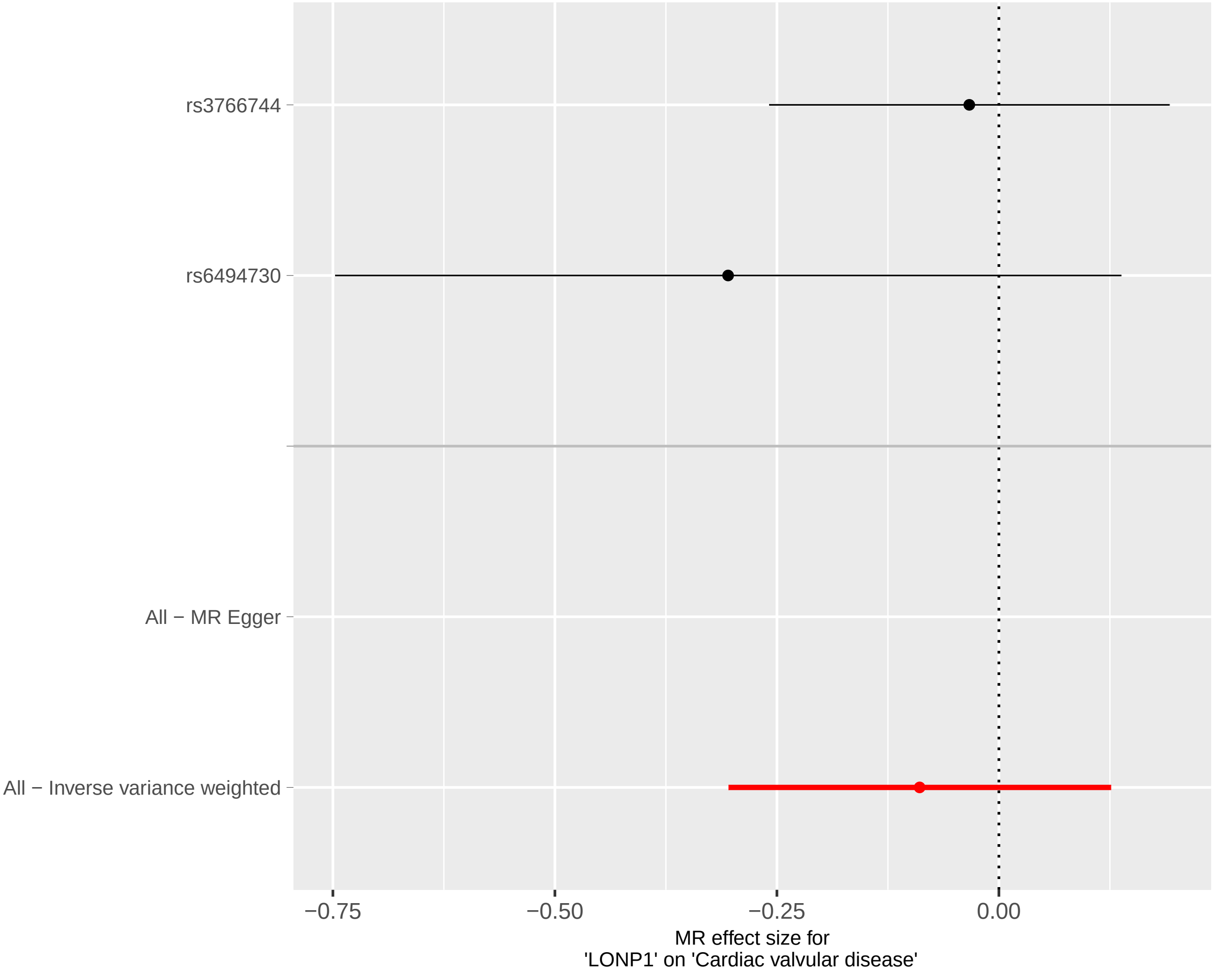
rs3766744
rs6494730
All − MR Egger
All − Inverse variance weighted
−0.75
−0.50
−0.25
0.00
MR effect size for
'LONP1' on 'Cardiac valvular disease'

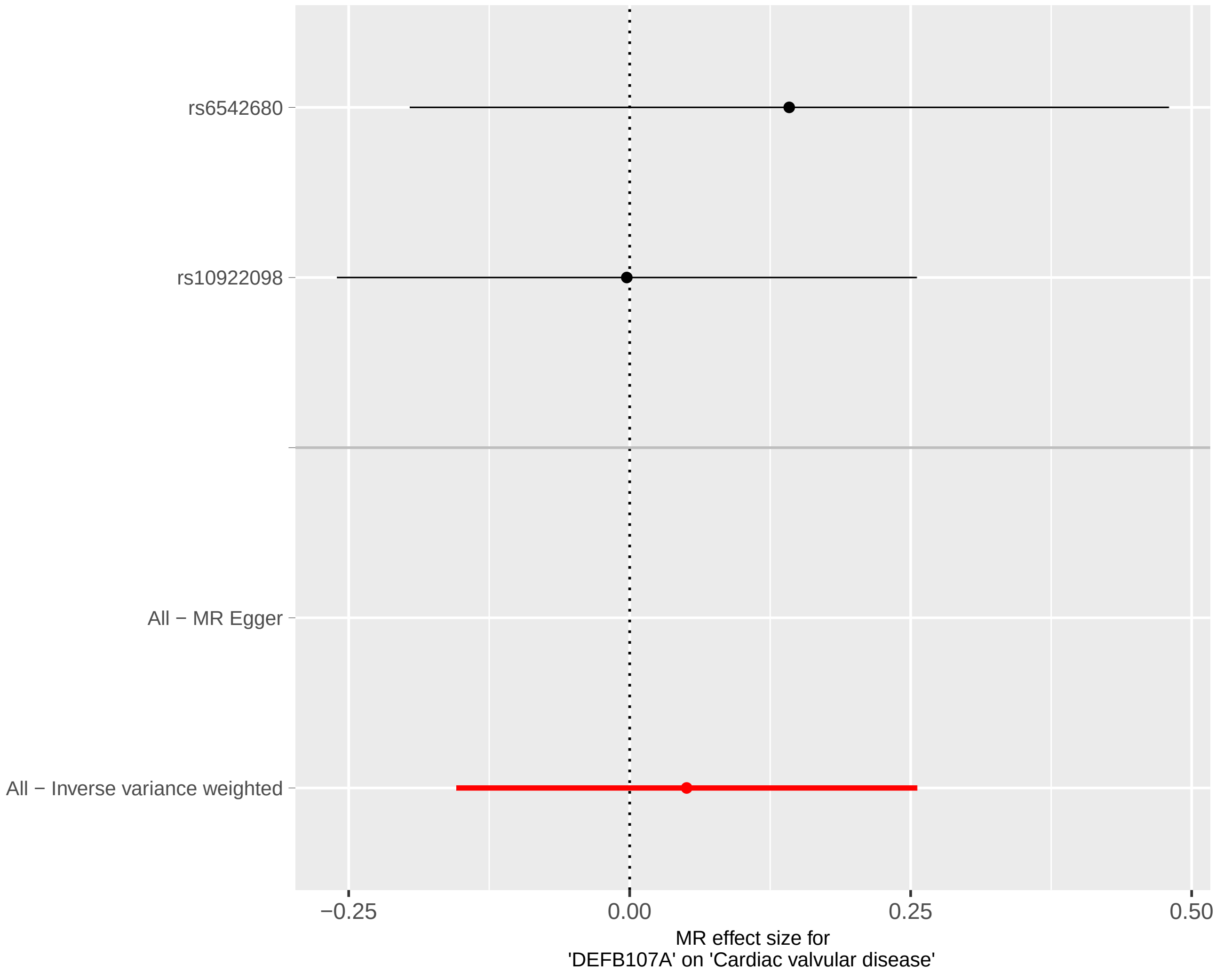
rs6542680
rs10922098
All − MR Egger
All − Inverse variance weighted
−0.25
0.00
0.25
0.50
MR effect size for
'DEFB107A' on 'Cardiac valvular disease'

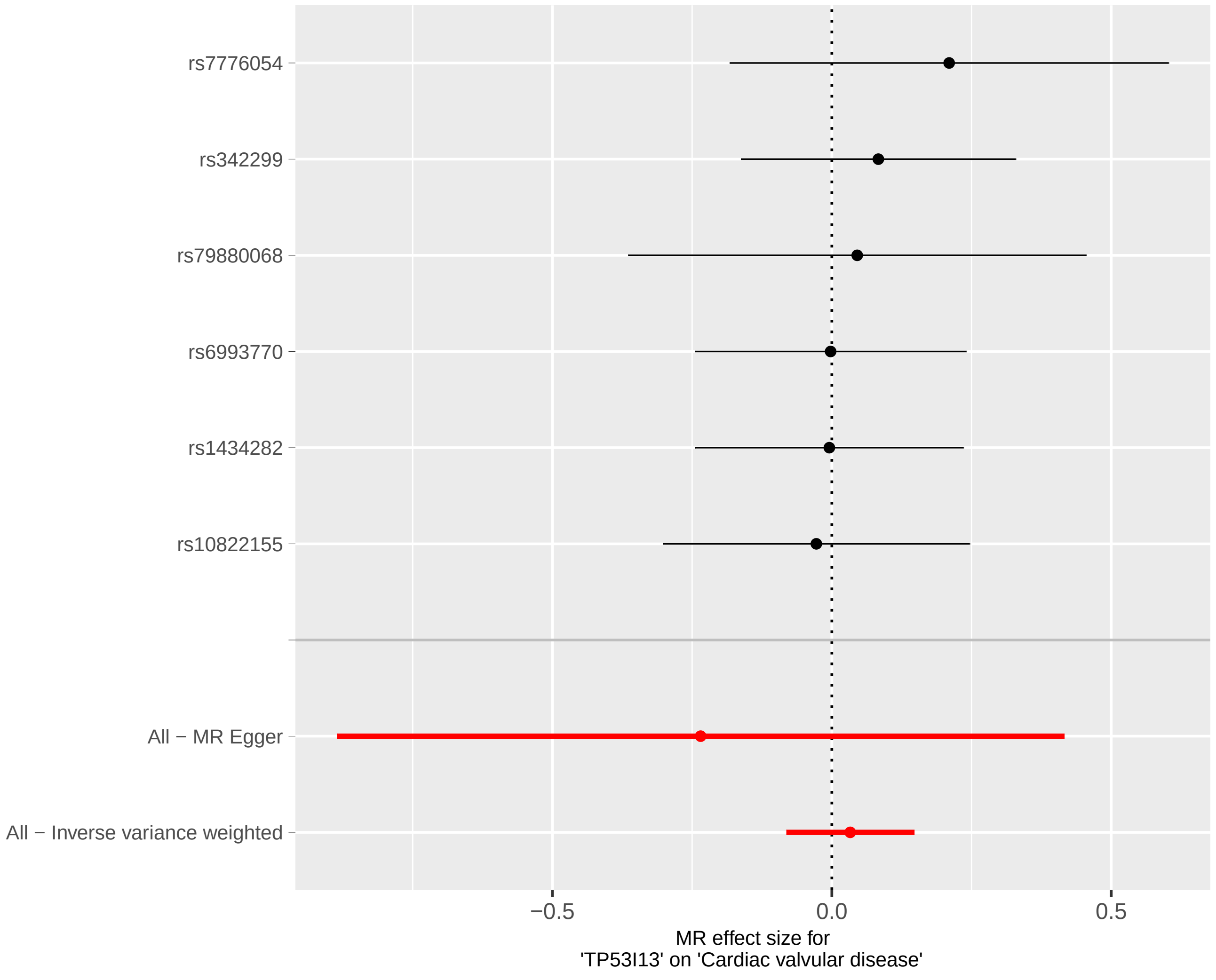
rs7776054
rs342299
rs79880068
rs6993770
rs1434282
rs10822155
All − MR Egger
All − Inverse variance weighted
−0.5
0.0
0.5
MR effect size for
'TP53I13' on 'Cardiac valvular disease'

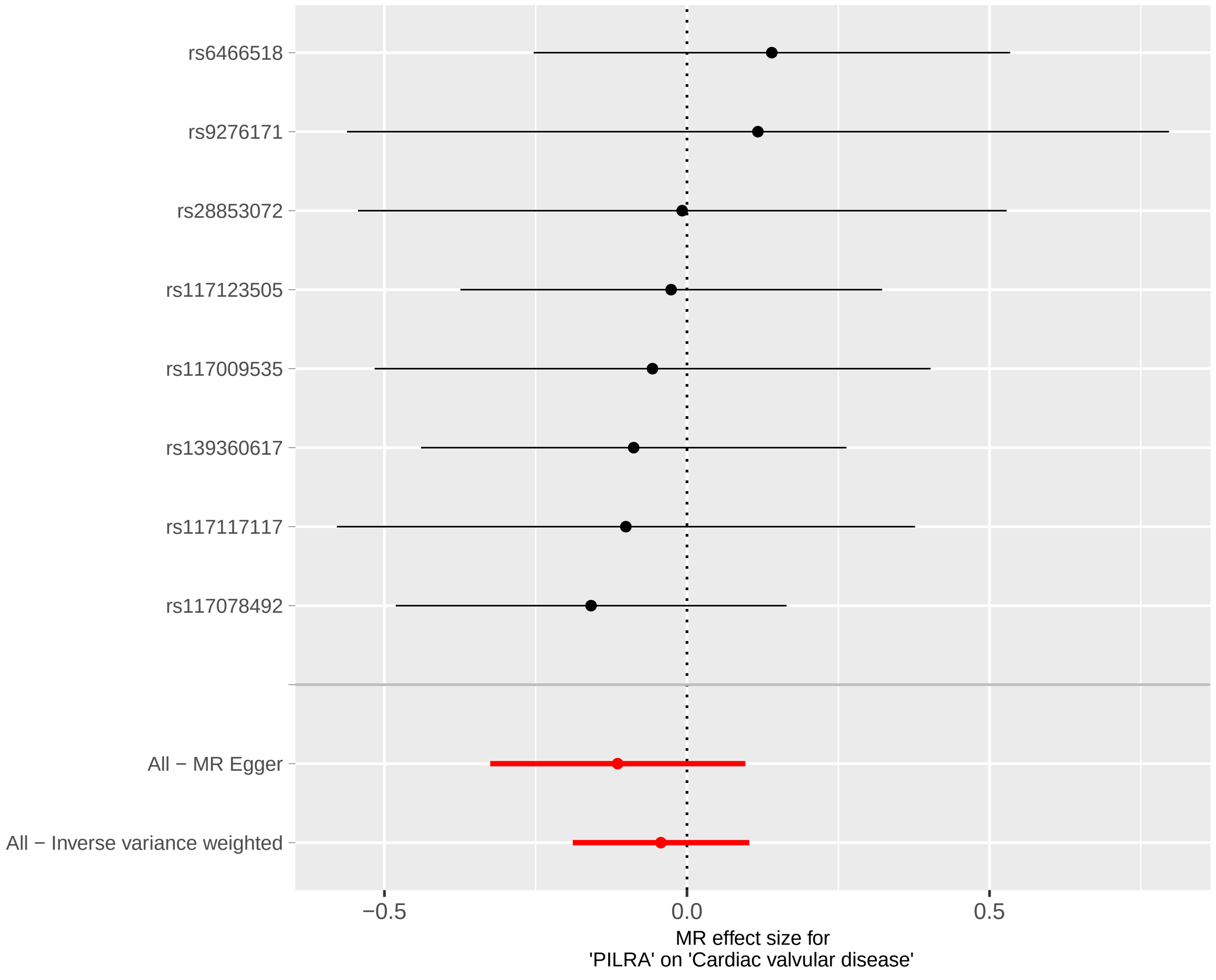
rs6466518
rs9276171
rs28853072
rs117123505
rs117009535
rs139360617
rs117117117
rs117078492
All − MR Egger
All − Inverse variance weighted
−0.5
0.0
0.5
MR effect size for
'PILRA' on 'Cardiac valvular disease'

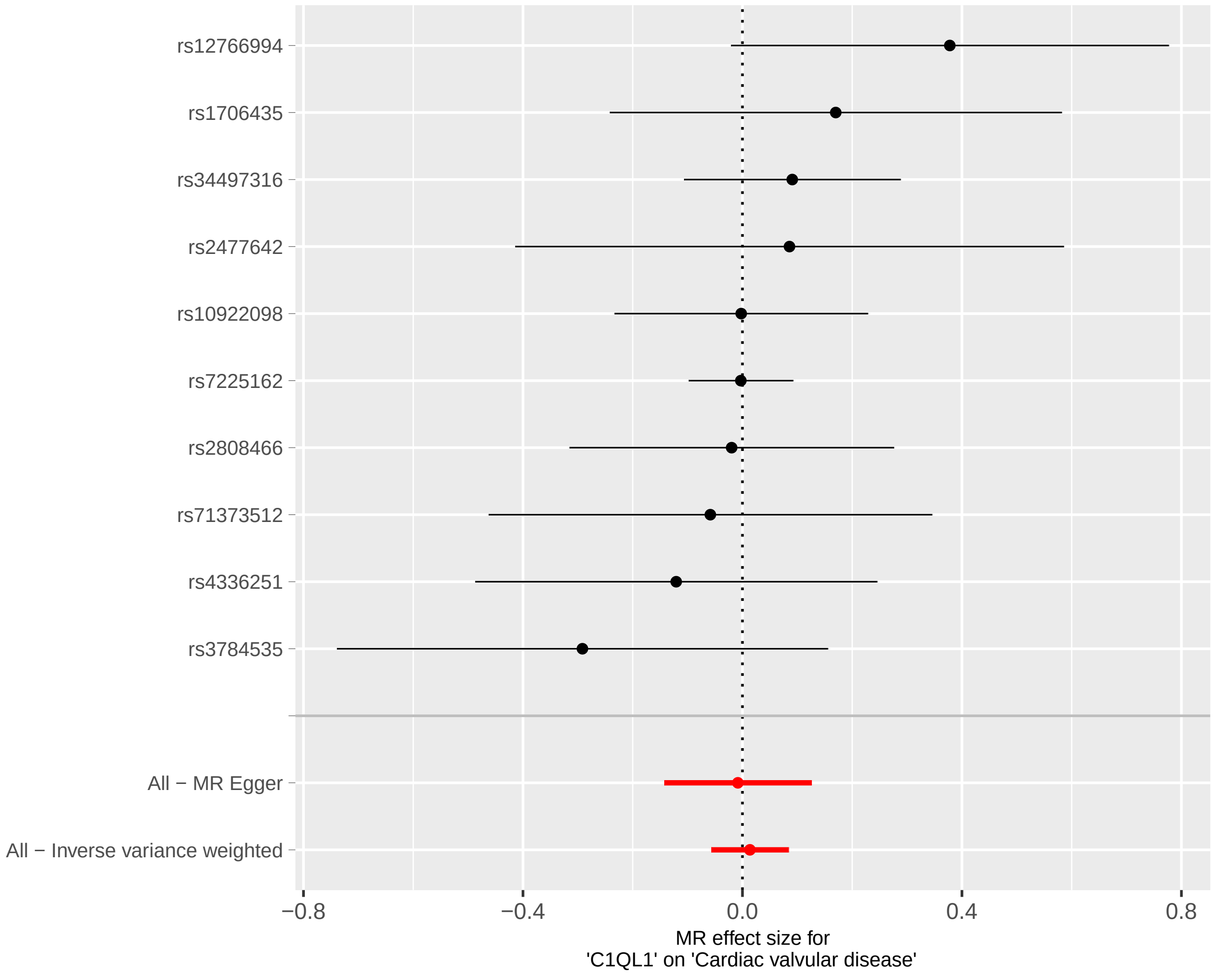
rs12766994
rs1706435
rs34497316
rs2477642
rs10922098
rs7225162
rs2808466
rs71373512
rs4336251
rs3784535
All − MR Egger
All − Inverse variance weighted
−0.8
−0.4
0.0
0.4
0.8
MR effect size for
'C1QL1' on 'Cardiac valvular disease'

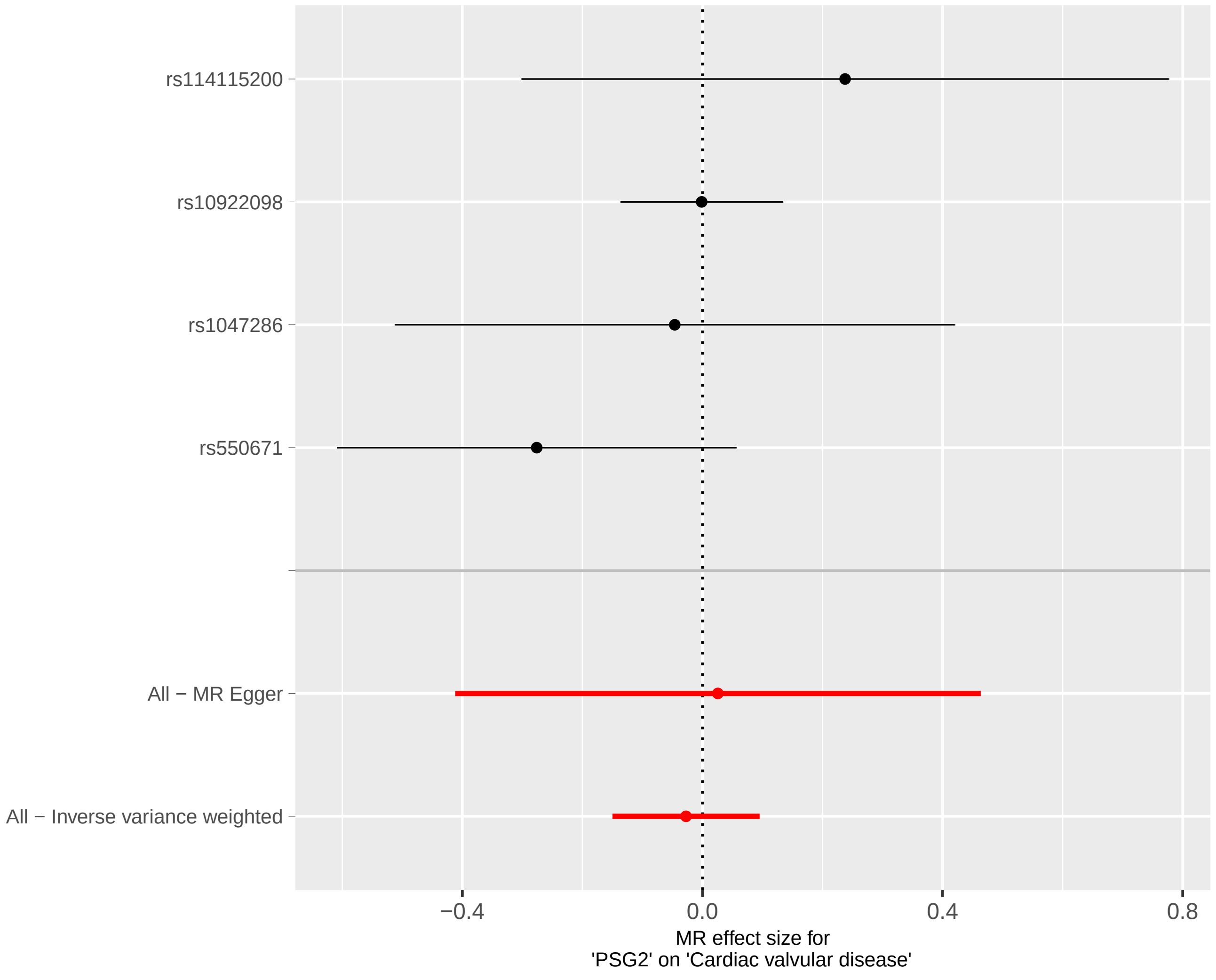
rs114115200
rs10922098
rs1047286
rs550671
All − MR Egger
All − Inverse variance weighted
−0.4
0.0
0.4
0.8
MR effect size for
'PSG2' on 'Cardiac valvular disease'

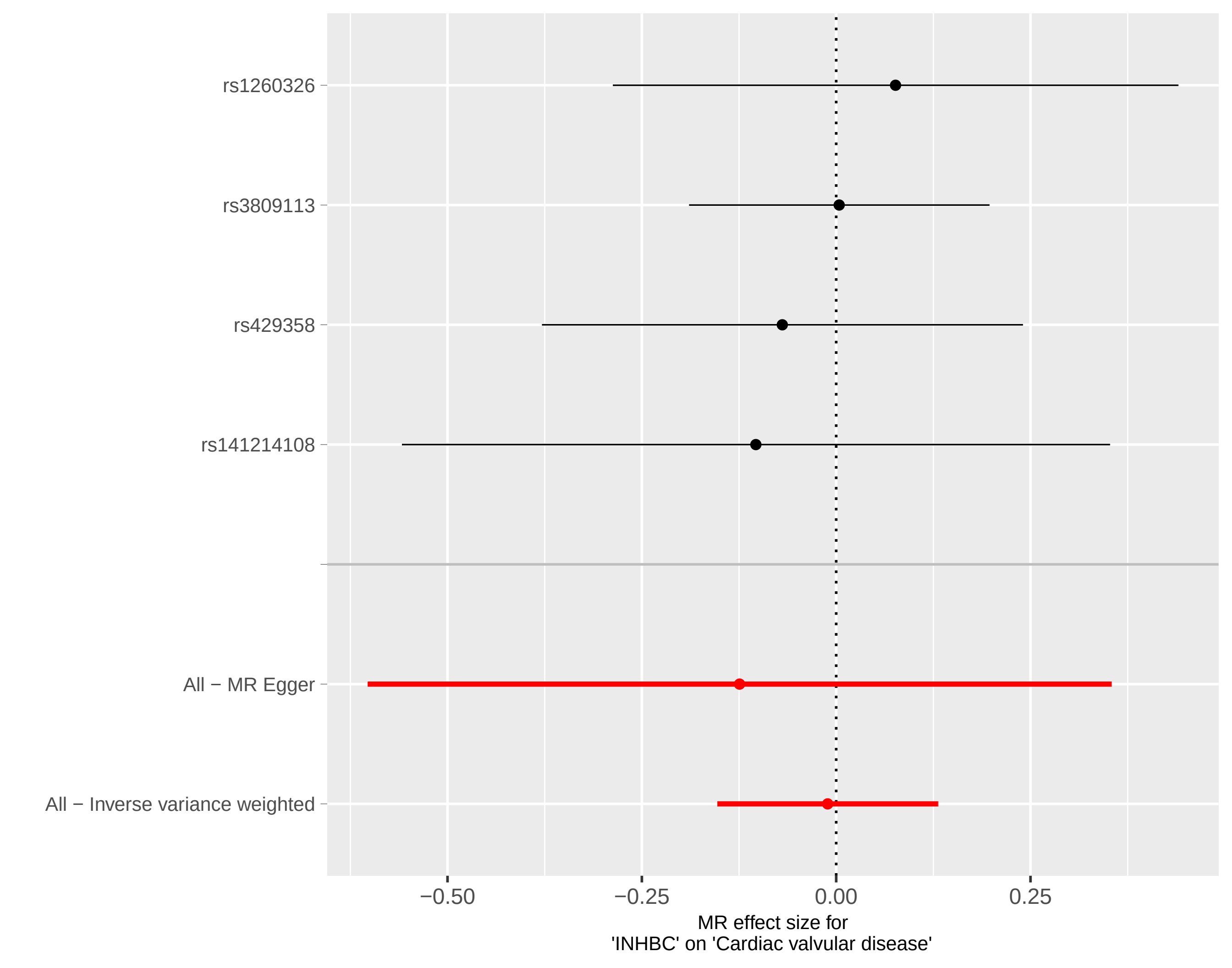
rs1260326
rs3809113
rs429358
rs141214108
All − MR Egger
All − Inverse variance weighted
−0.50
−0.25
0.00
0.25
MR effect size for
'INHBC' on 'Cardiac valvular disease'

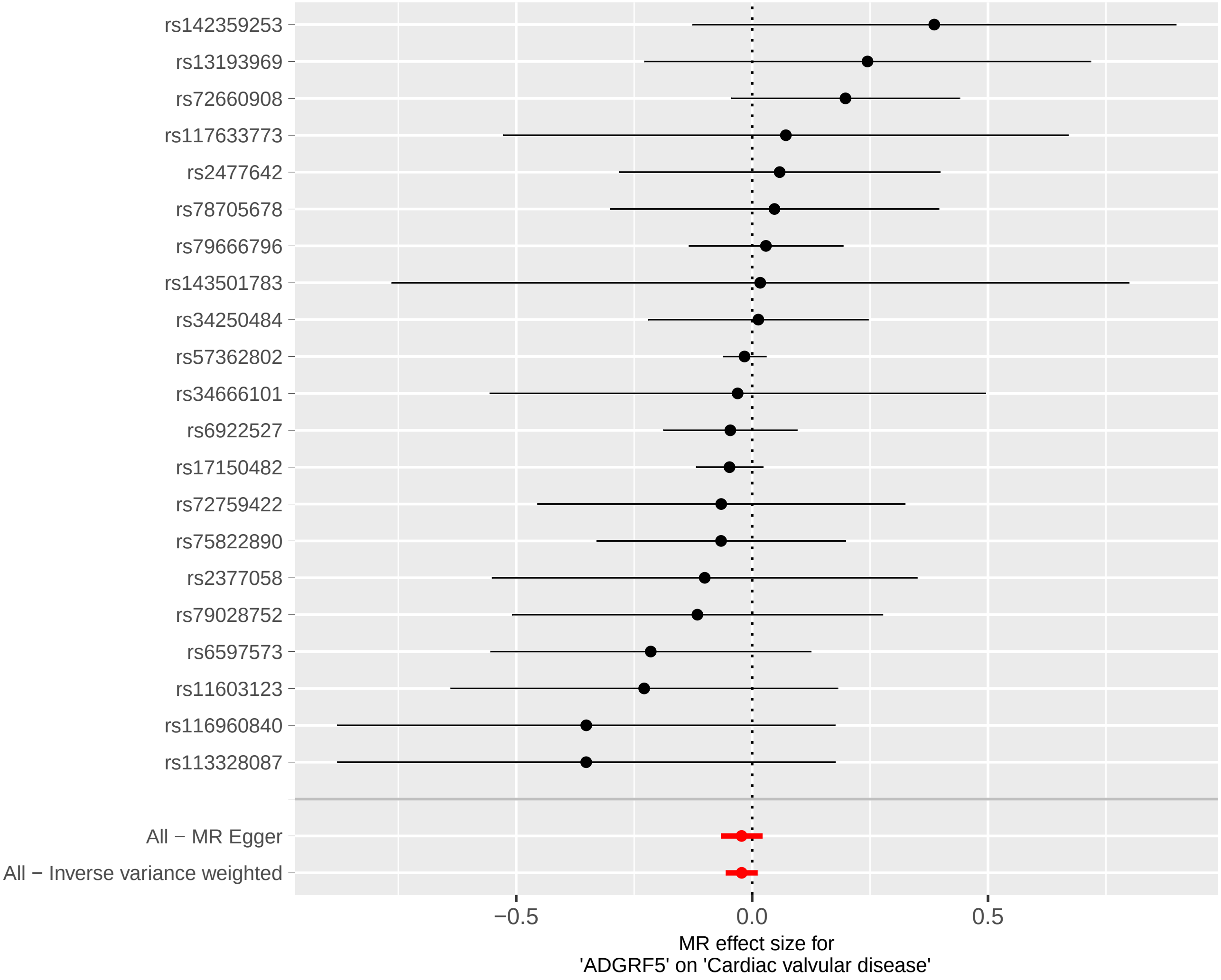
rs142359253
rs13193969
rs72660908
rs117633773
rs2477642
rs78705678
rs79666796
rs143501783
rs34250484
rs57362802
rs34666101
rs6922527
rs17150482
rs72759422
rs75822890
rs2377058
rs79028752
rs6597573
rs11603123
rs116960840
rs113328087
All − MR Egger
All − Inverse variance weighted
−0.5
0.0
0.5
MR effect size for
'ADGRF5' on 'Cardiac valvular disease'

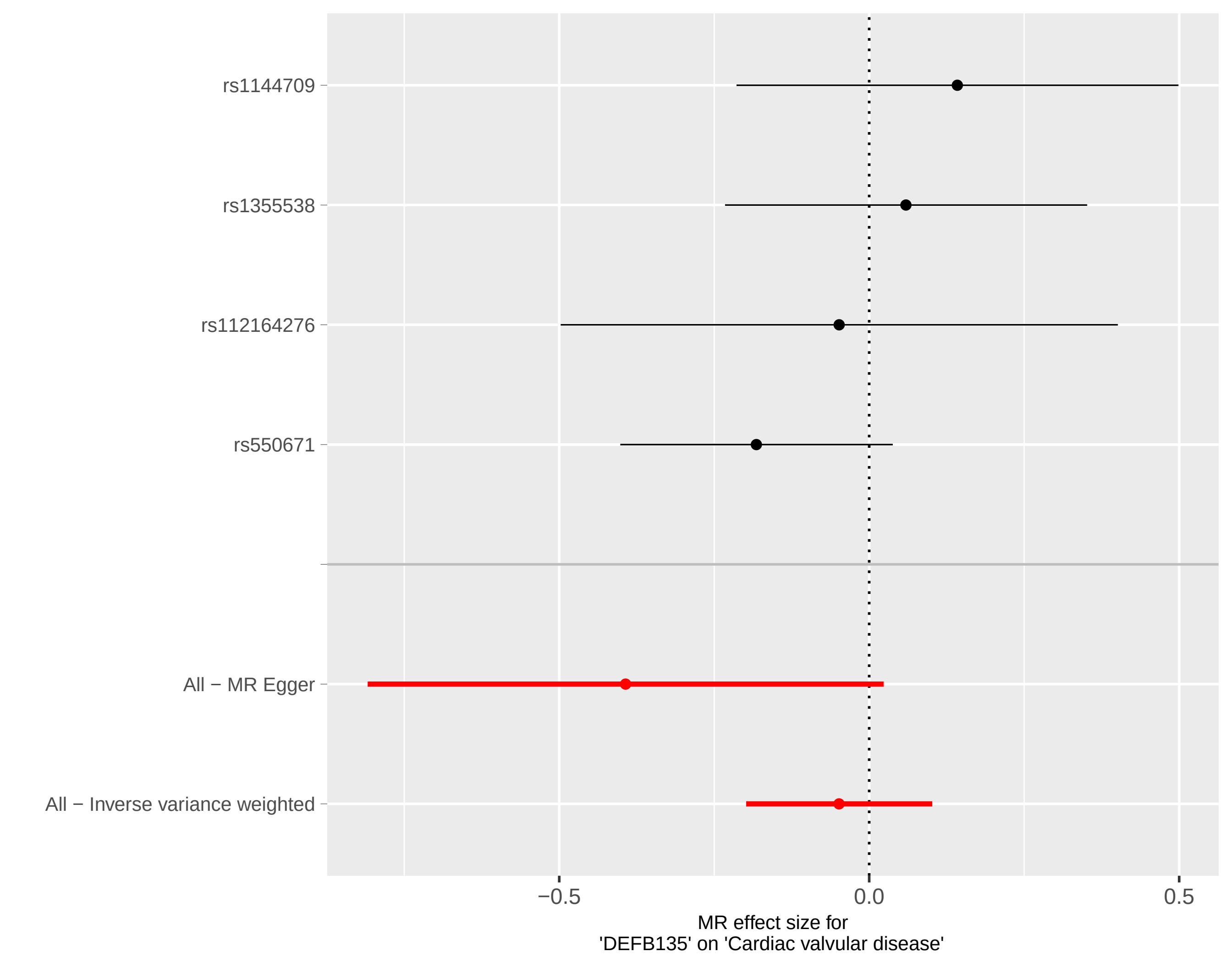

rs1144709
rs1355538
rs112164276
rs550671
All − MR Egger
All − Inverse variance weighted
−0.5
0.0
0.5
MR effect size for
'DEFB135' on 'Cardiac valvular disease'

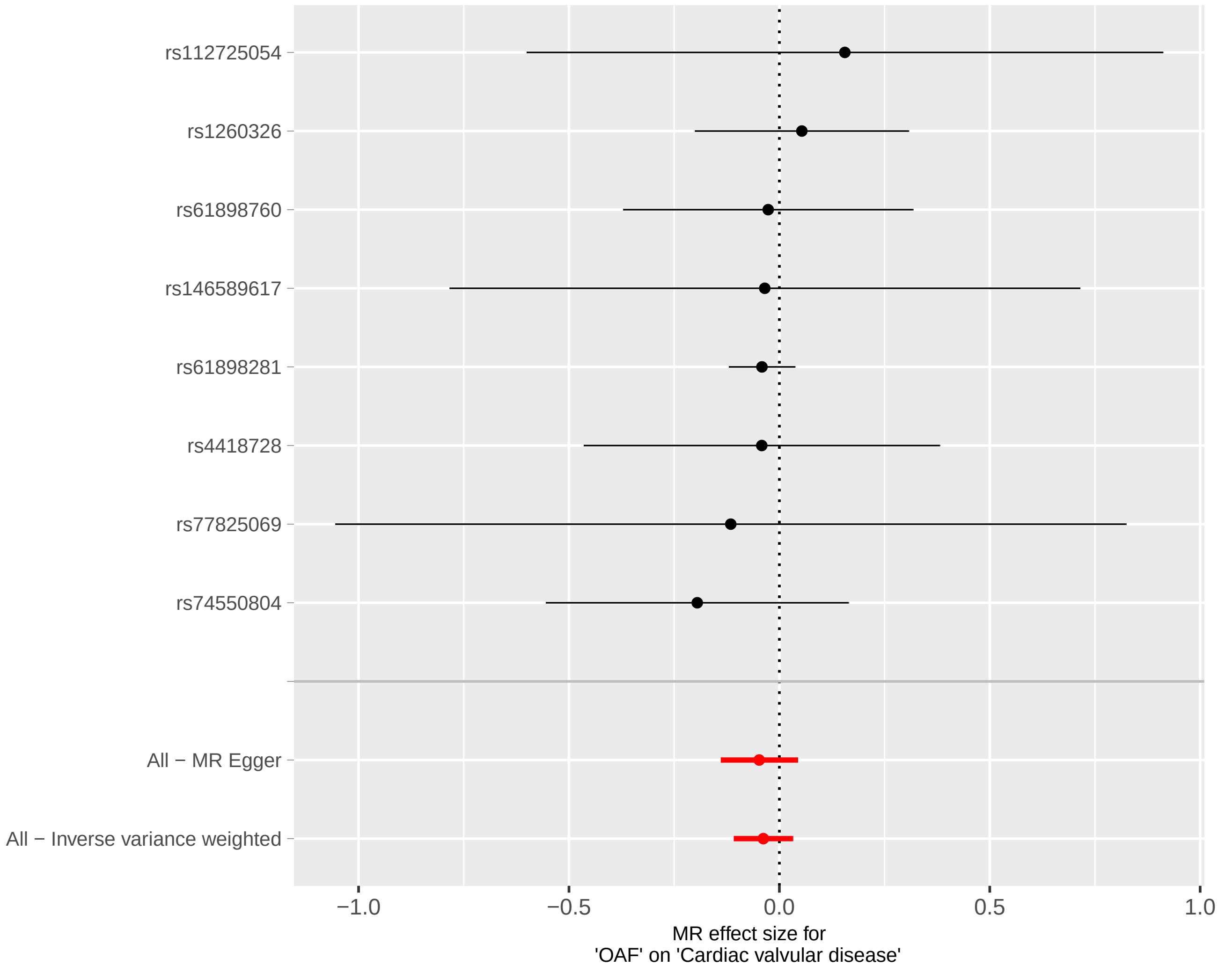
rs112725054
rs1260326
rs61898760
rs146589617
rs61898281
rs4418728
rs77825069
rs74550804
All − MR Egger
All − Inverse variance weighted
−1.0
−0.5
0.0
0.5
1.0
MR effect size for
'OAF' on 'Cardiac valvular disease'

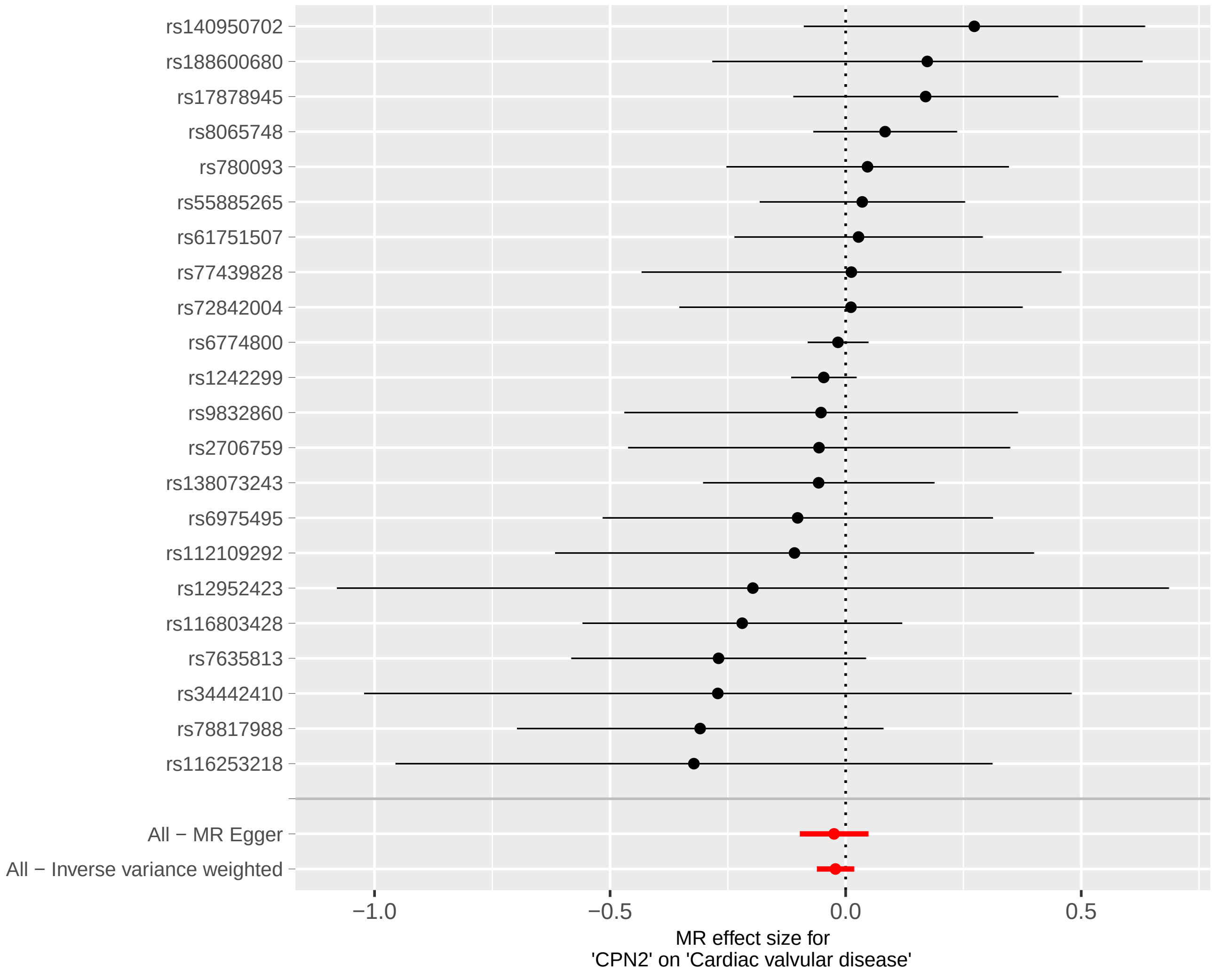
rs140950702
rs188600680
rs17878945
rs8065748
rs780093
rs55885265
rs61751507
rs77439828
rs72842004
rs6774800
rs1242299
rs9832860
rs2706759
rs138073243
rs6975495
rs112109292
rs12952423
rs116803428
rs7635813
rs34442410
rs78817988
rs116253218
All − MR Egger
All − Inverse variance weighted
−1.0
−0.5
0.0
0.5
MR effect size for
'CPN2' on 'Cardiac valvular disease'

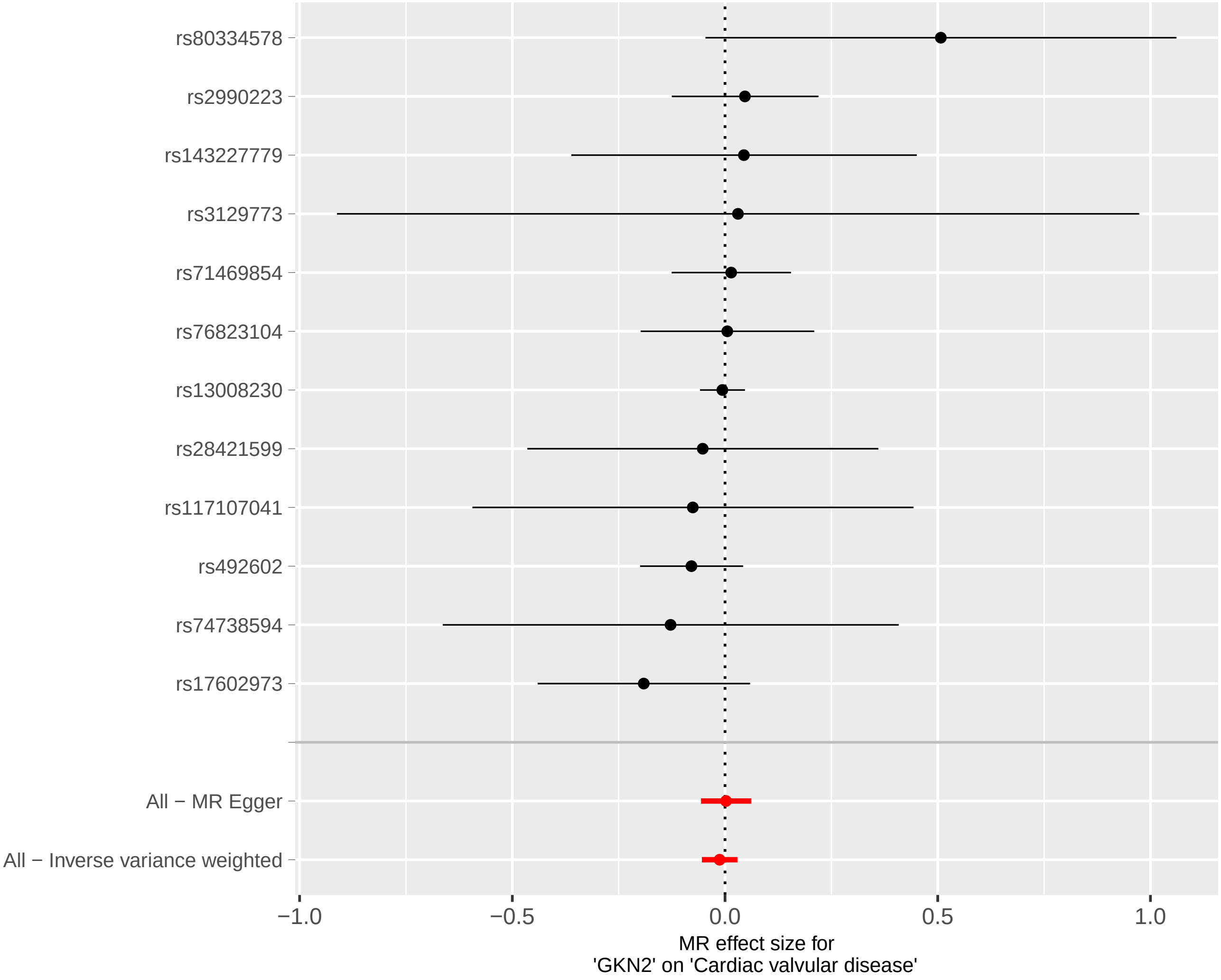
rs80334578
rs2990223
rs143227779
rs3129773
rs71469854
rs76823104
rs13008230
rs28421599
rs117107041
rs492602
rs74738594
rs17602973
All − MR Egger
All − Inverse variance weighted
−1.0
−0.5
0.0
0.5
1.0
MR effect size for
'GKN2' on 'Cardiac valvular disease'

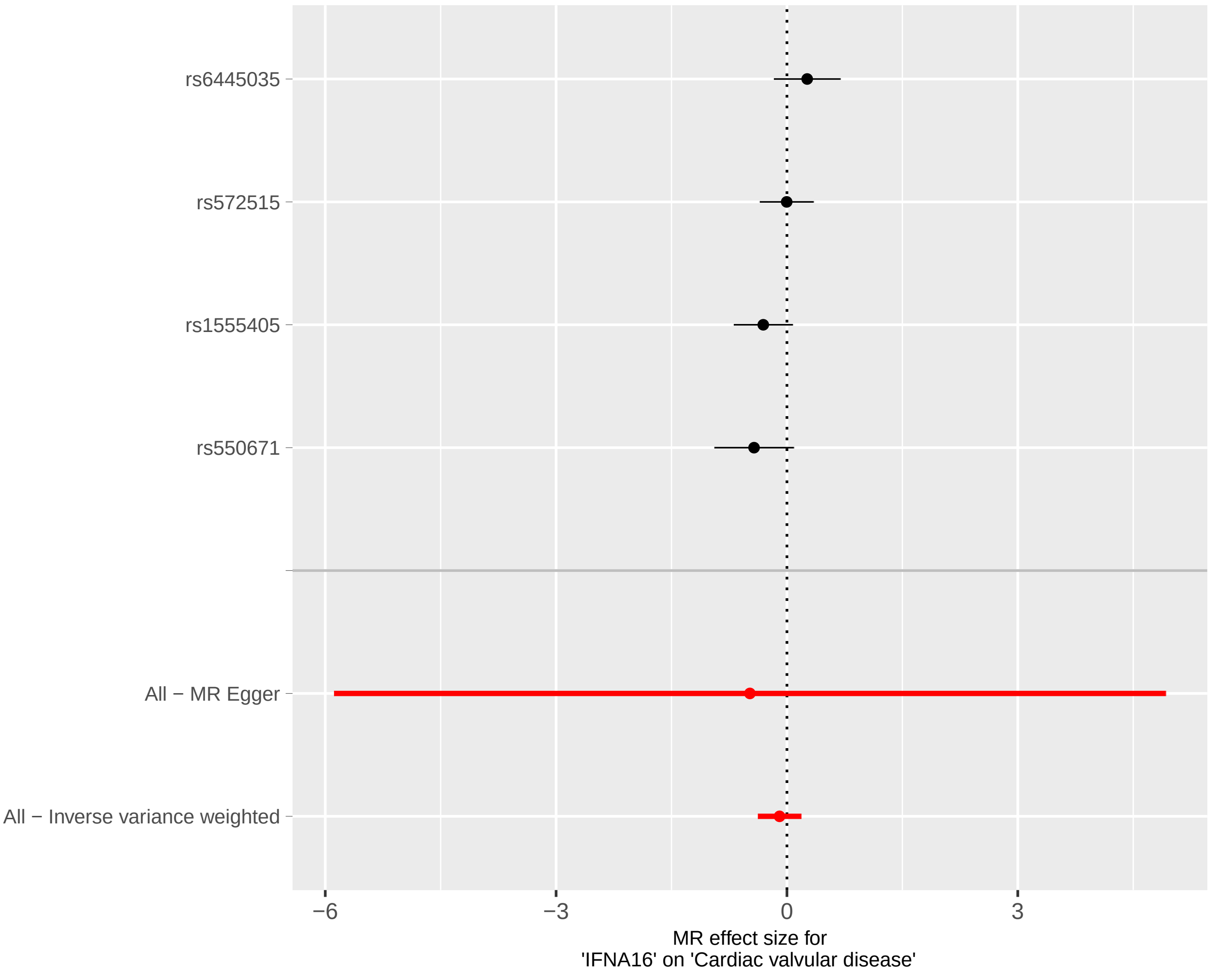
rs6445035
rs572515
rs1555405
rs550671
All − MR Egger
All − Inverse variance weighted
−6
−3
0
3
MR effect size for
'IFNA16' on 'Cardiac valvular disease'

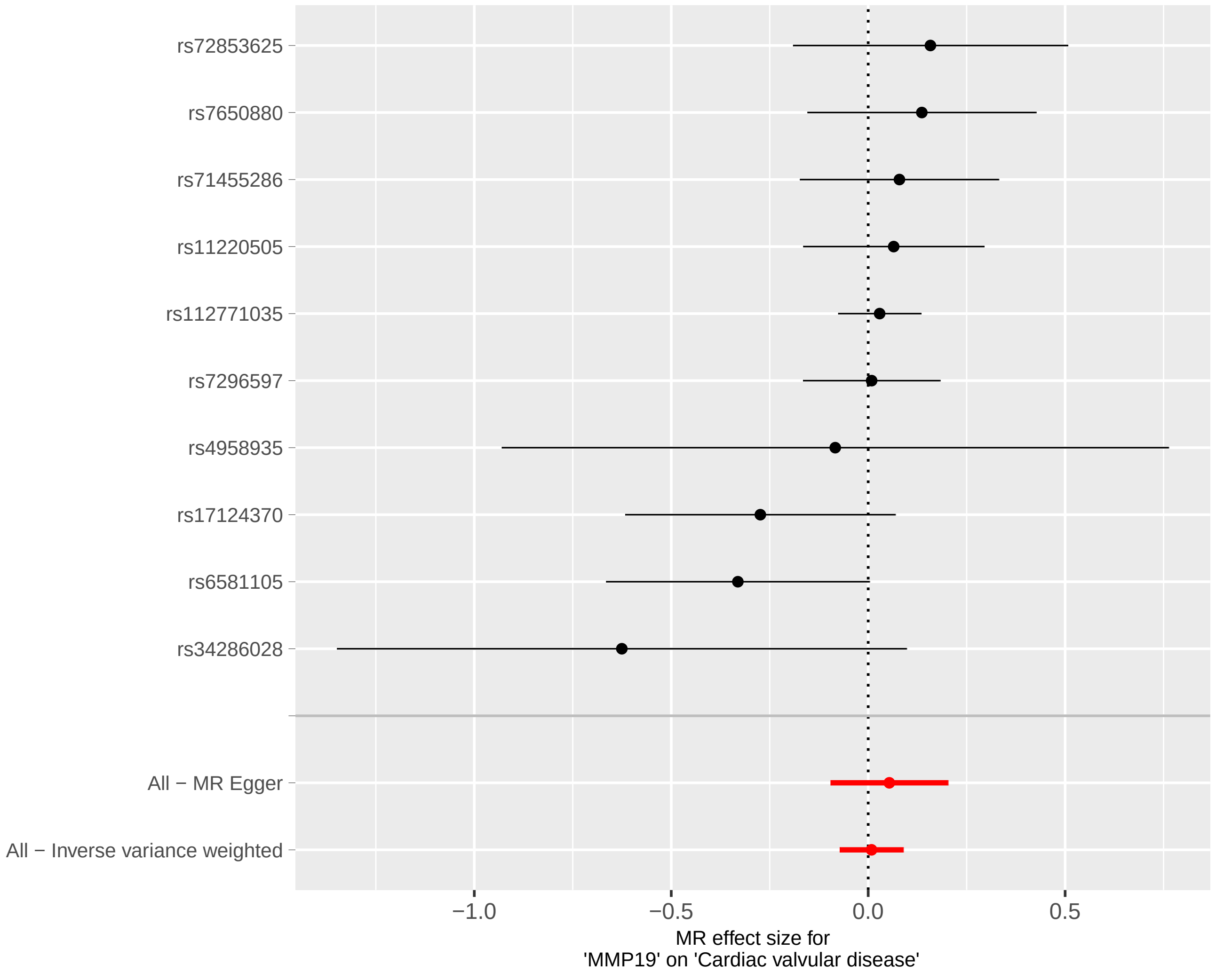
rs72853625
rs7650880
rs71455286
rs11220505
rs112771035
rs7296597
rs4958935
rs17124370
rs6581105
rs34286028
All − MR Egger
All − Inverse variance weighted
−1.0
−0.5
0.0
0.5
MR effect size for
'MMP19' on 'Cardiac valvular disease'

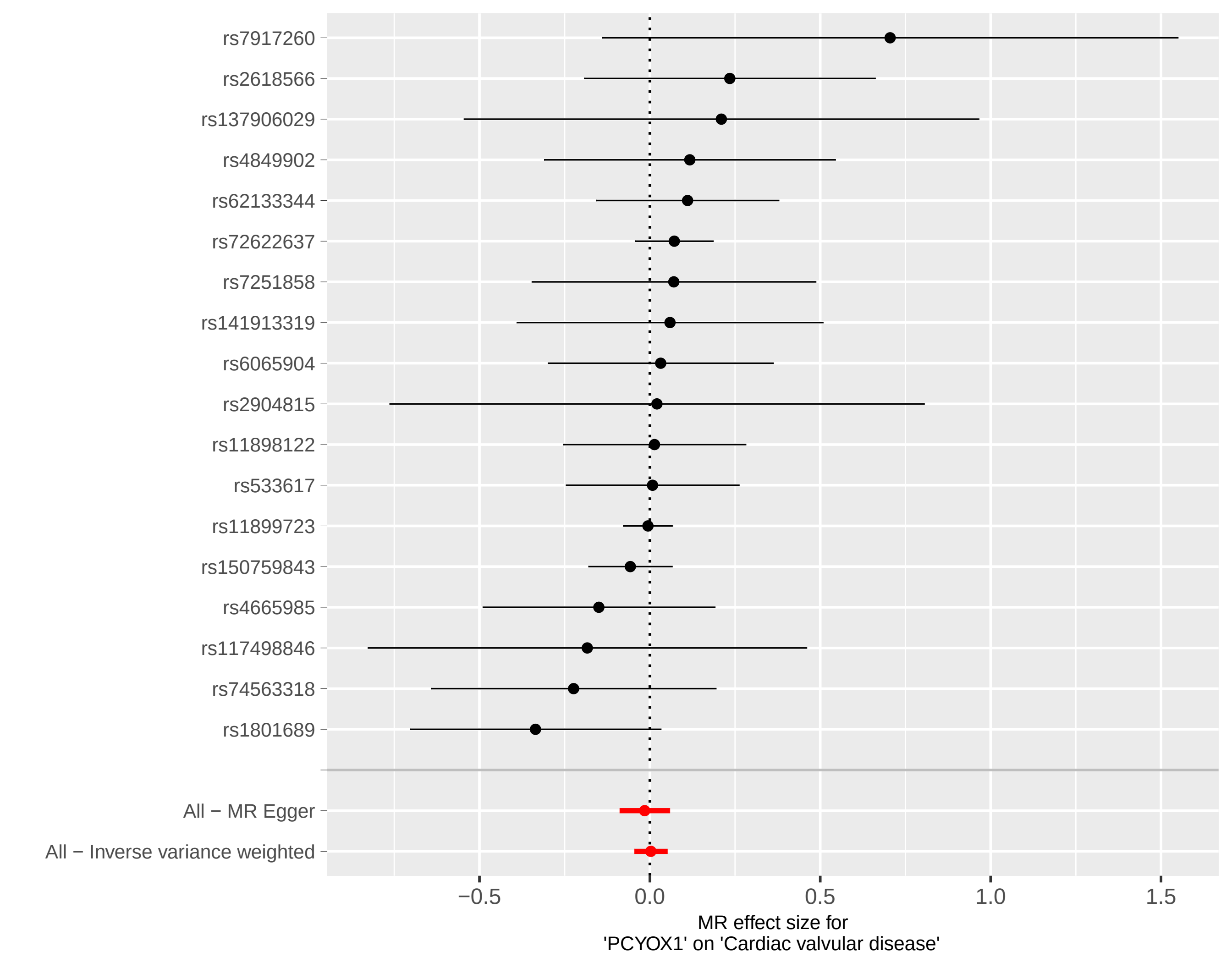

rs7917260
rs2618566
rs137906029
rs4849902
rs62133344
rs72622637
rs7251858
rs141913319
rs6065904
rs2904815
rs11898122
rs533617
rs11899723
rs150759843
rs4665985
rs117498846
rs74563318
rs1801689
All − MR Egger
All − Inverse variance weighted
−0.5
0.0
0.5
1.0
1.5
MR effect size for
'PCYOX1' on 'Cardiac valvular disease'

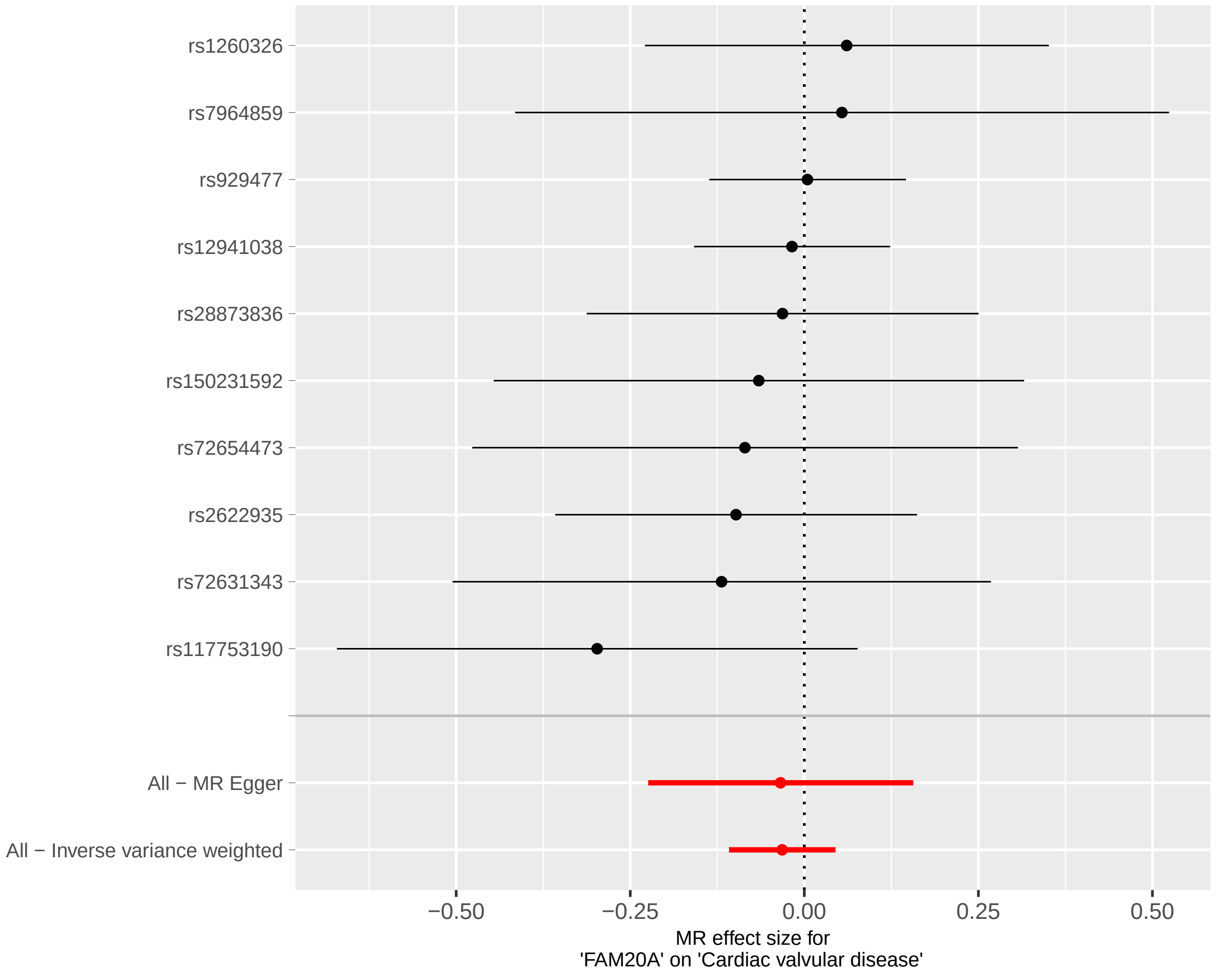
rs1260326
rs7964859
rs929477
rs12941038
rs28873836
rs150231592
rs72654473
rs2622935
rs72631343
rs117753190
All − MR Egger
All − Inverse variance weighted
−0.50
−0.25
0.00
0.25
0.50
MR effect size for
'FAM20A' on 'Cardiac valvular disease'

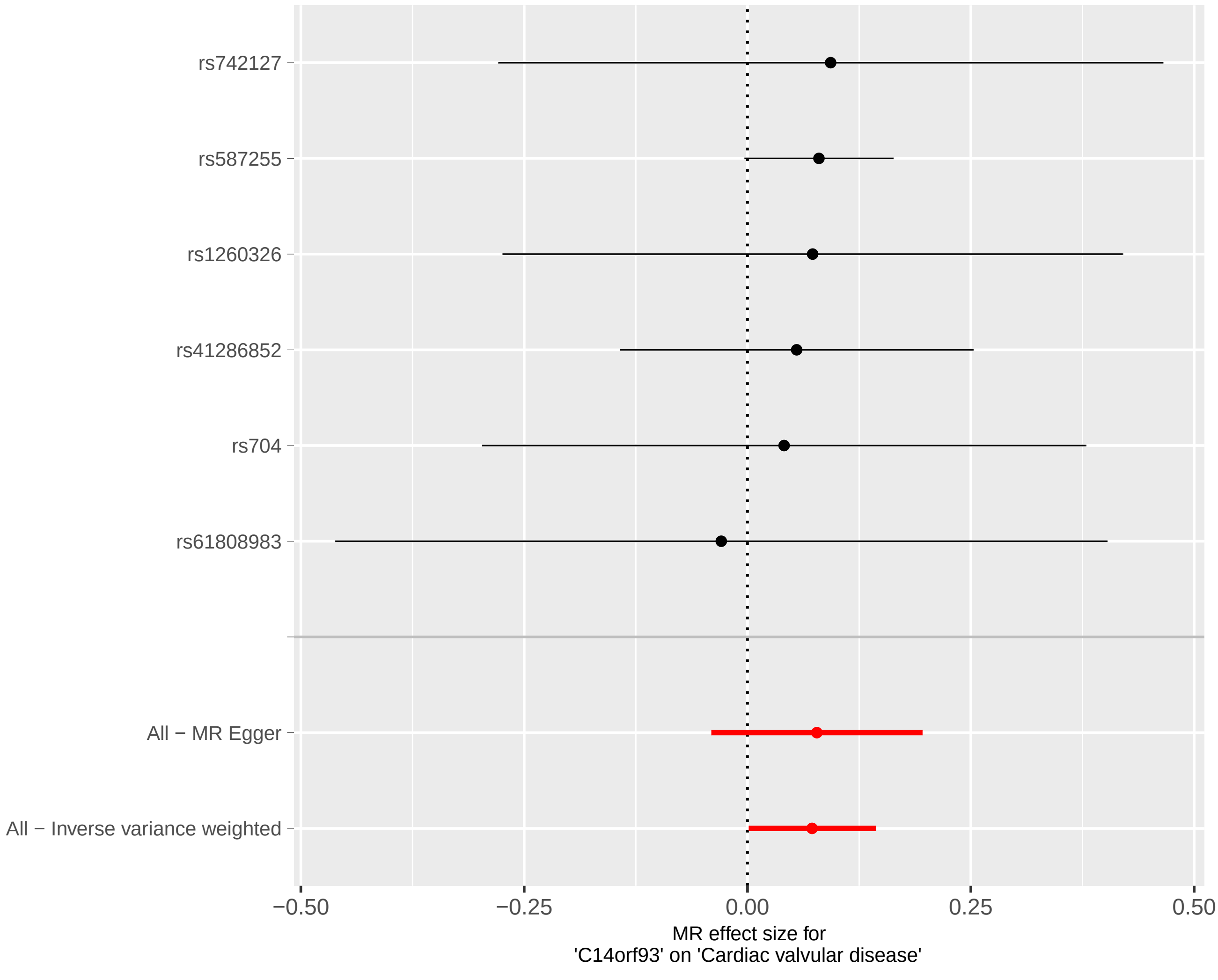

rs742127
rs587255
rs1260326
rs41286852
rs704
rs61808983
All − MR Egger
All − Inverse variance weighted
−0.50
−0.25
0.00
0.25
0.50
MR effect size for
'C14orf93' on 'Cardiac valvular disease'

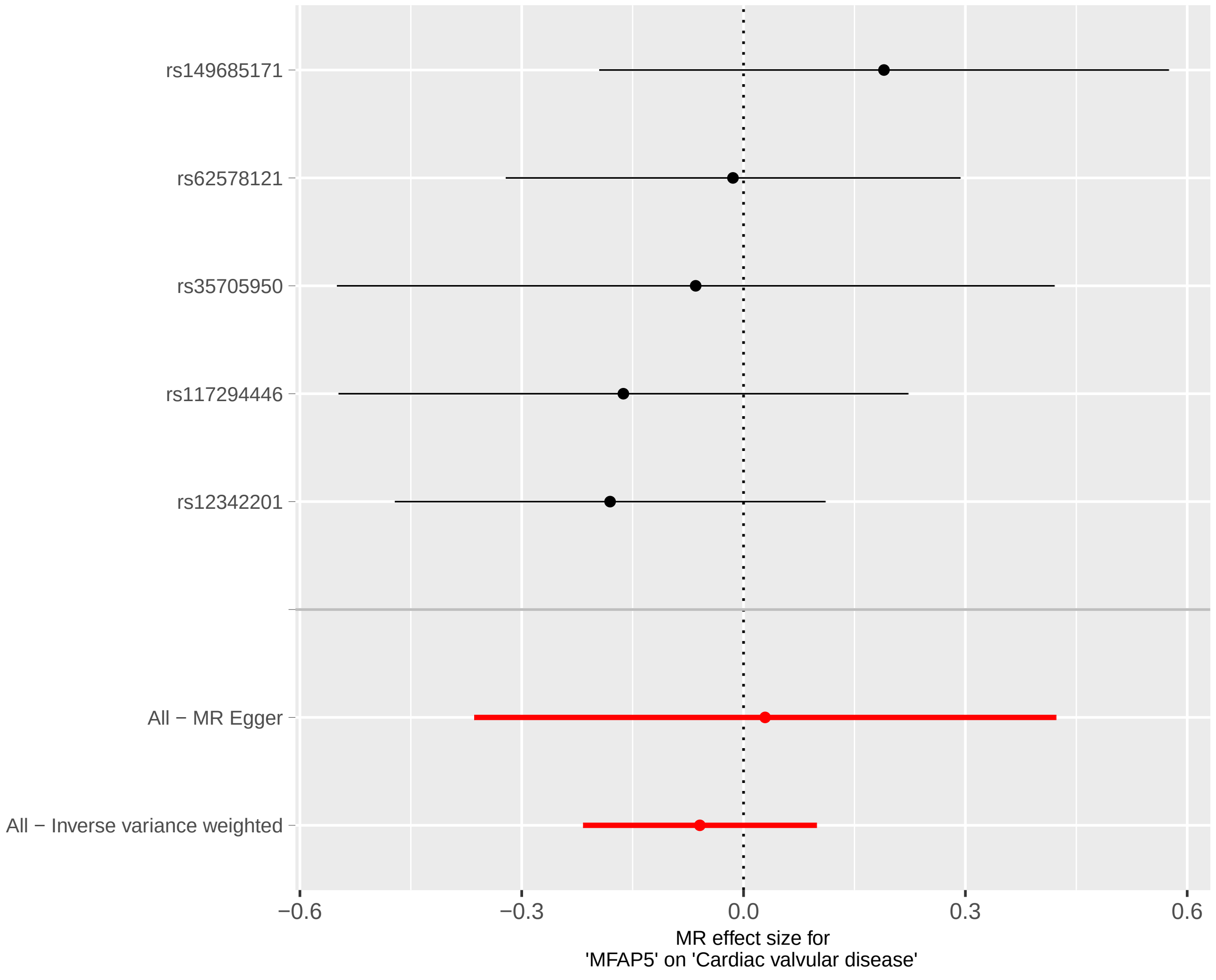
rs149685171
rs62578121
rs35705950
rs117294446
rs12342201
All − MR Egger
All − Inverse variance weighted
−0.6
−0.3
0.0
0.3
0.6
MR effect size for
'MFAP5' on 'Cardiac valvular disease'

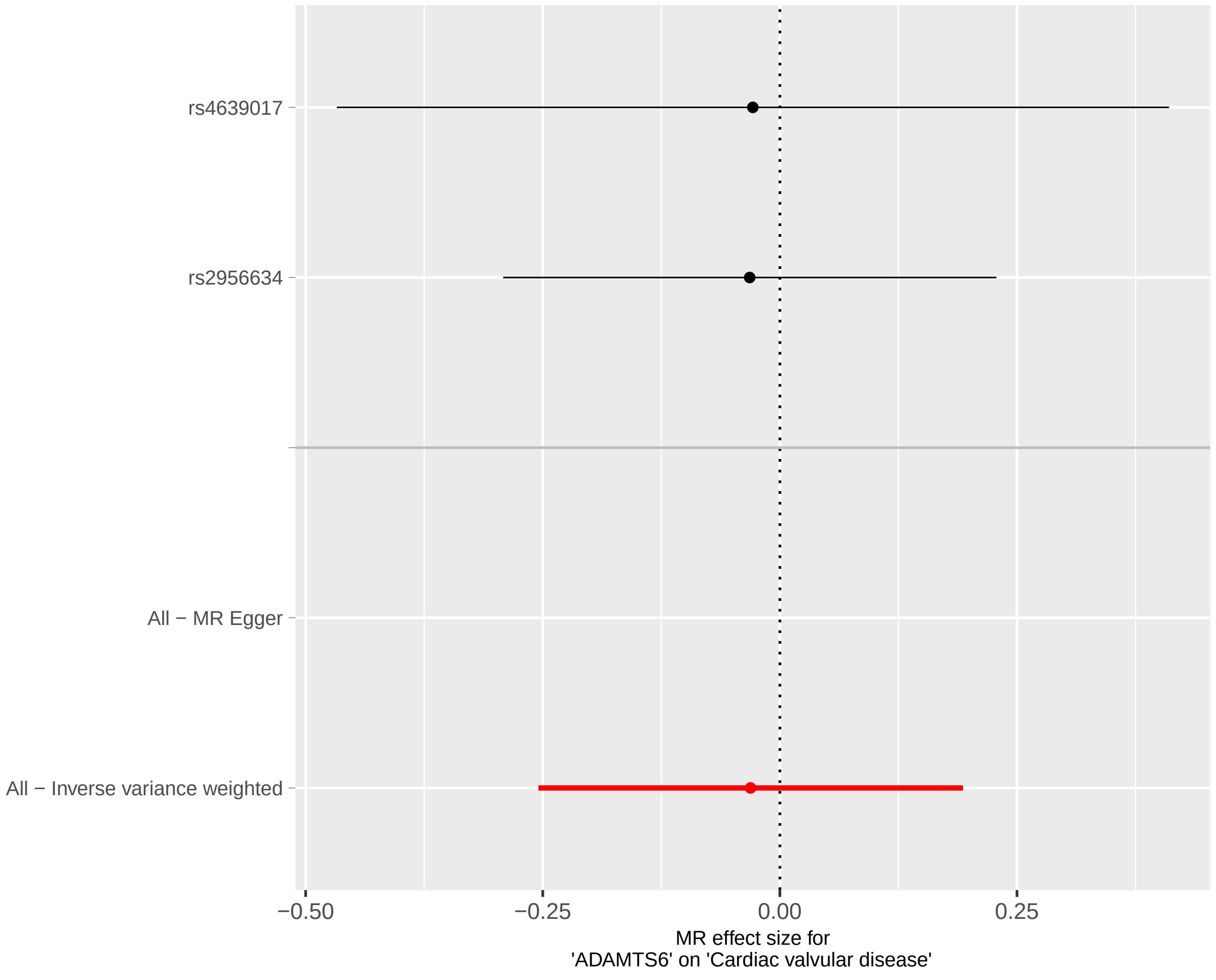
rs4639017
rs2956634
All − MR Egger
All − Inverse variance weighted
−0.50
−0.25
0.00
0.25
MR effect size for
'ADAMTS6' on 'Cardiac valvular disease'

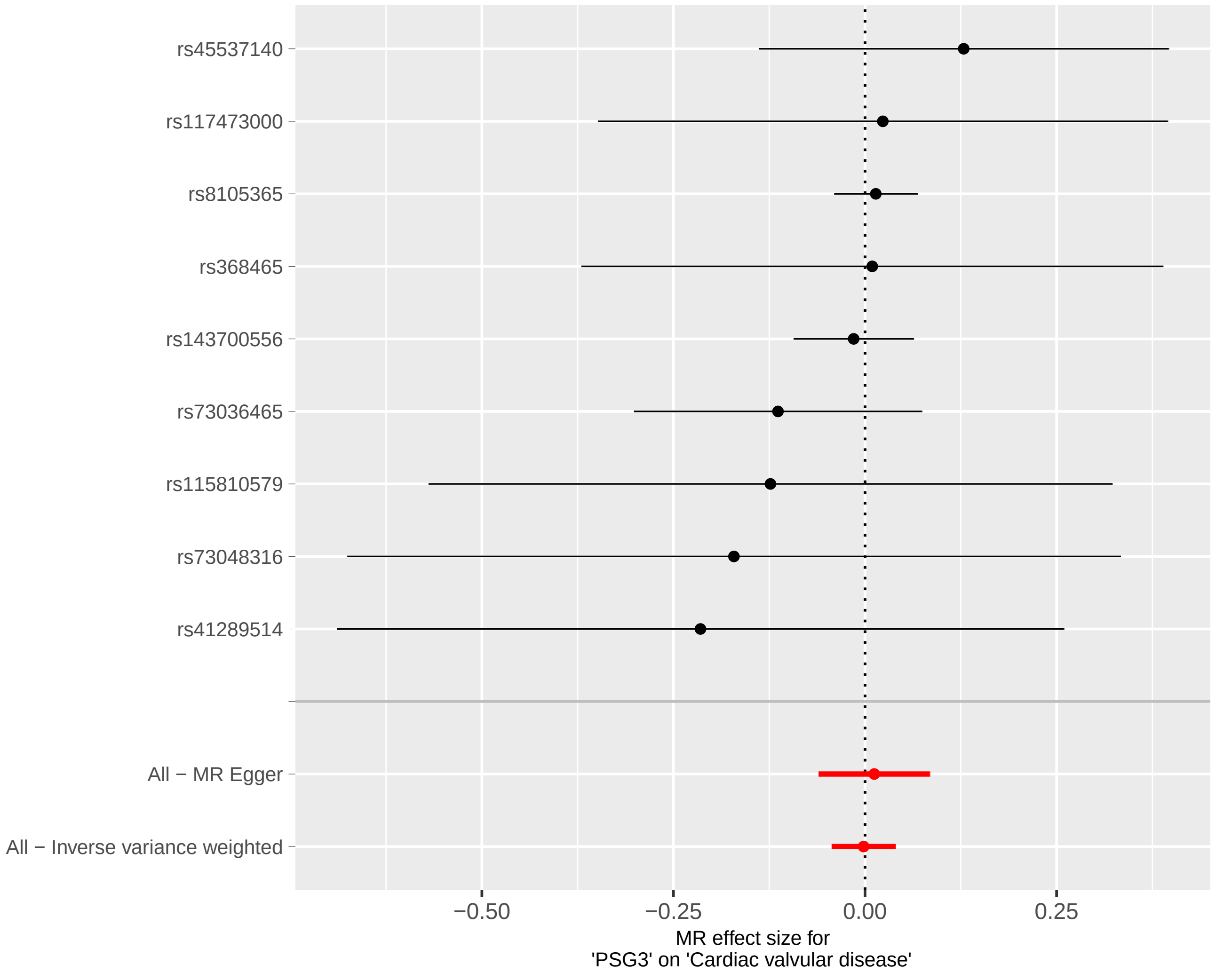
rs45537140
rs117473000
rs8105365
rs368465
rs143700556
rs73036465
rs115810579
rs73048316
rs41289514
All − MR Egger
All − Inverse variance weighted
−0.50
−0.25
0.00
0.25
MR effect size for
'PSG3' on 'Cardiac valvular disease'

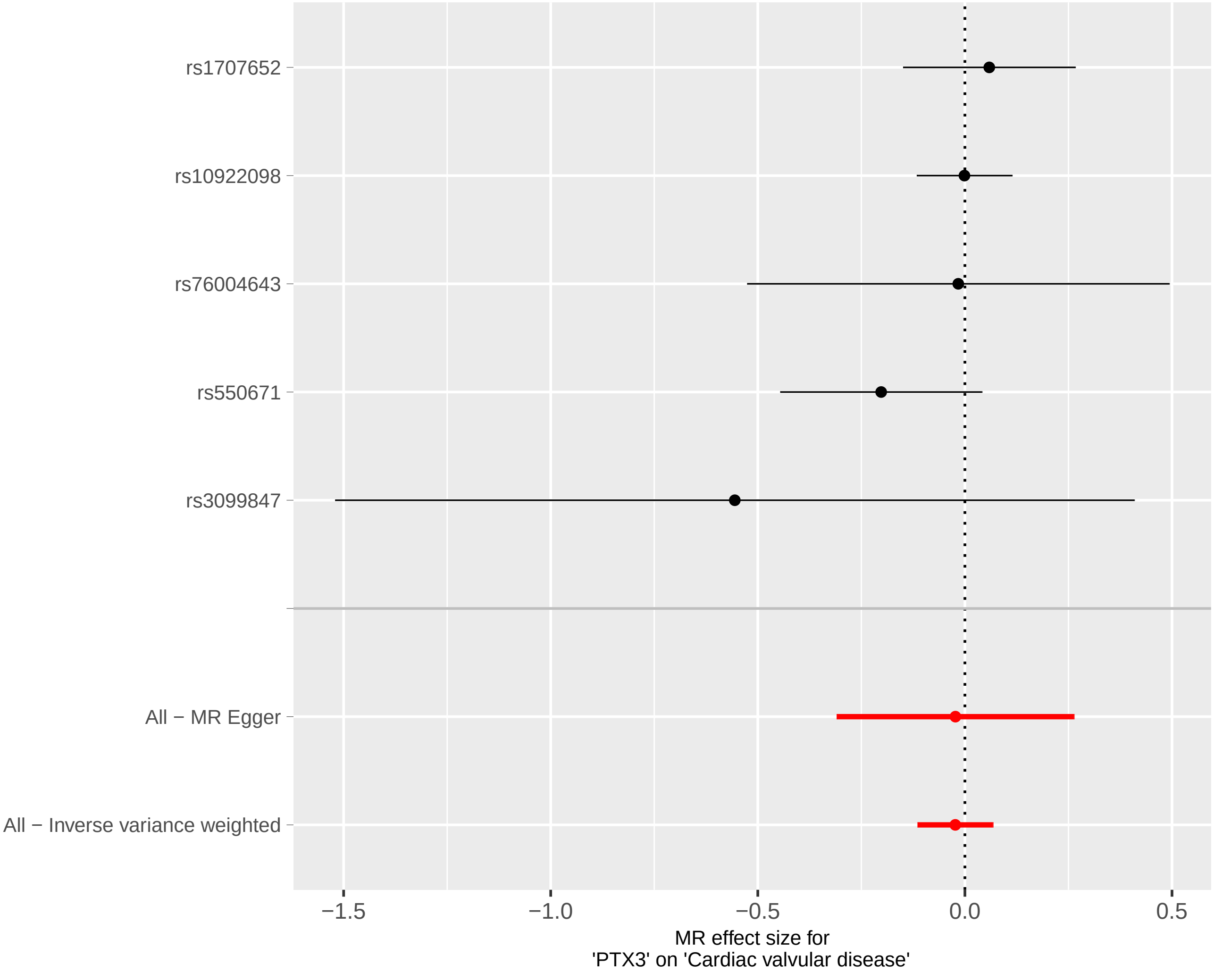

rs1707652
rs10922098
rs76004643
rs550671
rs3099847
All − MR Egger
All − Inverse variance weighted
−1.5
−1.0
−0.5
0.0
0.5
MR effect size for
'PTX3' on 'Cardiac valvular disease'

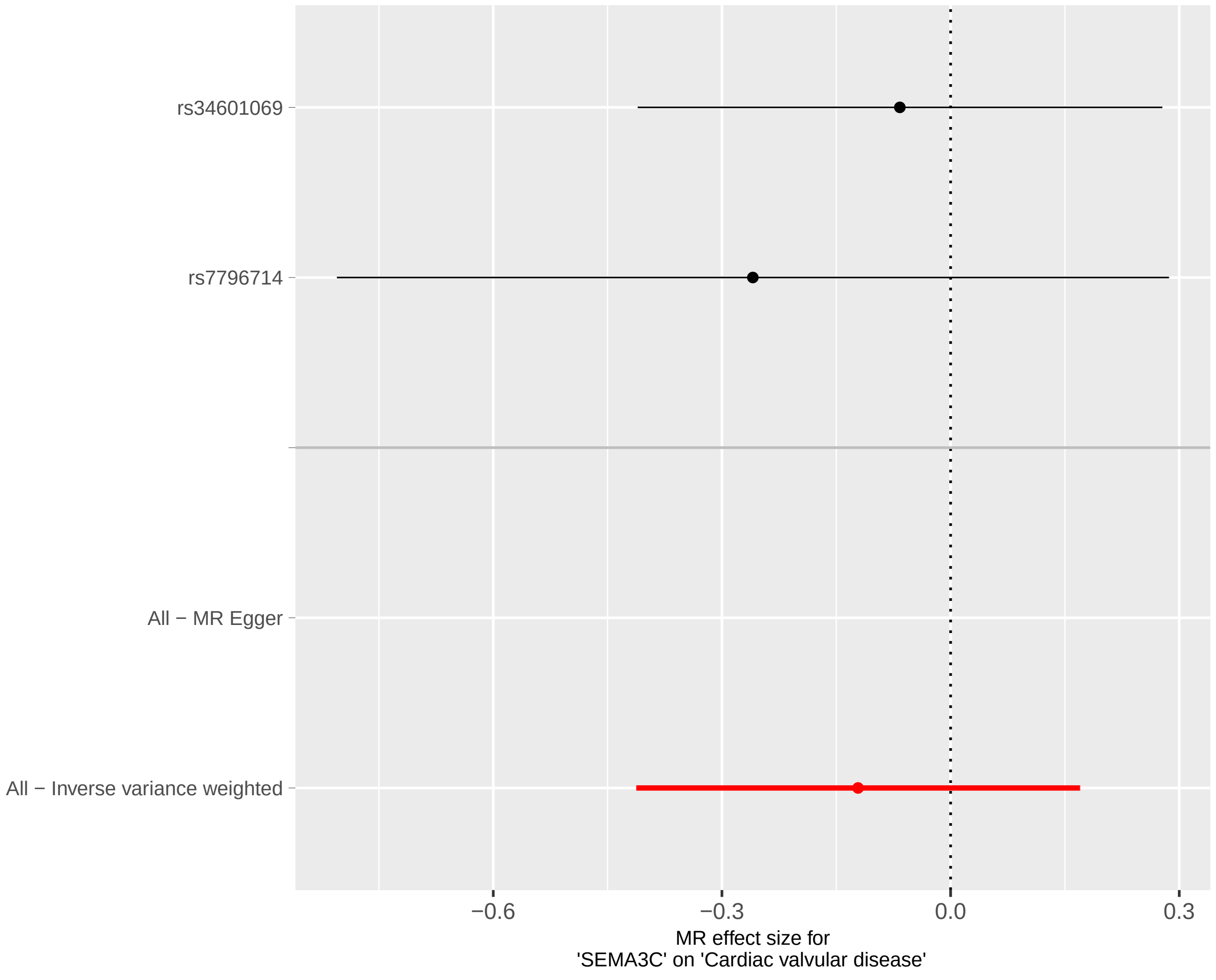
rs34601069
rs7796714
All − MR Egger
All − Inverse variance weighted
−0.6
−0.3
0.0
0.3
MR effect size for
'SEMA3C' on 'Cardiac valvular disease'

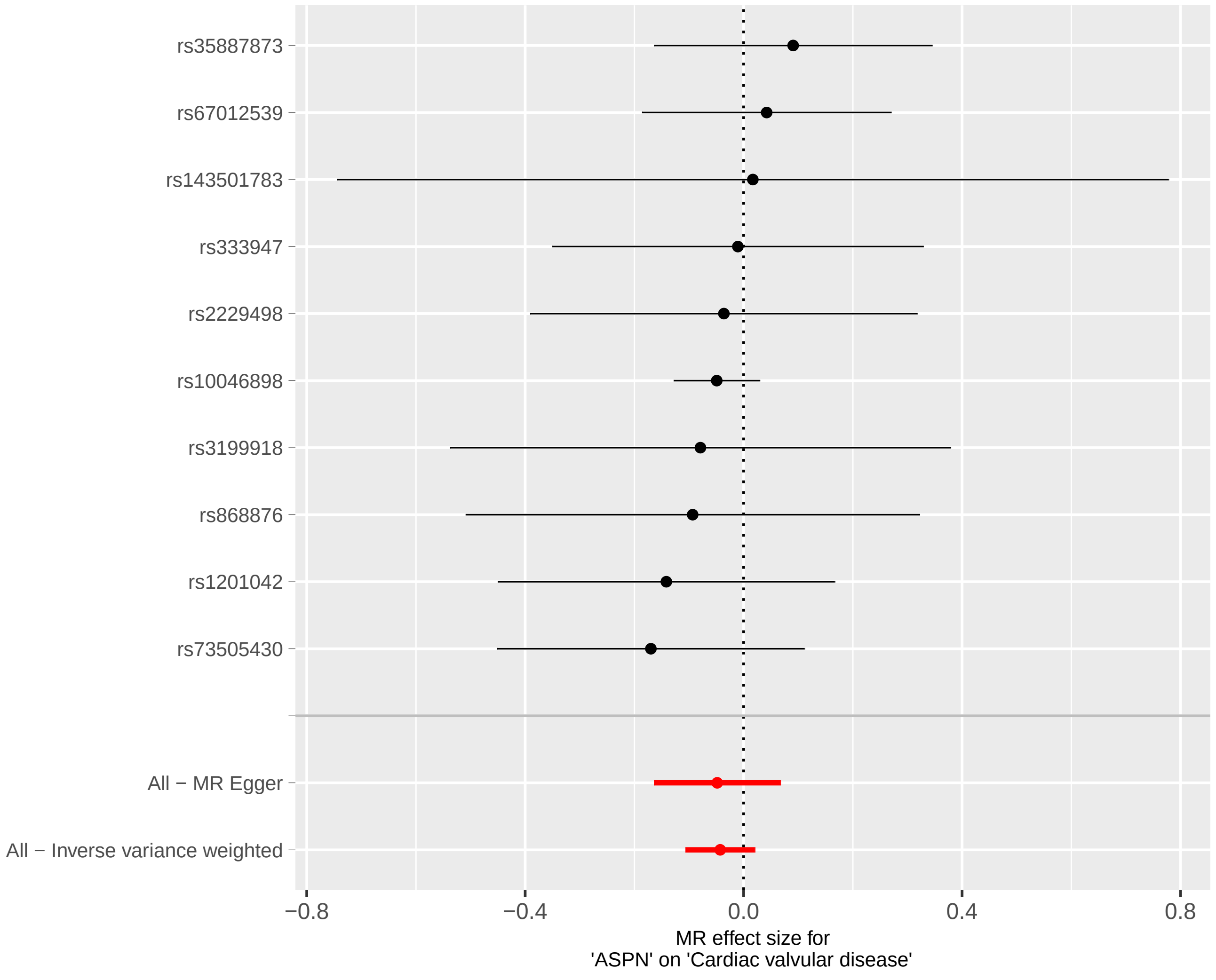
rs35887873
rs67012539
rs143501783
rs333947
rs2229498
rs10046898
rs3199918
rs868876
rs1201042
rs73505430
All − MR Egger
All − Inverse variance weighted
−0.8
−0.4
0.0
0.4
0.8
MR effect size for
'ASPN' on 'Cardiac valvular disease'

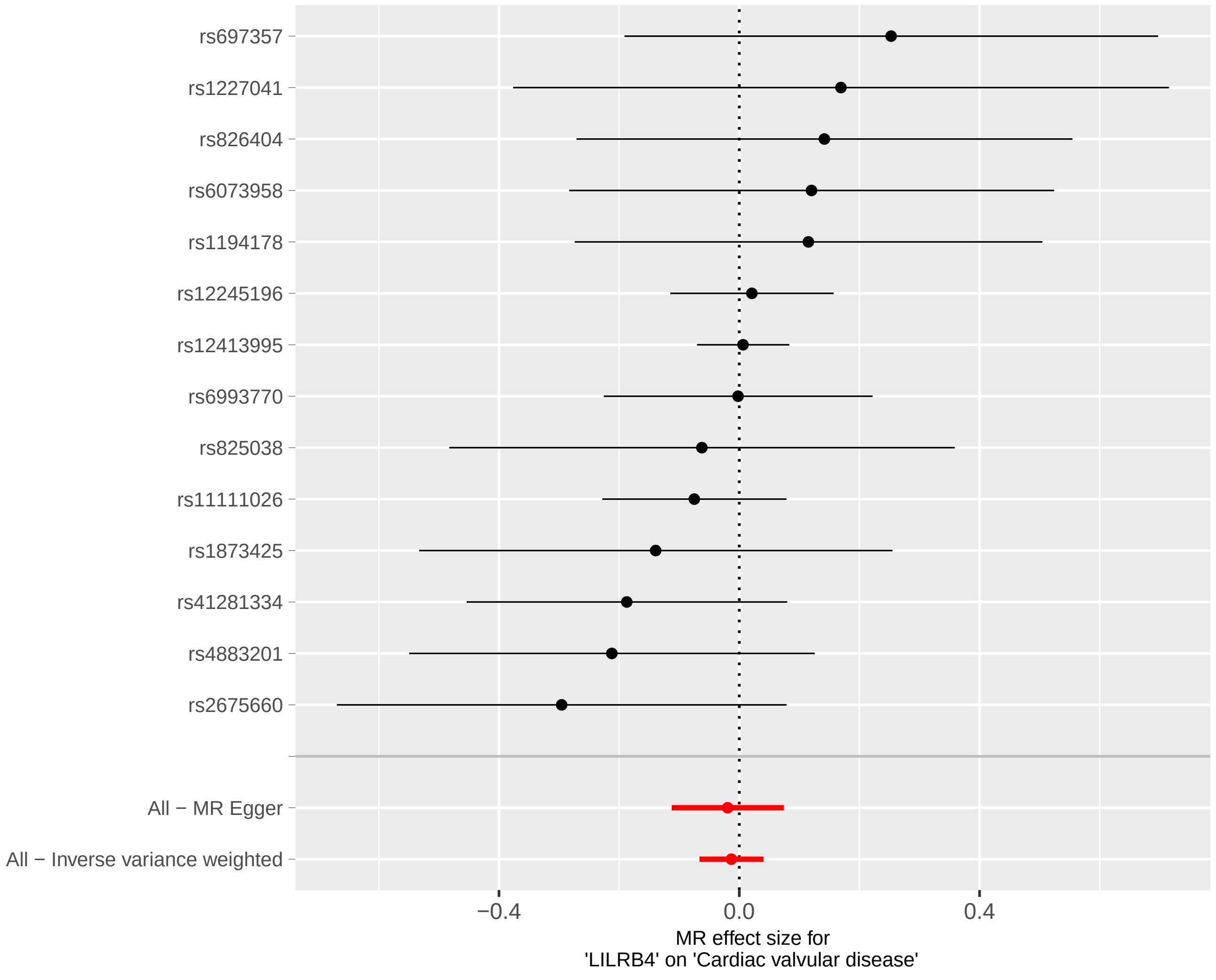

rs697357
rs1227041
rs826404
rs6073958
rs1194178
rs12245196
rs12413995
rs6993770
rs825038
rs11111026
rs1873425
rs41281334
rs4883201
rs2675660
All − MR Egger
All − Inverse variance weighted
−0.4
0.0
0.4
MR effect size for
'LILRB4' on 'Cardiac valvular disease'

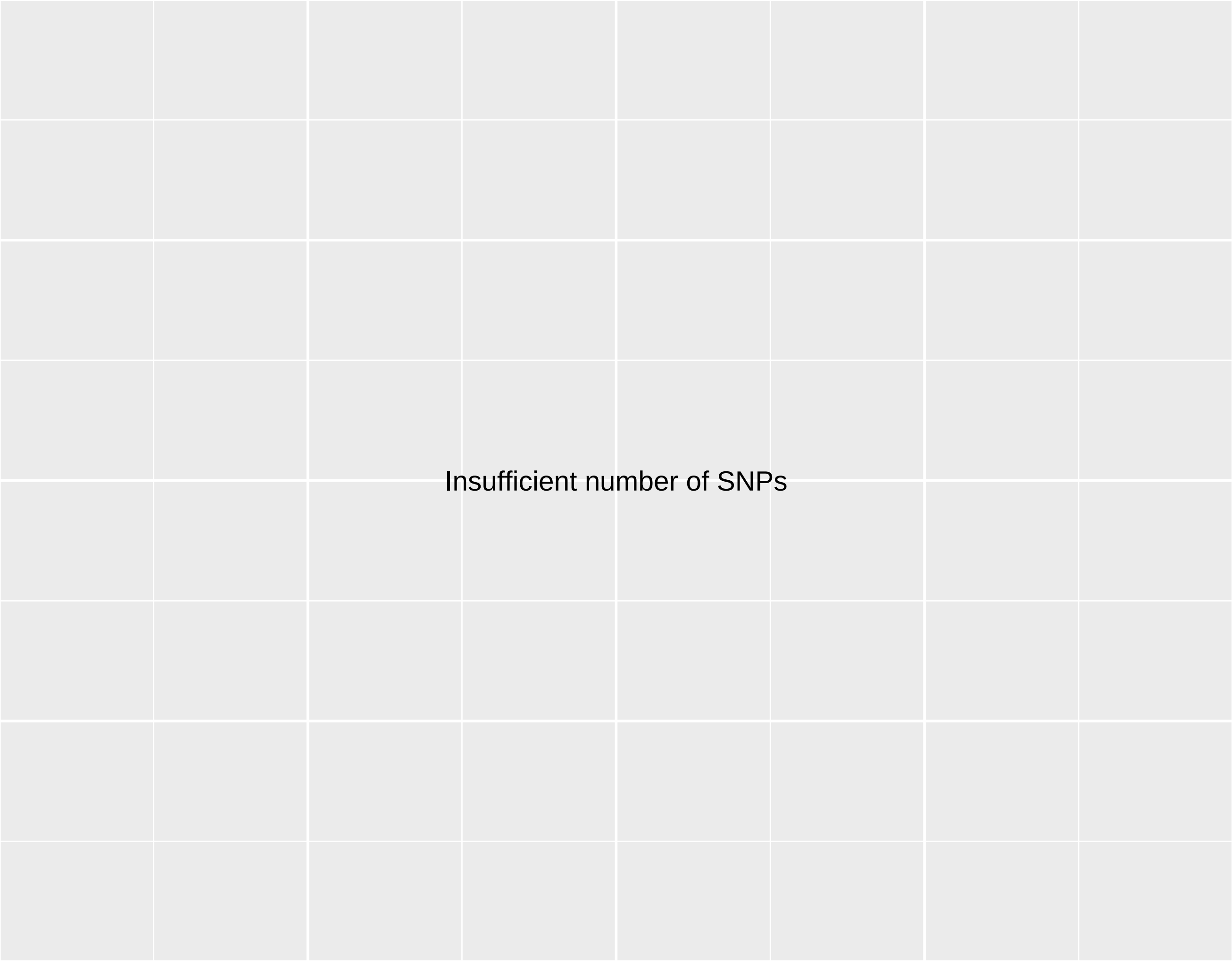
Insufficient number of SNPs

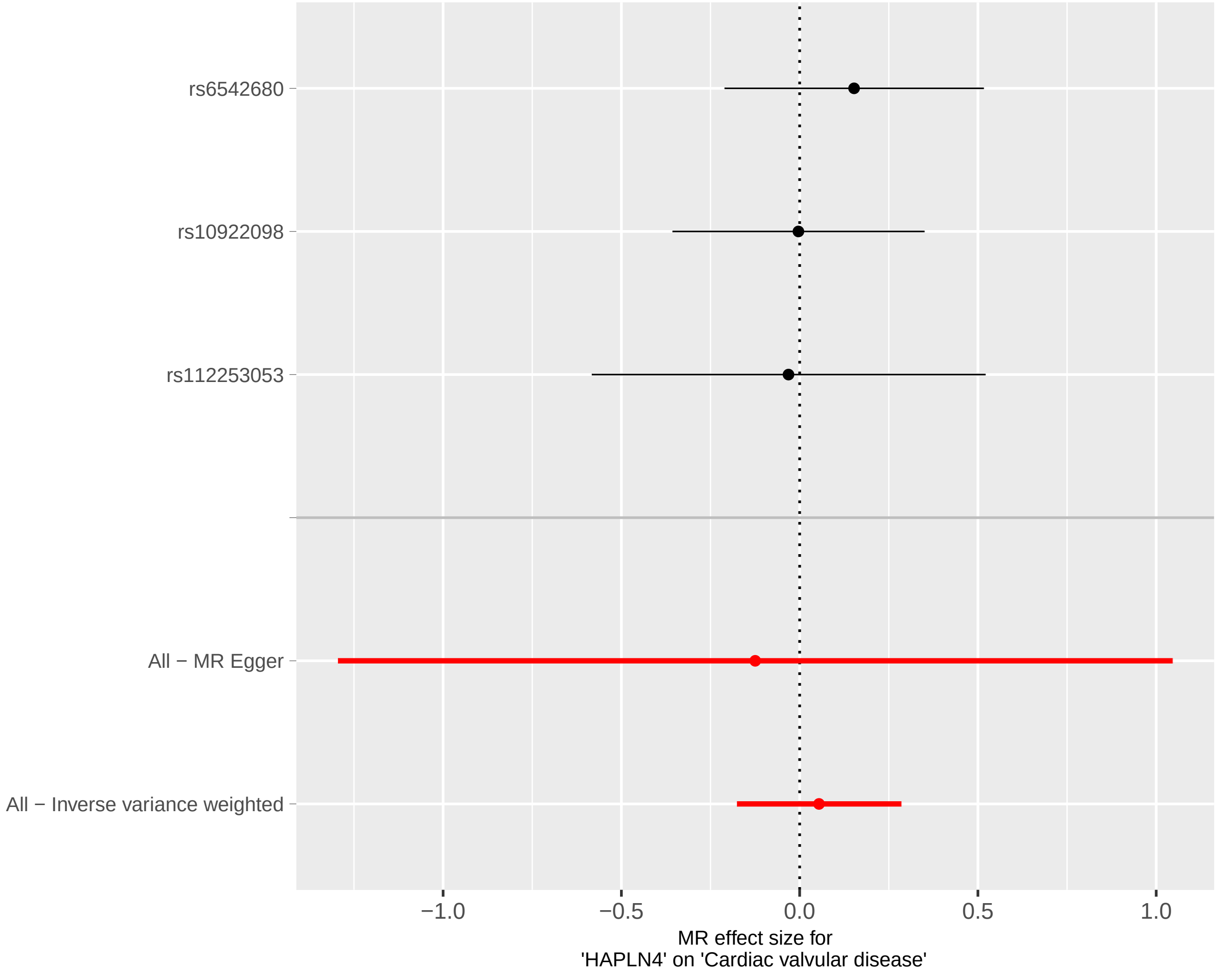
rs6542680
rs10922098
rs112253053
All − MR Egger
All − Inverse variance weighted
−1.0
−0.5
0.0
0.5
1.0
MR effect size for
'HAPLN4' on 'Cardiac valvular disease'

Insufficient number of SNPs

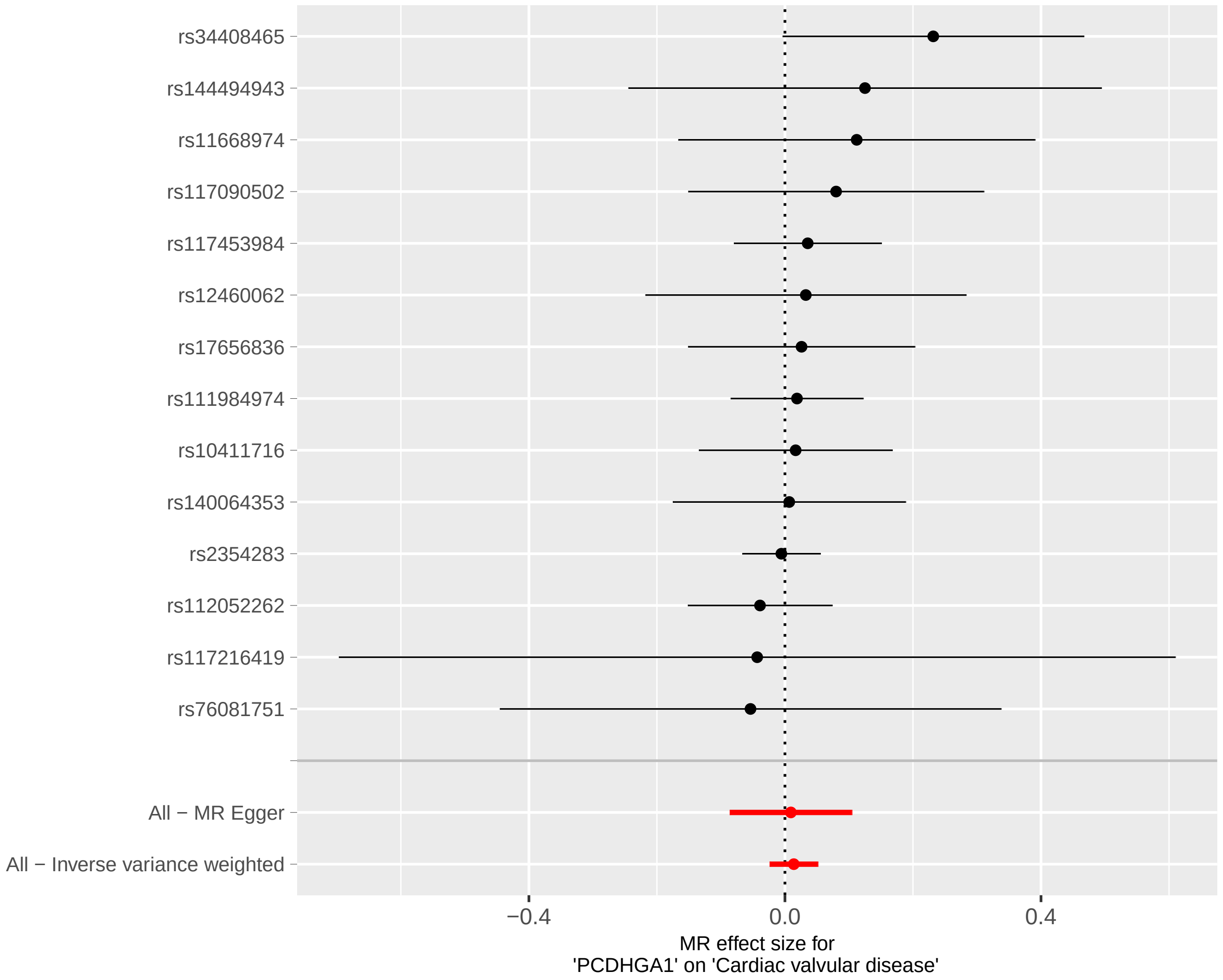
rs34408465
rs144494943
rs11668974
rs117090502
rs117453984
rs12460062
rs17656836
rs111984974
rs10411716
rs140064353
rs2354283
rs112052262
rs117216419
rs76081751
All − MR Egger
All − Inverse variance weighted
−0.4
0.0
0.4
MR effect size for
'PCDHGA1' on 'Cardiac valvular disease'

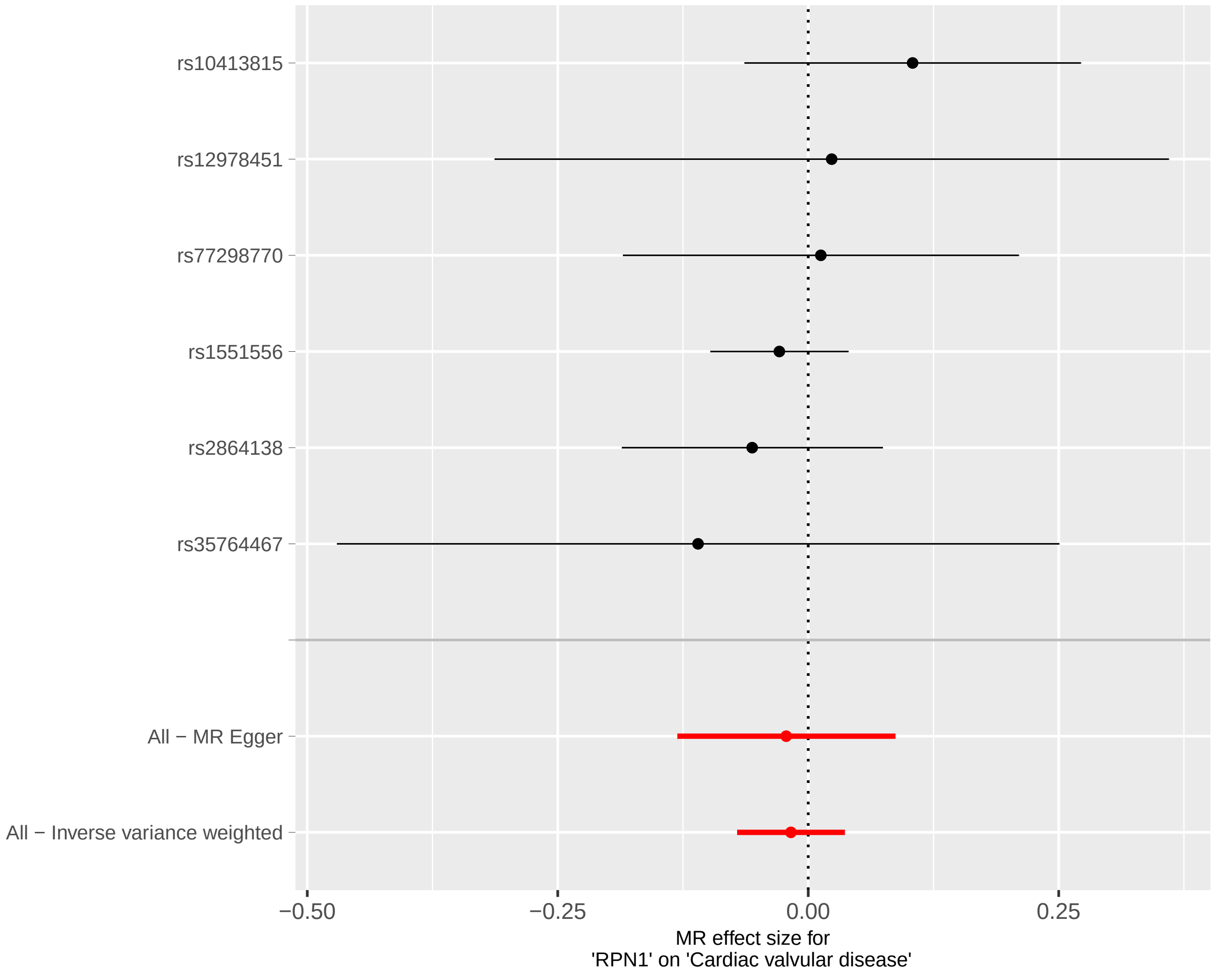
rs10413815
rs12978451
rs77298770
rs1551556
rs2864138
rs35764467
All − MR Egger
All − Inverse variance weighted
−0.50
−0.25
0.00
0.25
MR effect size for
'RPN1' on 'Cardiac valvular disease'

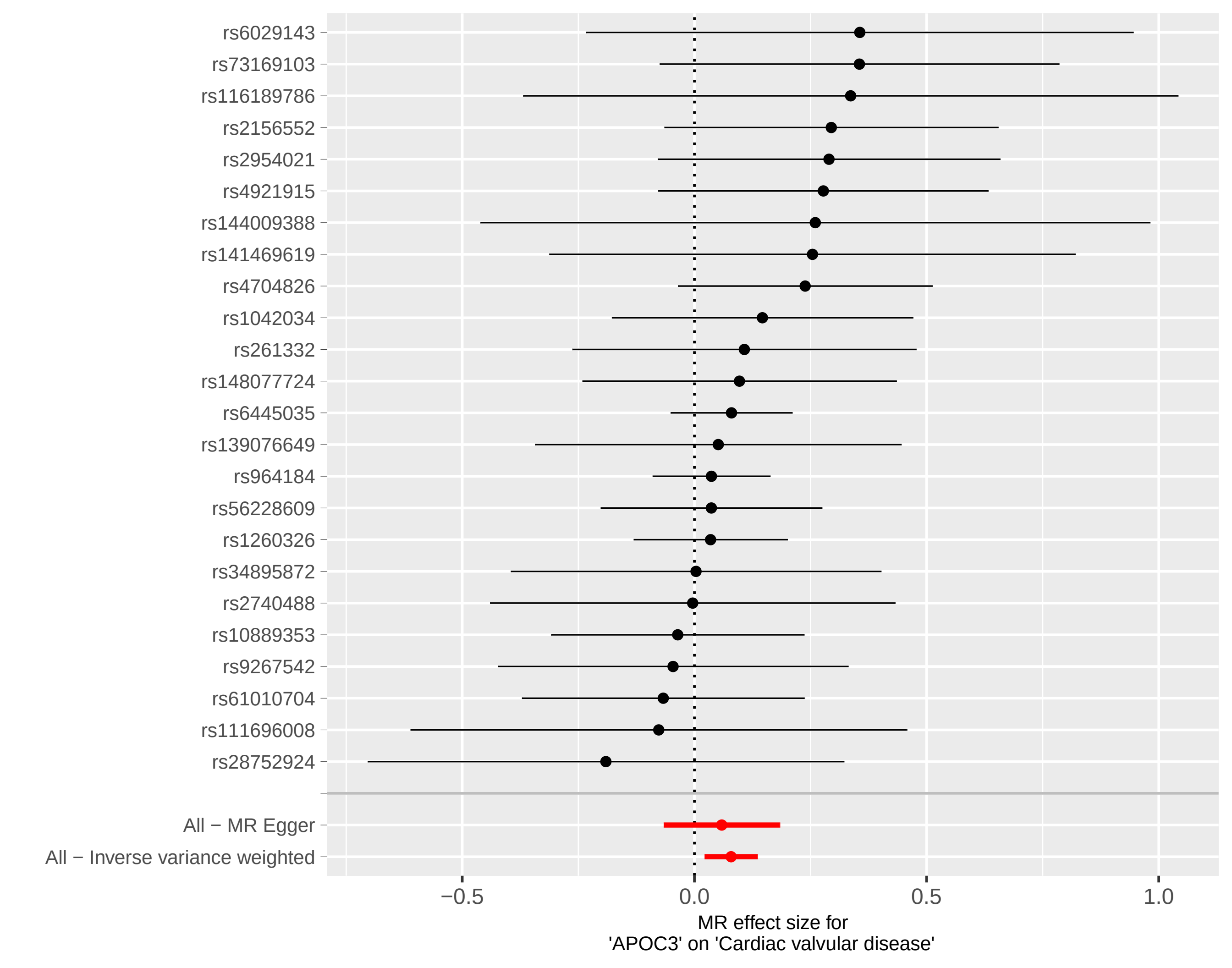
rs6029143
rs73169103
rs116189786
rs2156552
rs2954021
rs4921915
rs144009388
rs141469619
rs4704826
rs1042034
rs261332
rs148077724
rs6445035
rs139076649
rs964184
rs56228609
rs1260326
rs34895872
rs2740488
rs10889353
rs9267542
rs61010704
rs111696008
rs28752924
All − MR Egger
All − Inverse variance weighted
−0.5
0.0
0.5
1.0
MR effect size for
'APOC3' on 'Cardiac valvular disease'

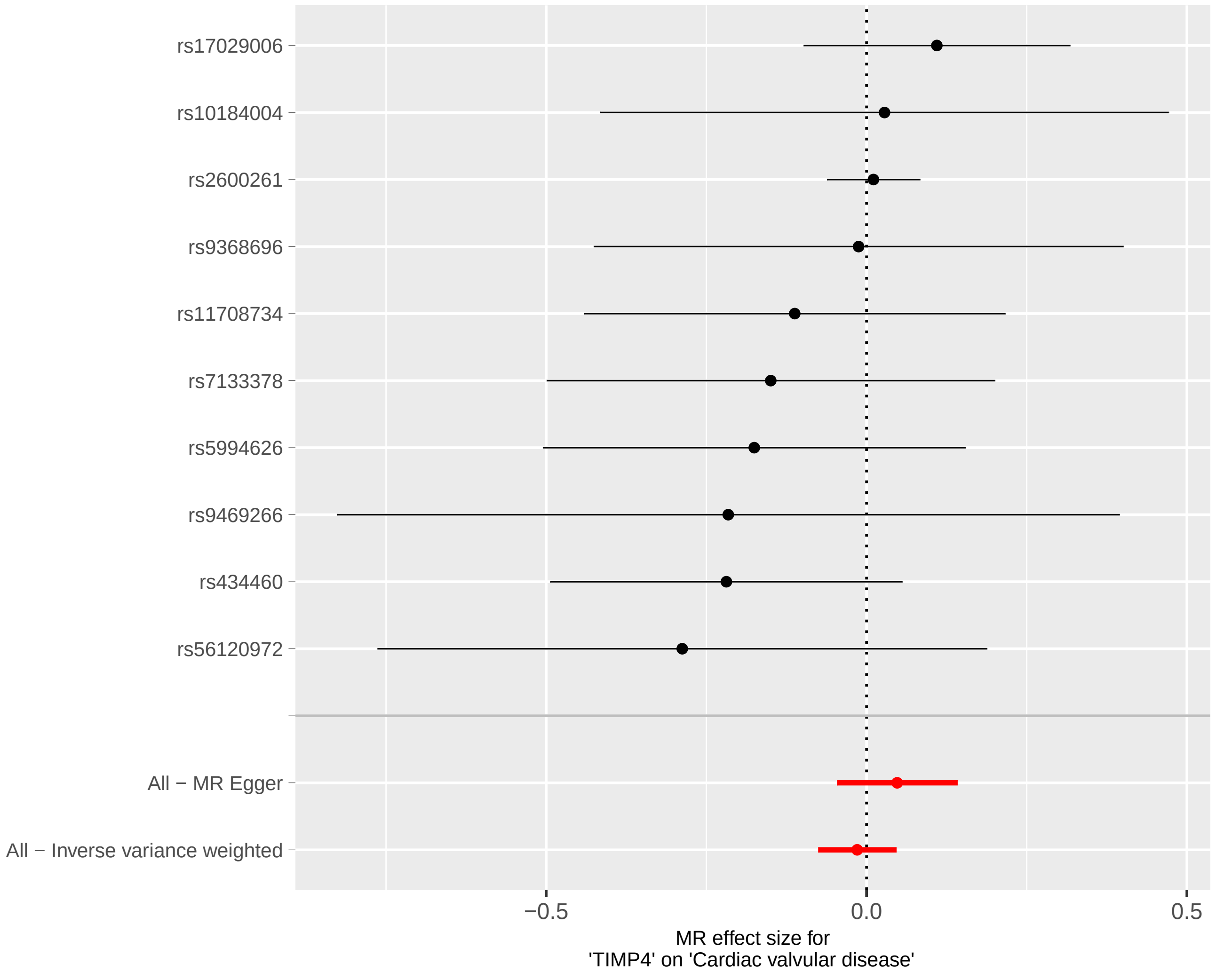
rs17029006
rs10184004
rs2600261
rs9368696
rs11708734
rs7133378
rs5994626
rs9469266
rs434460
rs56120972
All − MR Egger
All − Inverse variance weighted
−0.5
0.0
0.5
MR effect size for
'TIMP4' on 'Cardiac valvular disease'

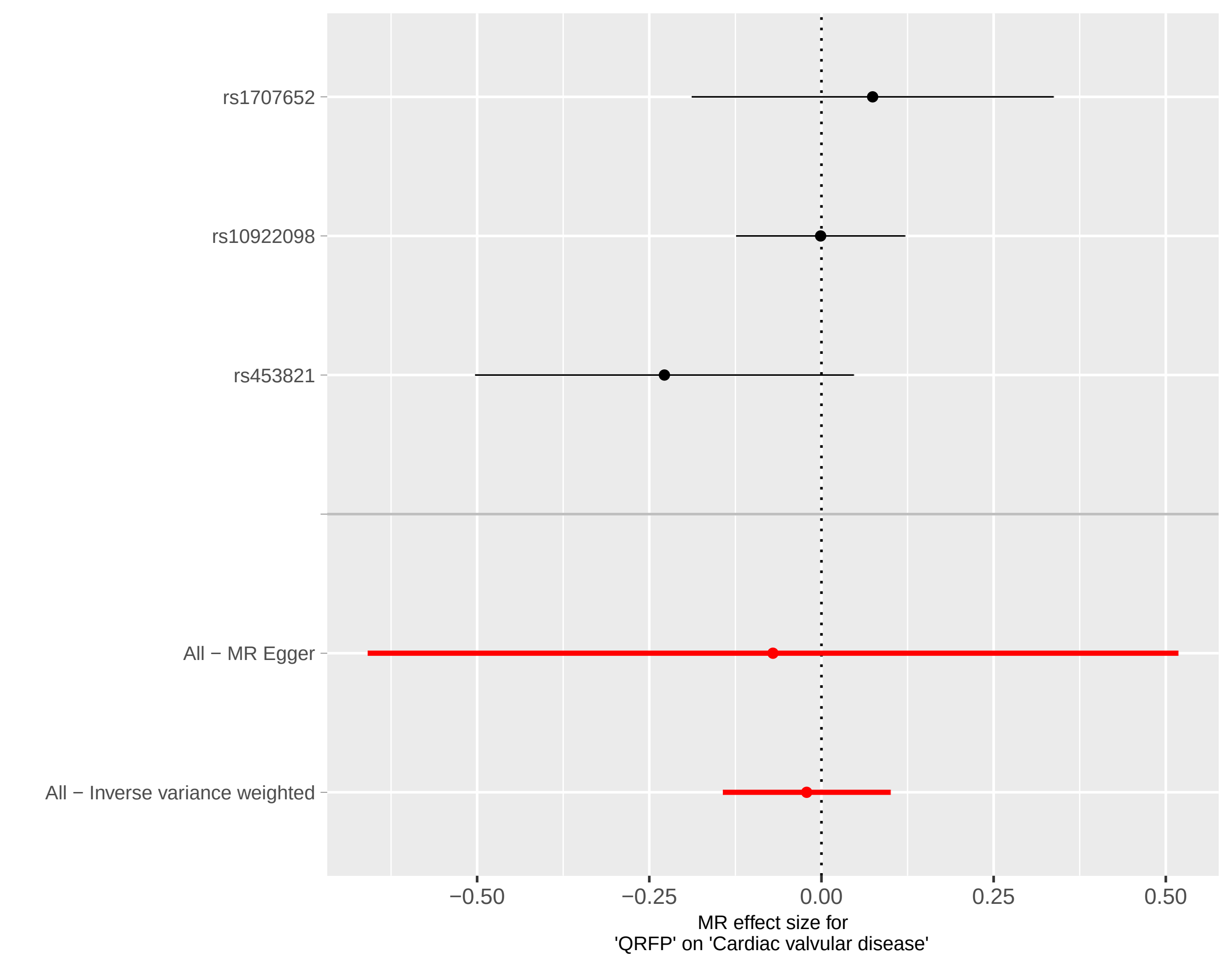
rs1707652
rs10922098
rs453821
All − MR Egger
All − Inverse variance weighted
−0.50
−0.25
0.00
0.25
0.50
MR effect size for
'QRFP' on 'Cardiac valvular disease'

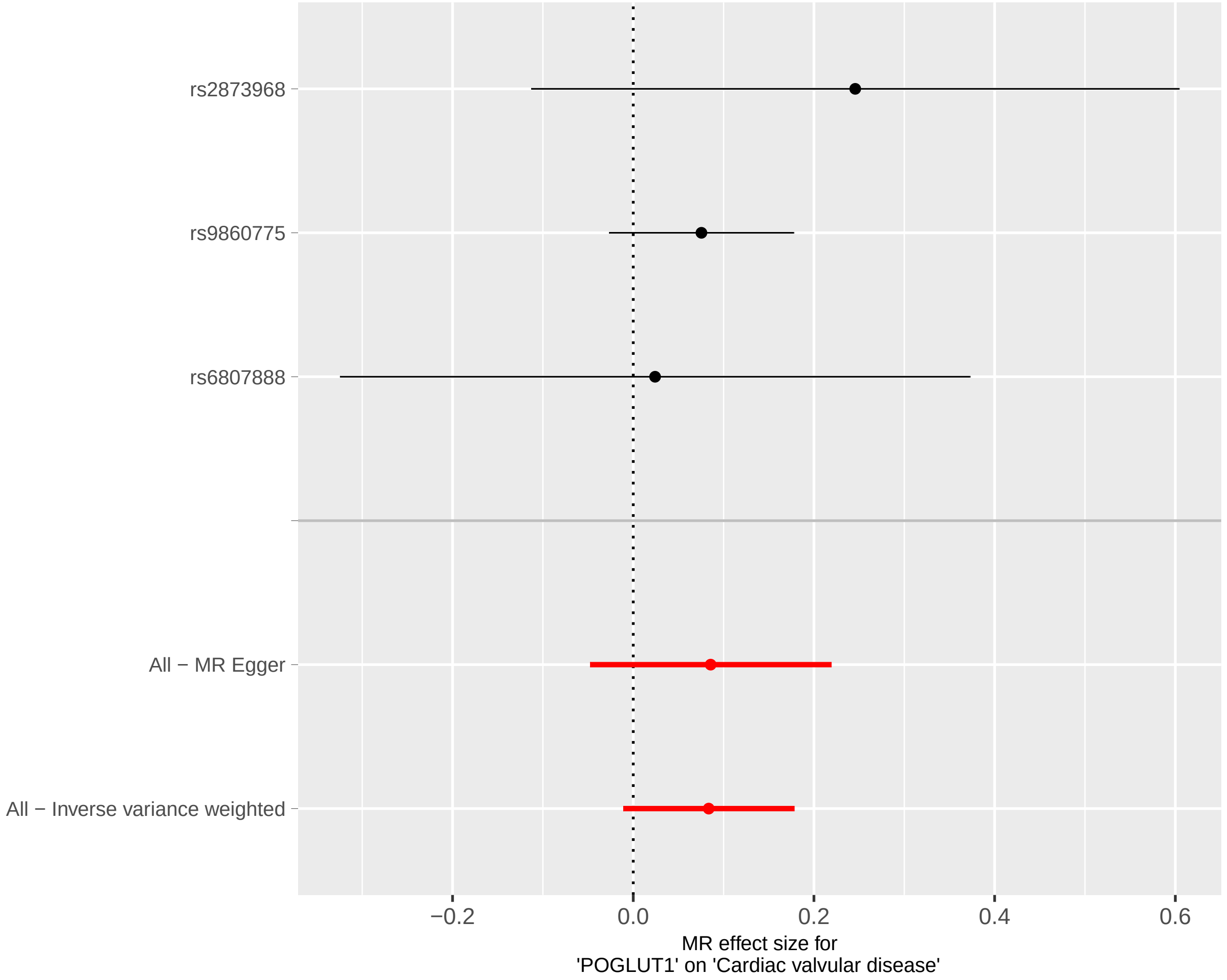

rs2873968
rs9860775
rs6807888
All − MR Egger
All − Inverse variance weighted
−0.2
0.0
0.2
0.4
0.6
MR effect size for
'POGLUT1' on 'Cardiac valvular disease'

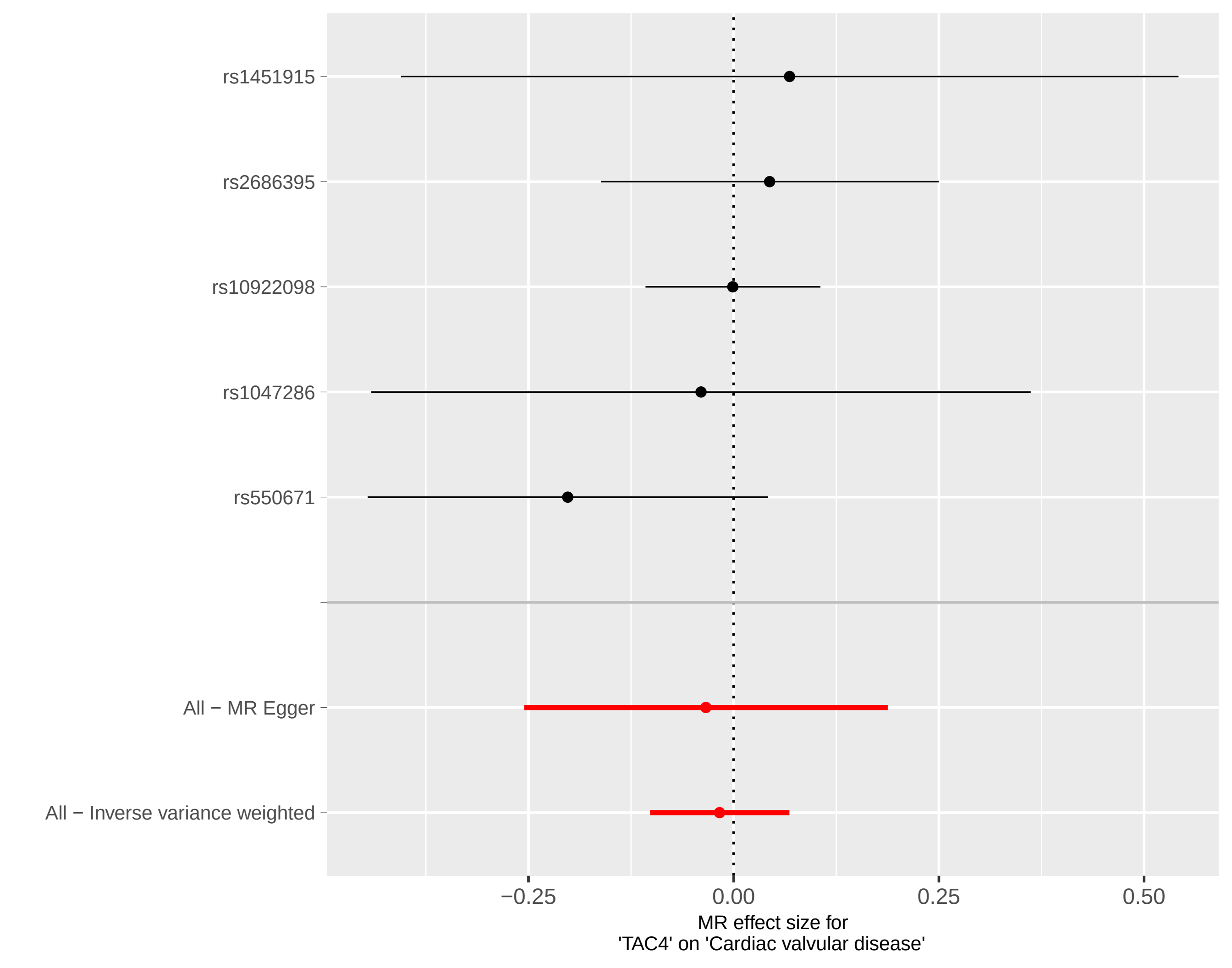
rs1451915
rs2686395
rs10922098
rs1047286
rs550671
All − MR Egger
All − Inverse variance weighted
−0.25
0.00
0.25
0.50
MR effect size for
'TAC4' on 'Cardiac valvular disease'

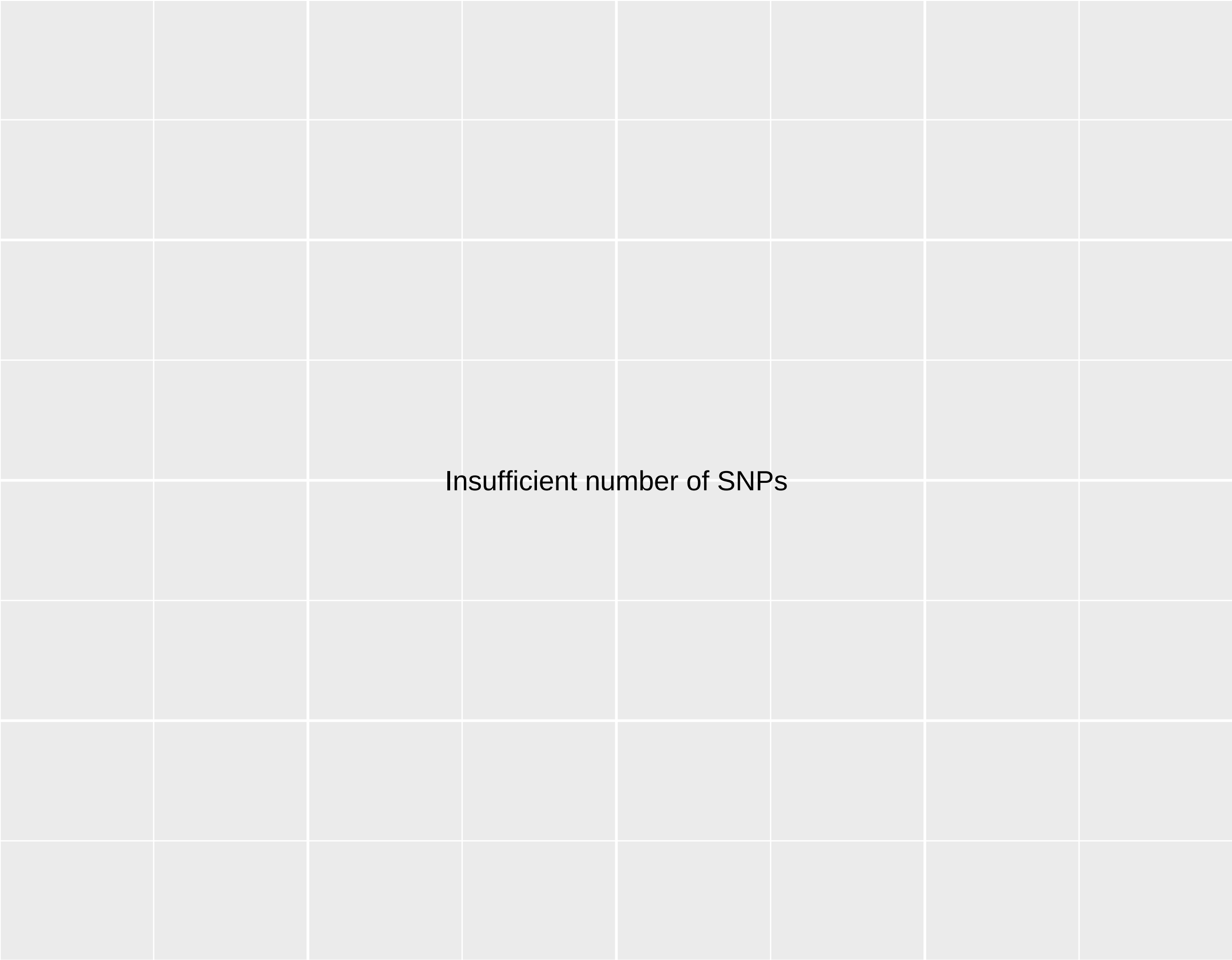
Insufficient number of SNPs

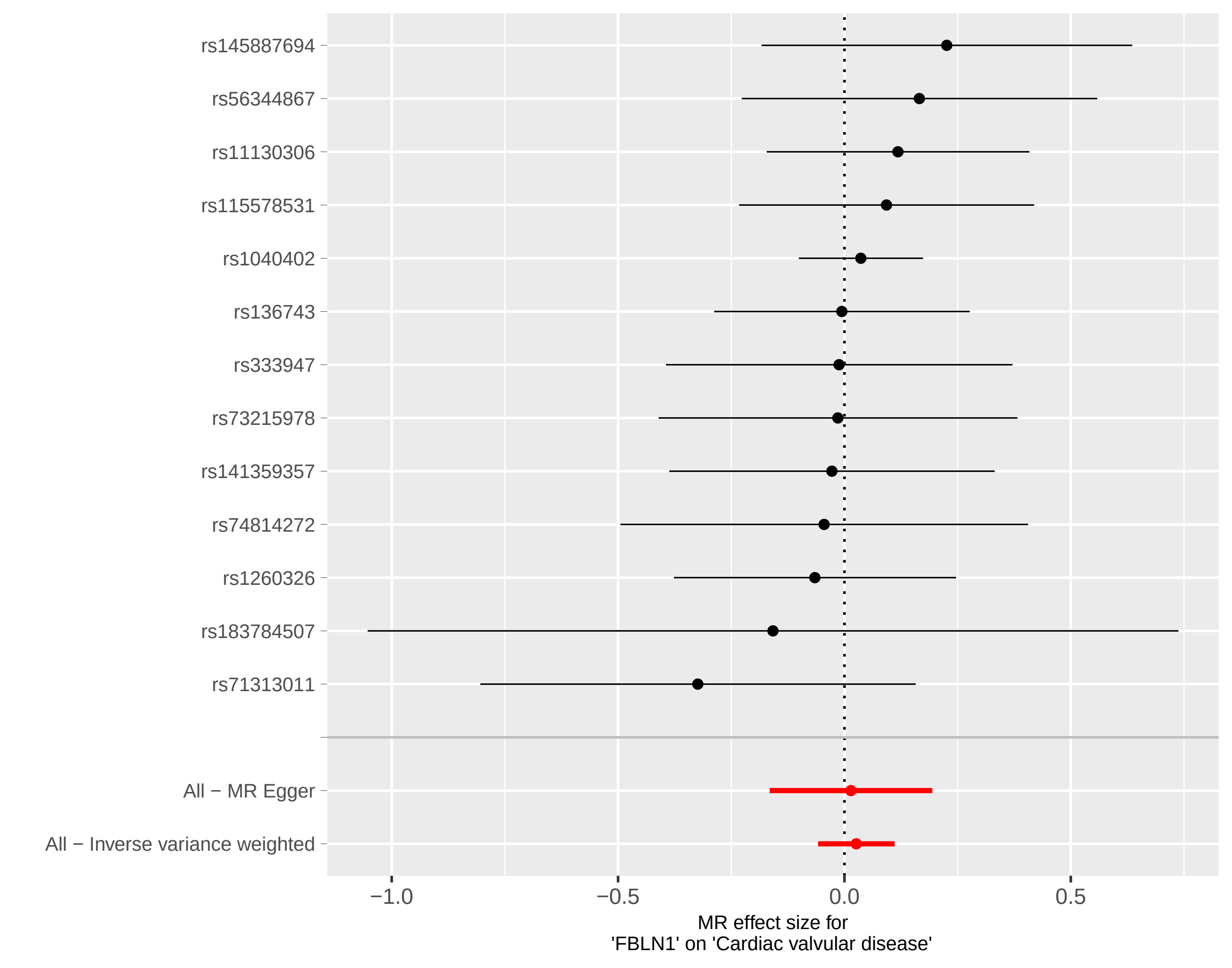

rs145887694
rs56344867
rs11130306
rs115578531
rs1040402
rs136743
rs333947
rs73215978
rs141359357
rs74814272
rs1260326
rs183784507
rs71313011
All − MR Egger
All − Inverse variance weighted
−1.0
−0.5
0.0
0.5
MR effect size for
'FBLN1' on 'Cardiac valvular disease'

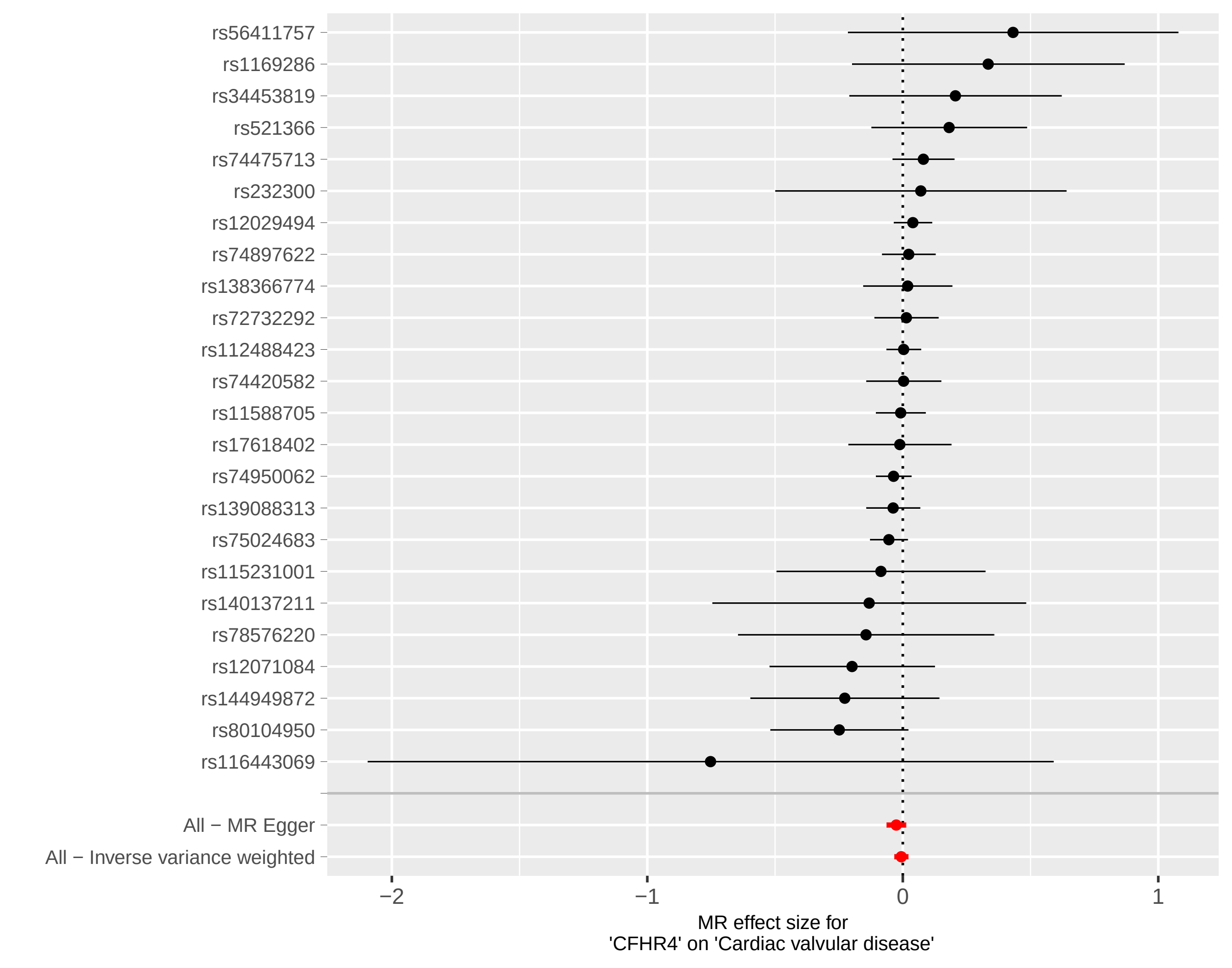
rs56411757
rs1169286
rs34453819
rs521366
rs74475713
rs232300
rs12029494
rs74897622
rs138366774
rs72732292
rs112488423
rs74420582
rs11588705
rs17618402
rs74950062
rs139088313
rs75024683
rs115231001
rs140137211
rs78576220
rs12071084
rs144949872
rs80104950
rs116443069
All − MR Egger
All − Inverse variance weighted
−2
−1
0
1
MR effect size for
'CFHR4' on 'Cardiac valvular disease'

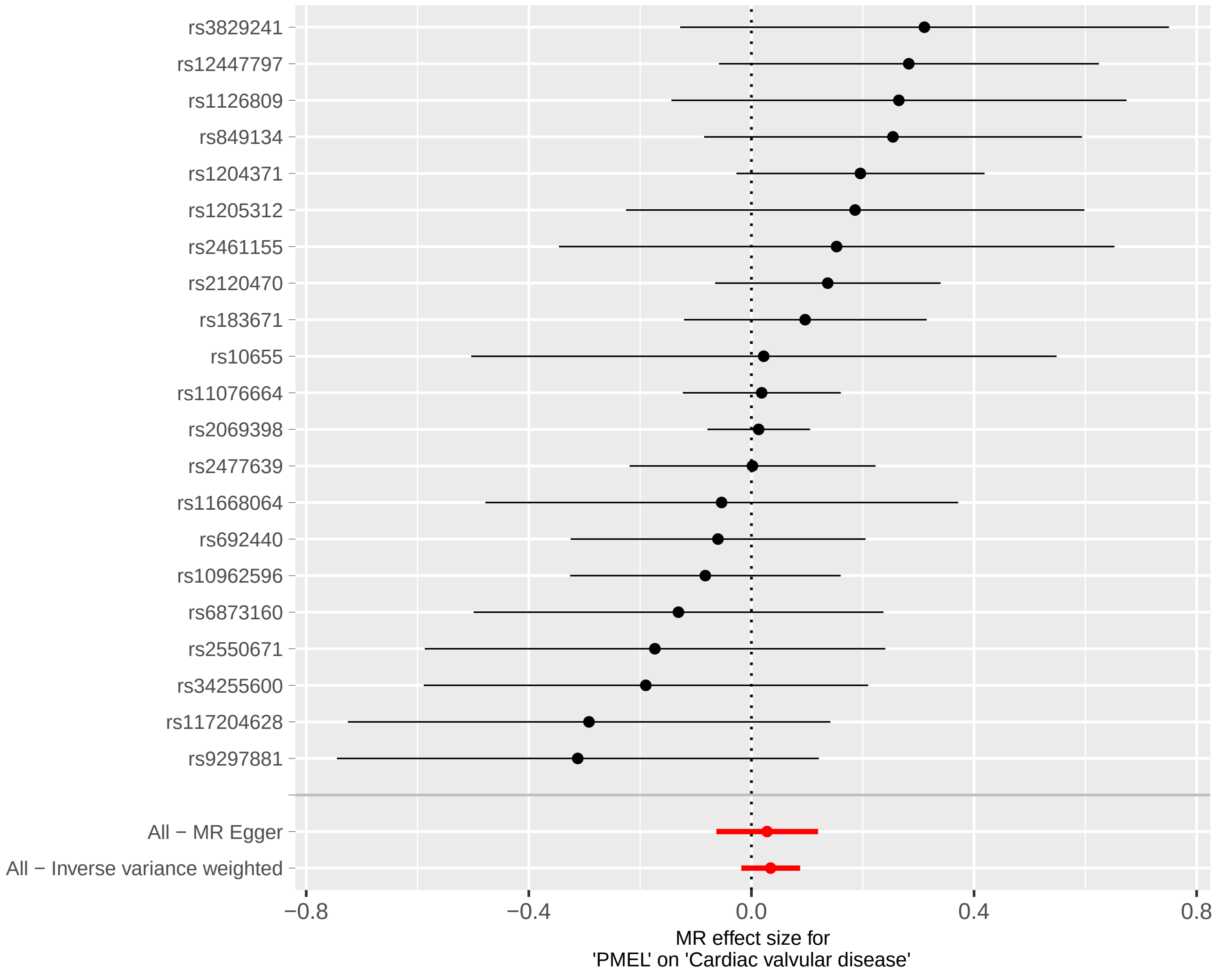
rs3829241
rs12447797
rs1126809
rs849134
rs1204371
rs1205312
rs2461155
rs2120470
rs183671
rs10655
rs11076664
rs2069398
rs2477639
rs11668064
rs692440
rs10962596
rs6873160
rs2550671
rs34255600
rs117204628
rs9297881
All − MR Egger
All − Inverse variance weighted
−0.8
−0.4
0.0
0.4
0.8
MR effect size for
'PMEL' on 'Cardiac valvular disease'

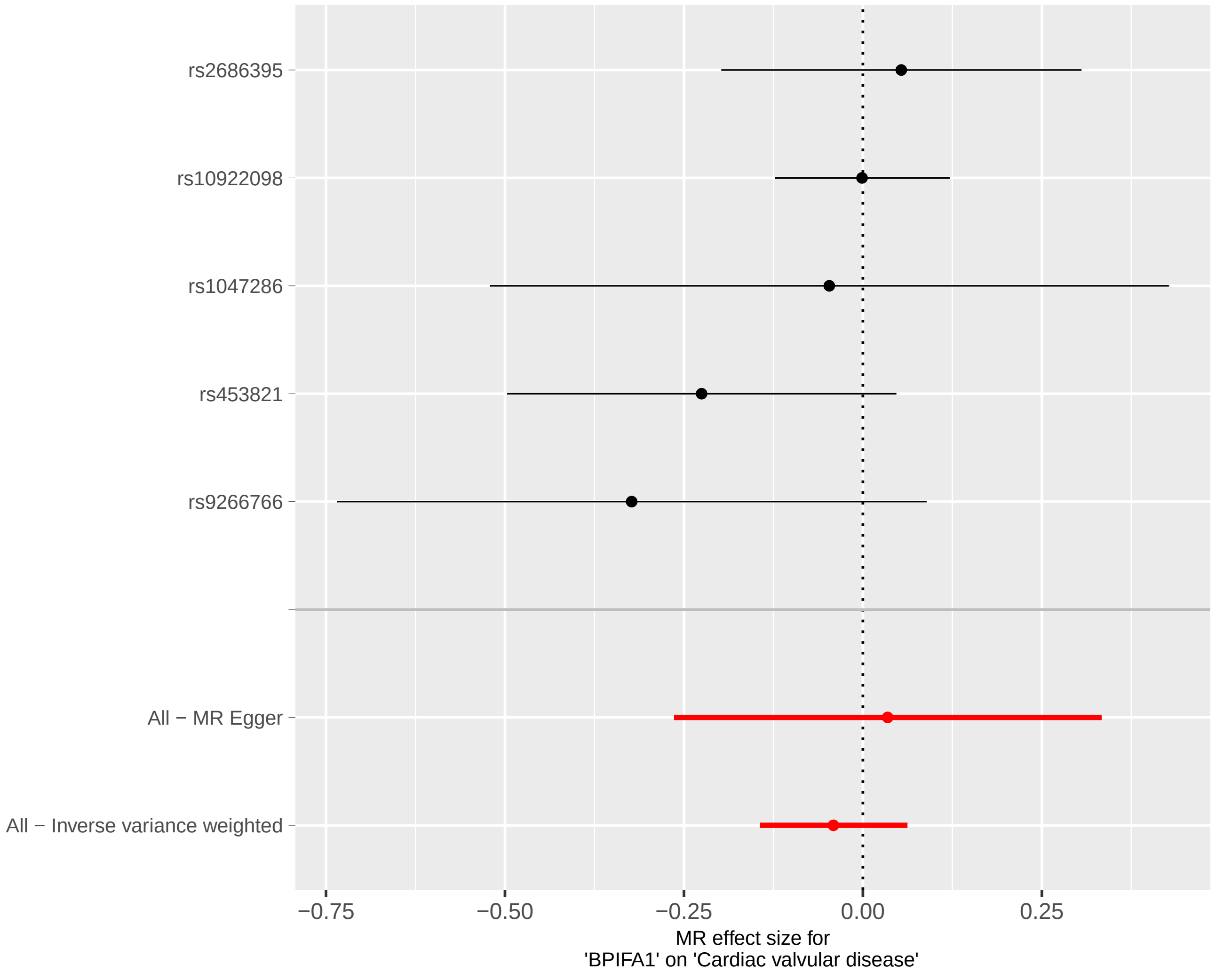

rs2686395
rs10922098
rs1047286
rs453821
rs9266766
All − MR Egger
All − Inverse variance weighted
−0.75
−0.50
−0.25
0.00
0.25
MR effect size for
'BPIFA1' on 'Cardiac valvular disease'

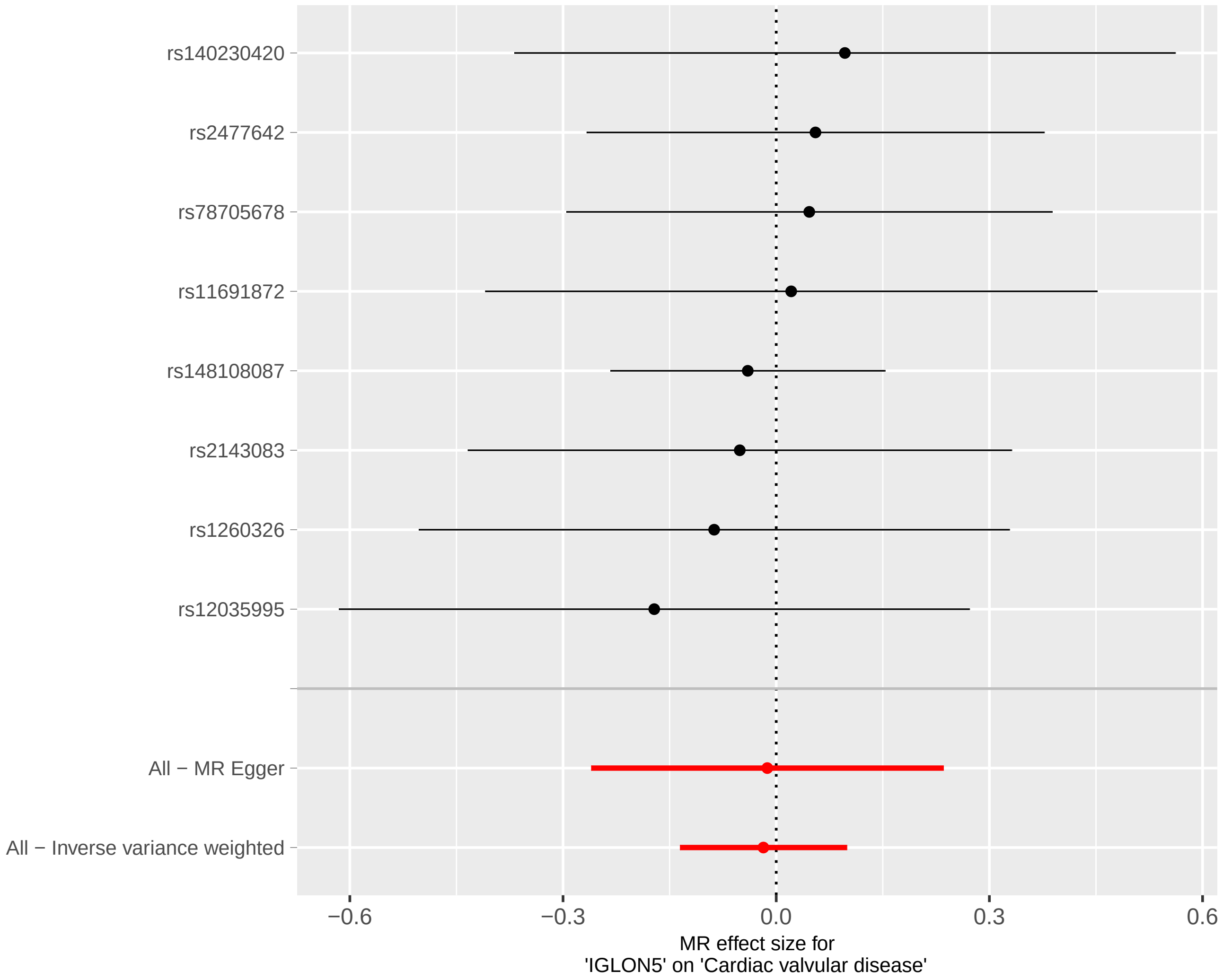
rs140230420
rs2477642
rs78705678
rs11691872
rs148108087
rs2143083
rs1260326
rs12035995
All − MR Egger
All − Inverse variance weighted
−0.6
−0.3
0.0
0.3
0.6
MR effect size for
'IGLON5' on 'Cardiac valvular disease'

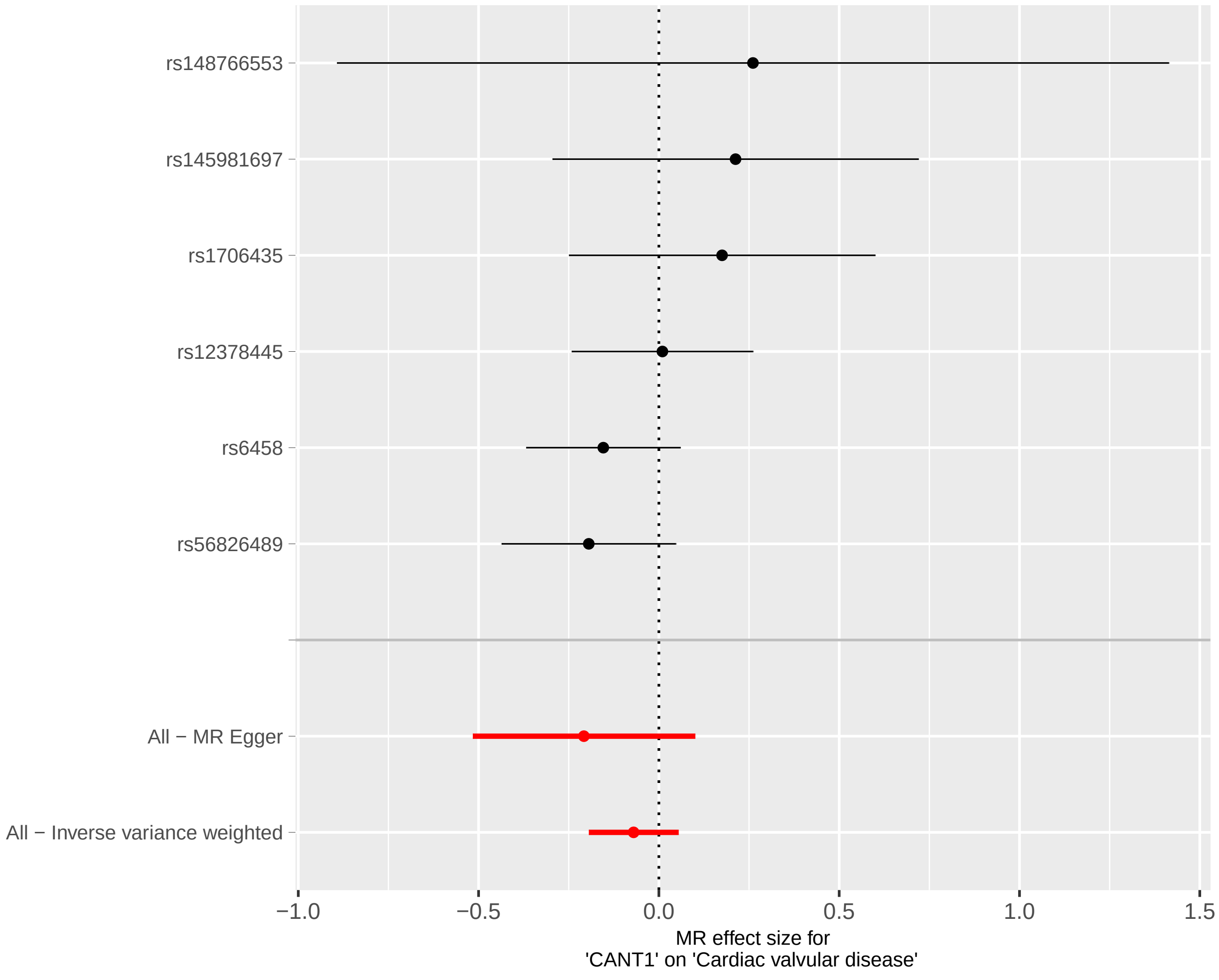
rs148766553
rs145981697
rs1706435
rs12378445
rs6458
rs56826489
All − MR Egger
All − Inverse variance weighted
−1.0
−0.5
0.0
0.5
1.0
1.5
MR effect size for
'CANT1' on 'Cardiac valvular disease'

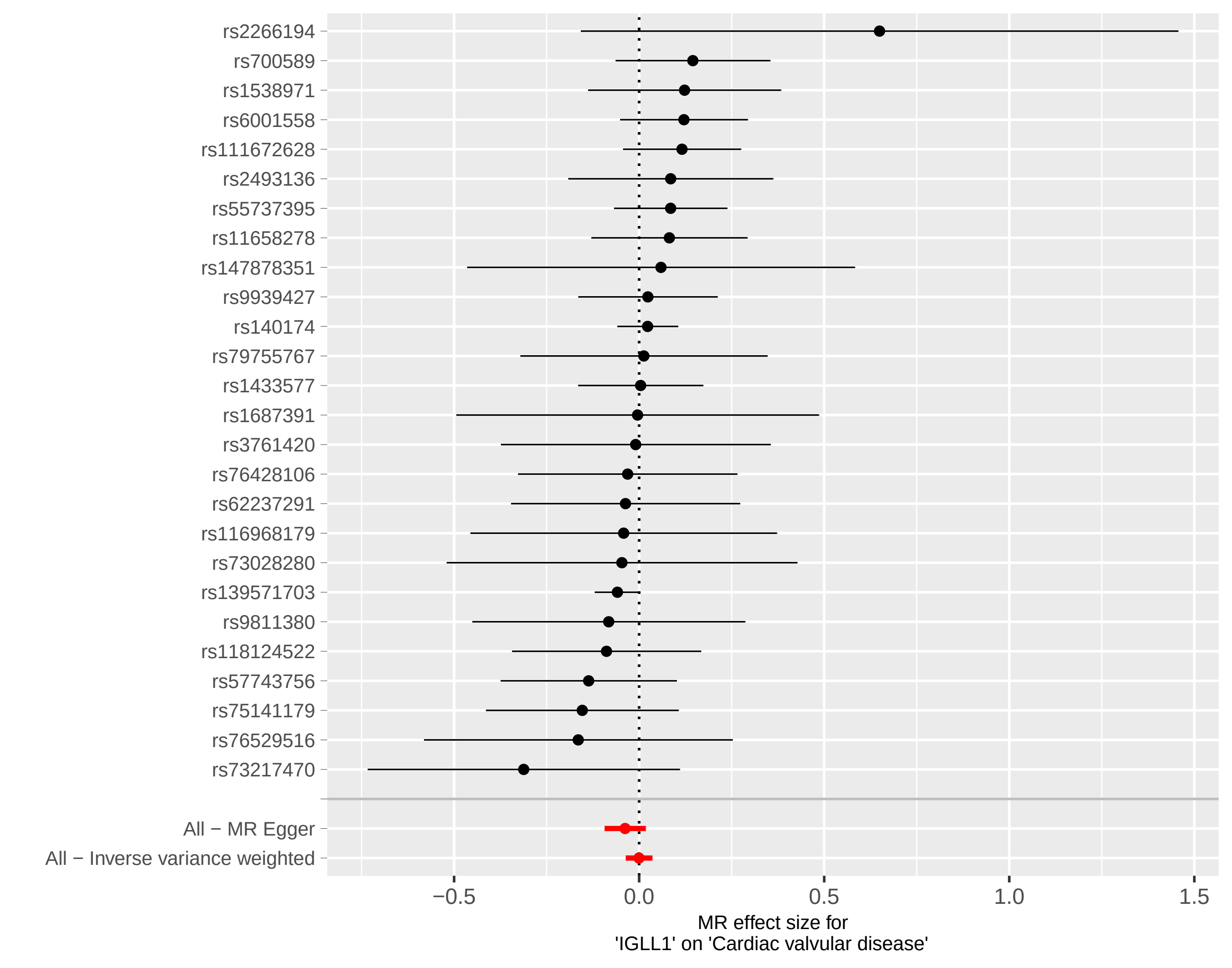
rs2266194
rs700589
rs1538971
rs6001558
rs111672628
rs2493136
rs55737395
rs11658278
rs147878351
rs9939427
rs140174
rs79755767
rs1433577
rs1687391
rs3761420
rs76428106
rs62237291
rs116968179
rs73028280
rs139571703
rs9811380
rs118124522
rs57743756
rs75141179
rs76529516
rs73217470
All − MR Egger
All − Inverse variance weighted
−0.5
0.0
0.5
1.0
1.5
MR effect size for
'IGLL1' on 'Cardiac valvular disease'

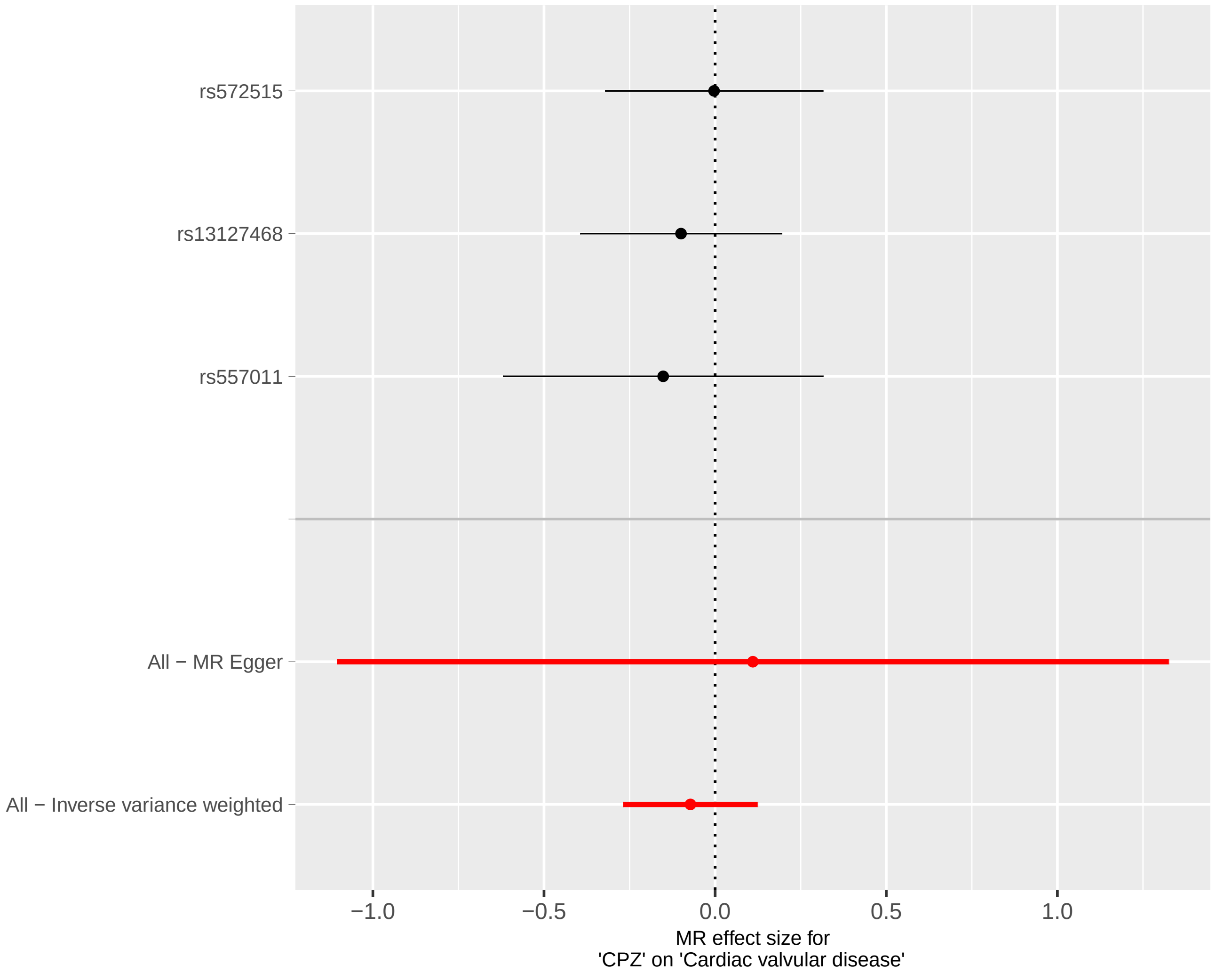

rs572515
rs13127468
rs557011
All − MR Egger
All − Inverse variance weighted
−1.0
−0.5
0.0
0.5
1.0
MR effect size for
'CPZ' on 'Cardiac valvular disease'

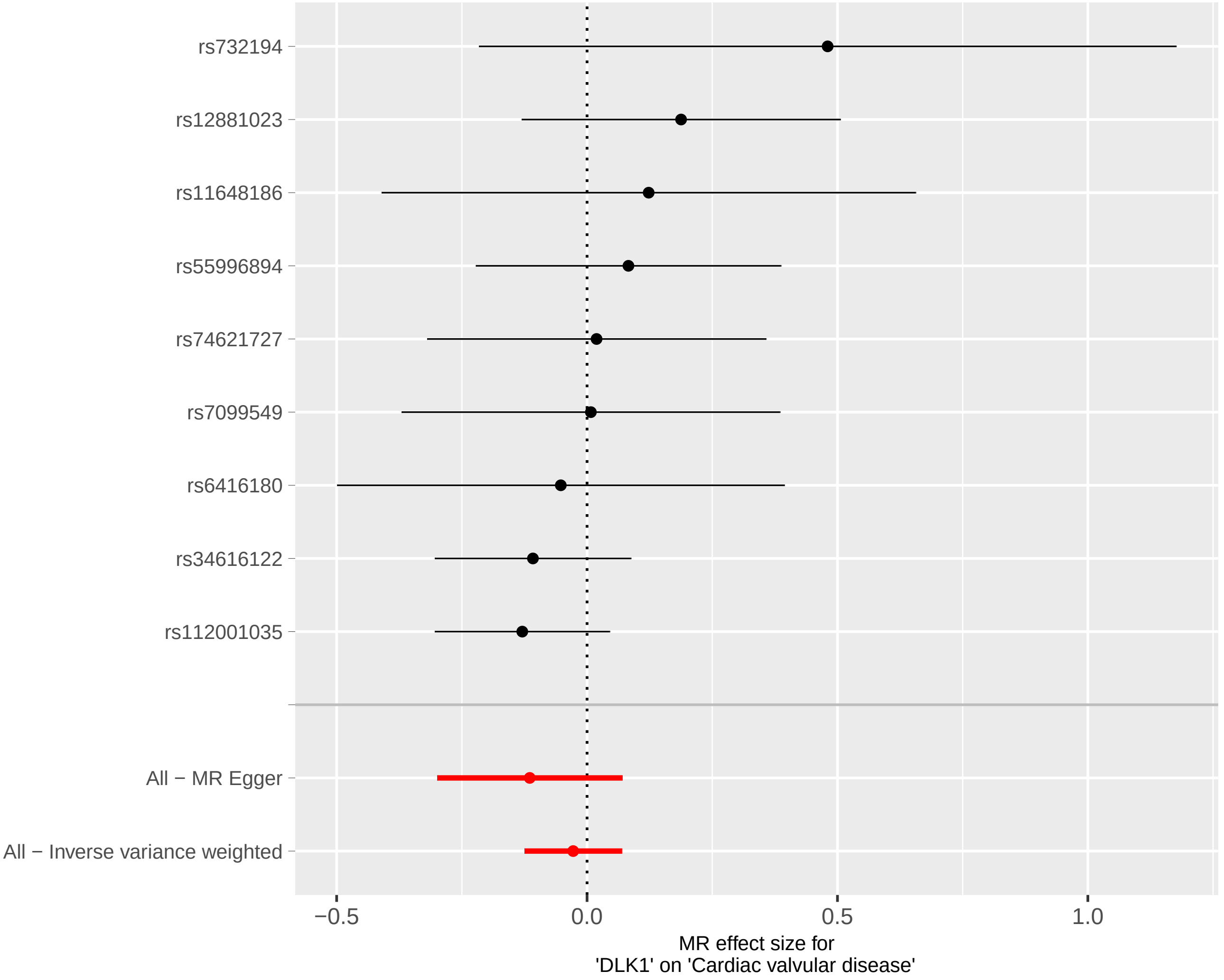
rs732194
rs12881023
rs11648186
rs55996894
rs74621727
rs7099549
rs6416180
rs34616122
rs112001035
All − MR Egger
All − Inverse variance weighted
−0.5
0.0
0.5
1.0
MR effect size for
'DLK1' on 'Cardiac valvular disease'

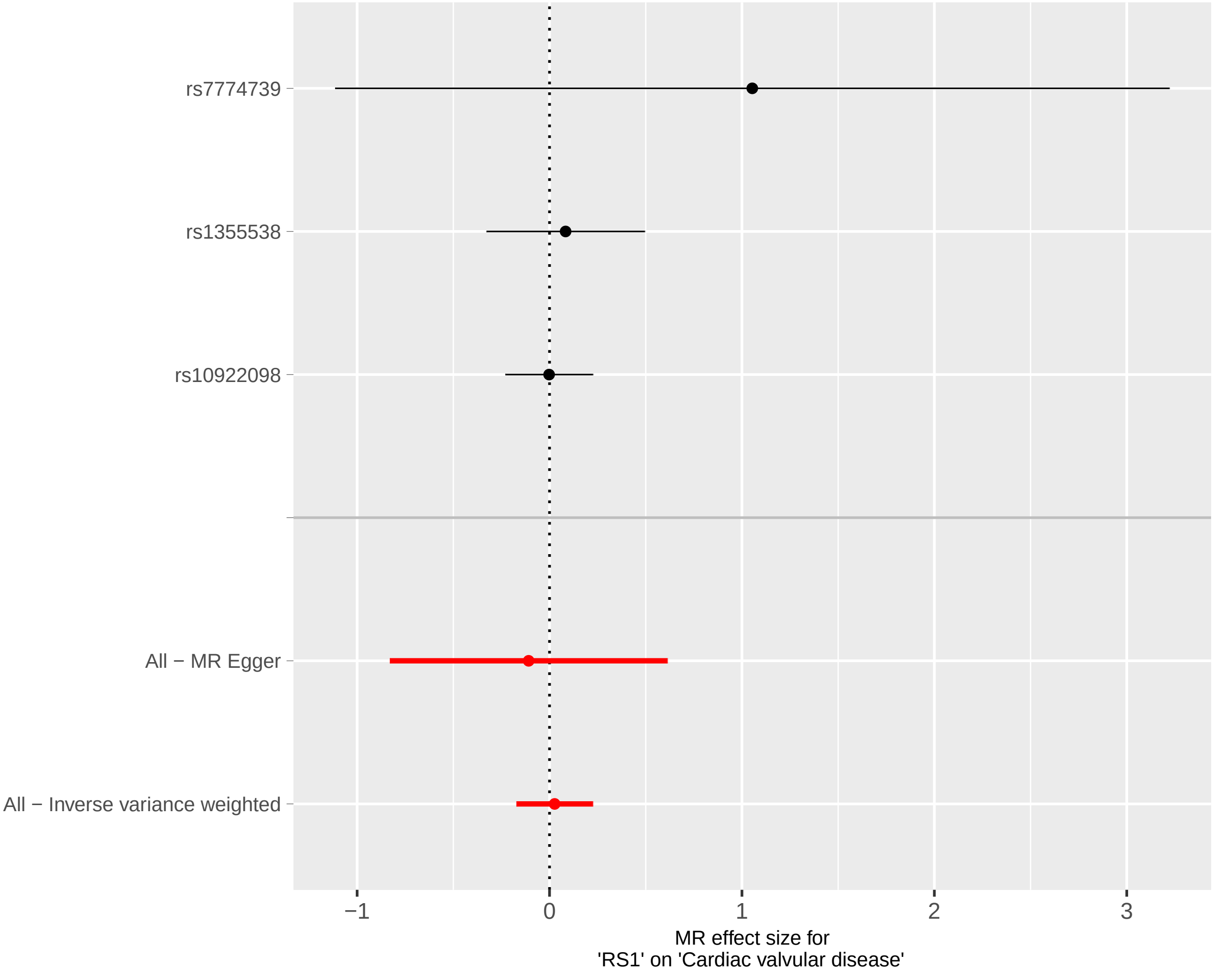
rs7774739
rs1355538
rs10922098
All − MR Egger
All − Inverse variance weighted
−1
0
1
2
3
MR effect size for
'RS1' on 'Cardiac valvular disease'

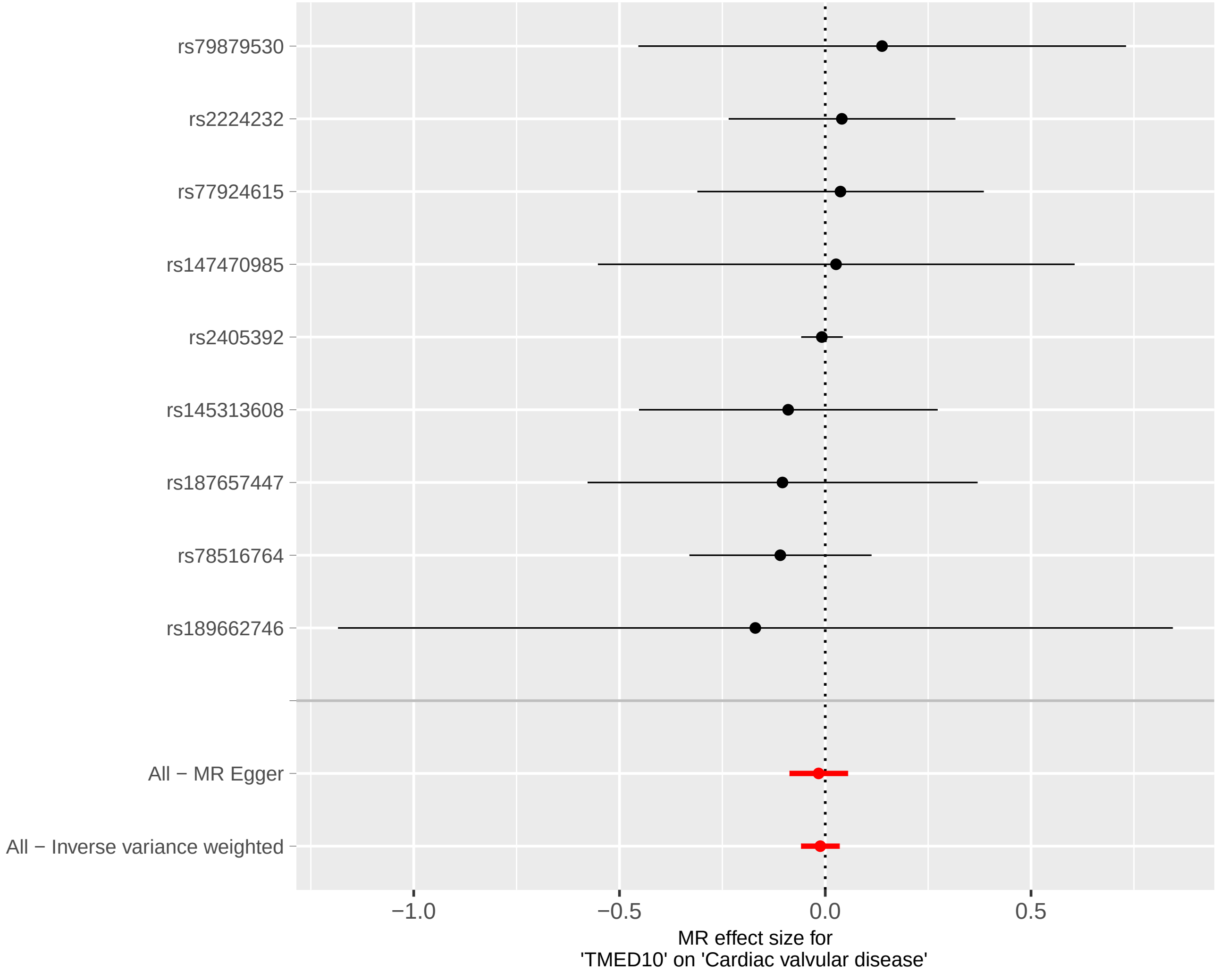
rs79879530
rs2224232
rs77924615
rs147470985
rs2405392
rs145313608
rs187657447
rs78516764
rs189662746
All − MR Egger
All − Inverse variance weighted
−1.0
−0.5
0.0
0.5
MR effect size for
'TMED10' on 'Cardiac valvular disease'

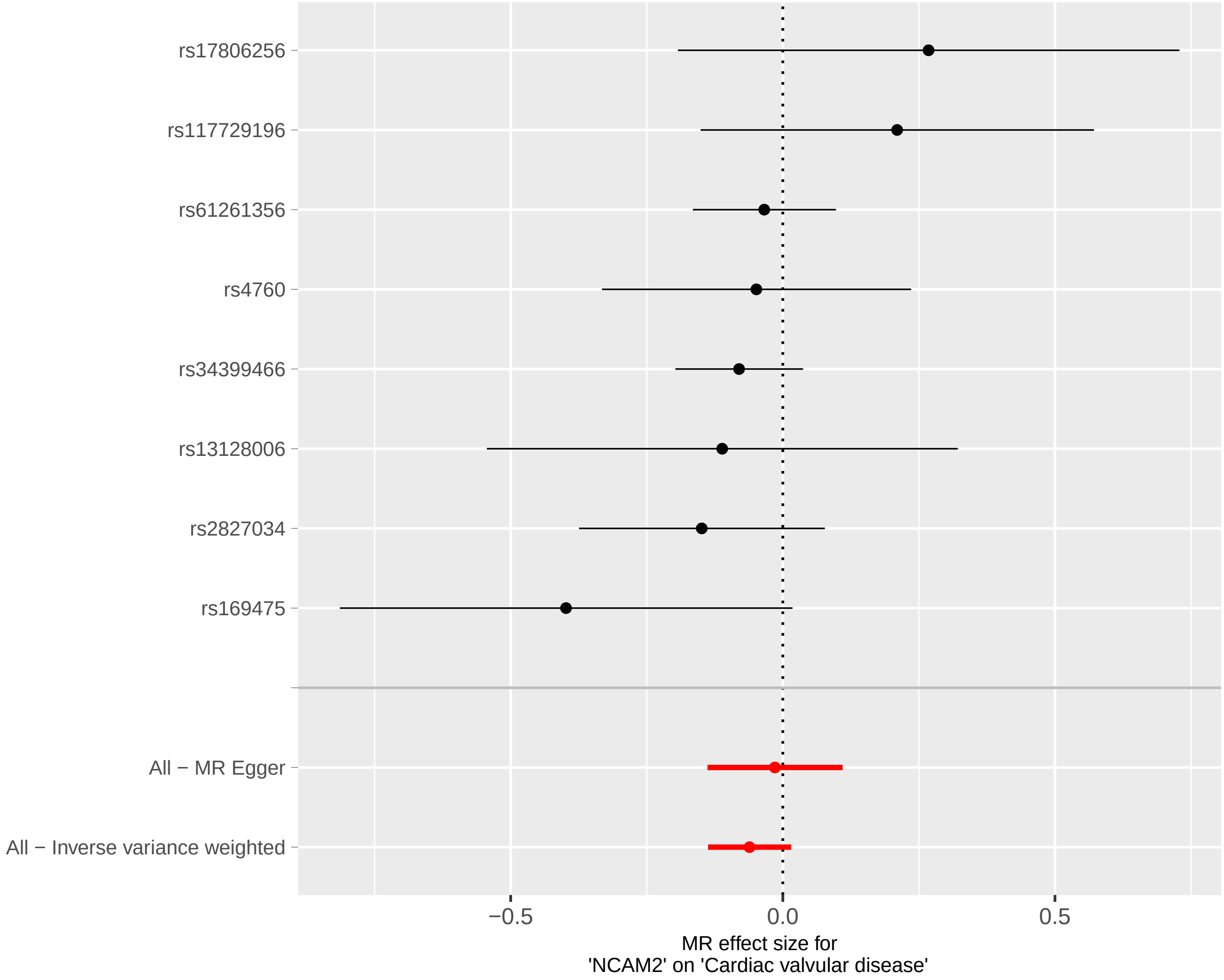
rs17806256
rs117729196
rs61261356
rs4760
rs34399466
rs13128006
rs2827034
rs169475
All − MR Egger
All − Inverse variance weighted
−0.5
0.0
0.5
MR effect size for
'NCAM2' on 'Cardiac valvular disease'

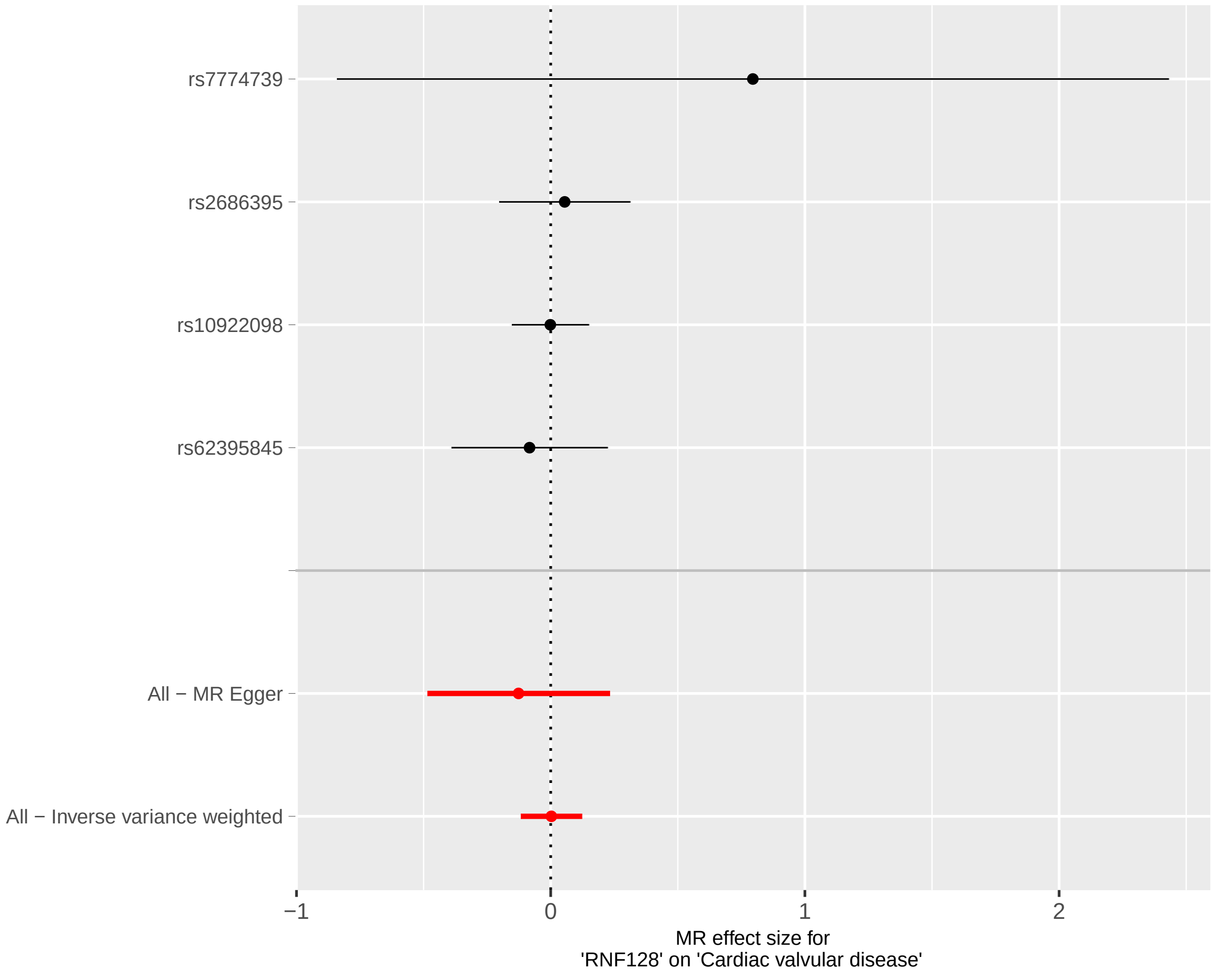
rs7774739
rs2686395
rs10922098
rs62395845
All − MR Egger
All − Inverse variance weighted
−1
0
1
2
MR effect size for
'RNF128' on 'Cardiac valvular disease'

Insufficient number of SNPs

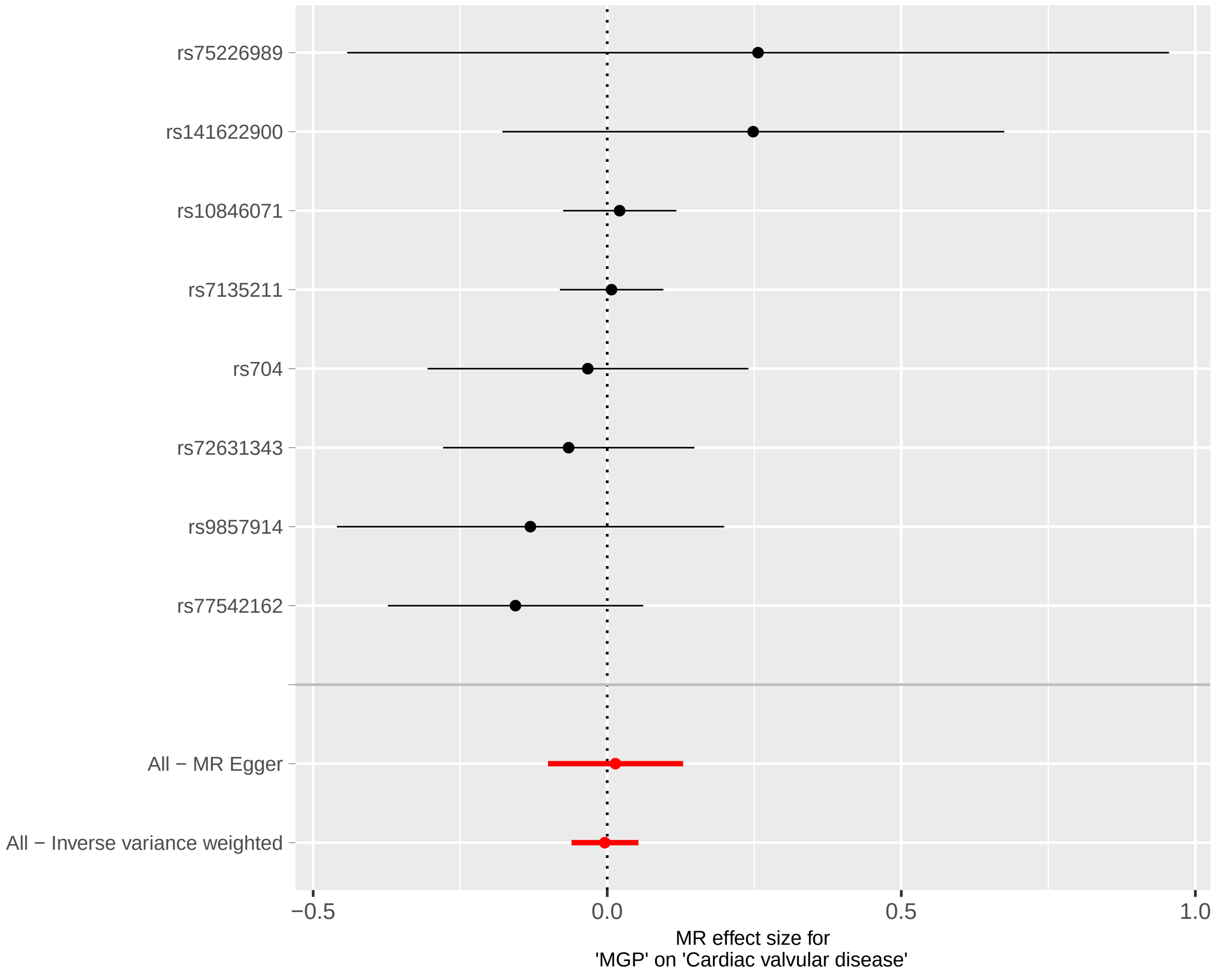
rs75226989
rs141622900
rs10846071
rs7135211
rs704
rs72631343
rs9857914
rs77542162
All − MR Egger
All − Inverse variance weighted
−0.5
0.0
0.5
1.0
MR effect size for
'MGP' on 'Cardiac valvular disease'

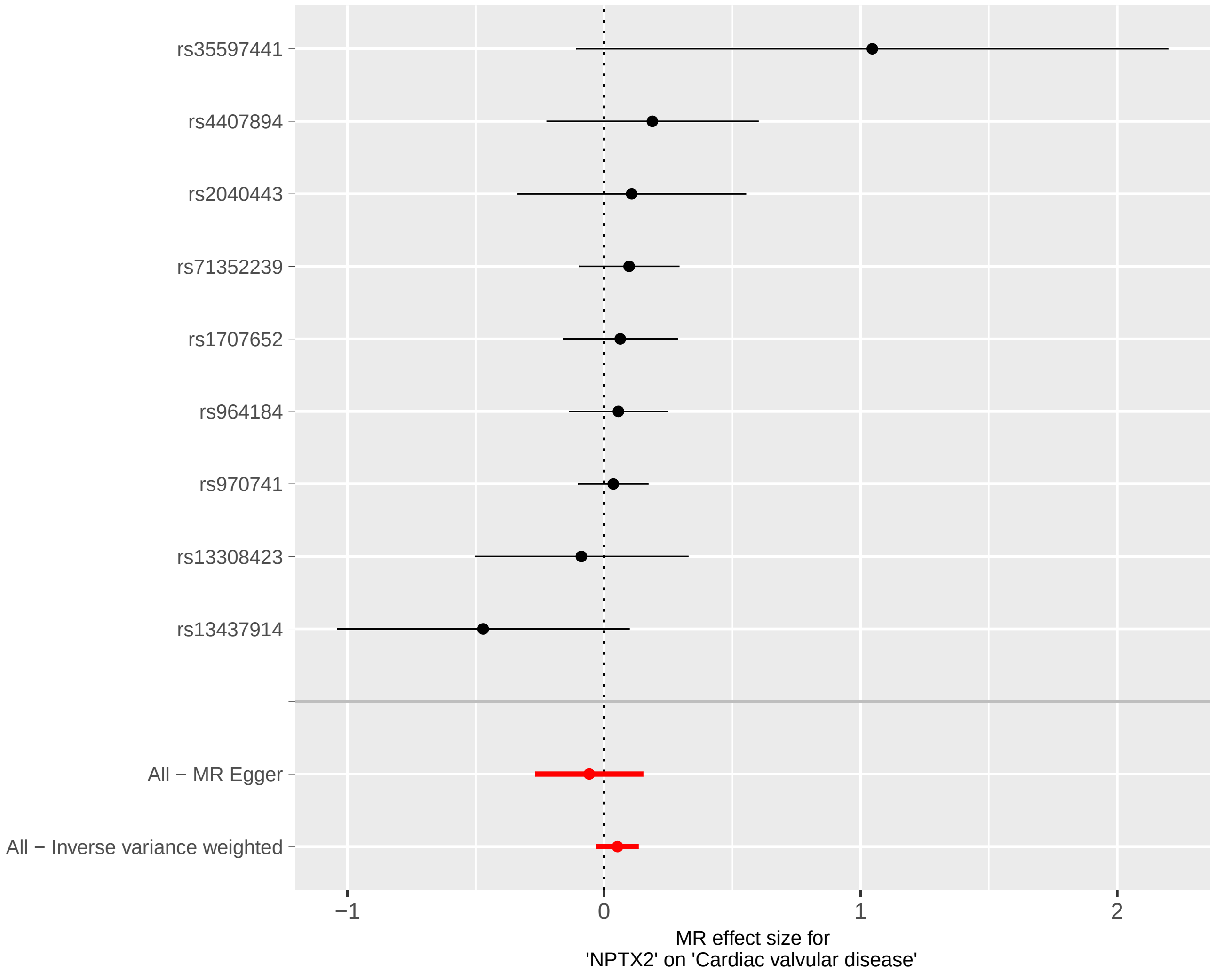
rs35597441
rs4407894
rs2040443
rs71352239
rs1707652
rs964184
rs970741
rs13308423
rs13437914
All − MR Egger
All − Inverse variance weighted
−1
0
1
2
MR effect size for
'NPTX2' on 'Cardiac valvular disease'

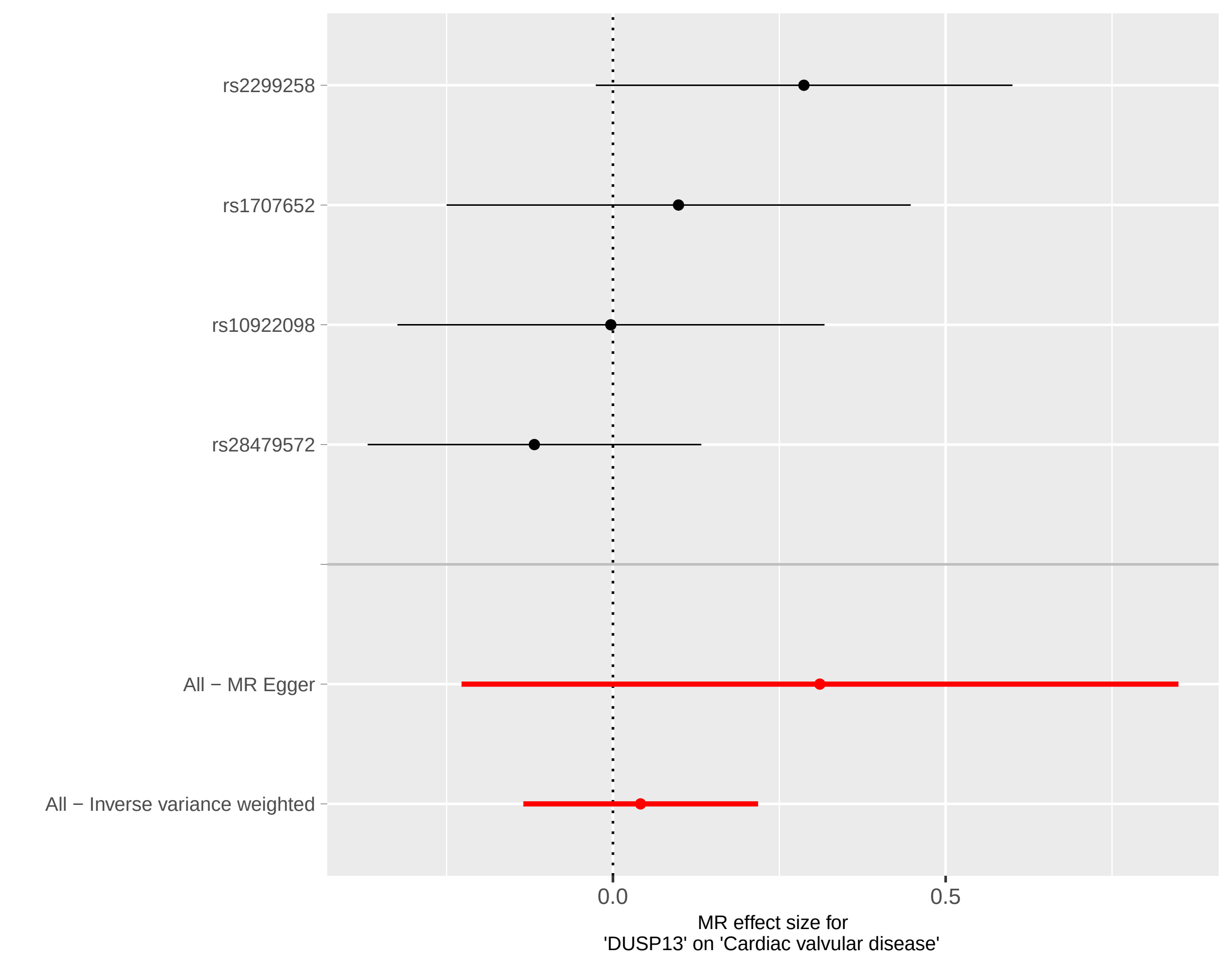

rs2299258
rs1707652
rs10922098
rs28479572
All − MR Egger
All − Inverse variance weighted
0.0
0.5
MR effect size for
'DUSP13' on 'Cardiac valvular disease'

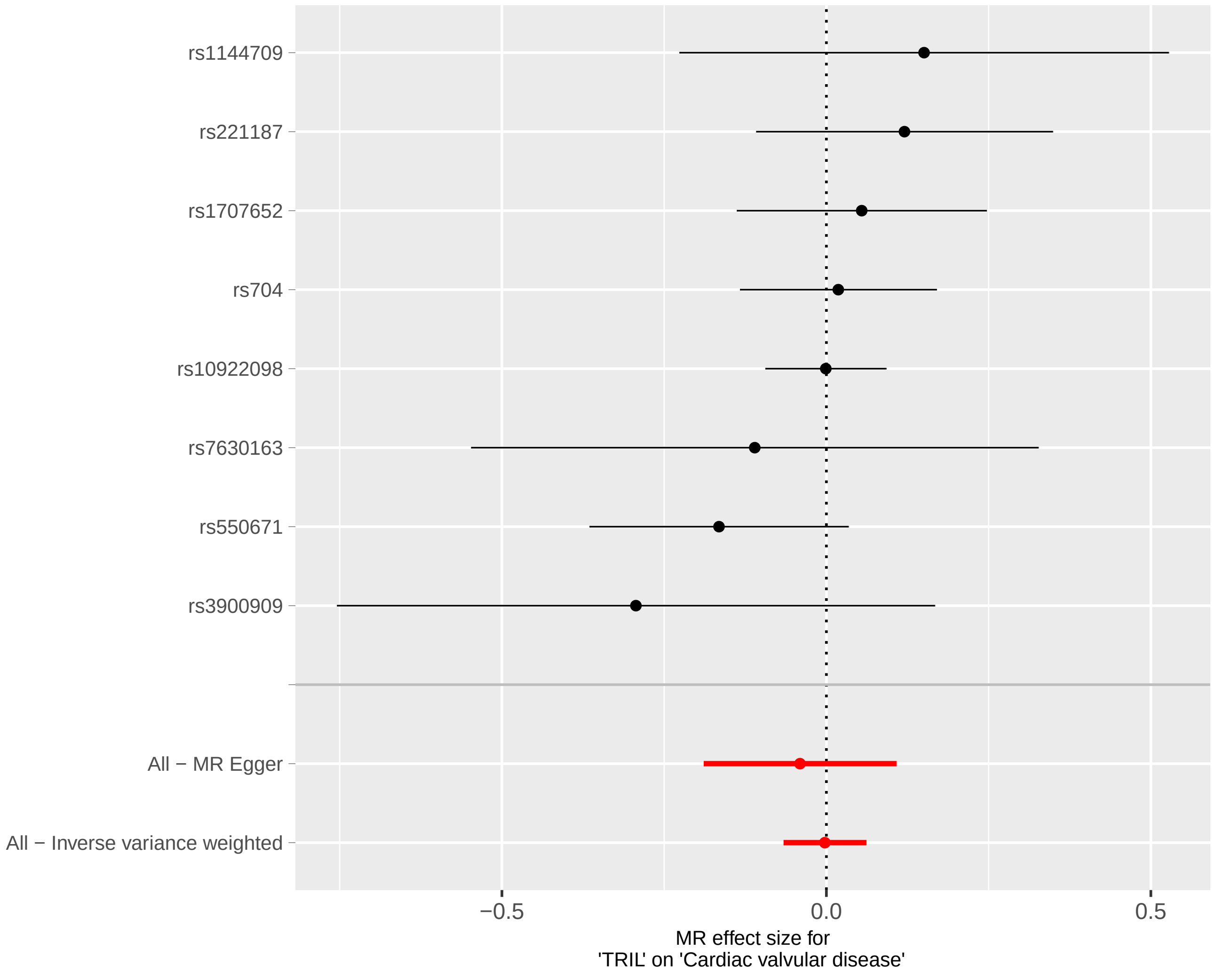
rs1144709
rs221187
rs1707652
rs704
rs10922098
rs7630163
rs550671
rs3900909
All − MR Egger
All − Inverse variance weighted
−0.5
0.0
0.5
MR effect size for
'TRIL' on 'Cardiac valvular disease'

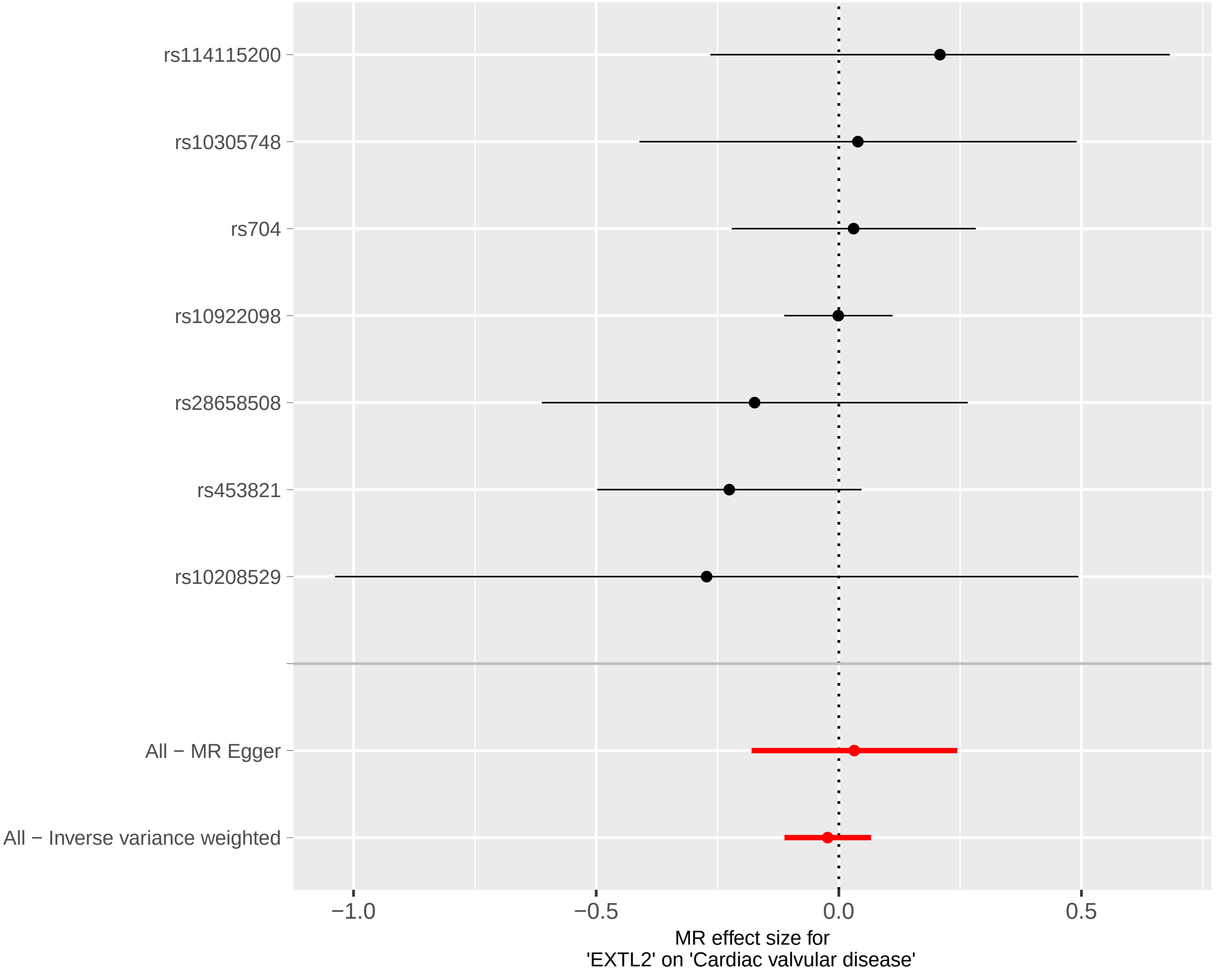

rs114115200
rs10305748
rs704
rs10922098
rs28658508
rs453821
rs10208529
All − MR Egger
All − Inverse variance weighted
−1.0
−0.5
0.0
0.5
MR effect size for
'EXTL2' on 'Cardiac valvular disease'

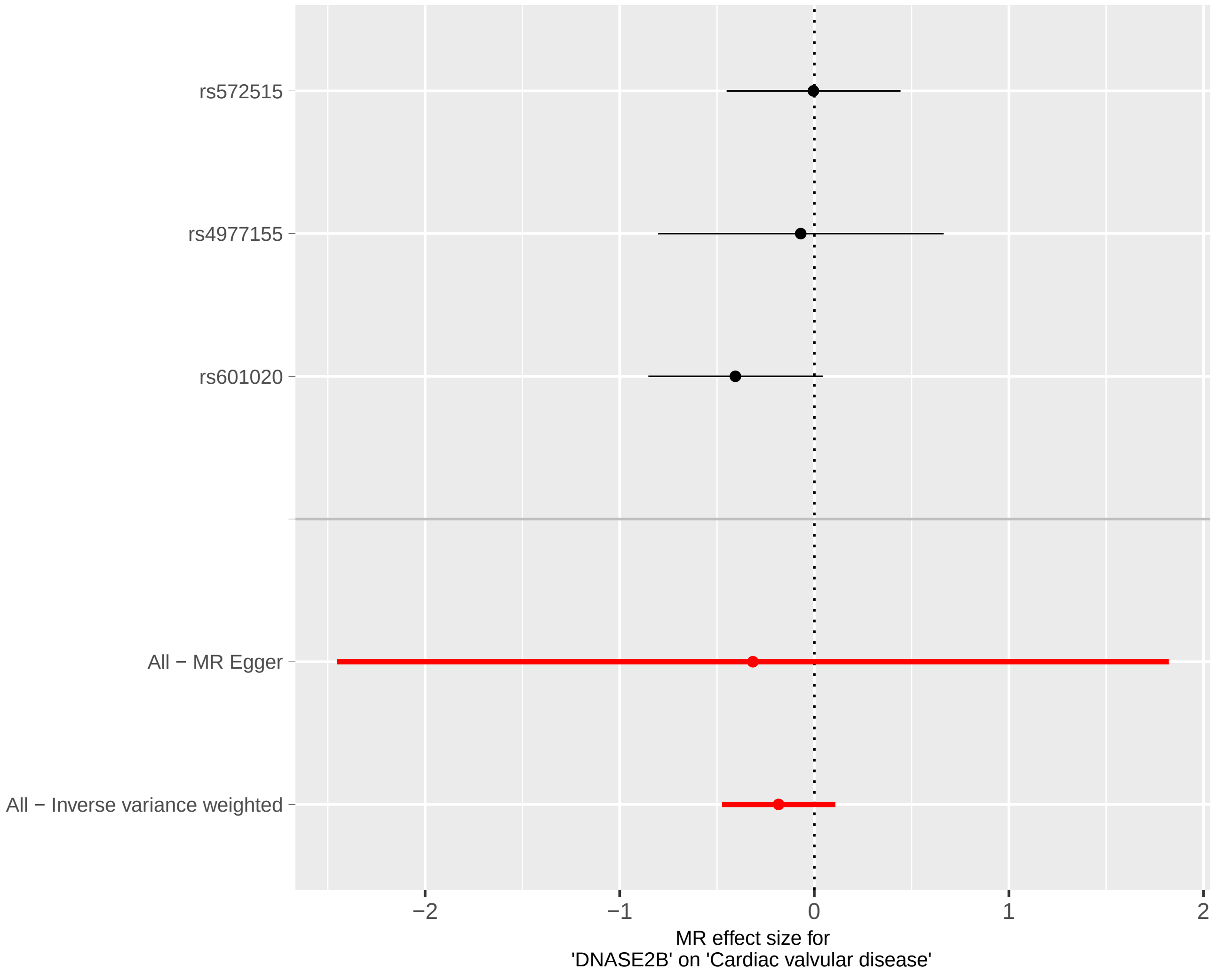
rs572515
rs4977155
rs601020
All − MR Egger
All − Inverse variance weighted
−2
−1
0
1
2
MR effect size for
'DNASE2B' on 'Cardiac valvular disease'

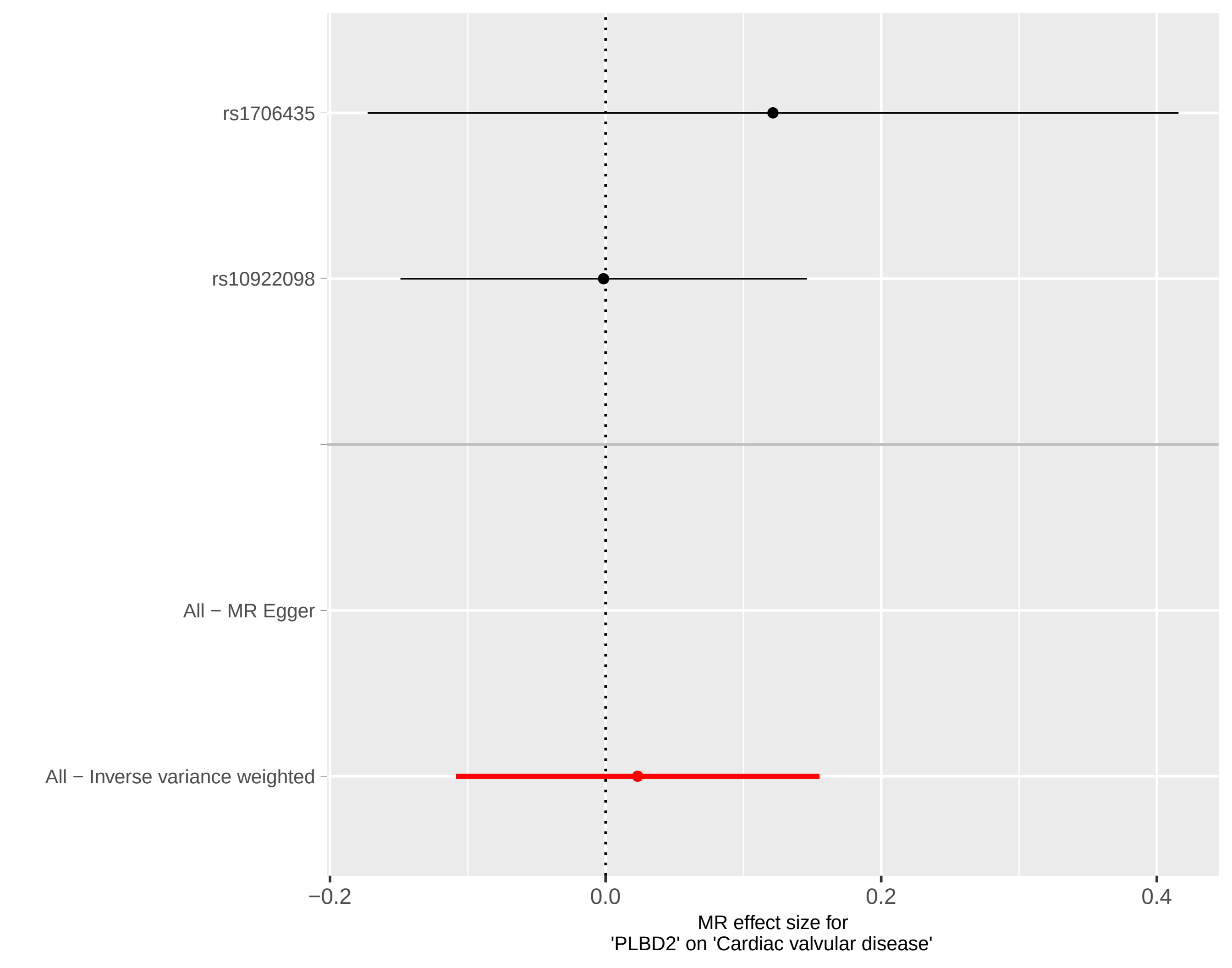
rs1706435
rs10922098
All − MR Egger
All − Inverse variance weighted
−0.2
0.0
0.2
0.4
MR effect size for
'PLBD2' on 'Cardiac valvular disease'

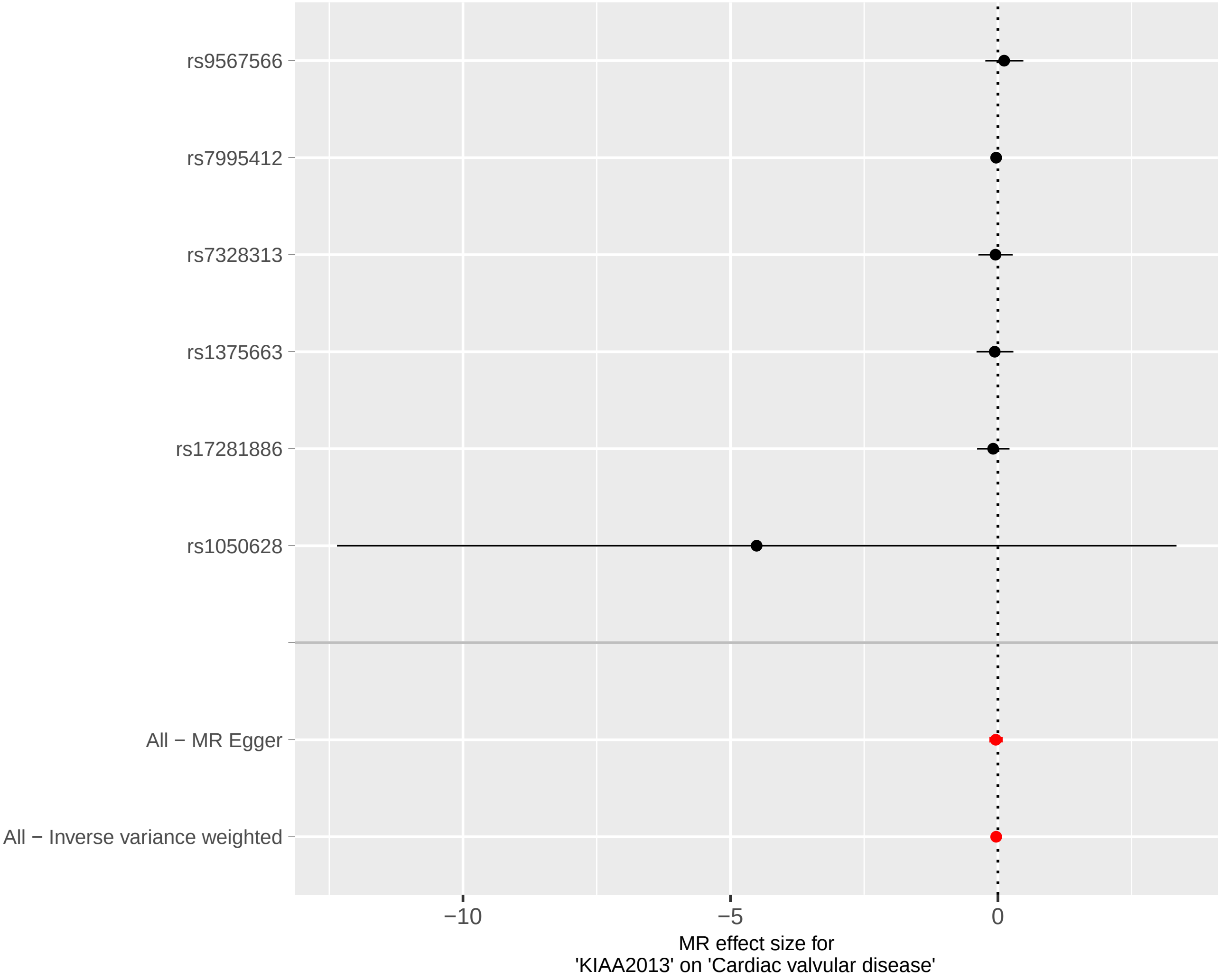
rs9567566
rs7995412
rs7328313
rs1375663
rs17281886
rs1050628
All − MR Egger
All − Inverse variance weighted
−10
−5
0
MR effect size for
'KIAA2013' on 'Cardiac valvular disease'

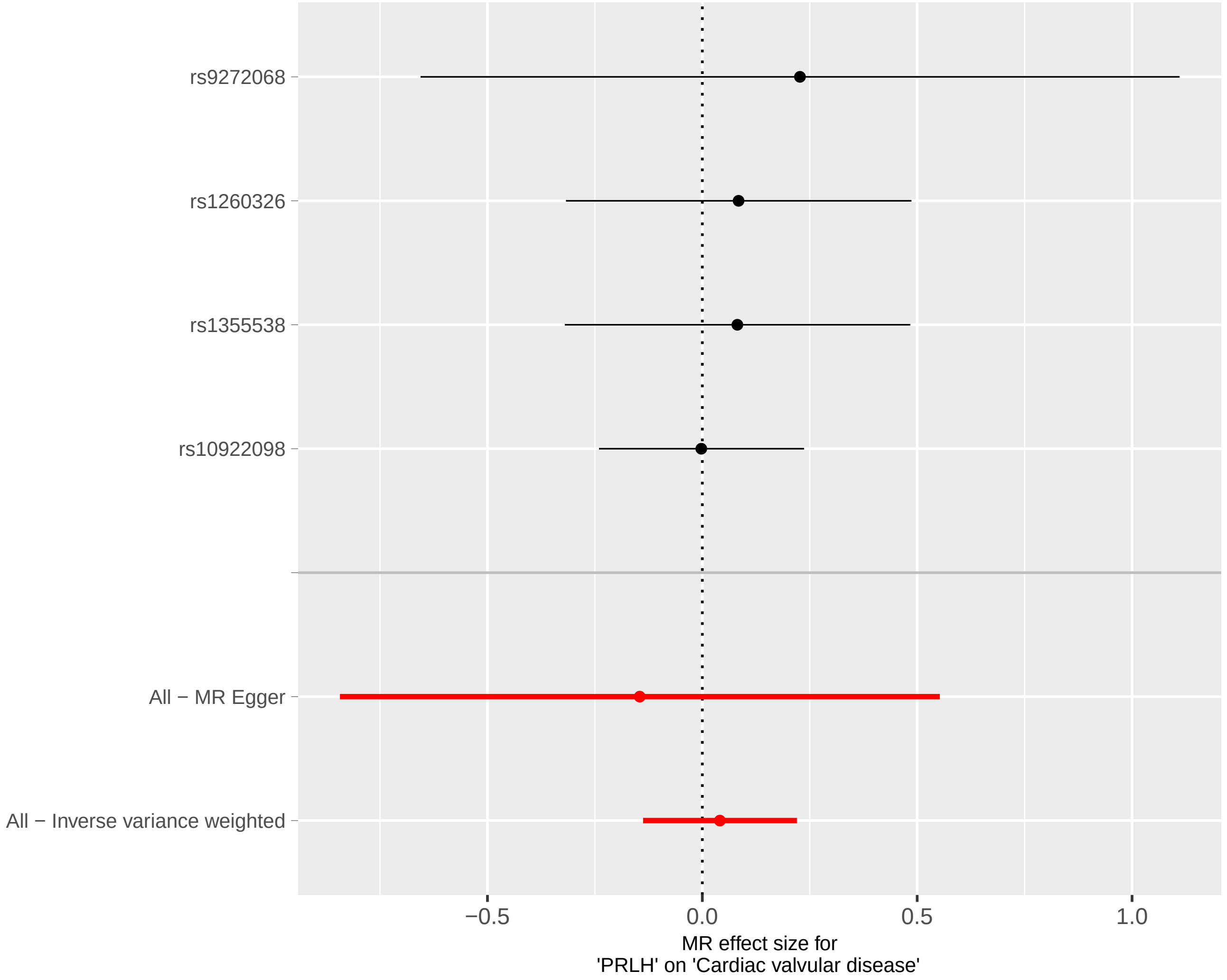
rs9272068
rs1260326
rs1355538
rs10922098
All − MR Egger
All − Inverse variance weighted
−0.5
0.0
0.5
1.0
MR effect size for
'PRLH' on 'Cardiac valvular disease'

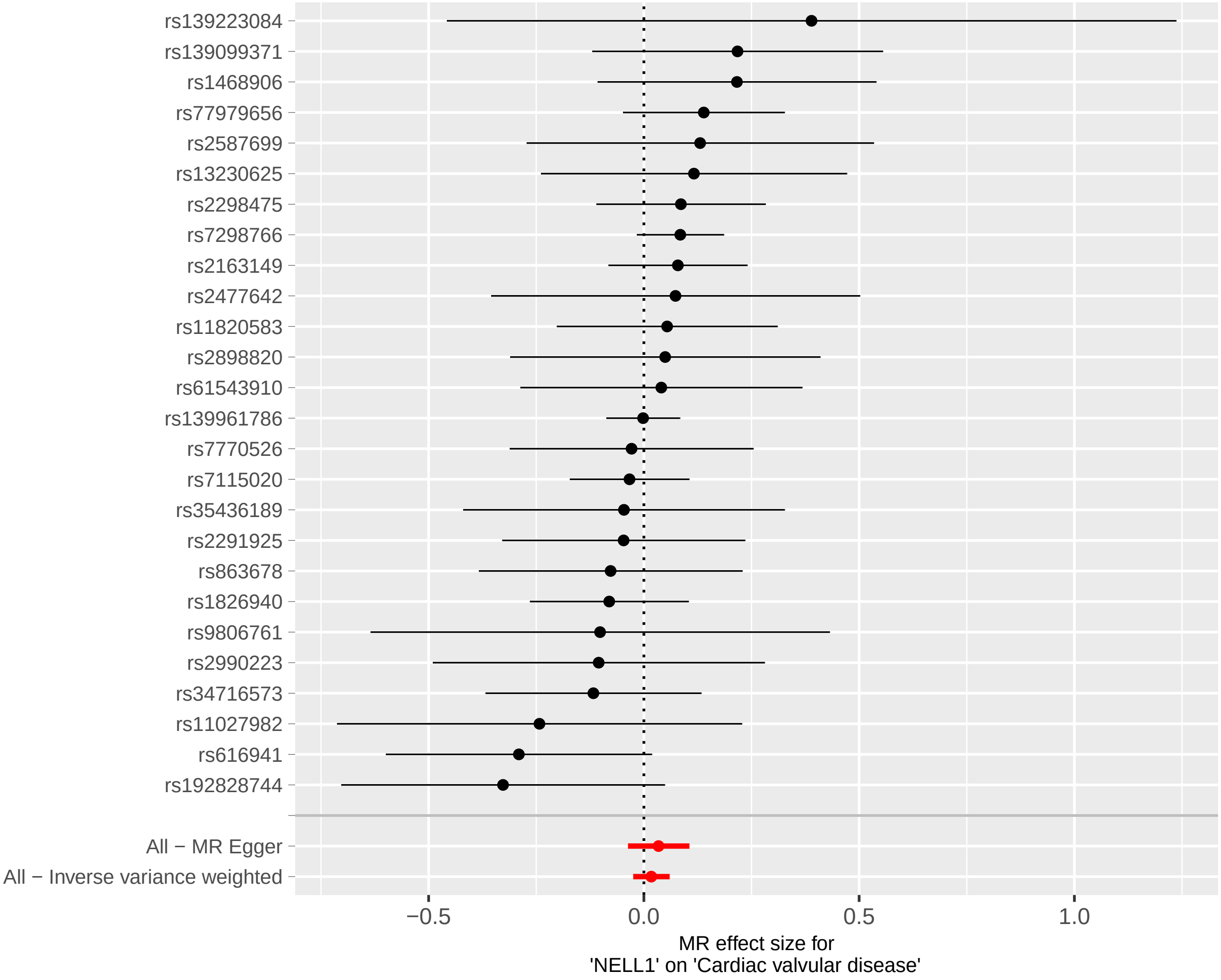

rs139223084
rs139099371
rs1468906
rs77979656
rs2587699
rs13230625
rs2298475
rs7298766
rs2163149
rs2477642
rs11820583
rs2898820
rs61543910
rs139961786
rs7770526
rs7115020
rs35436189
rs2291925
rs863678
rs1826940
rs9806761
rs2990223
rs34716573
rs11027982
rs616941
rs192828744
All − MR Egger
All − Inverse variance weighted
−0.5
0.0
0.5
1.0
MR effect size for
'NELL1' on 'Cardiac valvular disease'

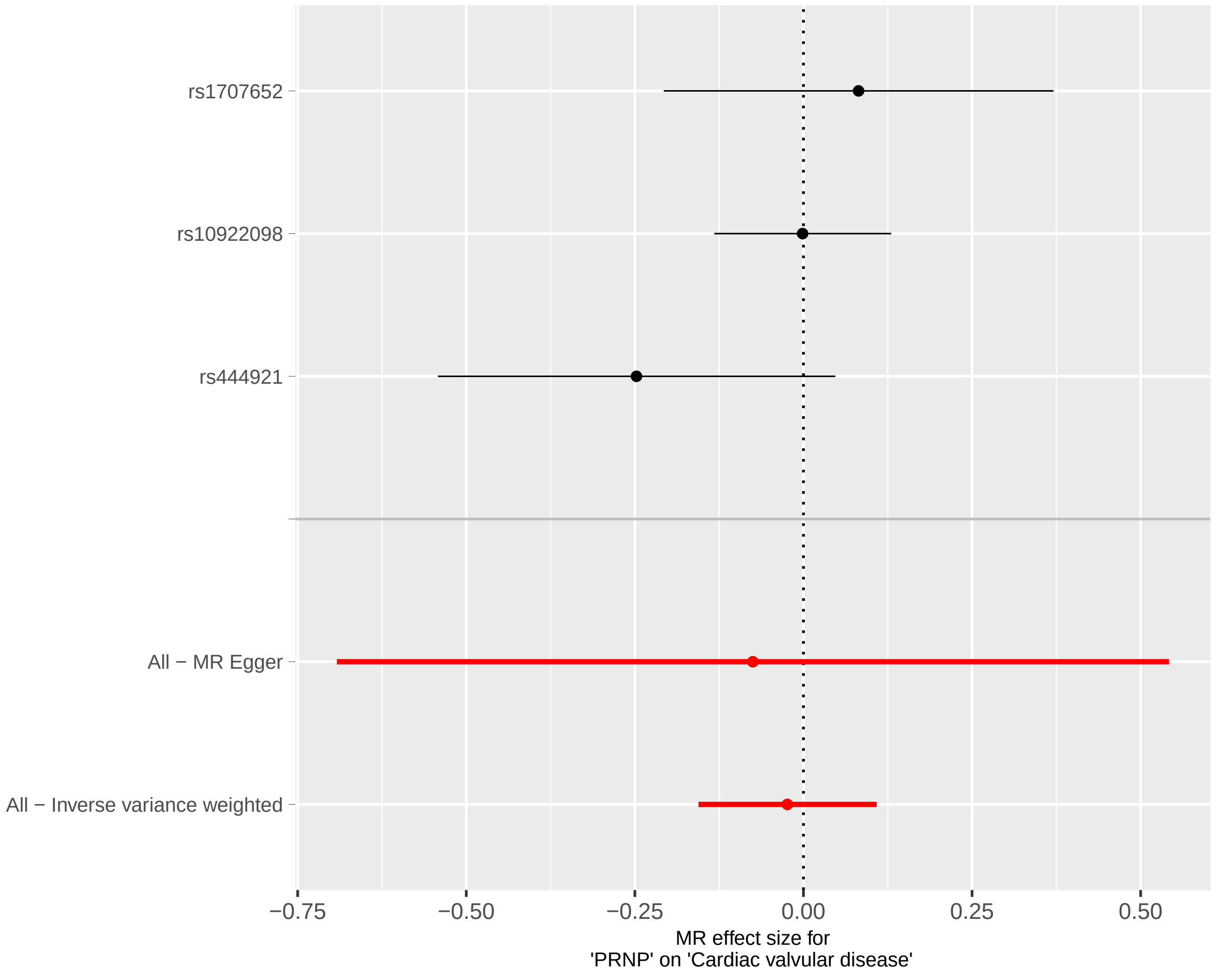

rs1707652
rs10922098
rs444921
All − MR Egger
All − Inverse variance weighted
−0.75
−0.50
−0.25
0.00
0.25
0.50
MR effect size for
'PRNP' on 'Cardiac valvular disease'

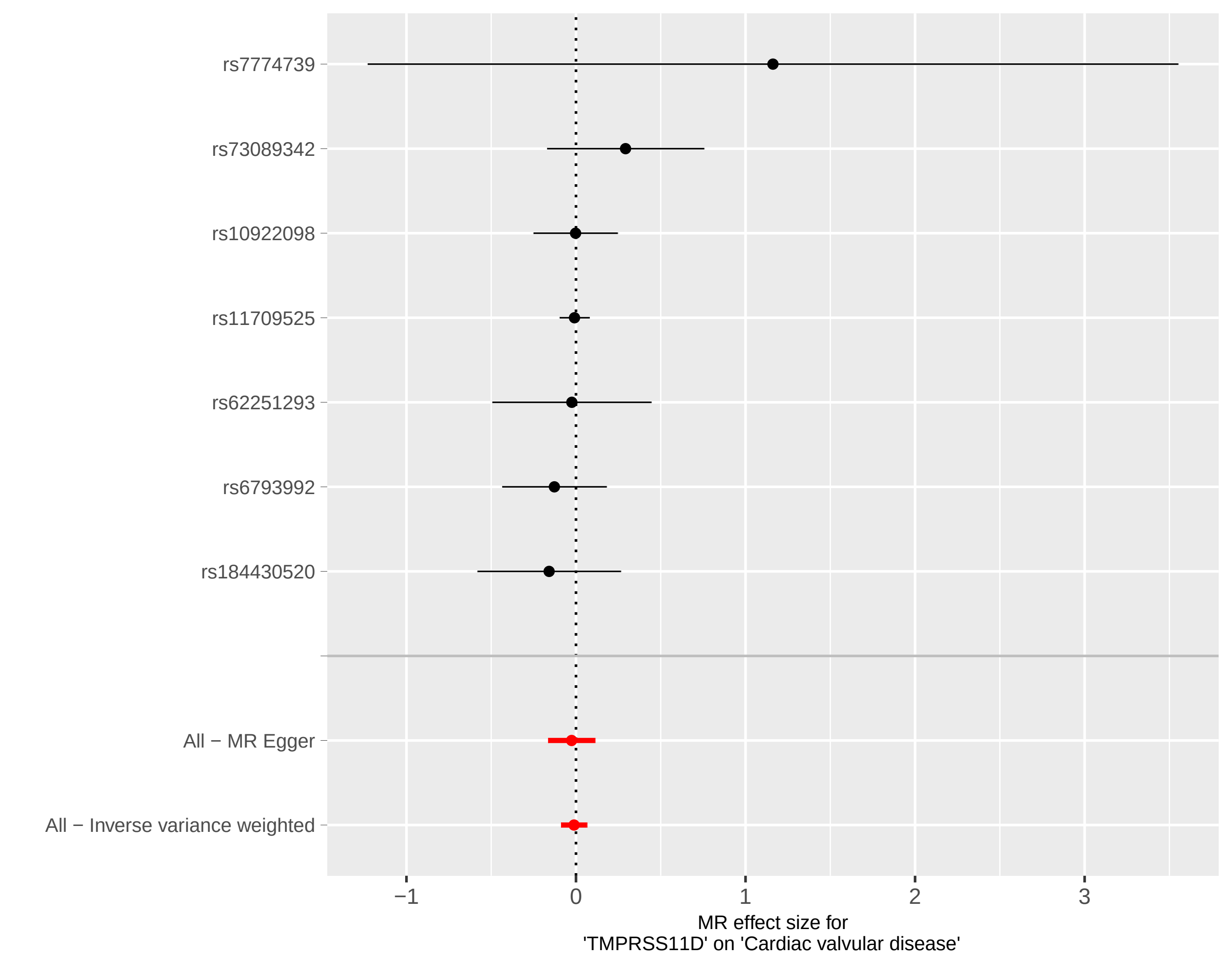
rs7774739
rs73089342
rs10922098
rs11709525
rs62251293
rs6793992
rs184430520
All − MR Egger
All − Inverse variance weighted
−1
0
1
2
3
MR effect size for
'TMPRSS11D' on 'Cardiac valvular disease'

Insufficient number of SNPs

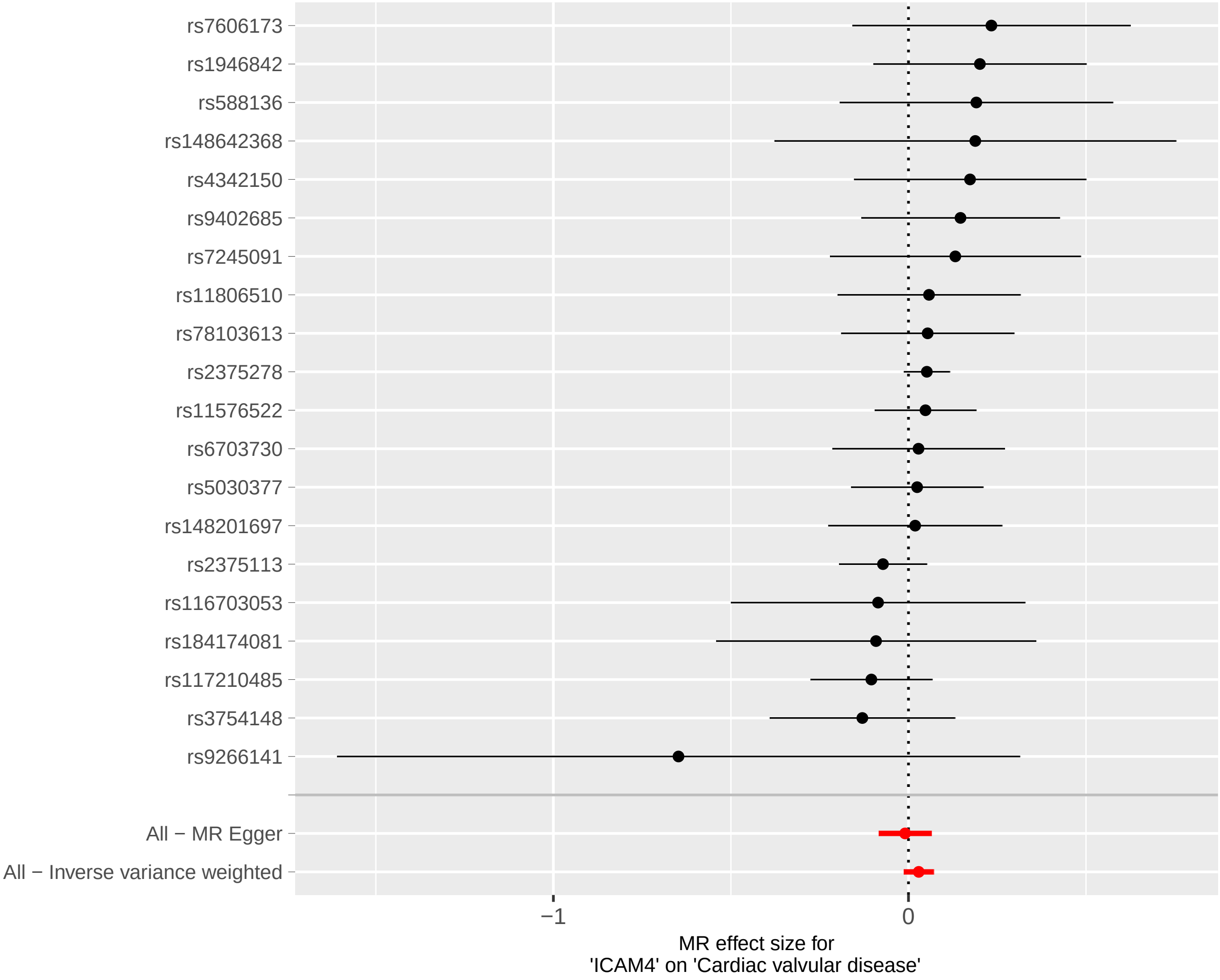
rs7606173
rs1946842
rs588136
rs148642368
rs4342150
rs9402685
rs7245091
rs11806510
rs78103613
rs2375278
rs11576522
rs6703730
rs5030377
rs148201697
rs2375113
rs116703053
rs184174081
rs117210485
rs3754148
rs9266141
All − MR Egger
All − Inverse variance weighted
−1
0
MR effect size for
'ICAM4' on 'Cardiac valvular disease'

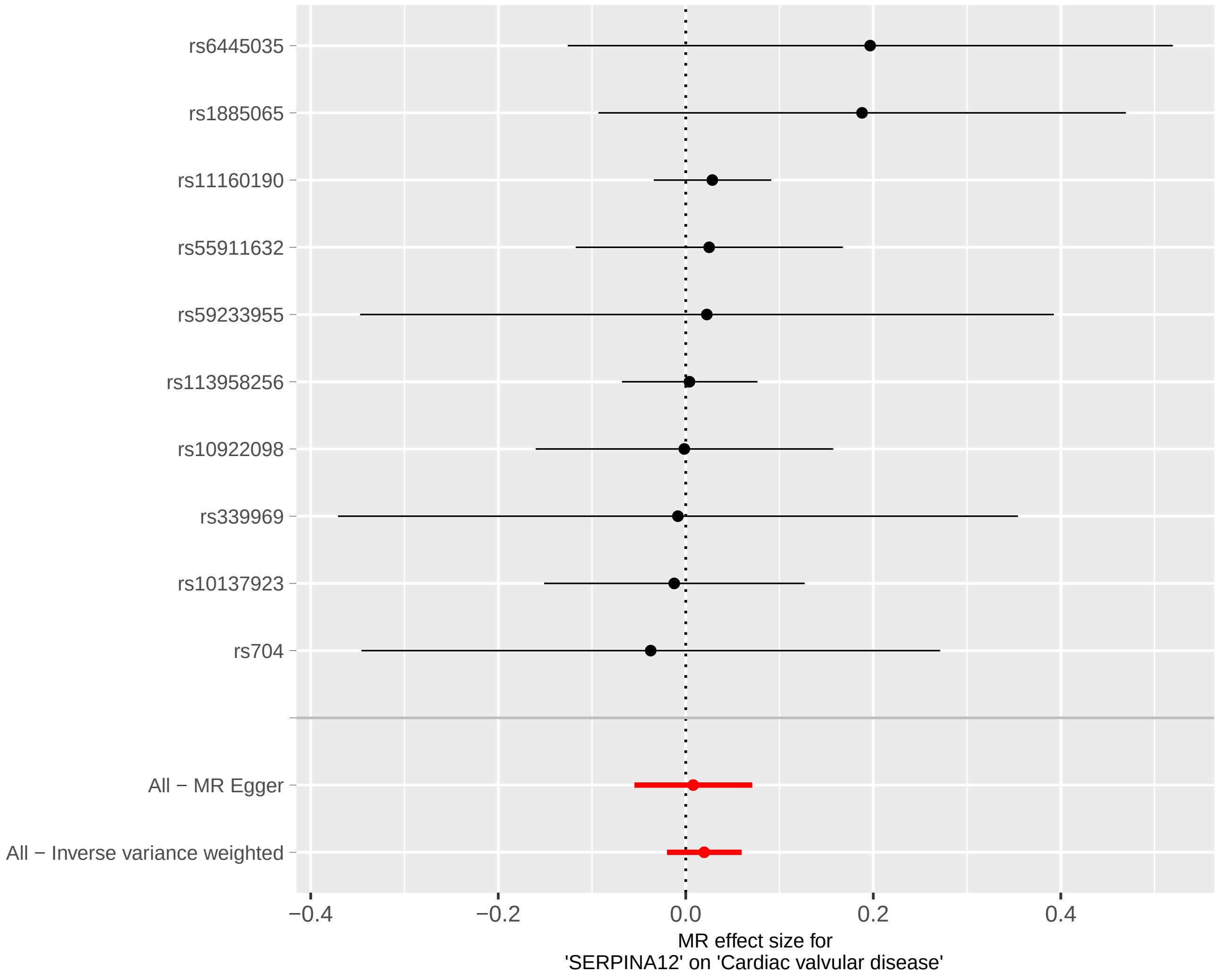
rs6445035
rs1885065
rs11160190
rs55911632
rs59233955
rs113958256
rs10922098
rs339969
rs10137923
rs704
All − MR Egger
All − Inverse variance weighted
−0.4
−0.2
0.0
0.2
0.4
MR effect size for
'SERPINA12' on 'Cardiac valvular disease'

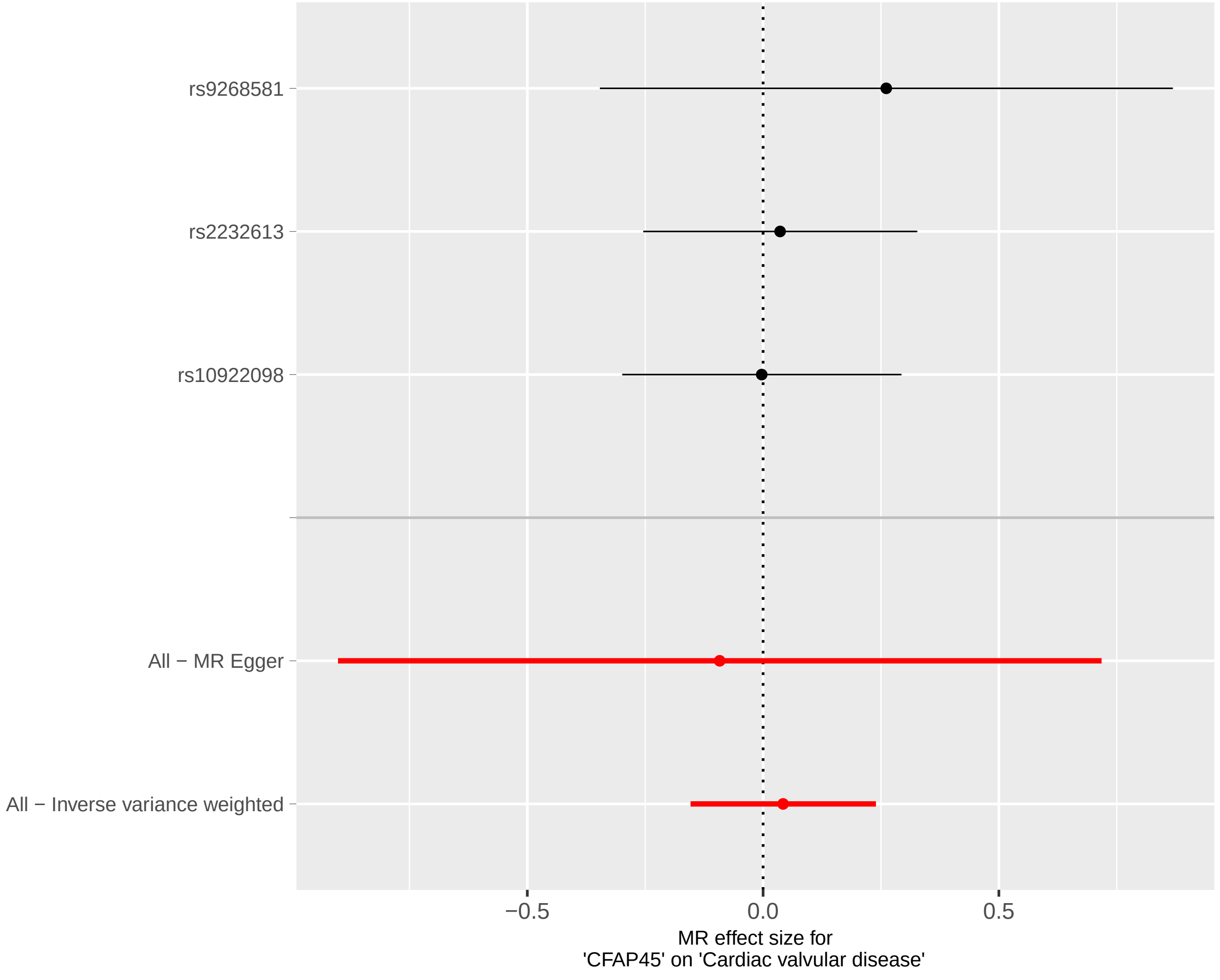
rs9268581
rs2232613
rs10922098
All − MR Egger
All − Inverse variance weighted
−0.5
0.0
0.5
MR effect size for
'CFAP45' on 'Cardiac valvular disease'

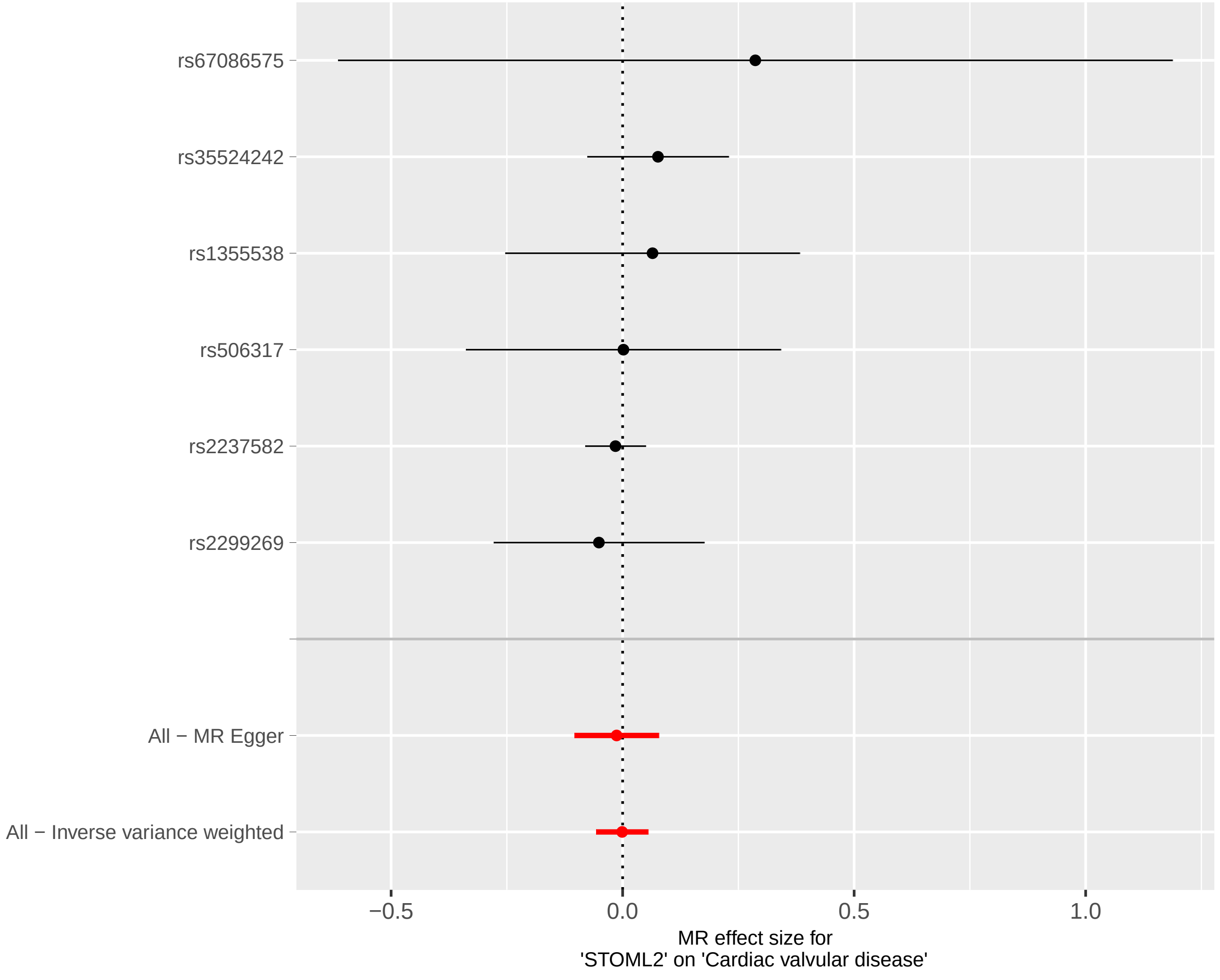
rs67086575
rs35524242
rs1355538
rs506317
rs2237582
rs2299269
All − MR Egger
All − Inverse variance weighted
−0.5
0.0
0.5
1.0
MR effect size for
'STOML2' on 'Cardiac valvular disease'

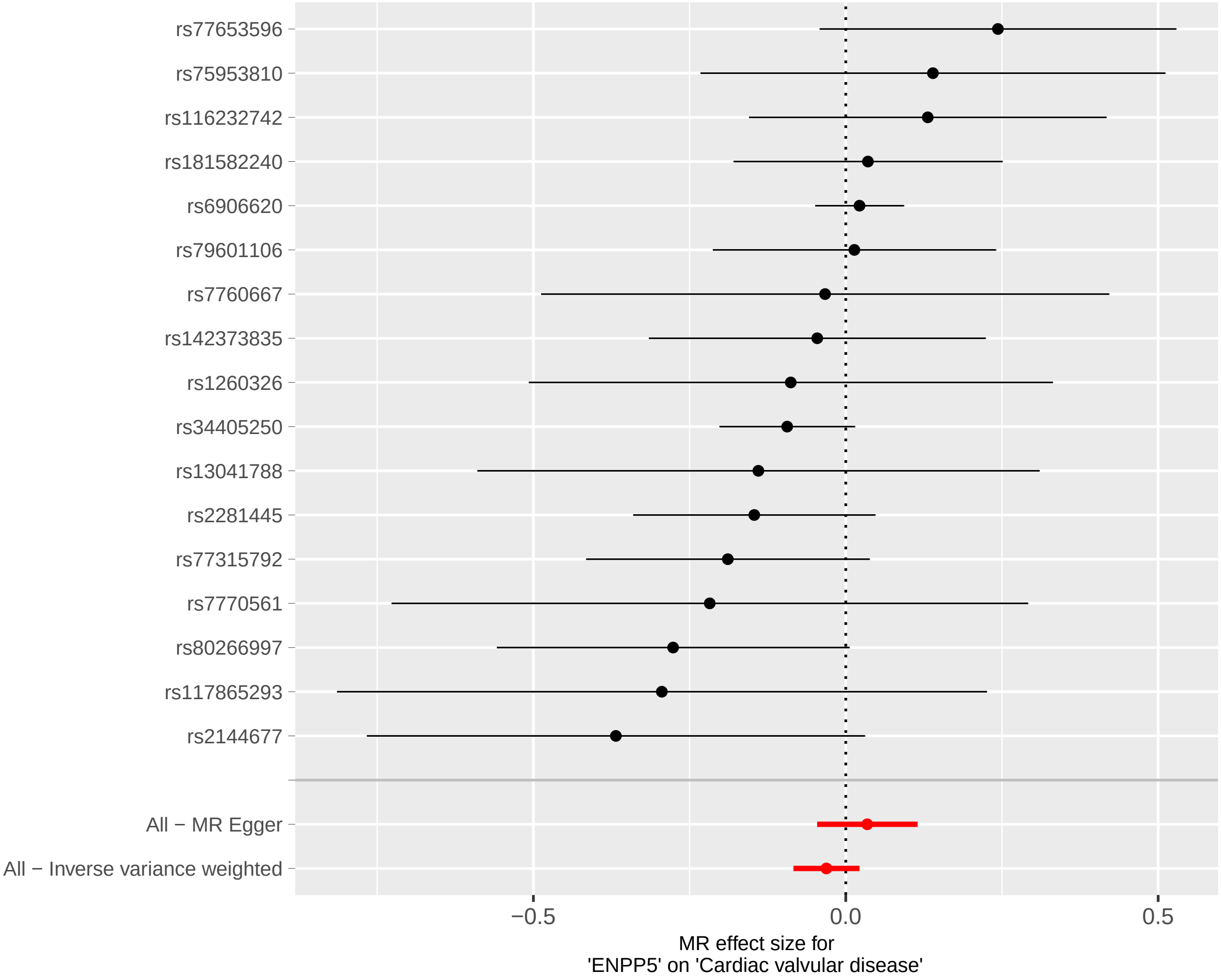
rs77653596
rs75953810
rs116232742
rs181582240
rs6906620
rs79601106
rs7760667
rs142373835
rs1260326
rs34405250
rs13041788
rs2281445
rs77315792
rs7770561
rs80266997
rs117865293
rs2144677
All − MR Egger
All − Inverse variance weighted
−0.5
0.0
0.5
MR effect size for
'ENPP5' on 'Cardiac valvular disease'

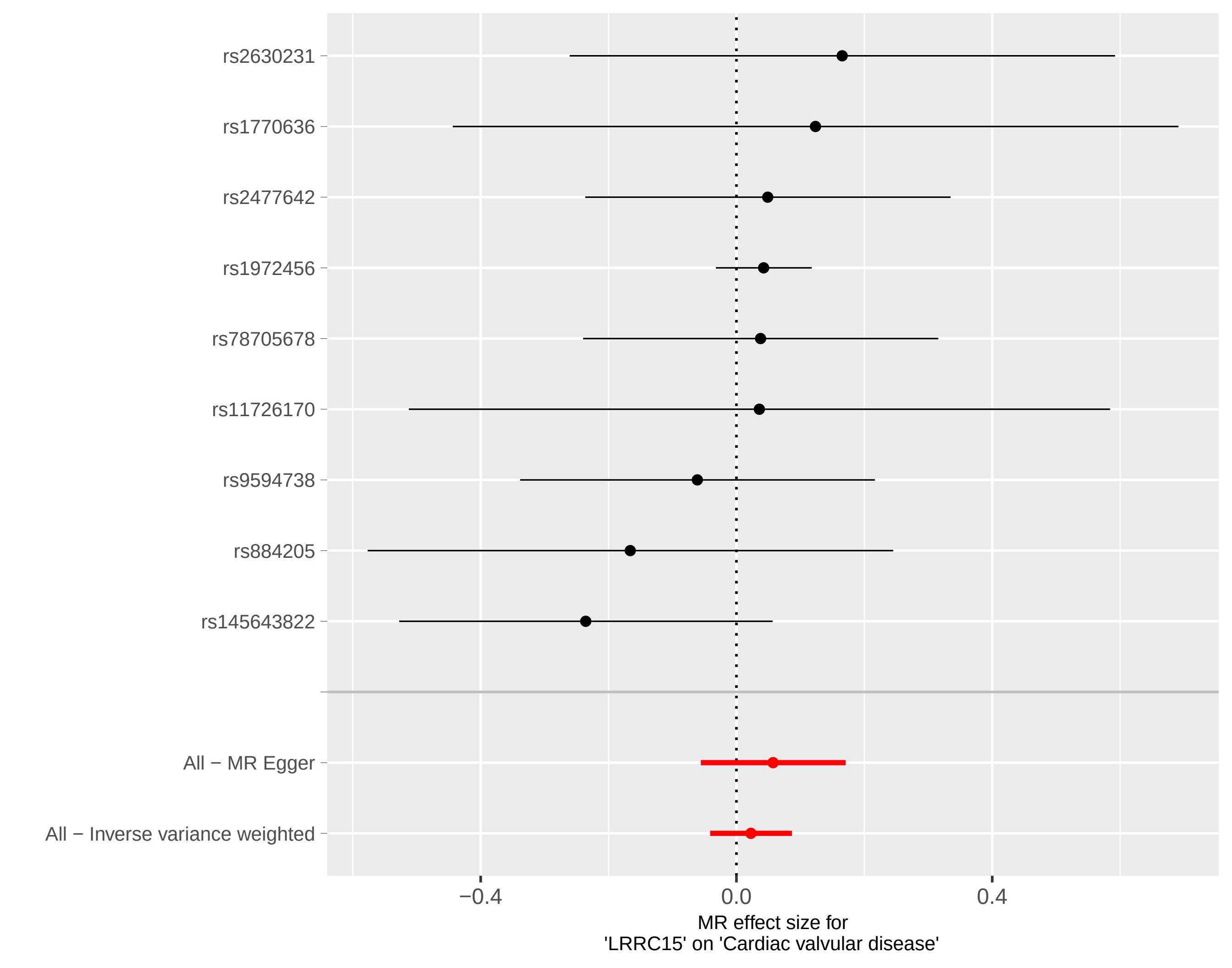
rs2630231
rs1770636
rs2477642
rs1972456
rs78705678
rs11726170
rs9594738
rs884205
rs145643822
All − MR Egger
All − Inverse variance weighted
−0.4
0.0
0.4
MR effect size for
'LRRC15' on 'Cardiac valvular disease'

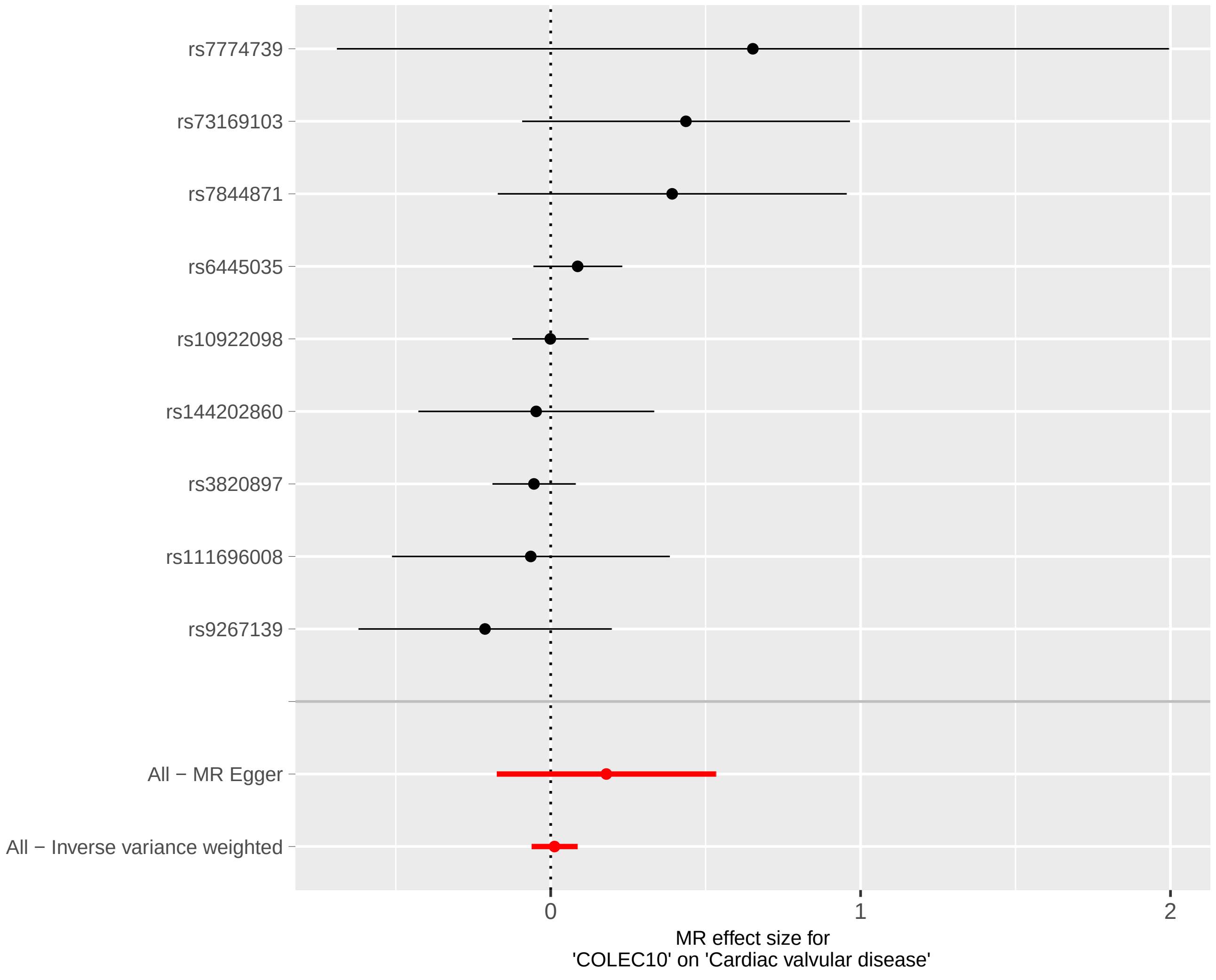
rs7774739
rs73169103
rs7844871
rs6445035
rs10922098
rs144202860
rs3820897
rs111696008
rs9267139
All − MR Egger
All − Inverse variance weighted
0
1
2
MR effect size for
'COLEC10' on 'Cardiac valvular disease'

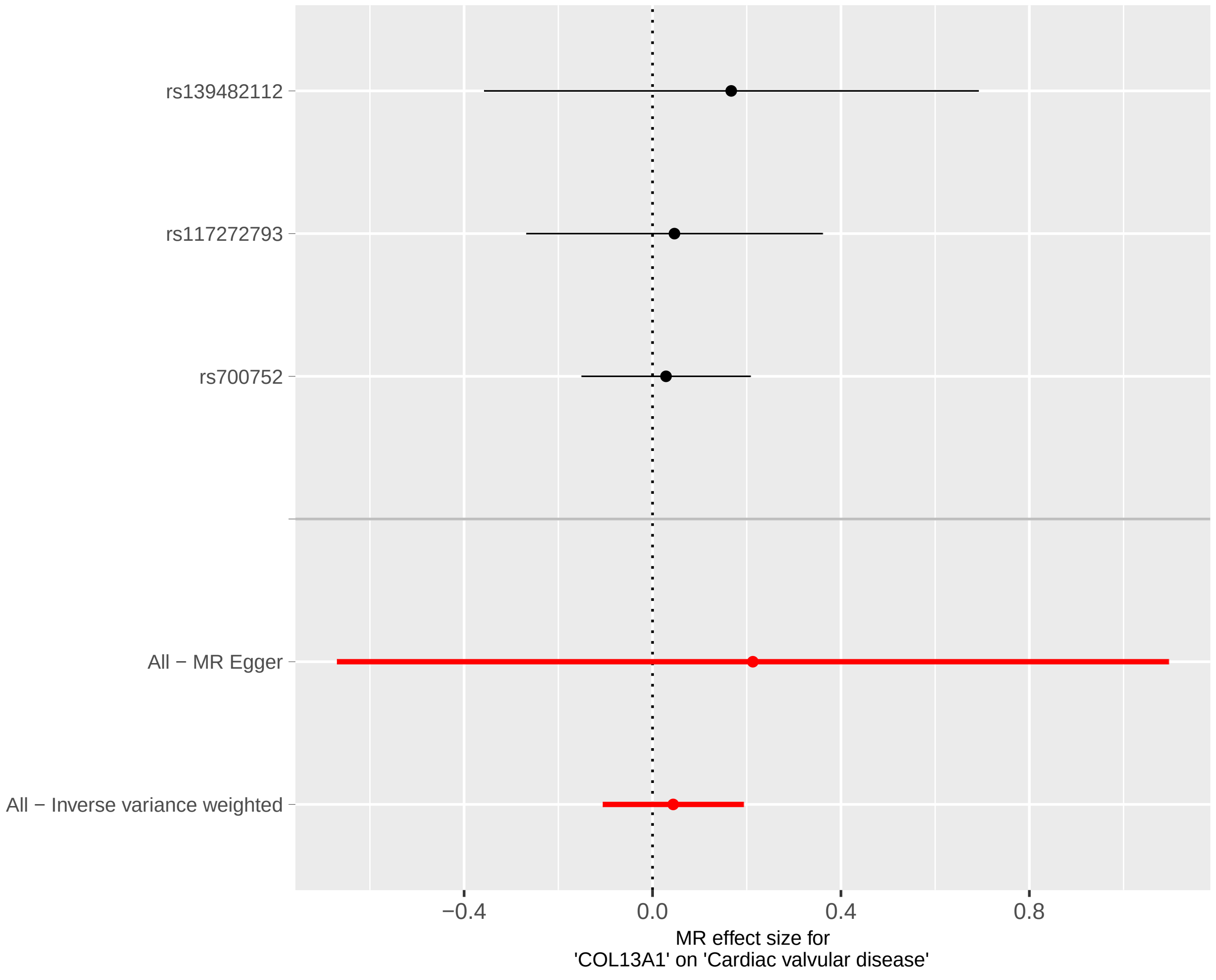
rs139482112
rs117272793
rs700752
All − MR Egger
All − Inverse variance weighted
−0.4
0.0
0.4
0.8
MR effect size for
'COL13A1' on 'Cardiac valvular disease'

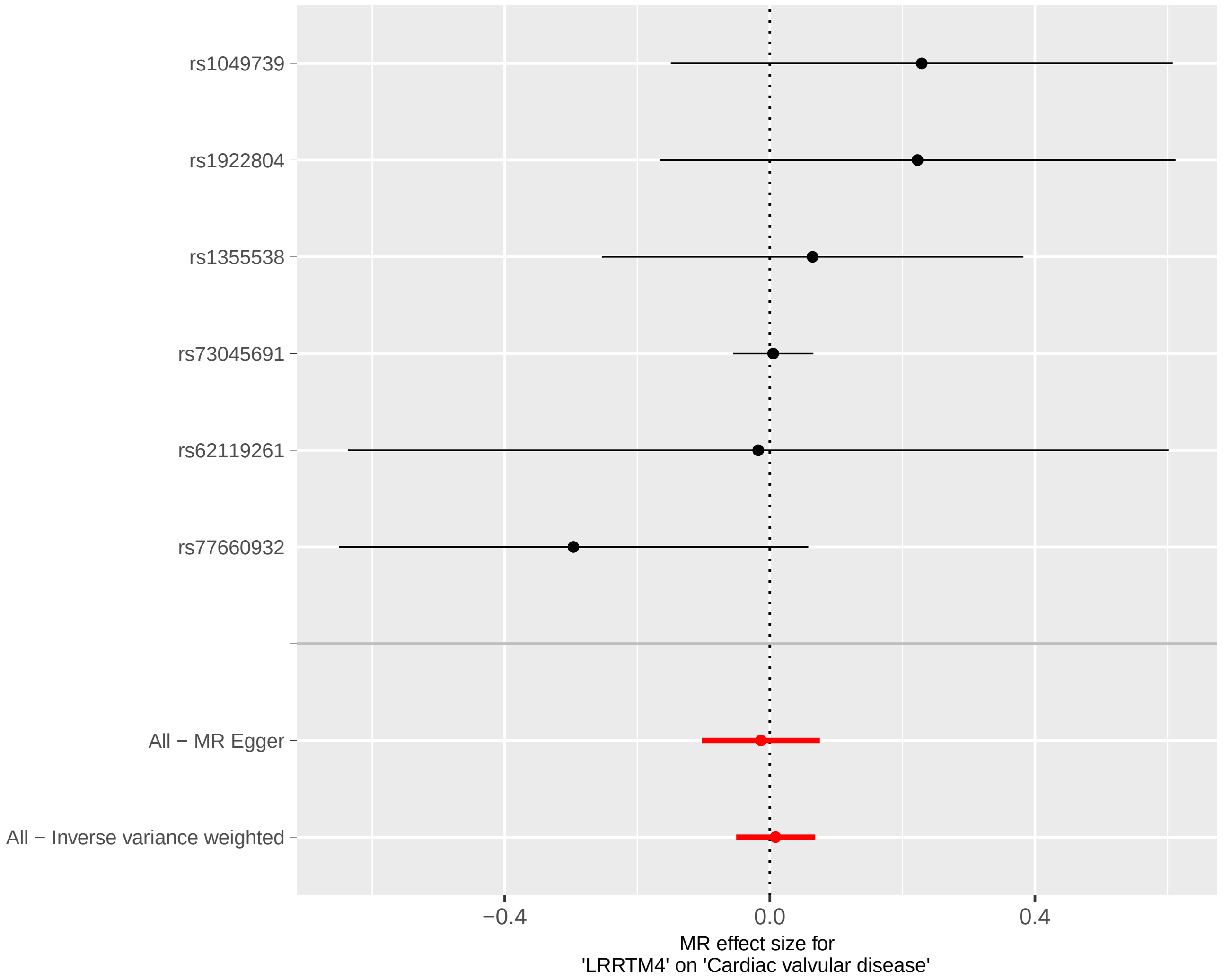
rs1049739
rs1922804
rs1355538
rs73045691
rs62119261
rs77660932
All − MR Egger
All − Inverse variance weighted
−0.4
0.0
0.4
MR effect size for
'LRRTM4' on 'Cardiac valvular disease'

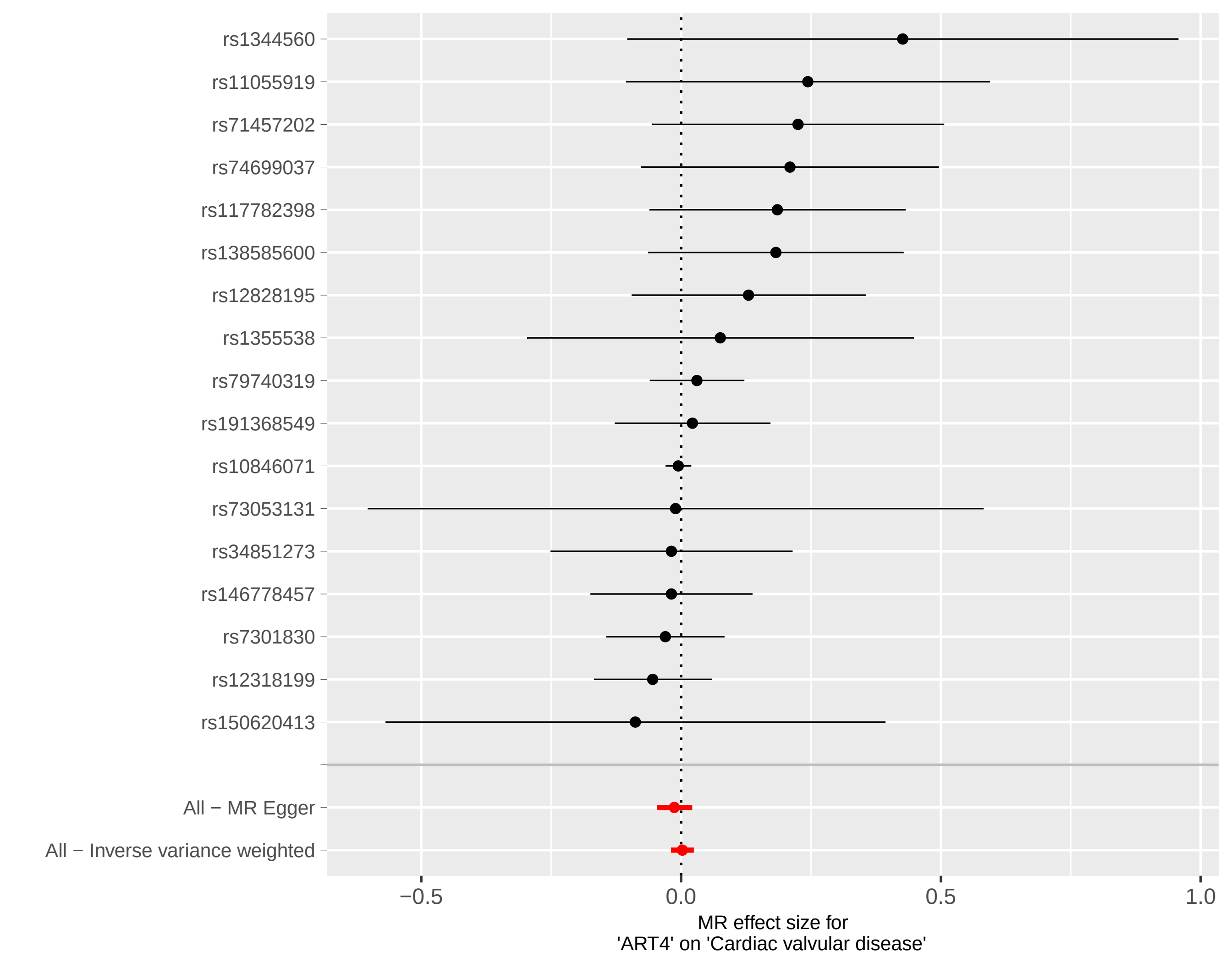
rs1344560
rs11055919
rs71457202
rs74699037
rs117782398
rs138585600
rs12828195
rs1355538
rs79740319
rs191368549
rs10846071
rs73053131
rs34851273
rs146778457
rs7301830
rs12318199
rs150620413
All − MR Egger
All − Inverse variance weighted
−0.5
0.0
0.5
1.0
MR effect size for
'ART4' on 'Cardiac valvular disease'

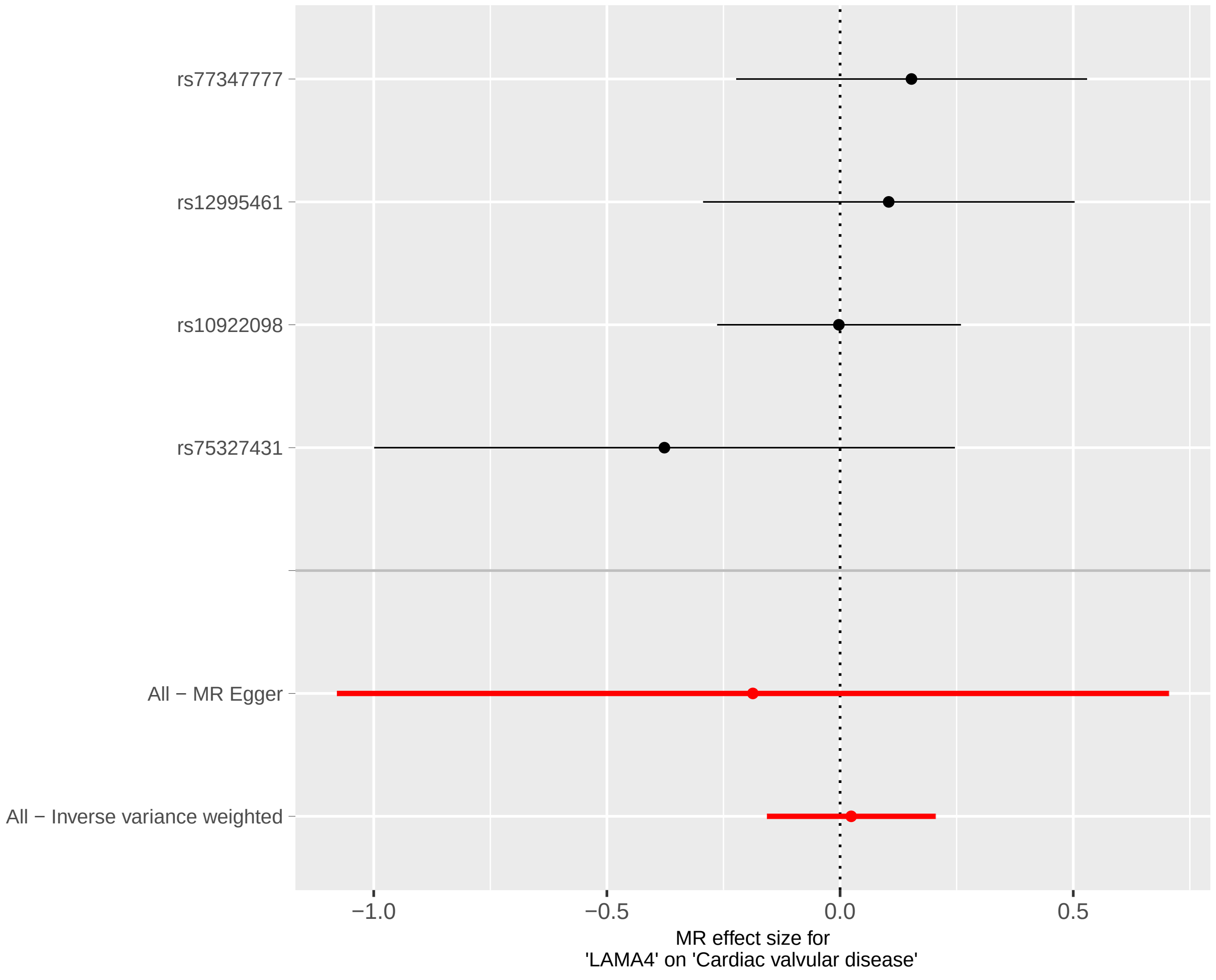
rs77347777
rs12995461
rs10922098
rs75327431
All − MR Egger
All − Inverse variance weighted
−1.0
−0.5
0.0
0.5
MR effect size for
'LAMA4' on 'Cardiac valvular disease'

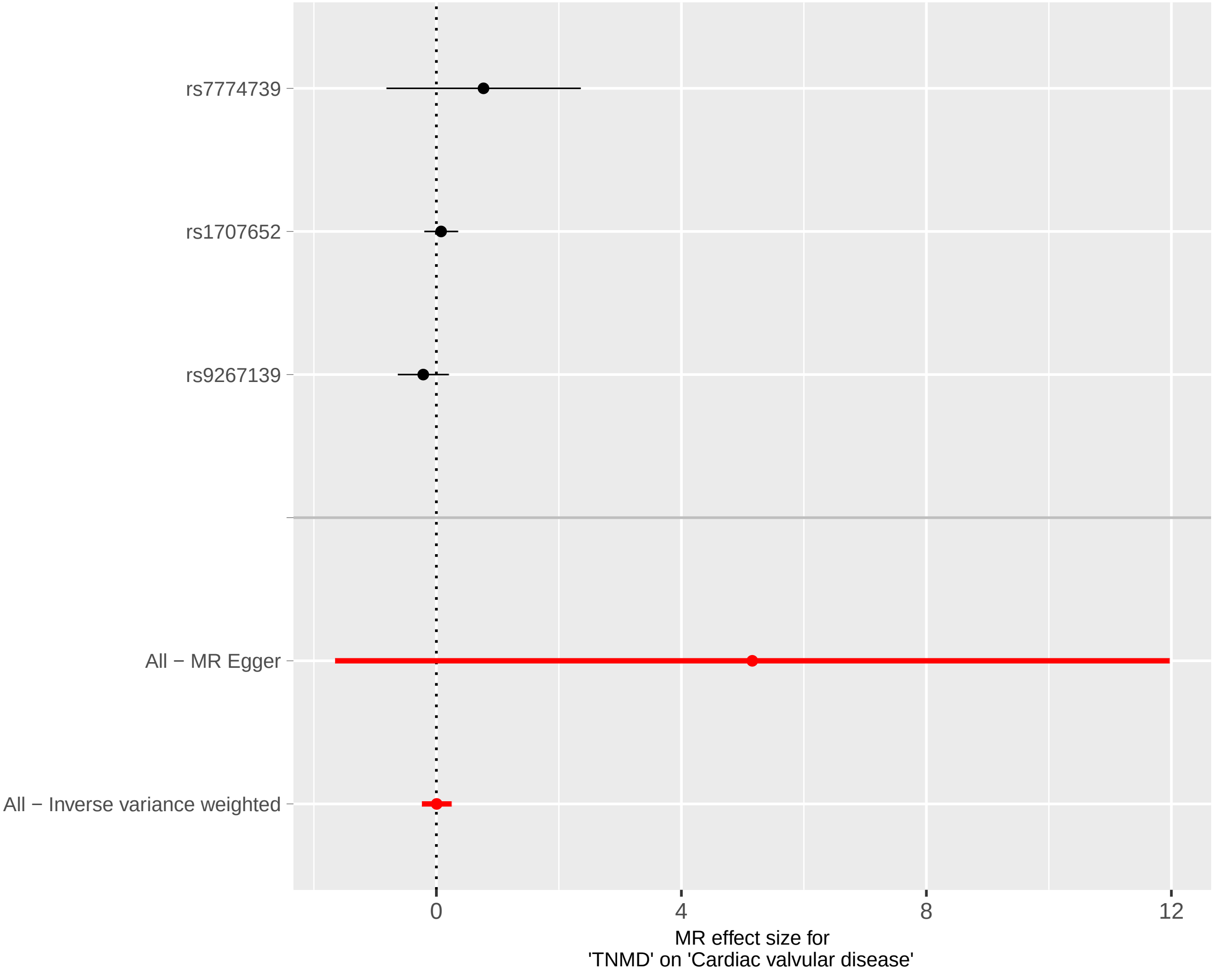
rs7774739
rs1707652
rs9267139
All − MR Egger
All − Inverse variance weighted
0
4
8
12
MR effect size for
'TNMD' on 'Cardiac valvular disease'

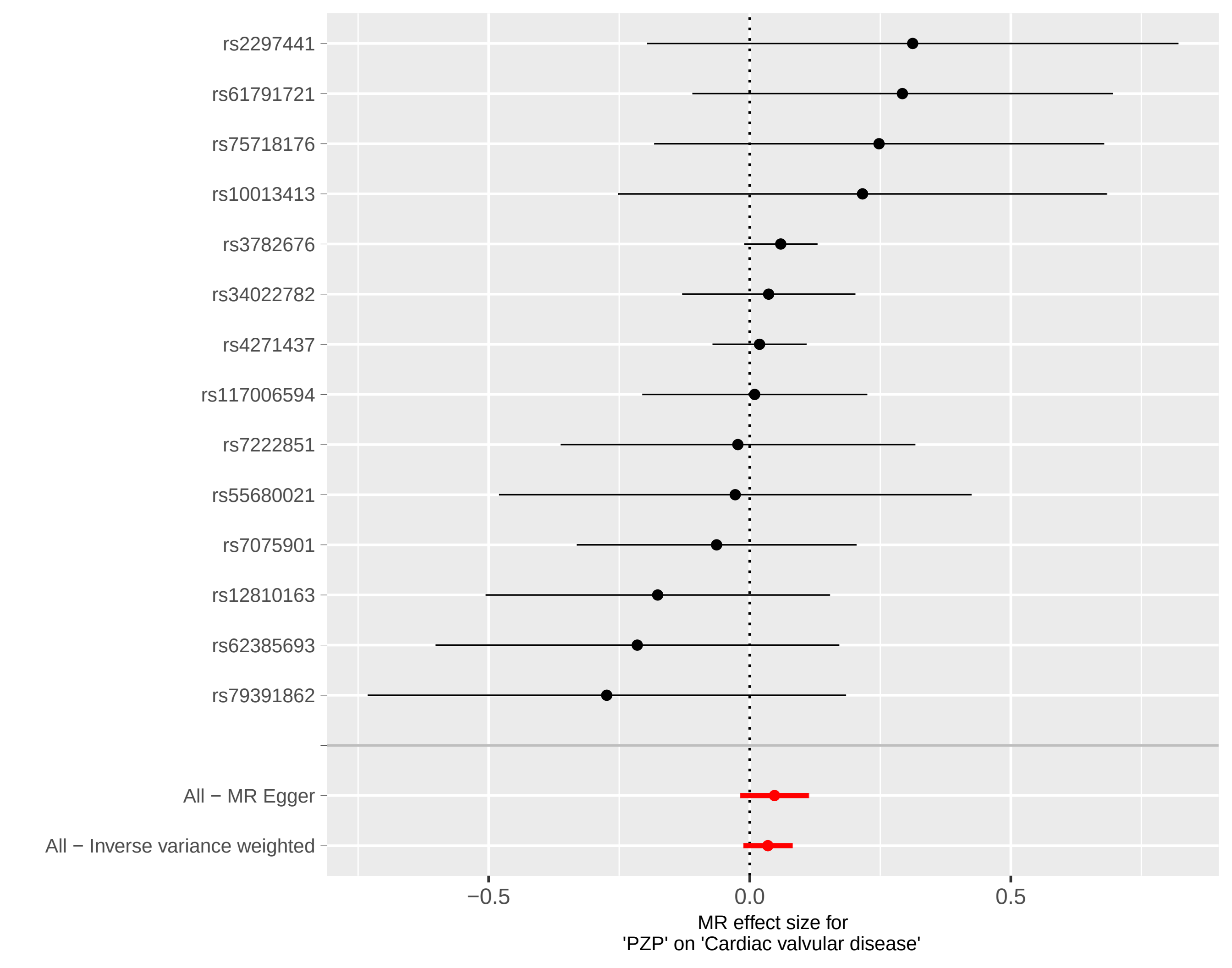
rs2297441
rs61791721
rs75718176
rs10013413
rs3782676
rs34022782
rs4271437
rs117006594
rs7222851
rs55680021
rs7075901
rs12810163
rs62385693
rs79391862
All − MR Egger
All − Inverse variance weighted
−0.5
0.0
0.5
MR effect size for
'PZP' on 'Cardiac valvular disease'

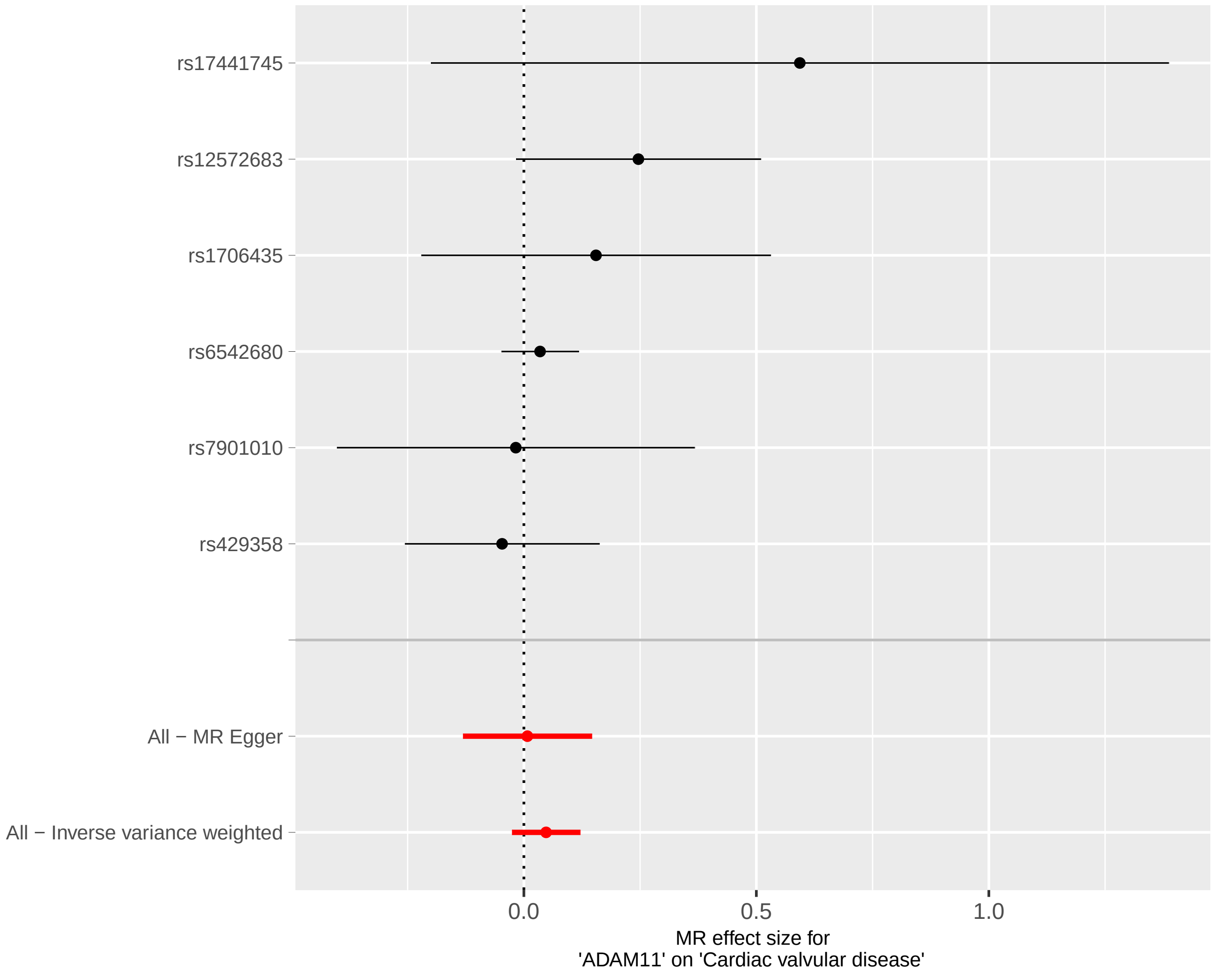
rs17441745
rs12572683
rs1706435
rs6542680
rs7901010
rs429358
All − MR Egger
All − Inverse variance weighted
0.0
0.5
1.0
MR effect size for
'ADAM11' on 'Cardiac valvular disease'

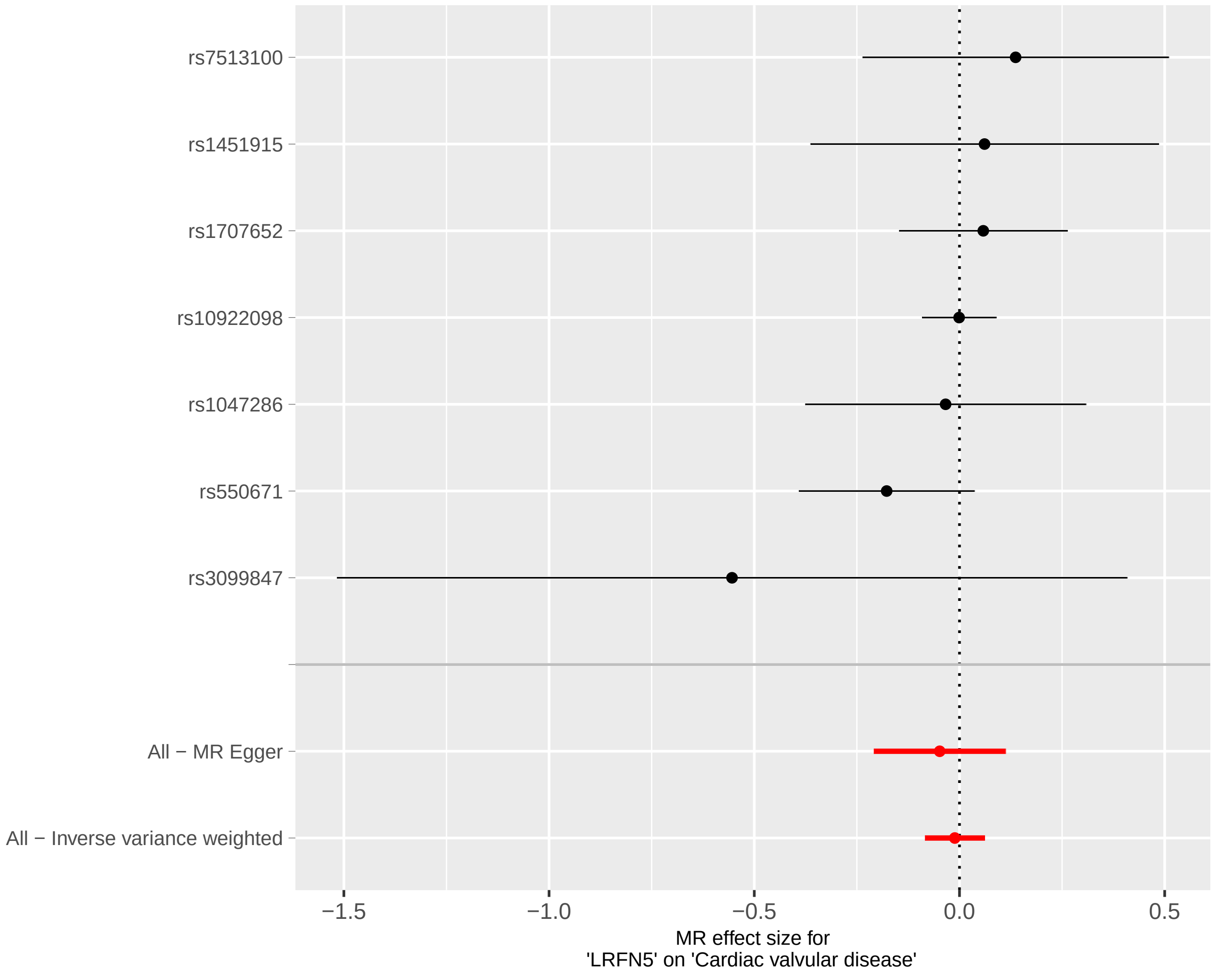

rs7513100
rs1451915
rs1707652
rs10922098
rs1047286
rs550671
rs3099847
All − MR Egger
All − Inverse variance weighted
−1.5
−1.0
−0.5
0.0
0.5
MR effect size for
'LRFN5' on 'Cardiac valvular disease'

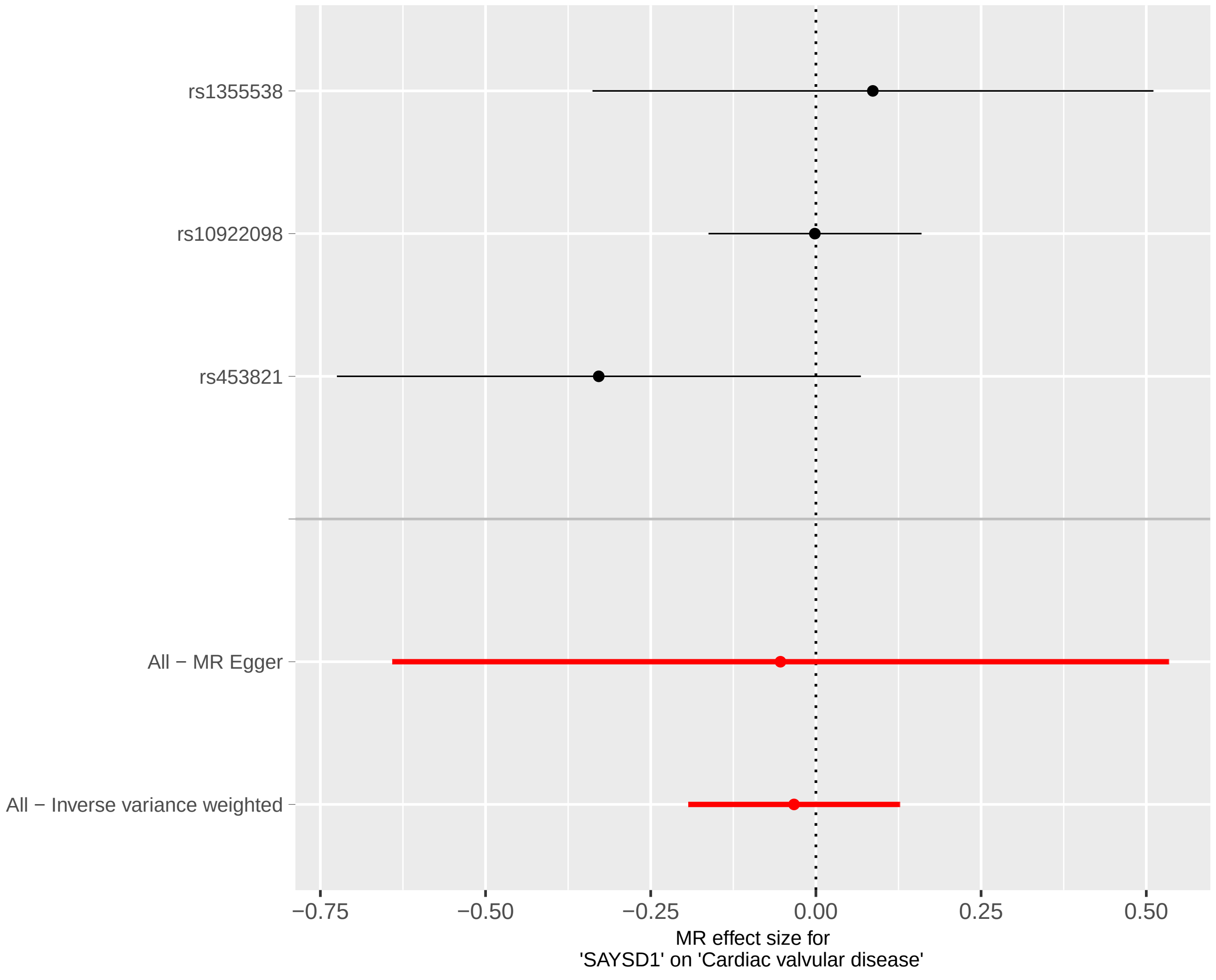
rs1355538
rs10922098
rs453821
All − MR Egger
All − Inverse variance weighted
−0.75
−0.50
−0.25
0.00
0.25
0.50
MR effect size for
'SAYSD1' on 'Cardiac valvular disease'

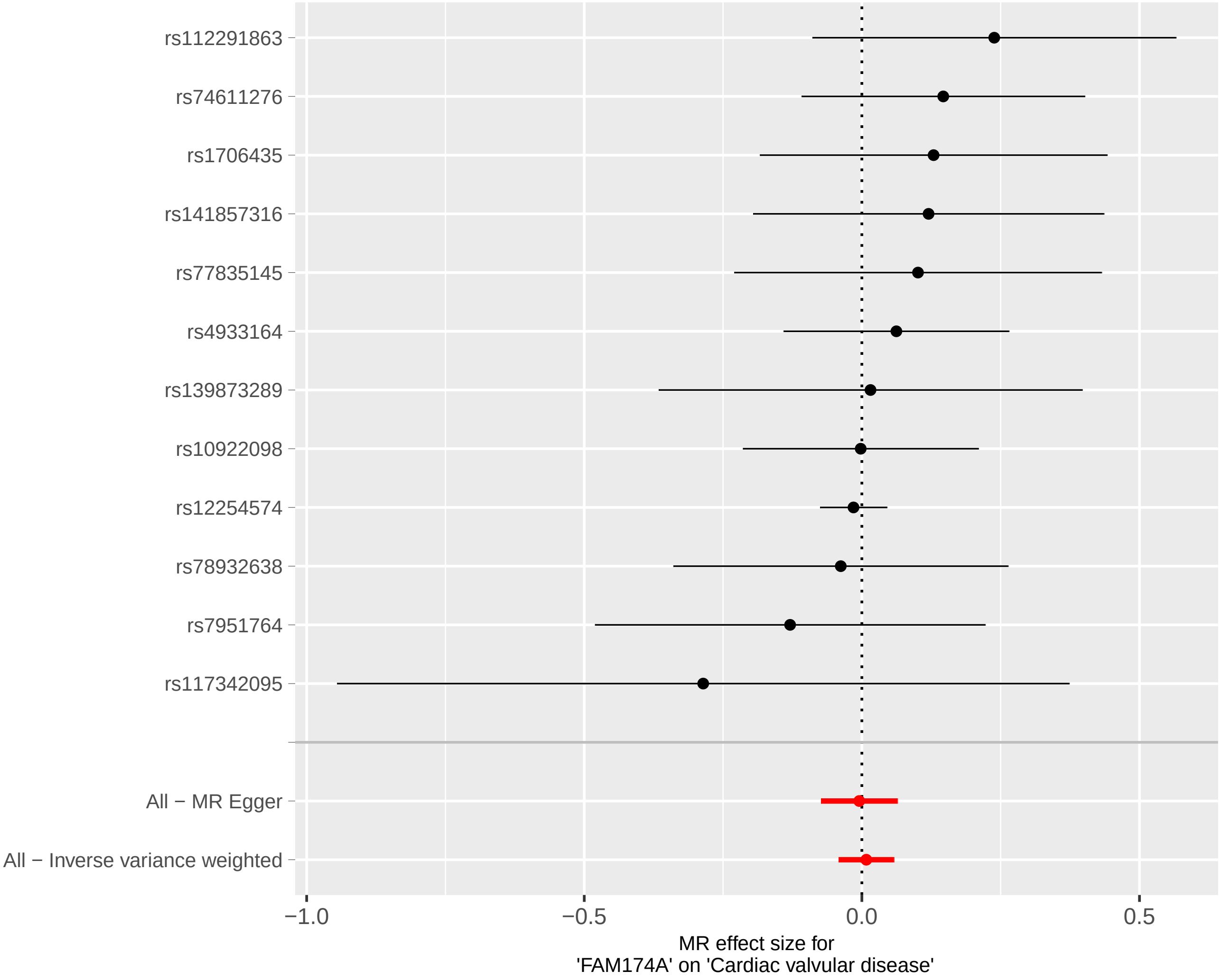
rs112291863
rs74611276
rs1706435
rs141857316
rs77835145
rs4933164
rs139873289
rs10922098
rs12254574
rs78932638
rs7951764
rs117342095
All − MR Egger
All − Inverse variance weighted
−1.0
−0.5
0.0
0.5
MR effect size for
'FAM174A' on 'Cardiac valvular disease'

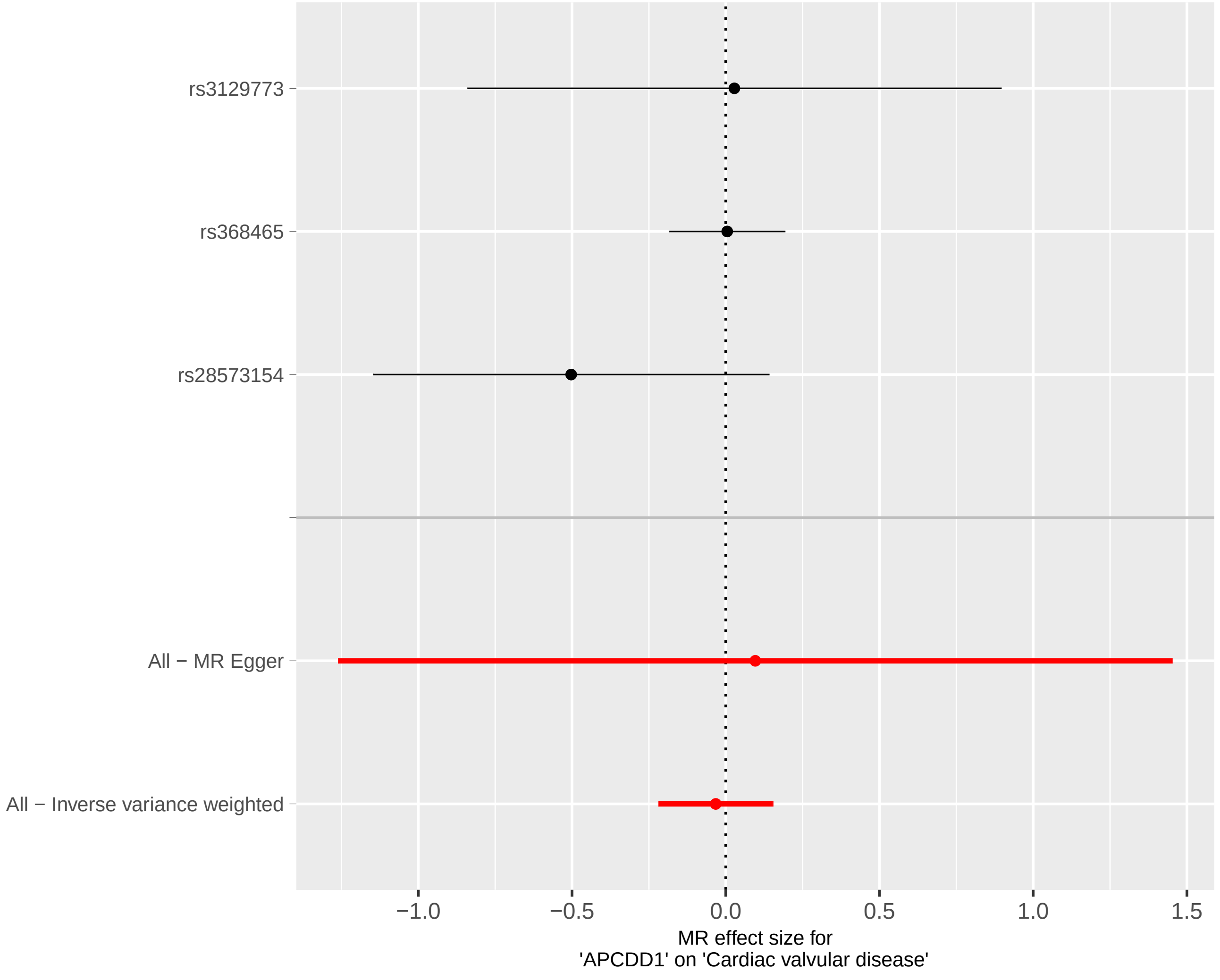
rs3129773
rs368465
rs28573154
All − MR Egger
All − Inverse variance weighted
−1.0
−0.5
0.0
0.5
1.0
1.5
MR effect size for
'APCDD1' on 'Cardiac valvular disease'

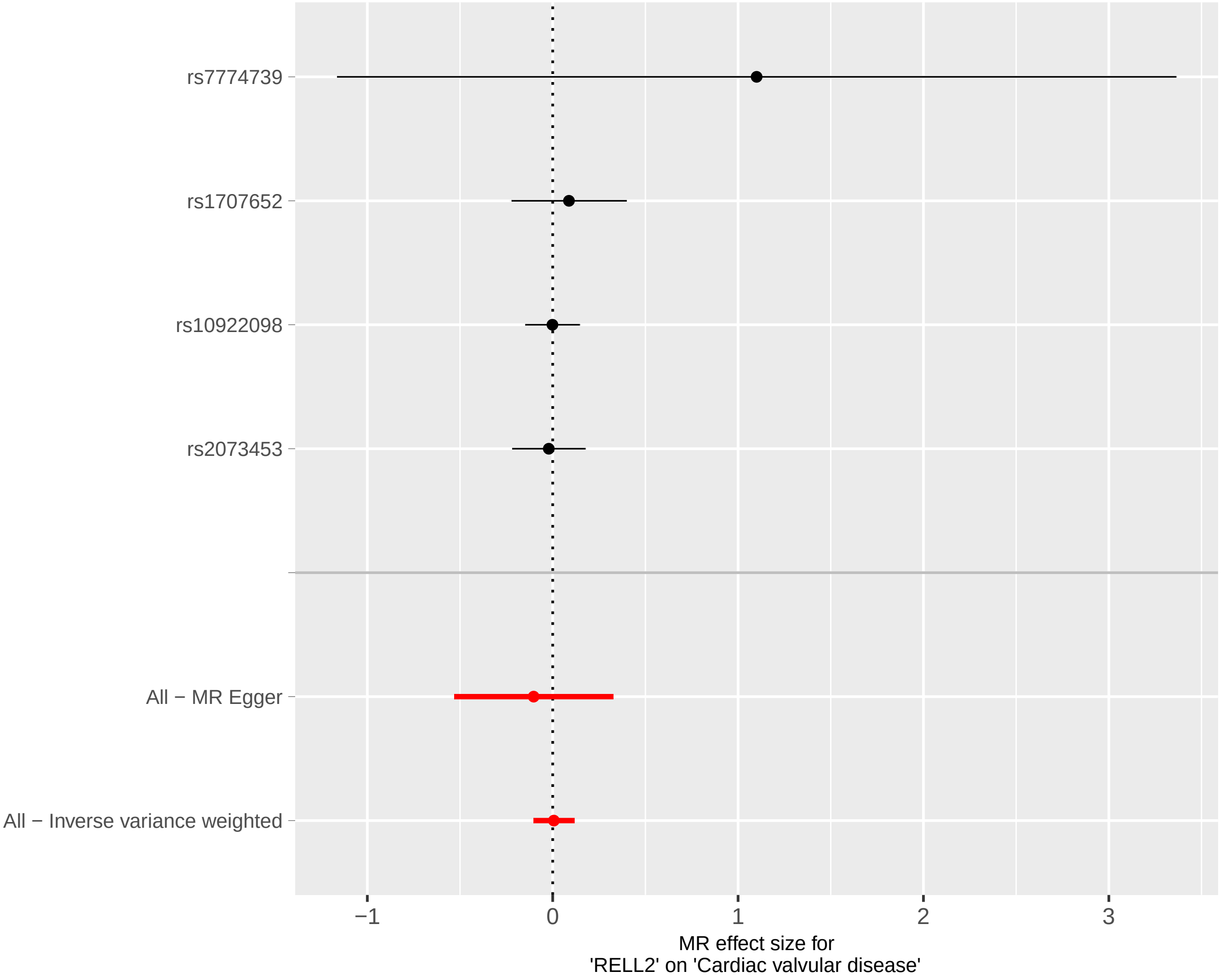
rs7774739
rs1707652
rs10922098
rs2073453
All − MR Egger
All − Inverse variance weighted
−1
0
1
2
3
MR effect size for
'RELL2' on 'Cardiac valvular disease'

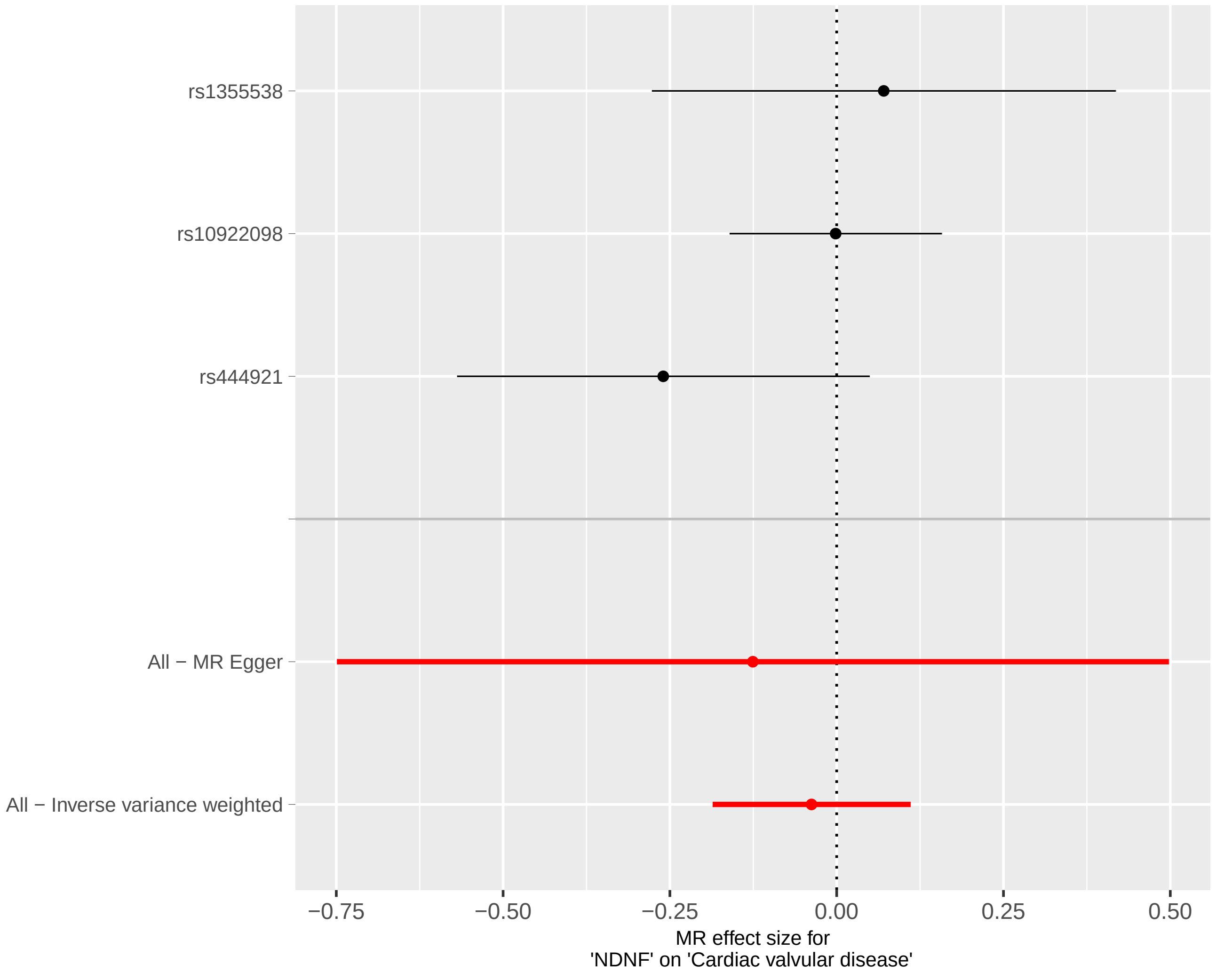
rs1355538
rs10922098
rs444921
All − MR Egger
All − Inverse variance weighted
−0.75
−0.50
−0.25
0.00
0.25
0.50
MR effect size for
'NDNF' on 'Cardiac valvular disease'

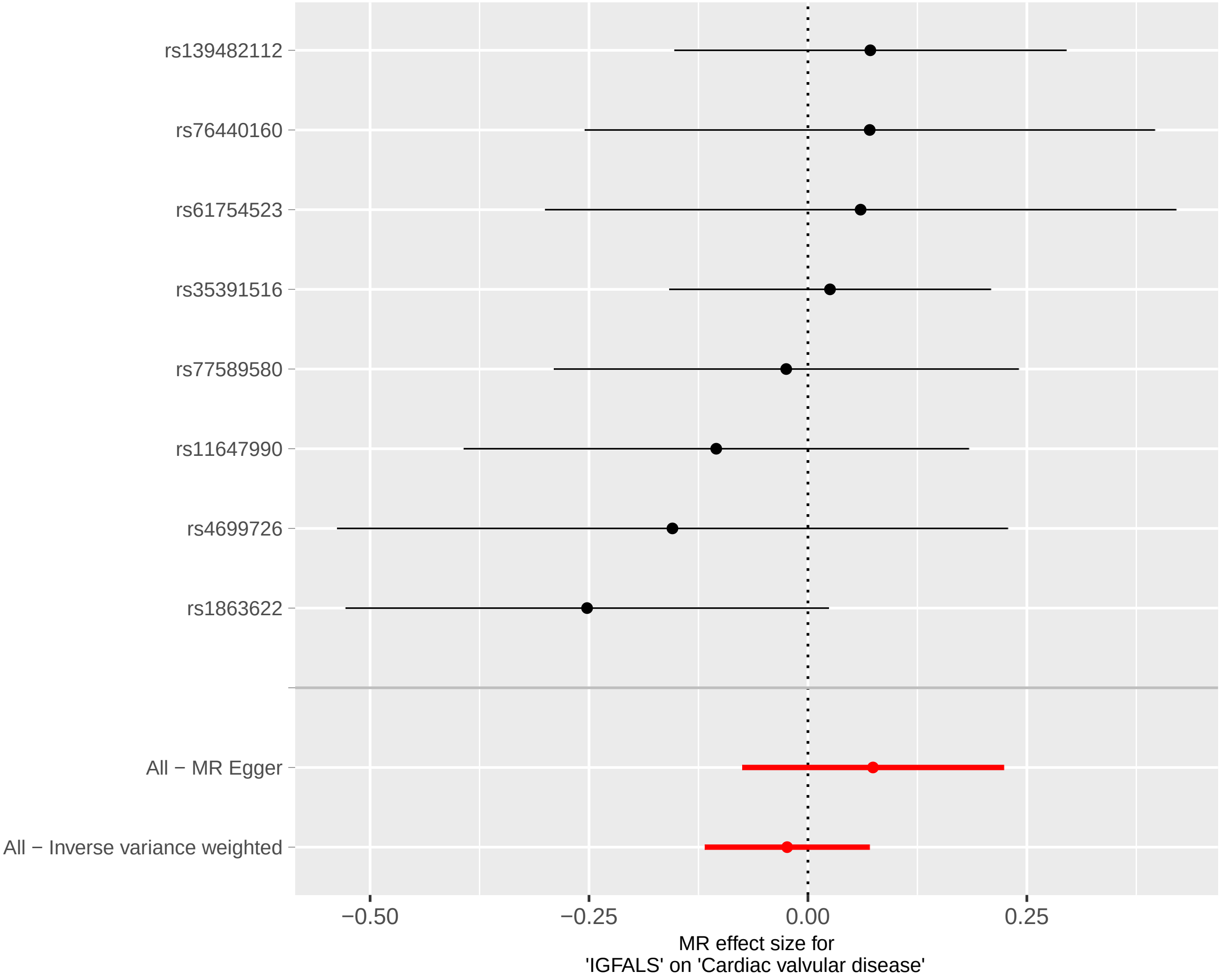
rs139482112
rs76440160
rs61754523
rs35391516
rs77589580
rs11647990
rs4699726
rs1863622
All − MR Egger
All − Inverse variance weighted
−0.50
−0.25
0.00
0.25
MR effect size for
'IGFALS' on 'Cardiac valvular disease'

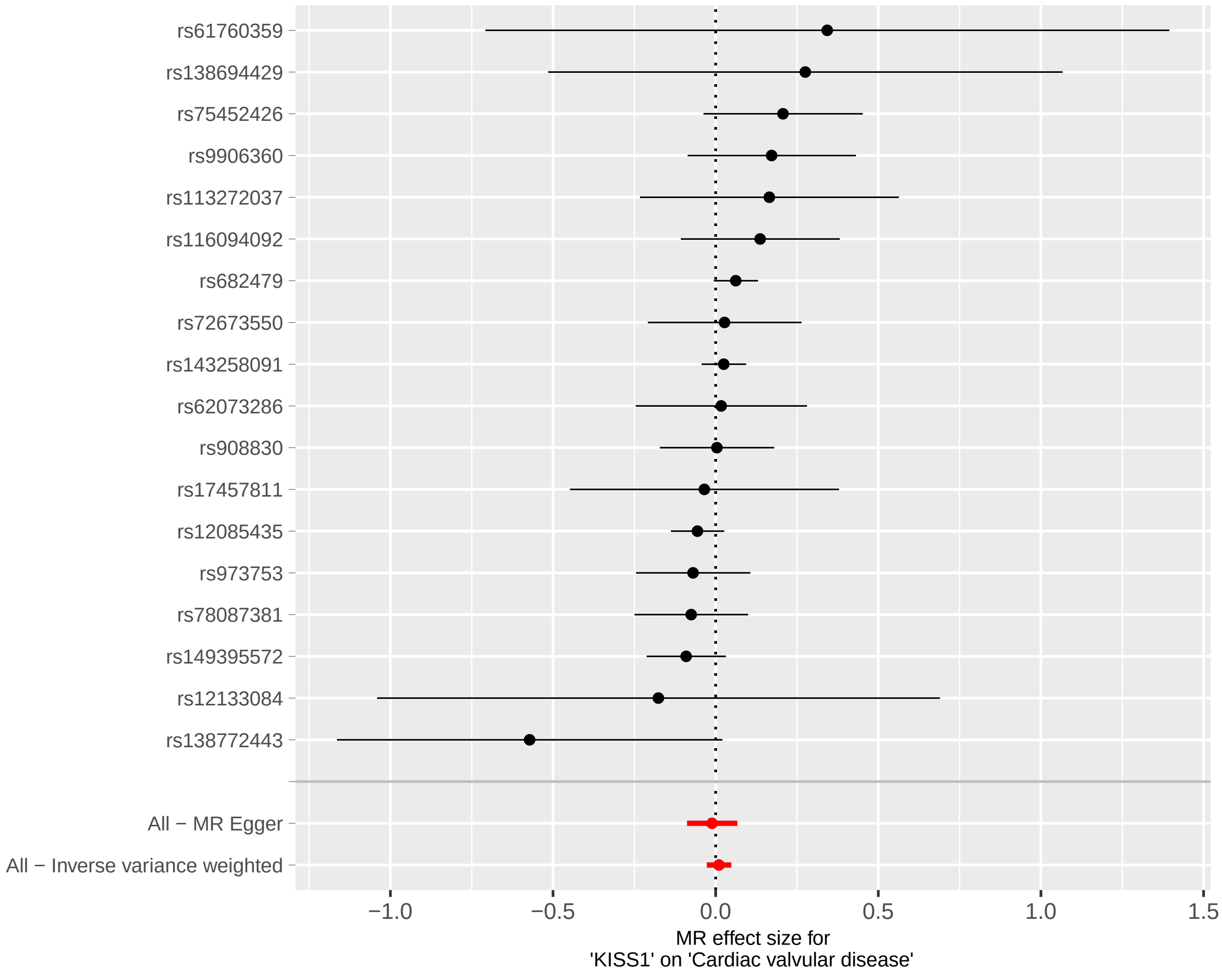

rs61760359
rs138694429
rs75452426
rs9906360
rs113272037
rs116094092
rs682479
rs72673550
rs143258091
rs62073286
rs908830
rs17457811
rs12085435
rs973753
rs78087381
rs149395572
rs12133084
rs138772443
All − MR Egger
All − Inverse variance weighted
−1.0
−0.5
0.0
0.5
1.0
1.5
MR effect size for
'KISS1' on 'Cardiac valvular disease'

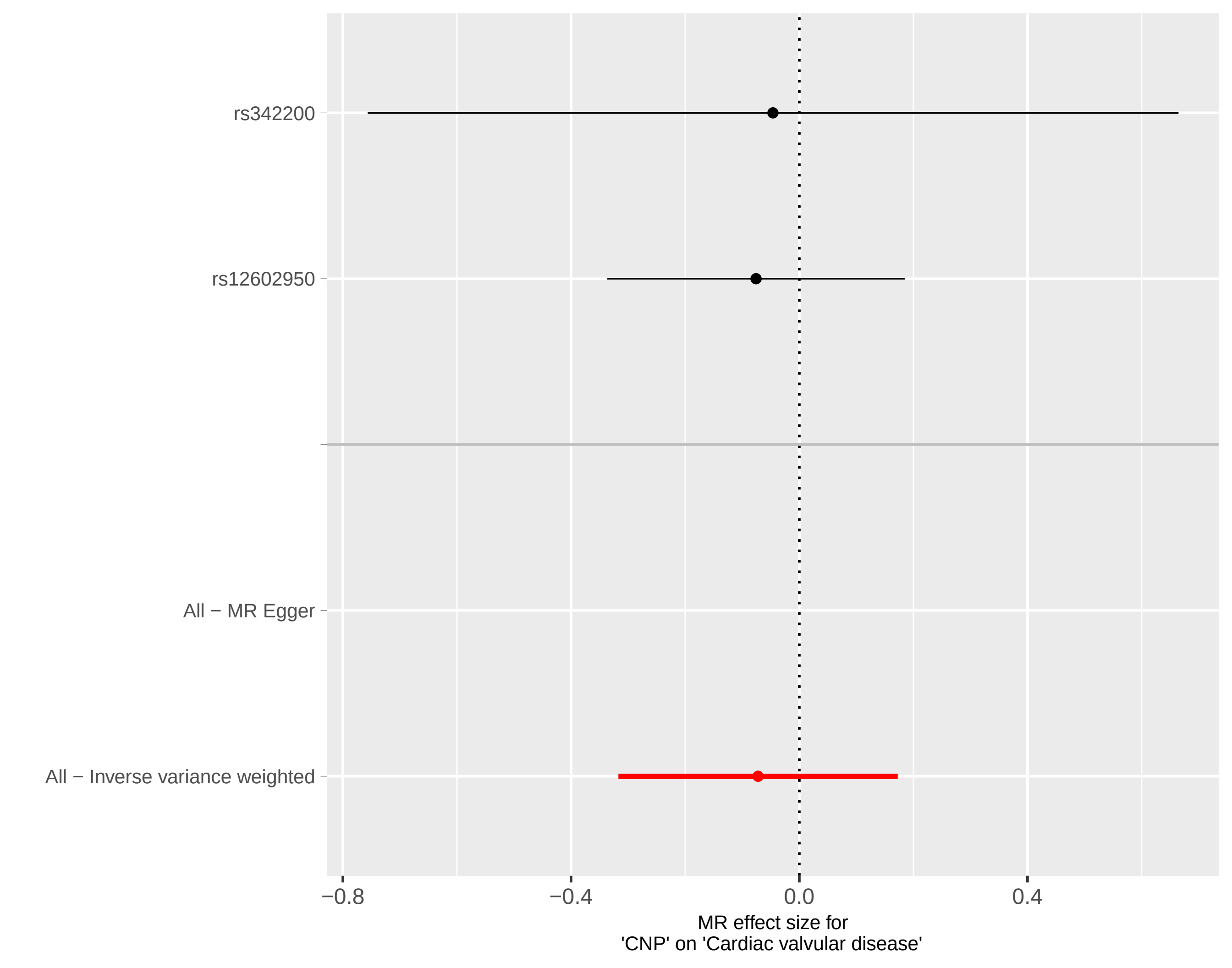
rs342200
rs12602950
All − MR Egger
All − Inverse variance weighted
−0.8
−0.4
0.0
0.4
MR effect size for
'CNP' on 'Cardiac valvular disease'

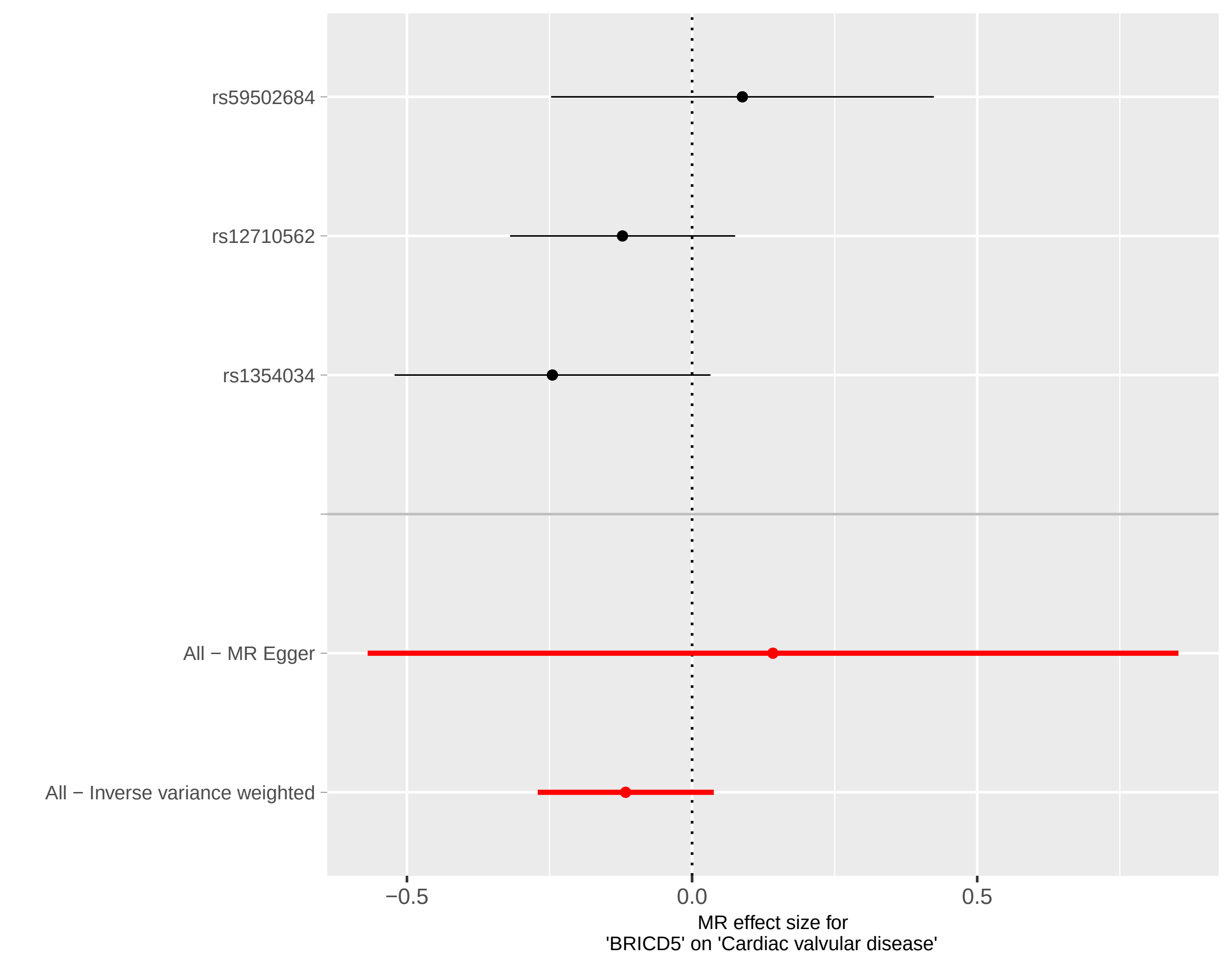

rs59502684
rs12710562
rs1354034
All − MR Egger
All − Inverse variance weighted
−0.5
0.0
0.5
MR effect size for
'BRICD5' on 'Cardiac valvular disease'

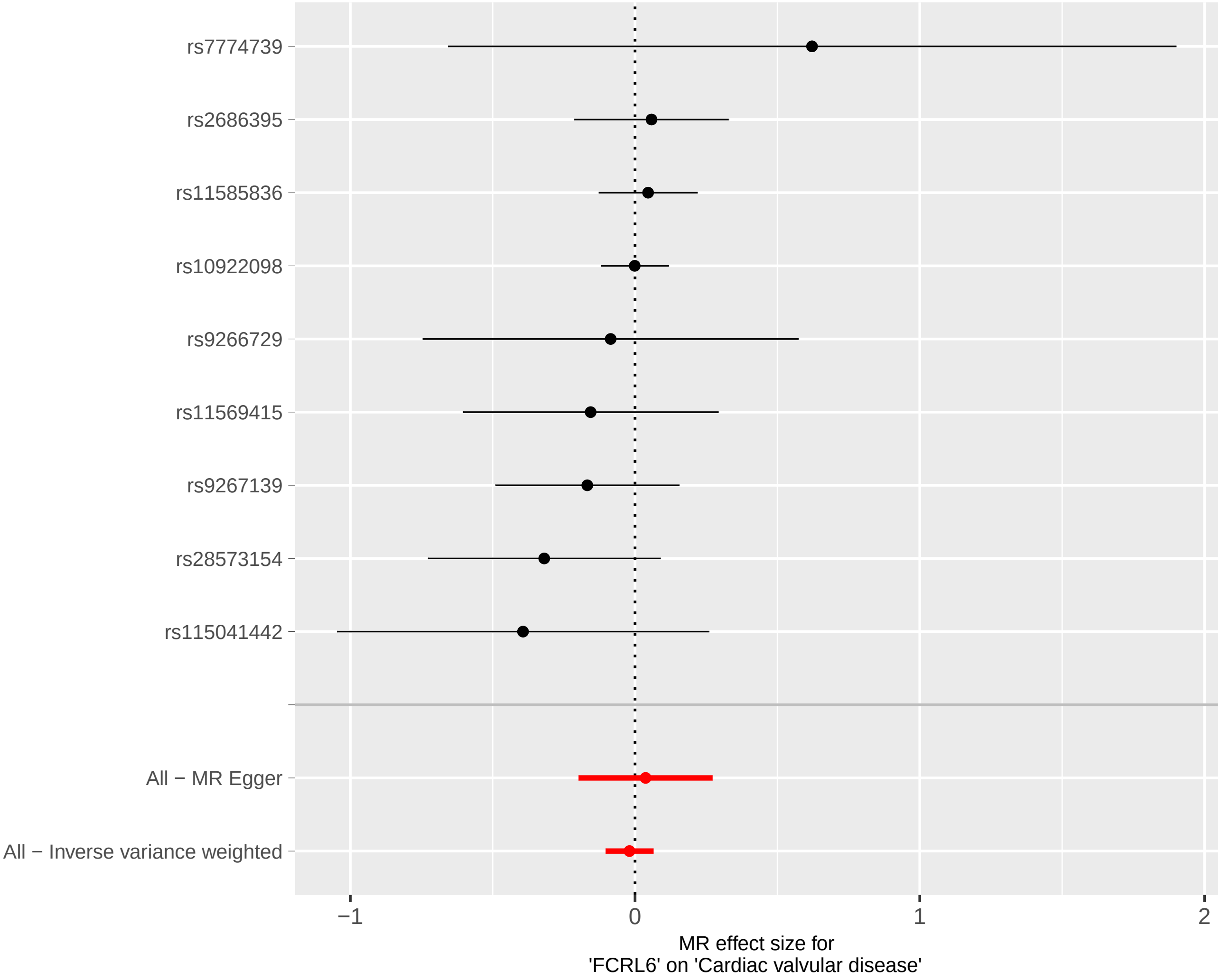
rs7774739
rs2686395
rs11585836
rs10922098
rs9266729
rs11569415
rs9267139
rs28573154
rs115041442
All − MR Egger
All − Inverse variance weighted
−1
0
1
2
MR effect size for
'FCRL6' on 'Cardiac valvular disease'

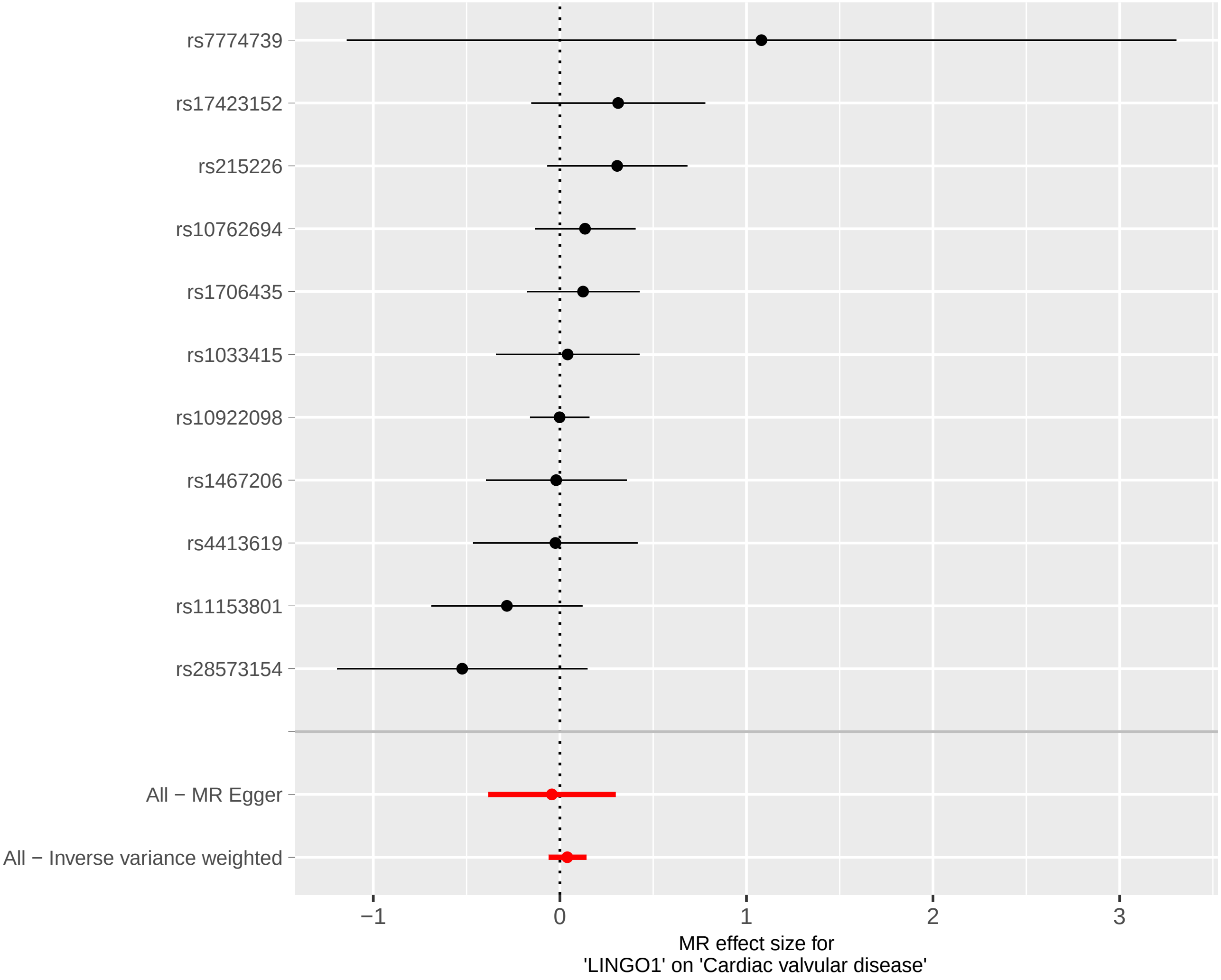
rs7774739
rs17423152
rs215226
rs10762694
rs1706435
rs1033415
rs10922098
rs1467206
rs4413619
rs11153801
rs28573154
All − MR Egger
All − Inverse variance weighted
−1
0
1
2
3
MR effect size for
'LINGO1' on 'Cardiac valvular disease'

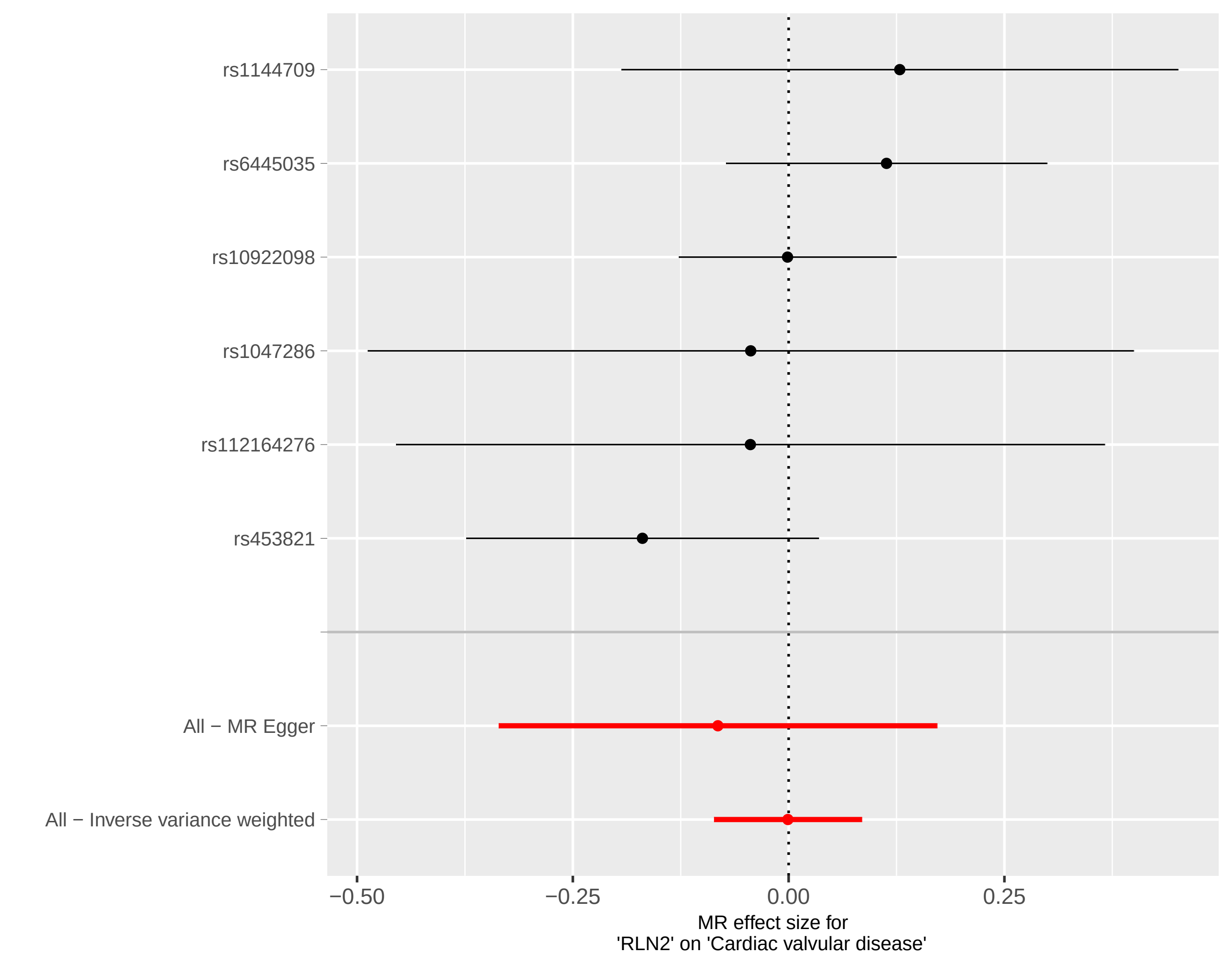

rs1144709
rs6445035
rs10922098
rs1047286
rs112164276
rs453821
All − MR Egger
All − Inverse variance weighted
−0.50
−0.25
0.00
0.25
MR effect size for
'RLN2' on 'Cardiac valvular disease'

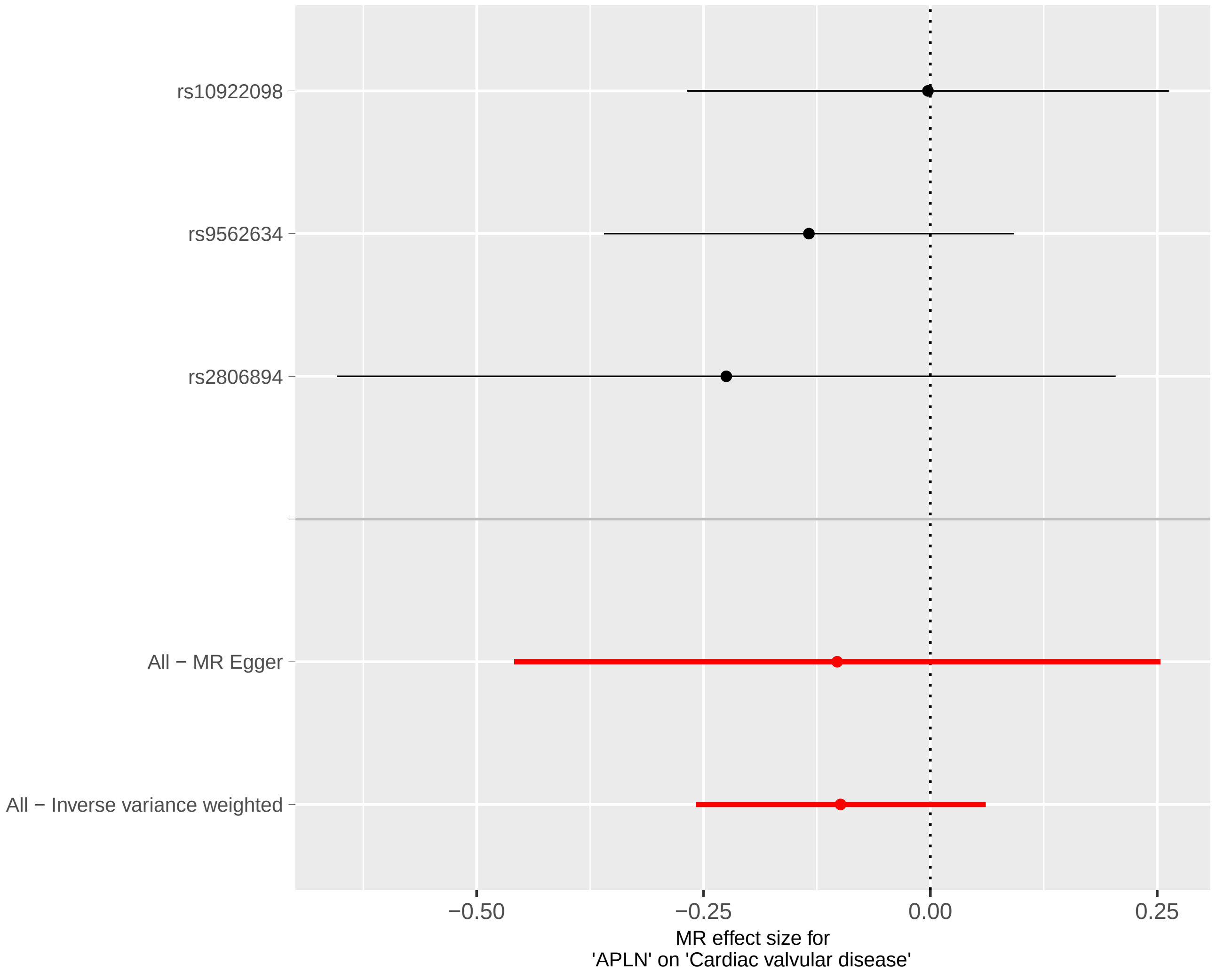
rs10922098
rs9562634
rs2806894
All − MR Egger
All − Inverse variance weighted
−0.50
−0.25
0.00
0.25
MR effect size for
'APLN' on 'Cardiac valvular disease'

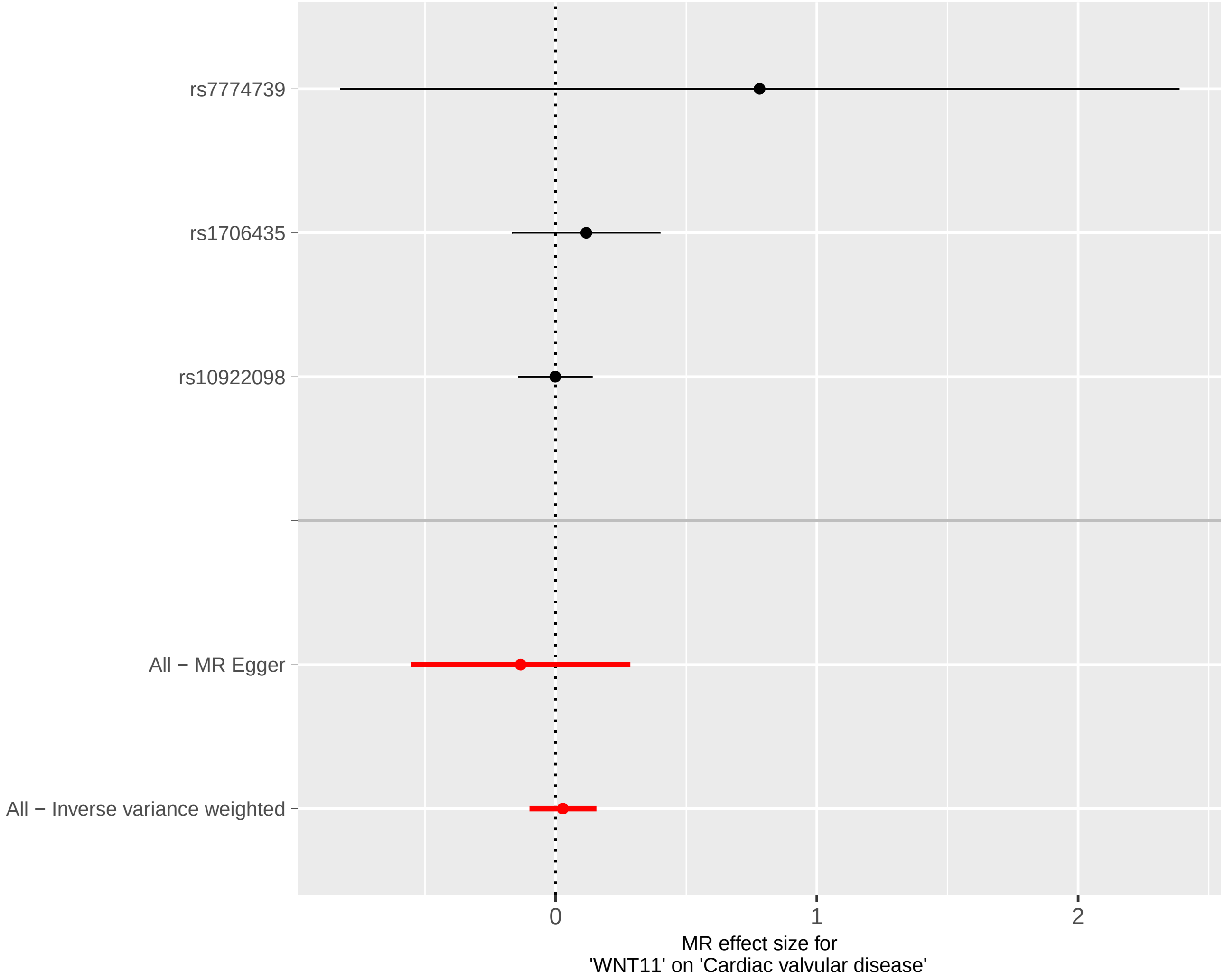

rs7774739
rs1706435
rs10922098
All − MR Egger
All − Inverse variance weighted
0
1
2
MR effect size for
'WNT11' on 'Cardiac valvular disease'

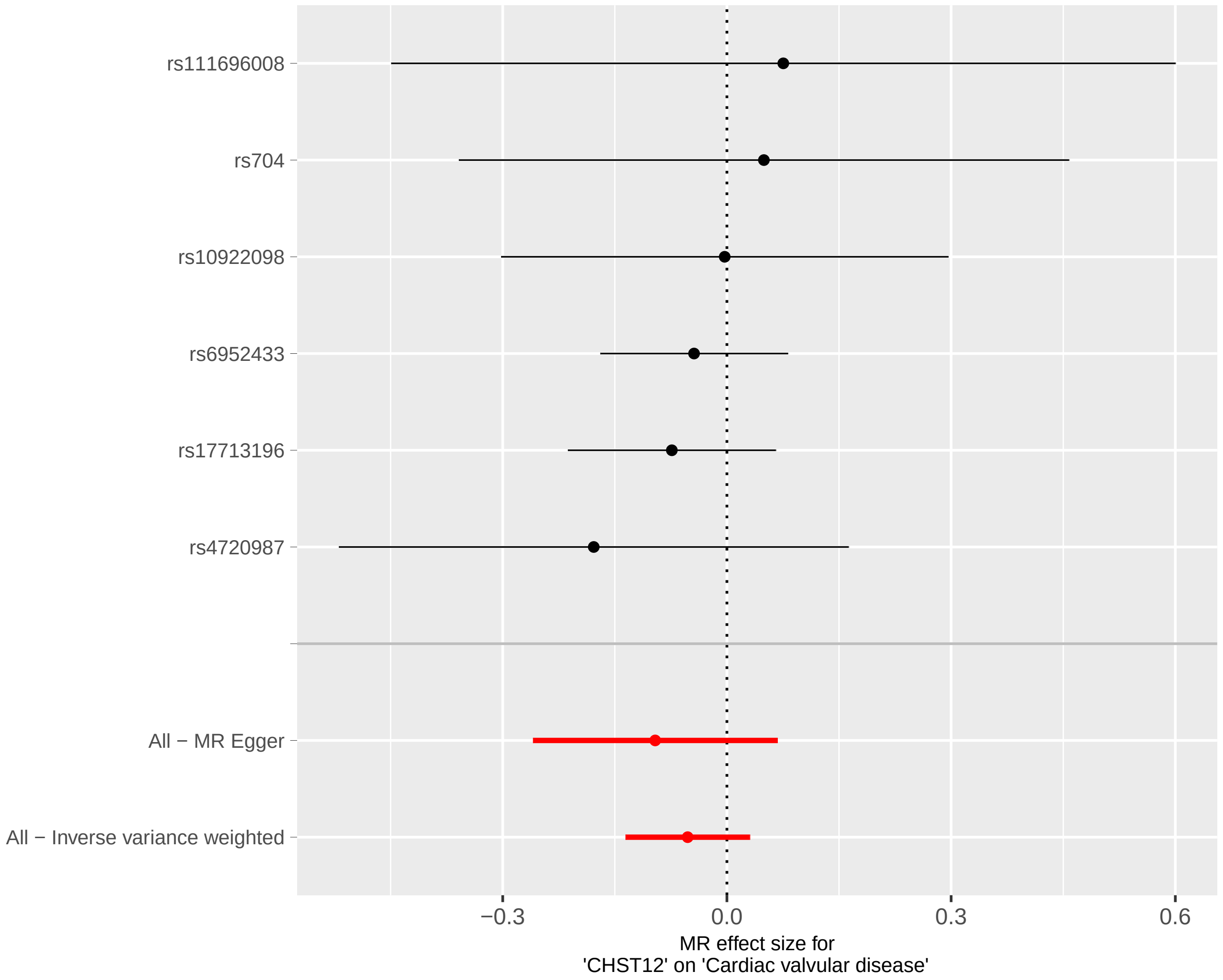

rs111696008
rs704
rs10922098
rs6952433
rs17713196
rs4720987
All − MR Egger
All − Inverse variance weighted
−0.3
0.0
0.3
0.6
MR effect size for
'CHST12' on 'Cardiac valvular disease'

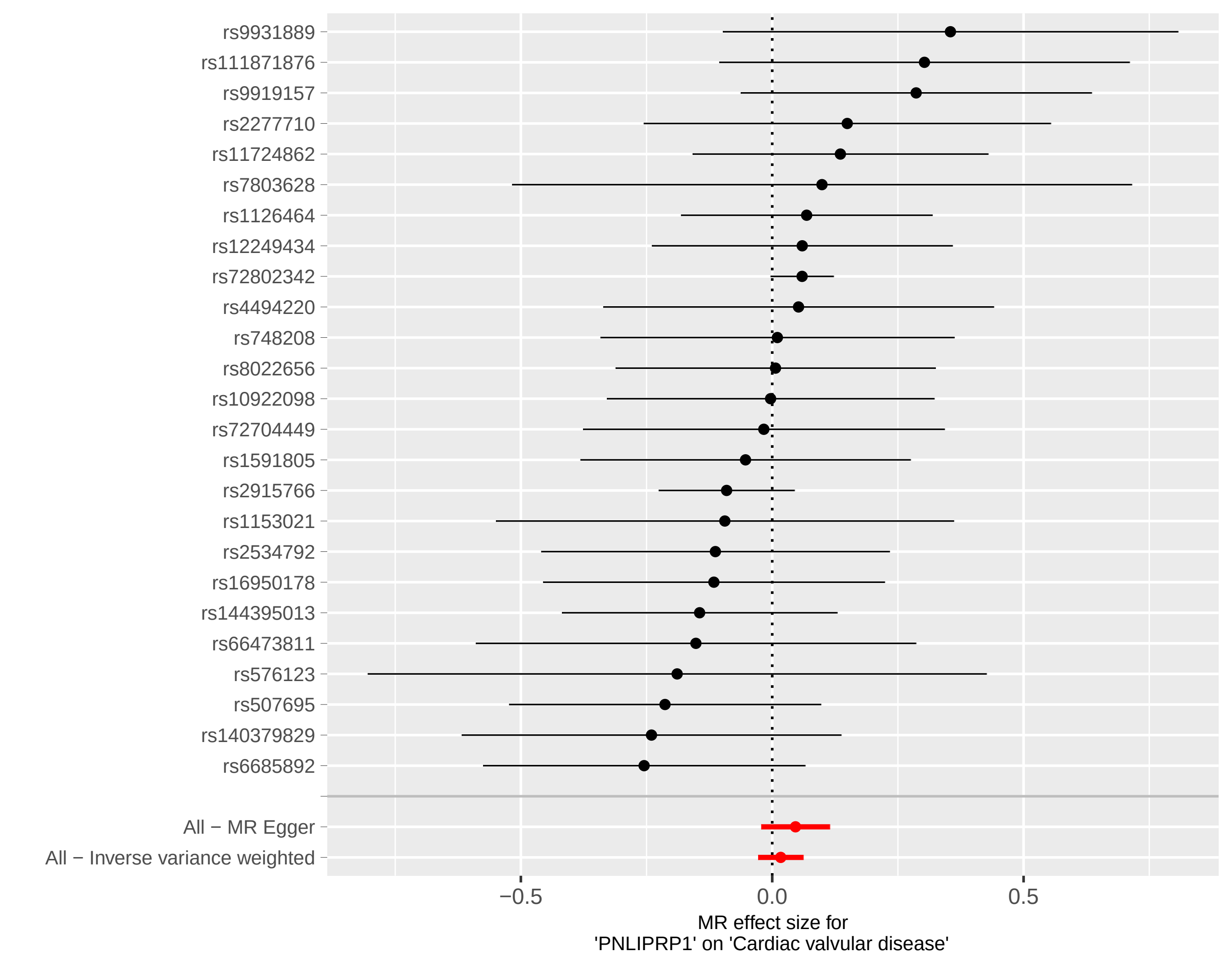
rs9931889
rs111871876
rs9919157
rs2277710
rs11724862
rs7803628
rs1126464
rs12249434
rs72802342
rs4494220
rs748208
rs8022656
rs10922098
rs72704449
rs1591805
rs2915766
rs1153021
rs2534792
rs16950178
rs144395013
rs66473811
rs576123
rs507695
rs140379829
rs6685892
All − MR Egger
All − Inverse variance weighted
−0.5
0.0
0.5
MR effect size for
'PNLIPRP1' on 'Cardiac valvular disease'

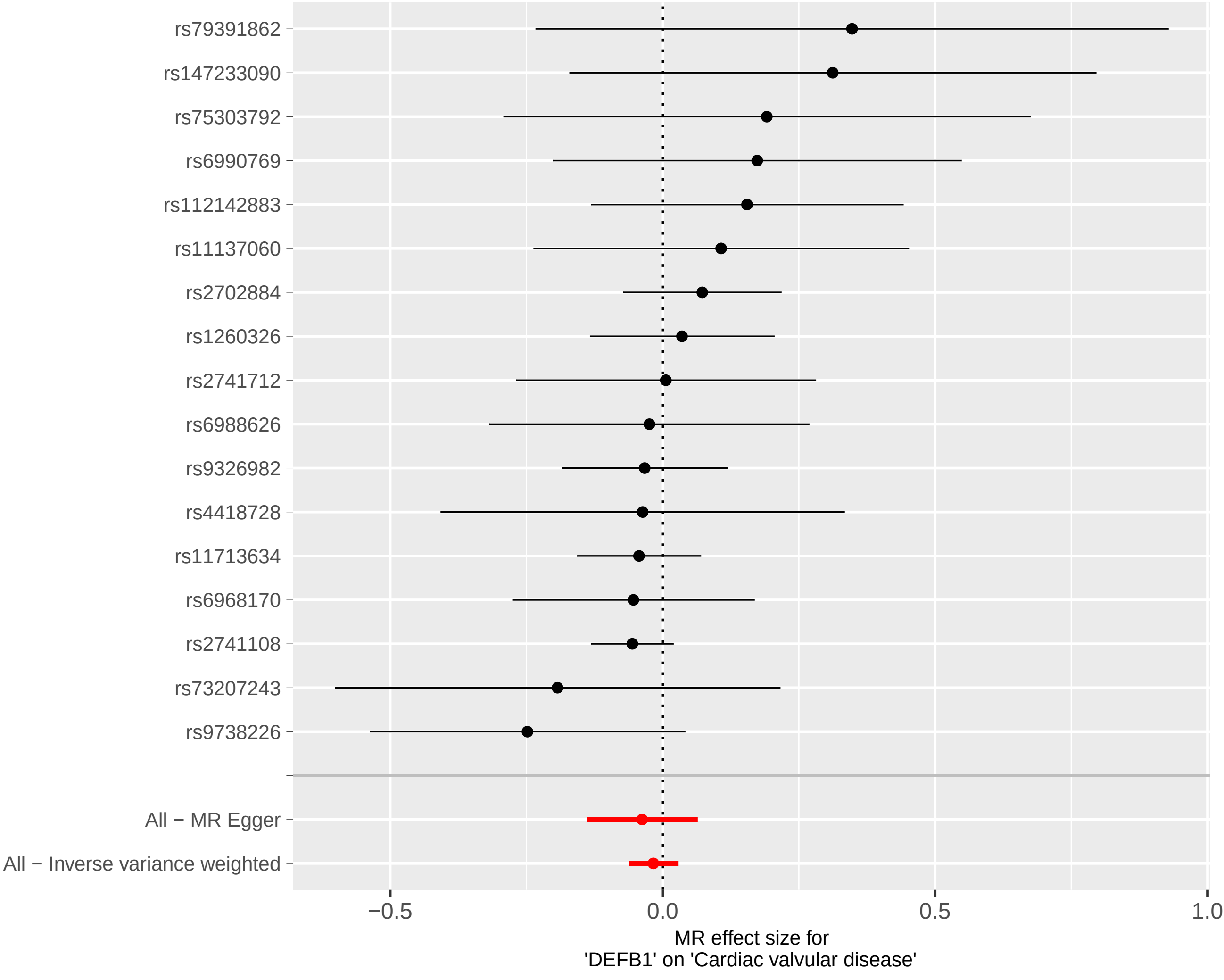
rs79391862
rs147233090
rs75303792
rs6990769
rs112142883
rs11137060
rs2702884
rs1260326
rs2741712
rs6988626
rs9326982
rs4418728
rs11713634
rs6968170
rs2741108
rs73207243
rs9738226
All − MR Egger
All − Inverse variance weighted
−0.5
0.0
0.5
1.0
MR effect size for
'DEFB1' on 'Cardiac valvular disease'

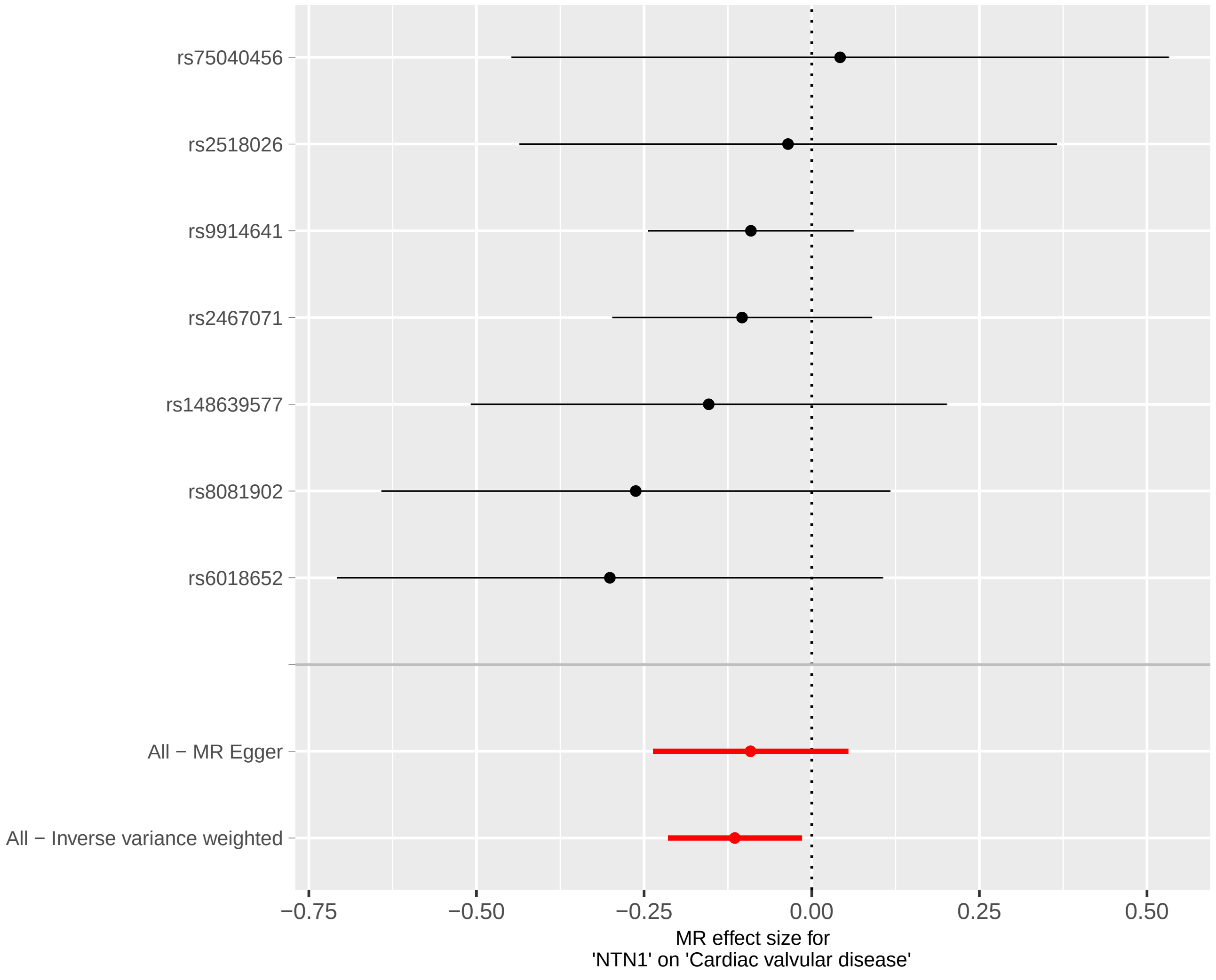
rs75040456
rs2518026
rs9914641
rs2467071
rs148639577
rs8081902
rs6018652
All − MR Egger
All − Inverse variance weighted
−0.75
−0.50
−0.25
0.00
0.25
0.50
MR effect size for
'NTN1' on 'Cardiac valvular disease'

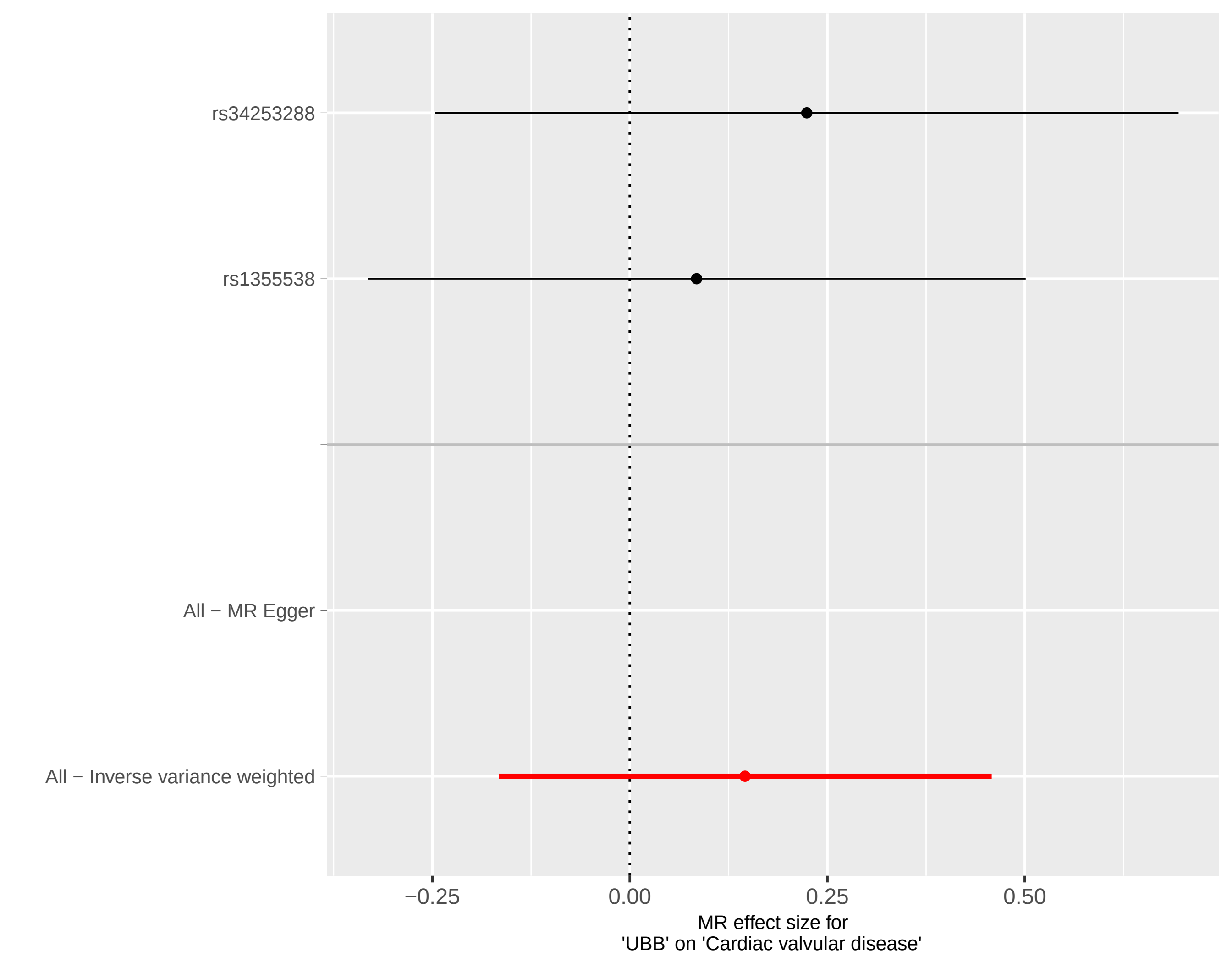
rs34253288
rs1355538
All − MR Egger
All − Inverse variance weighted
−0.25
0.00
0.25
0.50
MR effect size for
'UBB' on 'Cardiac valvular disease'

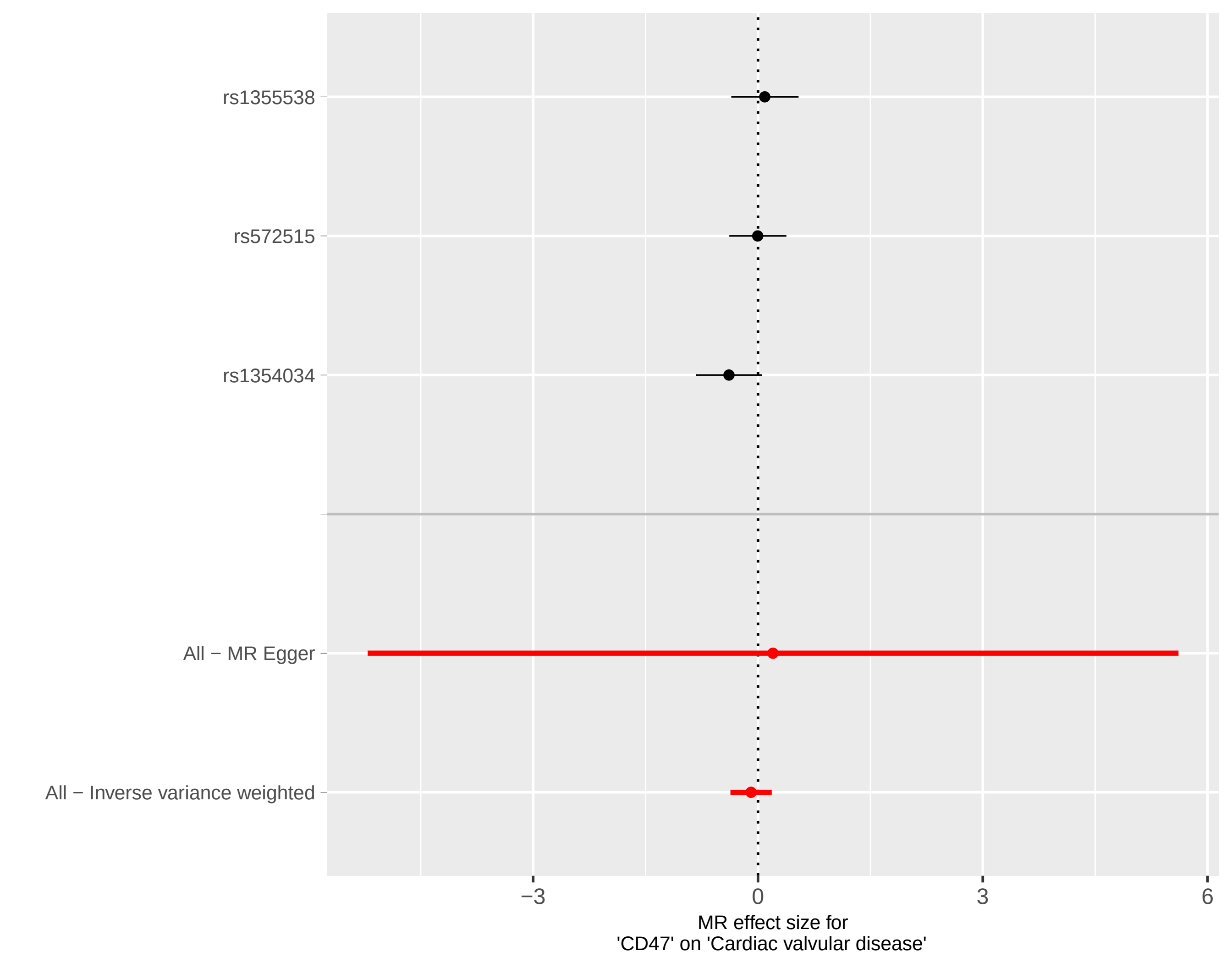

rs1355538
rs572515
rs1354034
All − MR Egger
All − Inverse variance weighted
−3
0
3
6
MR effect size for
'CD47' on 'Cardiac valvular disease'

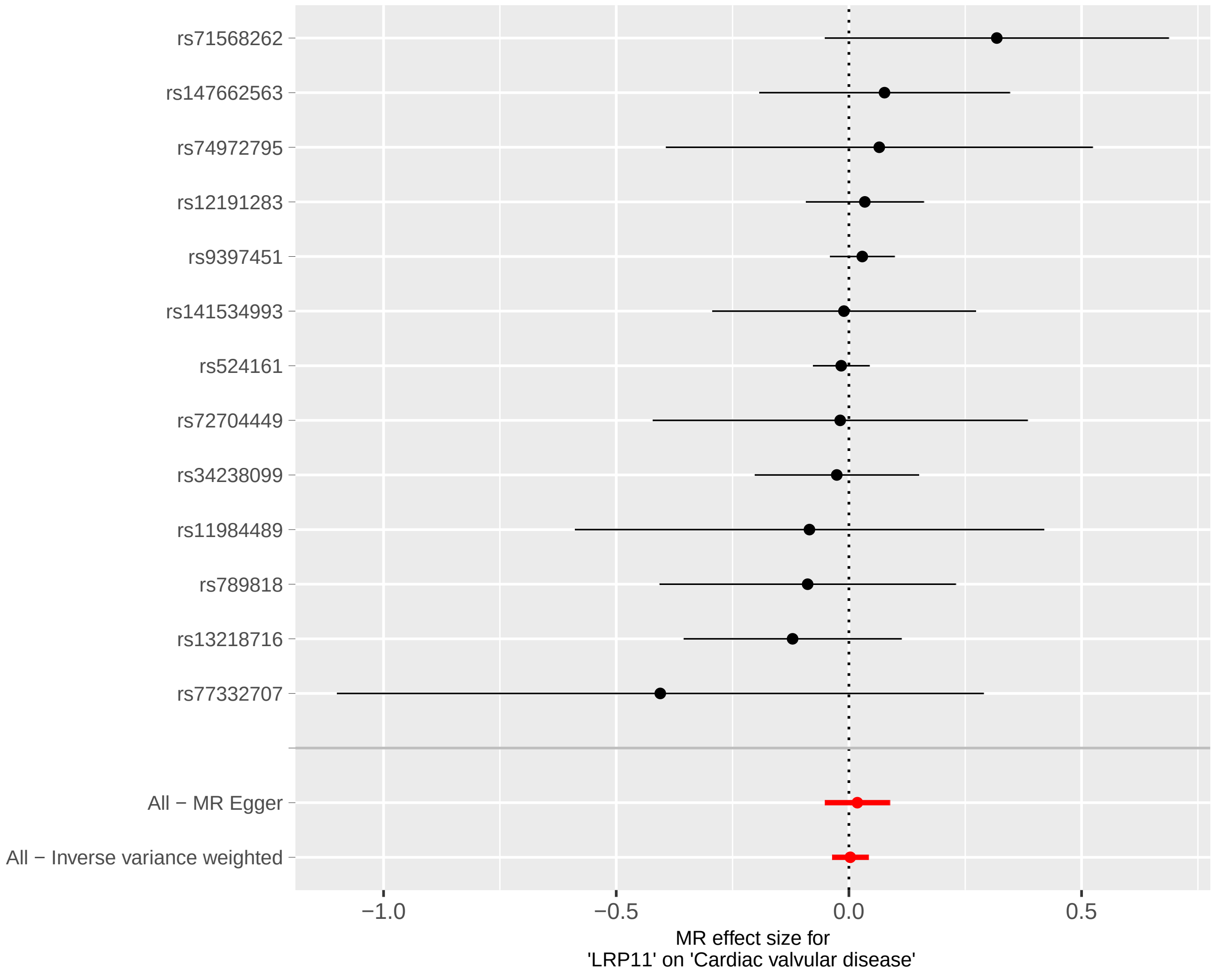
rs71568262
rs147662563
rs74972795
rs12191283
rs9397451
rs141534993
rs524161
rs72704449
rs34238099
rs11984489
rs789818
rs13218716
rs77332707
All − MR Egger
All − Inverse variance weighted
−1.0
−0.5
0.0
0.5
MR effect size for
'LRP11' on 'Cardiac valvular disease'

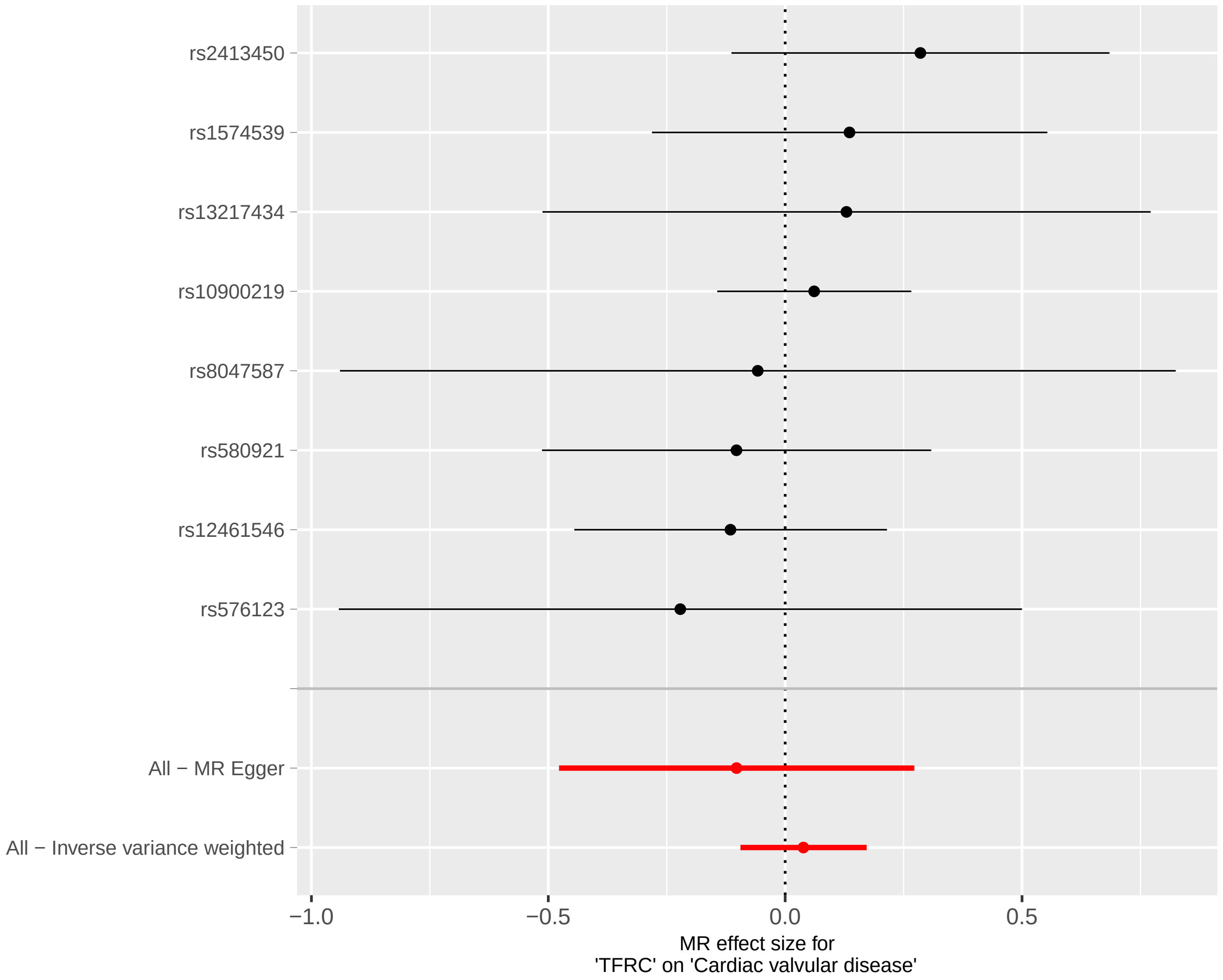
rs2413450
rs1574539
rs13217434
rs10900219
rs8047587
rs580921
rs12461546
rs576123
All − MR Egger
All − Inverse variance weighted
−1.0
−0.5
0.0
0.5
MR effect size for
'TFRC' on 'Cardiac valvular disease'

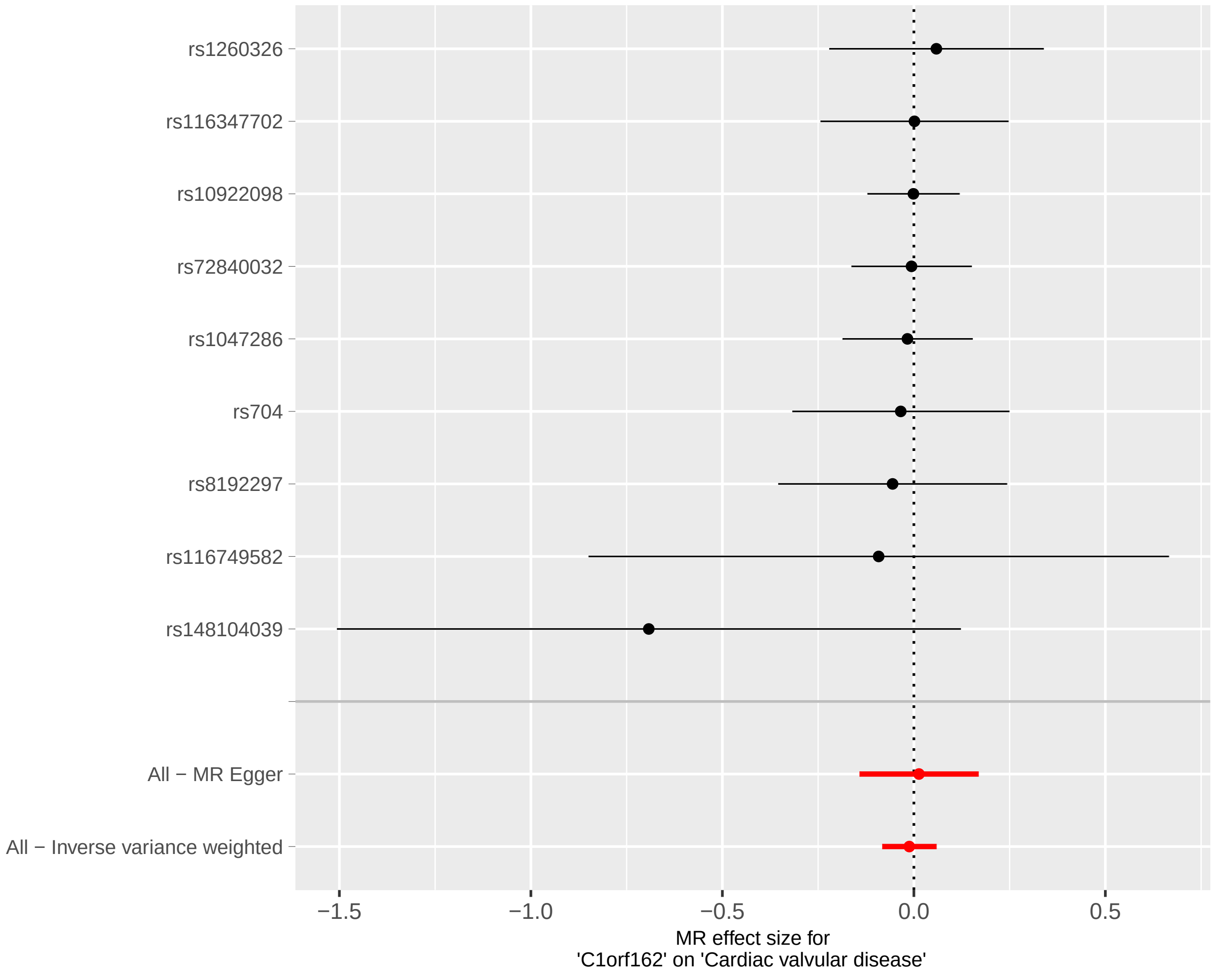
rs1260326
rs116347702
rs10922098
rs72840032
rs1047286
rs704
rs8192297
rs116749582
rs148104039
All − MR Egger
All − Inverse variance weighted
−1.5
−1.0
−0.5
0.0
0.5
MR effect size for
'C1orf162' on 'Cardiac valvular disease'

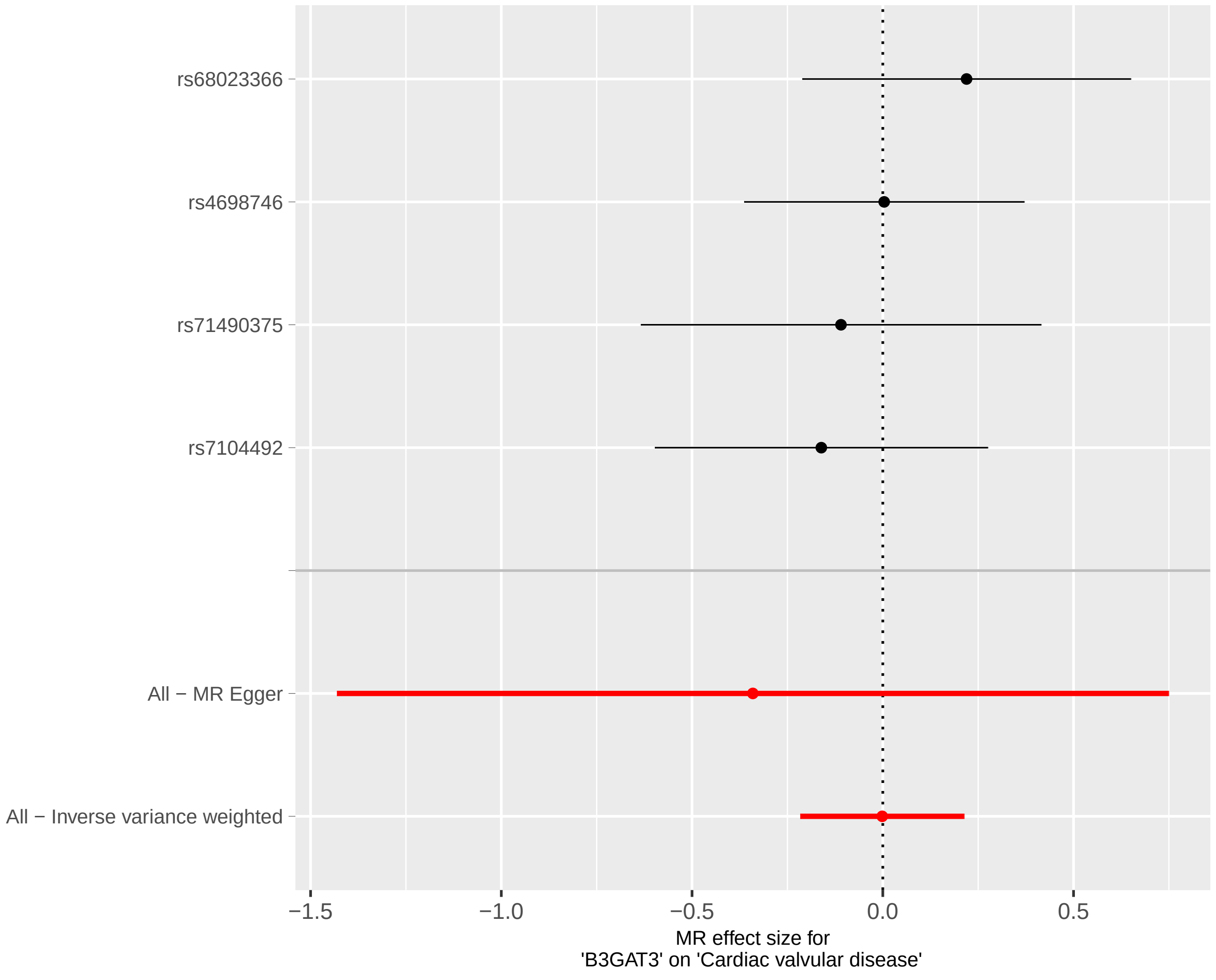

rs68023366
rs4698746
rs71490375
rs7104492
All − MR Egger
All − Inverse variance weighted
−1.5
−1.0
−0.5
0.0
0.5
MR effect size for
'B3GAT3' on 'Cardiac valvular disease'

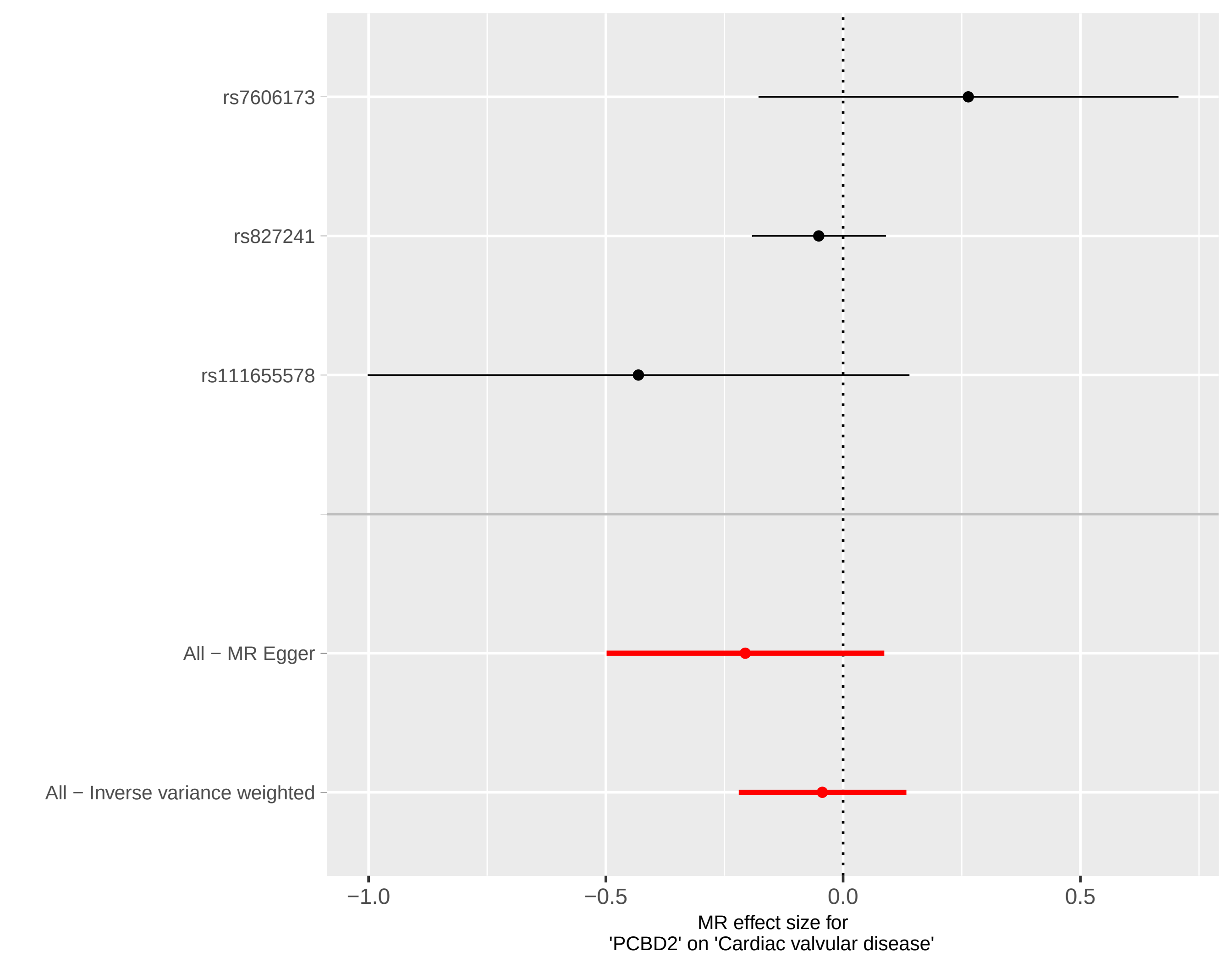

rs7606173
rs827241
rs111655578
All − MR Egger
All − Inverse variance weighted
−1.0
−0.5
0.0
0.5
MR effect size for
'PCBD2' on 'Cardiac valvular disease'

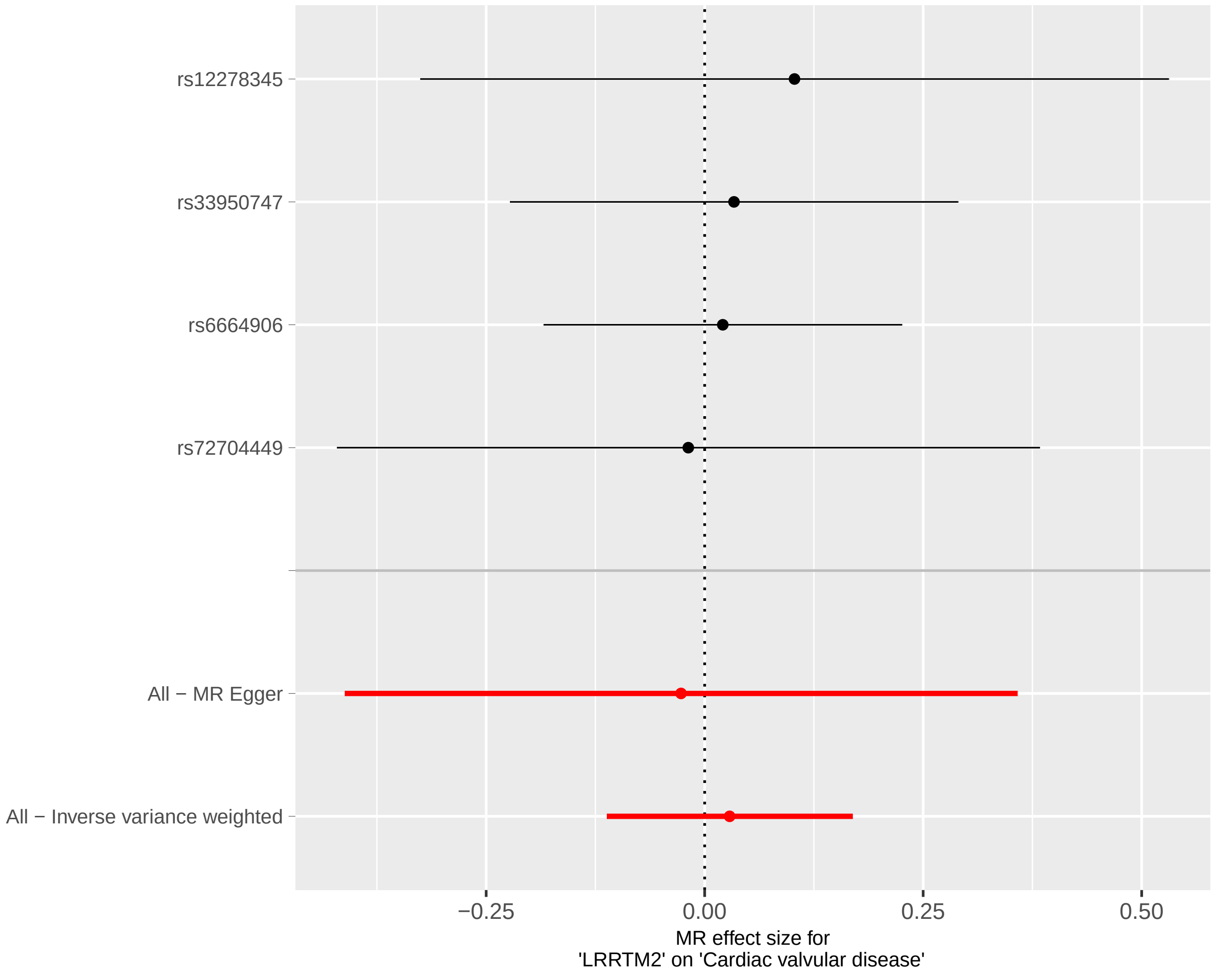
rs12278345
rs33950747
rs6664906
rs72704449
All − MR Egger
All − Inverse variance weighted
−0.25
0.00
0.25
0.50
MR effect size for
'LRRTM2' on 'Cardiac valvular disease'

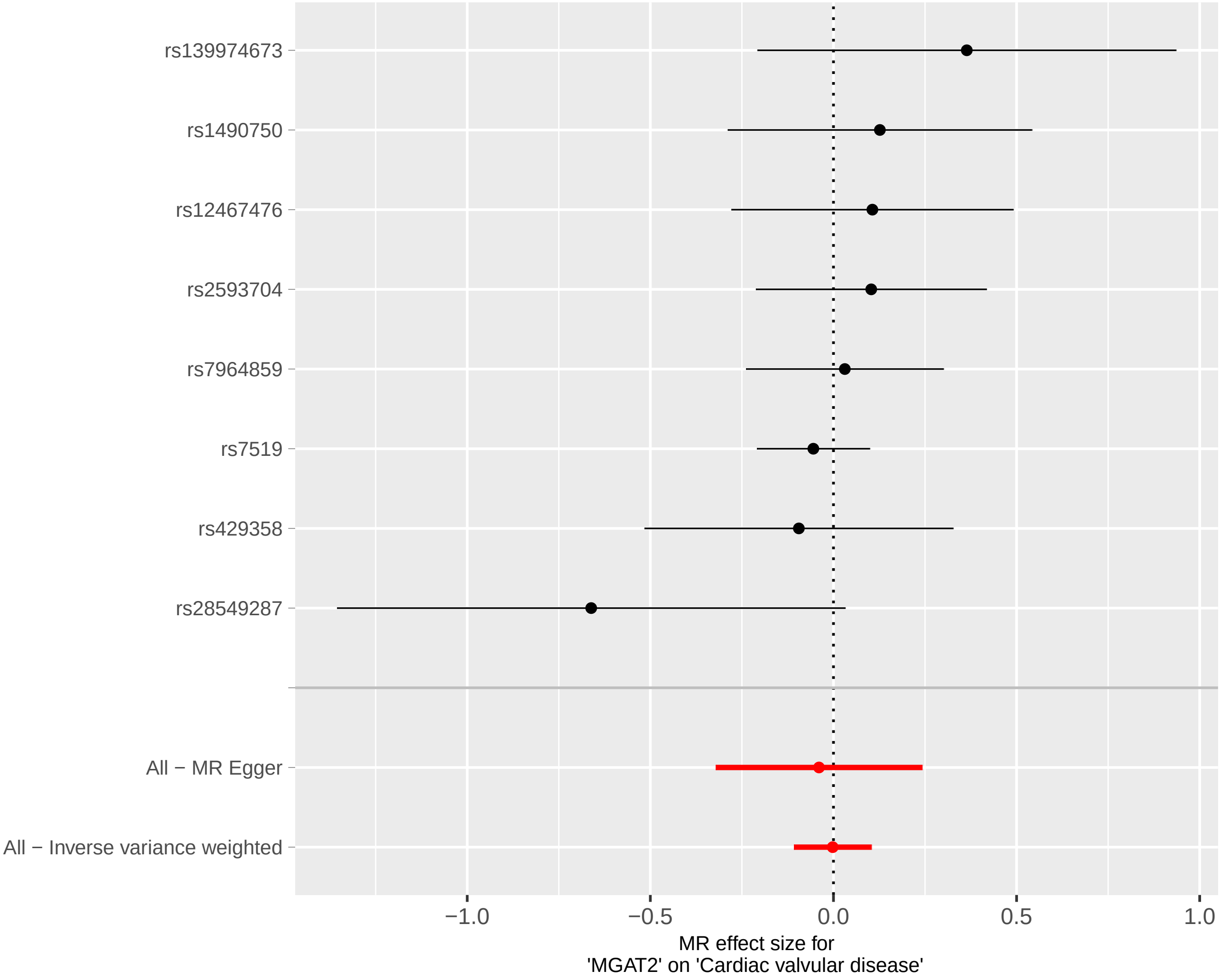
rs139974673
rs1490750
rs12467476
rs2593704
rs7964859
rs7519
rs429358
rs28549287
All − MR Egger
All − Inverse variance weighted
−1.0
−0.5
0.0
0.5
1.0
MR effect size for
'MGAT2' on 'Cardiac valvular disease'

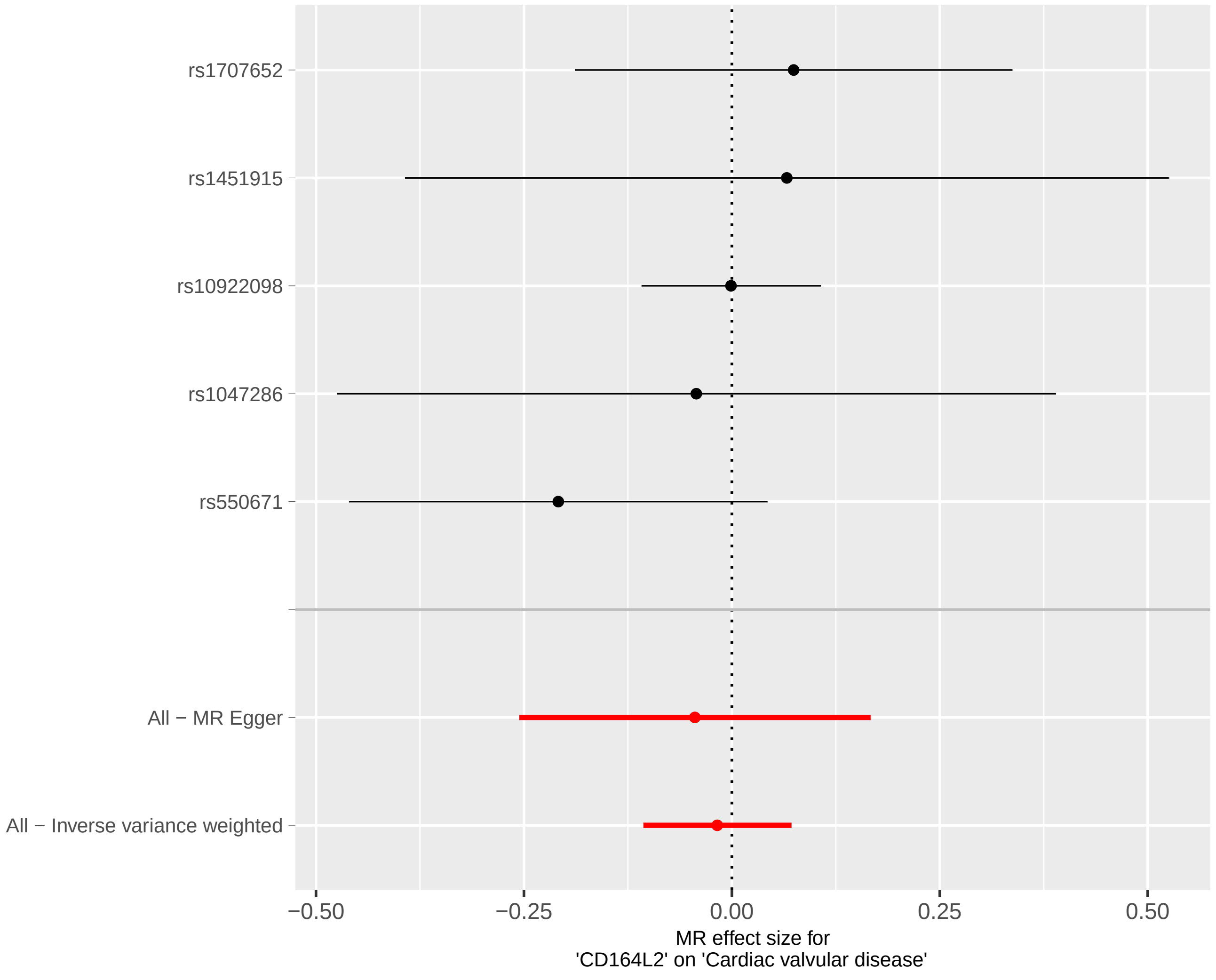

rs1707652
rs1451915
rs10922098
rs1047286
rs550671
All − MR Egger
All − Inverse variance weighted
−0.50
−0.25
0.00
0.25
0.50
MR effect size for
'CD164L2' on 'Cardiac valvular disease'

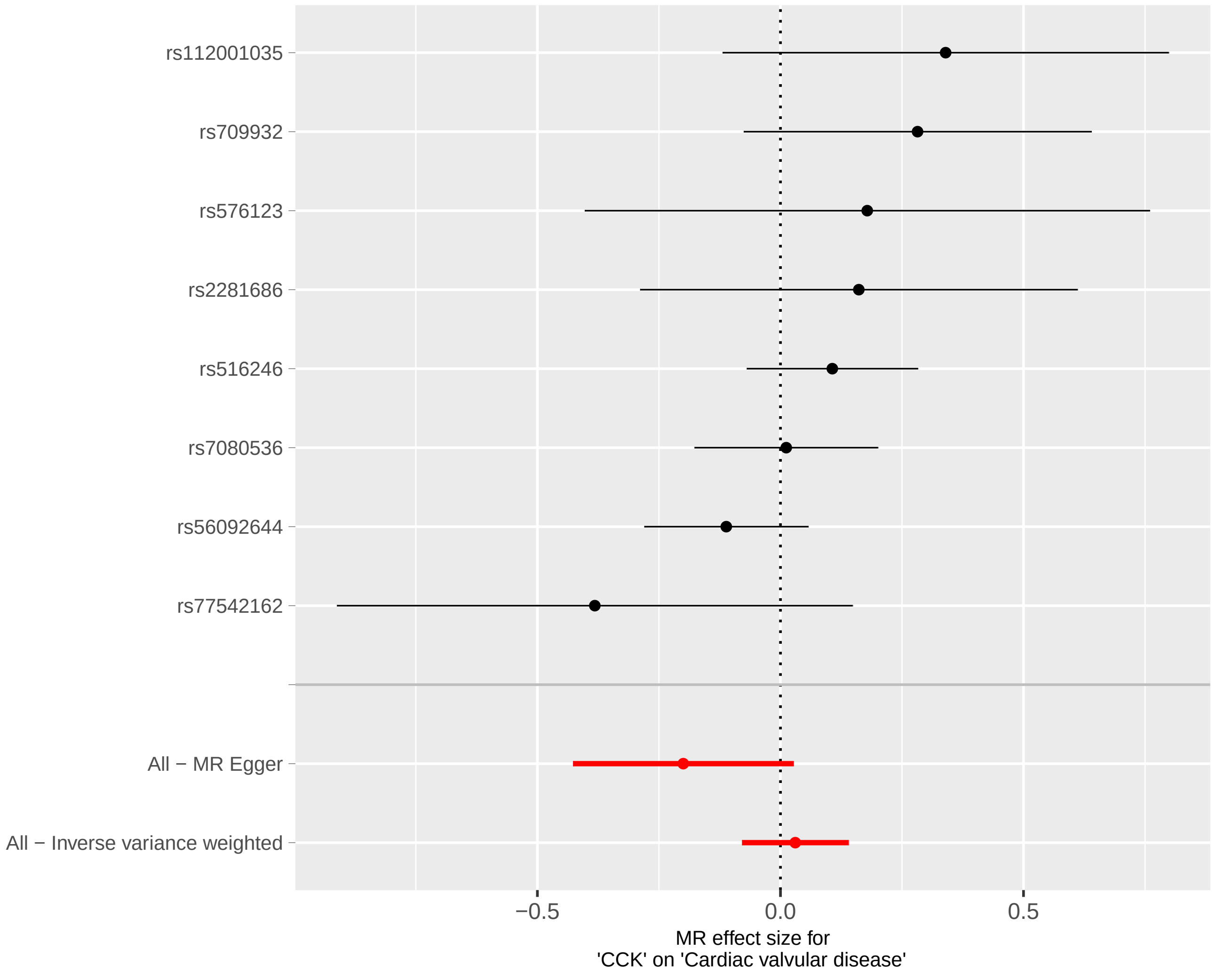
rs112001035
rs709932
rs576123
rs2281686
rs516246
rs7080536
rs56092644
rs77542162
All − MR Egger
All − Inverse variance weighted
−0.5
0.0
0.5
MR effect size for
'CCK' on 'Cardiac valvular disease'

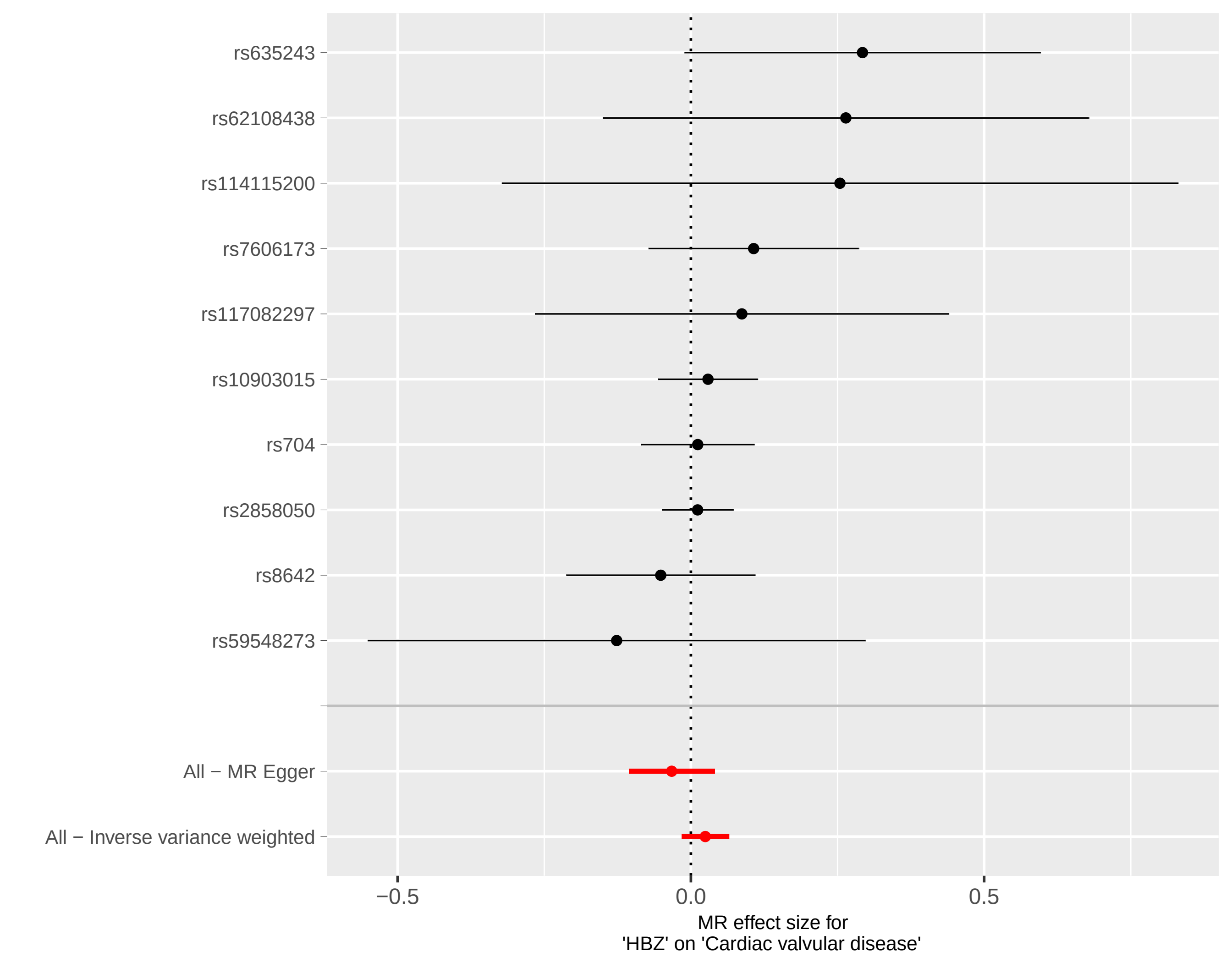
rs635243
rs62108438
rs114115200
rs7606173
rs117082297
rs10903015
rs704
rs2858050
rs8642
rs59548273
All − MR Egger
All − Inverse variance weighted
−0.5
0.0
0.5
MR effect size for
'HBZ' on 'Cardiac valvular disease'

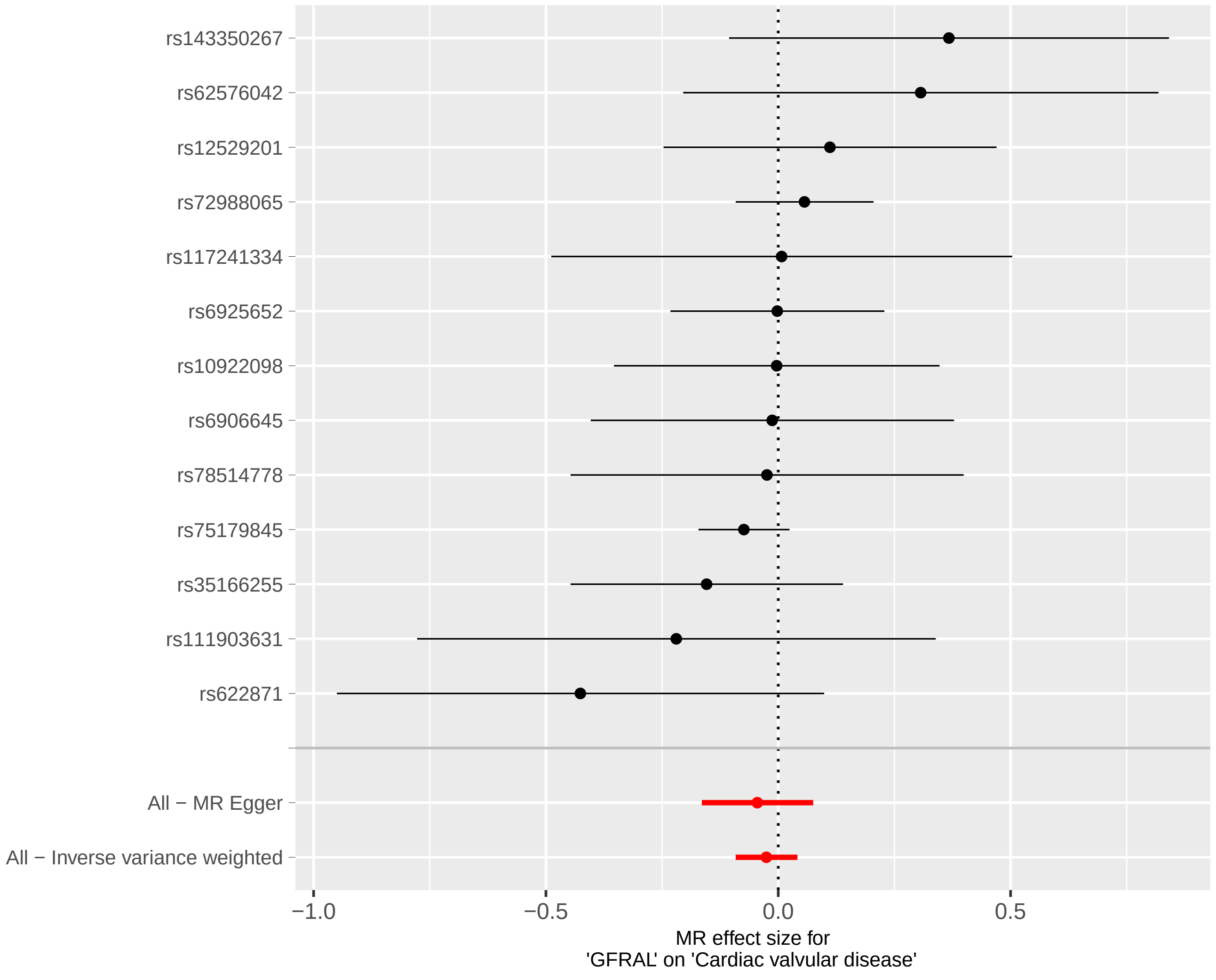
rs143350267
rs62576042
rs12529201
rs72988065
rs117241334
rs6925652
rs10922098
rs6906645
rs78514778
rs75179845
rs35166255
rs111903631
rs622871
All − MR Egger
All − Inverse variance weighted
−1.0
−0.5
0.0
0.5
MR effect size for
'GFRAL' on 'Cardiac valvular disease'

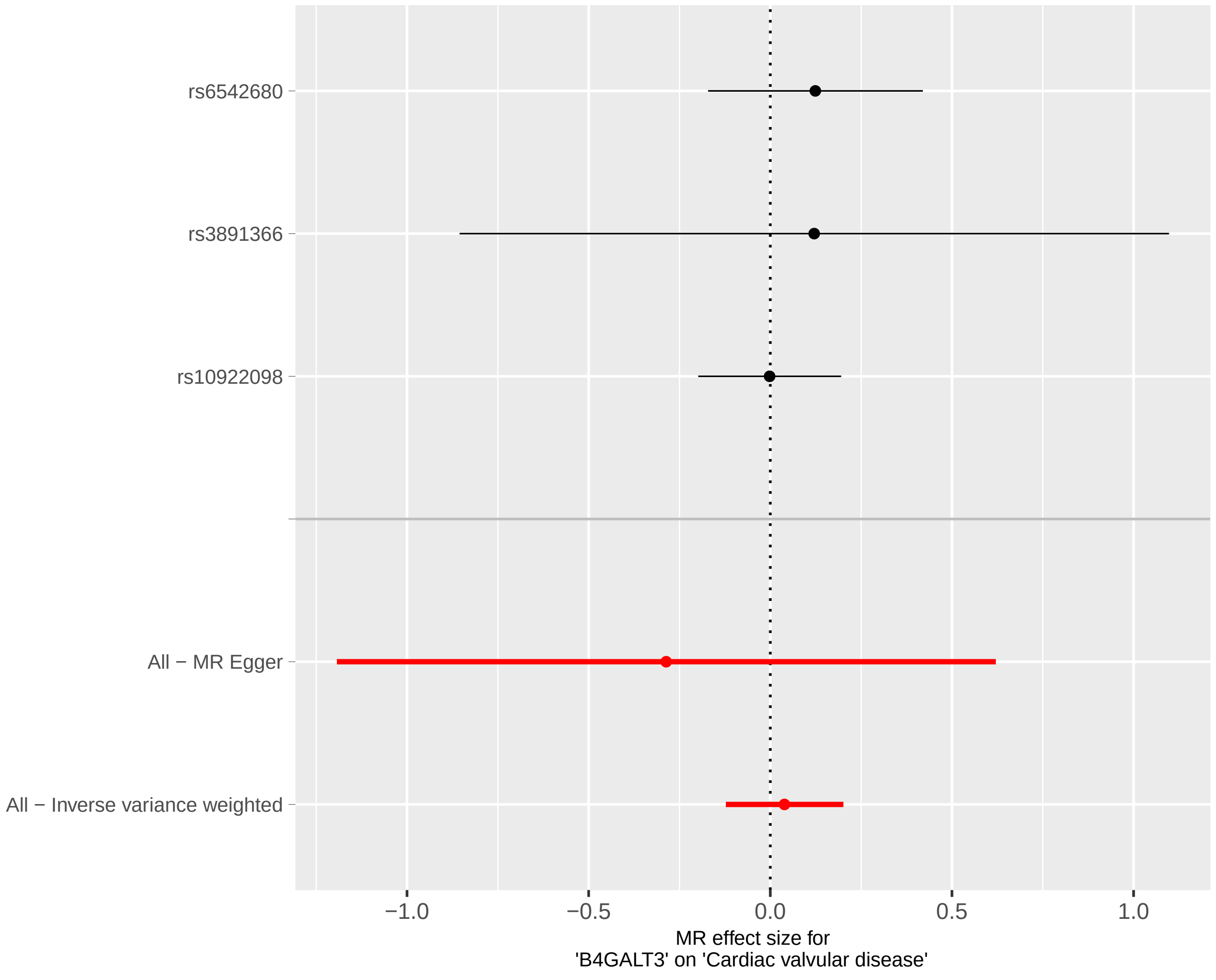
rs6542680
rs3891366
rs10922098
All − MR Egger
All − Inverse variance weighted
−1.0
−0.5
0.0
0.5
1.0
MR effect size for
'B4GALT3' on 'Cardiac valvular disease'

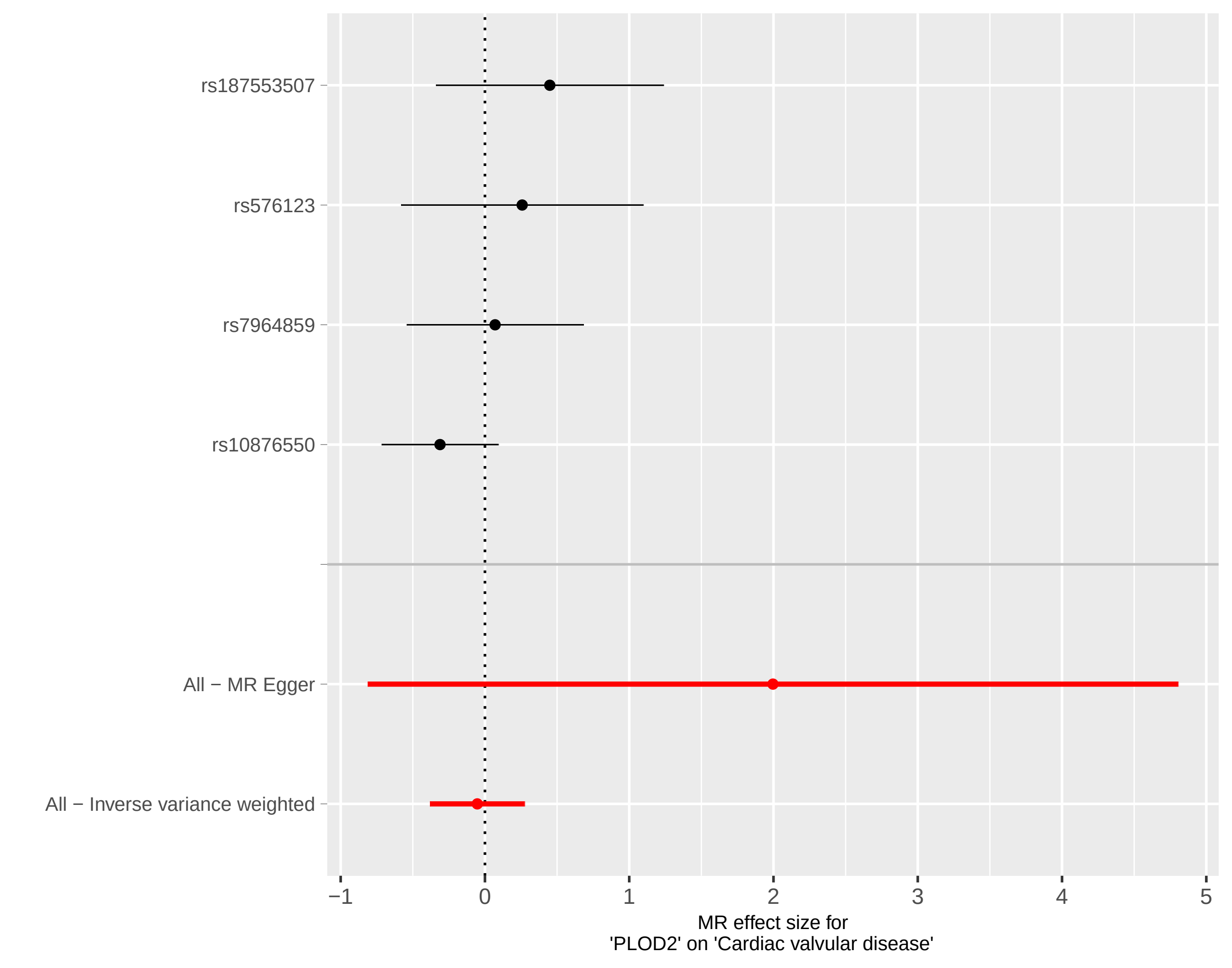

rs187553507
rs576123
rs7964859
rs10876550
All − MR Egger
All − Inverse variance weighted
−1
0
1
2
3
4
5
MR effect size for
'PLOD2' on 'Cardiac valvular disease'

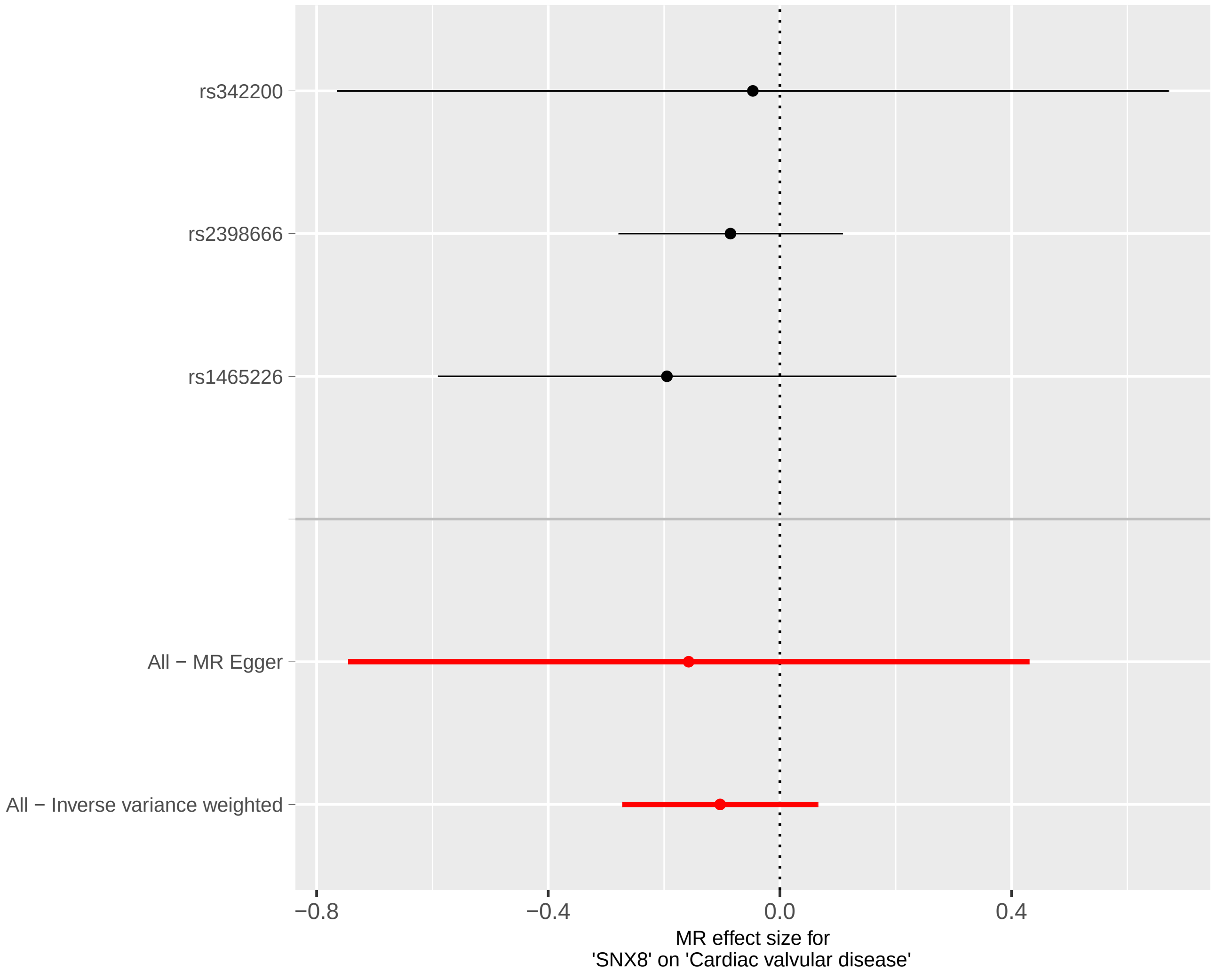
rs342200
rs2398666
rs1465226
All − MR Egger
All − Inverse variance weighted
−0.8
−0.4
0.0
0.4
MR effect size for
'SNX8' on 'Cardiac valvular disease'

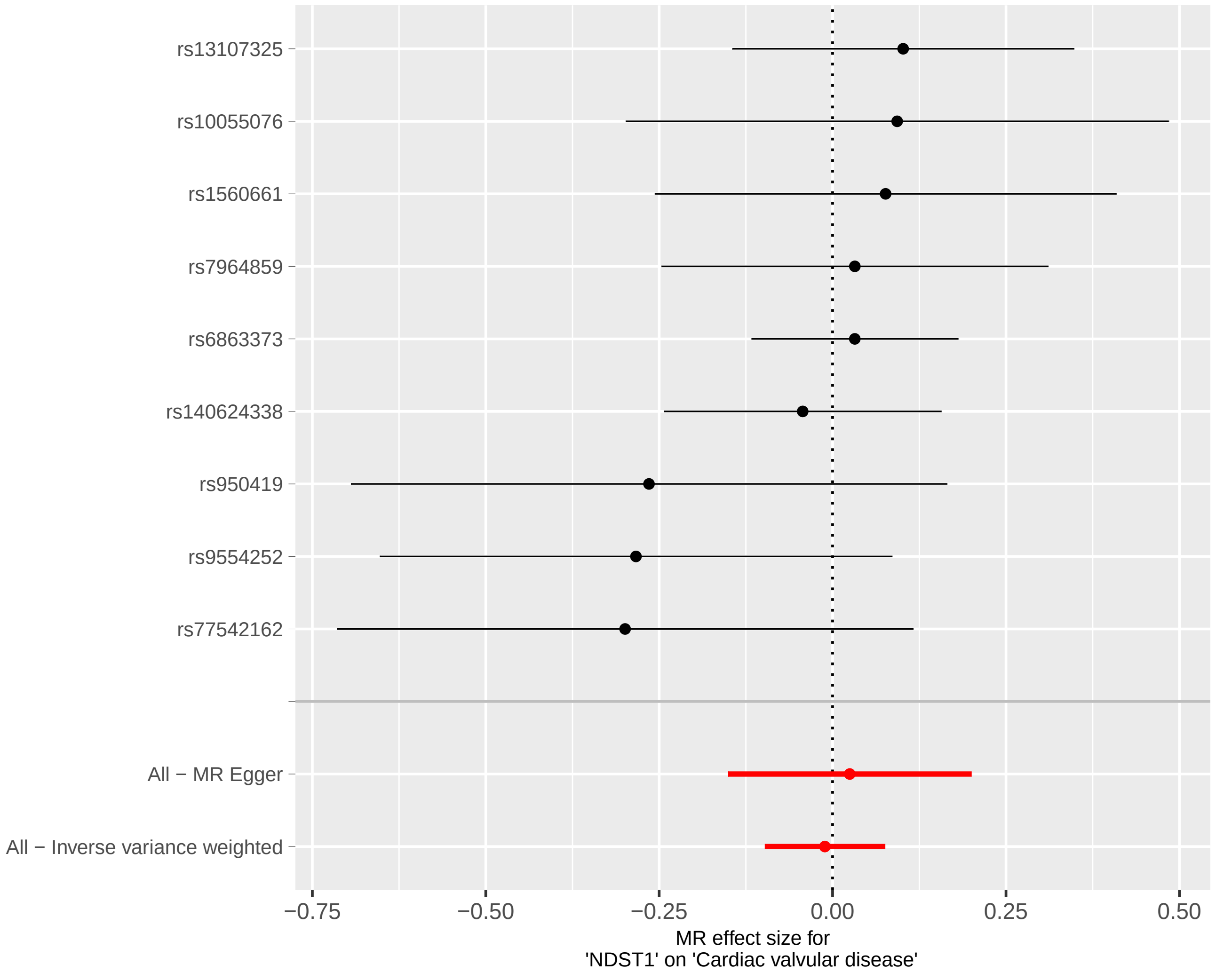
rs13107325
rs10055076
rs1560661
rs7964859
rs6863373
rs140624338
rs950419
rs9554252
rs77542162
All − MR Egger
All − Inverse variance weighted
−0.75
−0.50
−0.25
0.00
0.25
0.50
MR effect size for
'NDST1' on 'Cardiac valvular disease'

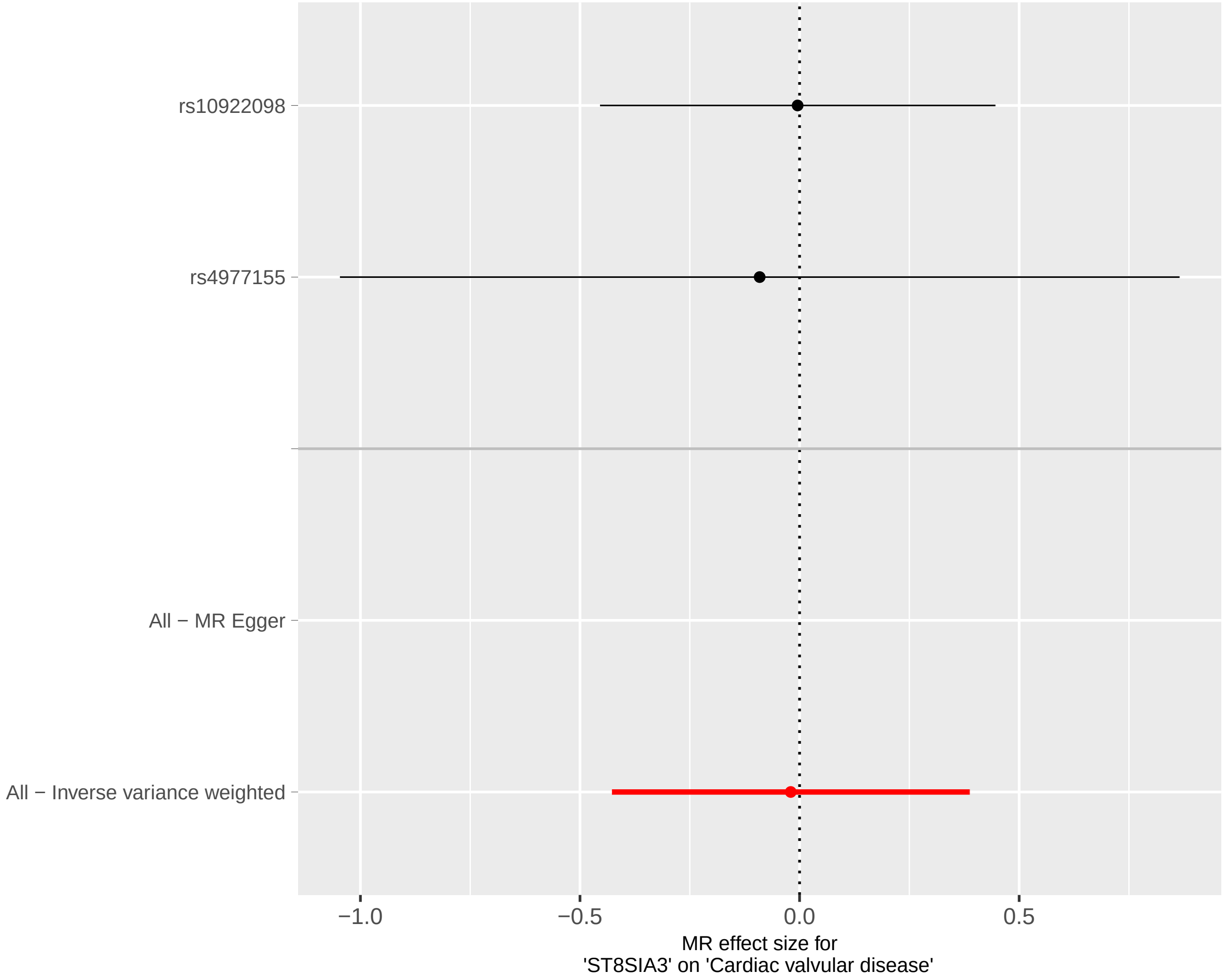

rs10922098
rs4977155
All − MR Egger
All − Inverse variance weighted
−1.0
−0.5
0.0
0.5
MR effect size for
'ST8SIA3' on 'Cardiac valvular disease'

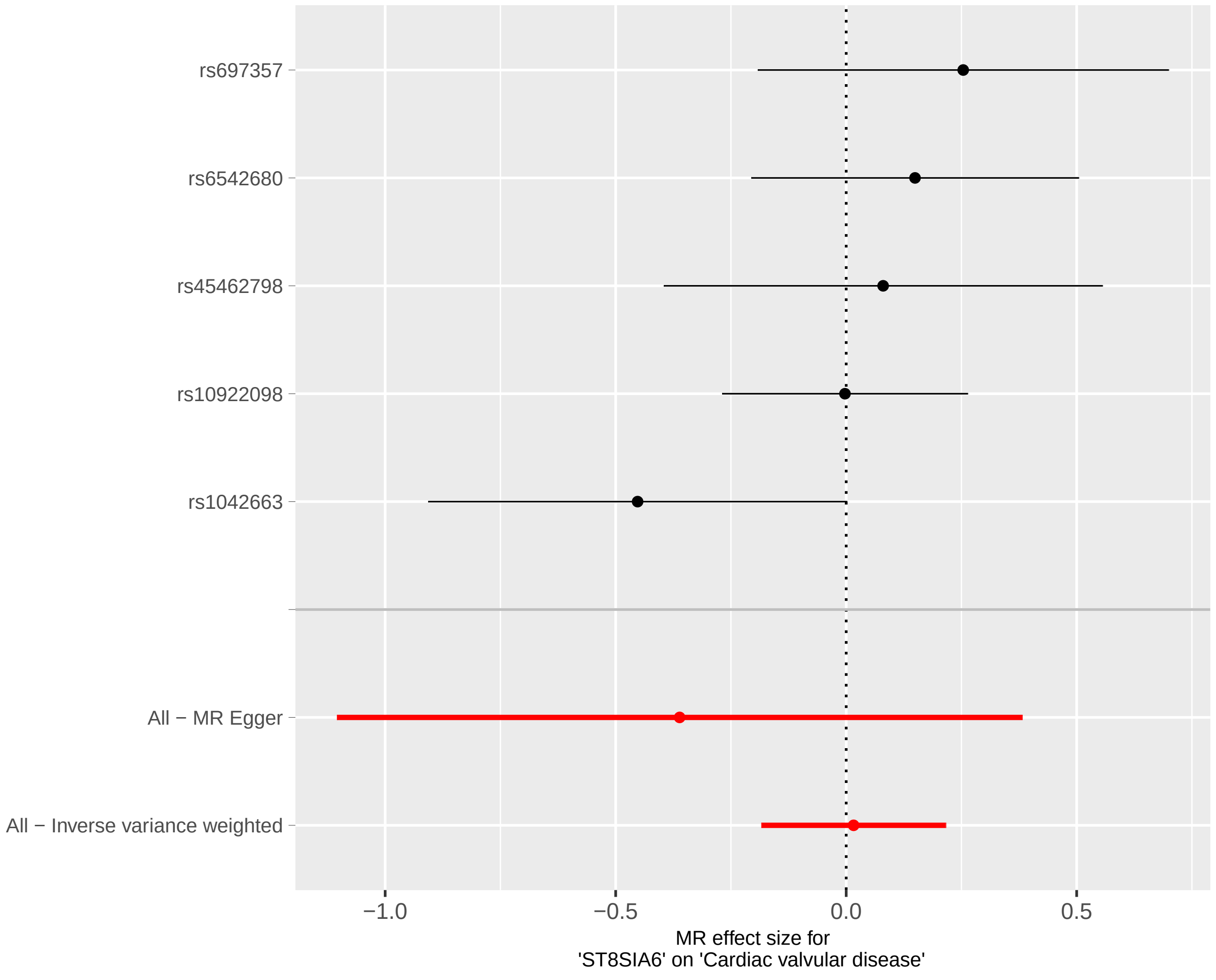

rs697357
rs6542680
rs45462798
rs10922098
rs1042663
All − MR Egger
All − Inverse variance weighted
−1.0
−0.5
0.0
0.5
MR effect size for
'ST8SIA6' on 'Cardiac valvular disease'

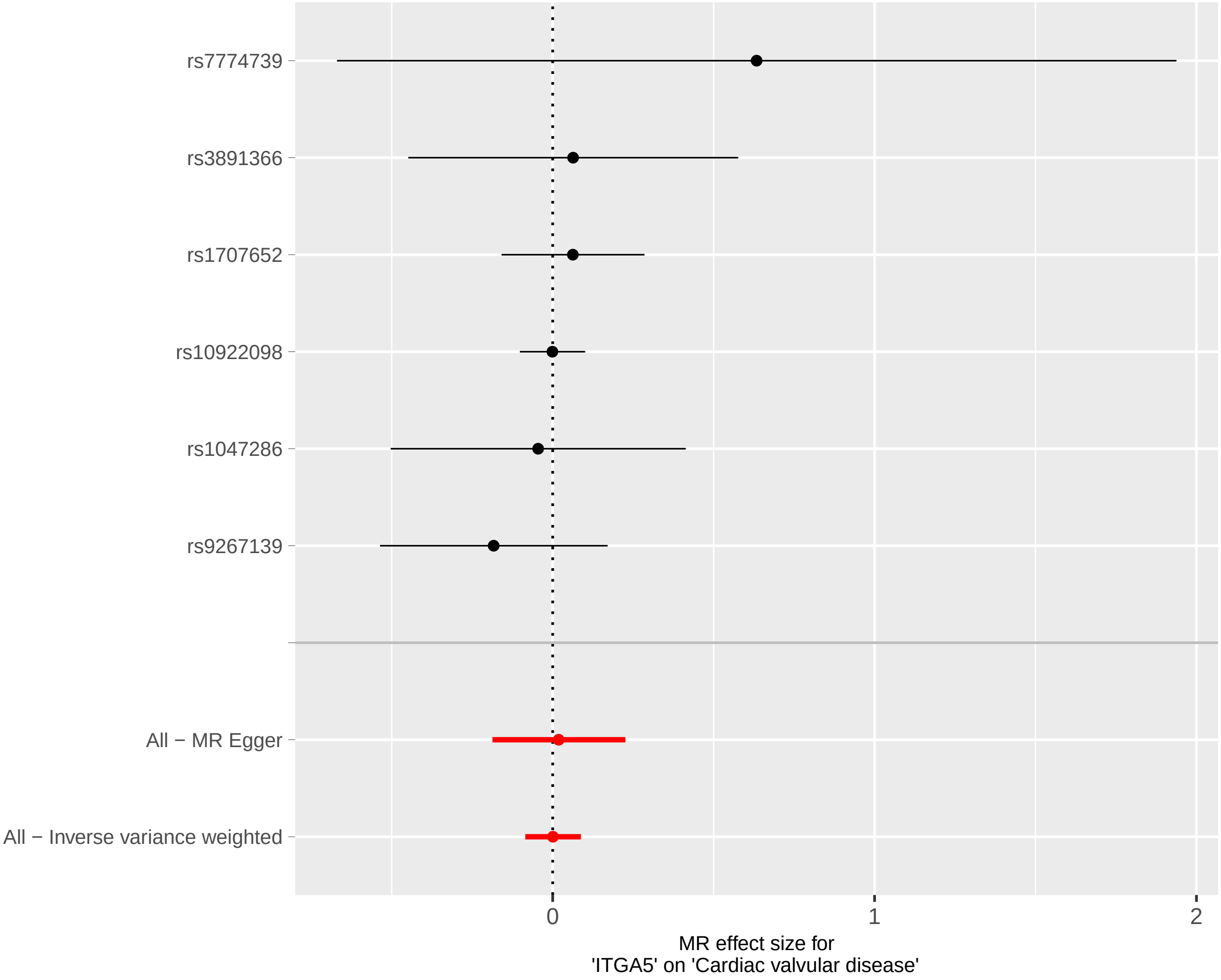
rs7774739
rs3891366
rs1707652
rs10922098
rs1047286
rs9267139
All − MR Egger
All − Inverse variance weighted
0
1
2
MR effect size for
'ITGA5' on 'Cardiac valvular disease'

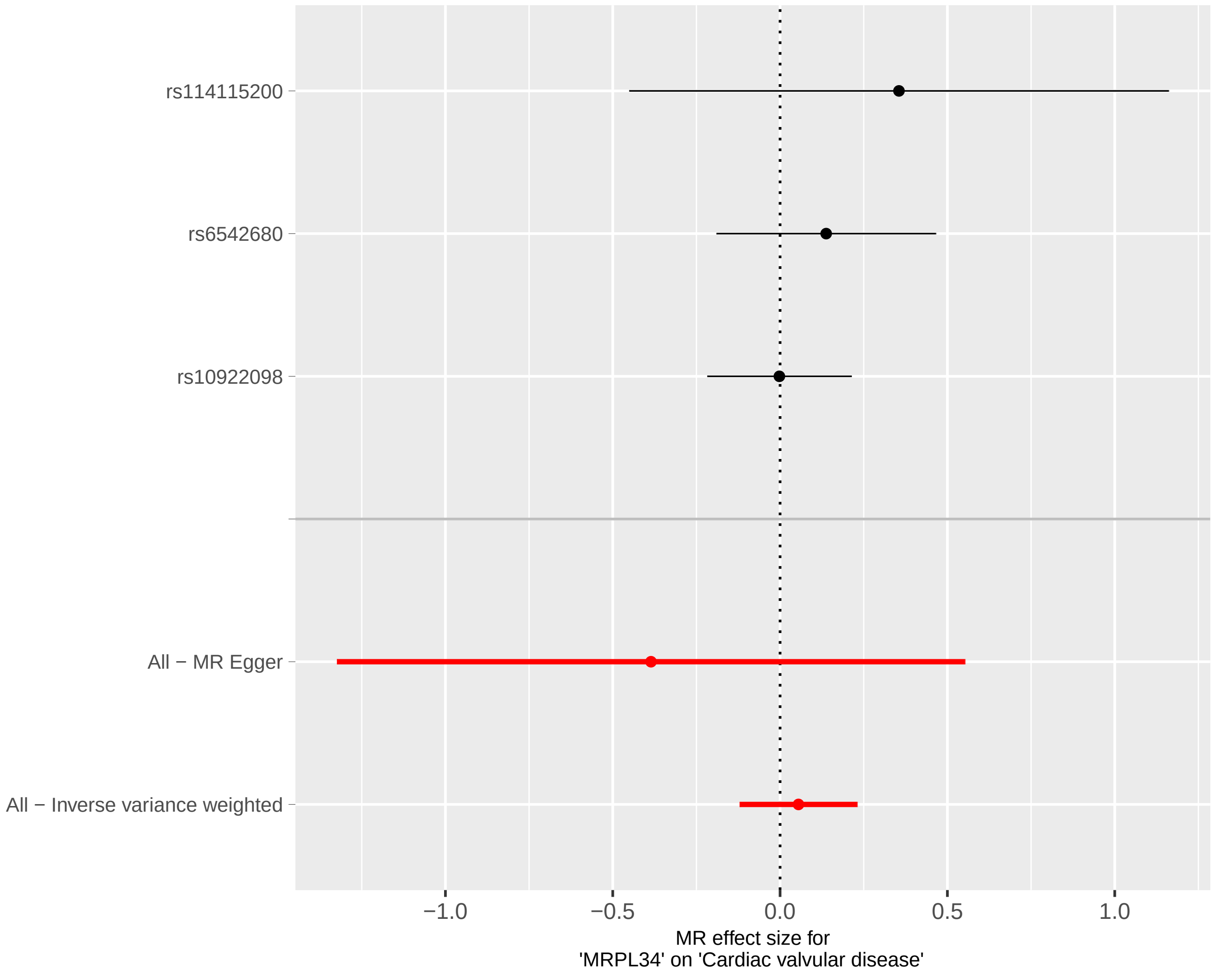
rs114115200
rs6542680
rs10922098
All − MR Egger
All − Inverse variance weighted
−1.0
−0.5
0.0
0.5
1.0
MR effect size for
'MRPL34' on 'Cardiac valvular disease'

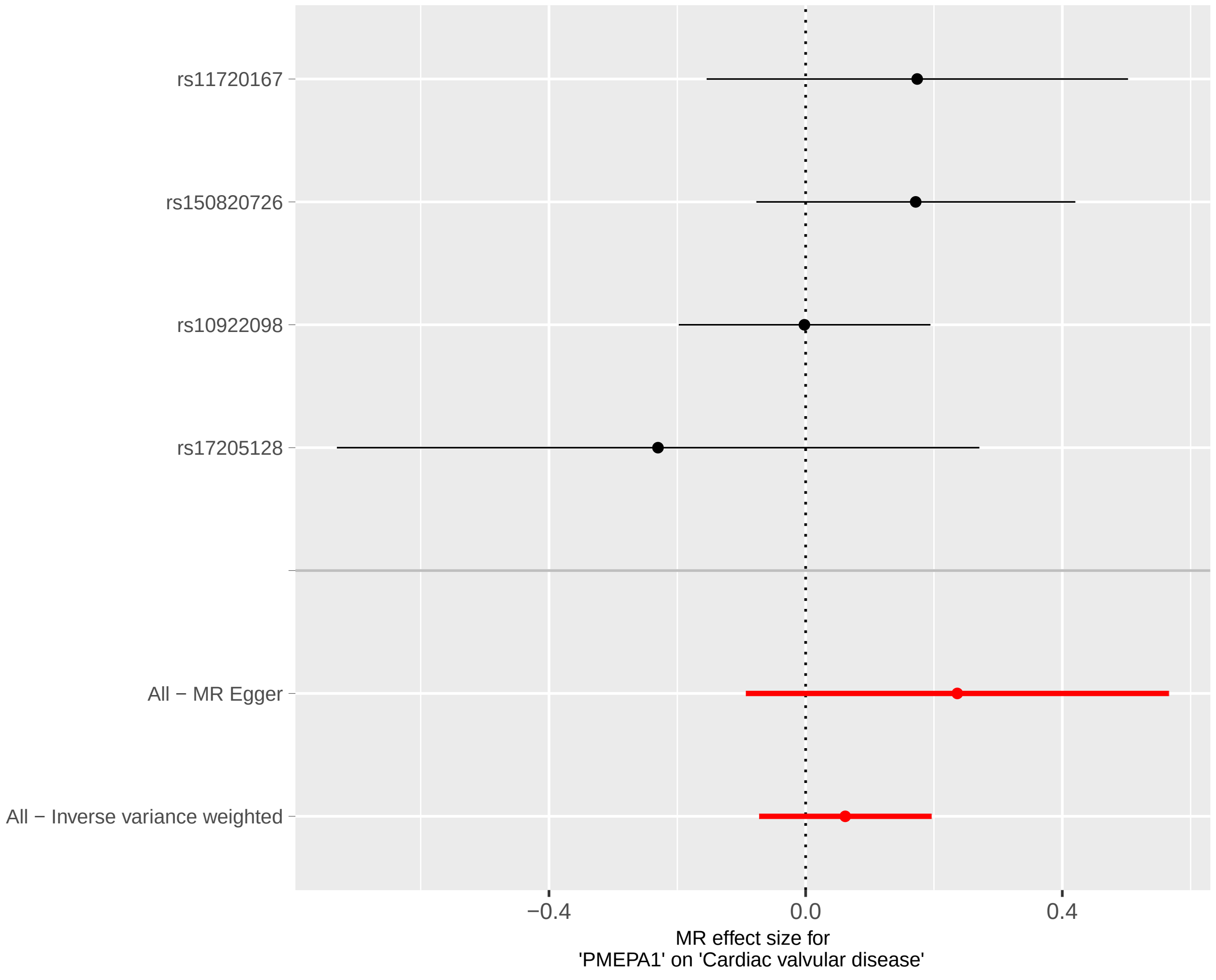

rs11720167
rs150820726
rs10922098
rs17205128
All − MR Egger
All − Inverse variance weighted
−0.4
0.0
0.4
MR effect size for
'PMEPA1' on 'Cardiac valvular disease'

Insufficient number of SNPs

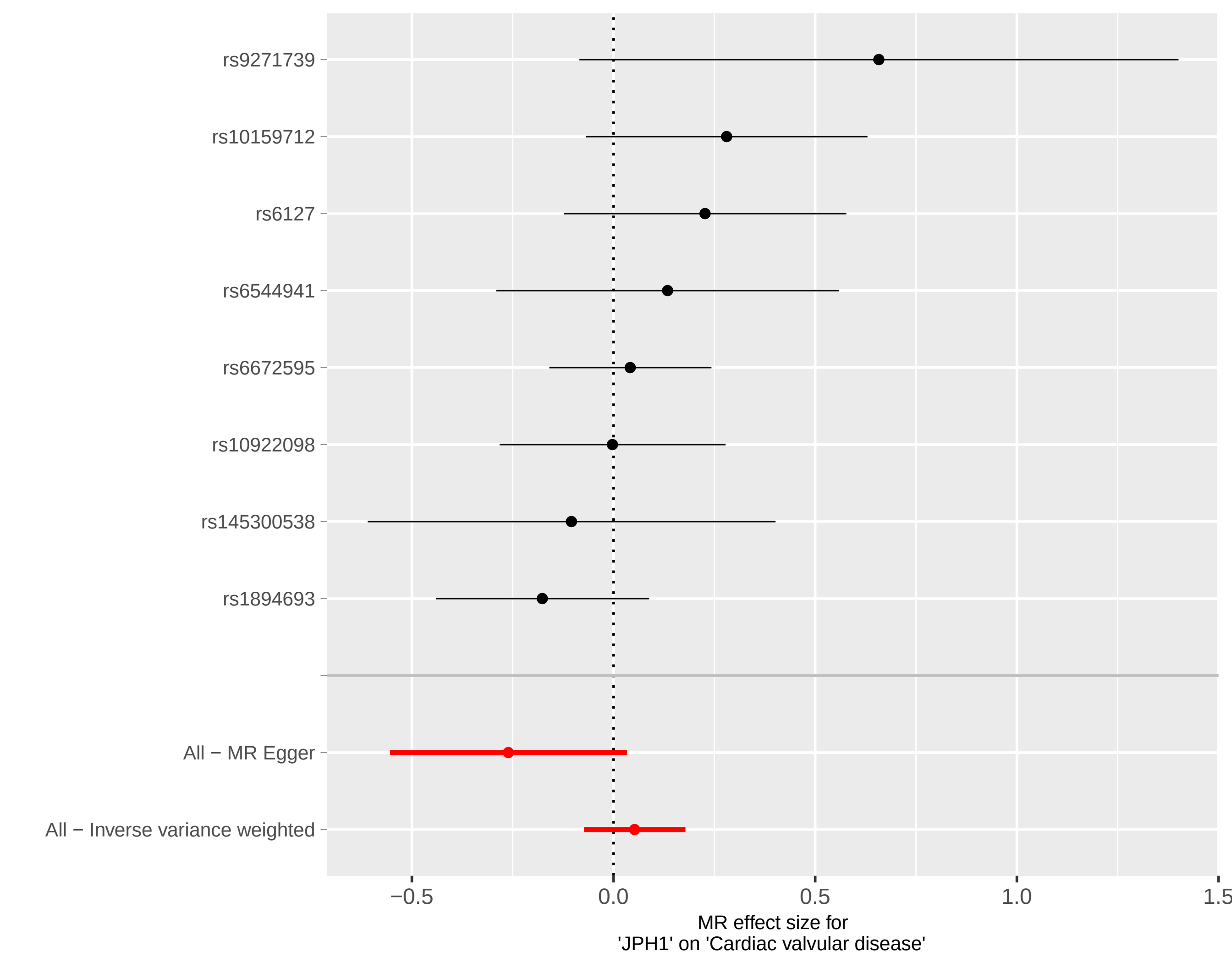
rs9271739
rs10159712
rs6127
rs6544941
rs6672595
rs10922098
rs145300538
rs1894693
All − MR Egger
All − Inverse variance weighted
−0.5
0.0
0.5
1.0
1.5
MR effect size for
'JPH1' on 'Cardiac valvular disease'

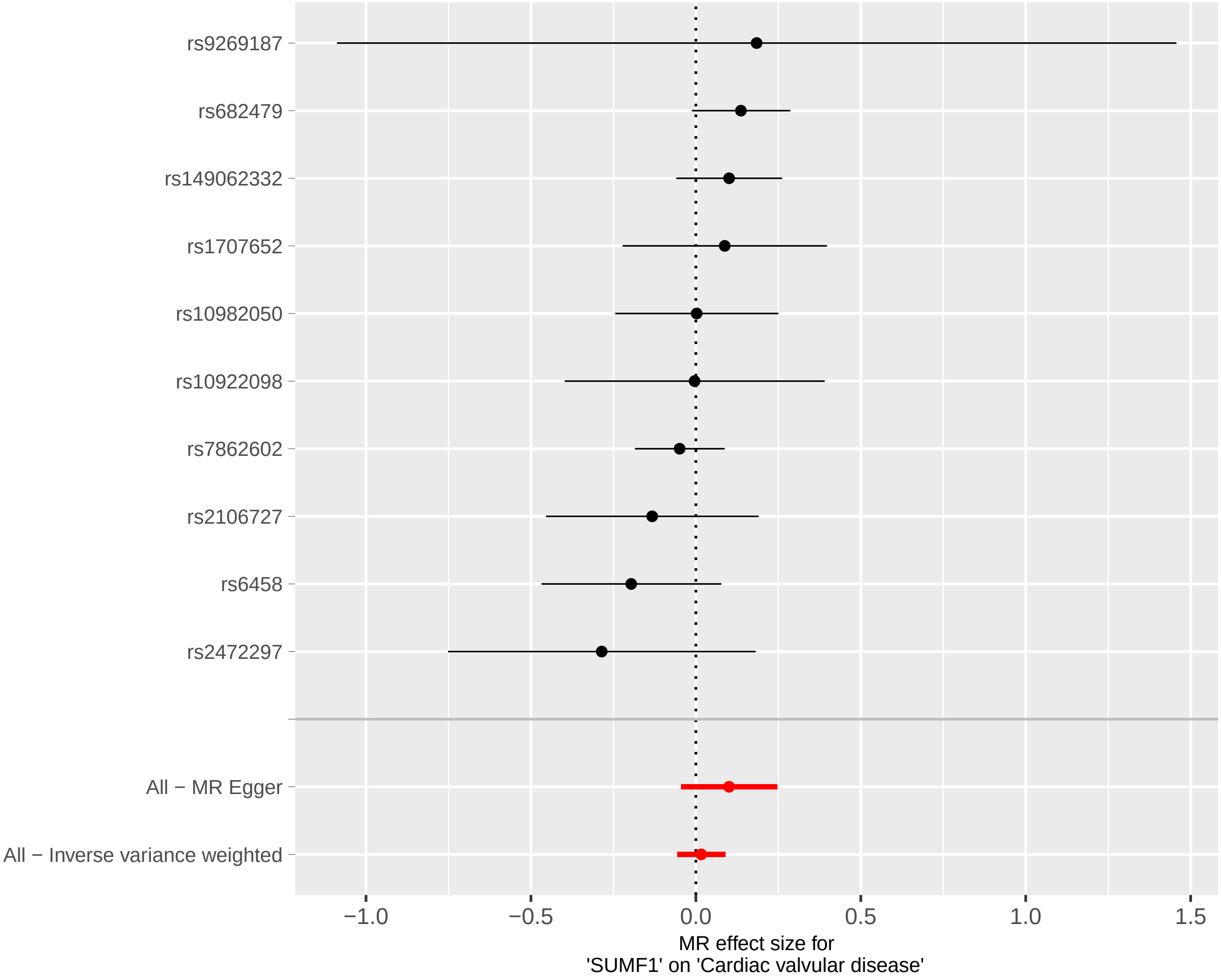
rs9269187
rs682479
rs149062332
rs1707652
rs10982050
rs10922098
rs7862602
rs2106727
rs6458
rs2472297
All − MR Egger
All − Inverse variance weighted
−1.0
−0.5
0.0
0.5
1.0
1.5
MR effect size for
'SUMF1' on 'Cardiac valvular disease'

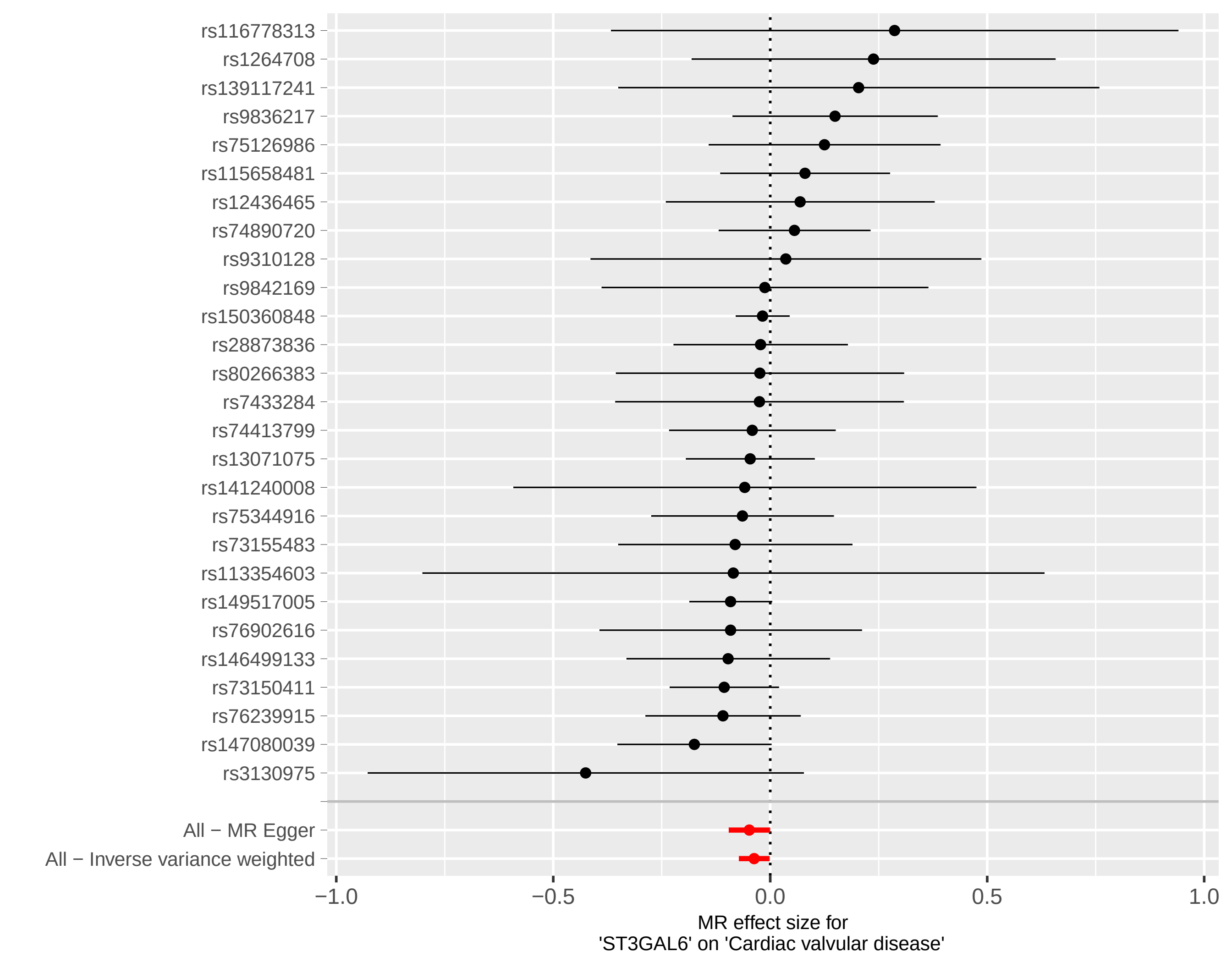
rs116778313
rs1264708
rs139117241
rs9836217
rs75126986
rs115658481
rs12436465
rs74890720
rs9310128
rs9842169
rs150360848
rs28873836
rs80266383
rs7433284
rs74413799
rs13071075
rs141240008
rs75344916
rs73155483
rs113354603
rs149517005
rs76902616
rs146499133
rs73150411
rs76239915
rs147080039
rs3130975
All − MR Egger
All − Inverse variance weighted
−1.0
−0.5
0.0
0.5
1.0
MR effect size for
'ST3GAL6' on 'Cardiac valvular disease'

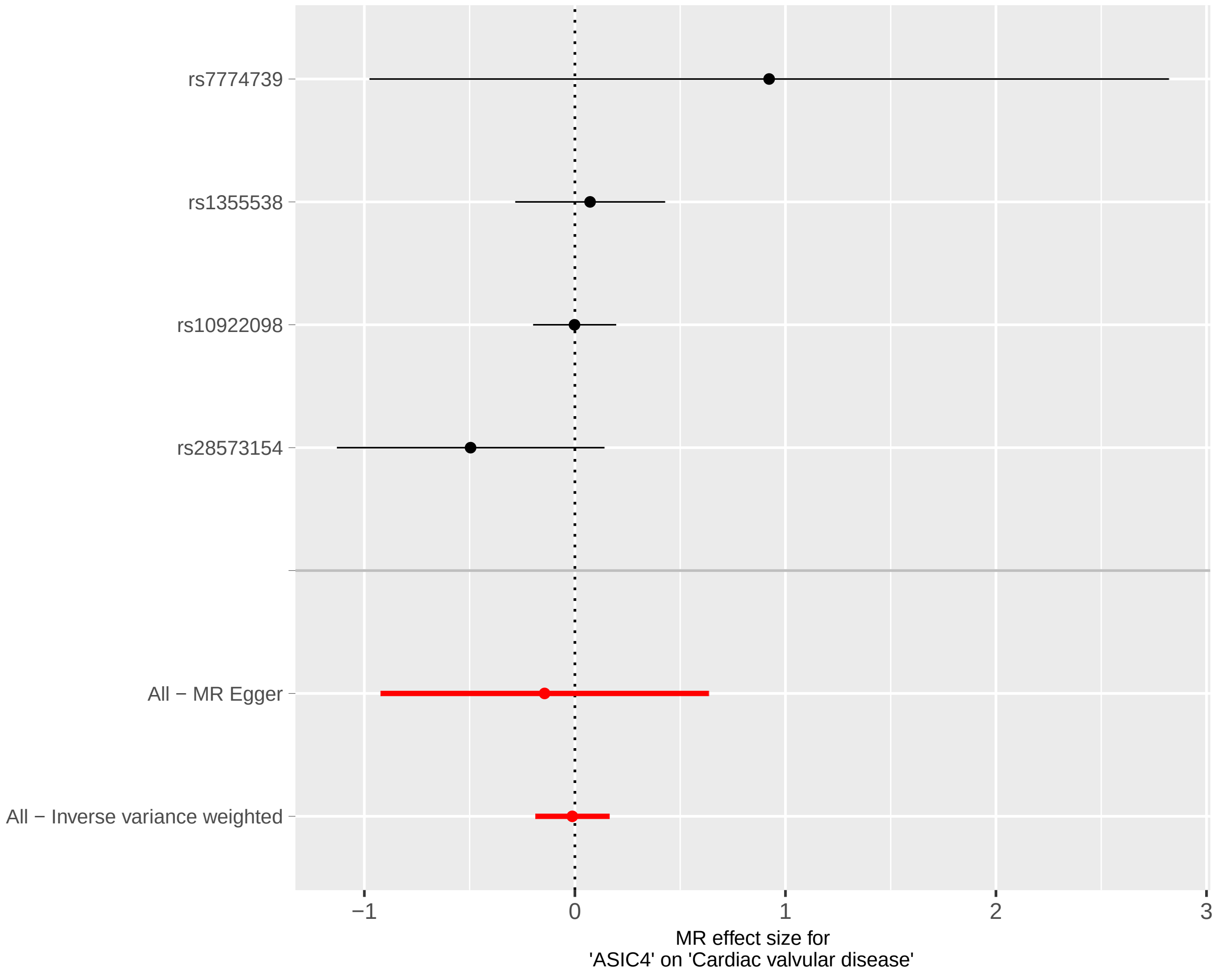

rs7774739
rs1355538
rs10922098
rs28573154
All − MR Egger
All − Inverse variance weighted
−1
0
1
2
3
MR effect size for
'ASIC4' on 'Cardiac valvular disease'

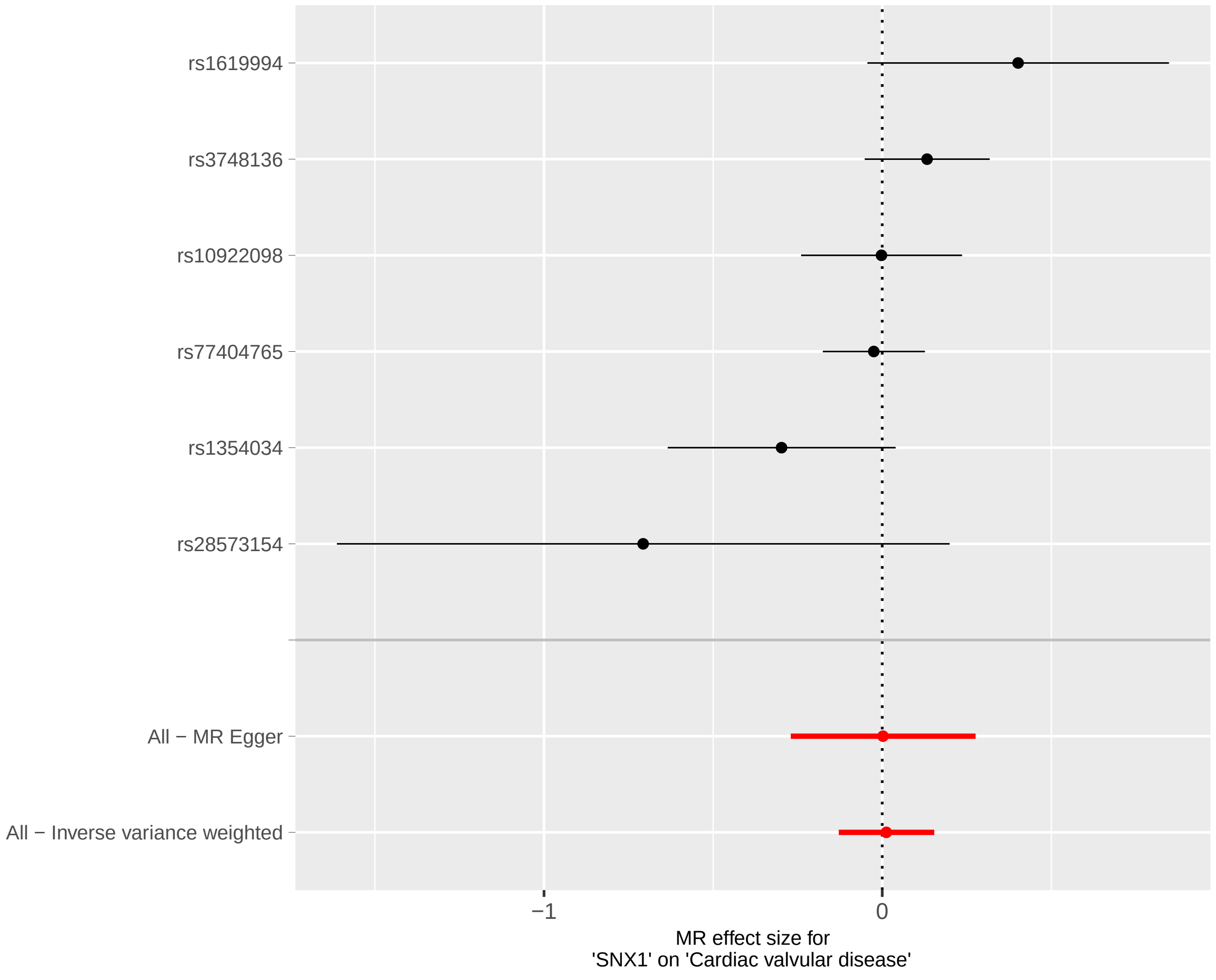
rs1619994
rs3748136
rs10922098
rs77404765
rs1354034
rs28573154
All − MR Egger
All − Inverse variance weighted
−1
0
MR effect size for
'SNX1' on 'Cardiac valvular disease'

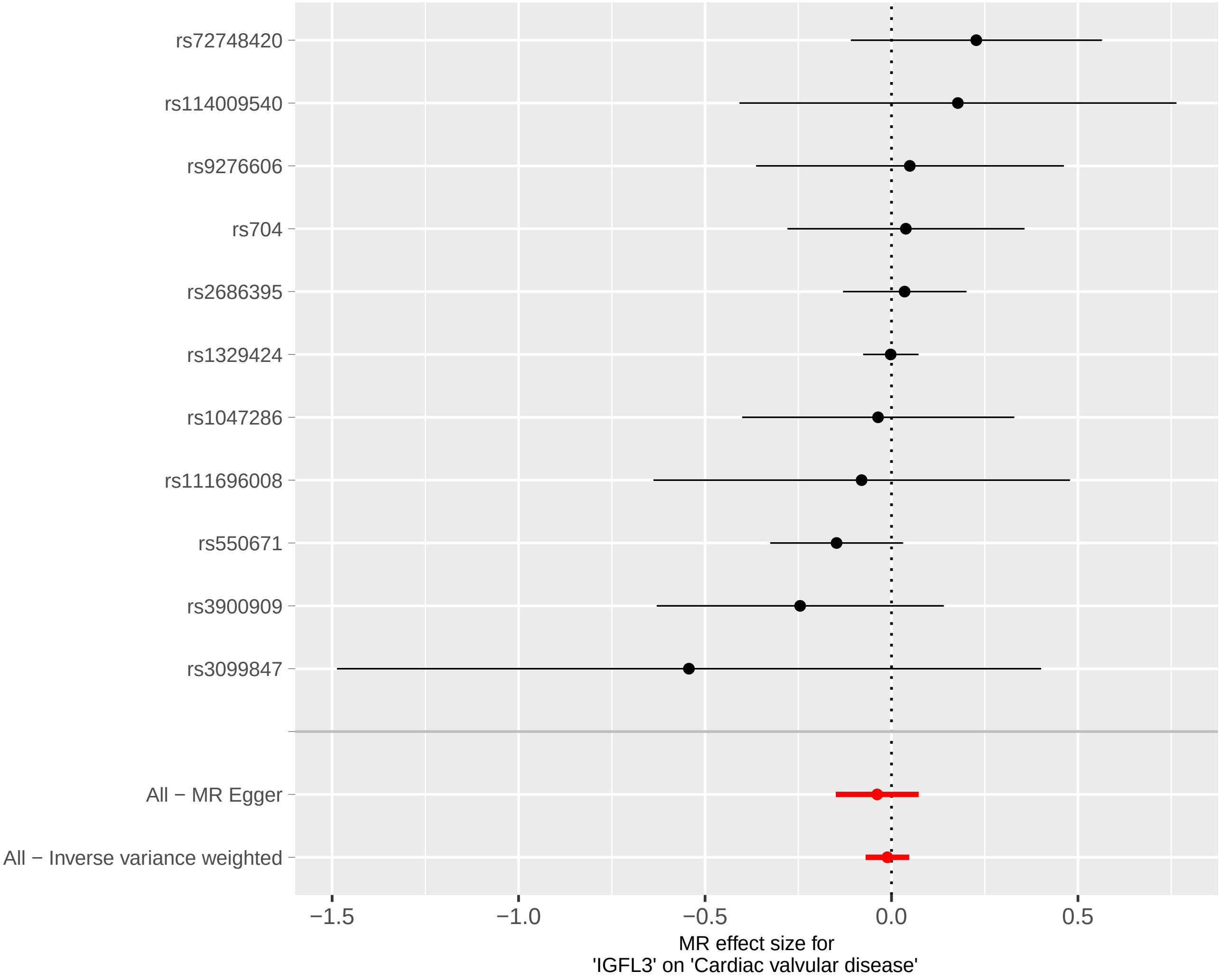
rs72748420
rs114009540
rs9276606
rs704
rs2686395
rs1329424
rs1047286
rs111696008
rs550671
rs3900909
rs3099847
All − MR Egger
All − Inverse variance weighted
−1.5
−1.0
−0.5
0.0
0.5
MR effect size for
'IGFL3' on 'Cardiac valvular disease'

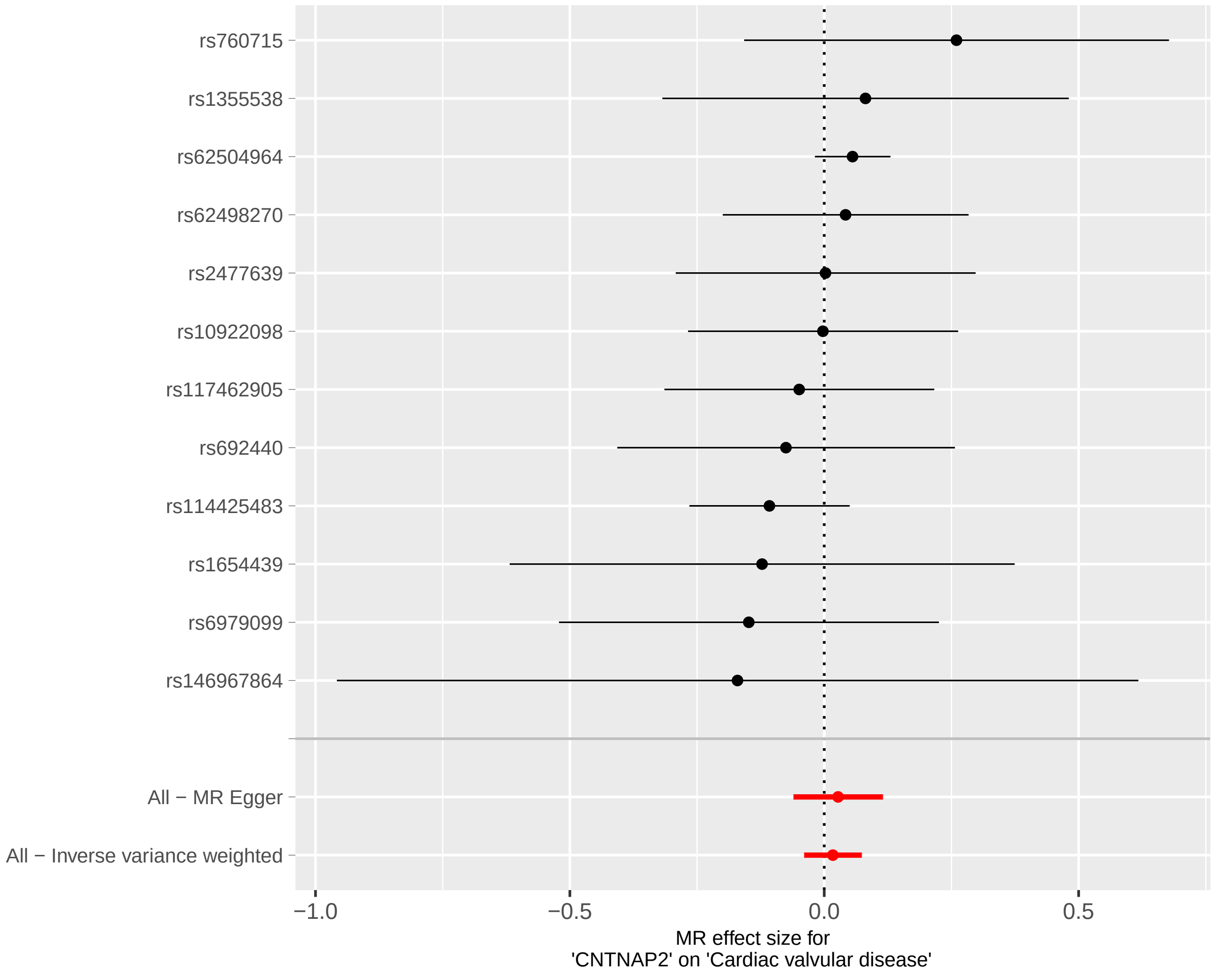
rs760715
rs1355538
rs62504964
rs62498270
rs2477639
rs10922098
rs117462905
rs692440
rs114425483
rs1654439
rs6979099
rs146967864
All − MR Egger
All − Inverse variance weighted
−1.0
−0.5
0.0
0.5
MR effect size for
'CNTNAP2' on 'Cardiac valvular disease'

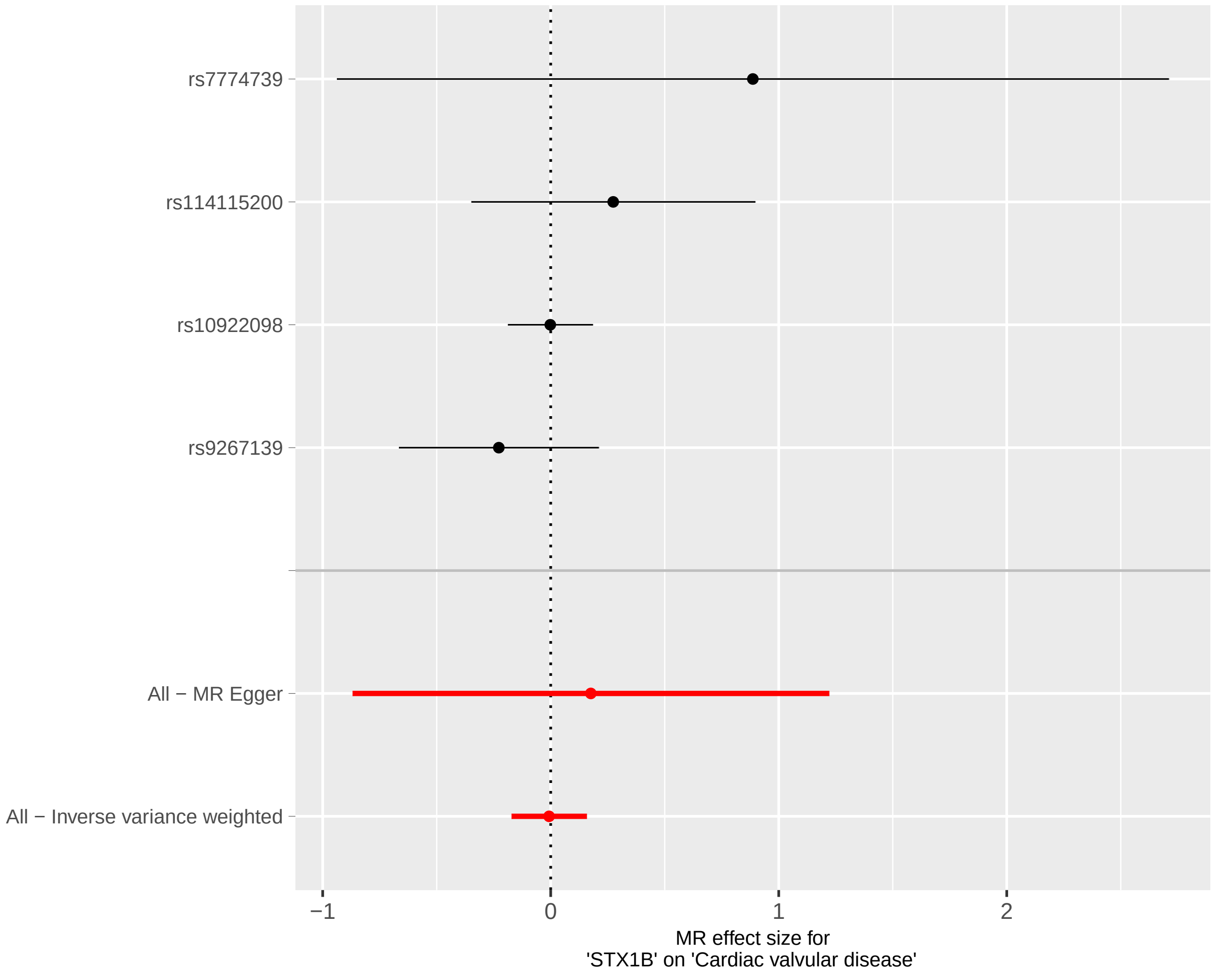
rs7774739
rs114115200
rs10922098
rs9267139
All − MR Egger
All − Inverse variance weighted
−1
0
1
2
MR effect size for
'STX1B' on 'Cardiac valvular disease'

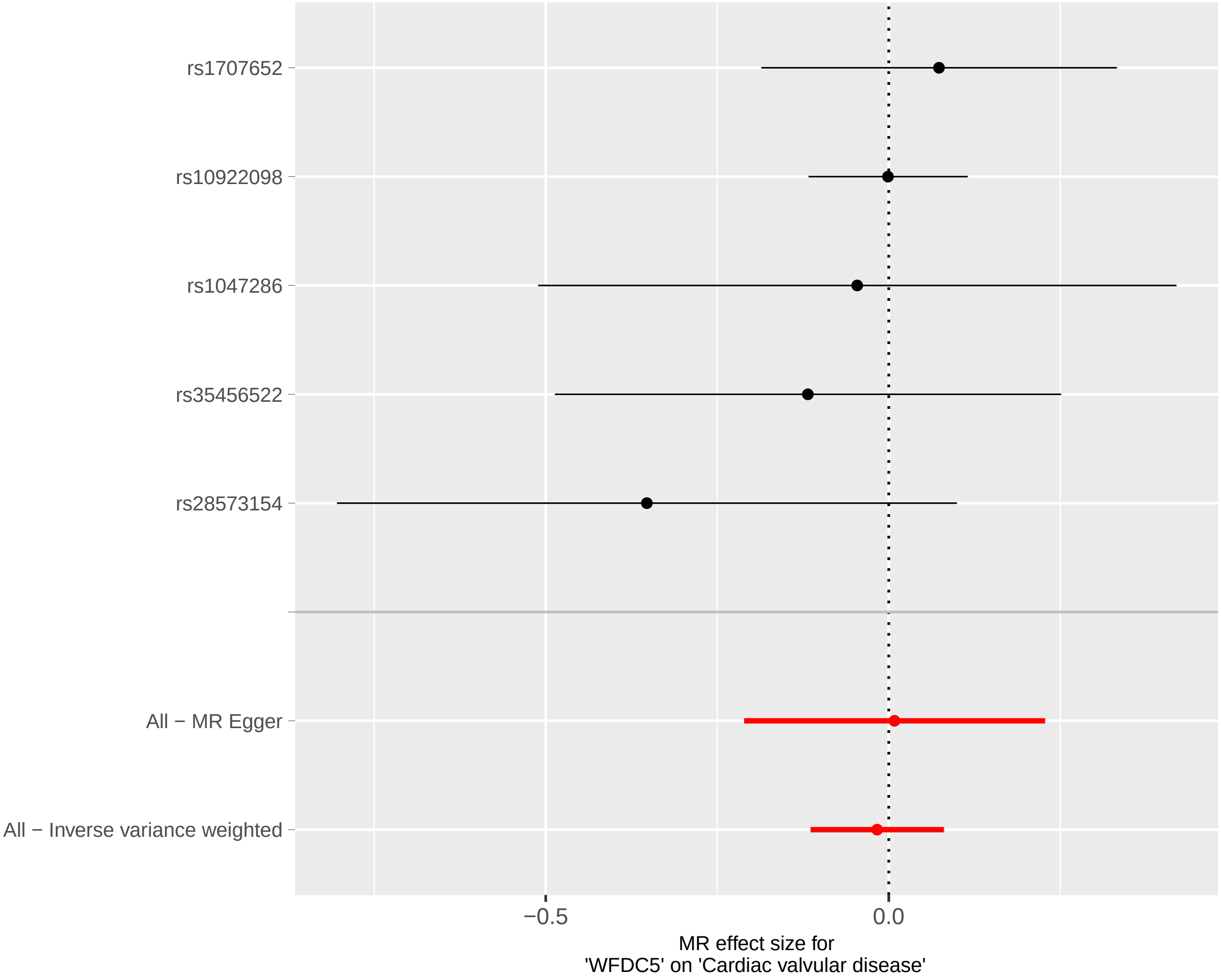

rs1707652
rs10922098
rs1047286
rs35456522
rs28573154
All − MR Egger
All − Inverse variance weighted
−0.5
0.0
MR effect size for
'WFDC5' on 'Cardiac valvular disease'

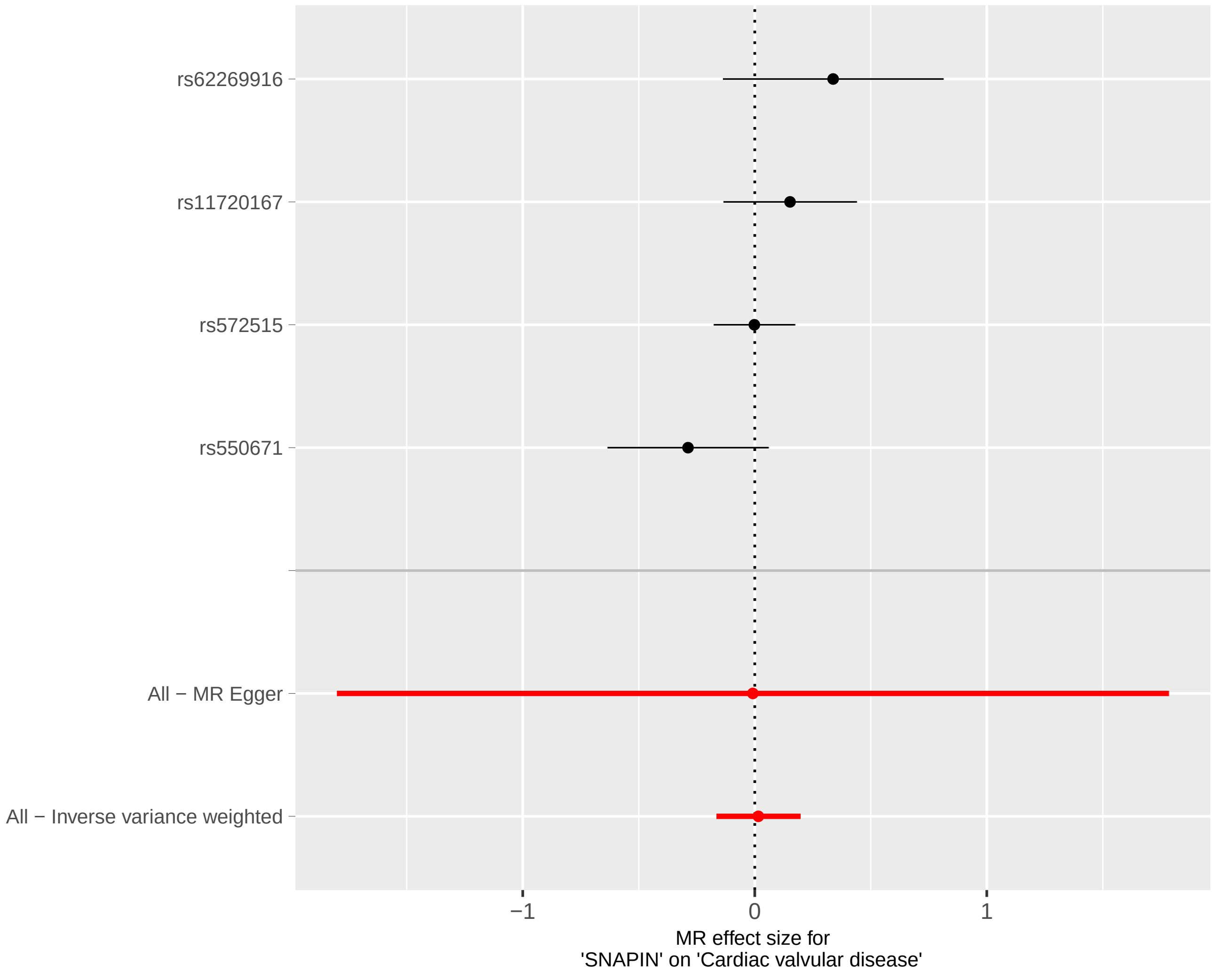
rs62269916
rs11720167
rs572515
rs550671
All − MR Egger
All − Inverse variance weighted
−1
0
1
MR effect size for
'SNAPIN' on 'Cardiac valvular disease'

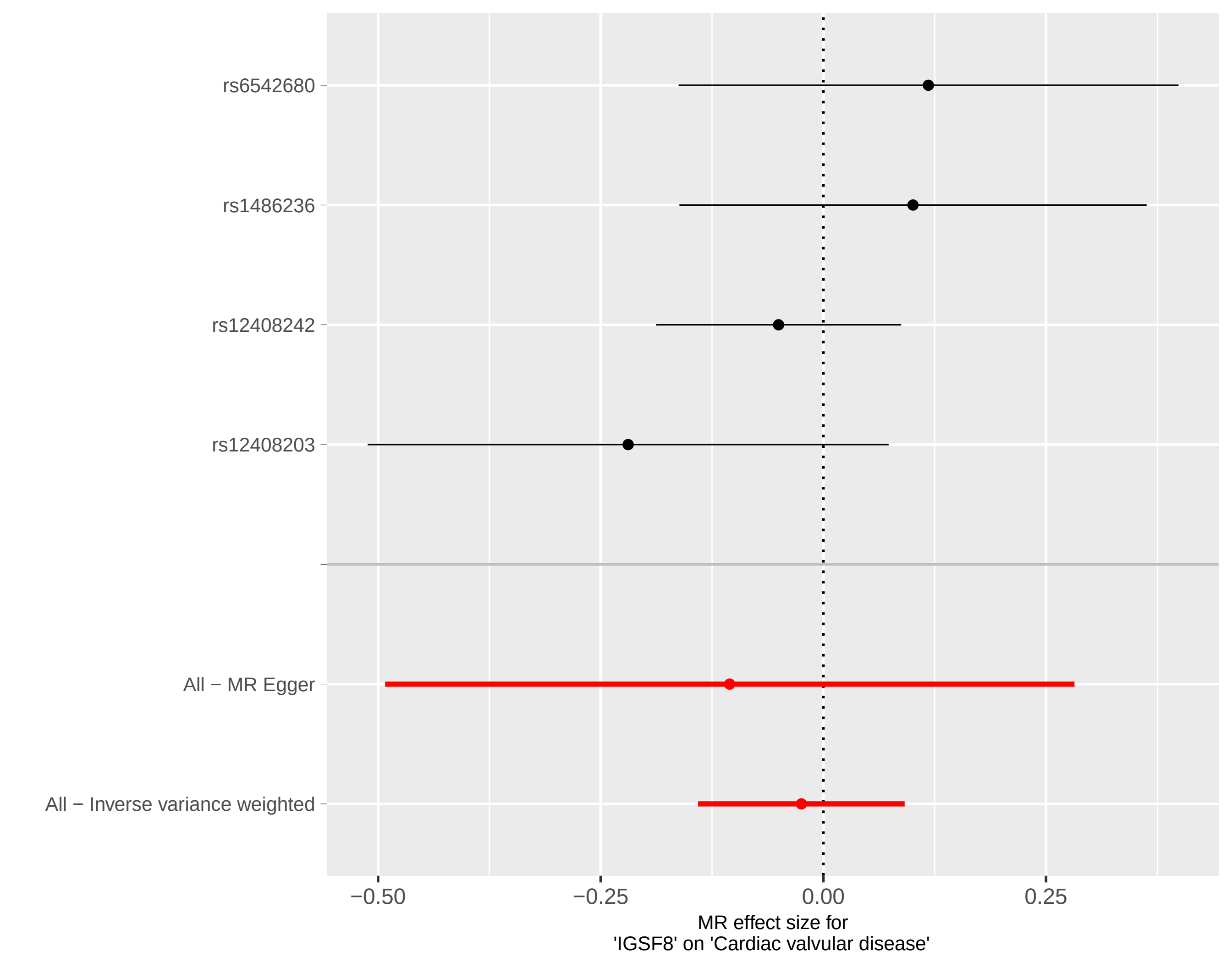
rs6542680
rs1486236
rs12408242
rs12408203
All − MR Egger
All − Inverse variance weighted
−0.50
−0.25
0.00
0.25
MR effect size for
'IGSF8' on 'Cardiac valvular disease'

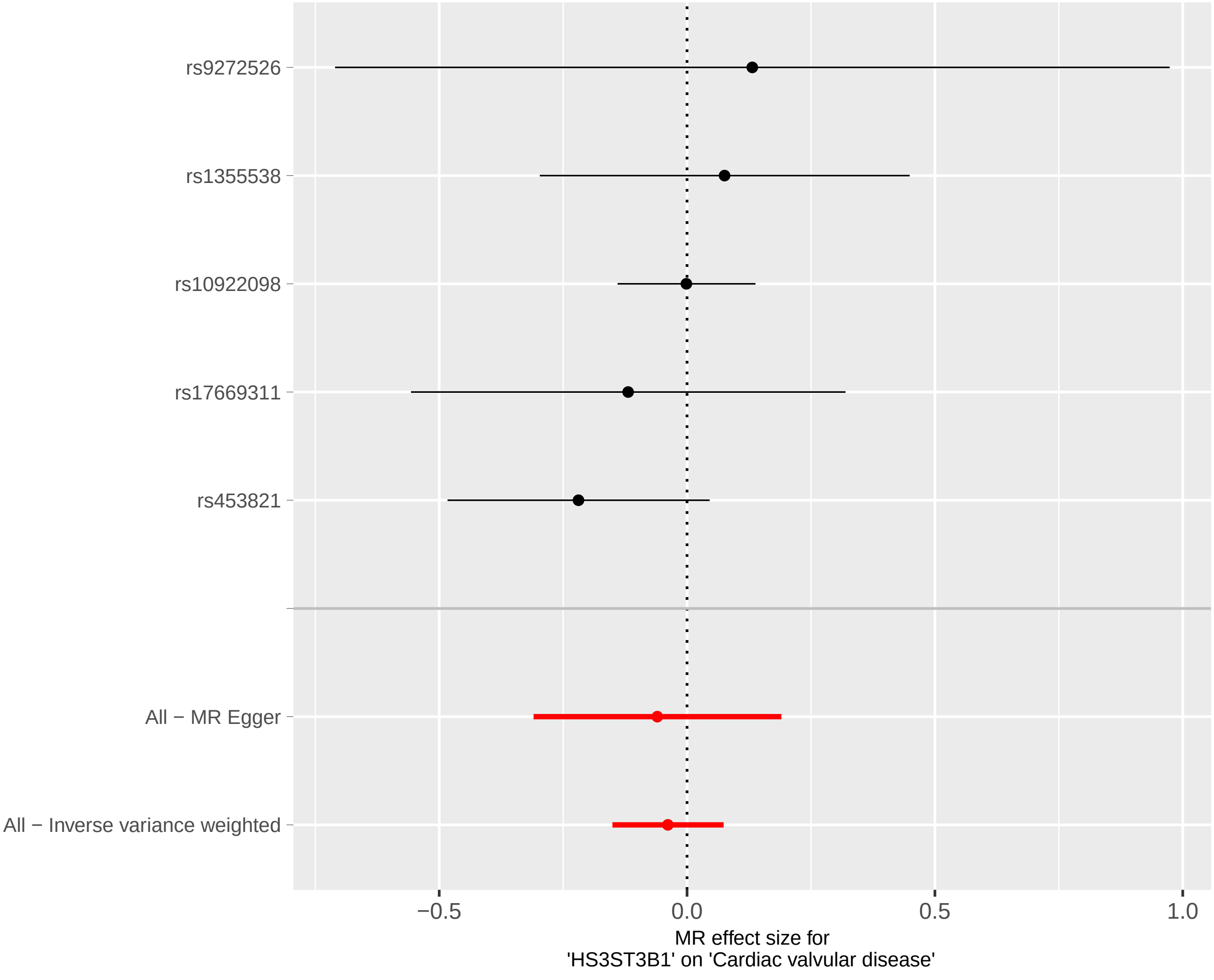
rs9272526
rs1355538
rs10922098
rs17669311
rs453821
All − MR Egger
All − Inverse variance weighted
−0.5
0.0
0.5
1.0
MR effect size for
'HS3ST3B1' on 'Cardiac valvular disease'

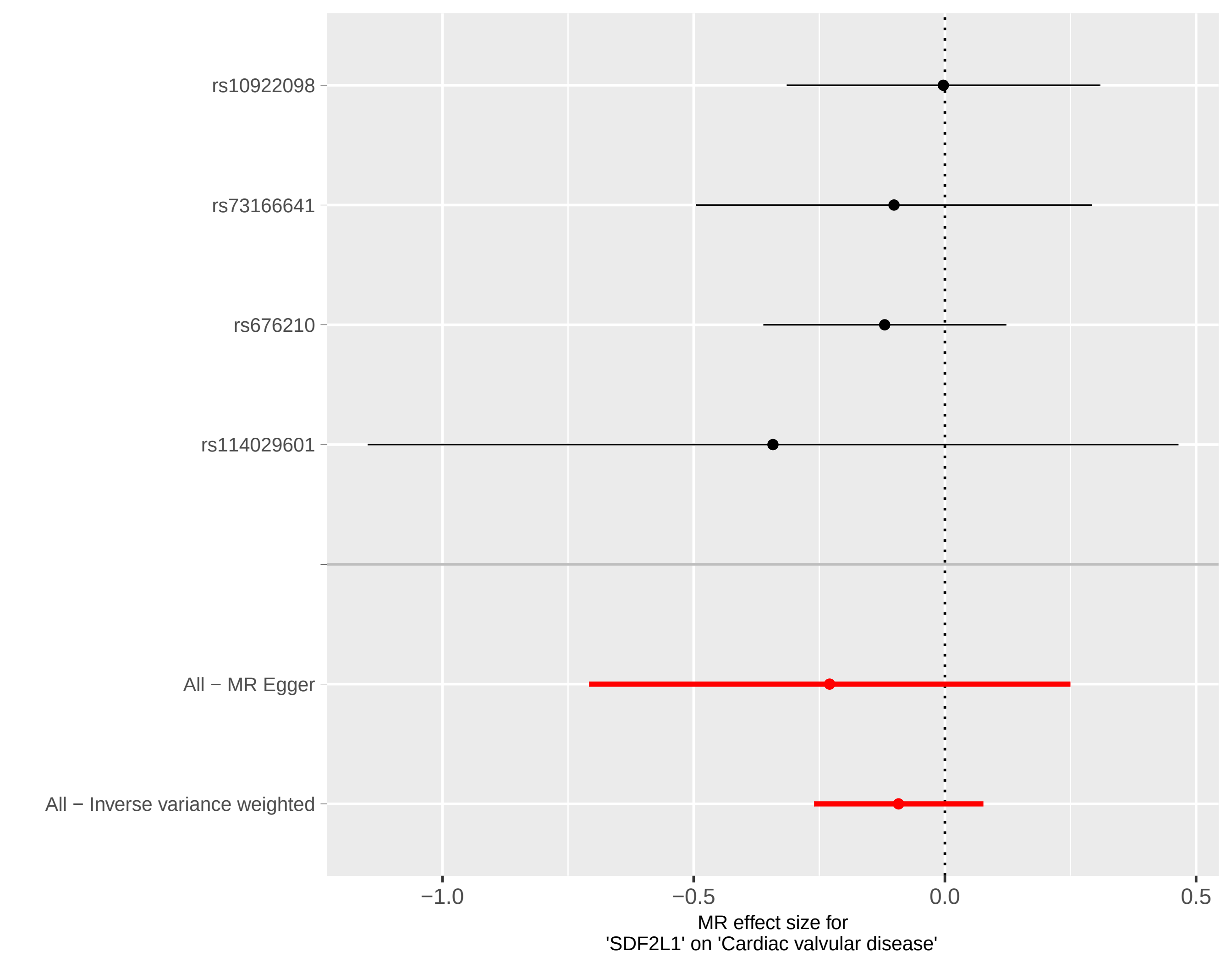

rs10922098
rs73166641
rs676210
rs114029601
All − MR Egger
All − Inverse variance weighted
−1.0
−0.5
0.0
0.5
MR effect size for
'SDF2L1' on 'Cardiac valvular disease'

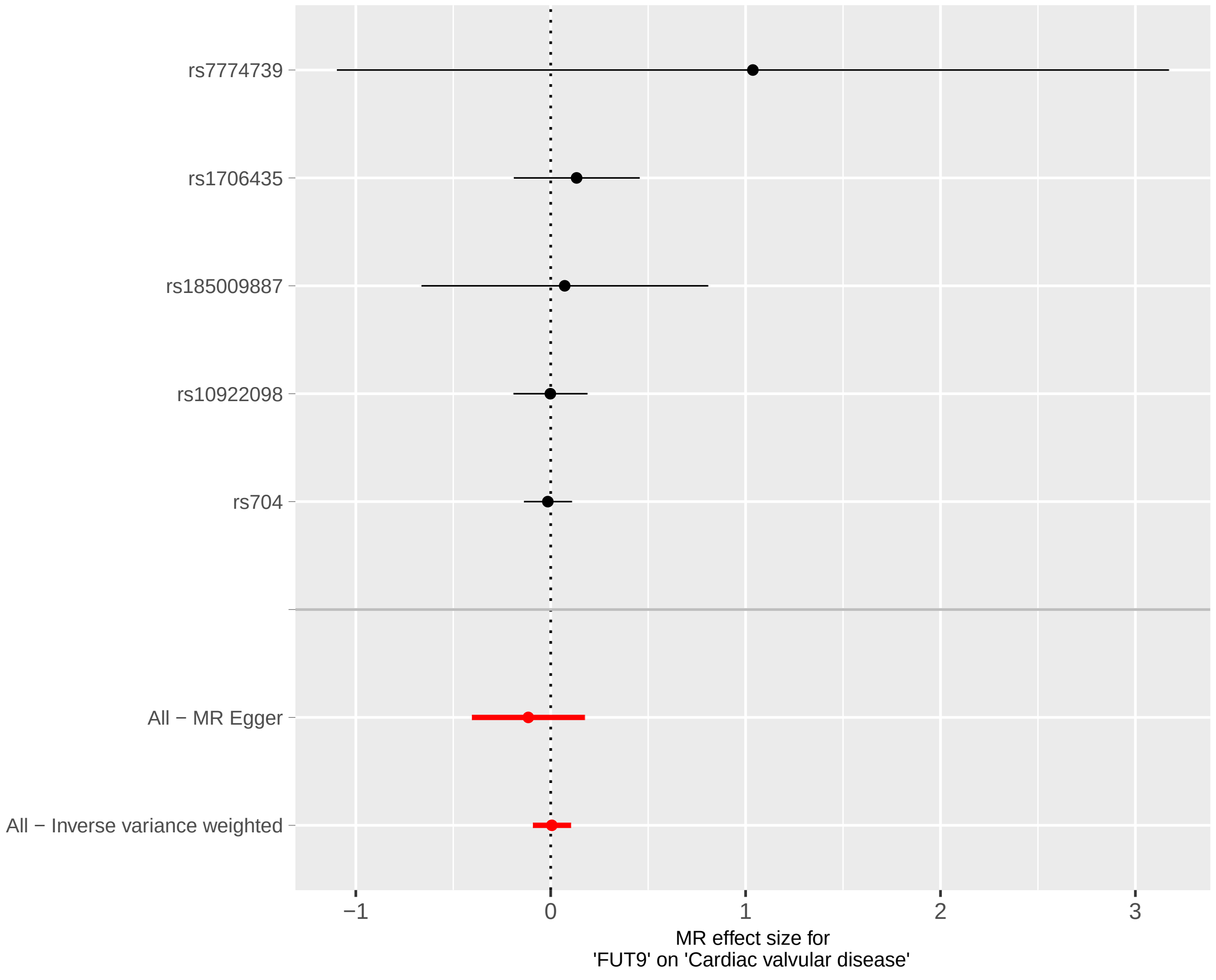
rs7774739
rs1706435
rs185009887
rs10922098
rs704
All − MR Egger
All − Inverse variance weighted
−1
0
1
2
3
MR effect size for
'FUT9' on 'Cardiac valvular disease'

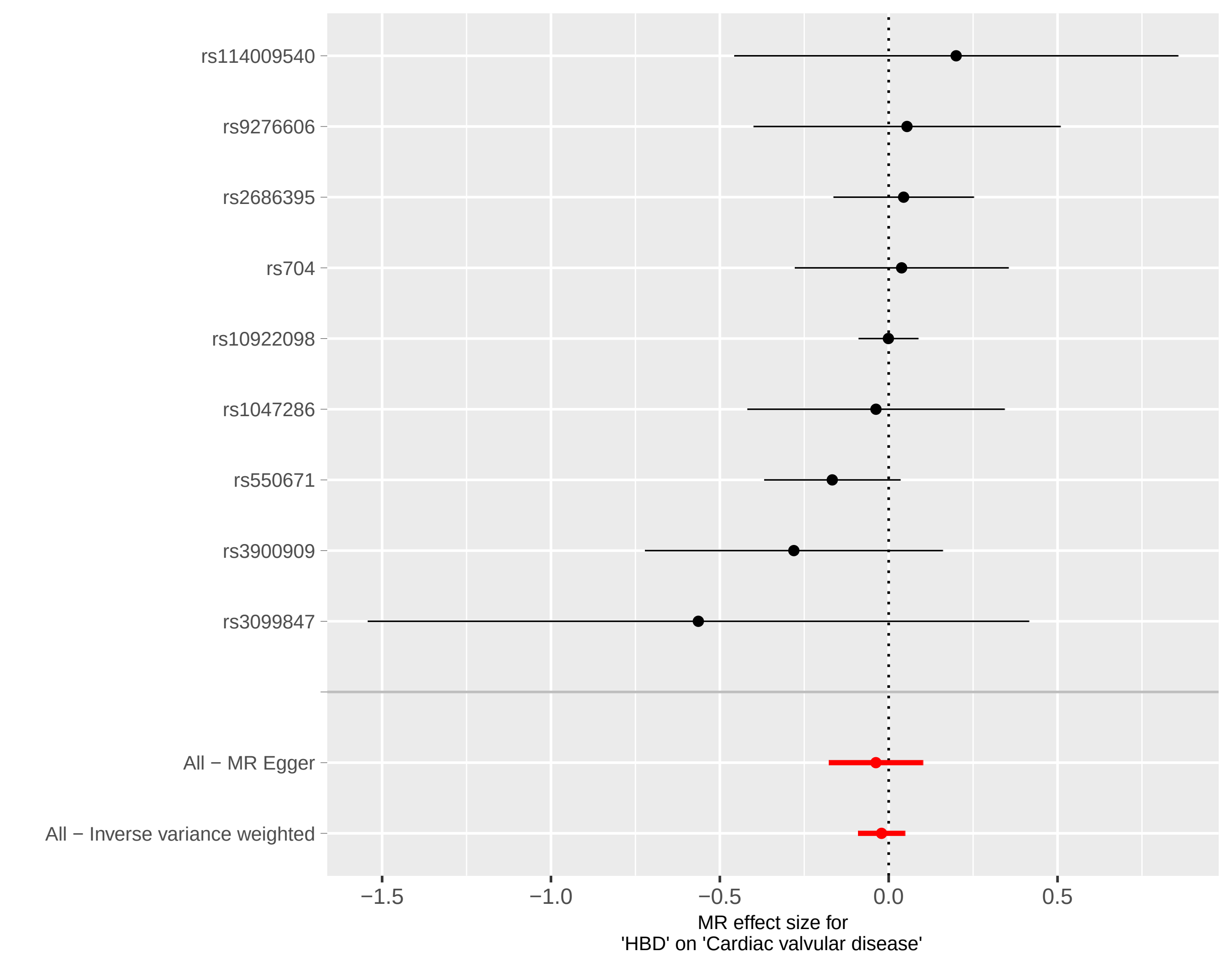

rs114009540
rs9276606
rs2686395
rs704
rs10922098
rs1047286
rs550671
rs3900909
rs3099847
All − MR Egger
All − Inverse variance weighted
−1.5
−1.0
−0.5
0.0
0.5
MR effect size for
'HBD' on 'Cardiac valvular disease'

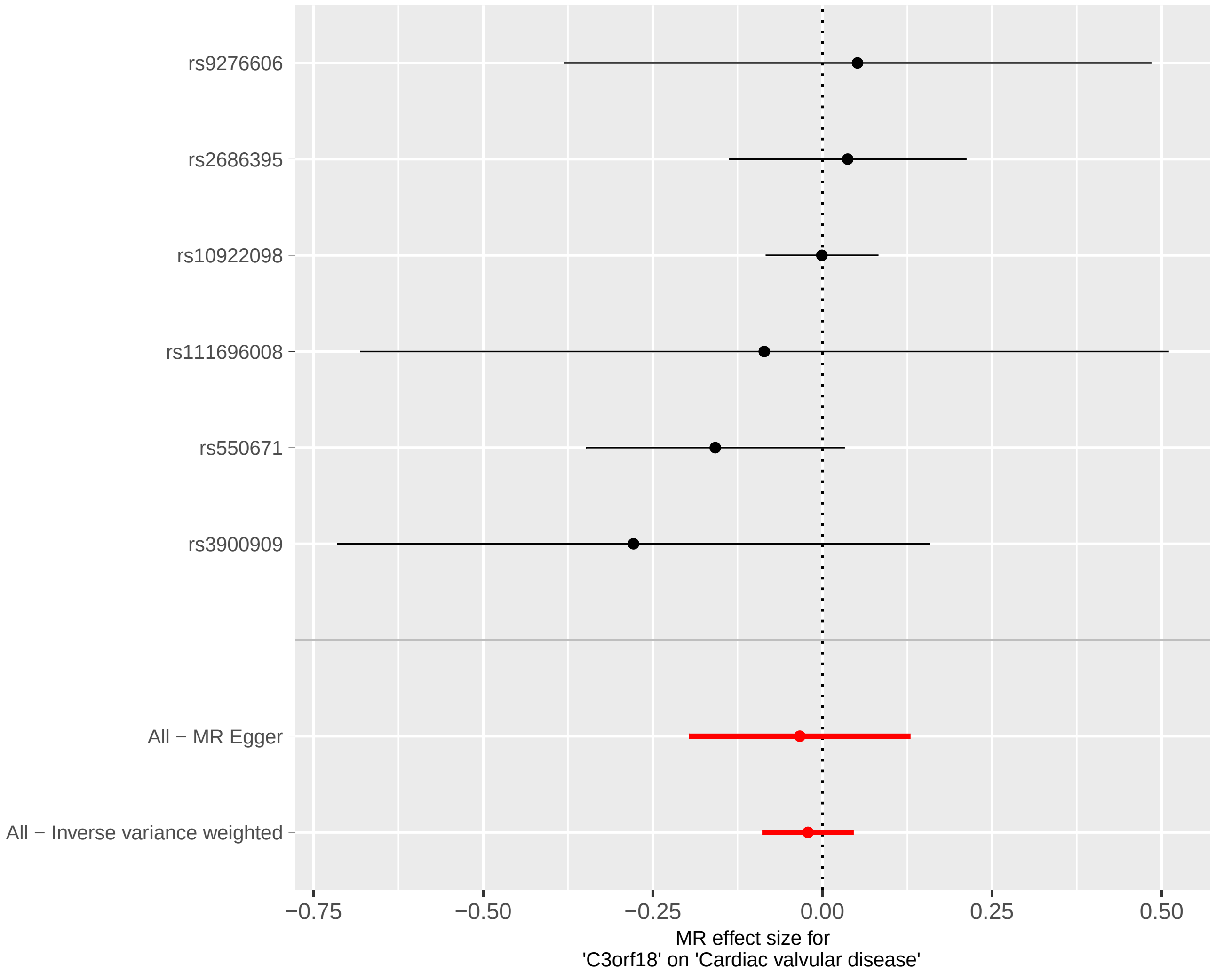
rs9276606
rs2686395
rs10922098
rs111696008
rs550671
rs3900909
All − MR Egger
All − Inverse variance weighted
−0.75
−0.50
−0.25
0.00
0.25
0.50
MR effect size for
'C3orf18' on 'Cardiac valvular disease'

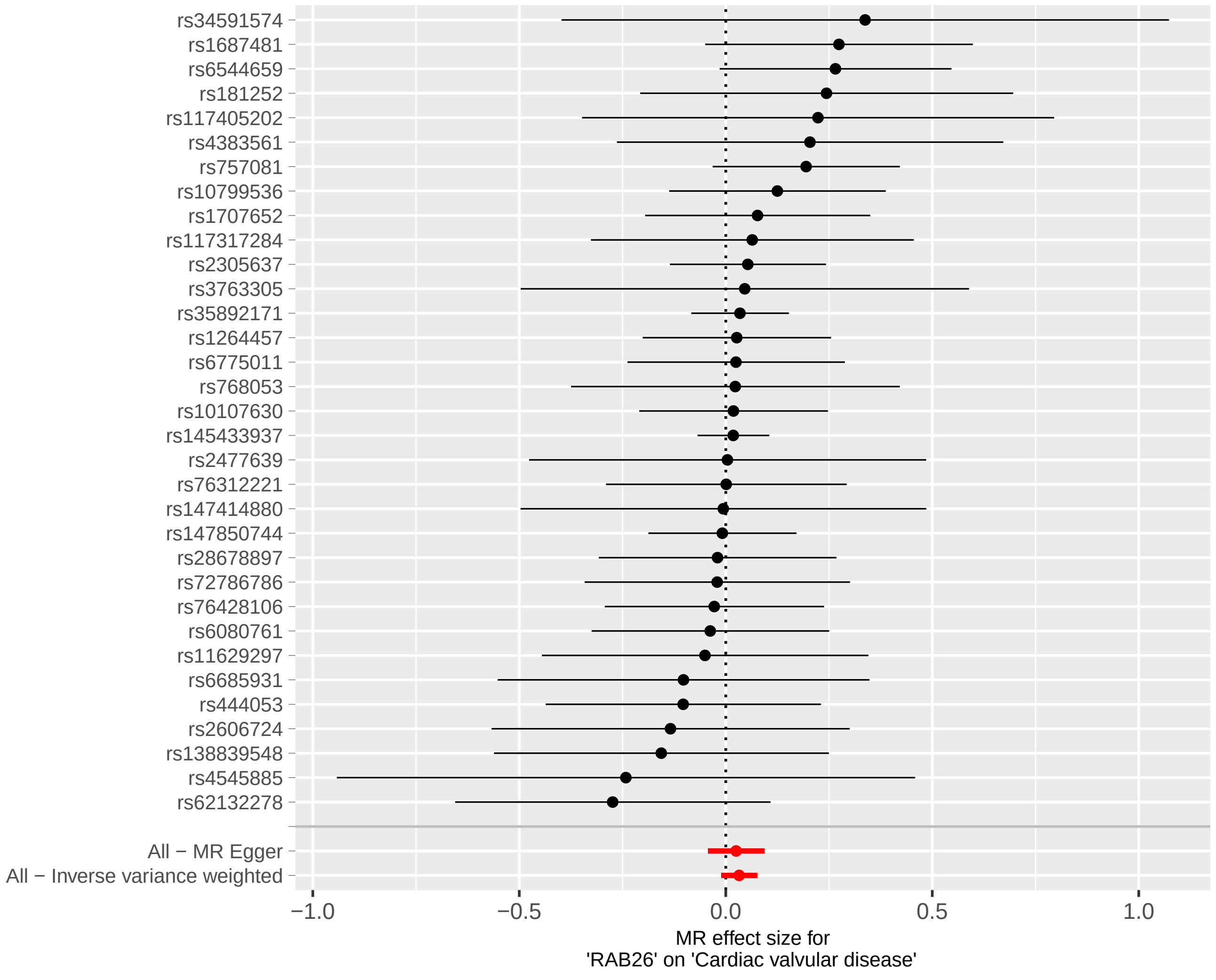
rs34591574
rs1687481
rs6544659
rs181252
rs117405202
rs4383561
rs757081
rs10799536
rs1707652
rs117317284
rs2305637
rs3763305
rs35892171
rs1264457
rs6775011
rs768053
rs10107630
rs145433937
rs2477639
rs76312221
rs147414880
rs147850744
rs28678897
rs72786786
rs76428106
rs6080761
rs11629297
rs6685931
rs444053
rs2606724
rs138839548
rs4545885
rs62132278
All − MR Egger
All − Inverse variance weighted
−1.0
−0.5
0.0
0.5
1.0
MR effect size for
'RAB26' on 'Cardiac valvular disease'

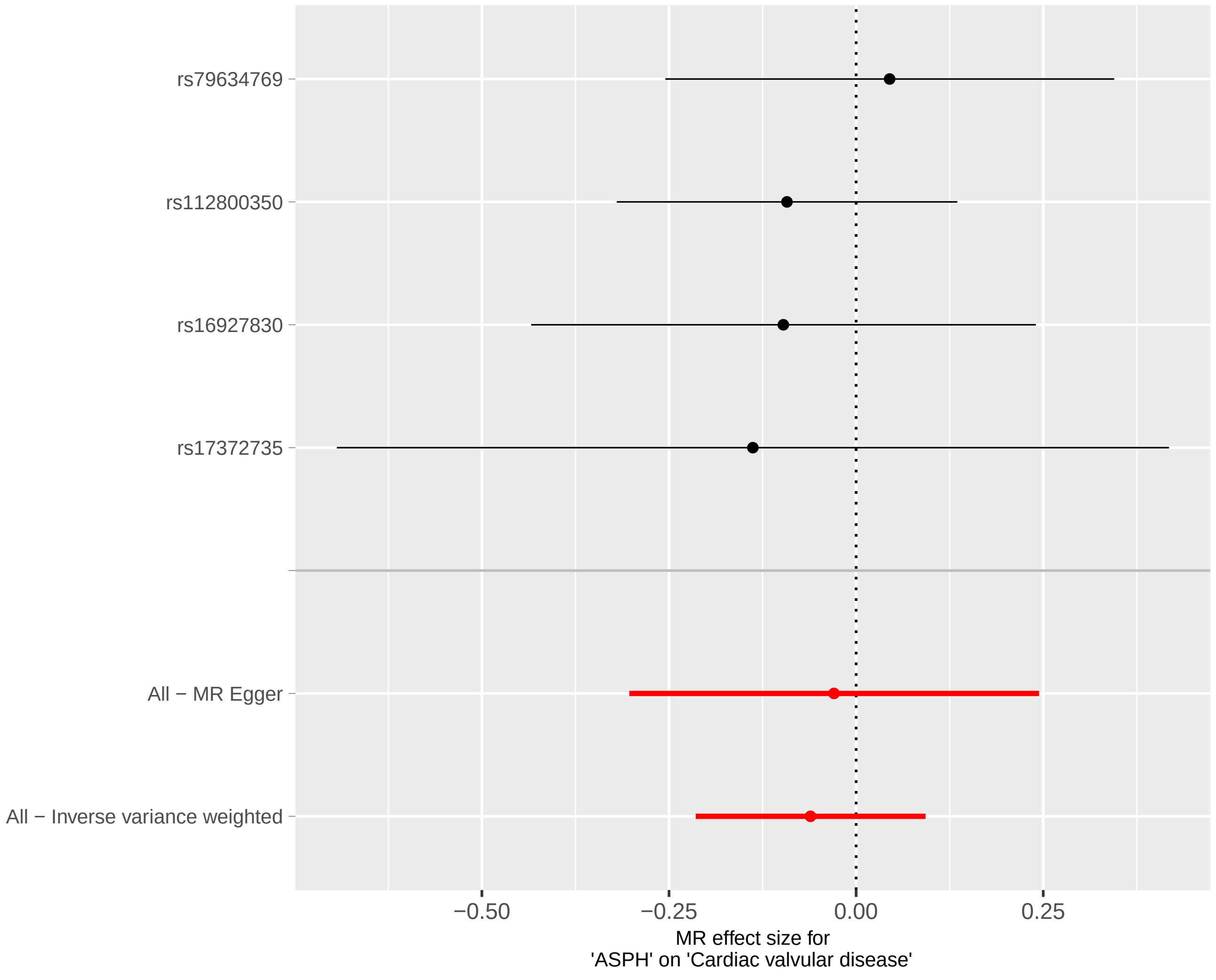
rs79634769
rs112800350
rs16927830
rs17372735
All − MR Egger
All − Inverse variance weighted
−0.50
−0.25
0.00
0.25
MR effect size for
'ASPH' on 'Cardiac valvular disease'

Insufficient number of SNPs

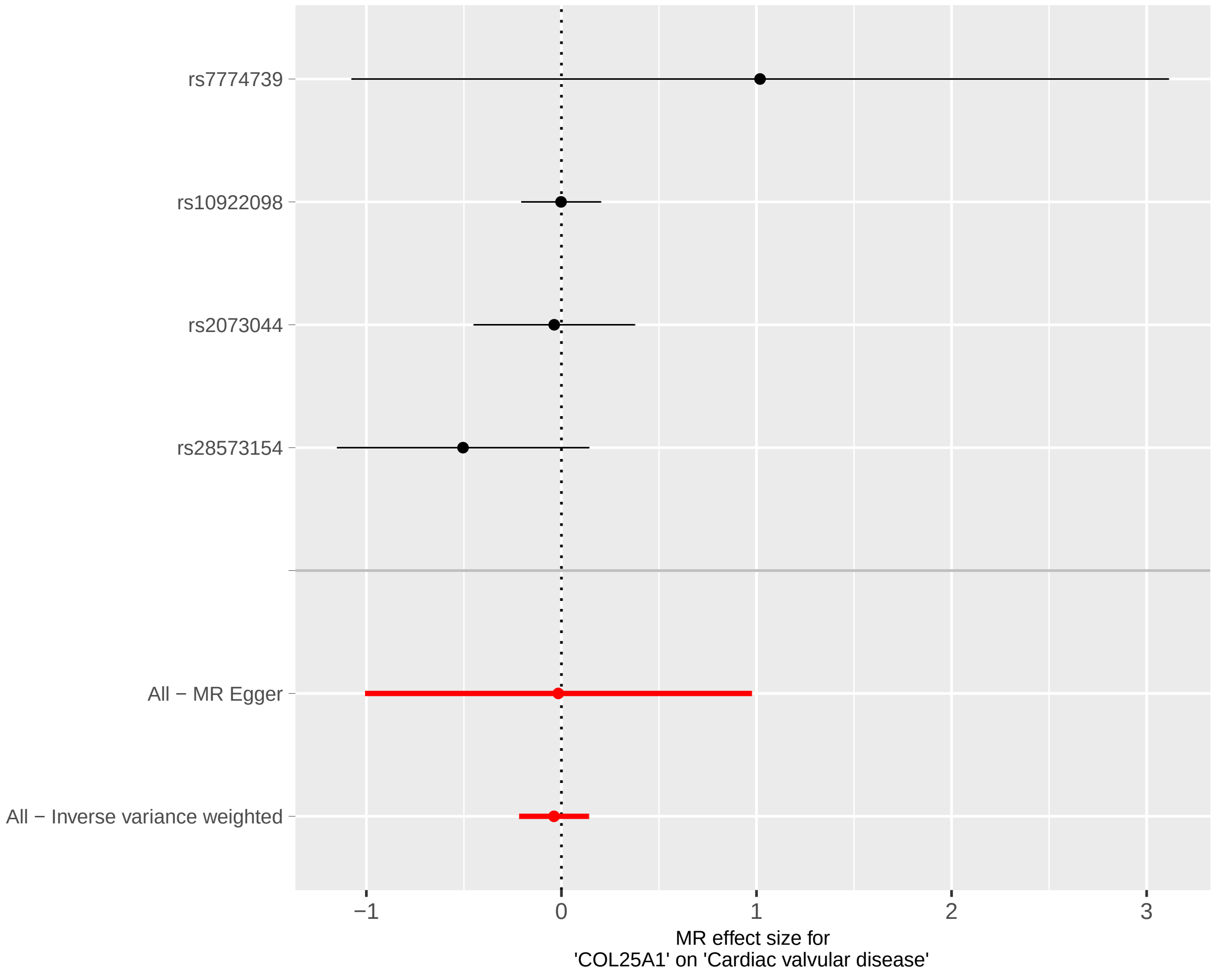
rs7774739
rs10922098
rs2073044
rs28573154
All − MR Egger
All − Inverse variance weighted
−1
0
1
2
3
MR effect size for
'COL25A1' on 'Cardiac valvular disease'

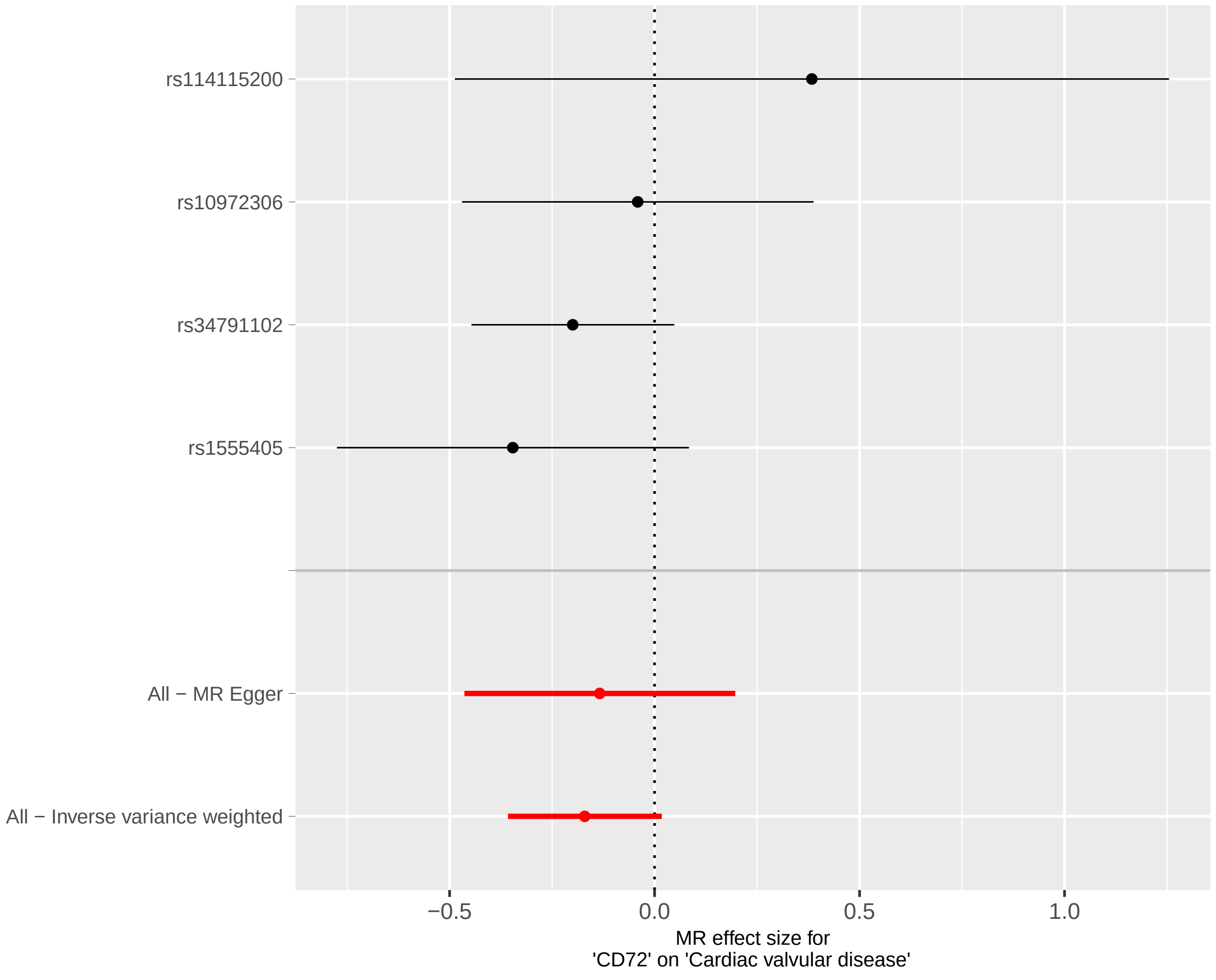
rs114115200
rs10972306
rs34791102
rs1555405
All − MR Egger
All − Inverse variance weighted
−0.5
0.0
0.5
1.0
MR effect size for
'CD72' on 'Cardiac valvular disease'

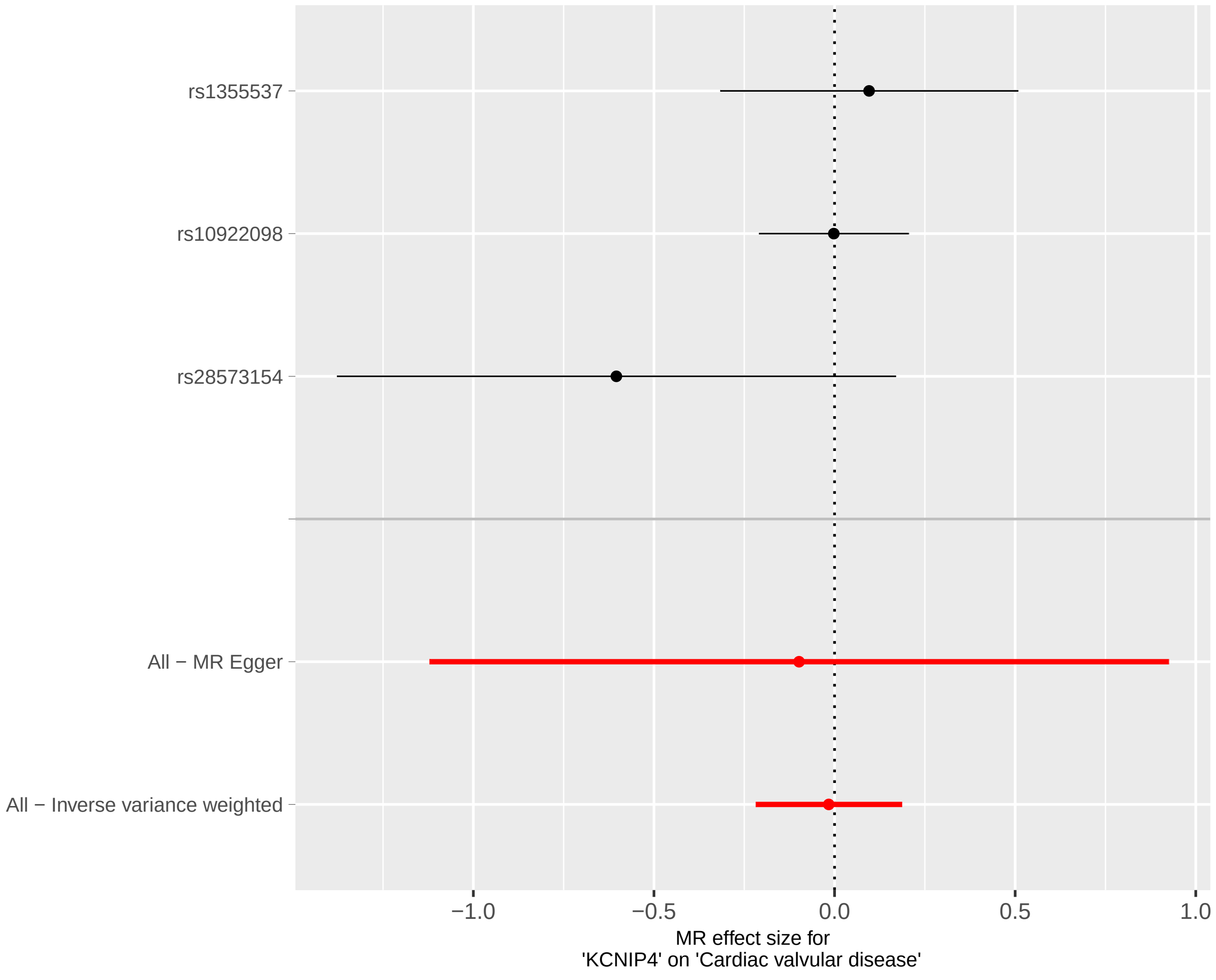
rs1355537
rs10922098
rs28573154
All − MR Egger
All − Inverse variance weighted
−1.0
−0.5
0.0
0.5
1.0
MR effect size for
'KCNIP4' on 'Cardiac valvular disease'

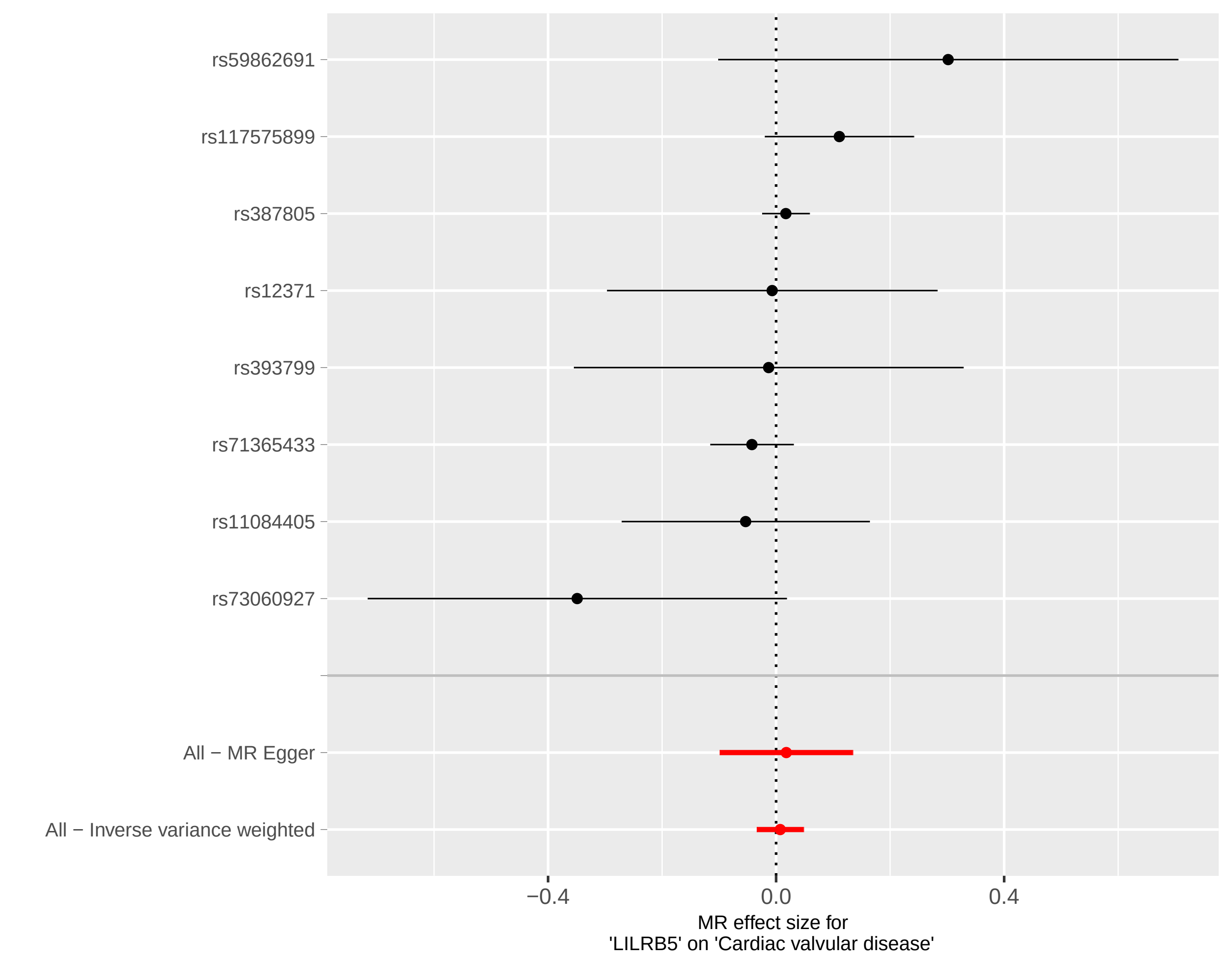
rs59862691
rs117575899
rs387805
rs12371
rs393799
rs71365433
rs11084405
rs73060927
All − MR Egger
All − Inverse variance weighted
−0.4
0.0
0.4
MR effect size for
'LILRB5' on 'Cardiac valvular disease'

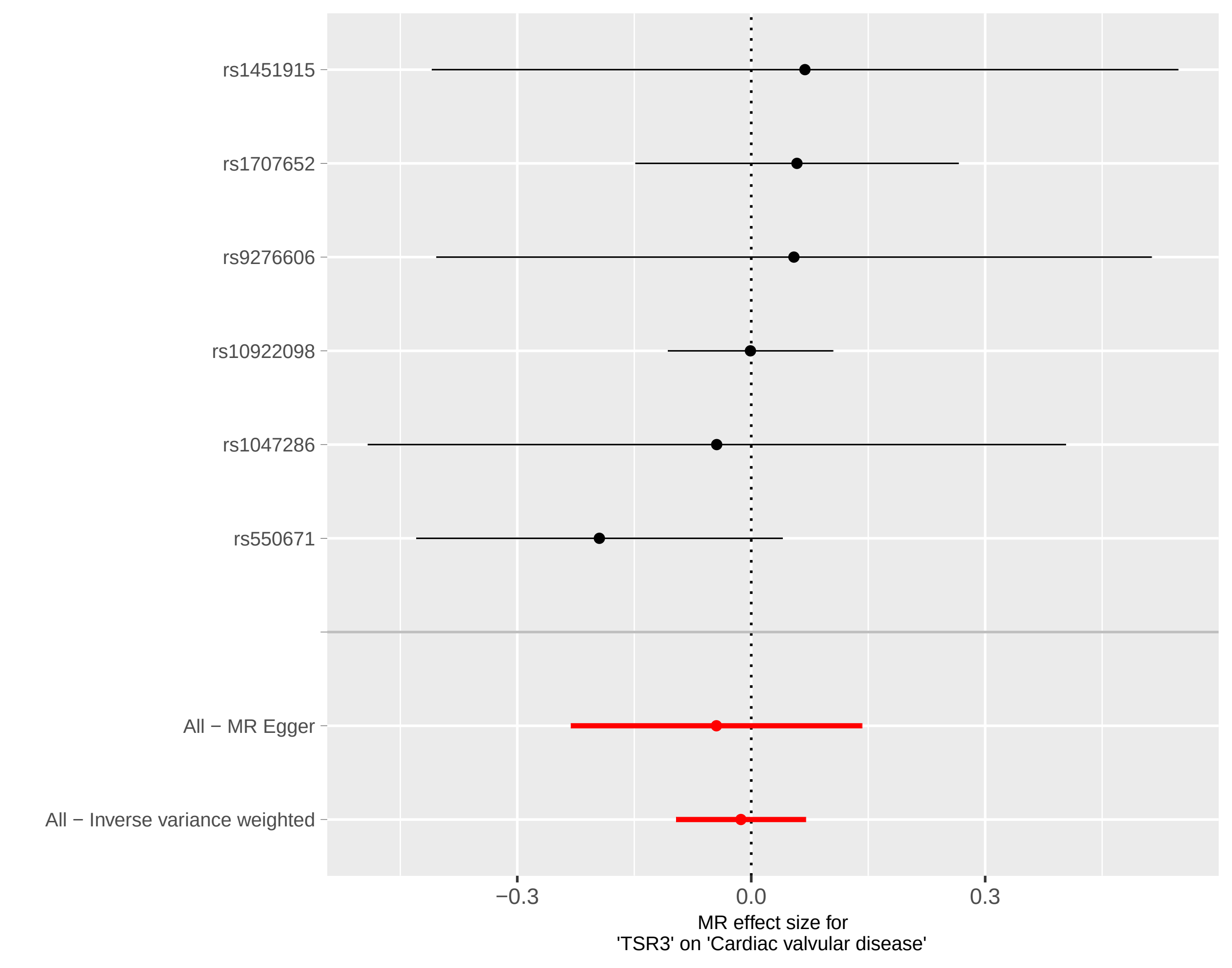
rs1451915
rs1707652
rs9276606
rs10922098
rs1047286
rs550671
All − MR Egger
All − Inverse variance weighted
−0.3
0.0
0.3
MR effect size for
'TSR3' on 'Cardiac valvular disease'

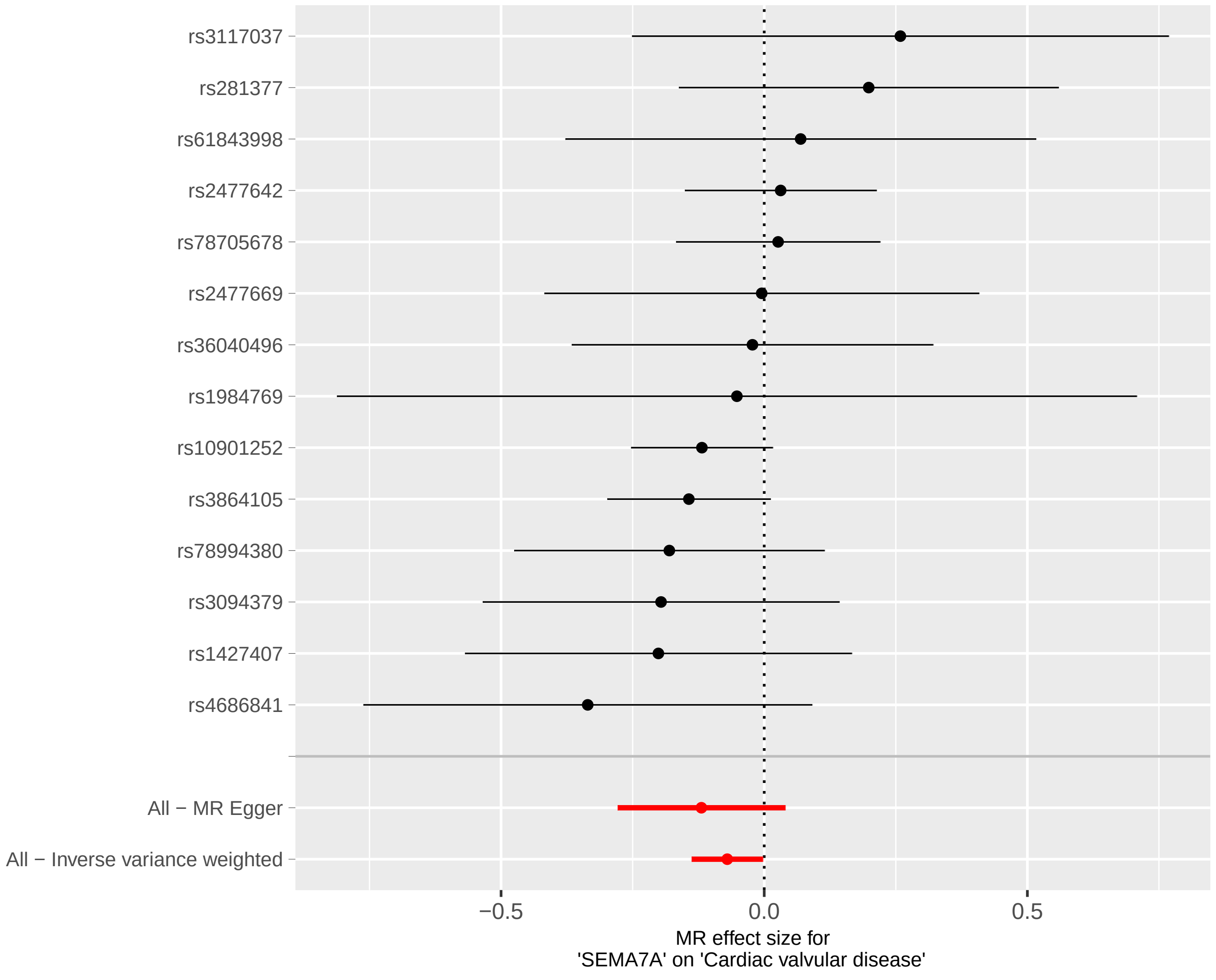
rs3117037
rs281377
rs61843998
rs2477642
rs78705678
rs2477669
rs36040496
rs1984769
rs10901252
rs3864105
rs78994380
rs3094379
rs1427407
rs4686841
All − MR Egger
All − Inverse variance weighted
−0.5
0.0
0.5
MR effect size for
'SEMA7A' on 'Cardiac valvular disease'

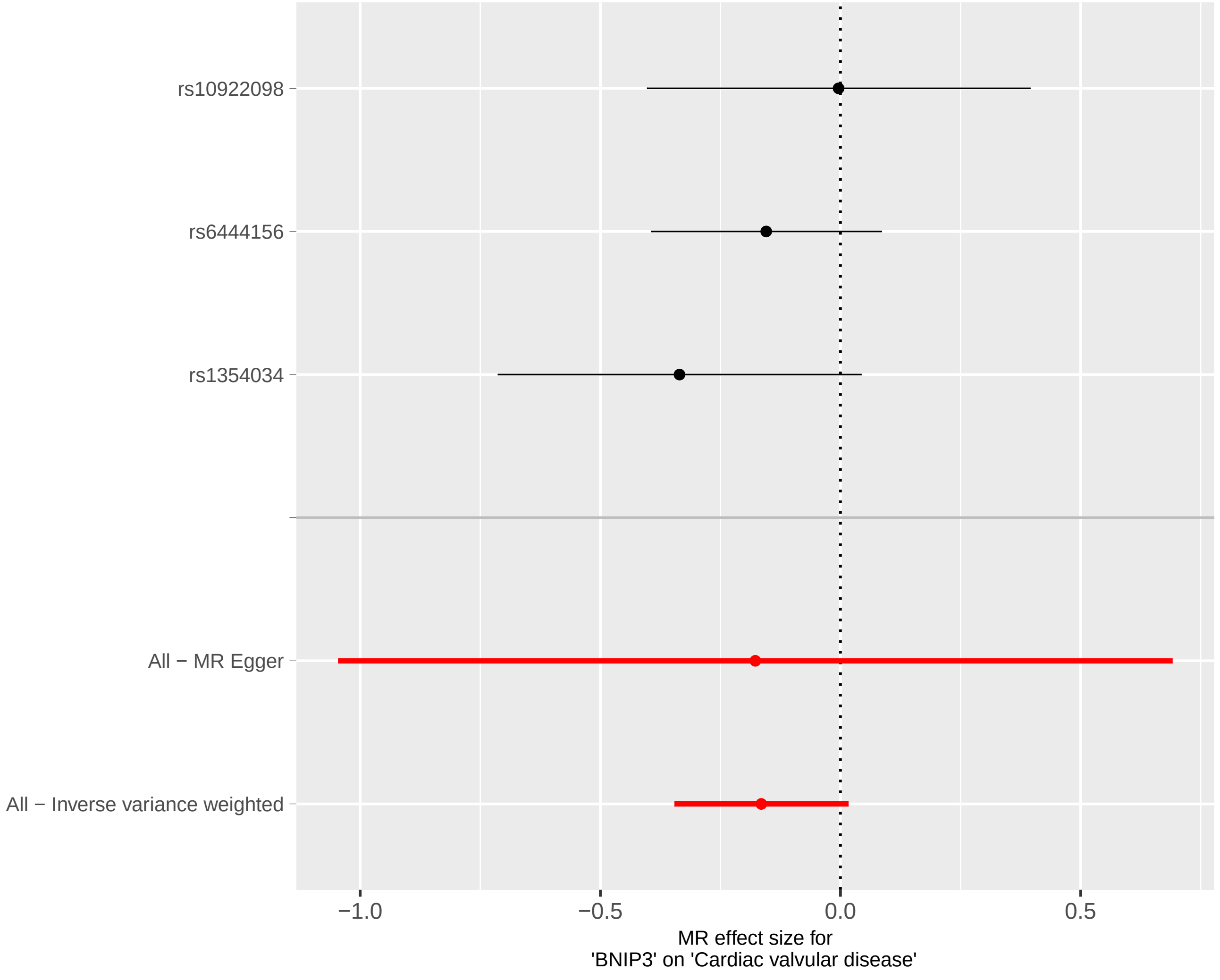

rs10922098
rs6444156
rs1354034
All − MR Egger
All − Inverse variance weighted
−1.0
−0.5
0.0
0.5
MR effect size for
'BNIP3' on 'Cardiac valvular disease'

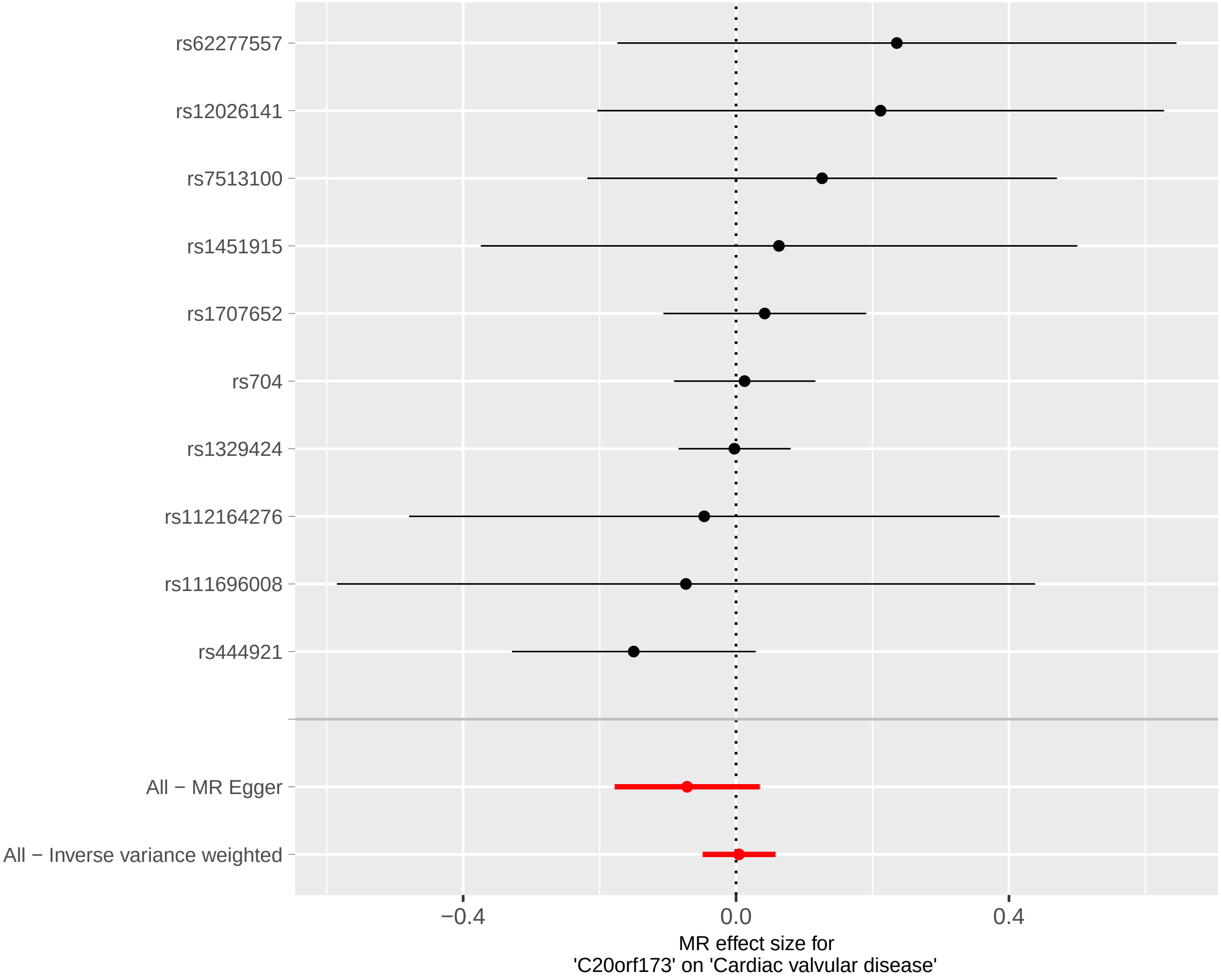
rs62277557
rs12026141
rs7513100
rs1451915
rs1707652
rs704
rs1329424
rs112164276
rs111696008
rs444921
All − MR Egger
All − Inverse variance weighted
−0.4
0.0
0.4
MR effect size for
'C20orf173' on 'Cardiac valvular disease'

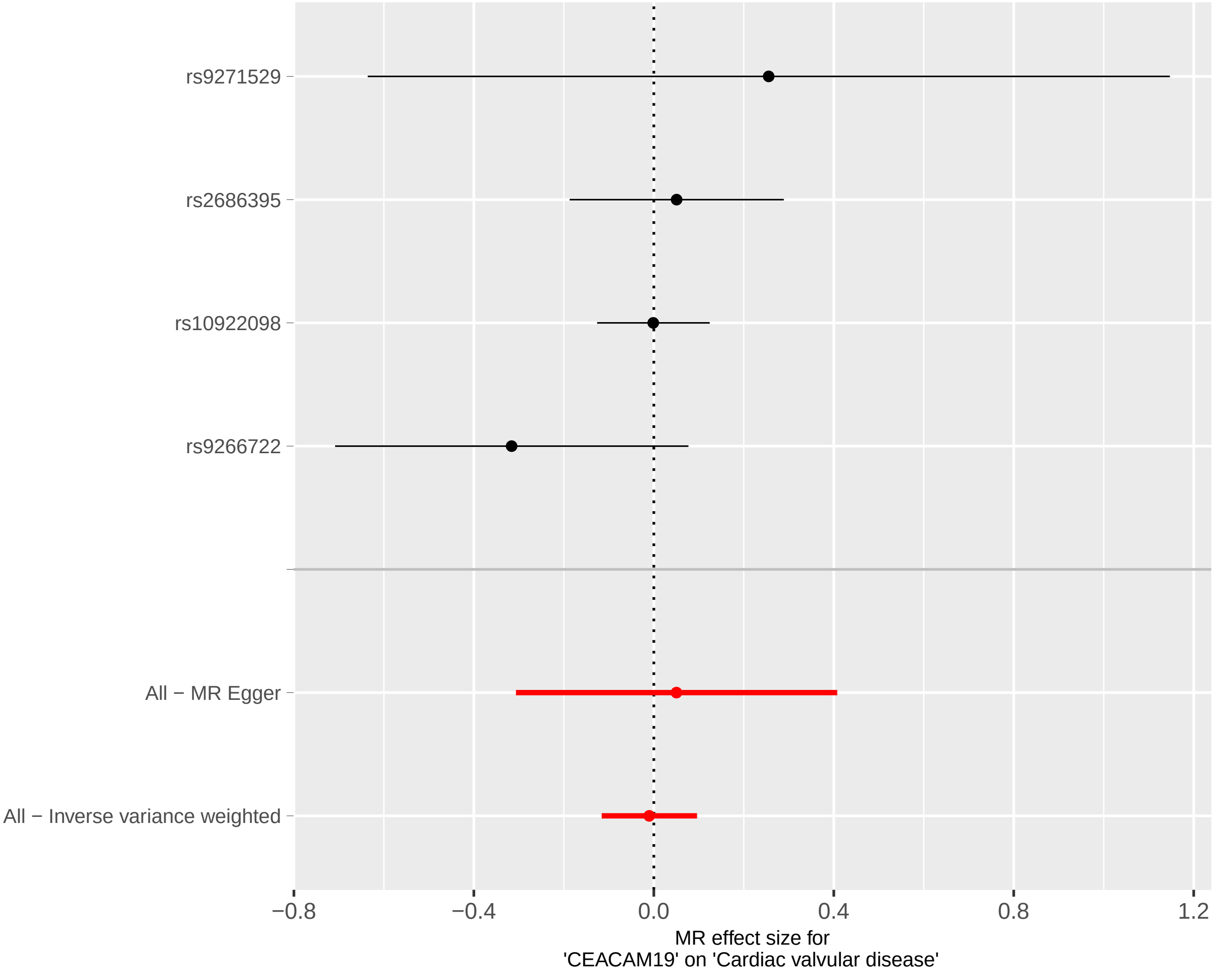
rs9271529
rs2686395
rs10922098
rs9266722
All − MR Egger
All − Inverse variance weighted
−0.8
−0.4
0.0
0.4
0.8
1.2
MR effect size for
'CEACAM19' on 'Cardiac valvular disease'

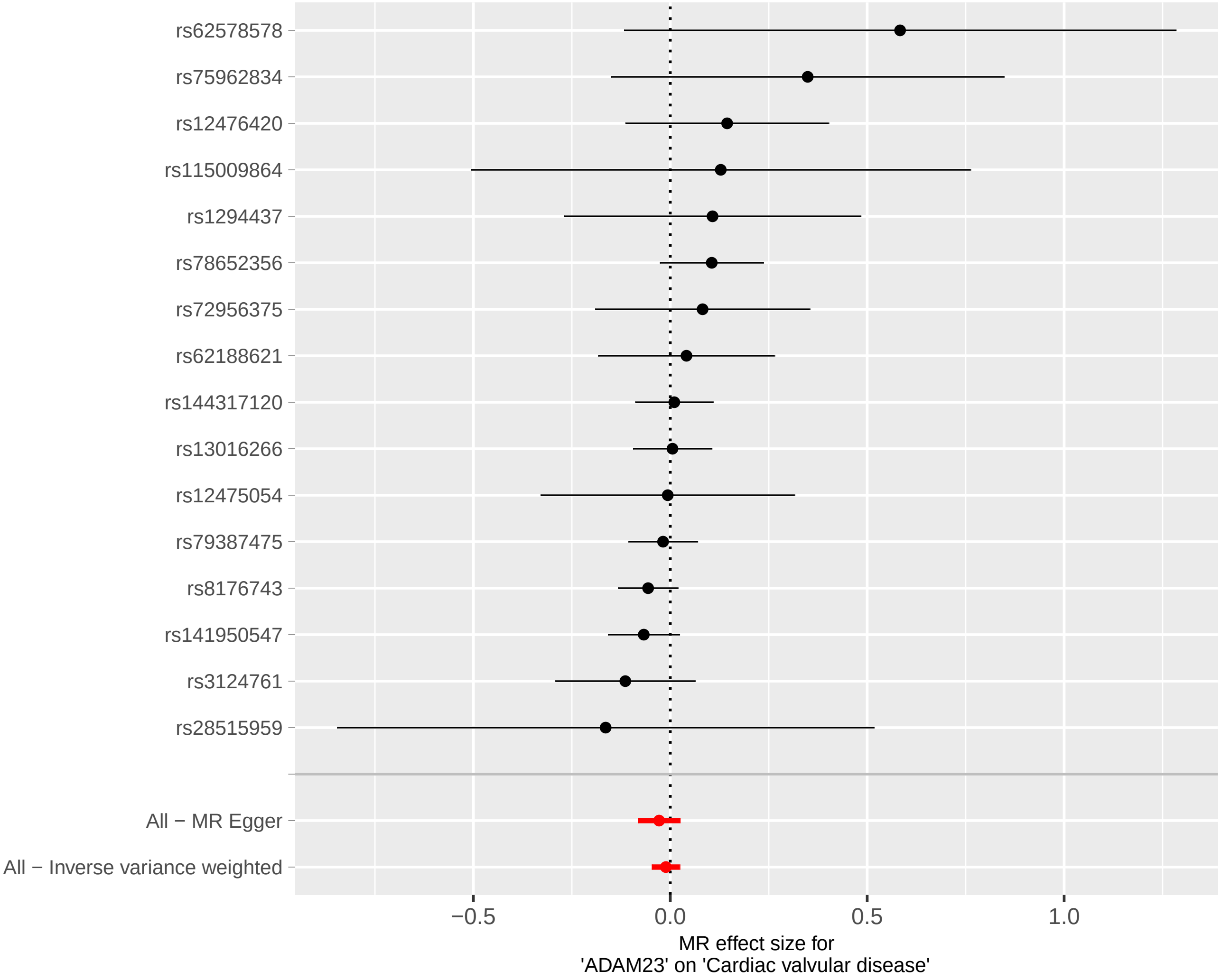

rs62578578
rs75962834
rs12476420
rs115009864
rs1294437
rs78652356
rs72956375
rs62188621
rs144317120
rs13016266
rs12475054
rs79387475
rs8176743
rs141950547
rs3124761
rs28515959
All − MR Egger
All − Inverse variance weighted
−0.5
0.0
0.5
1.0
MR effect size for
'ADAM23' on 'Cardiac valvular disease'

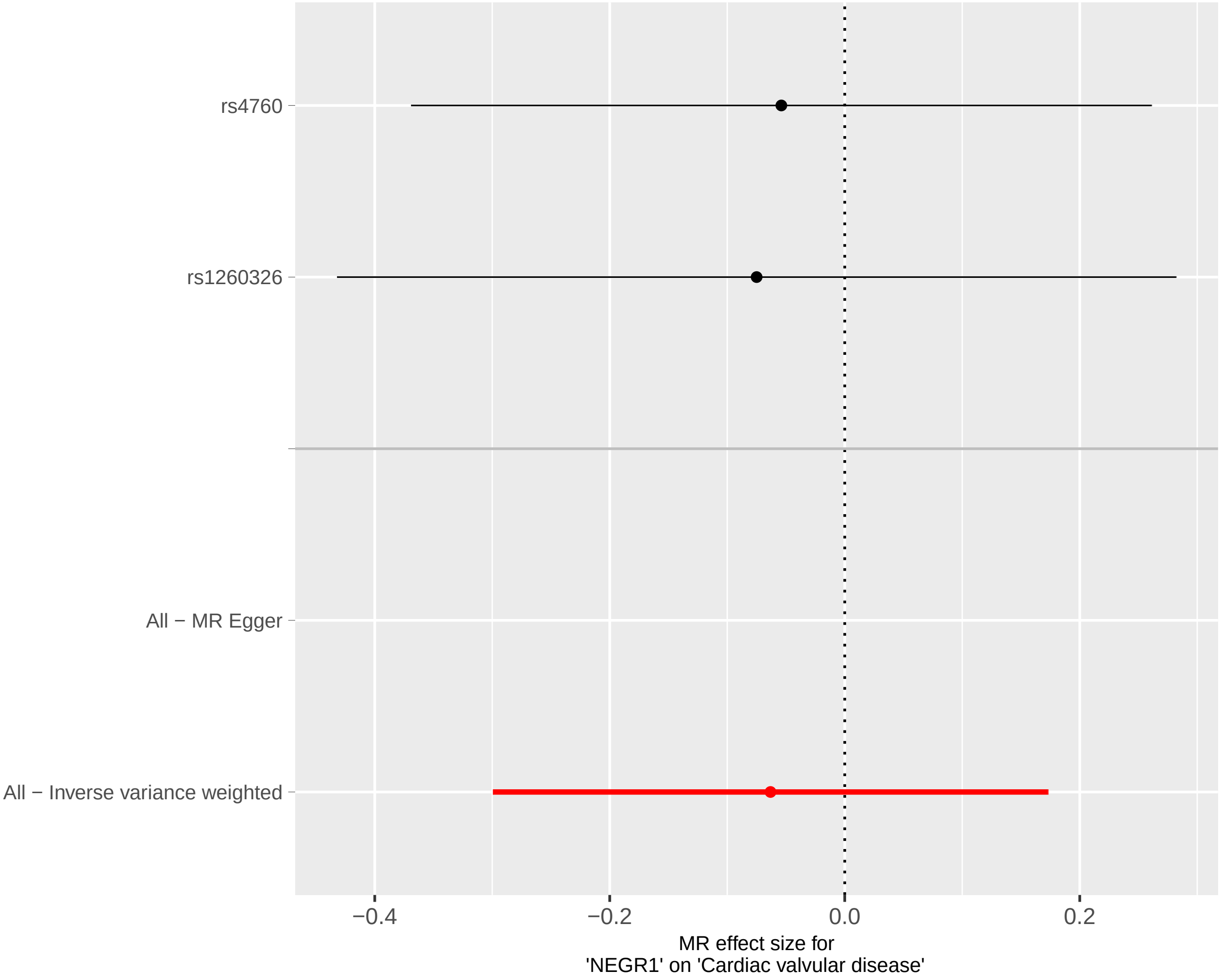
rs4760
rs1260326
All − MR Egger
All − Inverse variance weighted
−0.4
−0.2
0.0
0.2
MR effect size for
'NEGR1' on 'Cardiac valvular disease'

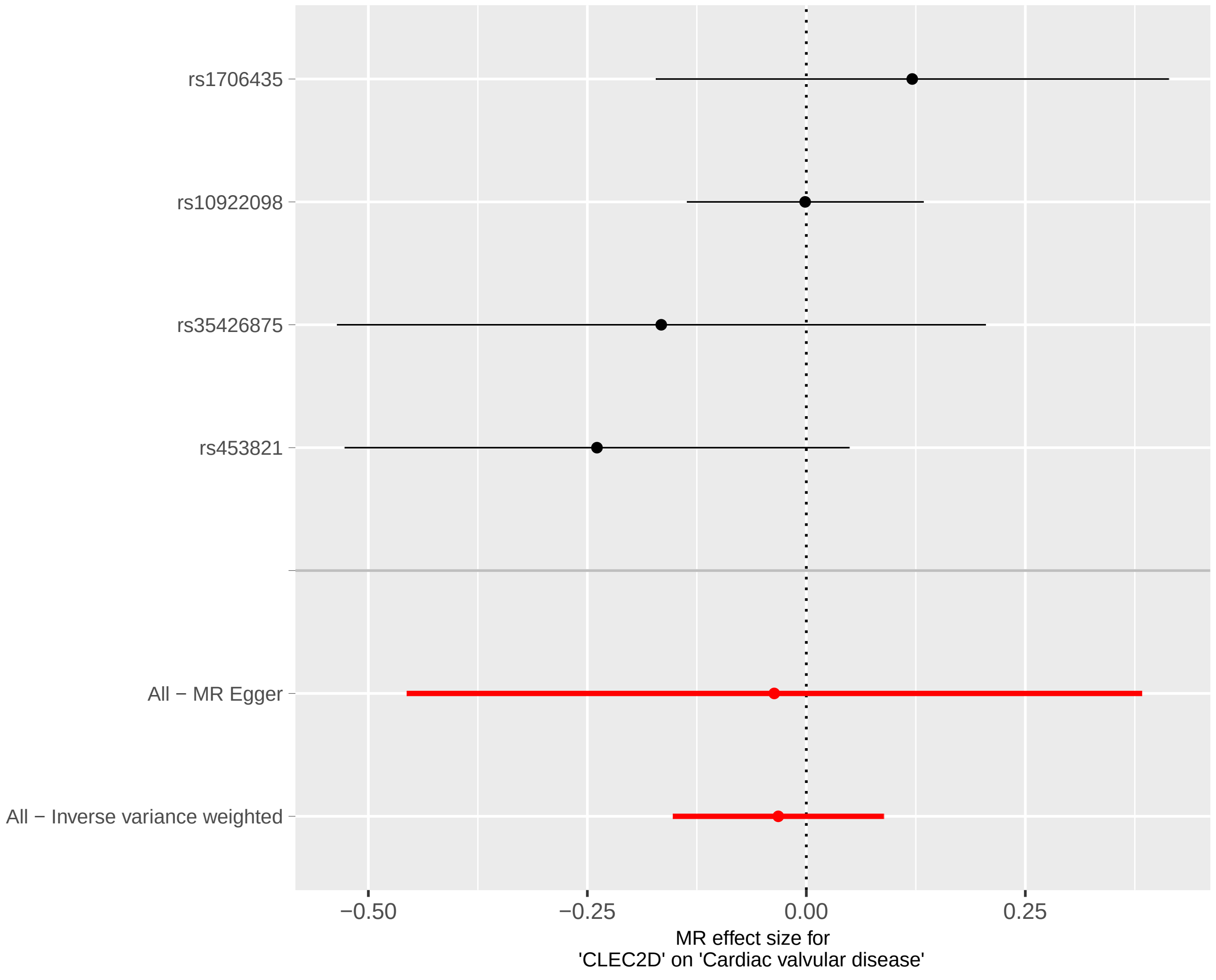
rs1706435
rs10922098
rs35426875
rs453821
All − MR Egger
All − Inverse variance weighted
−0.50
−0.25
0.00
0.25
MR effect size for
'CLEC2D' on 'Cardiac valvular disease'

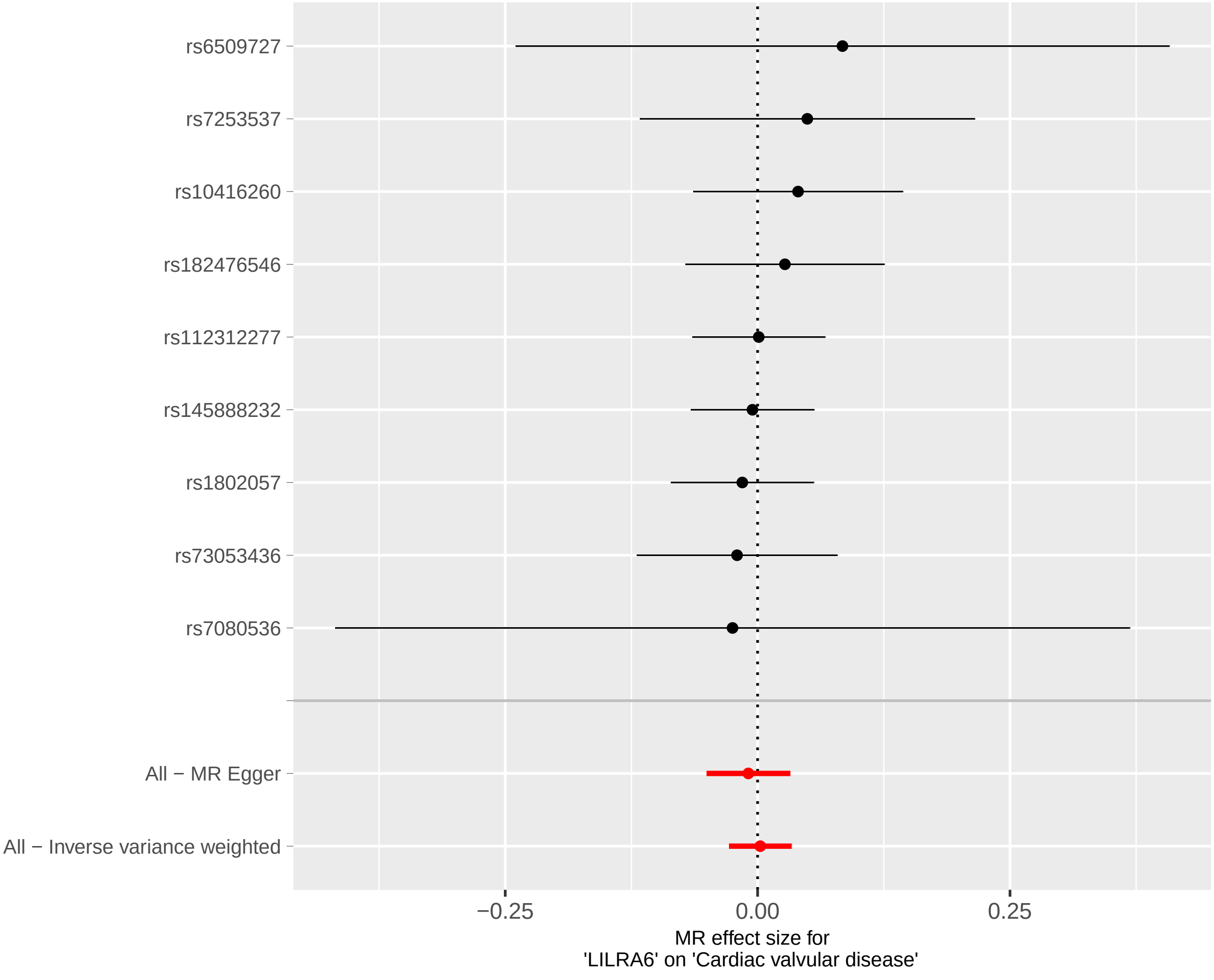
rs6509727
rs7253537
rs10416260
rs182476546
rs112312277
rs145888232
rs1802057
rs73053436
rs7080536
All − MR Egger
All − Inverse variance weighted
−0.25
0.00
0.25
MR effect size for
'LILRA6' on 'Cardiac valvular disease'

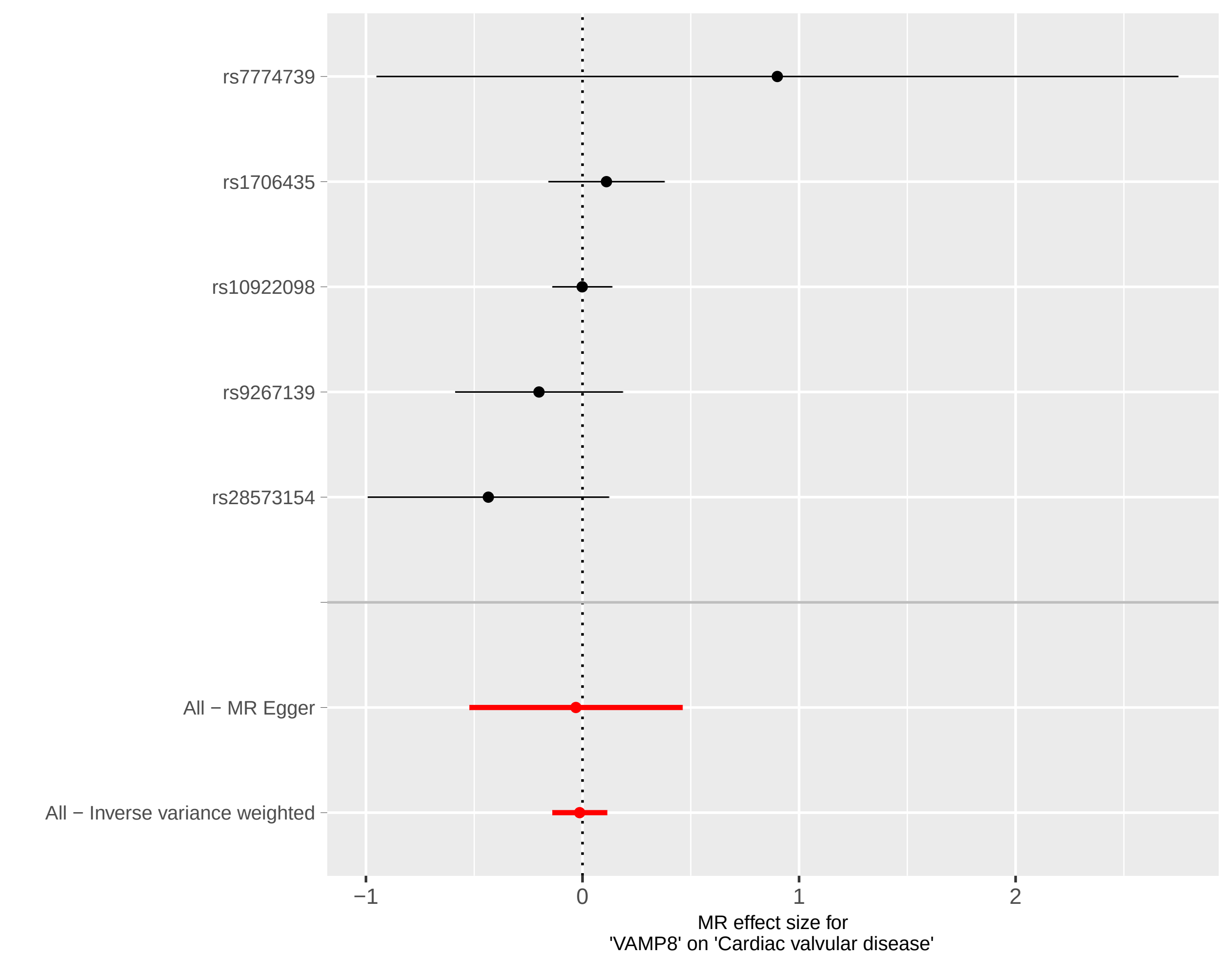
rs7774739
rs1706435
rs10922098
rs9267139
rs28573154
All − MR Egger
All − Inverse variance weighted
−1
0
1
2
MR effect size for
'VAMP8' on 'Cardiac valvular disease'

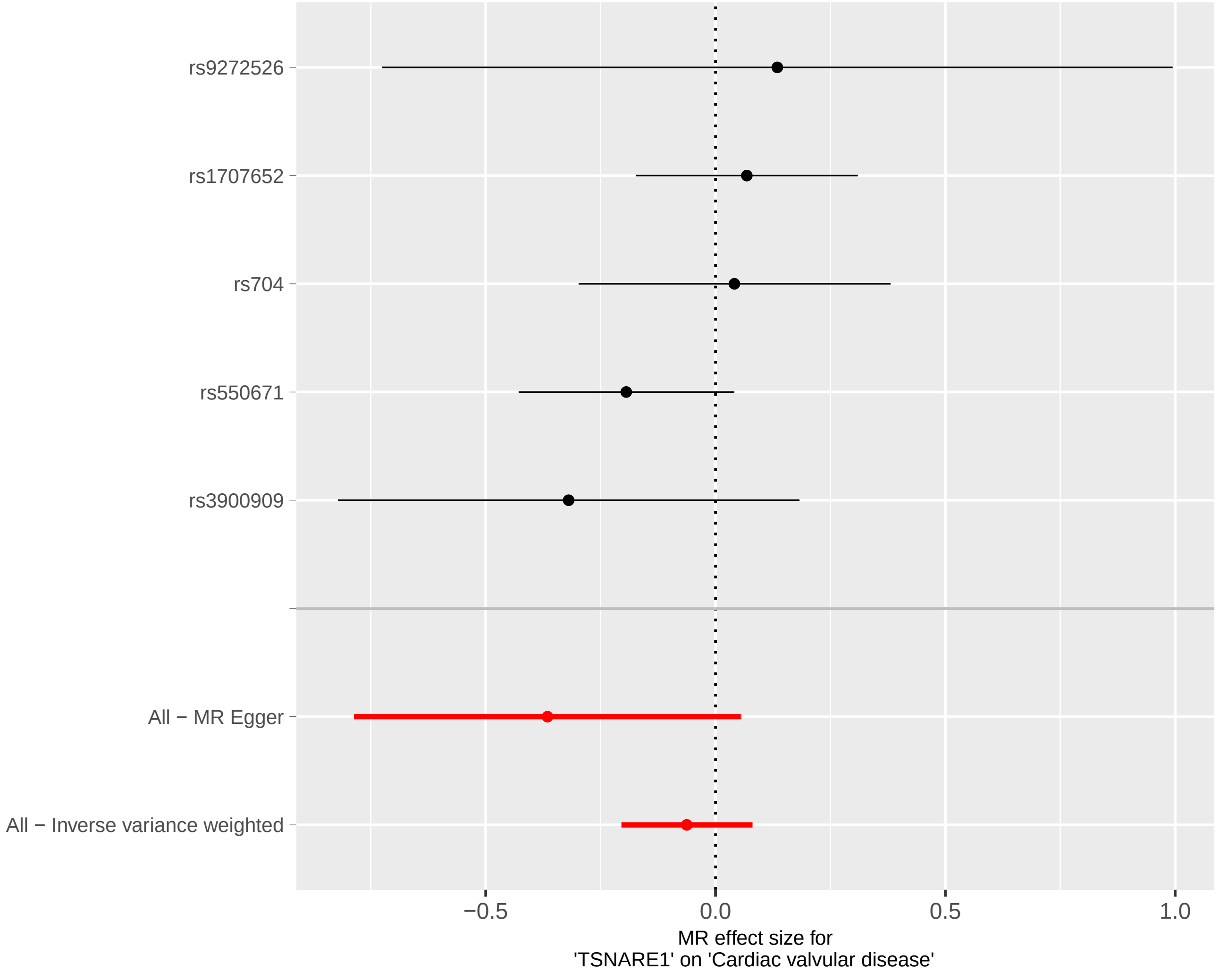

rs9272526
rs1707652
rs704
rs550671
rs3900909
All − MR Egger
All − Inverse variance weighted
−0.5
0.0
0.5
1.0
MR effect size for
'TSNARE1' on 'Cardiac valvular disease'

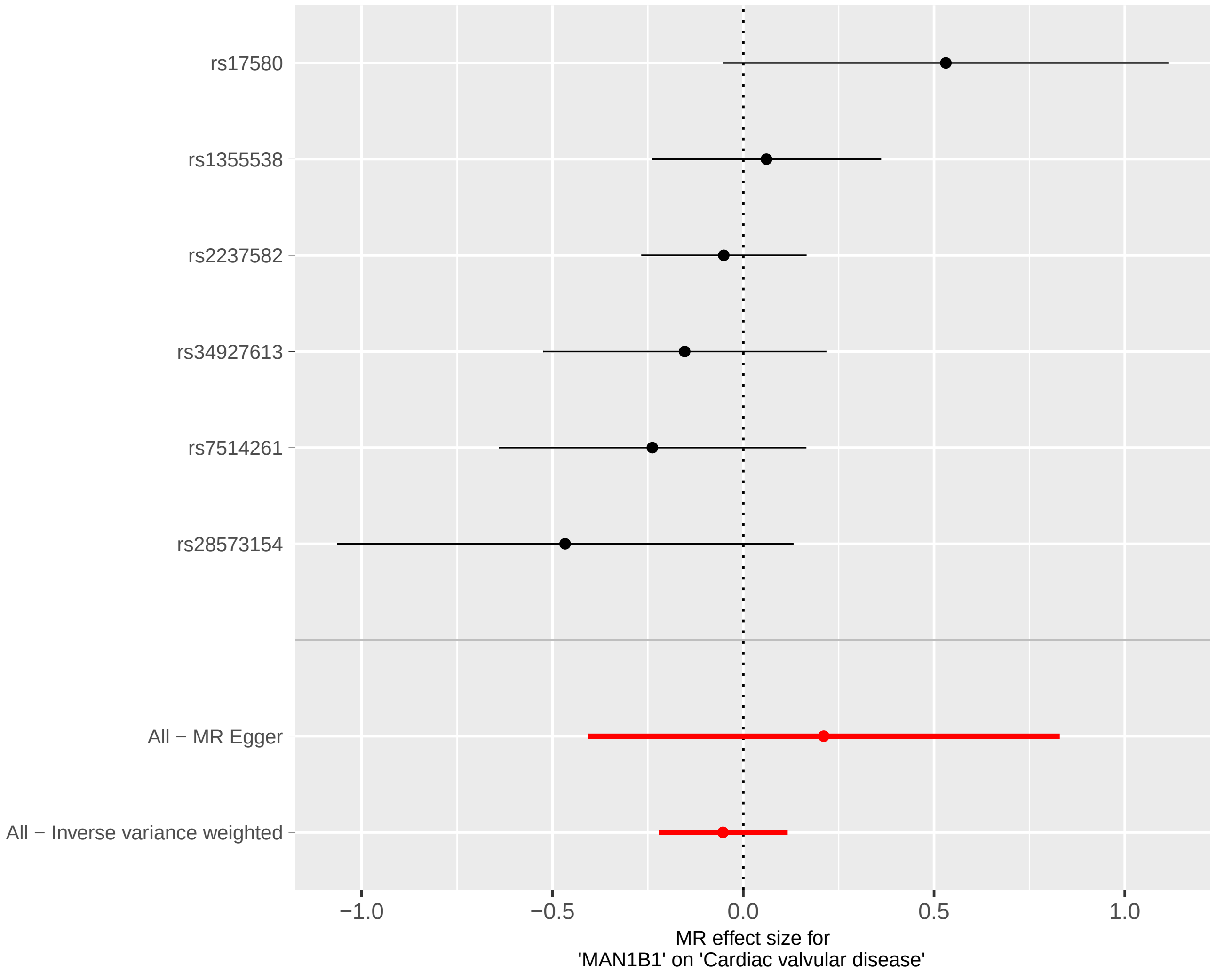
rs17580
rs1355538
rs2237582
rs34927613
rs7514261
rs28573154
All − MR Egger
All − Inverse variance weighted
−1.0
−0.5
0.0
0.5
1.0
MR effect size for
'MAN1B1' on 'Cardiac valvular disease'

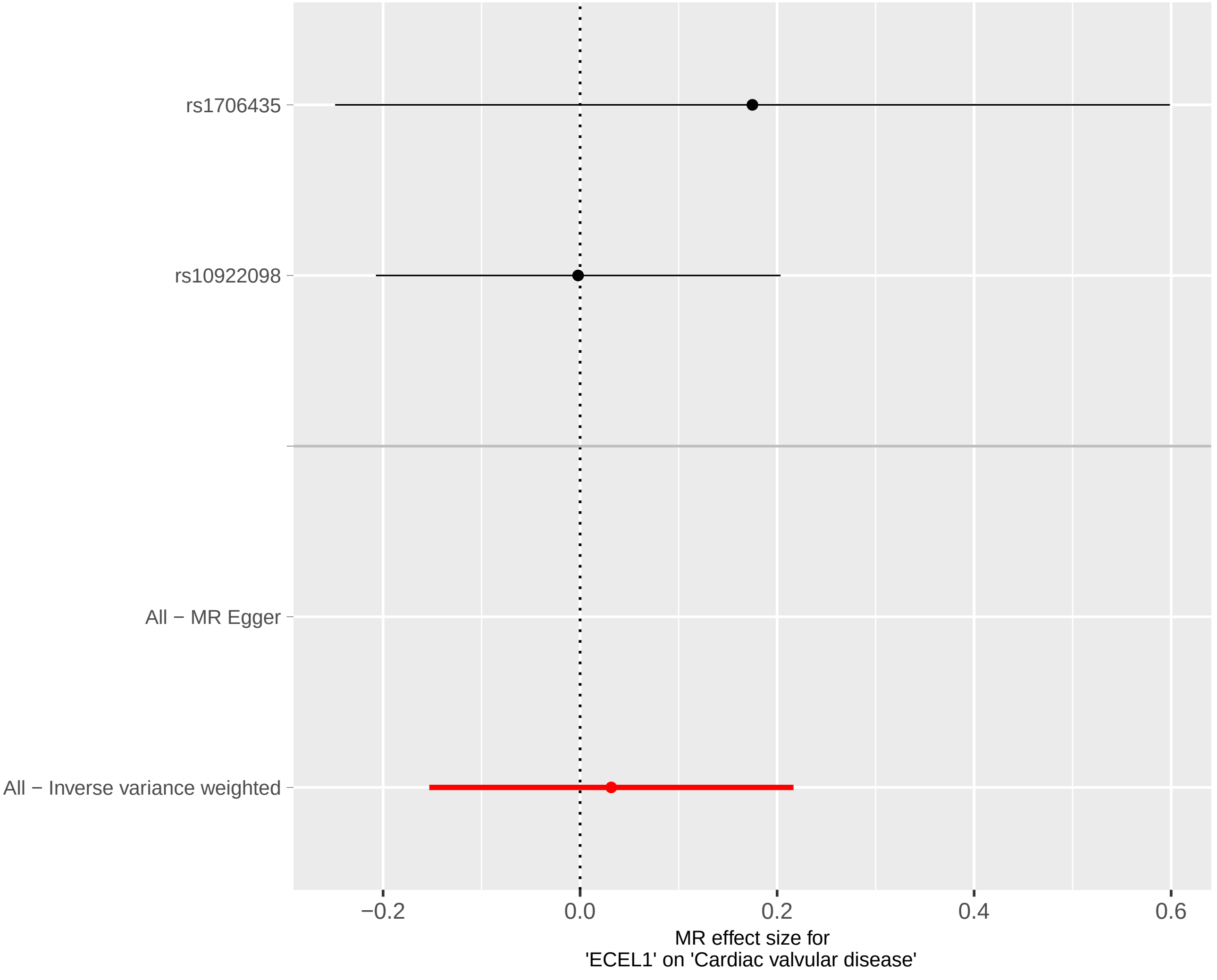
rs1706435
rs10922098
All − MR Egger
All − Inverse variance weighted
−0.2
0.0
0.2
0.4
0.6
MR effect size for
'ECEL1' on 'Cardiac valvular disease'

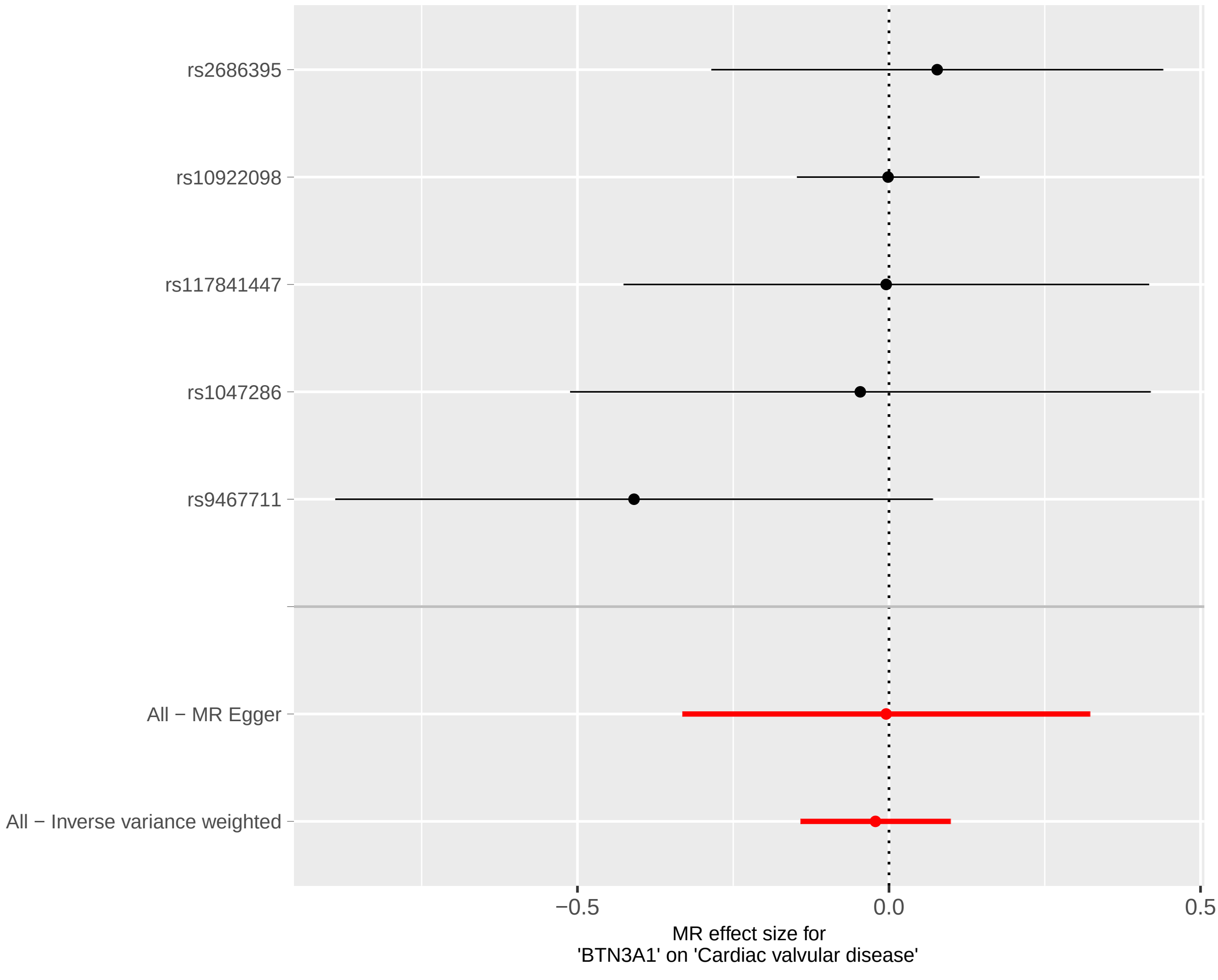
rs2686395
rs10922098
rs117841447
rs1047286
rs9467711
All − MR Egger
All − Inverse variance weighted
−0.5
0.0
0.5
MR effect size for
'BTN3A1' on 'Cardiac valvular disease'

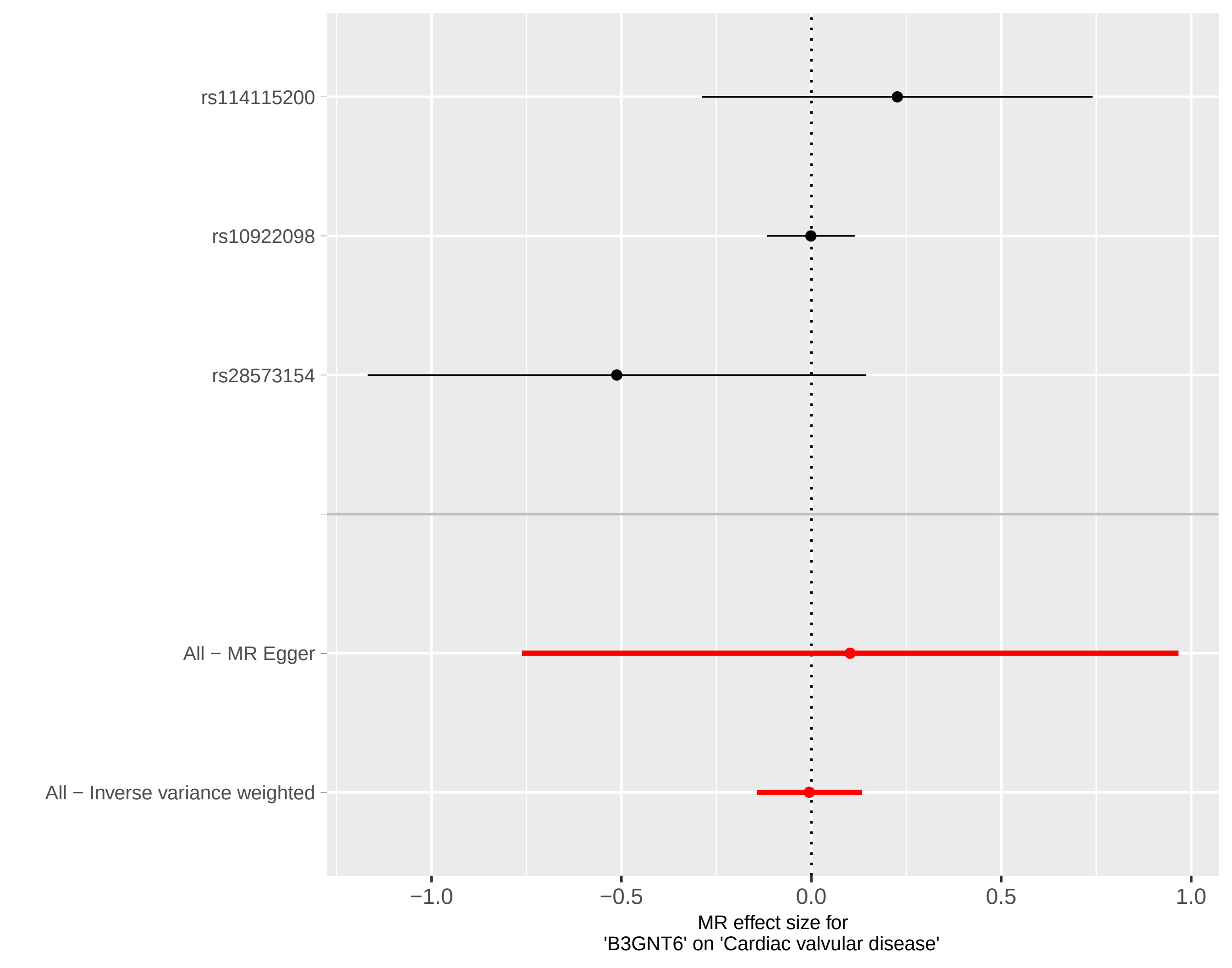
rs114115200
rs10922098
rs28573154
All − MR Egger
All − Inverse variance weighted
−1.0
−0.5
0.0
0.5
1.0
MR effect size for
'B3GNT6' on 'Cardiac valvular disease'

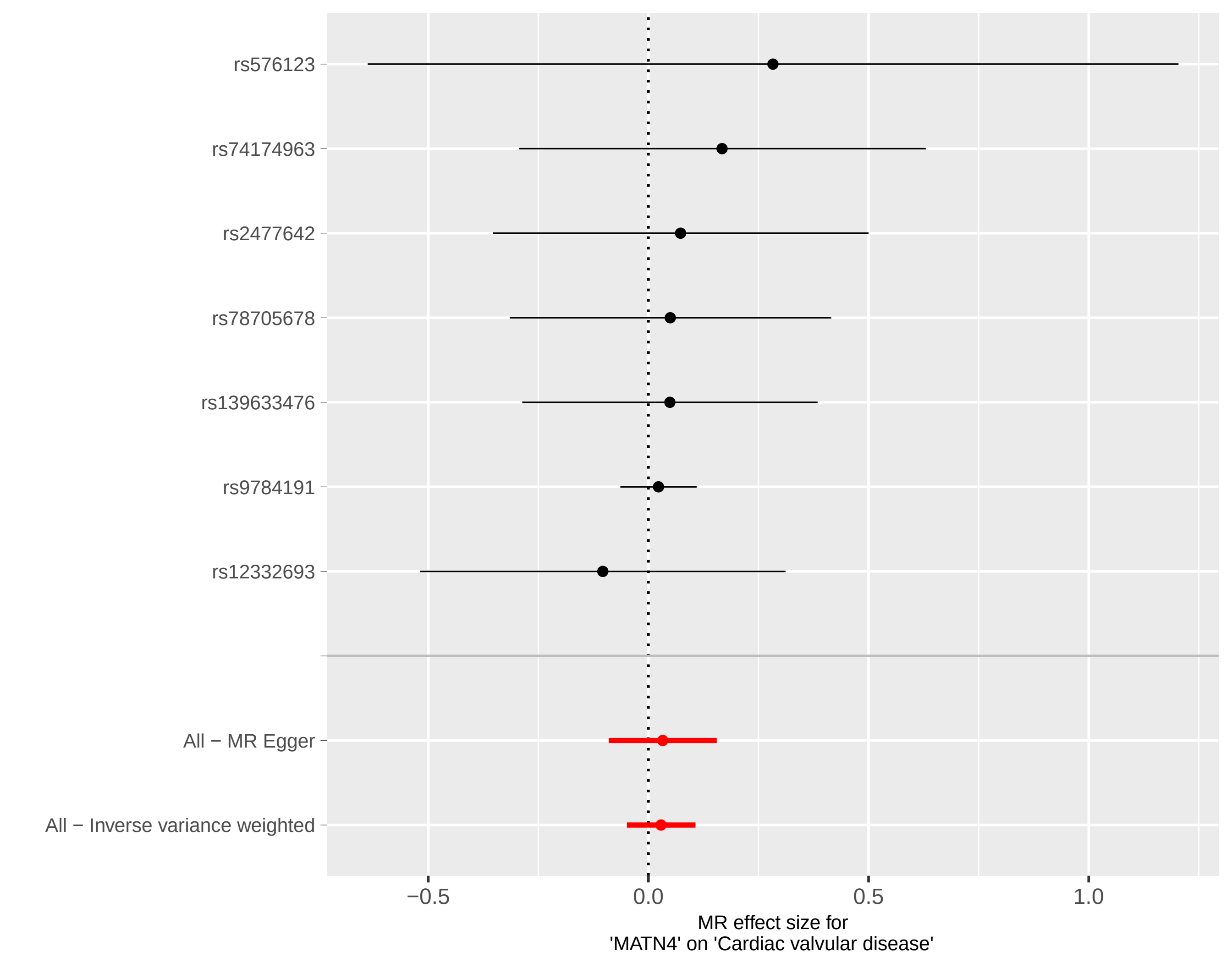
rs576123
rs74174963
rs2477642
rs78705678
rs139633476
rs9784191
rs12332693
All − MR Egger
All − Inverse variance weighted
−0.5
0.0
0.5
1.0
MR effect size for
'MATN4' on 'Cardiac valvular disease'

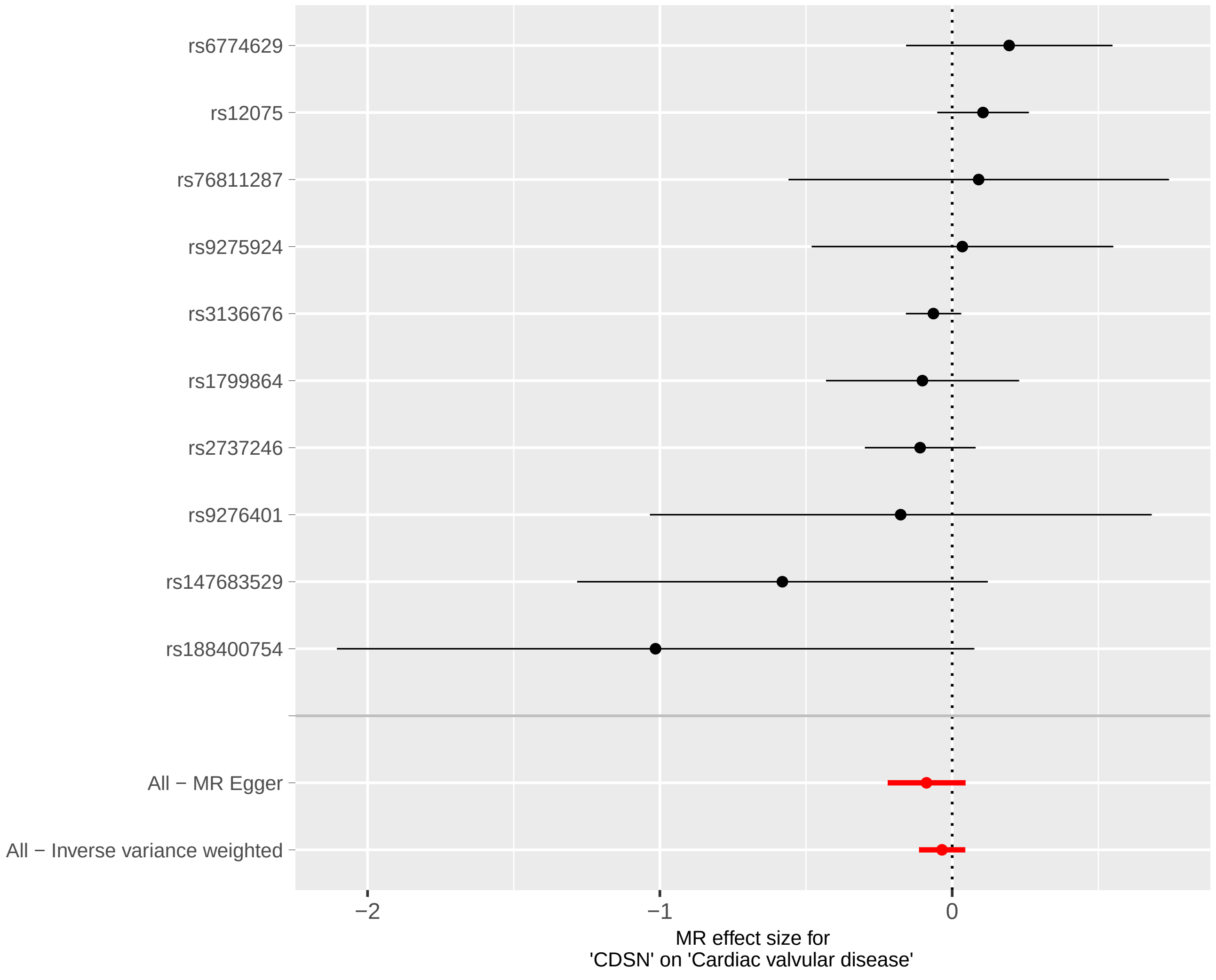
rs6774629
rs12075
rs76811287
rs9275924
rs3136676
rs1799864
rs2737246
rs9276401
rs147683529
rs188400754
All − MR Egger
All − Inverse variance weighted
−2
−1
0
MR effect size for
'CDSN' on 'Cardiac valvular disease'

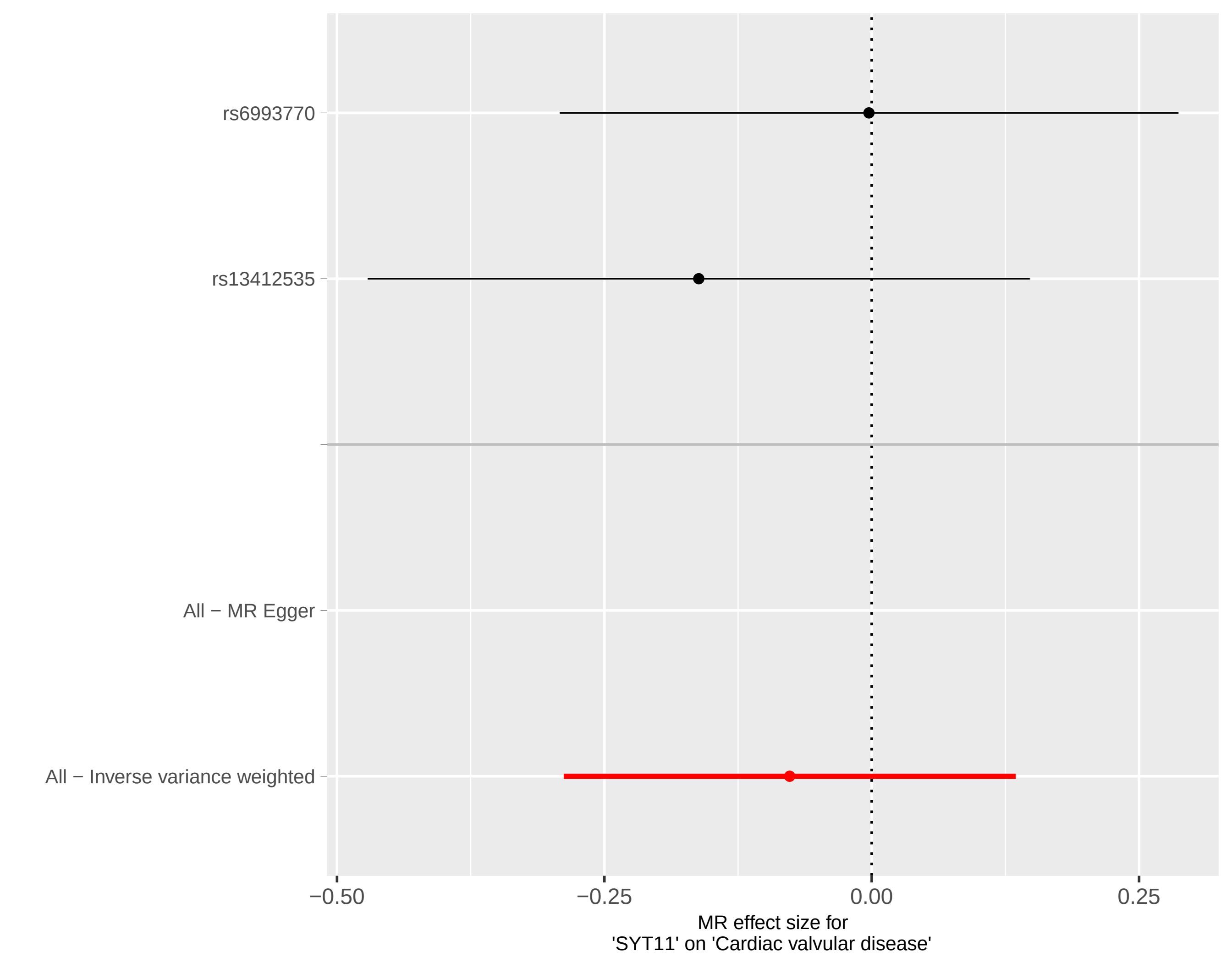
rs6993770
rs13412535
All − MR Egger
All − Inverse variance weighted
−0.50
−0.25
0.00
0.25
MR effect size for
'SYT11' on 'Cardiac valvular disease'

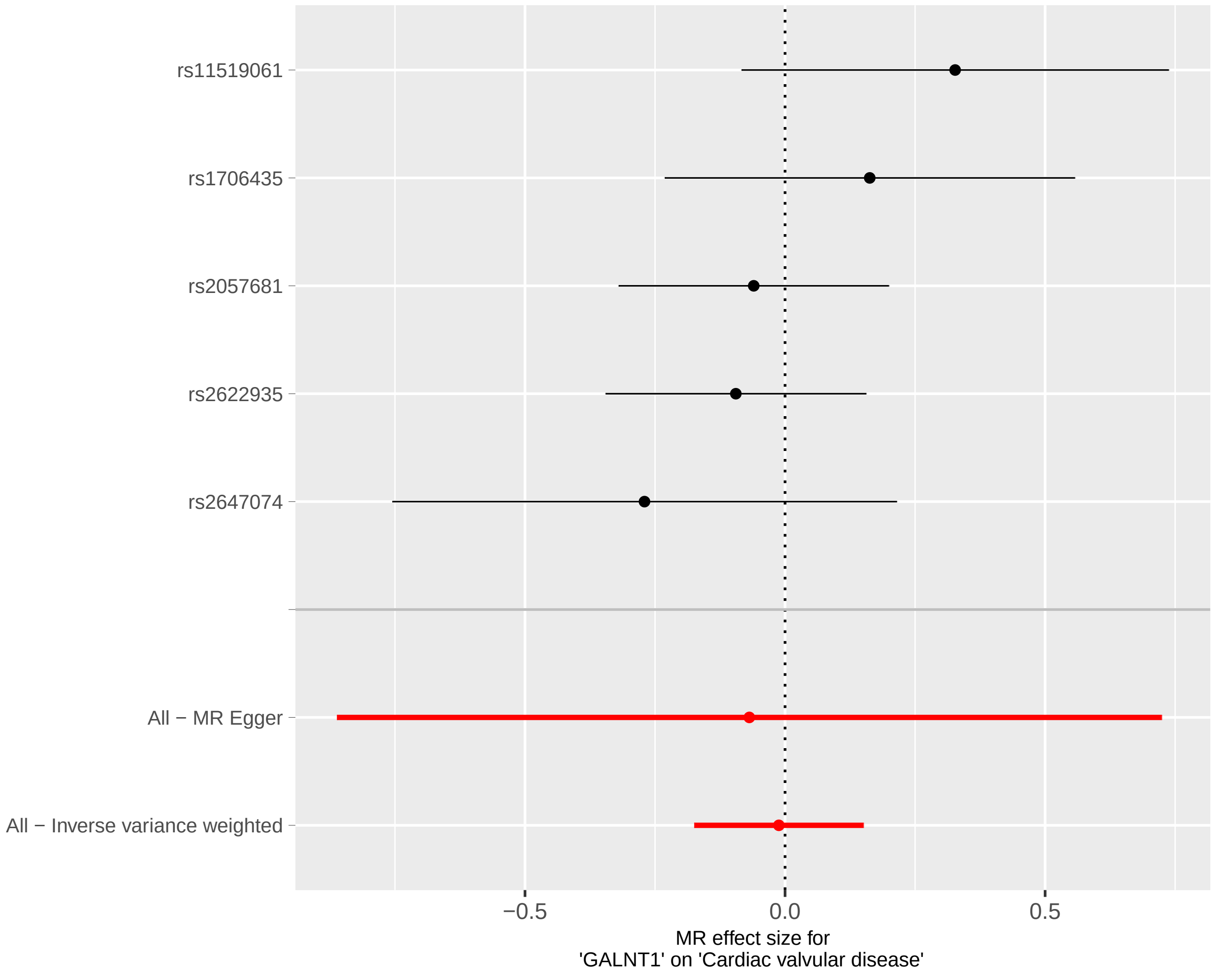
rs11519061
rs1706435
rs2057681
rs2622935
rs2647074
All − MR Egger
All − Inverse variance weighted
−0.5
0.0
0.5
MR effect size for
'GALNT1' on 'Cardiac valvular disease'

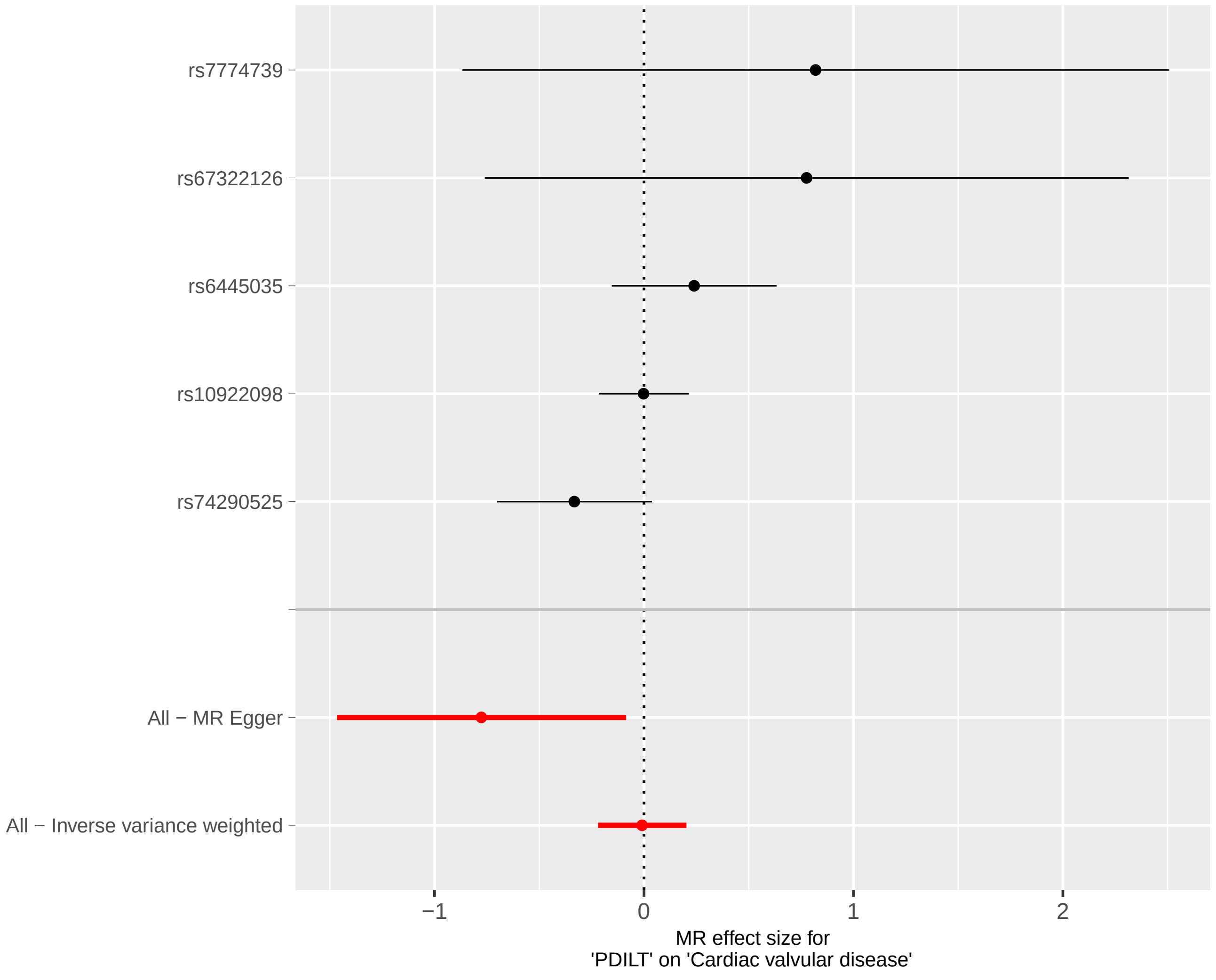

rs7774739
rs67322126
rs6445035
rs10922098
rs74290525
All − MR Egger
All − Inverse variance weighted
−1
0
1
2
MR effect size for
'PDILT' on 'Cardiac valvular disease'

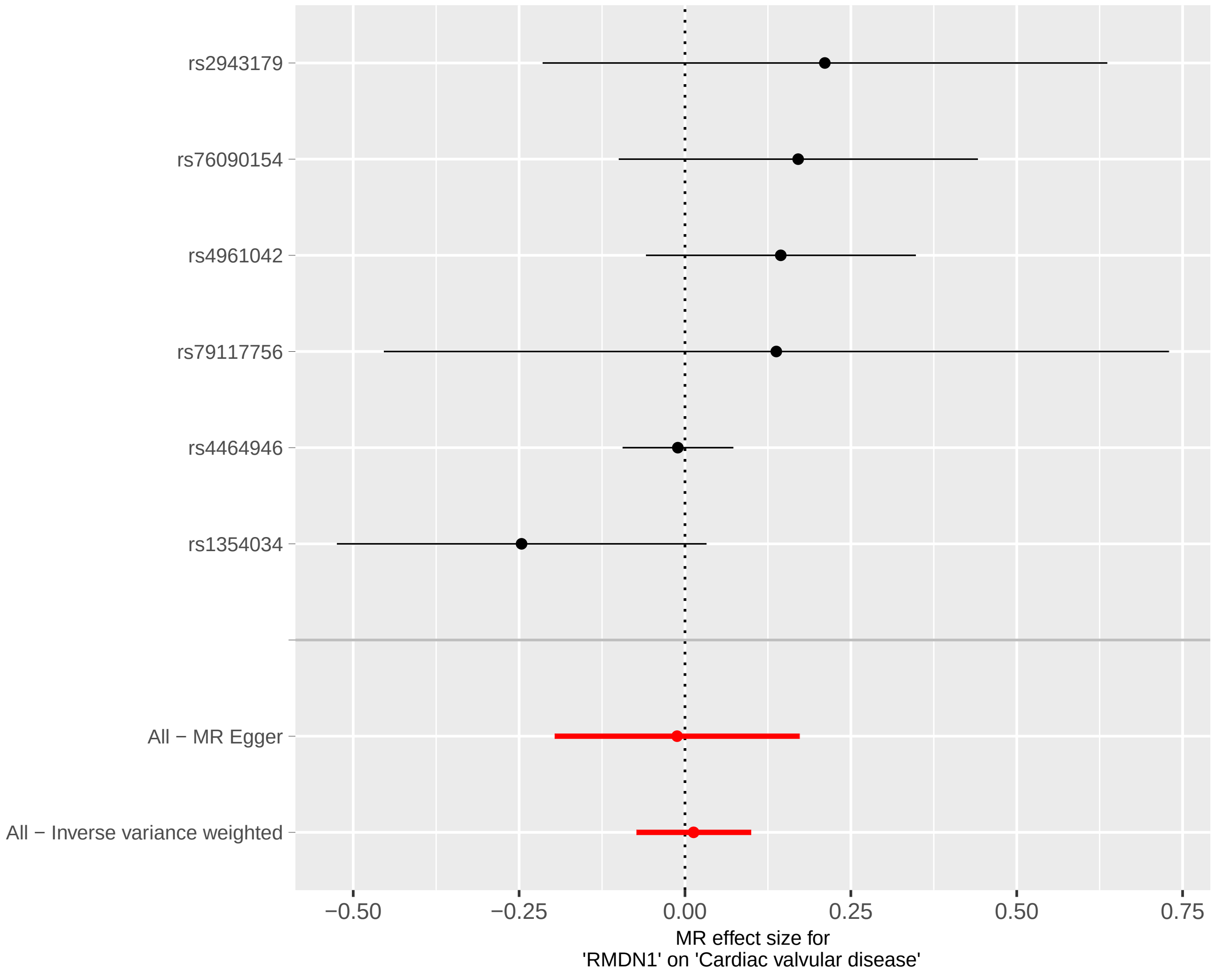
rs2943179
rs76090154
rs4961042
rs79117756
rs4464946
rs1354034
All − MR Egger
All − Inverse variance weighted
−0.50
−0.25
0.00
0.25
0.50
0.75
MR effect size for
'RMDN1' on 'Cardiac valvular disease'

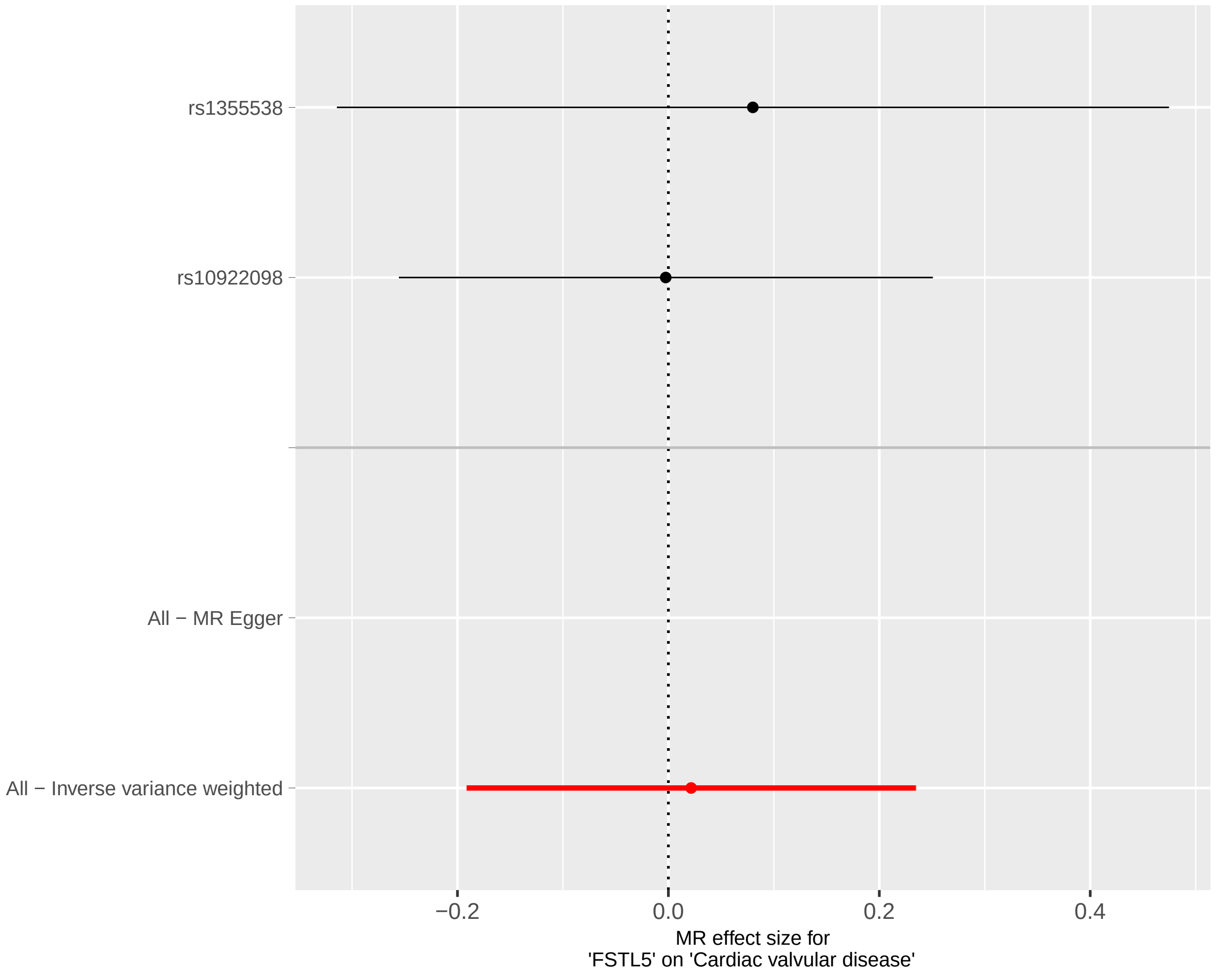
rs1355538
rs10922098
All − MR Egger
All − Inverse variance weighted
−0.2
0.0
0.2
0.4
MR effect size for
'FSTL5' on 'Cardiac valvular disease'

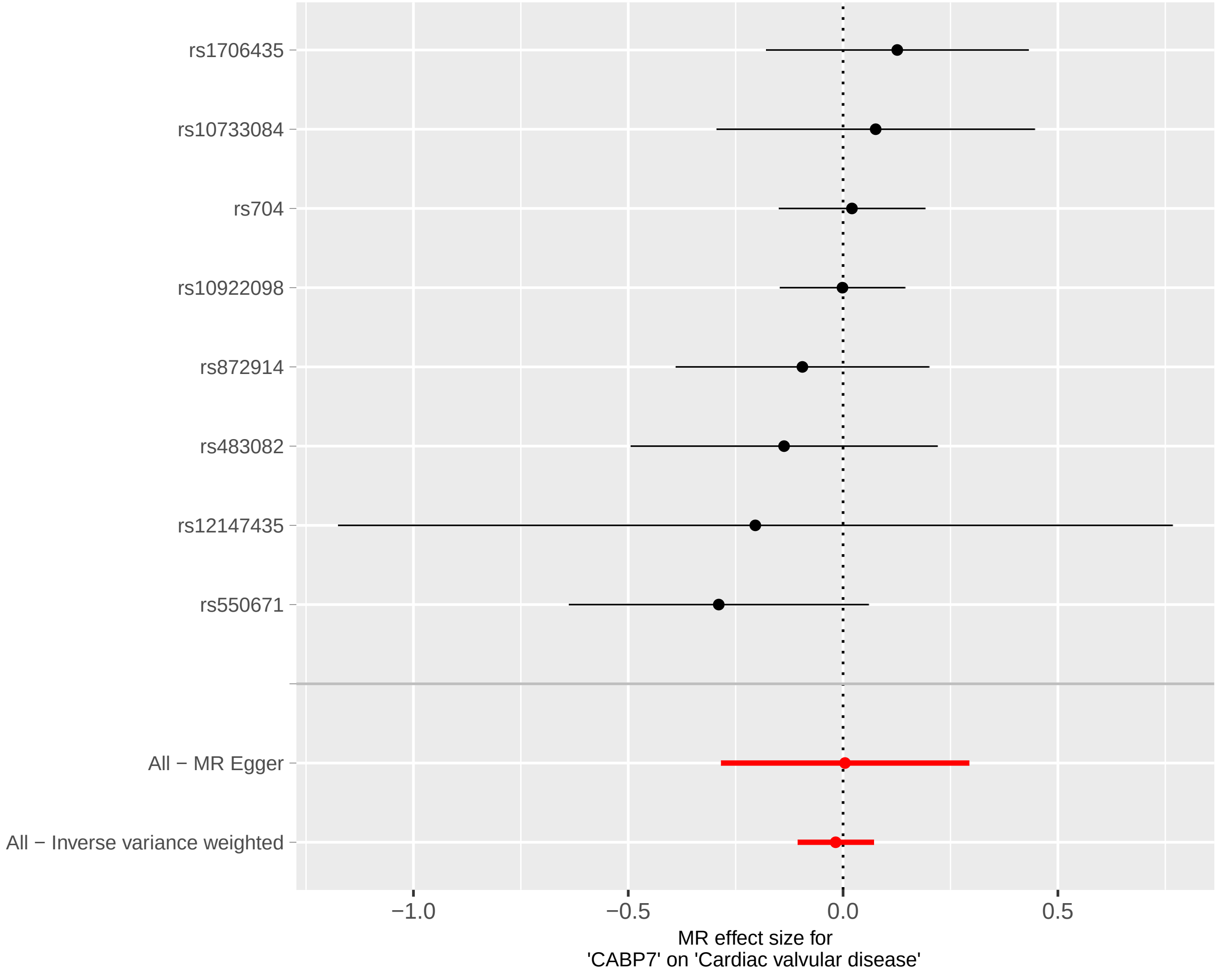
rs1706435
rs10733084
rs704
rs10922098
rs872914
rs483082
rs12147435
rs550671
All − MR Egger
All − Inverse variance weighted
−1.0
−0.5
0.0
0.5
MR effect size for
'CABP7' on 'Cardiac valvular disease'

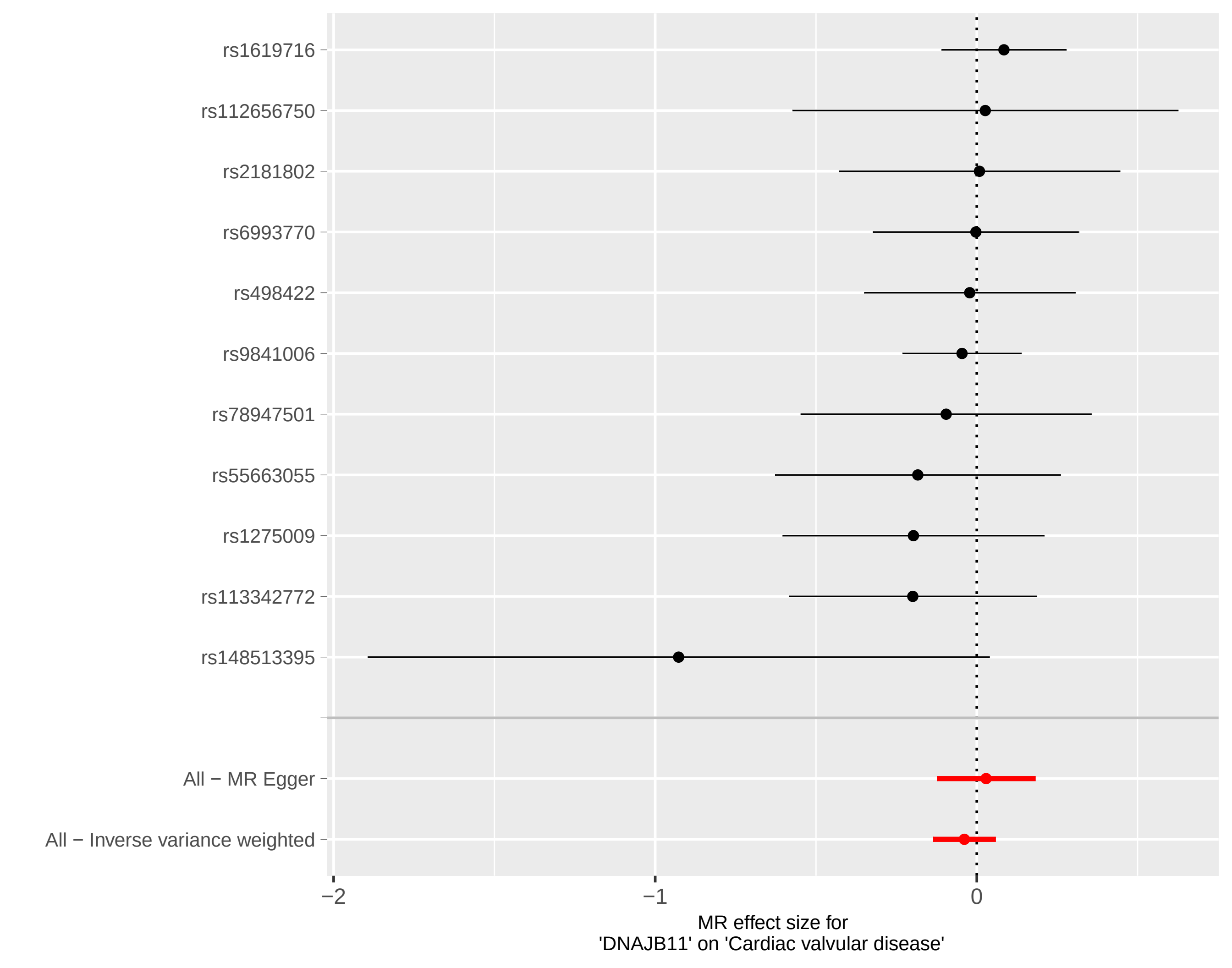
rs1619716
rs112656750
rs2181802
rs6993770
rs498422
rs9841006
rs78947501
rs55663055
rs1275009
rs113342772
rs148513395
All − MR Egger
All − Inverse variance weighted
−2
−1
0
MR effect size for
'DNAJB11' on 'Cardiac valvular disease'

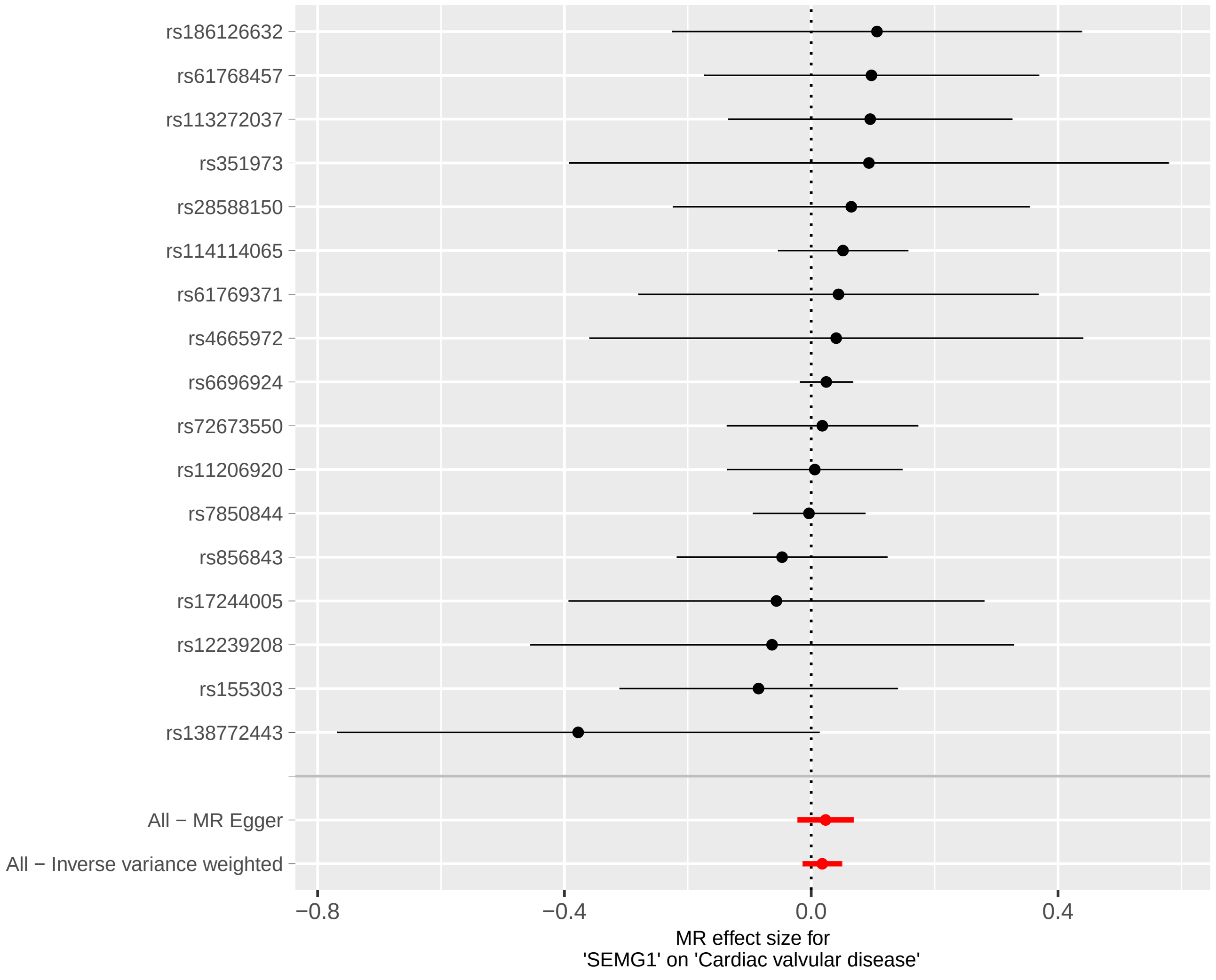
rs186126632
rs61768457
rs113272037
rs351973
rs28588150
rs114114065
rs61769371
rs4665972
rs6696924
rs72673550
rs11206920
rs7850844
rs856843
rs17244005
rs12239208
rs155303
rs138772443
All − MR Egger
All − Inverse variance weighted
−0.8
−0.4
0.0
0.4
MR effect size for
'SEMG1' on 'Cardiac valvular disease'

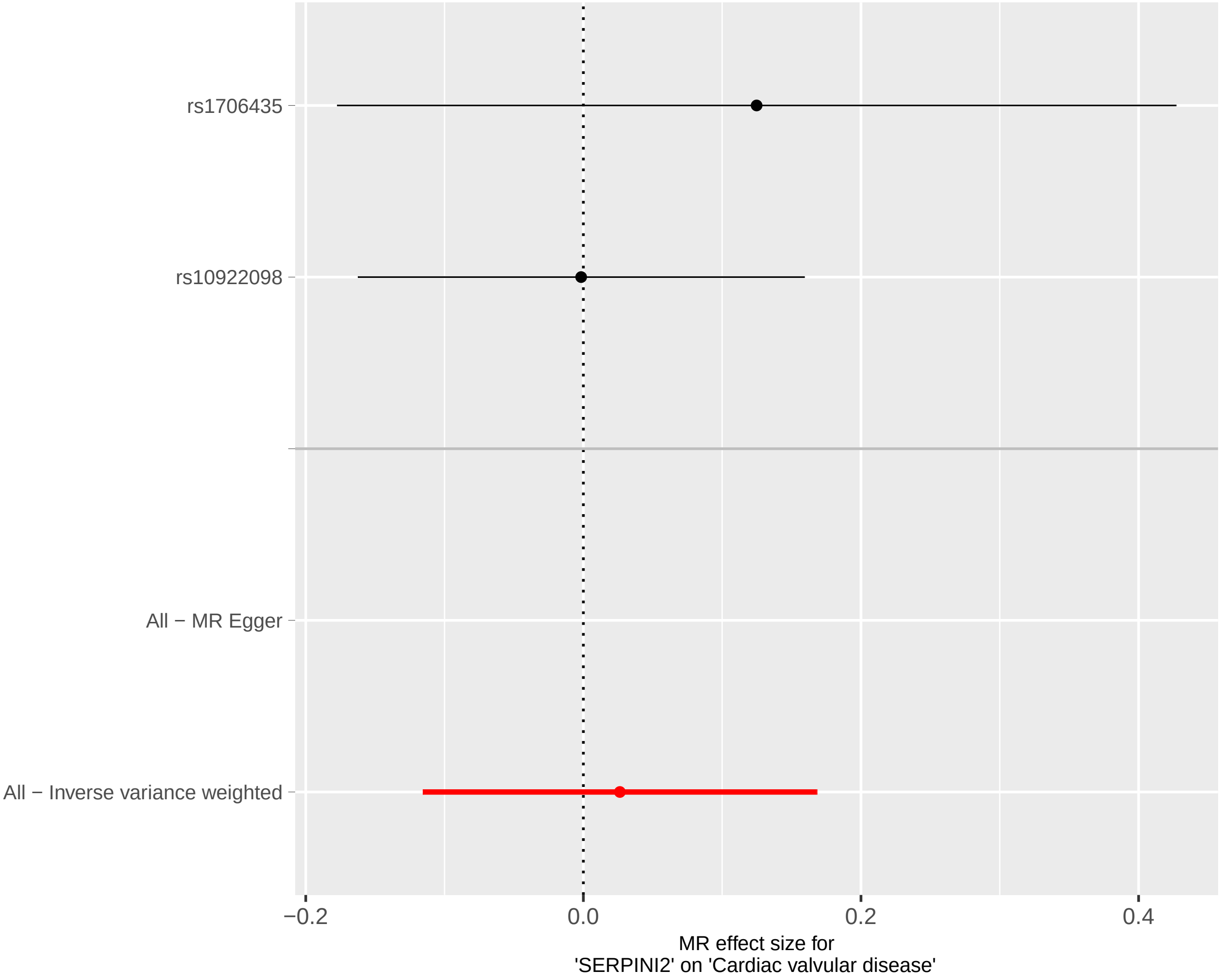
rs1706435
rs10922098
All − MR Egger
All − Inverse variance weighted
−0.2
0.0
0.2
0.4
MR effect size for
'SERPINI2' on 'Cardiac valvular disease'

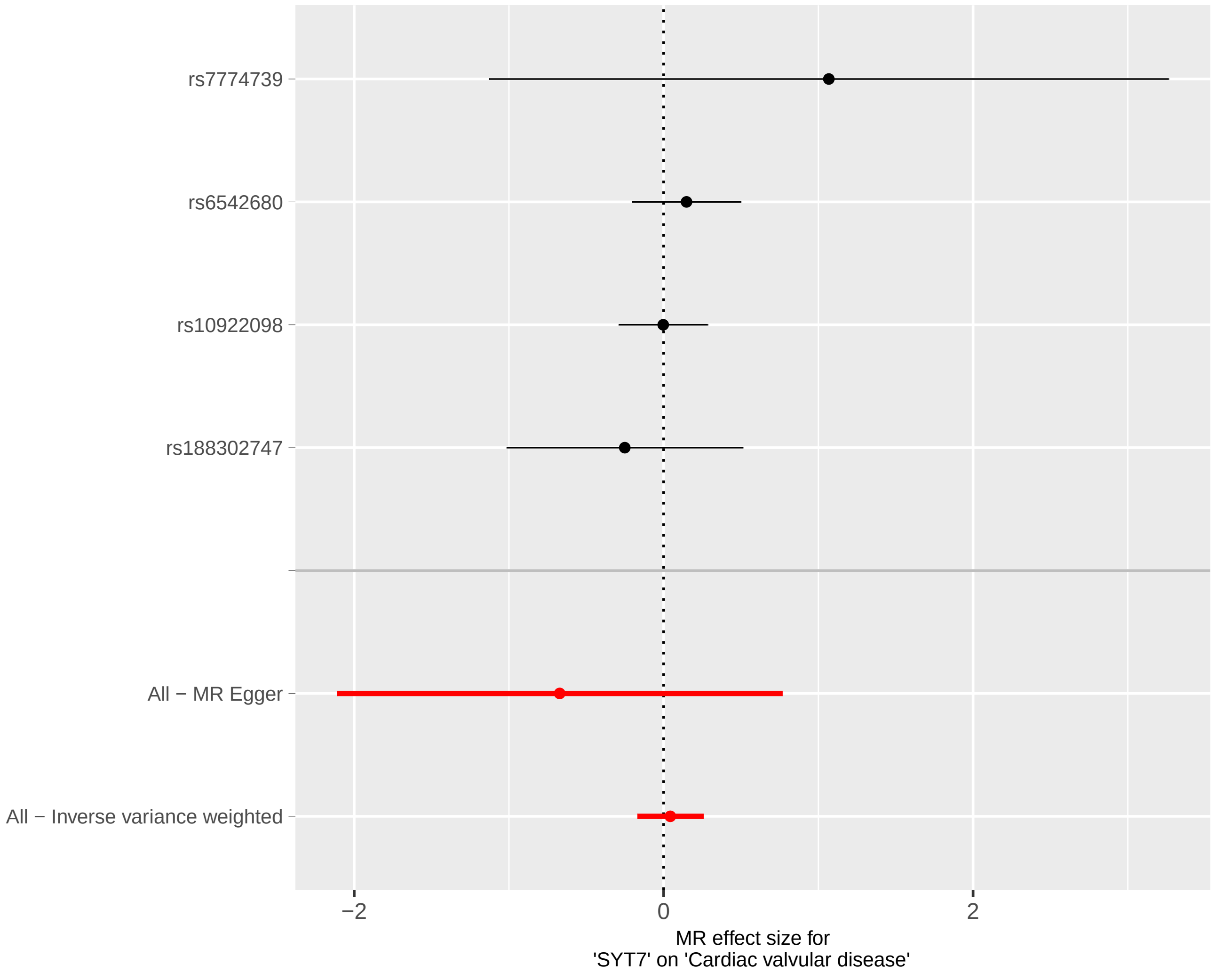
rs7774739
rs6542680
rs10922098
rs188302747
All − MR Egger
All − Inverse variance weighted
−2
0
2
MR effect size for
'SYT7' on 'Cardiac valvular disease'

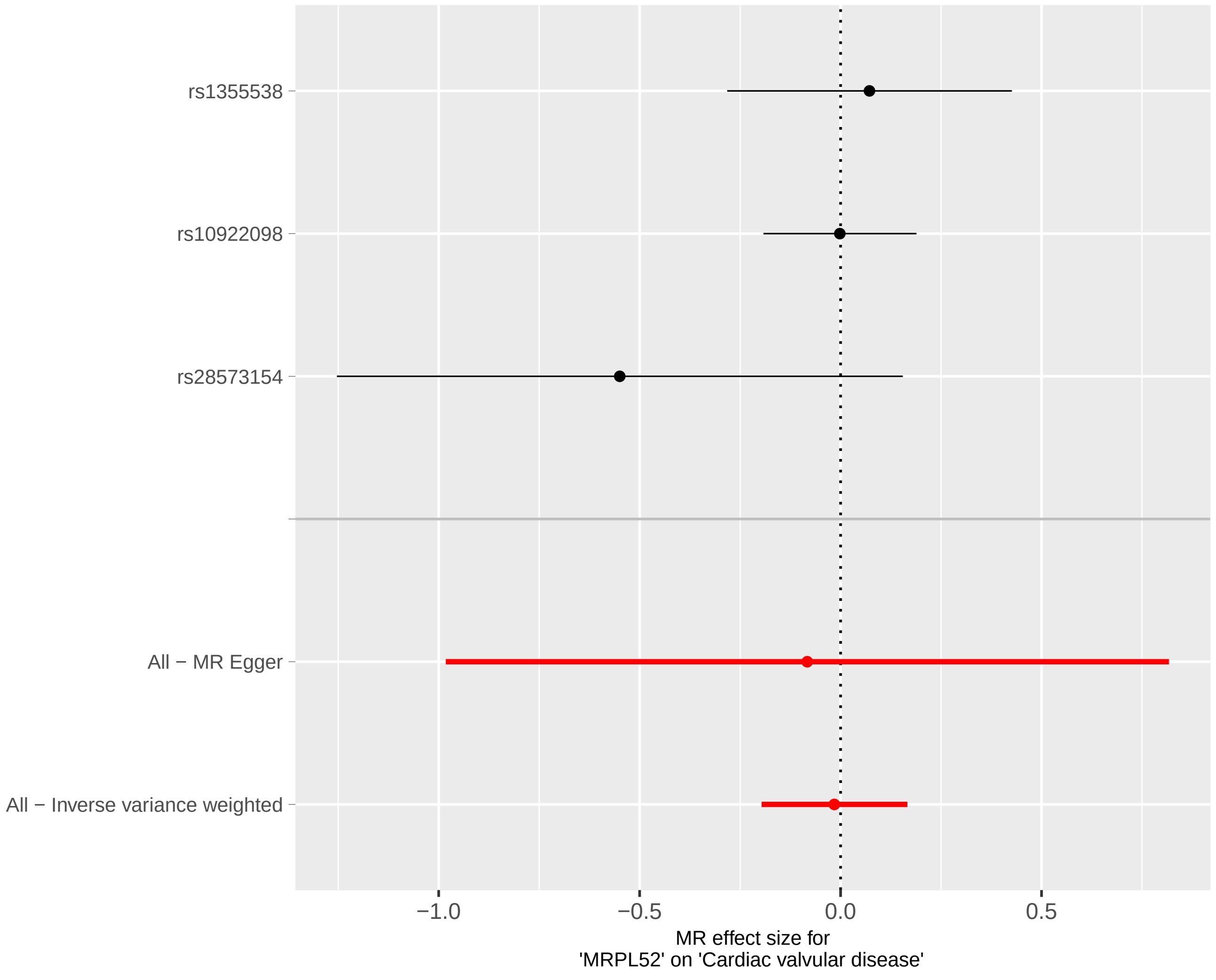
rs1355538
rs10922098
rs28573154
All − MR Egger
All − Inverse variance weighted
−1.0
−0.5
0.0
0.5
MR effect size for
'MRPL52' on 'Cardiac valvular disease'

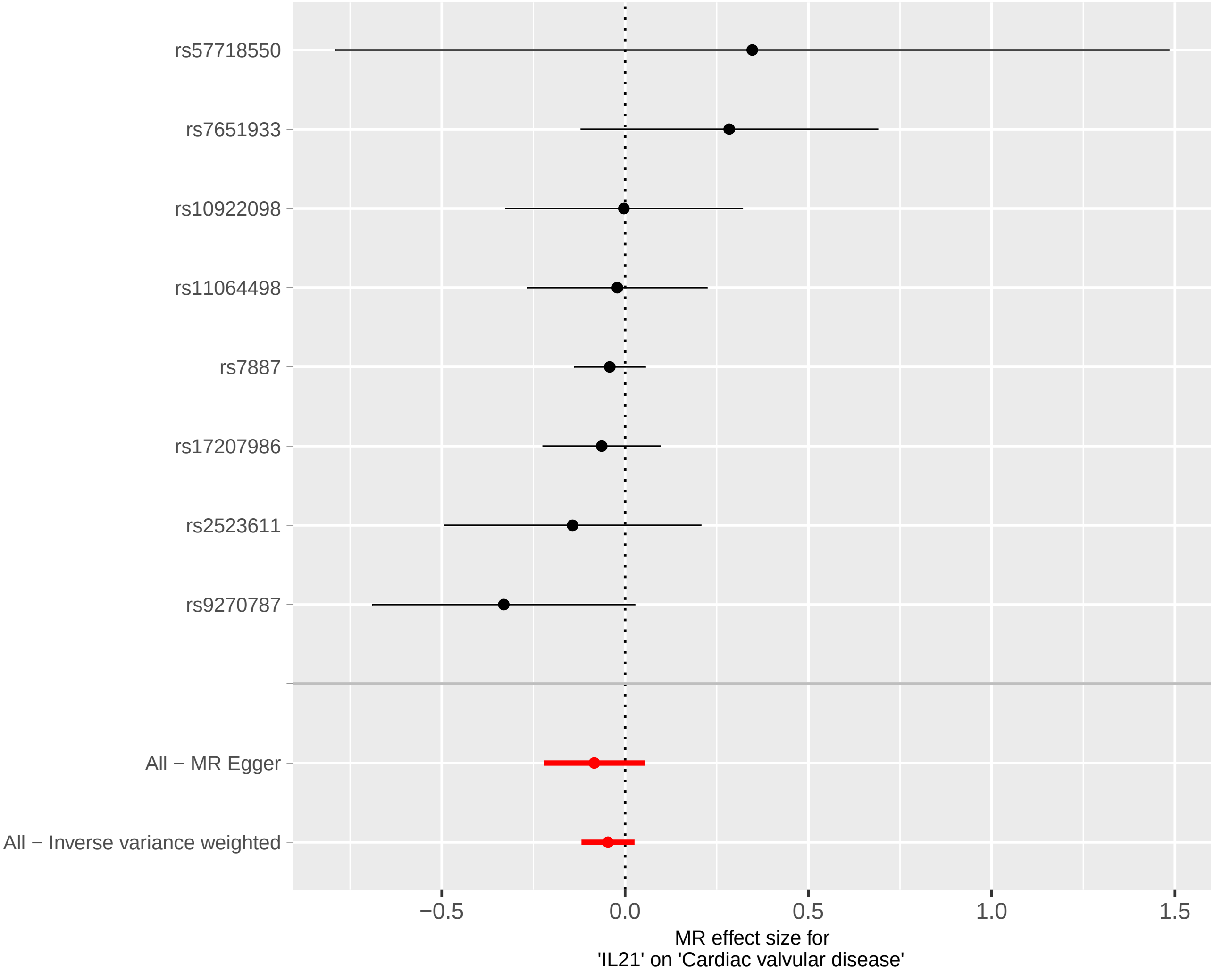
rs57718550
rs7651933
rs10922098
rs11064498
rs7887
rs17207986
rs2523611
rs9270787
All − MR Egger
All − Inverse variance weighted
−0.5
0.0
0.5
1.0
1.5
MR effect size for
'IL21' on 'Cardiac valvular disease'

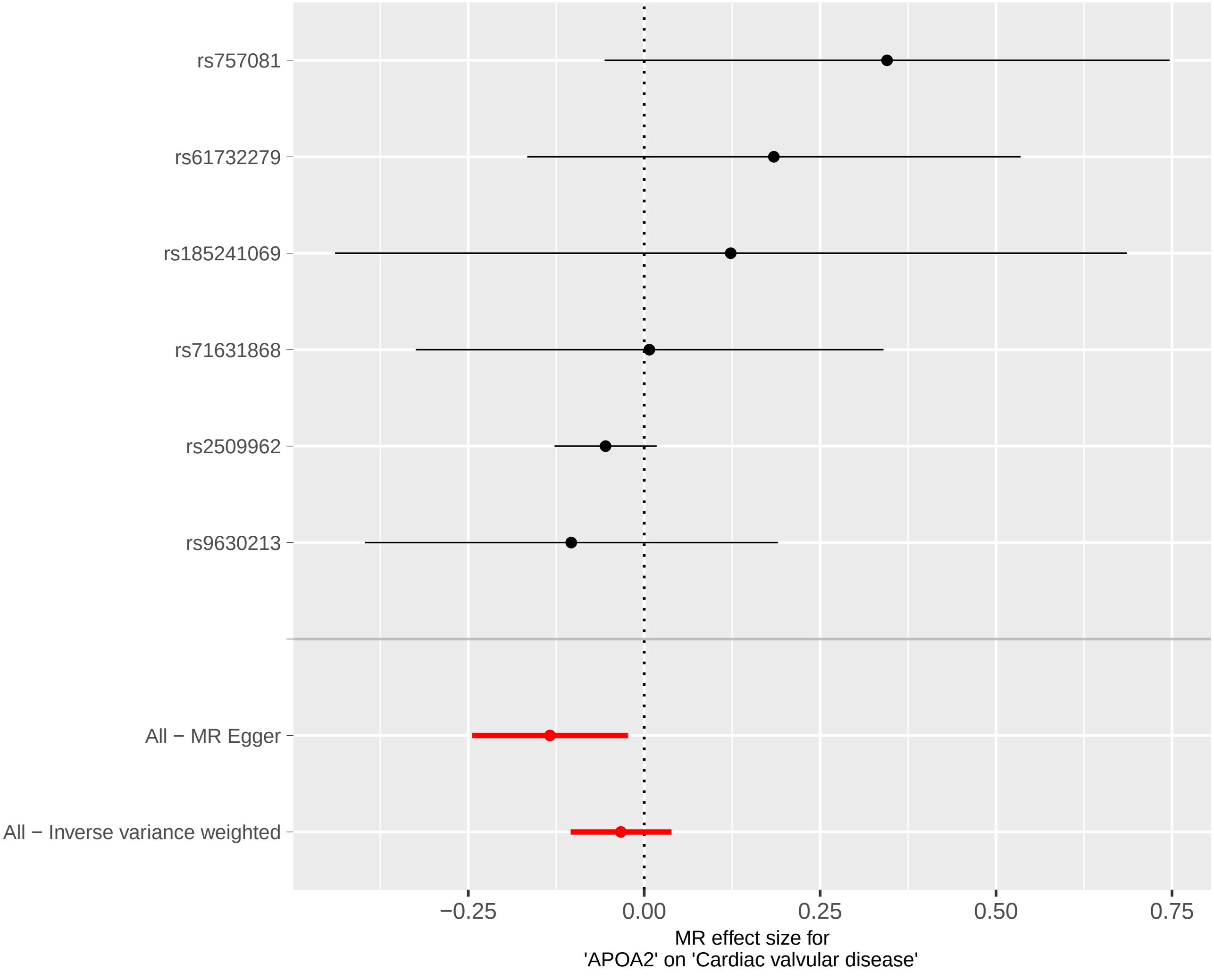

rs757081
rs61732279
rs185241069
rs71631868
rs2509962
rs9630213
All − MR Egger
All − Inverse variance weighted
−0.25
0.00
0.25
0.50
0.75
MR effect size for
'APOA2' on 'Cardiac valvular disease'

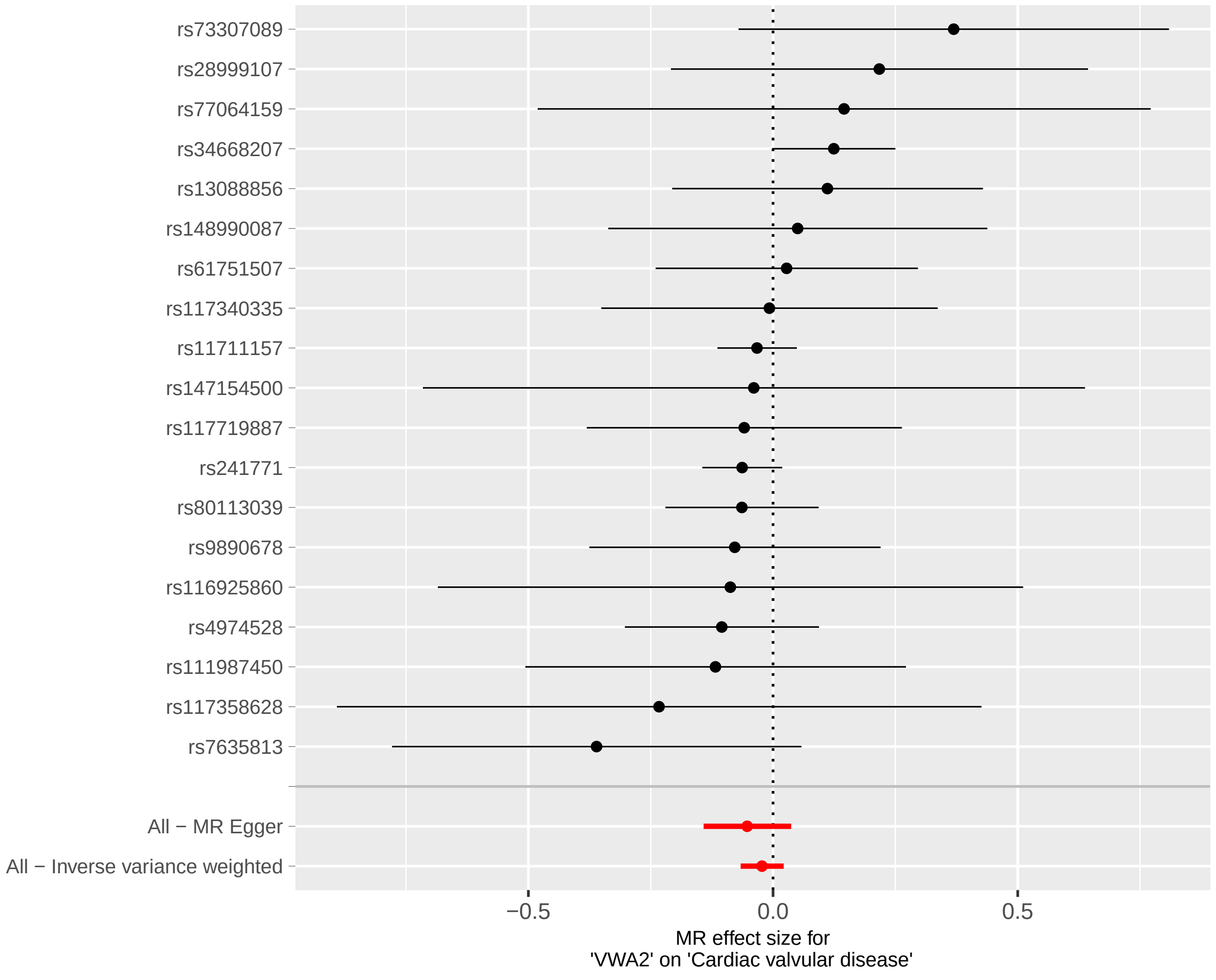
rs73307089
rs28999107
rs77064159
rs34668207
rs13088856
rs148990087
rs61751507
rs117340335
rs11711157
rs147154500
rs117719887
rs241771
rs80113039
rs9890678
rs116925860
rs4974528
rs111987450
rs117358628
rs7635813
All − MR Egger
All − Inverse variance weighted
−0.5
0.0
0.5
MR effect size for
'VWA2' on 'Cardiac valvular disease'

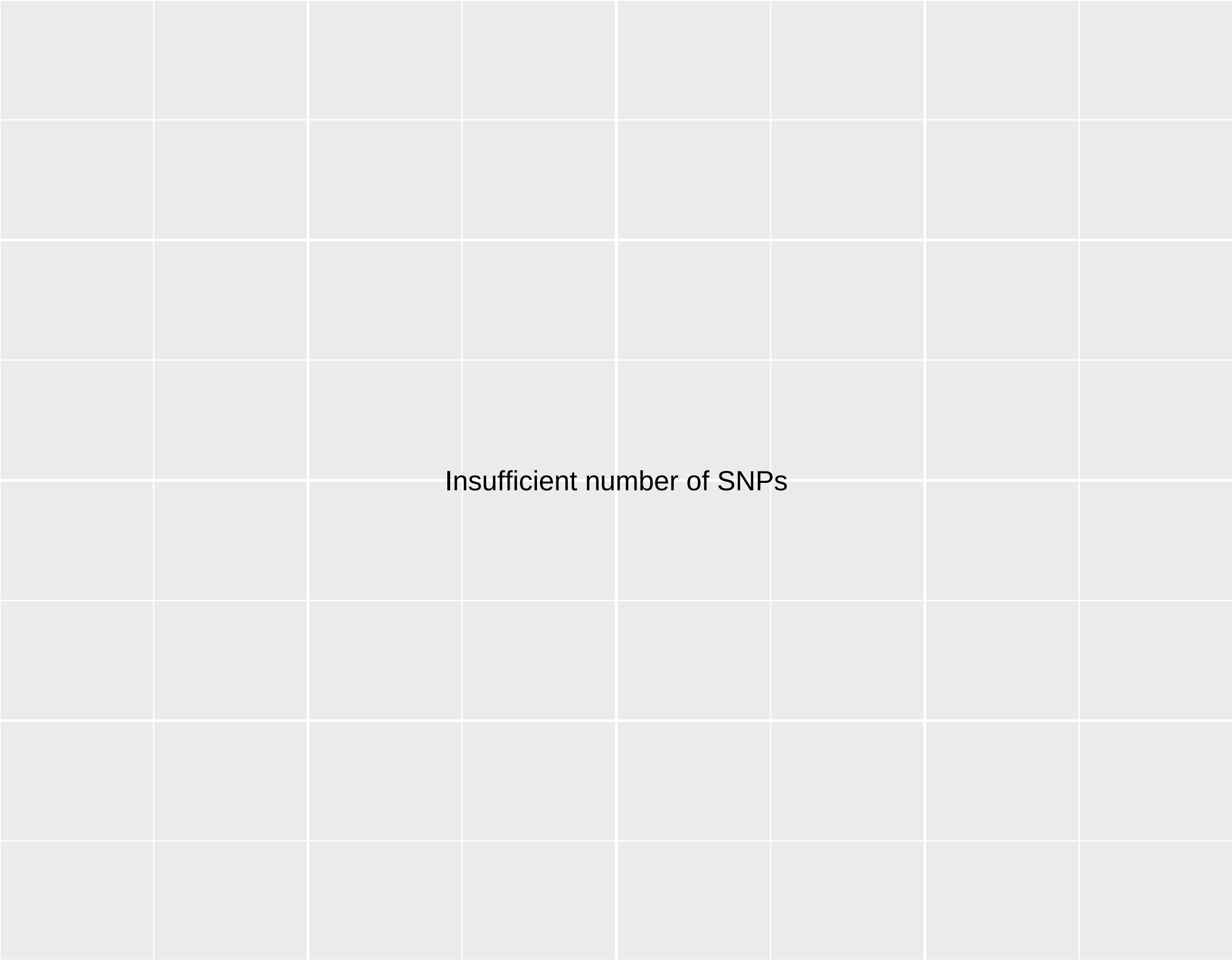
Insufficient number of SNPs

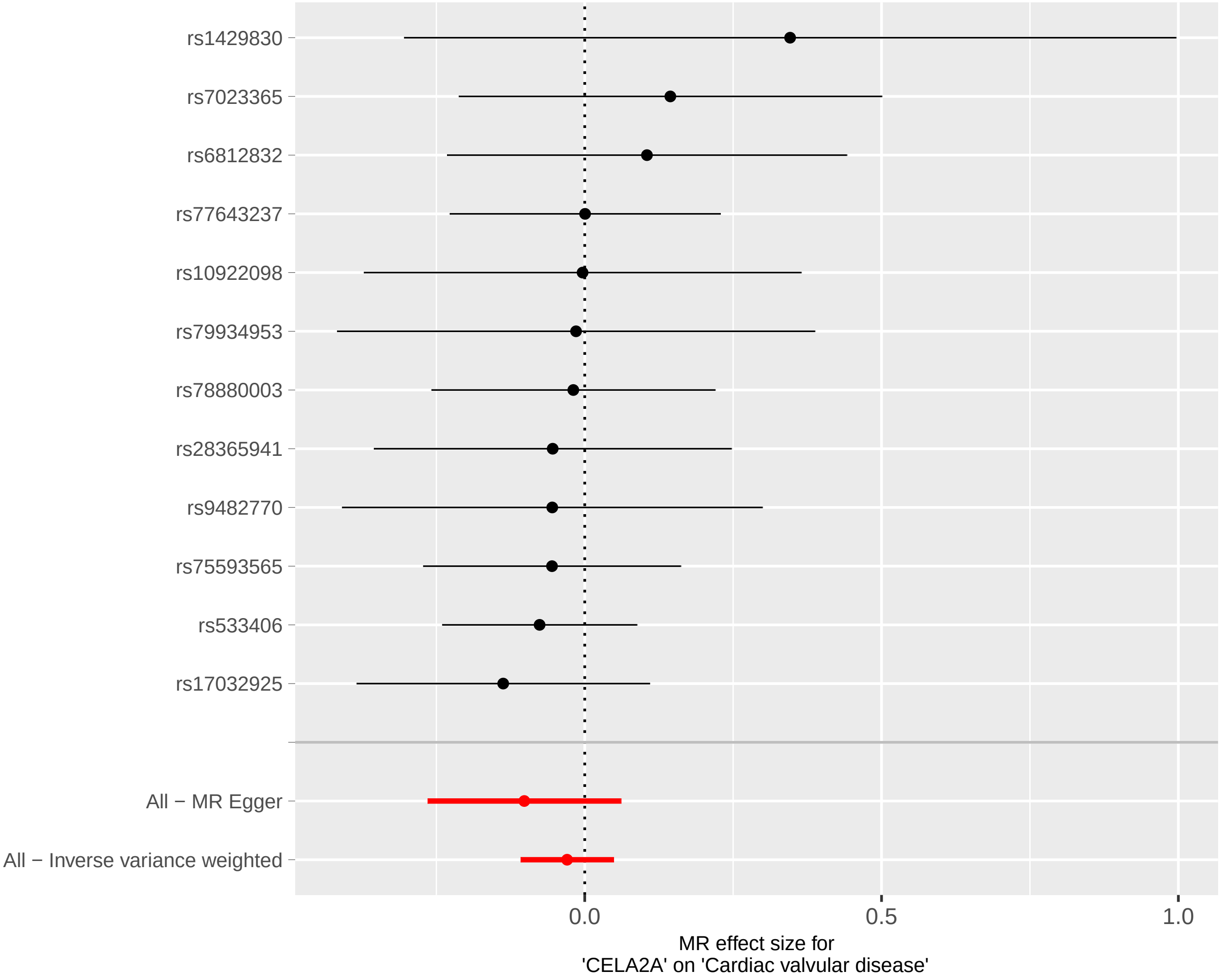
rs1429830
rs7023365
rs6812832
rs77643237
rs10922098
rs79934953
rs78880003
rs28365941
rs9482770
rs75593565
rs533406
rs17032925
All − MR Egger
All − Inverse variance weighted
0.0
0.5
1.0
MR effect size for
'CELA2A' on 'Cardiac valvular disease'

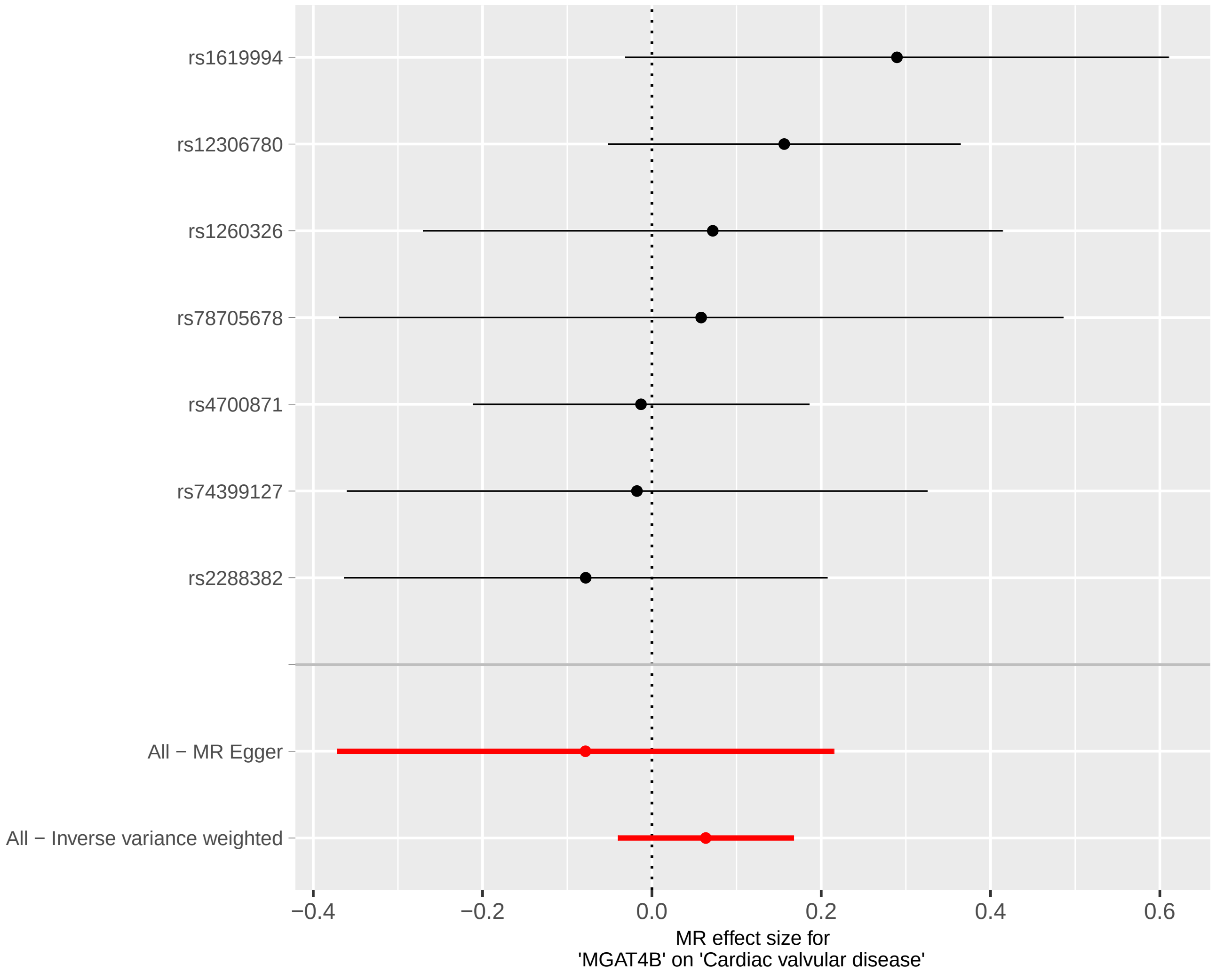

rs1619994
rs12306780
rs1260326
rs78705678
rs4700871
rs74399127
rs2288382
All − MR Egger
All − Inverse variance weighted
−0.4
−0.2
0.0
0.2
0.4
0.6
MR effect size for
'MGAT4B' on 'Cardiac valvular disease'

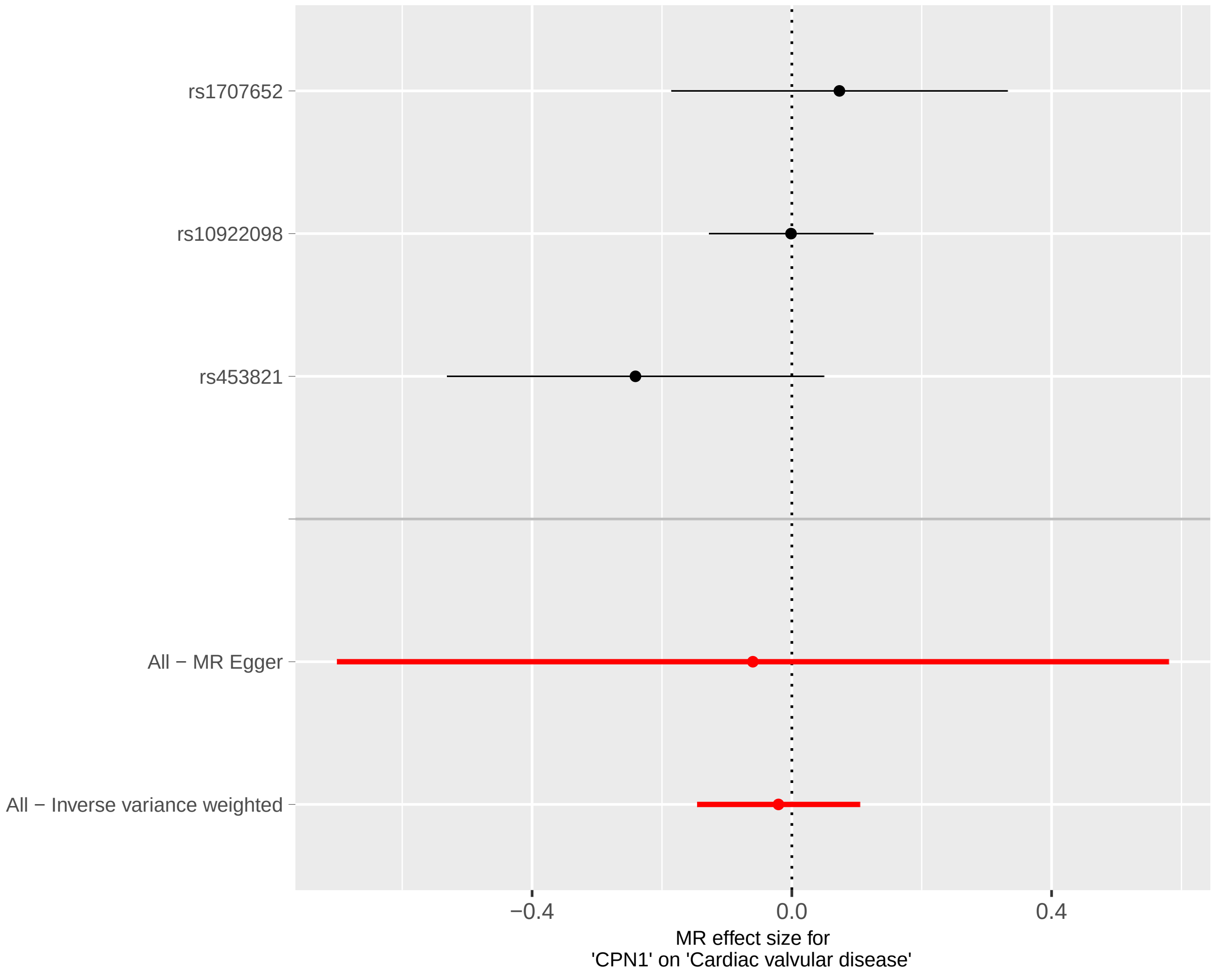
rs1707652
rs10922098
rs453821
All − MR Egger
All − Inverse variance weighted
−0.4
0.0
0.4
MR effect size for
'CPN1' on 'Cardiac valvular disease'

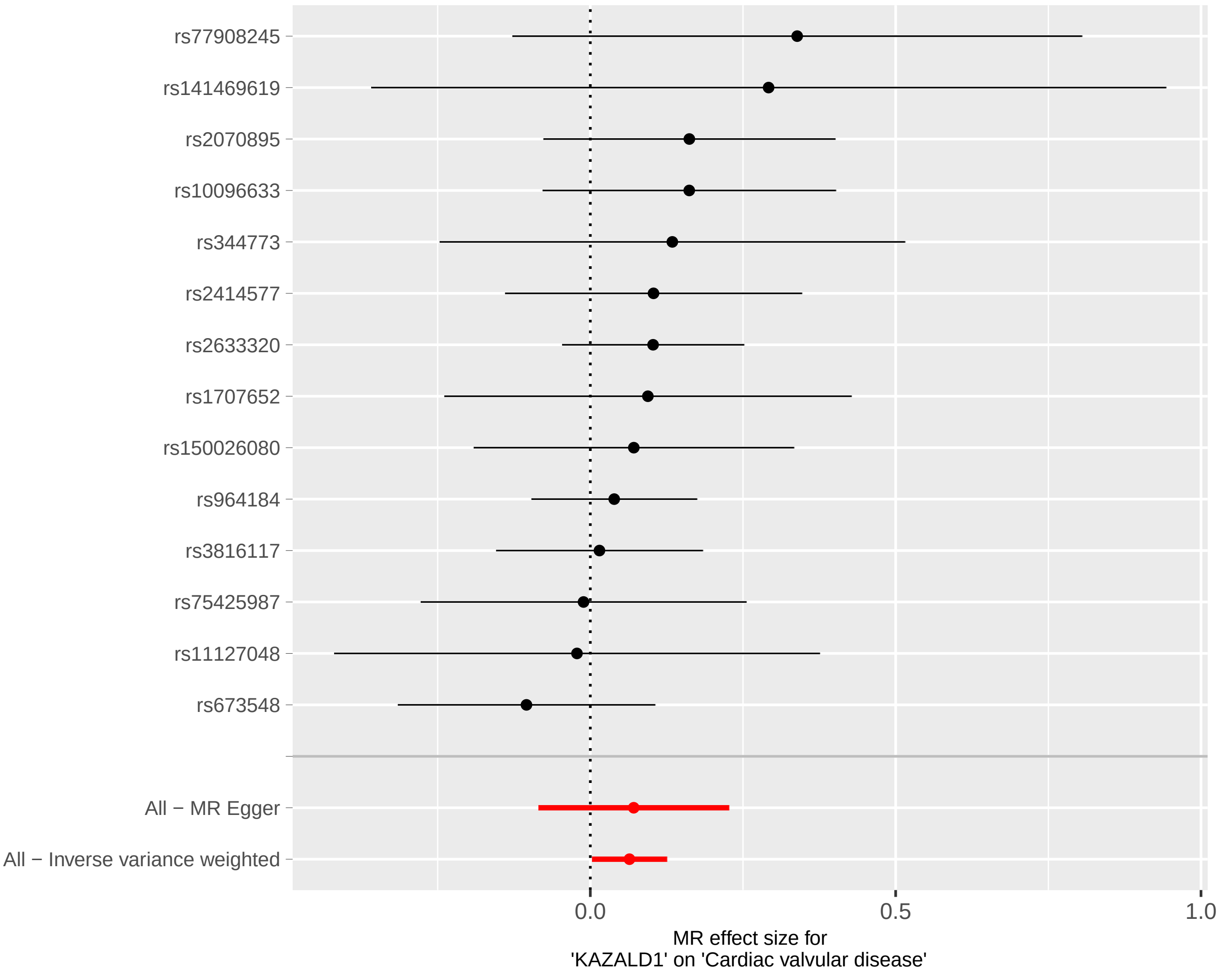

rs77908245
rs141469619
rs2070895
rs10096633
rs344773
rs2414577
rs2633320
rs1707652
rs150026080
rs964184
rs3816117
rs75425987
rs11127048
rs673548
All − MR Egger
All − Inverse variance weighted
0.0
0.5
1.0
MR effect size for
'KAZALD1' on 'Cardiac valvular disease'

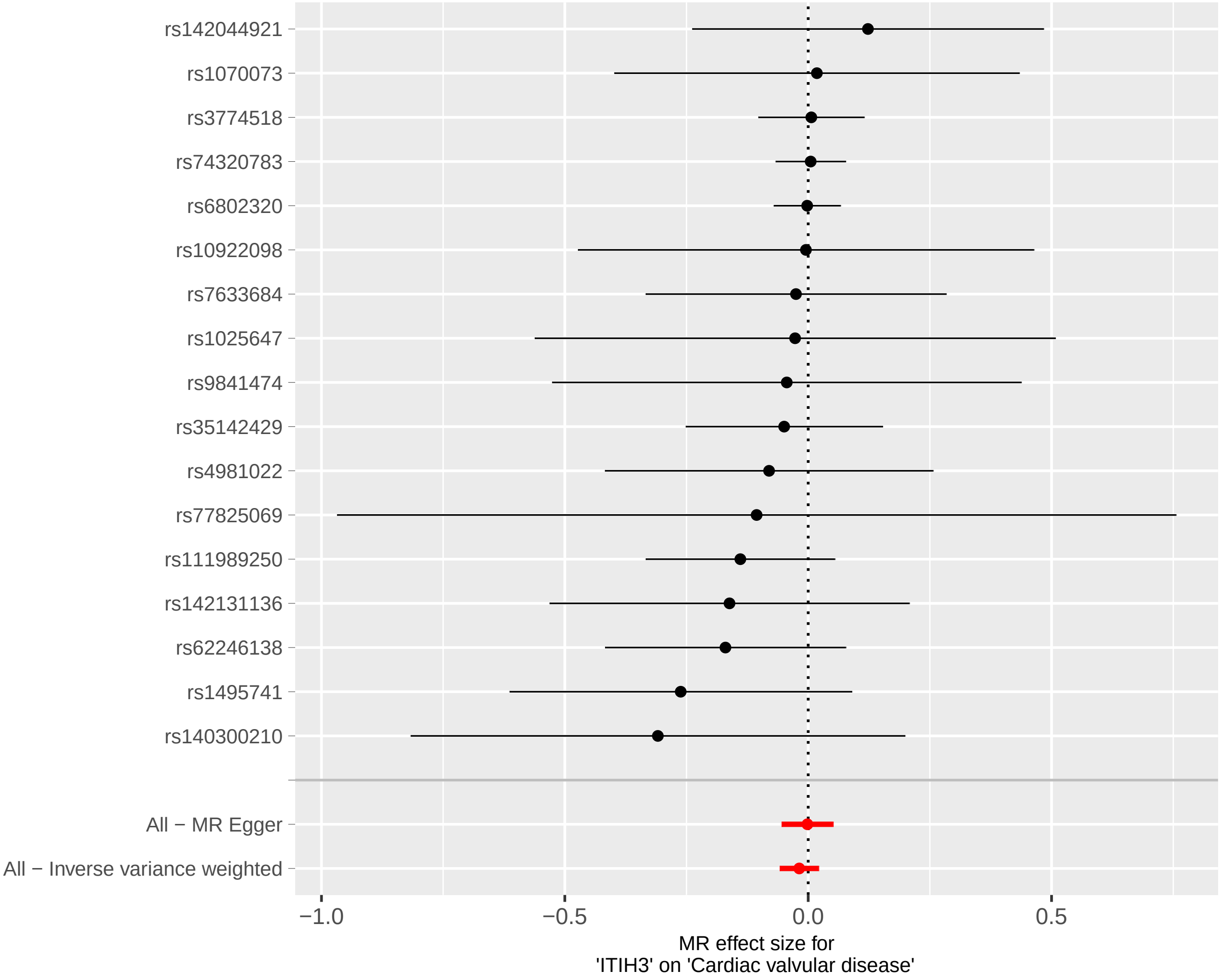
rs142044921
rs1070073
rs3774518
rs74320783
rs6802320
rs10922098
rs7633684
rs1025647
rs9841474
rs35142429
rs4981022
rs77825069
rs111989250
rs142131136
rs62246138
rs1495741
rs140300210
All − MR Egger
All − Inverse variance weighted
−1.0
−0.5
0.0
0.5
MR effect size for
'ITIH3' on 'Cardiac valvular disease'

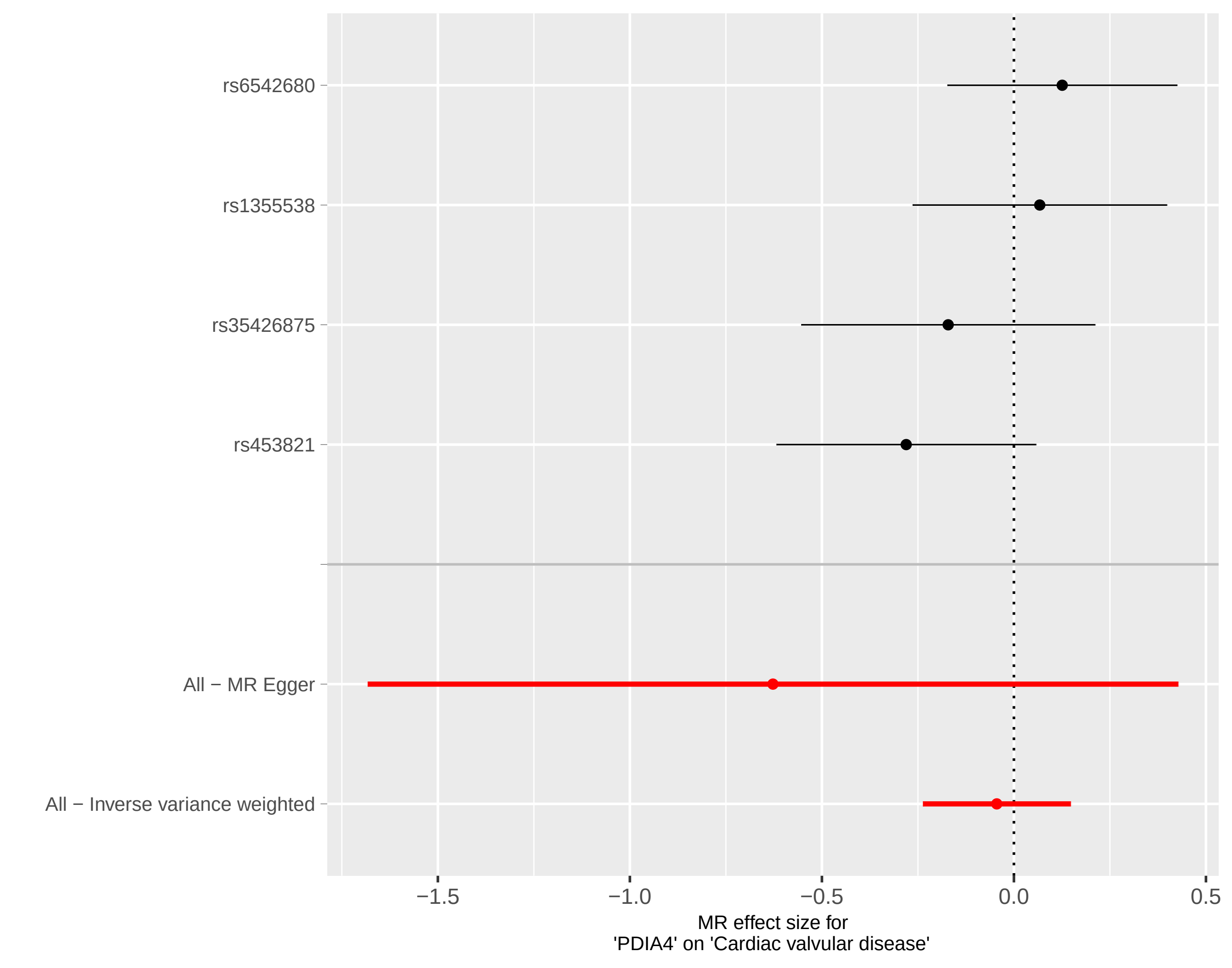
rs6542680
rs1355538
rs35426875
rs453821
All − MR Egger
All − Inverse variance weighted
−1.5
−1.0
−0.5
0.0
0.5
MR effect size for
'PDIA4' on 'Cardiac valvular disease'

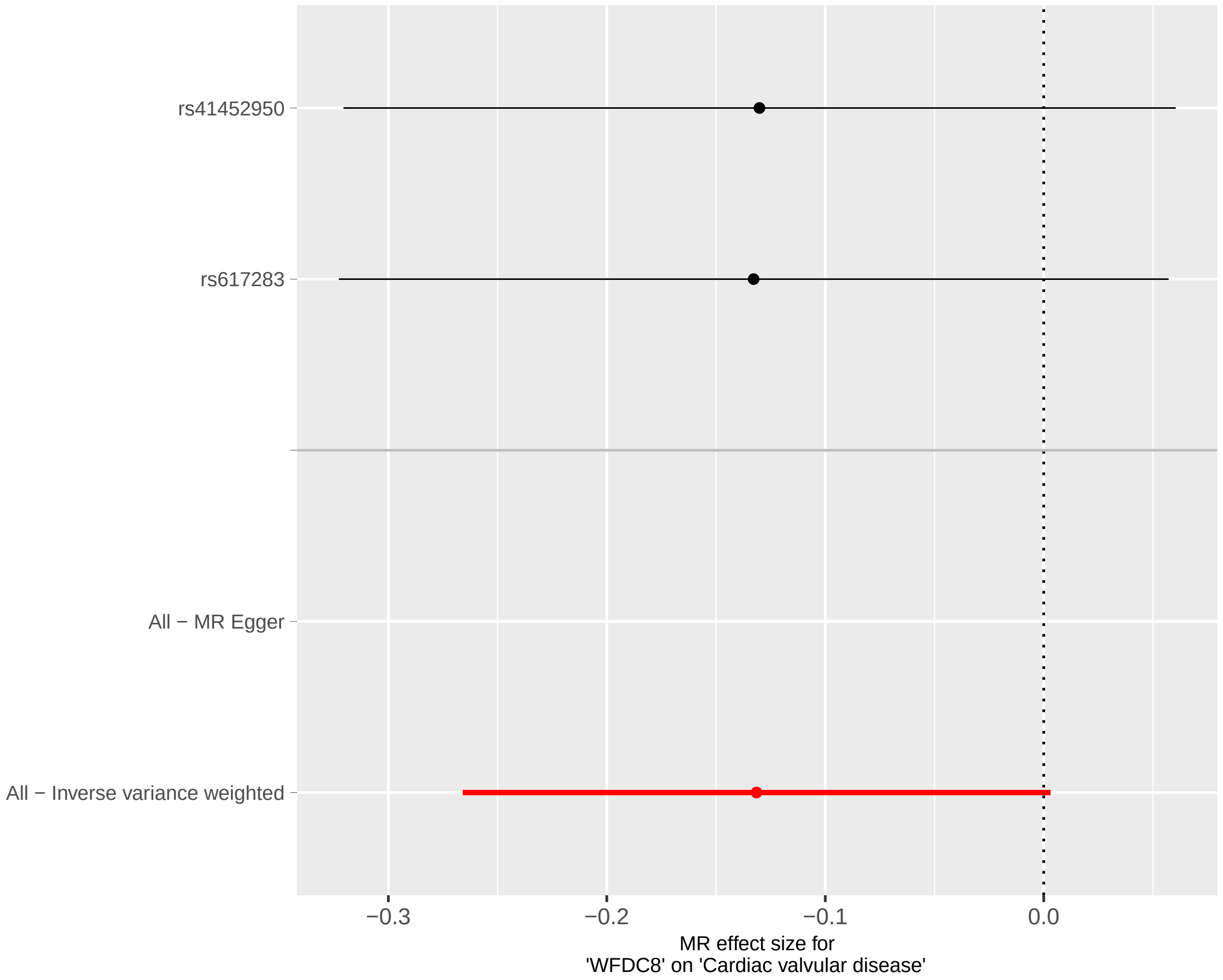

rs41452950
rs617283
All − MR Egger
All − Inverse variance weighted
−0.3
−0.2
−0.1
0.0
MR effect size for
'WFDC8' on 'Cardiac valvular disease'

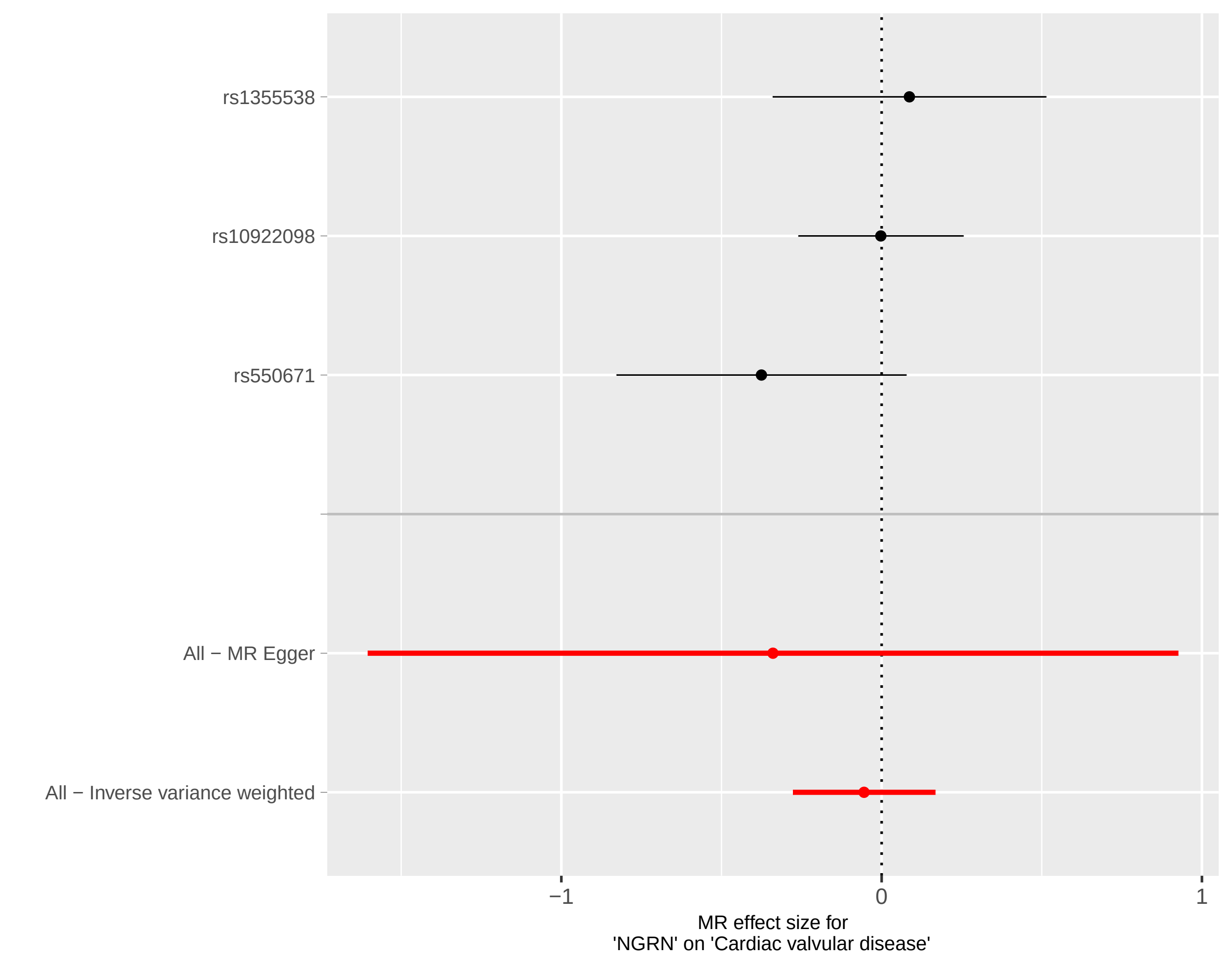
rs1355538
rs10922098
rs550671
All − MR Egger
All − Inverse variance weighted
−1
0
1
MR effect size for
'NGRN' on 'Cardiac valvular disease'

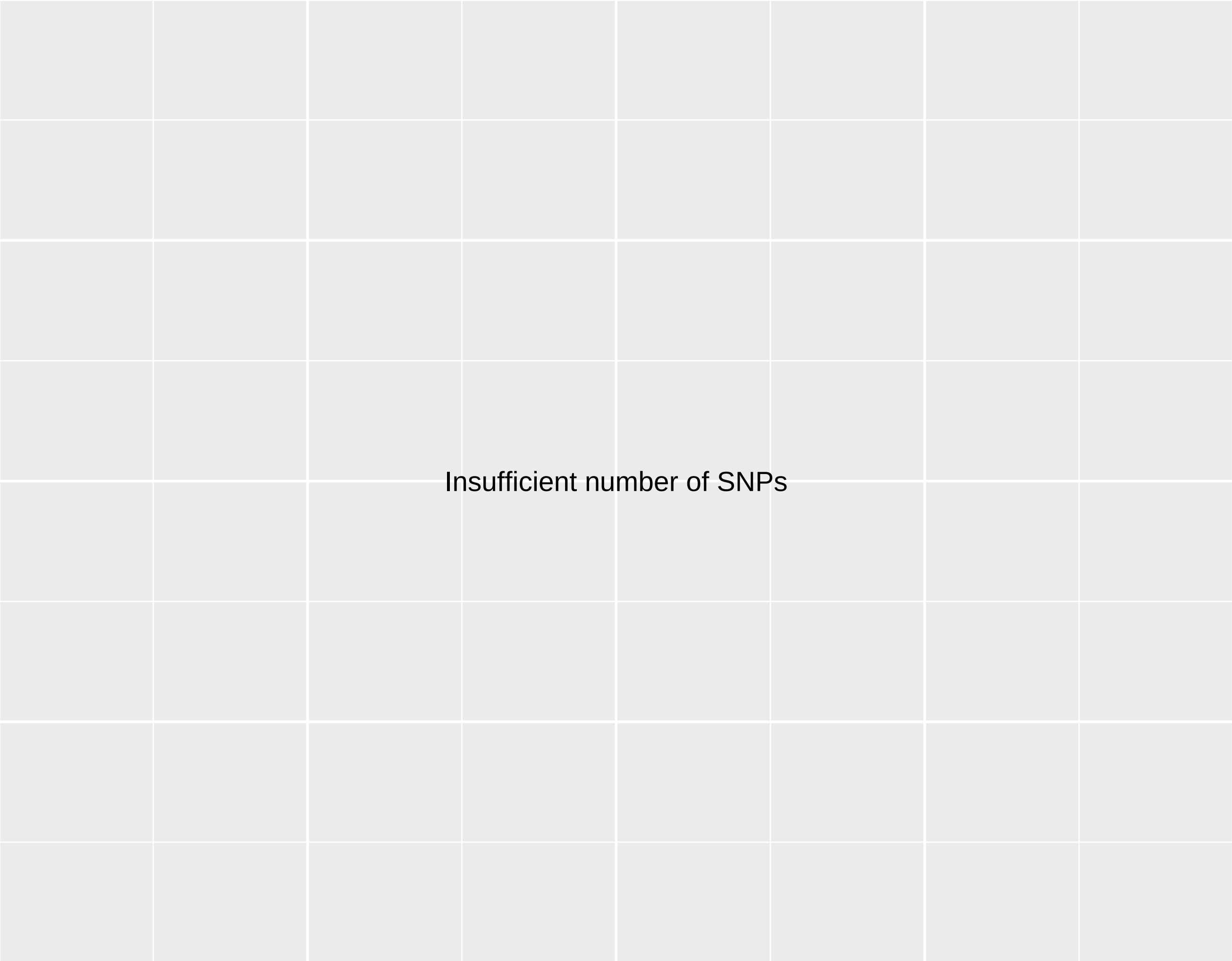
Insufficient number of SNPs

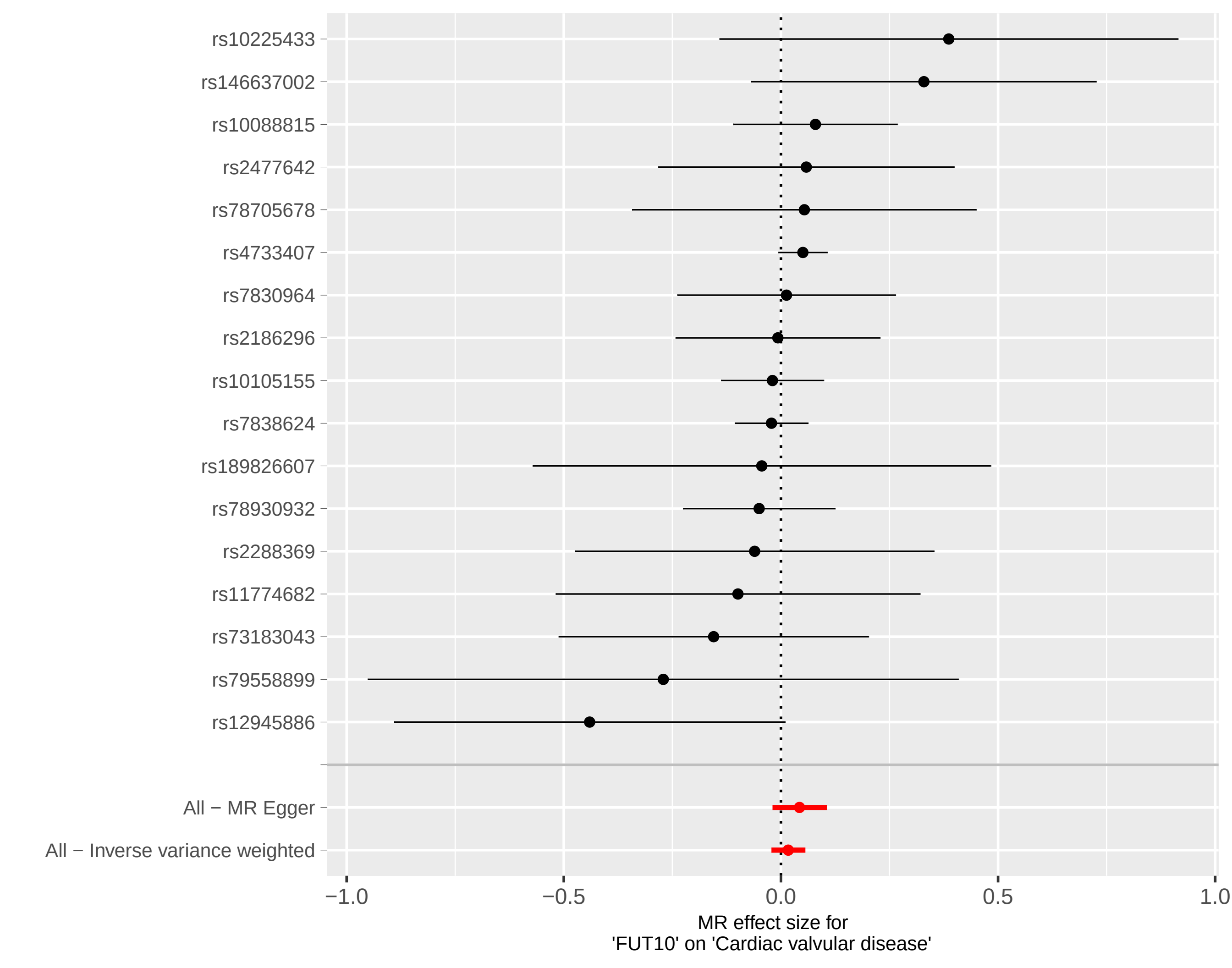

rs10225433
rs146637002
rs10088815
rs2477642
rs78705678
rs4733407
rs7830964
rs2186296
rs10105155
rs7838624
rs189826607
rs78930932
rs2288369
rs11774682
rs73183043
rs79558899
rs12945886
All − MR Egger
All − Inverse variance weighted
−1.0
−0.5
0.0
0.5
1.0
MR effect size for
'FUT10' on 'Cardiac valvular disease'

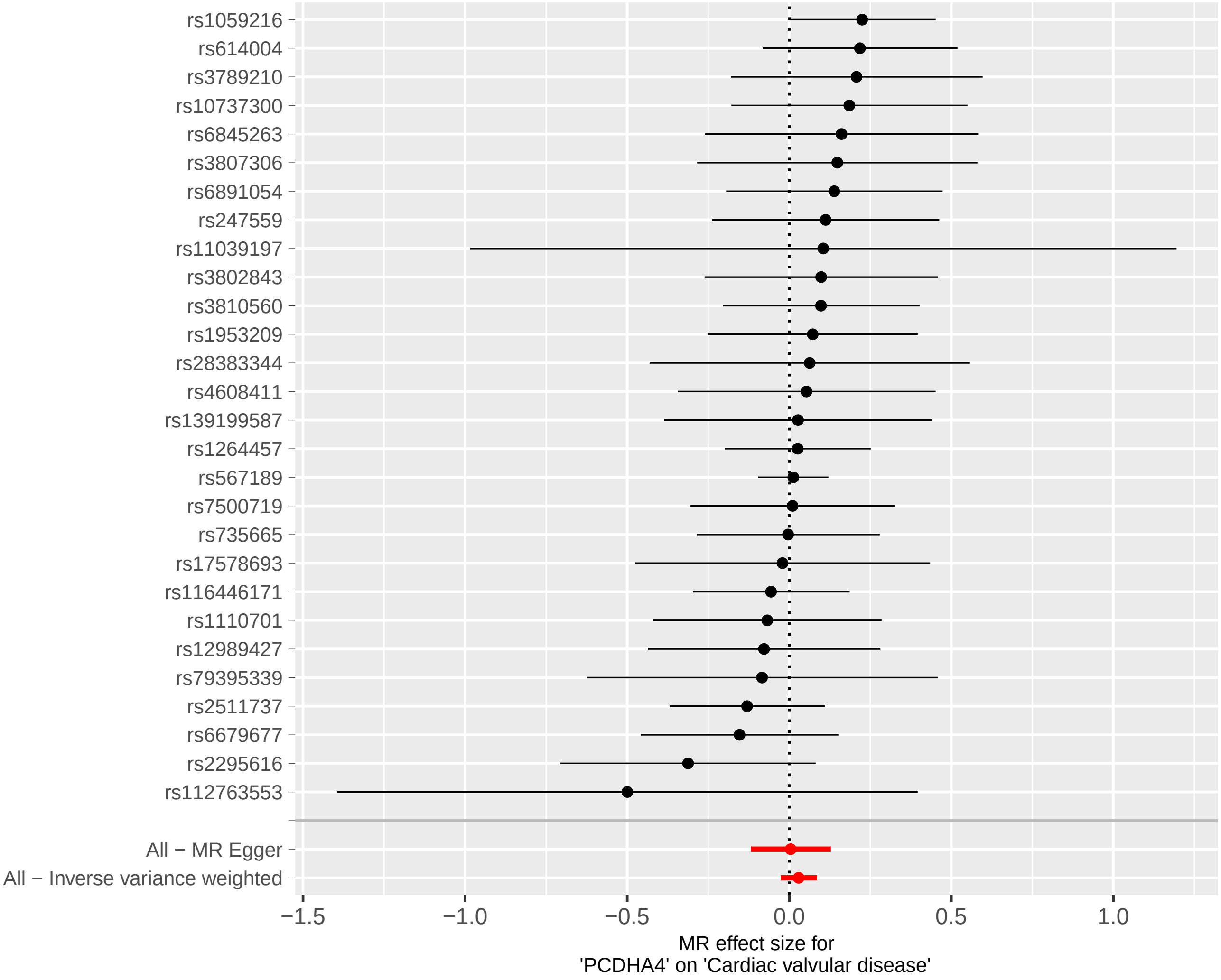
rs1059216
rs614004
rs3789210
rs10737300
rs6845263
rs3807306
rs6891054
rs247559
rs11039197
rs3802843
rs3810560
rs1953209
rs28383344
rs4608411
rs139199587
rs1264457
rs567189
rs7500719
rs735665
rs17578693
rs116446171
rs1110701
rs12989427
rs79395339
rs2511737
rs6679677
rs2295616
rs112763553
All − MR Egger
All − Inverse variance weighted
−1.5
−1.0
−0.5
0.0
0.5
1.0
MR effect size for
'PCDHA4' on 'Cardiac valvular disease'

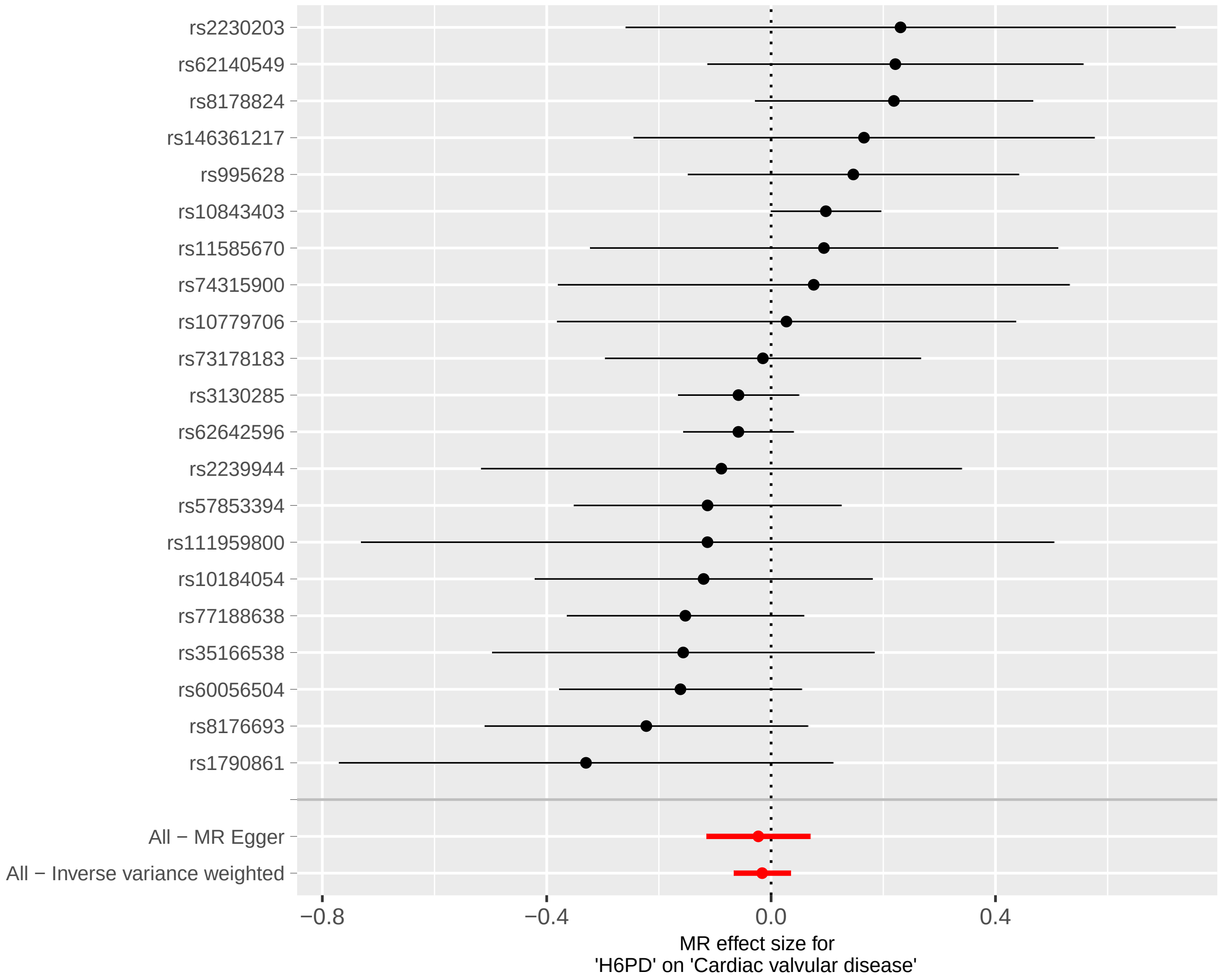
rs2230203
rs62140549
rs8178824
rs146361217
rs995628
rs10843403
rs11585670
rs74315900
rs10779706
rs73178183
rs3130285
rs62642596
rs2239944
rs57853394
rs111959800
rs10184054
rs77188638
rs35166538
rs60056504
rs8176693
rs1790861
All − MR Egger
All − Inverse variance weighted
−0.8
−0.4
0.0
0.4
MR effect size for
'H6PD' on 'Cardiac valvular disease'

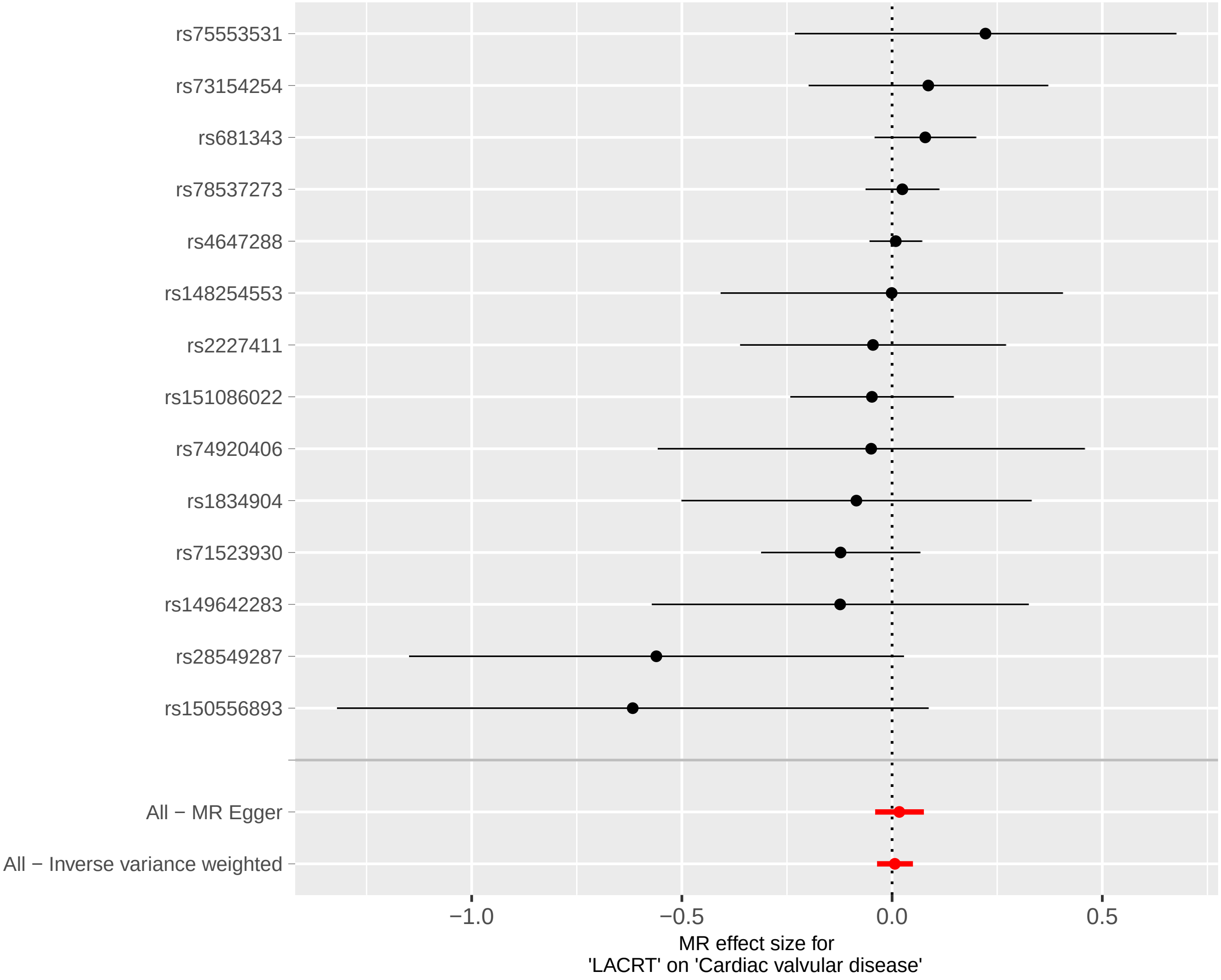

rs75553531
rs73154254
rs681343
rs78537273
rs4647288
rs148254553
rs2227411
rs151086022
rs74920406
rs1834904
rs71523930
rs149642283
rs28549287
rs150556893
All − MR Egger
All − Inverse variance weighted
−1.0
−0.5
0.0
0.5
MR effect size for
'LACRT' on 'Cardiac valvular disease'

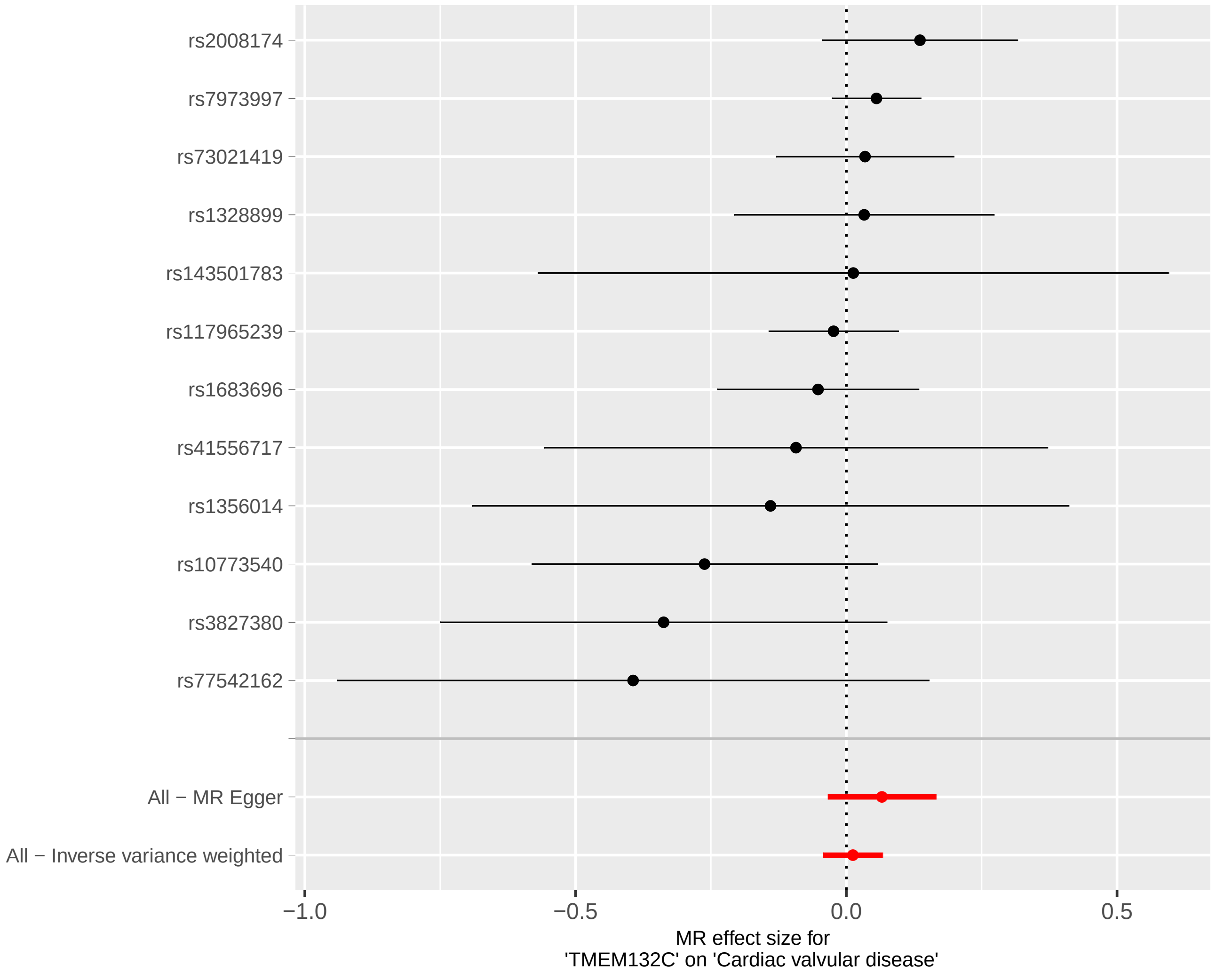
rs2008174
rs7973997
rs73021419
rs1328899
rs143501783
rs117965239
rs1683696
rs41556717
rs1356014
rs10773540
rs3827380
rs77542162
All − MR Egger
All − Inverse variance weighted
−1.0
−0.5
0.0
0.5
MR effect size for
'TMEM132C' on 'Cardiac valvular disease'

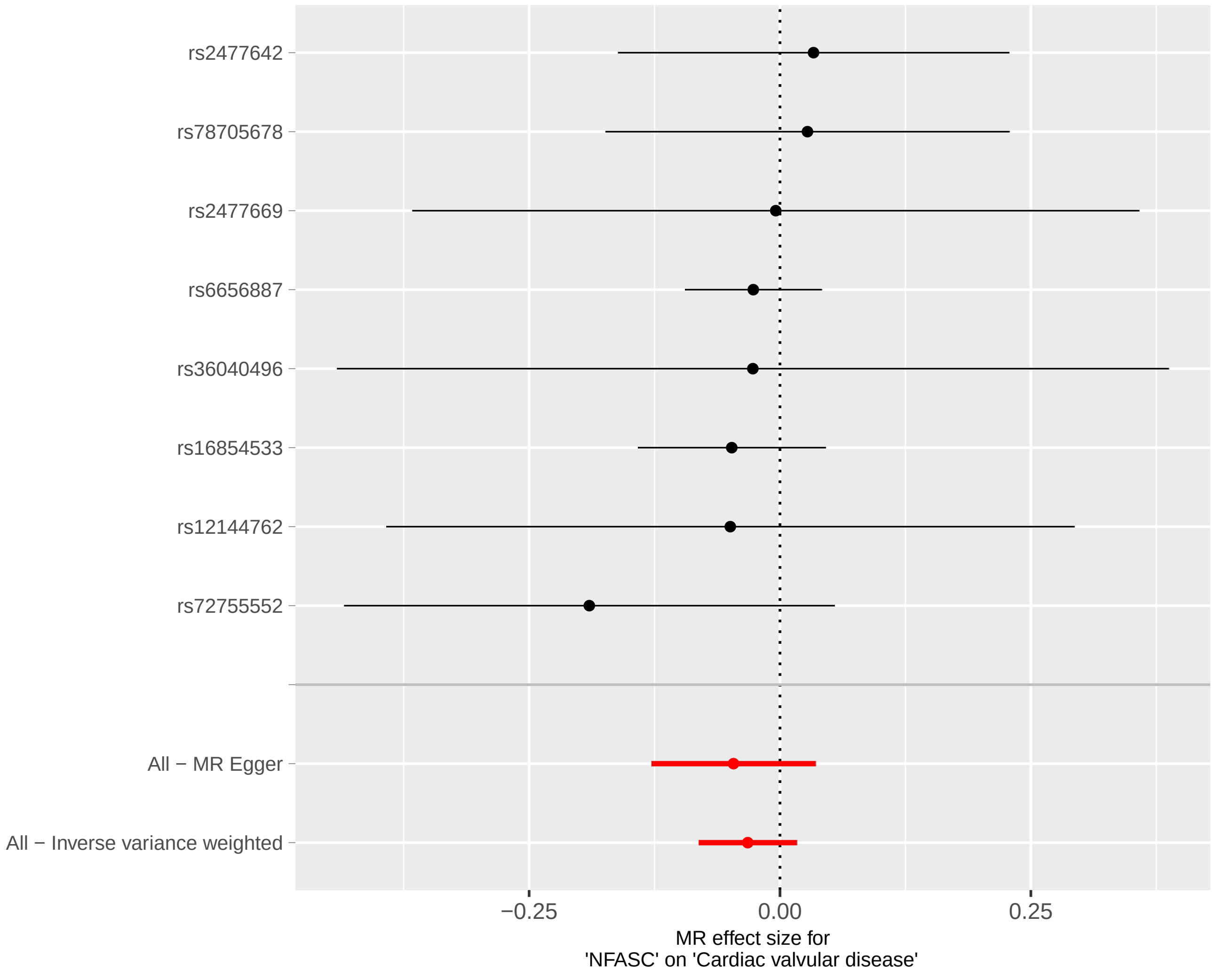

rs2477642
rs78705678
rs2477669
rs6656887
rs36040496
rs16854533
rs12144762
rs72755552
All − MR Egger
All − Inverse variance weighted
−0.25
0.00
0.25
MR effect size for
'NFASC' on 'Cardiac valvular disease'

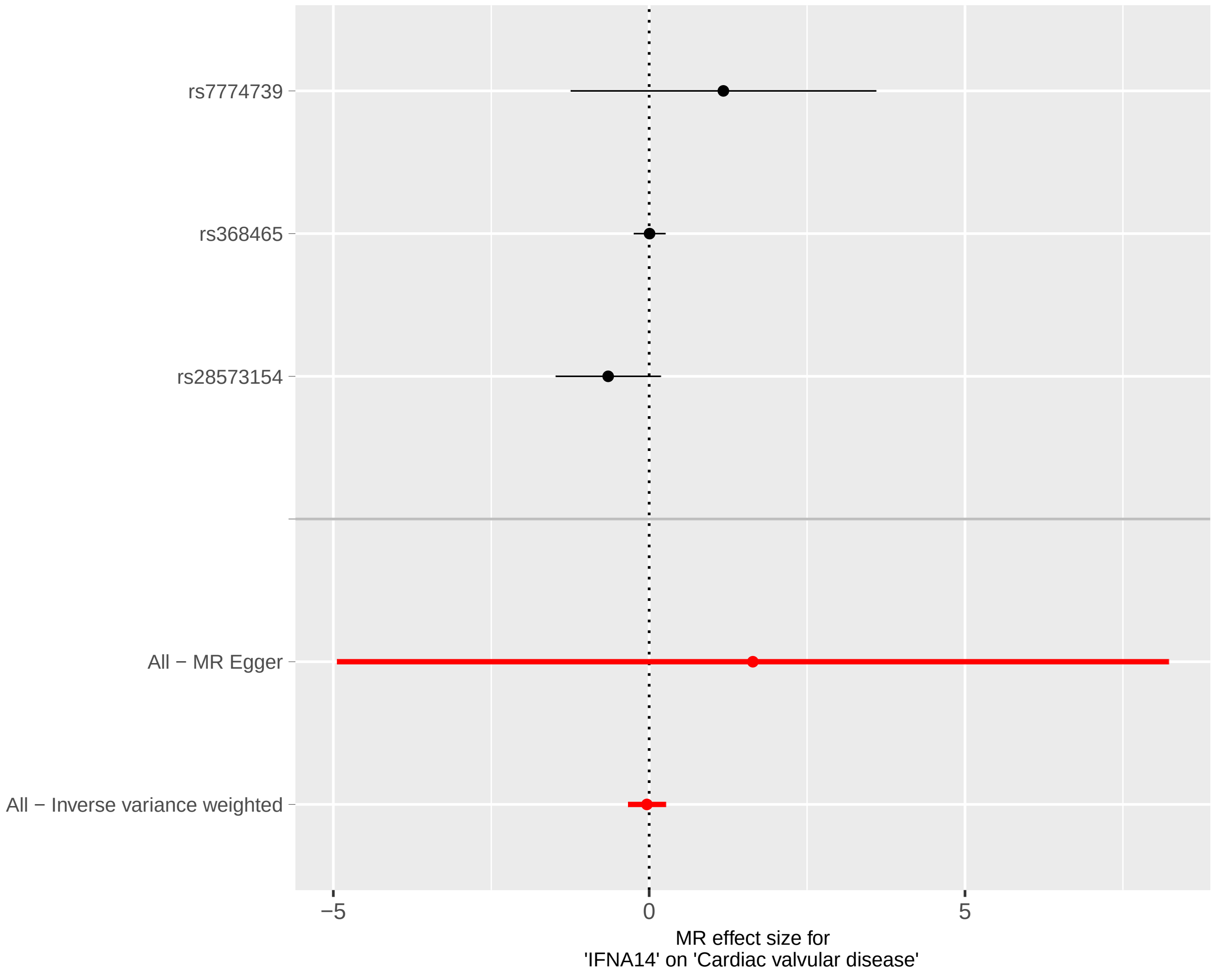
rs7774739
rs368465
rs28573154
All − MR Egger
All − Inverse variance weighted
−5
0
5
MR effect size for
'IFNA14' on 'Cardiac valvular disease'

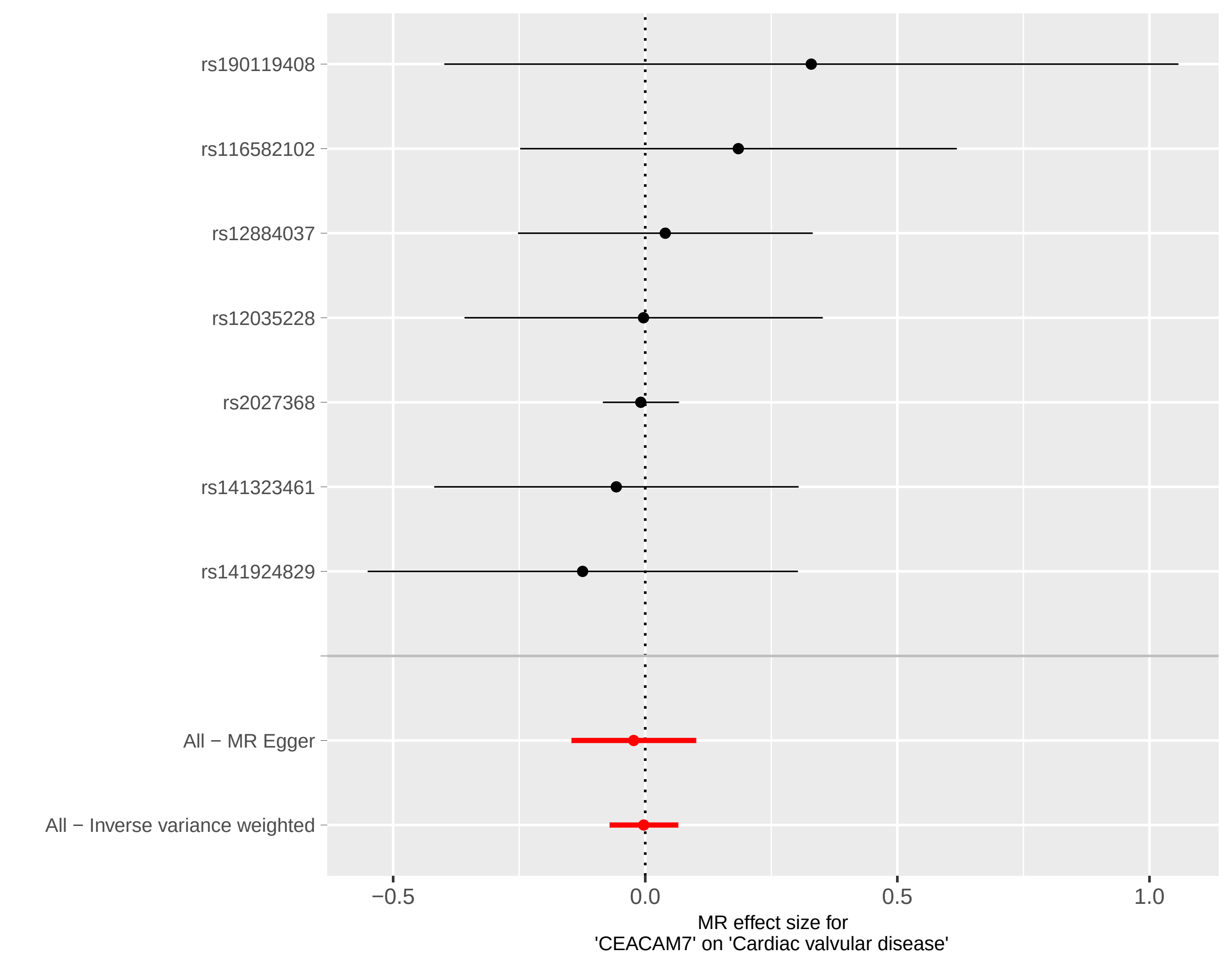

rs190119408
rs116582102
rs12884037
rs12035228
rs2027368
rs141323461
rs141924829
All − MR Egger
All − Inverse variance weighted
−0.5
0.0
0.5
1.0
MR effect size for
'CEACAM7' on 'Cardiac valvular disease'

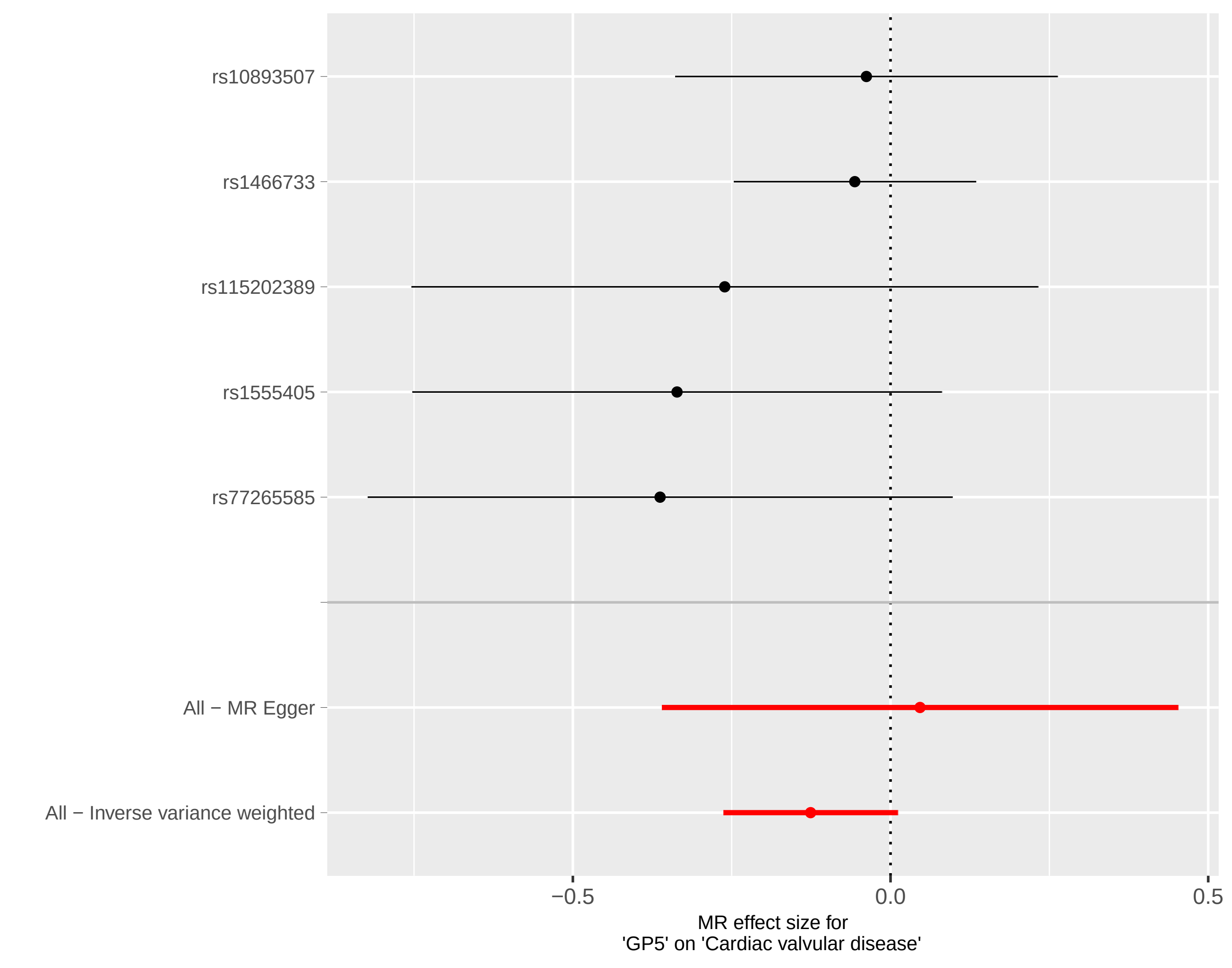
rs10893507
rs1466733
rs115202389
rs1555405
rs77265585
All − MR Egger
All − Inverse variance weighted
−0.5
0.0
0.5
MR effect size for
'GP5' on 'Cardiac valvular disease'

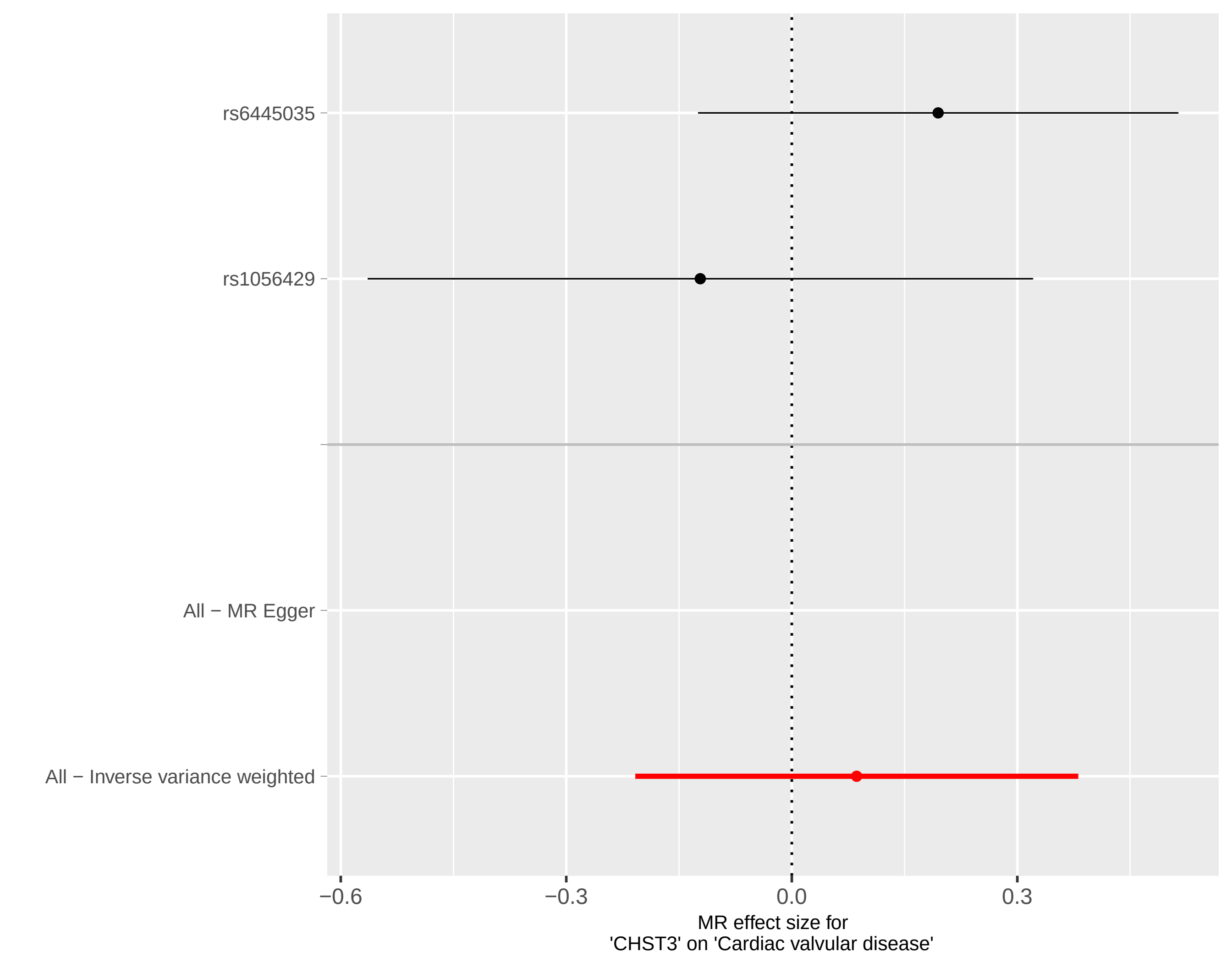
rs6445035
rs1056429
All − MR Egger
All − Inverse variance weighted
−0.6
−0.3
0.0
0.3
MR effect size for
'CHST3' on 'Cardiac valvular disease'

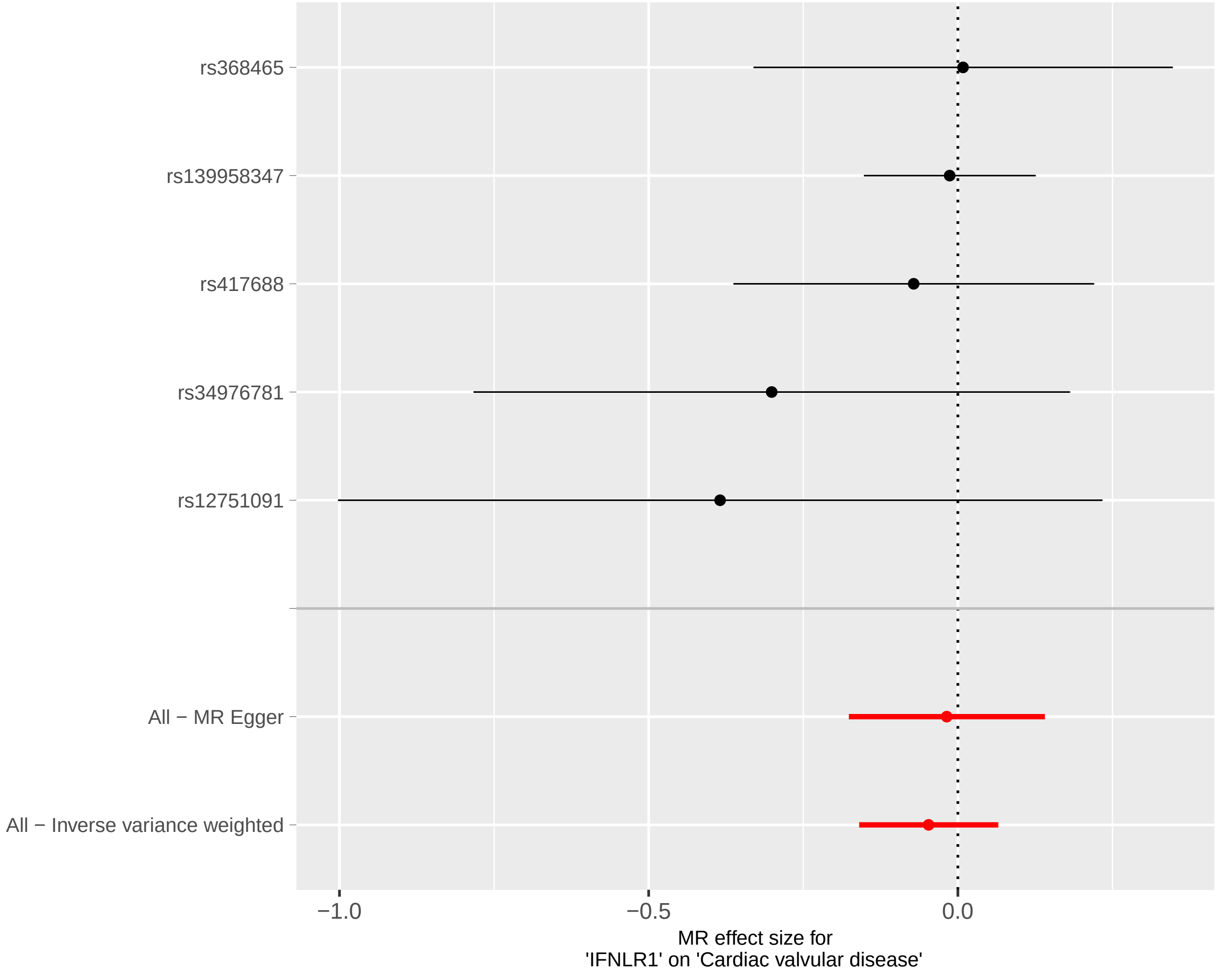
rs368465
rs139958347
rs417688
rs34976781
rs12751091
All − MR Egger
All − Inverse variance weighted
−1.0
−0.5
0.0
MR effect size for
'IFNLR1' on 'Cardiac valvular disease'

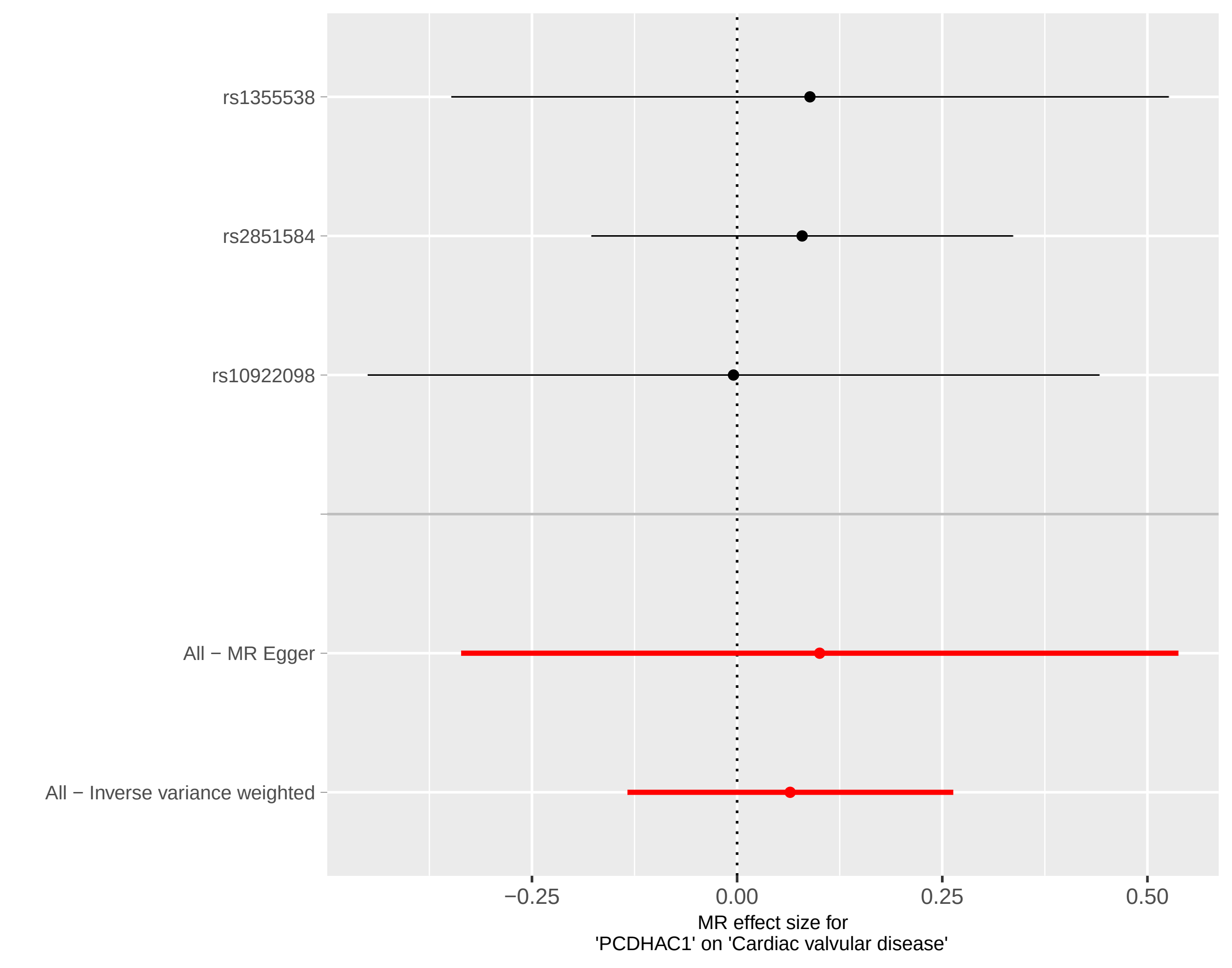

rs1355538
rs2851584
rs10922098
All − MR Egger
All − Inverse variance weighted
−0.25
0.00
0.25
0.50
MR effect size for
'PCDHAC1' on 'Cardiac valvular disease'

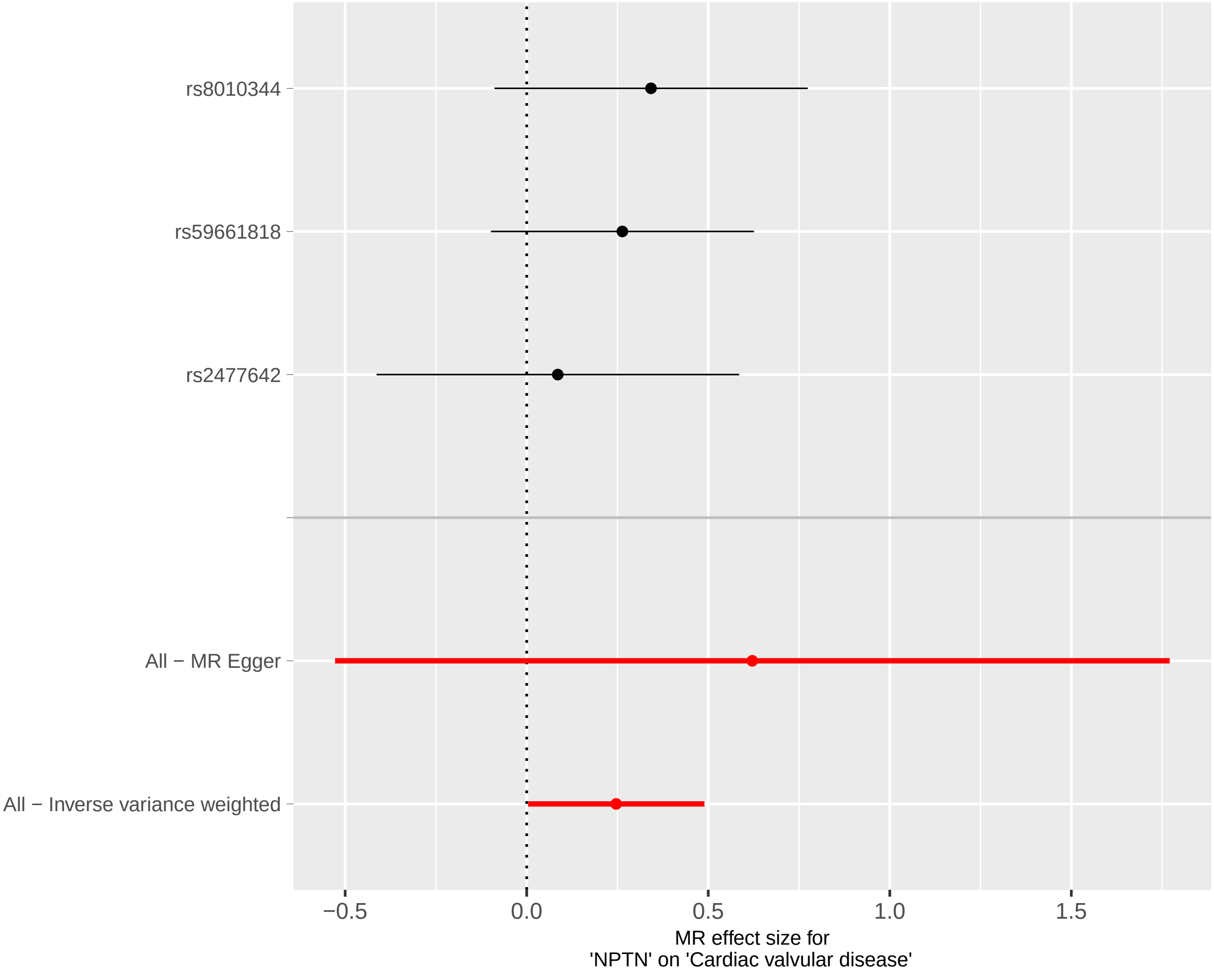
rs8010344
rs59661818
rs2477642
All − MR Egger
All − Inverse variance weighted
−0.5
0.0
0.5
1.0
1.5
MR effect size for
'NPTN' on 'Cardiac valvular disease'

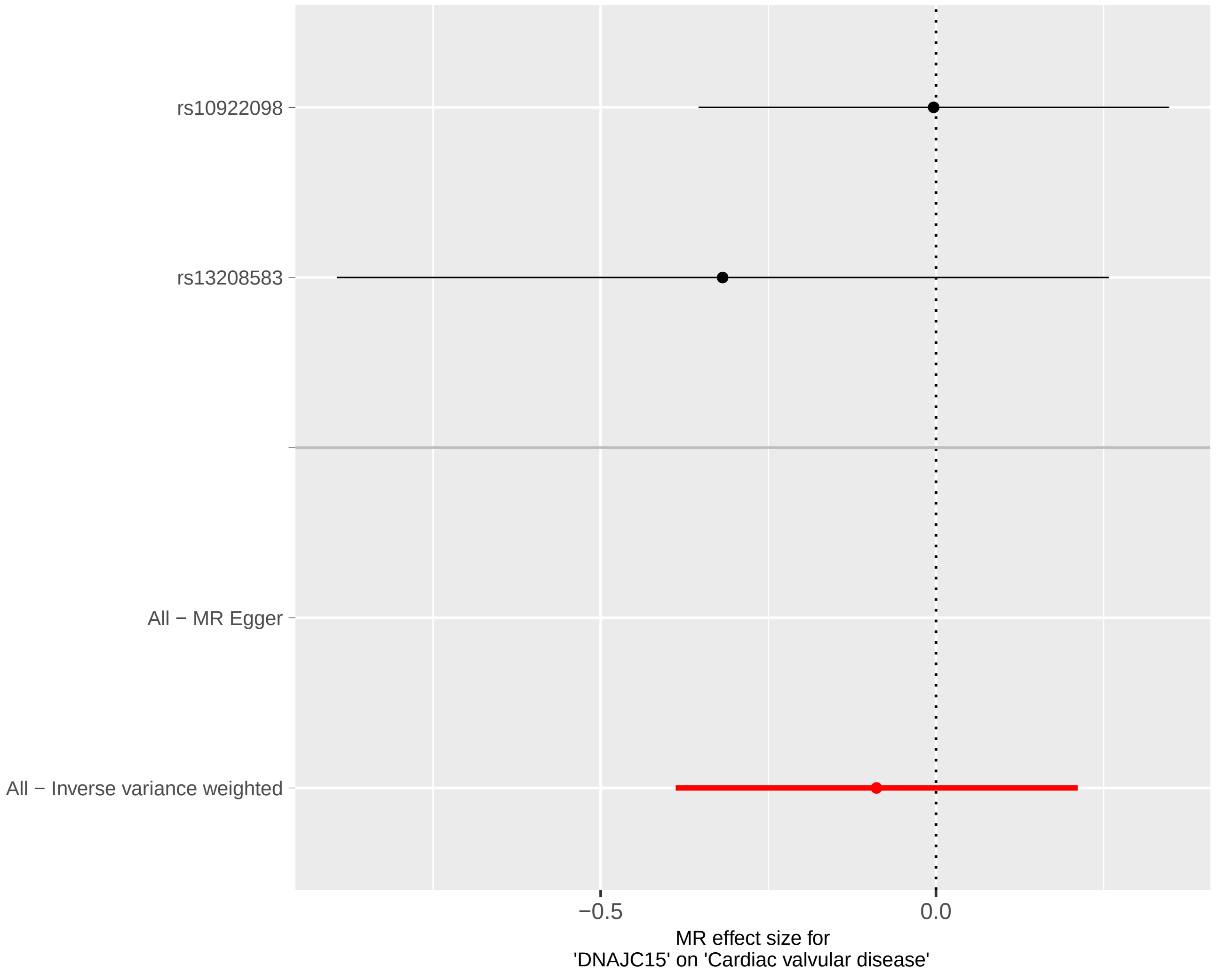
rs10922098
rs13208583
All − MR Egger
All − Inverse variance weighted
−0.5
0.0
MR effect size for
'DNAJC15' on 'Cardiac valvular disease'

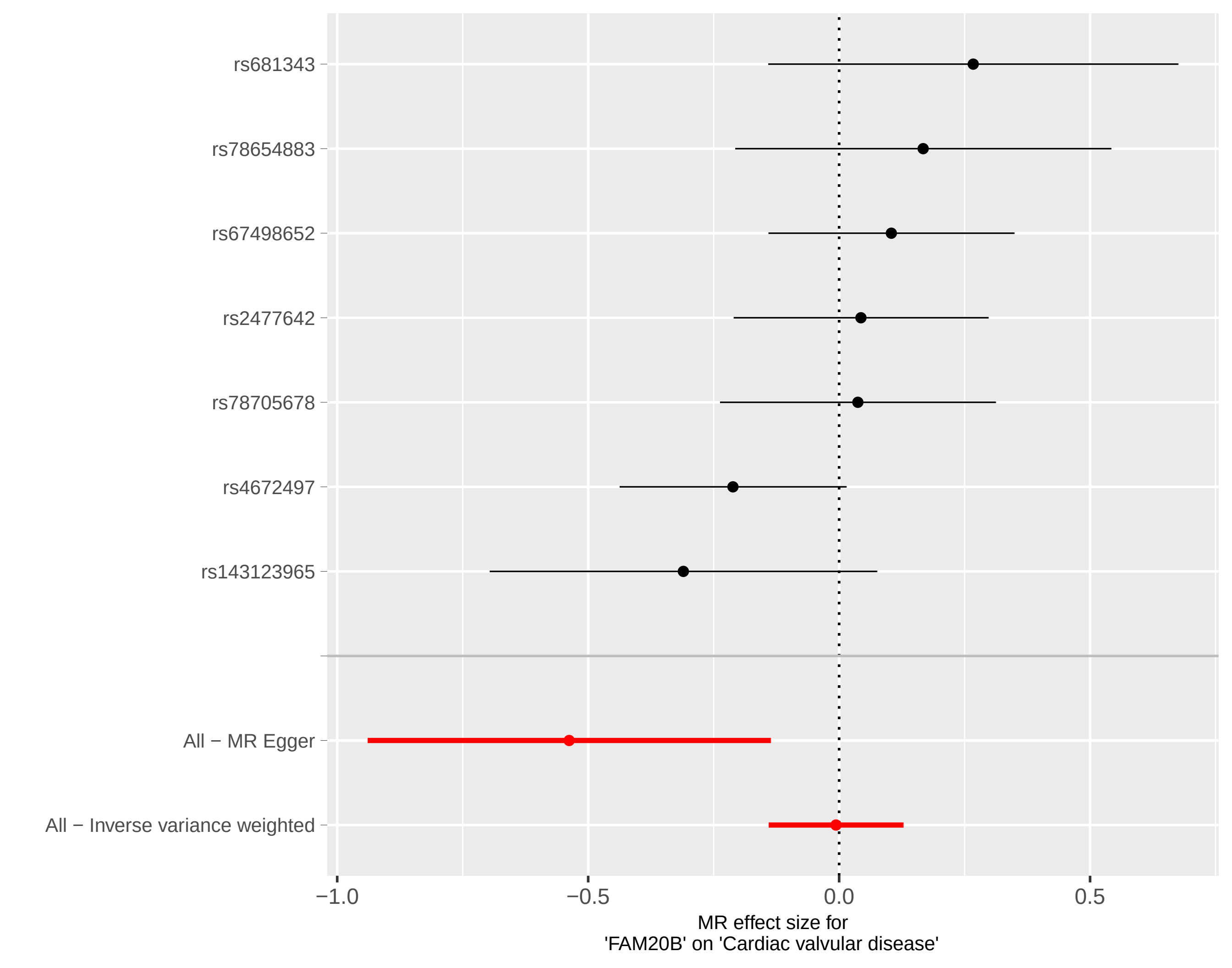

rs681343
rs78654883
rs67498652
rs2477642
rs78705678
rs4672497
rs143123965
All − MR Egger
All − Inverse variance weighted
−1.0
−0.5
0.0
0.5
MR effect size for
'FAM20B' on 'Cardiac valvular disease'

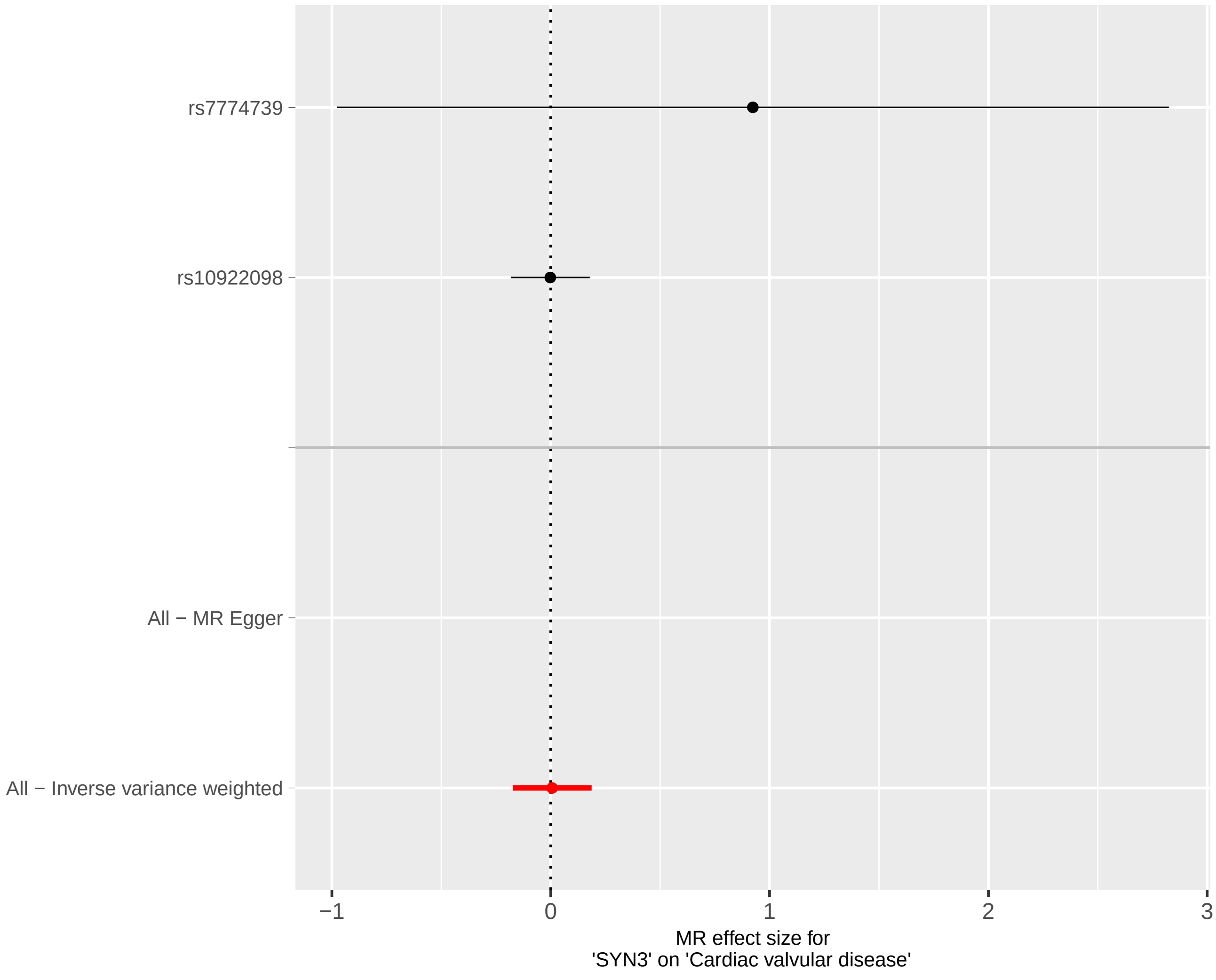

rs7774739
rs10922098
All − MR Egger
All − Inverse variance weighted
−1
0
1
2
3
MR effect size for
'SYN3' on 'Cardiac valvular disease'

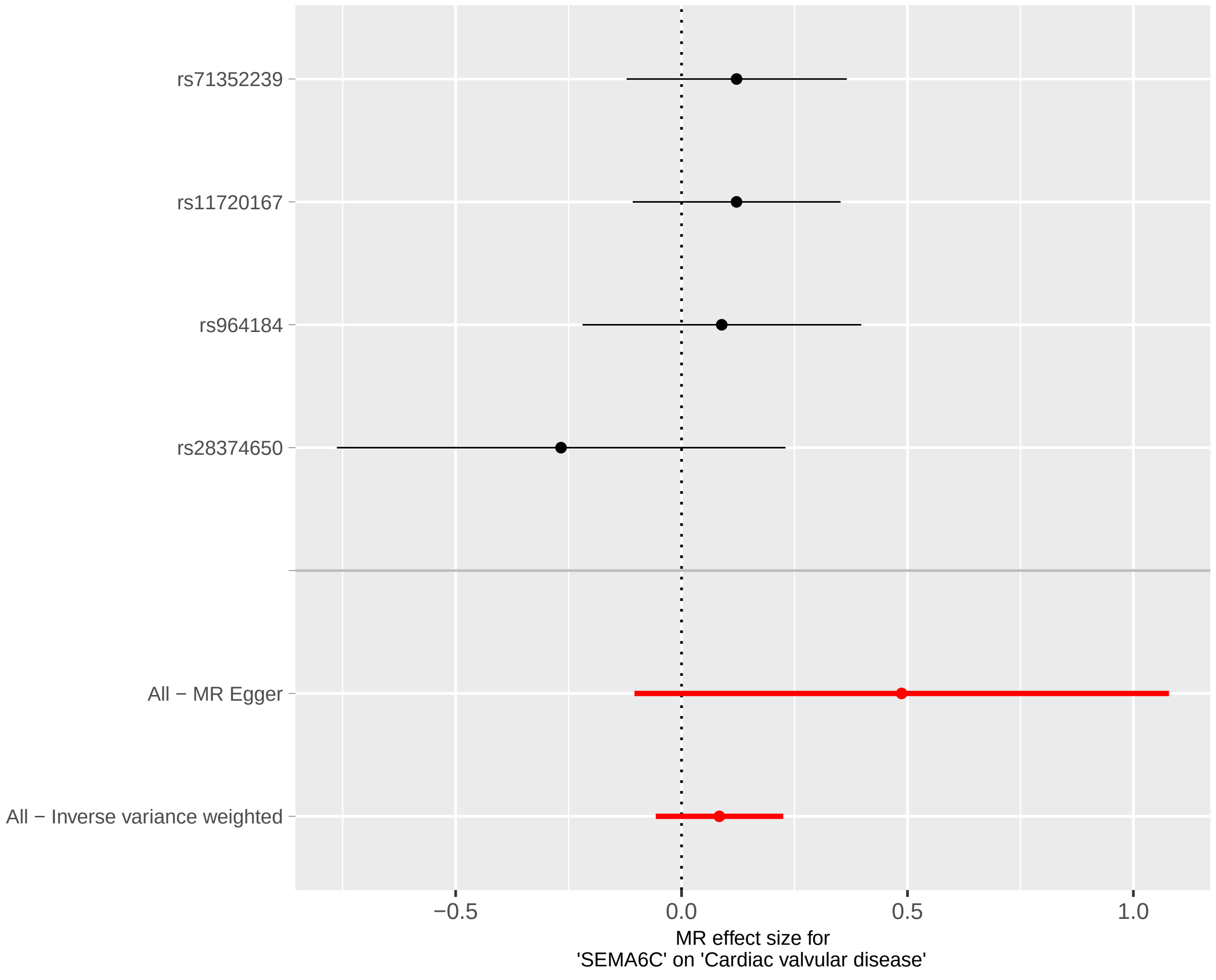

rs71352239
rs11720167
rs964184
rs28374650
All − MR Egger
All − Inverse variance weighted
−0.5
0.0
0.5
1.0
MR effect size for
'SEMA6C' on 'Cardiac valvular disease'

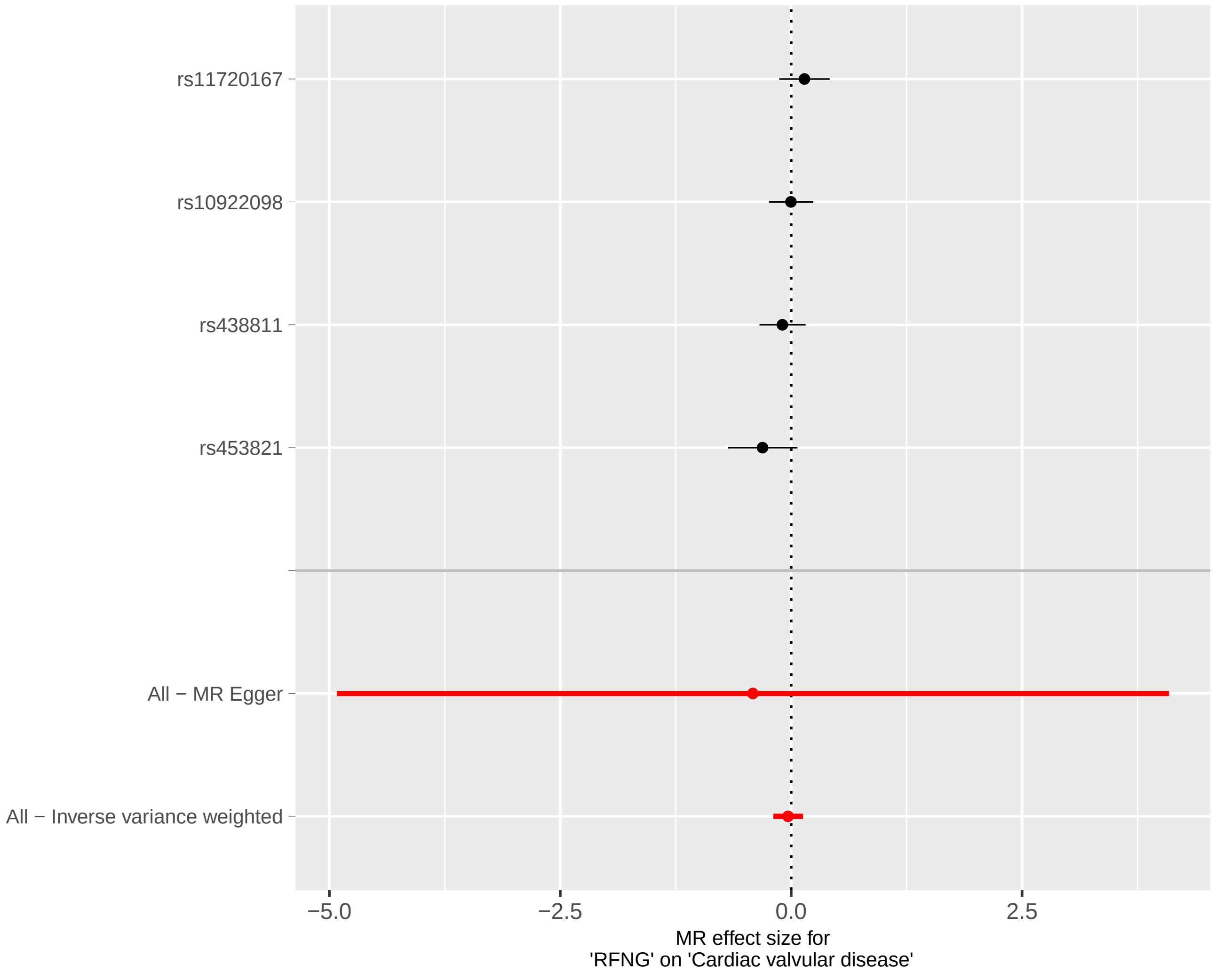
rs11720167
rs10922098
rs438811
rs453821
All − MR Egger
All − Inverse variance weighted
−5.0
−2.5
0.0
2.5
MR effect size for
'RFNG' on 'Cardiac valvular disease'

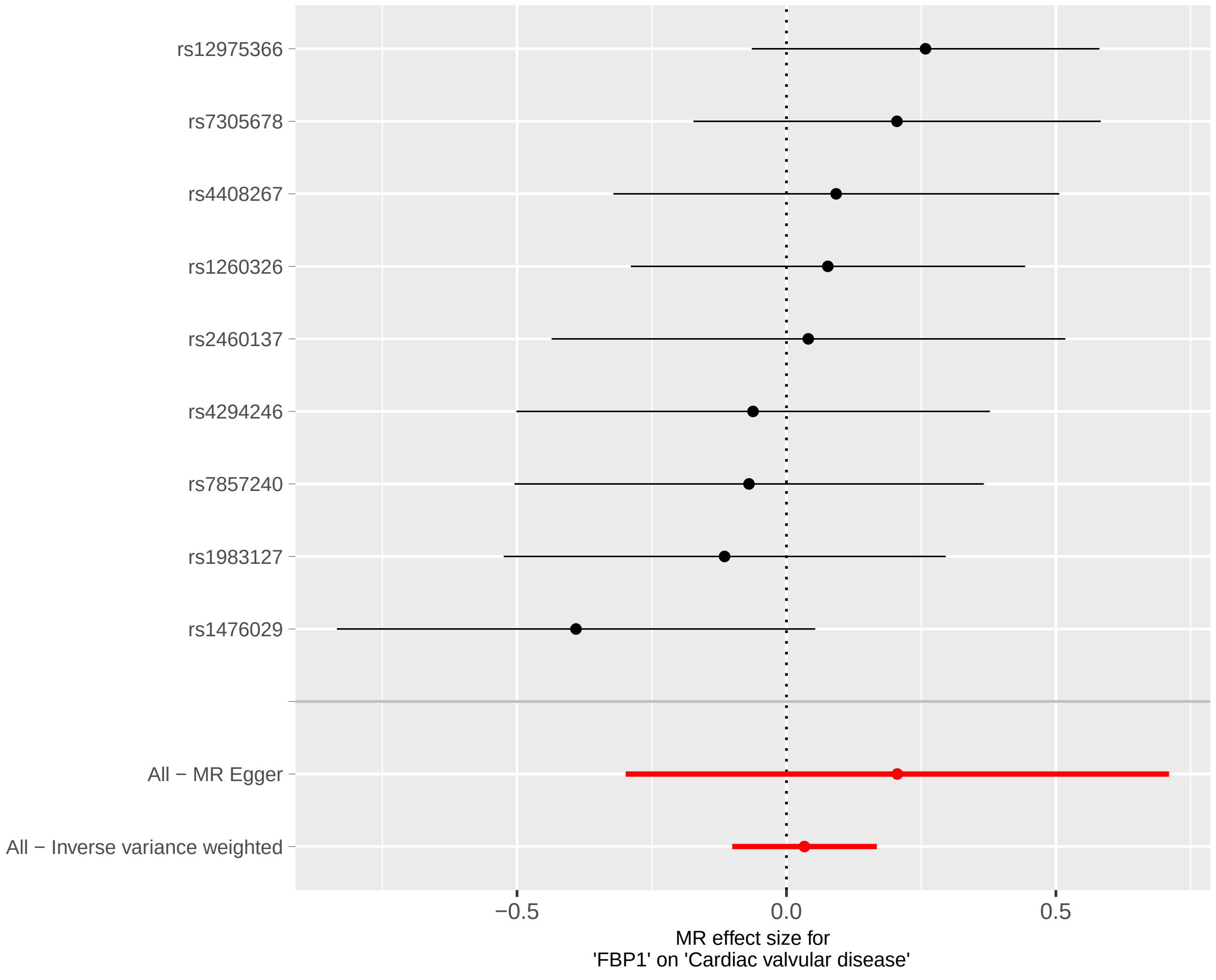

rs12975366
rs7305678
rs4408267
rs1260326
rs2460137
rs4294246
rs7857240
rs1983127
rs1476029
All − MR Egger
All − Inverse variance weighted
−0.5
0.0
0.5
MR effect size for
'FBP1' on 'Cardiac valvular disease'

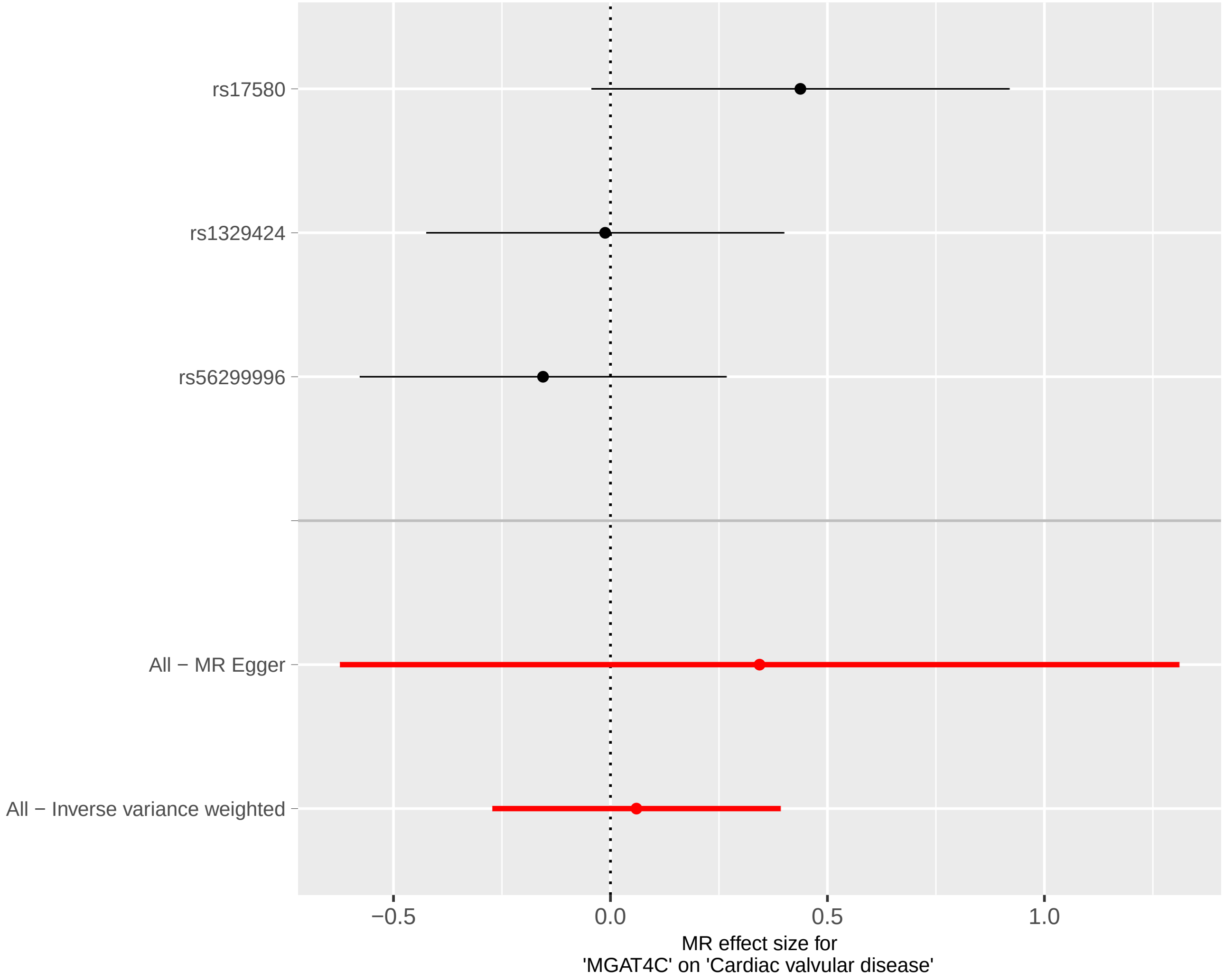
rs17580
rs1329424
rs56299996
All − MR Egger
All − Inverse variance weighted
−0.5
0.0
0.5
1.0
MR effect size for
'MGAT4C' on 'Cardiac valvular disease'

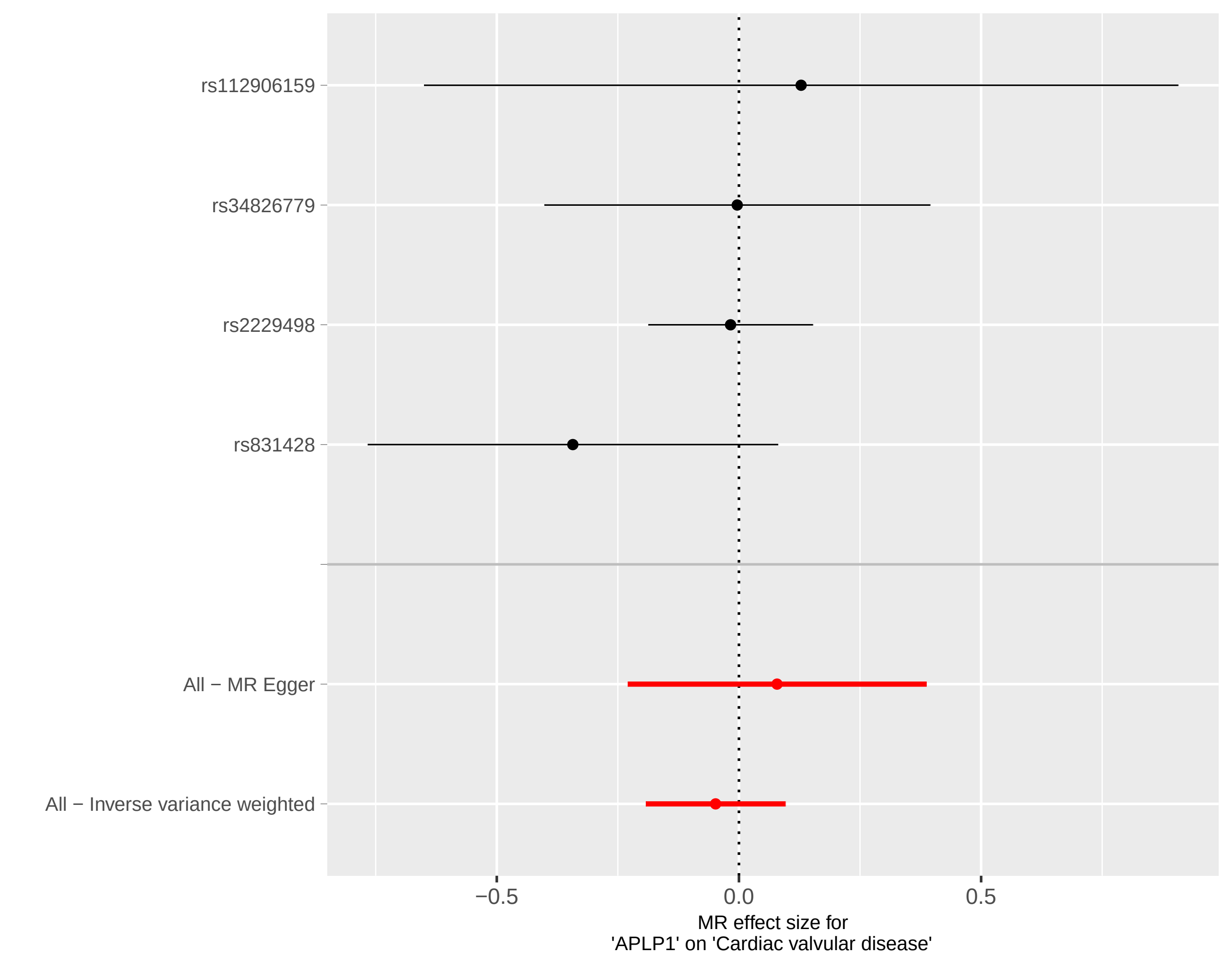
rs112906159
rs34826779
rs2229498
rs831428
All − MR Egger
All − Inverse variance weighted
−0.5
0.0
0.5
MR effect size for
'APLP1' on 'Cardiac valvular disease'

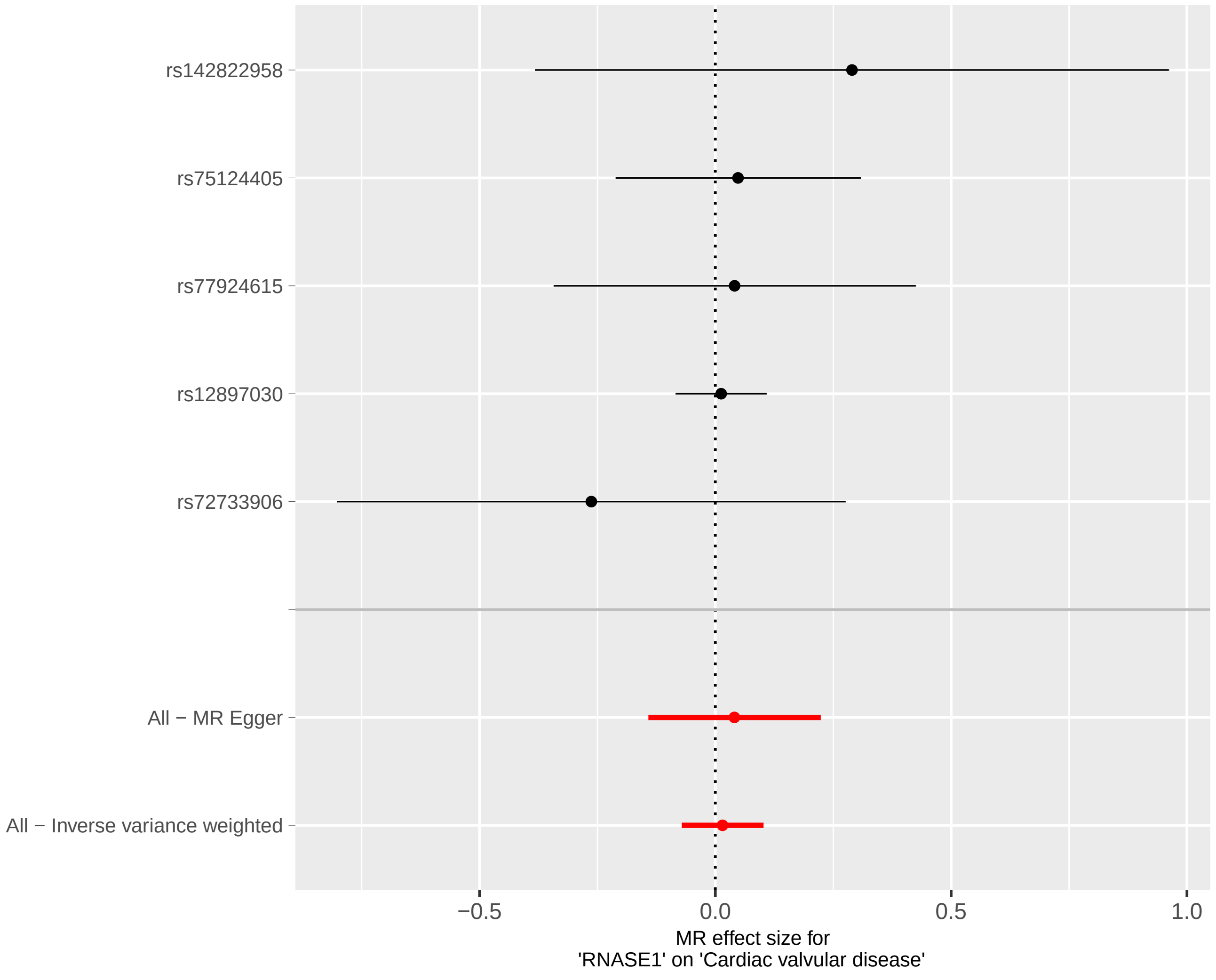

rs142822958
rs75124405
rs77924615
rs12897030
rs72733906
All − MR Egger
All − Inverse variance weighted
−0.5
0.0
0.5
1.0
MR effect size for
'RNASE1' on 'Cardiac valvular disease'

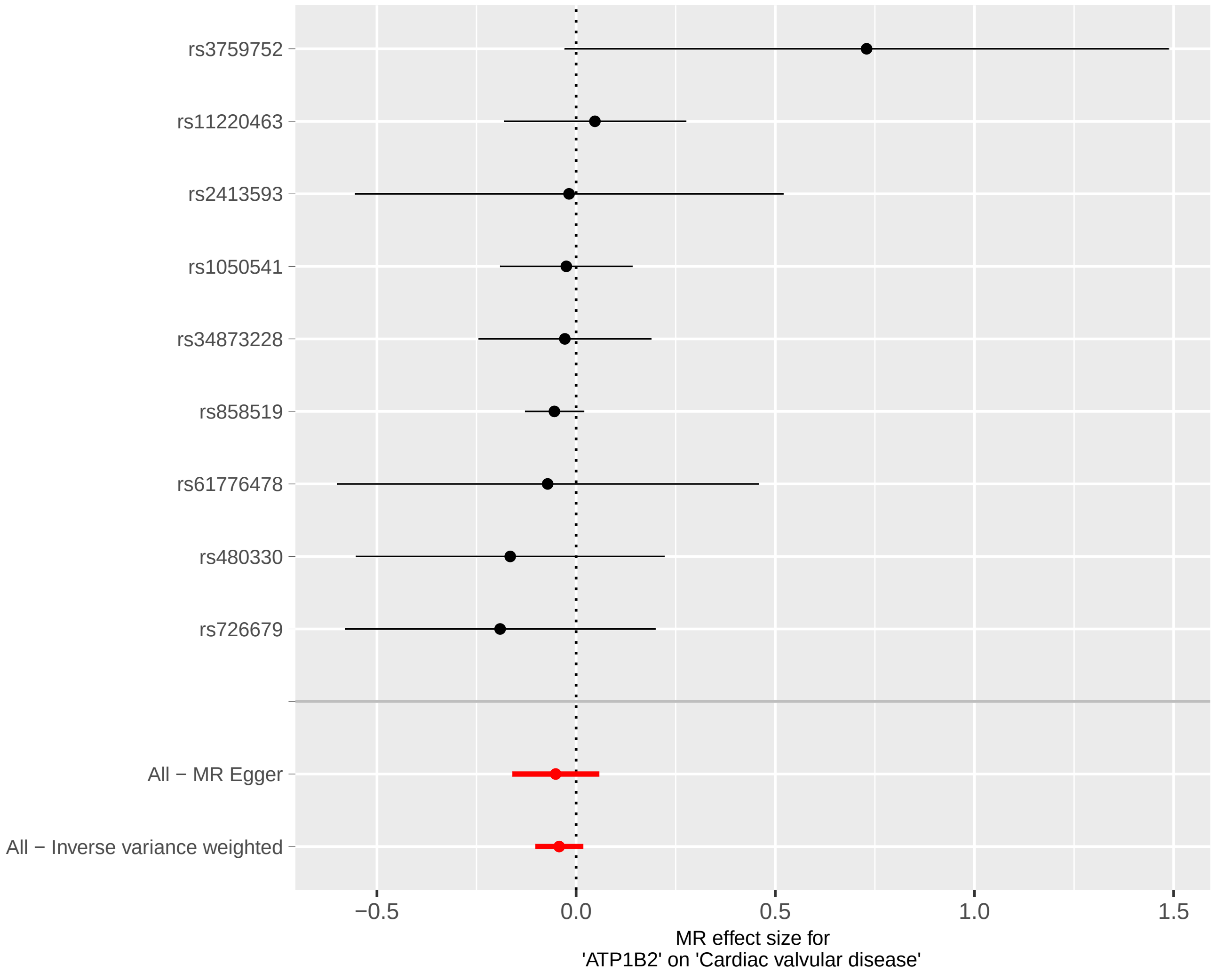
rs3759752
rs11220463
rs2413593
rs1050541
rs34873228
rs858519
rs61776478
rs480330
rs726679
All − MR Egger
All − Inverse variance weighted
−0.5
0.0
0.5
1.0
1.5
MR effect size for
'ATP1B2' on 'Cardiac valvular disease'

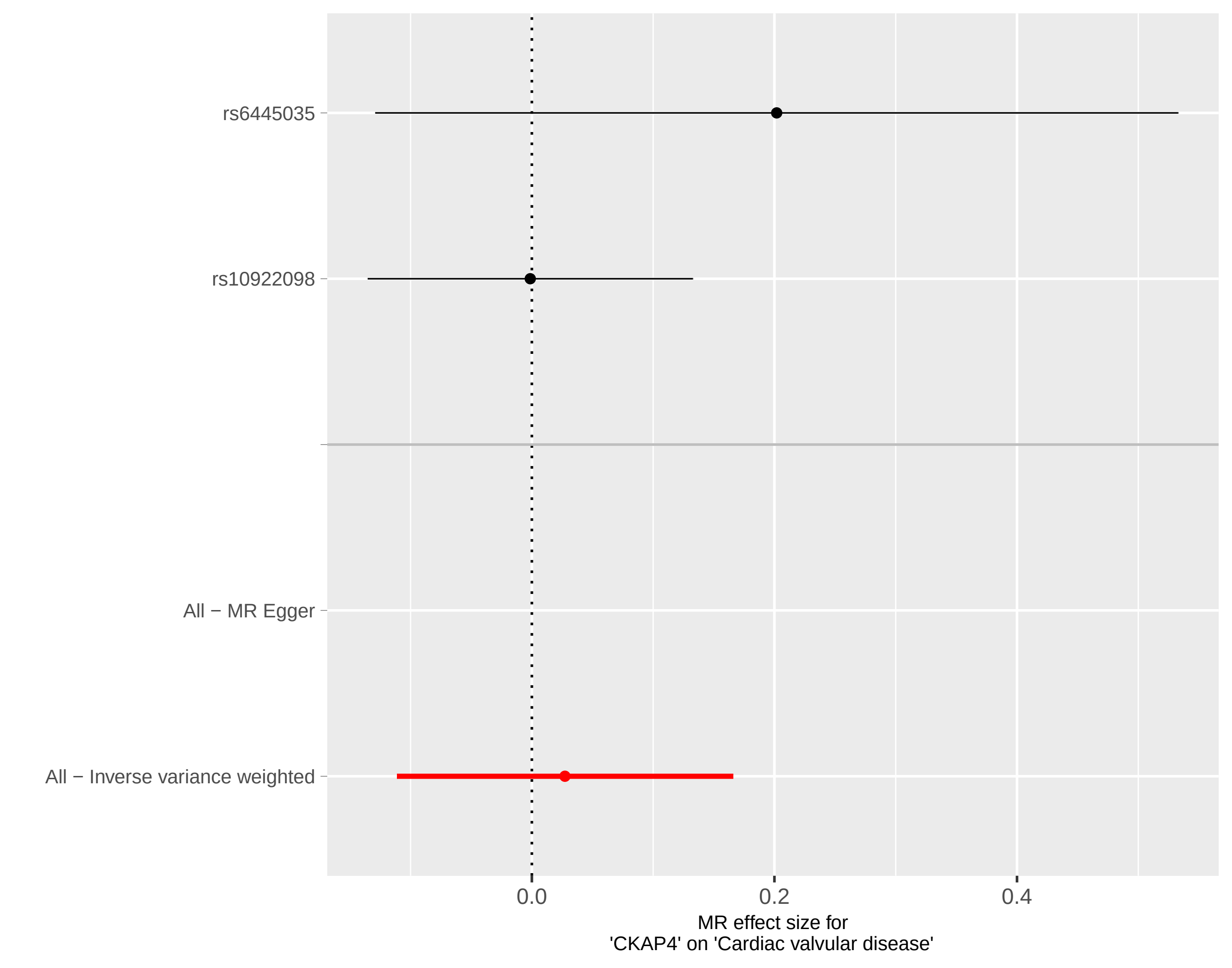
rs6445035
rs10922098
All − MR Egger
All − Inverse variance weighted
0.0
0.2
0.4
MR effect size for
'CKAP4' on 'Cardiac valvular disease'

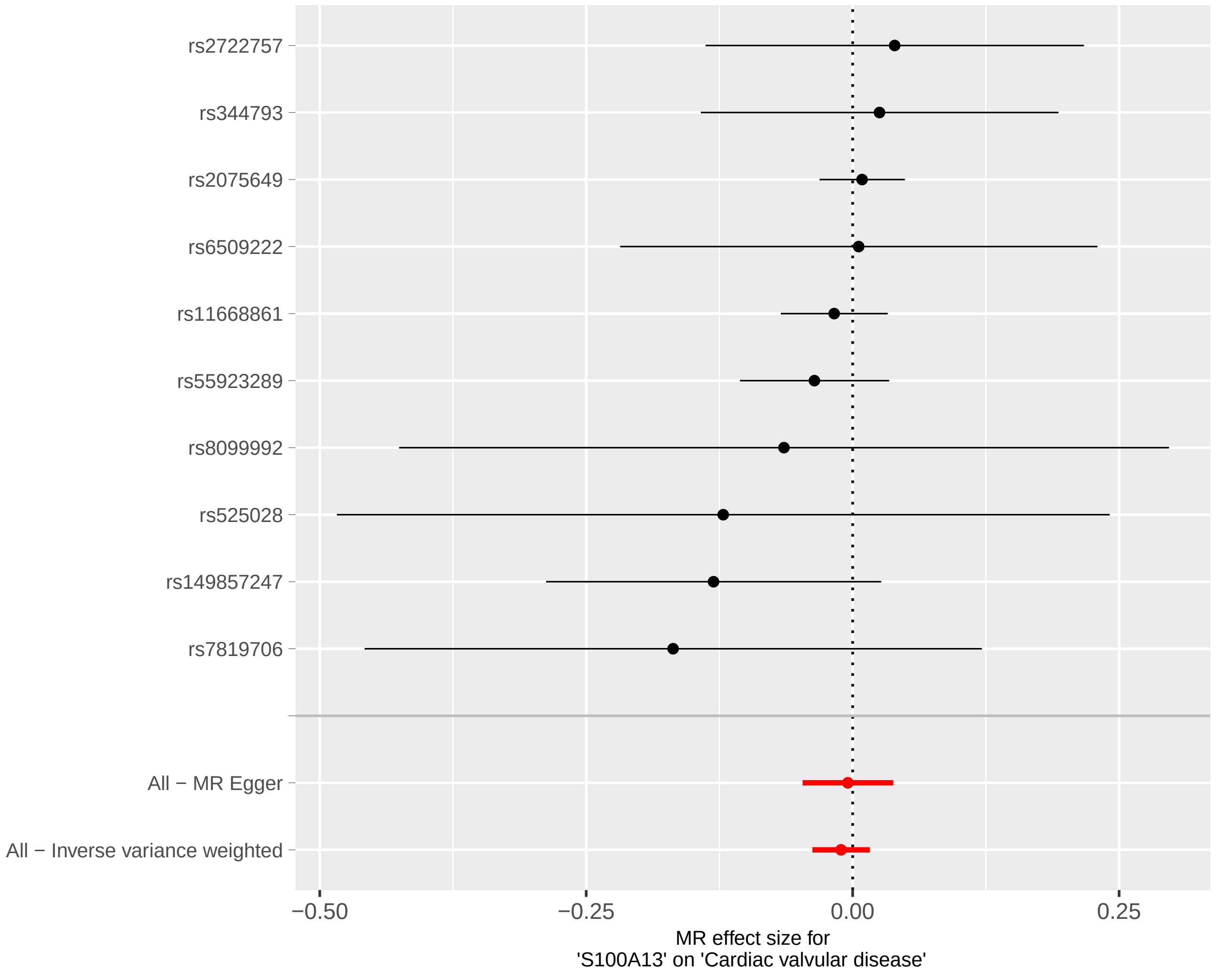

rs2722757
rs344793
rs2075649
rs6509222
rs11668861
rs55923289
rs8099992
rs525028
rs149857247
rs7819706
All − MR Egger
All − Inverse variance weighted
−0.50
−0.25
0.00
0.25
MR effect size for
'S100A13' on 'Cardiac valvular disease'

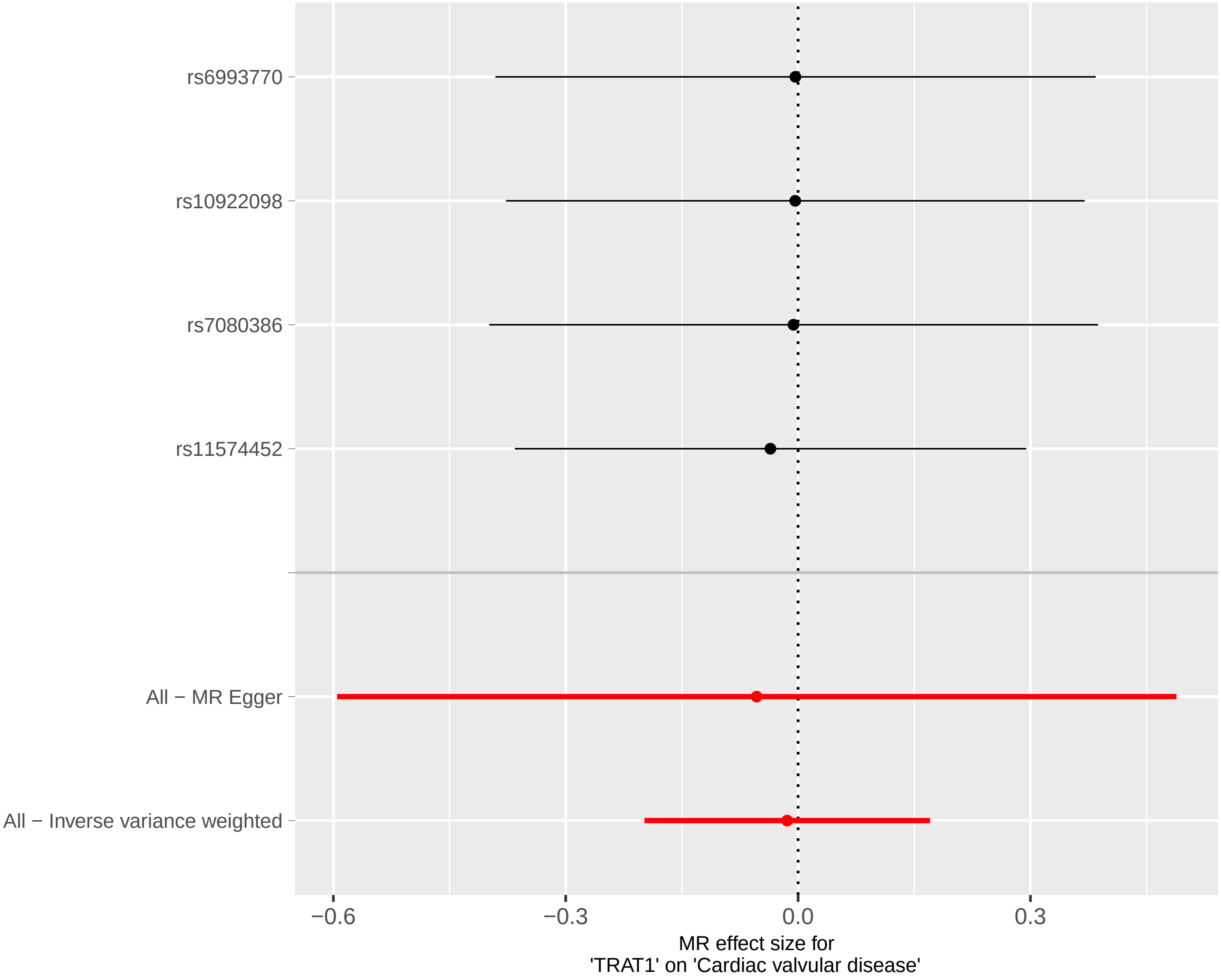
rs6993770
rs10922098
rs7080386
rs11574452
All − MR Egger
All − Inverse variance weighted
−0.6
−0.3
0.0
0.3
MR effect size for
'TRAT1' on 'Cardiac valvular disease'

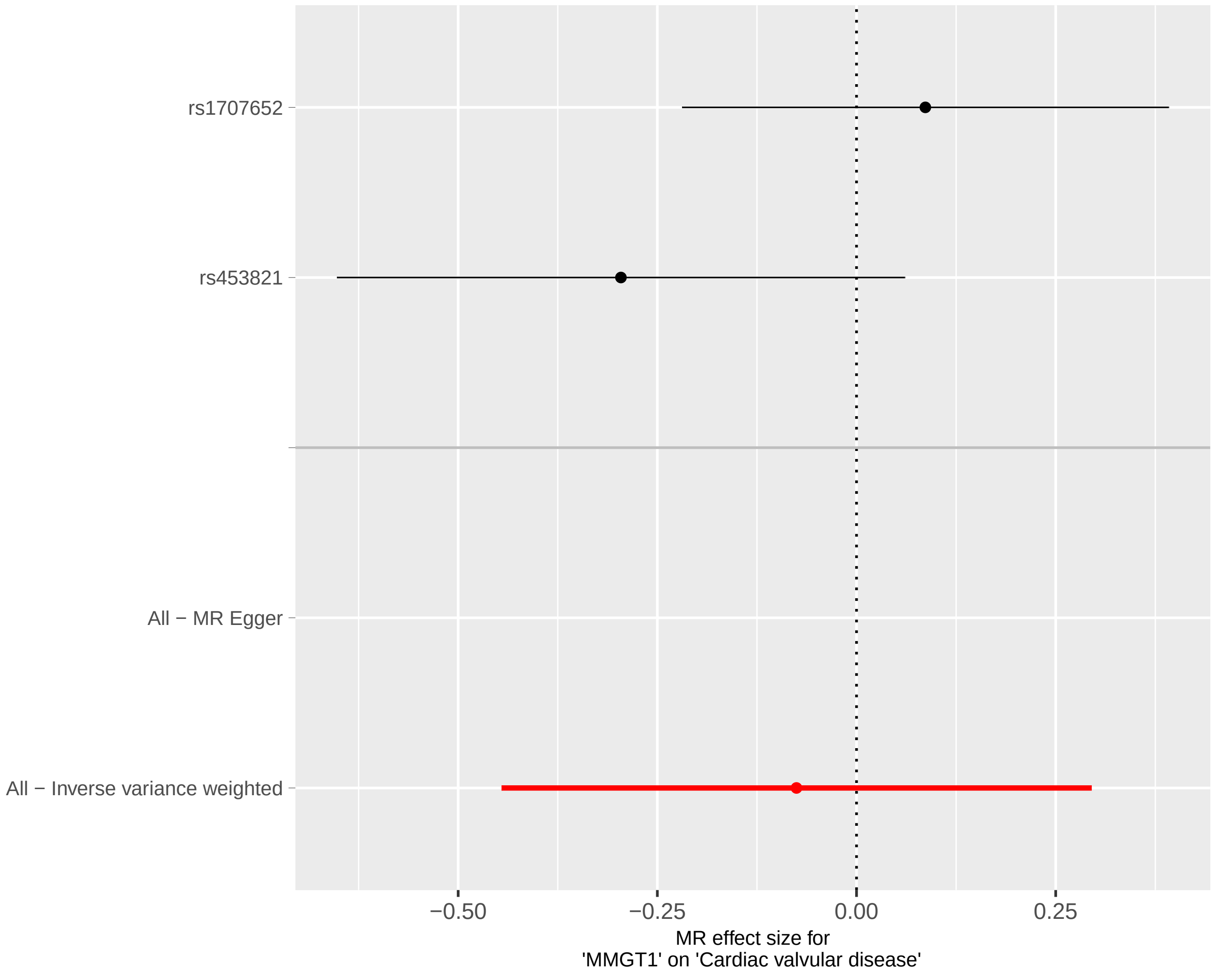
rs1707652
rs453821
All − MR Egger
All − Inverse variance weighted
−0.50
−0.25
0.00
0.25
MR effect size for
'MMGT1' on 'Cardiac valvular disease'

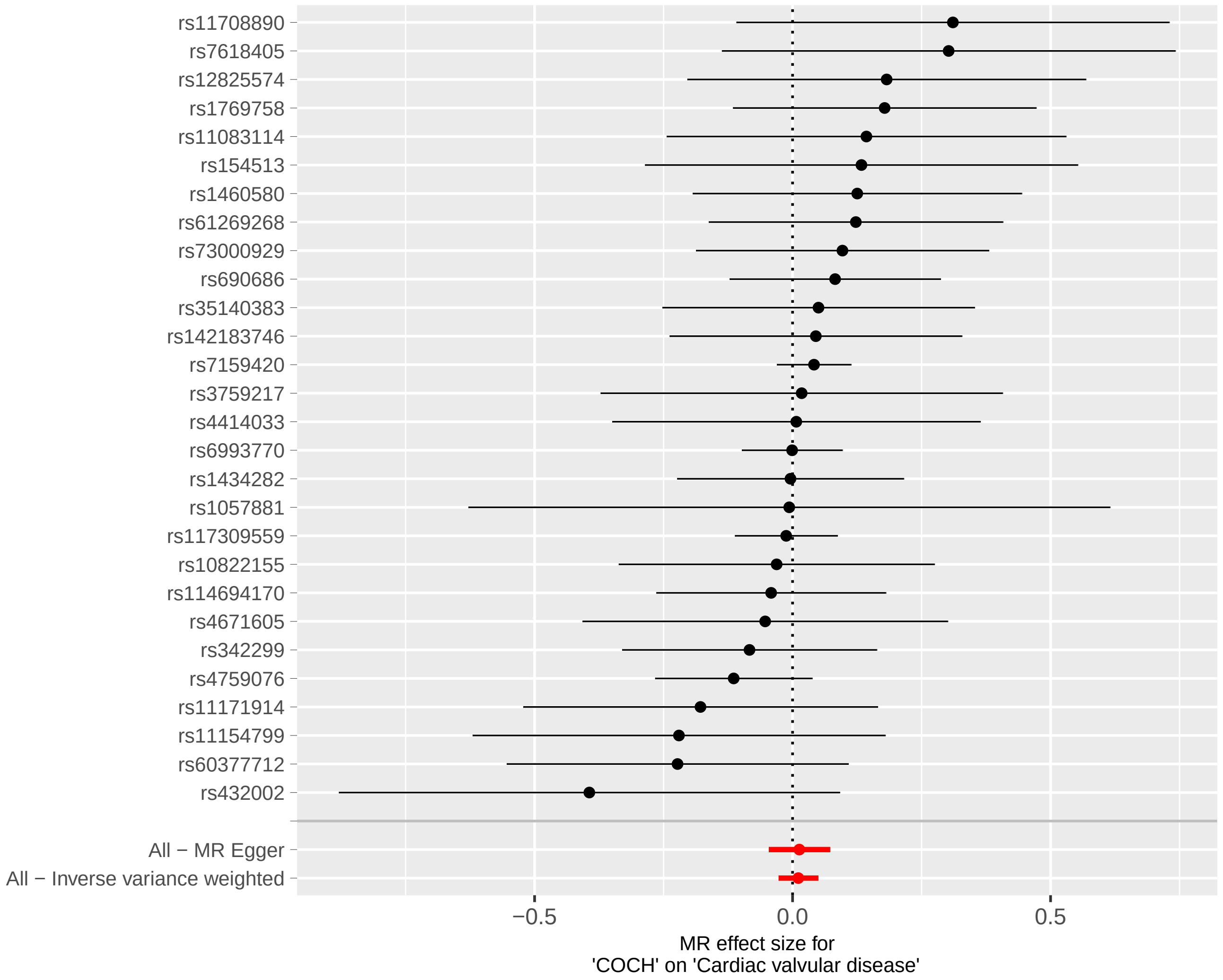

rs11708890
rs7618405
rs12825574
rs1769758
rs11083114
rs154513
rs1460580
rs61269268
rs73000929
rs690686
rs35140383
rs142183746
rs7159420
rs3759217
rs4414033
rs6993770
rs1434282
rs1057881
rs117309559
rs10822155
rs114694170
rs4671605
rs342299
rs4759076
rs11171914
rs11154799
rs60377712
rs432002
All − MR Egger
All − Inverse variance weighted
−0.5
0.0
0.5
MR effect size for
'COCH' on 'Cardiac valvular disease'

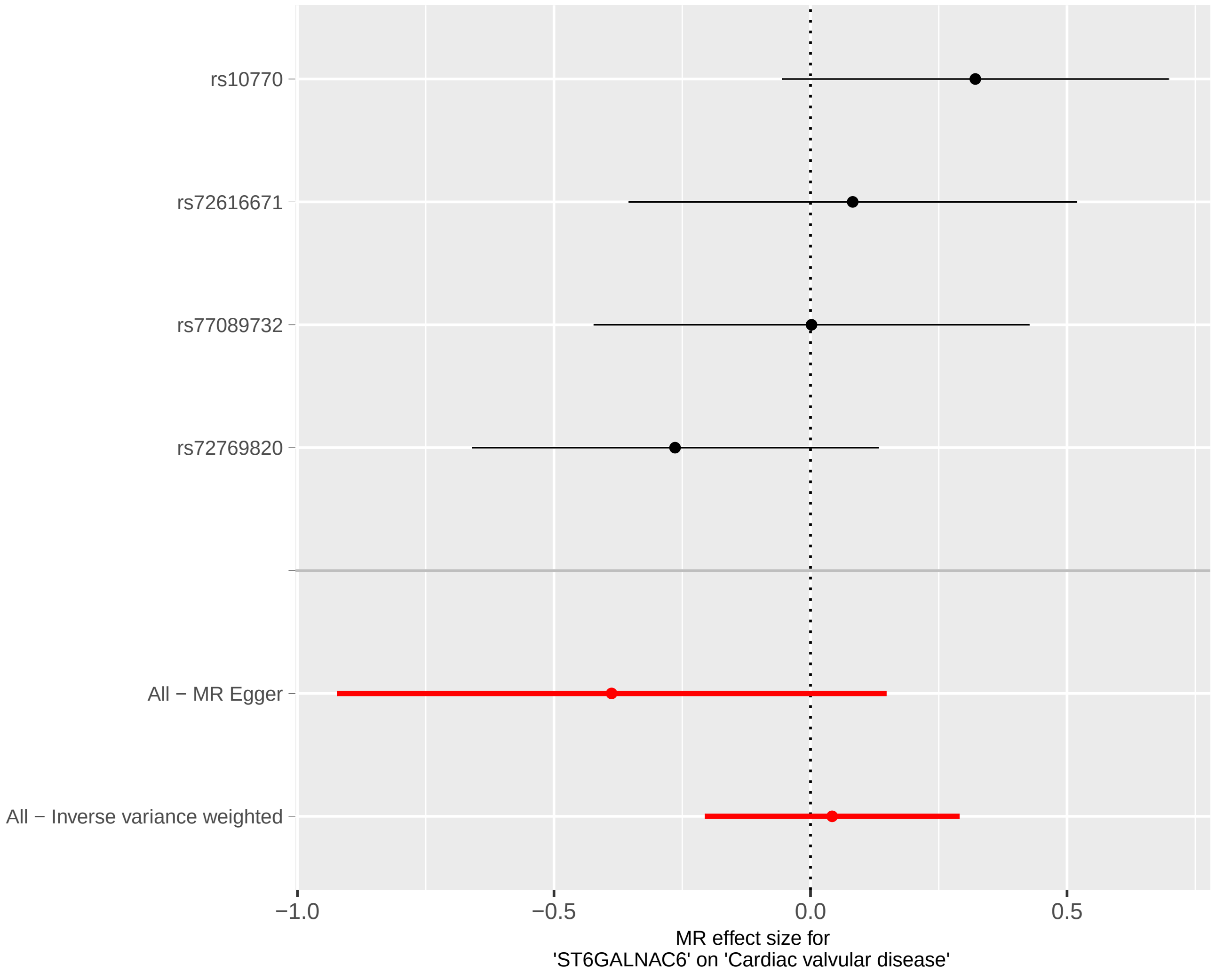
rs10770
rs72616671
rs77089732
rs72769820
All − MR Egger
All − Inverse variance weighted
−1.0
−0.5
0.0
0.5
MR effect size for
'ST6GALNAC6' on 'Cardiac valvular disease'

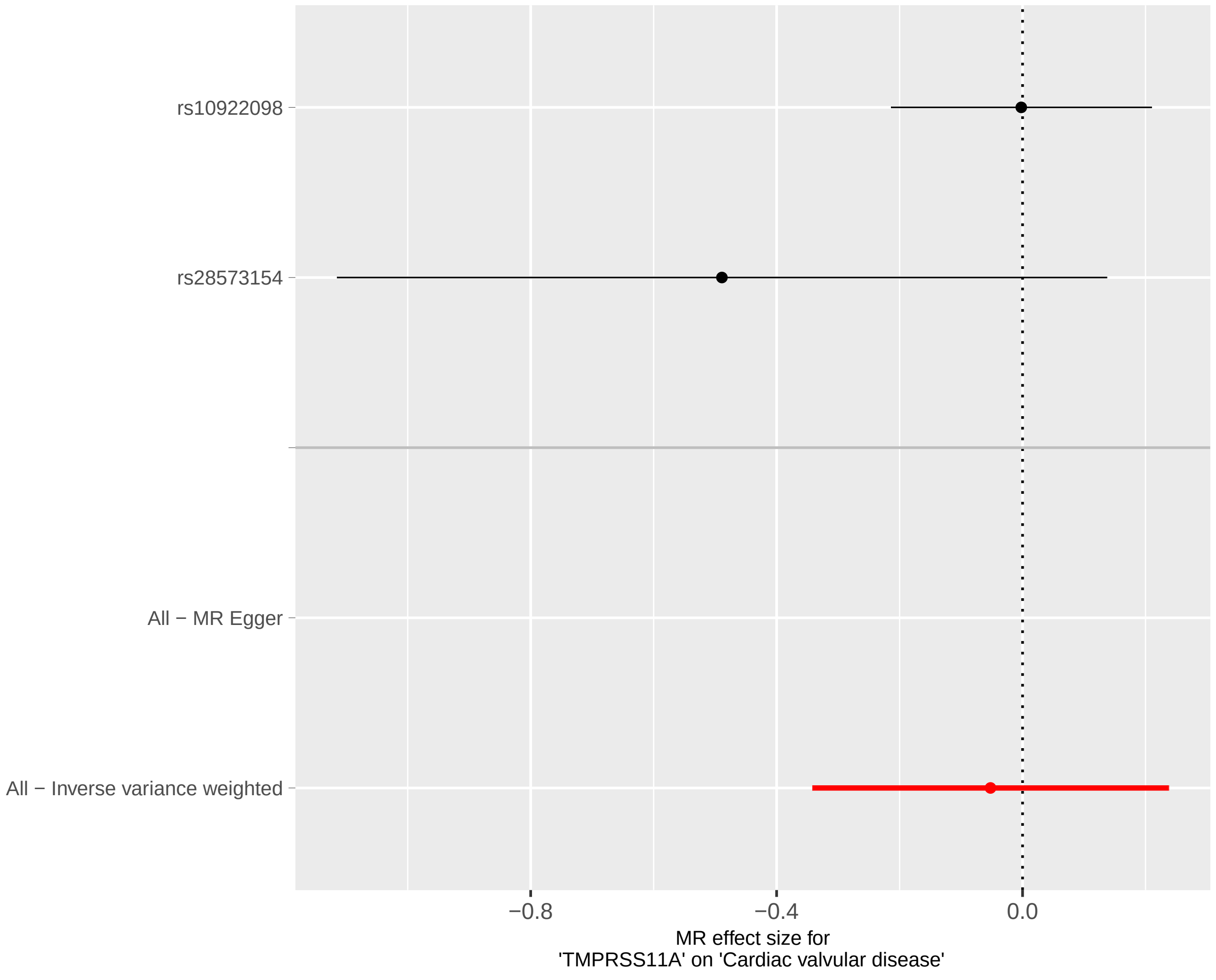
rs10922098
rs28573154
All − MR Egger
All − Inverse variance weighted
−0.8
−0.4
0.0
MR effect size for
'TMPRSS11A' on 'Cardiac valvular disease'

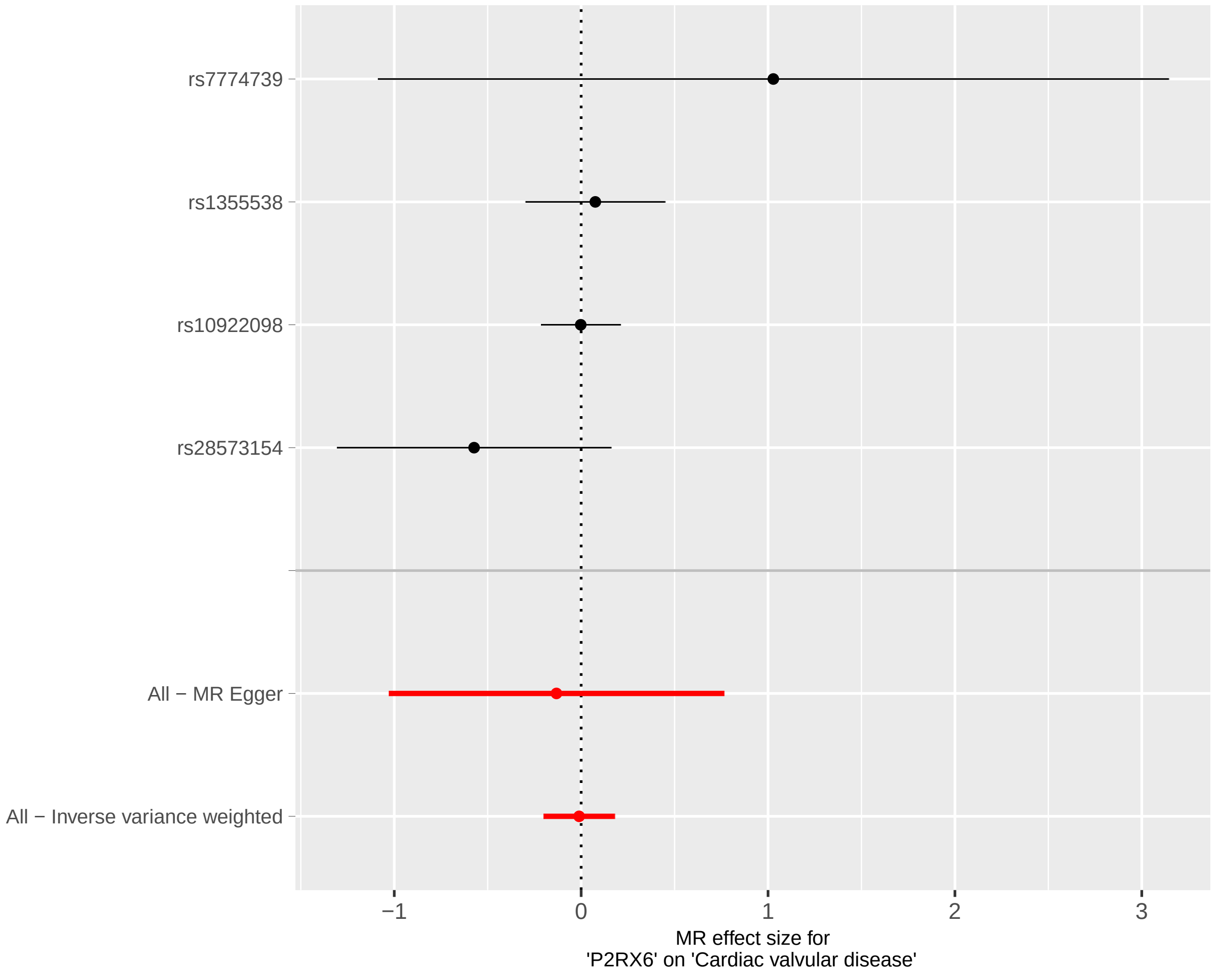

rs7774739
rs1355538
rs10922098
rs28573154
All − MR Egger
All − Inverse variance weighted
−1
0
1
2
3
MR effect size for
'P2RX6' on 'Cardiac valvular disease'

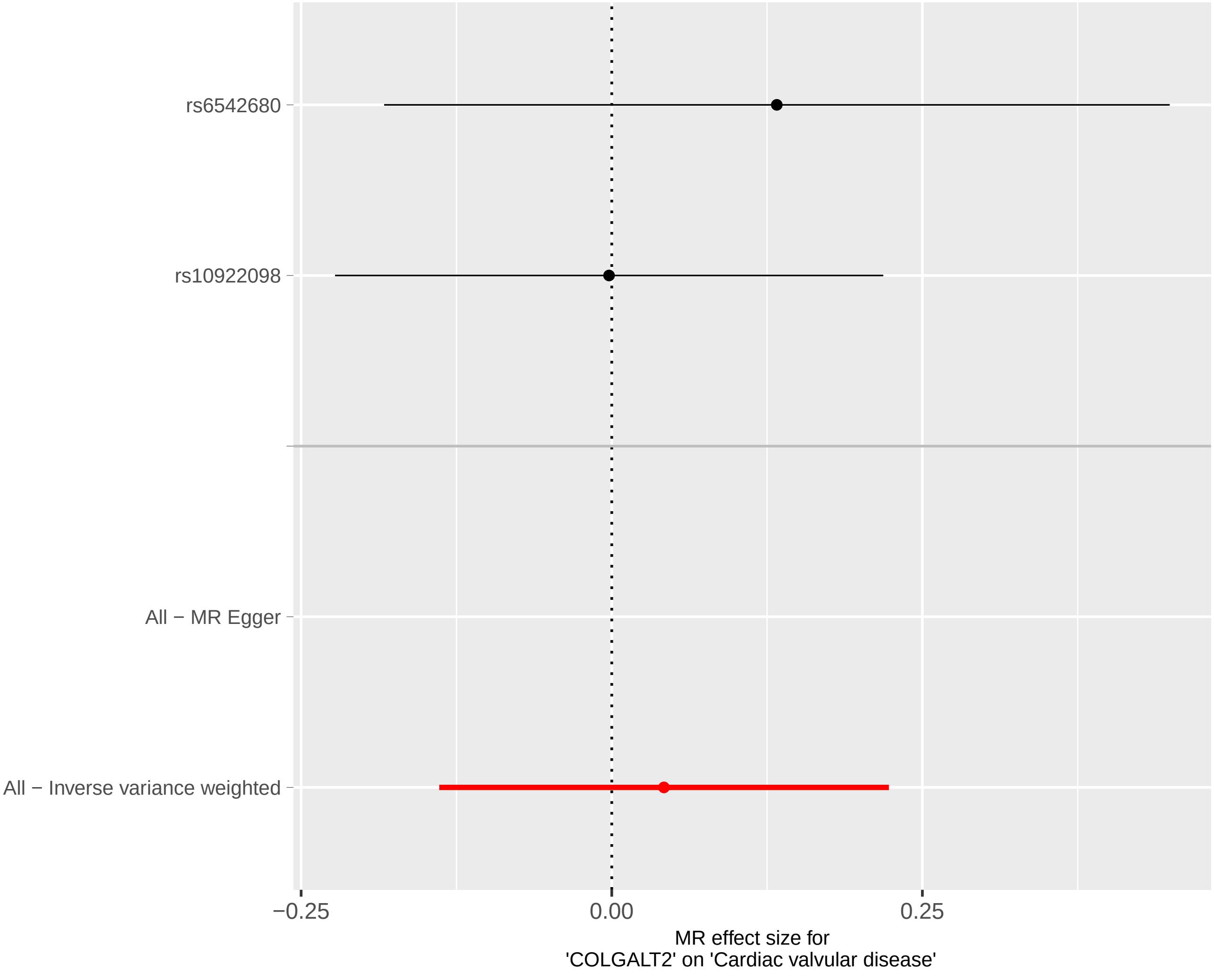
rs6542680
rs10922098
All − MR Egger
All − Inverse variance weighted
−0.25
0.00
0.25
MR effect size for
'COLGALT2' on 'Cardiac valvular disease'

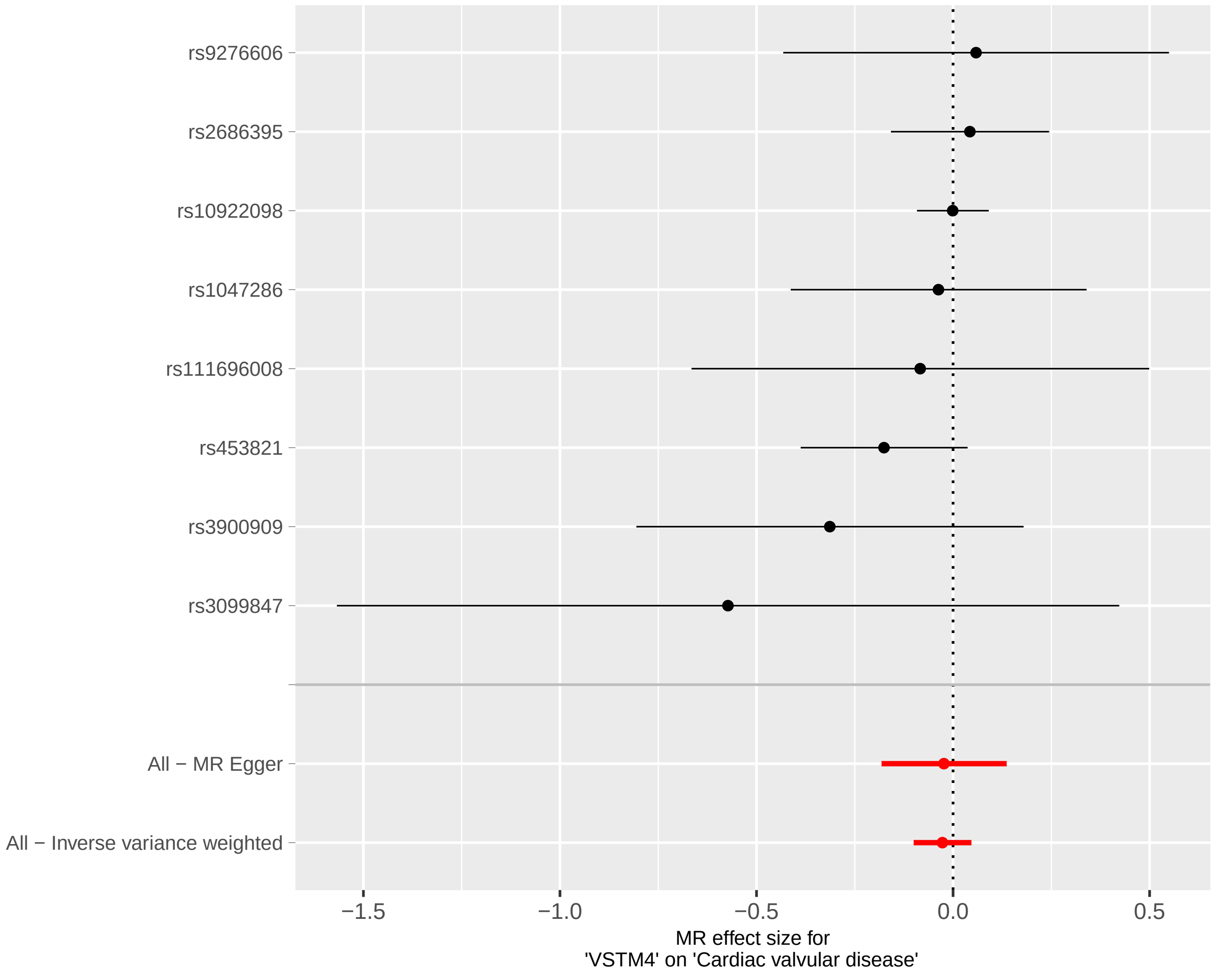

rs9276606
rs2686395
rs10922098
rs1047286
rs111696008
rs453821
rs3900909
rs3099847
All − MR Egger
All − Inverse variance weighted
−1.5
−1.0
−0.5
0.0
0.5
MR effect size for
'VSTM4' on 'Cardiac valvular disease'

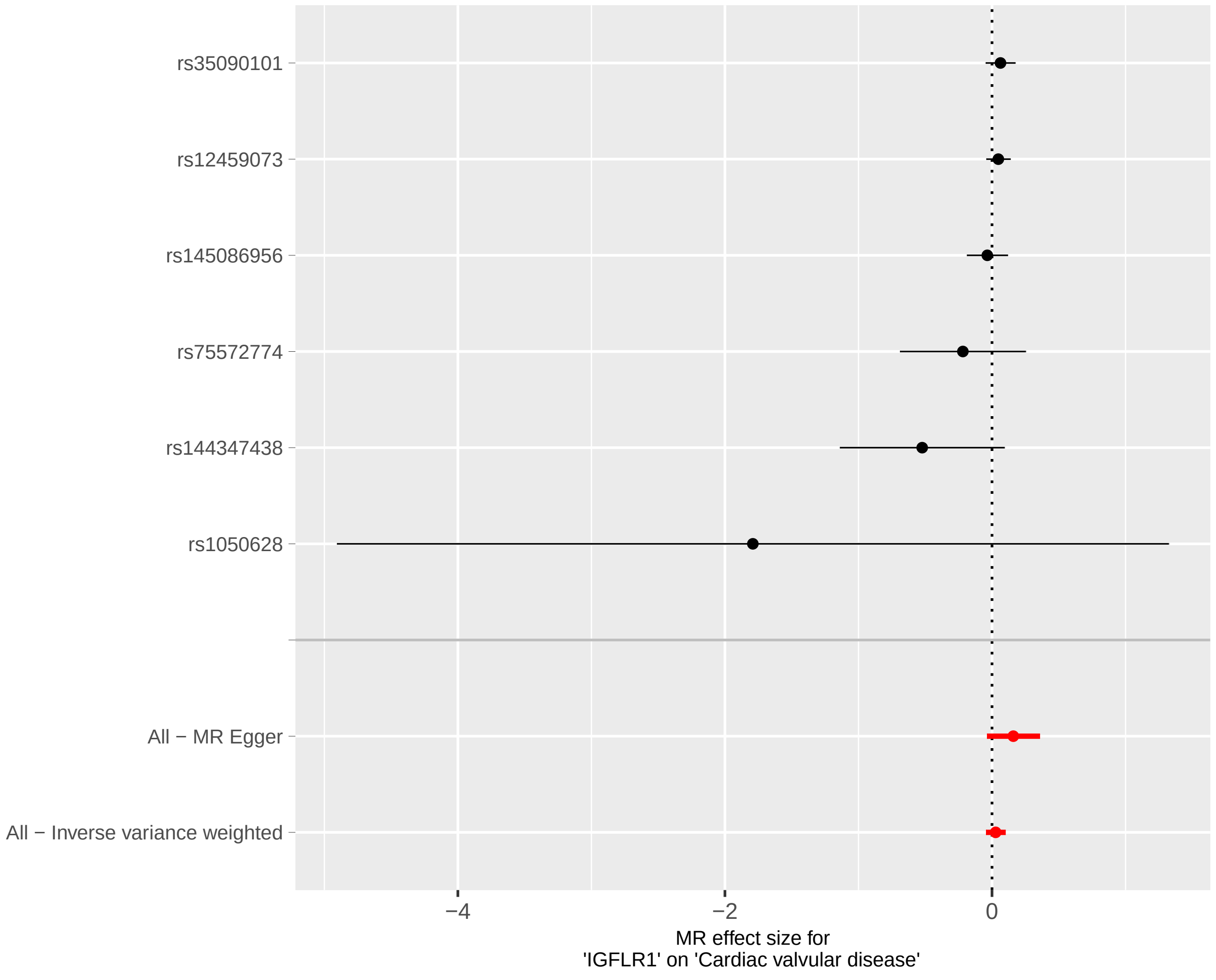

rs35090101
rs12459073
rs145086956
rs75572774
rs144347438
rs1050628
All − MR Egger
All − Inverse variance weighted
−4
−2
0
MR effect size for
'IGFLR1' on 'Cardiac valvular disease'

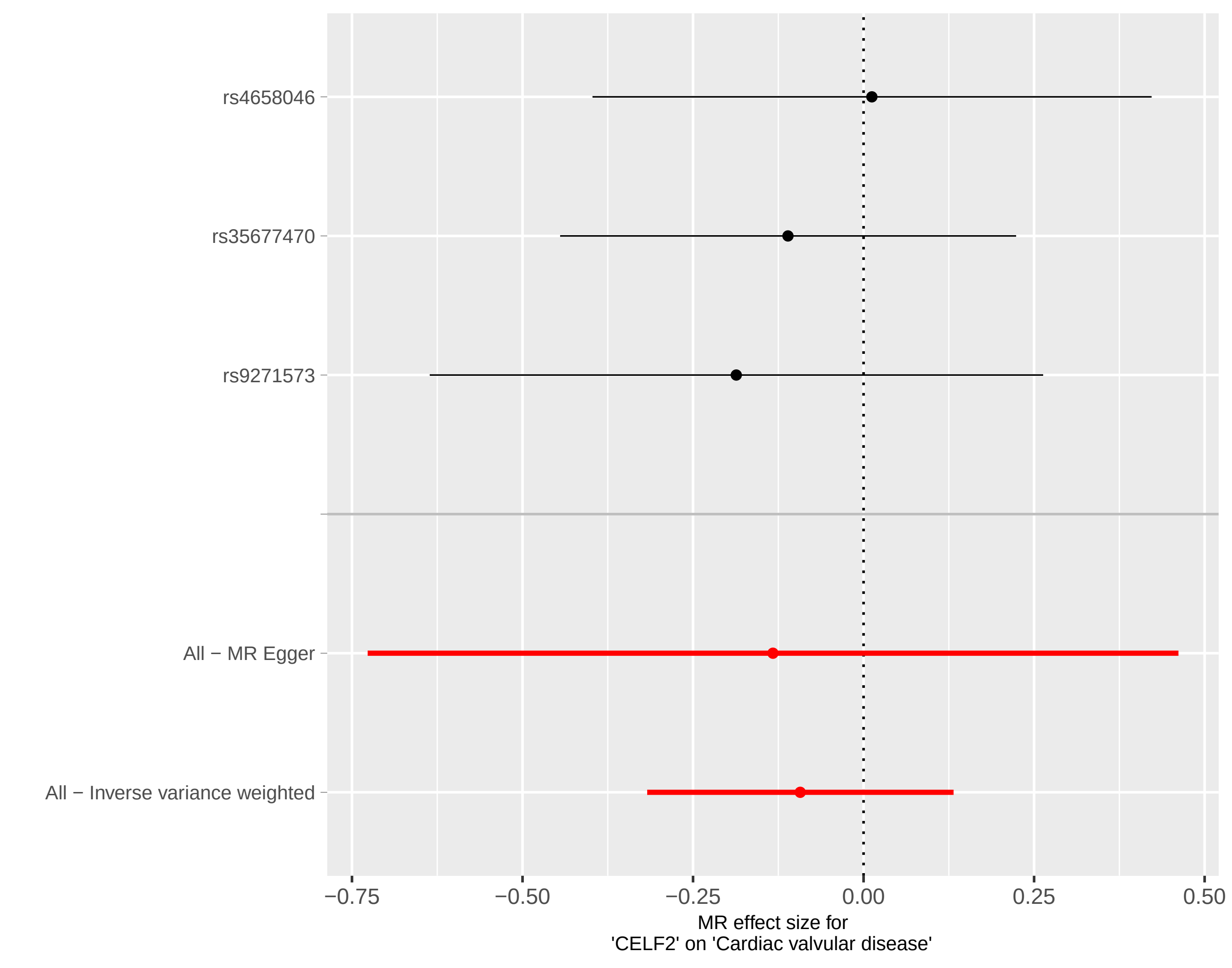
rs4658046
rs35677470
rs9271573
All − MR Egger
All − Inverse variance weighted
−0.75
−0.50
−0.25
0.00
0.25
0.50
MR effect size for
'CELF2' on 'Cardiac valvular disease'

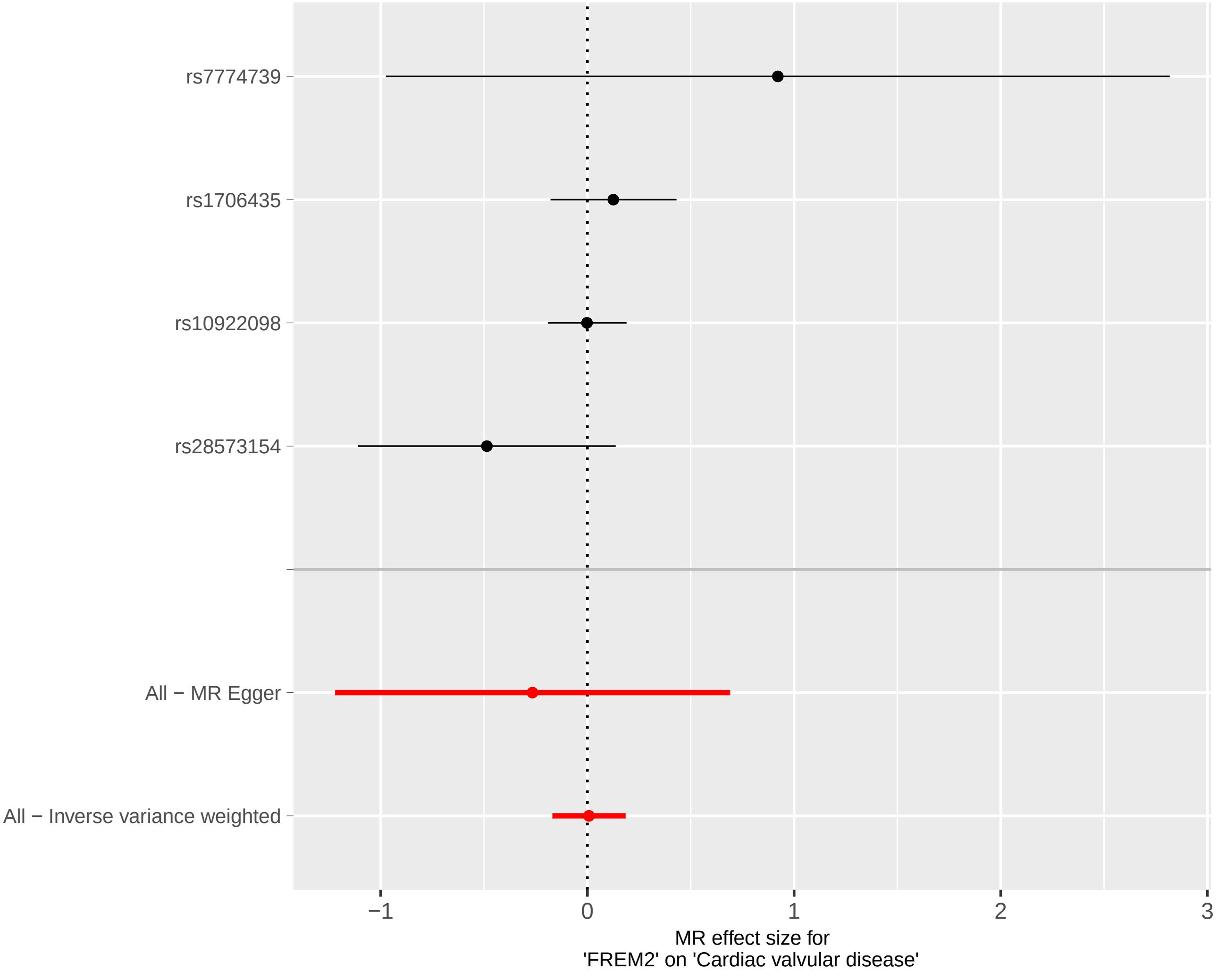
rs7774739
rs1706435
rs10922098
rs28573154
All − MR Egger
All − Inverse variance weighted
−1
0
1
2
3
MR effect size for
'FREM2' on 'Cardiac valvular disease'

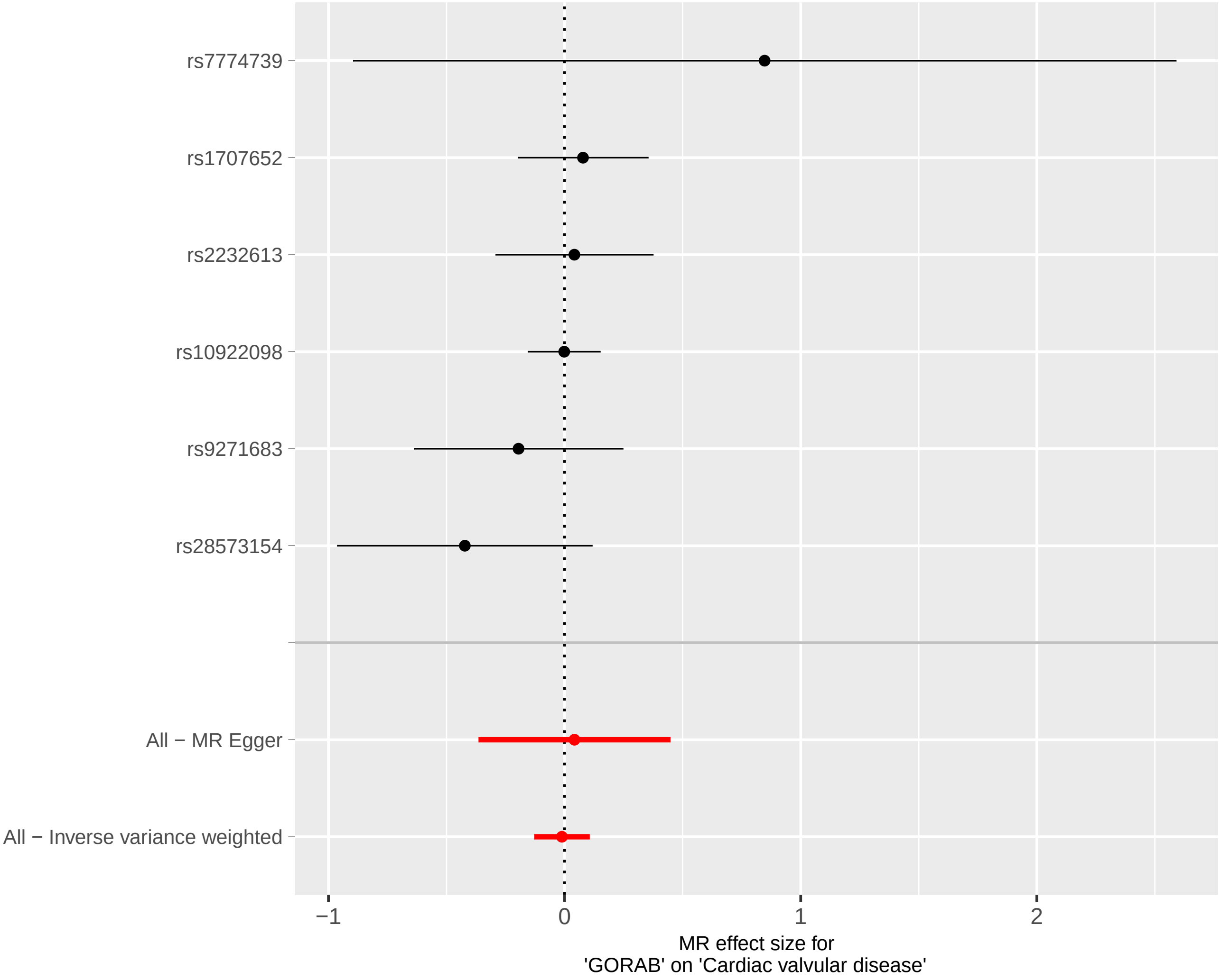
rs7774739
rs1707652
rs2232613
rs10922098
rs9271683
rs28573154
All − MR Egger
All − Inverse variance weighted
−1
0
1
2
MR effect size for
'GORAB' on 'Cardiac valvular disease'

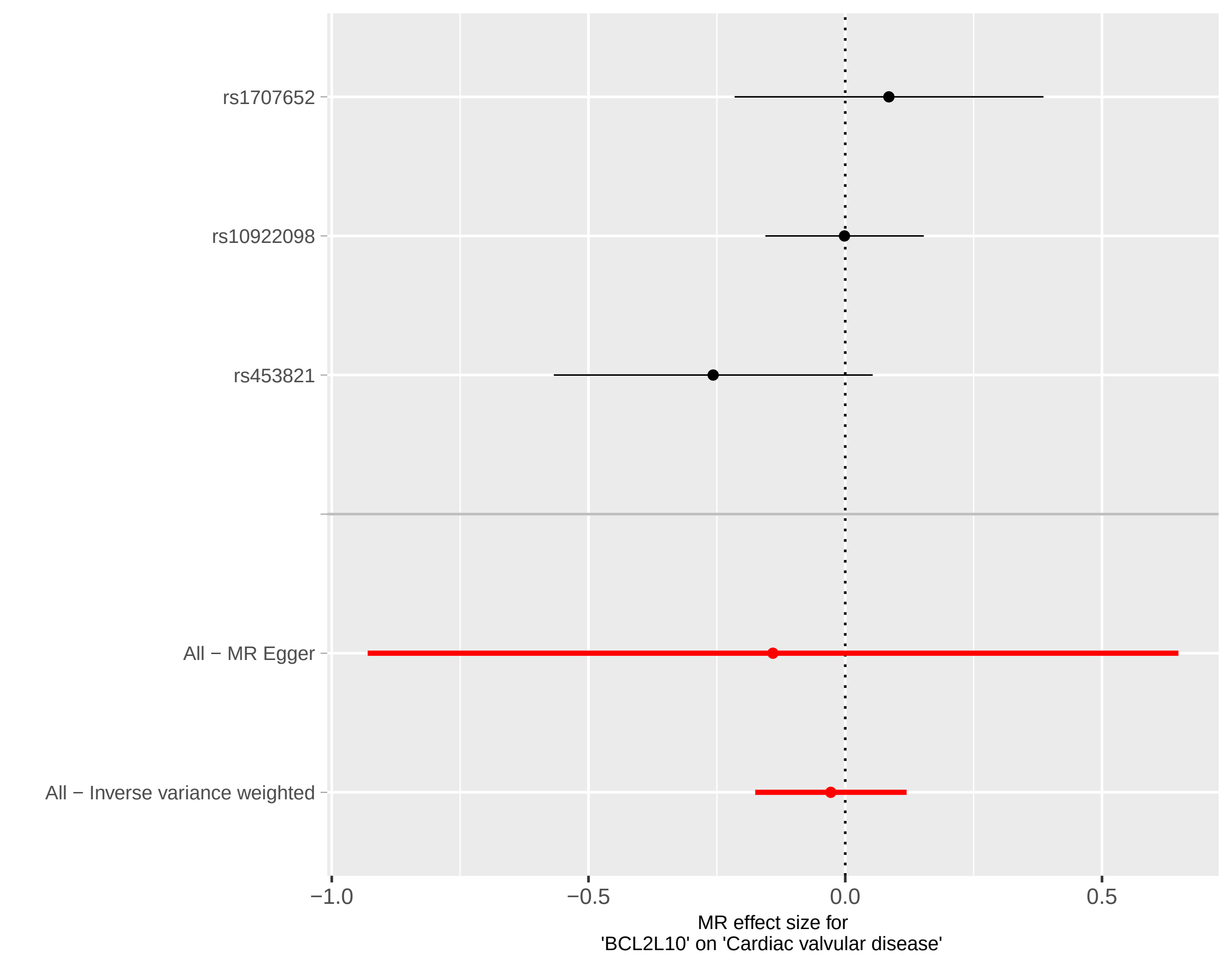
rs1707652
rs10922098
rs453821
All − MR Egger
All − Inverse variance weighted
−1.0
−0.5
0.0
0.5
MR effect size for
'BCL2L10' on 'Cardiac valvular disease'

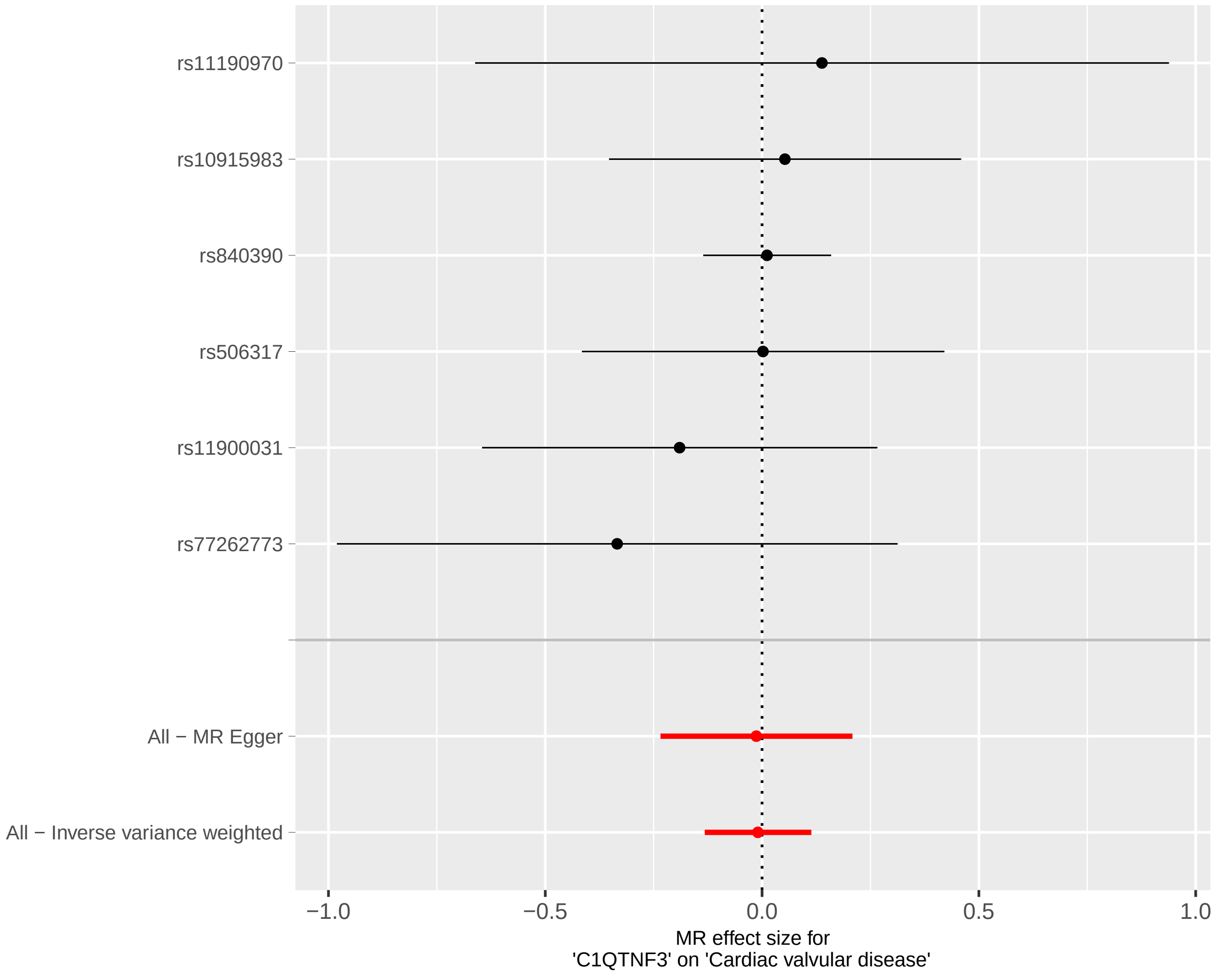
rs11190970
rs10915983
rs840390
rs506317
rs11900031
rs77262773
All − MR Egger
All − Inverse variance weighted
−1.0
−0.5
0.0
0.5
1.0
MR effect size for
'C1QTNF3' on 'Cardiac valvular disease'

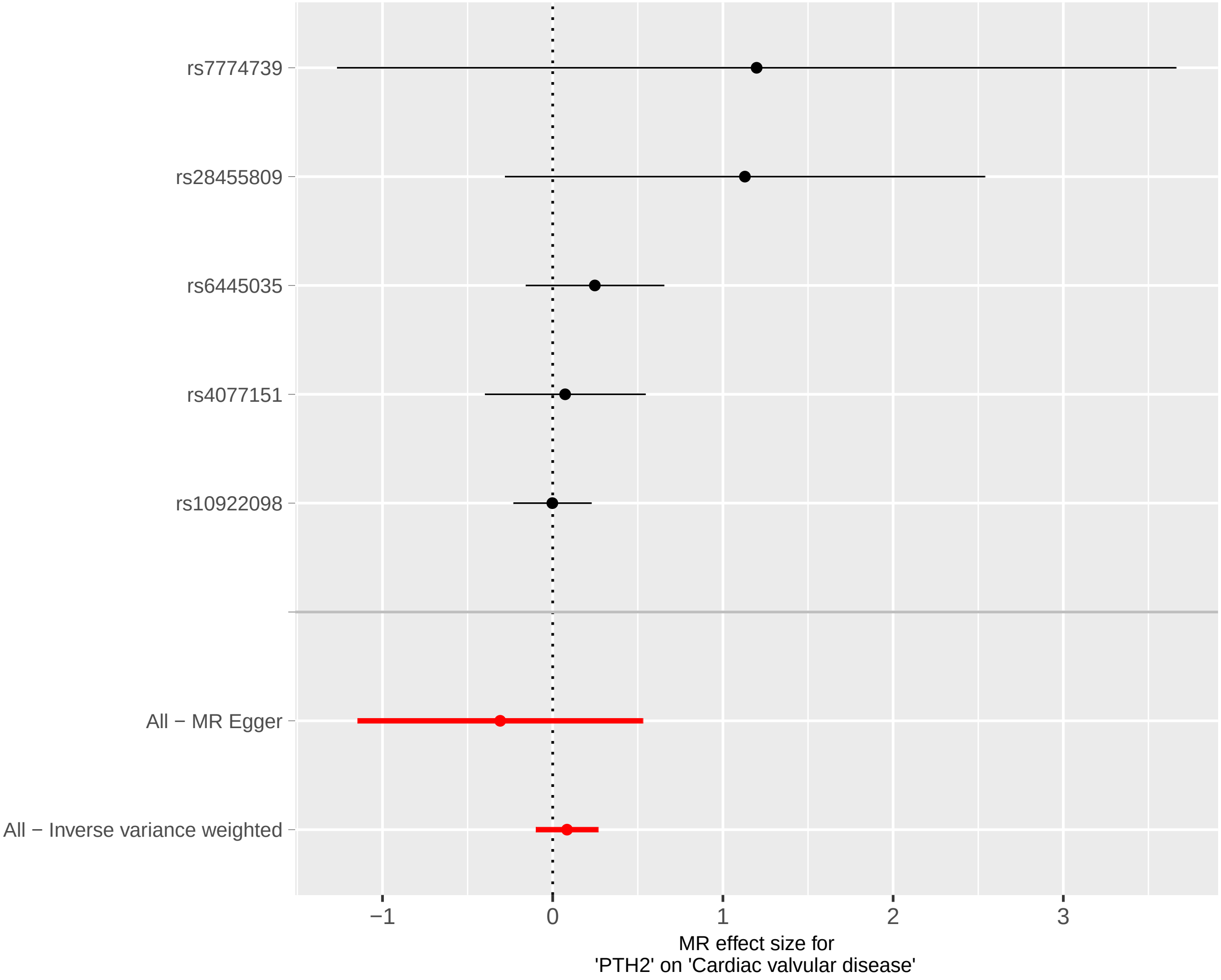
rs7774739
rs28455809
rs6445035
rs4077151
rs10922098
All − MR Egger
All − Inverse variance weighted
−1
0
1
2
3
MR effect size for
'PTH2' on 'Cardiac valvular disease'

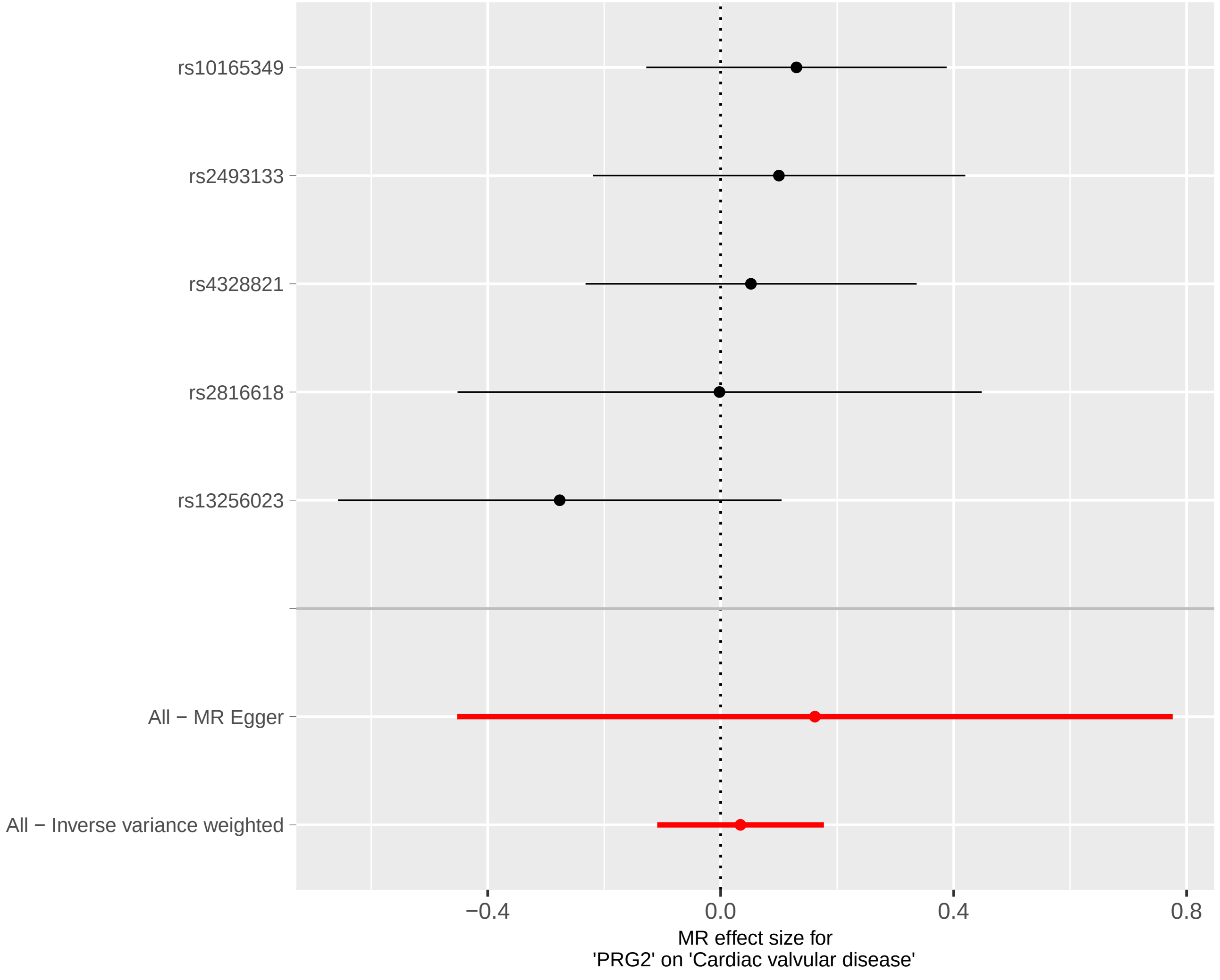

rs10165349
rs2493133
rs4328821
rs2816618
rs13256023
All − MR Egger
All − Inverse variance weighted
−0.4
0.0
0.4
0.8
MR effect size for
'PRG2' on 'Cardiac valvular disease'

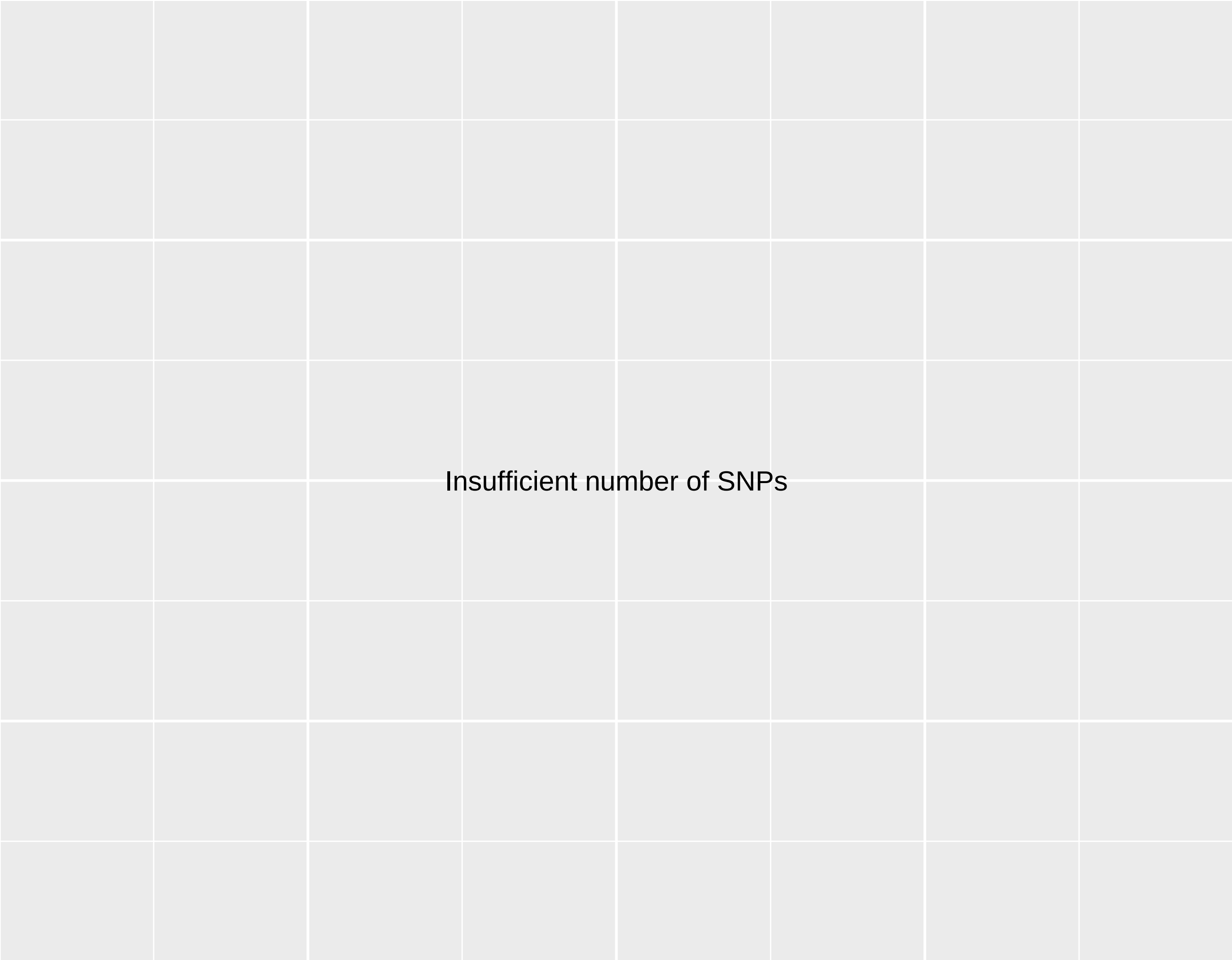
Insufficient number of SNPs

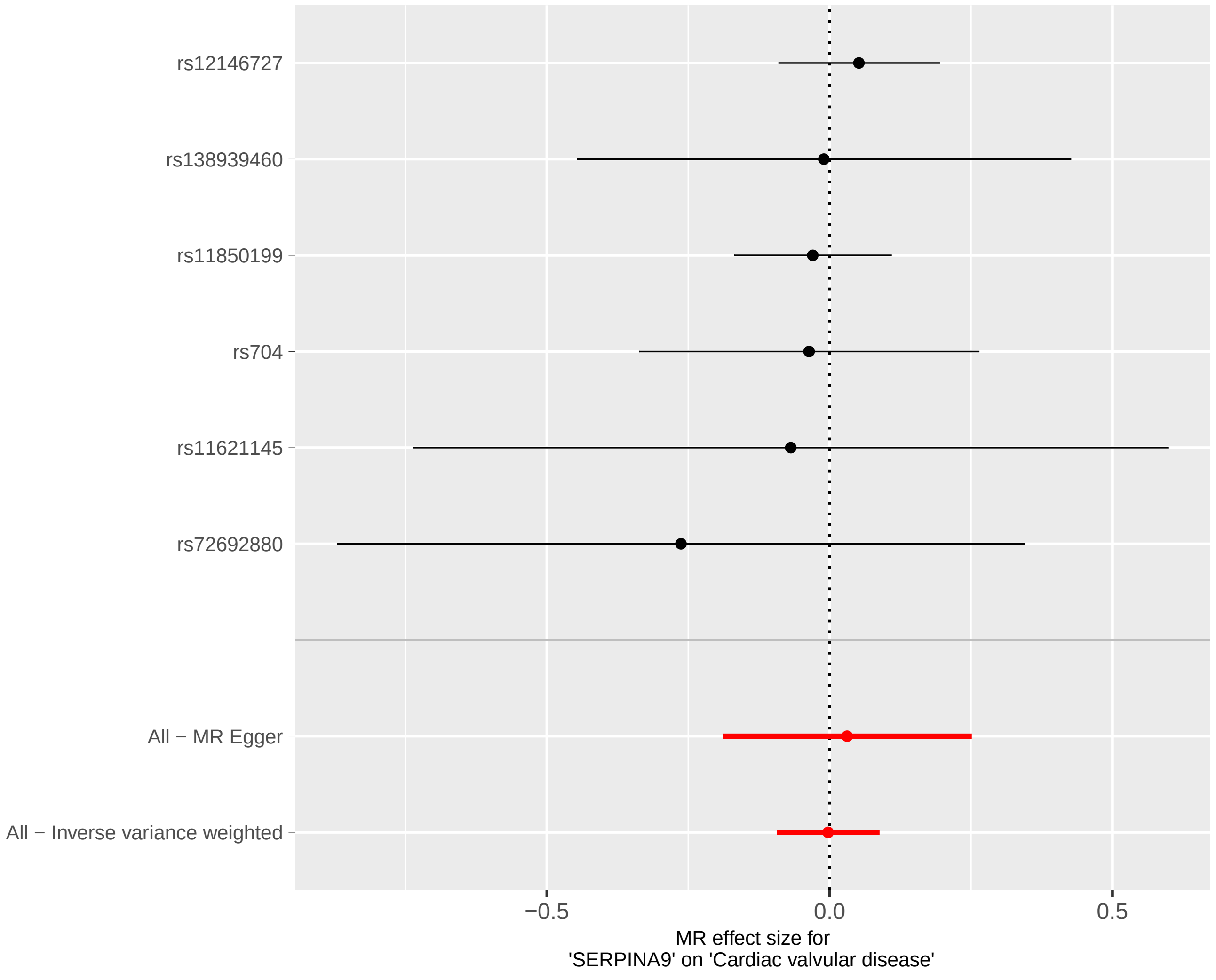

rs12146727
rs138939460
rs11850199
rs704
rs11621145
rs72692880
All − MR Egger
All − Inverse variance weighted
−0.5
0.0
0.5
MR effect size for
'SERPINA9' on 'Cardiac valvular disease'

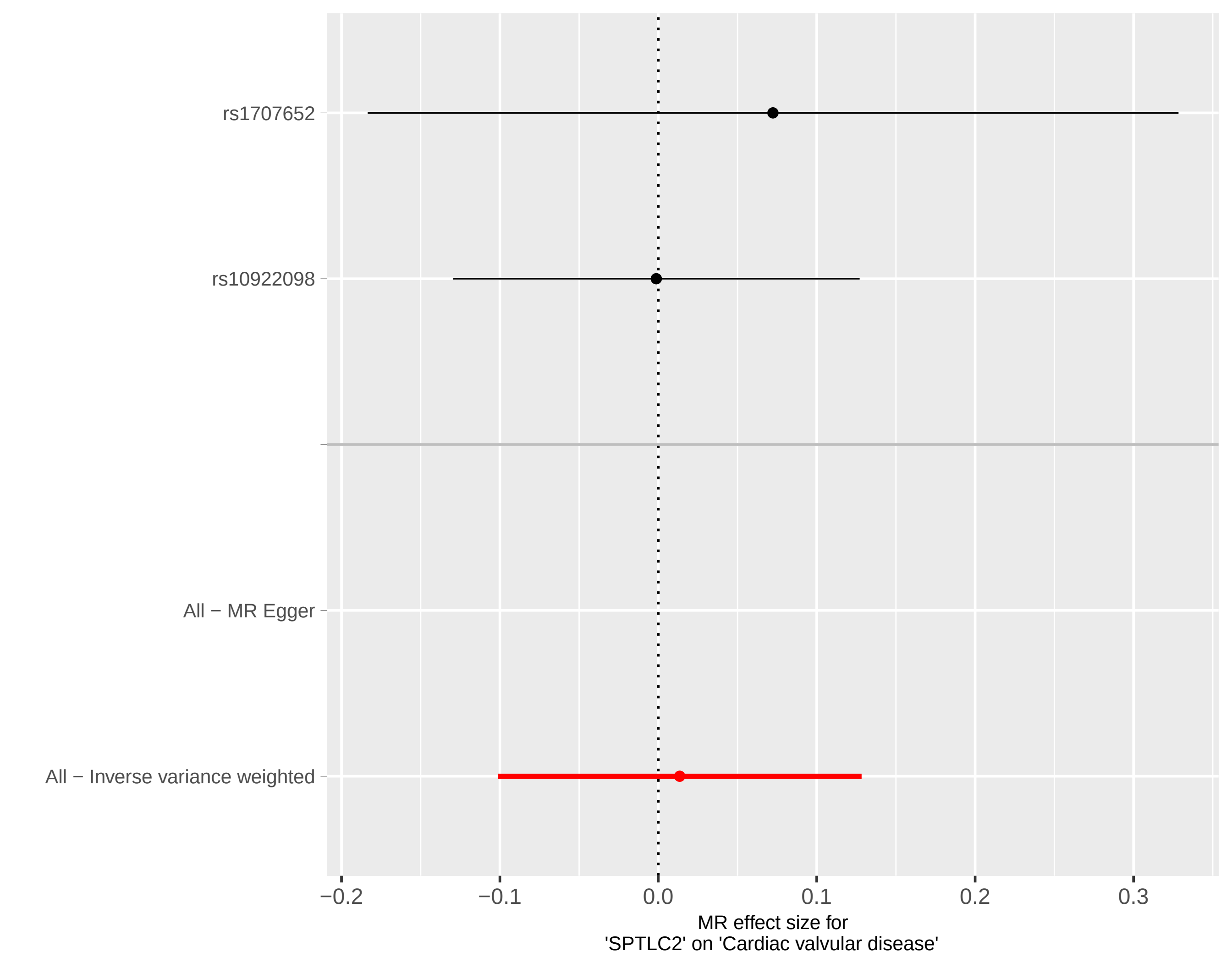

rs1707652
rs10922098
All − MR Egger
All − Inverse variance weighted
−0.2
−0.1
0.0
0.1
0.2
0.3
MR effect size for
'SPTLC2' on 'Cardiac valvular disease'

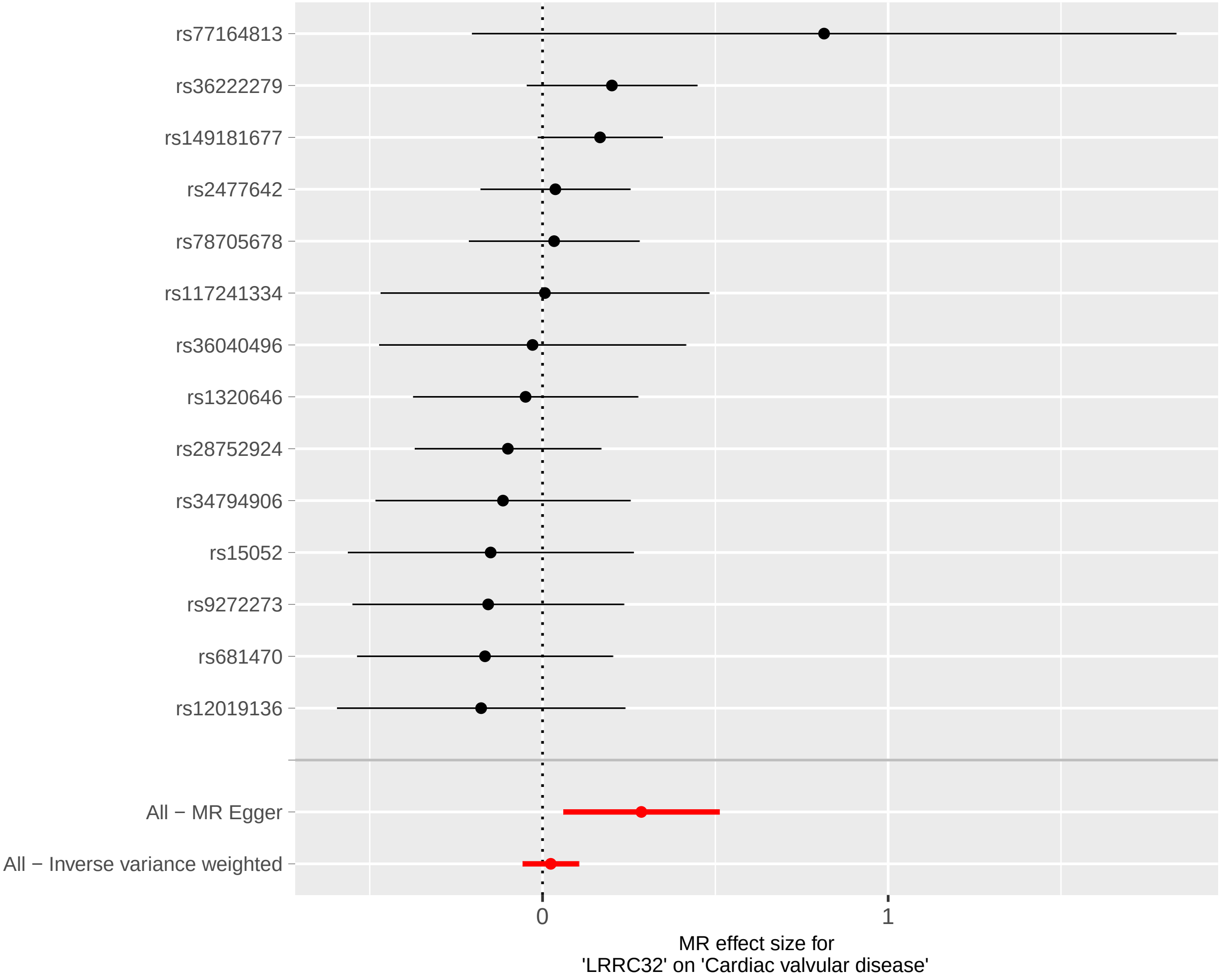
rs77164813
rs36222279
rs149181677
rs2477642
rs78705678
rs117241334
rs36040496
rs1320646
rs28752924
rs34794906
rs15052
rs9272273
rs681470
rs12019136
All − MR Egger
All − Inverse variance weighted
0
1
MR effect size for
'LRRC32' on 'Cardiac valvular disease'

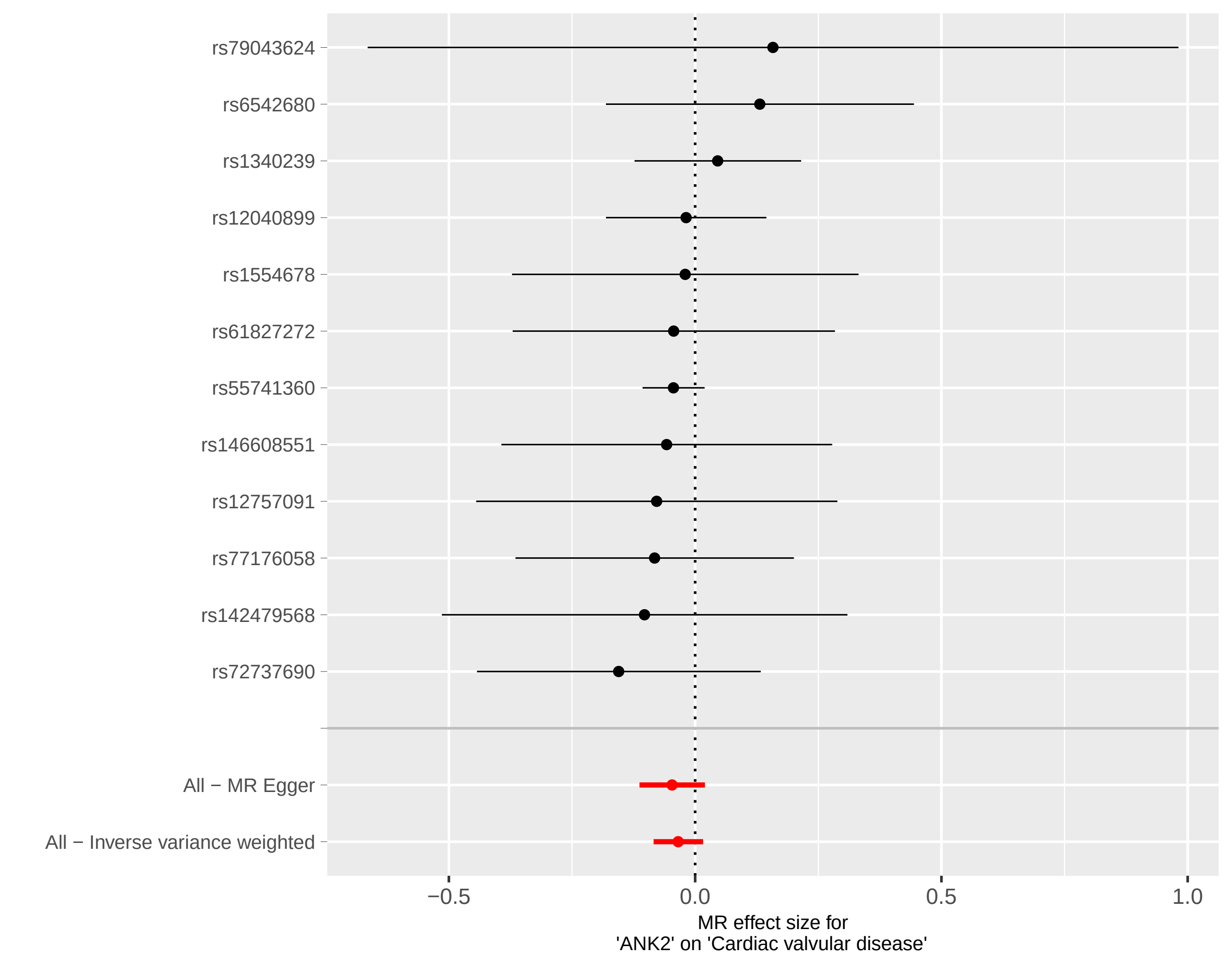
rs79043624
rs6542680
rs1340239
rs12040899
rs1554678
rs61827272
rs55741360
rs146608551
rs12757091
rs77176058
rs142479568
rs72737690
All − MR Egger
All − Inverse variance weighted
−0.5
0.0
0.5
1.0
MR effect size for
'ANK2' on 'Cardiac valvular disease'

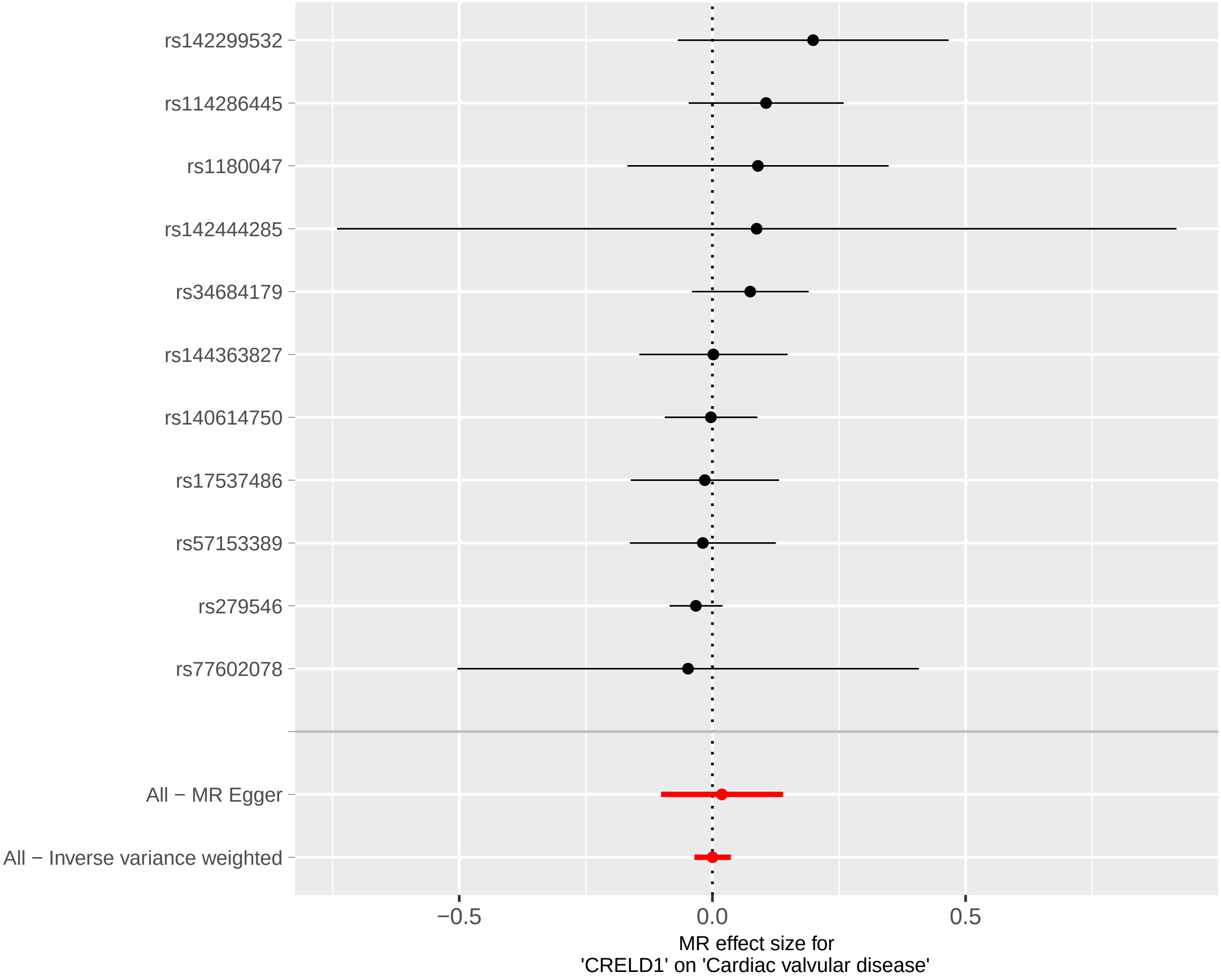
rs142299532
rs114286445
rs1180047
rs142444285
rs34684179
rs144363827
rs140614750
rs17537486
rs57153389
rs279546
rs77602078
All − MR Egger
All − Inverse variance weighted
−0.5
0.0
0.5
MR effect size for
'CRELD1' on 'Cardiac valvular disease'

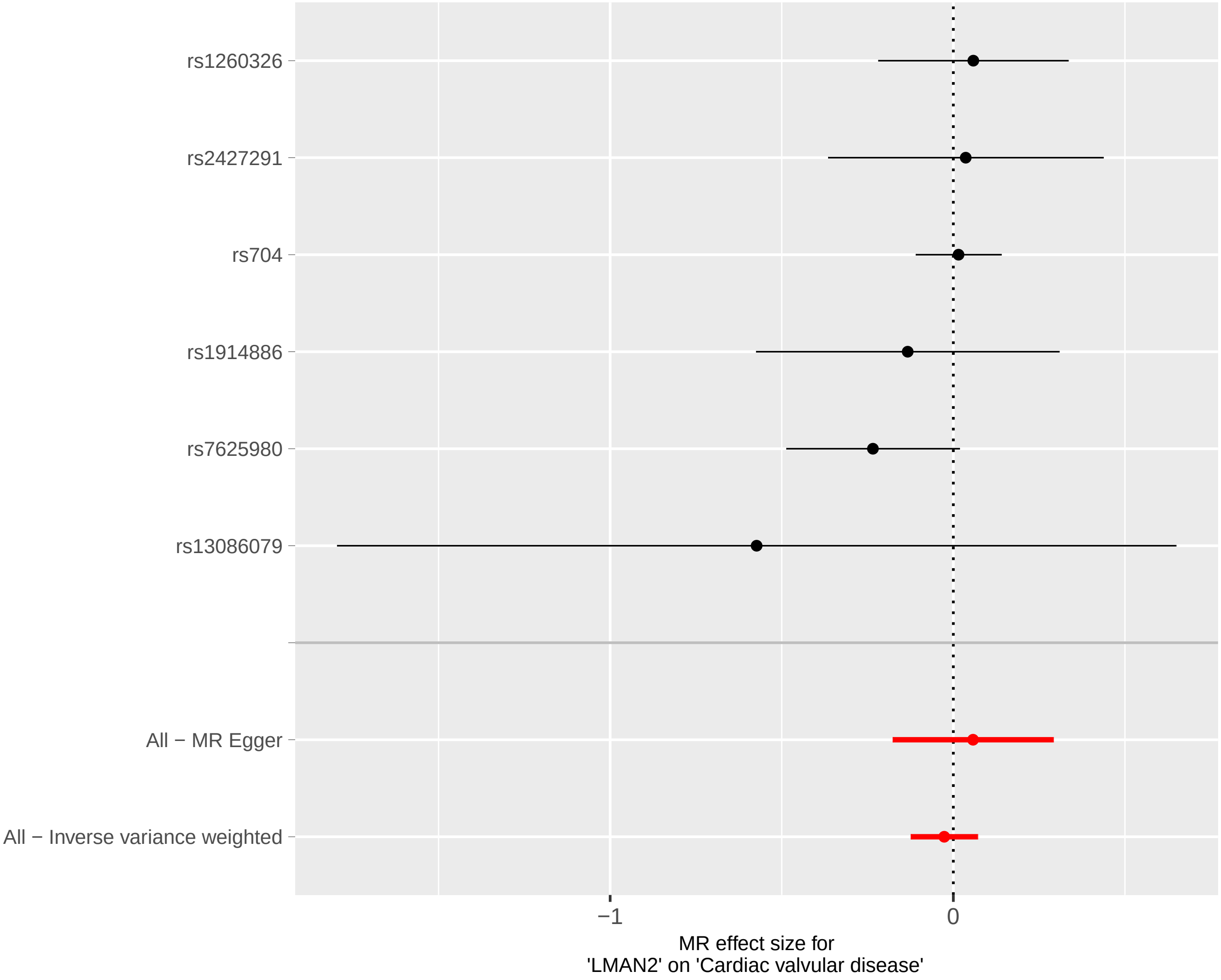

rs1260326
rs2427291
rs704
rs1914886
rs7625980
rs13086079
All − MR Egger
All − Inverse variance weighted
−1
0
MR effect size for
'LMAN2' on 'Cardiac valvular disease'

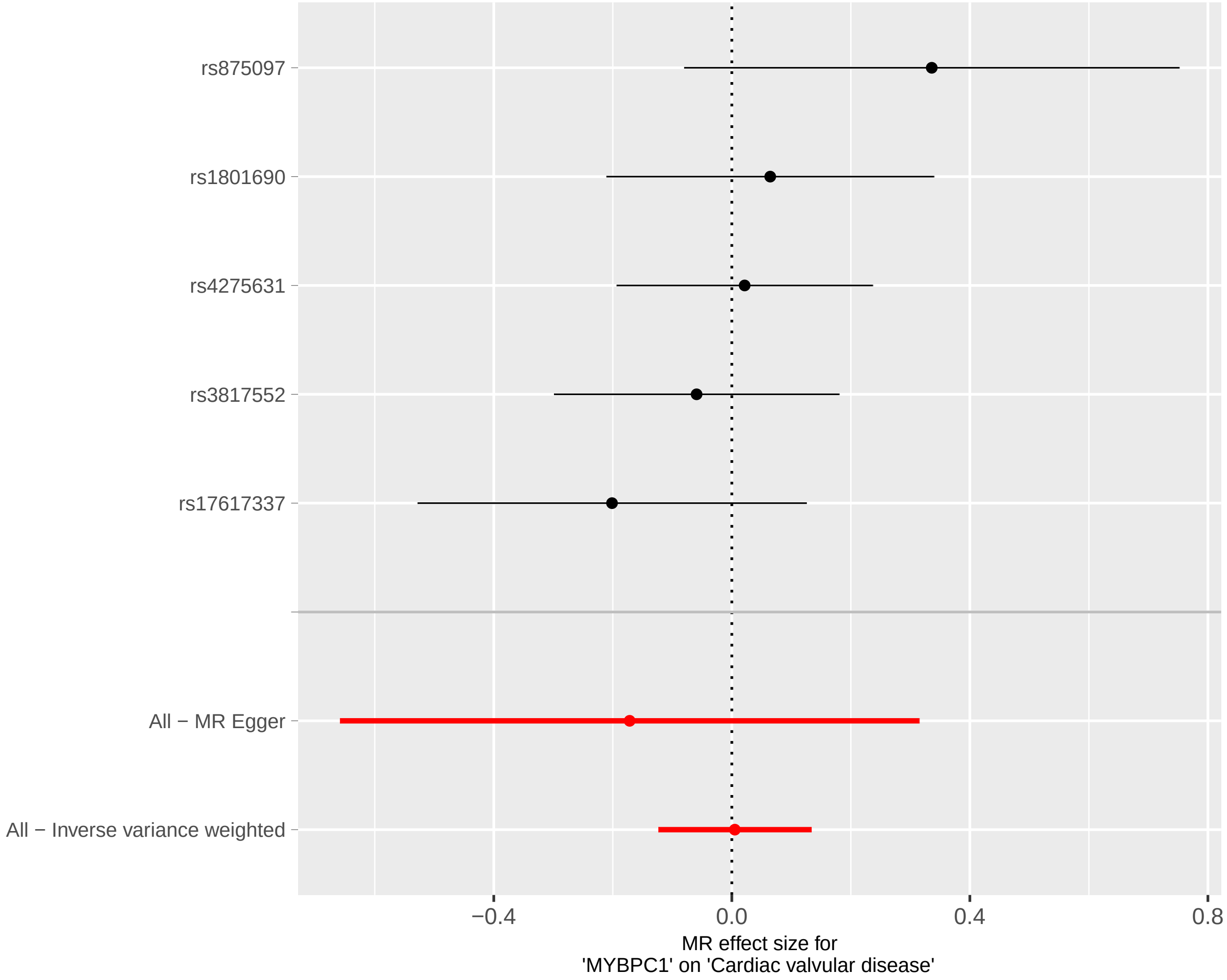
rs875097
rs1801690
rs4275631
rs3817552
rs17617337
All − MR Egger
All − Inverse variance weighted
−0.4
0.0
0.4
0.8
MR effect size for
'MYBPC1' on 'Cardiac valvular disease'

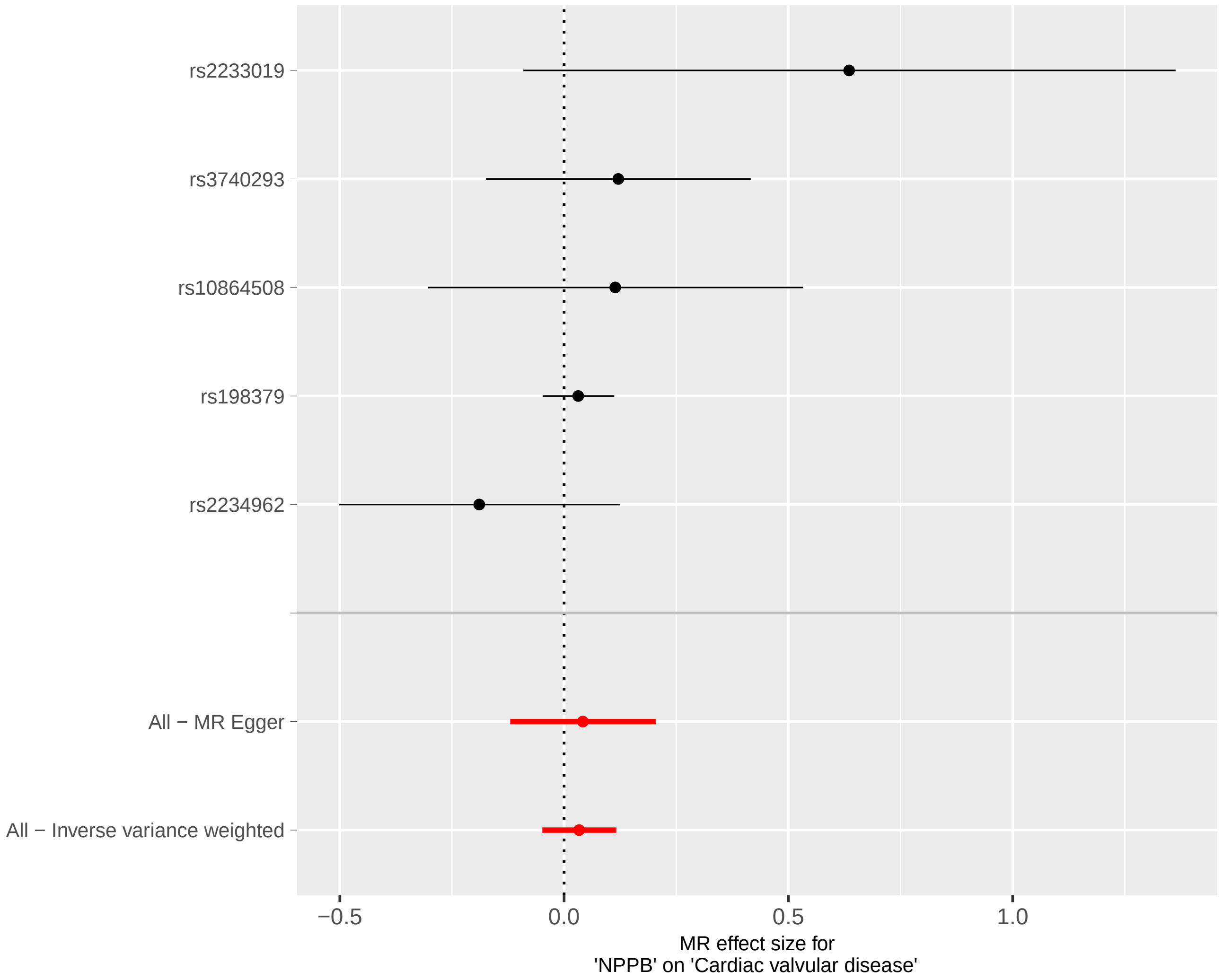
rs2233019
rs3740293
rs10864508
rs198379
rs2234962
All − MR Egger
All − Inverse variance weighted
−0.5
0.0
0.5
1.0
MR effect size for
'NPPB' on 'Cardiac valvular disease'

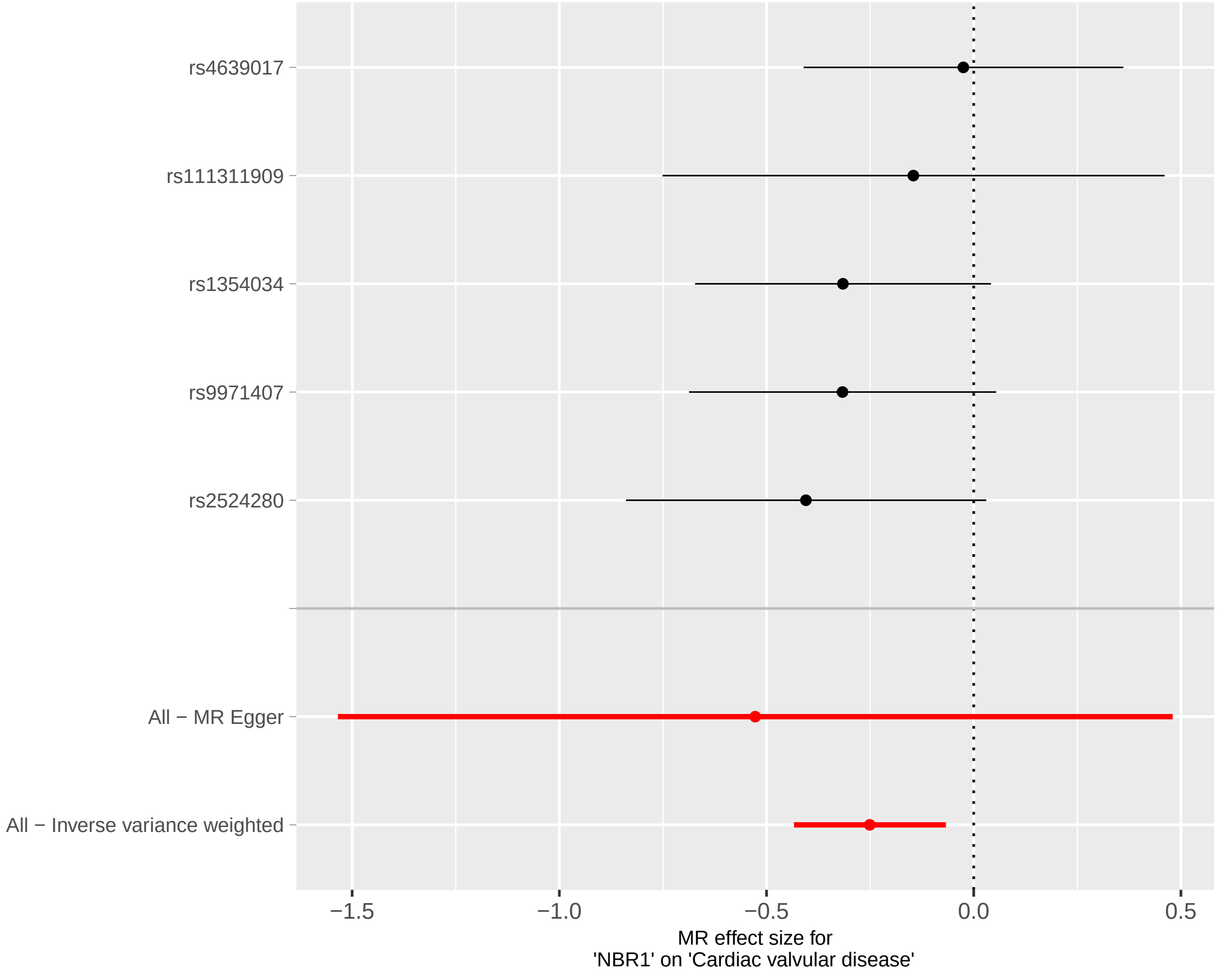

rs4639017
rs111311909
rs1354034
rs9971407
rs2524280
All − MR Egger
All − Inverse variance weighted
−1.5
−1.0
−0.5
0.0
0.5
MR effect size for
'NBR1' on 'Cardiac valvular disease'

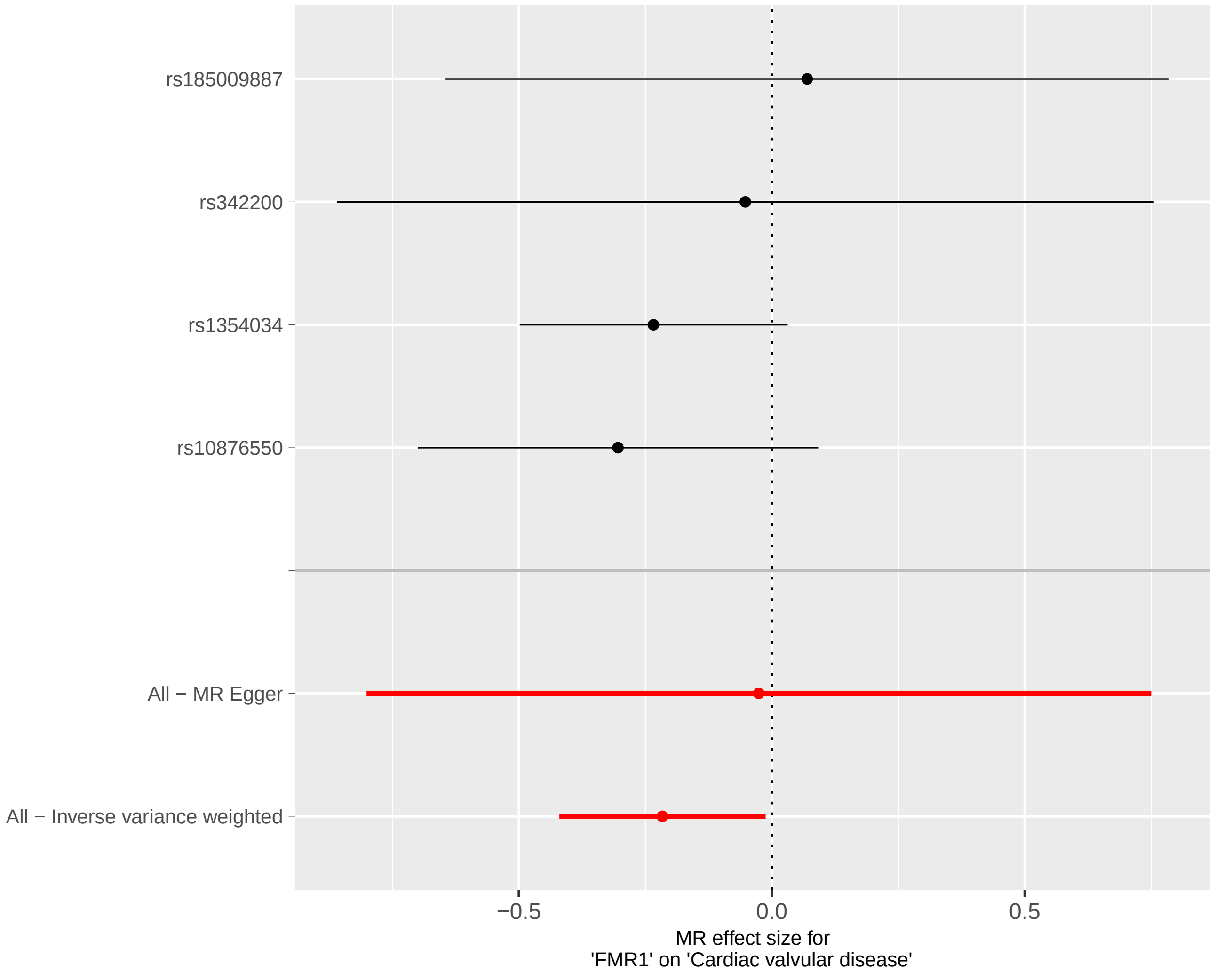
rs185009887
rs342200
rs1354034
rs10876550
All − MR Egger
All − Inverse variance weighted
−0.5
0.0
0.5
MR effect size for
'FMR1' on 'Cardiac valvular disease'

Insufficient number of SNPs

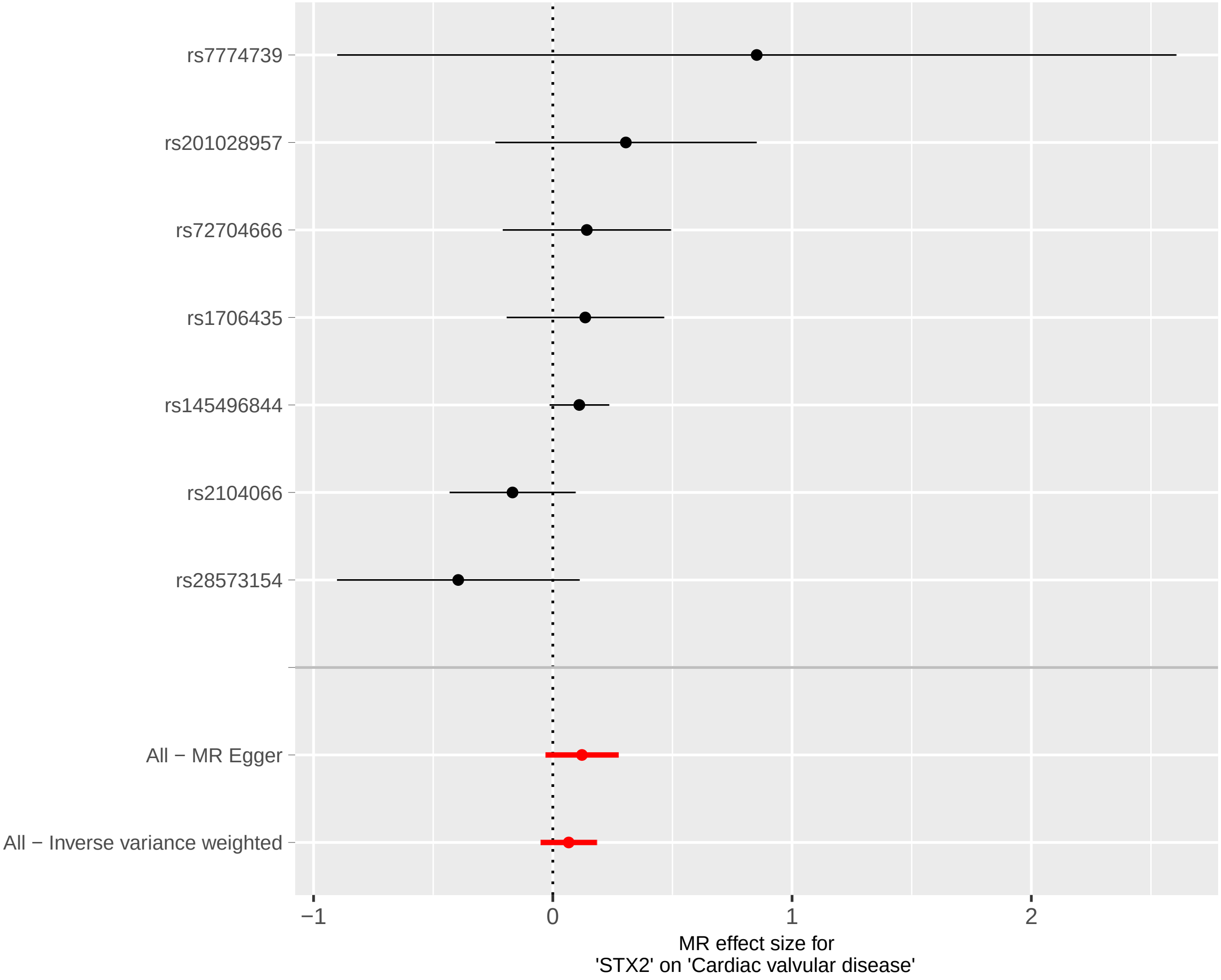

rs7774739
rs201028957
rs72704666
rs1706435
rs145496844
rs2104066
rs28573154
All − MR Egger
All − Inverse variance weighted
−1
0
1
2
MR effect size for
'STX2' on 'Cardiac valvular disease'

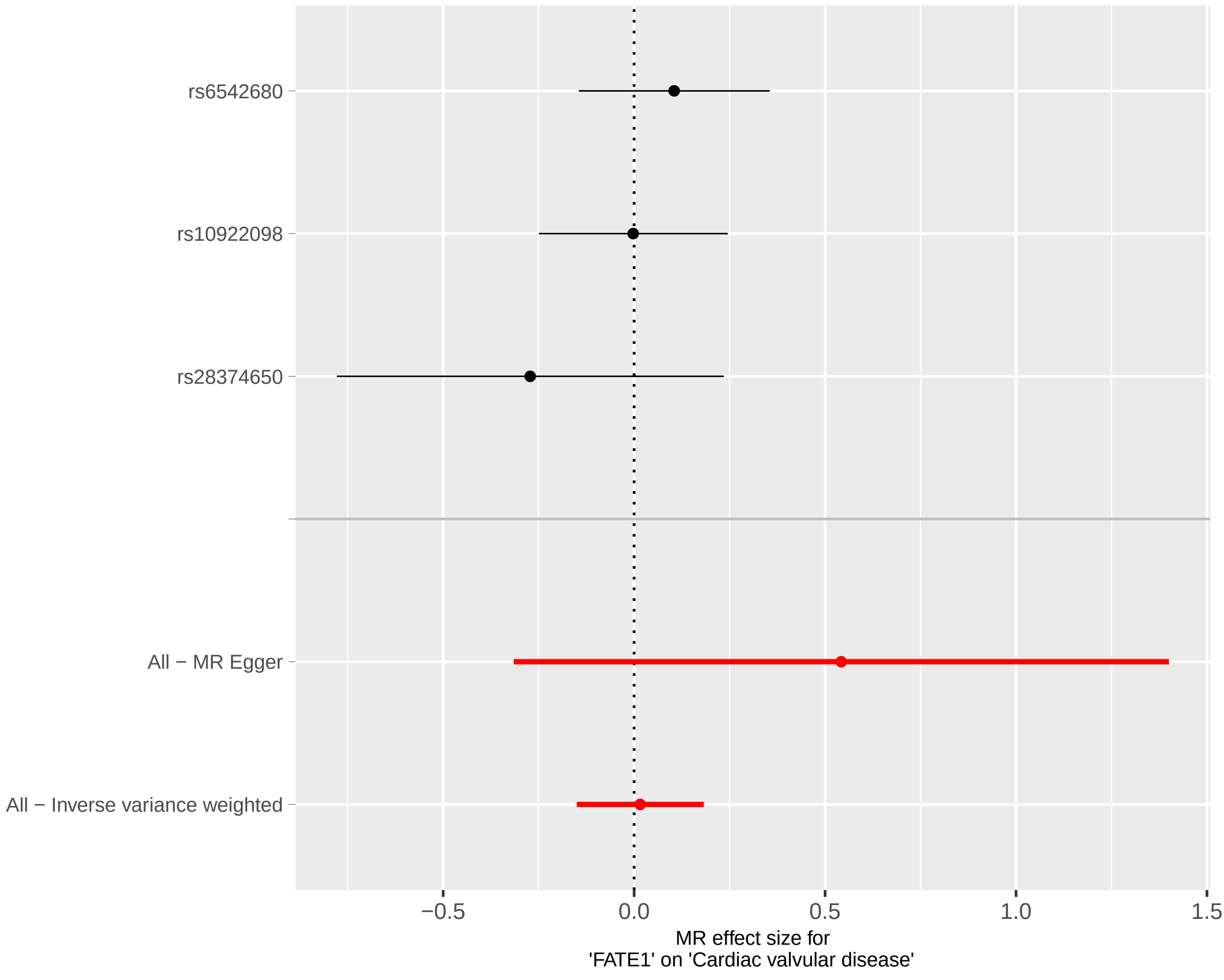

rs6542680
rs10922098
rs28374650
All − MR Egger
All − Inverse variance weighted
−0.5
0.0
0.5
1.0
1.5
MR effect size for
'FATE1' on 'Cardiac valvular disease'

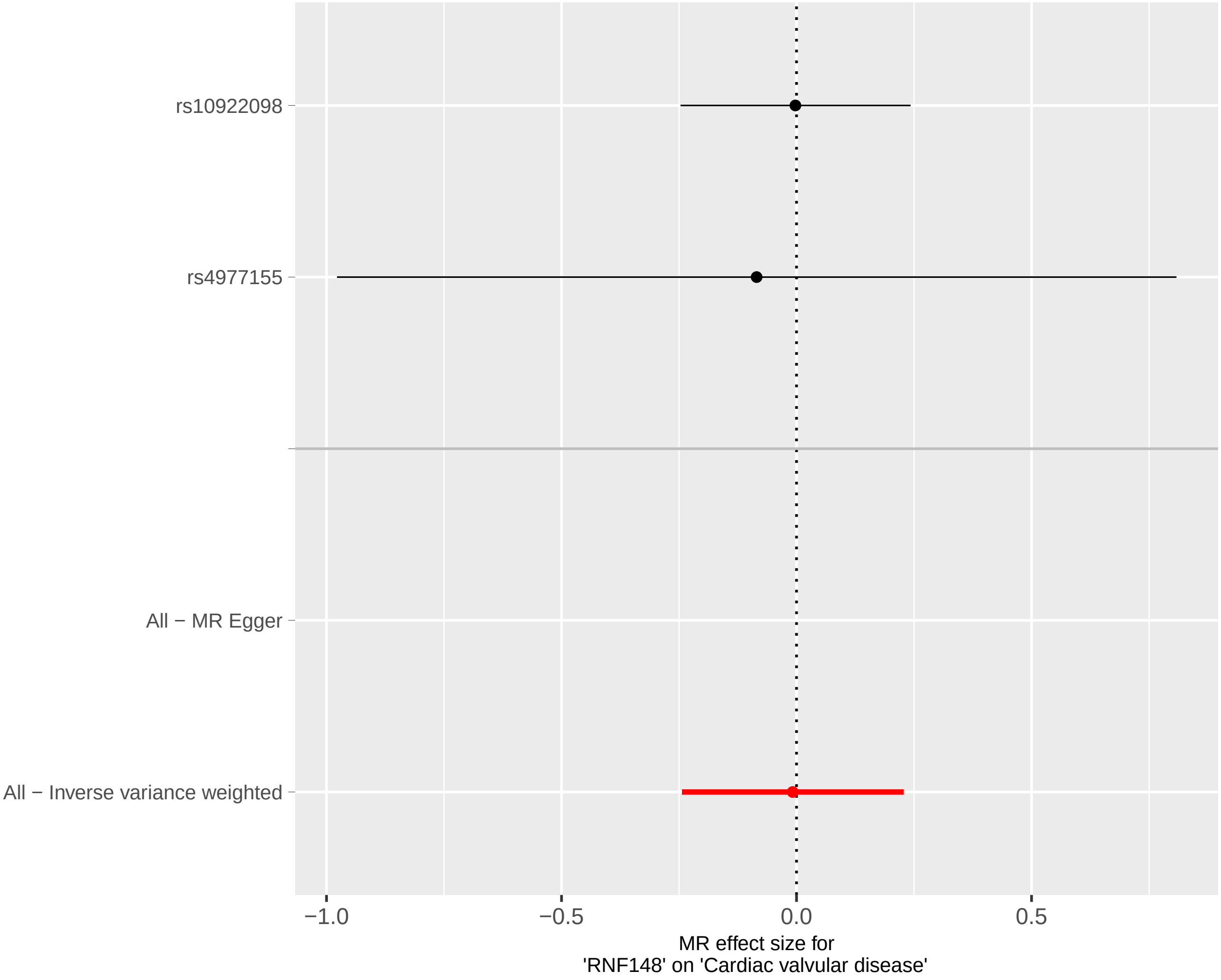

rs10922098
rs4977155
All − MR Egger
All − Inverse variance weighted
−1.0
−0.5
0.0
0.5
MR effect size for
'RNF148' on 'Cardiac valvular disease'

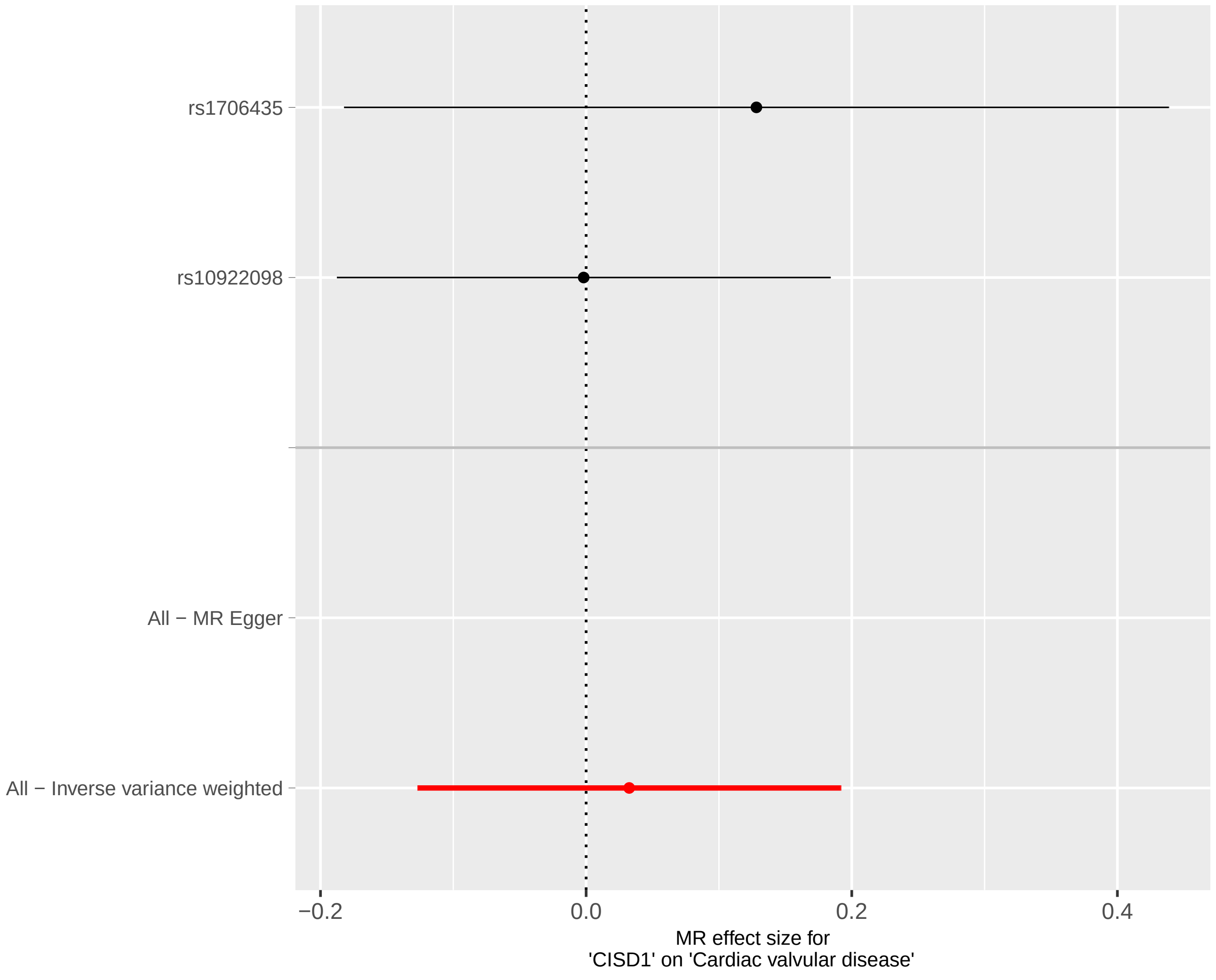
rs1706435
rs10922098
All − MR Egger
All − Inverse variance weighted
−0.2
0.0
0.2
0.4
MR effect size for
'CISD1' on 'Cardiac valvular disease'

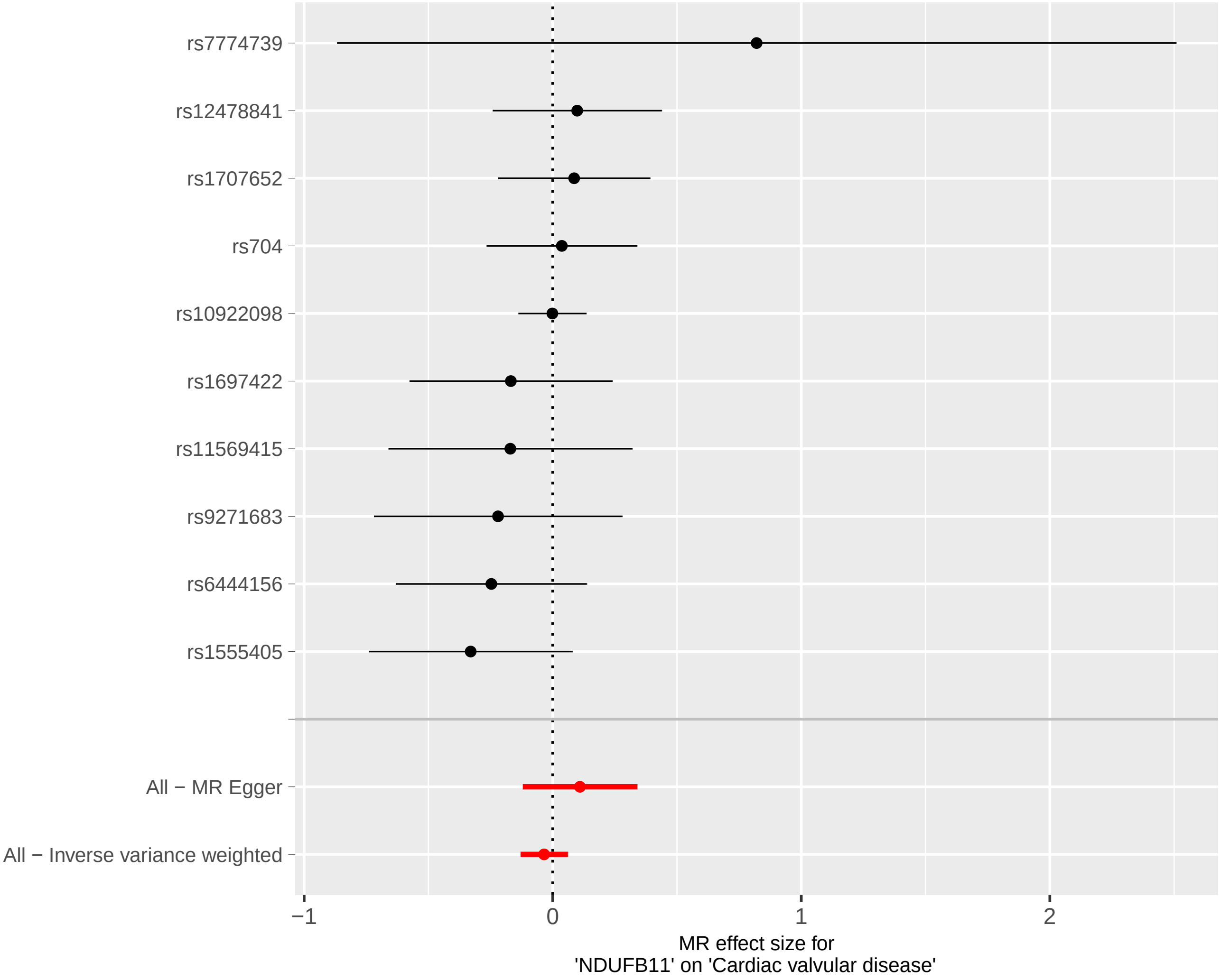
rs7774739
rs12478841
rs1707652
rs704
rs10922098
rs1697422
rs11569415
rs9271683
rs6444156
rs1555405
All − MR Egger
All − Inverse variance weighted
−1
0
1
2
MR effect size for
'NDUFB11' on 'Cardiac valvular disease'

Insufficient number of SNPs

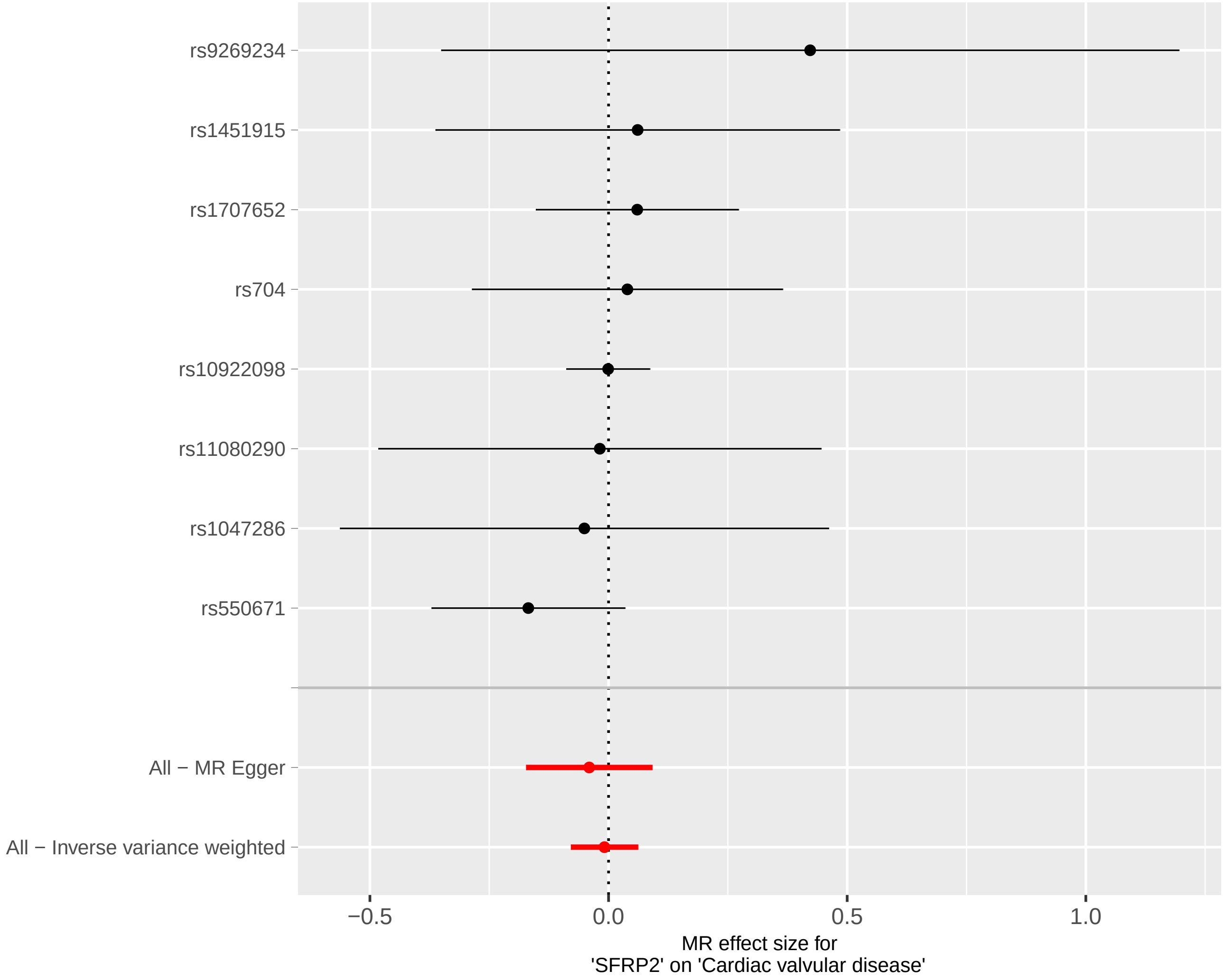

rs9269234
rs1451915
rs1707652
rs704
rs10922098
rs11080290
rs1047286
rs550671
All − MR Egger
All − Inverse variance weighted
−0.5
0.0
0.5
1.0
MR effect size for
'SFRP2' on 'Cardiac valvular disease'

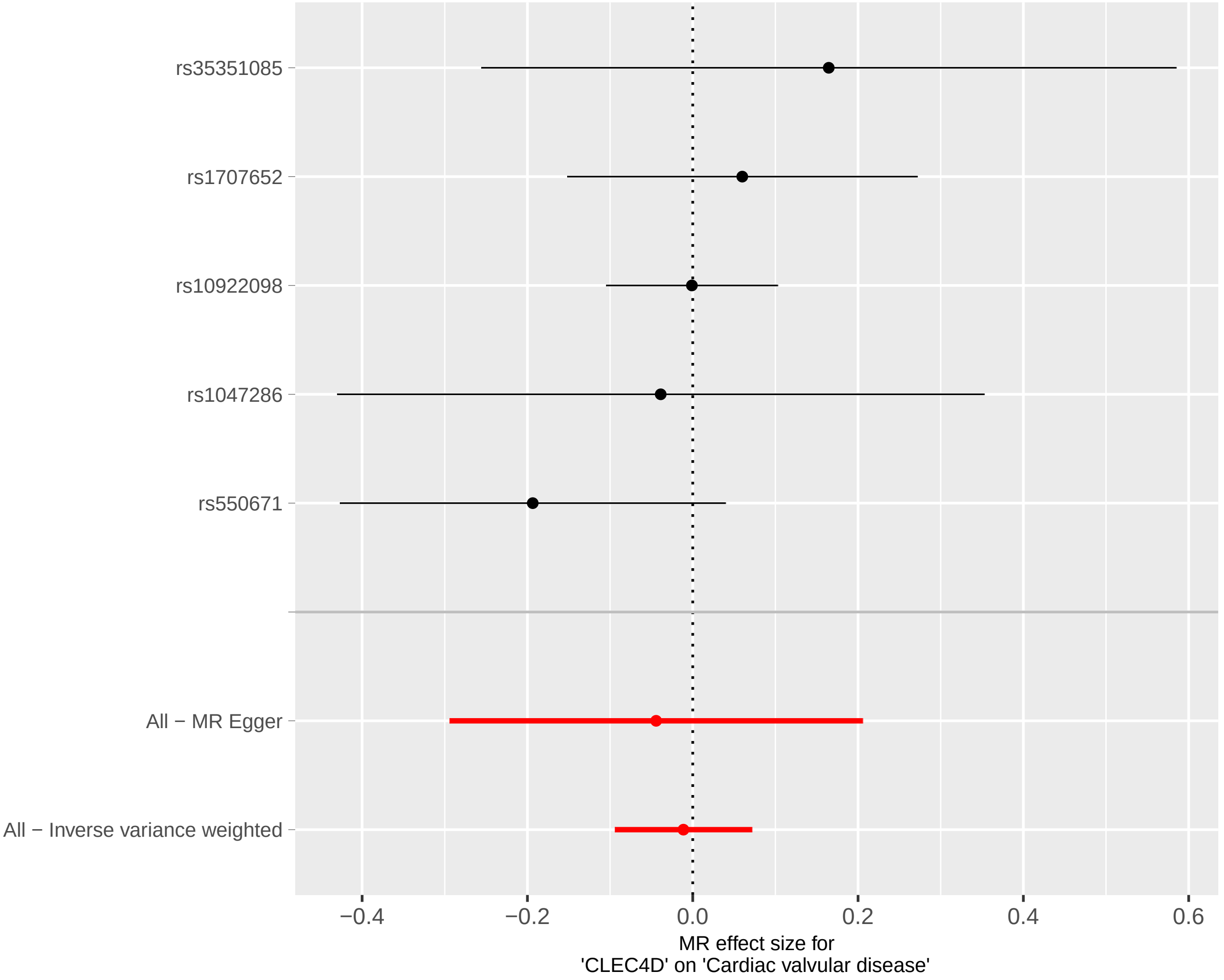
rs35351085
rs1707652
rs10922098
rs1047286
rs550671
All − MR Egger
All − Inverse variance weighted
−0.4
−0.2
0.0
0.2
0.4
0.6
MR effect size for
'CLEC4D' on 'Cardiac valvular disease'

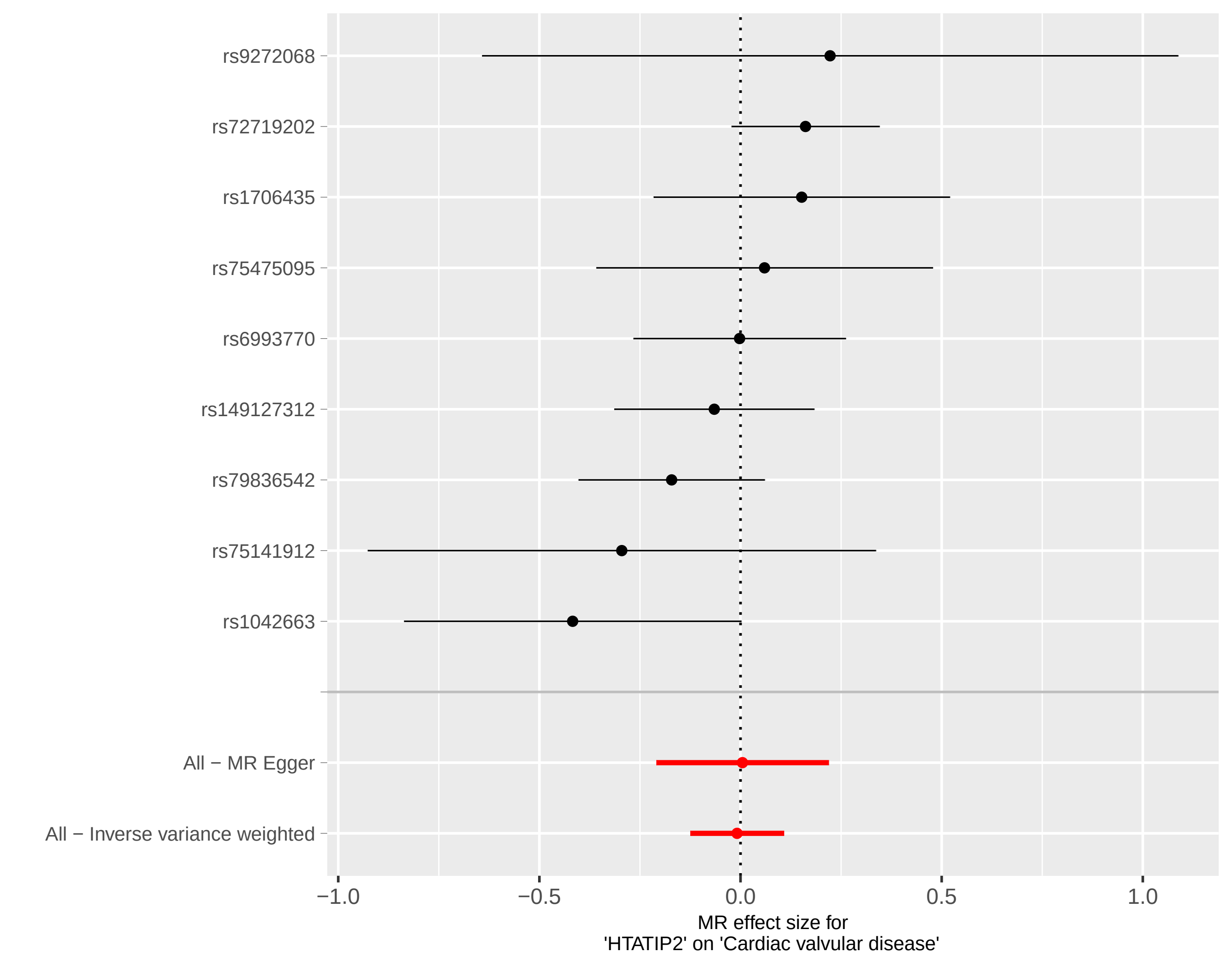
rs9272068
rs72719202
rs1706435
rs75475095
rs6993770
rs149127312
rs79836542
rs75141912
rs1042663
All − MR Egger
All − Inverse variance weighted
−1.0
−0.5
0.0
0.5
1.0
MR effect size for
'HTATIP2' on 'Cardiac valvular disease'

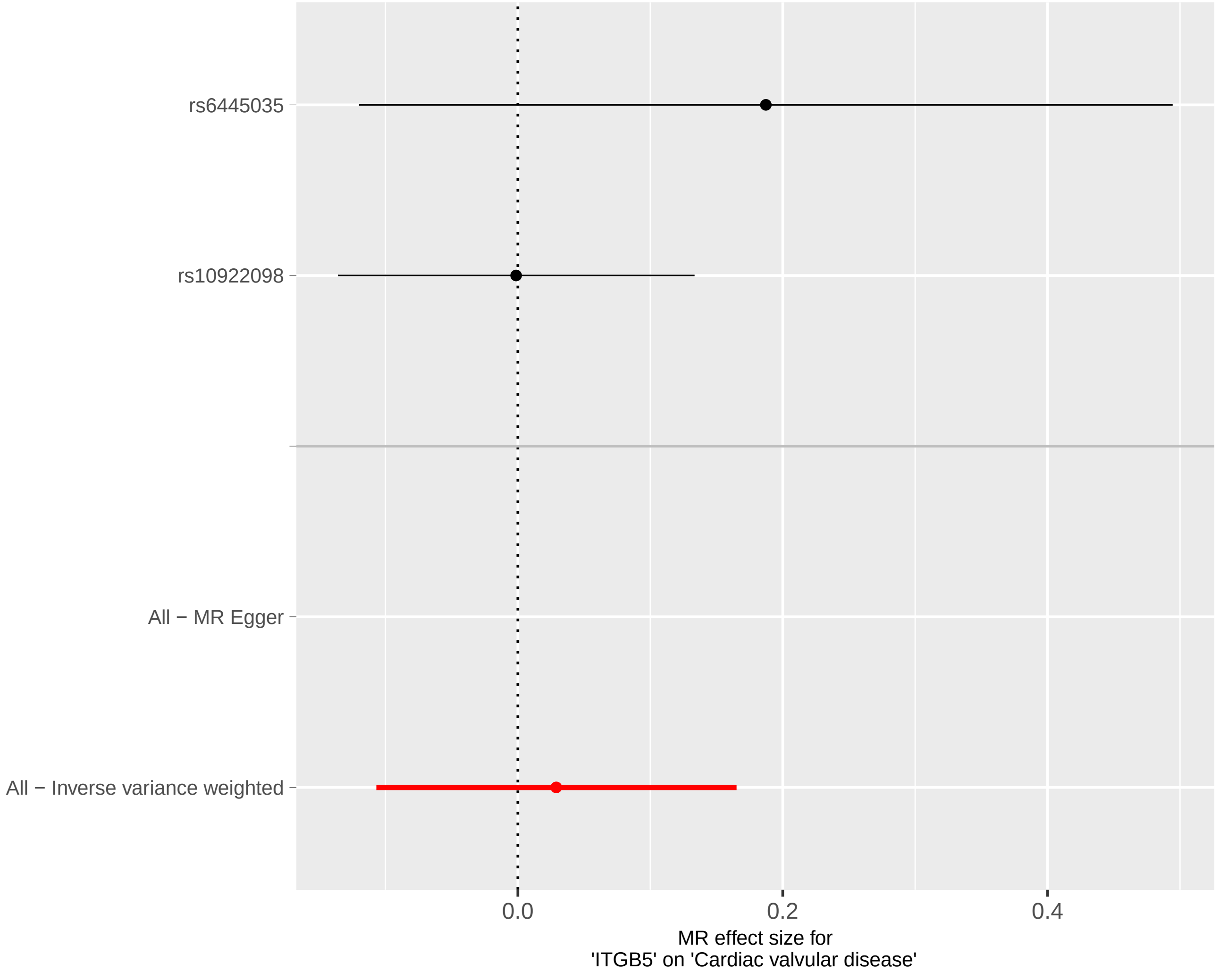

rs6445035
rs10922098
All − MR Egger
All − Inverse variance weighted
0.0
0.2
0.4
MR effect size for
'ITGB5' on 'Cardiac valvular disease'

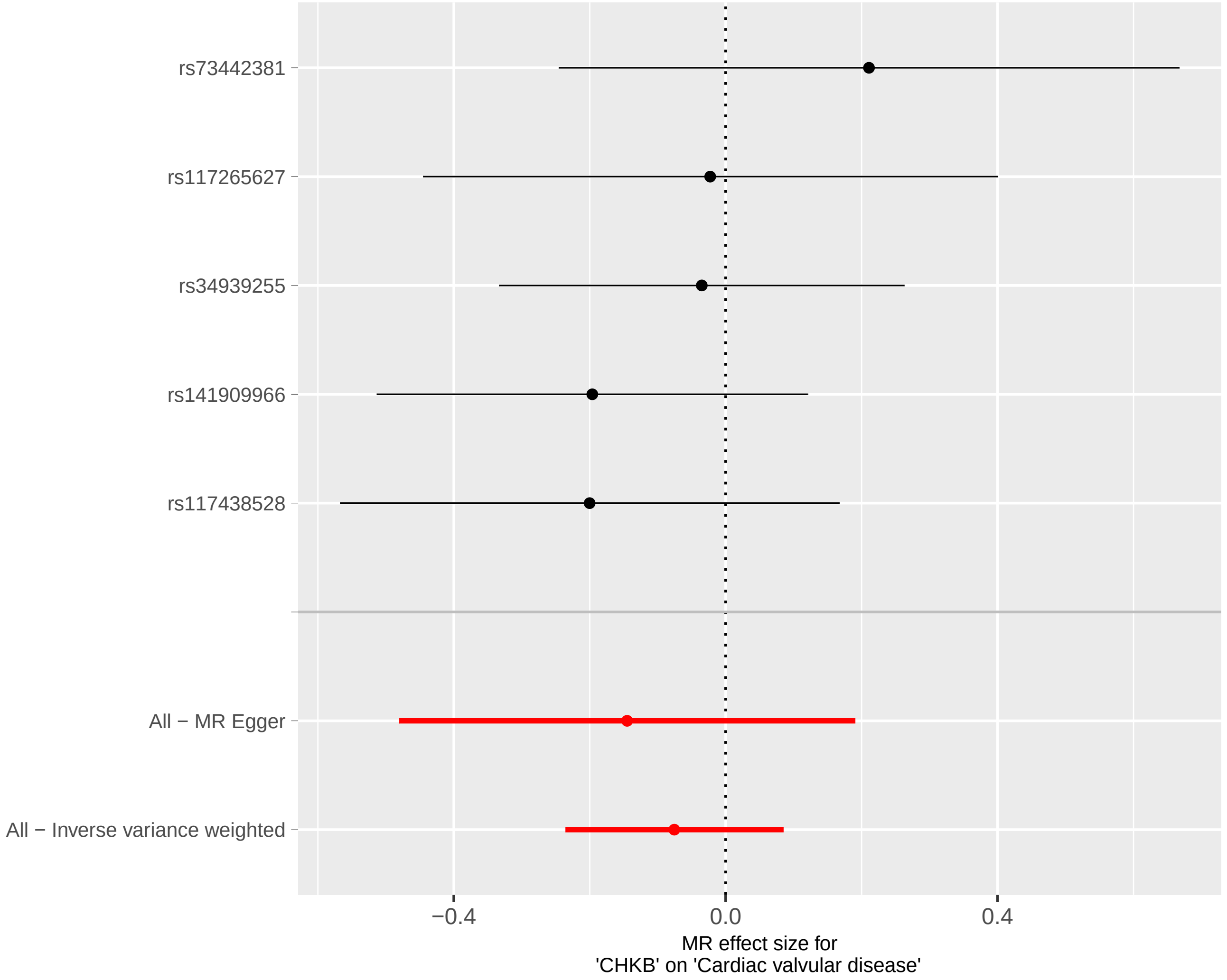
rs73442381
rs117265627
rs34939255
rs141909966
rs117438528
All − MR Egger
All − Inverse variance weighted
−0.4
0.0
0.4
MR effect size for
'CHKB' on 'Cardiac valvular disease'

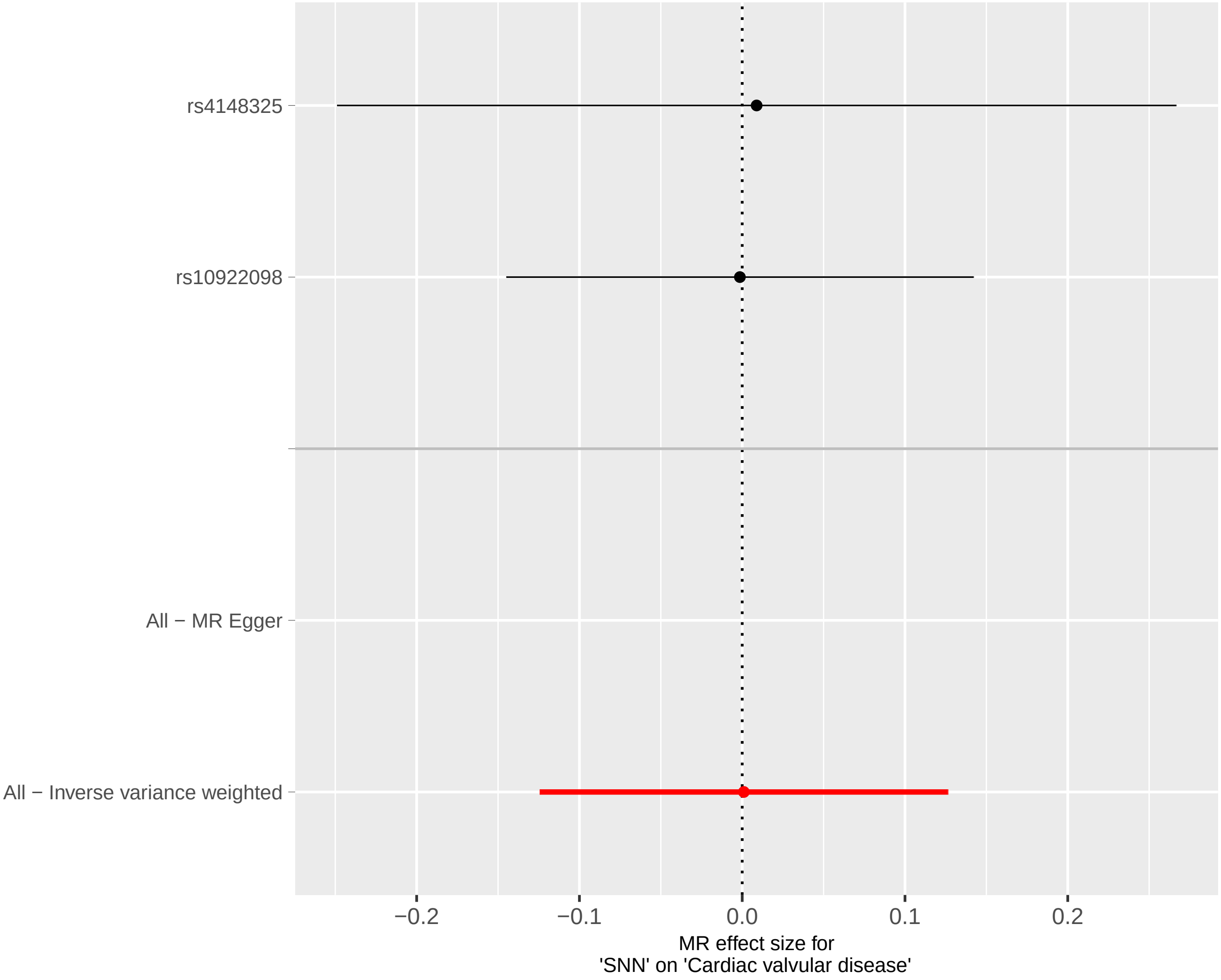
rs4148325
rs10922098
All − MR Egger
All − Inverse variance weighted
−0.2
−0.1
0.0
0.1
0.2
MR effect size for
'SNN' on 'Cardiac valvular disease'

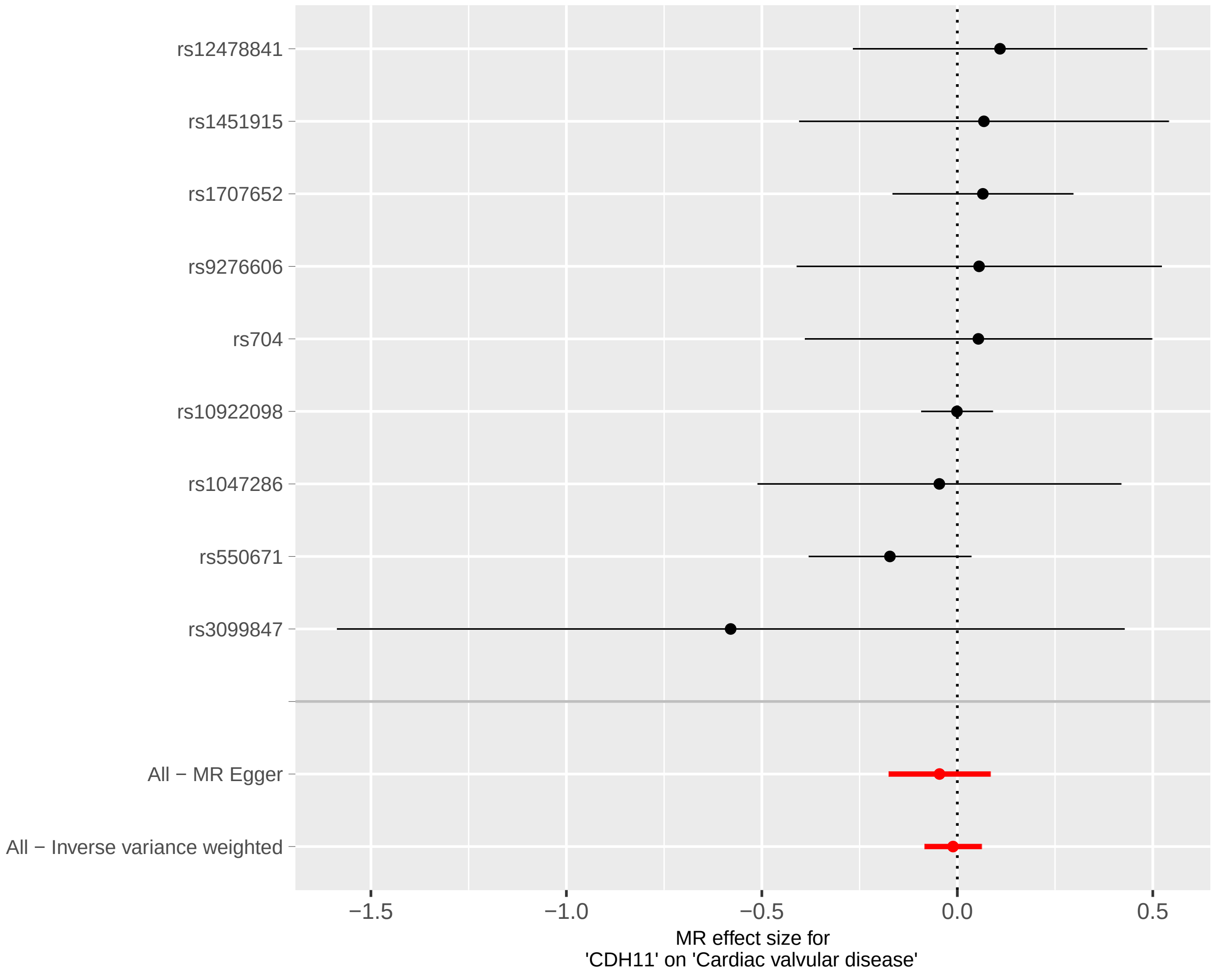
rs12478841
rs1451915
rs1707652
rs9276606
rs704
rs10922098
rs1047286
rs550671
rs3099847
All − MR Egger
All − Inverse variance weighted
−1.5
−1.0
−0.5
0.0
0.5
MR effect size for
'CDH11' on 'Cardiac valvular disease'

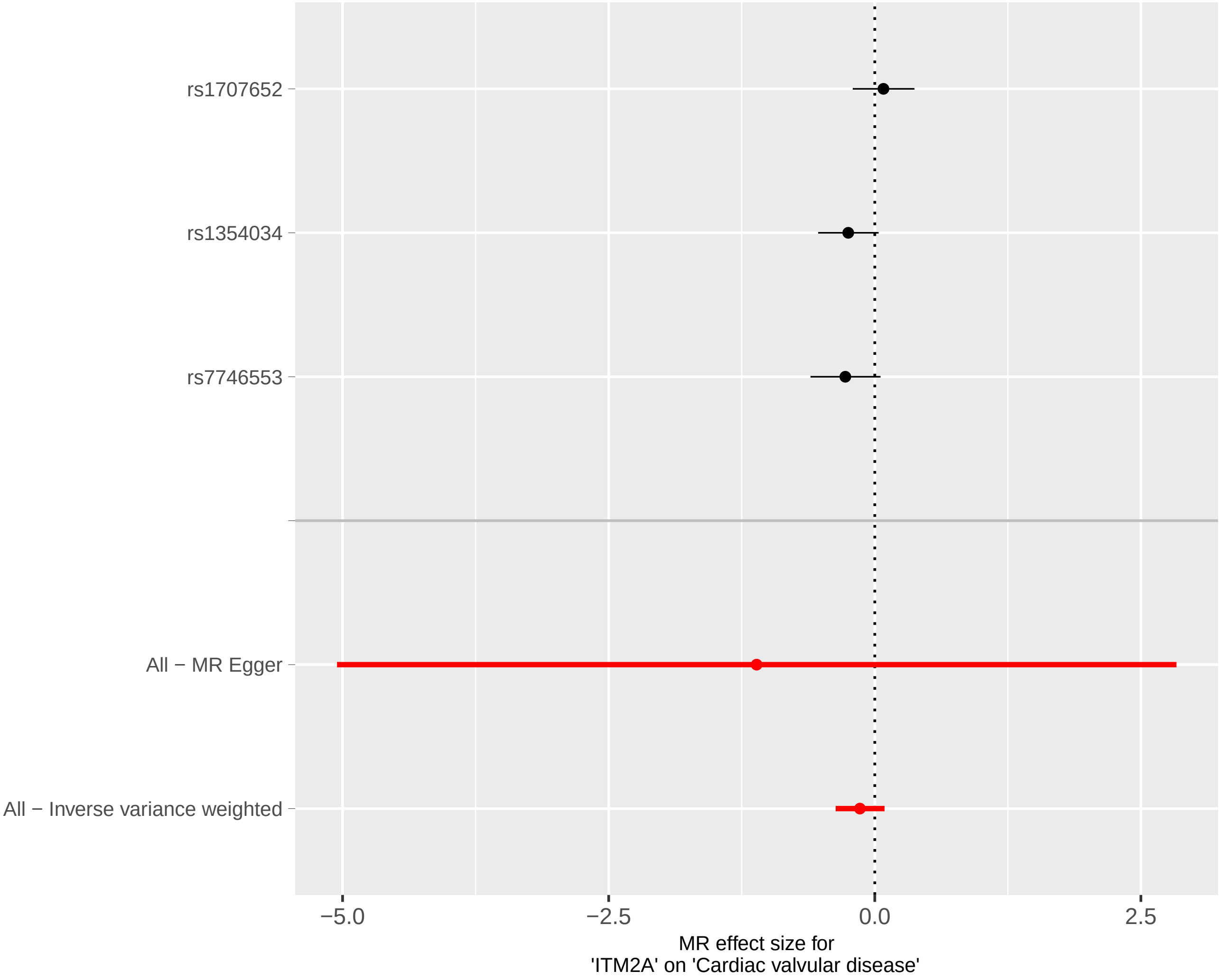
rs1707652
rs1354034
rs7746553
All − MR Egger
All − Inverse variance weighted
−5.0
−2.5
0.0
2.5
MR effect size for
'ITM2A' on 'Cardiac valvular disease'

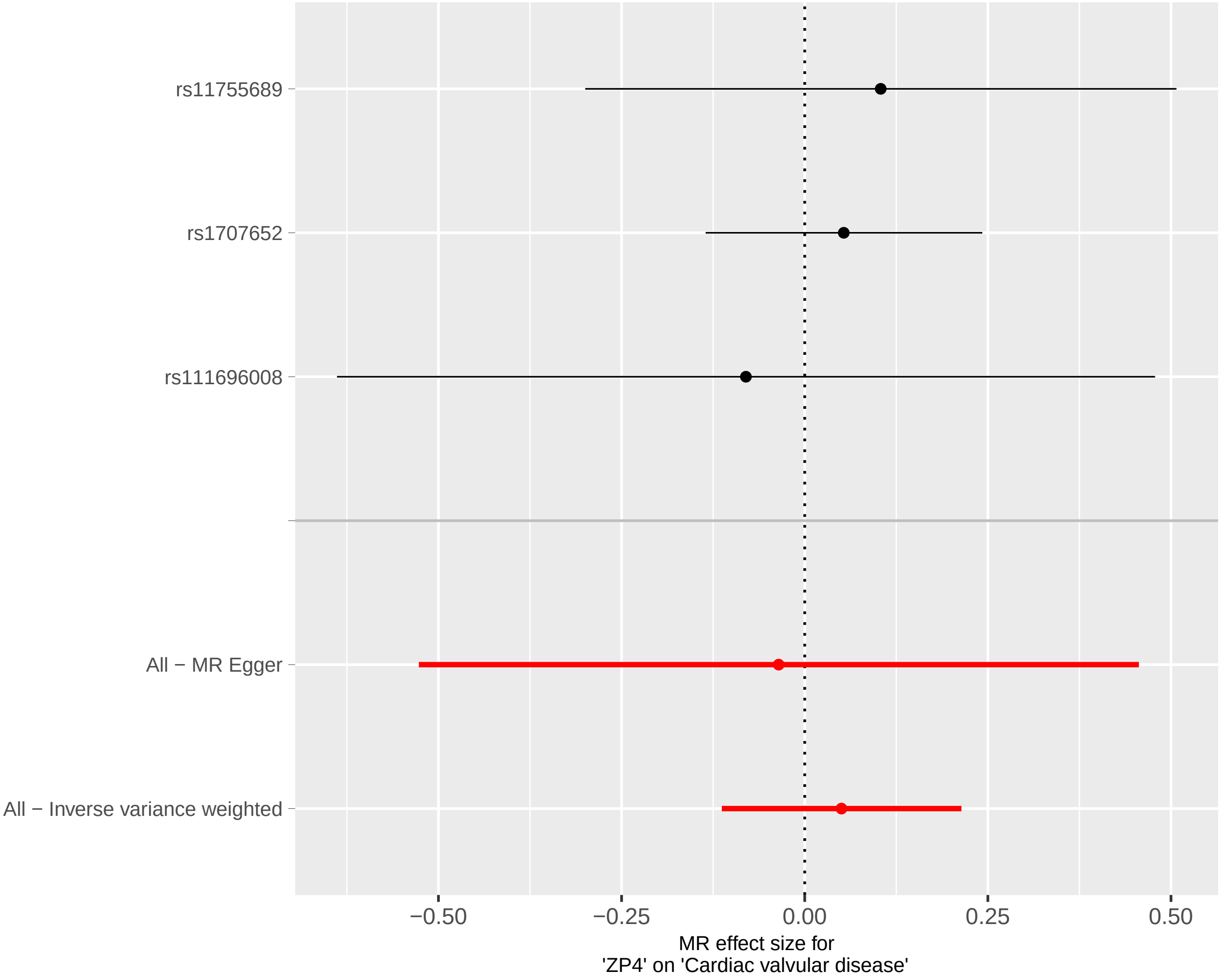
rs11755689
rs1707652
rs111696008
All − MR Egger
All − Inverse variance weighted
−0.50
−0.25
0.00
0.25
0.50
MR effect size for
'ZP4' on 'Cardiac valvular disease'

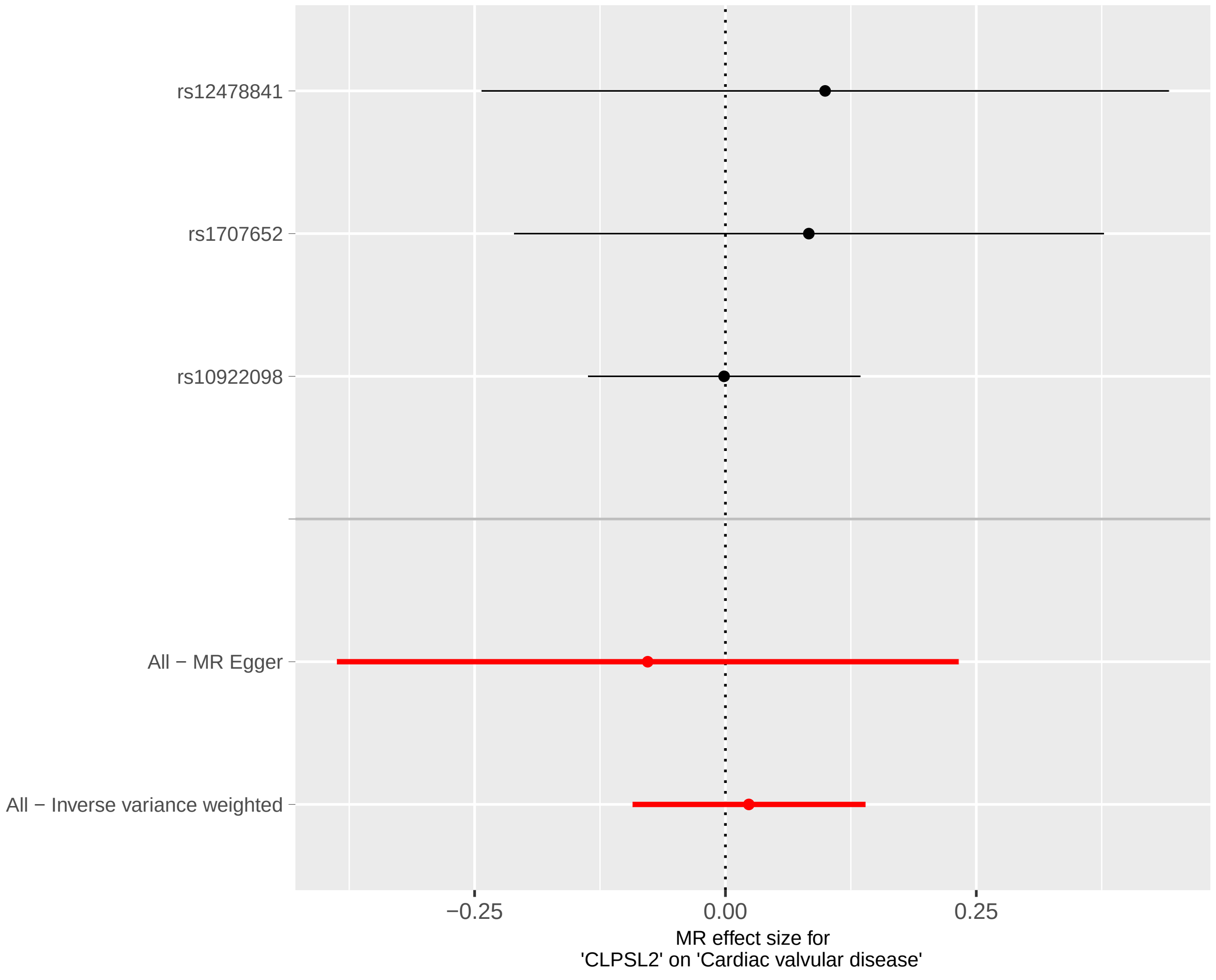
rs12478841
rs1707652
rs10922098
All − MR Egger
All − Inverse variance weighted
−0.25
0.00
0.25
MR effect size for
'CLPSL2' on 'Cardiac valvular disease'

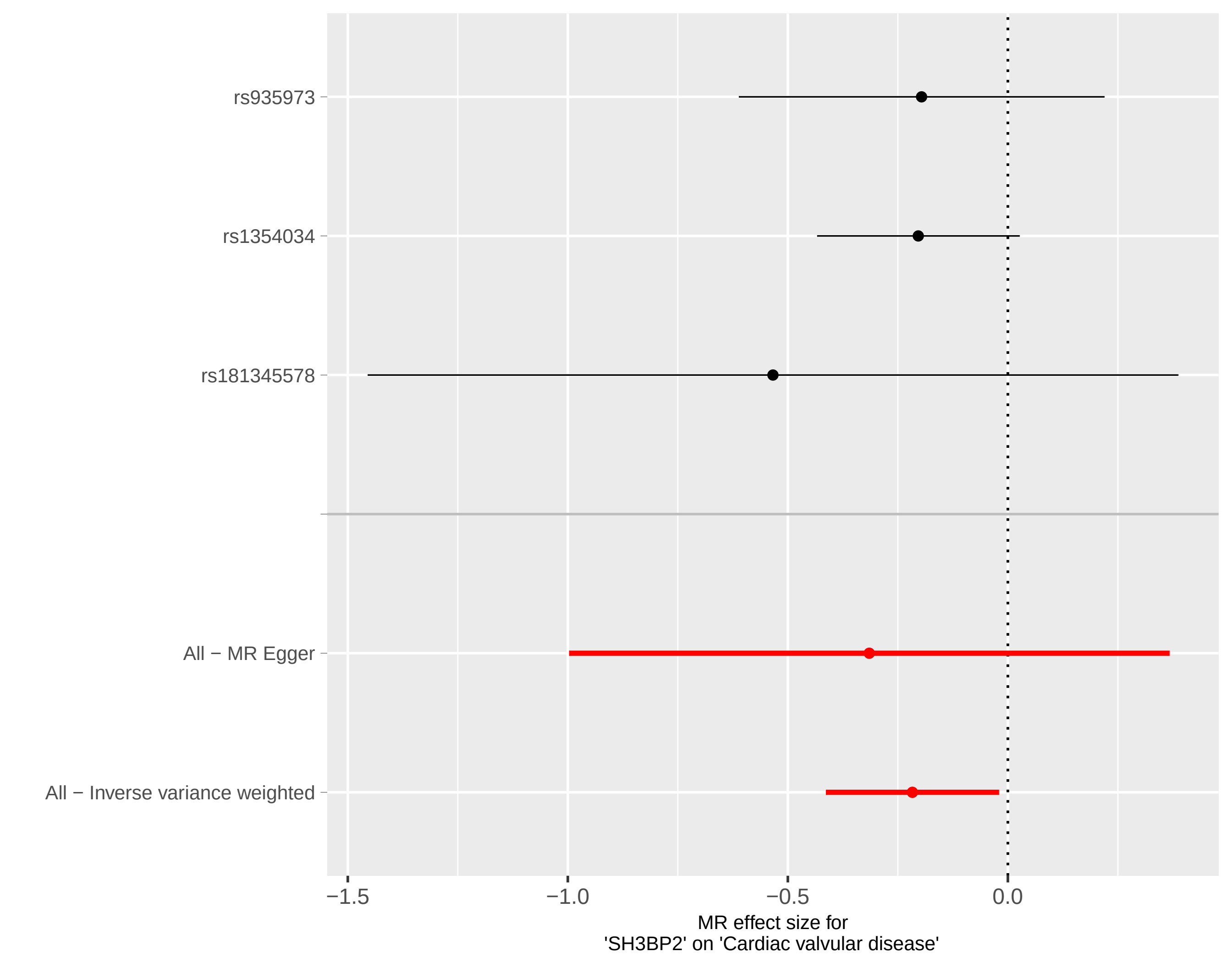
rs935973
rs1354034
rs181345578
All − MR Egger
All − Inverse variance weighted
−1.5
−1.0
−0.5
0.0
MR effect size for
'SH3BP2' on 'Cardiac valvular disease'

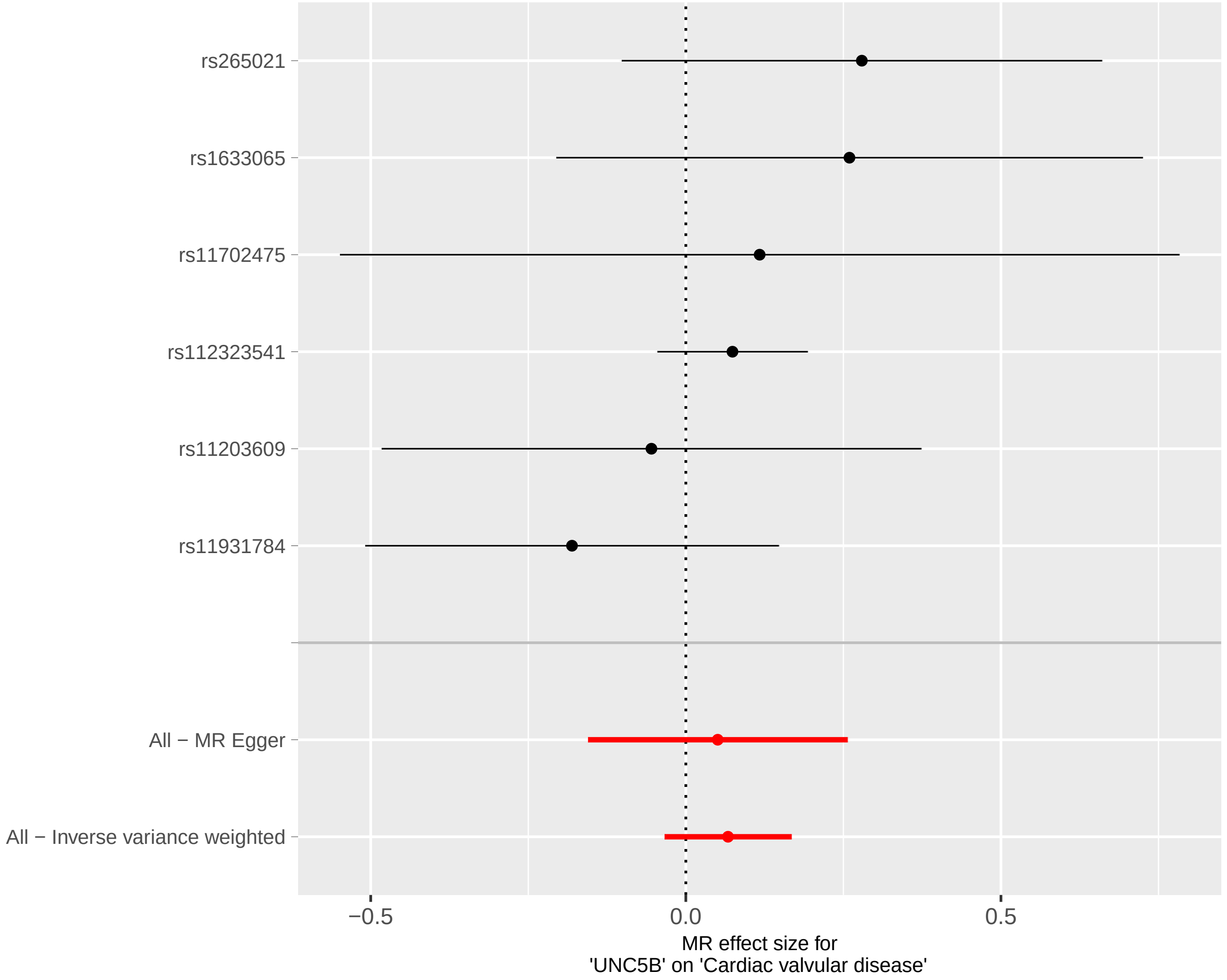
rs265021
rs1633065
rs11702475
rs112323541
rs11203609
rs11931784
All − MR Egger
All − Inverse variance weighted
−0.5
0.0
0.5
MR effect size for
'UNC5B' on 'Cardiac valvular disease'

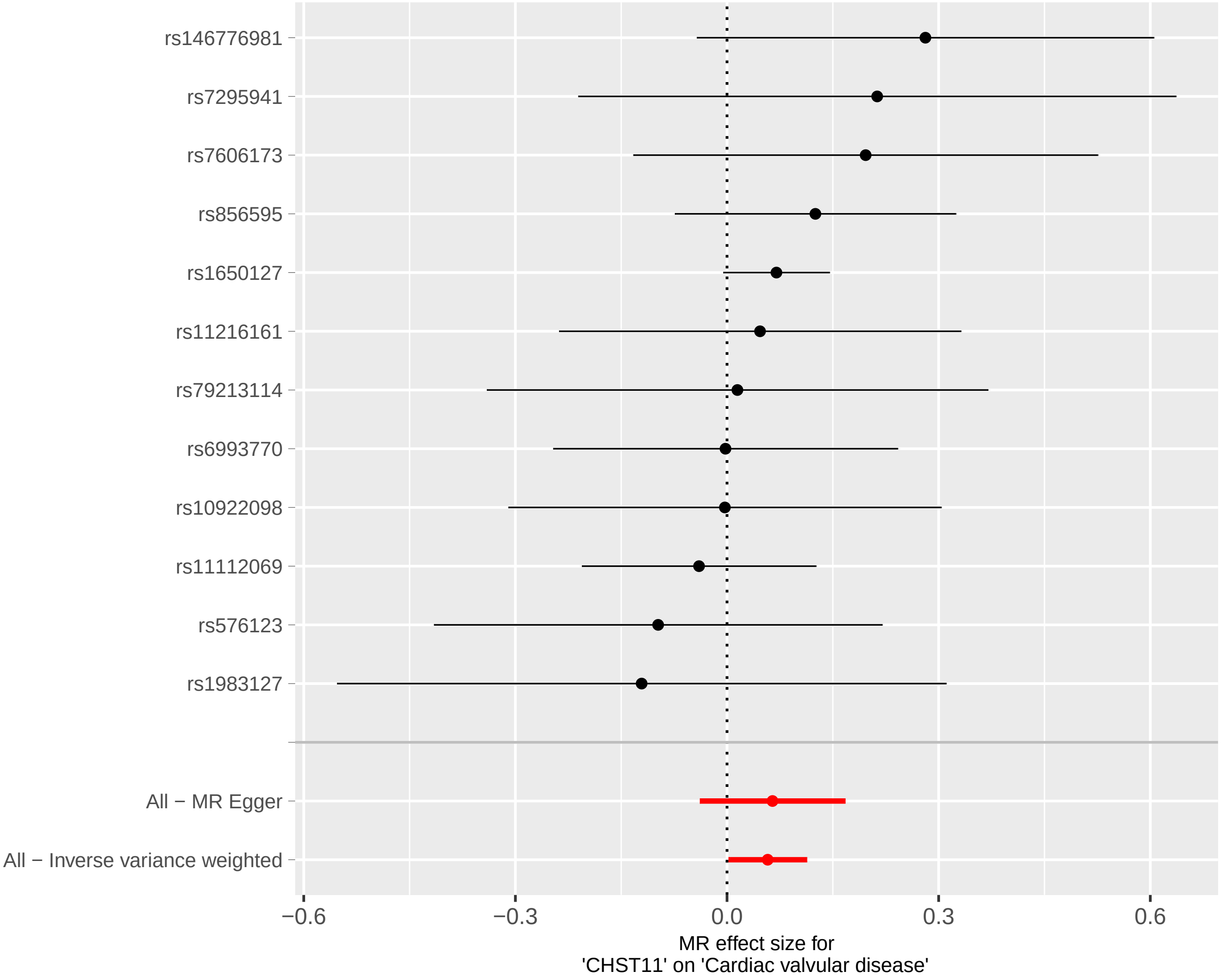
rs146776981
rs7295941
rs7606173
rs856595
rs1650127
rs11216161
rs79213114
rs6993770
rs10922098
rs11112069
rs576123
rs1983127
All − MR Egger
All − Inverse variance weighted
−0.6
−0.3
0.0
0.3
0.6
MR effect size for
'CHST11' on 'Cardiac valvular disease'

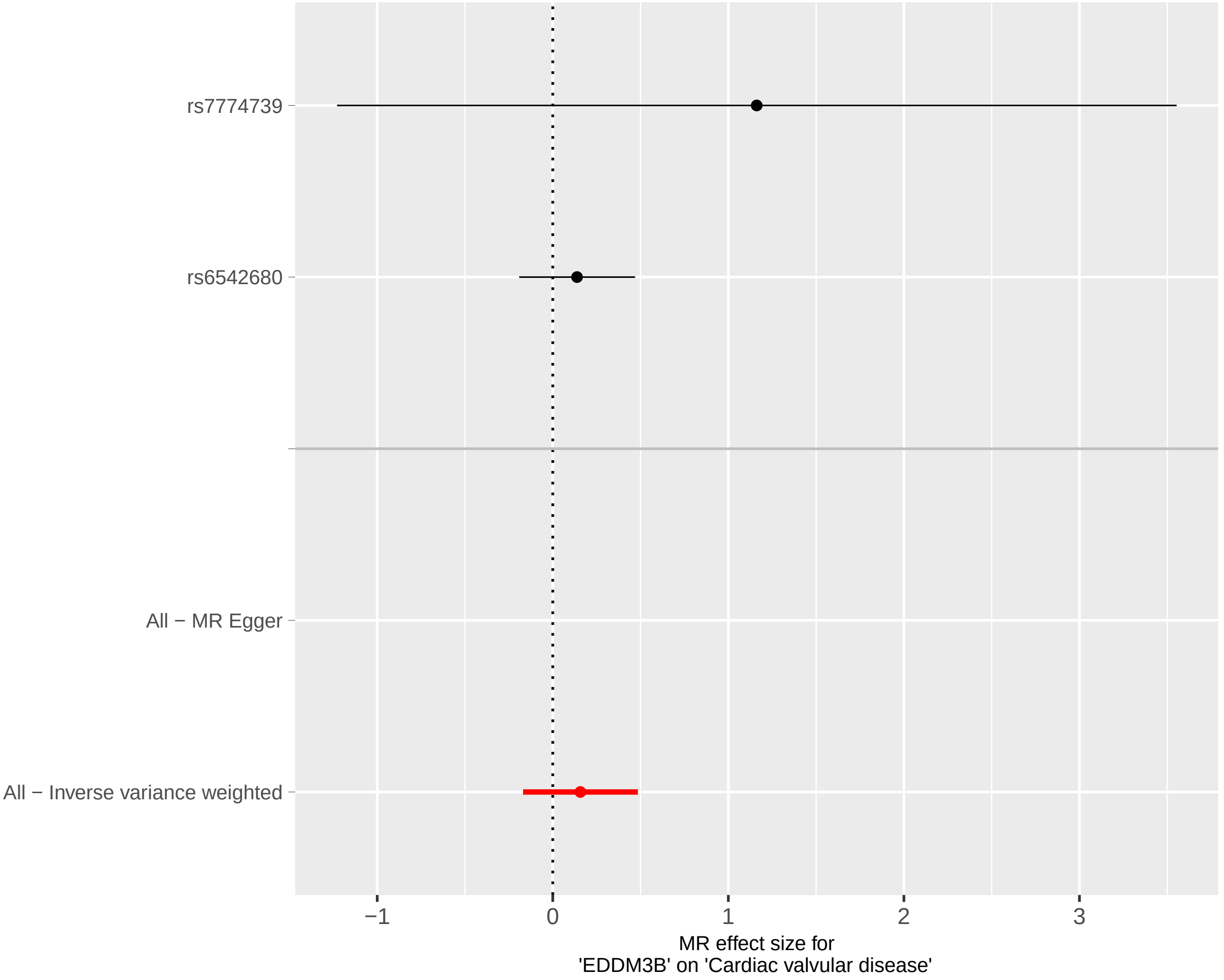

rs7774739
rs6542680
All − MR Egger
All − Inverse variance weighted
−1
0
1
2
3
MR effect size for
'EDDM3B' on 'Cardiac valvular disease'

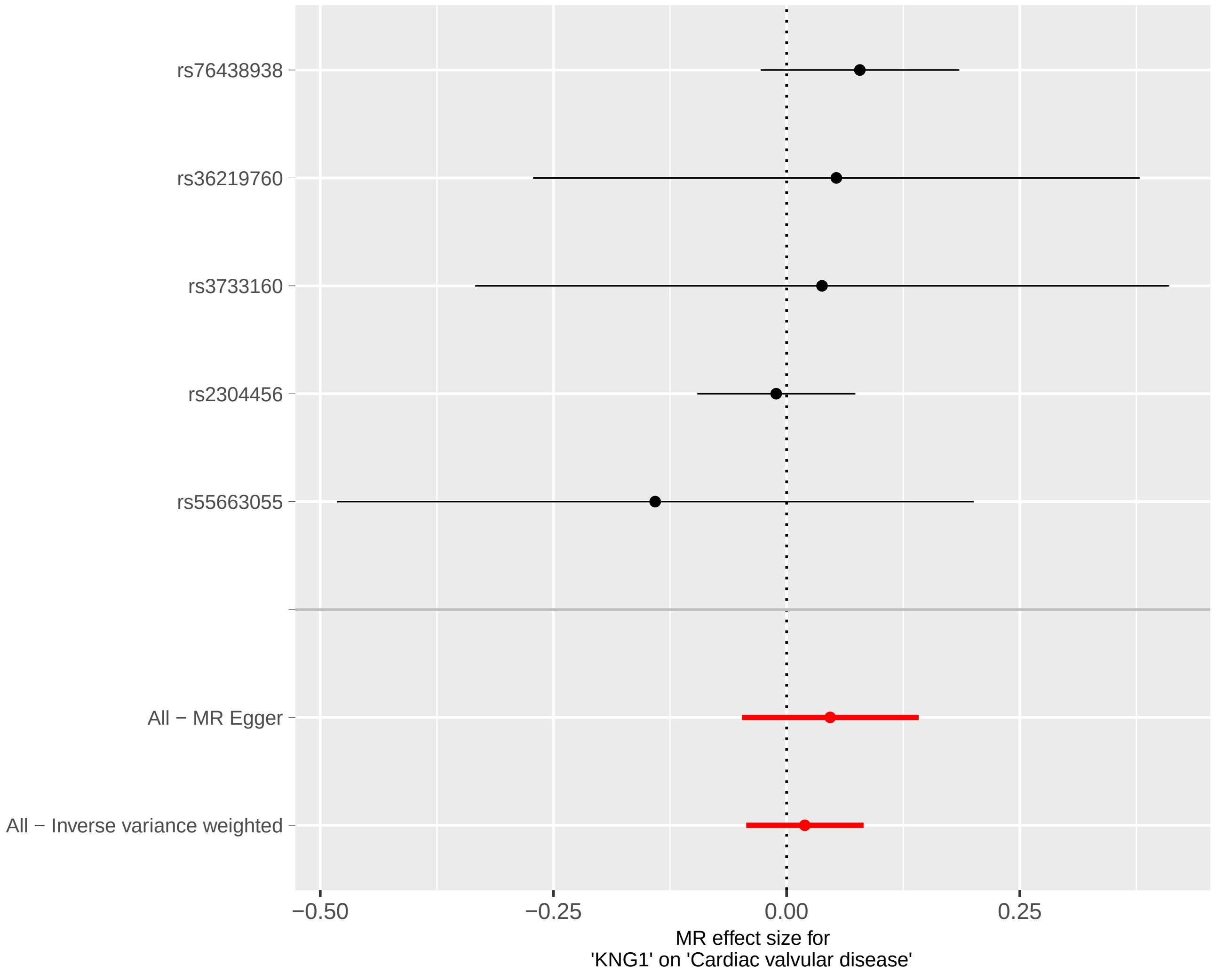

rs76438938
rs36219760
rs3733160
rs2304456
rs55663055
All − MR Egger
All − Inverse variance weighted
−0.50
−0.25
0.00
0.25
MR effect size for
'KNG1' on 'Cardiac valvular disease'

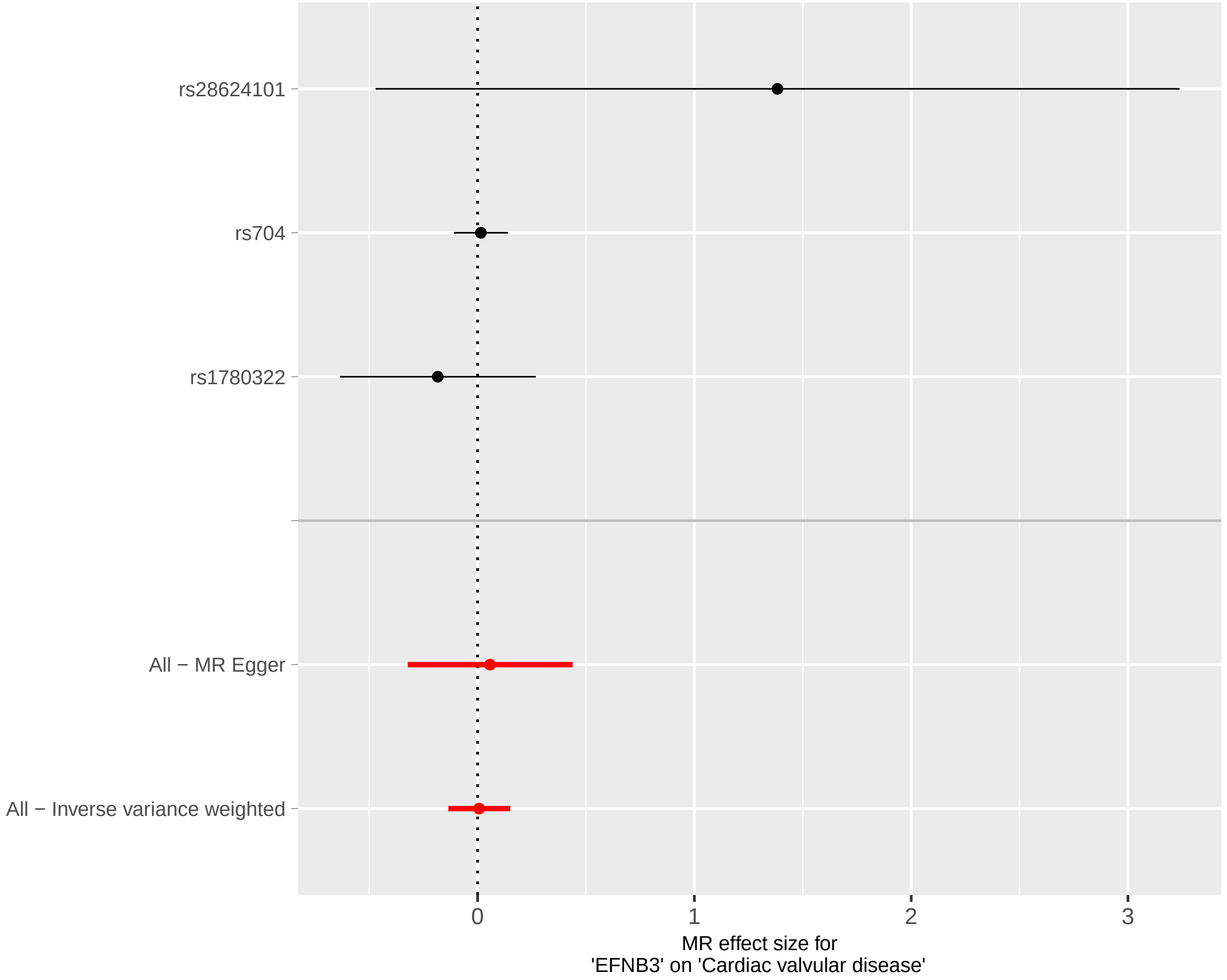
rs28624101
rs704
rs1780322
All − MR Egger
All − Inverse variance weighted
0
1
2
3
MR effect size for
'EFNB3' on 'Cardiac valvular disease'

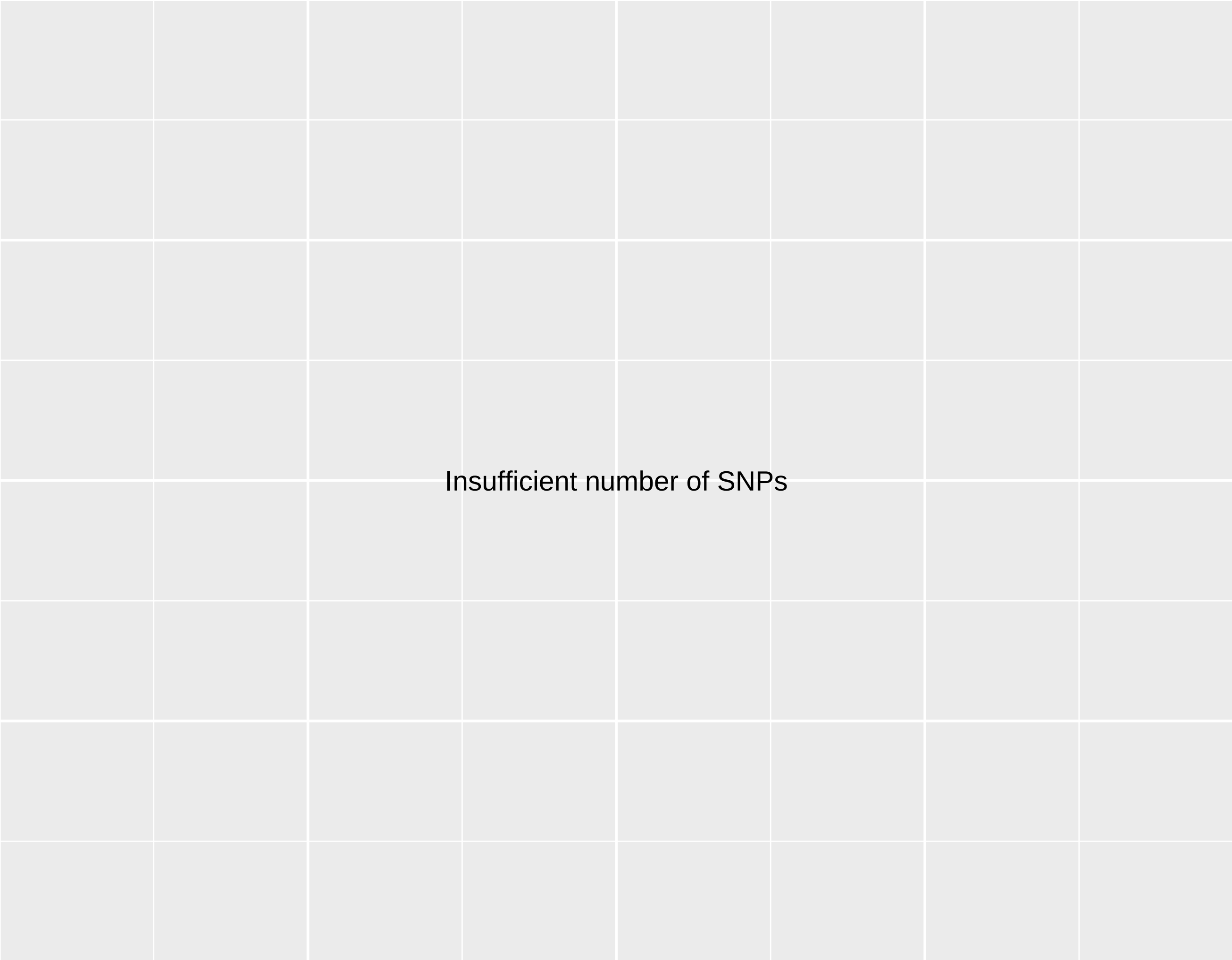
Insufficient number of SNPs

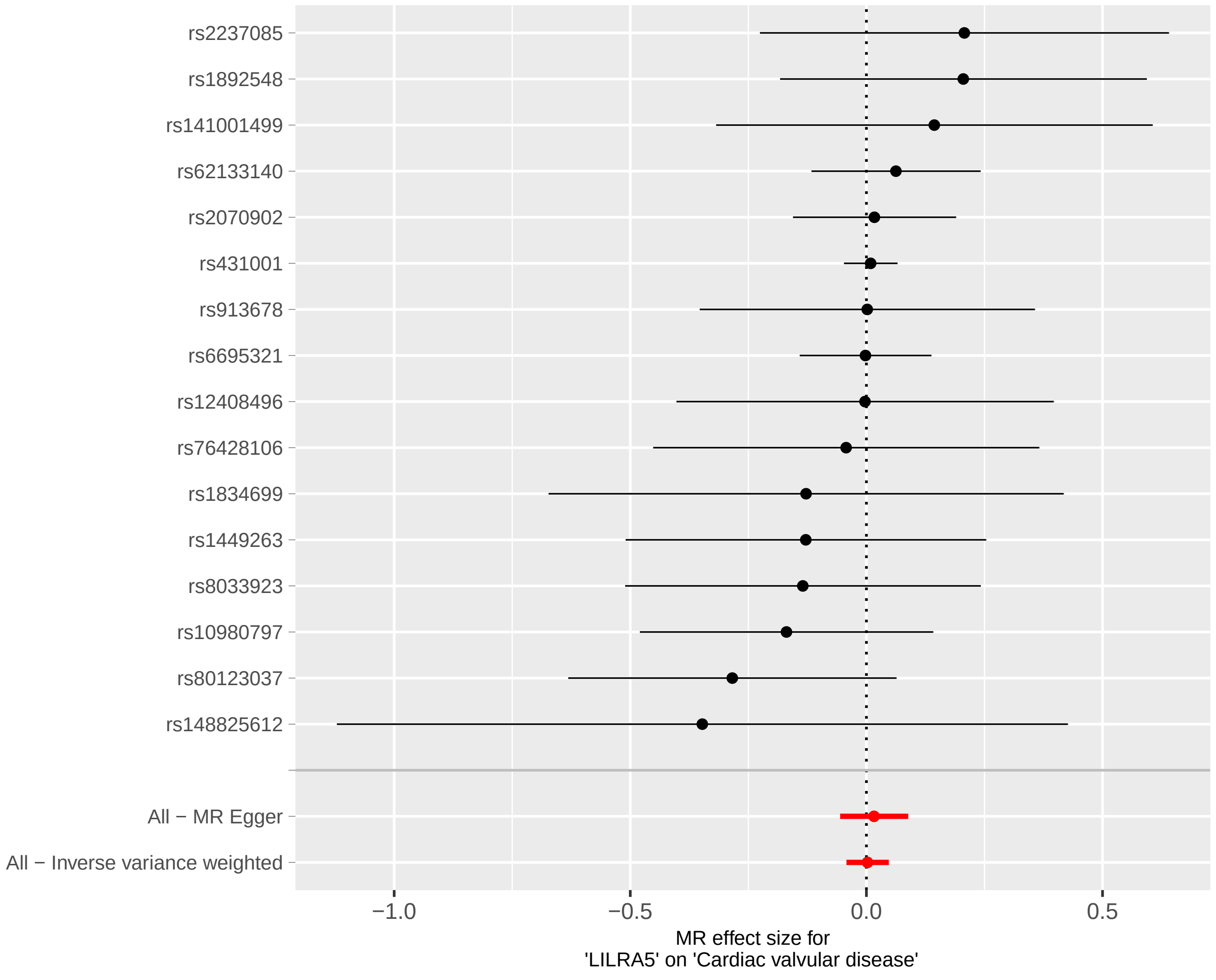
rs2237085
rs1892548
rs141001499
rs62133140
rs2070902
rs431001
rs913678
rs6695321
rs12408496
rs76428106
rs1834699
rs1449263
rs8033923
rs10980797
rs80123037
rs148825612
All − MR Egger
All − Inverse variance weighted
−1.0
−0.5
0.0
0.5
MR effect size for
'LILRA5' on 'Cardiac valvular disease'

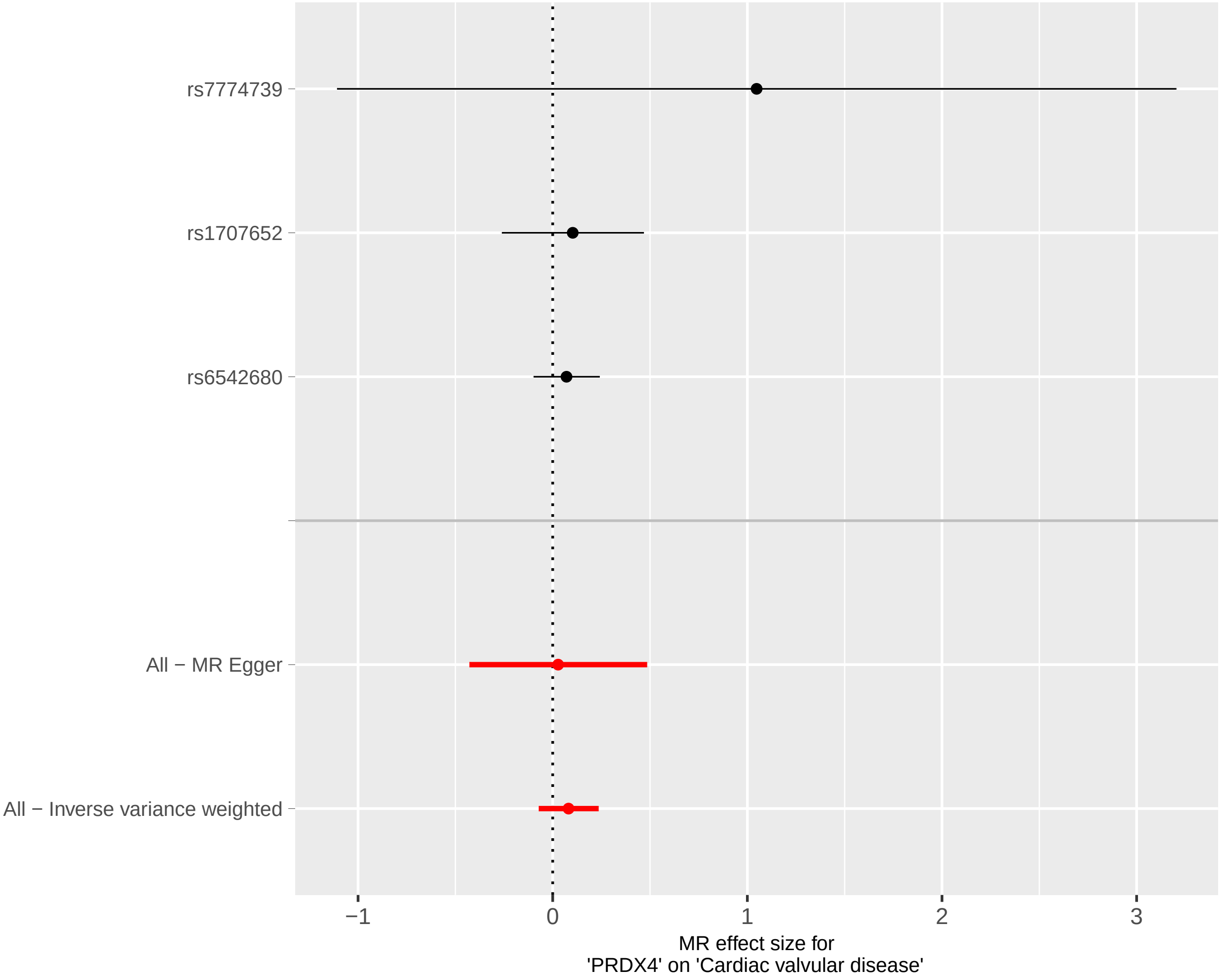
rs7774739
rs1707652
rs6542680
All − MR Egger
All − Inverse variance weighted
−1
0
1
2
3
MR effect size for
'PRDX4' on 'Cardiac valvular disease'

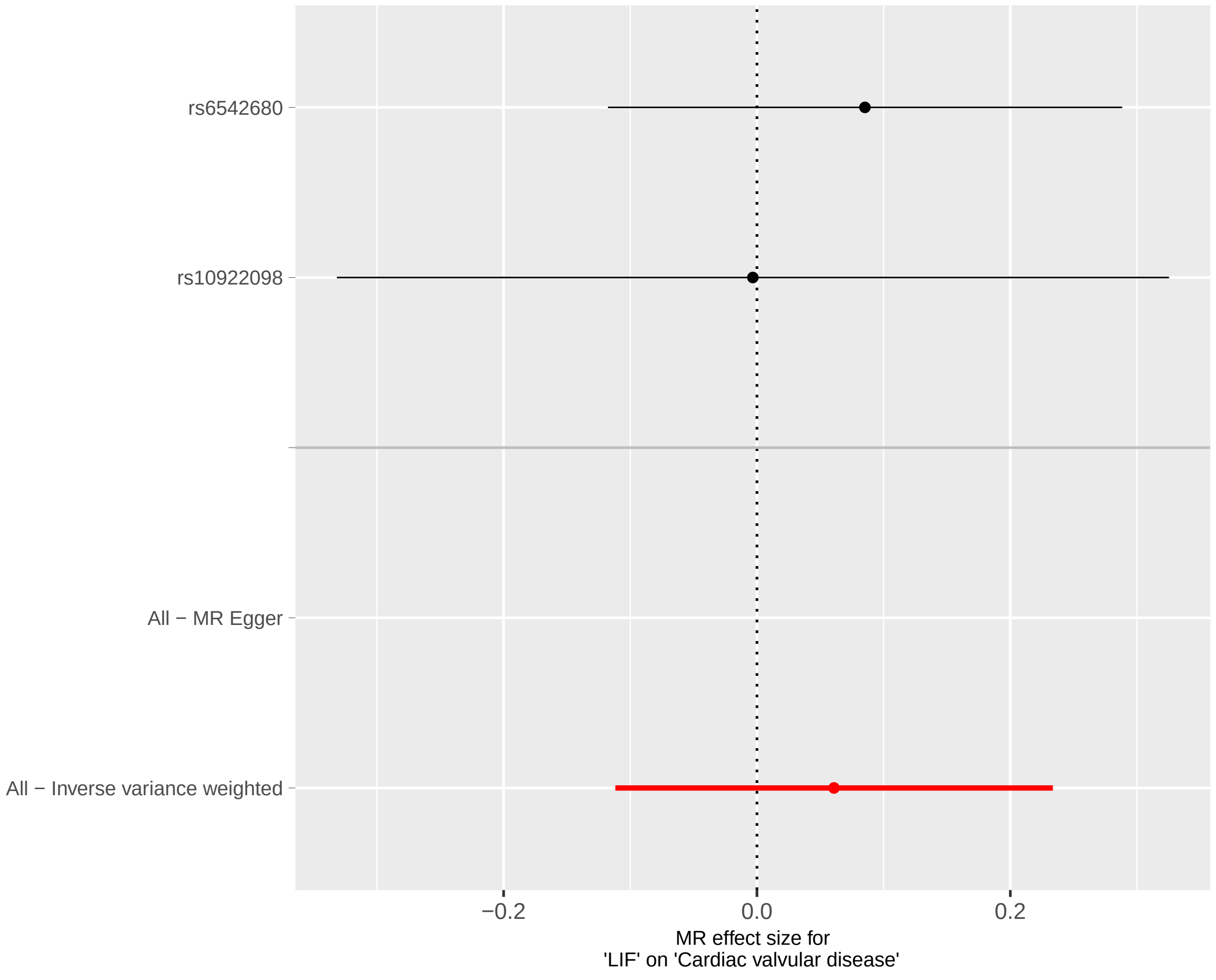
rs6542680
rs10922098
All − MR Egger
All − Inverse variance weighted
−0.2
0.0
0.2
MR effect size for
'LIF' on 'Cardiac valvular disease'

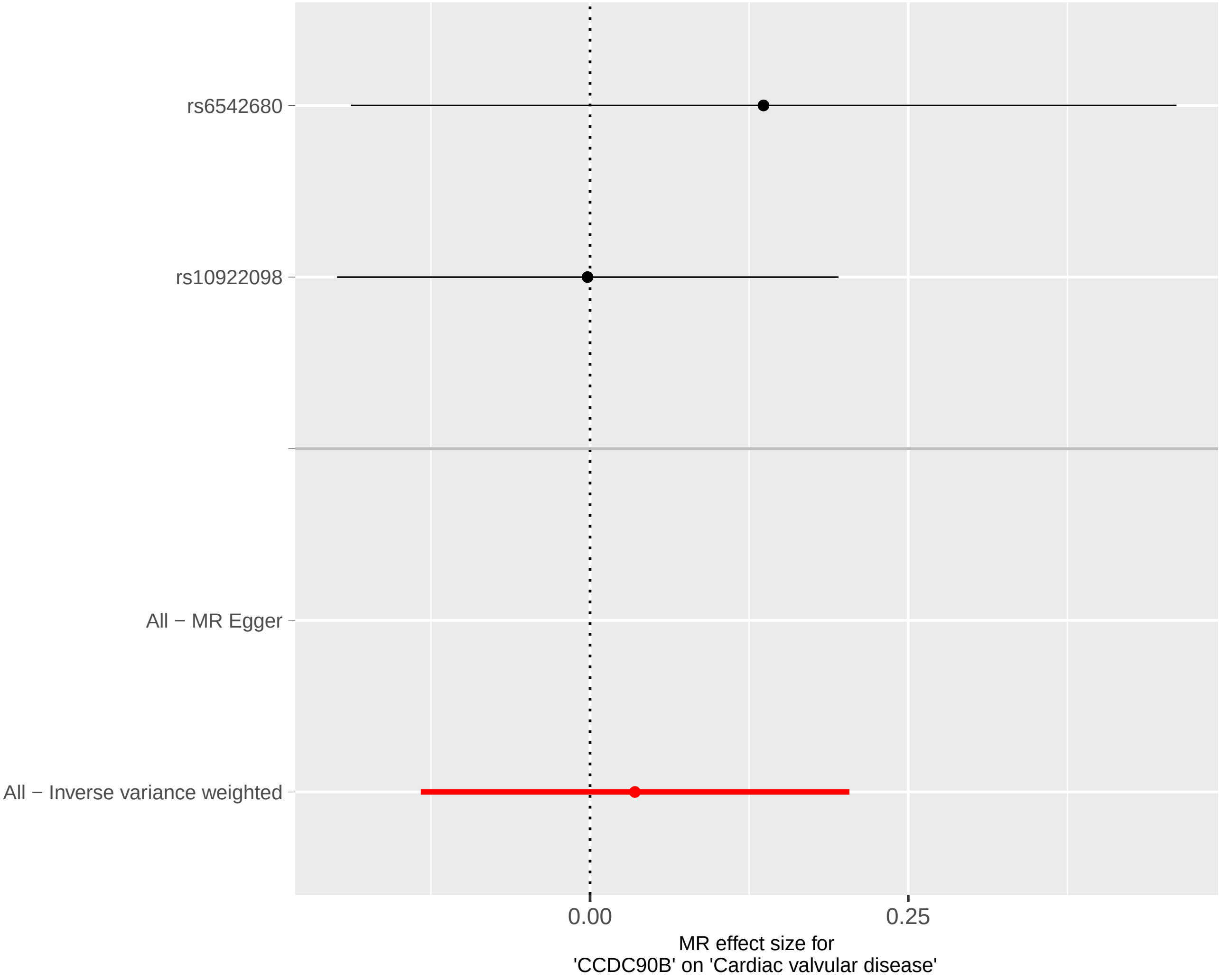
rs6542680
rs10922098
All − MR Egger
All − Inverse variance weighted
0.00
0.25
MR effect size for
'CCDC90B' on 'Cardiac valvular disease'

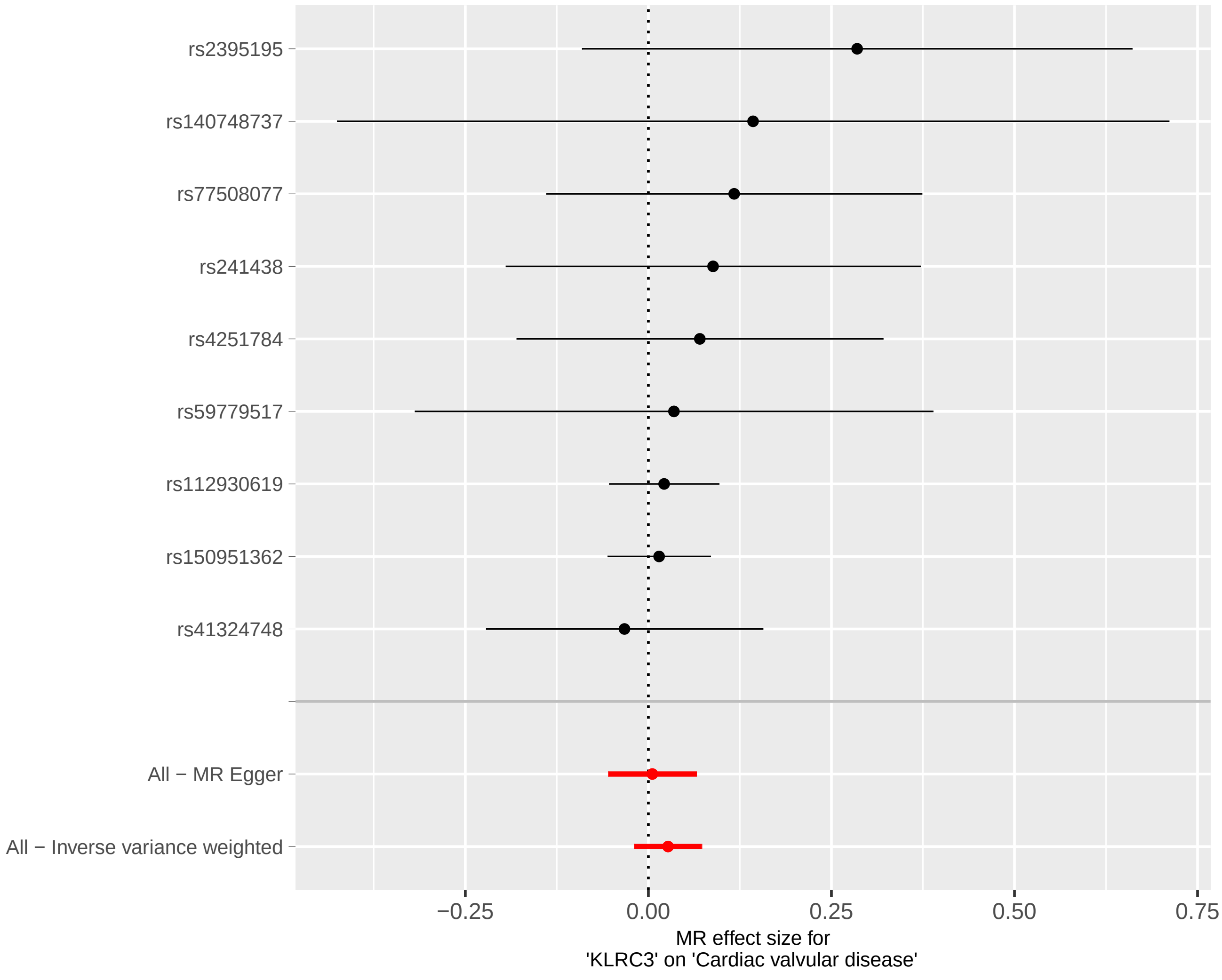

rs2395195
rs140748737
rs77508077
rs241438
rs4251784
rs59779517
rs112930619
rs150951362
rs41324748
All − MR Egger
All − Inverse variance weighted
−0.25
0.00
0.25
0.50
0.75
MR effect size for
'KLRC3' on 'Cardiac valvular disease'

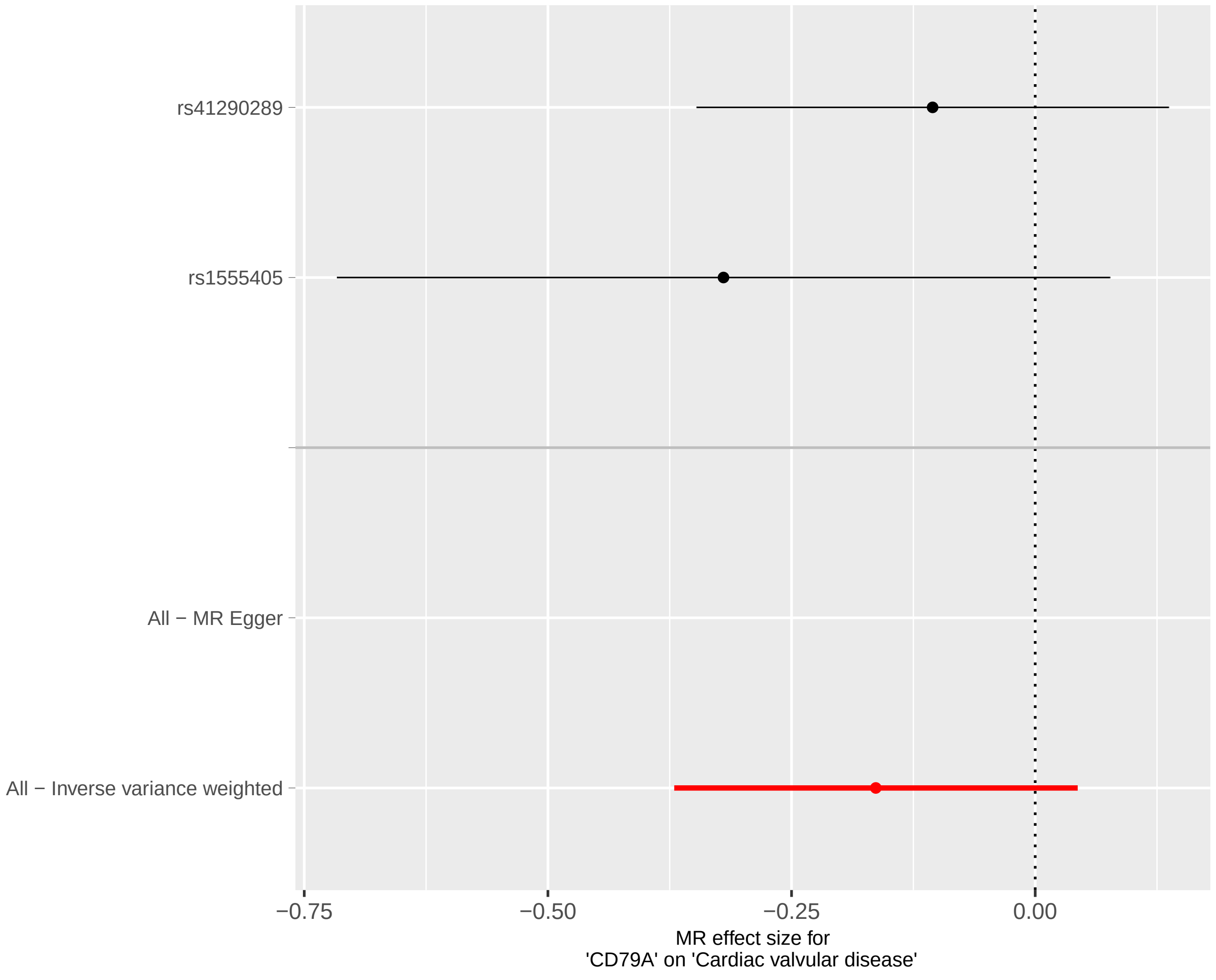

rs41290289
rs1555405
All − MR Egger
All − Inverse variance weighted
−0.75
−0.50
−0.25
0.00
MR effect size for
'CD79A' on 'Cardiac valvular disease'

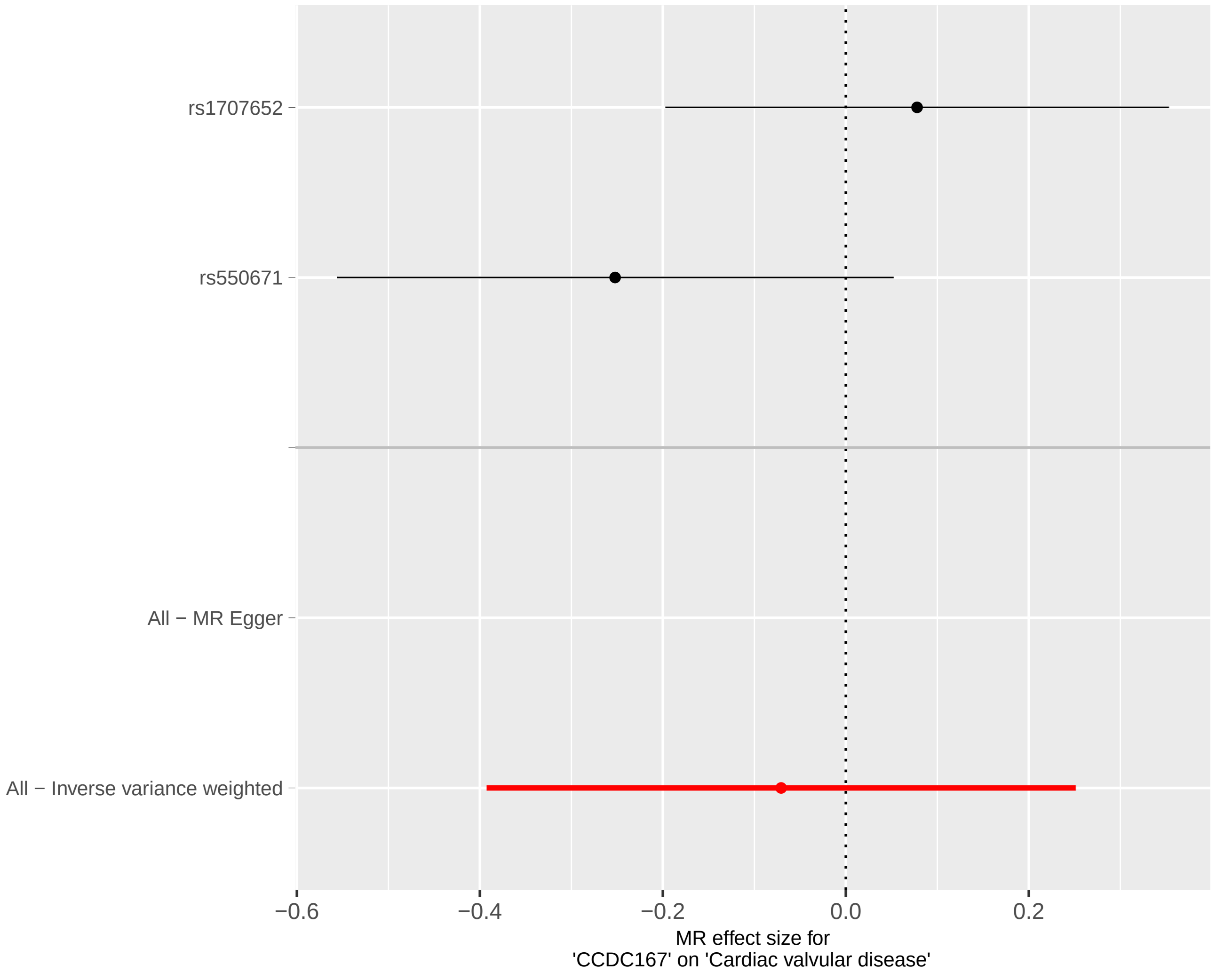

rs1707652
rs550671
All − MR Egger
All − Inverse variance weighted
−0.6
−0.4
−0.2
0.0
0.2
MR effect size for
'CCDC167' on 'Cardiac valvular disease'

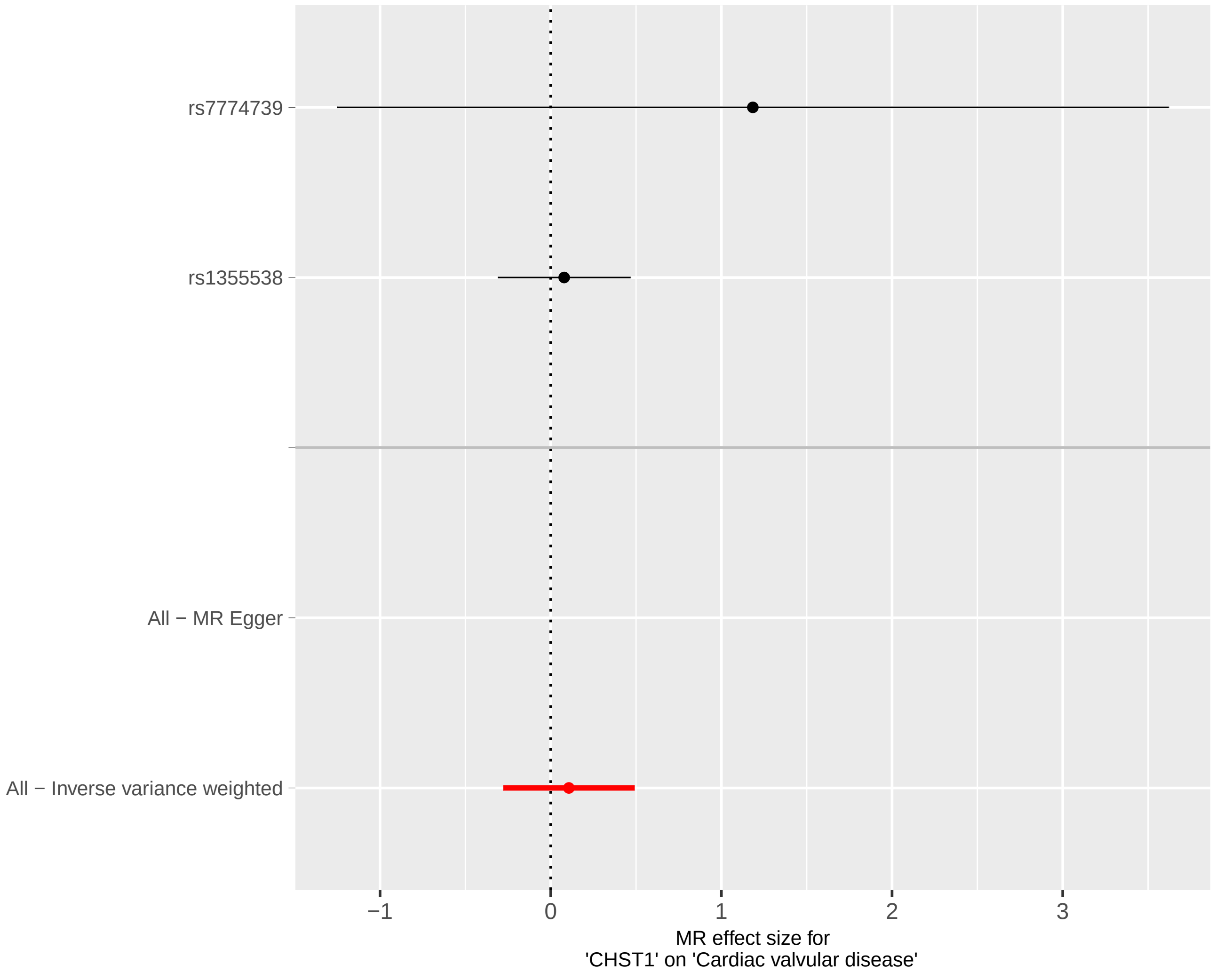
rs7774739
rs1355538
All − MR Egger
All − Inverse variance weighted
−1
0
1
2
3
MR effect size for
'CHST1' on 'Cardiac valvular disease'

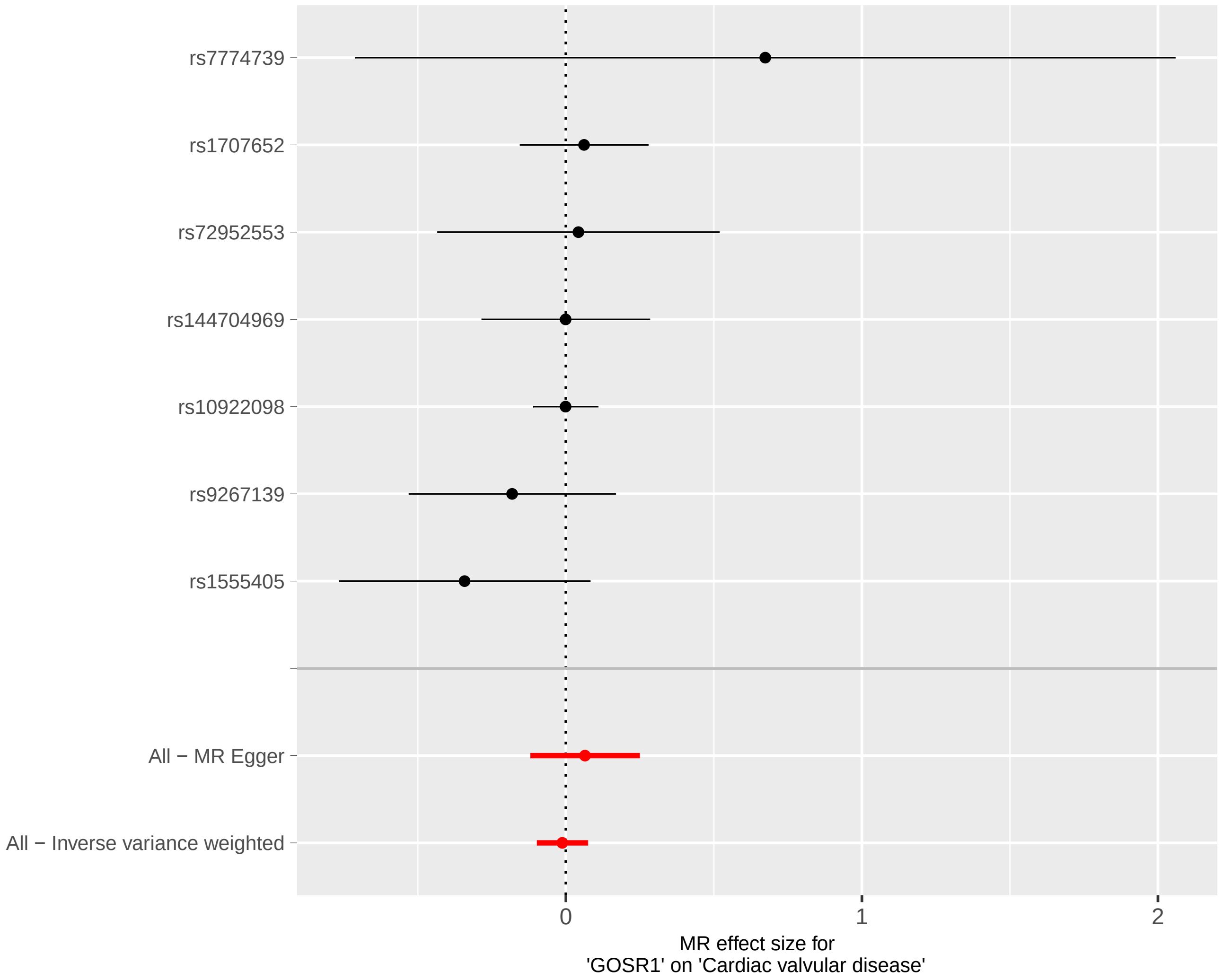
rs7774739
rs1707652
rs72952553
rs144704969
rs10922098
rs9267139
rs1555405
All − MR Egger
All − Inverse variance weighted
0
1
2
MR effect size for
'GOSR1' on 'Cardiac valvular disease'

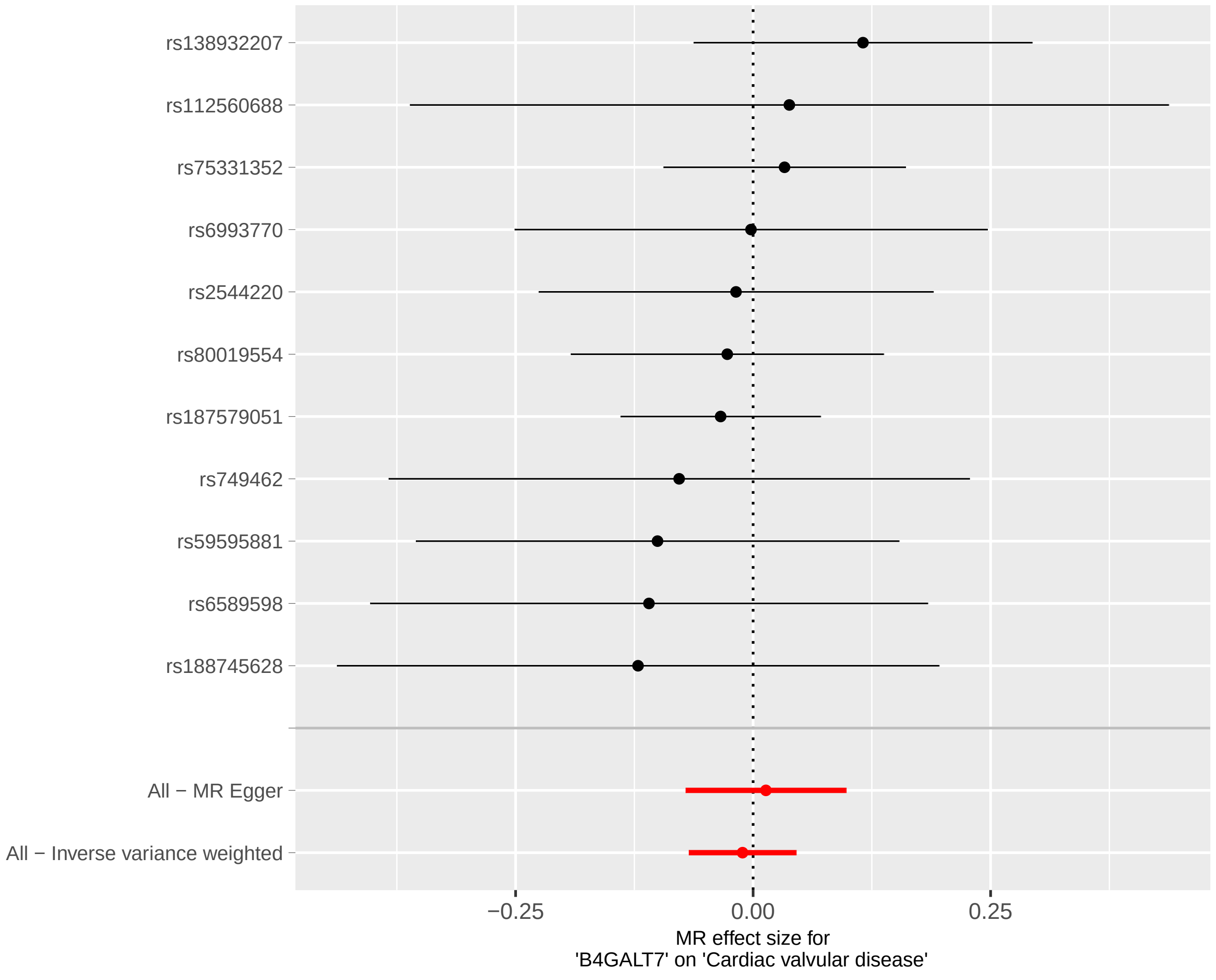

rs138932207
rs112560688
rs75331352
rs6993770
rs2544220
rs80019554
rs187579051
rs749462
rs59595881
rs6589598
rs188745628
All − MR Egger
All − Inverse variance weighted
−0.25
0.00
0.25
MR effect size for
'B4GALT7' on 'Cardiac valvular disease'

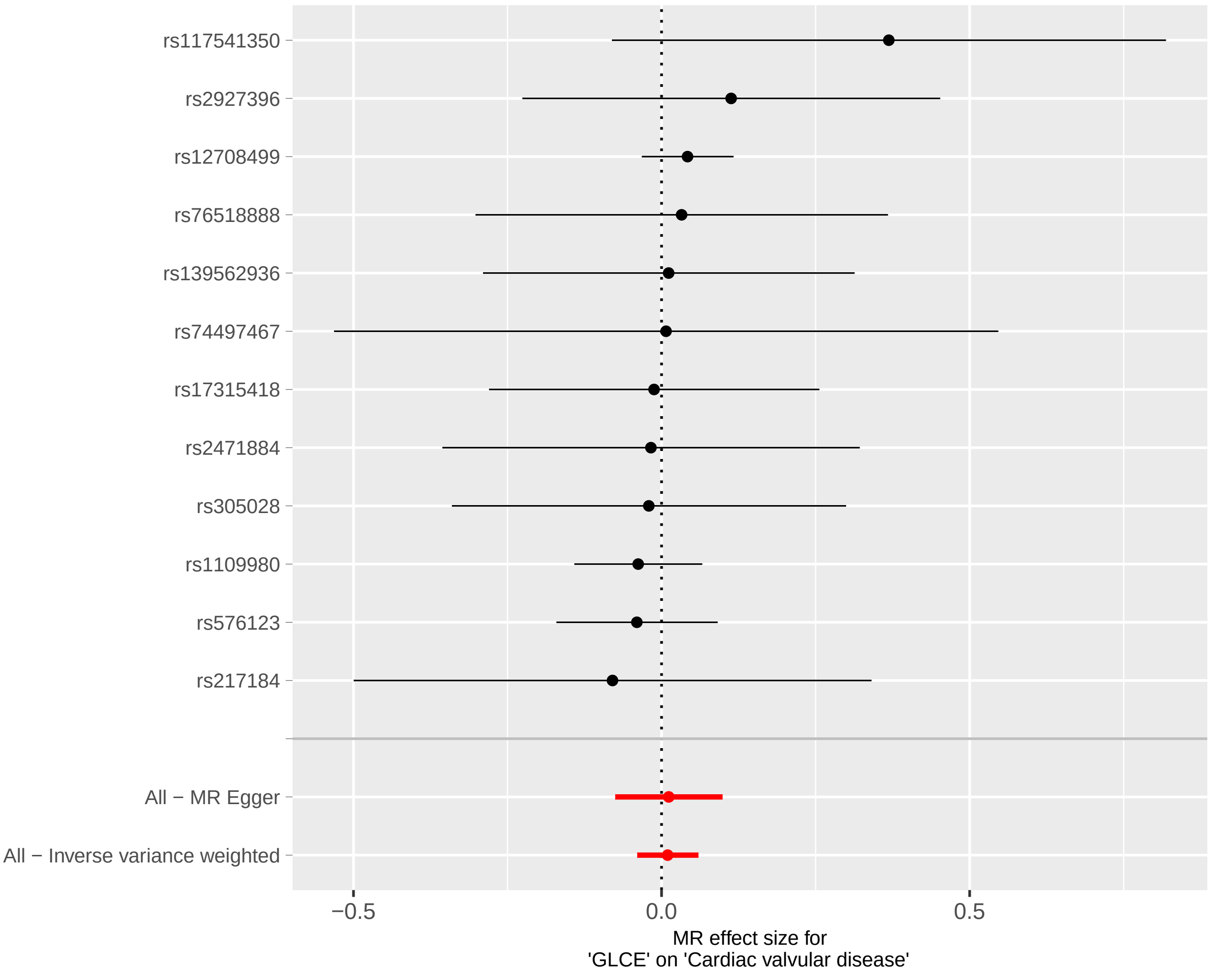

rs117541350
rs2927396
rs12708499
rs76518888
rs139562936
rs74497467
rs17315418
rs2471884
rs305028
rs1109980
rs576123
rs217184
All − MR Egger
All − Inverse variance weighted
−0.5
0.0
0.5
MR effect size for
'GLCE' on 'Cardiac valvular disease'

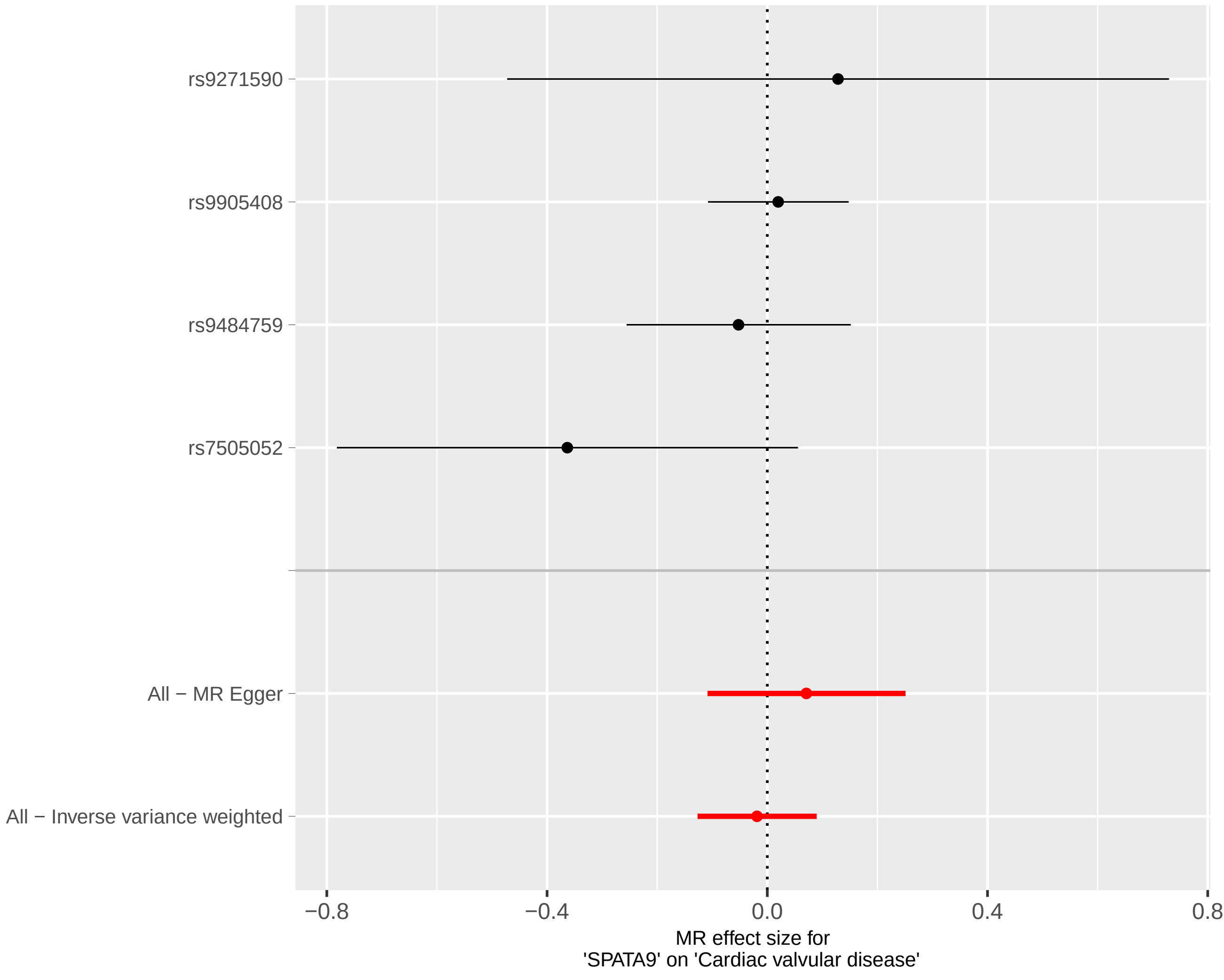
rs9271590
rs9905408
rs9484759
rs7505052
All − MR Egger
All − Inverse variance weighted
−0.8
−0.4
0.0
0.4
0.8
MR effect size for
'SPATA9' on 'Cardiac valvular disease'

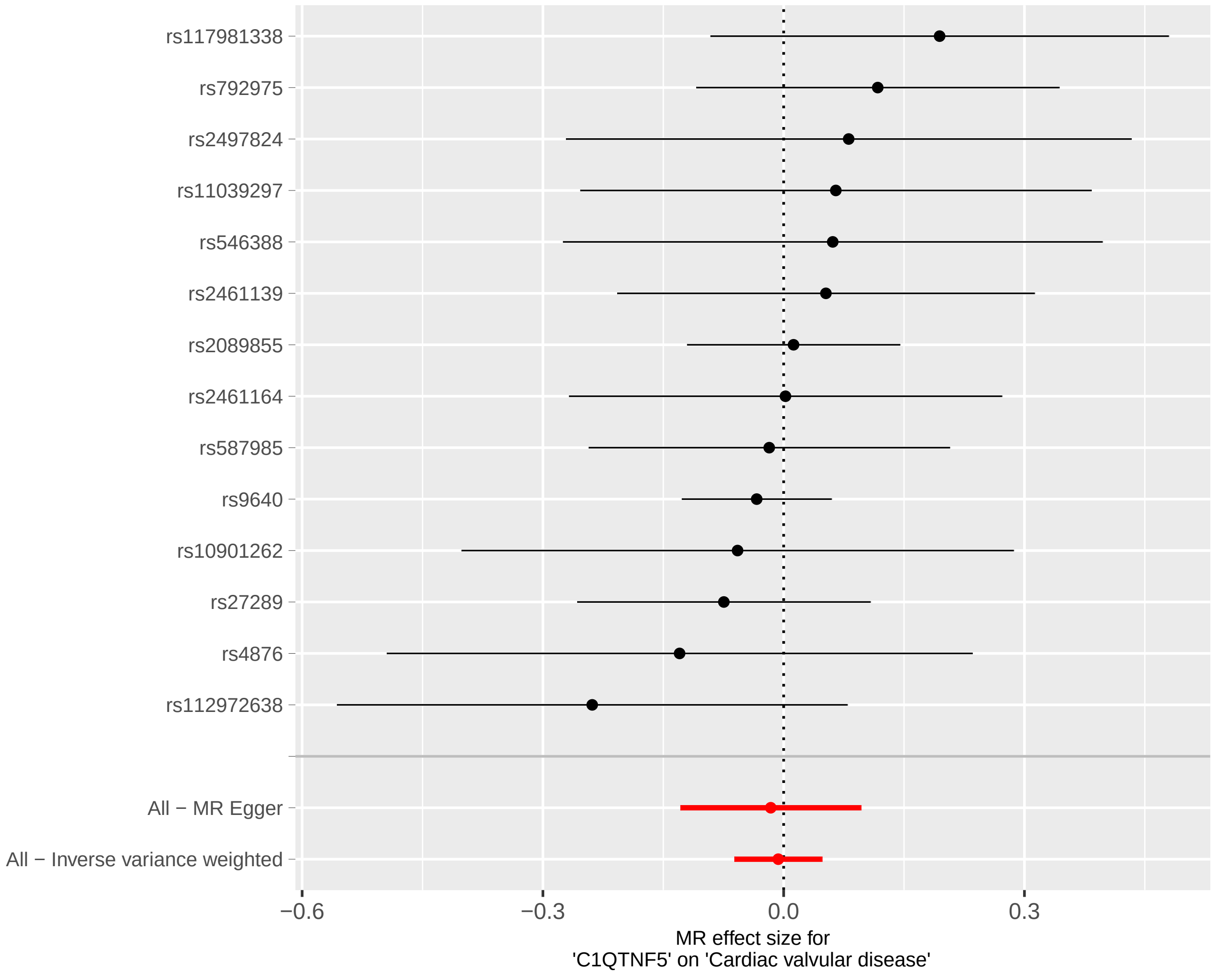
rs117981338
rs792975
rs2497824
rs11039297
rs546388
rs2461139
rs2089855
rs2461164
rs587985
rs9640
rs10901262
rs27289
rs4876
rs112972638
All − MR Egger
All − Inverse variance weighted
−0.6
−0.3
0.0
0.3
MR effect size for
'C1QTNF5' on 'Cardiac valvular disease'

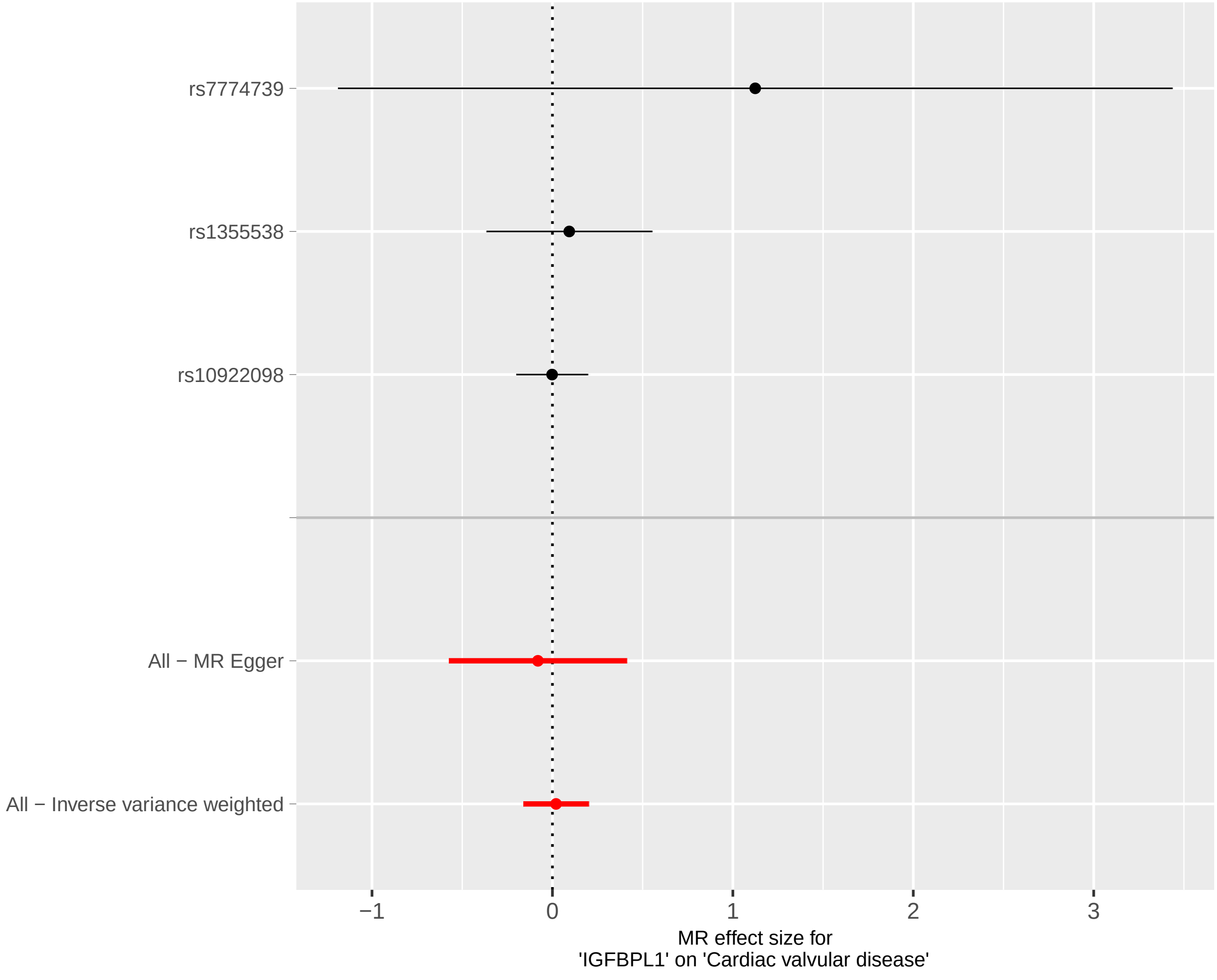
rs7774739
rs1355538
rs10922098
All − MR Egger
All − Inverse variance weighted
−1
0
1
2
3
MR effect size for
'IGFBPL1' on 'Cardiac valvular disease'

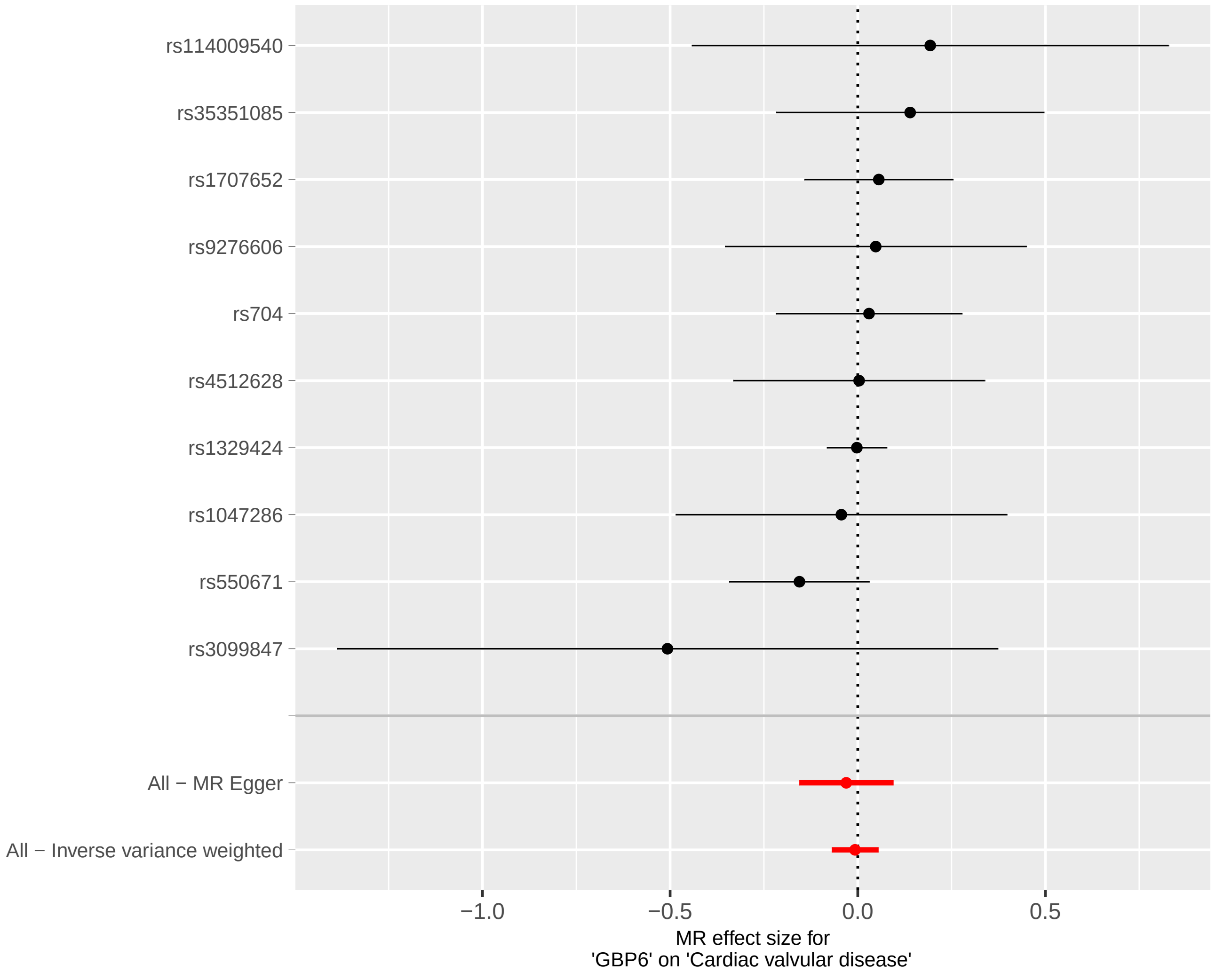

rs114009540
rs35351085
rs1707652
rs9276606
rs704
rs4512628
rs1329424
rs1047286
rs550671
rs3099847
All − MR Egger
All − Inverse variance weighted
−1.0
−0.5
0.0
0.5
MR effect size for
'GBP6' on 'Cardiac valvular disease'

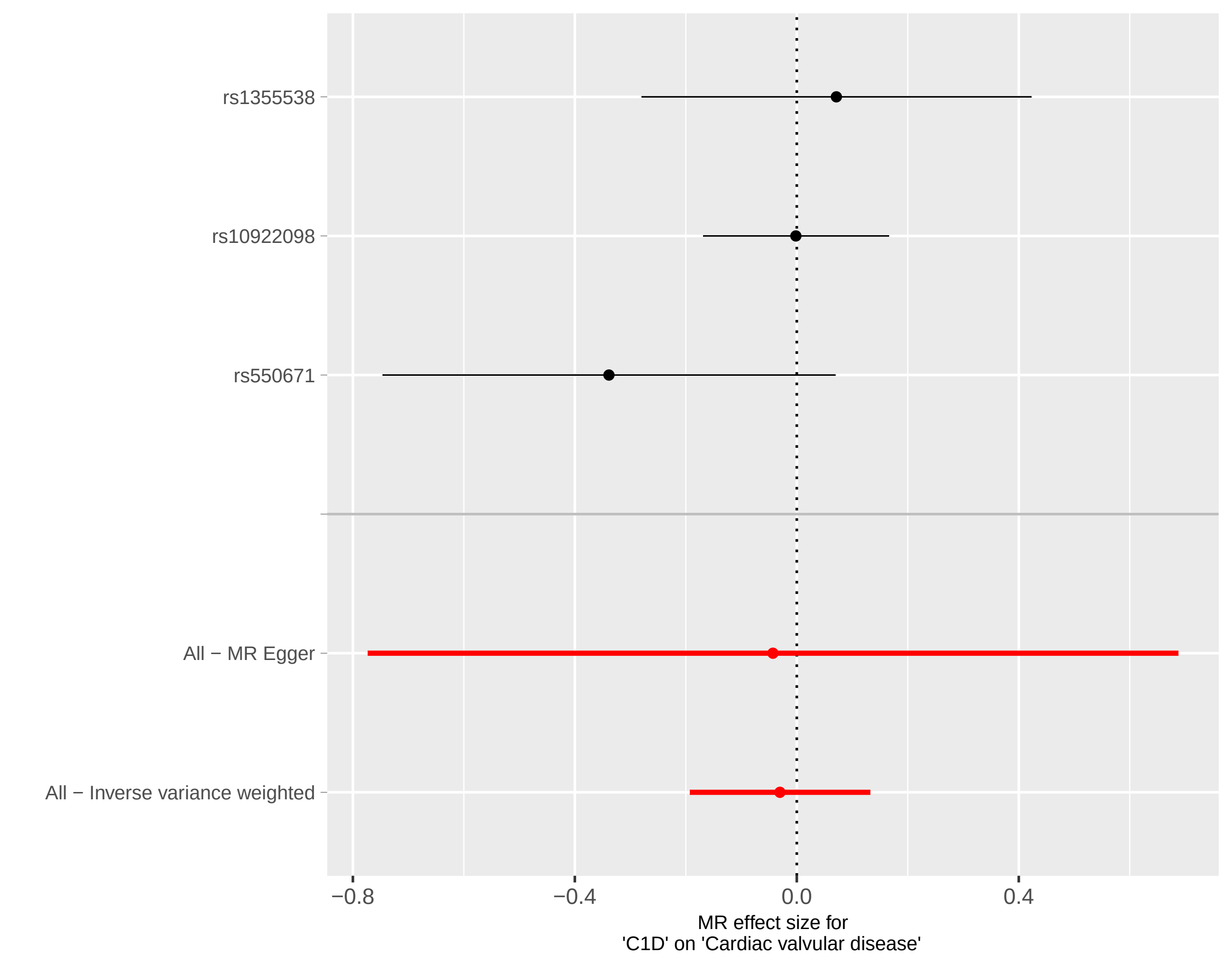
rs1355538
rs10922098
rs550671
All − MR Egger
All − Inverse variance weighted
−0.8
−0.4
0.0
0.4
MR effect size for
'C1D' on 'Cardiac valvular disease'

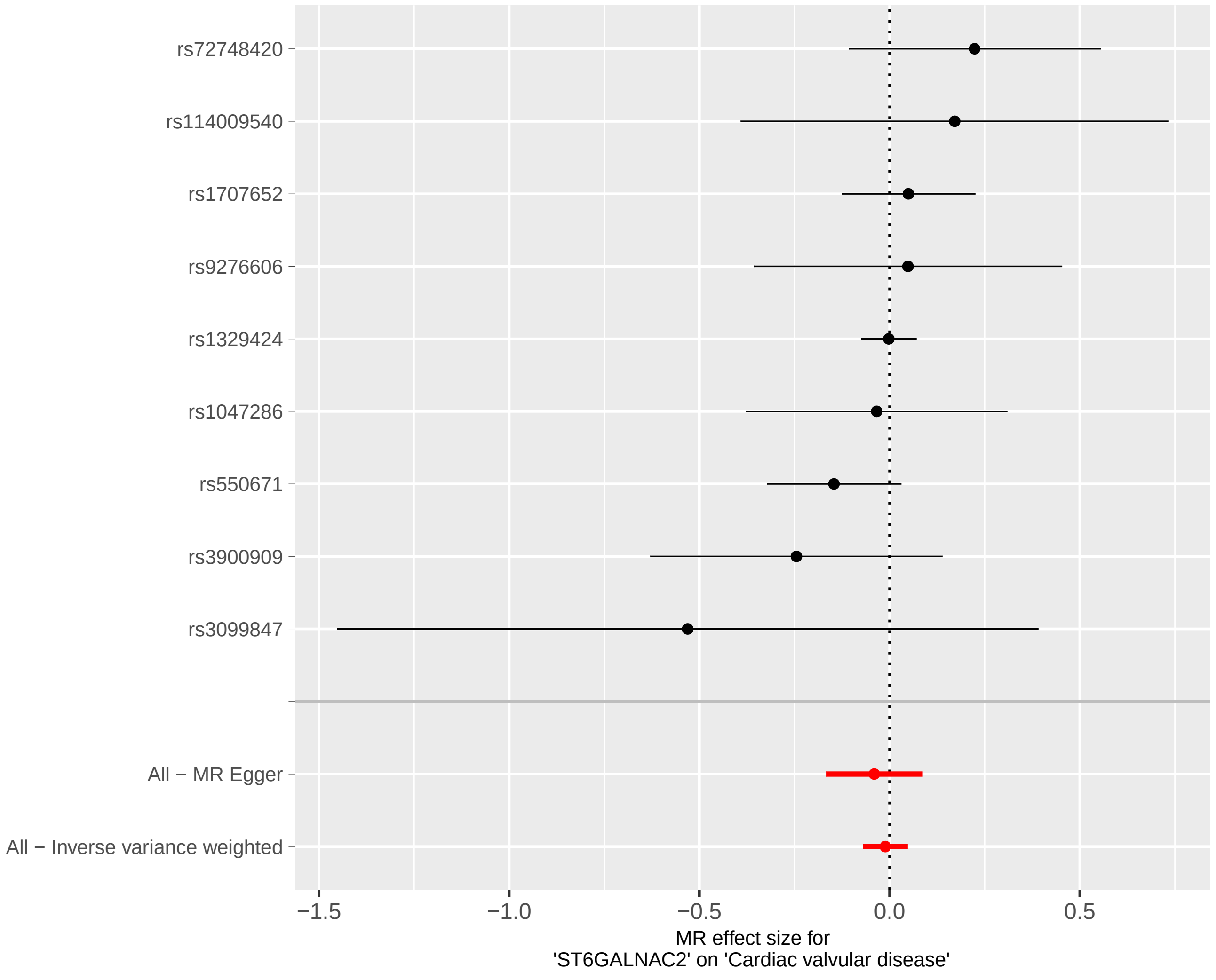

rs72748420
rs114009540
rs1707652
rs9276606
rs1329424
rs1047286
rs550671
rs3900909
rs3099847
All − MR Egger
All − Inverse variance weighted
−1.5
−1.0
−0.5
0.0
0.5
MR effect size for
'ST6GALNAC2' on 'Cardiac valvular disease'

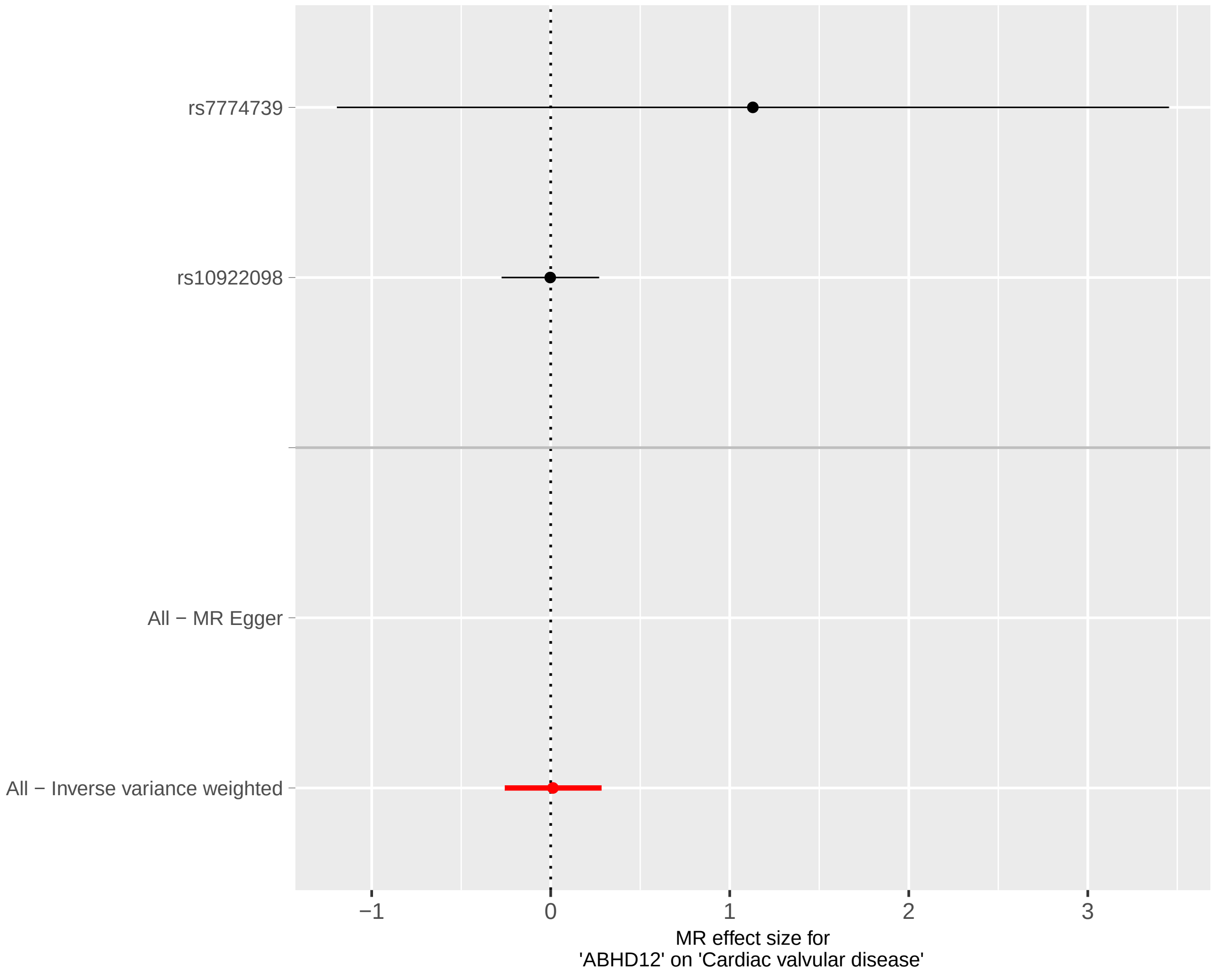

rs7774739
rs10922098
All − MR Egger
All − Inverse variance weighted
−1
0
1
2
3
MR effect size for
'ABHD12' on 'Cardiac valvular disease'

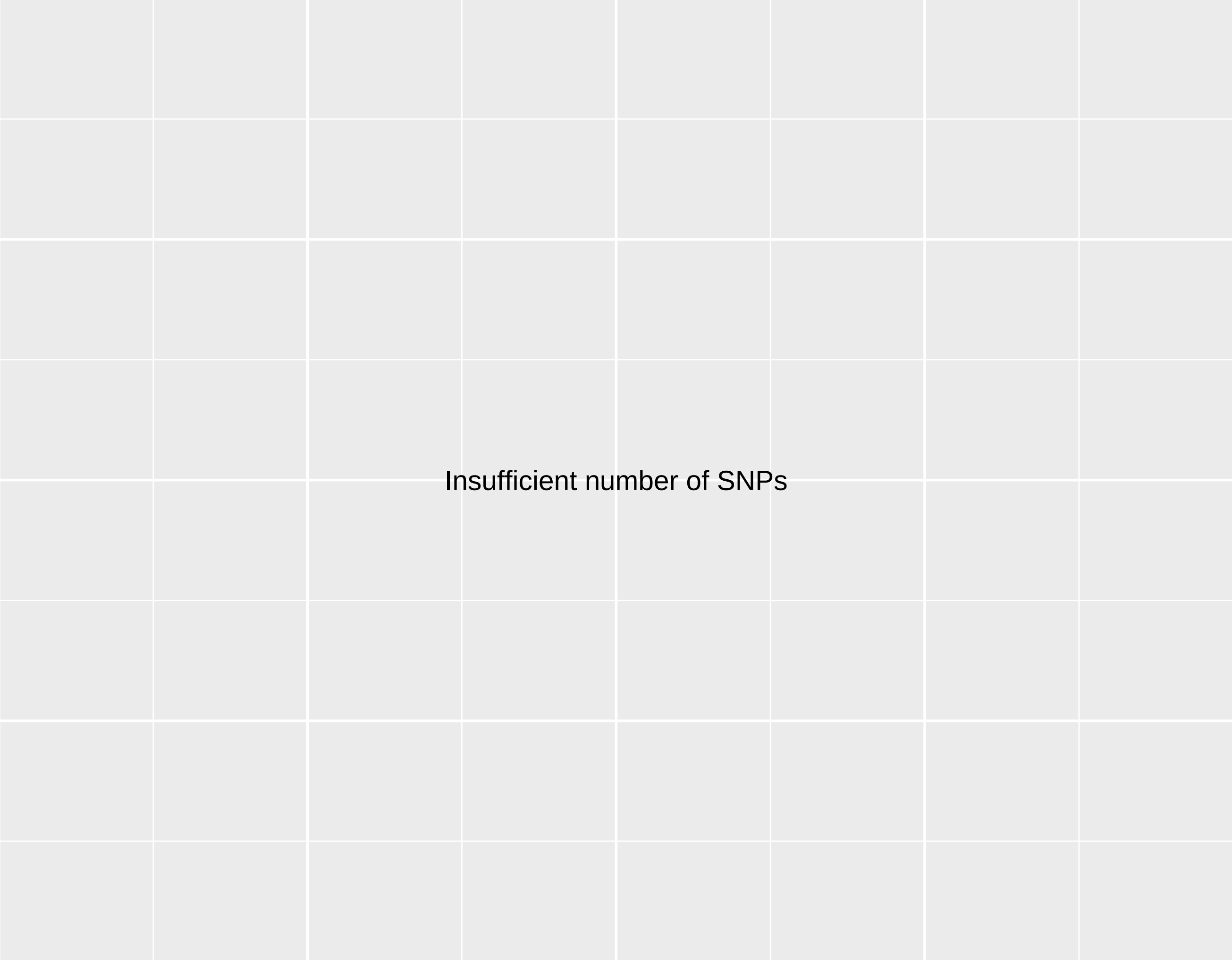
Insufficient number of SNPs

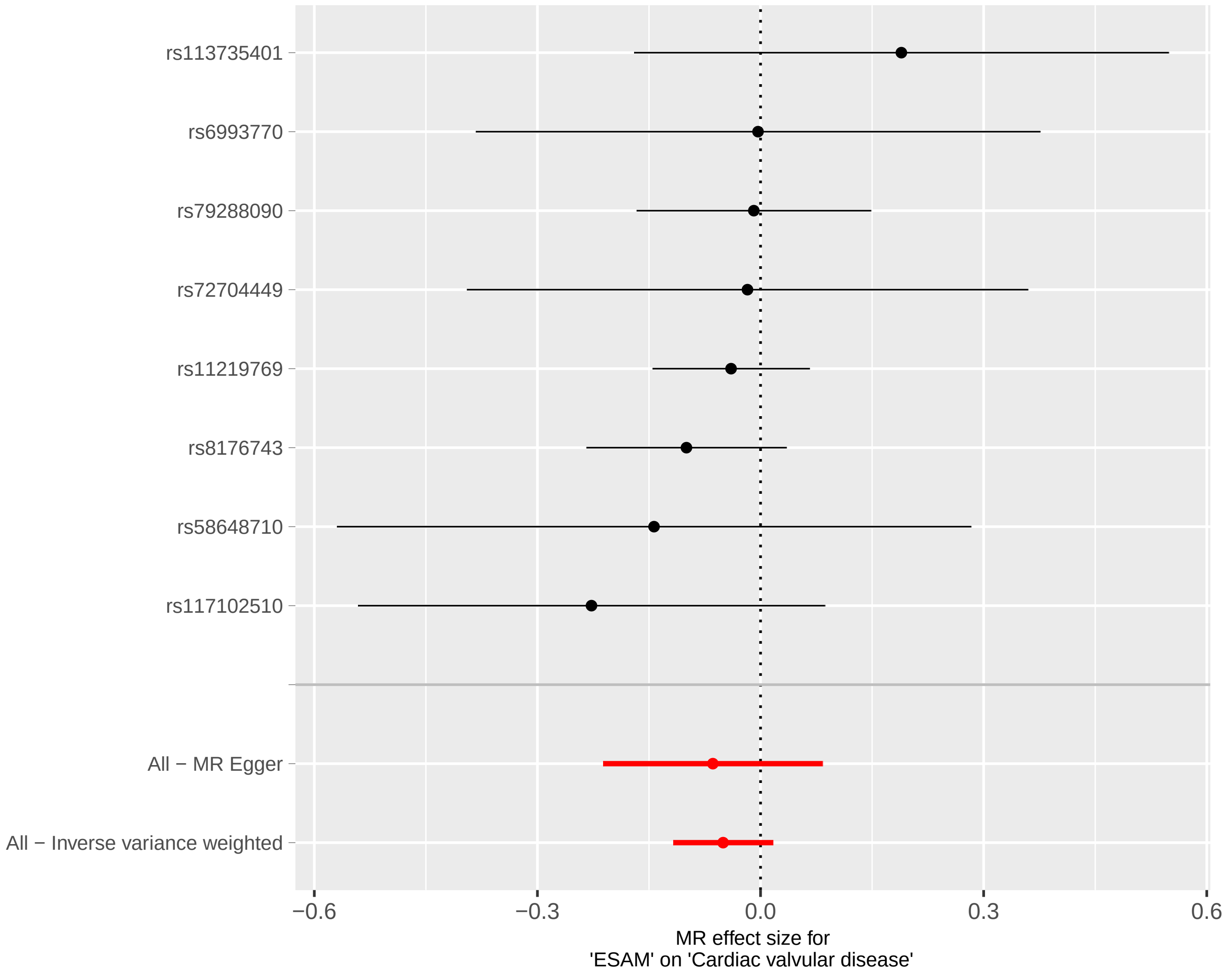
rs113735401
rs6993770
rs79288090
rs72704449
rs11219769
rs8176743
rs58648710
rs117102510
All − MR Egger
All − Inverse variance weighted
−0.6
−0.3
0.0
0.3
0.6
MR effect size for
'ESAM' on 'Cardiac valvular disease'

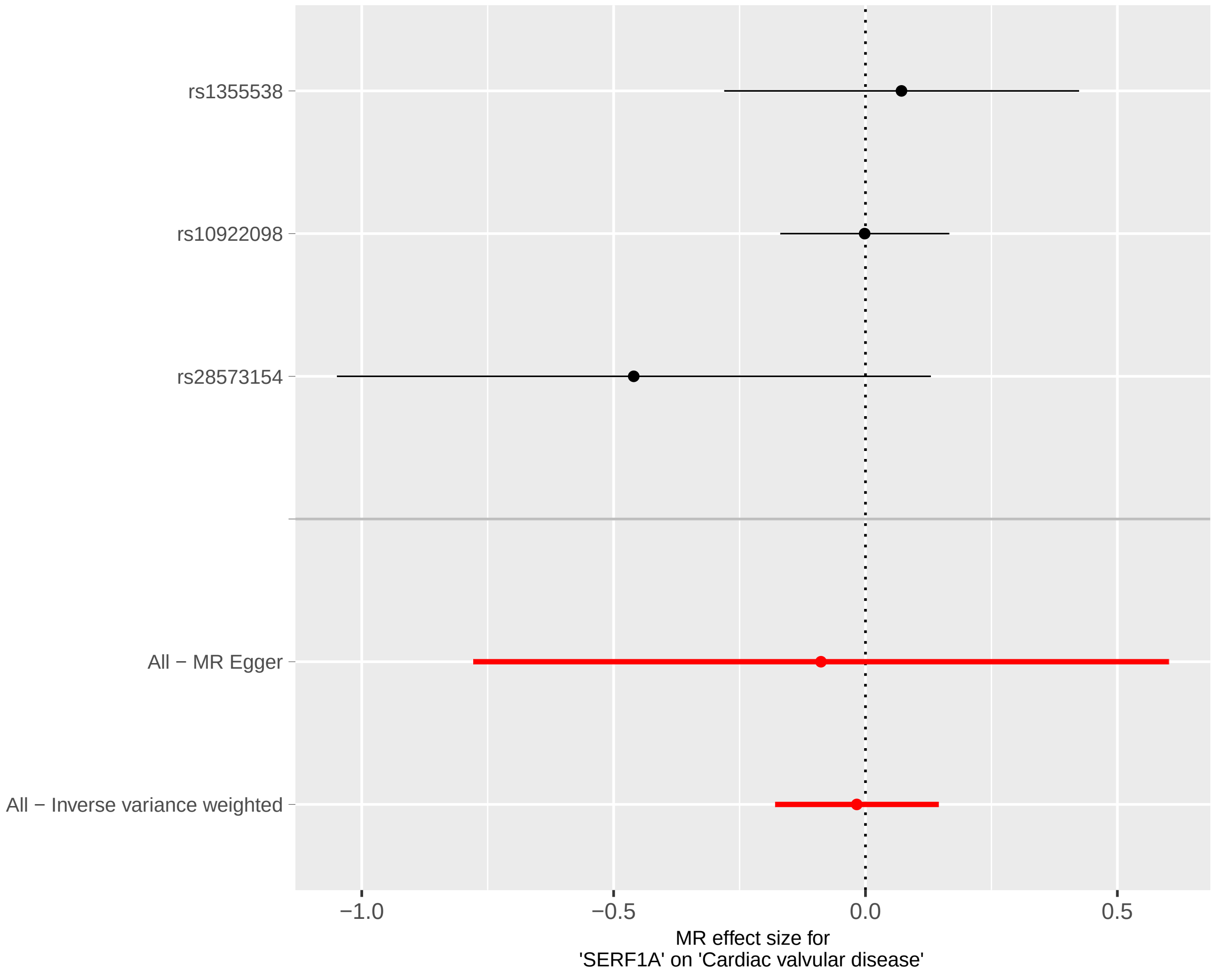
rs1355538
rs10922098
rs28573154
All − MR Egger
All − Inverse variance weighted
−1.0
−0.5
0.0
0.5
MR effect size for
'SERF1A' on 'Cardiac valvular disease'

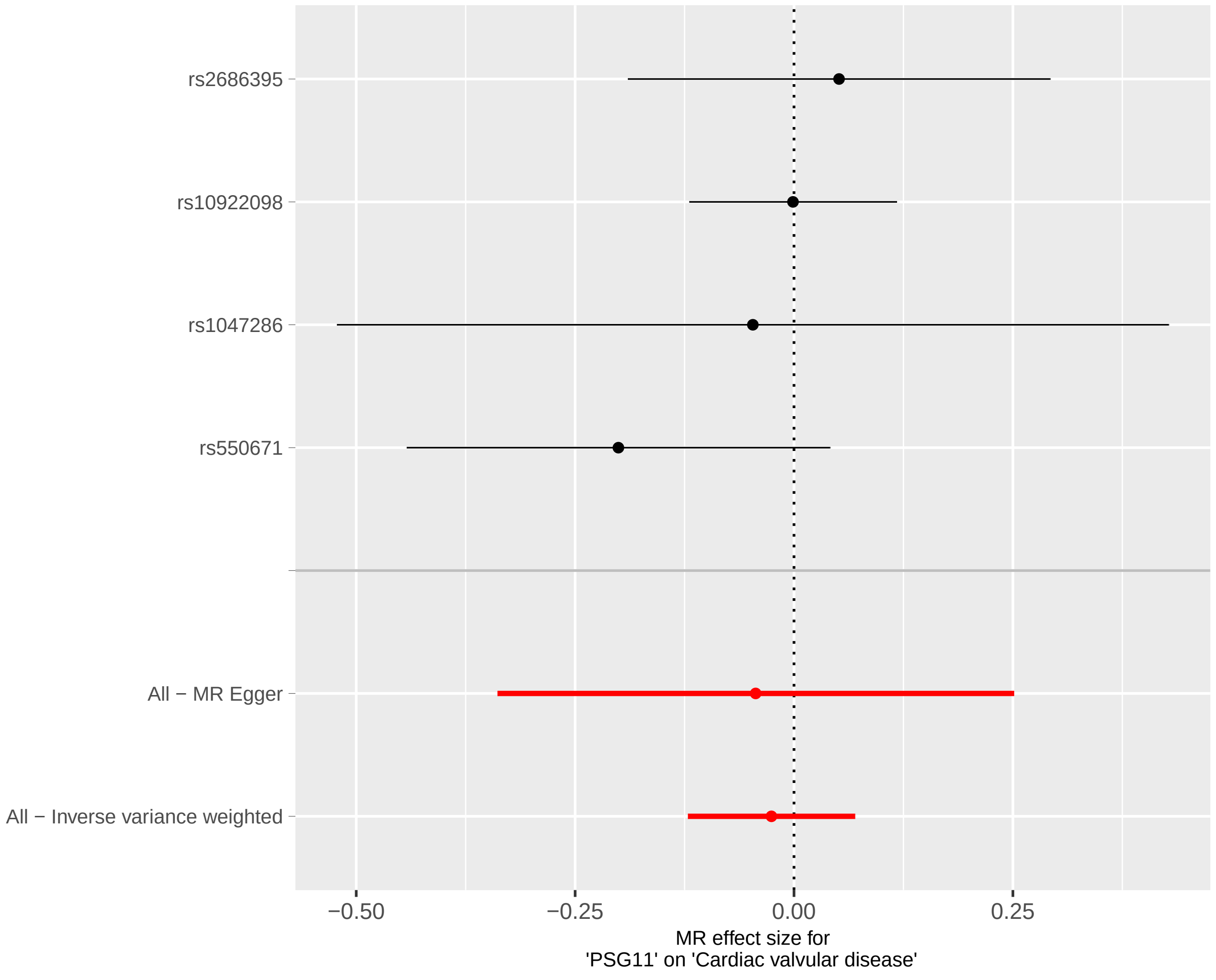
rs2686395
rs10922098
rs1047286
rs550671
All − MR Egger
All − Inverse variance weighted
−0.50
−0.25
0.00
0.25
MR effect size for
'PSG11' on 'Cardiac valvular disease'

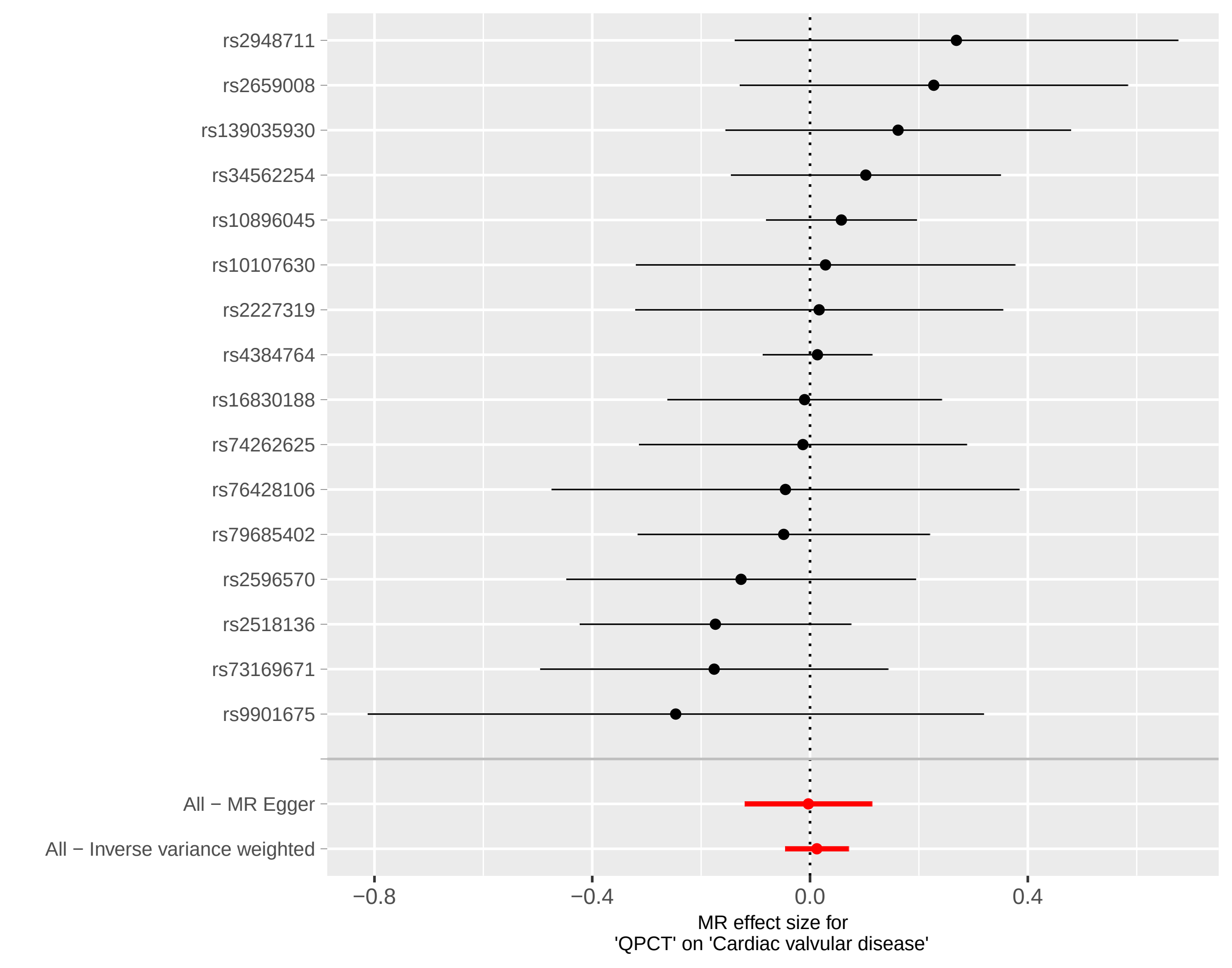

rs2948711
rs2659008
rs139035930
rs34562254
rs10896045
rs10107630
rs2227319
rs4384764
rs16830188
rs74262625
rs76428106
rs79685402
rs2596570
rs2518136
rs73169671
rs9901675
All − MR Egger
All − Inverse variance weighted
−0.8
−0.4
0.0
0.4
MR effect size for
'QPCT' on 'Cardiac valvular disease'

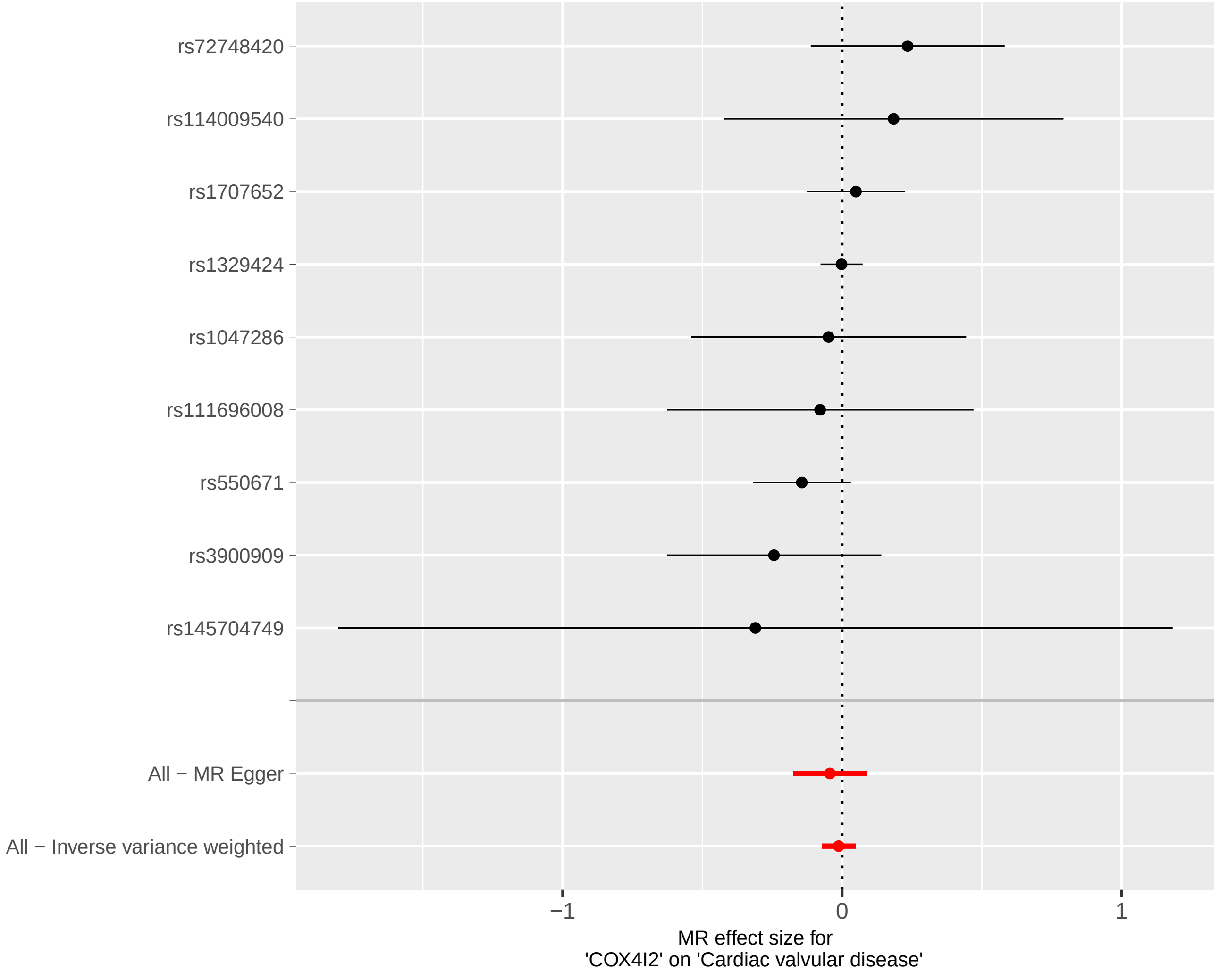
rs72748420
rs114009540
rs1707652
rs1329424
rs1047286
rs111696008
rs550671
rs3900909
rs145704749
All − MR Egger
All − Inverse variance weighted
−1
0
1
MR effect size for
'COX4I2' on 'Cardiac valvular disease'

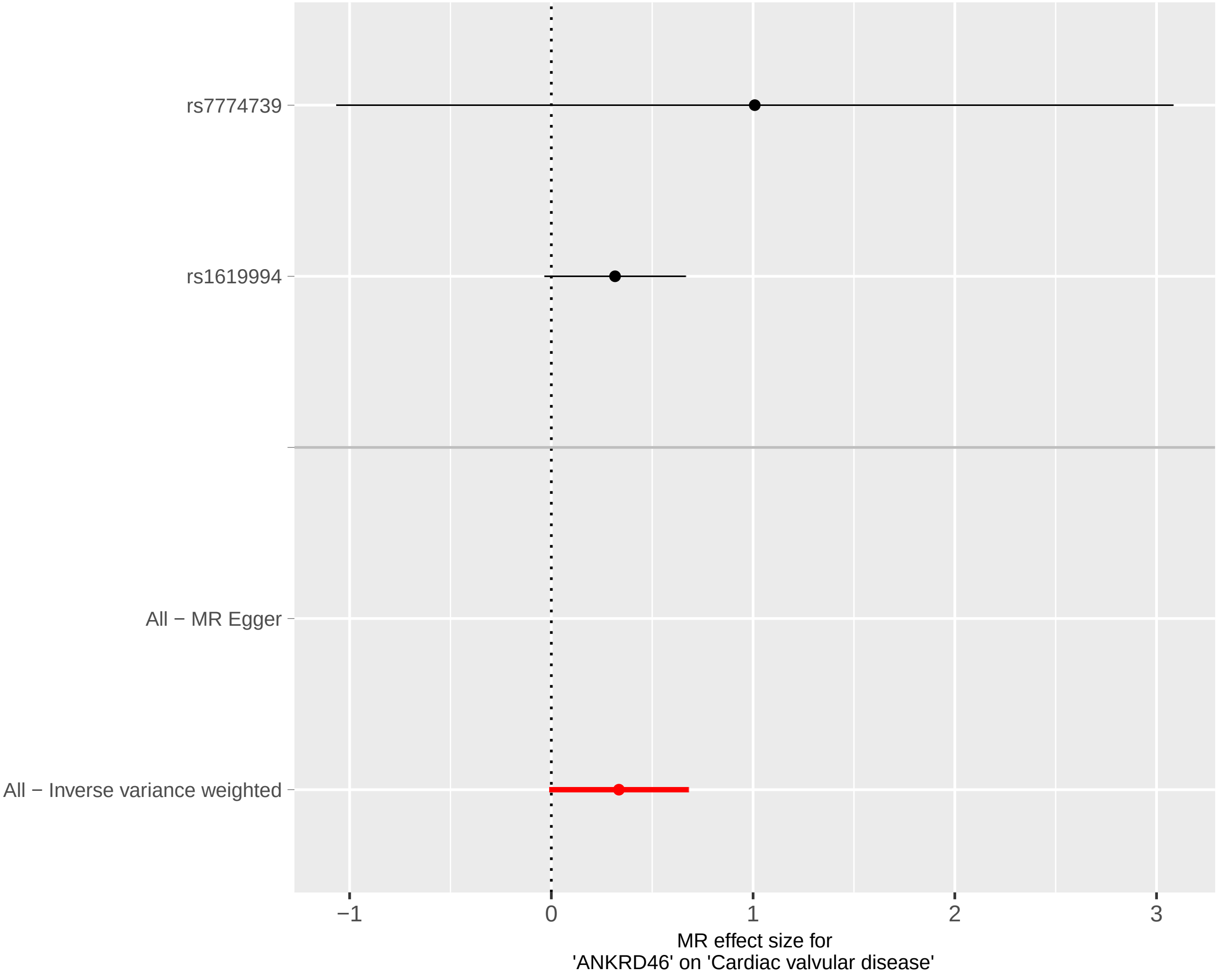
rs7774739
rs1619994
All − MR Egger
All − Inverse variance weighted
−1
0
1
2
3
MR effect size for
'ANKRD46' on 'Cardiac valvular disease'

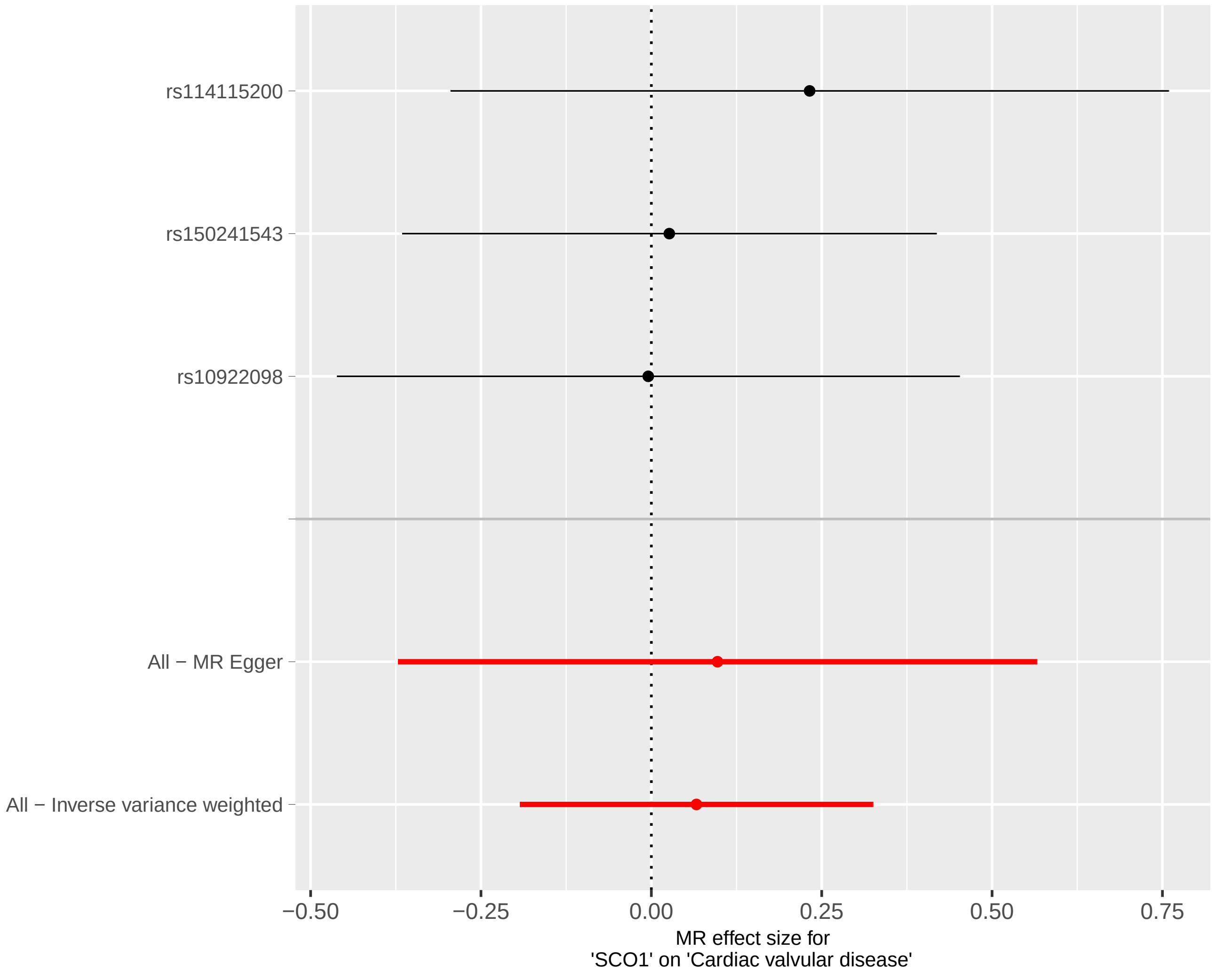
rs114115200
rs150241543
rs10922098
All − MR Egger
All − Inverse variance weighted
−0.50
−0.25
0.00
0.25
0.50
0.75
MR effect size for
'SCO1' on 'Cardiac valvular disease'

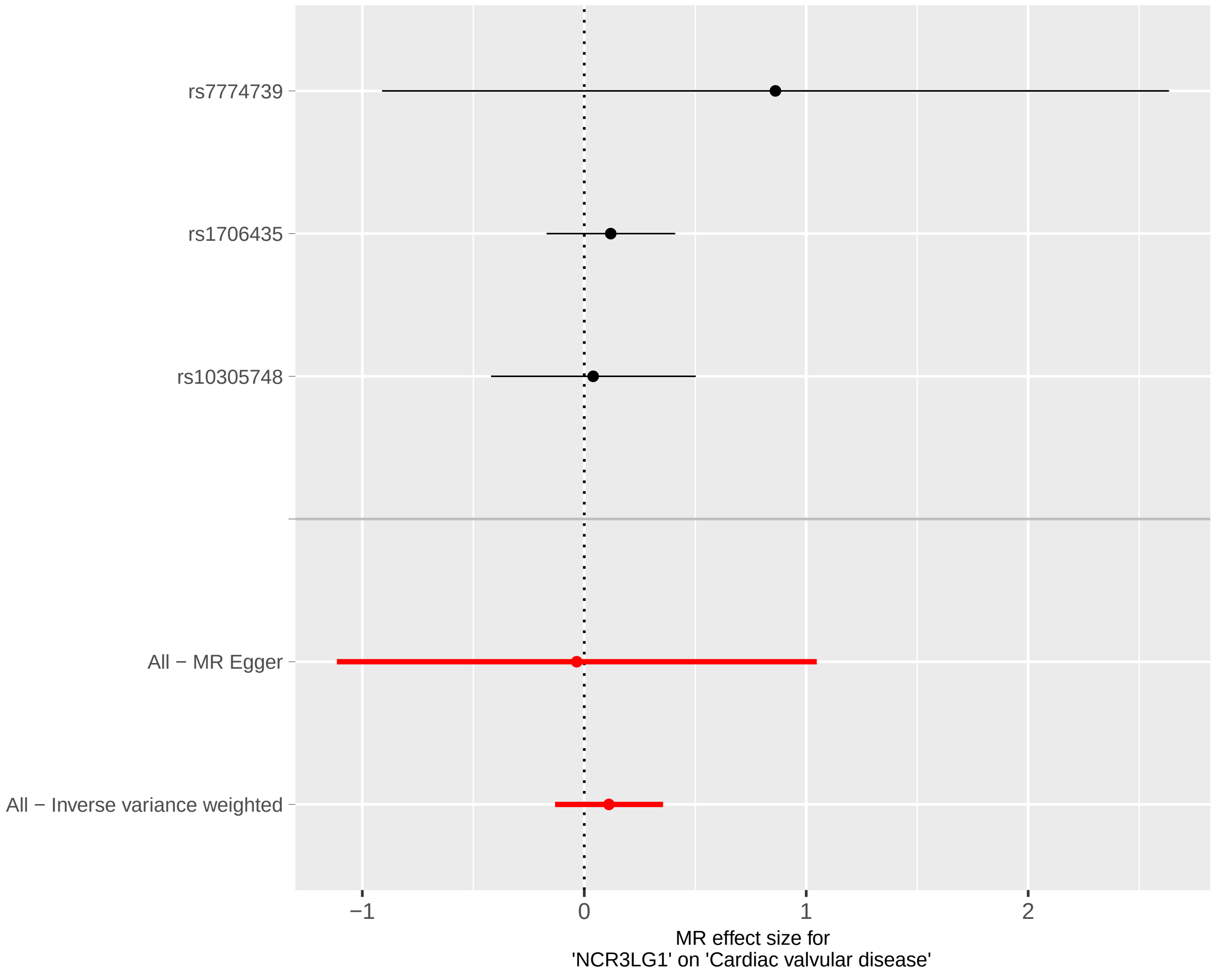
rs7774739
rs1706435
rs10305748
All − MR Egger
All − Inverse variance weighted
−1
0
1
2
MR effect size for
'NCR3LG1' on 'Cardiac valvular disease'

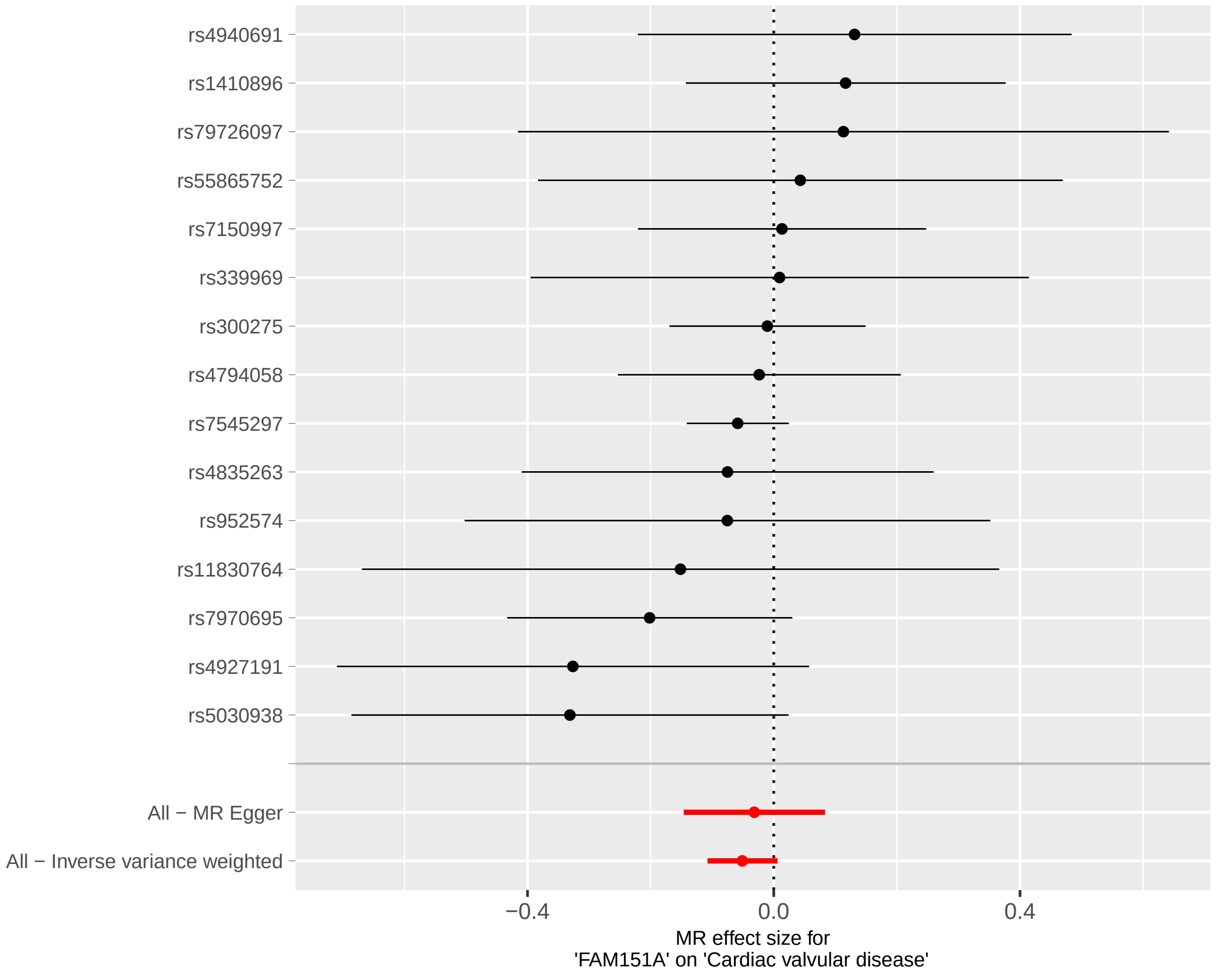
rs4940691
rs1410896
rs79726097
rs55865752
rs7150997
rs339969
rs300275
rs4794058
rs7545297
rs4835263
rs952574
rs11830764
rs7970695
rs4927191
rs5030938
All − MR Egger
All − Inverse variance weighted
−0.4
0.0
0.4
MR effect size for
'FAM151A' on 'Cardiac valvular disease'

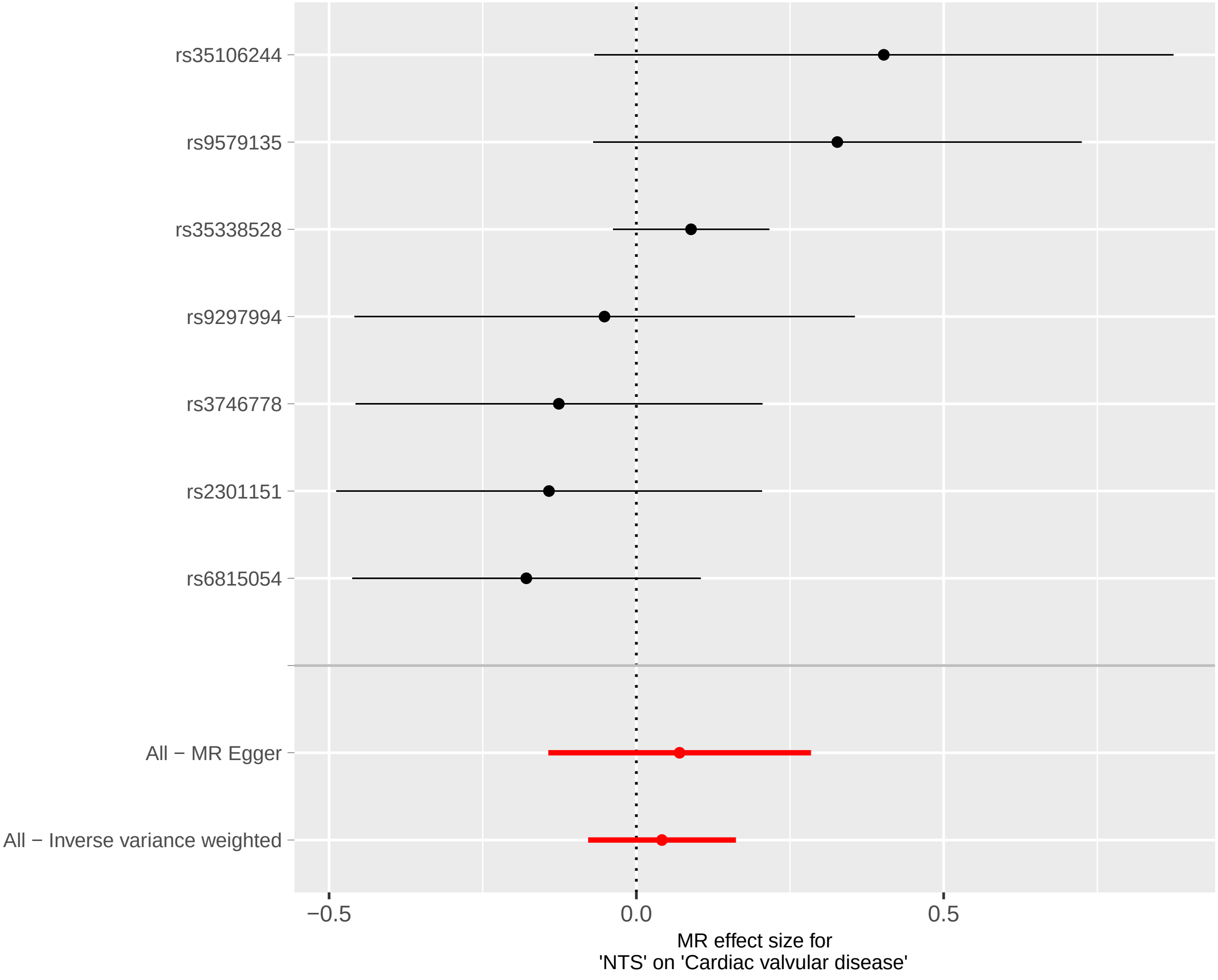

rs35106244
rs9579135
rs35338528
rs9297994
rs3746778
rs2301151
rs6815054
All − MR Egger
All − Inverse variance weighted
−0.5
0.0
0.5
MR effect size for
'NTS' on 'Cardiac valvular disease'

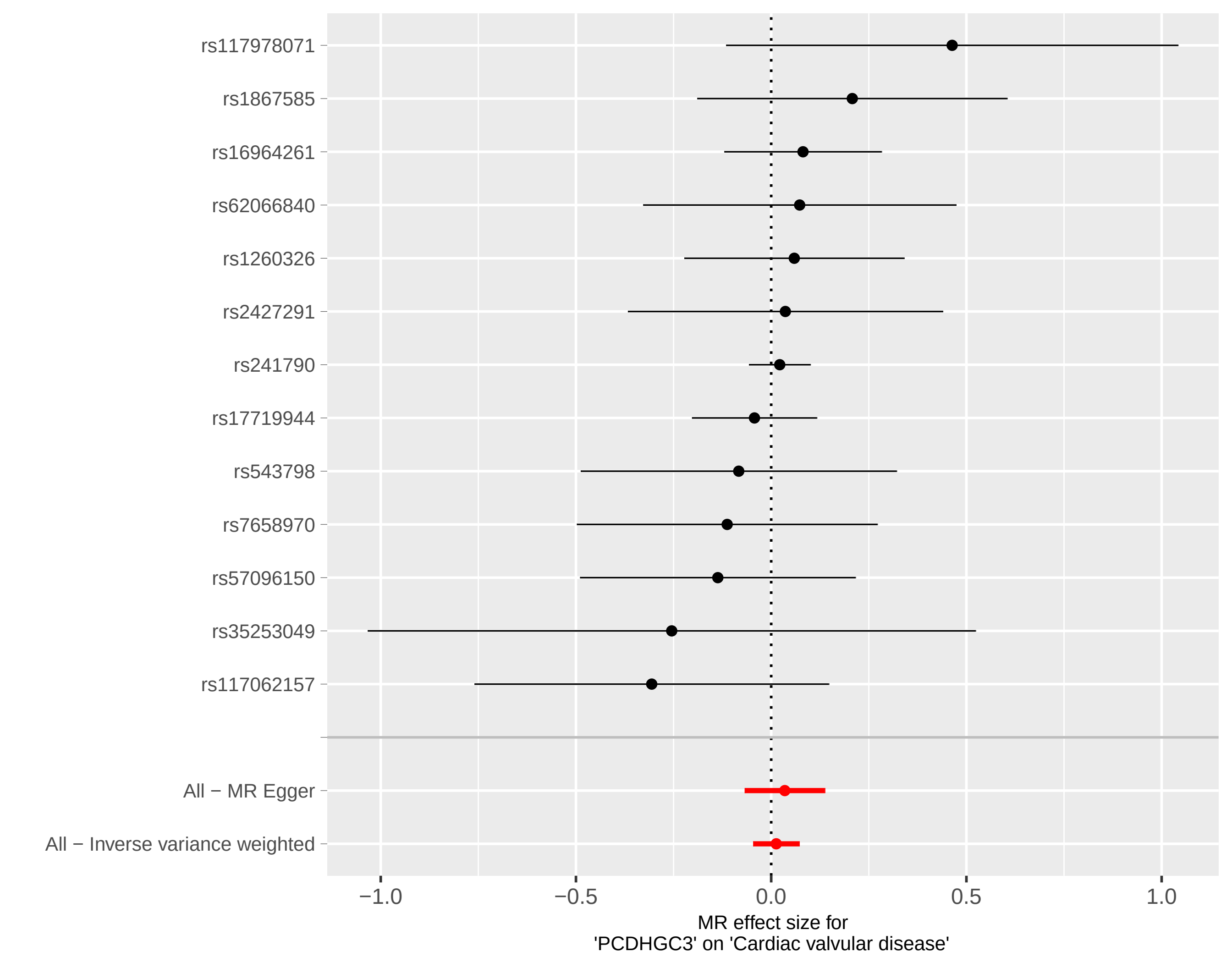
rs117978071
rs1867585
rs16964261
rs62066840
rs1260326
rs2427291
rs241790
rs17719944
rs543798
rs7658970
rs57096150
rs35253049
rs117062157
All − MR Egger
All − Inverse variance weighted
−1.0
−0.5
0.0
0.5
1.0
MR effect size for
'PCDHGC3' on 'Cardiac valvular disease'

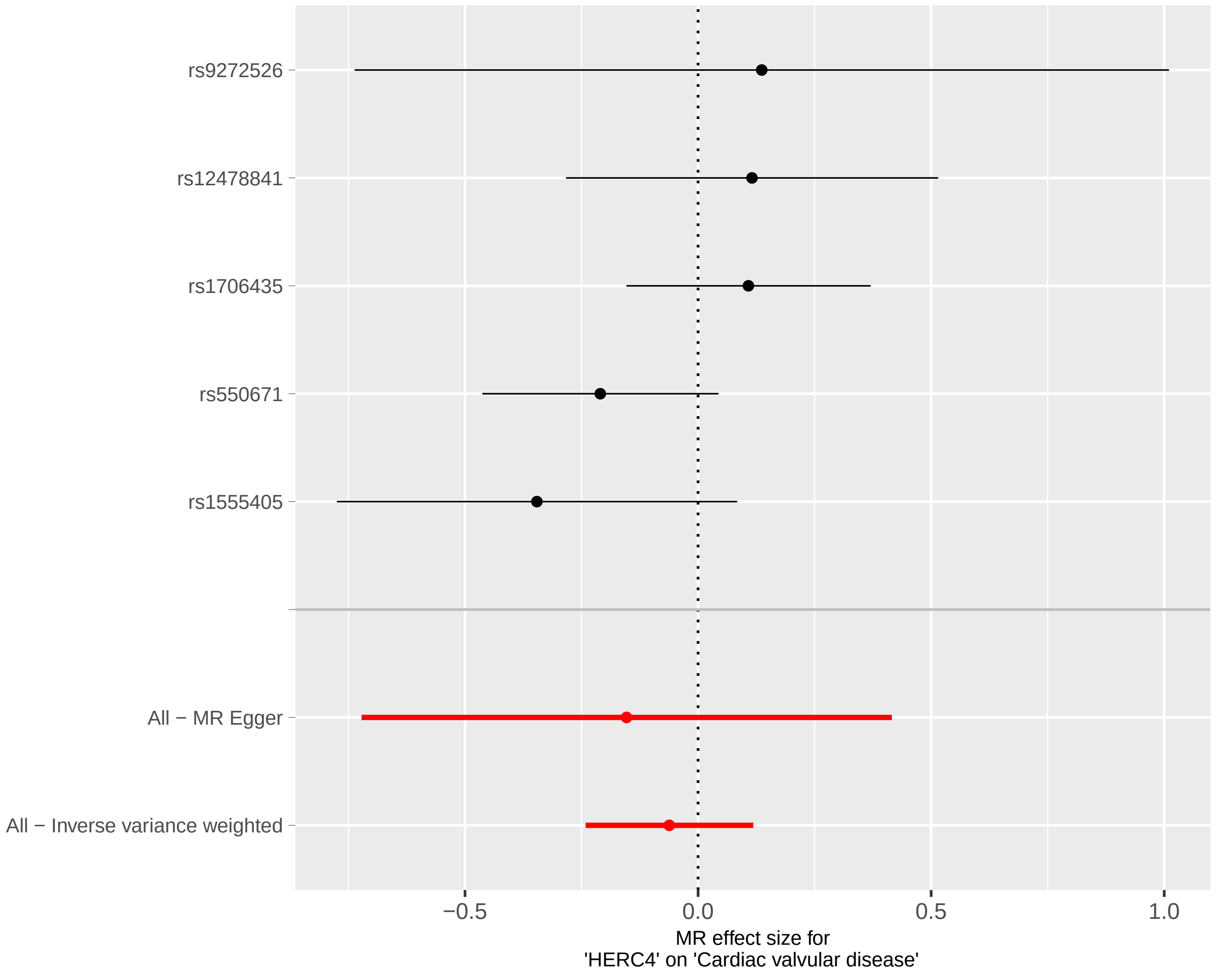
rs9272526
rs12478841
rs1706435
rs550671
rs1555405
All − MR Egger
All − Inverse variance weighted
−0.5
0.0
0.5
1.0
MR effect size for
'HERC4' on 'Cardiac valvular disease'

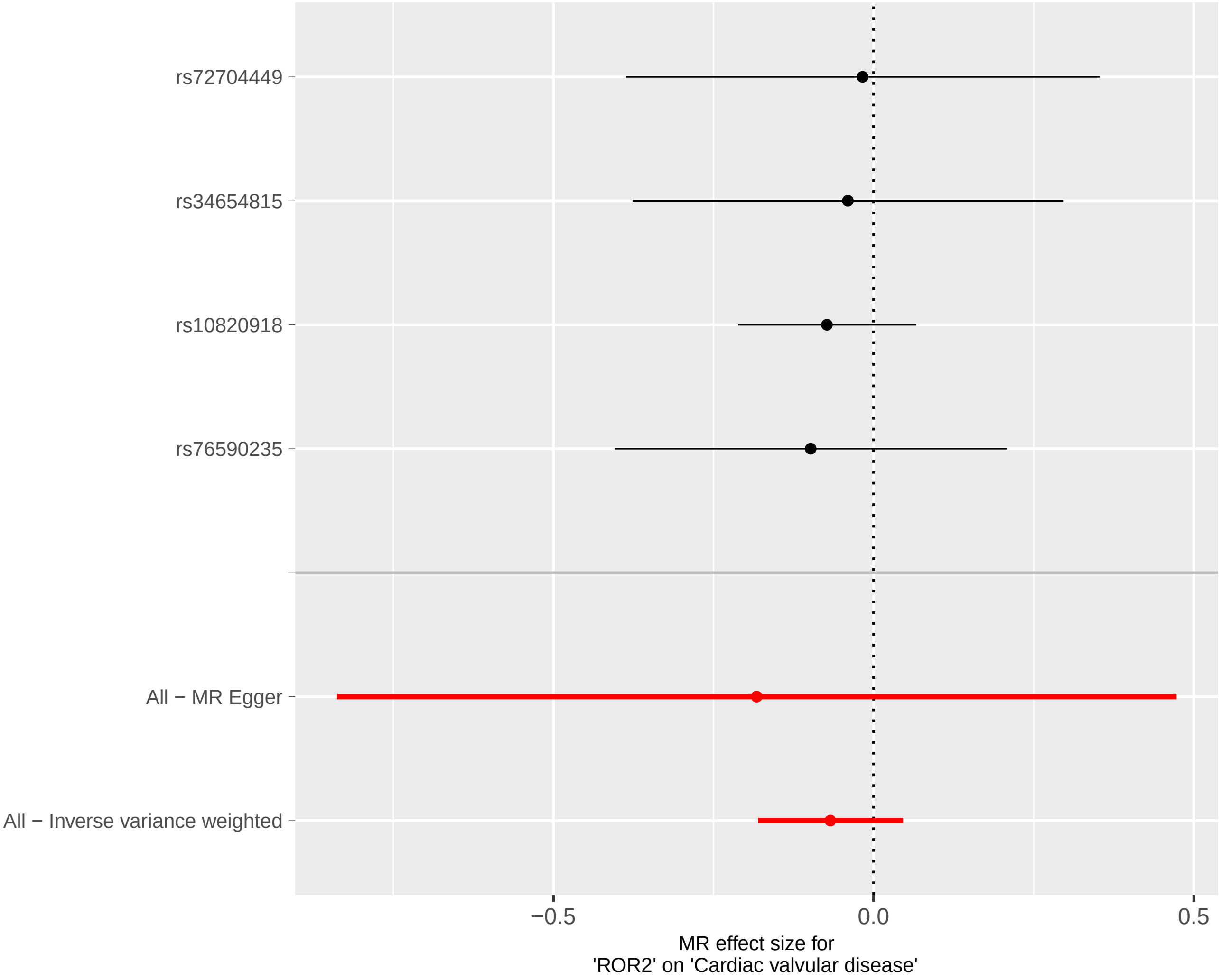
rs72704449
rs34654815
rs10820918
rs76590235
All − MR Egger
All − Inverse variance weighted
−0.5
0.0
0.5
MR effect size for
'ROR2' on 'Cardiac valvular disease'

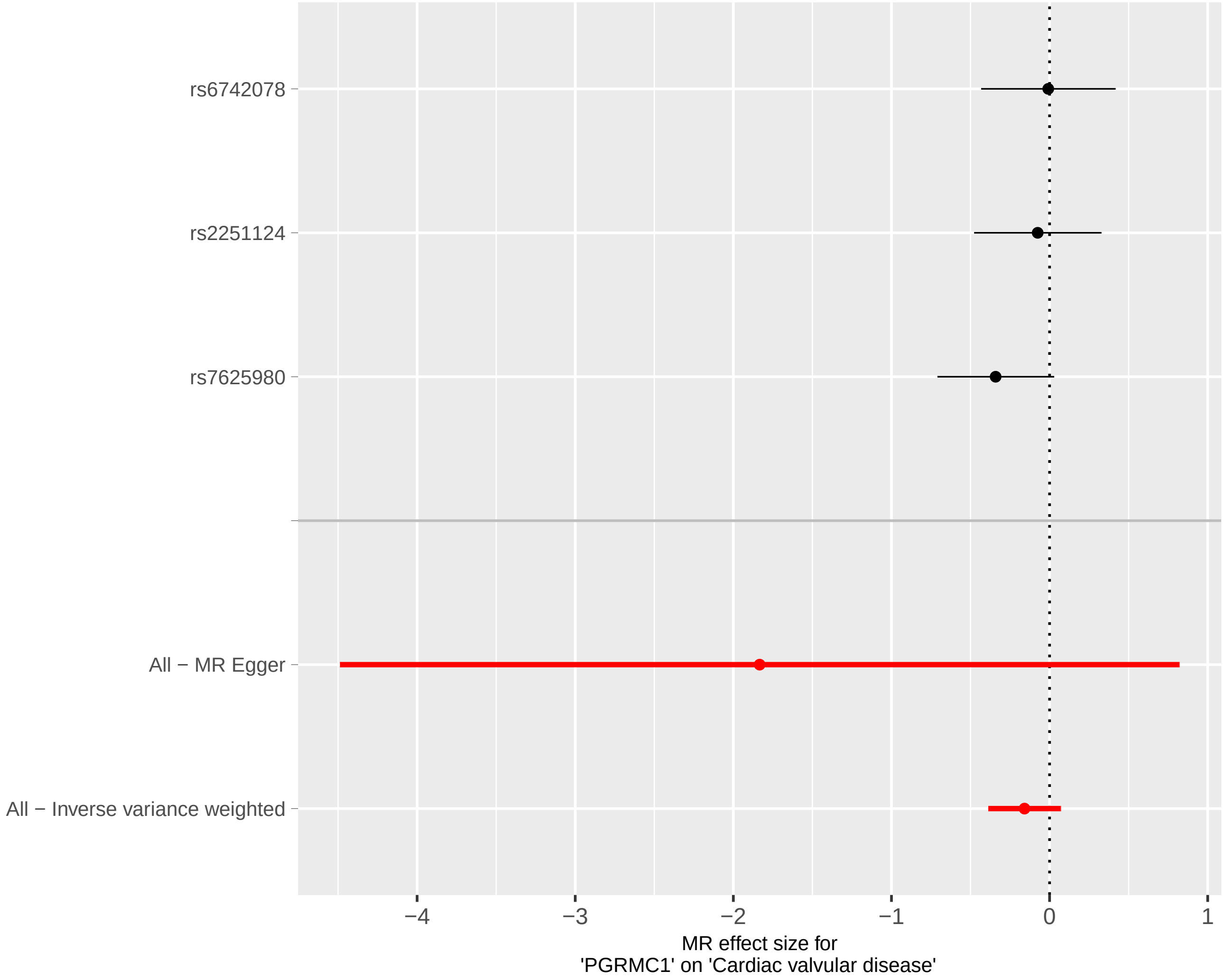
rs6742078
rs2251124
rs7625980
All − MR Egger
All − Inverse variance weighted
−4
−3
−2
−1
0
1
MR effect size for
'PGRMC1' on 'Cardiac valvular disease'

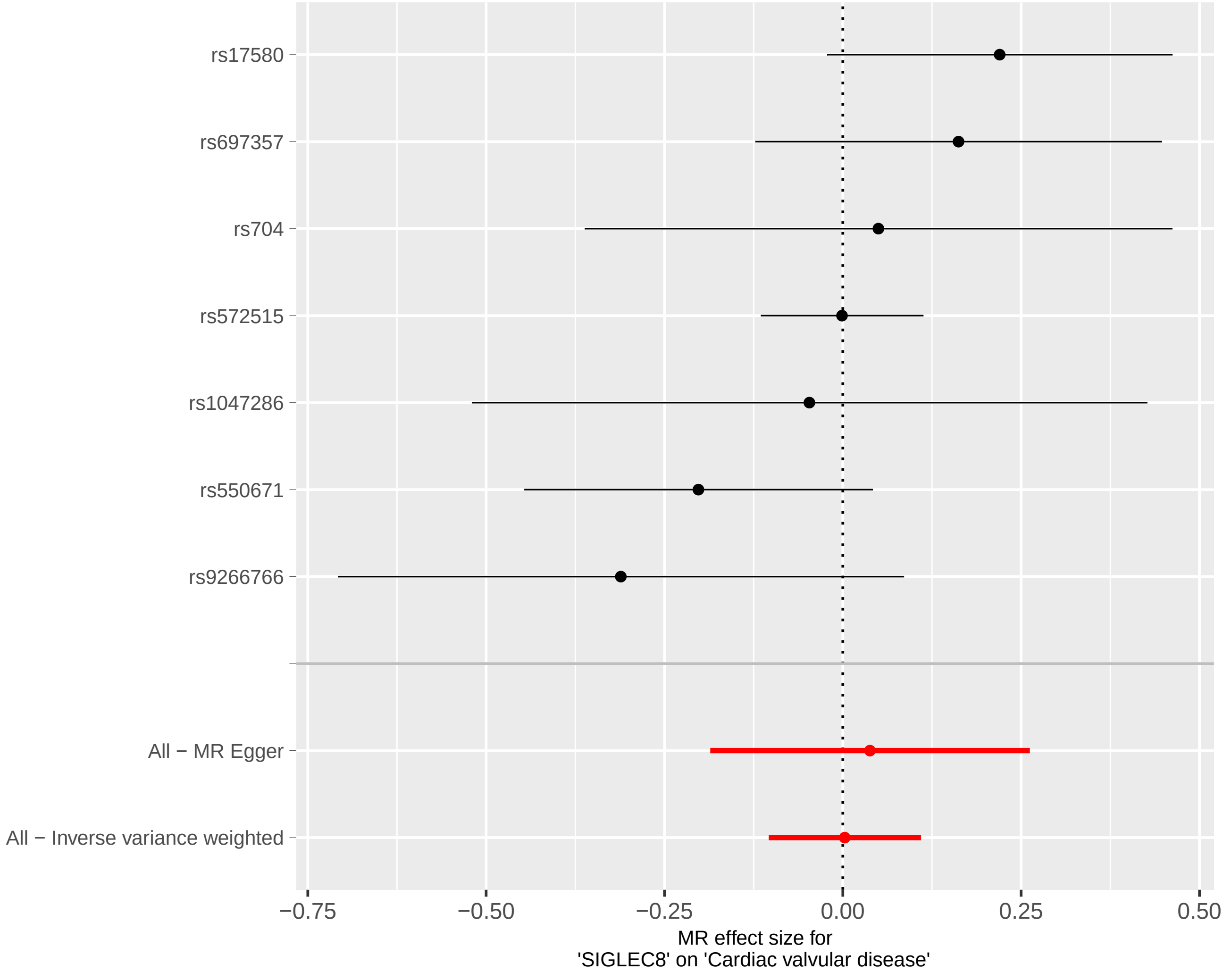
rs17580
rs697357
rs704
rs572515
rs1047286
rs550671
rs9266766
All − MR Egger
All − Inverse variance weighted
−0.75
−0.50
−0.25
0.00
0.25
0.50
MR effect size for
'SIGLEC8' on 'Cardiac valvular disease'

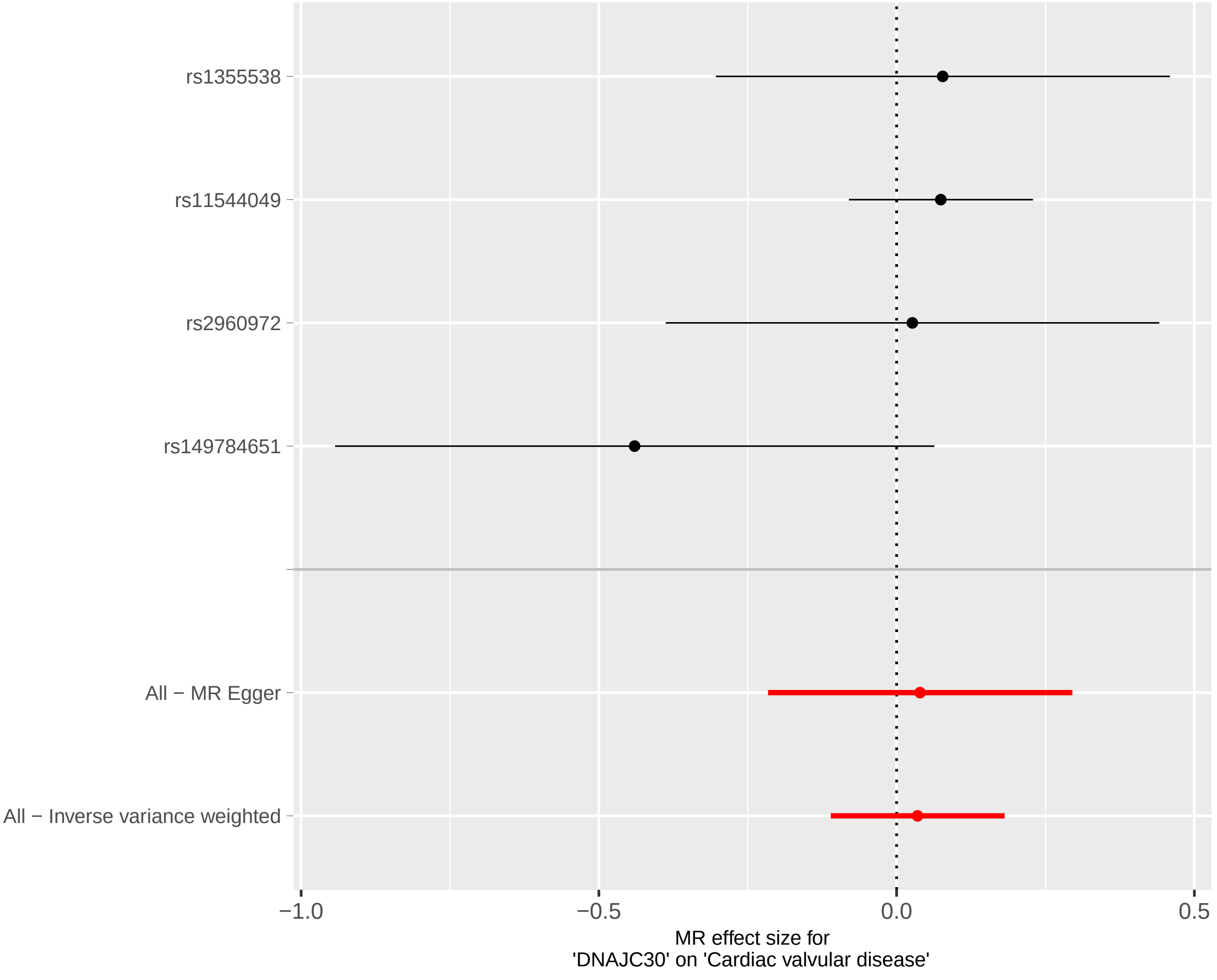
rs1355538
rs11544049
rs2960972
rs149784651
All − MR Egger
All − Inverse variance weighted
−1.0
−0.5
0.0
0.5
MR effect size for
'DNAJC30' on 'Cardiac valvular disease'

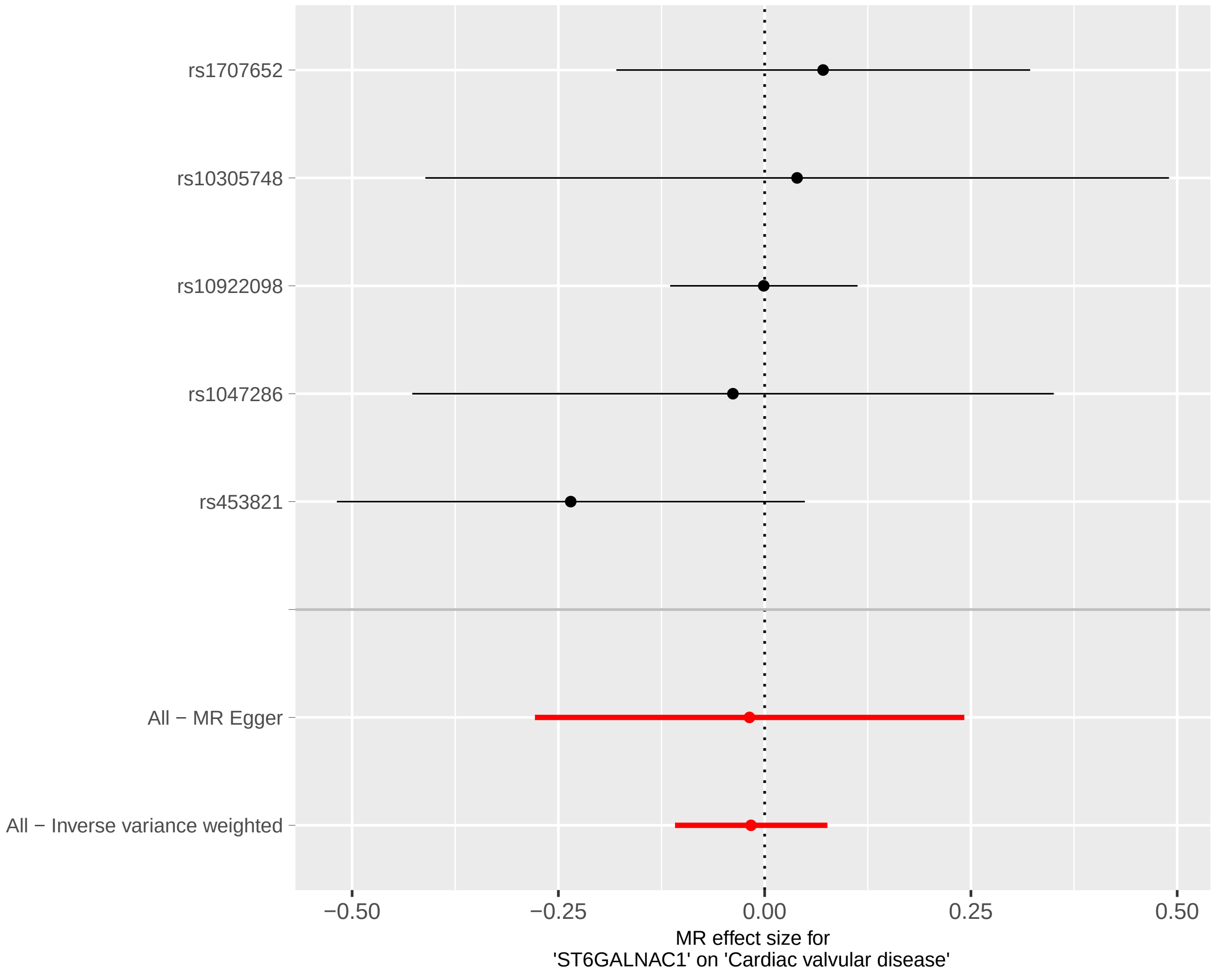
rs1707652
rs10305748
rs10922098
rs1047286
rs453821
All − MR Egger
All − Inverse variance weighted
−0.50
−0.25
0.00
0.25
0.50
MR effect size for
'ST6GALNAC1' on 'Cardiac valvular disease'

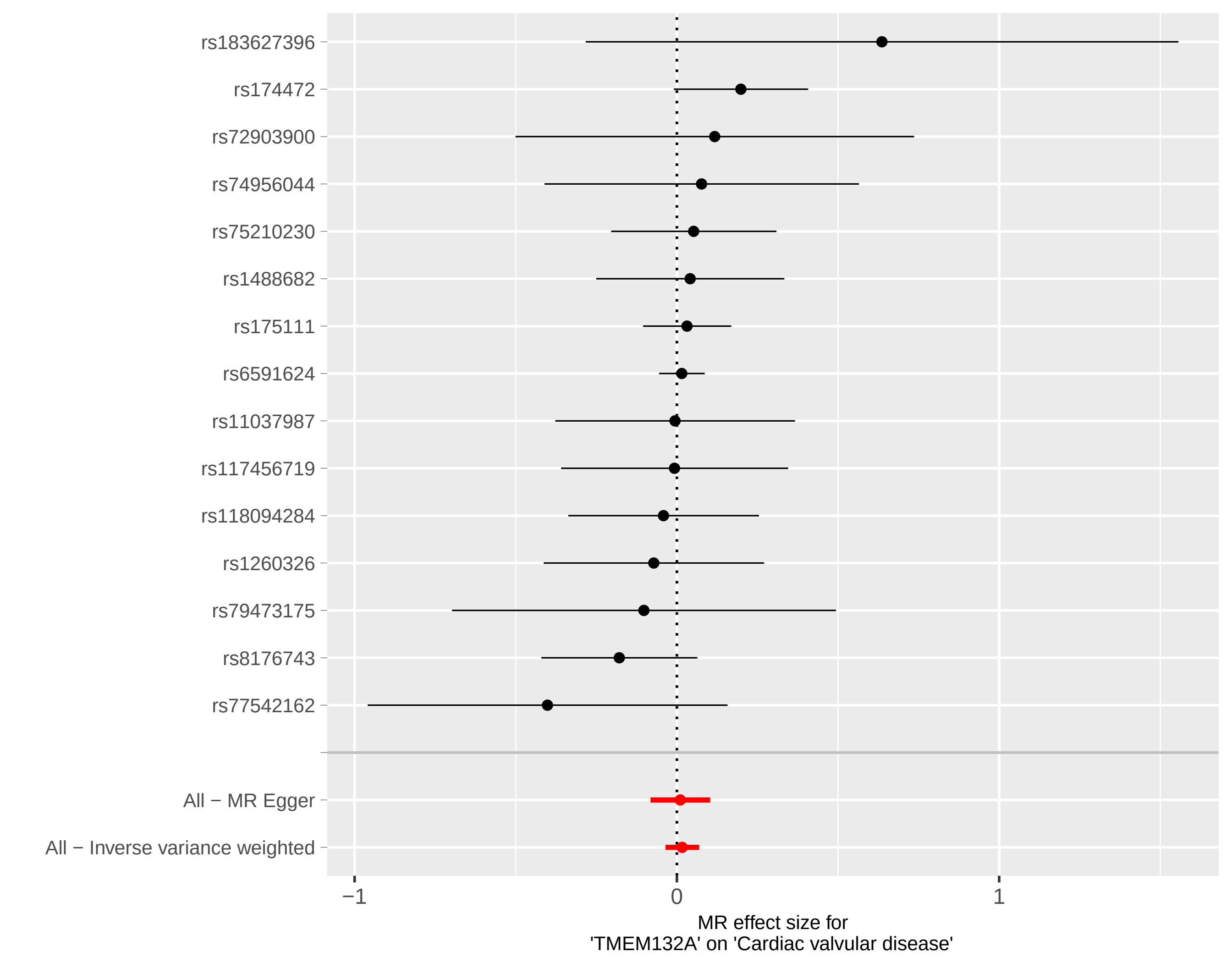
rs183627396
rs174472
rs72903900
rs74956044
rs75210230
rs1488682
rs175111
rs6591624
rs11037987
rs117456719
rs118094284
rs1260326
rs79473175
rs8176743
rs77542162
All − MR Egger
All − Inverse variance weighted
−1
0
1
MR effect size for
'TMEM132A' on 'Cardiac valvular disease'

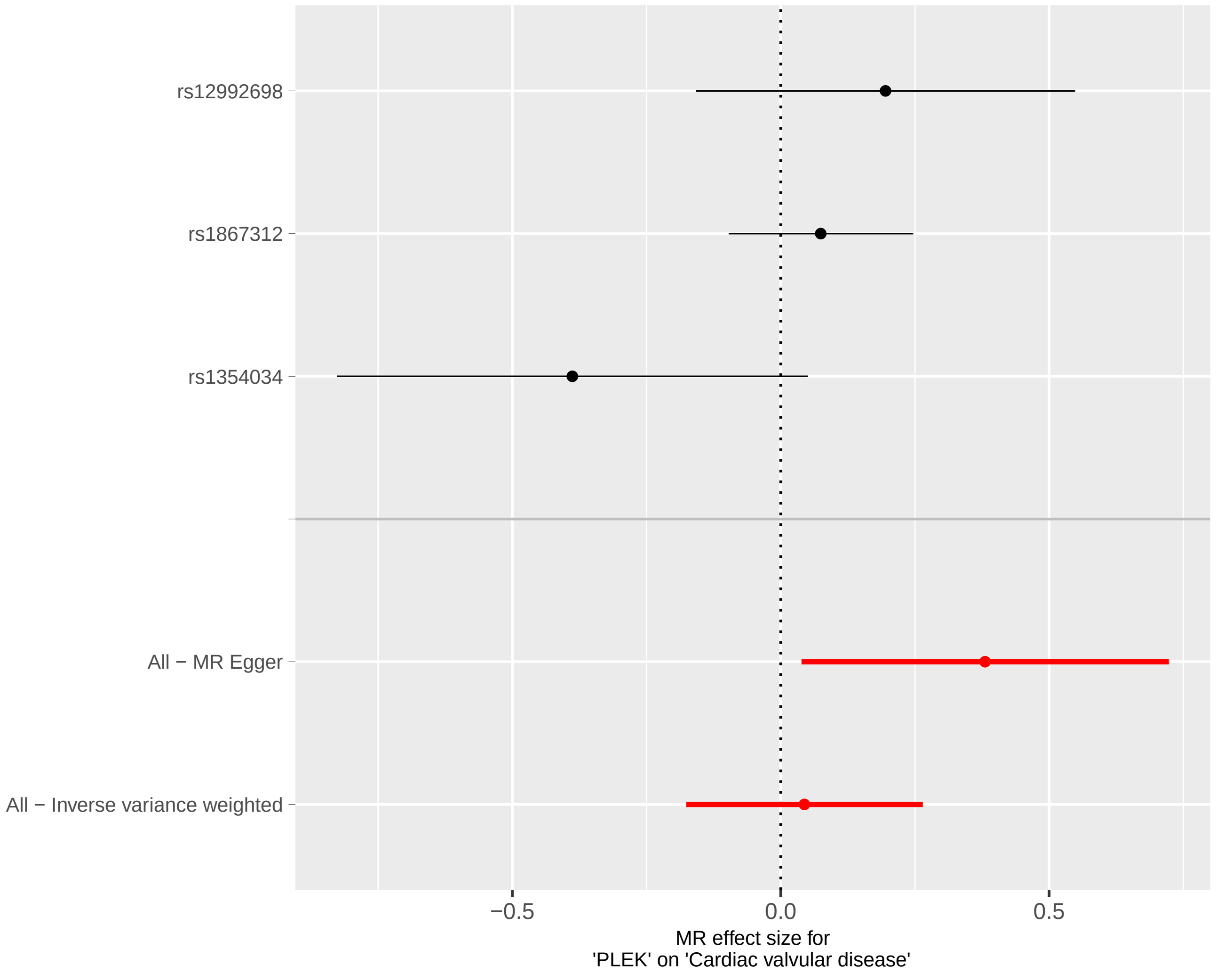
rs12992698
rs1867312
rs1354034
All − MR Egger
All − Inverse variance weighted
−0.5
0.0
0.5
MR effect size for
'PLEK' on 'Cardiac valvular disease'

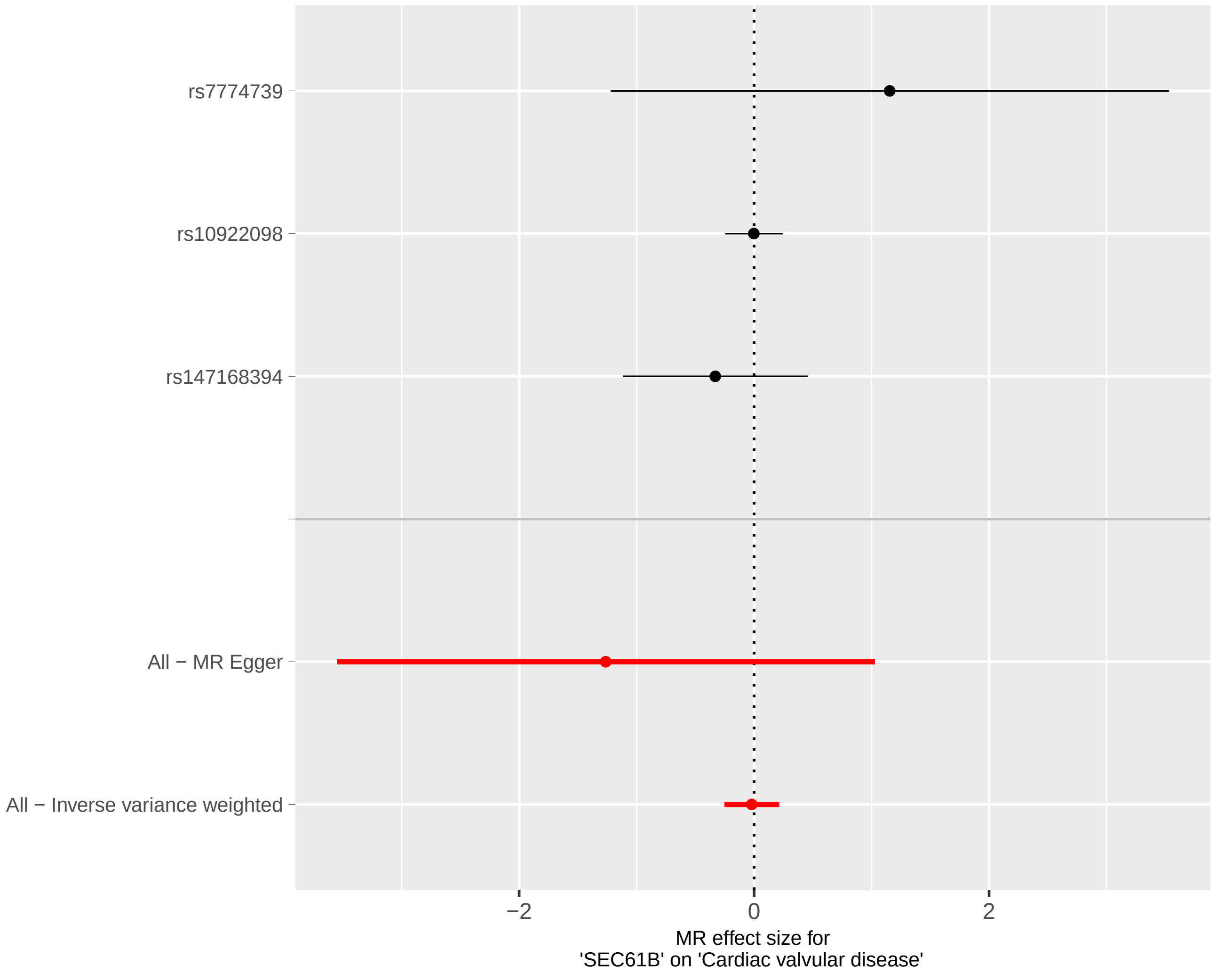
rs7774739
rs10922098
rs147168394
All − MR Egger
All − Inverse variance weighted
−2
0
2
MR effect size for
'SEC61B' on 'Cardiac valvular disease'

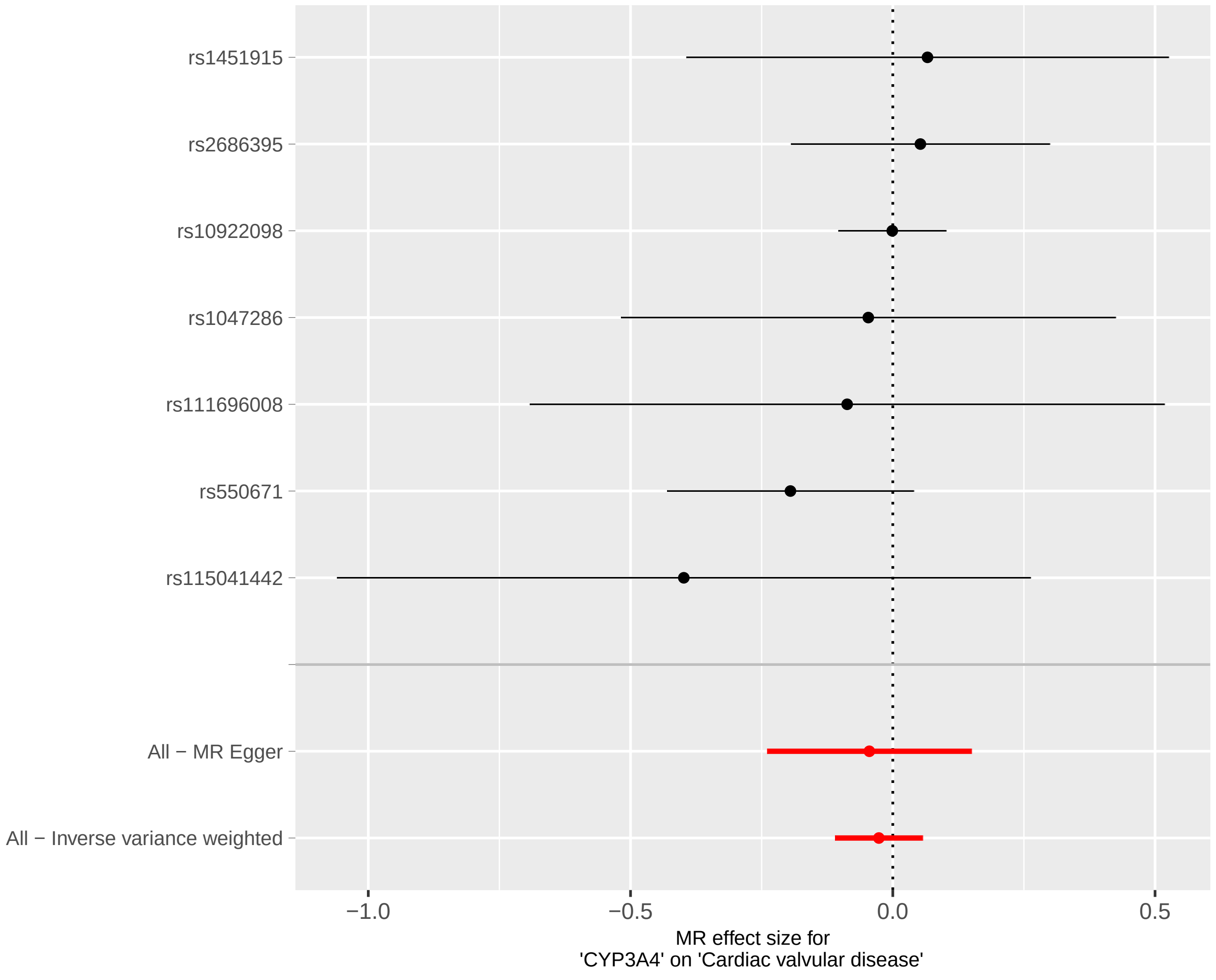

rs1451915
rs2686395
rs10922098
rs1047286
rs111696008
rs550671
rs115041442
All − MR Egger
All − Inverse variance weighted
−1.0
−0.5
0.0
0.5
MR effect size for
'CYP3A4' on 'Cardiac valvular disease'

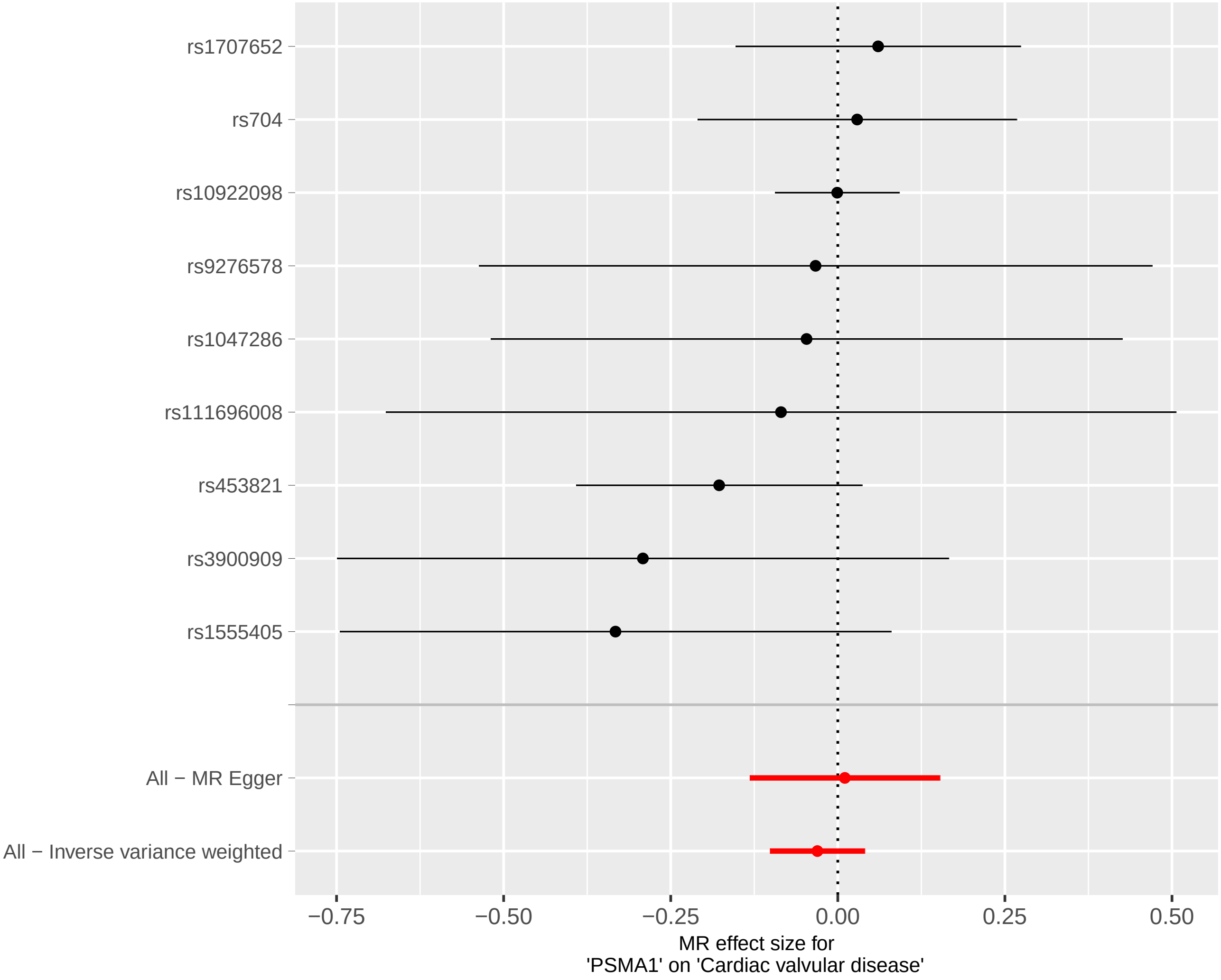
rs1707652
rs704
rs10922098
rs9276578
rs1047286
rs111696008
rs453821
rs3900909
rs1555405
All − MR Egger
All − Inverse variance weighted
−0.75
−0.50
−0.25
0.00
0.25
0.50
MR effect size for
'PSMA1' on 'Cardiac valvular disease'

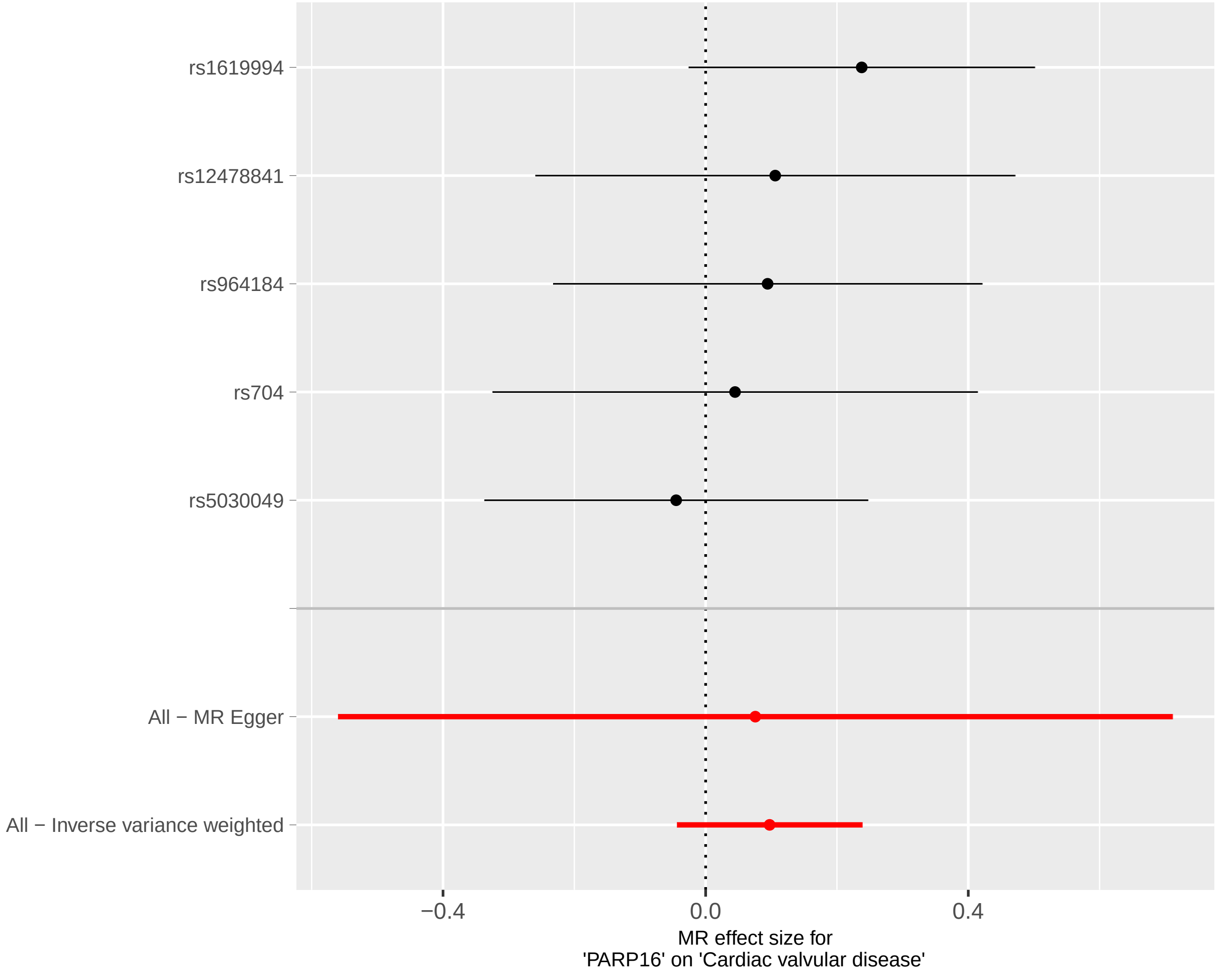
rs1619994
rs12478841
rs964184
rs704
rs5030049
All − MR Egger
All − Inverse variance weighted
−0.4
0.0
0.4
MR effect size for
'PARP16' on 'Cardiac valvular disease'

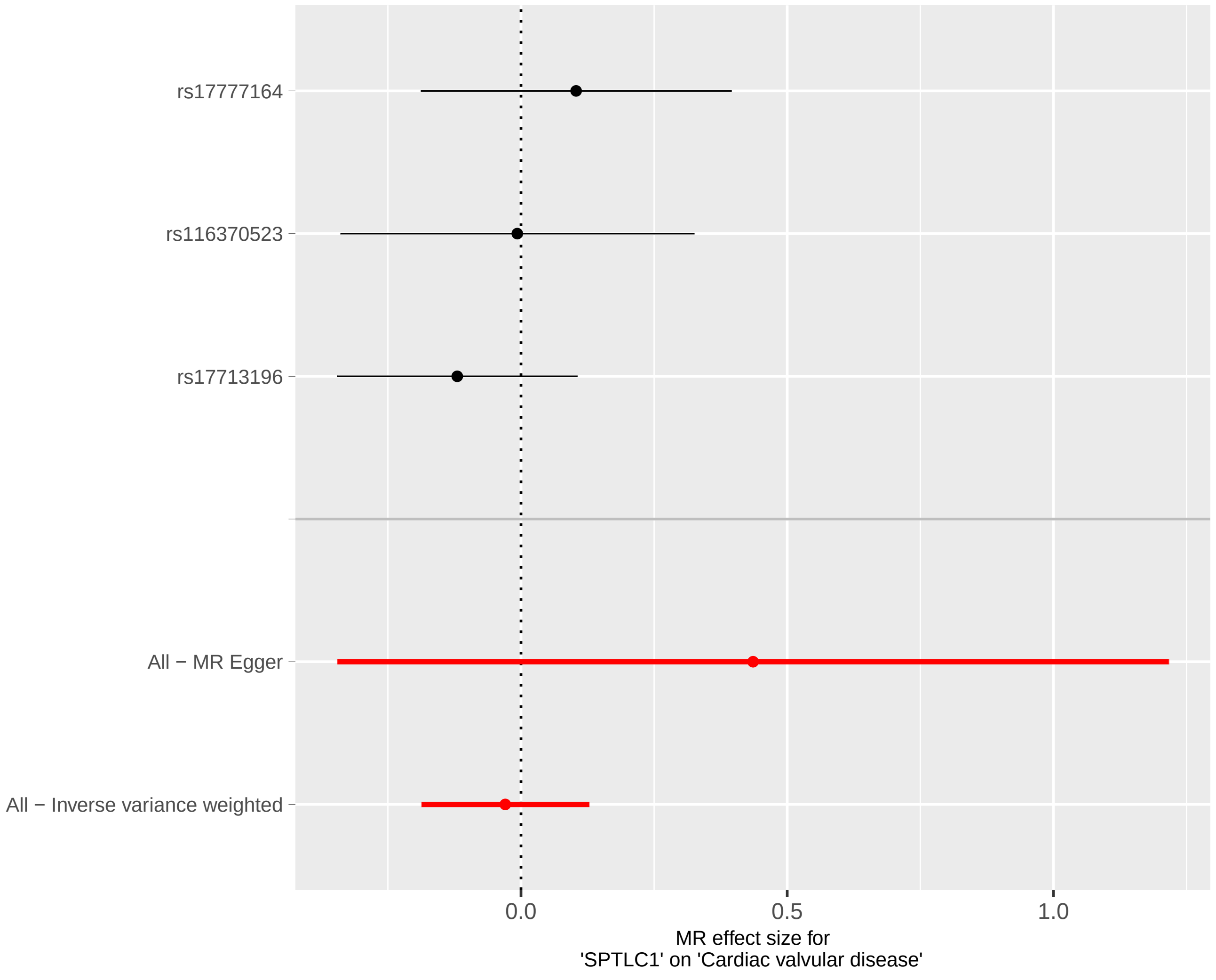
rs17777164
rs116370523
rs17713196
All − MR Egger
All − Inverse variance weighted
0.0
0.5
1.0
MR effect size for
'SPTLC1' on 'Cardiac valvular disease'

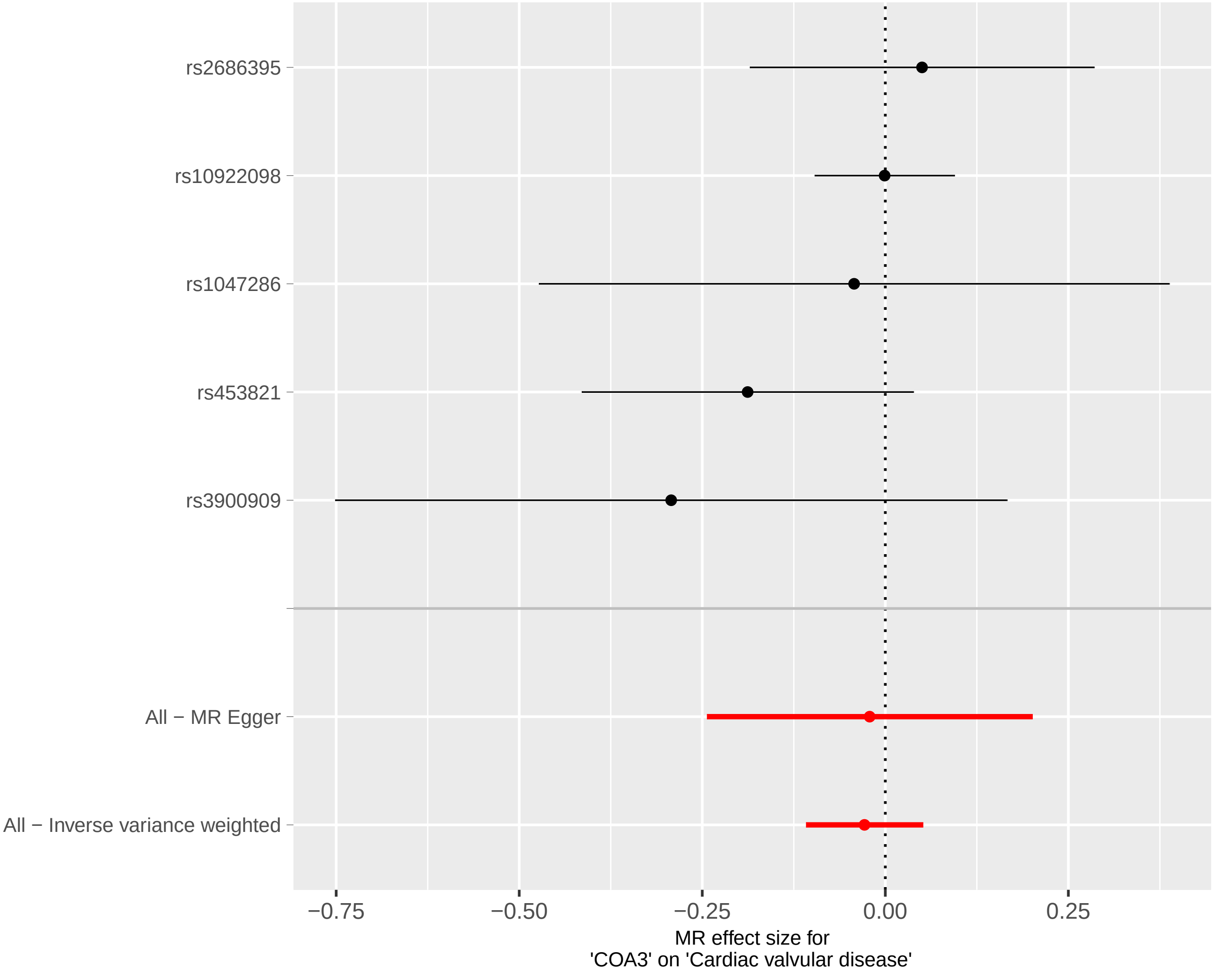
rs2686395
rs10922098
rs1047286
rs453821
rs3900909
All − MR Egger
All − Inverse variance weighted
−0.75
−0.50
−0.25
0.00
0.25
MR effect size for
'COA3' on 'Cardiac valvular disease'

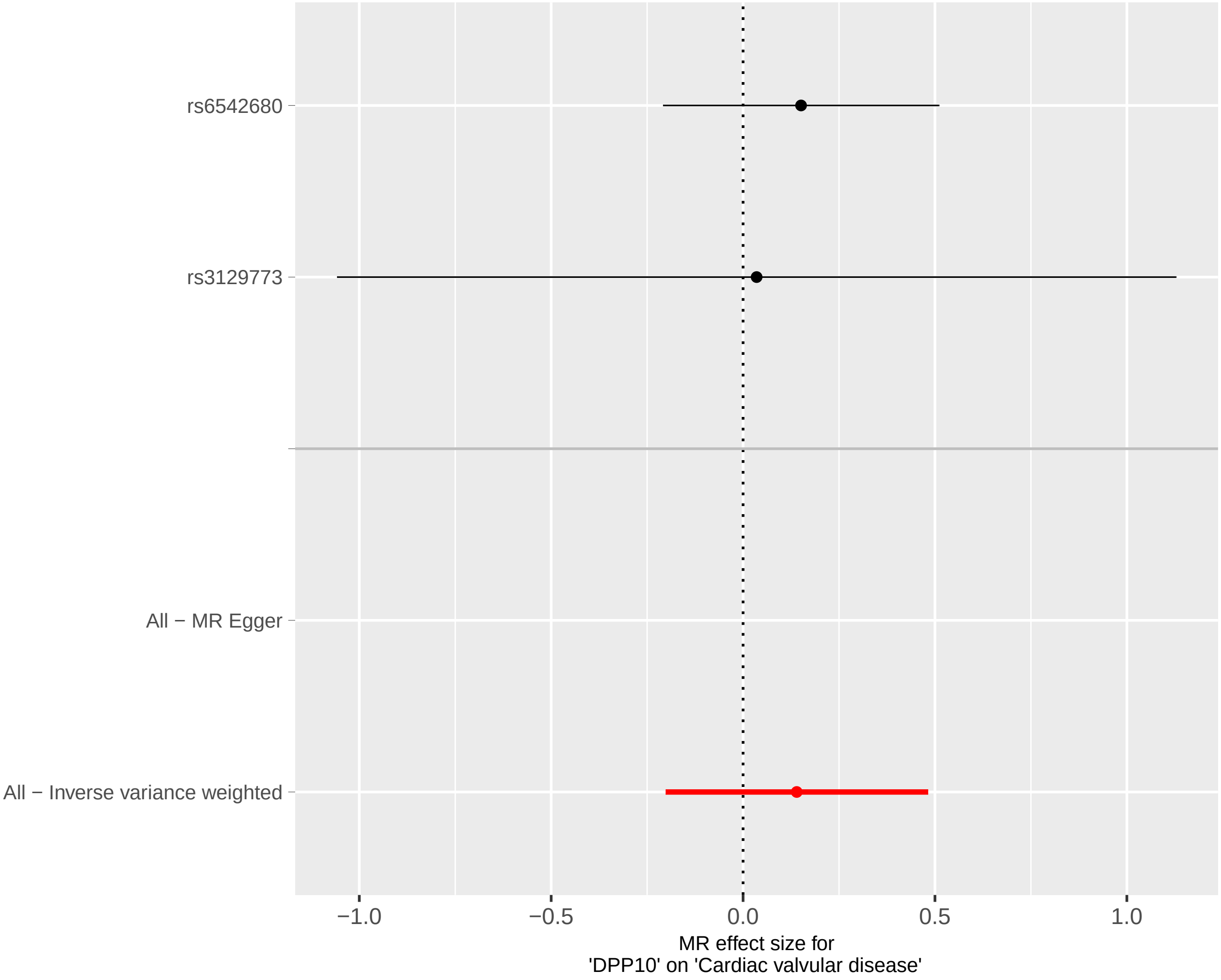

rs6542680
rs3129773
All − MR Egger
All − Inverse variance weighted
−1.0
−0.5
0.0
0.5
1.0
MR effect size for
'DPP10' on 'Cardiac valvular disease'

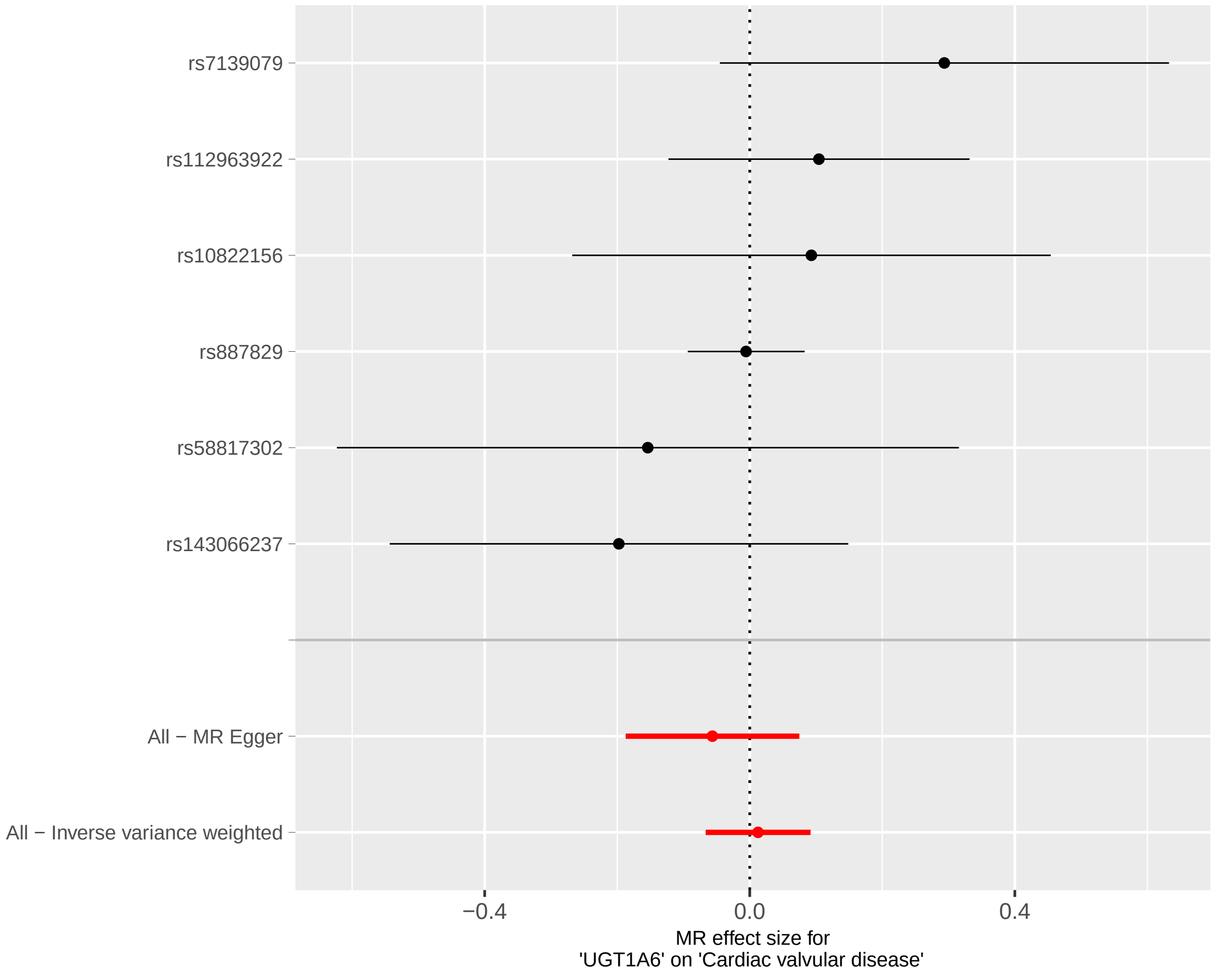
rs7139079
rs112963922
rs10822156
rs887829
rs58817302
rs143066237
All − MR Egger
All − Inverse variance weighted
−0.4
0.0
0.4
MR effect size for
'UGT1A6' on 'Cardiac valvular disease'

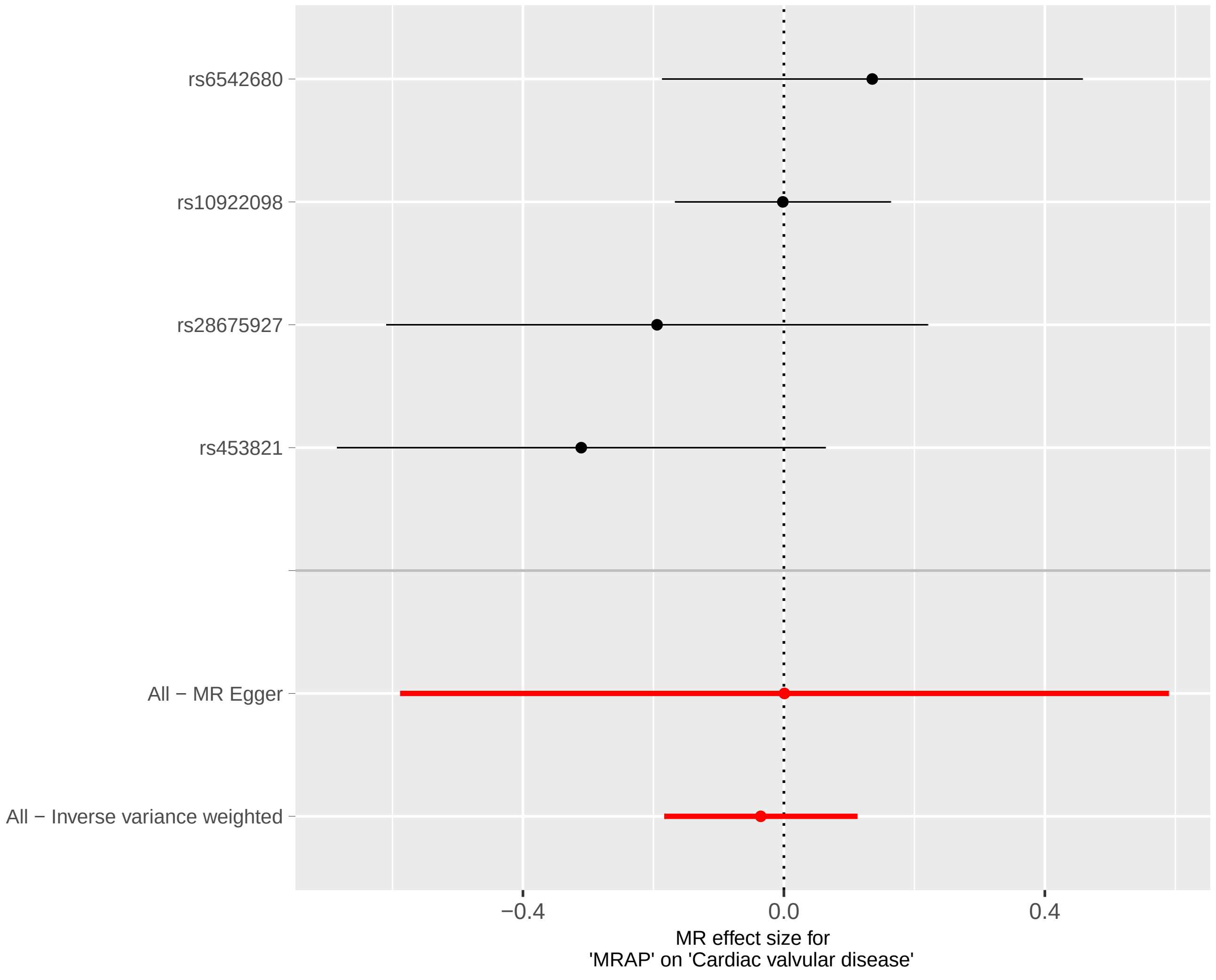
rs6542680
rs10922098
rs28675927
rs453821
All − MR Egger
All − Inverse variance weighted
−0.4
0.0
0.4
MR effect size for
'MRAP' on 'Cardiac valvular disease'

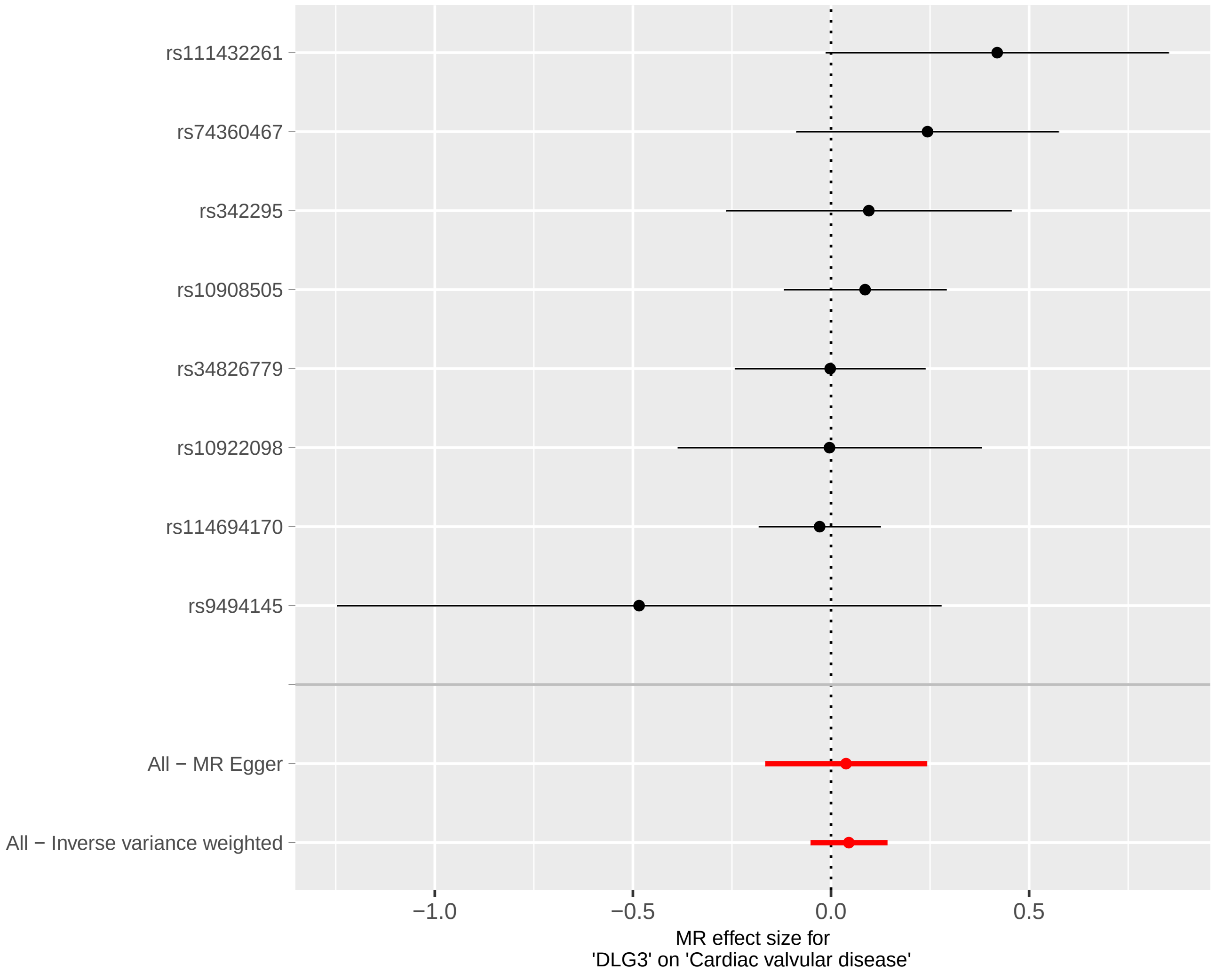
rs111432261
rs74360467
rs342295
rs10908505
rs34826779
rs10922098
rs114694170
rs9494145
All − MR Egger
All − Inverse variance weighted
−1.0
−0.5
0.0
0.5
MR effect size for
'DLG3' on 'Cardiac valvular disease'

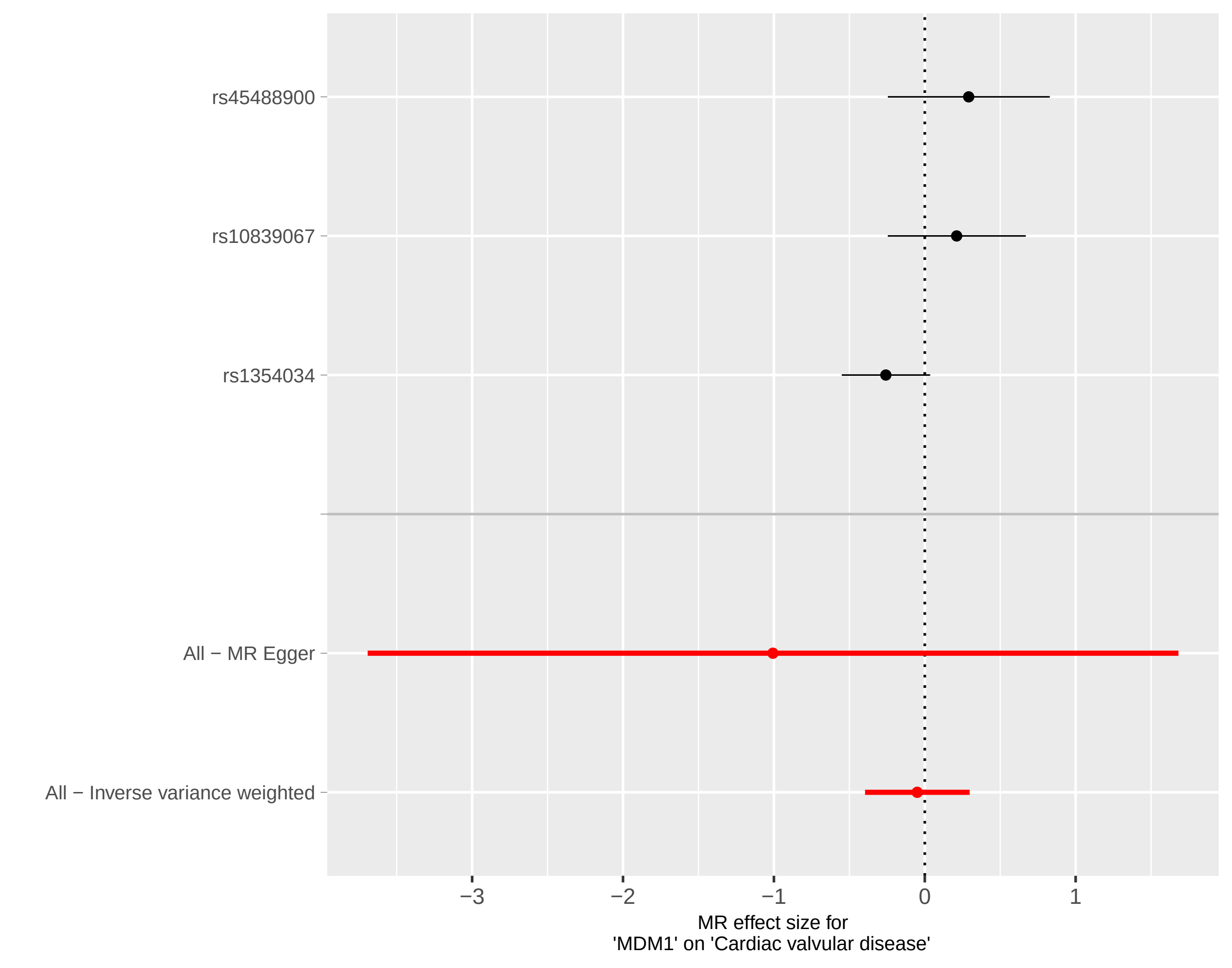

rs45488900
rs10839067
rs1354034
All − MR Egger
All − Inverse variance weighted
−3
−2
−1
0
1
MR effect size for
'MDM1' on 'Cardiac valvular disease'

Insufficient number of SNPs

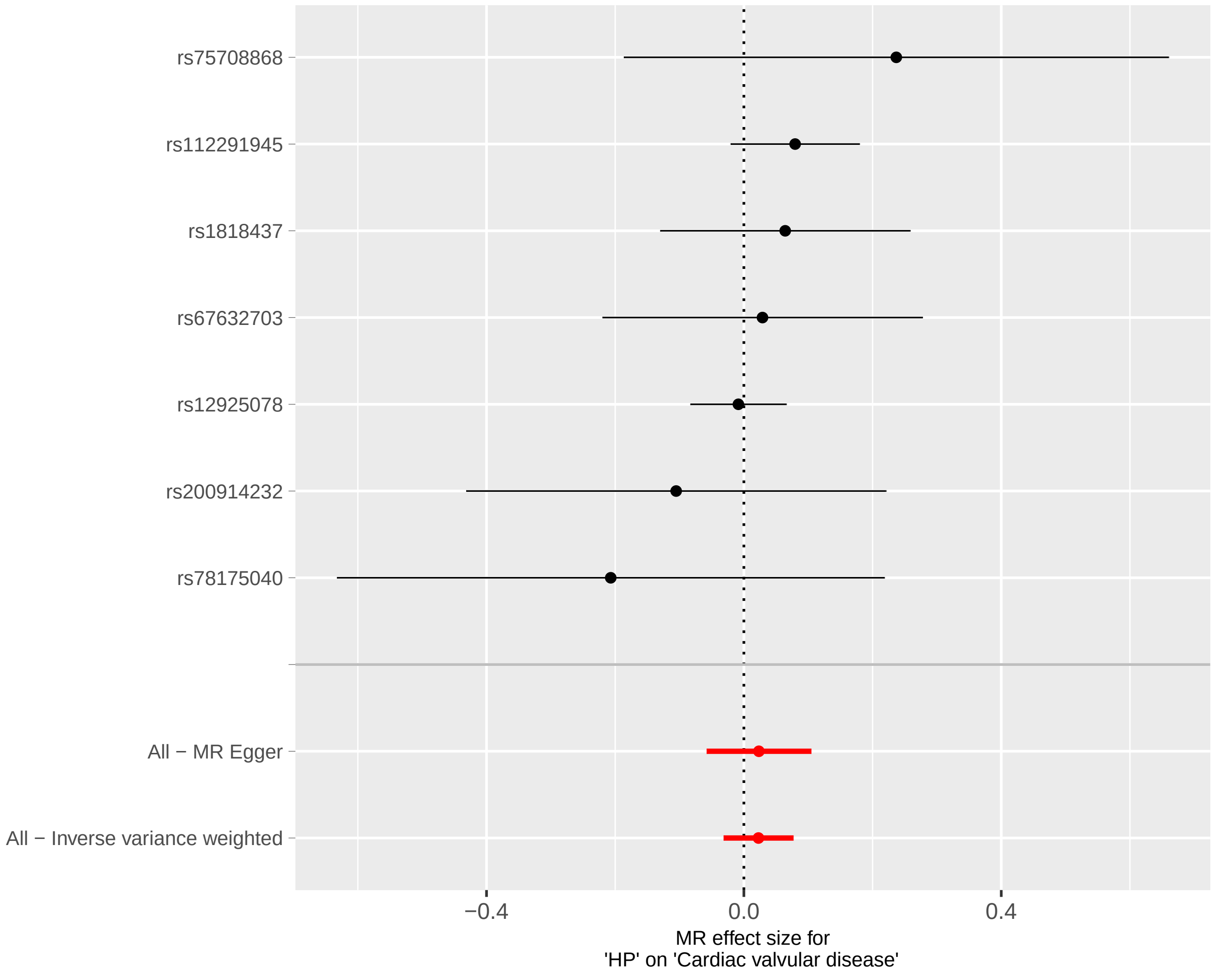
rs75708868
rs112291945
rs1818437
rs67632703
rs12925078
rs200914232
rs78175040
All − MR Egger
All − Inverse variance weighted
−0.4
0.0
0.4
MR effect size for
'HP' on 'Cardiac valvular disease'

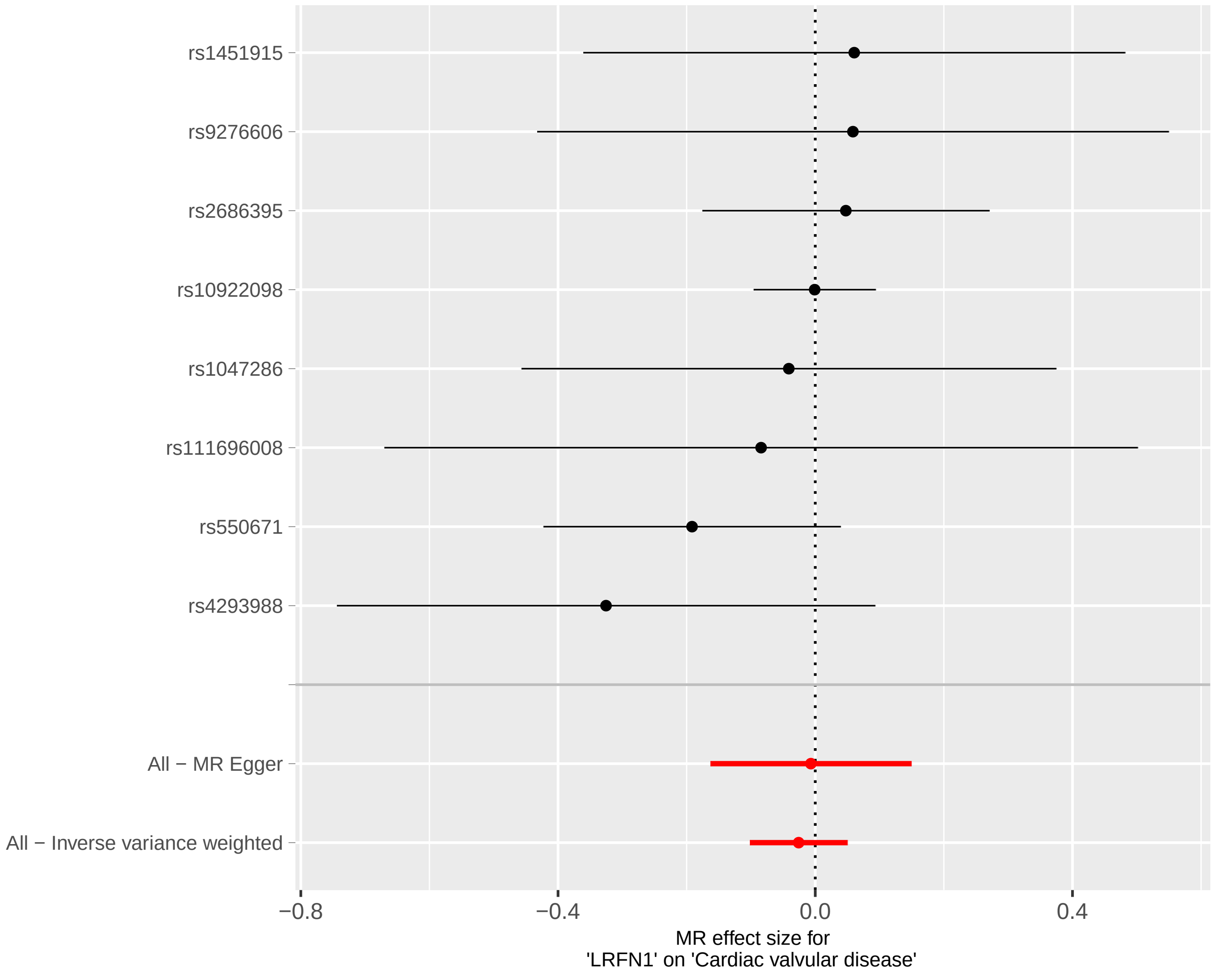
rs1451915
rs9276606
rs2686395
rs10922098
rs1047286
rs111696008
rs550671
rs4293988
All − MR Egger
All − Inverse variance weighted
−0.8
−0.4
0.0
0.4
MR effect size for
'LRFN1' on 'Cardiac valvular disease'

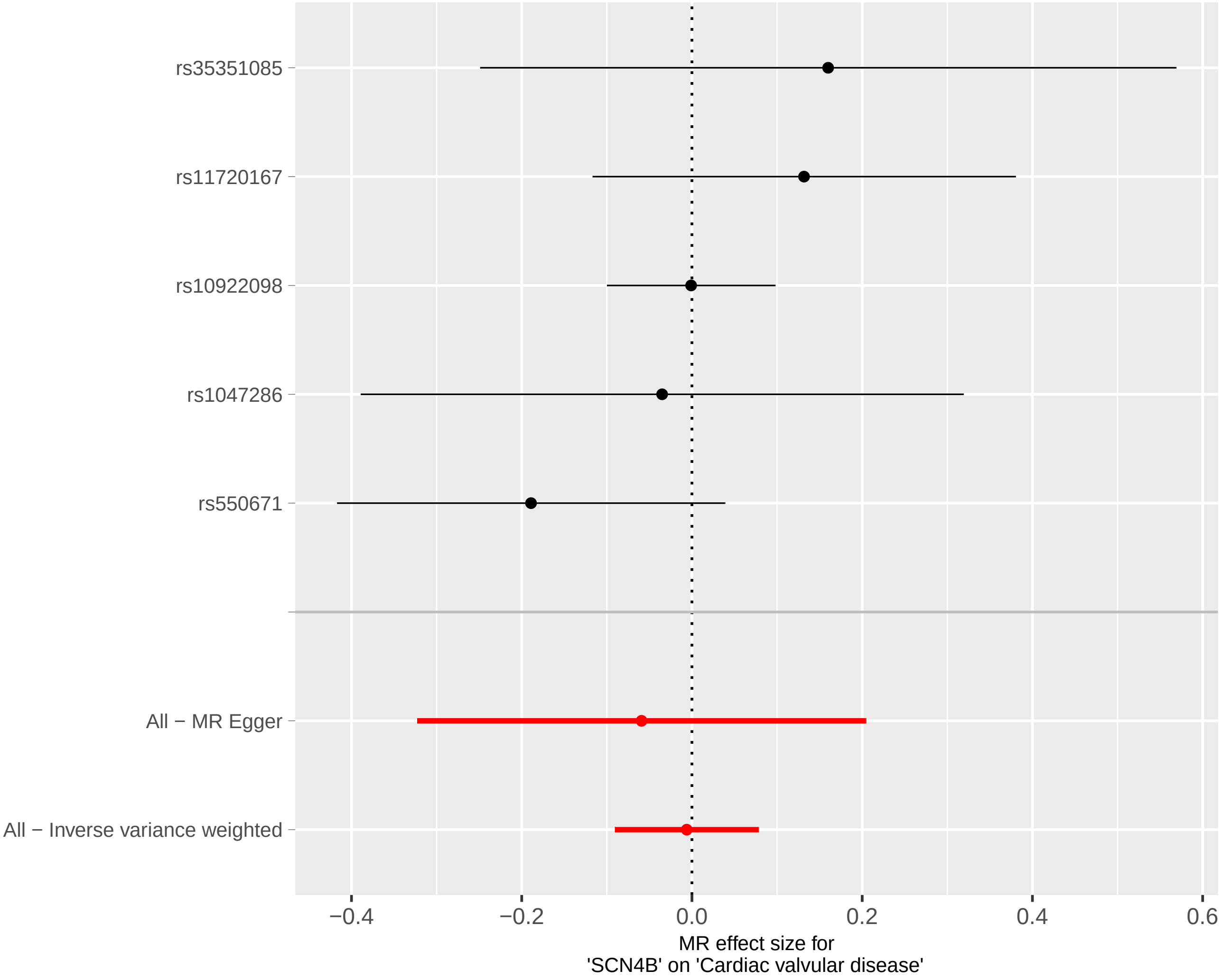
rs35351085
rs11720167
rs10922098
rs1047286
rs550671
All − MR Egger
All − Inverse variance weighted
−0.4
−0.2
0.0
0.2
0.4
0.6
MR effect size for
'SCN4B' on 'Cardiac valvular disease'

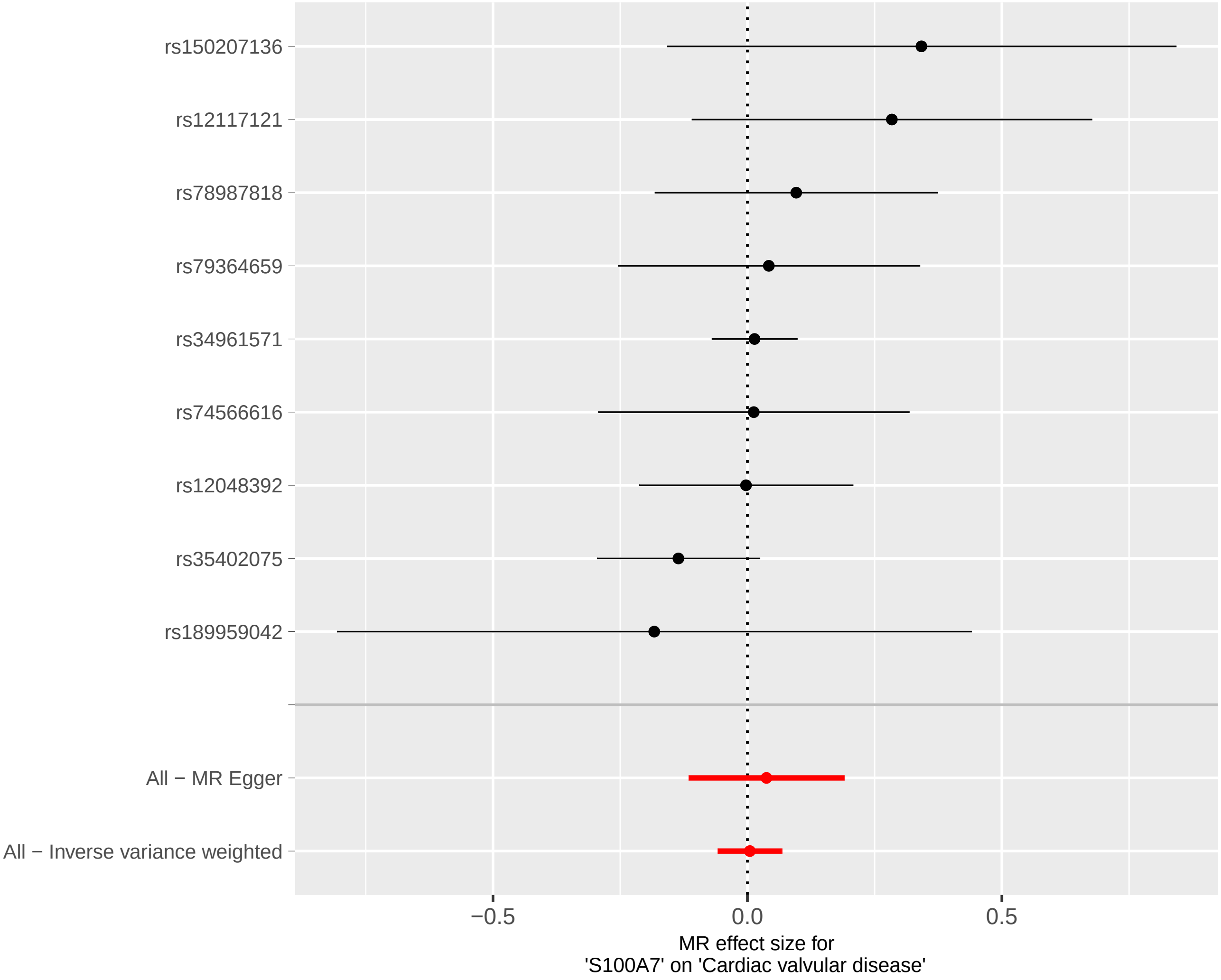
rs150207136
rs12117121
rs78987818
rs79364659
rs34961571
rs74566616
rs12048392
rs35402075
rs189959042
All − MR Egger
All − Inverse variance weighted
−0.5
0.0
0.5
MR effect size for
'S100A7' on 'Cardiac valvular disease'

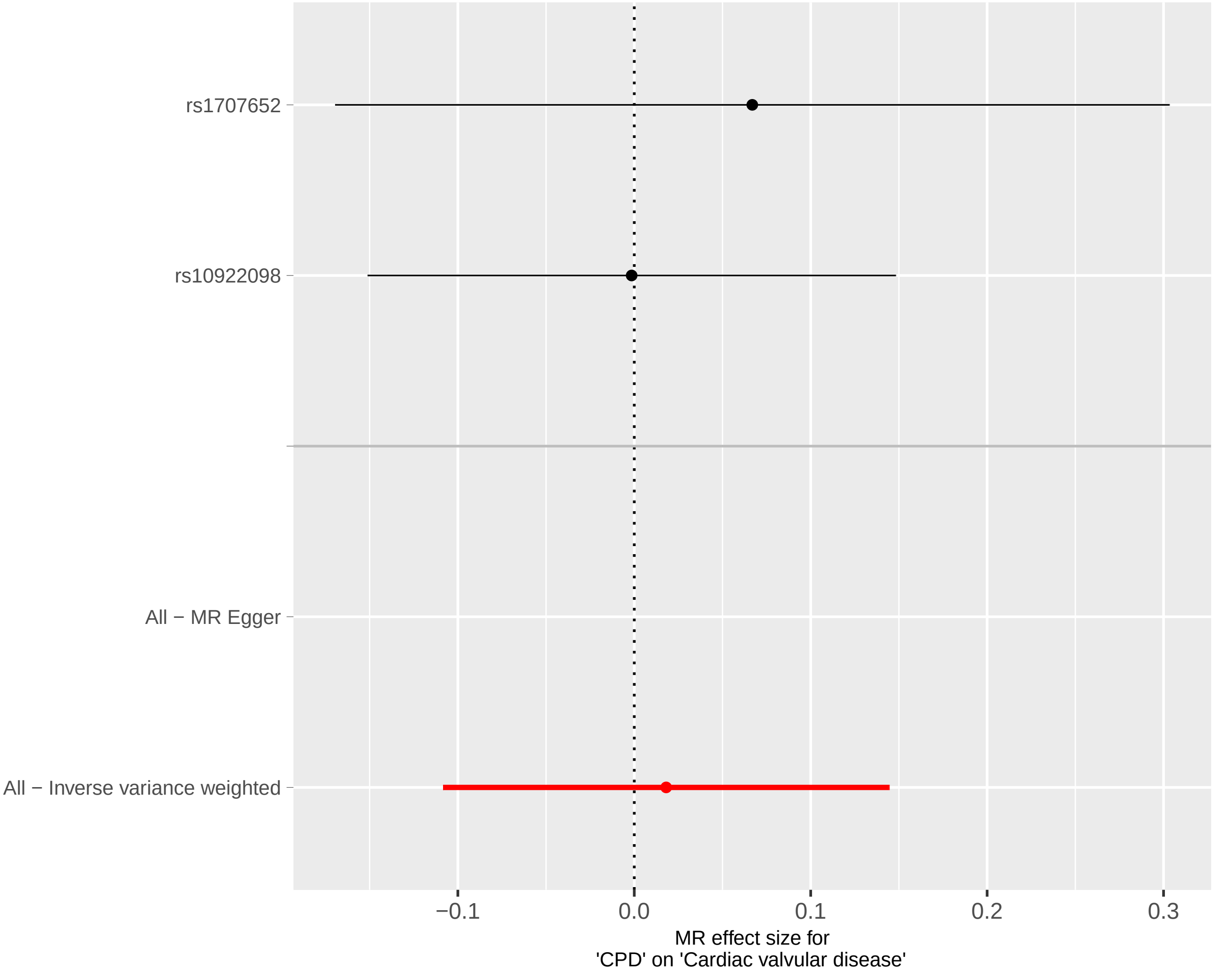

rs1707652
rs10922098
All − MR Egger
All − Inverse variance weighted
−0.1
0.0
0.1
0.2
0.3
MR effect size for
'CPD' on 'Cardiac valvular disease'

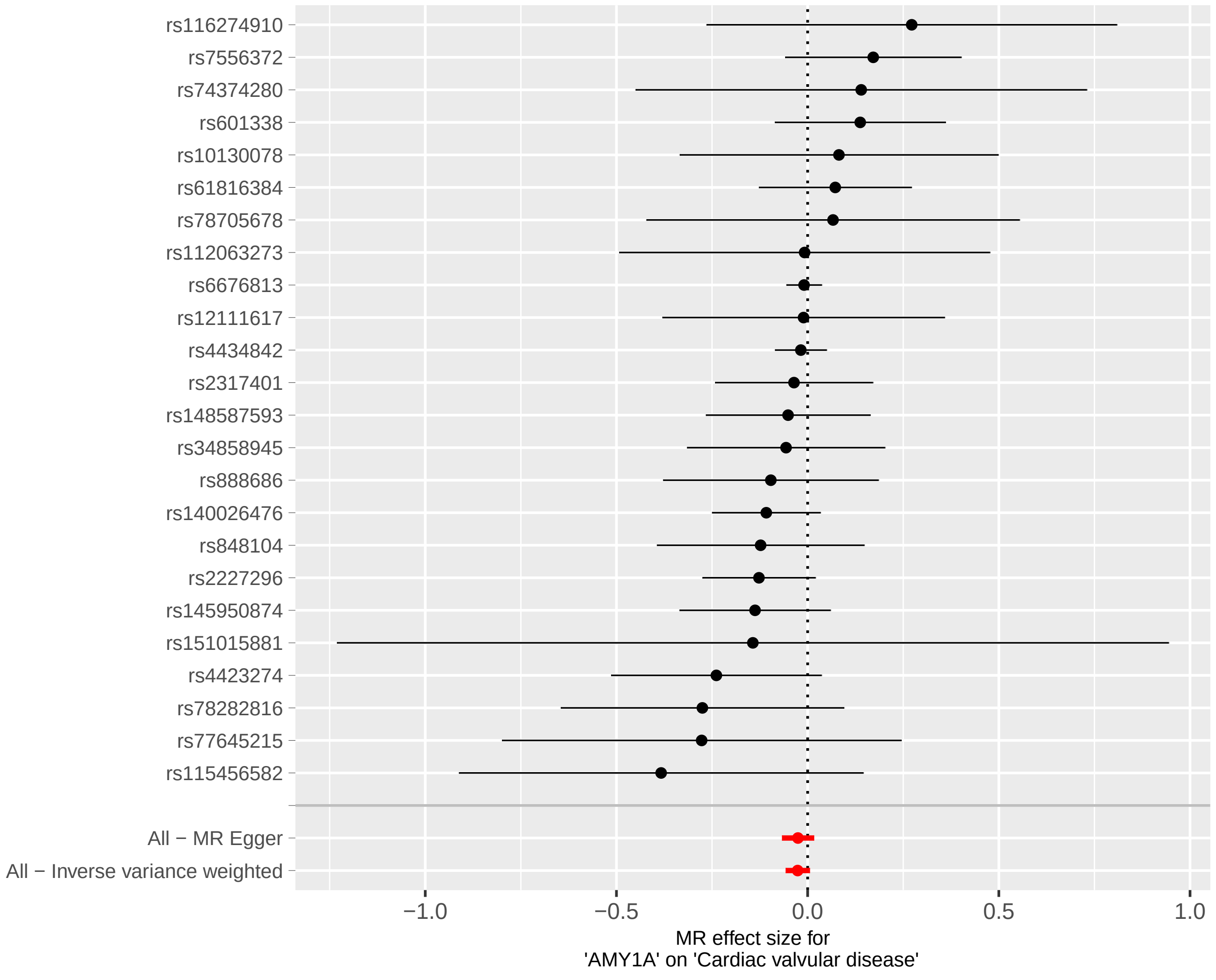
rs116274910
rs7556372
rs74374280
rs601338
rs10130078
rs61816384
rs78705678
rs112063273
rs6676813
rs12111617
rs4434842
rs2317401
rs148587593
rs34858945
rs888686
rs140026476
rs848104
rs2227296
rs145950874
rs151015881
rs4423274
rs78282816
rs77645215
rs115456582
All − MR Egger
All − Inverse variance weighted
−1.0
−0.5
0.0
0.5
1.0
MR effect size for
'AMY1A' on 'Cardiac valvular disease'

Insufficient number of SNPs

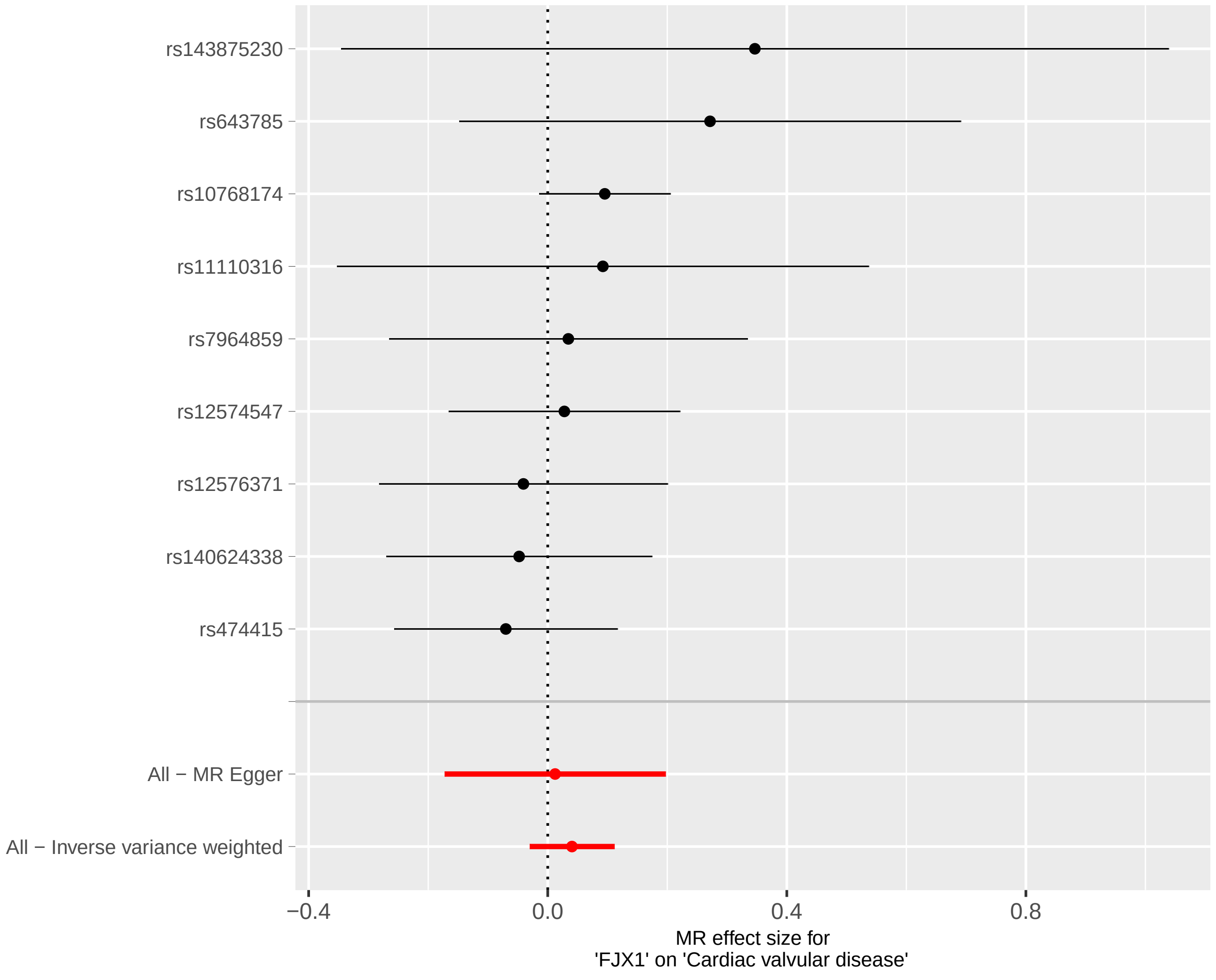
rs143875230
rs643785
rs10768174
rs11110316
rs7964859
rs12574547
rs12576371
rs140624338
rs474415
All − MR Egger
All − Inverse variance weighted
−0.4
0.0
0.4
0.8
MR effect size for
'FJX1' on 'Cardiac valvular disease'

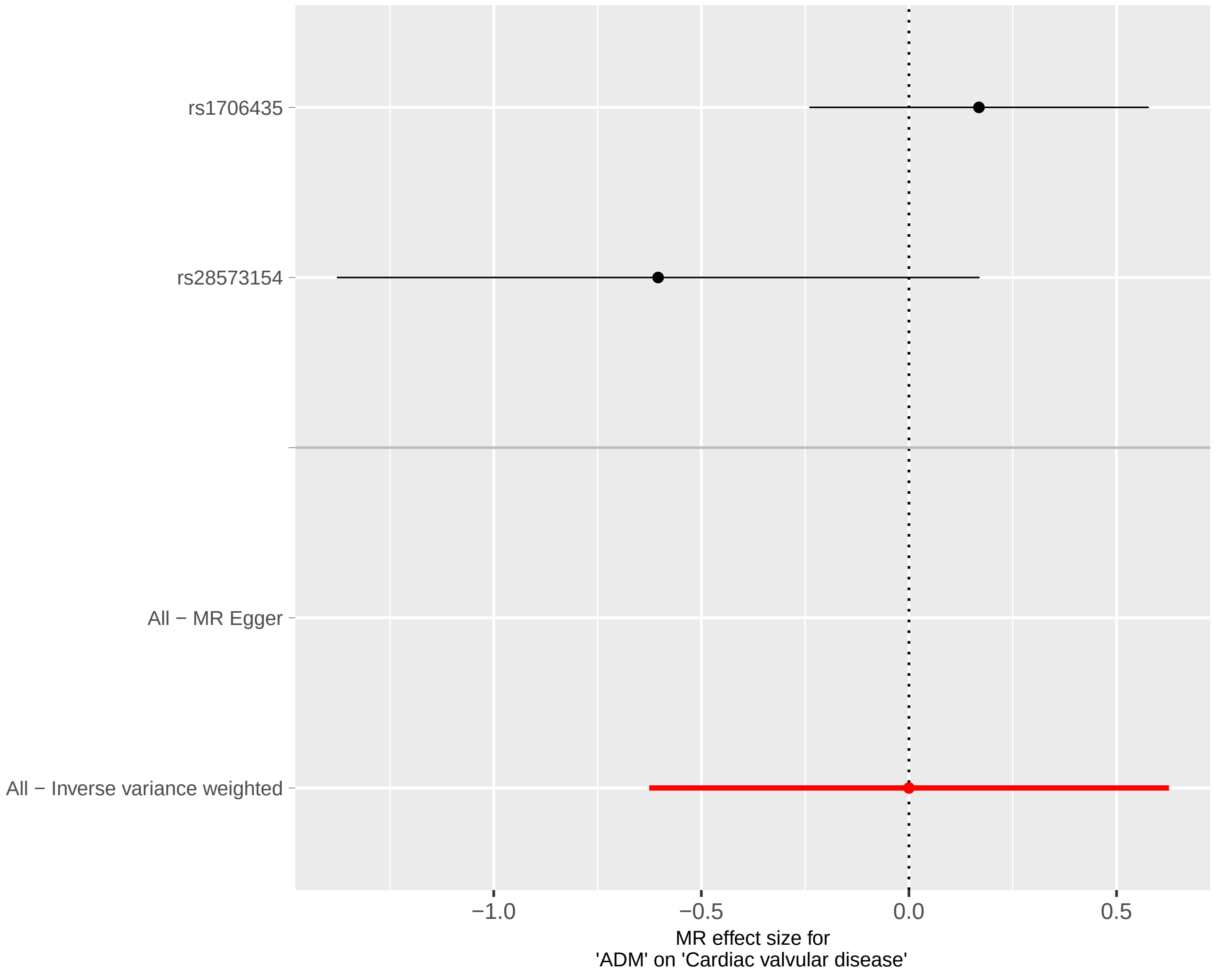
rs1706435
rs28573154
All − MR Egger
All − Inverse variance weighted
−1.0
−0.5
0.0
0.5
MR effect size for
'ADM' on 'Cardiac valvular disease'

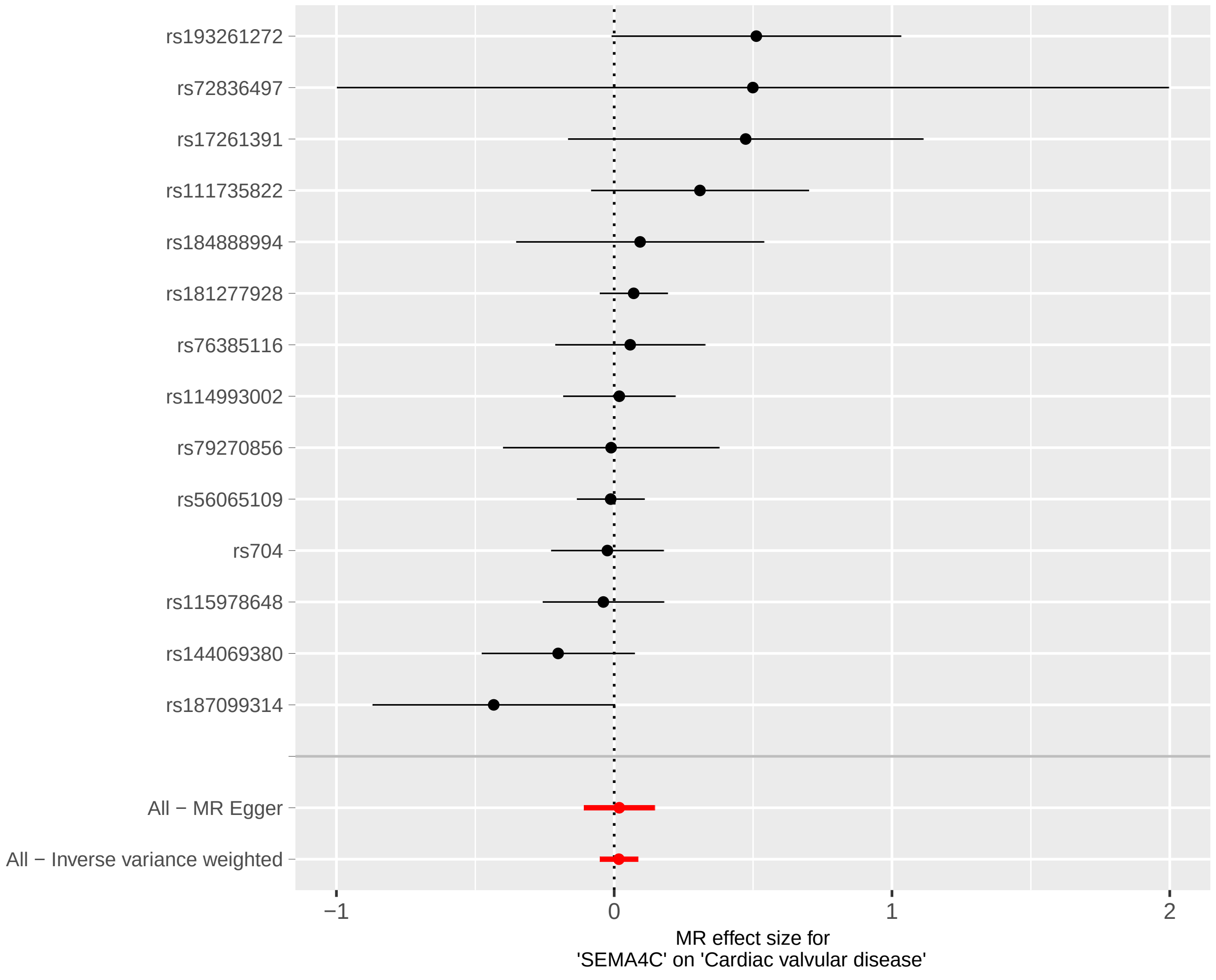

rs193261272
rs72836497
rs17261391
rs111735822
rs184888994
rs181277928
rs76385116
rs114993002
rs79270856
rs56065109
rs704
rs115978648
rs144069380
rs187099314
All − MR Egger
All − Inverse variance weighted
−1
0
1
2
MR effect size for
'SEMA4C' on 'Cardiac valvular disease'

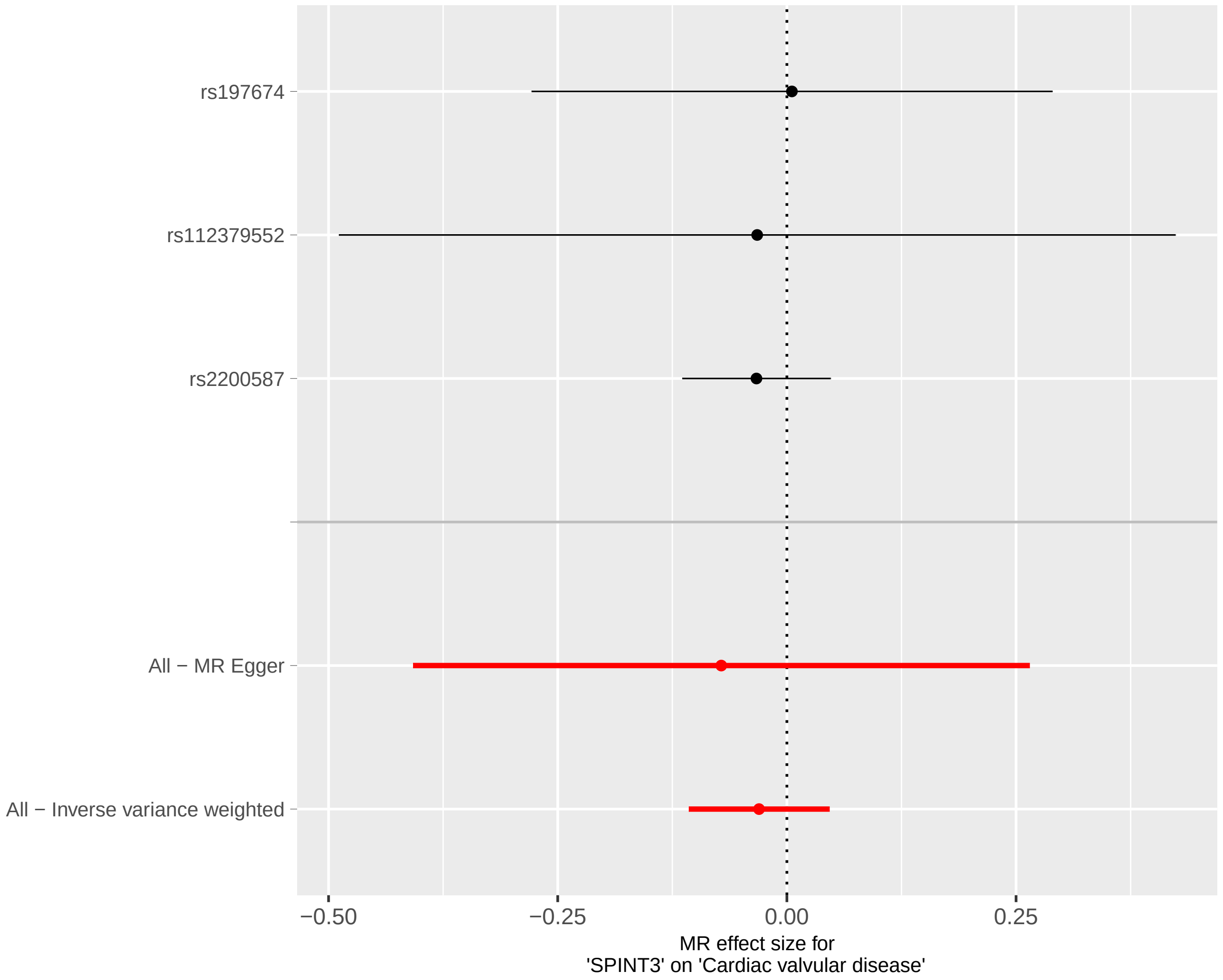
rs197674
rs112379552
rs2200587
All − MR Egger
All − Inverse variance weighted
−0.50
−0.25
0.00
0.25
MR effect size for
'SPINT3' on 'Cardiac valvular disease'

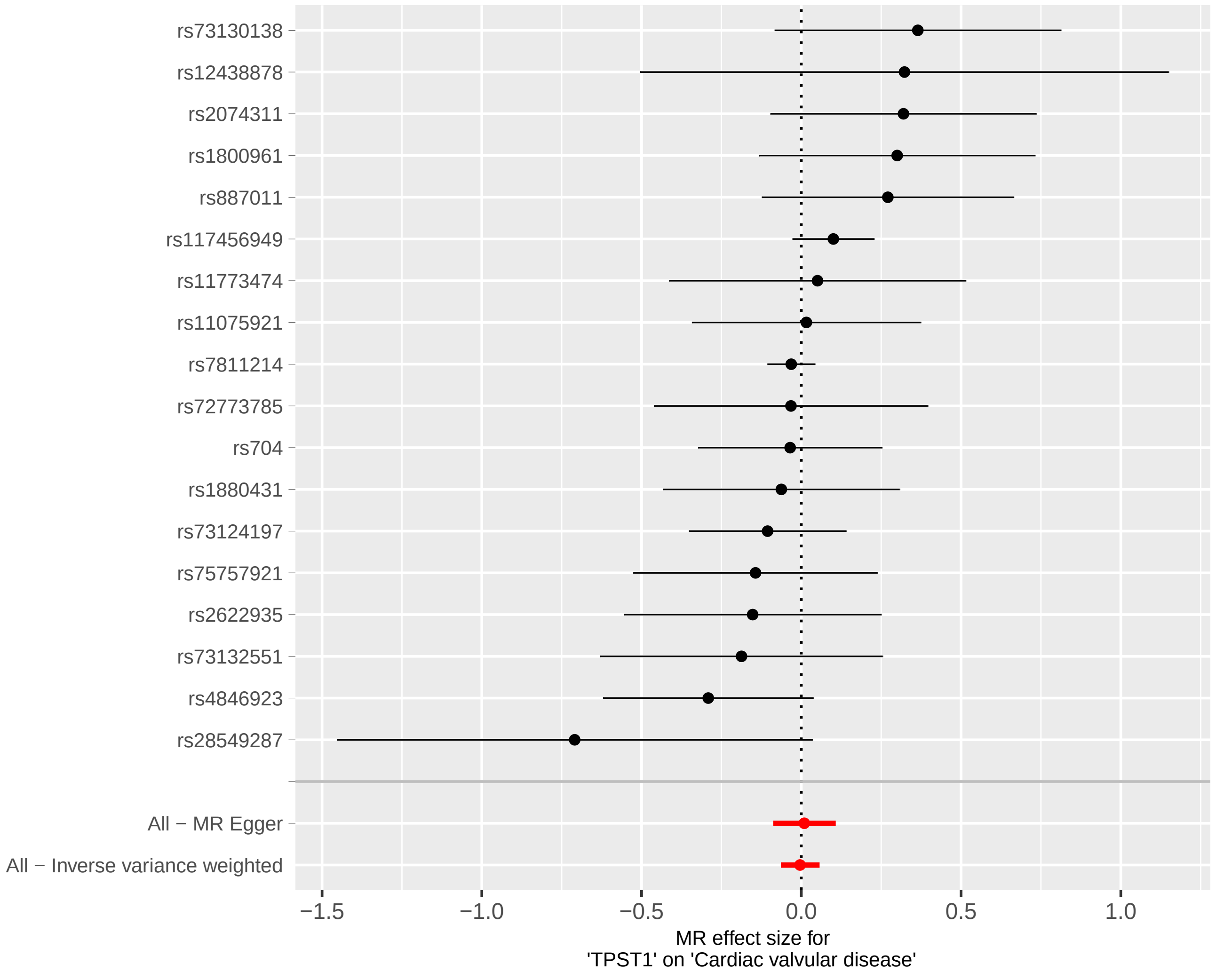
rs73130138
rs12438878
rs2074311
rs1800961
rs887011
rs117456949
rs11773474
rs11075921
rs7811214
rs72773785
rs704
rs1880431
rs73124197
rs75757921
rs2622935
rs73132551
rs4846923
rs28549287
All − MR Egger
All − Inverse variance weighted
−1.5
−1.0
−0.5
0.0
0.5
1.0
MR effect size for
'TPST1' on 'Cardiac valvular disease'

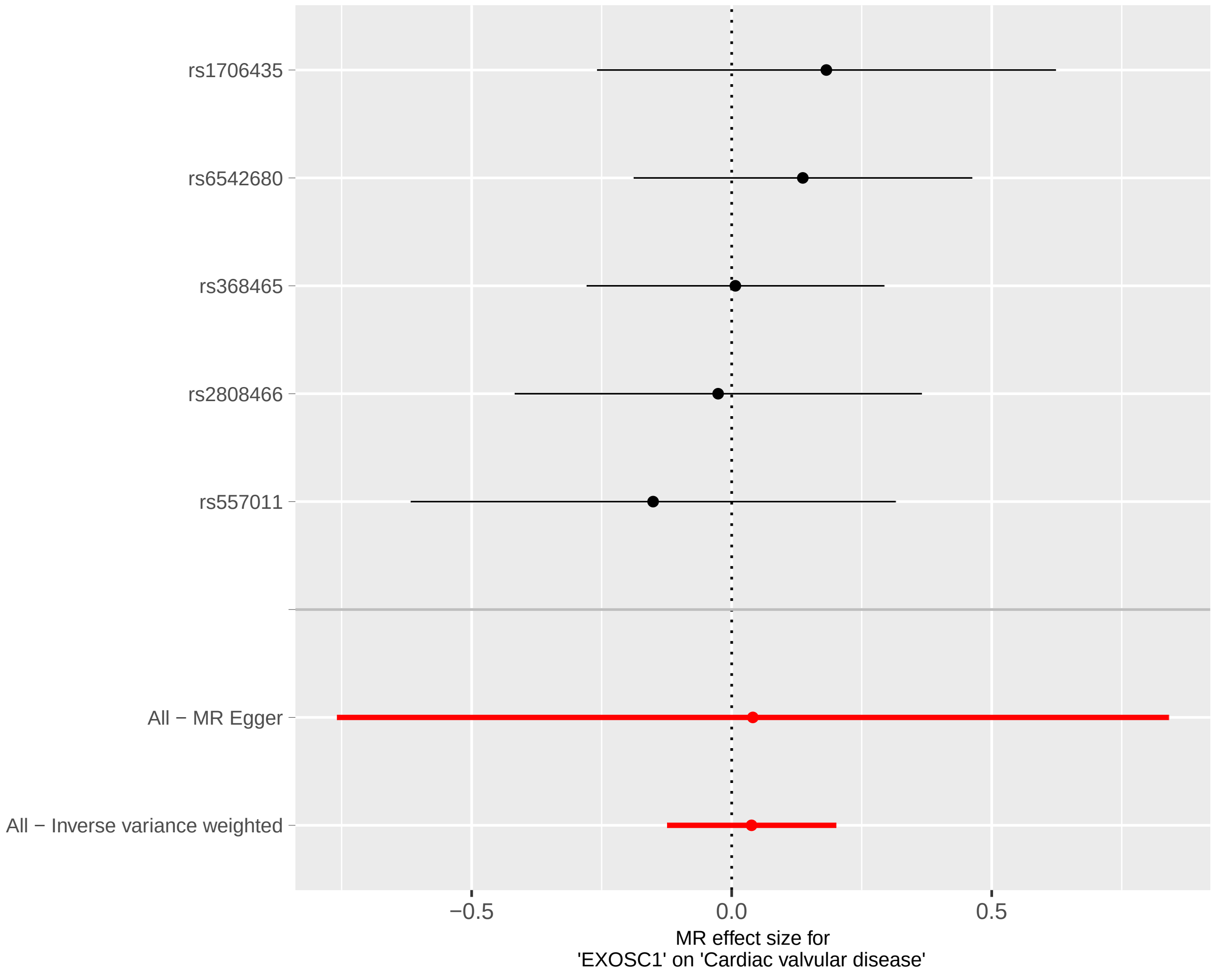
rs1706435
rs6542680
rs368465
rs2808466
rs557011
All − MR Egger
All − Inverse variance weighted
−0.5
0.0
0.5
MR effect size for
'EXOSC1' on 'Cardiac valvular disease'

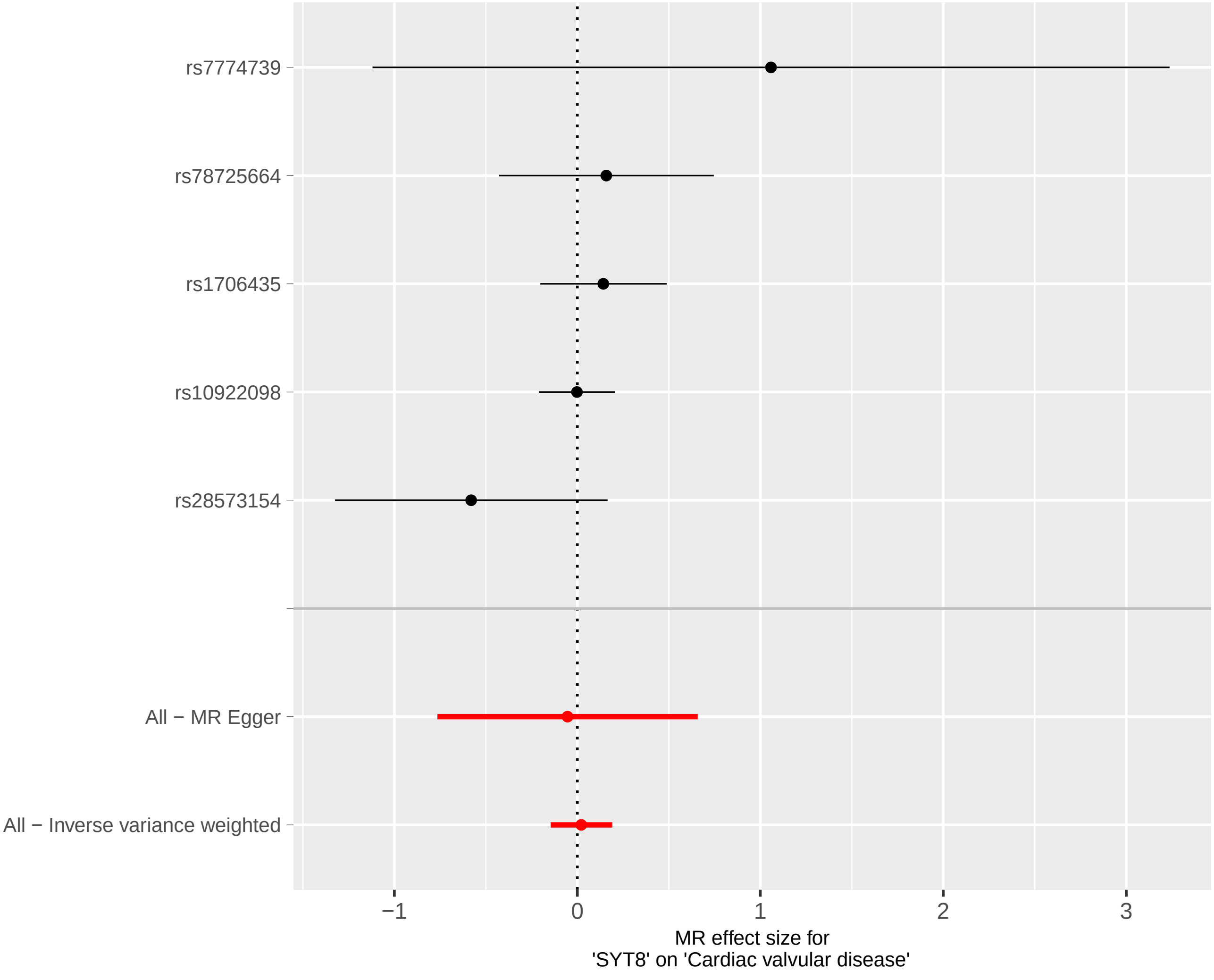
rs7774739
rs78725664
rs1706435
rs10922098
rs28573154
All − MR Egger
All − Inverse variance weighted
−1
0
1
2
3
MR effect size for
'SYT8' on 'Cardiac valvular disease'

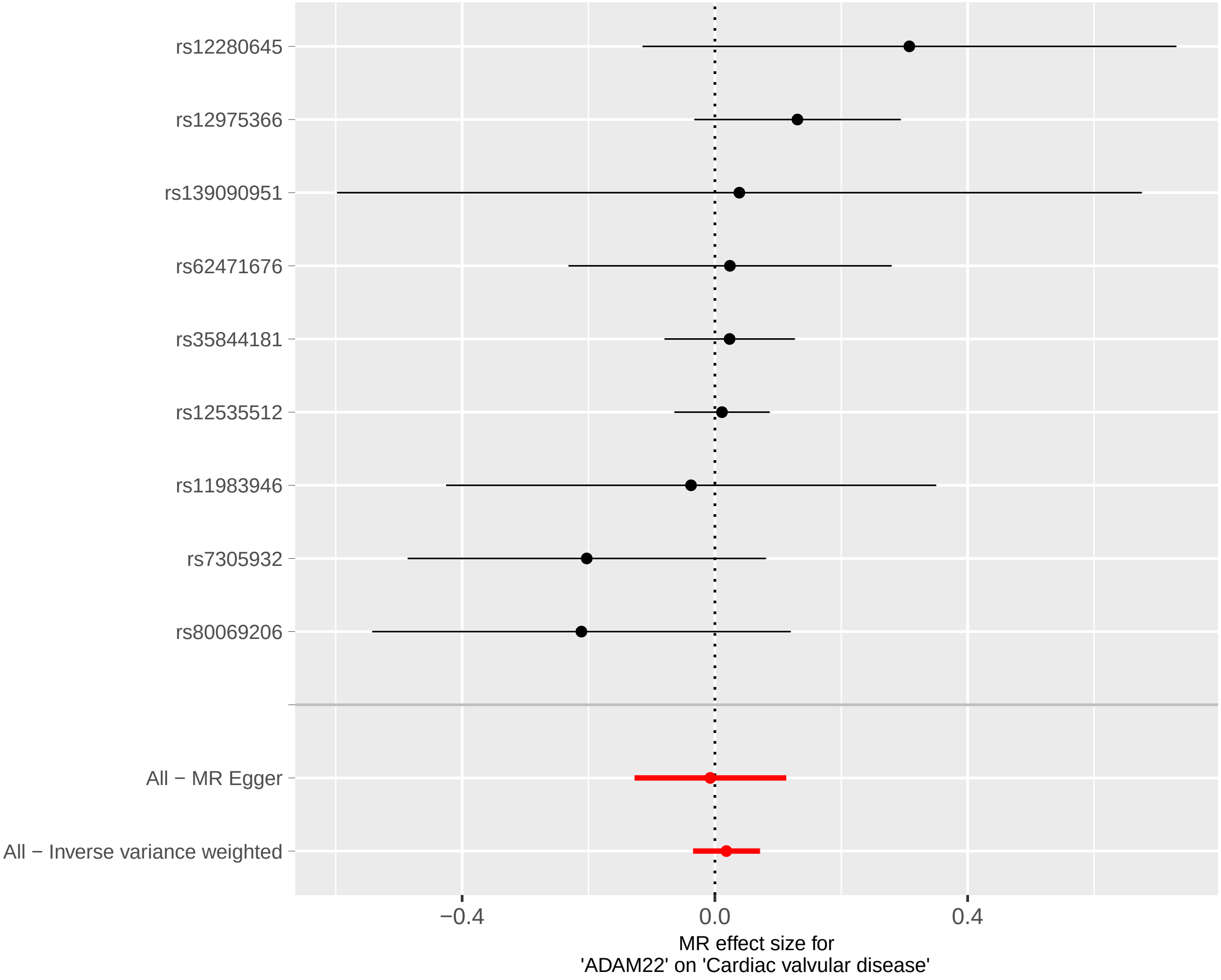
rs12280645
rs12975366
rs139090951
rs62471676
rs35844181
rs12535512
rs11983946
rs7305932
rs80069206
All − MR Egger
All − Inverse variance weighted
−0.4
0.0
0.4
MR effect size for
'ADAM22' on 'Cardiac valvular disease'

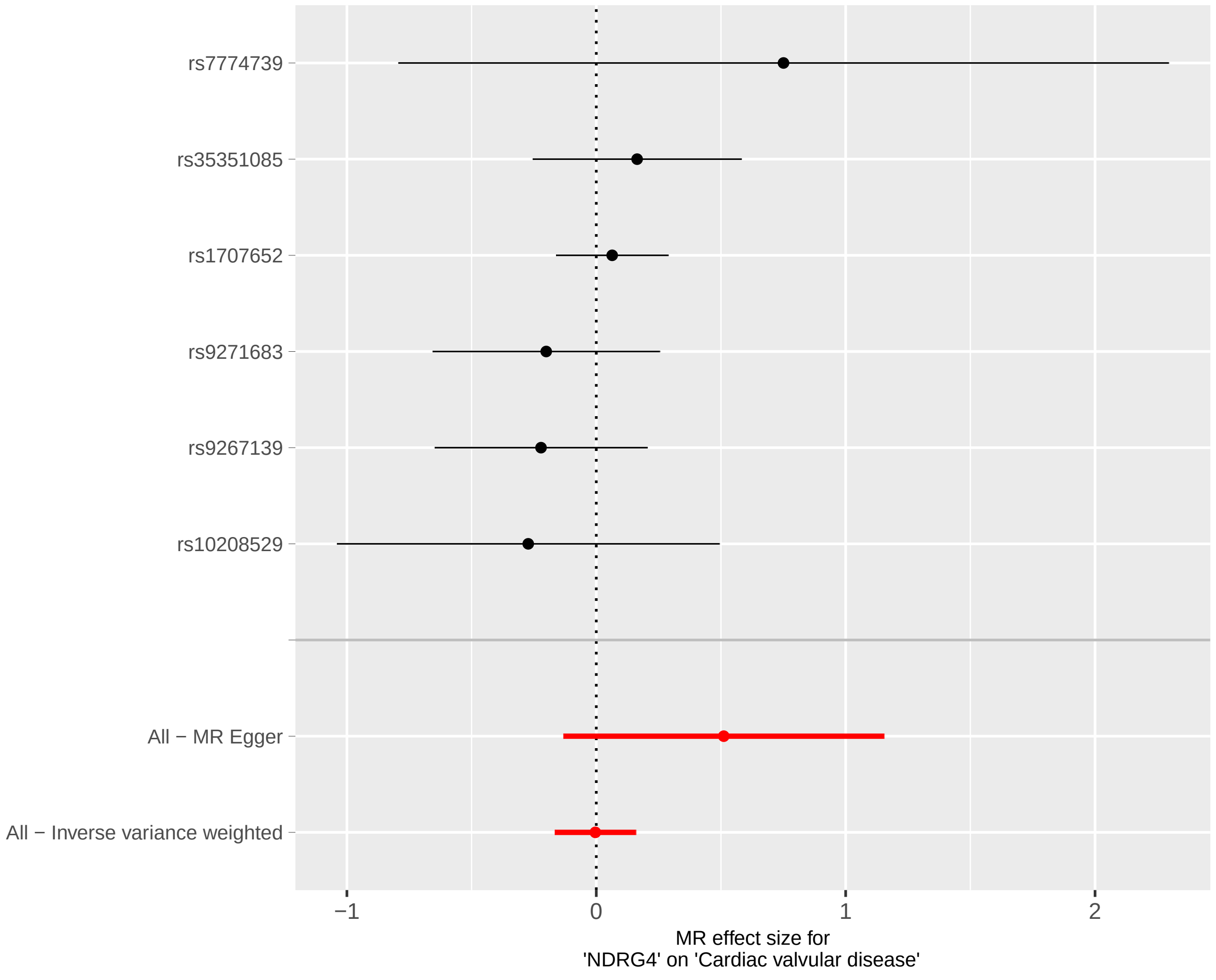
rs7774739
rs35351085
rs1707652
rs9271683
rs9267139
rs10208529
All − MR Egger
All − Inverse variance weighted
−1
0
1
2
MR effect size for
'NDRG4' on 'Cardiac valvular disease'

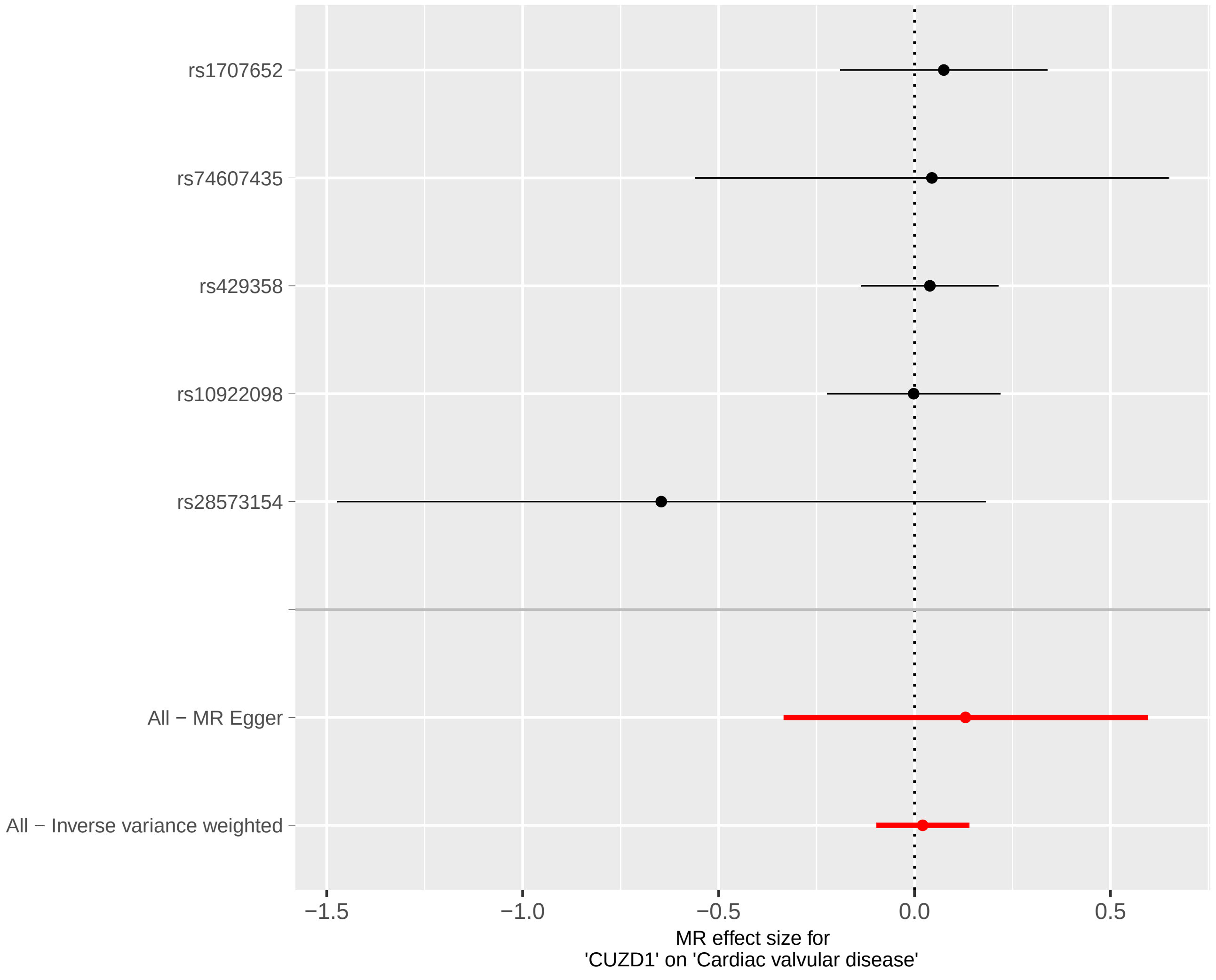
rs1707652
rs74607435
rs429358
rs10922098
rs28573154
All − MR Egger
All − Inverse variance weighted
−1.5
−1.0
−0.5
0.0
0.5
MR effect size for
'CUZD1' on 'Cardiac valvular disease'

Insufficient number of SNPs

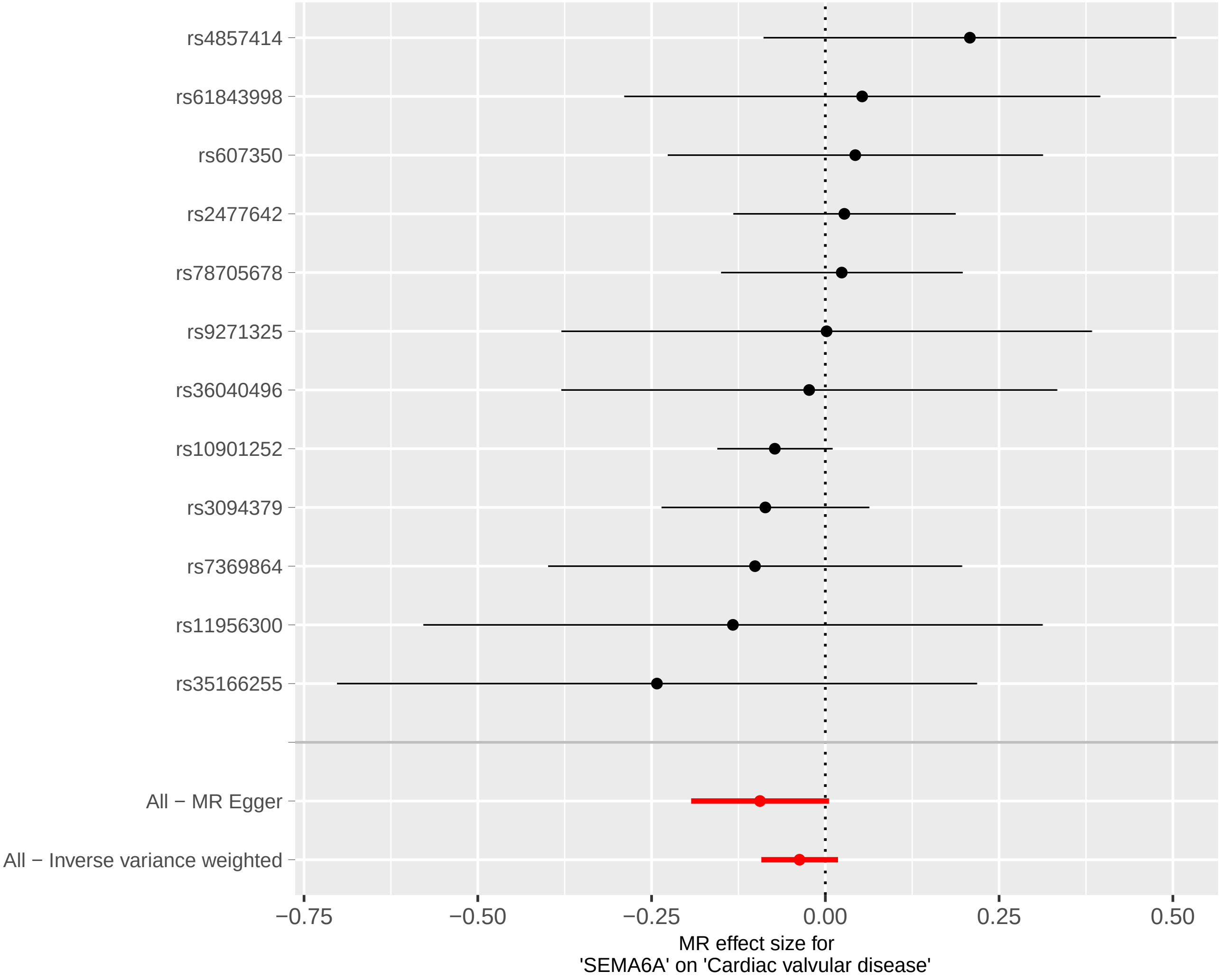
rs4857414
rs61843998
rs607350
rs2477642
rs78705678
rs9271325
rs36040496
rs10901252
rs3094379
rs7369864
rs11956300
rs35166255
All − MR Egger
All − Inverse variance weighted
−0.75
−0.50
−0.25
0.00
0.25
0.50
MR effect size for
'SEMA6A' on 'Cardiac valvular disease'

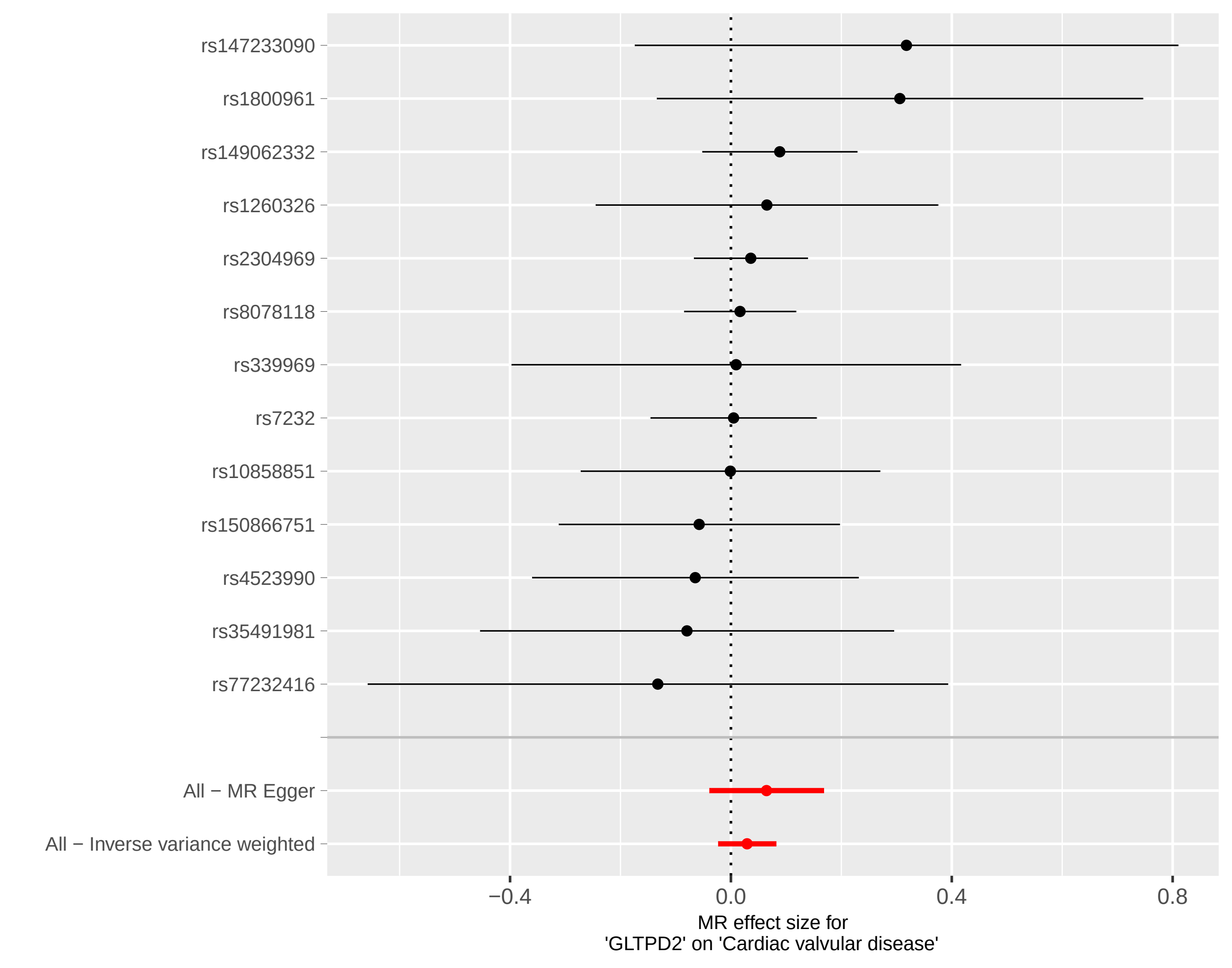
rs147233090
rs1800961
rs149062332
rs1260326
rs2304969
rs8078118
rs339969
rs7232
rs10858851
rs150866751
rs4523990
rs35491981
rs77232416
All − MR Egger
All − Inverse variance weighted
−0.4
0.0
0.4
0.8
MR effect size for
'GLTPD2' on 'Cardiac valvular disease'

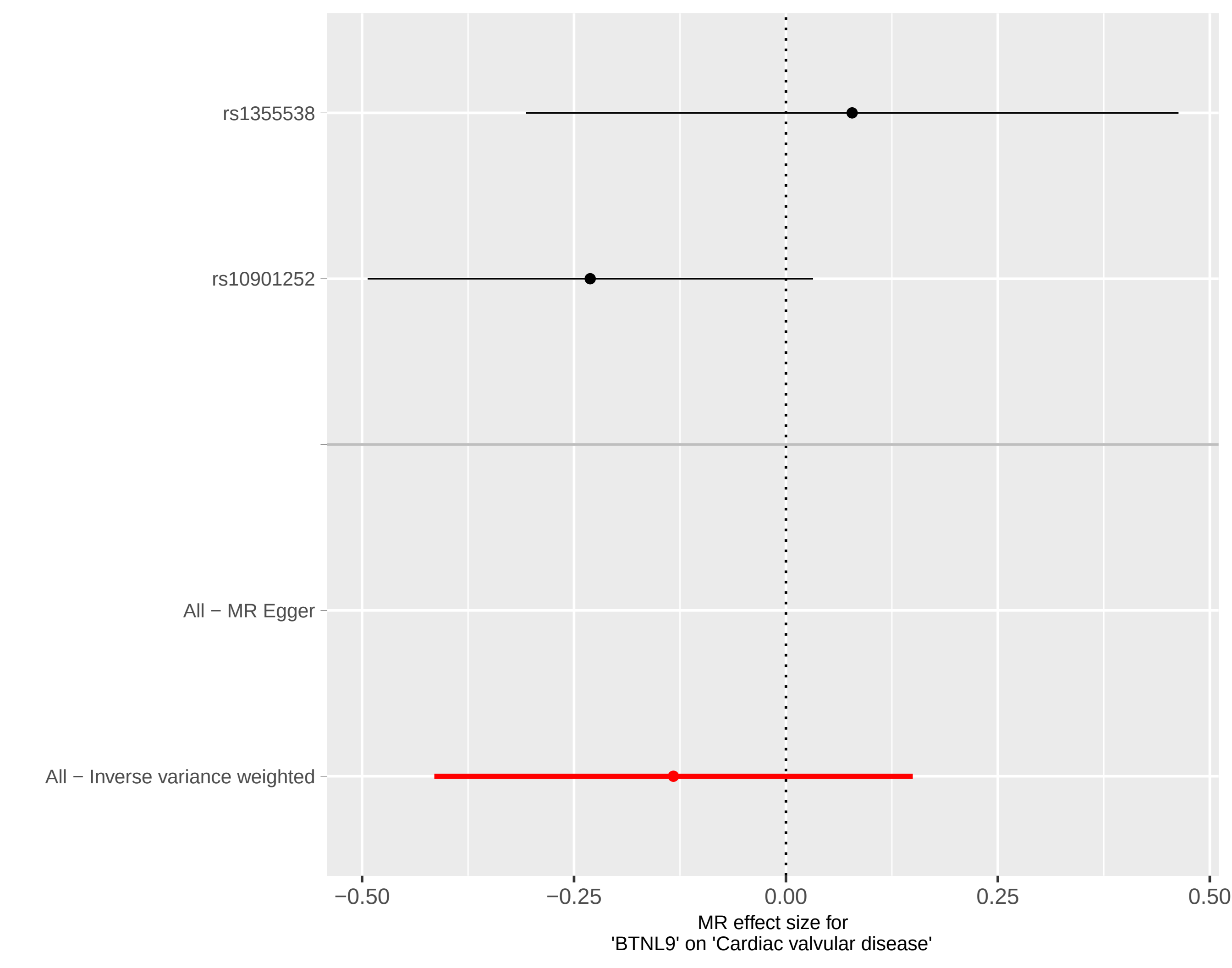

rs1355538
rs10901252
All − MR Egger
All − Inverse variance weighted
−0.50
−0.25
0.00
0.25
0.50
MR effect size for
'BTNL9' on 'Cardiac valvular disease'

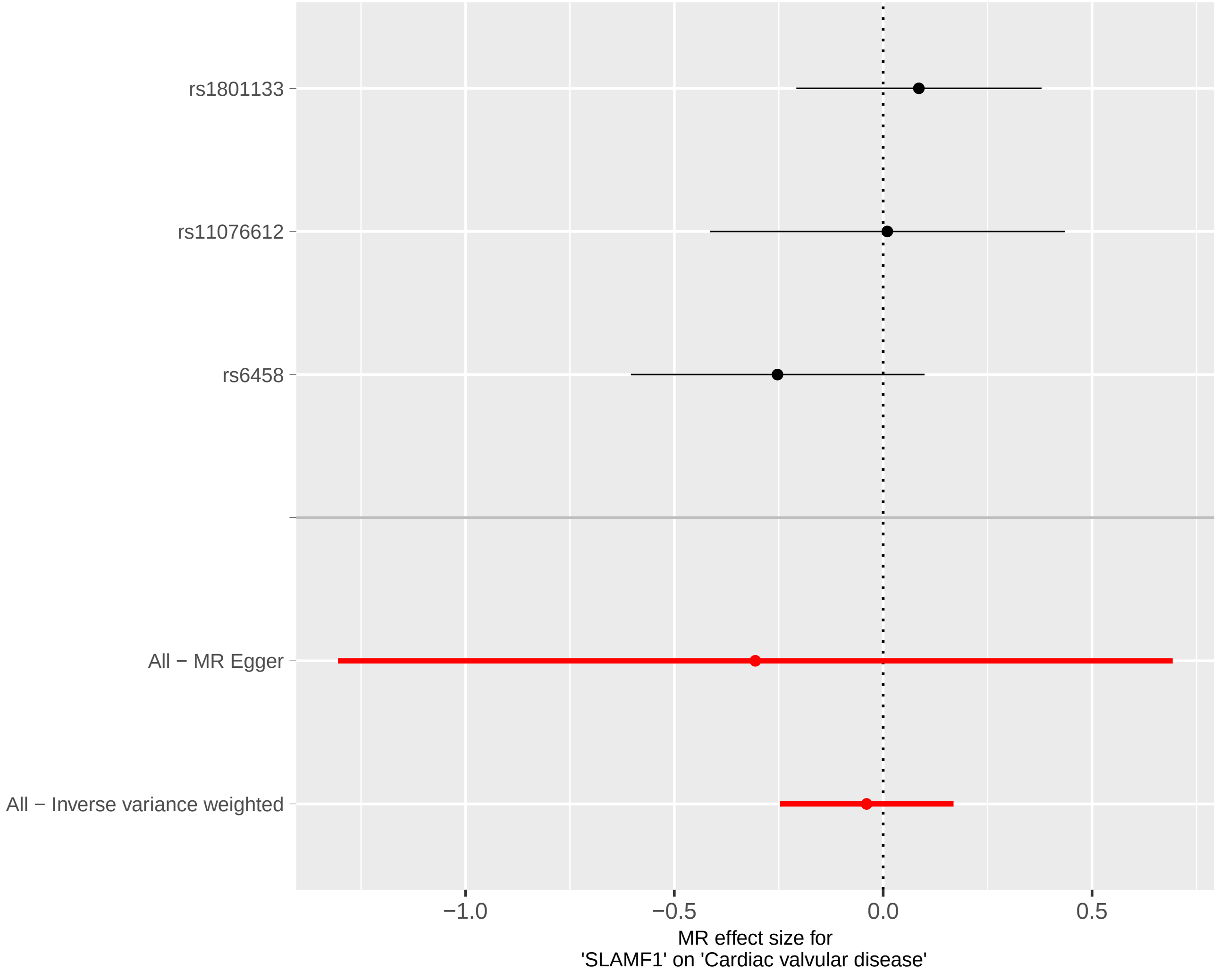
rs1801133
rs11076612
rs6458
All − MR Egger
All − Inverse variance weighted
−1.0
−0.5
0.0
0.5
MR effect size for
'SLAMF1' on 'Cardiac valvular disease'

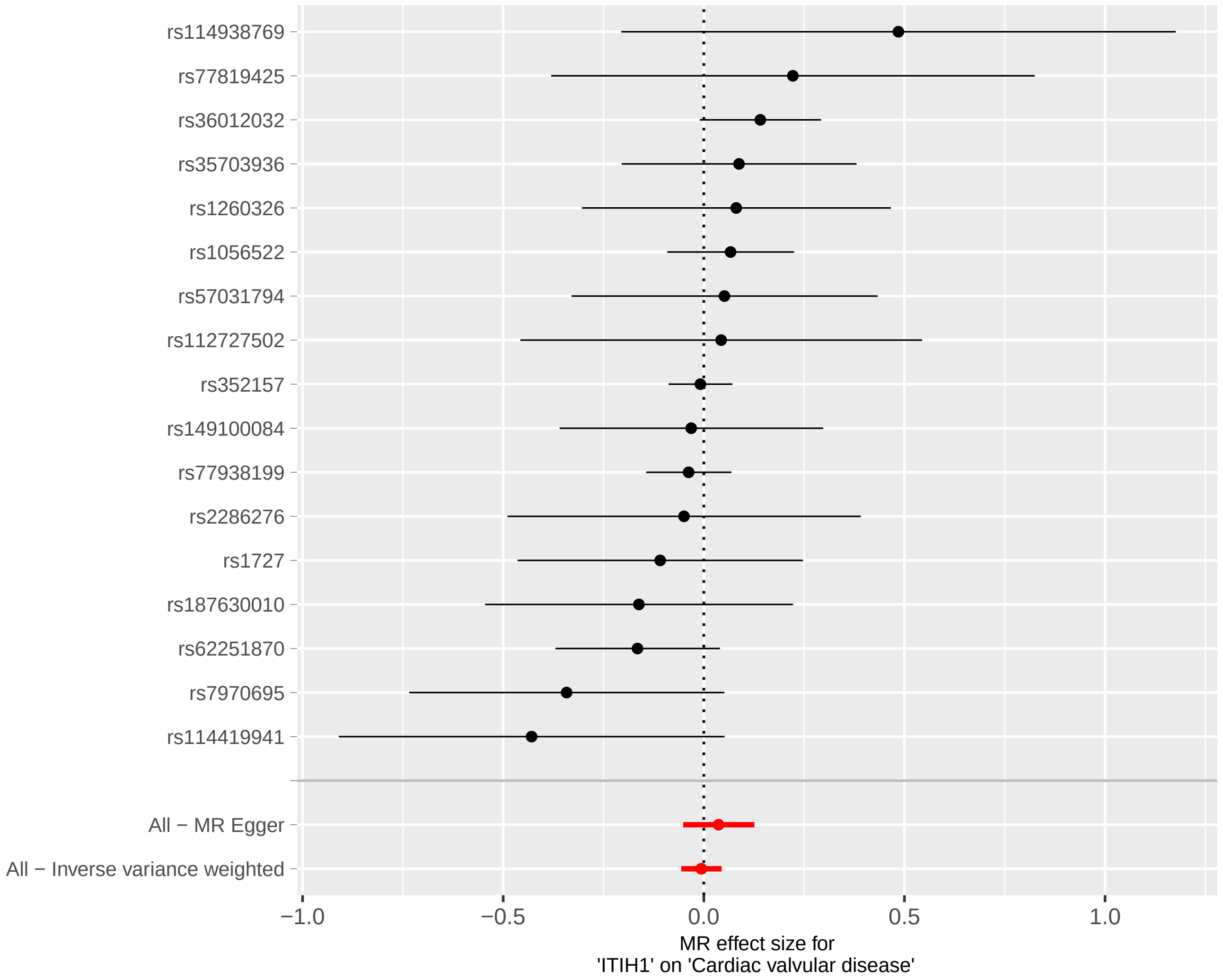
rs114938769
rs77819425
rs36012032
rs35703936
rs1260326
rs1056522
rs57031794
rs112727502
rs352157
rs149100084
rs77938199
rs2286276
rs1727
rs187630010
rs62251870
rs7970695
rs114419941
All − MR Egger
All − Inverse variance weighted
−1.0
−0.5
0.0
0.5
1.0
MR effect size for
'ITIH1' on 'Cardiac valvular disease'

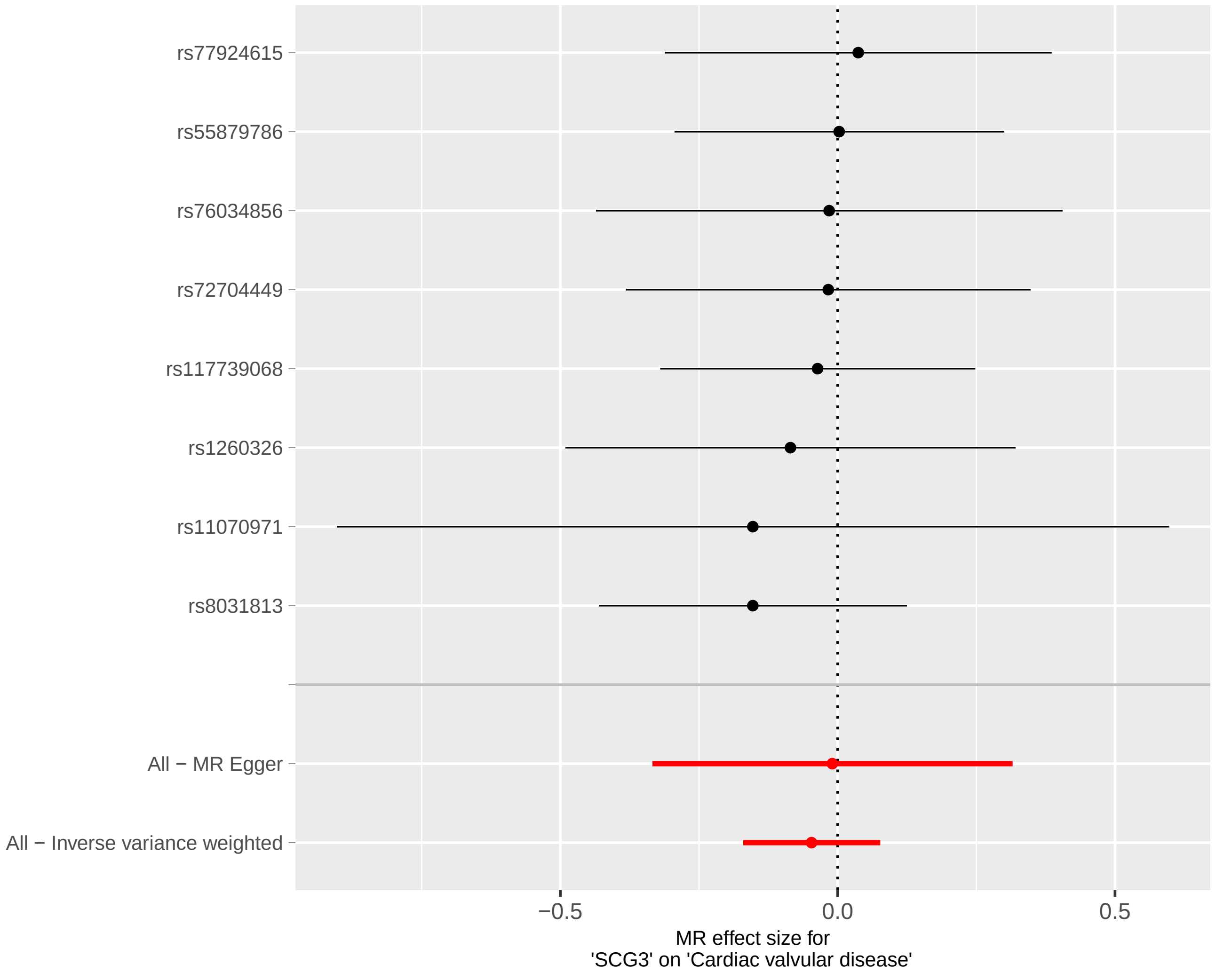

rs77924615
rs55879786
rs76034856
rs72704449
rs117739068
rs1260326
rs11070971
rs8031813
All − MR Egger
All − Inverse variance weighted
−0.5
0.0
0.5
MR effect size for
'SCG3' on 'Cardiac valvular disease'

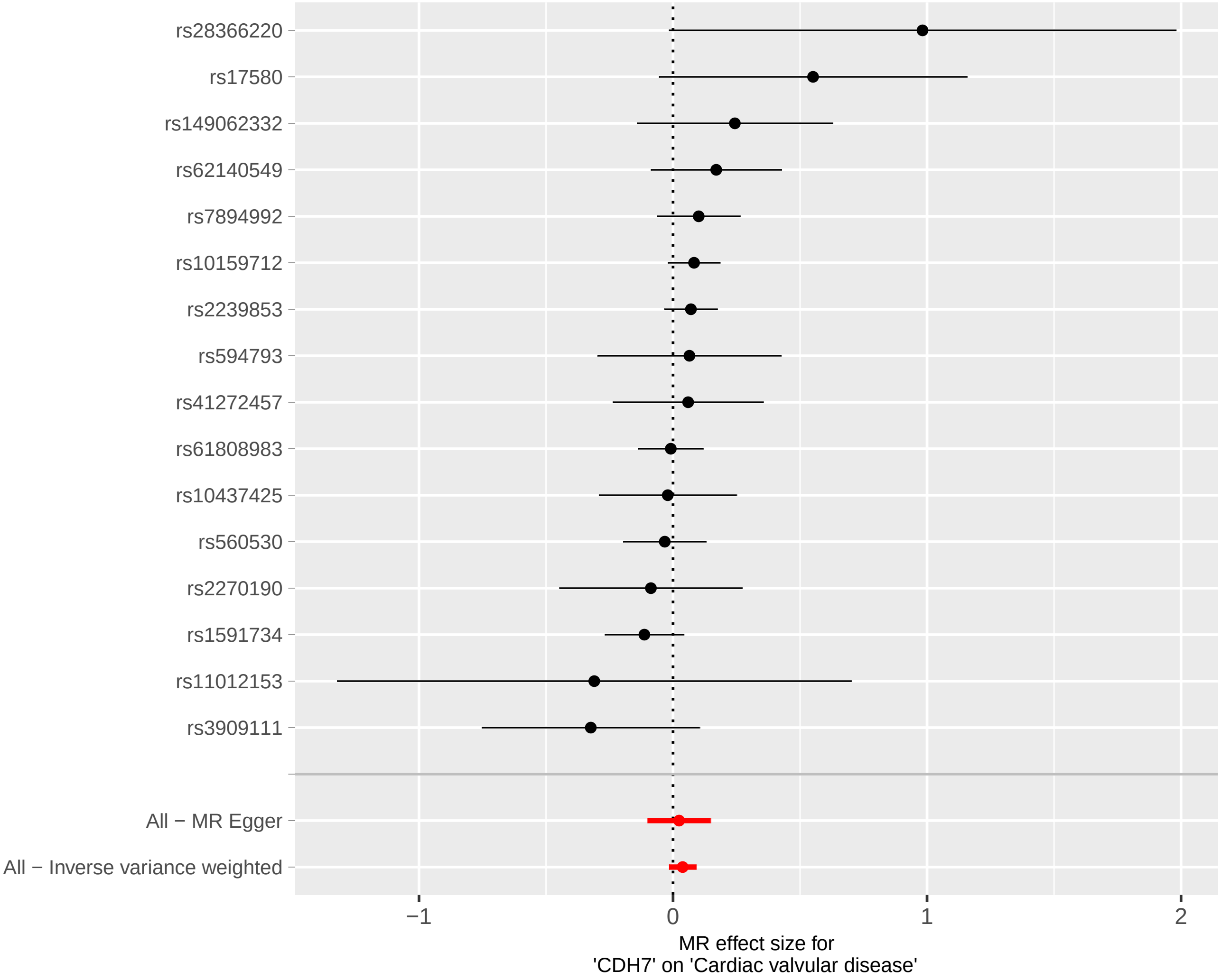
rs28366220
rs17580
rs149062332
rs62140549
rs7894992
rs10159712
rs2239853
rs594793
rs41272457
rs61808983
rs10437425
rs560530
rs2270190
rs1591734
rs11012153
rs3909111
All − MR Egger
All − Inverse variance weighted
−1
0
1
2
MR effect size for
'CDH7' on 'Cardiac valvular disease'

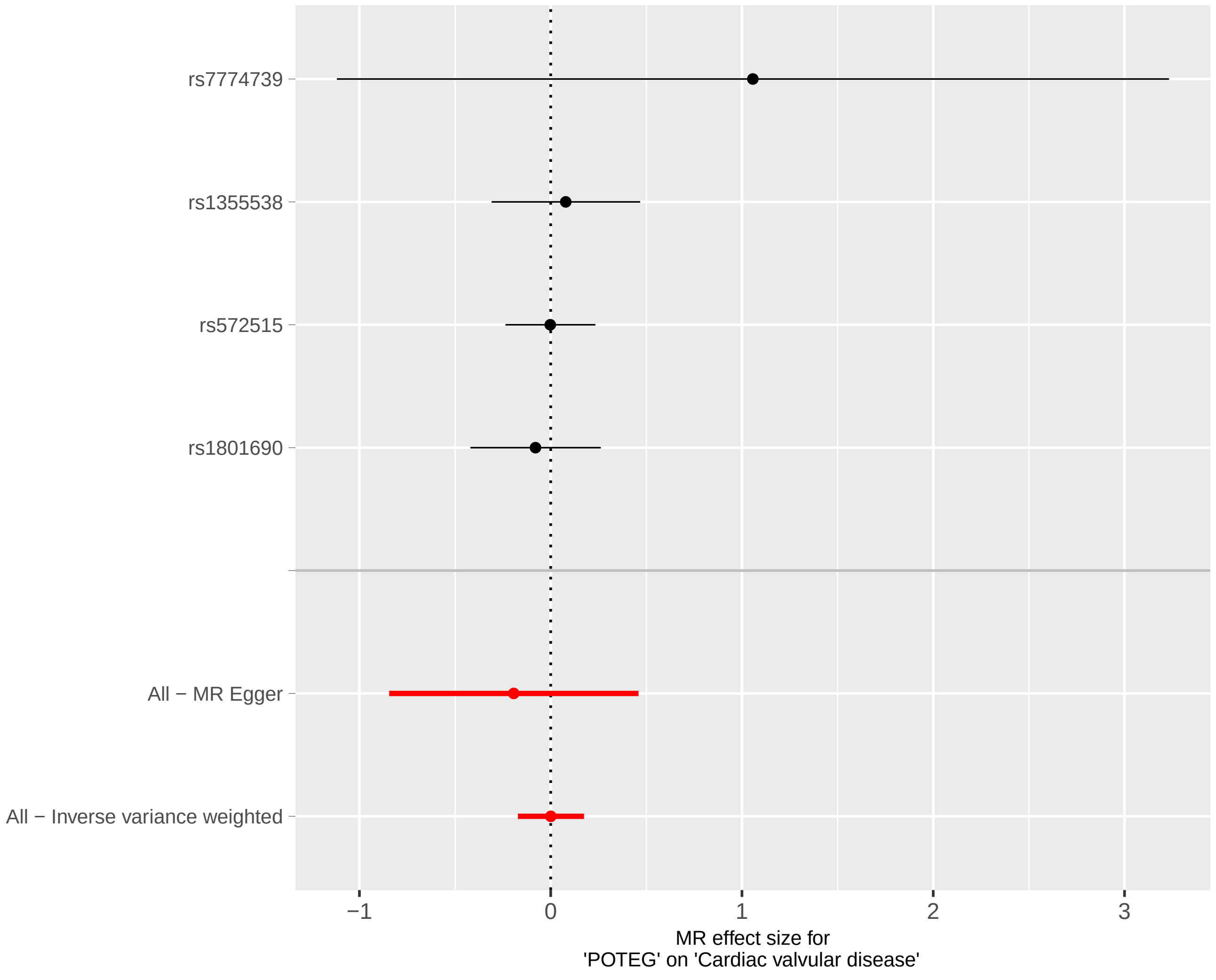
rs7774739
rs1355538
rs572515
rs1801690
All − MR Egger
All − Inverse variance weighted
−1
0
1
2
3
MR effect size for
'POTEG' on 'Cardiac valvular disease'

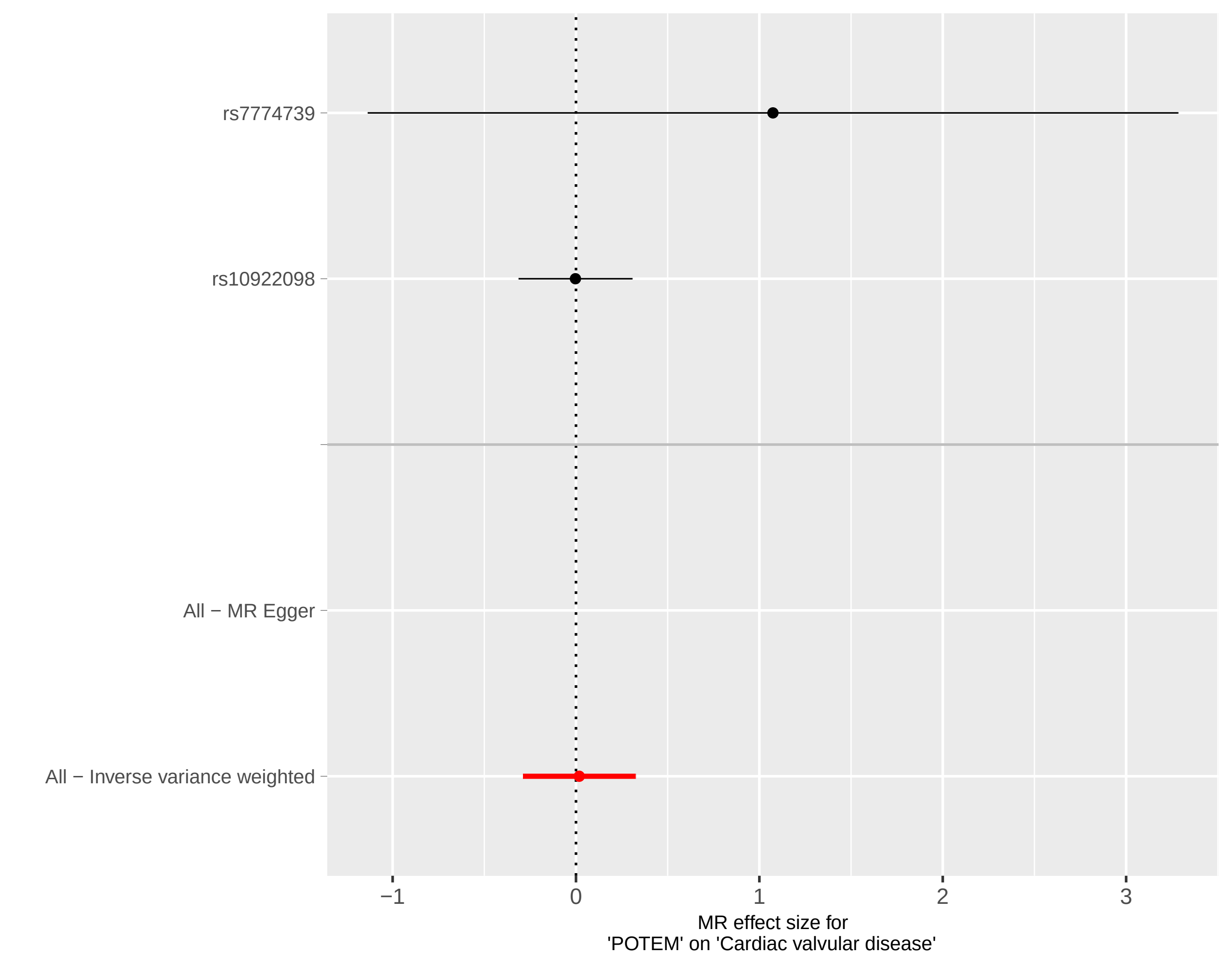

rs7774739
rs10922098
All − MR Egger
All − Inverse variance weighted
−1
0
1
2
3
MR effect size for
'POTEM' on 'Cardiac valvular disease'

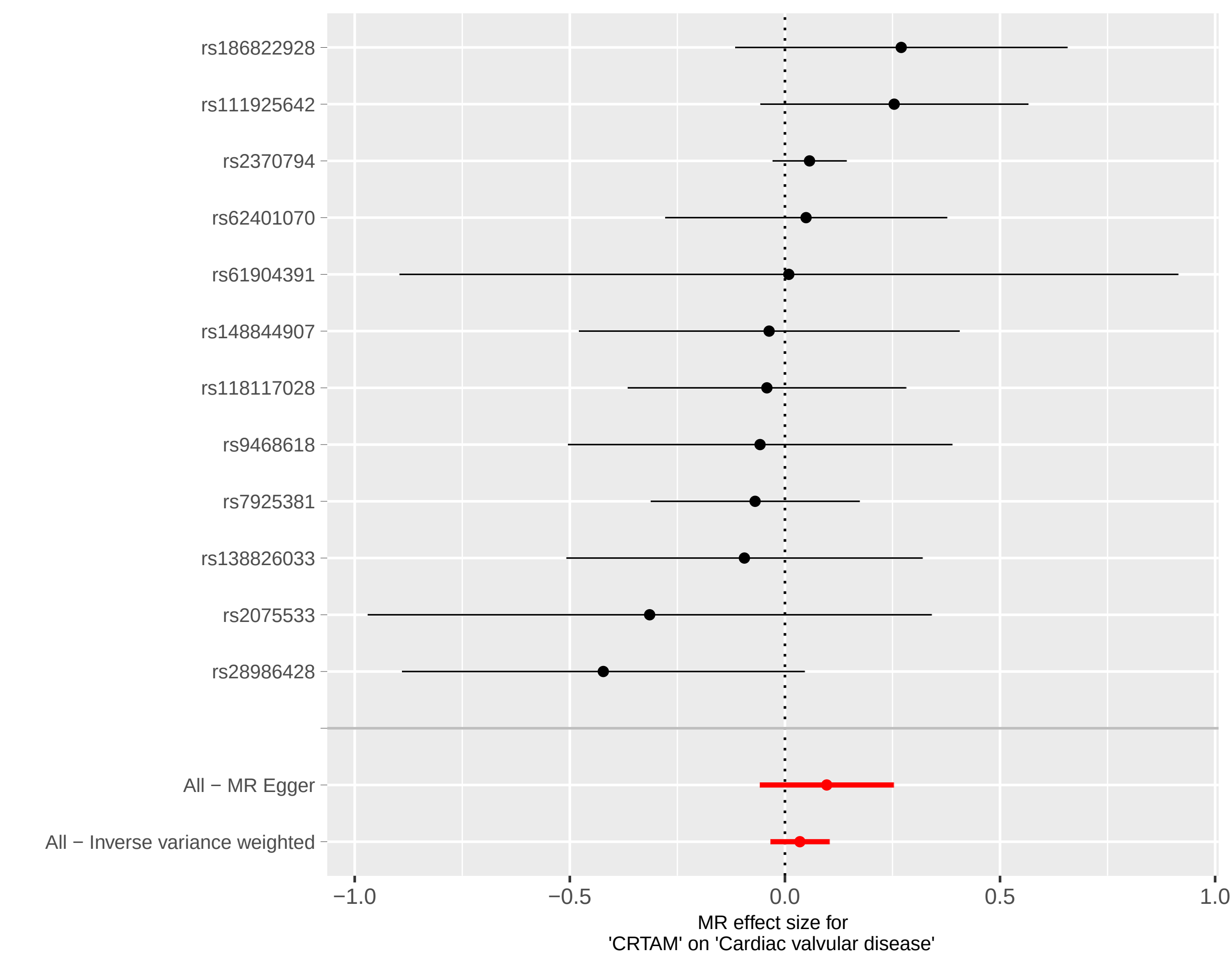
rs186822928
rs111925642
rs2370794
rs62401070
rs61904391
rs148844907
rs118117028
rs9468618
rs7925381
rs138826033
rs2075533
rs28986428
All − MR Egger
All − Inverse variance weighted
−1.0
−0.5
0.0
0.5
1.0
MR effect size for
'CRTAM' on 'Cardiac valvular disease'

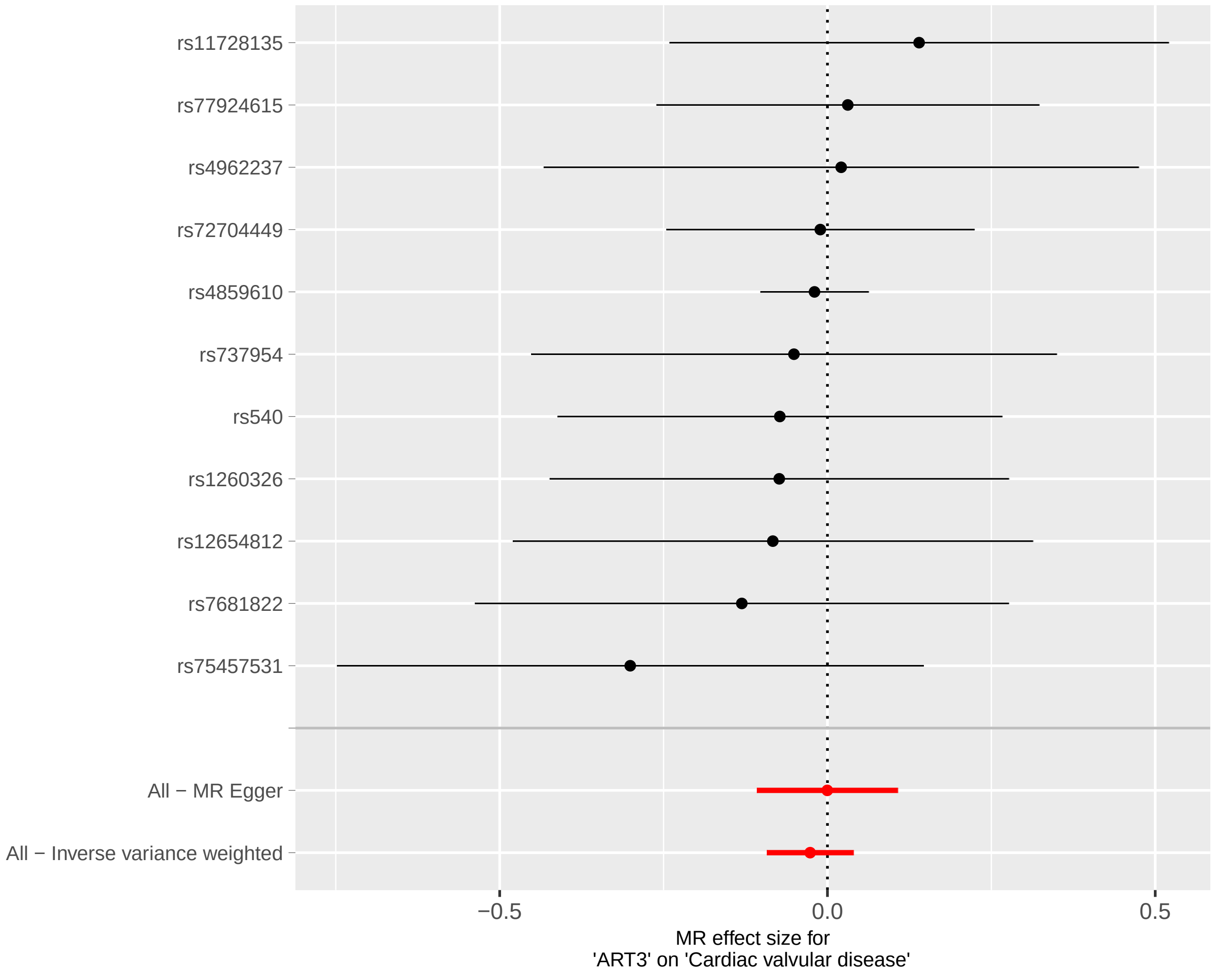

rs11728135
rs77924615
rs4962237
rs72704449
rs4859610
rs737954
rs540
rs1260326
rs12654812
rs7681822
rs75457531
All − MR Egger
All − Inverse variance weighted
−0.5
0.0
0.5
MR effect size for
'ART3' on 'Cardiac valvular disease'

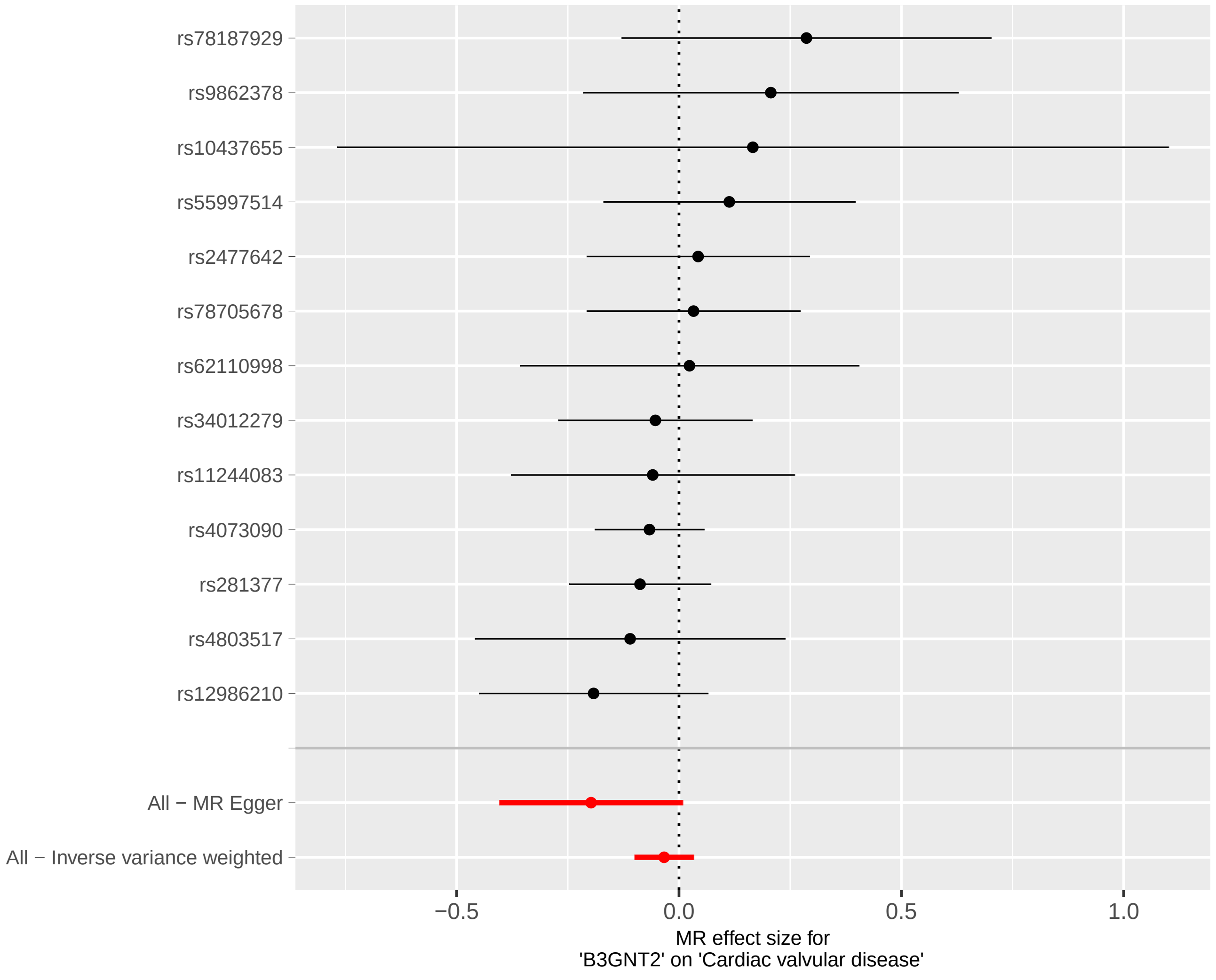
rs78187929
rs9862378
rs10437655
rs55997514
rs2477642
rs78705678
rs62110998
rs34012279
rs11244083
rs4073090
rs281377
rs4803517
rs12986210
All − MR Egger
All − Inverse variance weighted
−0.5
0.0
0.5
1.0
MR effect size for
'B3GNT2' on 'Cardiac valvular disease'

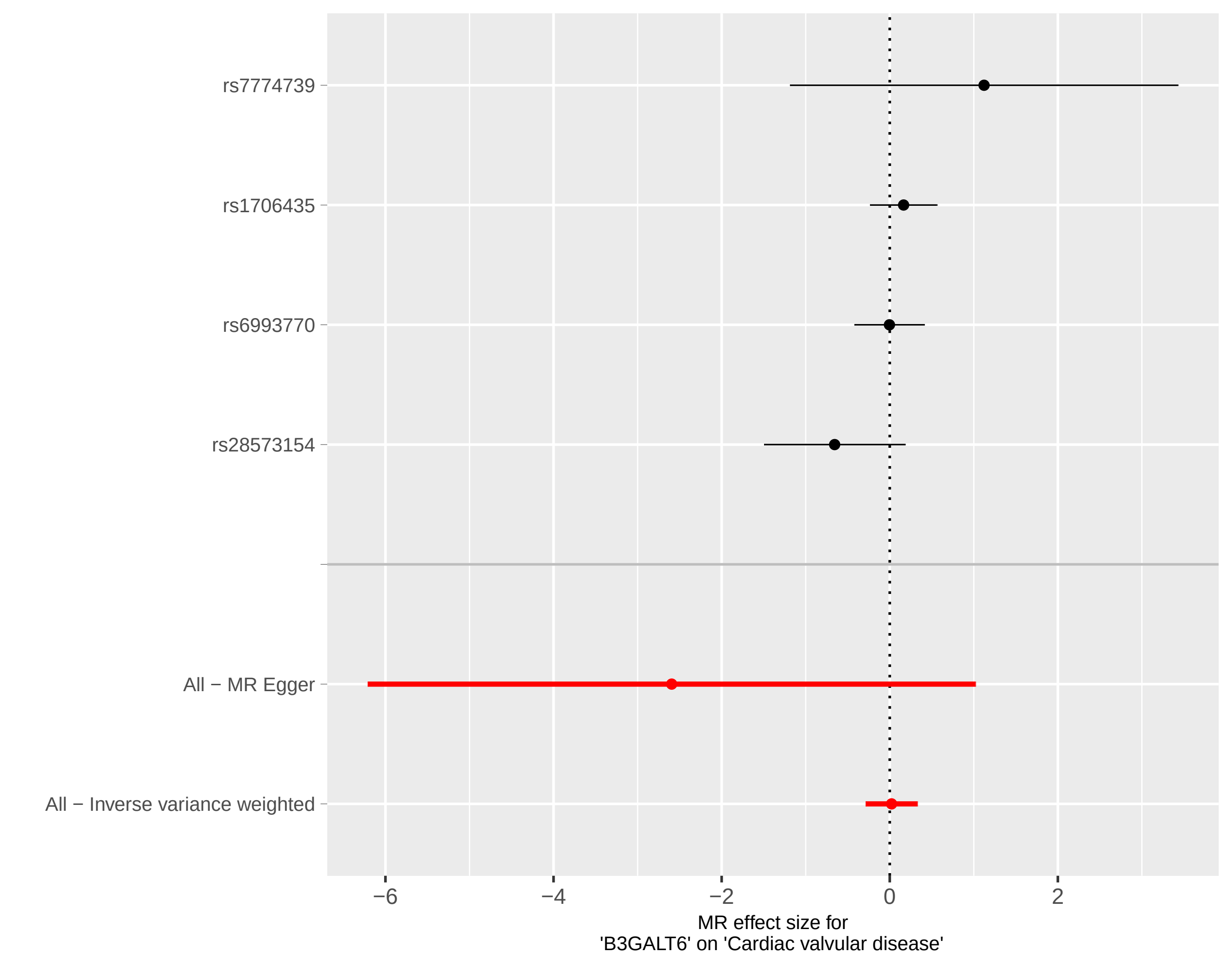
rs7774739
rs1706435
rs6993770
rs28573154
All − MR Egger
All − Inverse variance weighted
−6
−4
−2
0
2
MR effect size for
'B3GALT6' on 'Cardiac valvular disease'

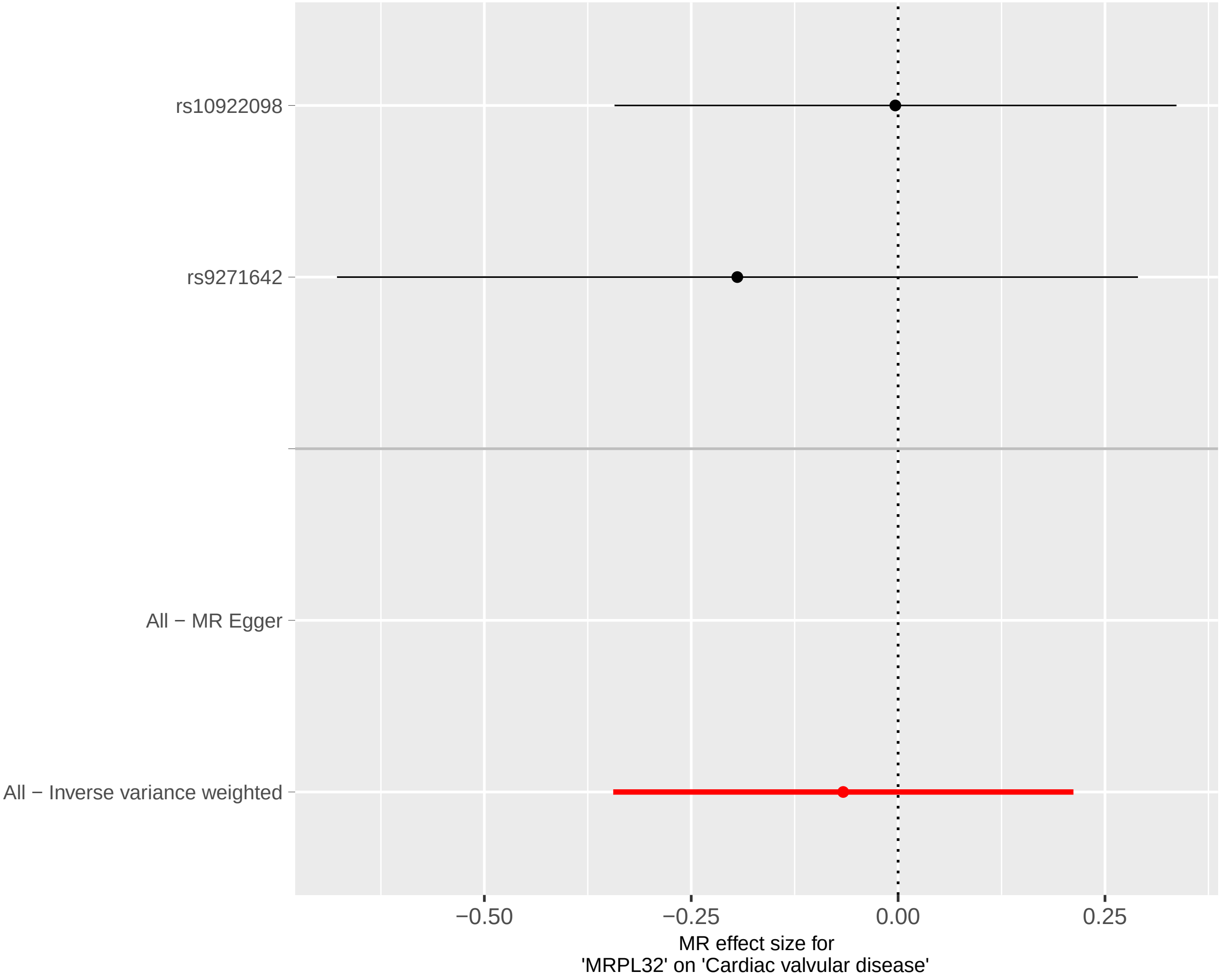
rs10922098
rs9271642
All − MR Egger
All − Inverse variance weighted
−0.50
−0.25
0.00
0.25
MR effect size for
'MRPL32' on 'Cardiac valvular disease'

Insufficient number of SNPs

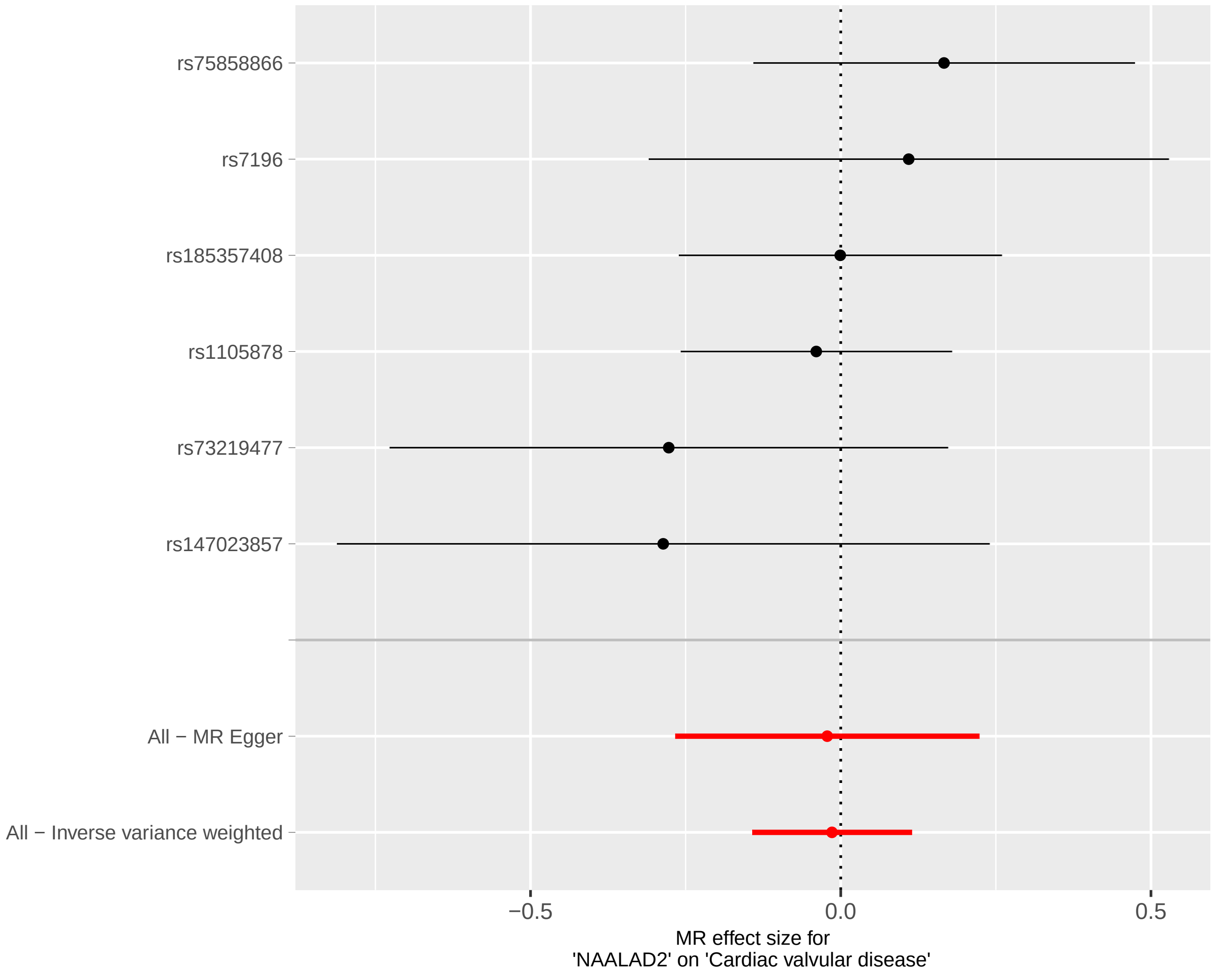
rs75858866
rs7196
rs185357408
rs1105878
rs73219477
rs147023857
All − MR Egger
All − Inverse variance weighted
−0.5
0.0
0.5
MR effect size for
'NAALAD2' on 'Cardiac valvular disease'

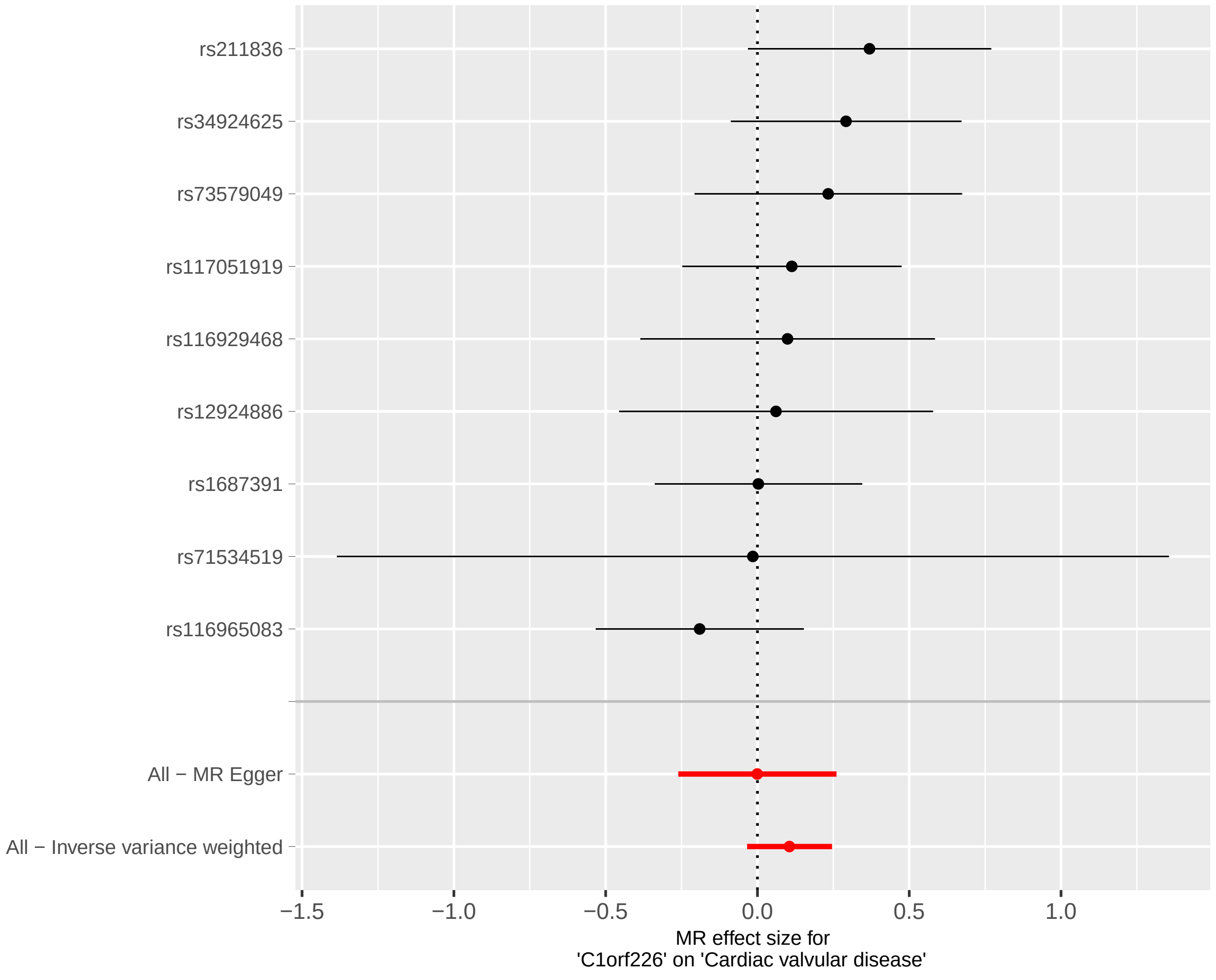
rs211836
rs34924625
rs73579049
rs117051919
rs116929468
rs12924886
rs1687391
rs71534519
rs116965083
All − MR Egger
All − Inverse variance weighted
−1.5
−1.0
−0.5
0.0
0.5
1.0
MR effect size for
'C1orf226' on 'Cardiac valvular disease'

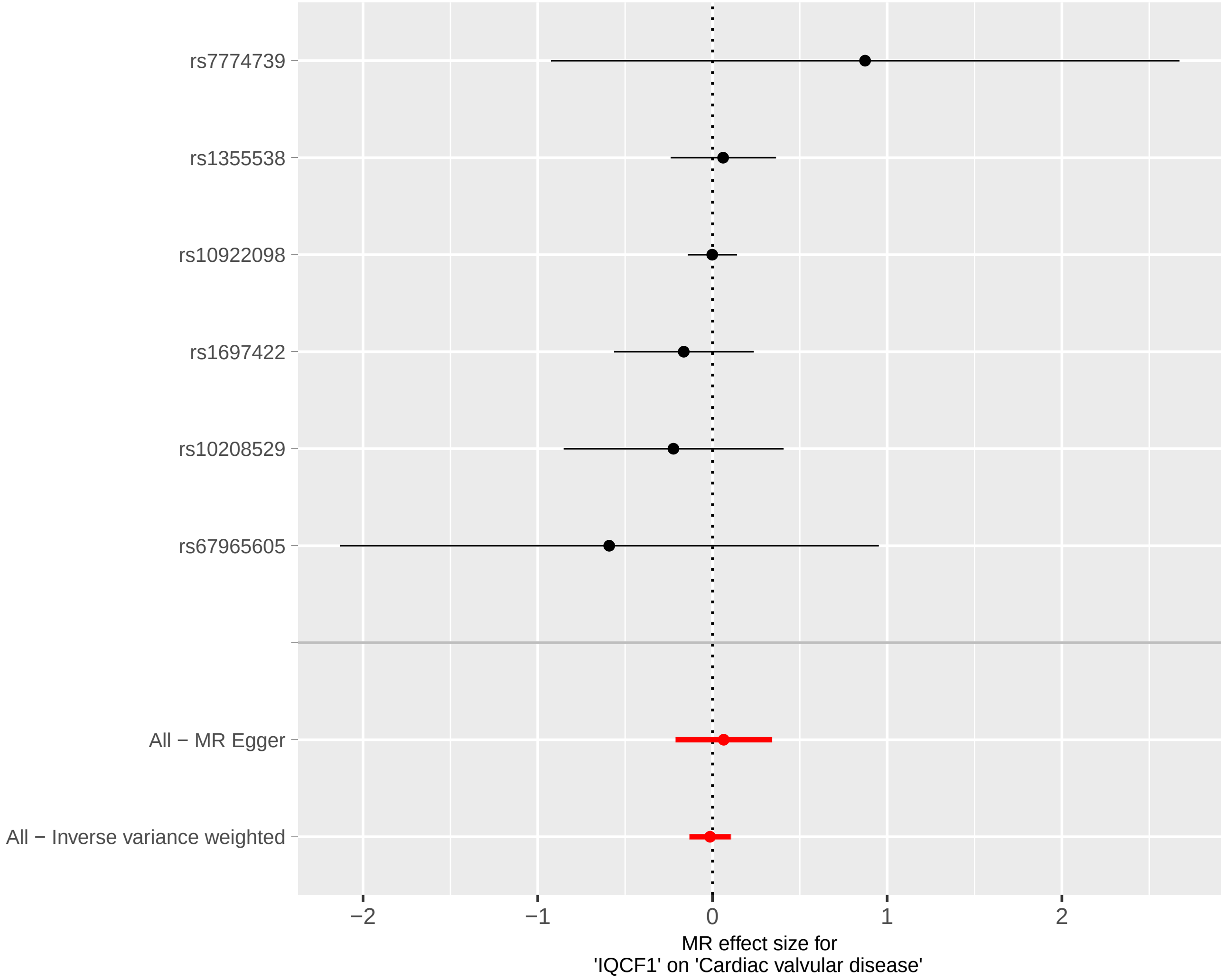
rs7774739
rs1355538
rs10922098
rs1697422
rs10208529
rs67965605
All − MR Egger
All − Inverse variance weighted
−2
−1
0
1
2
MR effect size for
'IQCF1' on 'Cardiac valvular disease'

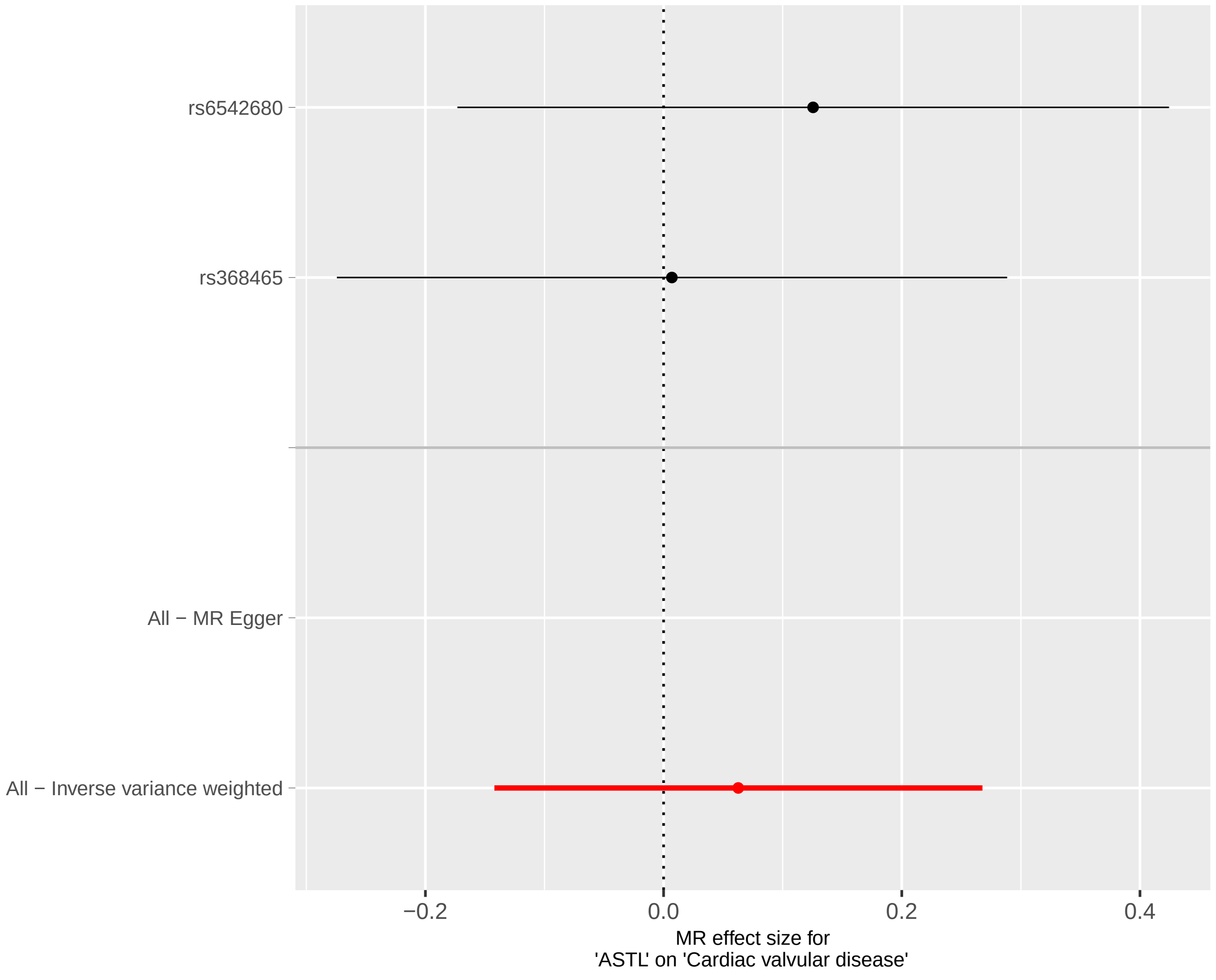
rs6542680
rs368465
All − MR Egger
All − Inverse variance weighted
−0.2
0.0
0.2
0.4
MR effect size for
'ASTL' on 'Cardiac valvular disease'

Insufficient number of SNPs

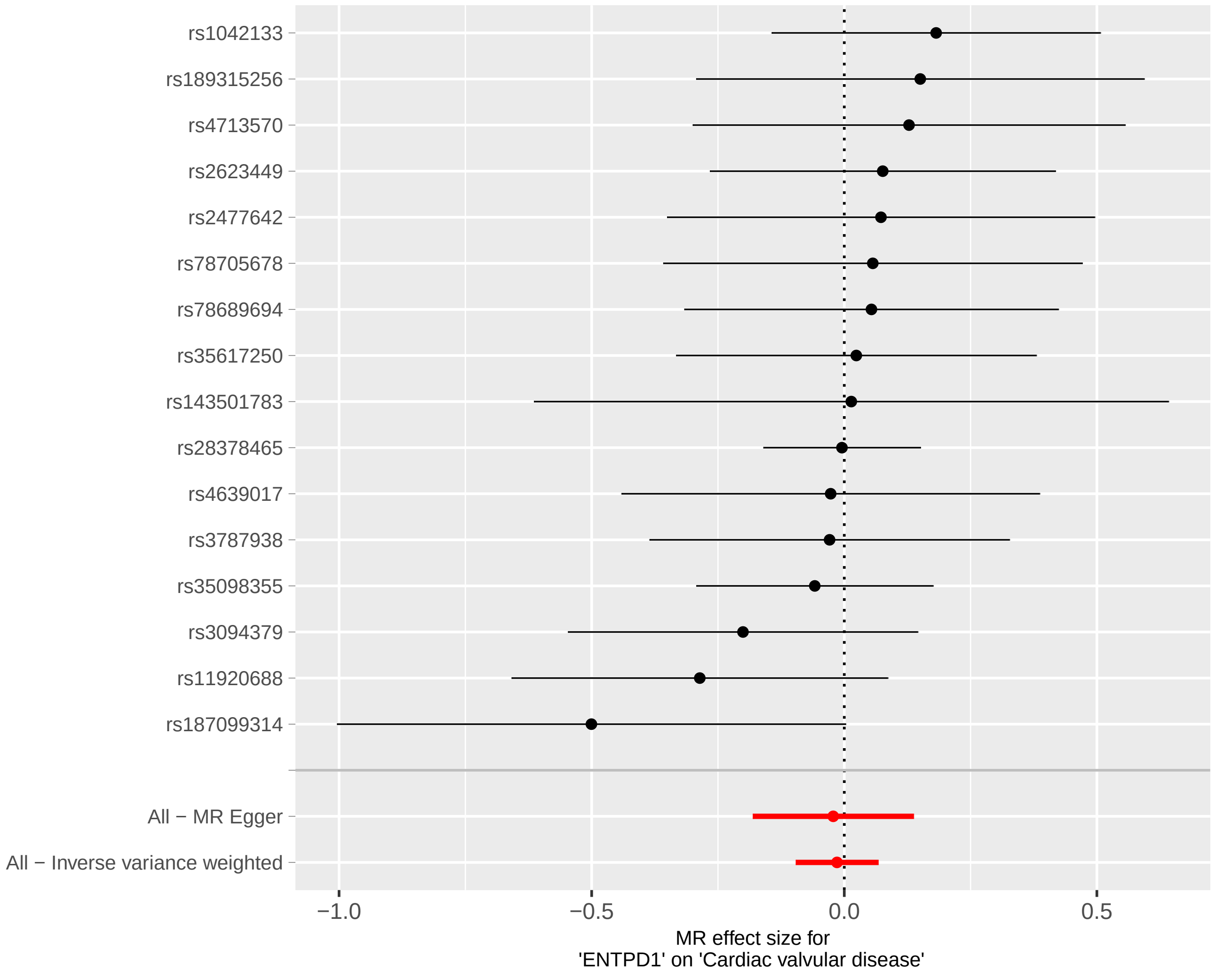

rs1042133
rs189315256
rs4713570
rs2623449
rs2477642
rs78705678
rs78689694
rs35617250
rs143501783
rs28378465
rs4639017
rs3787938
rs35098355
rs3094379
rs11920688
rs187099314
All − MR Egger
All − Inverse variance weighted
−1.0
−0.5
0.0
0.5
MR effect size for
'ENTPD1' on 'Cardiac valvular disease'

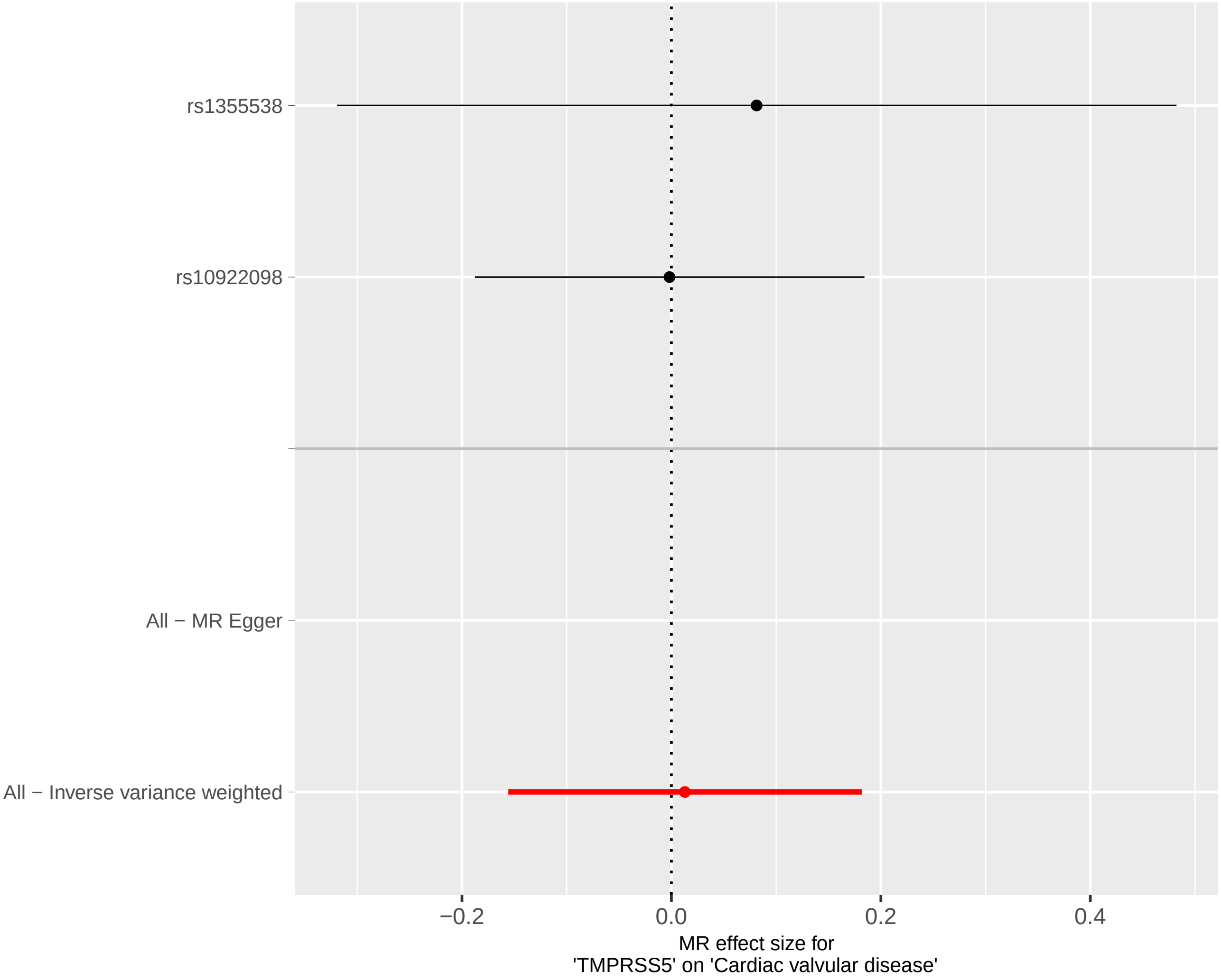
rs1355538
rs10922098
All − MR Egger
All − Inverse variance weighted
−0.2
0.0
0.2
0.4
MR effect size for
'TMPRSS5' on 'Cardiac valvular disease'

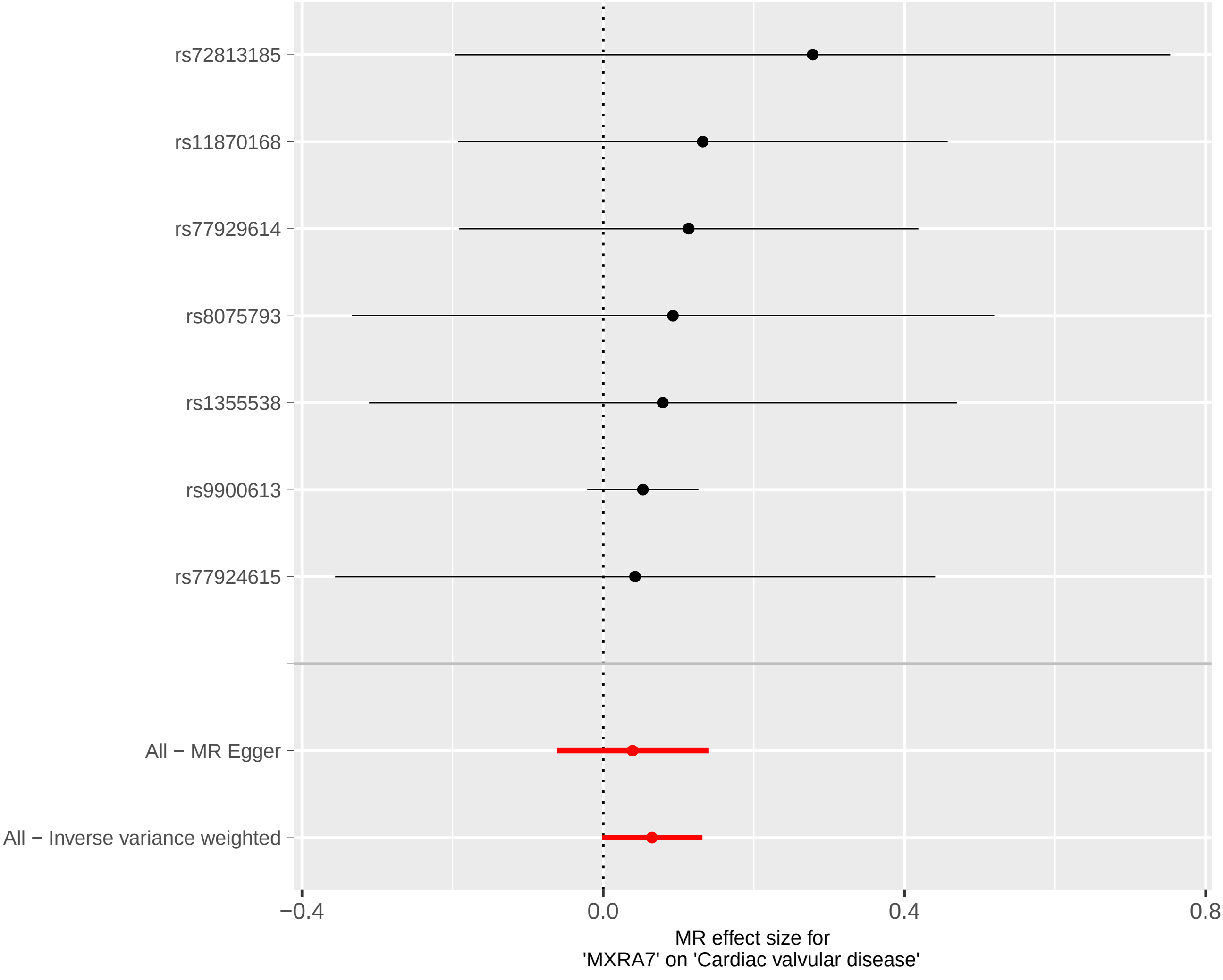
rs72813185
rs11870168
rs77929614
rs8075793
rs1355538
rs9900613
rs77924615
All − MR Egger
All − Inverse variance weighted
−0.4
0.0
0.4
0.8
MR effect size for
'MXRA7' on 'Cardiac valvular disease'

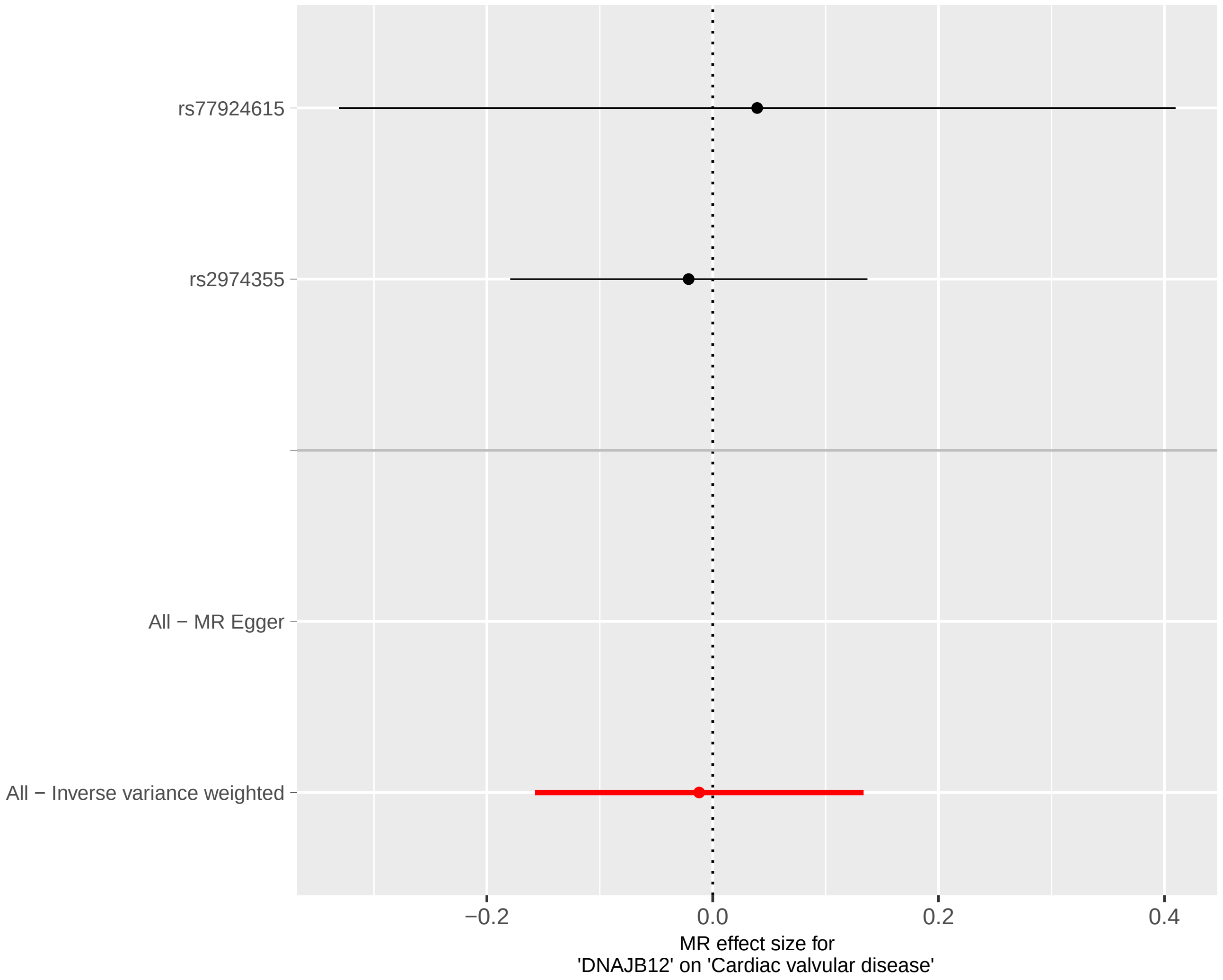
rs77924615
rs2974355
All − MR Egger
All − Inverse variance weighted
−0.2
0.0
0.2
0.4
MR effect size for
'DNAJB12' on 'Cardiac valvular disease'

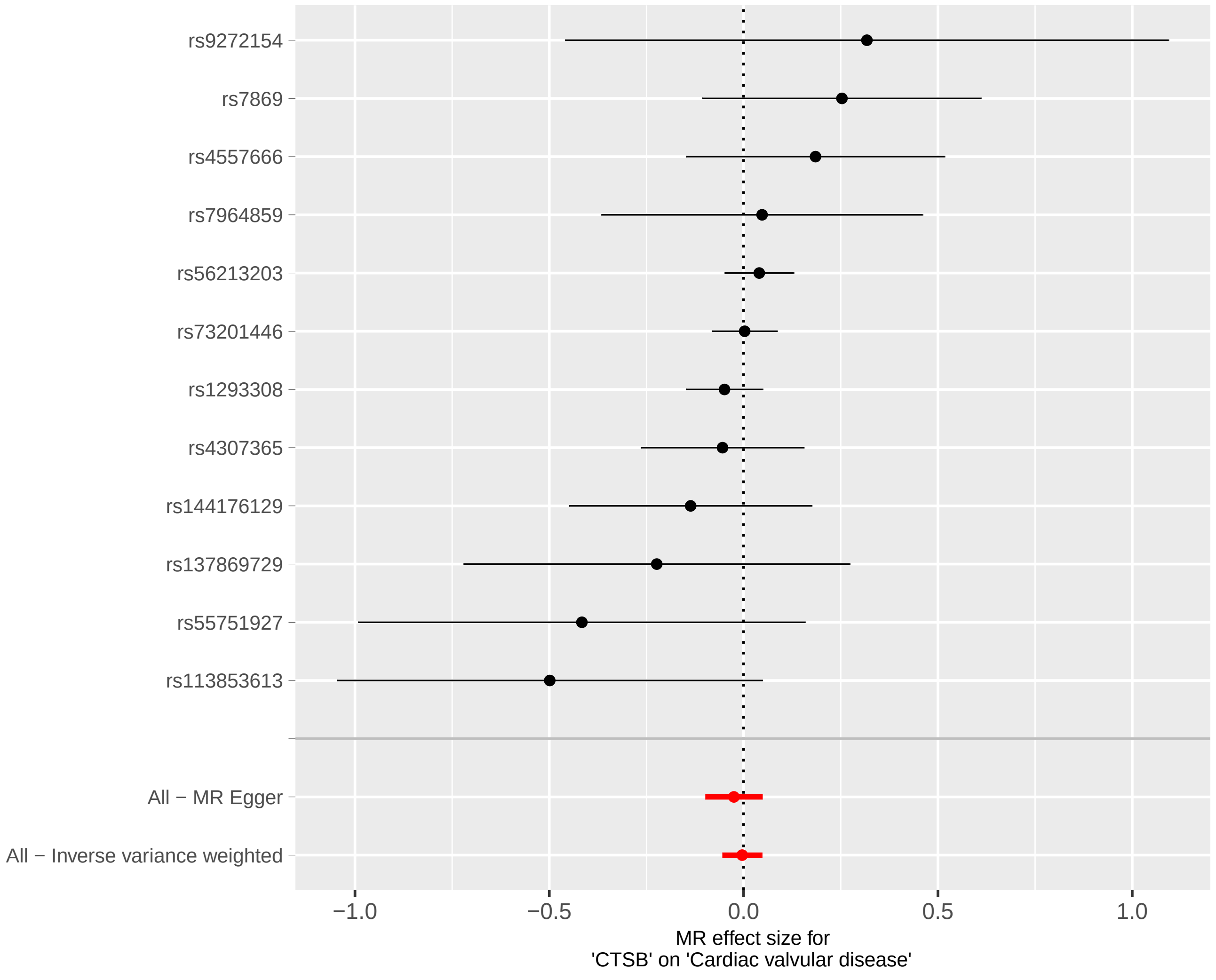
rs9272154
rs7869
rs4557666
rs7964859
rs56213203
rs73201446
rs1293308
rs4307365
rs144176129
rs137869729
rs55751927
rs113853613
All − MR Egger
All − Inverse variance weighted
−1.0
−0.5
0.0
0.5
1.0
MR effect size for
'CTSB' on 'Cardiac valvular disease'

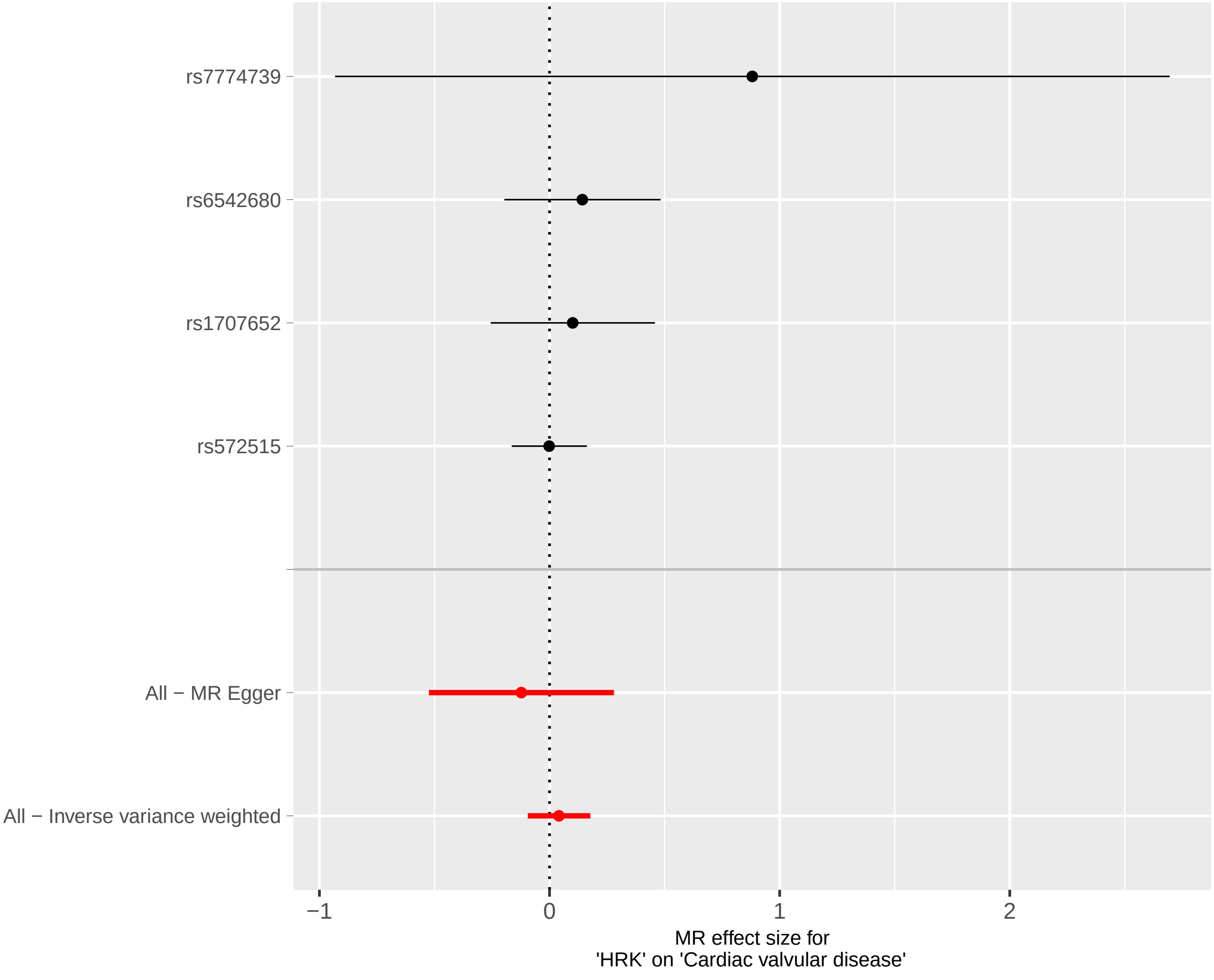
rs7774739
rs6542680
rs1707652
rs572515
All − MR Egger
All − Inverse variance weighted
−1
0
1
2
MR effect size for
'HRK' on 'Cardiac valvular disease'

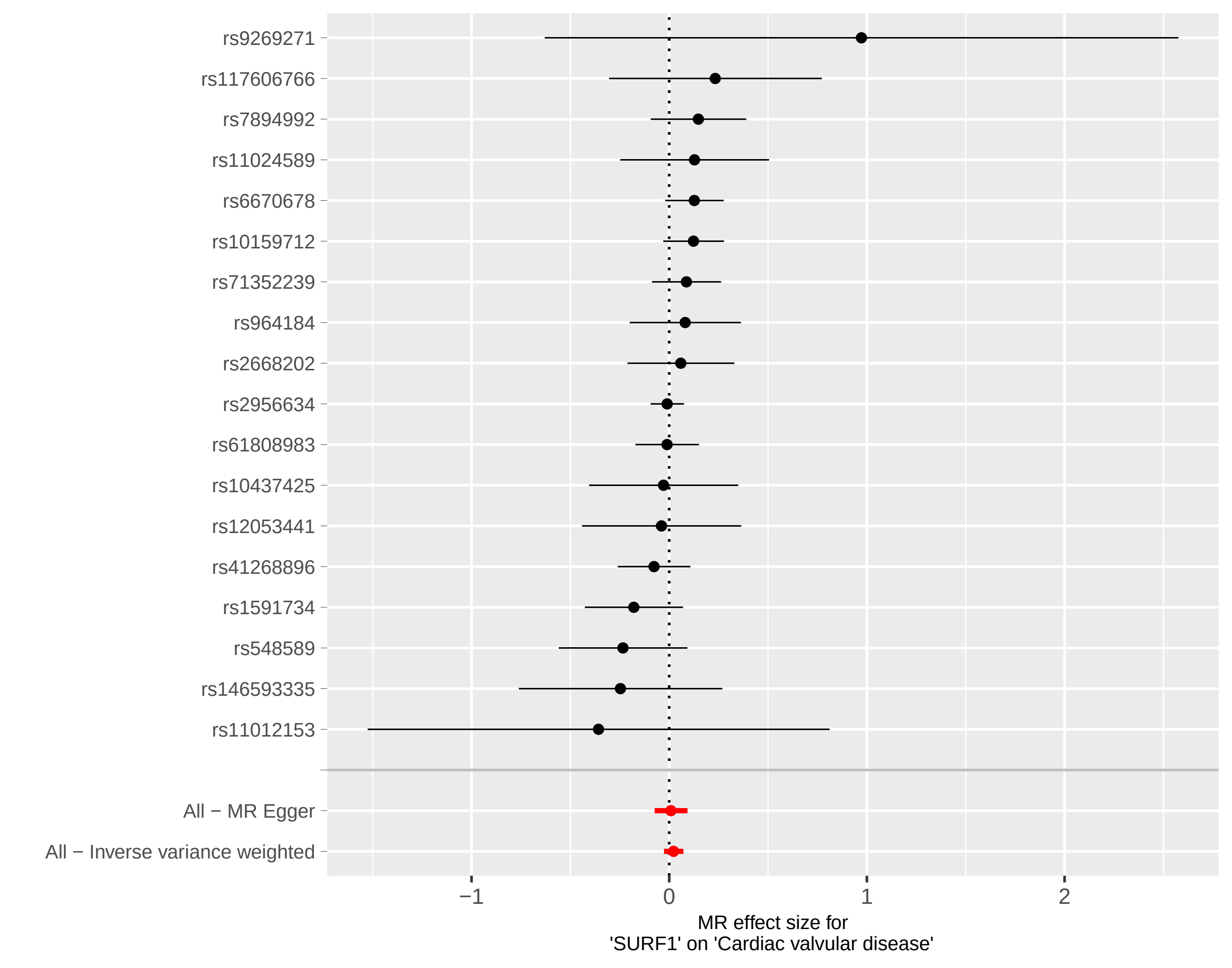

rs9269271
rs117606766
rs7894992
rs11024589
rs6670678
rs10159712
rs71352239
rs964184
rs2668202
rs2956634
rs61808983
rs10437425
rs12053441
rs41268896
rs1591734
rs548589
rs146593335
rs11012153
All − MR Egger
All − Inverse variance weighted
−1
0
1
2
MR effect size for
'SURF1' on 'Cardiac valvular disease'

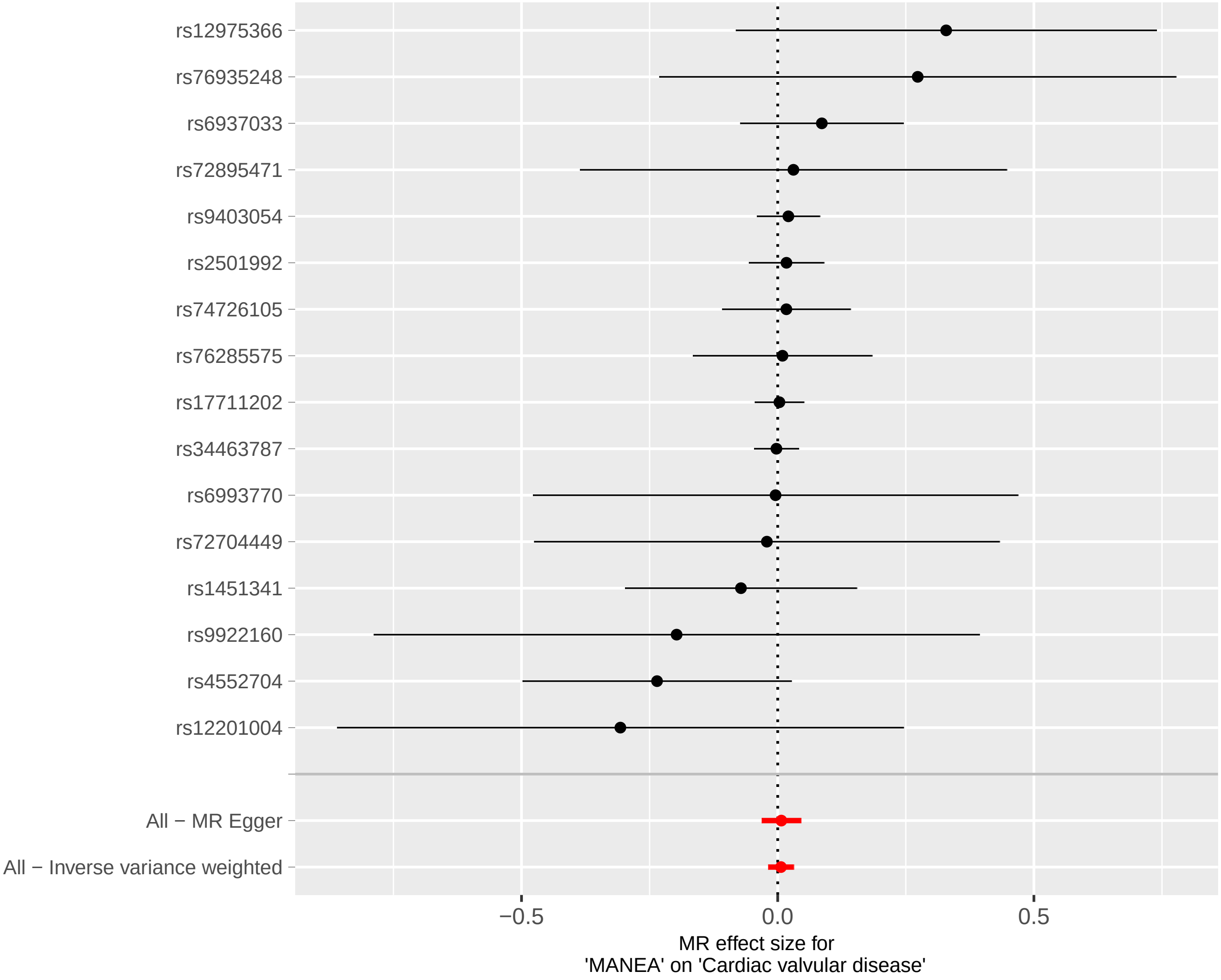

rs12975366
rs76935248
rs6937033
rs72895471
rs9403054
rs2501992
rs74726105
rs76285575
rs17711202
rs34463787
rs6993770
rs72704449
rs1451341
rs9922160
rs4552704
rs12201004
All − MR Egger
All − Inverse variance weighted
−0.5
0.0
0.5
MR effect size for
'MANEA' on 'Cardiac valvular disease'

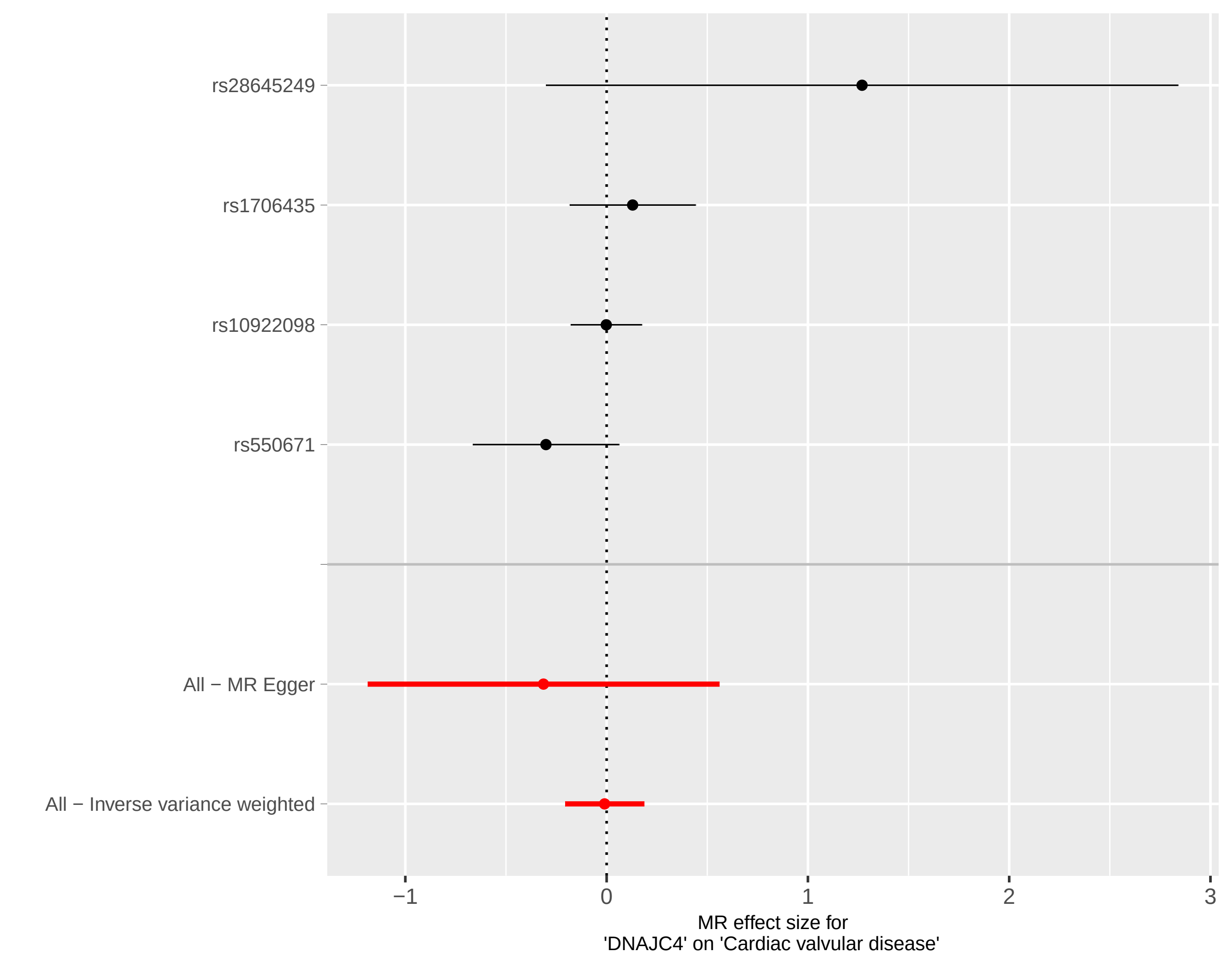
rs28645249
rs1706435
rs10922098
rs550671
All − MR Egger
All − Inverse variance weighted
−1
0
1
2
3
MR effect size for
'DNAJC4' on 'Cardiac valvular disease'

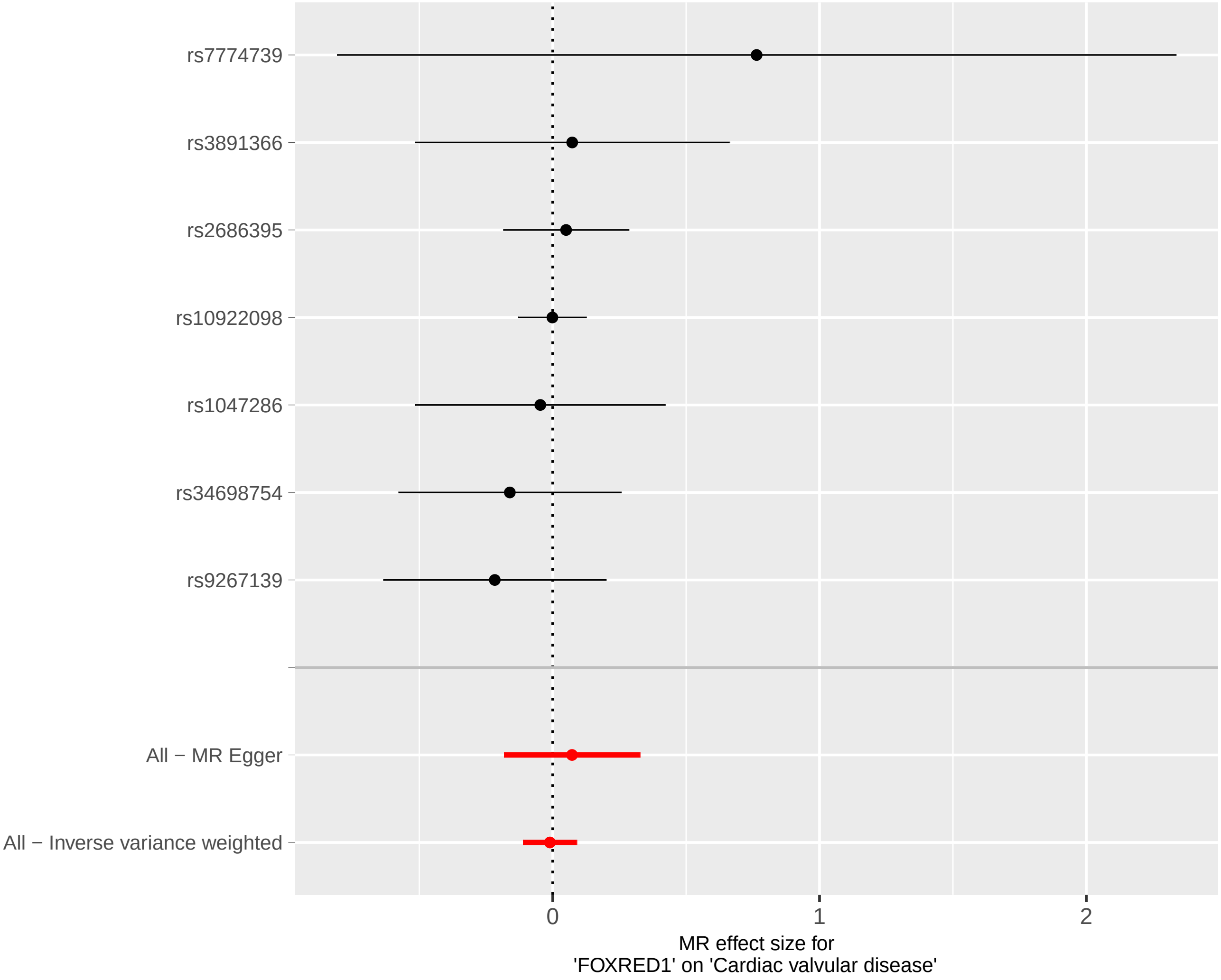

rs7774739
rs3891366
rs2686395
rs10922098
rs1047286
rs34698754
rs9267139
All − MR Egger
All − Inverse variance weighted
0
1
2
MR effect size for
'FOXRED1' on 'Cardiac valvular disease'

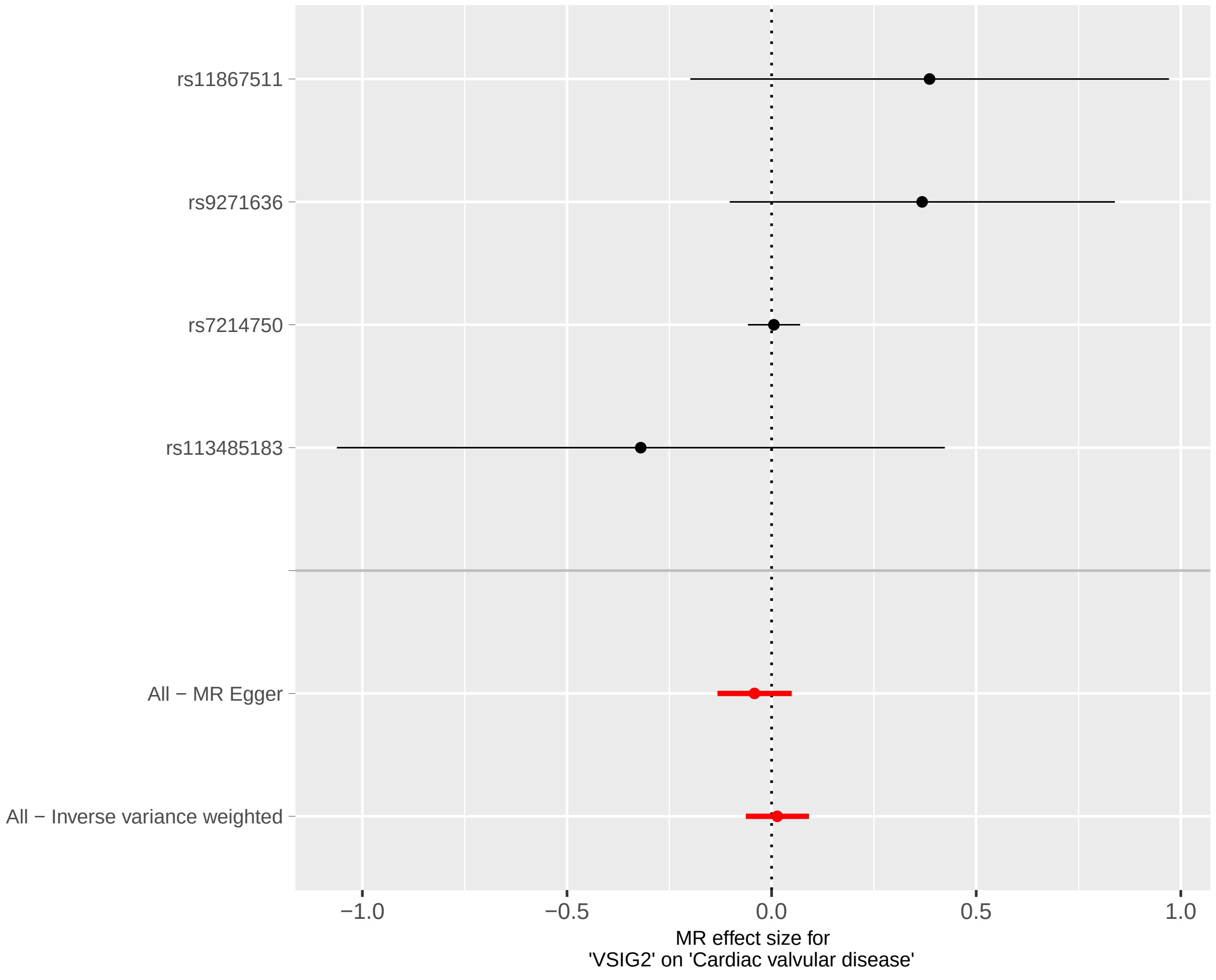

rs11867511
rs9271636
rs7214750
rs113485183
All − MR Egger
All − Inverse variance weighted
−1.0
−0.5
0.0
0.5
1.0
MR effect size for
'VSIG2' on 'Cardiac valvular disease'

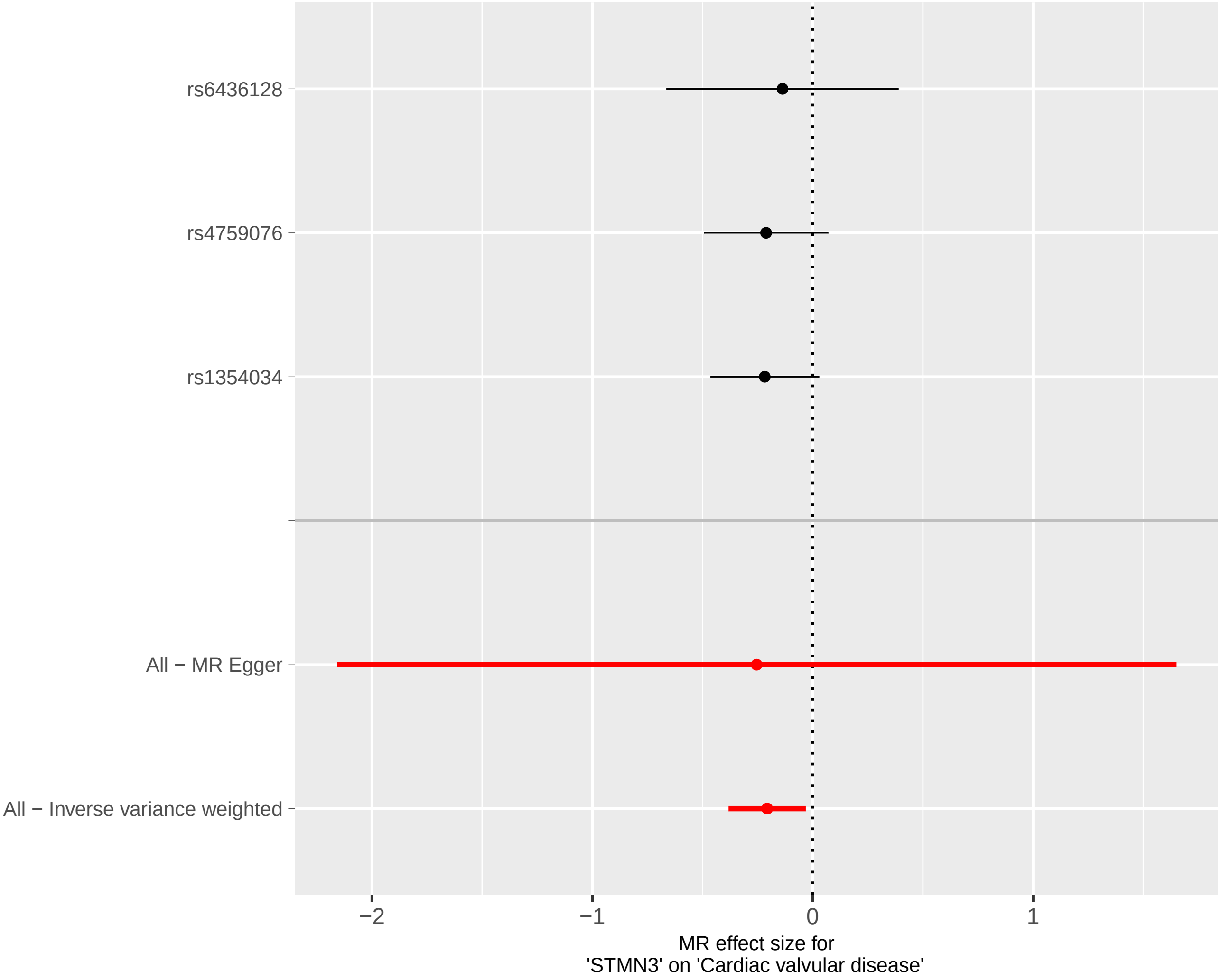
rs6436128
rs4759076
rs1354034
All − MR Egger
All − Inverse variance weighted
−2
−1
0
1
MR effect size for
'STMN3' on 'Cardiac valvular disease'

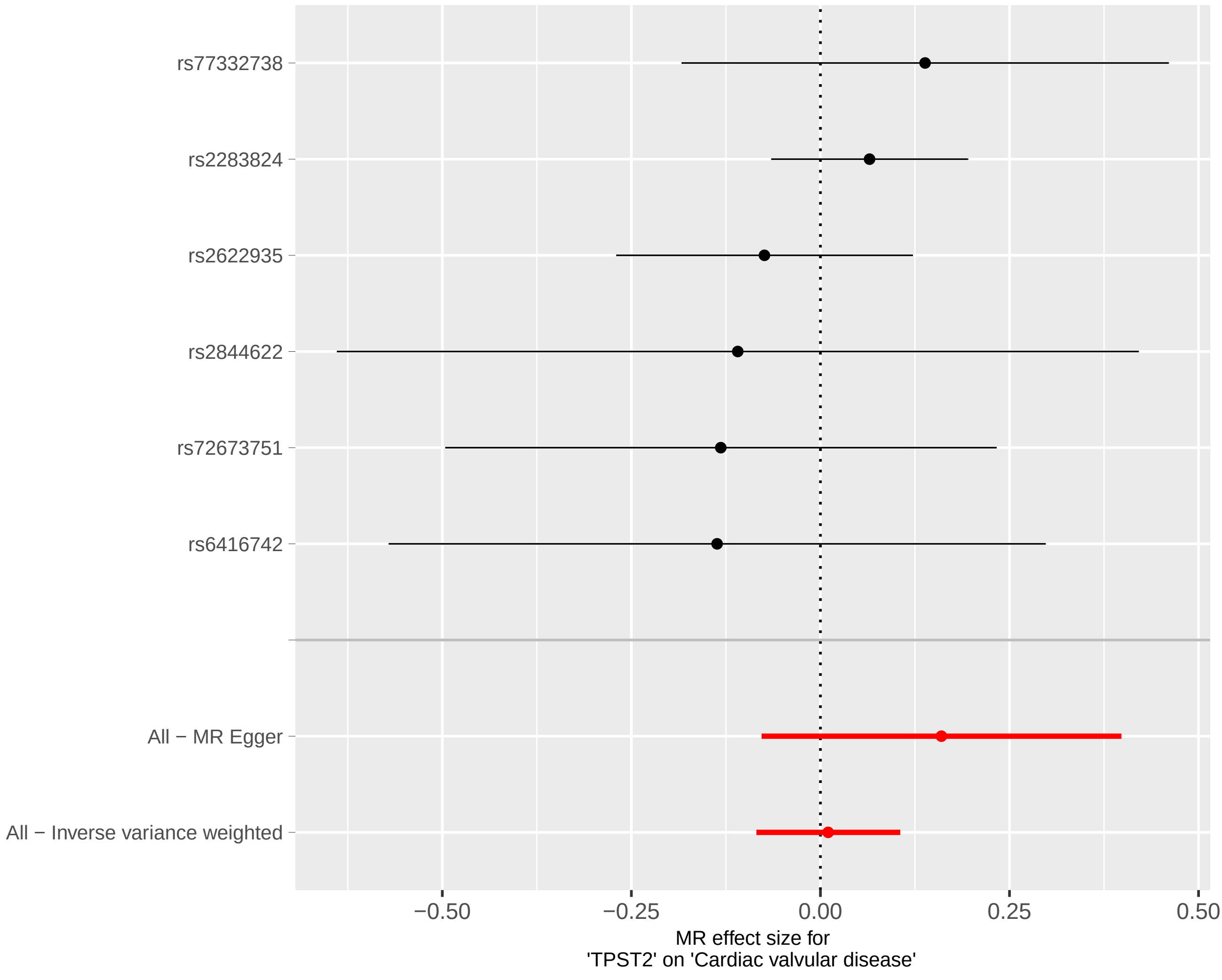

rs77332738
rs2283824
rs2622935
rs2844622
rs72673751
rs6416742
All − MR Egger
All − Inverse variance weighted
−0.50
−0.25
0.00
0.25
0.50
MR effect size for
'TPST2' on 'Cardiac valvular disease'

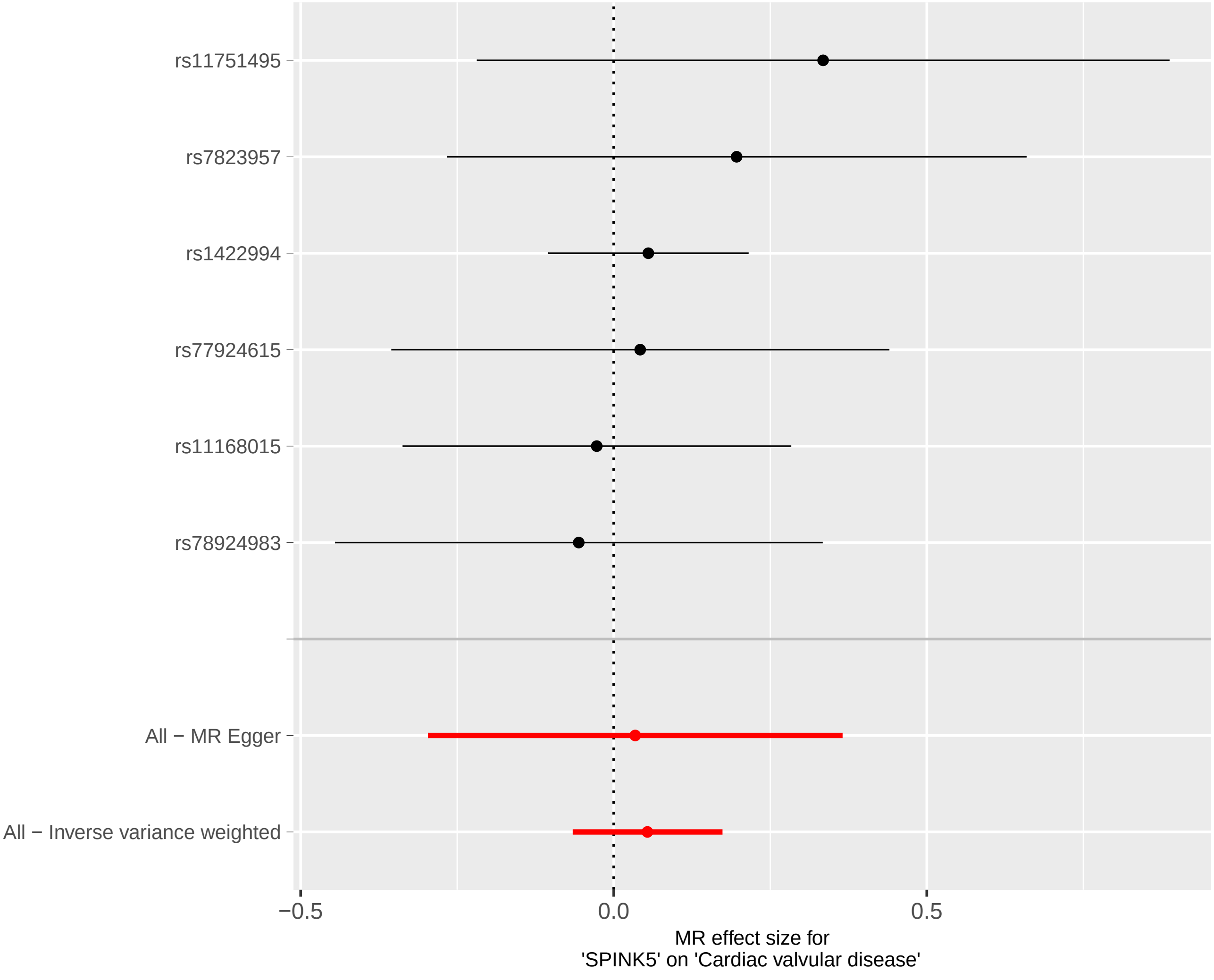
rs11751495
rs7823957
rs1422994
rs77924615
rs11168015
rs78924983
All − MR Egger
All − Inverse variance weighted
−0.5
0.0
0.5
MR effect size for
'SPINK5' on 'Cardiac valvular disease'

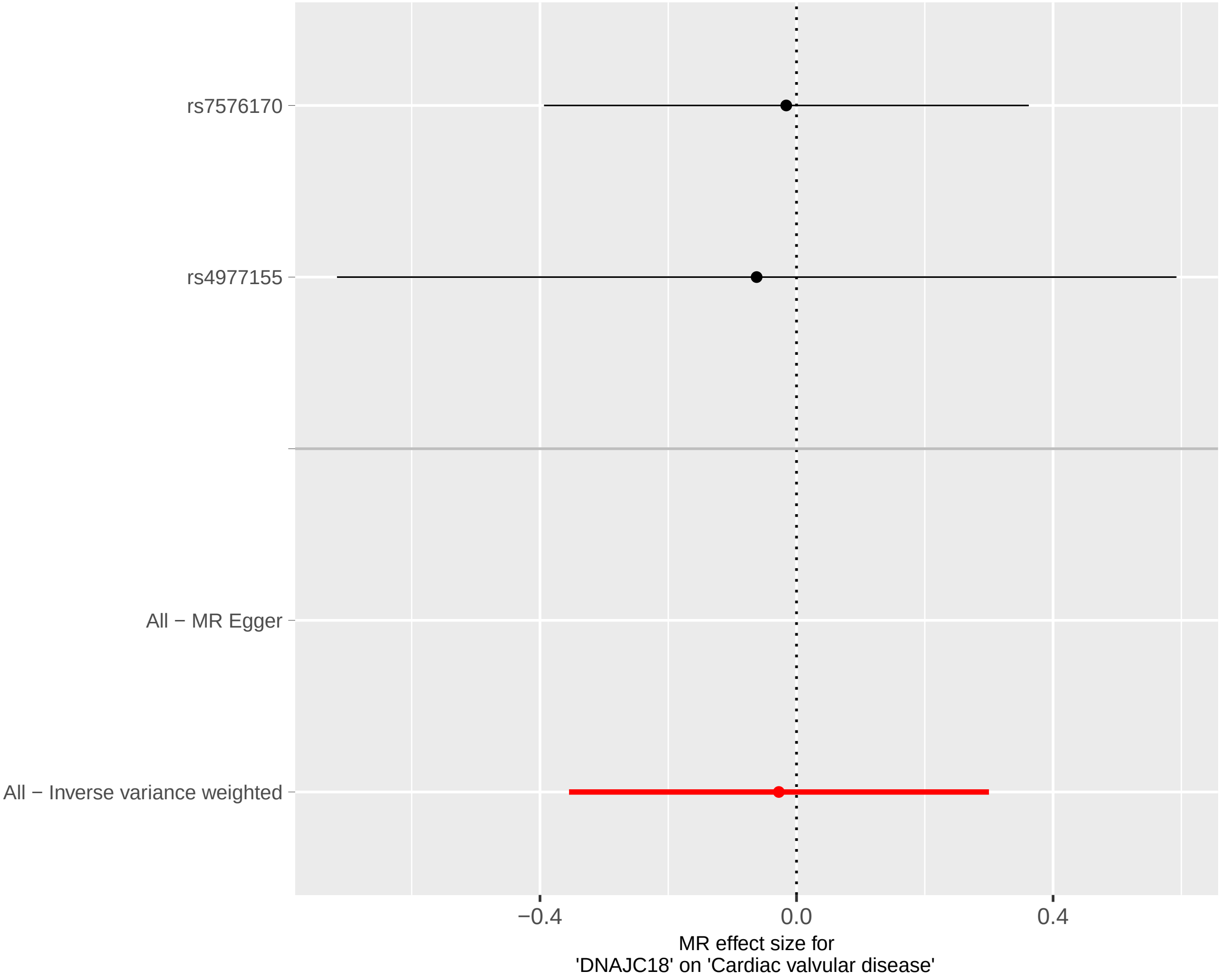
rs7576170
rs4977155
All − MR Egger
All − Inverse variance weighted
−0.4
0.0
0.4
MR effect size for
'DNAJC18' on 'Cardiac valvular disease'

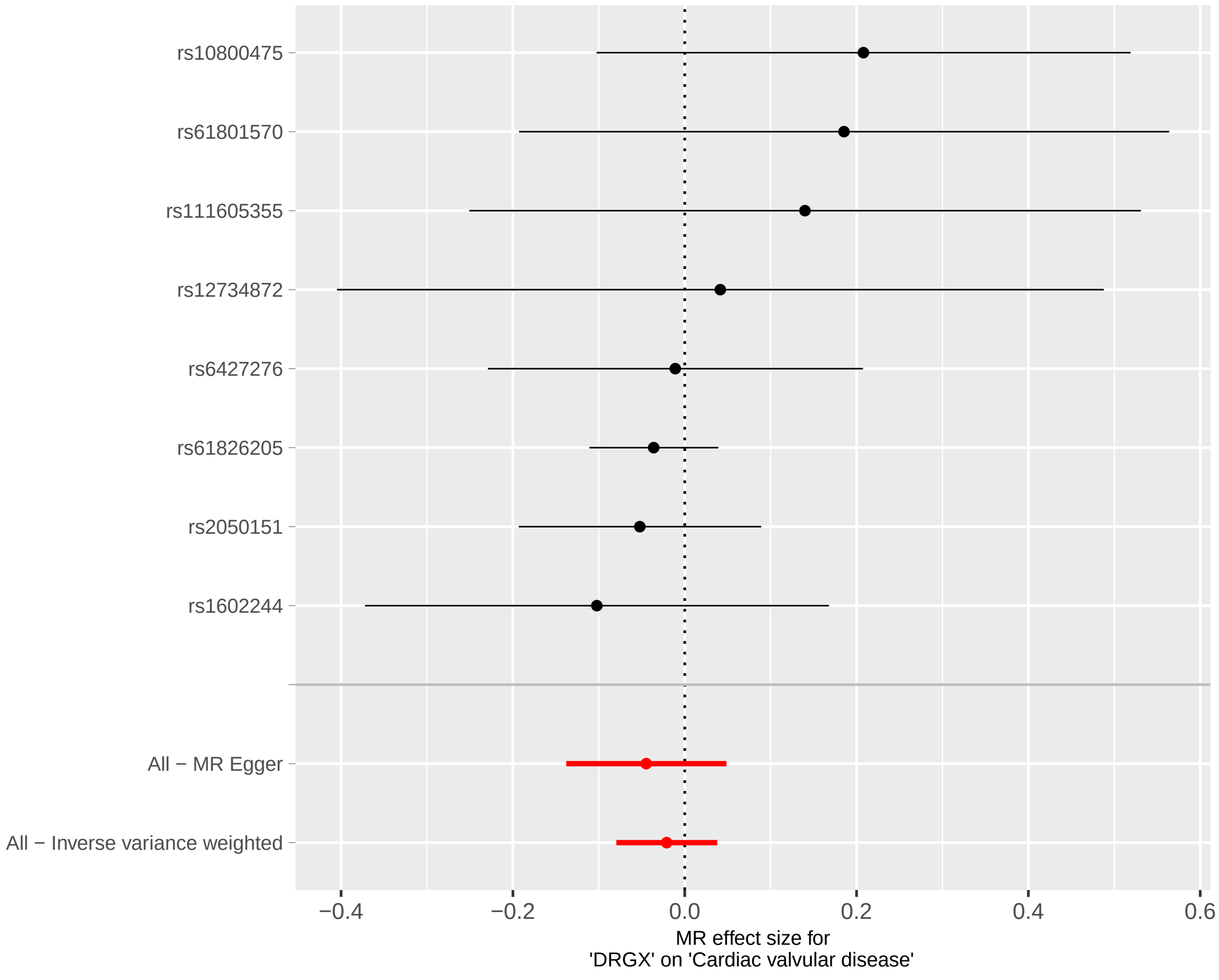
rs10800475
rs61801570
rs111605355
rs12734872
rs6427276
rs61826205
rs2050151
rs1602244
All − MR Egger
All − Inverse variance weighted
−0.4
−0.2
0.0
0.2
0.4
0.6
MR effect size for
'DRGX' on 'Cardiac valvular disease'

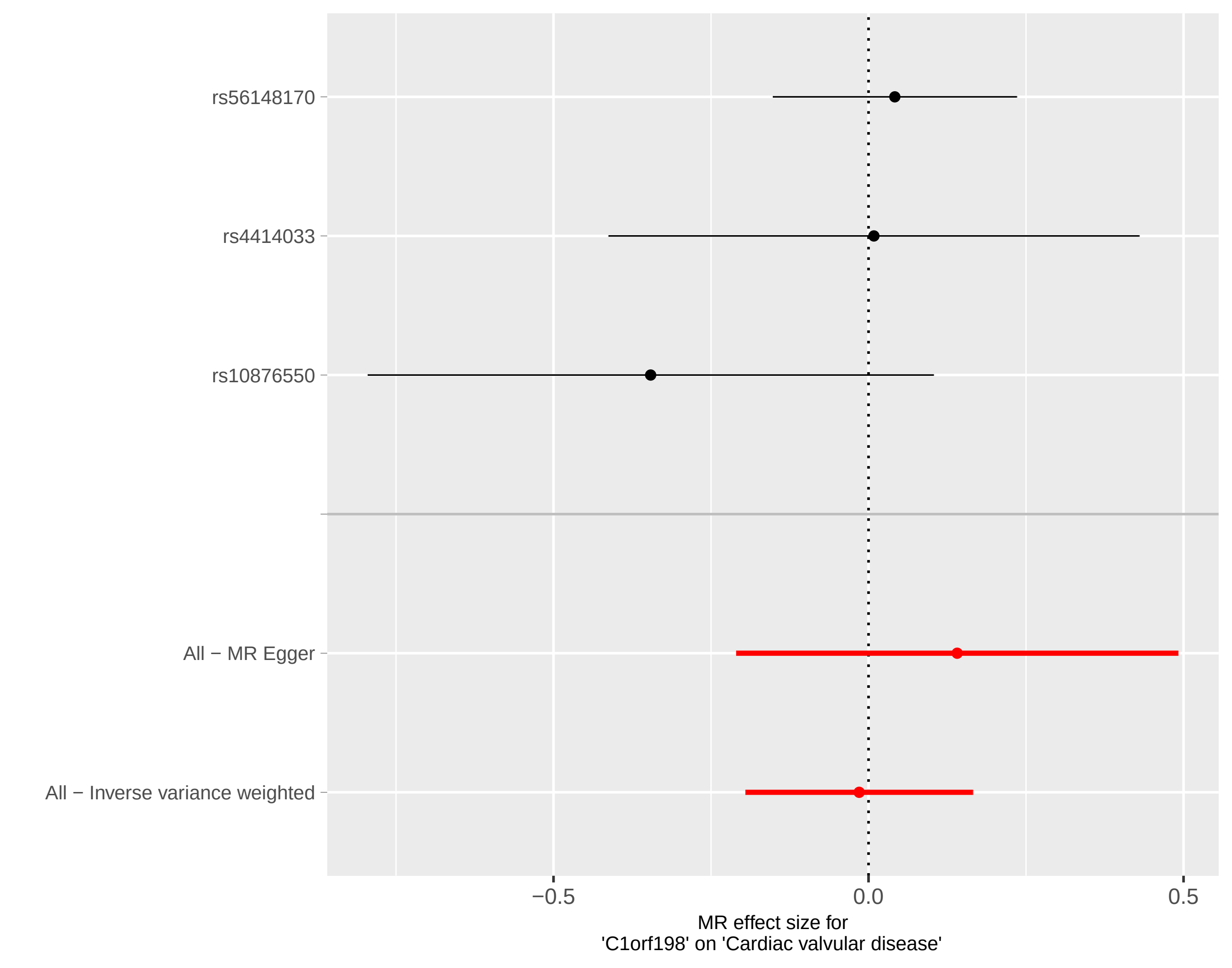
rs56148170
rs4414033
rs10876550
All − MR Egger
All − Inverse variance weighted
−0.5
0.0
0.5
MR effect size for
'C1orf198' on 'Cardiac valvular disease'

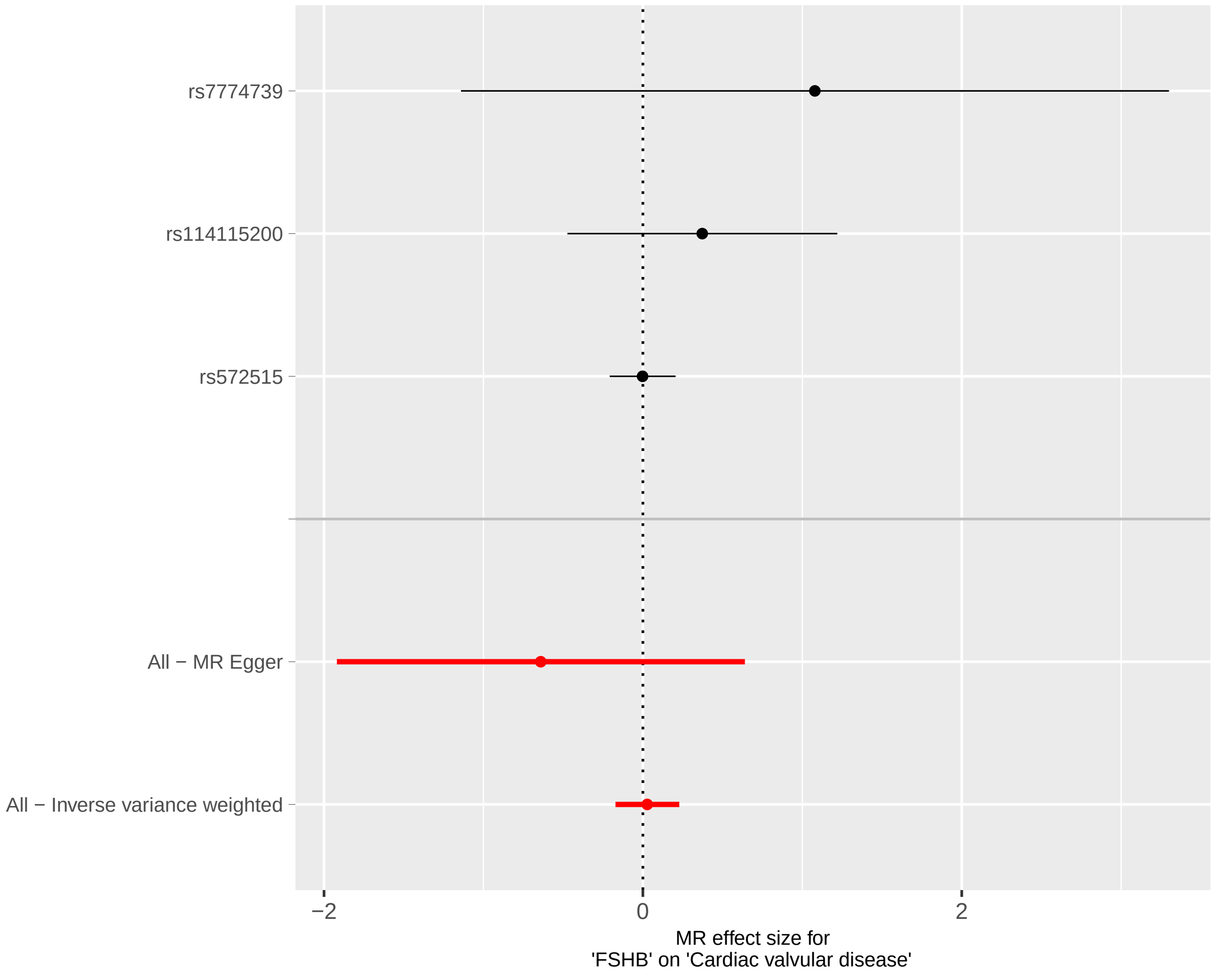
rs7774739
rs114115200
rs572515
All − MR Egger
All − Inverse variance weighted
−2
0
2
MR effect size for
'FSHB' on 'Cardiac valvular disease'

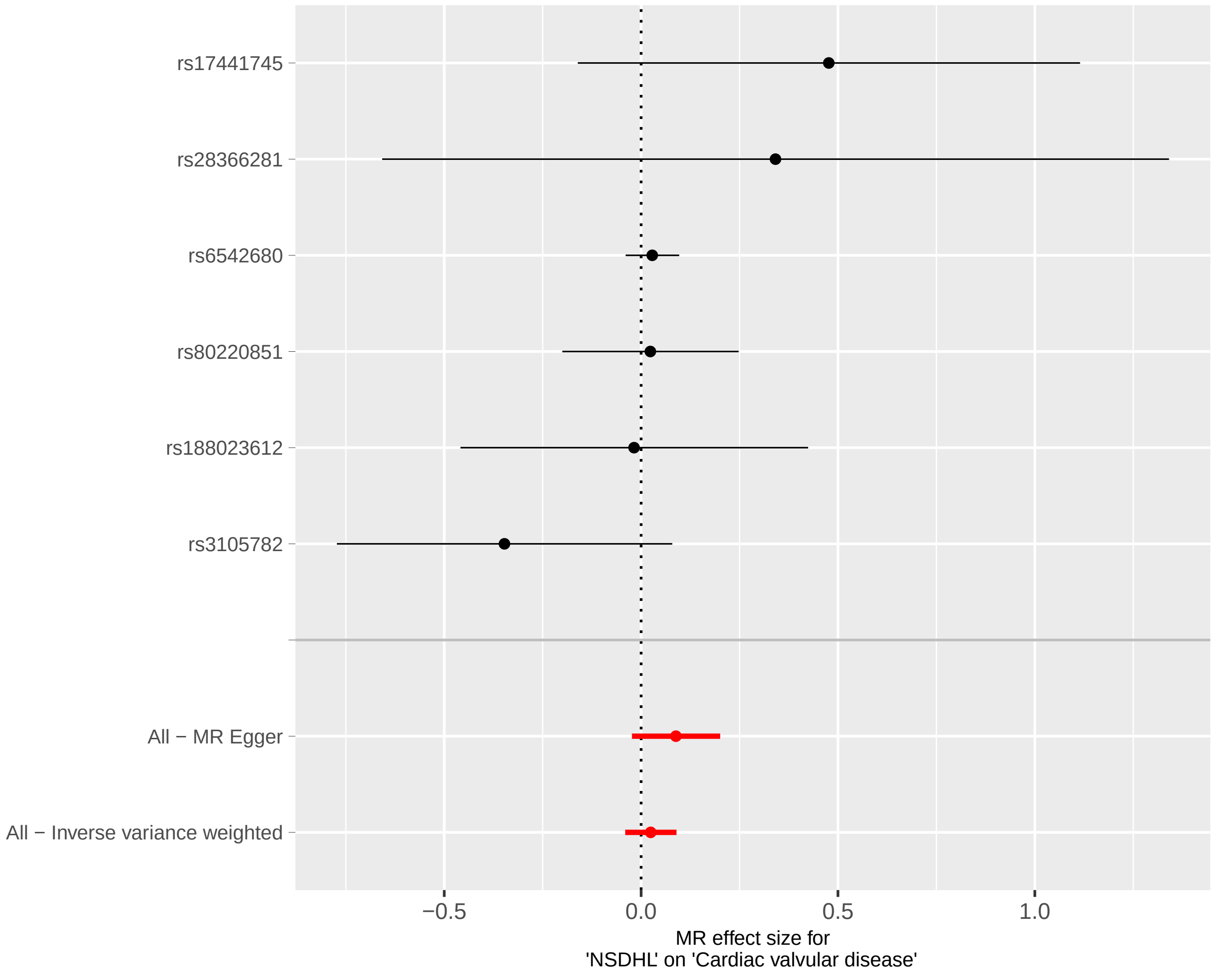
rs17441745
rs28366281
rs6542680
rs80220851
rs188023612
rs3105782
All − MR Egger
All − Inverse variance weighted
−0.5
0.0
0.5
1.0
MR effect size for
'NSDHL' on 'Cardiac valvular disease'

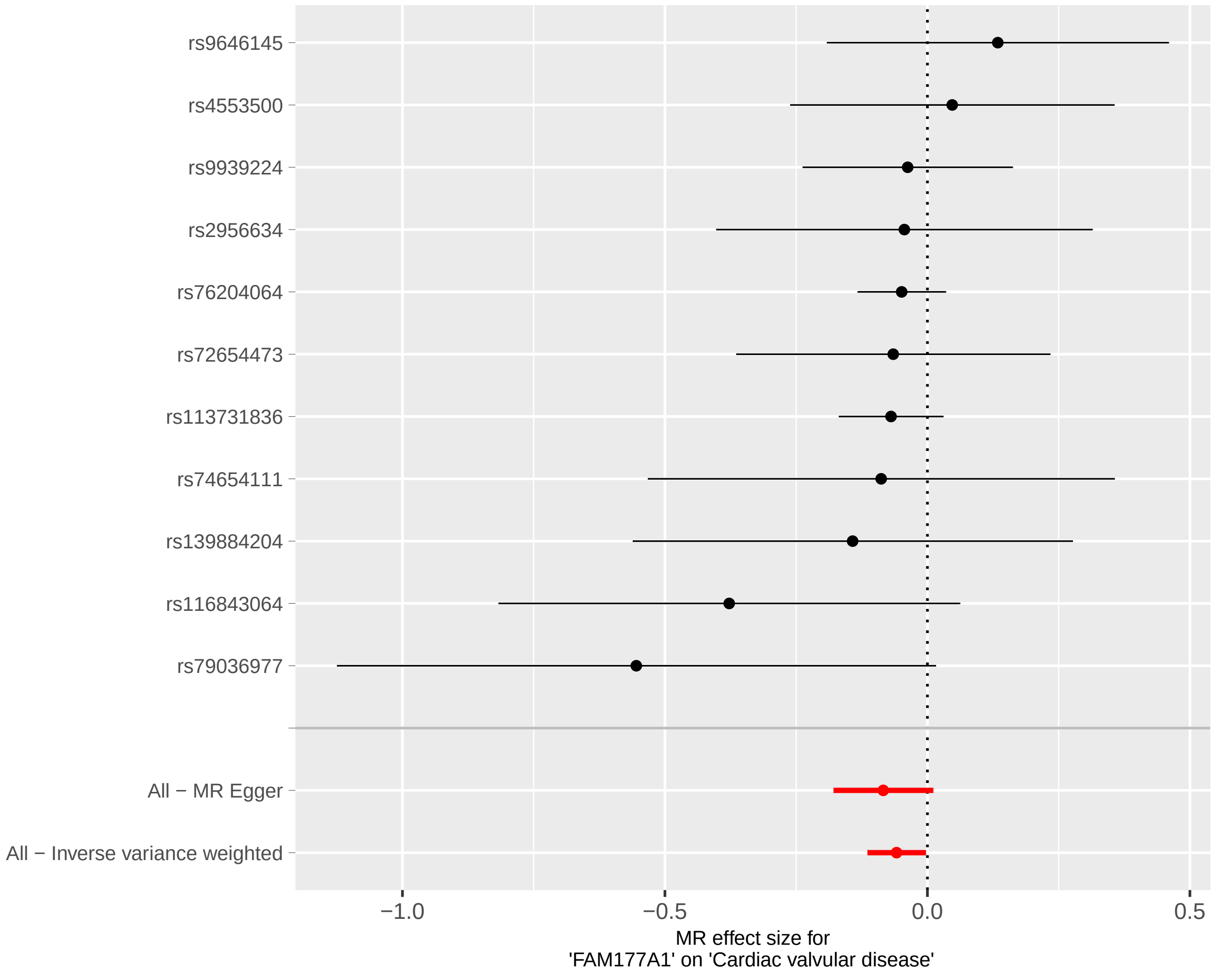
rs9646145
rs4553500
rs9939224
rs2956634
rs76204064
rs72654473
rs113731836
rs74654111
rs139884204
rs116843064
rs79036977
All − MR Egger
All − Inverse variance weighted
−1.0
−0.5
0.0
0.5
MR effect size for
'FAM177A1' on 'Cardiac valvular disease'

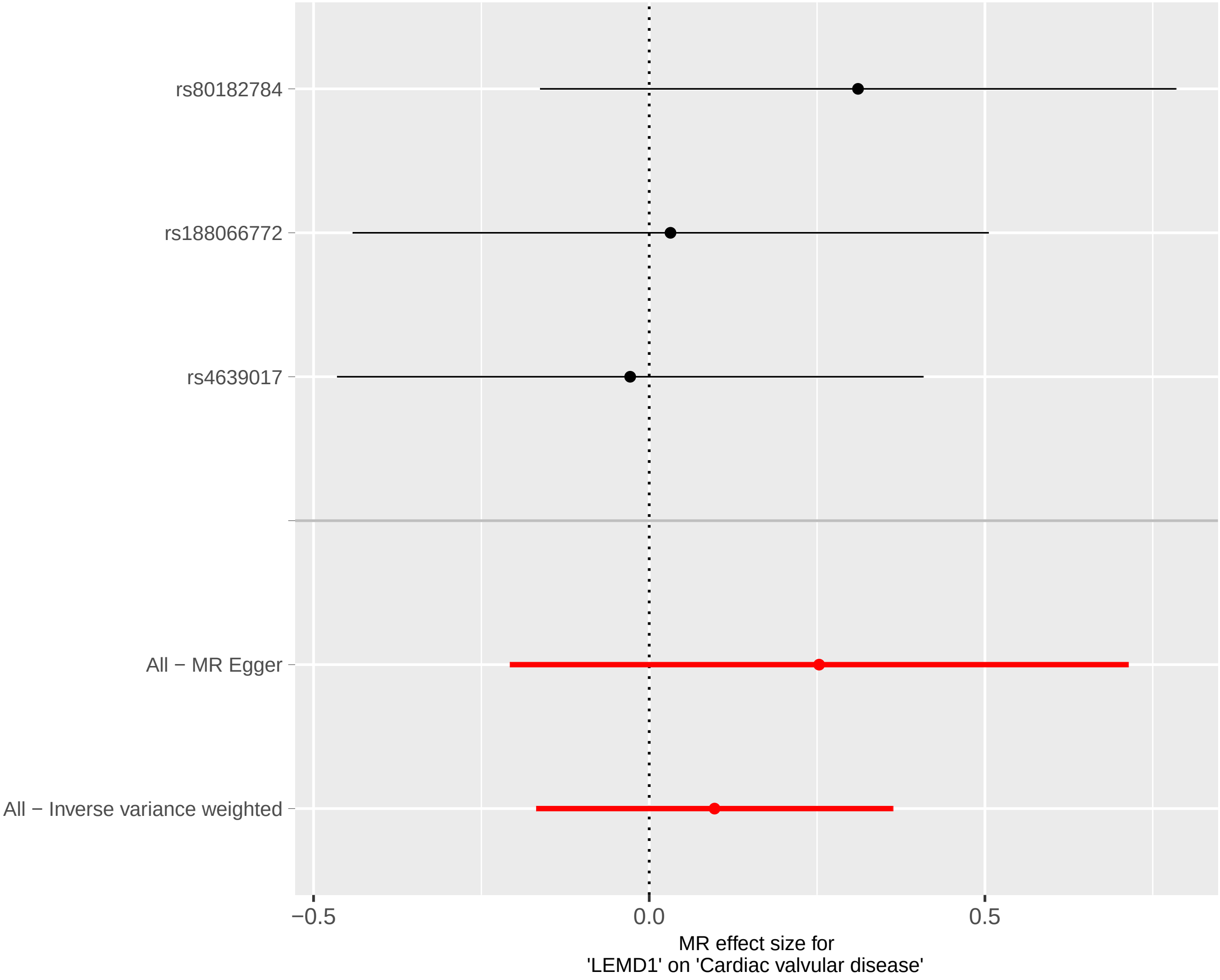
rs80182784
rs188066772
rs4639017
All − MR Egger
All − Inverse variance weighted
−0.5
0.0
0.5
MR effect size for
'LEMD1' on 'Cardiac valvular disease'

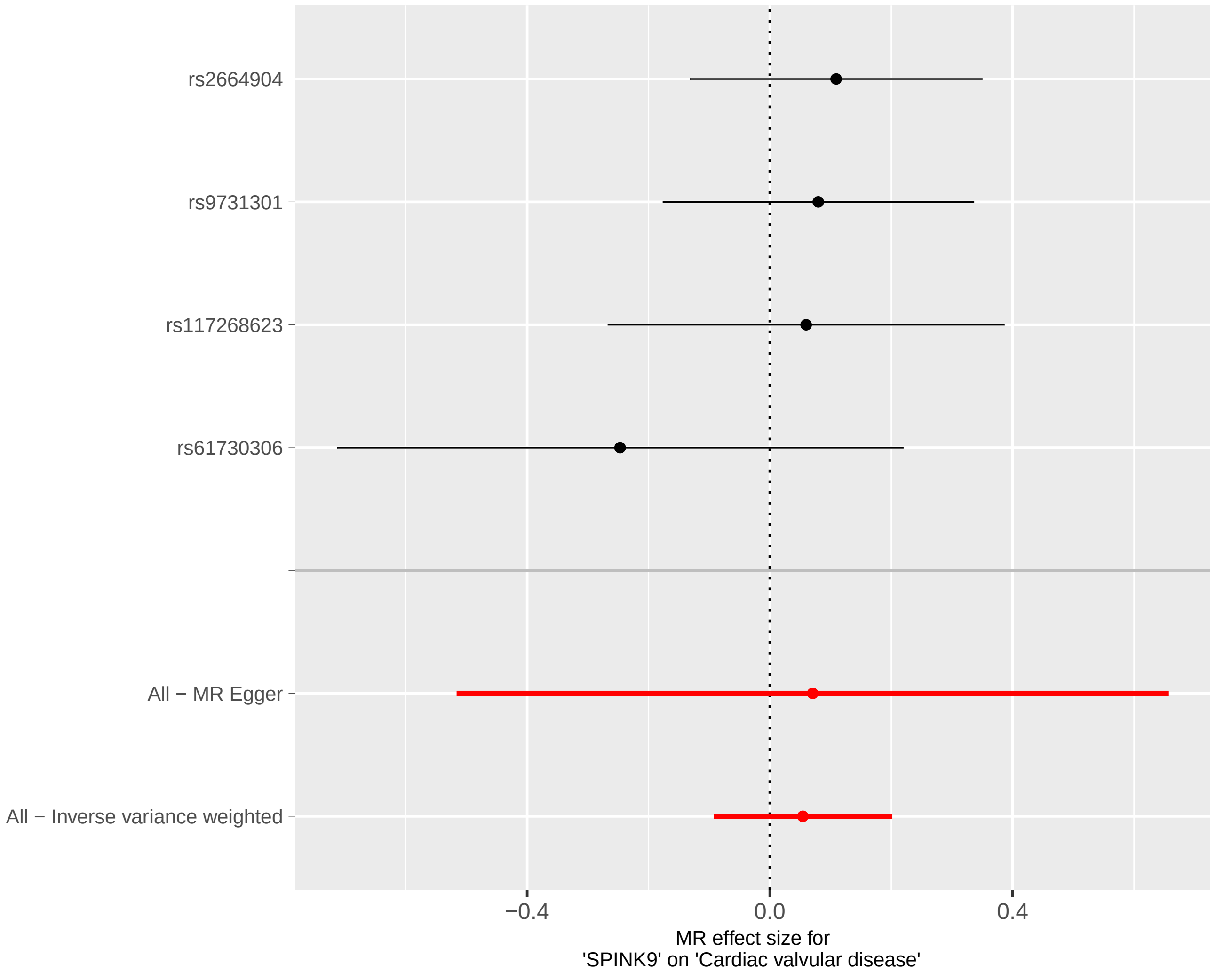
rs2664904
rs9731301
rs117268623
rs61730306
All − MR Egger
All − Inverse variance weighted
−0.4
0.0
0.4
MR effect size for
'SPINK9' on 'Cardiac valvular disease'

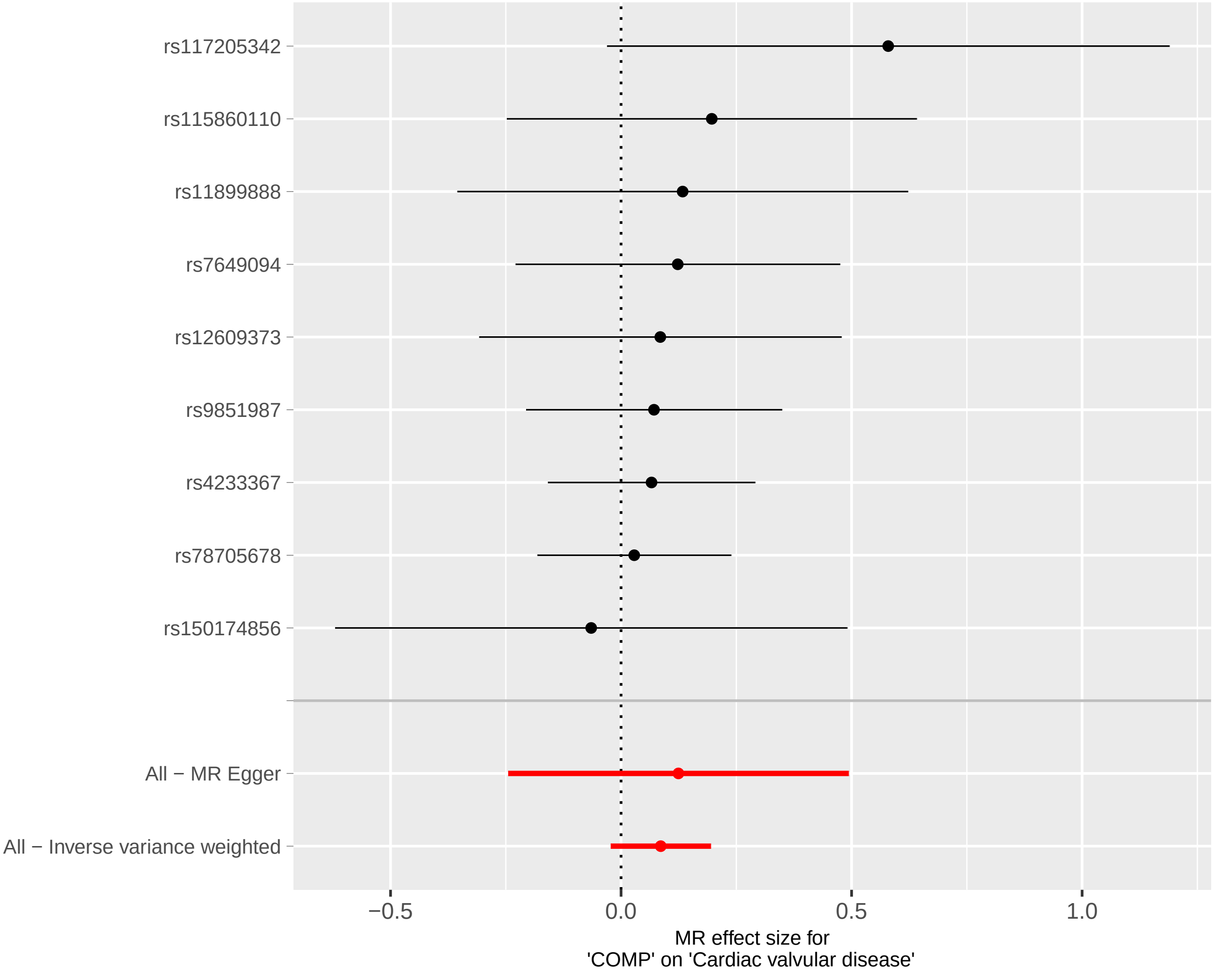

rs117205342
rs115860110
rs11899888
rs7649094
rs12609373
rs9851987
rs4233367
rs78705678
rs150174856
All − MR Egger
All − Inverse variance weighted
−0.5
0.0
0.5
1.0
MR effect size for
'COMP' on 'Cardiac valvular disease'

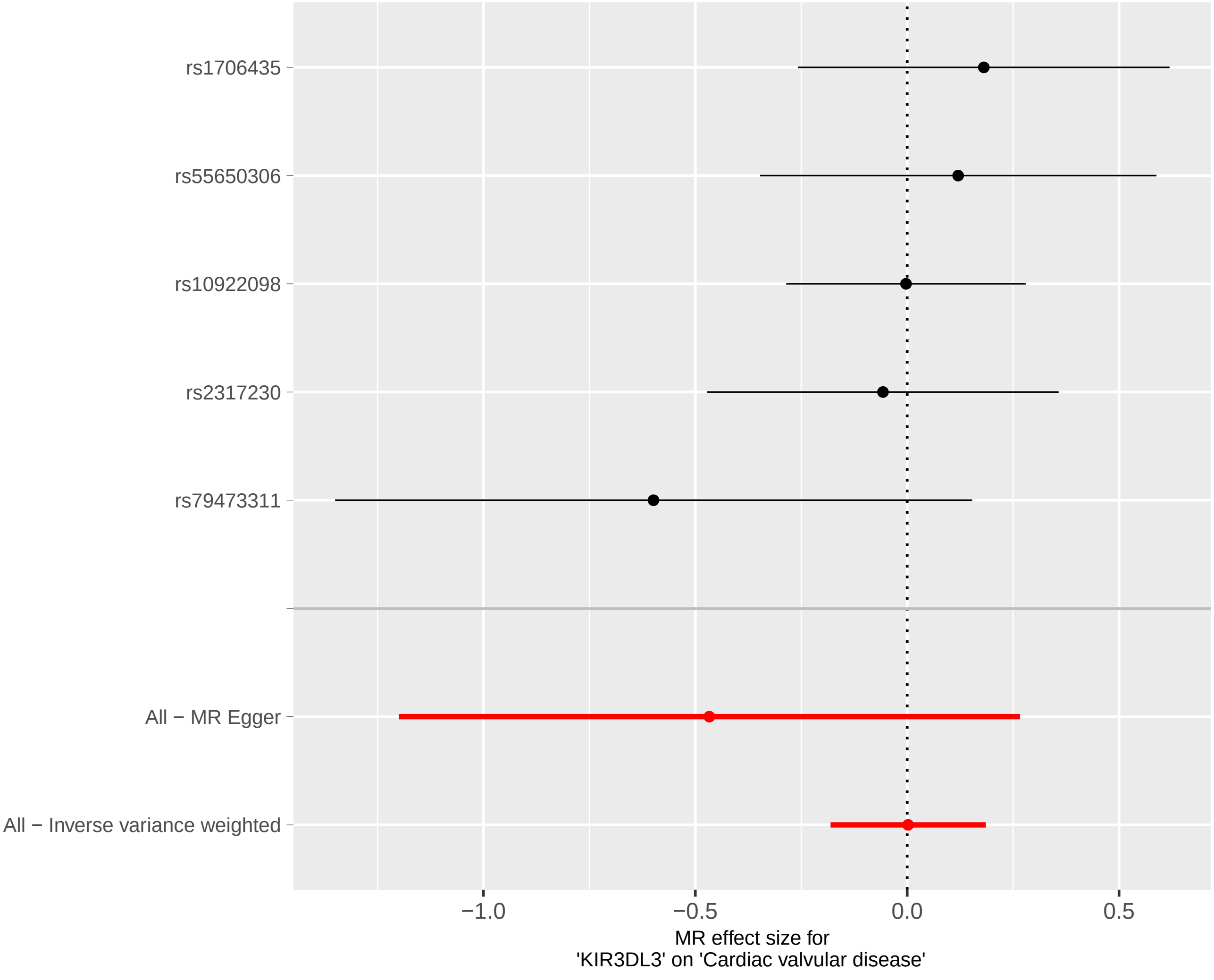
rs1706435
rs55650306
rs10922098
rs2317230
rs79473311
All − MR Egger
All − Inverse variance weighted
−1.0
−0.5
0.0
0.5
MR effect size for
'KIR3DL3' on 'Cardiac valvular disease'

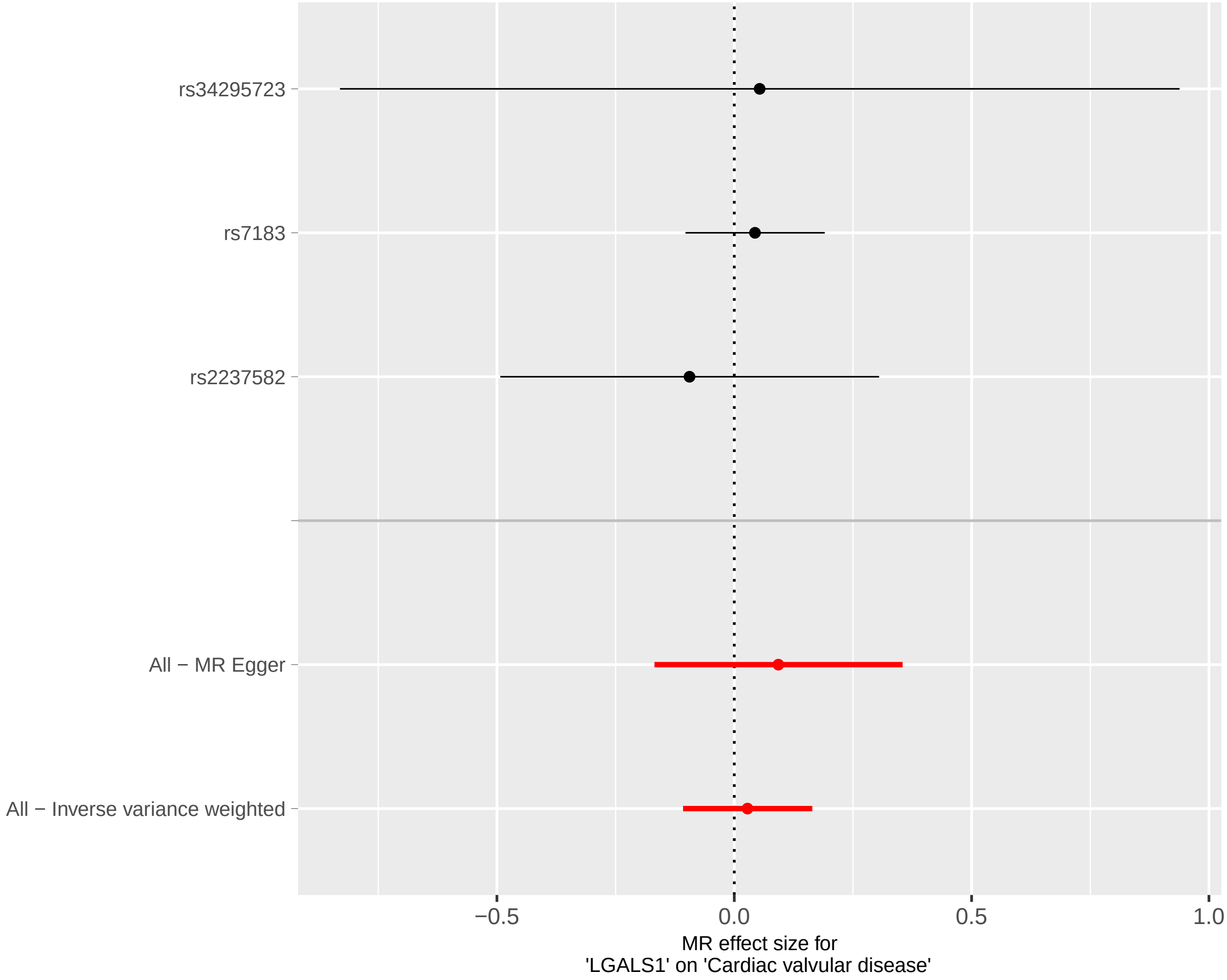
rs34295723
rs7183
rs2237582
All − MR Egger
All − Inverse variance weighted
−0.5
0.0
0.5
1.0
MR effect size for
'LGALS1' on 'Cardiac valvular disease'

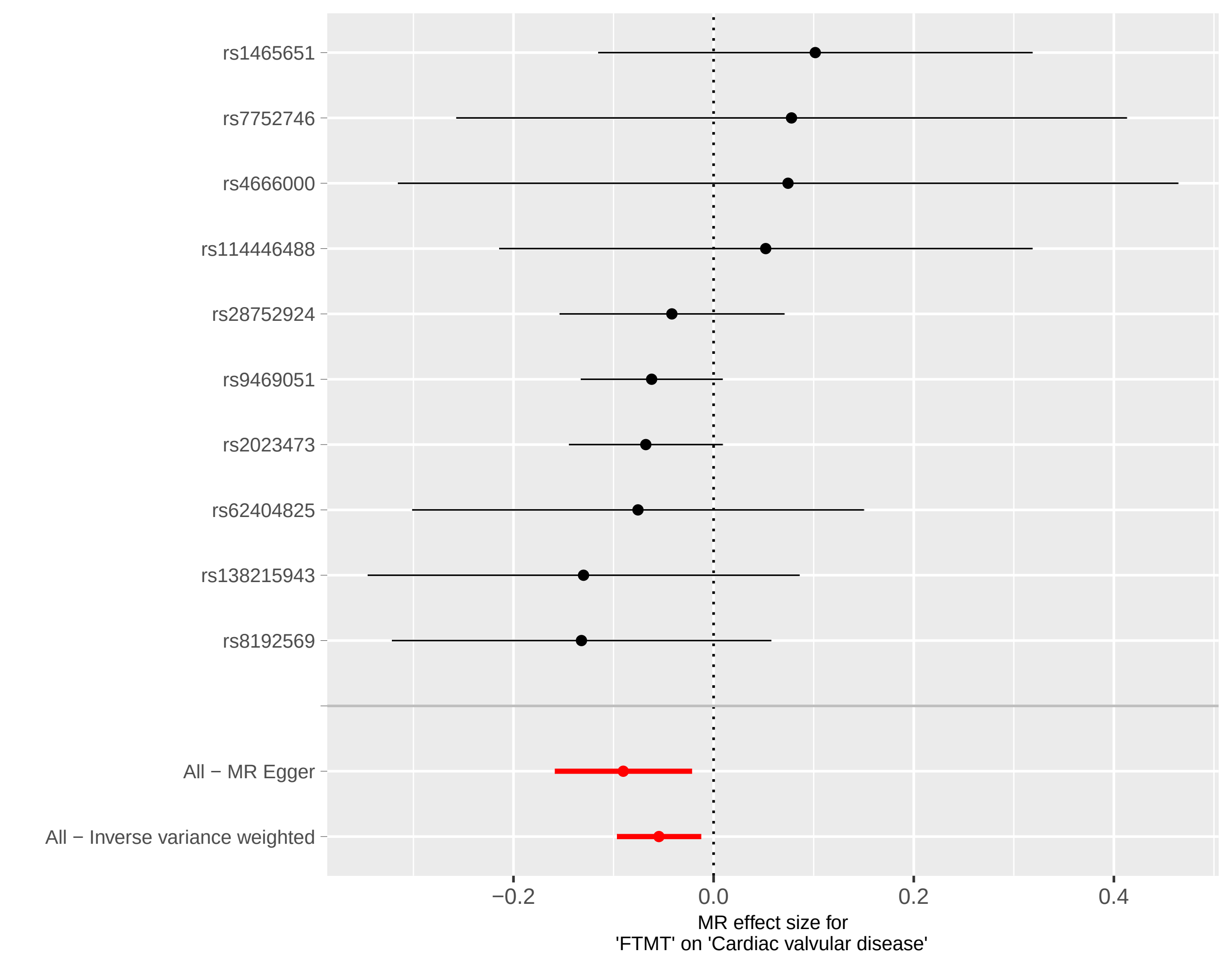
rs1465651
rs7752746
rs4666000
rs114446488
rs28752924
rs9469051
rs2023473
rs62404825
rs138215943
rs8192569
All − MR Egger
All − Inverse variance weighted
−0.2
0.0
0.2
0.4
MR effect size for
'FTMT' on 'Cardiac valvular disease'

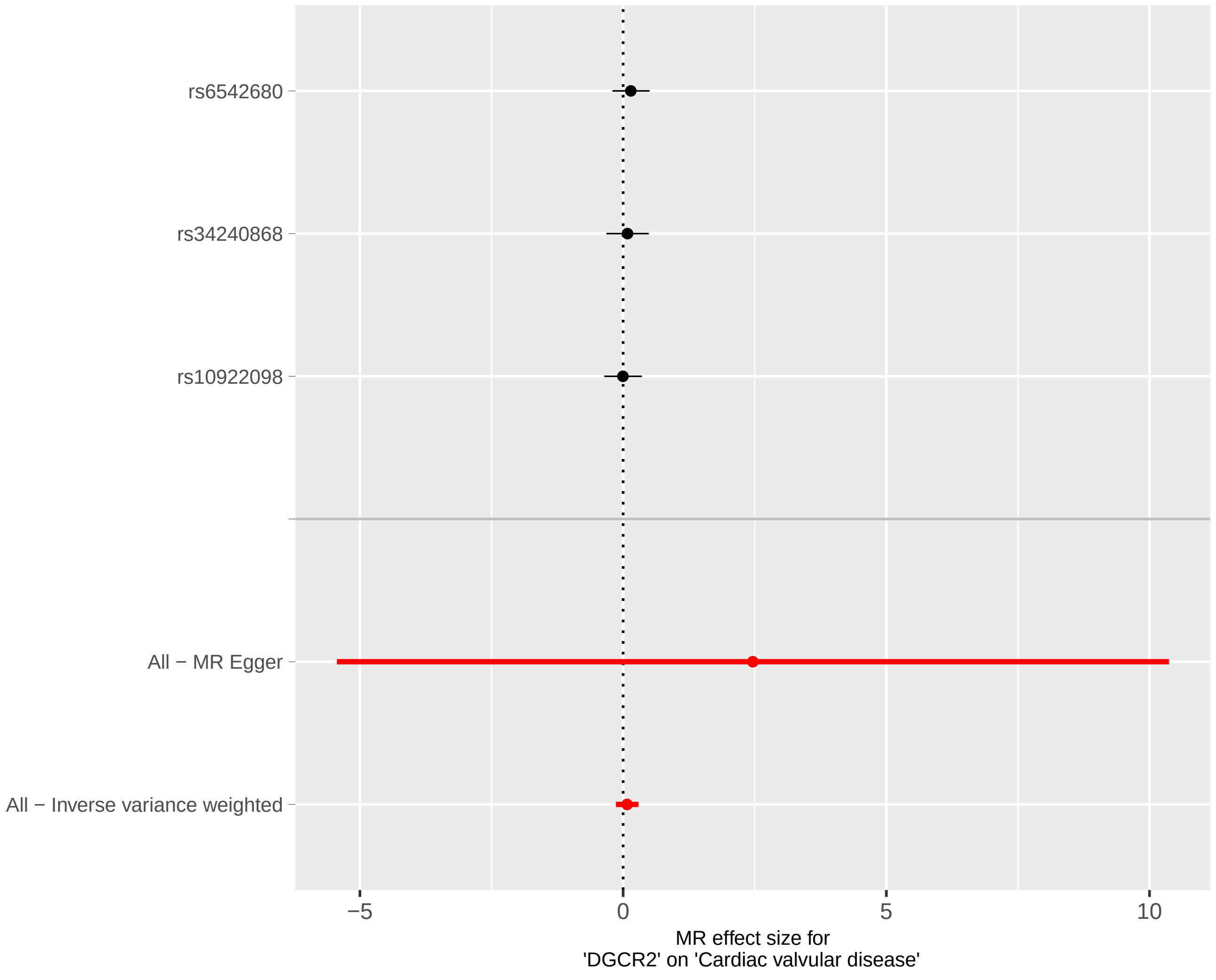
rs6542680
rs34240868
rs10922098
All − MR Egger
All − Inverse variance weighted
−5
0
5
10
MR effect size for
'DGCR2' on 'Cardiac valvular disease'

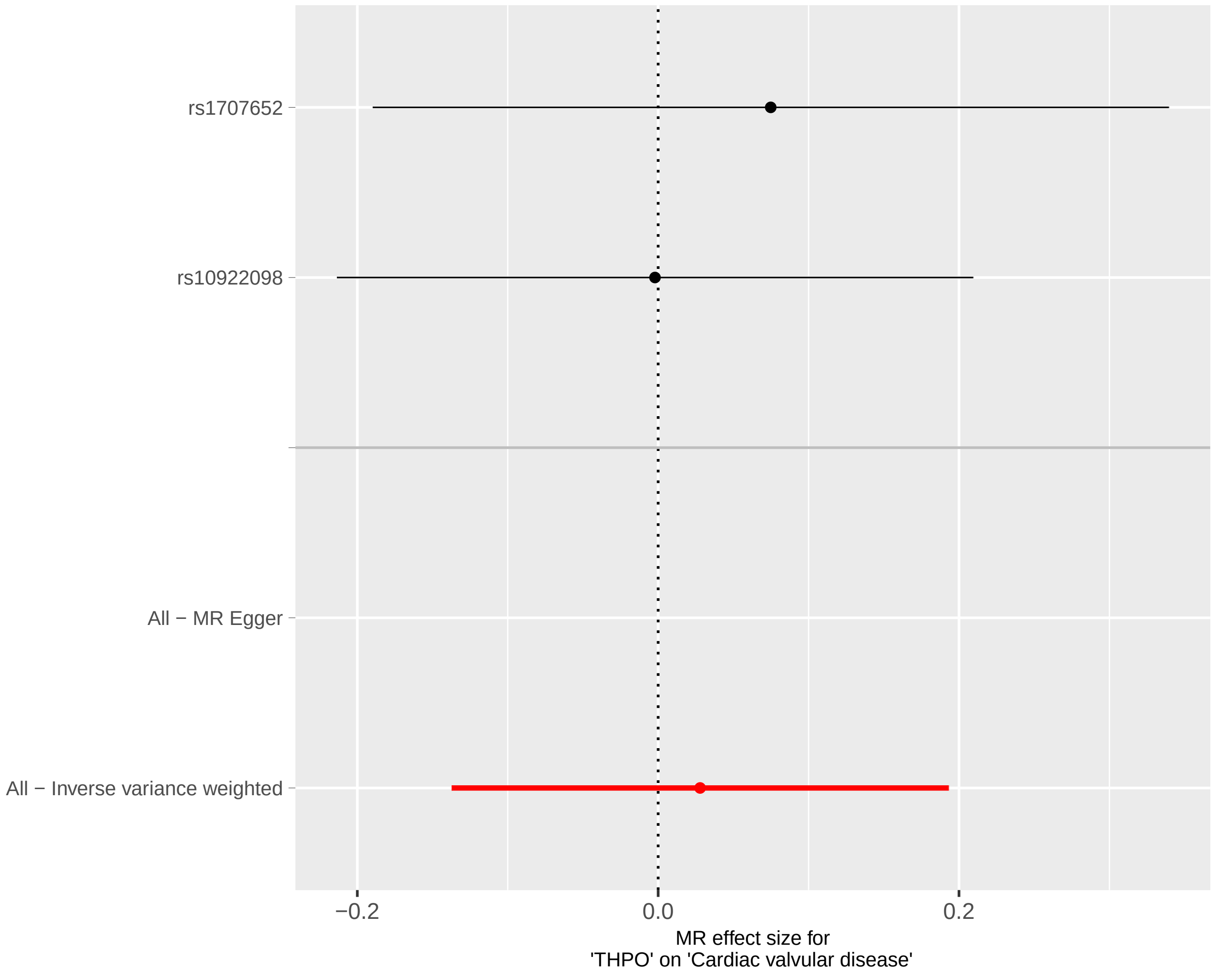
rs1707652
rs10922098
All − MR Egger
All − Inverse variance weighted
−0.2
0.0
0.2
MR effect size for
'THPO' on 'Cardiac valvular disease'

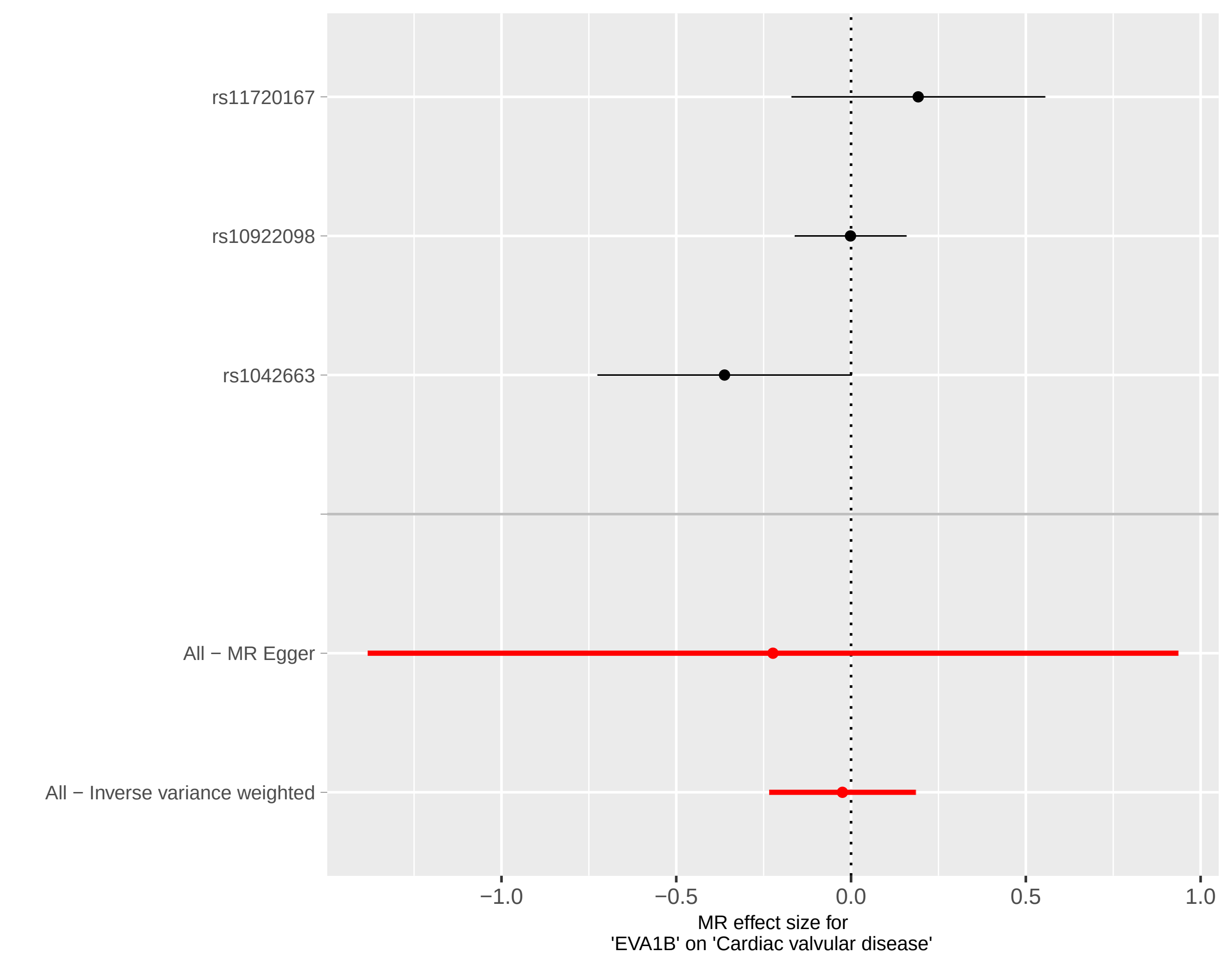
rs11720167
rs10922098
rs1042663
All − MR Egger
All − Inverse variance weighted
−1.0
−0.5
0.0
0.5
1.0
MR effect size for
'EVA1B' on 'Cardiac valvular disease'

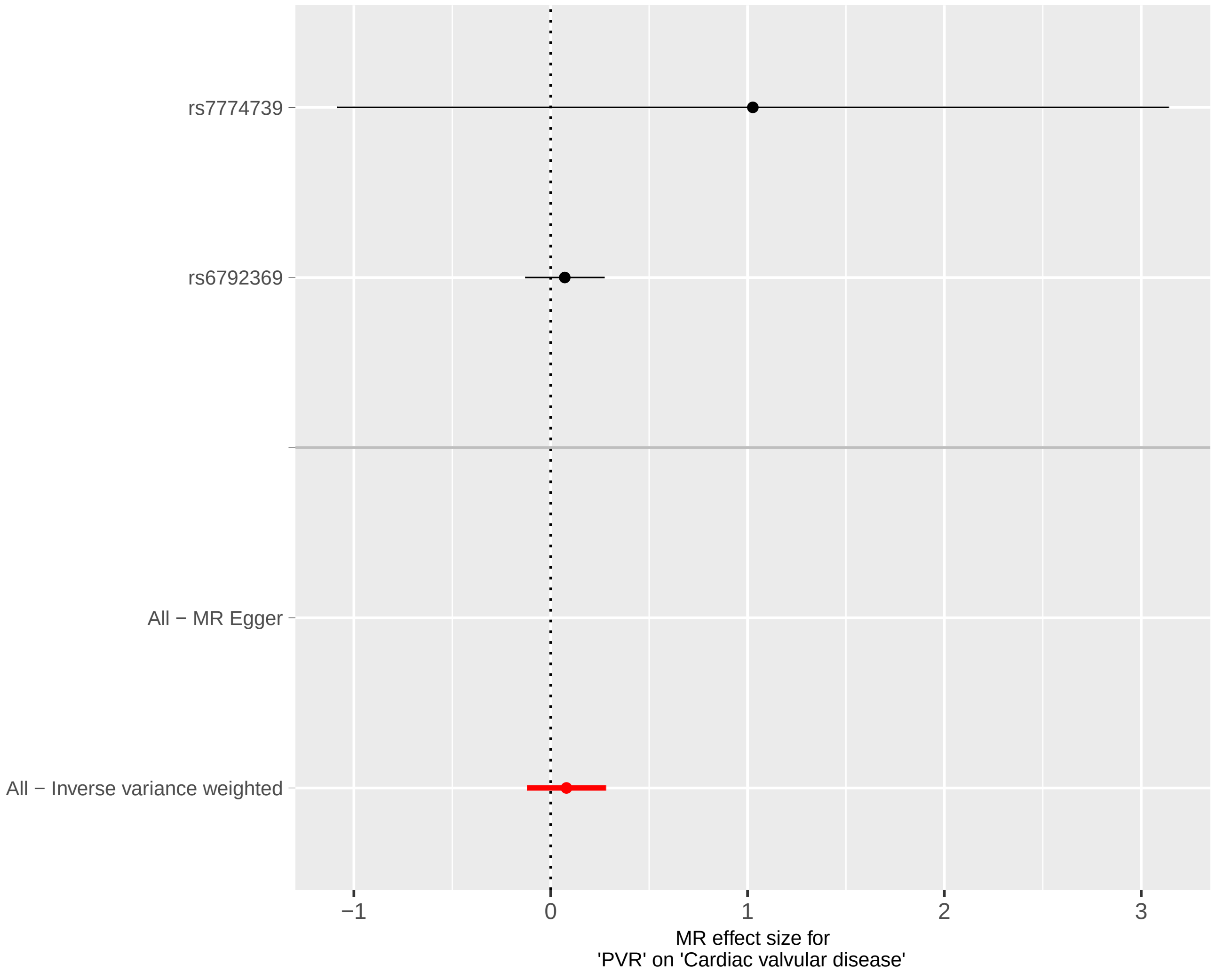
rs7774739
rs6792369
All − MR Egger
All − Inverse variance weighted
−1
0
1
2
3
MR effect size for
'PVR' on 'Cardiac valvular disease'

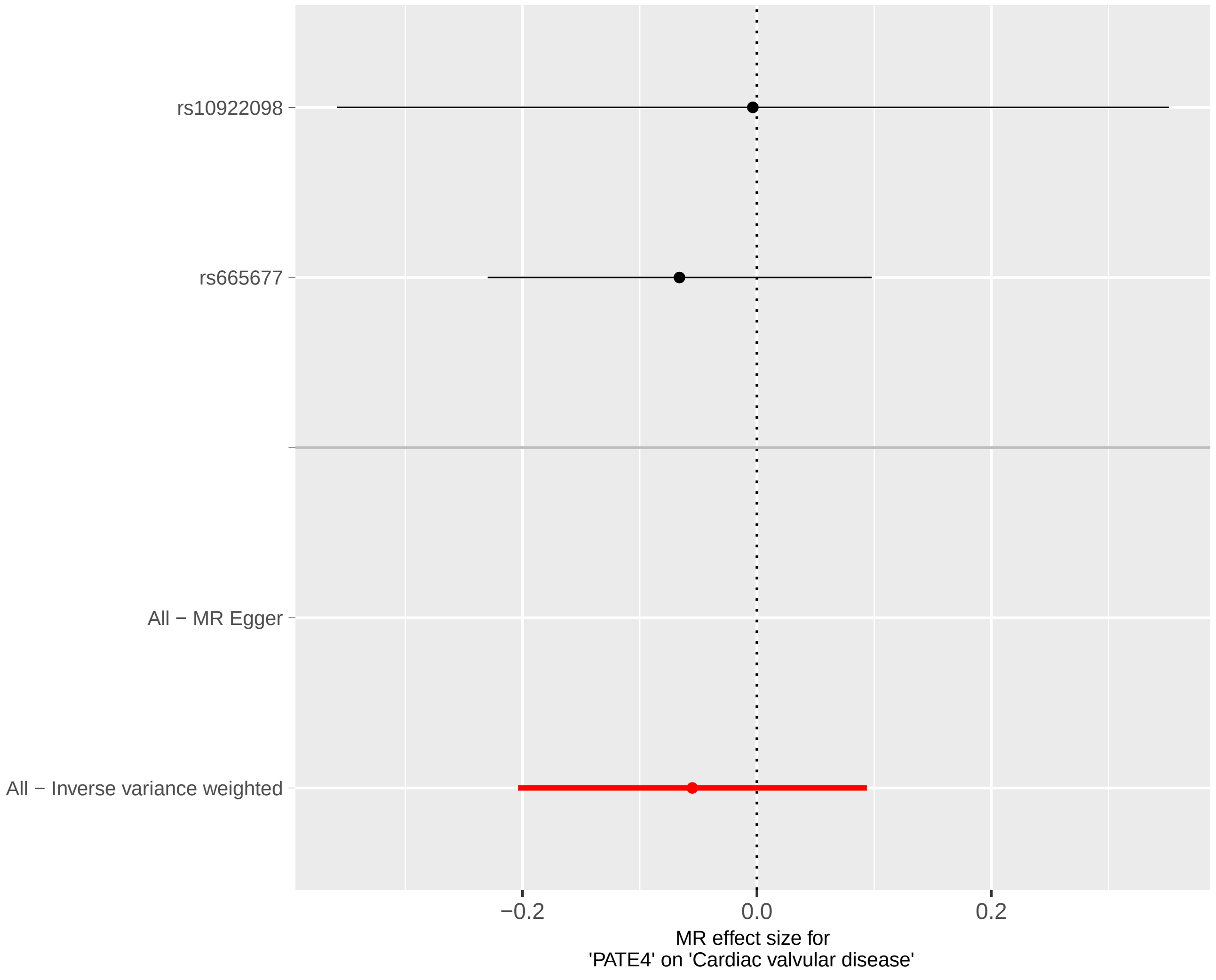

rs10922098
rs665677
All − MR Egger
All − Inverse variance weighted
−0.2
0.0
0.2
MR effect size for
'PATE4' on 'Cardiac valvular disease'

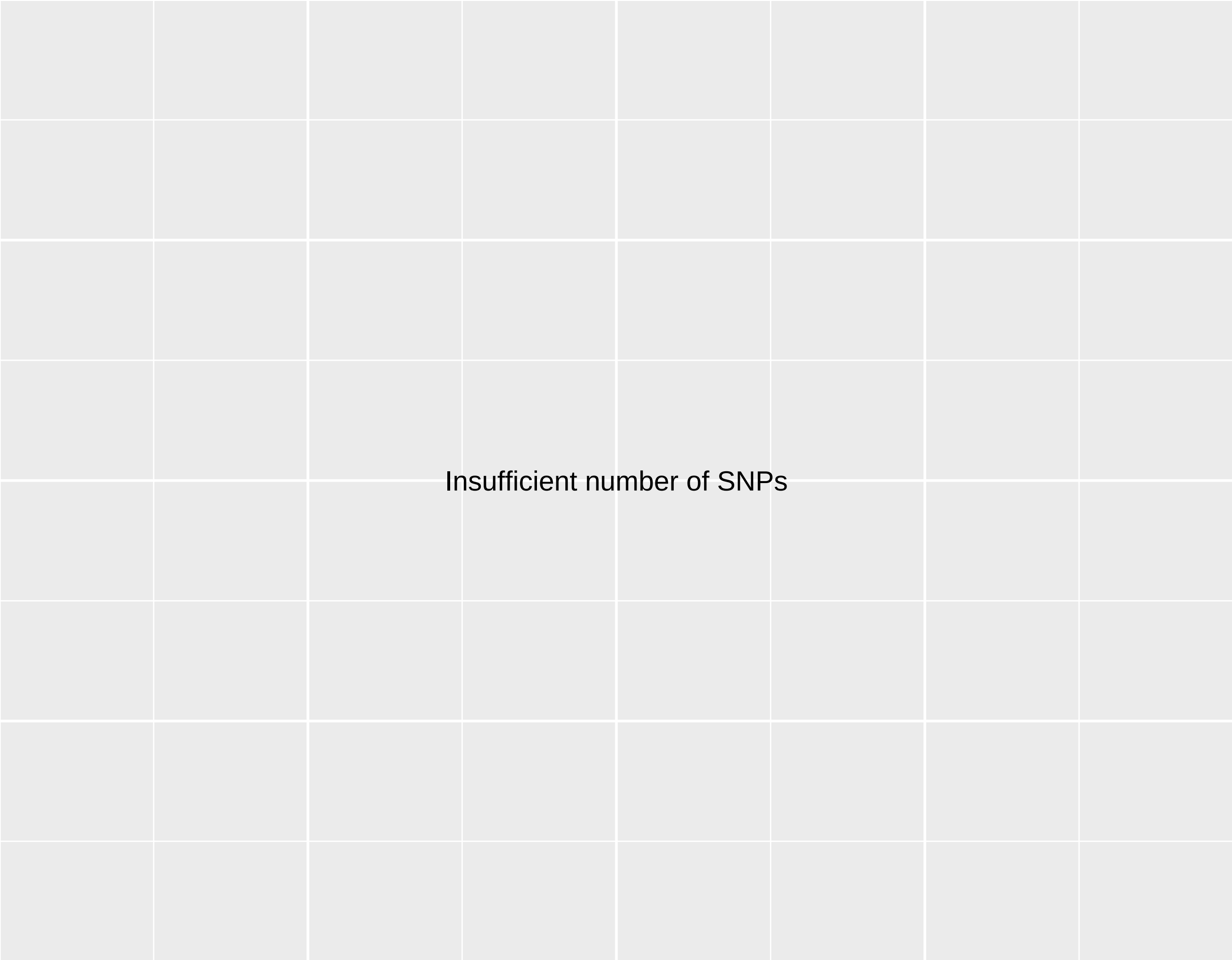
Insufficient number of SNPs

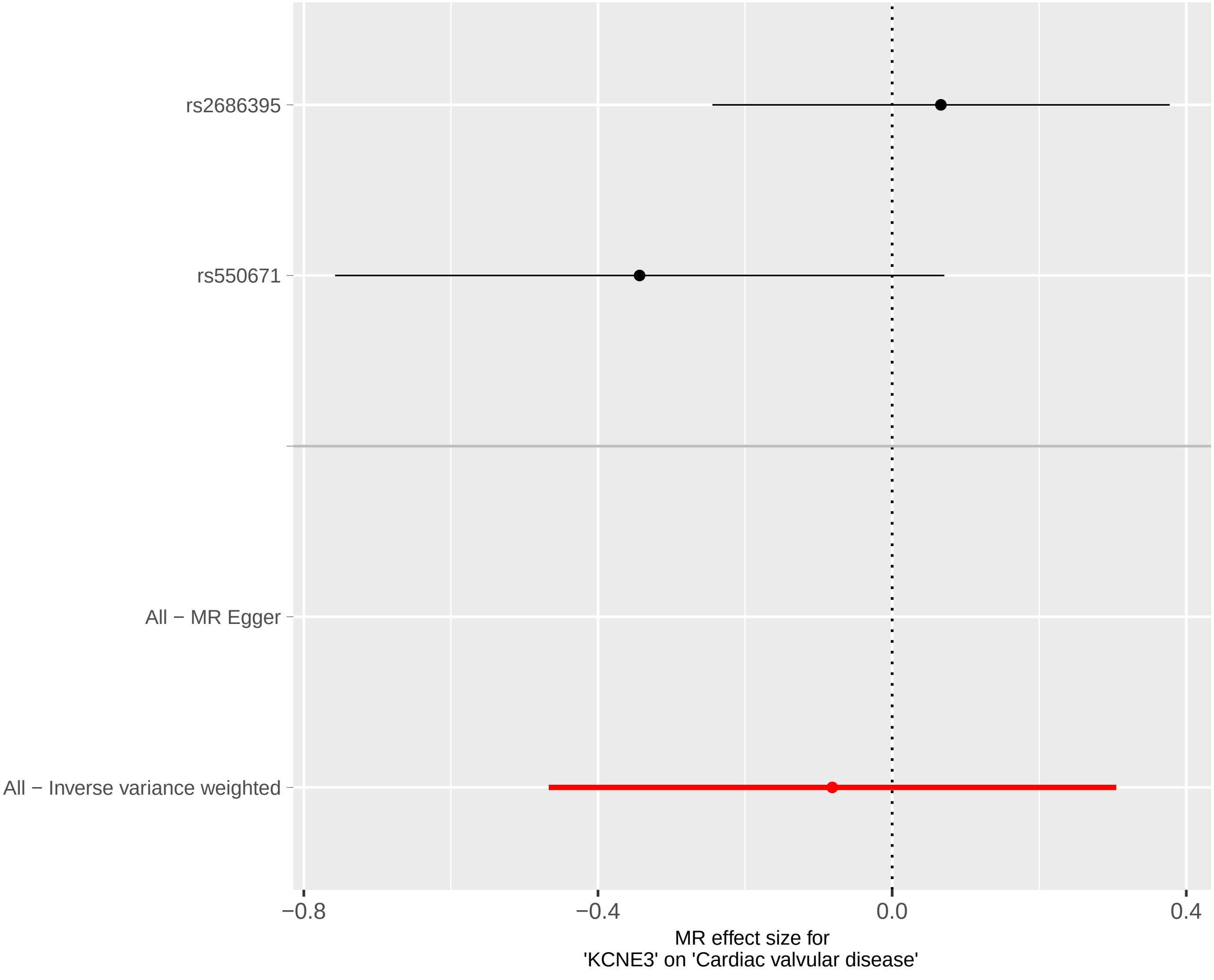

rs2686395
rs550671
All − MR Egger
All − Inverse variance weighted
−0.8
−0.4
0.0
0.4
MR effect size for
'KCNE3' on 'Cardiac valvular disease'

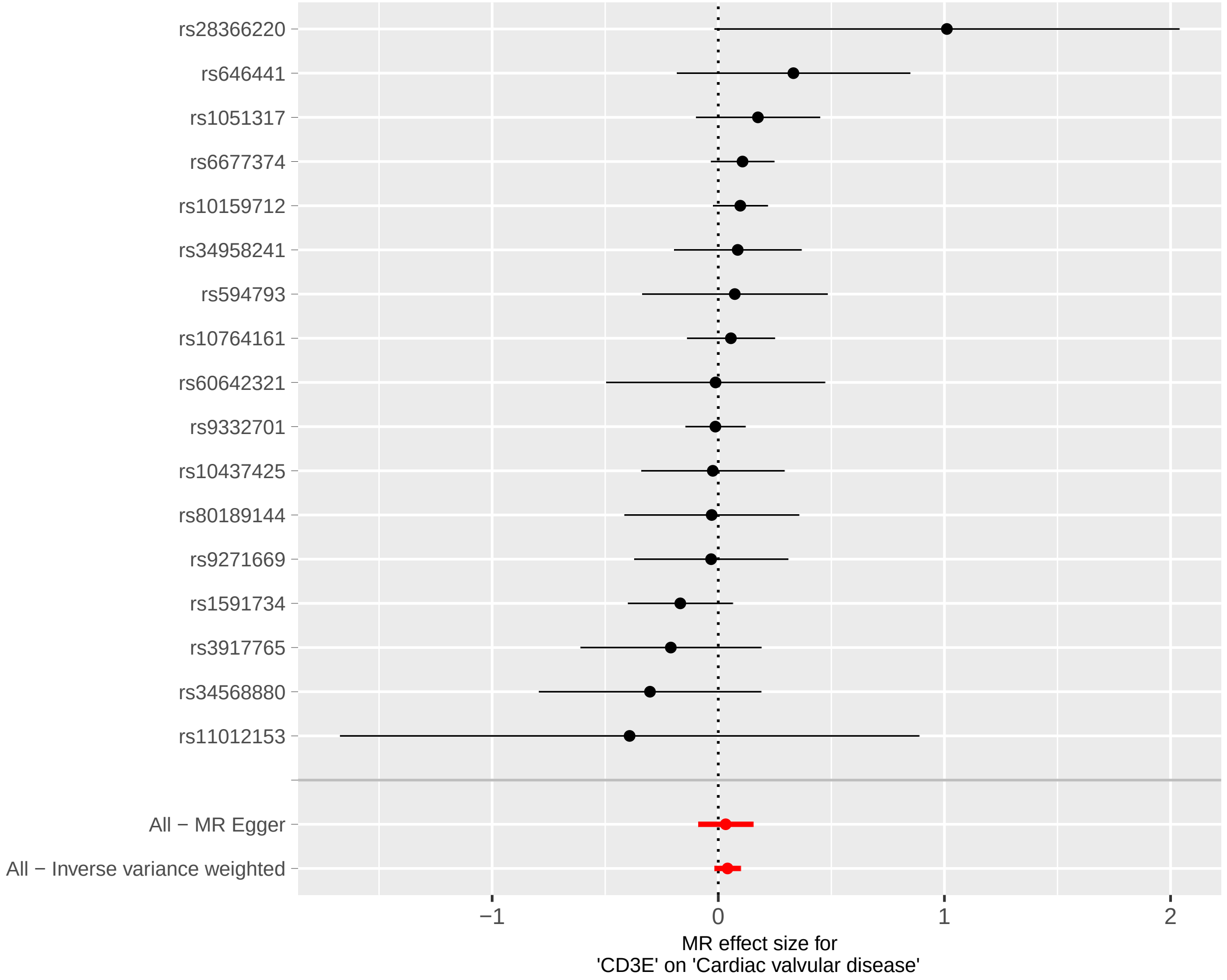
rs28366220
rs646441
rs1051317
rs6677374
rs10159712
rs34958241
rs594793
rs10764161
rs60642321
rs9332701
rs10437425
rs80189144
rs9271669
rs1591734
rs3917765
rs34568880
rs11012153
All − MR Egger
All − Inverse variance weighted
−1
0
1
2
MR effect size for
'CD3E' on 'Cardiac valvular disease'

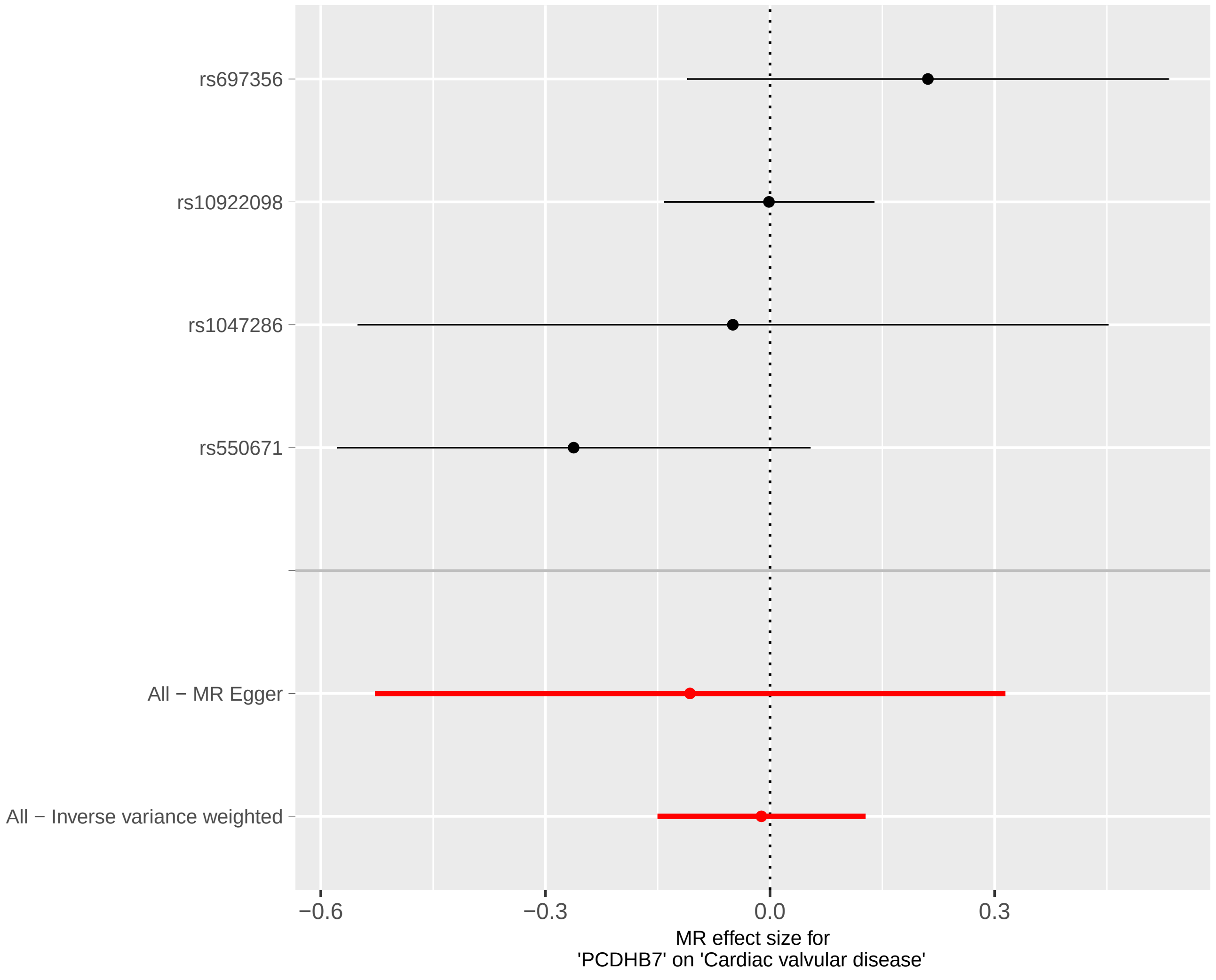
rs697356
rs10922098
rs1047286
rs550671
All − MR Egger
All − Inverse variance weighted
−0.6
−0.3
0.0
0.3
MR effect size for
'PCDHB7' on 'Cardiac valvular disease'

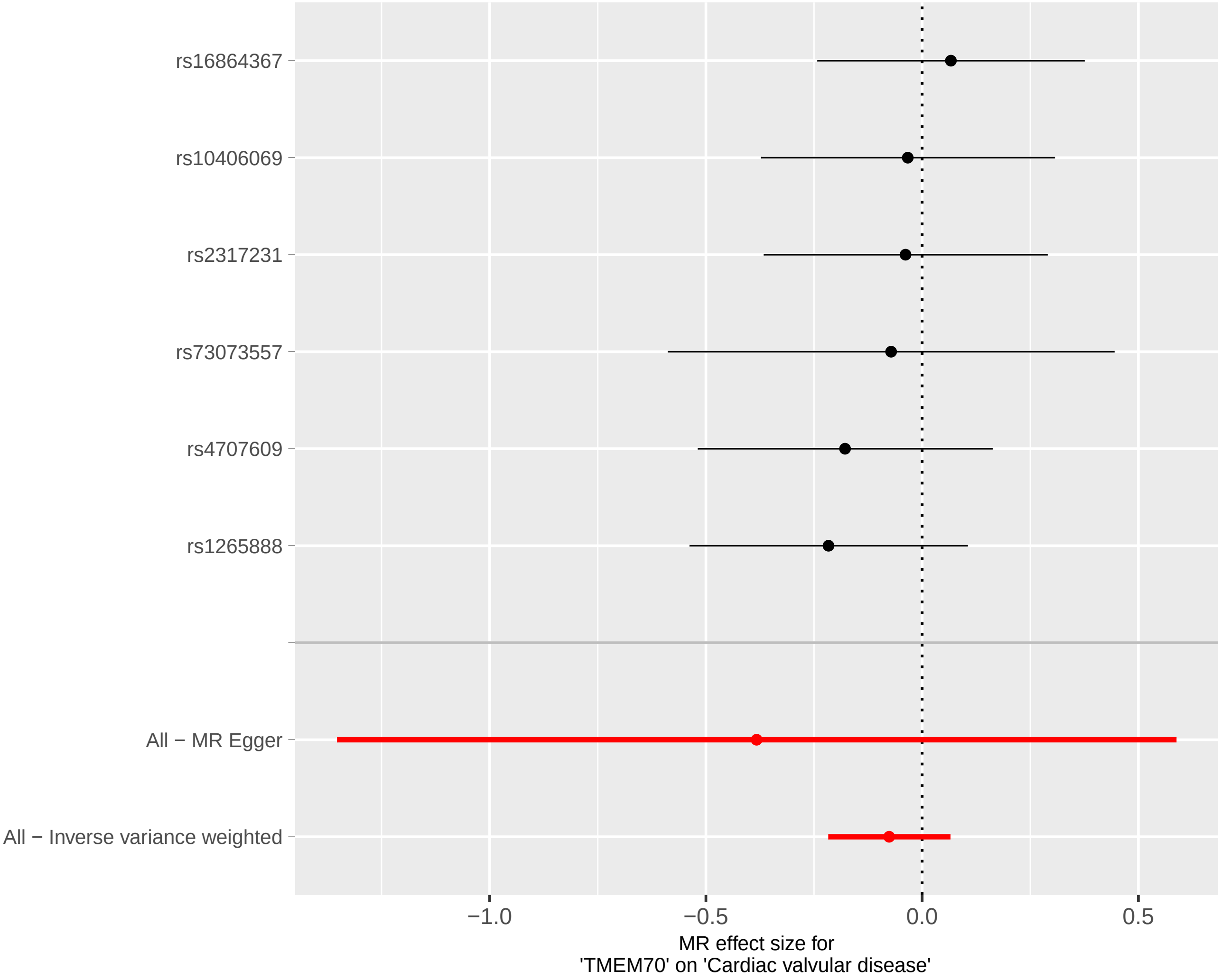
rs16864367
rs10406069
rs2317231
rs73073557
rs4707609
rs1265888
All − MR Egger
All − Inverse variance weighted
−1.0
−0.5
0.0
0.5
MR effect size for
'TMEM70' on 'Cardiac valvular disease'

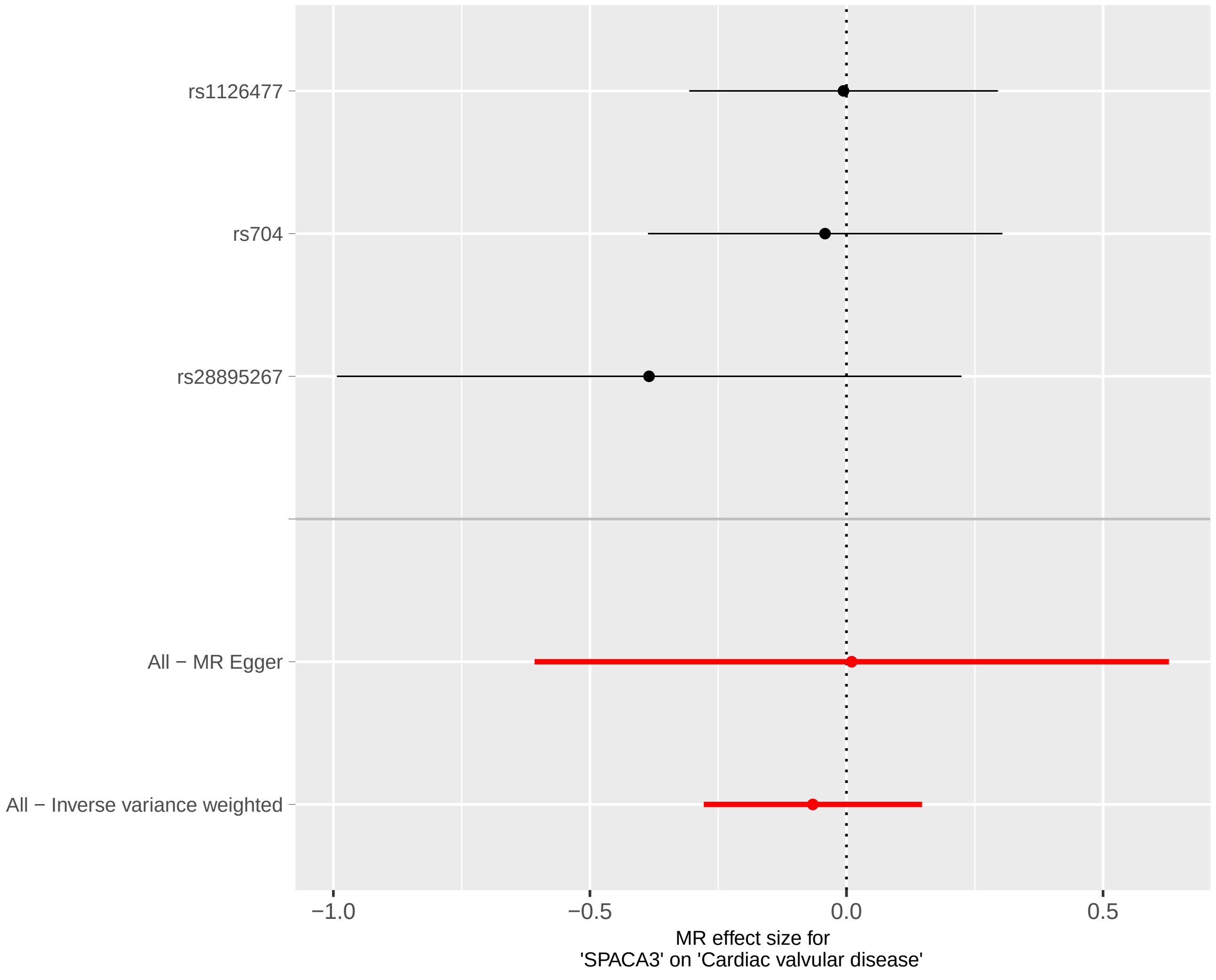
rs1126477
rs704
rs28895267
All − MR Egger
All − Inverse variance weighted
−1.0
−0.5
0.0
0.5
MR effect size for
'SPACA3' on 'Cardiac valvular disease'

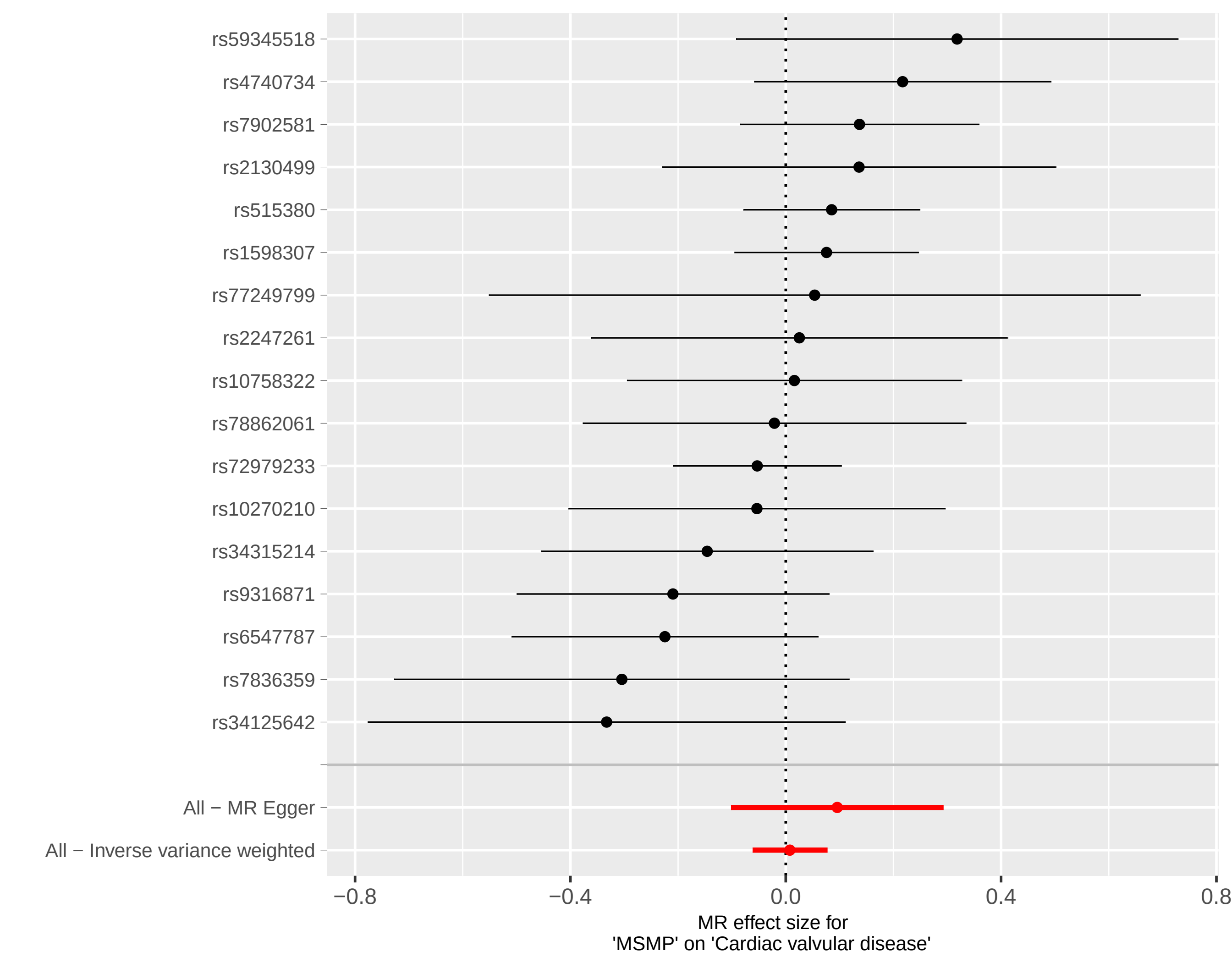
rs59345518
rs4740734
rs7902581
rs2130499
rs515380
rs1598307
rs77249799
rs2247261
rs10758322
rs78862061
rs72979233
rs10270210
rs34315214
rs9316871
rs6547787
rs7836359
rs34125642
All − MR Egger
All − Inverse variance weighted
−0.8
−0.4
0.0
0.4
0.8
MR effect size for
'MSMP' on 'Cardiac valvular disease'

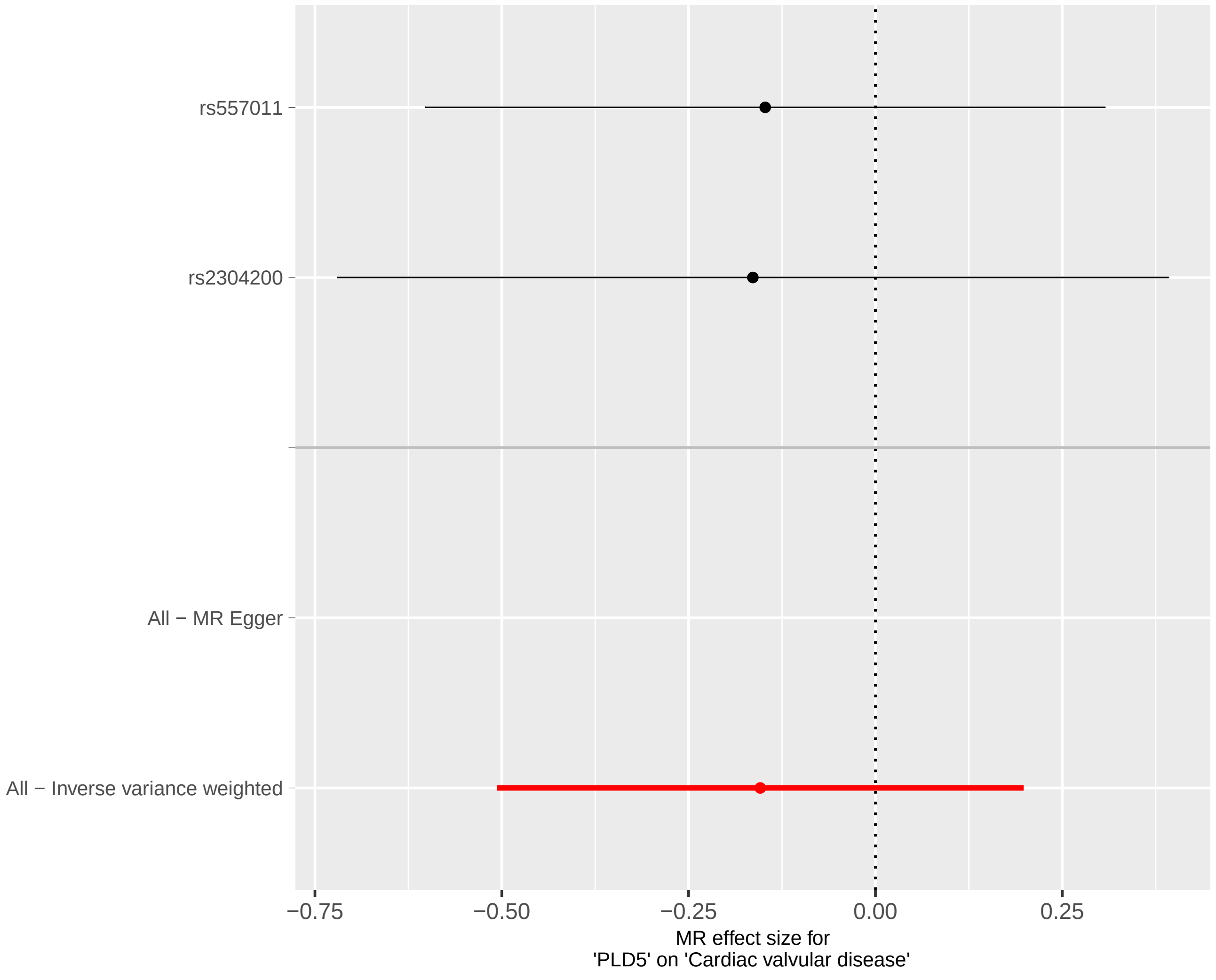
rs557011
rs2304200
All − MR Egger
All − Inverse variance weighted
−0.75
−0.50
−0.25
0.00
0.25
MR effect size for
'PLD5' on 'Cardiac valvular disease'

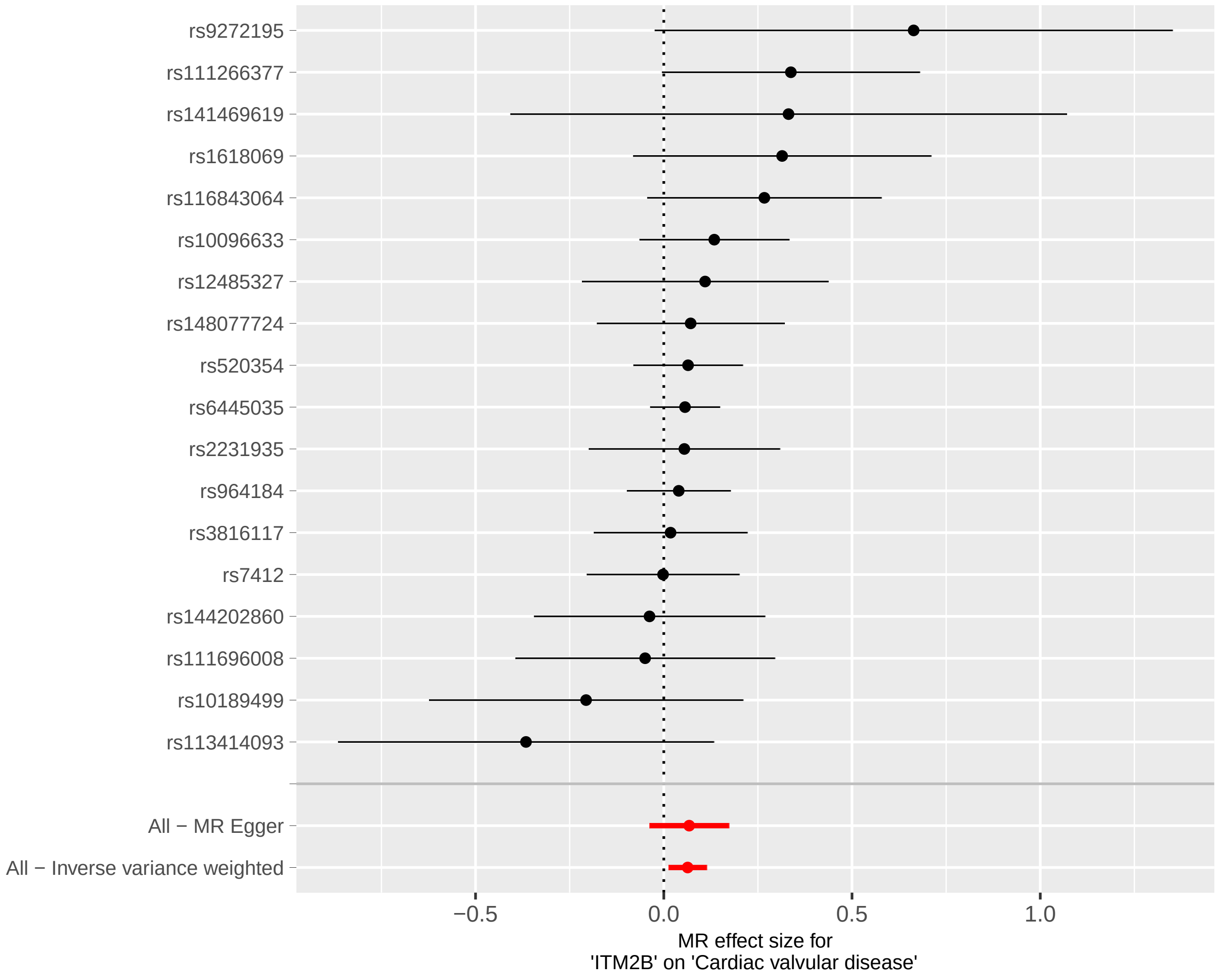
rs9272195
rs111266377
rs141469619
rs1618069
rs116843064
rs10096633
rs12485327
rs148077724
rs520354
rs6445035
rs2231935
rs964184
rs3816117
rs7412
rs144202860
rs111696008
rs10189499
rs113414093
All − MR Egger
All − Inverse variance weighted
−0.5
0.0
0.5
1.0
MR effect size for
'ITM2B' on 'Cardiac valvular disease'

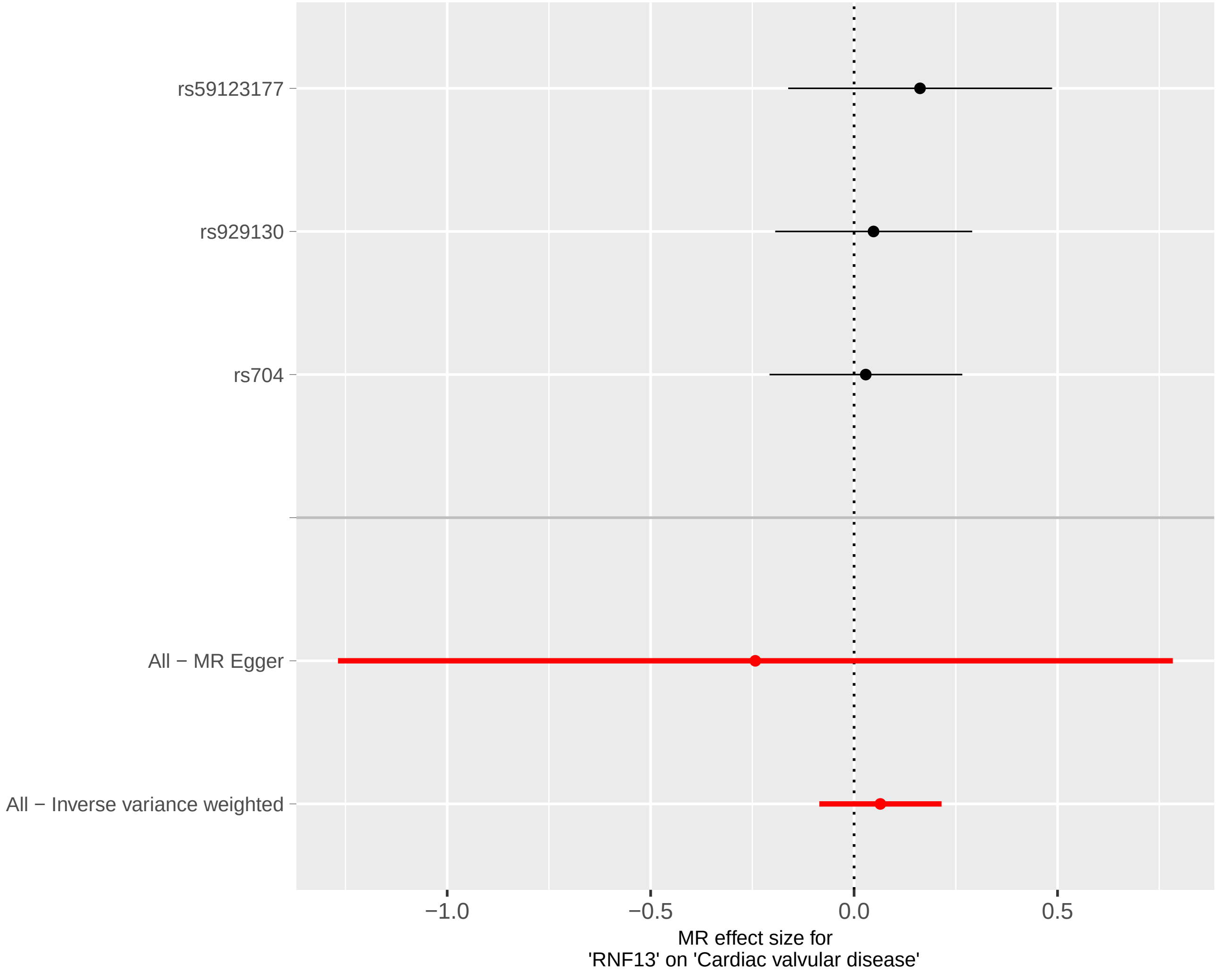
rs59123177
rs929130
rs704
All − MR Egger
All − Inverse variance weighted
−1.0
−0.5
0.0
0.5
MR effect size for
'RNF13' on 'Cardiac valvular disease'

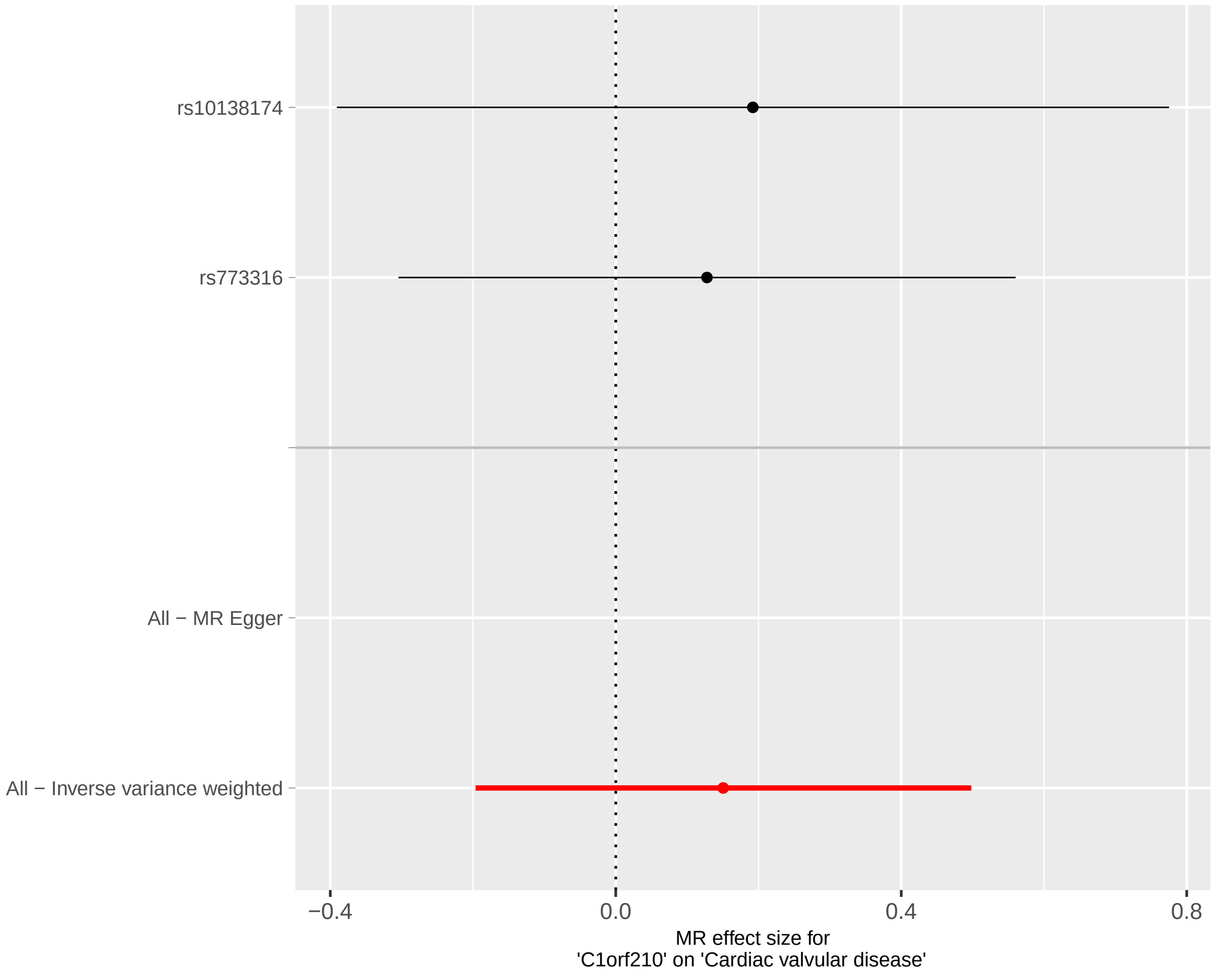
rs10138174
rs773316
All − MR Egger
All − Inverse variance weighted
−0.4
0.0
0.4
0.8
MR effect size for
'C1orf210' on 'Cardiac valvular disease'

Insufficient number of SNPs

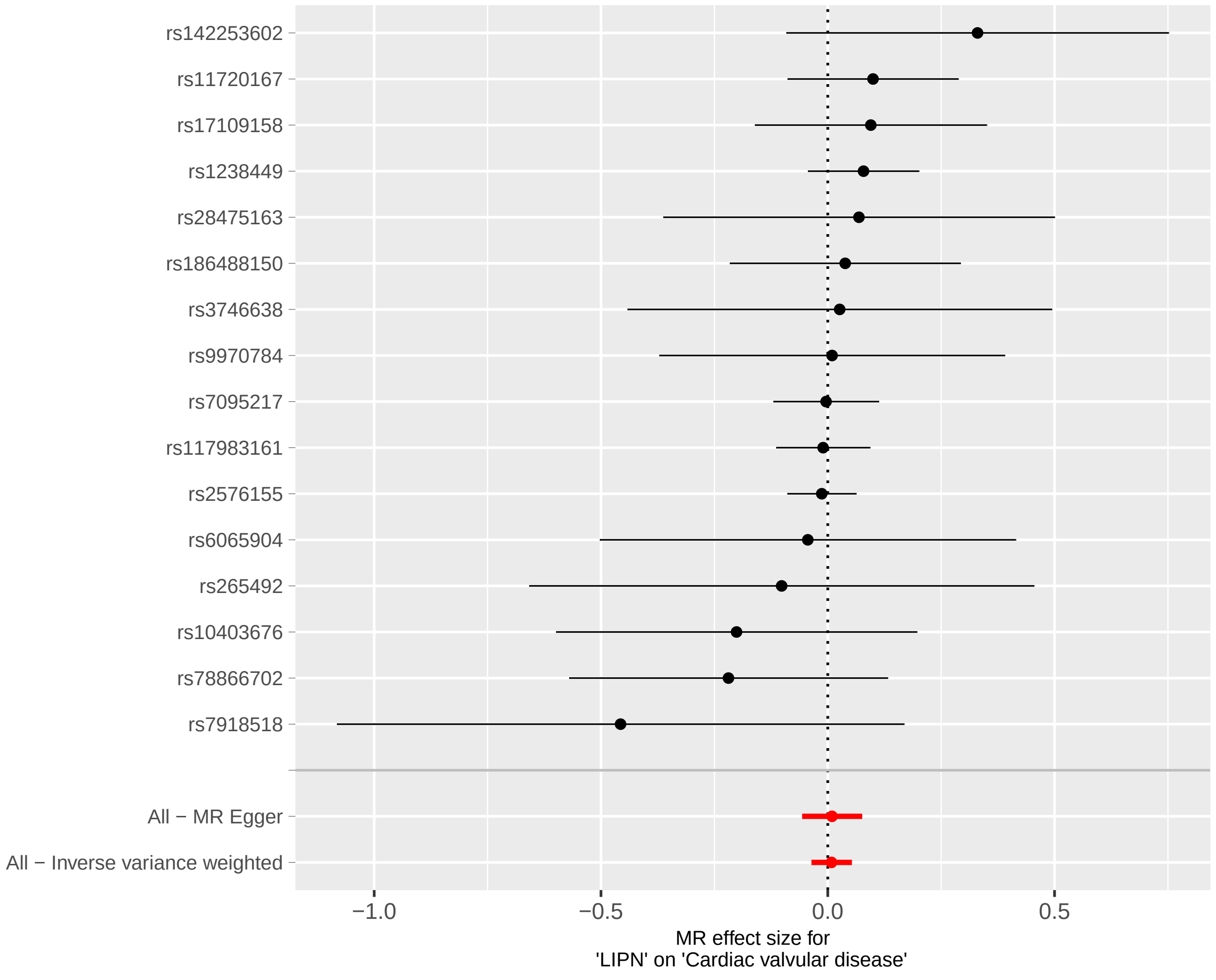
rs142253602
rs11720167
rs17109158
rs1238449
rs28475163
rs186488150
rs3746638
rs9970784
rs7095217
rs117983161
rs2576155
rs6065904
rs265492
rs10403676
rs78866702
rs7918518
All − MR Egger
All − Inverse variance weighted
−1.0
−0.5
0.0
0.5
MR effect size for
'LIPN' on 'Cardiac valvular disease'

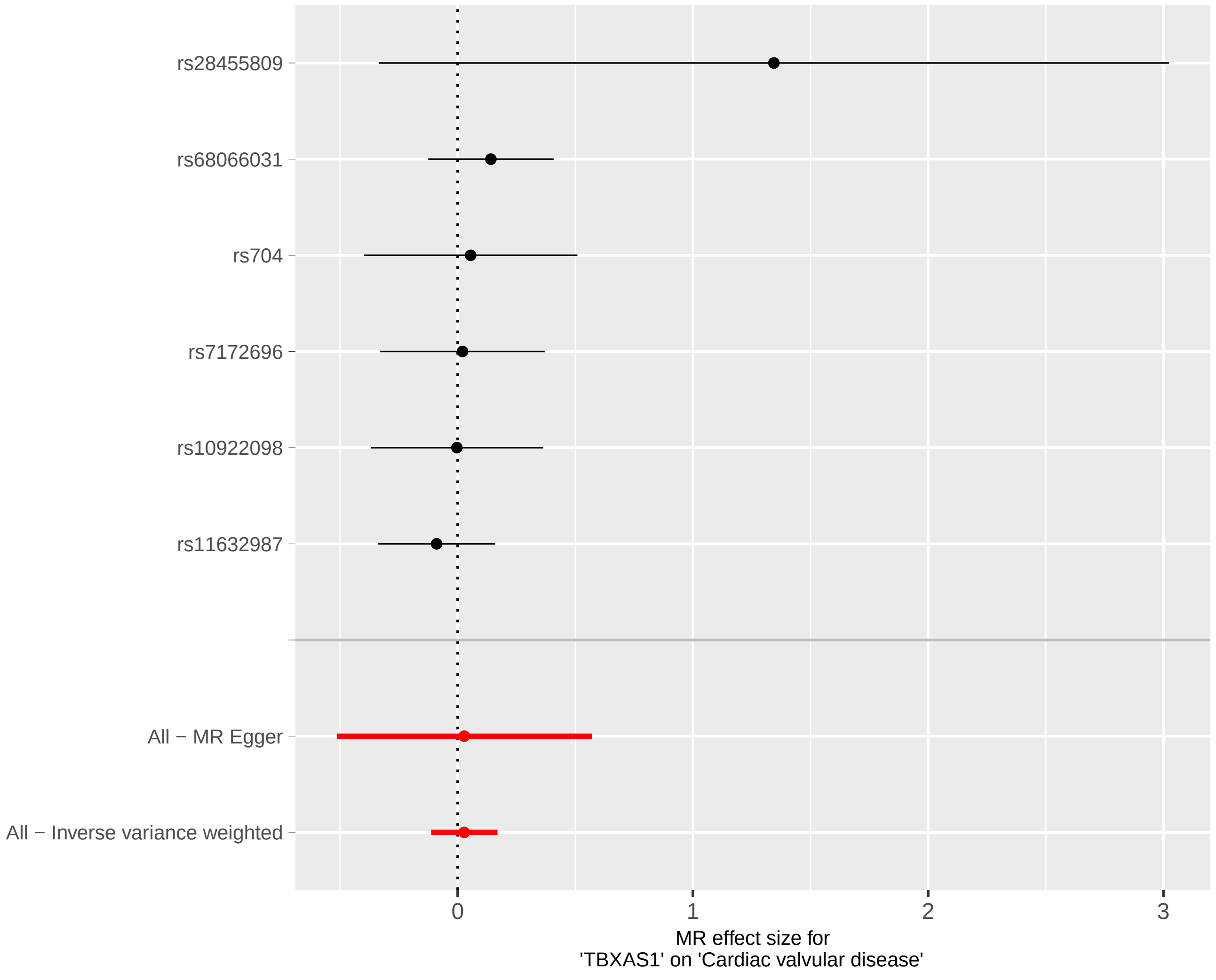

rs28455809
rs68066031
rs704
rs7172696
rs10922098
rs11632987
All − MR Egger
All − Inverse variance weighted
0
1
2
3
MR effect size for
'TBXAS1' on 'Cardiac valvular disease'

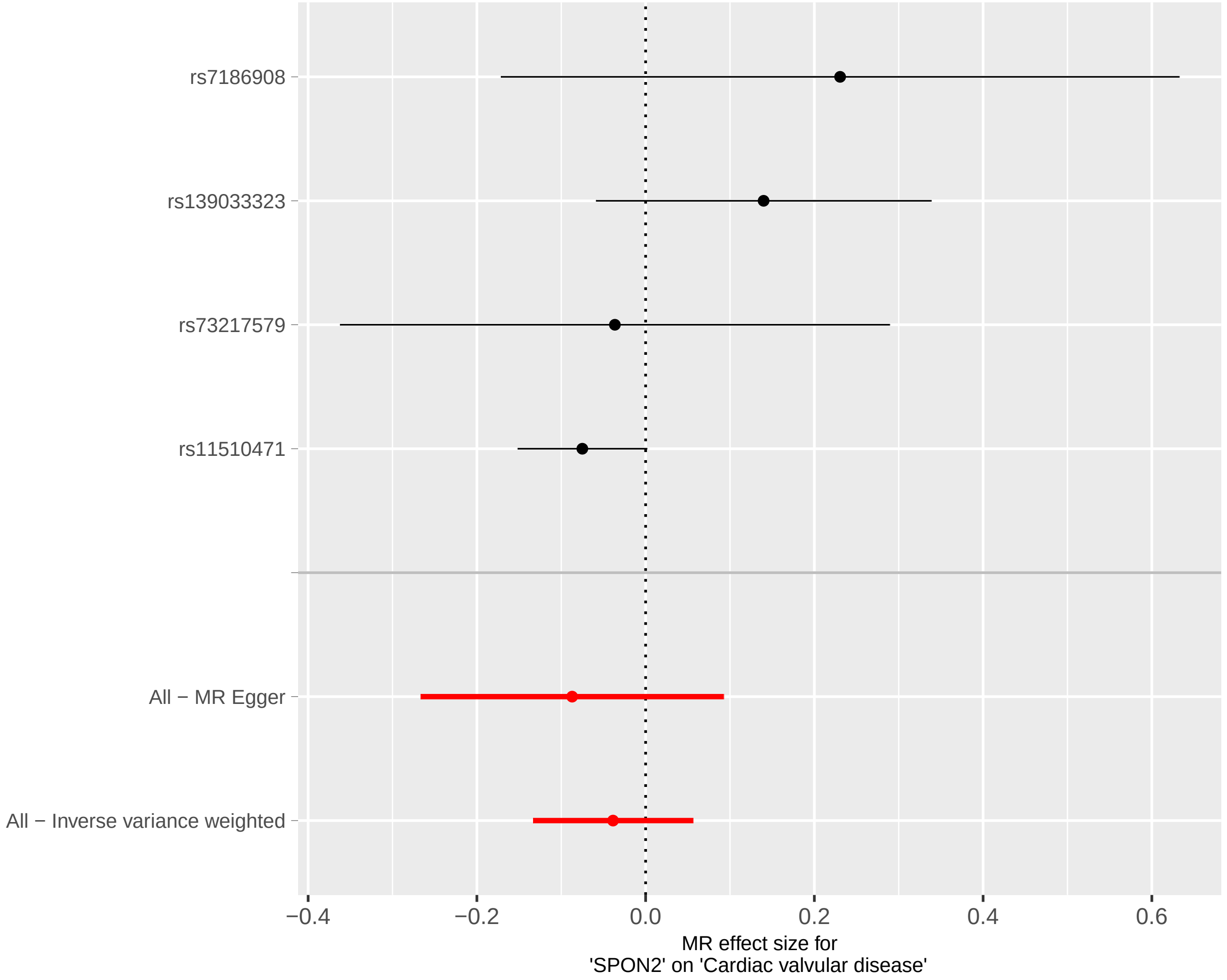
rs7186908
rs139033323
rs73217579
rs11510471
All − MR Egger
All − Inverse variance weighted
−0.4
−0.2
0.0
0.2
0.4
0.6
MR effect size for
'SPON2' on 'Cardiac valvular disease'

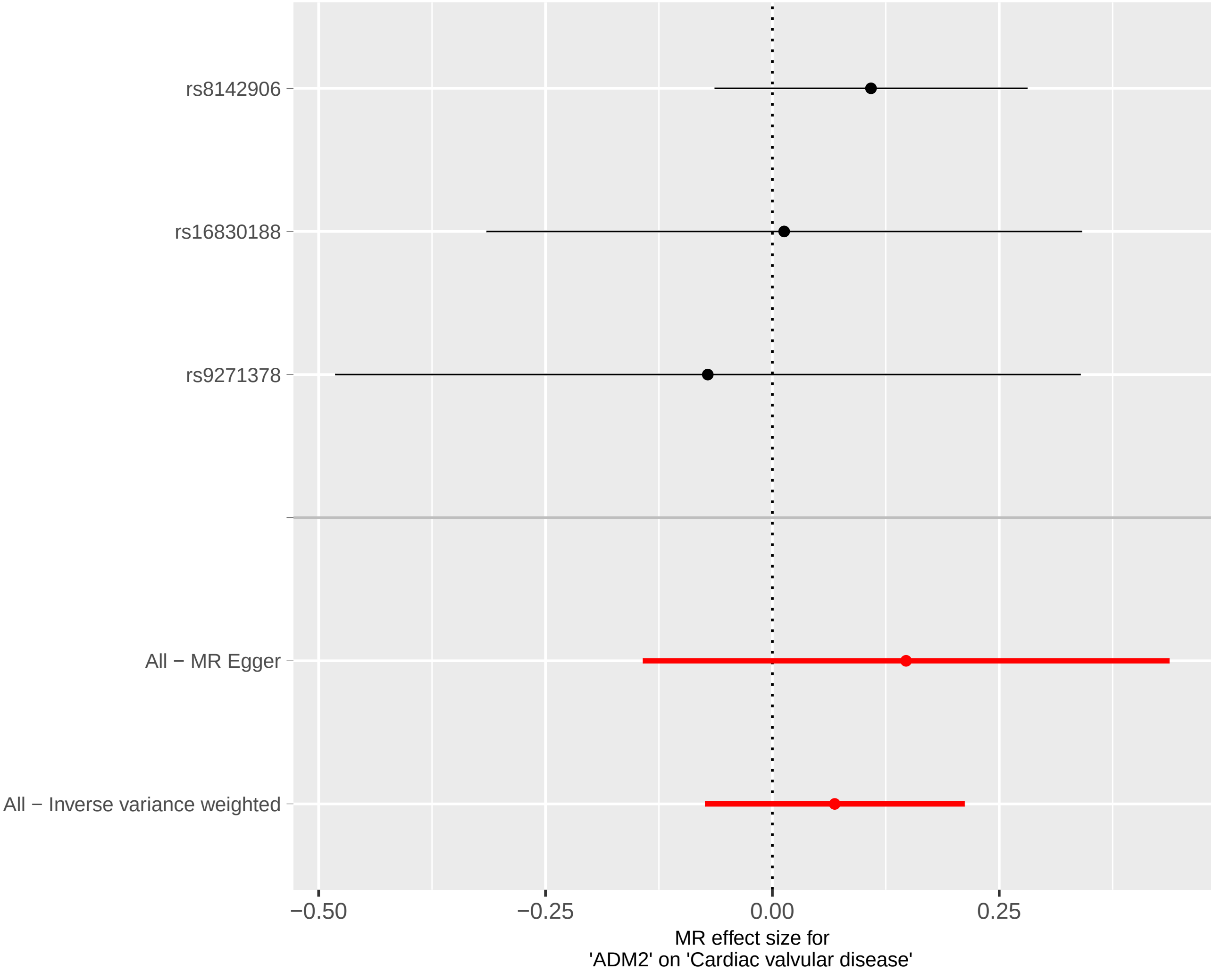
rs8142906
rs16830188
rs9271378
All − MR Egger
All − Inverse variance weighted
−0.50
−0.25
0.00
0.25
MR effect size for
'ADM2' on 'Cardiac valvular disease'

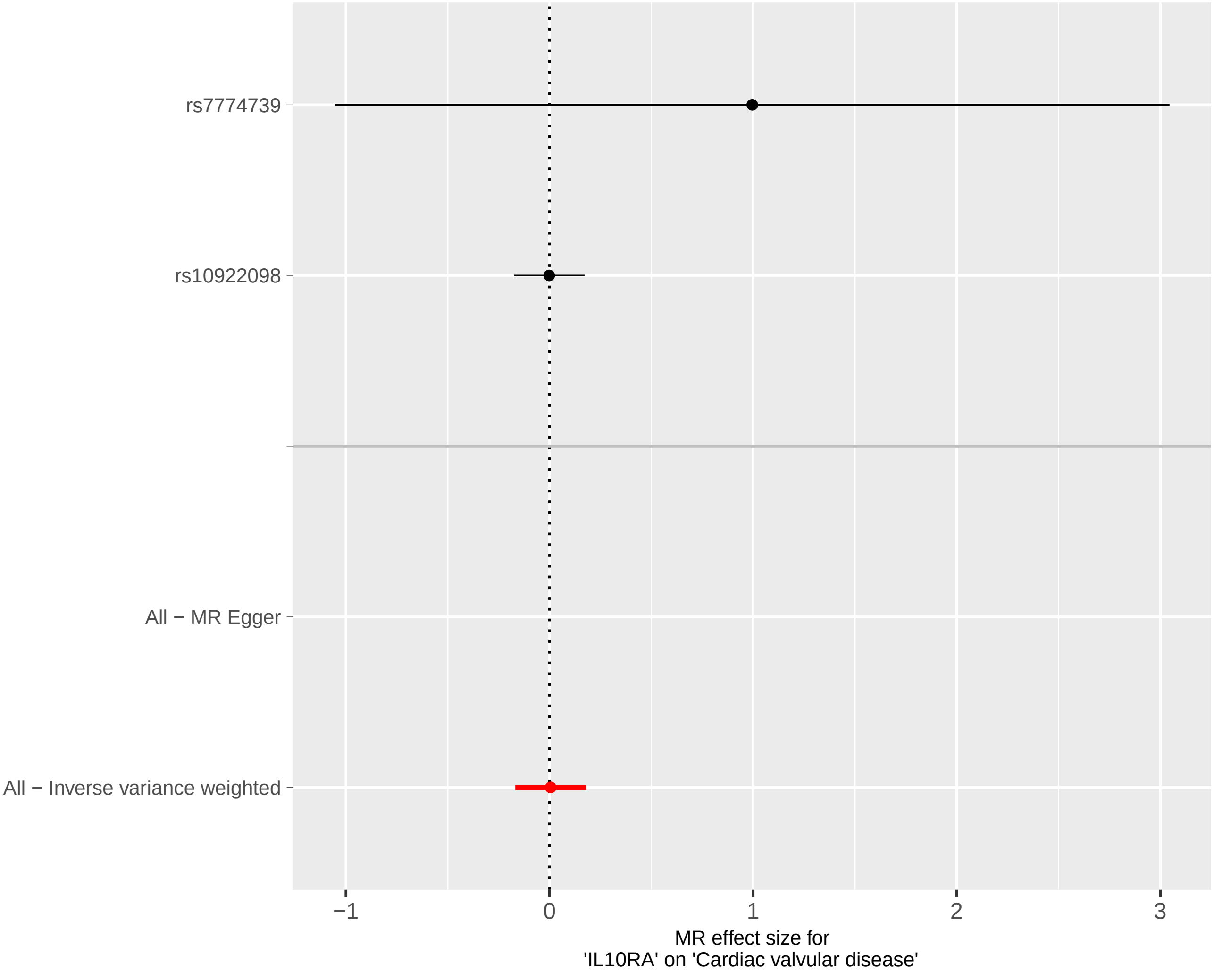
rs7774739
rs10922098
All − MR Egger
All − Inverse variance weighted
−1
0
1
2
3
MR effect size for
'IL10RA' on 'Cardiac valvular disease'

Insufficient number of SNPs

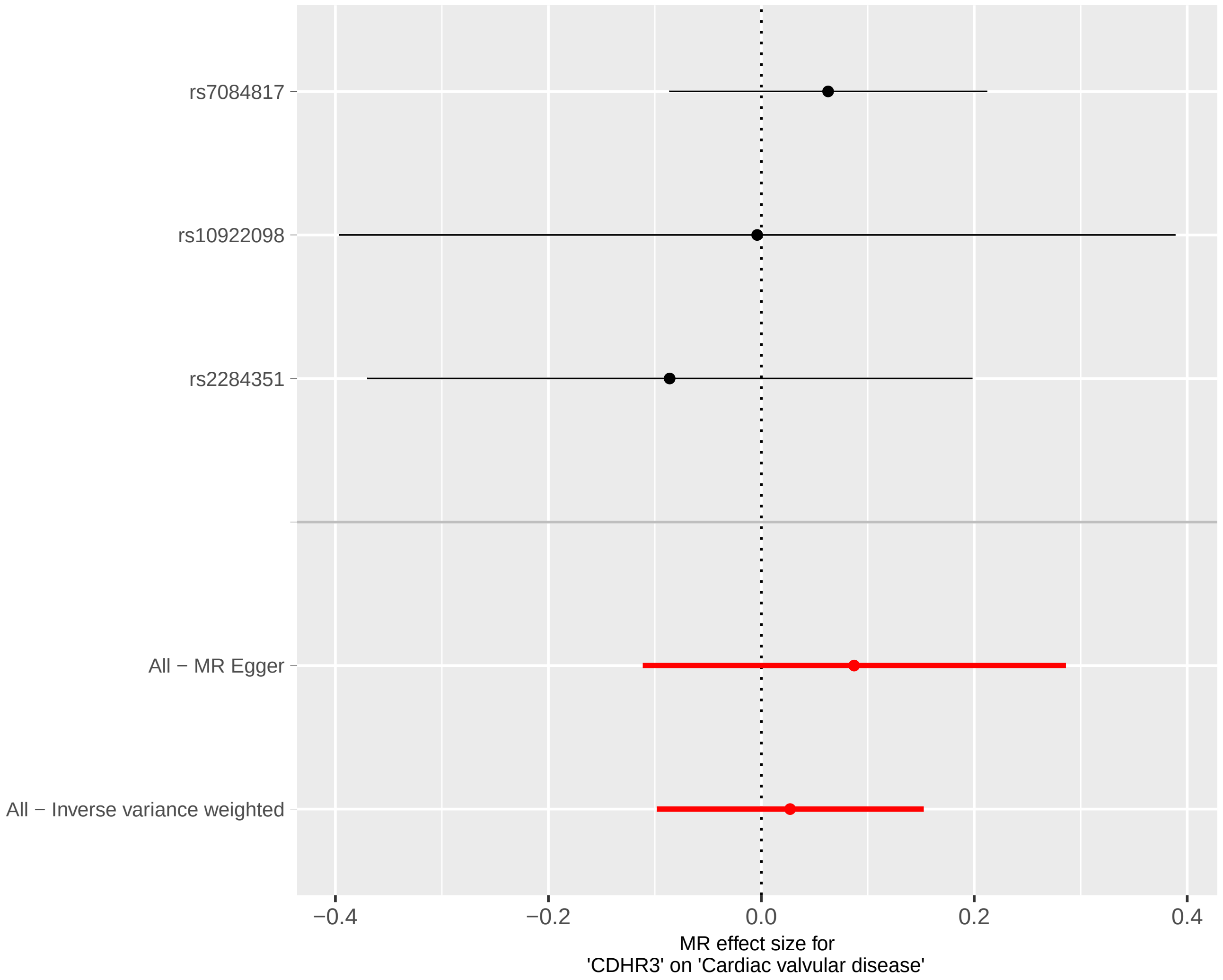

rs7084817
rs10922098
rs2284351
All − MR Egger
All − Inverse variance weighted
−0.4
−0.2
0.0
0.2
0.4
MR effect size for
'CDHR3' on 'Cardiac valvular disease'

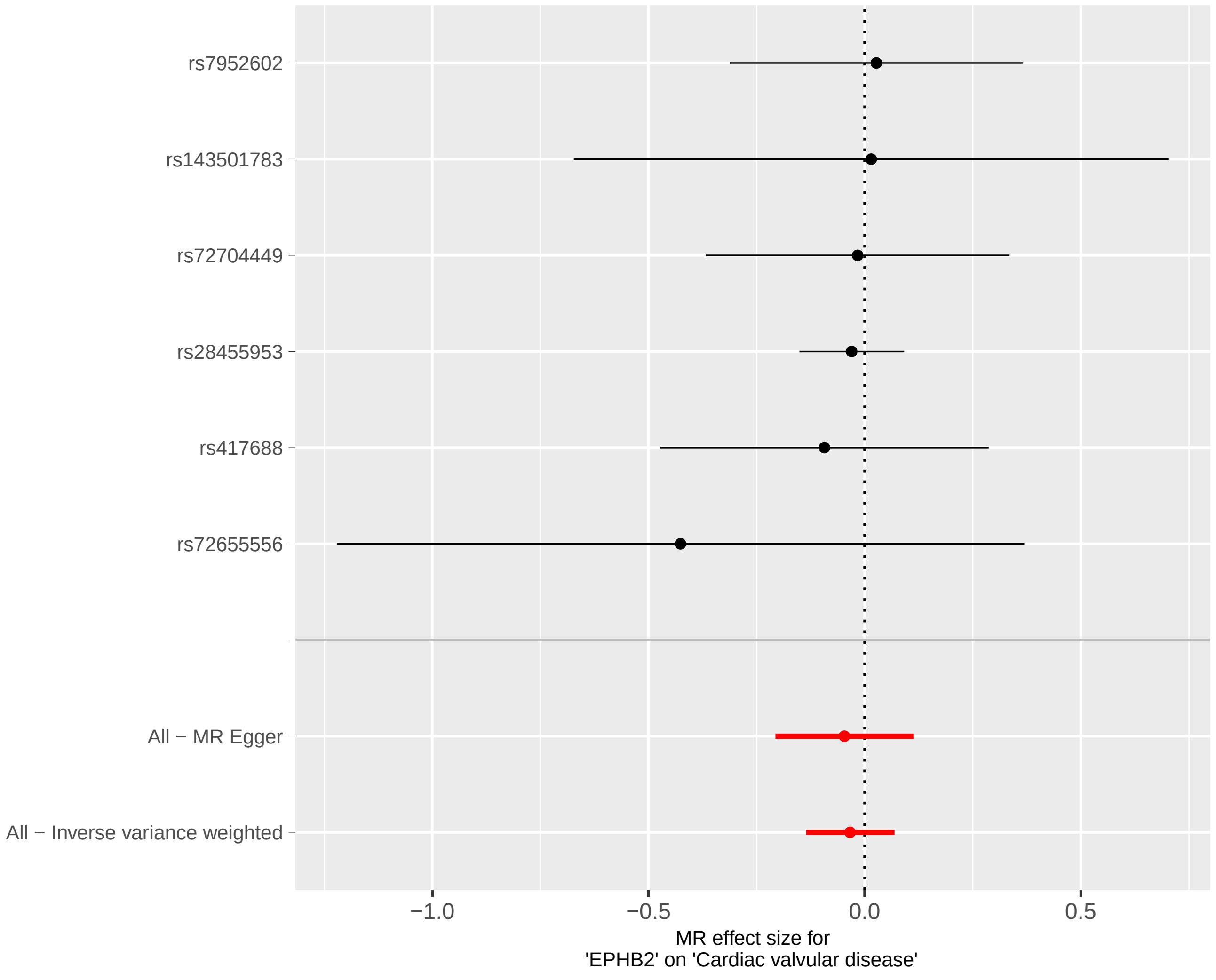
rs7952602
rs143501783
rs72704449
rs28455953
rs417688
rs72655556
All − MR Egger
All − Inverse variance weighted
−1.0
−0.5
0.0
0.5
MR effect size for
'EPHB2' on 'Cardiac valvular disease'

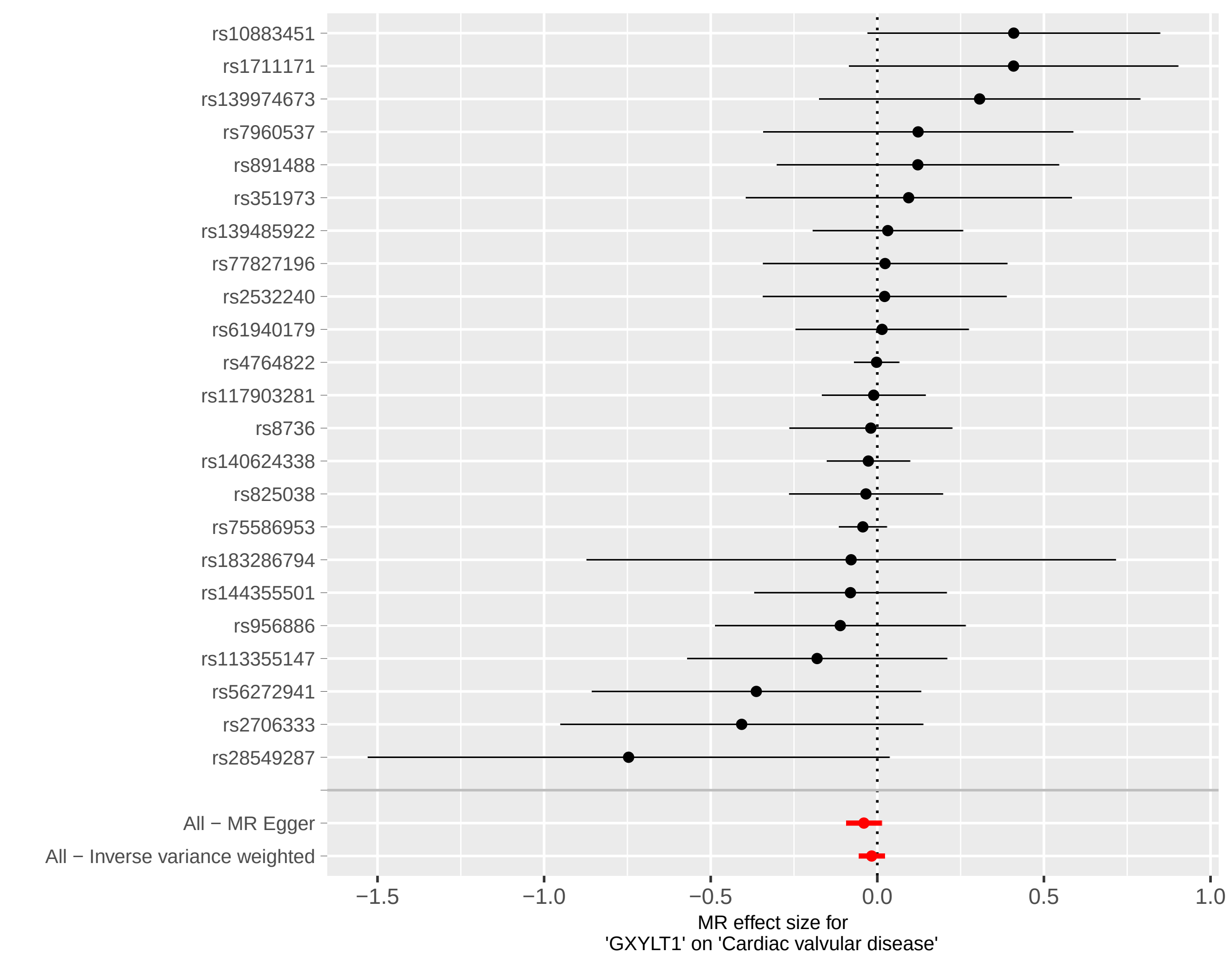
rs10883451
rs1711171
rs139974673
rs7960537
rs891488
rs351973
rs139485922
rs77827196
rs2532240
rs61940179
rs4764822
rs117903281
rs8736
rs140624338
rs825038
rs75586953
rs183286794
rs144355501
rs956886
rs113355147
rs56272941
rs2706333
rs28549287
All − MR Egger
All − Inverse variance weighted
−1.5
−1.0
−0.5
0.0
0.5
1.0
MR effect size for
'GXYLT1' on 'Cardiac valvular disease'

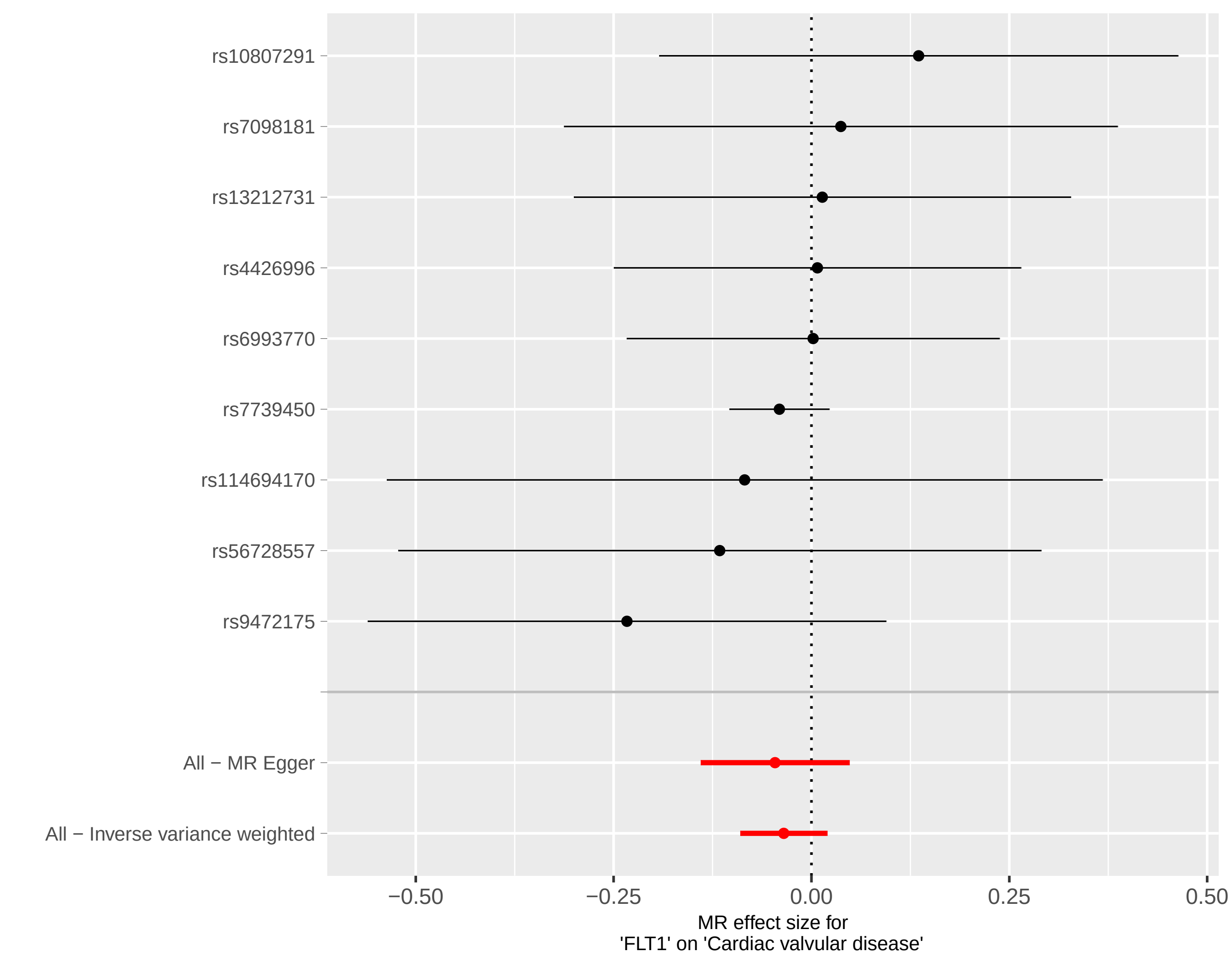
rs10807291
rs7098181
rs13212731
rs4426996
rs6993770
rs7739450
rs114694170
rs56728557
rs9472175
All − MR Egger
All − Inverse variance weighted
−0.50
−0.25
0.00
0.25
0.50
MR effect size for
'FLT1' on 'Cardiac valvular disease'

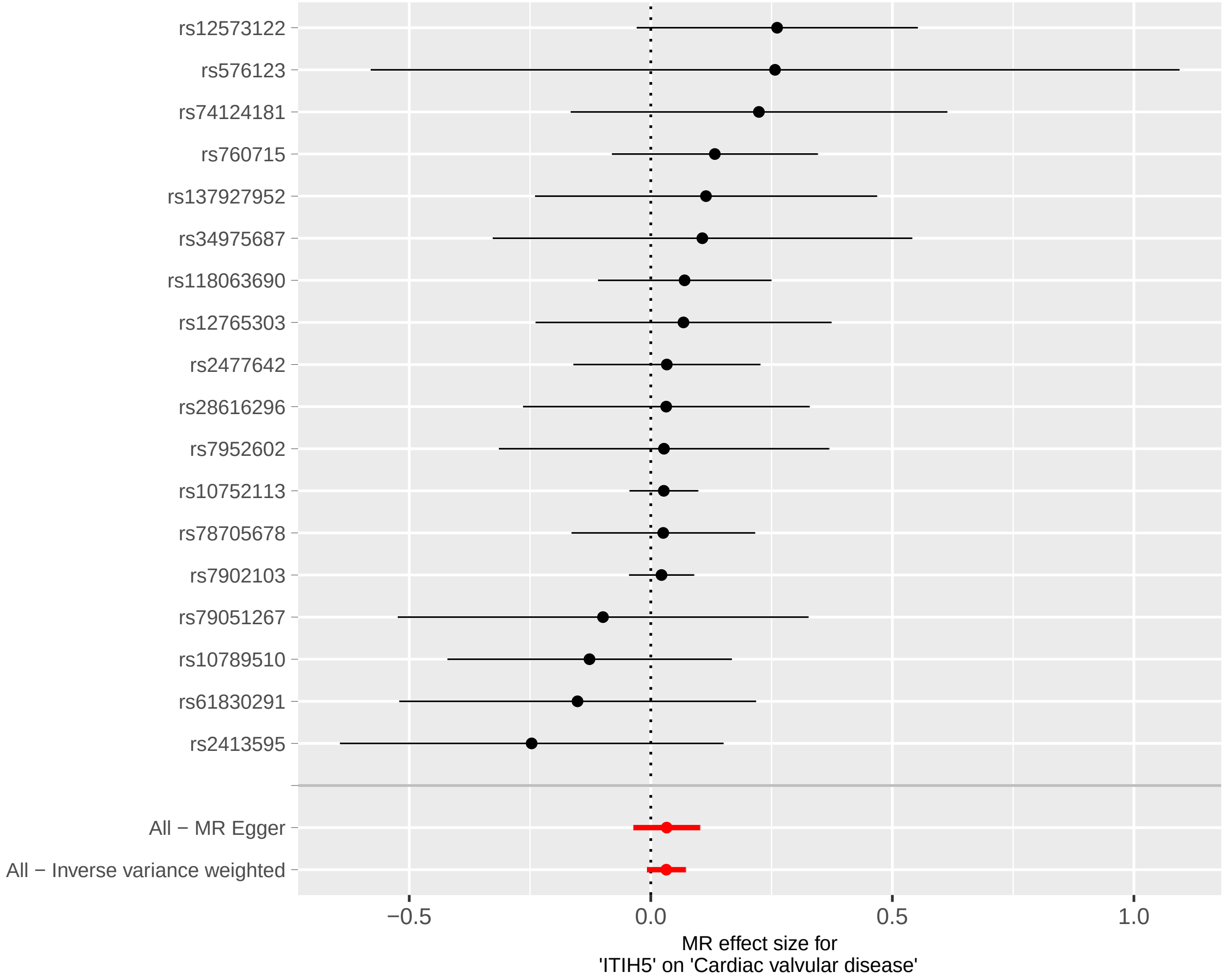

rs12573122
rs576123
rs74124181
rs760715
rs137927952
rs34975687
rs118063690
rs12765303
rs2477642
rs28616296
rs7952602
rs10752113
rs78705678
rs7902103
rs79051267
rs10789510
rs61830291
rs2413595
All − MR Egger
All − Inverse variance weighted
−0.5
0.0
0.5
1.0
MR effect size for
'ITIH5' on 'Cardiac valvular disease'

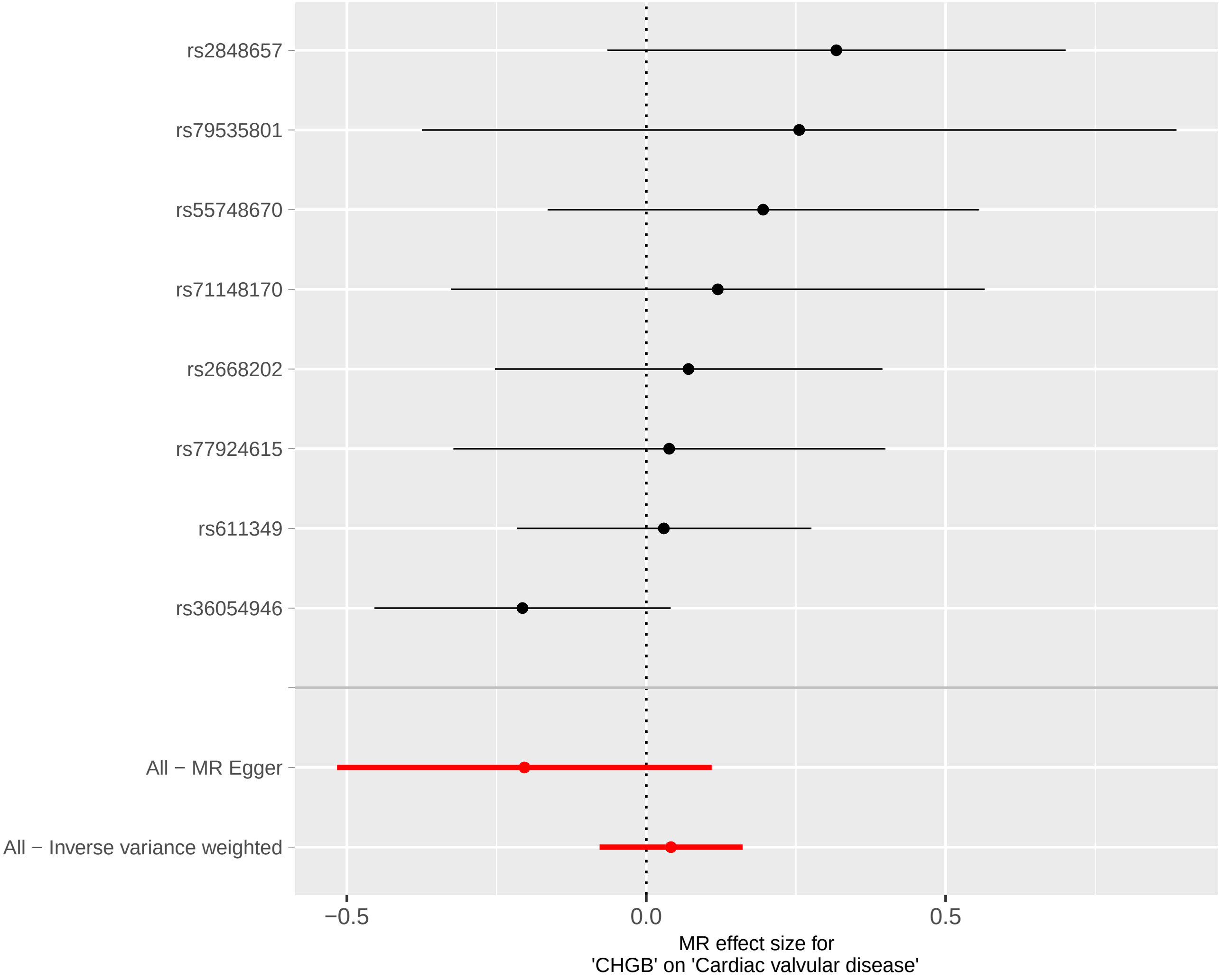
rs2848657
rs79535801
rs55748670
rs71148170
rs2668202
rs77924615
rs611349
rs36054946
All − MR Egger
All − Inverse variance weighted
−0.5
0.0
0.5
MR effect size for
'CHGB' on 'Cardiac valvular disease'

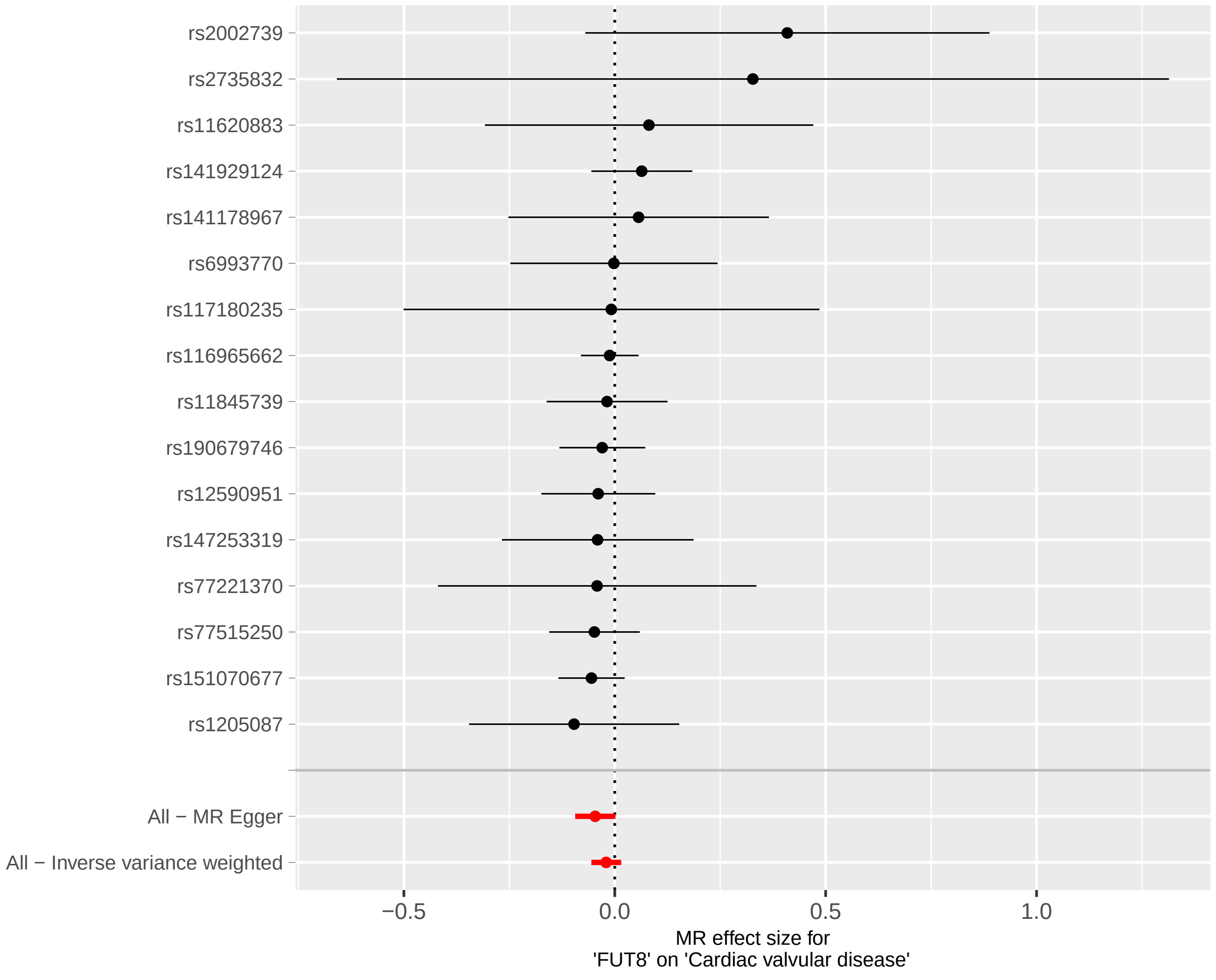

rs2002739
rs2735832
rs11620883
rs141929124
rs141178967
rs6993770
rs117180235
rs116965662
rs11845739
rs190679746
rs12590951
rs147253319
rs77221370
rs77515250
rs151070677
rs1205087
All − MR Egger
All − Inverse variance weighted
−0.5
0.0
0.5
1.0
MR effect size for
'FUT8' on 'Cardiac valvular disease'

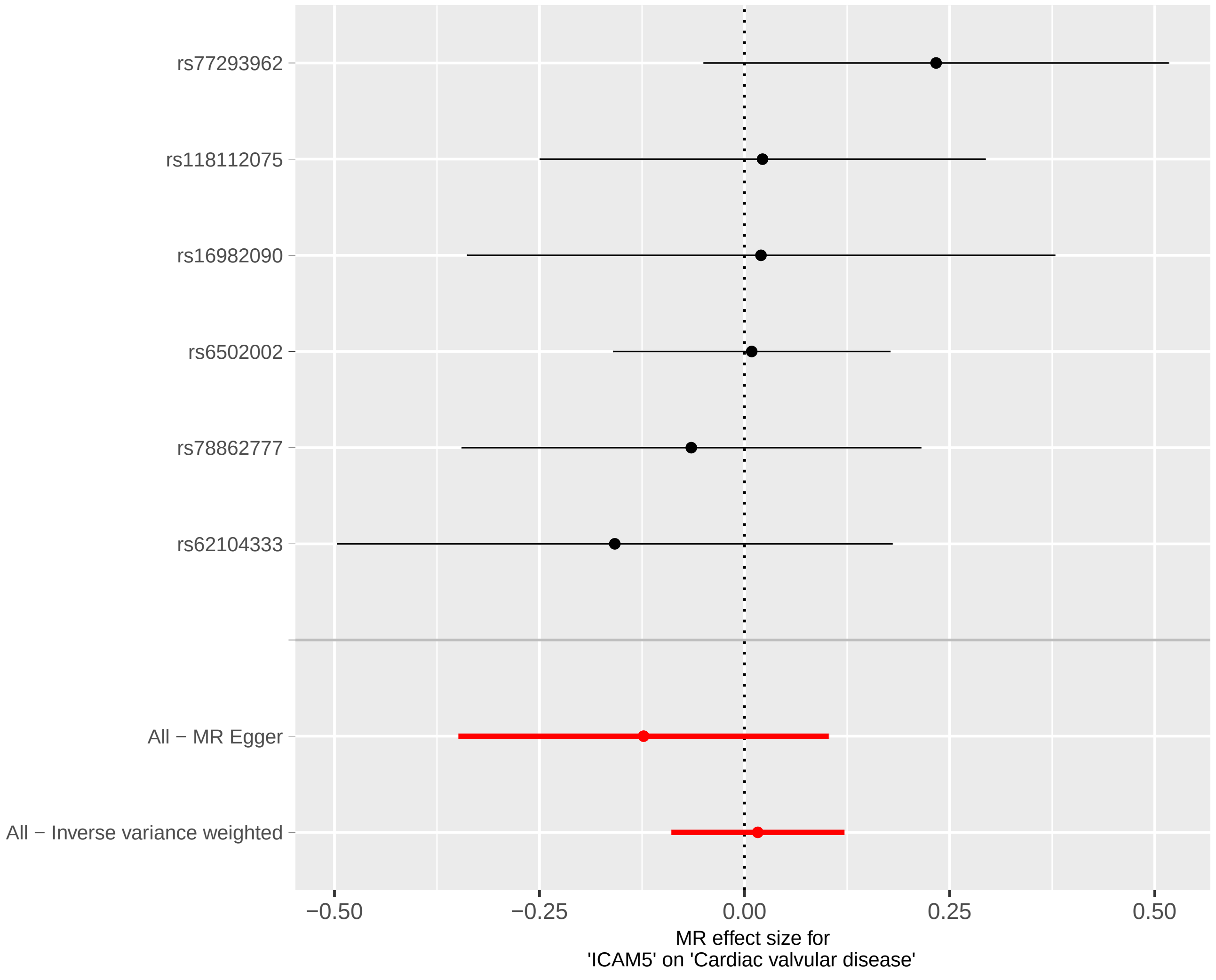
rs77293962
rs118112075
rs16982090
rs6502002
rs78862777
rs62104333
All − MR Egger
All − Inverse variance weighted
−0.50
−0.25
0.00
0.25
0.50
MR effect size for
'ICAM5' on 'Cardiac valvular disease'

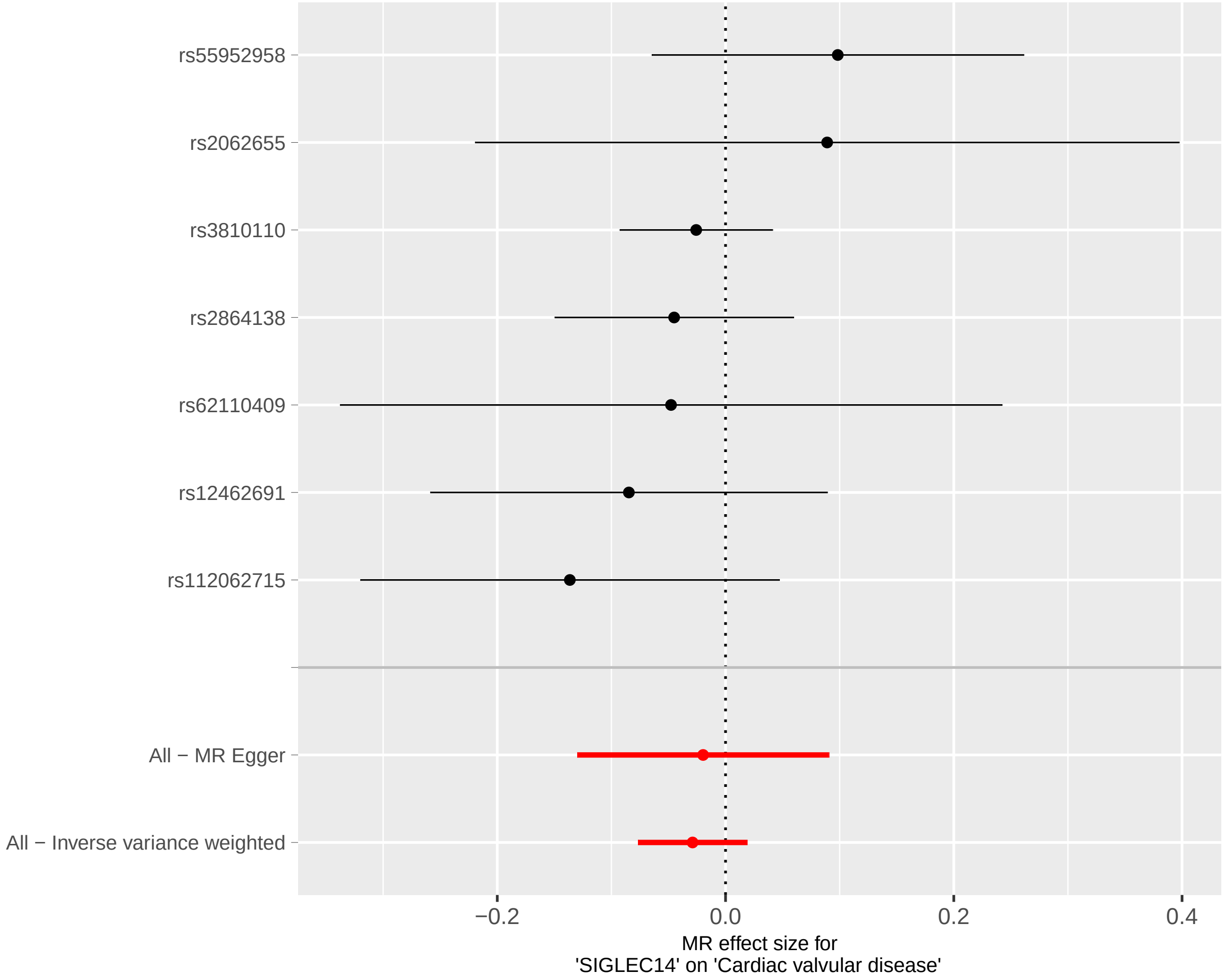

rs55952958
rs2062655
rs3810110
rs2864138
rs62110409
rs12462691
rs112062715
All − MR Egger
All − Inverse variance weighted
−0.2
0.0
0.2
0.4
MR effect size for
'SIGLEC14' on 'Cardiac valvular disease'

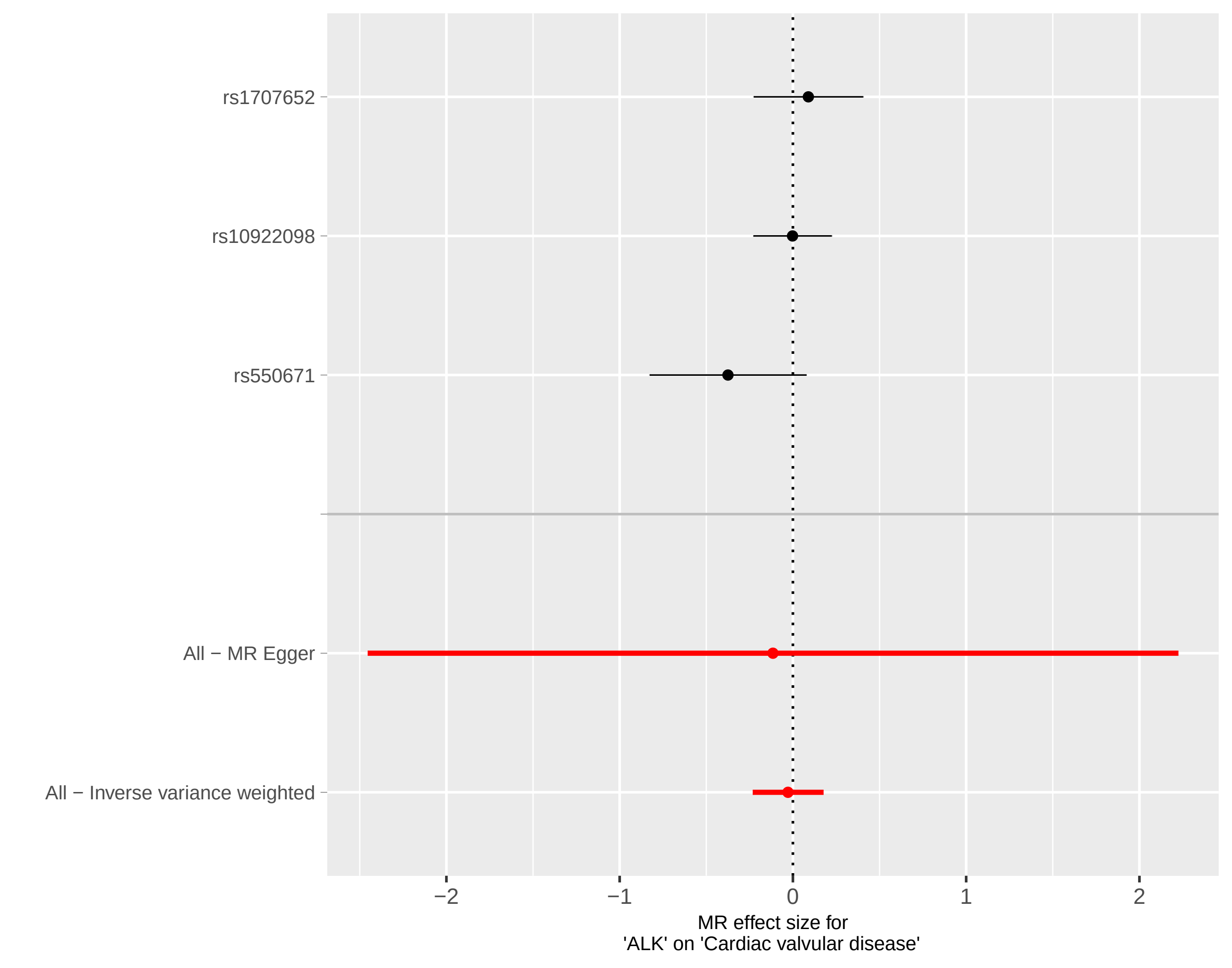
rs1707652
rs10922098
rs550671
All − MR Egger
All − Inverse variance weighted
−2
−1
0
1
2
MR effect size for
'ALK' on 'Cardiac valvular disease'

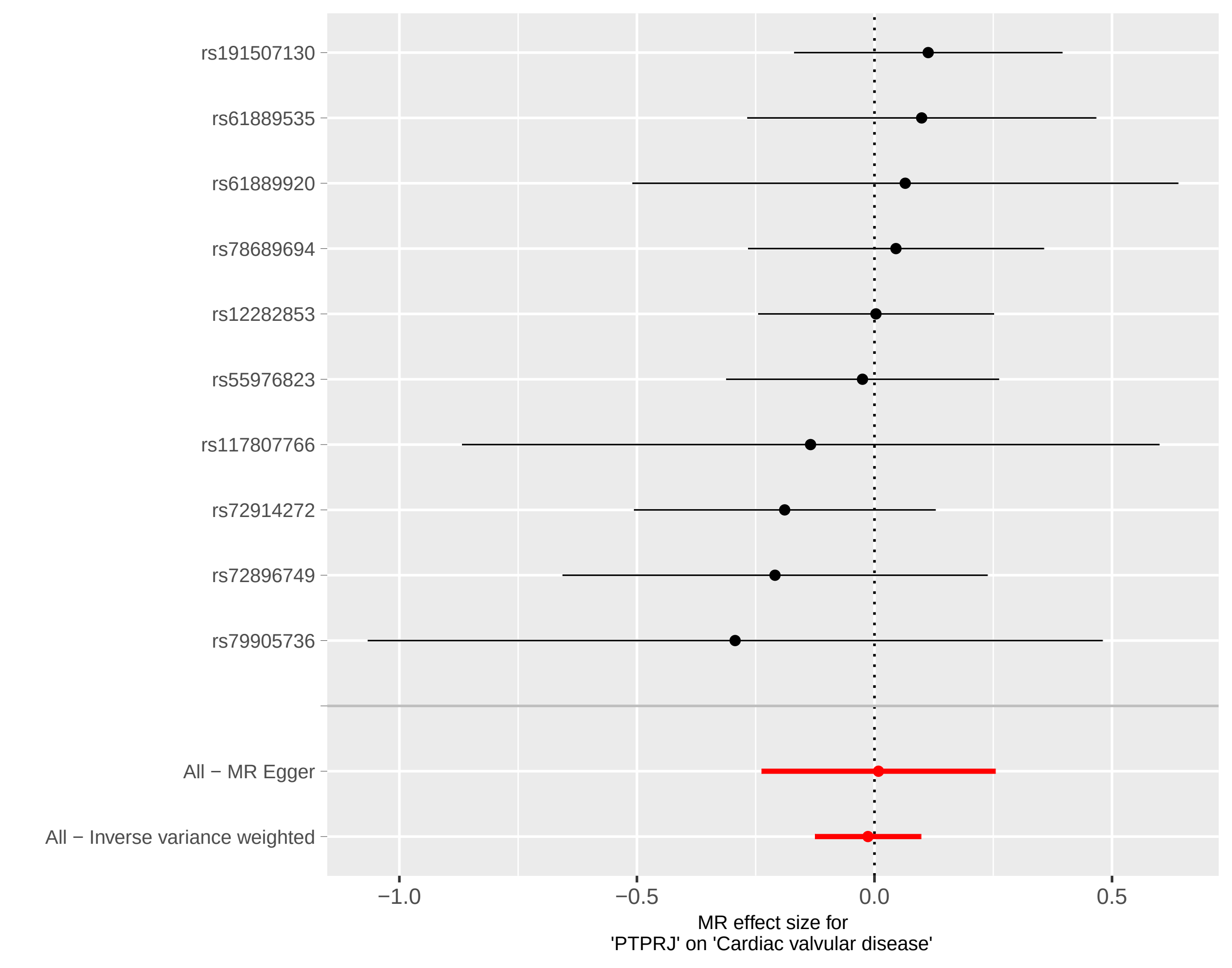
rs191507130
rs61889535
rs61889920
rs78689694
rs12282853
rs55976823
rs117807766
rs72914272
rs72896749
rs79905736
All − MR Egger
All − Inverse variance weighted
−1.0
−0.5
0.0
0.5
MR effect size for
'PTPRJ' on 'Cardiac valvular disease'

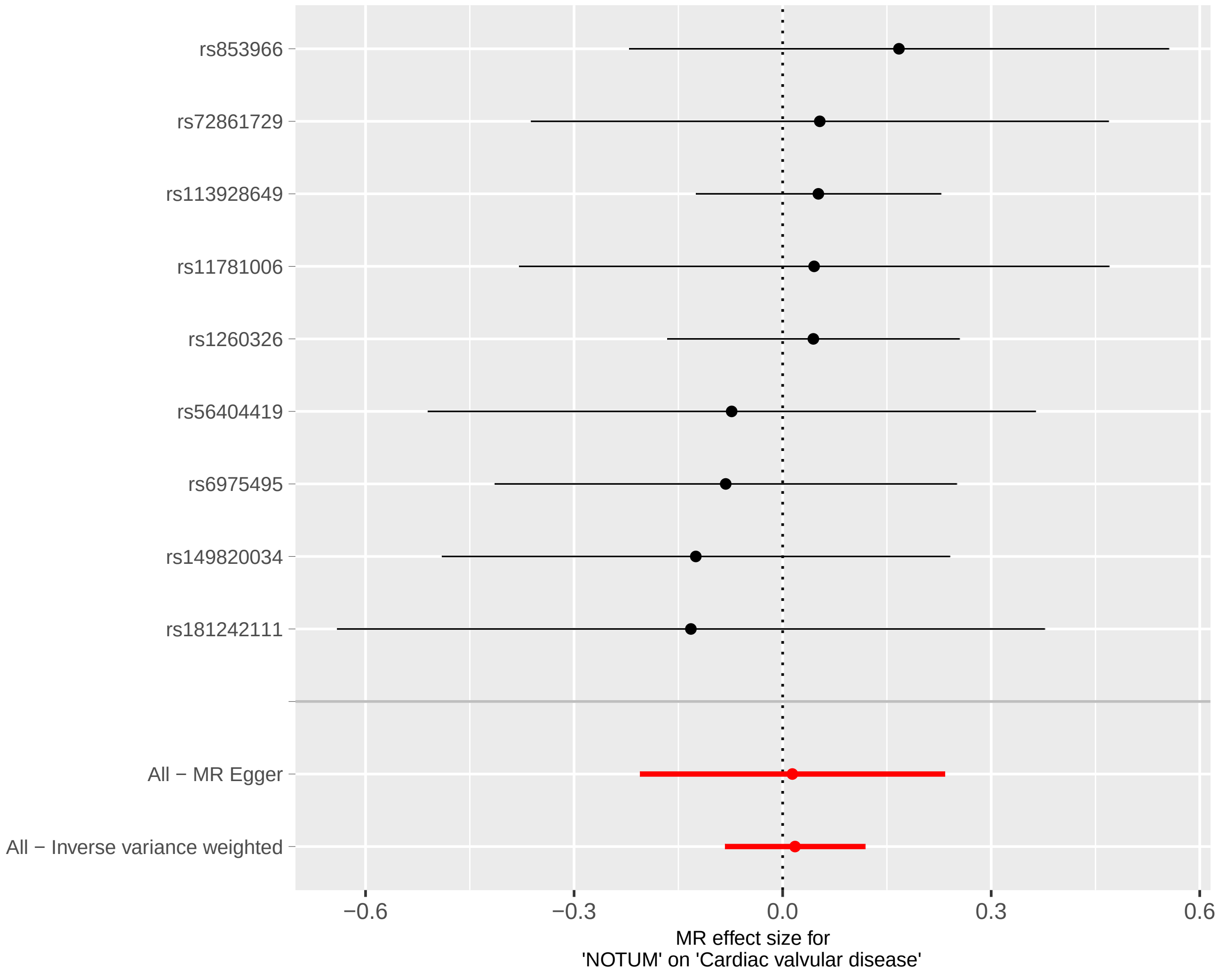

rs853966
rs72861729
rs113928649
rs11781006
rs1260326
rs56404419
rs6975495
rs149820034
rs181242111
All − MR Egger
All − Inverse variance weighted
−0.6
−0.3
0.0
0.3
0.6
MR effect size for
'NOTUM' on 'Cardiac valvular disease'

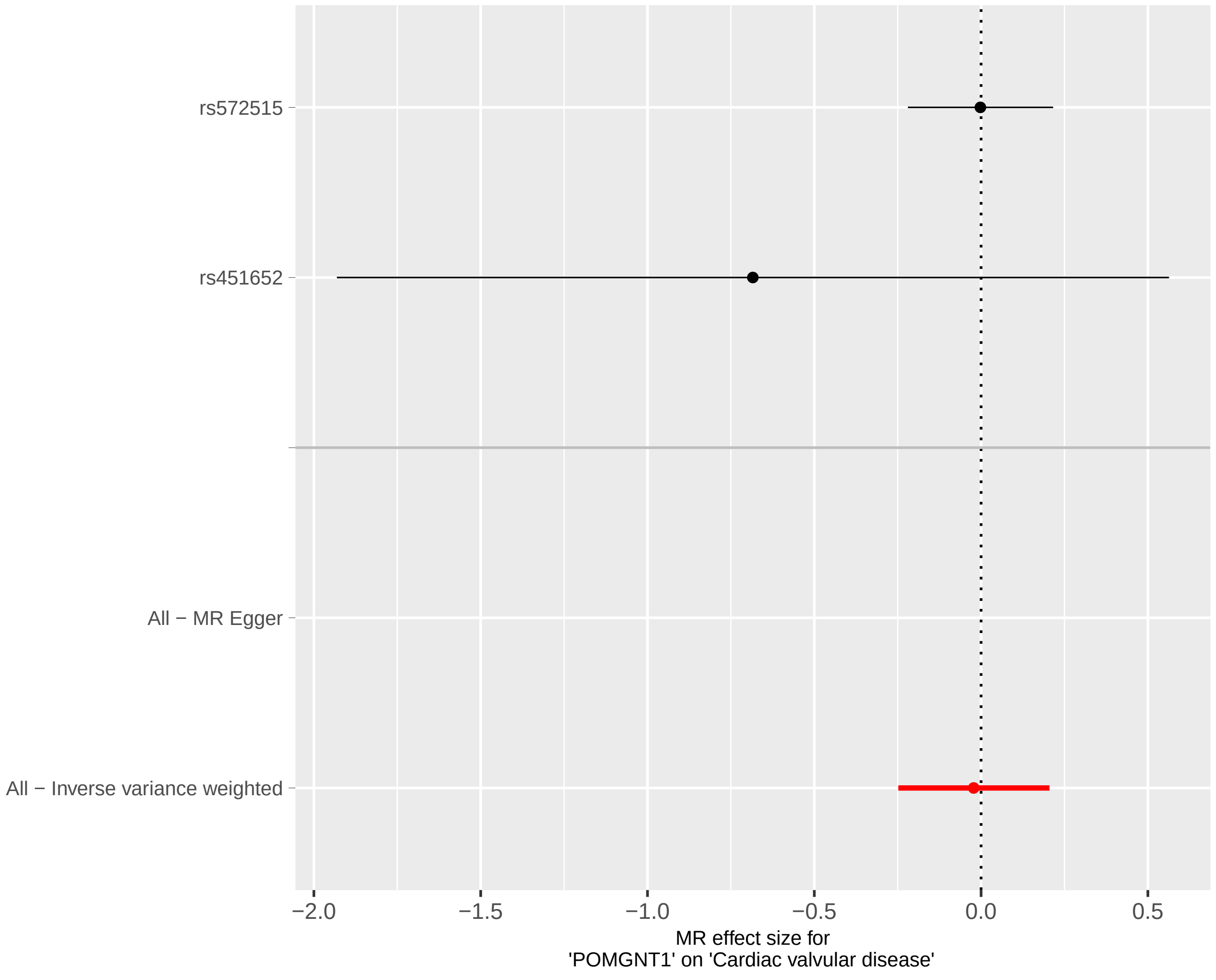

rs572515
rs451652
All − MR Egger
All − Inverse variance weighted
−2.0
−1.5
−1.0
−0.5
0.0
0.5
MR effect size for
'POMGNT1' on 'Cardiac valvular disease'

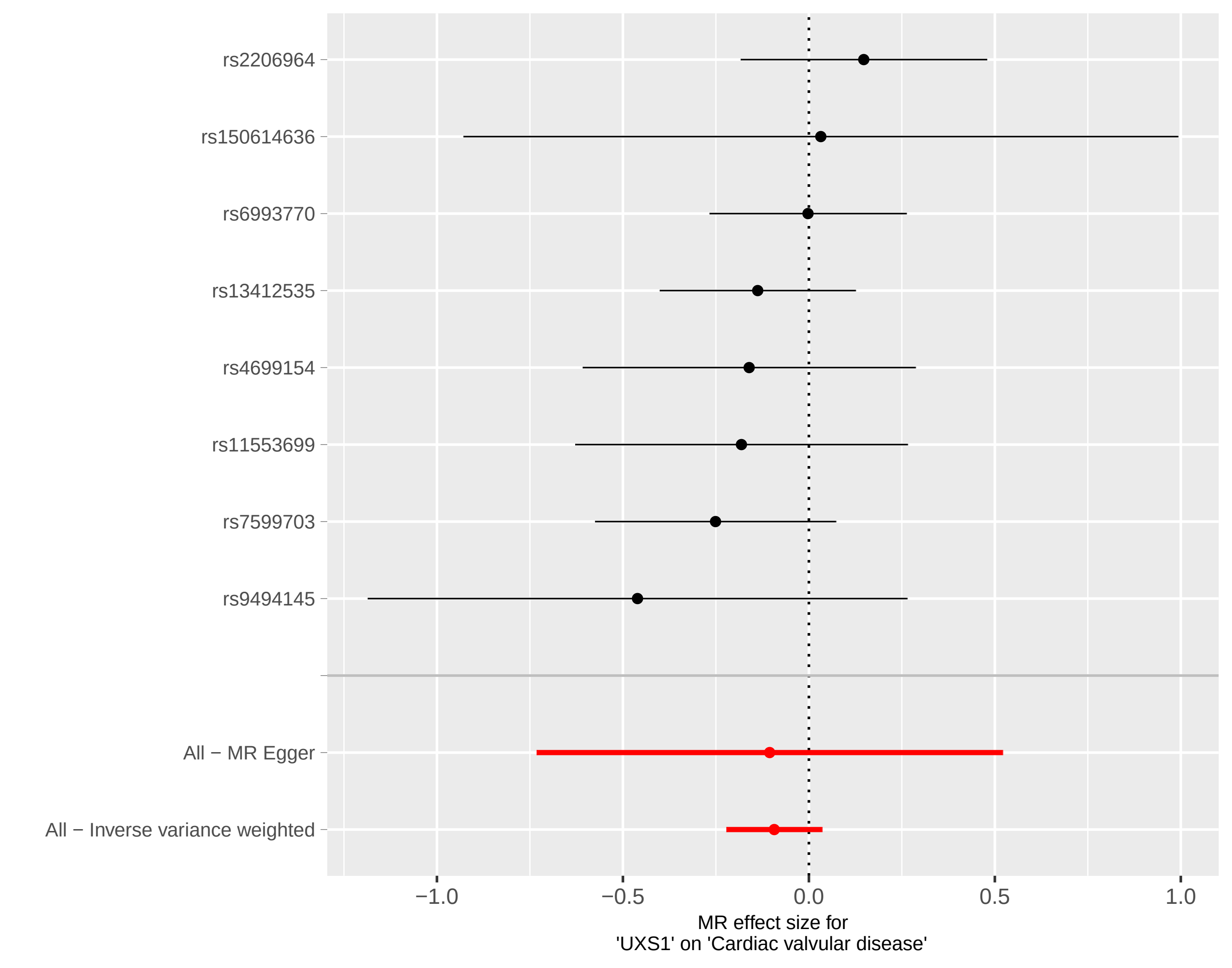
rs2206964
rs150614636
rs6993770
rs13412535
rs4699154
rs11553699
rs7599703
rs9494145
All − MR Egger
All − Inverse variance weighted
−1.0
−0.5
0.0
0.5
1.0
MR effect size for
'UXS1' on 'Cardiac valvular disease'

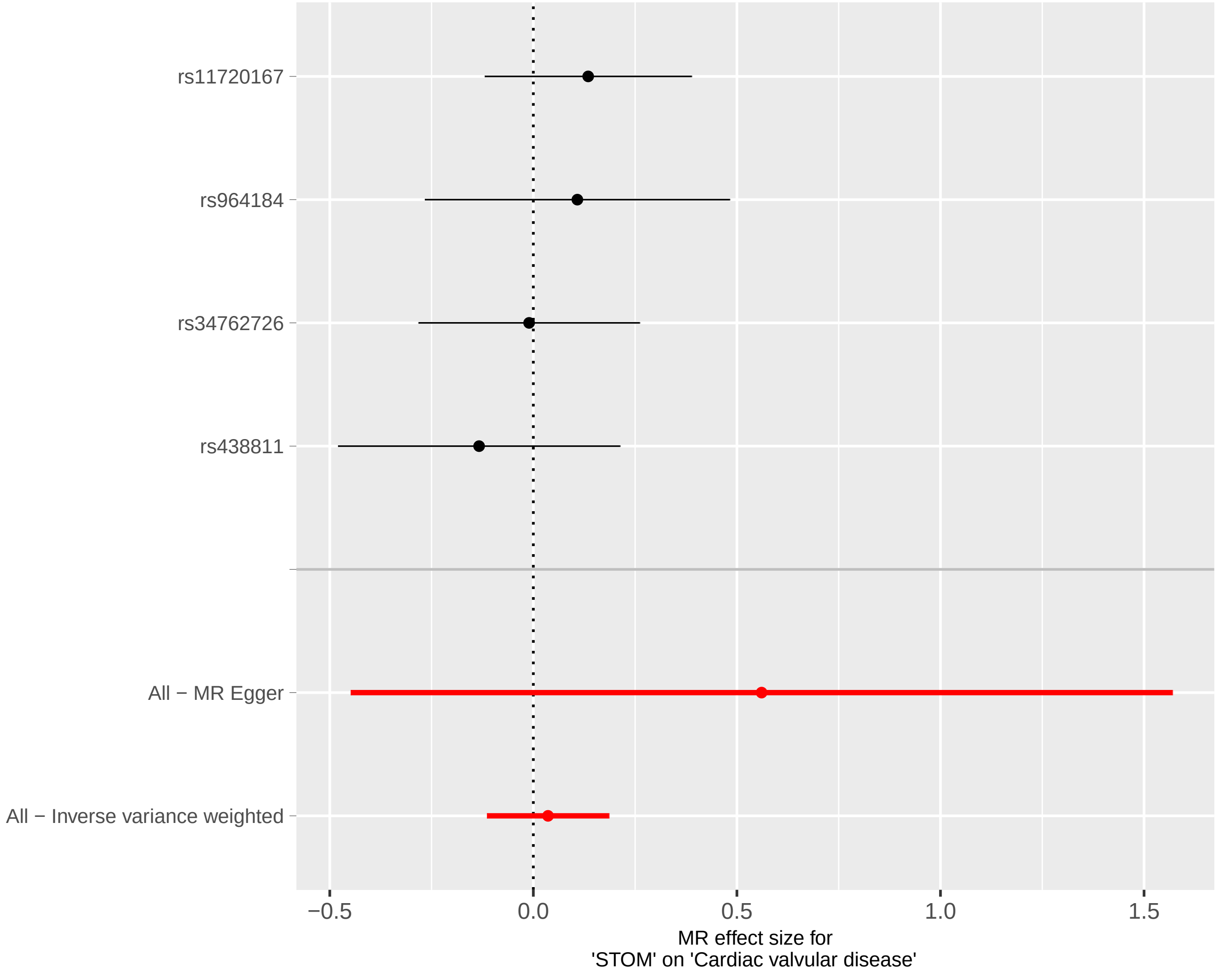
rs11720167
rs964184
rs34762726
rs438811
All − MR Egger
All − Inverse variance weighted
−0.5
0.0
0.5
1.0
1.5
MR effect size for
'STOM' on 'Cardiac valvular disease'

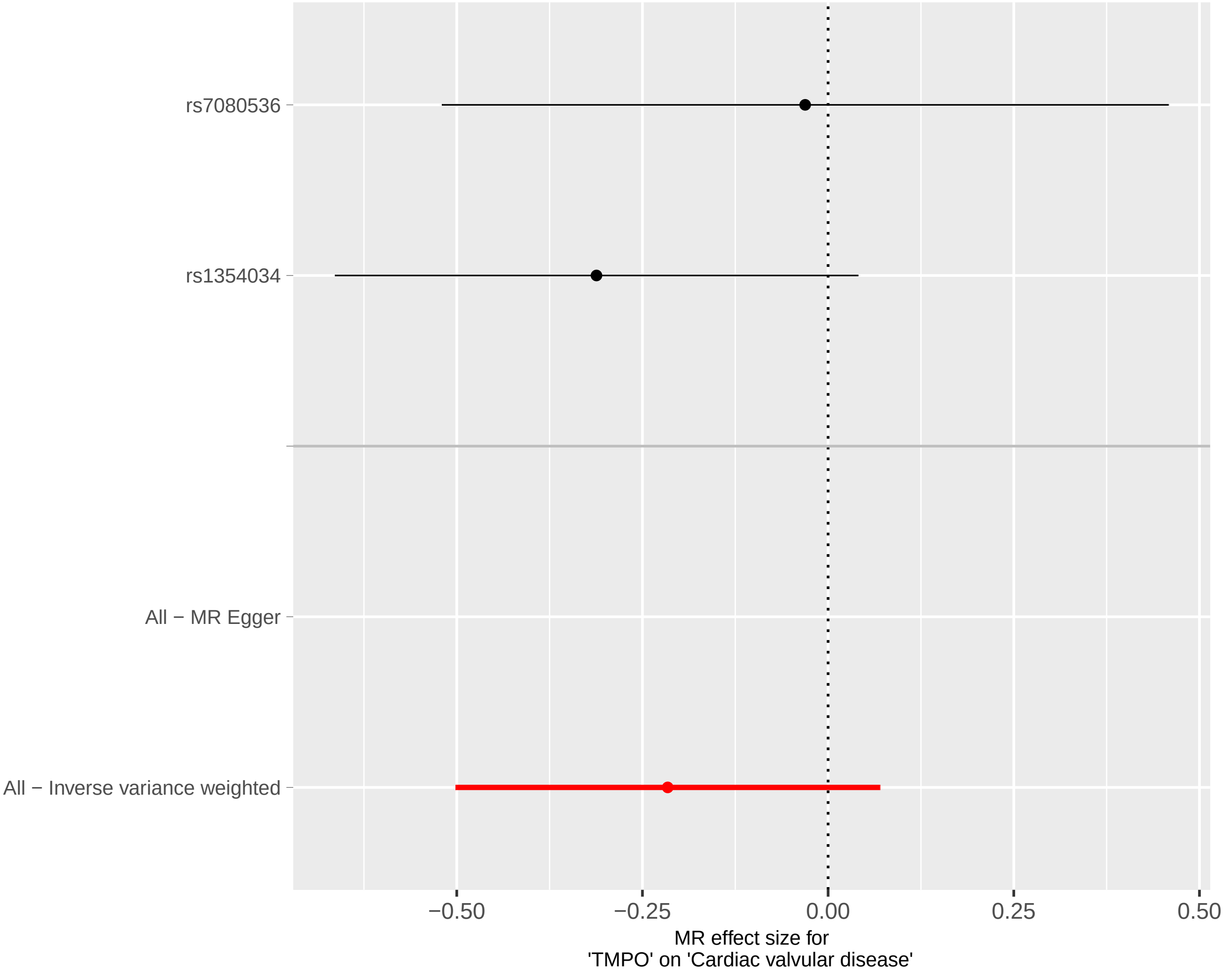
rs7080536
rs1354034
All − MR Egger
All − Inverse variance weighted
−0.50
−0.25
0.00
0.25
0.50
MR effect size for
'TMPO' on 'Cardiac valvular disease'

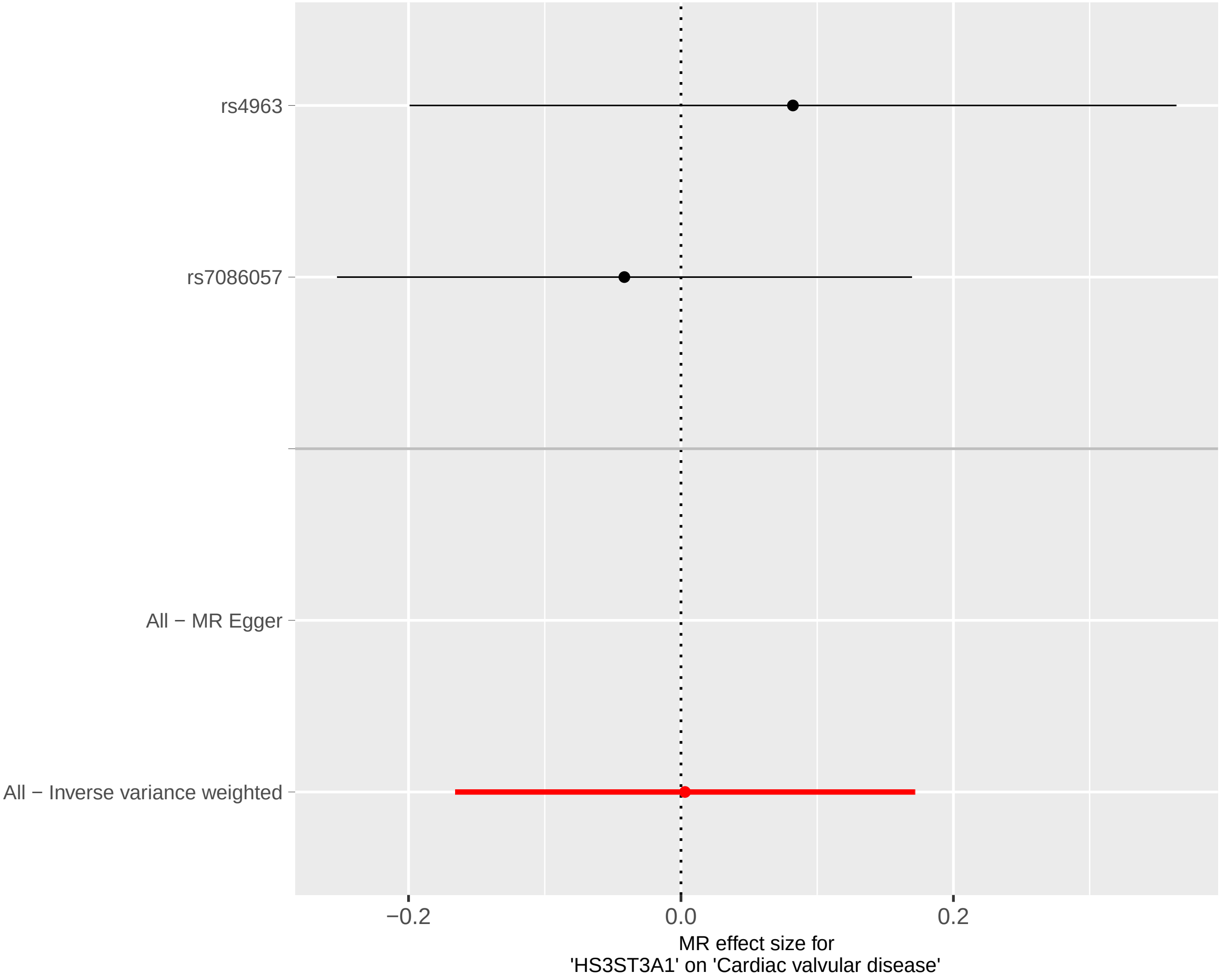
rs4963
rs7086057
All − MR Egger
All − Inverse variance weighted
−0.2
0.0
0.2
MR effect size for
'HS3ST3A1' on 'Cardiac valvular disease'

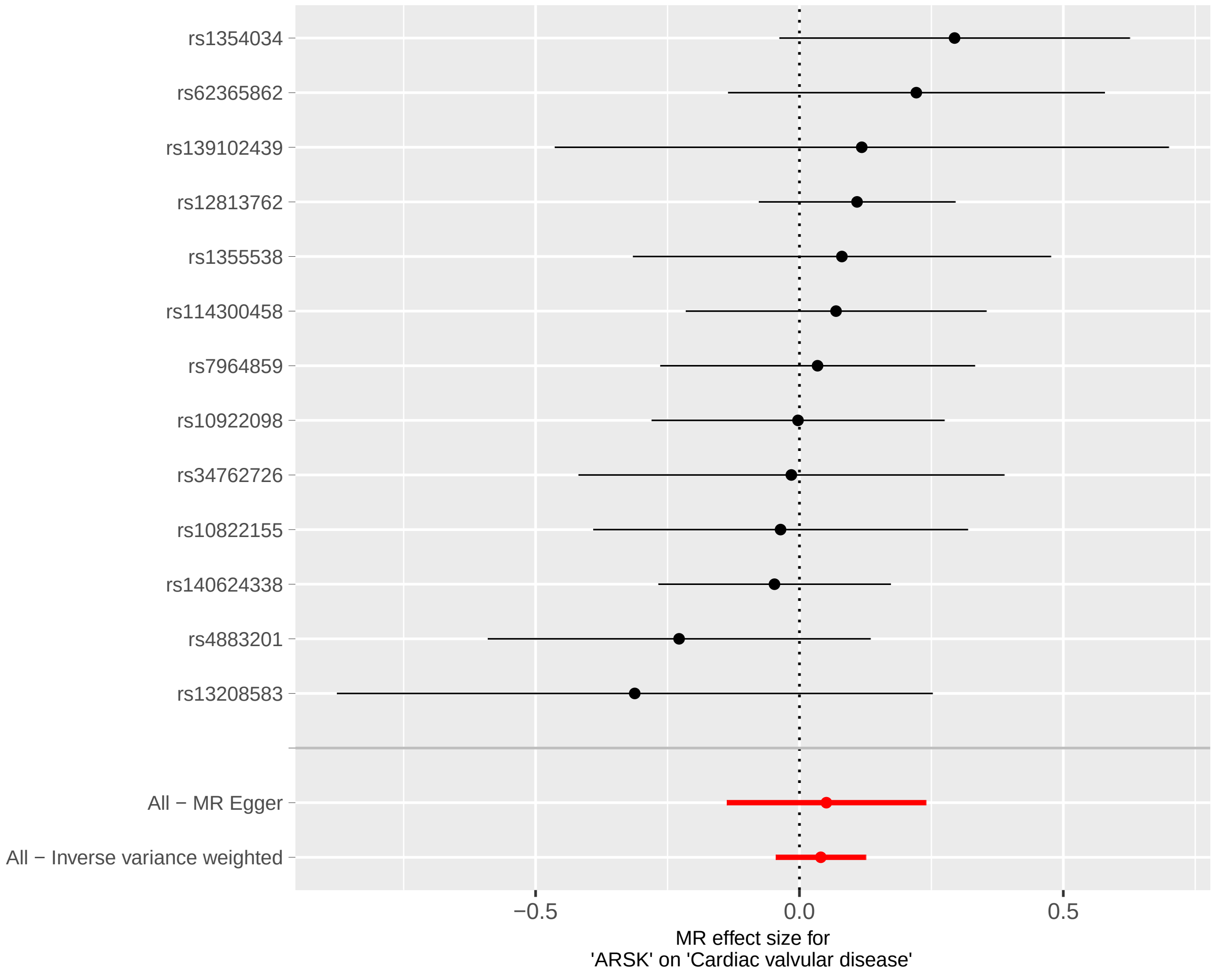

rs1354034
rs62365862
rs139102439
rs12813762
rs1355538
rs114300458
rs7964859
rs10922098
rs34762726
rs10822155
rs140624338
rs4883201
rs13208583
All − MR Egger
All − Inverse variance weighted
−0.5
0.0
0.5
MR effect size for
'ARSK' on 'Cardiac valvular disease'

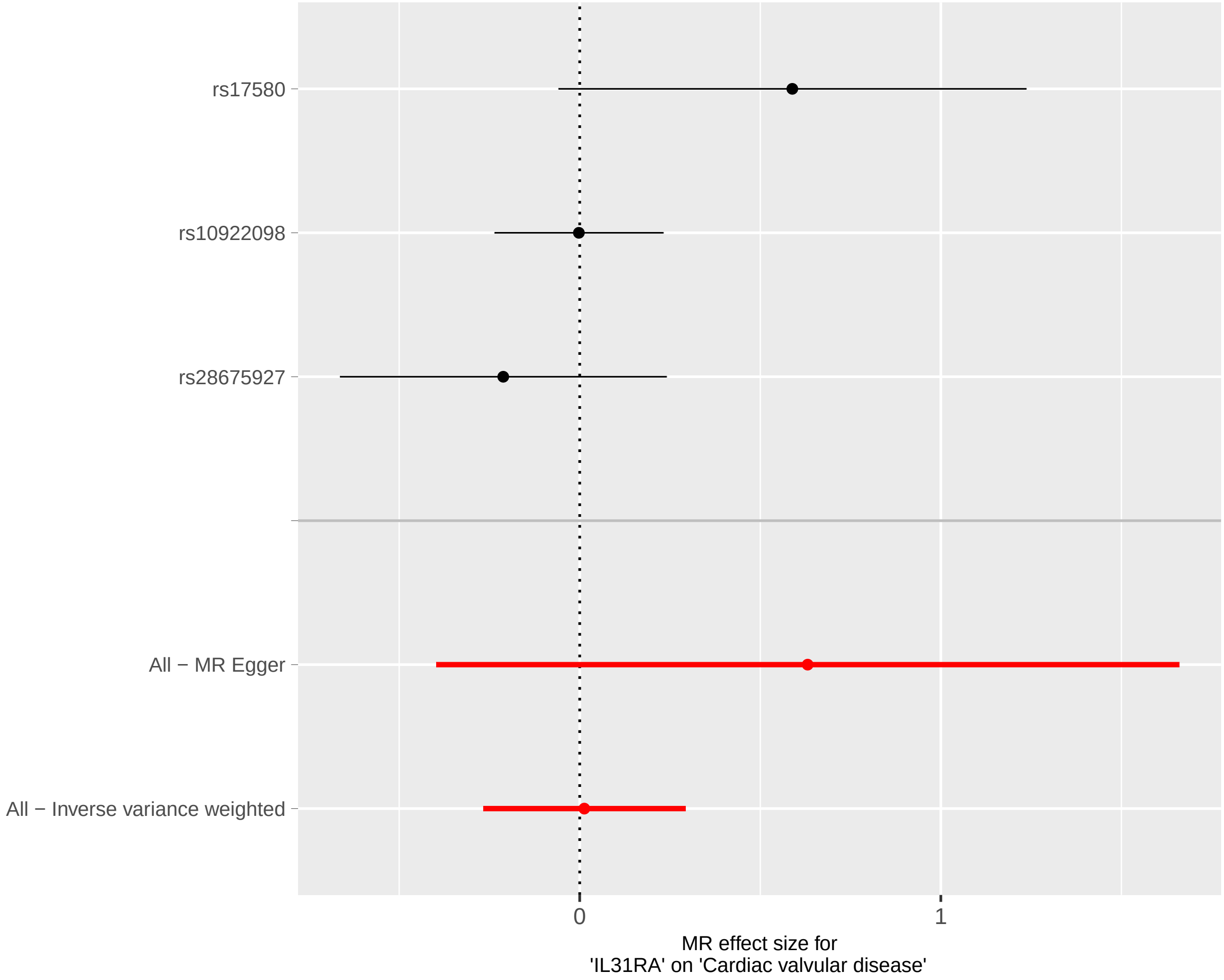
rs17580
rs10922098
rs28675927
All − MR Egger
All − Inverse variance weighted
0
1
MR effect size for
'IL31RA' on 'Cardiac valvular disease'

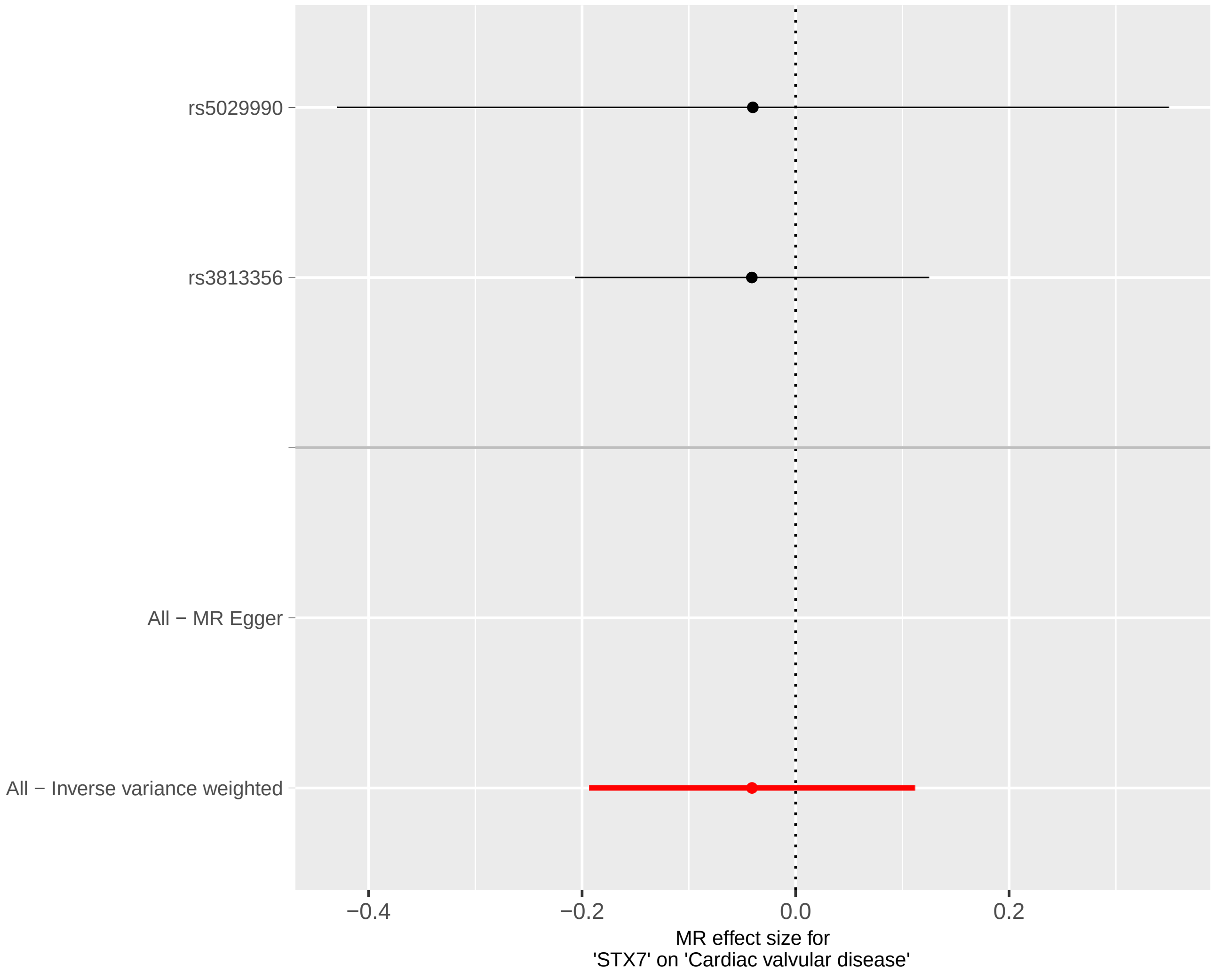
rs5029990
rs3813356
All − MR Egger
All − Inverse variance weighted
−0.4
−0.2
0.0
0.2
MR effect size for
'STX7' on 'Cardiac valvular disease'

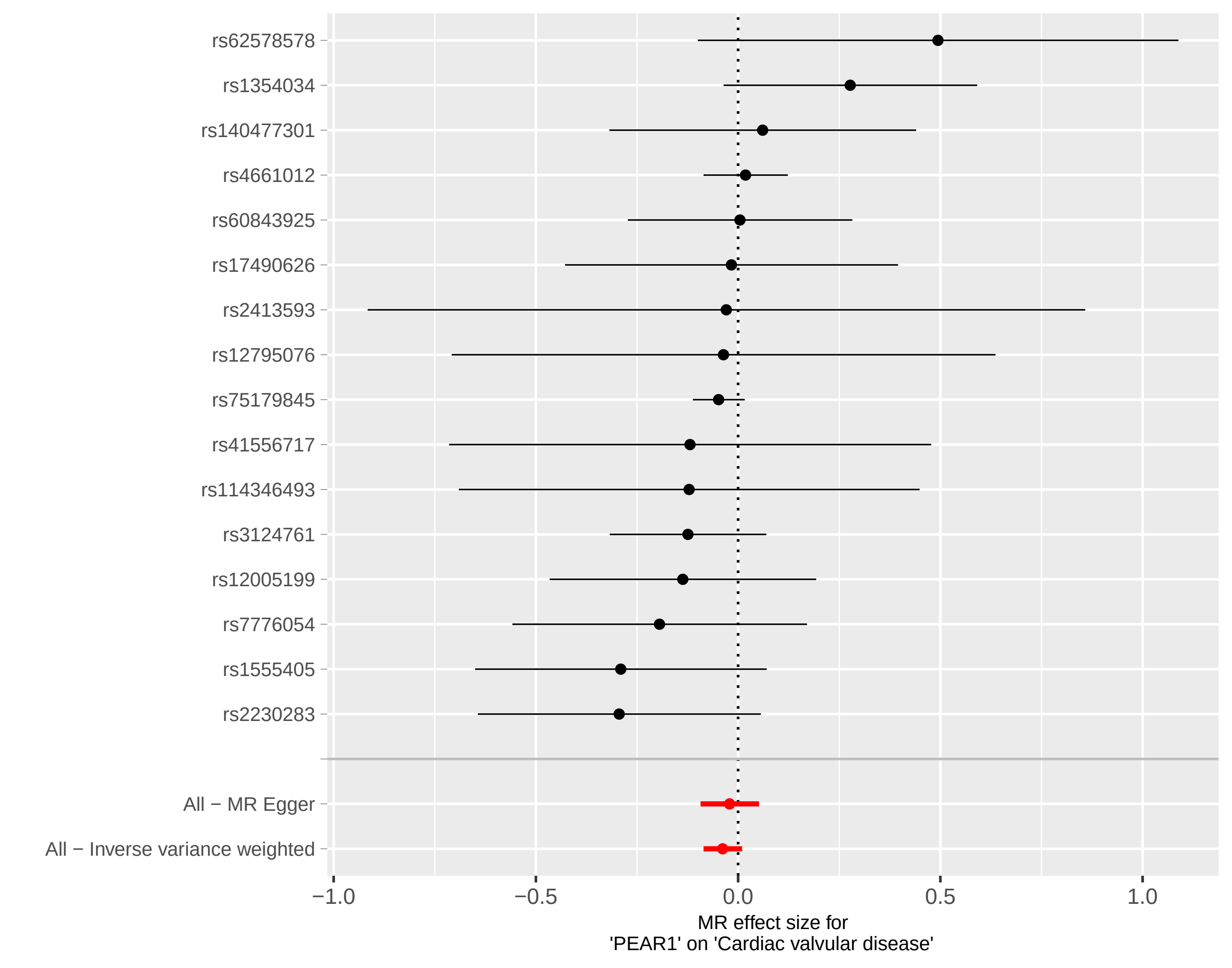
rs62578578
rs1354034
rs140477301
rs4661012
rs60843925
rs17490626
rs2413593
rs12795076
rs75179845
rs41556717
rs114346493
rs3124761
rs12005199
rs7776054
rs1555405
rs2230283
All − MR Egger
All − Inverse variance weighted
−1.0
−0.5
0.0
0.5
1.0
MR effect size for
'PEAR1' on 'Cardiac valvular disease'

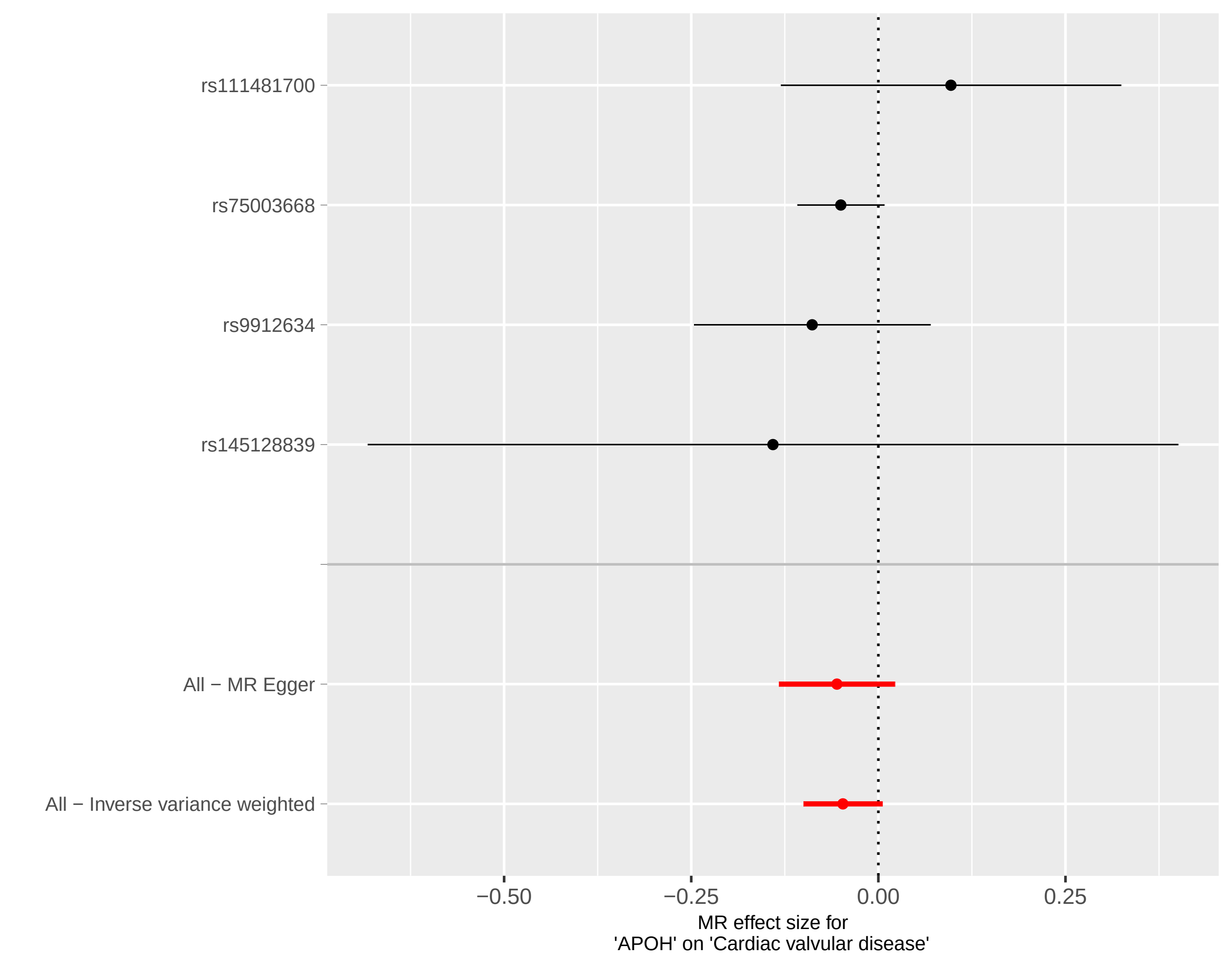
rs111481700
rs75003668
rs9912634
rs145128839
All − MR Egger
All − Inverse variance weighted
−0.50
−0.25
0.00
0.25
MR effect size for
'APOH' on 'Cardiac valvular disease'

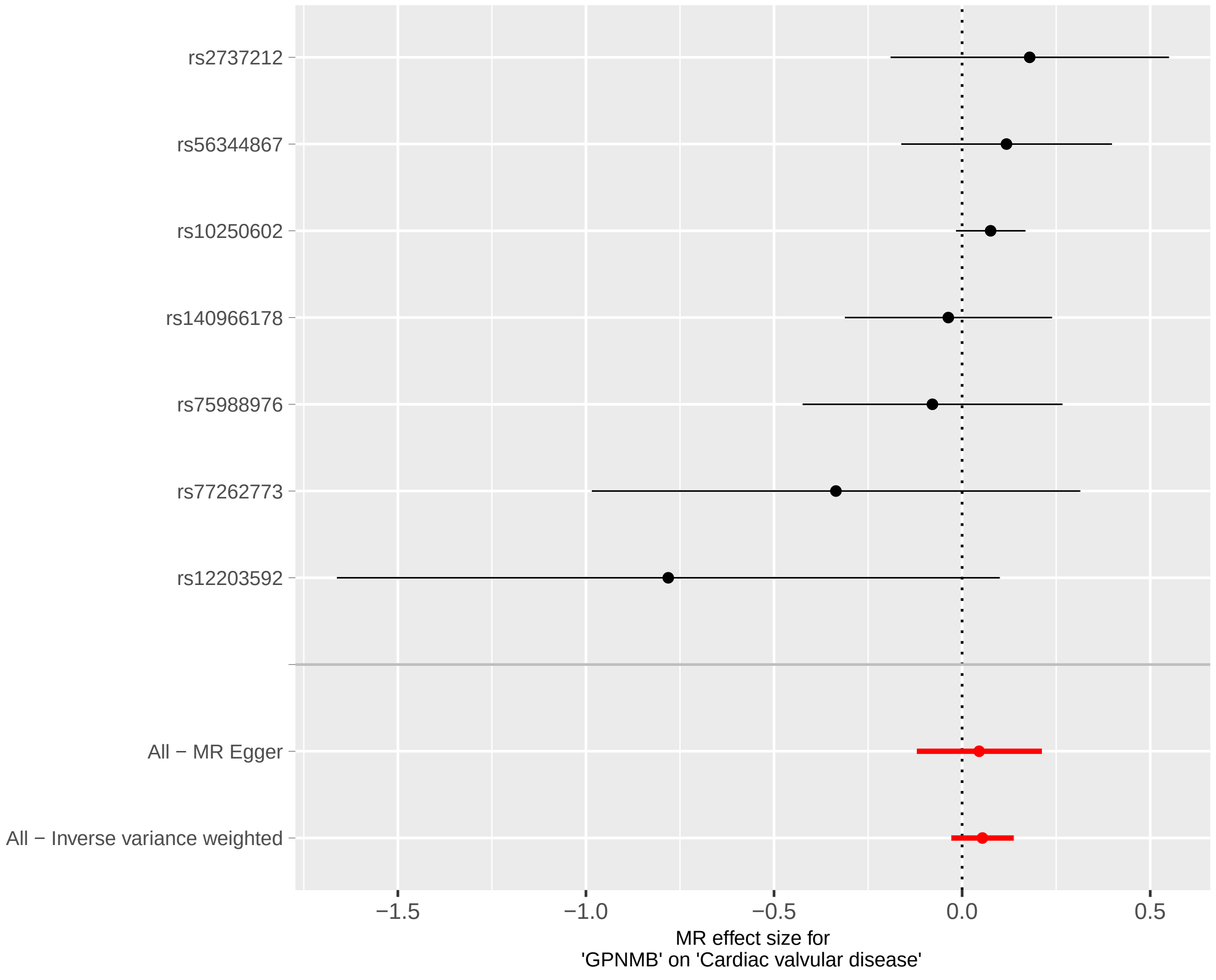
rs2737212
rs56344867
rs10250602
rs140966178
rs75988976
rs77262773
rs12203592
All − MR Egger
All − Inverse variance weighted
−1.5
−1.0
−0.5
0.0
0.5
MR effect size for
'GPNMB' on 'Cardiac valvular disease'

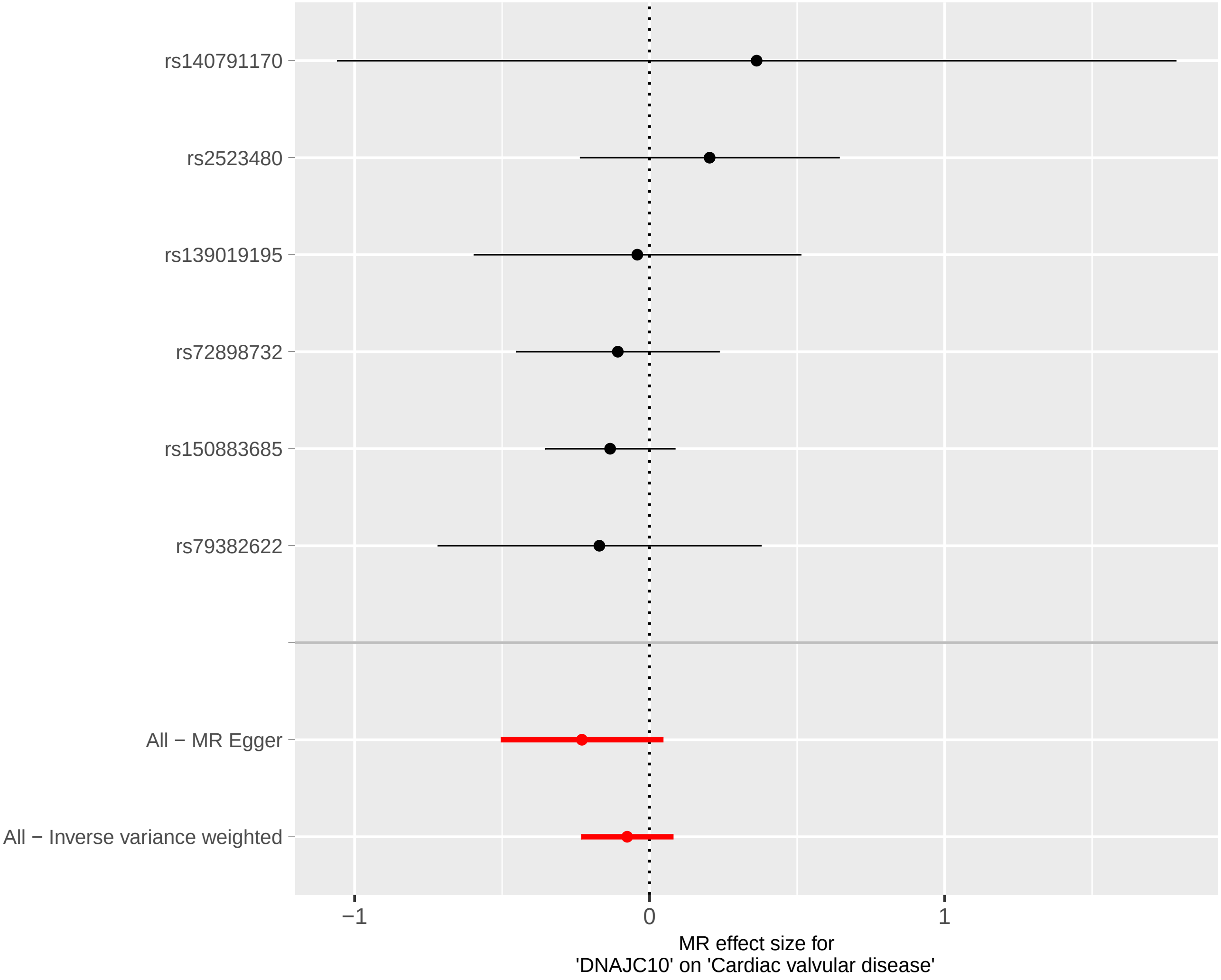
rs140791170
rs2523480
rs139019195
rs72898732
rs150883685
rs79382622
All − MR Egger
All − Inverse variance weighted
−1
0
1
MR effect size for
'DNAJC10' on 'Cardiac valvular disease'

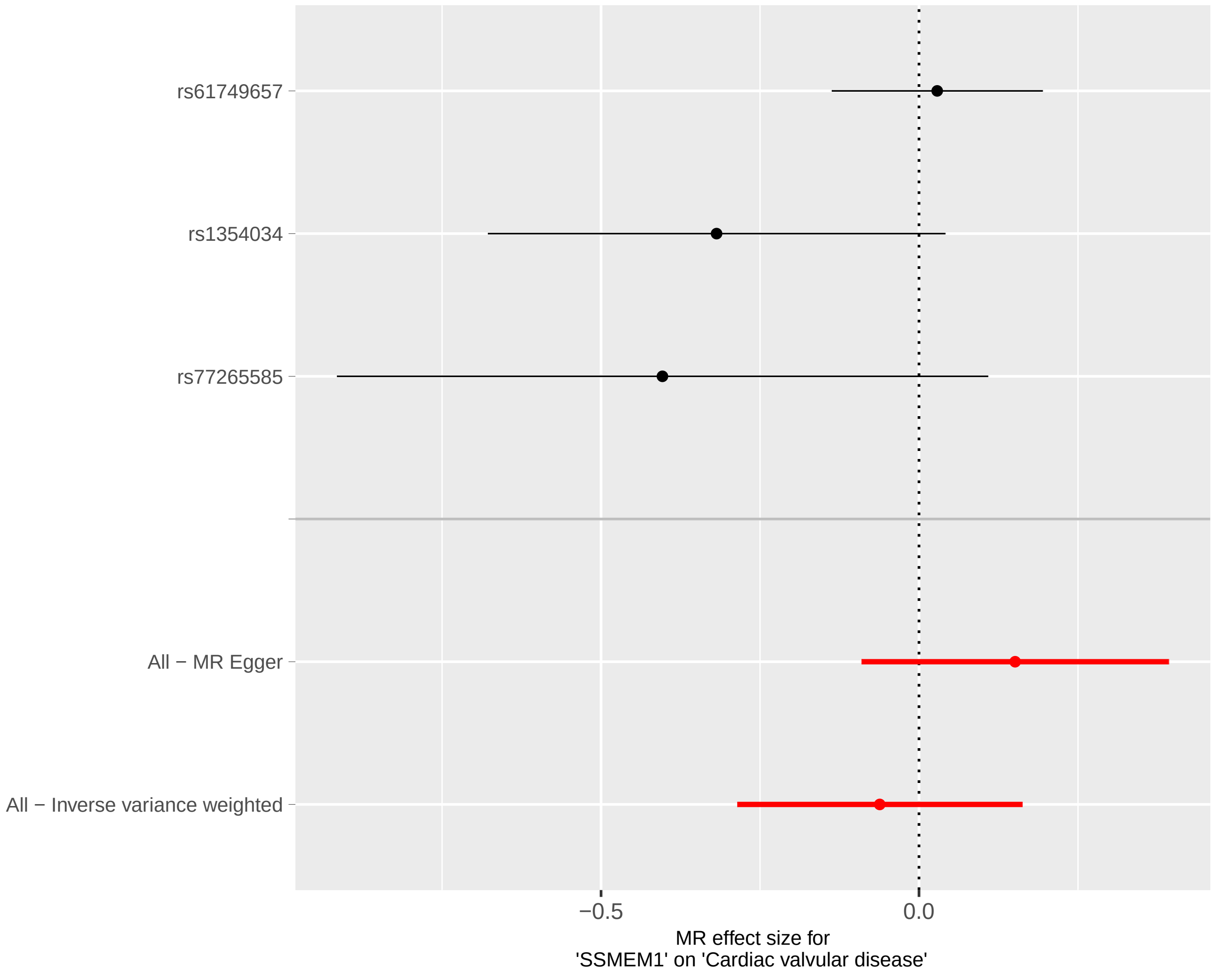

rs61749657
rs1354034
rs77265585
All − MR Egger
All − Inverse variance weighted
−0.5
0.0
MR effect size for
'SSMEM1' on 'Cardiac valvular disease'

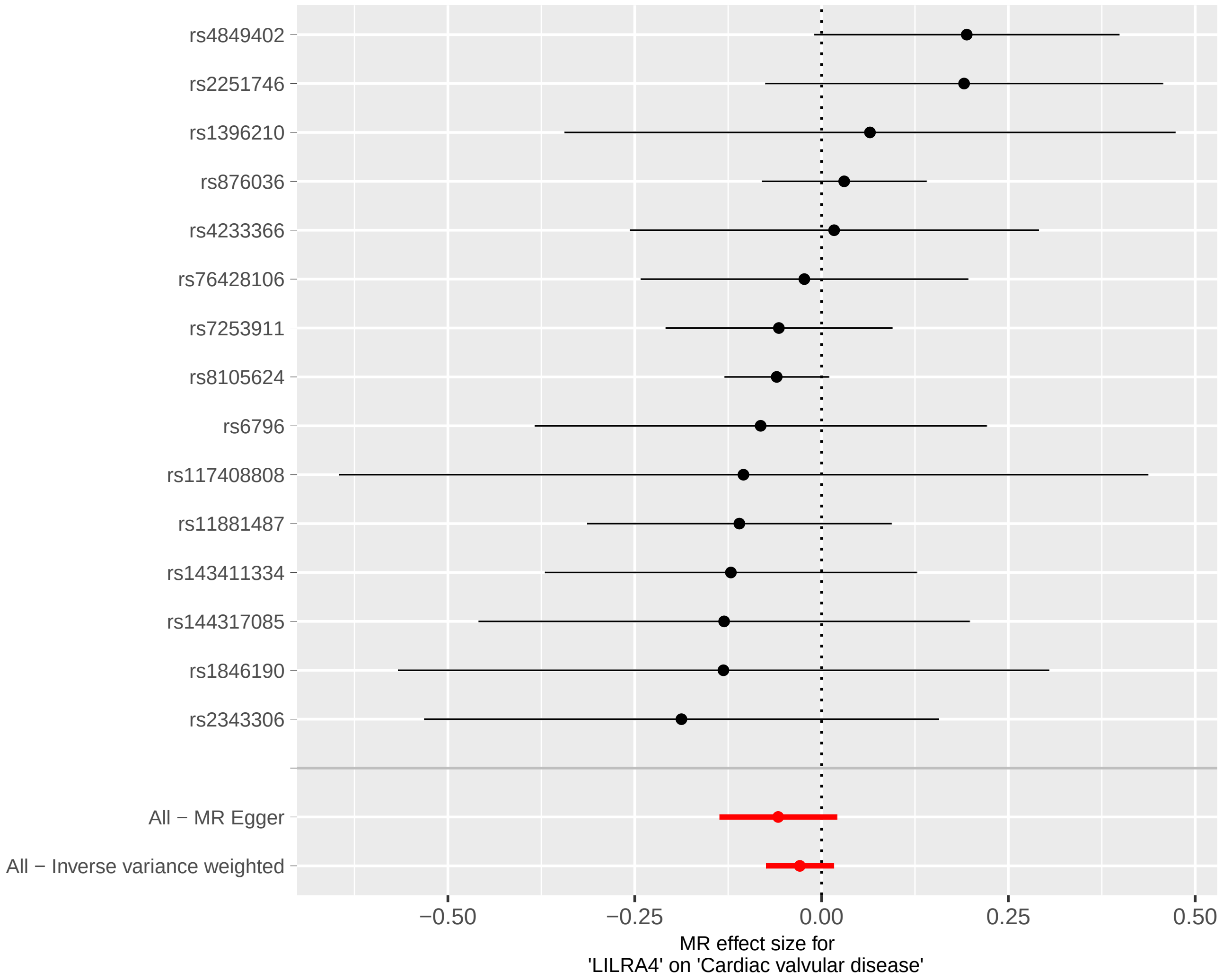
rs4849402
rs2251746
rs1396210
rs876036
rs4233366
rs76428106
rs7253911
rs8105624
rs6796
rs117408808
rs11881487
rs143411334
rs144317085
rs1846190
rs2343306
All − MR Egger
All − Inverse variance weighted
−0.50
−0.25
0.00
0.25
0.50
MR effect size for
'LILRA4' on 'Cardiac valvular disease'

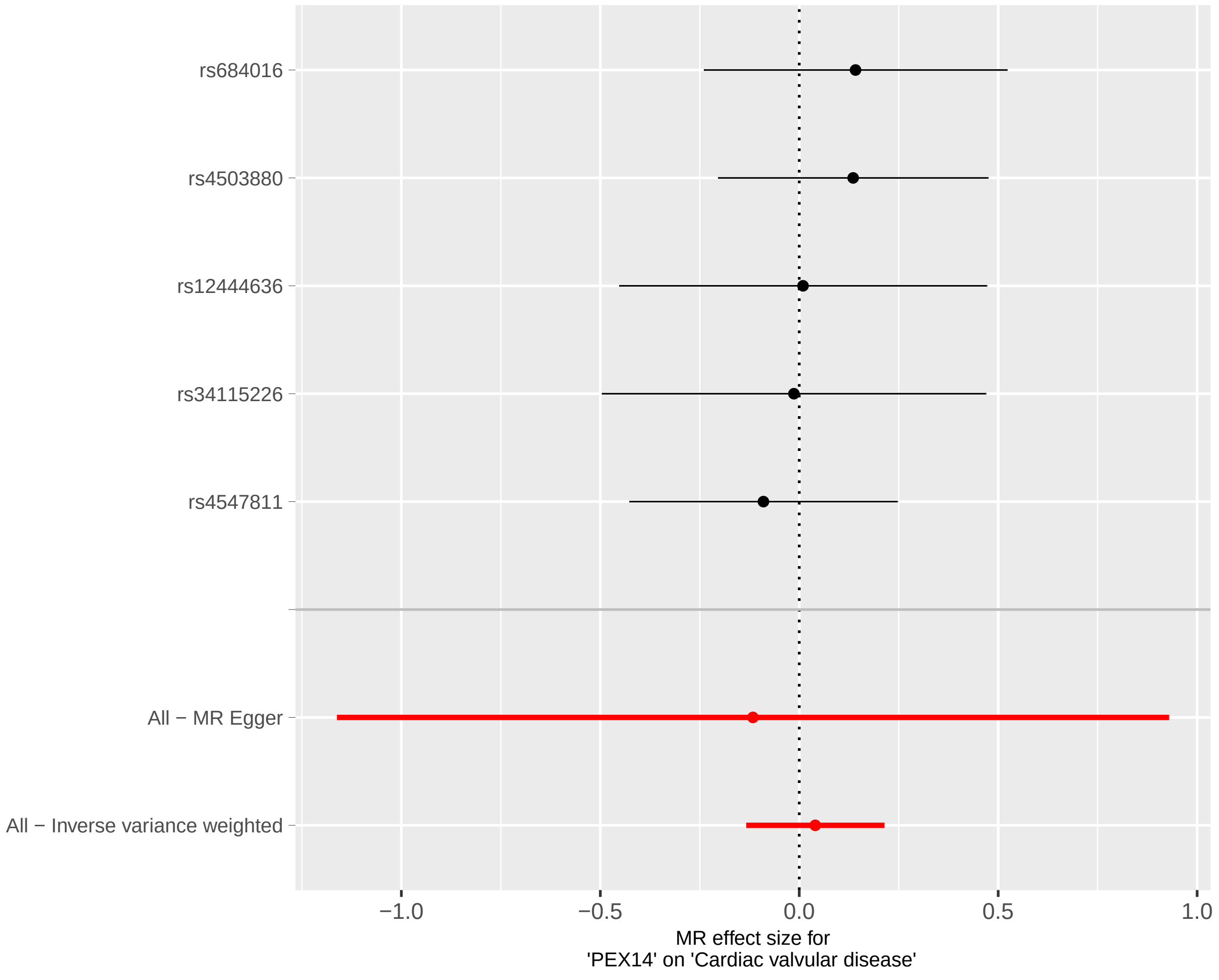
rs684016
rs4503880
rs12444636
rs34115226
rs4547811
All − MR Egger
All − Inverse variance weighted
−1.0
−0.5
0.0
0.5
1.0
MR effect size for
'PEX14' on 'Cardiac valvular disease'

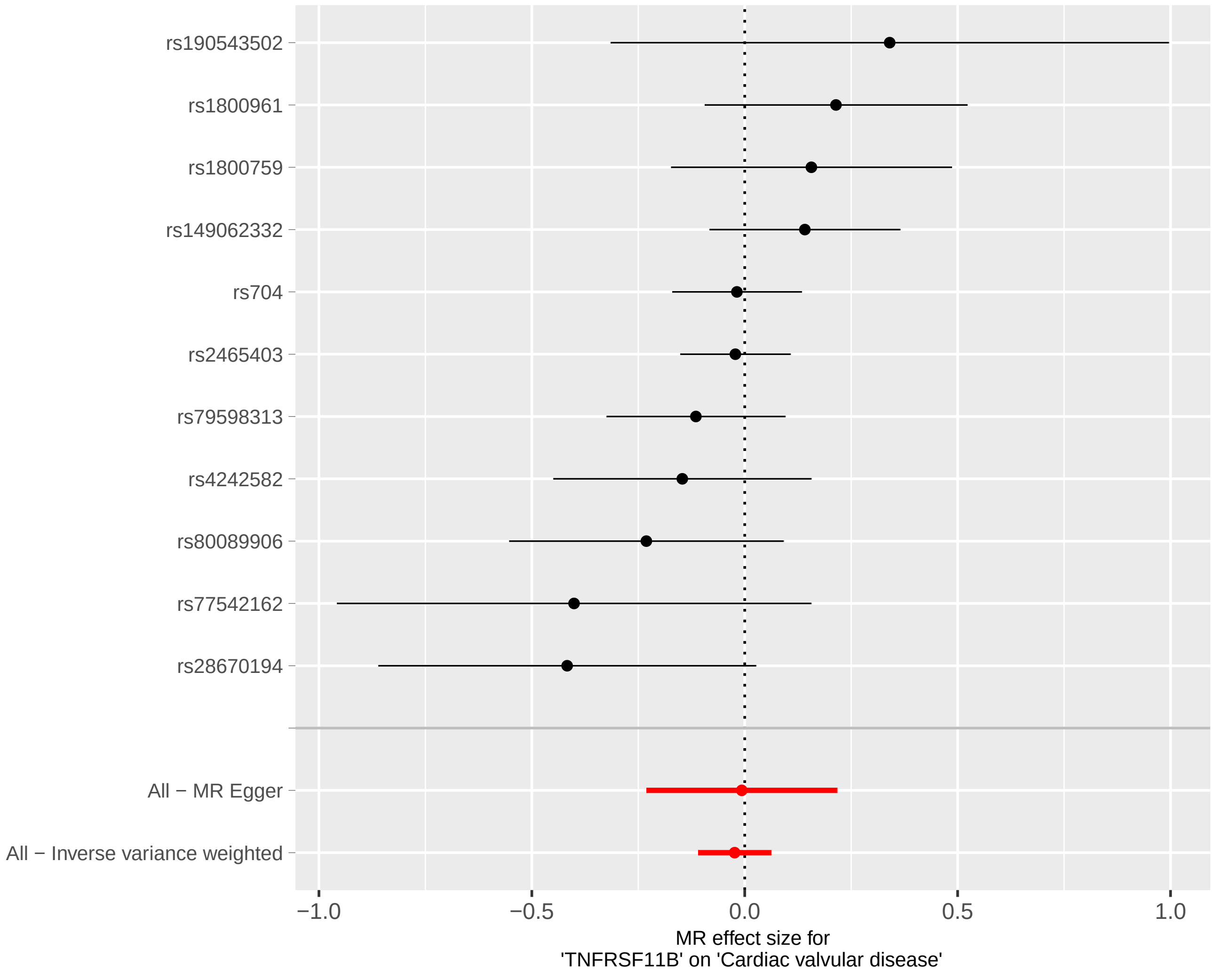

rs190543502
rs1800961
rs1800759
rs149062332
rs704
rs2465403
rs79598313
rs4242582
rs80089906
rs77542162
rs28670194
All − MR Egger
All − Inverse variance weighted
−1.0
−0.5
0.0
0.5
1.0
MR effect size for
'TNFRSF11B' on 'Cardiac valvular disease'

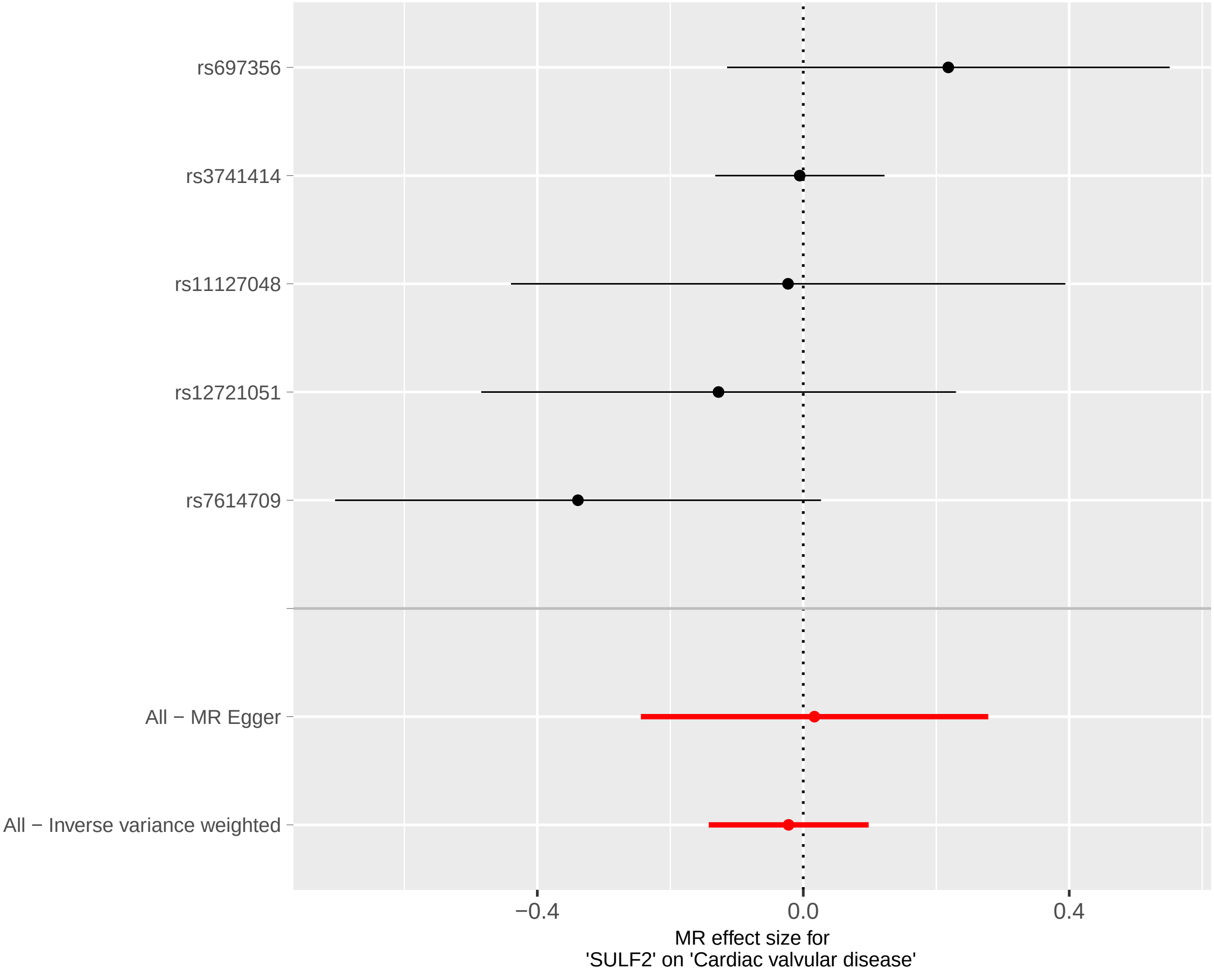
rs697356
rs3741414
rs11127048
rs12721051
rs7614709
All − MR Egger
All − Inverse variance weighted
−0.4
0.0
0.4
MR effect size for
'SULF2' on 'Cardiac valvular disease'

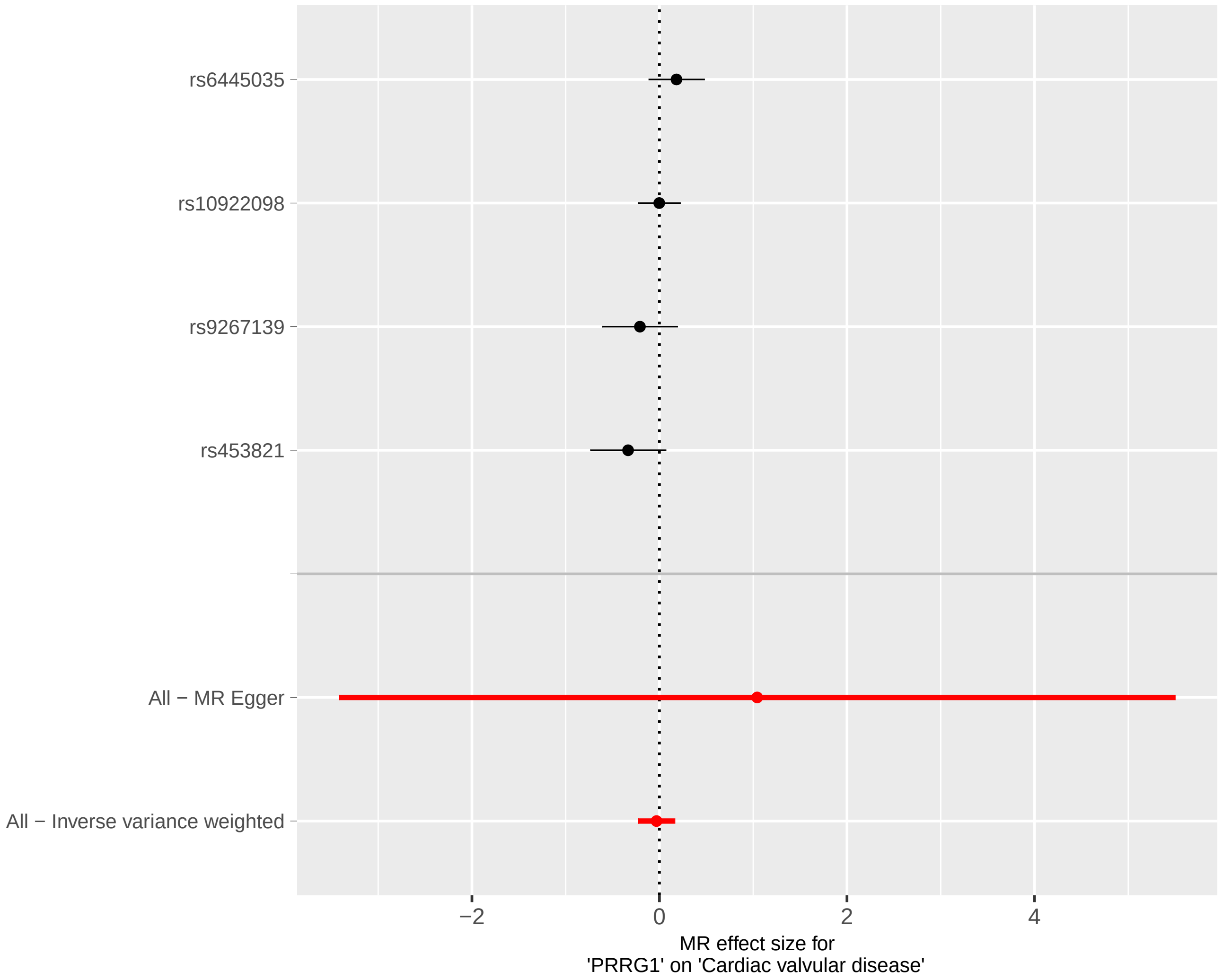
rs6445035
rs10922098
rs9267139
rs453821
All − MR Egger
All − Inverse variance weighted
−2
0
2
4
MR effect size for
'PRRG1' on 'Cardiac valvular disease'

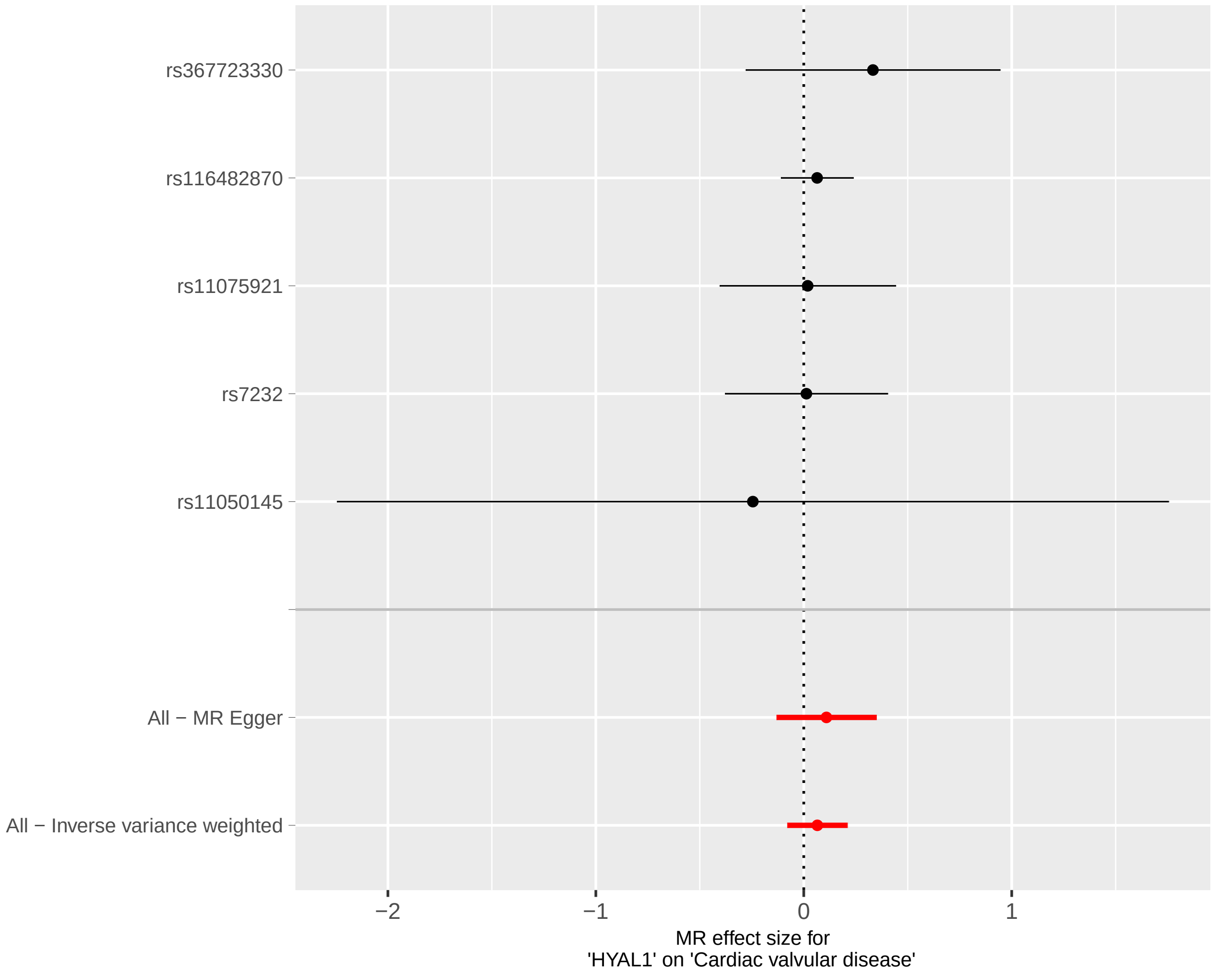

rs367723330
rs116482870
rs11075921
rs7232
rs11050145
All − MR Egger
All − Inverse variance weighted
−2
−1
0
1
MR effect size for
'HYAL1' on 'Cardiac valvular disease'

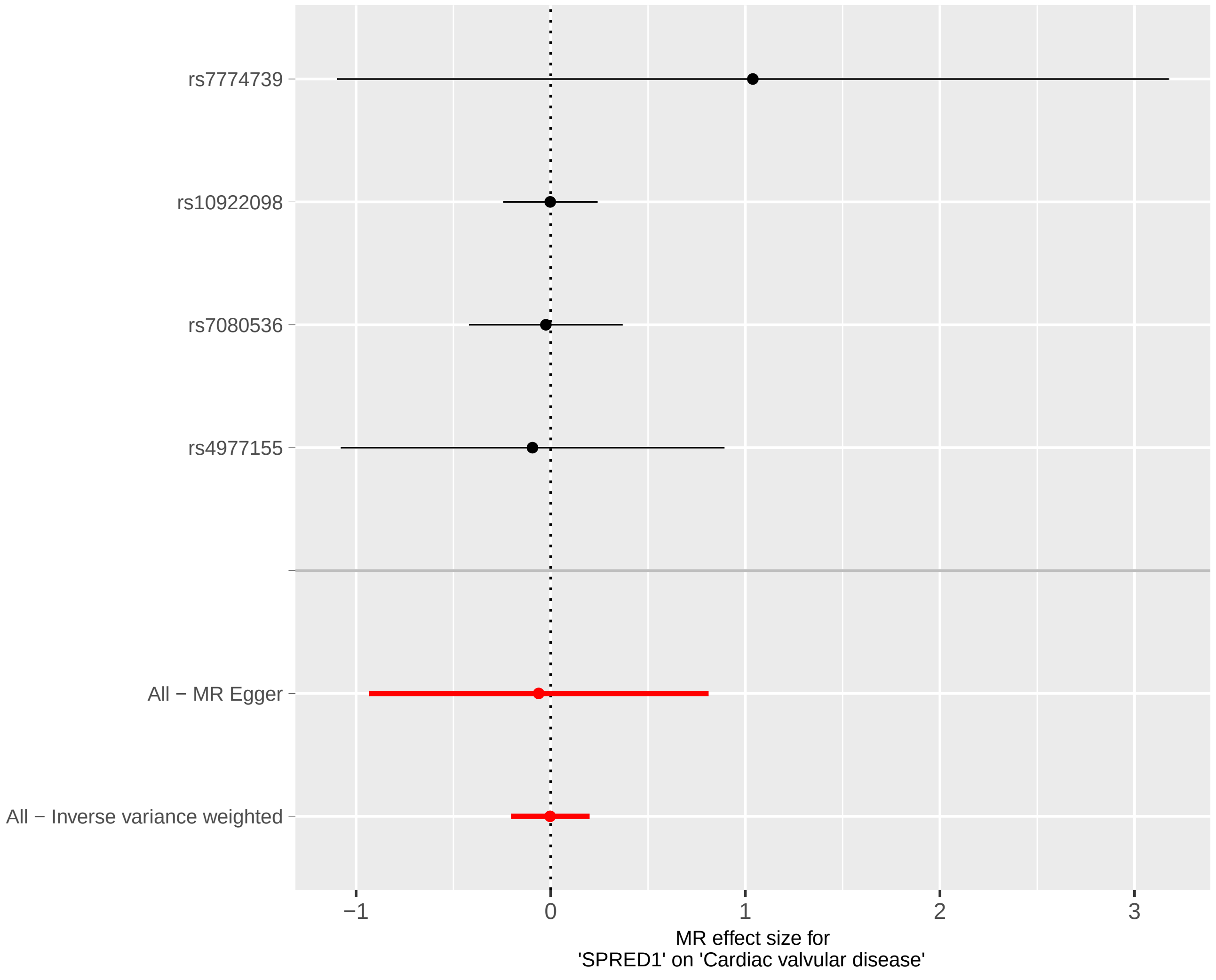
rs7774739
rs10922098
rs7080536
rs4977155
All − MR Egger
All − Inverse variance weighted
−1
0
1
2
3
MR effect size for
'SPRED1' on 'Cardiac valvular disease'

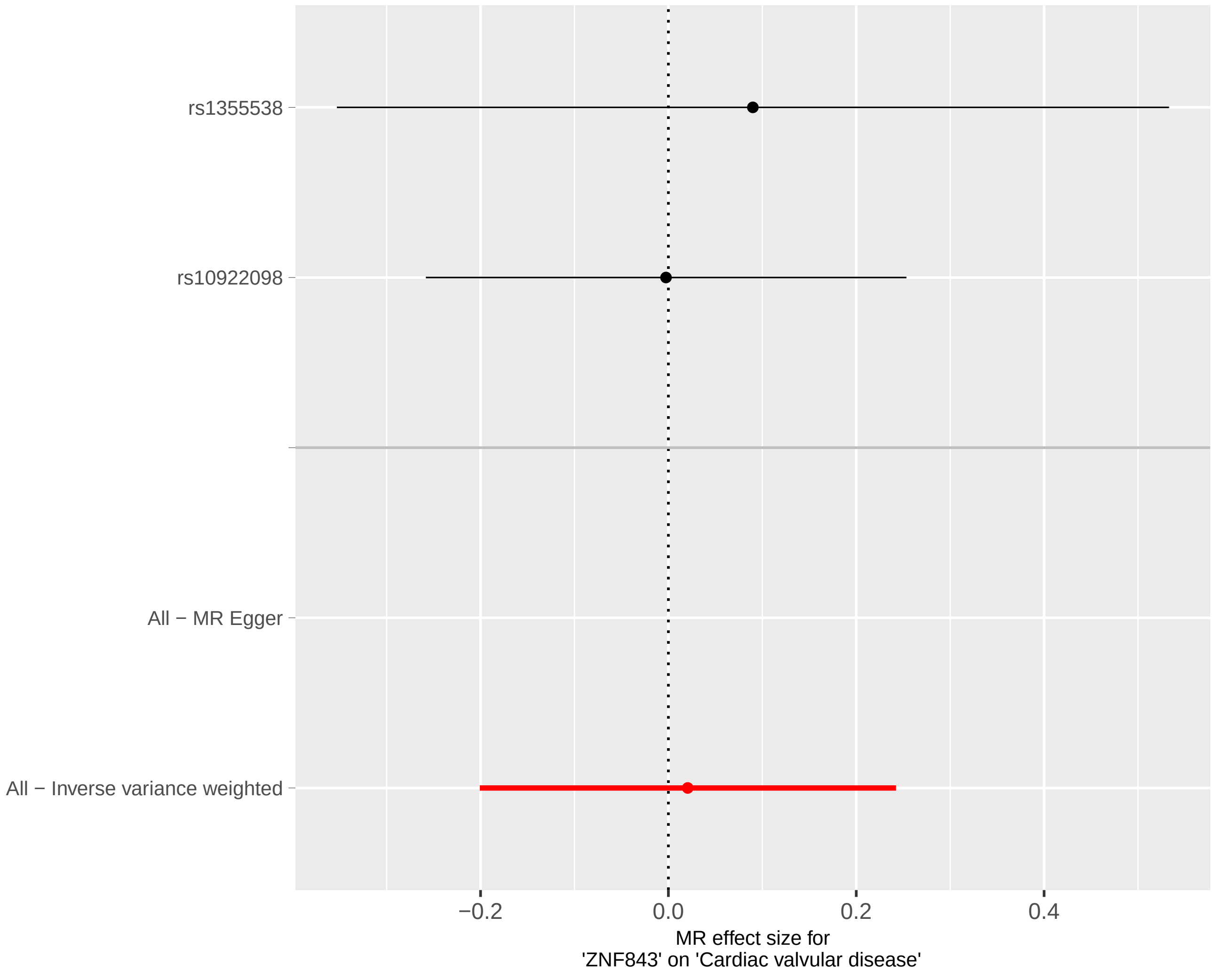
rs1355538
rs10922098
All − MR Egger
All − Inverse variance weighted
−0.2
0.0
0.2
0.4
MR effect size for
'ZNF843' on 'Cardiac valvular disease'

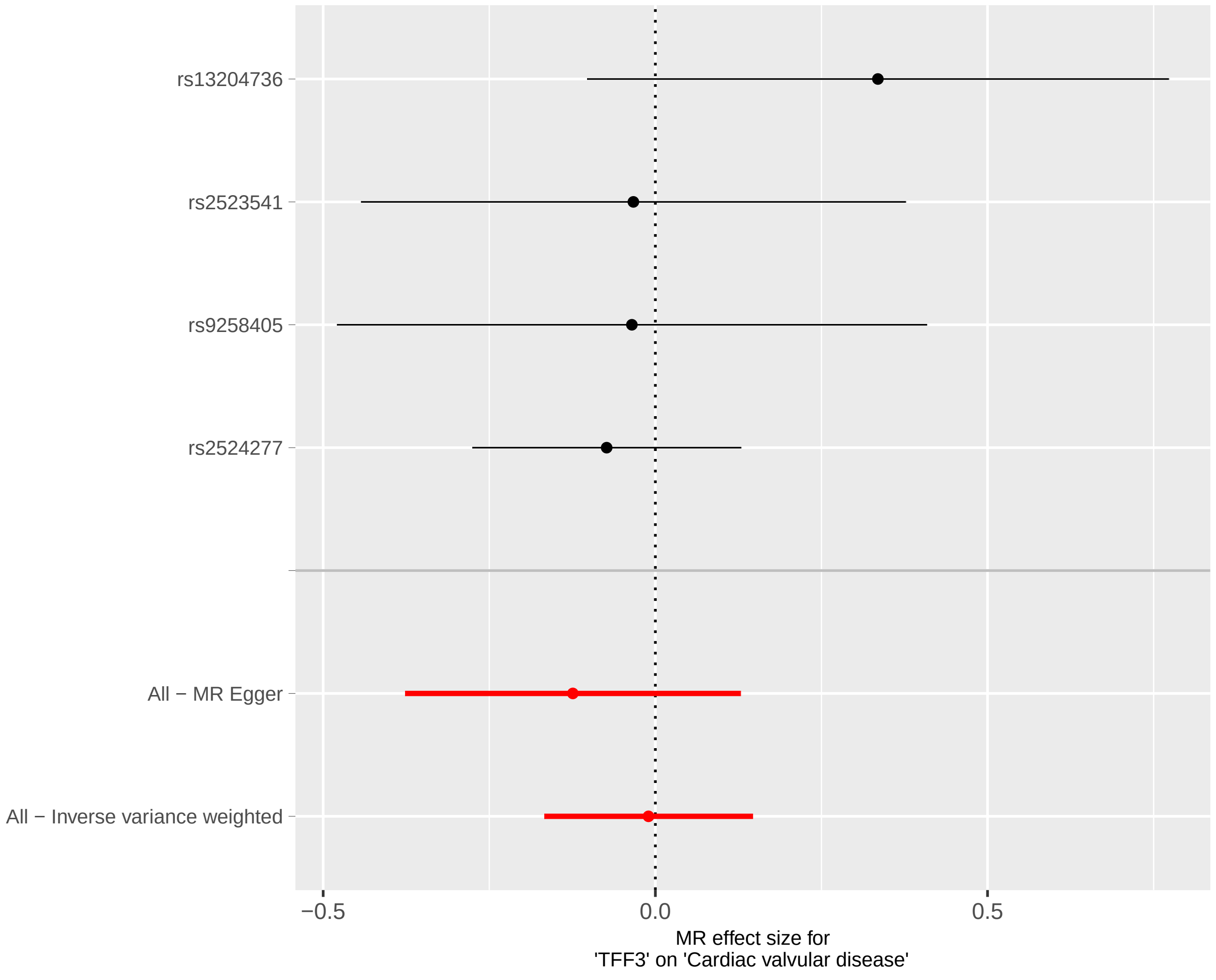

rs13204736
rs2523541
rs9258405
rs2524277
All − MR Egger
All − Inverse variance weighted
−0.5
0.0
0.5
MR effect size for
'TFF3' on 'Cardiac valvular disease'

Insufficient number of SNPs

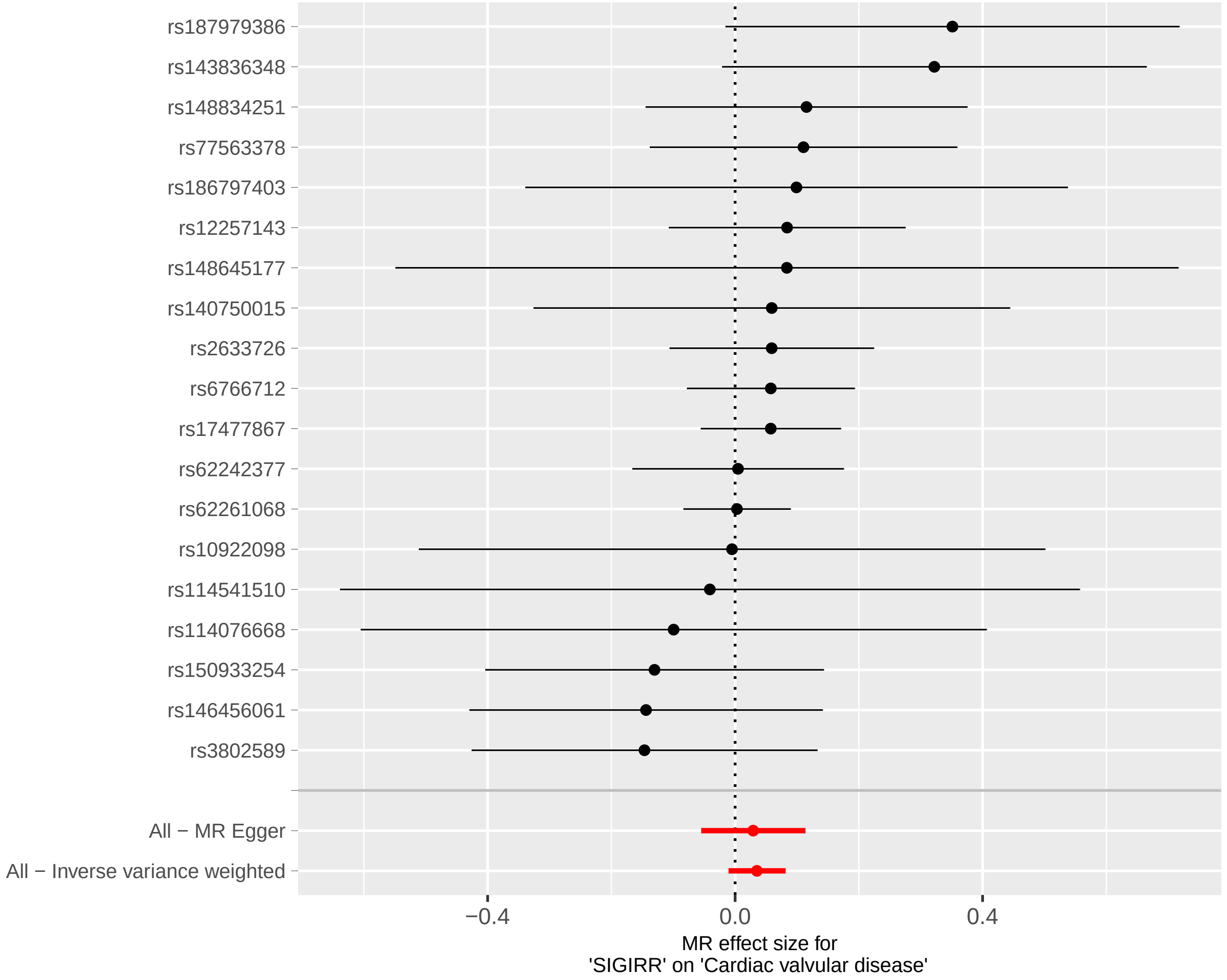
rs187979386
rs143836348
rs148834251
rs77563378
rs186797403
rs12257143
rs148645177
rs140750015
rs2633726
rs6766712
rs17477867
rs62242377
rs62261068
rs10922098
rs114541510
rs114076668
rs150933254
rs146456061
rs3802589
All − MR Egger
All − Inverse variance weighted
−0.4
0.0
0.4
MR effect size for
'SIGIRR' on 'Cardiac valvular disease'

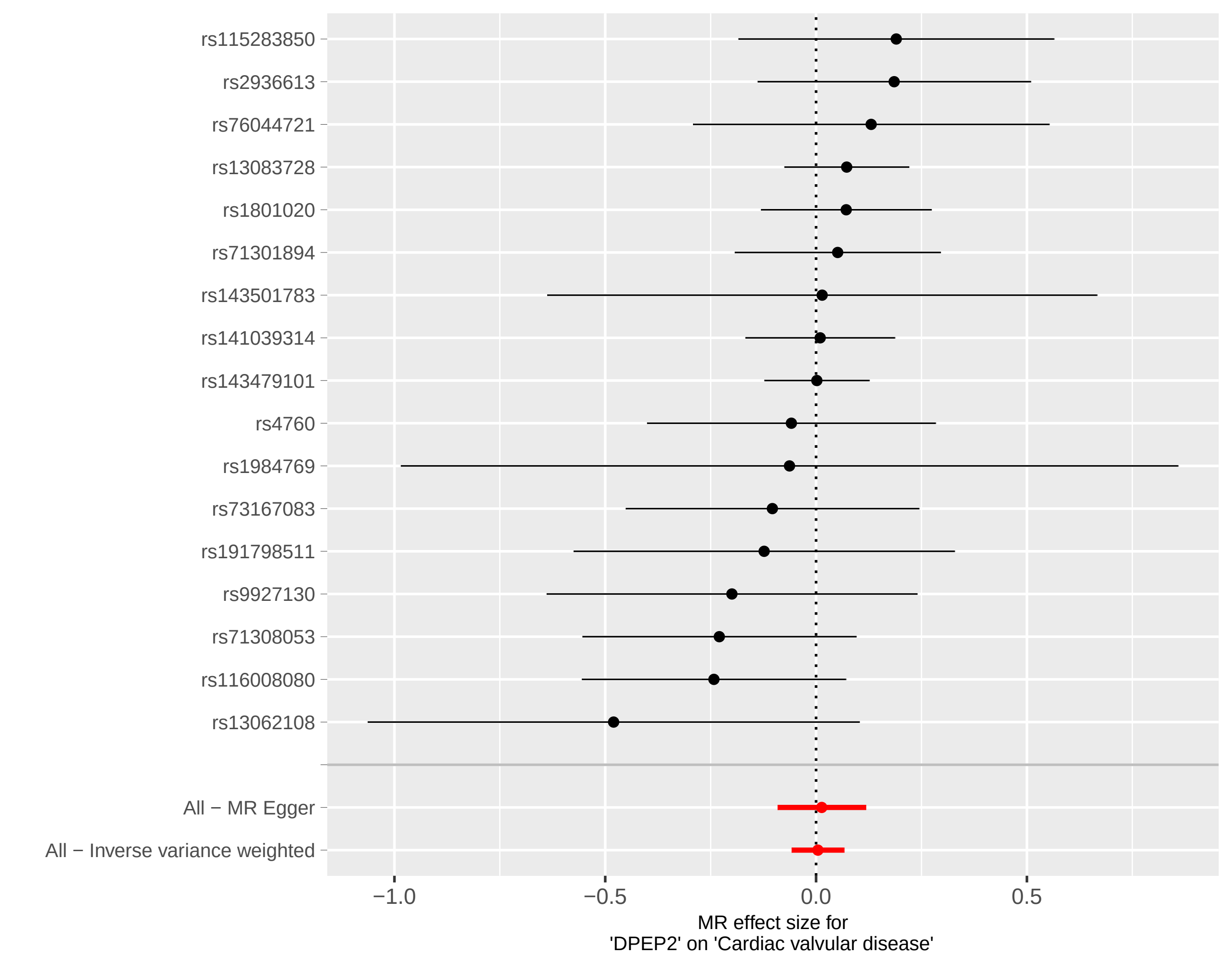
rs115283850
rs2936613
rs76044721
rs13083728
rs1801020
rs71301894
rs143501783
rs141039314
rs143479101
rs4760
rs1984769
rs73167083
rs191798511
rs9927130
rs71308053
rs116008080
rs13062108
All − MR Egger
All − Inverse variance weighted
−1.0
−0.5
0.0
0.5
MR effect size for
'DPEP2' on 'Cardiac valvular disease'

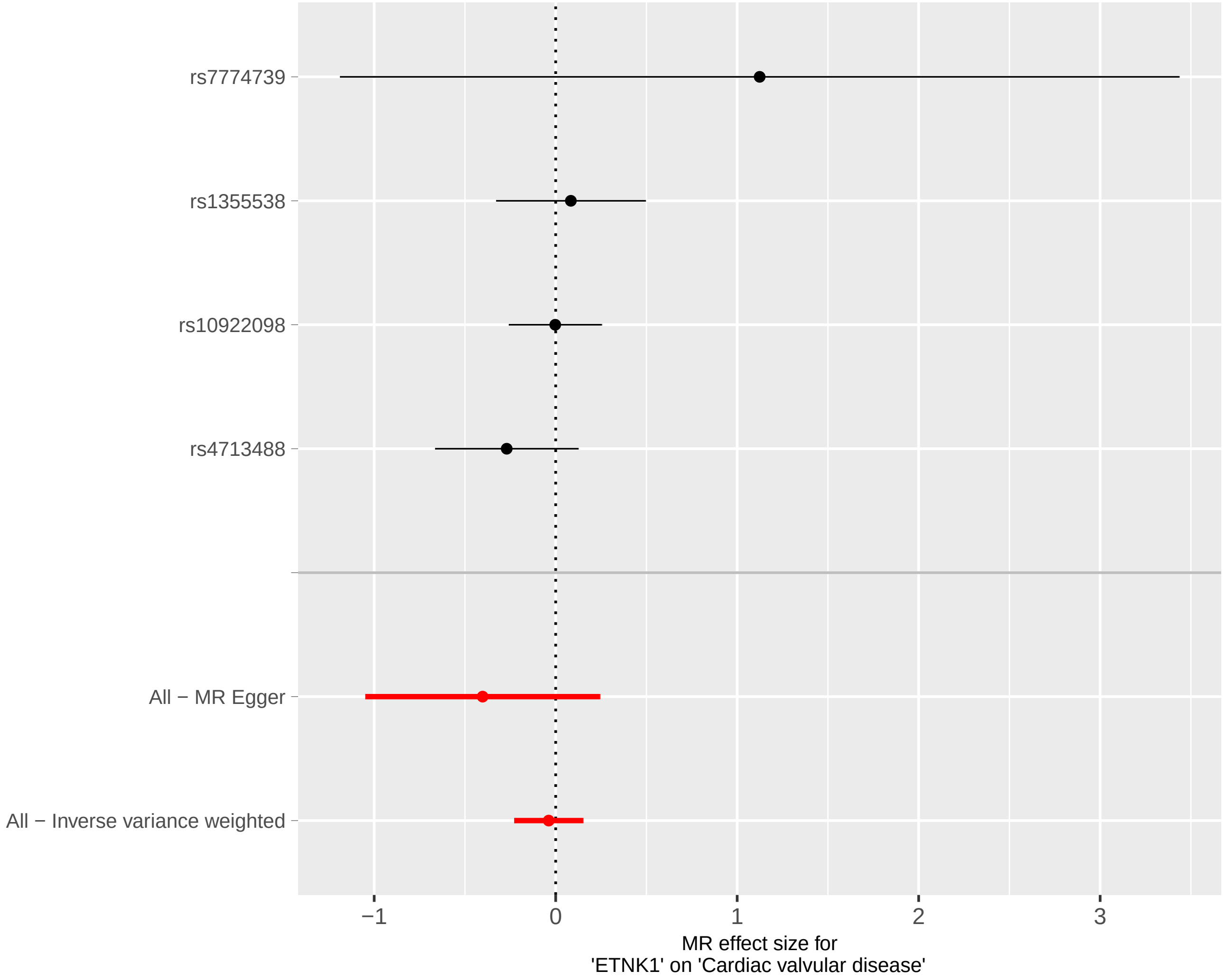
rs7774739
rs1355538
rs10922098
rs4713488
All − MR Egger
All − Inverse variance weighted
−1
0
1
2
3
MR effect size for
'ETNK1' on 'Cardiac valvular disease'

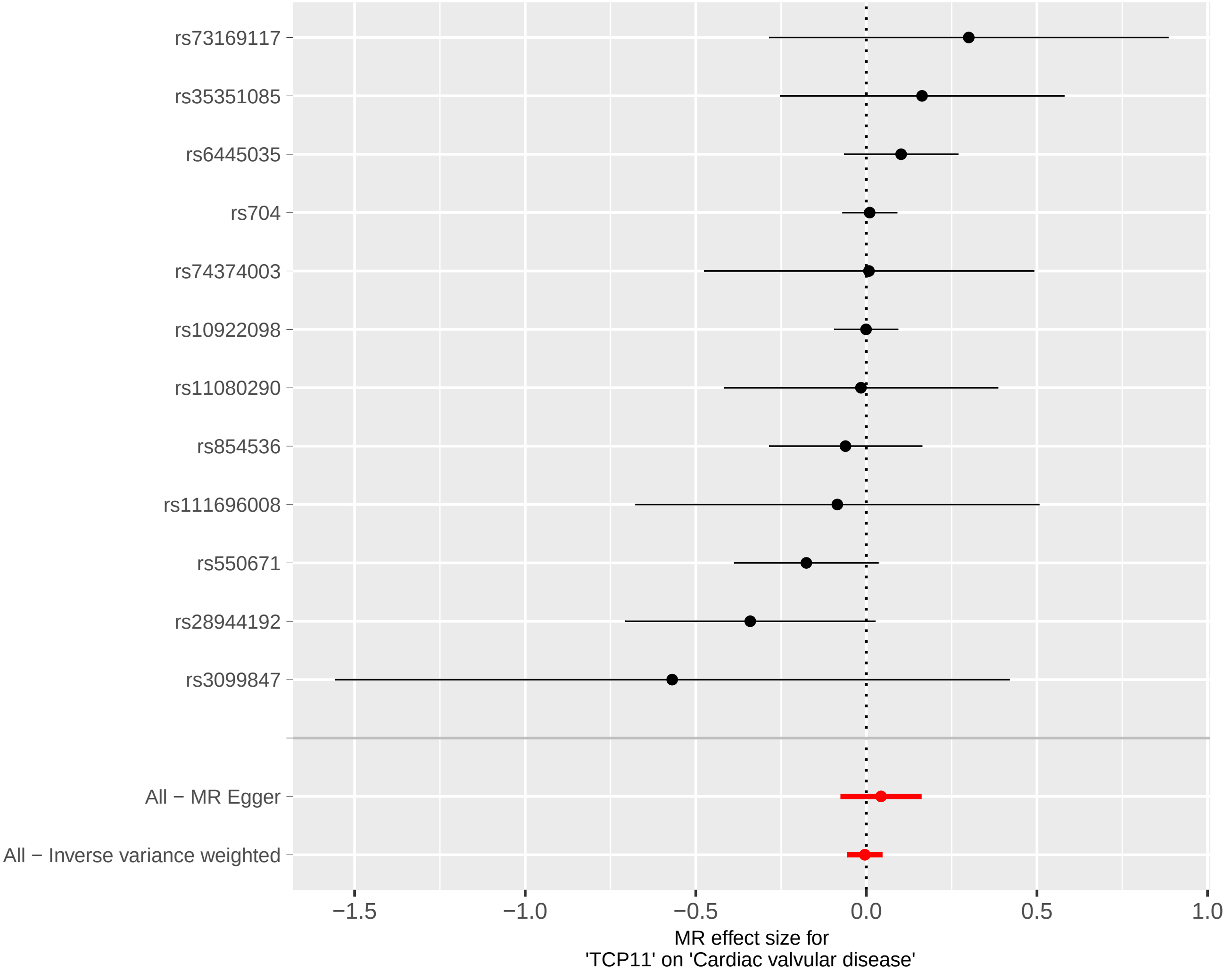
rs73169117
rs35351085
rs6445035
rs704
rs74374003
rs10922098
rs11080290
rs854536
rs111696008
rs550671
rs28944192
rs3099847
All − MR Egger
All − Inverse variance weighted
−1.5
−1.0
−0.5
0.0
0.5
1.0
MR effect size for
'TCP11' on 'Cardiac valvular disease'

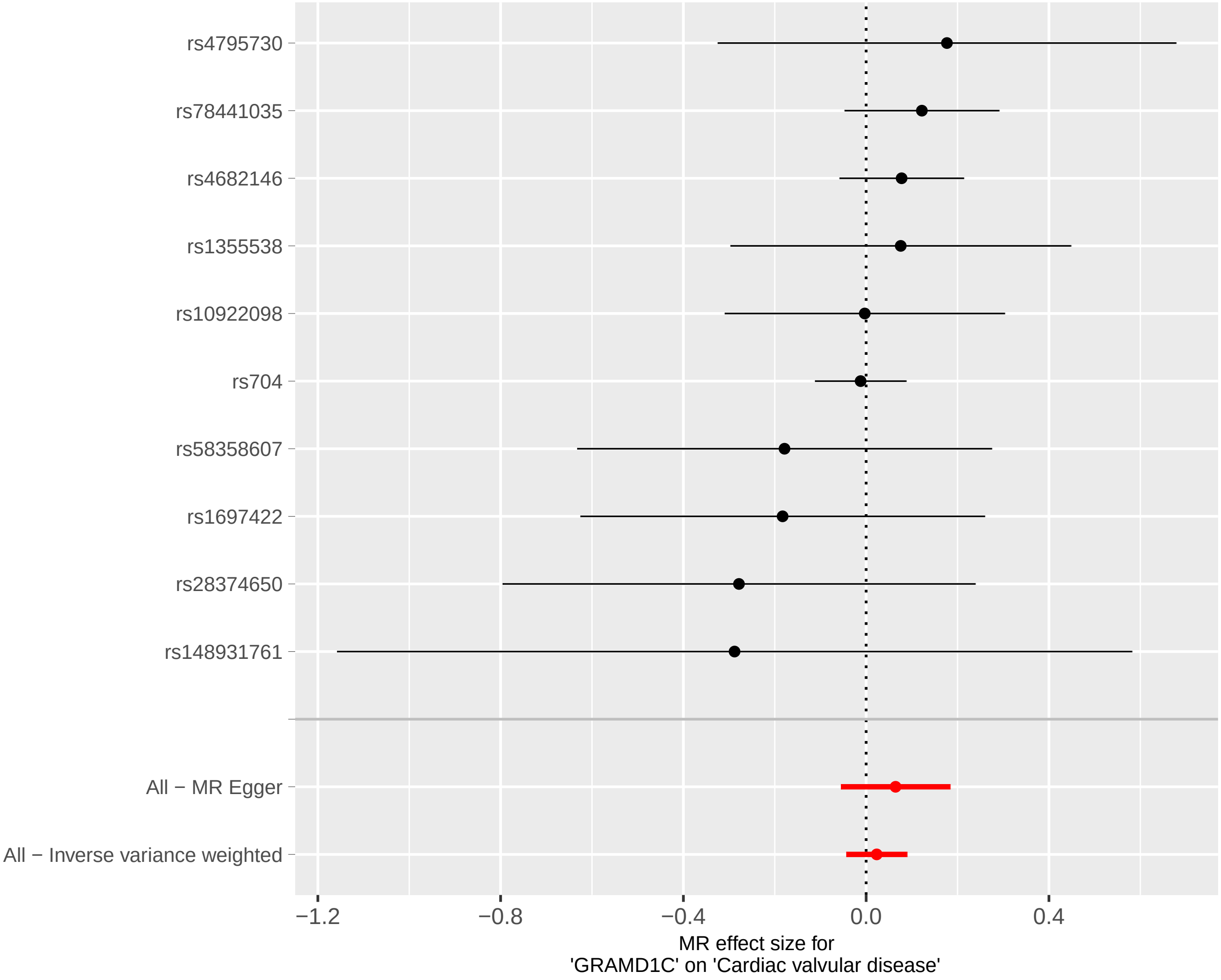

rs4795730
rs78441035
rs4682146
rs1355538
rs10922098
rs704
rs58358607
rs1697422
rs28374650
rs148931761
All − MR Egger
All − Inverse variance weighted
−1.2
−0.8
−0.4
0.0
0.4
MR effect size for
'GRAMD1C' on 'Cardiac valvular disease'

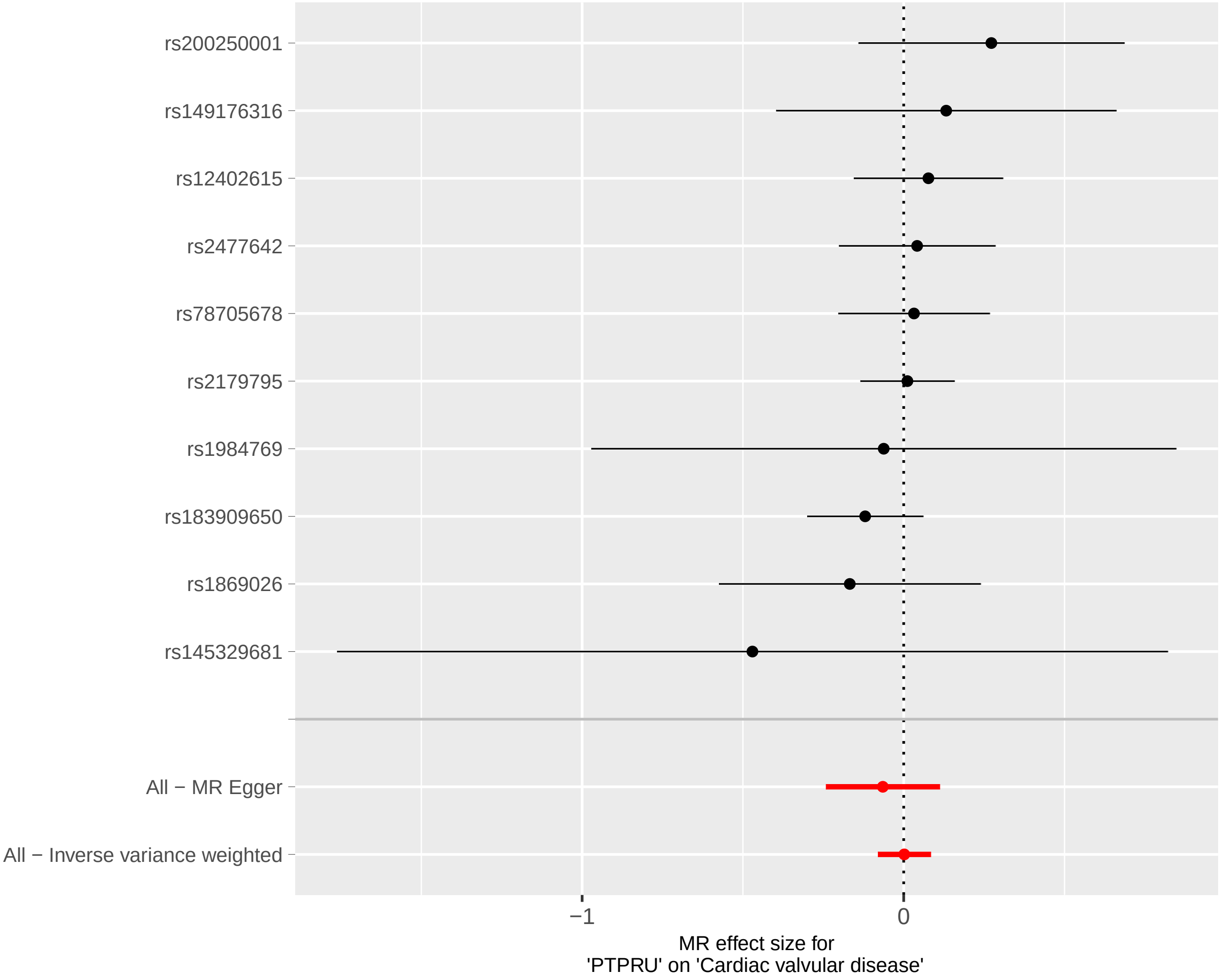
rs200250001
rs149176316
rs12402615
rs2477642
rs78705678
rs2179795
rs1984769
rs183909650
rs1869026
rs145329681
All − MR Egger
All − Inverse variance weighted
−1
0
MR effect size for
'PTPRU' on 'Cardiac valvular disease'

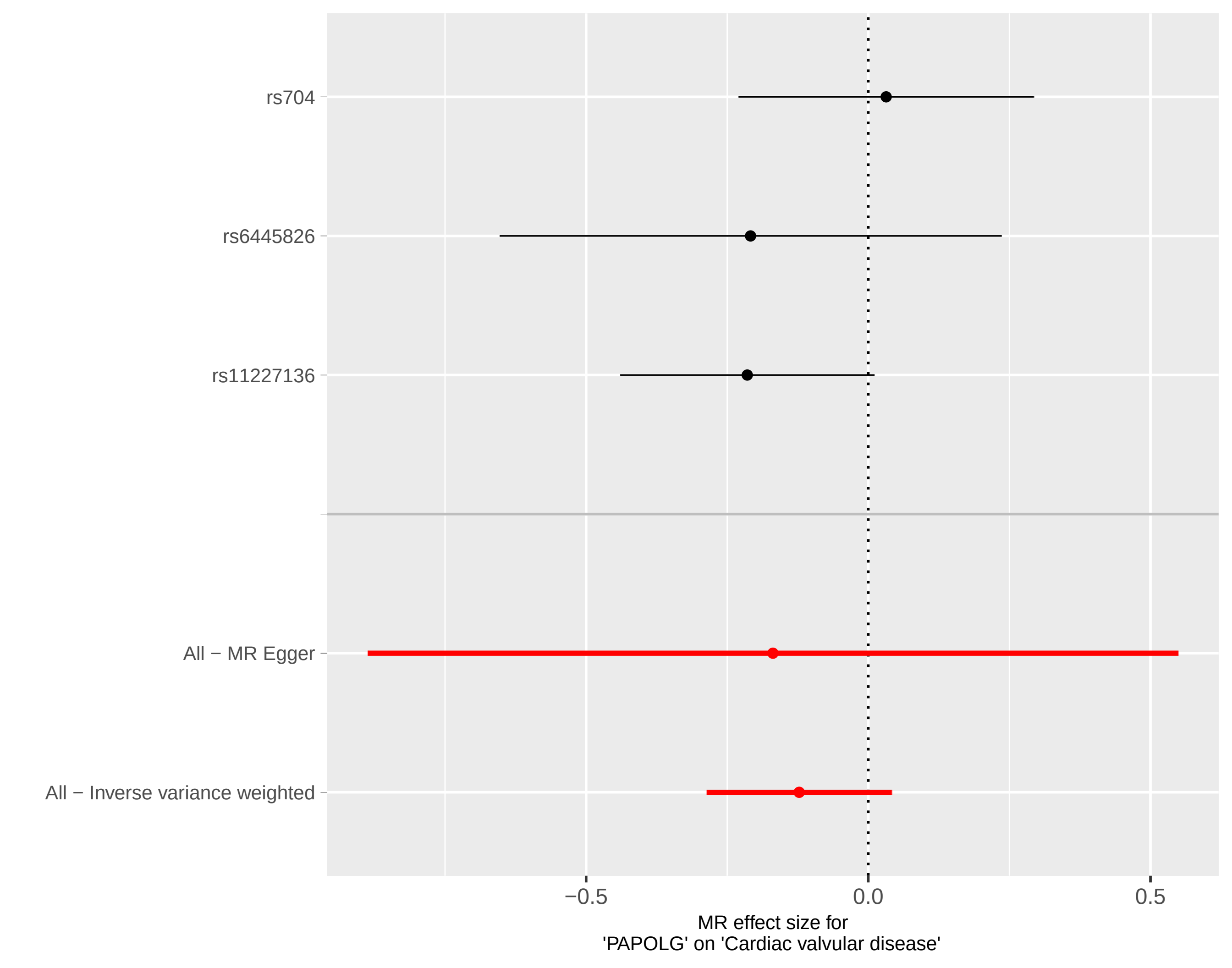
rs704
rs6445826
rs11227136
All − MR Egger
All − Inverse variance weighted
−0.5
0.0
0.5
MR effect size for
'PAPOLG' on 'Cardiac valvular disease'

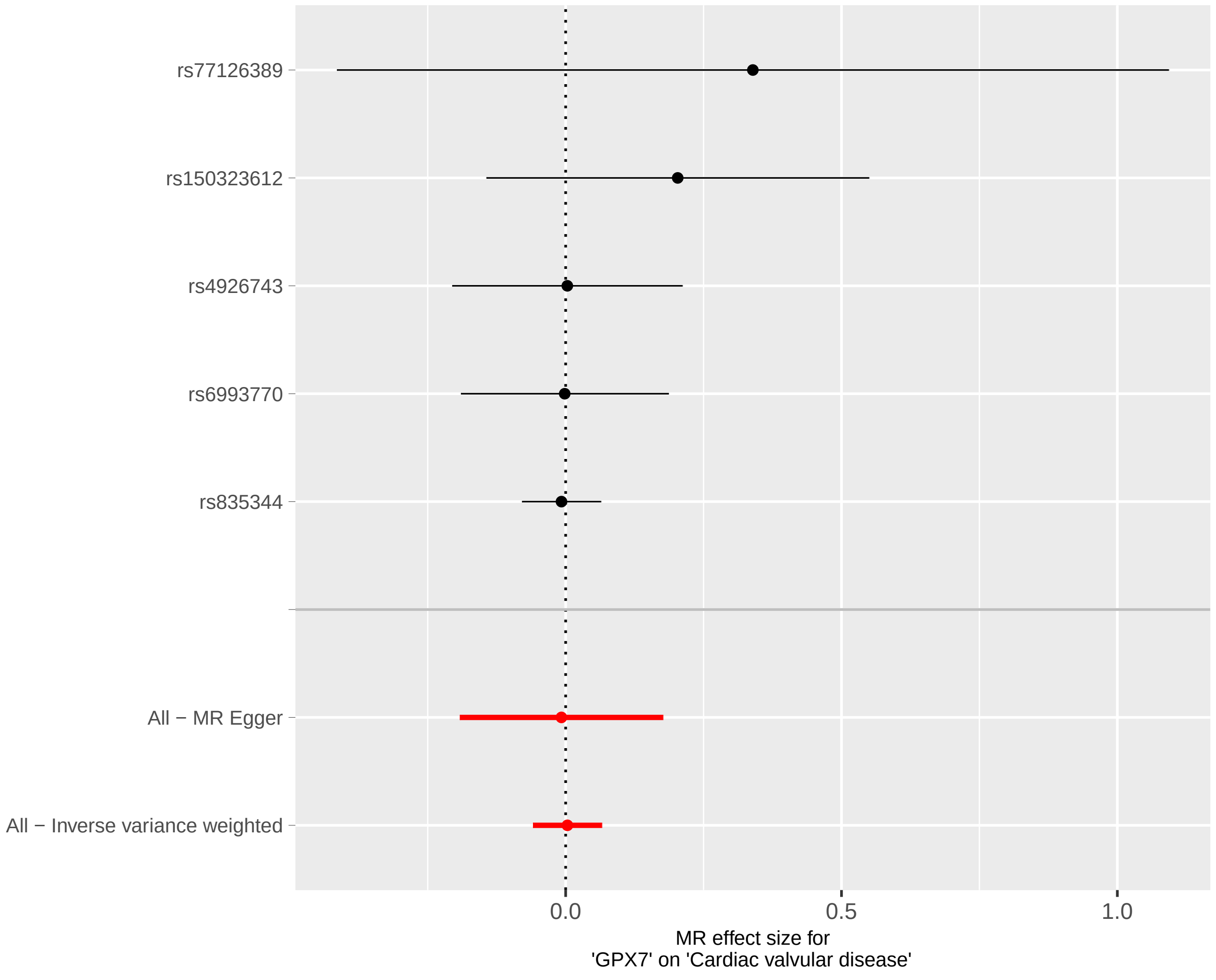
rs77126389
rs150323612
rs4926743
rs6993770
rs835344
All − MR Egger
All − Inverse variance weighted
0.0
0.5
1.0
MR effect size for
'GPX7' on 'Cardiac valvular disease'

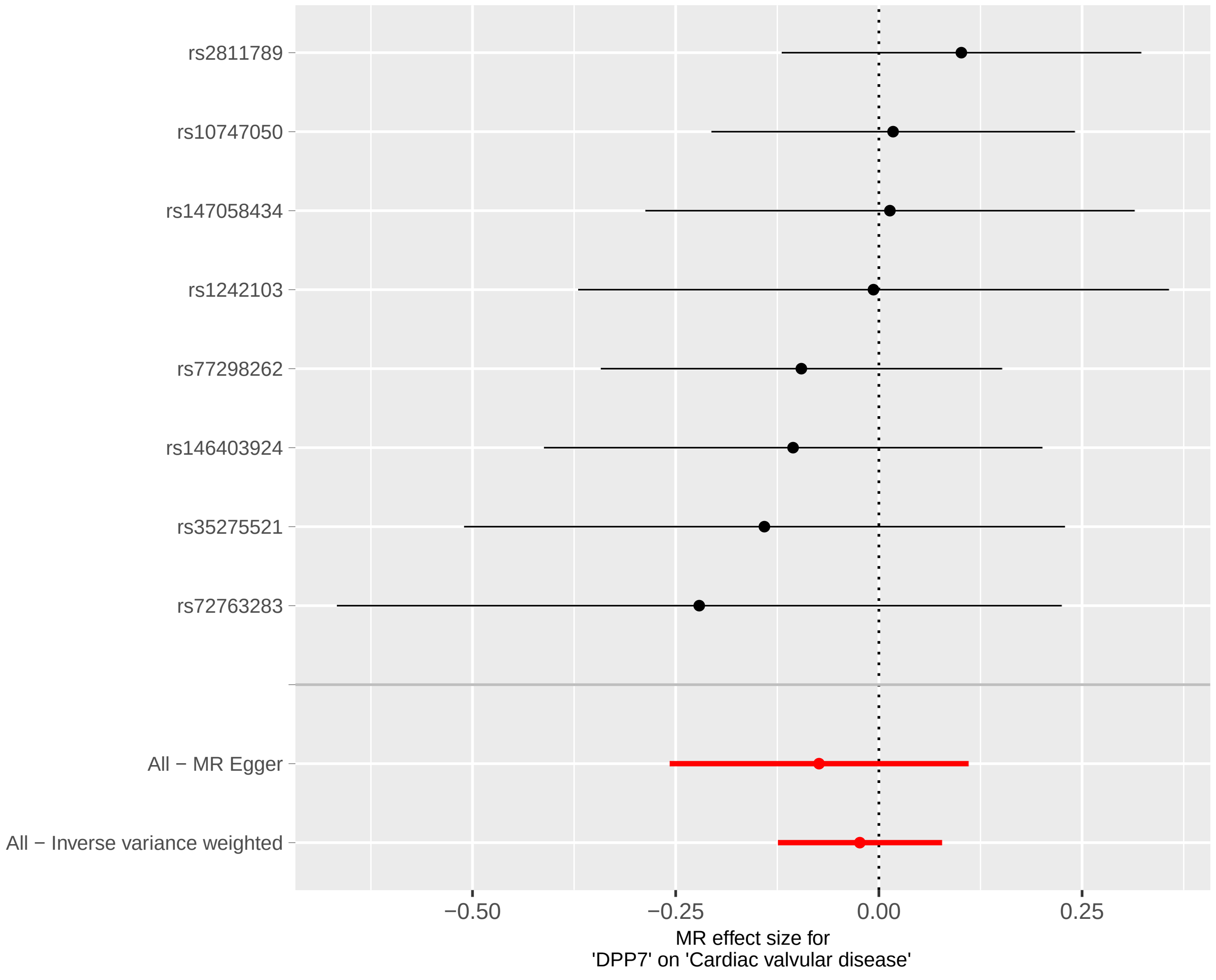

rs2811789
rs10747050
rs147058434
rs1242103
rs77298262
rs146403924
rs35275521
rs72763283
All − MR Egger
All − Inverse variance weighted
−0.50
−0.25
0.00
0.25
MR effect size for
'DPP7' on 'Cardiac valvular disease'

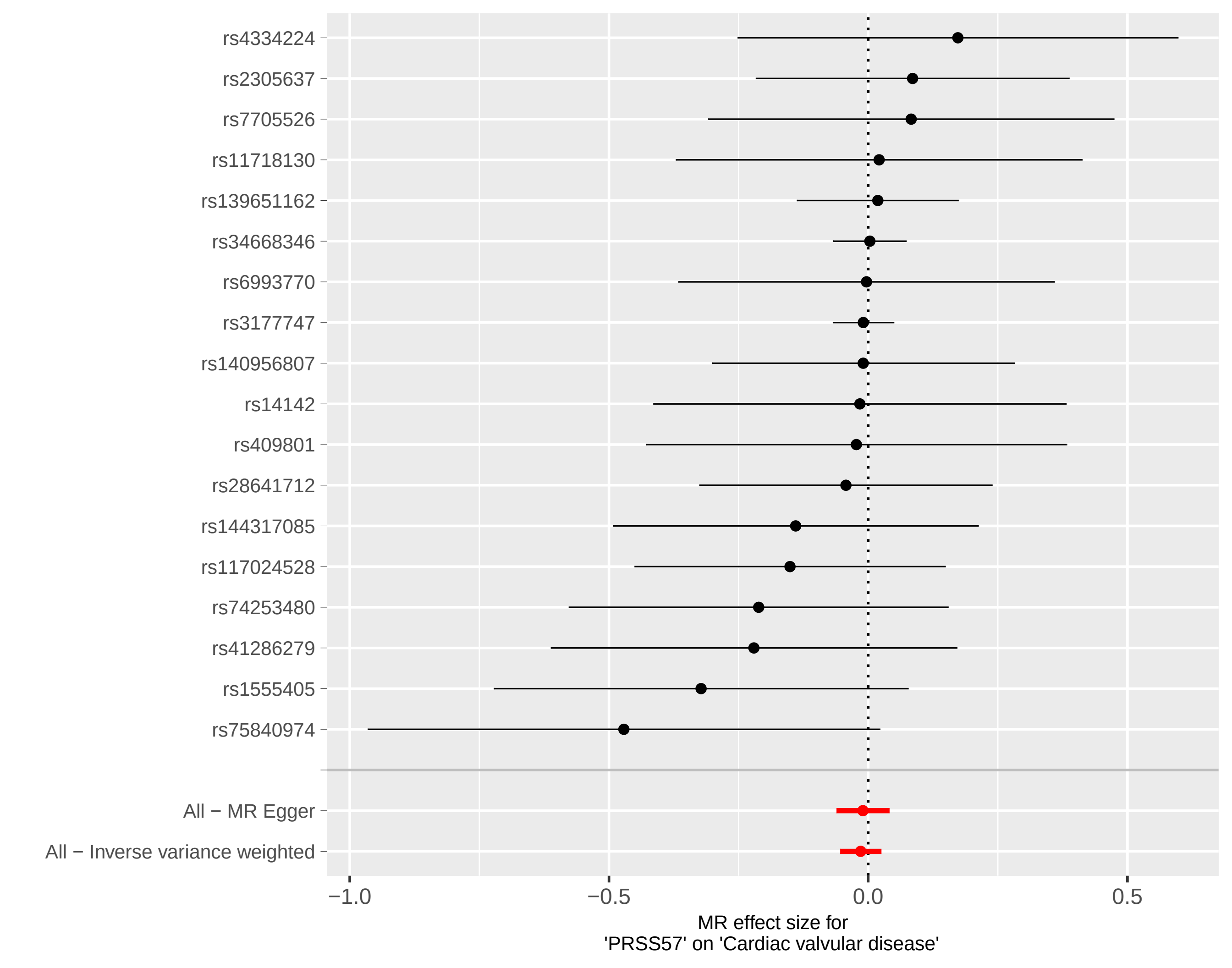

rs4334224
rs2305637
rs7705526
rs11718130
rs139651162
rs34668346
rs6993770
rs3177747
rs140956807
rs14142
rs409801
rs28641712
rs144317085
rs117024528
rs74253480
rs41286279
rs1555405
rs75840974
All − MR Egger
All − Inverse variance weighted
−1.0
−0.5
0.0
0.5
MR effect size for
'PRSS57' on 'Cardiac valvular disease'

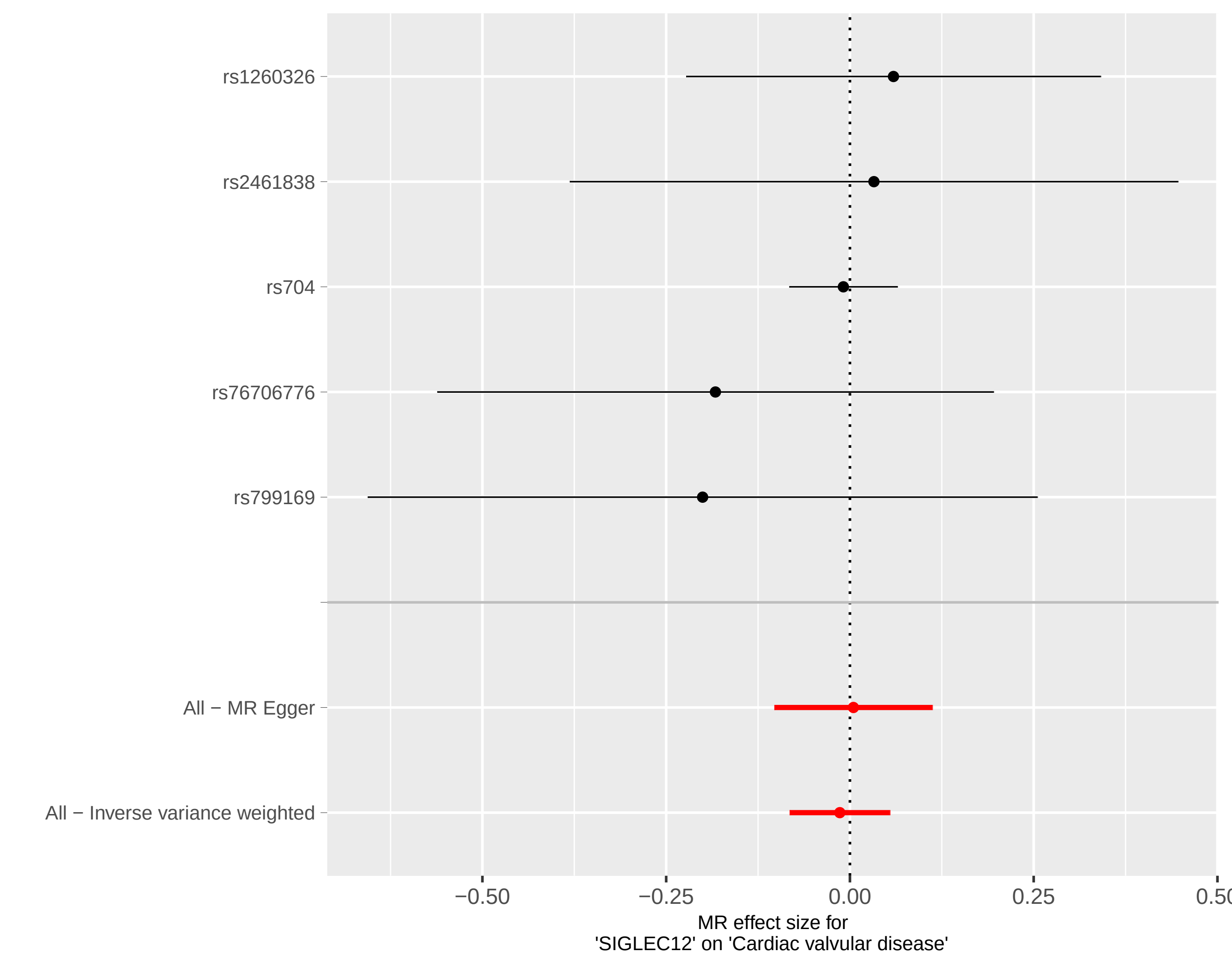
rs1260326
rs2461838
rs704
rs76706776
rs799169
All − MR Egger
All − Inverse variance weighted
−0.50
−0.25
0.00
0.25
0.50
MR effect size for
'SIGLEC12' on 'Cardiac valvular disease'

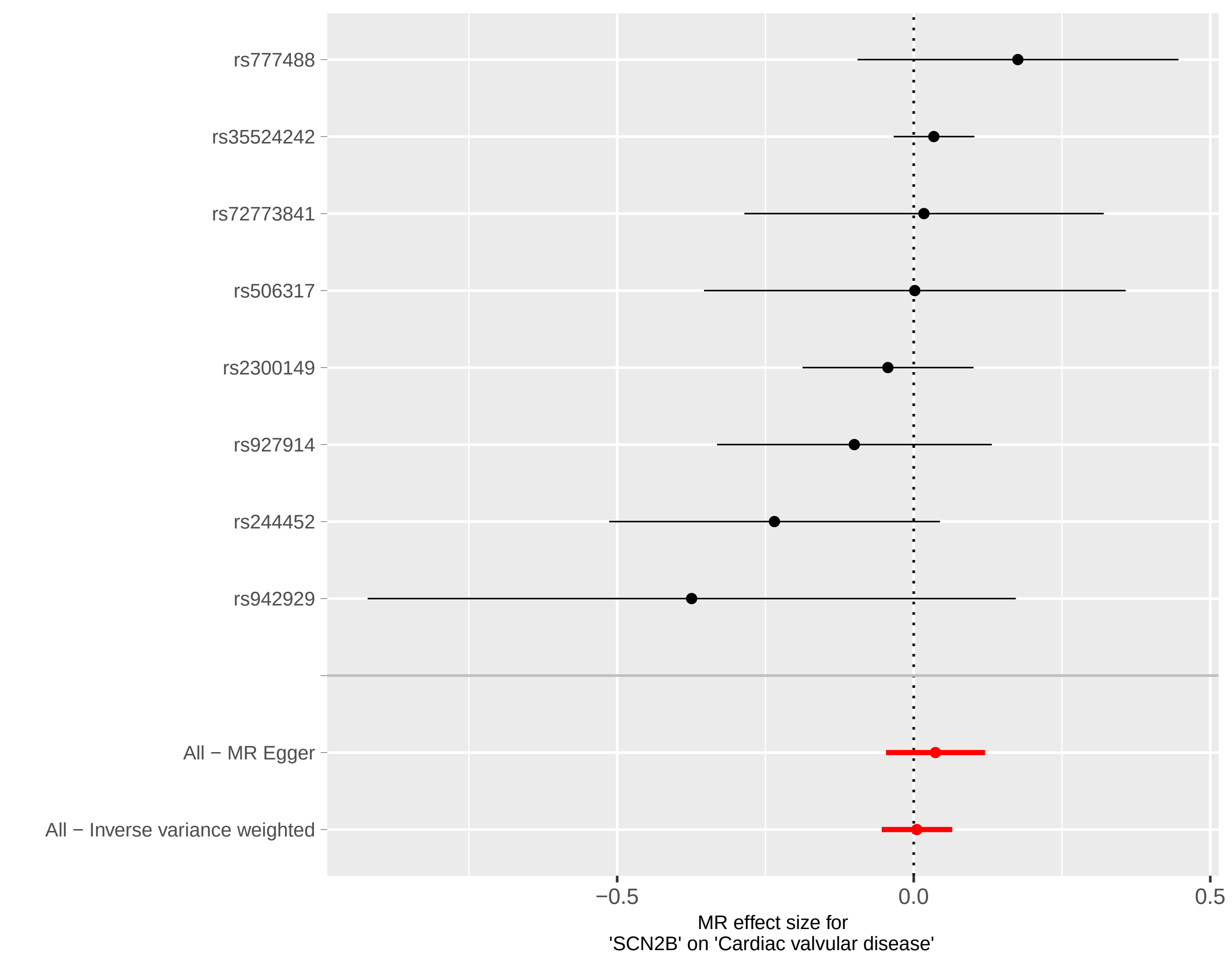
rs777488
rs35524242
rs72773841
rs506317
rs2300149
rs927914
rs244452
rs942929
All − MR Egger
All − Inverse variance weighted
−0.5
0.0
0.5
MR effect size for
'SCN2B' on 'Cardiac valvular disease'

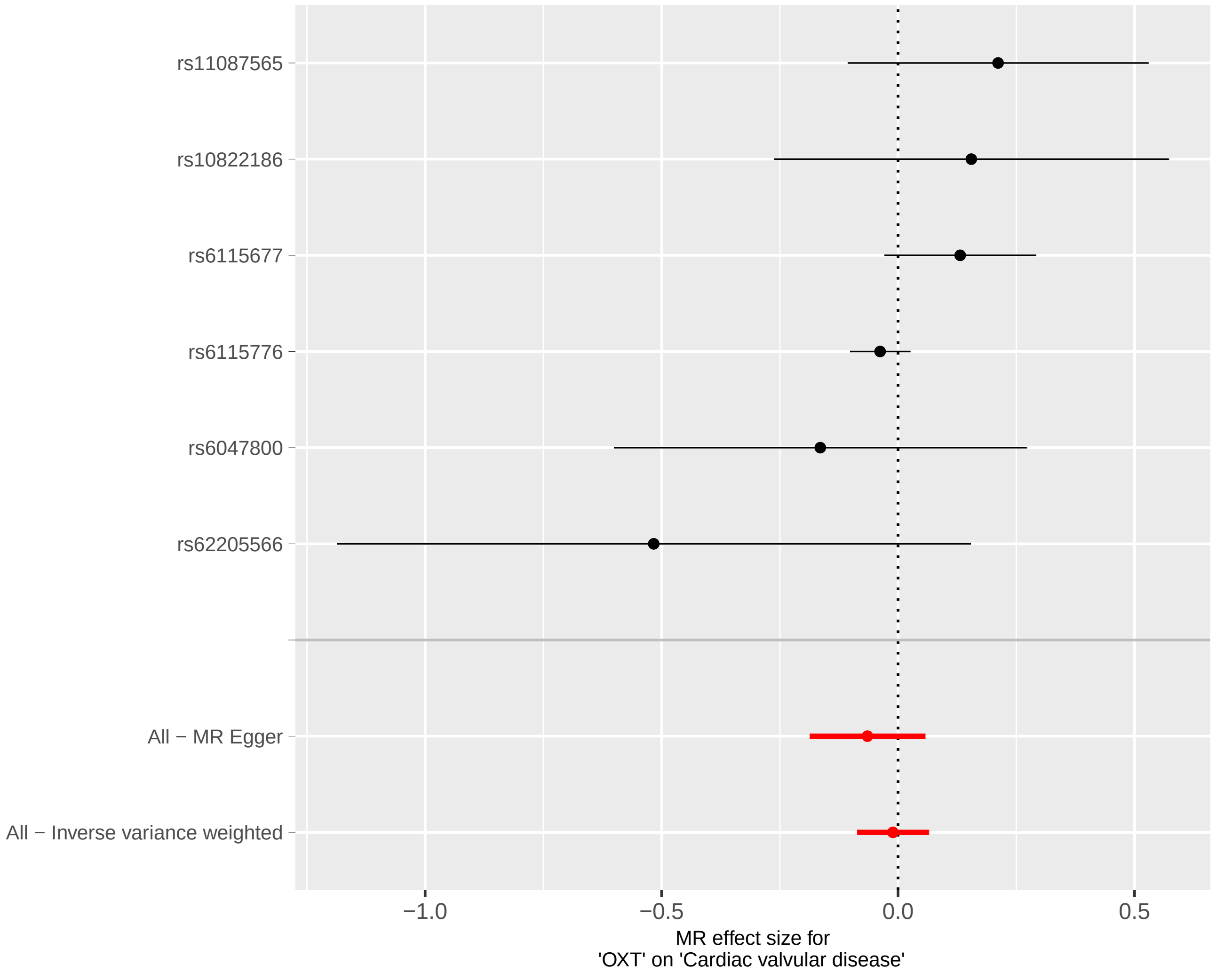
rs11087565
rs10822186
rs6115677
rs6115776
rs6047800
rs62205566
All − MR Egger
All − Inverse variance weighted
−1.0
−0.5
0.0
0.5
MR effect size for
'OXT' on 'Cardiac valvular disease'

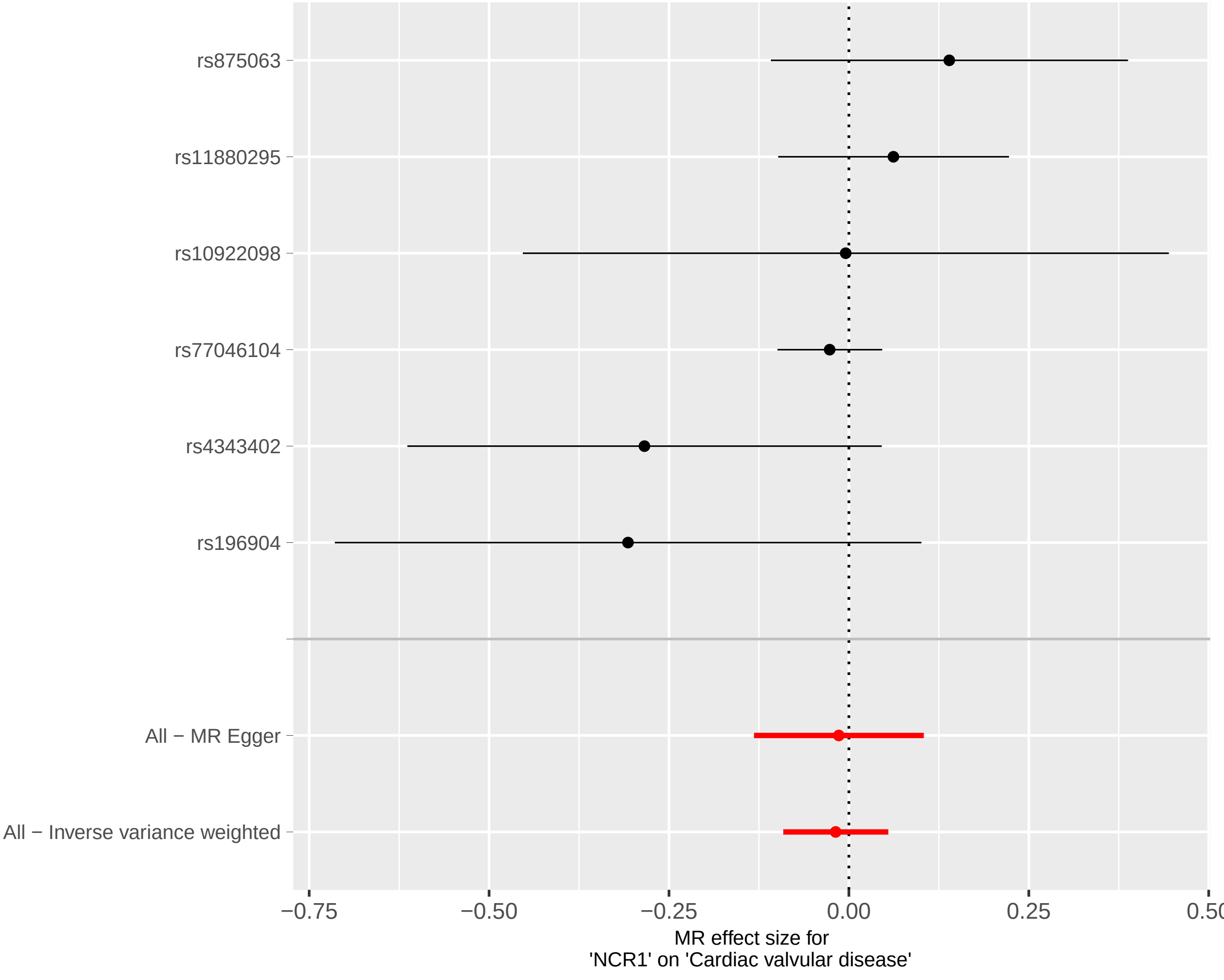
rs875063
rs11880295
rs10922098
rs77046104
rs4343402
rs196904
All − MR Egger
All − Inverse variance weighted
−0.75
−0.50
−0.25
0.00
0.25
0.50
MR effect size for
'NCR1' on 'Cardiac valvular disease'

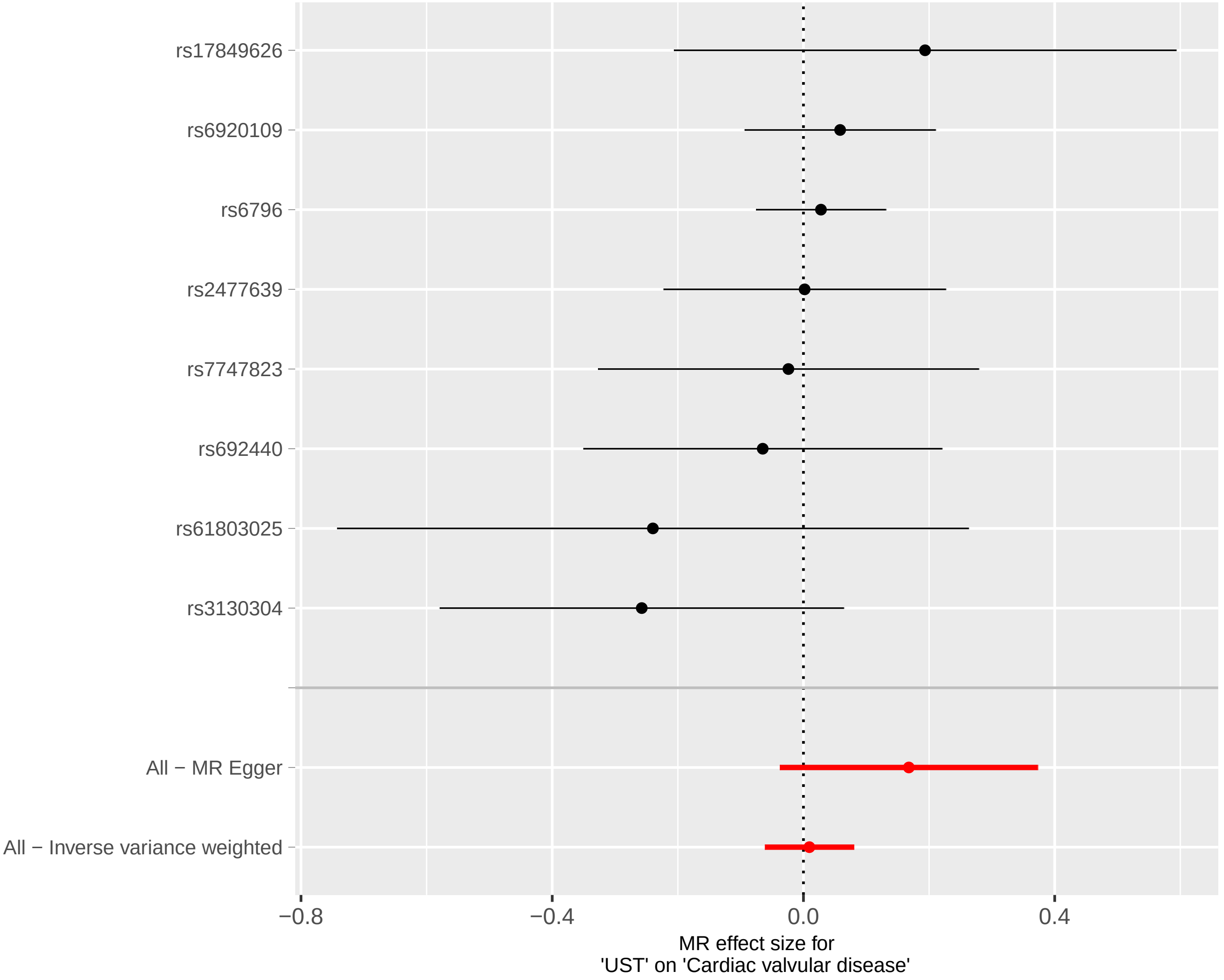

rs17849626
rs6920109
rs6796
rs2477639
rs7747823
rs692440
rs61803025
rs3130304
All − MR Egger
All − Inverse variance weighted
−0.8
−0.4
0.0
0.4
MR effect size for
'UST' on 'Cardiac valvular disease'

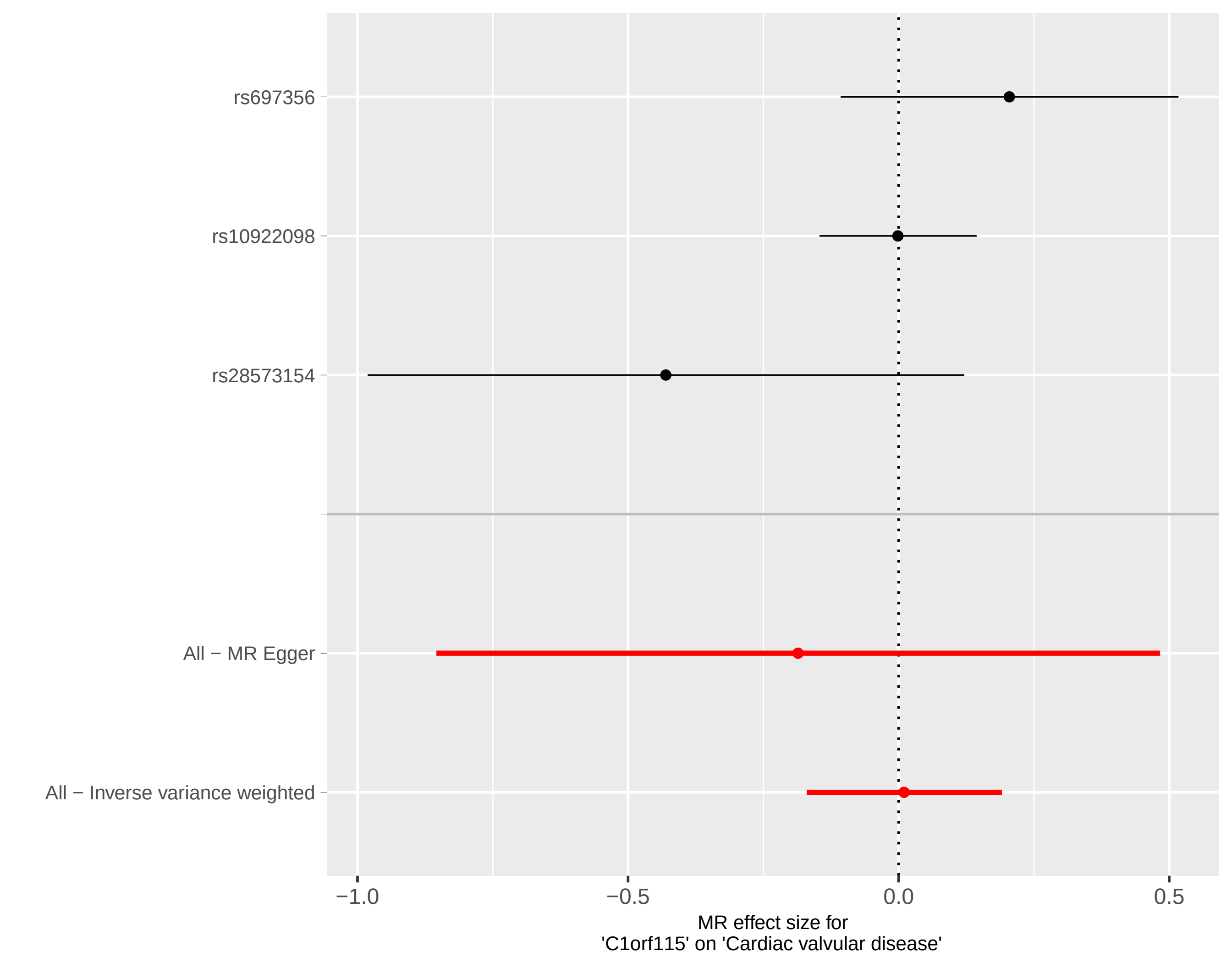

rs697356
rs10922098
rs28573154
All − MR Egger
All − Inverse variance weighted
−1.0
−0.5
0.0
0.5
MR effect size for
'C1orf115' on 'Cardiac valvular disease'

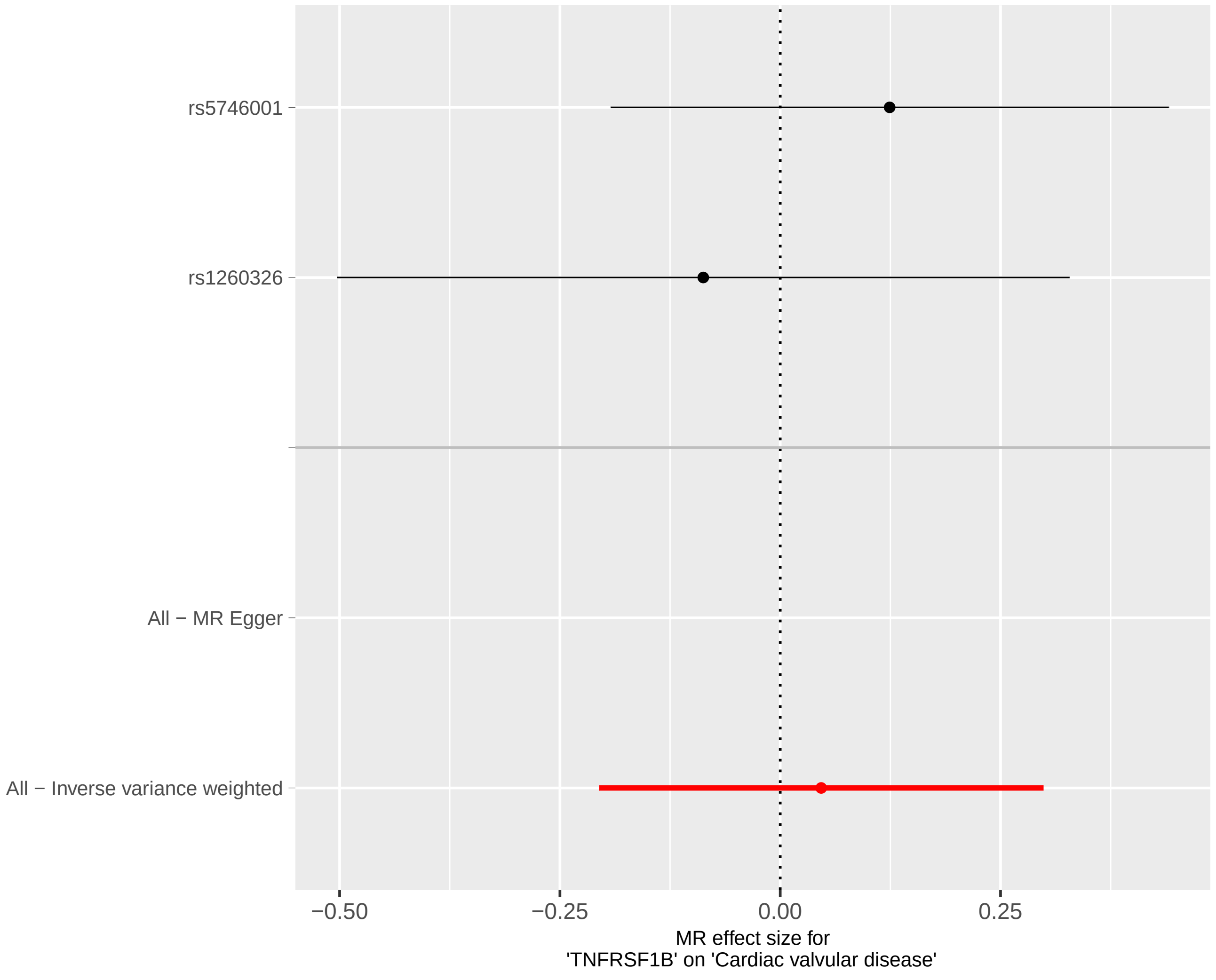
rs5746001
rs1260326
All − MR Egger
All − Inverse variance weighted
−0.50
−0.25
0.00
0.25
MR effect size for
'TNFRSF1B' on 'Cardiac valvular disease'

Insufficient number of SNPs

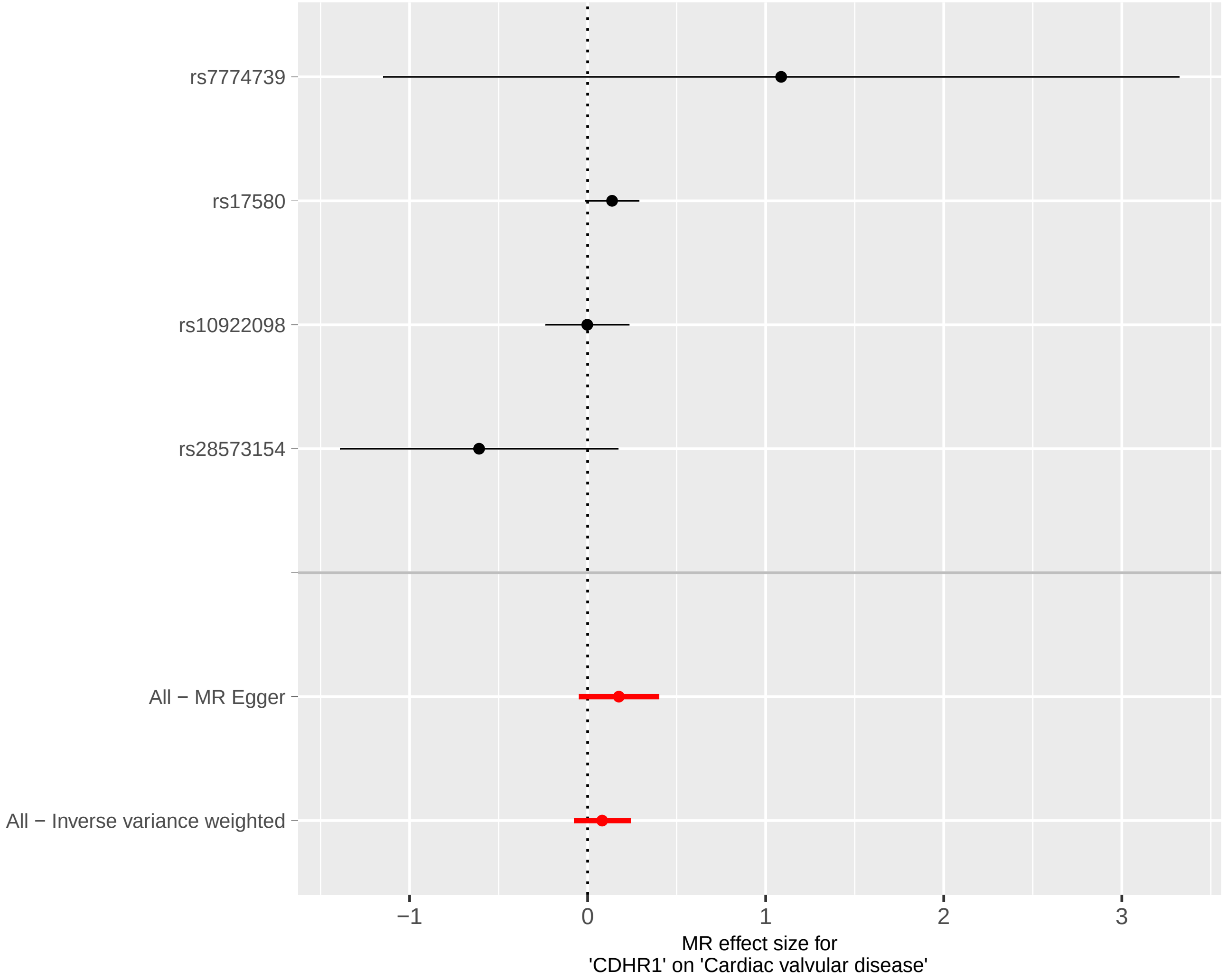

rs7774739
rs17580
rs10922098
rs28573154
All − MR Egger
All − Inverse variance weighted
−1
0
1
2
3
MR effect size for
'CDHR1' on 'Cardiac valvular disease'

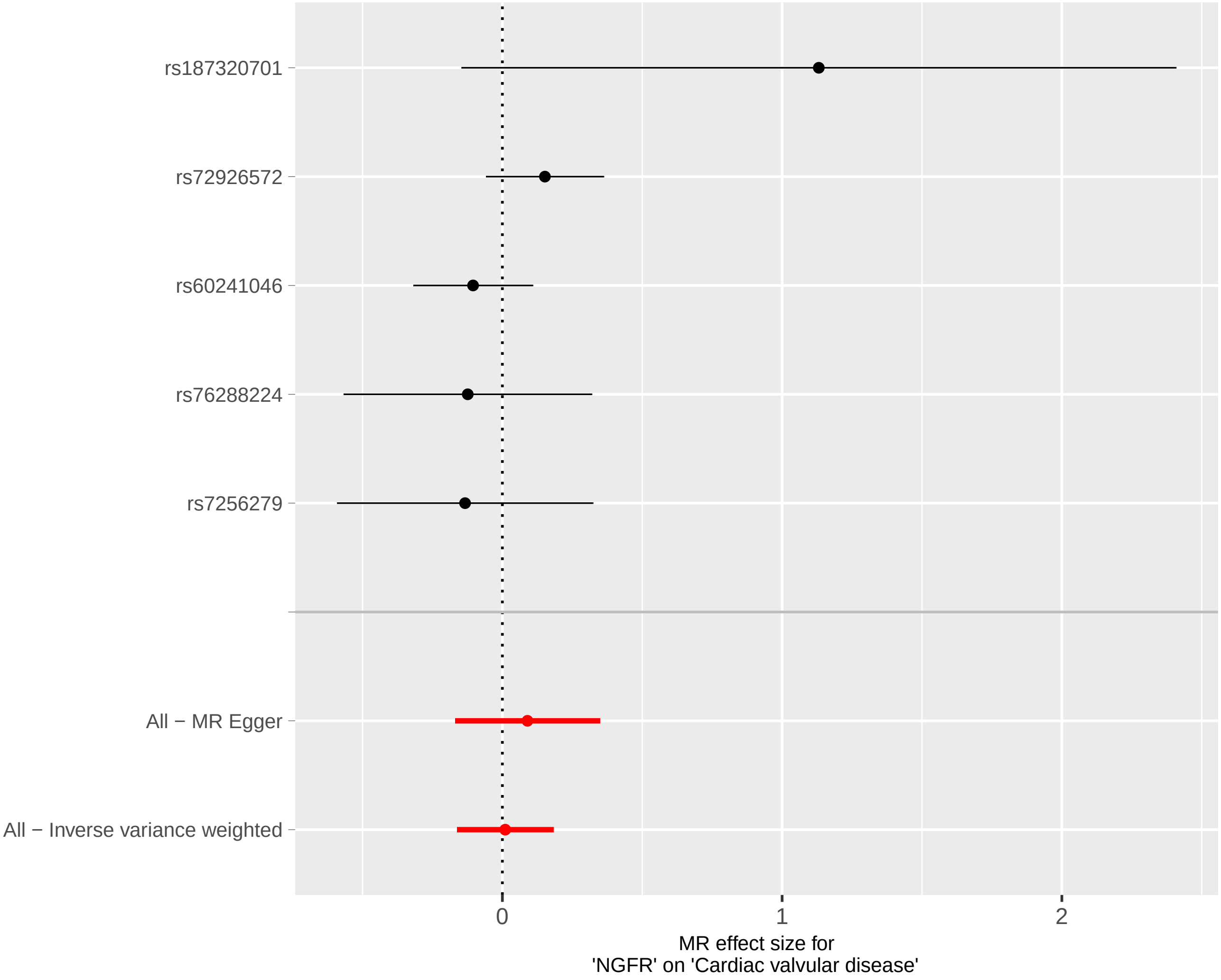
rs187320701
rs72926572
rs60241046
rs76288224
rs7256279
All − MR Egger
All − Inverse variance weighted
0
1
2
MR effect size for
'NGFR' on 'Cardiac valvular disease'

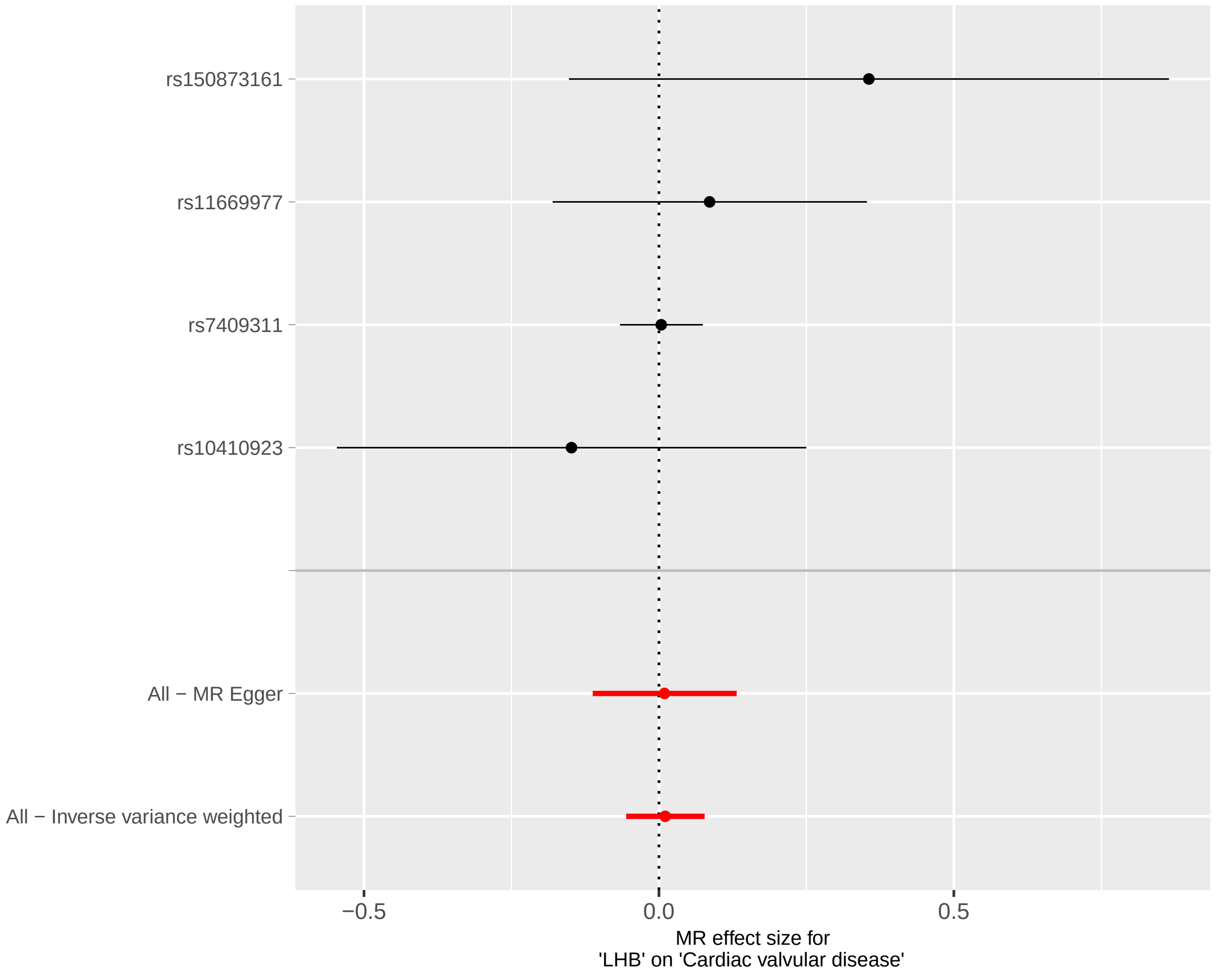
rs150873161
rs11669977
rs7409311
rs10410923
All − MR Egger
All − Inverse variance weighted
−0.5
0.0
0.5
MR effect size for
'LHB' on 'Cardiac valvular disease'

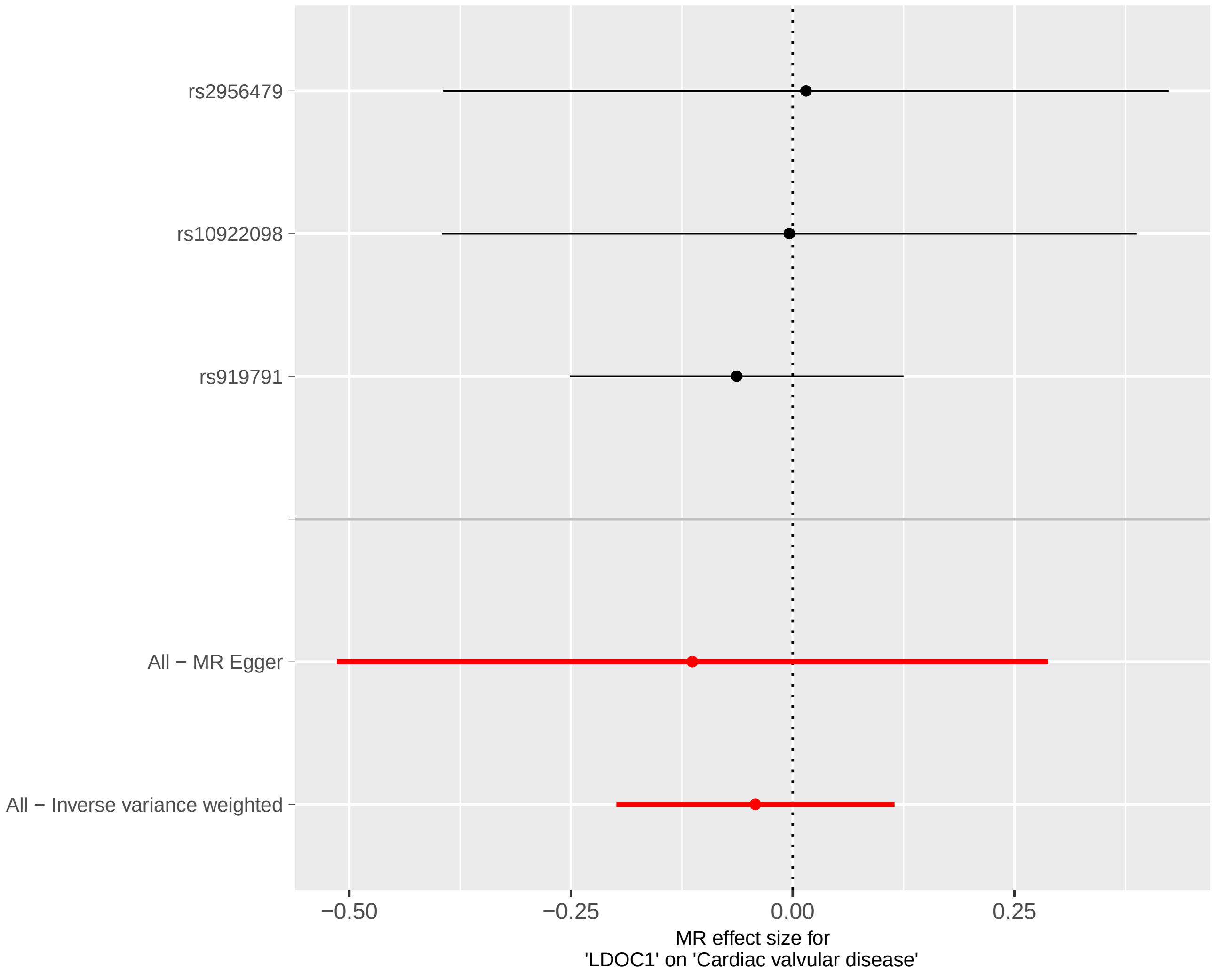
rs2956479
rs10922098
rs919791
All − MR Egger
All − Inverse variance weighted
−0.50
−0.25
0.00
0.25
MR effect size for
'LDOC1' on 'Cardiac valvular disease'

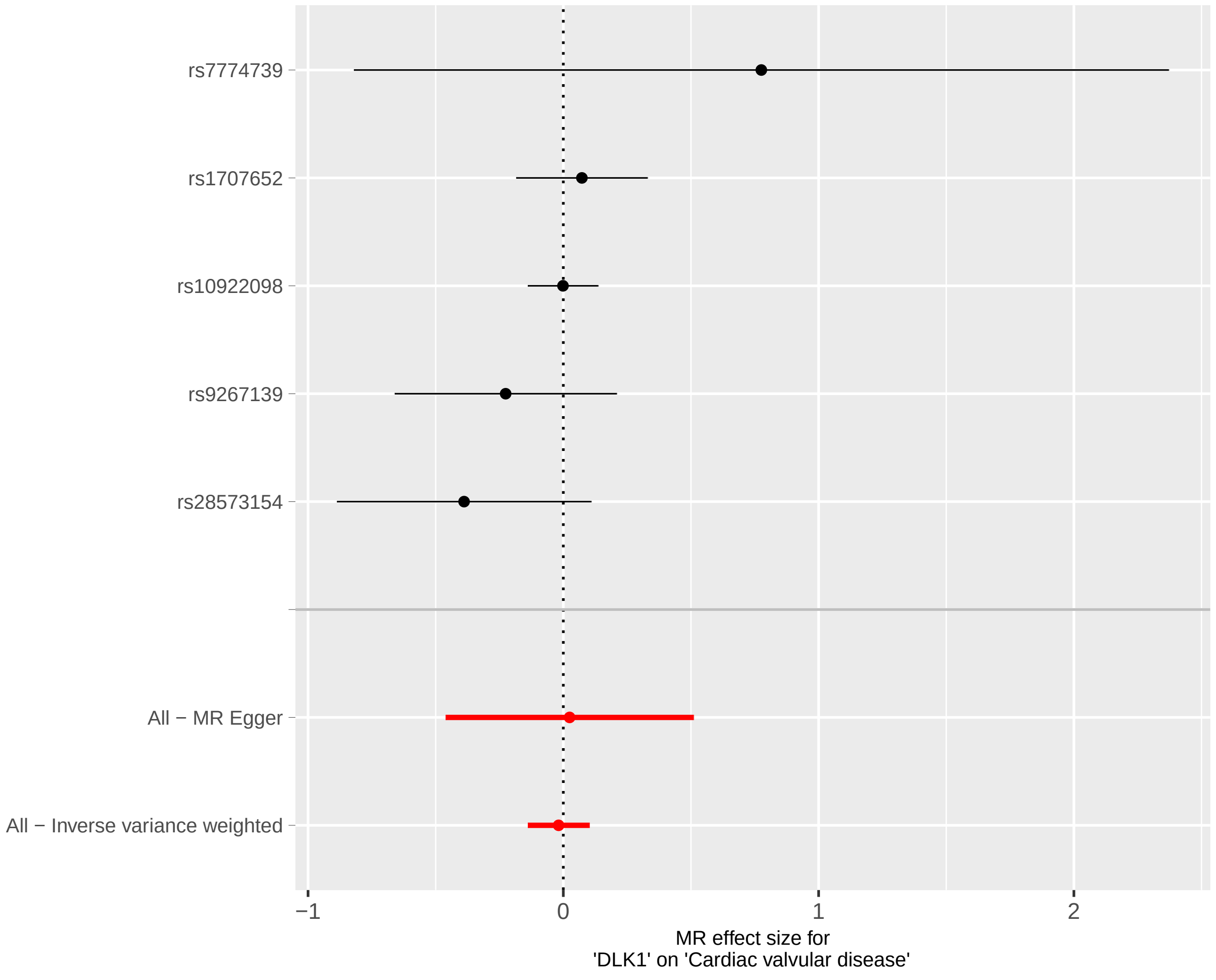
rs7774739
rs1707652
rs10922098
rs9267139
rs28573154
All − MR Egger
All − Inverse variance weighted
−1
0
1
2
MR effect size for
'DLK1' on 'Cardiac valvular disease'

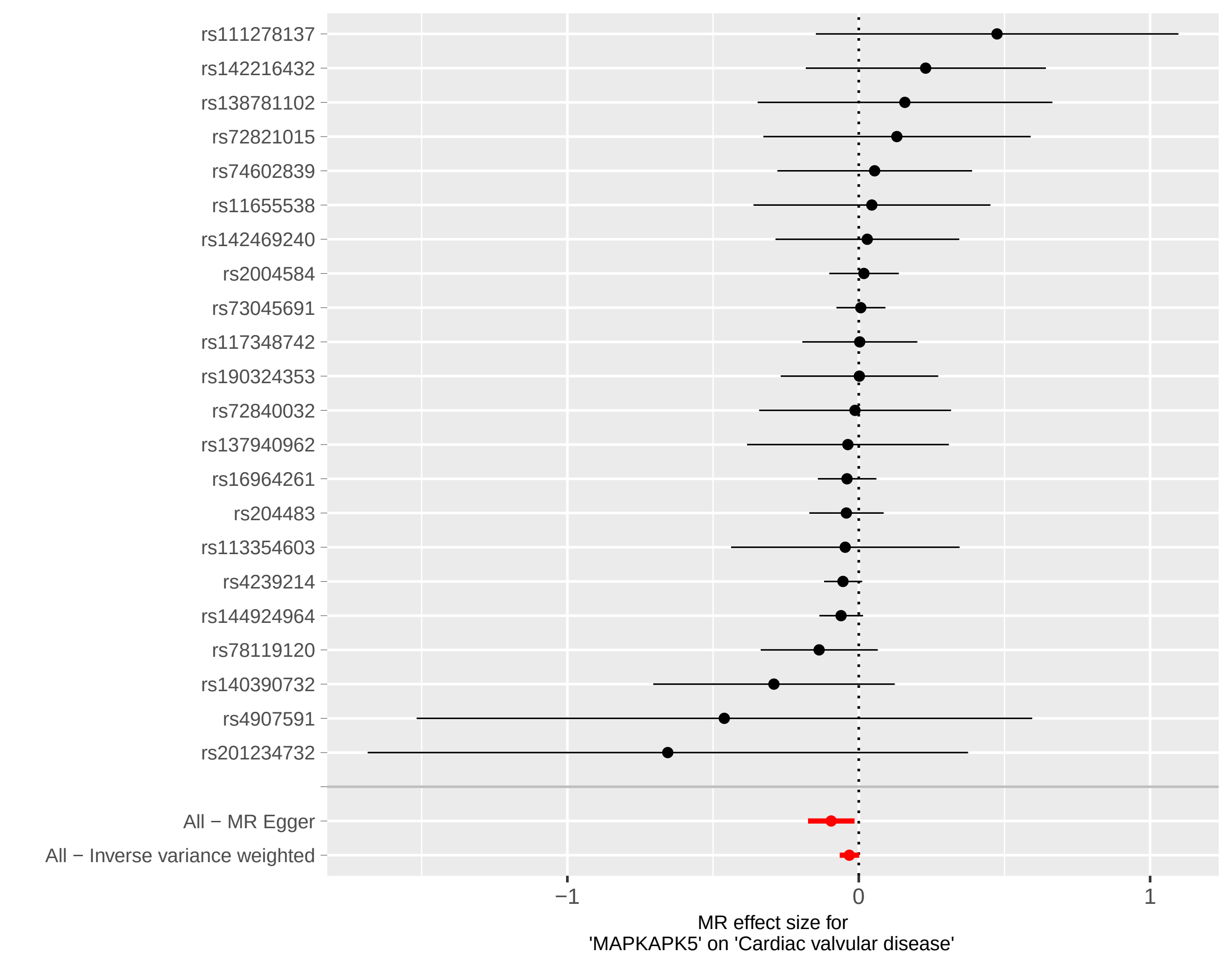
rs111278137
rs142216432
rs138781102
rs72821015
rs74602839
rs11655538
rs142469240
rs2004584
rs73045691
rs117348742
rs190324353
rs72840032
rs137940962
rs16964261
rs204483
rs113354603
rs4239214
rs144924964
rs78119120
rs140390732
rs4907591
rs201234732
All − MR Egger
All − Inverse variance weighted
−1
0
1
MR effect size for
'MAPKAPK5' on 'Cardiac valvular disease'

Insufficient number of SNPs

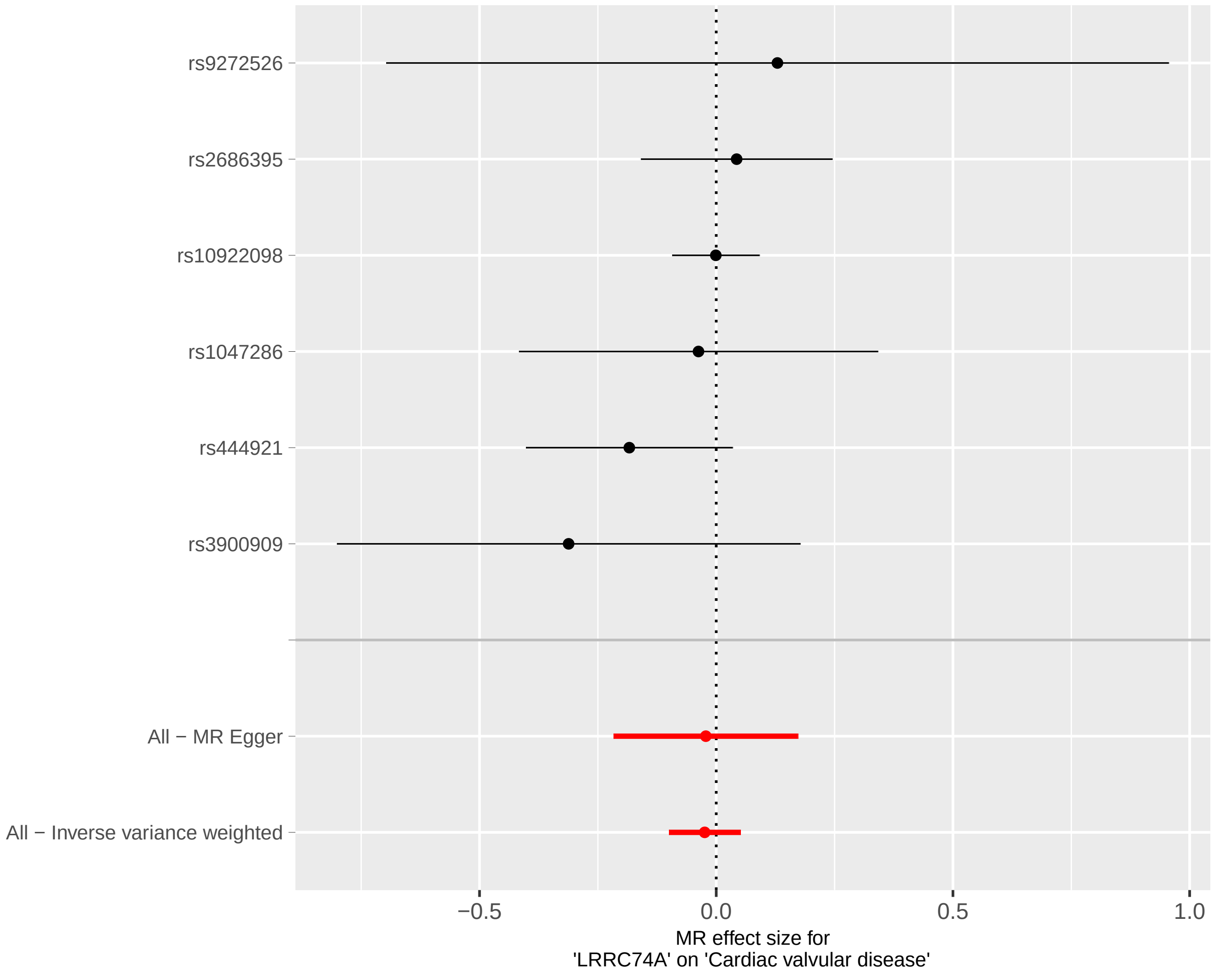
rs9272526
rs2686395
rs10922098
rs1047286
rs444921
rs3900909
All − MR Egger
All − Inverse variance weighted
−0.5
0.0
0.5
1.0
MR effect size for
'LRRC74A' on 'Cardiac valvular disease'

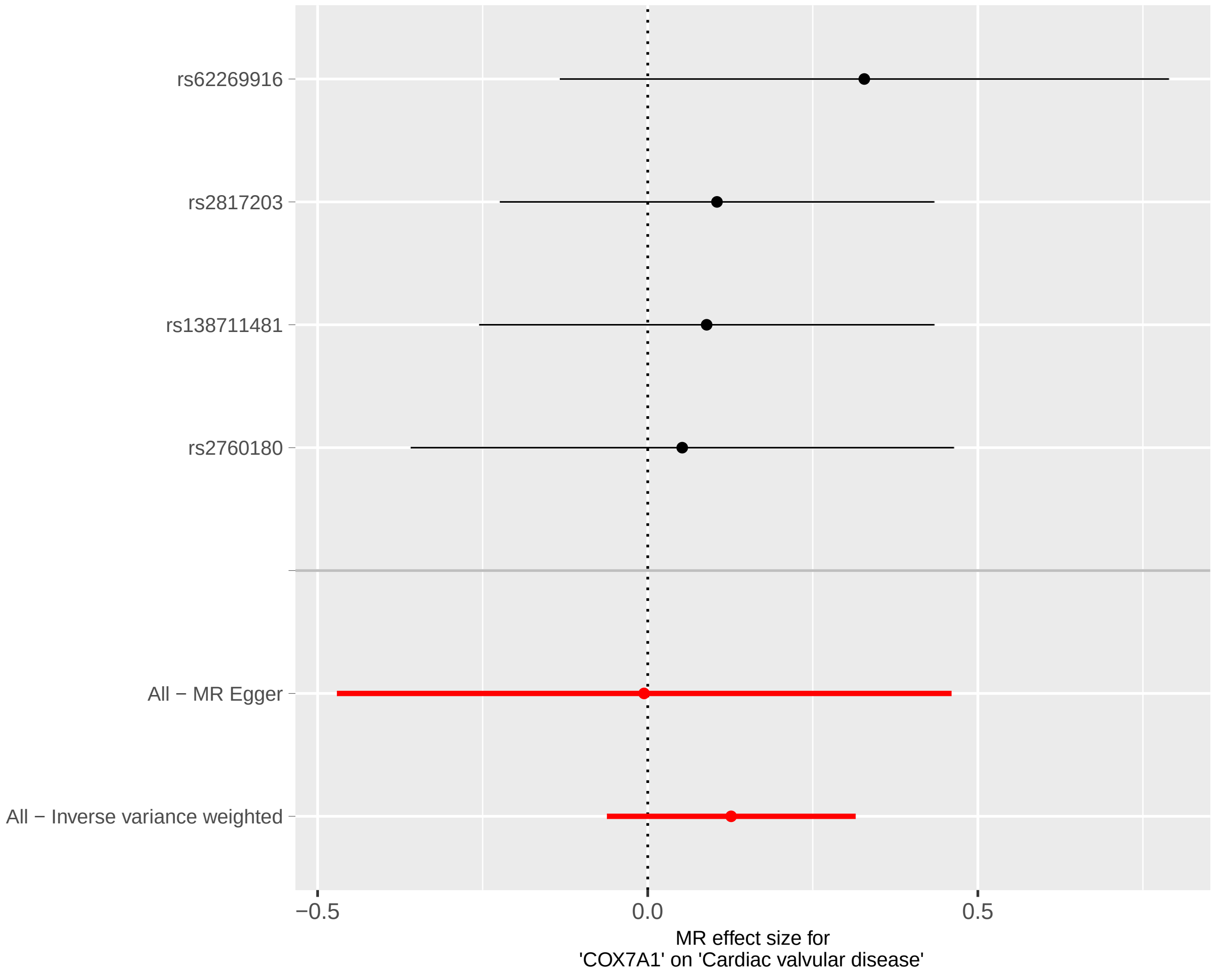

rs62269916
rs2817203
rs138711481
rs2760180
All − MR Egger
All − Inverse variance weighted
−0.5
0.0
0.5
MR effect size for
'COX7A1' on 'Cardiac valvular disease'

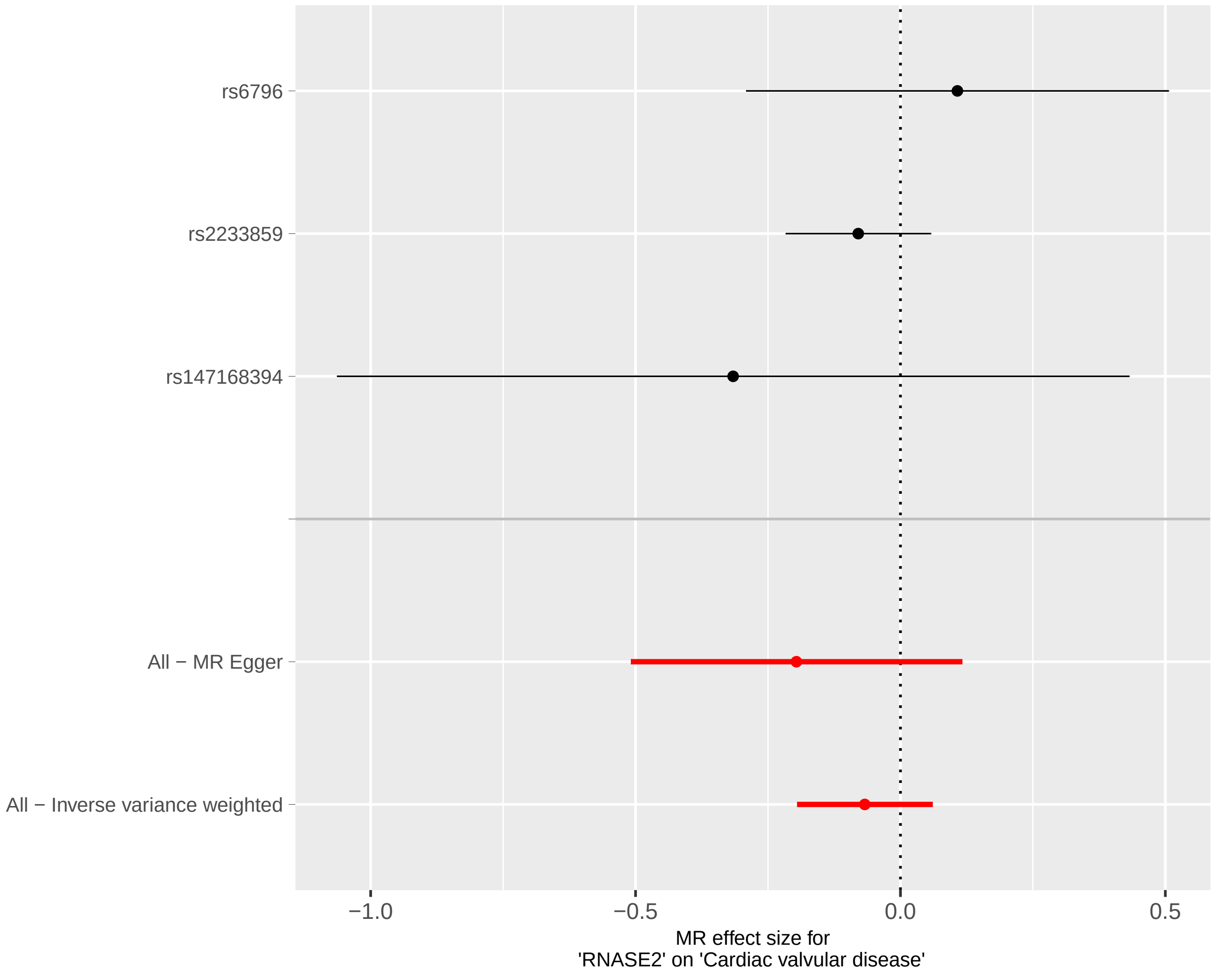
rs6796
rs2233859
rs147168394
All − MR Egger
All − Inverse variance weighted
−1.0
−0.5
0.0
0.5
MR effect size for
'RNASE2' on 'Cardiac valvular disease'

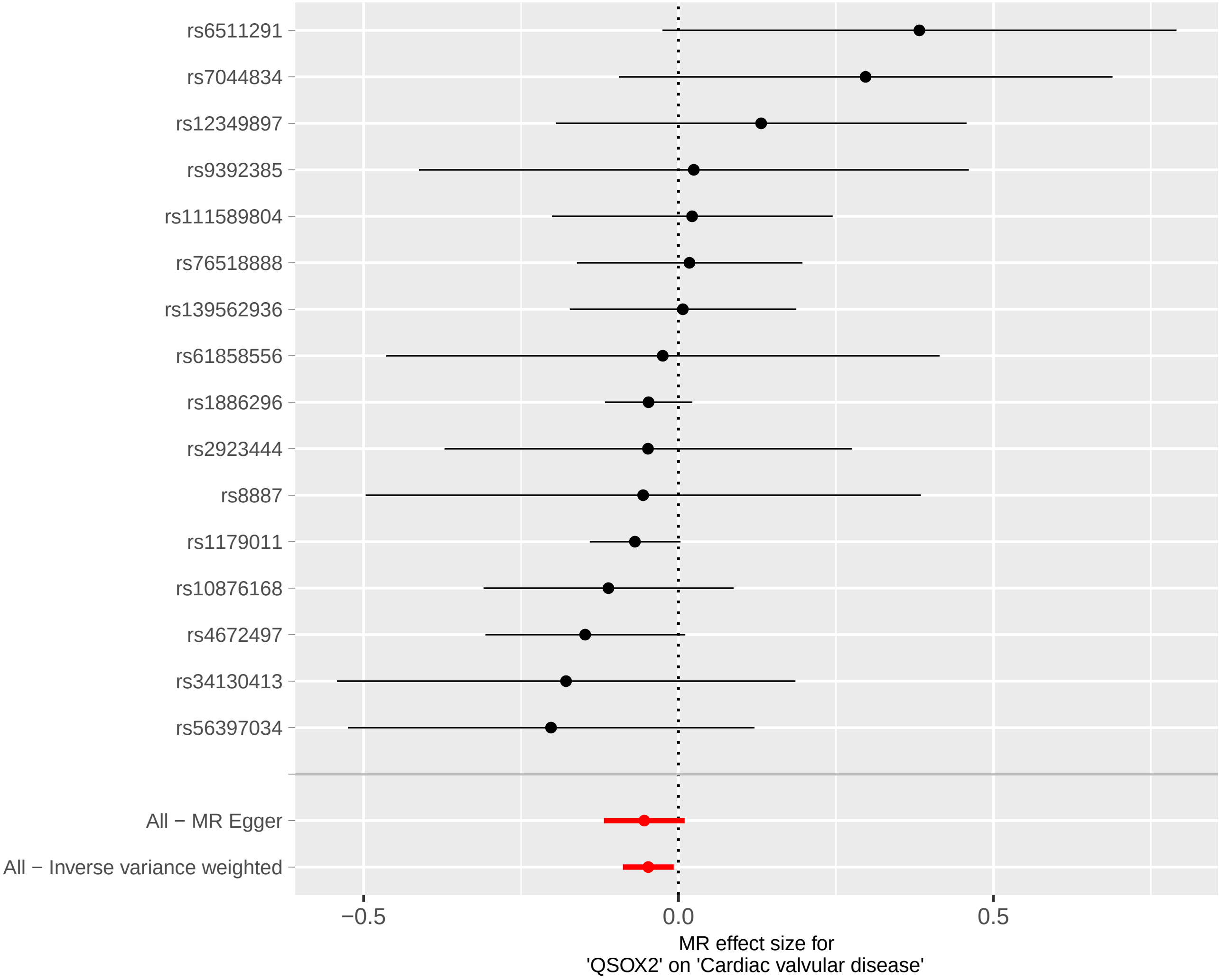
rs6511291
rs7044834
rs12349897
rs9392385
rs111589804
rs76518888
rs139562936
rs61858556
rs1886296
rs2923444
rs8887
rs1179011
rs10876168
rs4672497
rs34130413
rs56397034
All − MR Egger
All − Inverse variance weighted
−0.5
0.0
0.5
MR effect size for
'QSOX2' on 'Cardiac valvular disease'

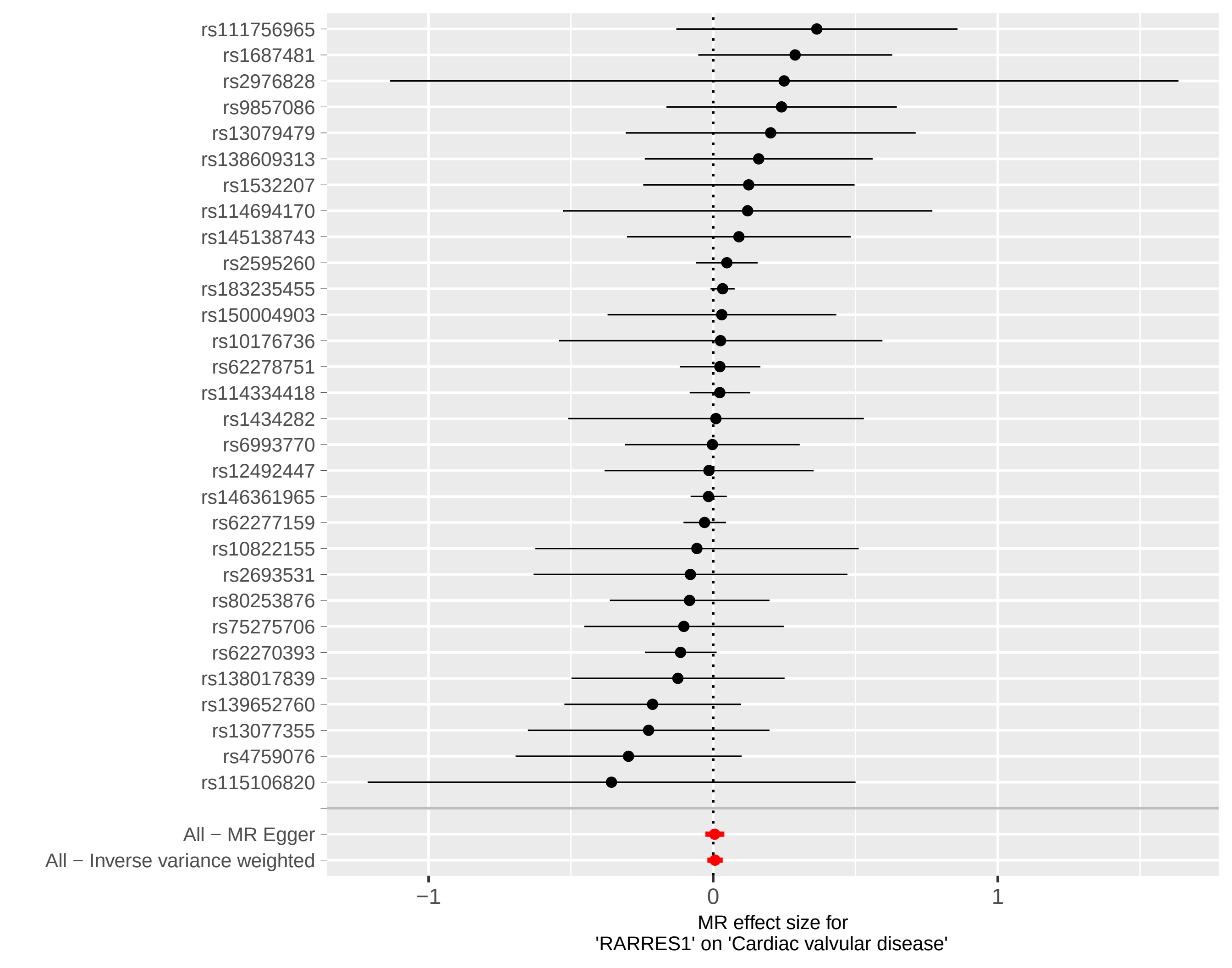
rs111756965
rs1687481
rs2976828
rs9857086
rs13079479
rs138609313
rs1532207
rs114694170
rs145138743
rs2595260
rs183235455
rs150004903
rs10176736
rs62278751
rs114334418
rs1434282
rs6993770
rs12492447
rs146361965
rs62277159
rs10822155
rs2693531
rs80253876
rs75275706
rs62270393
rs138017839
rs139652760
rs13077355
rs4759076
rs115106820
All − MR Egger
All − Inverse variance weighted
−1
0
1
MR effect size for
'RARRES1' on 'Cardiac valvular disease'

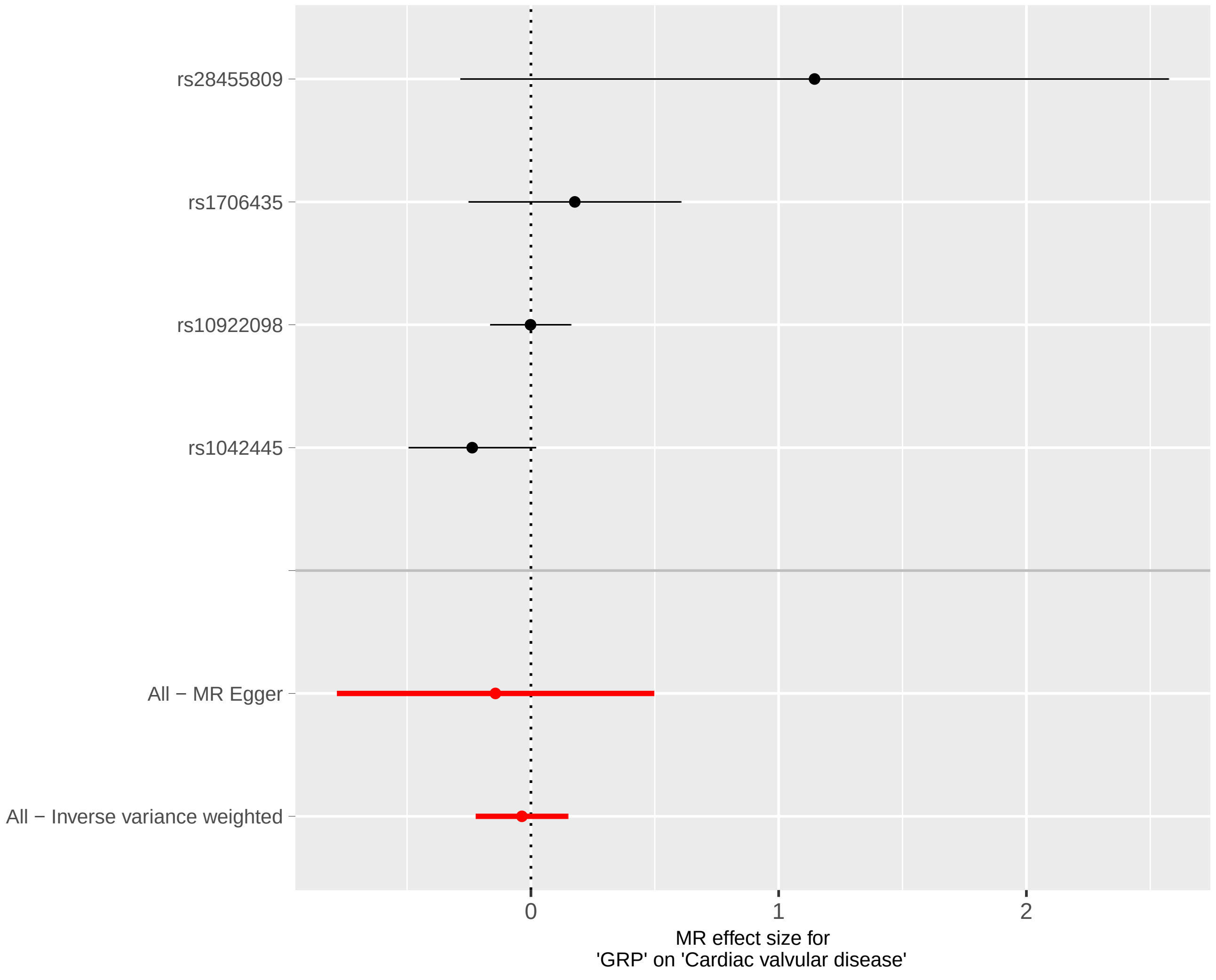
rs28455809
rs1706435
rs10922098
rs1042445
All − MR Egger
All − Inverse variance weighted
0
1
2
MR effect size for
'GRP' on 'Cardiac valvular disease'

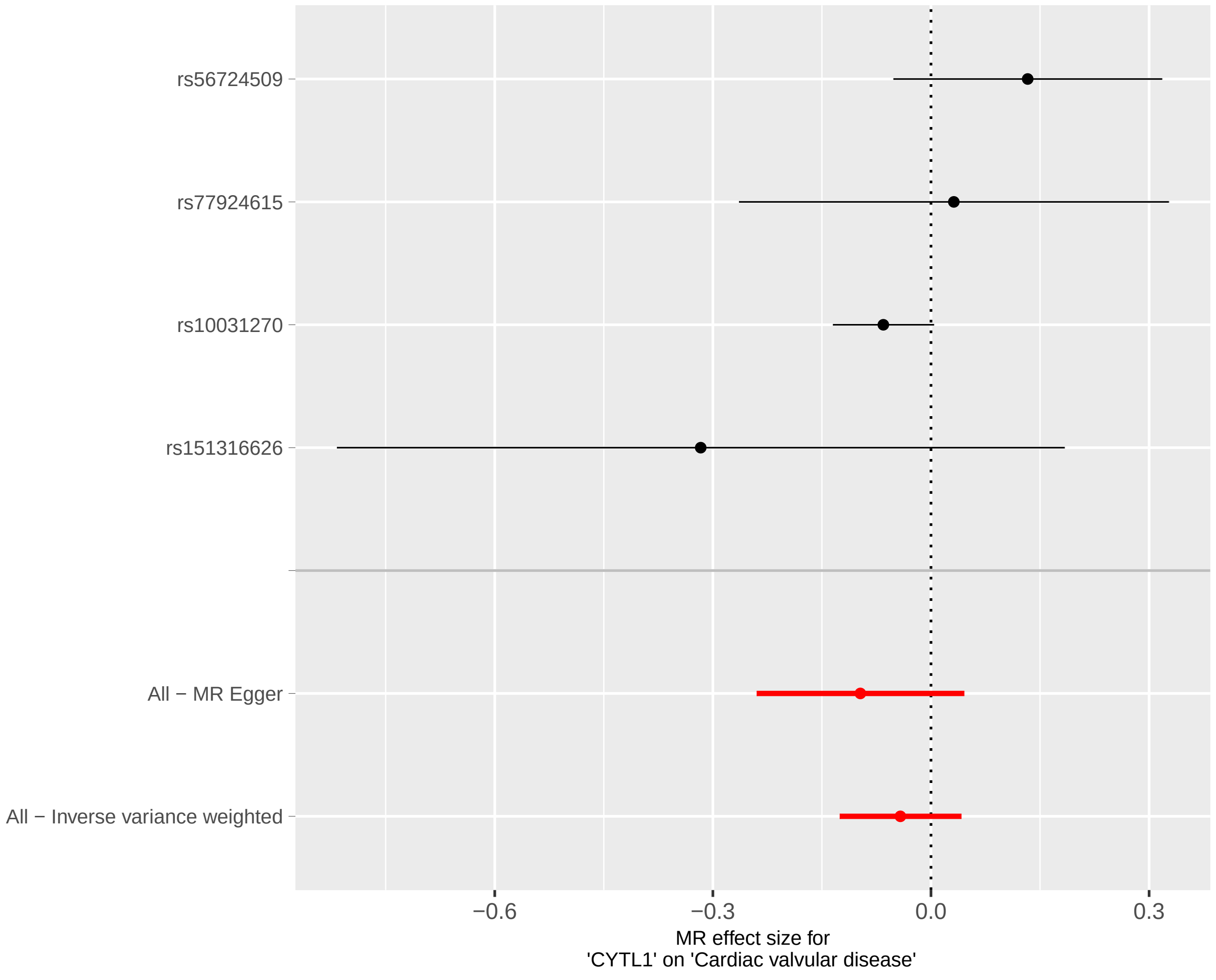
rs56724509
rs77924615
rs10031270
rs151316626
All − MR Egger
All − Inverse variance weighted
−0.6
−0.3
0.0
0.3
MR effect size for
'CYTL1' on 'Cardiac valvular disease'

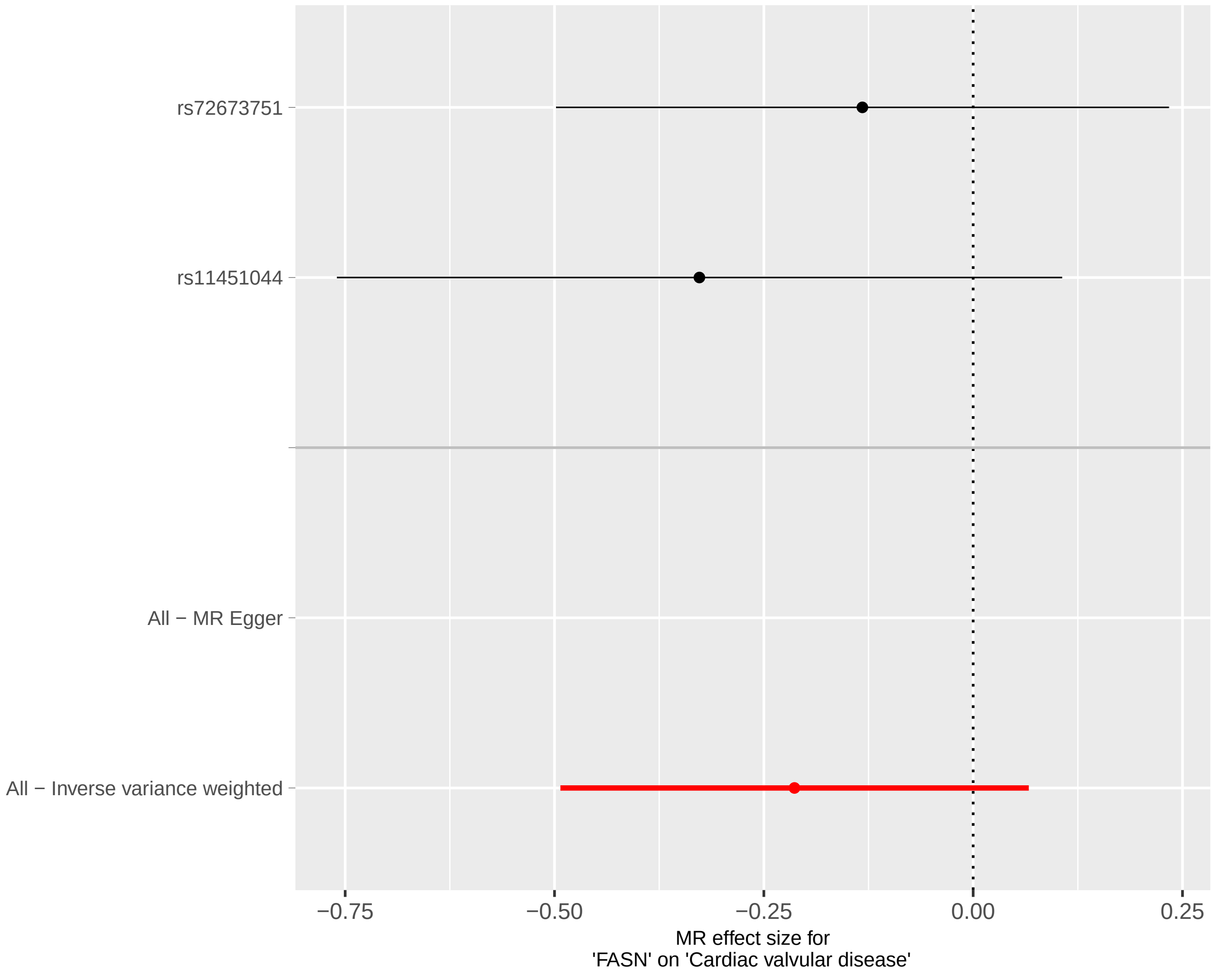

rs72673751
rs11451044
All − MR Egger
All − Inverse variance weighted
−0.75
−0.50
−0.25
0.00
0.25
MR effect size for
'FASN' on 'Cardiac valvular disease'

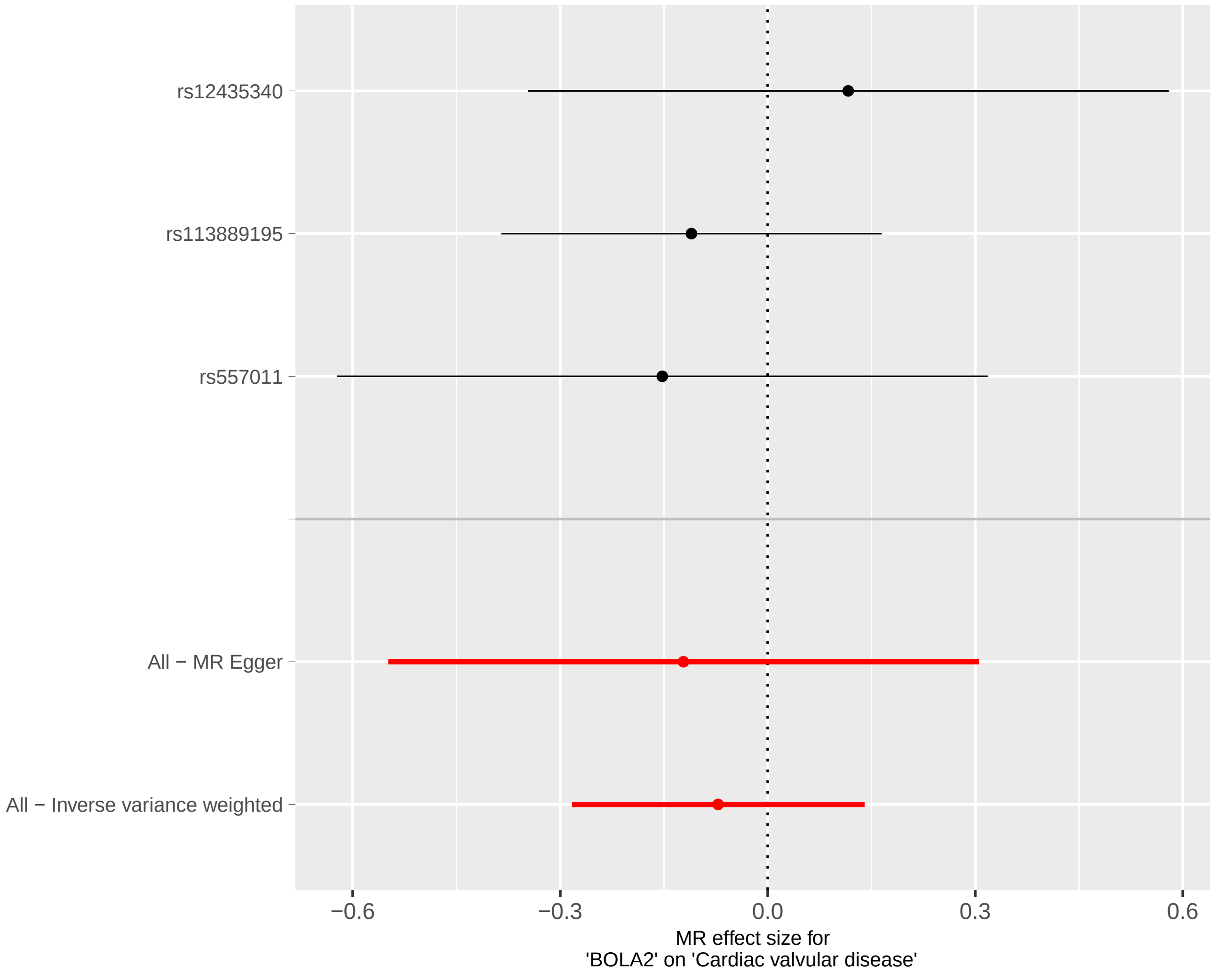
rs12435340
rs113889195
rs557011
All − MR Egger
All − Inverse variance weighted
−0.6
−0.3
0.0
0.3
0.6
MR effect size for
'BOLA2' on 'Cardiac valvular disease'

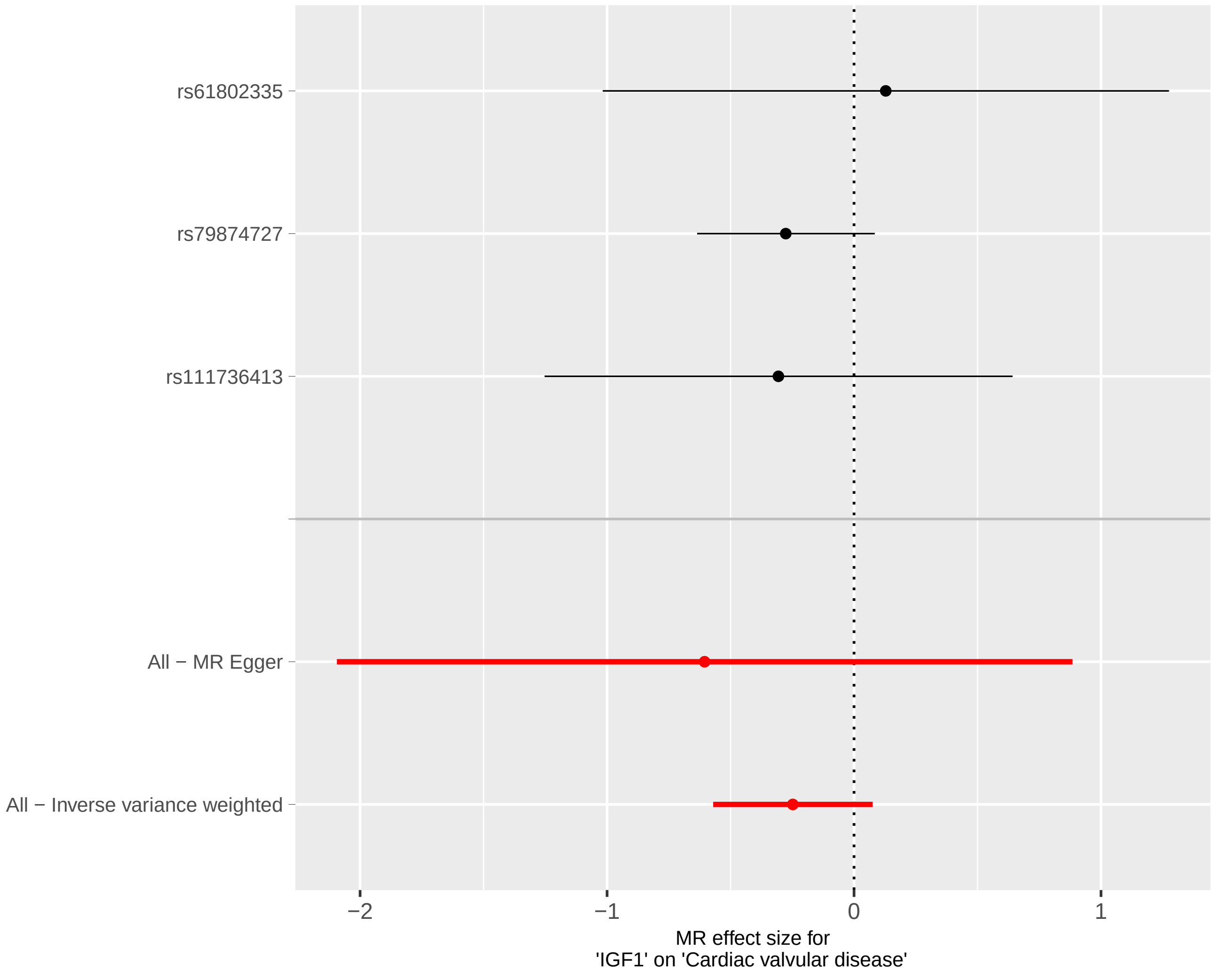
rs61802335
rs79874727
rs111736413
All − MR Egger
All − Inverse variance weighted
−2
−1
0
1
MR effect size for
'IGF1' on 'Cardiac valvular disease'

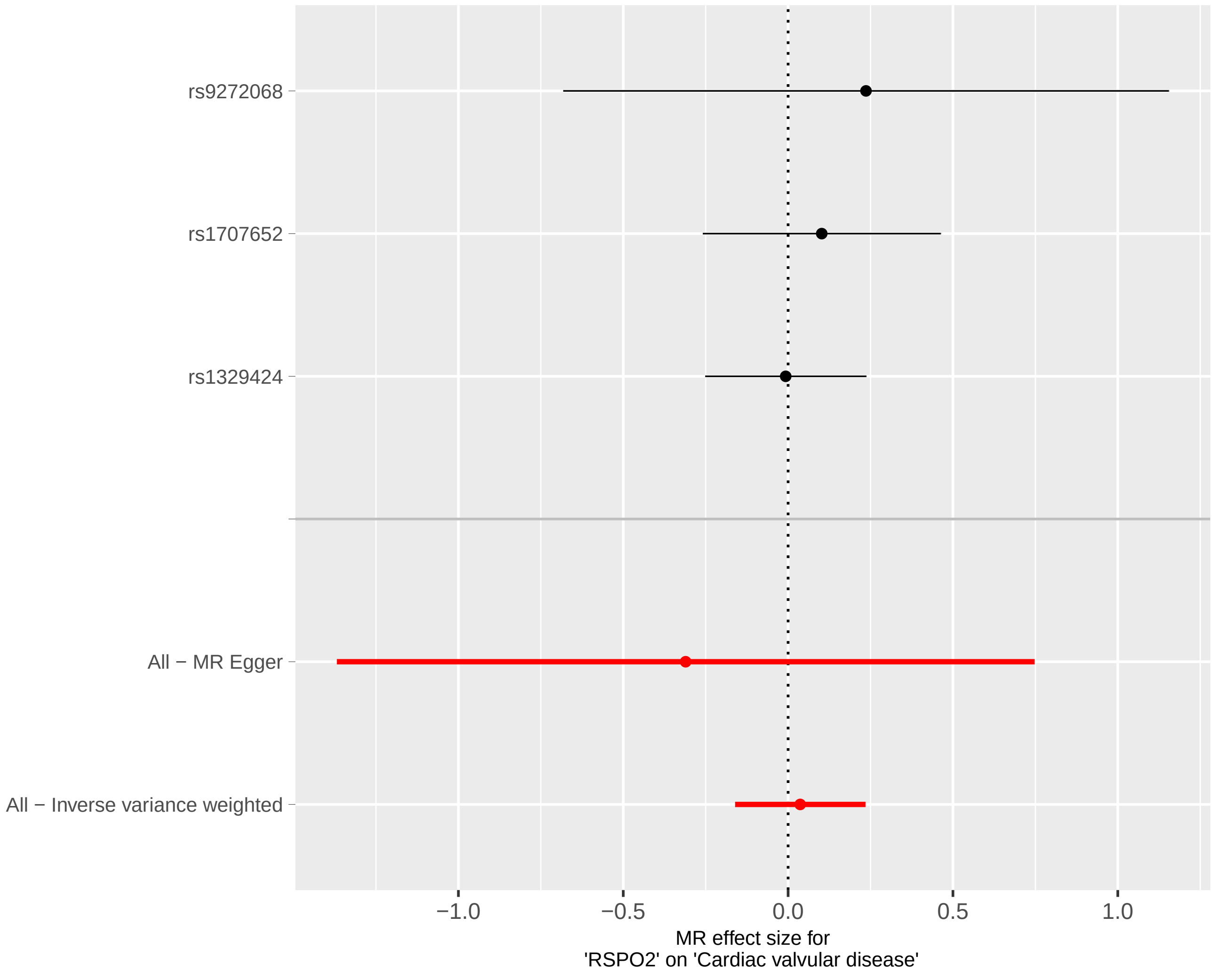
rs9272068
rs1707652
rs1329424
All − MR Egger
All − Inverse variance weighted
−1.0
−0.5
0.0
0.5
1.0
MR effect size for
'RSPO2' on 'Cardiac valvular disease'

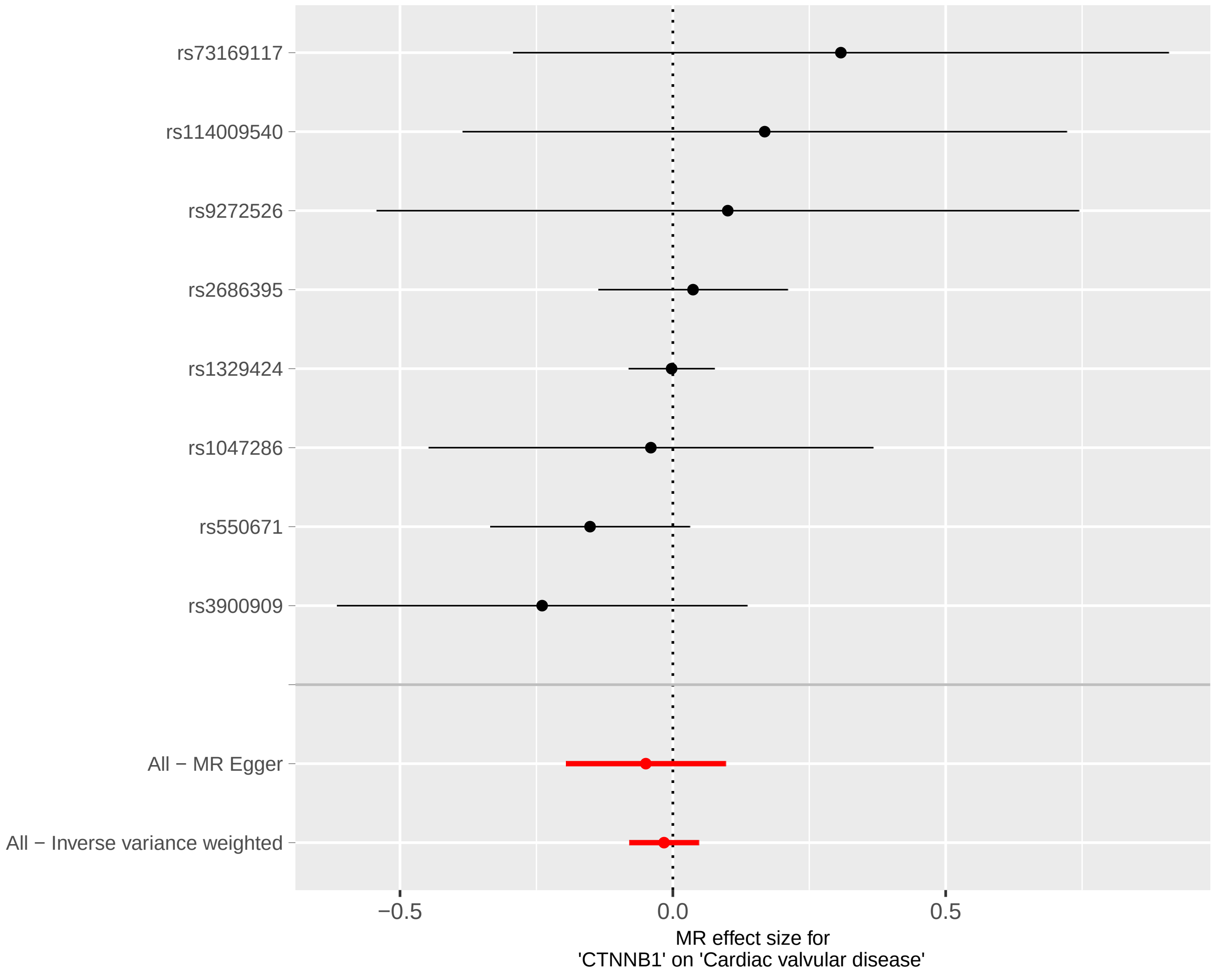
rs73169117
rs114009540
rs9272526
rs2686395
rs1329424
rs1047286
rs550671
rs3900909
All − MR Egger
All − Inverse variance weighted
−0.5
0.0
0.5
MR effect size for
'CTNNB1' on 'Cardiac valvular disease'

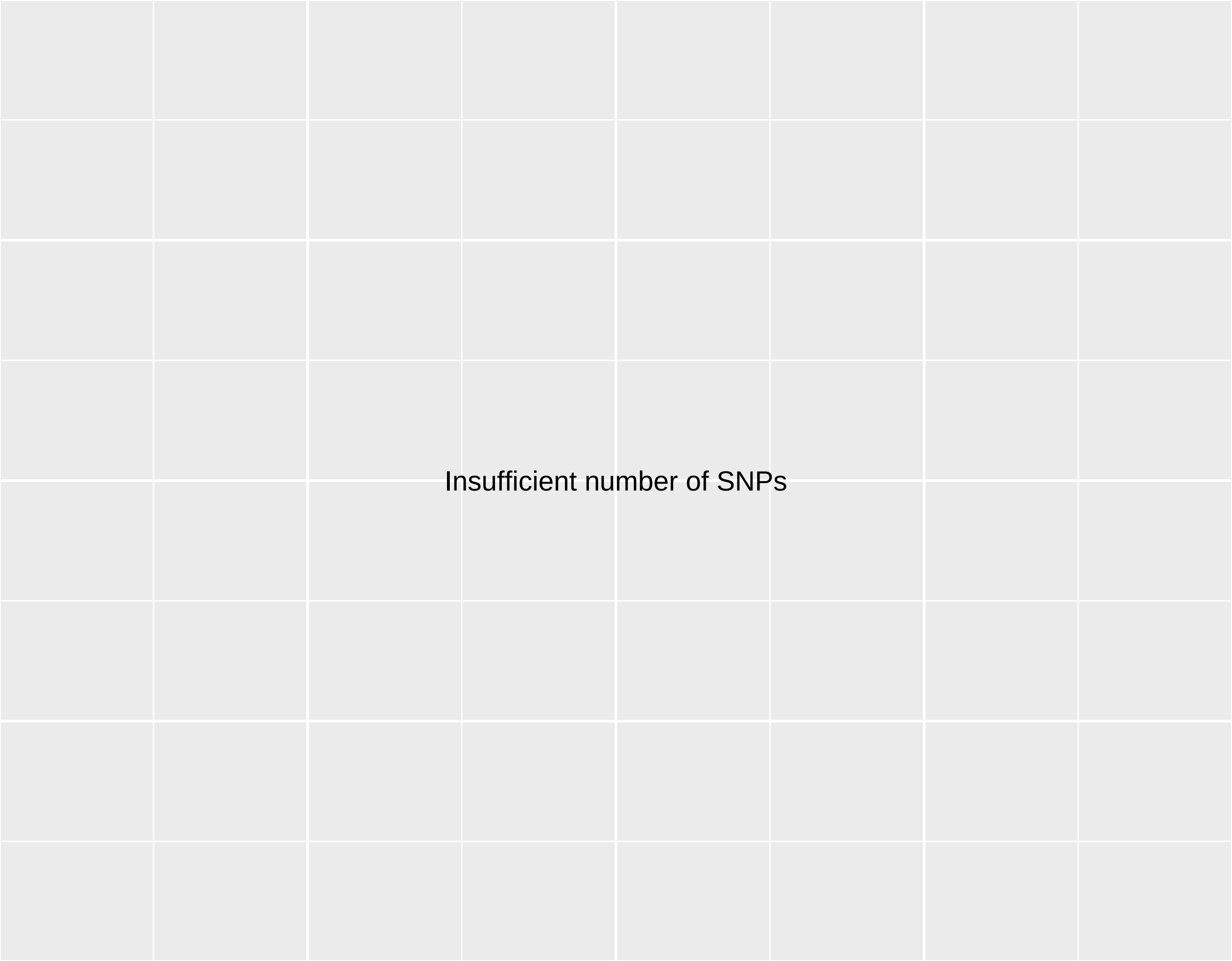
Insufficient number of SNPs

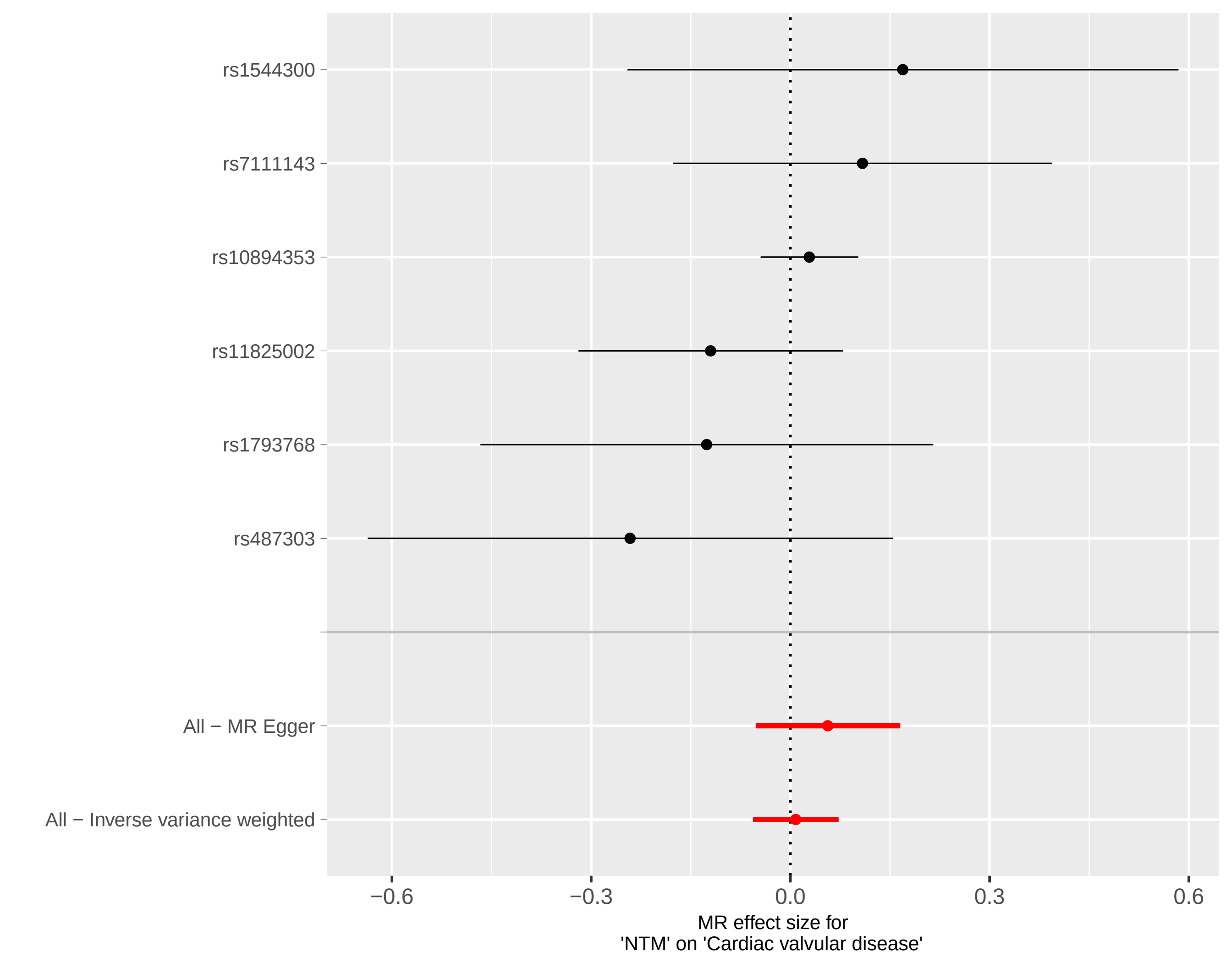
rs1544300
rs7111143
rs10894353
rs11825002
rs1793768
rs487303
All − MR Egger
All − Inverse variance weighted
−0.6
−0.3
0.0
0.3
0.6
MR effect size for
'NTM' on 'Cardiac valvular disease'

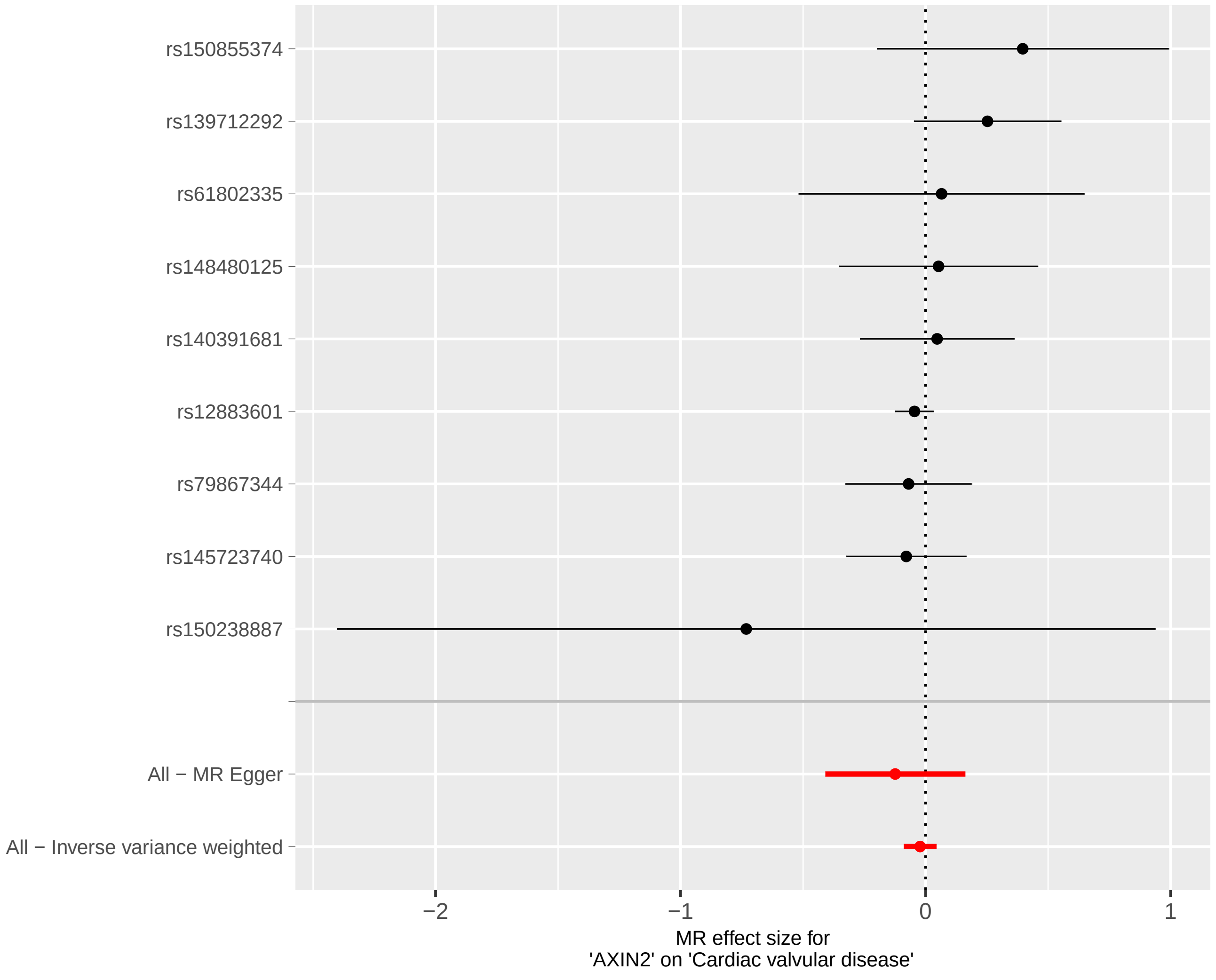

rs150855374
rs139712292
rs61802335
rs148480125
rs140391681
rs12883601
rs79867344
rs145723740
rs150238887
All − MR Egger
All − Inverse variance weighted
−2
−1
0
1
MR effect size for
'AXIN2' on 'Cardiac valvular disease'

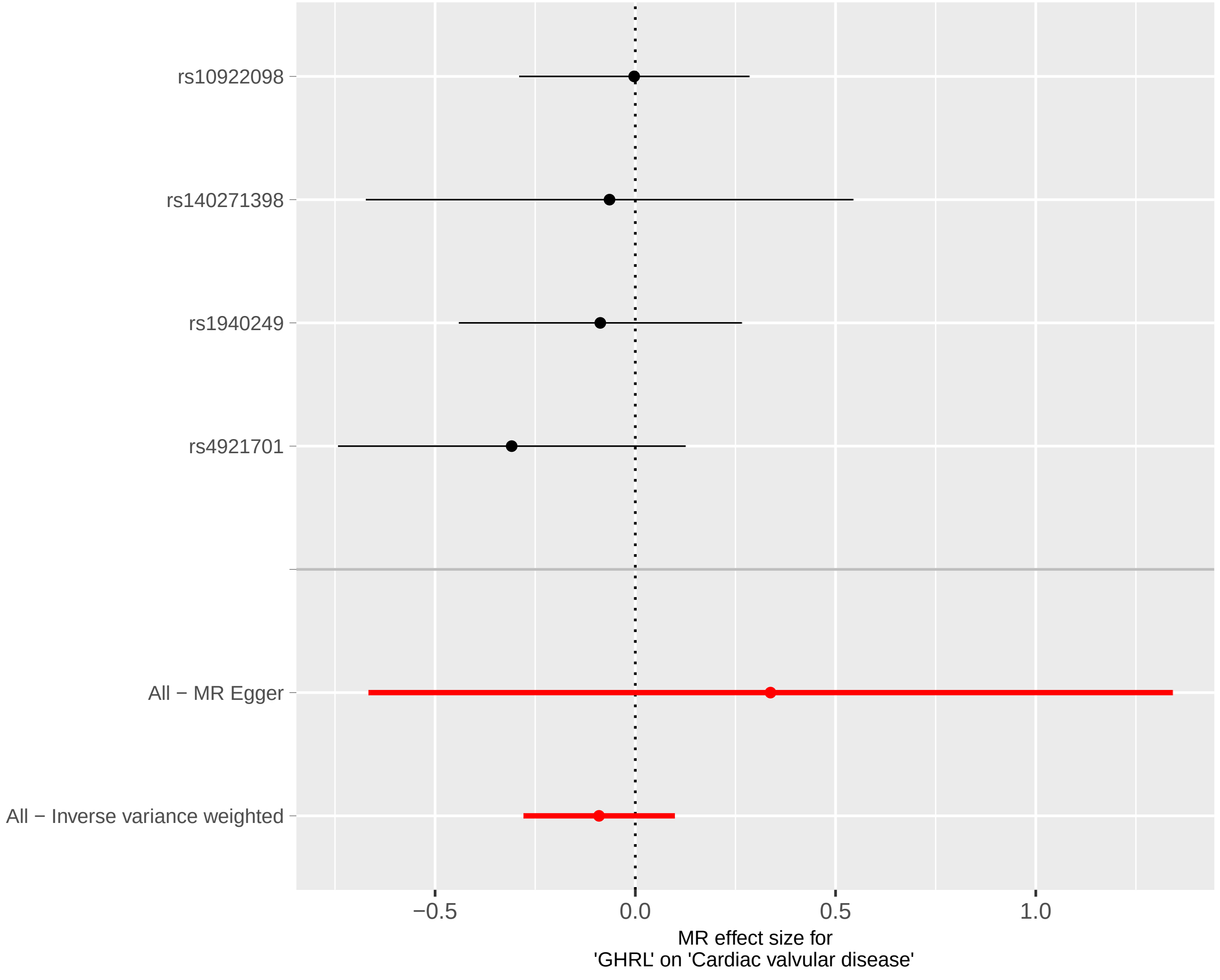
rs10922098
rs140271398
rs1940249
rs4921701
All − MR Egger
All − Inverse variance weighted
−0.5
0.0
0.5
1.0
MR effect size for
'GHRL' on 'Cardiac valvular disease'

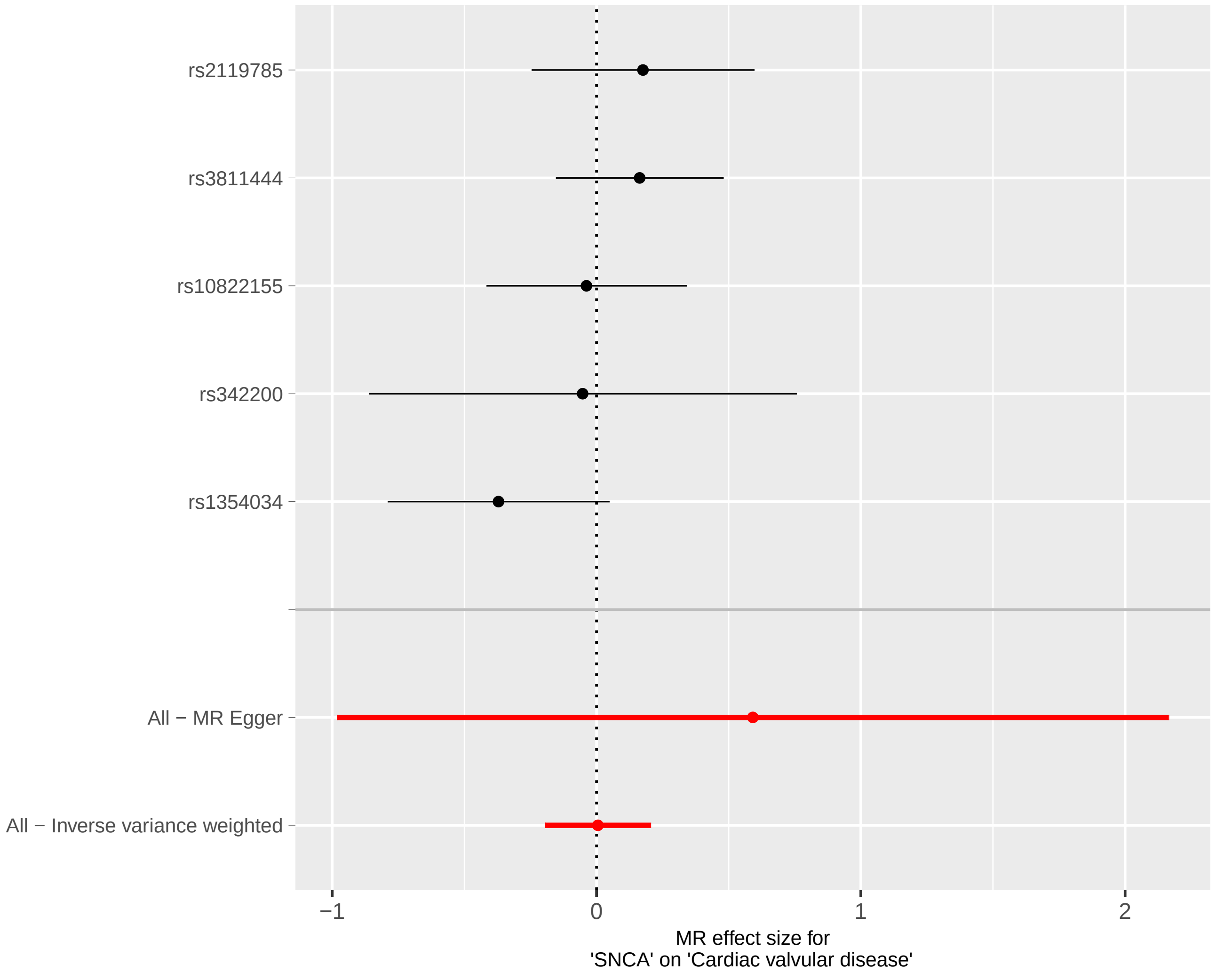
rs2119785
rs3811444
rs10822155
rs342200
rs1354034
All − MR Egger
All − Inverse variance weighted
−1
0
1
2
MR effect size for
'SNCA' on 'Cardiac valvular disease'

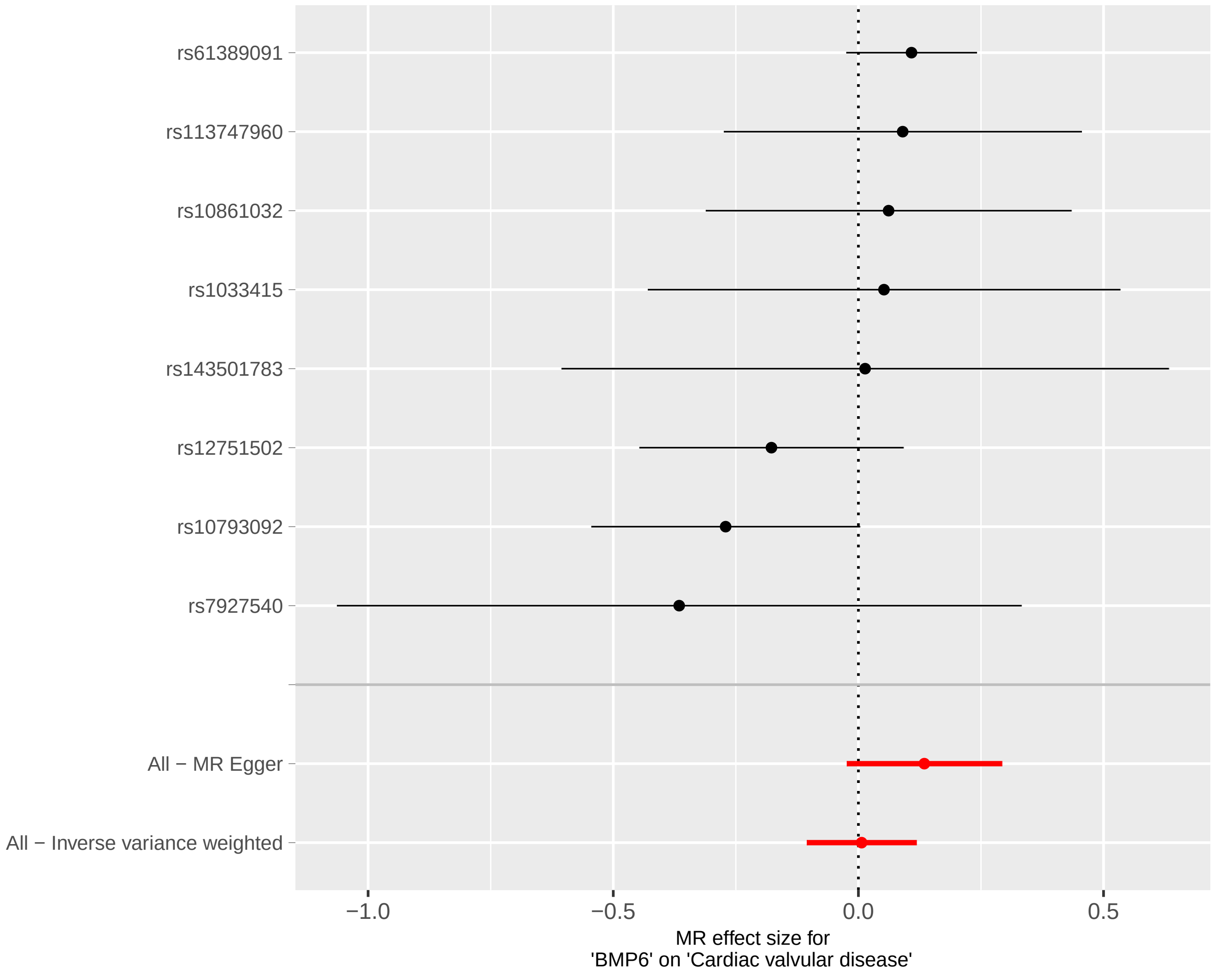
rs61389091
rs113747960
rs10861032
rs1033415
rs143501783
rs12751502
rs10793092
rs7927540
All − MR Egger
All − Inverse variance weighted
−1.0
−0.5
0.0
0.5
MR effect size for
'BMP6' on 'Cardiac valvular disease'

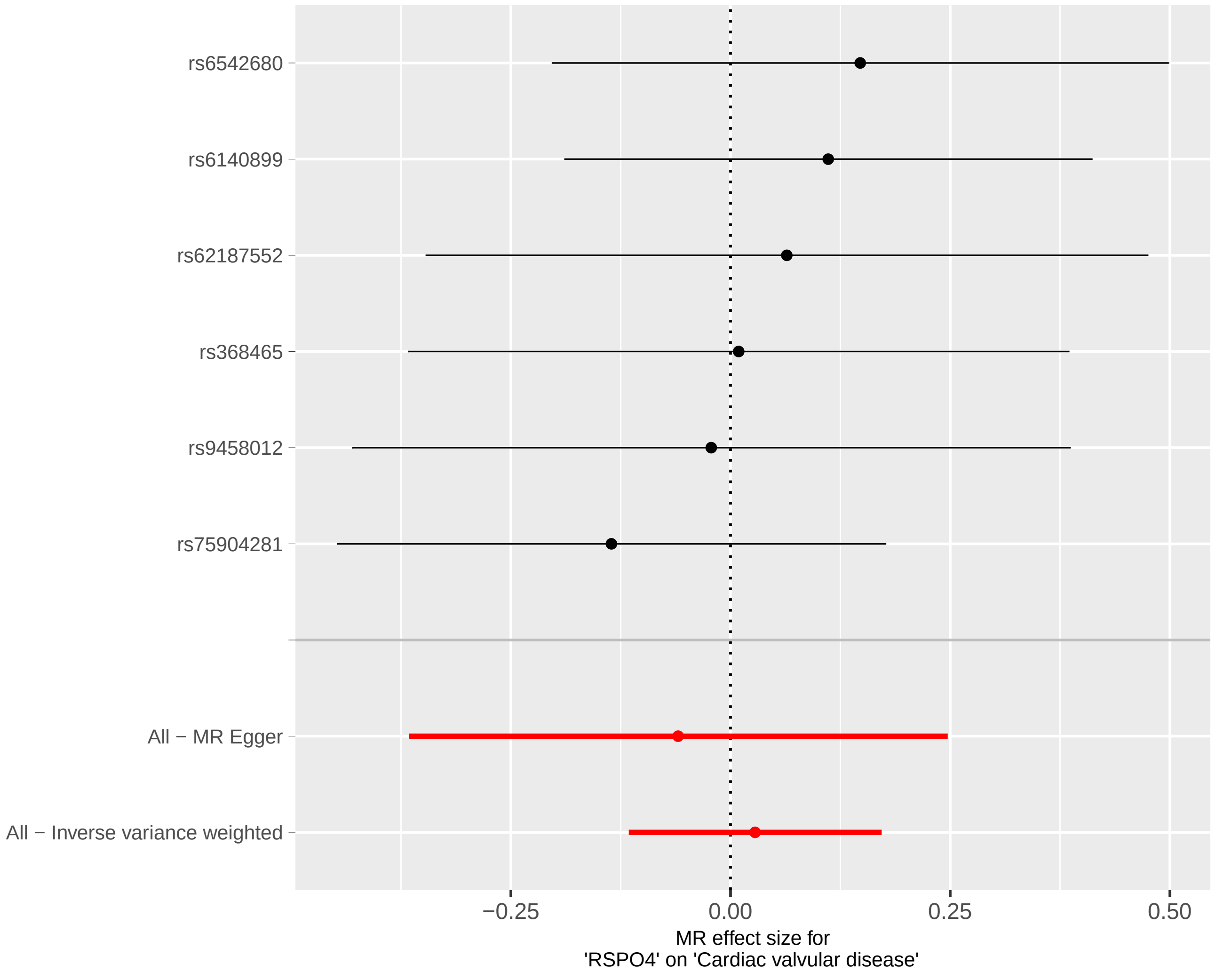

rs6542680
rs6140899
rs62187552
rs368465
rs9458012
rs75904281
All − MR Egger
All − Inverse variance weighted
−0.25
0.00
0.25
0.50
MR effect size for
'RSPO4' on 'Cardiac valvular disease'

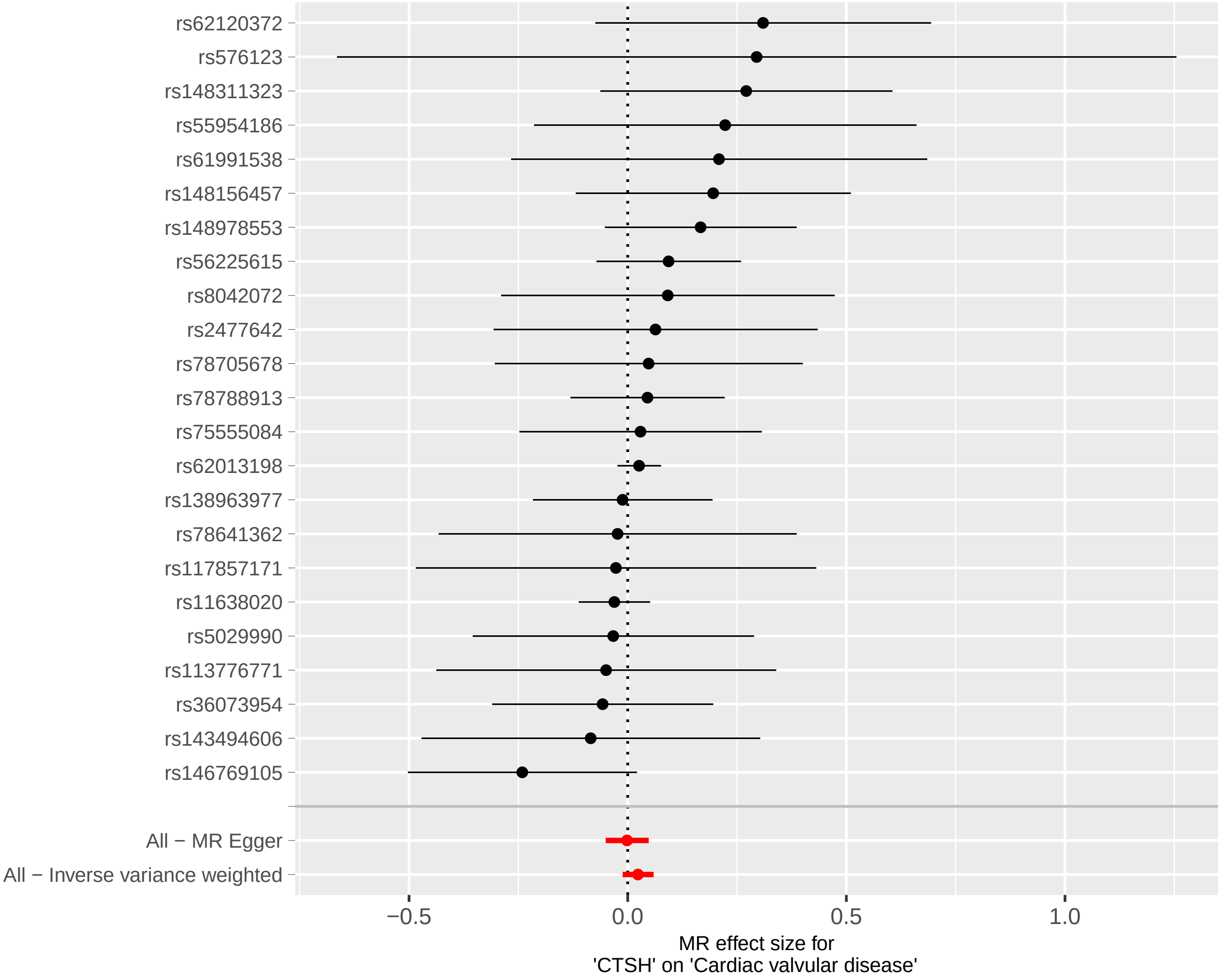
rs62120372
rs576123
rs148311323
rs55954186
rs61991538
rs148156457
rs148978553
rs56225615
rs8042072
rs2477642
rs78705678
rs78788913
rs75555084
rs62013198
rs138963977
rs78641362
rs117857171
rs11638020
rs5029990
rs113776771
rs36073954
rs143494606
rs146769105
All − MR Egger
All − Inverse variance weighted
−0.5
0.0
0.5
1.0
MR effect size for
'CTSH' on 'Cardiac valvular disease'

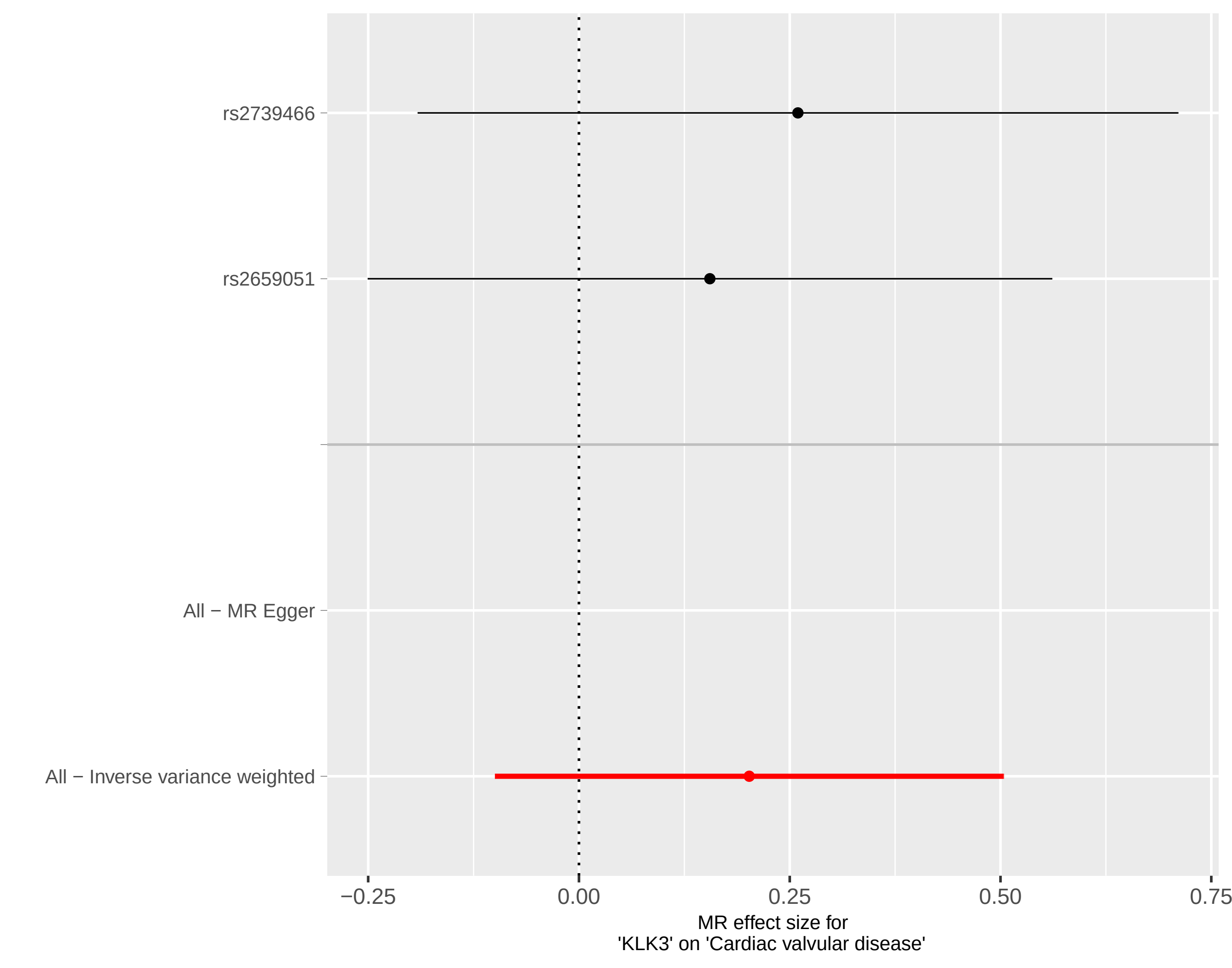
rs2739466
rs2659051
All − MR Egger
All − Inverse variance weighted
−0.25
0.00
0.25
0.50
0.75
MR effect size for
'KLK3' on 'Cardiac valvular disease'

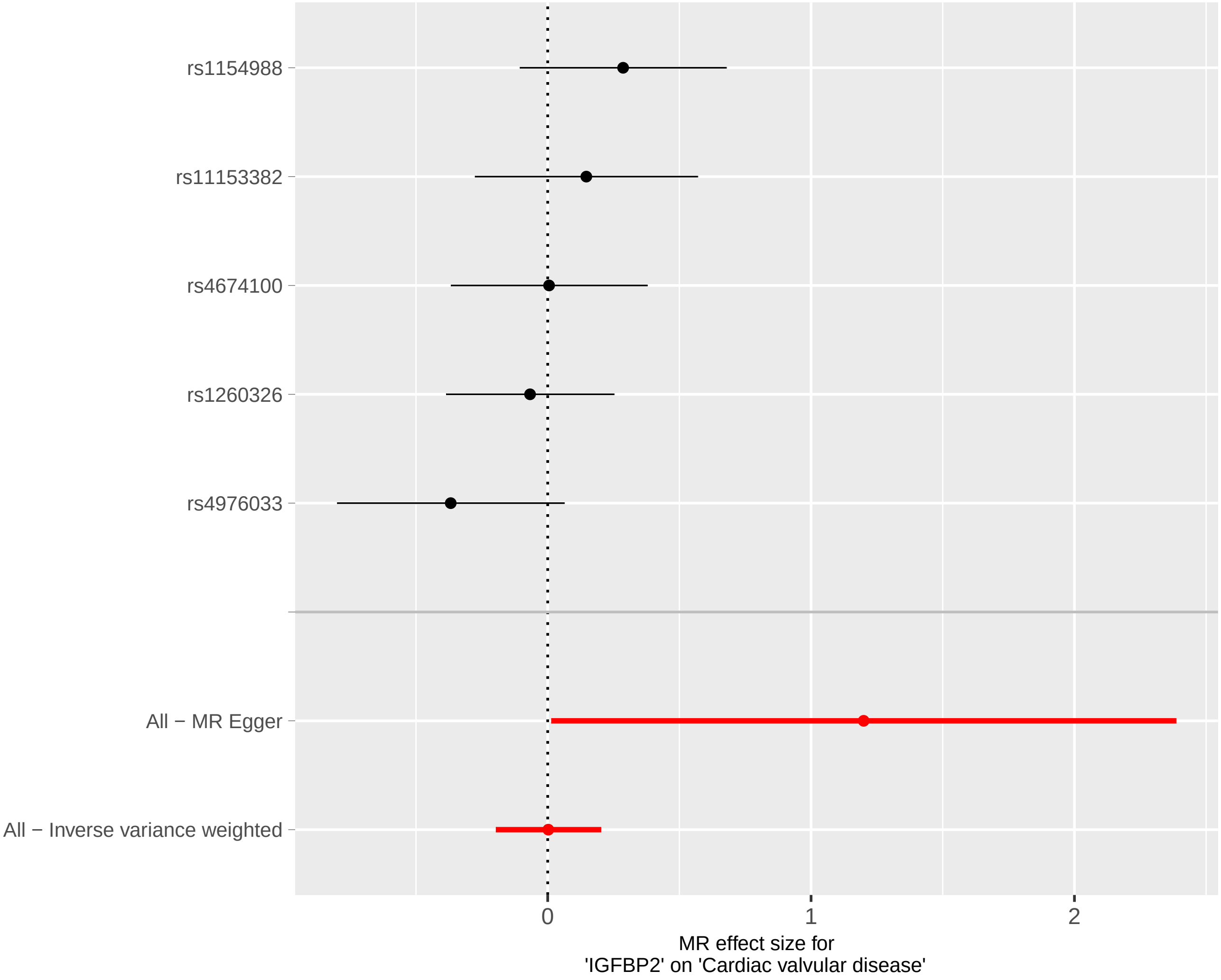
rs1154988
rs11153382
rs4674100
rs1260326
rs4976033
All − MR Egger
All − Inverse variance weighted
0
1
2
MR effect size for
'IGFBP2' on 'Cardiac valvular disease'

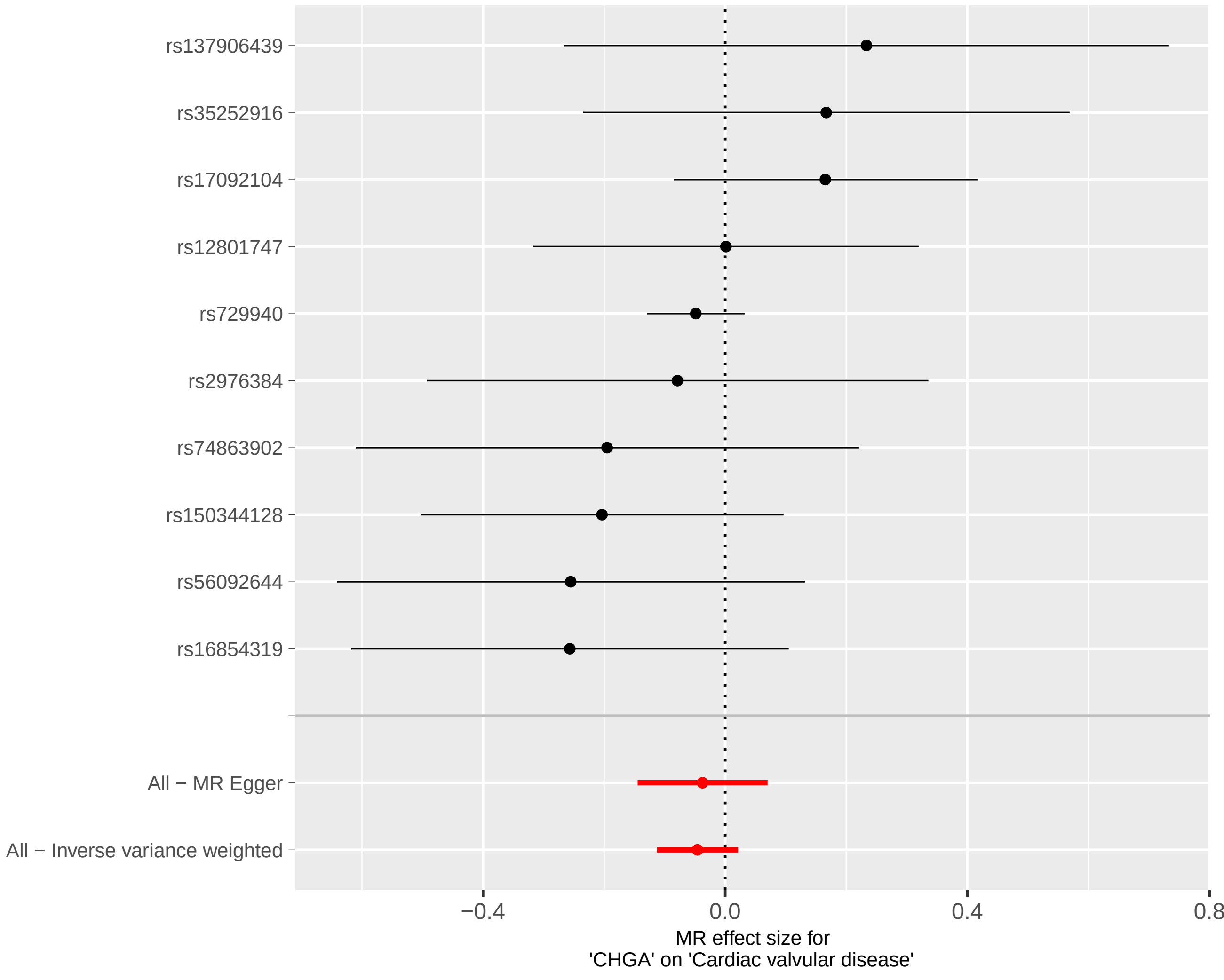
rs137906439
rs35252916
rs17092104
rs12801747
rs729940
rs2976384
rs74863902
rs150344128
rs56092644
rs16854319
All − MR Egger
All − Inverse variance weighted
−0.4
0.0
0.4
0.8
MR effect size for
'CHGA' on 'Cardiac valvular disease'

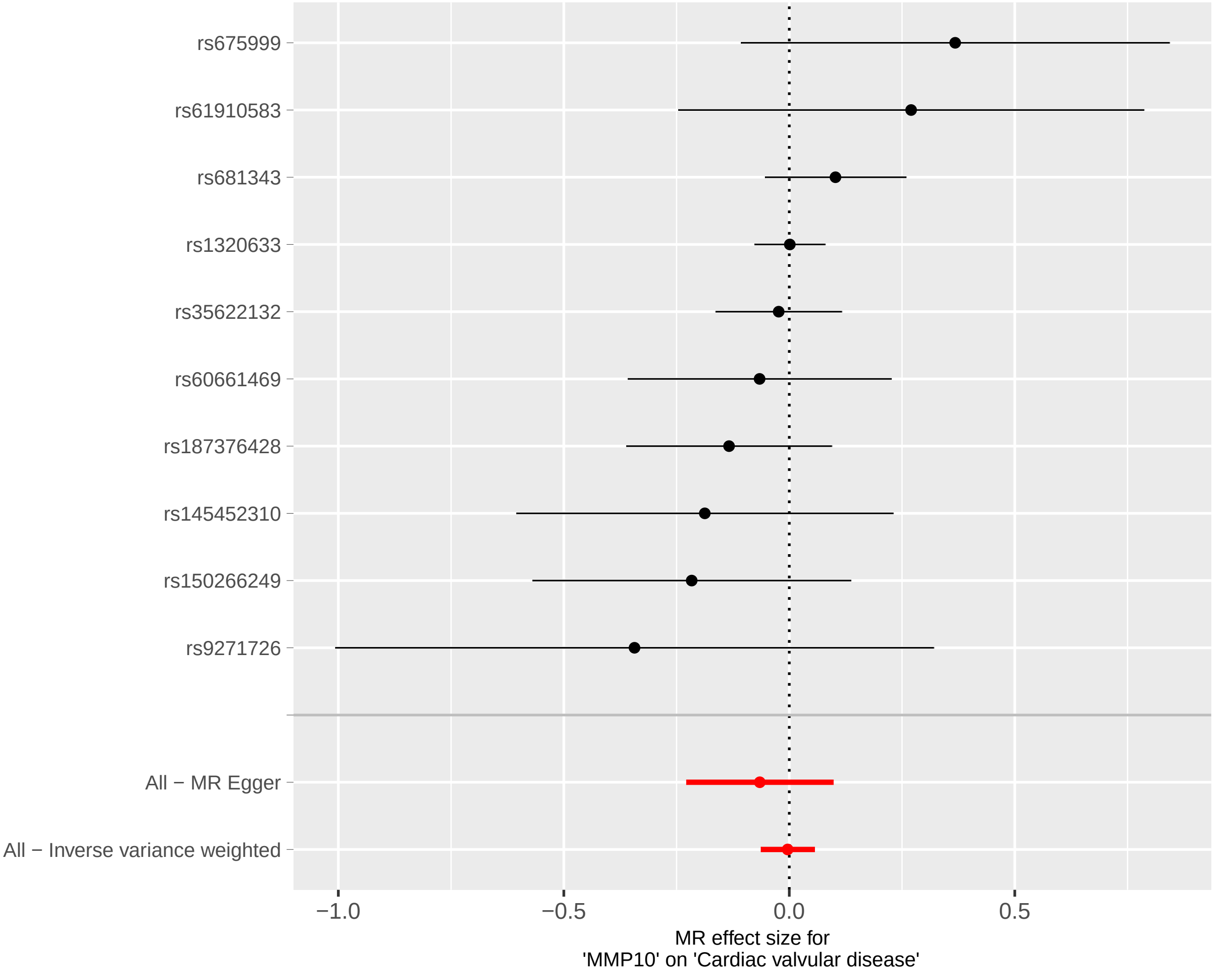
rs675999
rs61910583
rs681343
rs1320633
rs35622132
rs60661469
rs187376428
rs145452310
rs150266249
rs9271726
All − MR Egger
All − Inverse variance weighted
−1.0
−0.5
0.0
0.5
MR effect size for
'MMP10' on 'Cardiac valvular disease'

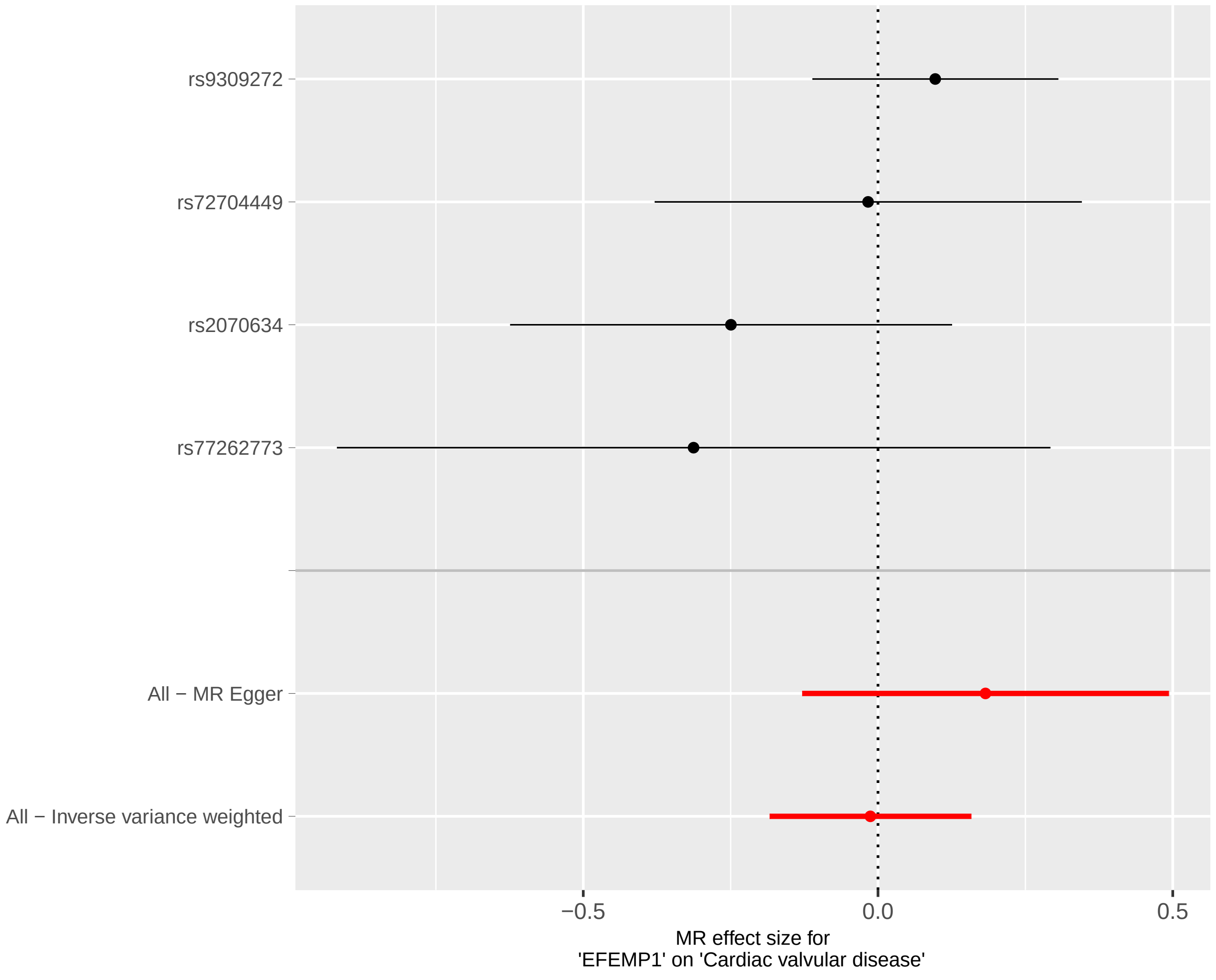
rs9309272
rs72704449
rs2070634
rs77262773
All − MR Egger
All − Inverse variance weighted
−0.5
0.0
0.5
MR effect size for
'EFEMP1' on 'Cardiac valvular disease'

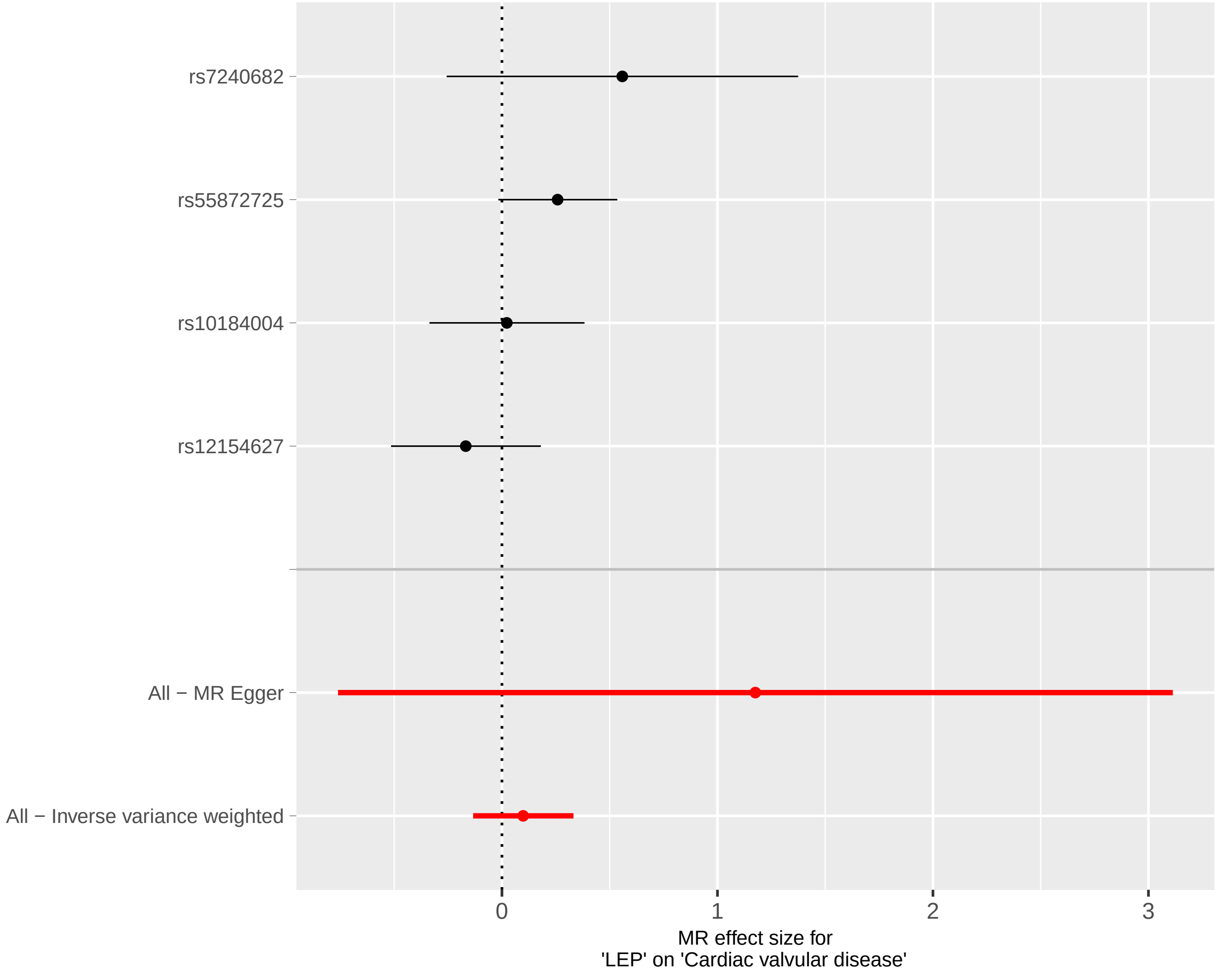
rs7240682
rs55872725
rs10184004
rs12154627
All − MR Egger
All − Inverse variance weighted
0
1
2
3
MR effect size for
'LEP' on 'Cardiac valvular disease'

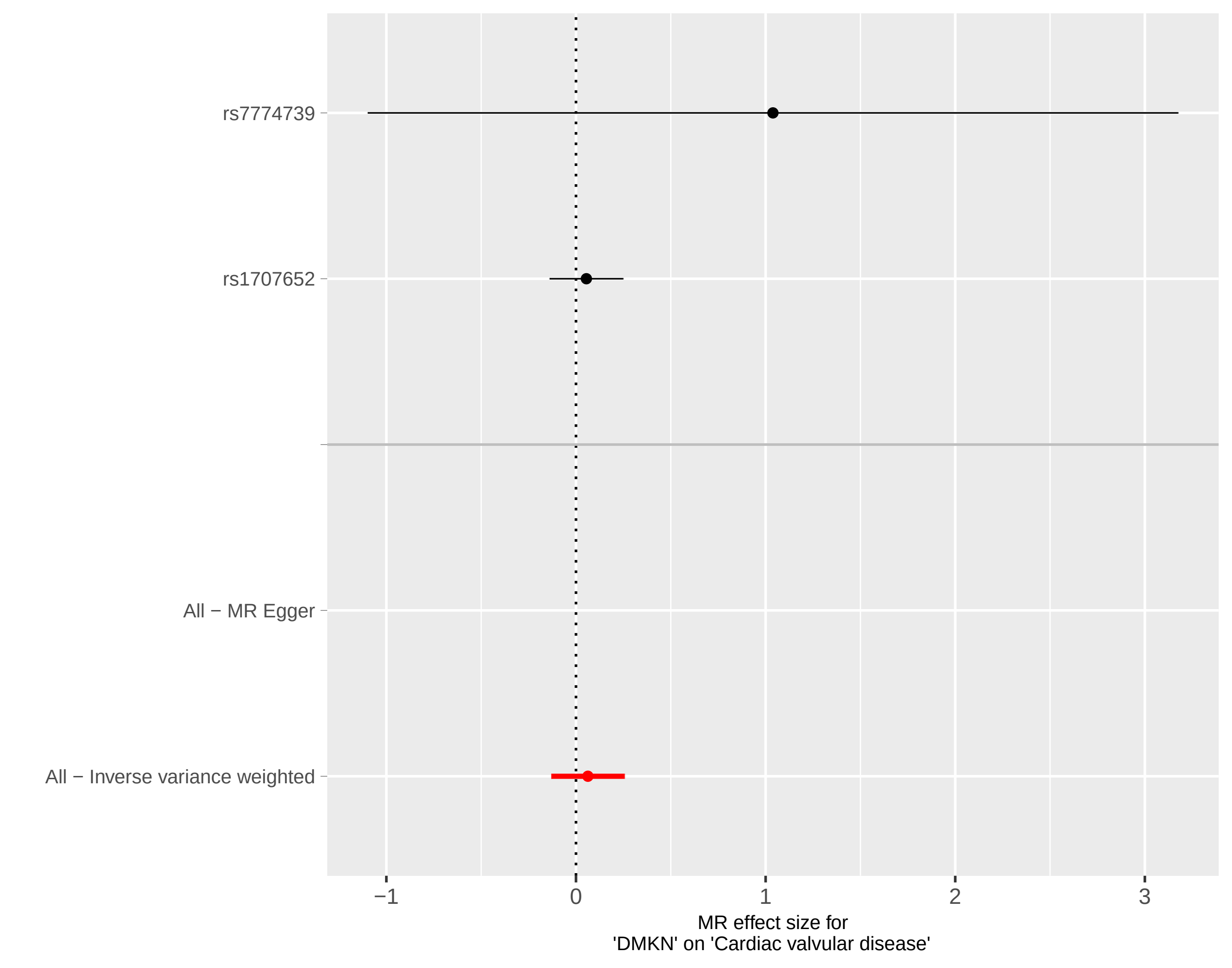
rs7774739
rs1707652
All − MR Egger
All − Inverse variance weighted
−1
0
1
2
3
MR effect size for
'DMKN' on 'Cardiac valvular disease'

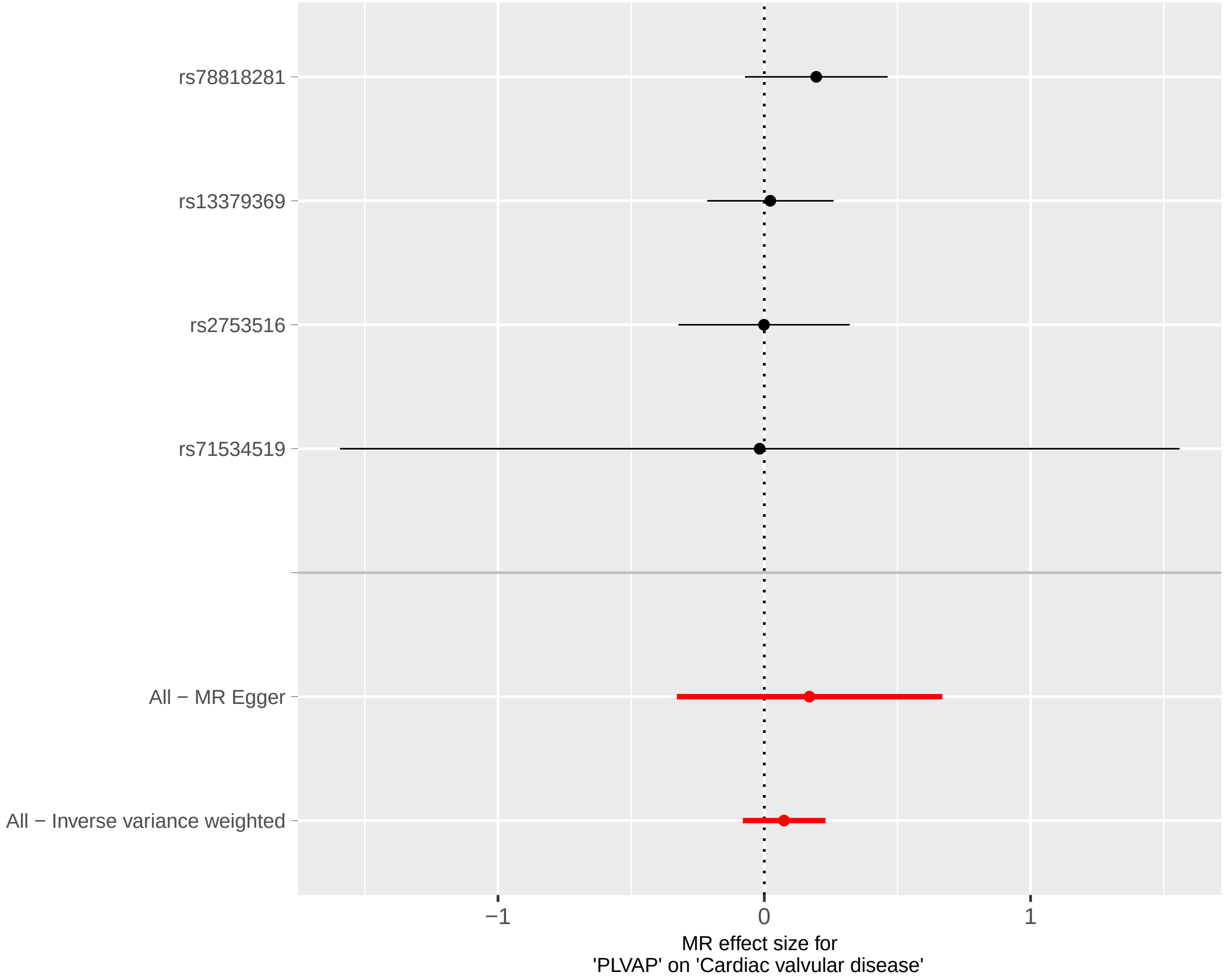

rs78818281
rs13379369
rs2753516
rs71534519
All − MR Egger
All − Inverse variance weighted
−1
0
1
MR effect size for
'PLVAP' on 'Cardiac valvular disease'

Insufficient number of SNPs

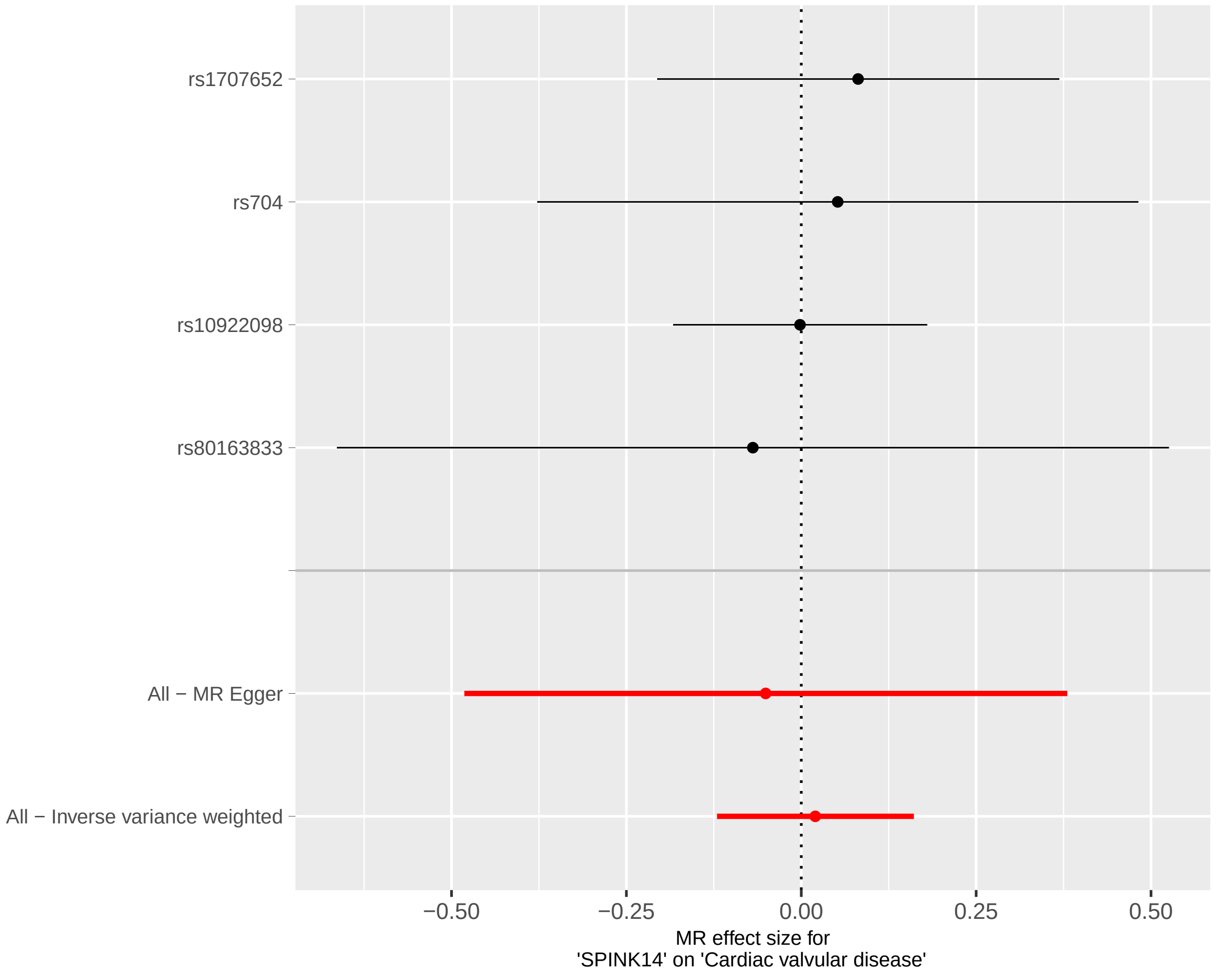

rs1707652
rs704
rs10922098
rs80163833
All − MR Egger
All − Inverse variance weighted
−0.50
−0.25
0.00
0.25
0.50
MR effect size for
'SPINK14' on 'Cardiac valvular disease'

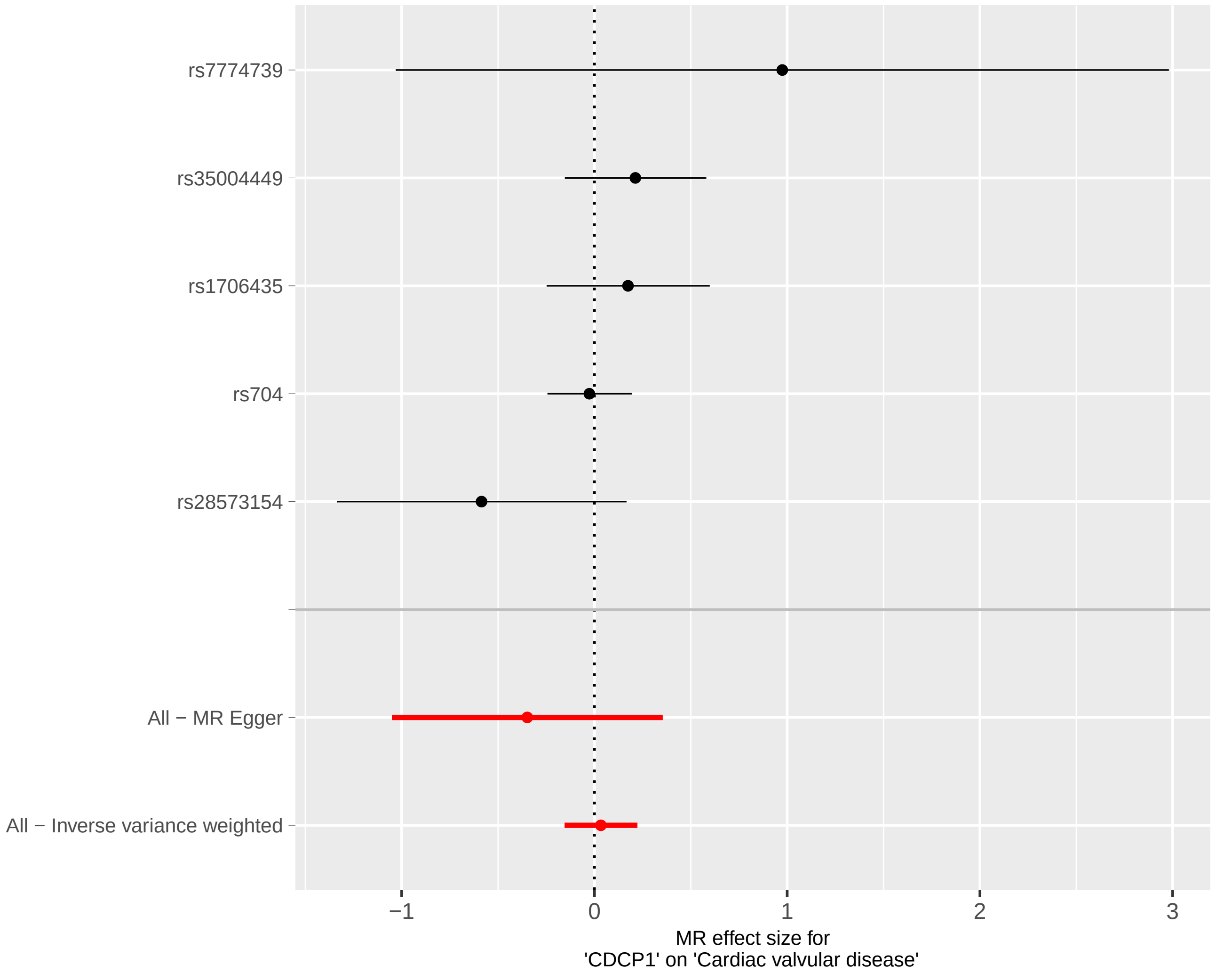
rs7774739
rs35004449
rs1706435
rs704
rs28573154
All − MR Egger
All − Inverse variance weighted
−1
0
1
2
3
MR effect size for
'CDCP1' on 'Cardiac valvular disease'

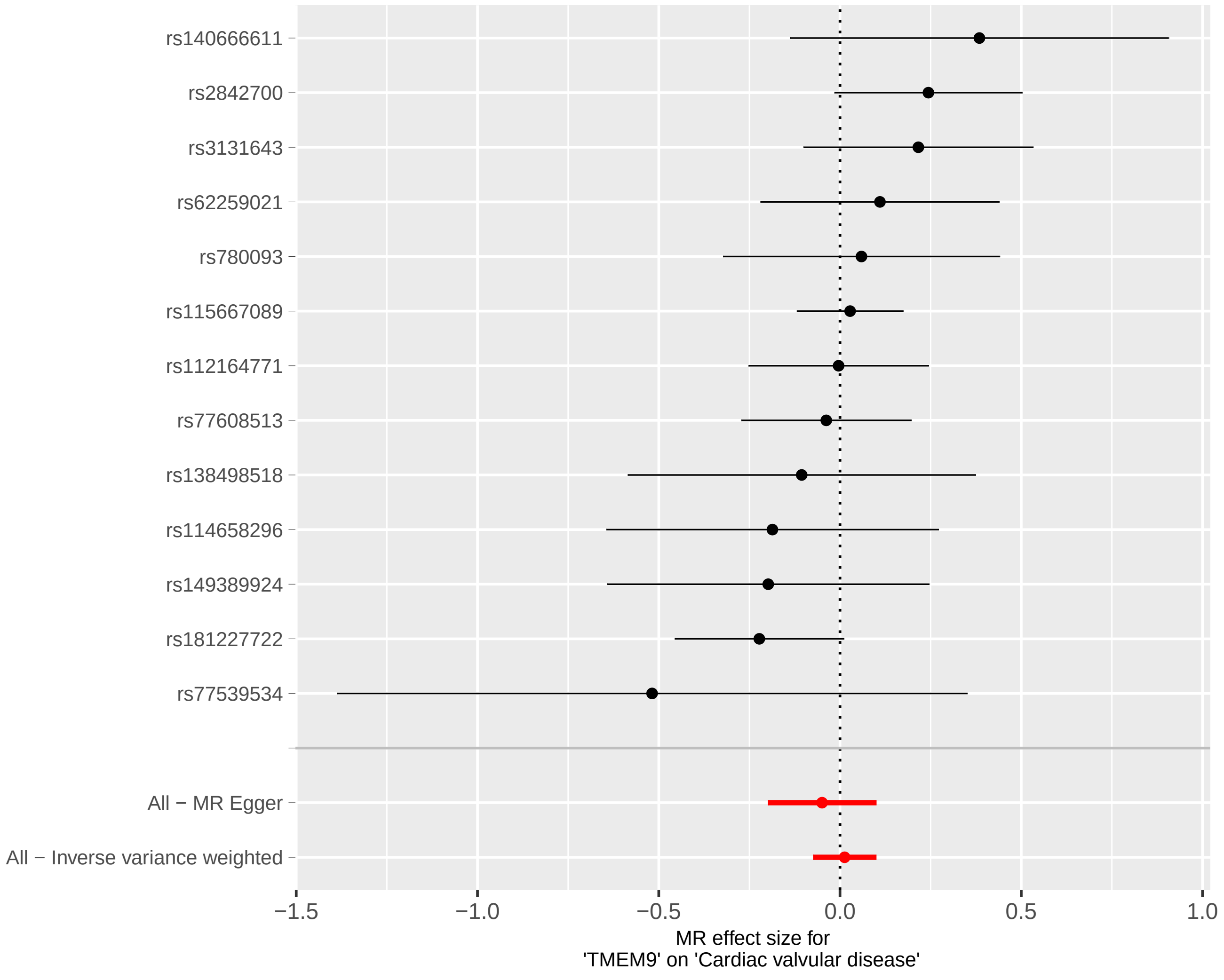
rs140666611
rs2842700
rs3131643
rs62259021
rs780093
rs115667089
rs112164771
rs77608513
rs138498518
rs114658296
rs149389924
rs181227722
rs77539534
All − MR Egger
All − Inverse variance weighted
−1.5
−1.0
−0.5
0.0
0.5
1.0
MR effect size for
'TMEM9' on 'Cardiac valvular disease'

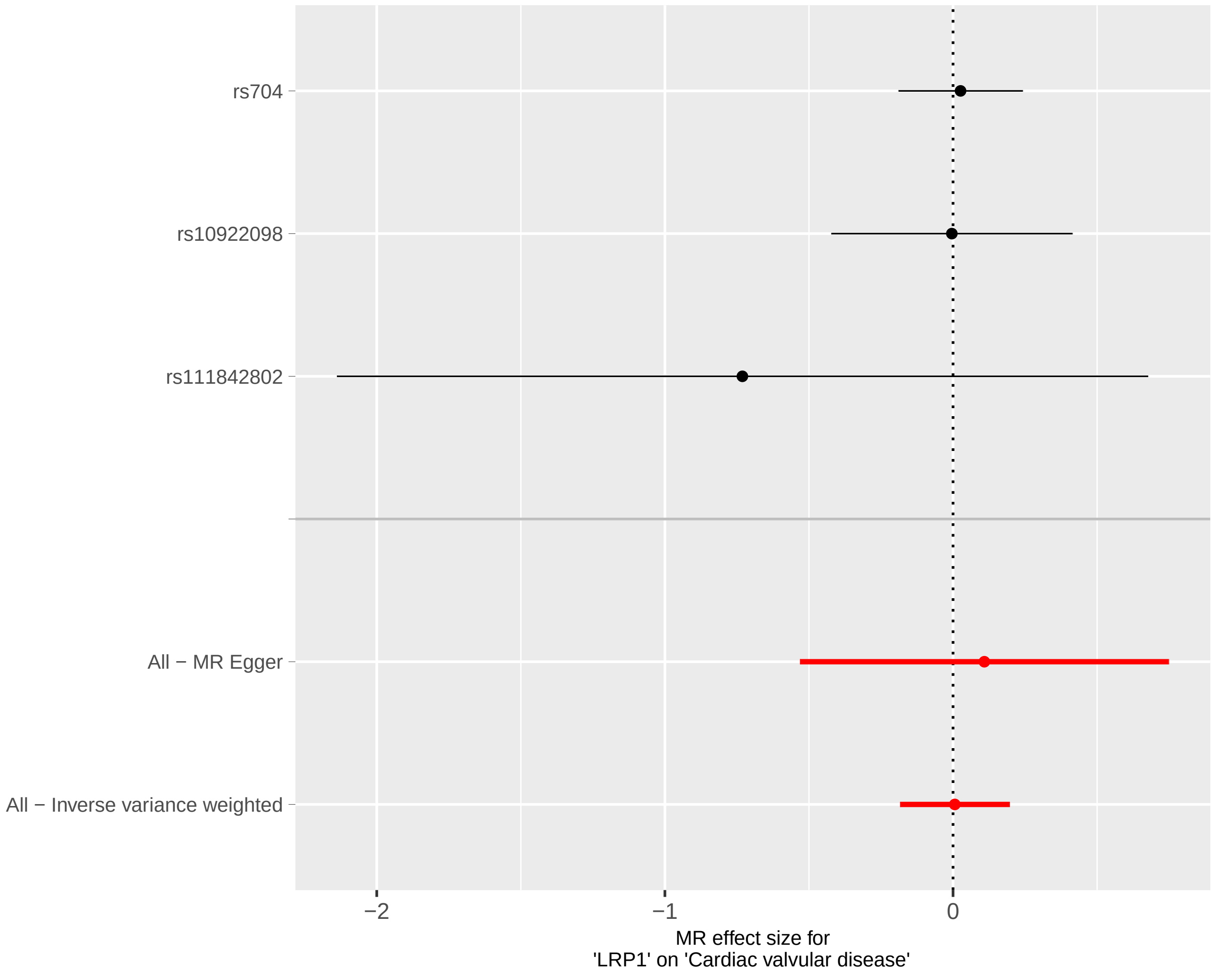
rs704
rs10922098
rs111842802
All − MR Egger
All − Inverse variance weighted
−2
−1
0
MR effect size for
'LRP1' on 'Cardiac valvular disease'

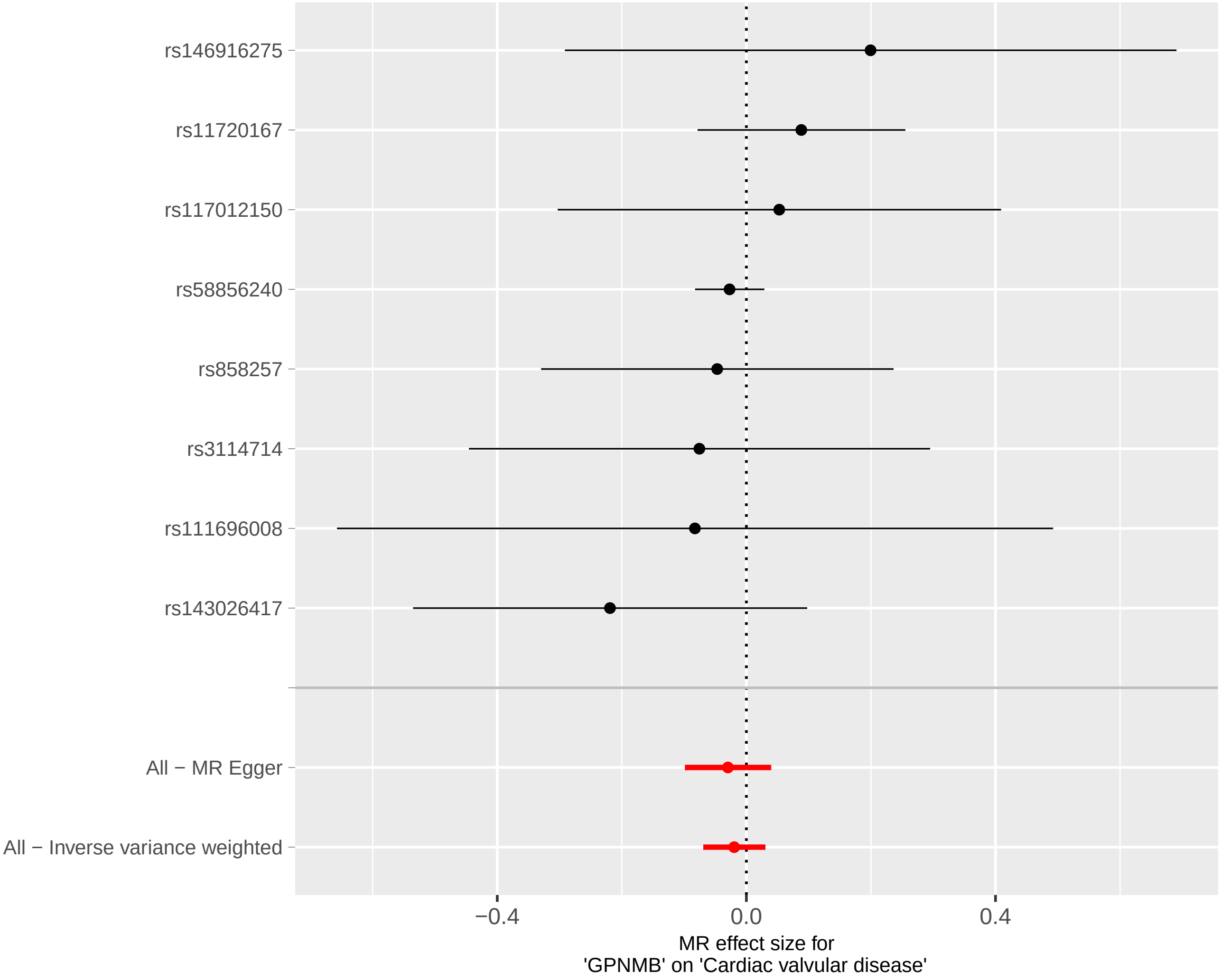

rs146916275
rs11720167
rs117012150
rs58856240
rs858257
rs3114714
rs111696008
rs143026417
All − MR Egger
All − Inverse variance weighted
−0.4
0.0
0.4
MR effect size for
'GPNMB' on 'Cardiac valvular disease'

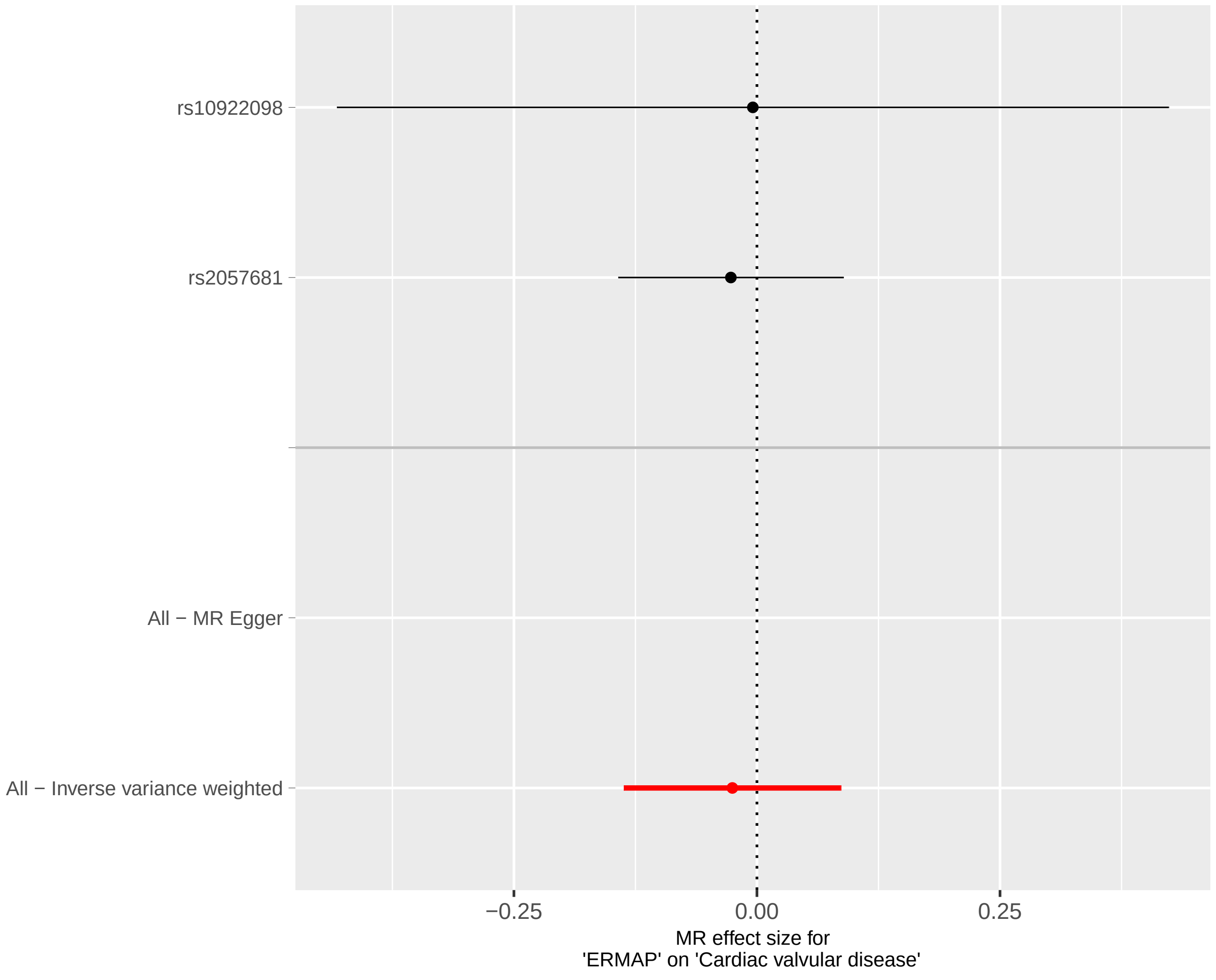

rs10922098
rs2057681
All − MR Egger
All − Inverse variance weighted
−0.25
0.00
0.25
MR effect size for
'ERMAP' on 'Cardiac valvular disease'

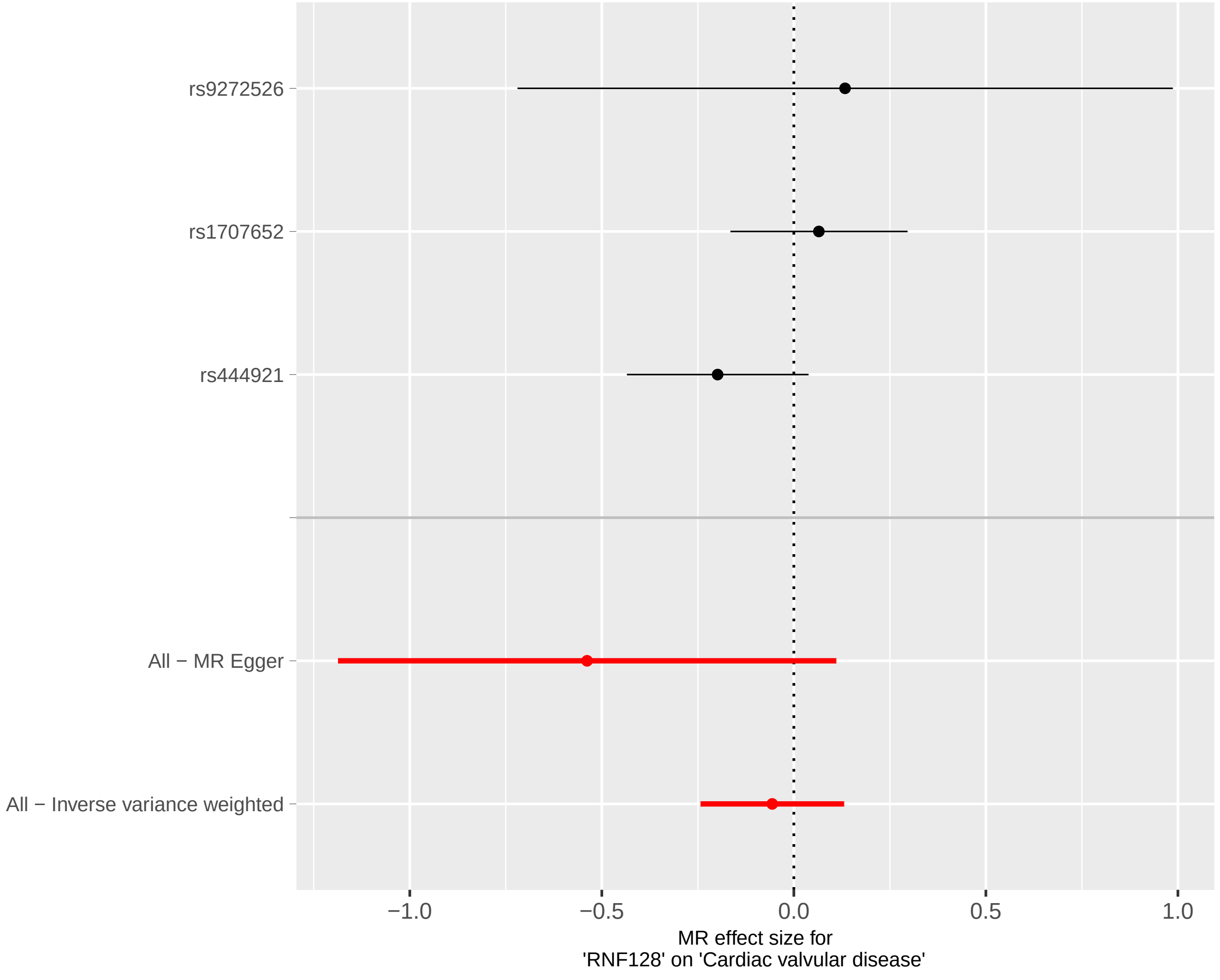
rs9272526
rs1707652
rs444921
All − MR Egger
All − Inverse variance weighted
−1.0
−0.5
0.0
0.5
1.0
MR effect size for
'RNF128' on 'Cardiac valvular disease'

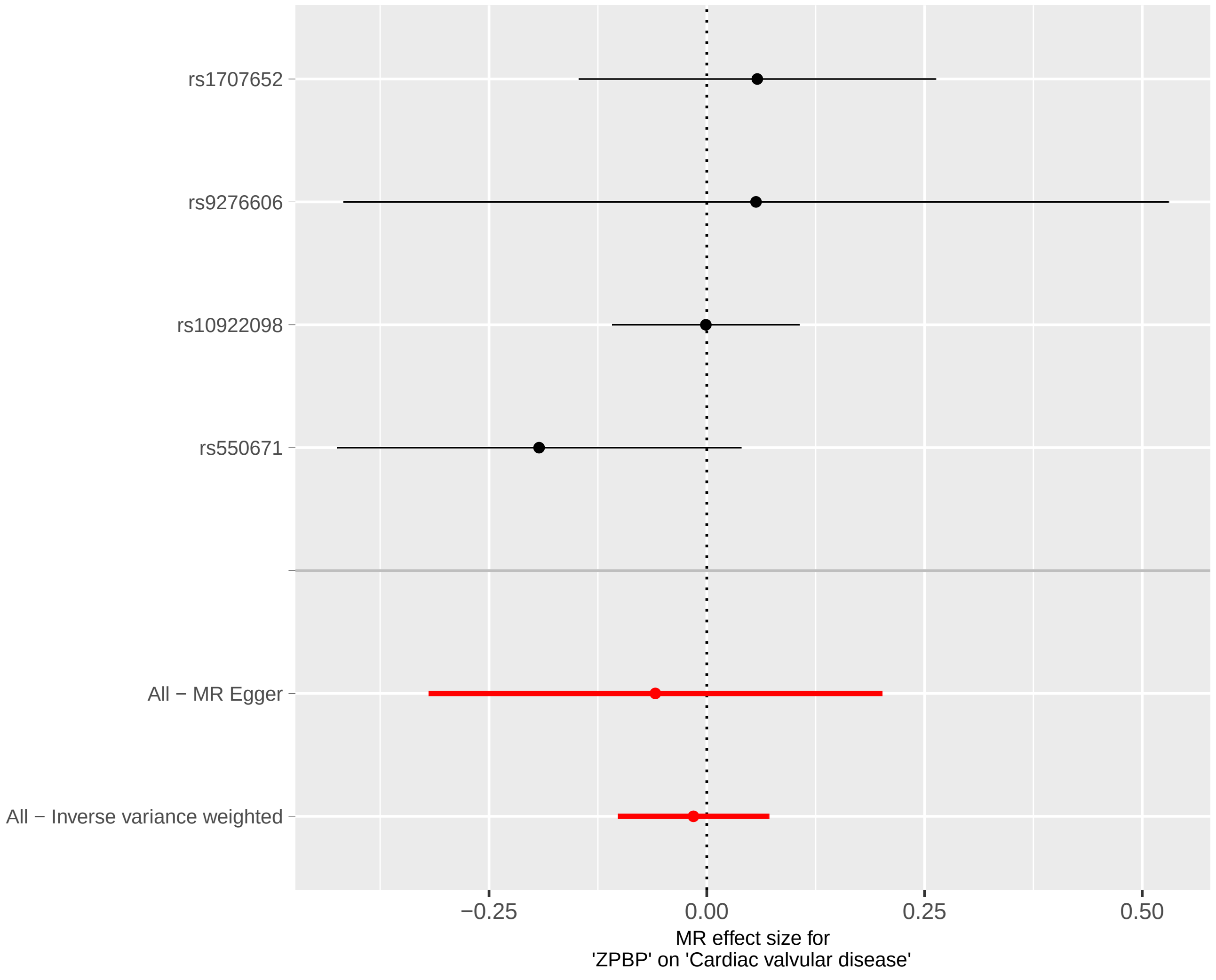
rs1707652
rs9276606
rs10922098
rs550671
All − MR Egger
All − Inverse variance weighted
−0.25
0.00
0.25
0.50
MR effect size for
'ZPBP' on 'Cardiac valvular disease'

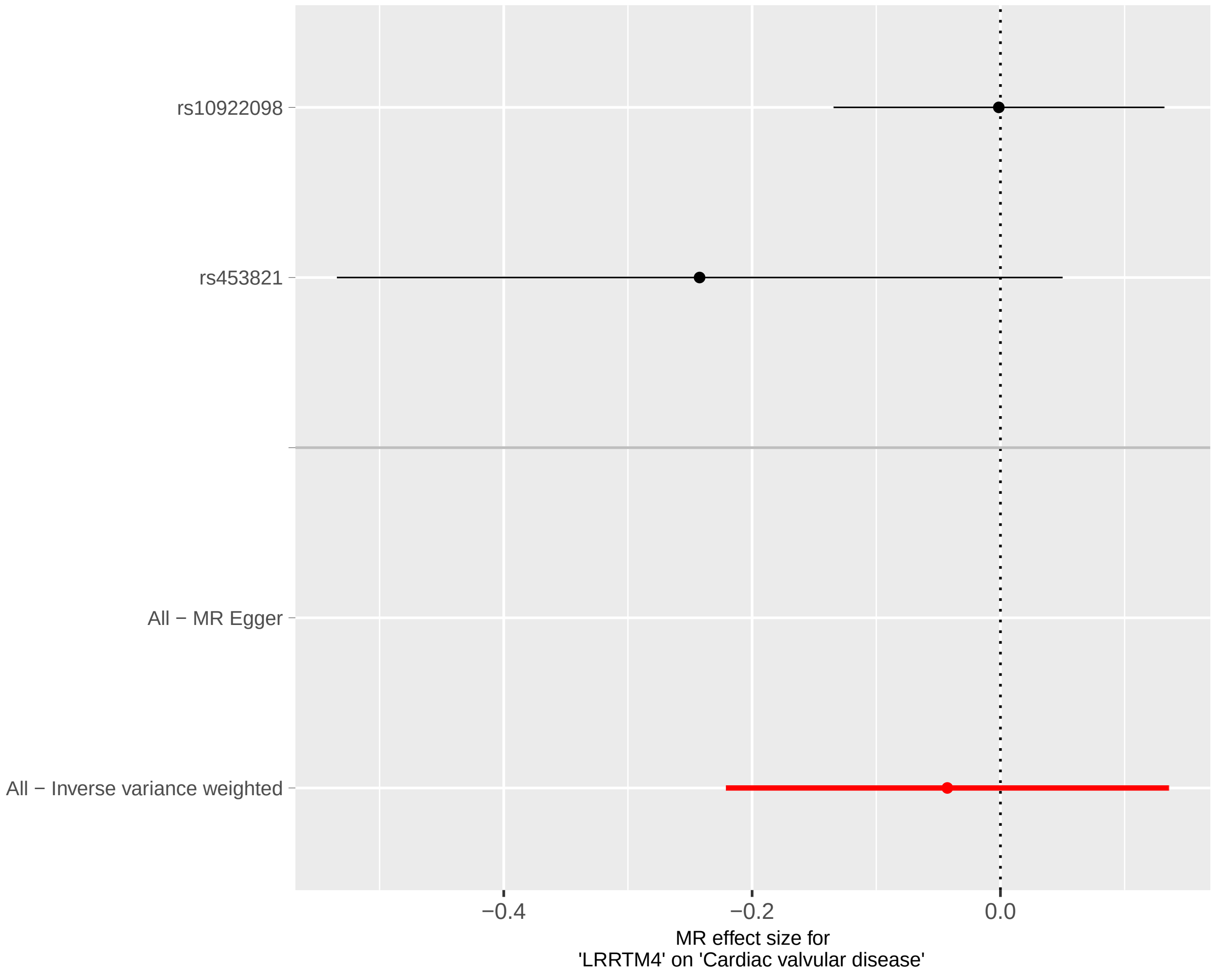
rs10922098
rs453821
All − MR Egger
All − Inverse variance weighted
−0.4
−0.2
0.0
MR effect size for
'LRRTM4' on 'Cardiac valvular disease'

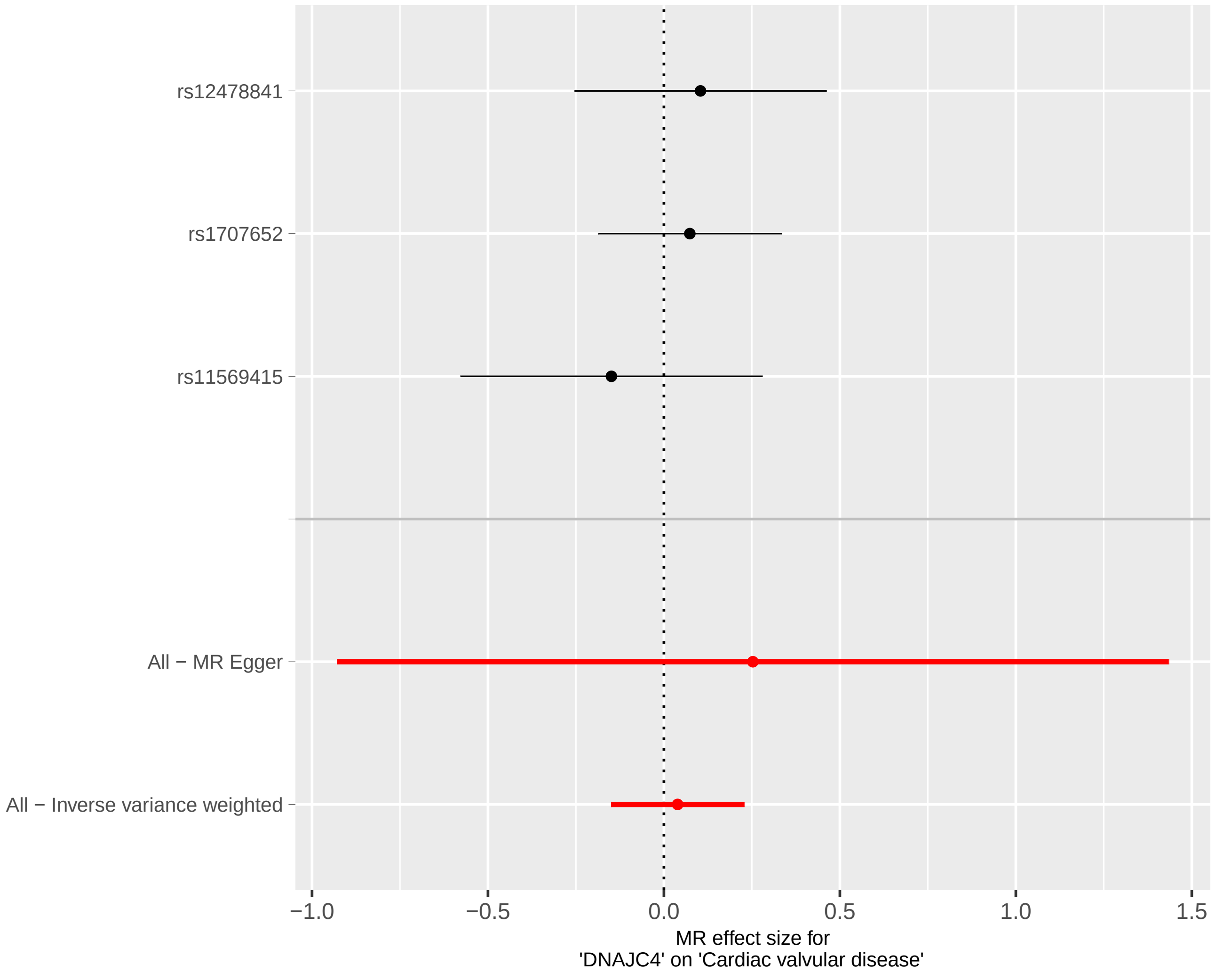
rs12478841
rs1707652
rs11569415
All − MR Egger
All − Inverse variance weighted
−1.0
−0.5
0.0
0.5
1.0
1.5
MR effect size for
'DNAJC4' on 'Cardiac valvular disease'

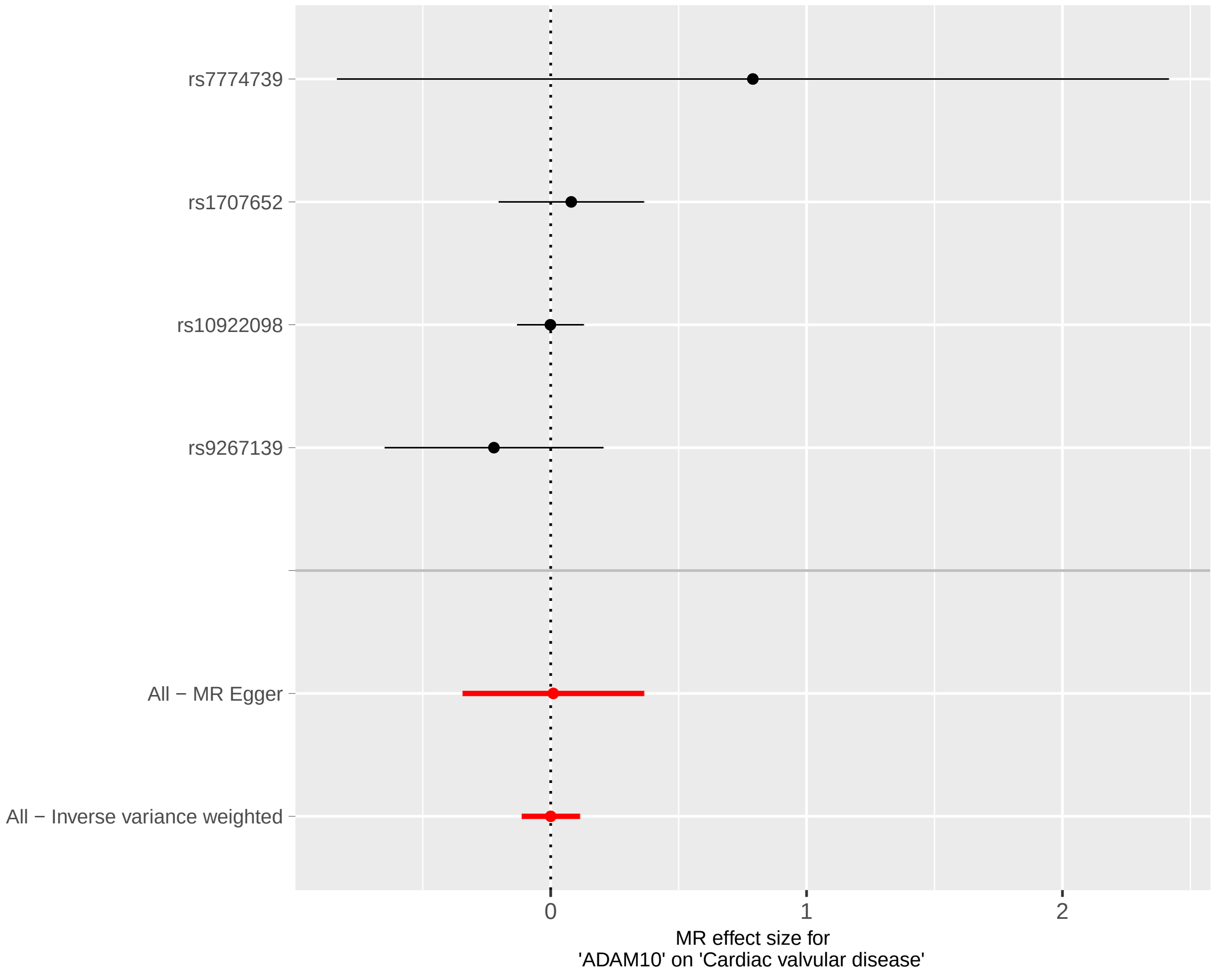
rs7774739
rs1707652
rs10922098
rs9267139
All − MR Egger
All − Inverse variance weighted
0
1
2
MR effect size for
'ADAM10' on 'Cardiac valvular disease'

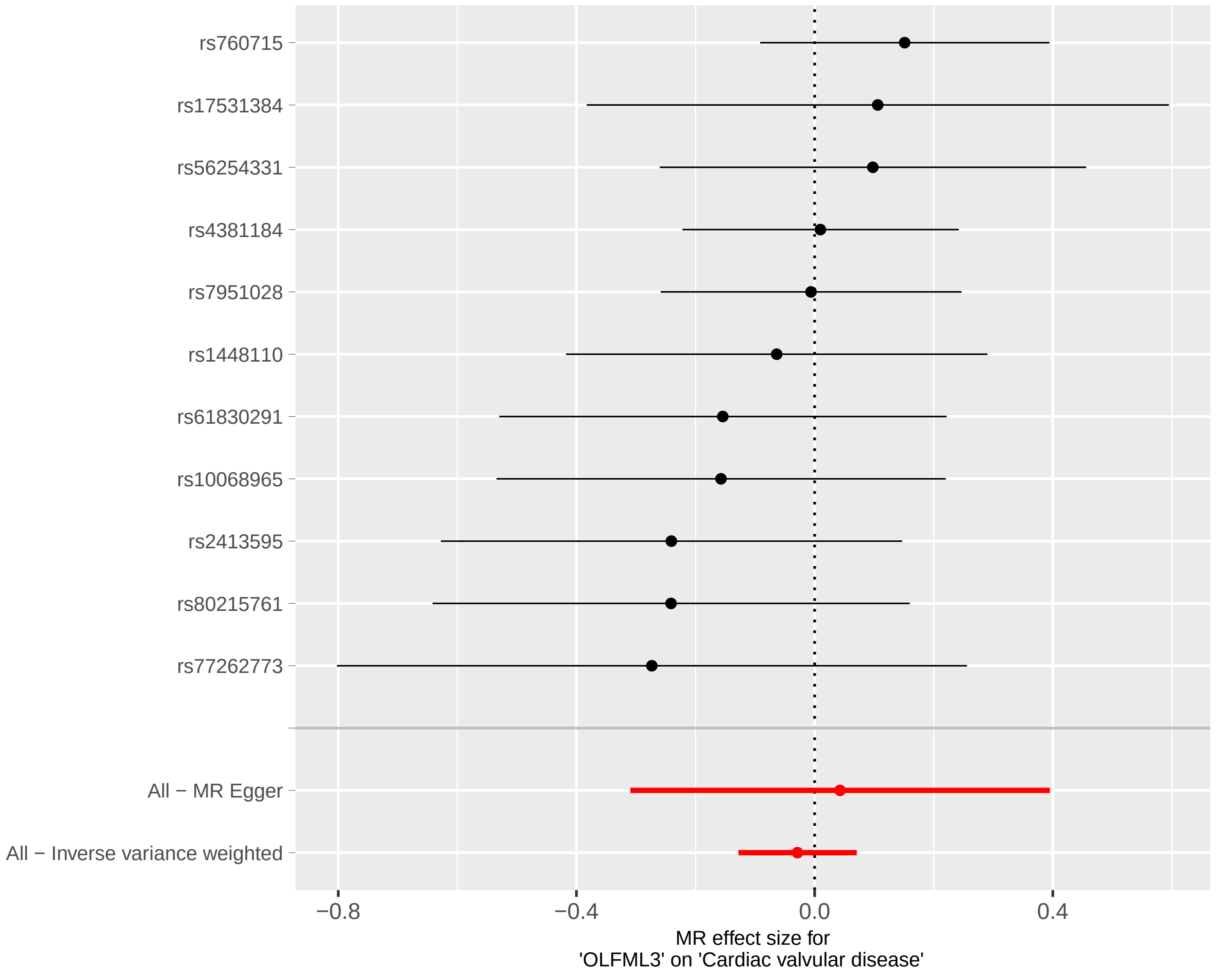
rs760715
rs17531384
rs56254331
rs4381184
rs7951028
rs1448110
rs61830291
rs10068965
rs2413595
rs80215761
rs77262773
All − MR Egger
All − Inverse variance weighted
−0.8
−0.4
0.0
0.4
MR effect size for
'OLFML3' on 'Cardiac valvular disease'

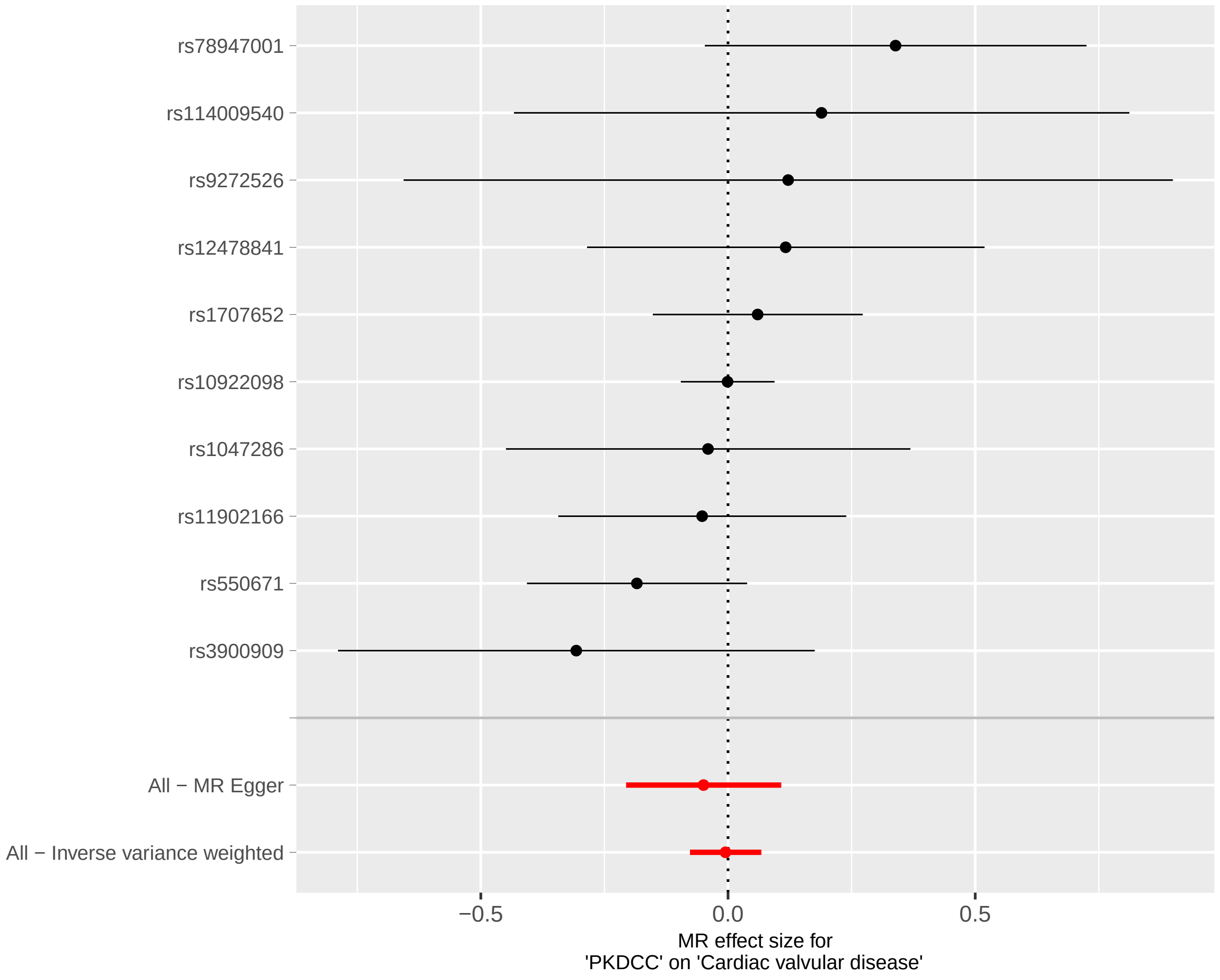
rs78947001
rs114009540
rs9272526
rs12478841
rs1707652
rs10922098
rs1047286
rs11902166
rs550671
rs3900909
All − MR Egger
All − Inverse variance weighted
−0.5
0.0
0.5
MR effect size for
'PKDCC' on 'Cardiac valvular disease'

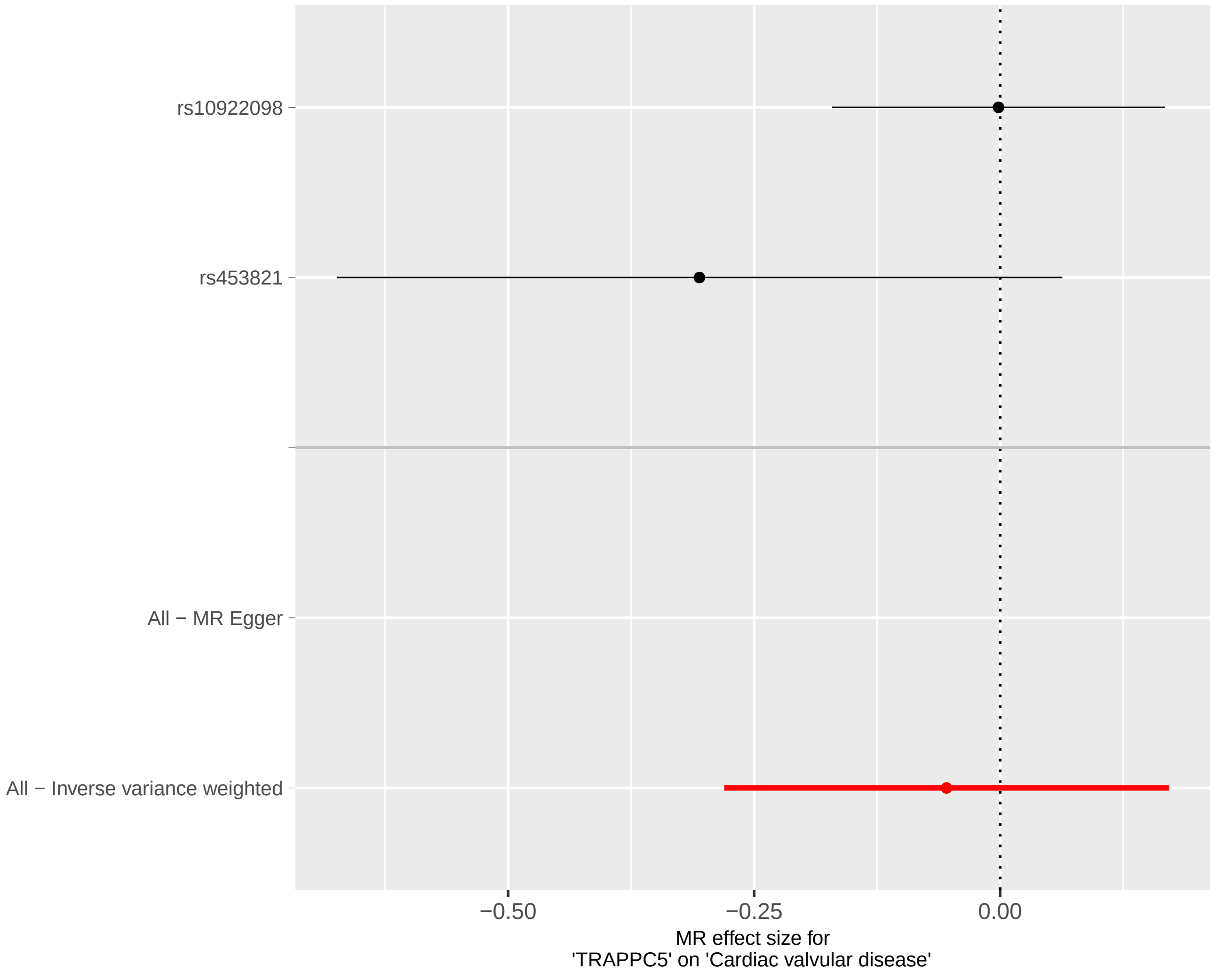
rs10922098
rs453821
All − MR Egger
All − Inverse variance weighted
−0.50
−0.25
0.00
MR effect size for
'TRAPPC5' on 'Cardiac valvular disease'

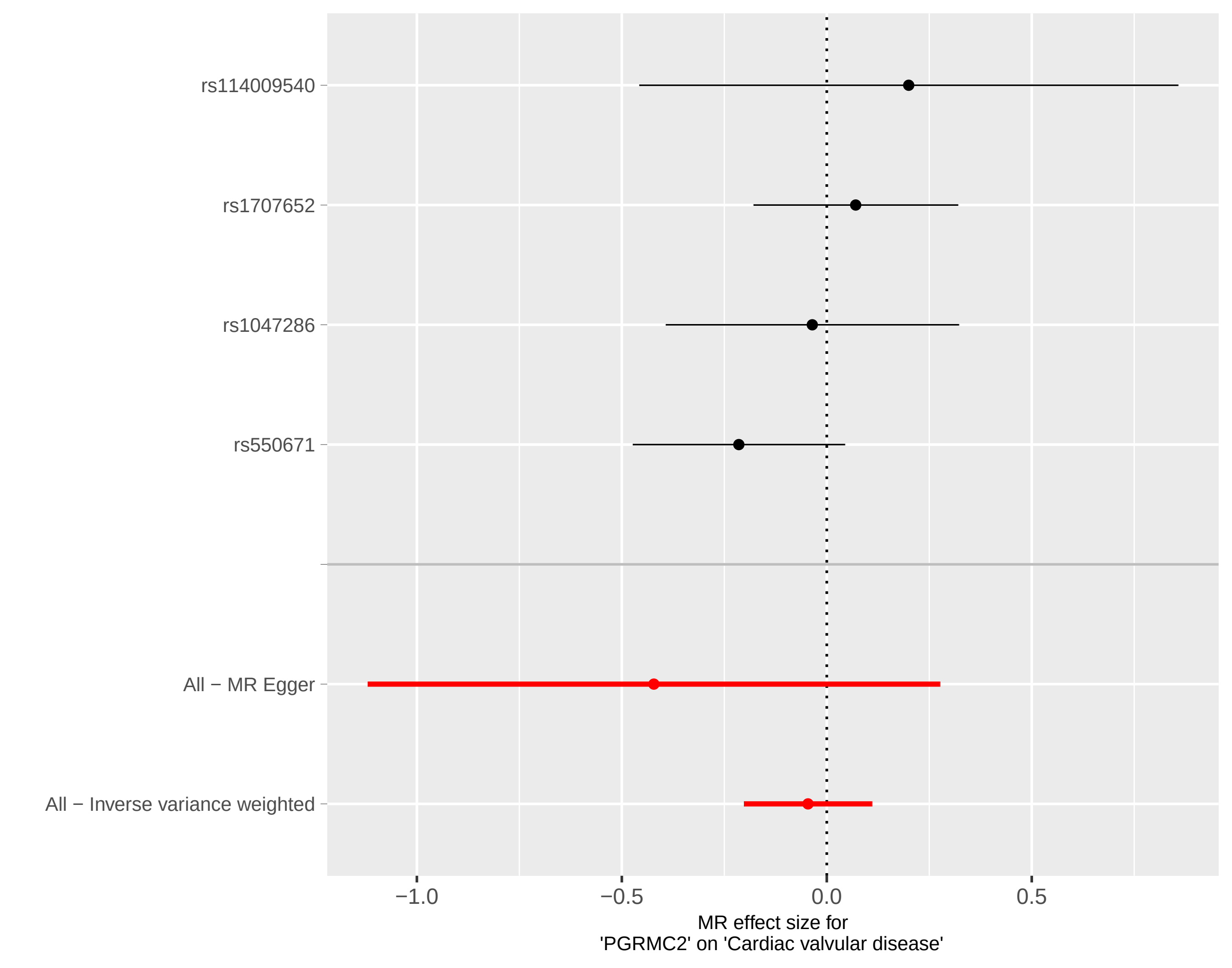
rs114009540
rs1707652
rs1047286
rs550671
All − MR Egger
All − Inverse variance weighted
−1.0
−0.5
0.0
0.5
MR effect size for
'PGRMC2' on 'Cardiac valvular disease'

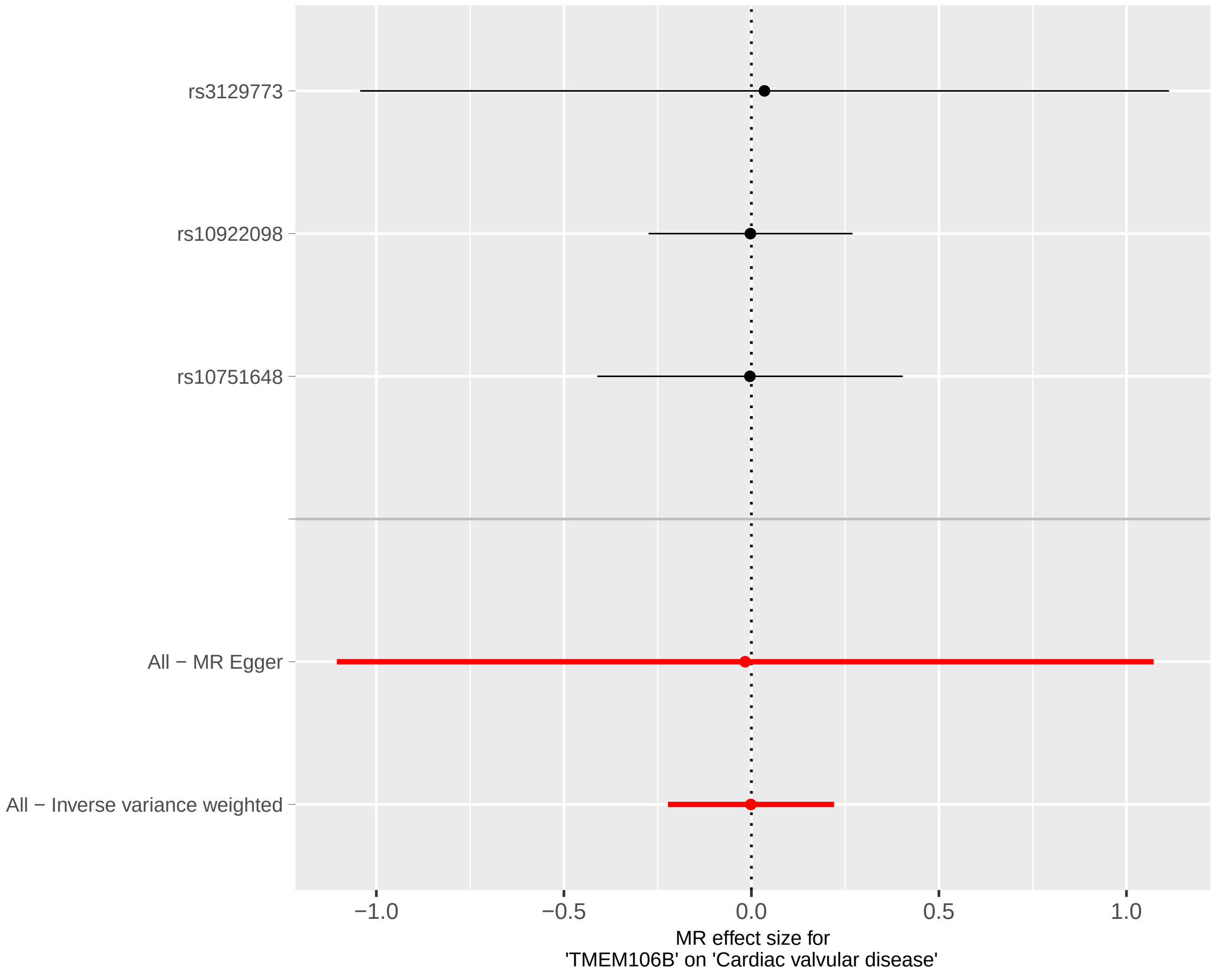
rs3129773
rs10922098
rs10751648
All − MR Egger
All − Inverse variance weighted
−1.0
−0.5
0.0
0.5
1.0
MR effect size for
'TMEM106B' on 'Cardiac valvular disease'

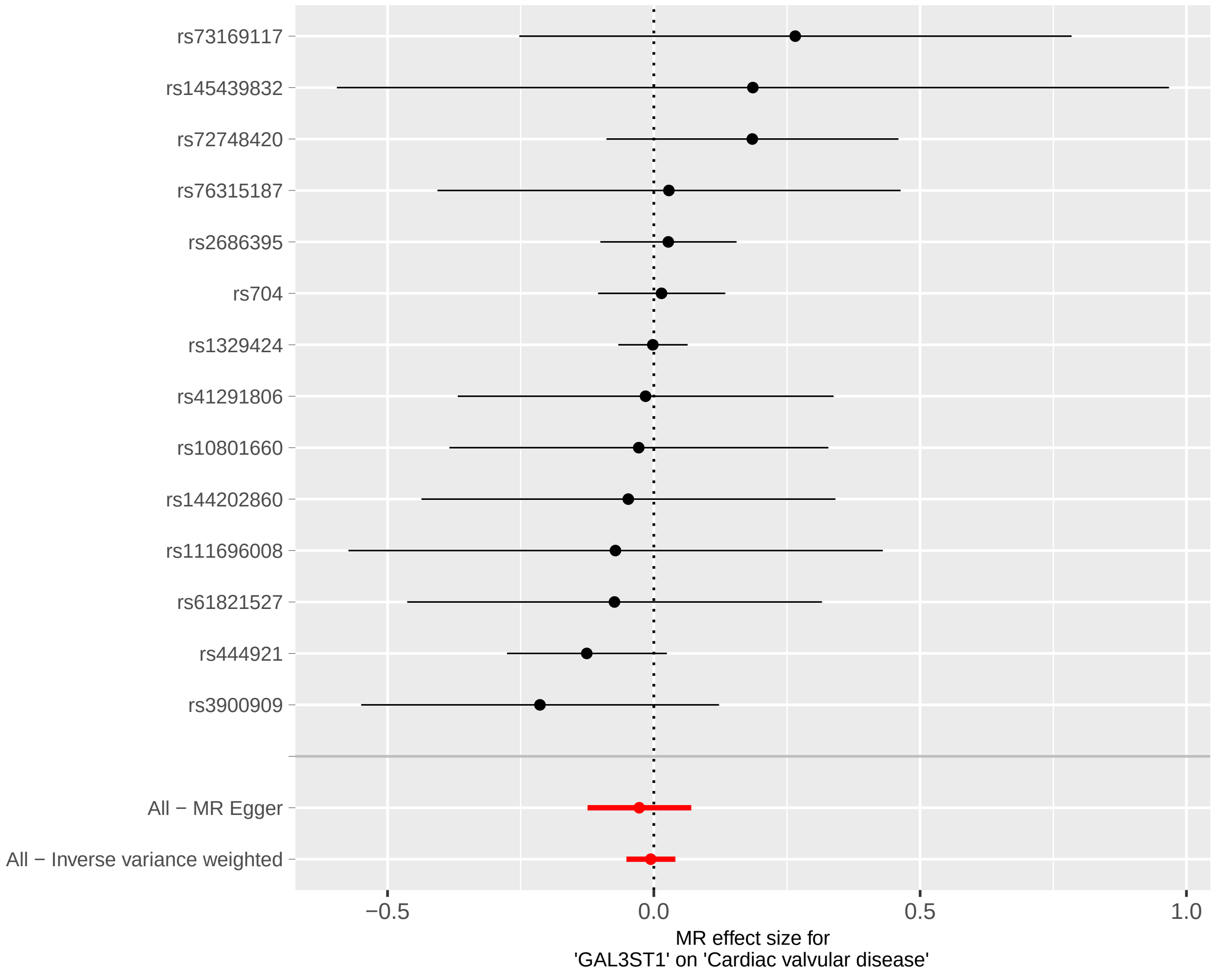
rs73169117
rs145439832
rs72748420
rs76315187
rs2686395
rs704
rs1329424
rs41291806
rs10801660
rs144202860
rs111696008
rs61821527
rs444921
rs3900909
All − MR Egger
All − Inverse variance weighted
−0.5
0.0
0.5
1.0
MR effect size for
'GAL3ST1' on 'Cardiac valvular disease'

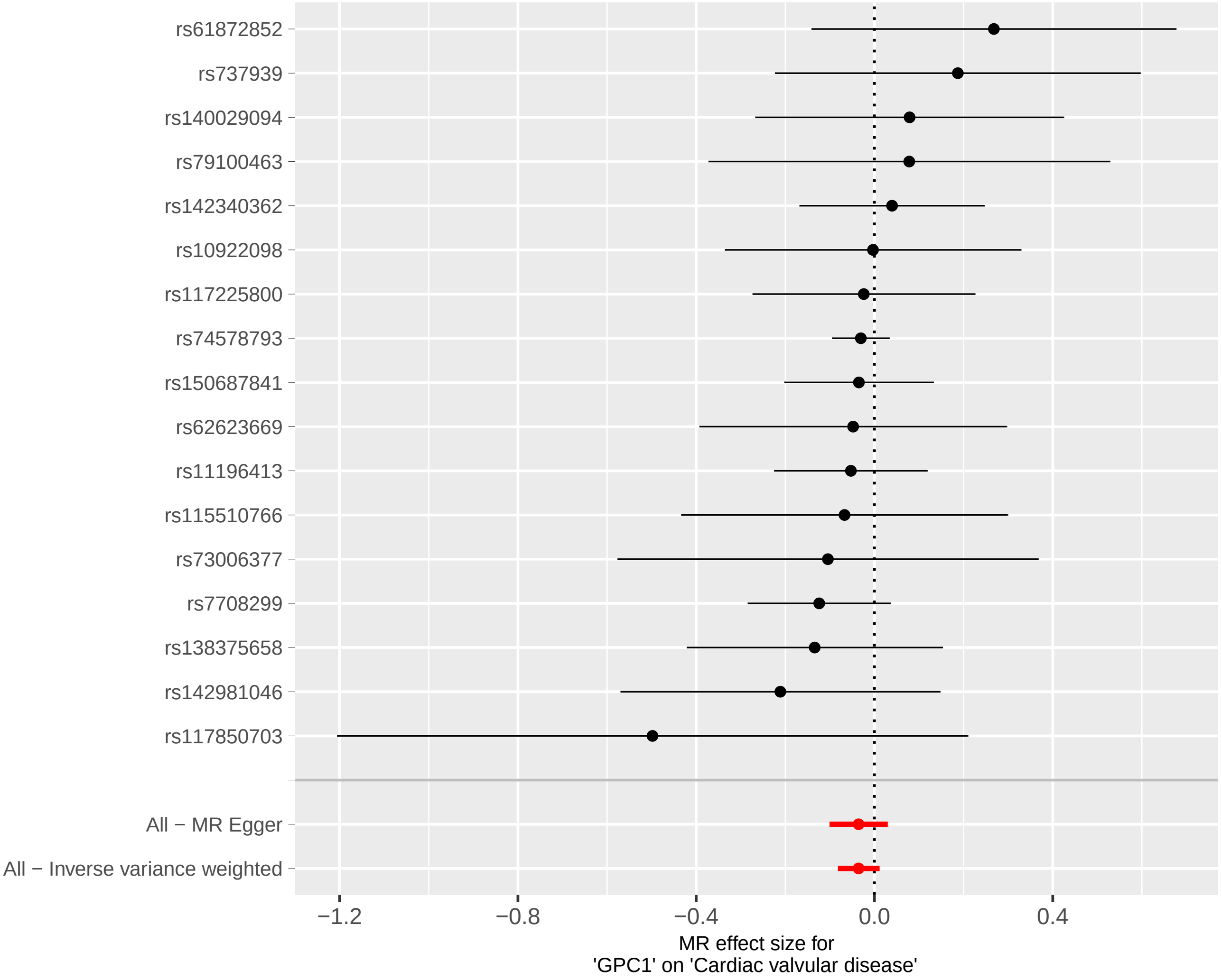
rs61872852
rs737939
rs140029094
rs79100463
rs142340362
rs10922098
rs117225800
rs74578793
rs150687841
rs62623669
rs11196413
rs115510766
rs73006377
rs7708299
rs138375658
rs142981046
rs117850703
All − MR Egger
All − Inverse variance weighted
−1.2
−0.8
−0.4
0.0
0.4
MR effect size for
'GPC1' on 'Cardiac valvular disease'

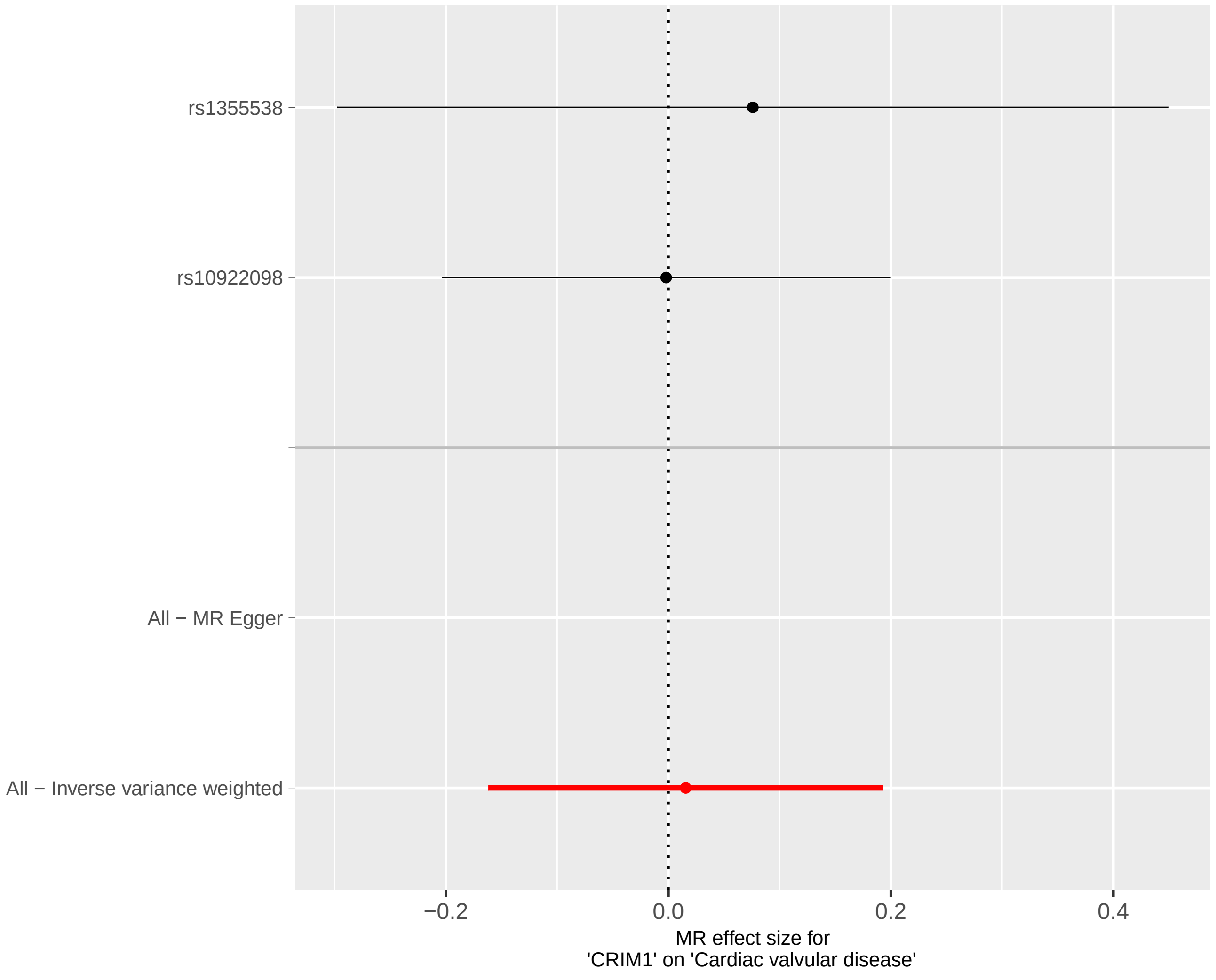
rs1355538
rs10922098
All − MR Egger
All − Inverse variance weighted
−0.2
0.0
0.2
0.4
MR effect size for
'CRIM1' on 'Cardiac valvular disease'

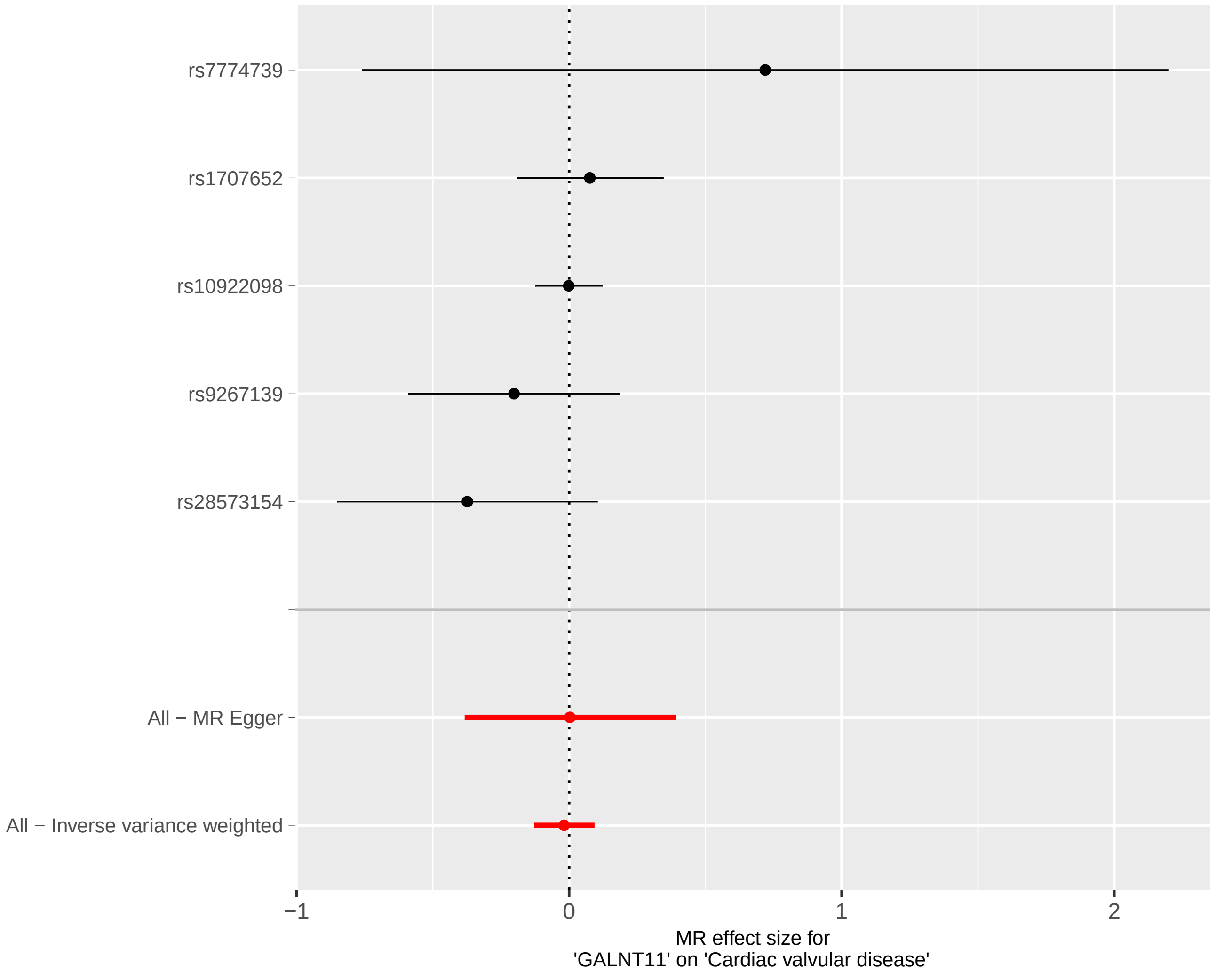
rs7774739
rs1707652
rs10922098
rs9267139
rs28573154
All − MR Egger
All − Inverse variance weighted
−1
0
1
2
MR effect size for
'GALNT11' on 'Cardiac valvular disease'

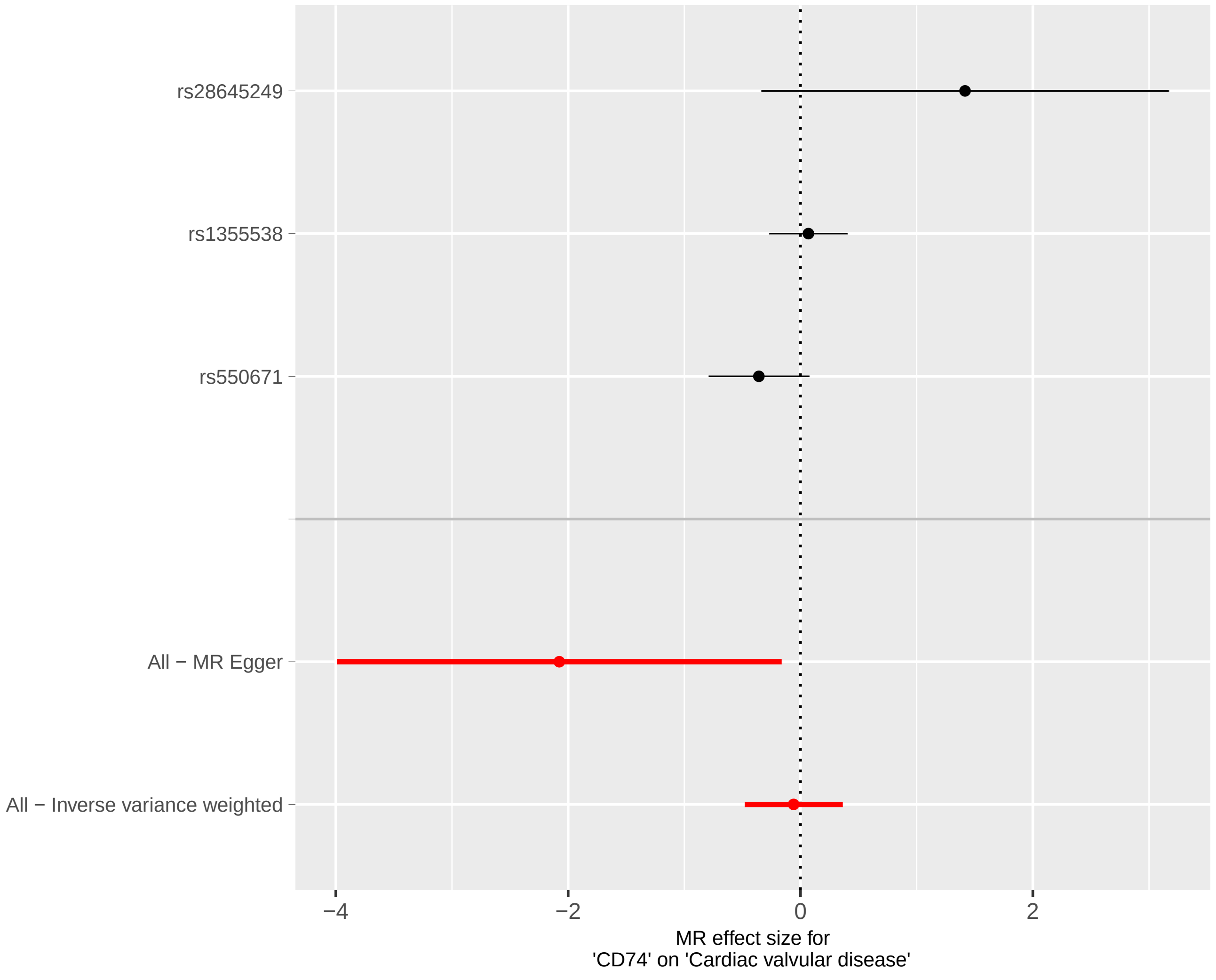

rs28645249
rs1355538
rs550671
All − MR Egger
All − Inverse variance weighted
−4
−2
0
2
MR effect size for
'CD74' on 'Cardiac valvular disease'

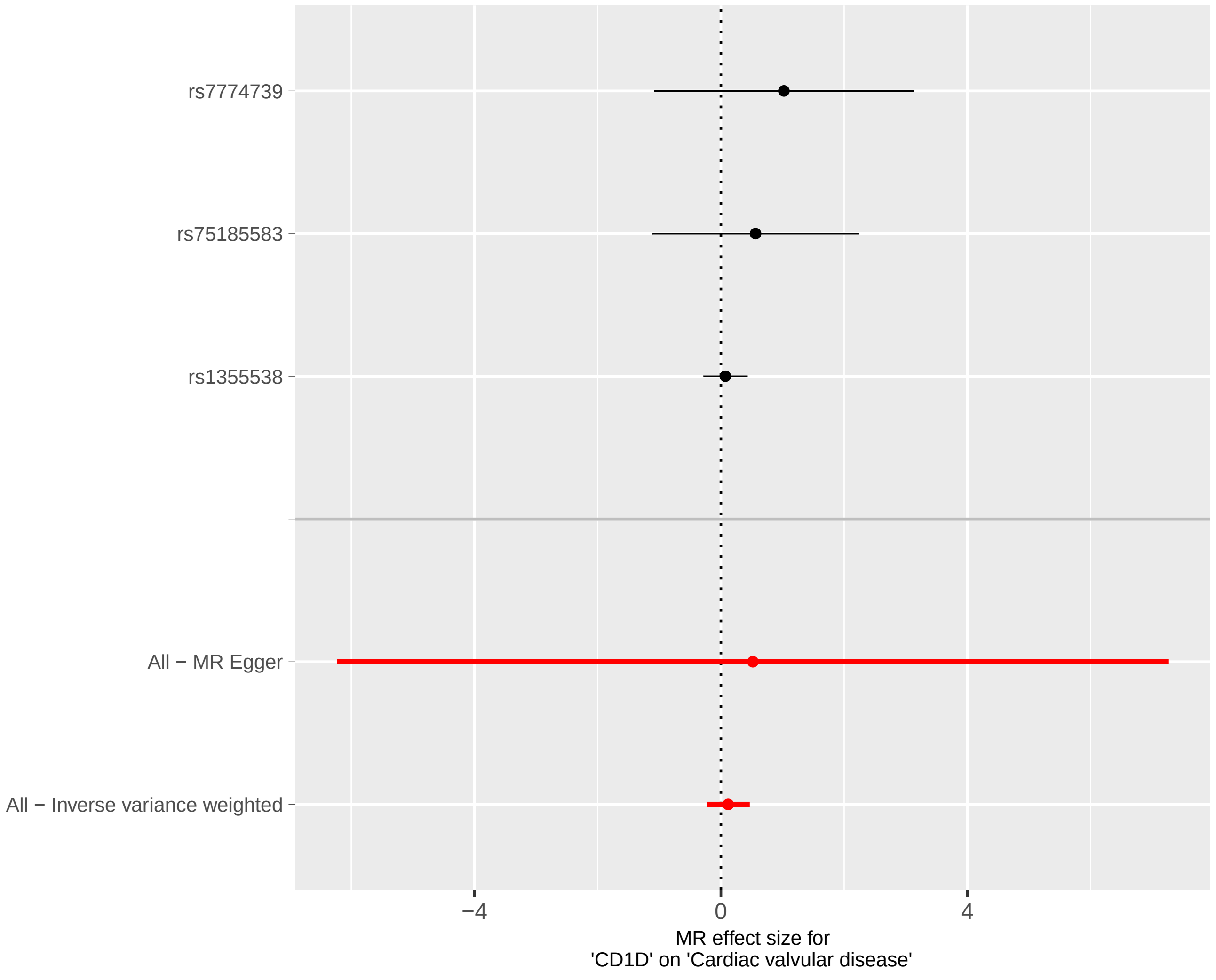
rs7774739
rs75185583
rs1355538
All − MR Egger
All − Inverse variance weighted
−4
0
4
MR effect size for
'CD1D' on 'Cardiac valvular disease'

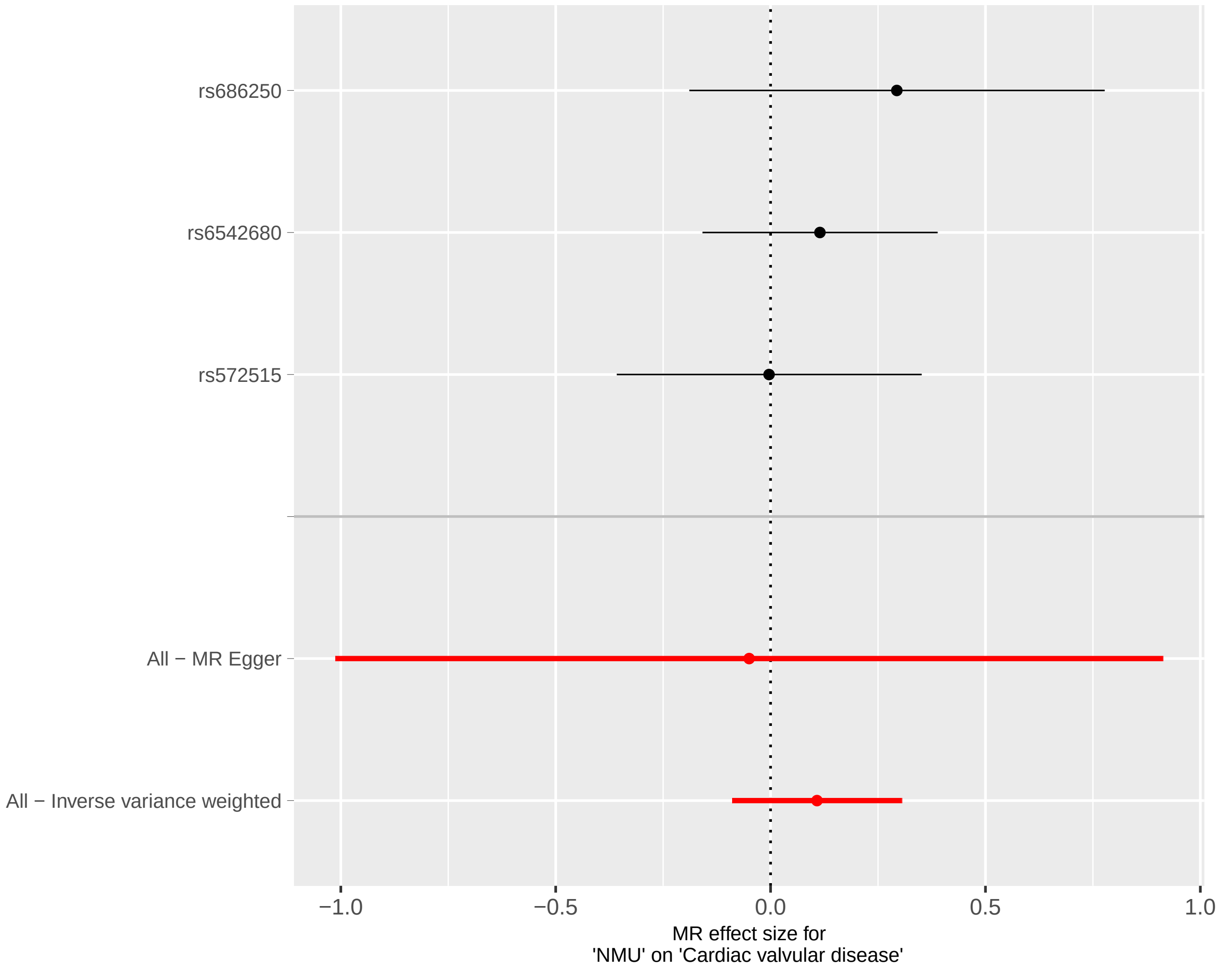
rs686250
rs6542680
rs572515
All − MR Egger
All − Inverse variance weighted
−1.0
−0.5
0.0
0.5
1.0
MR effect size for
'NMU' on 'Cardiac valvular disease'

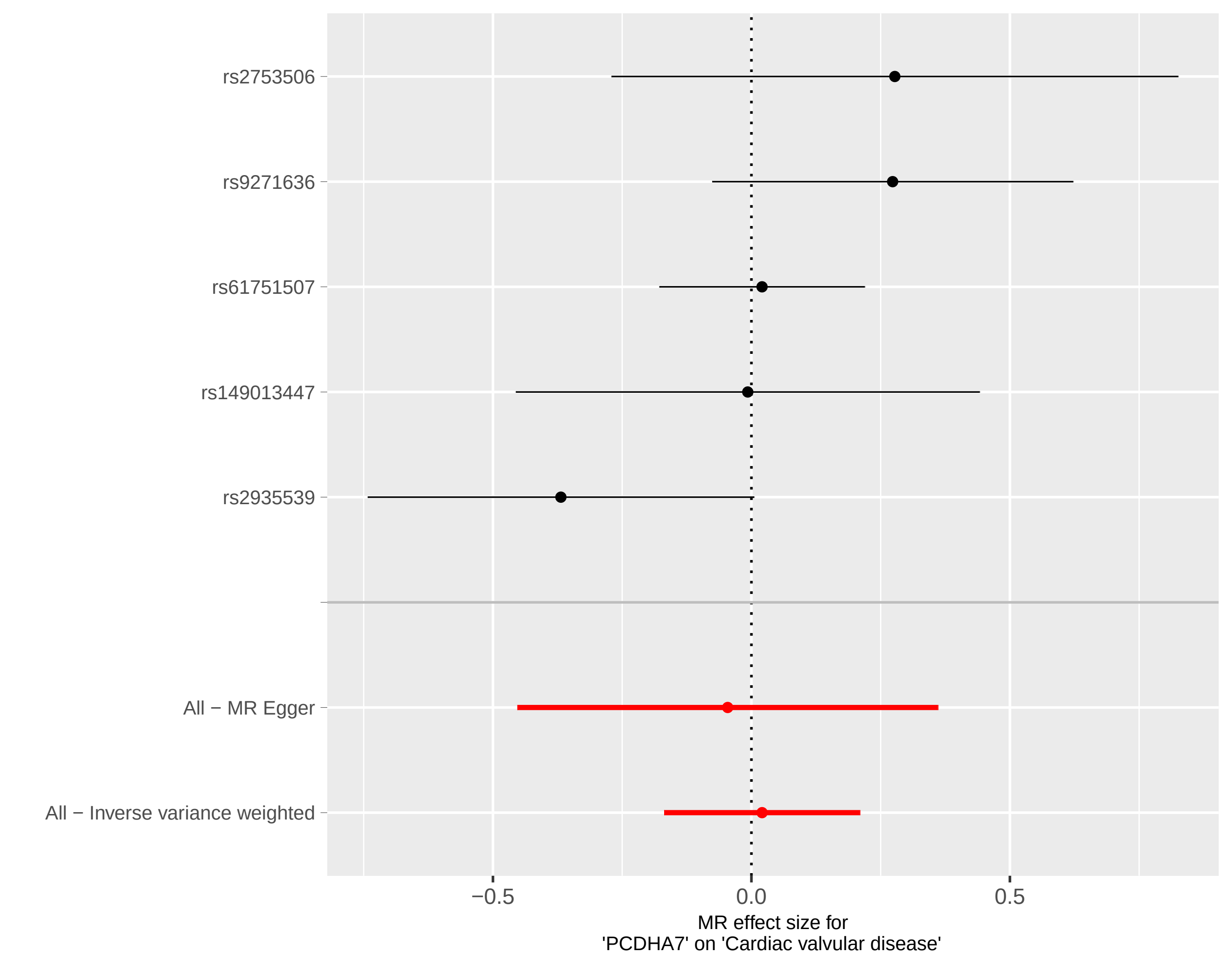

rs2753506
rs9271636
rs61751507
rs149013447
rs2935539
All − MR Egger
All − Inverse variance weighted
−0.5
0.0
0.5
MR effect size for
'PCDHA7' on 'Cardiac valvular disease'

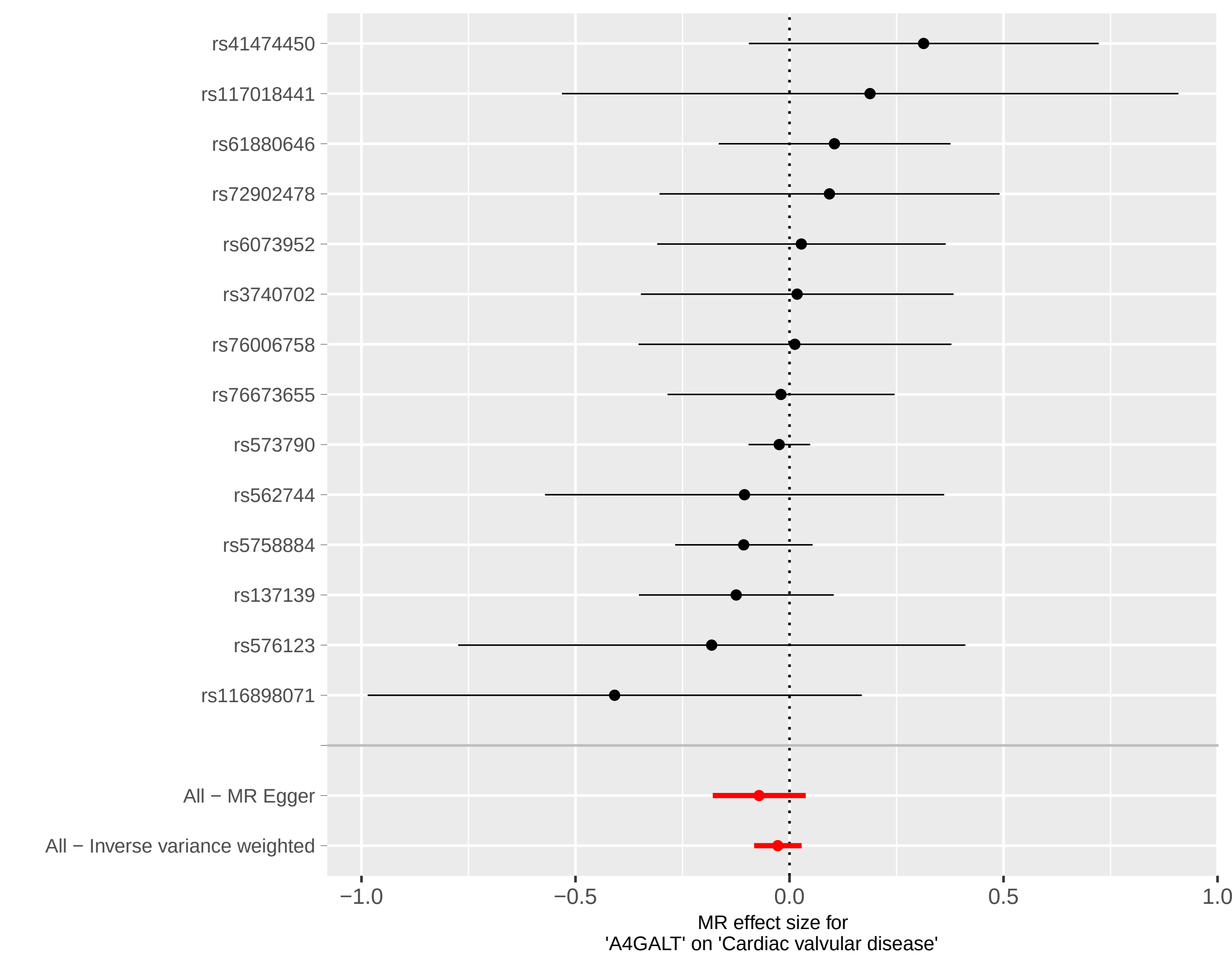
rs41474450
rs117018441
rs61880646
rs72902478
rs6073952
rs3740702
rs76006758
rs76673655
rs573790
rs562744
rs5758884
rs137139
rs576123
rs116898071
All − MR Egger
All − Inverse variance weighted
−1.0
−0.5
0.0
0.5
1.0
MR effect size for
'A4GALT' on 'Cardiac valvular disease'

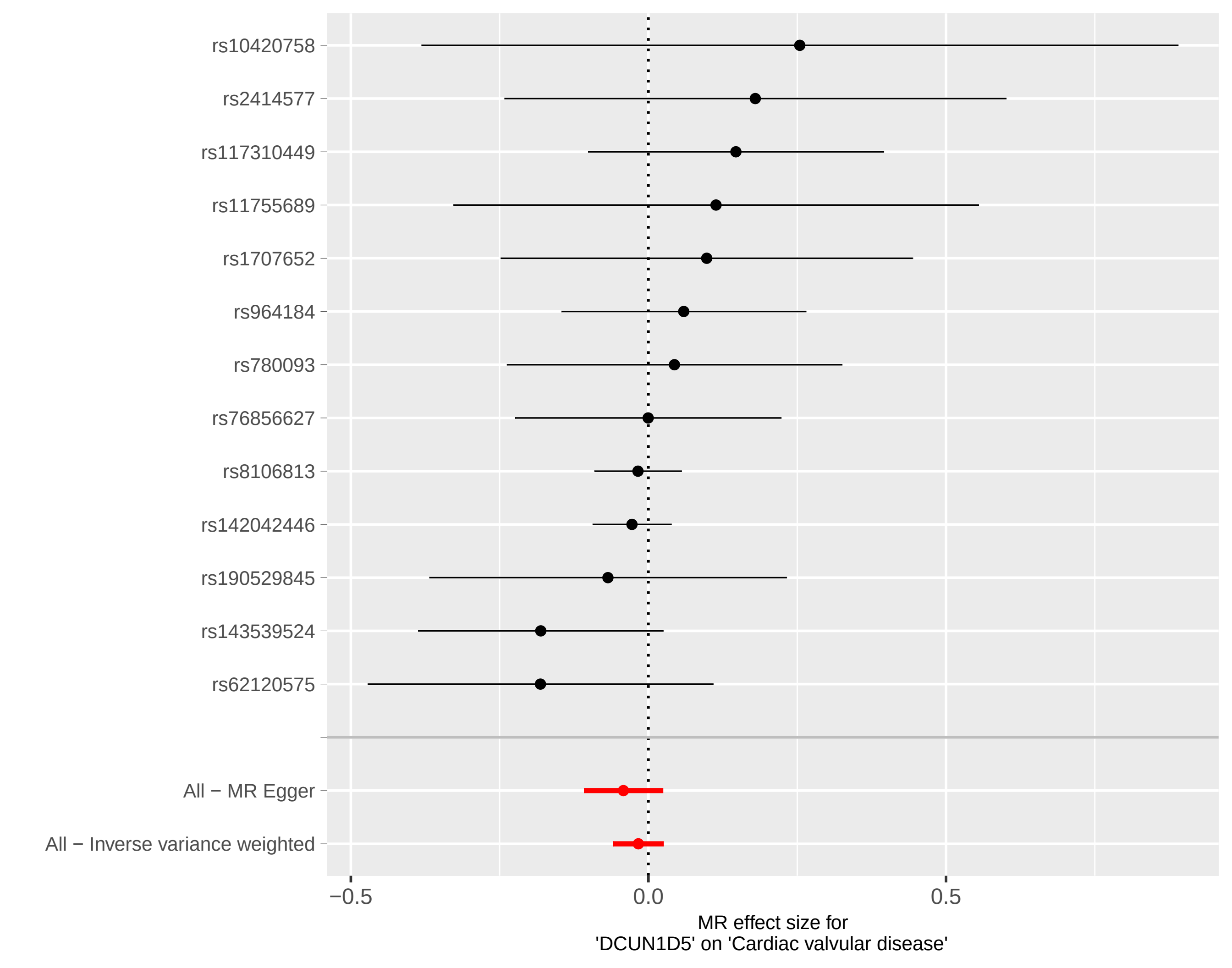
rs10420758
rs2414577
rs117310449
rs11755689
rs1707652
rs964184
rs780093
rs76856627
rs8106813
rs142042446
rs190529845
rs143539524
rs62120575
All − MR Egger
All − Inverse variance weighted
−0.5
0.0
0.5
MR effect size for
'DCUN1D5' on 'Cardiac valvular disease'

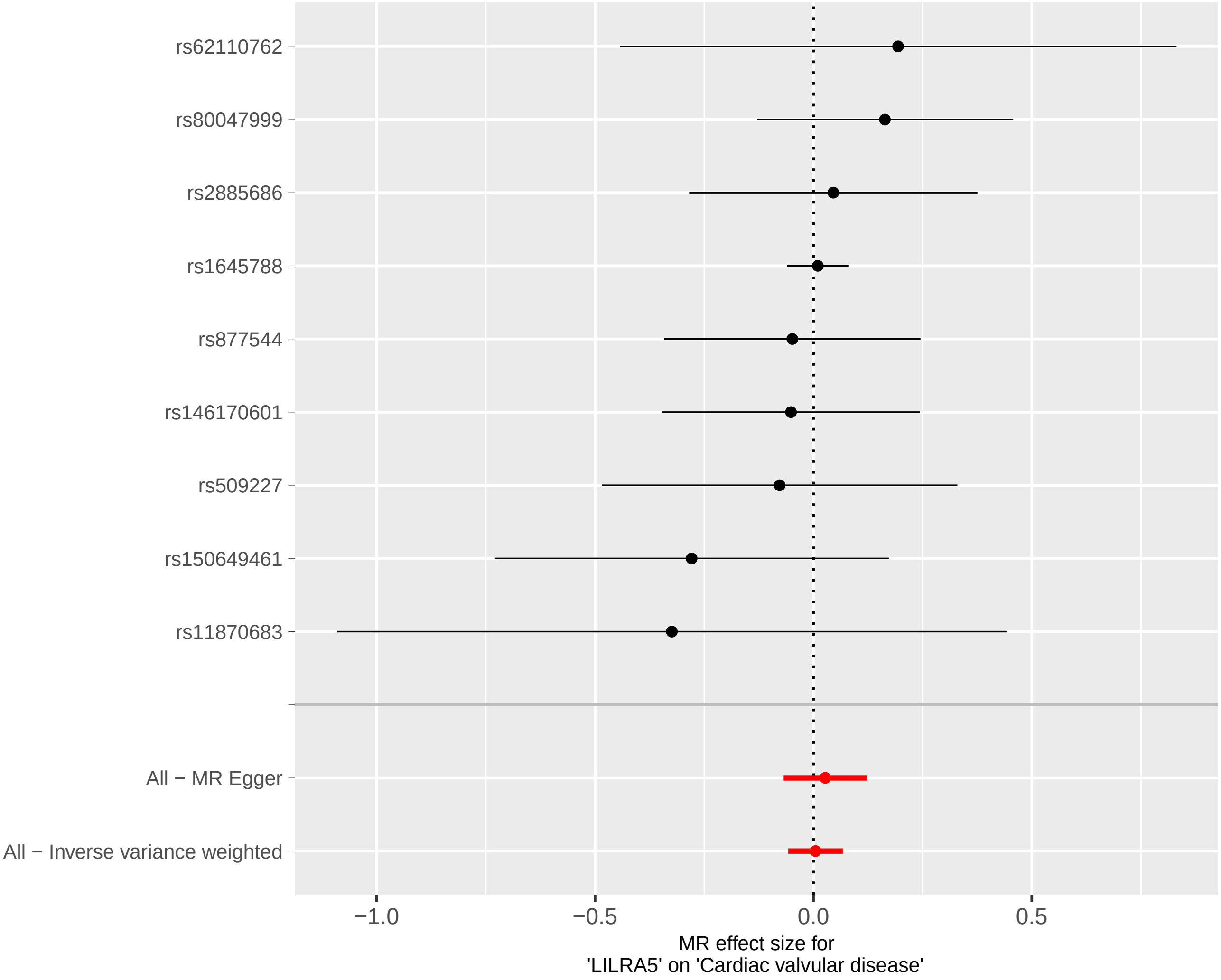
rs62110762
rs80047999
rs2885686
rs1645788
rs877544
rs146170601
rs509227
rs150649461
rs11870683
All − MR Egger
All − Inverse variance weighted
−1.0
−0.5
0.0
0.5
MR effect size for
'LILRA5' on 'Cardiac valvular disease'

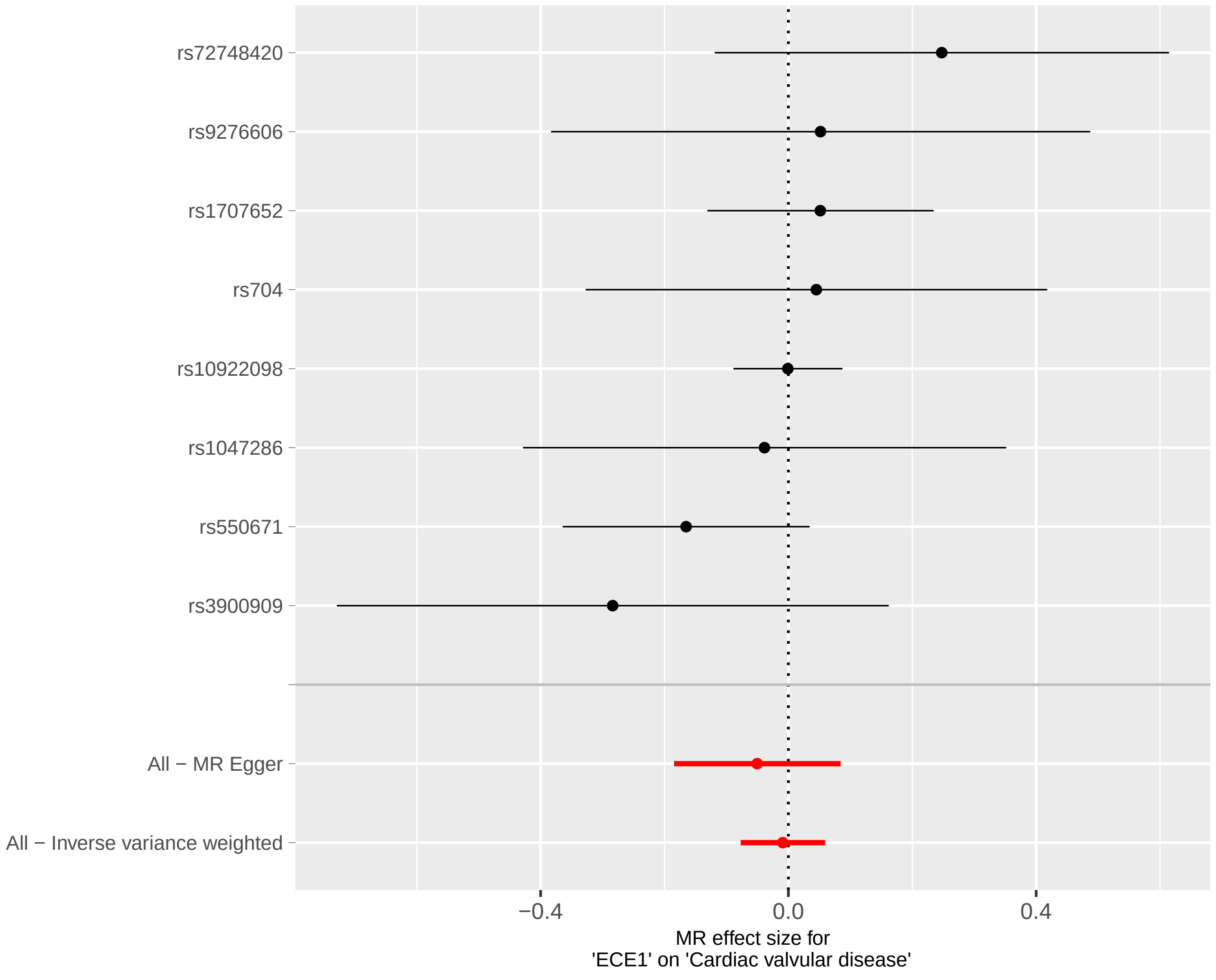
rs72748420
rs9276606
rs1707652
rs704
rs10922098
rs1047286
rs550671
rs3900909
All − MR Egger
All − Inverse variance weighted
−0.4
0.0
0.4
MR effect size for
'ECE1' on 'Cardiac valvular disease'

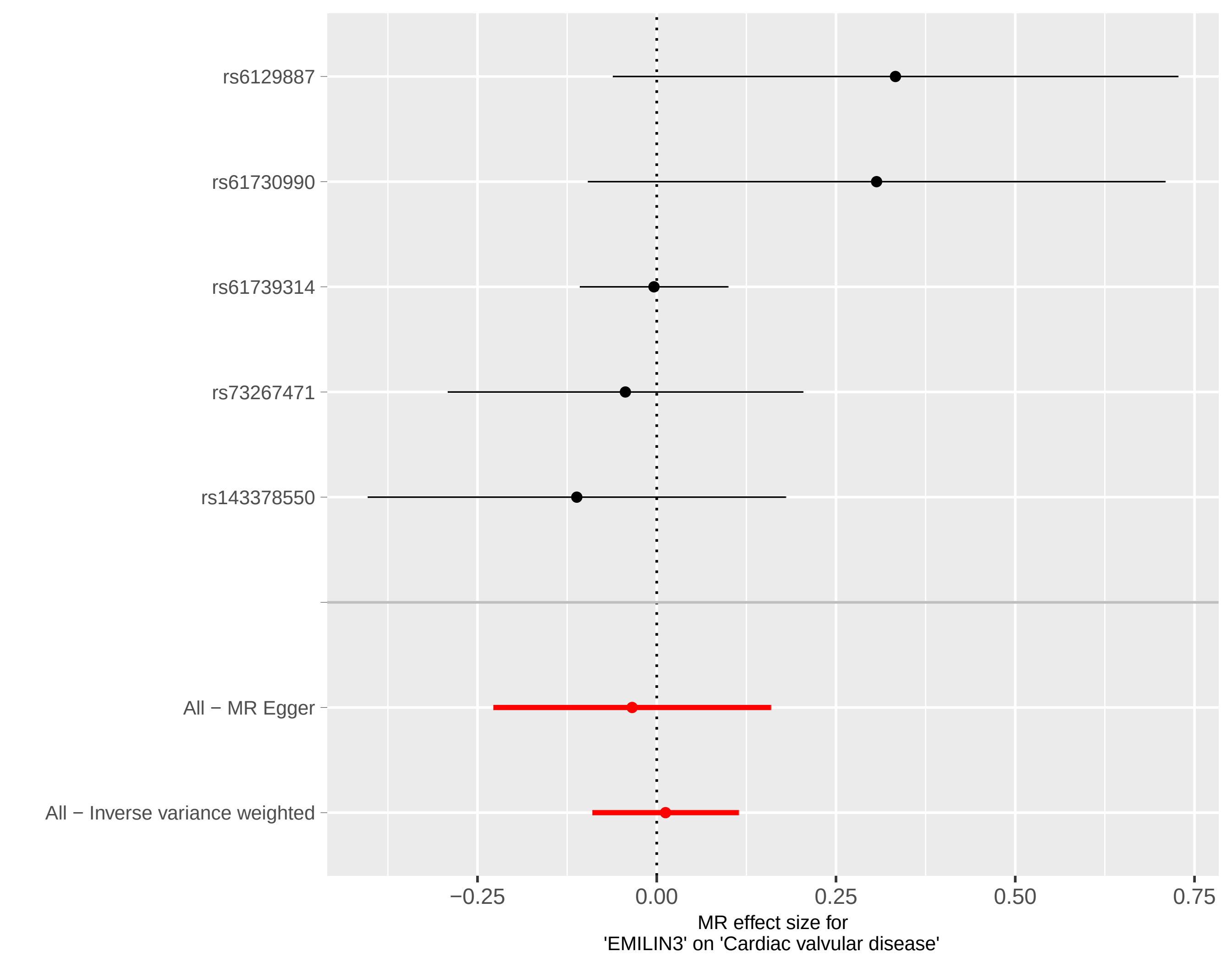
rs6129887
rs61730990
rs61739314
rs73267471
rs143378550
All − MR Egger
All − Inverse variance weighted
−0.25
0.00
0.25
0.50
0.75
MR effect size for
'EMILIN3' on 'Cardiac valvular disease'

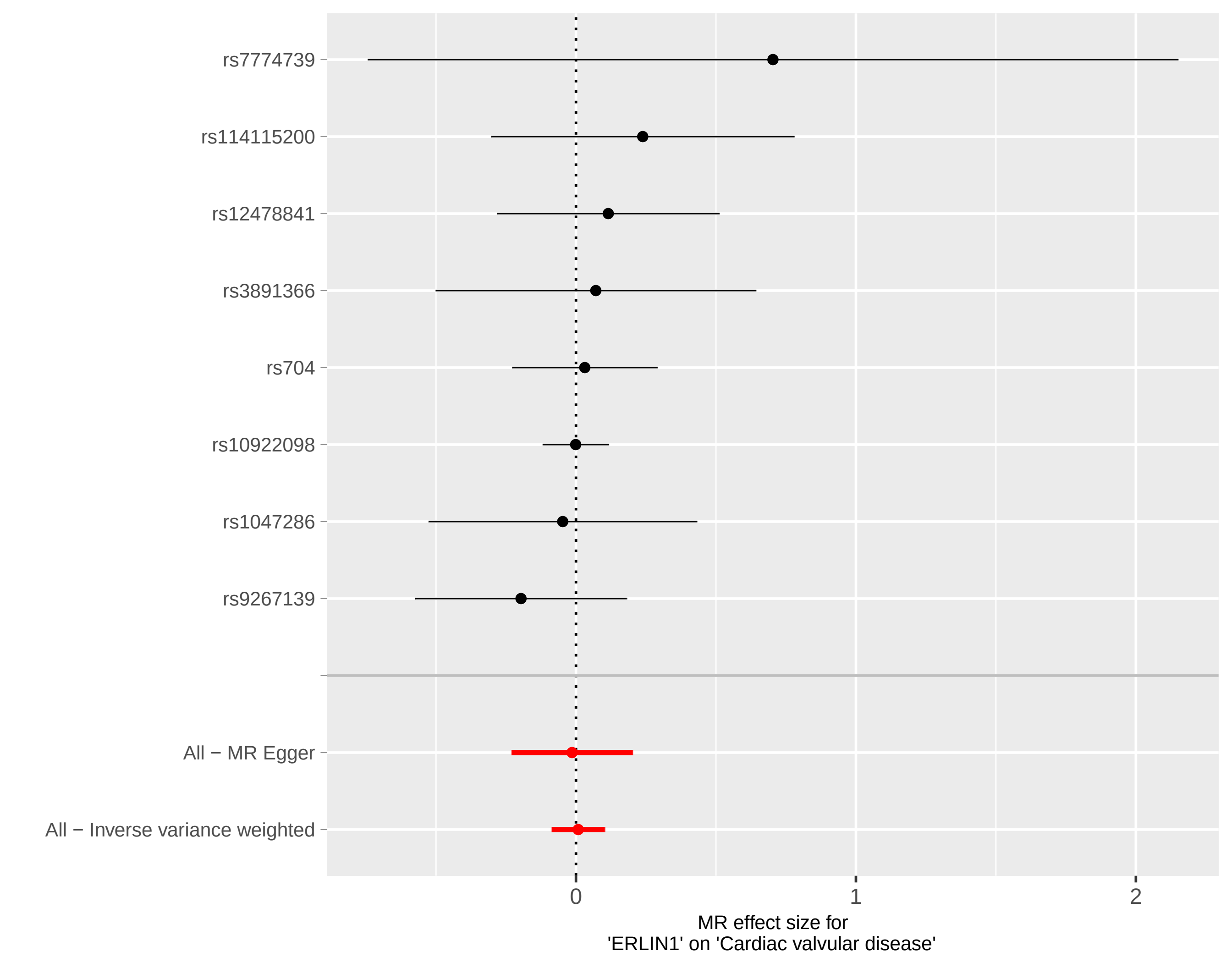
rs7774739
rs114115200
rs12478841
rs3891366
rs704
rs10922098
rs1047286
rs9267139
All − MR Egger
All − Inverse variance weighted
0
1
2
MR effect size for
'ERLIN1' on 'Cardiac valvular disease'

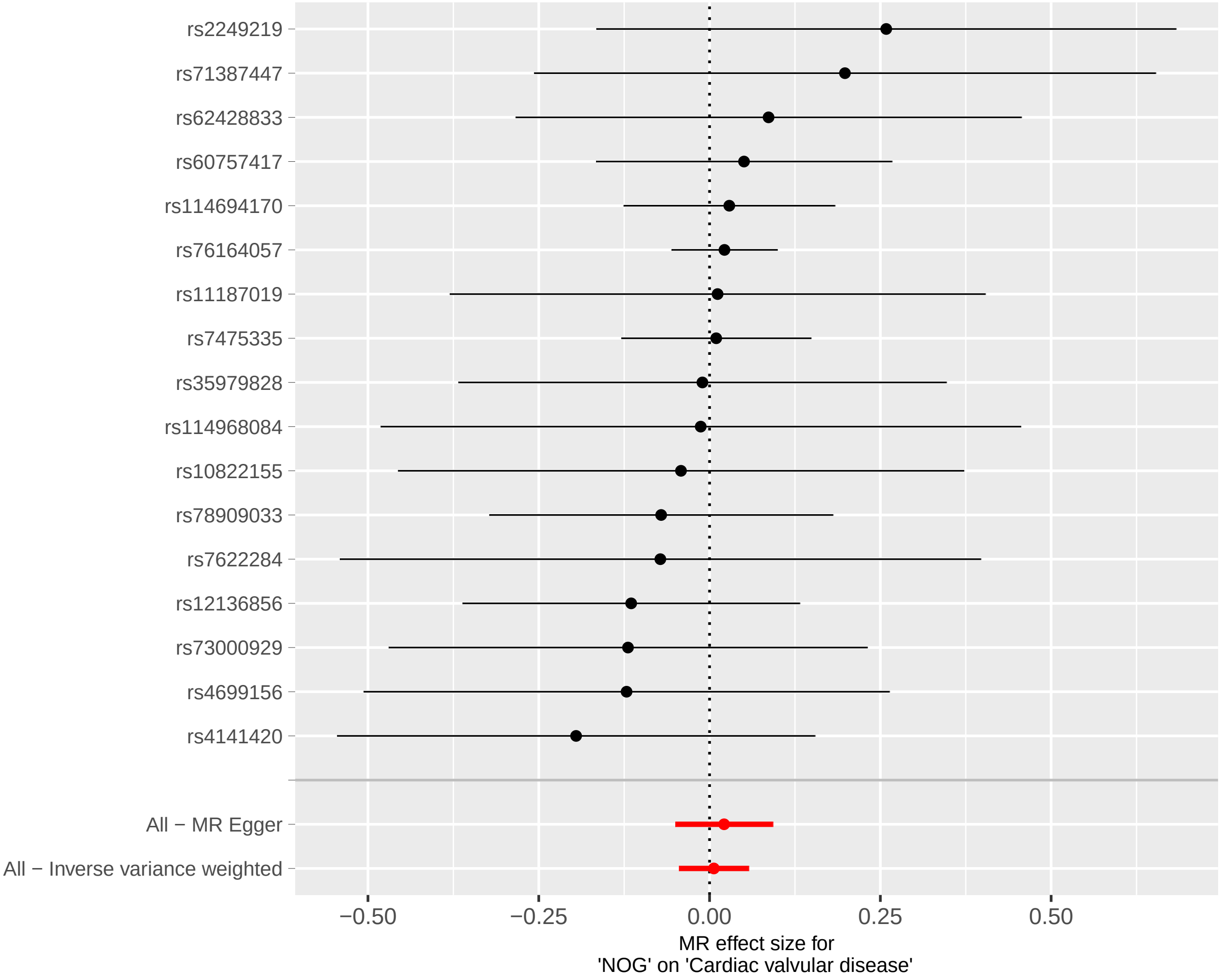

rs2249219
rs71387447
rs62428833
rs60757417
rs114694170
rs76164057
rs11187019
rs7475335
rs35979828
rs114968084
rs10822155
rs78909033
rs7622284
rs12136856
rs73000929
rs4699156
rs4141420
All − MR Egger
All − Inverse variance weighted
−0.50
−0.25
0.00
0.25
0.50
MR effect size for
'NOG' on 'Cardiac valvular disease'

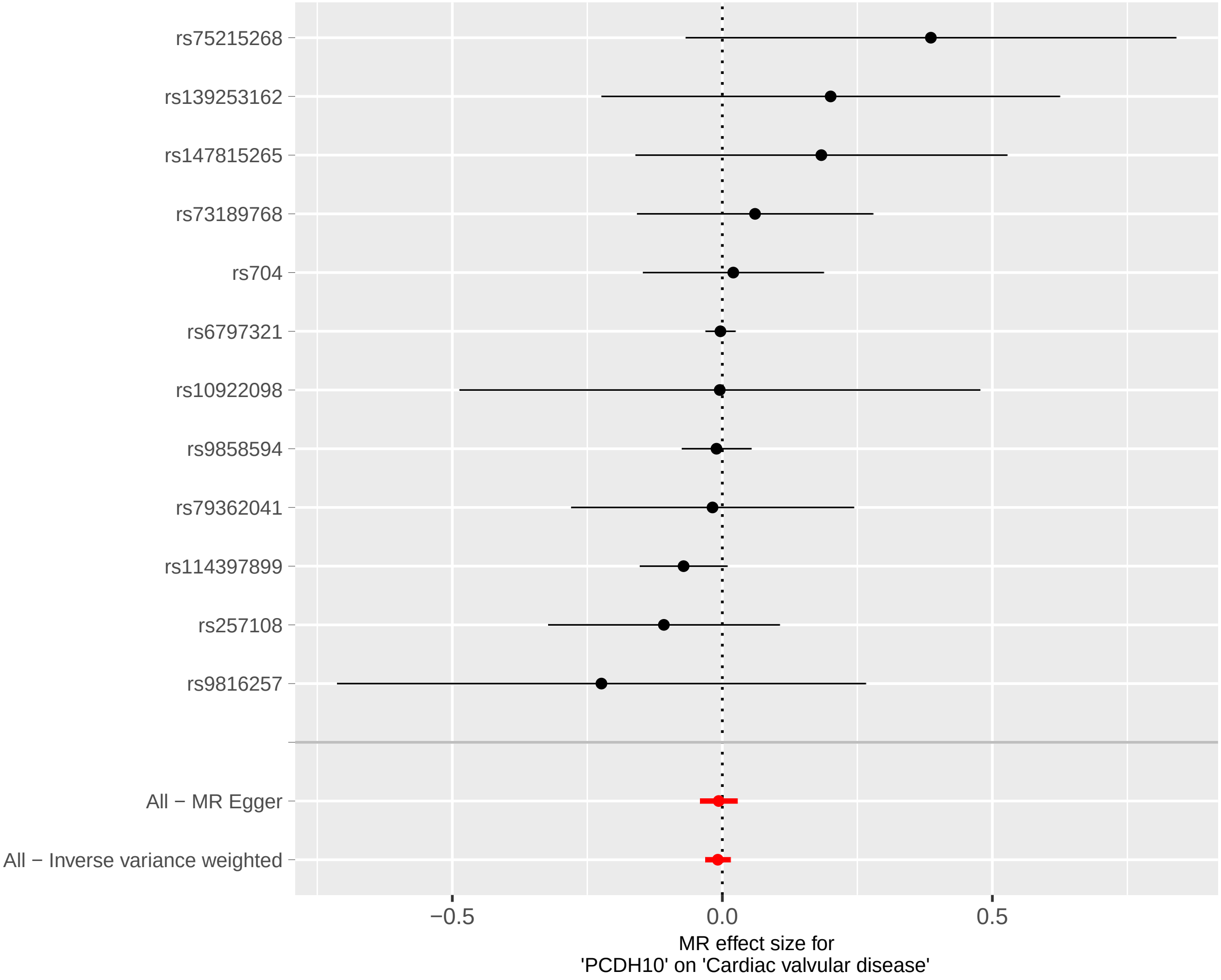
rs75215268
rs139253162
rs147815265
rs73189768
rs704
rs6797321
rs10922098
rs9858594
rs79362041
rs114397899
rs257108
rs9816257
All − MR Egger
All − Inverse variance weighted
−0.5
0.0
0.5
MR effect size for
'PCDH10' on 'Cardiac valvular disease'

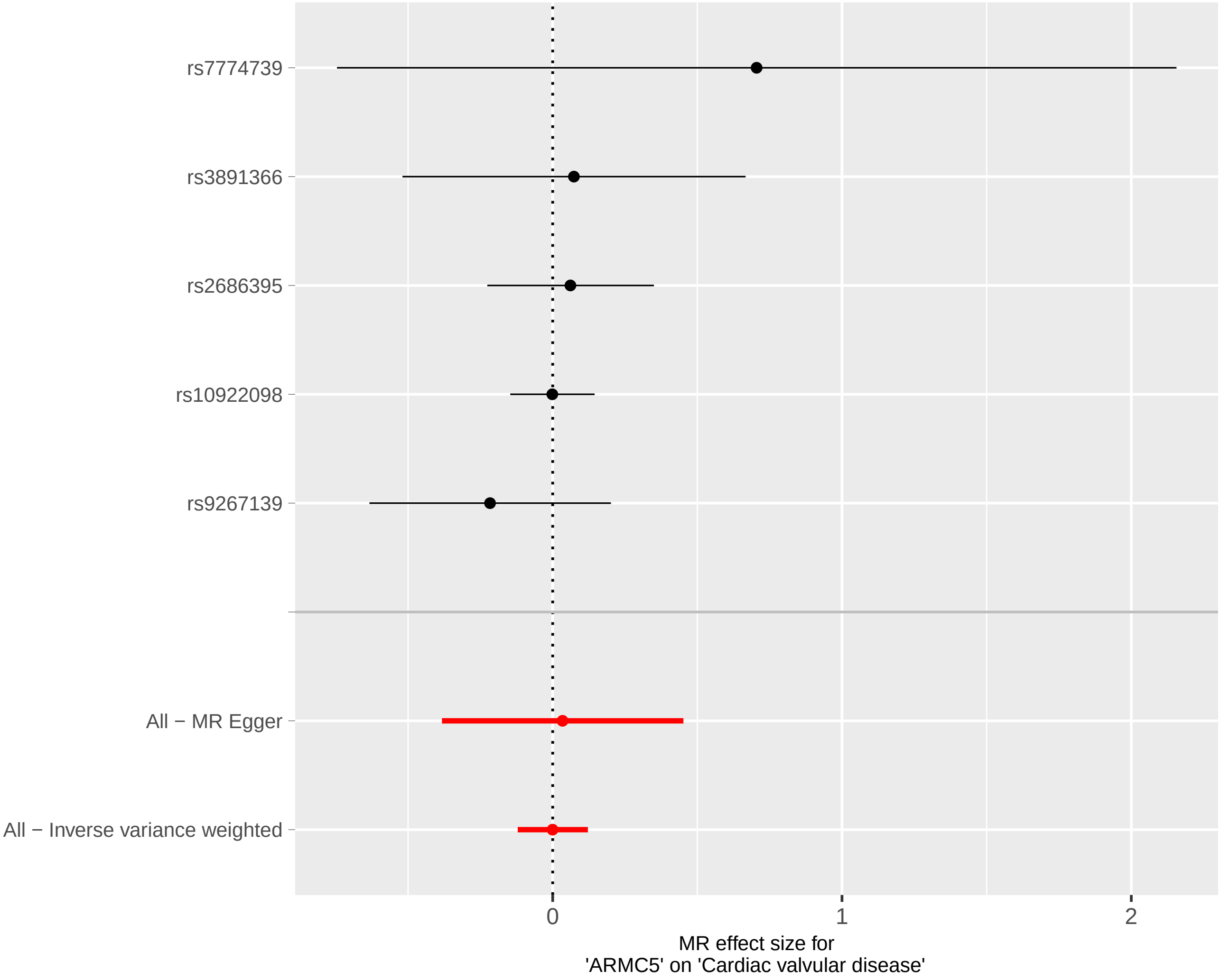
rs7774739
rs3891366
rs2686395
rs10922098
rs9267139
All − MR Egger
All − Inverse variance weighted
0
1
2
MR effect size for
'ARMC5' on 'Cardiac valvular disease'

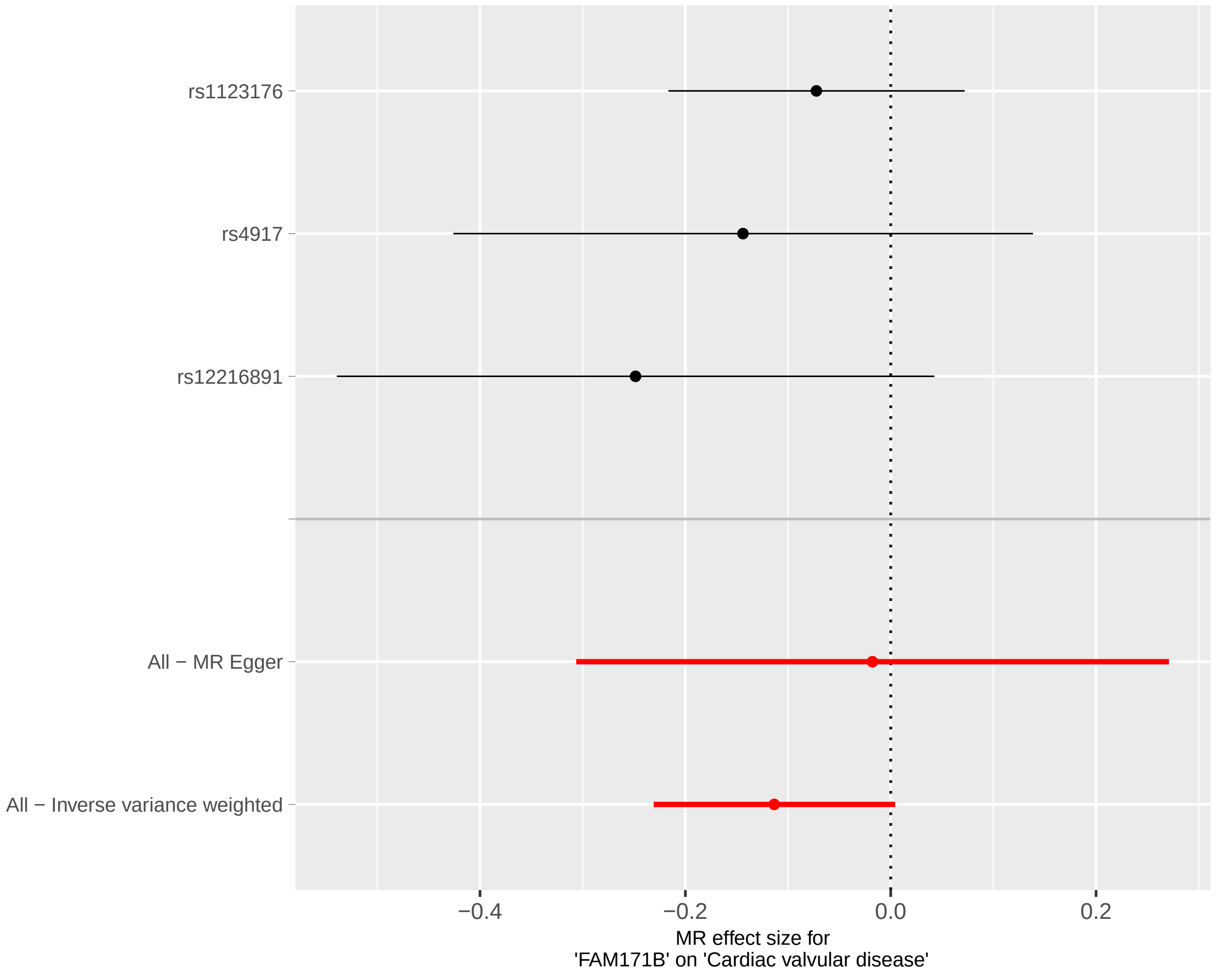
rs1123176
rs4917
rs12216891
All − MR Egger
All − Inverse variance weighted
−0.4
−0.2
0.0
0.2
MR effect size for
'FAM171B' on 'Cardiac valvular disease'

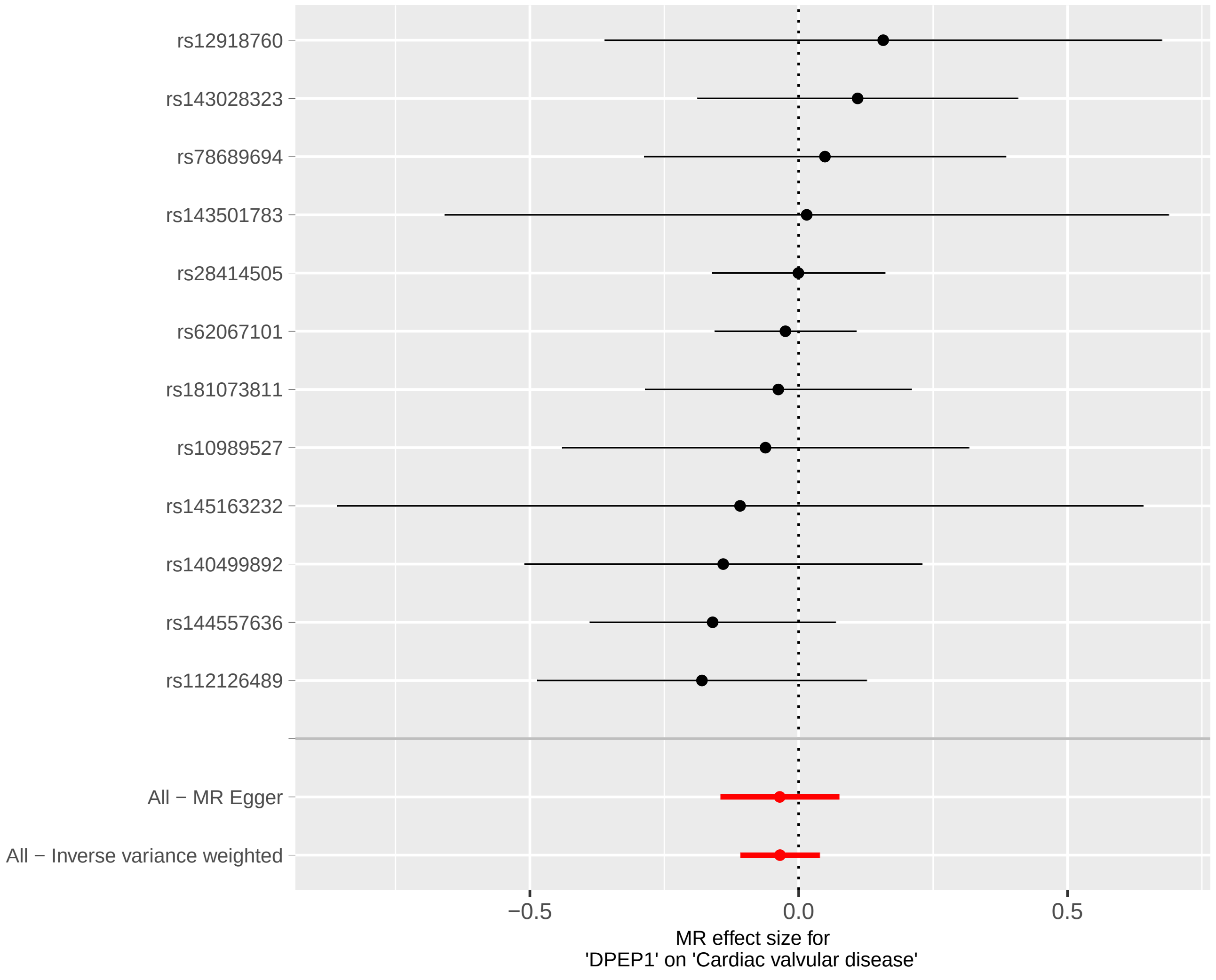
rs12918760
rs143028323
rs78689694
rs143501783
rs28414505
rs62067101
rs181073811
rs10989527
rs145163232
rs140499892
rs144557636
rs112126489
All − MR Egger
All − Inverse variance weighted
−0.5
0.0
0.5
MR effect size for
'DPEP1' on 'Cardiac valvular disease'

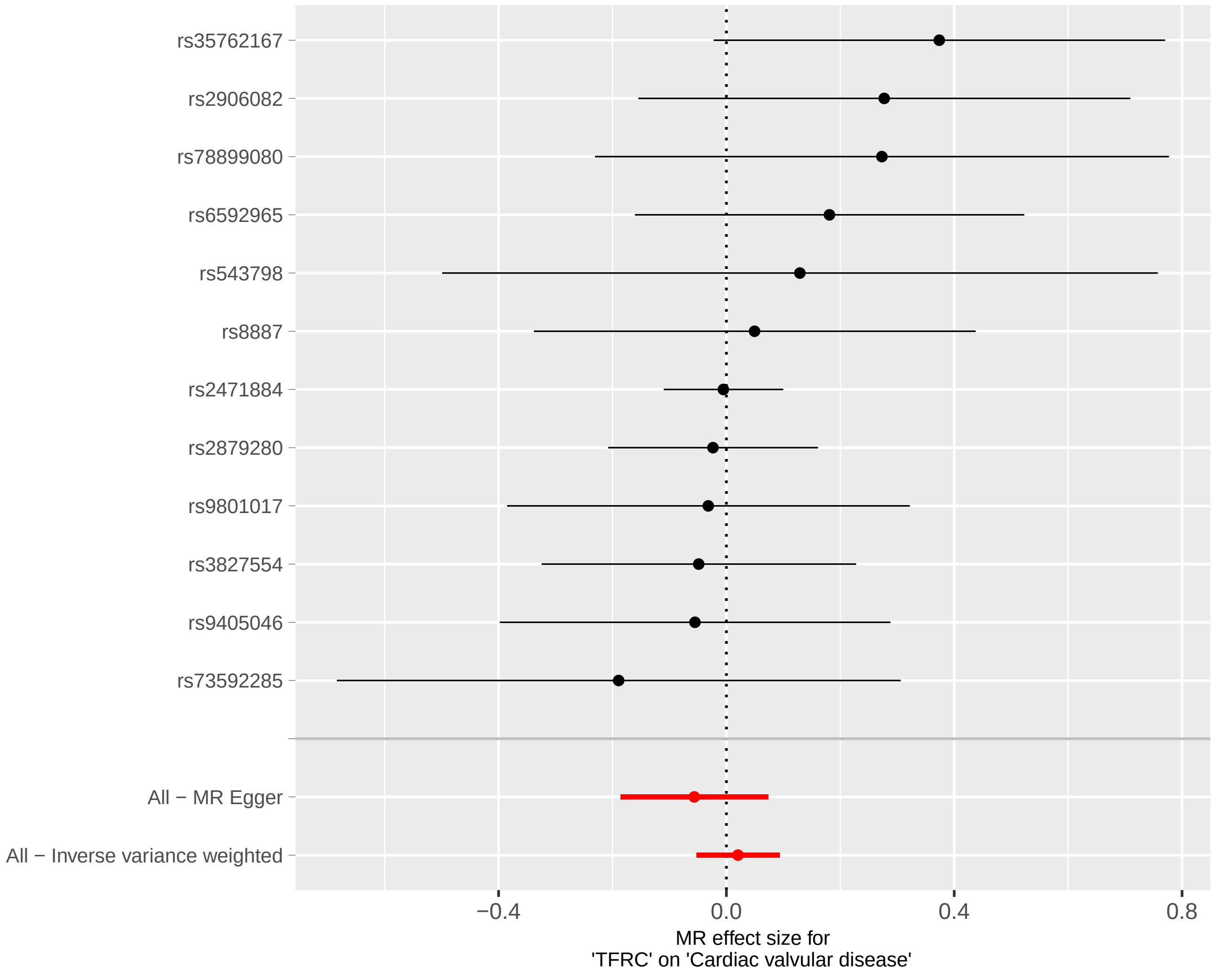
rs35762167
rs2906082
rs78899080
rs6592965
rs543798
rs8887
rs2471884
rs2879280
rs9801017
rs3827554
rs9405046
rs73592285
All − MR Egger
All − Inverse variance weighted
−0.4
0.0
0.4
0.8
MR effect size for
'TFRC' on 'Cardiac valvular disease'

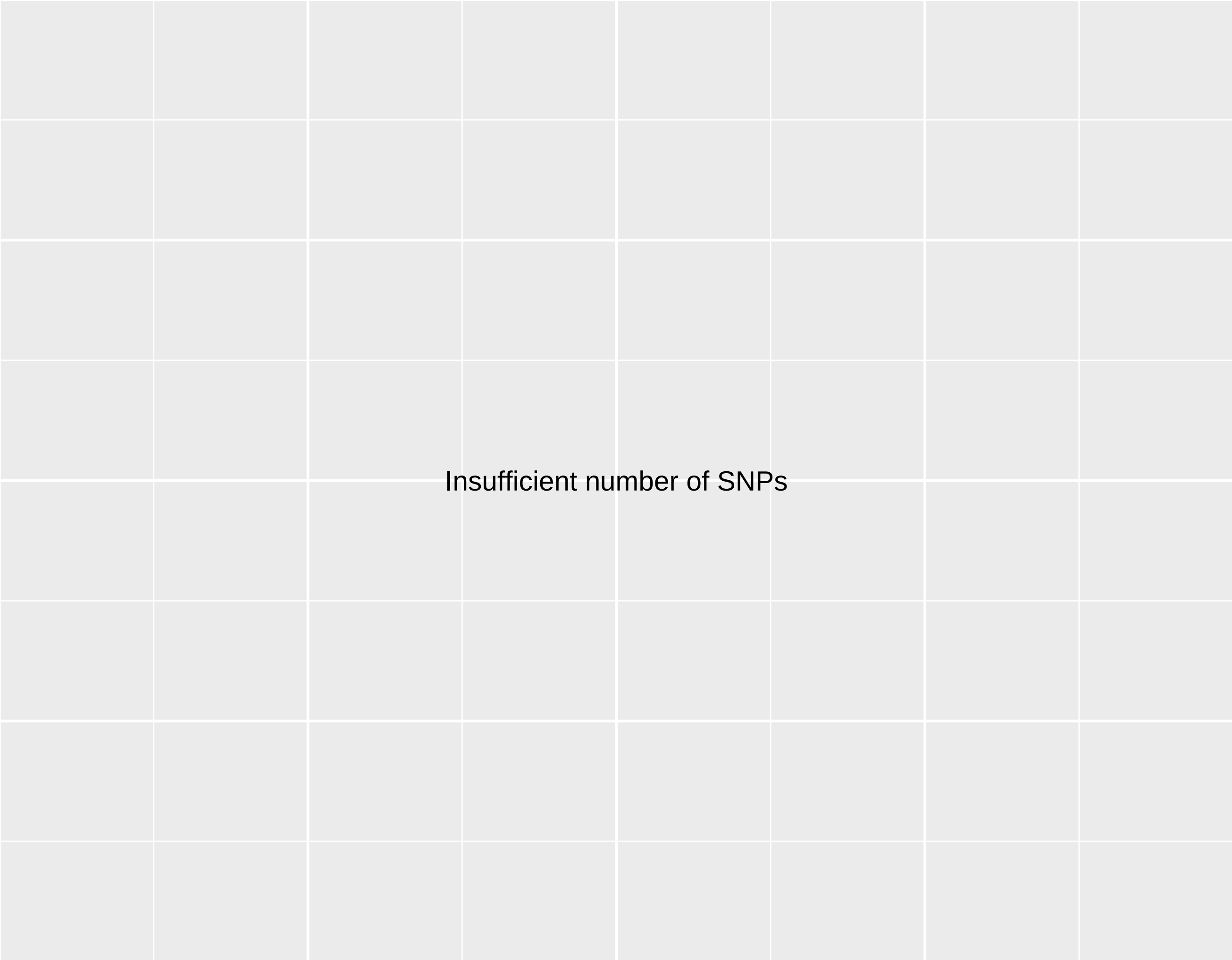
Insufficient number of SNPs

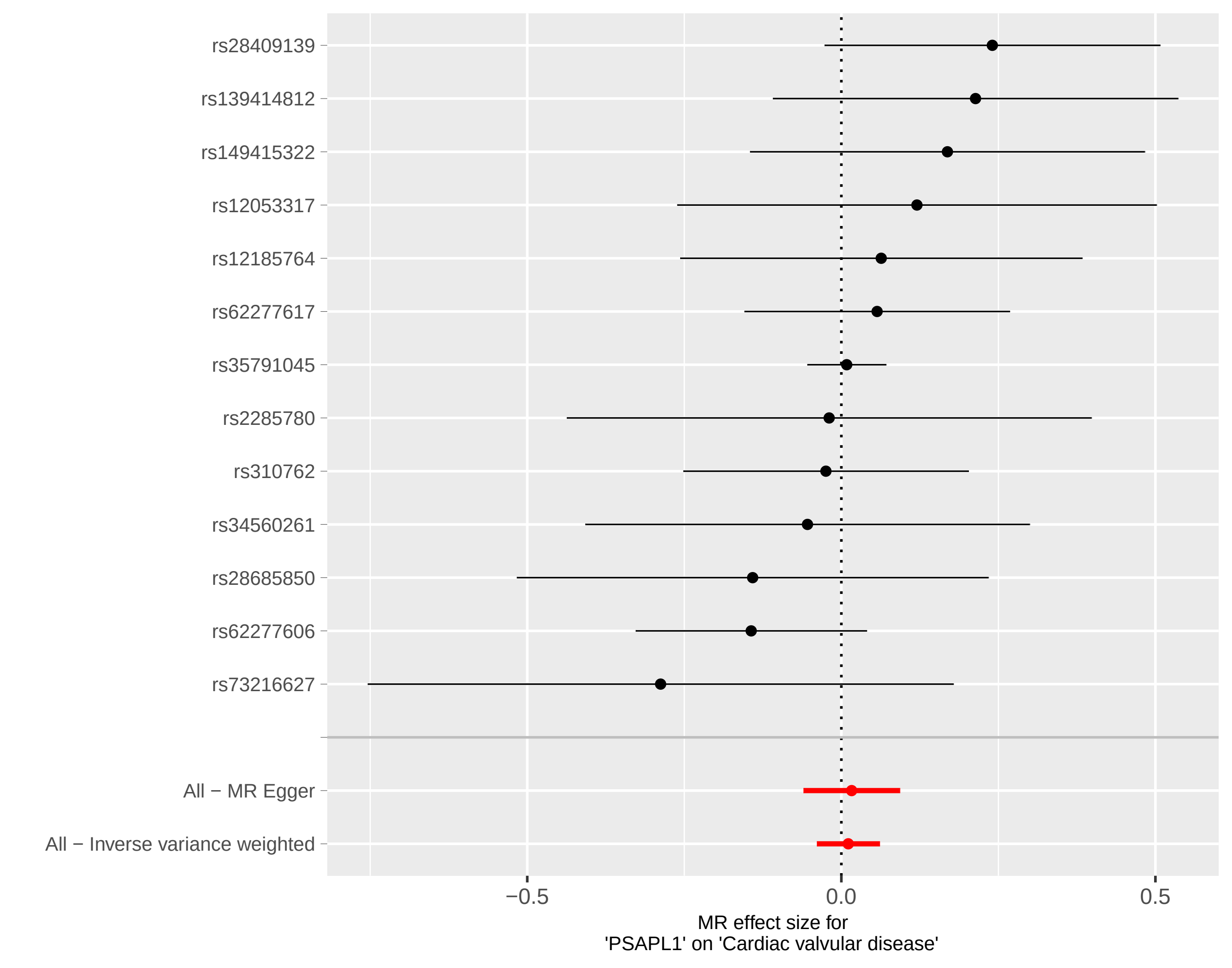

rs28409139
rs139414812
rs149415322
rs12053317
rs12185764
rs62277617
rs35791045
rs2285780
rs310762
rs34560261
rs28685850
rs62277606
rs73216627
All − MR Egger
All − Inverse variance weighted
−0.5
0.0
0.5
MR effect size for
'PSAPL1' on 'Cardiac valvular disease'

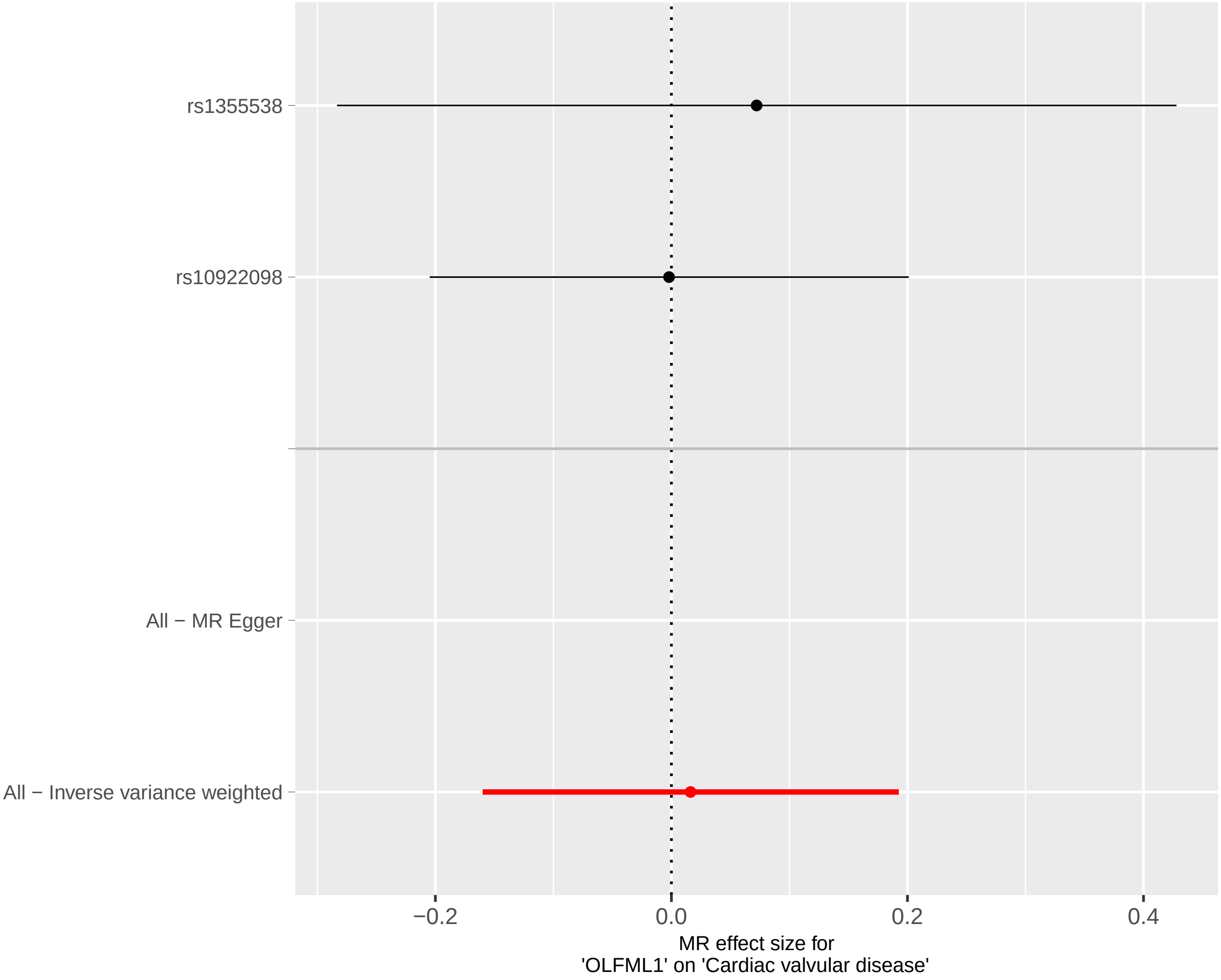
rs1355538
rs10922098
All − MR Egger
All − Inverse variance weighted
−0.2
0.0
0.2
0.4
MR effect size for
'OLFML1' on 'Cardiac valvular disease'

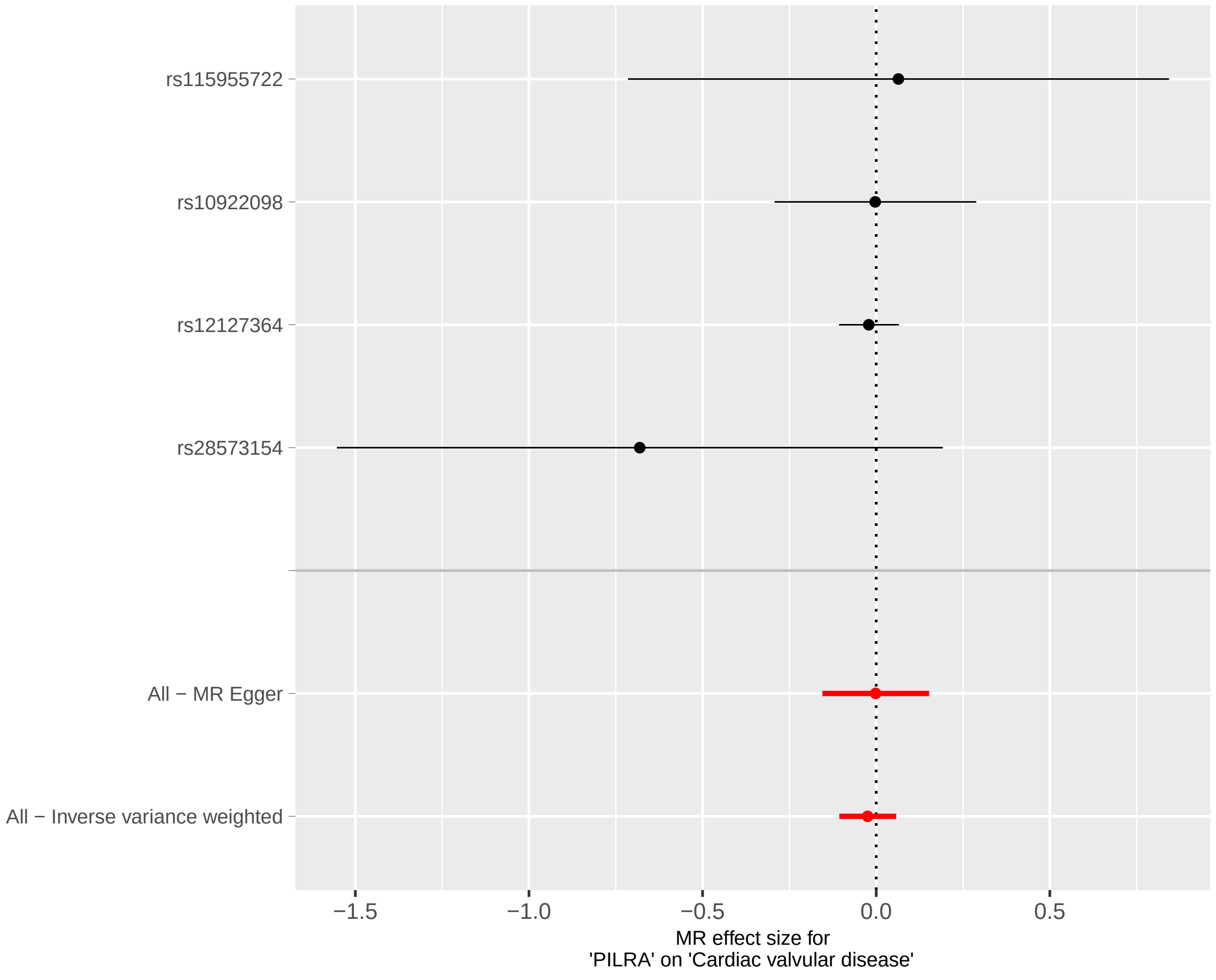
rs115955722
rs10922098
rs12127364
rs28573154
All − MR Egger
All − Inverse variance weighted
−1.5
−1.0
−0.5
0.0
0.5
MR effect size for
'PILRA' on 'Cardiac valvular disease'

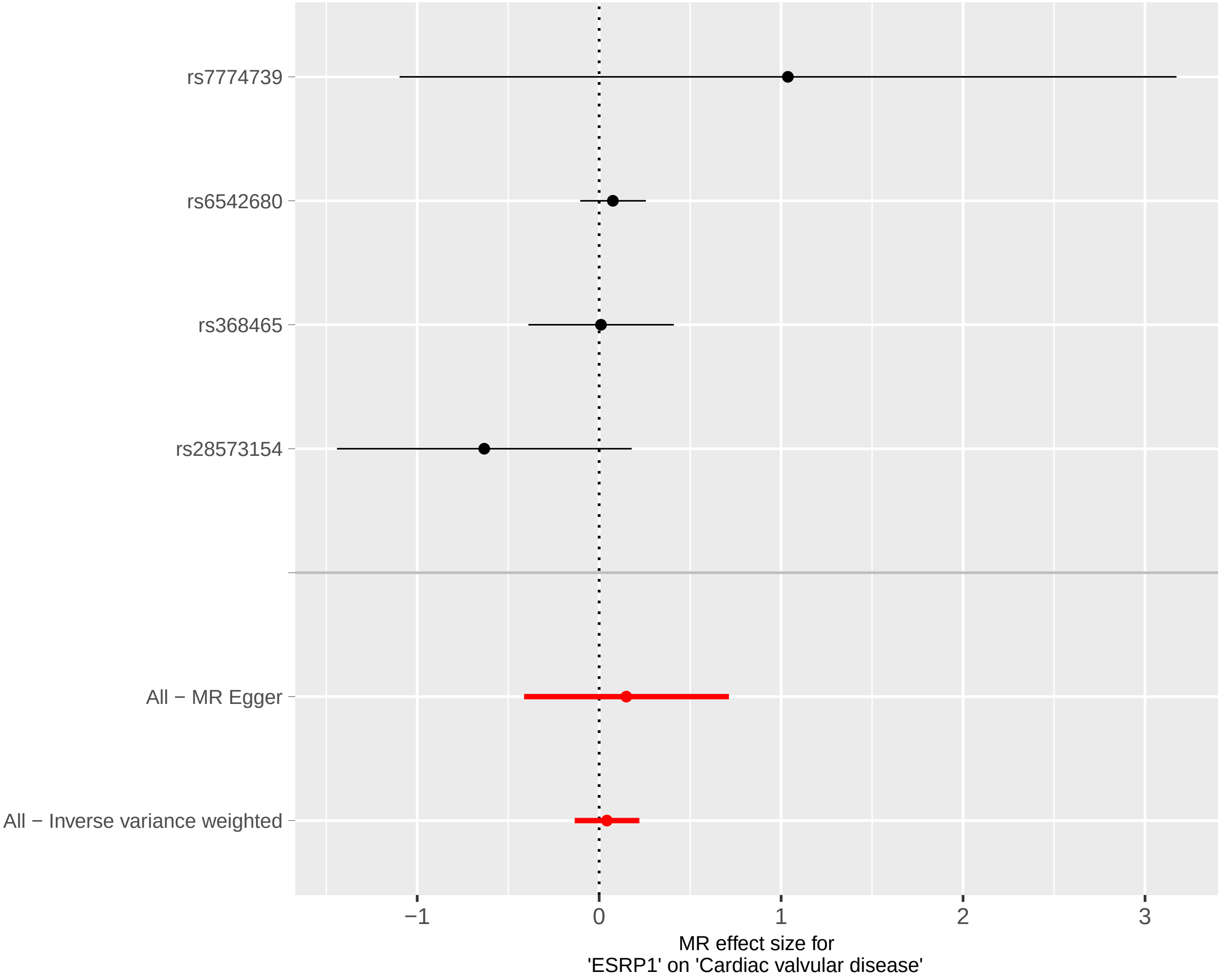
rs7774739
rs6542680
rs368465
rs28573154
All − MR Egger
All − Inverse variance weighted
−1
0
1
2
3
MR effect size for
'ESRP1' on 'Cardiac valvular disease'

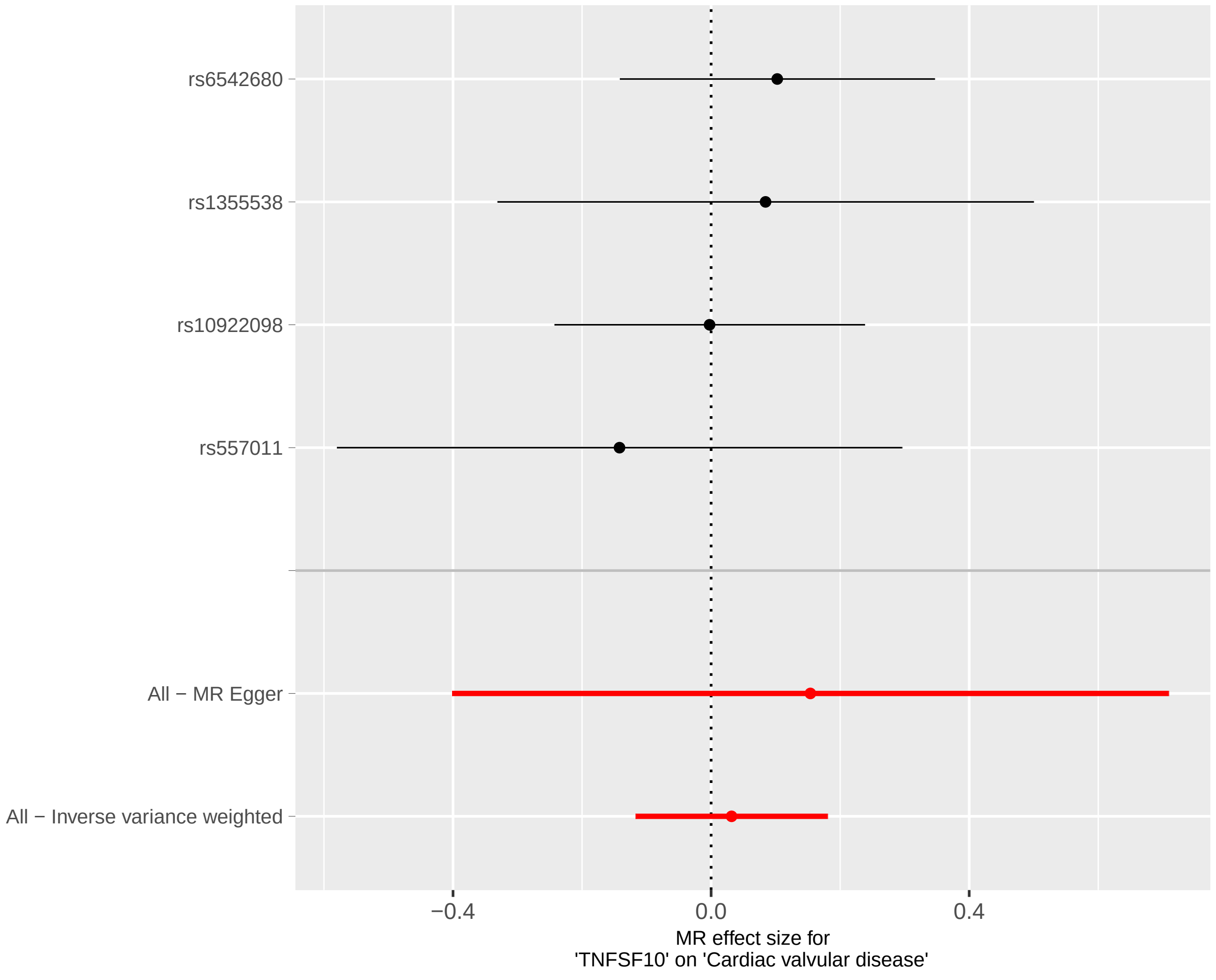
rs6542680
rs1355538
rs10922098
rs557011
All − MR Egger
All − Inverse variance weighted
−0.4
0.0
0.4
MR effect size for
'TNFSF10' on 'Cardiac valvular disease'

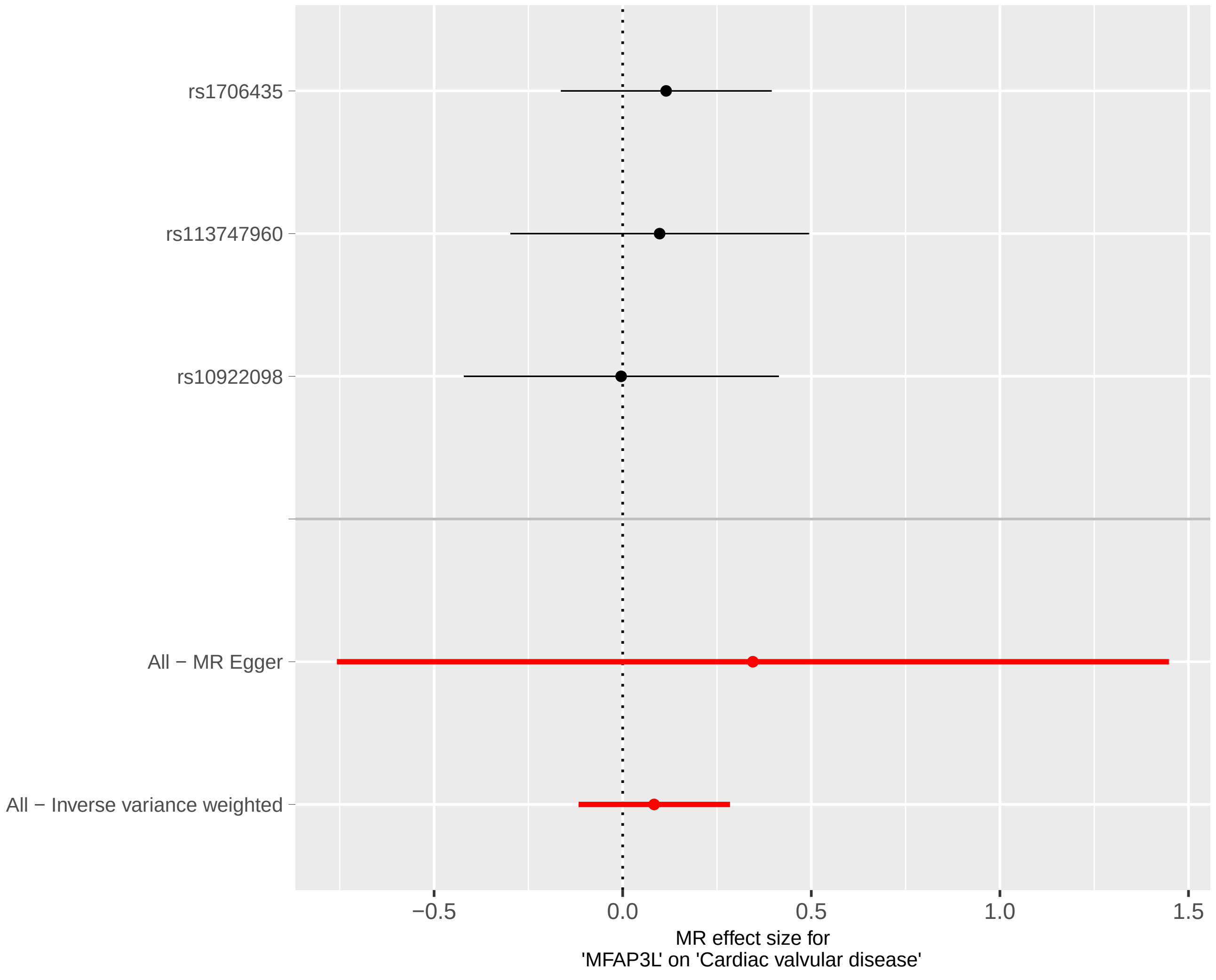

rs1706435
rs113747960
rs10922098
All − MR Egger
All − Inverse variance weighted
−0.5
0.0
0.5
1.0
1.5
MR effect size for
'MFAP3L' on 'Cardiac valvular disease'

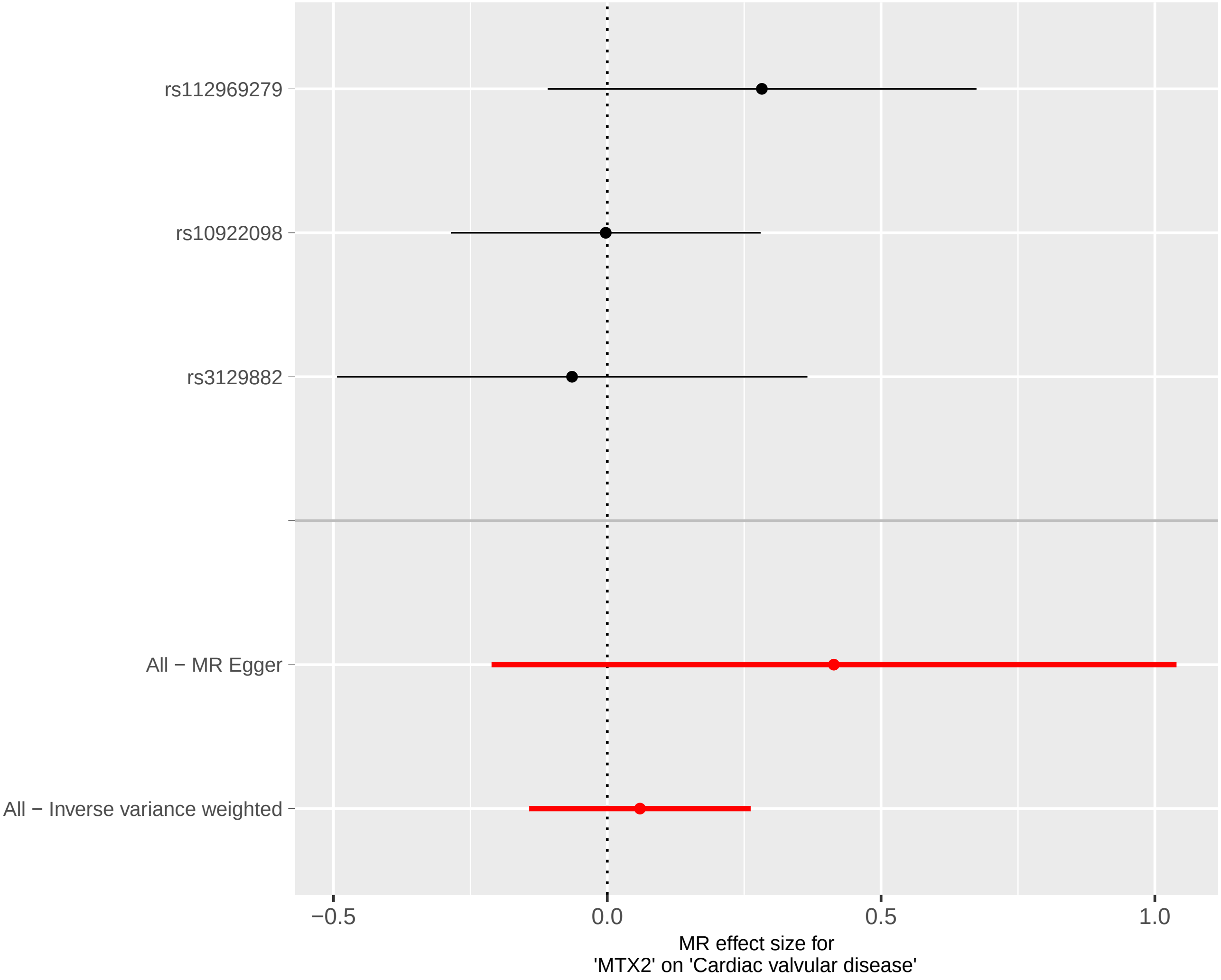
rs112969279
rs10922098
rs3129882
All − MR Egger
All − Inverse variance weighted
−0.5
0.0
0.5
1.0
MR effect size for
'MTX2' on 'Cardiac valvular disease'

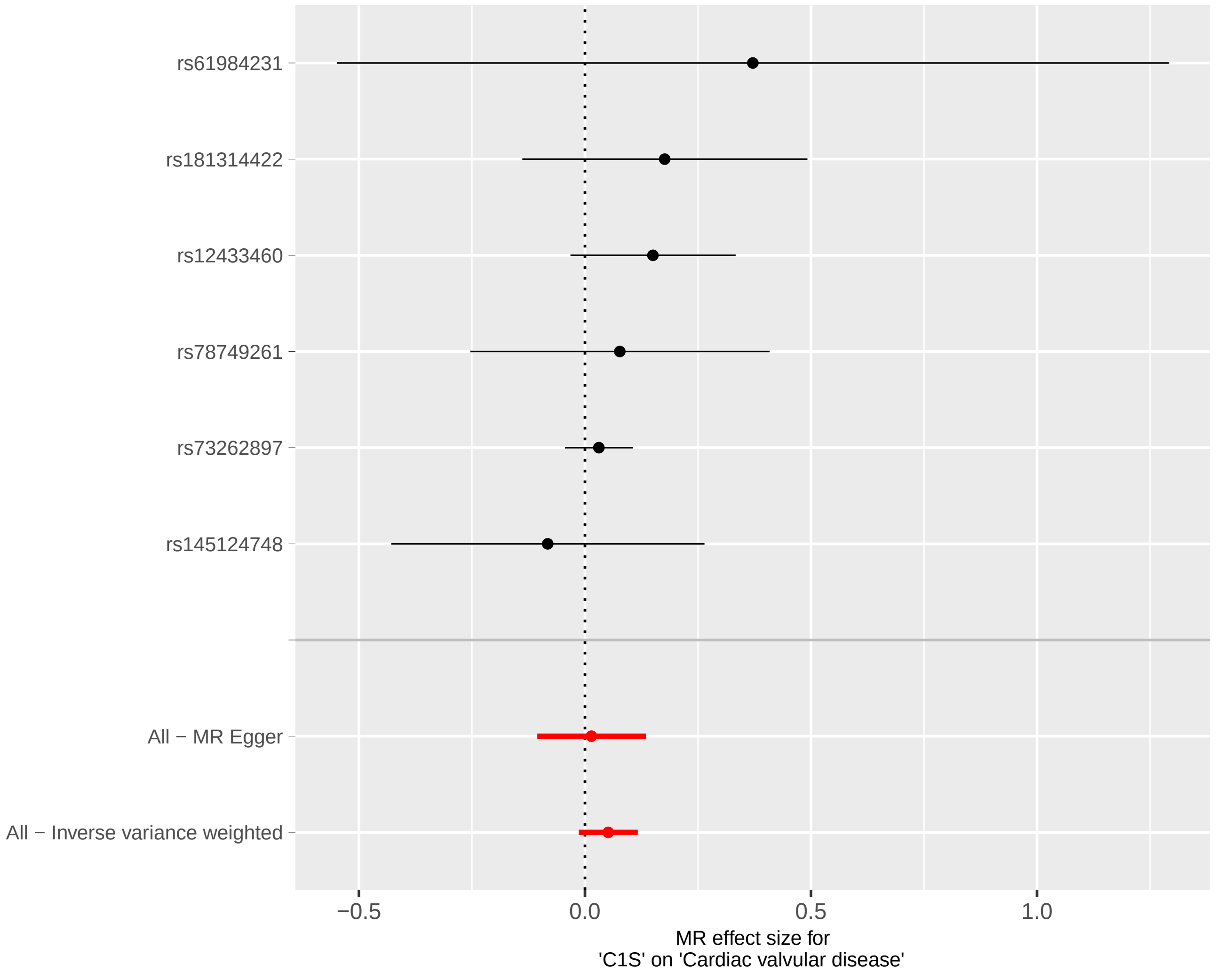

rs61984231
rs181314422
rs12433460
rs78749261
rs73262897
rs145124748
All − MR Egger
All − Inverse variance weighted
−0.5
0.0
0.5
1.0
MR effect size for
'C1S' on 'Cardiac valvular disease'

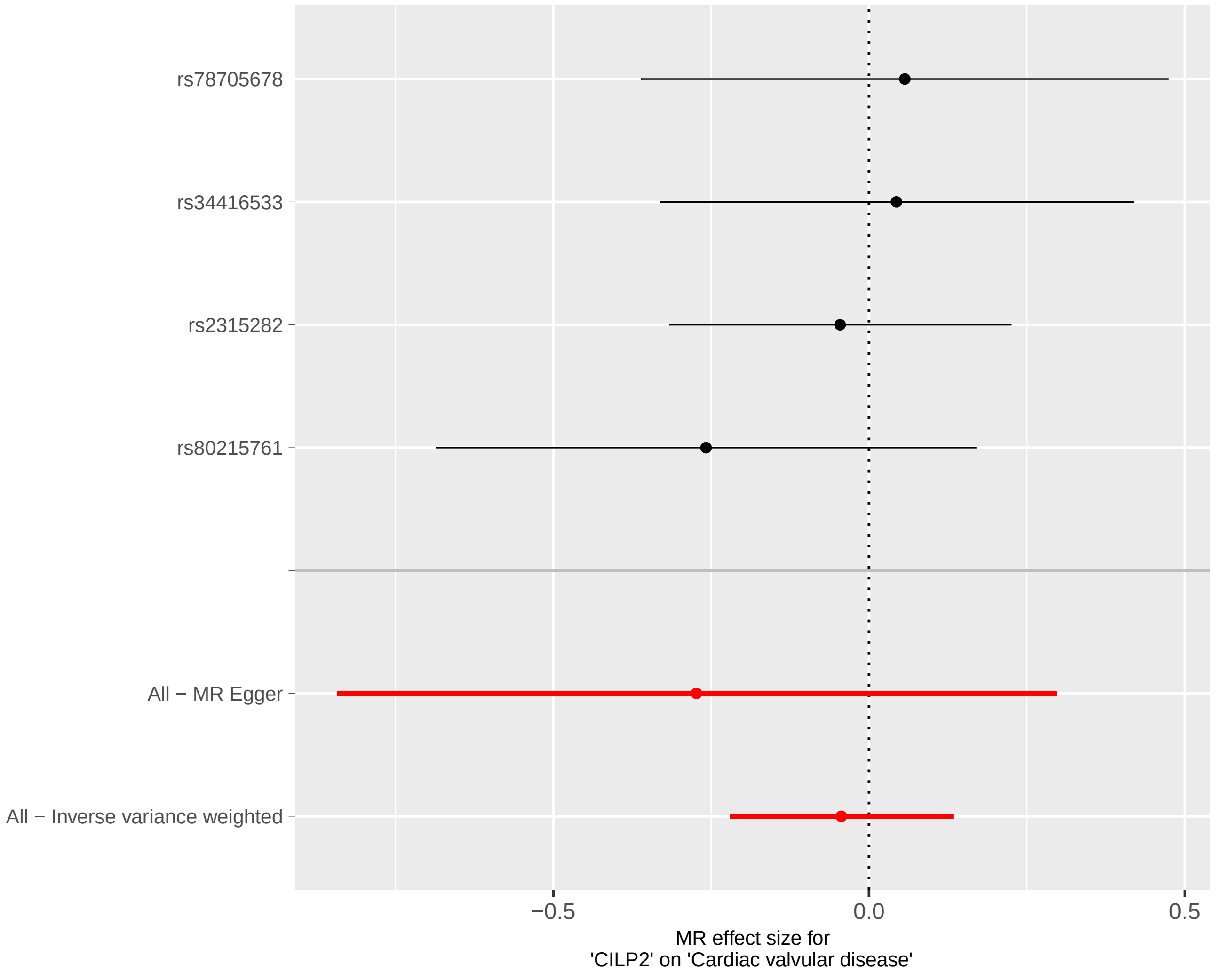
rs78705678
rs34416533
rs2315282
rs80215761
All − MR Egger
All − Inverse variance weighted
−0.5
0.0
0.5
MR effect size for
'CILP2' on 'Cardiac valvular disease'

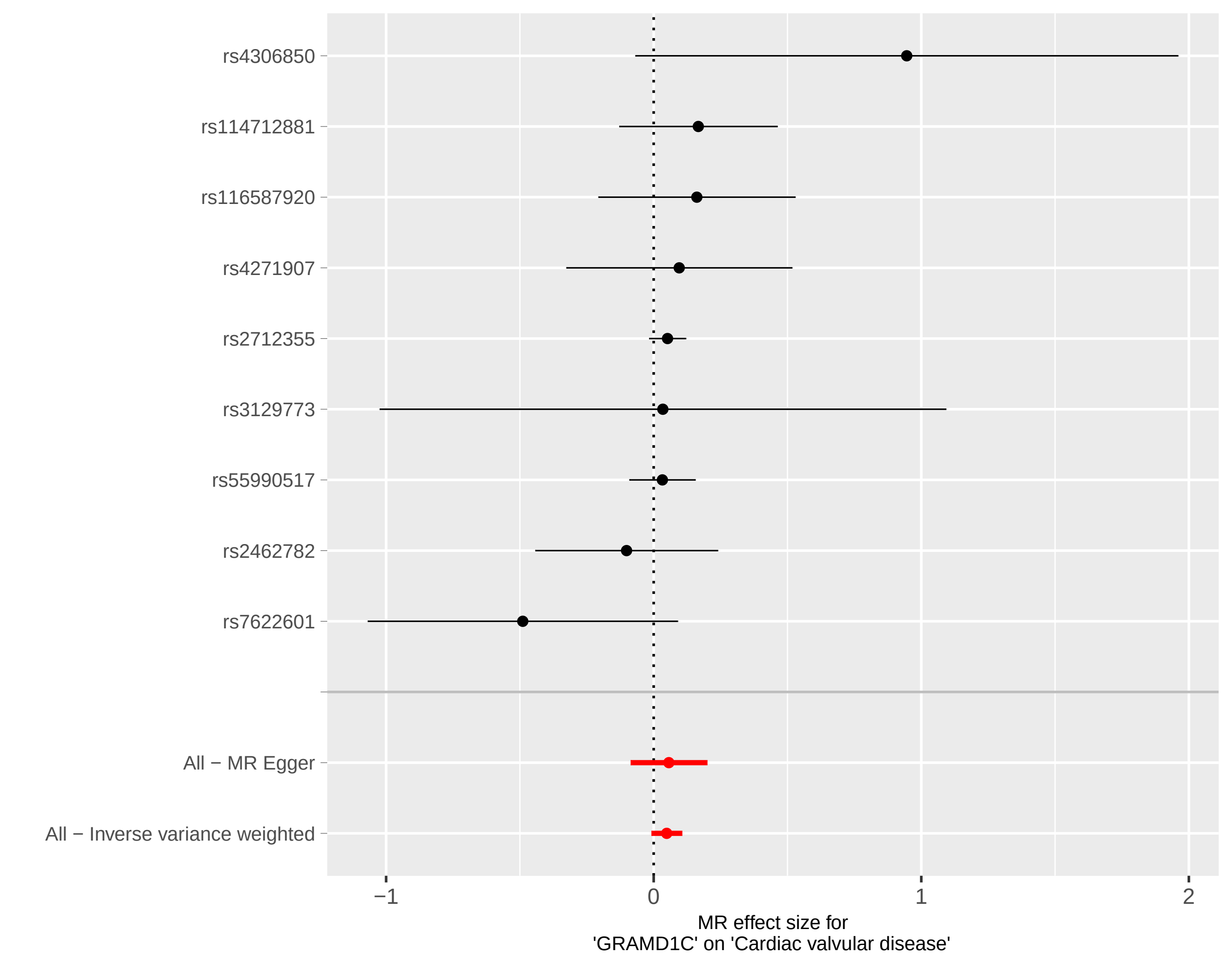
rs4306850
rs114712881
rs116587920
rs4271907
rs2712355
rs3129773
rs55990517
rs2462782
rs7622601
All − MR Egger
All − Inverse variance weighted
−1
0
1
2
MR effect size for
'GRAMD1C' on 'Cardiac valvular disease'

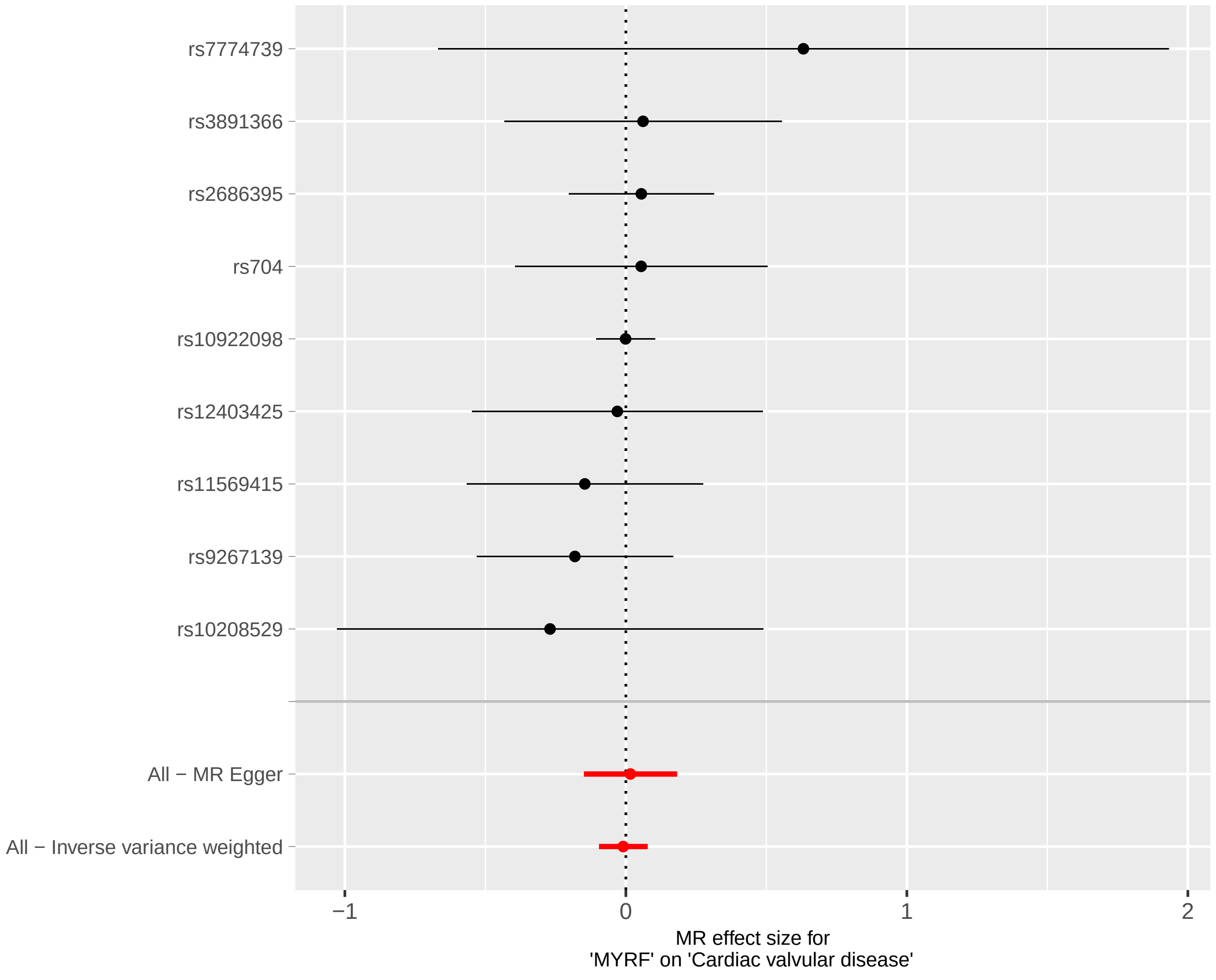
rs7774739
rs3891366
rs2686395
rs704
rs10922098
rs12403425
rs11569415
rs9267139
rs10208529
All − MR Egger
All − Inverse variance weighted
−1
0
1
2
MR effect size for
'MYRF' on 'Cardiac valvular disease'

Insufficient number of SNPs

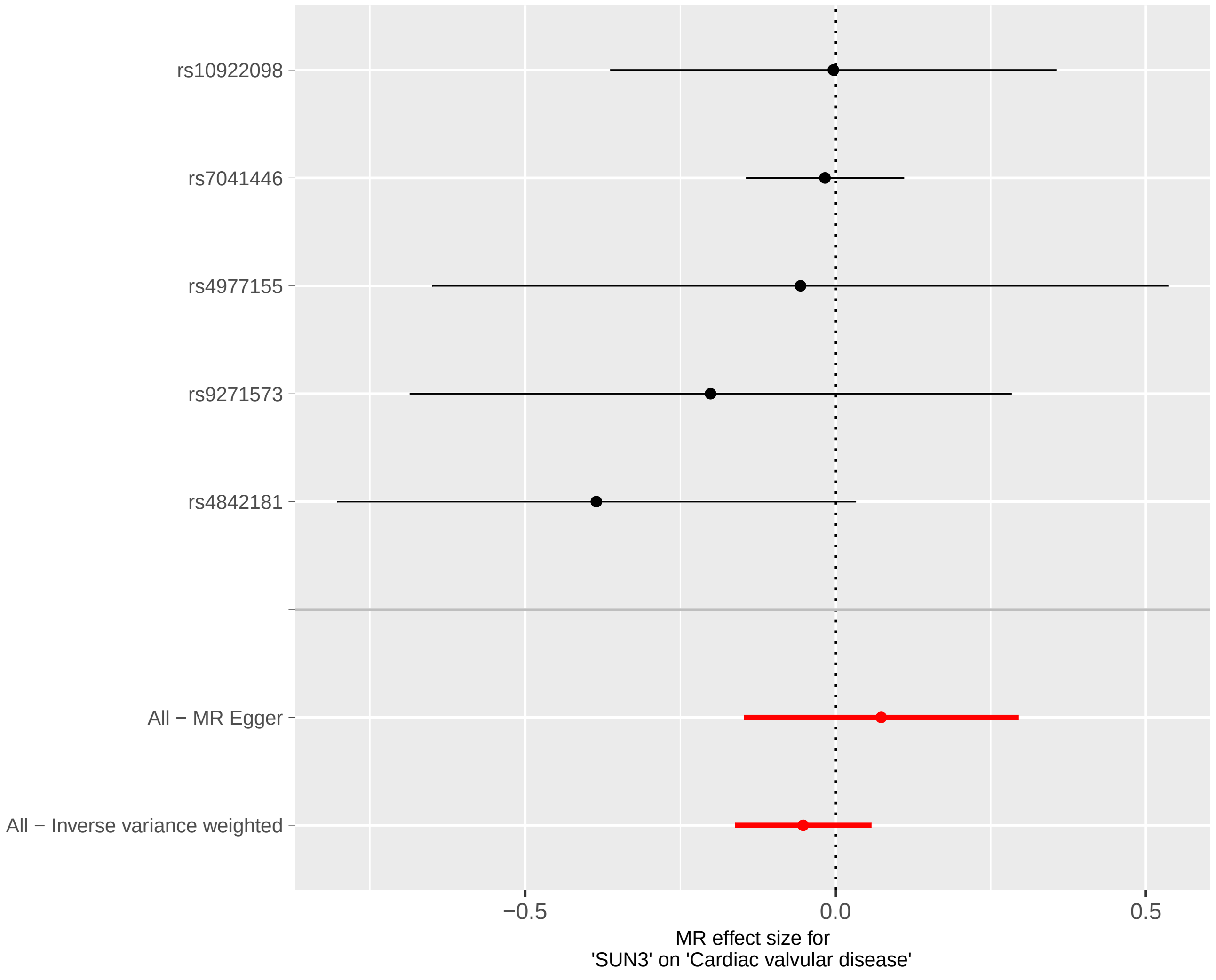

rs10922098
rs7041446
rs4977155
rs9271573
rs4842181
All − MR Egger
All − Inverse variance weighted
−0.5
0.0
0.5
MR effect size for
'SUN3' on 'Cardiac valvular disease'

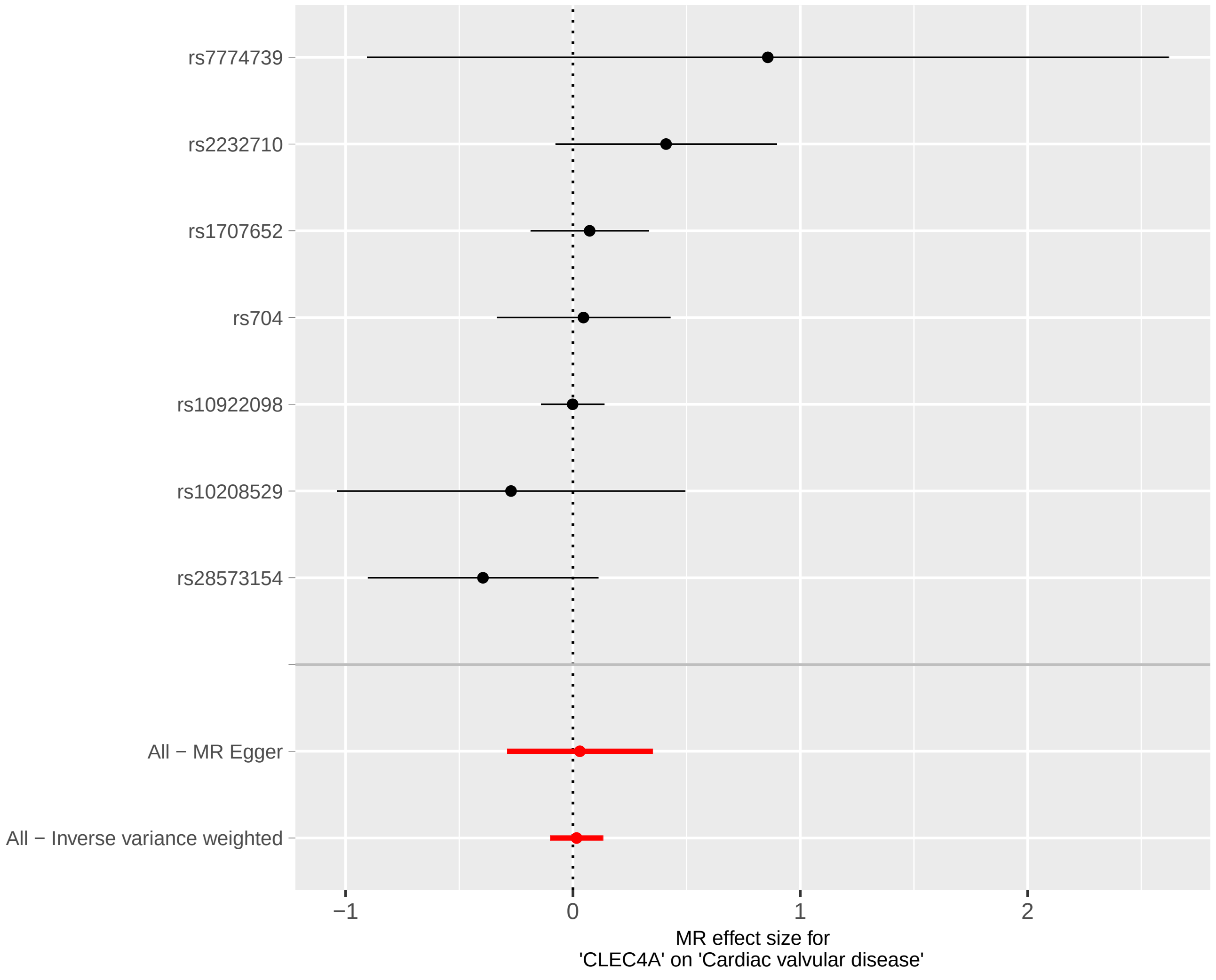

rs7774739
rs2232710
rs1707652
rs704
rs10922098
rs10208529
rs28573154
All − MR Egger
All − Inverse variance weighted
−1
0
1
2
MR effect size for
'CLEC4A' on 'Cardiac valvular disease'

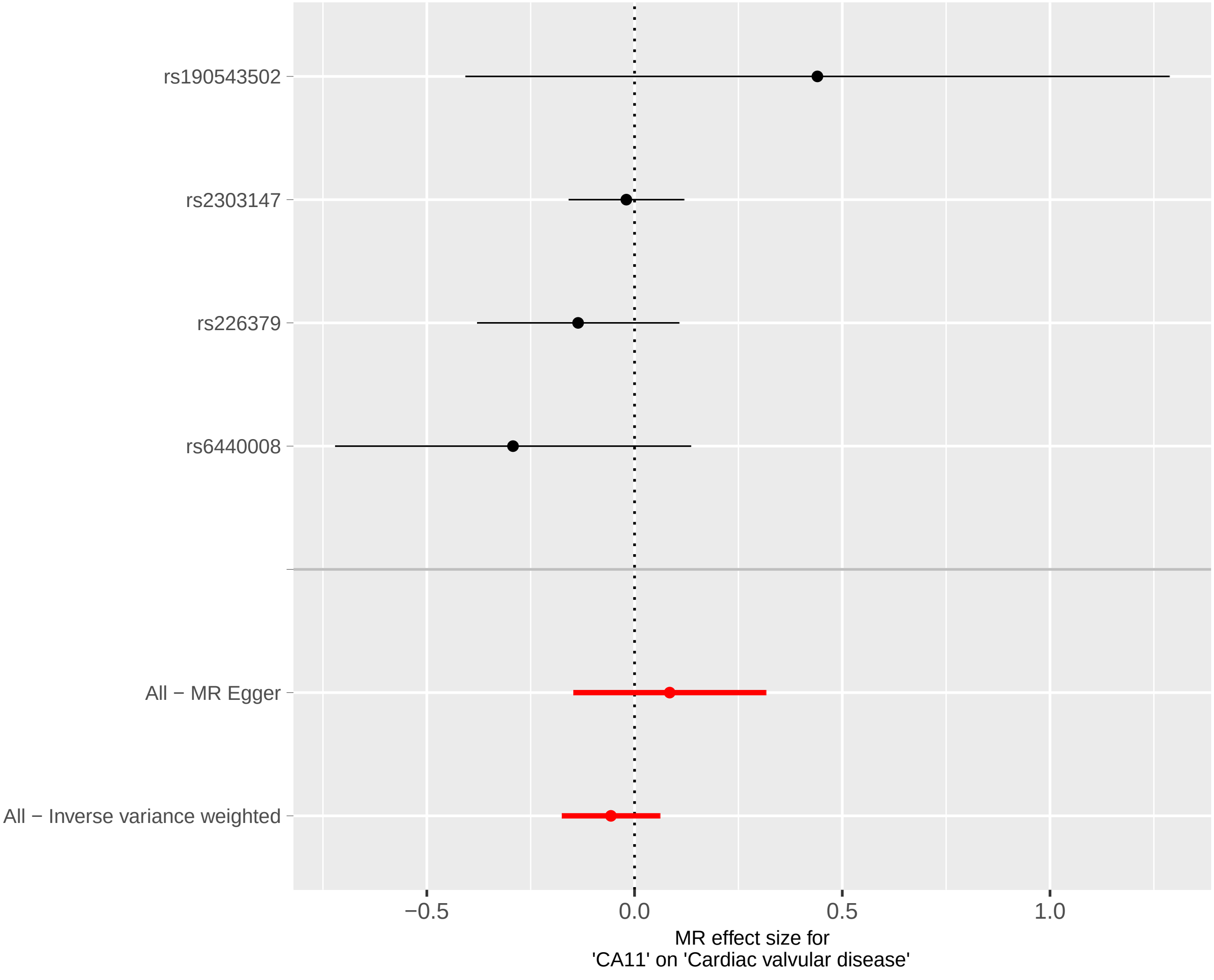
rs190543502
rs2303147
rs226379
rs6440008
All − MR Egger
All − Inverse variance weighted
−0.5
0.0
0.5
1.0
MR effect size for
'CA11' on 'Cardiac valvular disease'

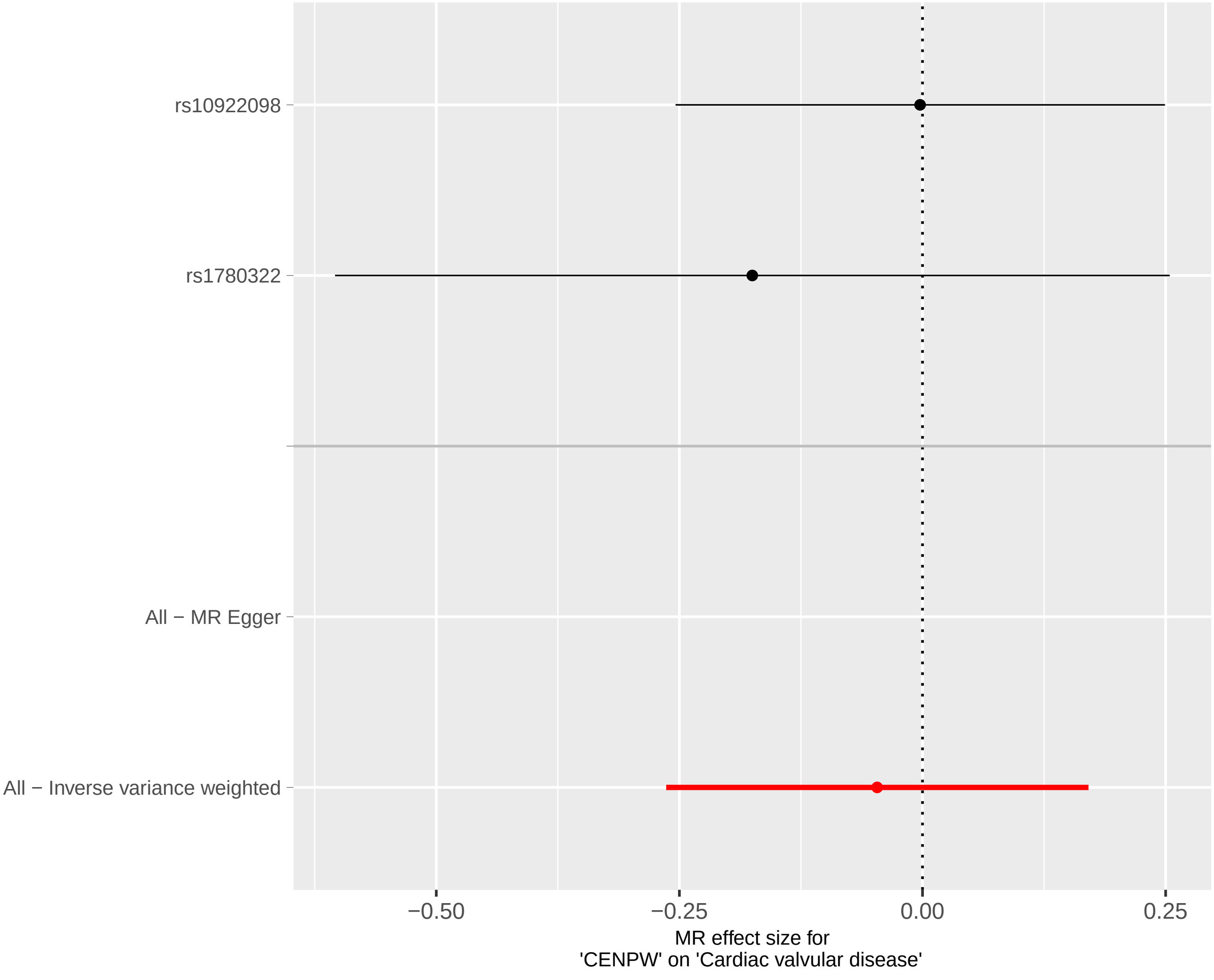

rs10922098
rs1780322
All − MR Egger
All − Inverse variance weighted
−0.50
−0.25
0.00
0.25
MR effect size for
'CENPW' on 'Cardiac valvular disease'

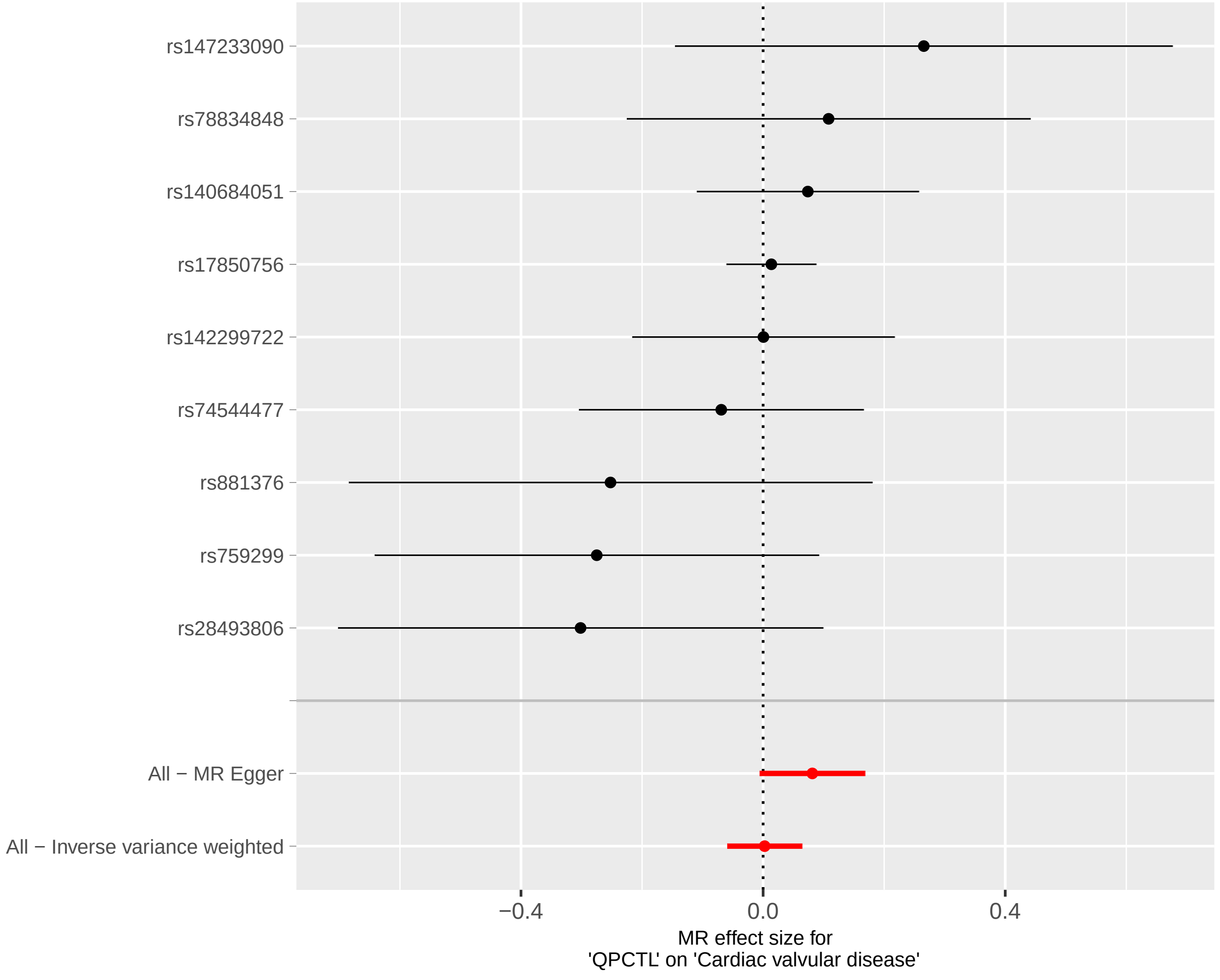

rs147233090
rs78834848
rs140684051
rs17850756
rs142299722
rs74544477
rs881376
rs759299
rs28493806
All − MR Egger
All − Inverse variance weighted
−0.4
0.0
0.4
MR effect size for
'QPCTL' on 'Cardiac valvular disease'

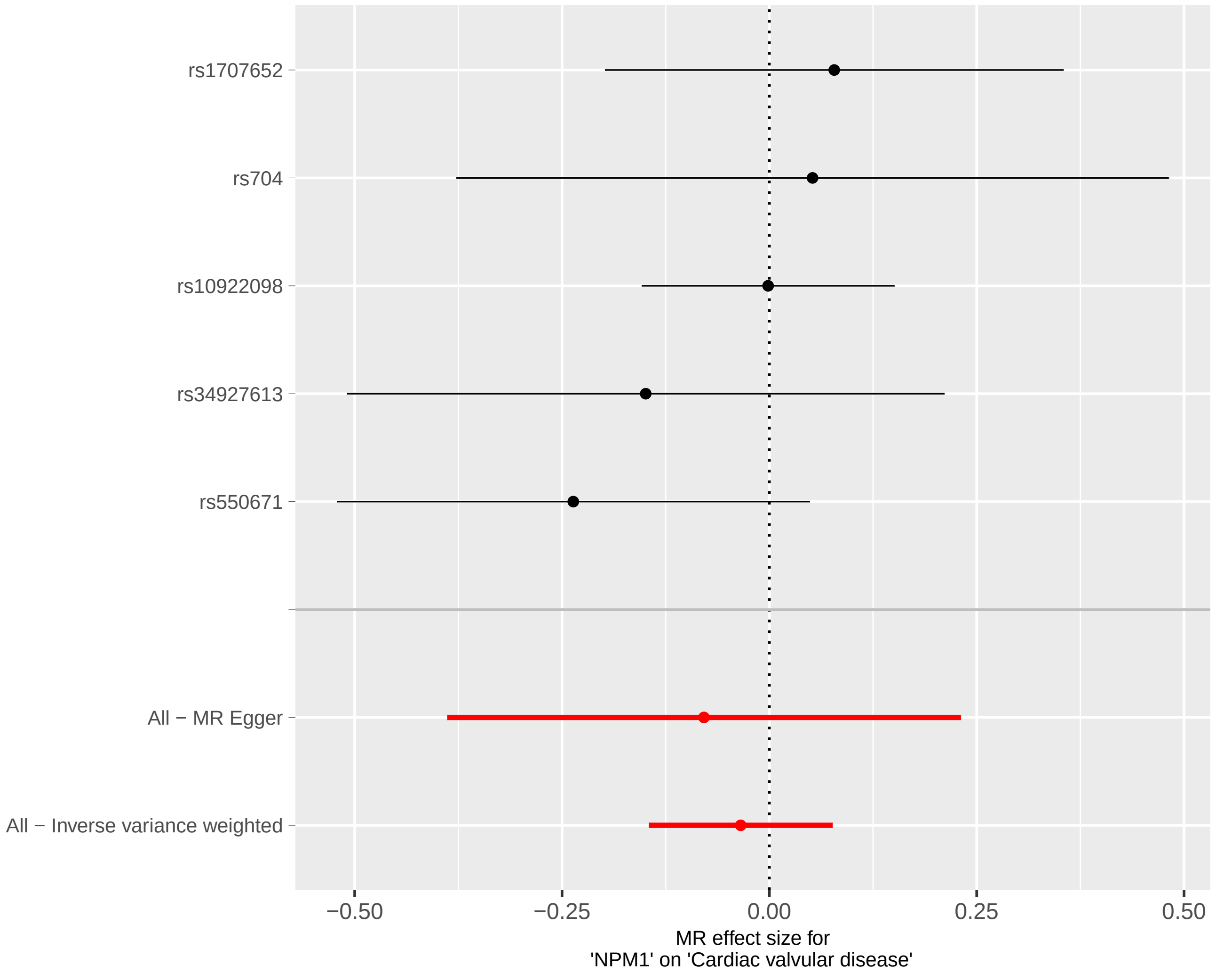

rs1707652
rs704
rs10922098
rs34927613
rs550671
All − MR Egger
All − Inverse variance weighted
−0.50
−0.25
0.00
0.25
0.50
MR effect size for
'NPM1' on 'Cardiac valvular disease'

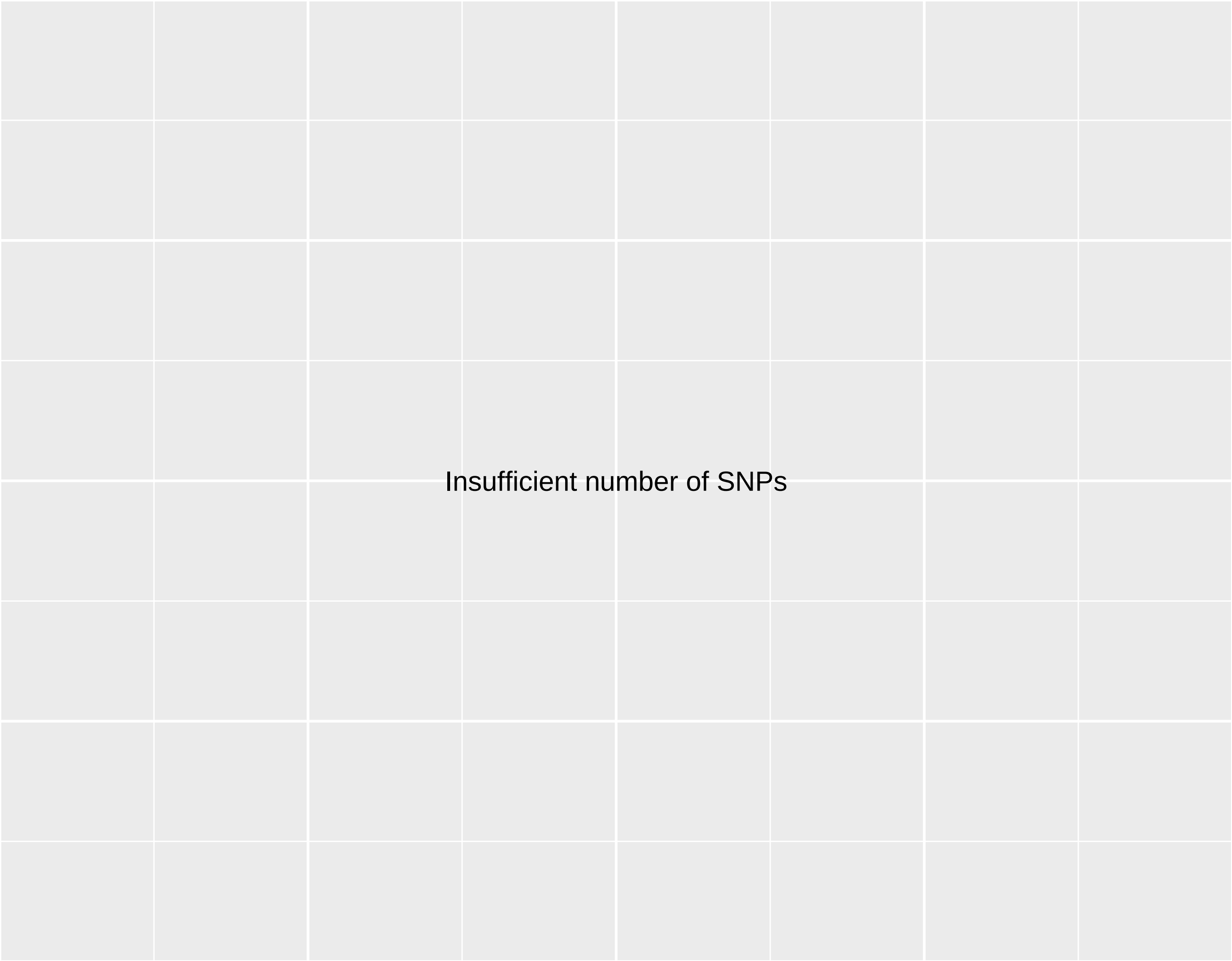
Insufficient number of SNPs

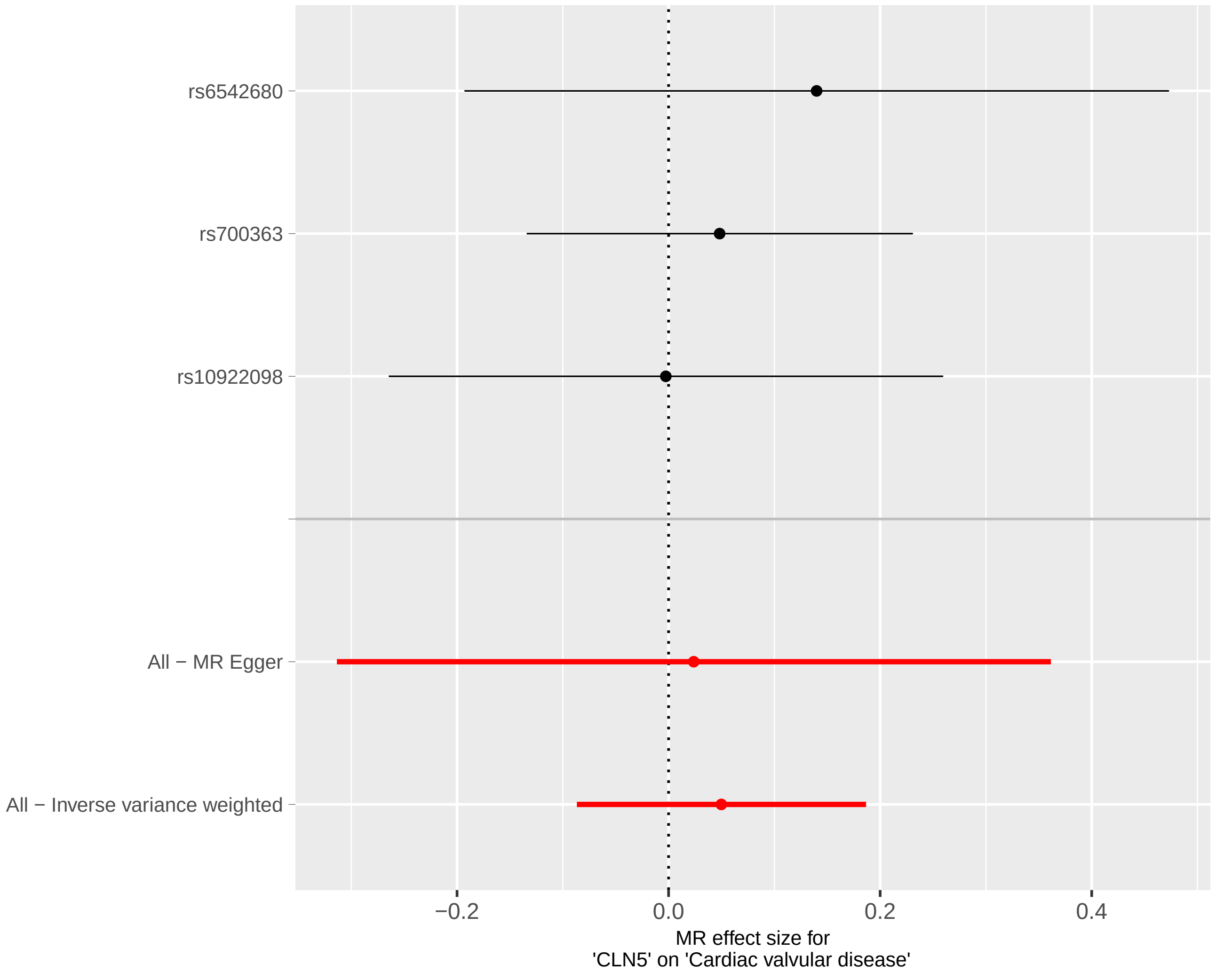
rs6542680
rs700363
rs10922098
All − MR Egger
All − Inverse variance weighted
−0.2
0.0
0.2
0.4
MR effect size for
'CLN5' on 'Cardiac valvular disease'

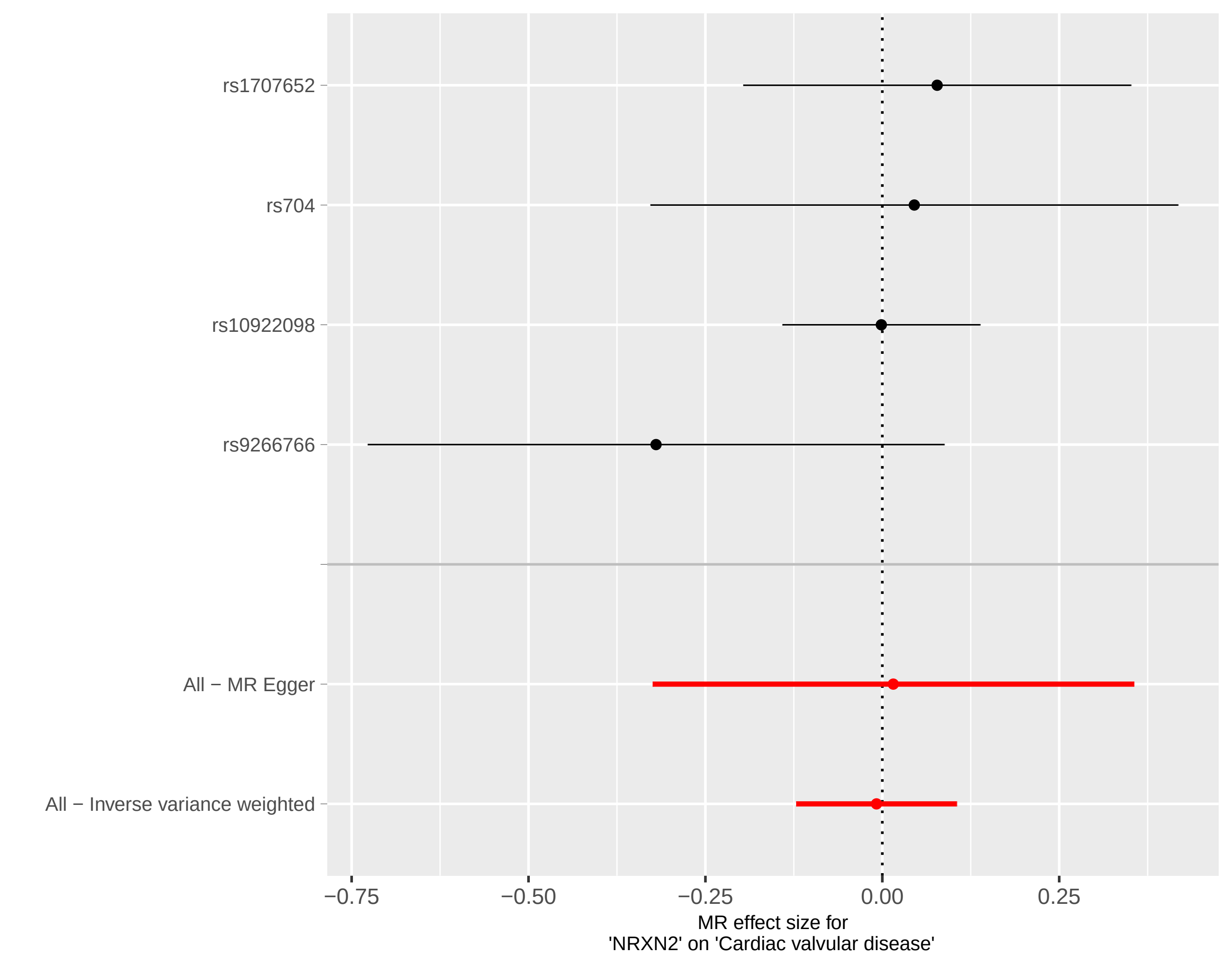

rs1707652
rs704
rs10922098
rs9266766
All − MR Egger
All − Inverse variance weighted
−0.75
−0.50
−0.25
0.00
0.25
MR effect size for
'NRXN2' on 'Cardiac valvular disease'

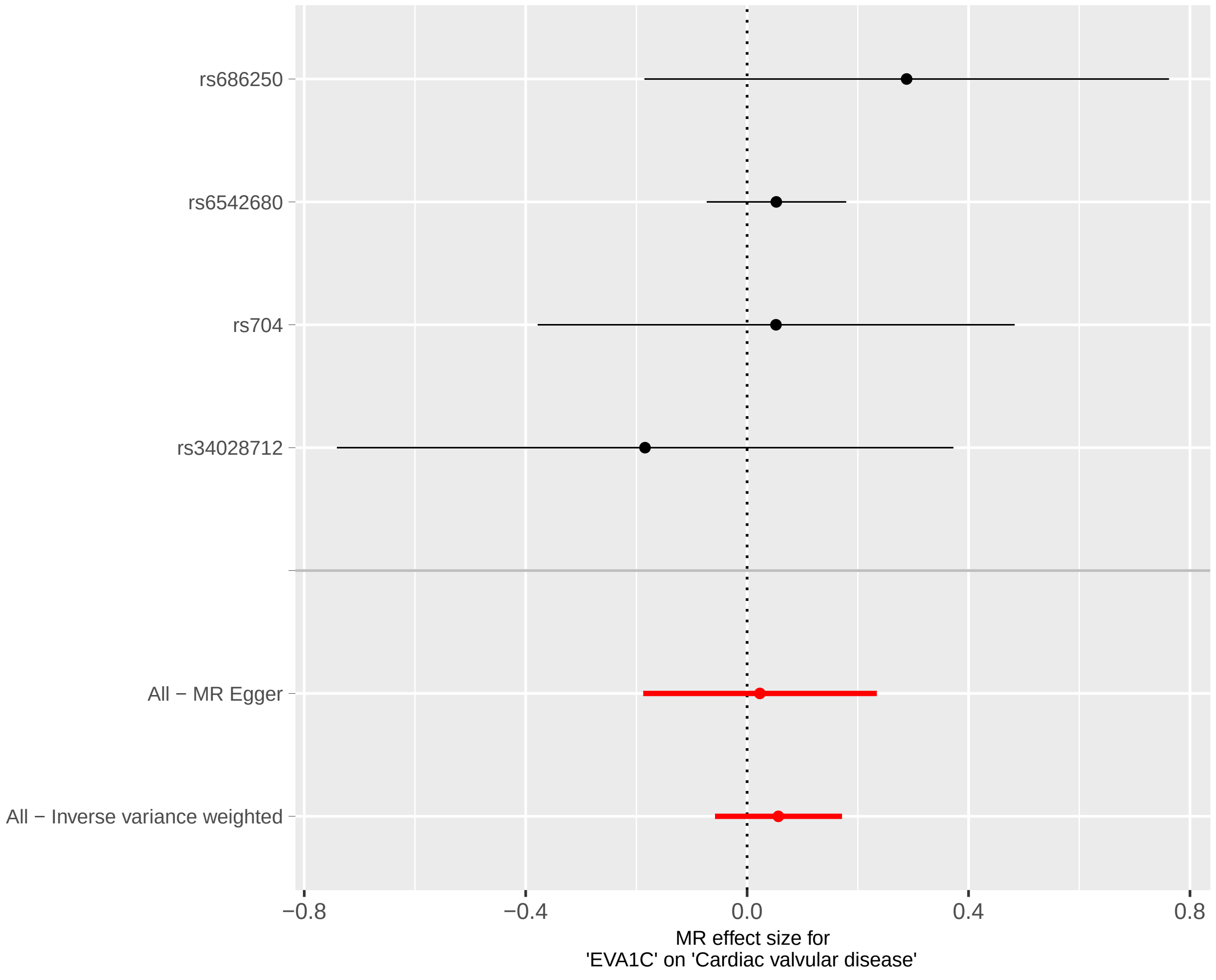
rs686250
rs6542680
rs704
rs34028712
All − MR Egger
All − Inverse variance weighted
−0.8
−0.4
0.0
0.4
0.8
MR effect size for
'EVA1C' on 'Cardiac valvular disease'

Insufficient number of SNPs

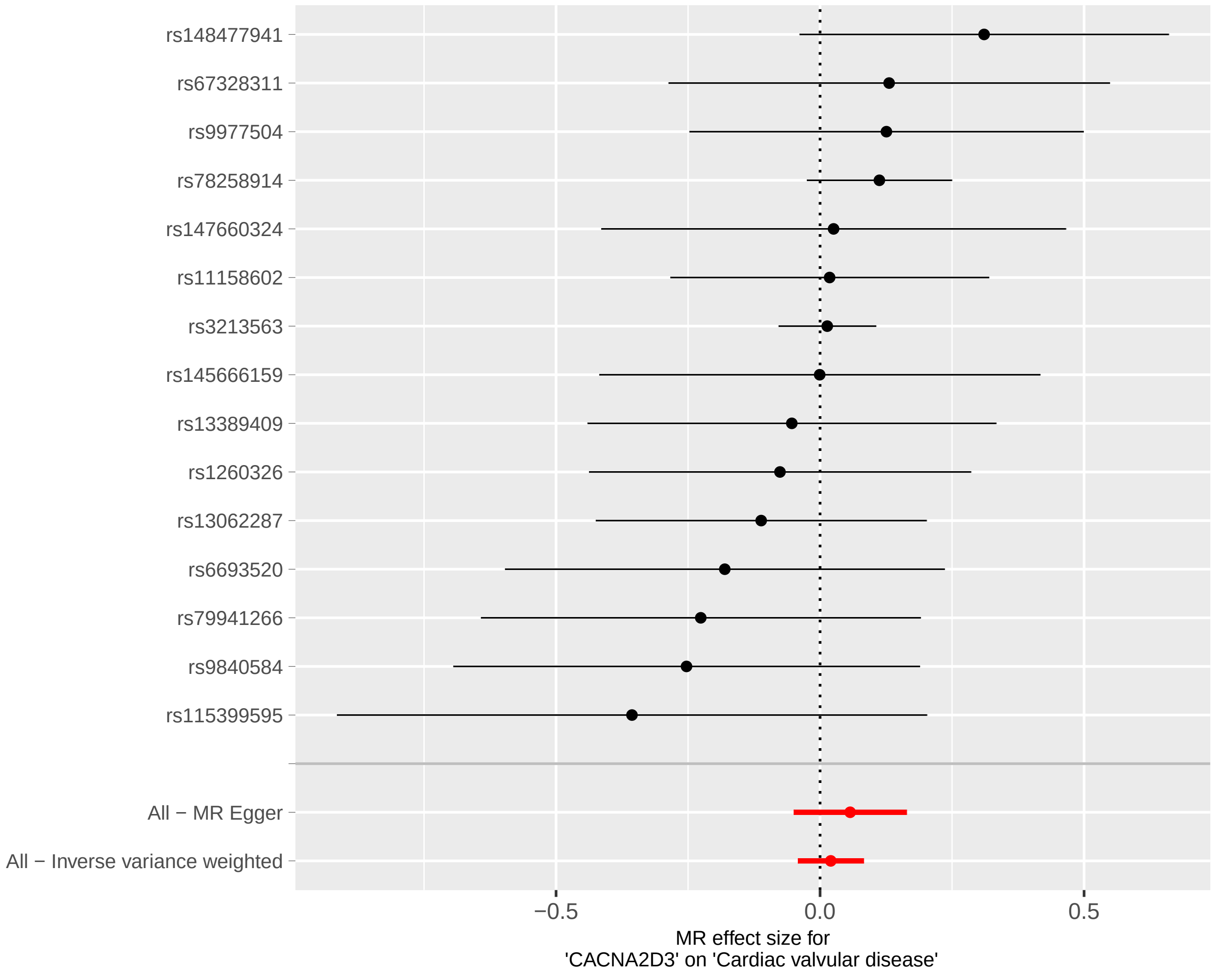

rs148477941
rs67328311
rs9977504
rs78258914
rs147660324
rs11158602
rs3213563
rs145666159
rs13389409
rs1260326
rs13062287
rs6693520
rs79941266
rs9840584
rs115399595
All − MR Egger
All − Inverse variance weighted
−0.5
0.0
0.5
MR effect size for
'CACNA2D3' on 'Cardiac valvular disease'

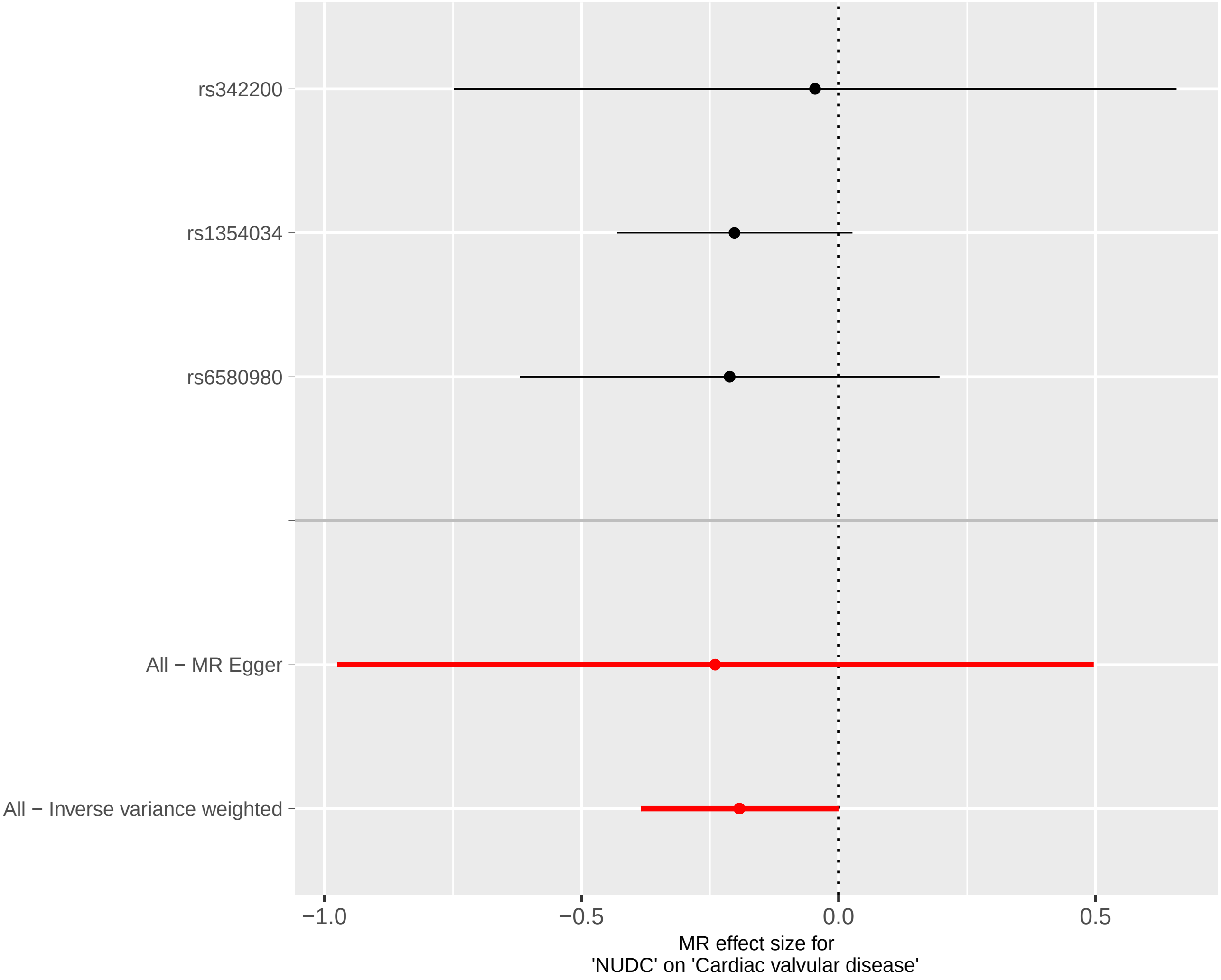
rs342200
rs1354034
rs6580980
All − MR Egger
All − Inverse variance weighted
−1.0
−0.5
0.0
0.5
MR effect size for
'NUDC' on 'Cardiac valvular disease'

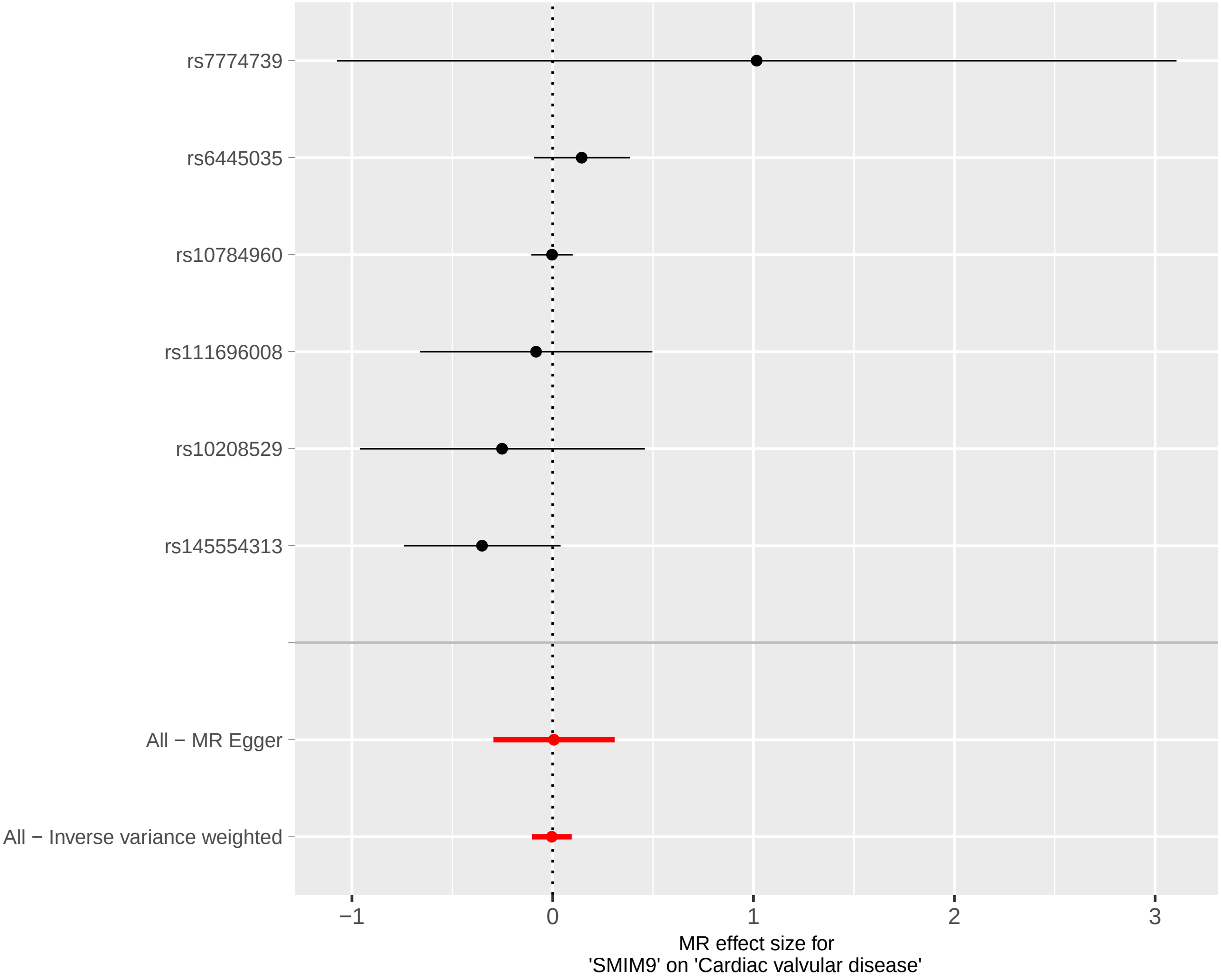
rs7774739
rs6445035
rs10784960
rs111696008
rs10208529
rs145554313
All − MR Egger
All − Inverse variance weighted
−1
0
1
2
3
MR effect size for
'SMIM9' on 'Cardiac valvular disease'

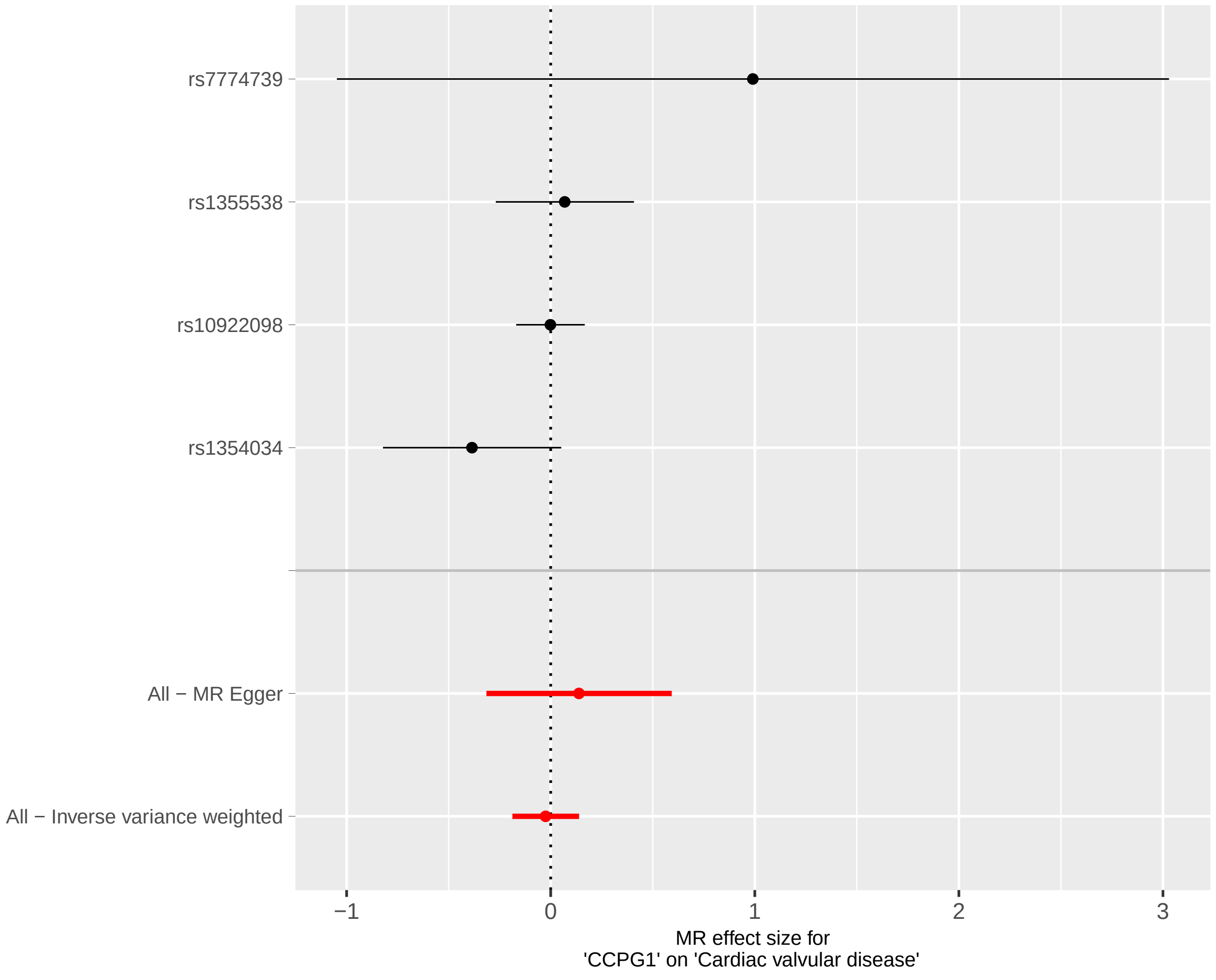

rs7774739
rs1355538
rs10922098
rs1354034
All − MR Egger
All − Inverse variance weighted
−1
0
1
2
3
MR effect size for
'CCPG1' on 'Cardiac valvular disease'

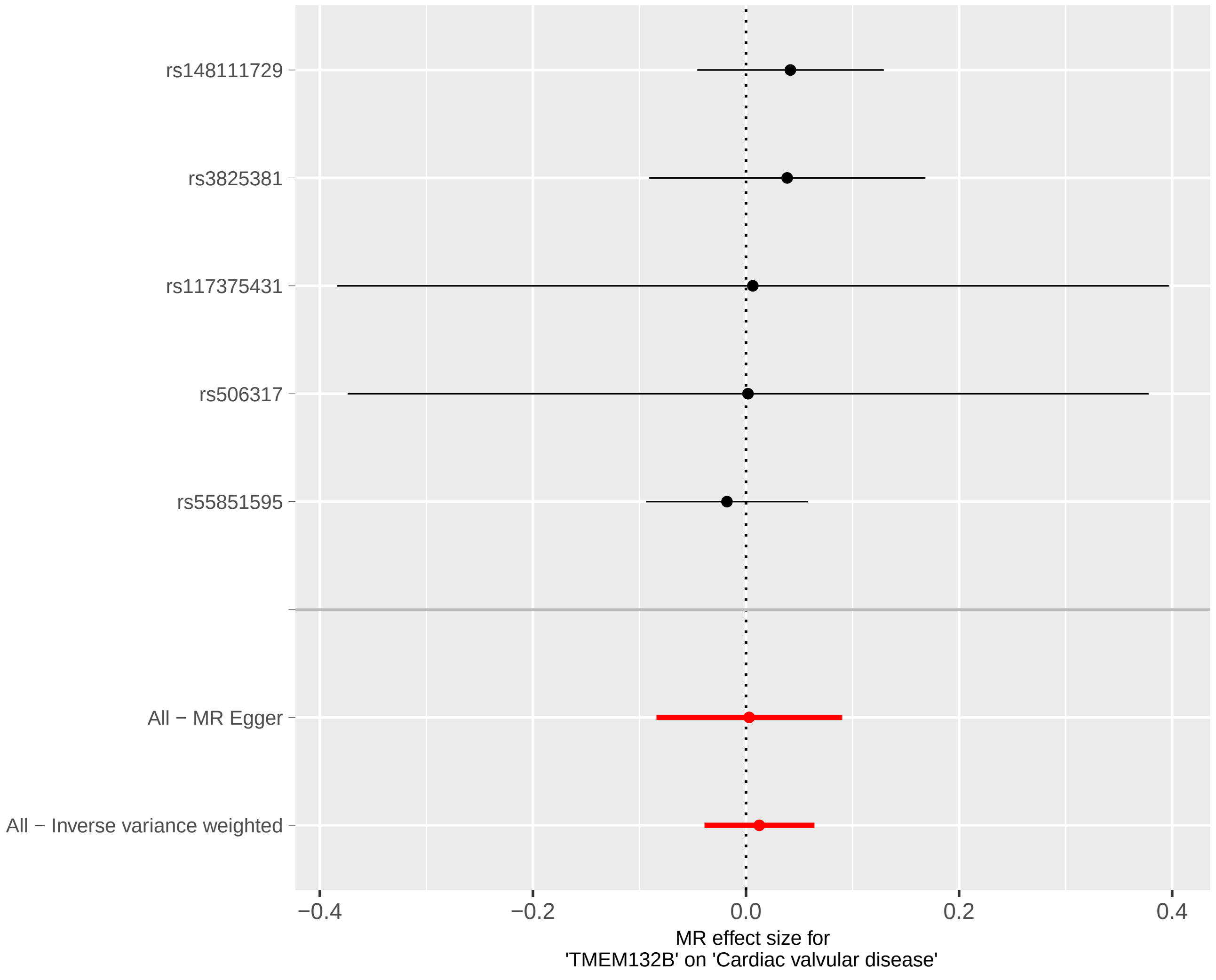
rs148111729
rs3825381
rs117375431
rs506317
rs55851595
All − MR Egger
All − Inverse variance weighted
−0.4
−0.2
0.0
0.2
0.4
MR effect size for
'TMEM132B' on 'Cardiac valvular disease'

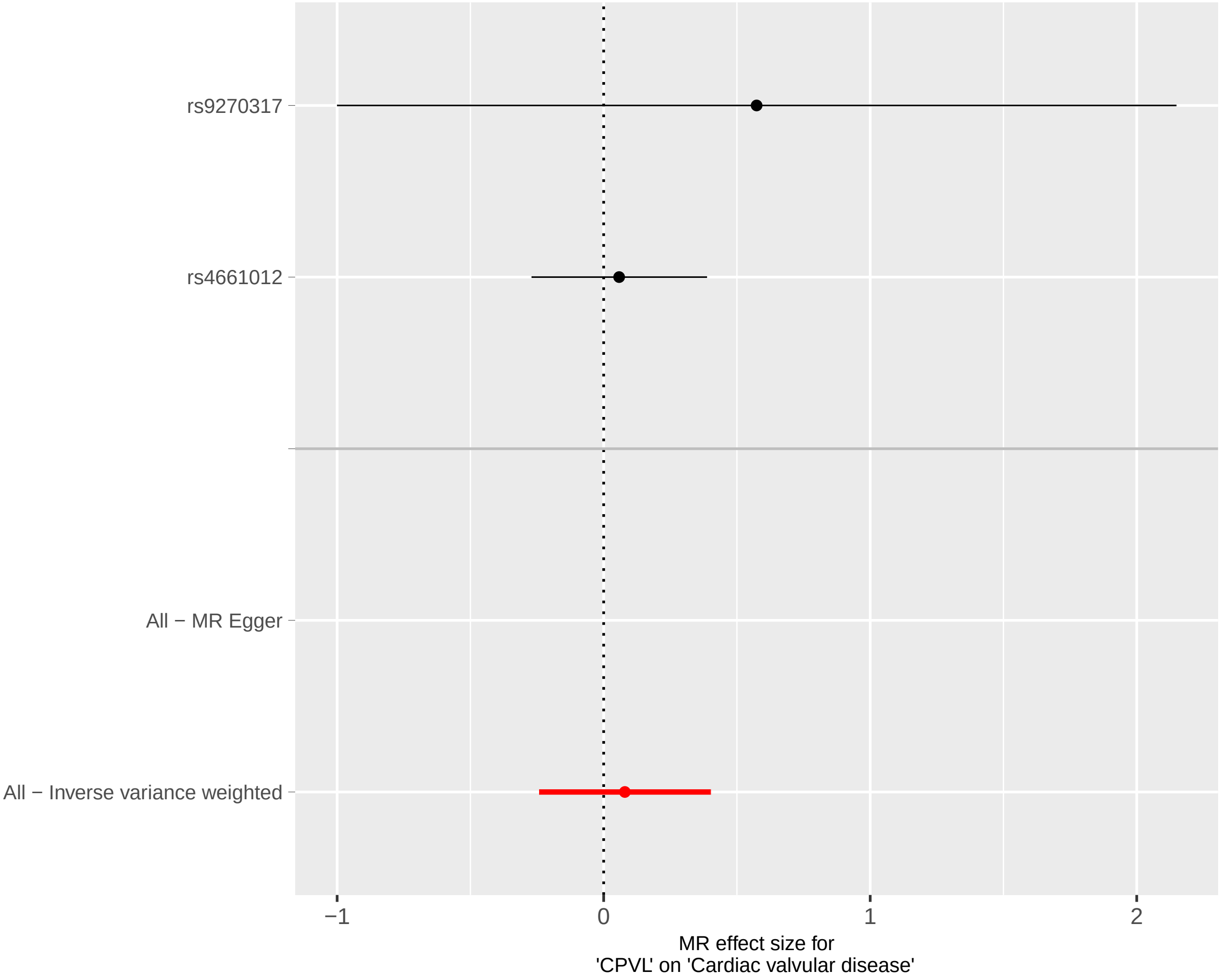
rs9270317
rs4661012
All − MR Egger
All − Inverse variance weighted
−1
0
1
2
MR effect size for
'CPVL' on 'Cardiac valvular disease'

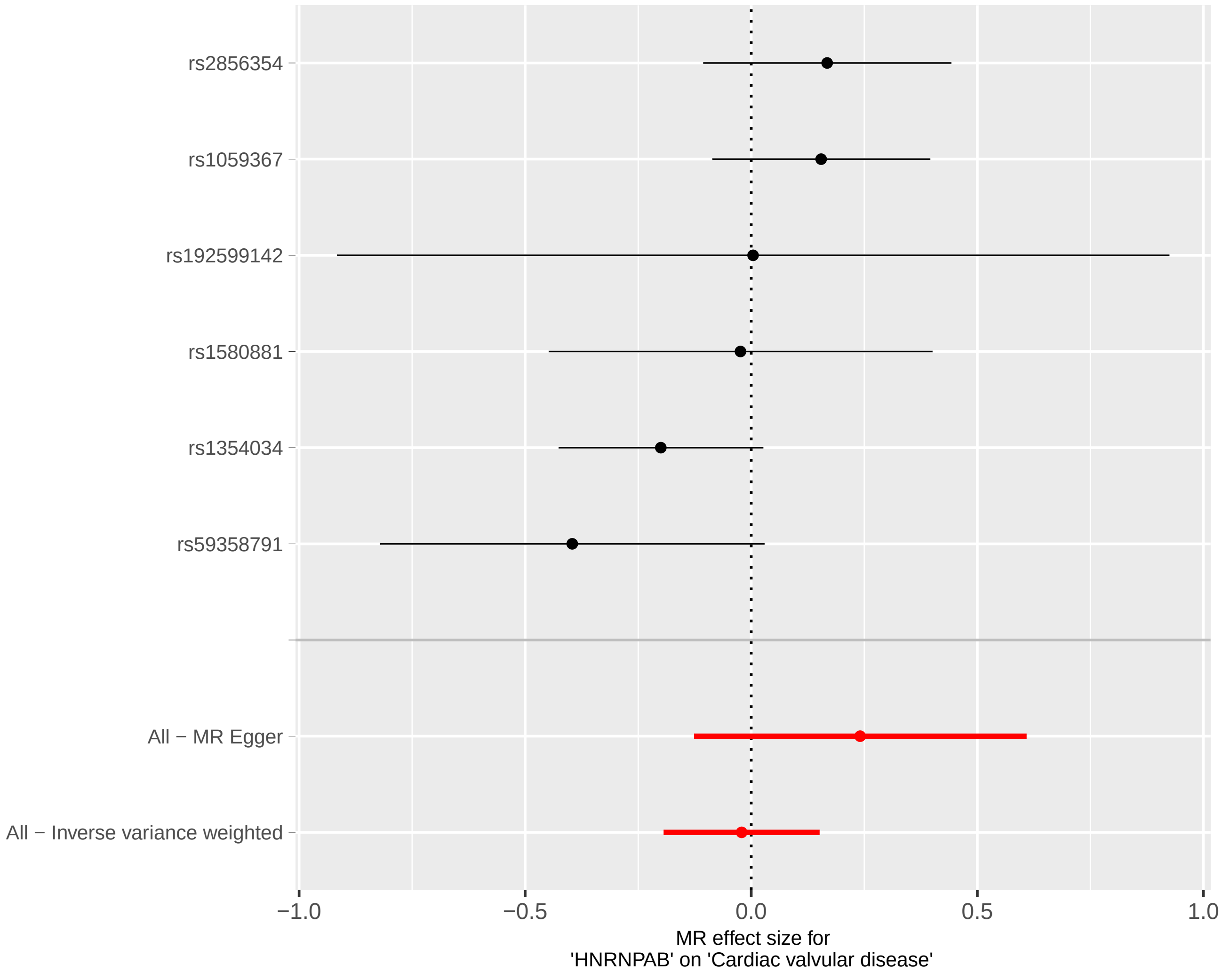
rs2856354
rs1059367
rs192599142
rs1580881
rs1354034
rs59358791
All − MR Egger
All − Inverse variance weighted
−1.0
−0.5
0.0
0.5
1.0
MR effect size for
'HNRNPAB' on 'Cardiac valvular disease'

Insufficient number of SNPs

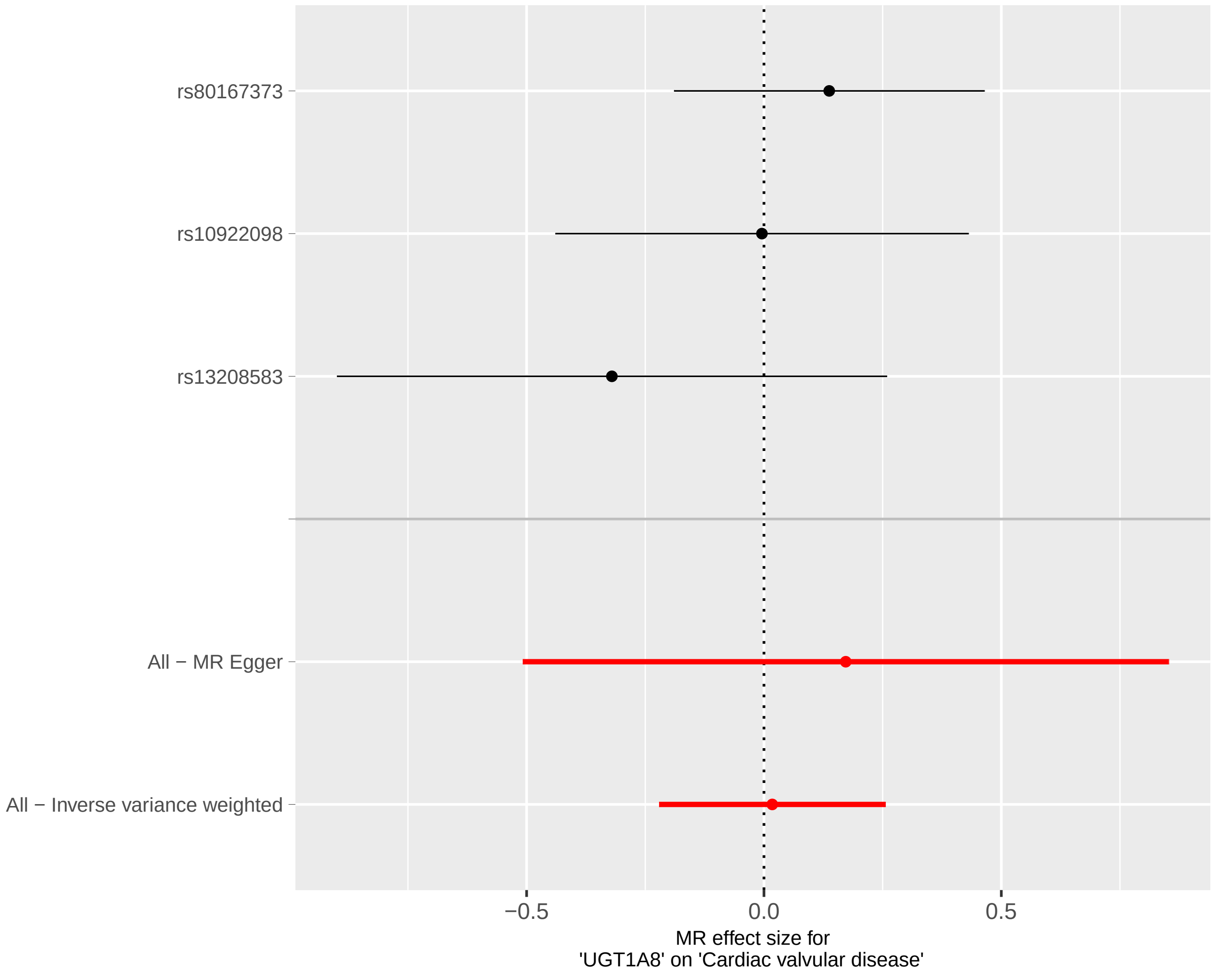

rs80167373
rs10922098
rs13208583
All − MR Egger
All − Inverse variance weighted
−0.5
0.0
0.5
MR effect size for
'UGT1A8' on 'Cardiac valvular disease'

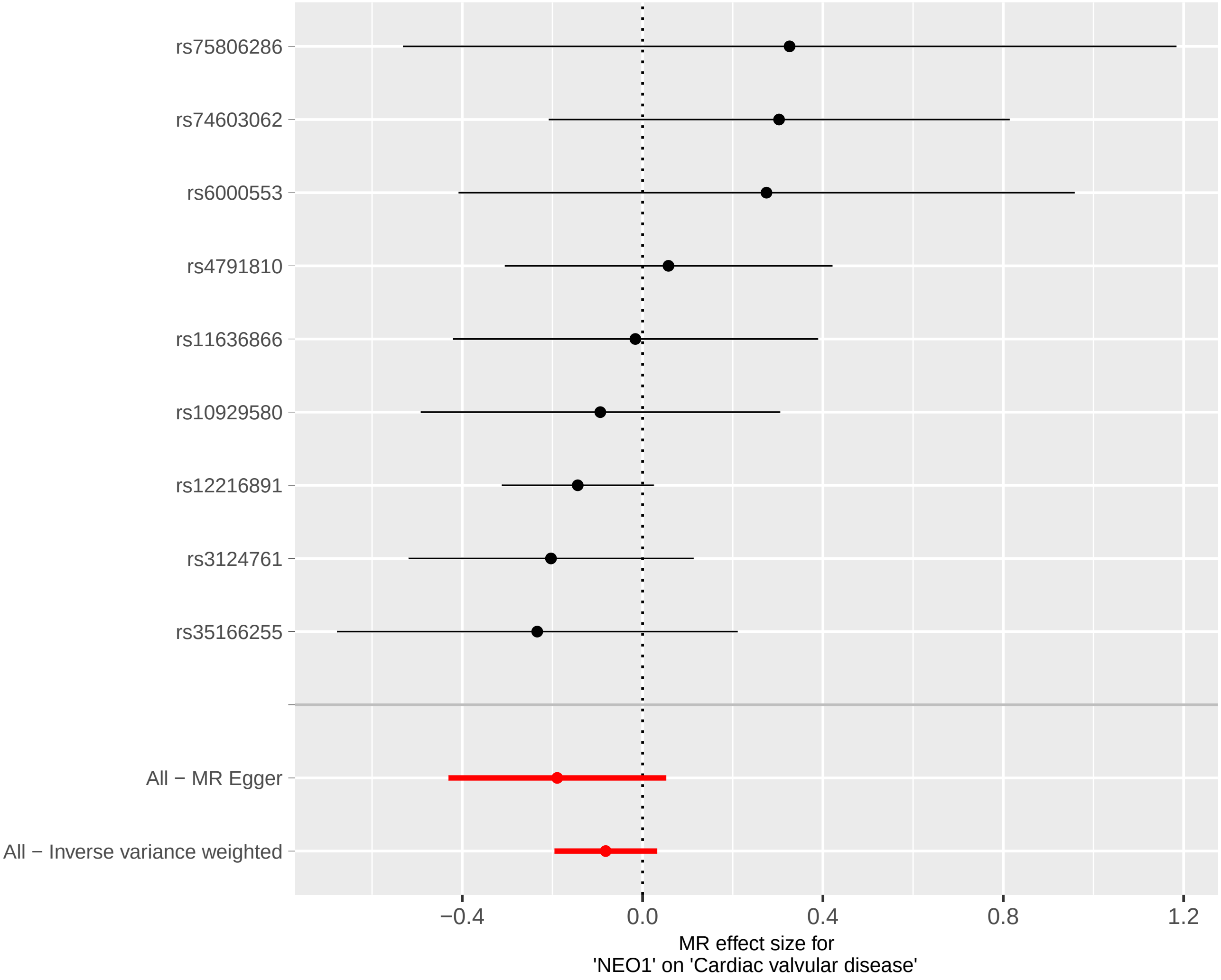
rs75806286
rs74603062
rs6000553
rs4791810
rs11636866
rs10929580
rs12216891
rs3124761
rs35166255
All − MR Egger
All − Inverse variance weighted
−0.4
0.0
0.4
0.8
1.2
MR effect size for
'NEO1' on 'Cardiac valvular disease'

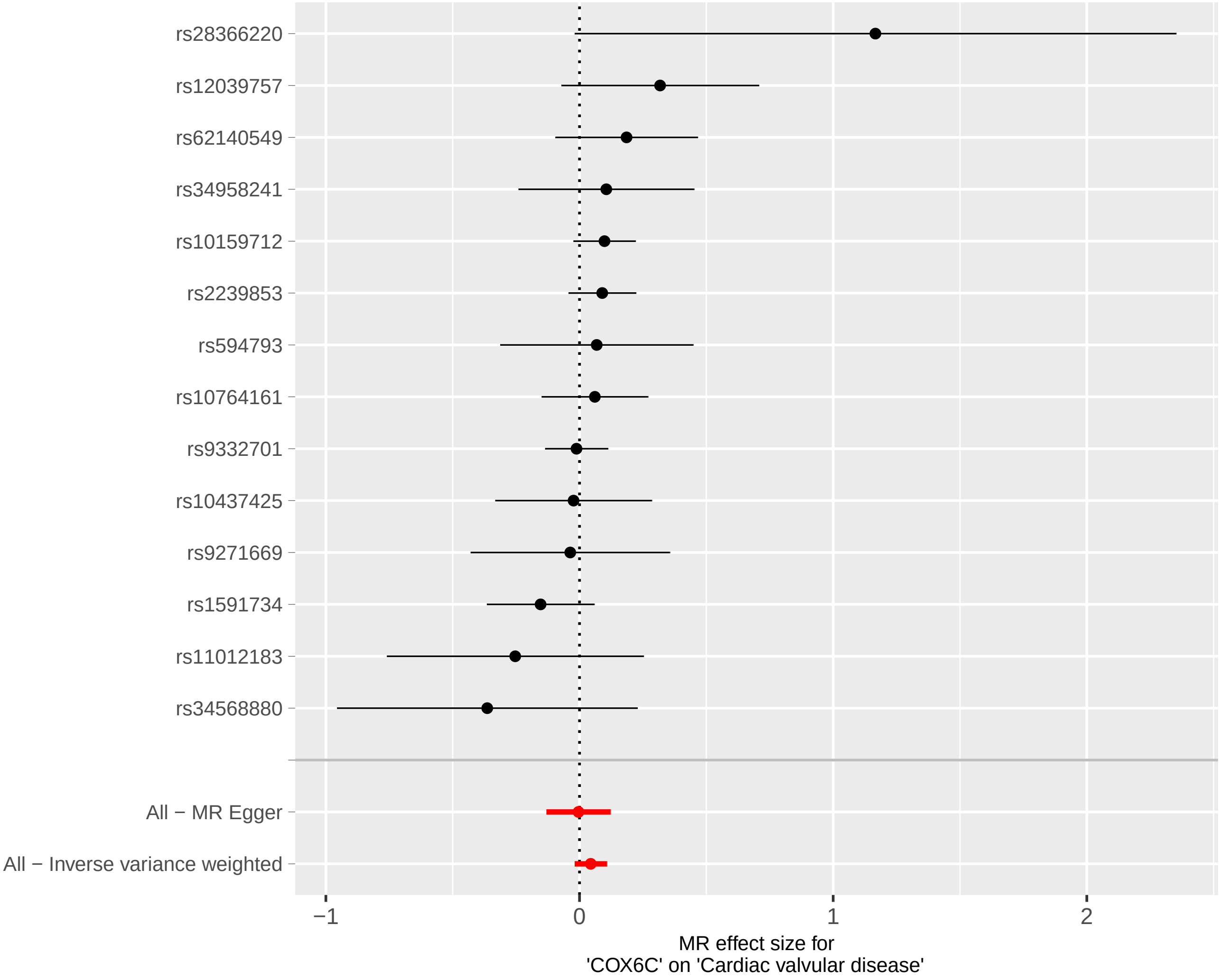

rs28366220
rs12039757
rs62140549
rs34958241
rs10159712
rs2239853
rs594793
rs10764161
rs9332701
rs10437425
rs9271669
rs1591734
rs11012183
rs34568880
All − MR Egger
All − Inverse variance weighted
−1
0
1
2
MR effect size for
'COX6C' on 'Cardiac valvular disease'

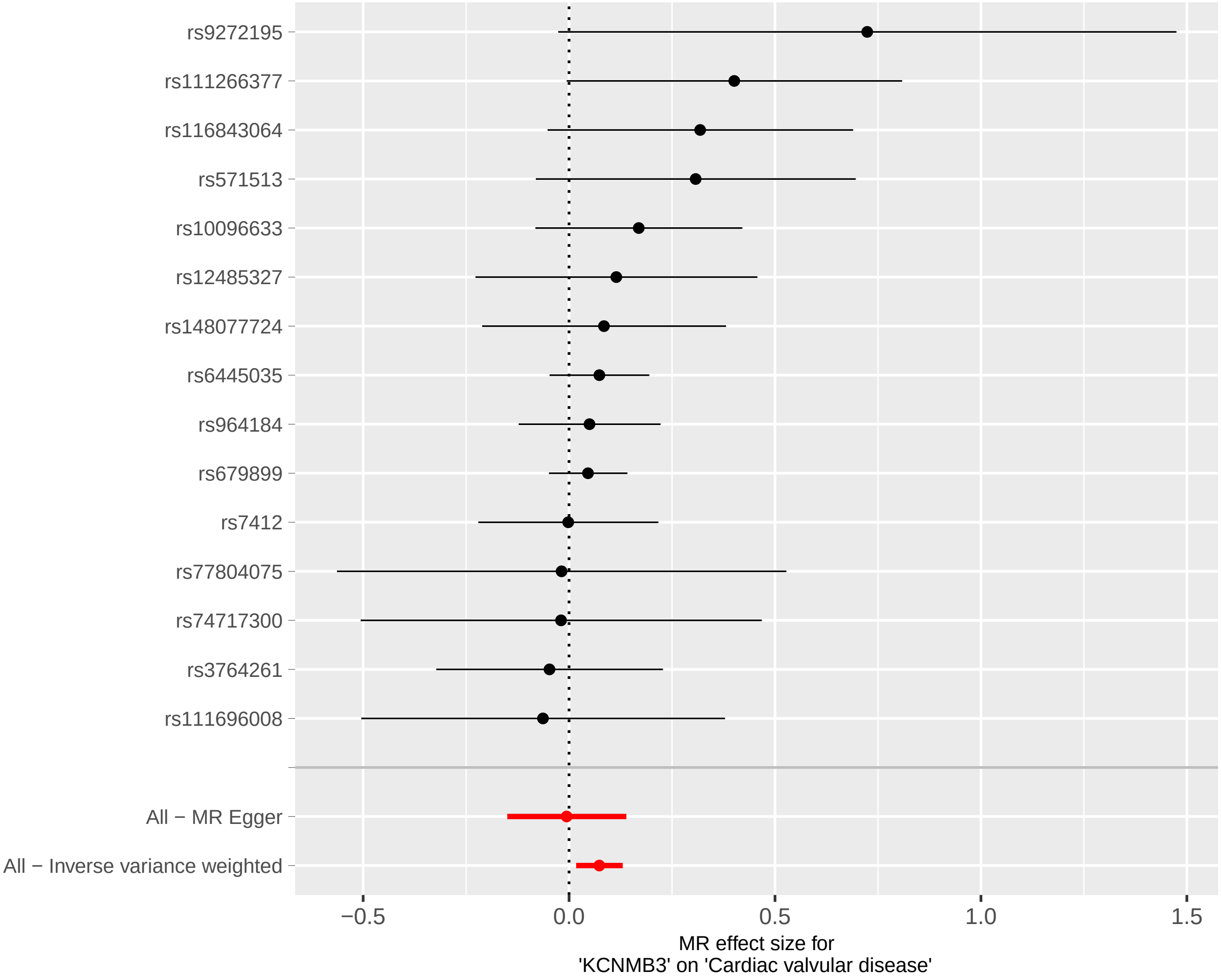

rs9272195
rs111266377
rs116843064
rs571513
rs10096633
rs12485327
rs148077724
rs6445035
rs964184
rs679899
rs7412
rs77804075
rs74717300
rs3764261
rs111696008
All − MR Egger
All − Inverse variance weighted
−0.5
0.0
0.5
1.0
1.5
MR effect size for
'KCNMB3' on 'Cardiac valvular disease'

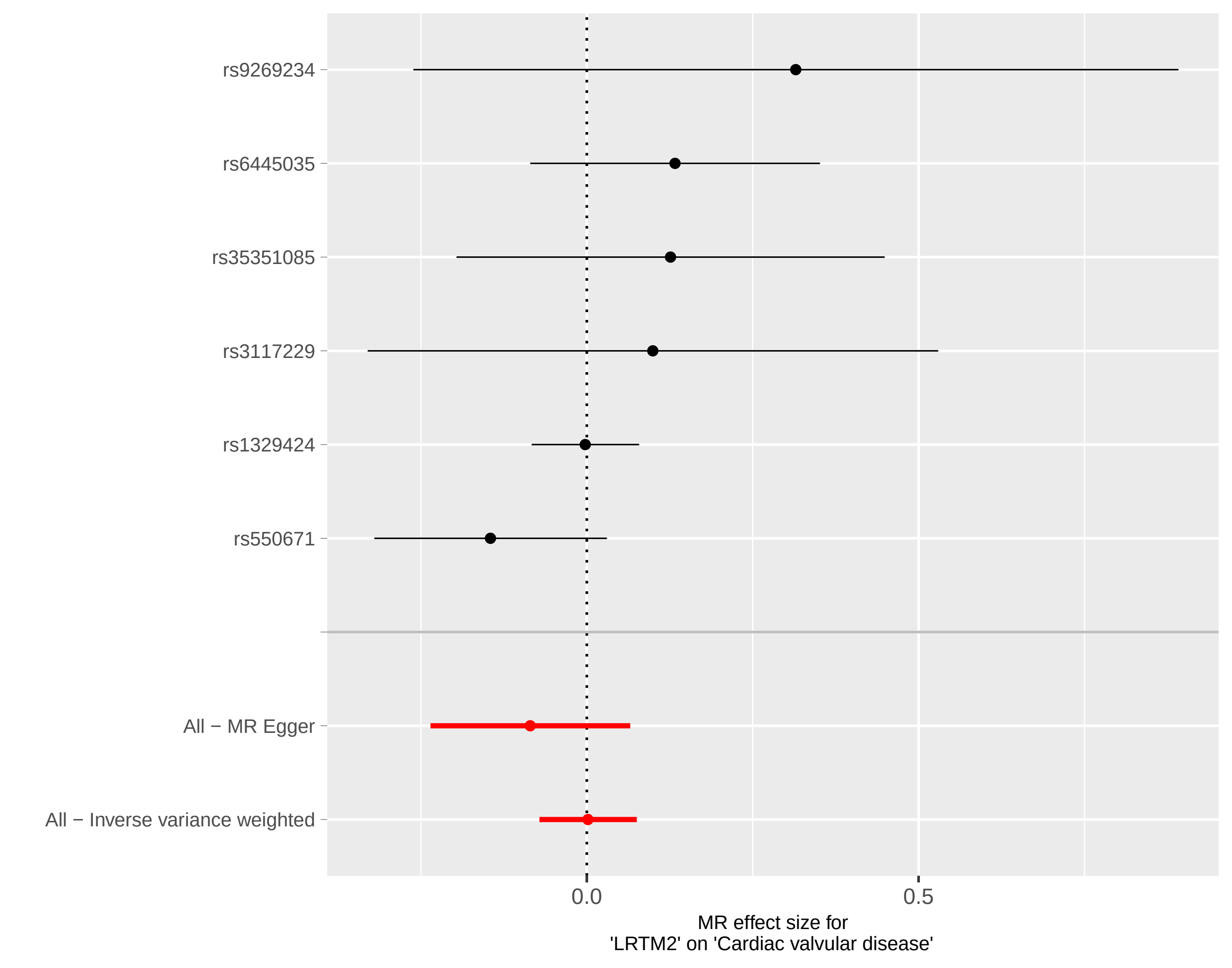

rs9269234
rs6445035
rs35351085
rs3117229
rs1329424
rs550671
All − MR Egger
All − Inverse variance weighted
0.0
0.5
MR effect size for
'LRTM2' on 'Cardiac valvular disease'

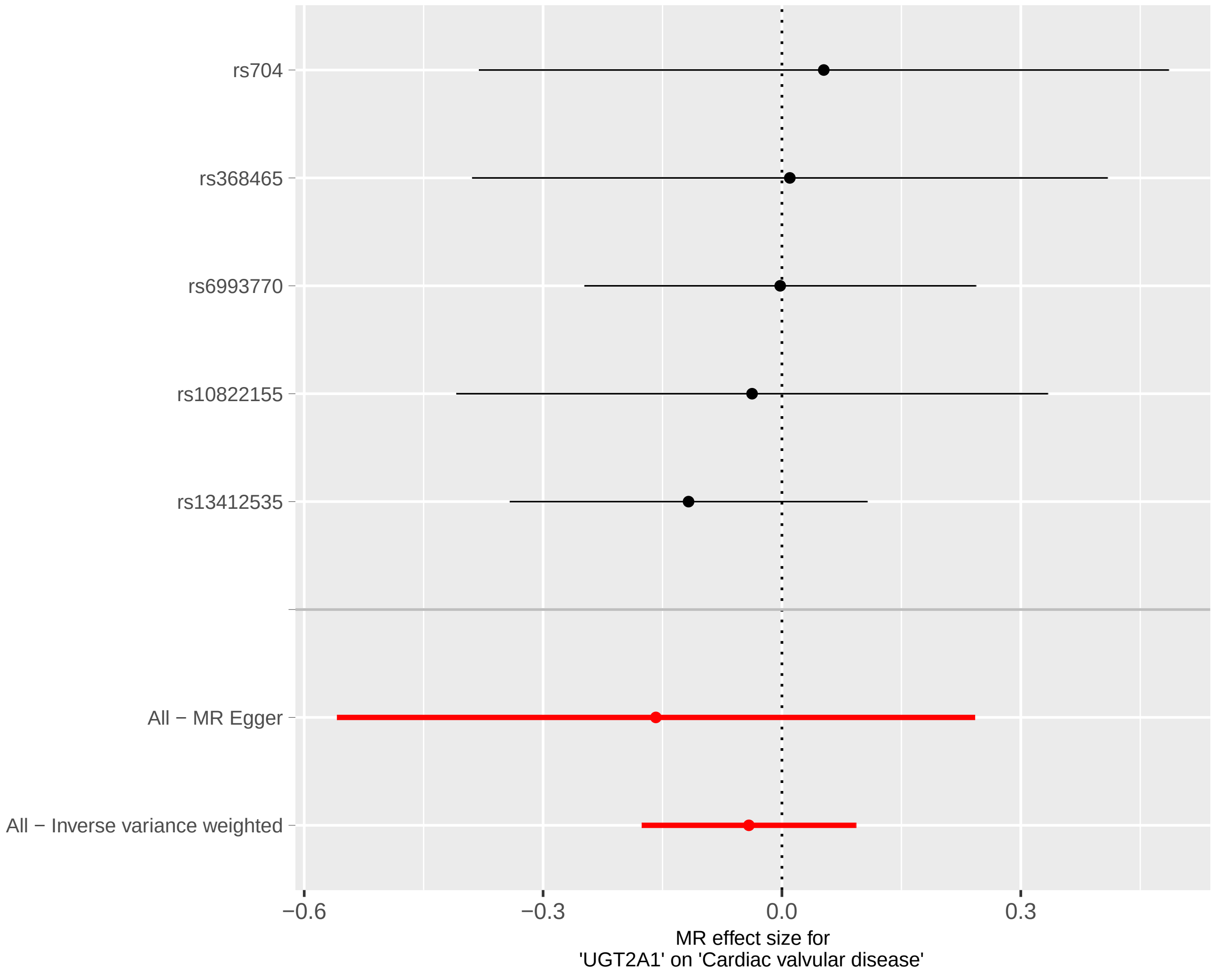

rs704
rs368465
rs6993770
rs10822155
rs13412535
All − MR Egger
All − Inverse variance weighted
−0.6
−0.3
0.0
0.3
MR effect size for
'UGT2A1' on 'Cardiac valvular disease'

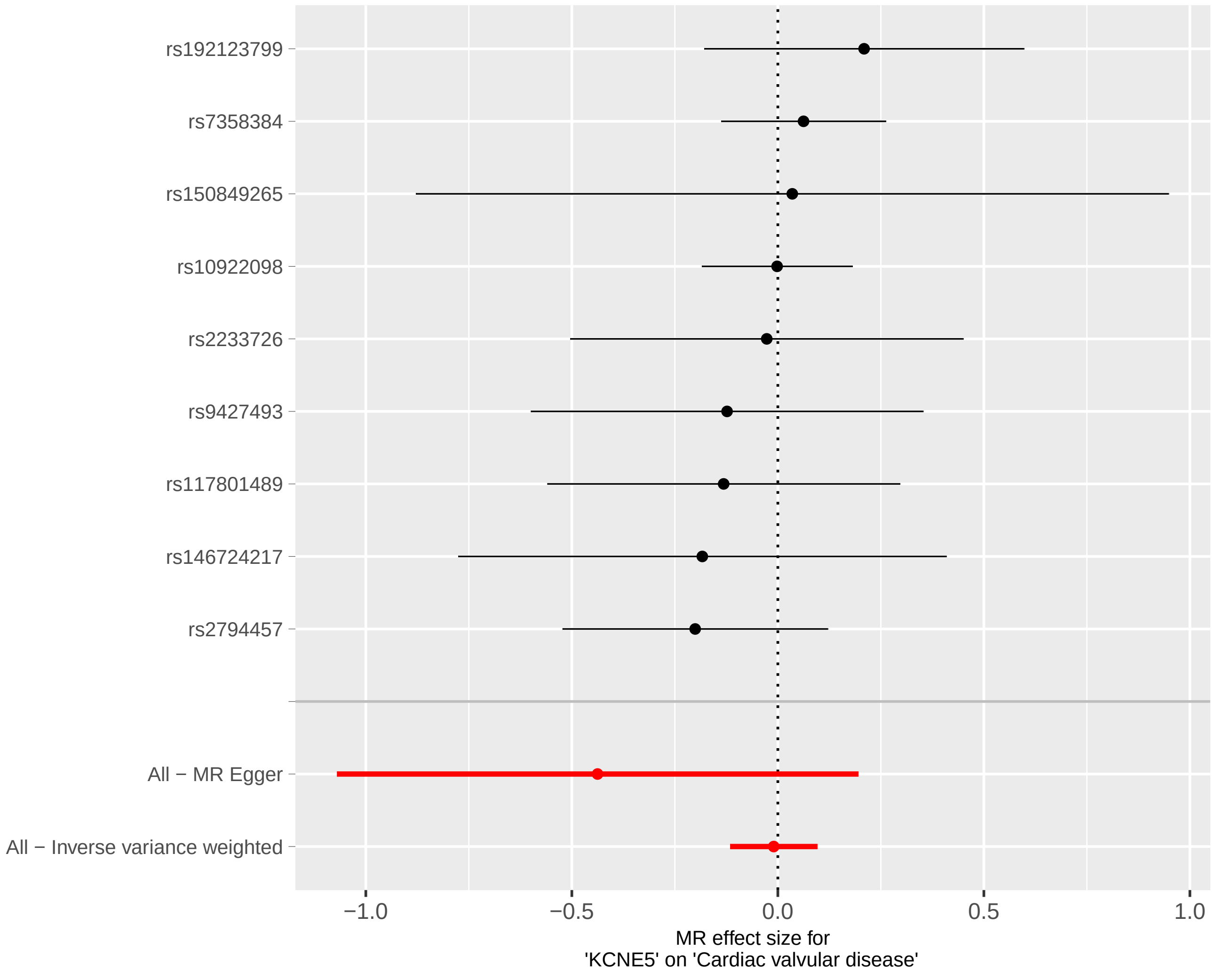
rs192123799
rs7358384
rs150849265
rs10922098
rs2233726
rs9427493
rs117801489
rs146724217
rs2794457
All − MR Egger
All − Inverse variance weighted
−1.0
−0.5
0.0
0.5
1.0
MR effect size for
'KCNE5' on 'Cardiac valvular disease'

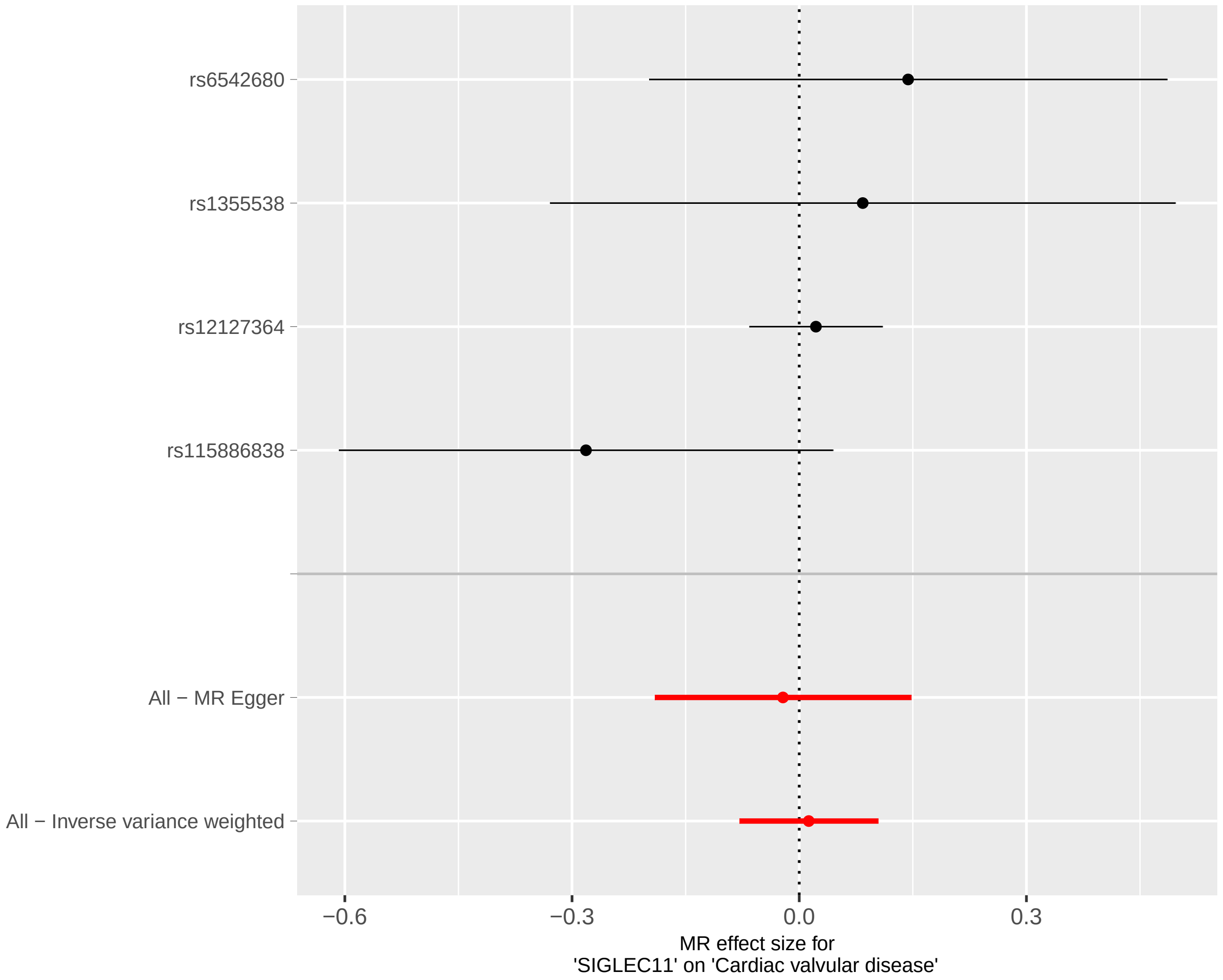
rs6542680
rs1355538
rs12127364
rs115886838
All − MR Egger
All − Inverse variance weighted
−0.6
−0.3
0.0
0.3
MR effect size for
'SIGLEC11' on 'Cardiac valvular disease'

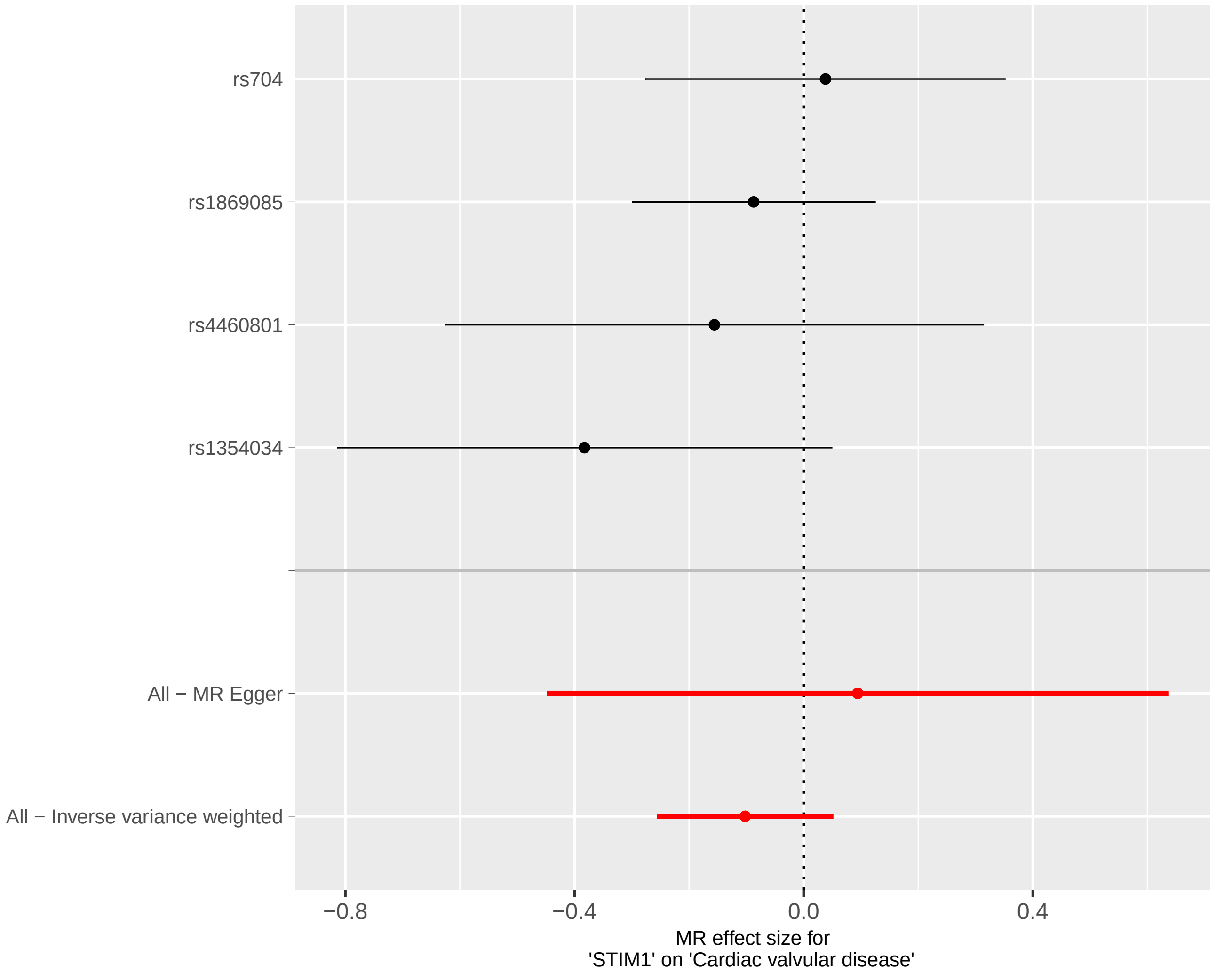
rs704
rs1869085
rs4460801
rs1354034
All − MR Egger
All − Inverse variance weighted
−0.8
−0.4
0.0
0.4
MR effect size for
'STIM1' on 'Cardiac valvular disease'

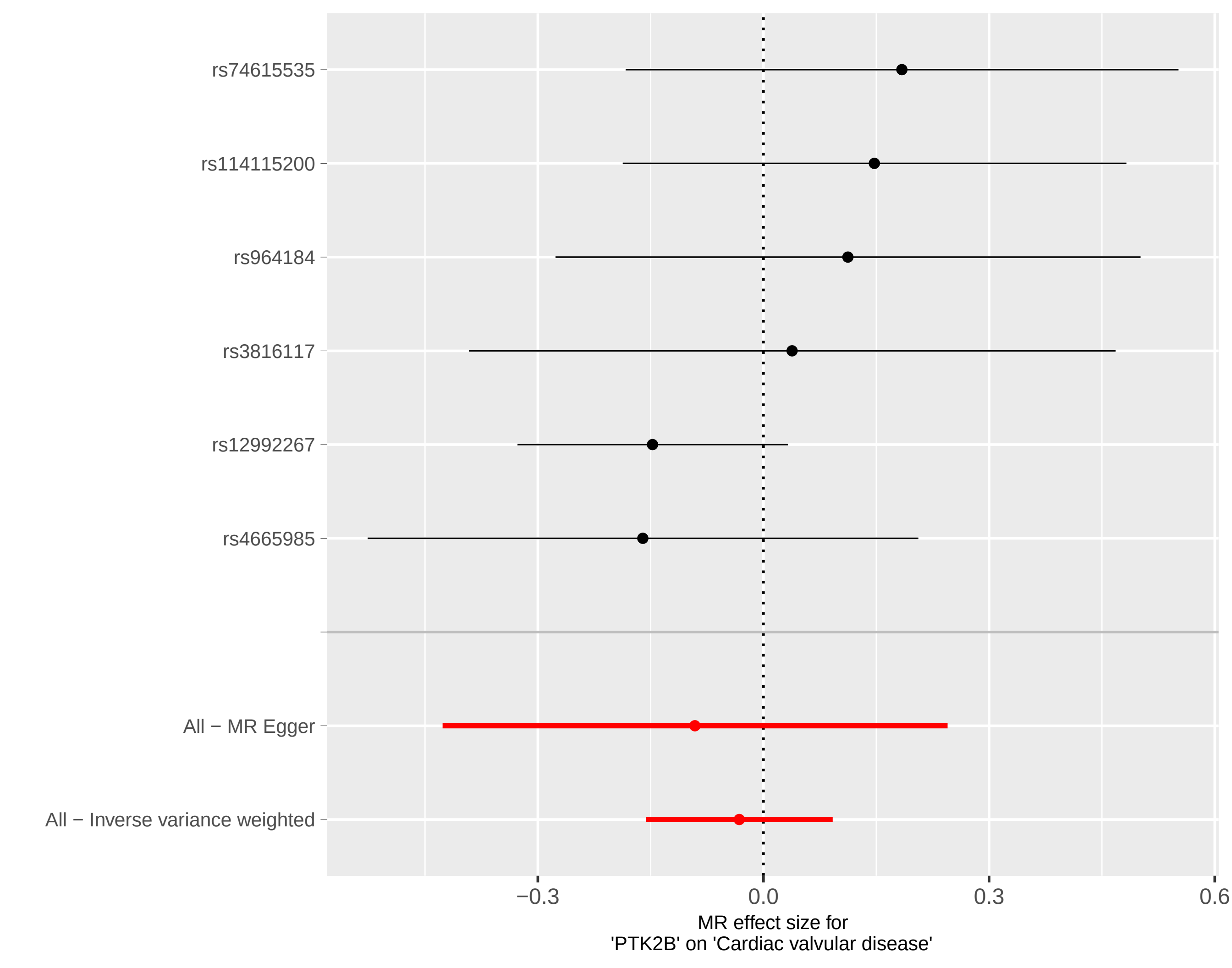
rs74615535
rs114115200
rs964184
rs3816117
rs12992267
rs4665985
All − MR Egger
All − Inverse variance weighted
−0.3
0.0
0.3
0.6
MR effect size for
'PTK2B' on 'Cardiac valvular disease'

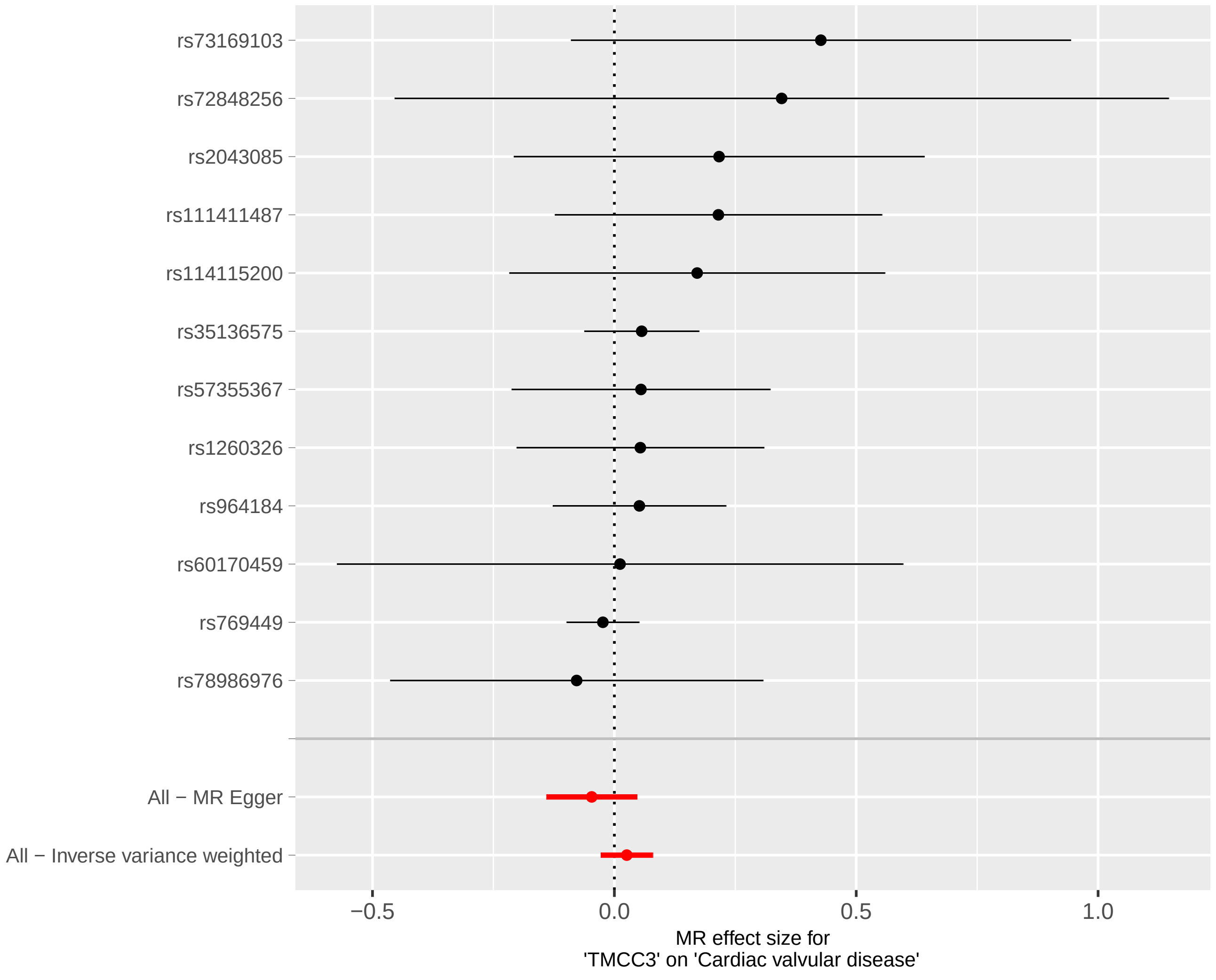

rs73169103
rs72848256
rs2043085
rs111411487
rs114115200
rs35136575
rs57355367
rs1260326
rs964184
rs60170459
rs769449
rs78986976
All − MR Egger
All − Inverse variance weighted
−0.5
0.0
0.5
1.0
MR effect size for
'TMCC3' on 'Cardiac valvular disease'

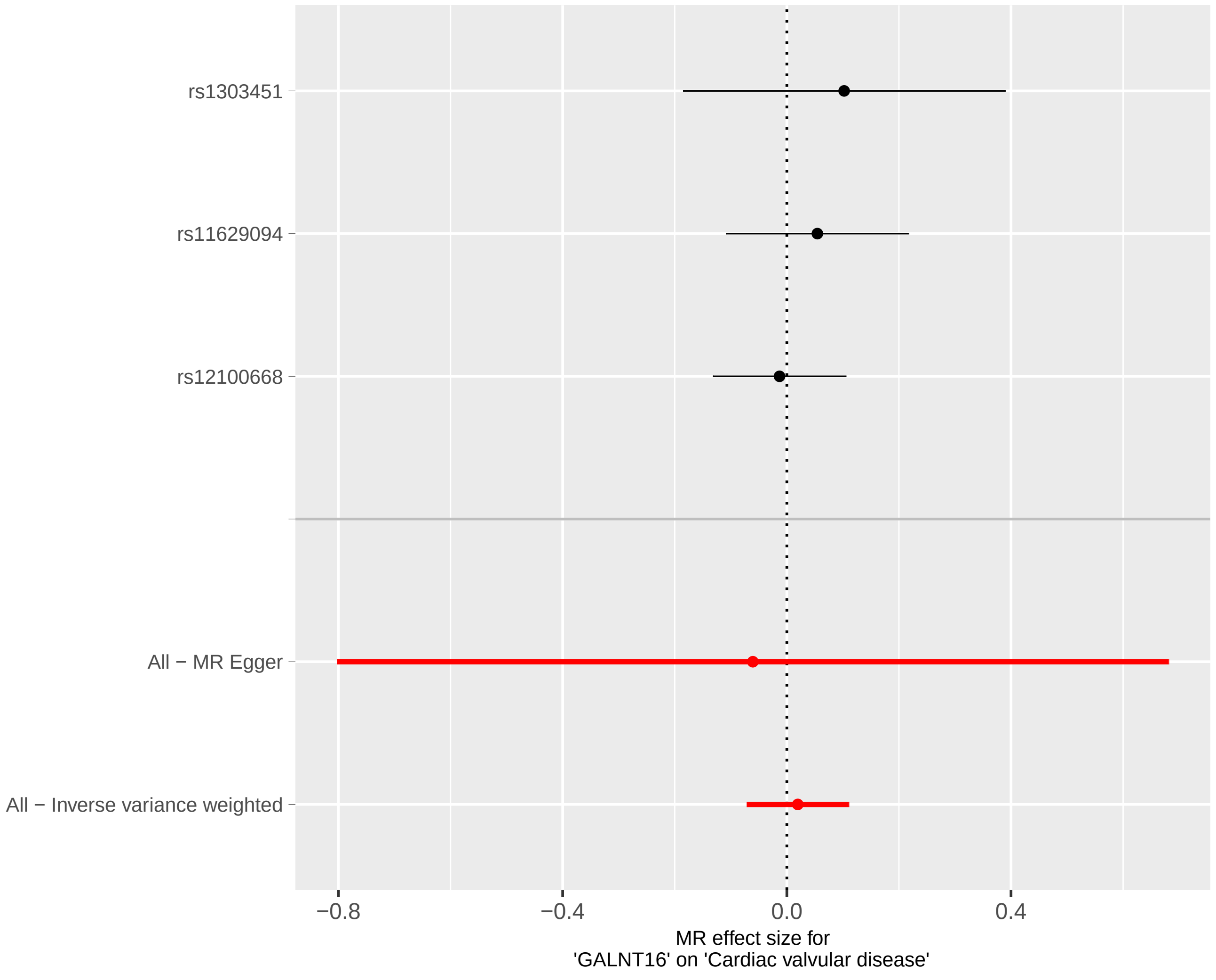
rs1303451
rs11629094
rs12100668
All − MR Egger
All − Inverse variance weighted
−0.8
−0.4
0.0
0.4
MR effect size for
'GALNT16' on 'Cardiac valvular disease'

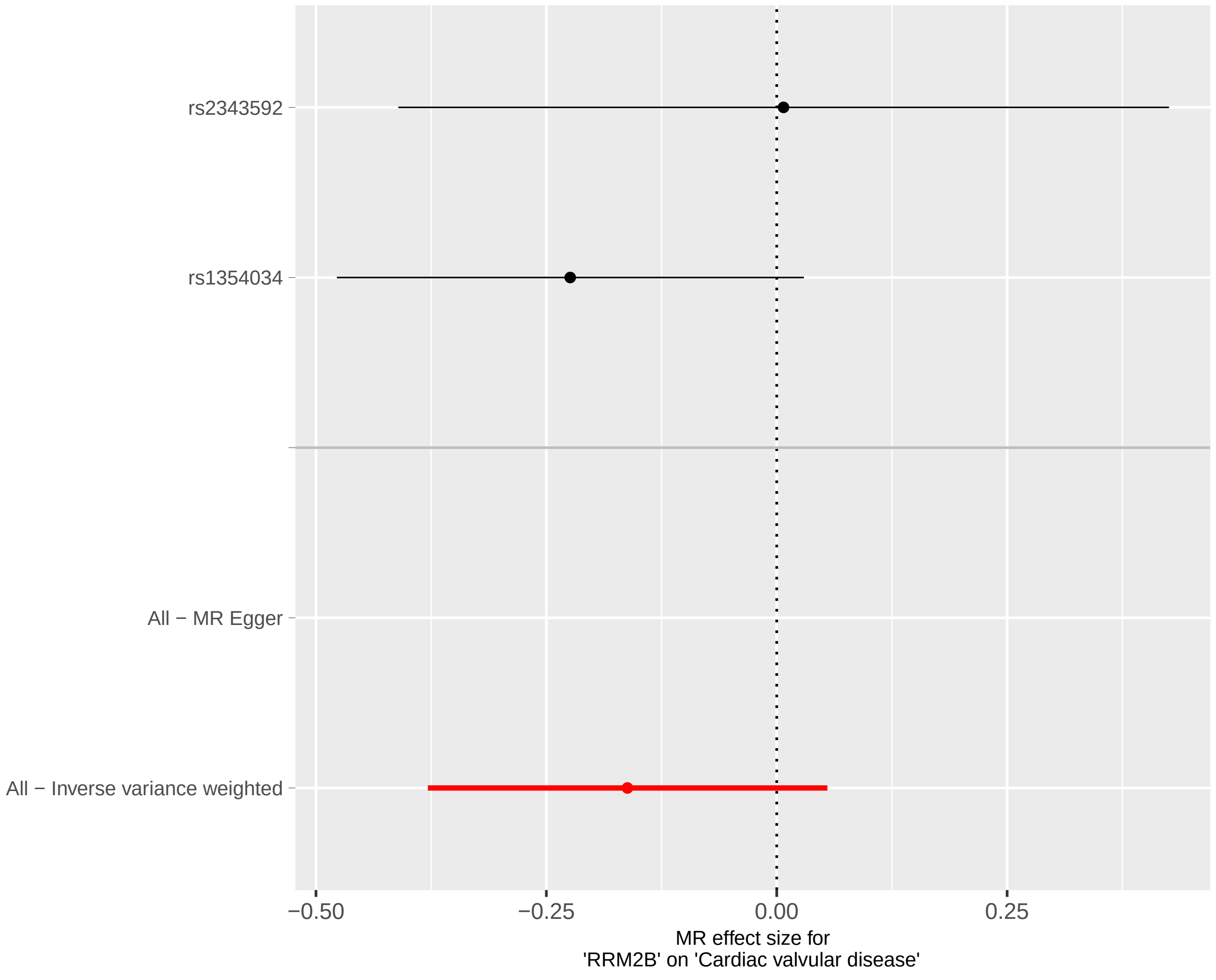
rs2343592
rs1354034
All − MR Egger
All − Inverse variance weighted
−0.50
−0.25
0.00
0.25
MR effect size for
'RRM2B' on 'Cardiac valvular disease'

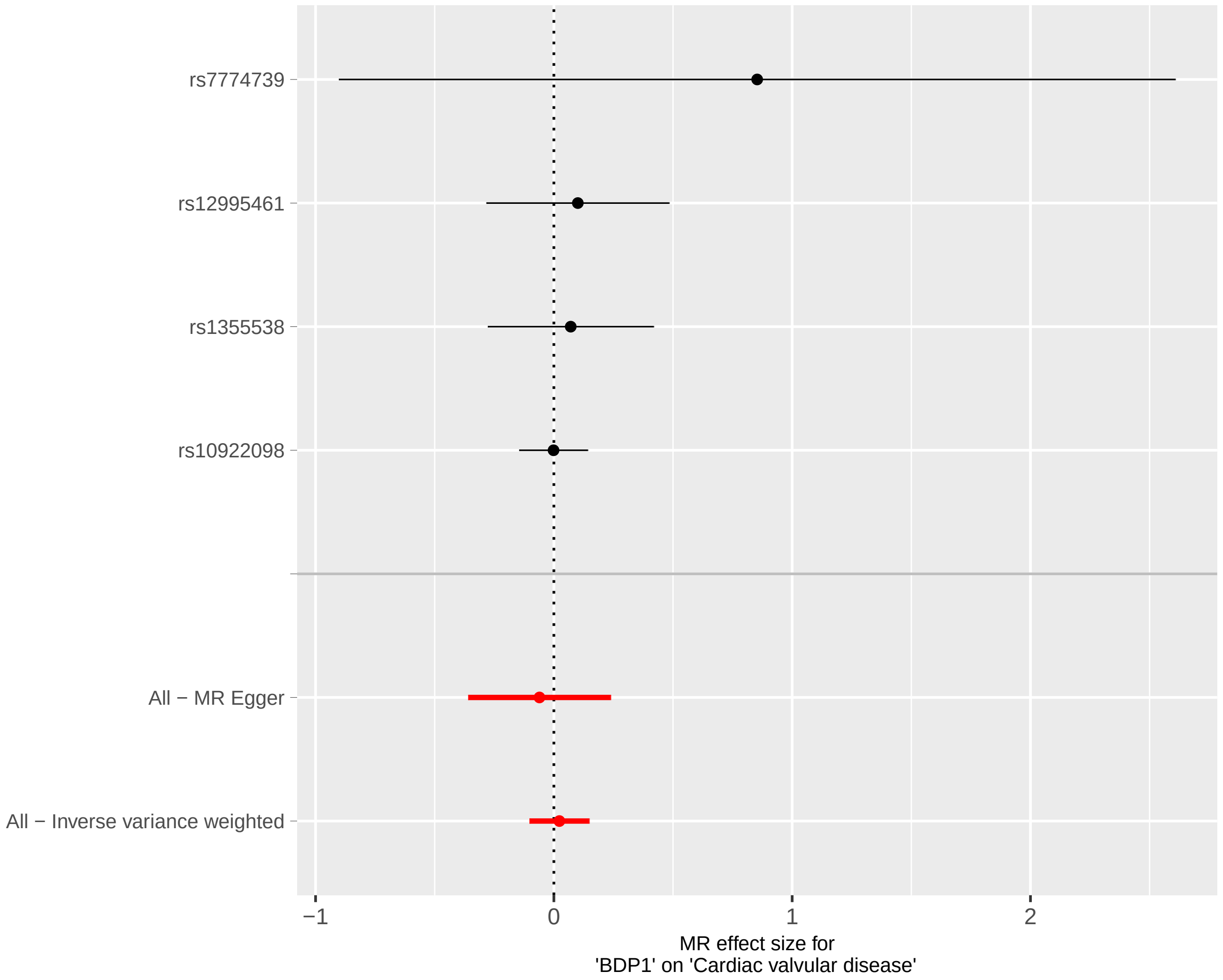

rs7774739
rs12995461
rs1355538
rs10922098
All − MR Egger
All − Inverse variance weighted
−1
0
1
2
MR effect size for
'BDP1' on 'Cardiac valvular disease'

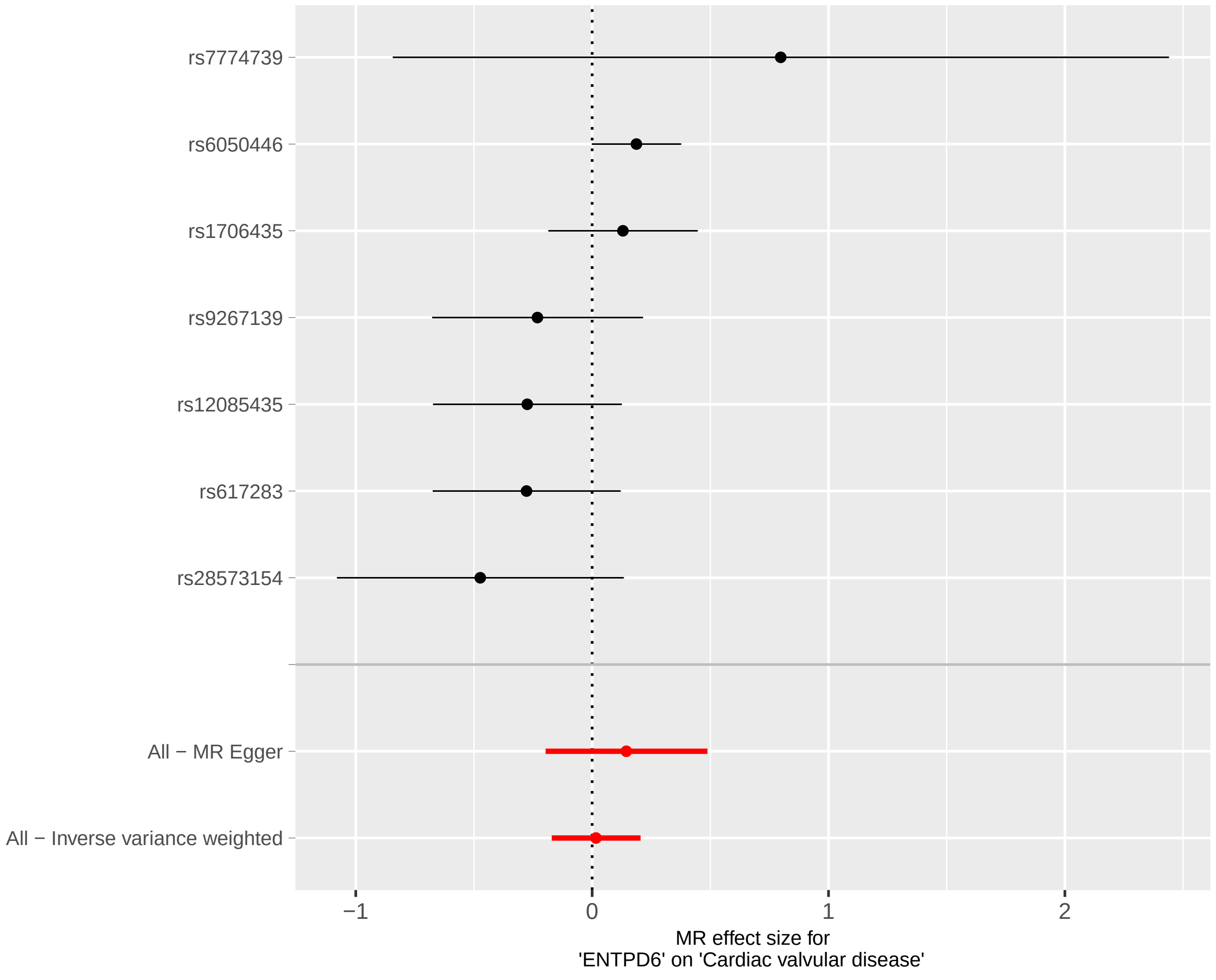
rs7774739
rs6050446
rs1706435
rs9267139
rs12085435
rs617283
rs28573154
All − MR Egger
All − Inverse variance weighted
−1
0
1
2
MR effect size for
'ENTPD6' on 'Cardiac valvular disease'

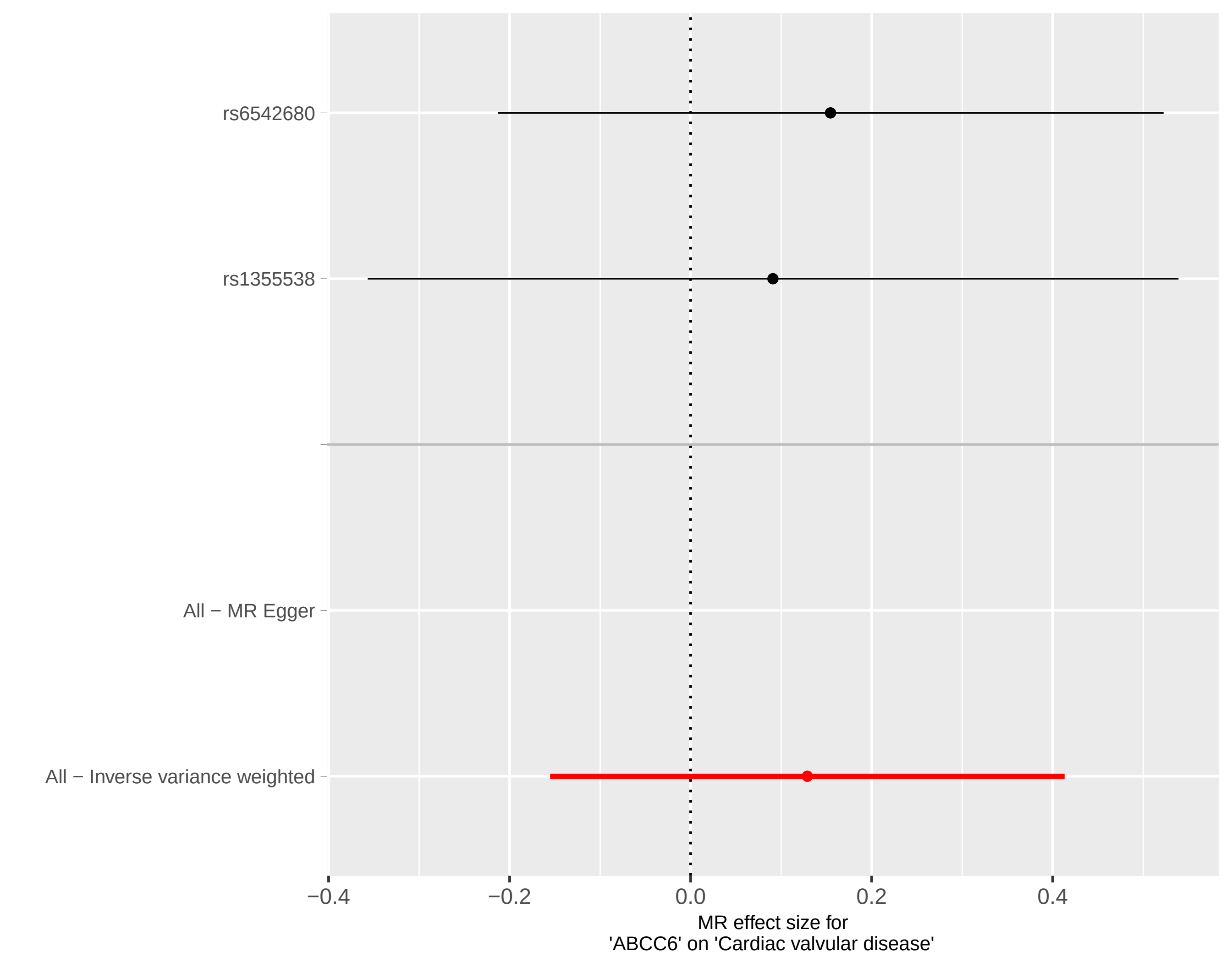

rs6542680
rs1355538
All − MR Egger
All − Inverse variance weighted
−0.4
−0.2
0.0
0.2
0.4
MR effect size for
'ABCC6' on 'Cardiac valvular disease'

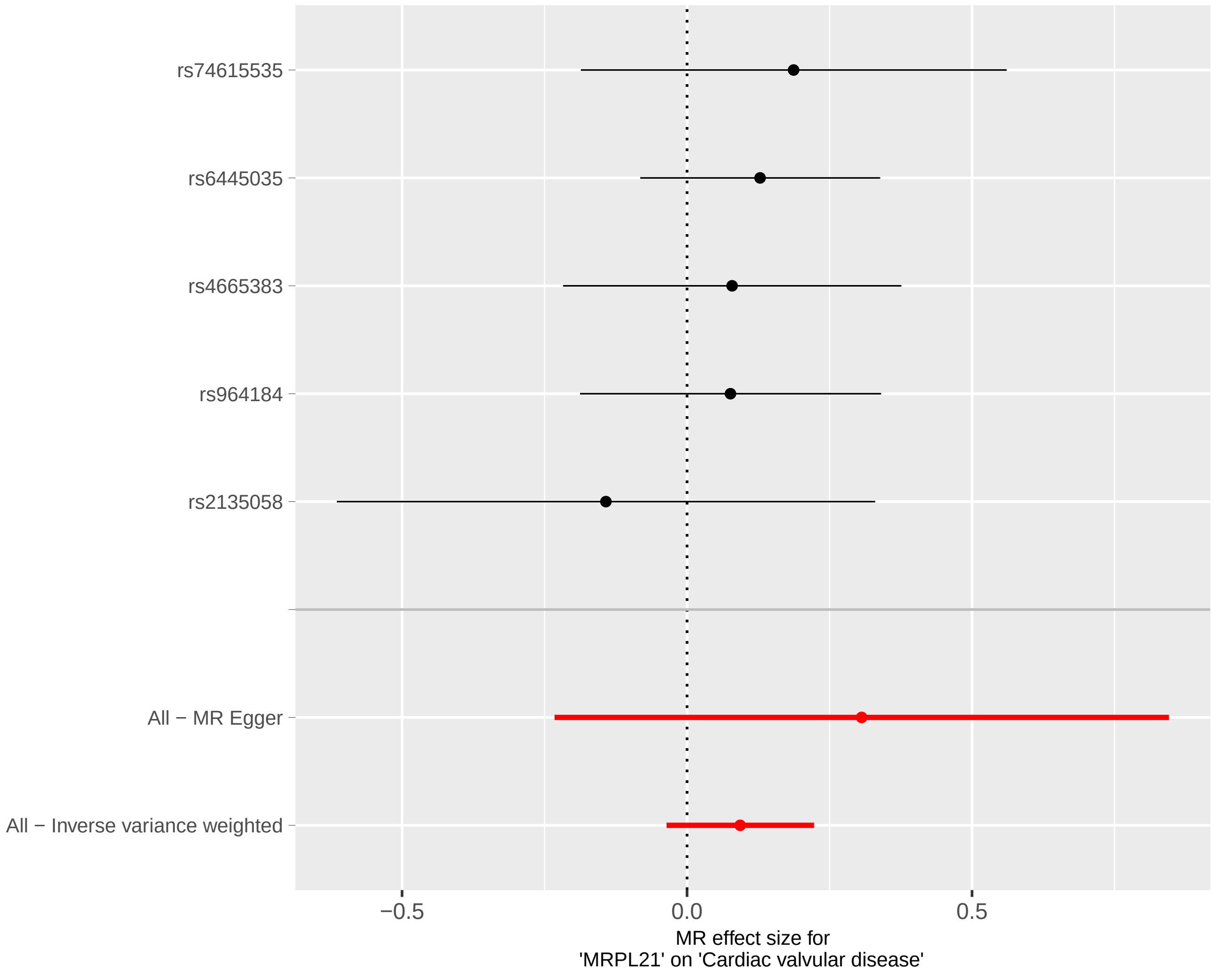
rs74615535
rs6445035
rs4665383
rs964184
rs2135058
All − MR Egger
All − Inverse variance weighted
−0.5
0.0
0.5
MR effect size for
'MRPL21' on 'Cardiac valvular disease'

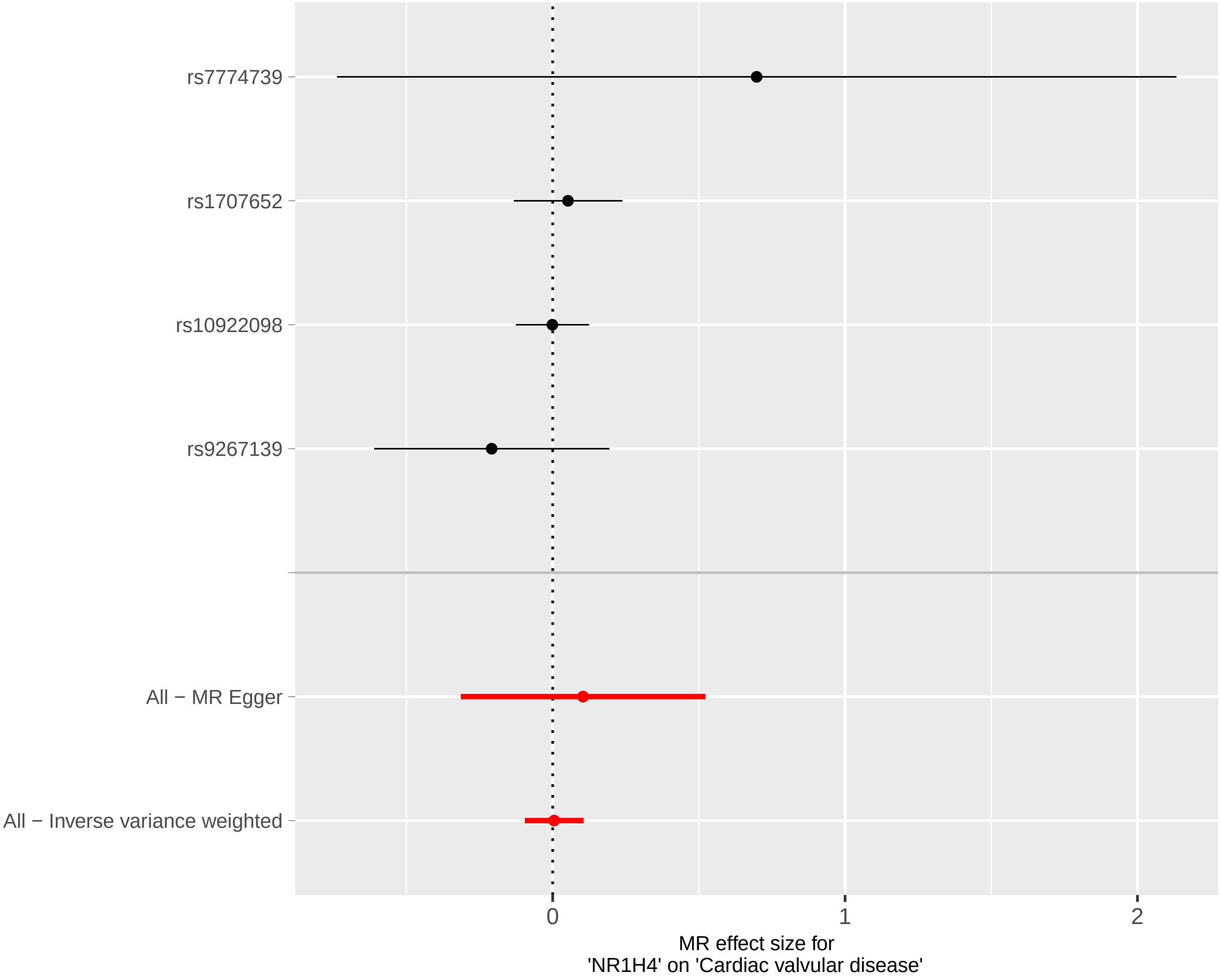
rs7774739
rs1707652
rs10922098
rs9267139
All − MR Egger
All − Inverse variance weighted
0
1
2
MR effect size for
'NR1H4' on 'Cardiac valvular disease'

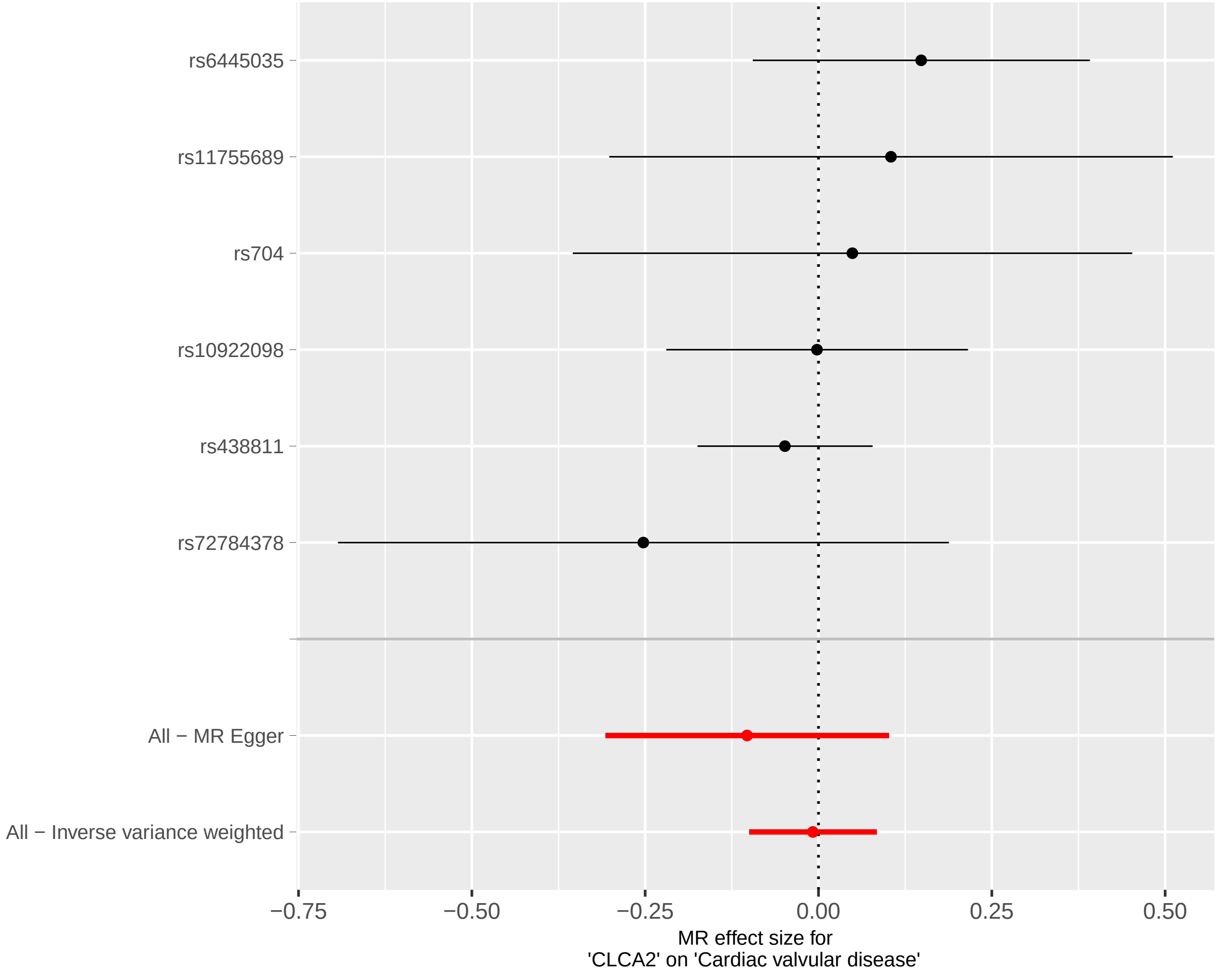

rs6445035
rs11755689
rs704
rs10922098
rs438811
rs72784378
All − MR Egger
All − Inverse variance weighted
−0.75
−0.50
−0.25
0.00
0.25
0.50
MR effect size for
'CLCA2' on 'Cardiac valvular disease'

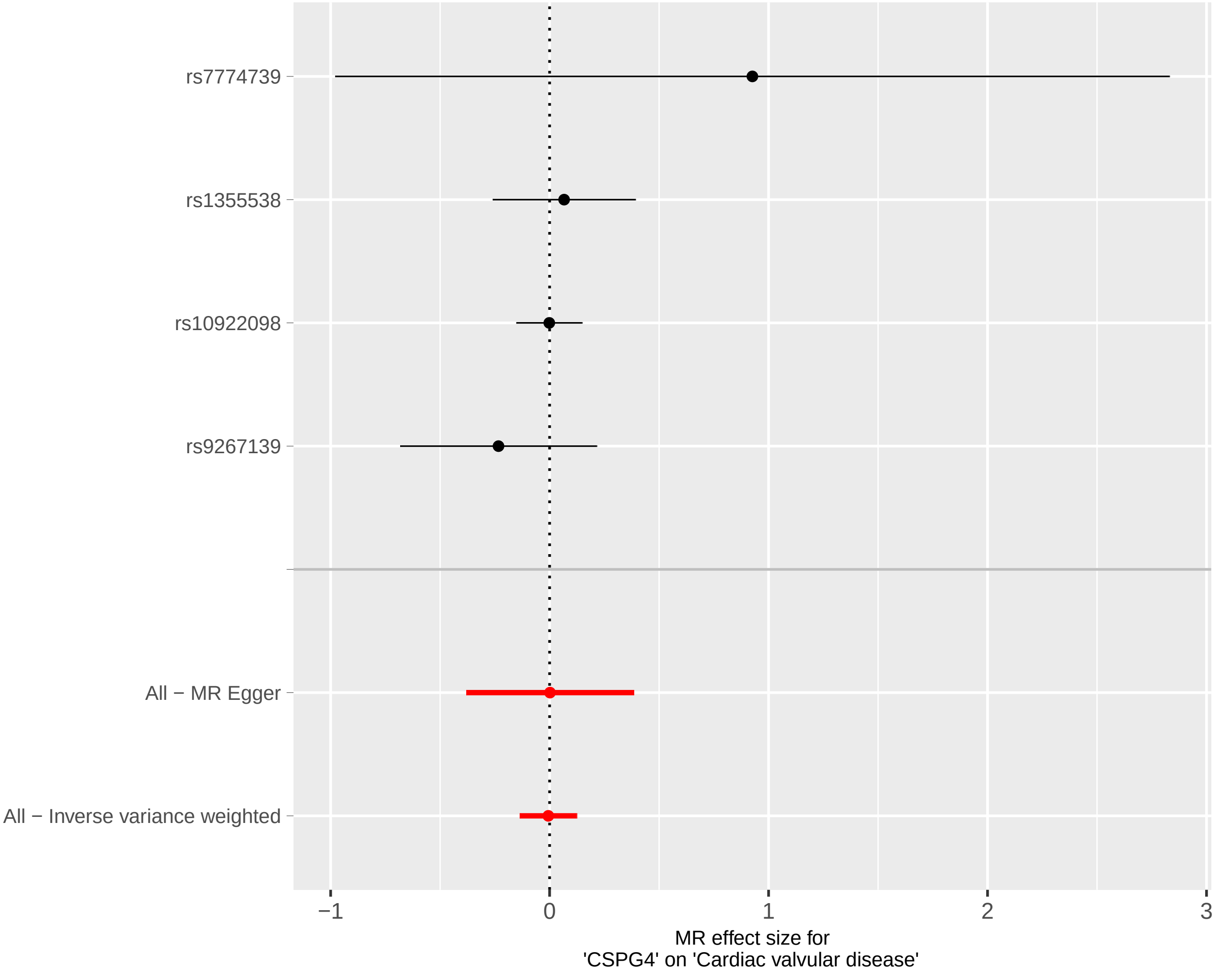
rs7774739
rs1355538
rs10922098
rs9267139
All − MR Egger
All − Inverse variance weighted
−1
0
1
2
3
MR effect size for
'CSPG4' on 'Cardiac valvular disease'

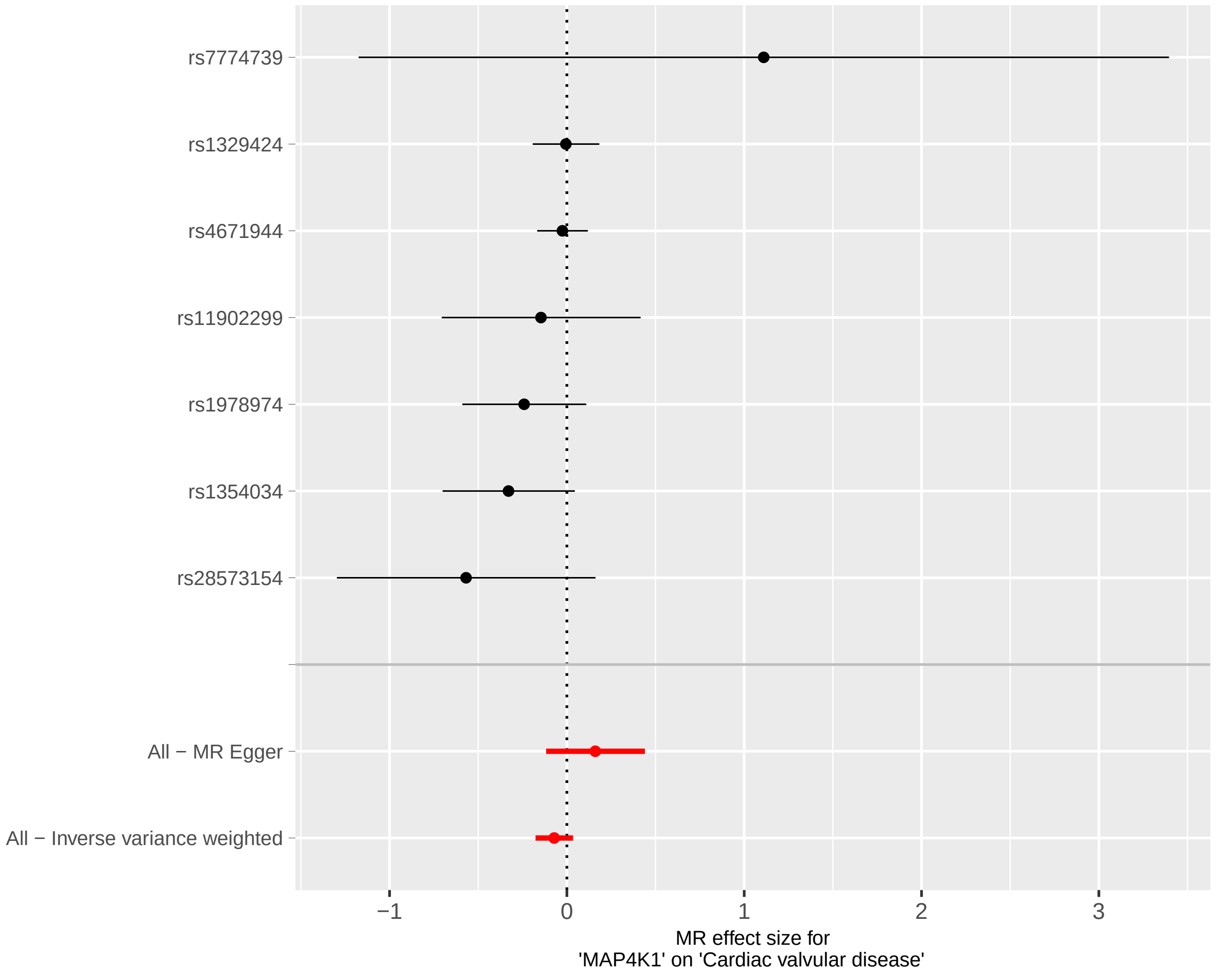
rs7774739
rs1329424
rs4671944
rs11902299
rs1978974
rs1354034
rs28573154
All − MR Egger
All − Inverse variance weighted
−1
0
1
2
3
MR effect size for
'MAP4K1' on 'Cardiac valvular disease'

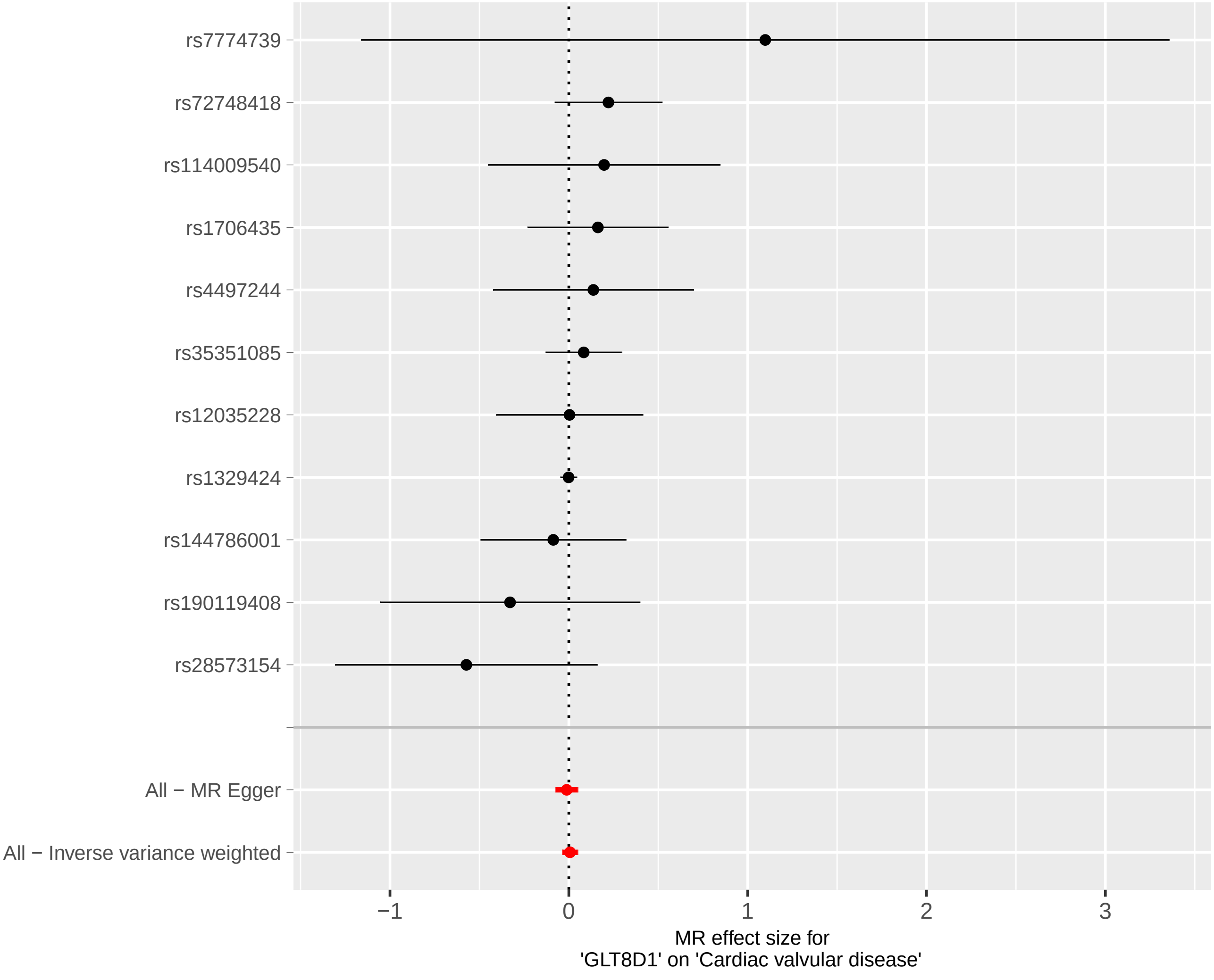
rs7774739
rs72748418
rs114009540
rs1706435
rs4497244
rs35351085
rs12035228
rs1329424
rs144786001
rs190119408
rs28573154
All − MR Egger
All − Inverse variance weighted
−1
0
1
2
3
MR effect size for
'GLT8D1' on 'Cardiac valvular disease'

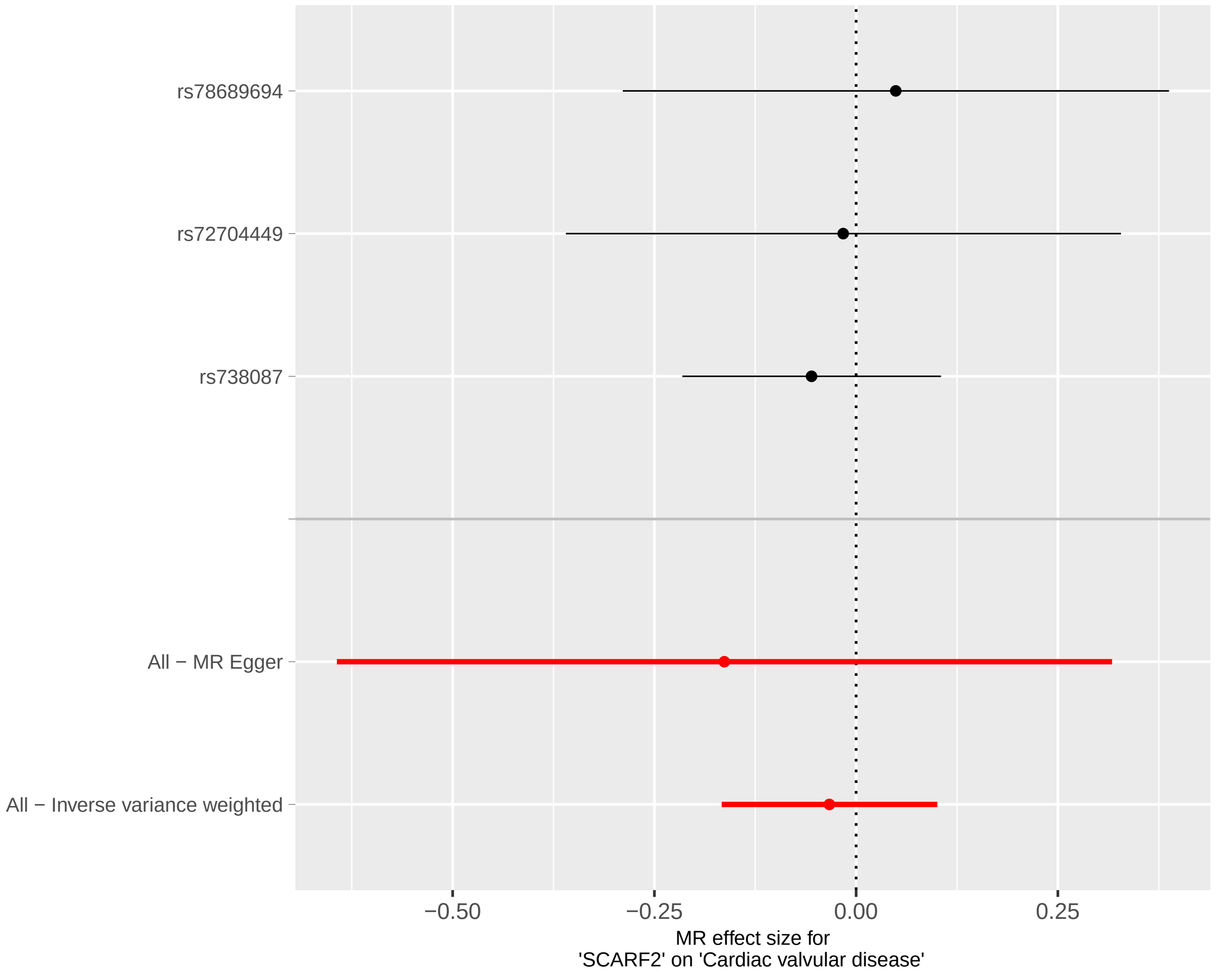
rs78689694
rs72704449
rs738087
All − MR Egger
All − Inverse variance weighted
−0.50
−0.25
0.00
0.25
MR effect size for
'SCARF2' on 'Cardiac valvular disease'

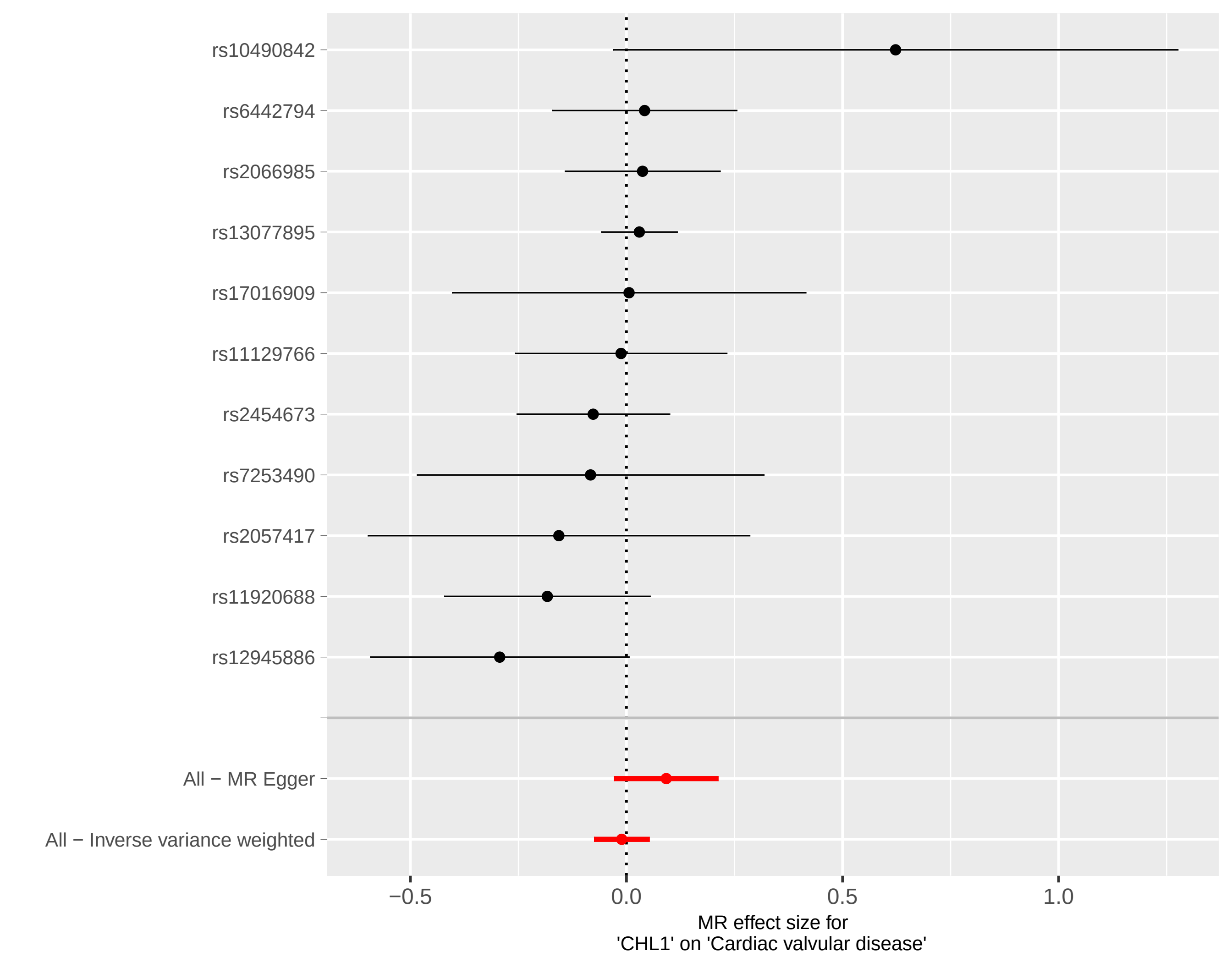
rs10490842
rs6442794
rs2066985
rs13077895
rs17016909
rs11129766
rs2454673
rs7253490
rs2057417
rs11920688
rs12945886
All − MR Egger
All − Inverse variance weighted
−0.5
0.0
0.5
1.0
MR effect size for
'CHL1' on 'Cardiac valvular disease'

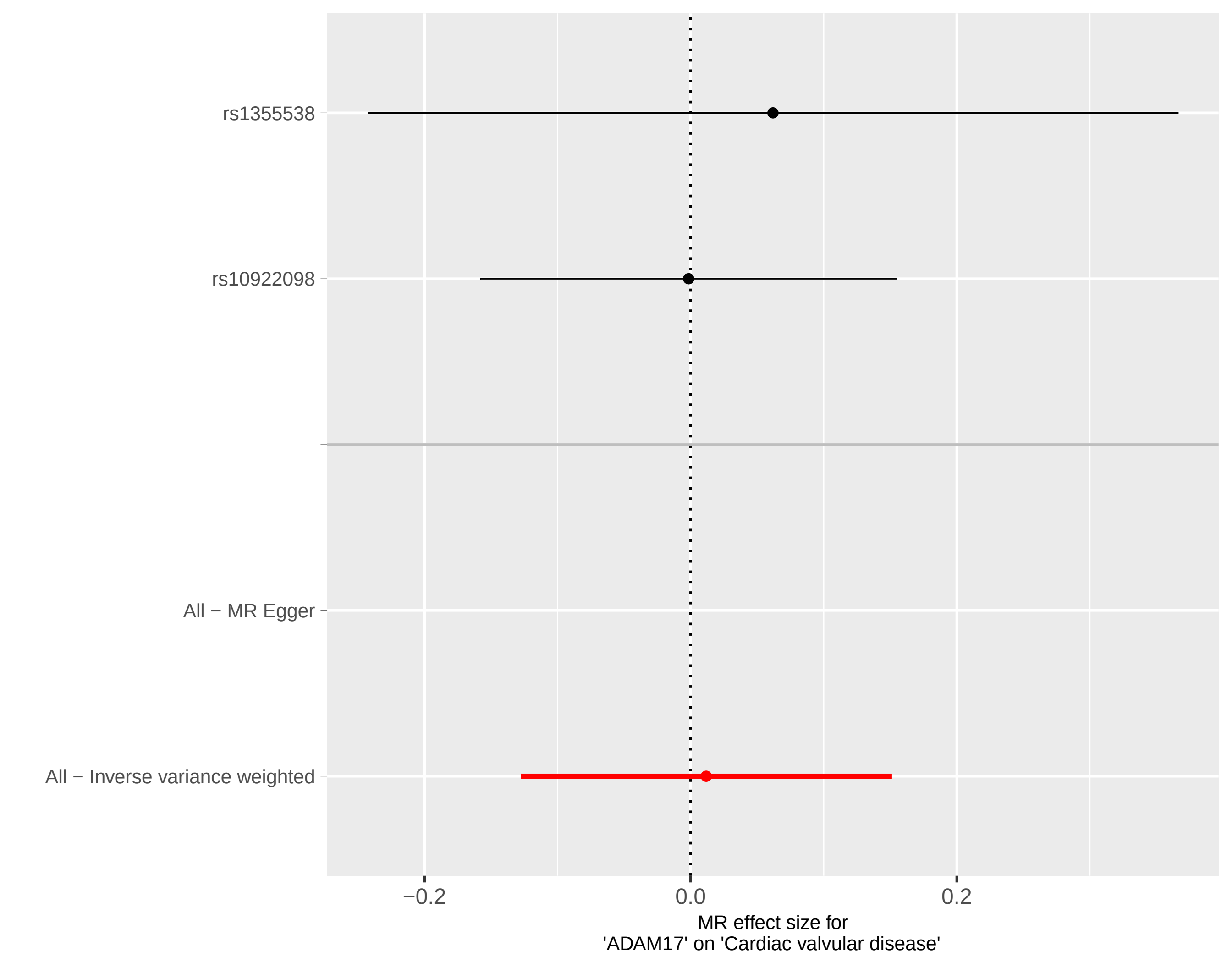

rs1355538
rs10922098
All − MR Egger
All − Inverse variance weighted
−0.2
0.0
0.2
MR effect size for
'ADAM17' on 'Cardiac valvular disease'

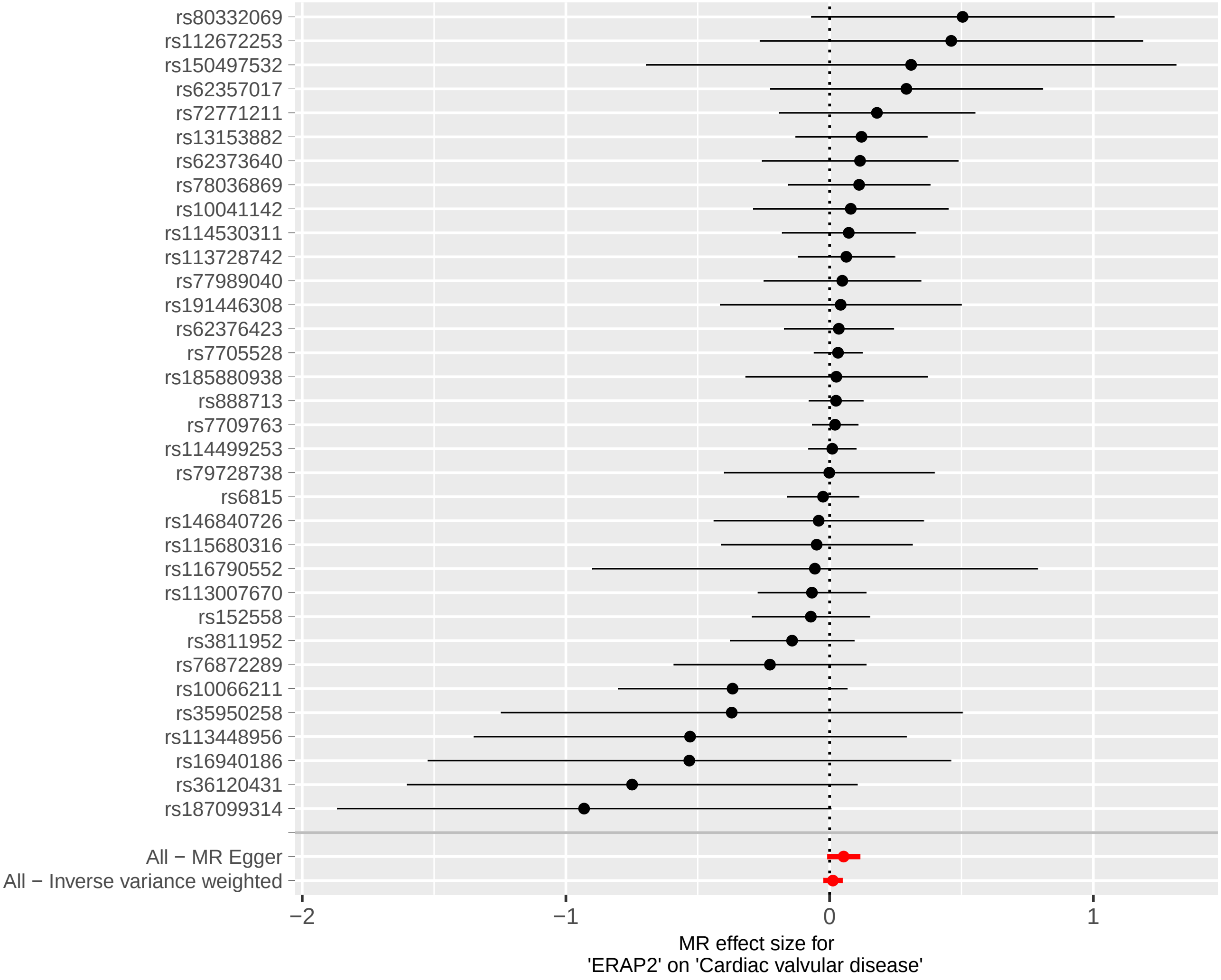

rs80332069
rs112672253
rs150497532
rs62357017
rs72771211
rs13153882
rs62373640
rs78036869
rs10041142
rs114530311
rs113728742
rs77989040
rs191446308
rs62376423
rs7705528
rs185880938
rs888713
rs7709763
rs114499253
rs79728738
rs6815
rs146840726
rs115680316
rs116790552
rs113007670
rs152558
rs3811952
rs76872289
rs10066211
rs35950258
rs113448956
rs16940186
rs36120431
rs187099314
All − MR Egger
All − Inverse variance weighted
−2
−1
0
1
MR effect size for
'ERAP2' on 'Cardiac valvular disease'

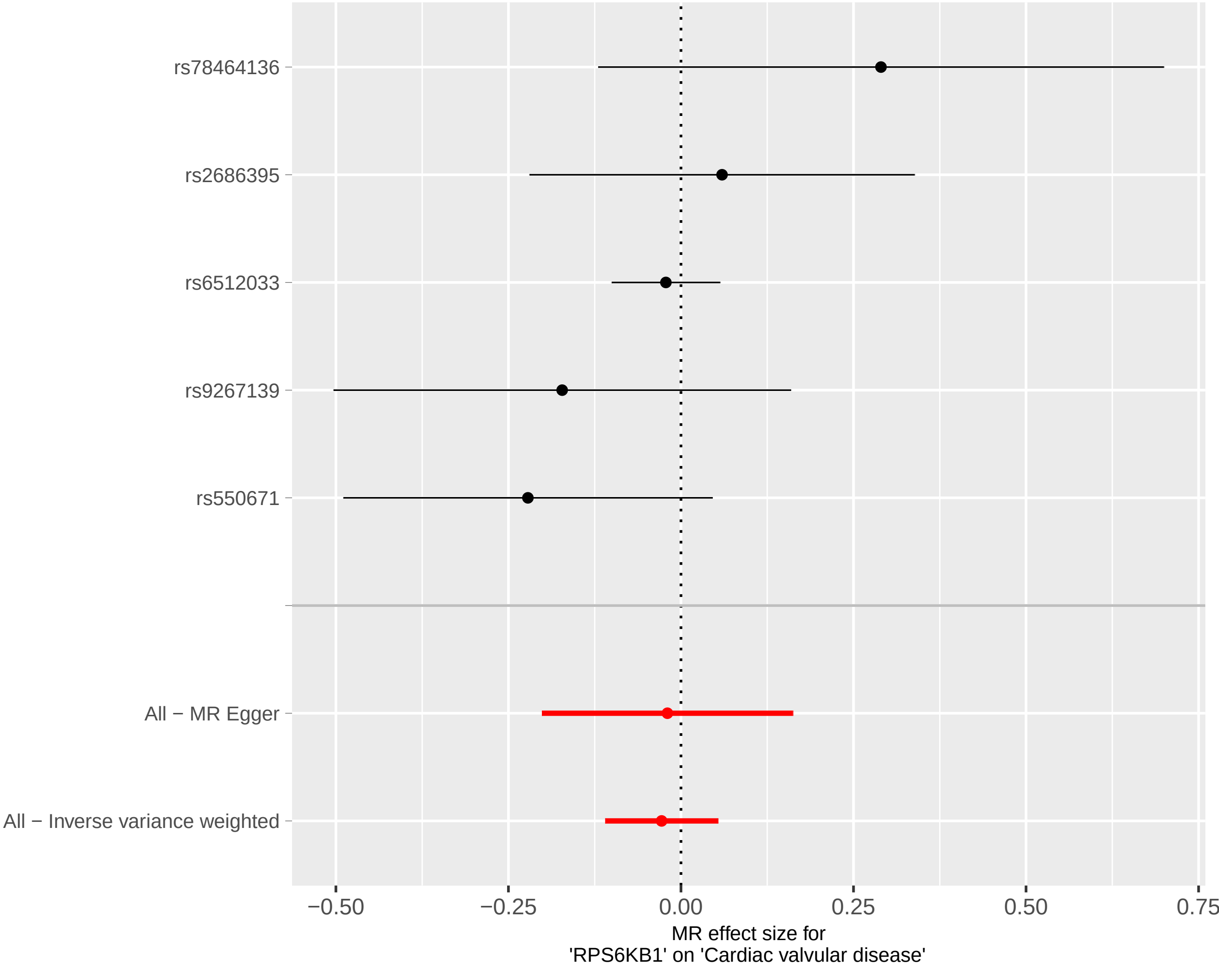
rs78464136
rs2686395
rs6512033
rs9267139
rs550671
All − MR Egger
All − Inverse variance weighted
−0.50
−0.25
0.00
0.25
0.50
0.75
MR effect size for
'RPS6KB1' on 'Cardiac valvular disease'

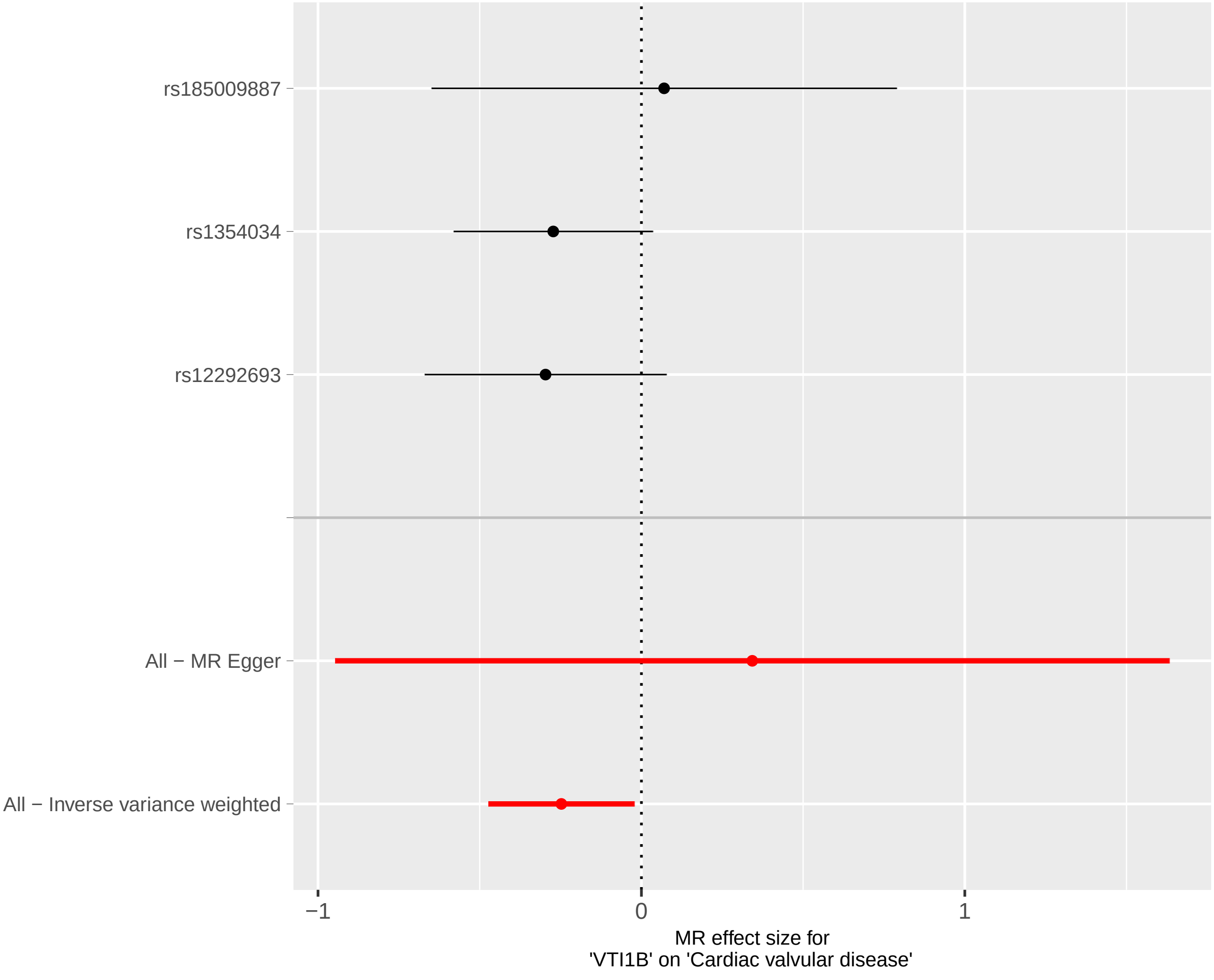
rs185009887
rs1354034
rs12292693
All − MR Egger
All − Inverse variance weighted
−1
0
1
MR effect size for
'VTI1B' on 'Cardiac valvular disease'

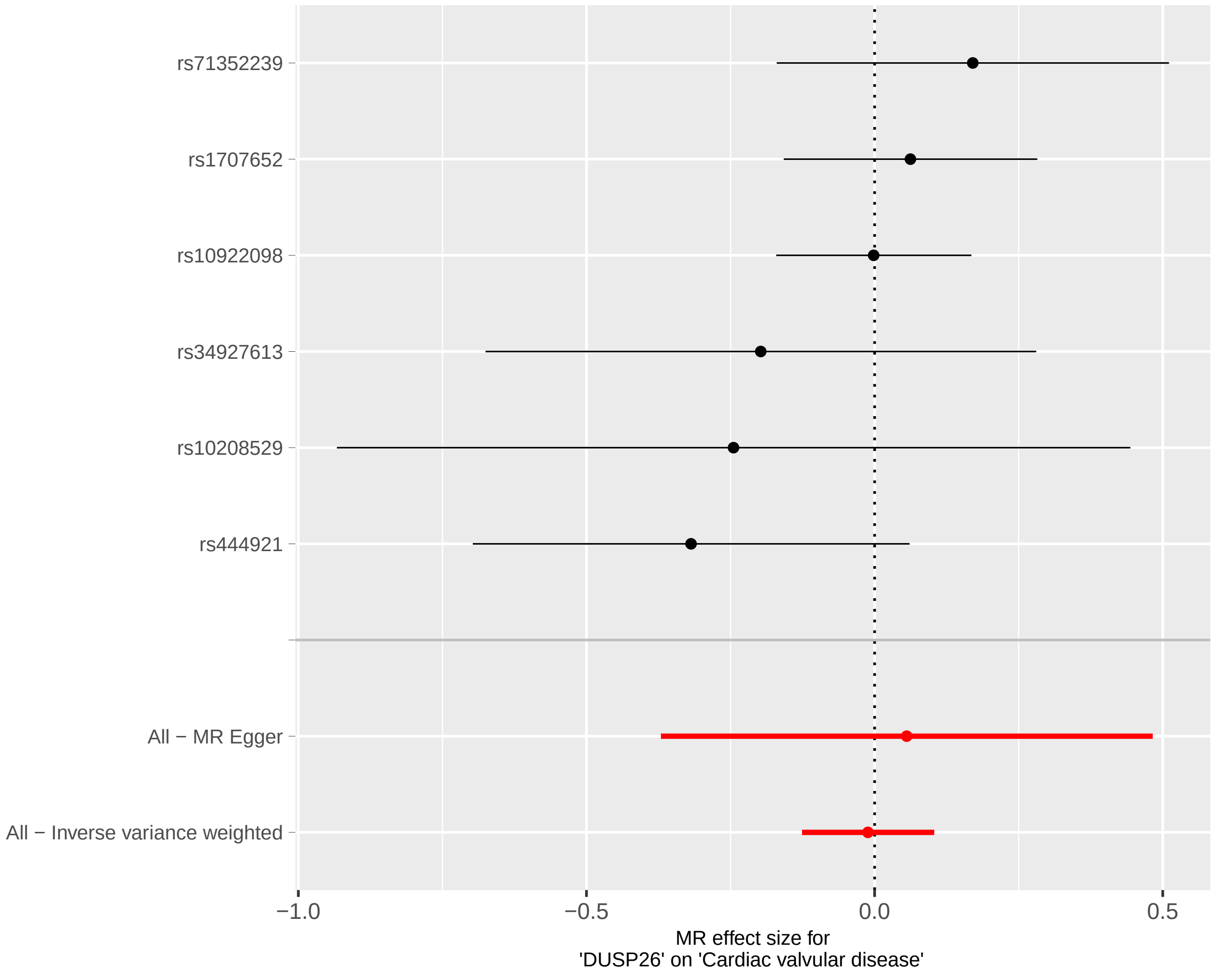
rs71352239
rs1707652
rs10922098
rs34927613
rs10208529
rs444921
All − MR Egger
All − Inverse variance weighted
−1.0
−0.5
0.0
0.5
MR effect size for
'DUSP26' on 'Cardiac valvular disease'

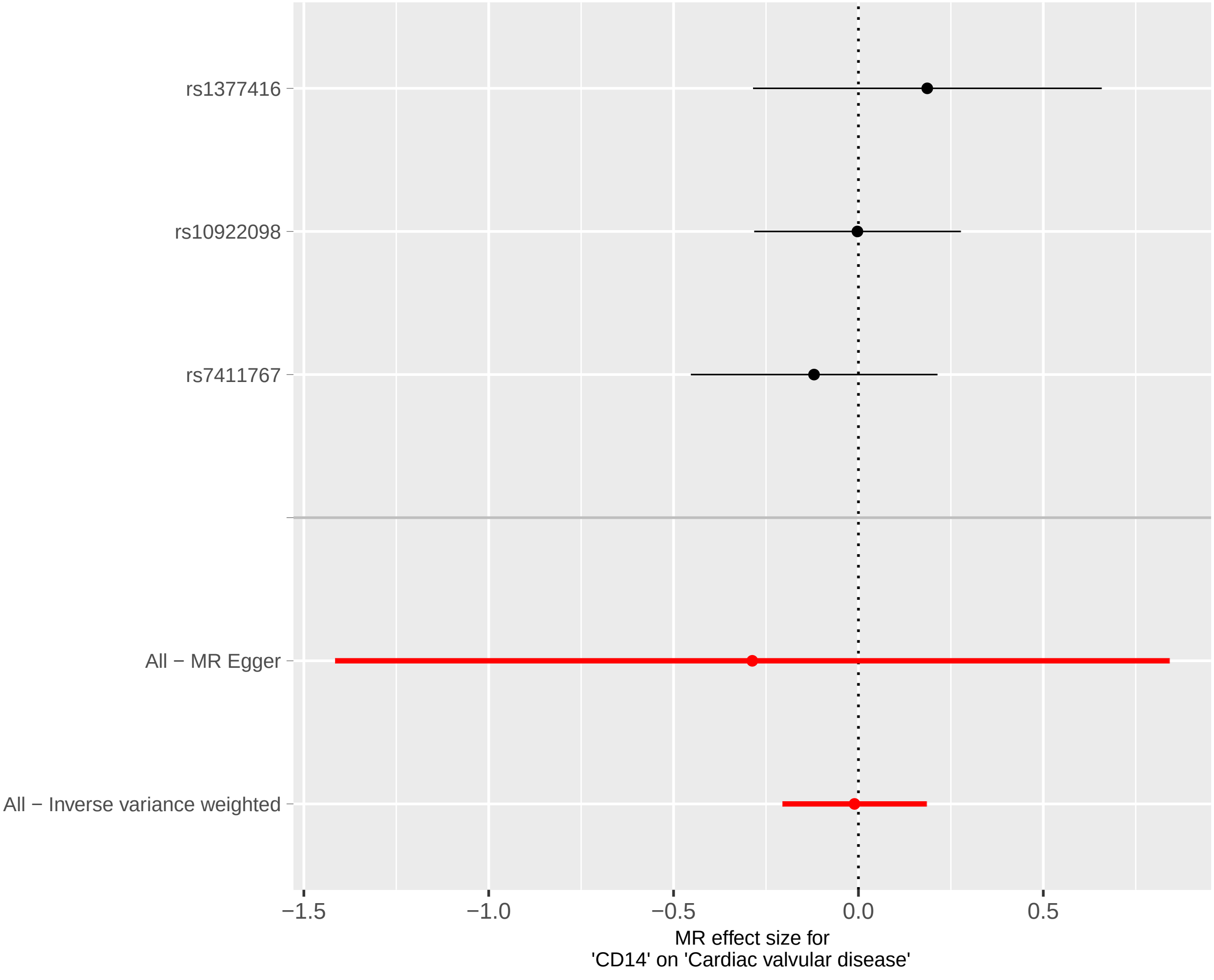
rs1377416
rs10922098
rs7411767
All − MR Egger
All − Inverse variance weighted
−1.5
−1.0
−0.5
0.0
0.5
MR effect size for
'CD14' on 'Cardiac valvular disease'

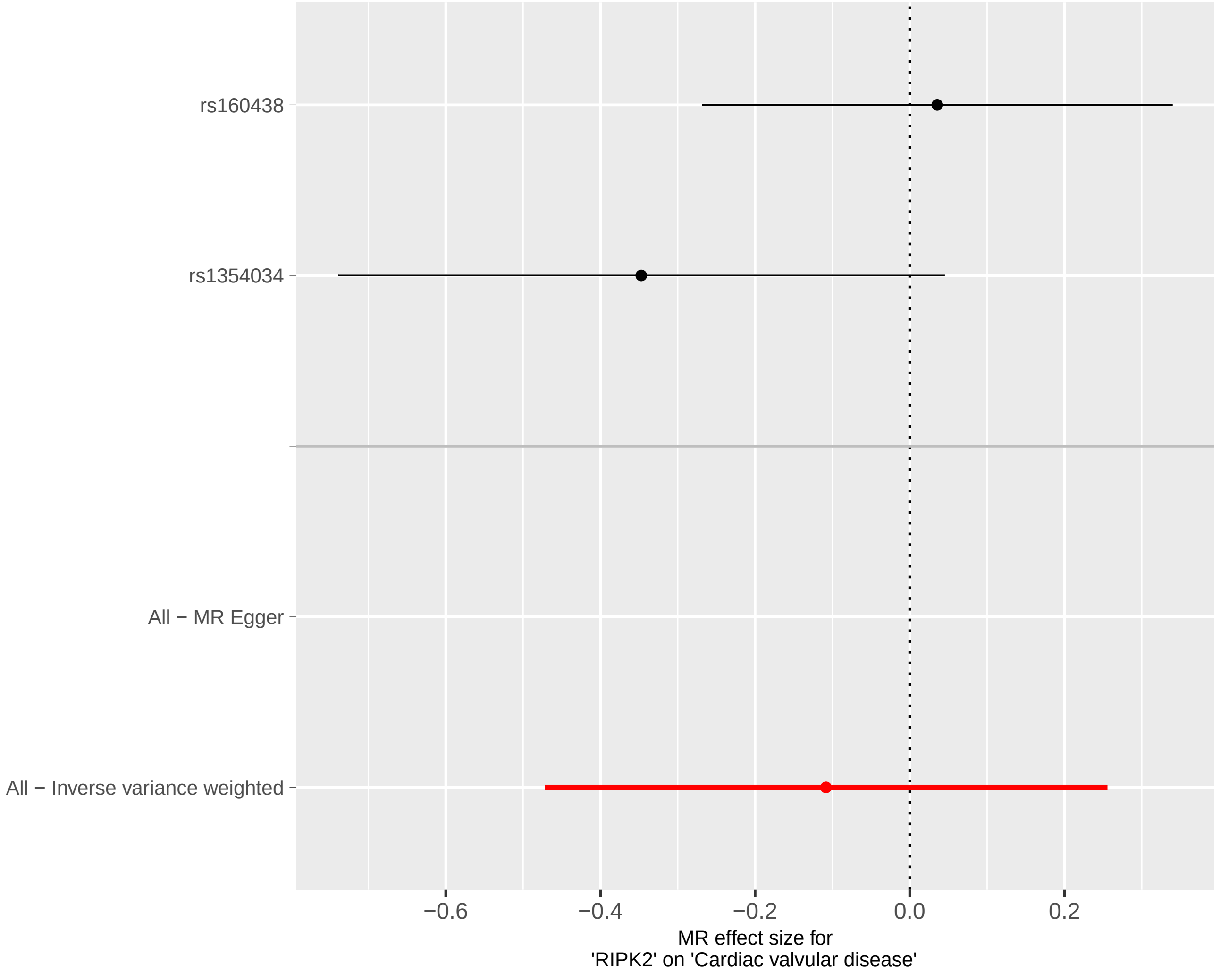
rs160438
rs1354034
All − MR Egger
All − Inverse variance weighted
−0.6
−0.4
−0.2
0.0
0.2
MR effect size for
'RIPK2' on 'Cardiac valvular disease'

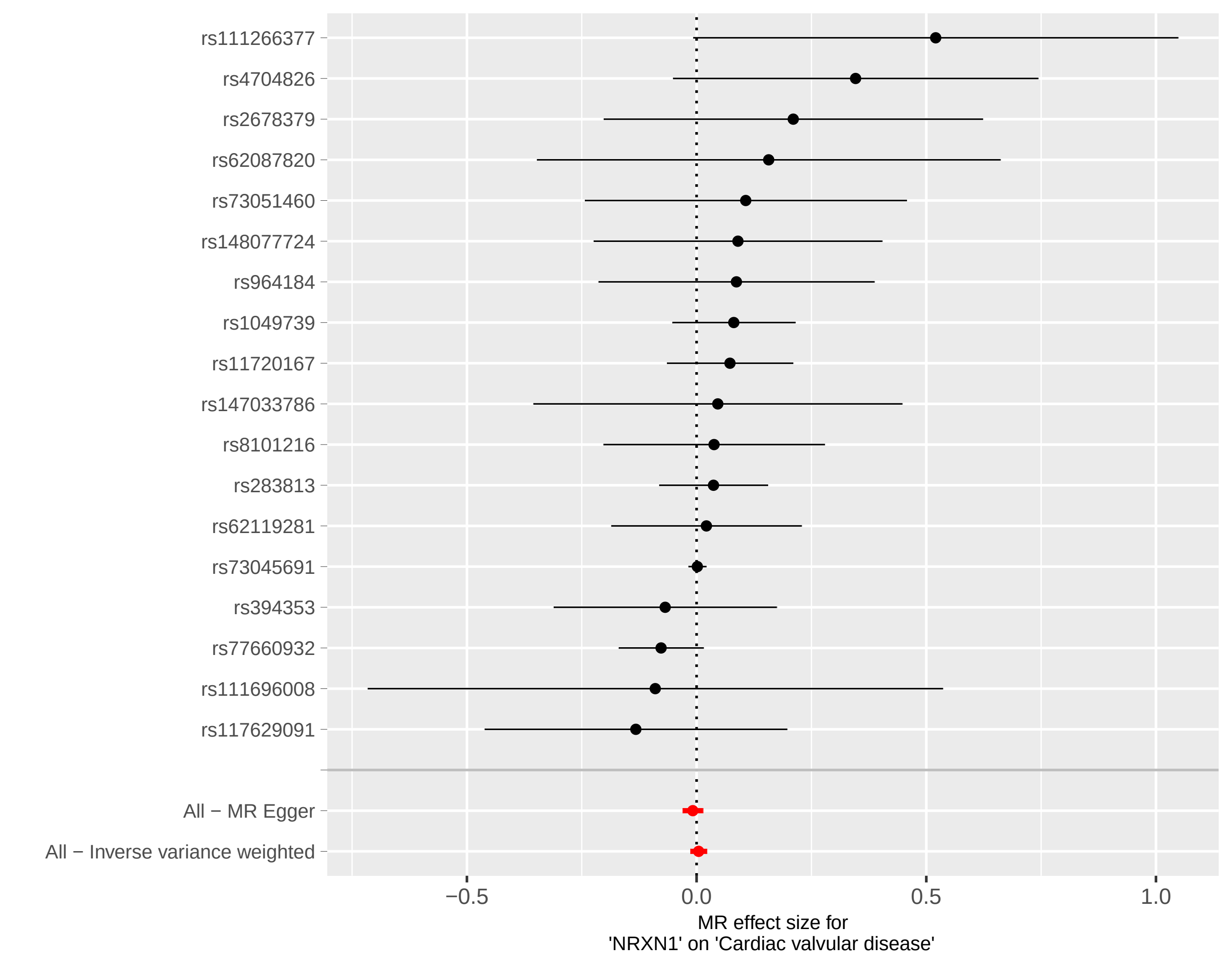

rs111266377
rs4704826
rs2678379
rs62087820
rs73051460
rs148077724
rs964184
rs1049739
rs11720167
rs147033786
rs8101216
rs283813
rs62119281
rs73045691
rs394353
rs77660932
rs111696008
rs117629091
All − MR Egger
All − Inverse variance weighted
−0.5
0.0
0.5
1.0
MR effect size for
'NRXN1' on 'Cardiac valvular disease'

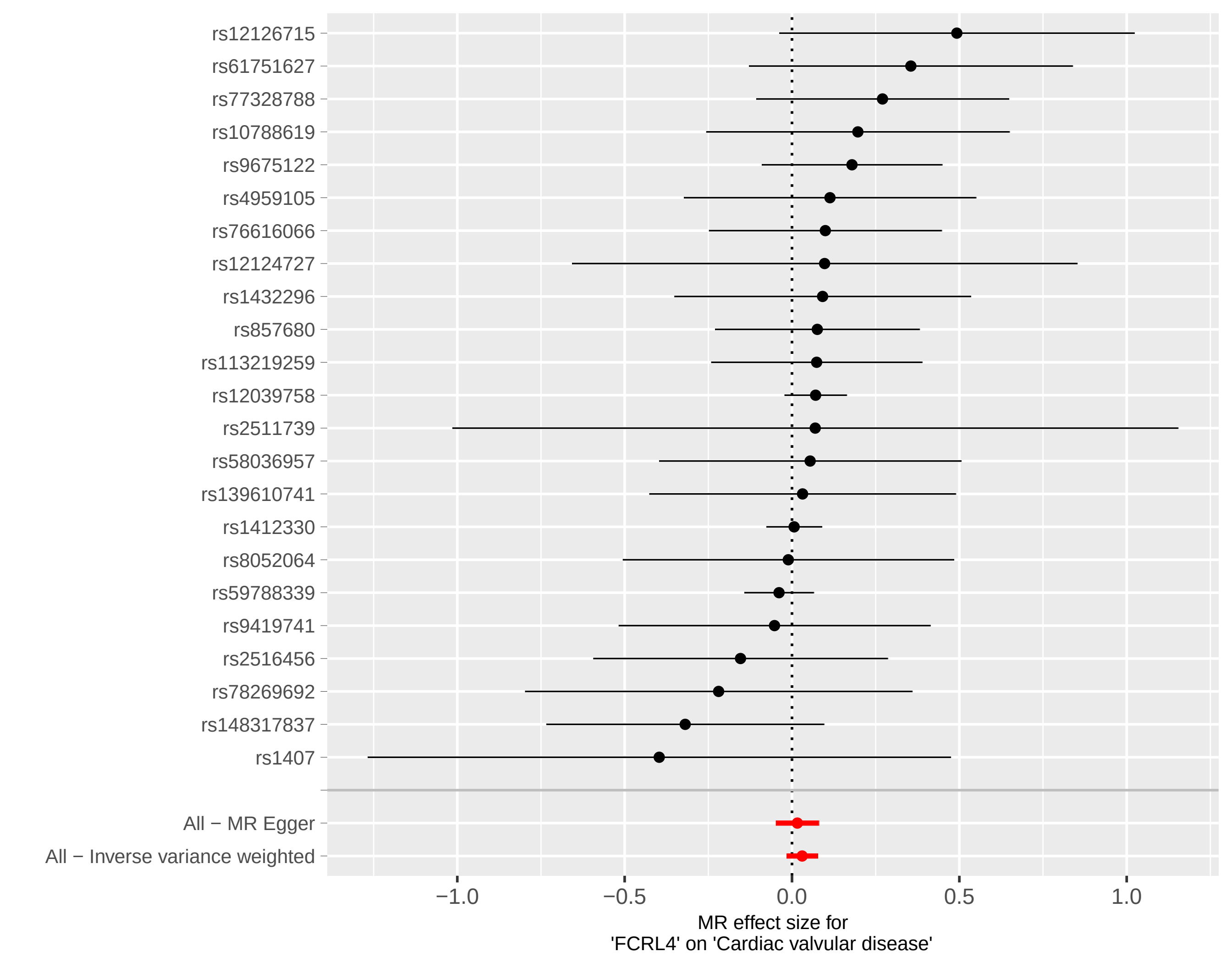
rs12126715
rs61751627
rs77328788
rs10788619
rs9675122
rs4959105
rs76616066
rs12124727
rs1432296
rs857680
rs113219259
rs12039758
rs2511739
rs58036957
rs139610741
rs1412330
rs8052064
rs59788339
rs9419741
rs2516456
rs78269692
rs148317837
rs1407
All − MR Egger
All − Inverse variance weighted
−1.0
−0.5
0.0
0.5
1.0
MR effect size for
'FCRL4' on 'Cardiac valvular disease'

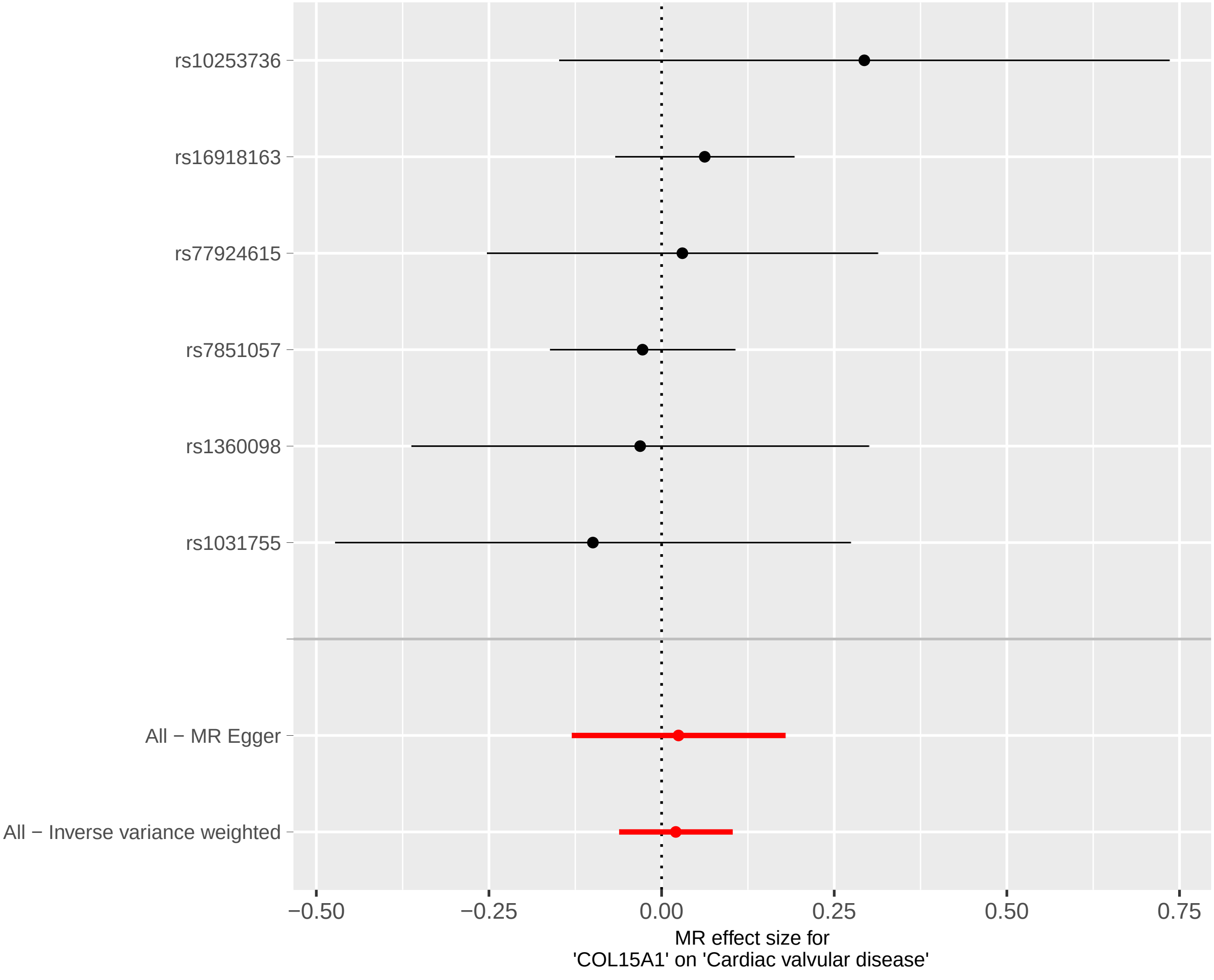
rs10253736
rs16918163
rs77924615
rs7851057
rs1360098
rs1031755
All − MR Egger
All − Inverse variance weighted
−0.50
−0.25
0.00
0.25
0.50
0.75
MR effect size for
'COL15A1' on 'Cardiac valvular disease'

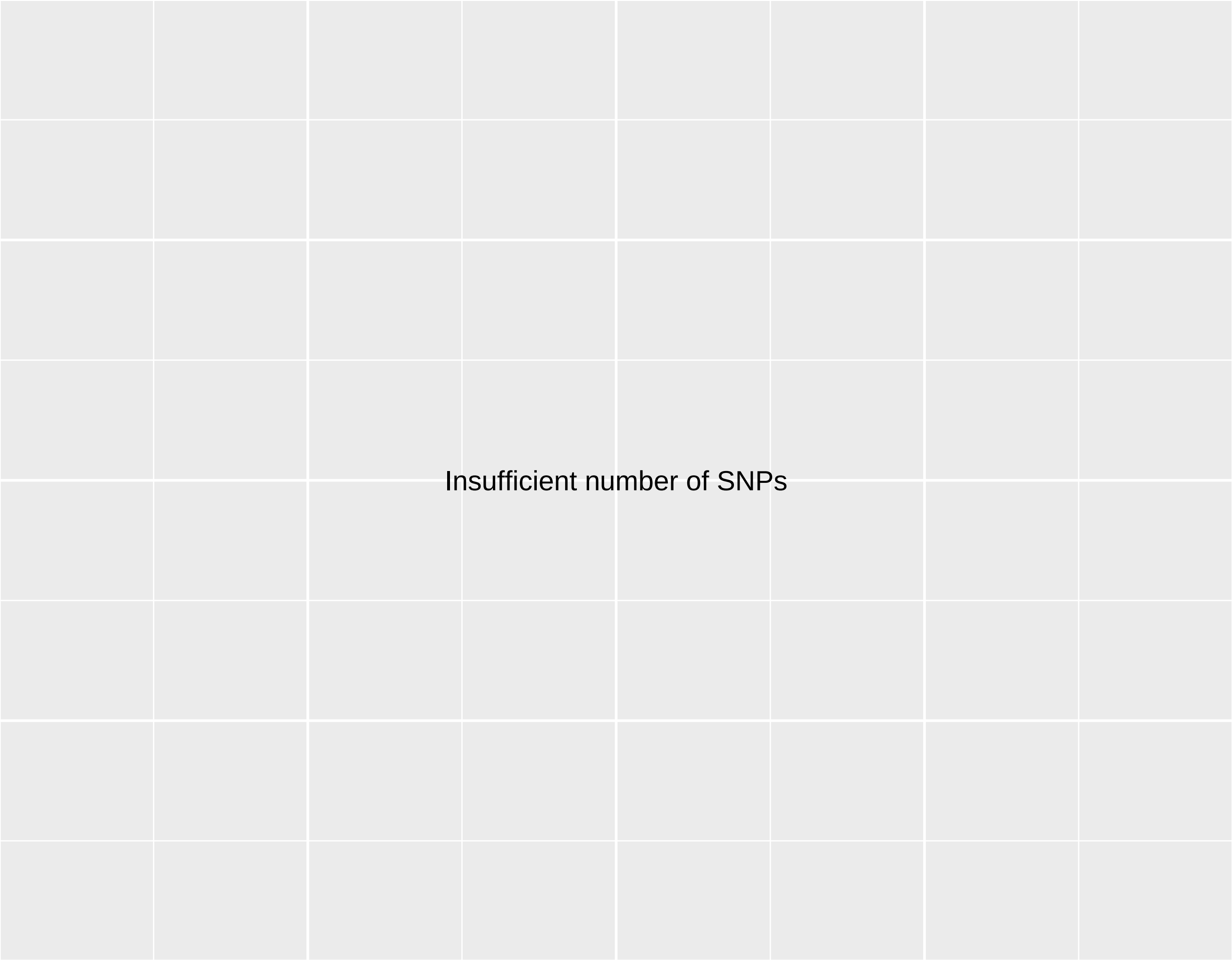
Insufficient number of SNPs

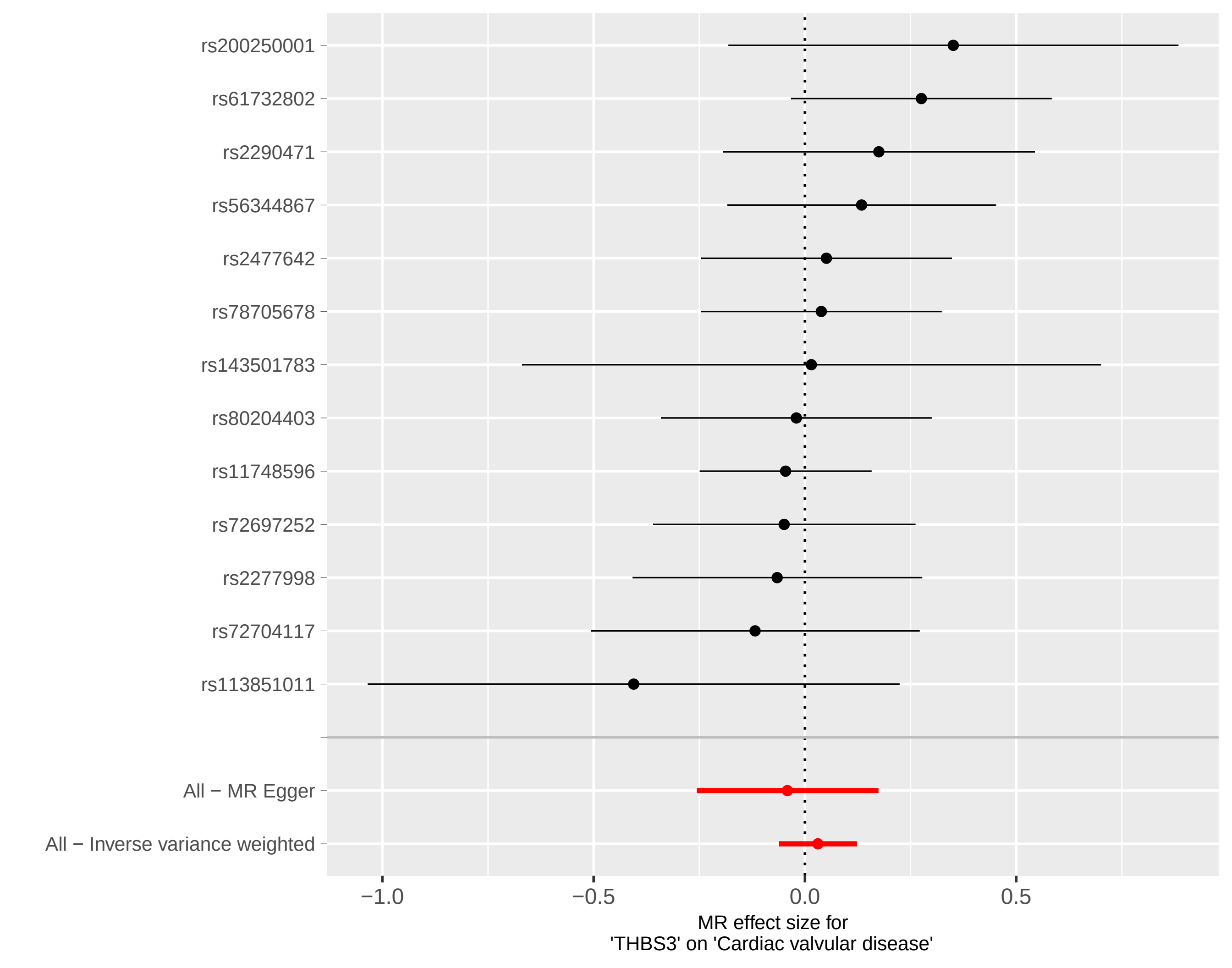
rs200250001
rs61732802
rs2290471
rs56344867
rs2477642
rs78705678
rs143501783
rs80204403
rs11748596
rs72697252
rs2277998
rs72704117
rs113851011
All − MR Egger
All − Inverse variance weighted
−1.0
−0.5
0.0
0.5
MR effect size for
'THBS3' on 'Cardiac valvular disease'

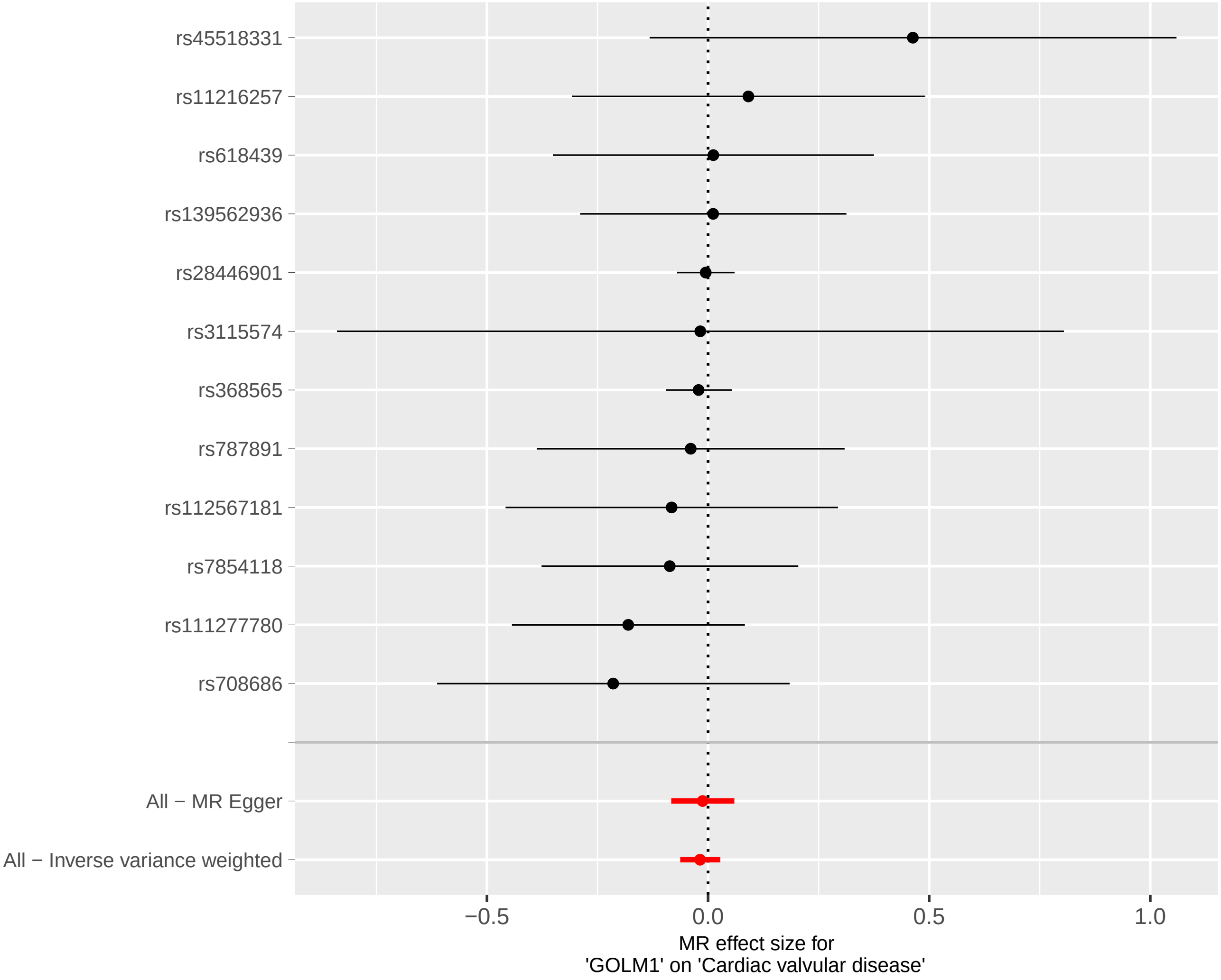
rs45518331
rs11216257
rs618439
rs139562936
rs28446901
rs3115574
rs368565
rs787891
rs112567181
rs7854118
rs111277780
rs708686
All − MR Egger
All − Inverse variance weighted
−0.5
0.0
0.5
1.0
MR effect size for
'GOLM1' on 'Cardiac valvular disease'

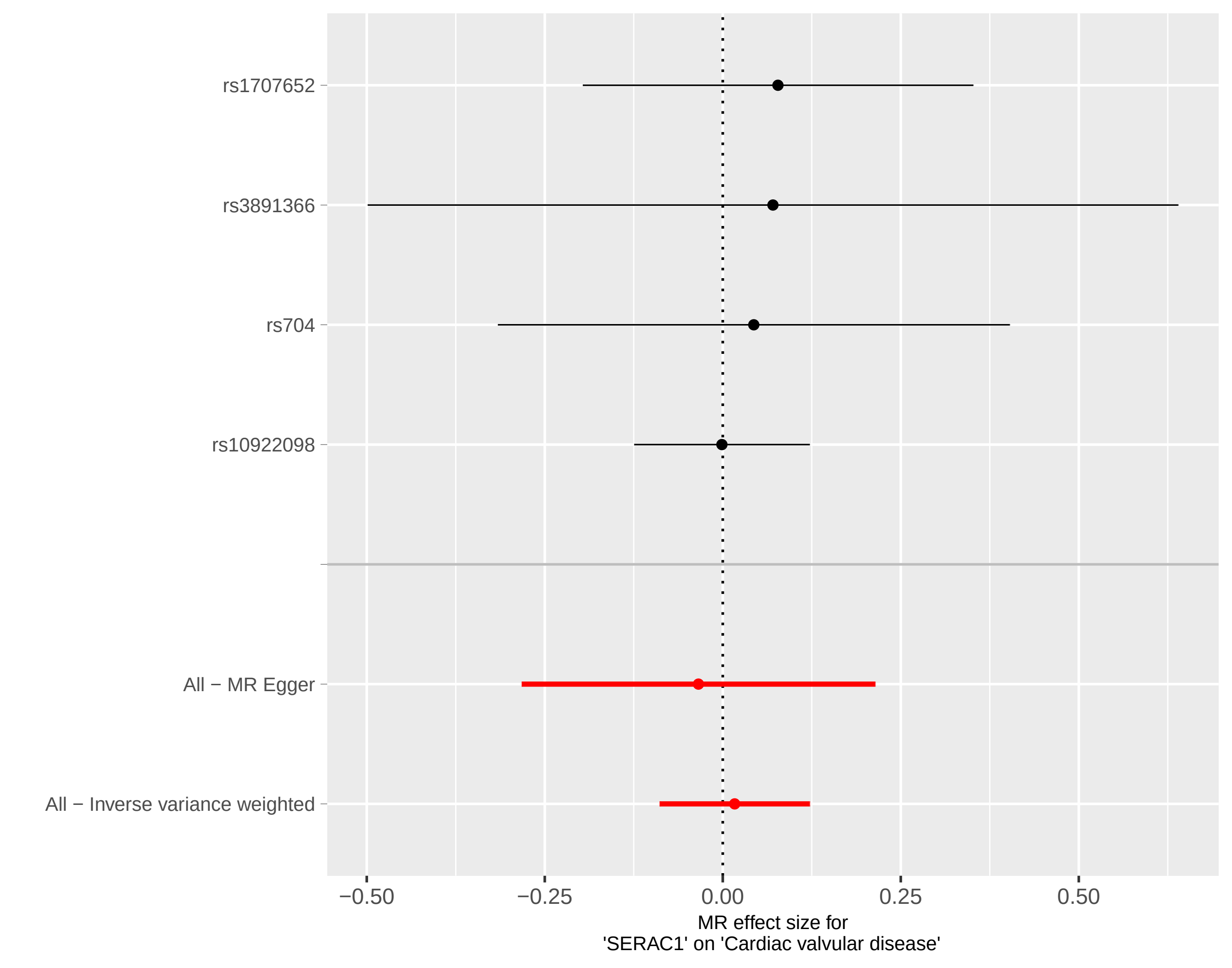
rs1707652
rs3891366
rs704
rs10922098
All − MR Egger
All − Inverse variance weighted
−0.50
−0.25
0.00
0.25
0.50
MR effect size for
'SERAC1' on 'Cardiac valvular disease'

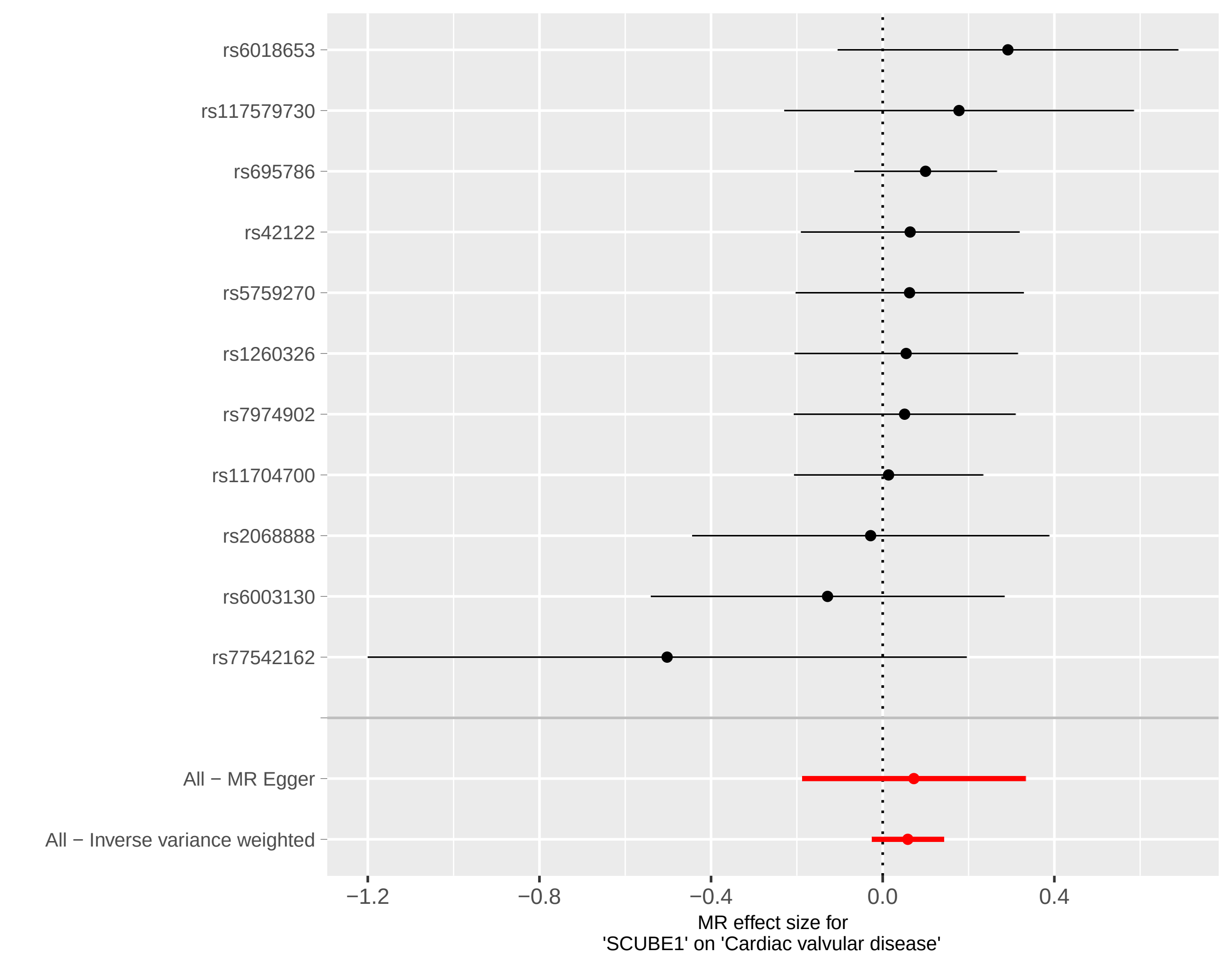

rs6018653
rs117579730
rs695786
rs42122
rs5759270
rs1260326
rs7974902
rs11704700
rs2068888
rs6003130
rs77542162
All − MR Egger
All − Inverse variance weighted
−1.2
−0.8
−0.4
0.0
0.4
MR effect size for
'SCUBE1' on 'Cardiac valvular disease'

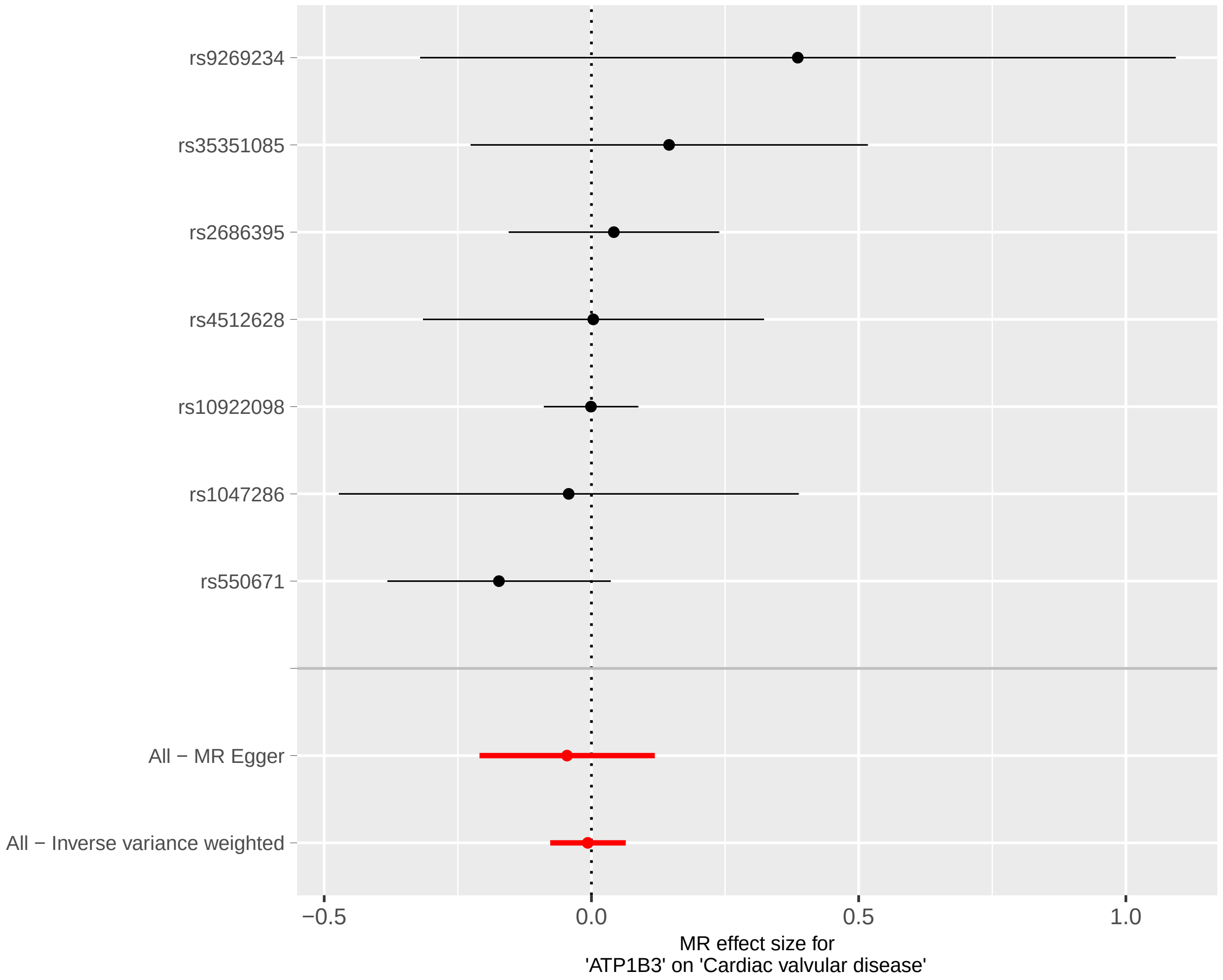

rs9269234
rs35351085
rs2686395
rs4512628
rs10922098
rs1047286
rs550671
All − MR Egger
All − Inverse variance weighted
−0.5
0.0
0.5
1.0
MR effect size for
'ATP1B3' on 'Cardiac valvular disease'

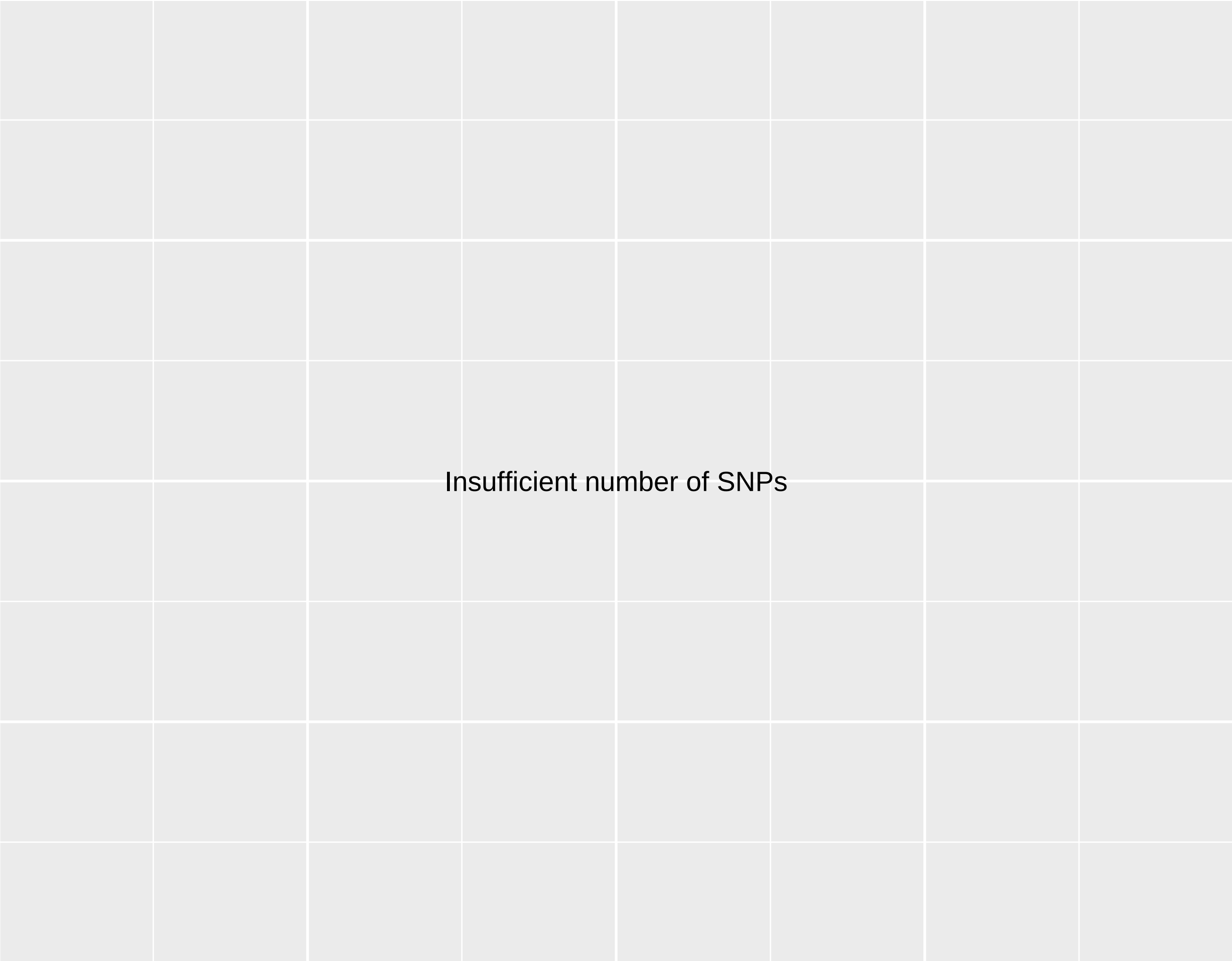
Insufficient number of SNPs

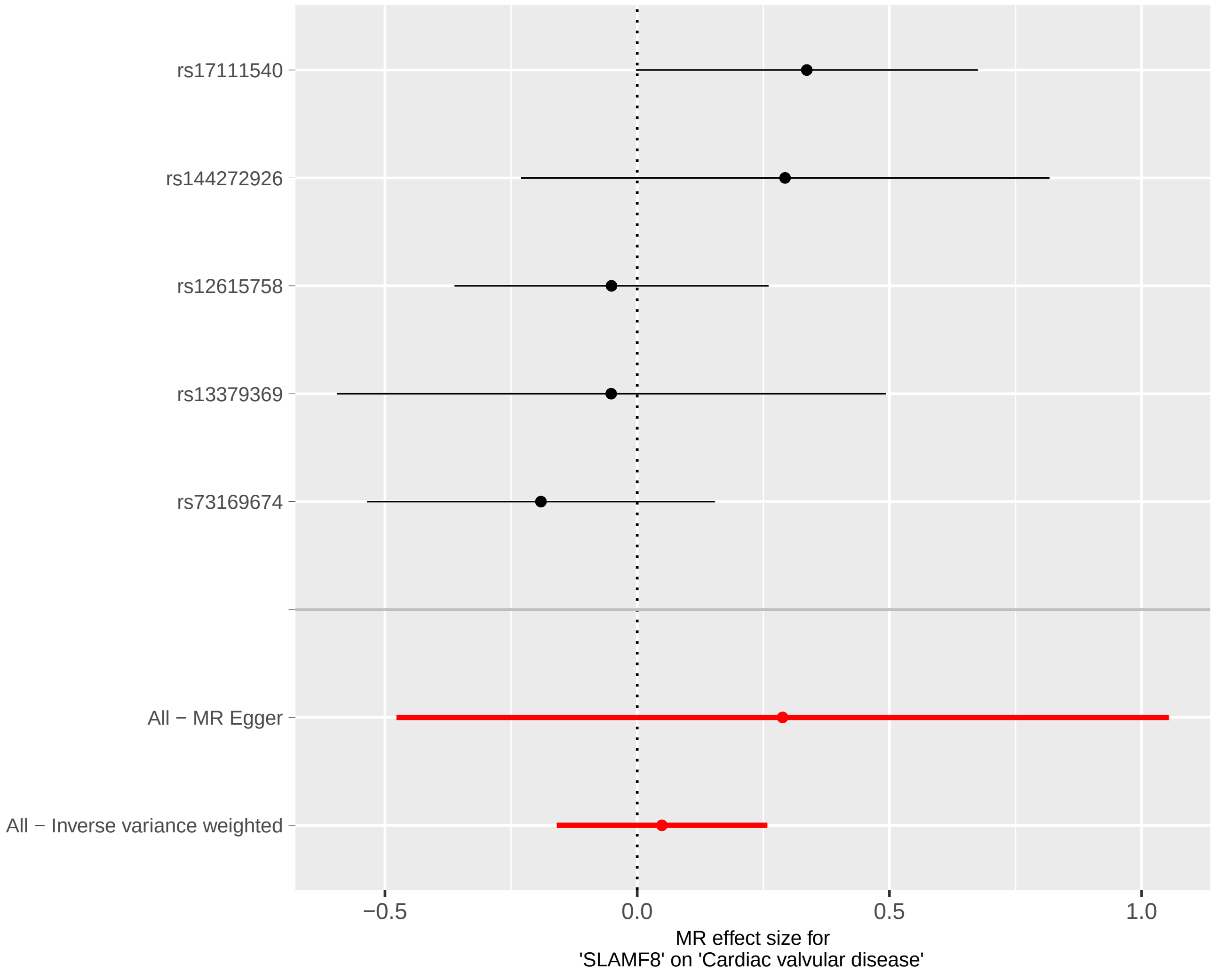
rs17111540
rs144272926
rs12615758
rs13379369
rs73169674
All − MR Egger
All − Inverse variance weighted
−0.5
0.0
0.5
1.0
MR effect size for
'SLAMF8' on 'Cardiac valvular disease'

Insufficient number of SNPs

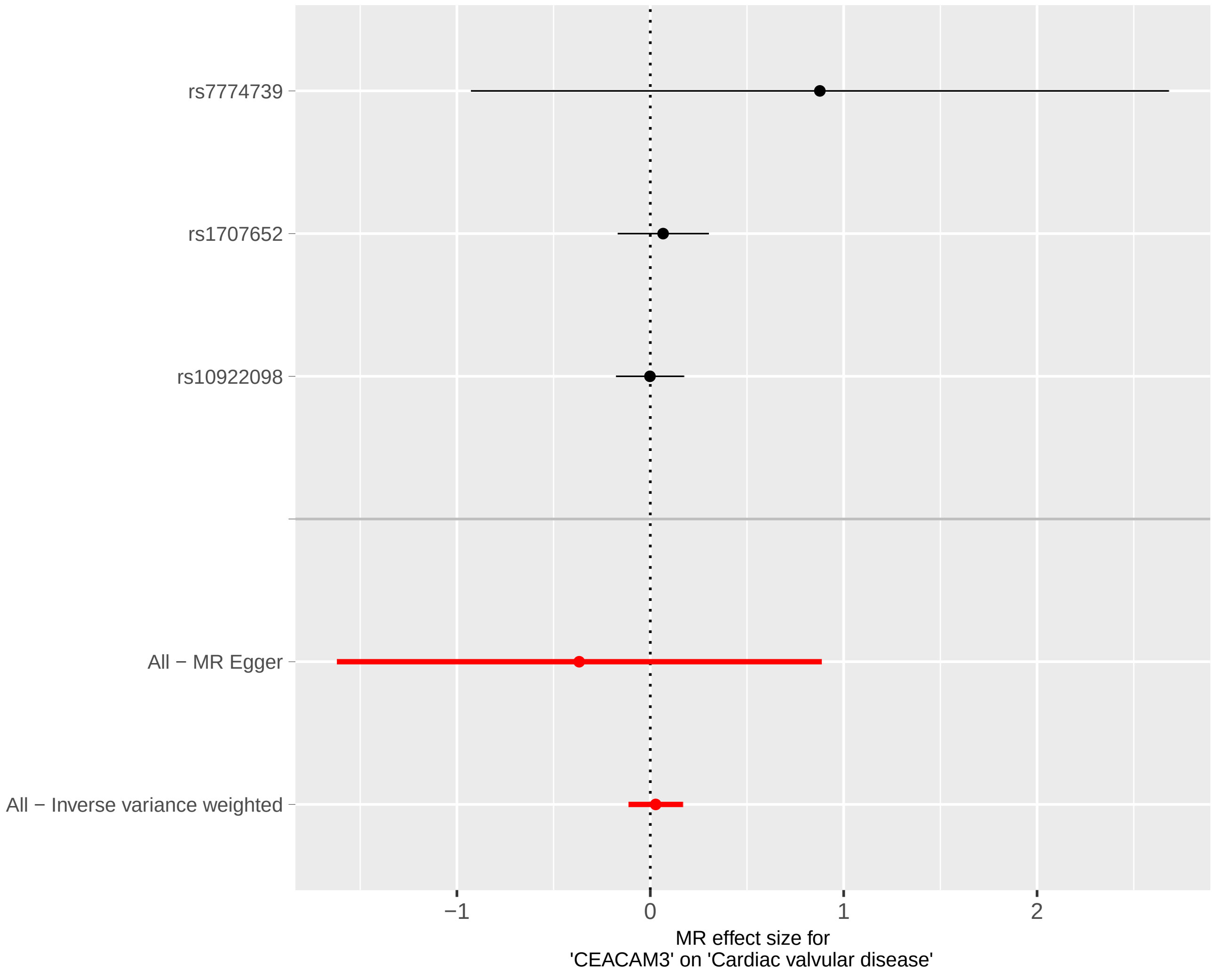
rs7774739
rs1707652
rs10922098
All − MR Egger
All − Inverse variance weighted
−1
0
1
2
MR effect size for
'CEACAM3' on 'Cardiac valvular disease'

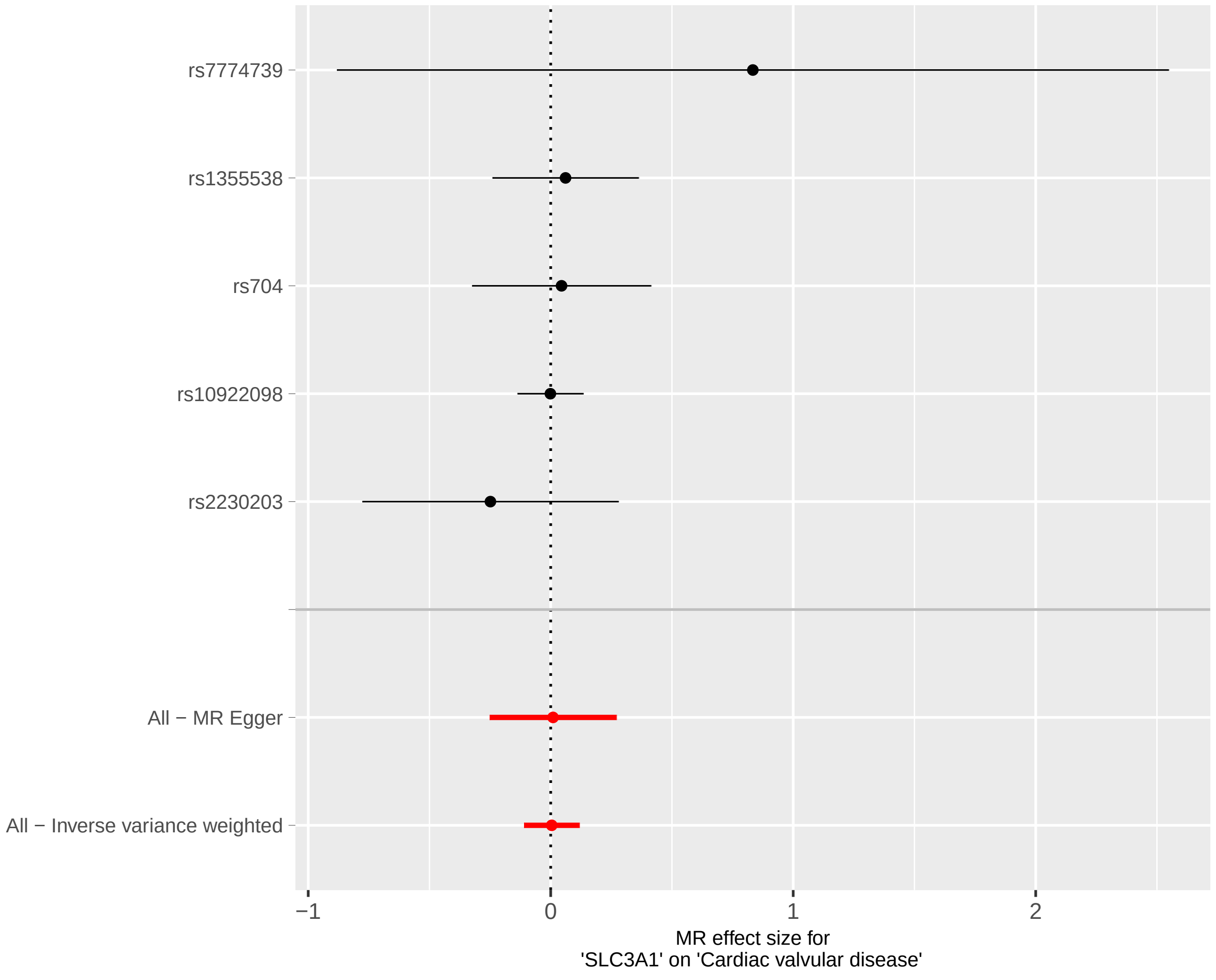
rs7774739
rs1355538
rs704
rs10922098
rs2230203
All − MR Egger
All − Inverse variance weighted
−1
0
1
2
MR effect size for
'SLC3A1' on 'Cardiac valvular disease'

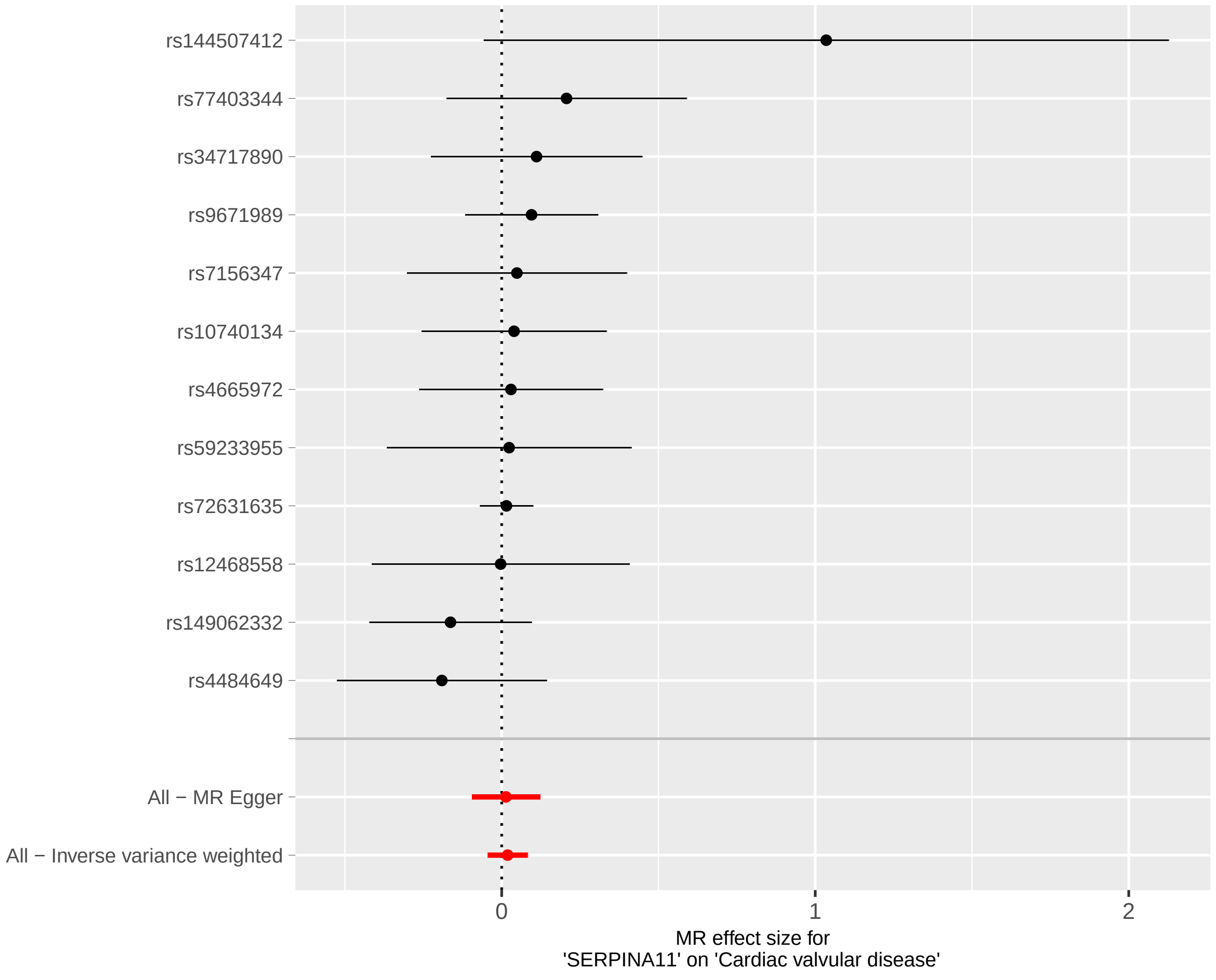
rs144507412
rs77403344
rs34717890
rs9671989
rs7156347
rs10740134
rs4665972
rs59233955
rs72631635
rs12468558
rs149062332
rs4484649
All − MR Egger
All − Inverse variance weighted
0
1
2
MR effect size for
'SERPINA11' on 'Cardiac valvular disease'

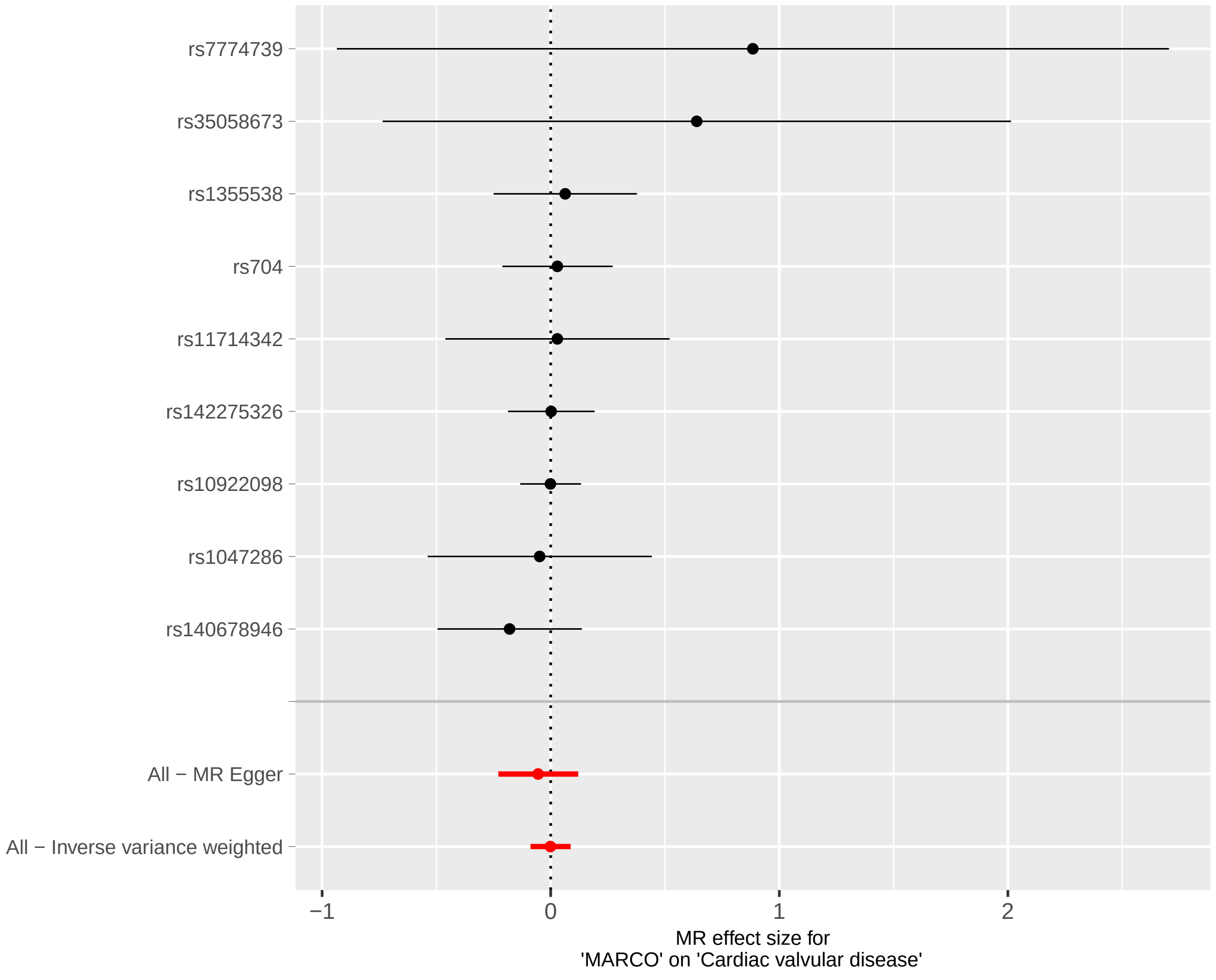
rs7774739
rs35058673
rs1355538
rs704
rs11714342
rs142275326
rs10922098
rs1047286
rs140678946
All − MR Egger
All − Inverse variance weighted
−1
0
1
2
MR effect size for
'MARCO' on 'Cardiac valvular disease'

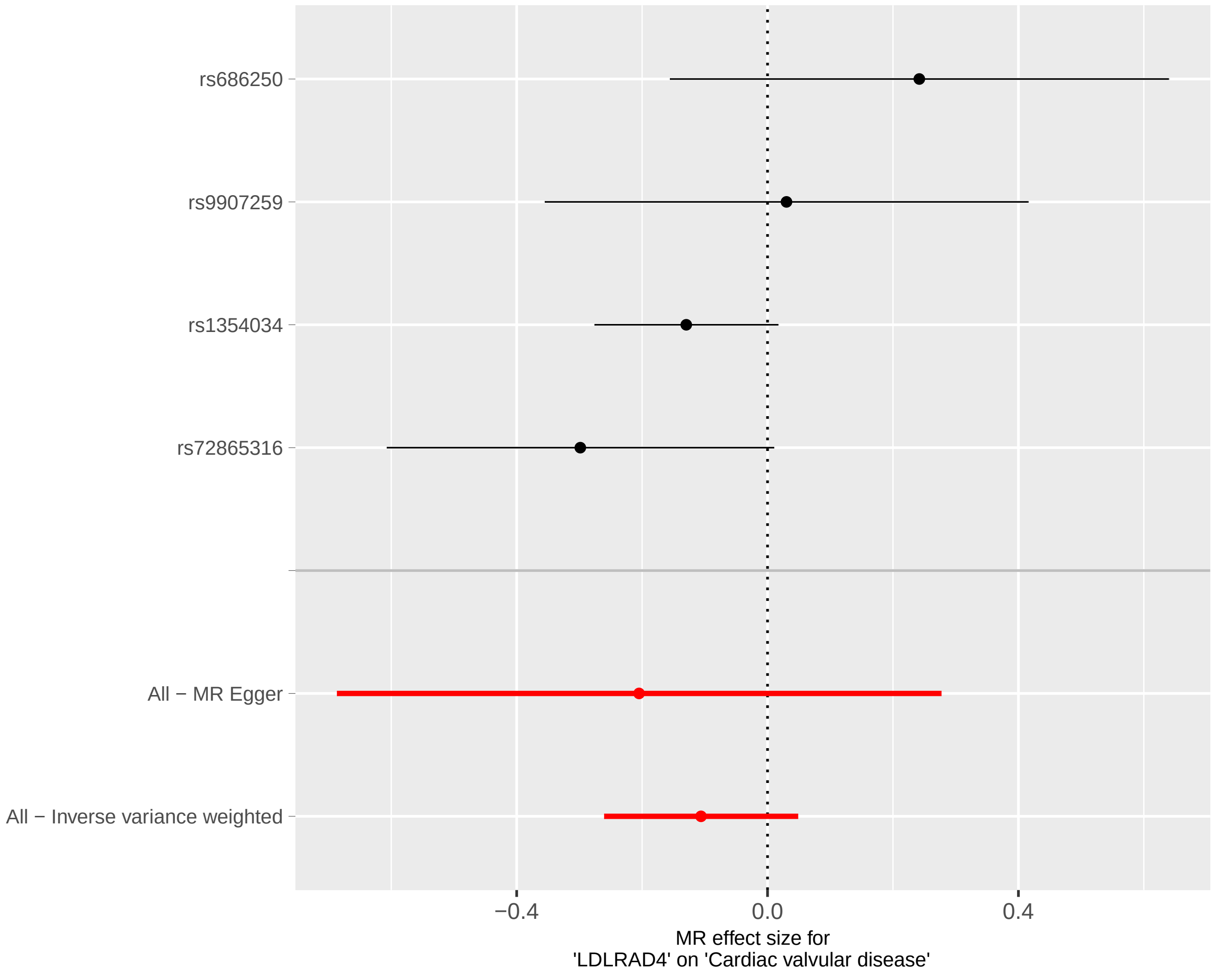
rs686250
rs9907259
rs1354034
rs72865316
All − MR Egger
All − Inverse variance weighted
−0.4
0.0
0.4
MR effect size for
'LDLRAD4' on 'Cardiac valvular disease'

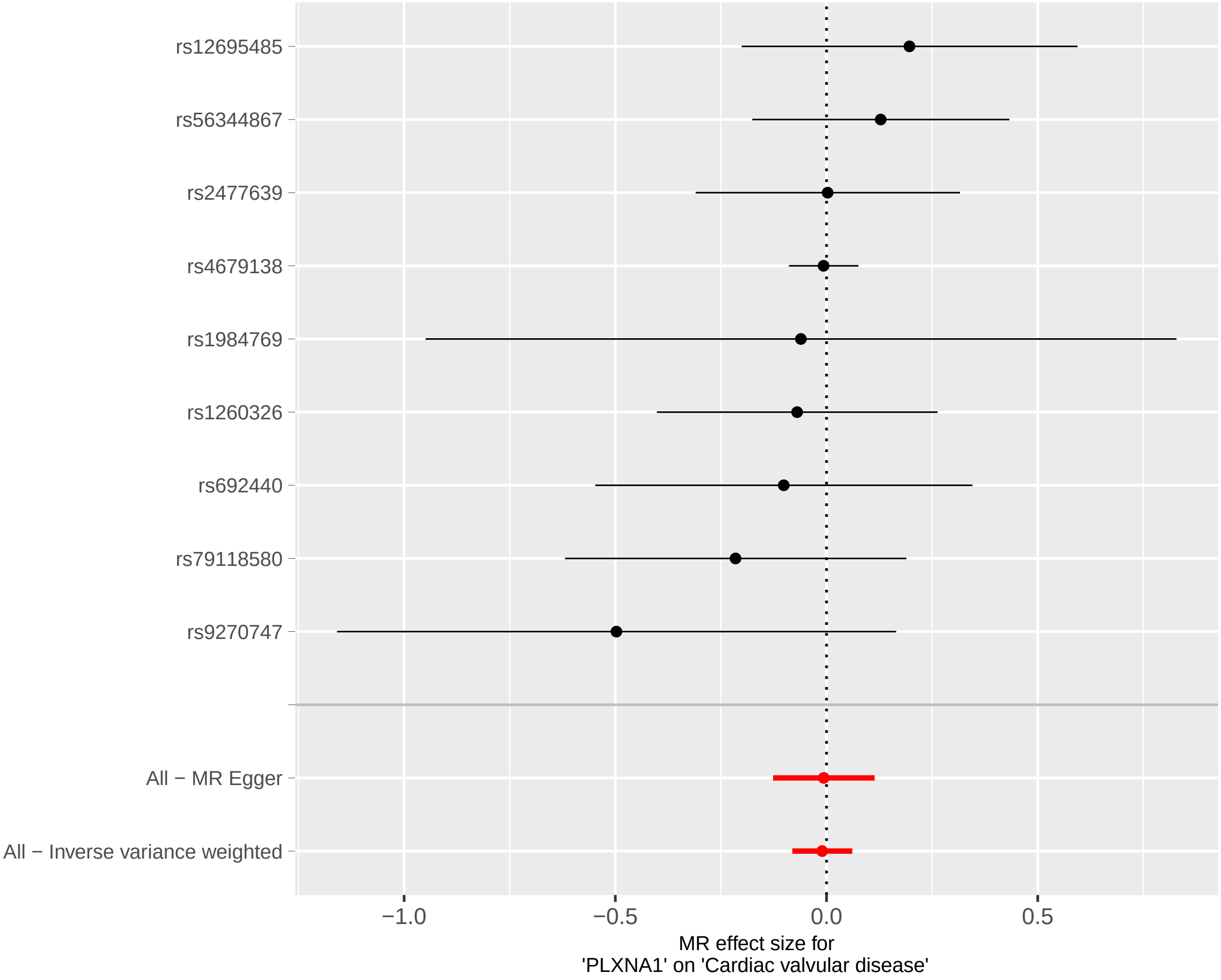
rs12695485
rs56344867
rs2477639
rs4679138
rs1984769
rs1260326
rs692440
rs79118580
rs9270747
All − MR Egger
All − Inverse variance weighted
−1.0
−0.5
0.0
0.5
MR effect size for
'PLXNA1' on 'Cardiac valvular disease'

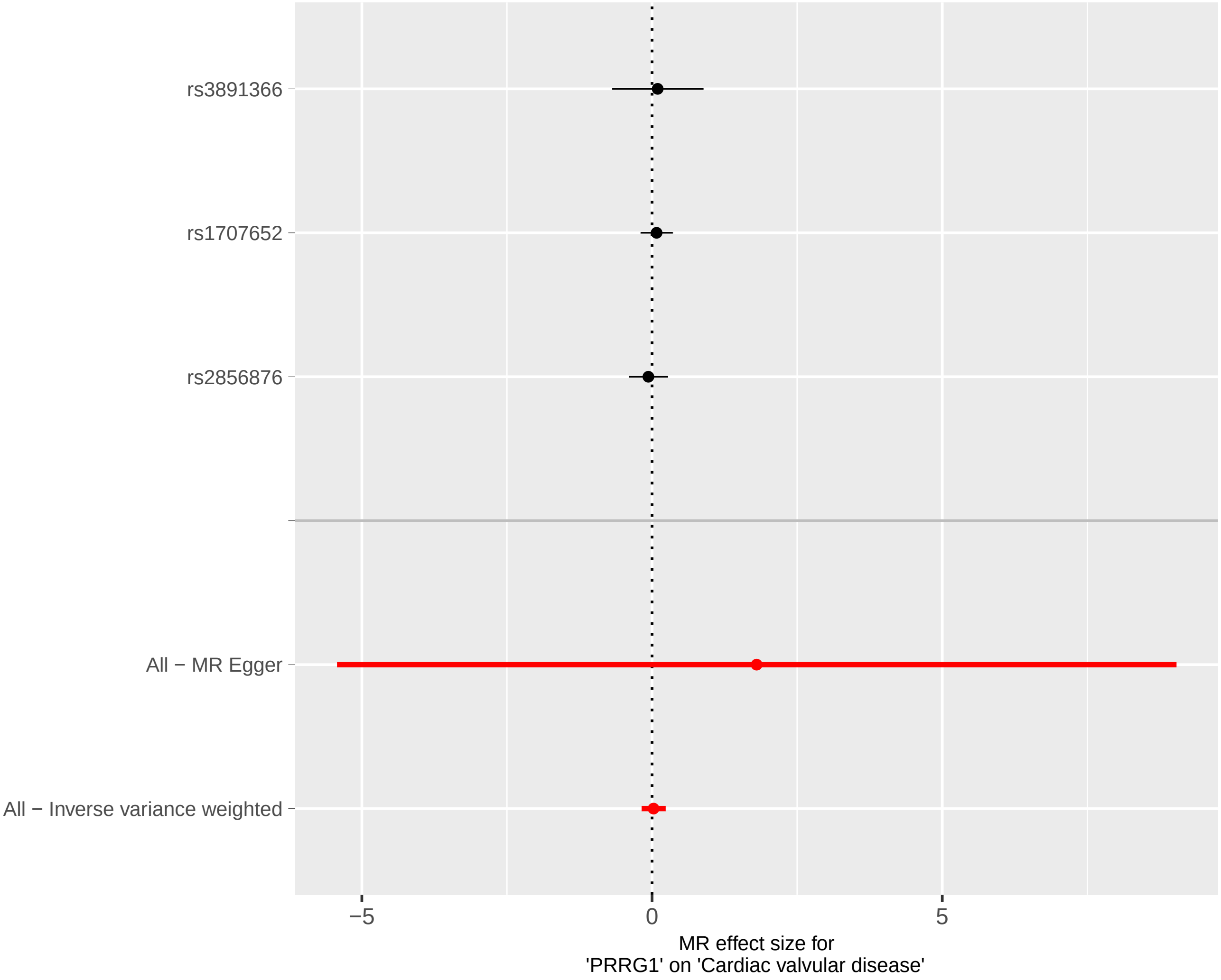
rs3891366
rs1707652
rs2856876
All − MR Egger
All − Inverse variance weighted
−5
0
5
MR effect size for
'PRRG1' on 'Cardiac valvular disease'

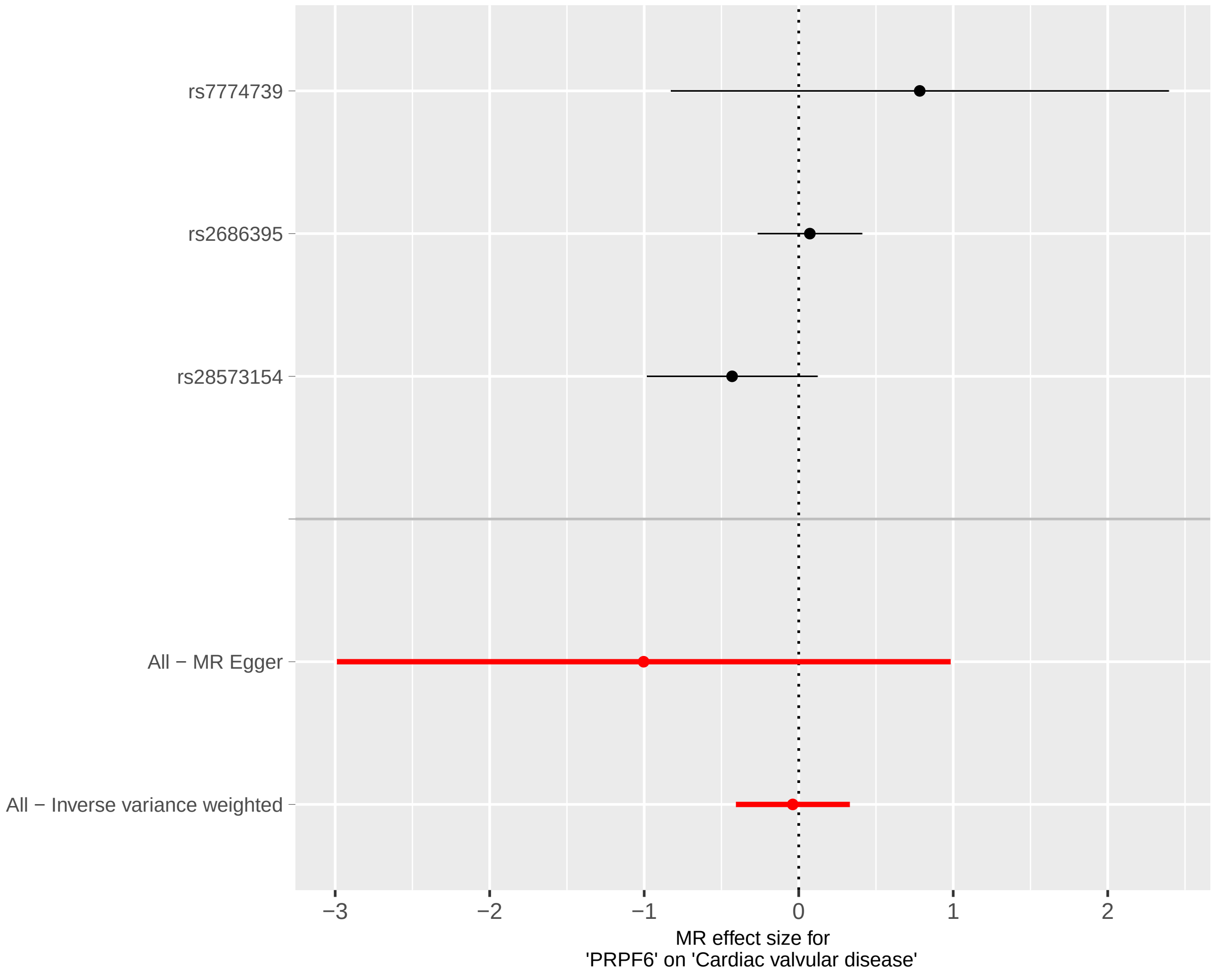
rs7774739
rs2686395
rs28573154
All − MR Egger
All − Inverse variance weighted
−3
−2
−1
0
1
2
MR effect size for
'PRPF6' on 'Cardiac valvular disease'

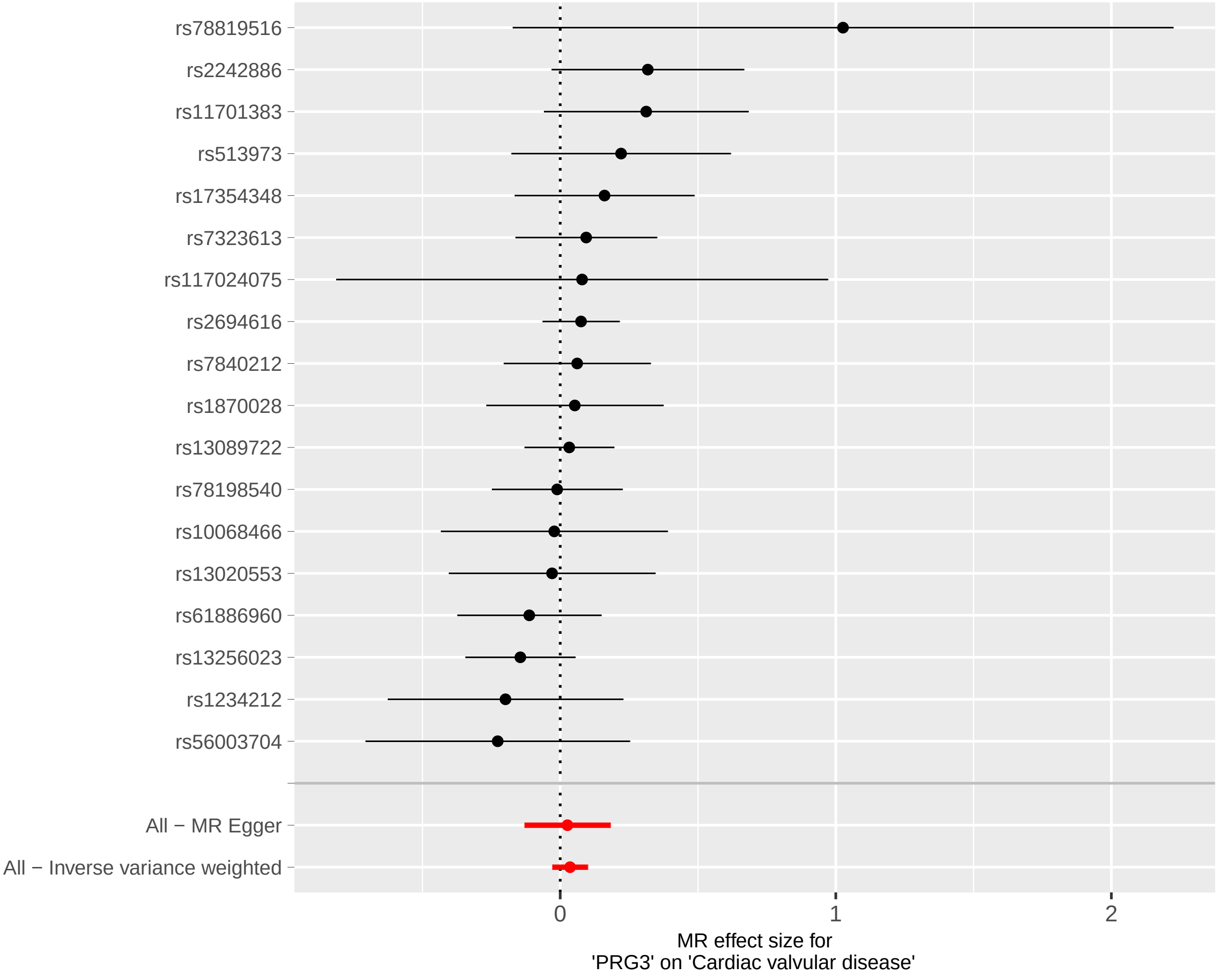
rs78819516
rs2242886
rs11701383
rs513973
rs17354348
rs7323613
rs117024075
rs2694616
rs7840212
rs1870028
rs13089722
rs78198540
rs10068466
rs13020553
rs61886960
rs13256023
rs1234212
rs56003704
All − MR Egger
All − Inverse variance weighted
0
1
2
MR effect size for
'PRG3' on 'Cardiac valvular disease'

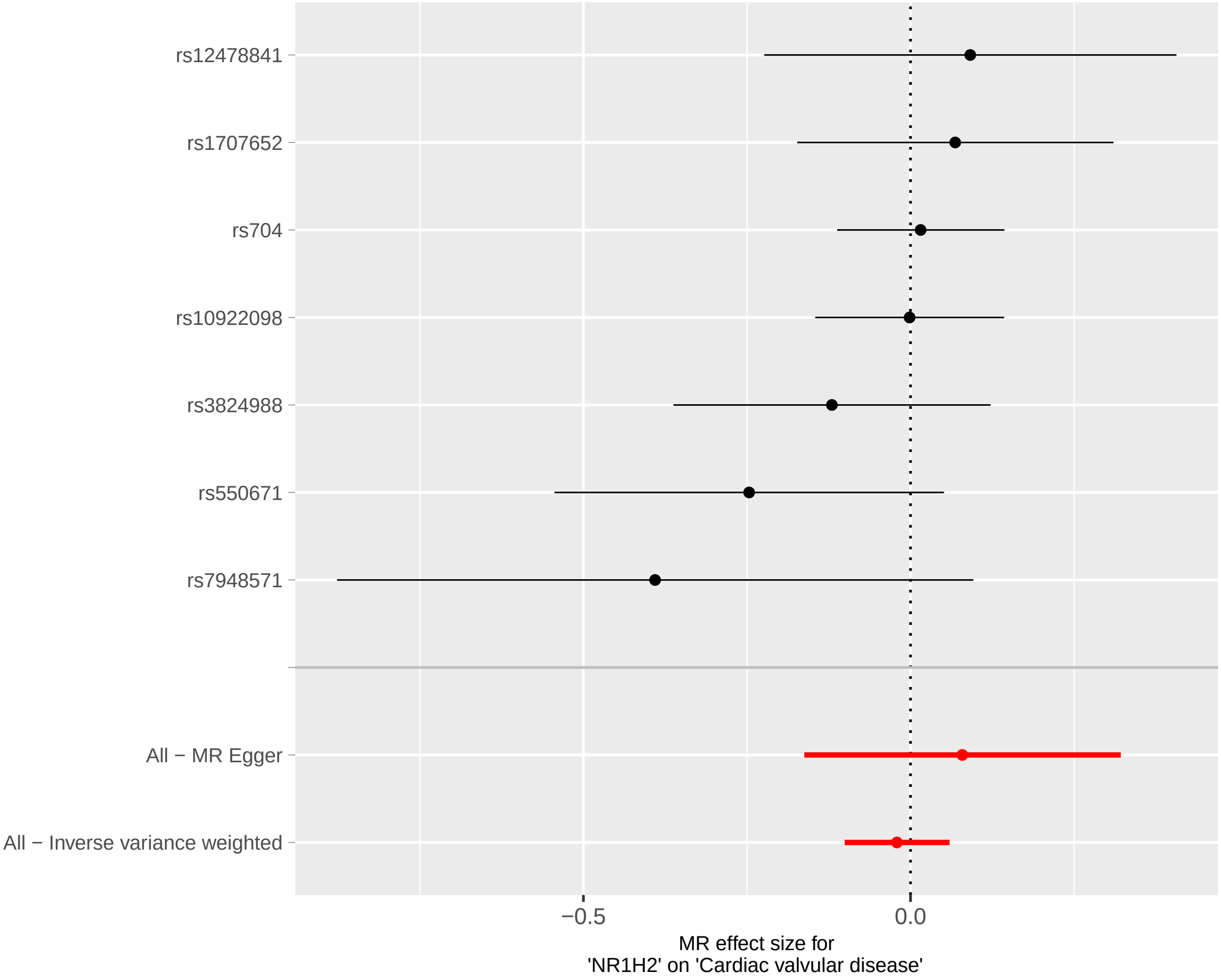
rs12478841
rs1707652
rs704
rs10922098
rs3824988
rs550671
rs7948571
All − MR Egger
All − Inverse variance weighted
−0.5
0.0
MR effect size for
'NR1H2' on 'Cardiac valvular disease'

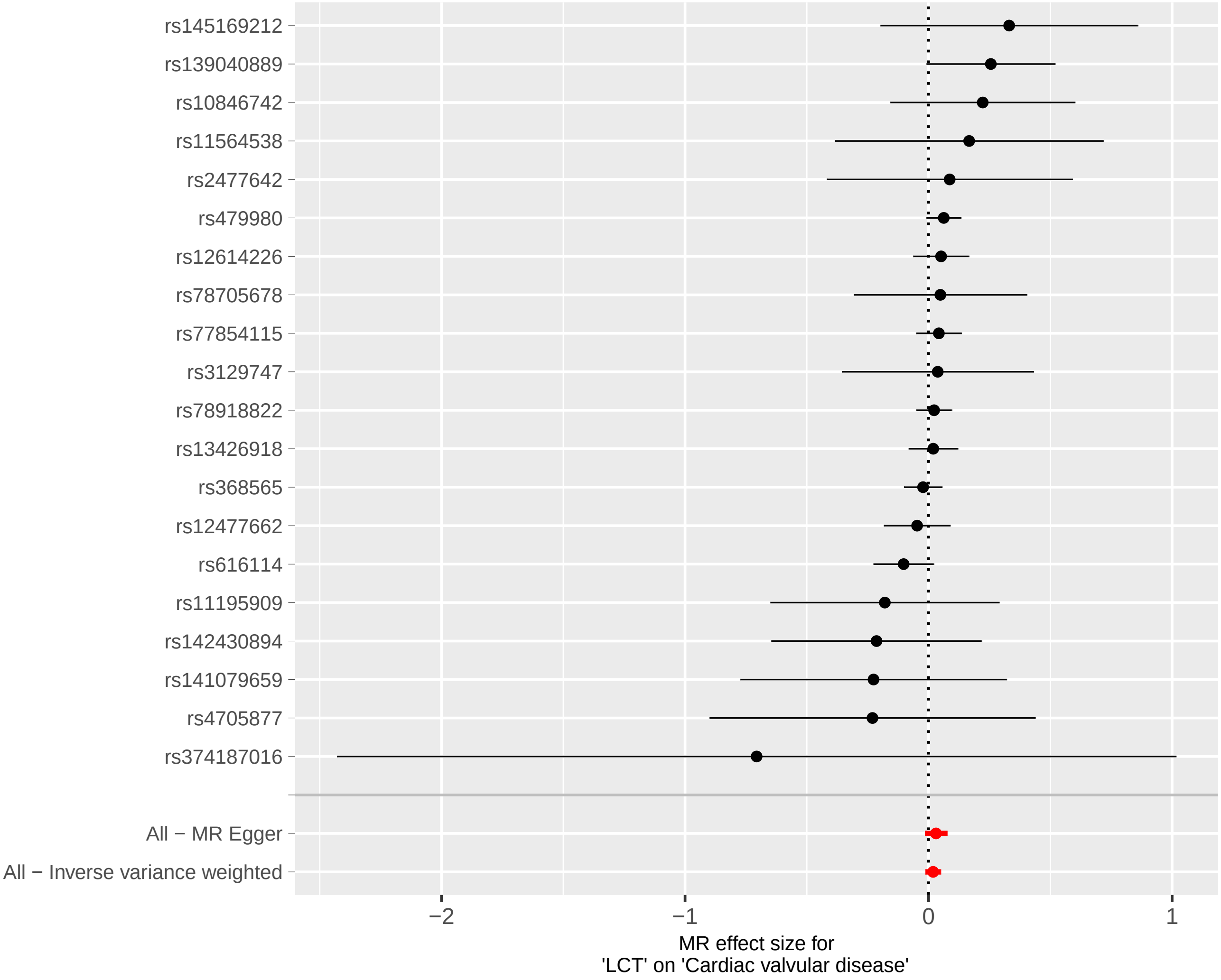
rs145169212
rs139040889
rs10846742
rs11564538
rs2477642
rs479980
rs12614226
rs78705678
rs77854115
rs3129747
rs78918822
rs13426918
rs368565
rs12477662
rs616114
rs11195909
rs142430894
rs141079659
rs4705877
rs374187016
All − MR Egger
All − Inverse variance weighted
−2
−1
0
1
MR effect size for
'LCT' on 'Cardiac valvular disease'

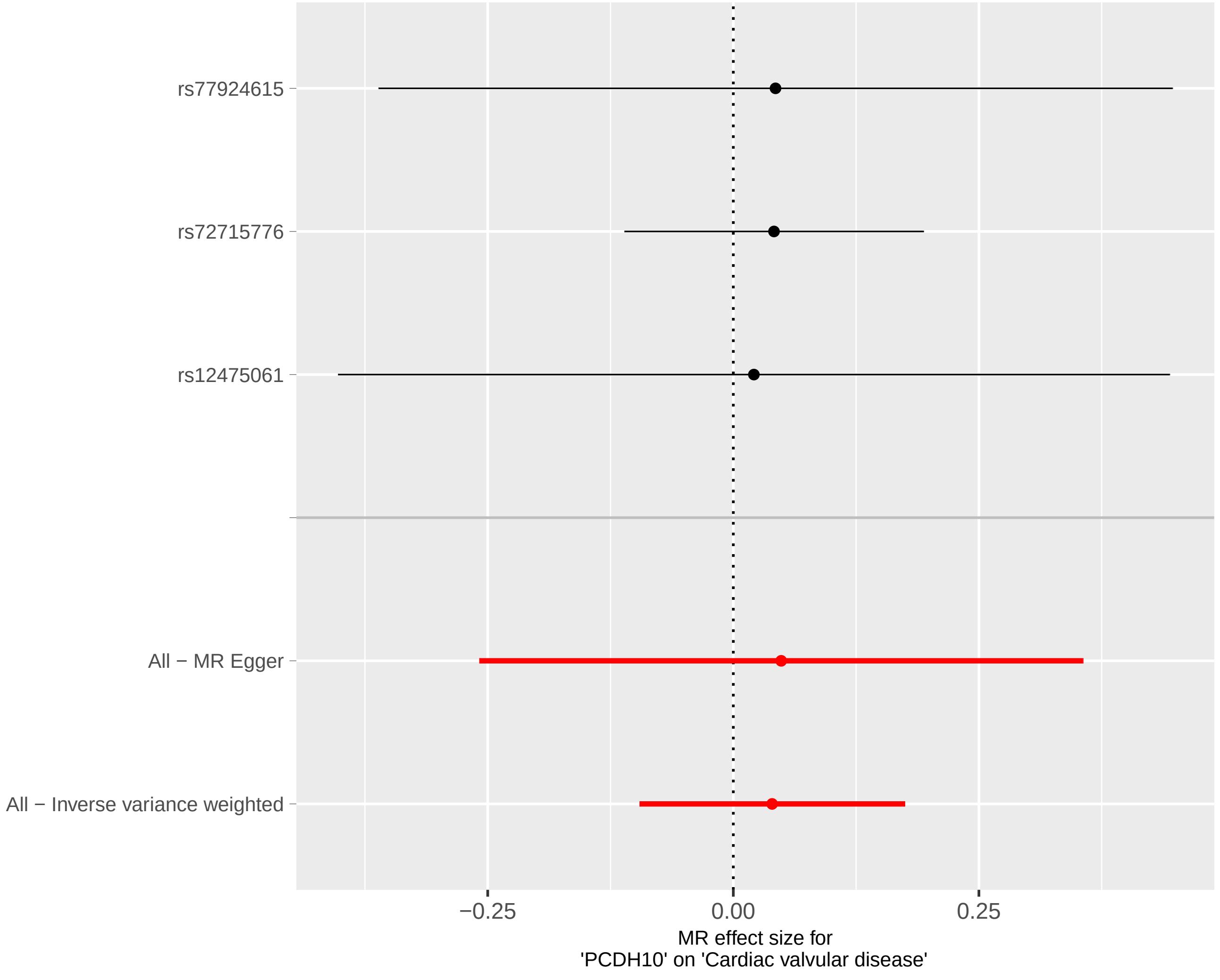

rs77924615
rs72715776
rs12475061
All − MR Egger
All − Inverse variance weighted
−0.25
0.00
0.25
MR effect size for
'PCDH10' on 'Cardiac valvular disease'

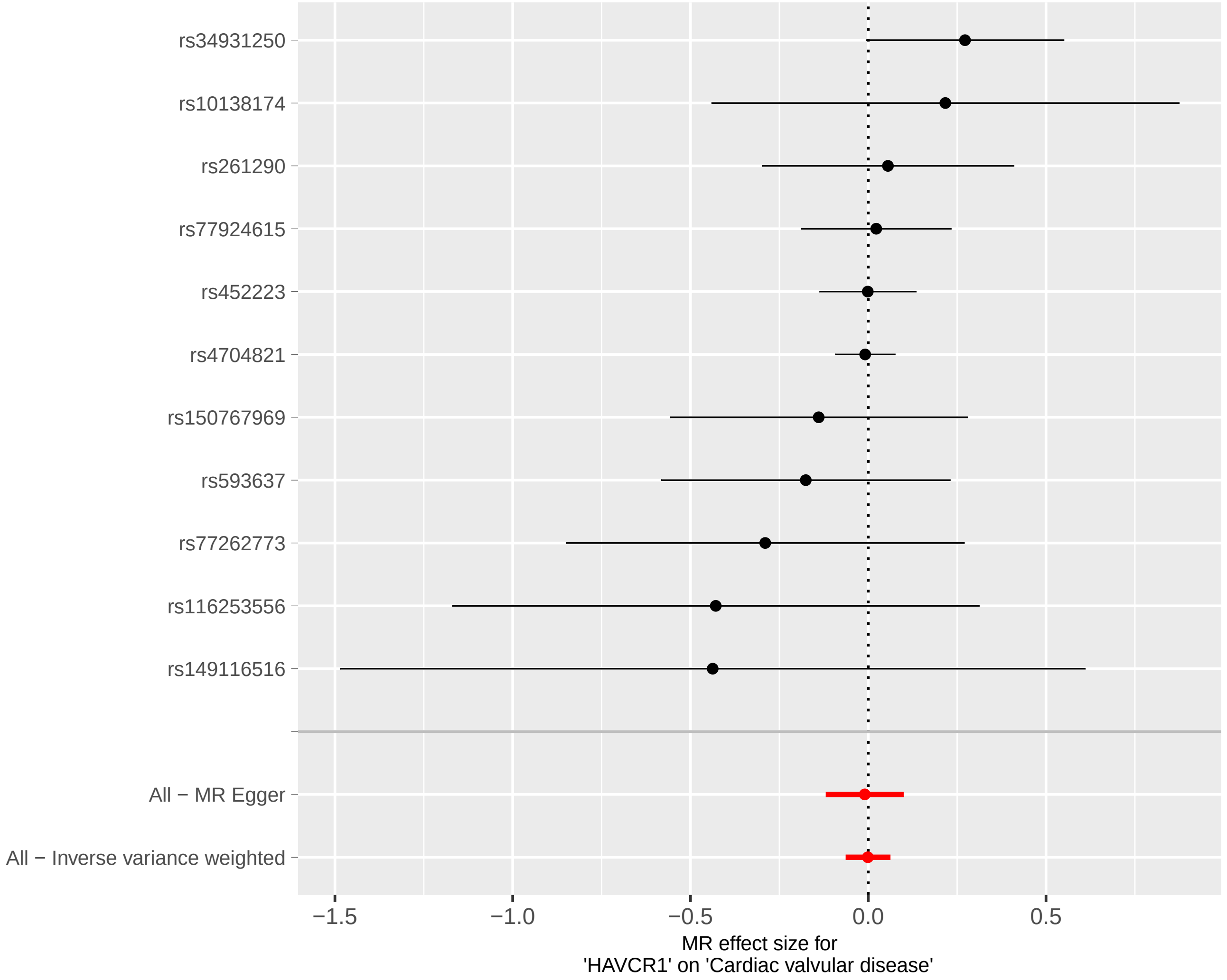
rs34931250
rs10138174
rs261290
rs77924615
rs452223
rs4704821
rs150767969
rs593637
rs77262773
rs116253556
rs149116516
All − MR Egger
All − Inverse variance weighted
−1.5
−1.0
−0.5
0.0
0.5
MR effect size for
'HAVCR1' on 'Cardiac valvular disease'

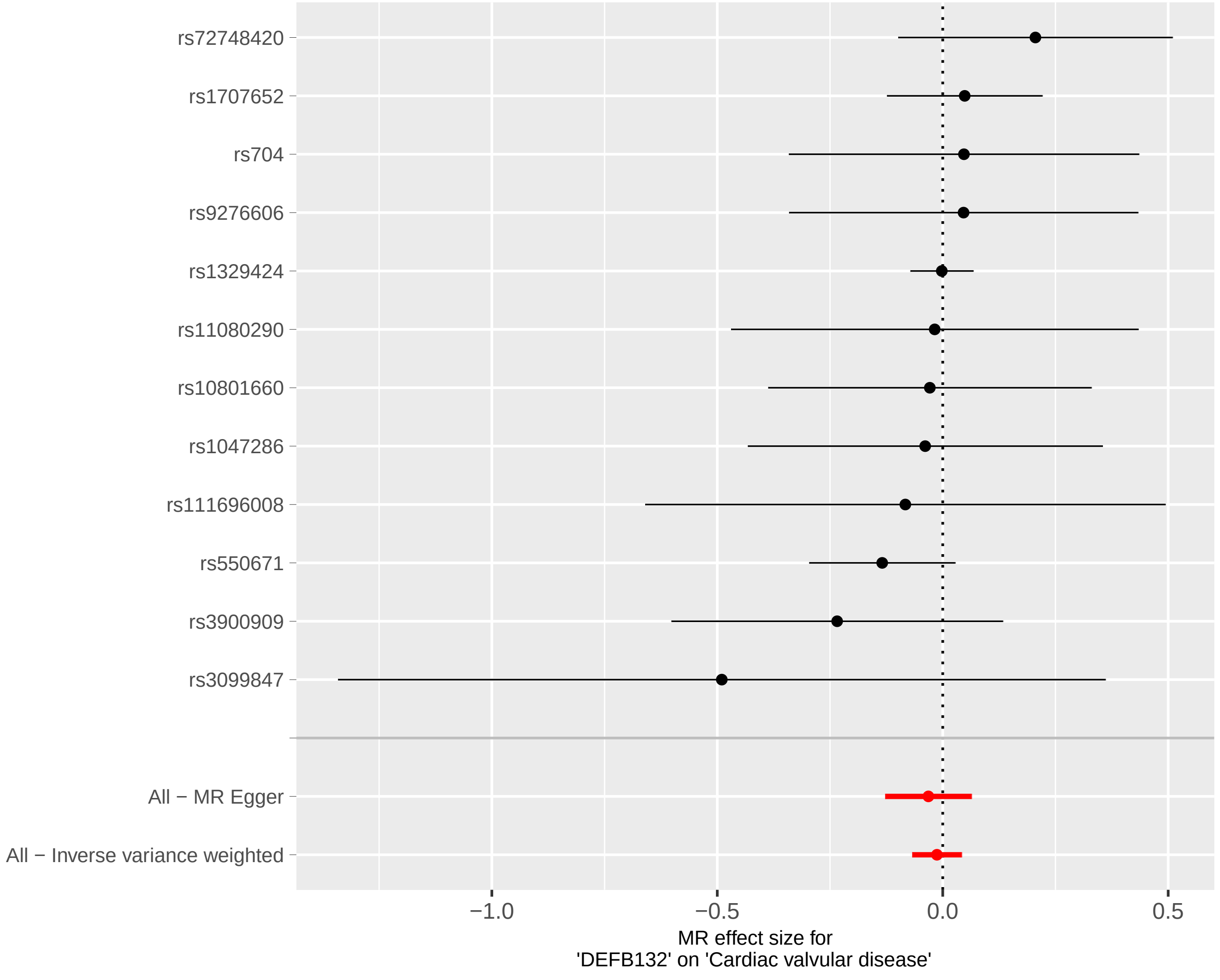
rs72748420
rs1707652
rs704
rs9276606
rs1329424
rs11080290
rs10801660
rs1047286
rs111696008
rs550671
rs3900909
rs3099847
All − MR Egger
All − Inverse variance weighted
−1.0
−0.5
0.0
0.5
MR effect size for
'DEFB132' on 'Cardiac valvular disease'

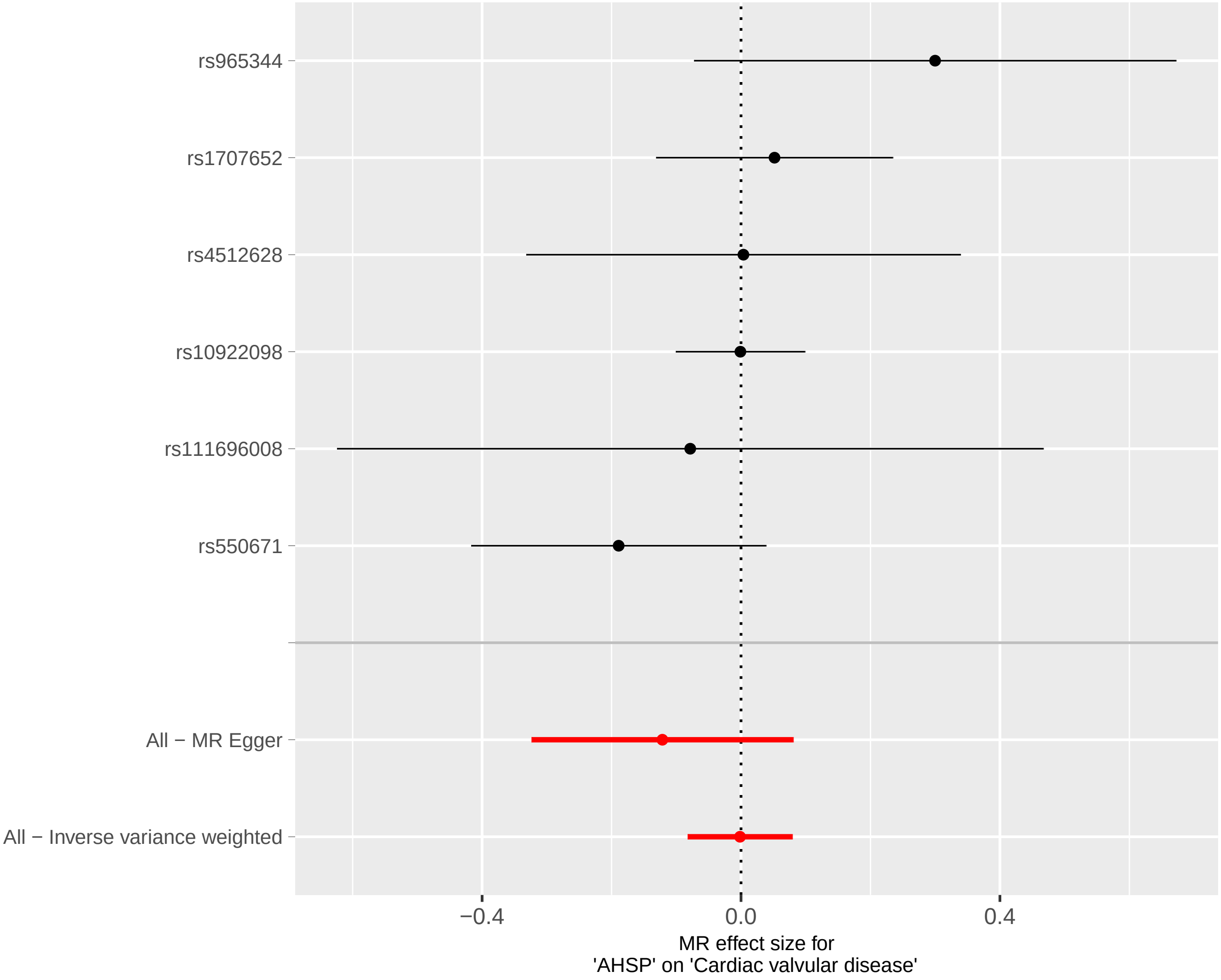
rs965344
rs1707652
rs4512628
rs10922098
rs111696008
rs550671
All − MR Egger
All − Inverse variance weighted
−0.4
0.0
0.4
MR effect size for
'AHSP' on 'Cardiac valvular disease'

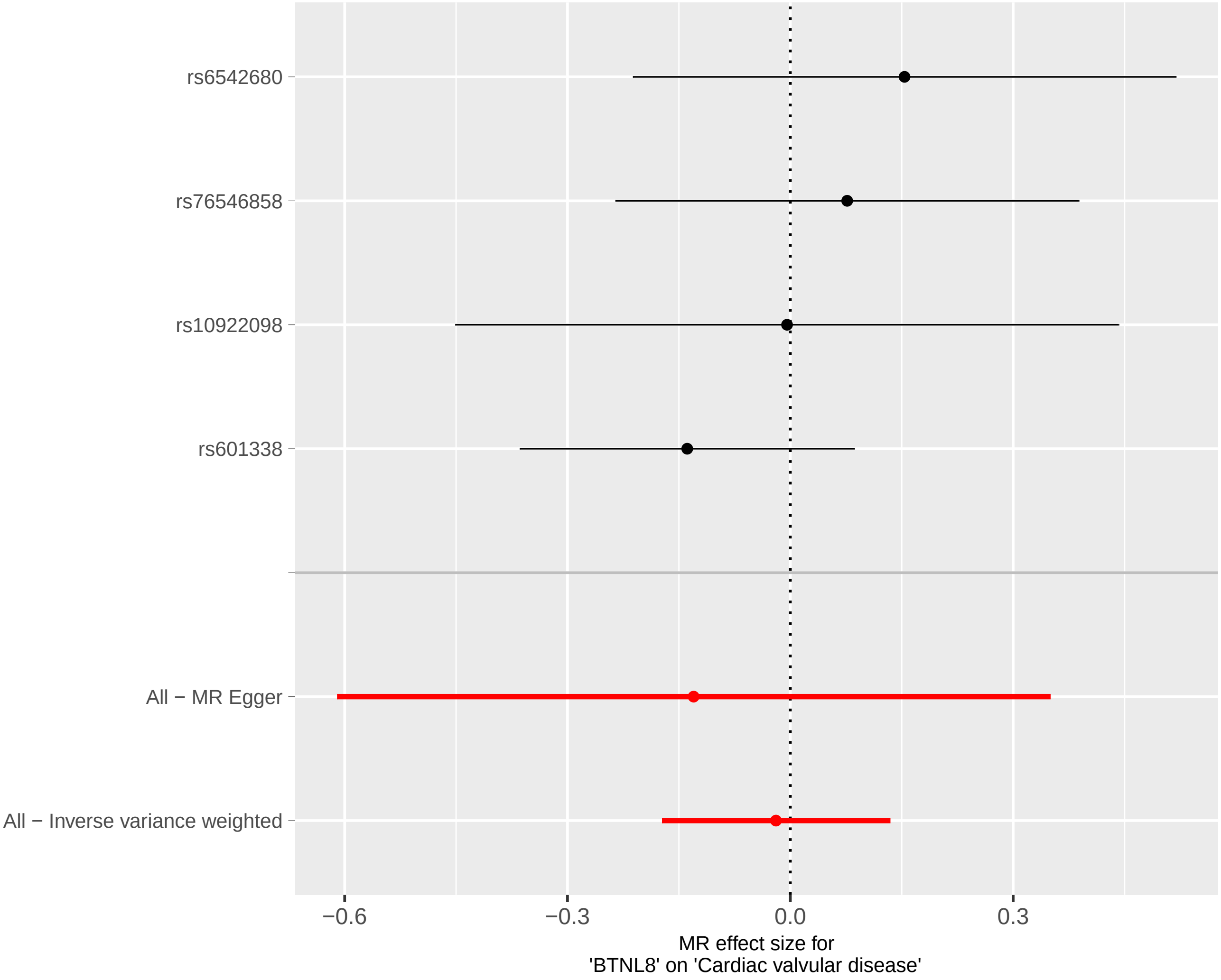
rs6542680
rs76546858
rs10922098
rs601338
All − MR Egger
All − Inverse variance weighted
−0.6
−0.3
0.0
0.3
MR effect size for
'BTNL8' on 'Cardiac valvular disease'

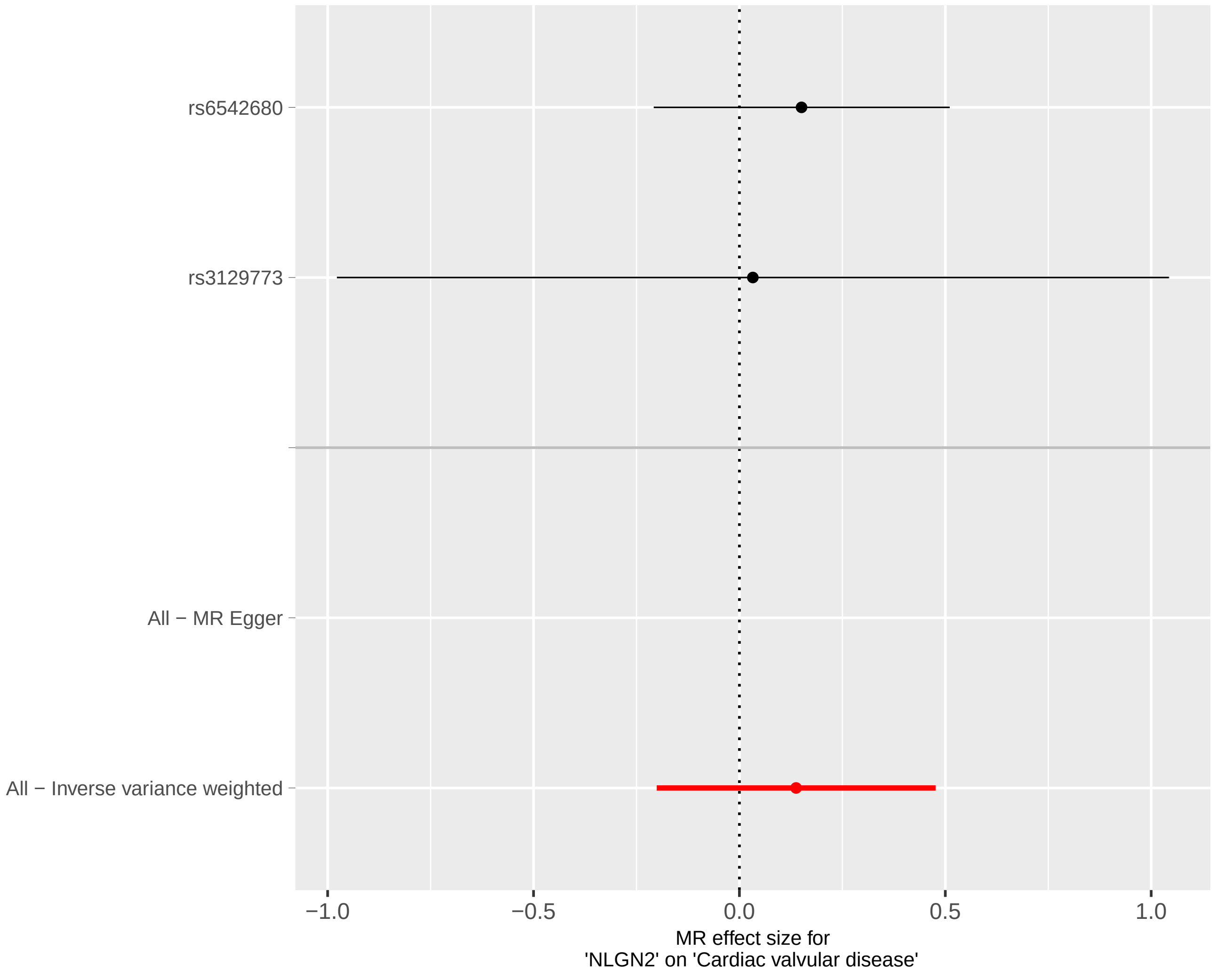
rs6542680
rs3129773
All − MR Egger
All − Inverse variance weighted
−1.0
−0.5
0.0
0.5
1.0
MR effect size for
'NLGN2' on 'Cardiac valvular disease'

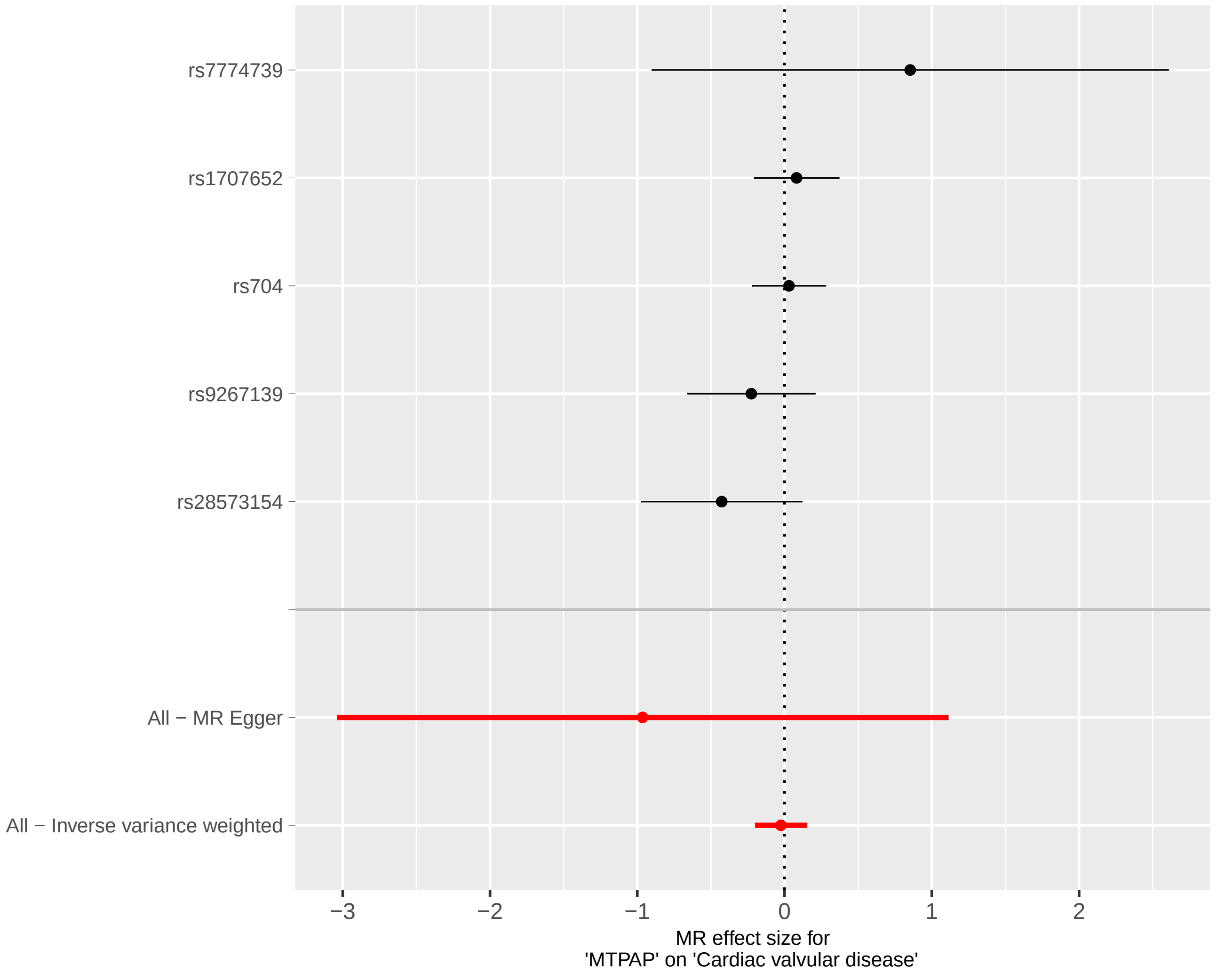
rs7774739
rs1707652
rs704
rs9267139
rs28573154
All − MR Egger
All − Inverse variance weighted
−3
−2
−1
0
1
2
MR effect size for
'MTPAP' on 'Cardiac valvular disease'

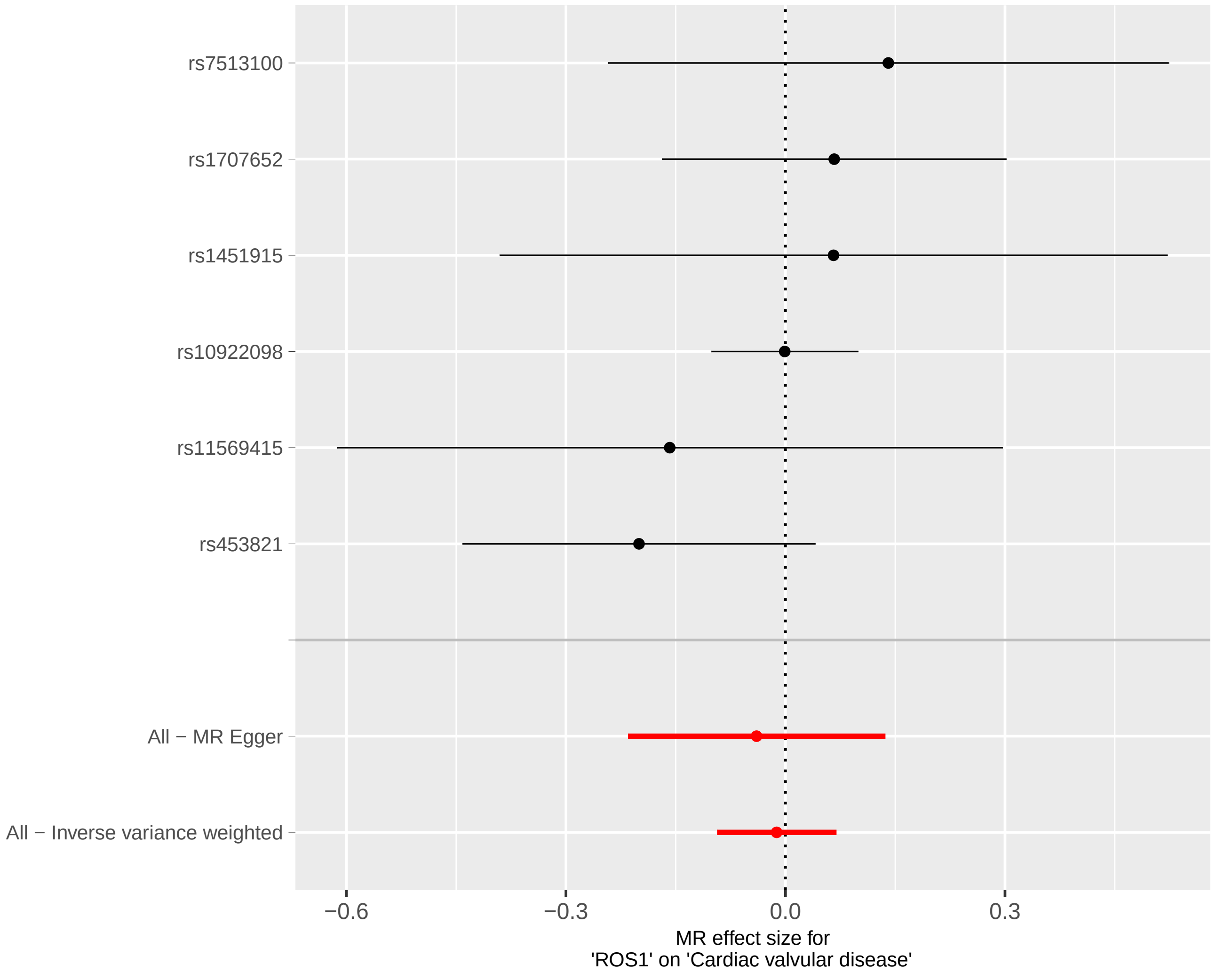
rs7513100
rs1707652
rs1451915
rs10922098
rs11569415
rs453821
All − MR Egger
All − Inverse variance weighted
−0.6
−0.3
0.0
0.3
MR effect size for
'ROS1' on 'Cardiac valvular disease'

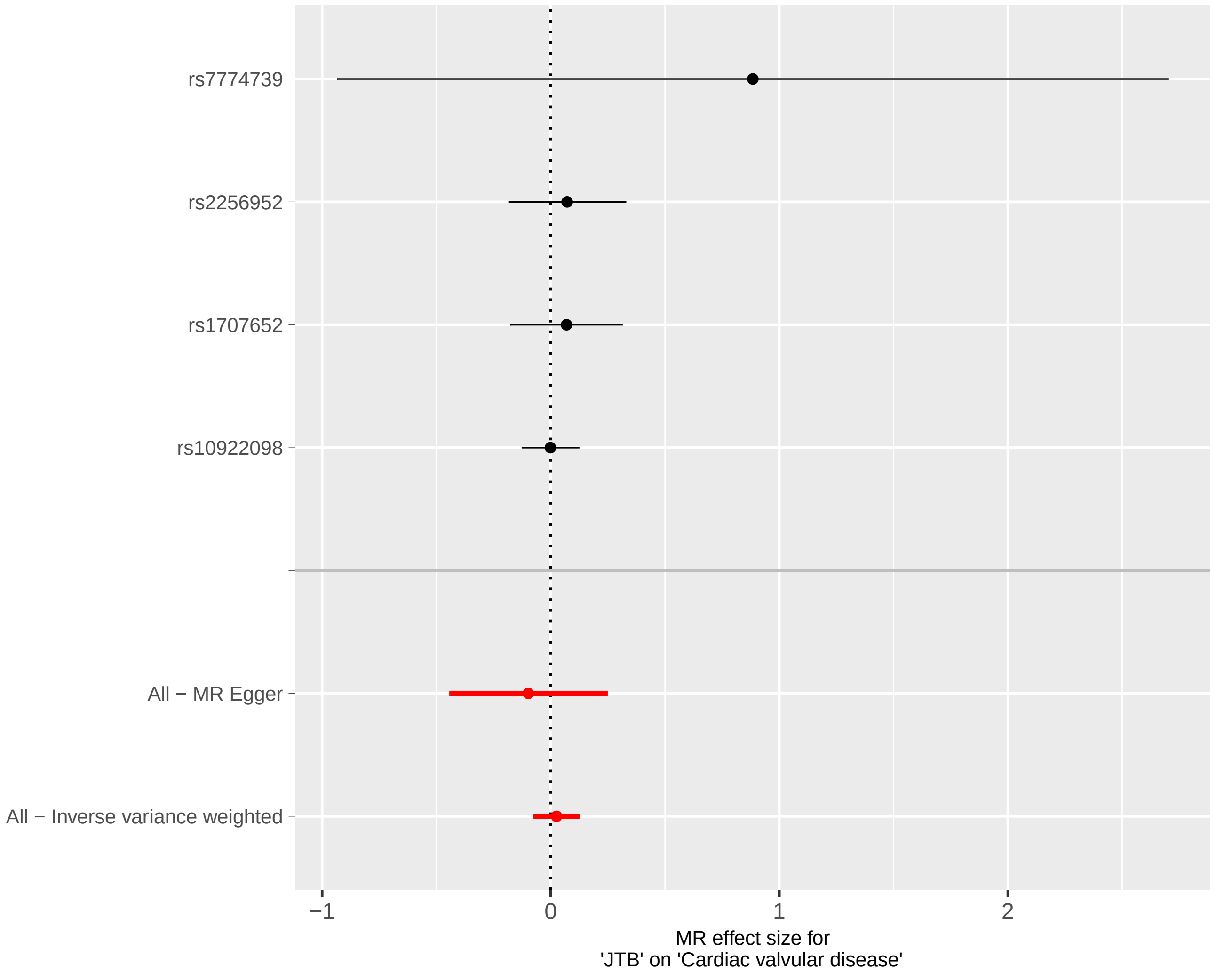
rs7774739
rs2256952
rs1707652
rs10922098
All − MR Egger
All − Inverse variance weighted
−1
0
1
2
MR effect size for
'JTB' on 'Cardiac valvular disease'

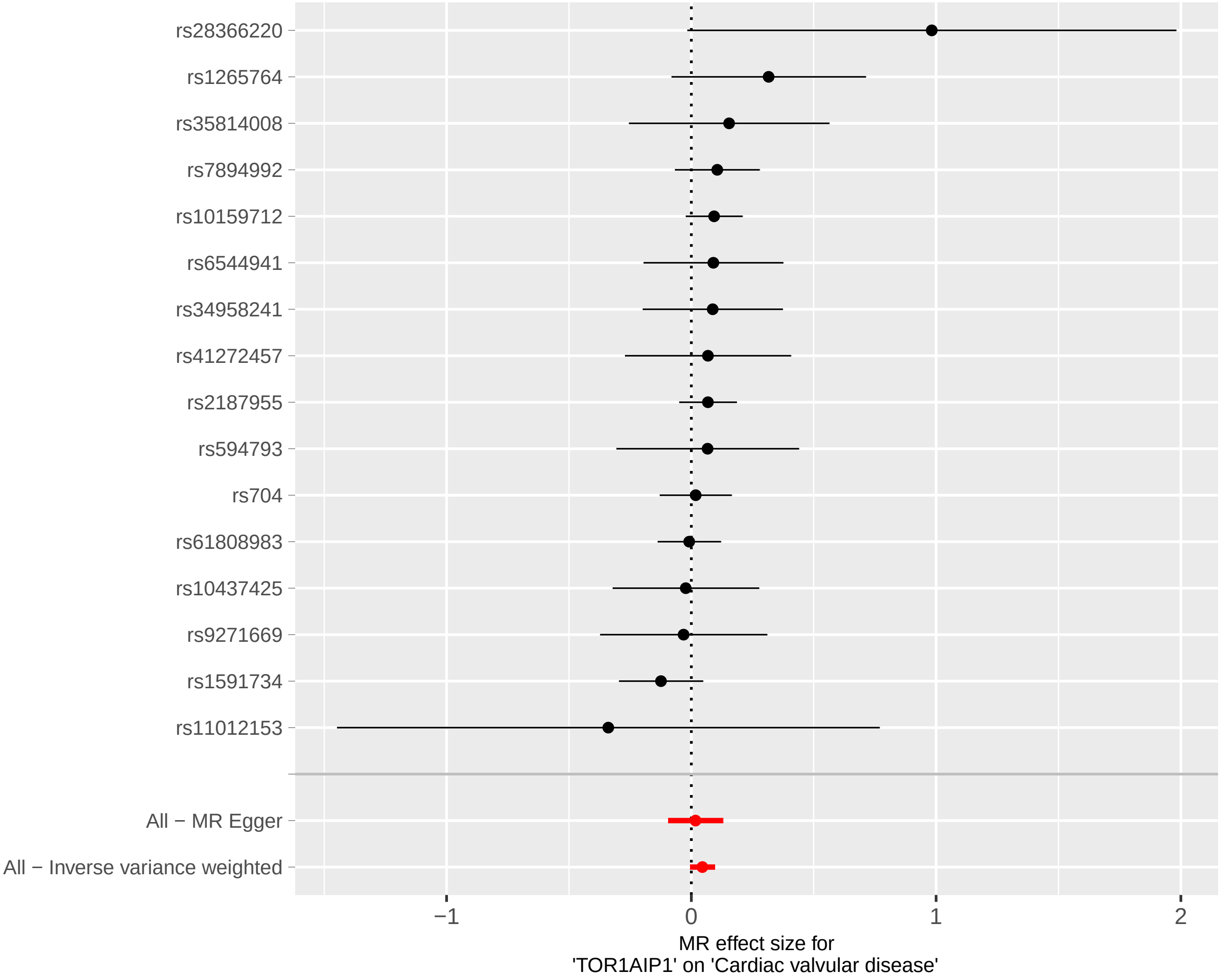

rs28366220
rs1265764
rs35814008
rs7894992
rs10159712
rs6544941
rs34958241
rs41272457
rs2187955
rs594793
rs704
rs61808983
rs10437425
rs9271669
rs1591734
rs11012153
All − MR Egger
All − Inverse variance weighted
−1
0
1
2
MR effect size for
'TOR1AIP1' on 'Cardiac valvular disease'

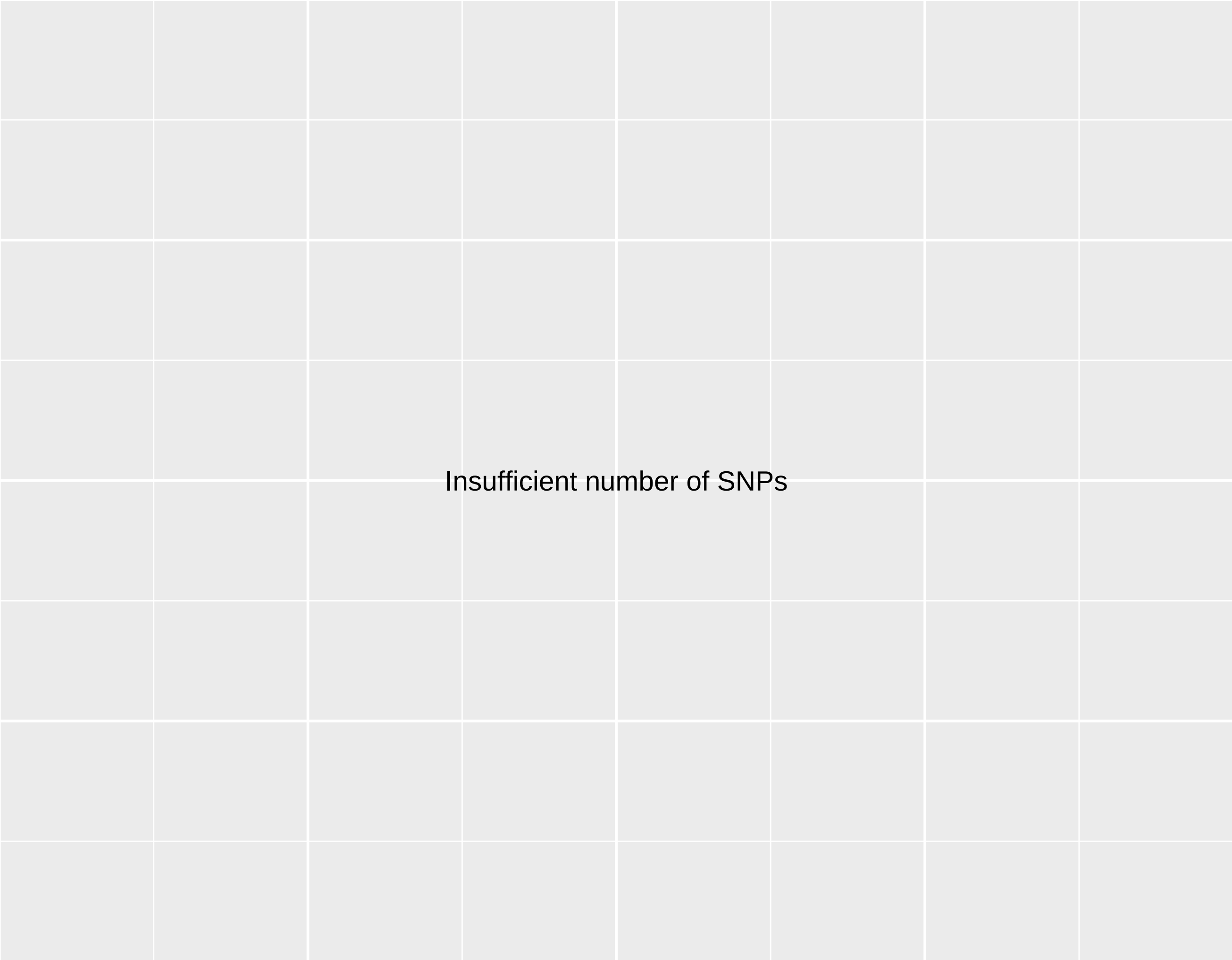
Insufficient number of SNPs

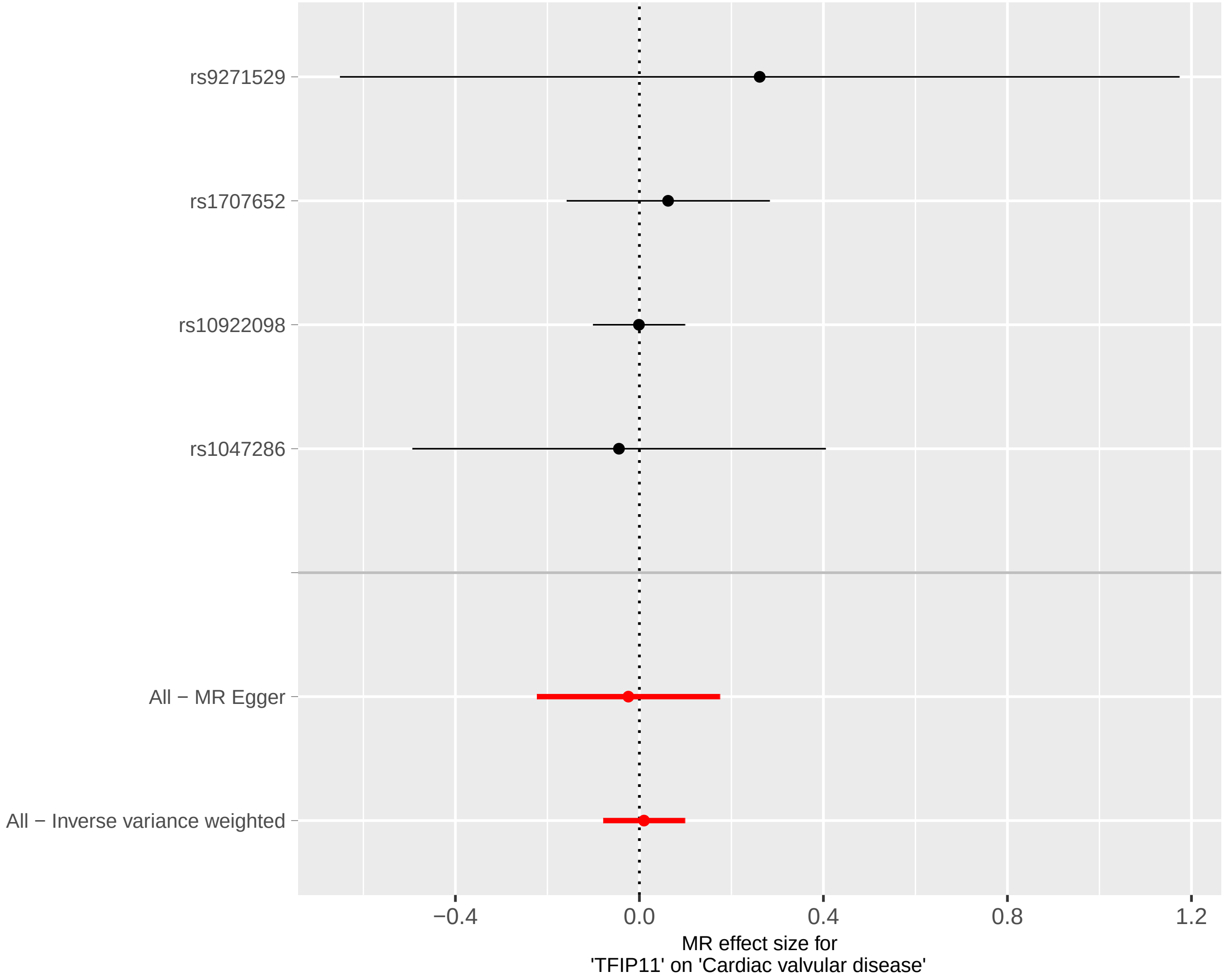
rs9271529
rs1707652
rs10922098
rs1047286
All − MR Egger
All − Inverse variance weighted
−0.4
0.0
0.4
0.8
1.2
MR effect size for
'TFIP11' on 'Cardiac valvular disease'

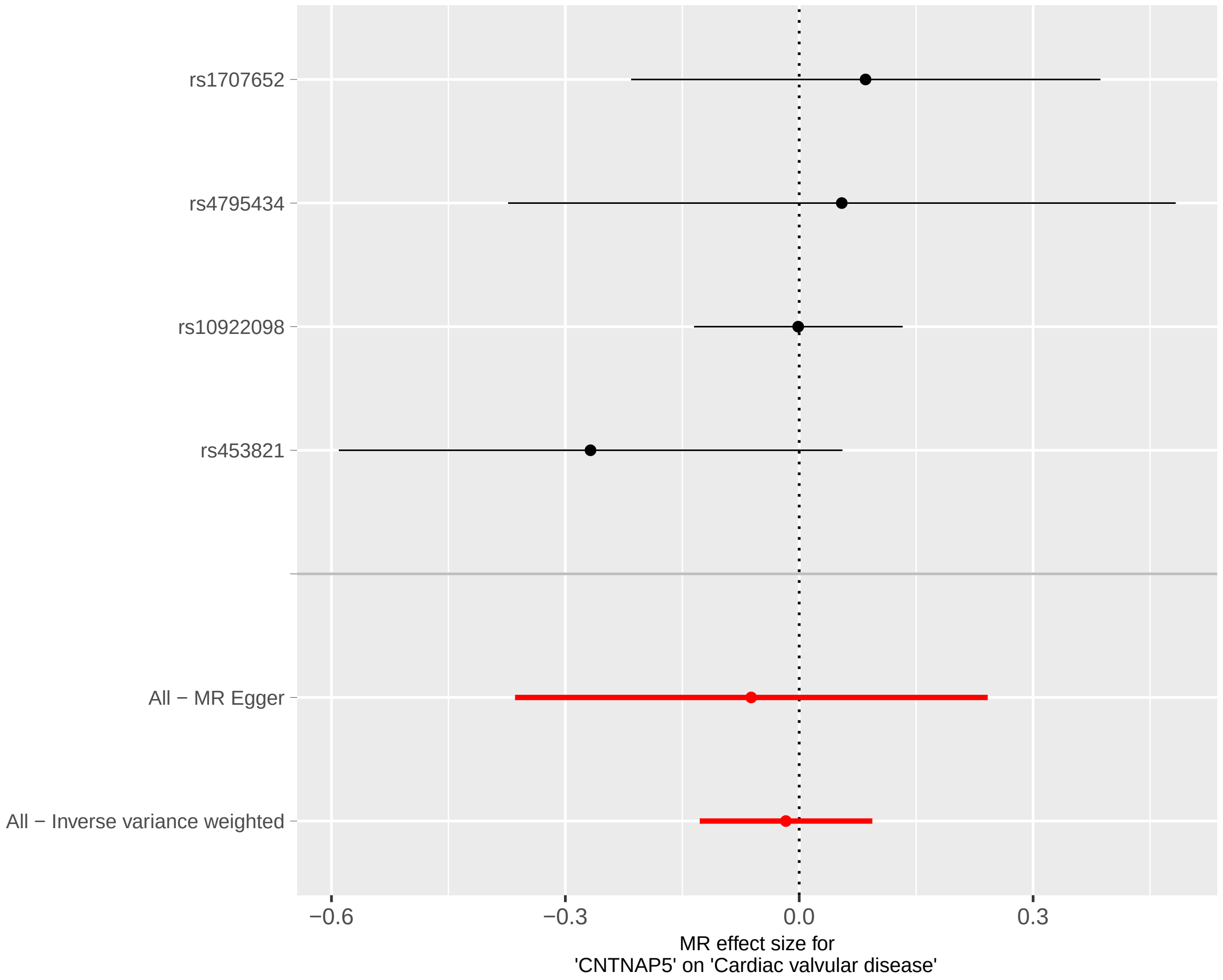
rs1707652
rs4795434
rs10922098
rs453821
All − MR Egger
All − Inverse variance weighted
−0.6
−0.3
0.0
0.3
MR effect size for
'CNTNAP5' on 'Cardiac valvular disease'

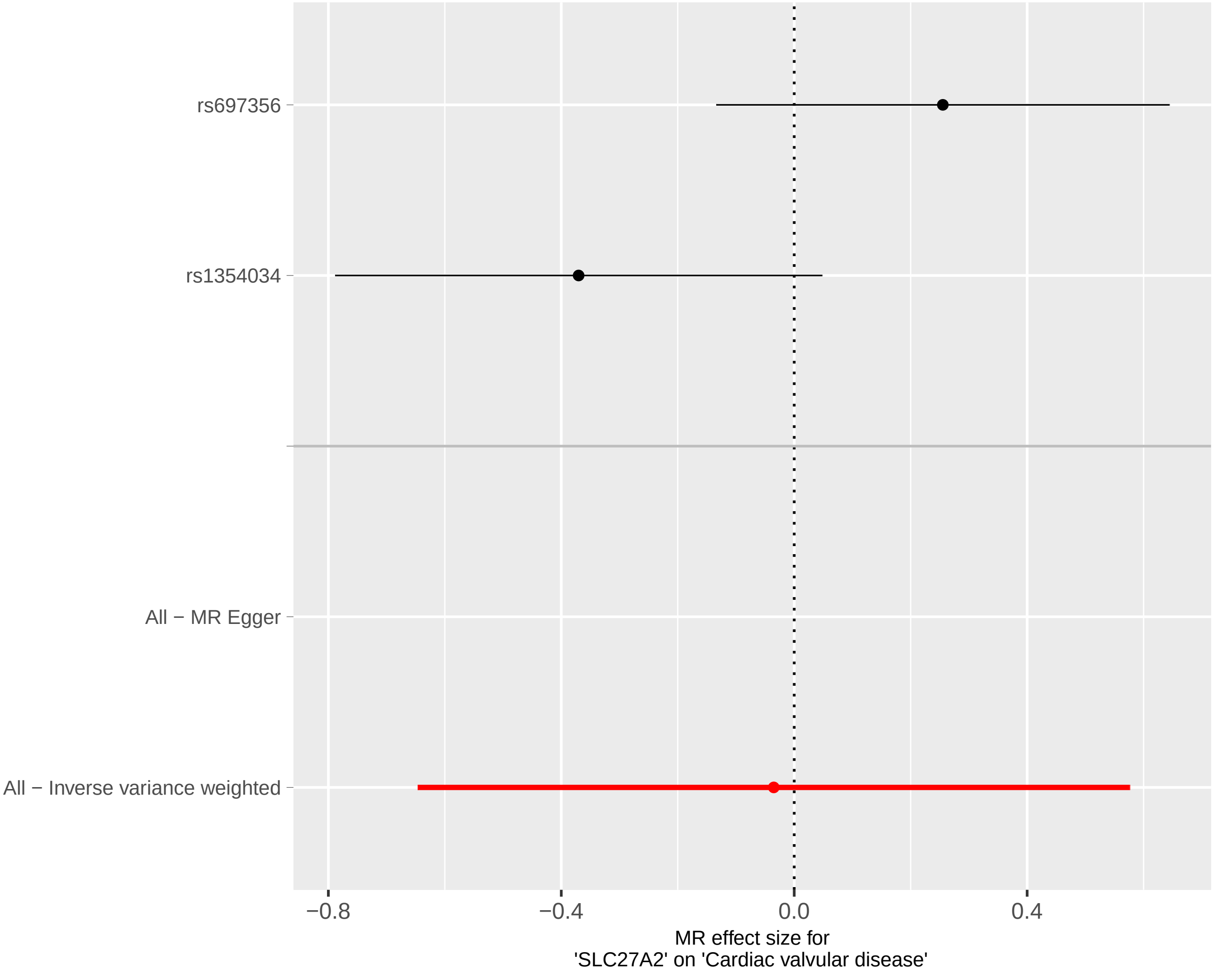
rs697356
rs1354034
All − MR Egger
All − Inverse variance weighted
−0.8
−0.4
0.0
0.4
MR effect size for
'SLC27A2' on 'Cardiac valvular disease'

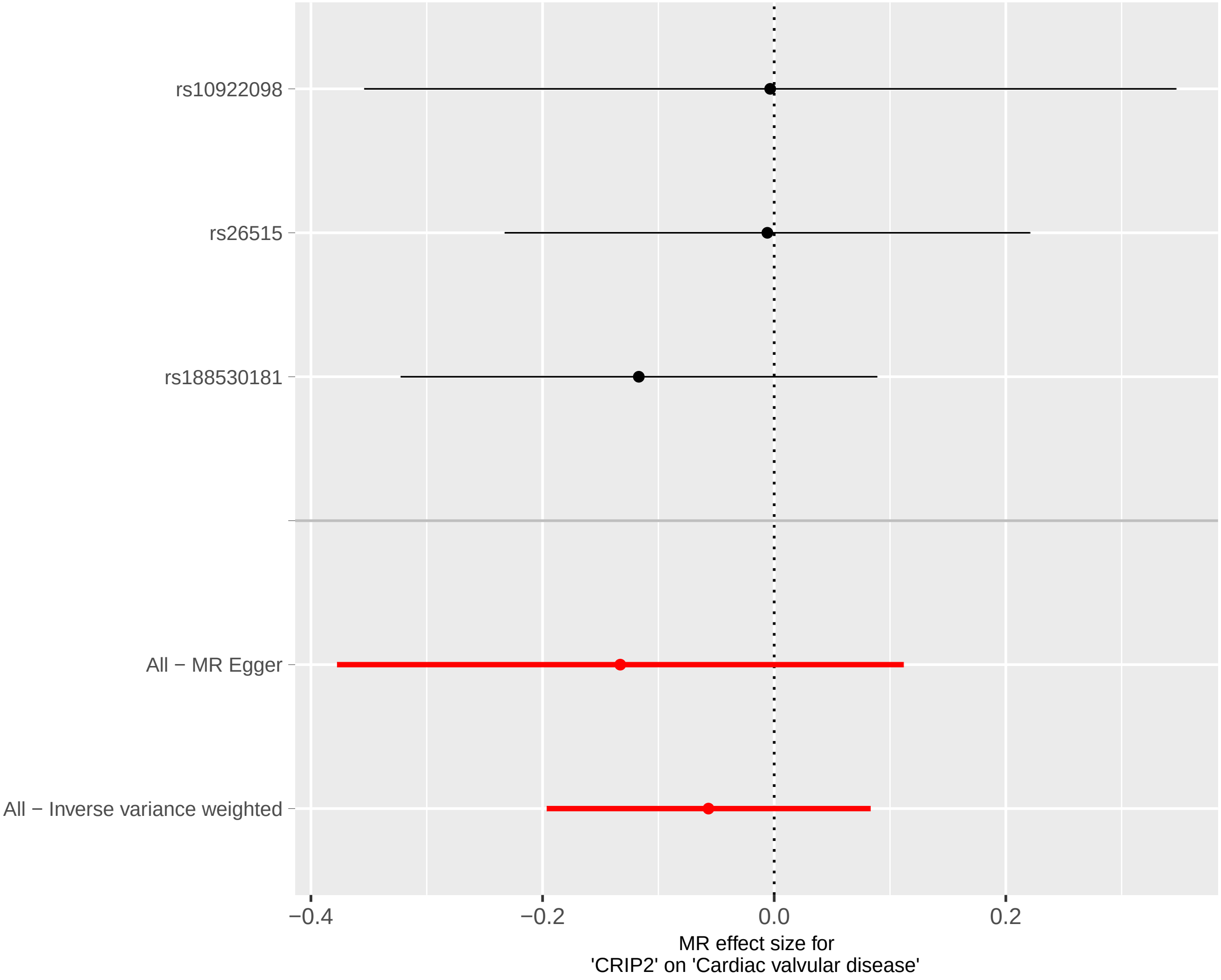
rs10922098
rs26515
rs188530181
All − MR Egger
All − Inverse variance weighted
−0.4
−0.2
0.0
0.2
MR effect size for
'CRIP2' on 'Cardiac valvular disease'

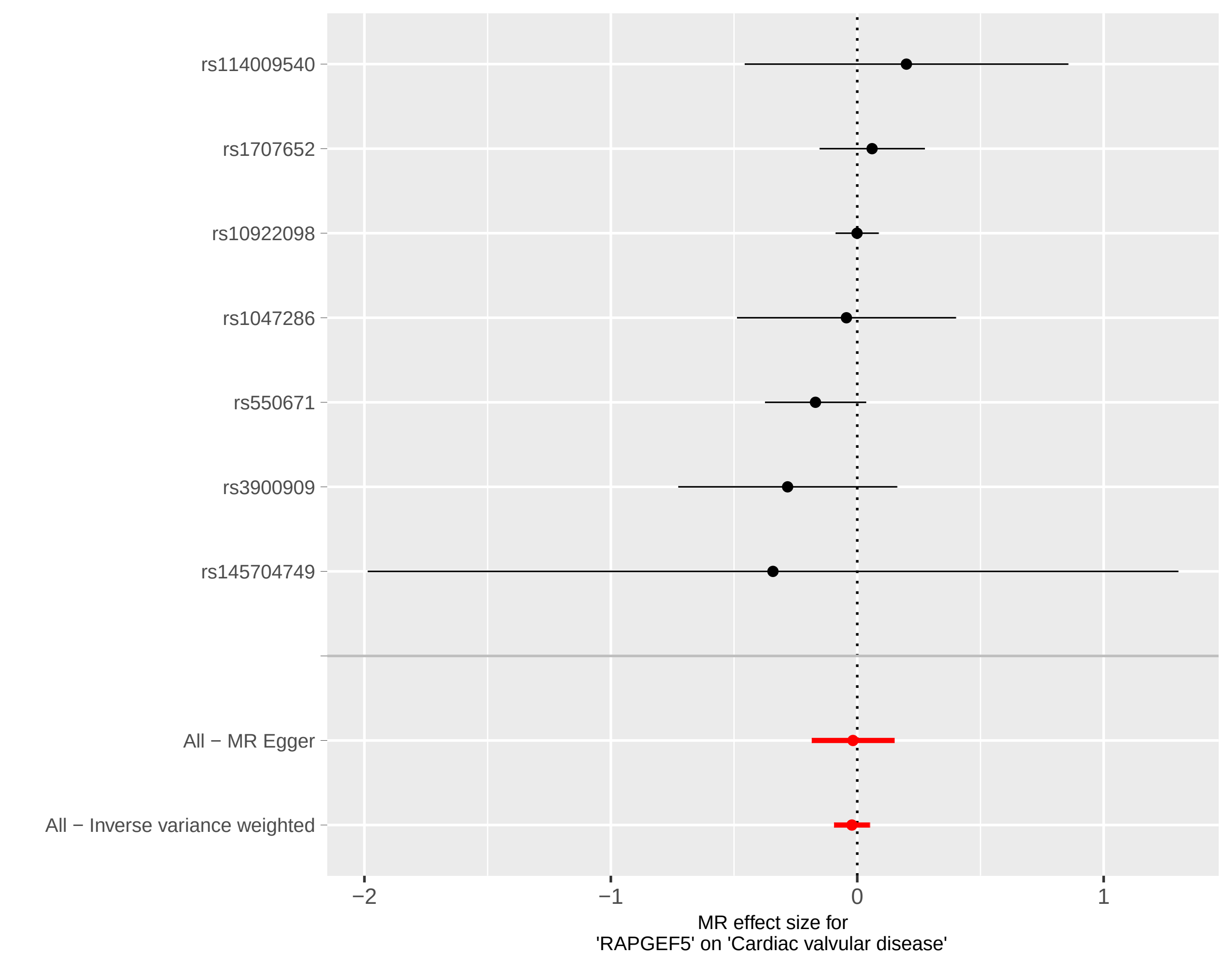
rs114009540
rs1707652
rs10922098
rs1047286
rs550671
rs3900909
rs145704749
All − MR Egger
All − Inverse variance weighted
−2
−1
0
1
MR effect size for
'RAPGEF5' on 'Cardiac valvular disease'

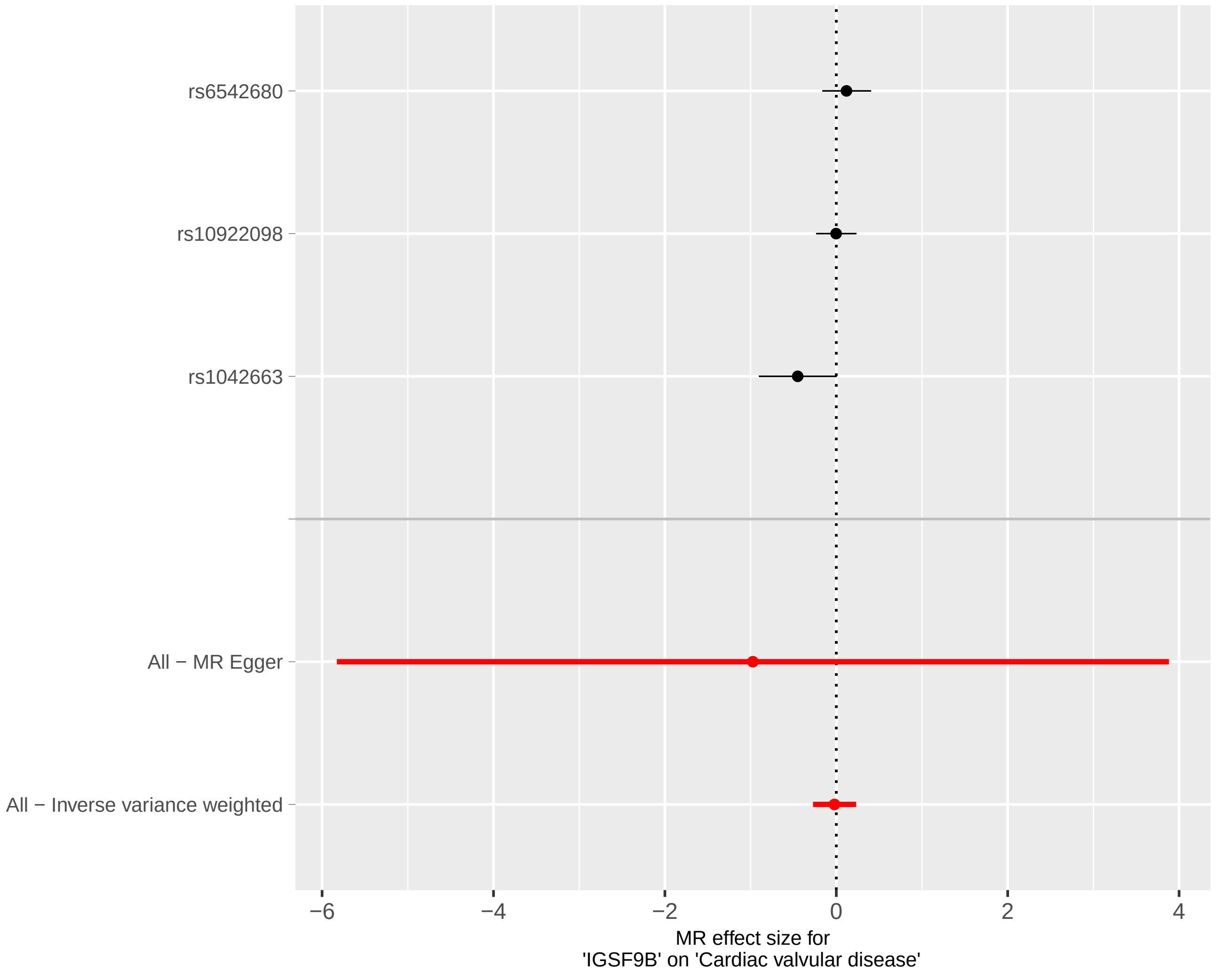

rs6542680
rs10922098
rs1042663
All − MR Egger
All − Inverse variance weighted
−6
−4
−2
0
2
4
MR effect size for
'IGSF9B' on 'Cardiac valvular disease'

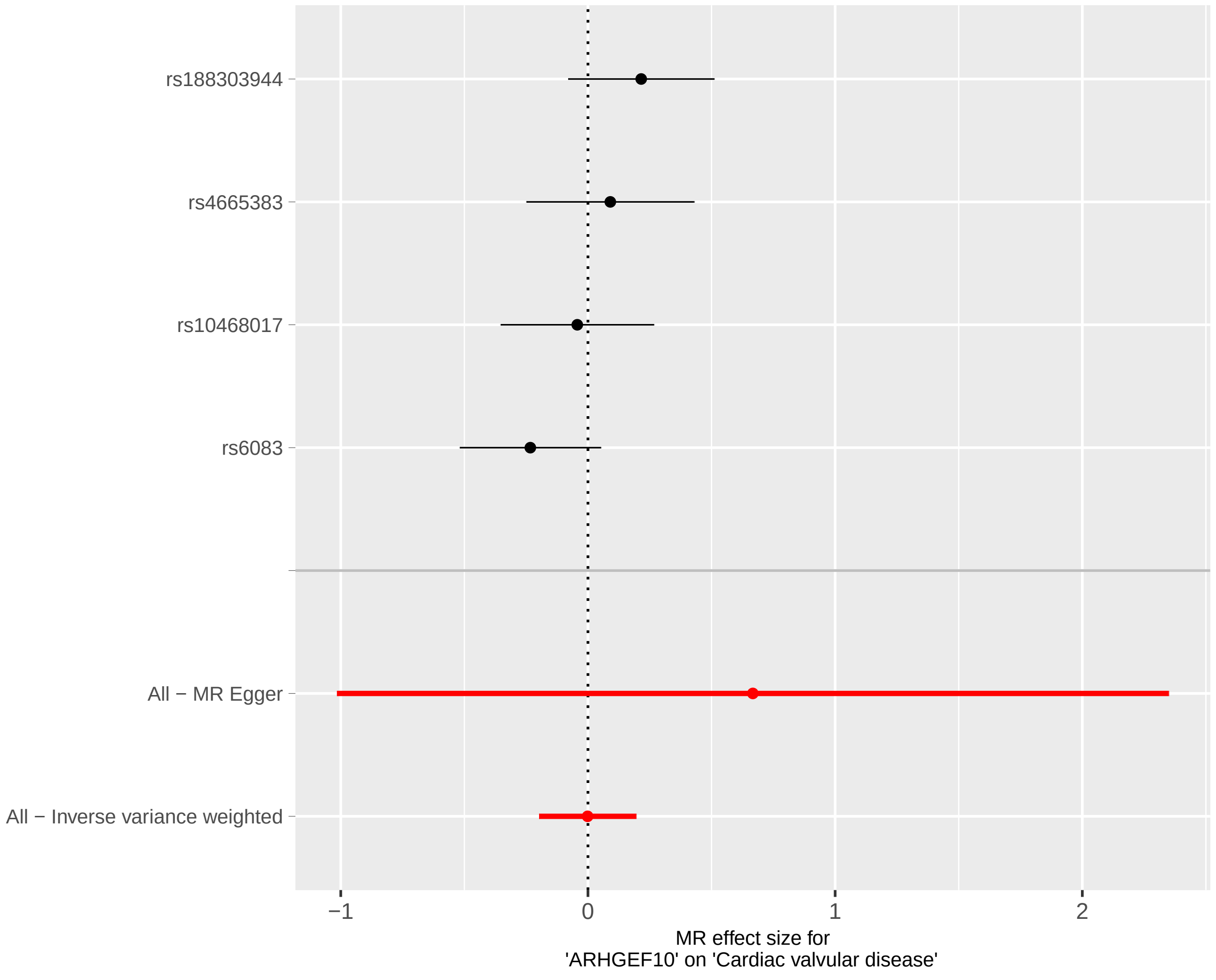
rs188303944
rs4665383
rs10468017
rs6083
All − MR Egger
All − Inverse variance weighted
−1
0
1
2
MR effect size for
'ARHGEF10' on 'Cardiac valvular disease'

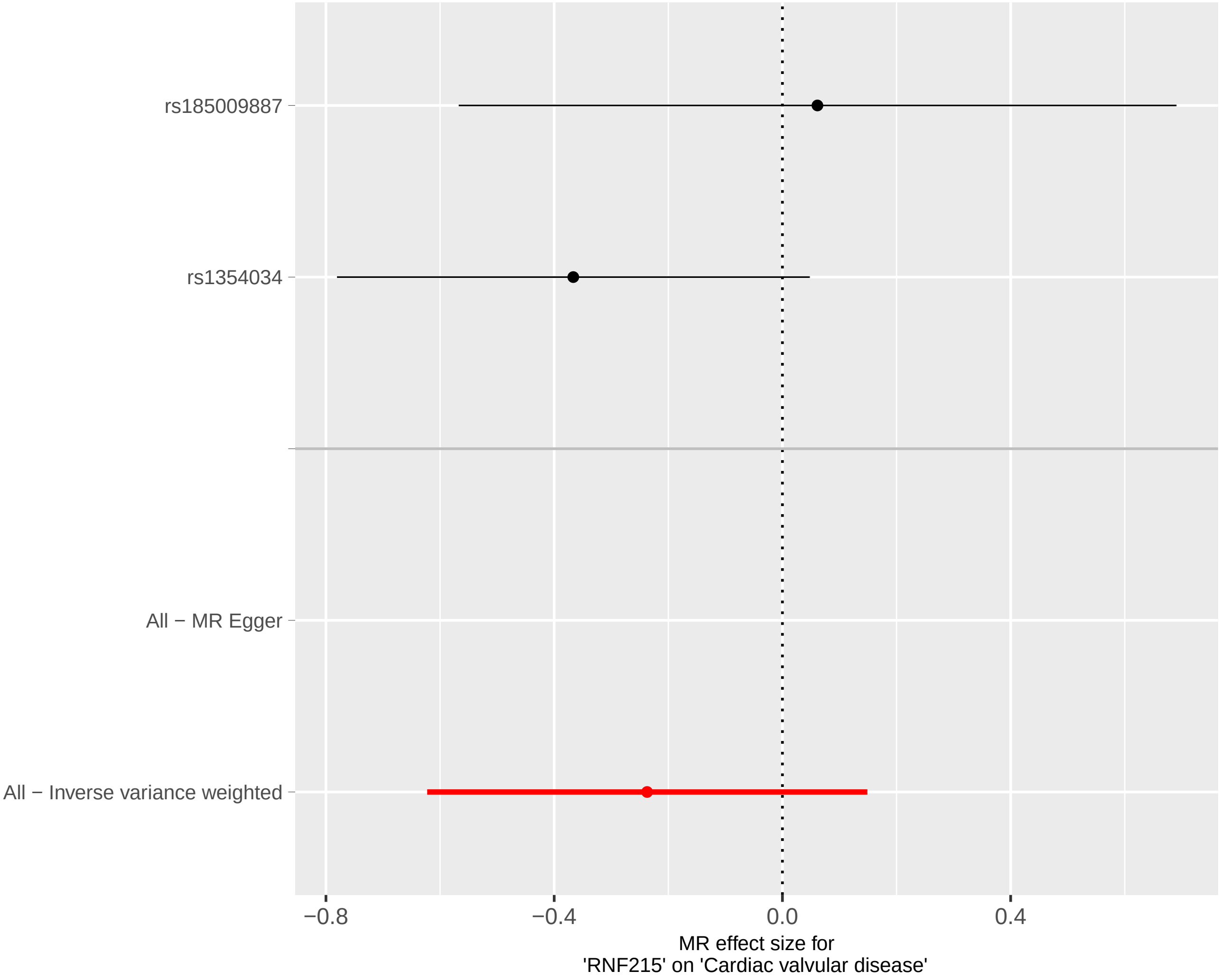
rs185009887
rs1354034
All − MR Egger
All − Inverse variance weighted
−0.8
−0.4
0.0
0.4
MR effect size for
'RNF215' on 'Cardiac valvular disease'

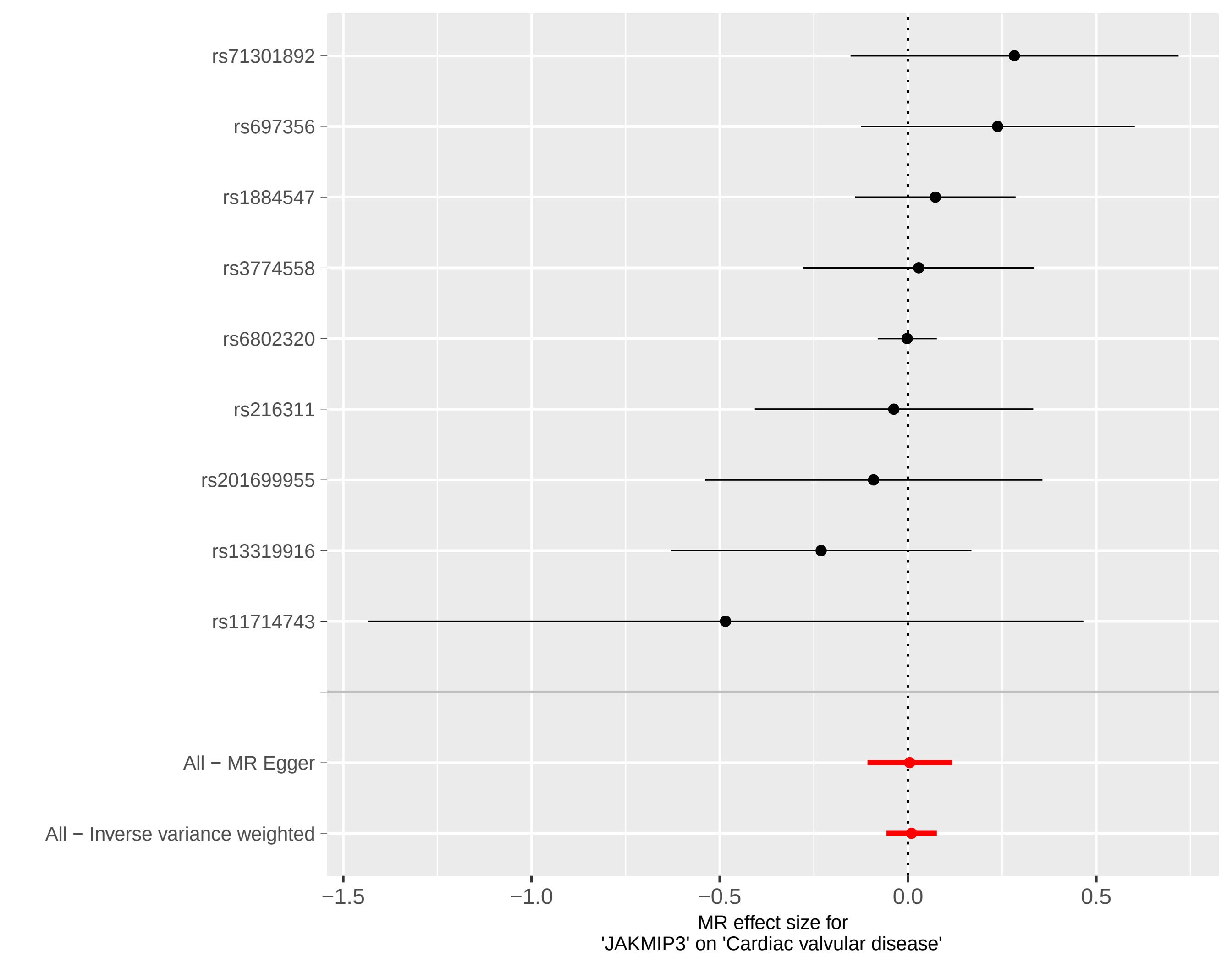
rs71301892
rs697356
rs1884547
rs3774558
rs6802320
rs216311
rs201699955
rs13319916
rs11714743
All − MR Egger
All − Inverse variance weighted
−1.5
−1.0
−0.5
0.0
0.5
MR effect size for
'JAKMIP3' on 'Cardiac valvular disease'

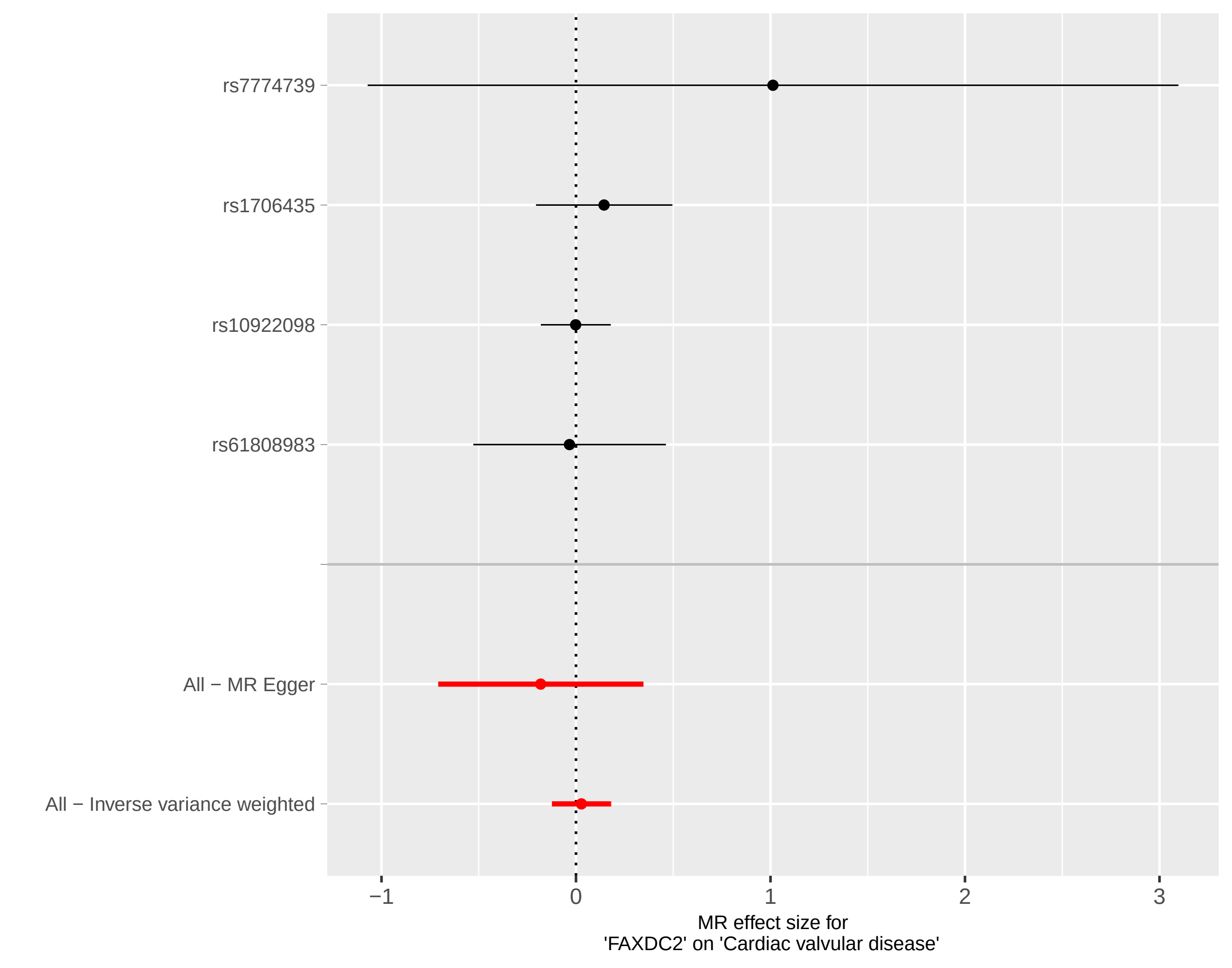
rs7774739
rs1706435
rs10922098
rs61808983
All − MR Egger
All − Inverse variance weighted
−1
0
1
2
3
MR effect size for
'FAXDC2' on 'Cardiac valvular disease'

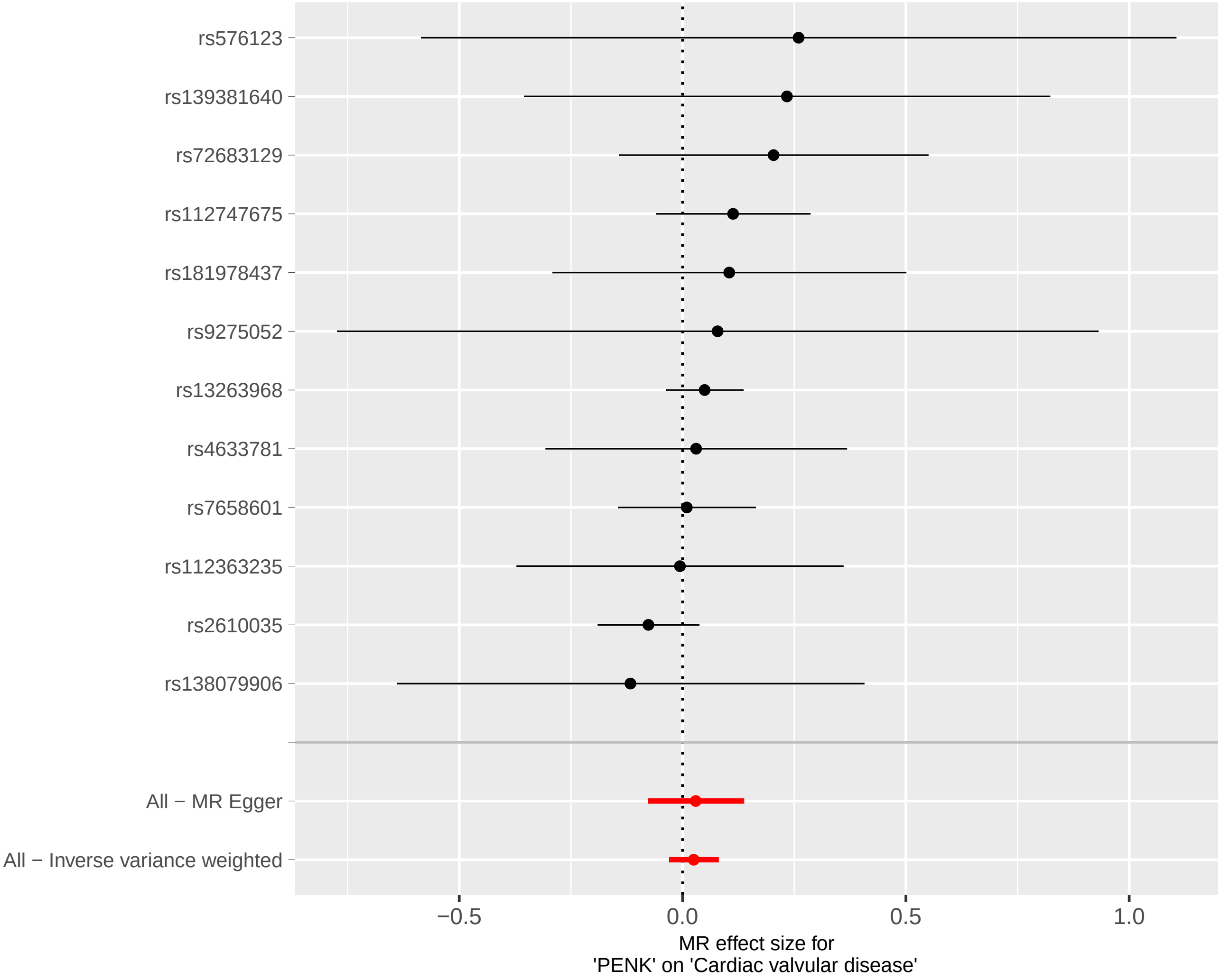
rs576123
rs139381640
rs72683129
rs112747675
rs181978437
rs9275052
rs13263968
rs4633781
rs7658601
rs112363235
rs2610035
rs138079906
All − MR Egger
All − Inverse variance weighted
−0.5
0.0
0.5
1.0
MR effect size for
'PENK' on 'Cardiac valvular disease'

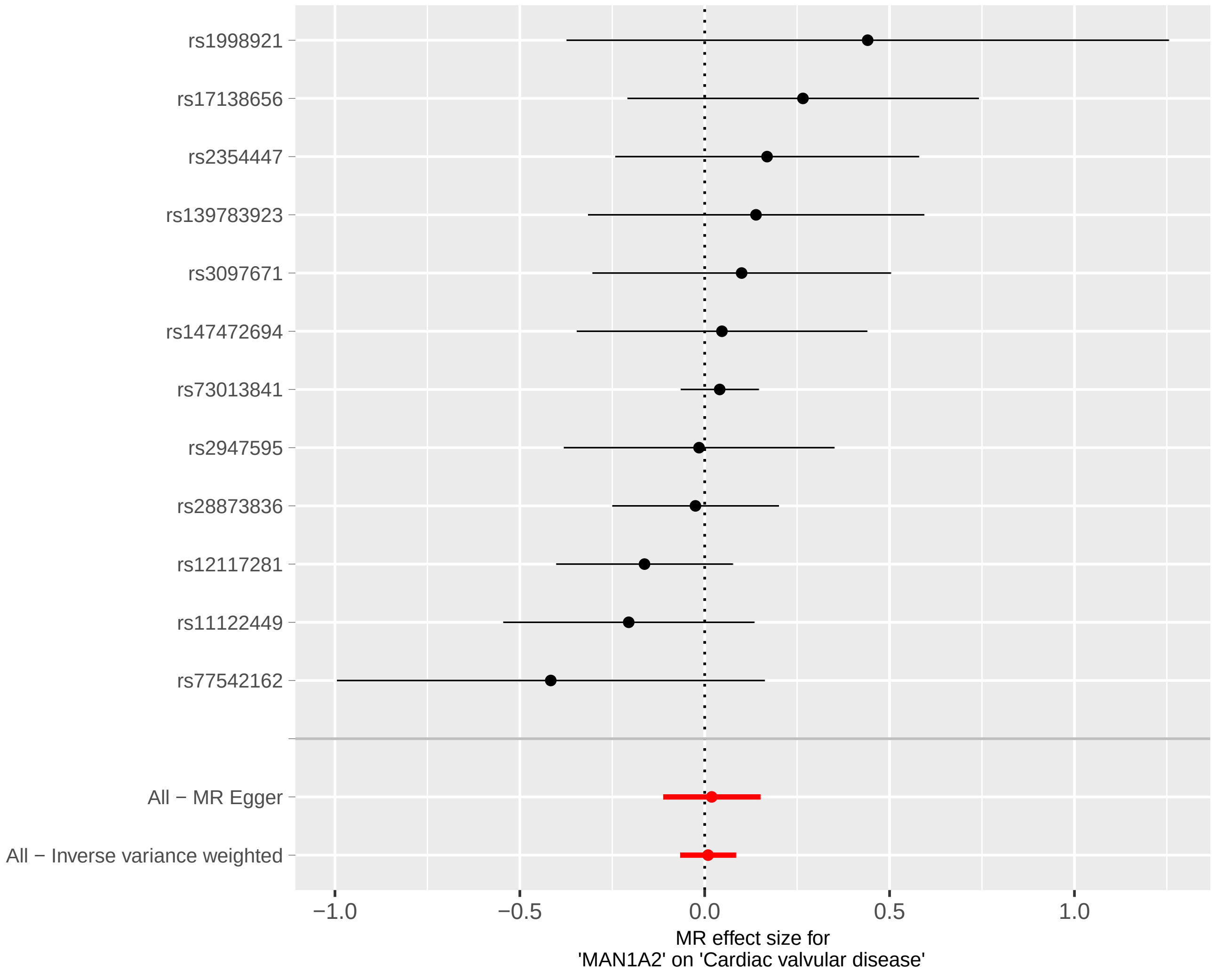
rs1998921
rs17138656
rs2354447
rs139783923
rs3097671
rs147472694
rs73013841
rs2947595
rs28873836
rs12117281
rs11122449
rs77542162
All − MR Egger
All − Inverse variance weighted
−1.0
−0.5
0.0
0.5
1.0
MR effect size for
'MAN1A2' on 'Cardiac valvular disease'

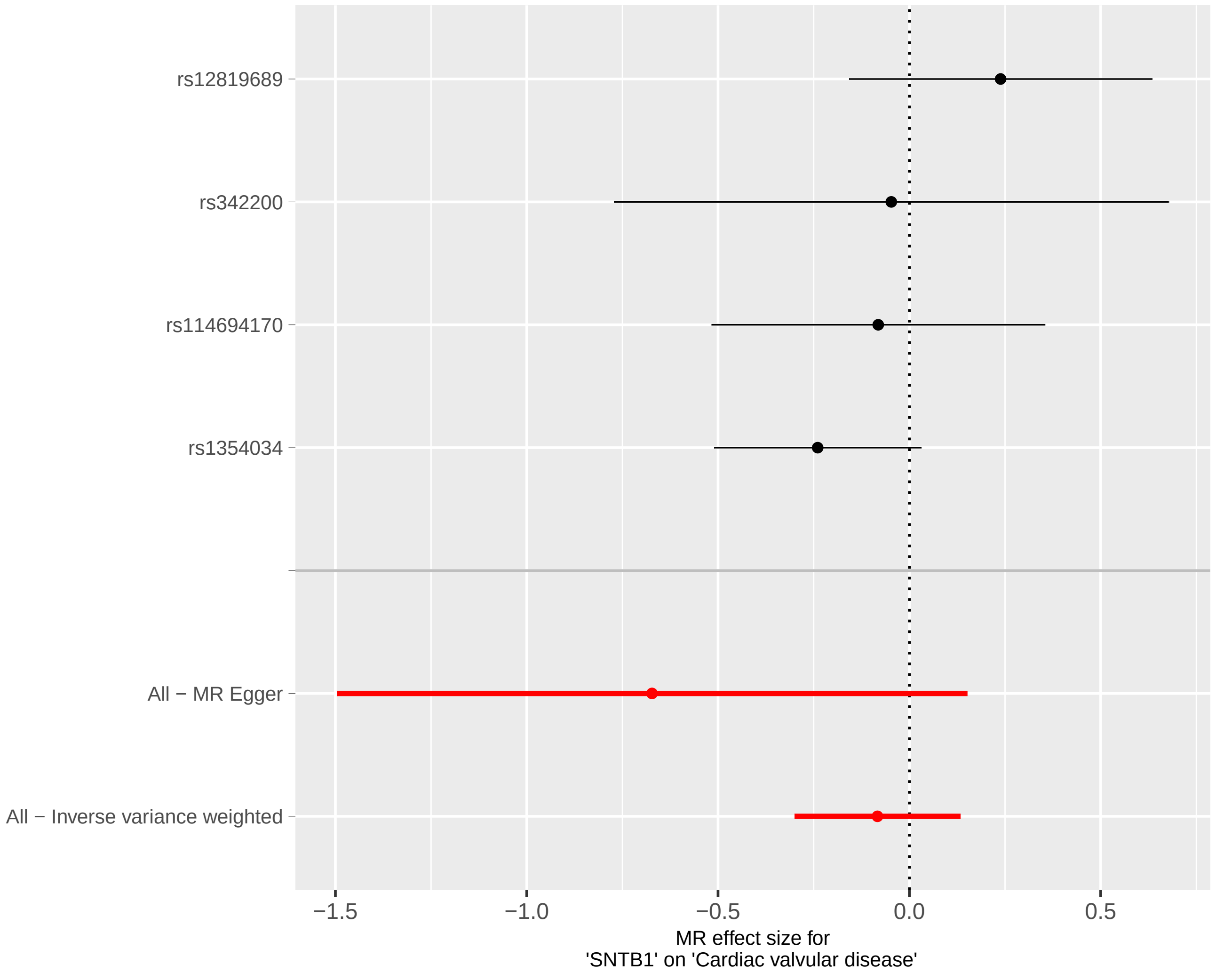
rs12819689
rs342200
rs114694170
rs1354034
All − MR Egger
All − Inverse variance weighted
−1.5
−1.0
−0.5
0.0
0.5
MR effect size for
'SNTB1' on 'Cardiac valvular disease'

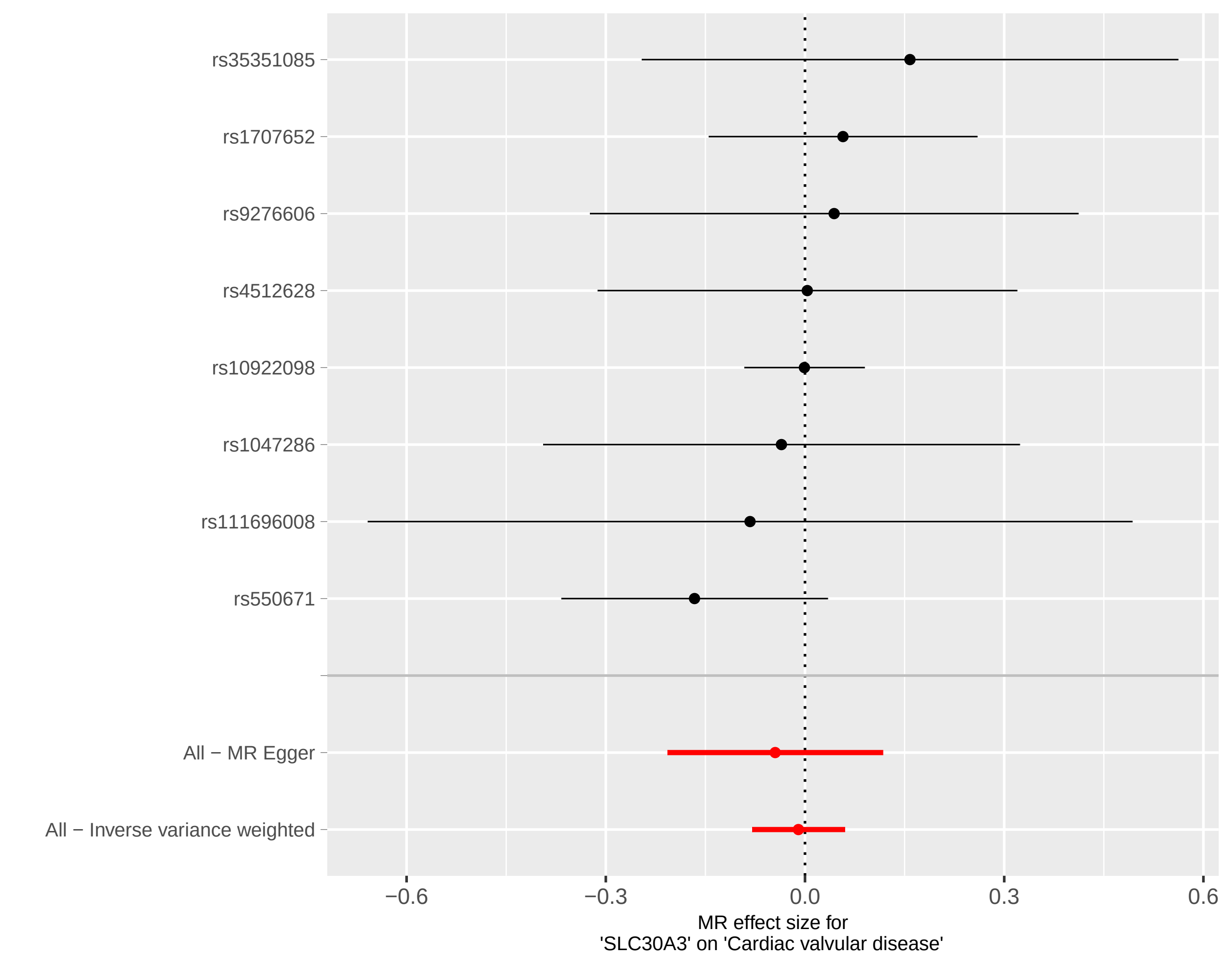

rs35351085
rs1707652
rs9276606
rs4512628
rs10922098
rs1047286
rs111696008
rs550671
All − MR Egger
All − Inverse variance weighted
−0.6
−0.3
0.0
0.3
0.6
MR effect size for
'SLC30A3' on 'Cardiac valvular disease'

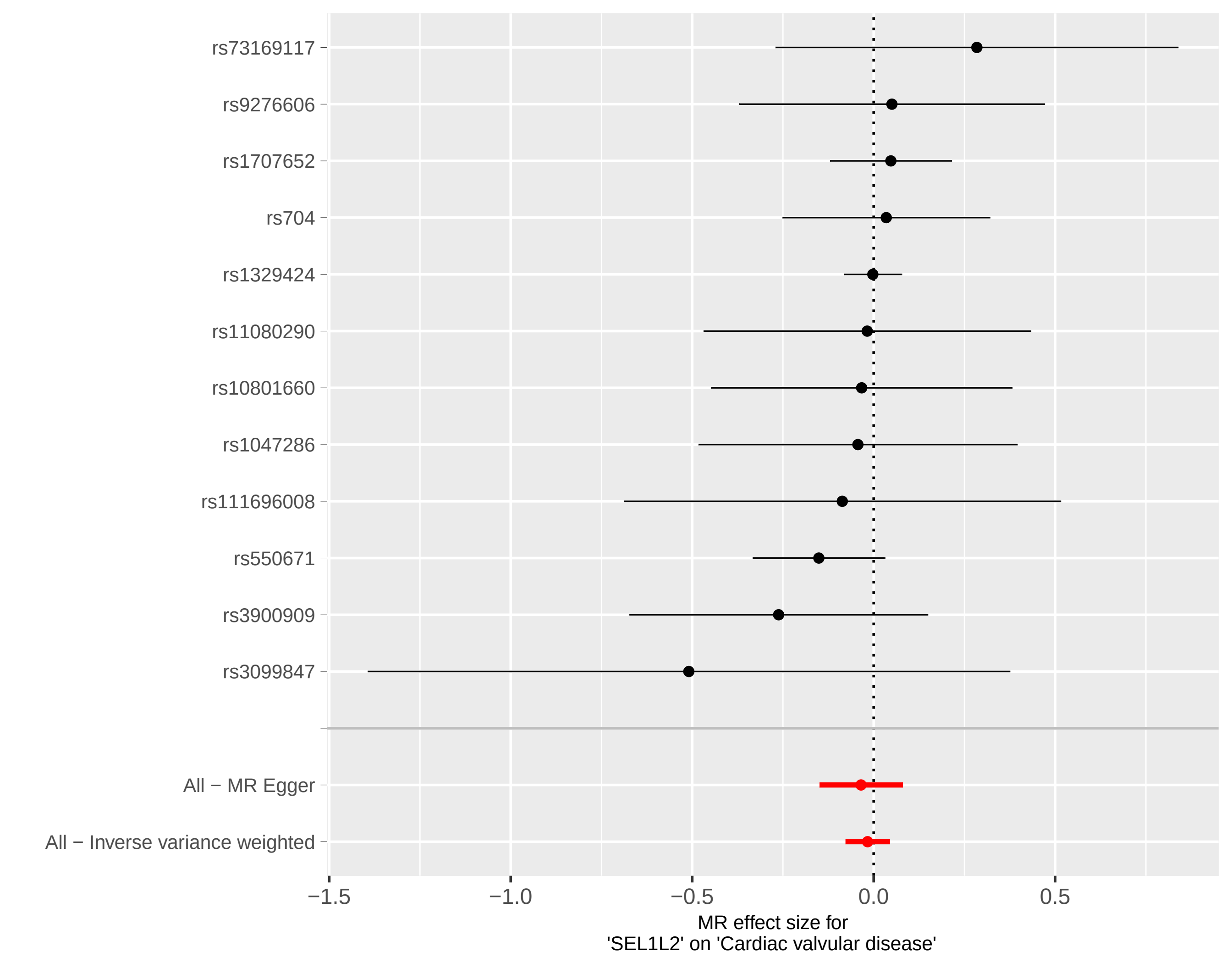
rs73169117
rs9276606
rs1707652
rs704
rs1329424
rs11080290
rs10801660
rs1047286
rs111696008
rs550671
rs3900909
rs3099847
All − MR Egger
All − Inverse variance weighted
−1.5
−1.0
−0.5
0.0
0.5
MR effect size for
'SEL1L2' on 'Cardiac valvular disease'

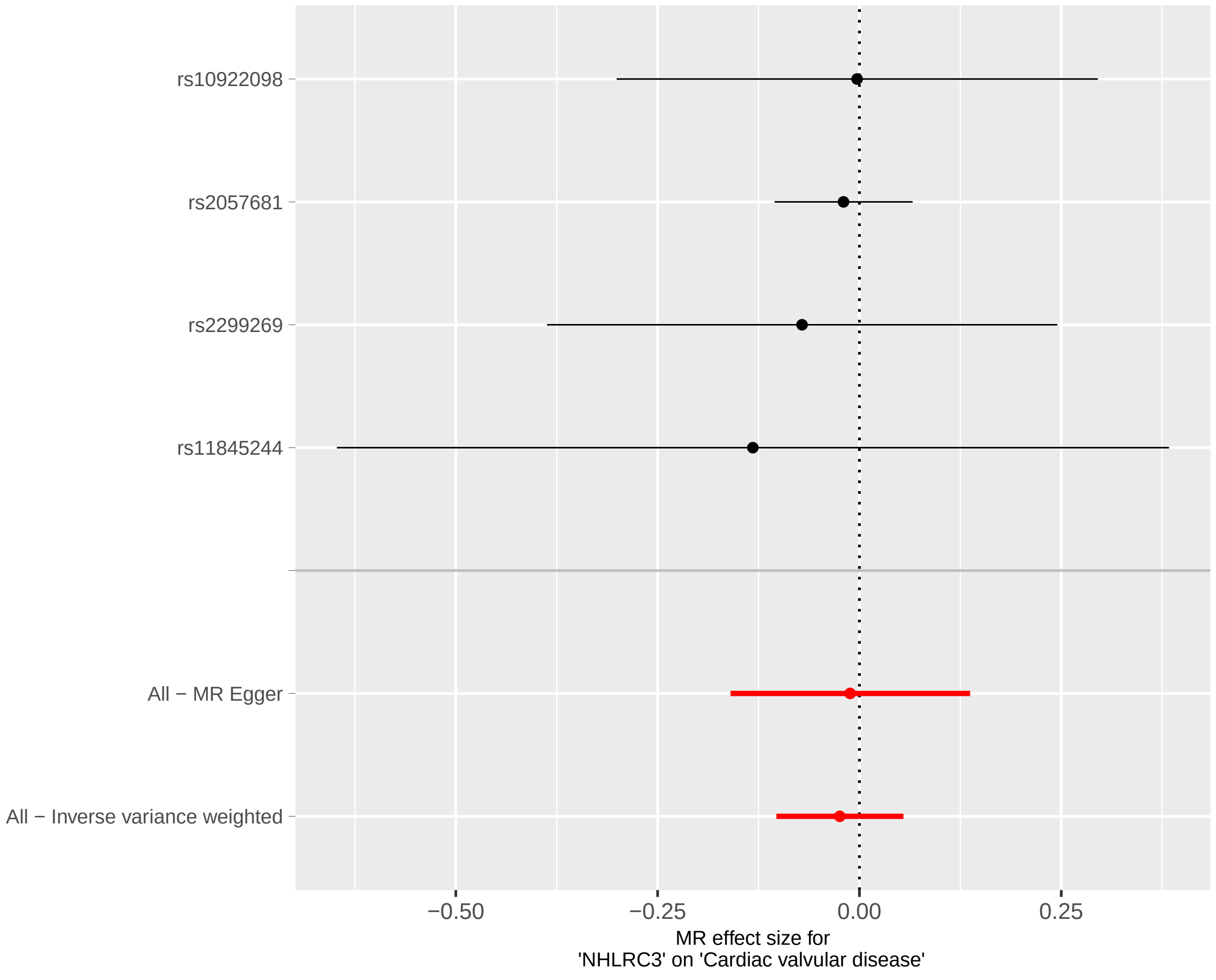

rs10922098
rs2057681
rs2299269
rs11845244
All − MR Egger
All − Inverse variance weighted
−0.50
−0.25
0.00
0.25
MR effect size for
'NHLRC3' on 'Cardiac valvular disease'

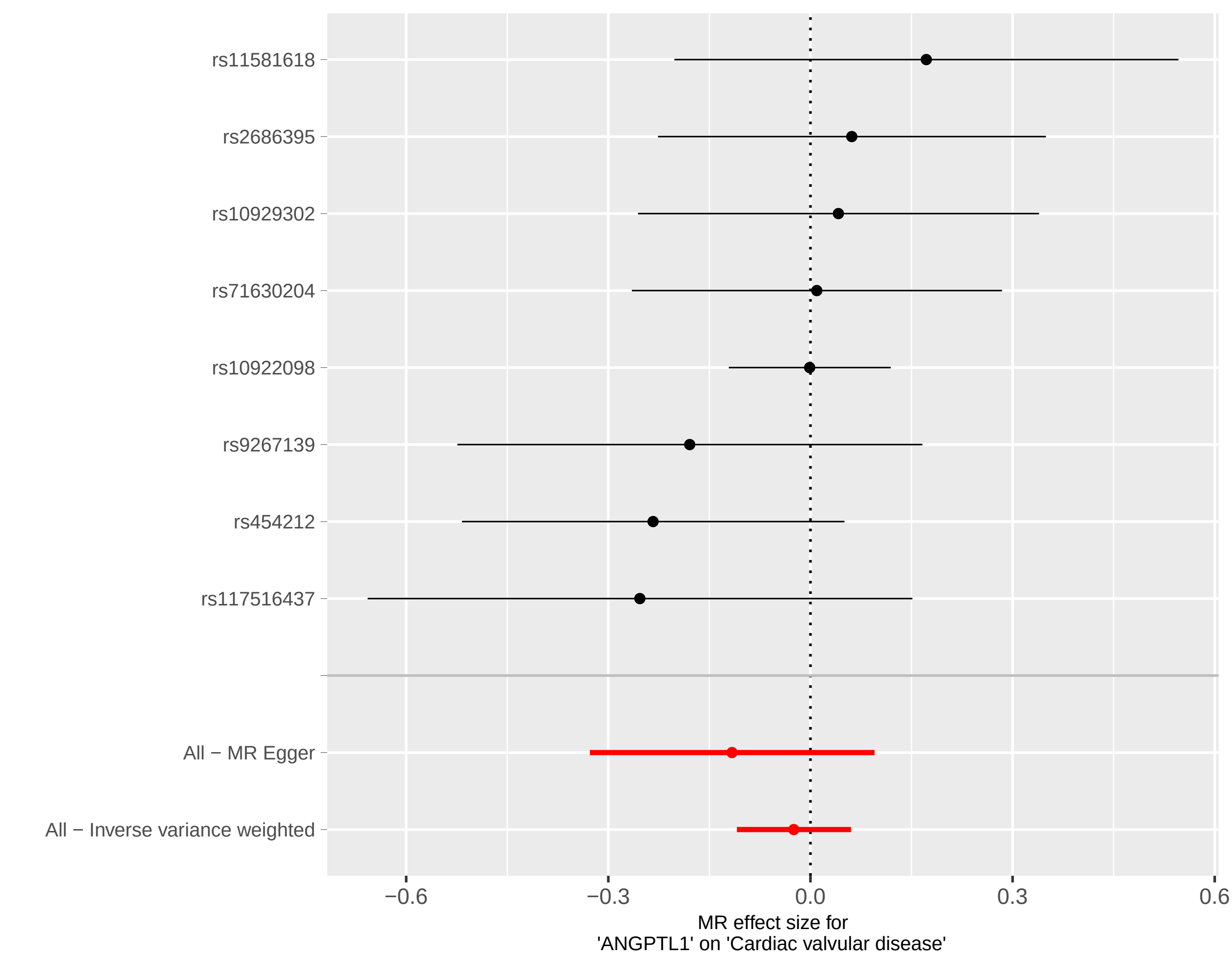
rs11581618
rs2686395
rs10929302
rs71630204
rs10922098
rs9267139
rs454212
rs117516437
All − MR Egger
All − Inverse variance weighted
−0.6
−0.3
0.0
0.3
0.6
MR effect size for
'ANGPTL1' on 'Cardiac valvular disease'

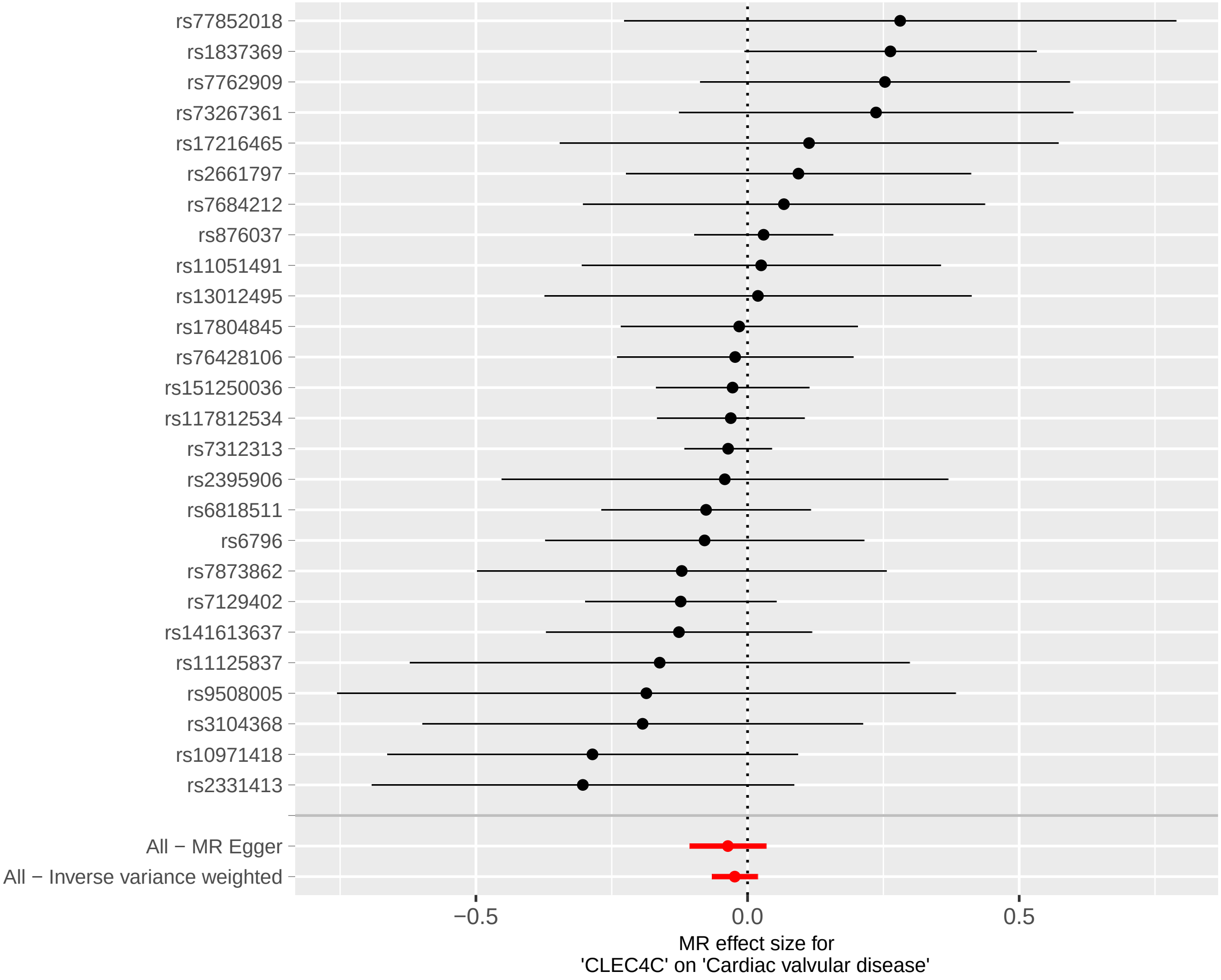
rs77852018
rs1837369
rs7762909
rs73267361
rs17216465
rs2661797
rs7684212
rs876037
rs11051491
rs13012495
rs17804845
rs76428106
rs151250036
rs117812534
rs7312313
rs2395906
rs6818511
rs6796
rs7873862
rs7129402
rs141613637
rs11125837
rs9508005
rs3104368
rs10971418
rs2331413
All − MR Egger
All − Inverse variance weighted
−0.5
0.0
0.5
MR effect size for
'CLEC4C' on 'Cardiac valvular disease'

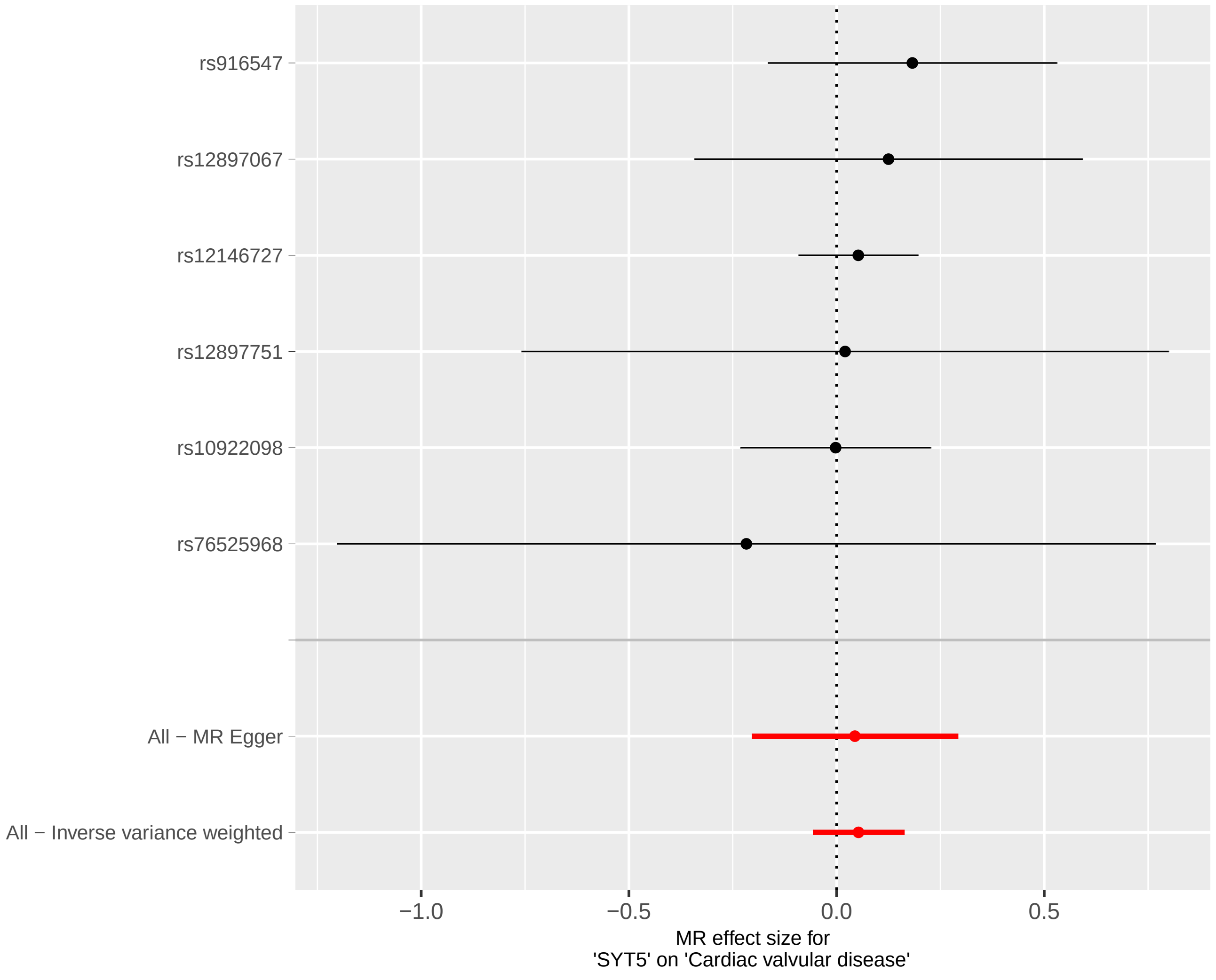

rs916547
rs12897067
rs12146727
rs12897751
rs10922098
rs76525968
All − MR Egger
All − Inverse variance weighted
−1.0
−0.5
0.0
0.5
MR effect size for
'SYT5' on 'Cardiac valvular disease'

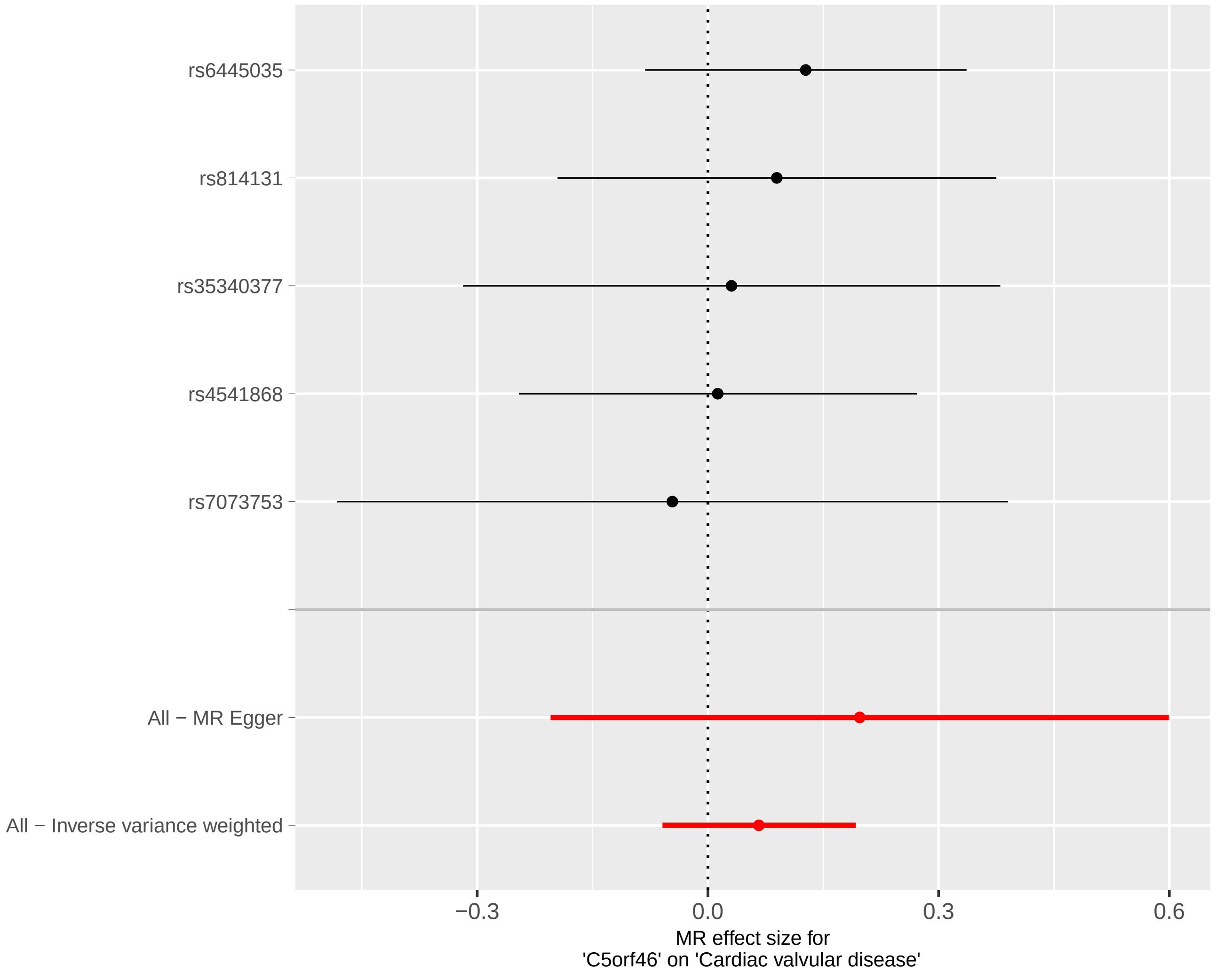
rs6445035
rs814131
rs35340377
rs4541868
rs7073753
All − MR Egger
All − Inverse variance weighted
−0.3
0.0
0.3
0.6
MR effect size for
'C5orf46' on 'Cardiac valvular disease'

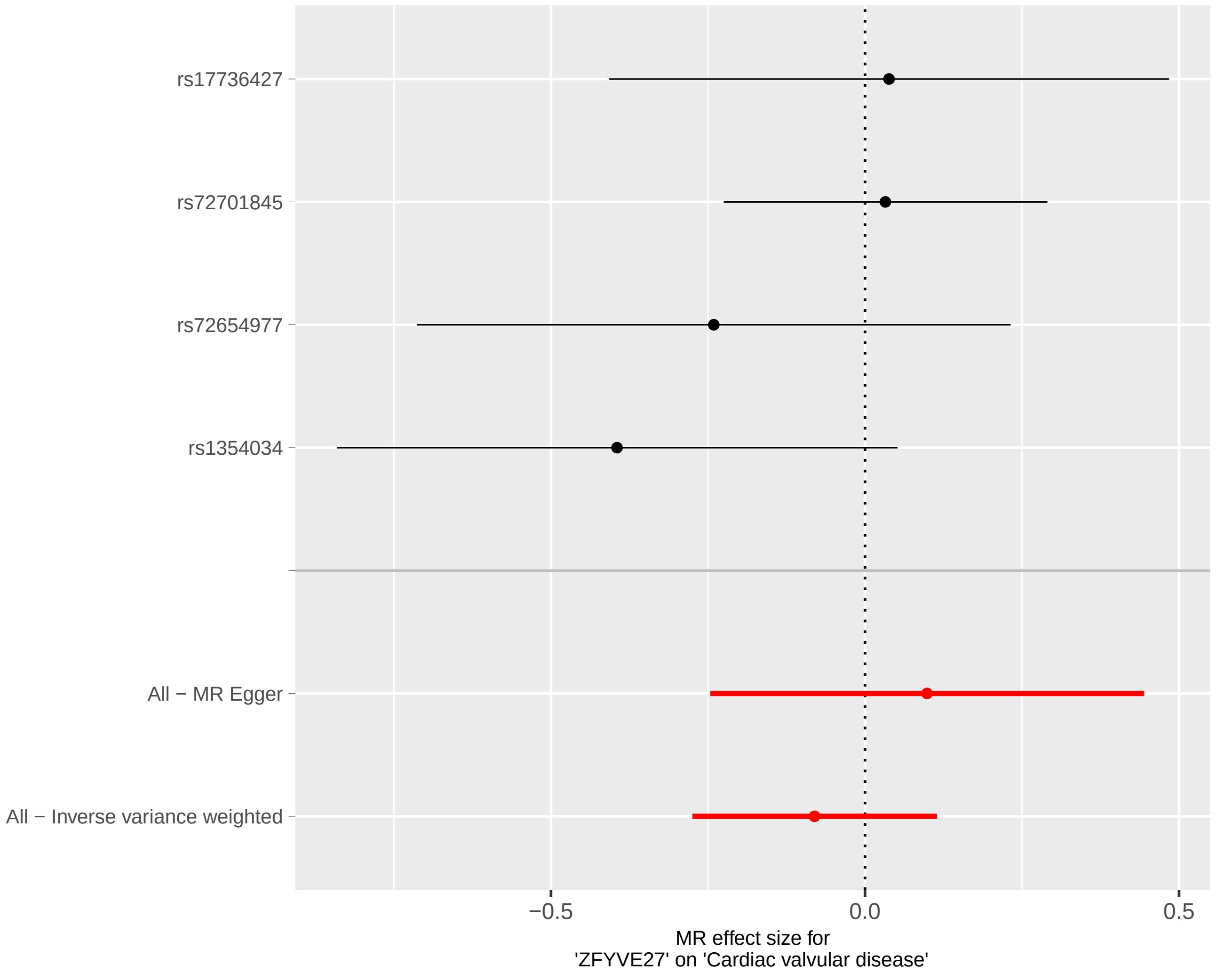
rs17736427
rs72701845
rs72654977
rs1354034
All − MR Egger
All − Inverse variance weighted
−0.5
0.0
0.5
MR effect size for
'ZFYVE27' on 'Cardiac valvular disease'

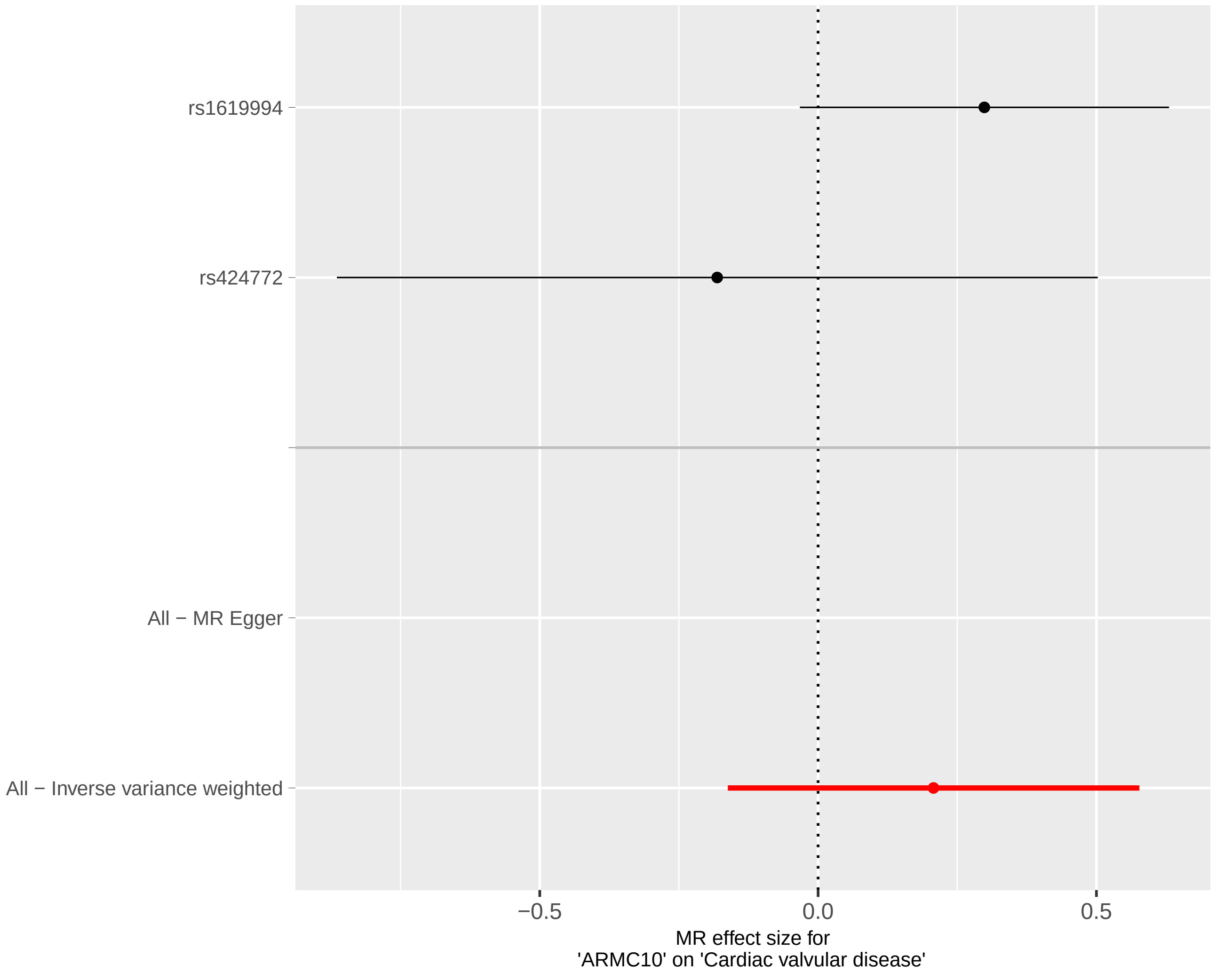
rs1619994
rs424772
All − MR Egger
All − Inverse variance weighted
−0.5
0.0
0.5
MR effect size for
'ARMC10' on 'Cardiac valvular disease'

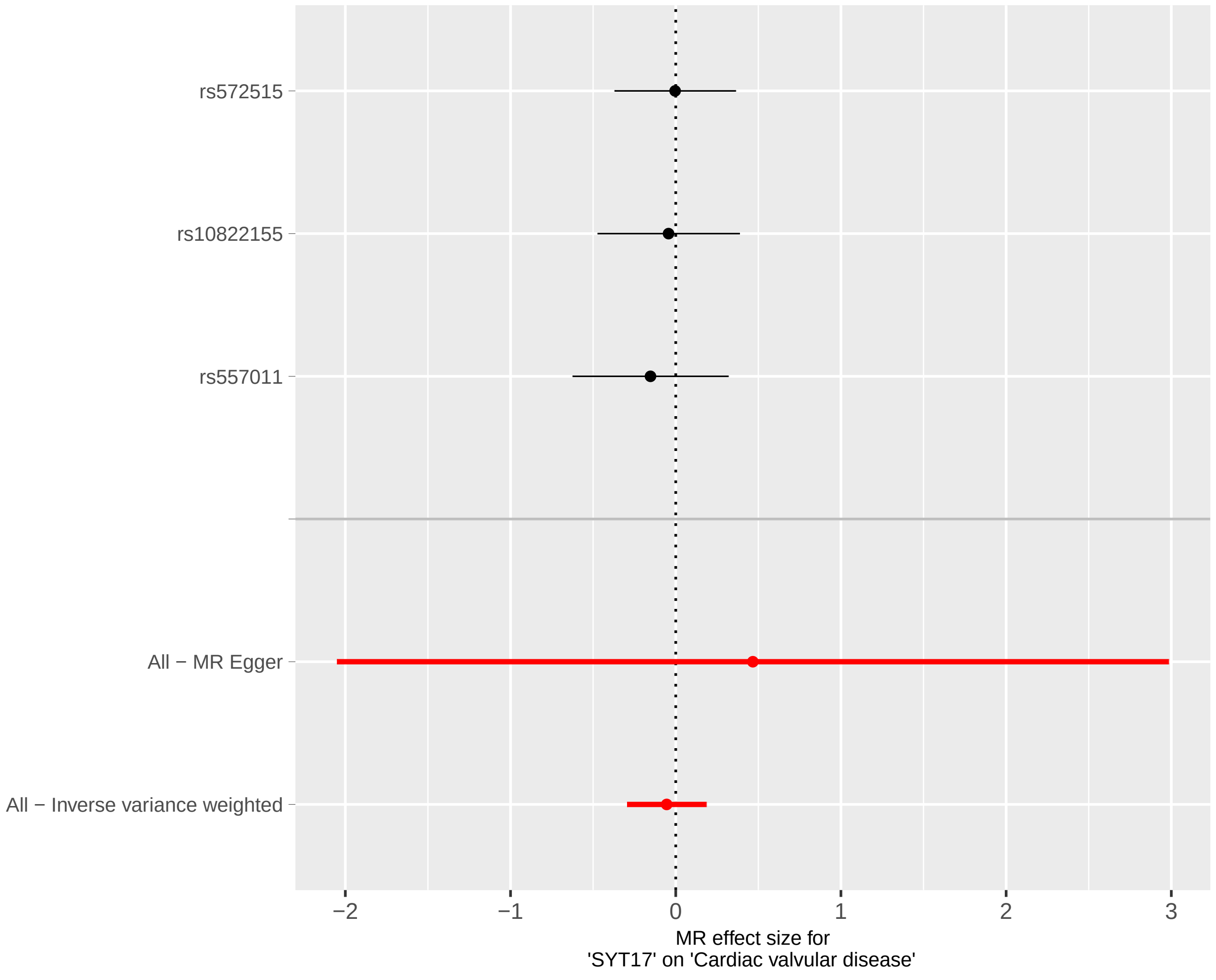
rs572515
rs10822155
rs557011
All − MR Egger
All − Inverse variance weighted
−2
−1
0
1
2
3
MR effect size for
'SYT17' on 'Cardiac valvular disease'

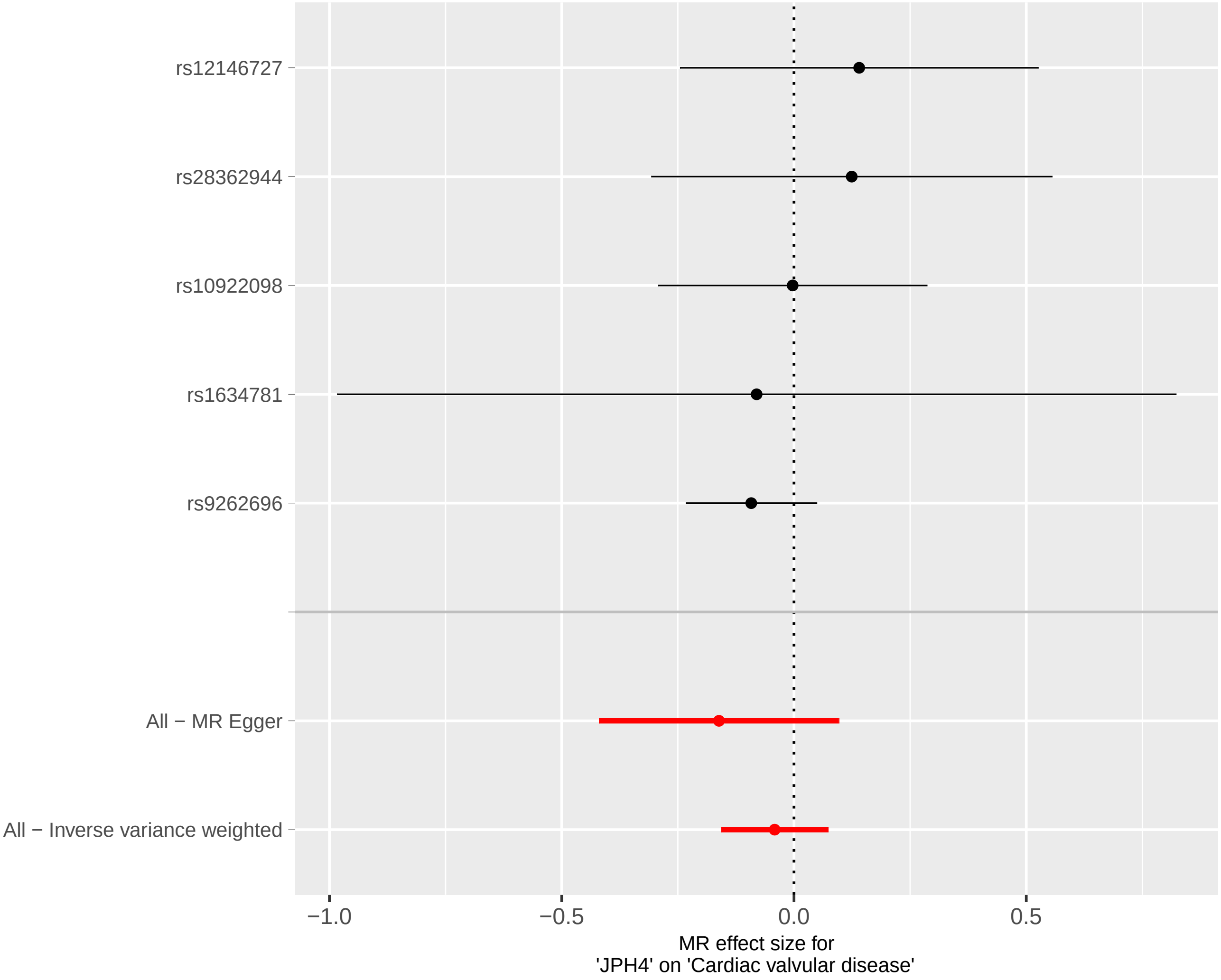
rs12146727
rs28362944
rs10922098
rs1634781
rs9262696
All − MR Egger
All − Inverse variance weighted
−1.0
−0.5
0.0
0.5
MR effect size for
'JPH4' on 'Cardiac valvular disease'

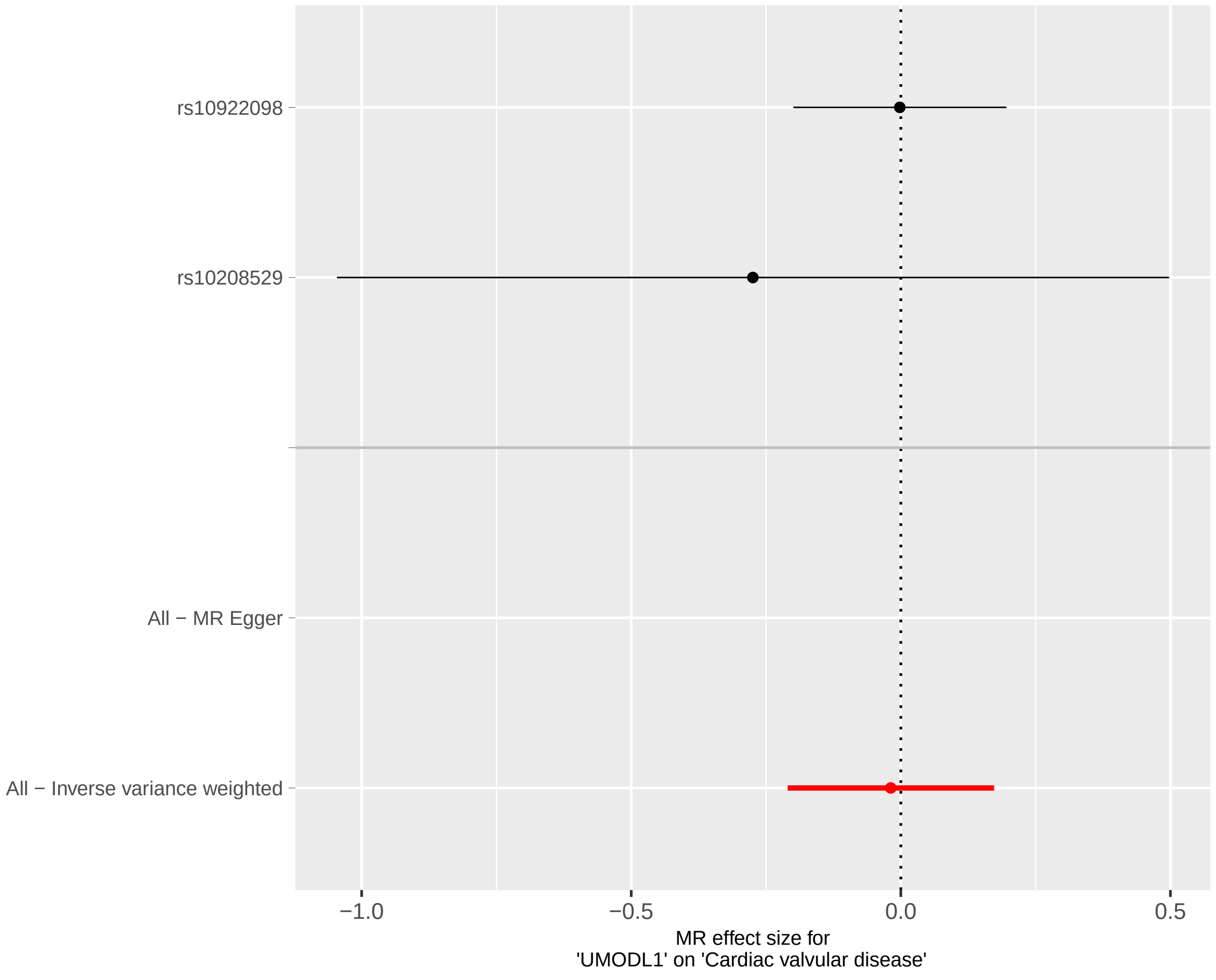
rs10922098
rs10208529
All − MR Egger
All − Inverse variance weighted
−1.0
−0.5
0.0
0.5
MR effect size for
'UMODL1' on 'Cardiac valvular disease'

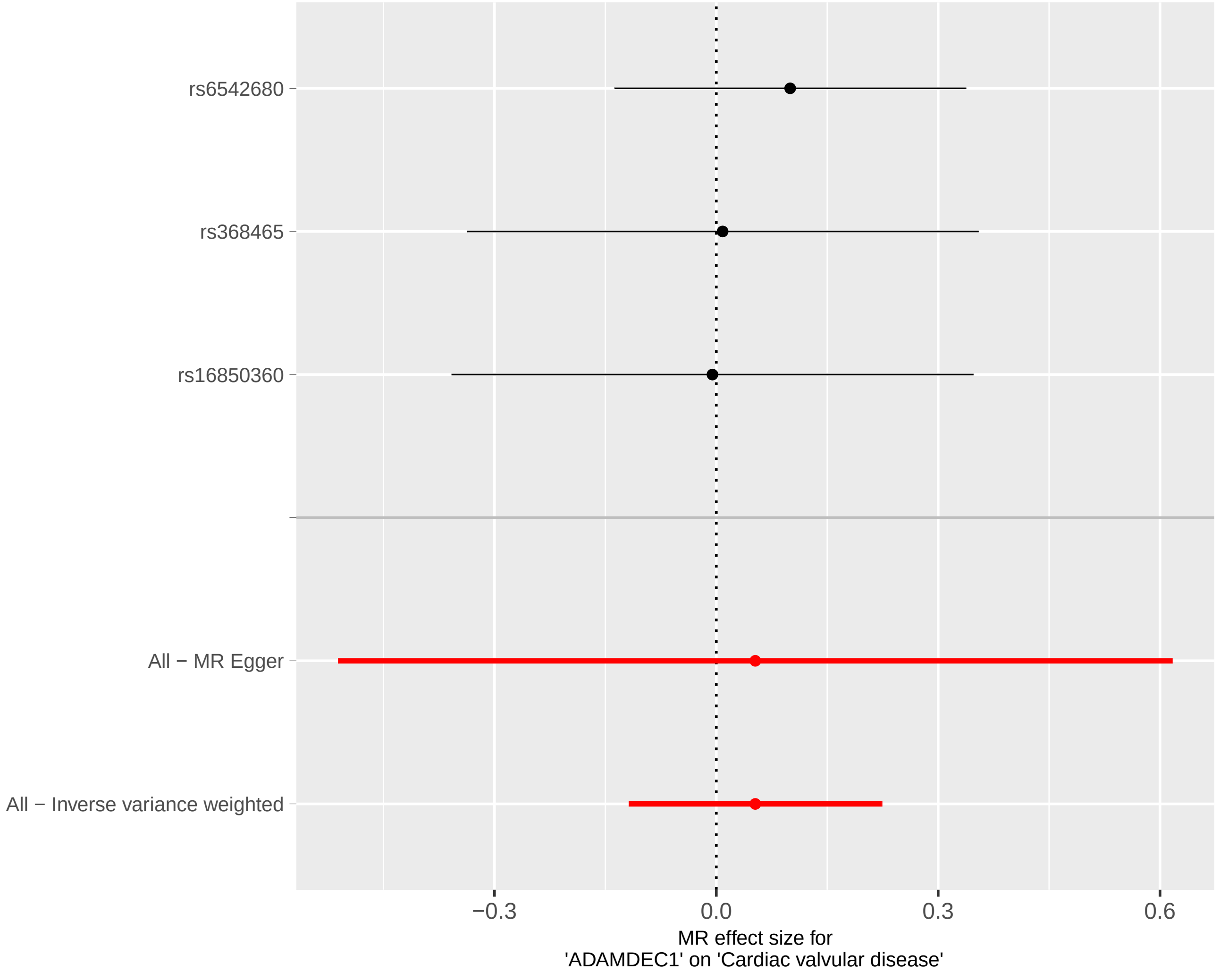

rs6542680
rs368465
rs16850360
All − MR Egger
All − Inverse variance weighted
−0.3
0.0
0.3
0.6
MR effect size for
'ADAMDEC1' on 'Cardiac valvular disease'

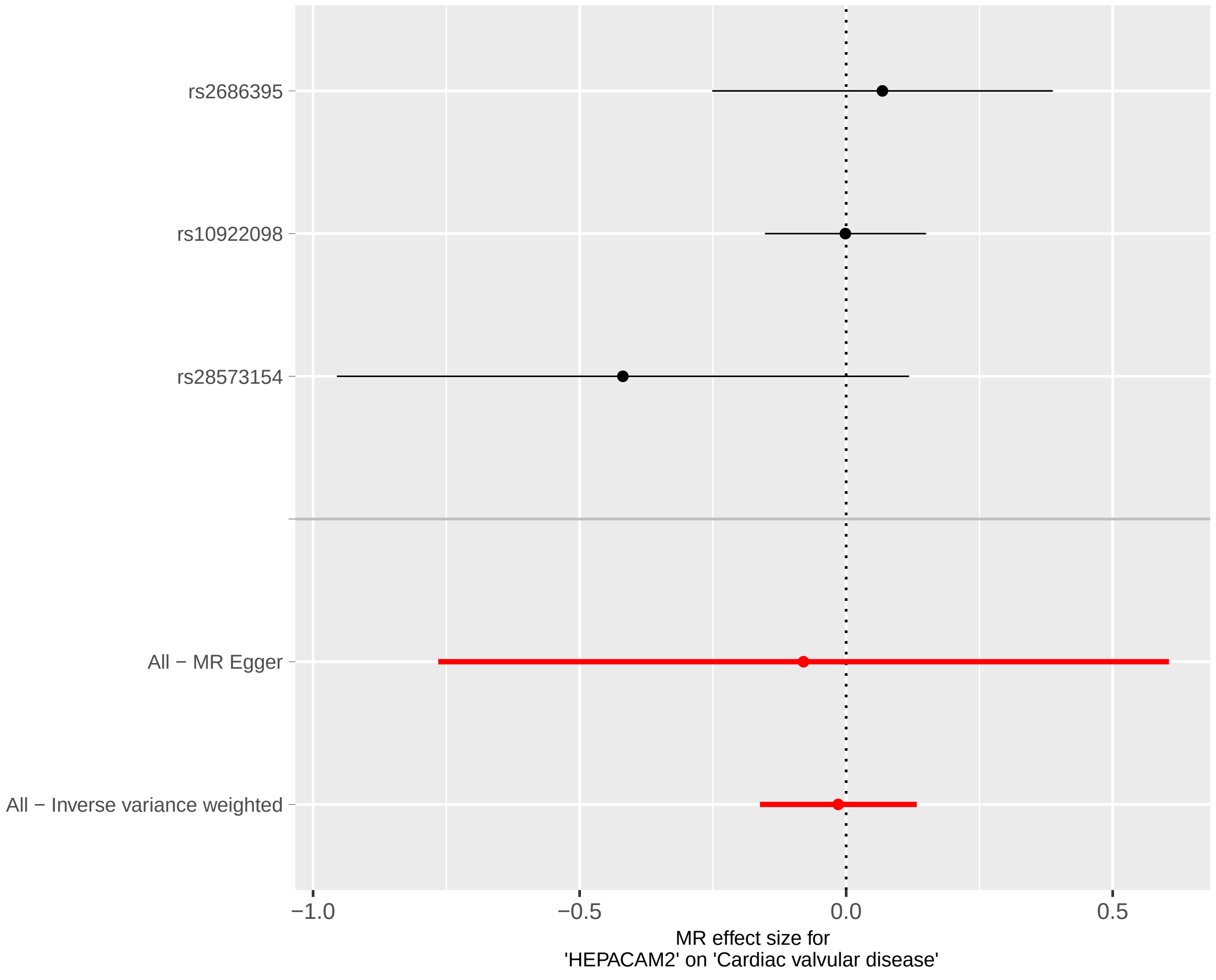
rs2686395
rs10922098
rs28573154
All − MR Egger
All − Inverse variance weighted
−1.0
−0.5
0.0
0.5
MR effect size for
'HEPACAM2' on 'Cardiac valvular disease'

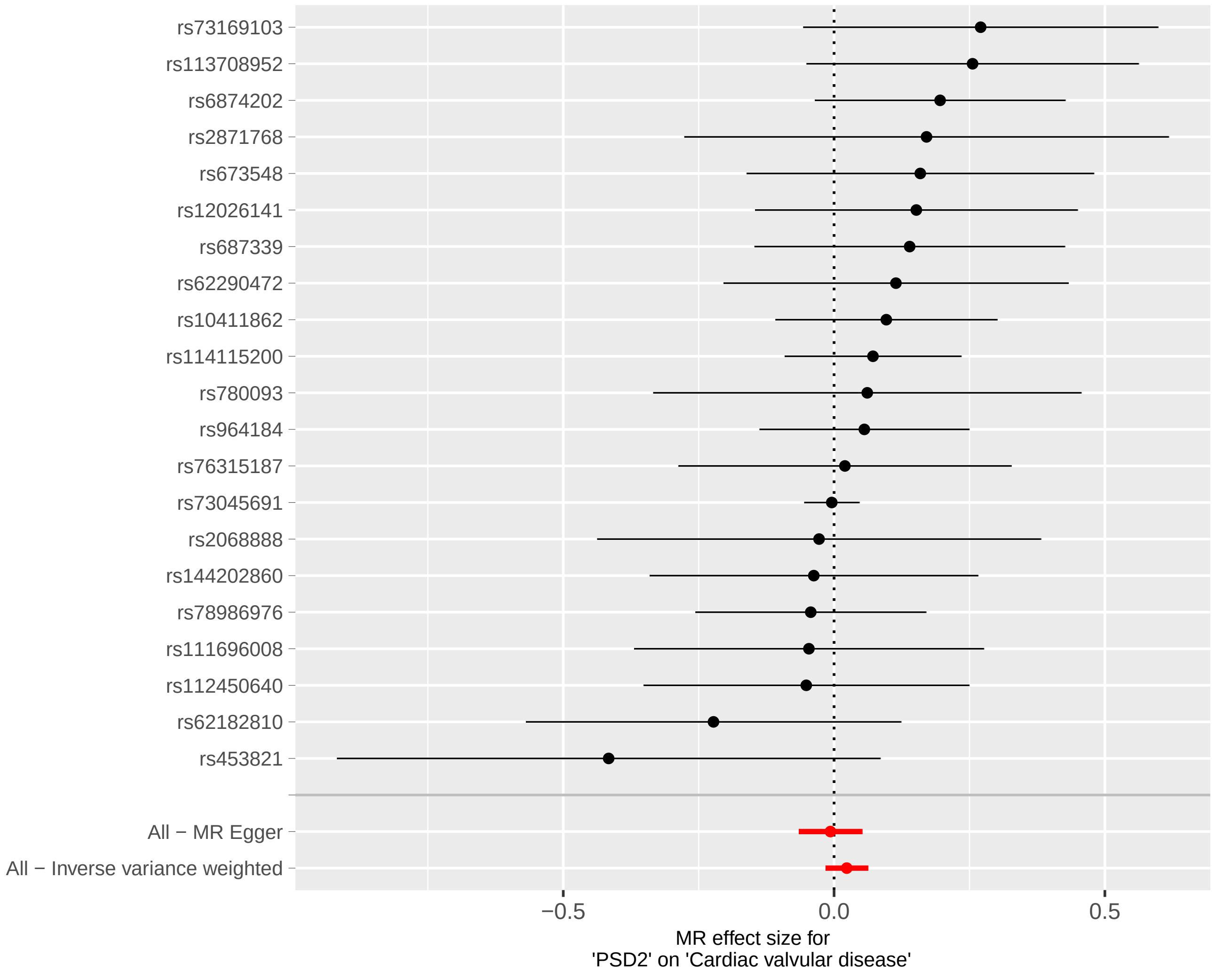
rs73169103
rs113708952
rs6874202
rs2871768
rs673548
rs12026141
rs687339
rs62290472
rs10411862
rs114115200
rs780093
rs964184
rs76315187
rs73045691
rs2068888
rs144202860
rs78986976
rs111696008
rs112450640
rs62182810
rs453821
All − MR Egger
All − Inverse variance weighted
−0.5
0.0
0.5
MR effect size for
'PSD2' on 'Cardiac valvular disease'

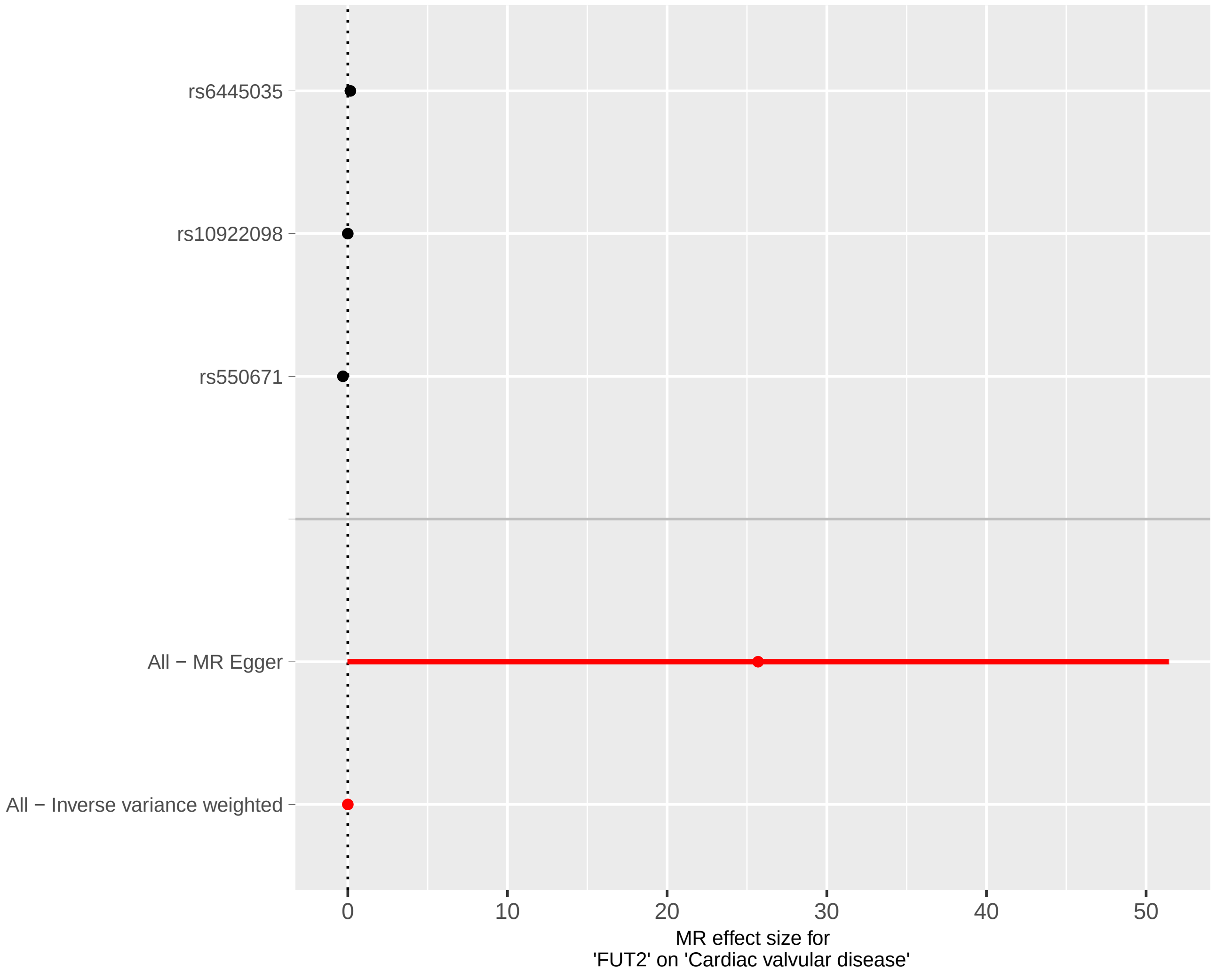
rs6445035
rs10922098
rs550671
All − MR Egger
All − Inverse variance weighted
0
10
20
30
40
50
MR effect size for
'FUT2' on 'Cardiac valvular disease'

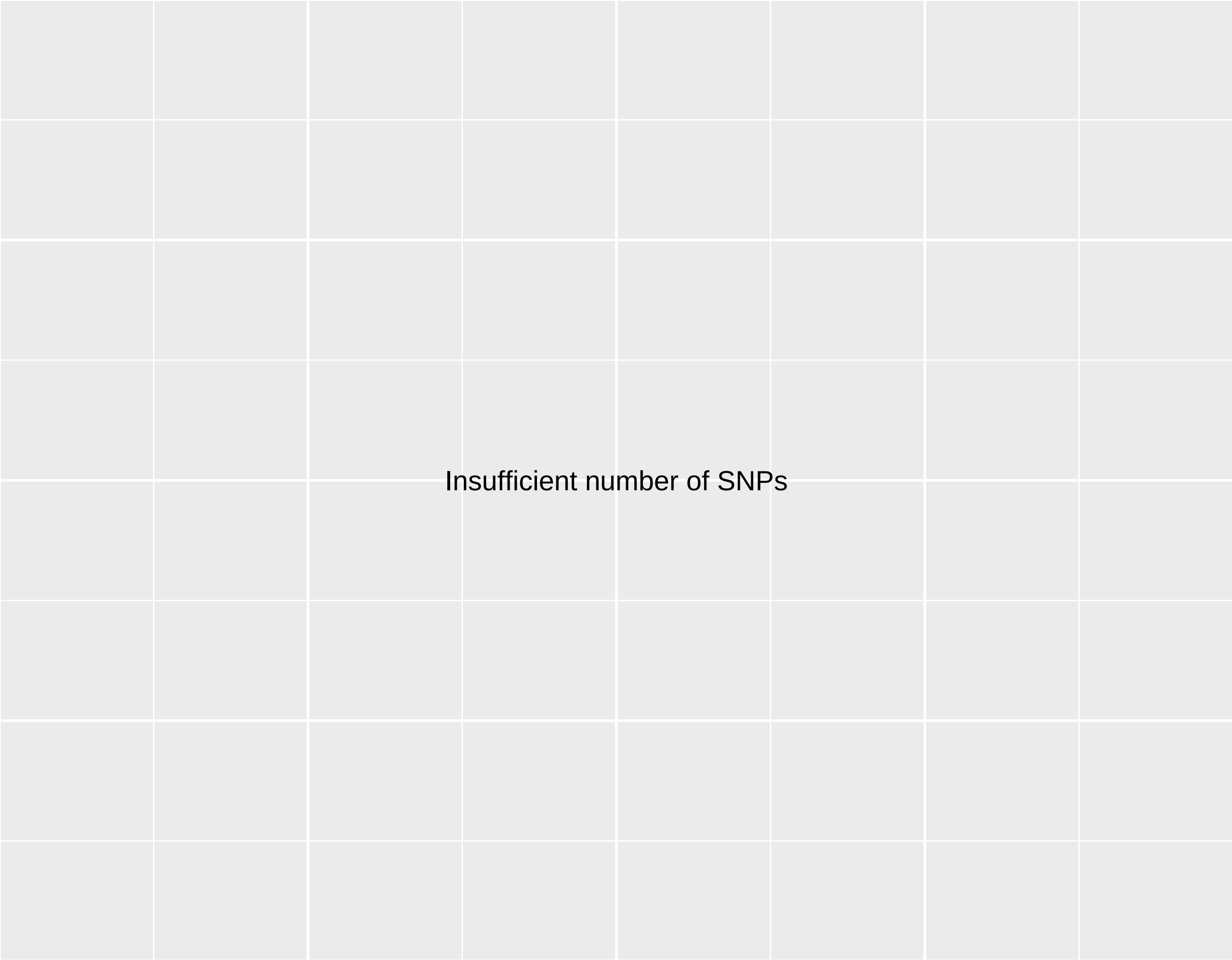
Insufficient number of SNPs

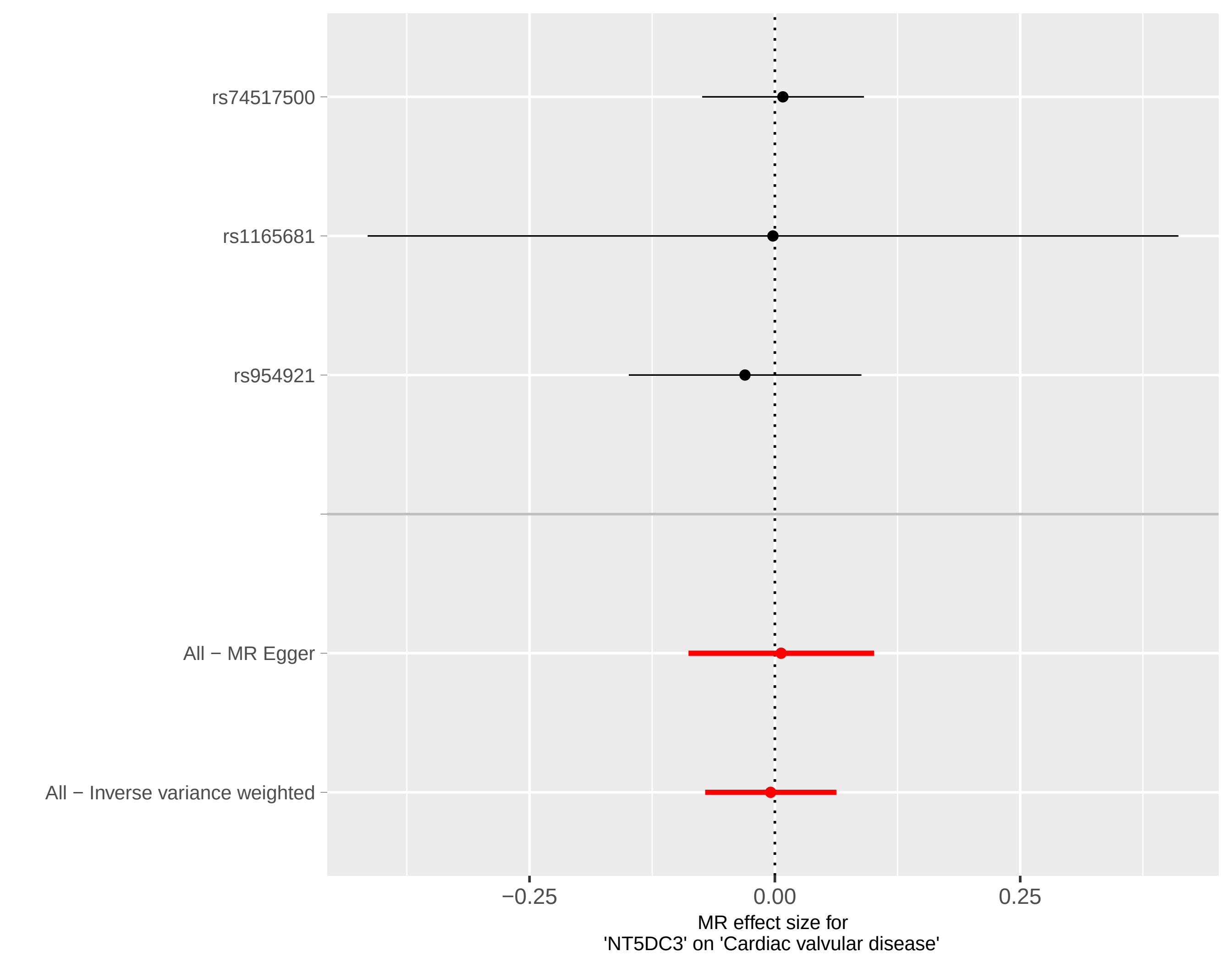
rs74517500
rs1165681
rs954921
All − MR Egger
All − Inverse variance weighted
−0.25
0.00
0.25
MR effect size for
'NT5DC3' on 'Cardiac valvular disease'

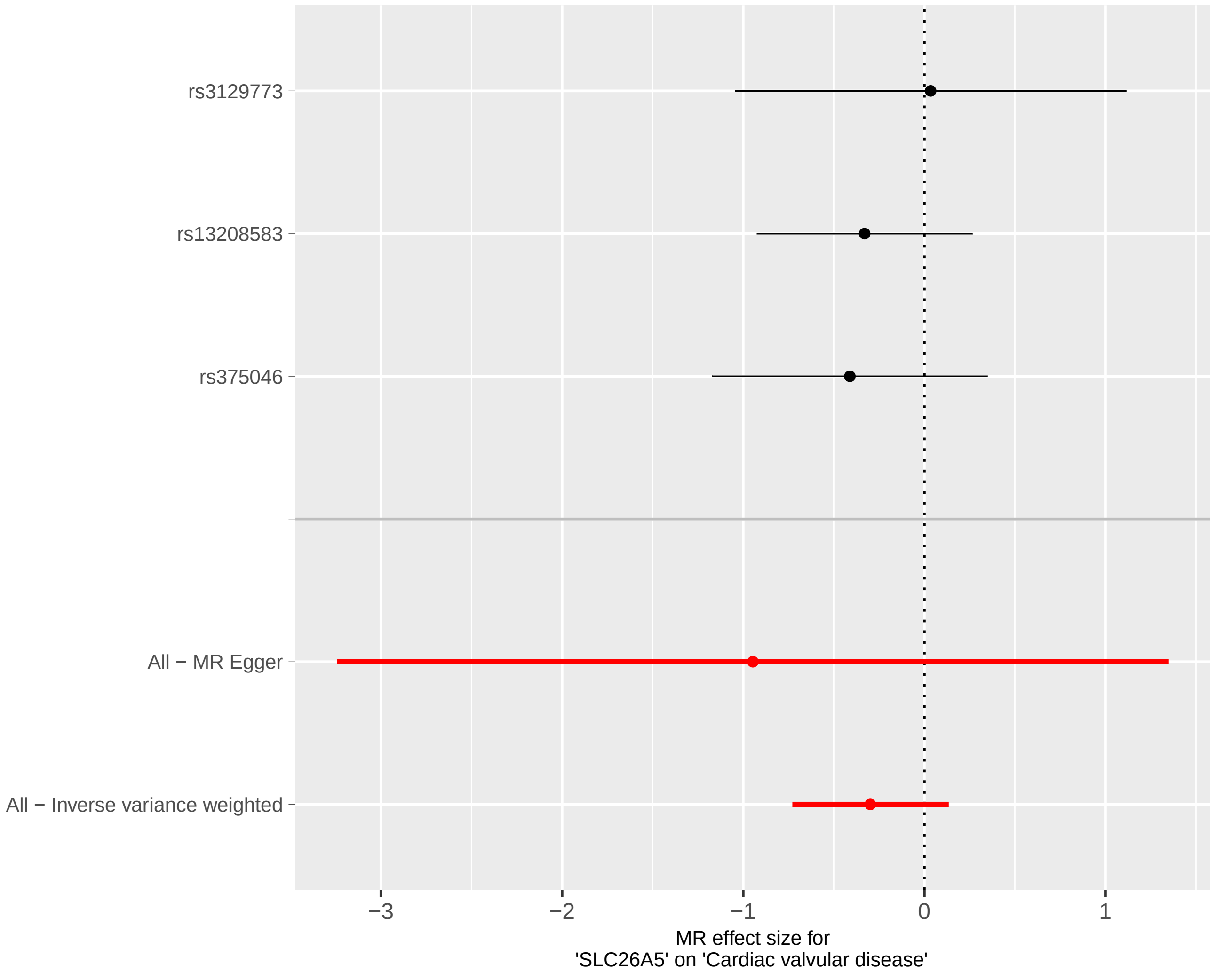

rs3129773
rs13208583
rs375046
All − MR Egger
All − Inverse variance weighted
−3
−2
−1
0
1
MR effect size for
'SLC26A5' on 'Cardiac valvular disease'

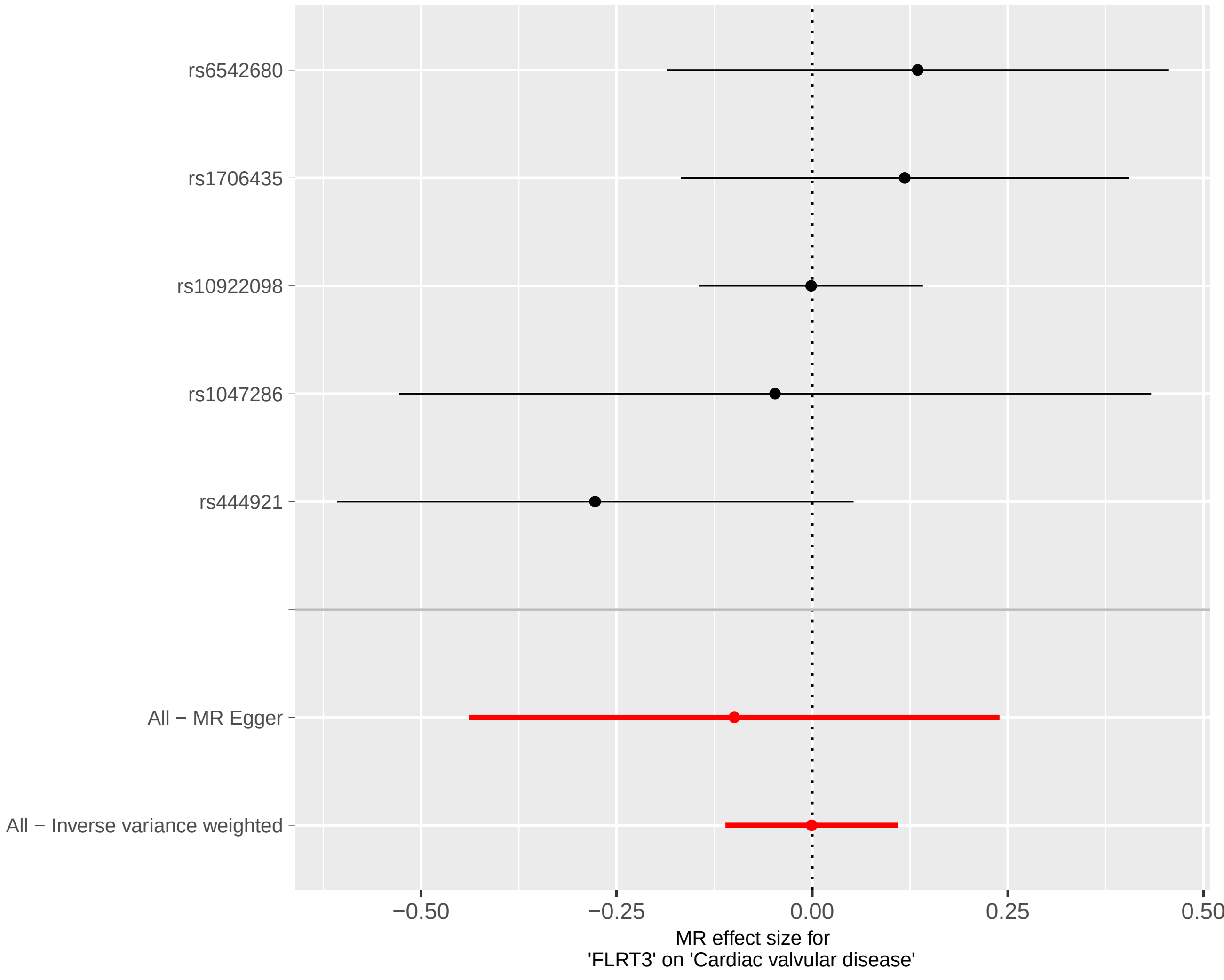

rs6542680
rs1706435
rs10922098
rs1047286
rs444921
All − MR Egger
All − Inverse variance weighted
−0.50
−0.25
0.00
0.25
0.50
MR effect size for
'FLRT3' on 'Cardiac valvular disease'

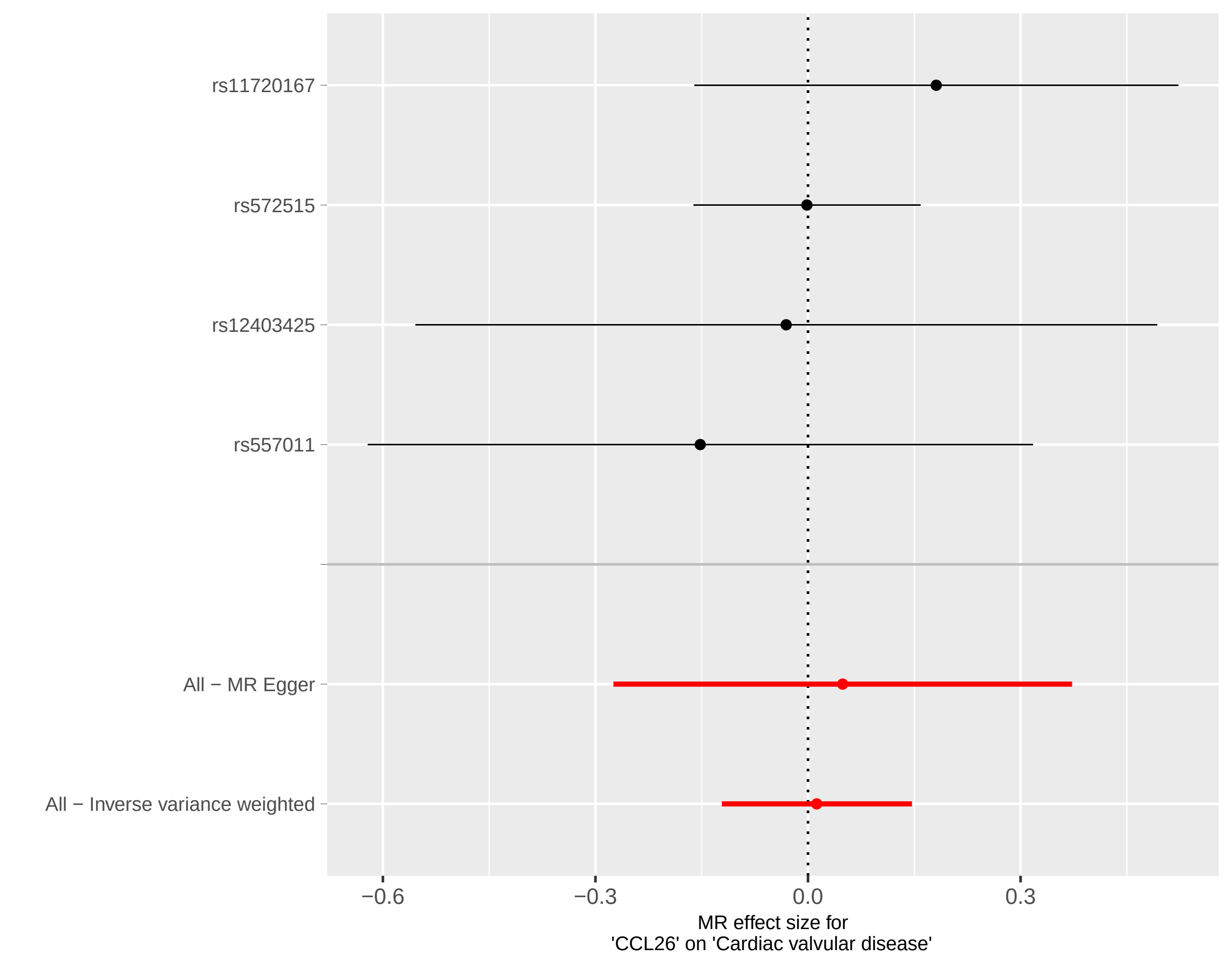

rs11720167
rs572515
rs12403425
rs557011
All − MR Egger
All − Inverse variance weighted
−0.6
−0.3
0.0
0.3
MR effect size for
'CCL26' on 'Cardiac valvular disease'

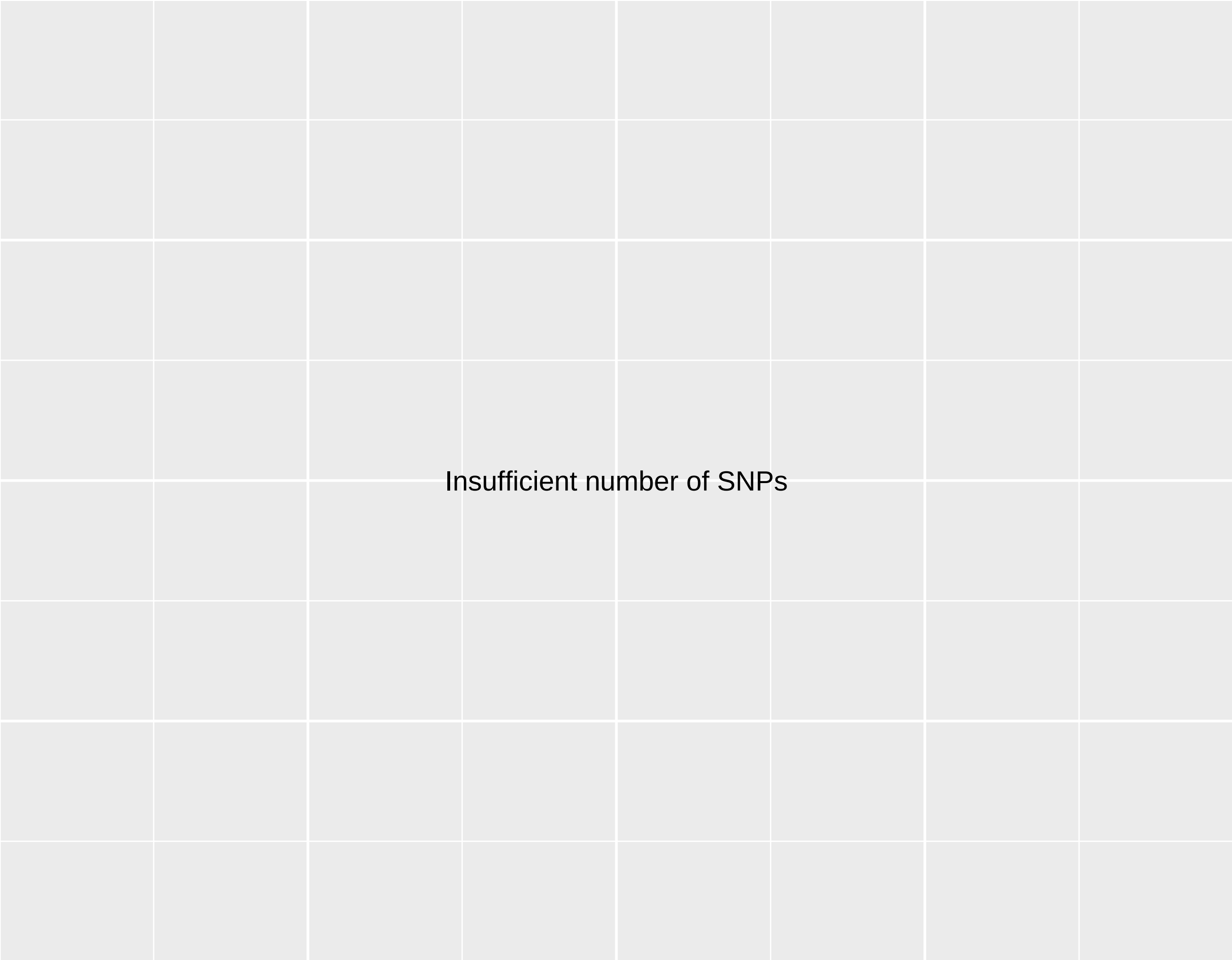
Insufficient number of SNPs

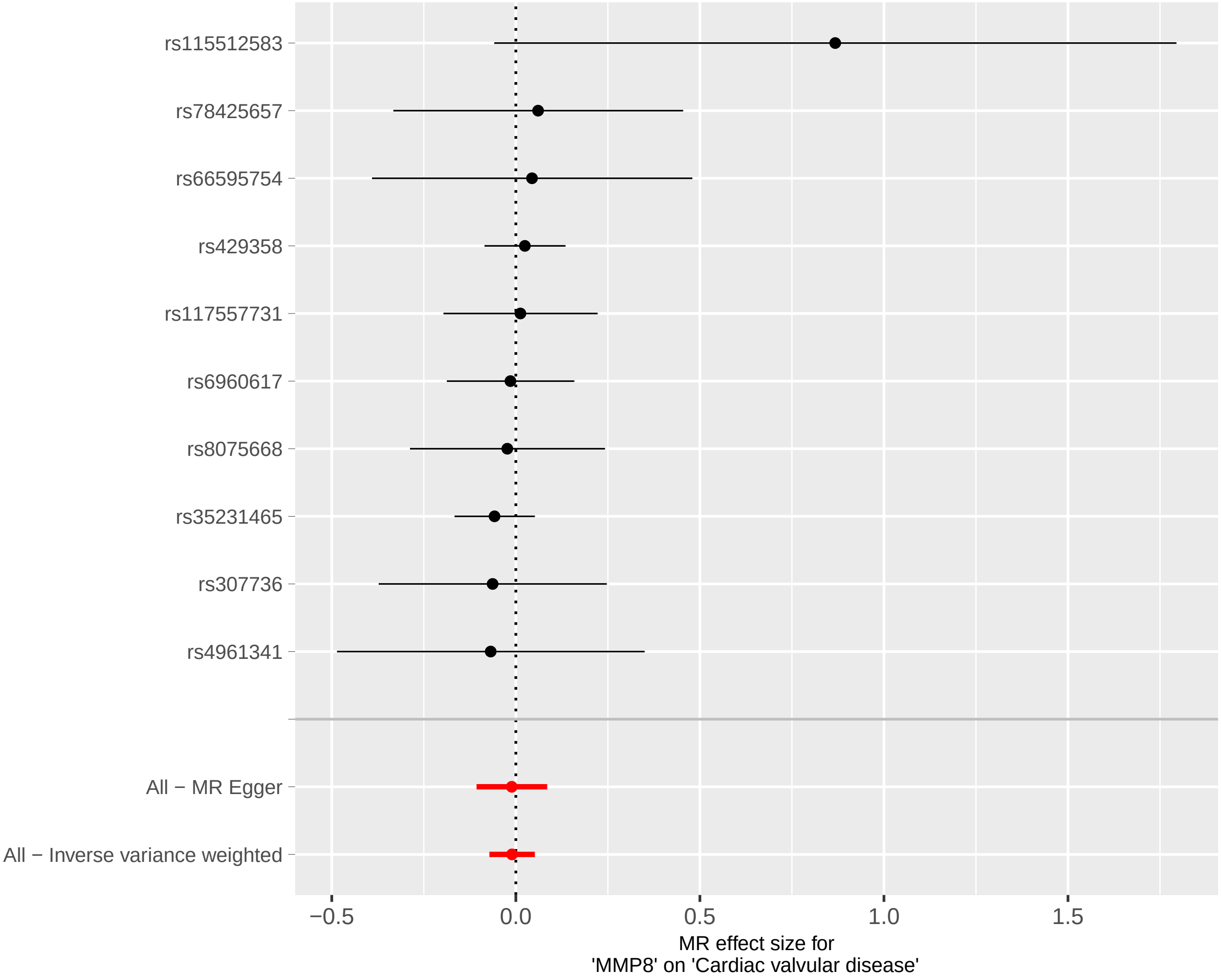
rs115512583
rs78425657
rs66595754
rs429358
rs117557731
rs6960617
rs8075668
rs35231465
rs307736
rs4961341
All − MR Egger
All − Inverse variance weighted
−0.5
0.0
0.5
1.0
1.5
MR effect size for
'MMP8' on 'Cardiac valvular disease'

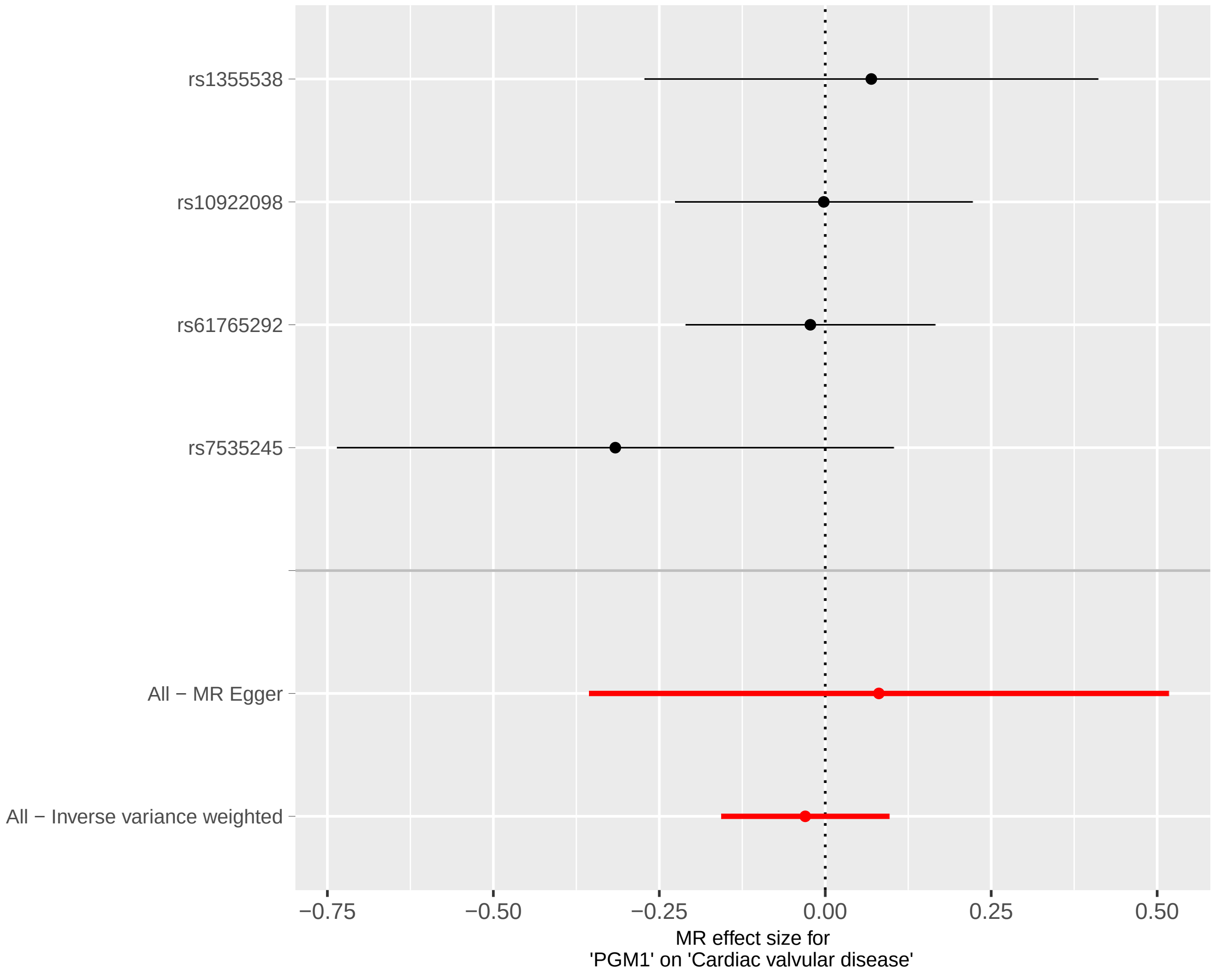
rs1355538
rs10922098
rs61765292
rs7535245
All − MR Egger
All − Inverse variance weighted
−0.75
−0.50
−0.25
0.00
0.25
0.50
MR effect size for
'PGM1' on 'Cardiac valvular disease'

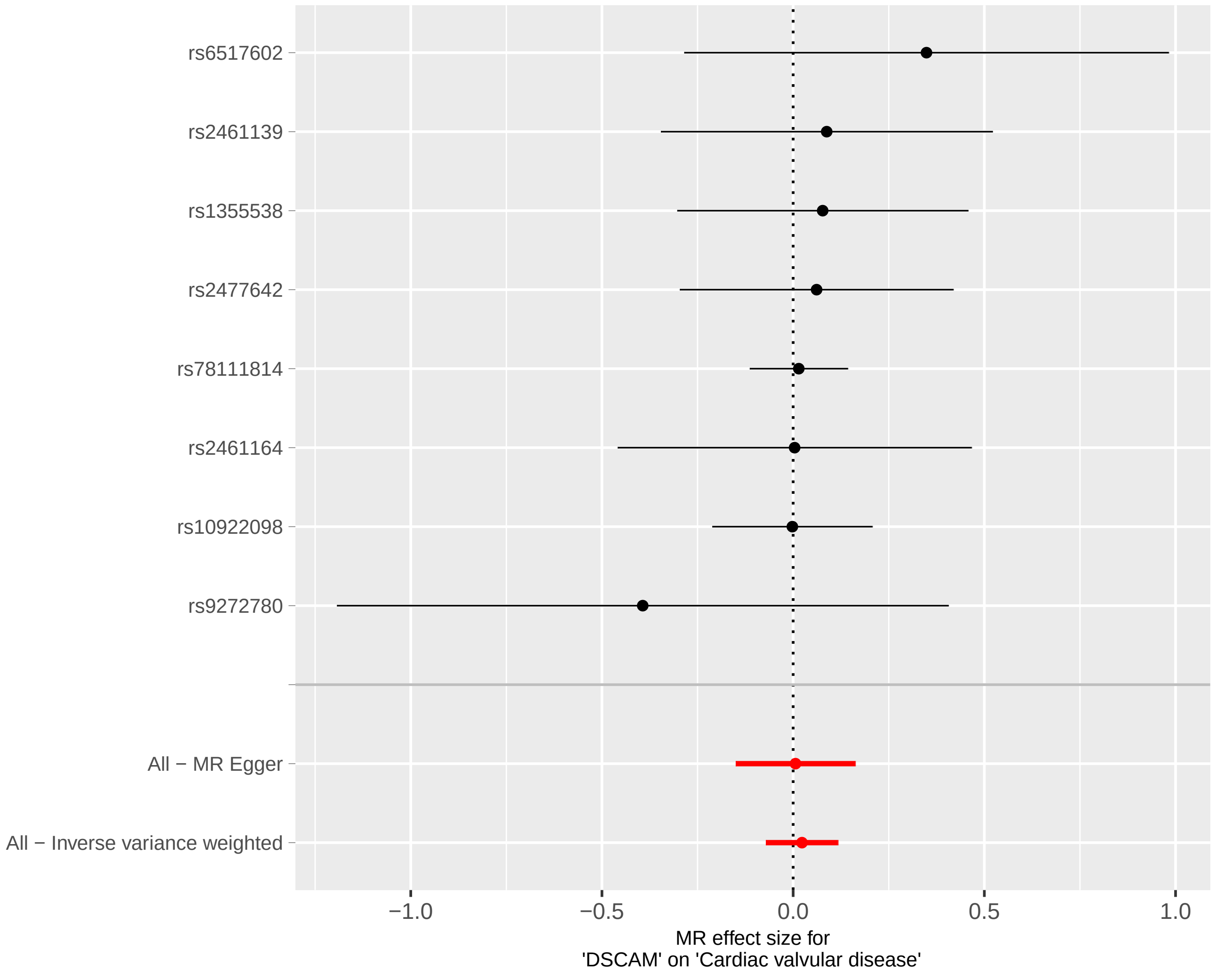
rs6517602
rs2461139
rs1355538
rs2477642
rs78111814
rs2461164
rs10922098
rs9272780
All − MR Egger
All − Inverse variance weighted
−1.0
−0.5
0.0
0.5
1.0
MR effect size for
'DSCAM' on 'Cardiac valvular disease'

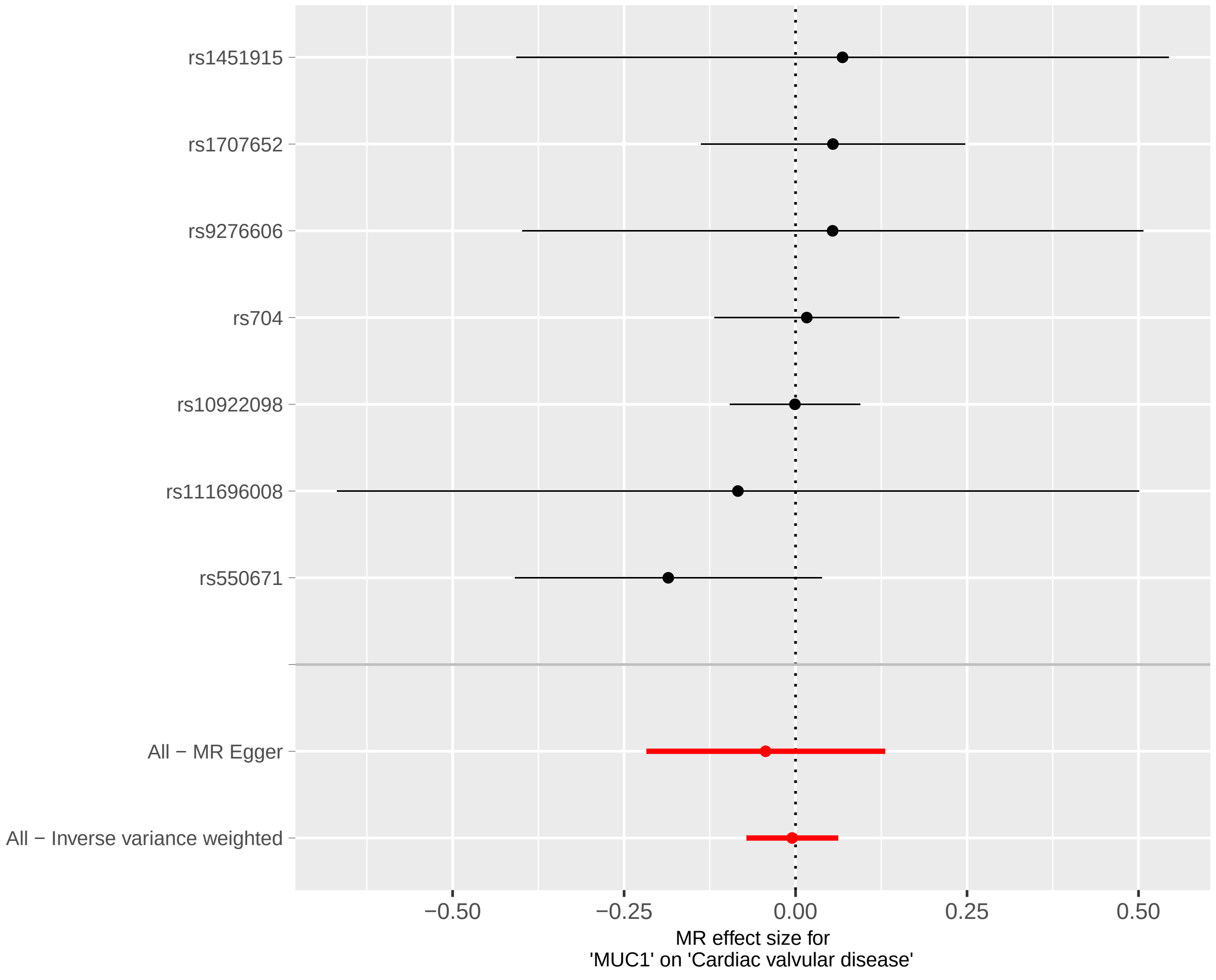
rs1451915
rs1707652
rs9276606
rs704
rs10922098
rs111696008
rs550671
All − MR Egger
All − Inverse variance weighted
−0.50
−0.25
0.00
0.25
0.50
MR effect size for
'MUC1' on 'Cardiac valvular disease'

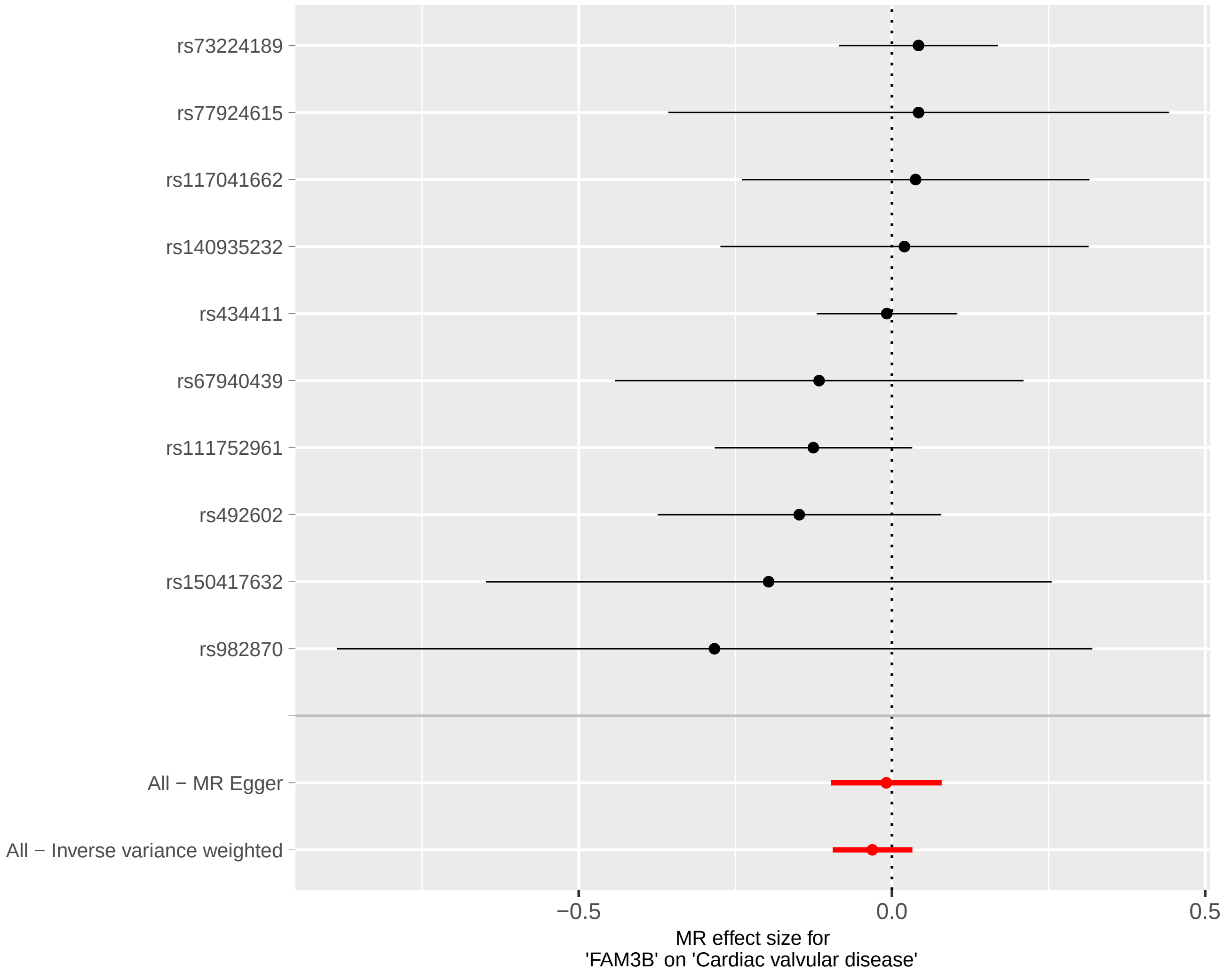
rs73224189
rs77924615
rs117041662
rs140935232
rs434411
rs67940439
rs111752961
rs492602
rs150417632
rs982870
All − MR Egger
All − Inverse variance weighted
−0.5
0.0
0.5
MR effect size for
'FAM3B' on 'Cardiac valvular disease'

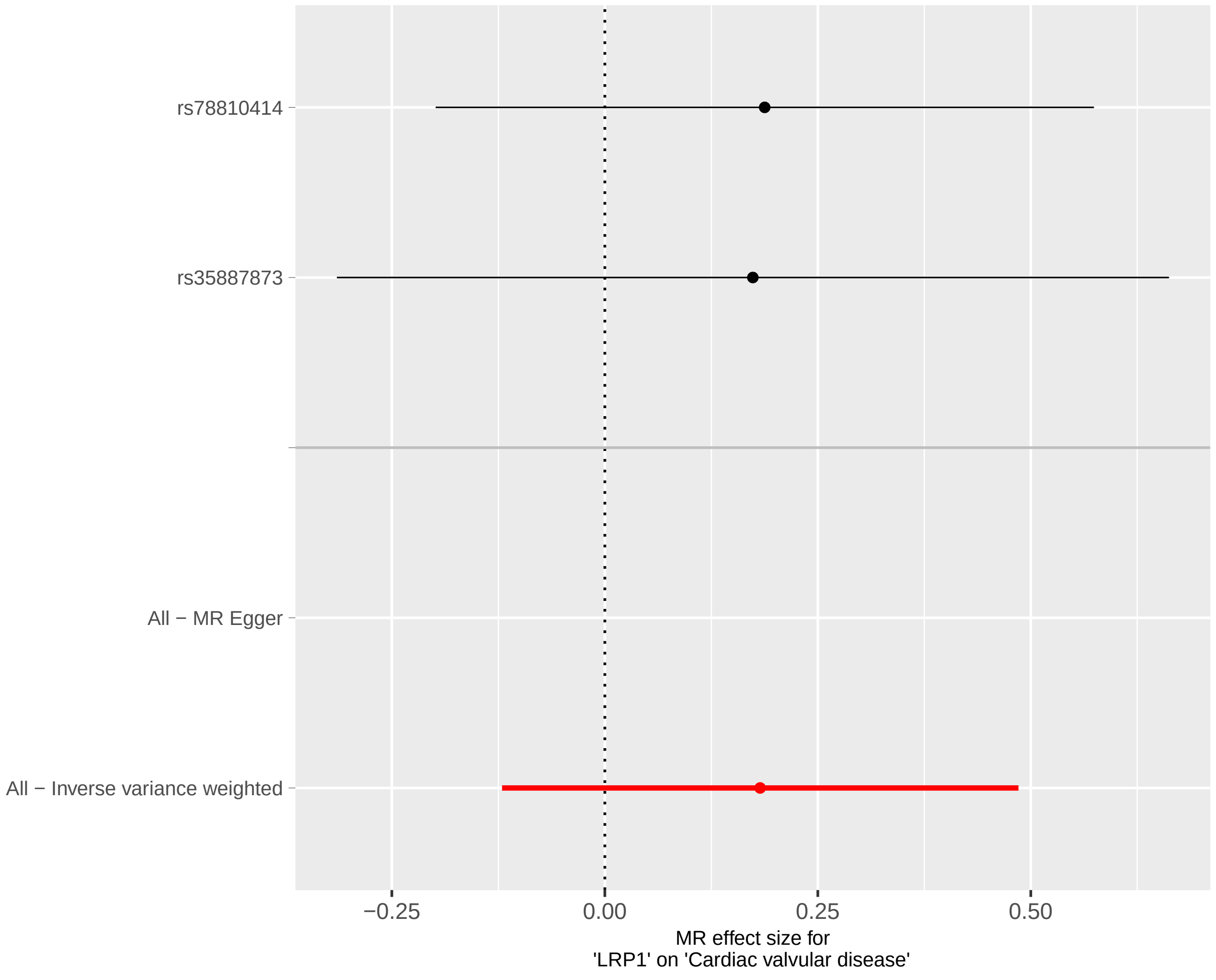

rs78810414
rs35887873
All − MR Egger
All − Inverse variance weighted
−0.25
0.00
0.25
0.50
MR effect size for
'LRP1' on 'Cardiac valvular disease'

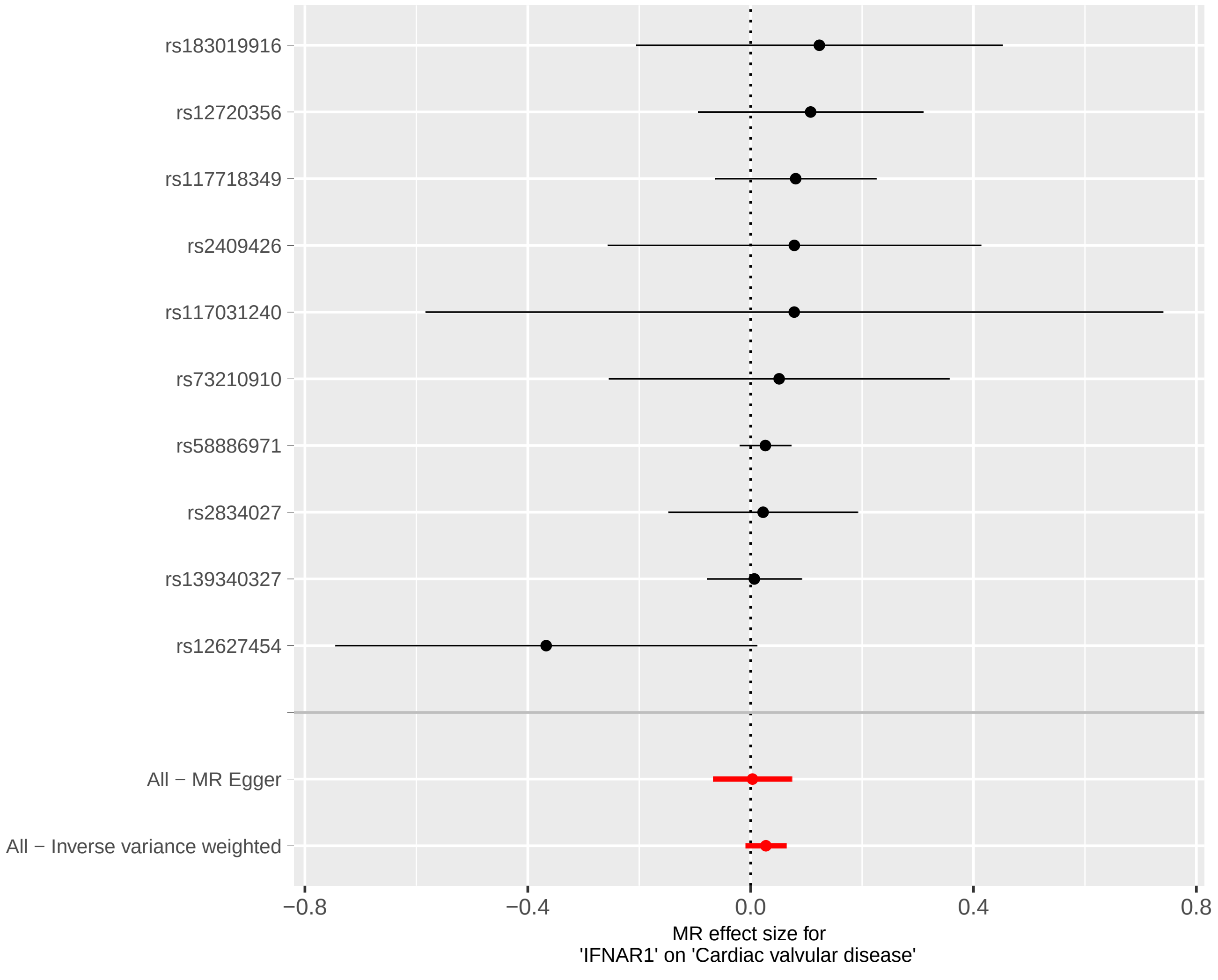

rs183019916
rs12720356
rs117718349
rs2409426
rs117031240
rs73210910
rs58886971
rs2834027
rs139340327
rs12627454
All − MR Egger
All − Inverse variance weighted
−0.8
−0.4
0.0
0.4
0.8
MR effect size for
'IFNAR1' on 'Cardiac valvular disease'

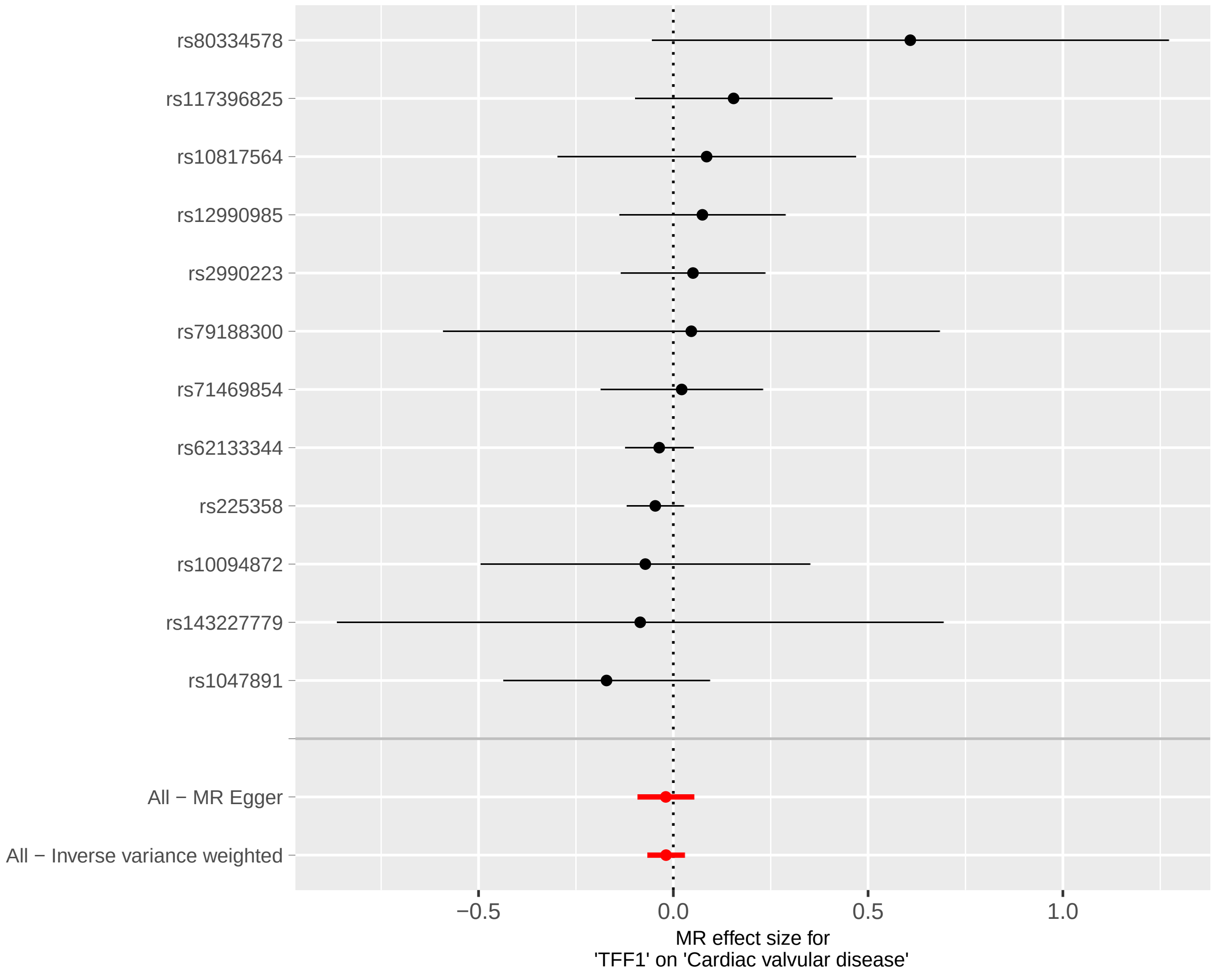
rs80334578
rs117396825
rs10817564
rs12990985
rs2990223
rs79188300
rs71469854
rs62133344
rs225358
rs10094872
rs143227779
rs1047891
All − MR Egger
All − Inverse variance weighted
−0.5
0.0
0.5
1.0
MR effect size for
'TFF1' on 'Cardiac valvular disease'

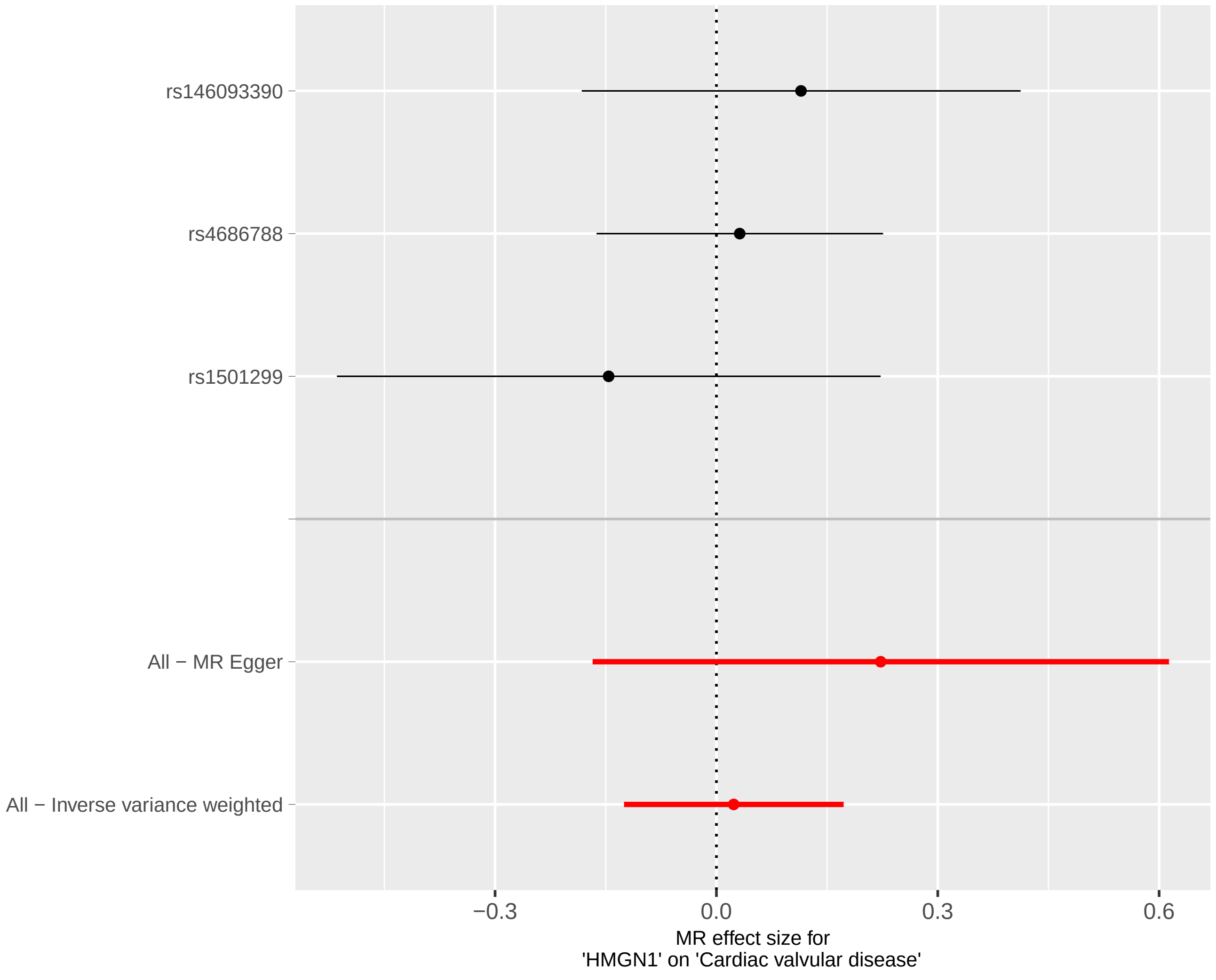

rs146093390
rs4686788
rs1501299
All − MR Egger
All − Inverse variance weighted
−0.3
0.0
0.3
0.6
MR effect size for
'HMGN1' on 'Cardiac valvular disease'

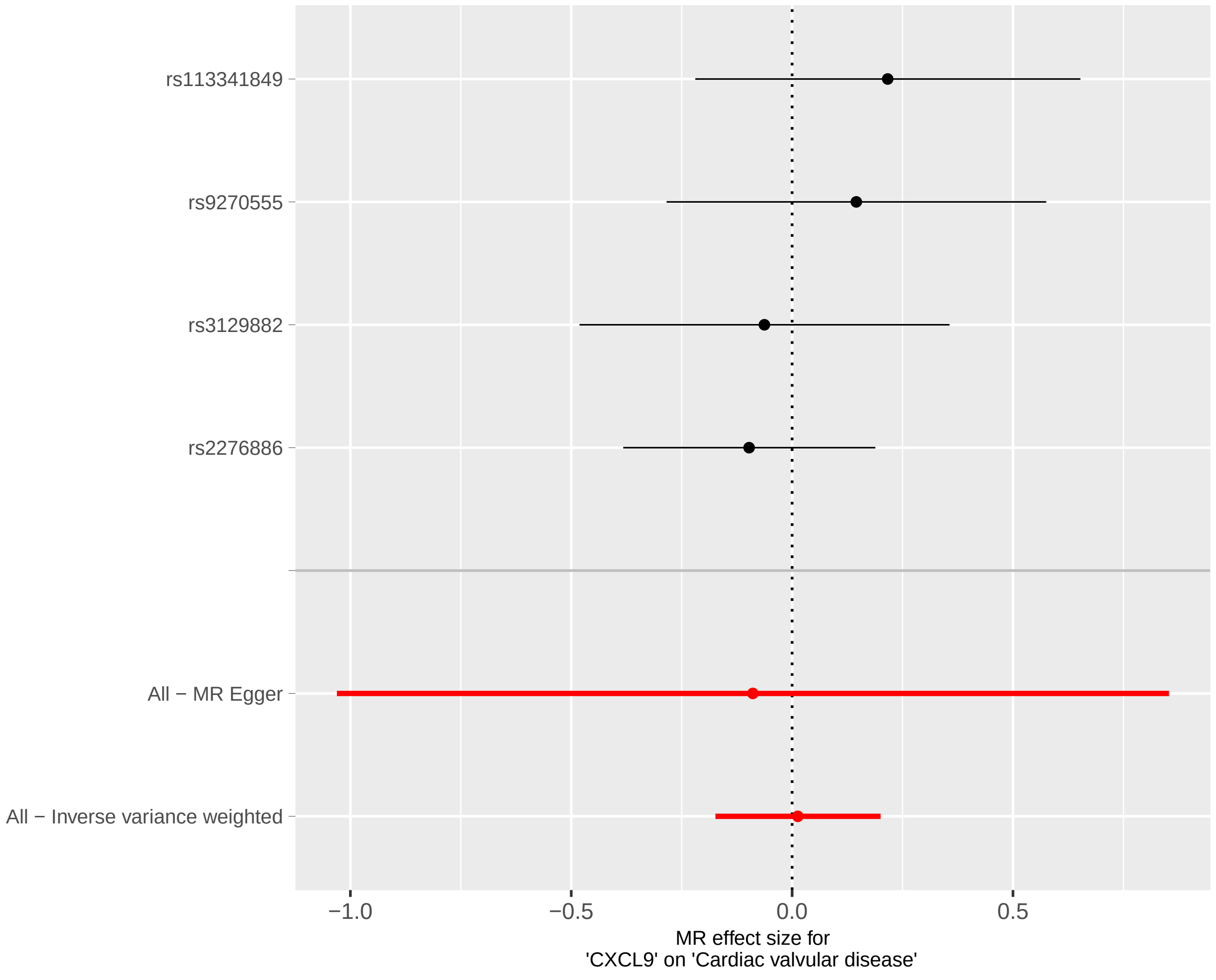
rs113341849
rs9270555
rs3129882
rs2276886
All − MR Egger
All − Inverse variance weighted
−1.0
−0.5
0.0
0.5
MR effect size for
'CXCL9' on 'Cardiac valvular disease'

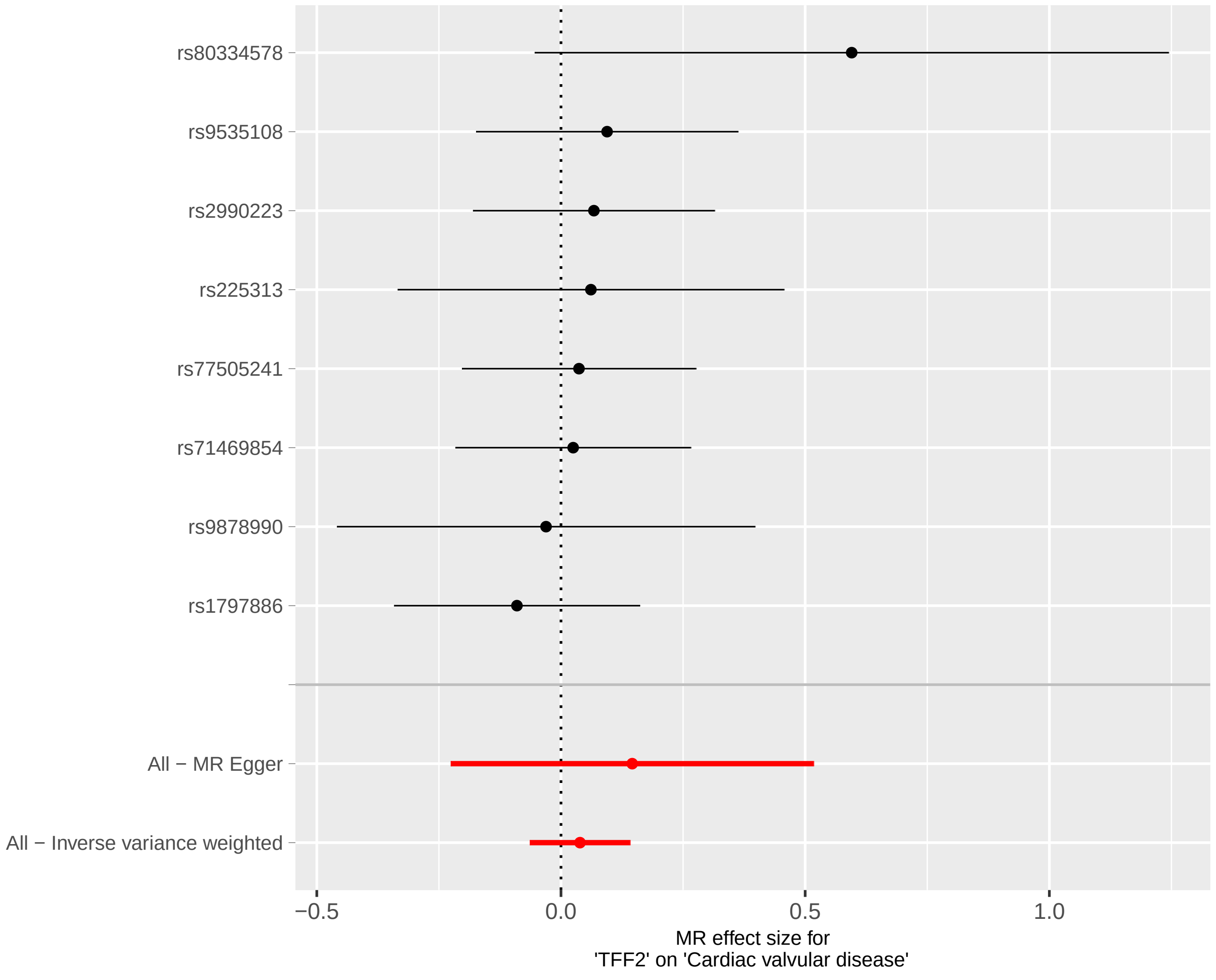
rs80334578
rs9535108
rs2990223
rs225313
rs77505241
rs71469854
rs9878990
rs1797886
All − MR Egger
All − Inverse variance weighted
−0.5
0.0
0.5
1.0
MR effect size for
'TFF2' on 'Cardiac valvular disease'

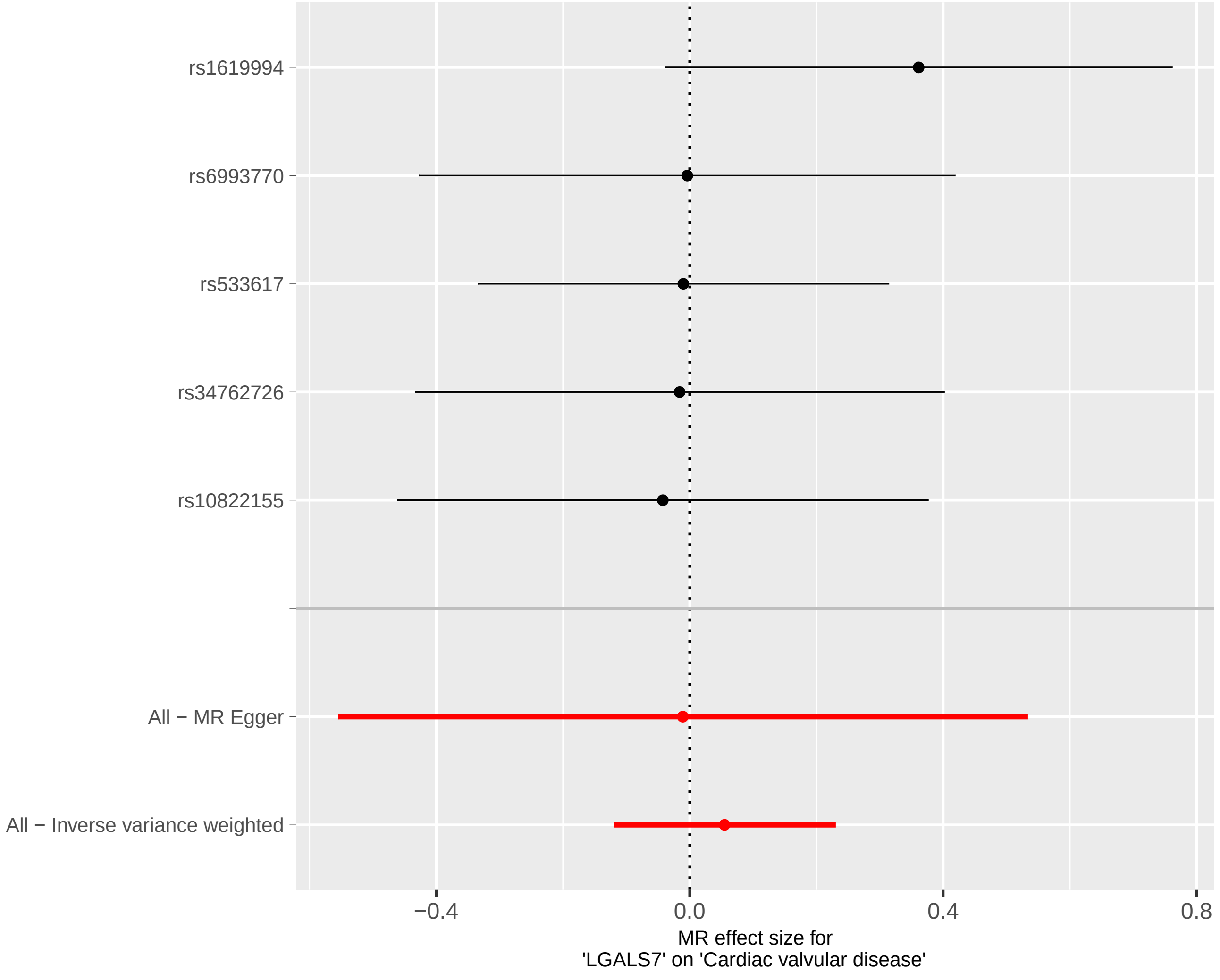
rs1619994
rs6993770
rs533617
rs34762726
rs10822155
All − MR Egger
All − Inverse variance weighted
−0.4
0.0
0.4
0.8
MR effect size for
'LGALS7' on 'Cardiac valvular disease'

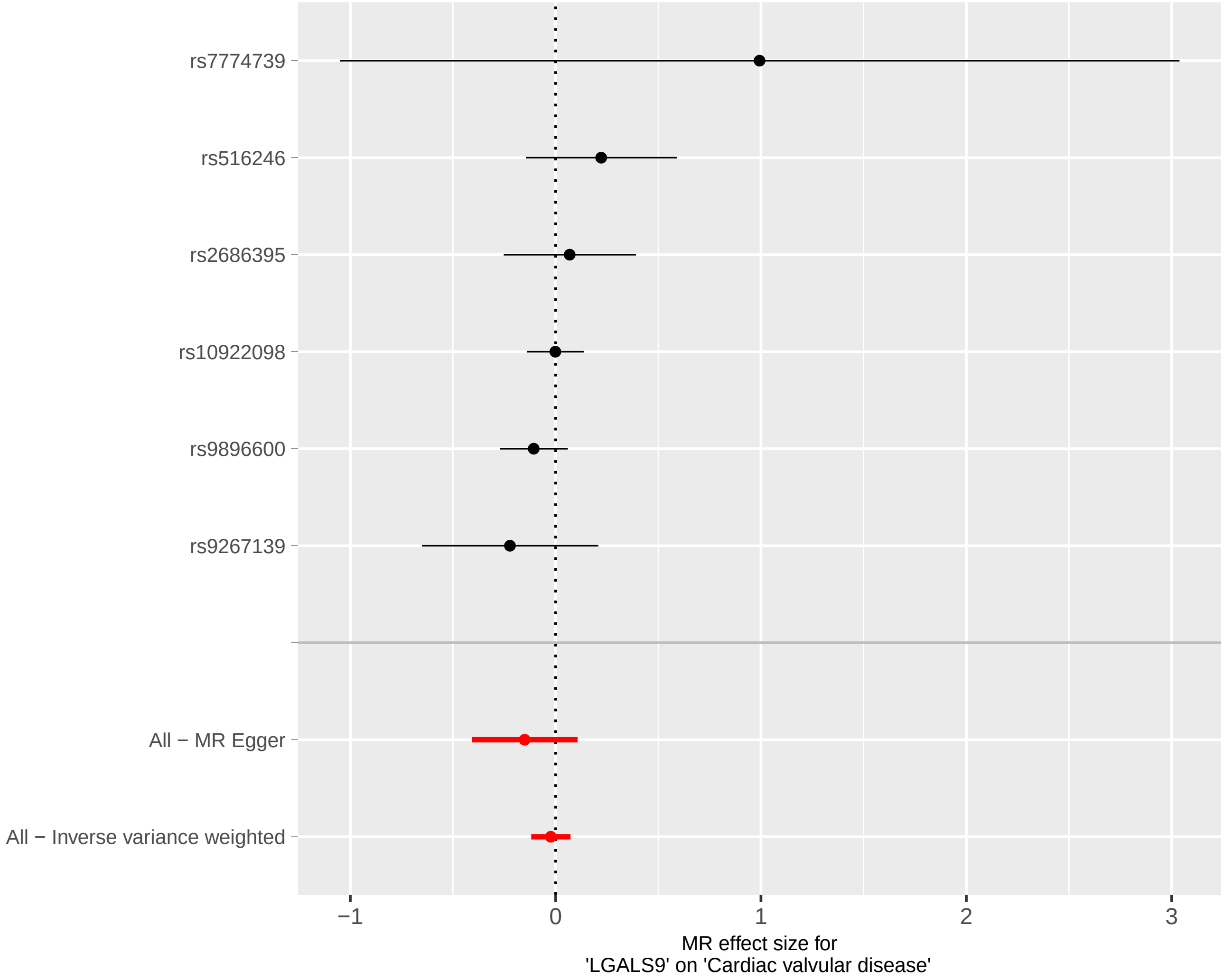
rs7774739
rs516246
rs2686395
rs10922098
rs9896600
rs9267139
All − MR Egger
All − Inverse variance weighted
−1
0
1
2
3
MR effect size for
'LGALS9' on 'Cardiac valvular disease'

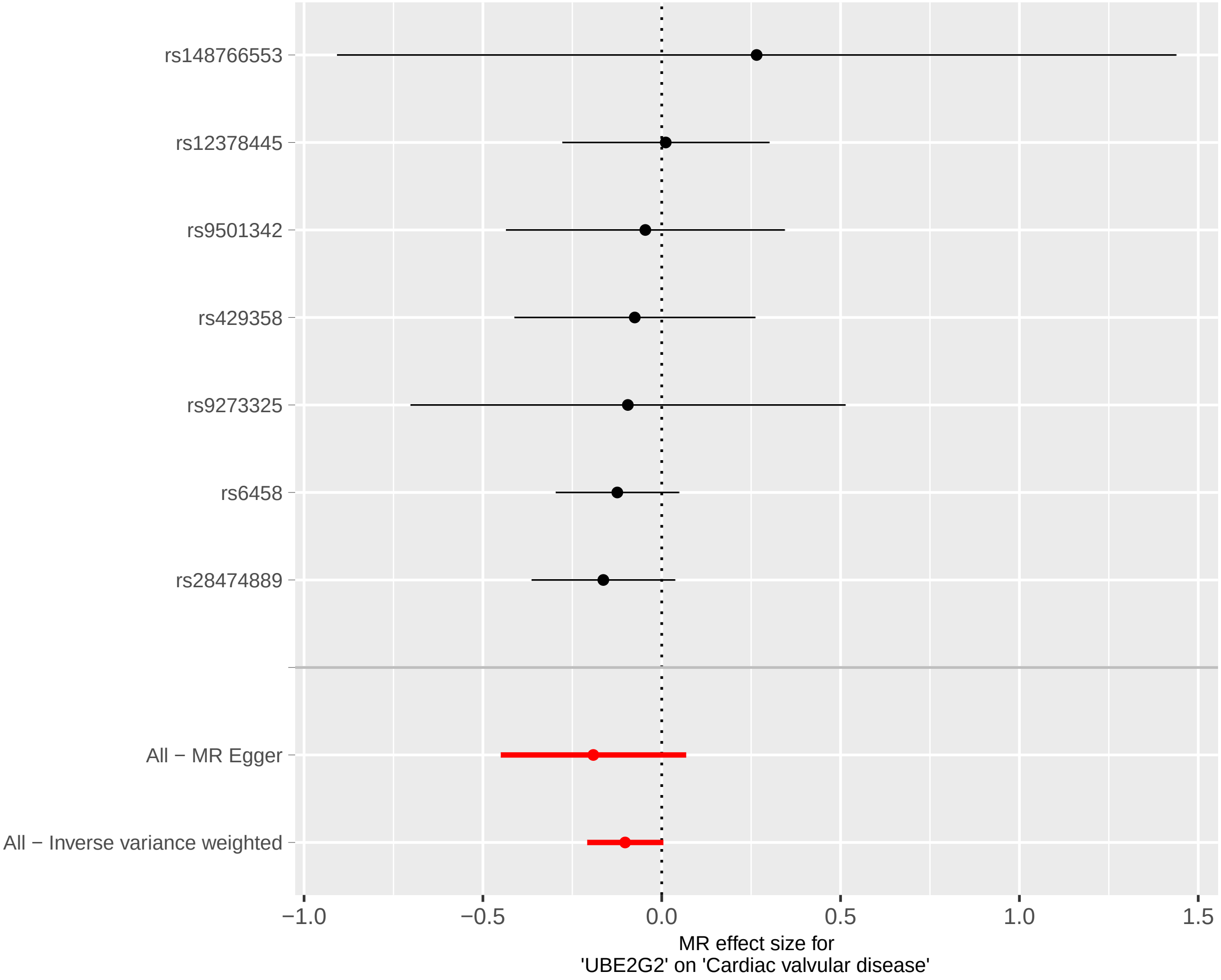
rs148766553
rs12378445
rs9501342
rs429358
rs9273325
rs6458
rs28474889
All − MR Egger
All − Inverse variance weighted
−1.0
−0.5
0.0
0.5
1.0
1.5
MR effect size for
'UBE2G2' on 'Cardiac valvular disease'

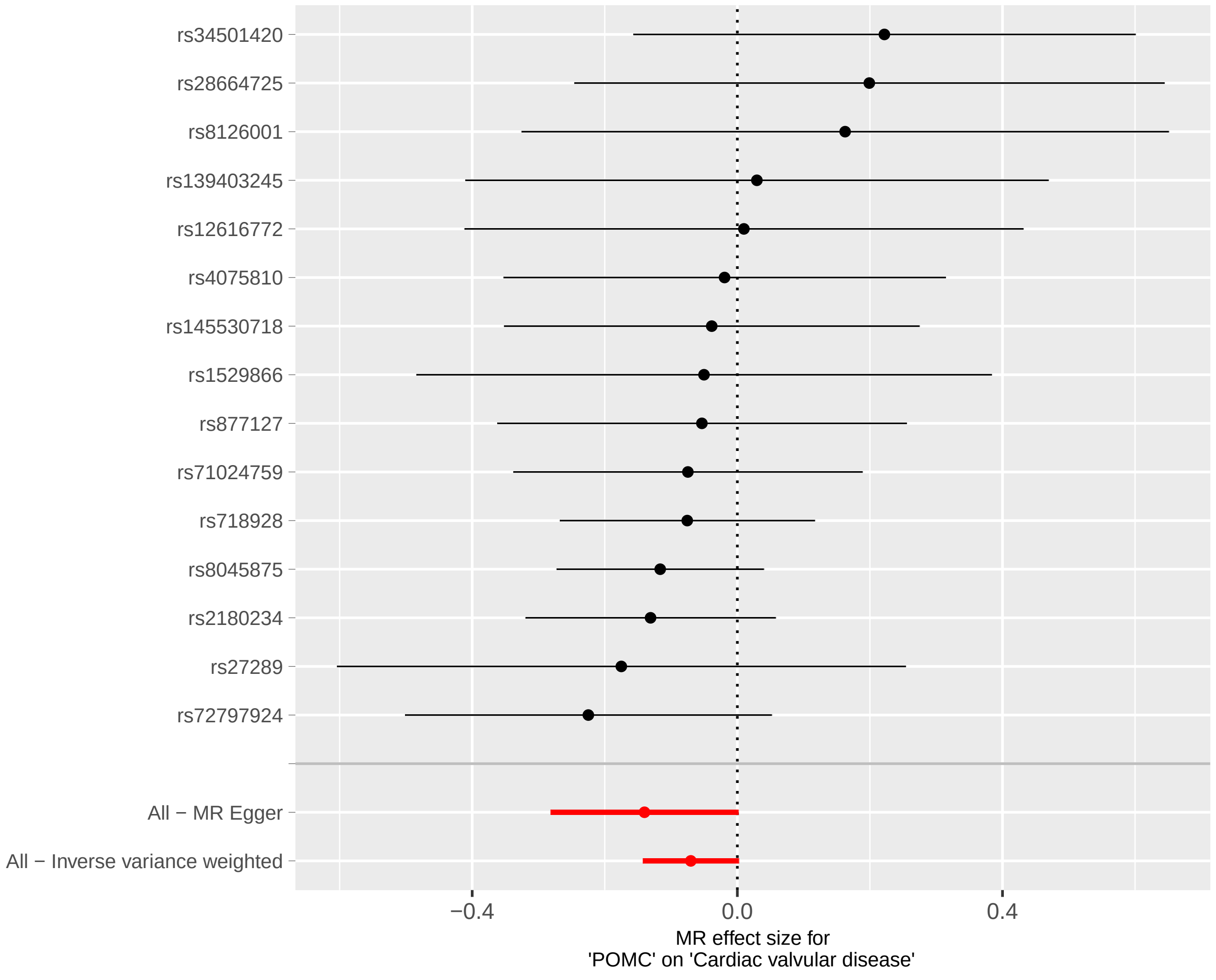
rs34501420
rs28664725
rs8126001
rs139403245
rs12616772
rs4075810
rs145530718
rs1529866
rs877127
rs71024759
rs718928
rs8045875
rs2180234
rs27289
rs72797924
All − MR Egger
All − Inverse variance weighted
−0.4
0.0
0.4
MR effect size for
'POMC' on 'Cardiac valvular disease'

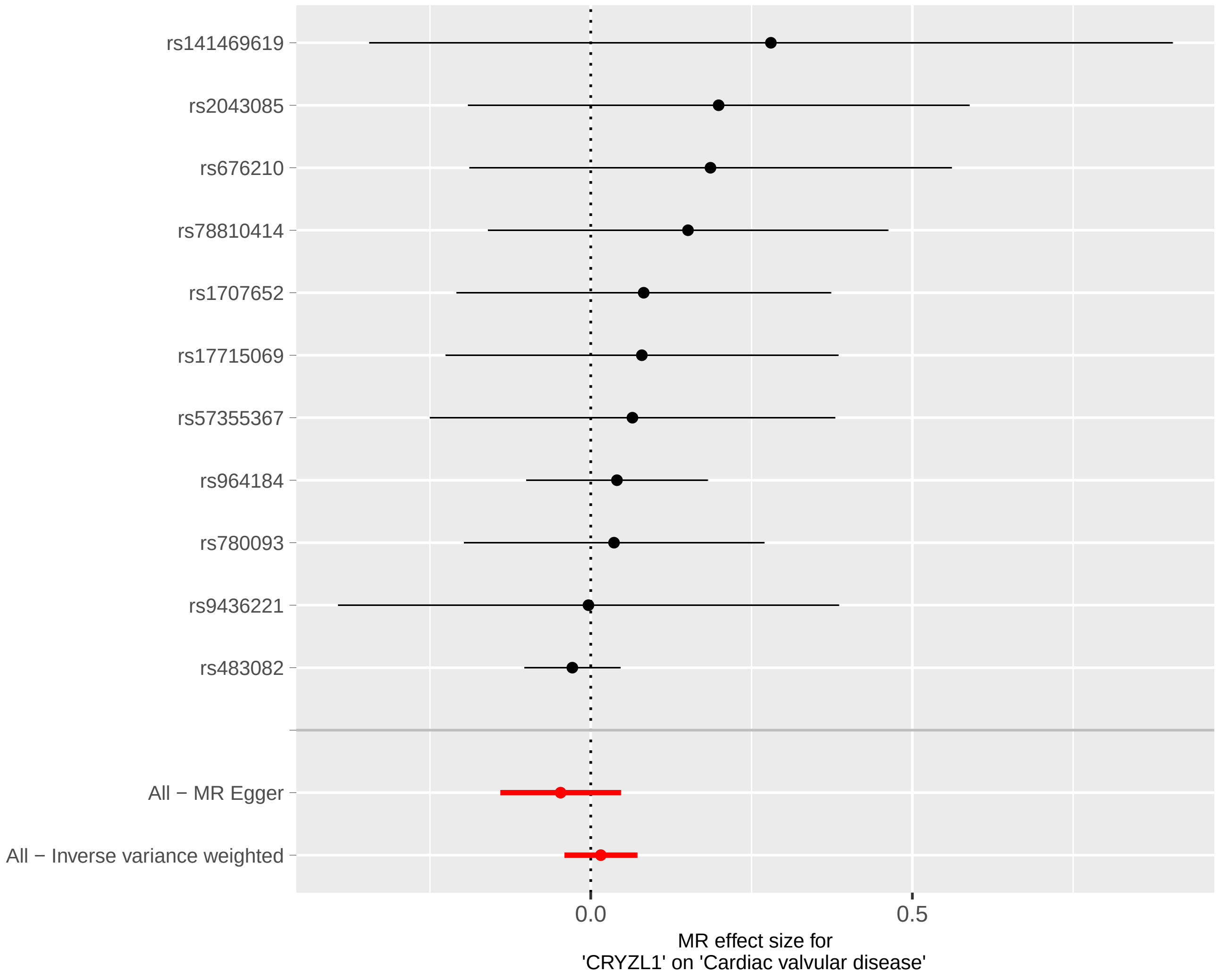

rs141469619
rs2043085
rs676210
rs78810414
rs1707652
rs17715069
rs57355367
rs964184
rs780093
rs9436221
rs483082
All − MR Egger
All − Inverse variance weighted
0.0
0.5
MR effect size for
'CRYZL1' on 'Cardiac valvular disease'

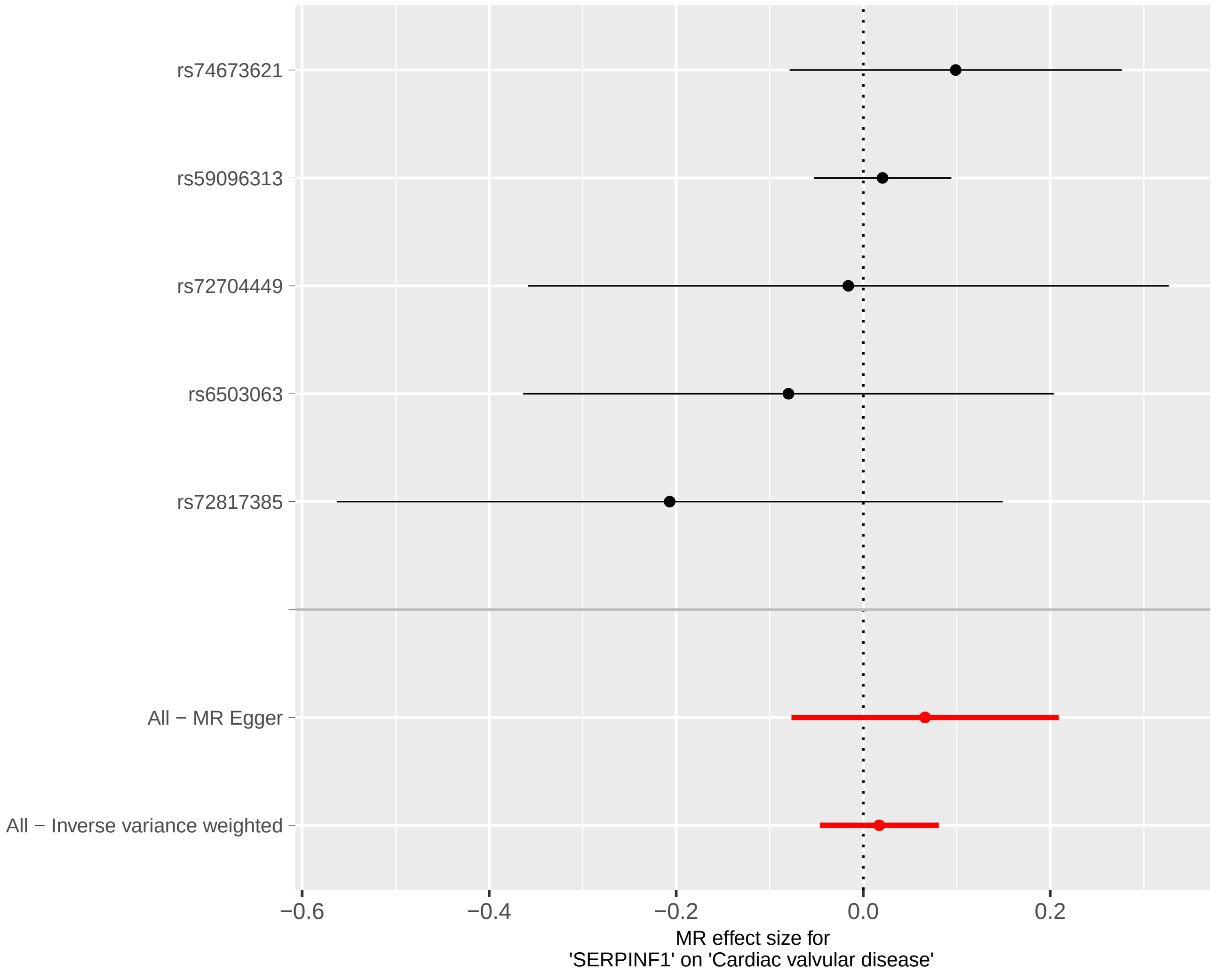

rs74673621
rs59096313
rs72704449
rs6503063
rs72817385
All − MR Egger
All − Inverse variance weighted
−0.6
−0.4
−0.2
0.0
0.2
MR effect size for
'SERPINF1' on 'Cardiac valvular disease'

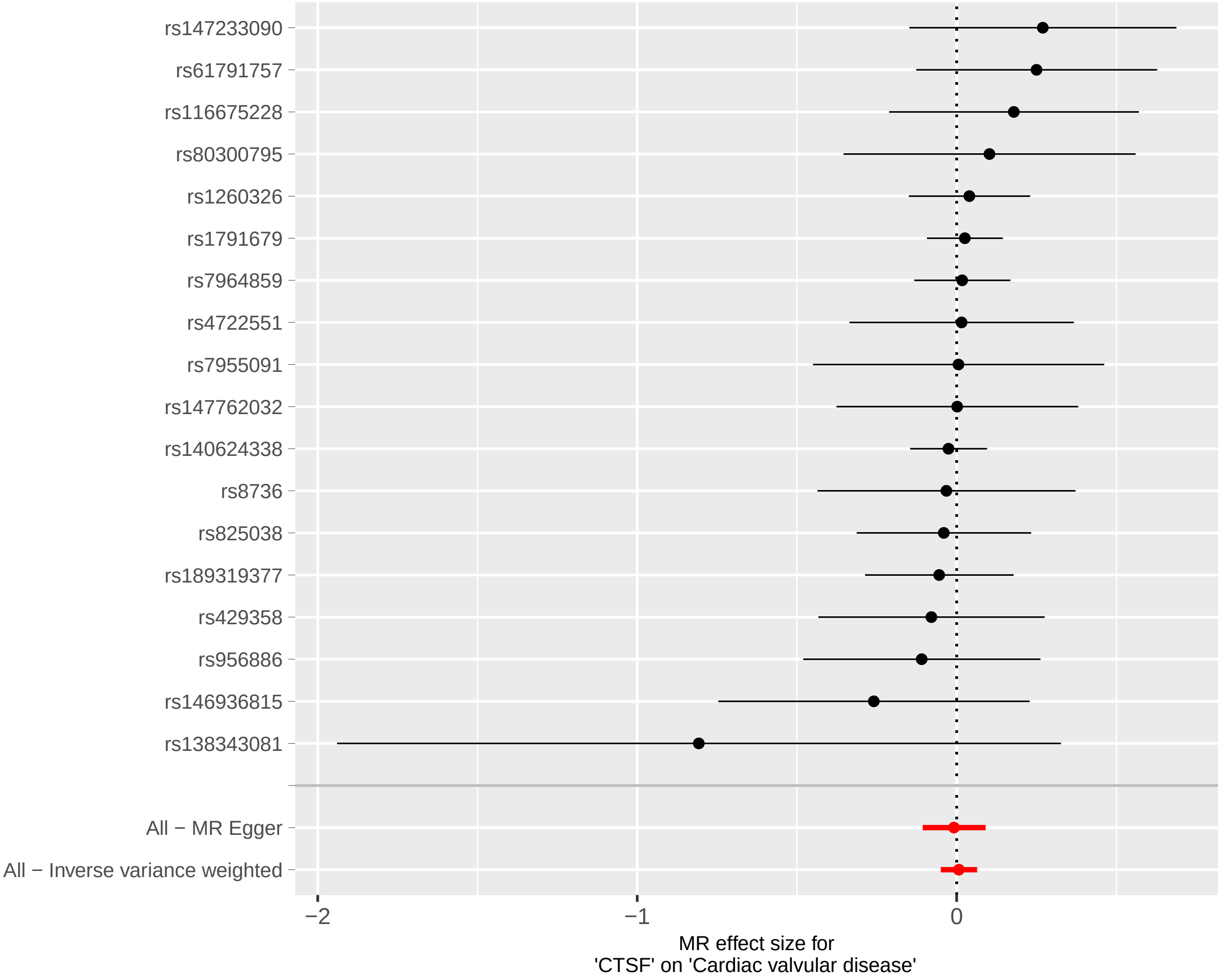
rs147233090
rs61791757
rs116675228
rs80300795
rs1260326
rs1791679
rs7964859
rs4722551
rs7955091
rs147762032
rs140624338
rs8736
rs825038
rs189319377
rs429358
rs956886
rs146936815
rs138343081
All − MR Egger
All − Inverse variance weighted
−2
−1
0
MR effect size for
'CTSF' on 'Cardiac valvular disease'

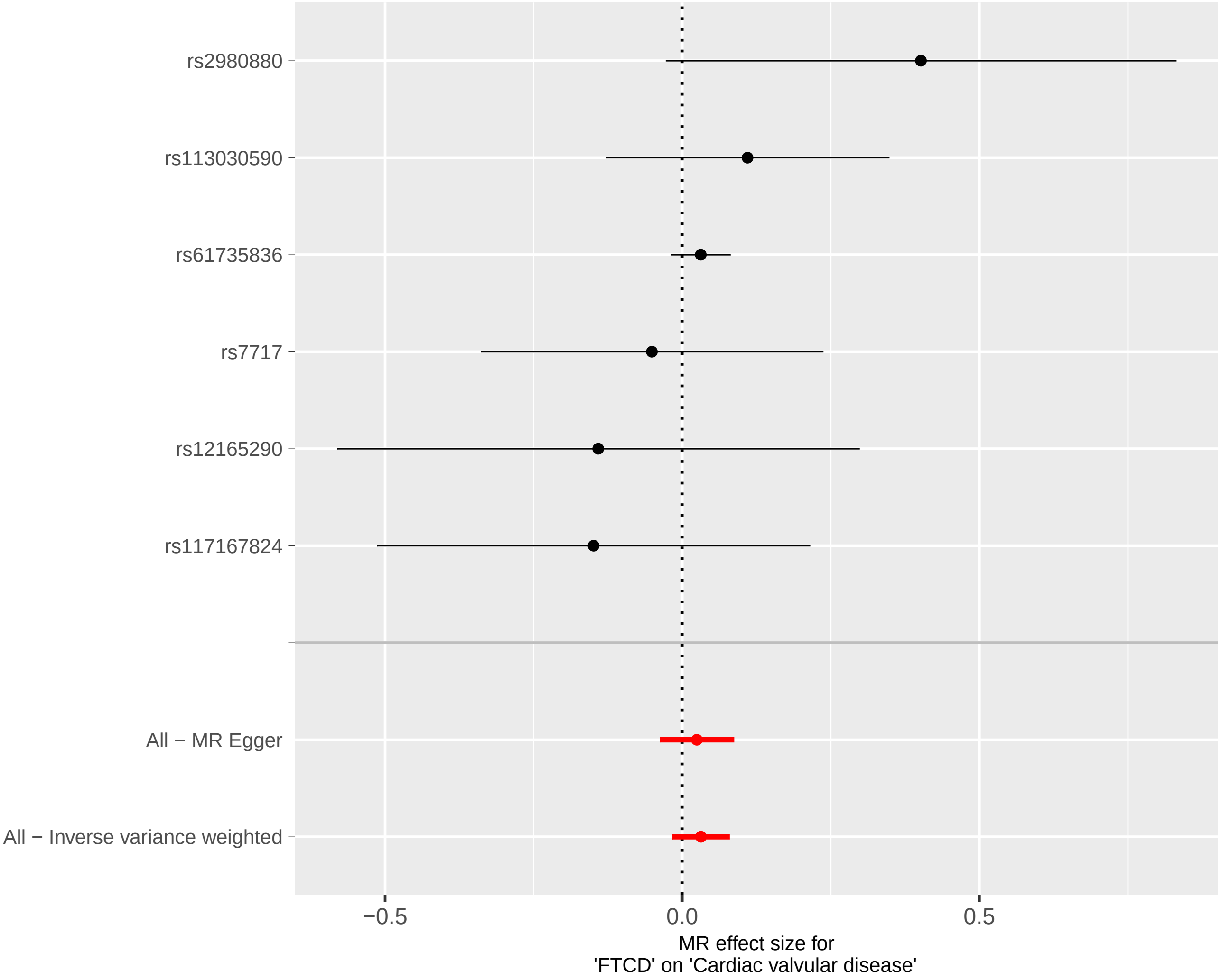
rs2980880
rs113030590
rs61735836
rs7717
rs12165290
rs117167824
All − MR Egger
All − Inverse variance weighted
−0.5
0.0
0.5
MR effect size for
'FTCD' on 'Cardiac valvular disease'

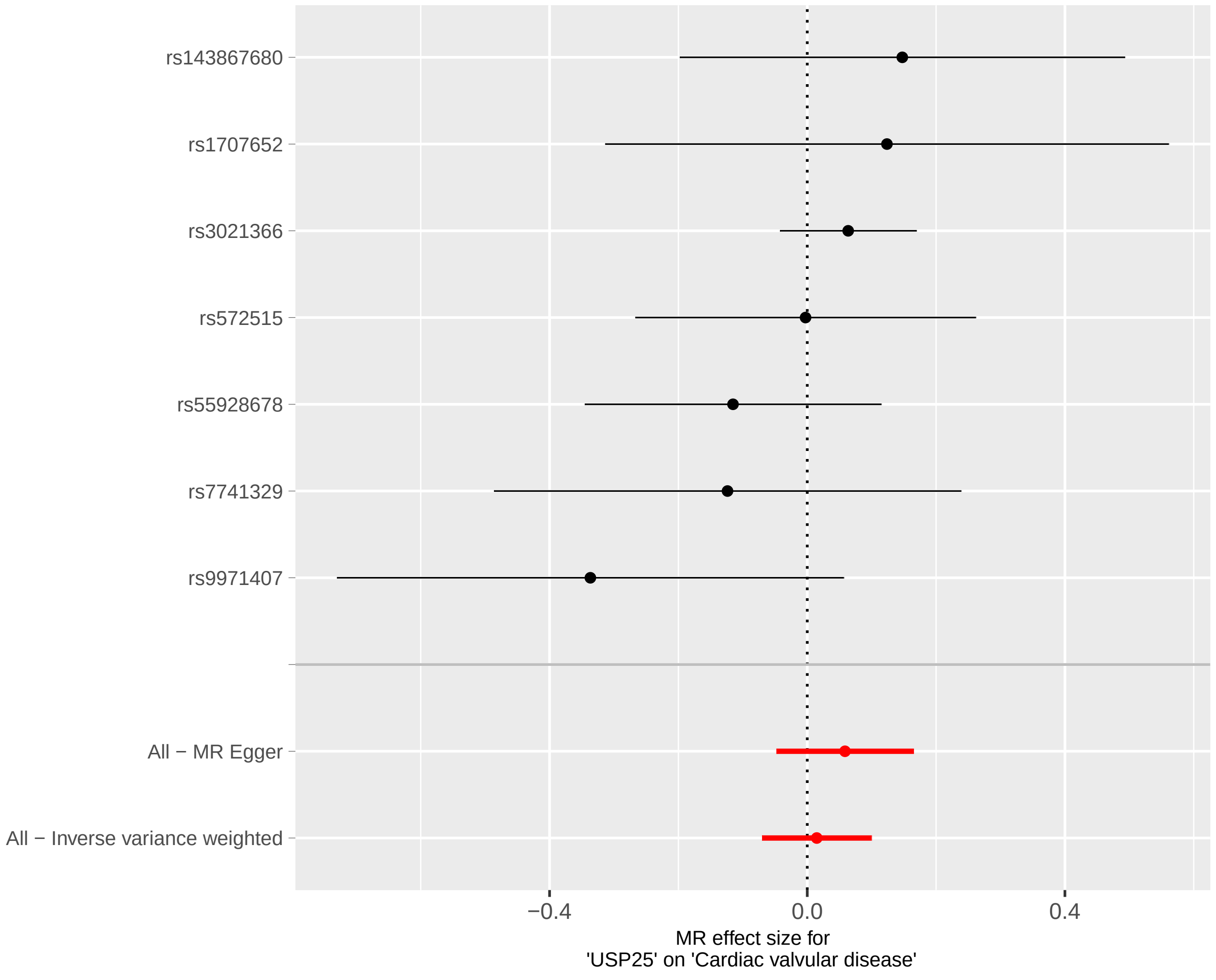

rs143867680
rs1707652
rs3021366
rs572515
rs55928678
rs7741329
rs9971407
All − MR Egger
All − Inverse variance weighted
−0.4
0.0
0.4
MR effect size for
'USP25' on 'Cardiac valvular disease'

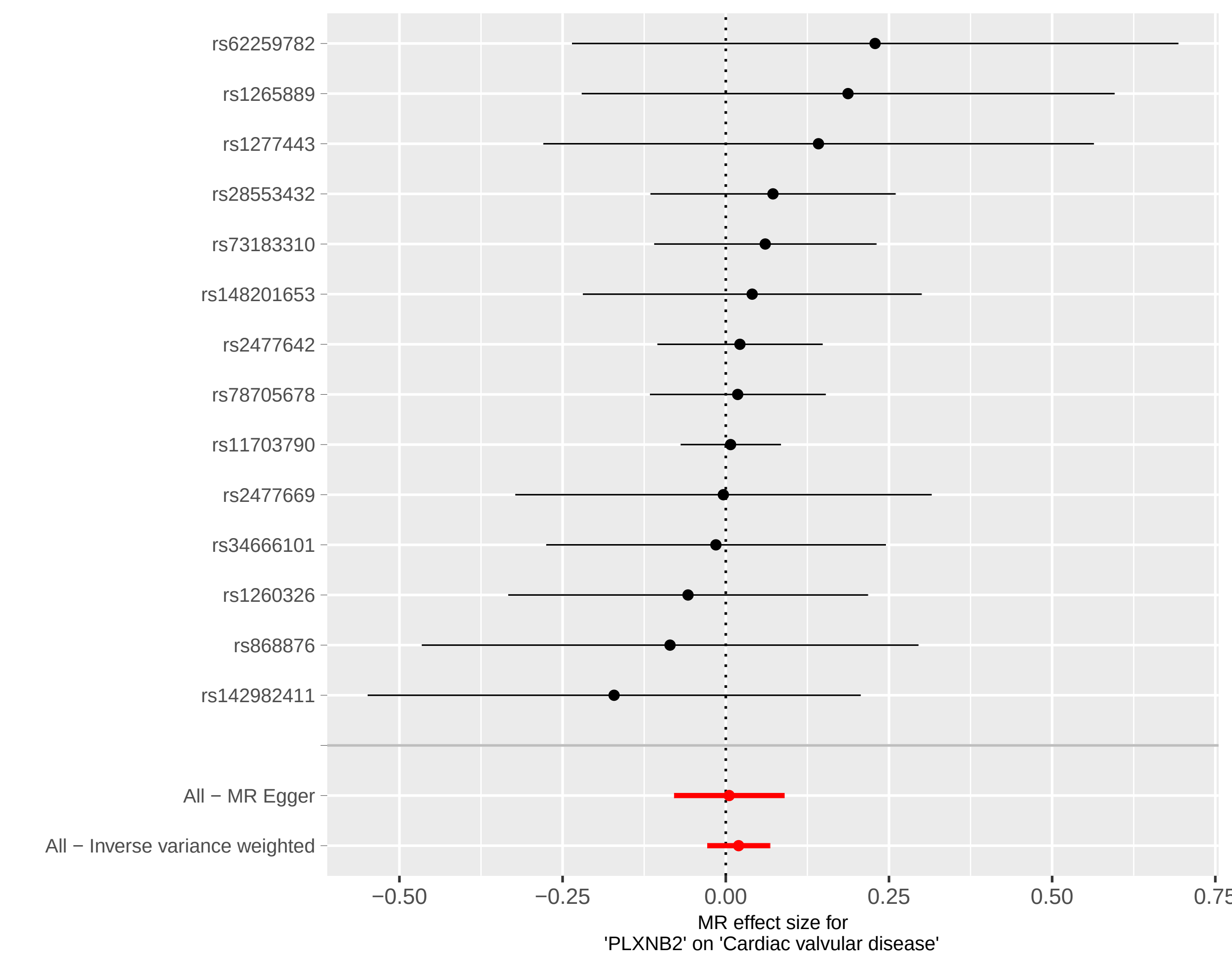
rs62259782
rs1265889
rs1277443
rs28553432
rs73183310
rs148201653
rs2477642
rs78705678
rs11703790
rs2477669
rs34666101
rs1260326
rs868876
rs142982411
All − MR Egger
All − Inverse variance weighted
−0.50
−0.25
0.00
0.25
0.50
0.75
MR effect size for
'PLXNB2' on 'Cardiac valvular disease'

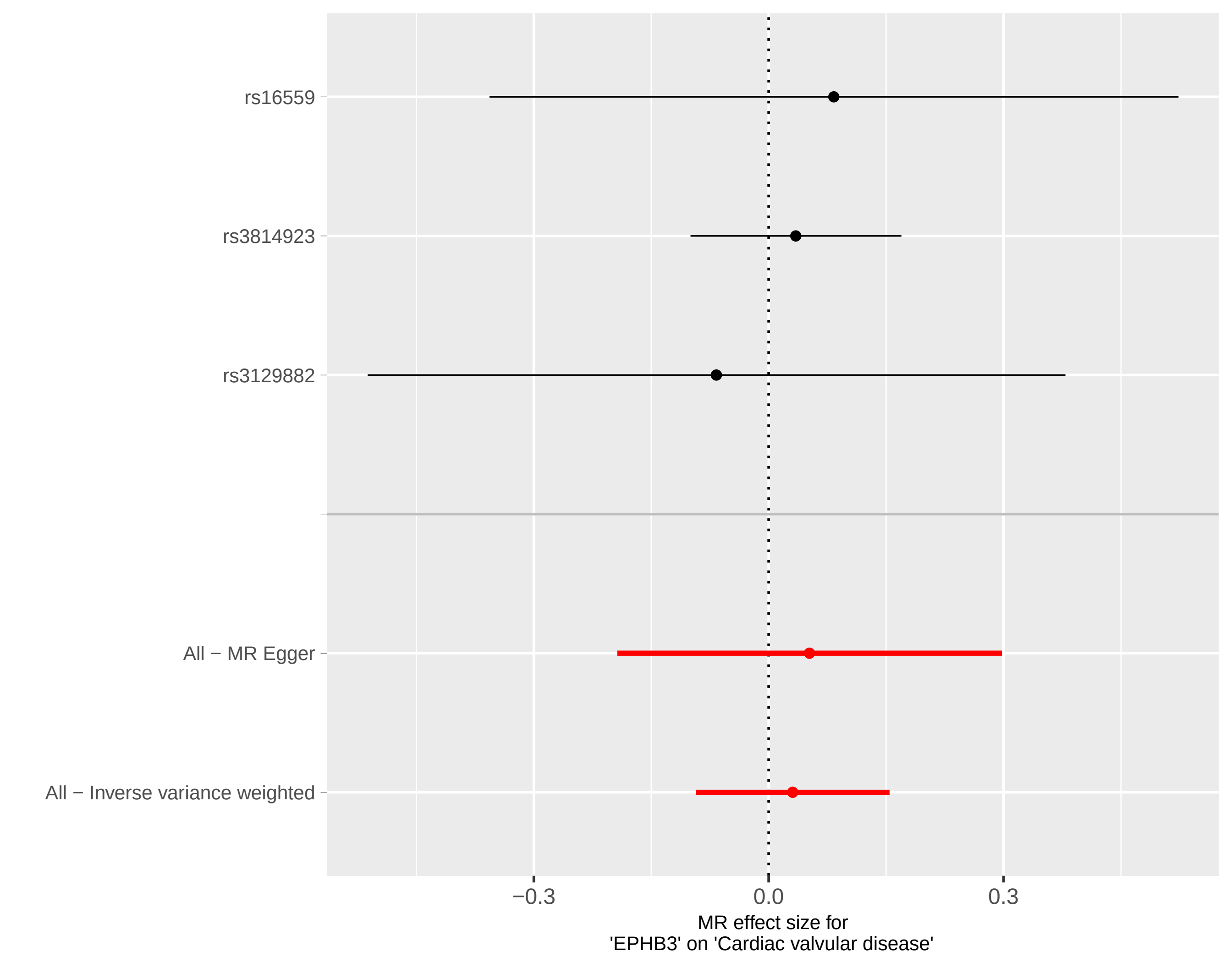
rs16559
rs3814923
rs3129882
All − MR Egger
All − Inverse variance weighted
−0.3
0.0
0.3
MR effect size for
'EPHB3' on 'Cardiac valvular disease'

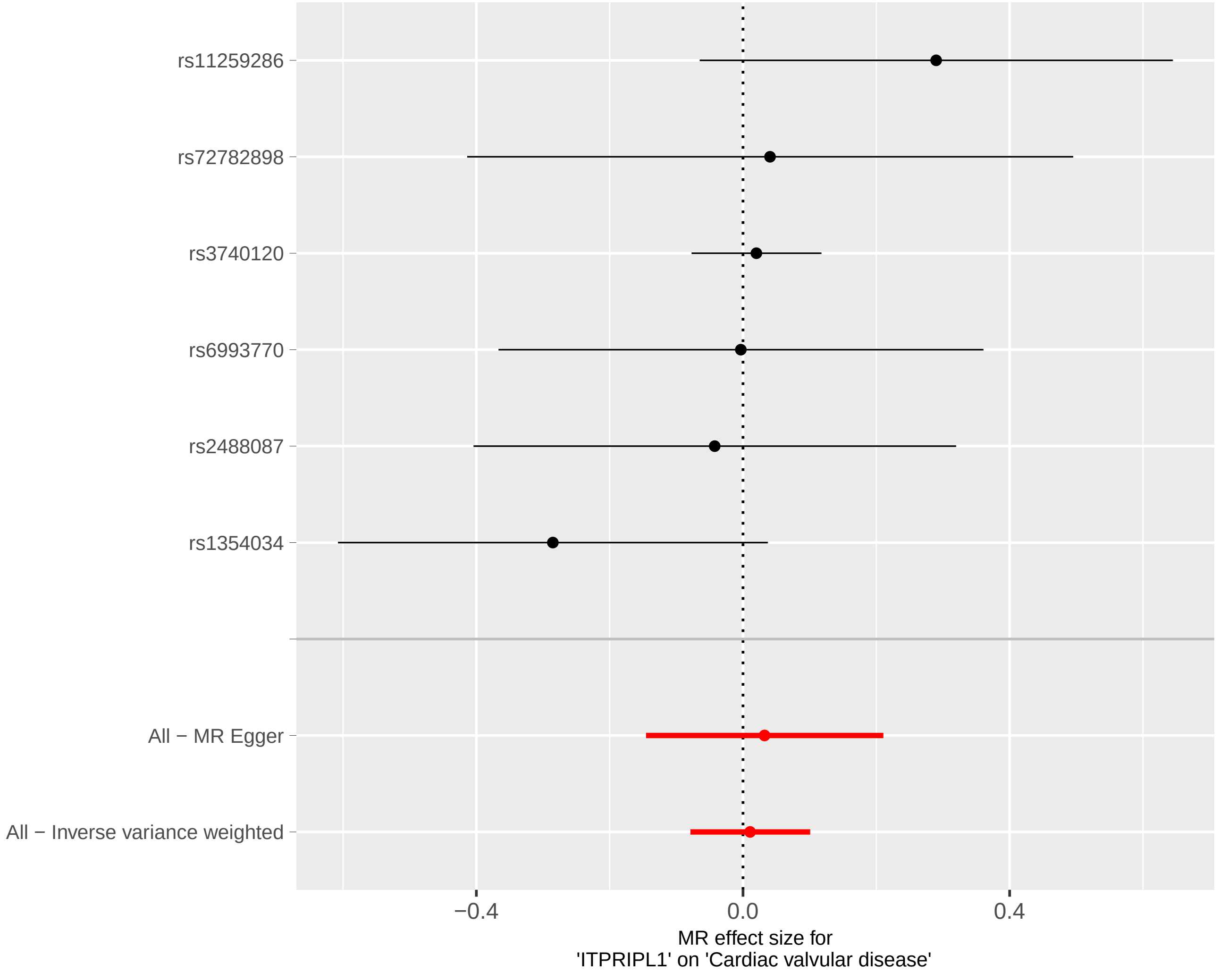
rs11259286
rs72782898
rs3740120
rs6993770
rs2488087
rs1354034
All − MR Egger
All − Inverse variance weighted
−0.4
0.0
0.4
MR effect size for
'ITPRIPL1' on 'Cardiac valvular disease'

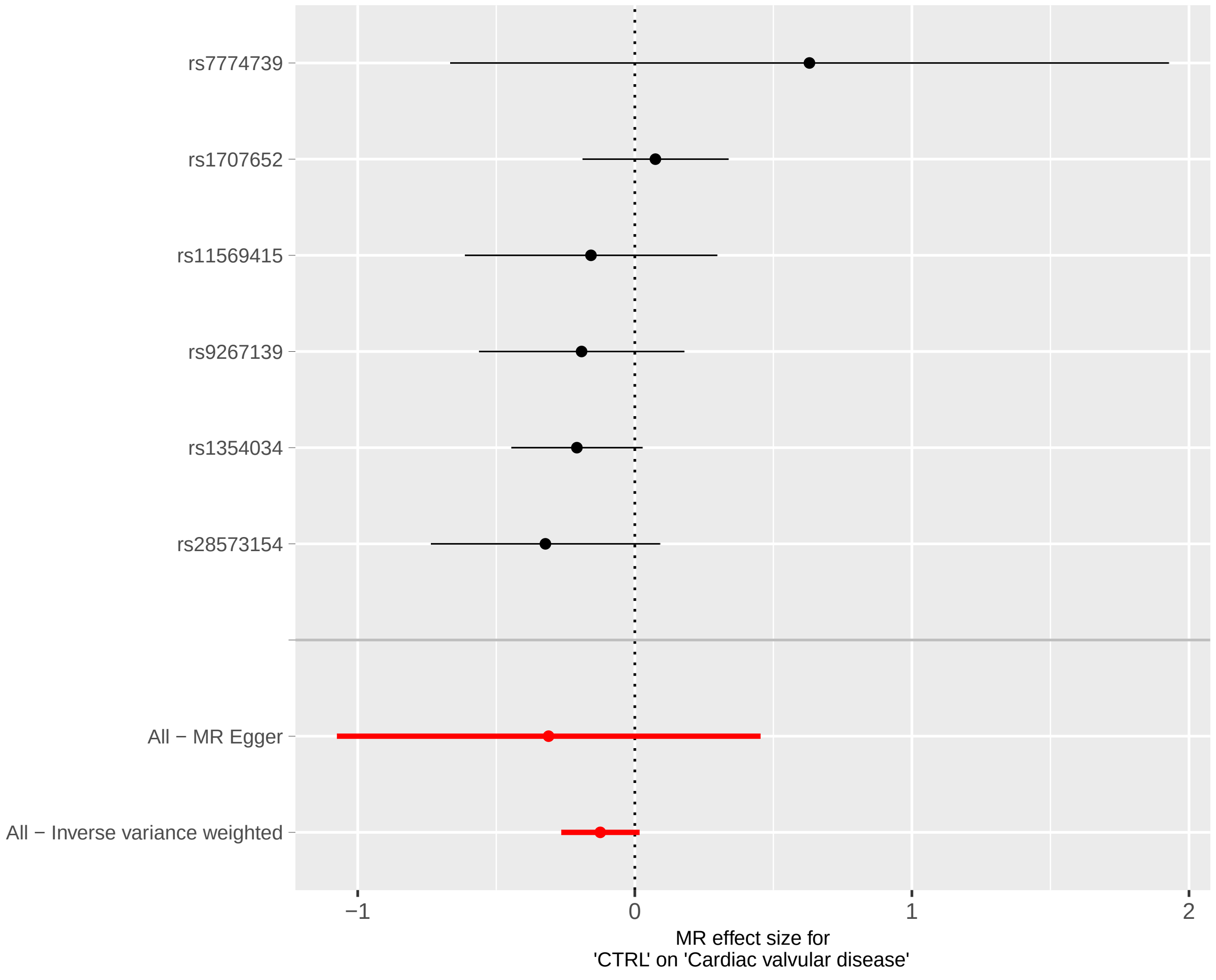
rs7774739
rs1707652
rs11569415
rs9267139
rs1354034
rs28573154
All − MR Egger
All − Inverse variance weighted
−1
0
1
2
MR effect size for
'CTRL' on 'Cardiac valvular disease'

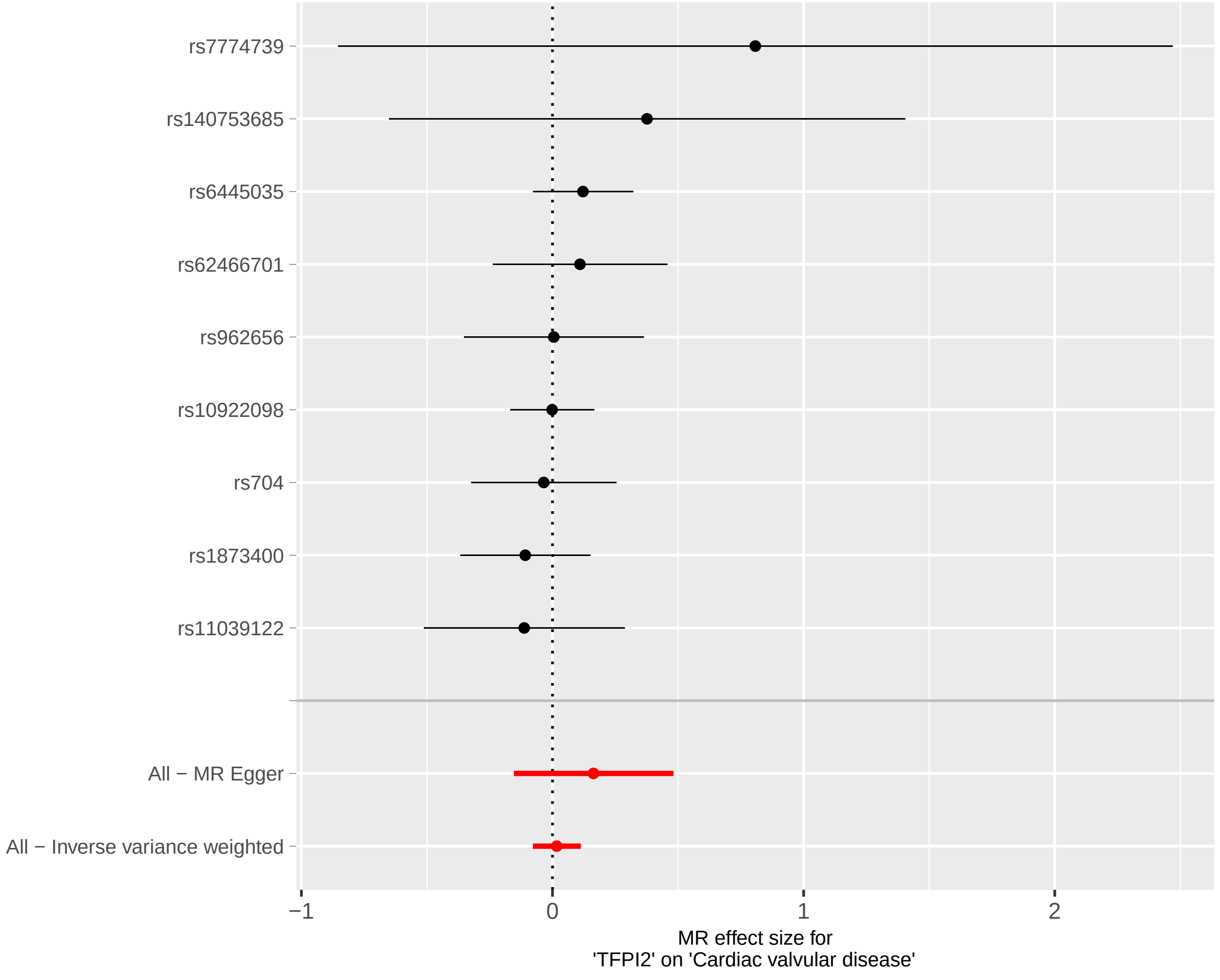

rs7774739
rs140753685
rs6445035
rs62466701
rs962656
rs10922098
rs704
rs1873400
rs11039122
All − MR Egger
All − Inverse variance weighted
−1
0
1
2
MR effect size for
'TFPI2' on 'Cardiac valvular disease'

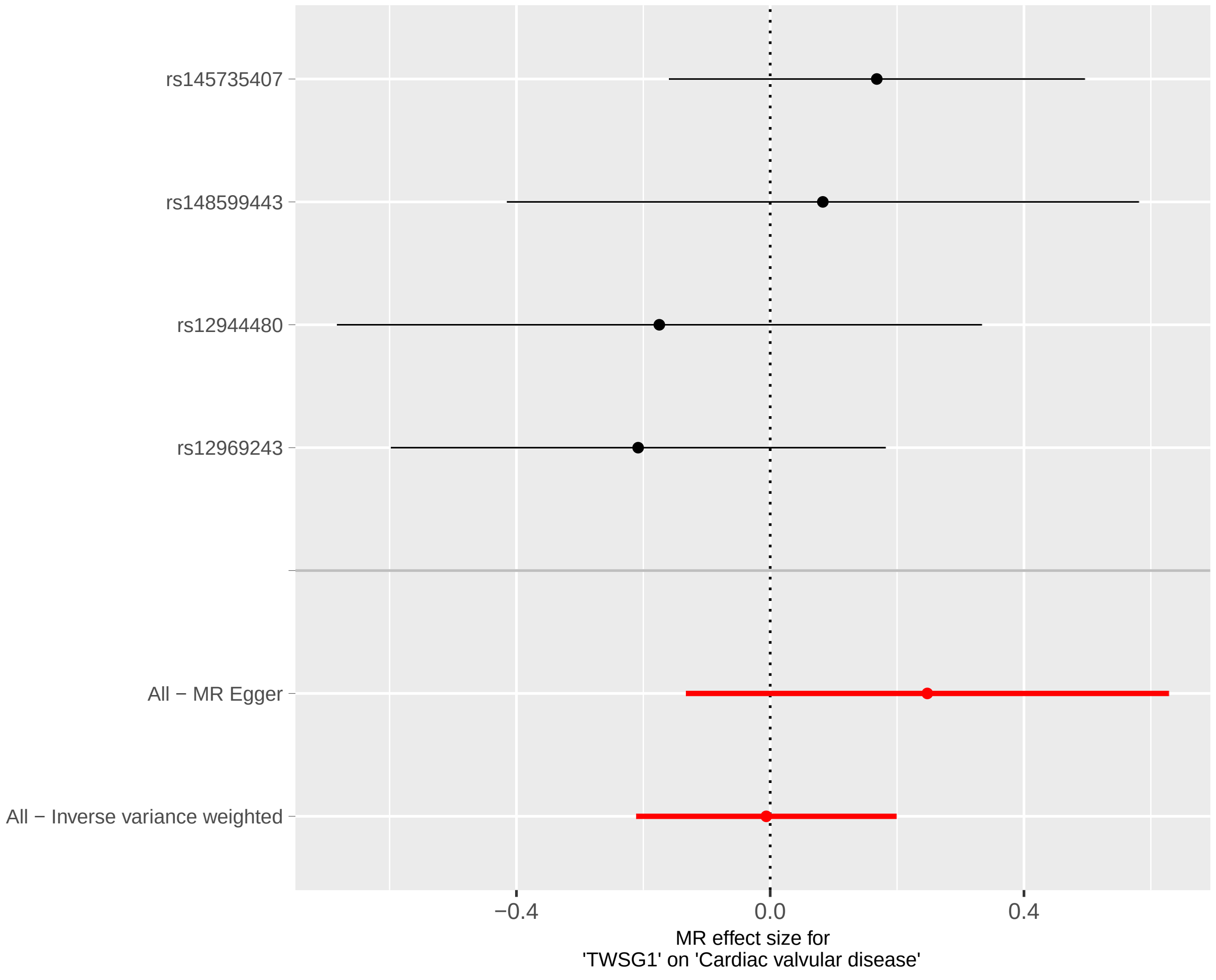
rs145735407
rs148599443
rs12944480
rs12969243
All − MR Egger
All − Inverse variance weighted
−0.4
0.0
0.4
MR effect size for
'TWSG1' on 'Cardiac valvular disease'

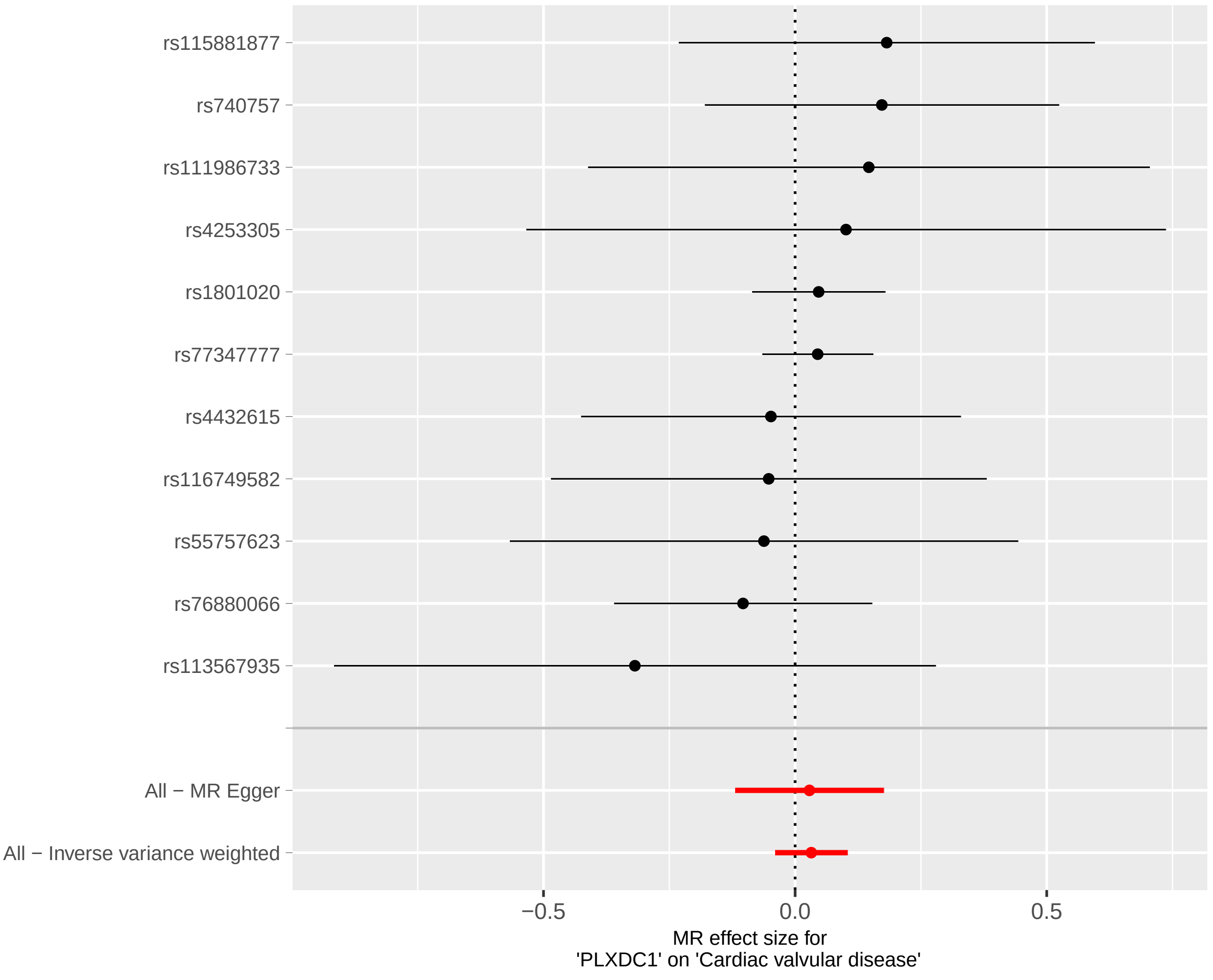
rs115881877
rs740757
rs111986733
rs4253305
rs1801020
rs77347777
rs4432615
rs116749582
rs55757623
rs76880066
rs113567935
All − MR Egger
All − Inverse variance weighted
−0.5
0.0
0.5
MR effect size for
'PLXDC1' on 'Cardiac valvular disease'

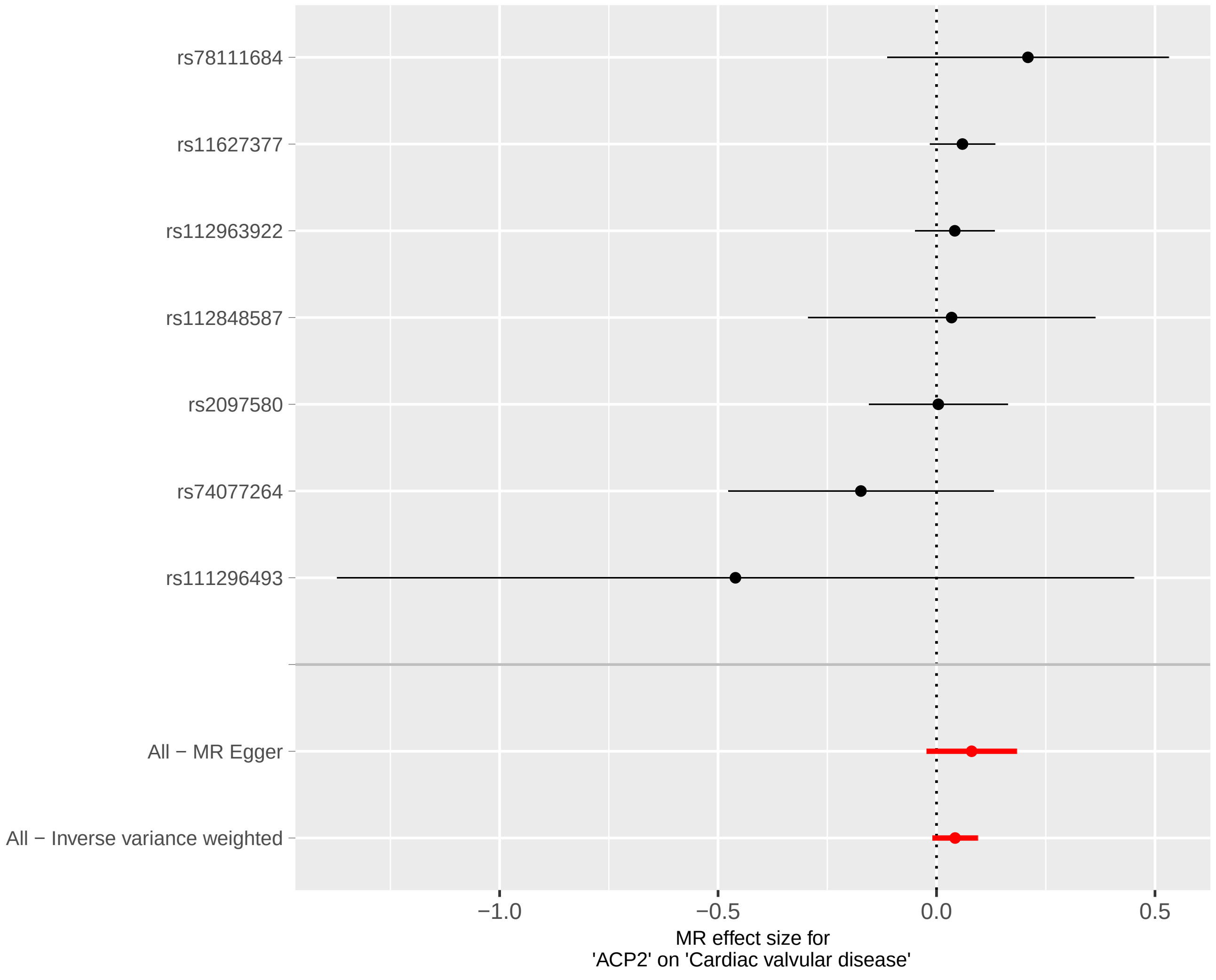
rs78111684
rs11627377
rs112963922
rs112848587
rs2097580
rs74077264
rs111296493
All − MR Egger
All − Inverse variance weighted
−1.0
−0.5
0.0
0.5
MR effect size for
'ACP2' on 'Cardiac valvular disease'

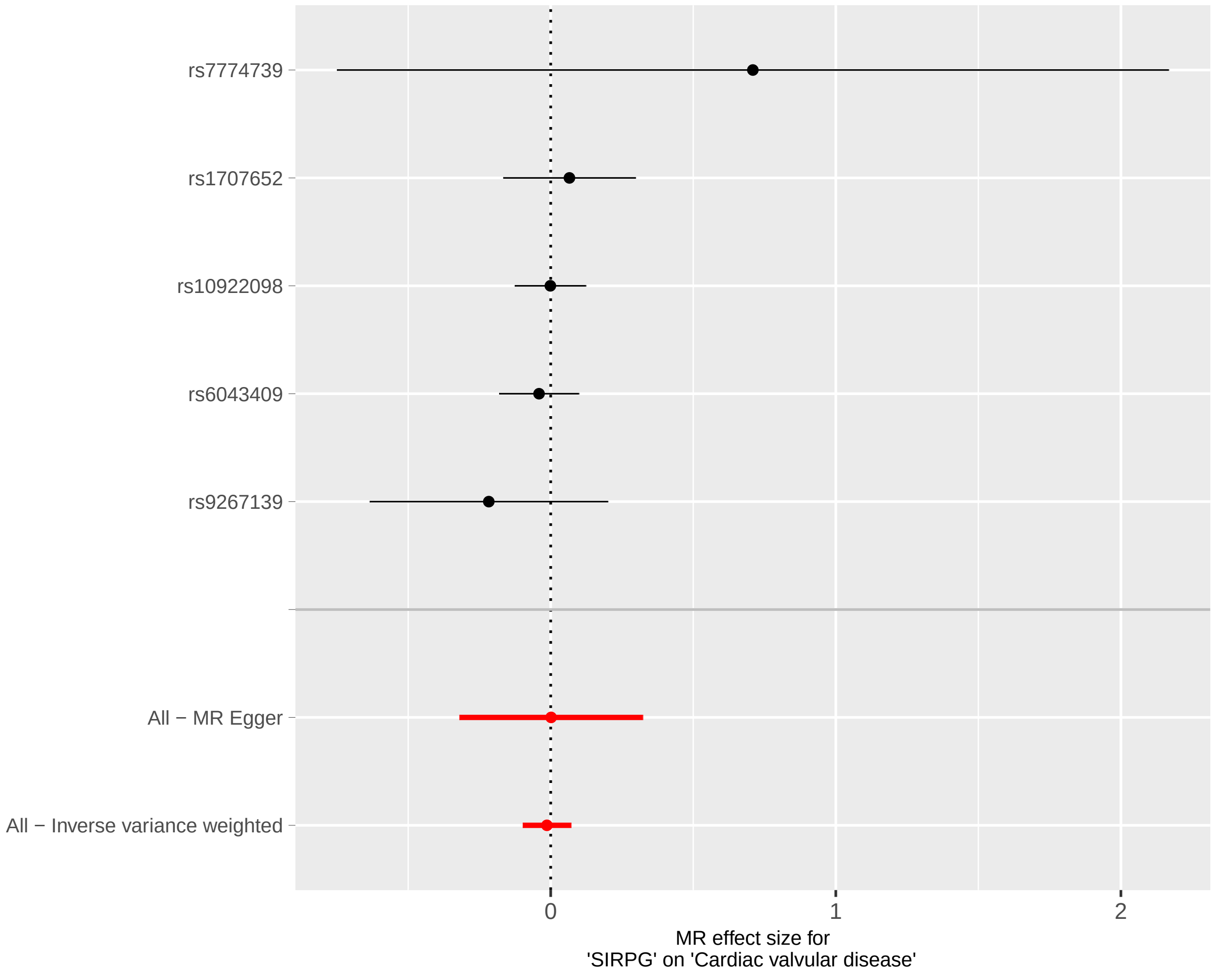
rs7774739
rs1707652
rs10922098
rs6043409
rs9267139
All − MR Egger
All − Inverse variance weighted
0
1
2
MR effect size for
'SIRPG' on 'Cardiac valvular disease'

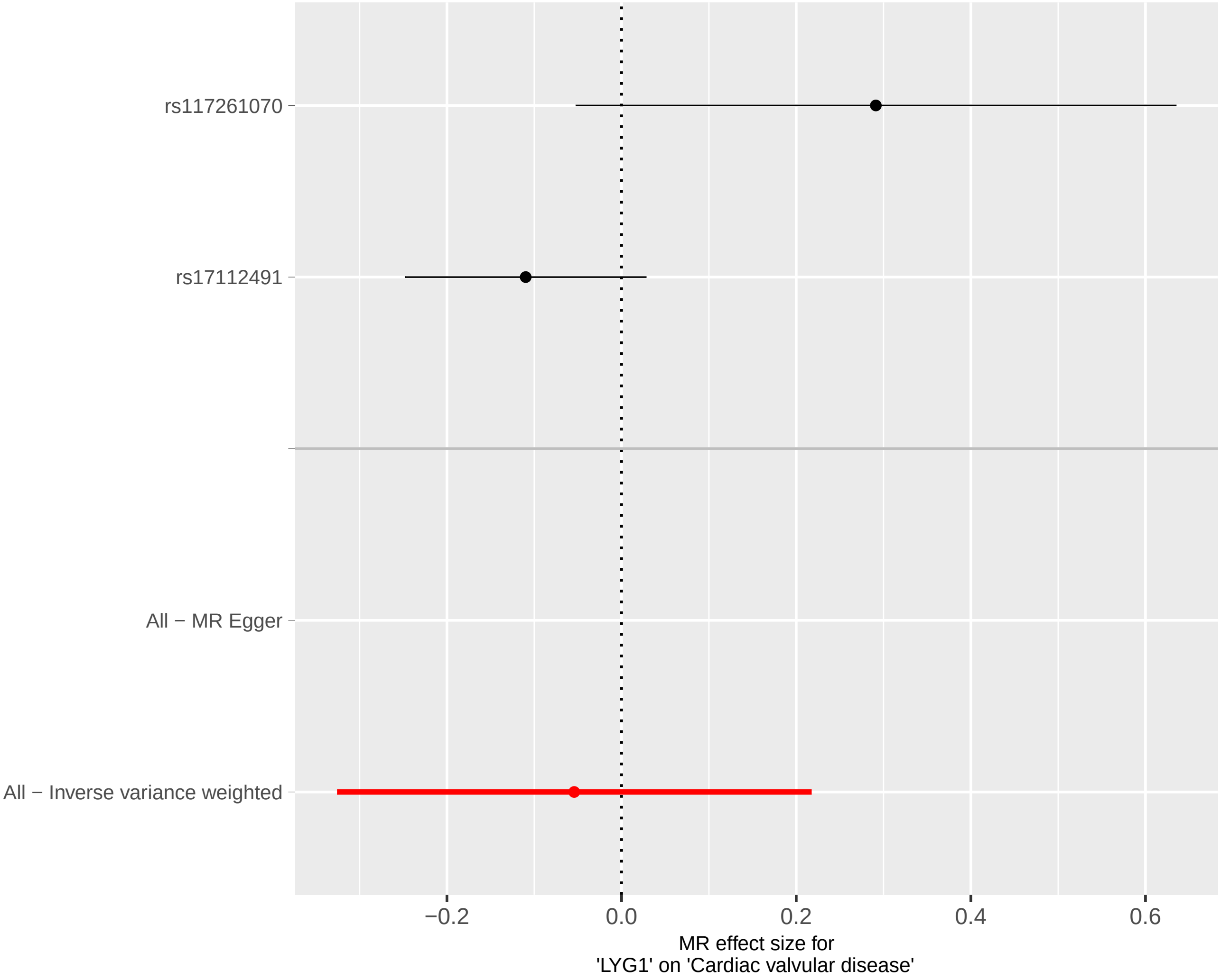
rs117261070
rs17112491
All − MR Egger
All − Inverse variance weighted
−0.2
0.0
0.2
0.4
0.6
MR effect size for
'LYG1' on 'Cardiac valvular disease'

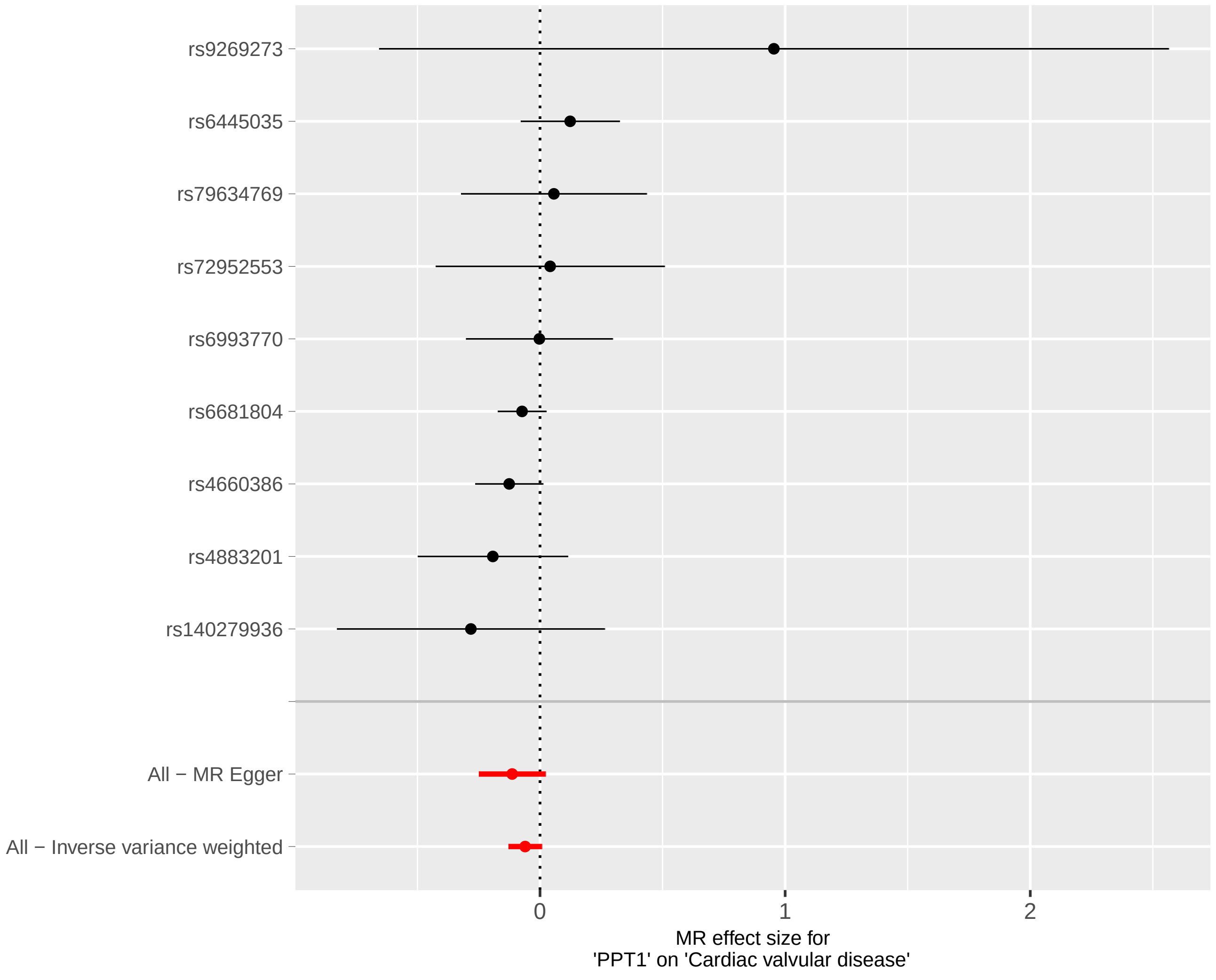

rs9269273
rs6445035
rs79634769
rs72952553
rs6993770
rs6681804
rs4660386
rs4883201
rs140279936
All − MR Egger
All − Inverse variance weighted
0
1
2
MR effect size for
'PPT1' on 'Cardiac valvular disease'

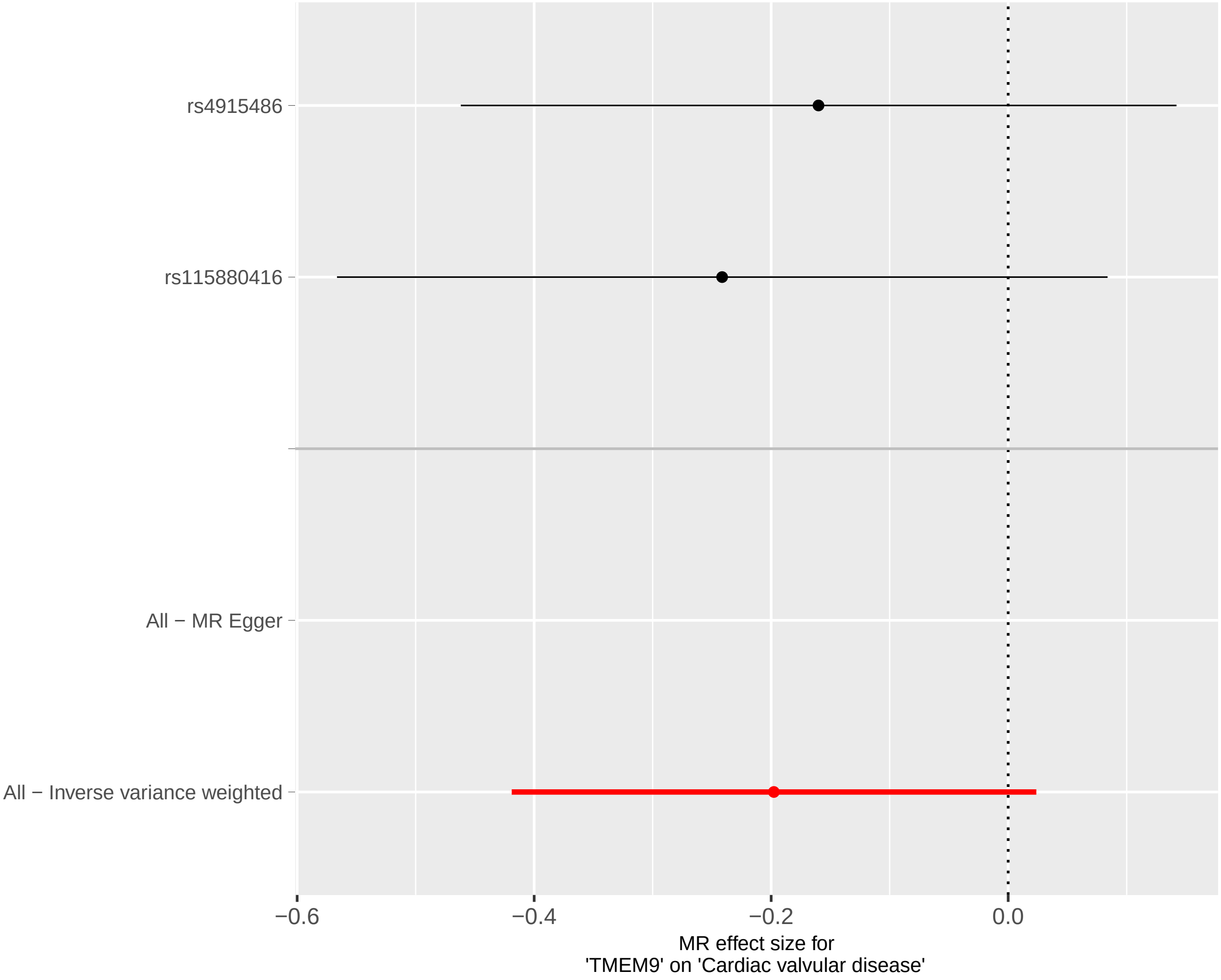

rs4915486
rs115880416
All − MR Egger
All − Inverse variance weighted
−0.6
−0.4
−0.2
0.0
MR effect size for
'TMEM9' on 'Cardiac valvular disease'

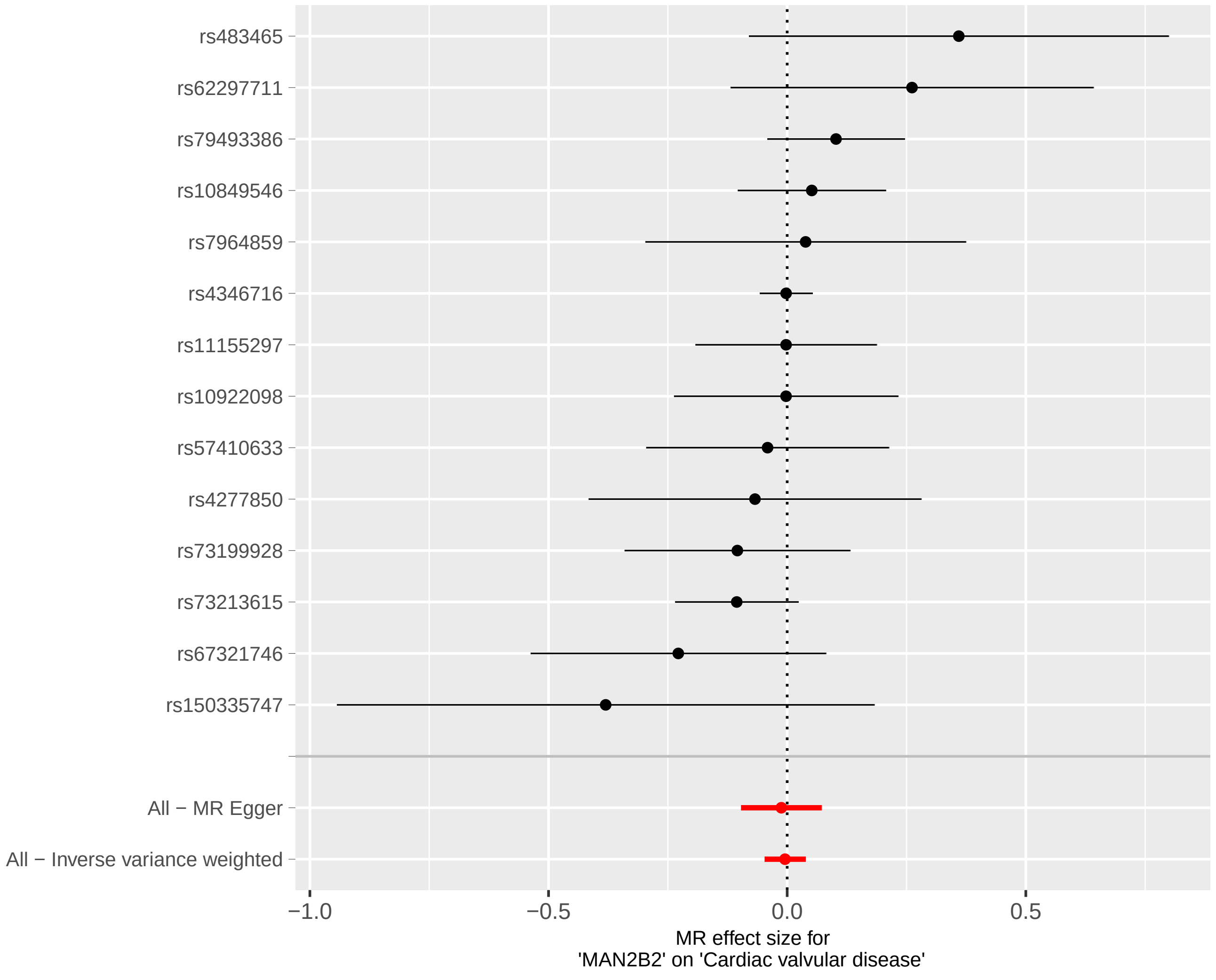
rs483465
rs62297711
rs79493386
rs10849546
rs7964859
rs4346716
rs11155297
rs10922098
rs57410633
rs4277850
rs73199928
rs73213615
rs67321746
rs150335747
All − MR Egger
All − Inverse variance weighted
−1.0
−0.5
0.0
0.5
MR effect size for
'MAN2B2' on 'Cardiac valvular disease'

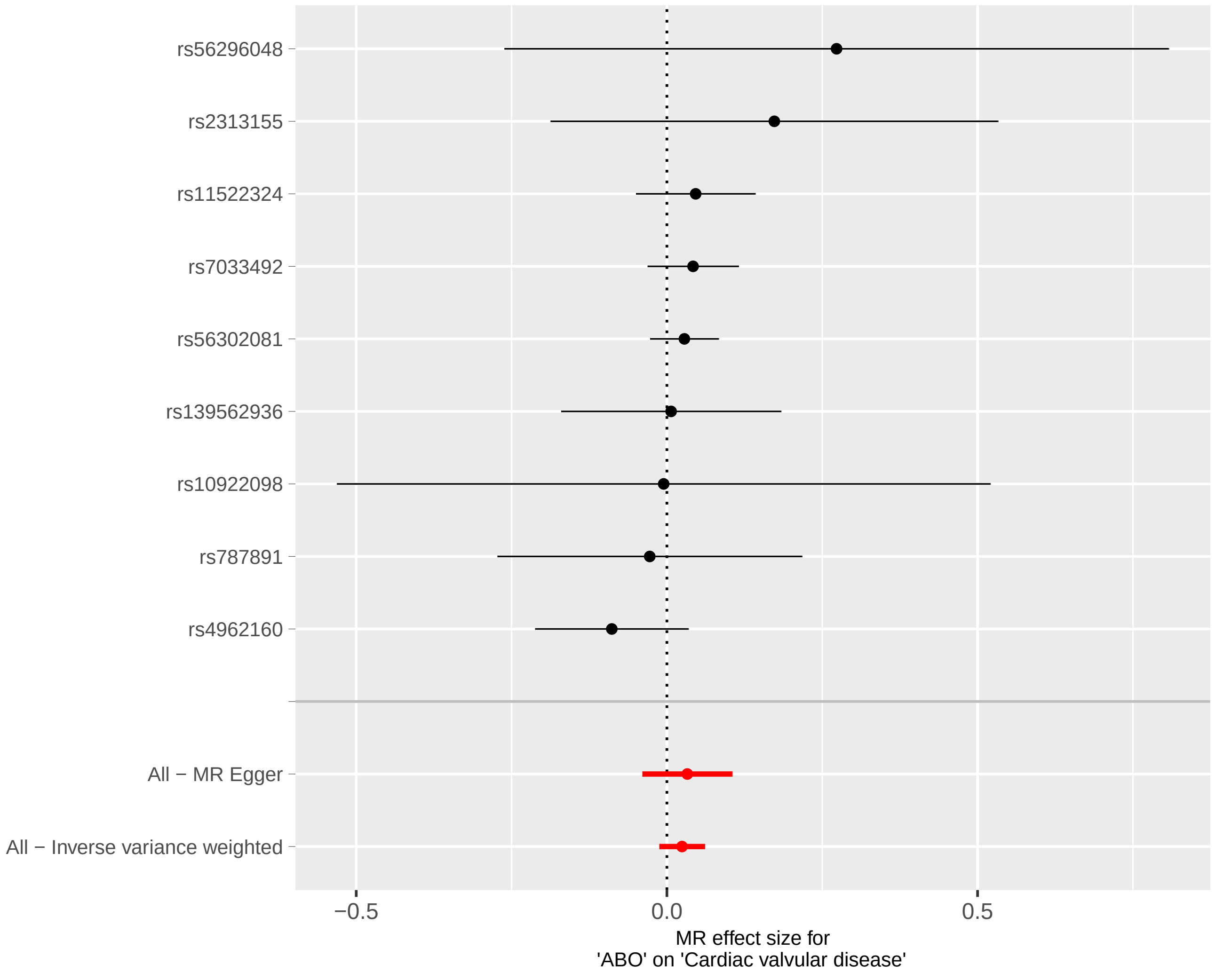
rs56296048
rs2313155
rs11522324
rs7033492
rs56302081
rs139562936
rs10922098
rs787891
rs4962160
All − MR Egger
All − Inverse variance weighted
−0.5
0.0
0.5
MR effect size for
'ABO' on 'Cardiac valvular disease'

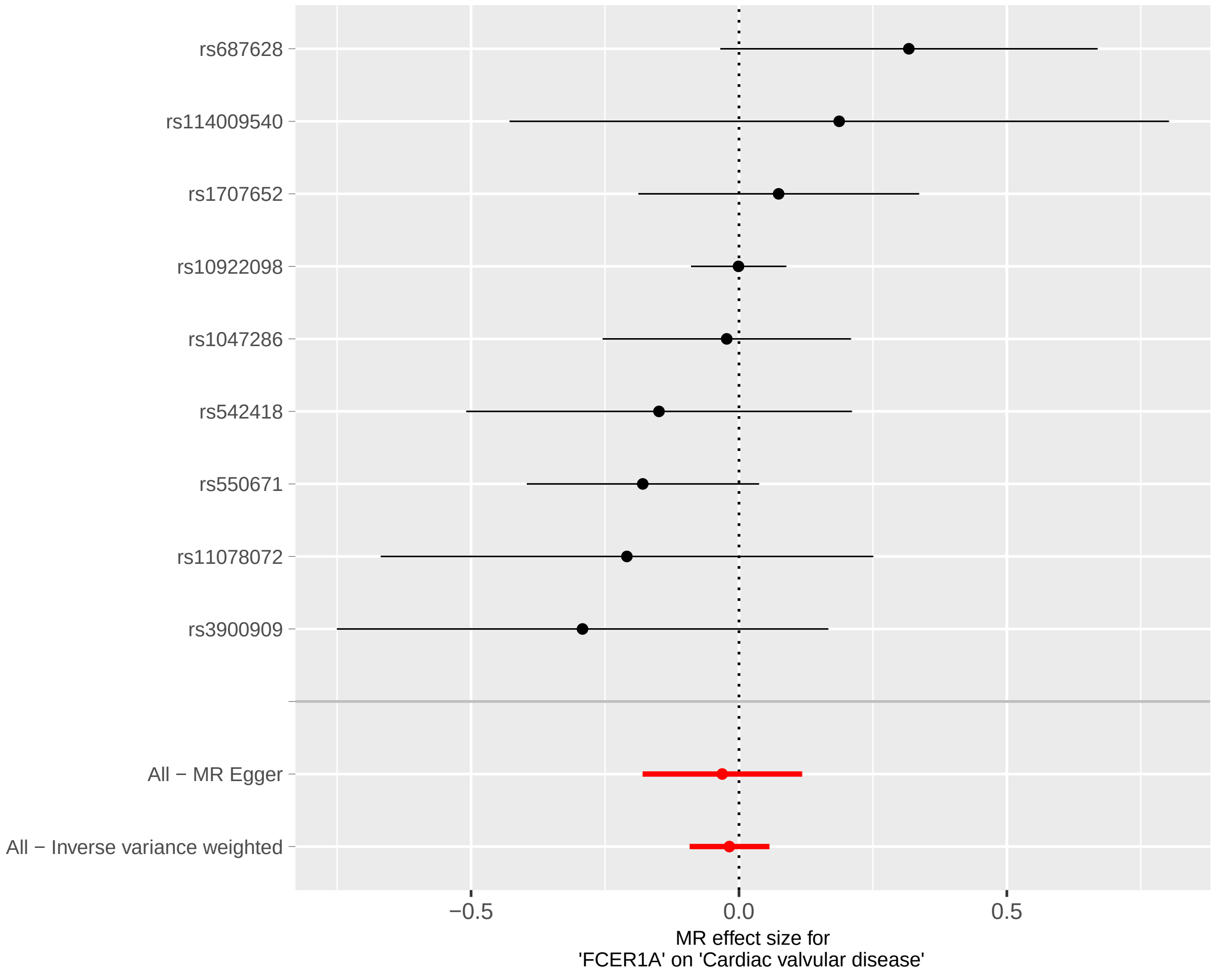
rs687628
rs114009540
rs1707652
rs10922098
rs1047286
rs542418
rs550671
rs11078072
rs3900909
All − MR Egger
All − Inverse variance weighted
−0.5
0.0
0.5
MR effect size for
'FCER1A' on 'Cardiac valvular disease'

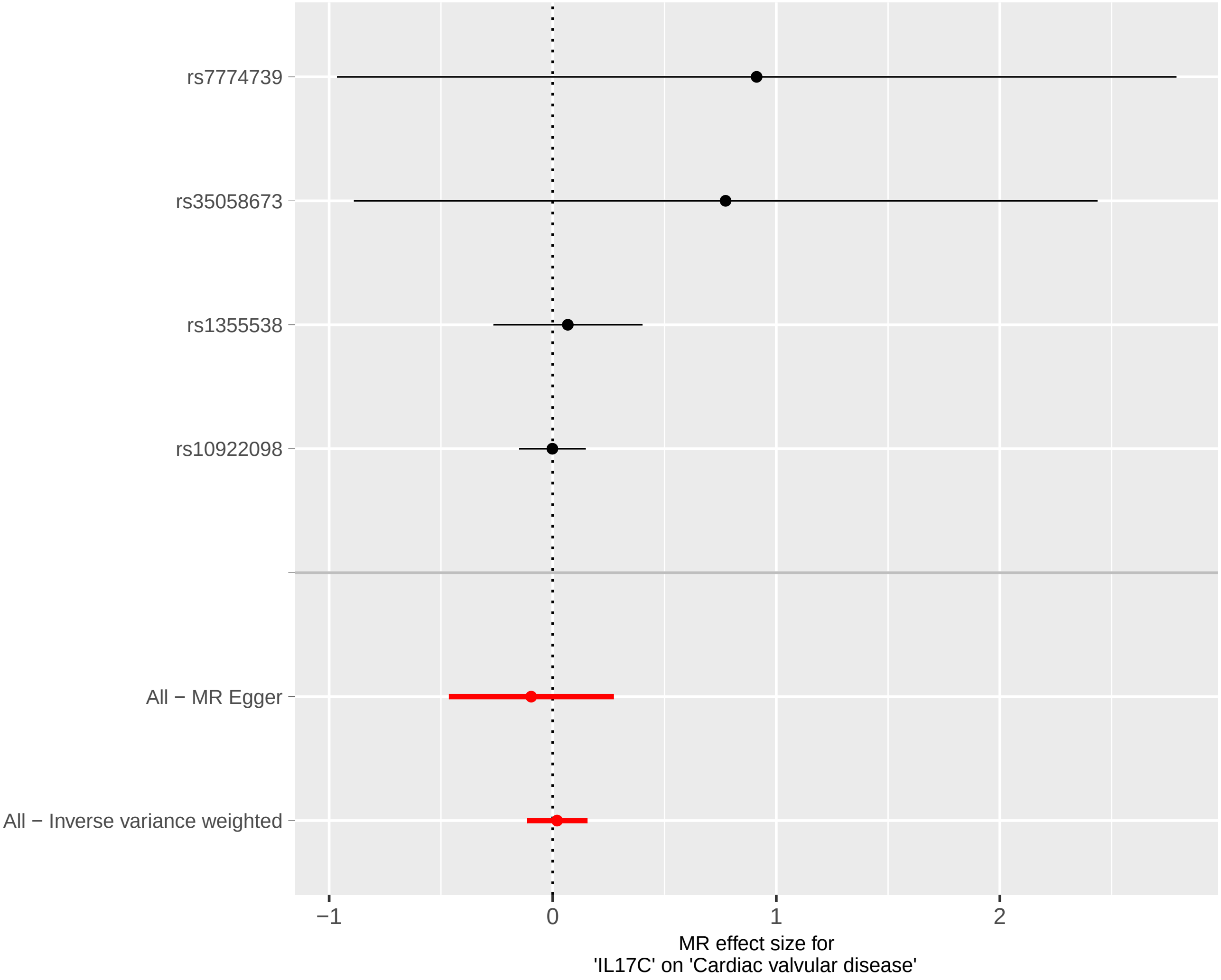
rs7774739
rs35058673
rs1355538
rs10922098
All − MR Egger
All − Inverse variance weighted
−1
0
1
2
MR effect size for
'IL17C' on 'Cardiac valvular disease'

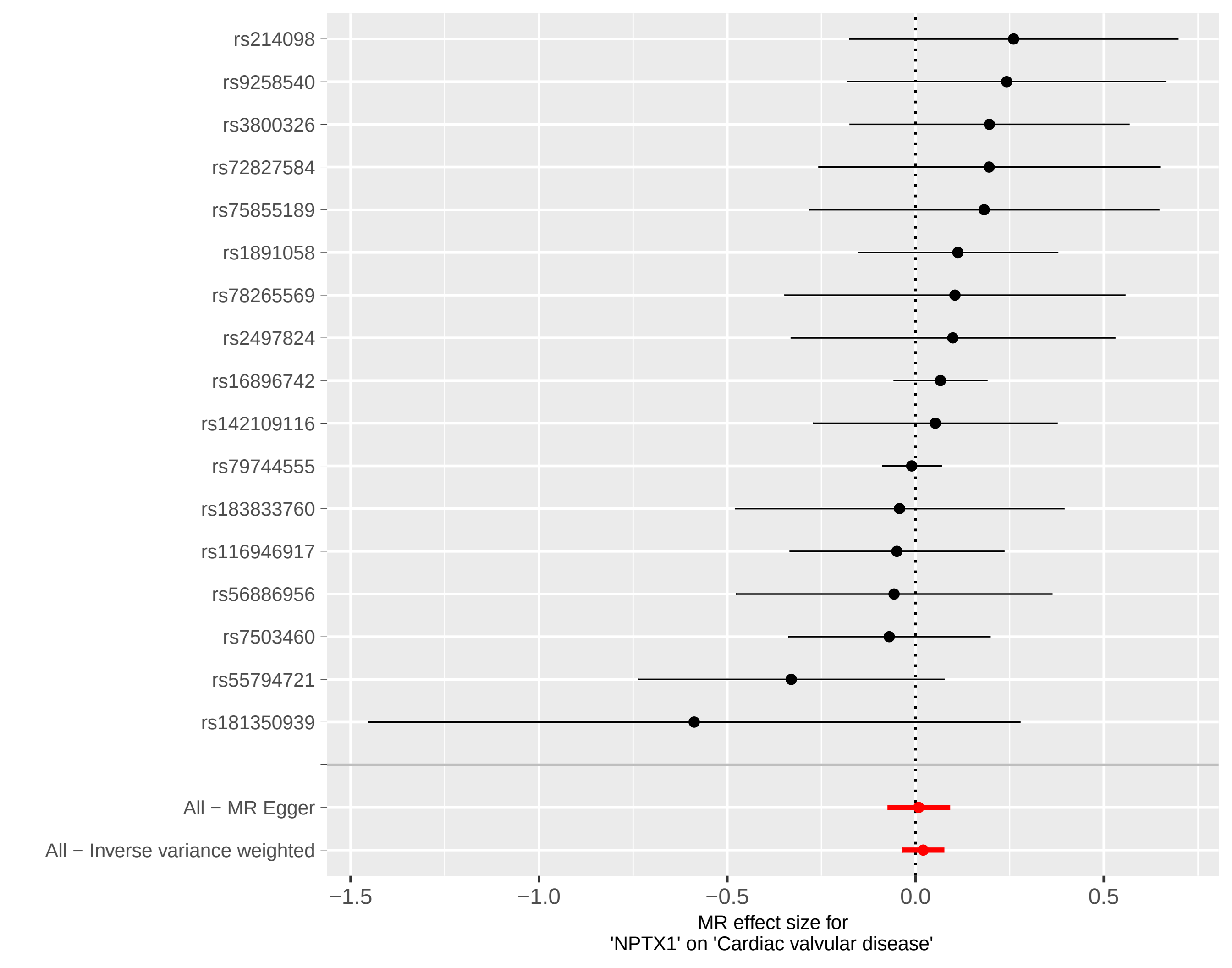
rs214098
rs9258540
rs3800326
rs72827584
rs75855189
rs1891058
rs78265569
rs2497824
rs16896742
rs142109116
rs79744555
rs183833760
rs116946917
rs56886956
rs7503460
rs55794721
rs181350939
All − MR Egger
All − Inverse variance weighted
−1.5
−1.0
−0.5
0.0
0.5
MR effect size for
'NPTX1' on 'Cardiac valvular disease'

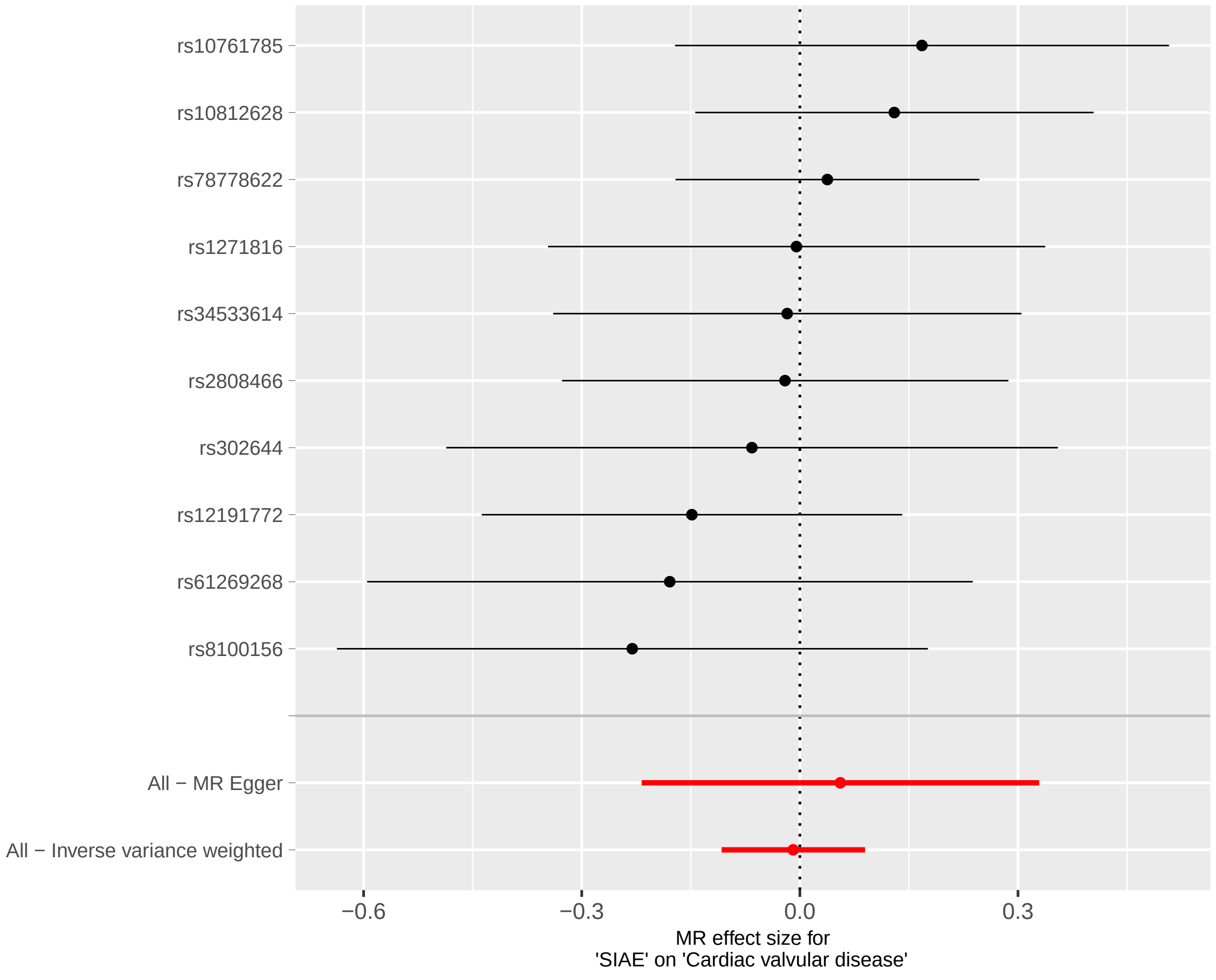
rs10761785
rs10812628
rs78778622
rs1271816
rs34533614
rs2808466
rs302644
rs12191772
rs61269268
rs8100156
All − MR Egger
All − Inverse variance weighted
−0.6
−0.3
0.0
0.3
MR effect size for
'SIAE' on 'Cardiac valvular disease'

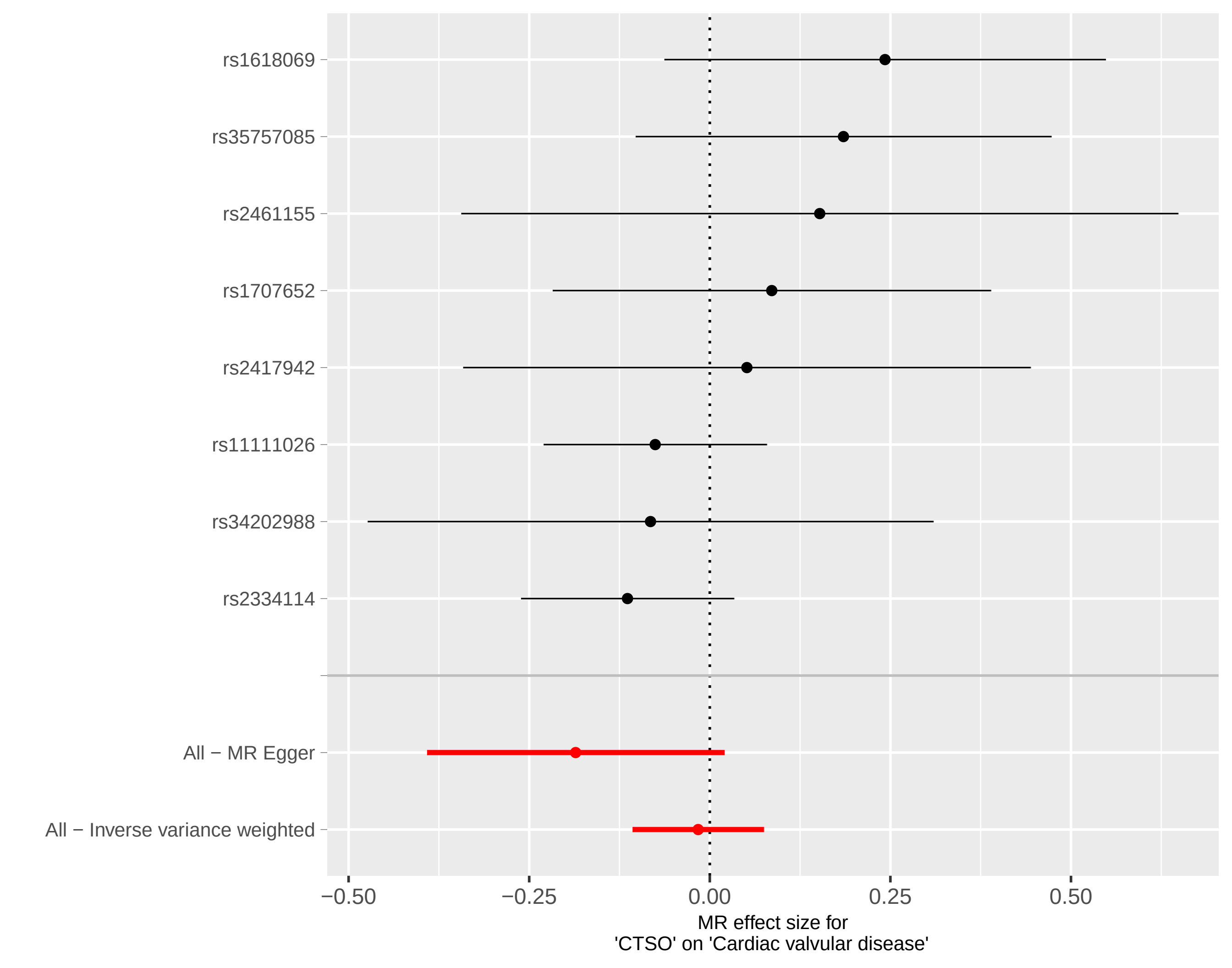
rs1618069
rs35757085
rs2461155
rs1707652
rs2417942
rs11111026
rs34202988
rs2334114
All − MR Egger
All − Inverse variance weighted
−0.50
−0.25
0.00
0.25
0.50
MR effect size for
'CTSO' on 'Cardiac valvular disease'

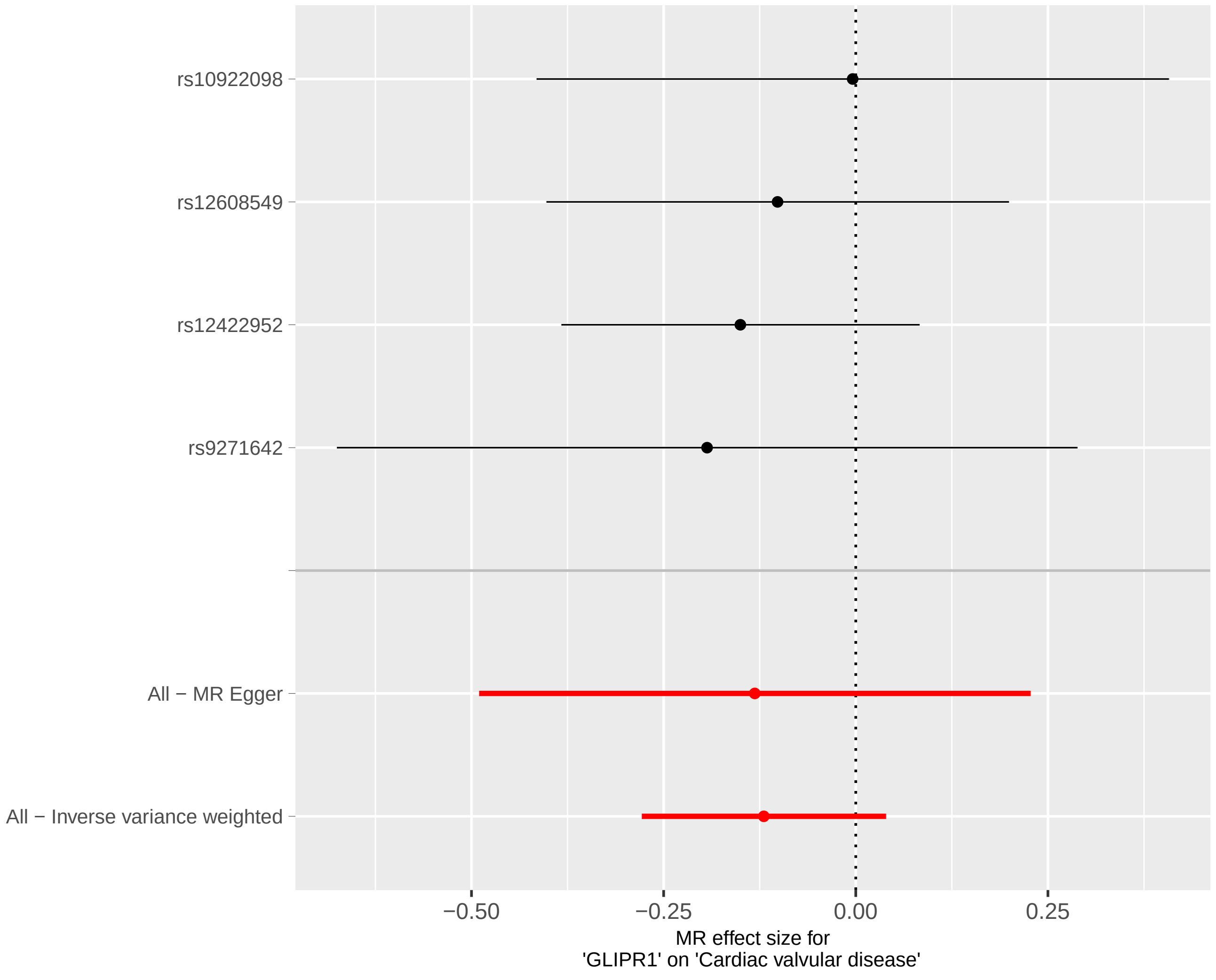
rs10922098
rs12608549
rs12422952
rs9271642
All − MR Egger
All − Inverse variance weighted
−0.50
−0.25
0.00
0.25
MR effect size for
'GLIPR1' on 'Cardiac valvular disease'

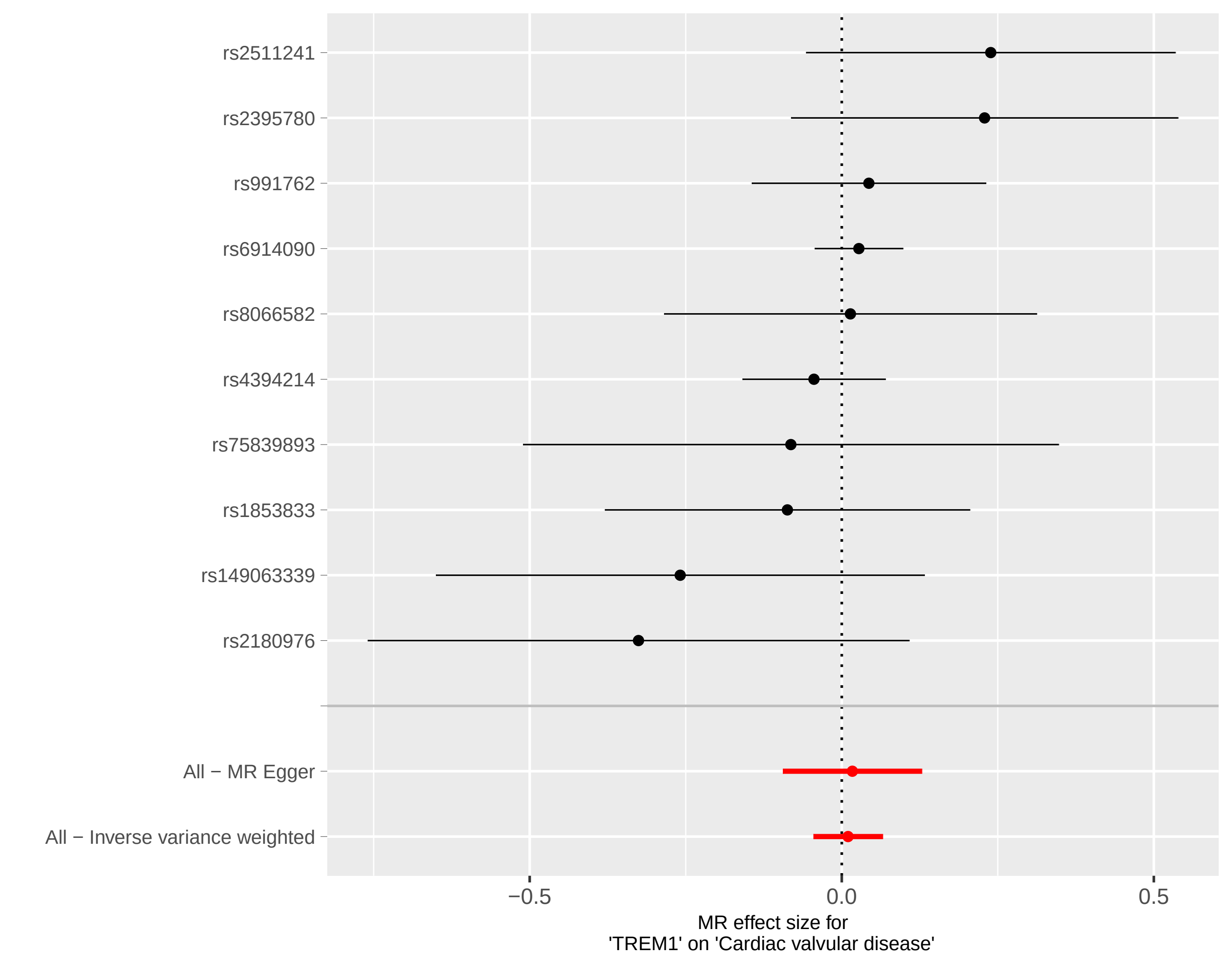
rs2511241
rs2395780
rs991762
rs6914090
rs8066582
rs4394214
rs75839893
rs1853833
rs149063339
rs2180976
All − MR Egger
All − Inverse variance weighted
−0.5
0.0
0.5
MR effect size for
'TREM1' on 'Cardiac valvular disease'

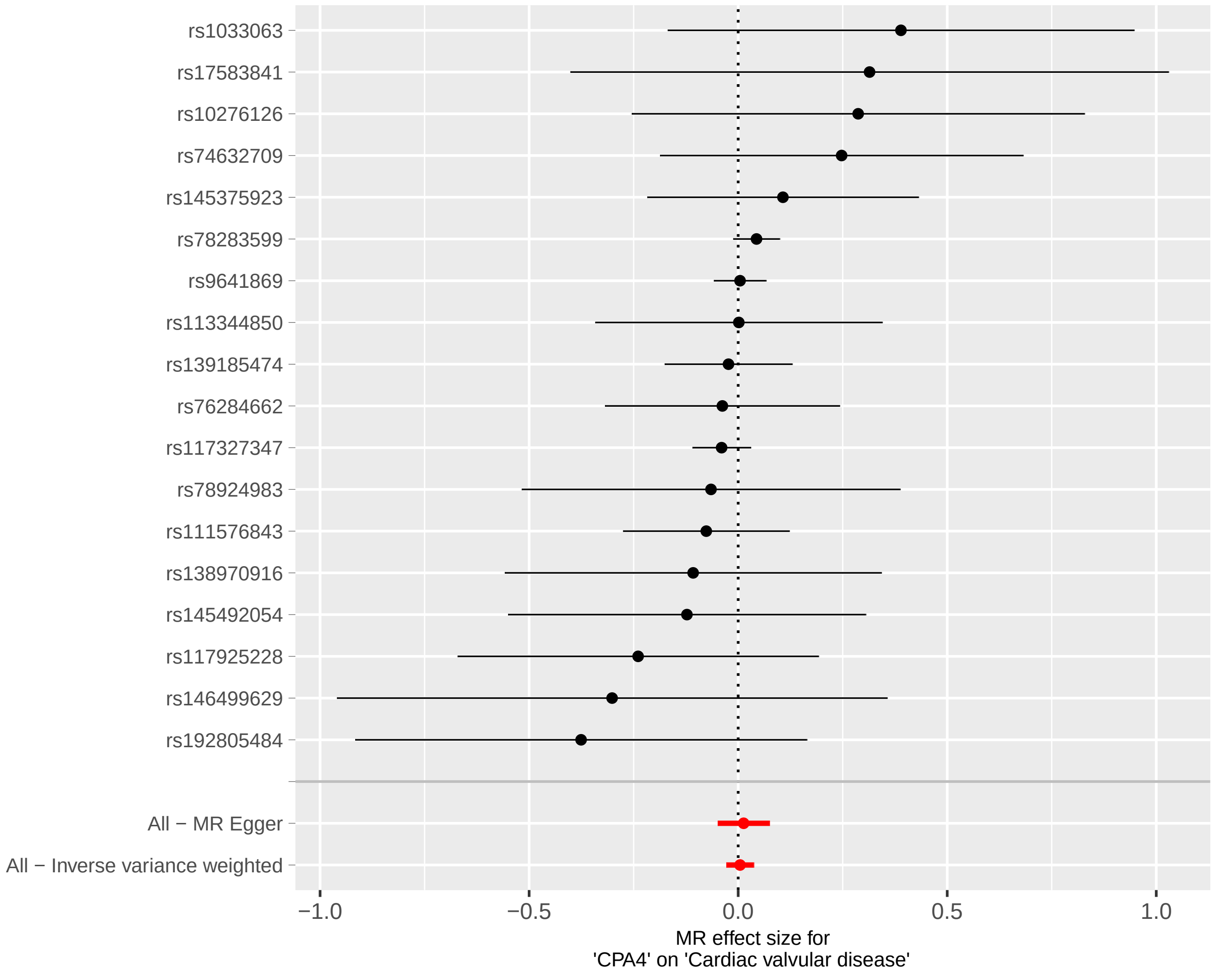
rs1033063
rs17583841
rs10276126
rs74632709
rs145375923
rs78283599
rs9641869
rs113344850
rs139185474
rs76284662
rs117327347
rs78924983
rs111576843
rs138970916
rs145492054
rs117925228
rs146499629
rs192805484
All − MR Egger
All − Inverse variance weighted
−1.0
−0.5
0.0
0.5
1.0
MR effect size for
'CPA4' on 'Cardiac valvular disease'

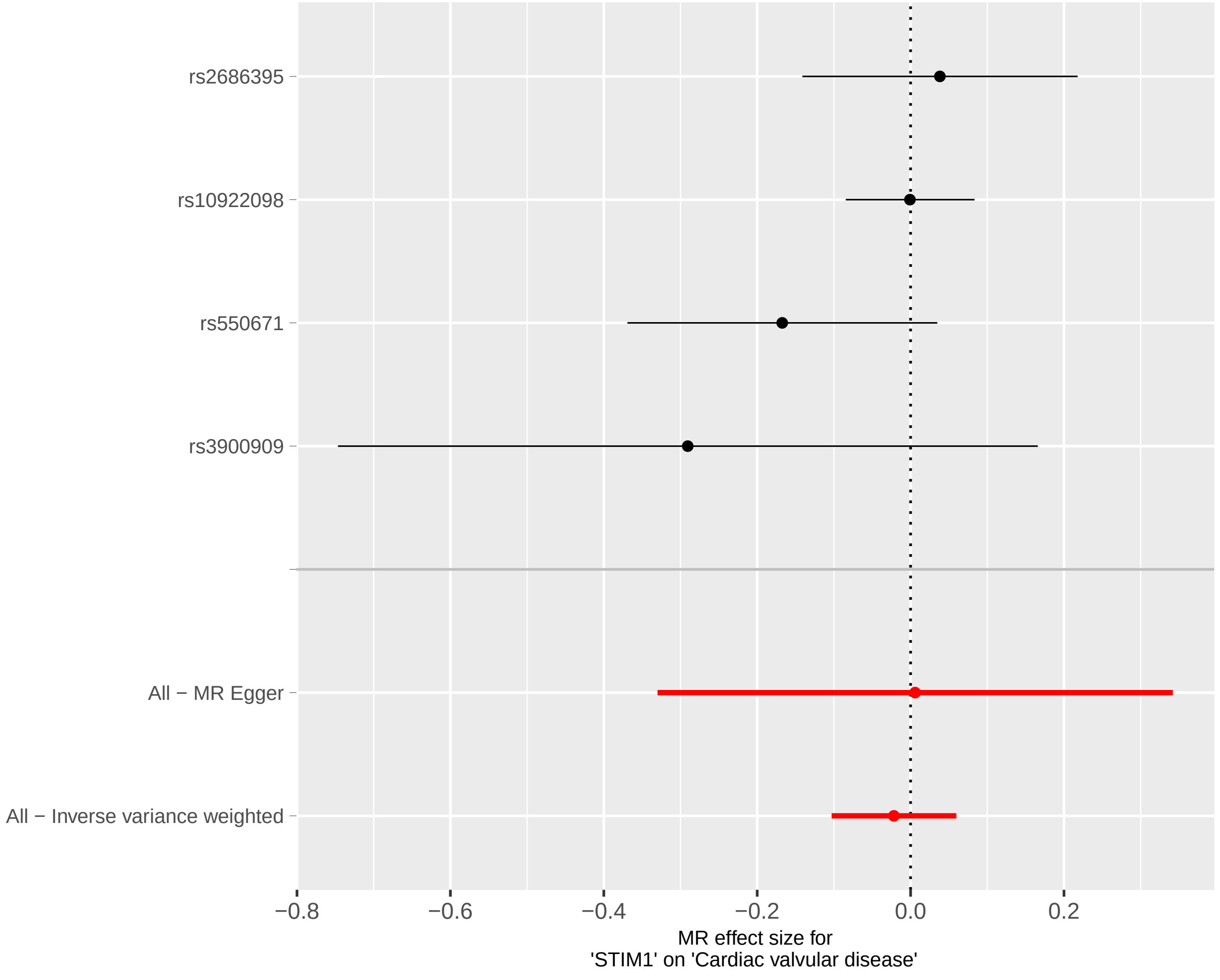
rs2686395
rs10922098
rs550671
rs3900909
All − MR Egger
All − Inverse variance weighted
−0.8
−0.6
−0.4
−0.2
0.0
0.2
MR effect size for
'STIM1' on 'Cardiac valvular disease'

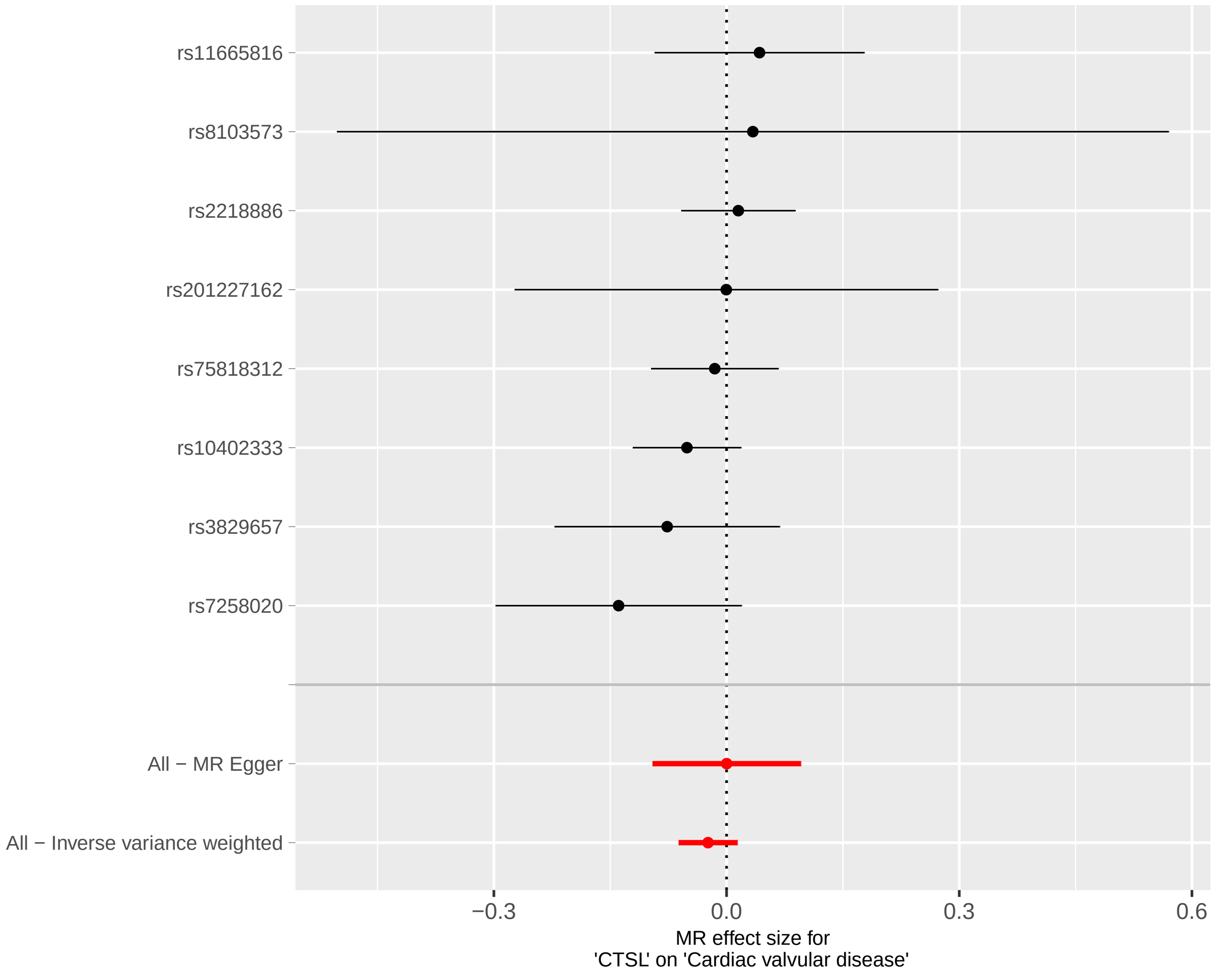
rs11665816
rs8103573
rs2218886
rs201227162
rs75818312
rs10402333
rs3829657
rs7258020
All − MR Egger
All − Inverse variance weighted
−0.3
0.0
0.3
0.6
MR effect size for
'CTSL' on 'Cardiac valvular disease'

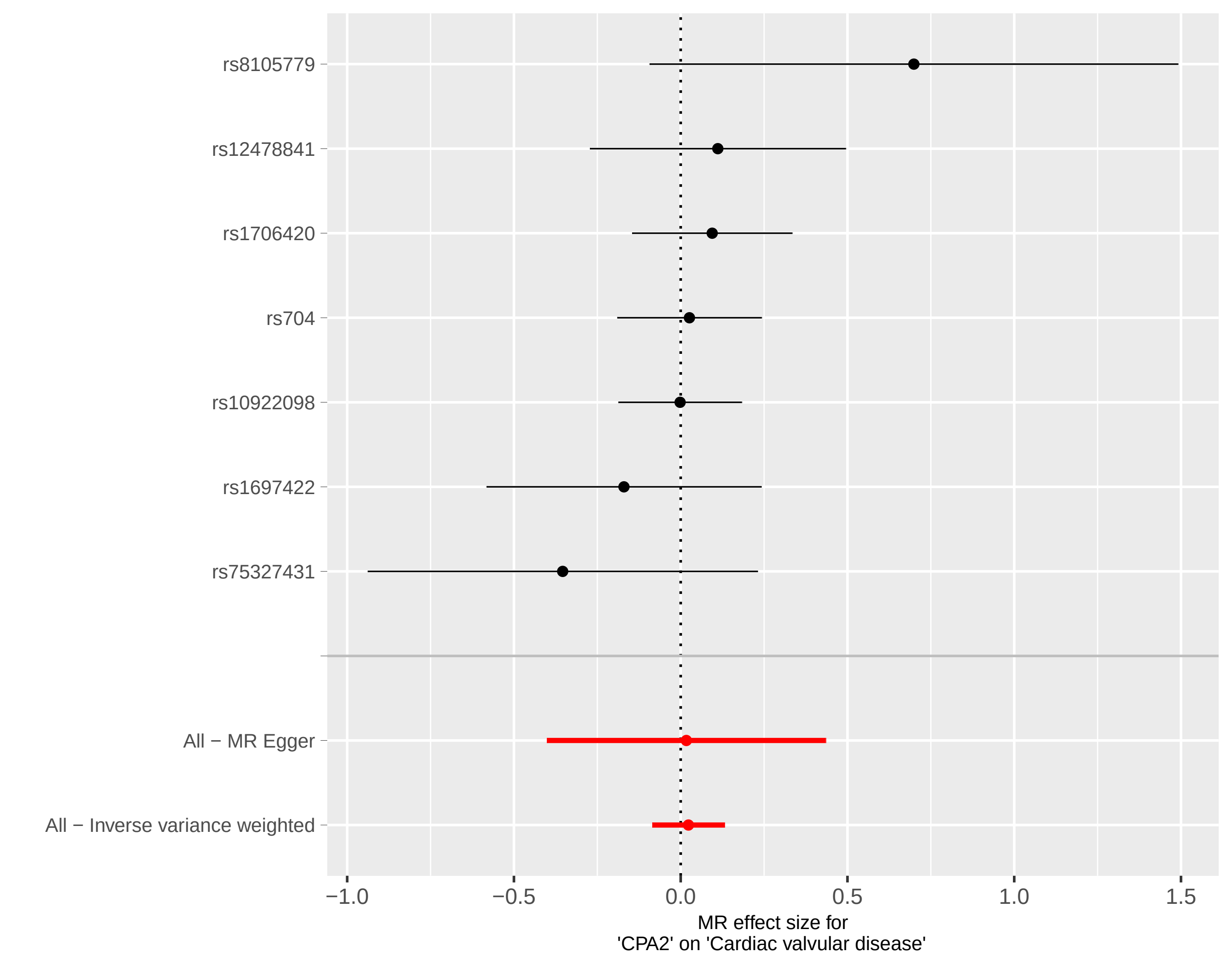
rs8105779
rs12478841
rs1706420
rs704
rs10922098
rs1697422
rs75327431
All − MR Egger
All − Inverse variance weighted
−1.0
−0.5
0.0
0.5
1.0
1.5
MR effect size for
'CPA2' on 'Cardiac valvular disease'

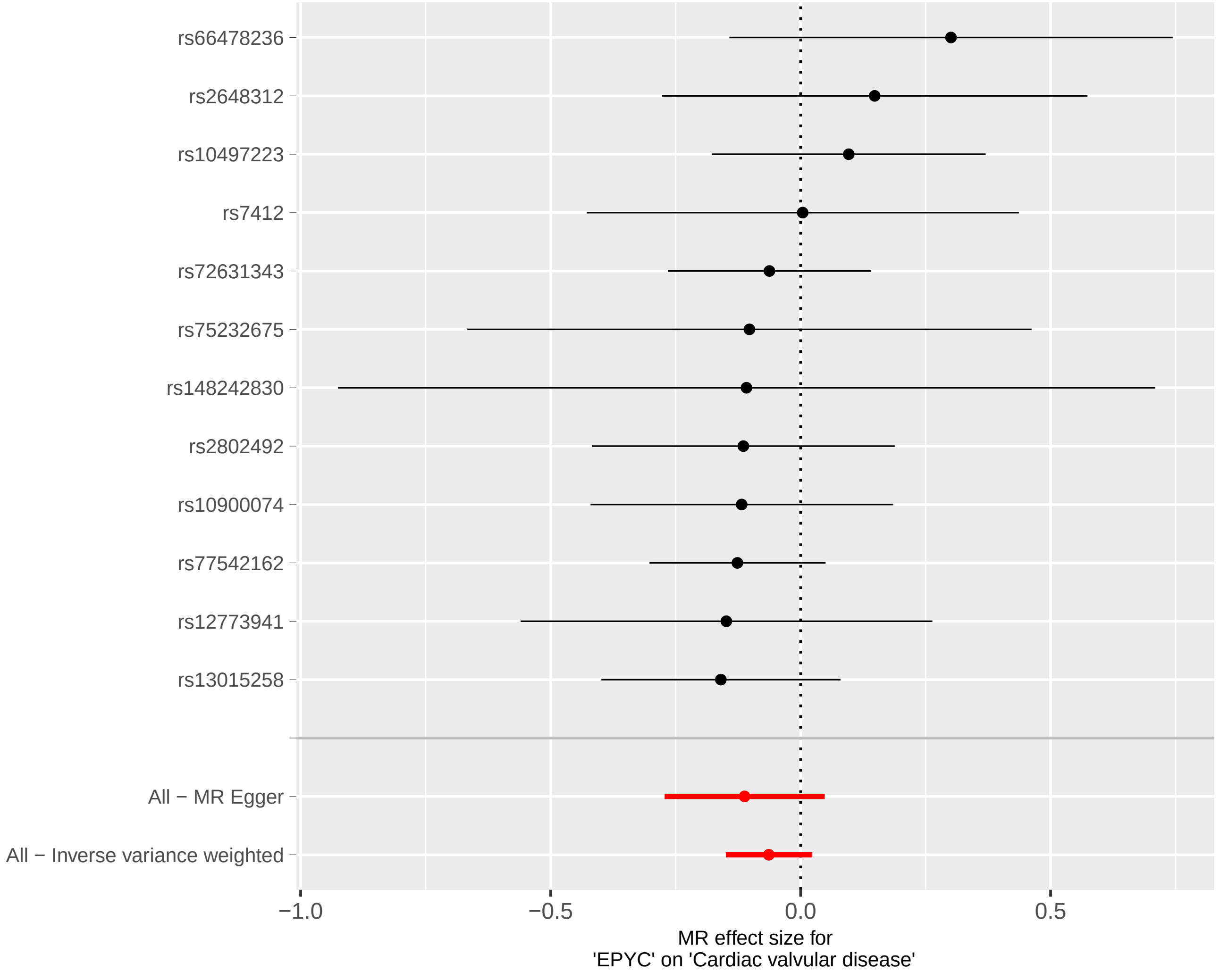

rs66478236
rs2648312
rs10497223
rs7412
rs72631343
rs75232675
rs148242830
rs2802492
rs10900074
rs77542162
rs12773941
rs13015258
All − MR Egger
All − Inverse variance weighted
−1.0
−0.5
0.0
0.5
MR effect size for
'EPYC' on 'Cardiac valvular disease'

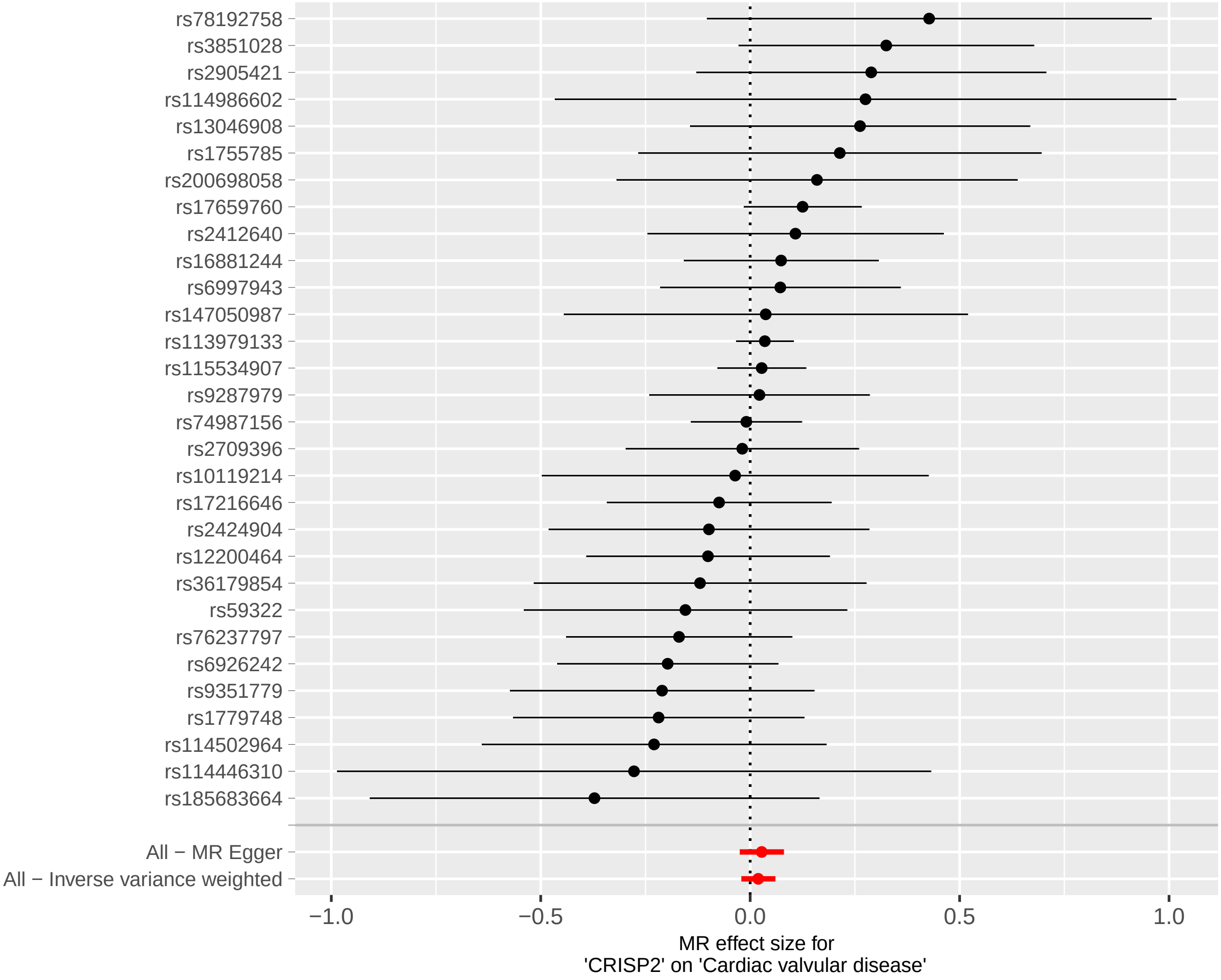
rs78192758
rs3851028
rs2905421
rs114986602
rs13046908
rs1755785
rs200698058
rs17659760
rs2412640
rs16881244
rs6997943
rs147050987
rs113979133
rs115534907
rs9287979
rs74987156
rs2709396
rs10119214
rs17216646
rs2424904
rs12200464
rs36179854
rs59322
rs76237797
rs6926242
rs9351779
rs1779748
rs114502964
rs114446310
rs185683664
All − MR Egger
All − Inverse variance weighted
−1.0
−0.5
0.0
0.5
1.0
MR effect size for
'CRISP2' on 'Cardiac valvular disease'

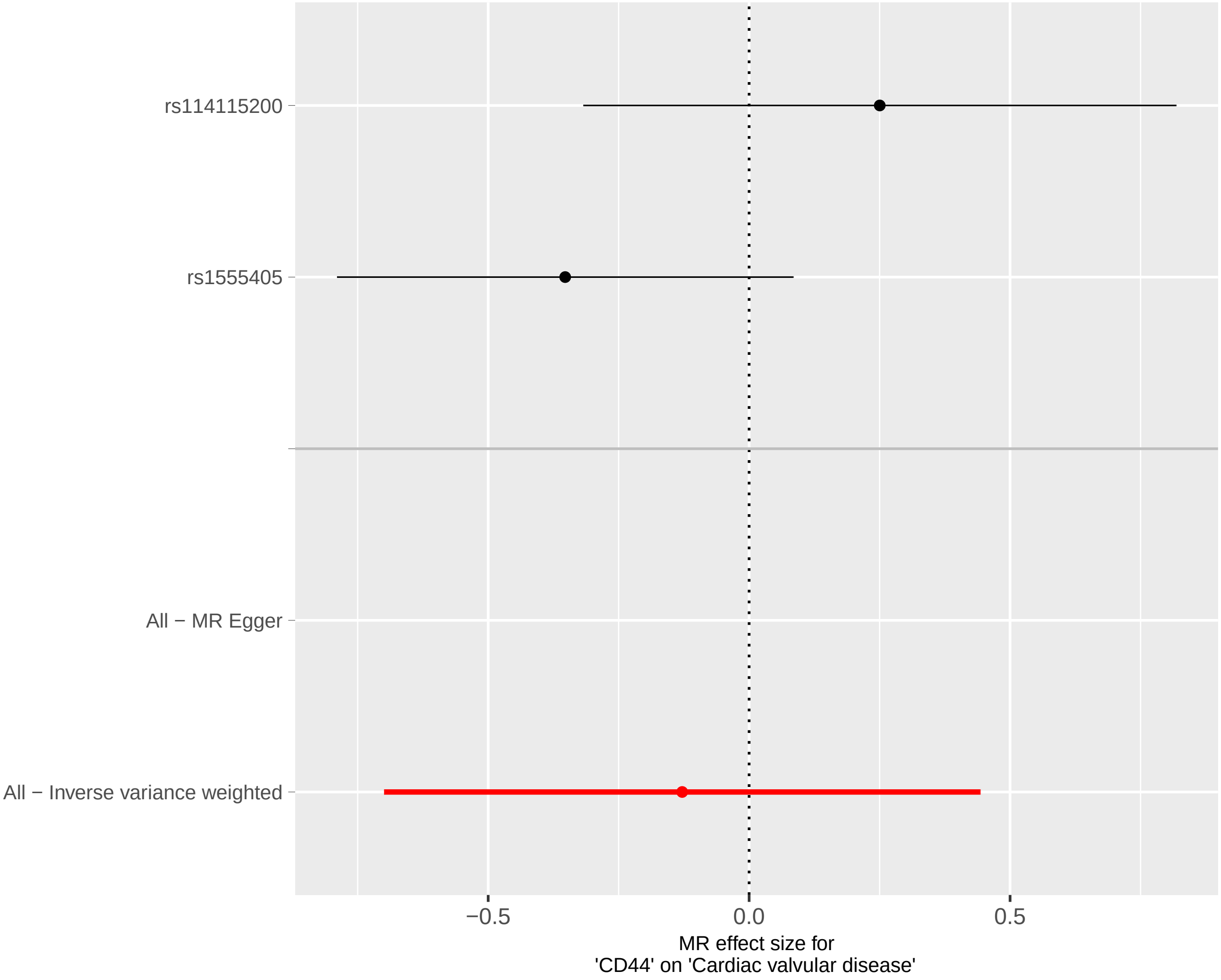
rs114115200
rs1555405
All − MR Egger
All − Inverse variance weighted
−0.5
0.0
0.5
MR effect size for
'CD44' on 'Cardiac valvular disease'

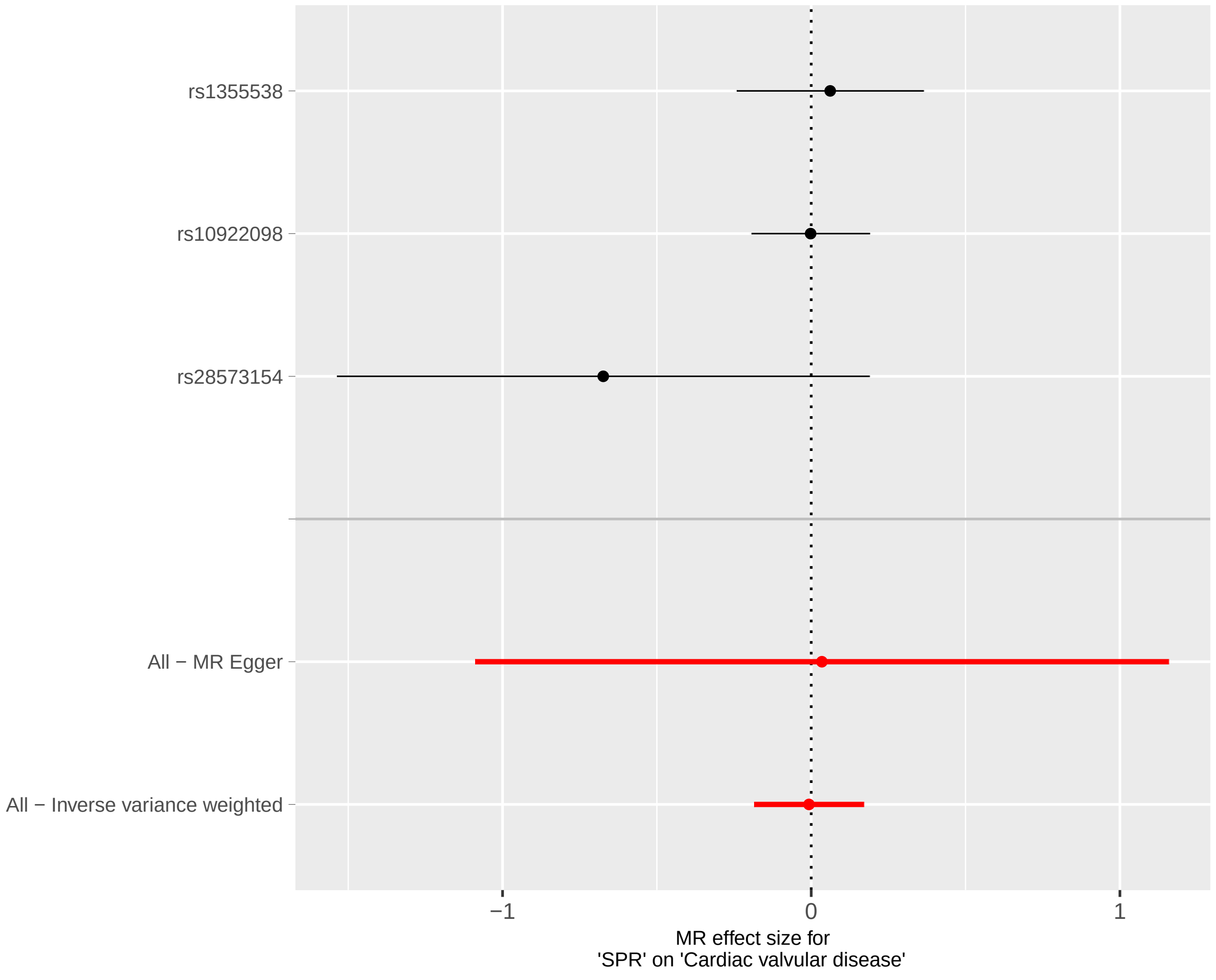
rs1355538
rs10922098
rs28573154
All − MR Egger
All − Inverse variance weighted
−1
0
1
MR effect size for
'SPR' on 'Cardiac valvular disease'

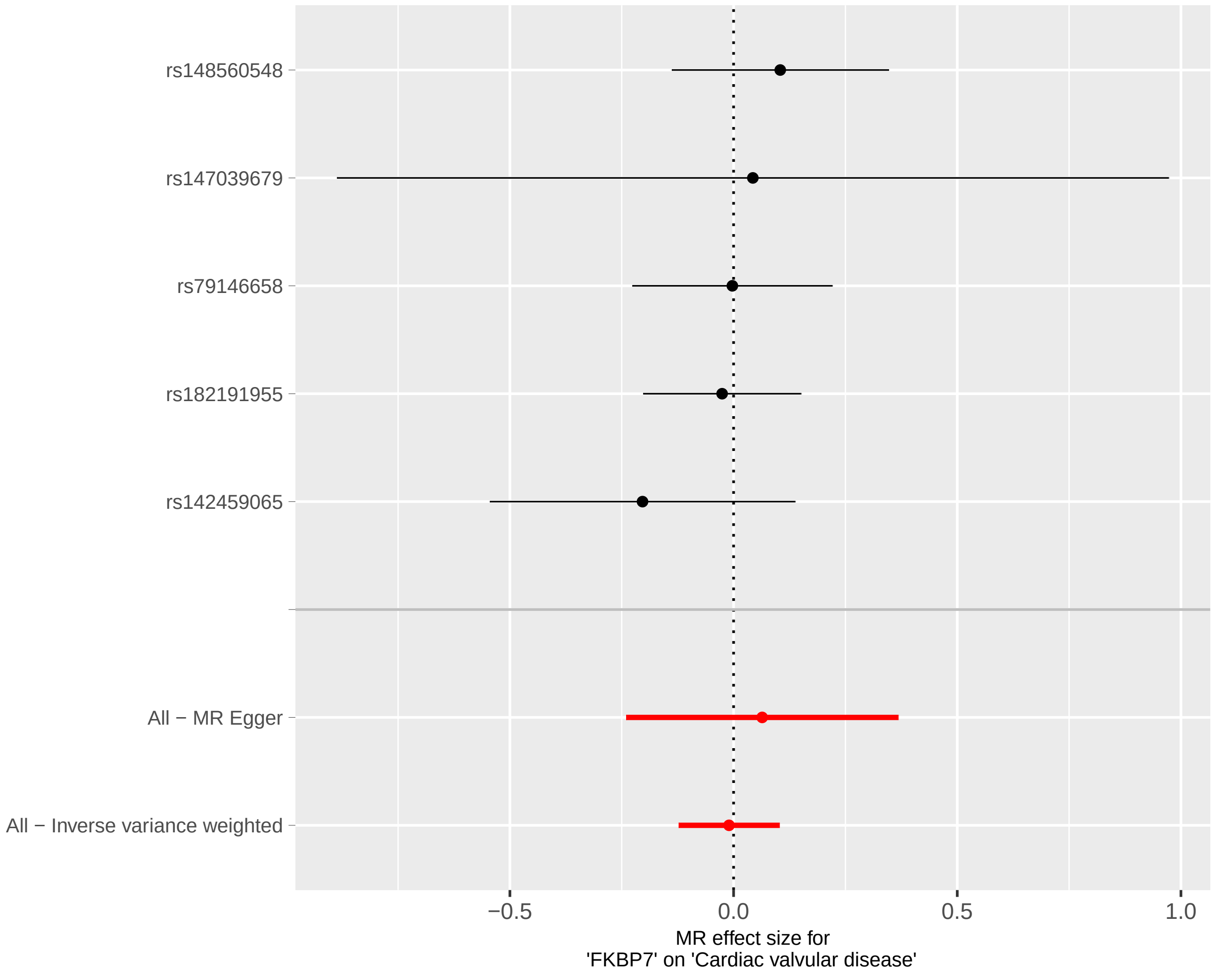

rs148560548
rs147039679
rs79146658
rs182191955
rs142459065
All − MR Egger
All − Inverse variance weighted
−0.5
0.0
0.5
1.0
MR effect size for
'FKBP7' on 'Cardiac valvular disease'

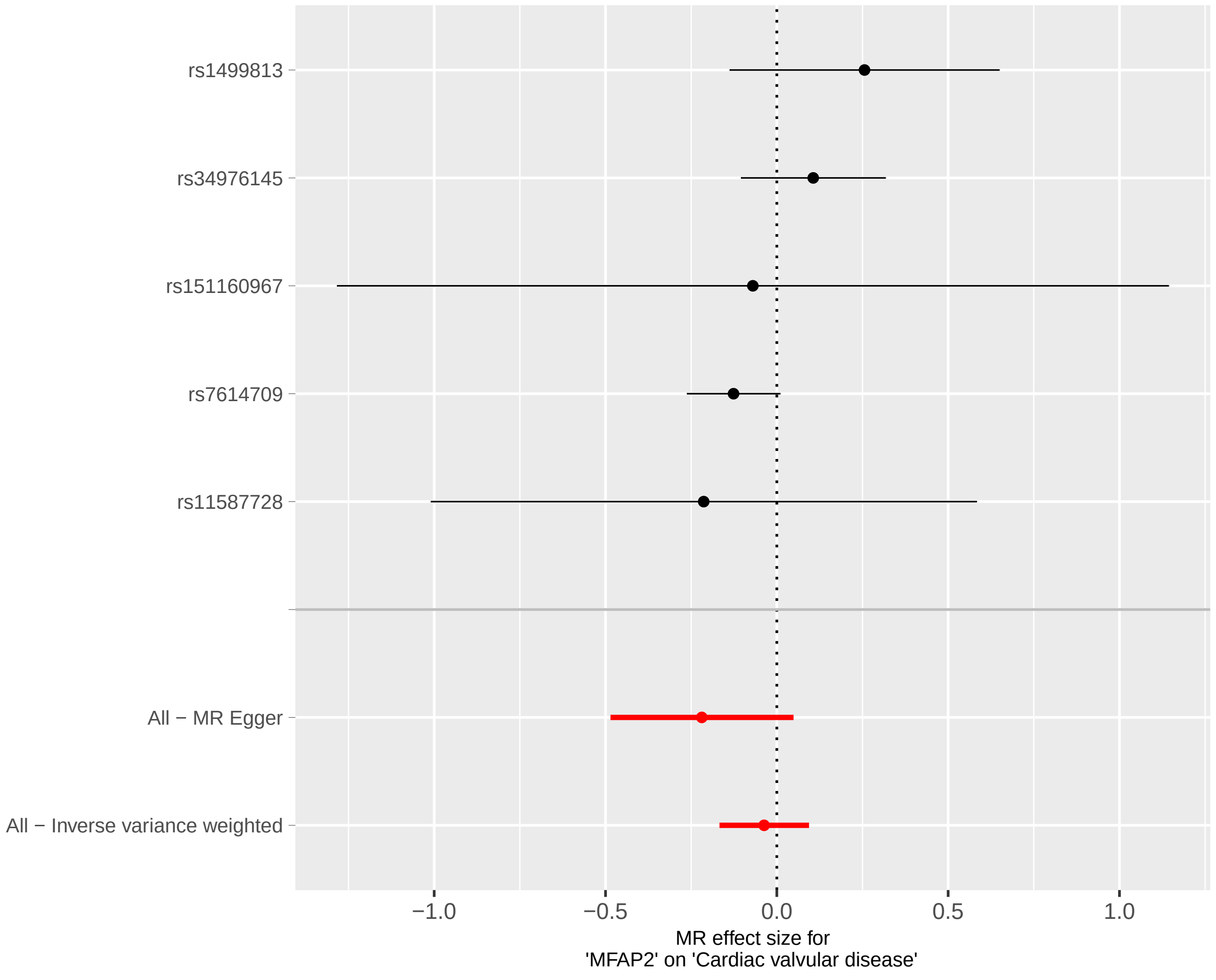
rs1499813
rs34976145
rs151160967
rs7614709
rs11587728
All − MR Egger
All − Inverse variance weighted
−1.0
−0.5
0.0
0.5
1.0
MR effect size for
'MFAP2' on 'Cardiac valvular disease'

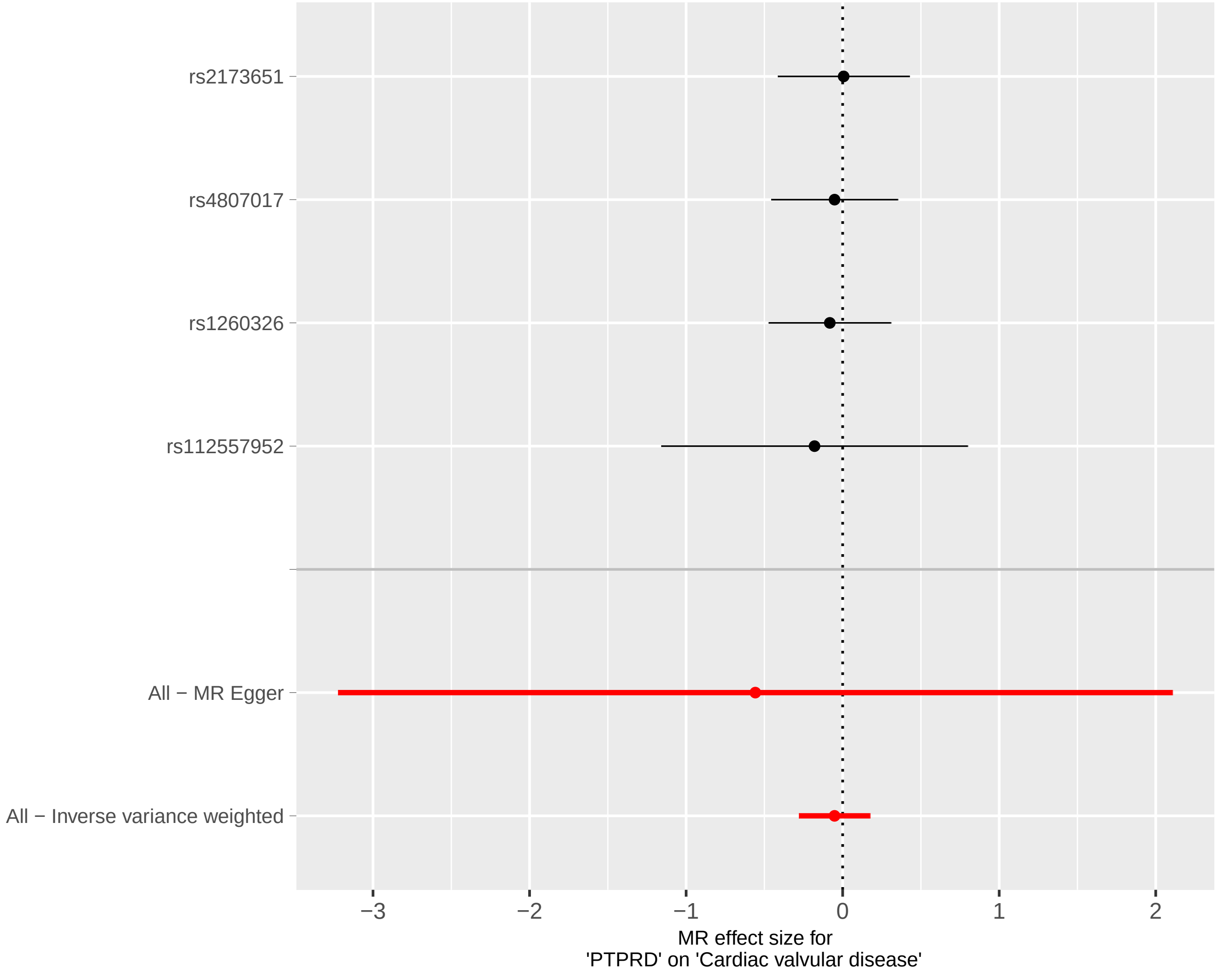
rs2173651
rs4807017
rs1260326
rs112557952
All − MR Egger
All − Inverse variance weighted
−3
−2
−1
0
1
2
MR effect size for
'PTPRD' on 'Cardiac valvular disease'

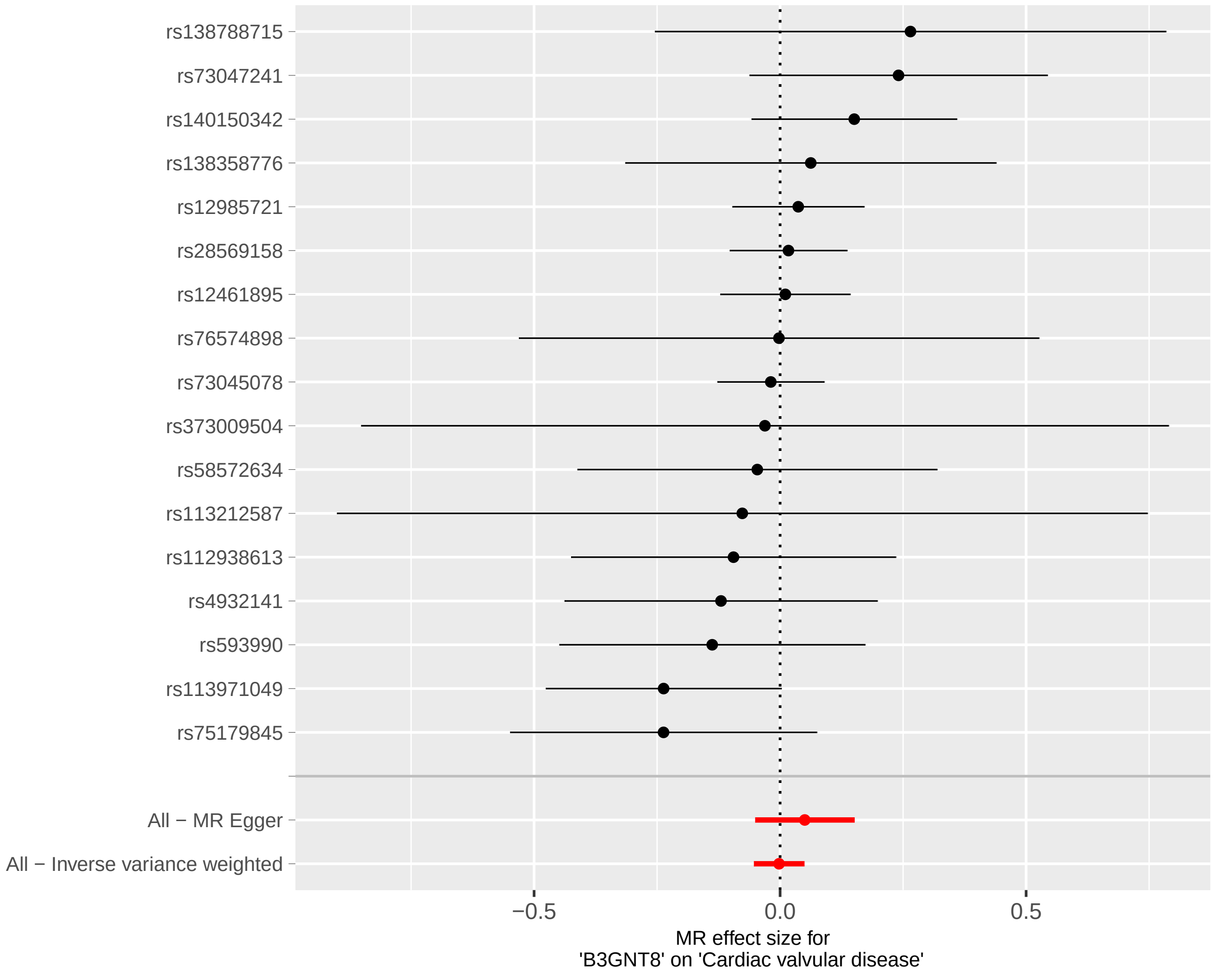
rs138788715
rs73047241
rs140150342
rs138358776
rs12985721
rs28569158
rs12461895
rs76574898
rs73045078
rs373009504
rs58572634
rs113212587
rs112938613
rs4932141
rs593990
rs113971049
rs75179845
All − MR Egger
All − Inverse variance weighted
−0.5
0.0
0.5
MR effect size for
'B3GNT8' on 'Cardiac valvular disease'

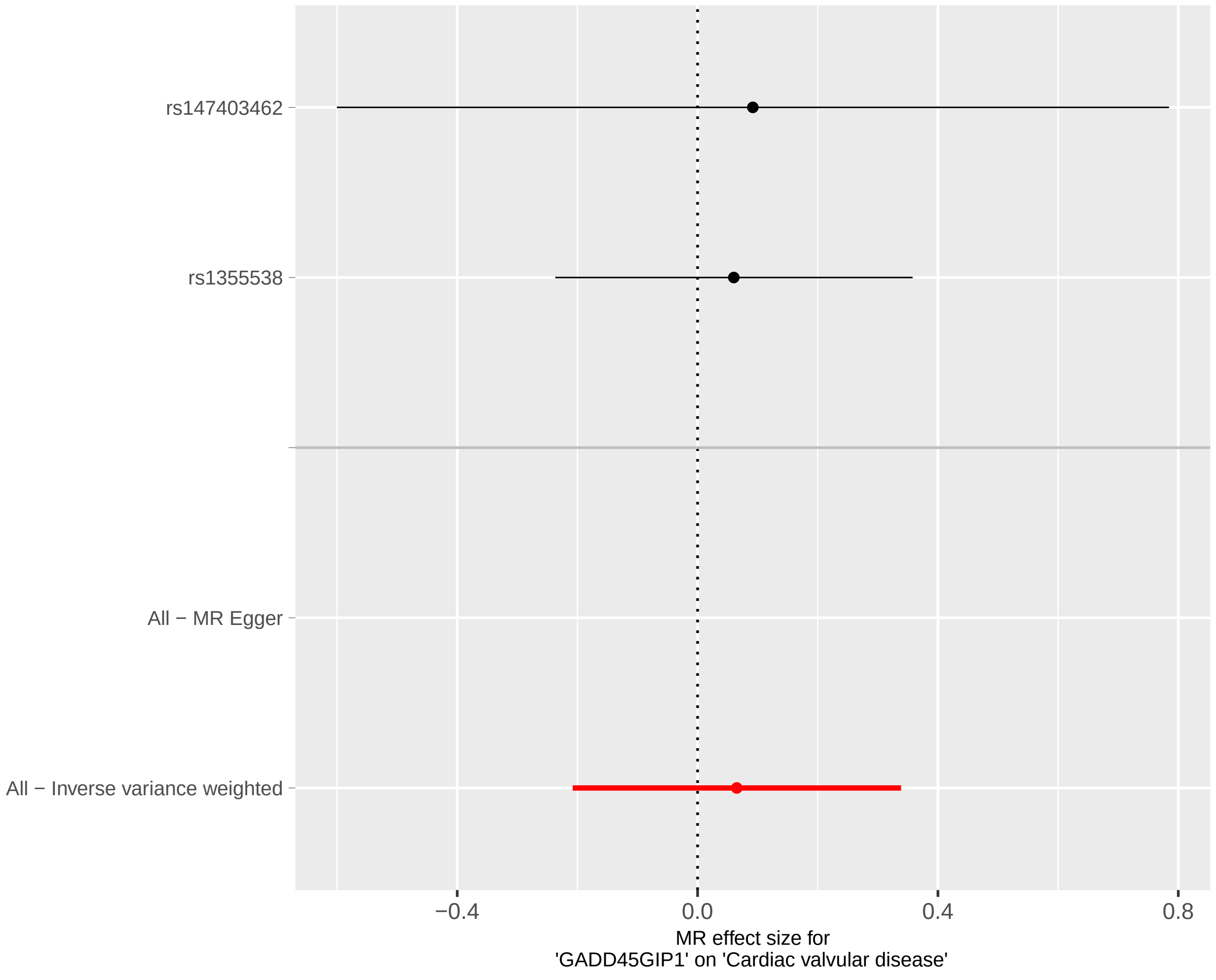
rs147403462
rs1355538
All − MR Egger
All − Inverse variance weighted
−0.4
0.0
0.4
0.8
MR effect size for
'GADD45GIP1' on 'Cardiac valvular disease'

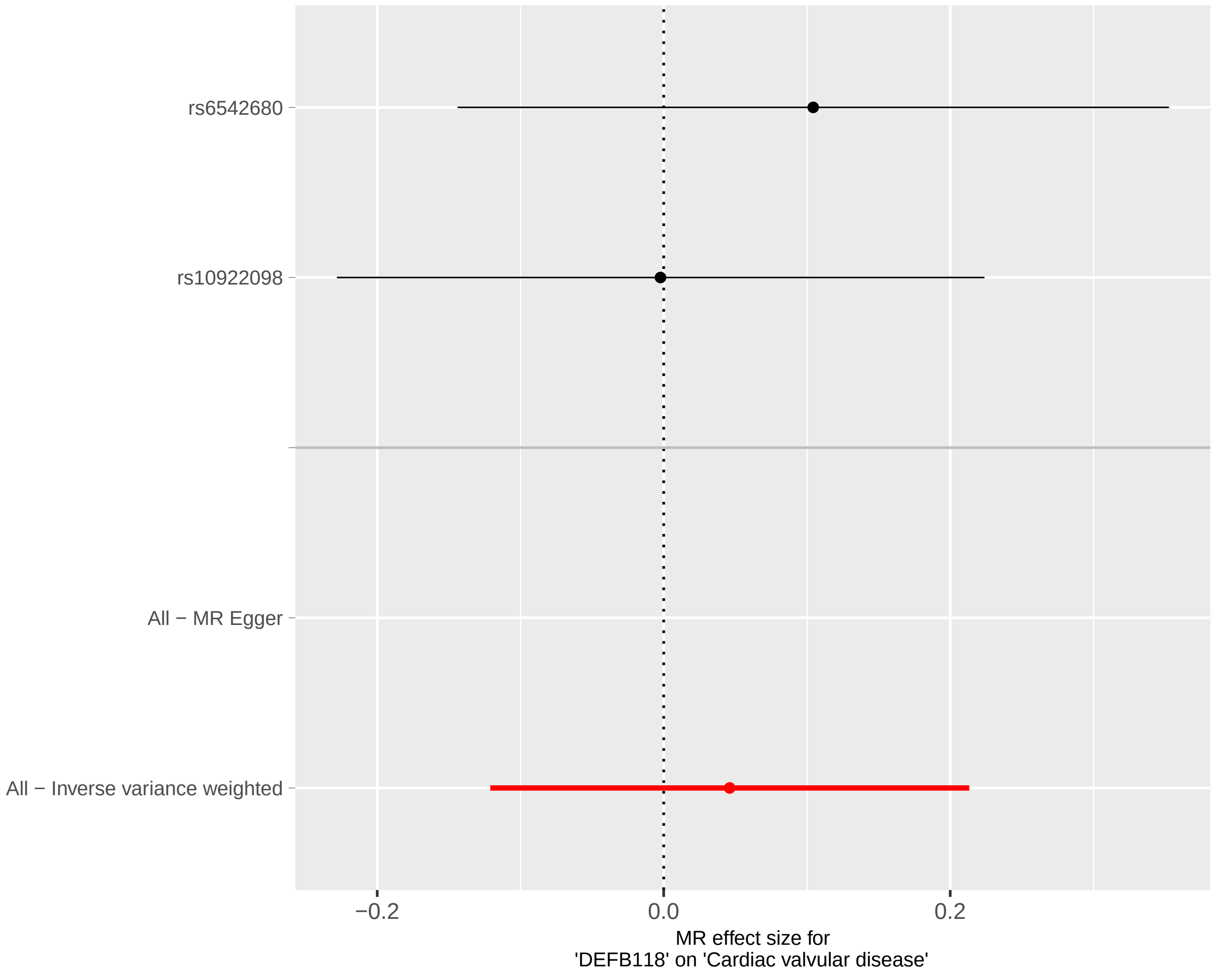
rs6542680
rs10922098
All − MR Egger
All − Inverse variance weighted
−0.2
0.0
0.2
MR effect size for
'DEFB118' on 'Cardiac valvular disease'

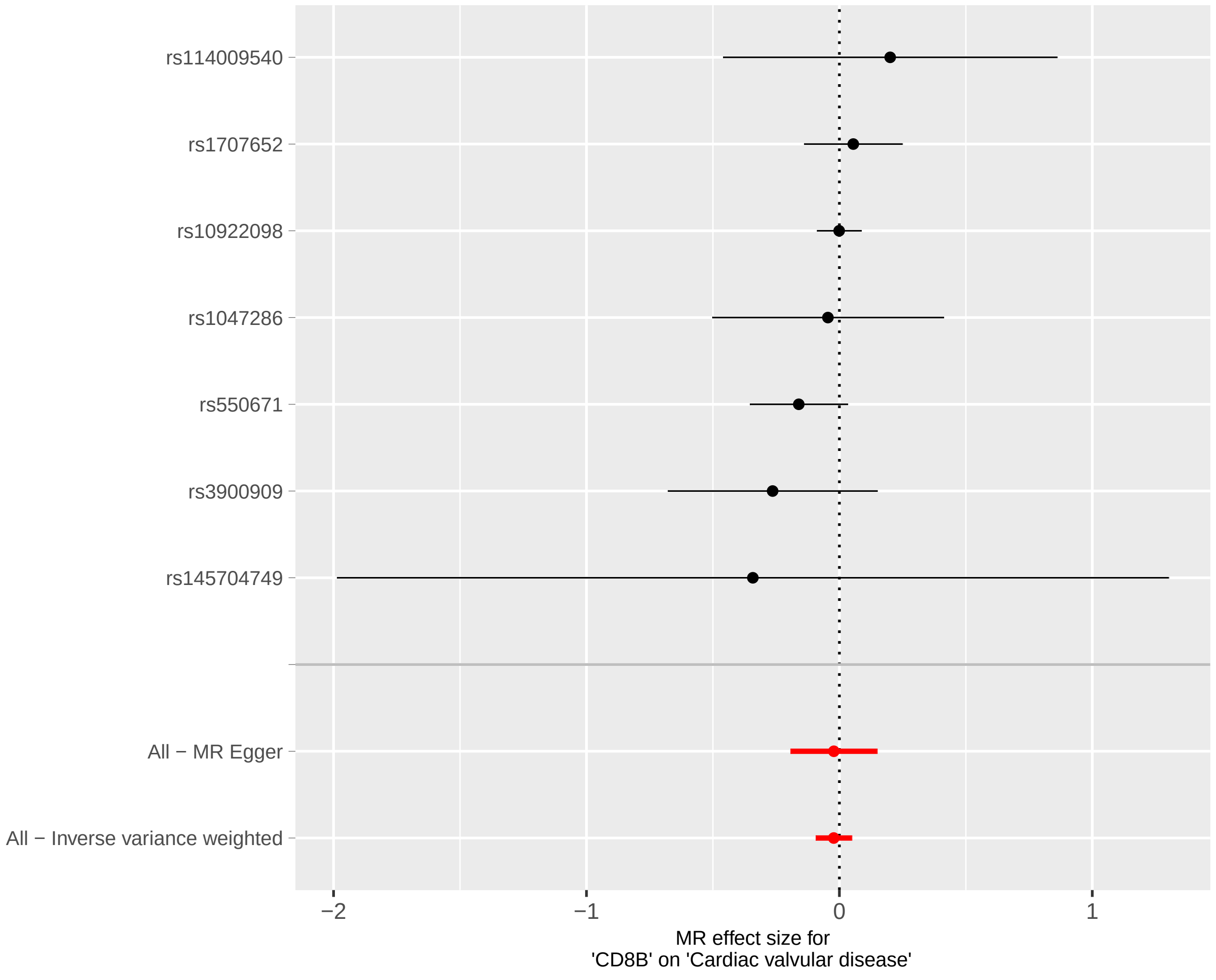
rs114009540
rs1707652
rs10922098
rs1047286
rs550671
rs3900909
rs145704749
All − MR Egger
All − Inverse variance weighted
−2
−1
0
1
MR effect size for
'CD8B' on 'Cardiac valvular disease'

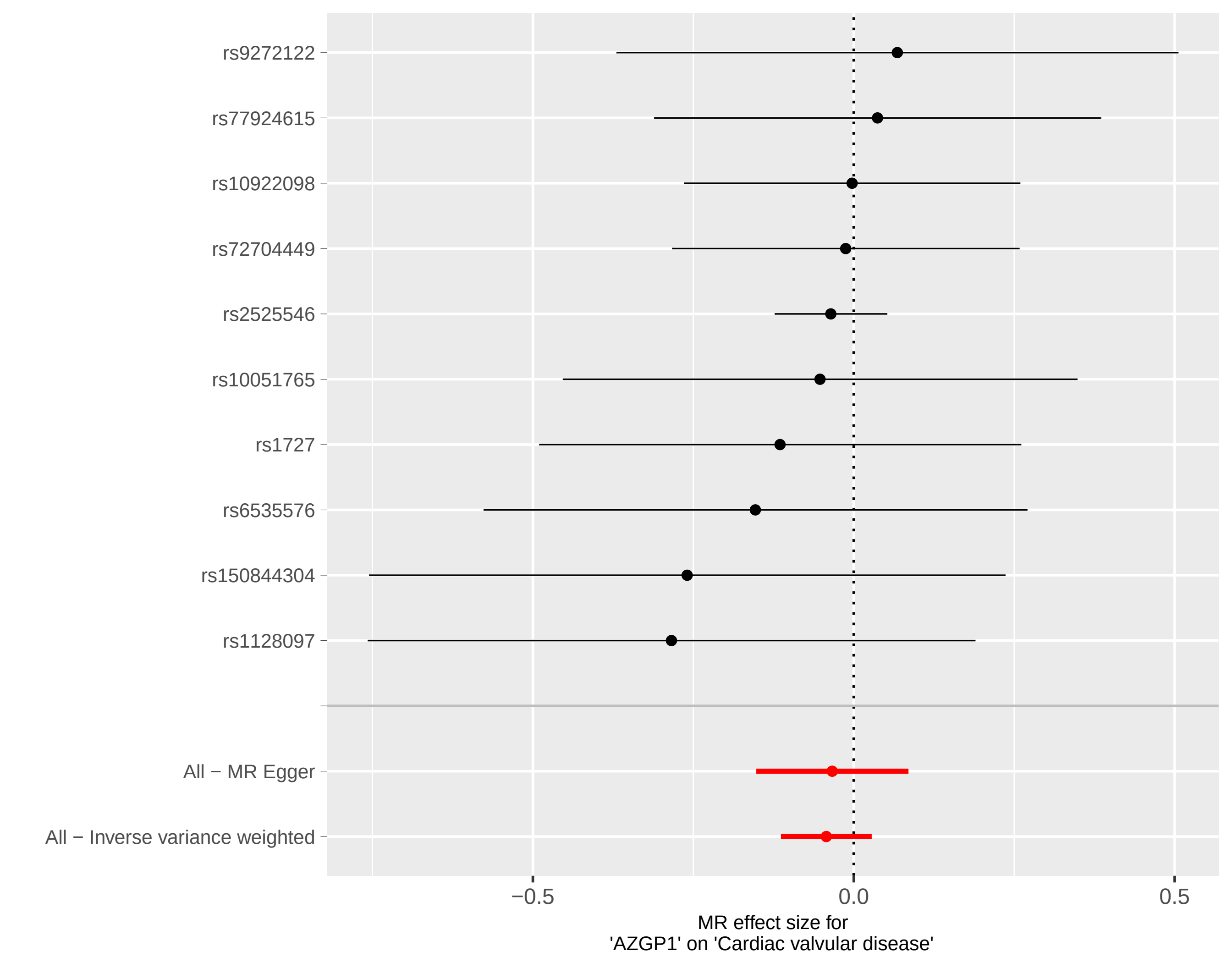
rs9272122
rs77924615
rs10922098
rs72704449
rs2525546
rs10051765
rs1727
rs6535576
rs150844304
rs1128097
All − MR Egger
All − Inverse variance weighted
−0.5
0.0
0.5
MR effect size for
'AZGP1' on 'Cardiac valvular disease'

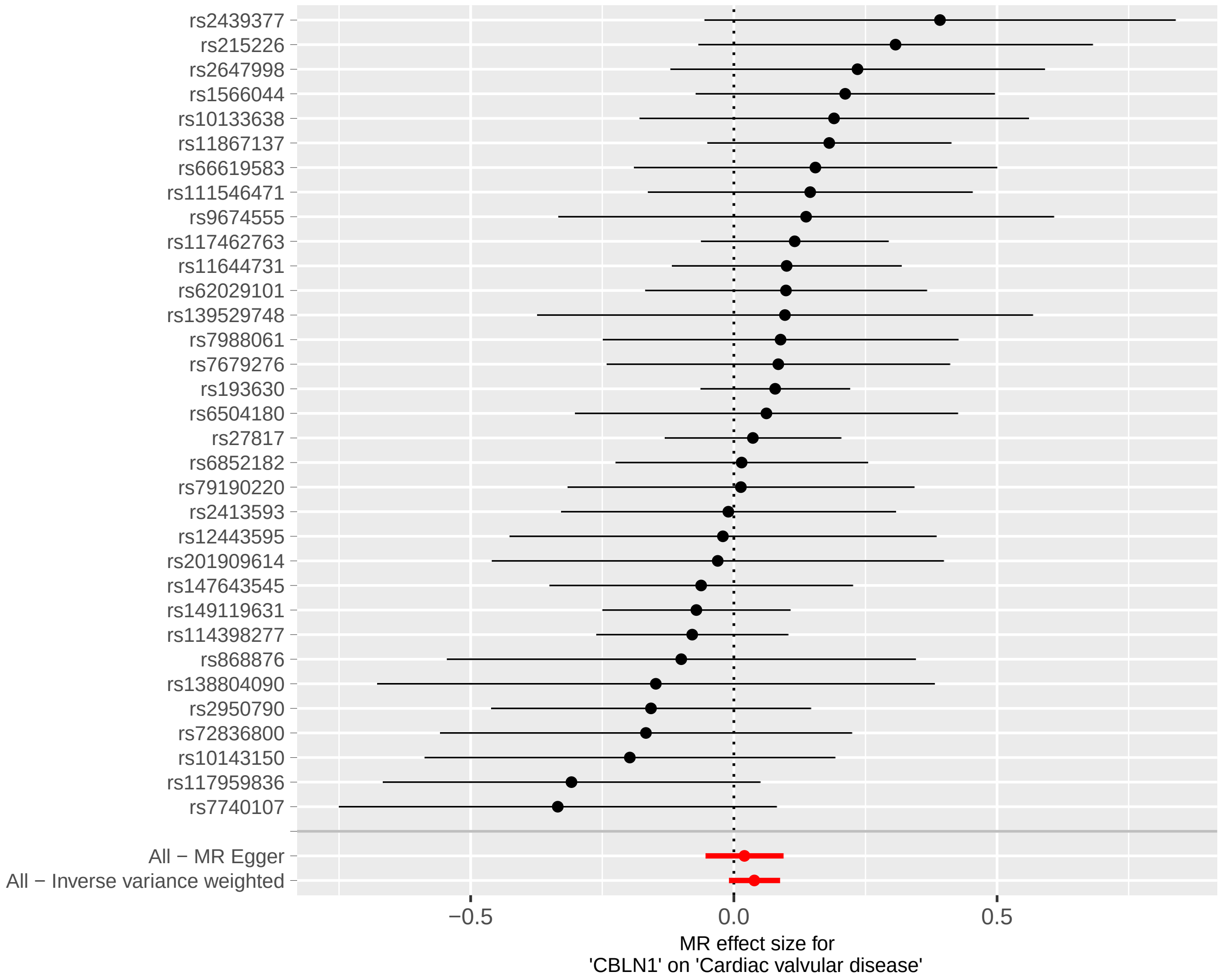
rs2439377
rs215226
rs2647998
rs1566044
rs10133638
rs11867137
rs66619583
rs111546471
rs9674555
rs117462763
rs11644731
rs62029101
rs139529748
rs7988061
rs7679276
rs193630
rs6504180
rs27817
rs6852182
rs79190220
rs2413593
rs12443595
rs201909614
rs147643545
rs149119631
rs114398277
rs868876
rs138804090
rs2950790
rs72836800
rs10143150
rs117959836
rs7740107
All − MR Egger
All − Inverse variance weighted
−0.5
0.0
0.5
MR effect size for
'CBLN1' on 'Cardiac valvular disease'

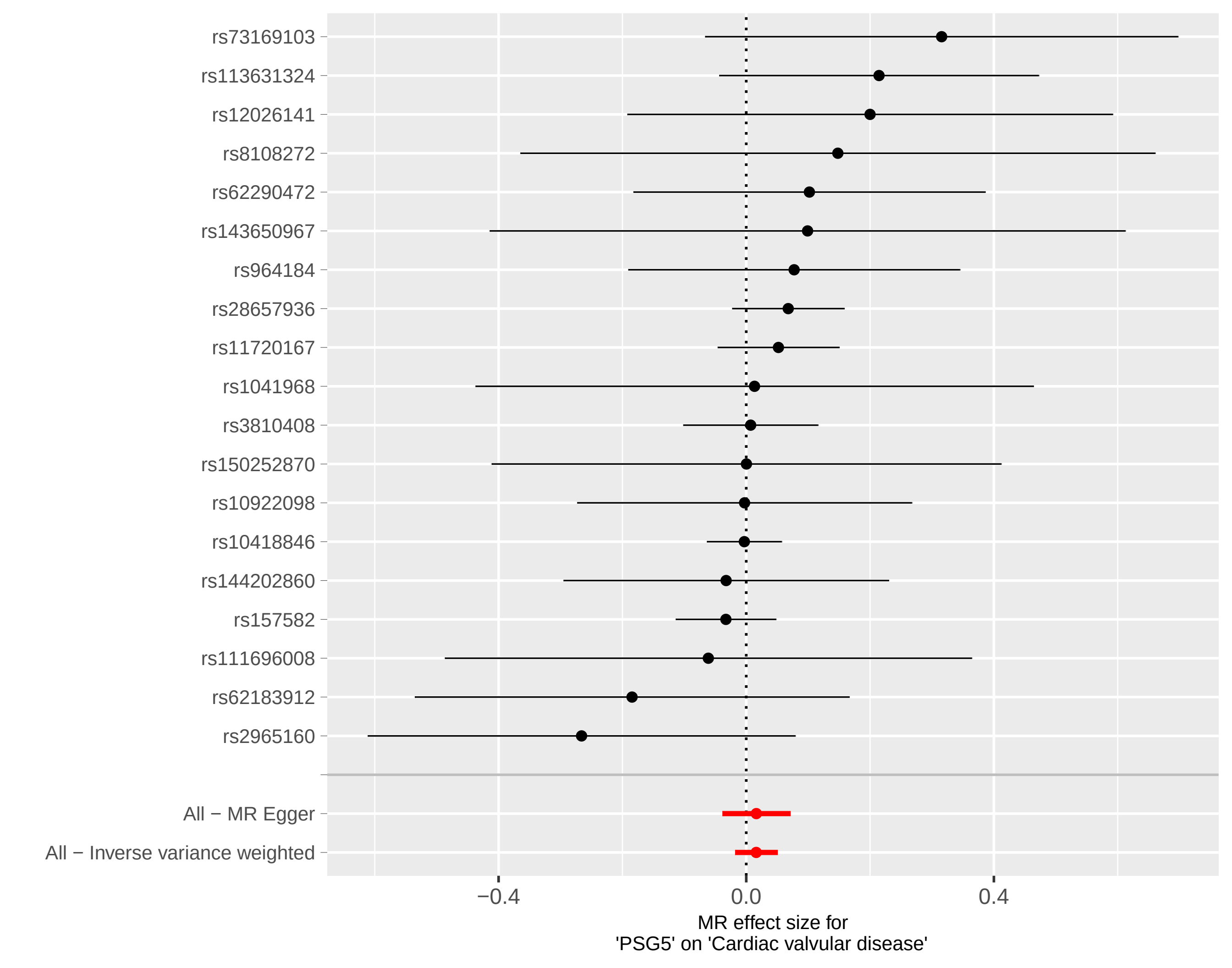
rs73169103
rs113631324
rs12026141
rs8108272
rs62290472
rs143650967
rs964184
rs28657936
rs11720167
rs1041968
rs3810408
rs150252870
rs10922098
rs10418846
rs144202860
rs157582
rs111696008
rs62183912
rs2965160
All − MR Egger
All − Inverse variance weighted
−0.4
0.0
0.4
MR effect size for
'PSG5' on 'Cardiac valvular disease'

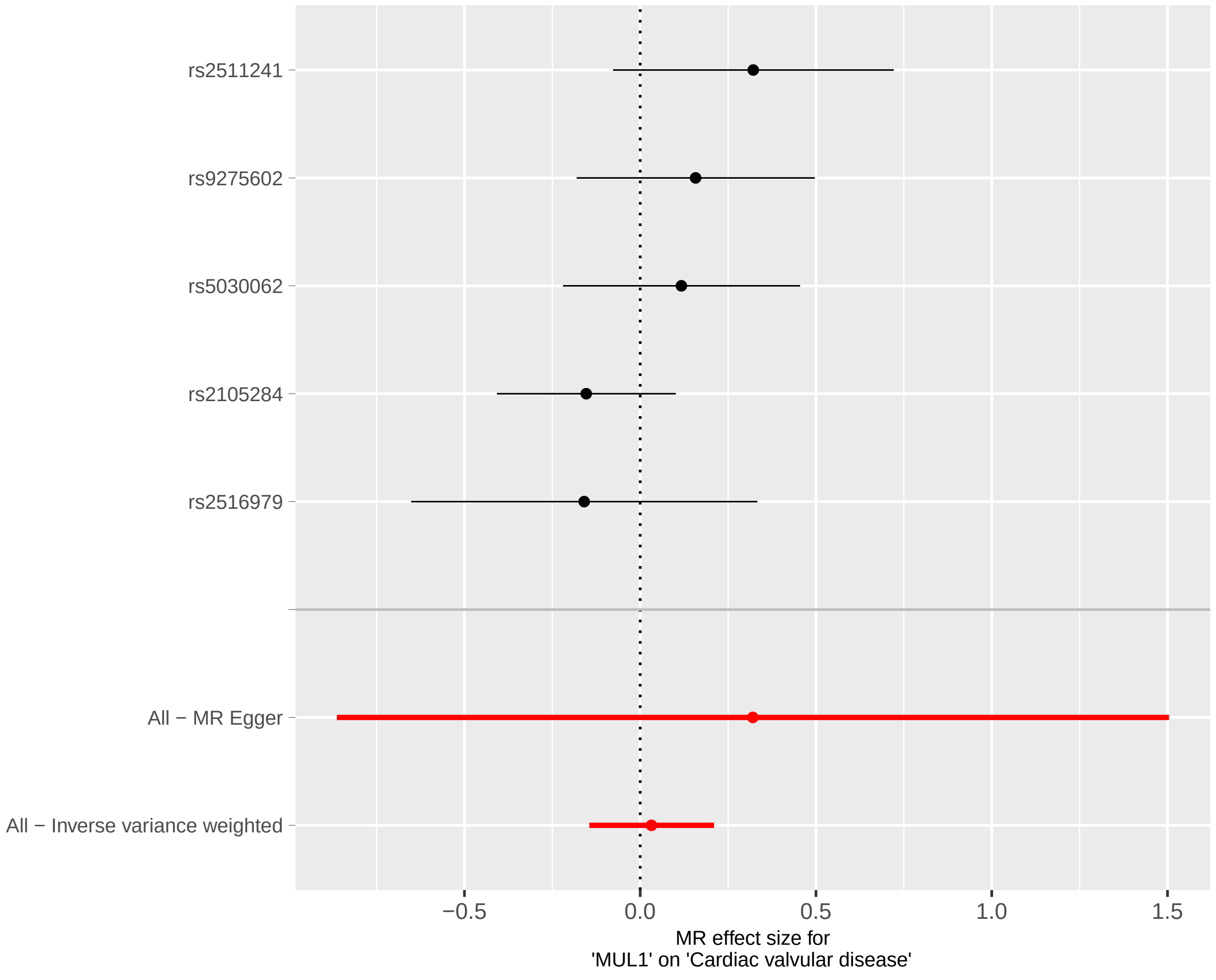

rs2511241
rs9275602
rs5030062
rs2105284
rs2516979
All − MR Egger
All − Inverse variance weighted
−0.5
0.0
0.5
1.0
1.5
MR effect size for
'MUL1' on 'Cardiac valvular disease'

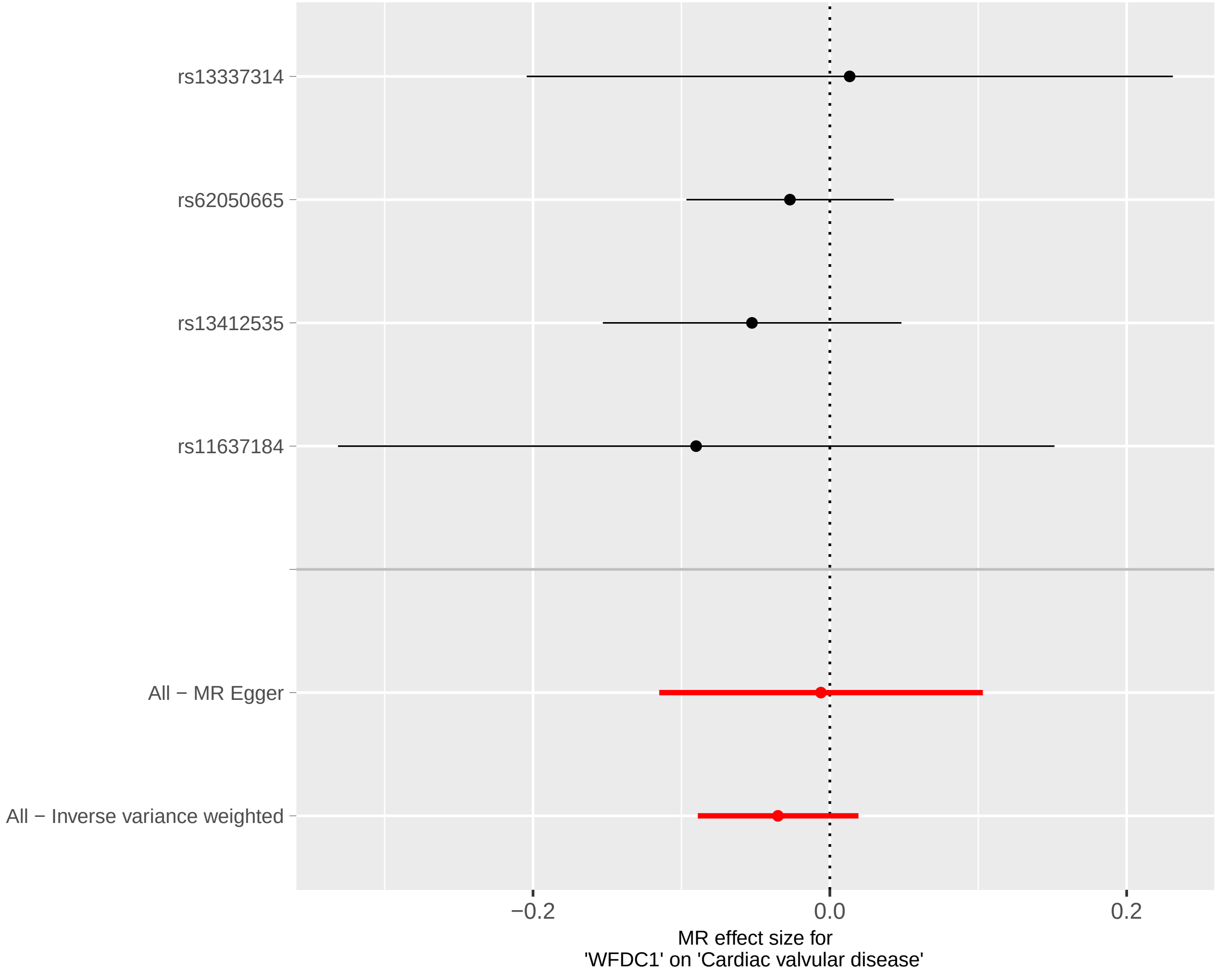
rs13337314
rs62050665
rs13412535
rs11637184
All − MR Egger
All − Inverse variance weighted
−0.2
0.0
0.2
MR effect size for
'WFDC1' on 'Cardiac valvular disease'

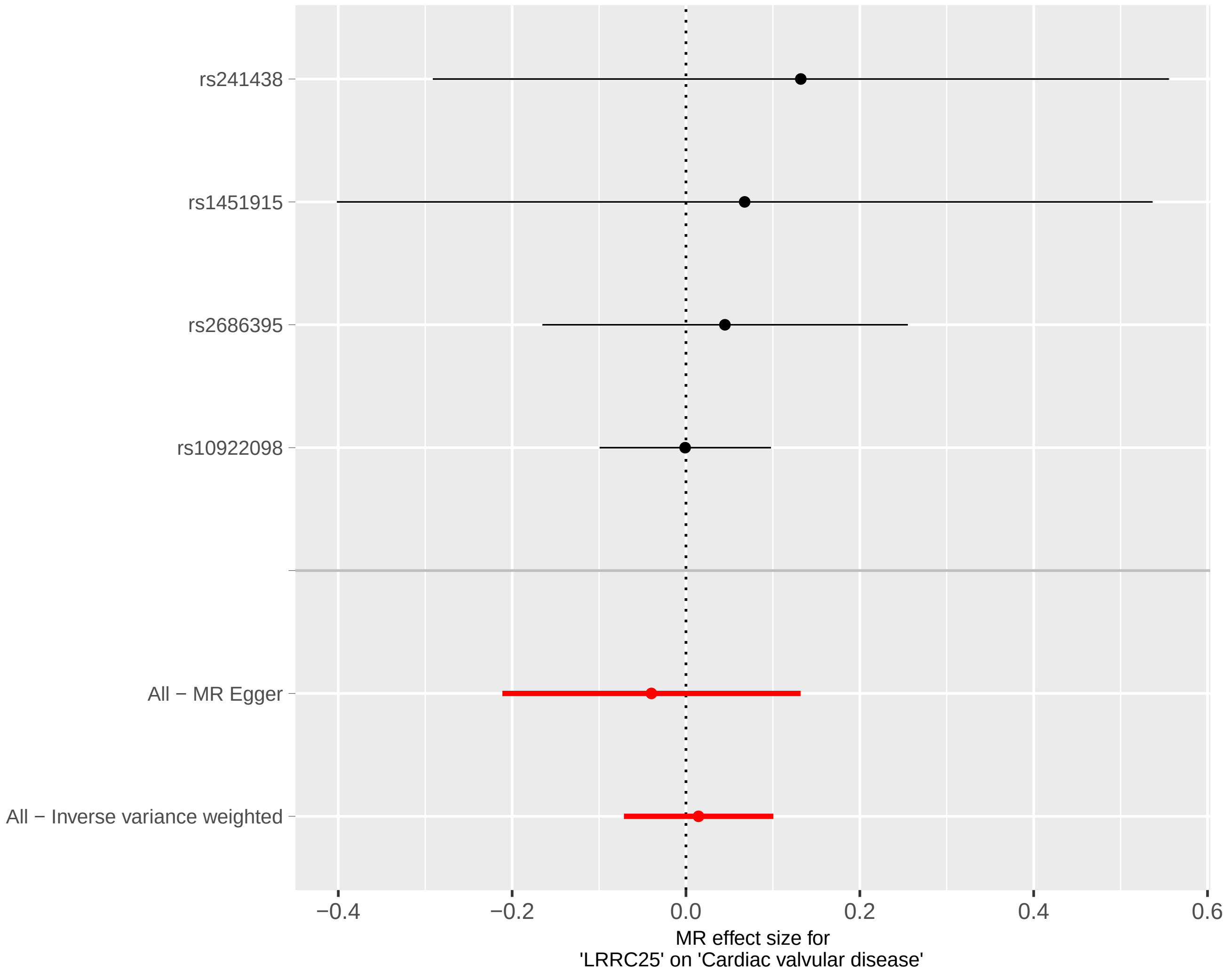
rs241438
rs1451915
rs2686395
rs10922098
All − MR Egger
All − Inverse variance weighted
−0.4
−0.2
0.0
0.2
0.4
0.6
MR effect size for
'LRRC25' on 'Cardiac valvular disease'

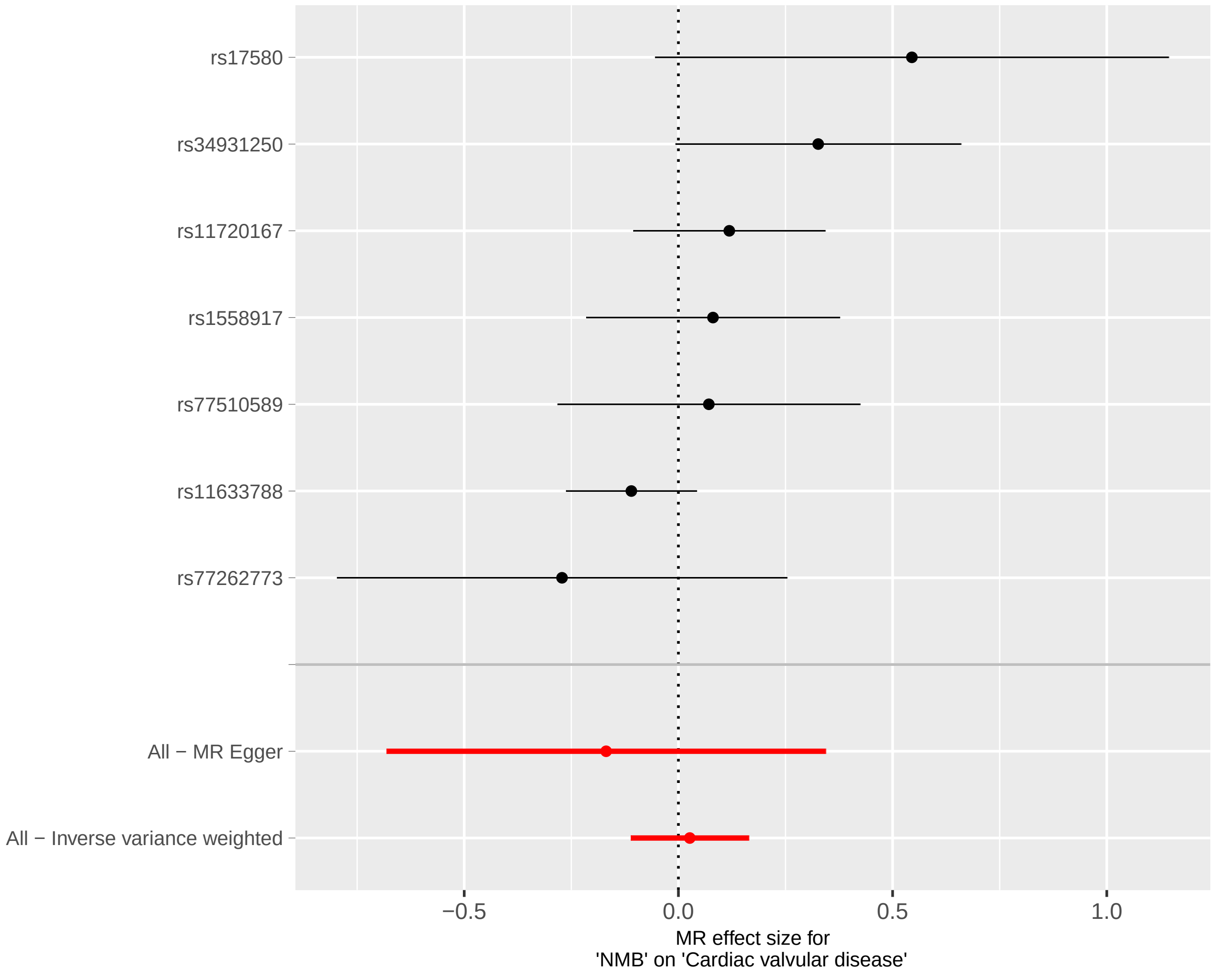
rs17580
rs34931250
rs11720167
rs1558917
rs77510589
rs11633788
rs77262773
All − MR Egger
All − Inverse variance weighted
−0.5
0.0
0.5
1.0
MR effect size for
'NMB' on 'Cardiac valvular disease'

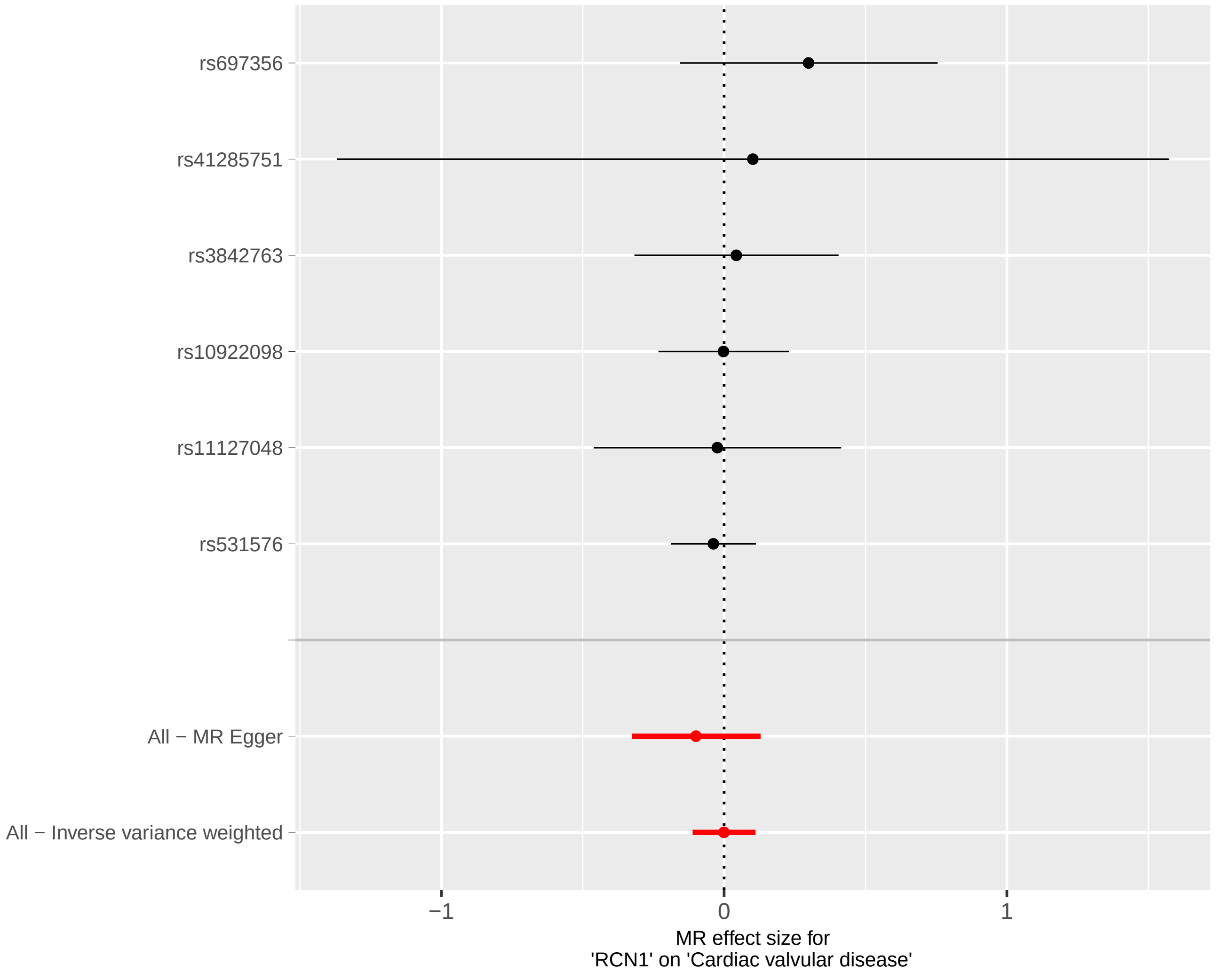
rs697356
rs41285751
rs3842763
rs10922098
rs11127048
rs531576
All − MR Egger
All − Inverse variance weighted
−1
0
1
MR effect size for
'RCN1' on 'Cardiac valvular disease'

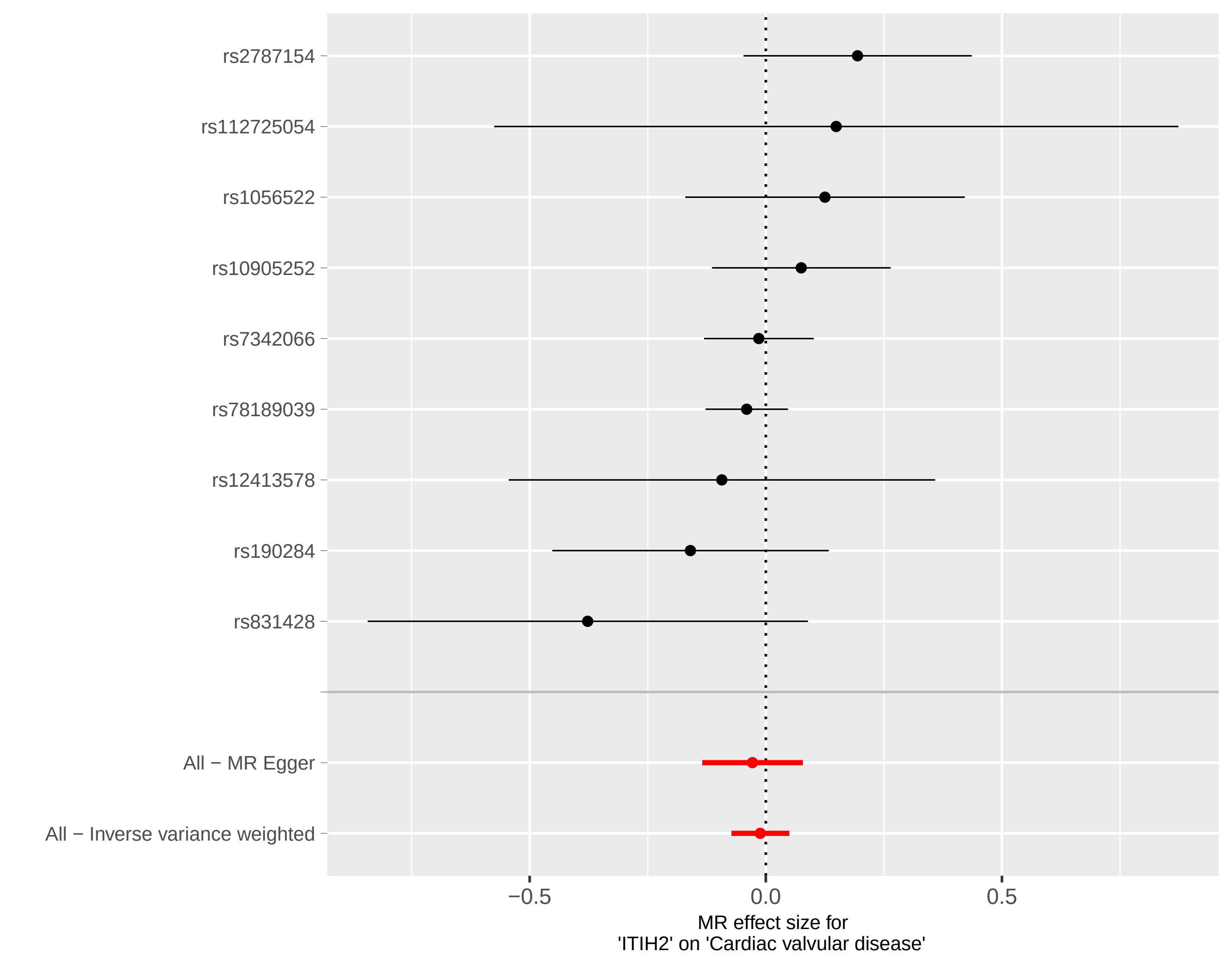
rs2787154
rs112725054
rs1056522
rs10905252
rs7342066
rs78189039
rs12413578
rs190284
rs831428
All − MR Egger
All − Inverse variance weighted
−0.5
0.0
0.5
MR effect size for
'ITIH2' on 'Cardiac valvular disease'

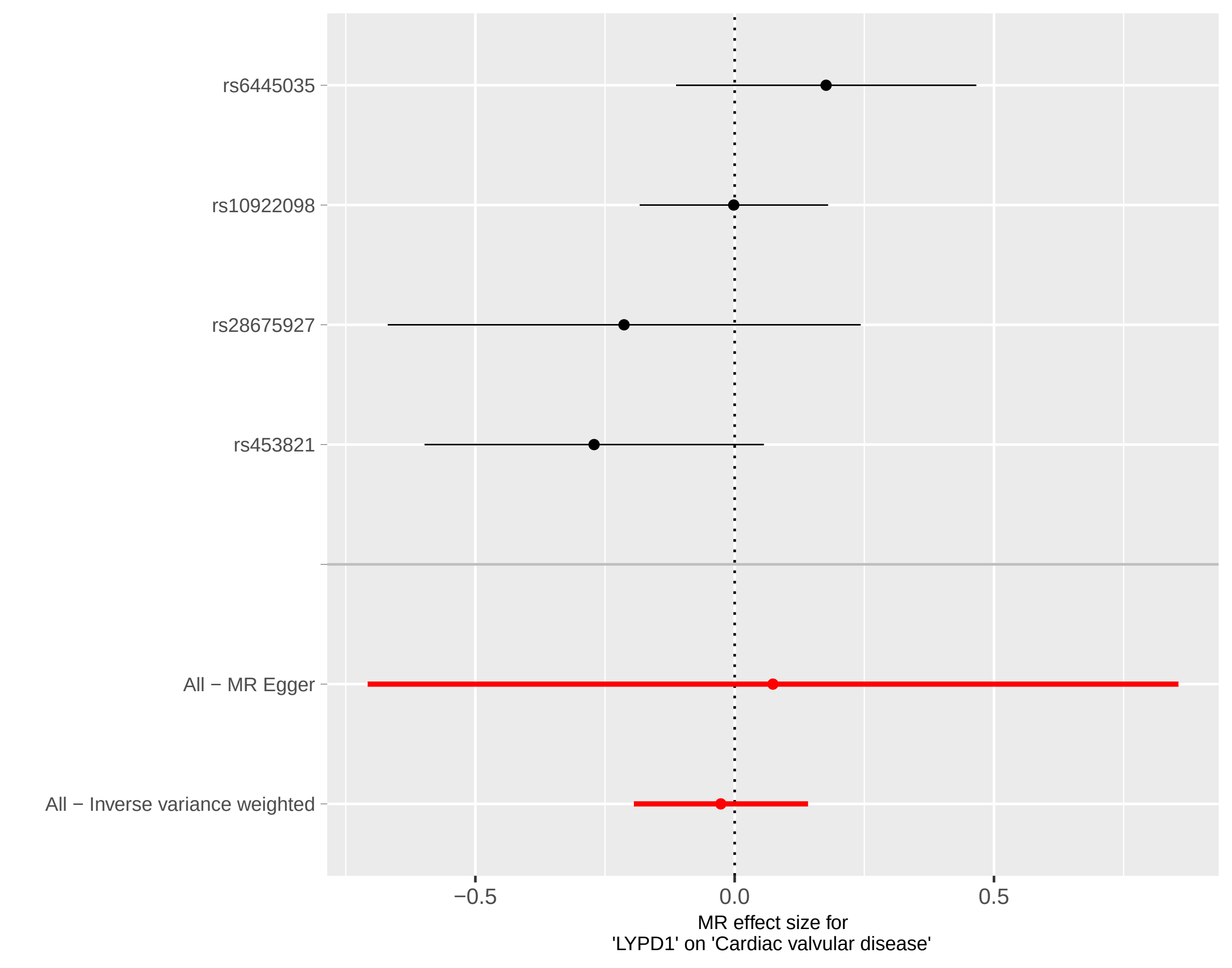
rs6445035
rs10922098
rs28675927
rs453821
All − MR Egger
All − Inverse variance weighted
−0.5
0.0
0.5
MR effect size for
'LYPD1' on 'Cardiac valvular disease'

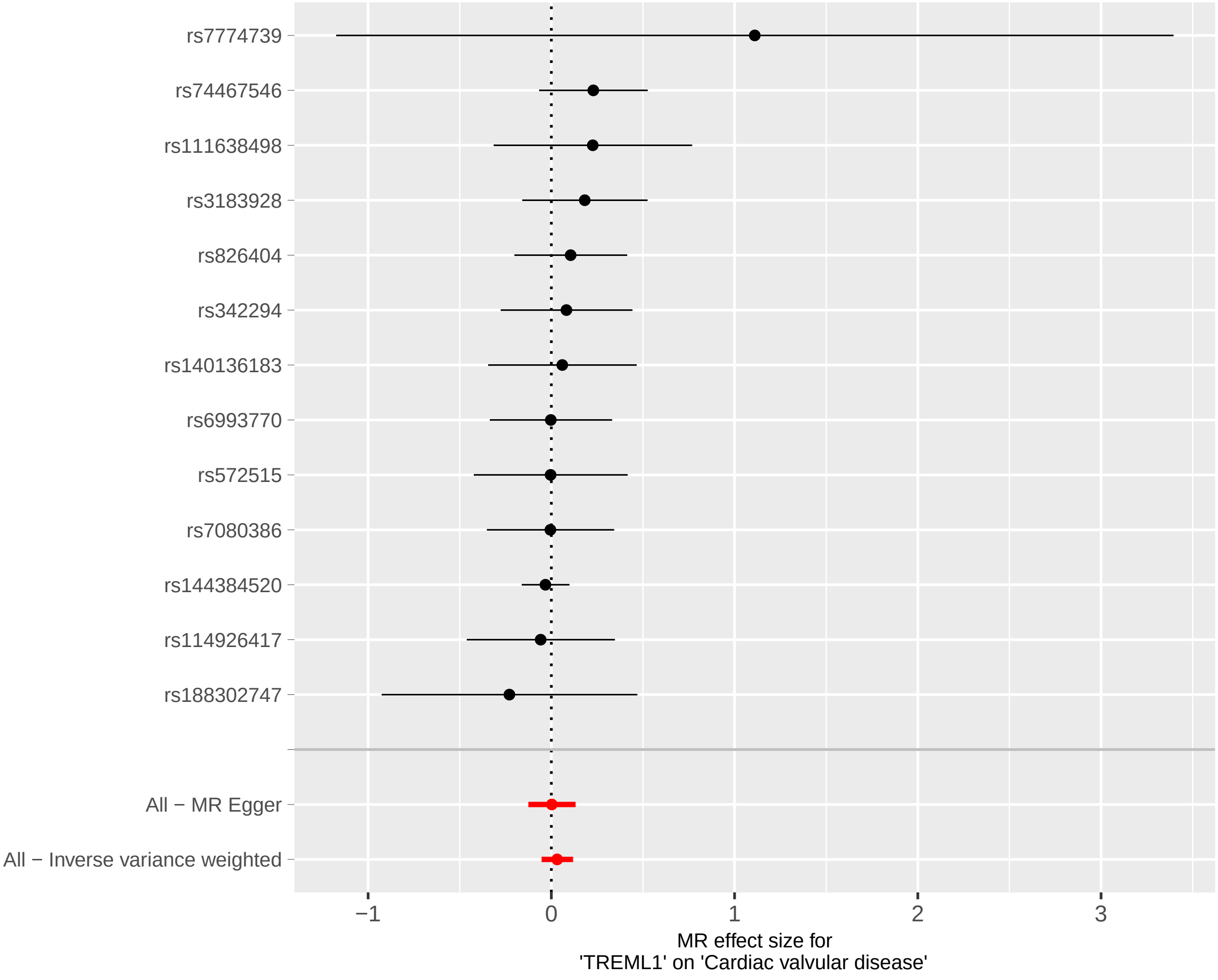
rs7774739
rs74467546
rs111638498
rs3183928
rs826404
rs342294
rs140136183
rs6993770
rs572515
rs7080386
rs144384520
rs114926417
rs188302747
All − MR Egger
All − Inverse variance weighted
−1
0
1
2
3
MR effect size for
'TREML1' on 'Cardiac valvular disease'

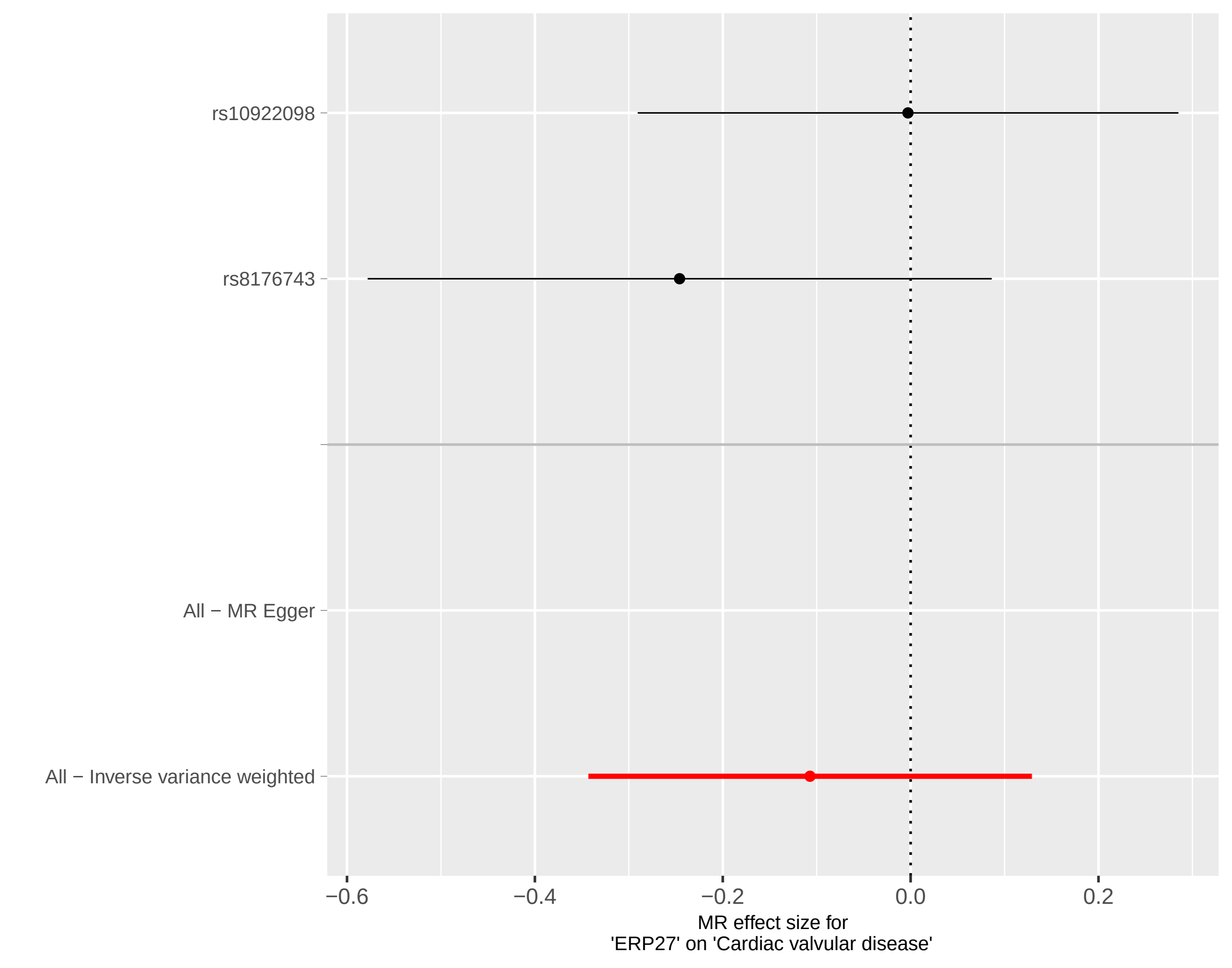
rs10922098
rs8176743
All − MR Egger
All − Inverse variance weighted
−0.6
−0.4
−0.2
0.0
0.2
MR effect size for
'ERP27' on 'Cardiac valvular disease'

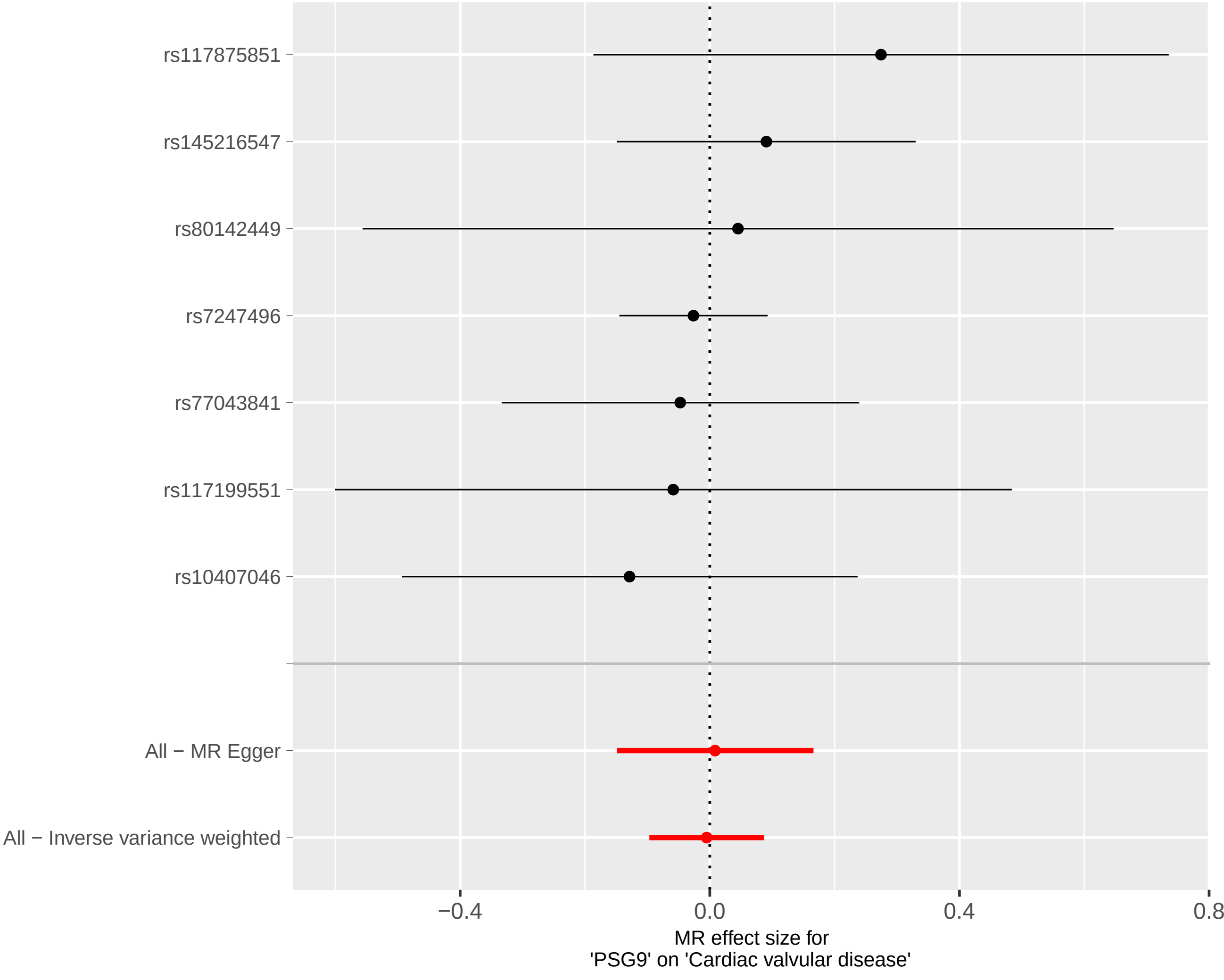

rs117875851
rs145216547
rs80142449
rs7247496
rs77043841
rs117199551
rs10407046
All − MR Egger
All − Inverse variance weighted
−0.4
0.0
0.4
0.8
MR effect size for
'PSG9' on 'Cardiac valvular disease'

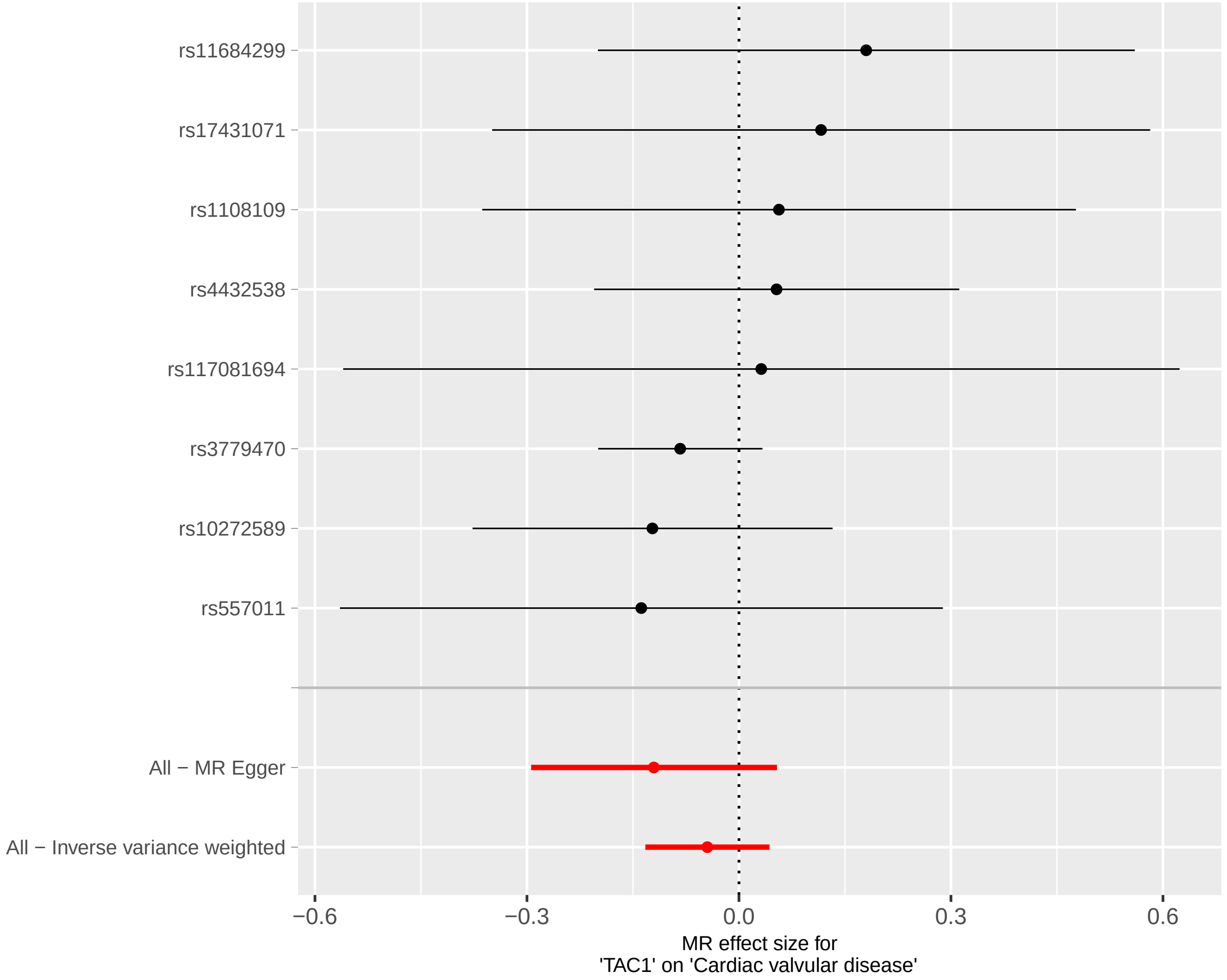

rs11684299
rs17431071
rs1108109
rs4432538
rs117081694
rs3779470
rs10272589
rs557011
All − MR Egger
All − Inverse variance weighted
−0.6
−0.3
0.0
0.3
0.6
MR effect size for
'TAC1' on 'Cardiac valvular disease'

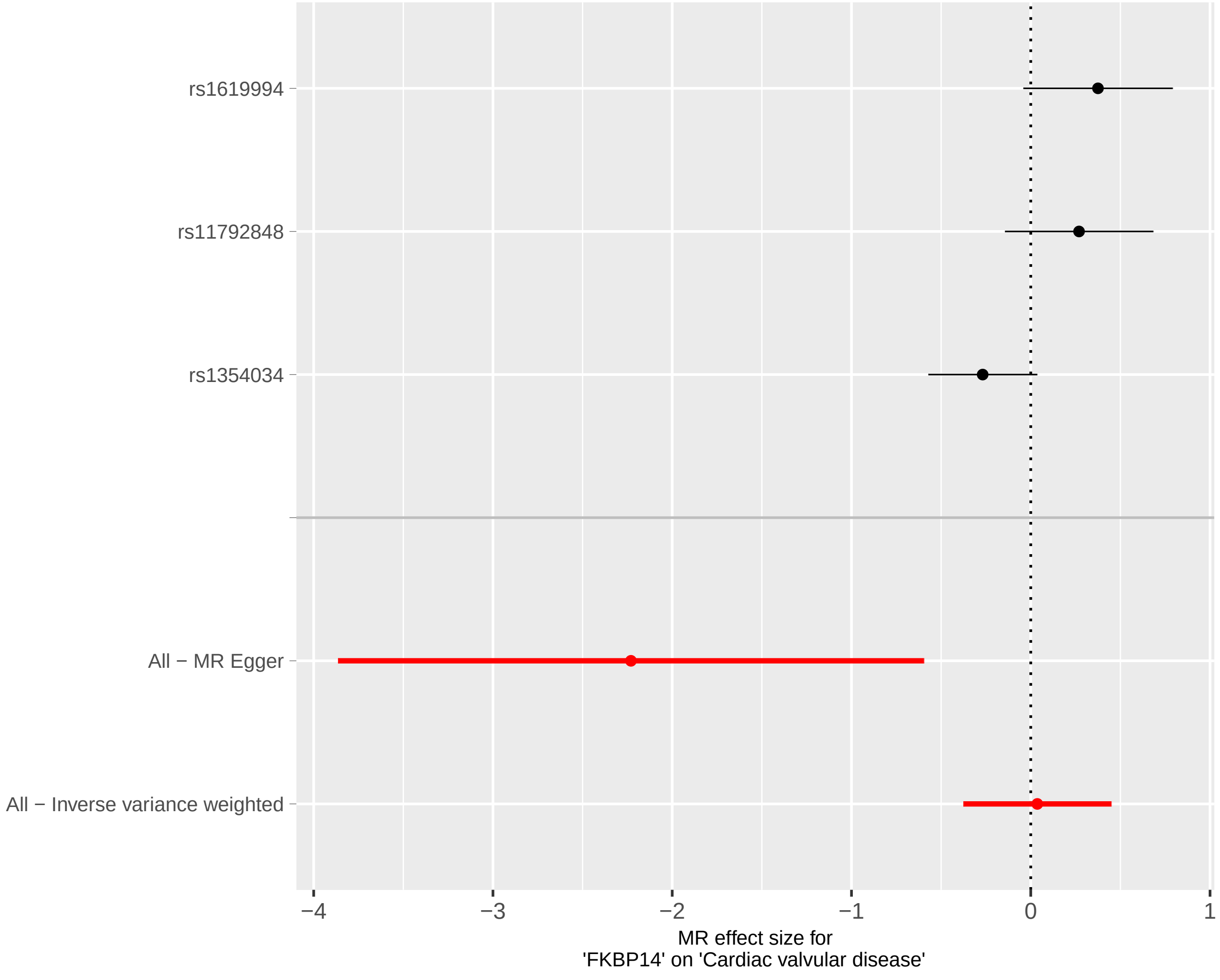
rs1619994
rs11792848
rs1354034
All − MR Egger
All − Inverse variance weighted
−4
−3
−2
−1
0
1
MR effect size for
'FKBP14' on 'Cardiac valvular disease'

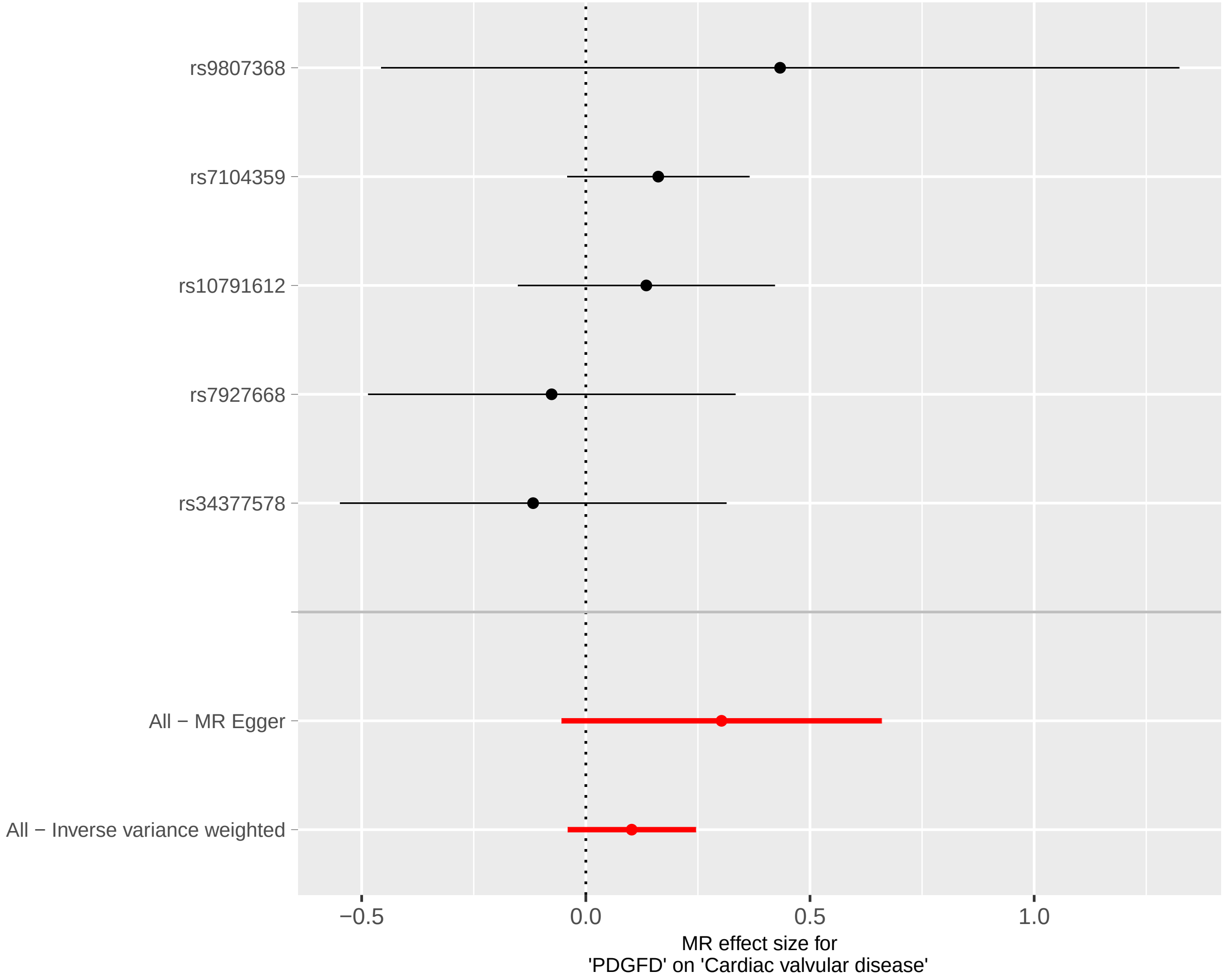
rs9807368
rs7104359
rs10791612
rs7927668
rs34377578
All − MR Egger
All − Inverse variance weighted
−0.5
0.0
0.5
1.0
MR effect size for
'PDGFD' on 'Cardiac valvular disease'

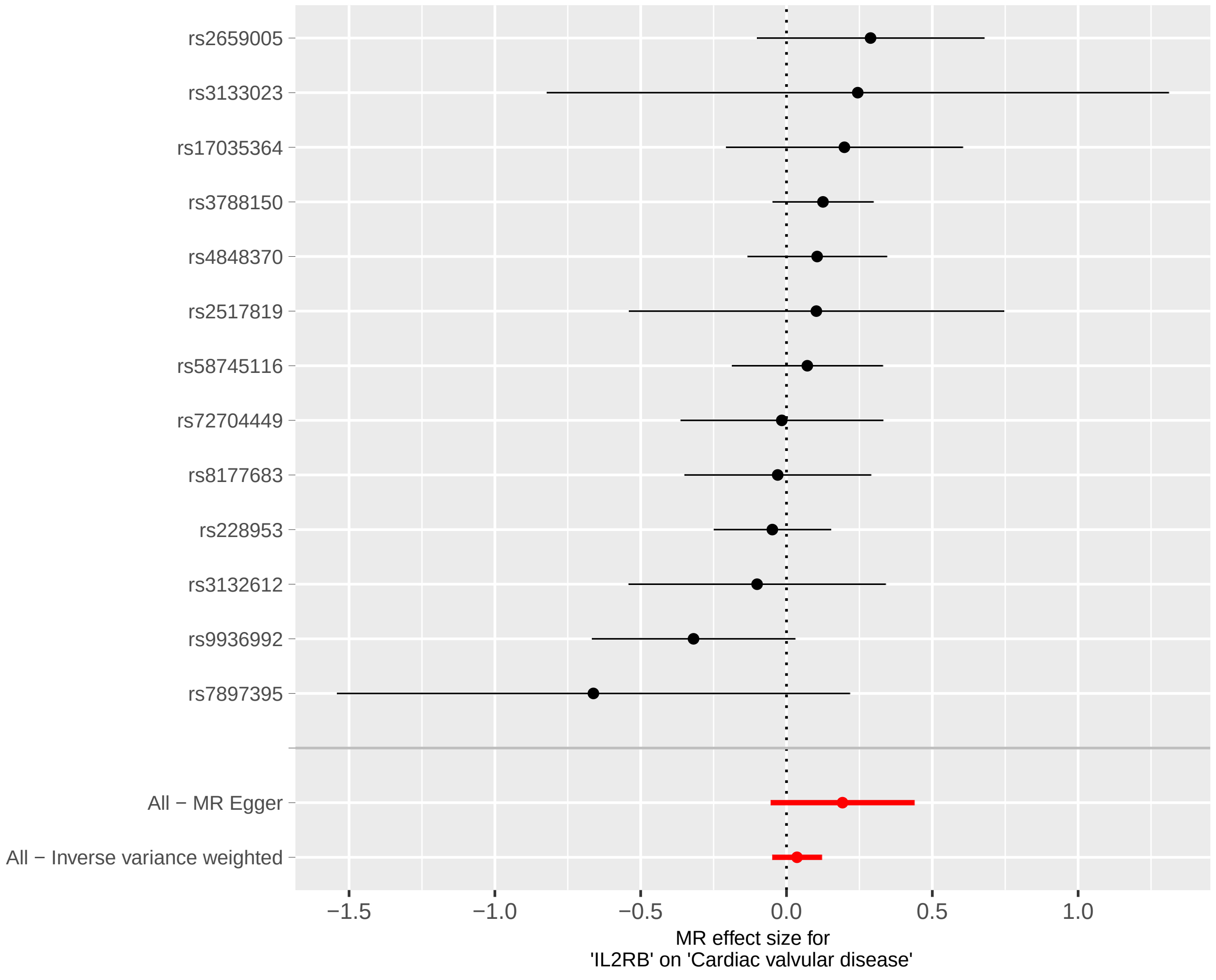

rs2659005
rs3133023
rs17035364
rs3788150
rs4848370
rs2517819
rs58745116
rs72704449
rs8177683
rs228953
rs3132612
rs9936992
rs7897395
All − MR Egger
All − Inverse variance weighted
−1.5
−1.0
−0.5
0.0
0.5
1.0
MR effect size for
'IL2RB' on 'Cardiac valvular disease'

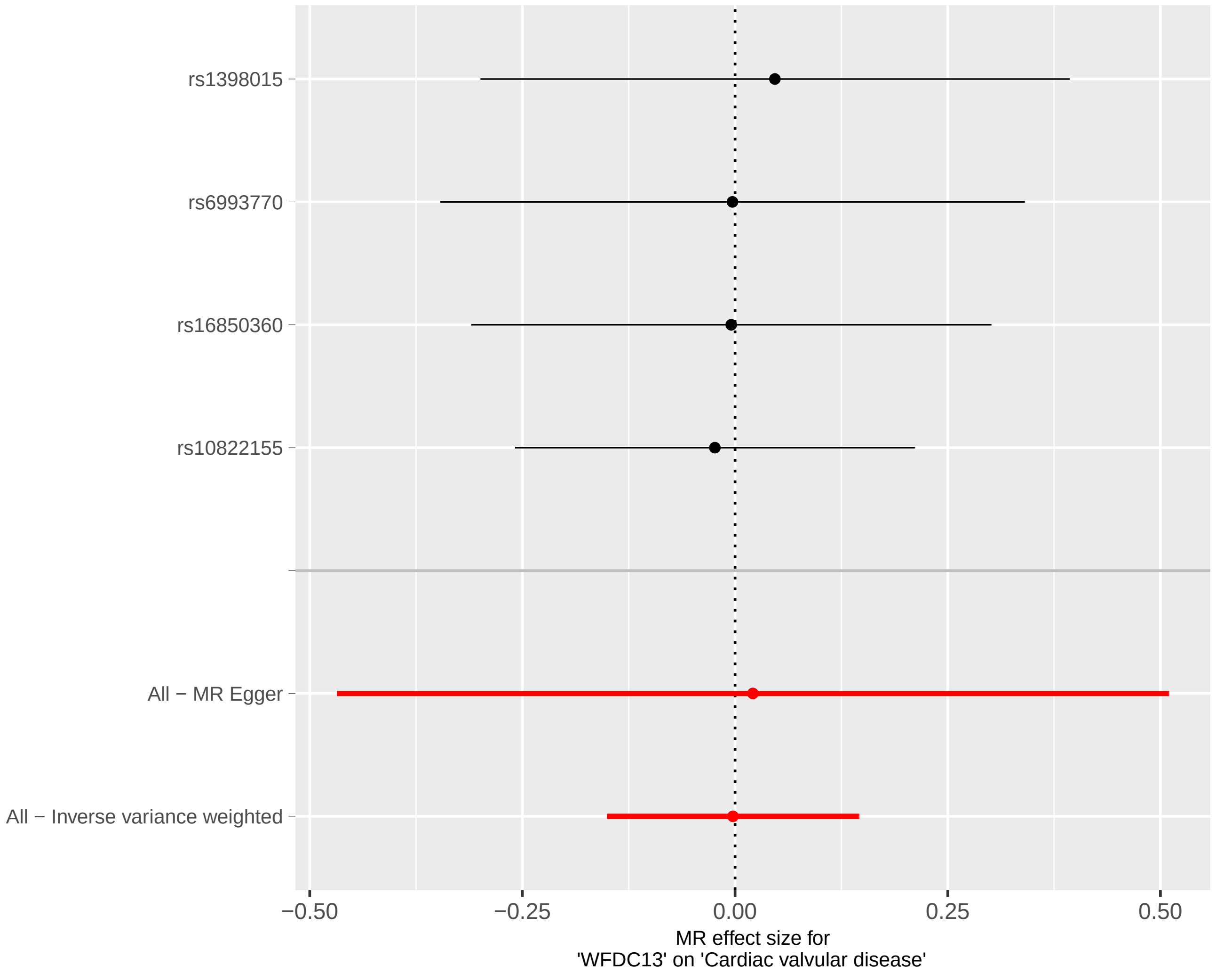
rs1398015
rs6993770
rs16850360
rs10822155
All − MR Egger
All − Inverse variance weighted
−0.50
−0.25
0.00
0.25
0.50
MR effect size for
'WFDC13' on 'Cardiac valvular disease'

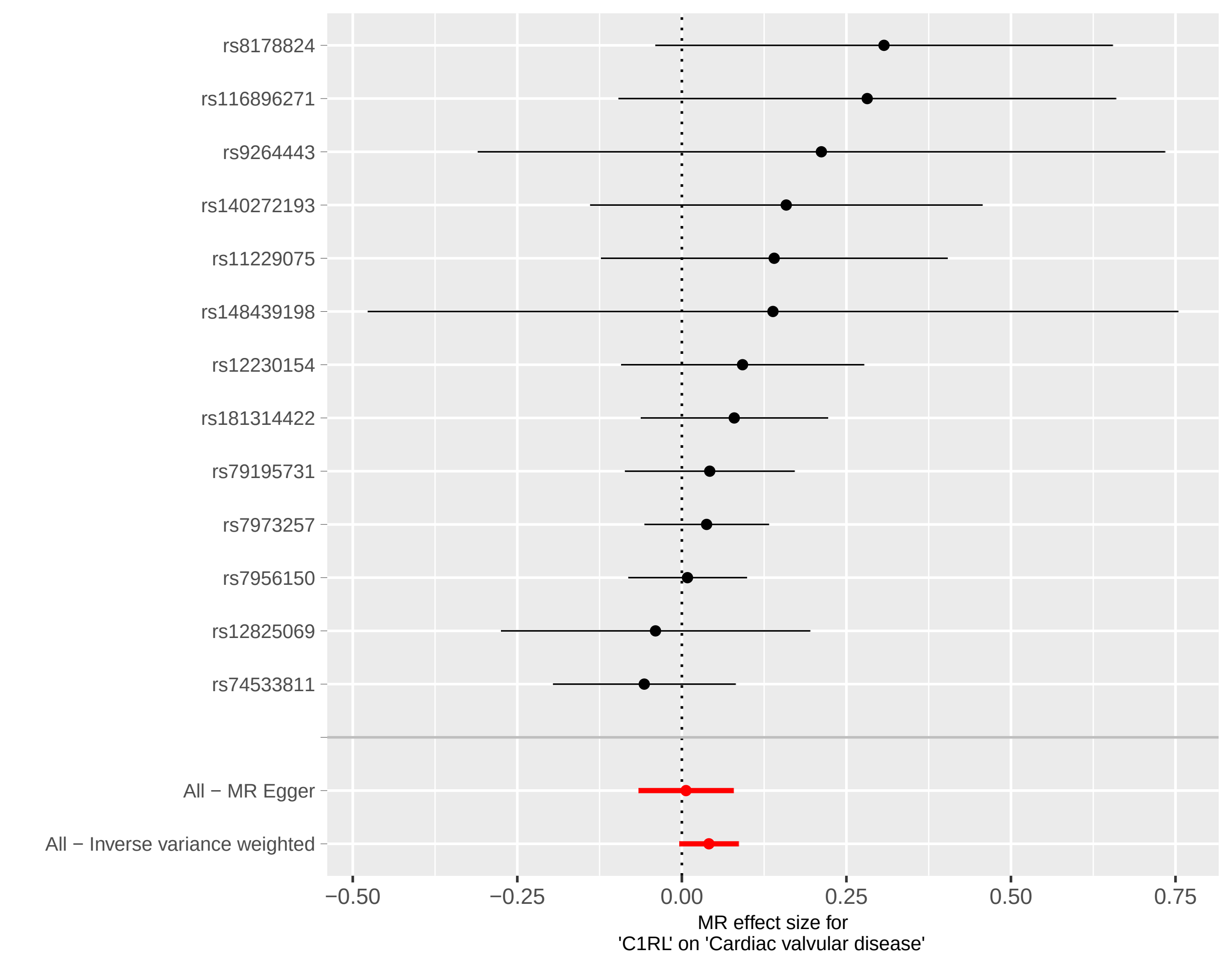
rs8178824
rs116896271
rs9264443
rs140272193
rs11229075
rs148439198
rs12230154
rs181314422
rs79195731
rs7973257
rs7956150
rs12825069
rs74533811
All − MR Egger
All − Inverse variance weighted
−0.50
−0.25
0.00
0.25
0.50
0.75
MR effect size for
'C1RL' on 'Cardiac valvular disease'

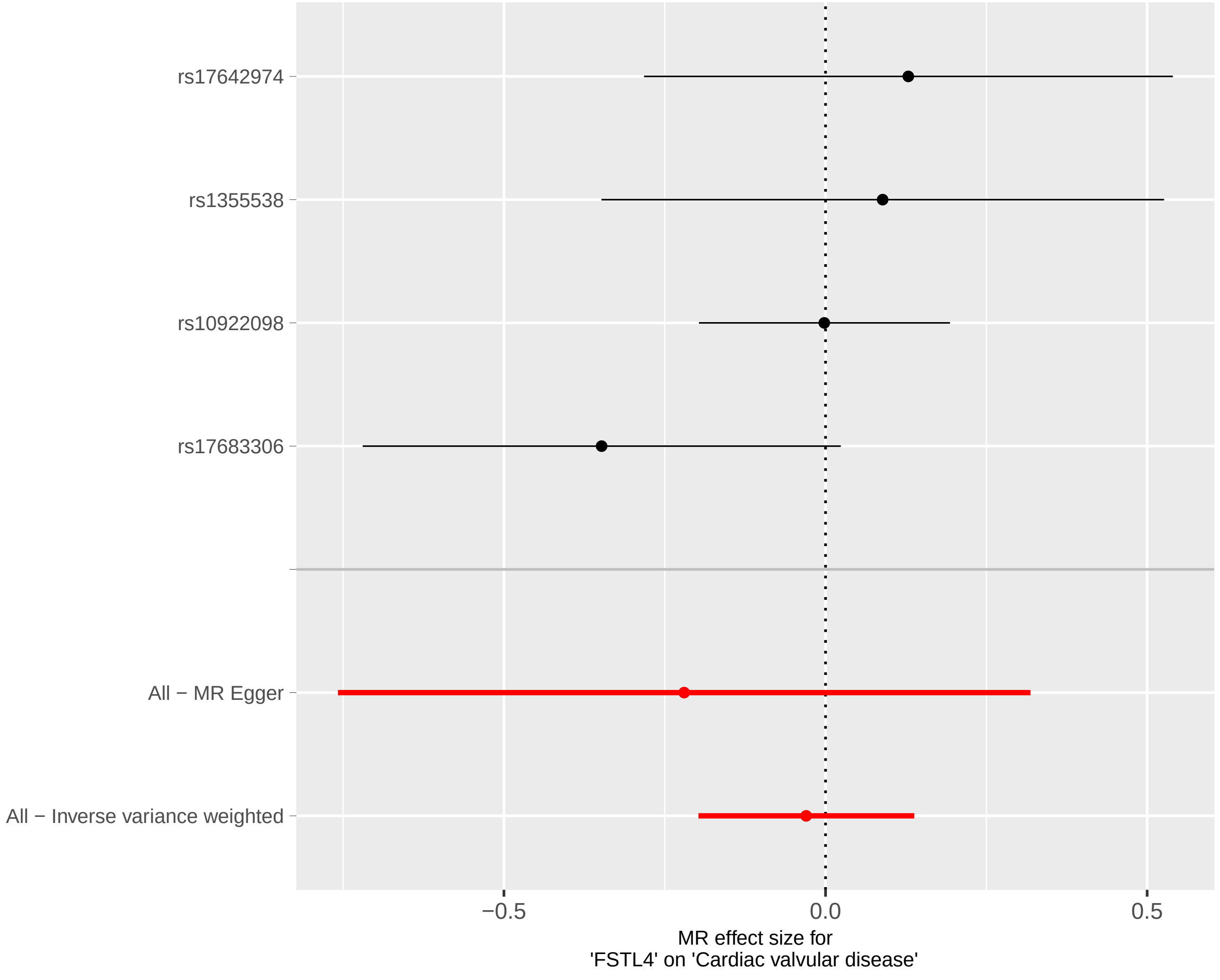
rs17642974
rs1355538
rs10922098
rs17683306
All − MR Egger
All − Inverse variance weighted
−0.5
0.0
0.5
MR effect size for
'FSTL4' on 'Cardiac valvular disease'

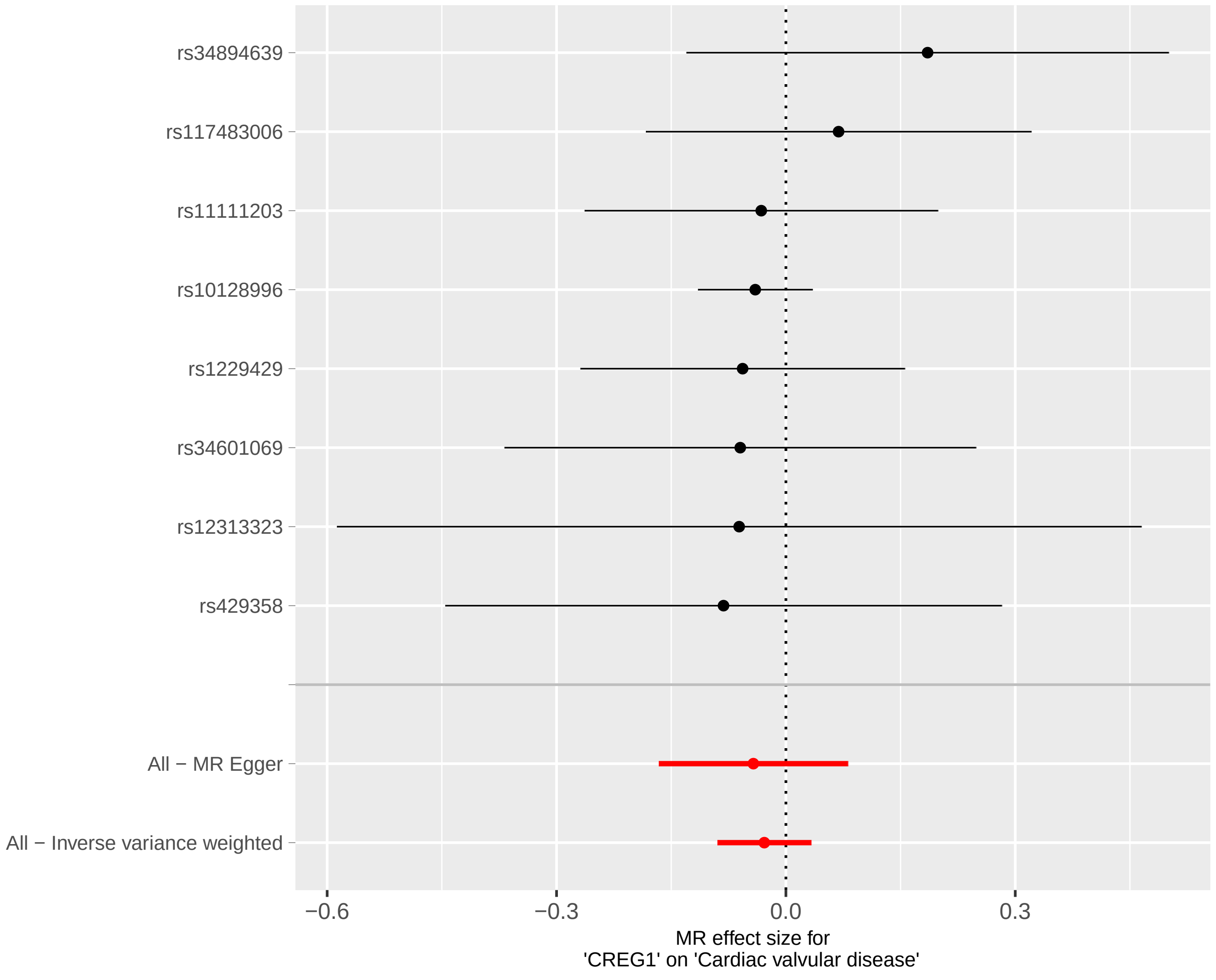

rs34894639
rs117483006
rs11111203
rs10128996
rs1229429
rs34601069
rs12313323
rs429358
All − MR Egger
All − Inverse variance weighted
−0.6
−0.3
0.0
0.3
MR effect size for
'CREG1' on 'Cardiac valvular disease'

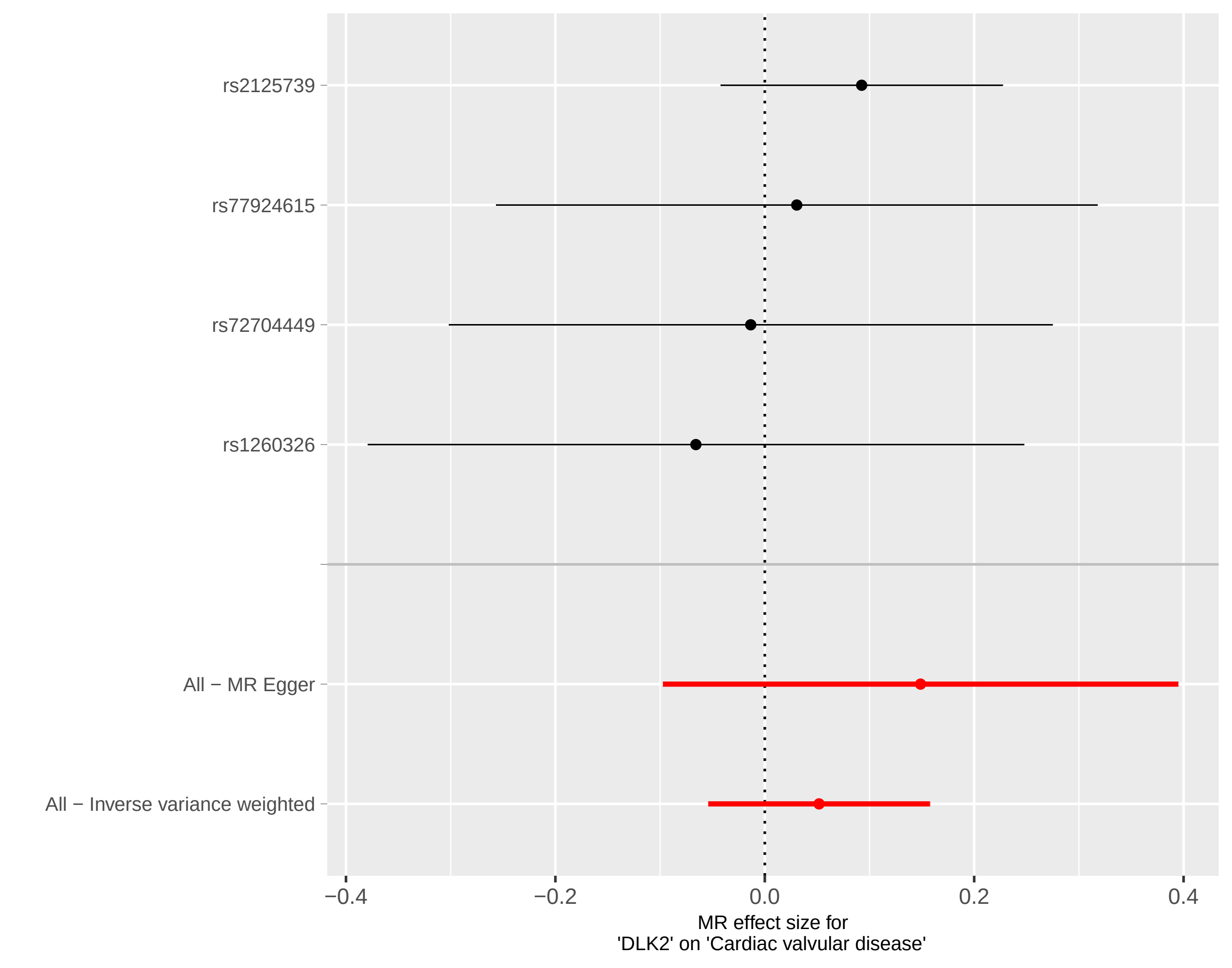
rs2125739
rs77924615
rs72704449
rs1260326
All − MR Egger
All − Inverse variance weighted
−0.4
−0.2
0.0
0.2
0.4
MR effect size for
'DLK2' on 'Cardiac valvular disease'

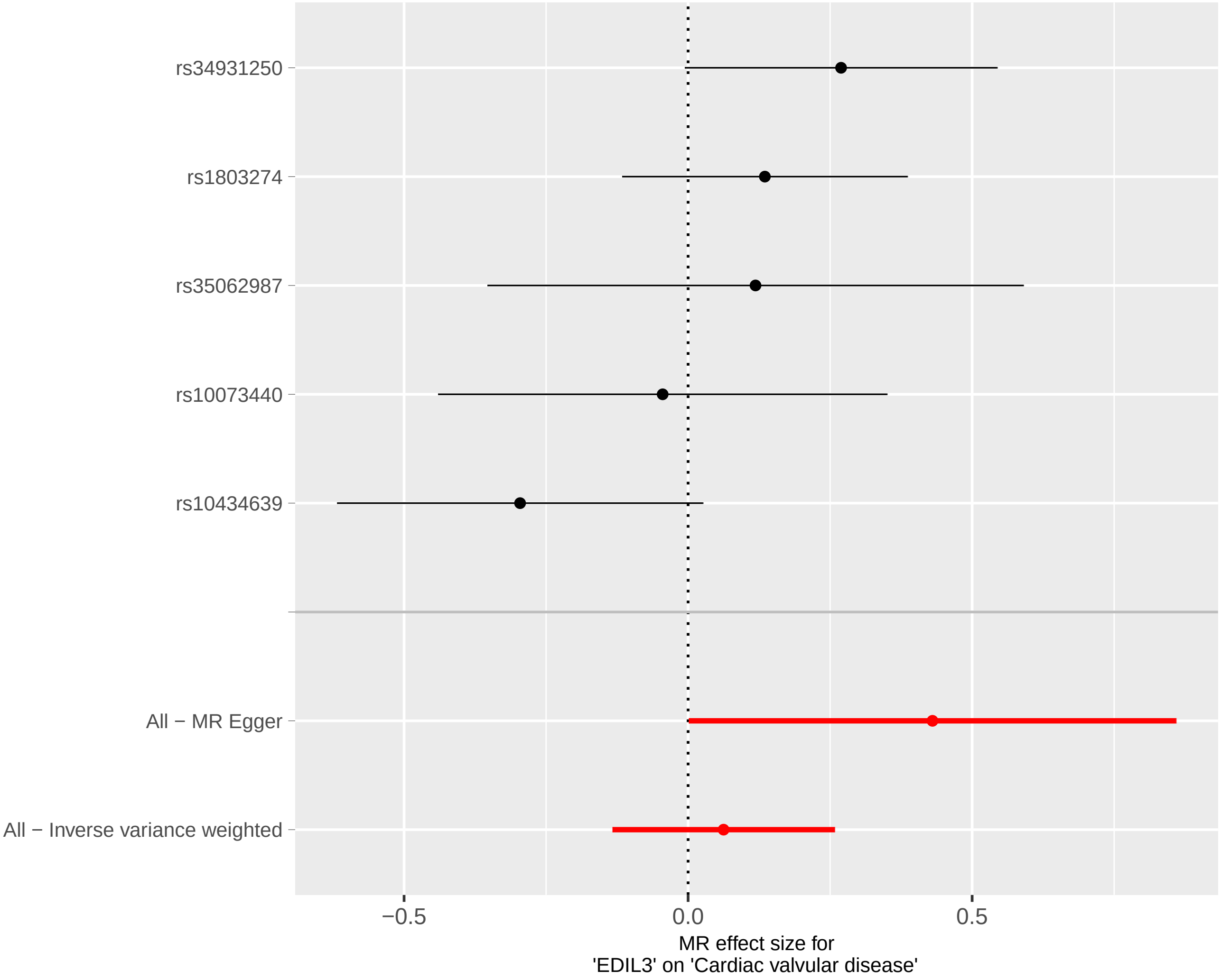

rs34931250
rs1803274
rs35062987
rs10073440
rs10434639
All − MR Egger
All − Inverse variance weighted
−0.5
0.0
0.5
MR effect size for
'EDIL3' on 'Cardiac valvular disease'

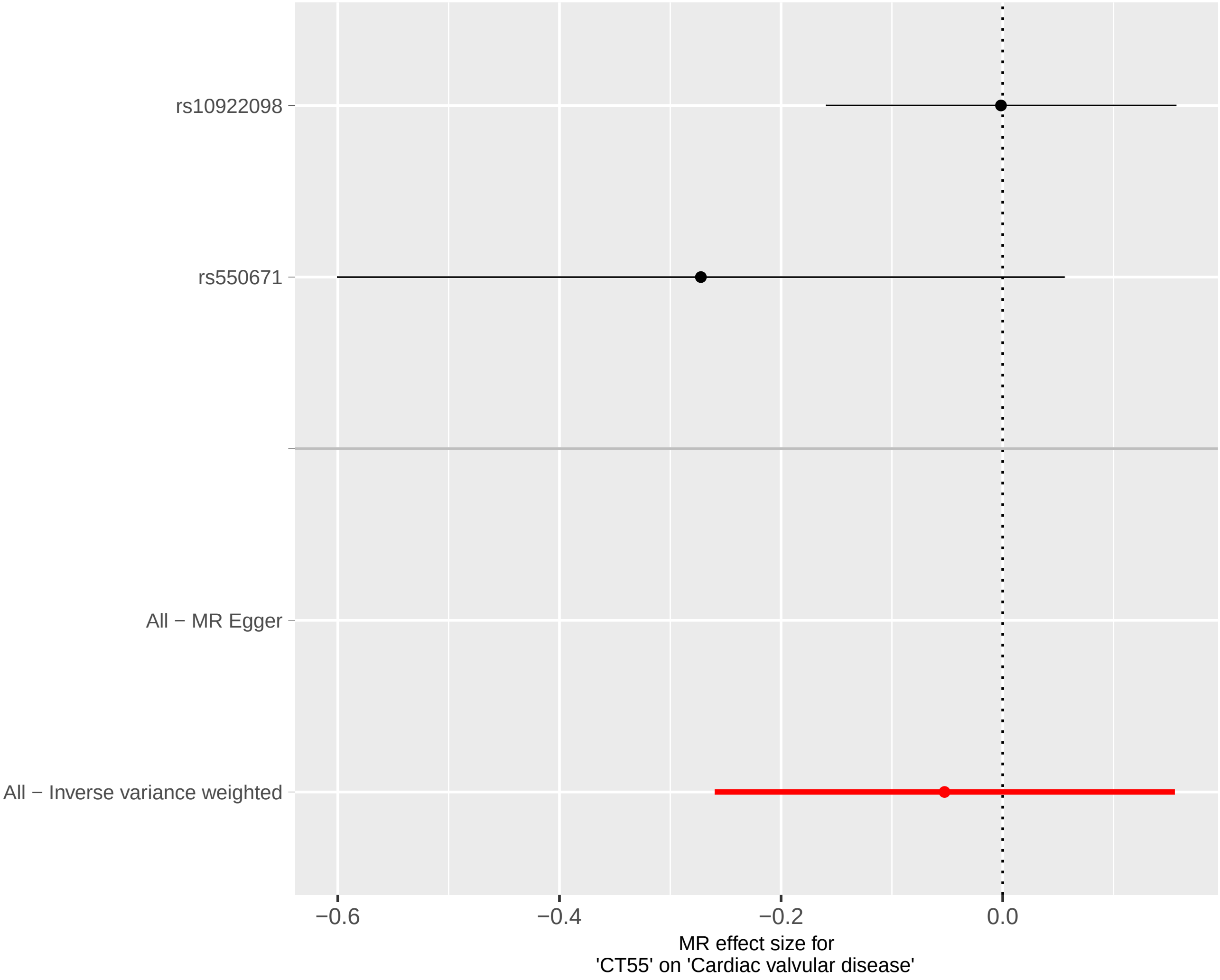

rs10922098
rs550671
All − MR Egger
All − Inverse variance weighted
−0.6
−0.4
−0.2
0.0
MR effect size for
'CT55' on 'Cardiac valvular disease'

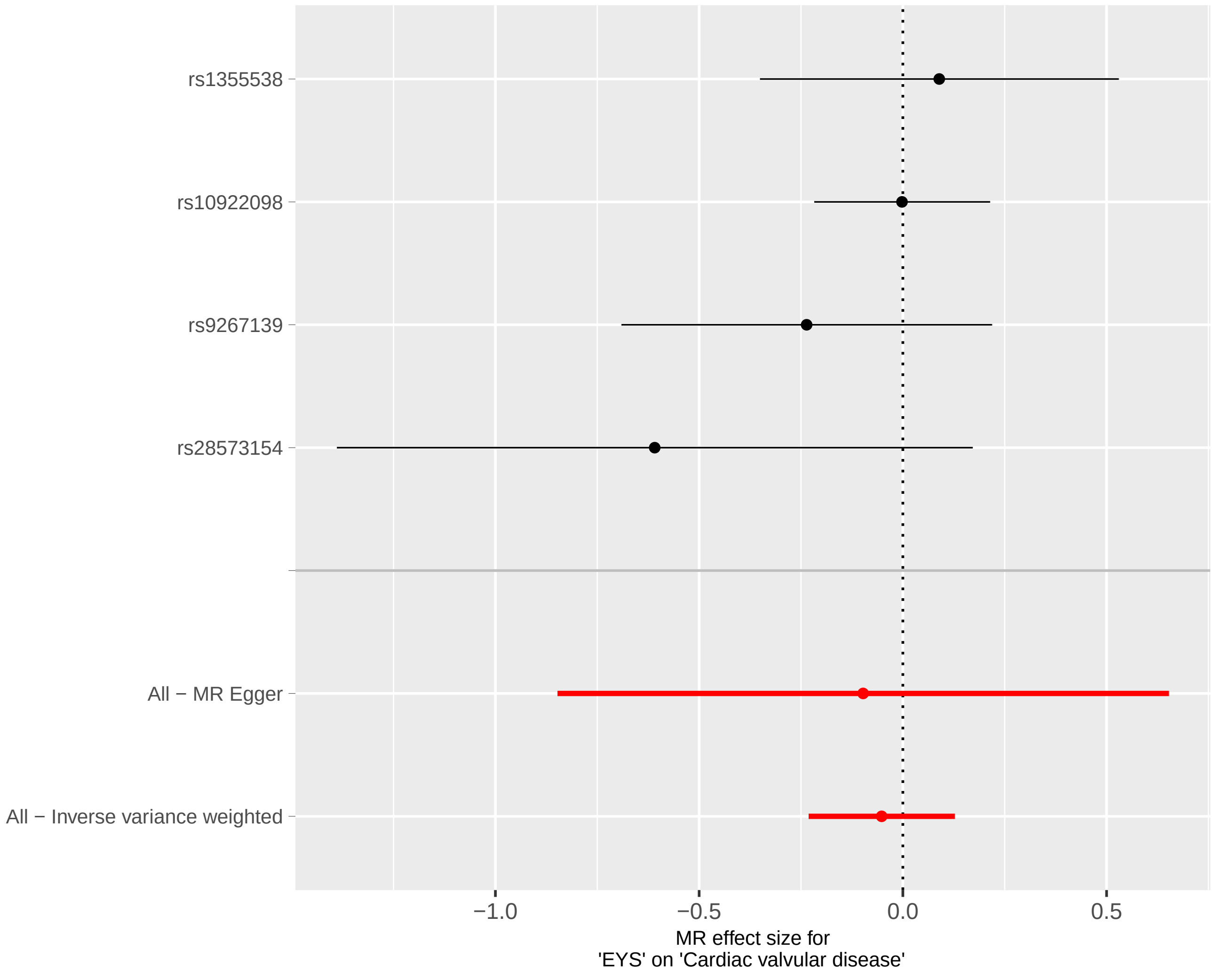
rs1355538
rs10922098
rs9267139
rs28573154
All − MR Egger
All − Inverse variance weighted
−1.0
−0.5
0.0
0.5
MR effect size for
'EYS' on 'Cardiac valvular disease'

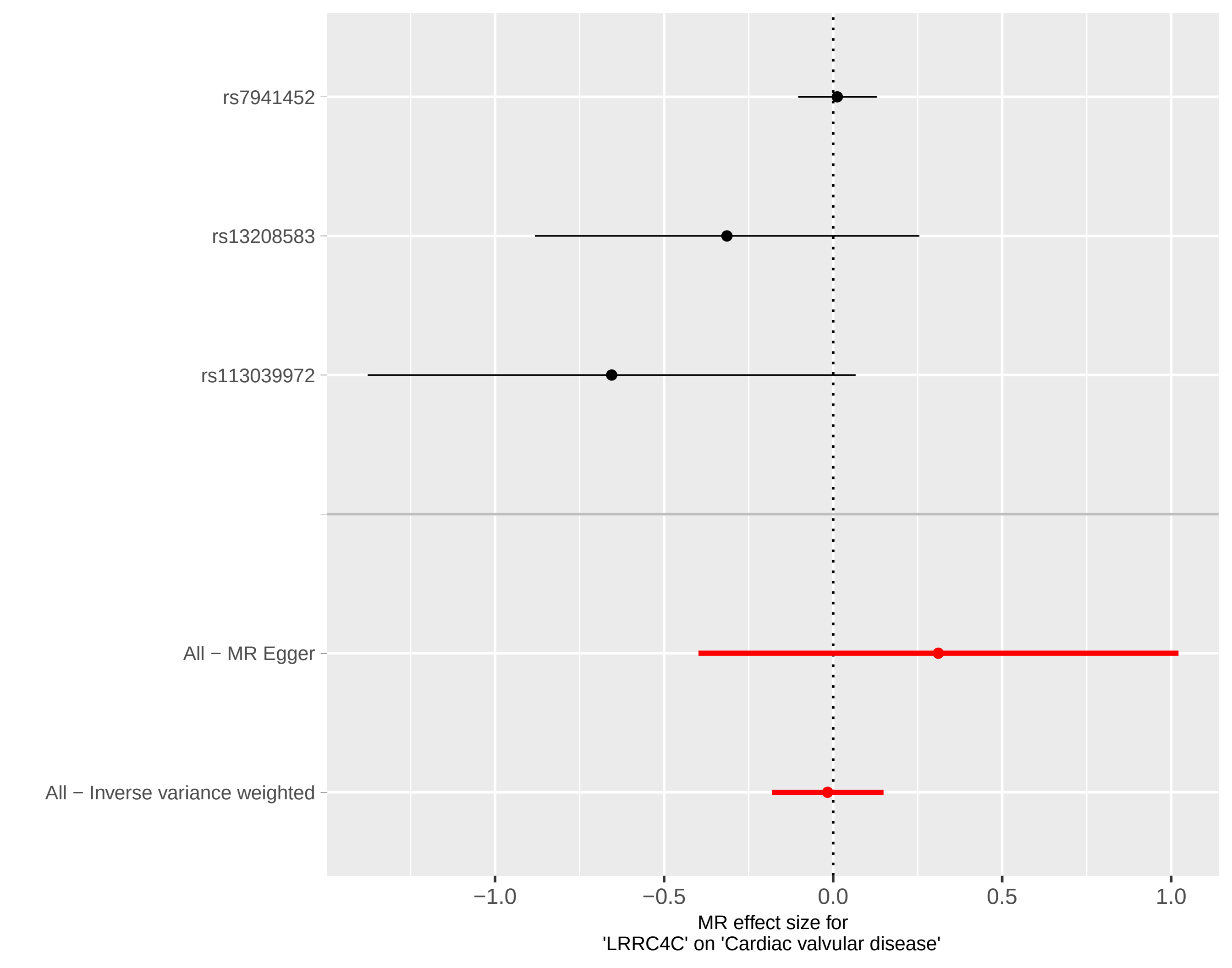
rs7941452
rs13208583
rs113039972
All − MR Egger
All − Inverse variance weighted
−1.0
−0.5
0.0
0.5
1.0
MR effect size for
'LRRC4C' on 'Cardiac valvular disease'

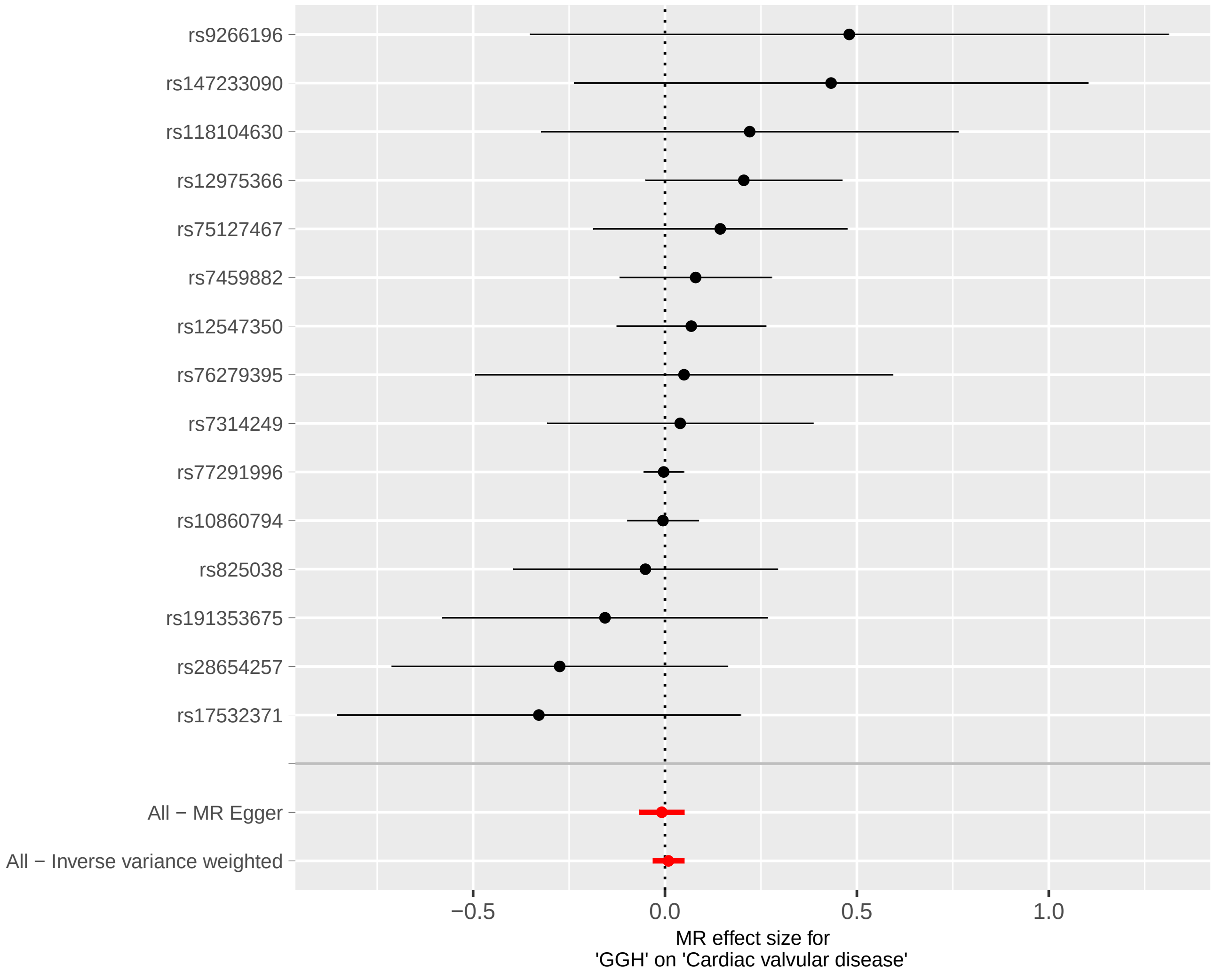
rs9266196
rs147233090
rs118104630
rs12975366
rs75127467
rs7459882
rs12547350
rs76279395
rs7314249
rs77291996
rs10860794
rs825038
rs191353675
rs28654257
rs17532371
All − MR Egger
All − Inverse variance weighted
−0.5
0.0
0.5
1.0
MR effect size for
'GGH' on 'Cardiac valvular disease'

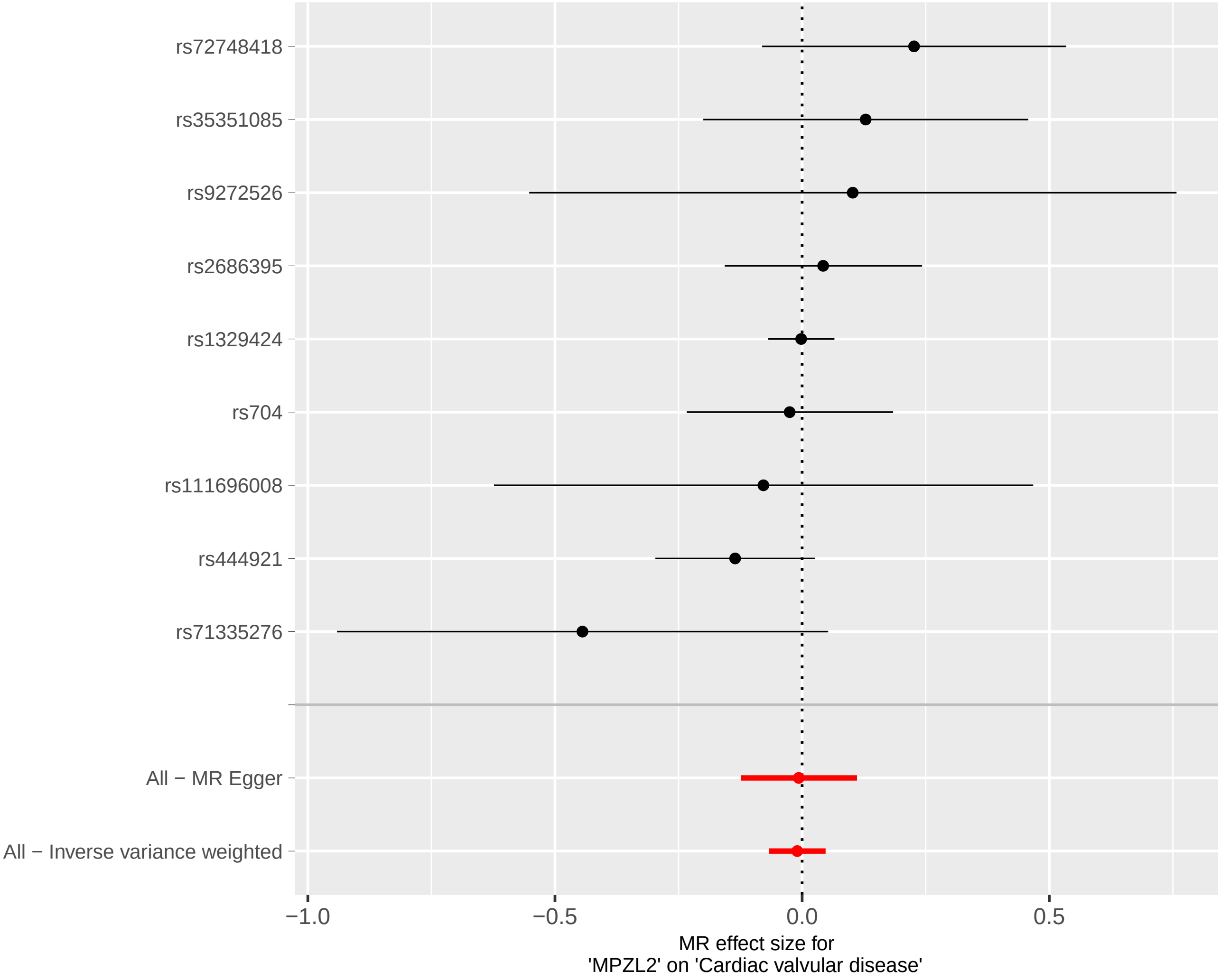
rs72748418
rs35351085
rs9272526
rs2686395
rs1329424
rs704
rs111696008
rs444921
rs71335276
All − MR Egger
All − Inverse variance weighted
−1.0
−0.5
0.0
0.5
MR effect size for
'MPZL2' on 'Cardiac valvular disease'

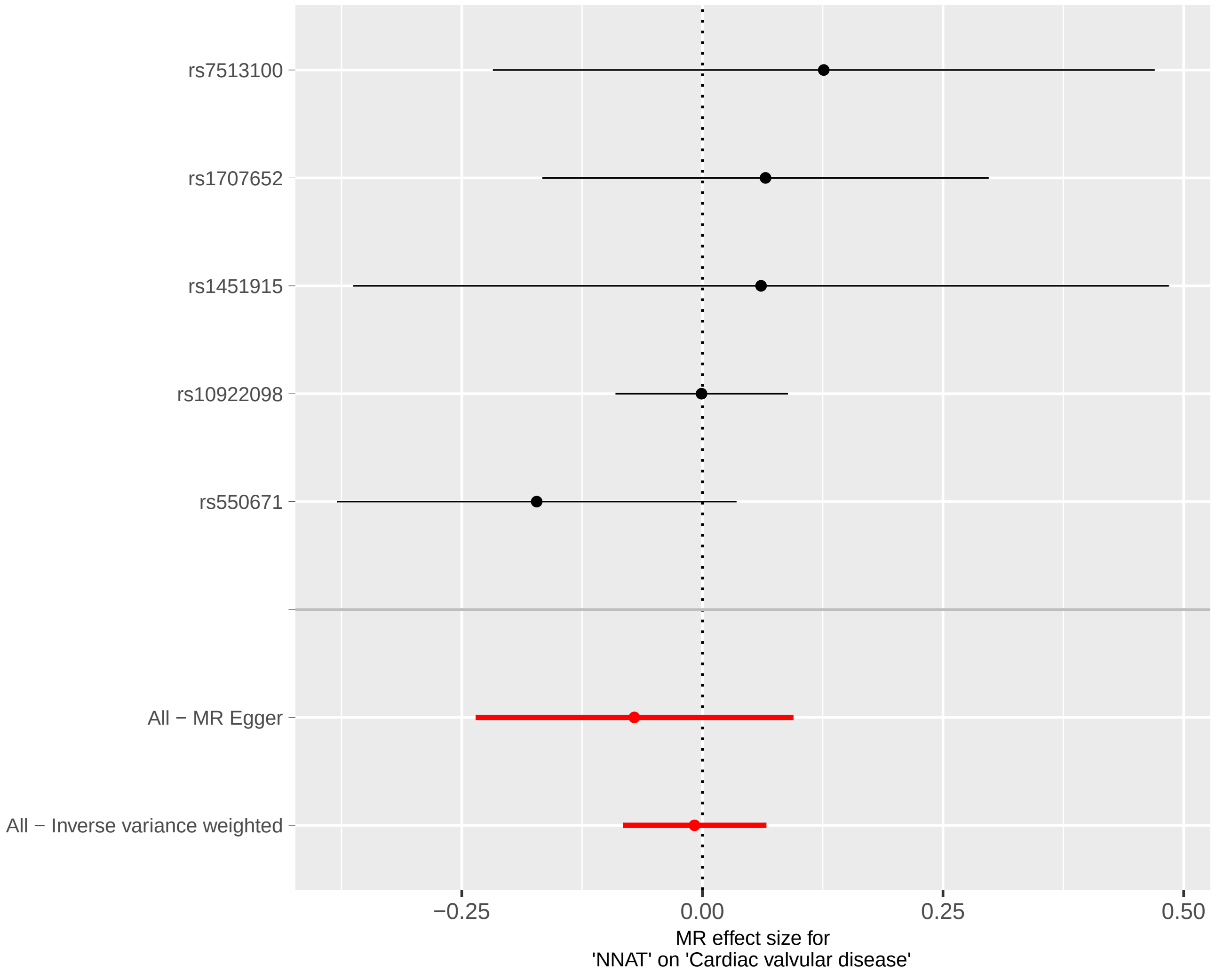
rs7513100
rs1707652
rs1451915
rs10922098
rs550671
All − MR Egger
All − Inverse variance weighted
−0.25
0.00
0.25
0.50
MR effect size for
'NNAT' on 'Cardiac valvular disease'

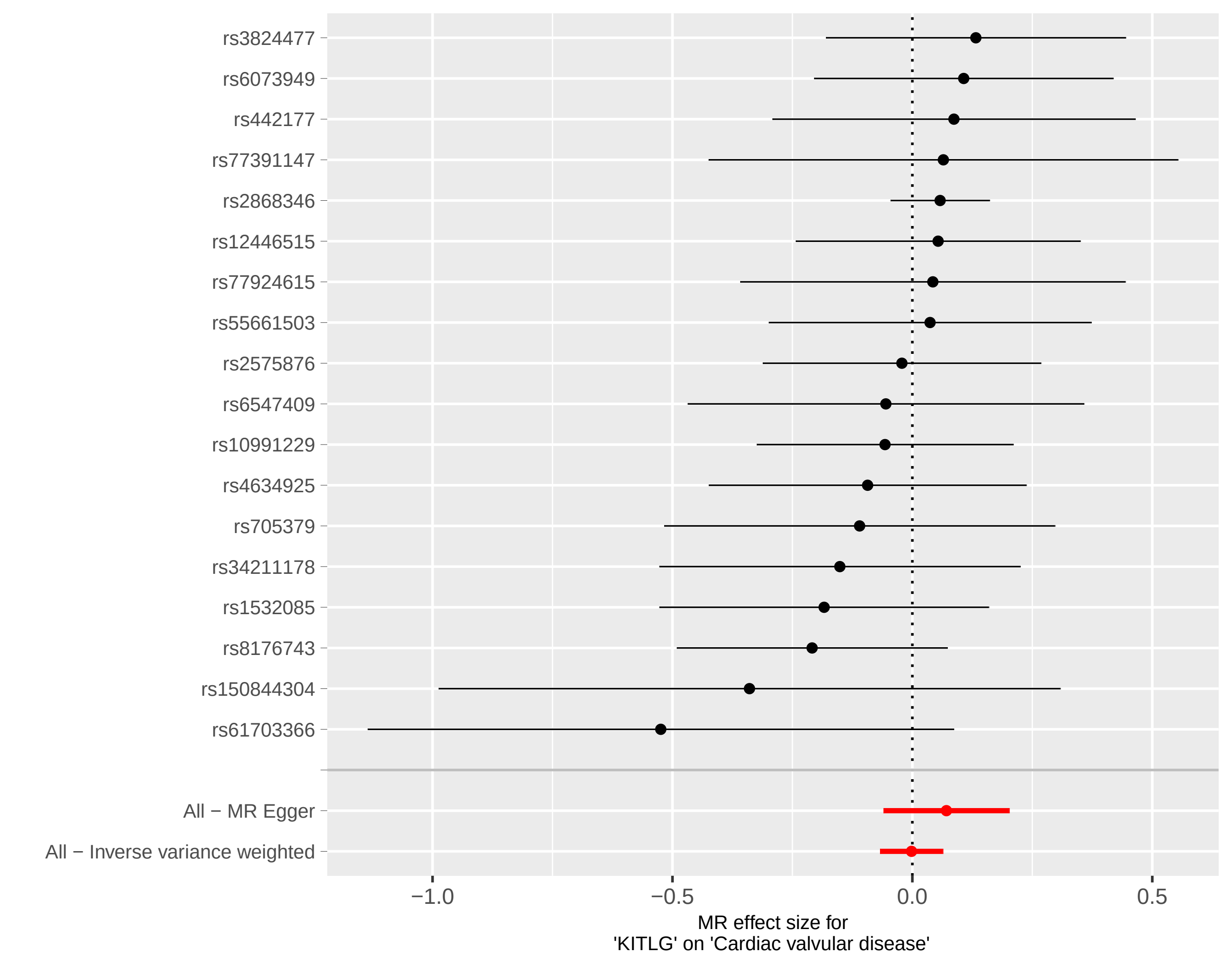
rs3824477
rs6073949
rs442177
rs77391147
rs2868346
rs12446515
rs77924615
rs55661503
rs2575876
rs6547409
rs10991229
rs4634925
rs705379
rs34211178
rs1532085
rs8176743
rs150844304
rs61703366
All − MR Egger
All − Inverse variance weighted
−1.0
−0.5
0.0
0.5
MR effect size for
'KITLG' on 'Cardiac valvular disease'

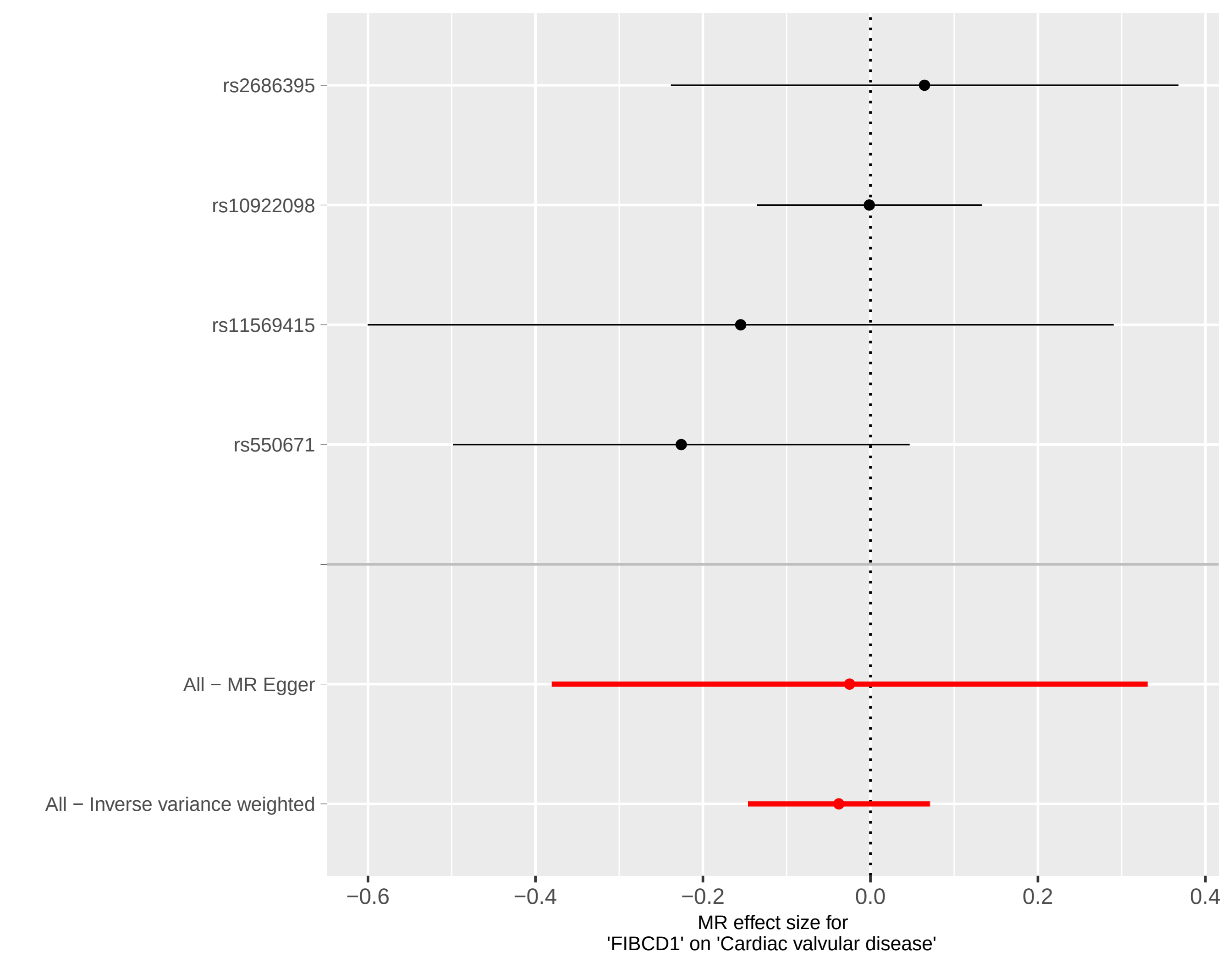

rs2686395
rs10922098
rs11569415
rs550671
All − MR Egger
All − Inverse variance weighted
−0.6
−0.4
−0.2
0.0
0.2
0.4
MR effect size for
'FIBCD1' on 'Cardiac valvular disease'

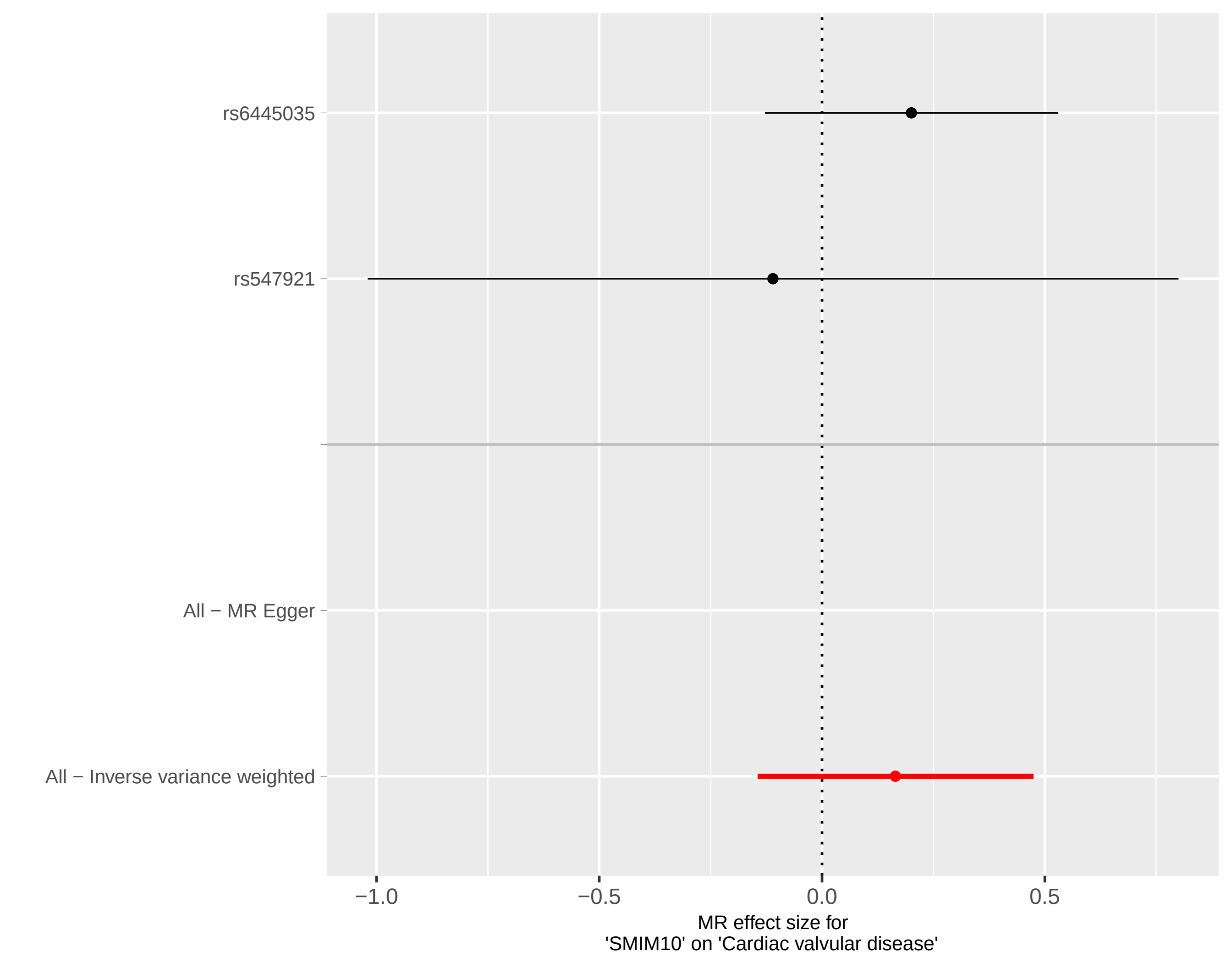
rs6445035
rs547921
All − MR Egger
All − Inverse variance weighted
−1.0
−0.5
0.0
0.5
MR effect size for
'SMIM10' on 'Cardiac valvular disease'

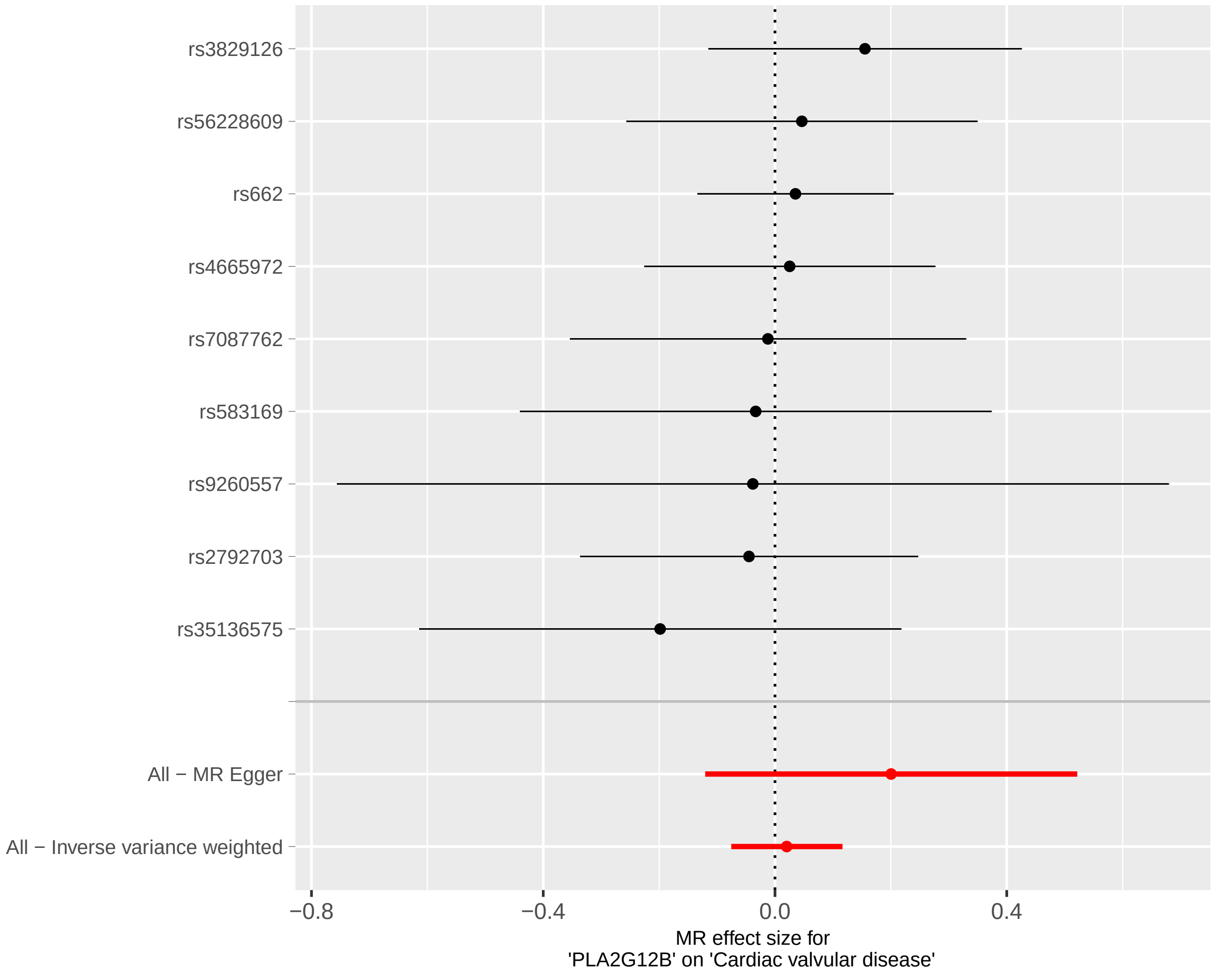
rs3829126
rs56228609
rs662
rs4665972
rs7087762
rs583169
rs9260557
rs2792703
rs35136575
All − MR Egger
All − Inverse variance weighted
−0.8
−0.4
0.0
0.4
MR effect size for
'PLA2G12B' on 'Cardiac valvular disease'

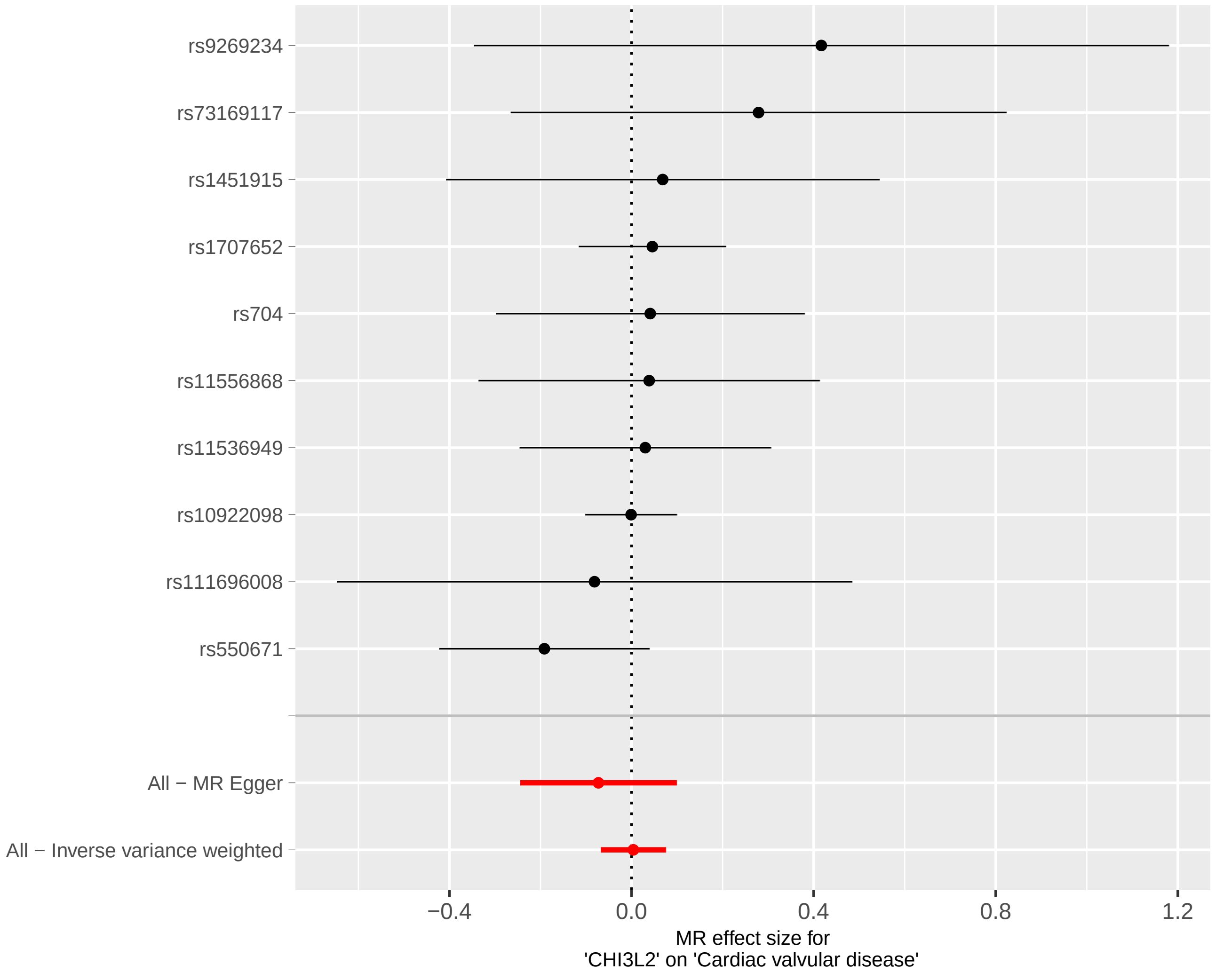
rs9269234
rs73169117
rs1451915
rs1707652
rs704
rs11556868
rs11536949
rs10922098
rs111696008
rs550671
All − MR Egger
All − Inverse variance weighted
−0.4
0.0
0.4
0.8
1.2
MR effect size for
'CHI3L2' on 'Cardiac valvular disease'

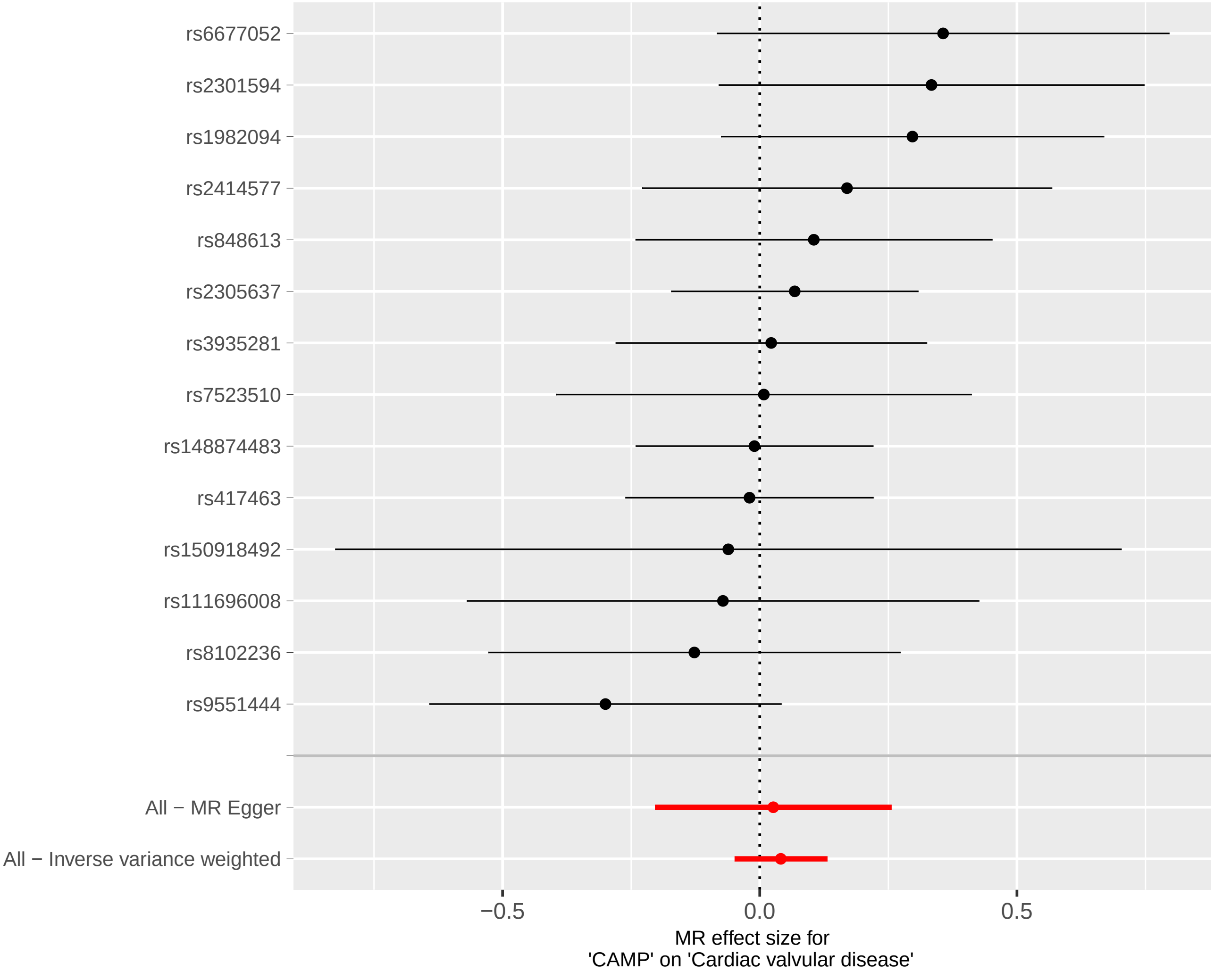
rs6677052
rs2301594
rs1982094
rs2414577
rs848613
rs2305637
rs3935281
rs7523510
rs148874483
rs417463
rs150918492
rs111696008
rs8102236
rs9551444
All − MR Egger
All − Inverse variance weighted
−0.5
0.0
0.5
MR effect size for
'CAMP' on 'Cardiac valvular disease'

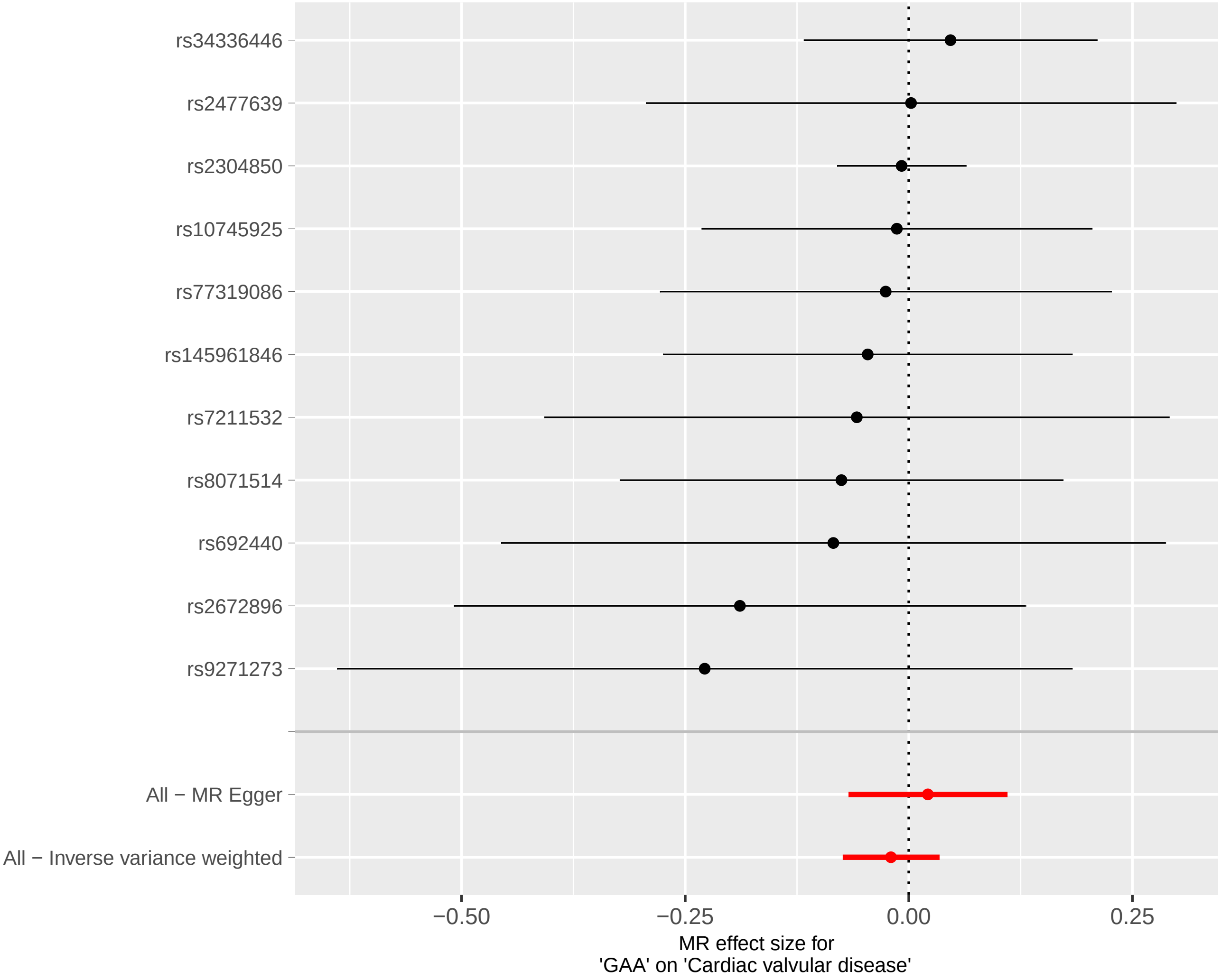

rs34336446
rs2477639
rs2304850
rs10745925
rs77319086
rs145961846
rs7211532
rs8071514
rs692440
rs2672896
rs9271273
All − MR Egger
All − Inverse variance weighted
−0.50
−0.25
0.00
0.25
MR effect size for
'GAA' on 'Cardiac valvular disease'

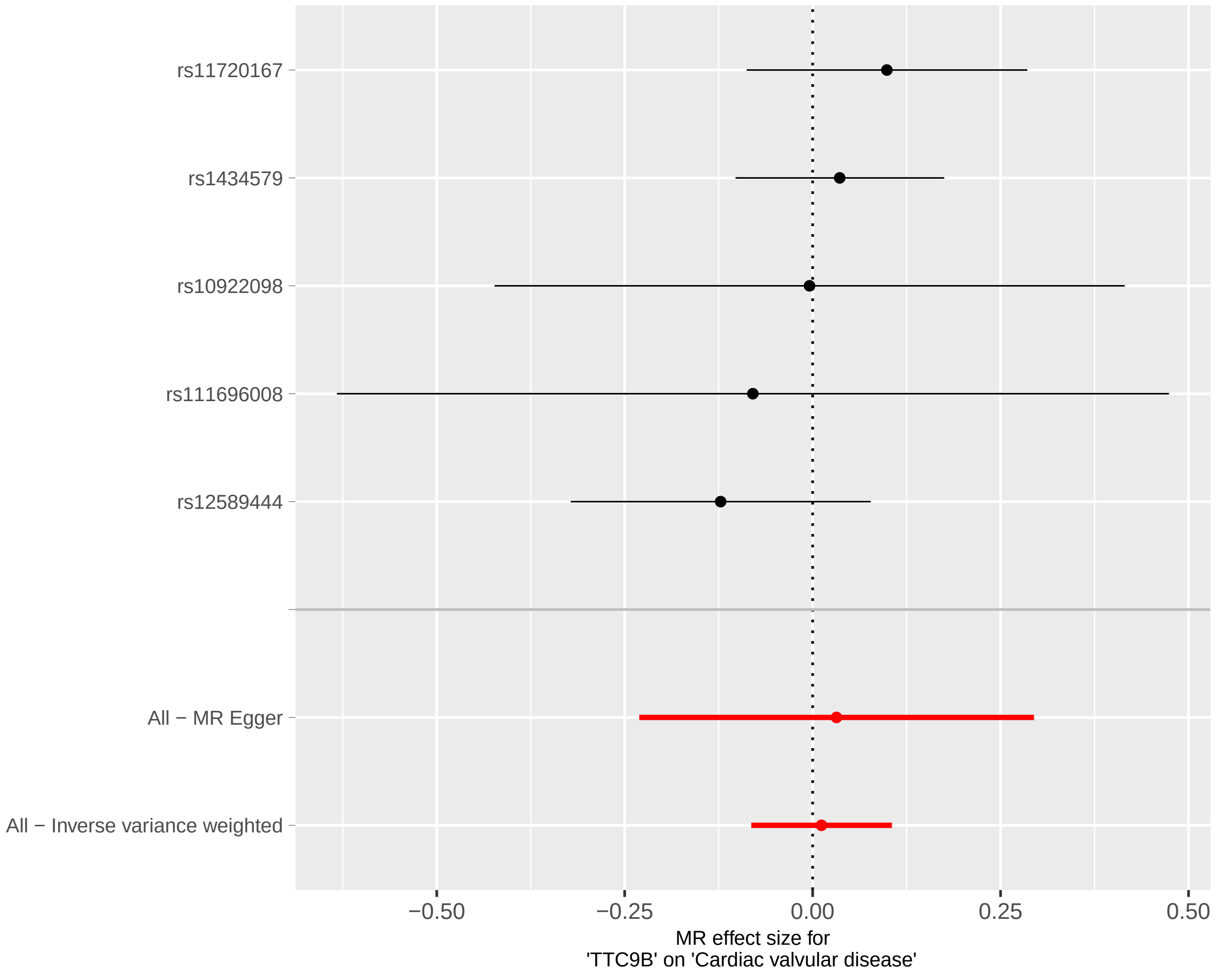
rs11720167
rs1434579
rs10922098
rs111696008
rs12589444
All − MR Egger
All − Inverse variance weighted
−0.50
−0.25
0.00
0.25
0.50
MR effect size for
'TTC9B' on 'Cardiac valvular disease'

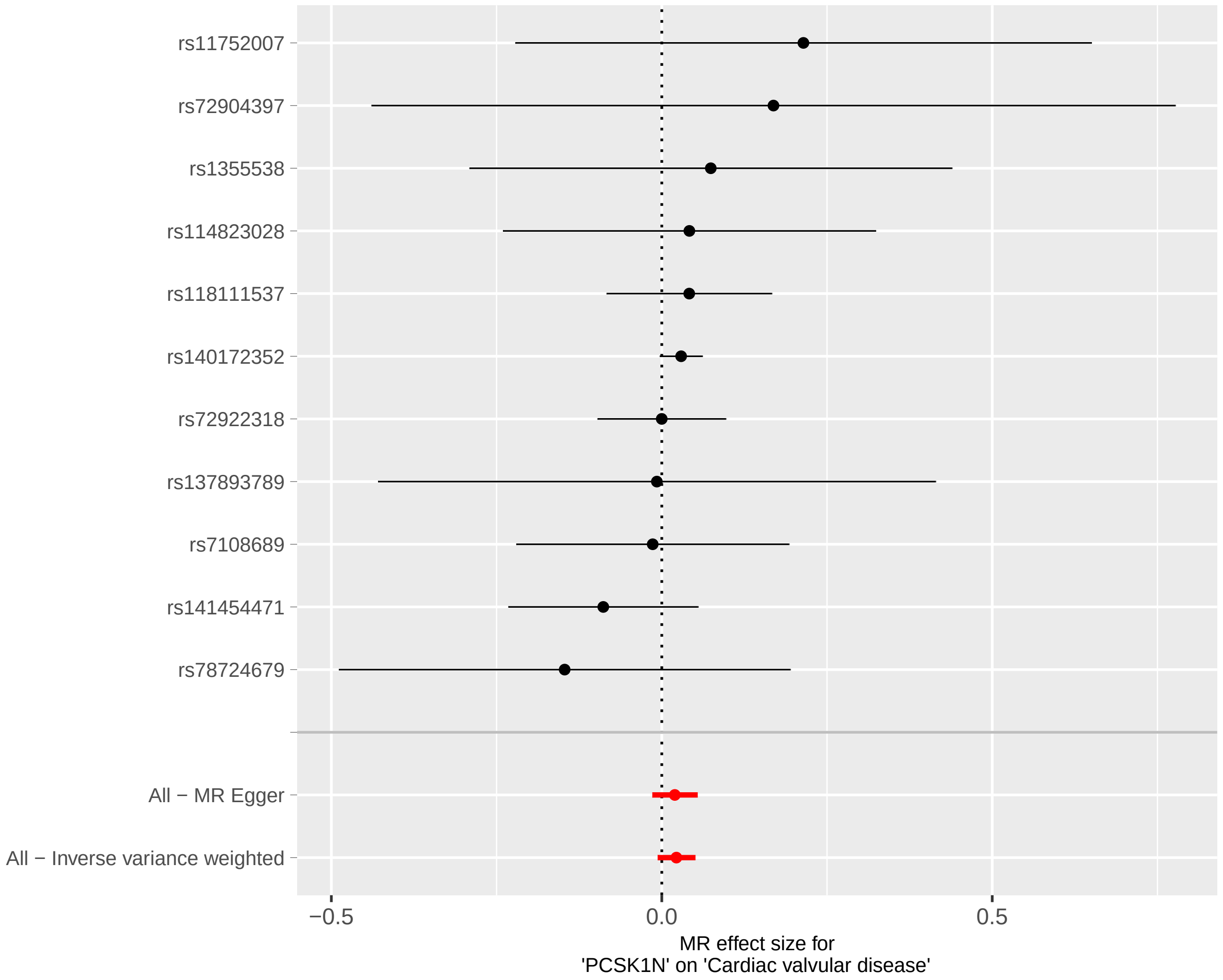

rs11752007
rs72904397
rs1355538
rs114823028
rs118111537
rs140172352
rs72922318
rs137893789
rs7108689
rs141454471
rs78724679
All − MR Egger
All − Inverse variance weighted
−0.5
0.0
0.5
MR effect size for
'PCSK1N' on 'Cardiac valvular disease'

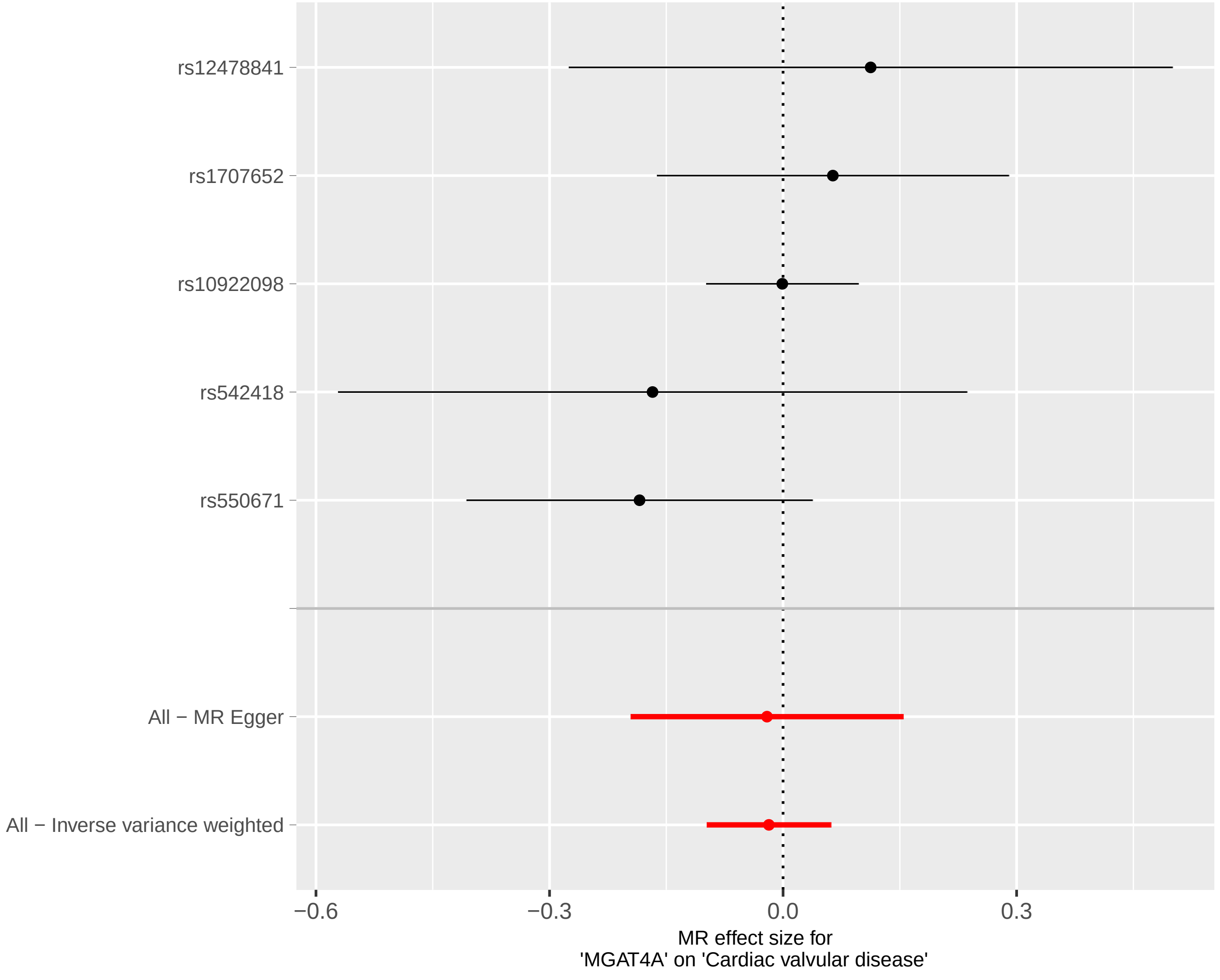
rs12478841
rs1707652
rs10922098
rs542418
rs550671
All − MR Egger
All − Inverse variance weighted
−0.6
−0.3
0.0
0.3
MR effect size for
'MGAT4A' on 'Cardiac valvular disease'

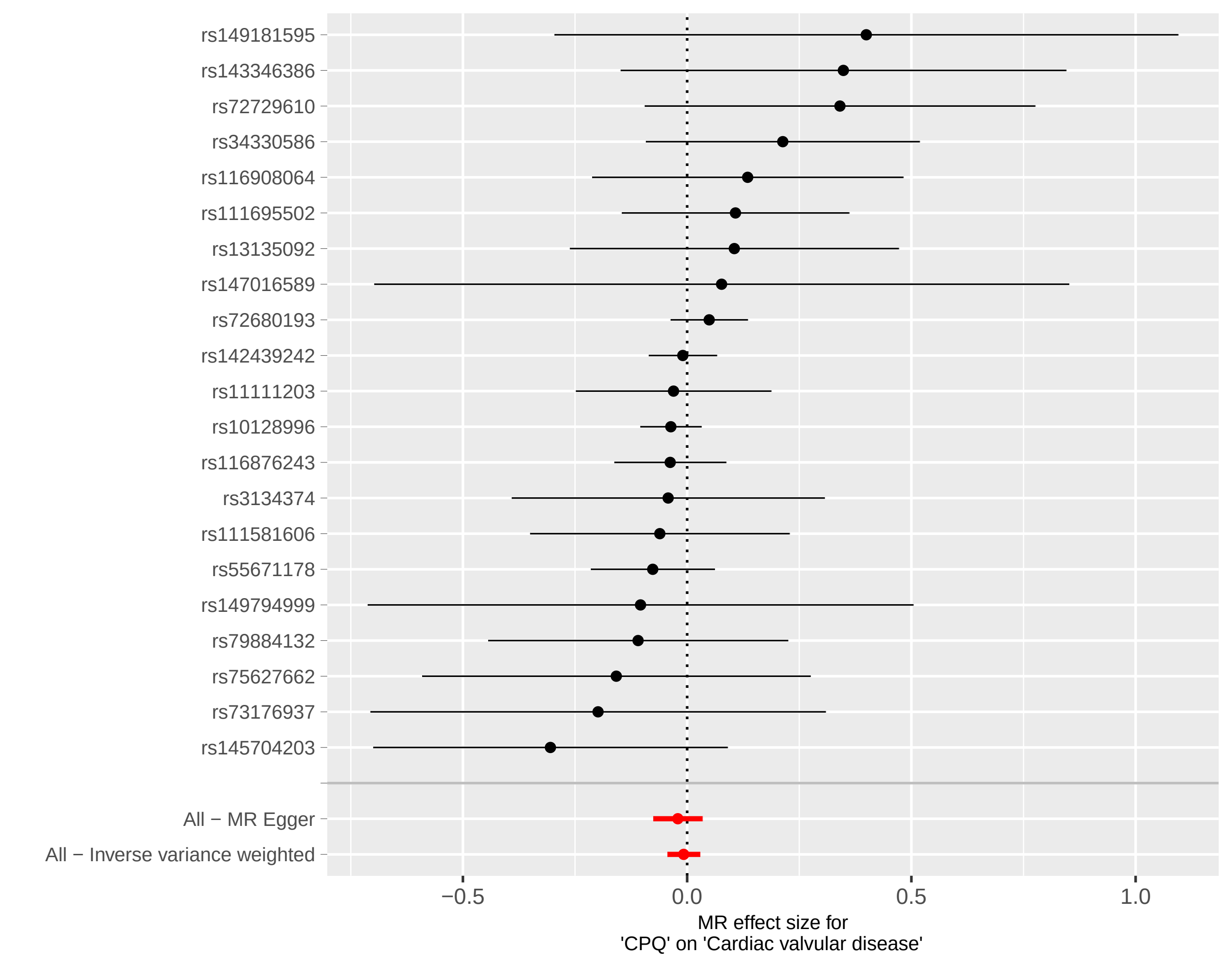
rs149181595
rs143346386
rs72729610
rs34330586
rs116908064
rs111695502
rs13135092
rs147016589
rs72680193
rs142439242
rs11111203
rs10128996
rs116876243
rs3134374
rs111581606
rs55671178
rs149794999
rs79884132
rs75627662
rs73176937
rs145704203
All − MR Egger
All − Inverse variance weighted
−0.5
0.0
0.5
1.0
MR effect size for
'CPQ' on 'Cardiac valvular disease'

Insufficient number of SNPs

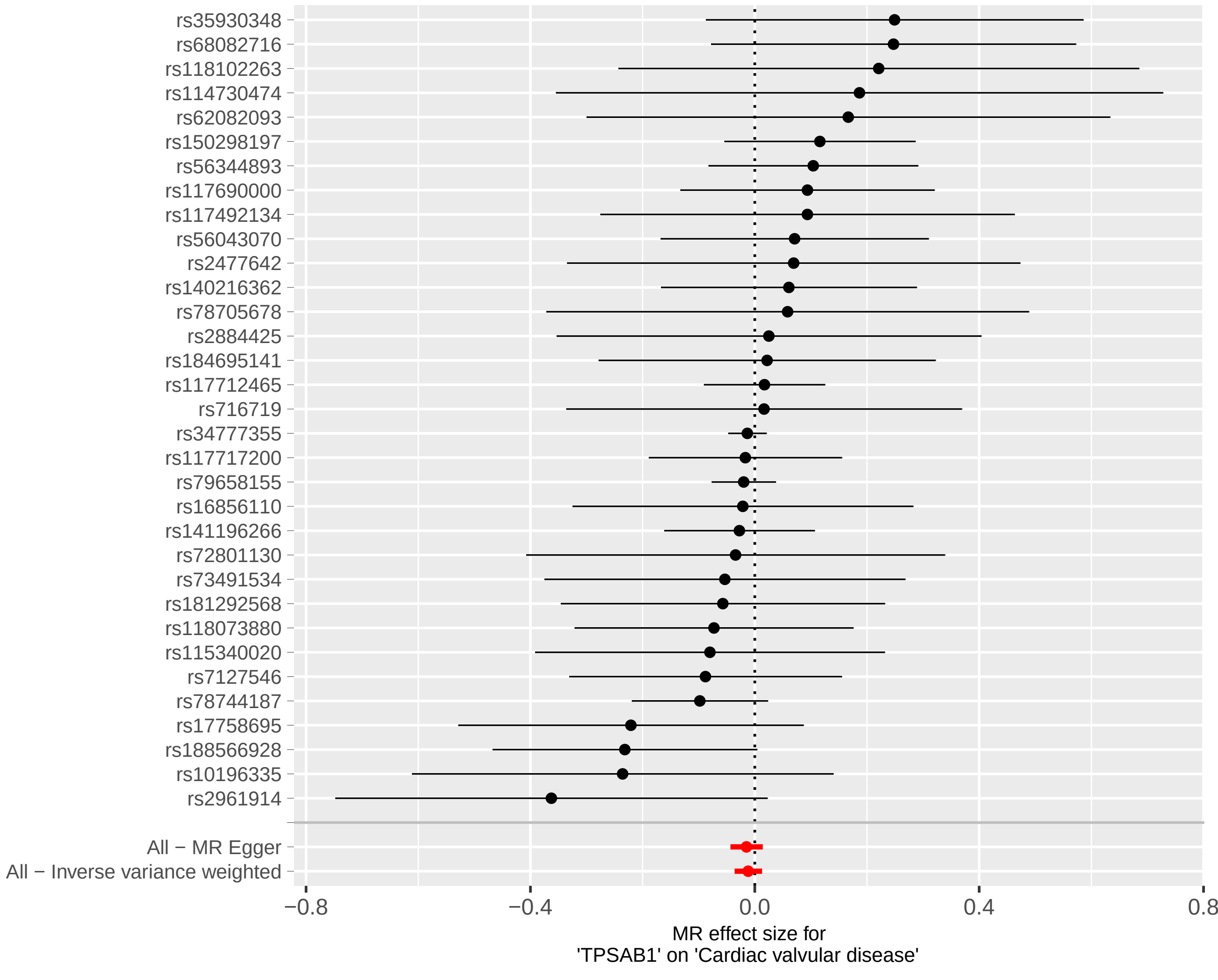

rs35930348
rs68082716
rs118102263
rs114730474
rs62082093
rs150298197
rs56344893
rs117690000
rs117492134
rs56043070
rs2477642
rs140216362
rs78705678
rs2884425
rs184695141
rs117712465
rs716719
rs34777355
rs117717200
rs79658155
rs16856110
rs141196266
rs72801130
rs73491534
rs181292568
rs118073880
rs115340020
rs7127546
rs78744187
rs17758695
rs188566928
rs10196335
rs2961914
All − MR Egger
All − Inverse variance weighted
−0.8
−0.4
0.0
0.4
0.8
MR effect size for
'TPSAB1' on 'Cardiac valvular disease'

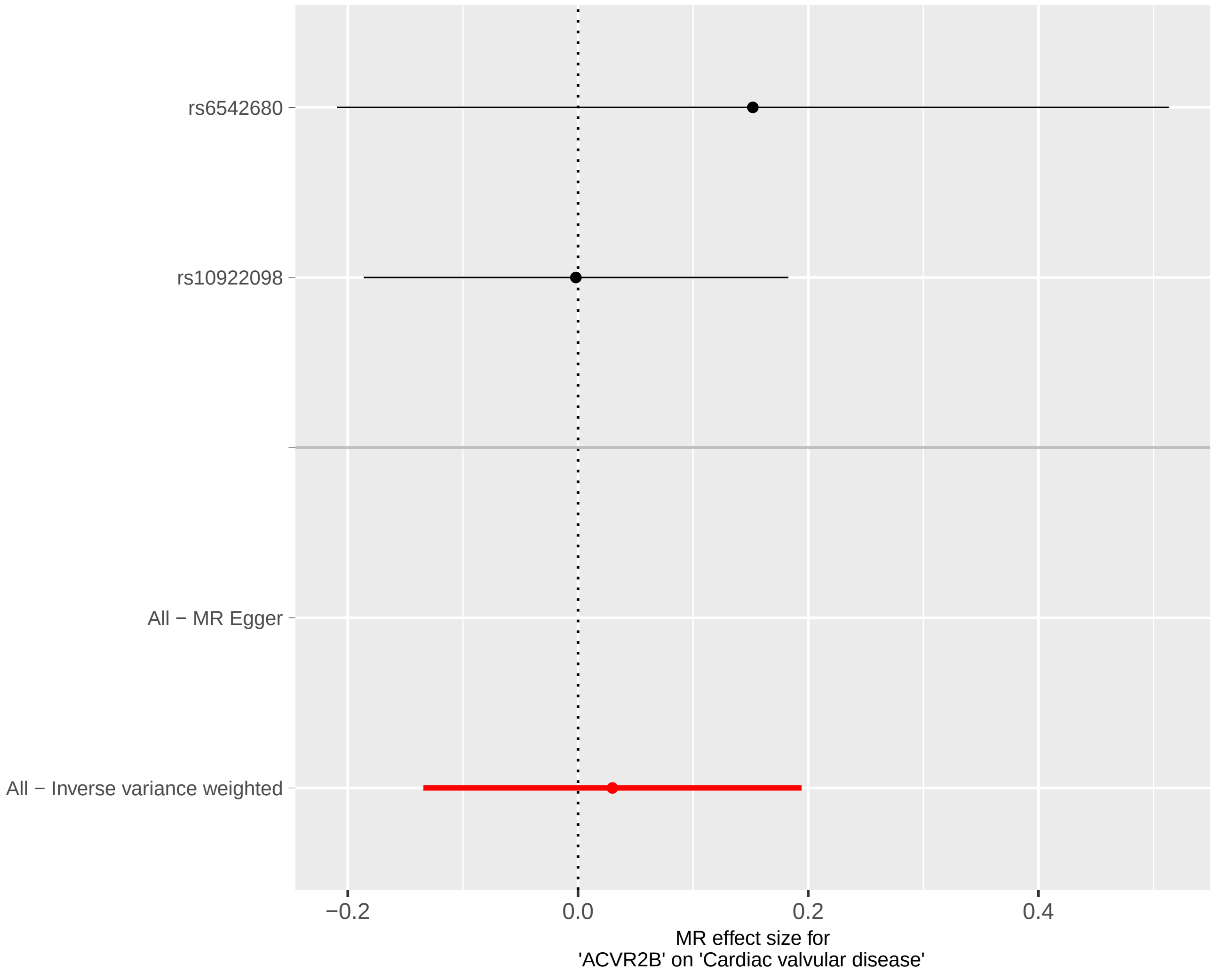
rs6542680
rs10922098
All − MR Egger
All − Inverse variance weighted
−0.2
0.0
0.2
0.4
MR effect size for
'ACVR2B' on 'Cardiac valvular disease'

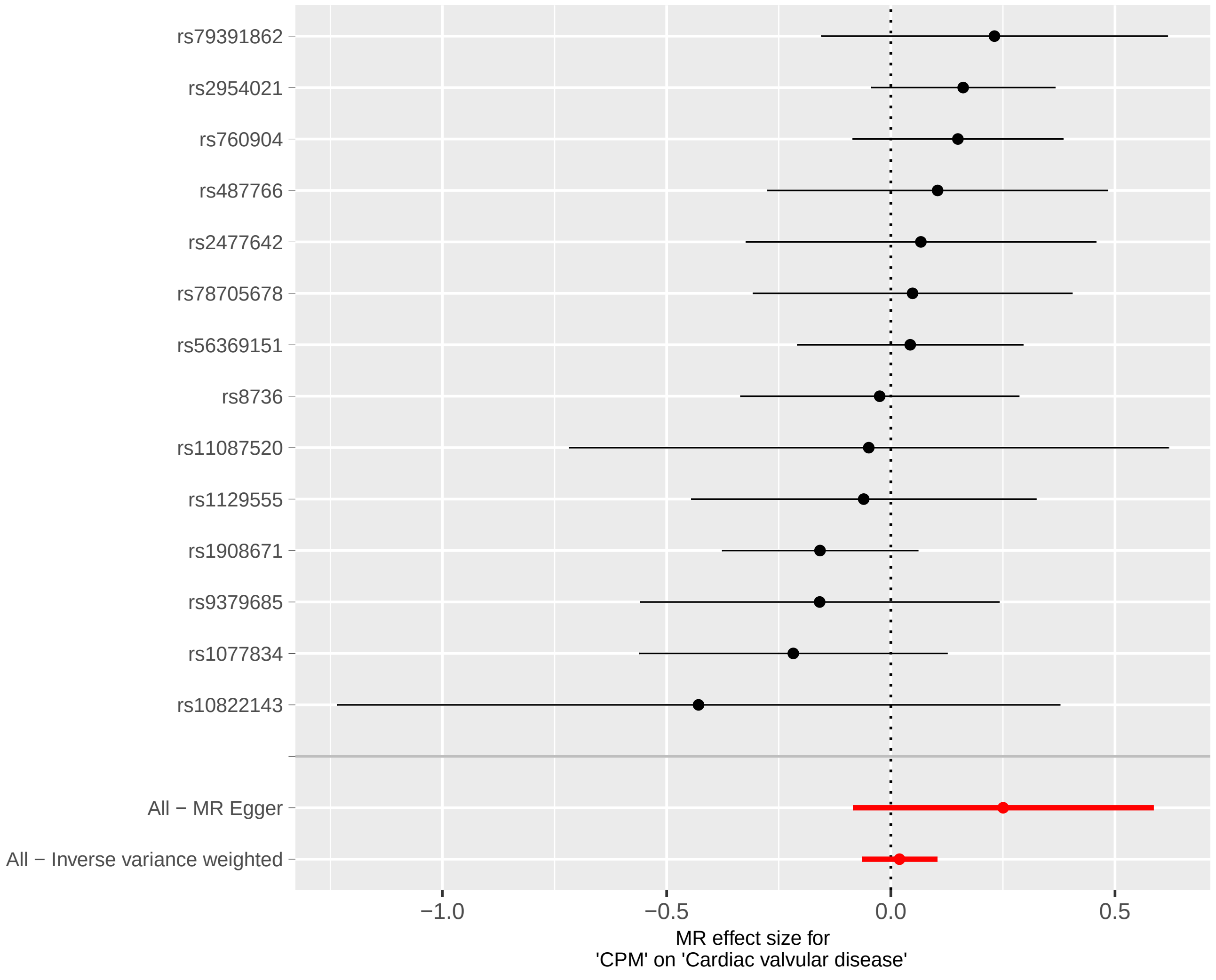

rs79391862
rs2954021
rs760904
rs487766
rs2477642
rs78705678
rs56369151
rs8736
rs11087520
rs1129555
rs1908671
rs9379685
rs1077834
rs10822143
All − MR Egger
All − Inverse variance weighted
−1.0
−0.5
0.0
0.5
MR effect size for
'CPM' on 'Cardiac valvular disease'

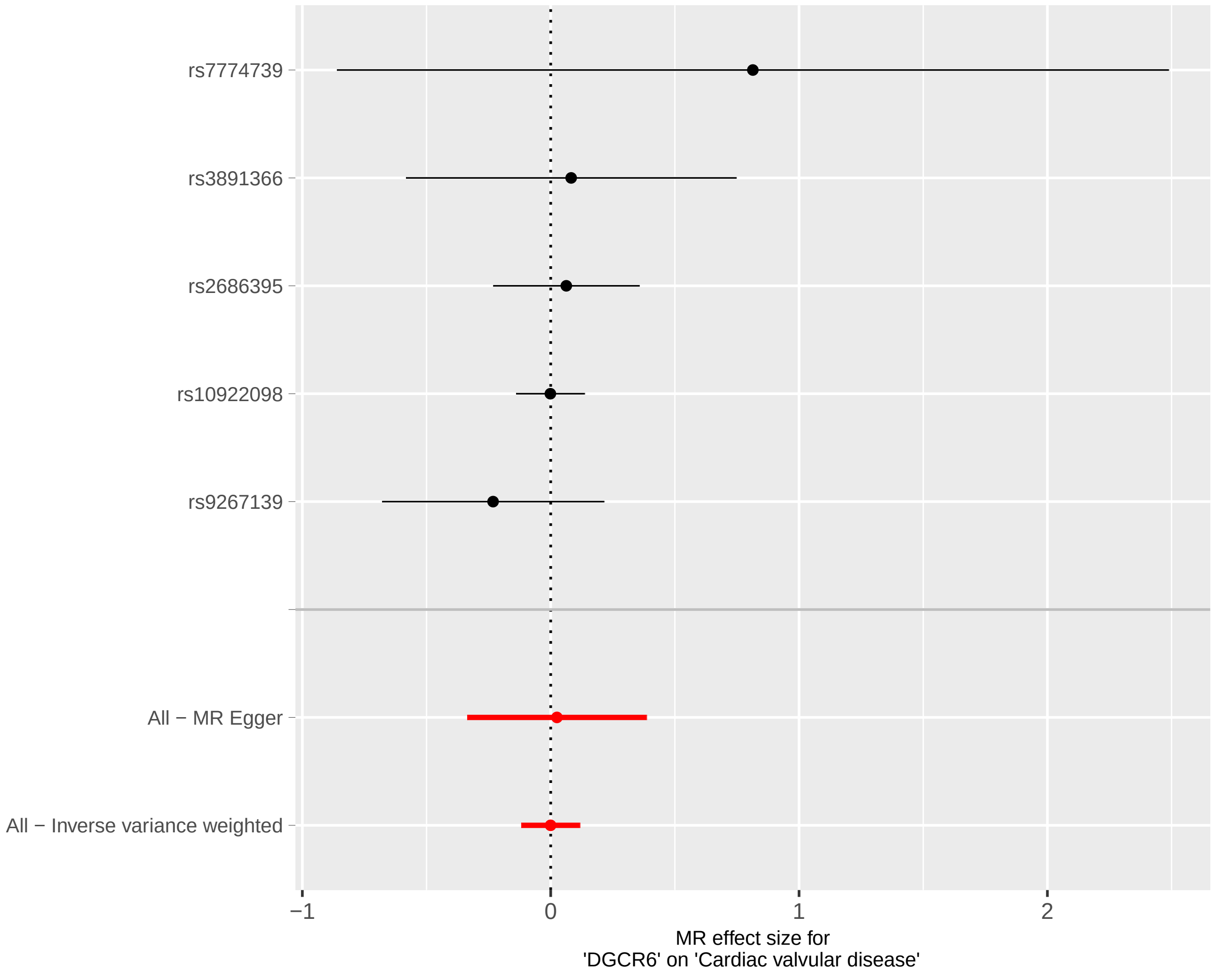

rs7774739
rs3891366
rs2686395
rs10922098
rs9267139
All − MR Egger
All − Inverse variance weighted
−1
0
1
2
MR effect size for
'DGCR6' on 'Cardiac valvular disease'

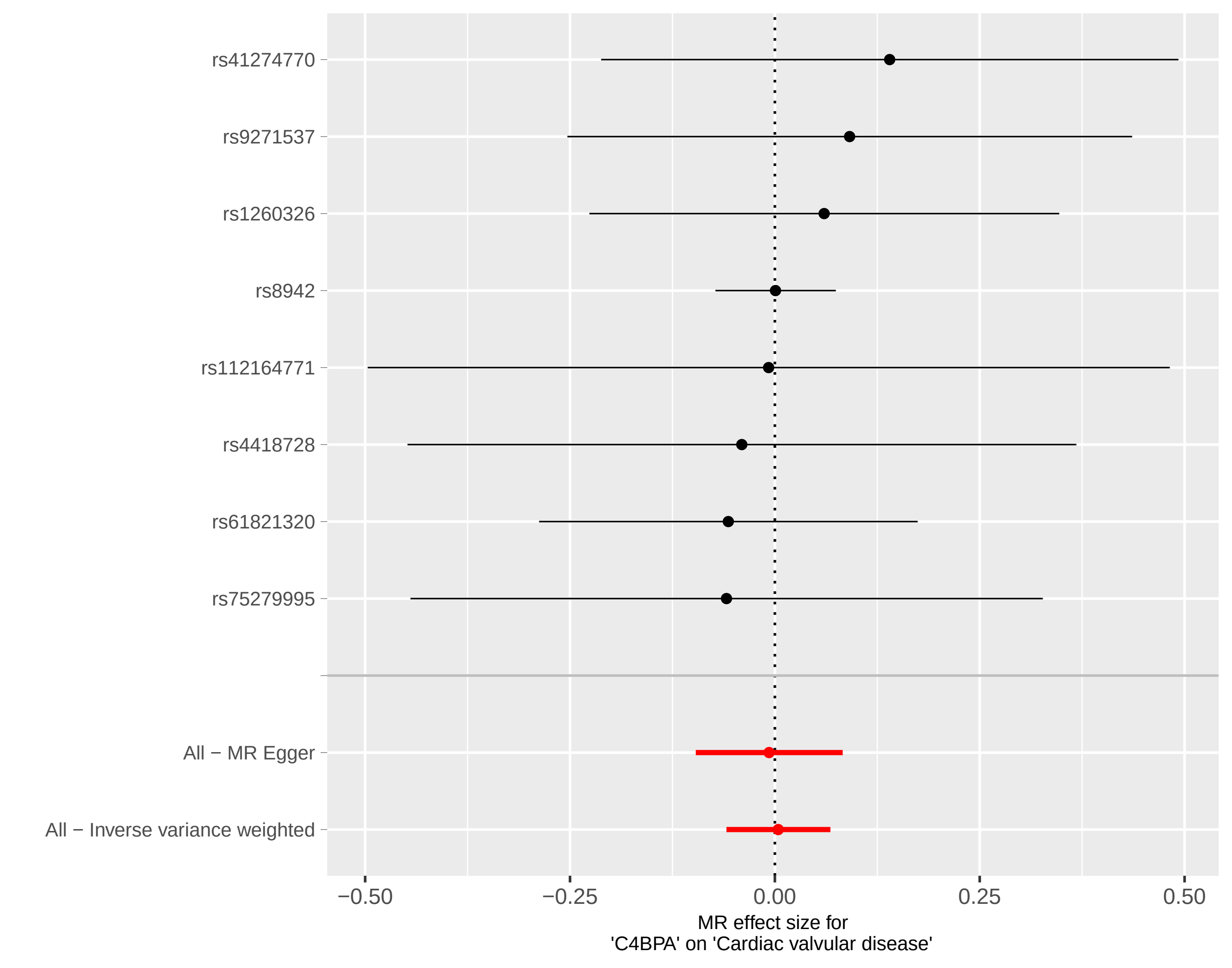
rs41274770
rs9271537
rs1260326
rs8942
rs112164771
rs4418728
rs61821320
rs75279995
All − MR Egger
All − Inverse variance weighted
−0.50
−0.25
0.00
0.25
0.50
MR effect size for
'C4BPA' on 'Cardiac valvular disease'

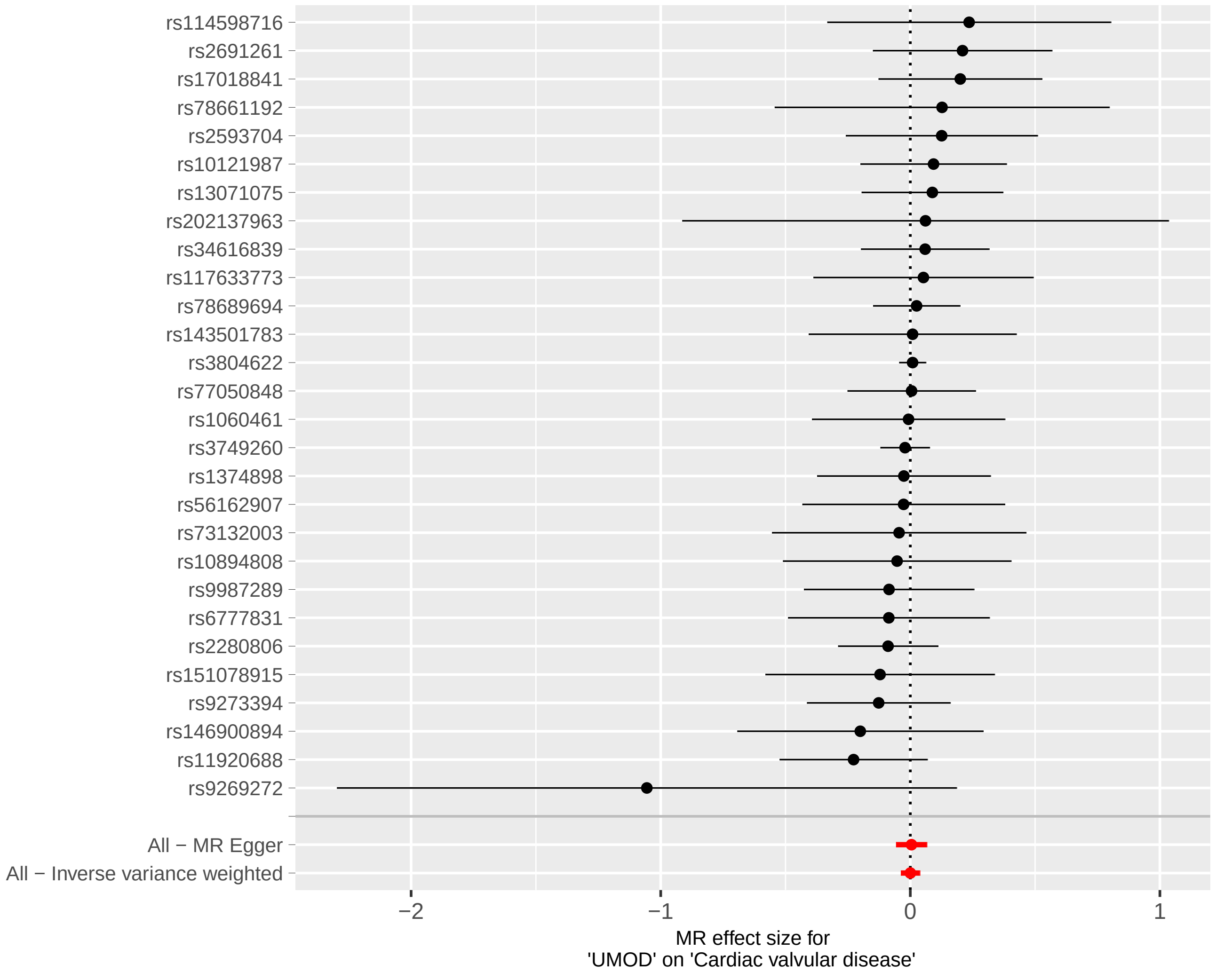
rs114598716
rs2691261
rs17018841
rs78661192
rs2593704
rs10121987
rs13071075
rs202137963
rs34616839
rs117633773
rs78689694
rs143501783
rs3804622
rs77050848
rs1060461
rs3749260
rs1374898
rs56162907
rs73132003
rs10894808
rs9987289
rs6777831
rs2280806
rs151078915
rs9273394
rs146900894
rs11920688
rs9269272
All − MR Egger
All − Inverse variance weighted
−2
−1
0
1
MR effect size for
'UMOD' on 'Cardiac valvular disease'

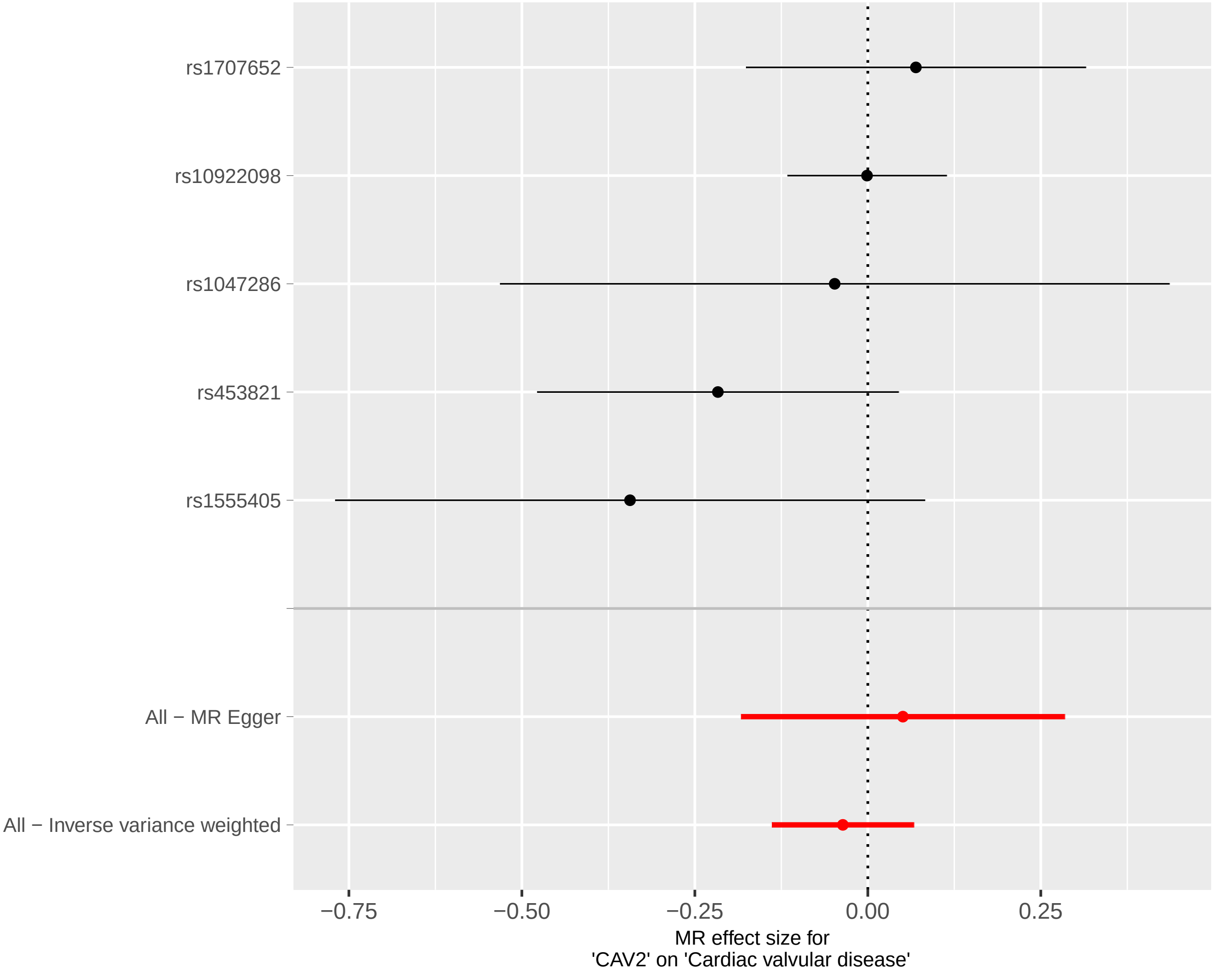

rs1707652
rs10922098
rs1047286
rs453821
rs1555405
All − MR Egger
All − Inverse variance weighted
−0.75
−0.50
−0.25
0.00
0.25
MR effect size for
'CAV2' on 'Cardiac valvular disease'

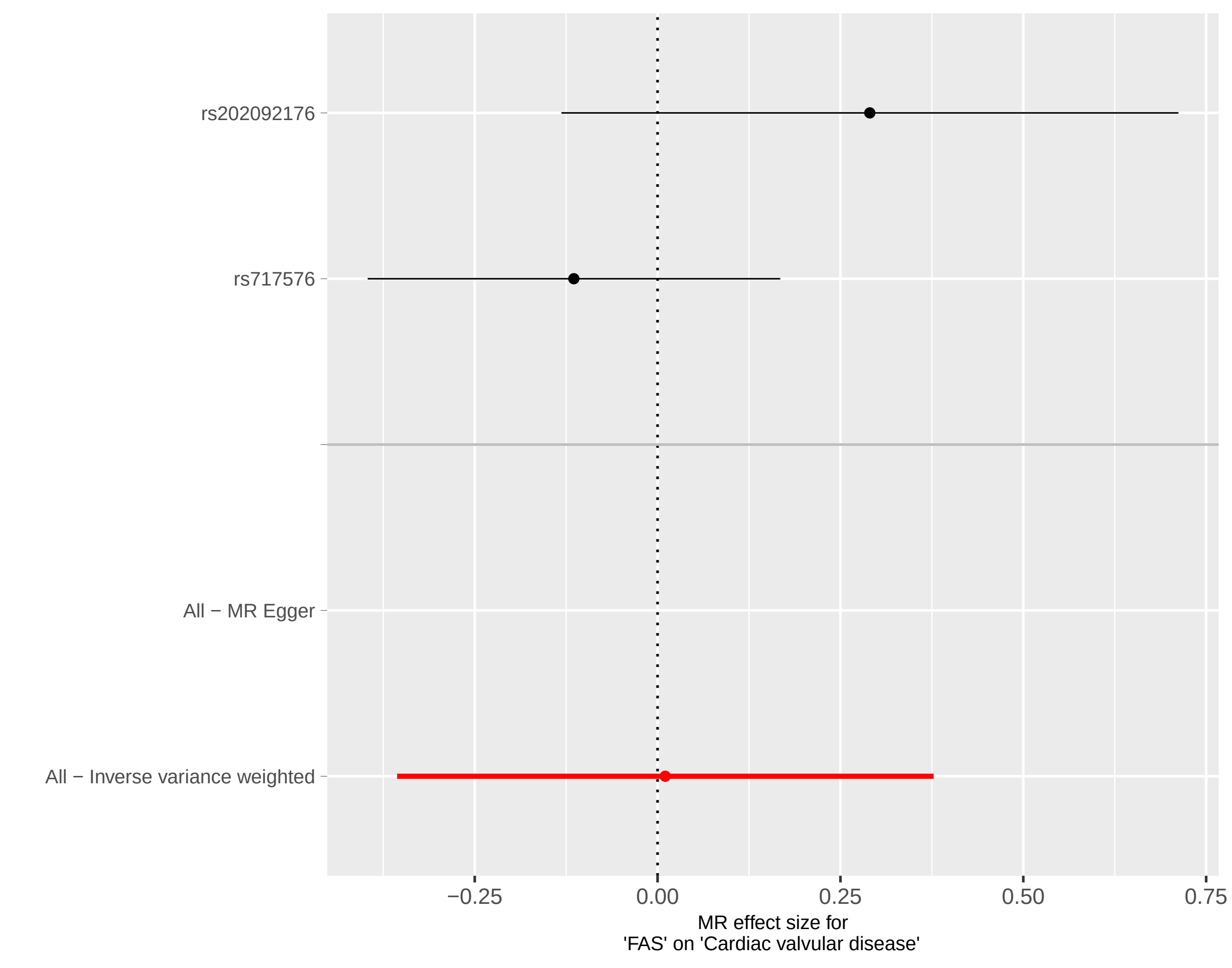
rs202092176
rs717576
All − MR Egger
All − Inverse variance weighted
−0.25
0.00
0.25
0.50
0.75
MR effect size for
'FAS' on 'Cardiac valvular disease'

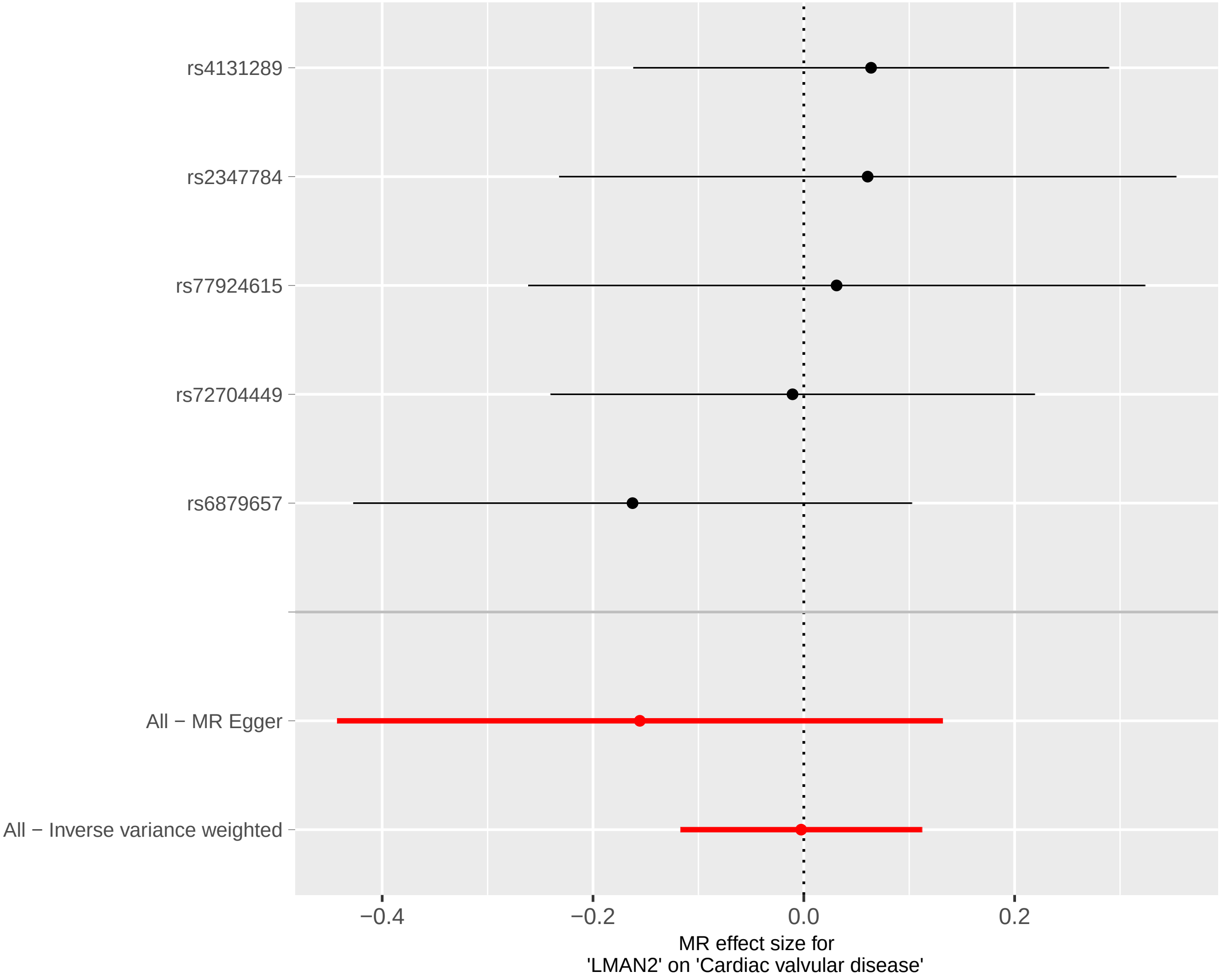
rs4131289
rs2347784
rs77924615
rs72704449
rs6879657
All − MR Egger
All − Inverse variance weighted
−0.4
−0.2
0.0
0.2
MR effect size for
'LMAN2' on 'Cardiac valvular disease'

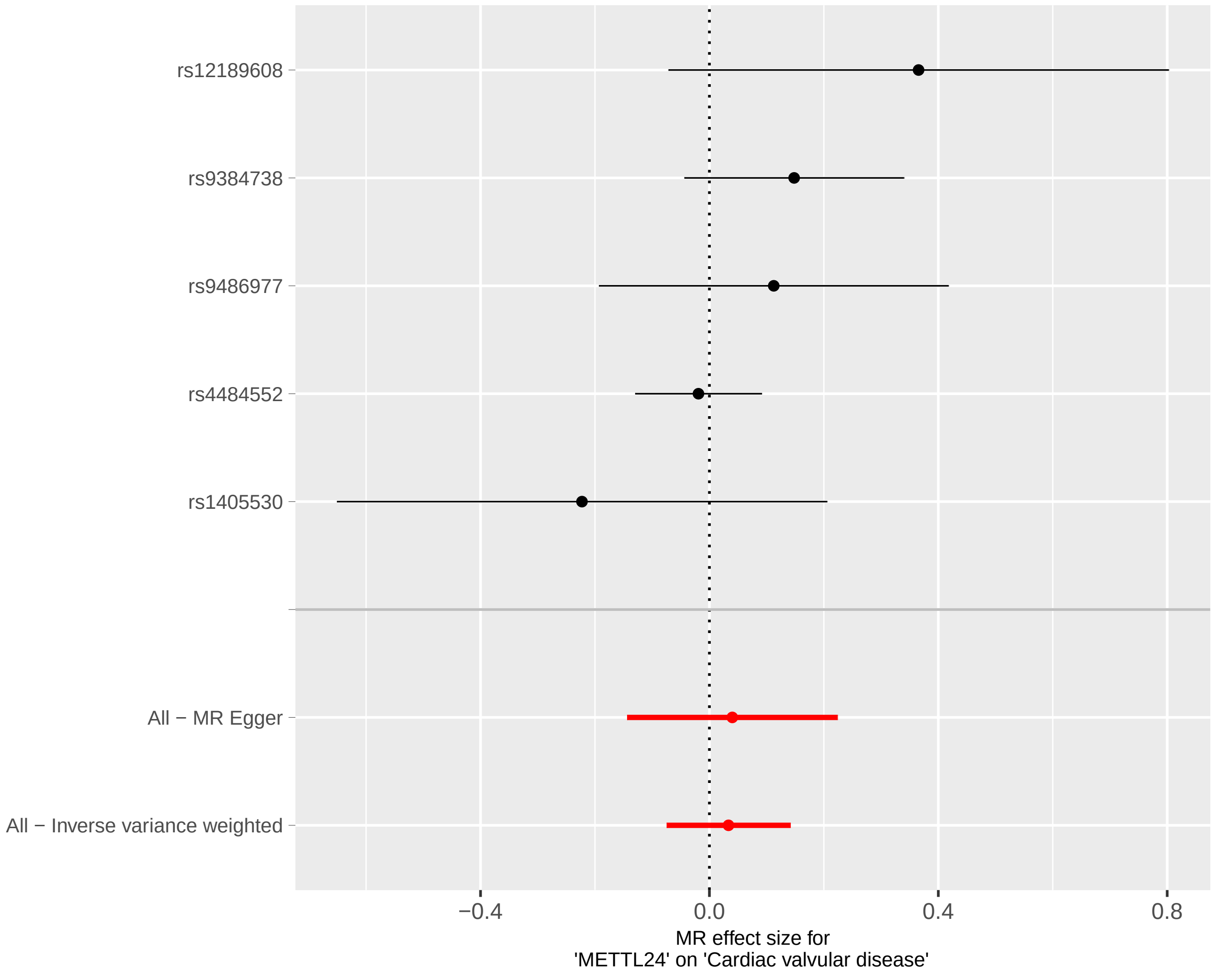

rs12189608
rs9384738
rs9486977
rs4484552
rs1405530
All − MR Egger
All − Inverse variance weighted
−0.4
0.0
0.4
0.8
MR effect size for
'METTL24' on 'Cardiac valvular disease'

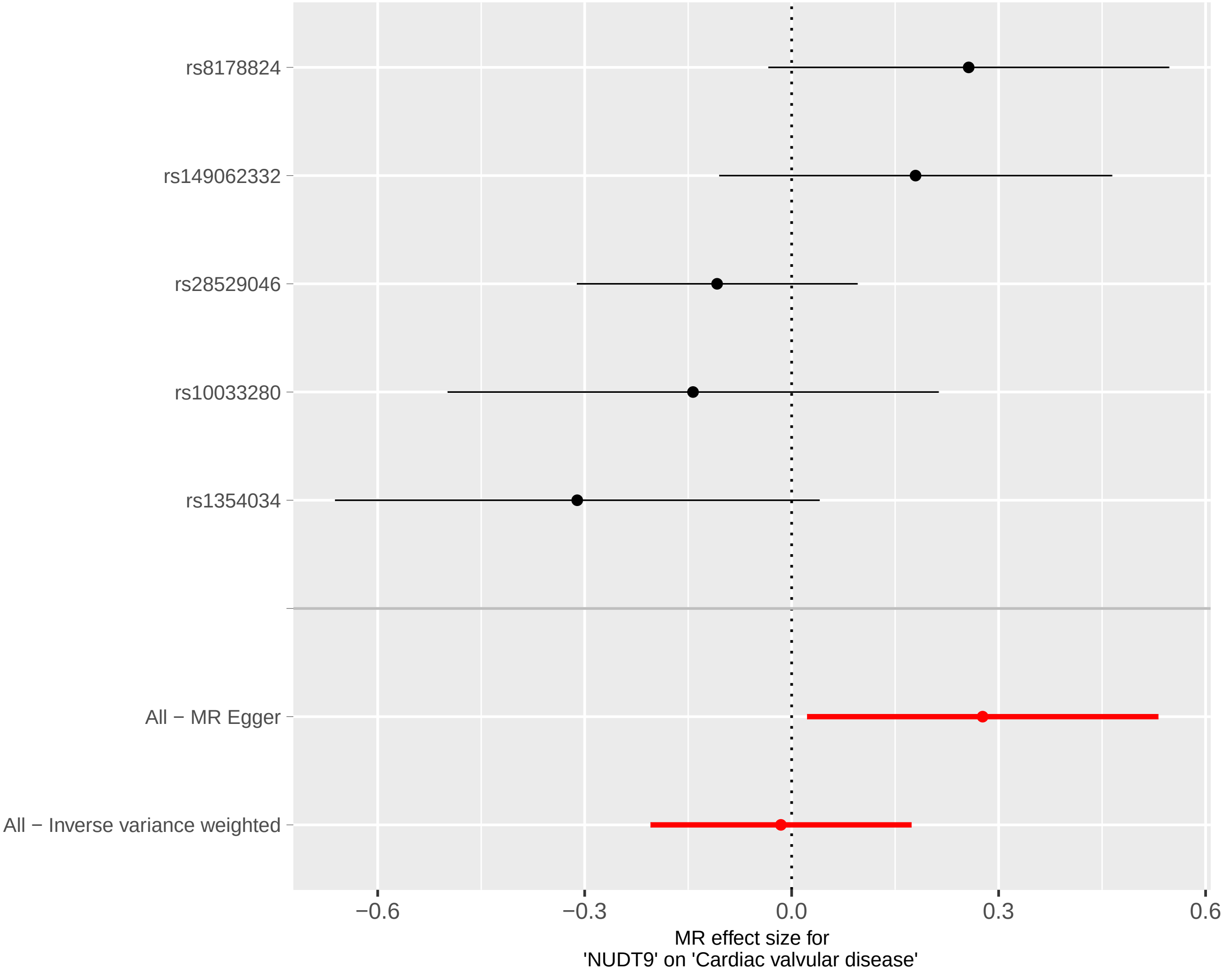

rs8178824
rs149062332
rs28529046
rs10033280
rs1354034
All − MR Egger
All − Inverse variance weighted
−0.6
−0.3
0.0
0.3
0.6
MR effect size for
'NUDT9' on 'Cardiac valvular disease'

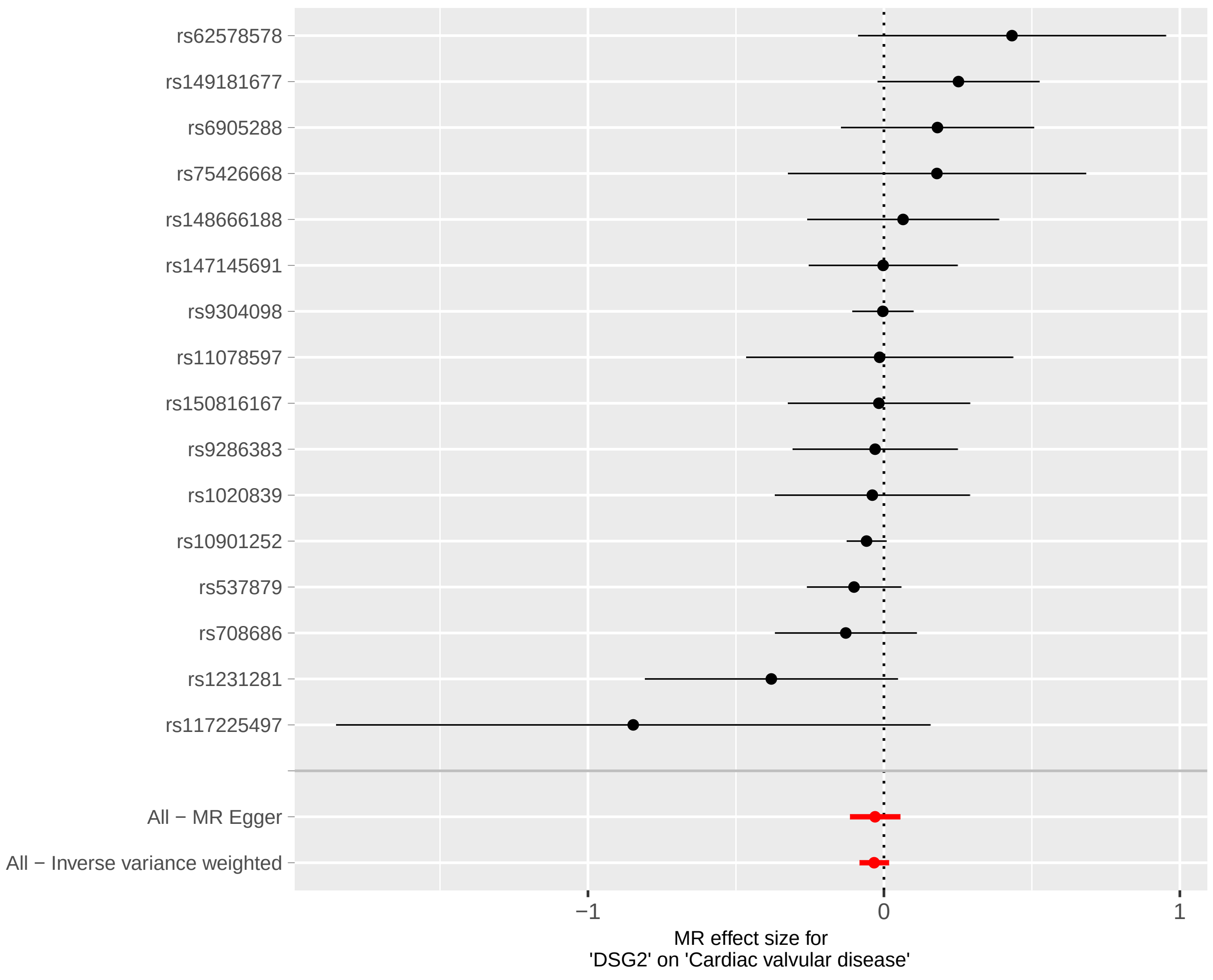

rs62578578
rs149181677
rs6905288
rs75426668
rs148666188
rs147145691
rs9304098
rs11078597
rs150816167
rs9286383
rs1020839
rs10901252
rs537879
rs708686
rs1231281
rs117225497
All − MR Egger
All − Inverse variance weighted
−1
0
1
MR effect size for
'DSG2' on 'Cardiac valvular disease'

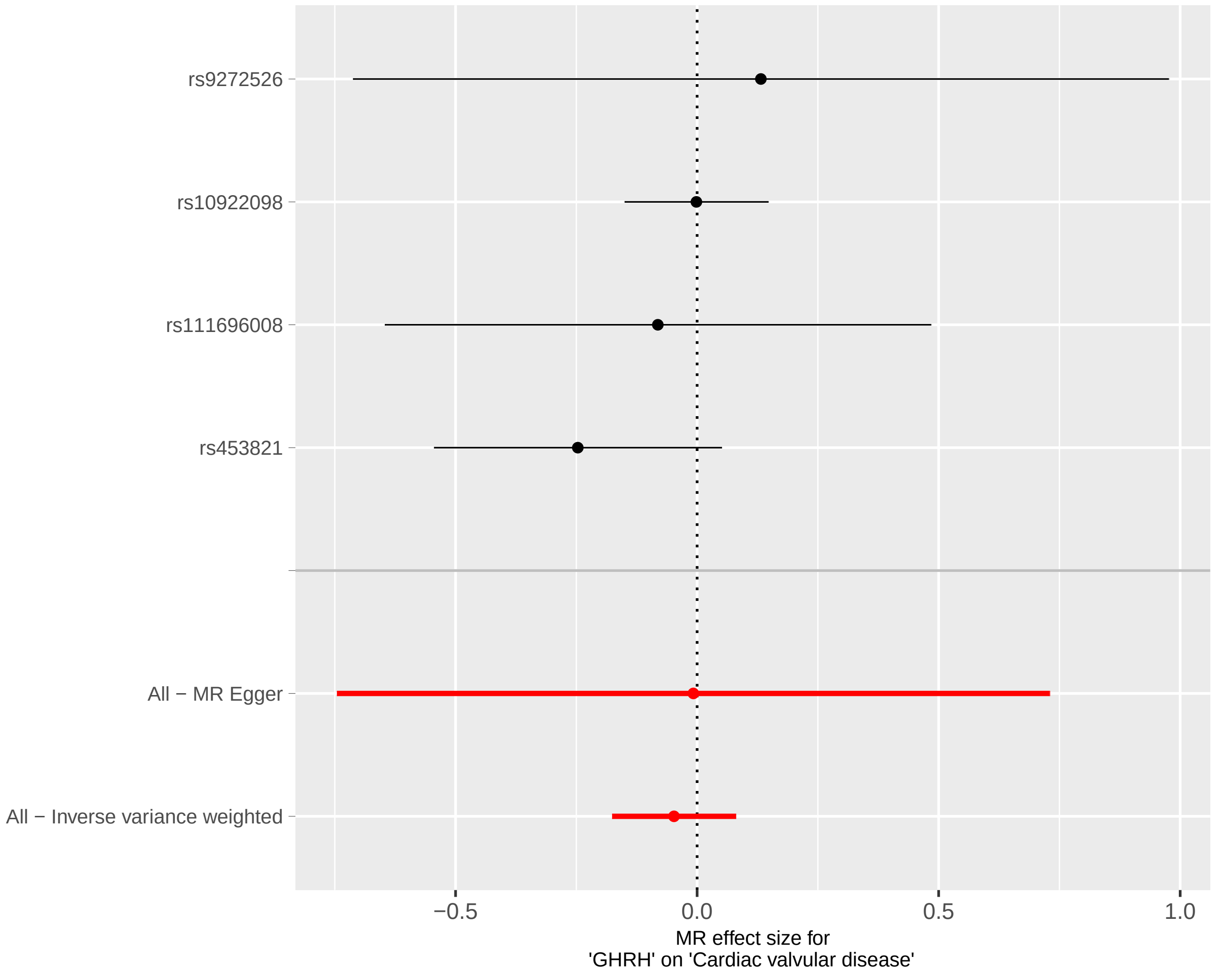
rs9272526
rs10922098
rs111696008
rs453821
All − MR Egger
All − Inverse variance weighted
−0.5
0.0
0.5
1.0
MR effect size for
'GHRH' on 'Cardiac valvular disease'

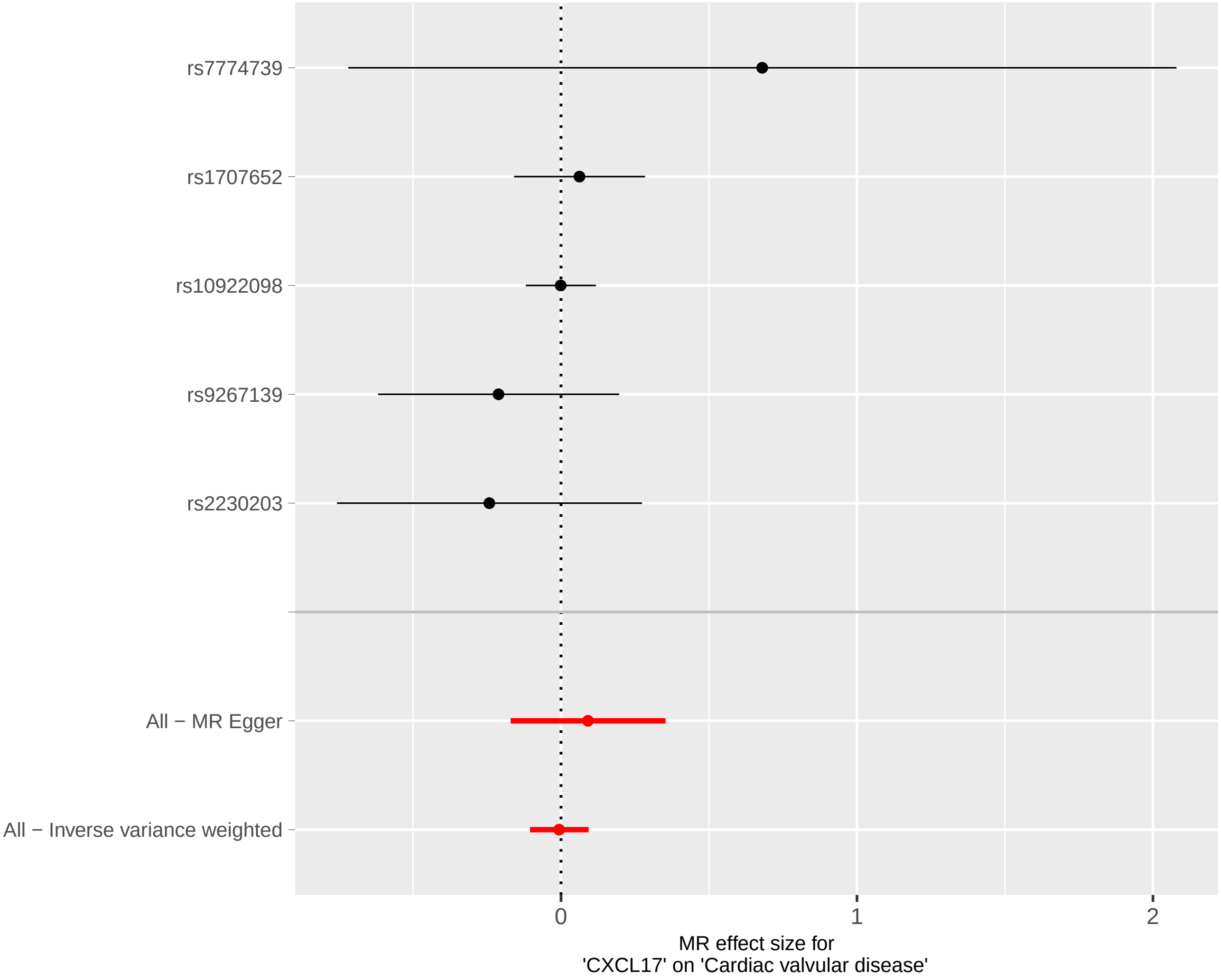

rs7774739
rs1707652
rs10922098
rs9267139
rs2230203
All − MR Egger
All − Inverse variance weighted
0
1
2
MR effect size for
'CXCL17' on 'Cardiac valvular disease'

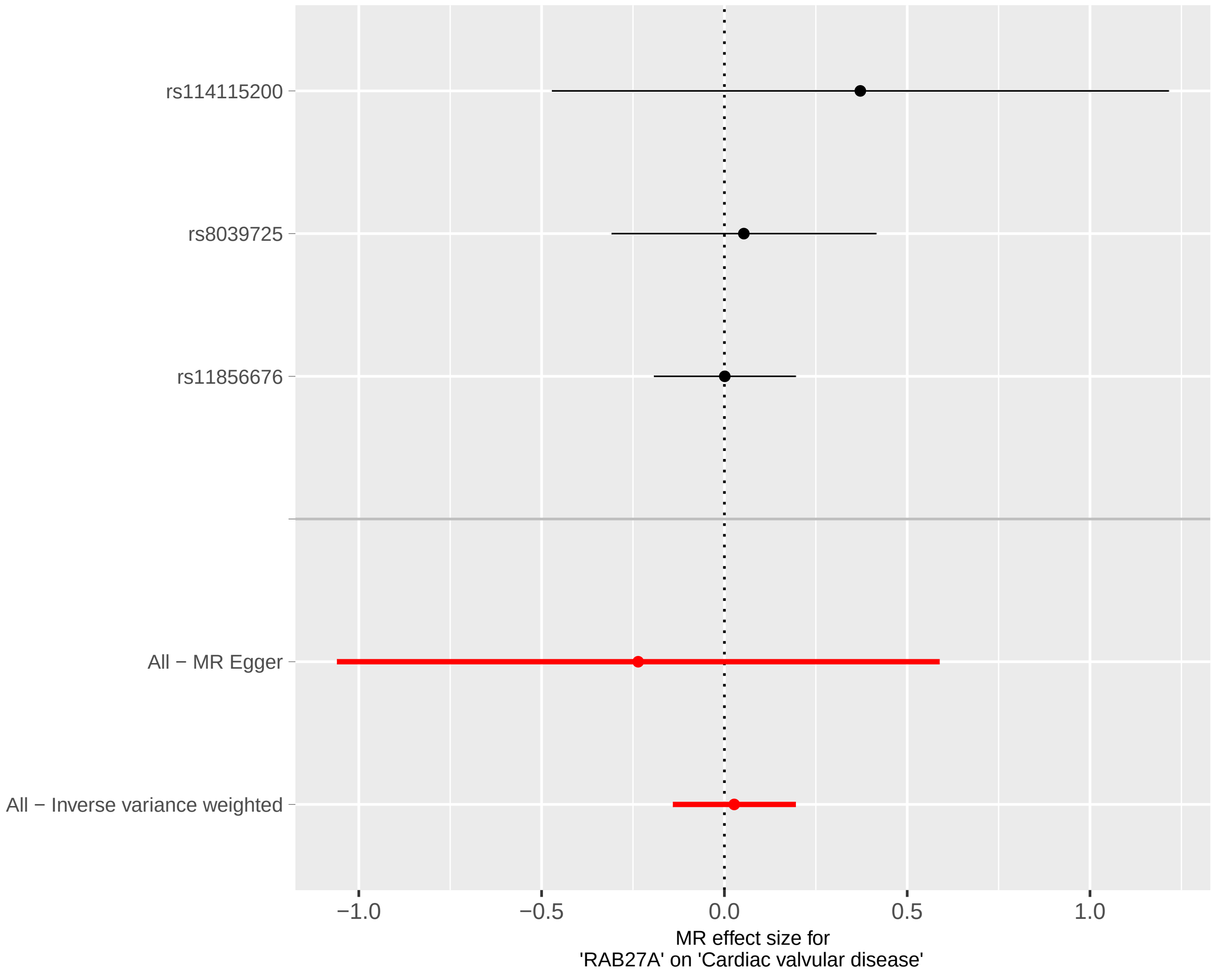
rs114115200
rs8039725
rs11856676
All − MR Egger
All − Inverse variance weighted
−1.0
−0.5
0.0
0.5
1.0
MR effect size for
'RAB27A' on 'Cardiac valvular disease'

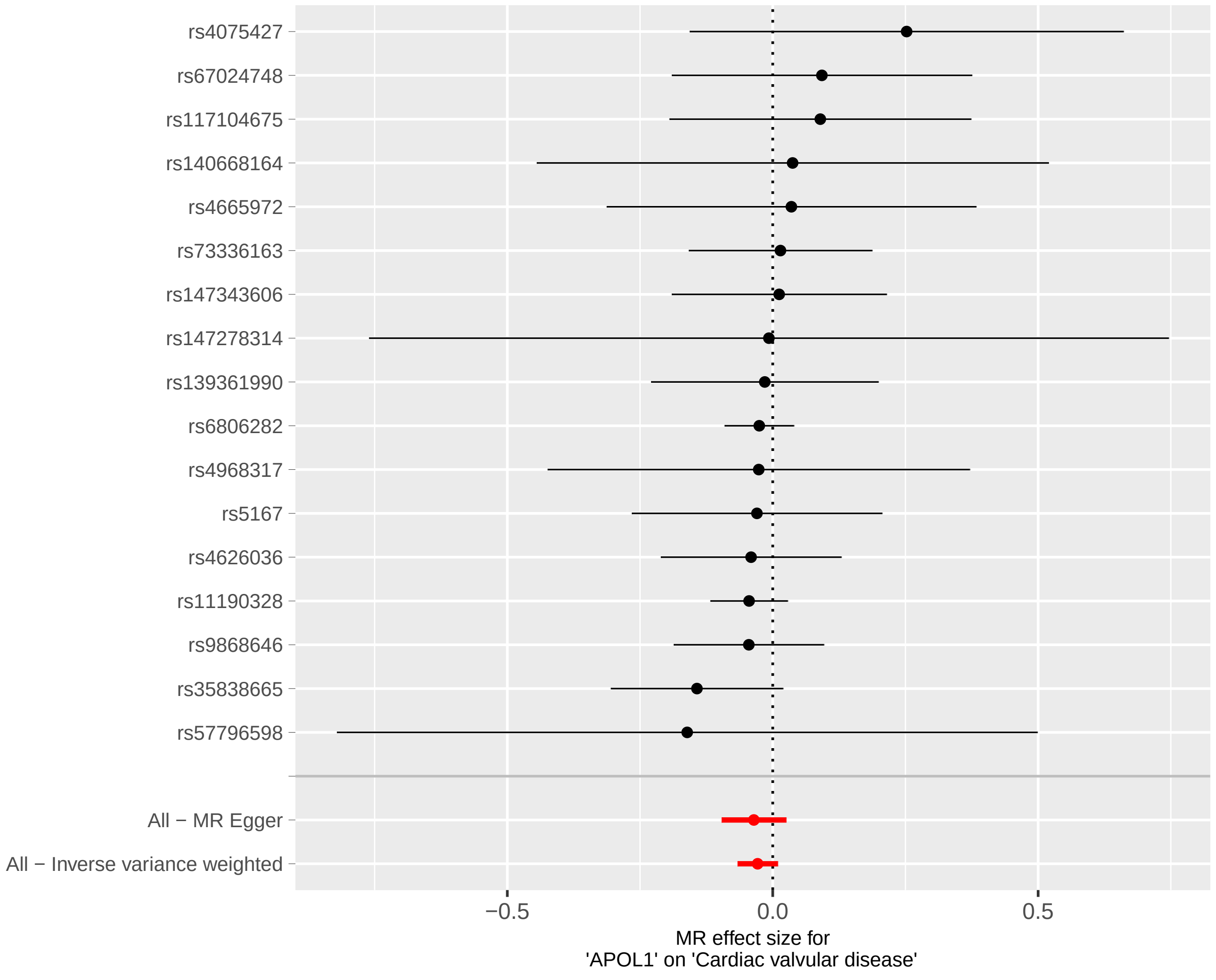
rs4075427
rs67024748
rs117104675
rs140668164
rs4665972
rs73336163
rs147343606
rs147278314
rs139361990
rs6806282
rs4968317
rs5167
rs4626036
rs11190328
rs9868646
rs35838665
rs57796598
All − MR Egger
All − Inverse variance weighted
−0.5
0.0
0.5
MR effect size for
'APOL1' on 'Cardiac valvular disease'

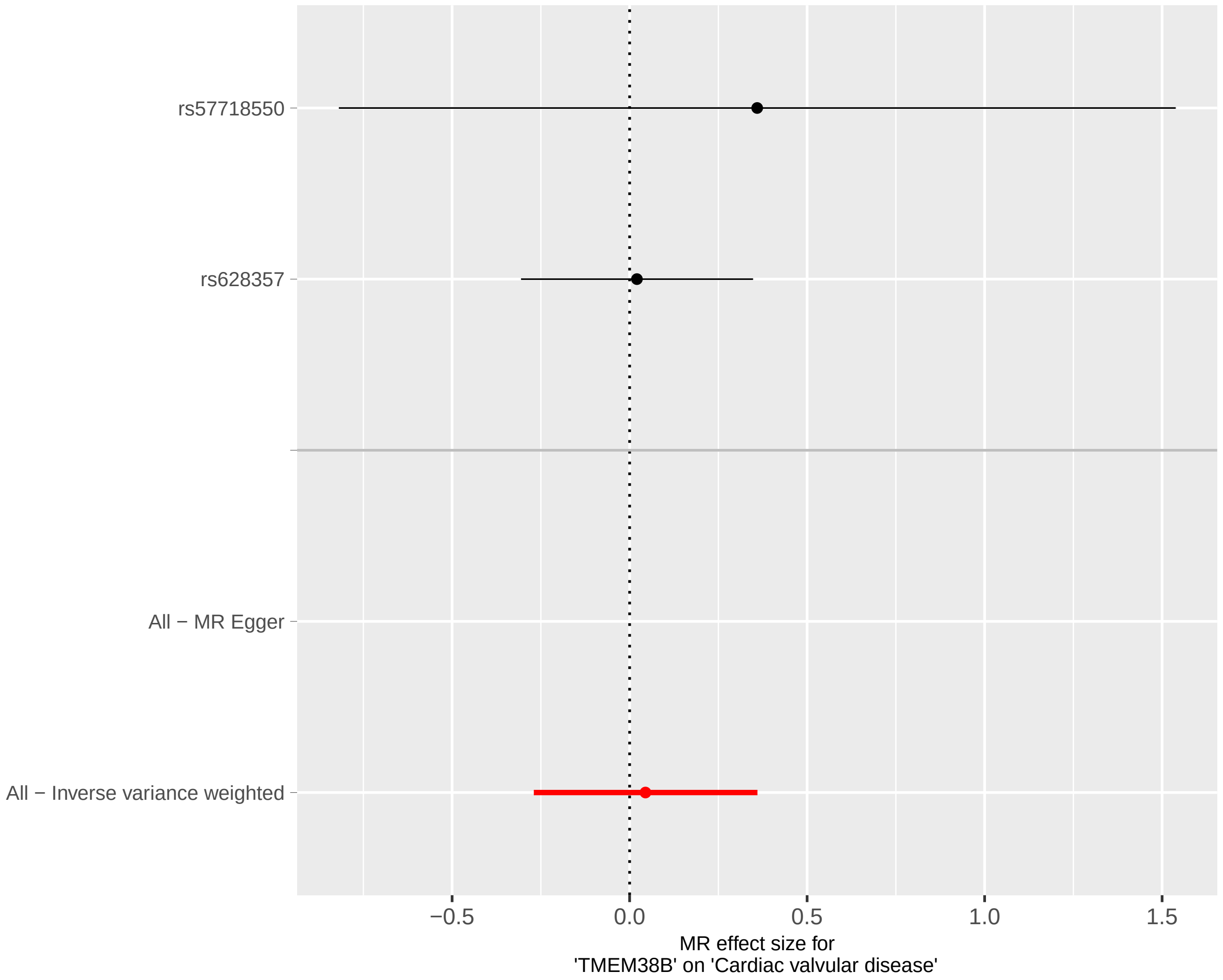
rs57718550
rs628357
All − MR Egger
All − Inverse variance weighted
−0.5
0.0
0.5
1.0
1.5
MR effect size for
'TMEM38B' on 'Cardiac valvular disease'

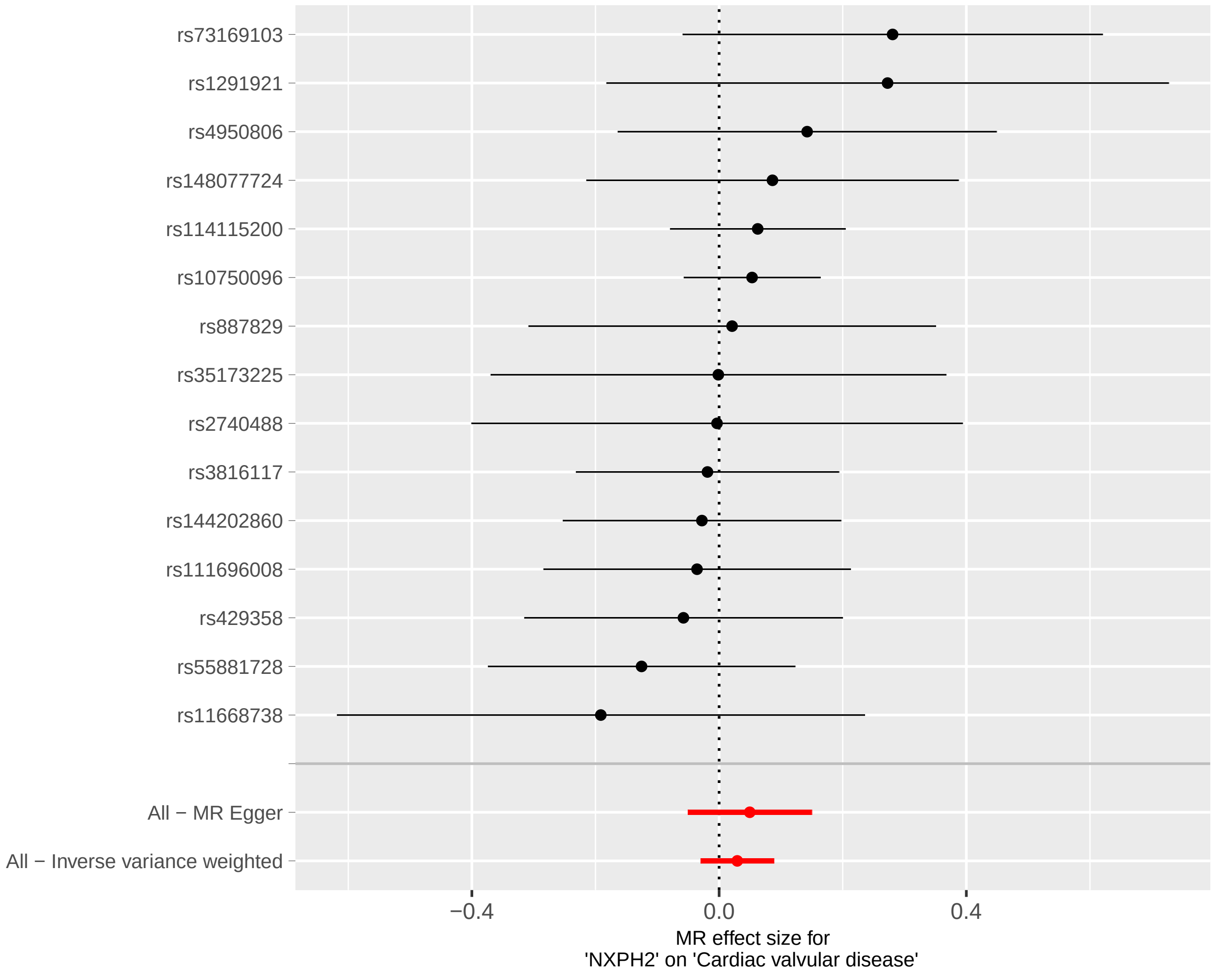

rs73169103
rs1291921
rs4950806
rs148077724
rs114115200
rs10750096
rs887829
rs35173225
rs2740488
rs3816117
rs144202860
rs111696008
rs429358
rs55881728
rs11668738
All − MR Egger
All − Inverse variance weighted
−0.4
0.0
0.4
MR effect size for
'NXPH2' on 'Cardiac valvular disease'

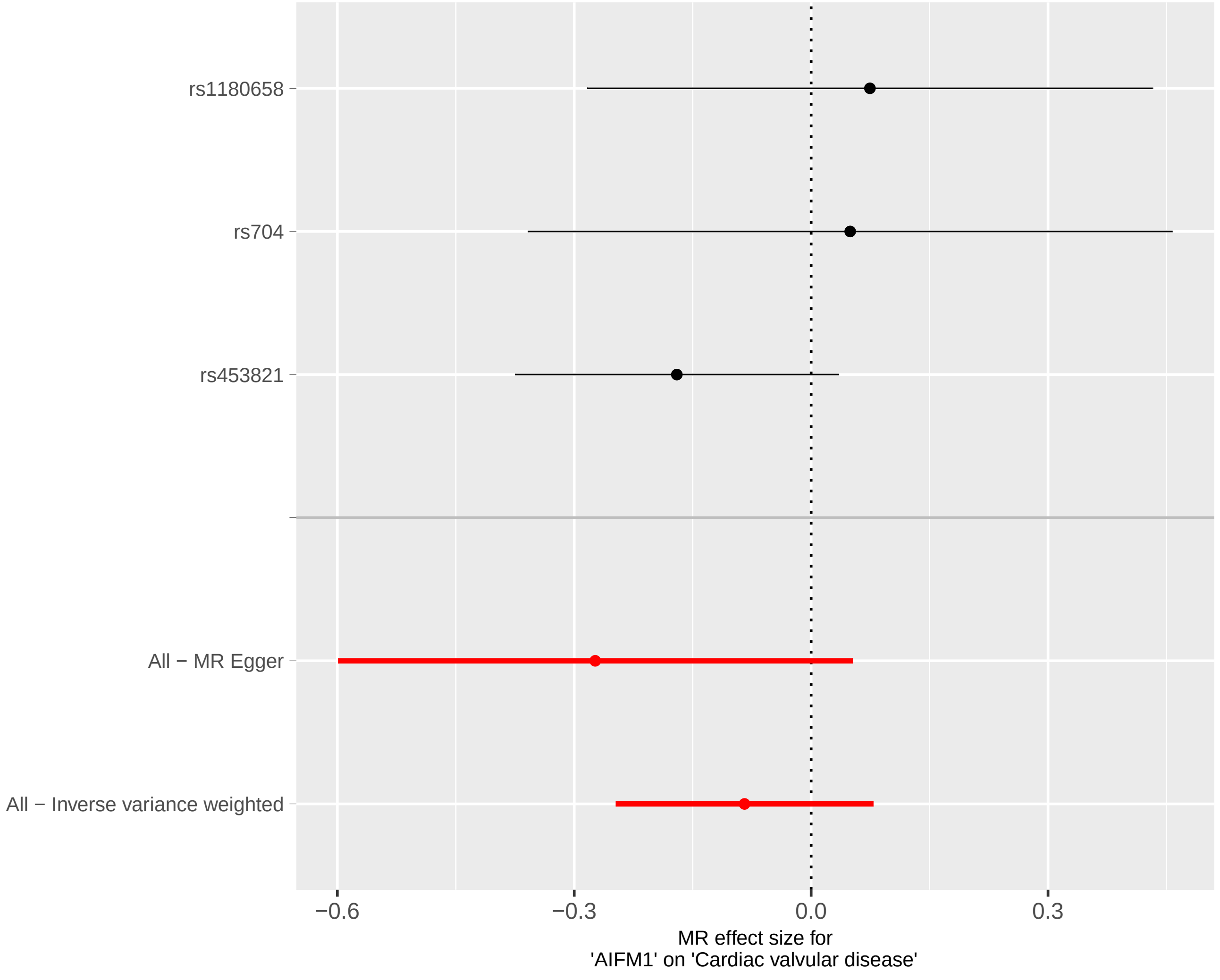
rs1180658
rs704
rs453821
All − MR Egger
All − Inverse variance weighted
−0.6
−0.3
0.0
0.3
MR effect size for
'AIFM1' on 'Cardiac valvular disease'

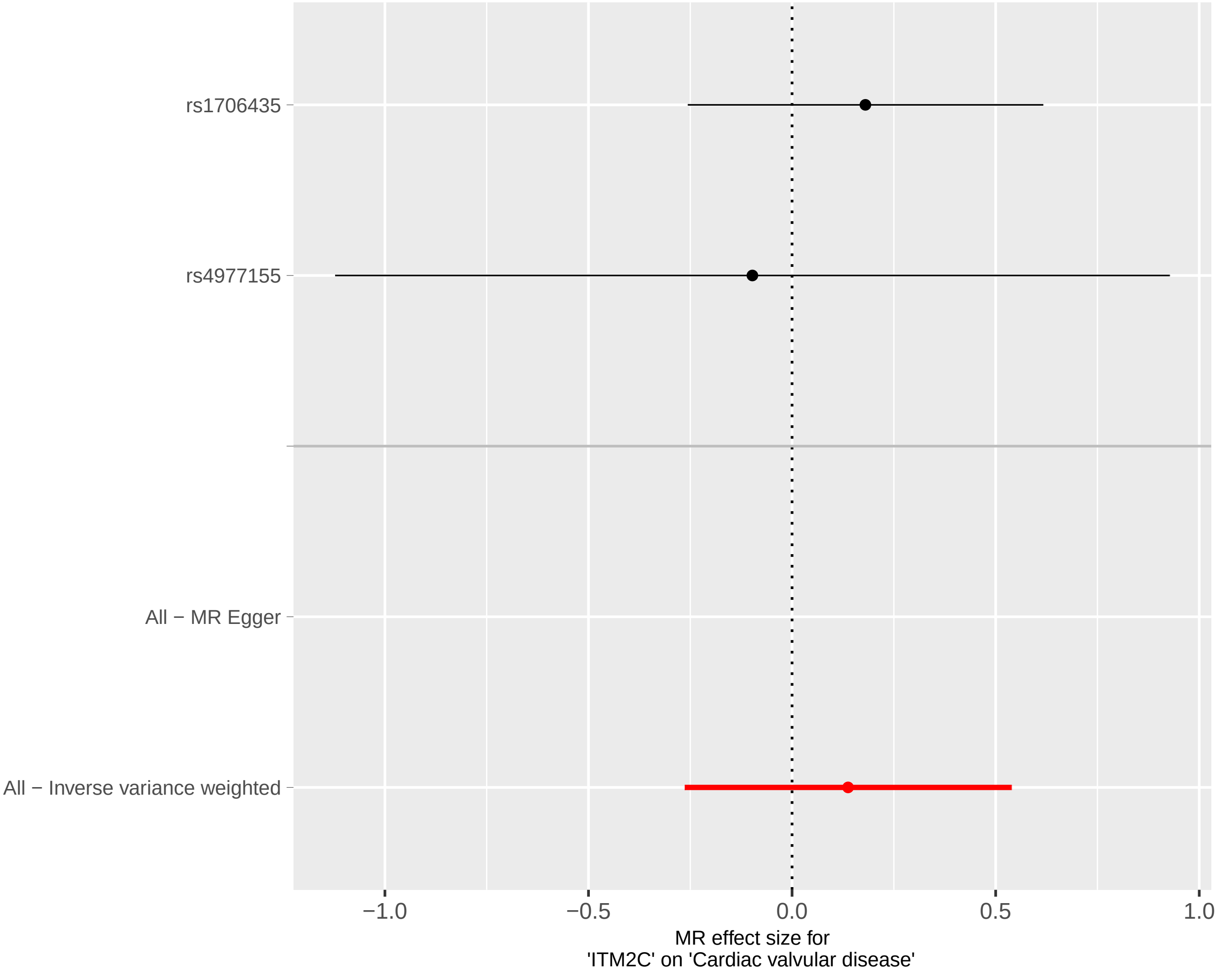

rs1706435
rs4977155
All − MR Egger
All − Inverse variance weighted
−1.0
−0.5
0.0
0.5
1.0
MR effect size for
'ITM2C' on 'Cardiac valvular disease'

Insufficient number of SNPs

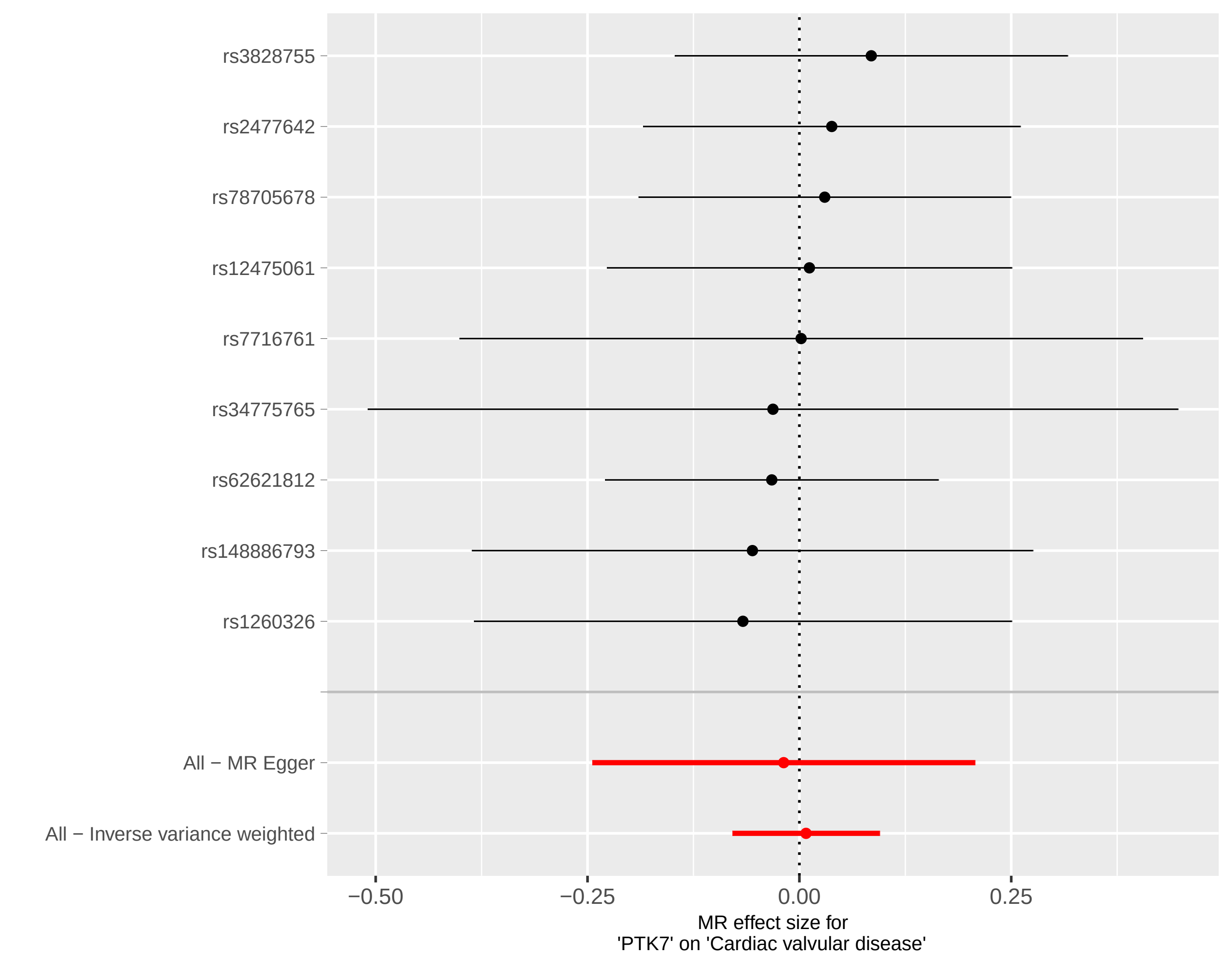
rs3828755
rs2477642
rs78705678
rs12475061
rs7716761
rs34775765
rs62621812
rs148886793
rs1260326
All − MR Egger
All − Inverse variance weighted
−0.50
−0.25
0.00
0.25
MR effect size for
'PTK7' on 'Cardiac valvular disease'

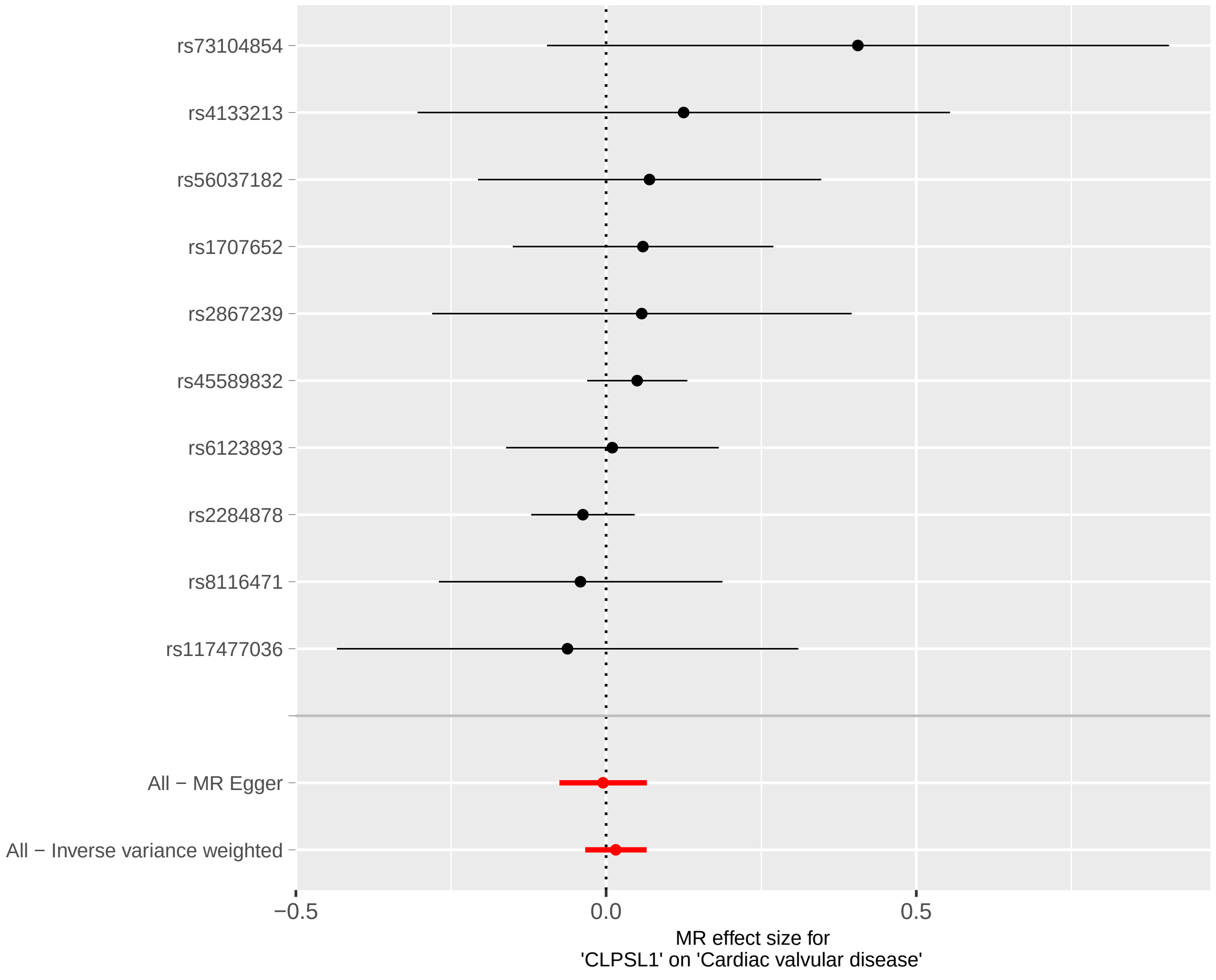
rs73104854
rs4133213
rs56037182
rs1707652
rs2867239
rs45589832
rs6123893
rs2284878
rs8116471
rs117477036
All − MR Egger
All − Inverse variance weighted
−0.5
0.0
0.5
MR effect size for
'CLPSL1' on 'Cardiac valvular disease'

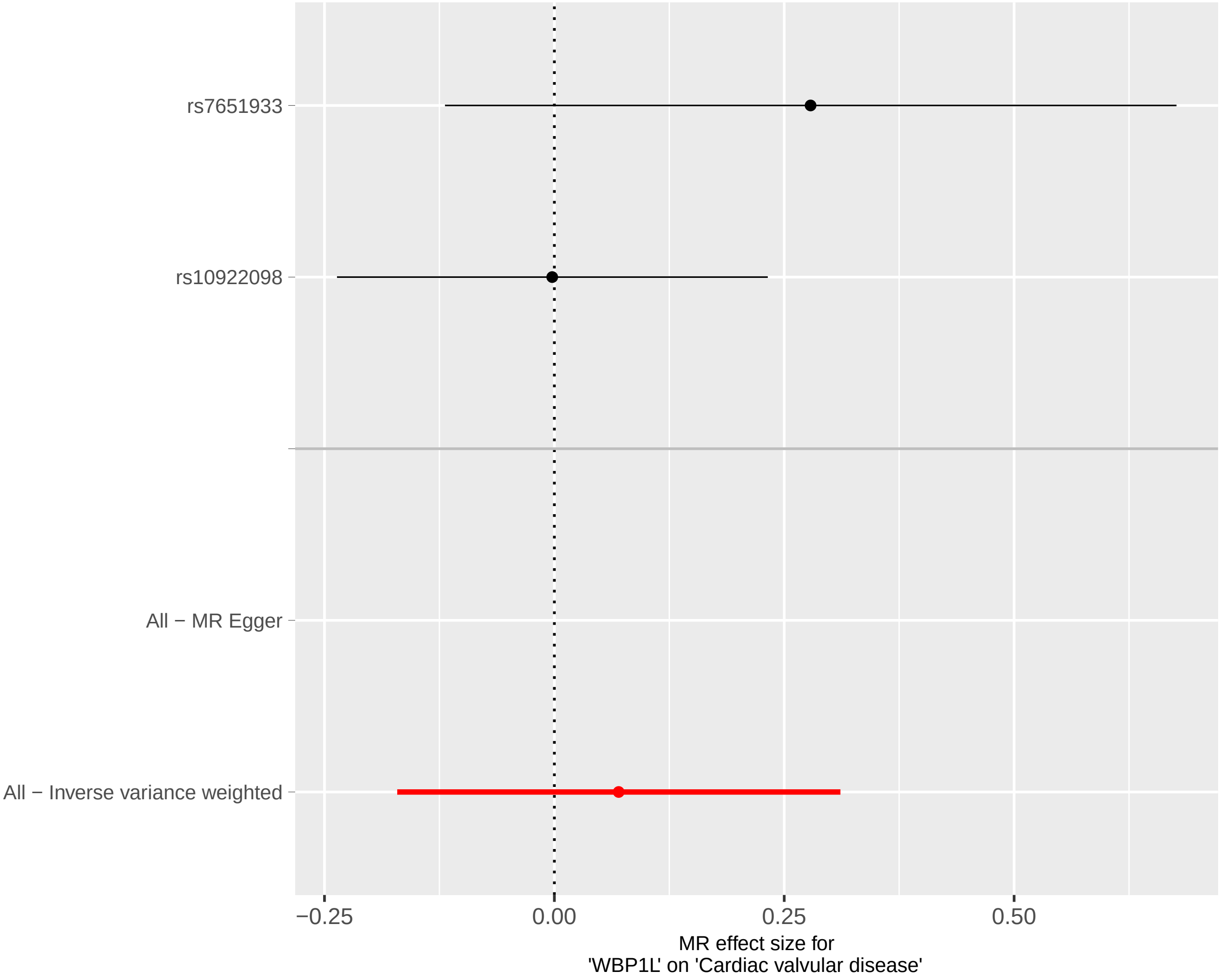
rs7651933
rs10922098
All − MR Egger
All − Inverse variance weighted
−0.25
0.00
0.25
0.50
MR effect size for
'WBP1L' on 'Cardiac valvular disease'

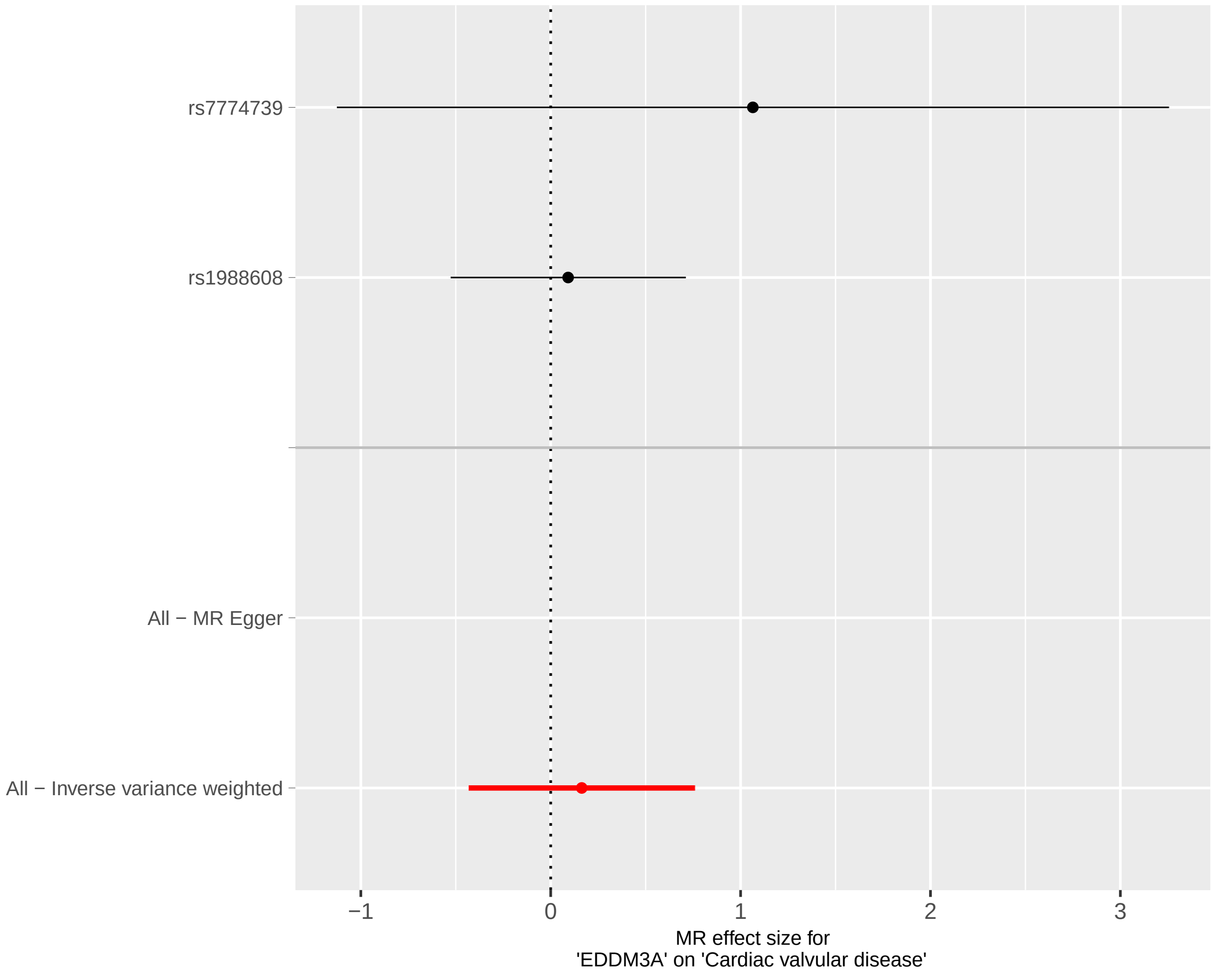

rs7774739
rs1988608
All − MR Egger
All − Inverse variance weighted
−1
0
1
2
3
MR effect size for
'EDDM3A' on 'Cardiac valvular disease'

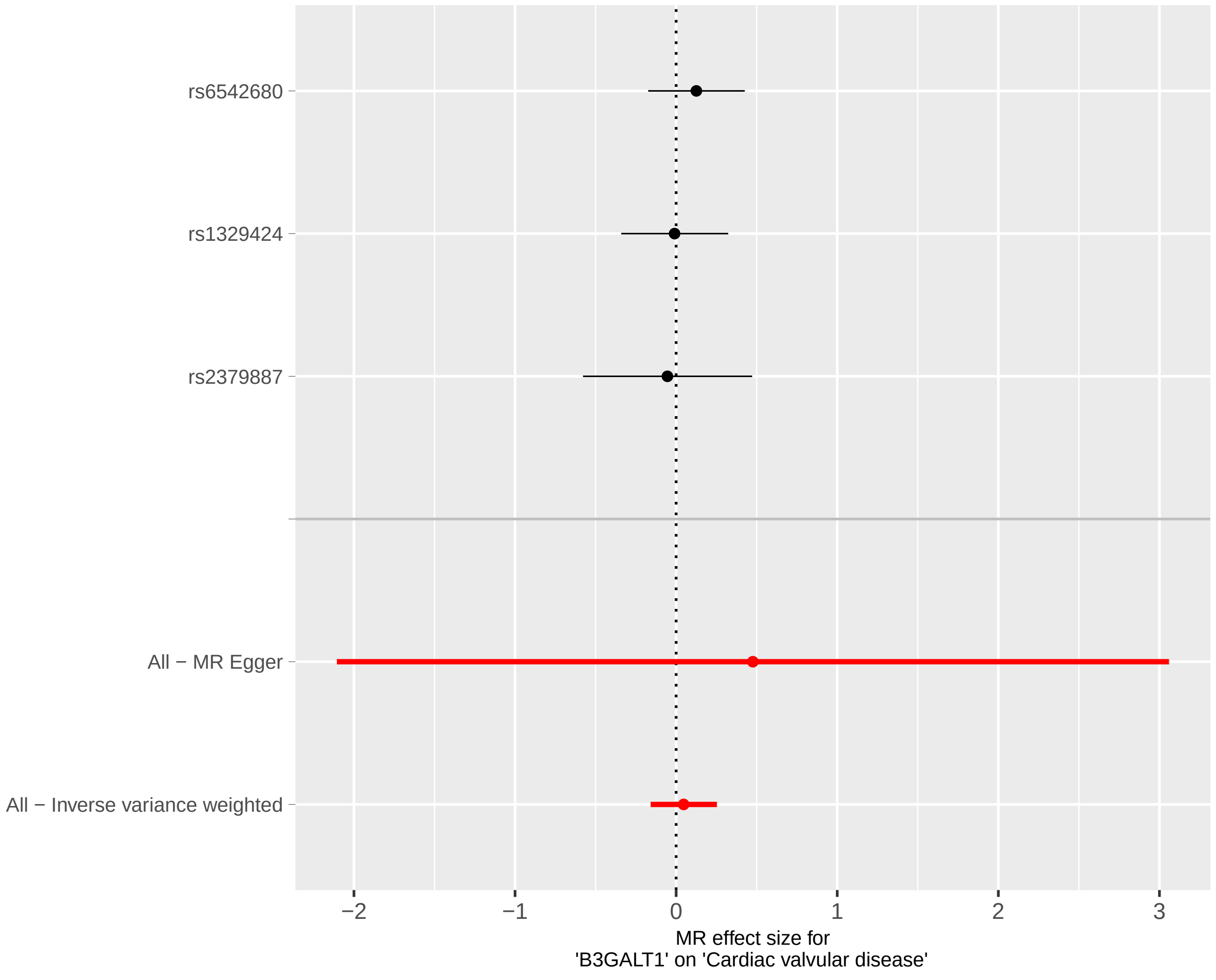

rs6542680
rs1329424
rs2379887
All − MR Egger
All − Inverse variance weighted
−2
−1
0
1
2
3
MR effect size for
'B3GALT1' on 'Cardiac valvular disease'

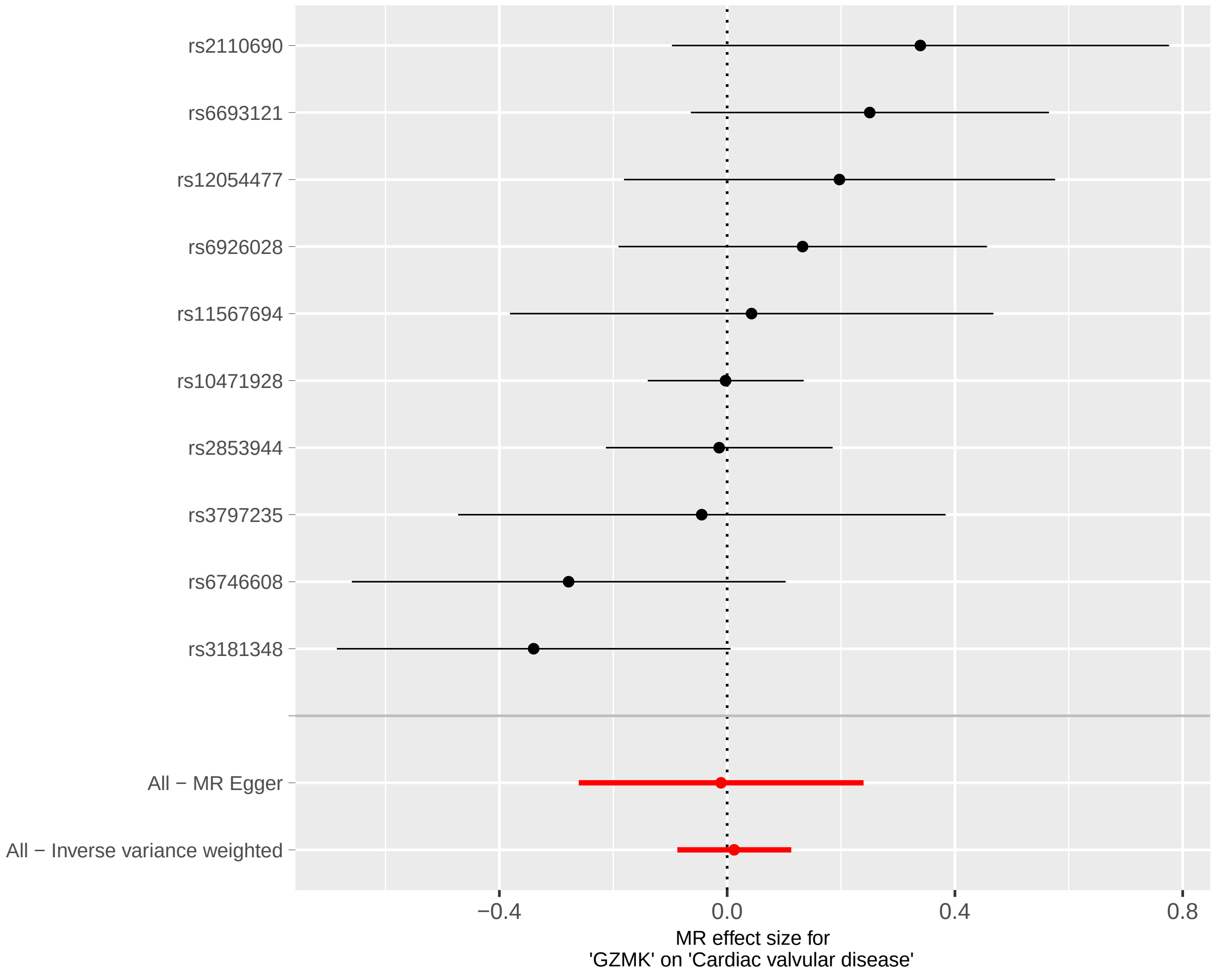

rs2110690
rs6693121
rs12054477
rs6926028
rs11567694
rs10471928
rs2853944
rs3797235
rs6746608
rs3181348
All − MR Egger
All − Inverse variance weighted
−0.4
0.0
0.4
0.8
MR effect size for
'GZMK' on 'Cardiac valvular disease'

Insufficient number of SNPs

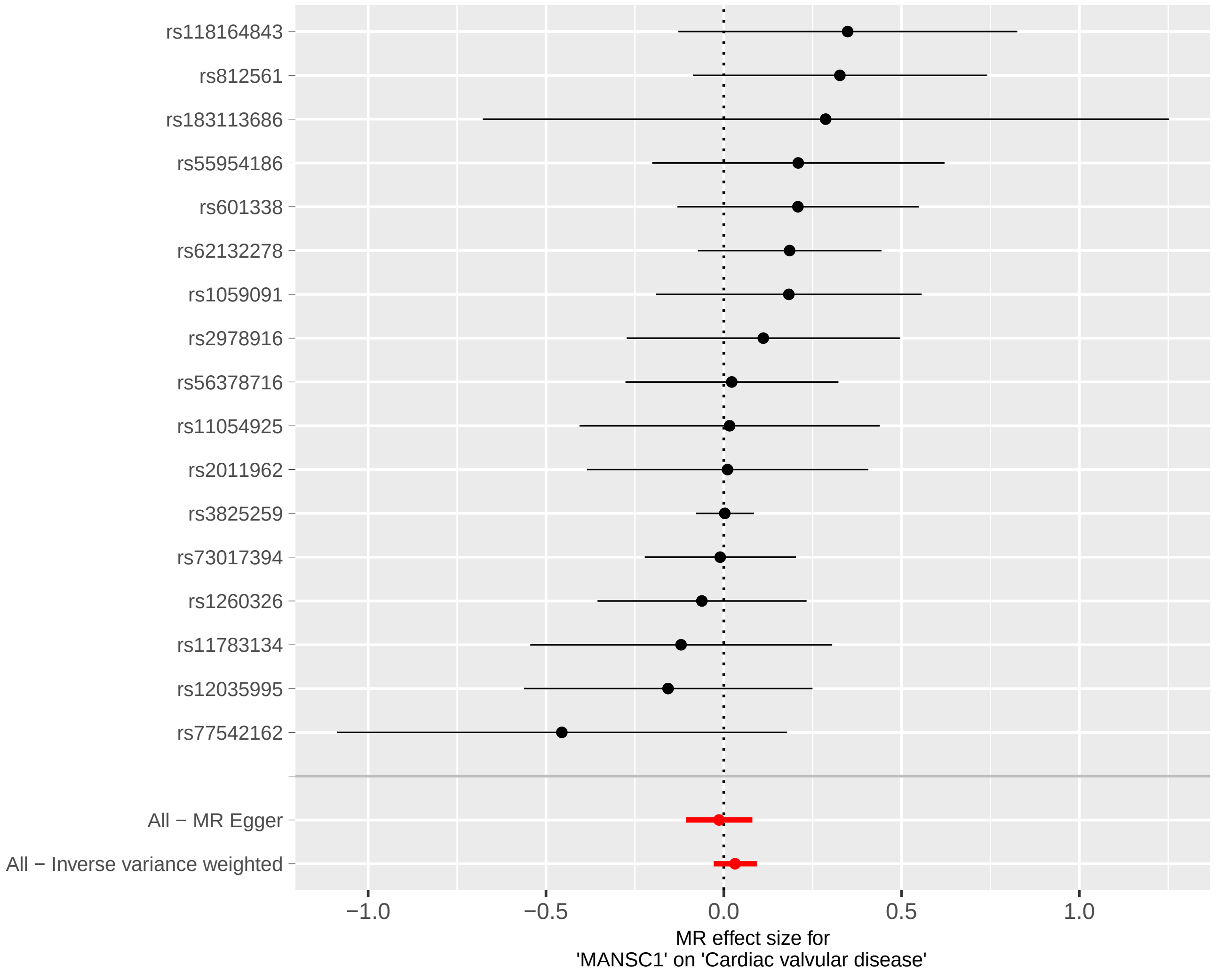
rs118164843
rs812561
rs183113686
rs55954186
rs601338
rs62132278
rs1059091
rs2978916
rs56378716
rs11054925
rs2011962
rs3825259
rs73017394
rs1260326
rs11783134
rs12035995
rs77542162
All − MR Egger
All − Inverse variance weighted
−1.0
−0.5
0.0
0.5
1.0
MR effect size for
'MANSC1' on 'Cardiac valvular disease'

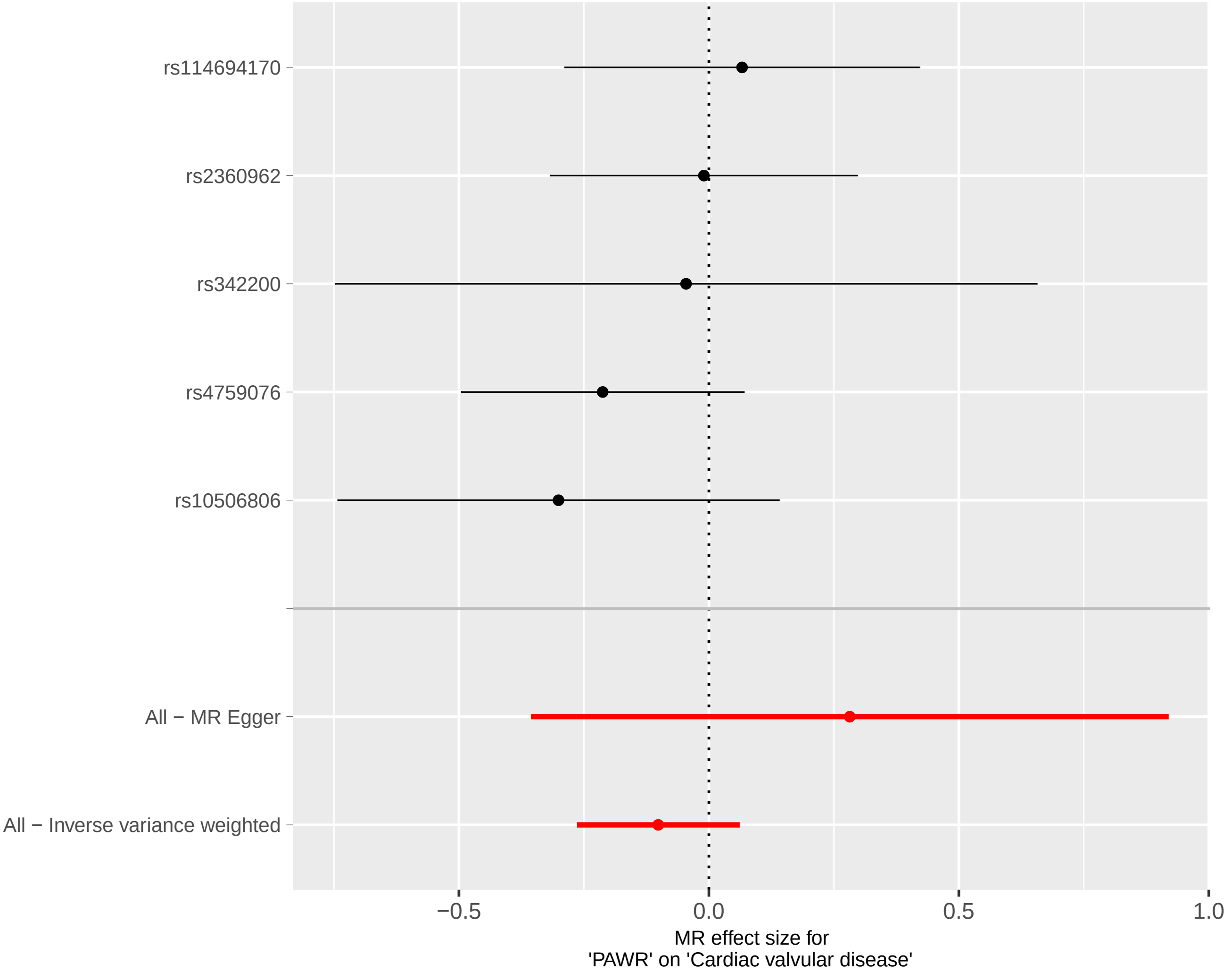
rs114694170
rs2360962
rs342200
rs4759076
rs10506806
All − MR Egger
All − Inverse variance weighted
−0.5
0.0
0.5
1.0
MR effect size for
'PAWR' on 'Cardiac valvular disease'

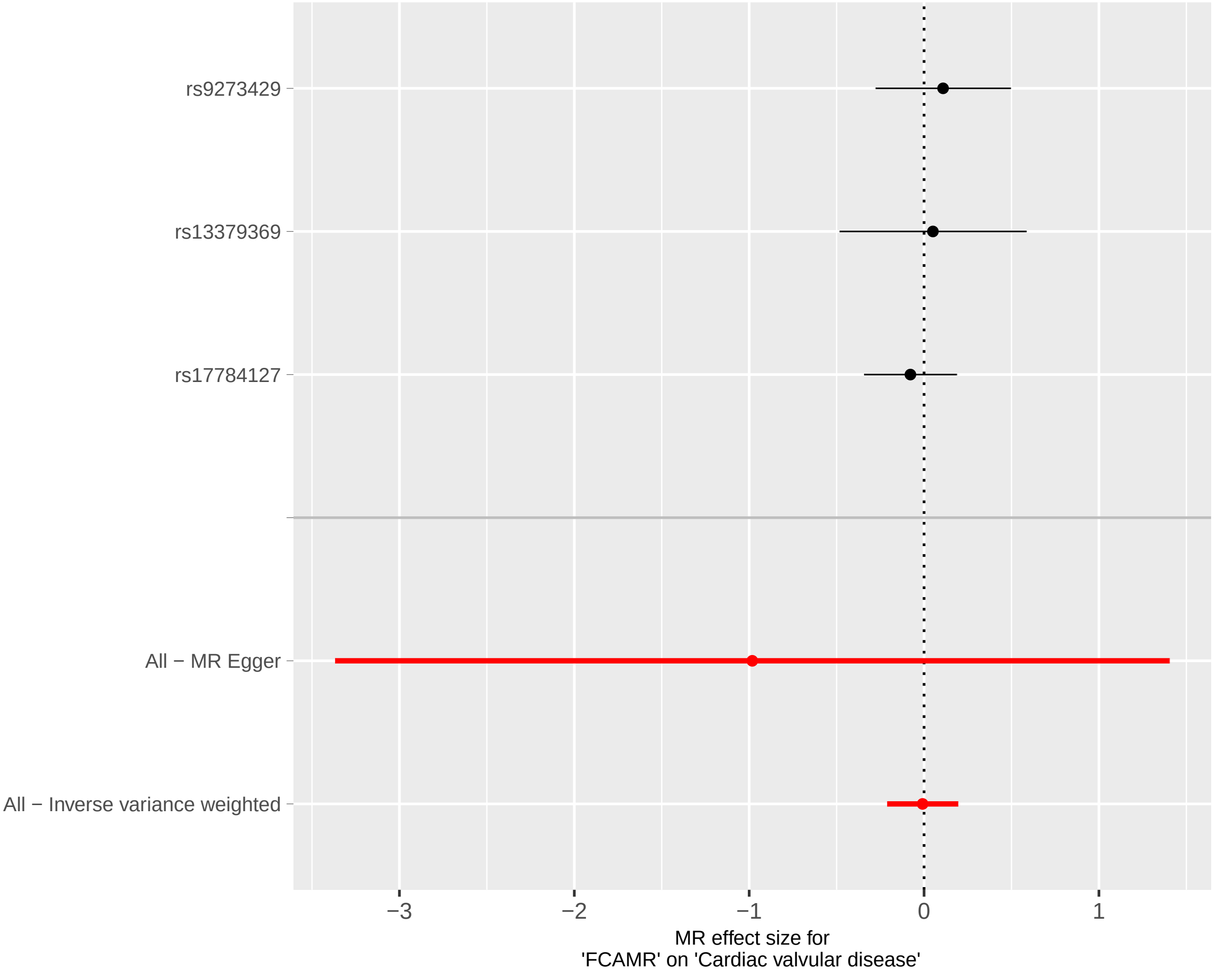

rs9273429
rs13379369
rs17784127
All − MR Egger
All − Inverse variance weighted
−3
−2
−1
0
1
MR effect size for
'FCAMR' on 'Cardiac valvular disease'

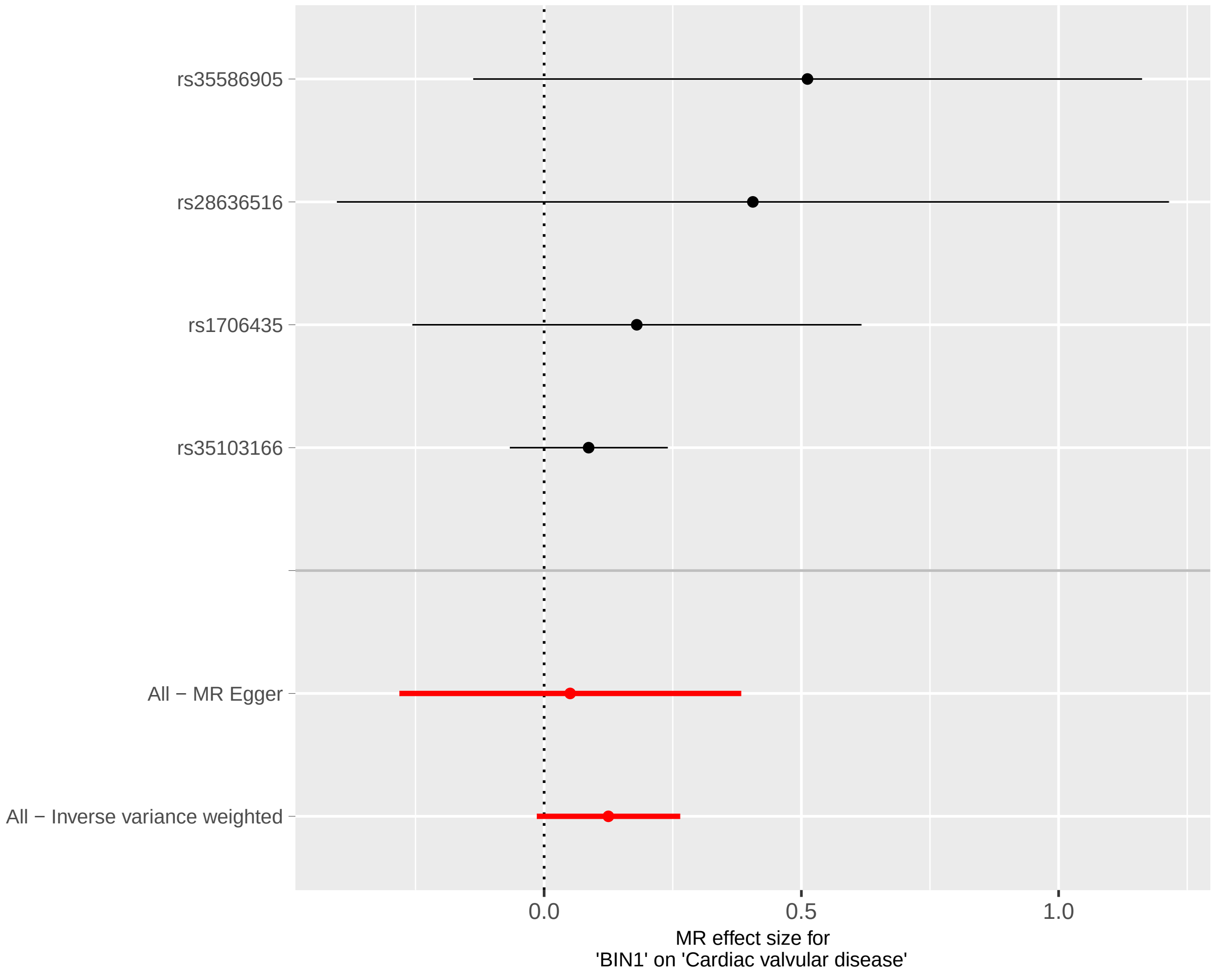
rs35586905
rs28636516
rs1706435
rs35103166
All − MR Egger
All − Inverse variance weighted
0.0
0.5
1.0
MR effect size for
'BIN1' on 'Cardiac valvular disease'

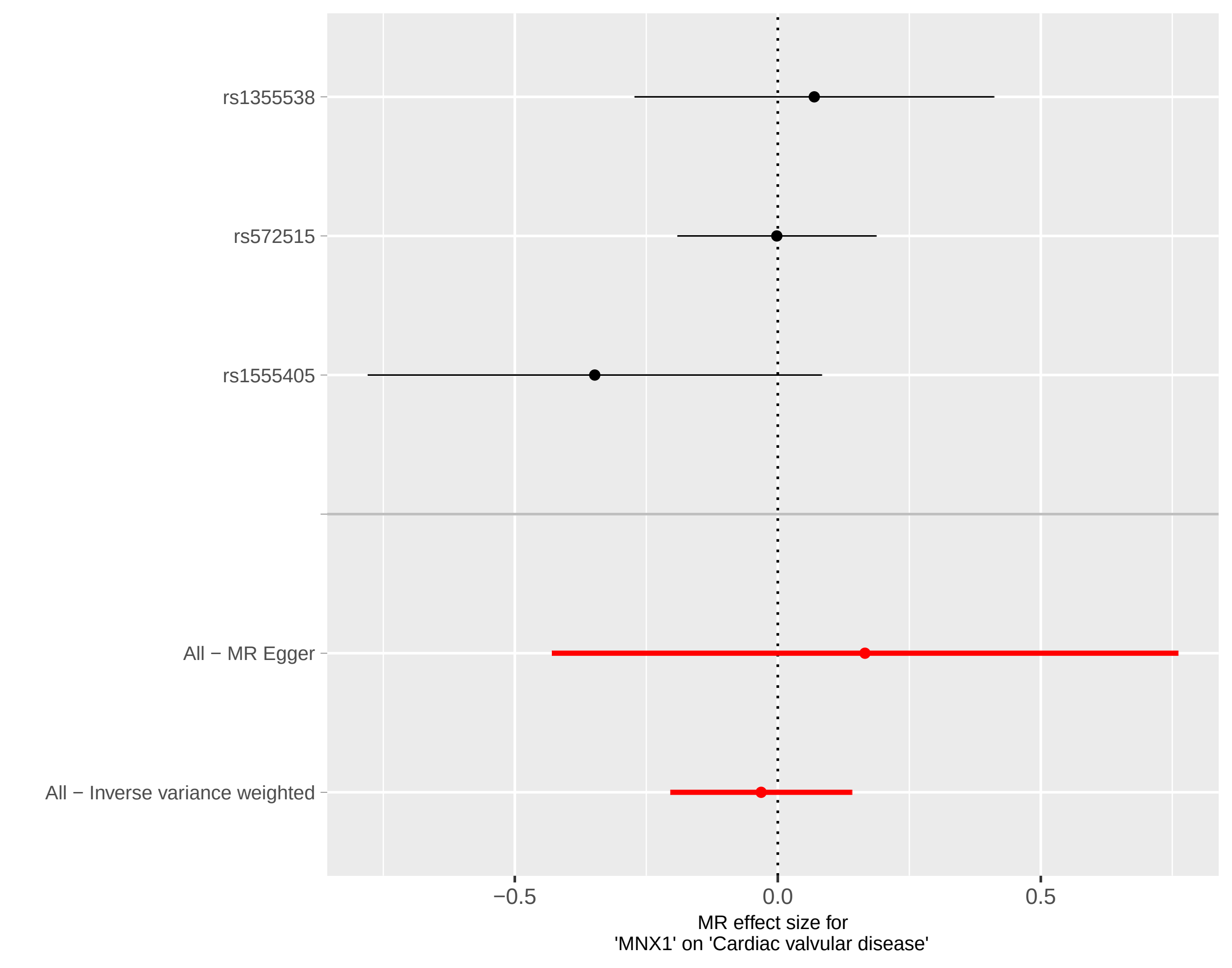
rs1355538
rs572515
rs1555405
All − MR Egger
All − Inverse variance weighted
−0.5
0.0
0.5
MR effect size for
'MNX1' on 'Cardiac valvular disease'

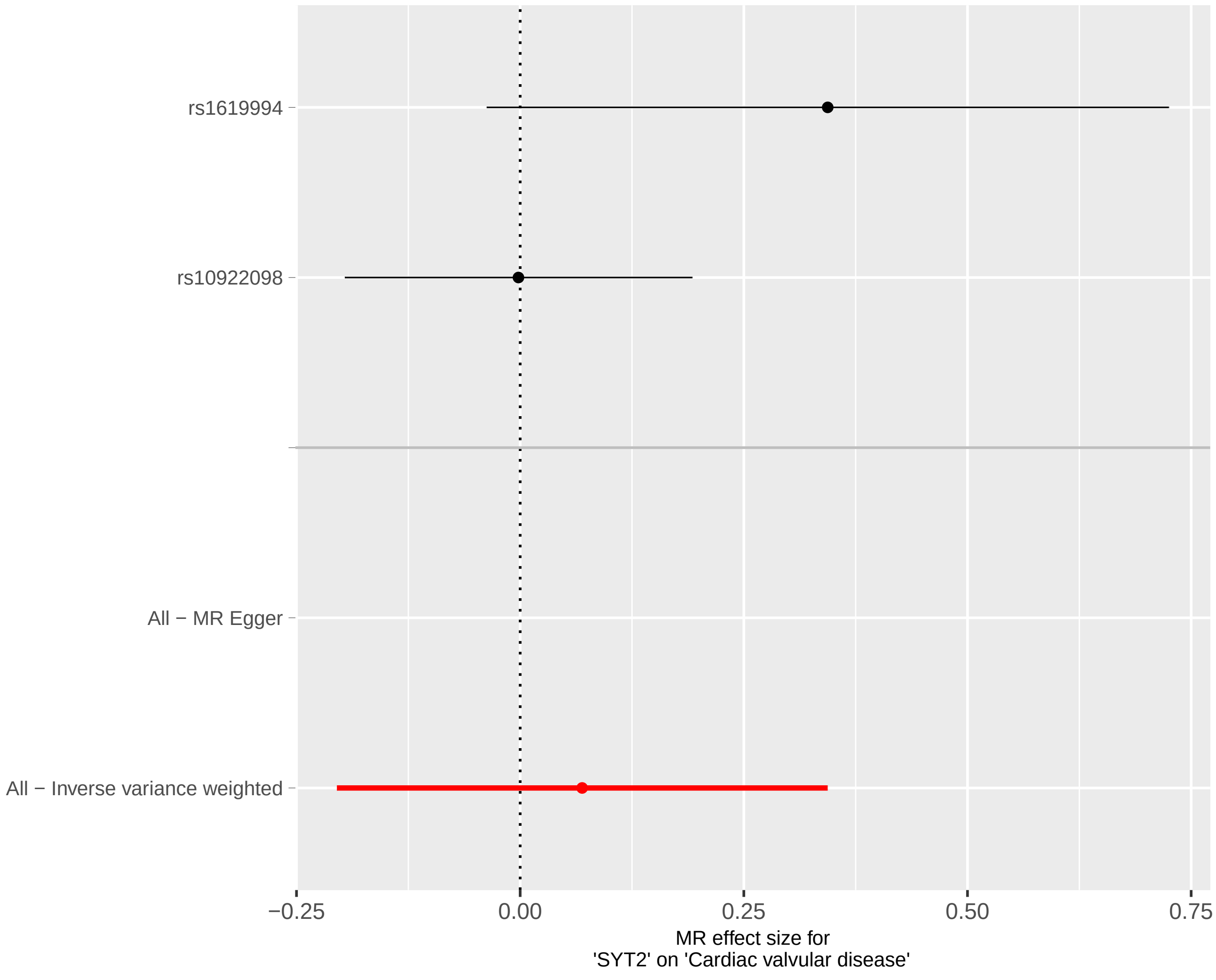
rs1619994
rs10922098
All − MR Egger
All − Inverse variance weighted
−0.25
0.00
0.25
0.50
0.75
MR effect size for
'SYT2' on 'Cardiac valvular disease'

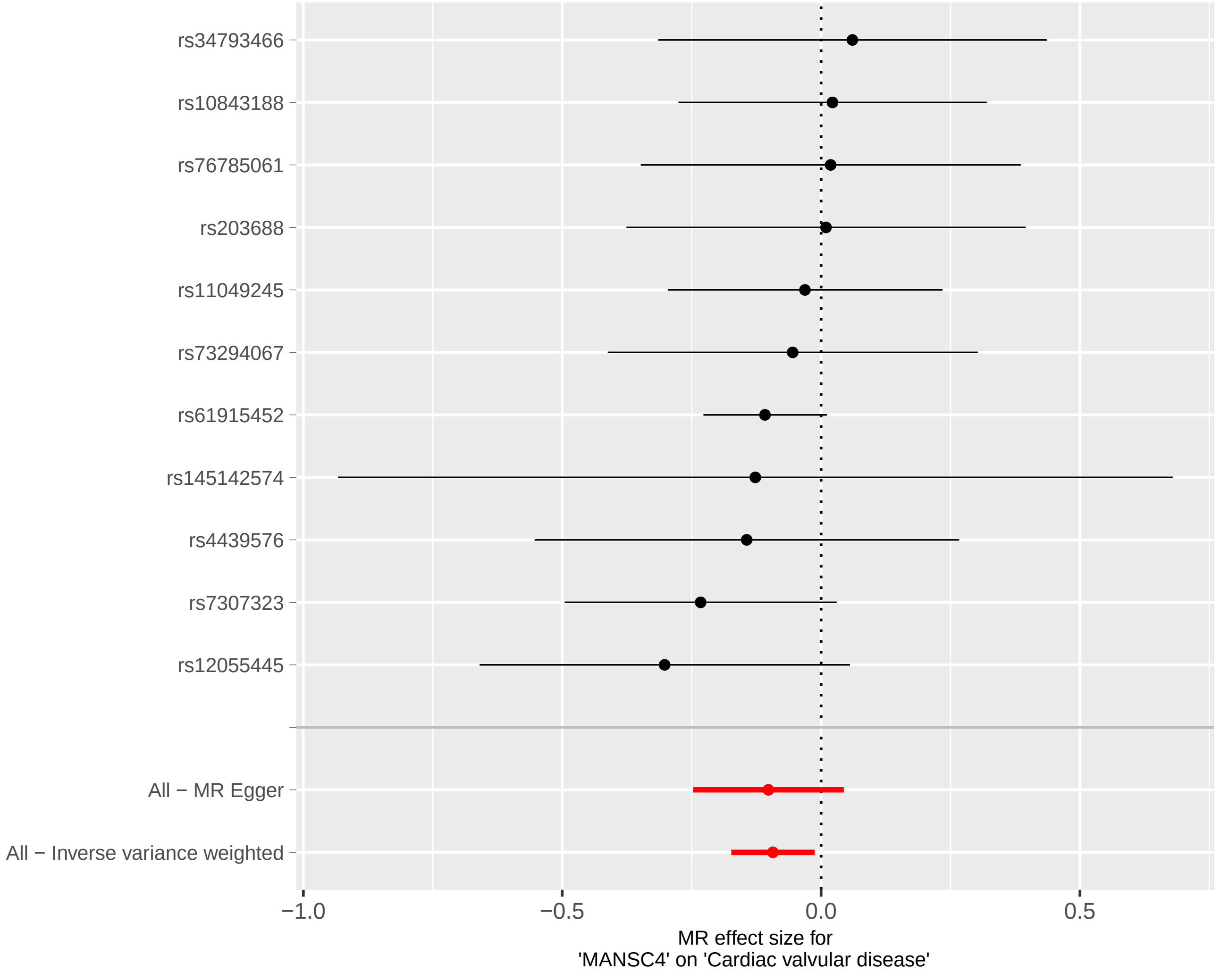
rs34793466
rs10843188
rs76785061
rs203688
rs11049245
rs73294067
rs61915452
rs145142574
rs4439576
rs7307323
rs12055445
All − MR Egger
All − Inverse variance weighted
−1.0
−0.5
0.0
0.5
MR effect size for
'MANSC4' on 'Cardiac valvular disease'

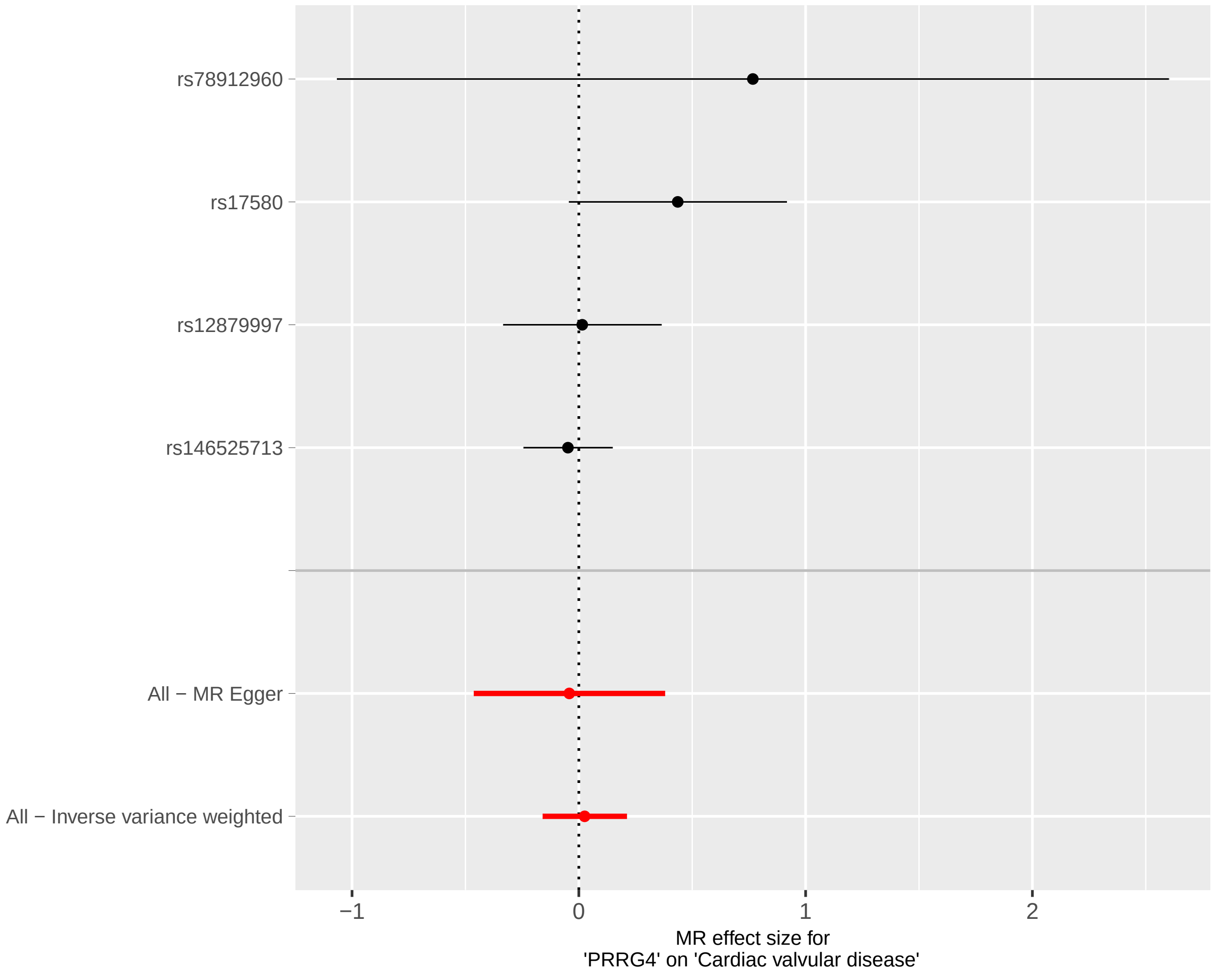
rs78912960
rs17580
rs12879997
rs146525713
All − MR Egger
All − Inverse variance weighted
−1
0
1
2
MR effect size for
'PRRG4' on 'Cardiac valvular disease'

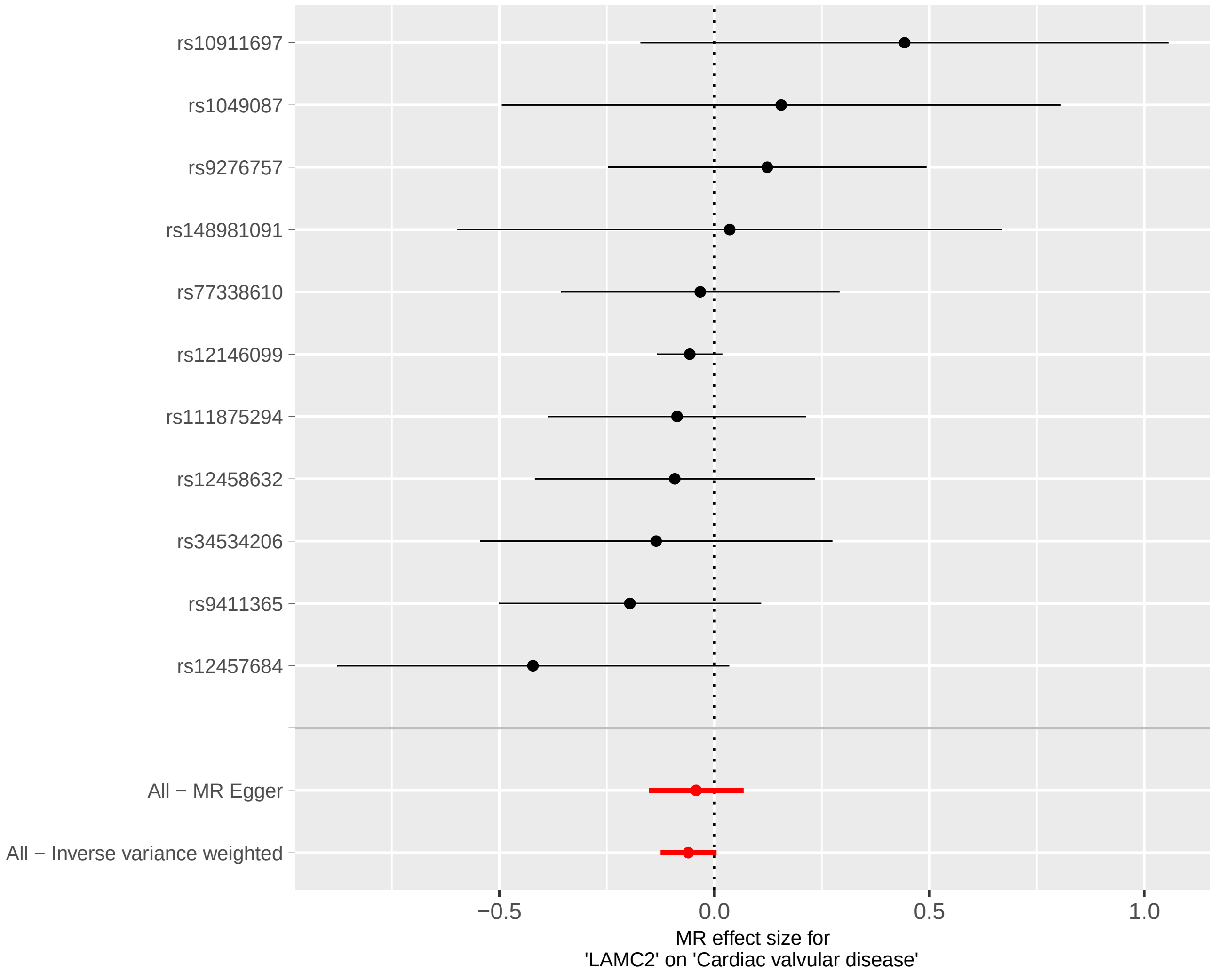
rs10911697
rs1049087
rs9276757
rs148981091
rs77338610
rs12146099
rs111875294
rs12458632
rs34534206
rs9411365
rs12457684
All − MR Egger
All − Inverse variance weighted
−0.5
0.0
0.5
1.0
MR effect size for
'LAMC2' on 'Cardiac valvular disease'

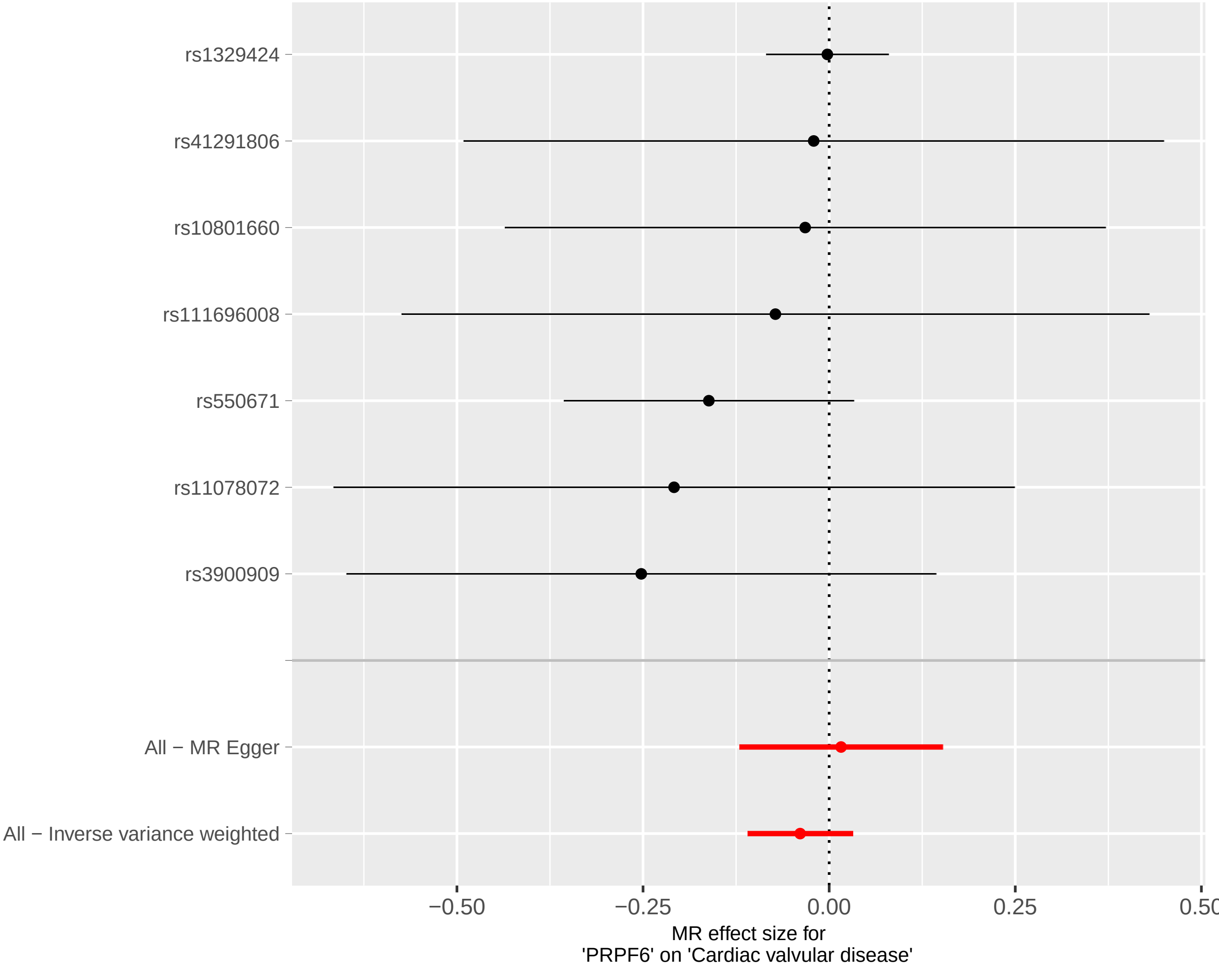
rs1329424
rs41291806
rs10801660
rs111696008
rs550671
rs11078072
rs3900909
All − MR Egger
All − Inverse variance weighted
−0.50
−0.25
0.00
0.25
0.50
MR effect size for
'PRPF6' on 'Cardiac valvular disease'

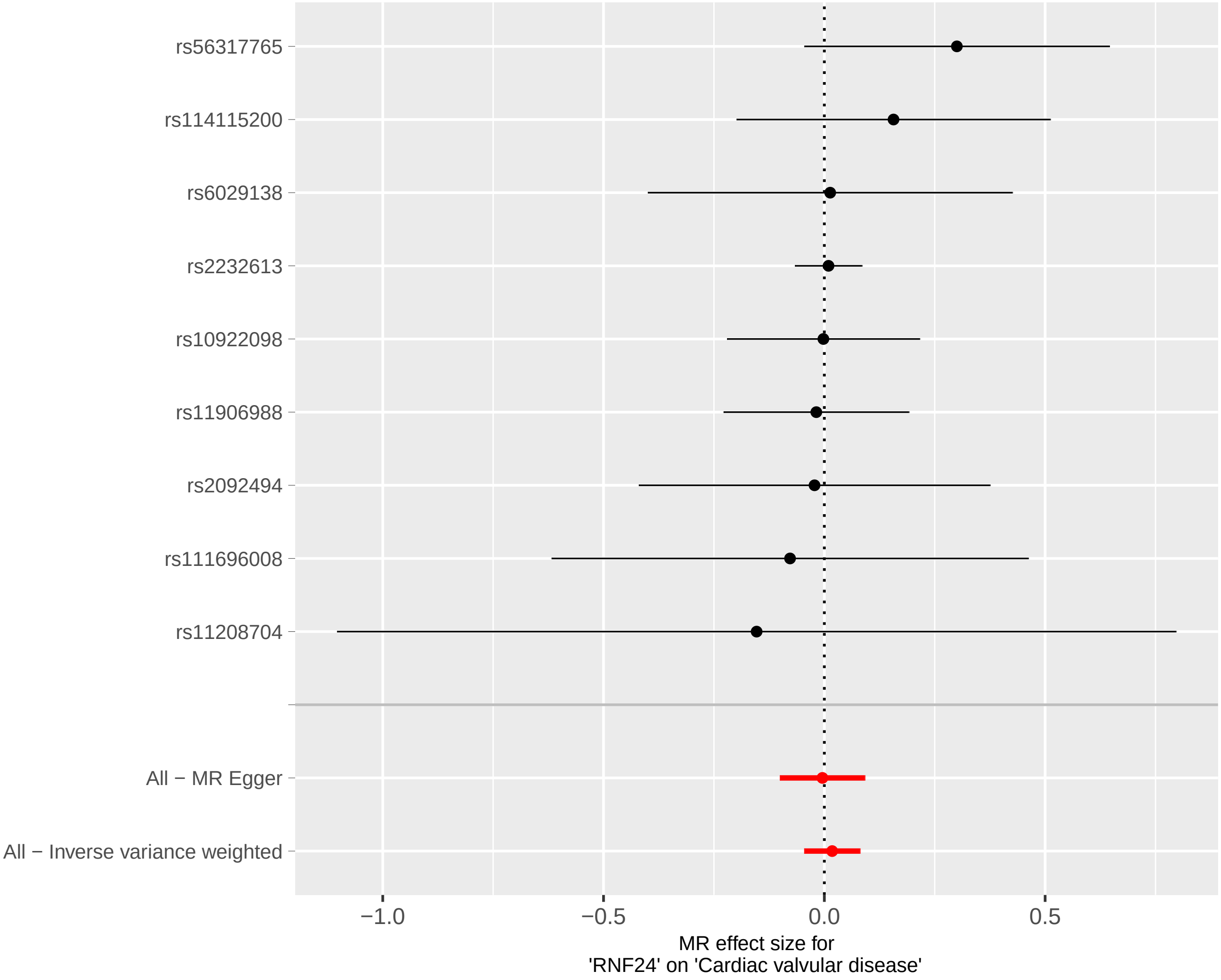
rs56317765
rs114115200
rs6029138
rs2232613
rs10922098
rs11906988
rs2092494
rs111696008
rs11208704
All − MR Egger
All − Inverse variance weighted
−1.0
−0.5
0.0
0.5
MR effect size for
'RNF24' on 'Cardiac valvular disease'

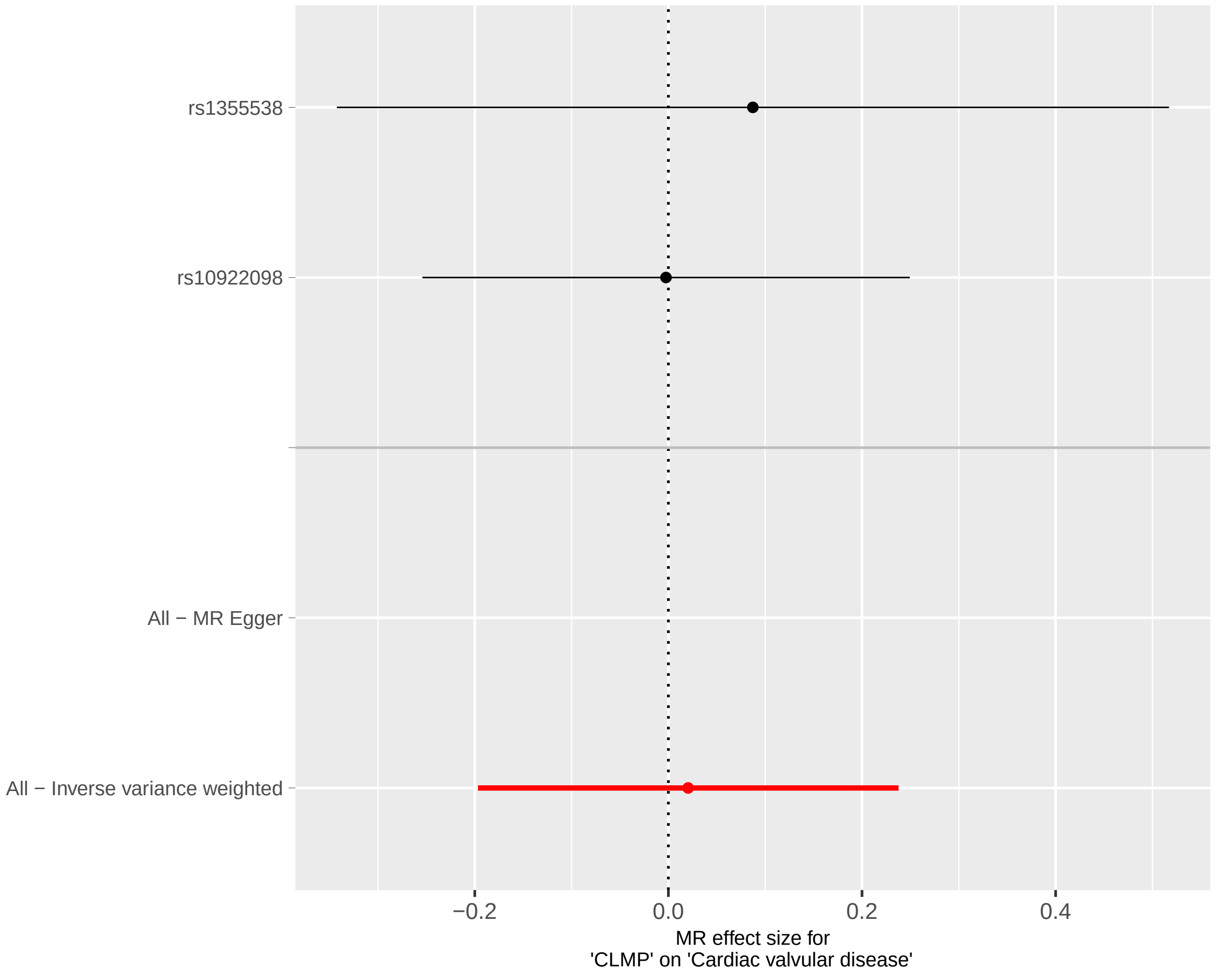
rs1355538
rs10922098
All − MR Egger
All − Inverse variance weighted
−0.2
0.0
0.2
0.4
MR effect size for
'CLMP' on 'Cardiac valvular disease'

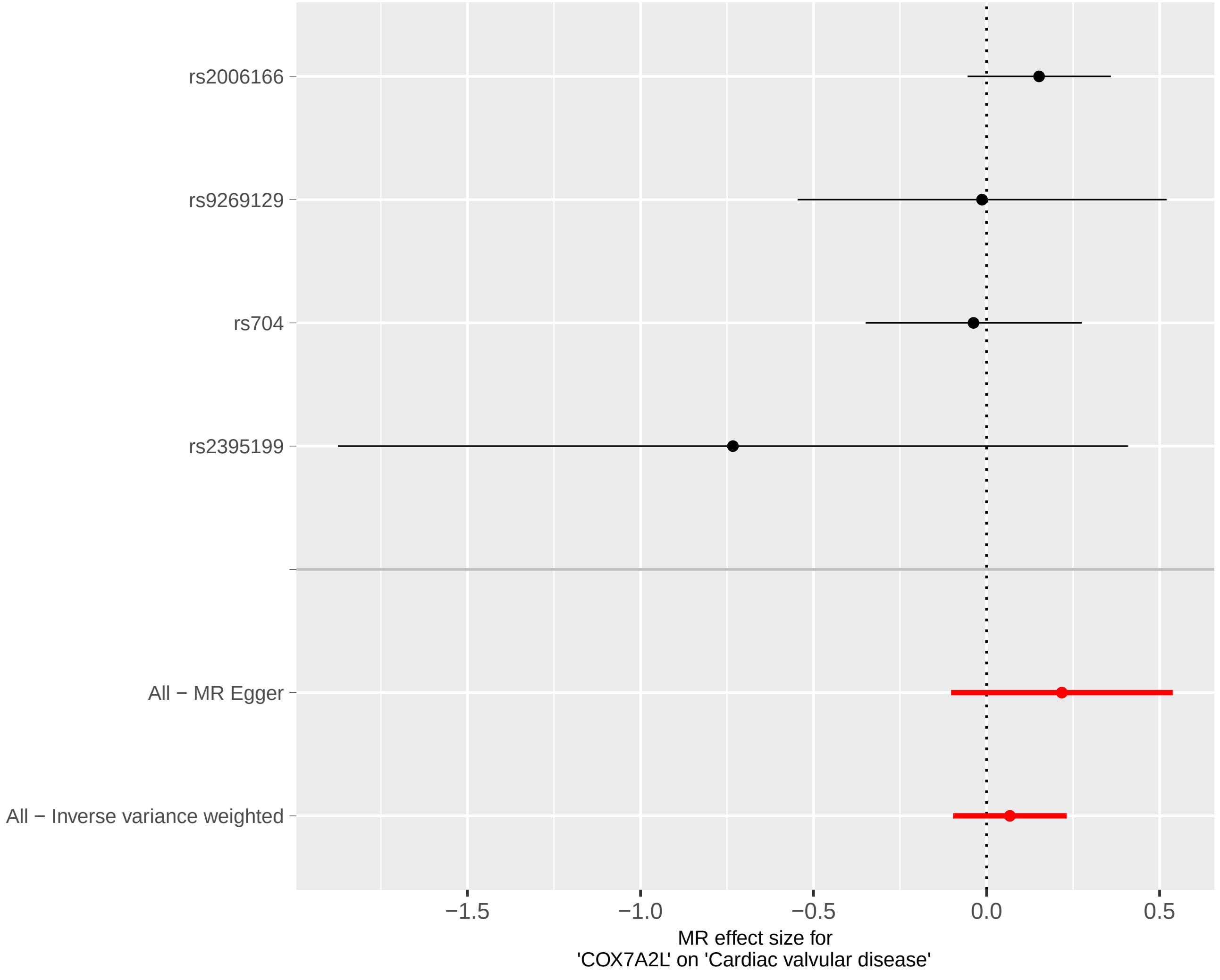

rs2006166
rs9269129
rs704
rs2395199
All − MR Egger
All − Inverse variance weighted
−1.5
−1.0
−0.5
0.0
0.5
MR effect size for
'COX7A2L' on 'Cardiac valvular disease'

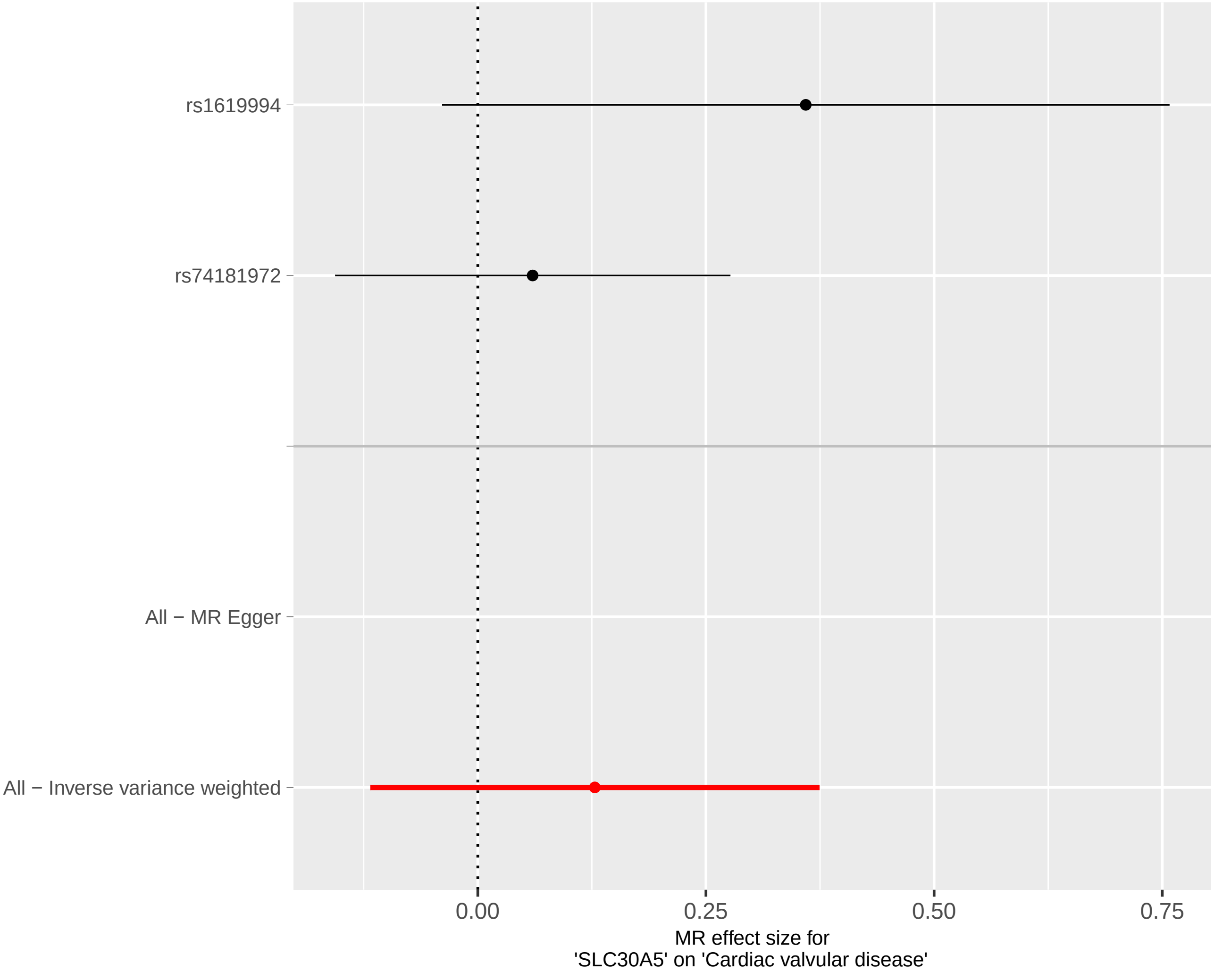

rs1619994
rs74181972
All − MR Egger
All − Inverse variance weighted
0.00
0.25
0.50
0.75
MR effect size for
'SLC30A5' on 'Cardiac valvular disease'

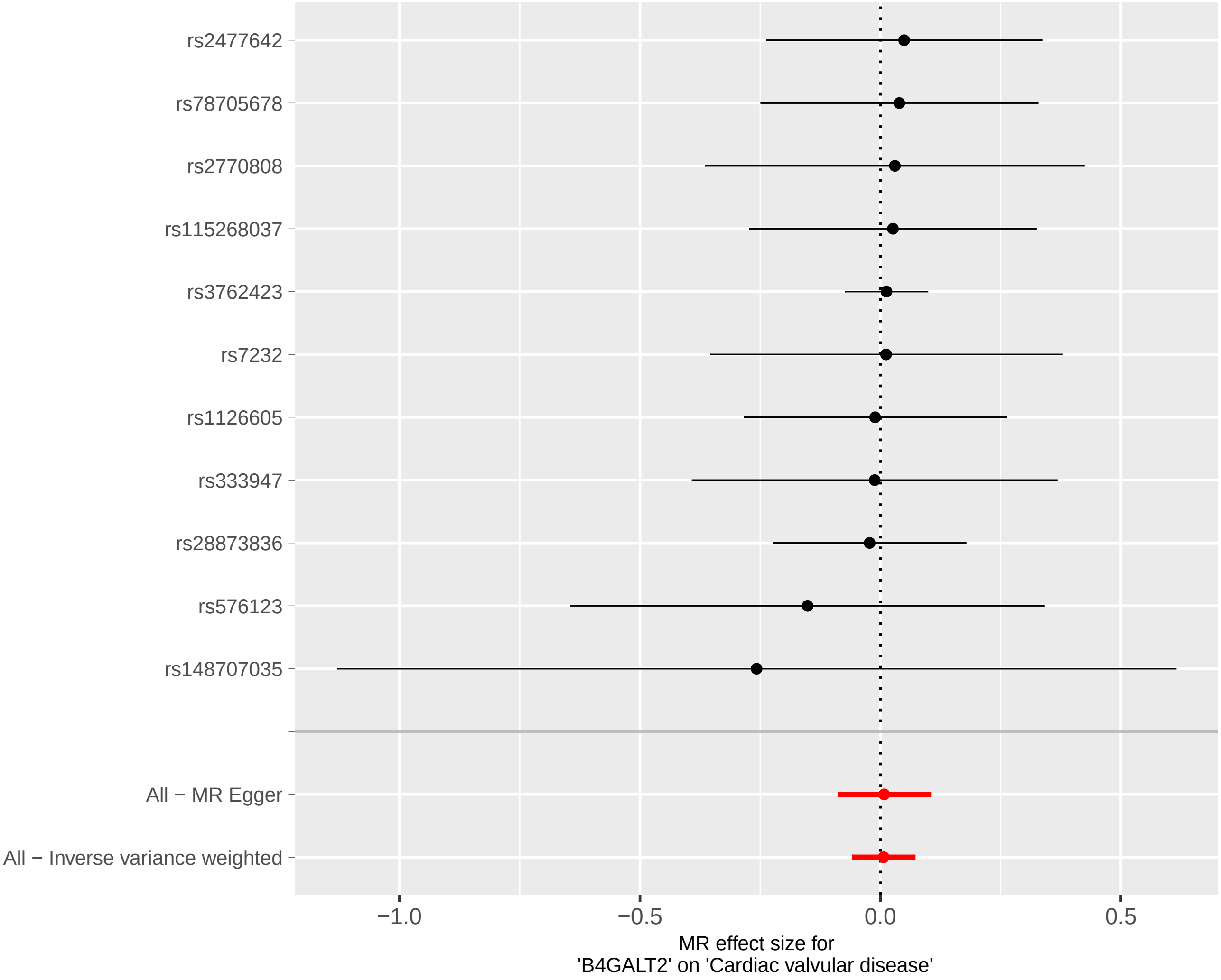
rs2477642
rs78705678
rs2770808
rs115268037
rs3762423
rs7232
rs1126605
rs333947
rs28873836
rs576123
rs148707035
All − MR Egger
All − Inverse variance weighted
−1.0
−0.5
0.0
0.5
MR effect size for
'B4GALT2' on 'Cardiac valvular disease'

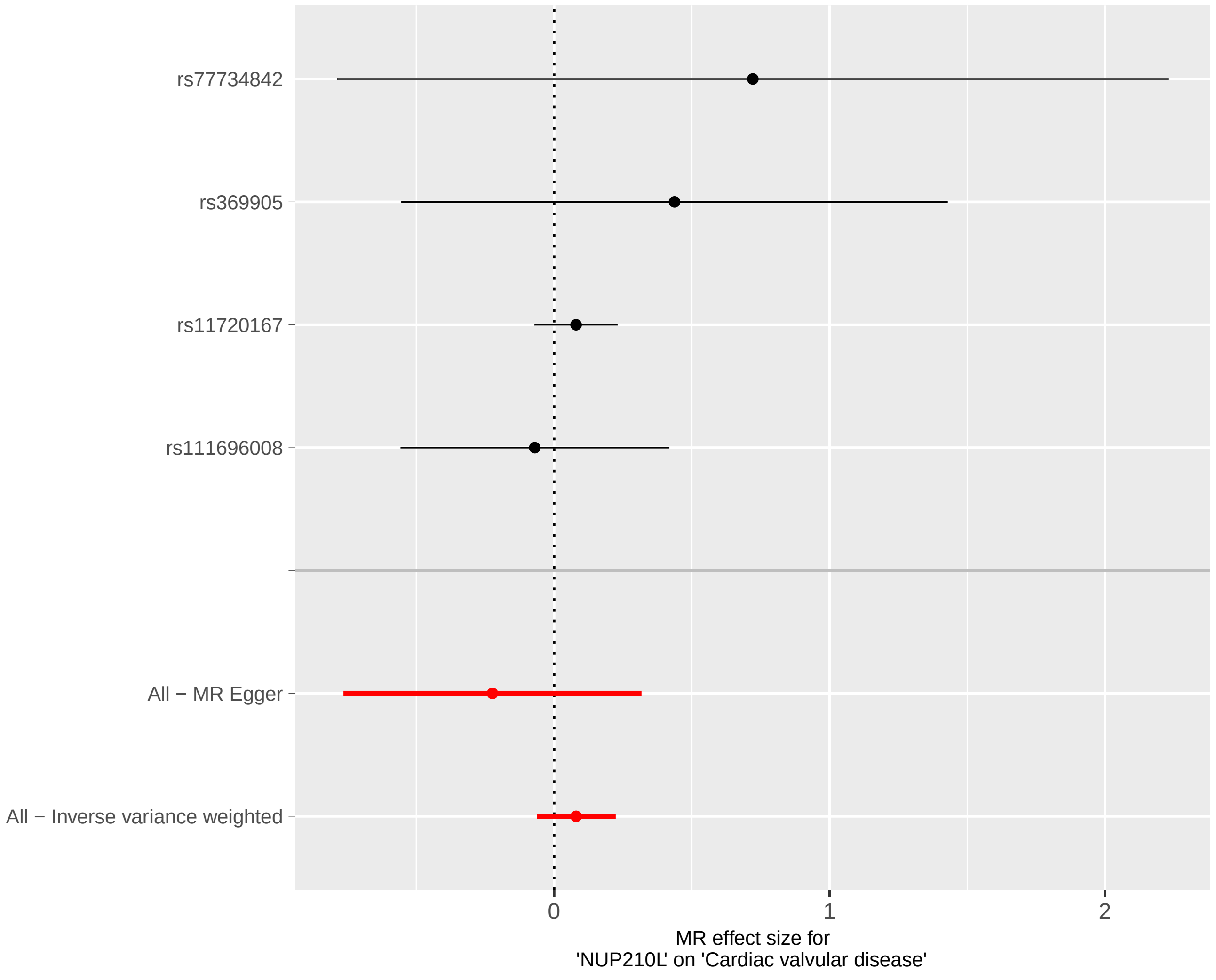
rs77734842
rs369905
rs11720167
rs111696008
All − MR Egger
All − Inverse variance weighted
0
1
2
MR effect size for
'NUP210L' on 'Cardiac valvular disease'

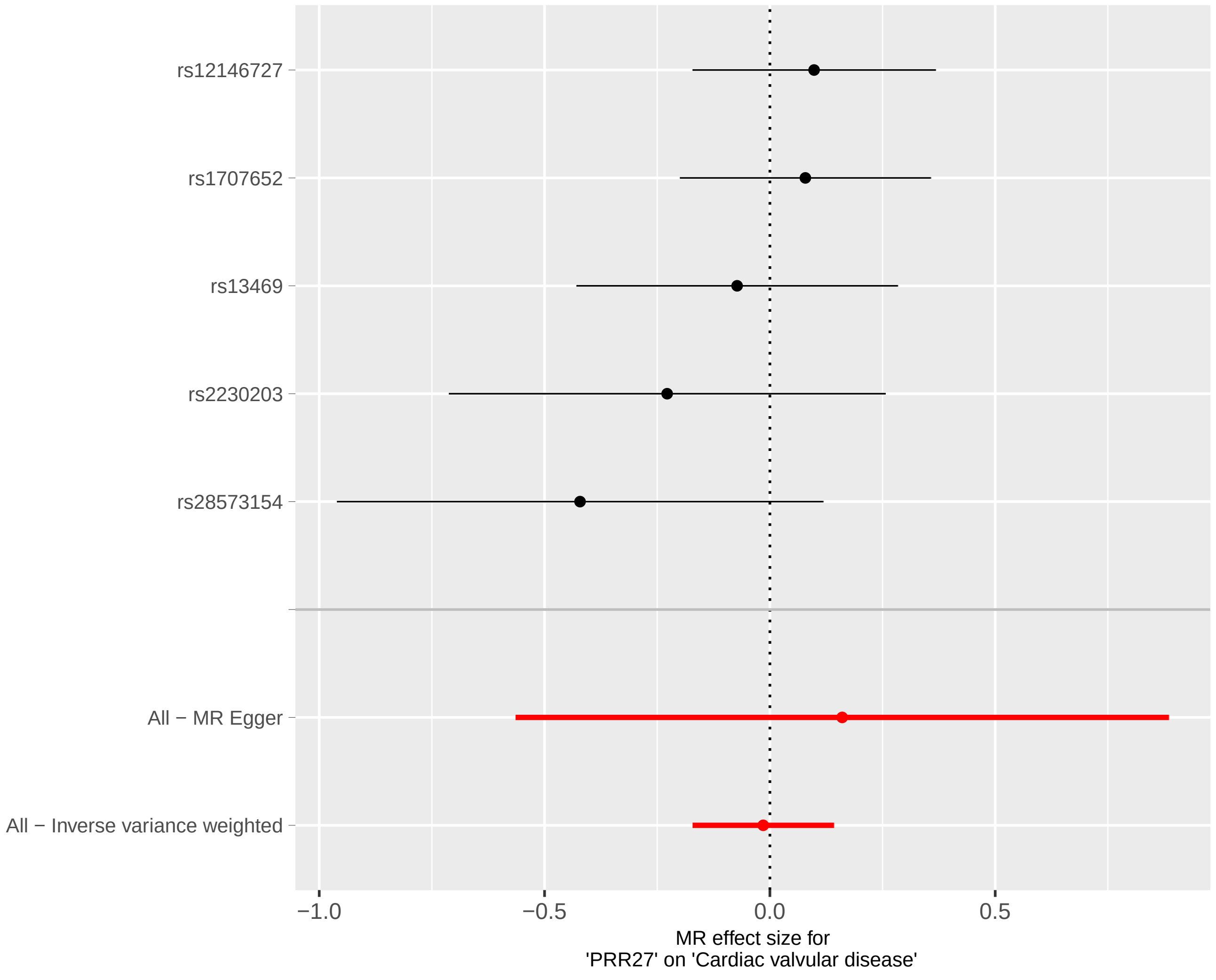
rs12146727
rs1707652
rs13469
rs2230203
rs28573154
All − MR Egger
All − Inverse variance weighted
−1.0
−0.5
0.0
0.5
MR effect size for
'PRR27' on 'Cardiac valvular disease'

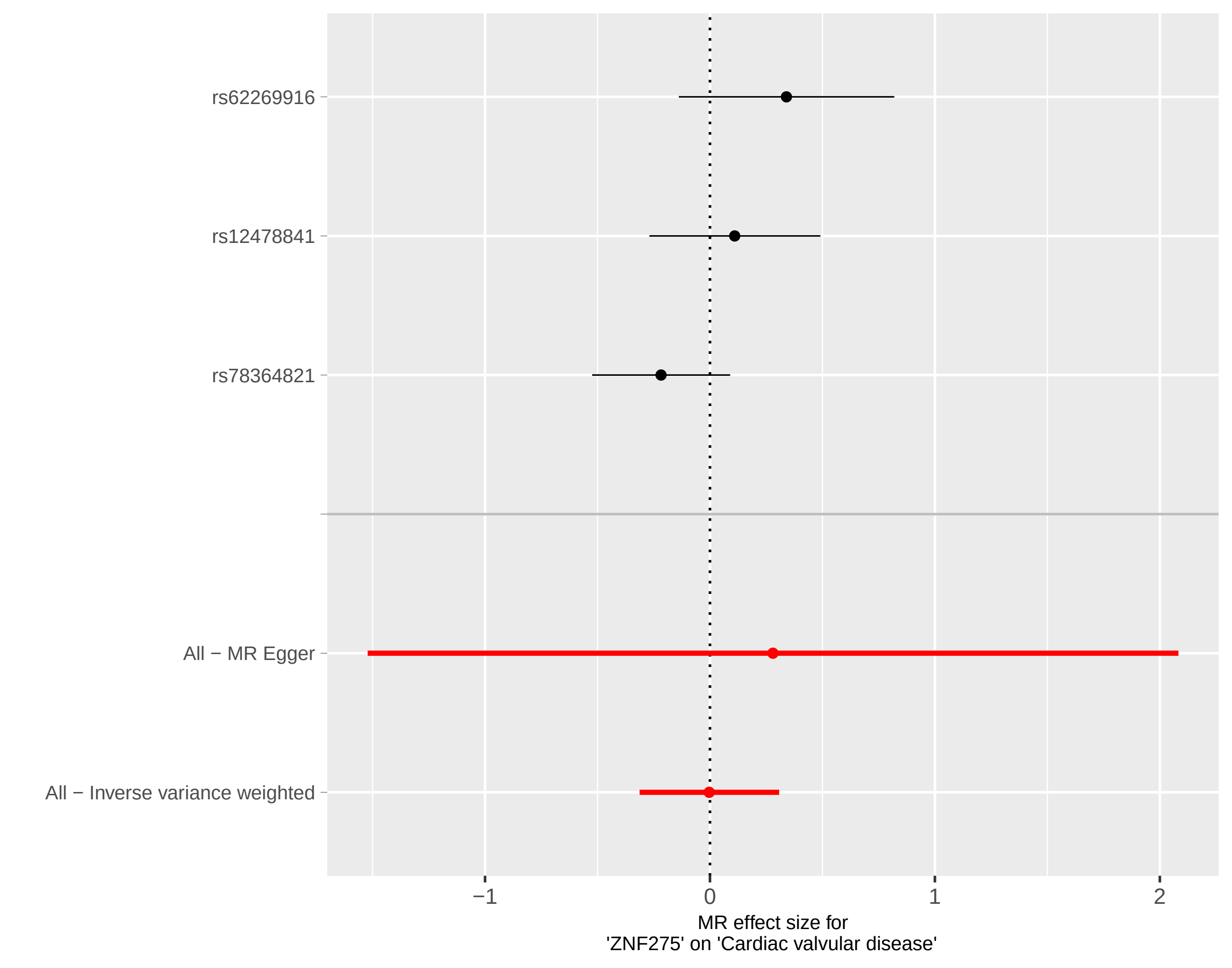
rs62269916
rs12478841
rs78364821
All − MR Egger
All − Inverse variance weighted
−1
0
1
2
MR effect size for
'ZNF275' on 'Cardiac valvular disease'

Insufficient number of SNPs

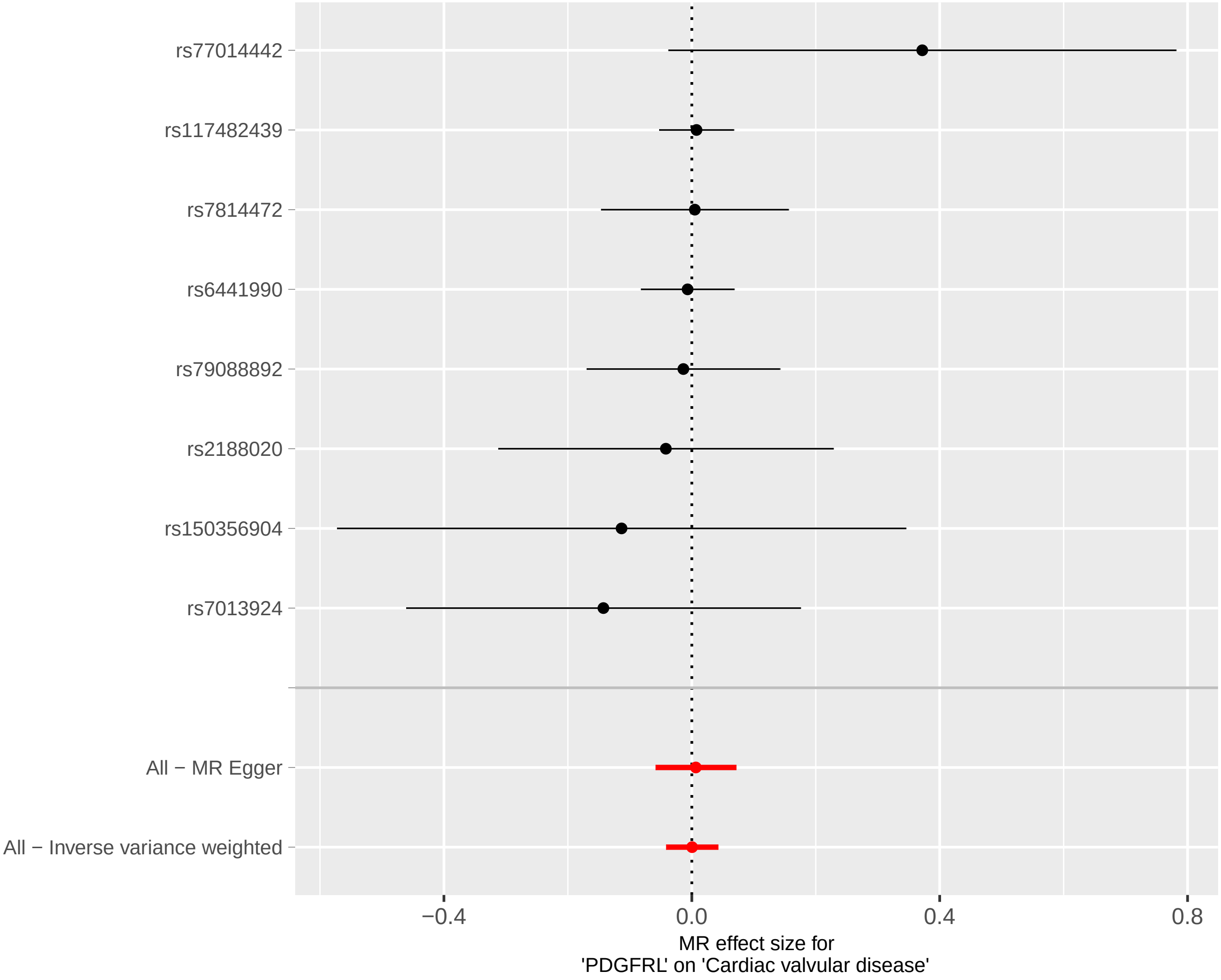
rs77014442
rs117482439
rs7814472
rs6441990
rs79088892
rs2188020
rs150356904
rs7013924
All − MR Egger
All − Inverse variance weighted
−0.4
0.0
0.4
0.8
MR effect size for
'PDGFRL' on 'Cardiac valvular disease'

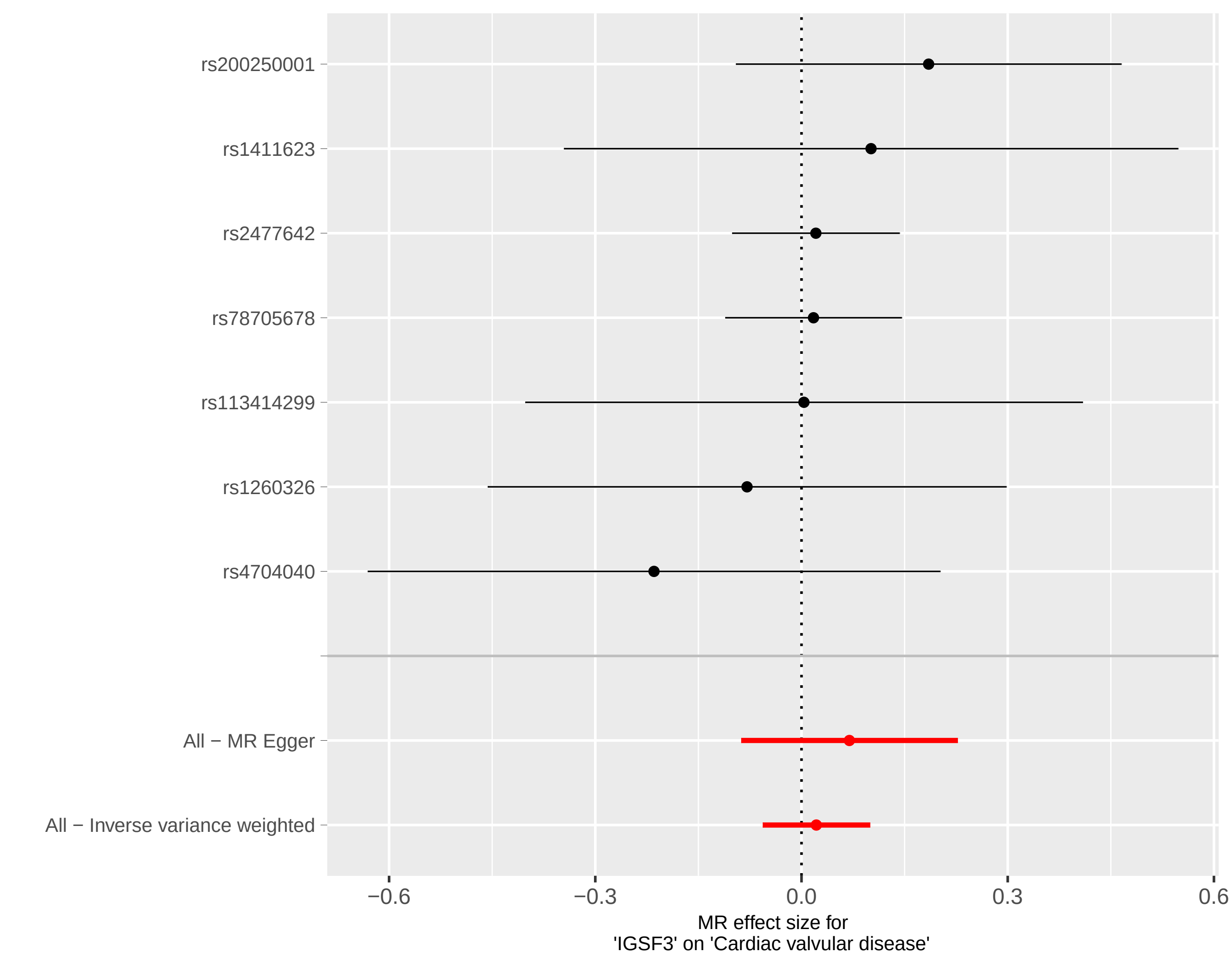
rs200250001
rs1411623
rs2477642
rs78705678
rs113414299
rs1260326
rs4704040
All − MR Egger
All − Inverse variance weighted
−0.6
−0.3
0.0
0.3
0.6
MR effect size for
'IGSF3' on 'Cardiac valvular disease'

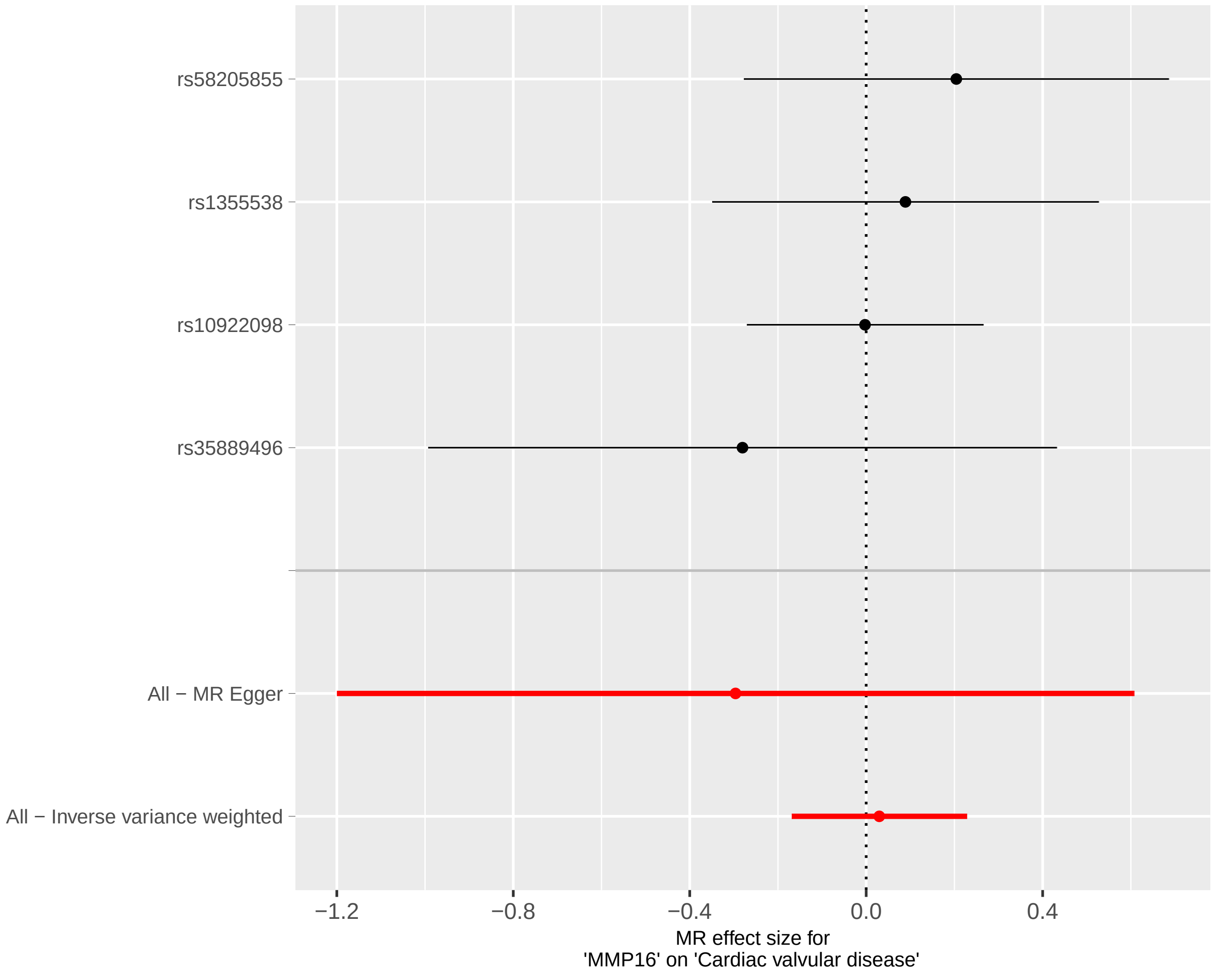
rs58205855
rs1355538
rs10922098
rs35889496
All − MR Egger
All − Inverse variance weighted
−1.2
−0.8
−0.4
0.0
0.4
MR effect size for
'MMP16' on 'Cardiac valvular disease'

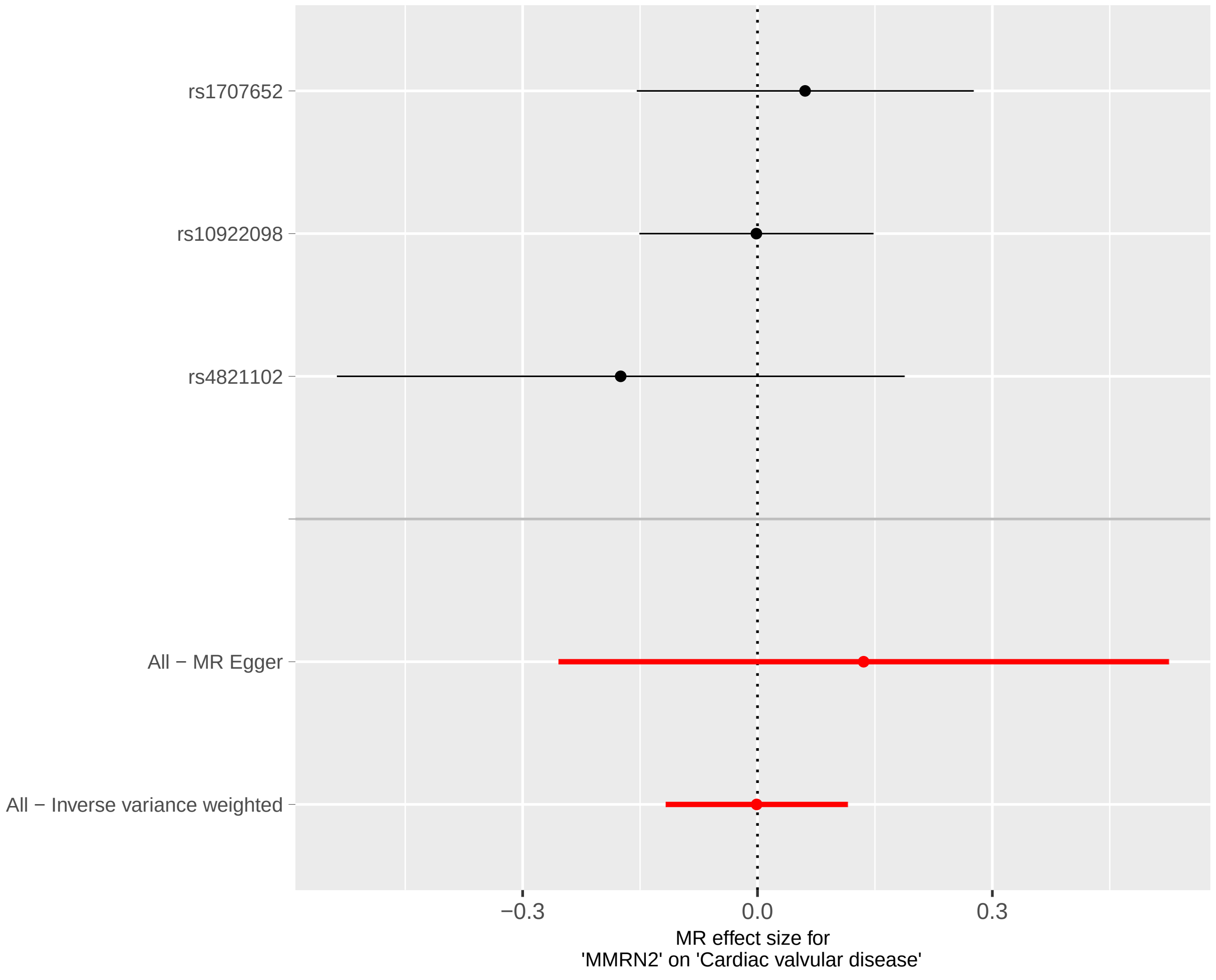
rs1707652
rs10922098
rs4821102
All − MR Egger
All − Inverse variance weighted
−0.3
0.0
0.3
MR effect size for
'MMRN2' on 'Cardiac valvular disease'

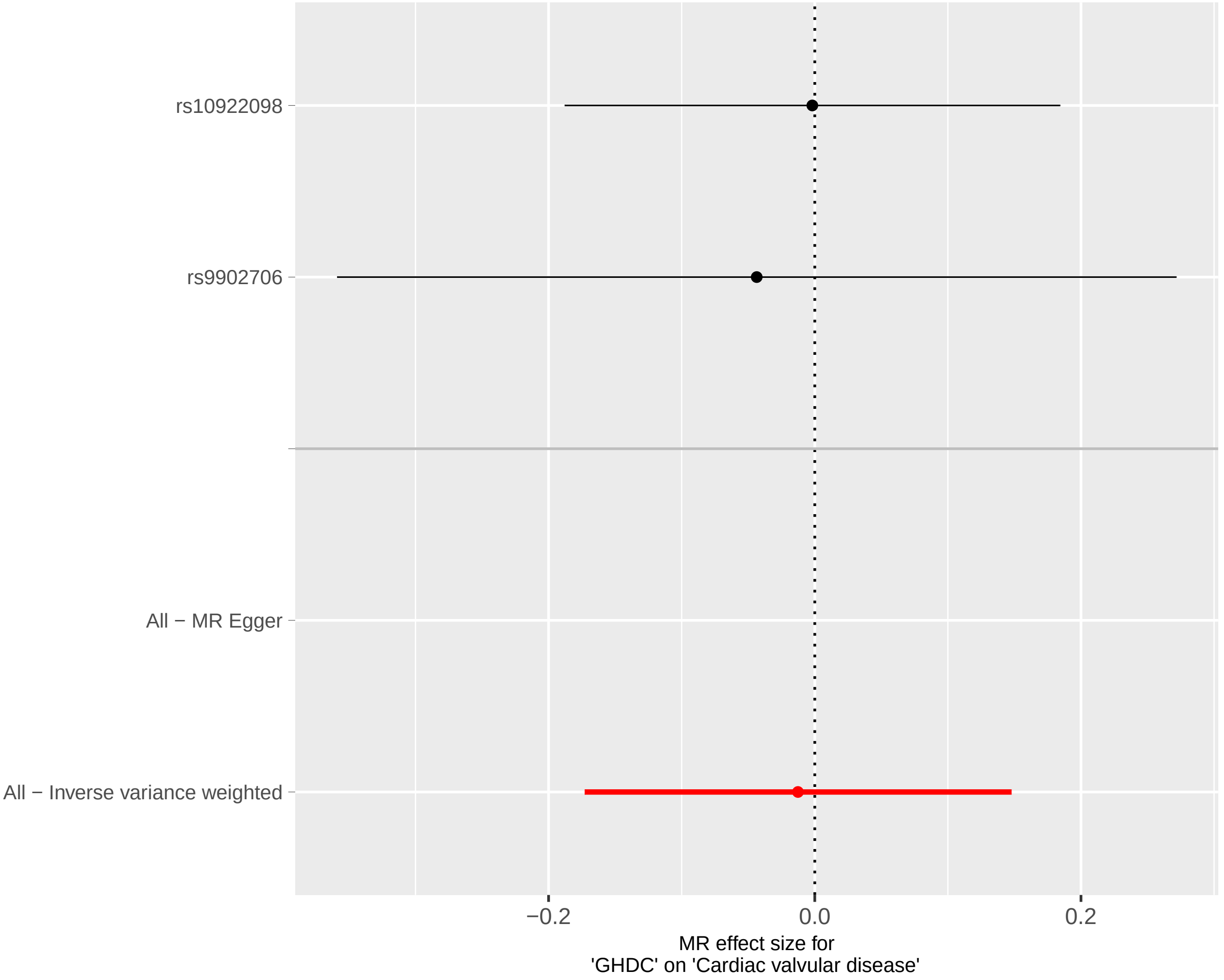

rs10922098
rs9902706
All − MR Egger
All − Inverse variance weighted
−0.2
0.0
0.2
MR effect size for
'GHDC' on 'Cardiac valvular disease'

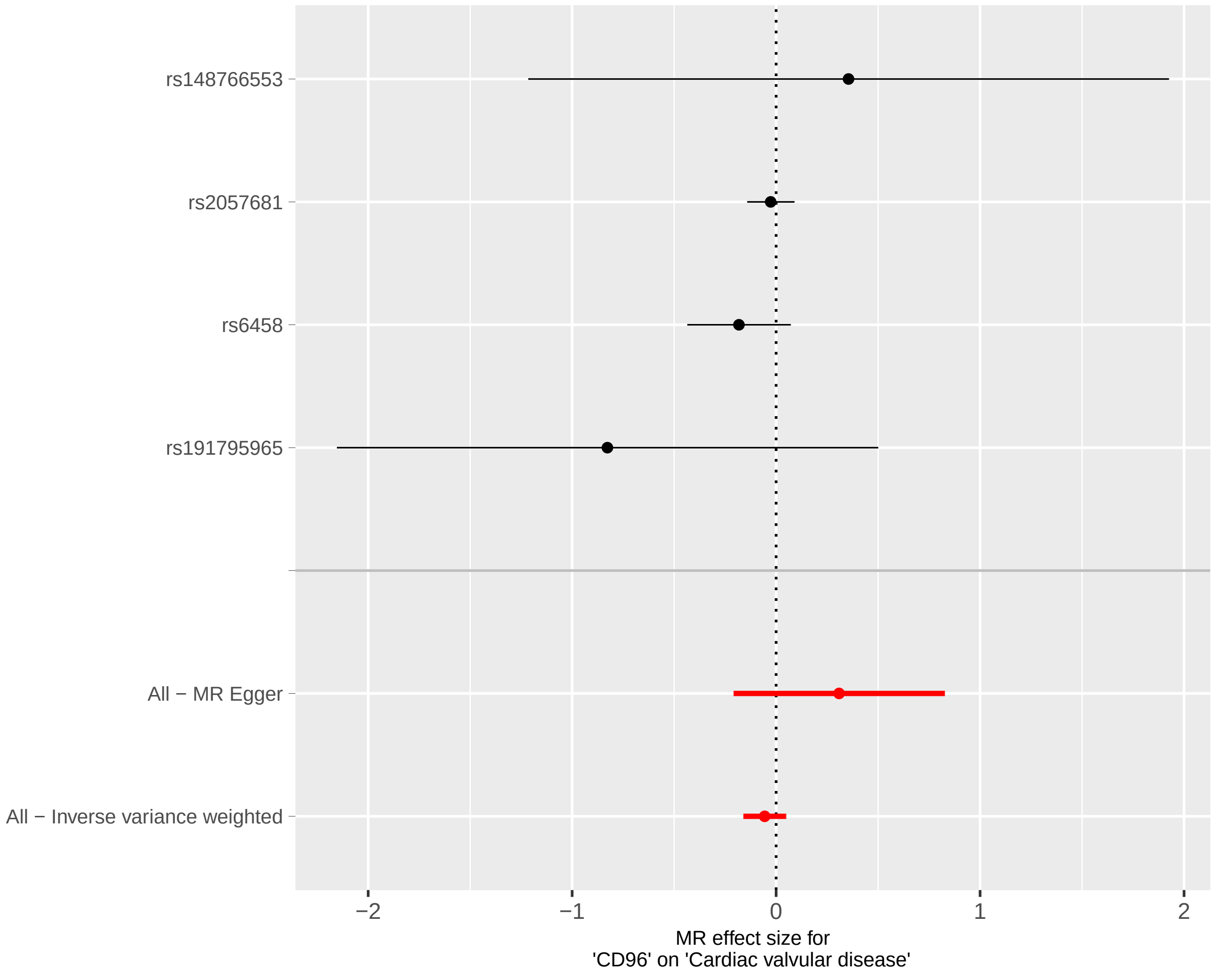
rs148766553
rs2057681
rs6458
rs191795965
All − MR Egger
All − Inverse variance weighted
−2
−1
0
1
2
MR effect size for
'CD96' on 'Cardiac valvular disease'

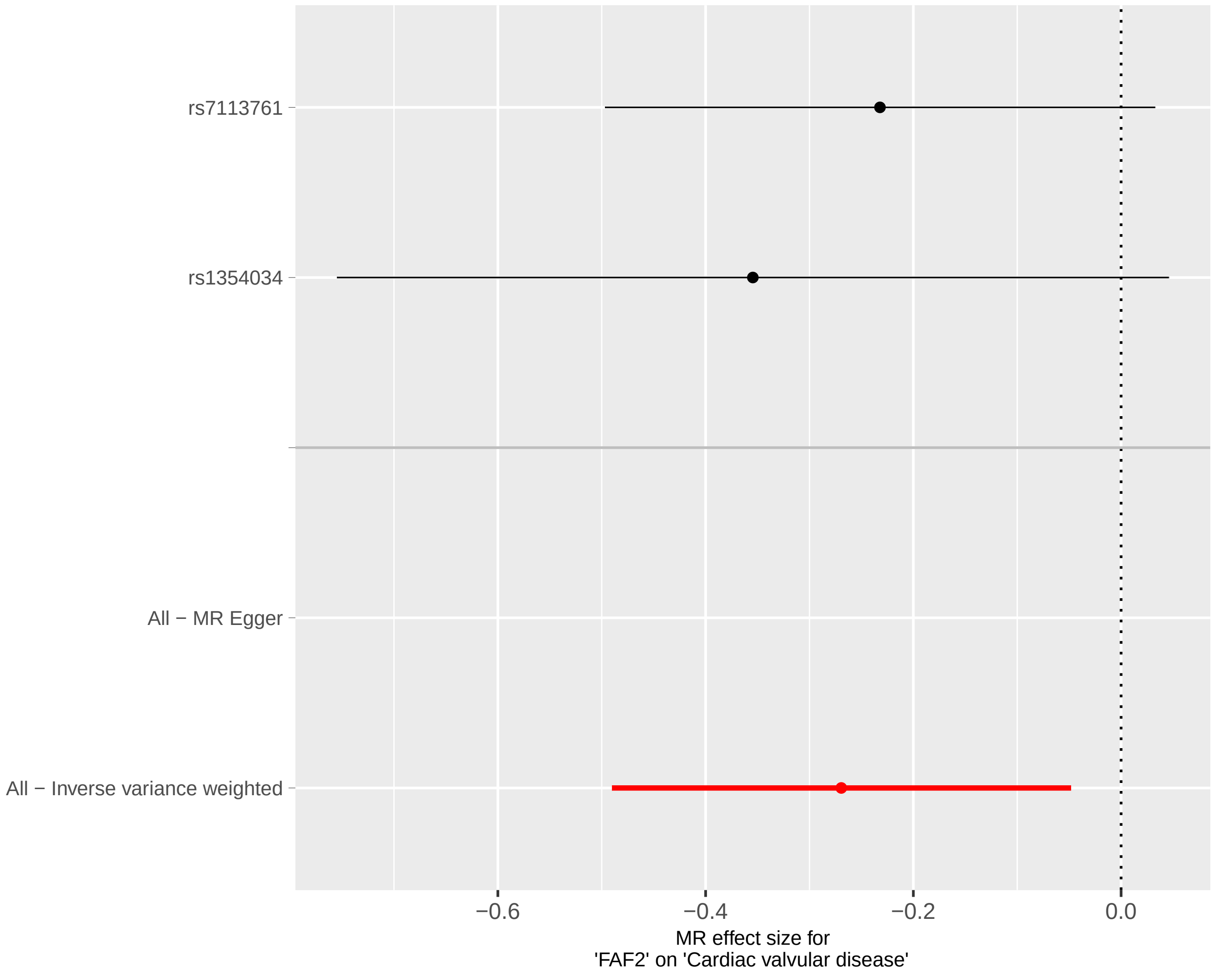
rs7113761
rs1354034
All − MR Egger
All − Inverse variance weighted
−0.6
−0.4
−0.2
0.0
MR effect size for
'FAF2' on 'Cardiac valvular disease'

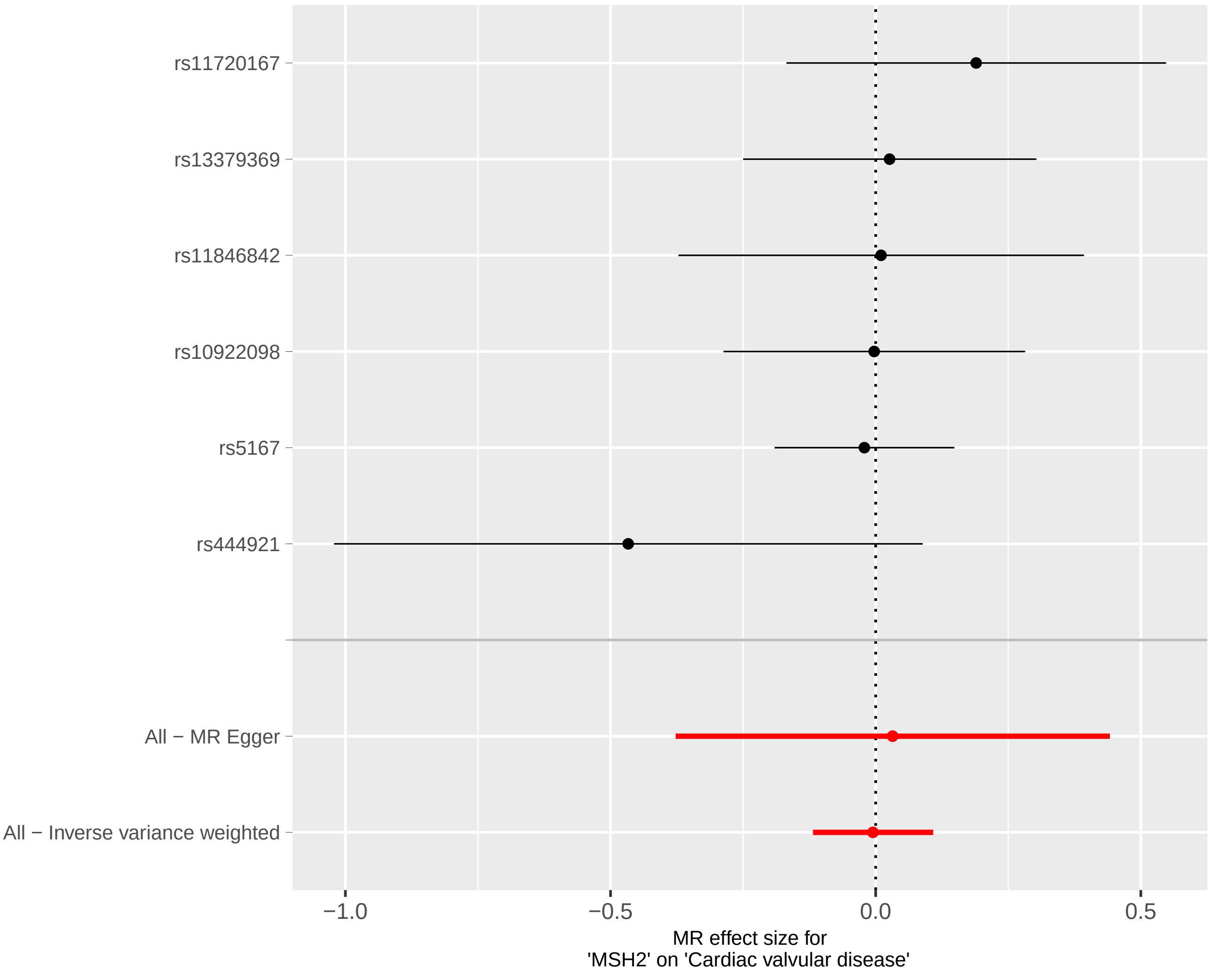

rs11720167
rs13379369
rs11846842
rs10922098
rs5167
rs444921
All − MR Egger
All − Inverse variance weighted
−1.0
−0.5
0.0
0.5
MR effect size for
'MSH2' on 'Cardiac valvular disease'

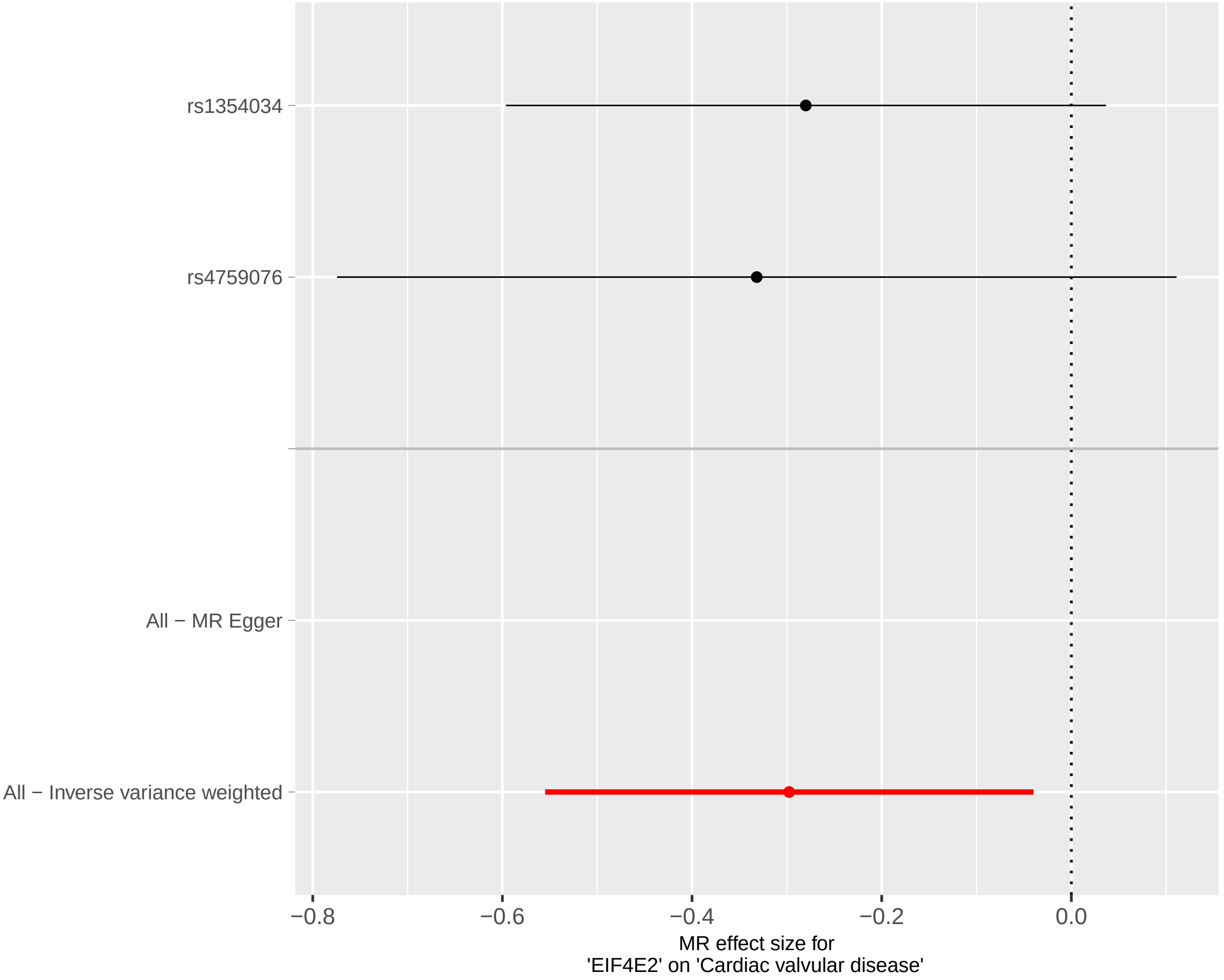

rs1354034
rs4759076
All − MR Egger
All − Inverse variance weighted
−0.8
−0.6
−0.4
−0.2
0.0
MR effect size for
'EIF4E2' on 'Cardiac valvular disease'

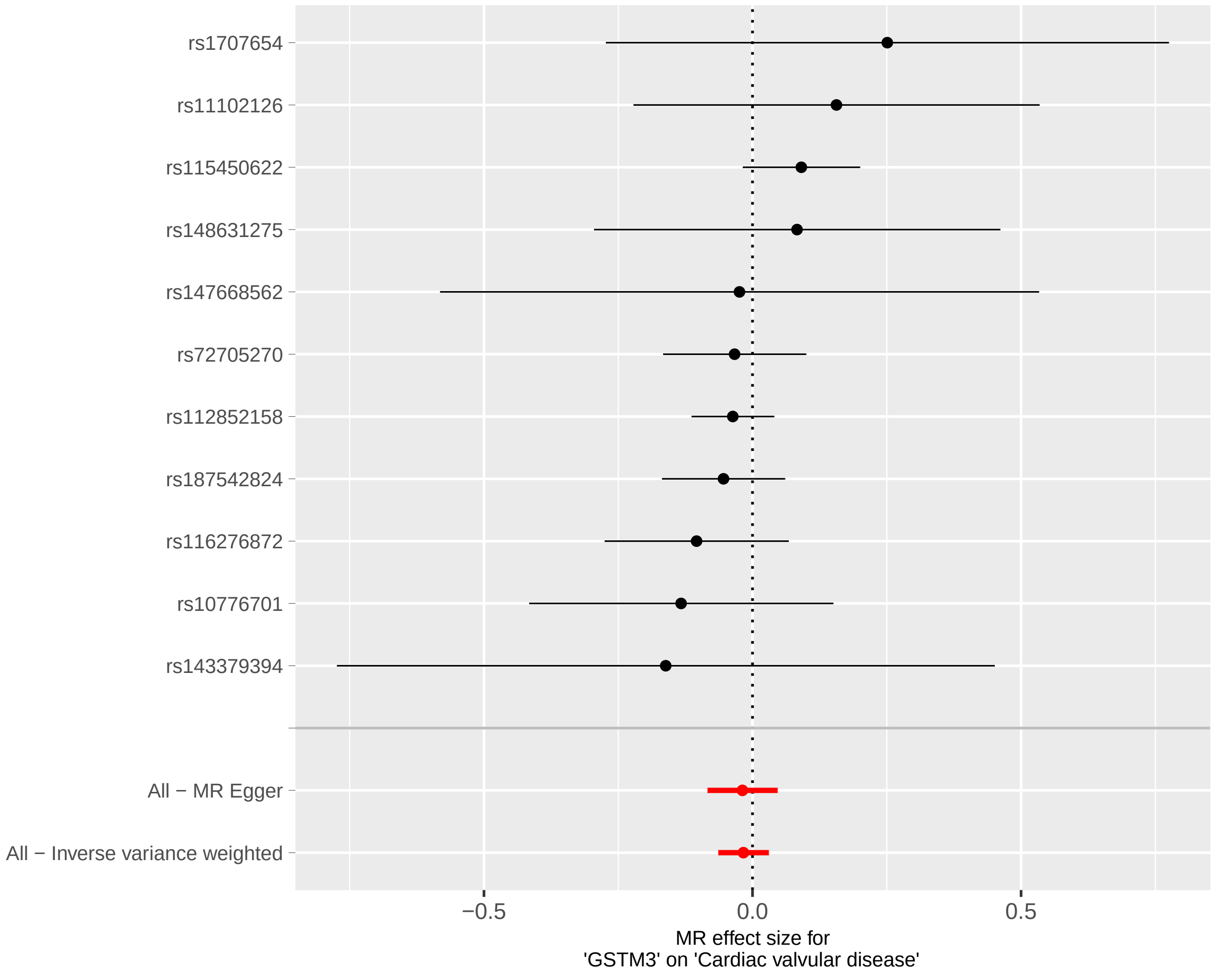
rs1707654
rs11102126
rs115450622
rs148631275
rs147668562
rs72705270
rs112852158
rs187542824
rs116276872
rs10776701
rs143379394
All − MR Egger
All − Inverse variance weighted
−0.5
0.0
0.5
MR effect size for
'GSTM3' on 'Cardiac valvular disease'

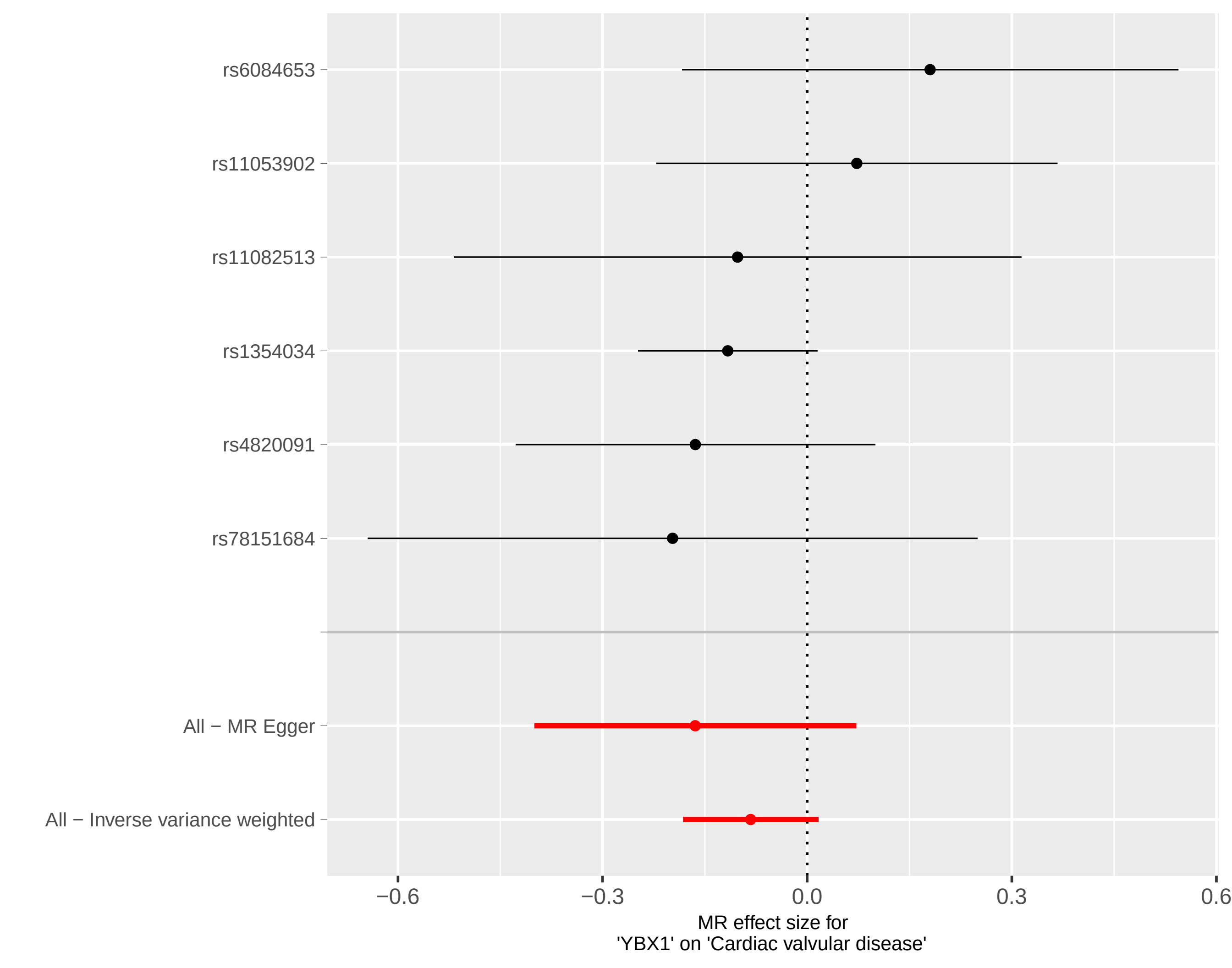
rs6084653
rs11053902
rs11082513
rs1354034
rs4820091
rs78151684
All − MR Egger
All − Inverse variance weighted
−0.6
−0.3
0.0
0.3
0.6
MR effect size for
'YBX1' on 'Cardiac valvular disease'

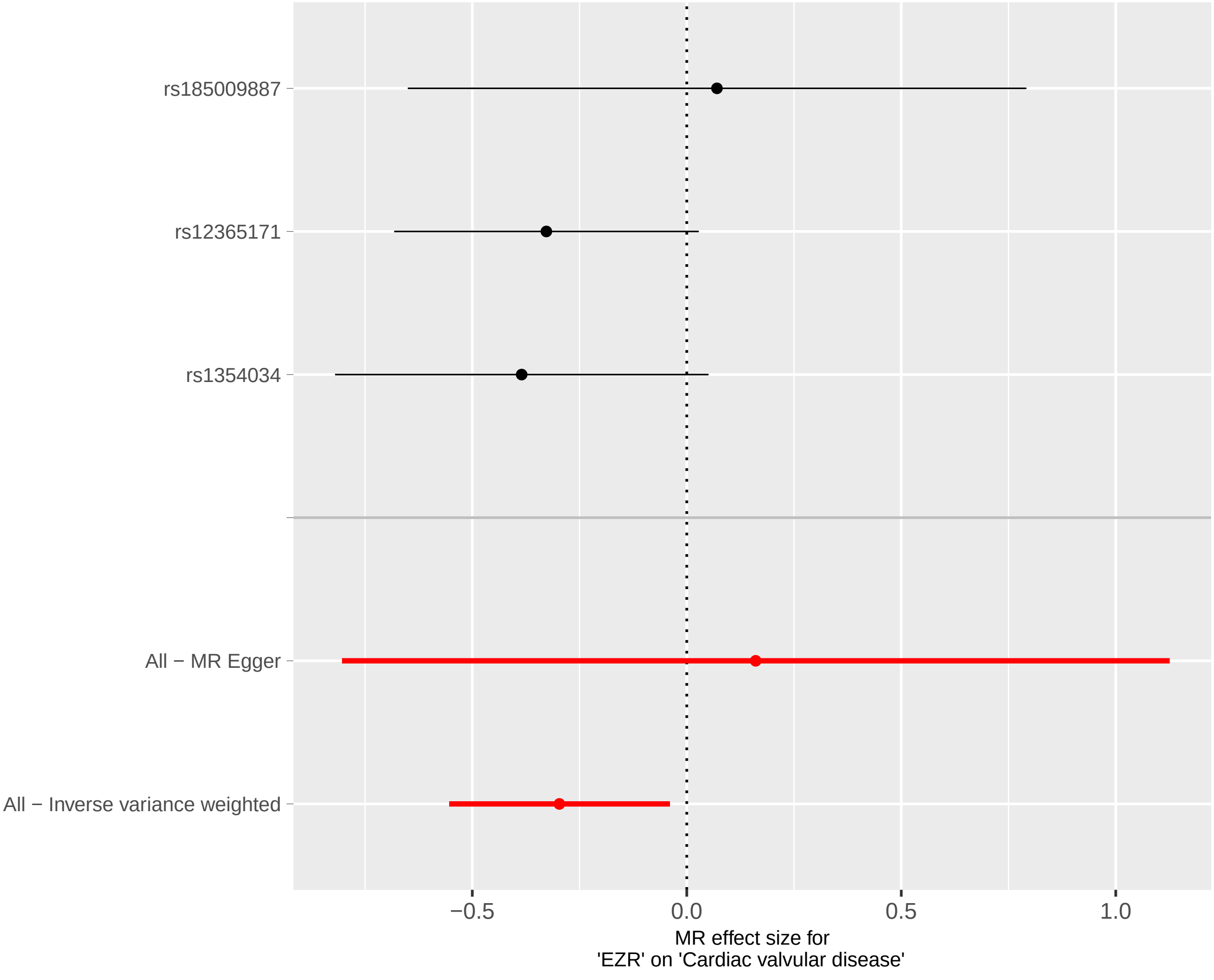
rs185009887
rs12365171
rs1354034
All − MR Egger
All − Inverse variance weighted
−0.5
0.0
0.5
1.0
MR effect size for
'EZR' on 'Cardiac valvular disease'

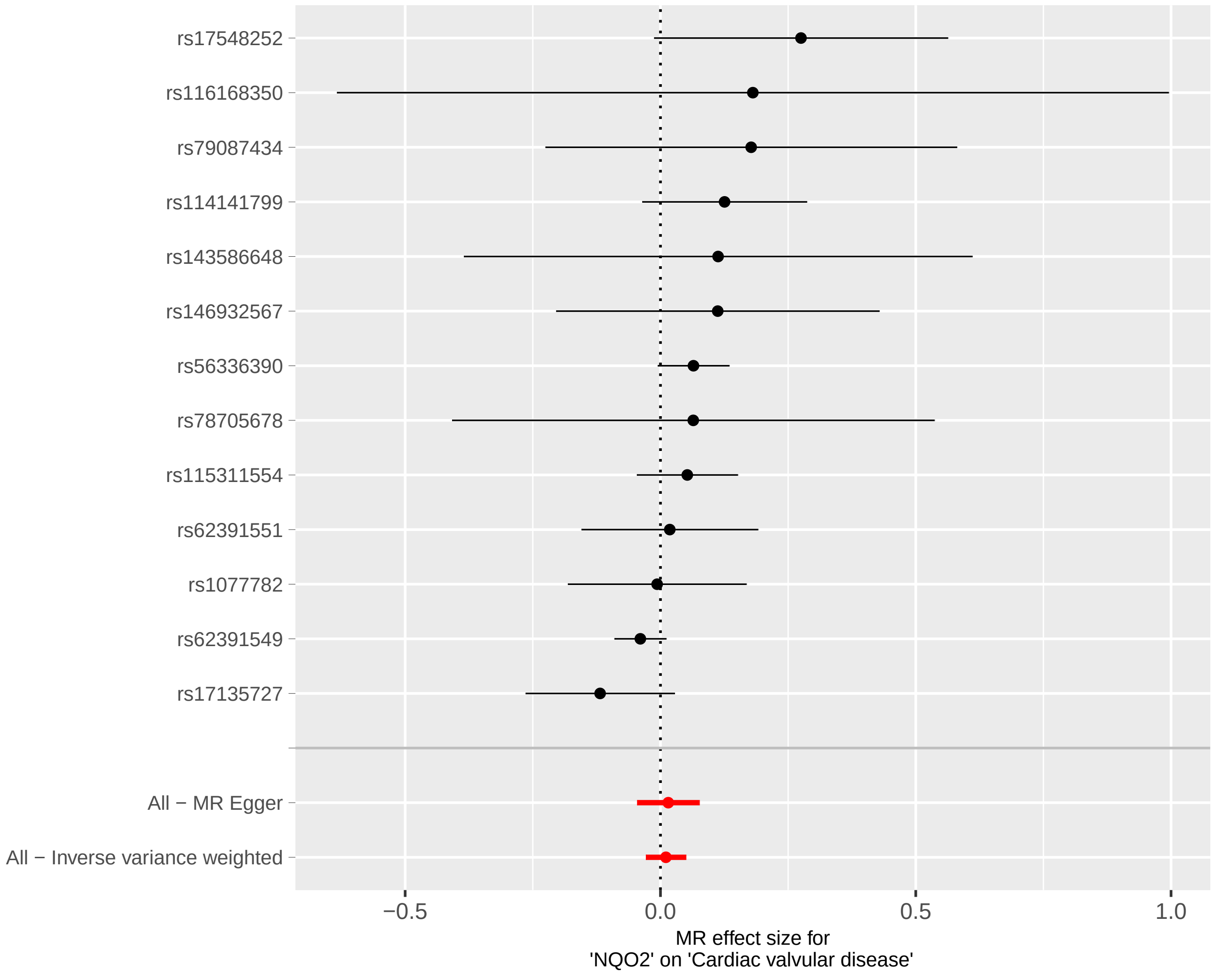

rs17548252
rs116168350
rs79087434
rs114141799
rs143586648
rs146932567
rs56336390
rs78705678
rs115311554
rs62391551
rs1077782
rs62391549
rs17135727
All − MR Egger
All − Inverse variance weighted
−0.5
0.0
0.5
1.0
MR effect size for
'NQO2' on 'Cardiac valvular disease'

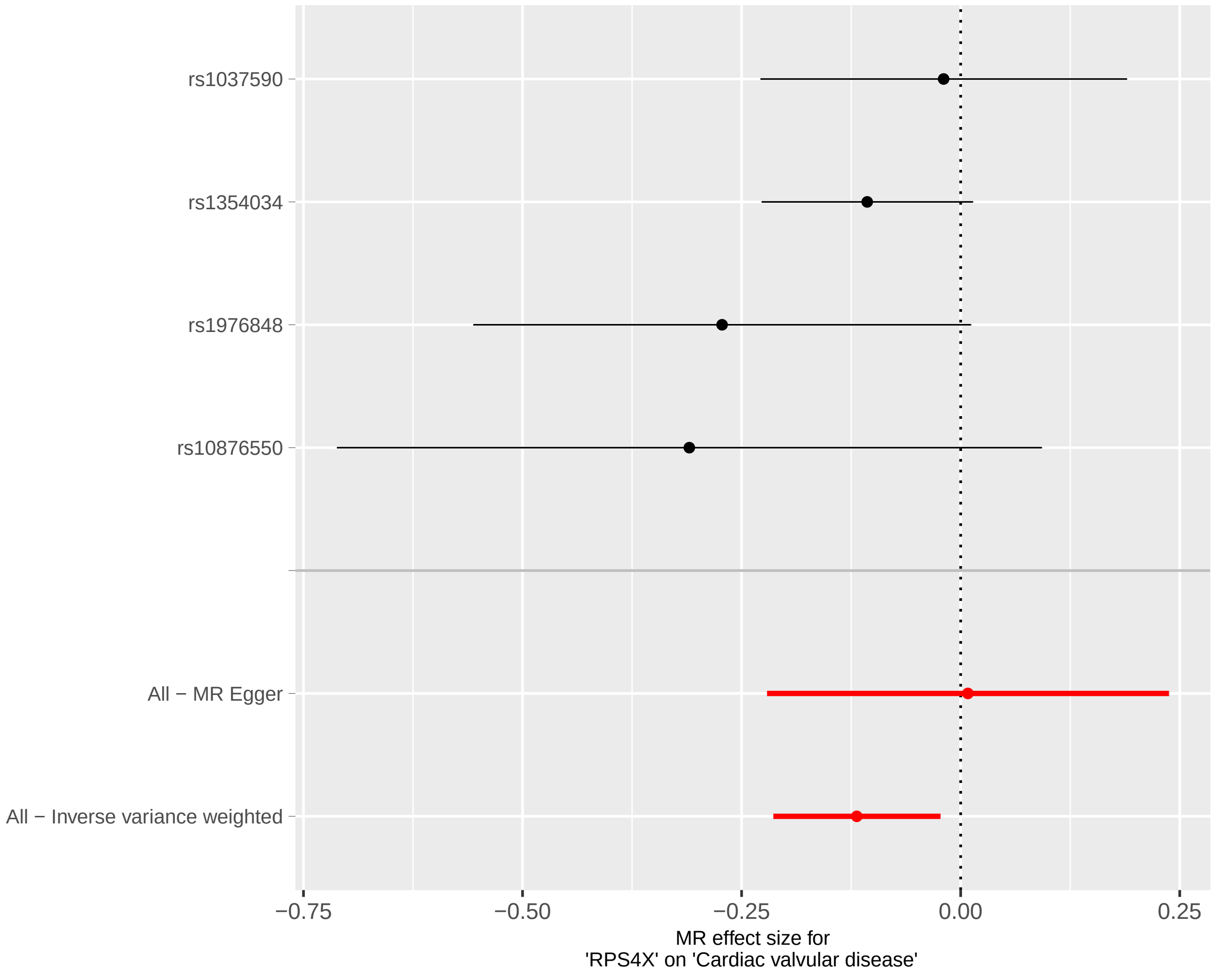

rs1037590
rs1354034
rs1976848
rs10876550
All − MR Egger
All − Inverse variance weighted
−0.75
−0.50
−0.25
0.00
0.25
MR effect size for
'RPS4X' on 'Cardiac valvular disease'

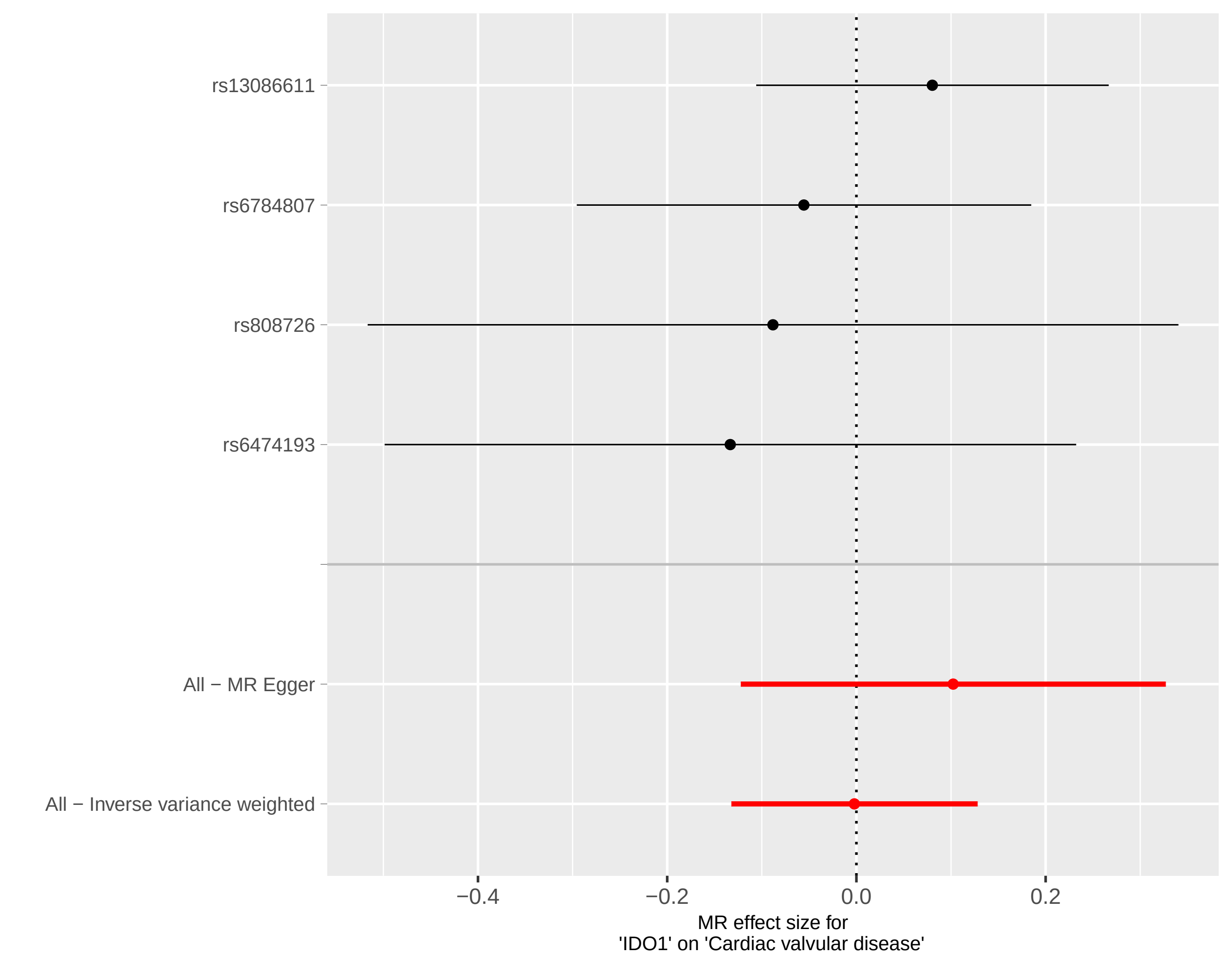

rs13086611
rs6784807
rs808726
rs6474193
All − MR Egger
All − Inverse variance weighted
−0.4
−0.2
0.0
0.2
MR effect size for
'IDO1' on 'Cardiac valvular disease'

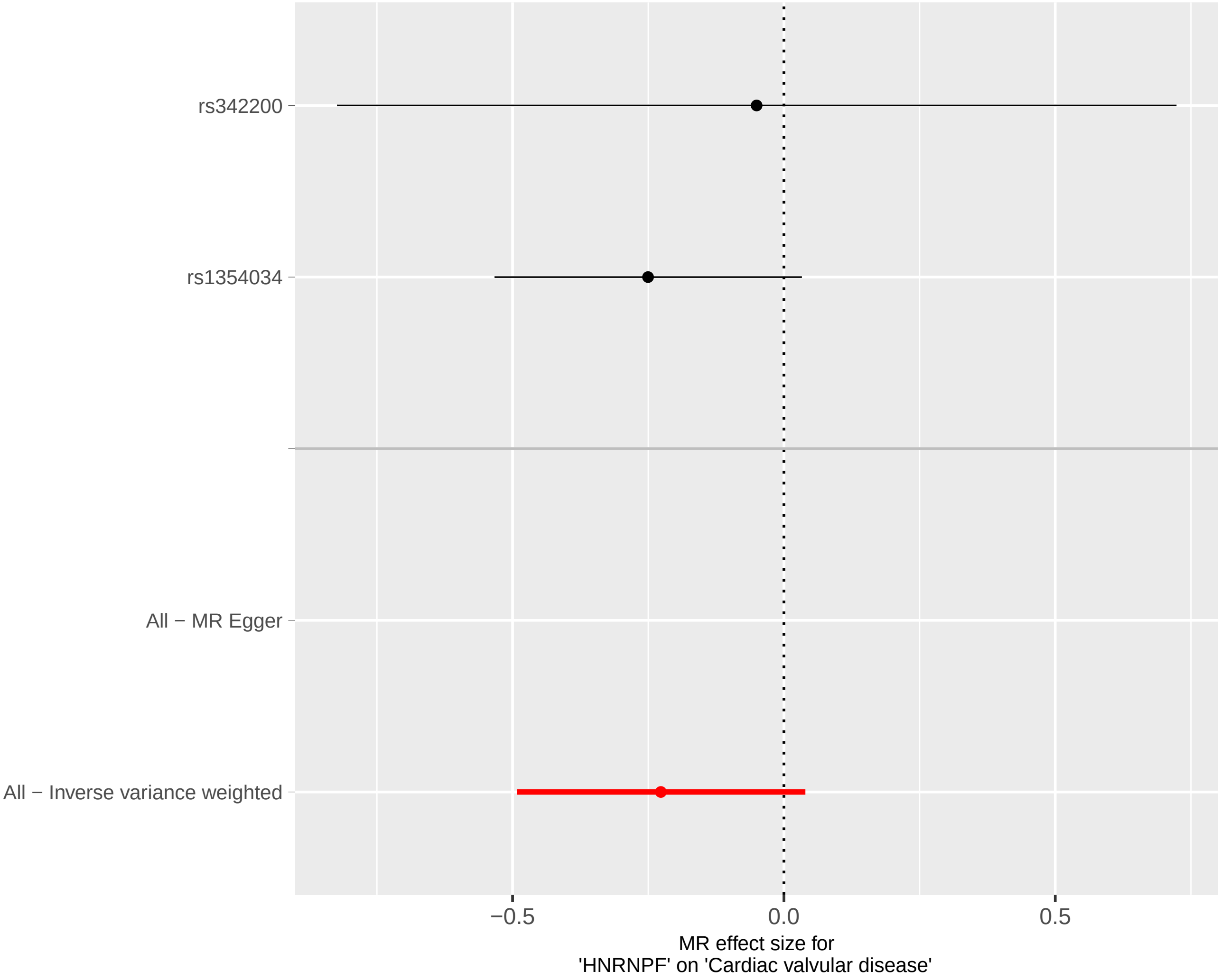
rs342200
rs1354034
All − MR Egger
All − Inverse variance weighted
−0.5
0.0
0.5
MR effect size for
'HNRNPF' on 'Cardiac valvular disease'

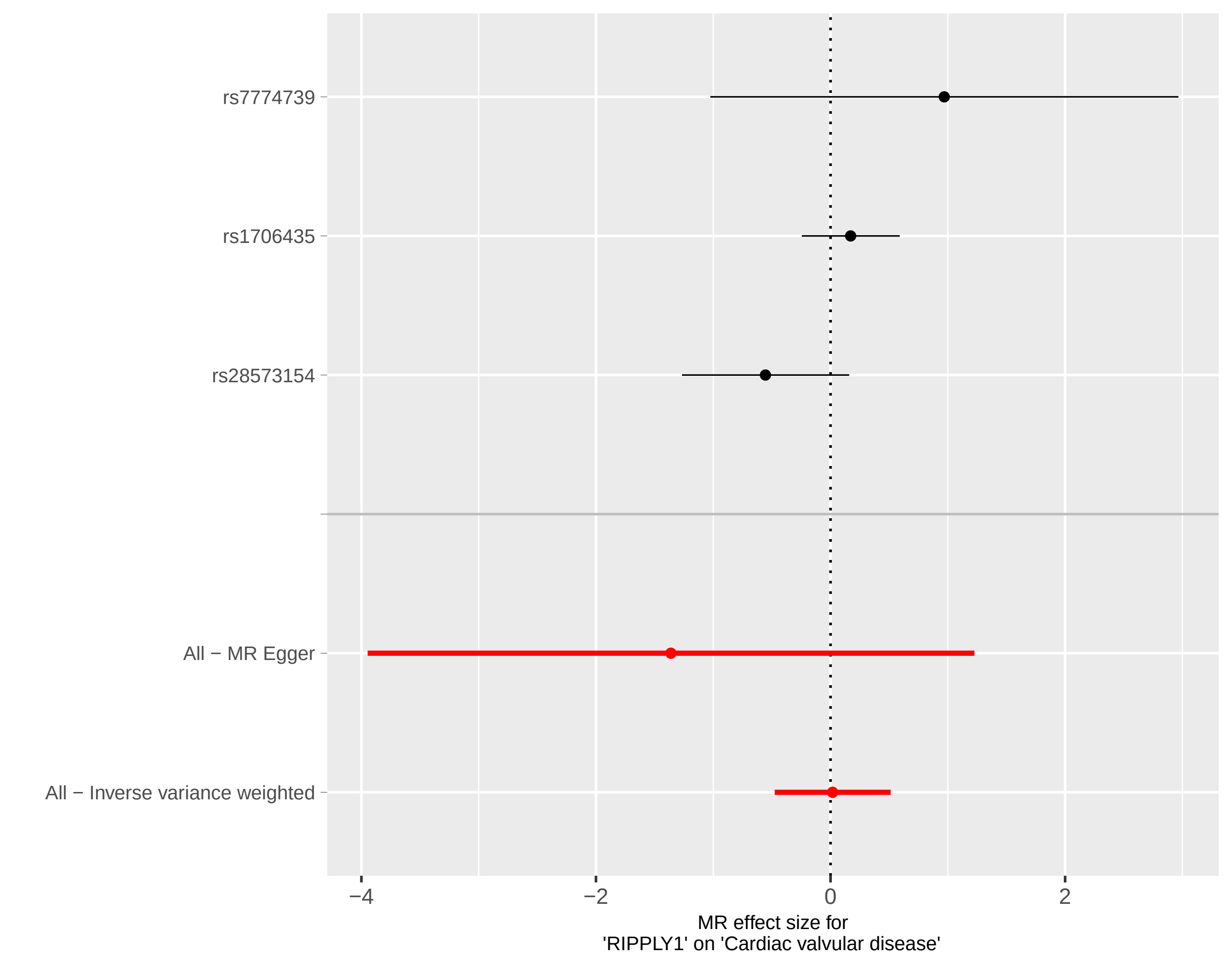
rs7774739
rs1706435
rs28573154
All − MR Egger
All − Inverse variance weighted
−4
−2
0
2
MR effect size for
'RIPPLY1' on 'Cardiac valvular disease'

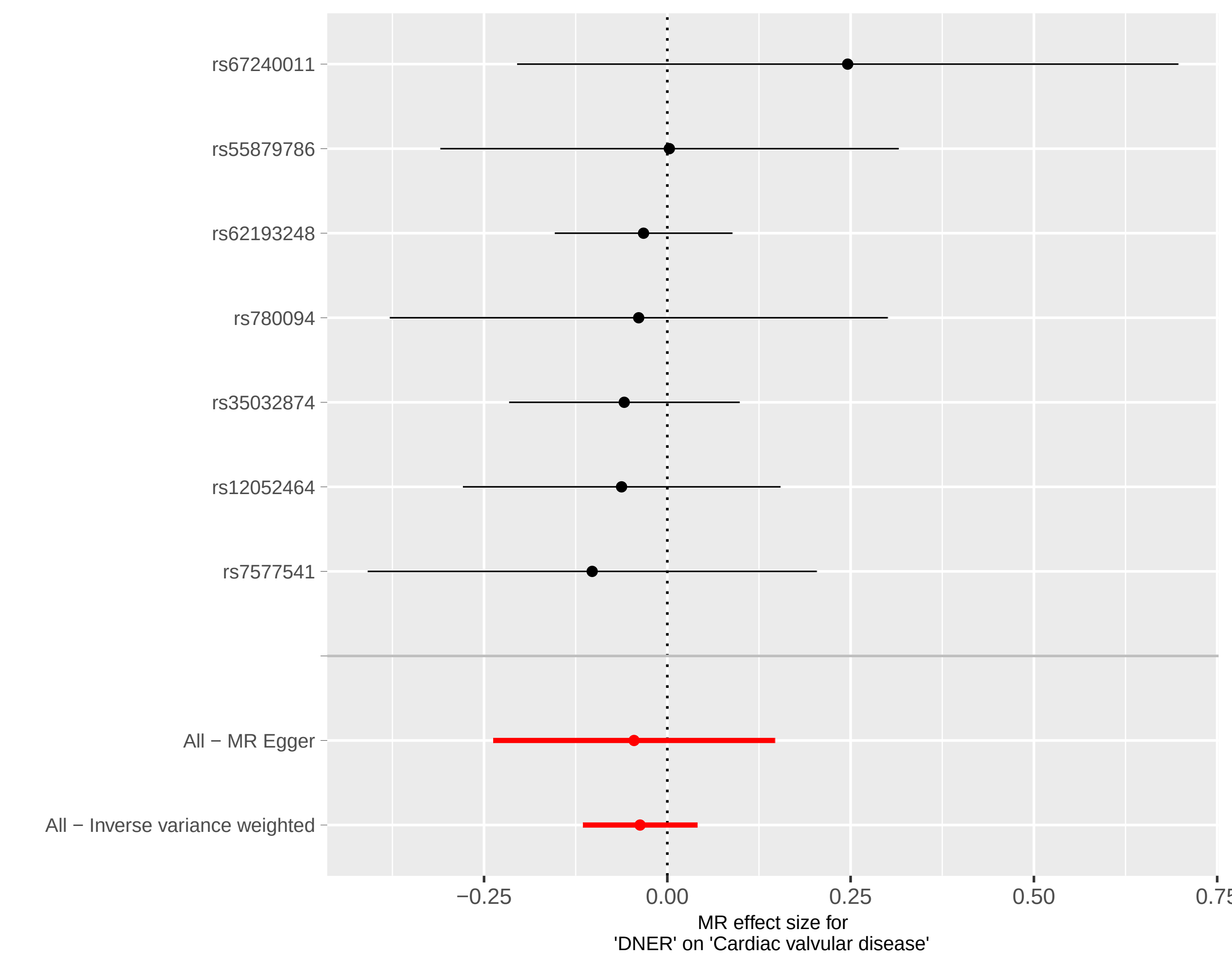
rs67240011
rs55879786
rs62193248
rs780094
rs35032874
rs12052464
rs7577541
All − MR Egger
All − Inverse variance weighted
−0.25
0.00
0.25
0.50
0.75
MR effect size for
'DNER' on 'Cardiac valvular disease'

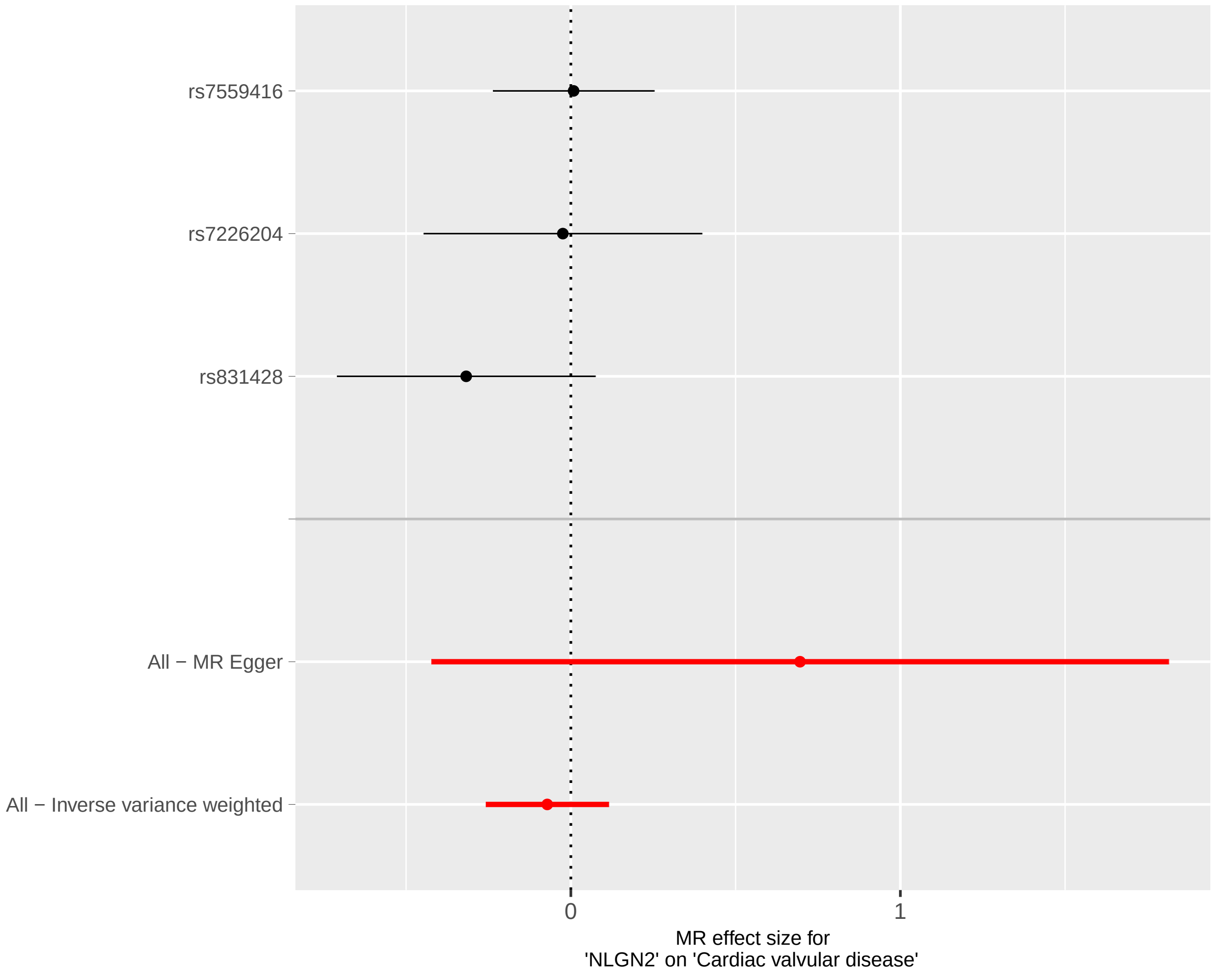

rs7559416
rs7226204
rs831428
All − MR Egger
All − Inverse variance weighted
0
1
MR effect size for
'NLGN2' on 'Cardiac valvular disease'

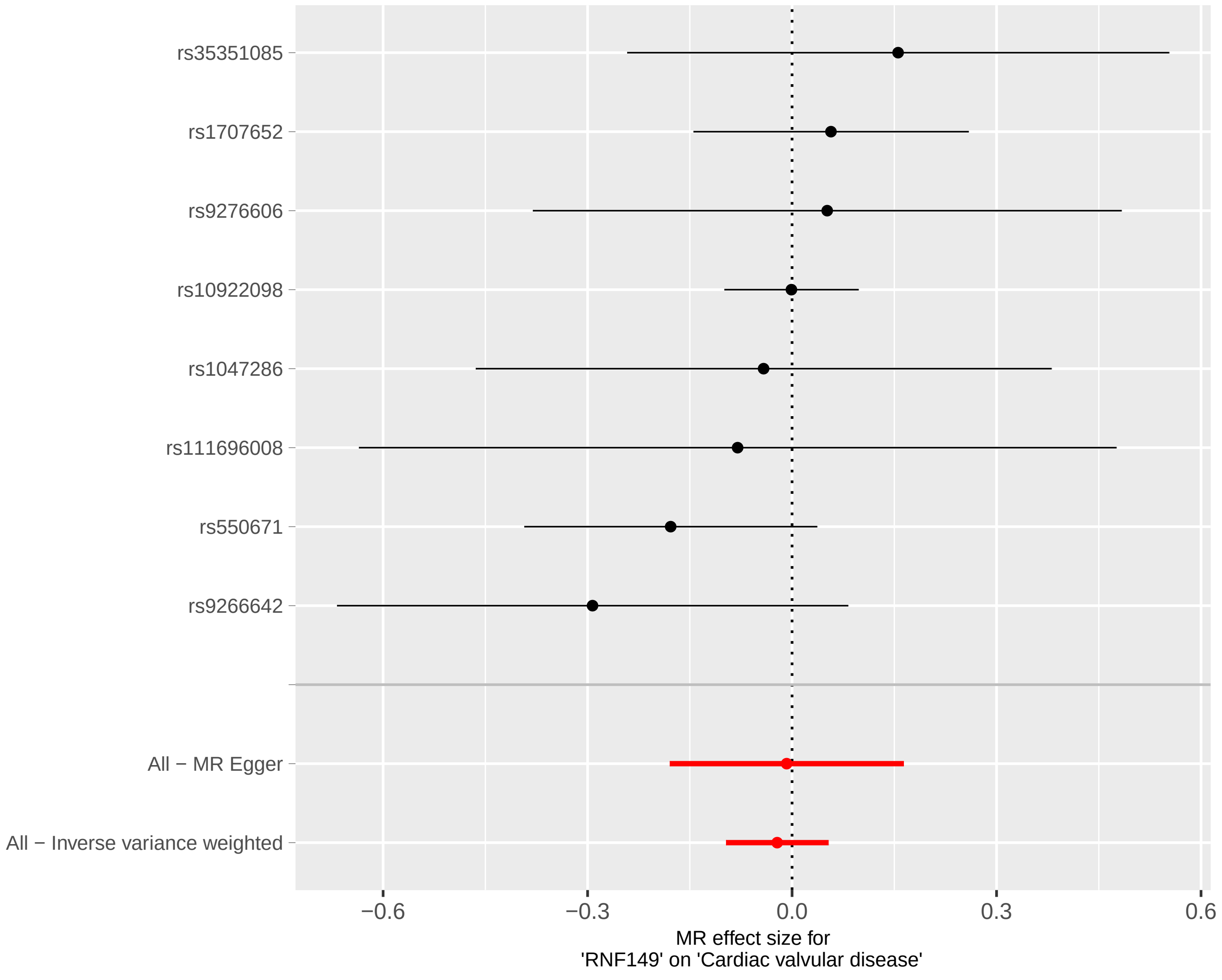

rs35351085
rs1707652
rs9276606
rs10922098
rs1047286
rs111696008
rs550671
rs9266642
All − MR Egger
All − Inverse variance weighted
−0.6
−0.3
0.0
0.3
0.6
MR effect size for
'RNF149' on 'Cardiac valvular disease'

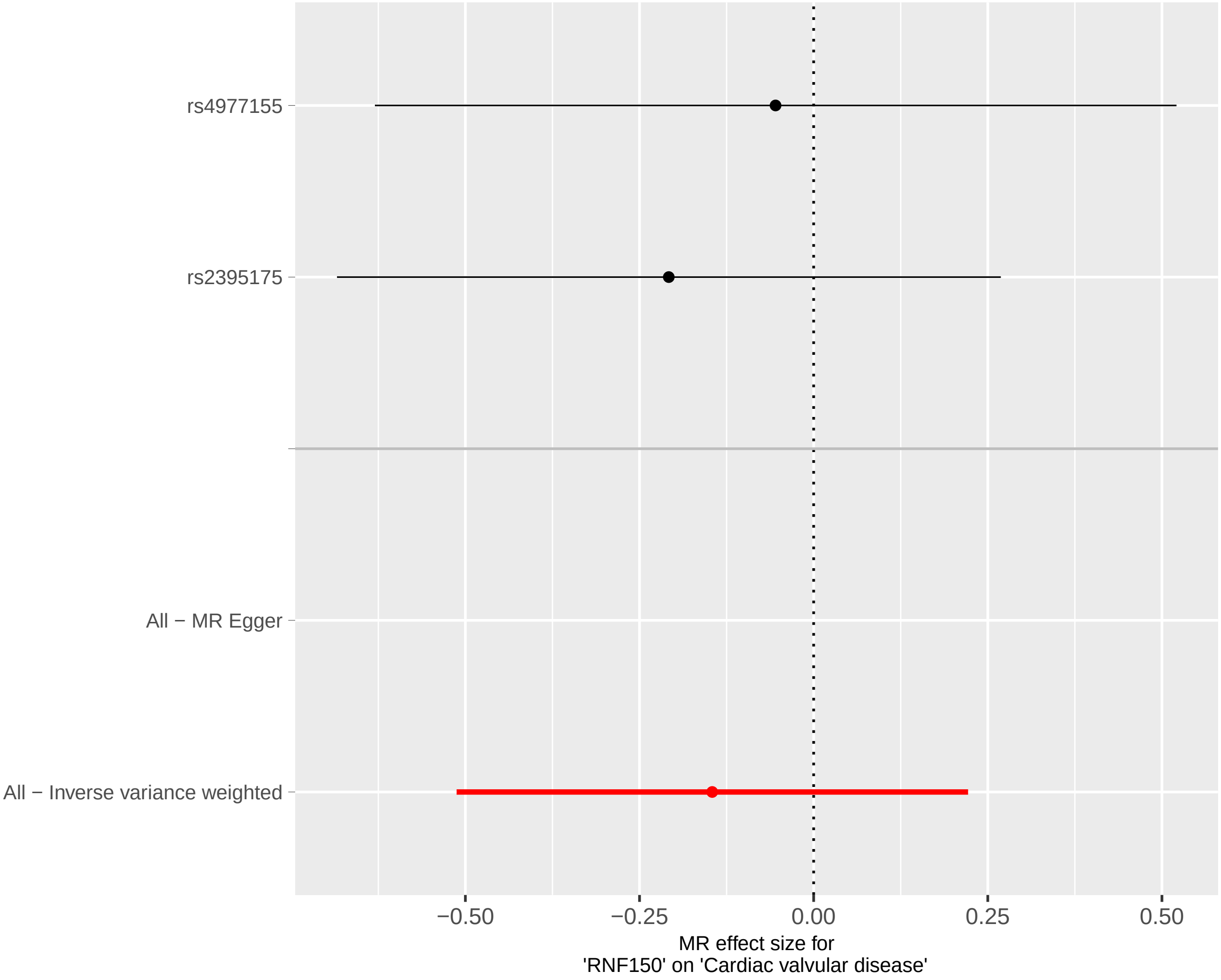
rs4977155
rs2395175
All − MR Egger
All − Inverse variance weighted
−0.50
−0.25
0.00
0.25
0.50
MR effect size for
'RNF150' on 'Cardiac valvular disease'

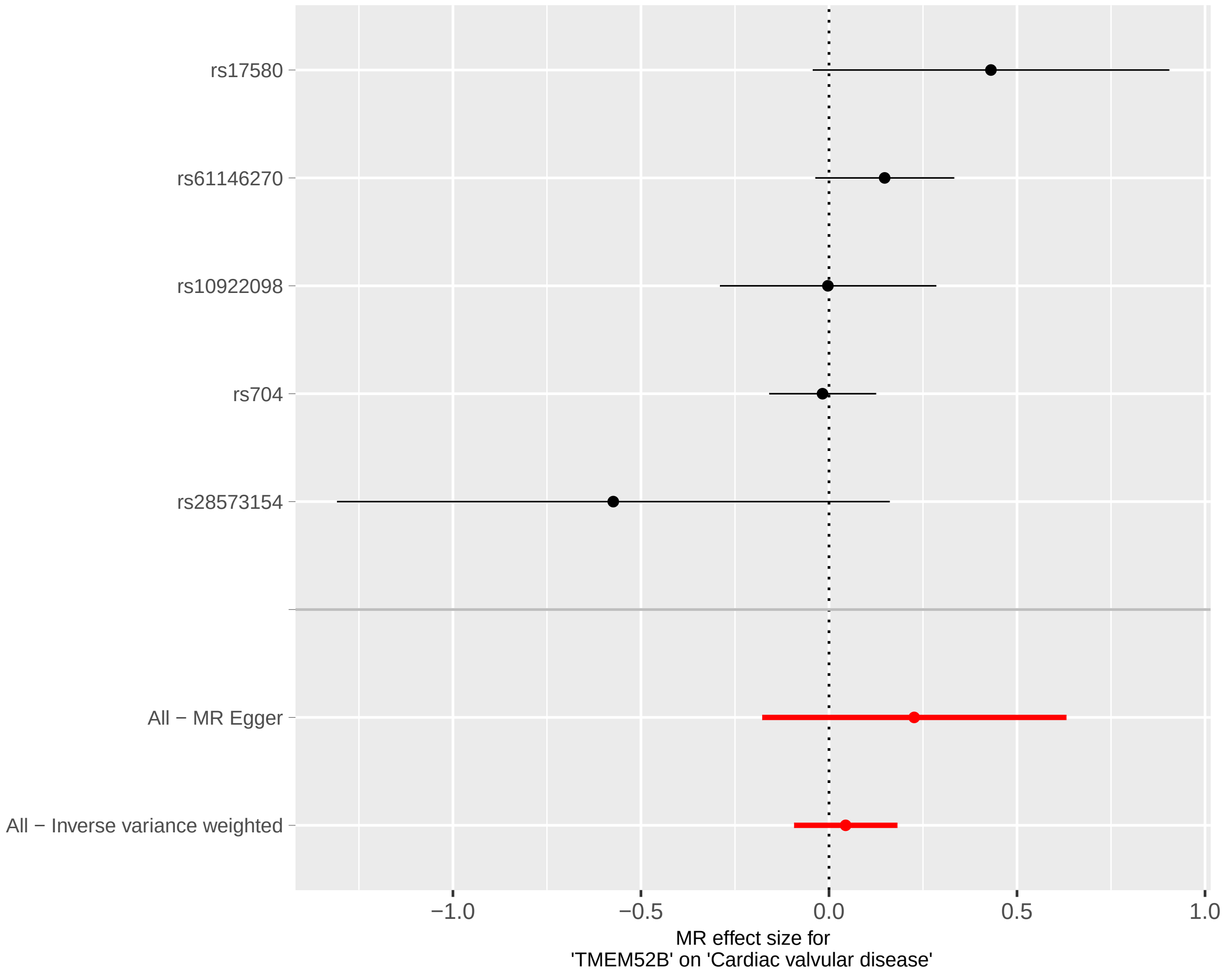
rs17580
rs61146270
rs10922098
rs704
rs28573154
All − MR Egger
All − Inverse variance weighted
−1.0
−0.5
0.0
0.5
1.0
MR effect size for
'TMEM52B' on 'Cardiac valvular disease'

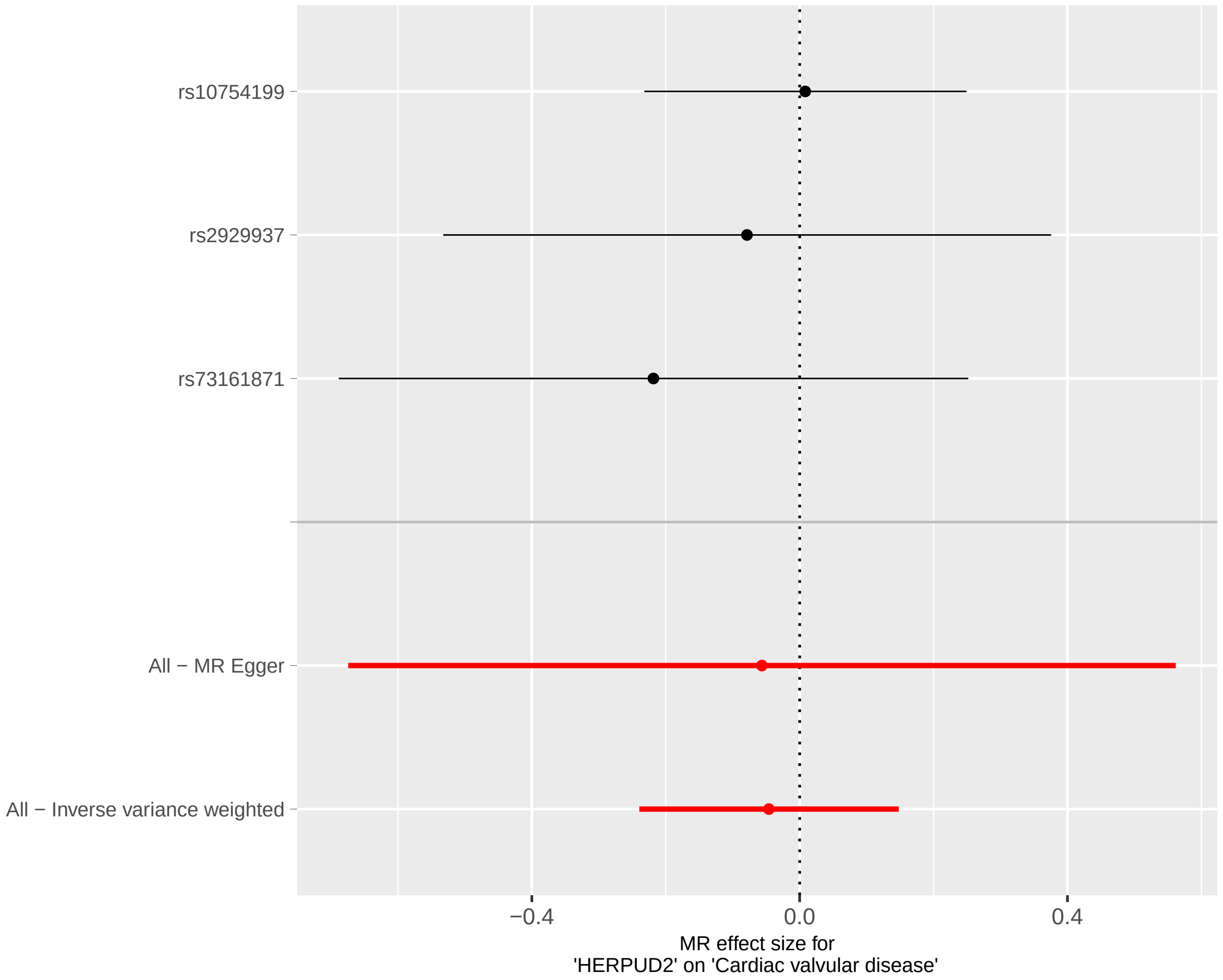
rs10754199
rs2929937
rs73161871
All − MR Egger
All − Inverse variance weighted
−0.4
0.0
0.4
MR effect size for
'HERPUD2' on 'Cardiac valvular disease'

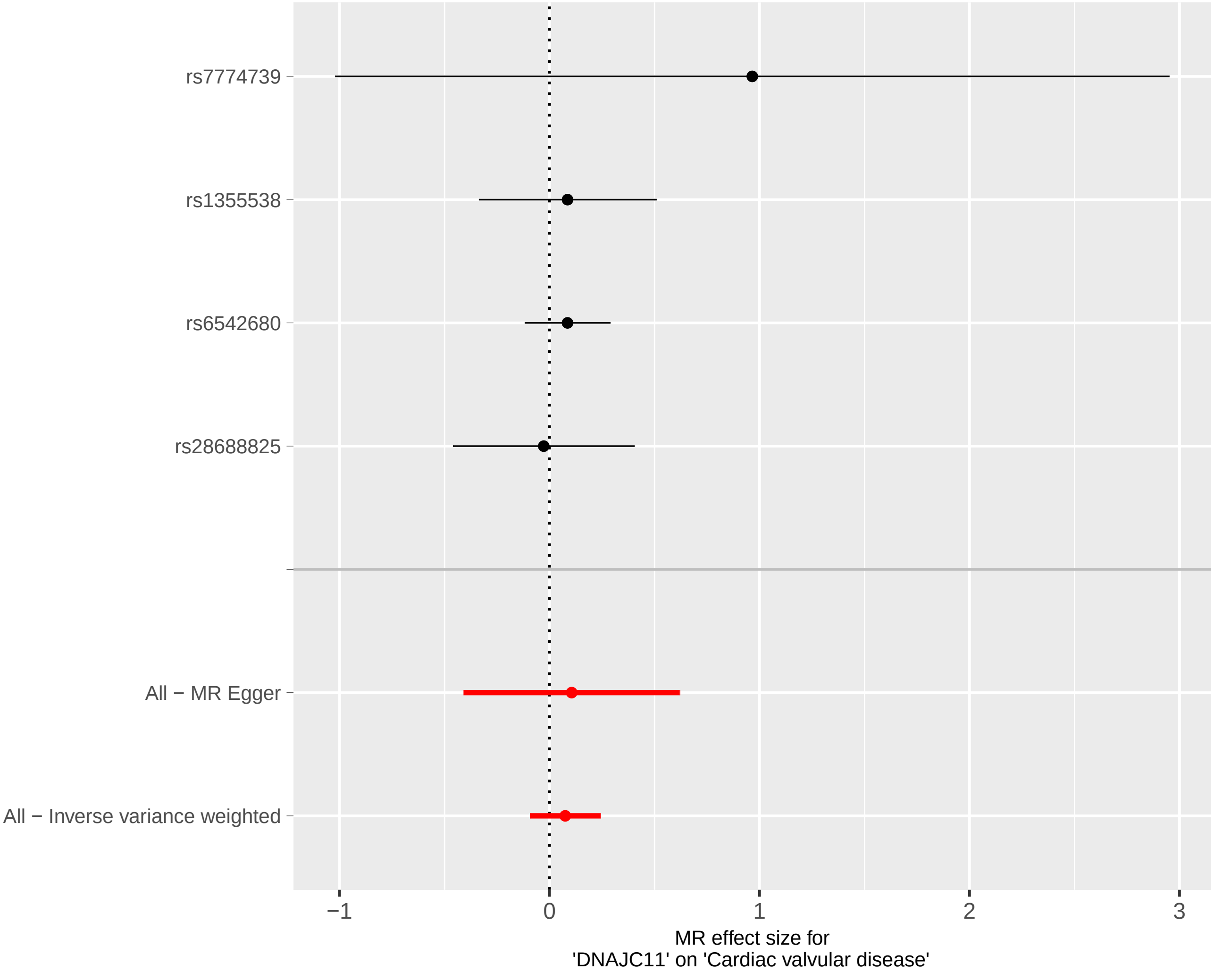
rs7774739
rs1355538
rs6542680
rs28688825
All − MR Egger
All − Inverse variance weighted
−1
0
1
2
3
MR effect size for
'DNAJC11' on 'Cardiac valvular disease'

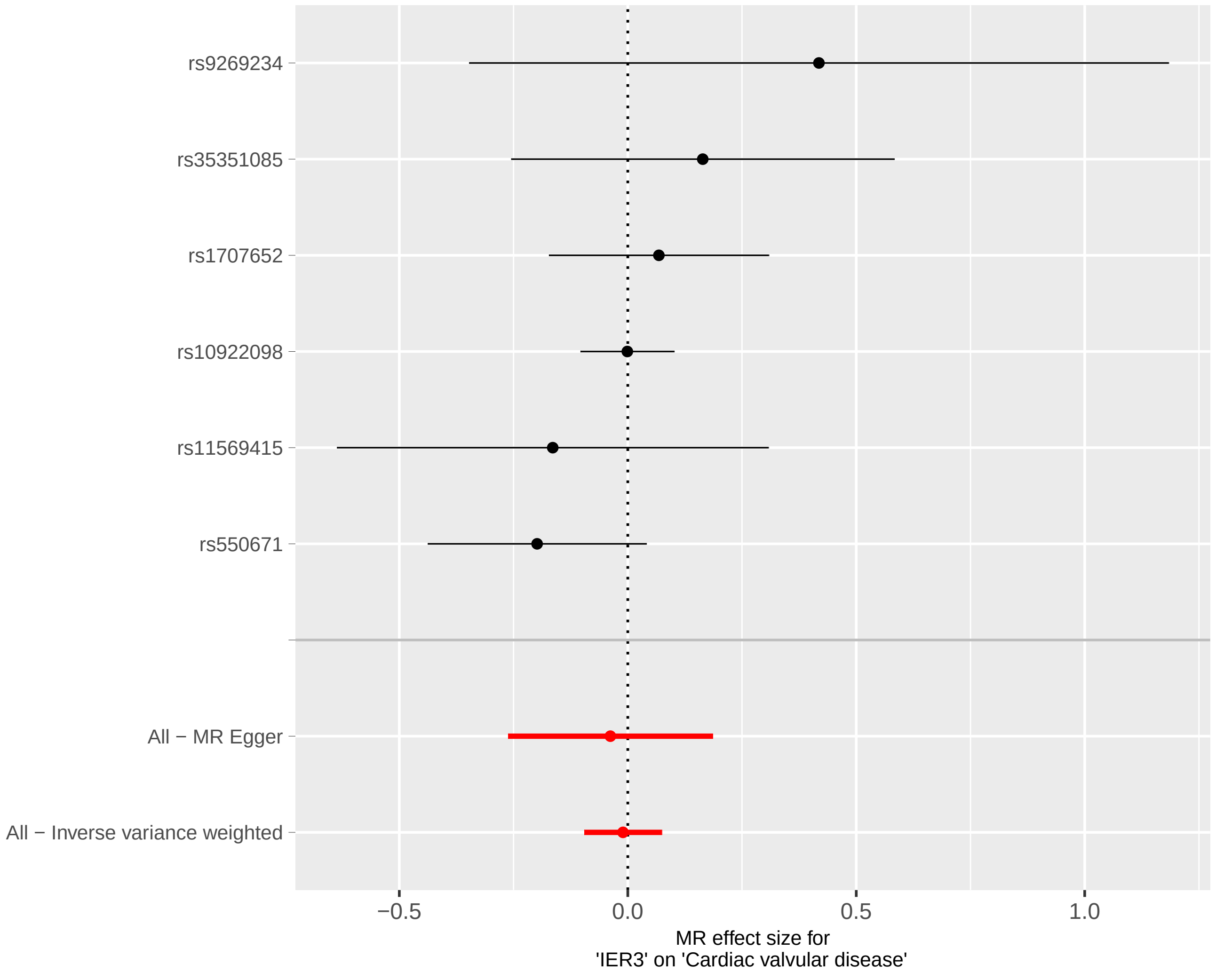
rs9269234
rs35351085
rs1707652
rs10922098
rs11569415
rs550671
All − MR Egger
All − Inverse variance weighted
−0.5
0.0
0.5
1.0
MR effect size for
'IER3' on 'Cardiac valvular disease'

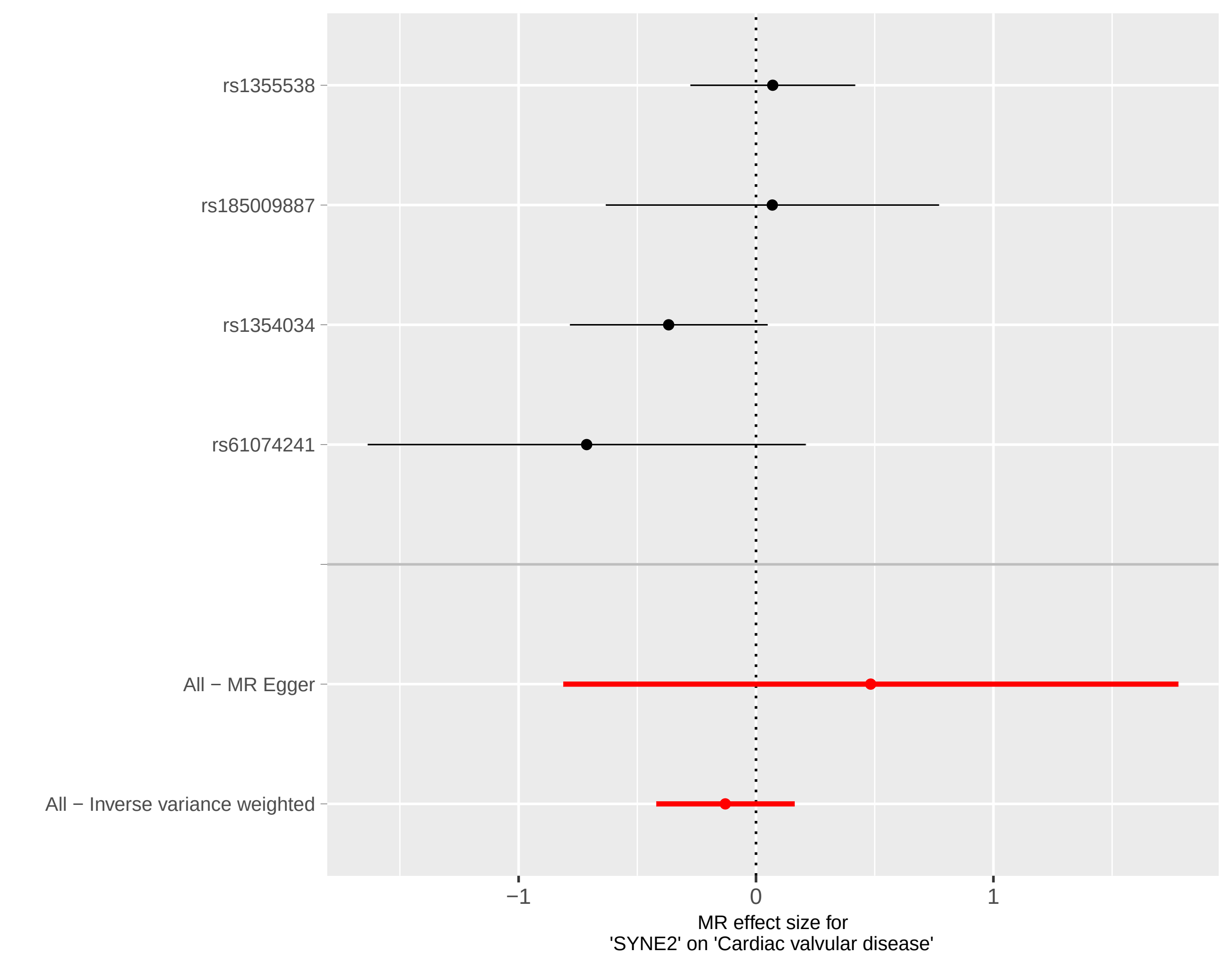

rs1355538
rs185009887
rs1354034
rs61074241
All − MR Egger
All − Inverse variance weighted
−1
0
1
MR effect size for
'SYNE2' on 'Cardiac valvular disease'

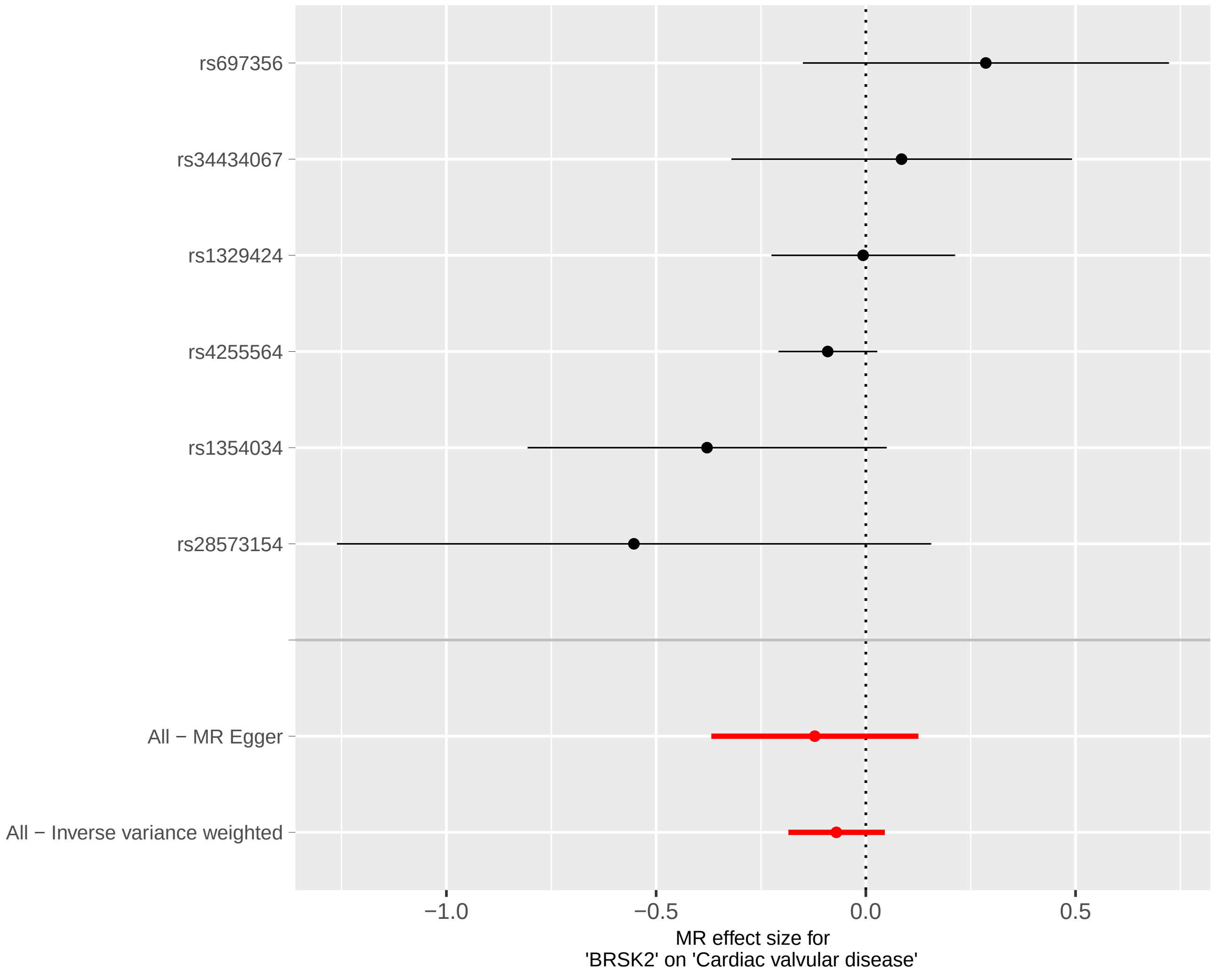
rs697356
rs34434067
rs1329424
rs4255564
rs1354034
rs28573154
All − MR Egger
All − Inverse variance weighted
−1.0
−0.5
0.0
0.5
MR effect size for
'BRSK2' on 'Cardiac valvular disease'

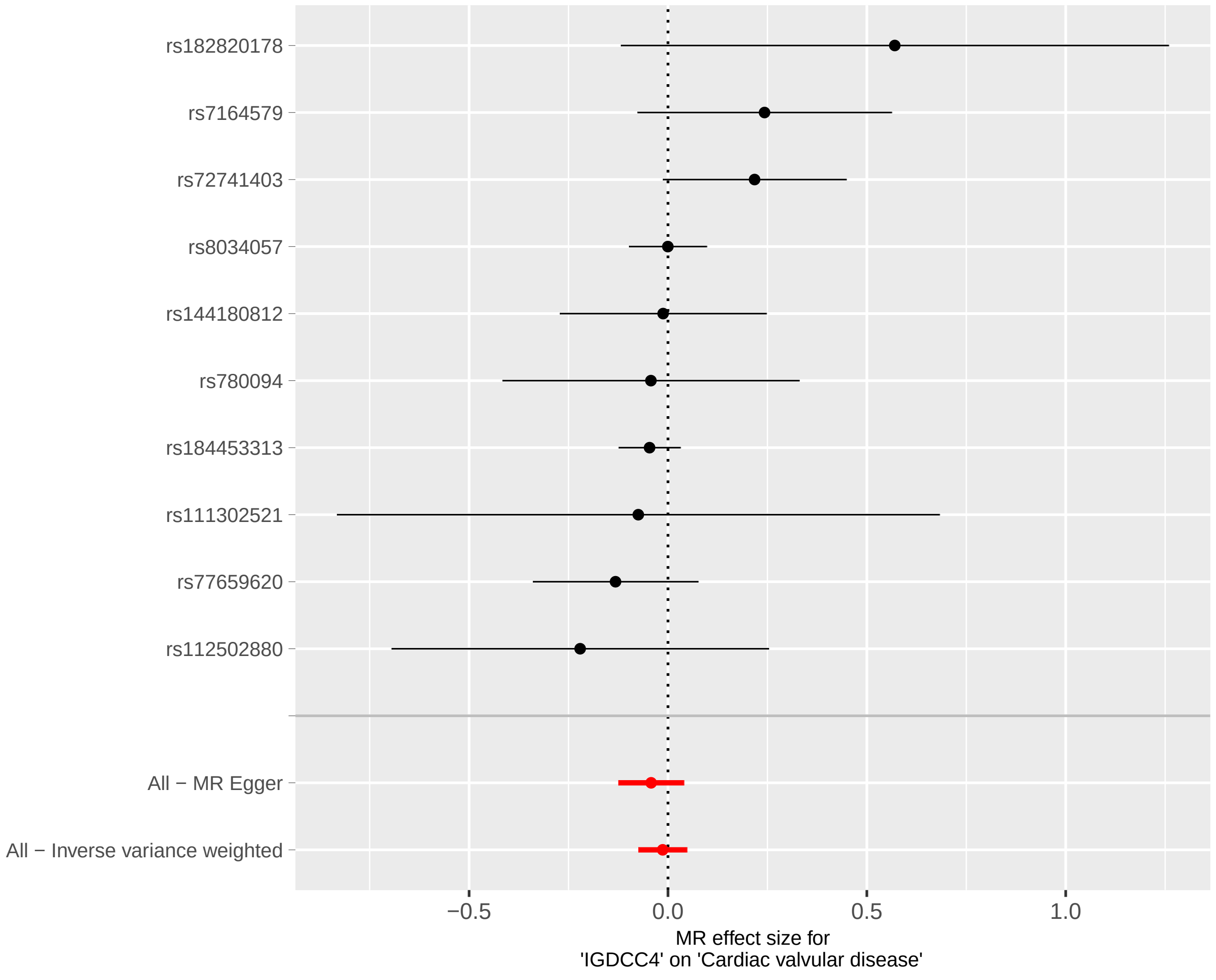
rs182820178
rs7164579
rs72741403
rs8034057
rs144180812
rs780094
rs184453313
rs111302521
rs77659620
rs112502880
All − MR Egger
All − Inverse variance weighted
−0.5
0.0
0.5
1.0
MR effect size for
'IGDCC4' on 'Cardiac valvular disease'

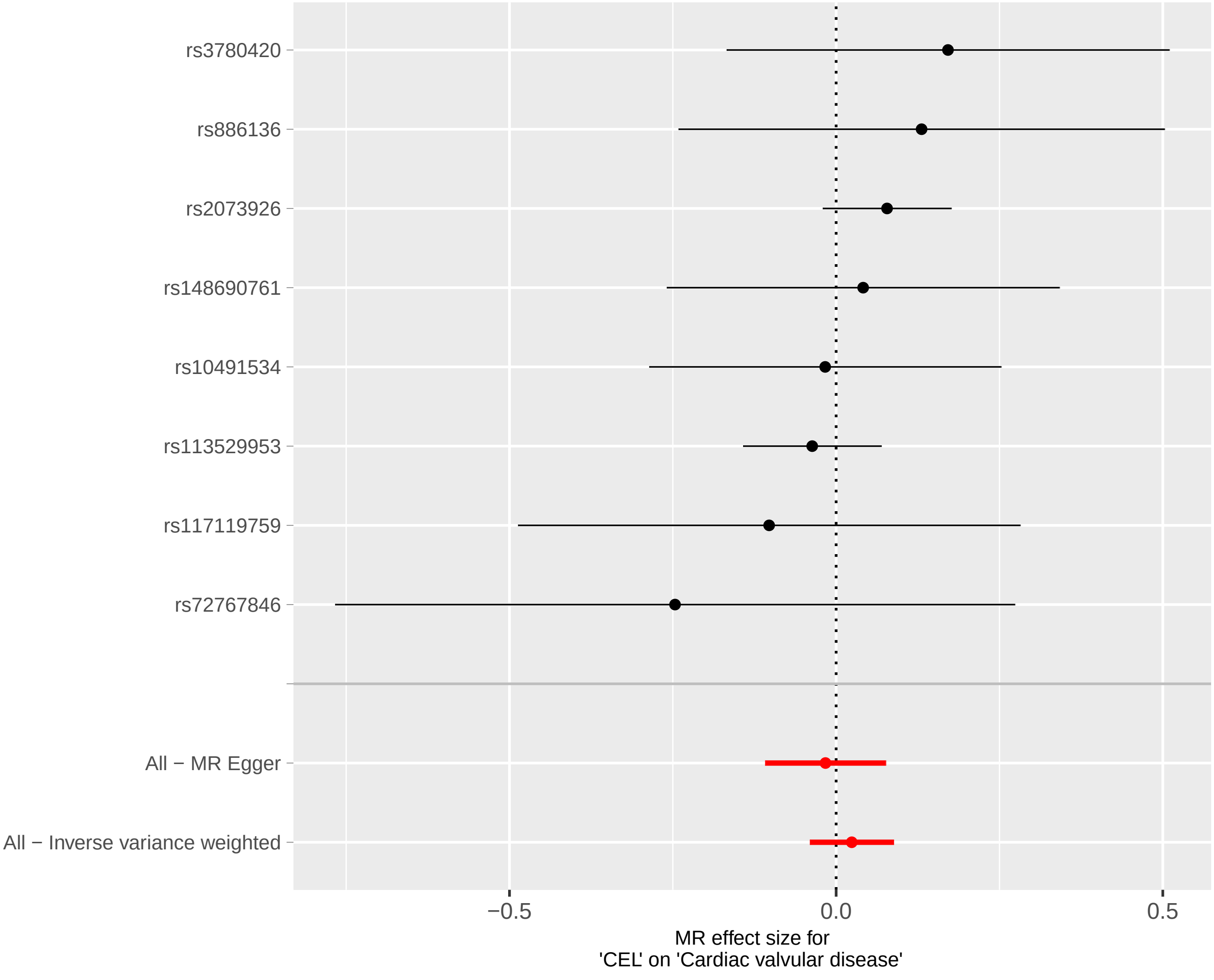
rs3780420
rs886136
rs2073926
rs148690761
rs10491534
rs113529953
rs117119759
rs72767846
All − MR Egger
All − Inverse variance weighted
−0.5
0.0
0.5
MR effect size for
'CEL' on 'Cardiac valvular disease'

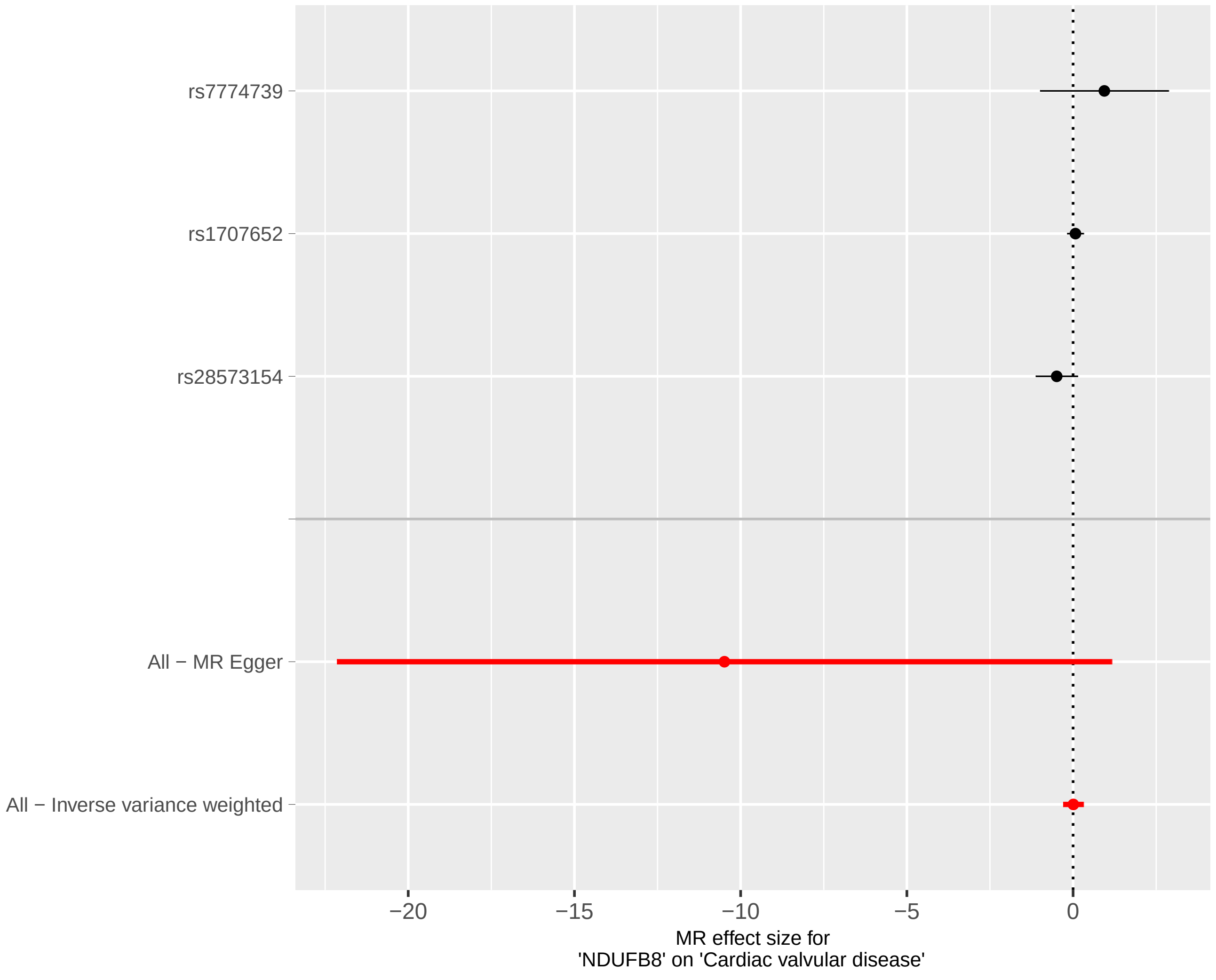
rs7774739
rs1707652
rs28573154
All − MR Egger
All − Inverse variance weighted
−20
−15
−10
−5
0
MR effect size for
'NDUFB8' on 'Cardiac valvular disease'

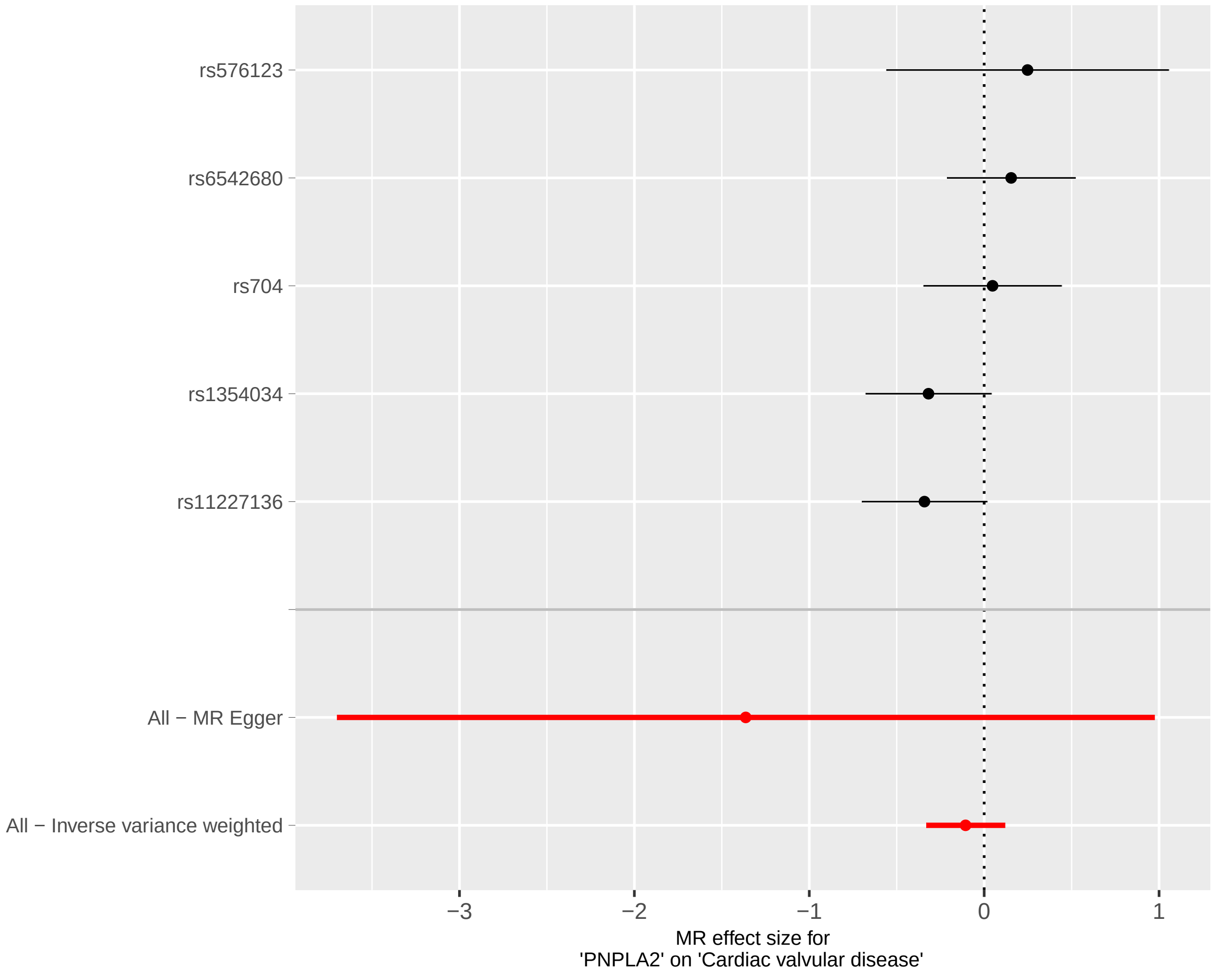
rs576123
rs6542680
rs704
rs1354034
rs11227136
All − MR Egger
All − Inverse variance weighted
−3
−2
−1
0
1
MR effect size for
'PNPLA2' on 'Cardiac valvular disease'

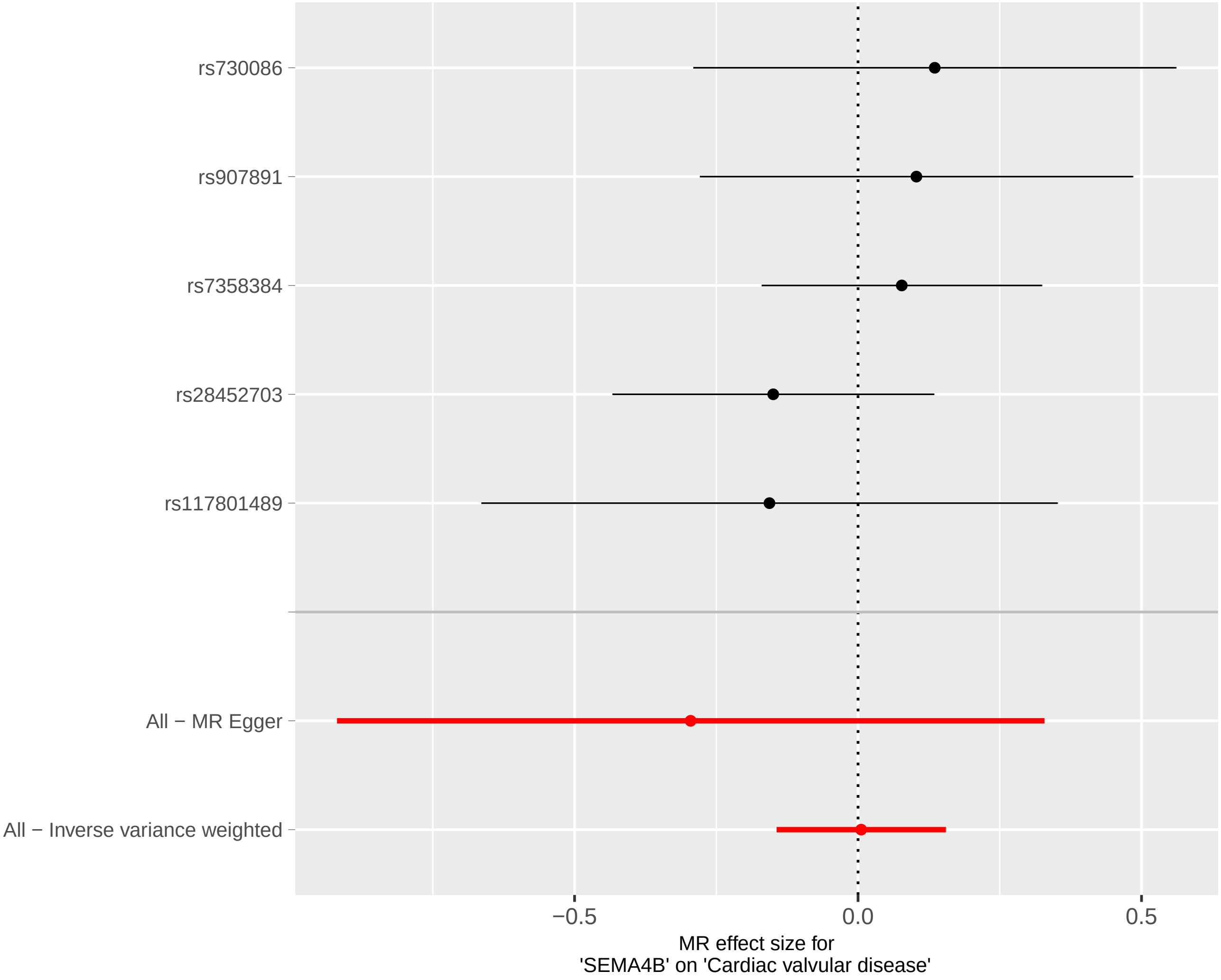
rs730086
rs907891
rs7358384
rs28452703
rs117801489
All − MR Egger
All − Inverse variance weighted
−0.5
0.0
0.5
MR effect size for
'SEMA4B' on 'Cardiac valvular disease'

Insufficient number of SNPs

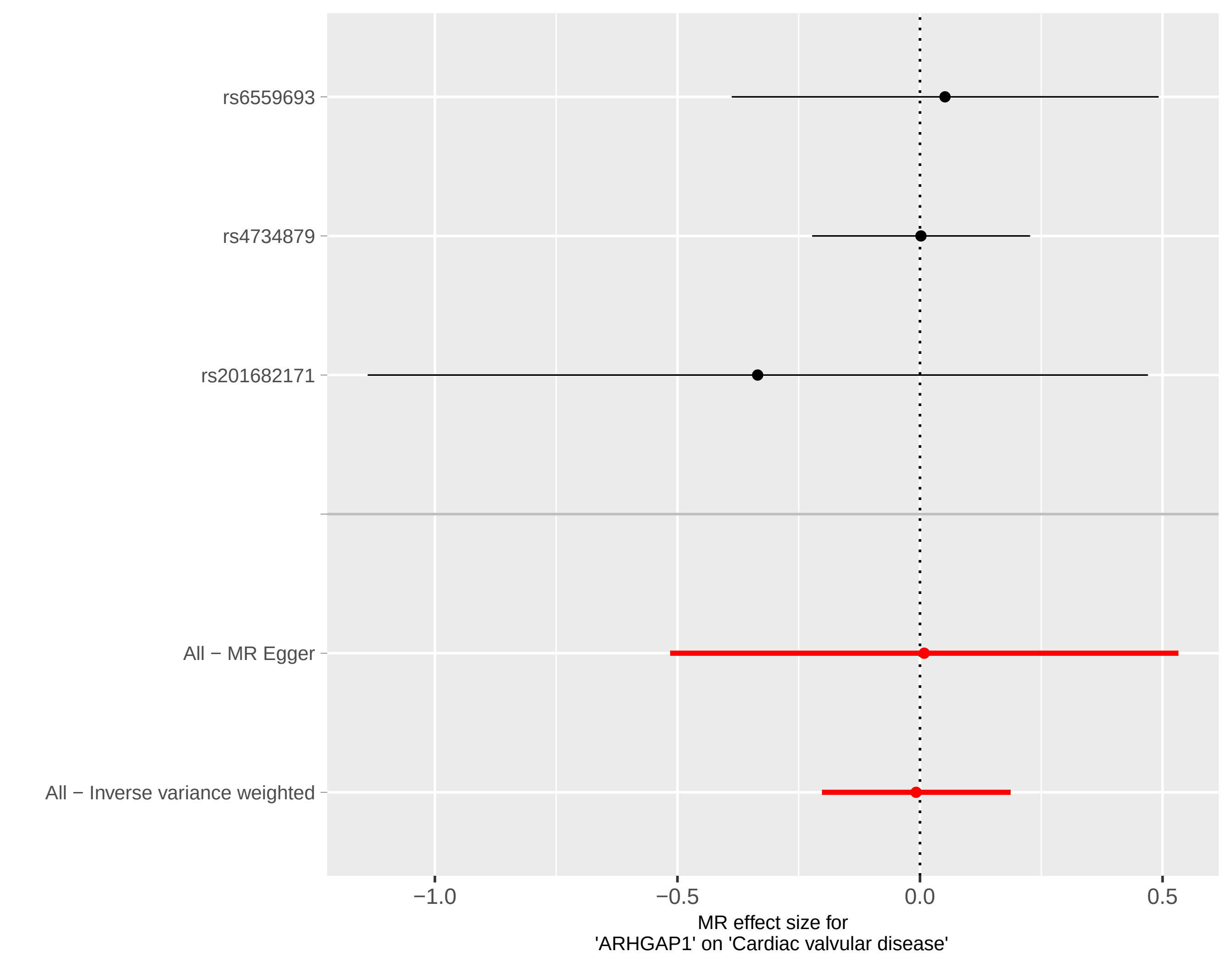
rs6559693
rs4734879
rs201682171
All − MR Egger
All − Inverse variance weighted
−1.0
−0.5
0.0
0.5
MR effect size for
'ARHGAP1' on 'Cardiac valvular disease'

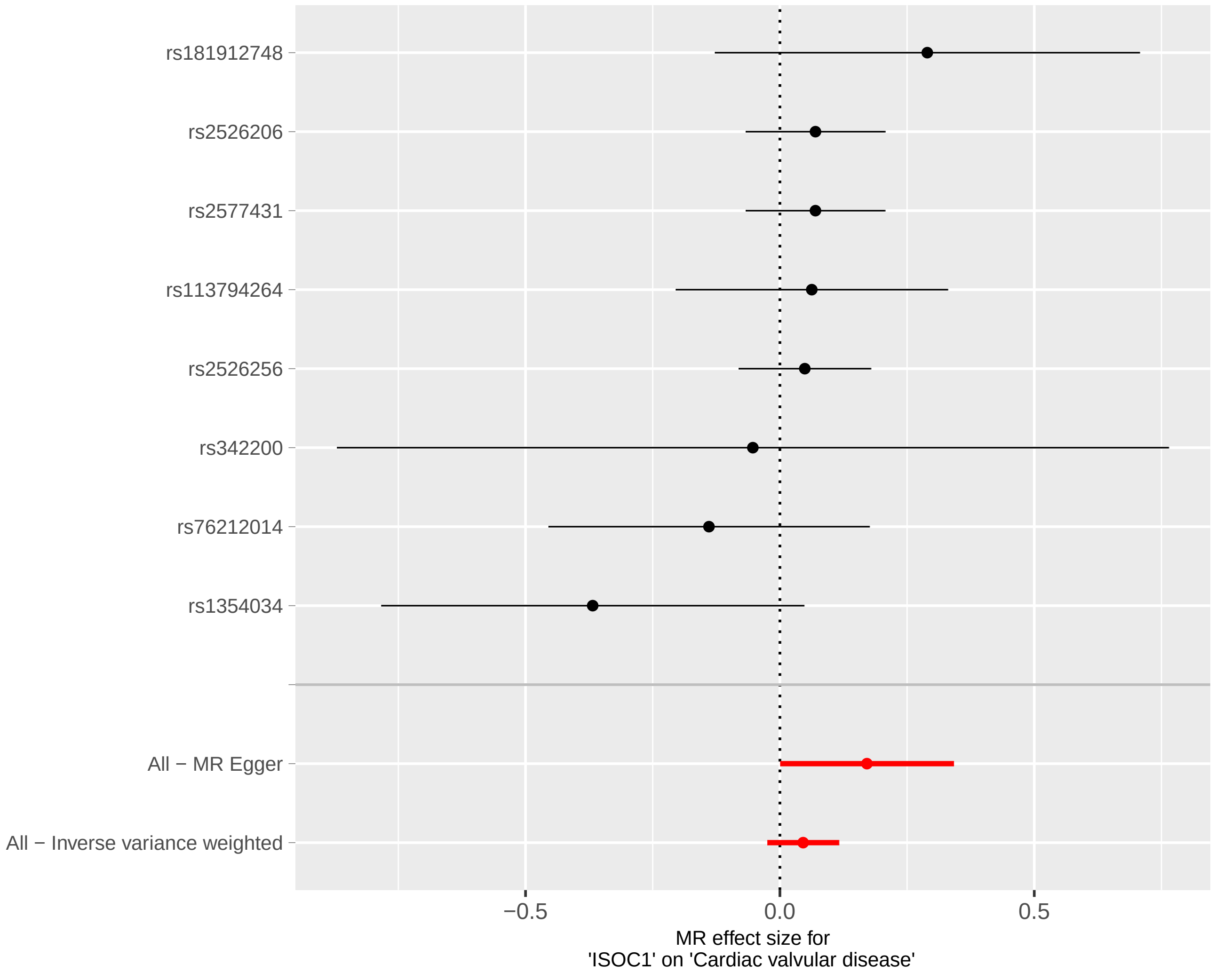
rs181912748
rs2526206
rs2577431
rs113794264
rs2526256
rs342200
rs76212014
rs1354034
All − MR Egger
All − Inverse variance weighted
−0.5
0.0
0.5
MR effect size for
'ISOC1' on 'Cardiac valvular disease'

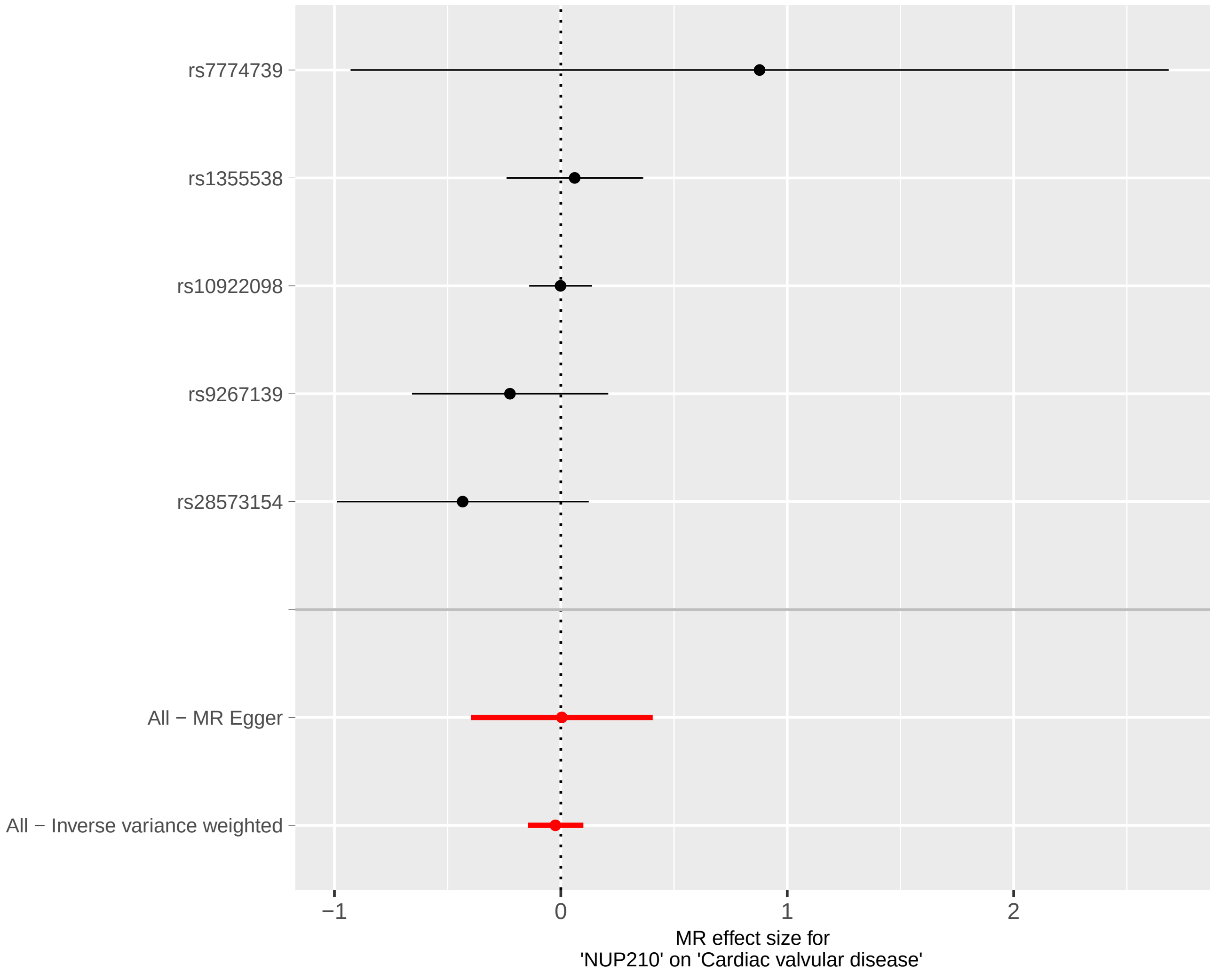

rs7774739
rs1355538
rs10922098
rs9267139
rs28573154
All − MR Egger
All − Inverse variance weighted
−1
0
1
2
MR effect size for
'NUP210' on 'Cardiac valvular disease'

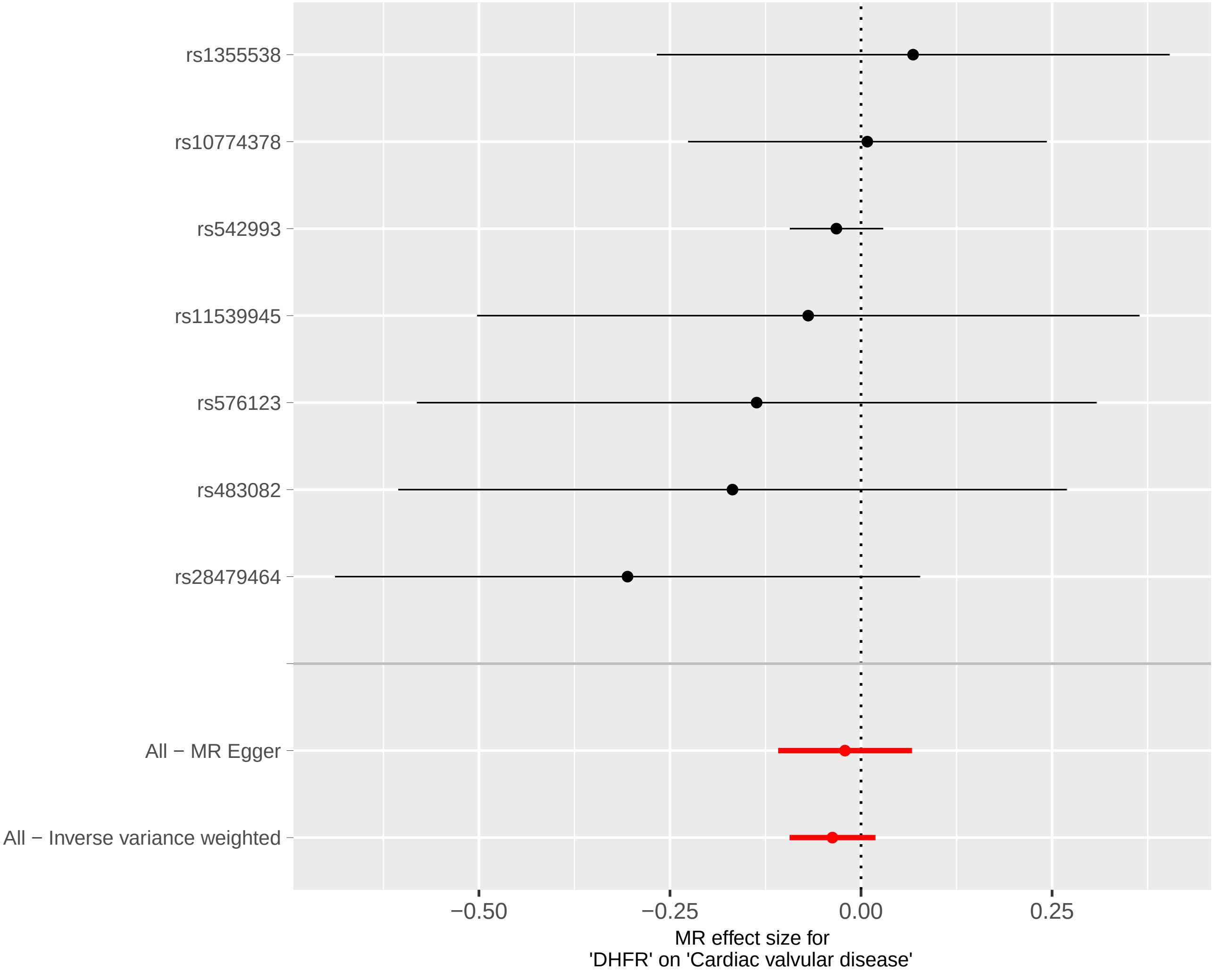
rs1355538
rs10774378
rs542993
rs11539945
rs576123
rs483082
rs28479464
All − MR Egger
All − Inverse variance weighted
−0.50
−0.25
0.00
0.25
MR effect size for
'DHFR' on 'Cardiac valvular disease'

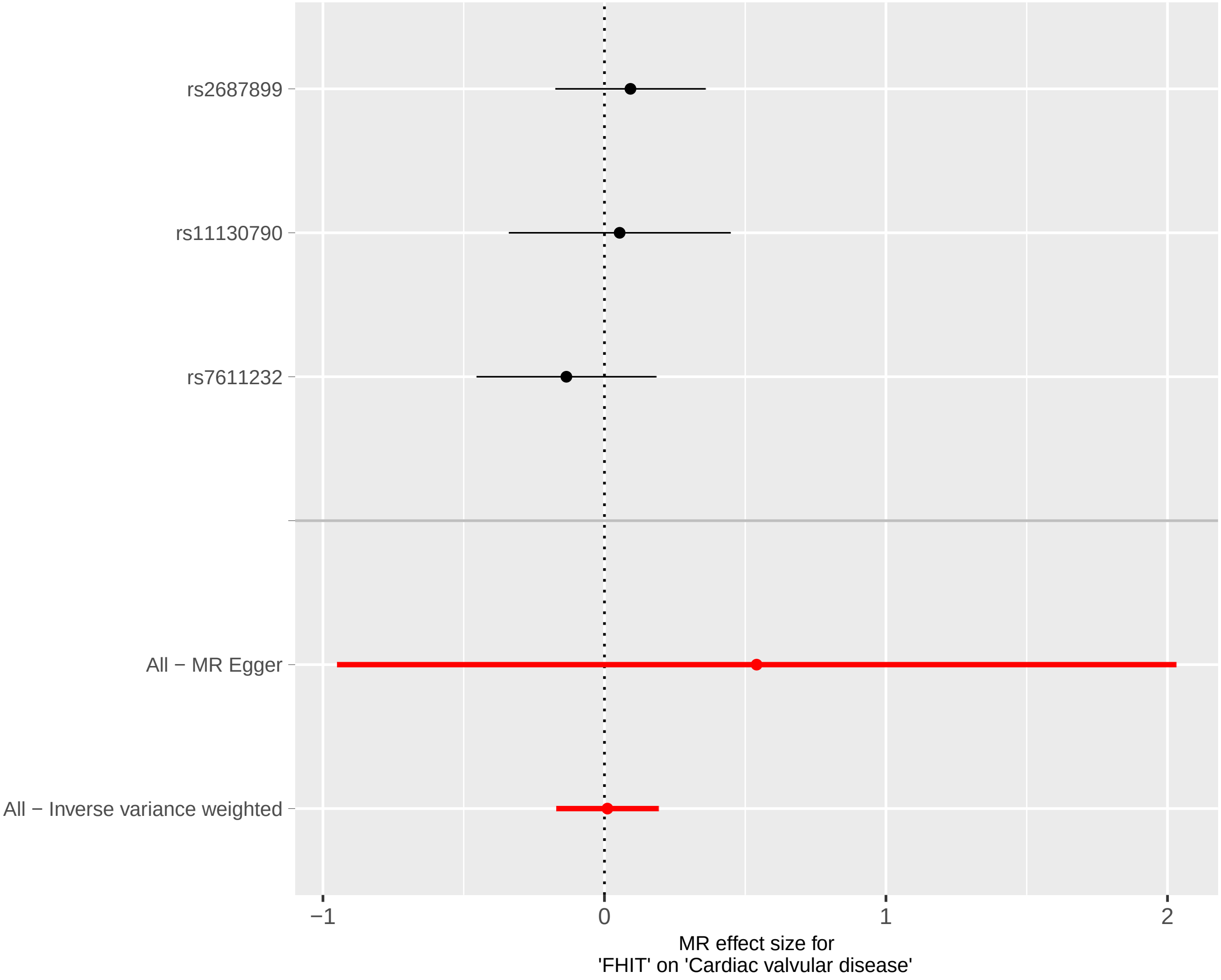
rs2687899
rs11130790
rs7611232
All − MR Egger
All − Inverse variance weighted
−1
0
1
2
MR effect size for
'FHIT' on 'Cardiac valvular disease'

Insufficient number of SNPs

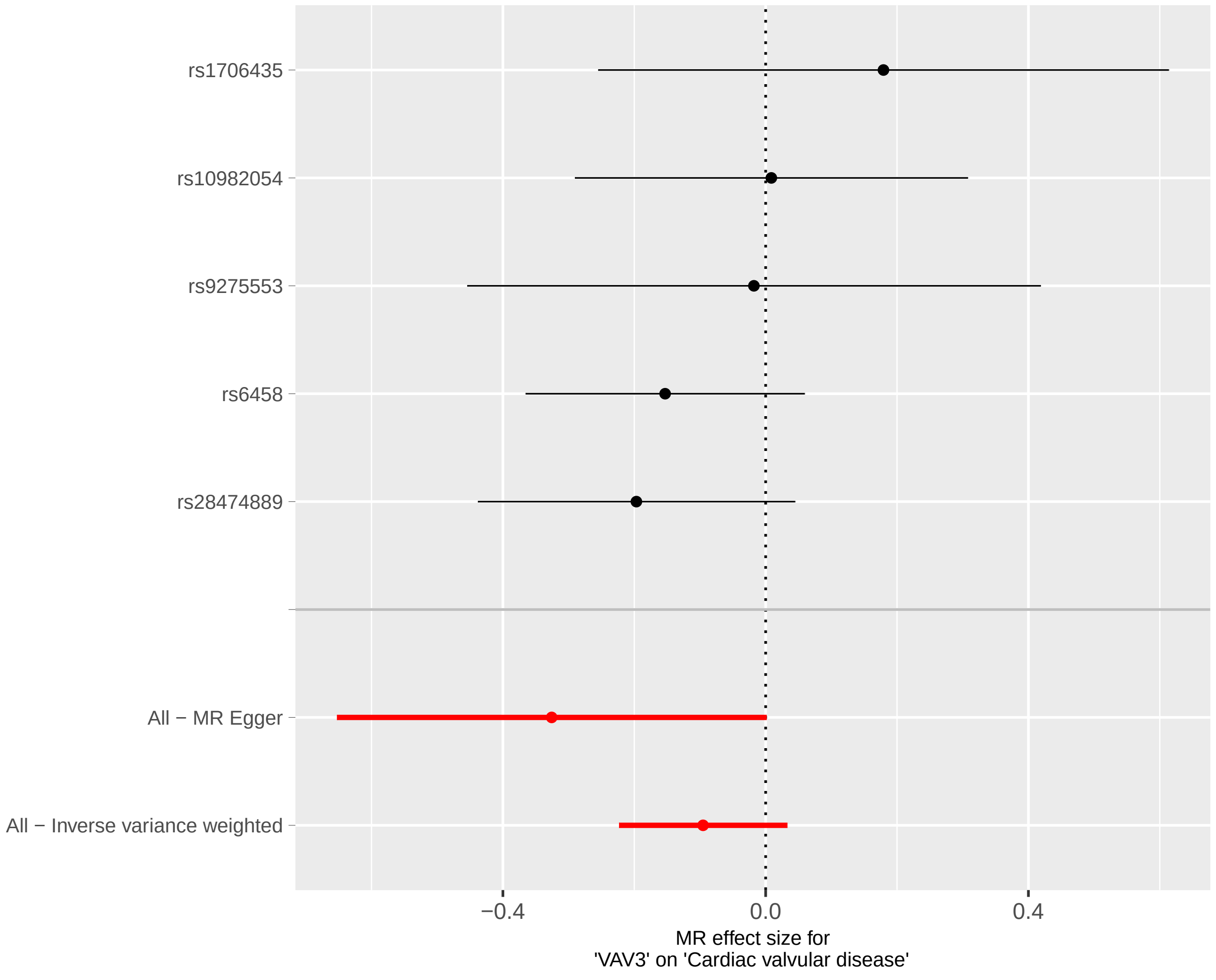

rs1706435
rs10982054
rs9275553
rs6458
rs28474889
All − MR Egger
All − Inverse variance weighted
−0.4
0.0
0.4
MR effect size for
'VAV3' on 'Cardiac valvular disease'

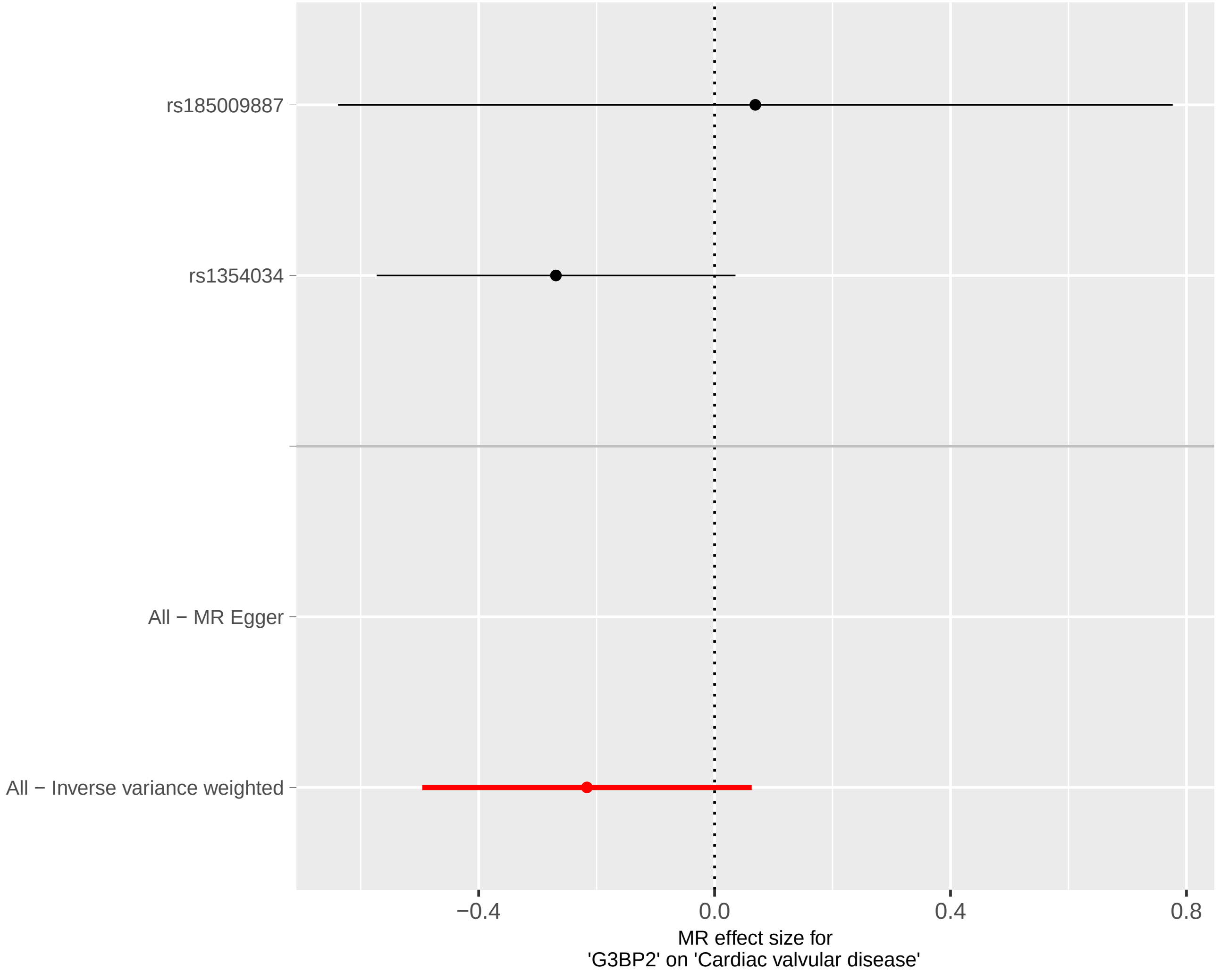

rs185009887
rs1354034
All − MR Egger
All − Inverse variance weighted
−0.4
0.0
0.4
0.8
MR effect size for
'G3BP2' on 'Cardiac valvular disease'

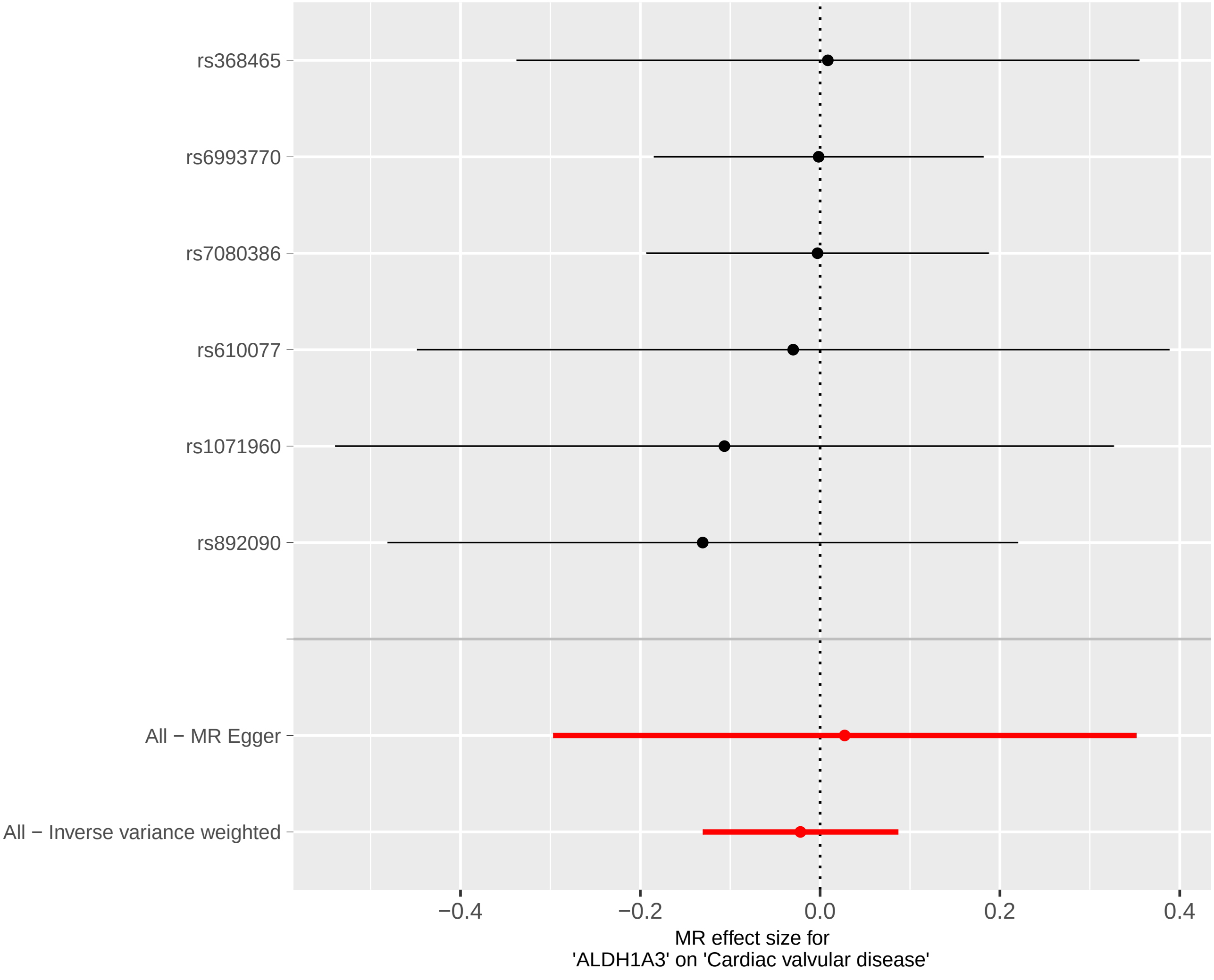
rs368465
rs6993770
rs7080386
rs610077
rs1071960
rs892090
All − MR Egger
All − Inverse variance weighted
−0.4
−0.2
0.0
0.2
0.4
MR effect size for
'ALDH1A3' on 'Cardiac valvular disease'

Insufficient number of SNPs

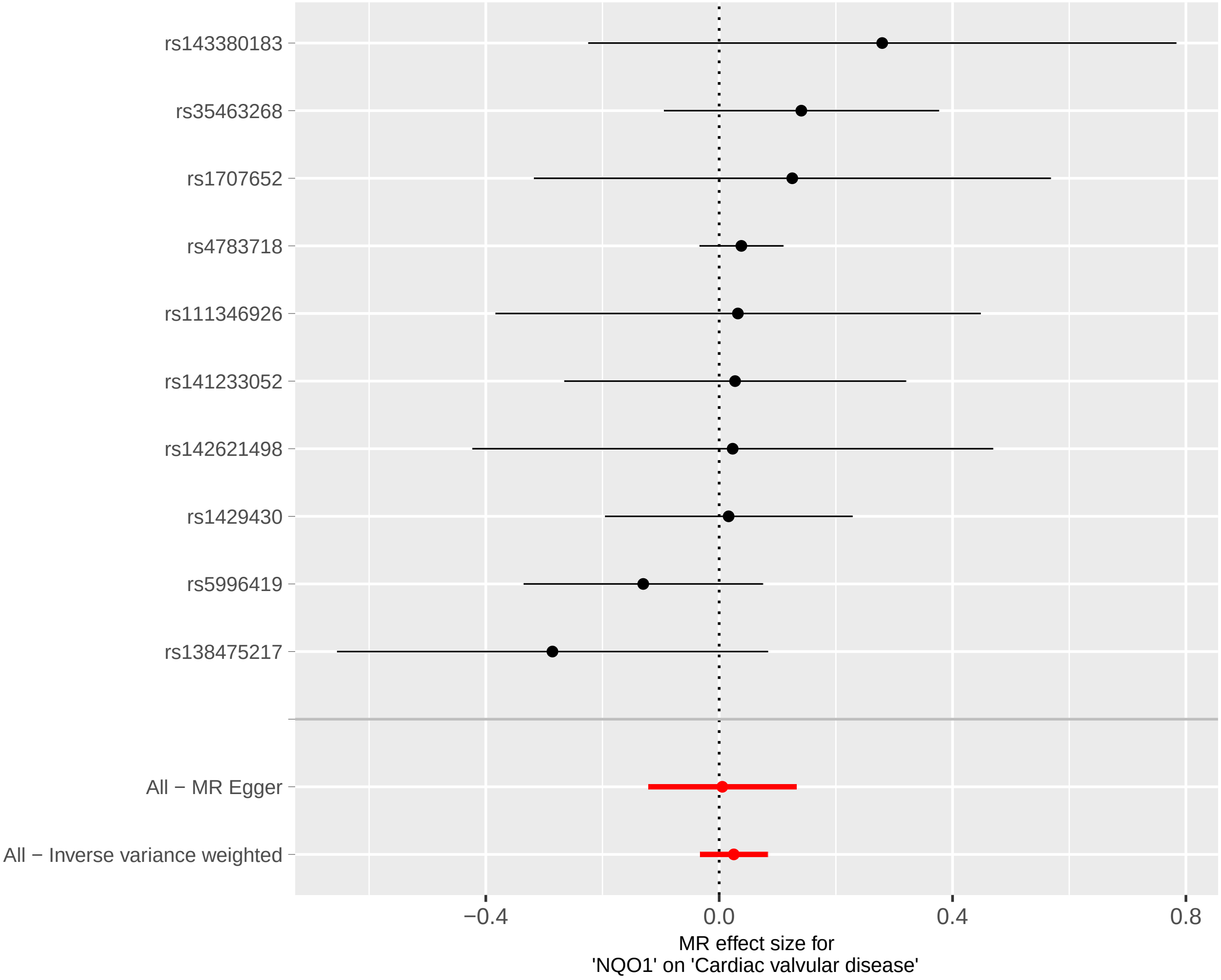
rs143380183
rs35463268
rs1707652
rs4783718
rs111346926
rs141233052
rs142621498
rs1429430
rs5996419
rs138475217
All − MR Egger
All − Inverse variance weighted
−0.4
0.0
0.4
0.8
MR effect size for
'NQO1' on 'Cardiac valvular disease'

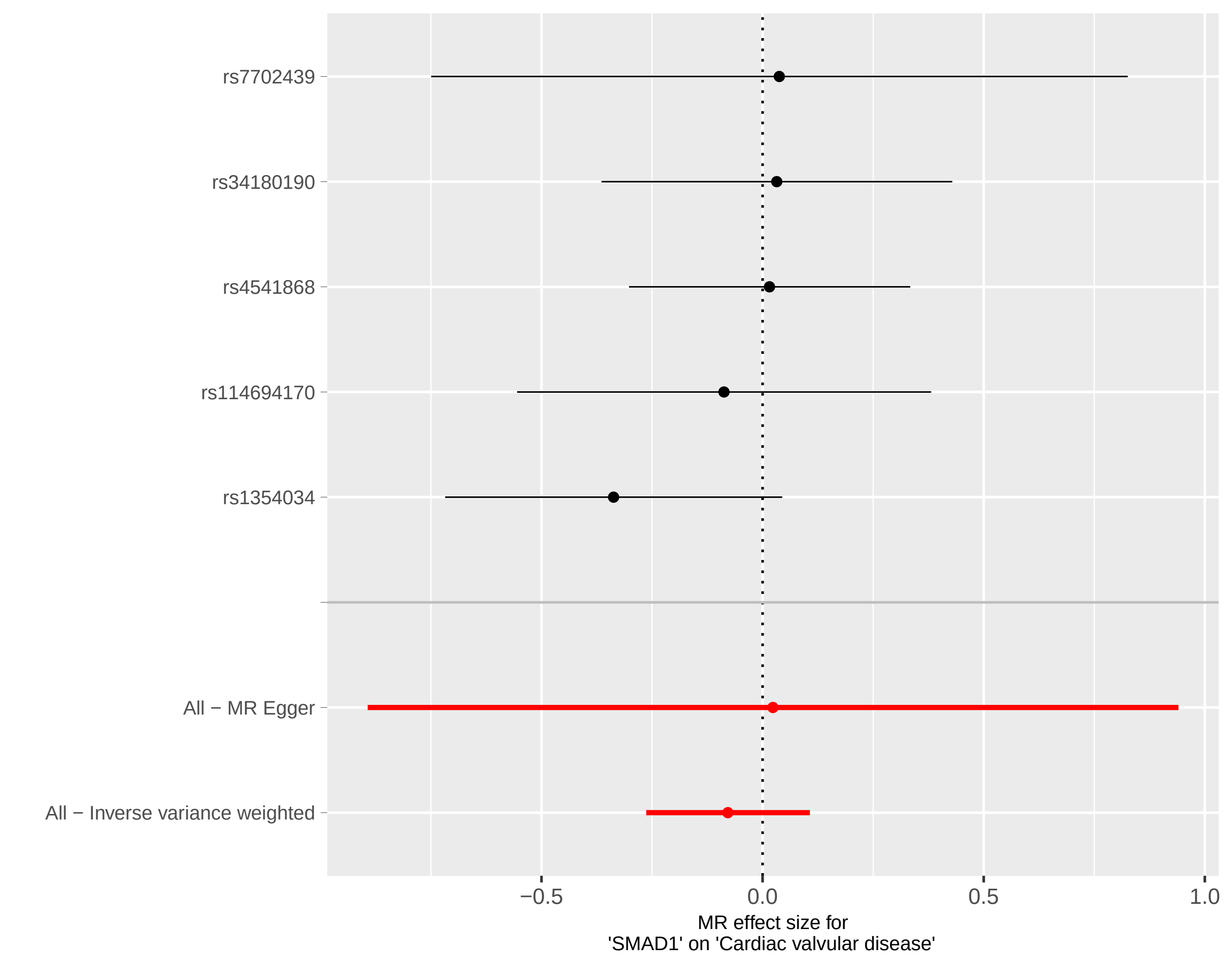
rs7702439
rs34180190
rs4541868
rs114694170
rs1354034
All − MR Egger
All − Inverse variance weighted
−0.5
0.0
0.5
1.0
MR effect size for
'SMAD1' on 'Cardiac valvular disease'

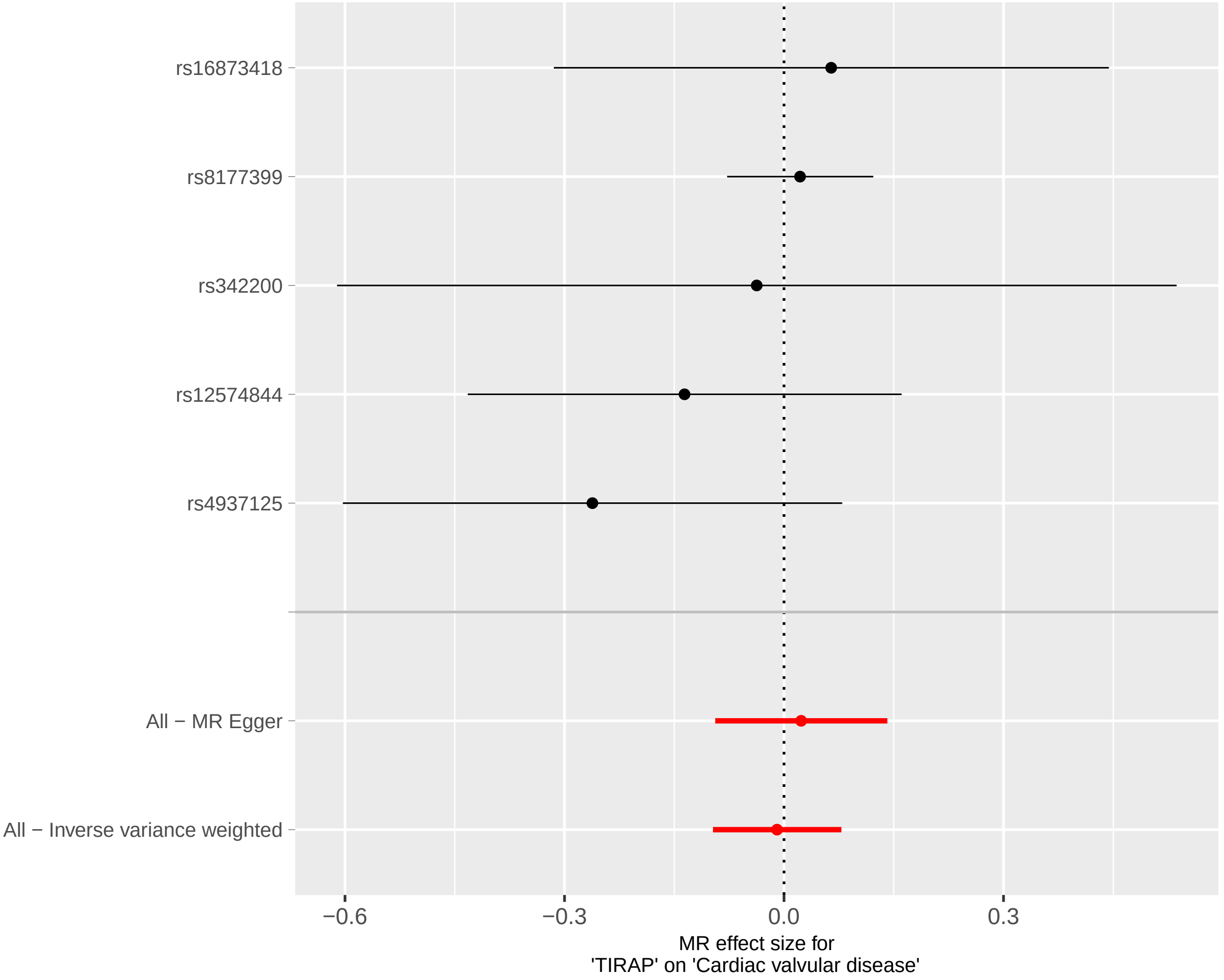
rs16873418
rs8177399
rs342200
rs12574844
rs4937125
All − MR Egger
All − Inverse variance weighted
−0.6
−0.3
0.0
0.3
MR effect size for
'TIRAP' on 'Cardiac valvular disease'

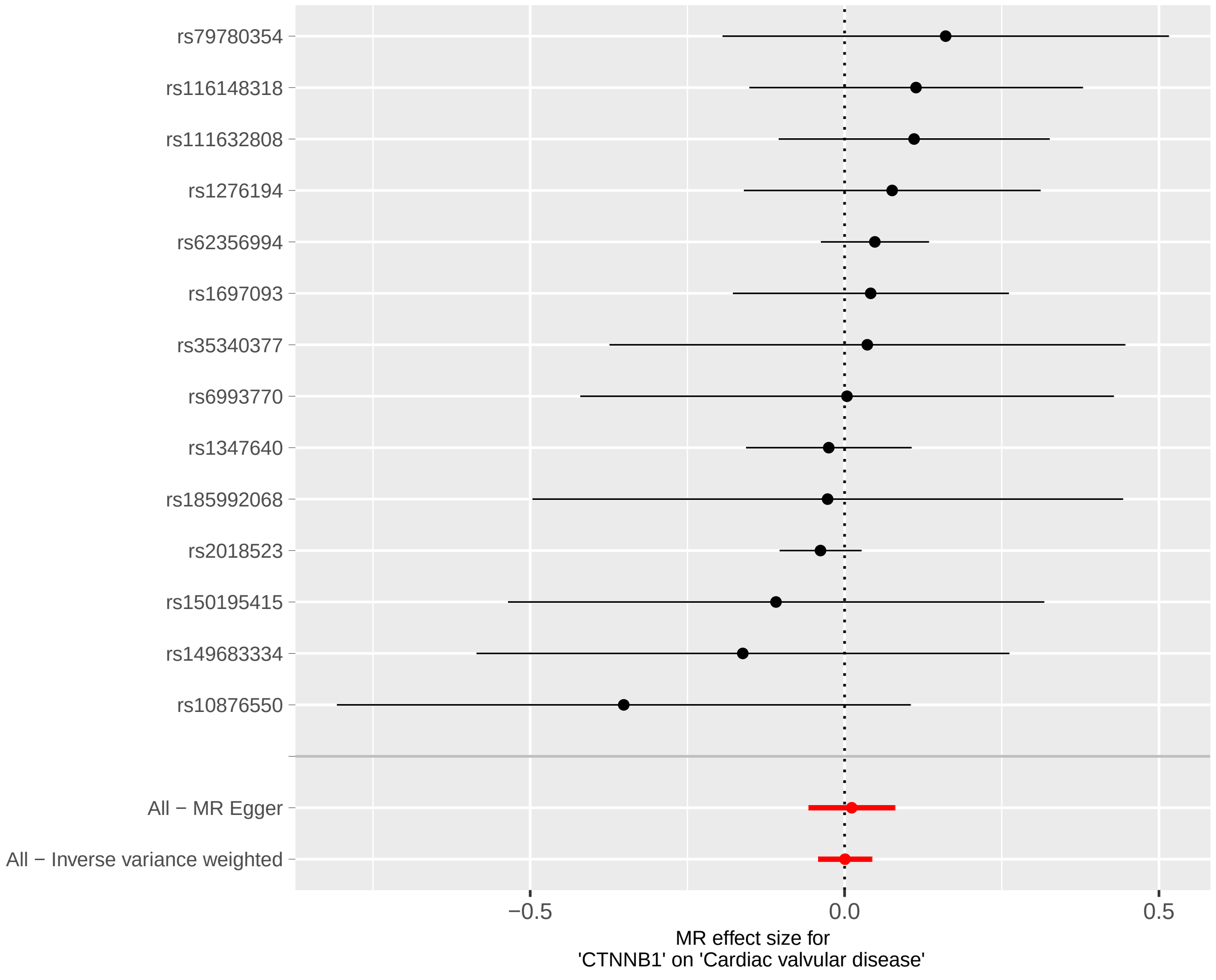

rs79780354
rs116148318
rs111632808
rs1276194
rs62356994
rs1697093
rs35340377
rs6993770
rs1347640
rs185992068
rs2018523
rs150195415
rs149683334
rs10876550
All − MR Egger
All − Inverse variance weighted
−0.5
0.0
0.5
MR effect size for
'CTNNB1' on 'Cardiac valvular disease'

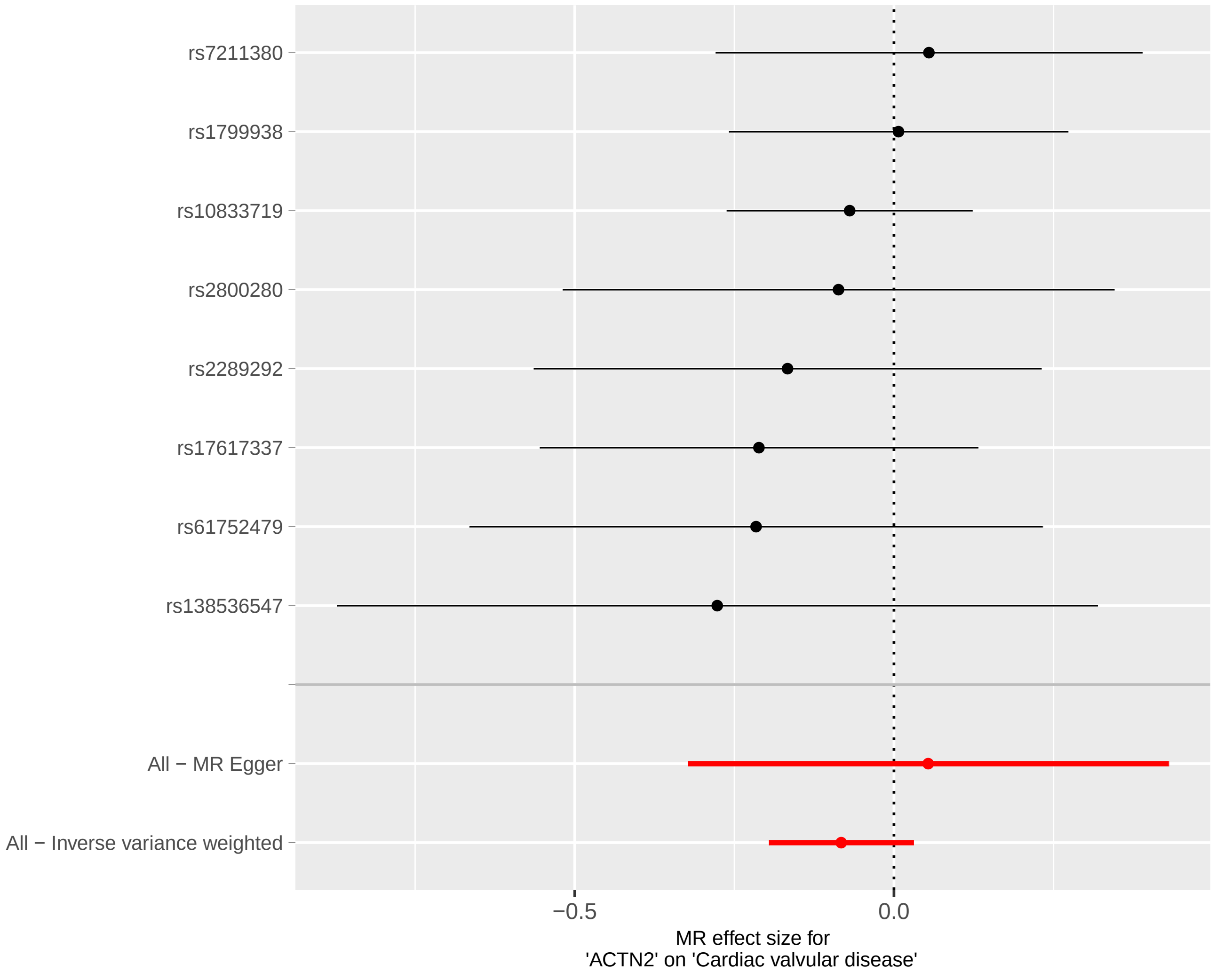
rs7211380
rs1799938
rs10833719
rs2800280
rs2289292
rs17617337
rs61752479
rs138536547
All − MR Egger
All − Inverse variance weighted
−0.5
0.0
MR effect size for
'ACTN2' on 'Cardiac valvular disease'

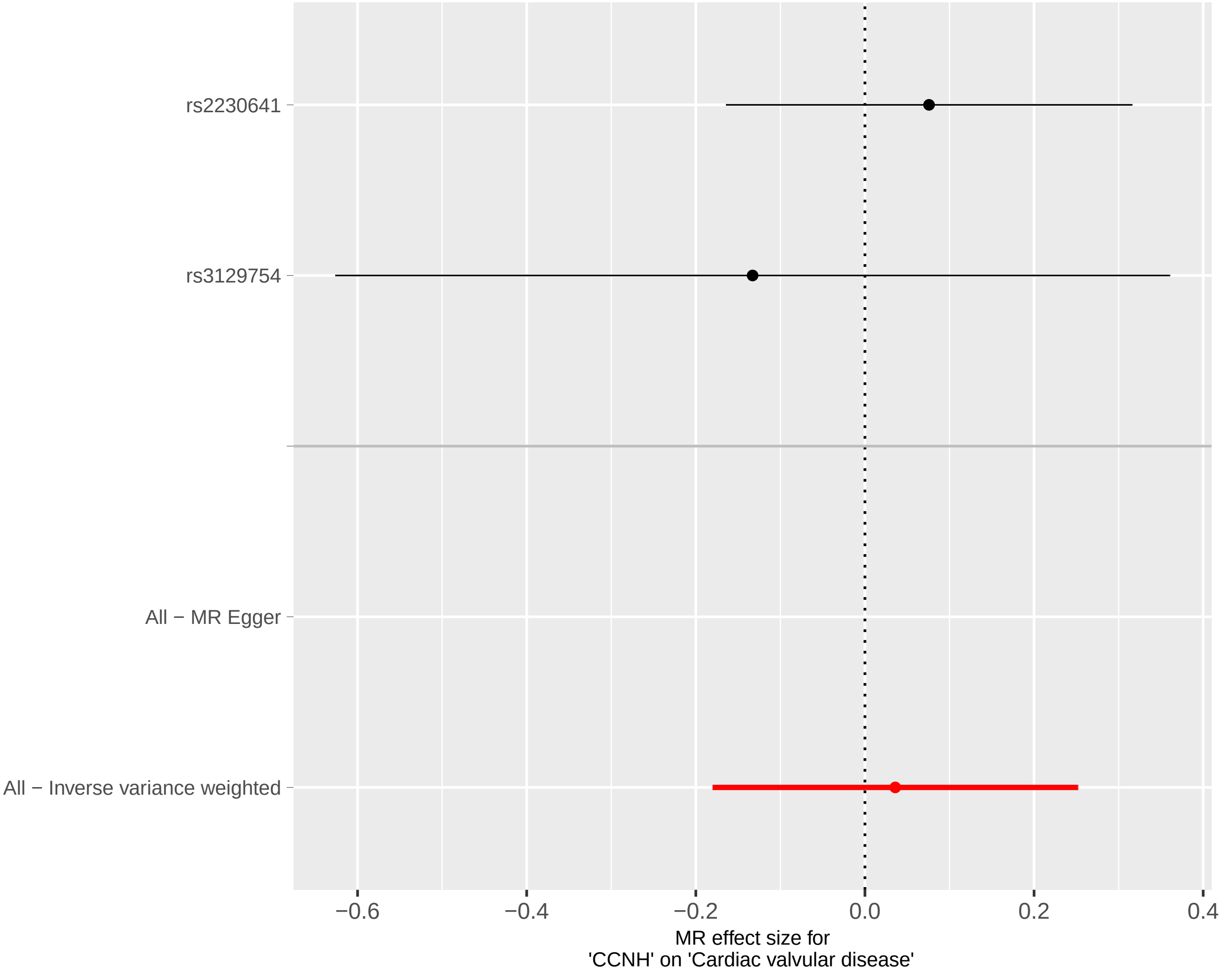

rs2230641
rs3129754
All − MR Egger
All − Inverse variance weighted
−0.6
−0.4
−0.2
0.0
0.2
0.4
MR effect size for
'CCNH' on 'Cardiac valvular disease'

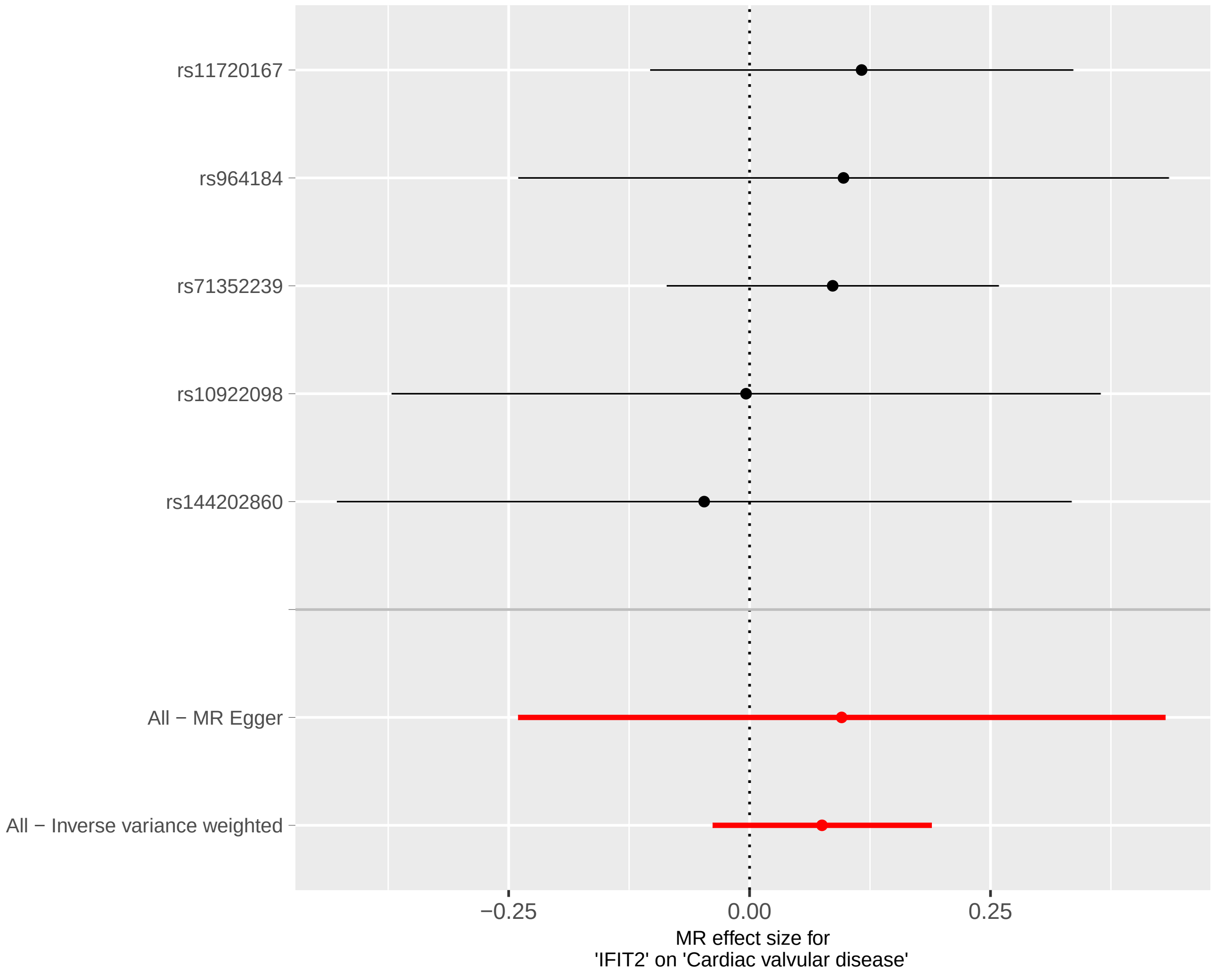

rs11720167
rs964184
rs71352239
rs10922098
rs144202860
All − MR Egger
All − Inverse variance weighted
−0.25
0.00
0.25
MR effect size for
'IFIT2' on 'Cardiac valvular disease'

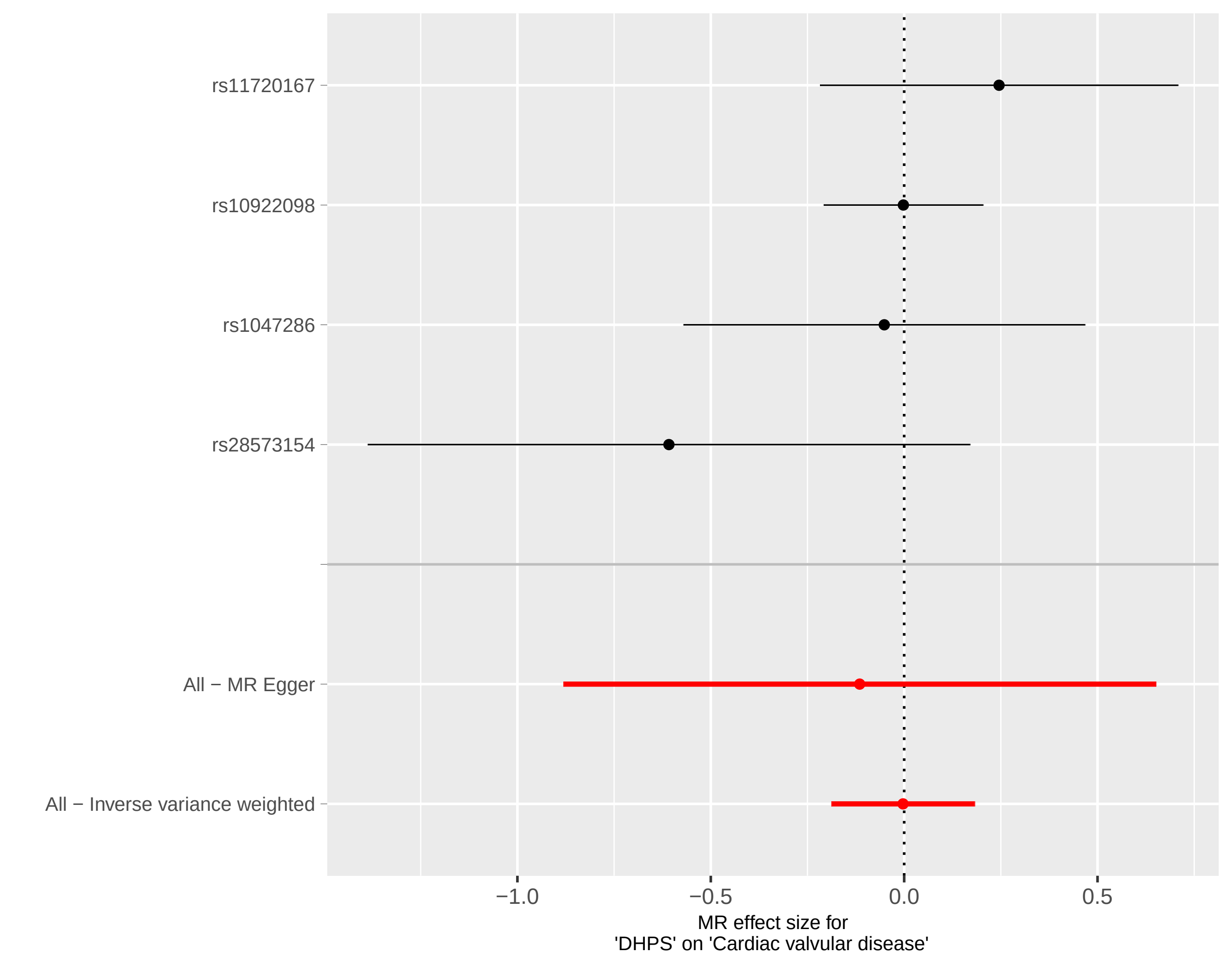
rs11720167
rs10922098
rs1047286
rs28573154
All − MR Egger
All − Inverse variance weighted
−1.0
−0.5
0.0
0.5
MR effect size for
'DHPS' on 'Cardiac valvular disease'

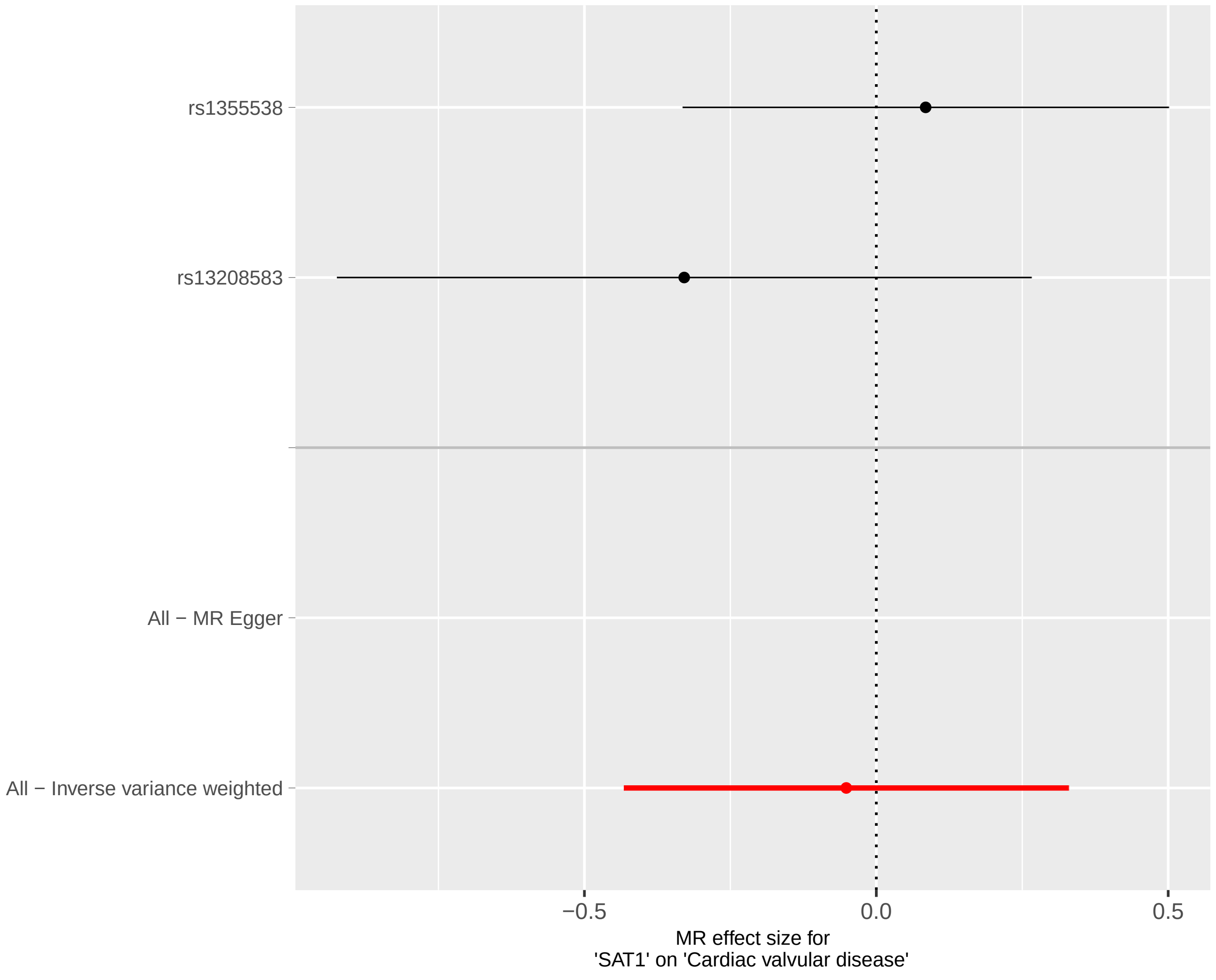
rs1355538
rs13208583
All − MR Egger
All − Inverse variance weighted
−0.5
0.0
0.5
MR effect size for
'SAT1' on 'Cardiac valvular disease'

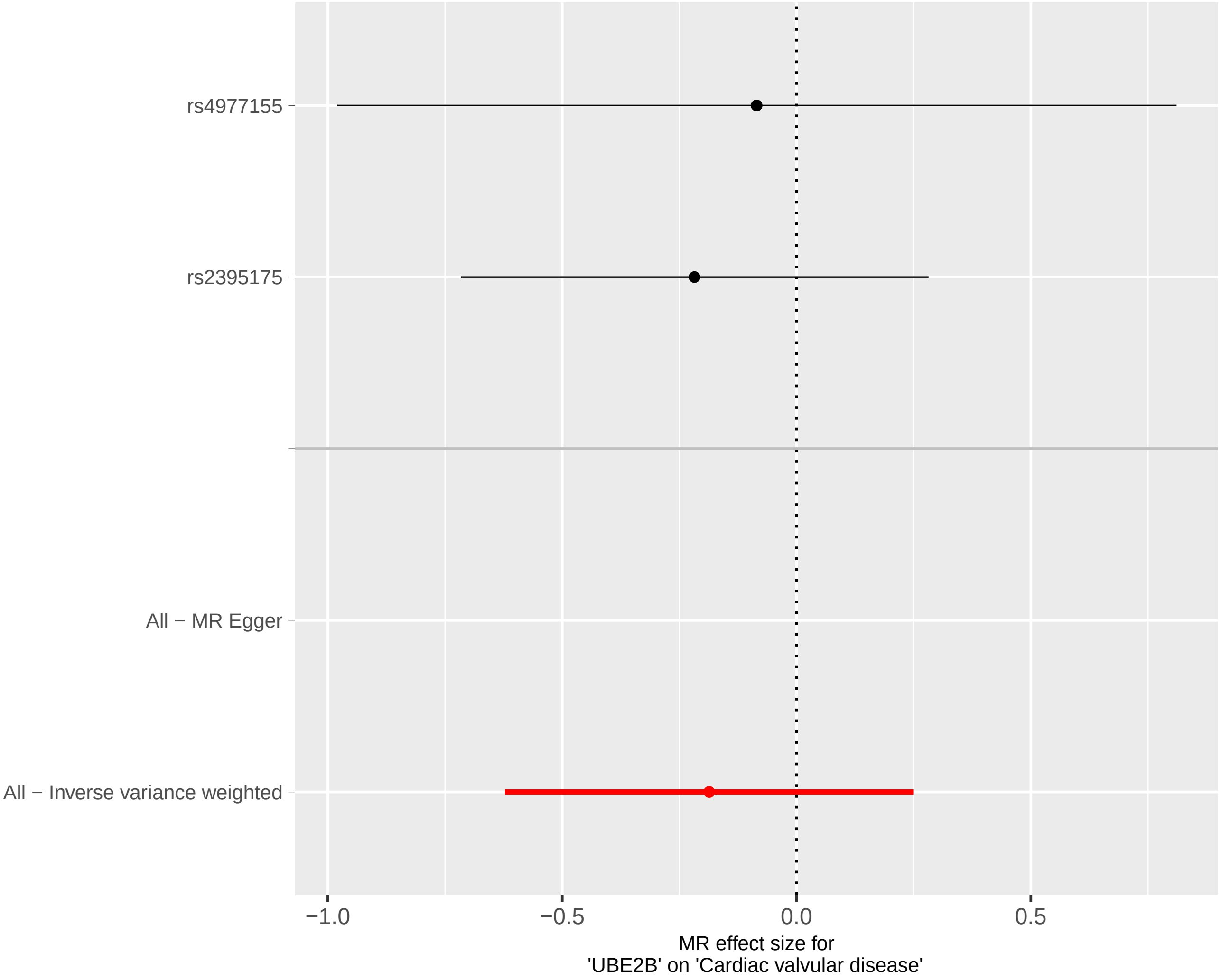
rs4977155
rs2395175
All − MR Egger
All − Inverse variance weighted
−1.0
−0.5
0.0
0.5
MR effect size for
'UBE2B' on 'Cardiac valvular disease'

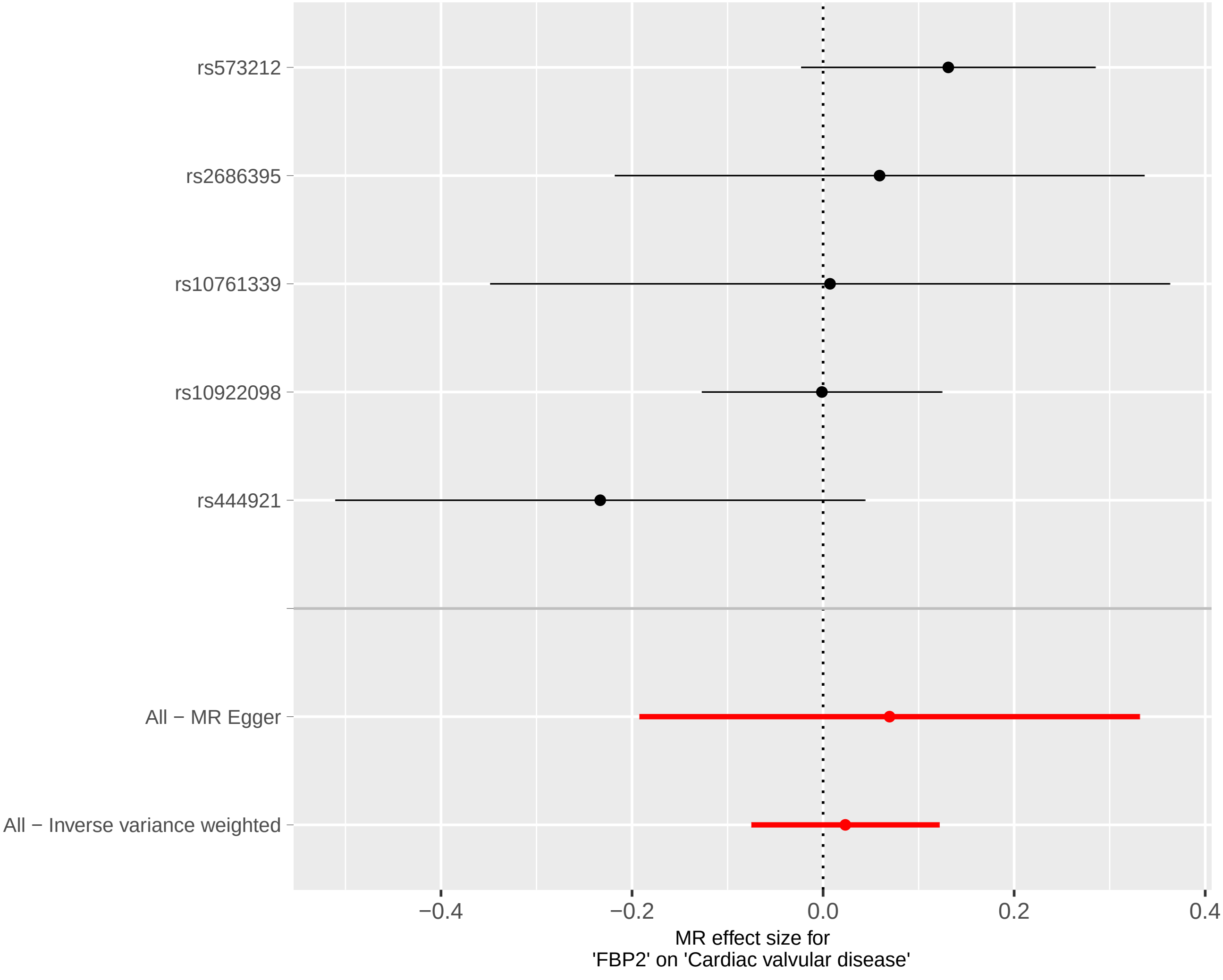
rs573212
rs2686395
rs10761339
rs10922098
rs444921
All − MR Egger
All − Inverse variance weighted
−0.4
−0.2
0.0
0.2
0.4
MR effect size for
'FBP2' on 'Cardiac valvular disease'

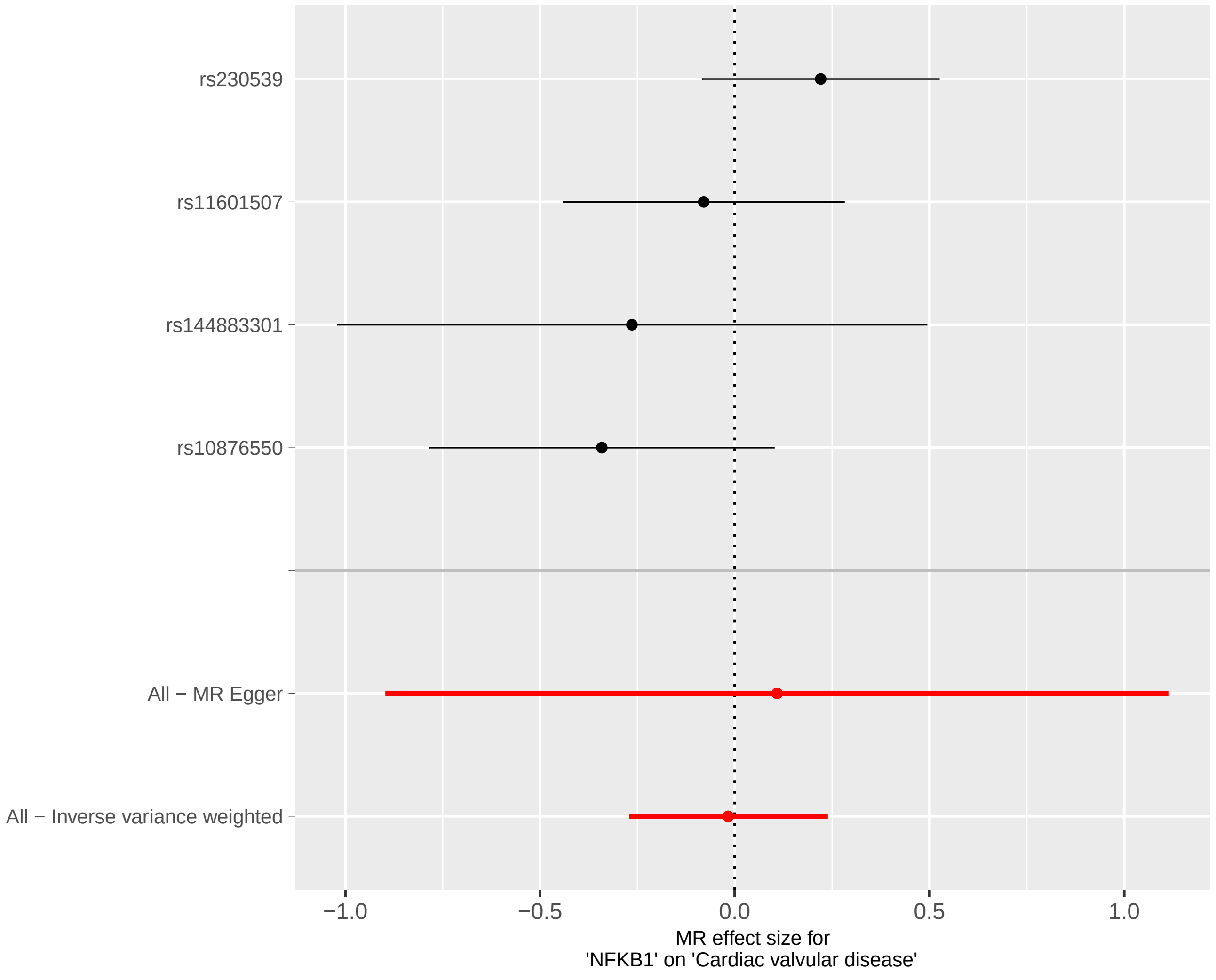
rs230539
rs11601507
rs144883301
rs10876550
All − MR Egger
All − Inverse variance weighted
−1.0
−0.5
0.0
0.5
1.0
MR effect size for
'NFKB1' on 'Cardiac valvular disease'

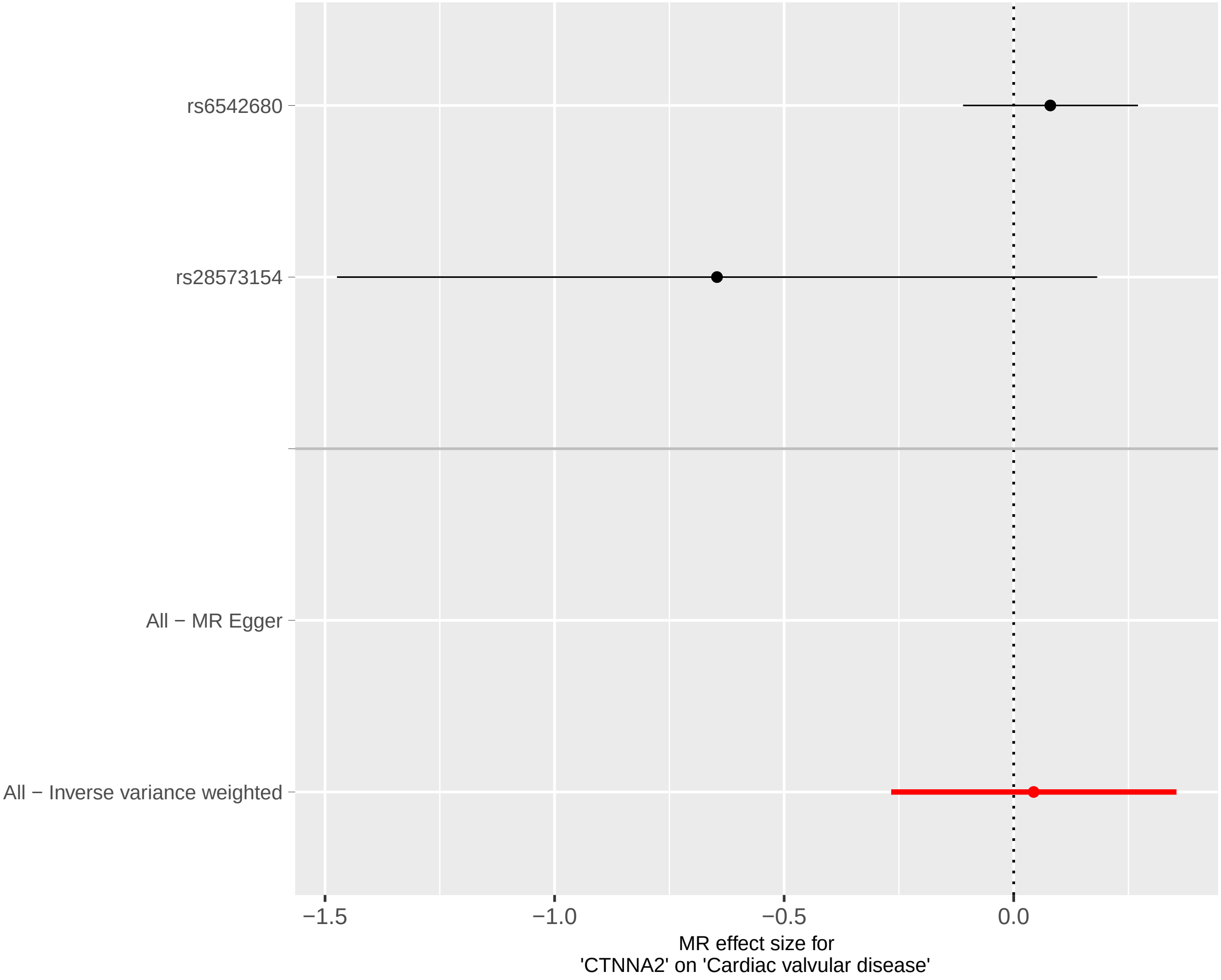
rs6542680
rs28573154
All − MR Egger
All − Inverse variance weighted
−1.5
−1.0
−0.5
0.0
MR effect size for
'CTNNA2' on 'Cardiac valvular disease'

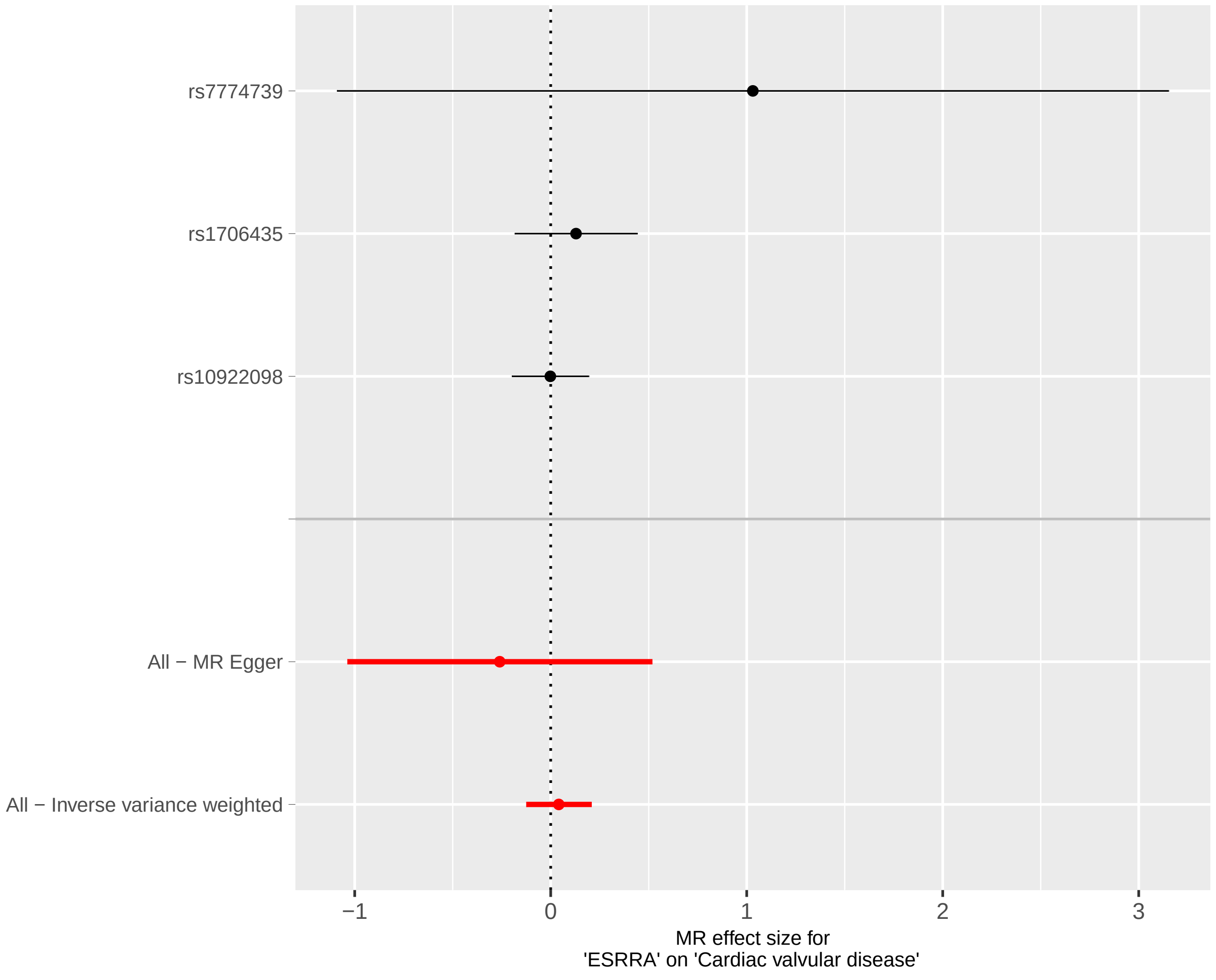
rs7774739
rs1706435
rs10922098
All − MR Egger
All − Inverse variance weighted
−1
0
1
2
3
MR effect size for
'ESRRA' on 'Cardiac valvular disease'

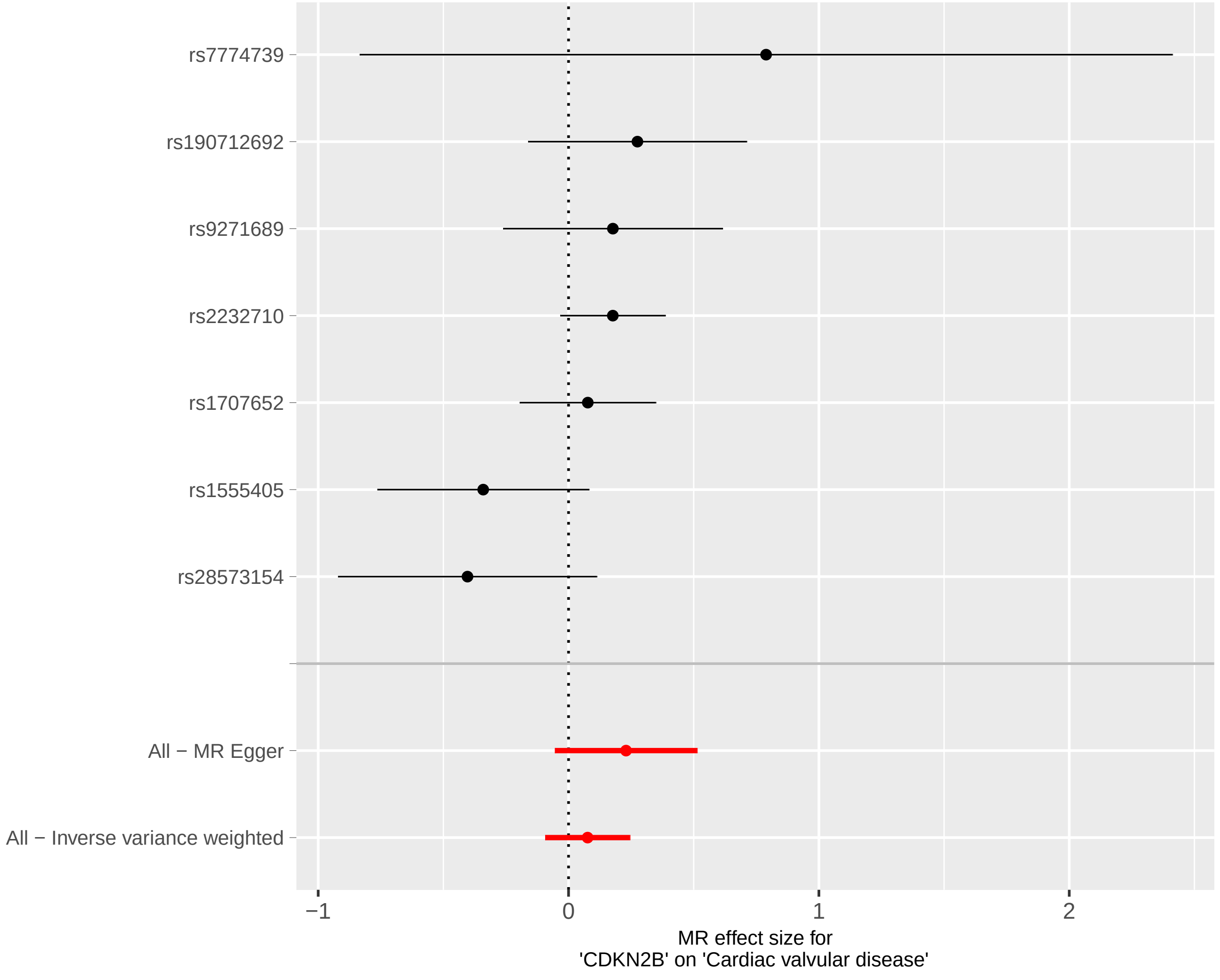

rs7774739
rs190712692
rs9271689
rs2232710
rs1707652
rs1555405
rs28573154
All − MR Egger
All − Inverse variance weighted
−1
0
1
2
MR effect size for
'CDKN2B' on 'Cardiac valvular disease'

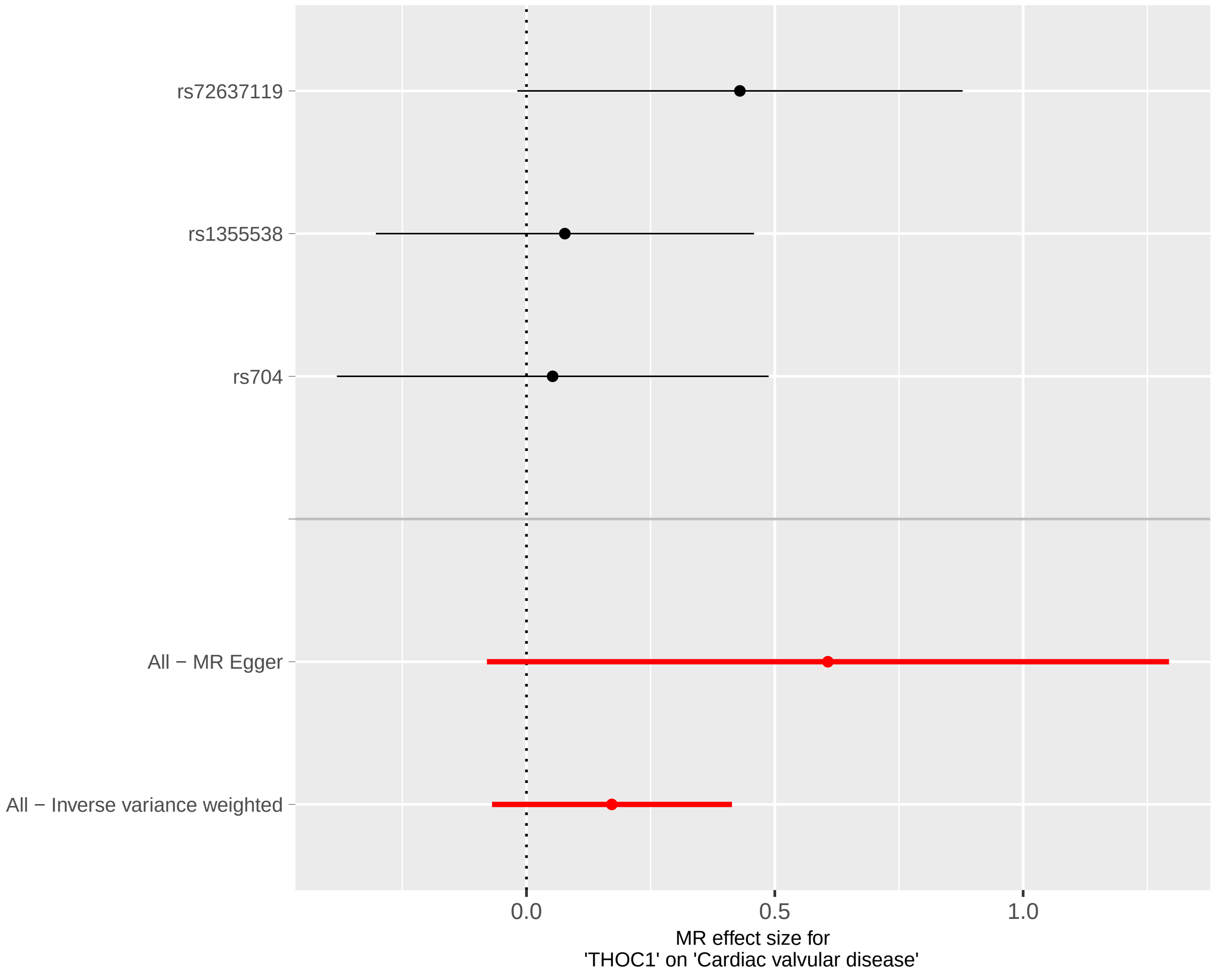
rs72637119
rs1355538
rs704
All − MR Egger
All − Inverse variance weighted
0.0
0.5
1.0
MR effect size for
'THOC1' on 'Cardiac valvular disease'

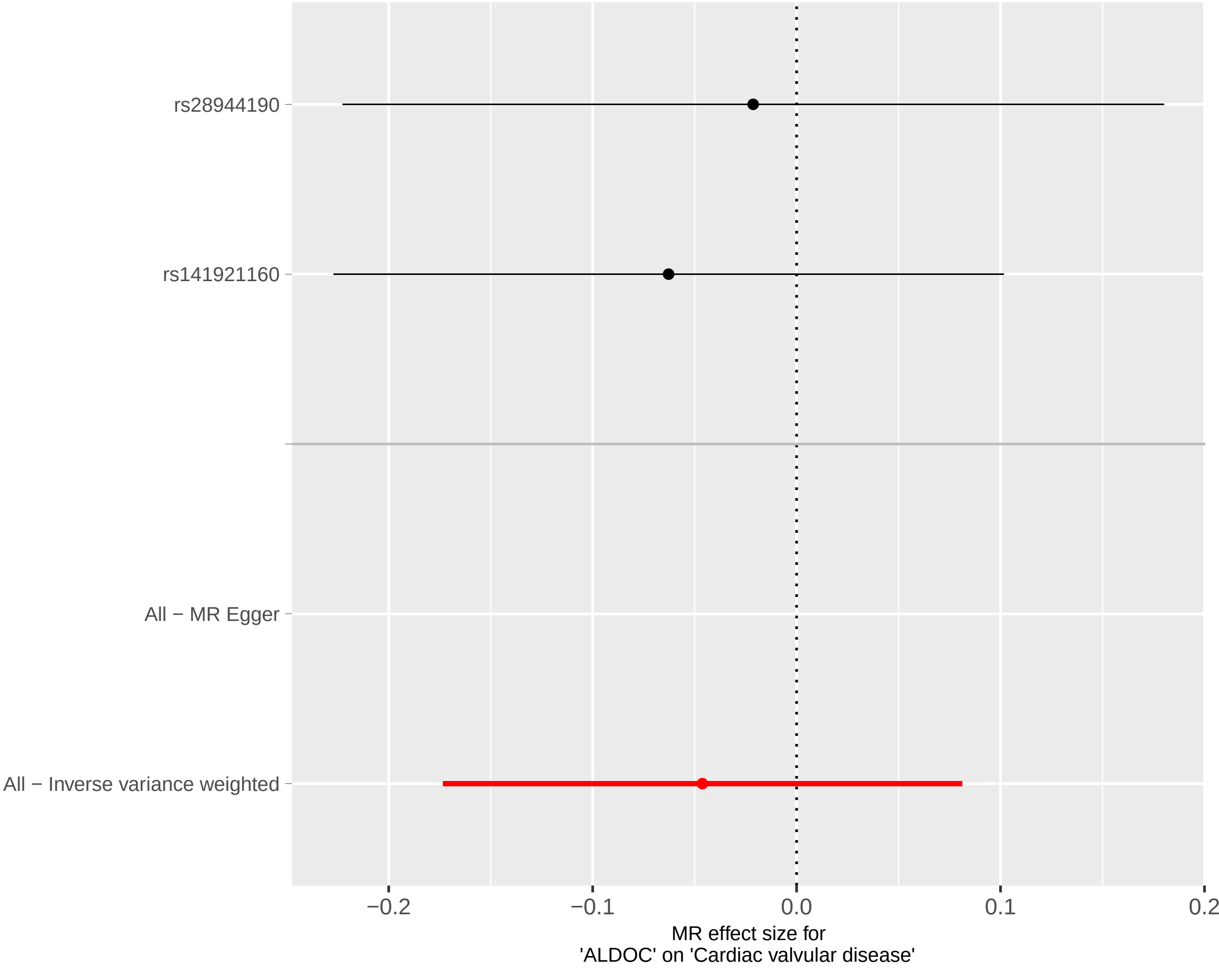
rs28944190
rs141921160
All − MR Egger
All − Inverse variance weighted
−0.2
−0.1
0.0
0.1
0.2
MR effect size for
'ALDOC' on 'Cardiac valvular disease'

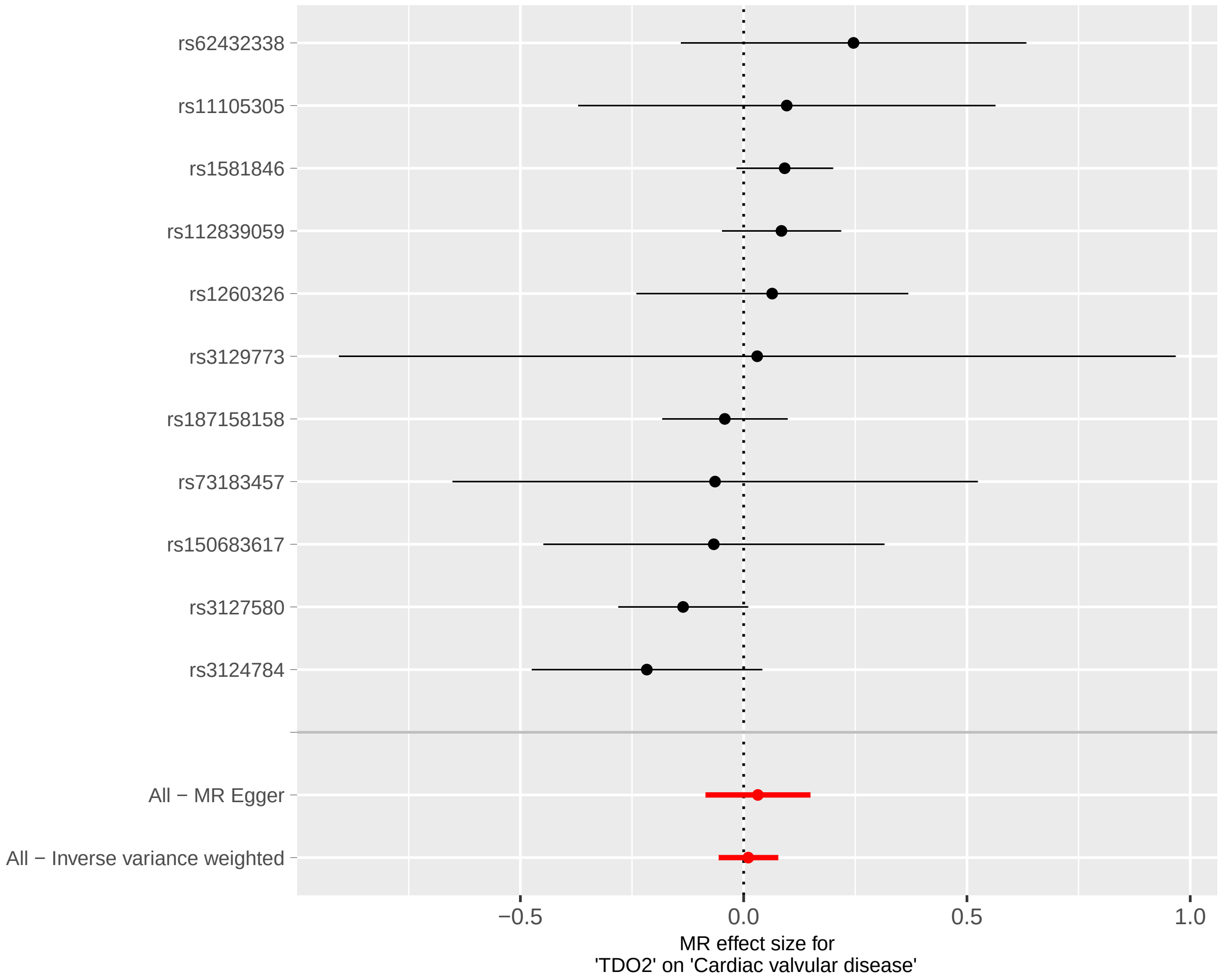

rs62432338
rs11105305
rs1581846
rs112839059
rs1260326
rs3129773
rs187158158
rs73183457
rs150683617
rs3127580
rs3124784
All − MR Egger
All − Inverse variance weighted
−0.5
0.0
0.5
1.0
MR effect size for
'TDO2' on 'Cardiac valvular disease'

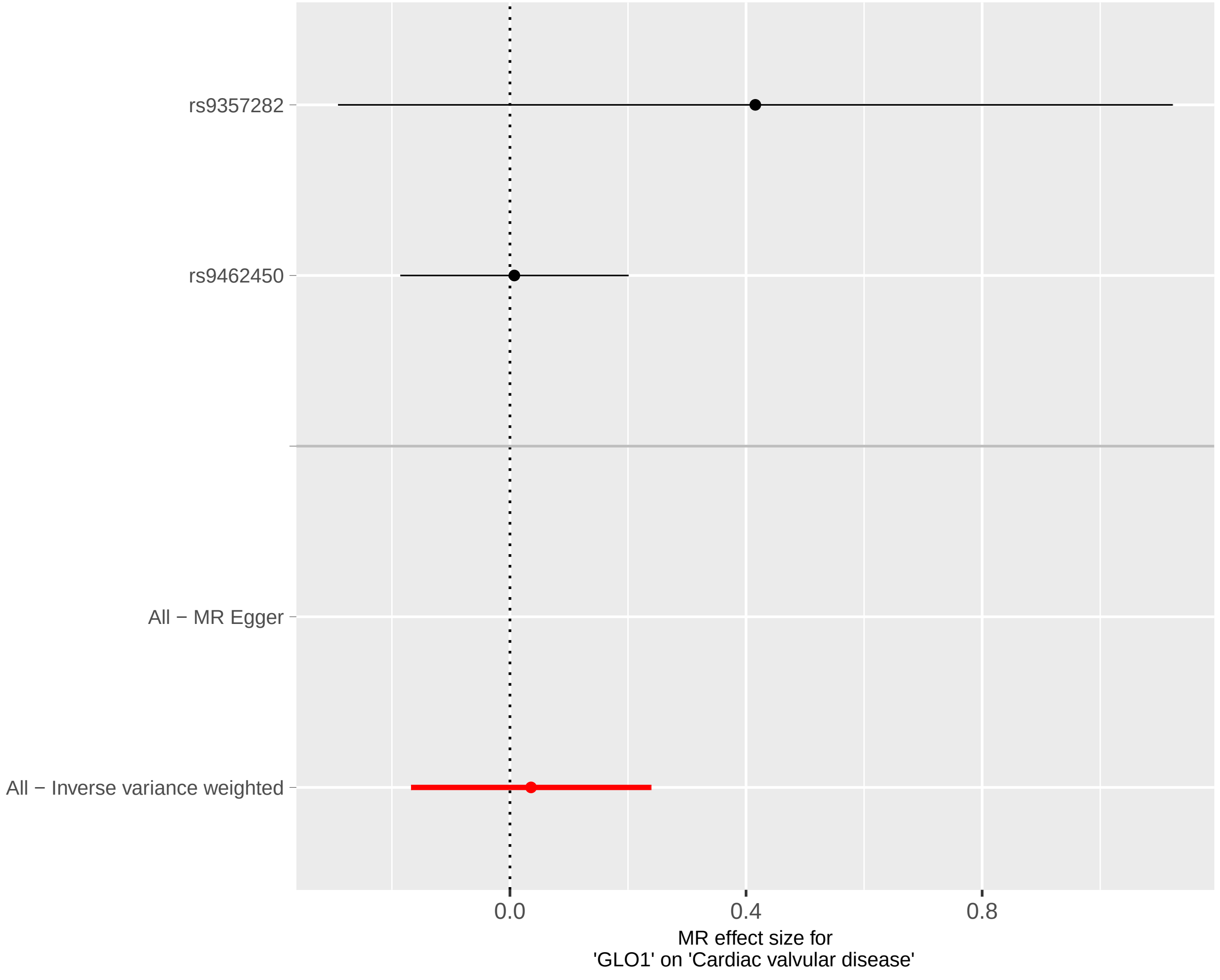

rs9357282
rs9462450
All − MR Egger
All − Inverse variance weighted
0.0
0.4
0.8
MR effect size for
'GLO1' on 'Cardiac valvular disease'

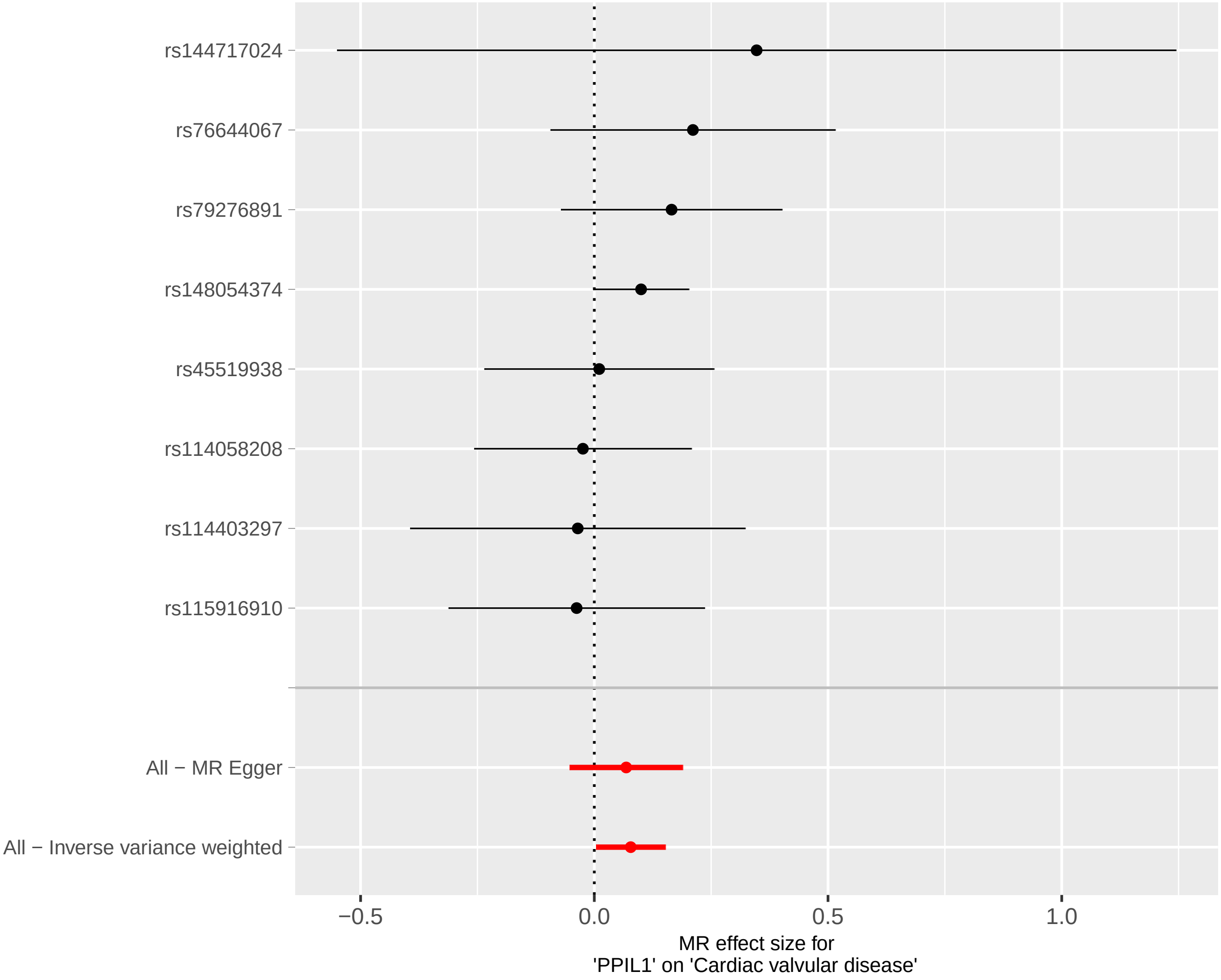
rs144717024
rs76644067
rs79276891
rs148054374
rs45519938
rs114058208
rs114403297
rs115916910
All − MR Egger
All − Inverse variance weighted
−0.5
0.0
0.5
1.0
MR effect size for
'PPIL1' on 'Cardiac valvular disease'

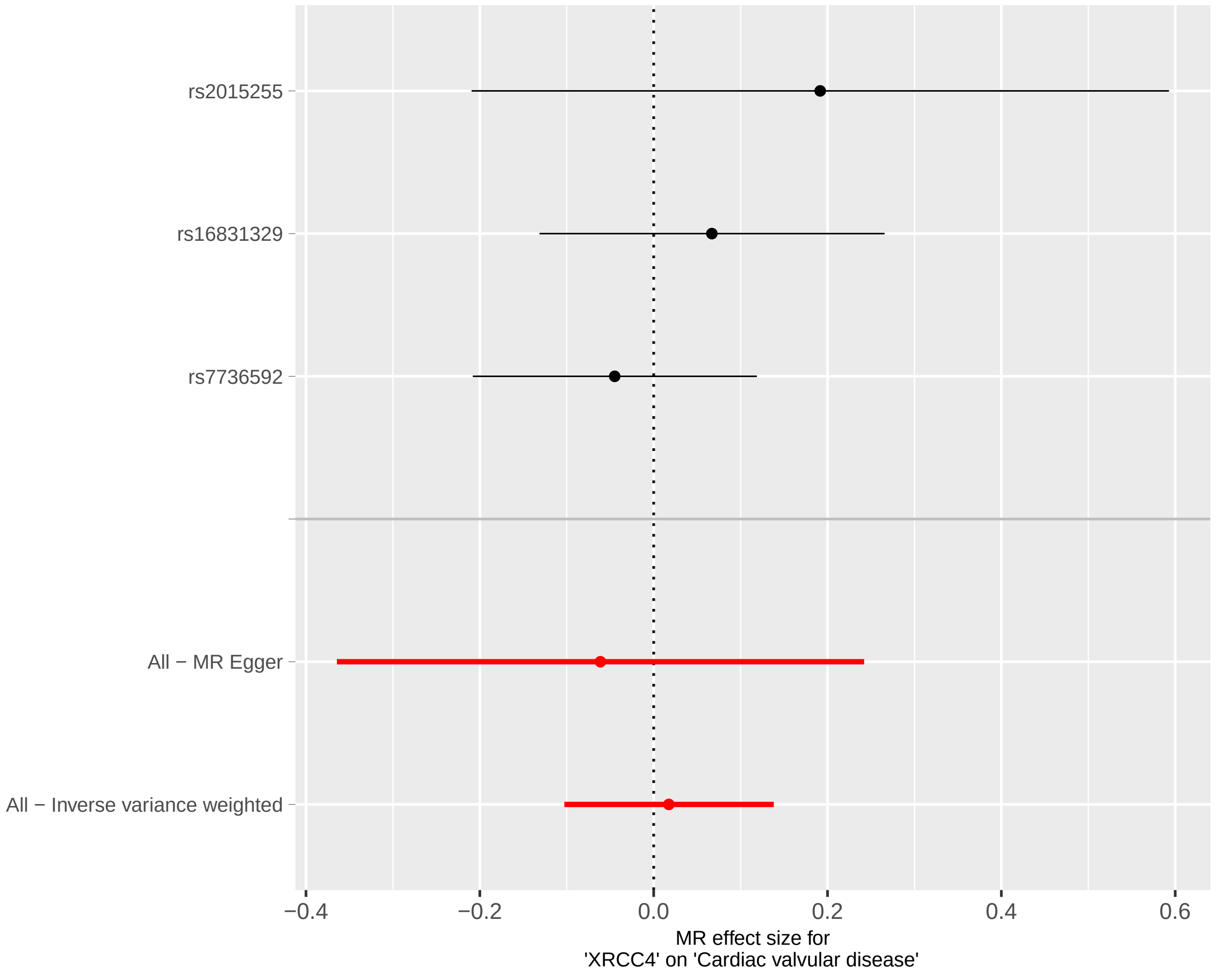
rs2015255
rs16831329
rs7736592
All − MR Egger
All − Inverse variance weighted
−0.4
−0.2
0.0
0.2
0.4
0.6
MR effect size for
'XRCC4' on 'Cardiac valvular disease'

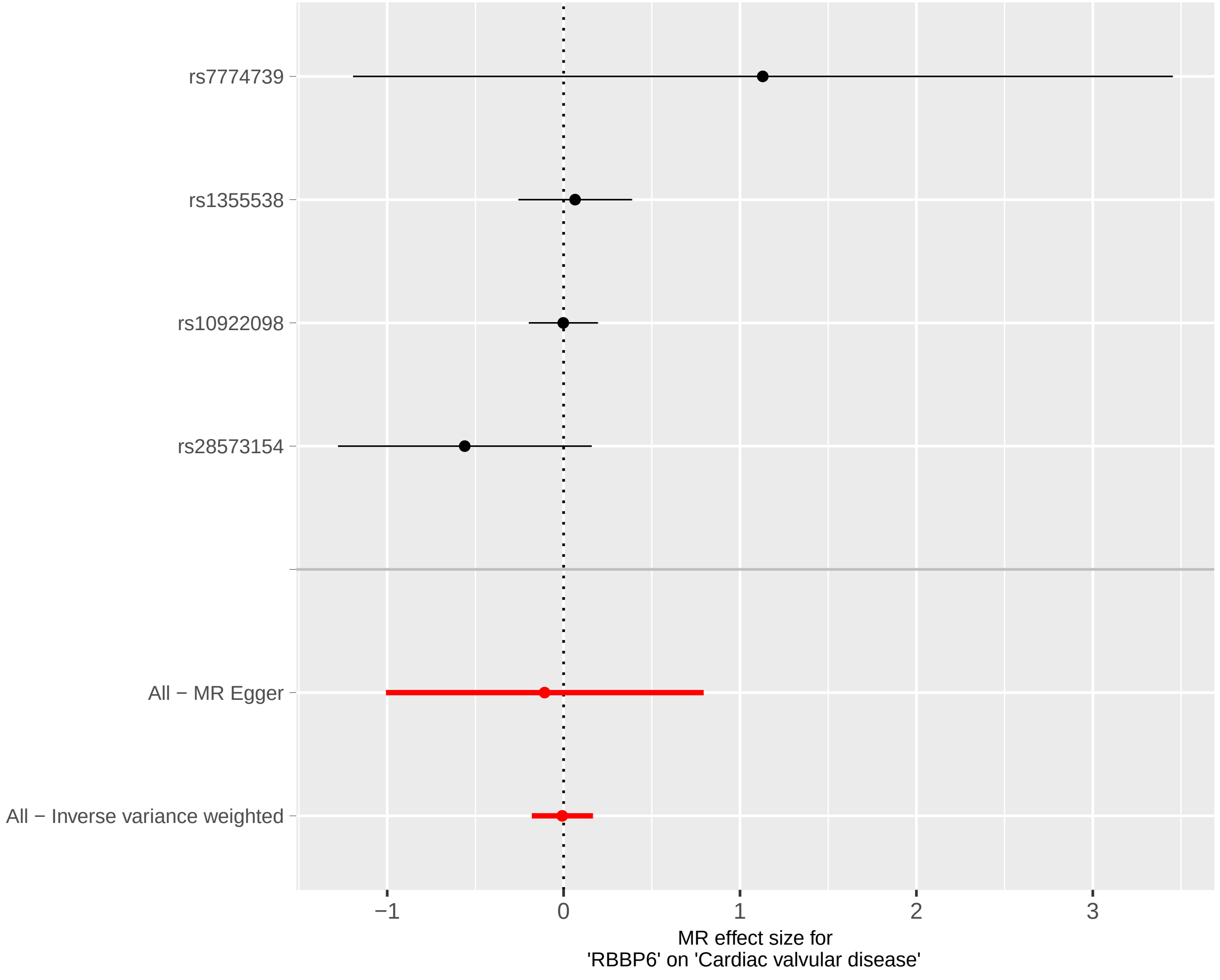
rs7774739
rs1355538
rs10922098
rs28573154
All − MR Egger
All − Inverse variance weighted
−1
0
1
2
3
MR effect size for
'RBBP6' on 'Cardiac valvular disease'

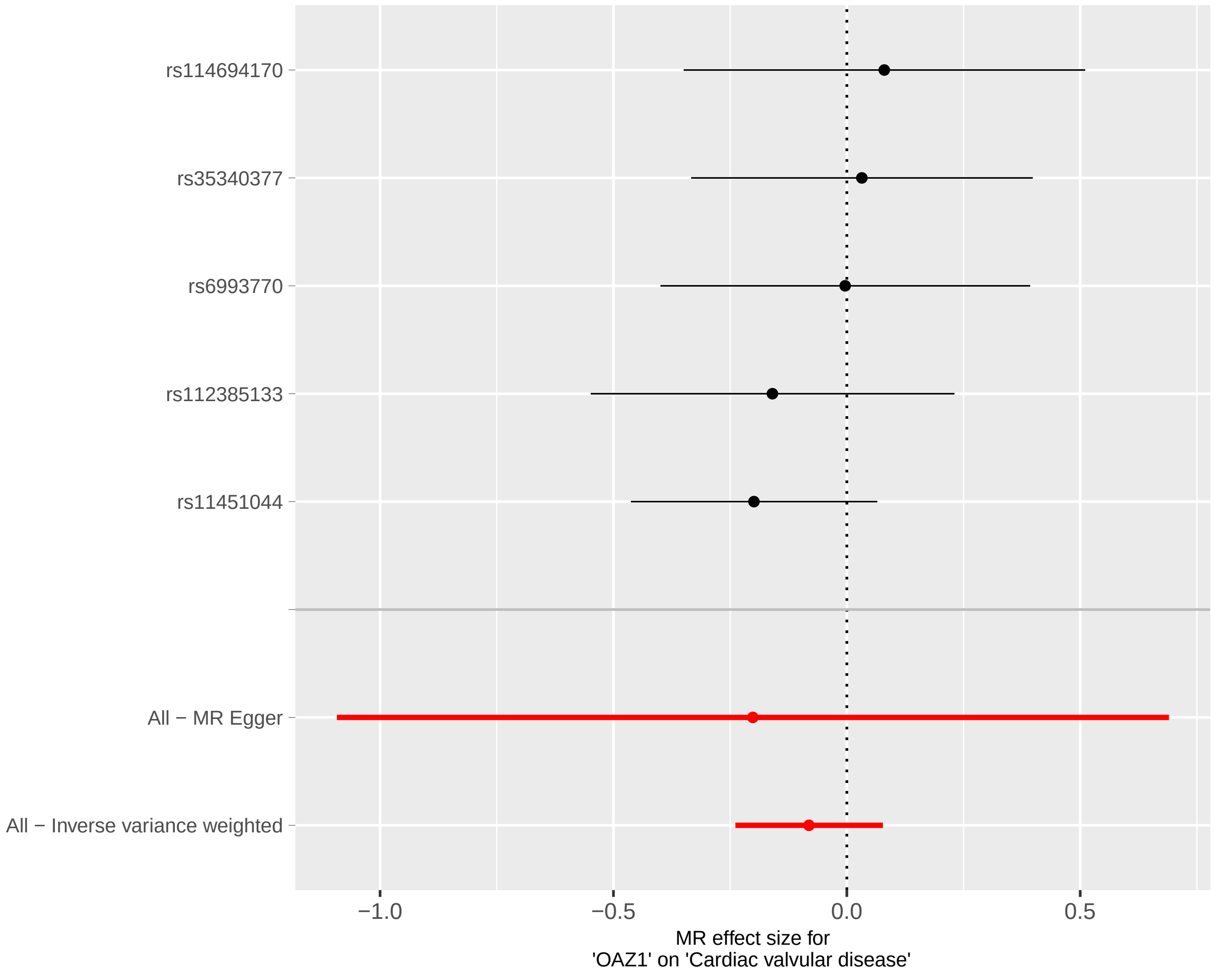
rs114694170
rs35340377
rs6993770
rs112385133
rs11451044
All − MR Egger
All − Inverse variance weighted
−1.0
−0.5
0.0
0.5
MR effect size for
'OAZ1' on 'Cardiac valvular disease'

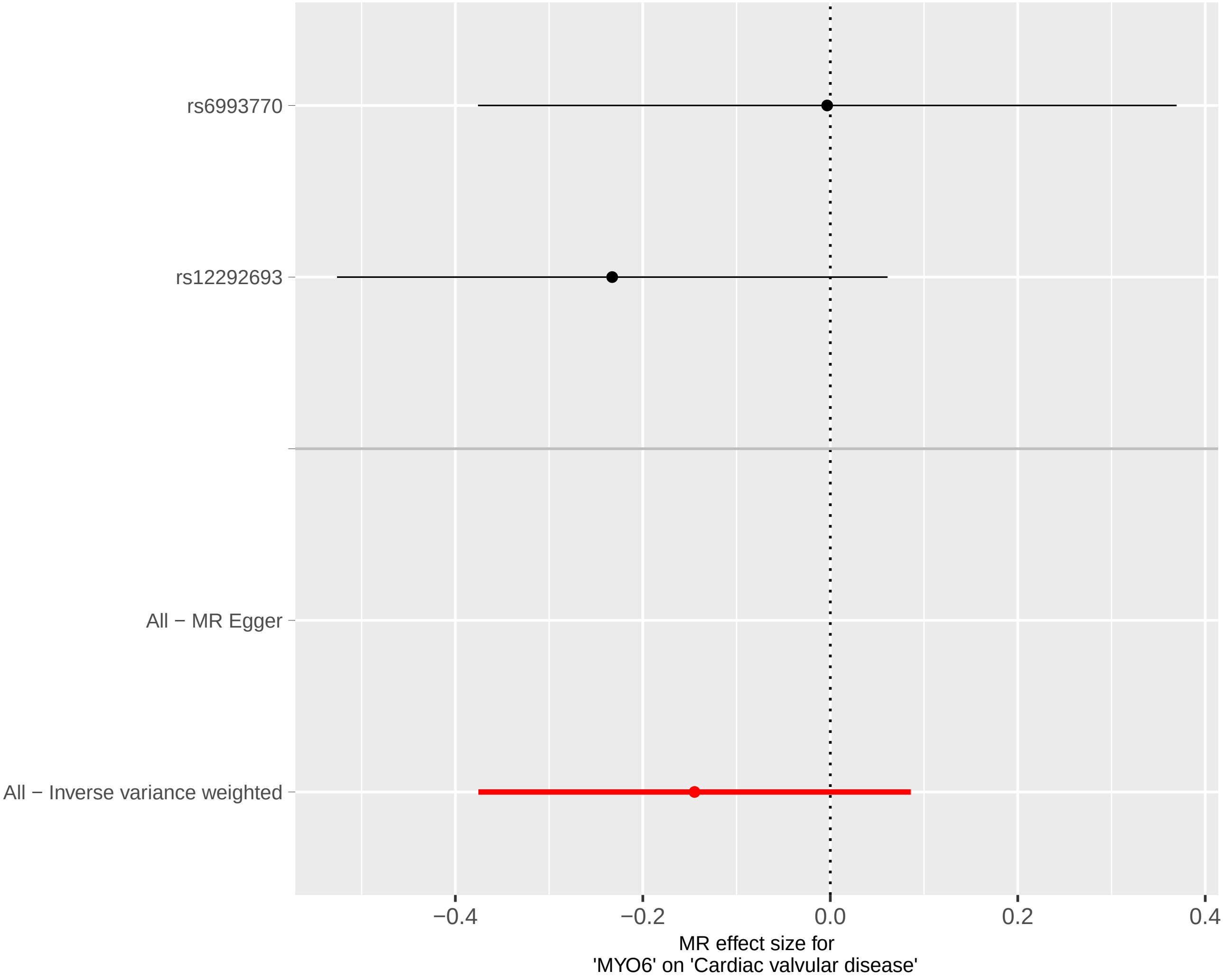

rs6993770
rs12292693
All − MR Egger
All − Inverse variance weighted
−0.4
−0.2
0.0
0.2
0.4
MR effect size for
'MYO6' on 'Cardiac valvular disease'

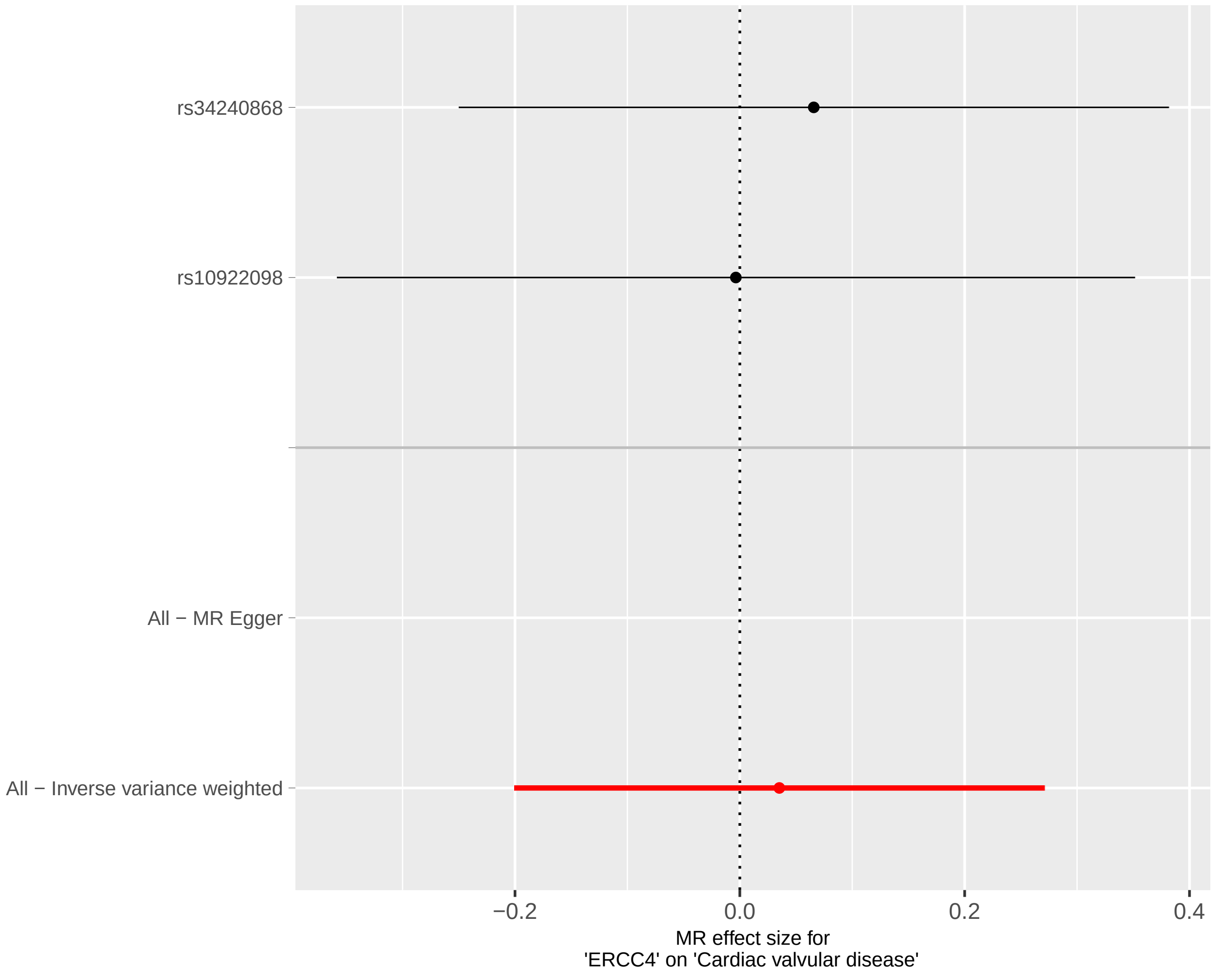
rs34240868
rs10922098
All − MR Egger
All − Inverse variance weighted
−0.2
0.0
0.2
0.4
MR effect size for
'ERCC4' on 'Cardiac valvular disease'

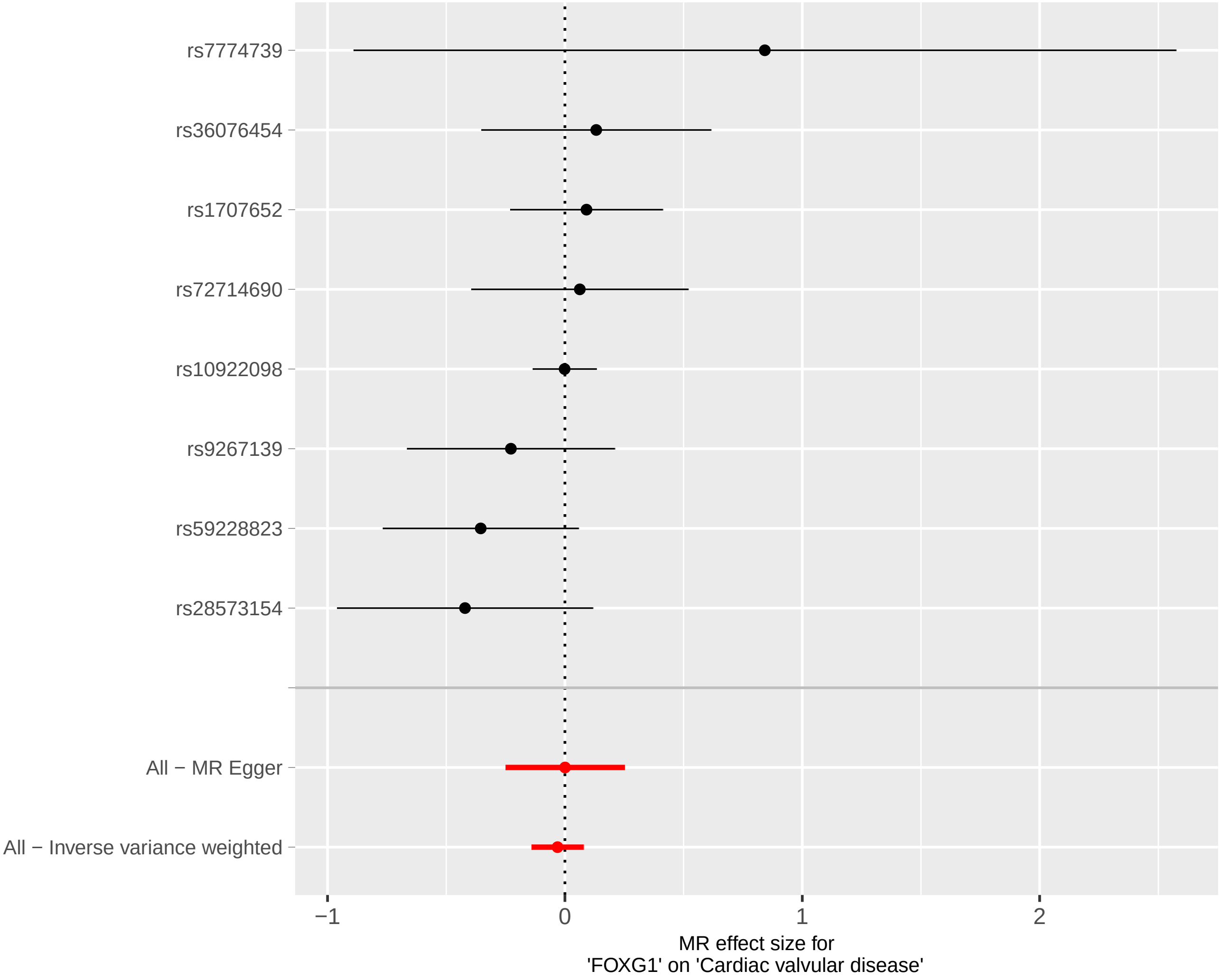

rs7774739
rs36076454
rs1707652
rs72714690
rs10922098
rs9267139
rs59228823
rs28573154
All − MR Egger
All − Inverse variance weighted
−1
0
1
2
MR effect size for
'FOXG1' on 'Cardiac valvular disease'

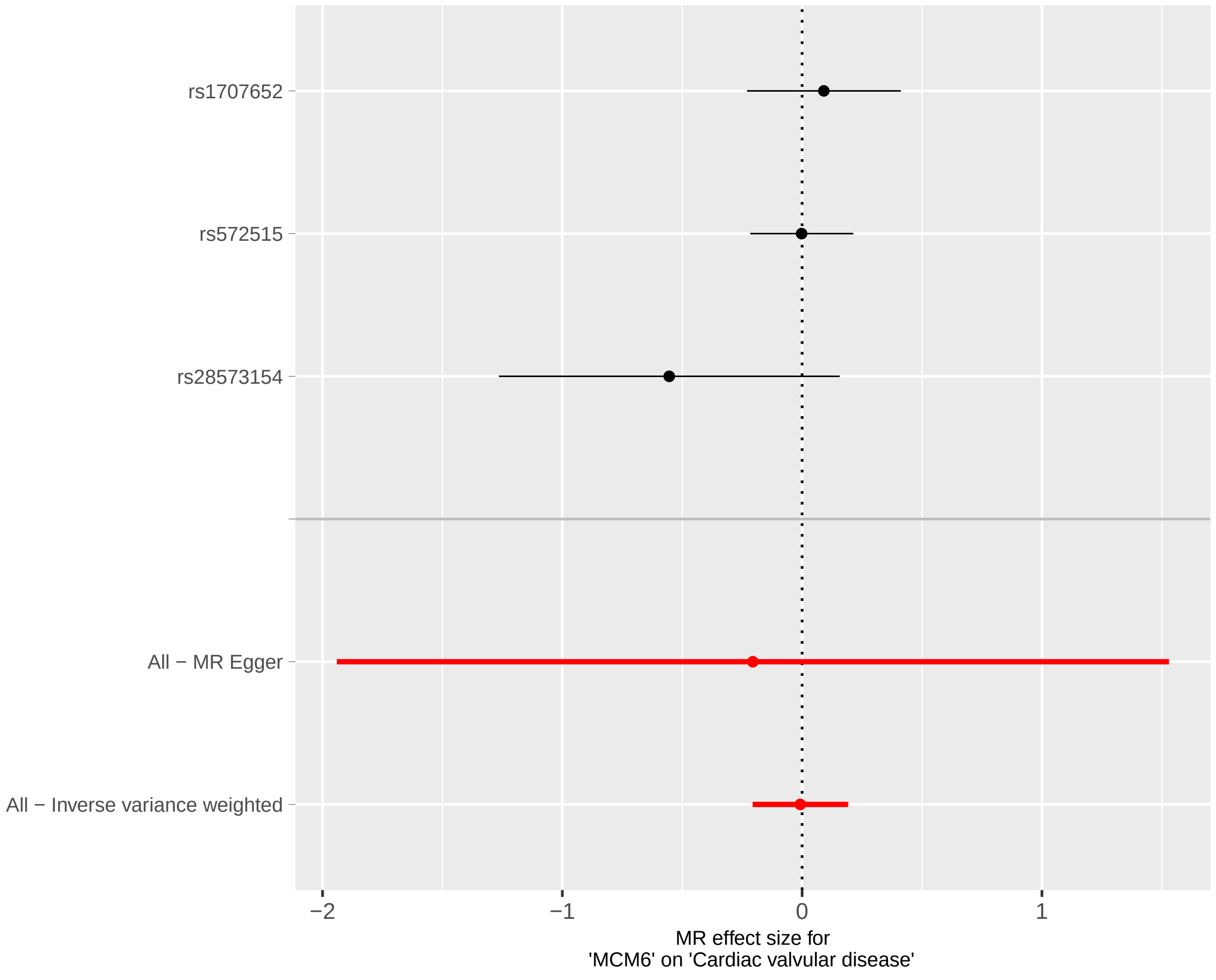
rs1707652
rs572515
rs28573154
All − MR Egger
All − Inverse variance weighted
−2
−1
0
1
MR effect size for
'MCM6' on 'Cardiac valvular disease'

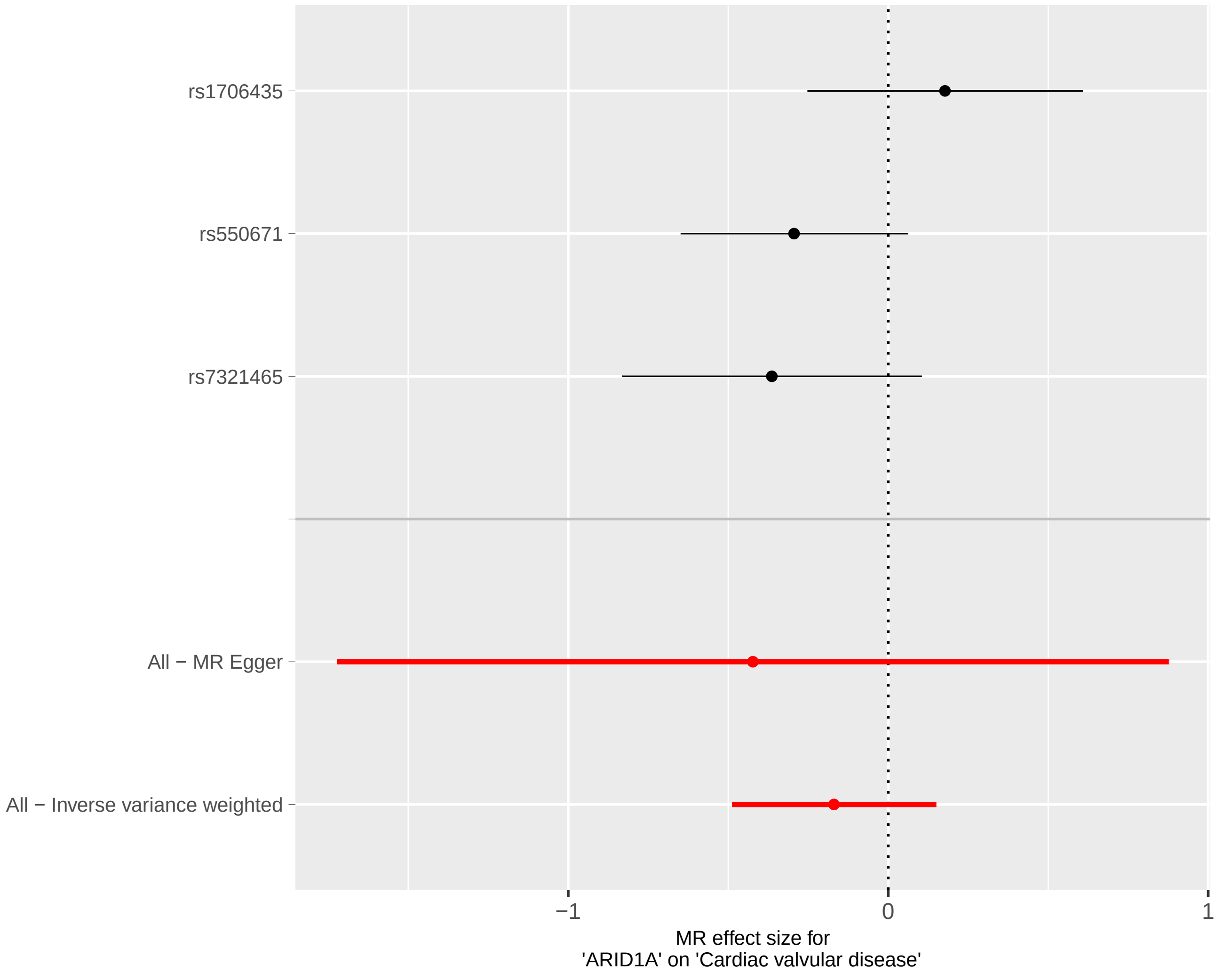

rs1706435
rs550671
rs7321465
All − MR Egger
All − Inverse variance weighted
−1
0
1
MR effect size for
'ARID1A' on 'Cardiac valvular disease'

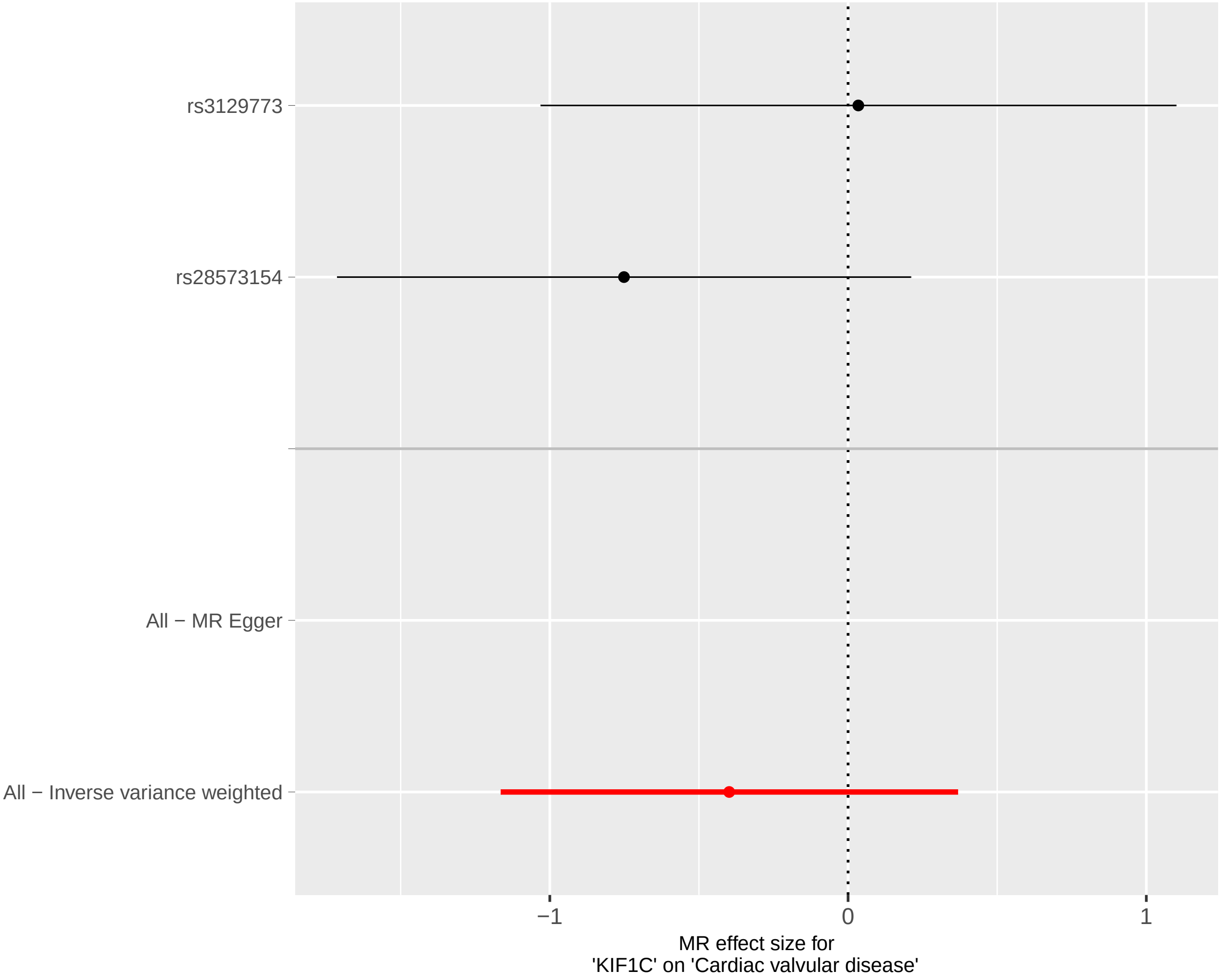

rs3129773
rs28573154
All − MR Egger
All − Inverse variance weighted
−1
0
1
MR effect size for
'KIF1C' on 'Cardiac valvular disease'

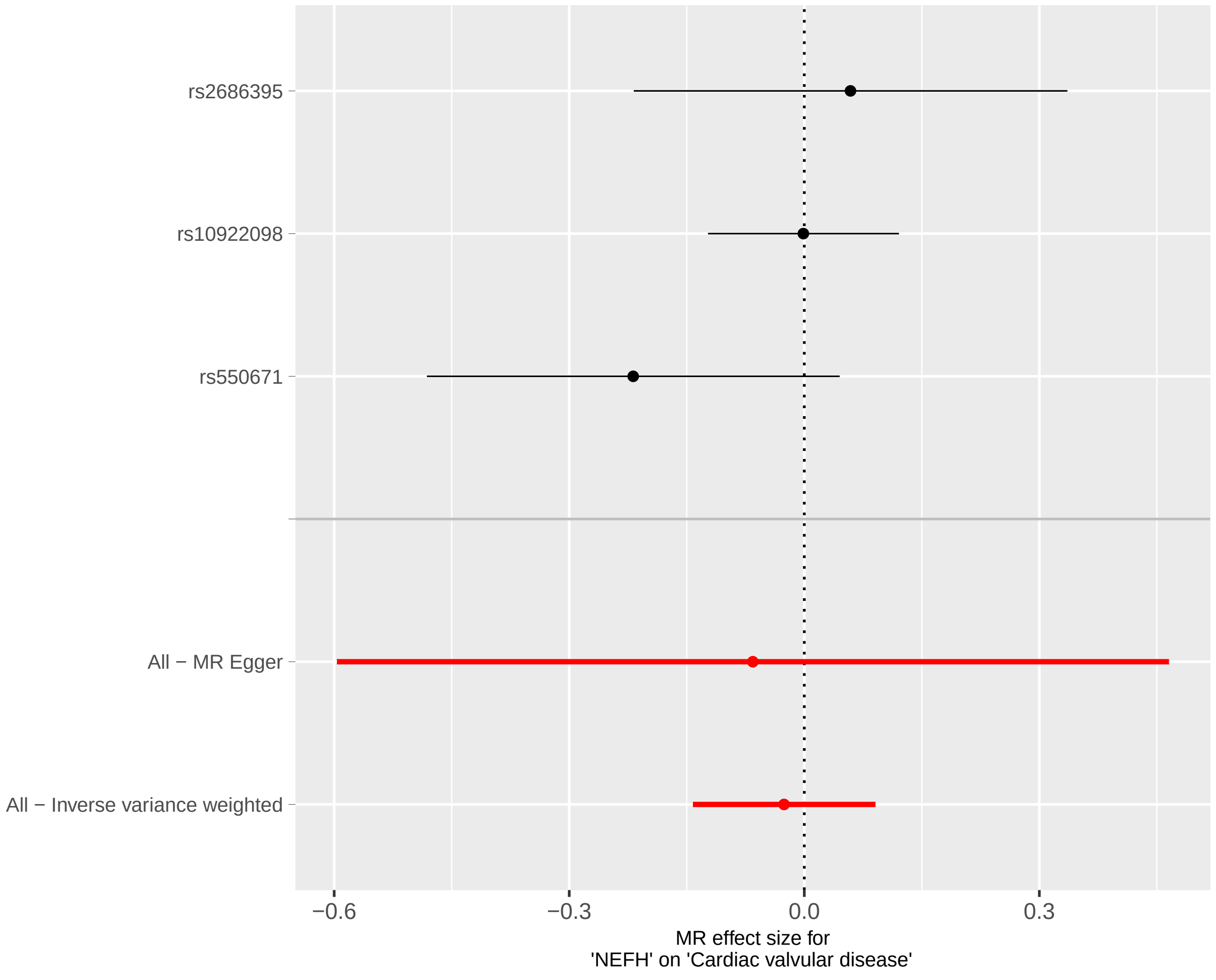
rs2686395
rs10922098
rs550671
All − MR Egger
All − Inverse variance weighted
−0.6
−0.3
0.0
0.3
MR effect size for
'NEFH' on 'Cardiac valvular disease'

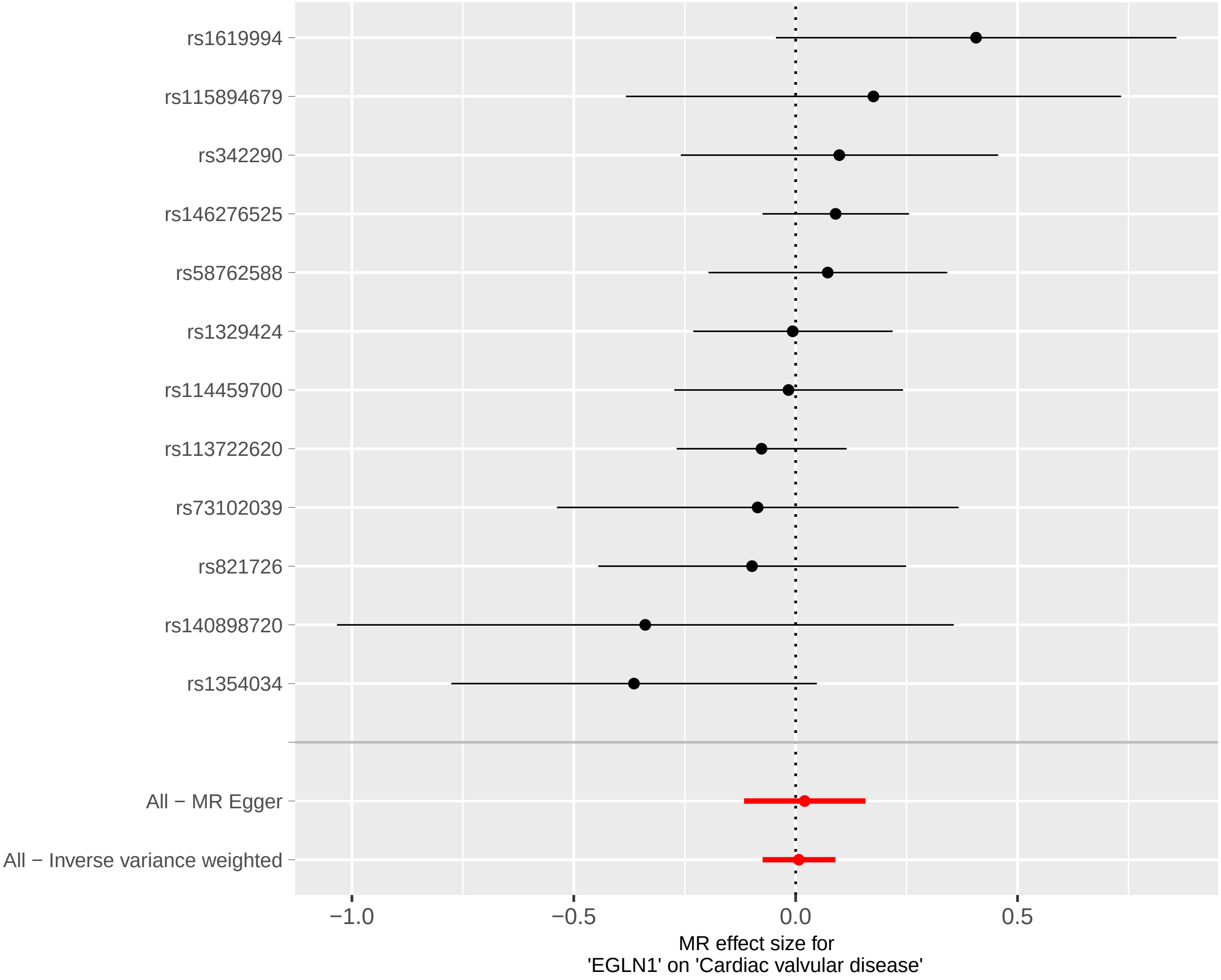
rs1619994
rs115894679
rs342290
rs146276525
rs58762588
rs1329424
rs114459700
rs113722620
rs73102039
rs821726
rs140898720
rs1354034
All − MR Egger
All − Inverse variance weighted
−1.0
−0.5
0.0
0.5
MR effect size for
'EGLN1' on 'Cardiac valvular disease'

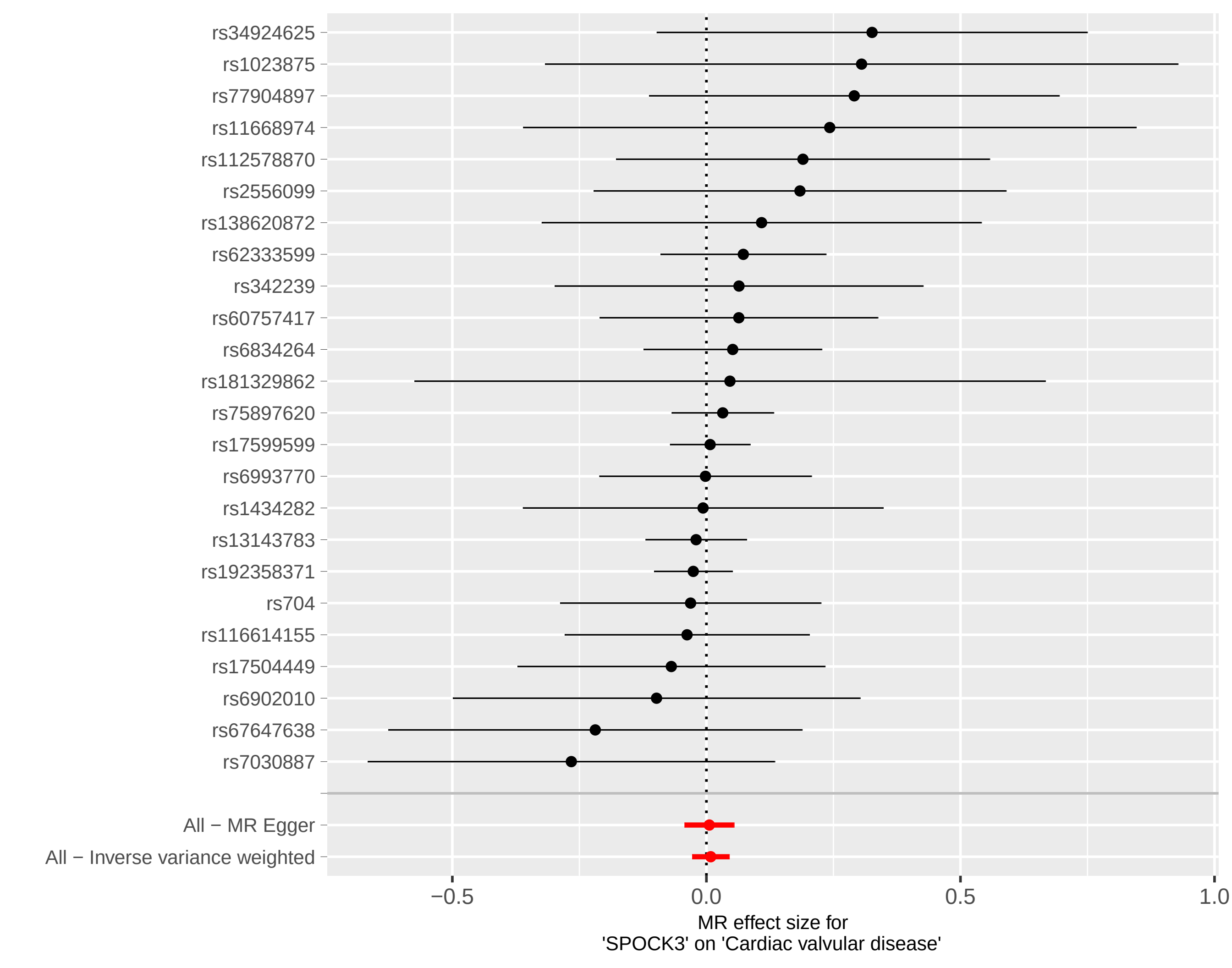
rs34924625
rs1023875
rs77904897
rs11668974
rs112578870
rs2556099
rs138620872
rs62333599
rs342239
rs60757417
rs6834264
rs181329862
rs75897620
rs17599599
rs6993770
rs1434282
rs13143783
rs192358371
rs704
rs116614155
rs17504449
rs6902010
rs67647638
rs7030887
All − MR Egger
All − Inverse variance weighted
−0.5
0.0
0.5
1.0
MR effect size for
'SPOCK3' on 'Cardiac valvular disease'

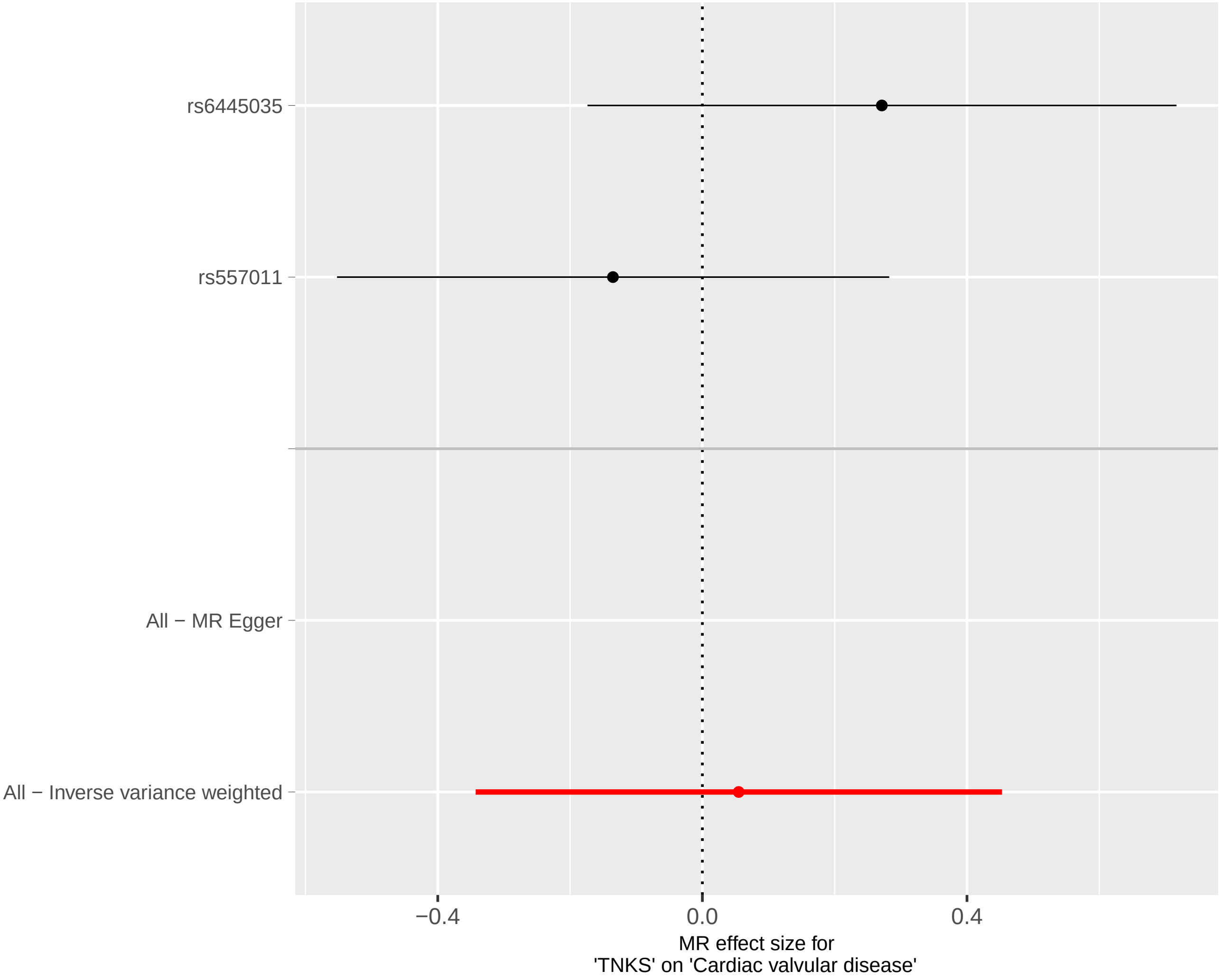
rs6445035
rs557011
All − MR Egger
All − Inverse variance weighted
−0.4
0.0
0.4
MR effect size for
'TNKS' on 'Cardiac valvular disease'

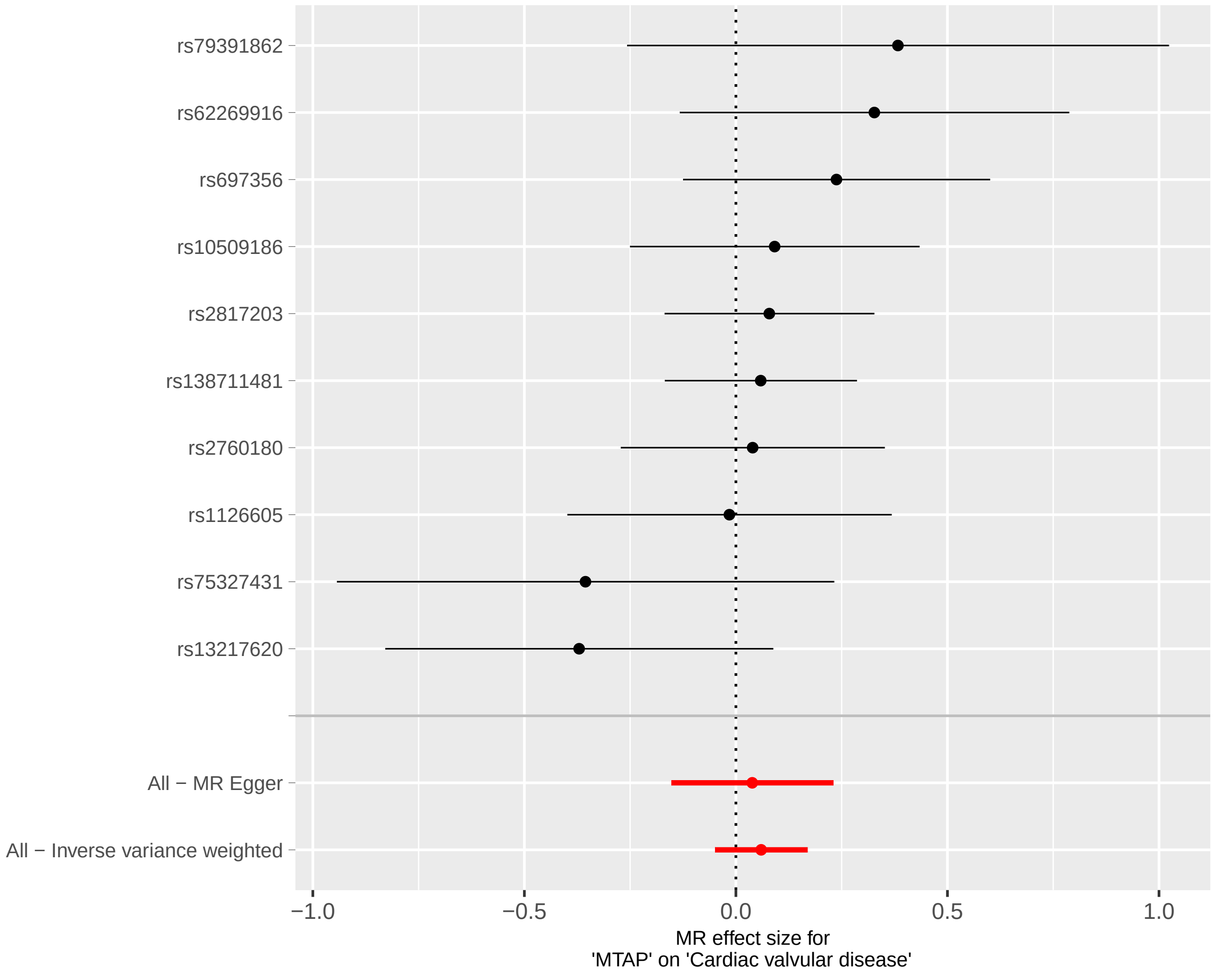
rs79391862
rs62269916
rs697356
rs10509186
rs2817203
rs138711481
rs2760180
rs1126605
rs75327431
rs13217620
All − MR Egger
All − Inverse variance weighted
−1.0
−0.5
0.0
0.5
1.0
MR effect size for
'MTAP' on 'Cardiac valvular disease'

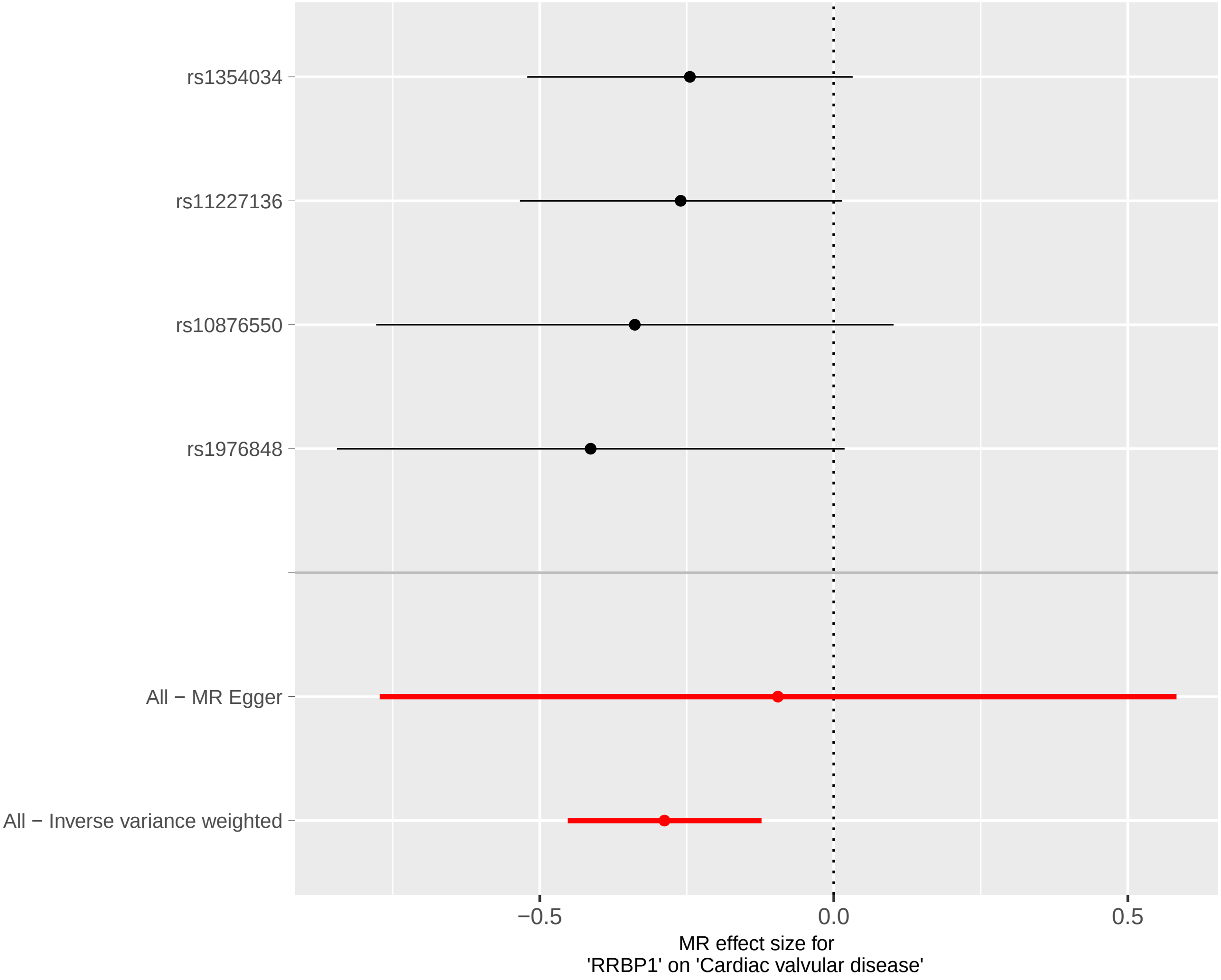
rs1354034
rs11227136
rs10876550
rs1976848
All − MR Egger
All − Inverse variance weighted
−0.5
0.0
0.5
MR effect size for
'RRBP1' on 'Cardiac valvular disease'

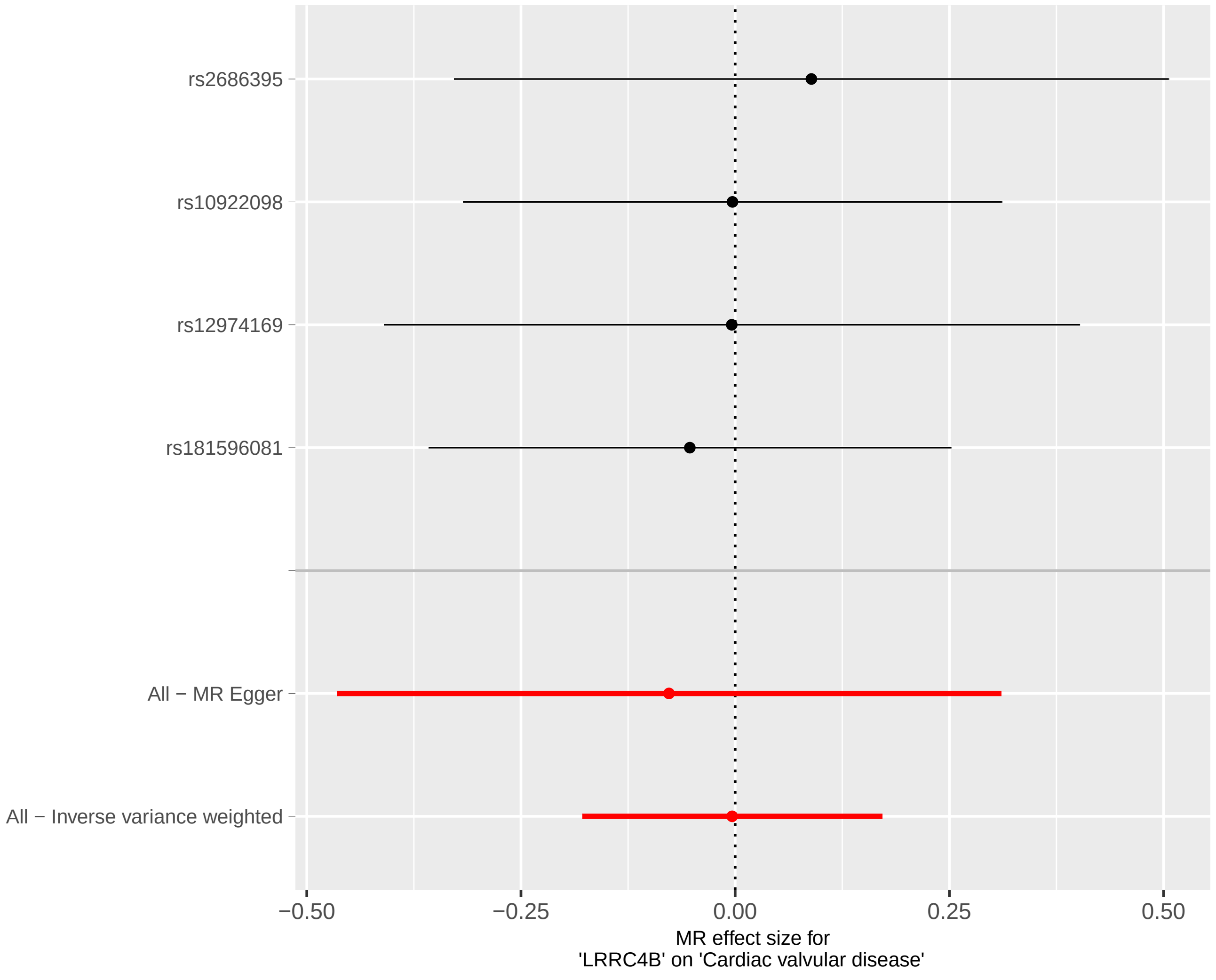
rs2686395
rs10922098
rs12974169
rs181596081
All − MR Egger
All − Inverse variance weighted
−0.50
−0.25
0.00
0.25
0.50
MR effect size for
'LRRC4B' on 'Cardiac valvular disease'

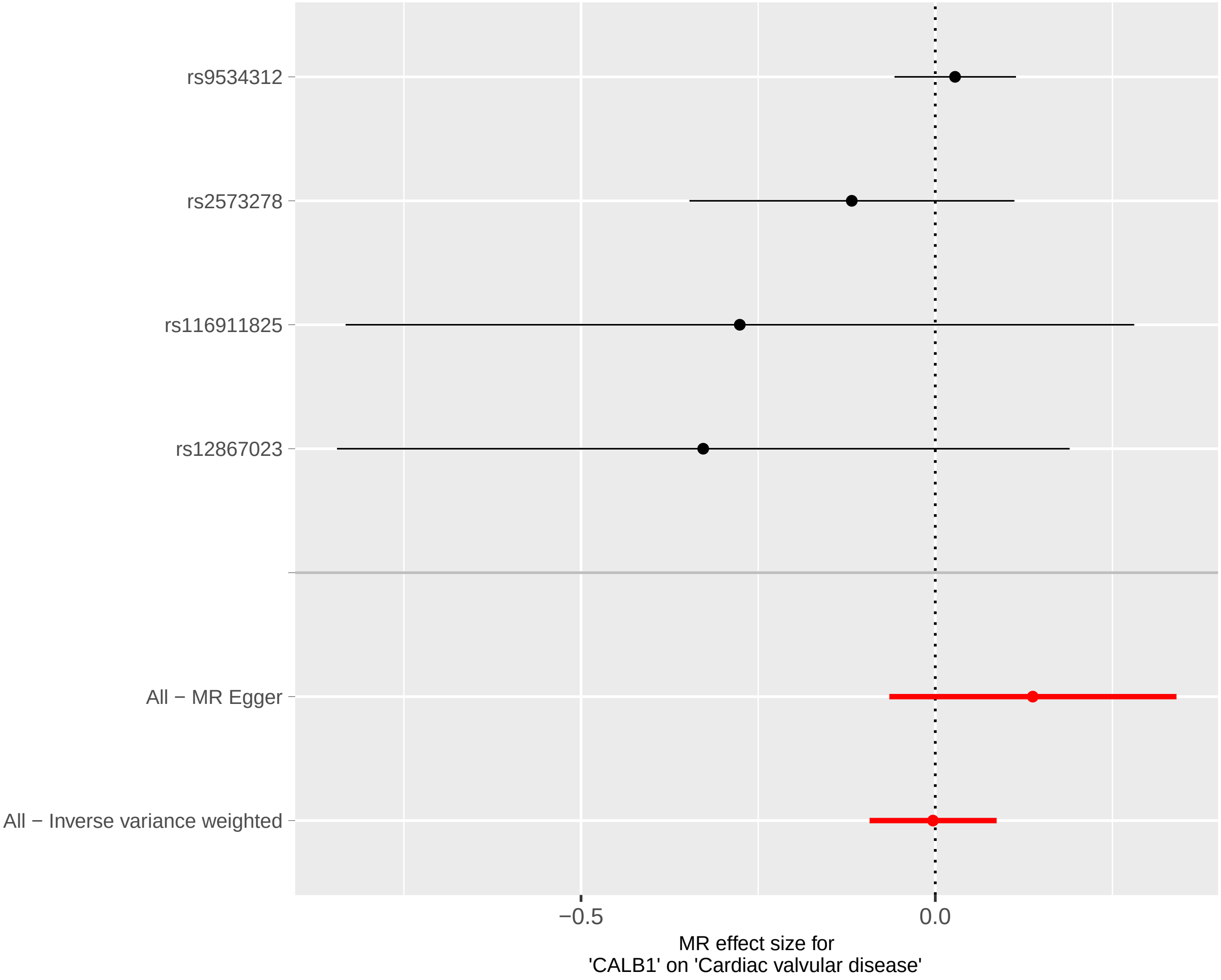
rs9534312
rs2573278
rs116911825
rs12867023
All − MR Egger
All − Inverse variance weighted
−0.5
0.0
MR effect size for
'CALB1' on 'Cardiac valvular disease'

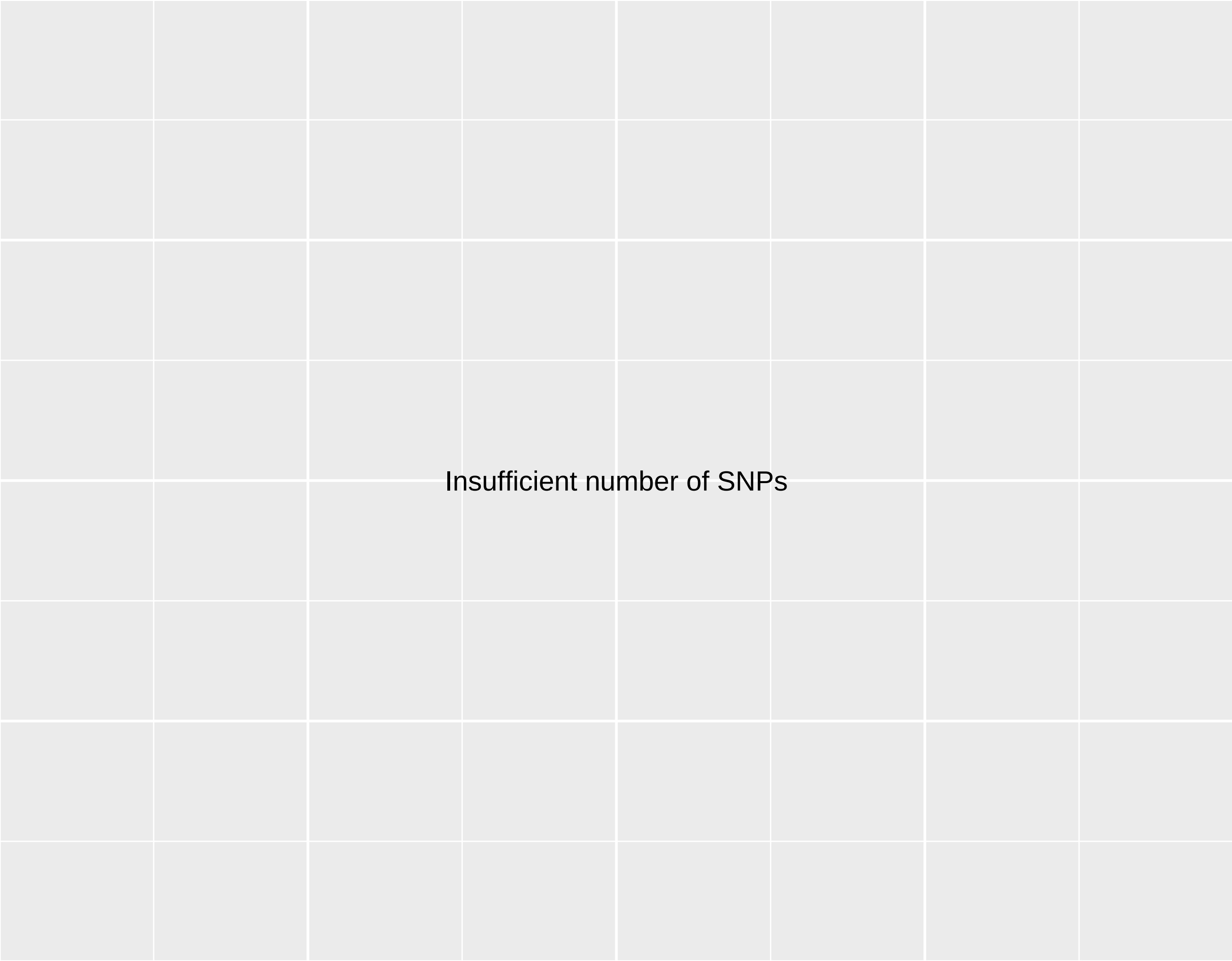
Insufficient number of SNPs

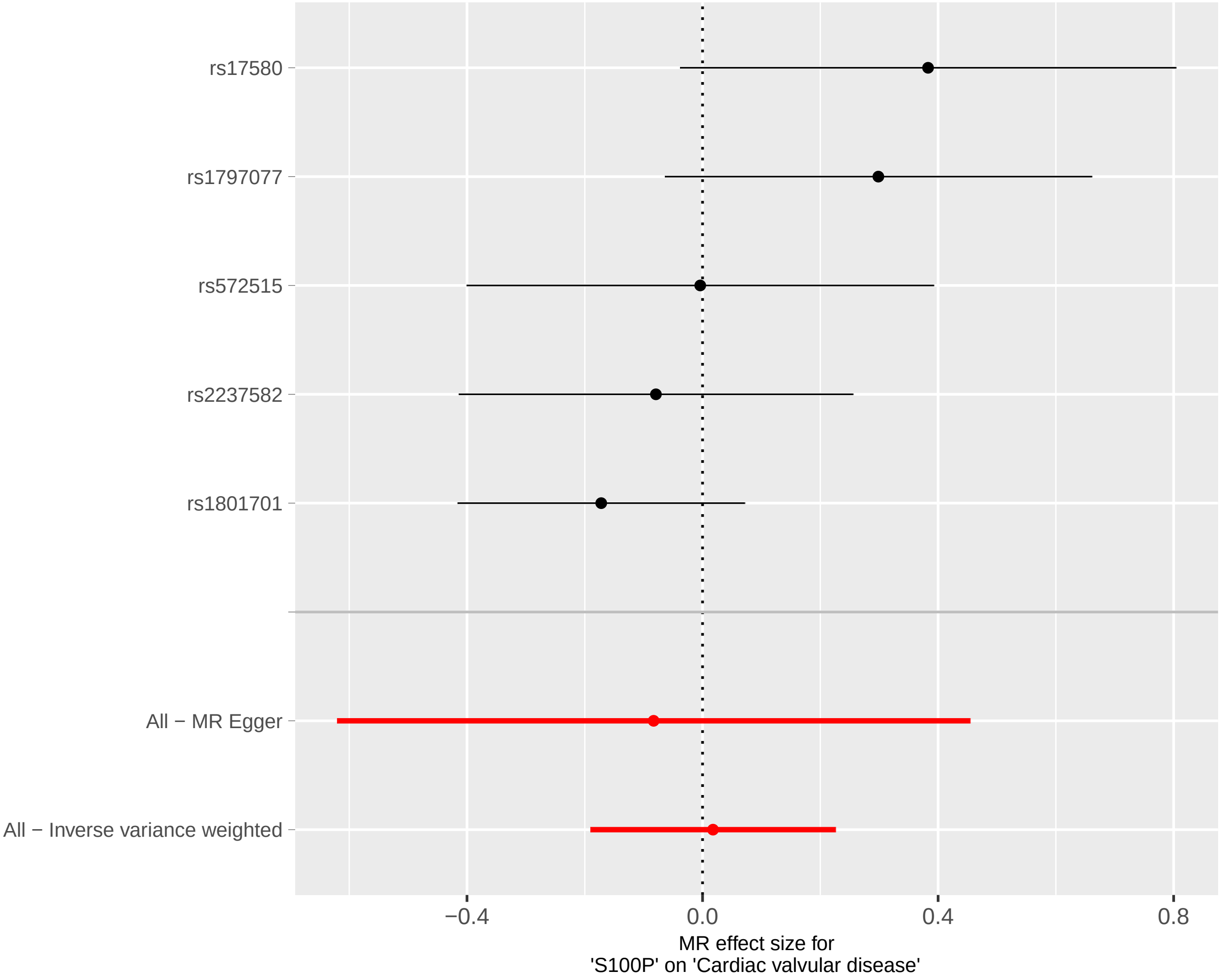
rs17580
rs1797077
rs572515
rs2237582
rs1801701
All − MR Egger
All − Inverse variance weighted
−0.4
0.0
0.4
0.8
MR effect size for
'S100P' on 'Cardiac valvular disease'

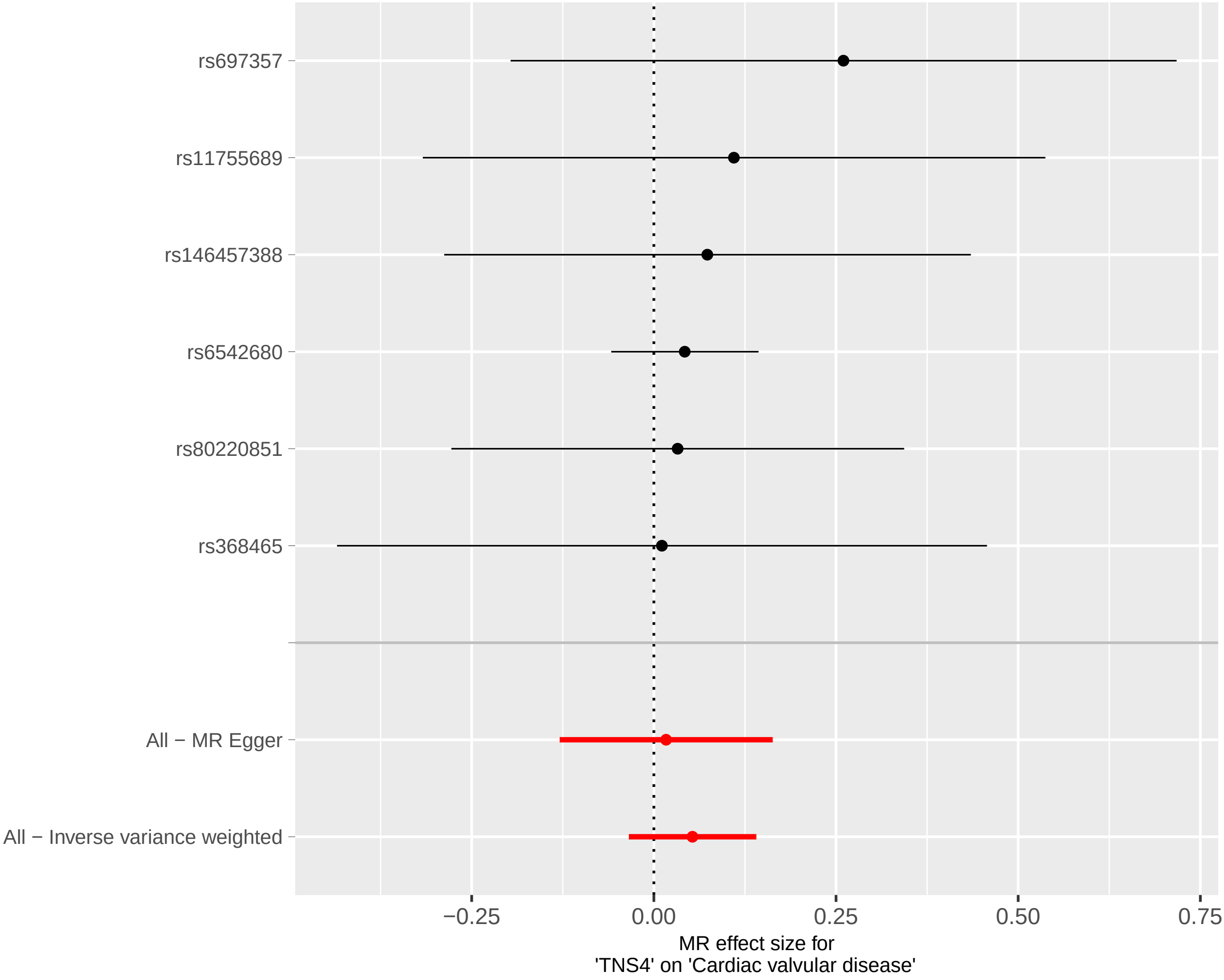
rs697357
rs11755689
rs146457388
rs6542680
rs80220851
rs368465
All − MR Egger
All − Inverse variance weighted
−0.25
0.00
0.25
0.50
0.75
MR effect size for
'TNS4' on 'Cardiac valvular disease'

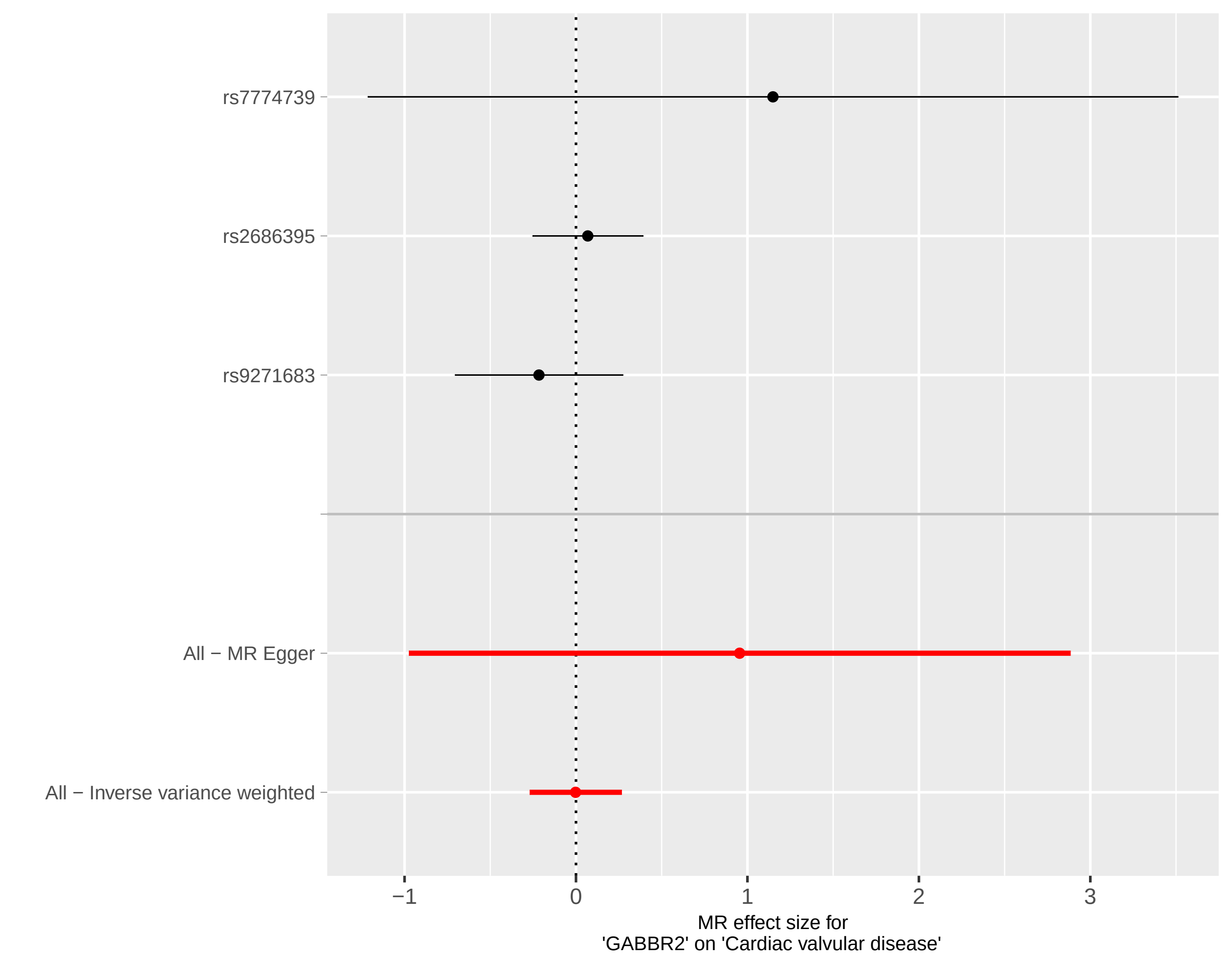
rs7774739
rs2686395
rs9271683
All − MR Egger
All − Inverse variance weighted
−1
0
1
2
3
MR effect size for
'GABBR2' on 'Cardiac valvular disease'

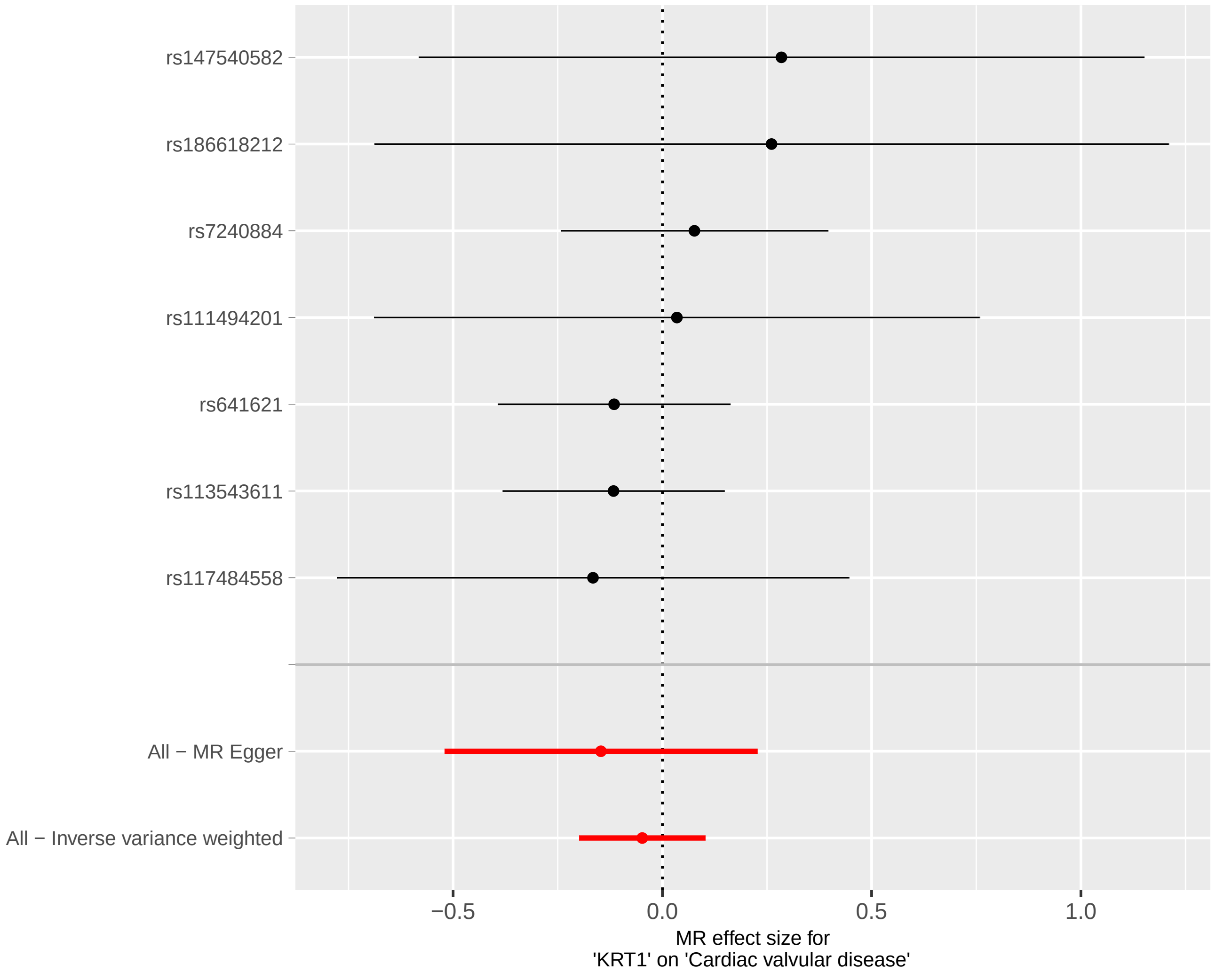
rs147540582
rs186618212
rs7240884
rs111494201
rs641621
rs113543611
rs117484558
All − MR Egger
All − Inverse variance weighted
−0.5
0.0
0.5
1.0
MR effect size for
'KRT1' on 'Cardiac valvular disease'

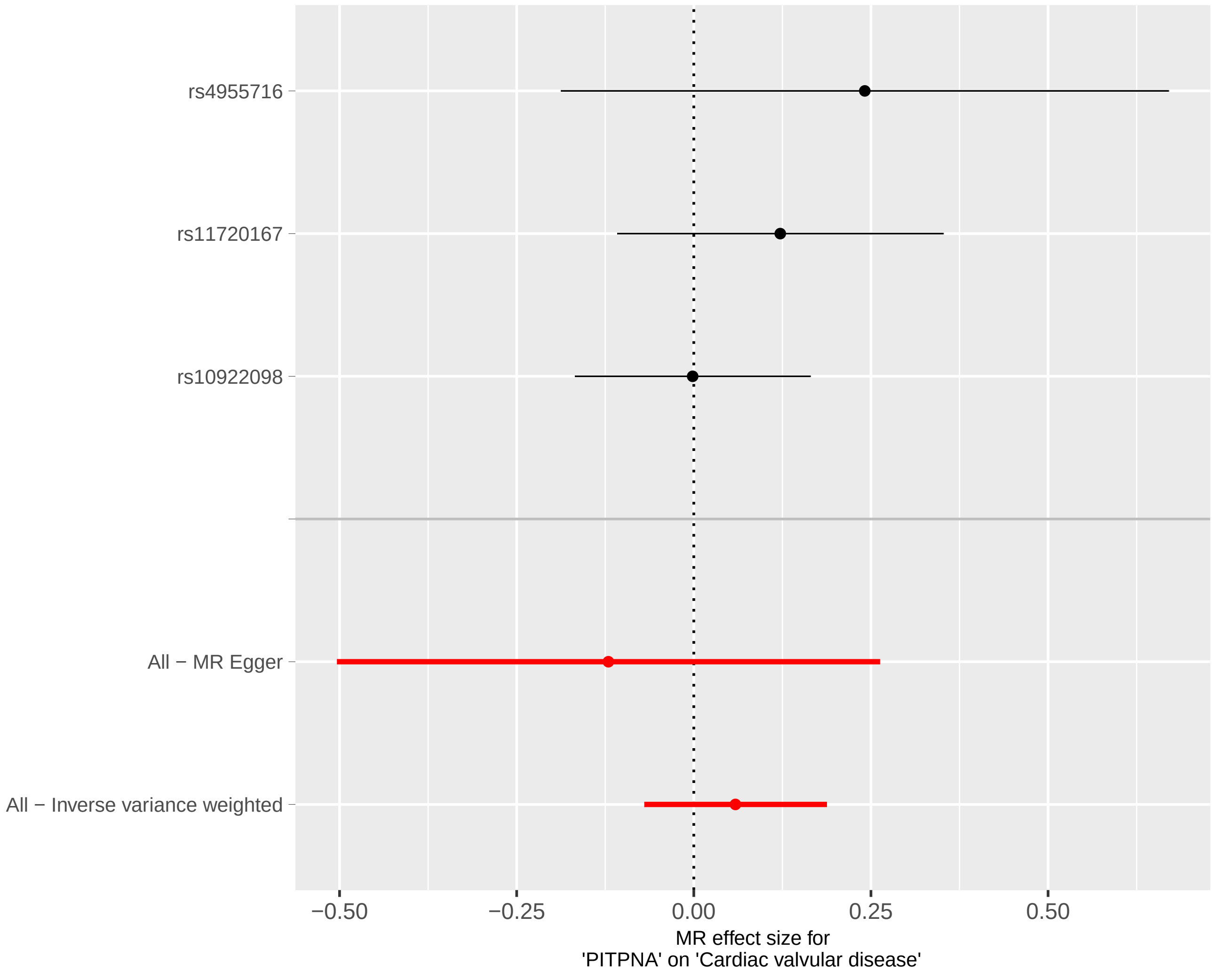
rs4955716
rs11720167
rs10922098
All − MR Egger
All − Inverse variance weighted
−0.50
−0.25
0.00
0.25
0.50
MR effect size for
'PITPNA' on 'Cardiac valvular disease'

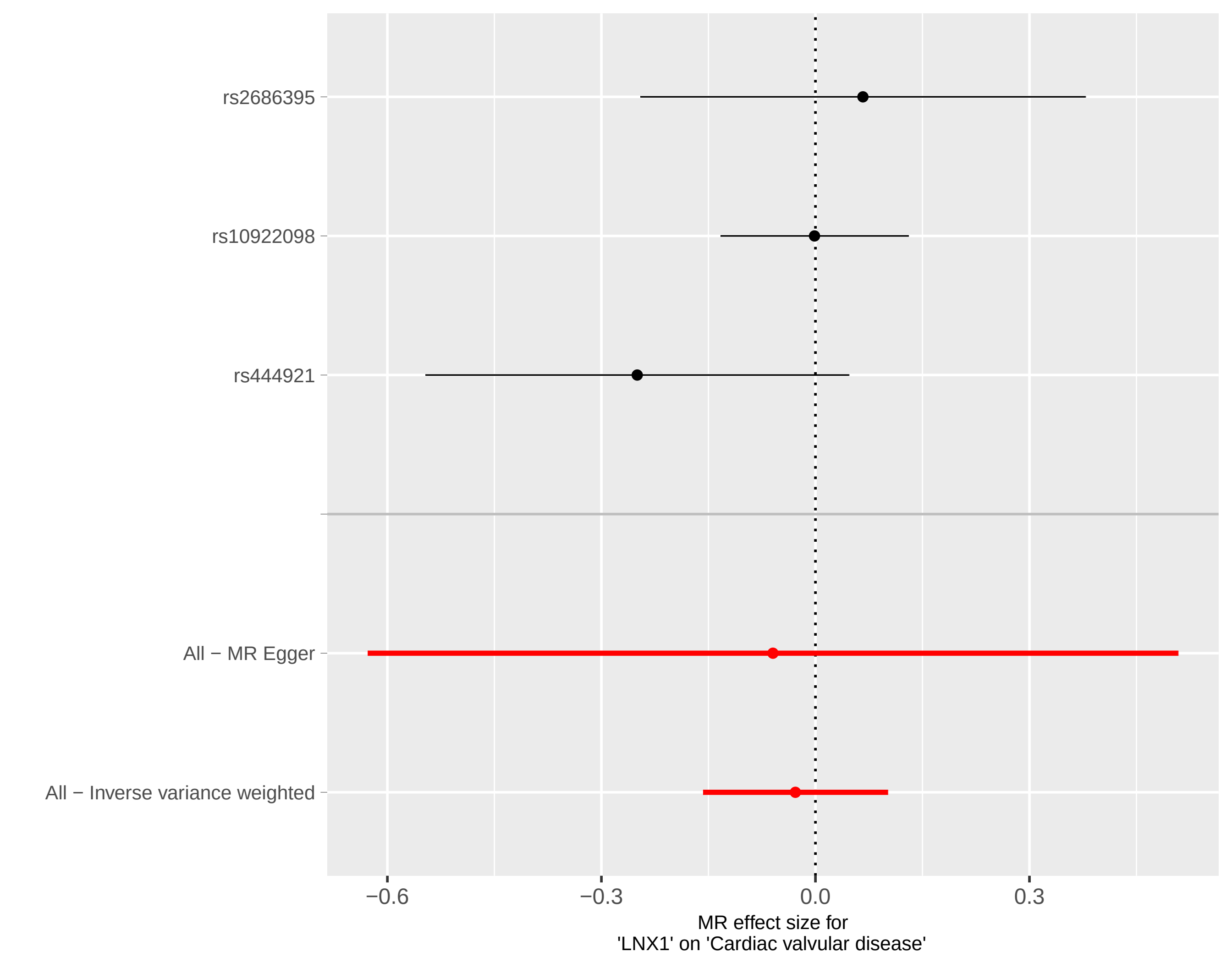

rs2686395
rs10922098
rs444921
All − MR Egger
All − Inverse variance weighted
−0.6
−0.3
0.0
0.3
MR effect size for
'LNX1' on 'Cardiac valvular disease'

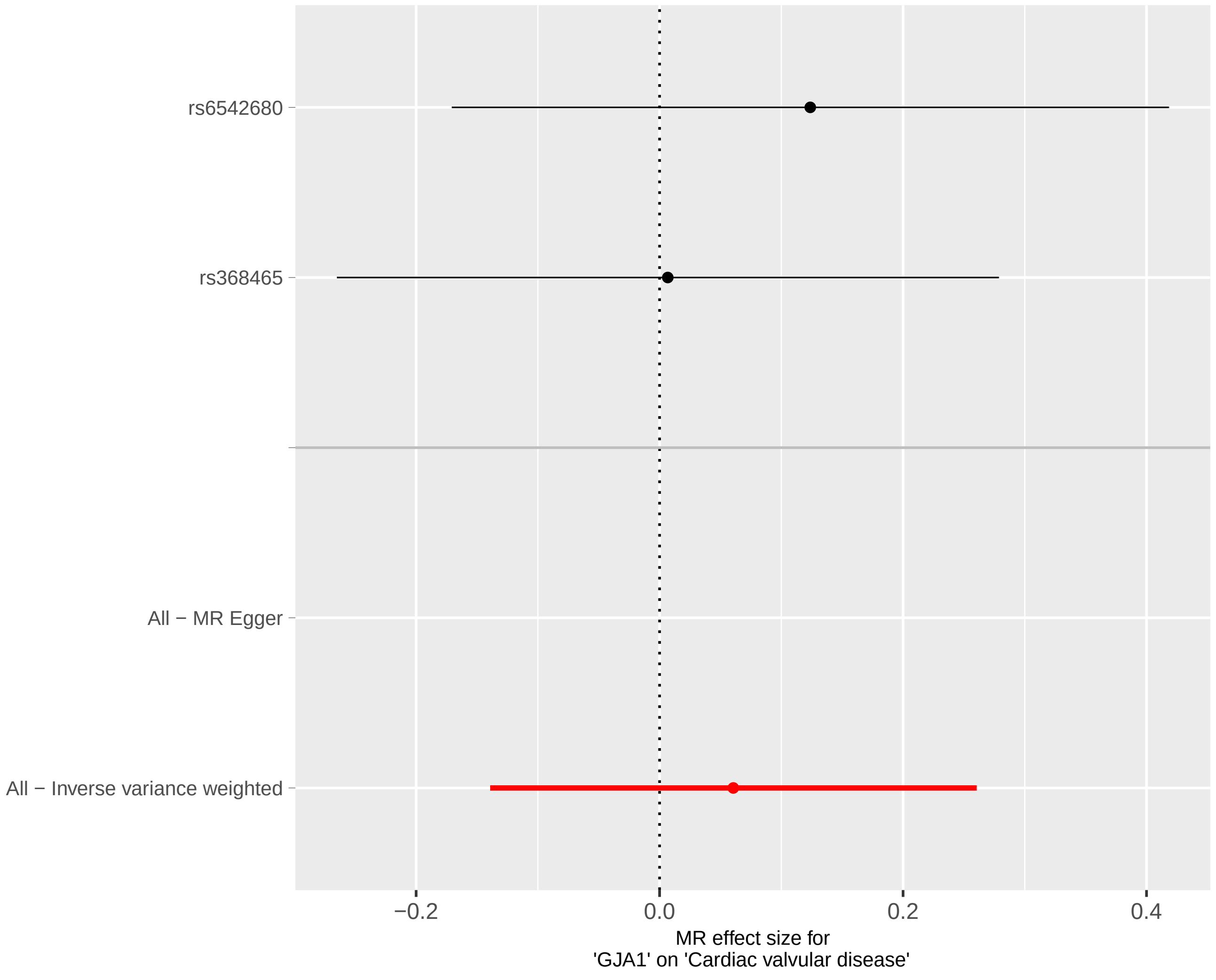
rs6542680
rs368465
All − MR Egger
All − Inverse variance weighted
−0.2
0.0
0.2
0.4
MR effect size for
'GJA1' on 'Cardiac valvular disease'

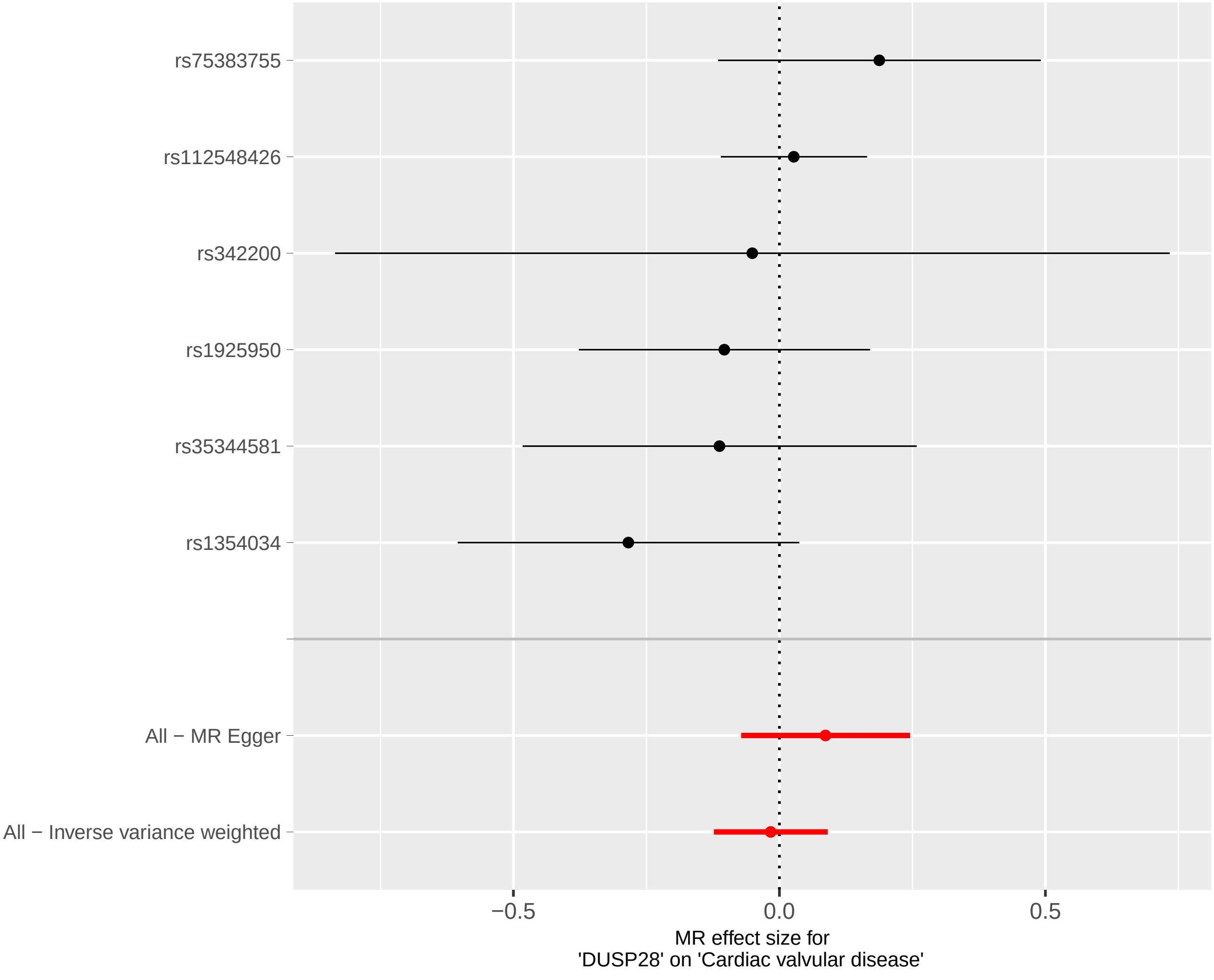
rs75383755
rs112548426
rs342200
rs1925950
rs35344581
rs1354034
All − MR Egger
All − Inverse variance weighted
−0.5
0.0
0.5
MR effect size for
'DUSP28' on 'Cardiac valvular disease'

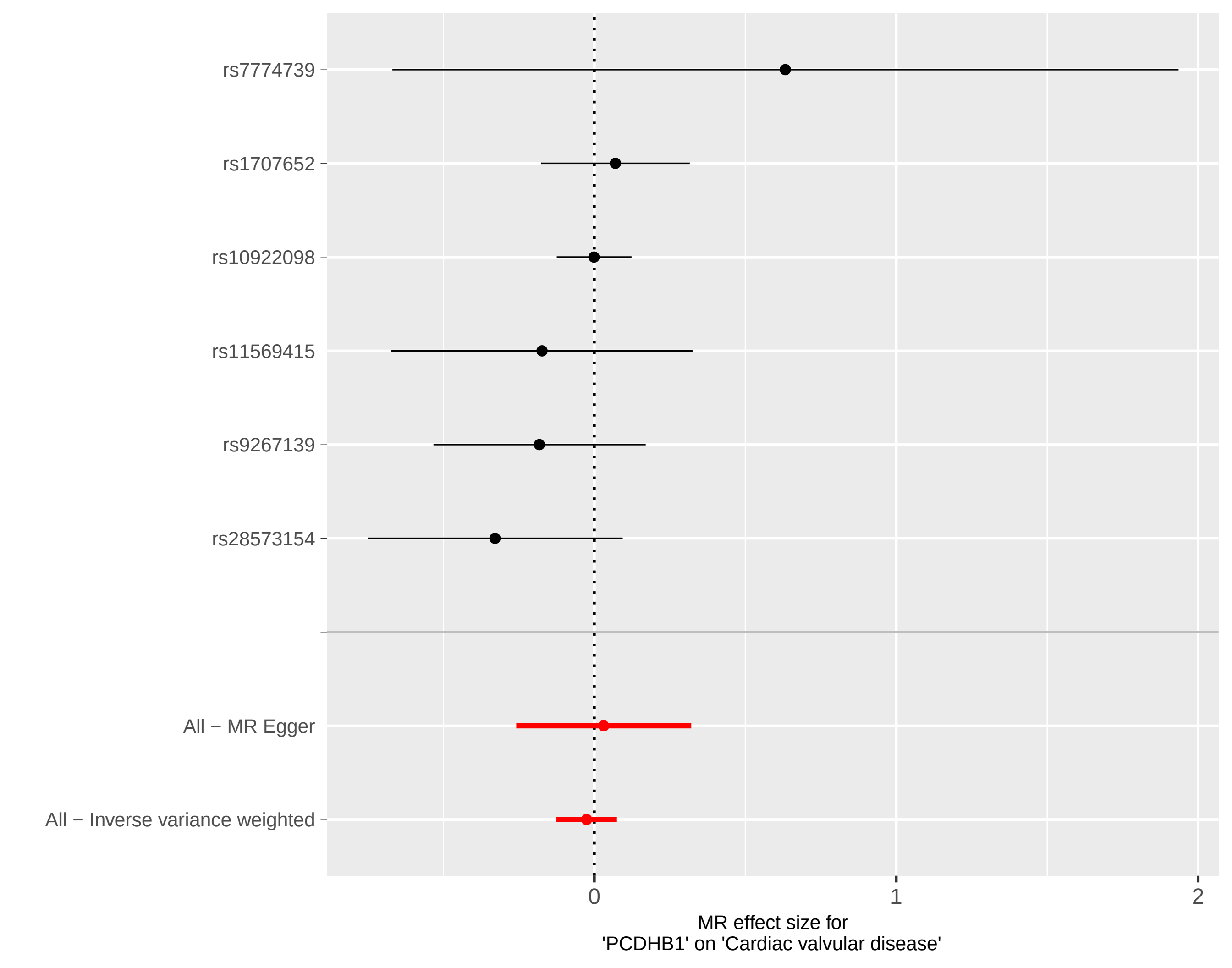
rs7774739
rs1707652
rs10922098
rs11569415
rs9267139
rs28573154
All − MR Egger
All − Inverse variance weighted
0
1
2
MR effect size for
'PCDHB1' on 'Cardiac valvular disease'

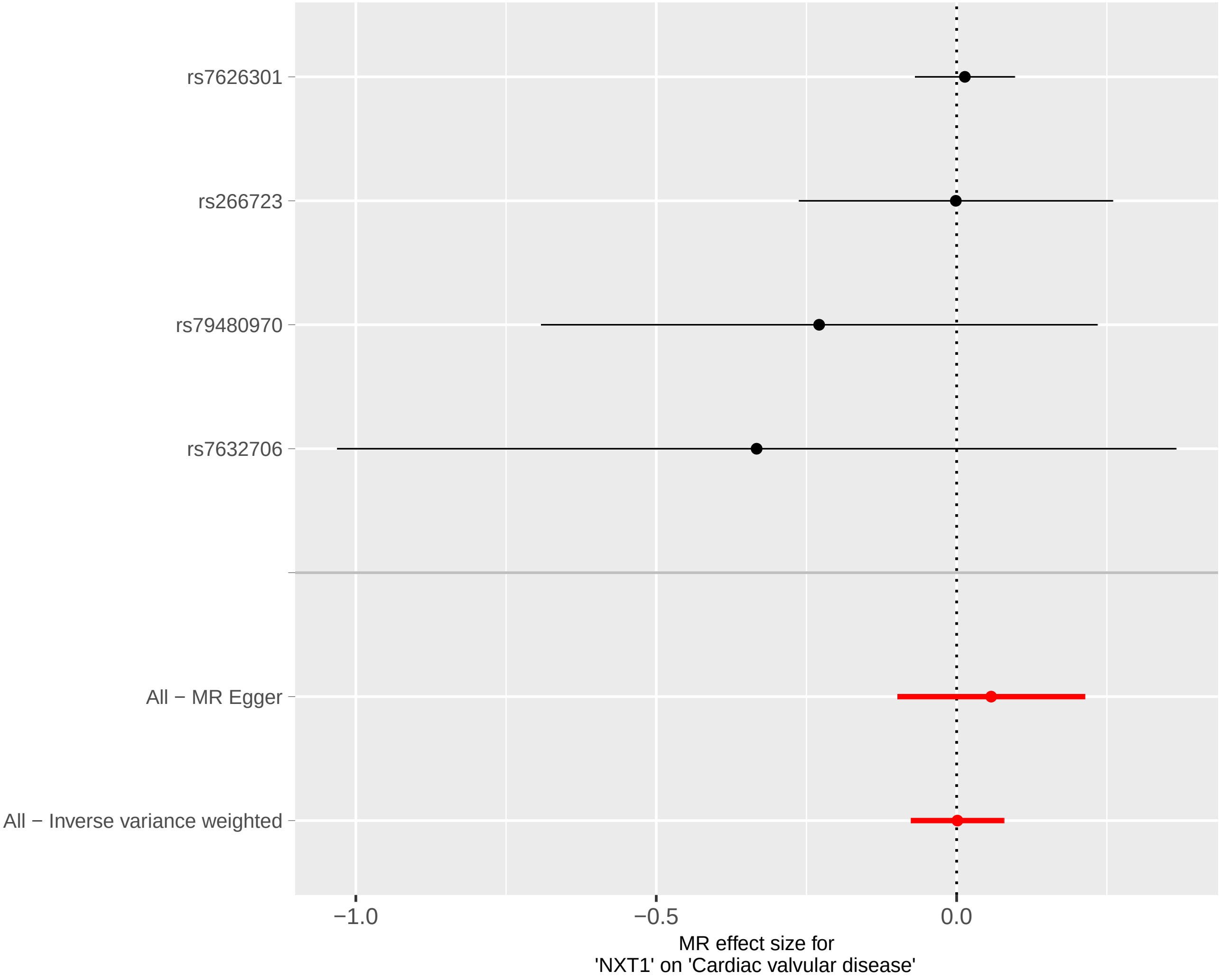

rs7626301
rs266723
rs79480970
rs7632706
All − MR Egger
All − Inverse variance weighted
−1.0
−0.5
0.0
MR effect size for
'NXT1' on 'Cardiac valvular disease'

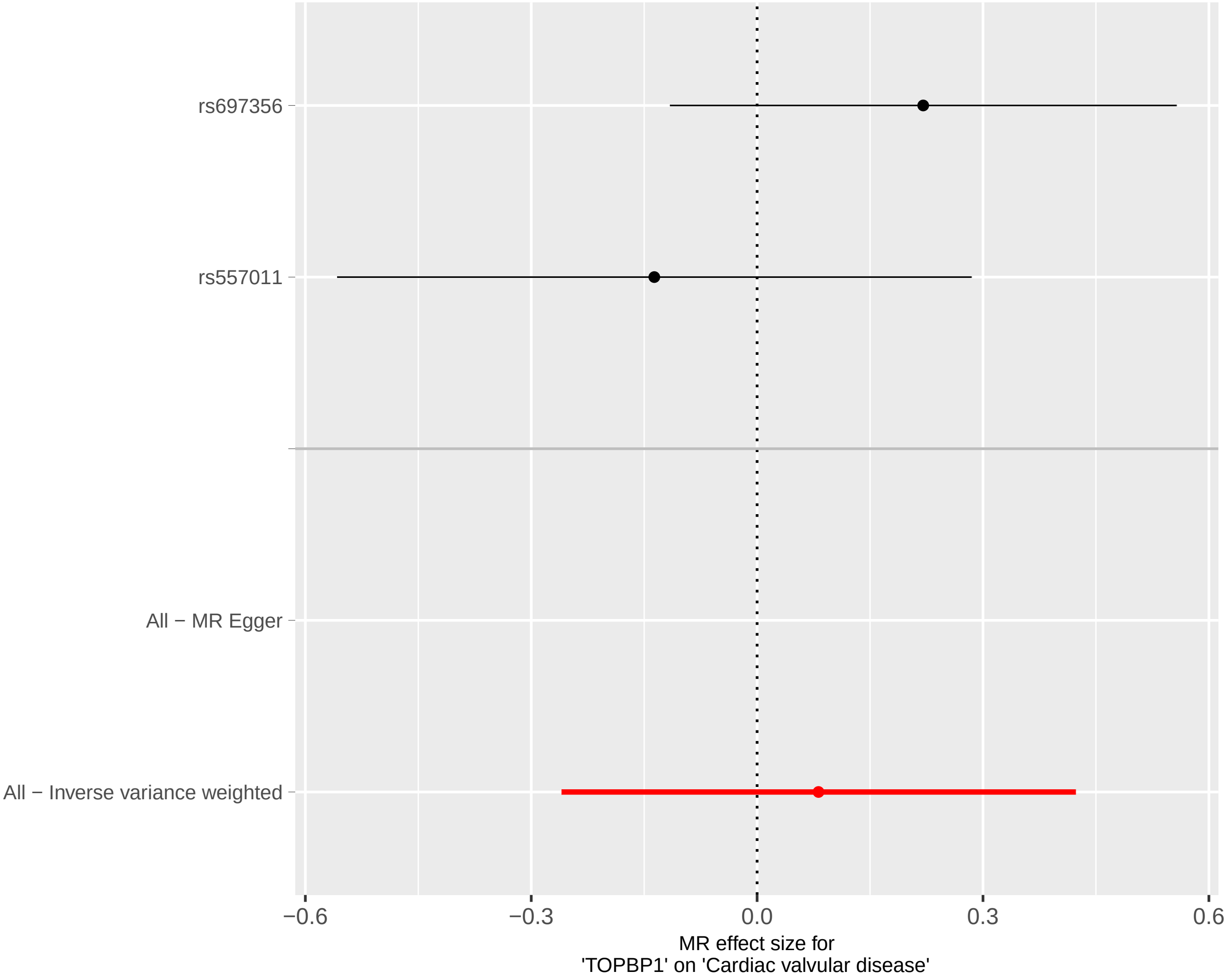
rs697356
rs557011
All − MR Egger
All − Inverse variance weighted
−0.6
−0.3
0.0
0.3
0.6
MR effect size for
'TOPBP1' on 'Cardiac valvular disease'

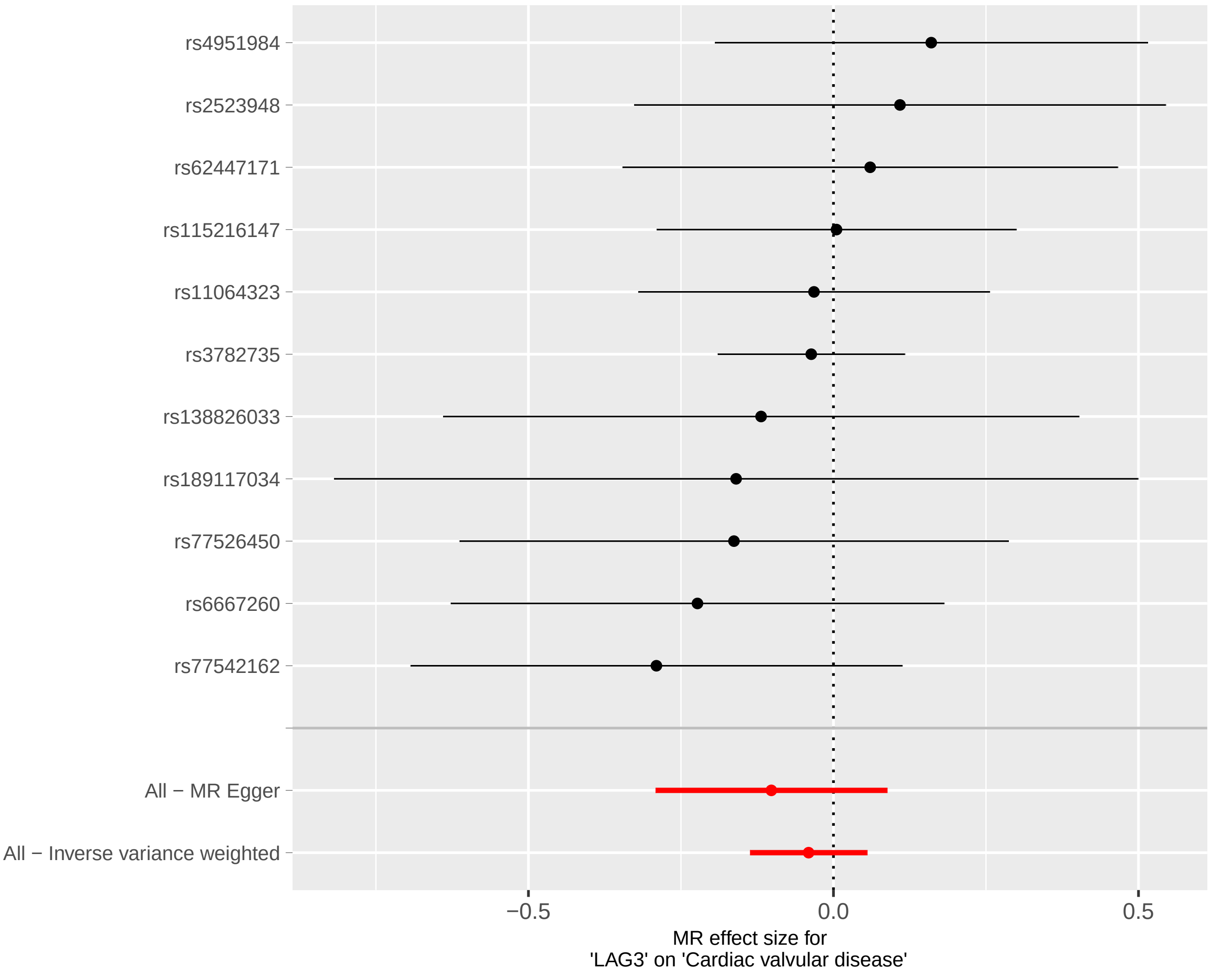
rs4951984
rs2523948
rs62447171
rs115216147
rs11064323
rs3782735
rs138826033
rs189117034
rs77526450
rs6667260
rs77542162
All − MR Egger
All − Inverse variance weighted
−0.5
0.0
0.5
MR effect size for
'LAG3' on 'Cardiac valvular disease'

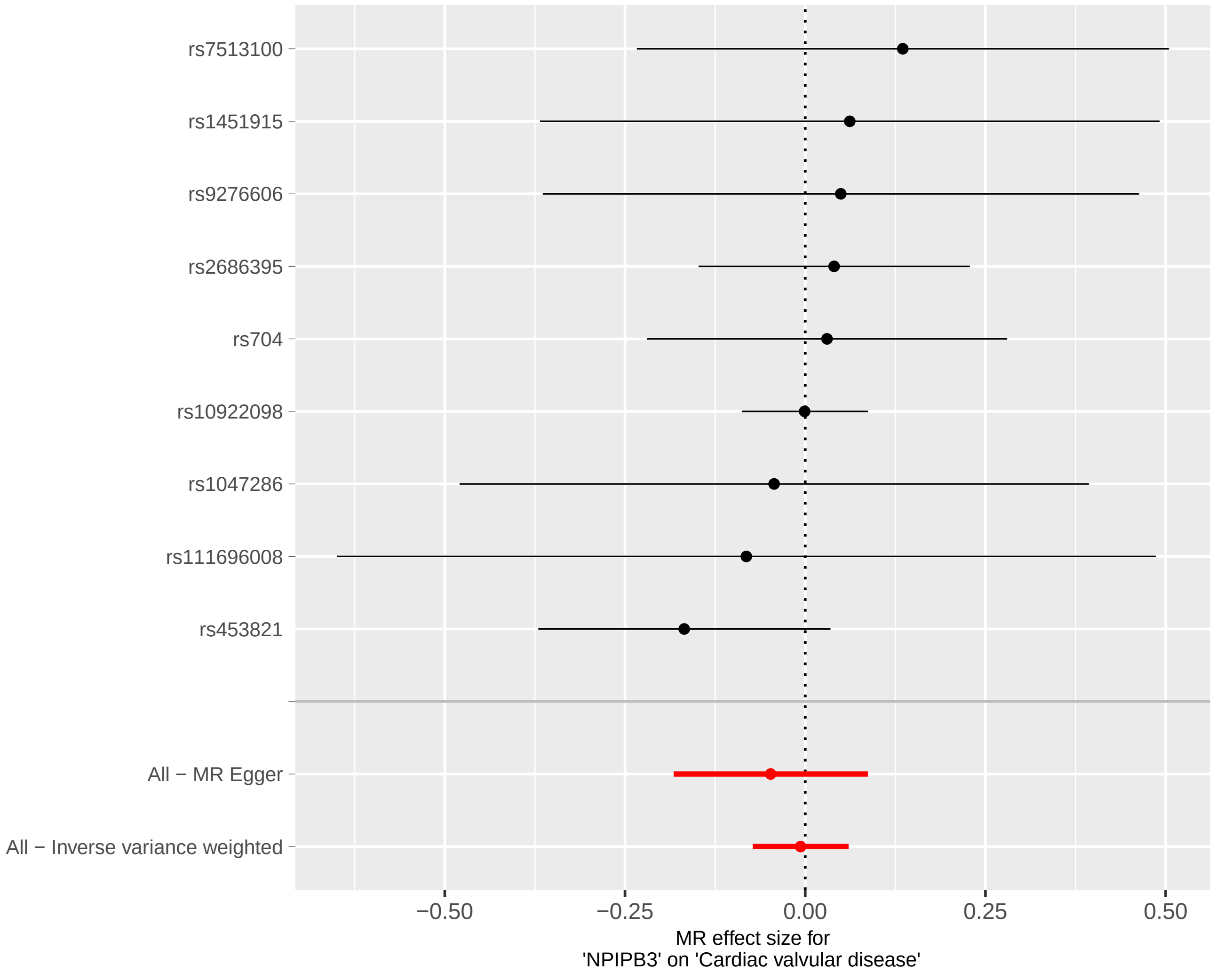
rs7513100
rs1451915
rs9276606
rs2686395
rs704
rs10922098
rs1047286
rs111696008
rs453821
All − MR Egger
All − Inverse variance weighted
−0.50
−0.25
0.00
0.25
0.50
MR effect size for
'NPIPB3' on 'Cardiac valvular disease'

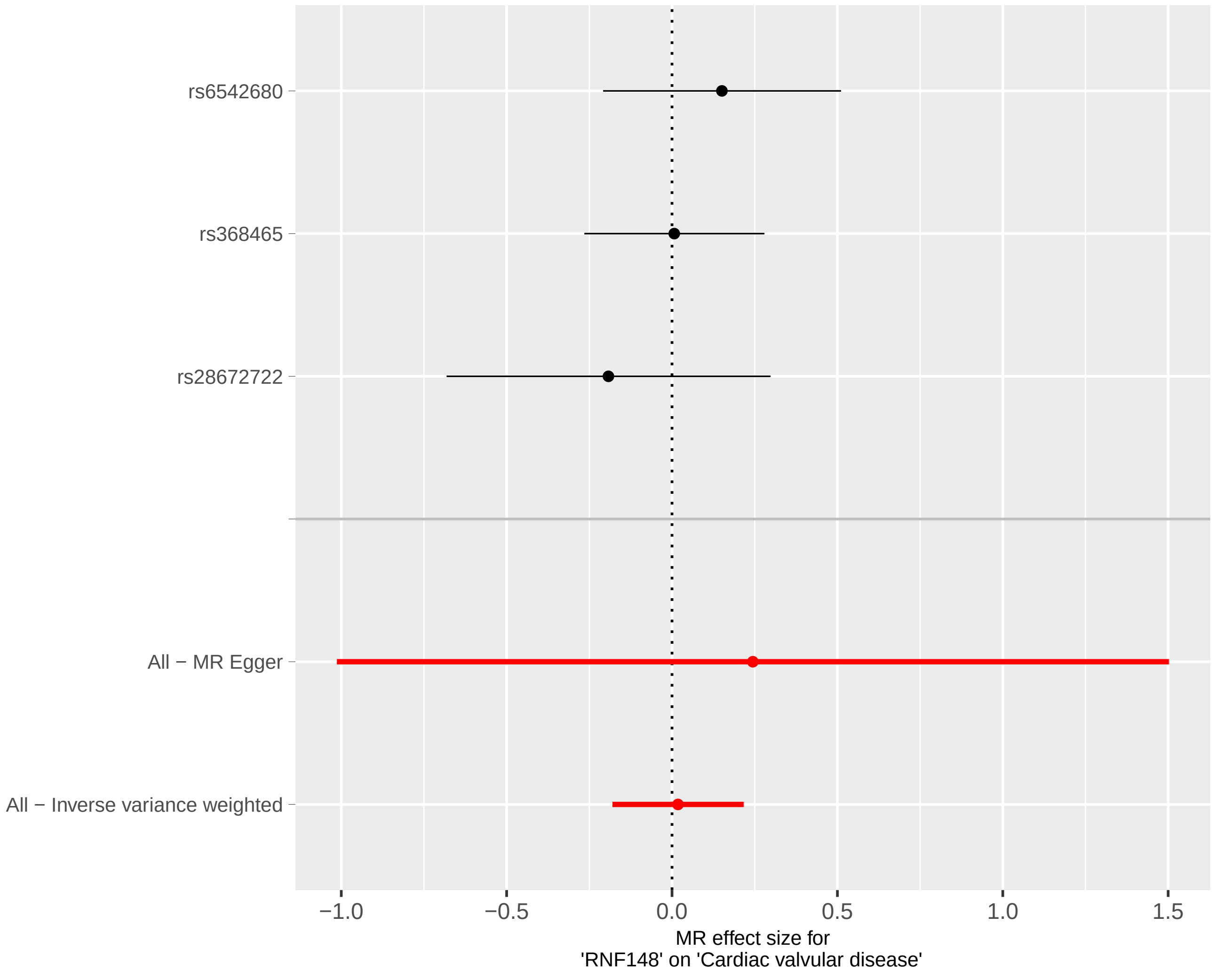

rs6542680
rs368465
rs28672722
All − MR Egger
All − Inverse variance weighted
−1.0
−0.5
0.0
0.5
1.0
1.5
MR effect size for
'RNF148' on 'Cardiac valvular disease'

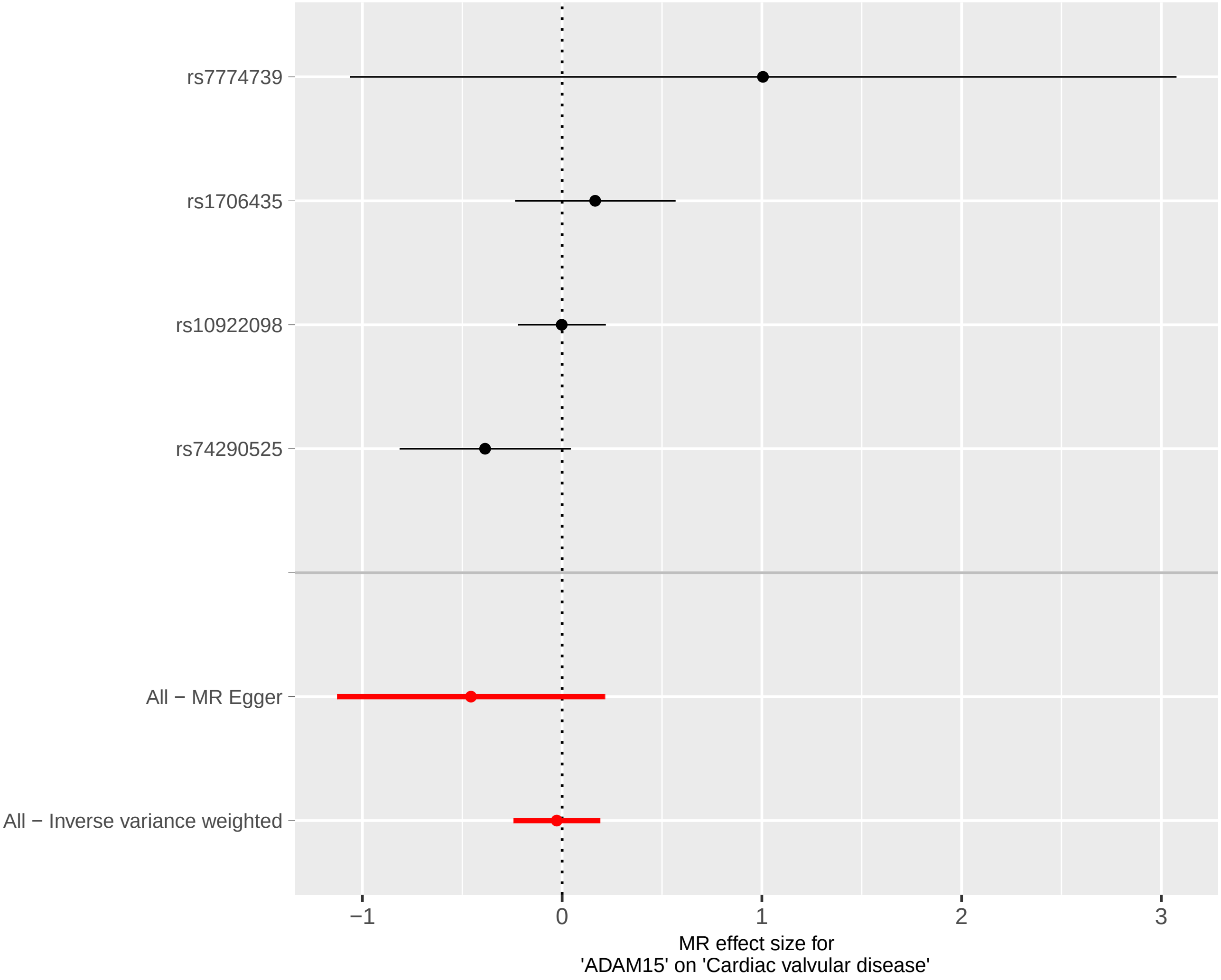

rs7774739
rs1706435
rs10922098
rs74290525
All − MR Egger
All − Inverse variance weighted
−1
0
1
2
3
MR effect size for
'ADAM15' on 'Cardiac valvular disease'

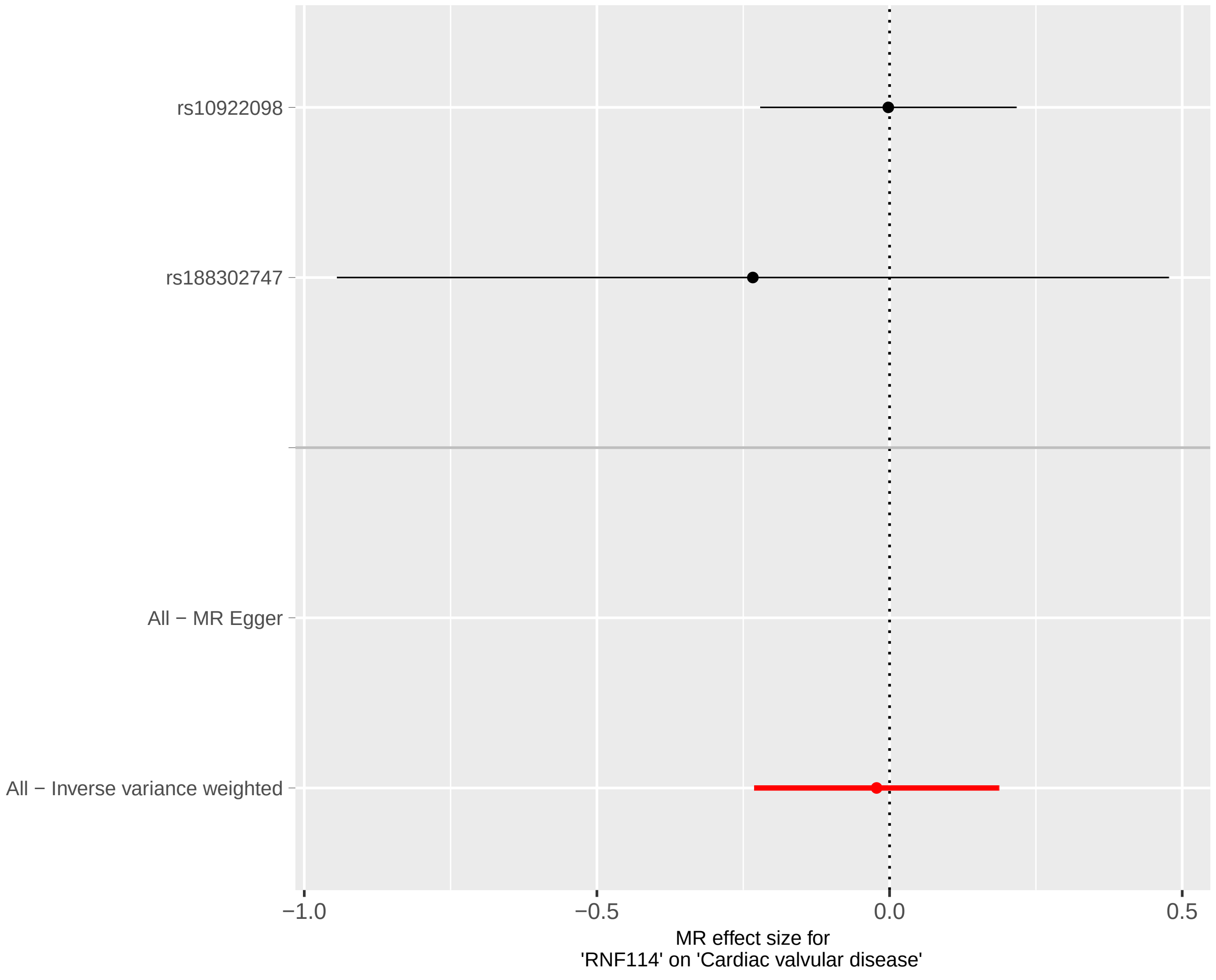
rs10922098
rs188302747
All − MR Egger
All − Inverse variance weighted
−1.0
−0.5
0.0
0.5
MR effect size for
'RNF114' on 'Cardiac valvular disease'

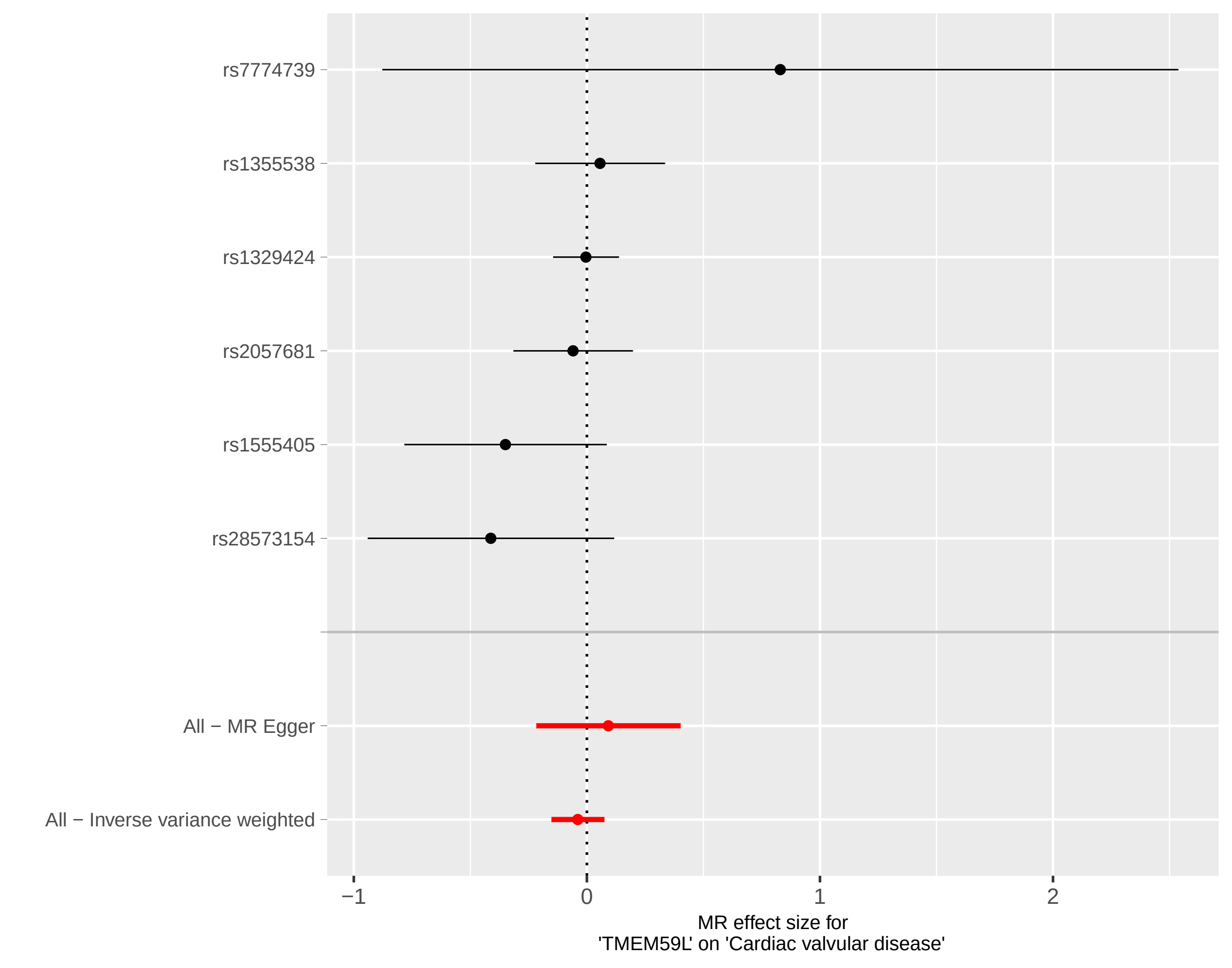

rs7774739
rs1355538
rs1329424
rs2057681
rs1555405
rs28573154
All − MR Egger
All − Inverse variance weighted
−1
0
1
2
MR effect size for
'TMEM59L' on 'Cardiac valvular disease'

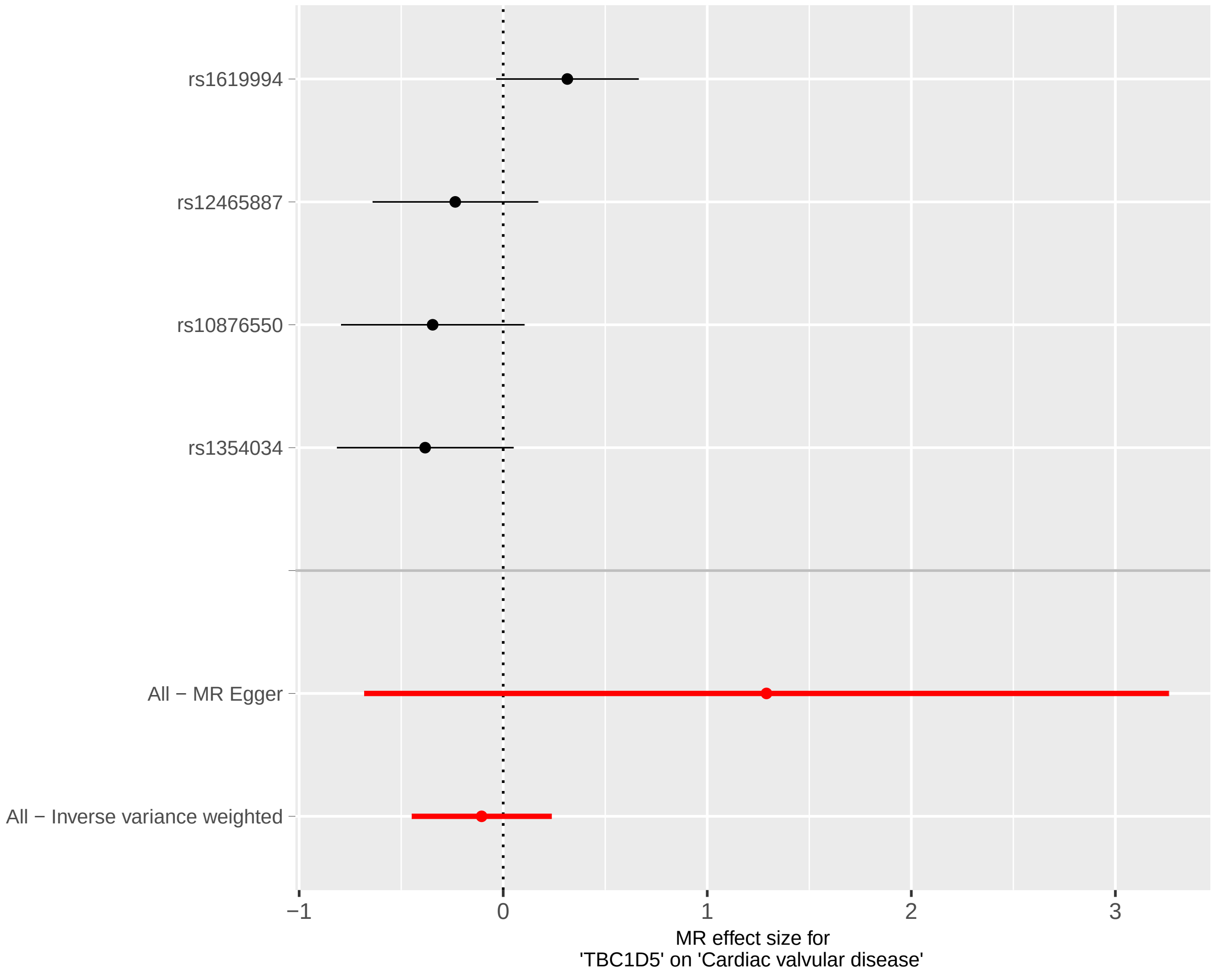
rs1619994
rs12465887
rs10876550
rs1354034
All − MR Egger
All − Inverse variance weighted
−1
0
1
2
3
MR effect size for
'TBC1D5' on 'Cardiac valvular disease'

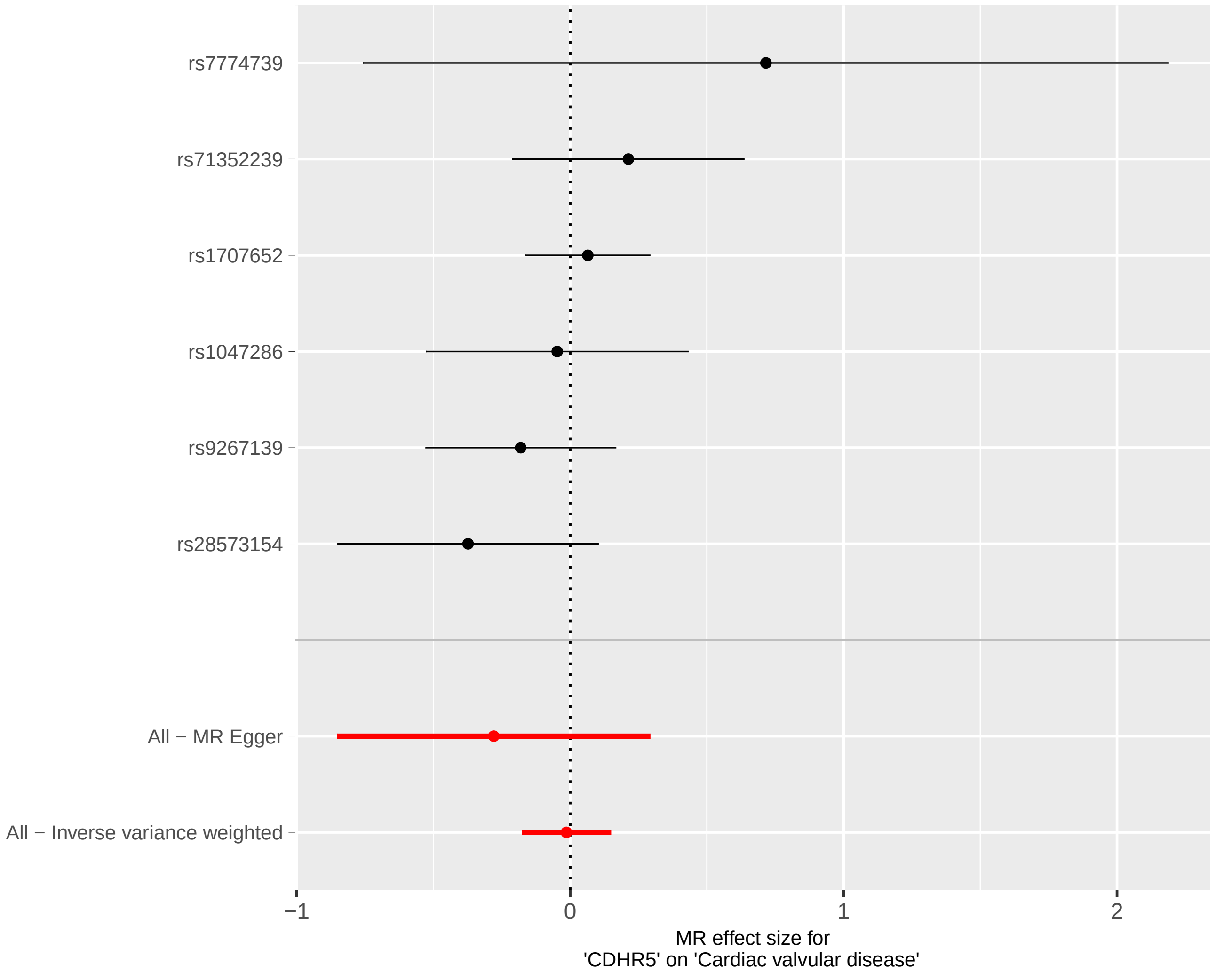
rs7774739
rs71352239
rs1707652
rs1047286
rs9267139
rs28573154
All − MR Egger
All − Inverse variance weighted
−1
0
1
2
MR effect size for
'CDHR5' on 'Cardiac valvular disease'

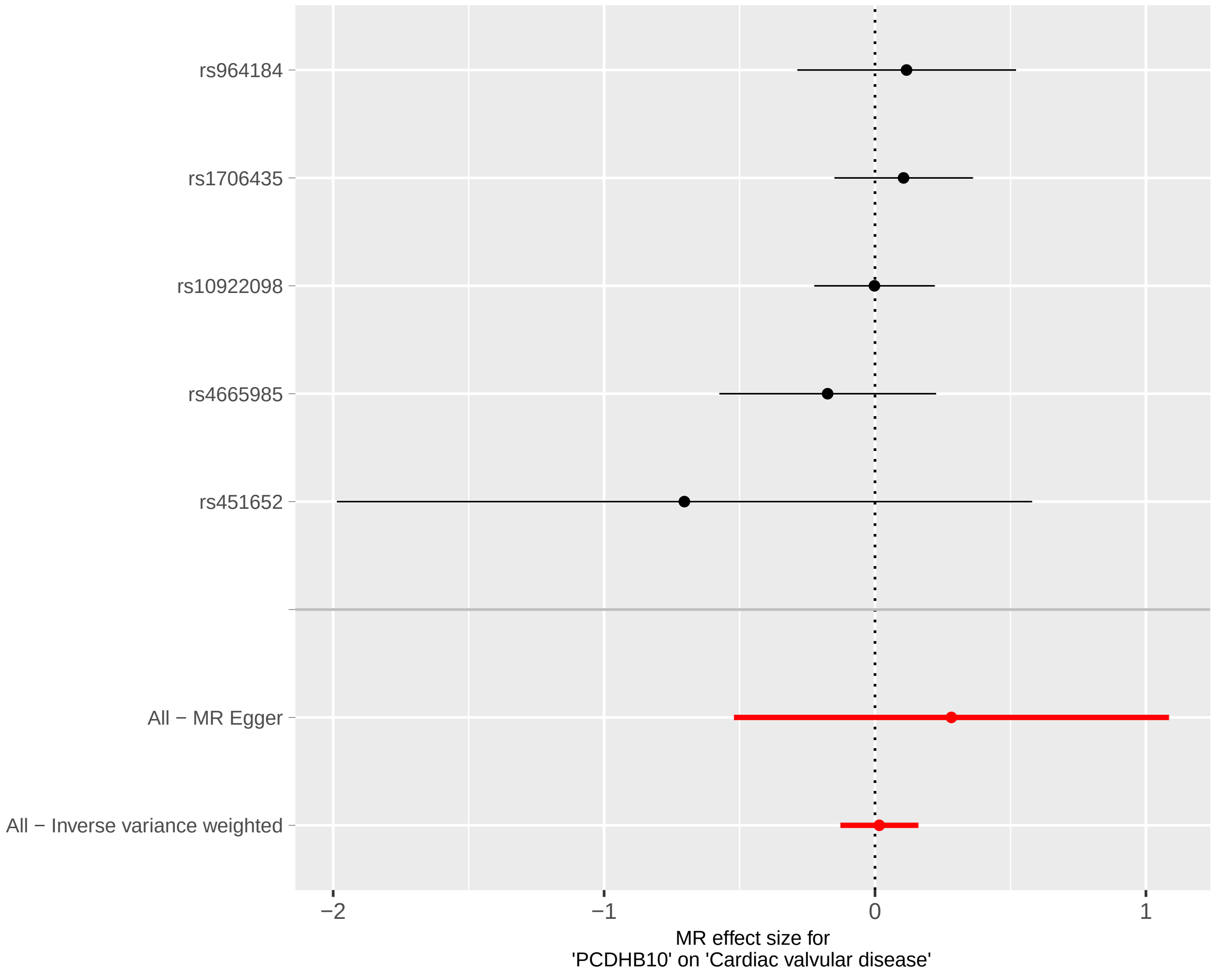
rs964184
rs1706435
rs10922098
rs4665985
rs451652
All − MR Egger
All − Inverse variance weighted
−2
−1
0
1
MR effect size for
'PCDHB10' on 'Cardiac valvular disease'

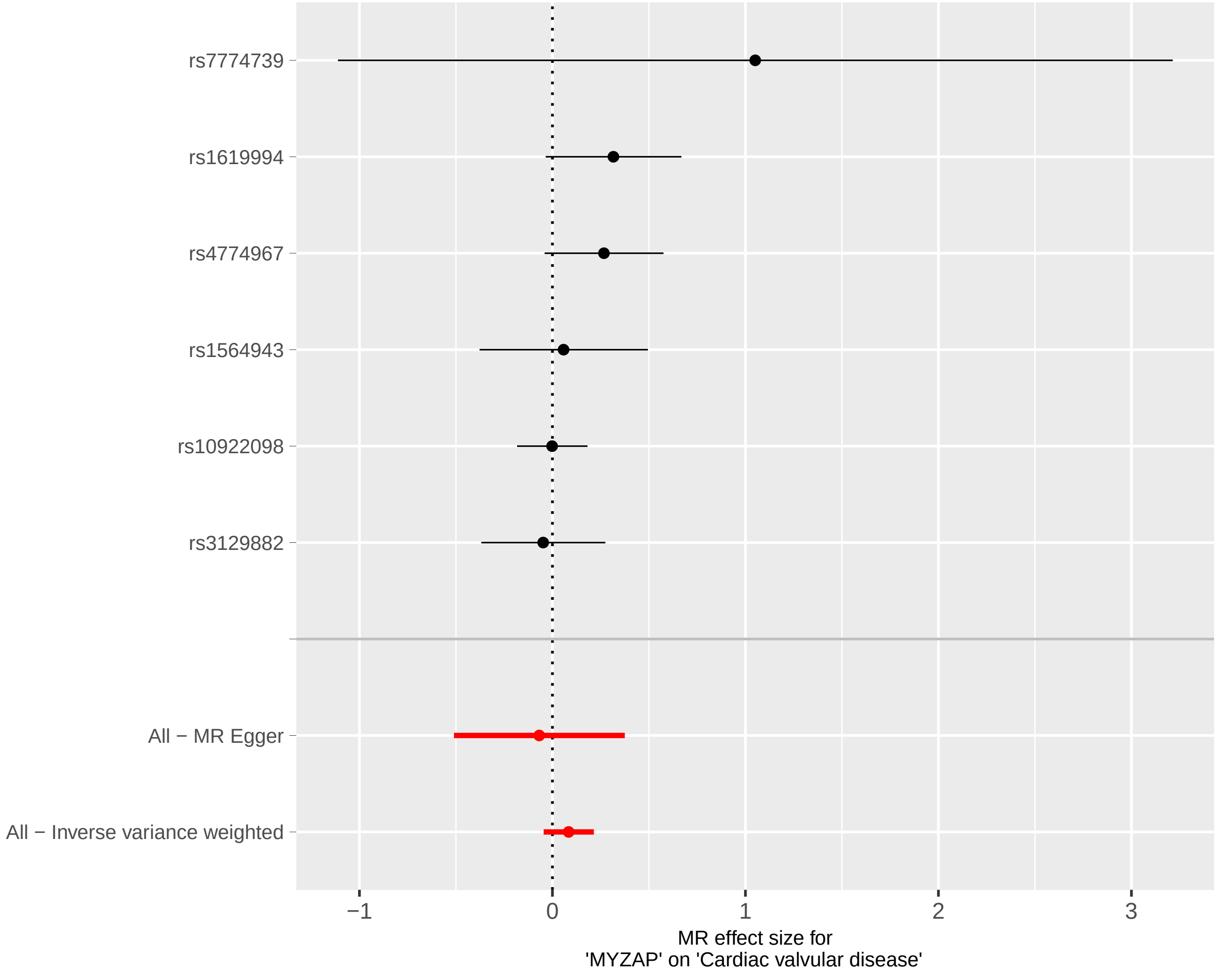
rs7774739
rs1619994
rs4774967
rs1564943
rs10922098
rs3129882
All − MR Egger
All − Inverse variance weighted
−1
0
1
2
3
MR effect size for
'MYZAP' on 'Cardiac valvular disease'

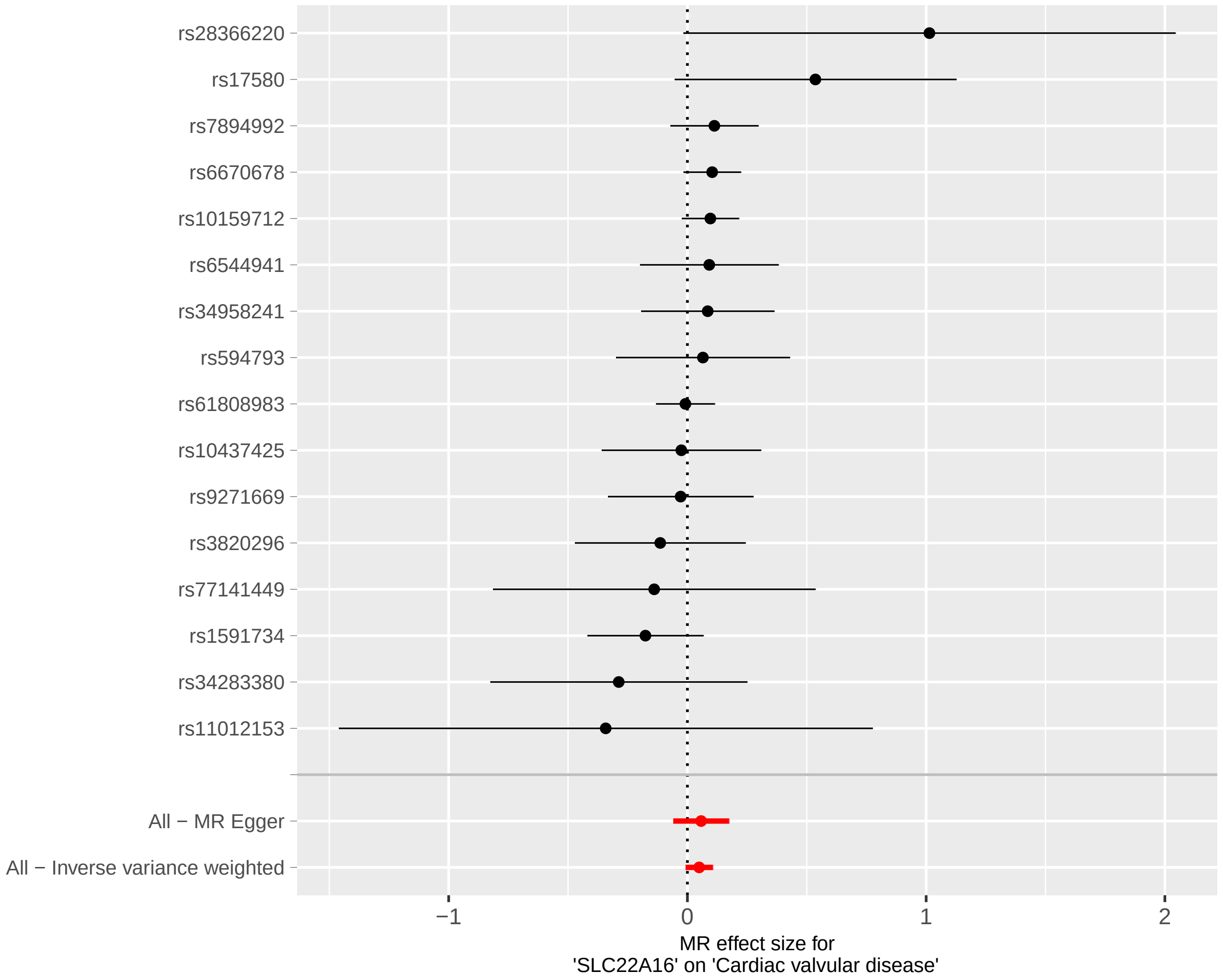

rs28366220
rs17580
rs7894992
rs6670678
rs10159712
rs6544941
rs34958241
rs594793
rs61808983
rs10437425
rs9271669
rs3820296
rs77141449
rs1591734
rs34283380
rs11012153
All − MR Egger
All − Inverse variance weighted
−1
0
1
2
MR effect size for
'SLC22A16' on 'Cardiac valvular disease'

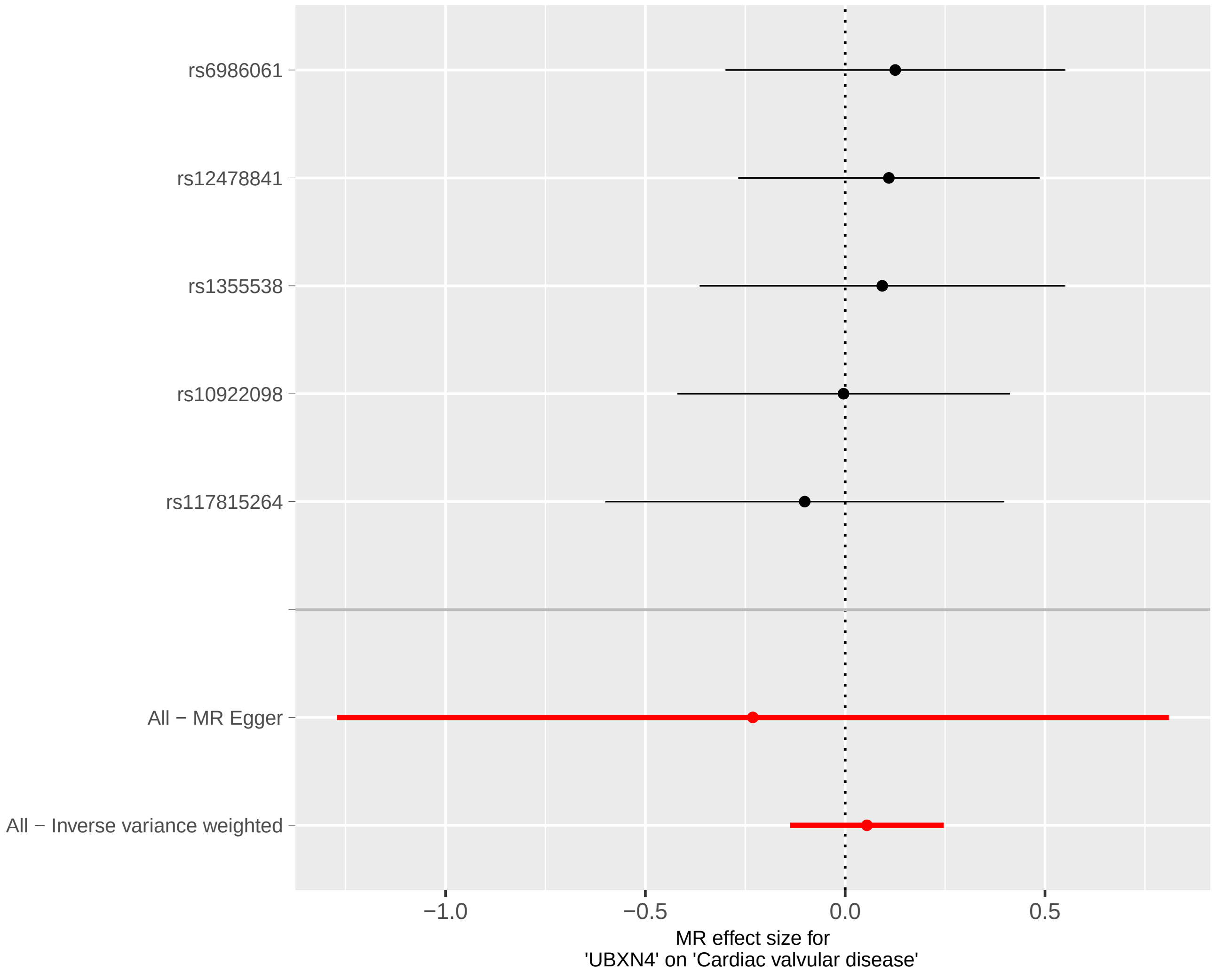
rs6986061
rs12478841
rs1355538
rs10922098
rs117815264
All − MR Egger
All − Inverse variance weighted
−1.0
−0.5
0.0
0.5
MR effect size for
'UBXN4' on 'Cardiac valvular disease'

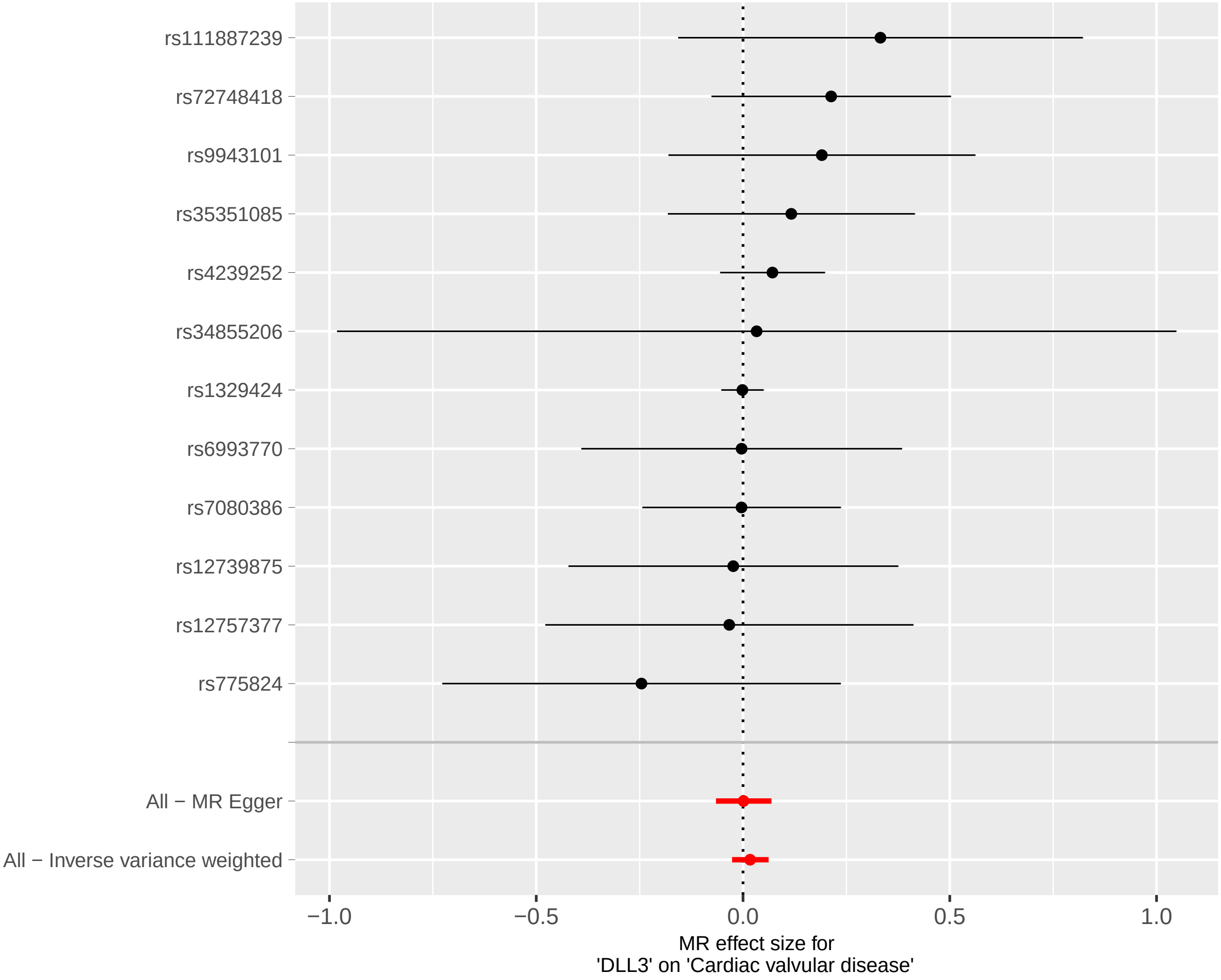

rs111887239
rs72748418
rs9943101
rs35351085
rs4239252
rs34855206
rs1329424
rs6993770
rs7080386
rs12739875
rs12757377
rs775824
All − MR Egger
All − Inverse variance weighted
−1.0
−0.5
0.0
0.5
1.0
MR effect size for
'DLL3' on 'Cardiac valvular disease'

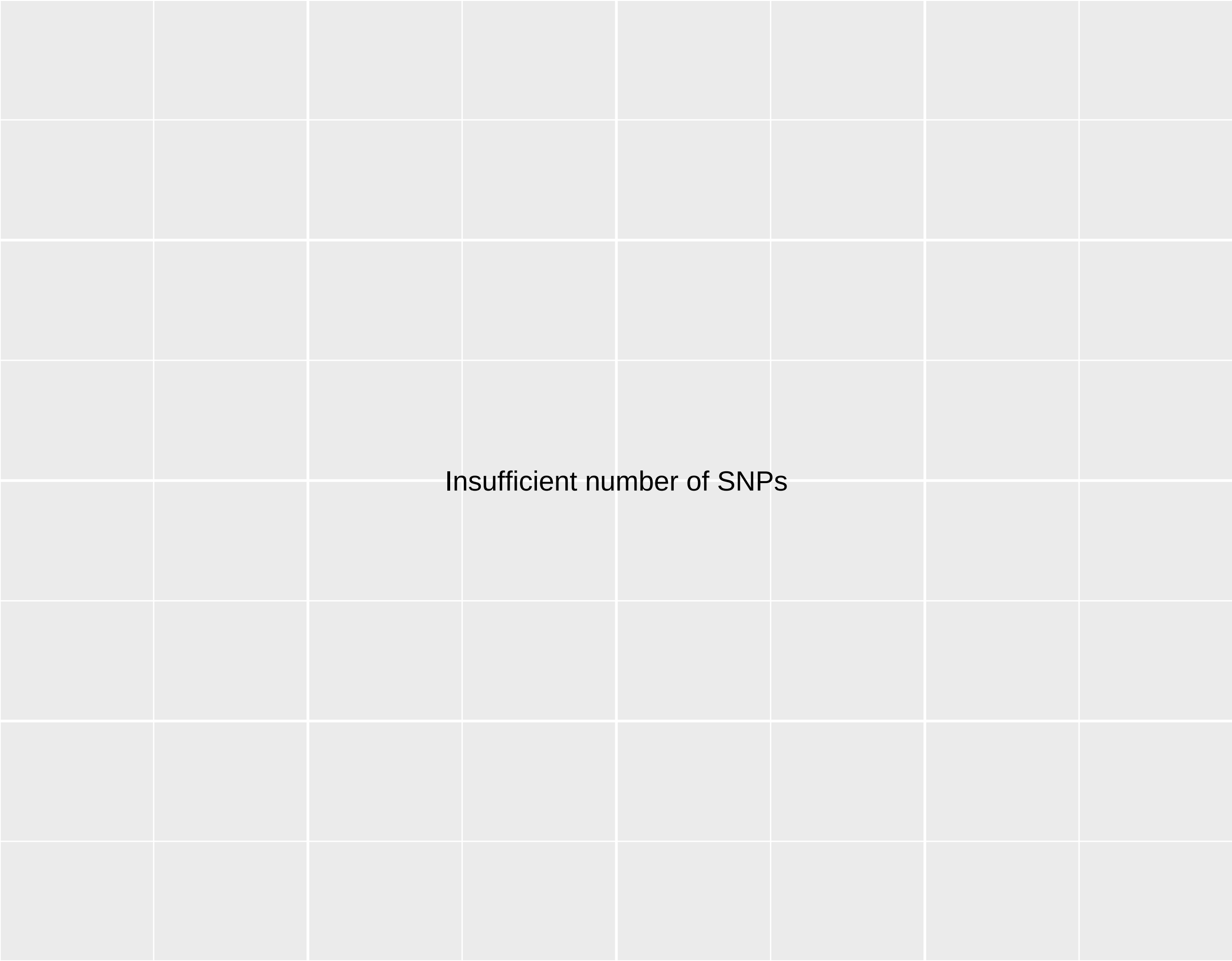
Insufficient number of SNPs

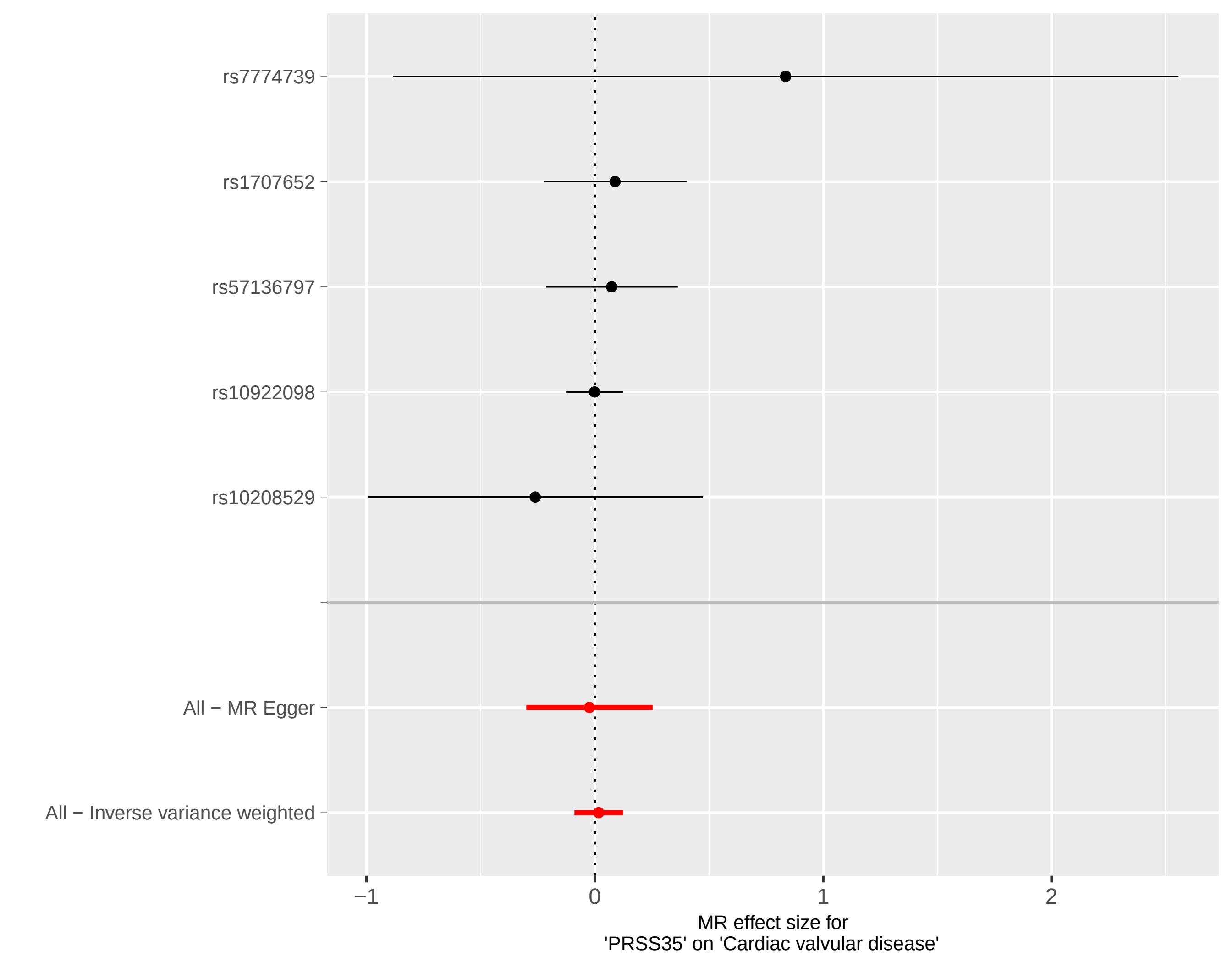
rs7774739
rs1707652
rs57136797
rs10922098
rs10208529
All − MR Egger
All − Inverse variance weighted
−1
0
1
2
MR effect size for
'PRSS35' on 'Cardiac valvular disease'

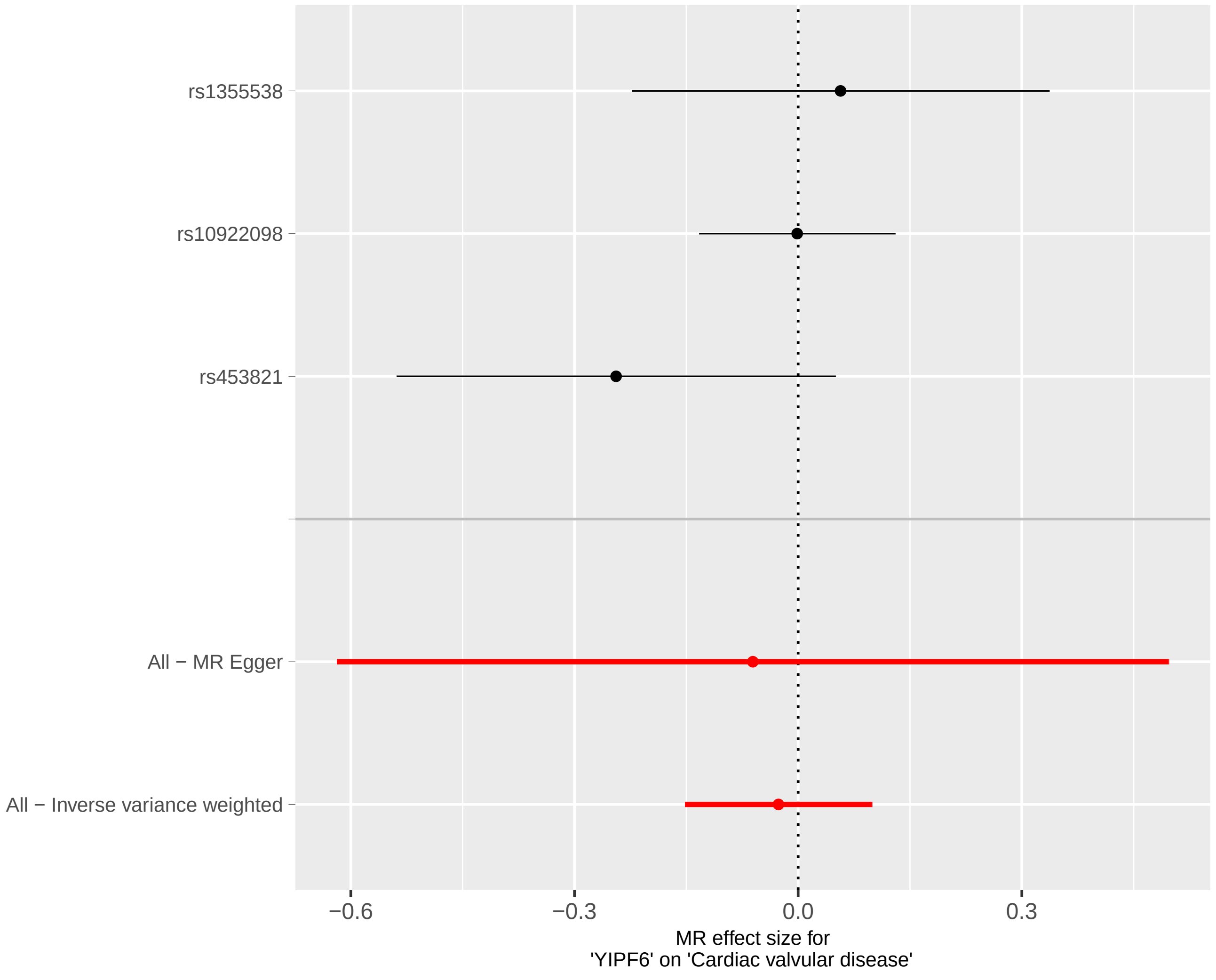

rs1355538
rs10922098
rs453821
All − MR Egger
All − Inverse variance weighted
−0.6
−0.3
0.0
0.3
MR effect size for
'YIPF6' on 'Cardiac valvular disease'

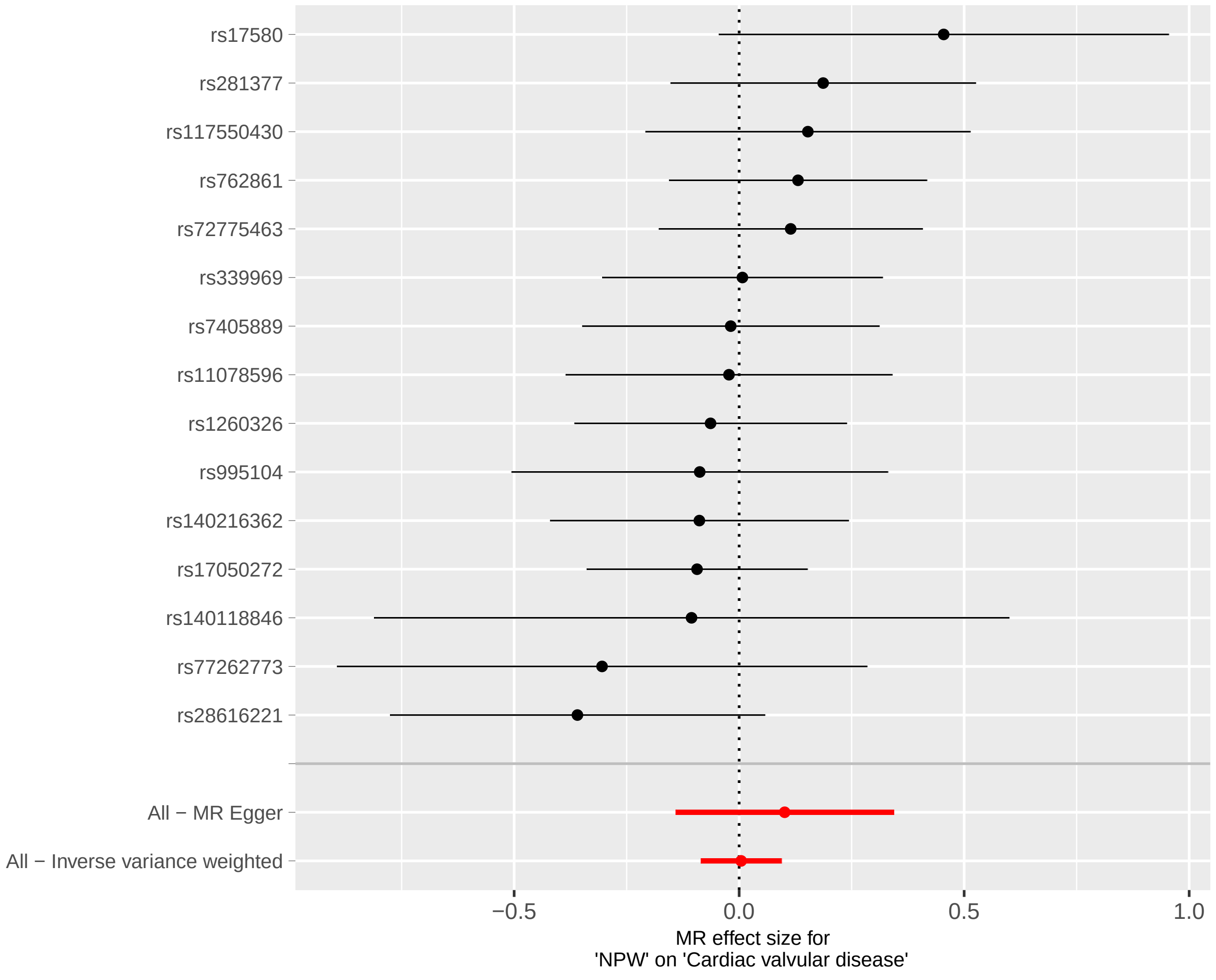
rs17580
rs281377
rs117550430
rs762861
rs72775463
rs339969
rs7405889
rs11078596
rs1260326
rs995104
rs140216362
rs17050272
rs140118846
rs77262773
rs28616221
All − MR Egger
All − Inverse variance weighted
−0.5
0.0
0.5
1.0
MR effect size for
'NPW' on 'Cardiac valvular disease'

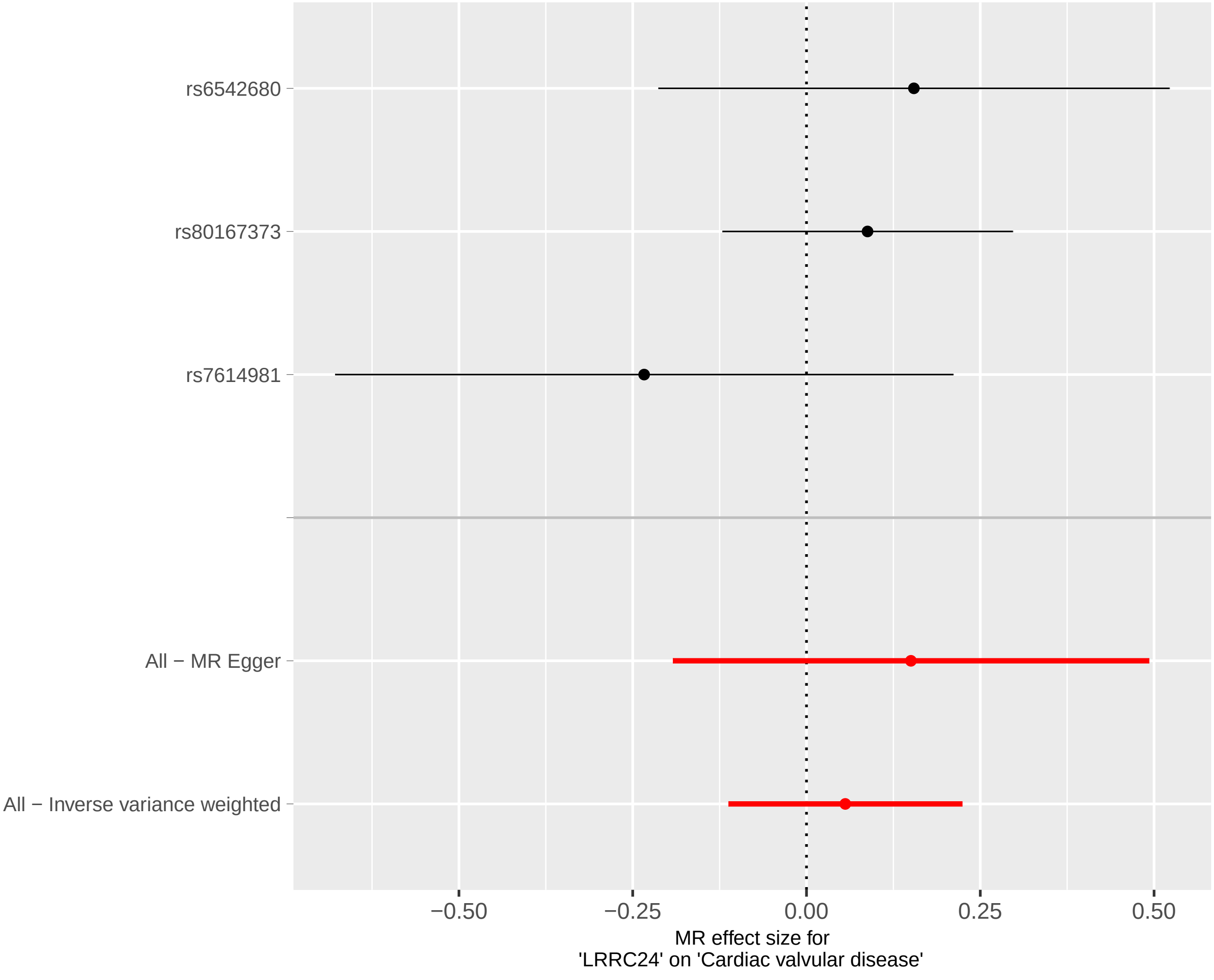
rs6542680
rs80167373
rs7614981
All − MR Egger
All − Inverse variance weighted
−0.50
−0.25
0.00
0.25
0.50
MR effect size for
'LRRC24' on 'Cardiac valvular disease'

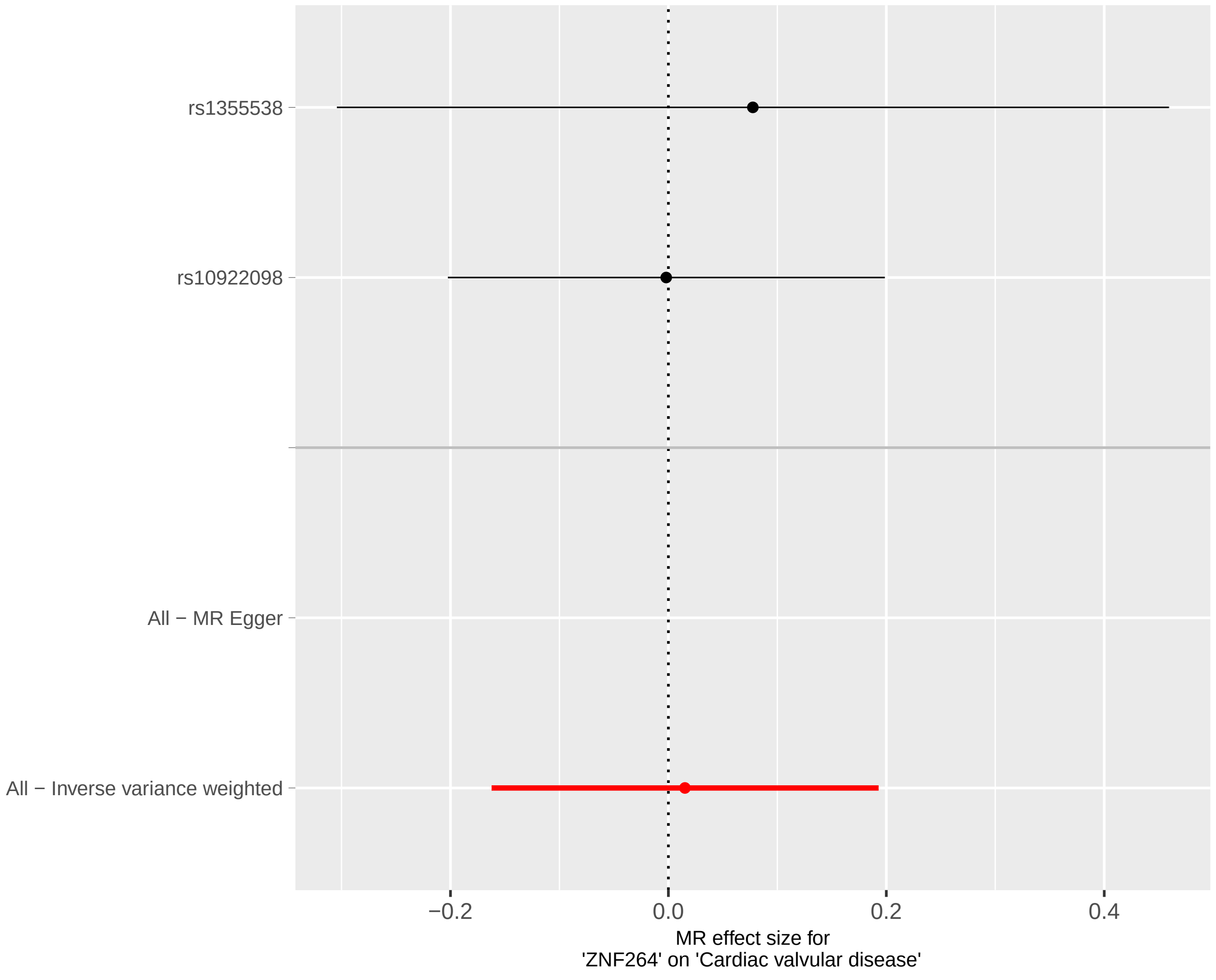
rs1355538
rs10922098
All − MR Egger
All − Inverse variance weighted
−0.2
0.0
0.2
0.4
MR effect size for
'ZNF264' on 'Cardiac valvular disease'

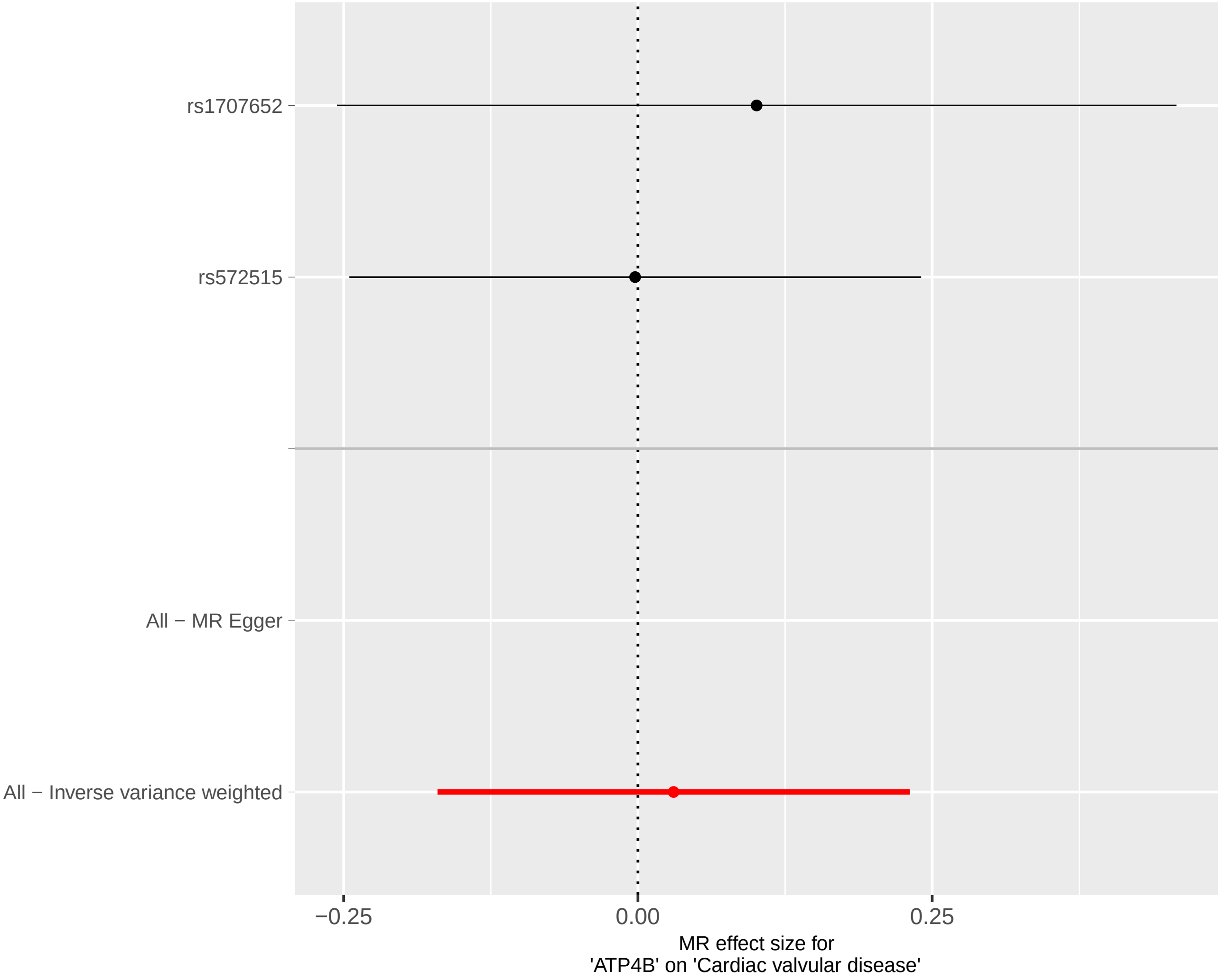

rs1707652
rs572515
All − MR Egger
All − Inverse variance weighted
−0.25
0.00
0.25
MR effect size for
'ATP4B' on 'Cardiac valvular disease'

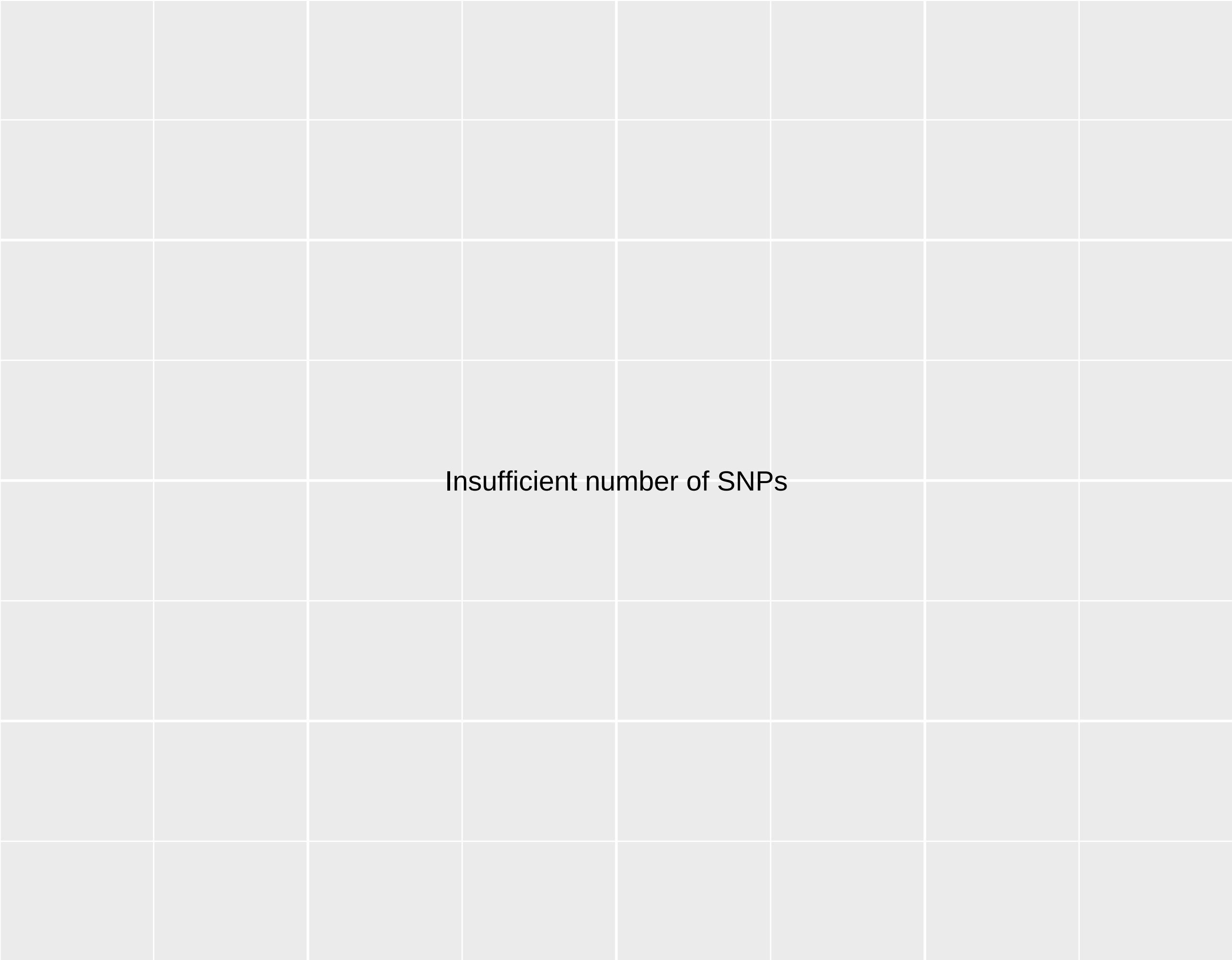
Insufficient number of SNPs
