# Supplementary material for: New perspectives on the mechanisms and treatment of valvular heart disease: Mendelian randomization and systematic analysis based on plasma proteins
Source: Medicine (Baltimore). 2026 Apr 3;105(14):e48210. doi: 10.1097/MD.0000000000048210 (PMC13052936; doi:10.1097/MD.0000000000048210)
Supplement: Supplementary file 3 [file medi-105-e48210-s003.pdf]

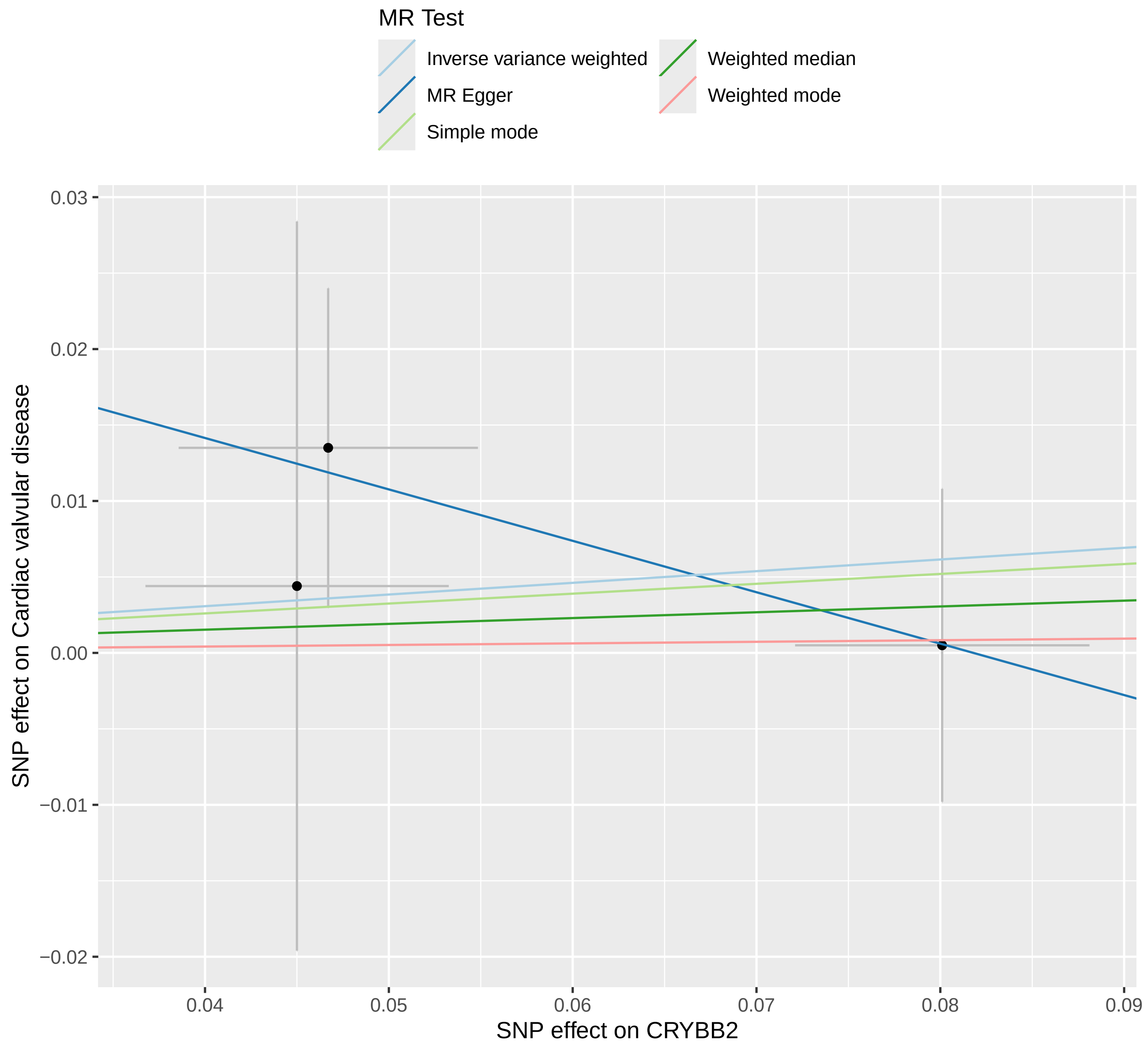
MR Test
Inverse variance weighted
MR Egger
Simple mode
Weighted median
Weighted mode
0.03
0.02
0.01
0.00
−0.01
−0.02
SNP effect on Cardiac valvular disease
0.04
0.05
0.06
0.07
0.08
0.09
SNP effect on CRYBB2

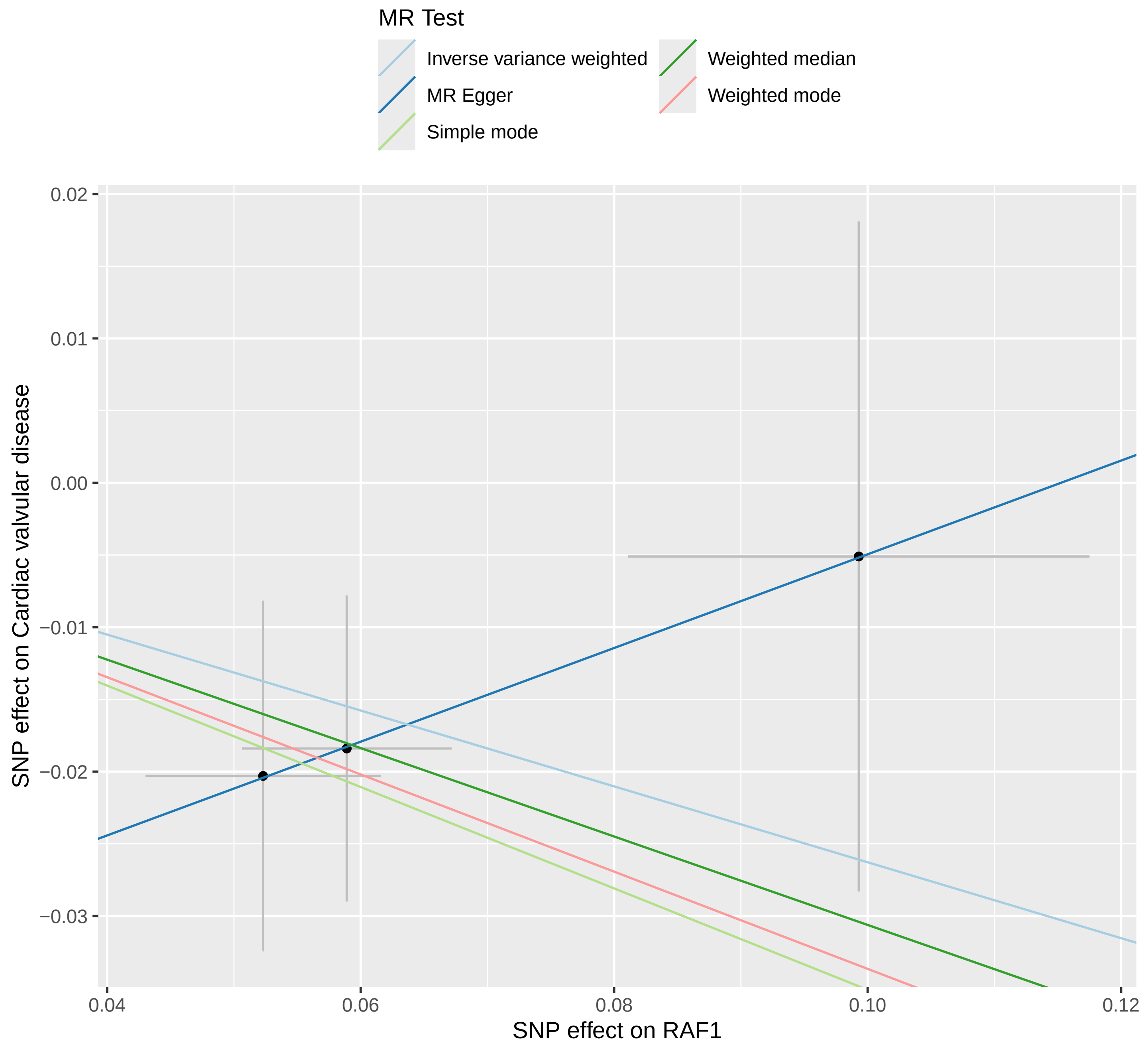

MR Test
Inverse variance weighted
MR Egger
Simple mode
Weighted median
Weighted mode
0.02
0.01
0.00
−0.01
−0.02
−0.03
SNP effect on Cardiac valvular disease
0.04
0.06
0.08
0.10
0.12
SNP effect on RAF1

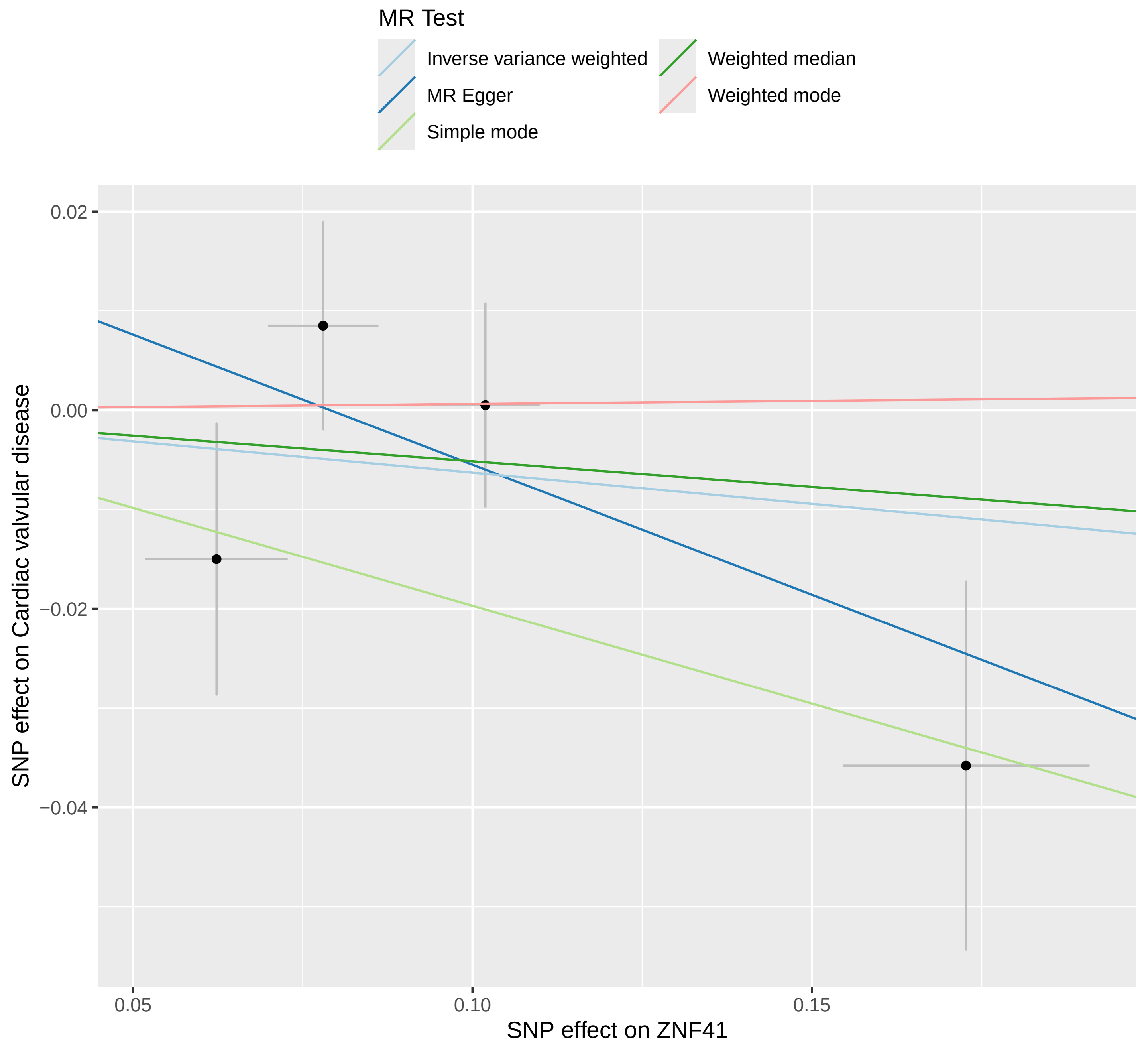

MR Test
Inverse variance weighted
MR Egger
Simple mode
Weighted median
Weighted mode
0.02
0.00
−0.02
−0.04
SNP effect on Cardiac valvular disease
0.05
0.10
0.15
SNP effect on ZNF41

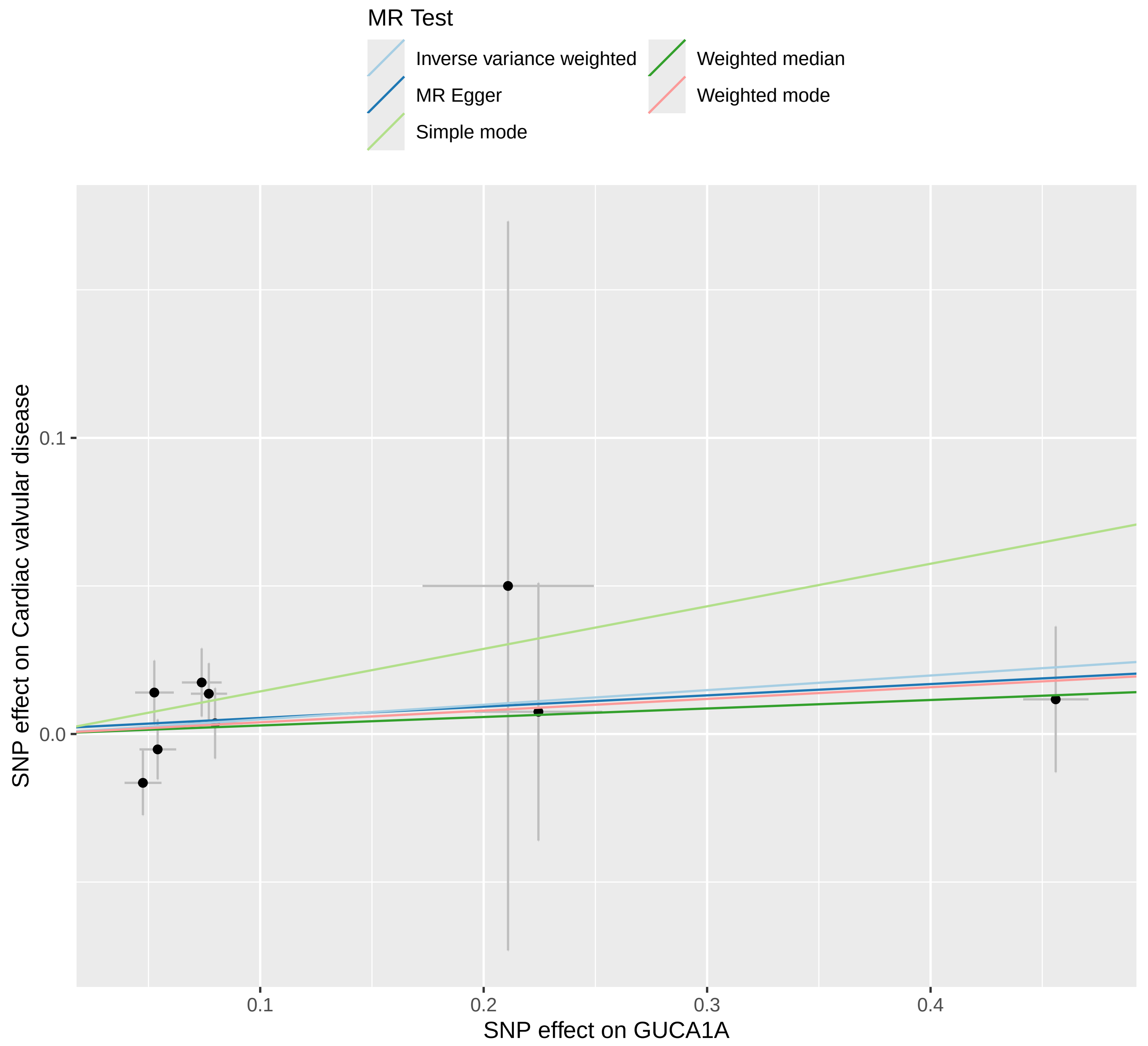

MR Test
Inverse variance weighted
MR Egger
Simple mode
Weighted median
Weighted mode
SNP effect on Cardiac valvular disease
0.1
0.0
0.1
0.2
0.3
0.4
SNP effect on GUCA1A

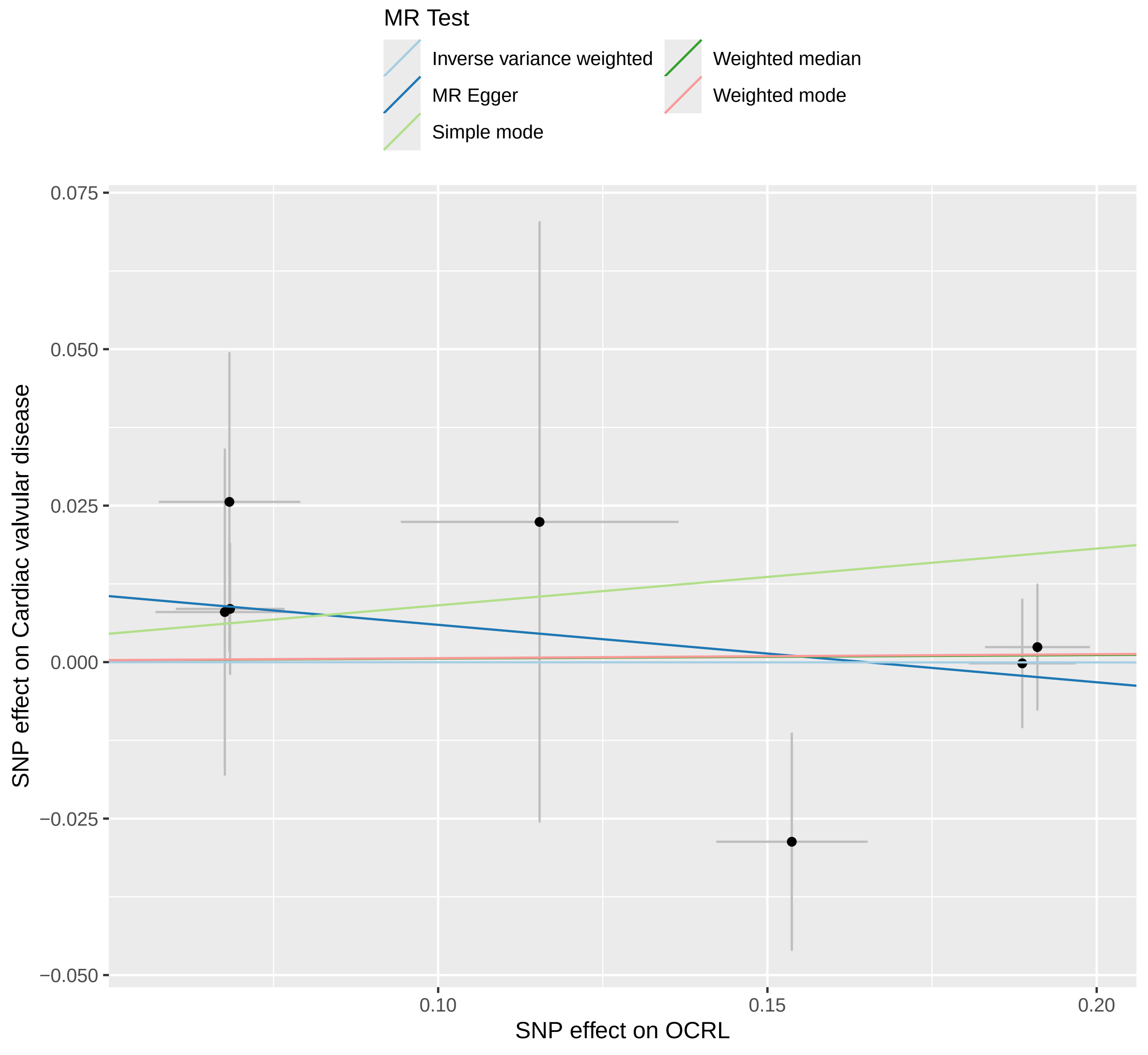

MR Test
Inverse variance weighted
MR Egger
Simple mode
Weighted median
Weighted mode
0.075
0.050
0.025
0.000
−0.025
−0.050
0.10
0.15
0.20
SNP effect on Cardiac valvular disease
SNP effect on OCRL

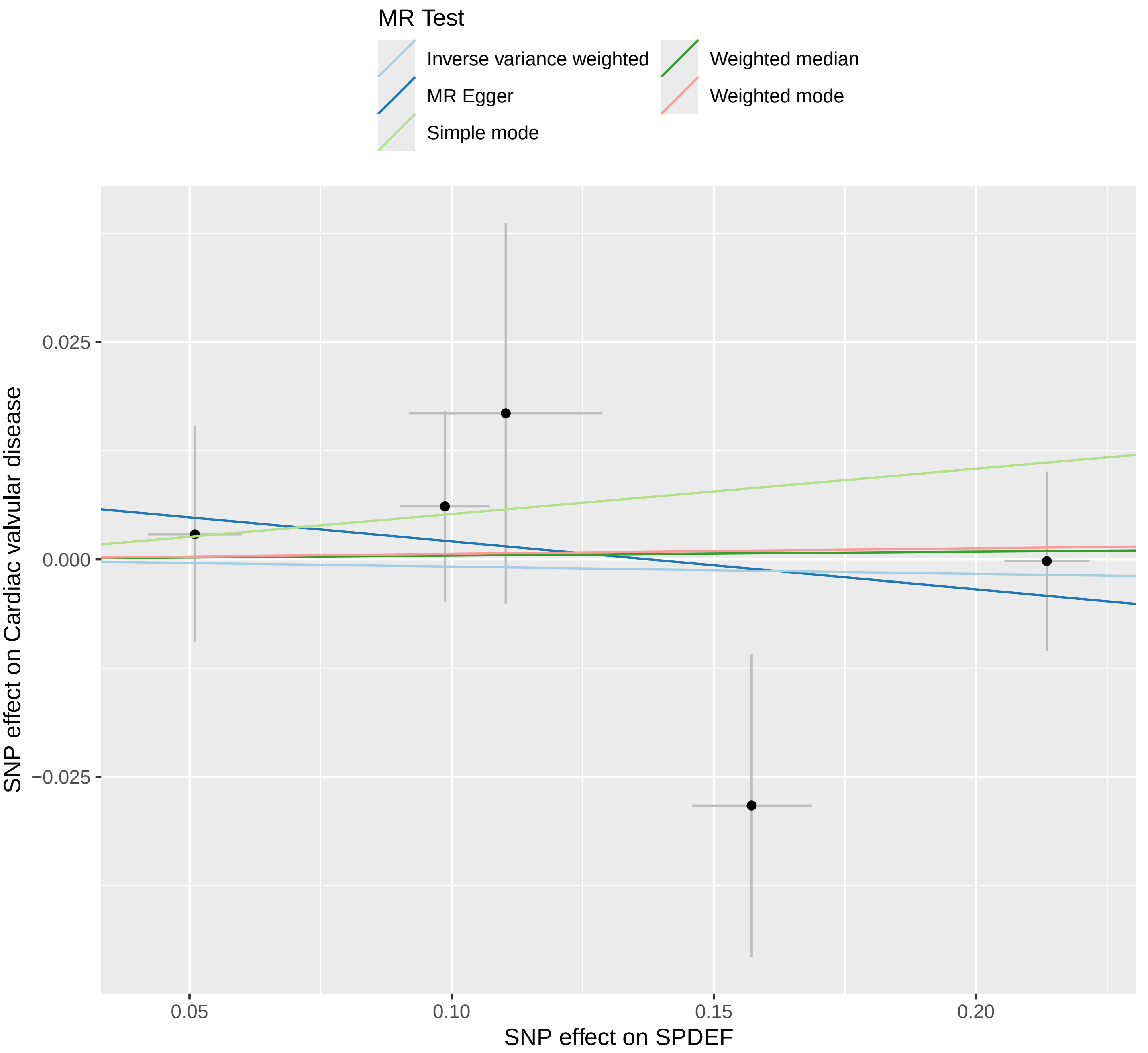

MR Test
Inverse variance weighted
MR Egger
Simple mode
Weighted median
Weighted mode
0.025
0.000
−0.025
0.05
0.10
0.15
0.20
SNP effect on Cardiac valvular disease
SNP effect on SPDEF

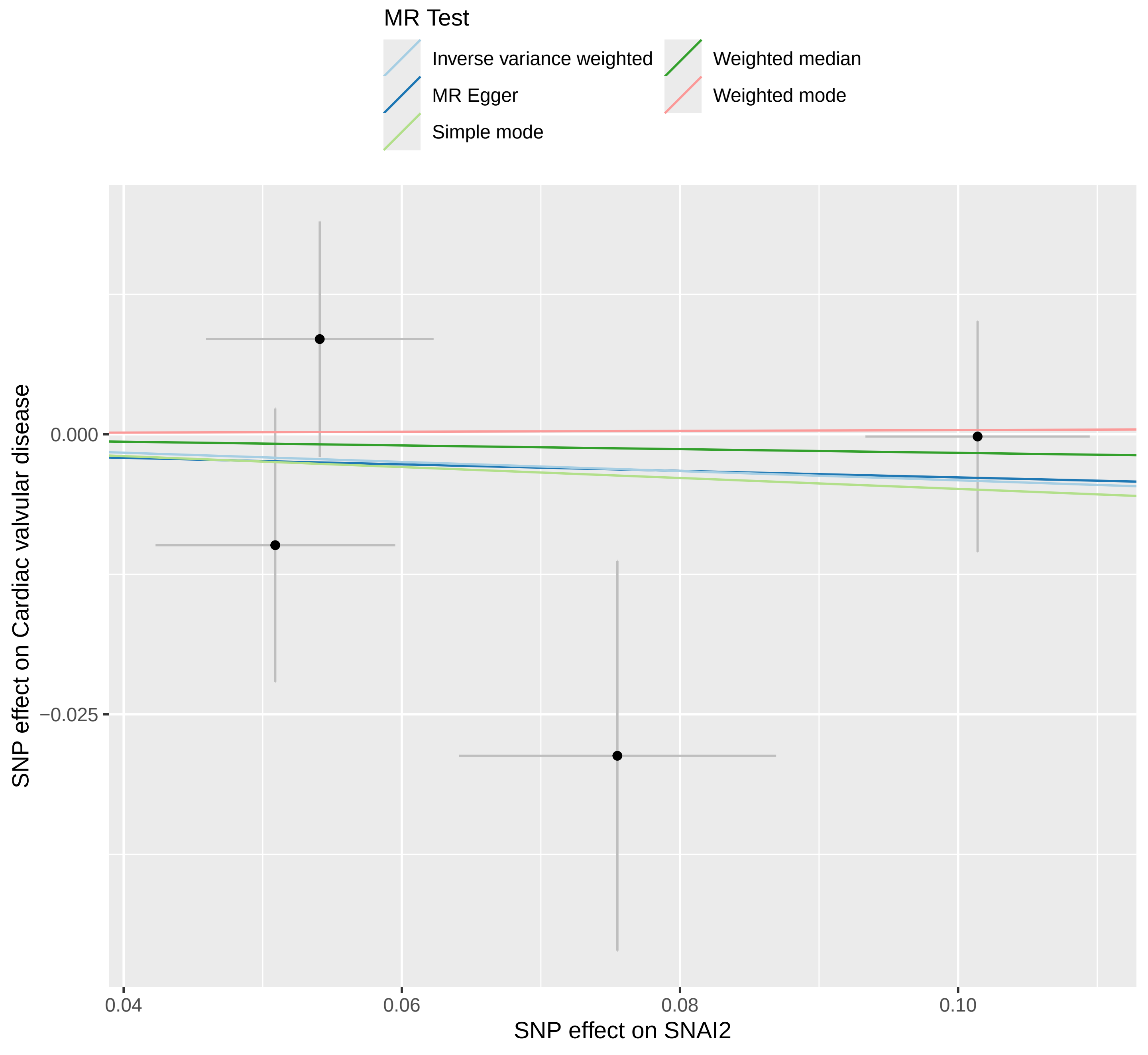
MR Test
Inverse variance weighted
MR Egger
Simple mode
Weighted median
Weighted mode
0.000
−0.025
0.04
0.06
0.08
0.10
SNP effect on SNAI2
SNP effect on Cardiac valvular disease

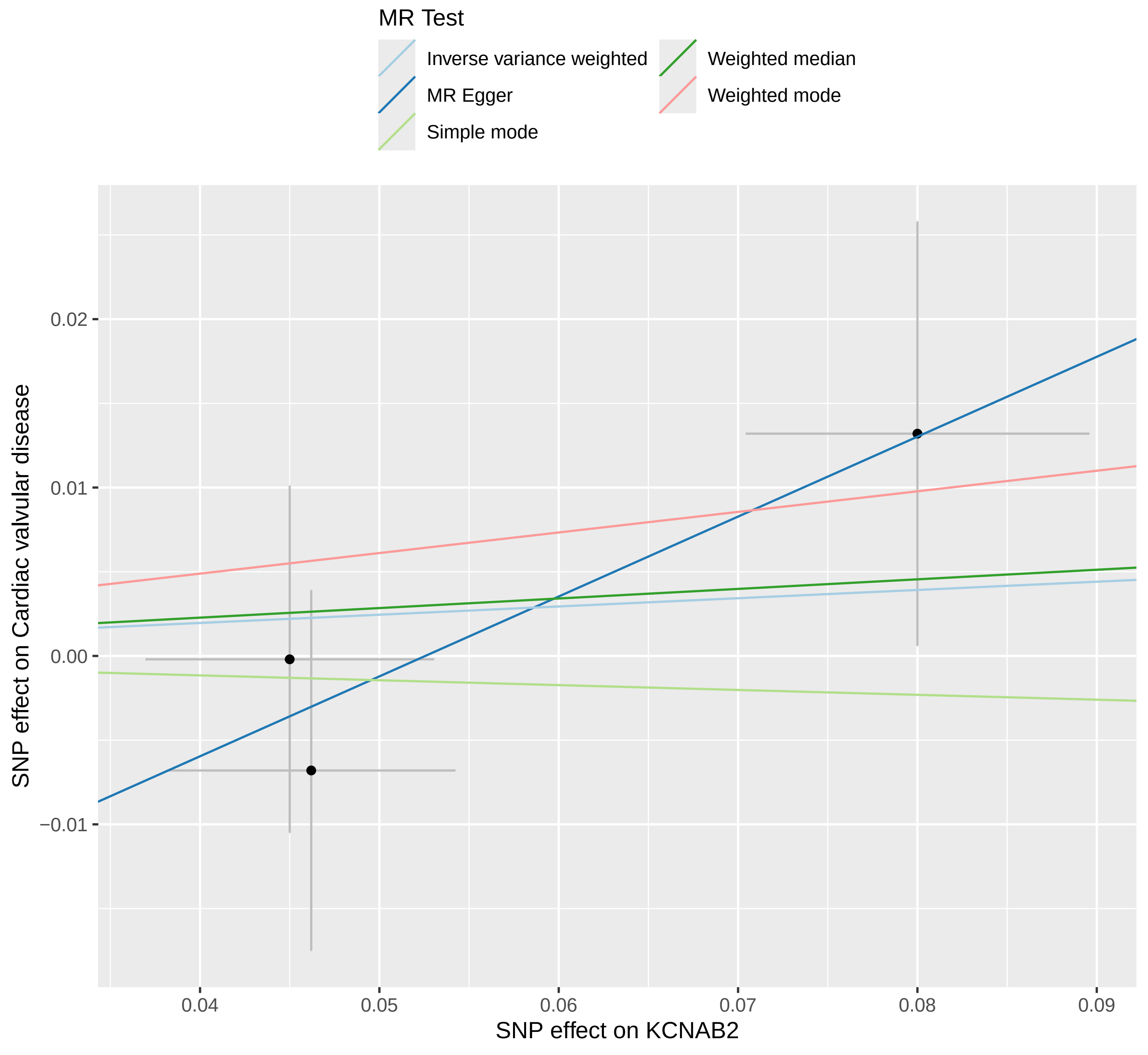
MR Test
Inverse variance weighted
MR Egger
Simple mode
Weighted median
Weighted mode
0.02
0.01
0.00
−0.01
0.04
0.05
0.06
0.07
0.08
0.09
SNP effect on Cardiac valvular disease
SNP effect on KCNAB2

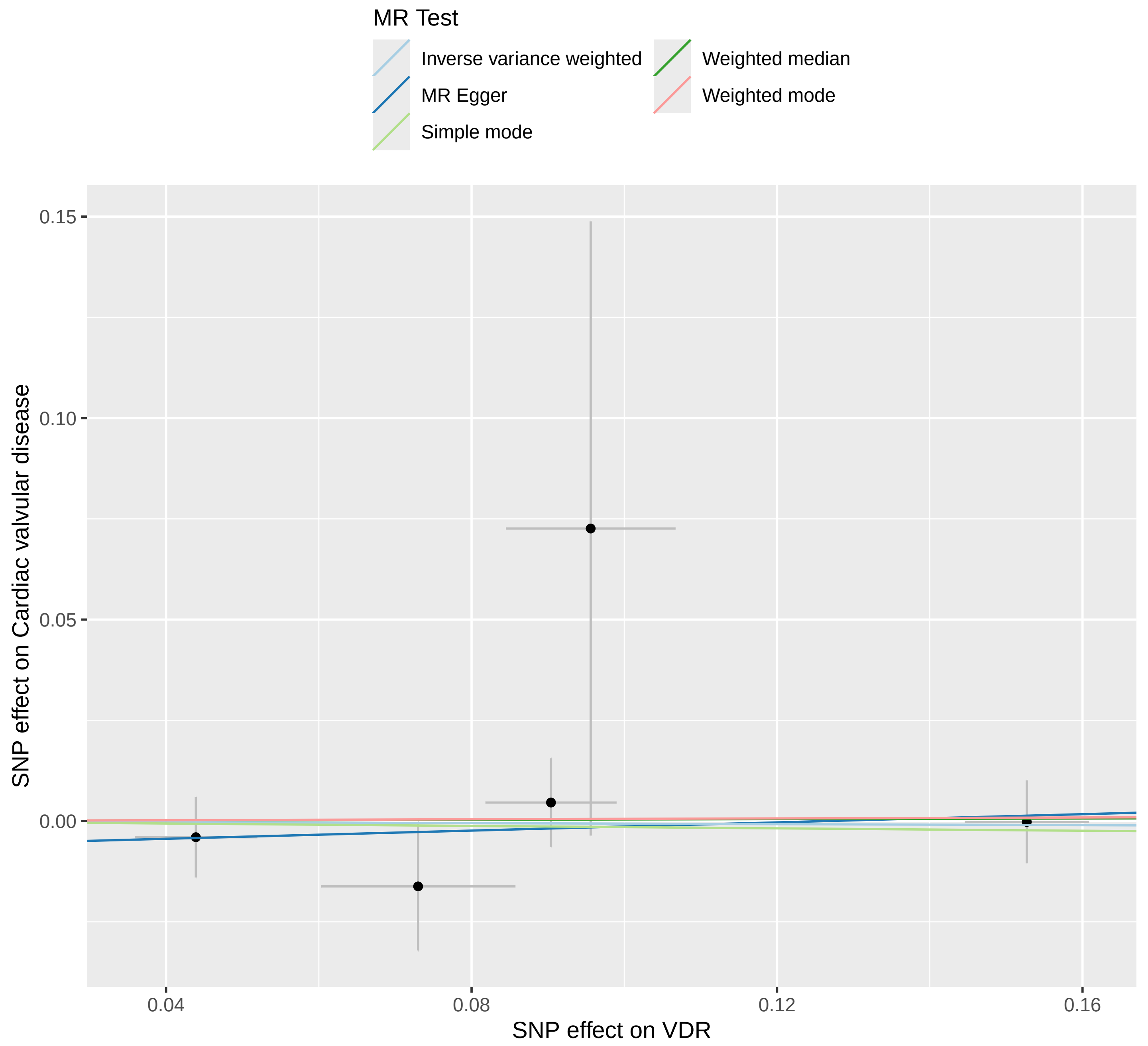
MR Test
Inverse variance weighted
Weighted median
MR Egger
Weighted mode
Simple mode
0.15
0.10
0.05
0.00
SNP effect on Cardiac valvular disease
0.04
0.08
0.12
0.16
SNP effect on VDR

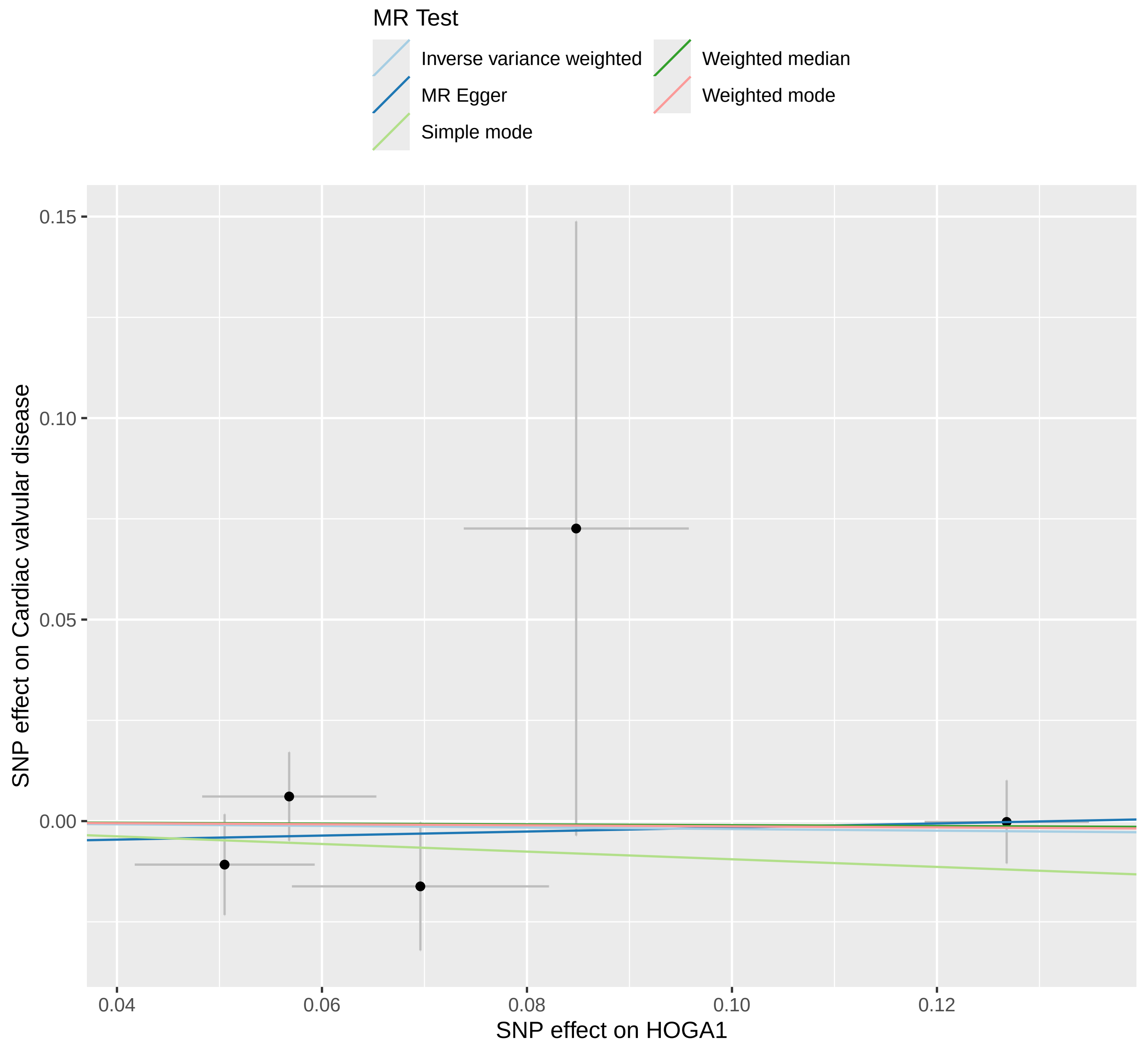
MR Test
Inverse variance weighted
MR Egger
Simple mode
Weighted median
Weighted mode
0.15
0.10
0.05
0.00
SNP effect on Cardiac valvular disease
0.04
0.06
0.08
0.10
0.12
SNP effect on HOGA1

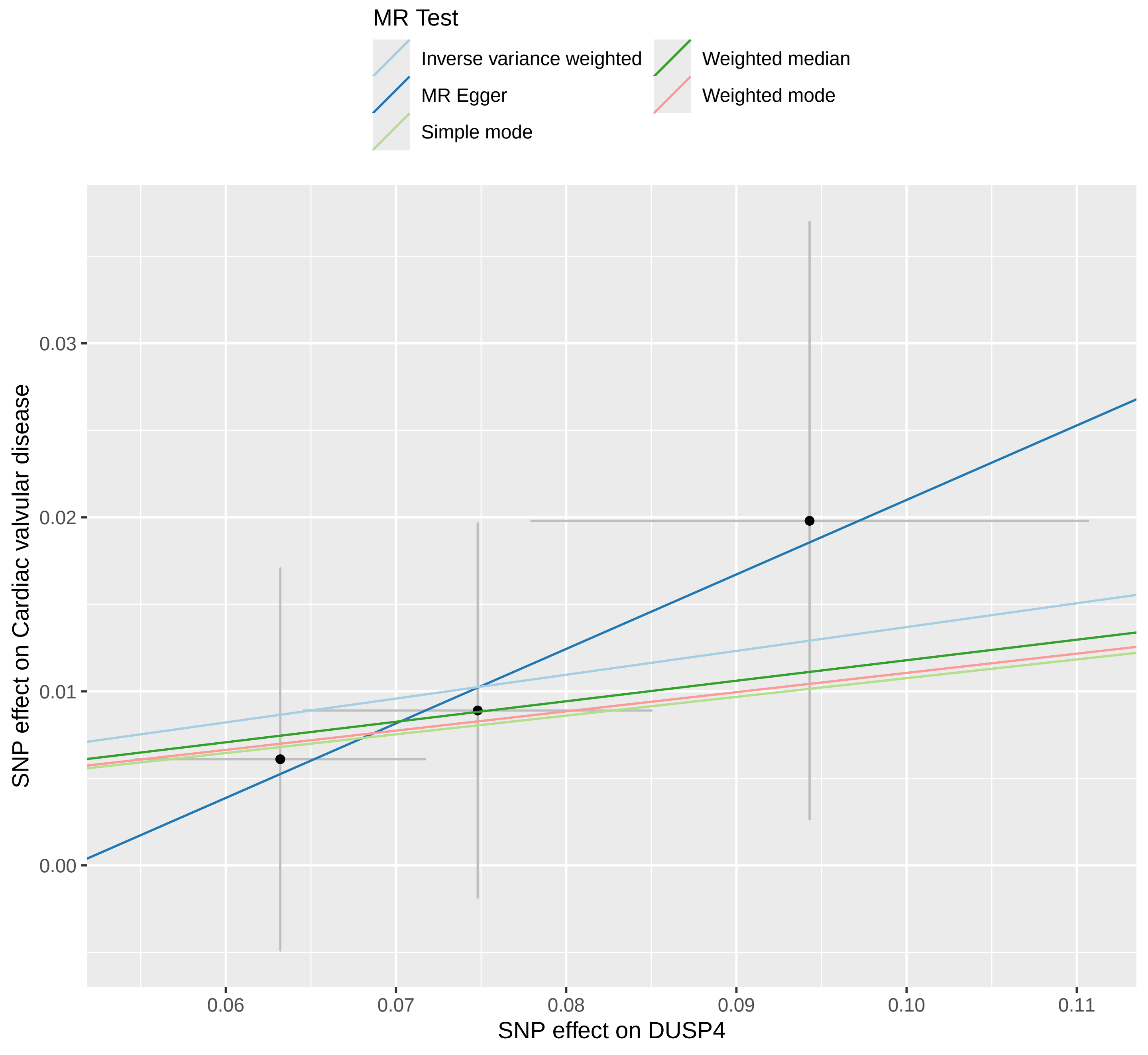
MR Test
Inverse variance weighted
MR Egger
Simple mode
Weighted median
Weighted mode
0.03
0.02
0.01
0.00
SNP effect on Cardiac valvular disease
0.06
0.07
0.08
0.09
0.10
0.11
SNP effect on DUSP4

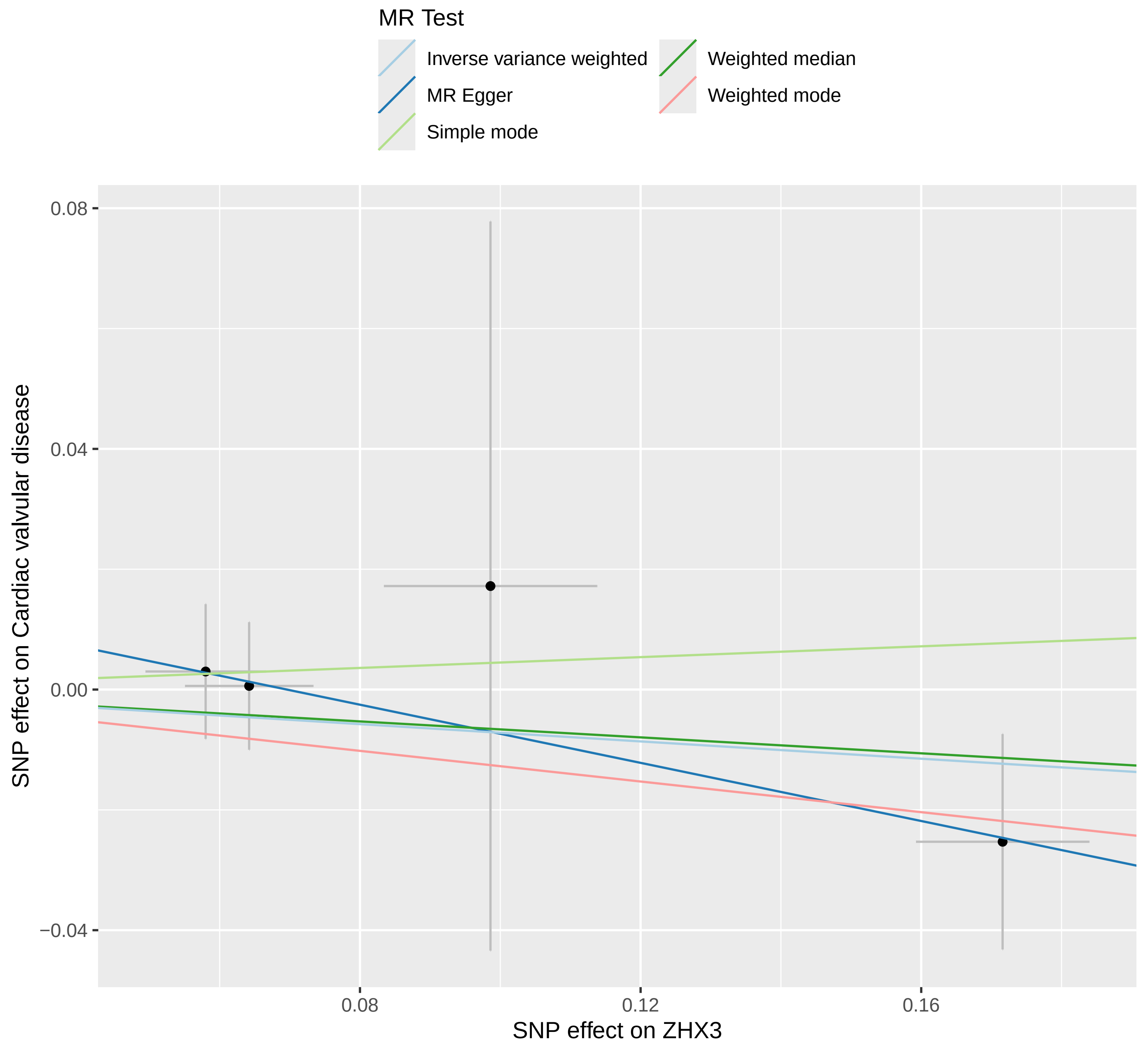

MR Test
Inverse variance weighted
MR Egger
Simple mode
Weighted median
Weighted mode
0.08
0.04
0.00
−0.04
0.08
0.12
0.16
SNP effect on Cardiac valvular disease
SNP effect on ZHX3

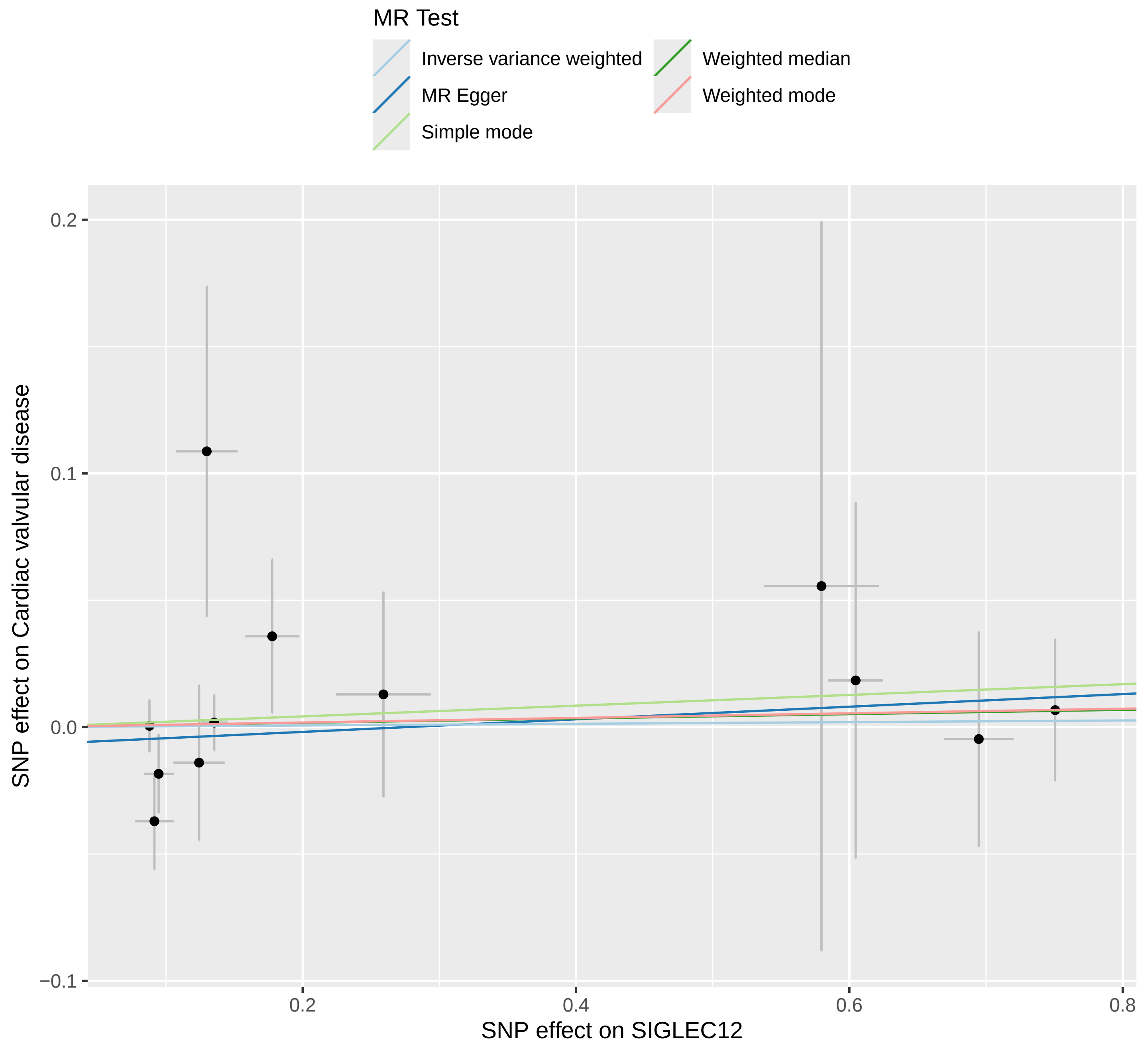
MR Test
Inverse variance weighted
MR Egger
Simple mode
Weighted median
Weighted mode
0.2
0.1
0.0
−0.1
0.2
0.4
0.6
0.8
SNP effect on Cardiac valvular disease
SNP effect on SIGLEC12

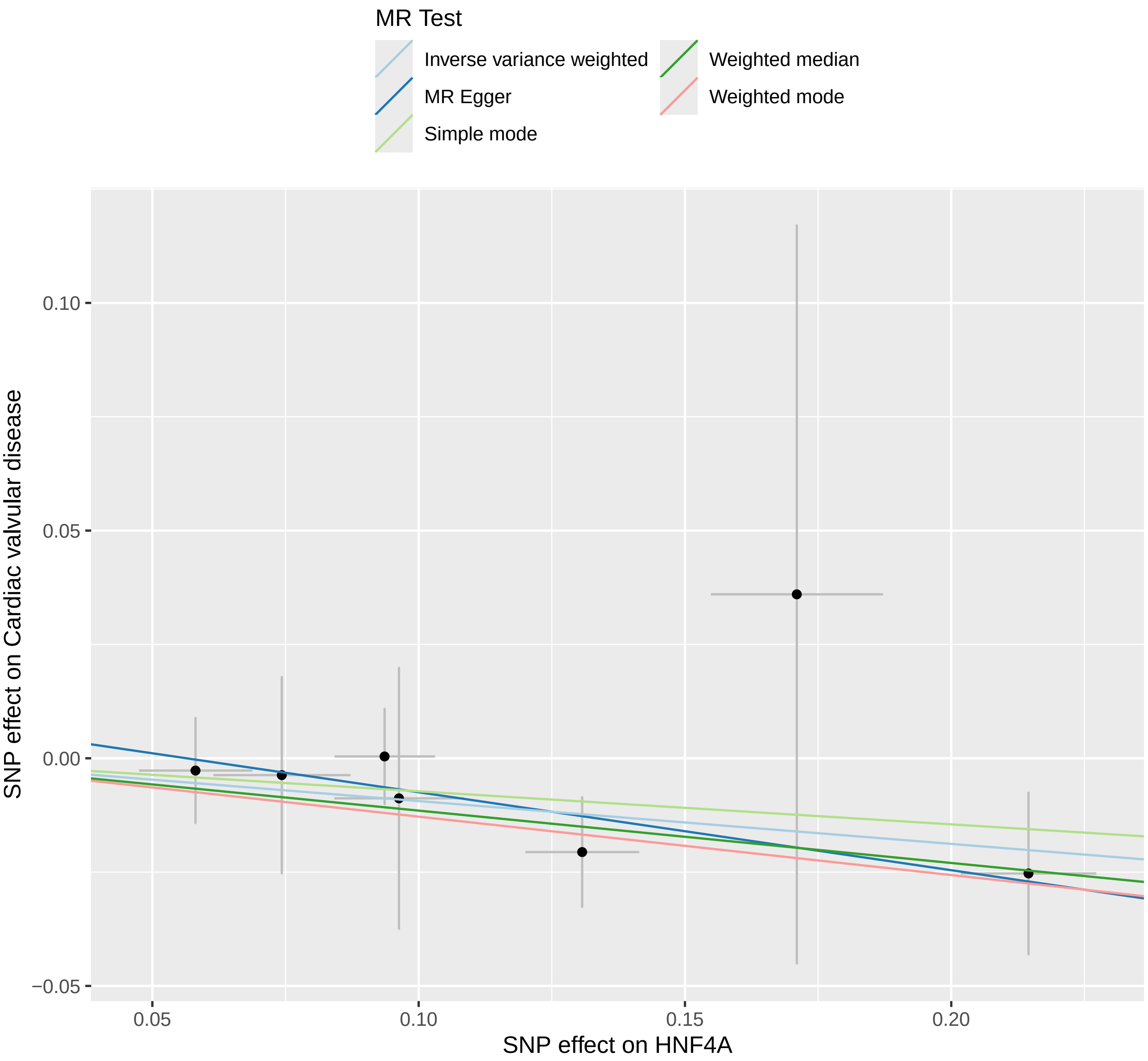
MR Test
Inverse variance weighted
MR Egger
Simple mode
Weighted median
Weighted mode
SNP effect on Cardiac valvular disease
0.10
0.05
0.00
−0.05
0.05
0.10
0.15
0.20
SNP effect on HNF4A

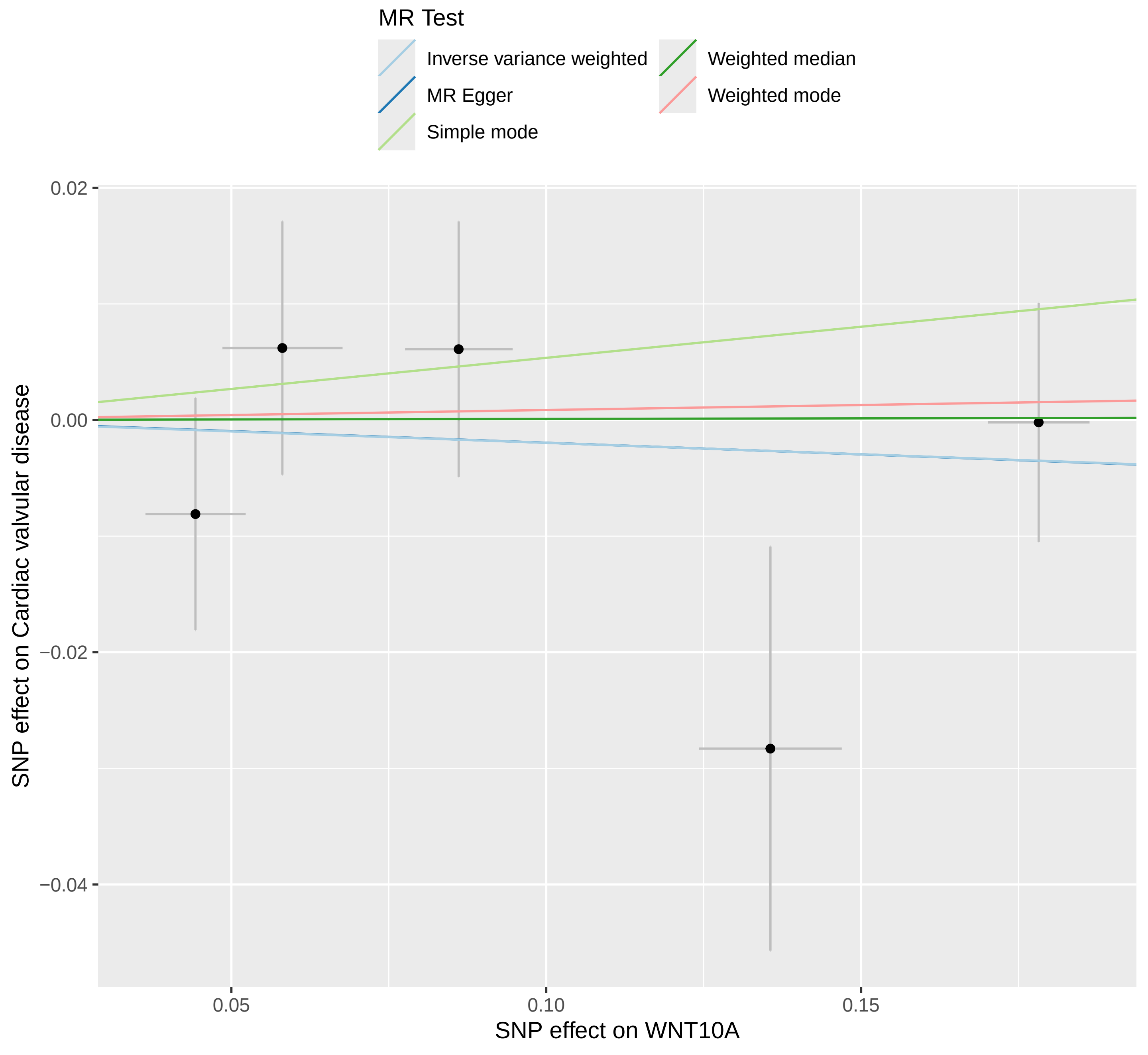
MR Test
Inverse variance weighted
MR Egger
Simple mode
Weighted median
Weighted mode
0.02
0.00
−0.02
−0.04
0.05
0.10
0.15
SNP effect on Cardiac valvular disease
SNP effect on WNT10A

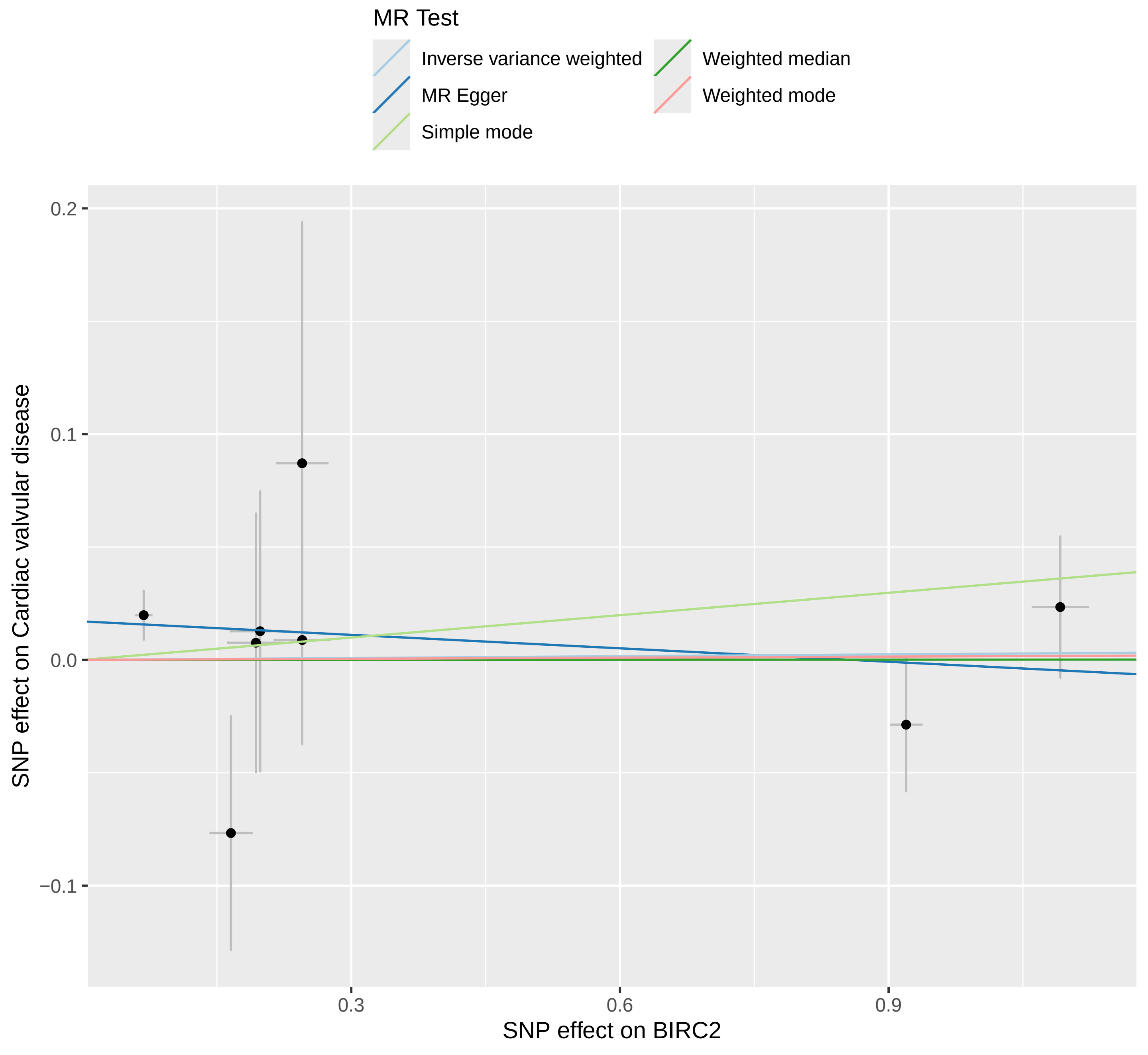

MR Test
Inverse variance weighted
MR Egger
Simple mode
Weighted median
Weighted mode
0.2
0.1
0.0
−0.1
0.3
0.6
0.9
SNP effect on Cardiac valvular disease
SNP effect on BIRC2

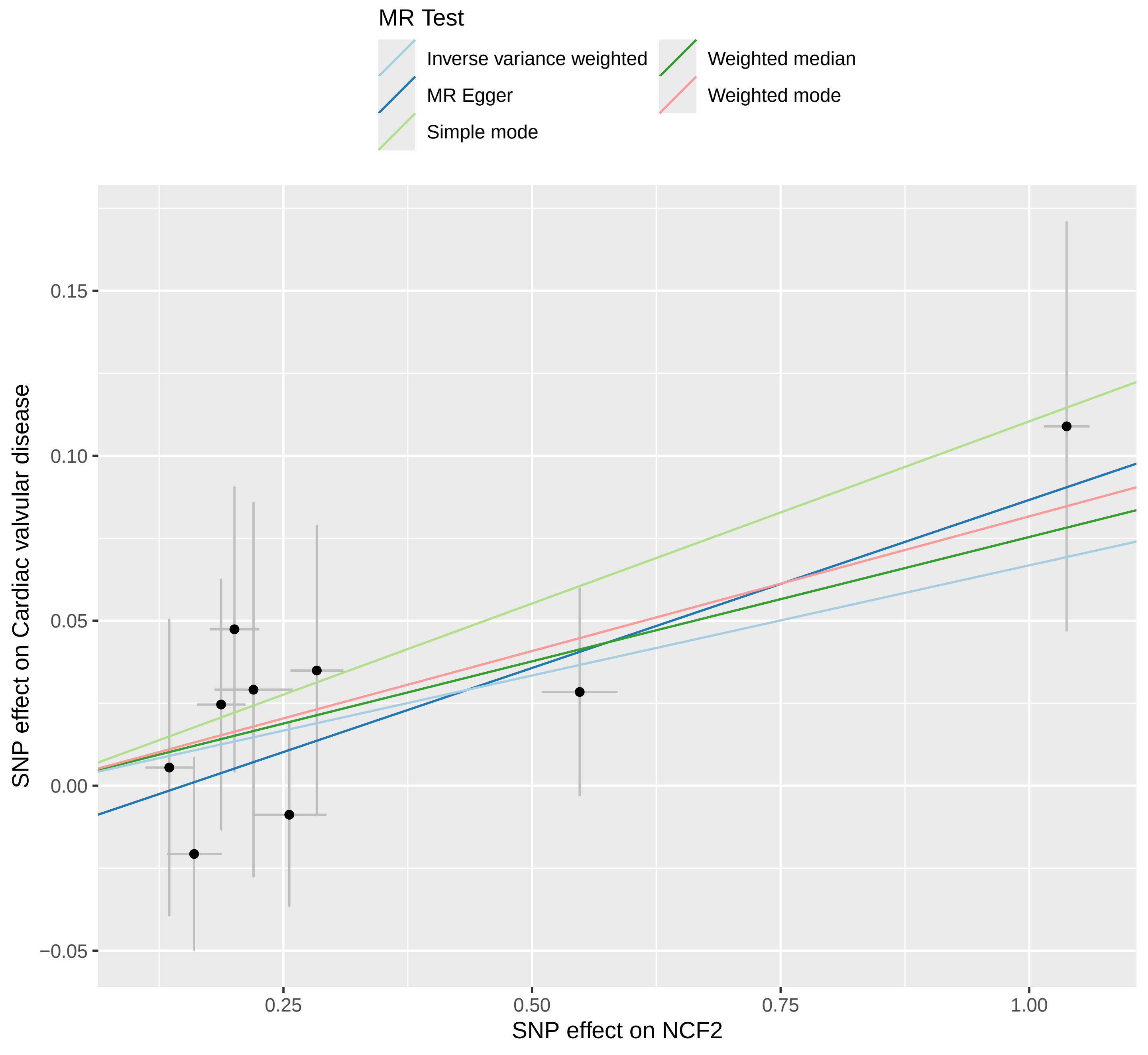
MR Test
Inverse variance weighted
MR Egger
Simple mode
Weighted median
Weighted mode
SNP effect on Cardiac valvular disease
0.15
0.10
0.05
0.00
−0.05
0.25
0.50
0.75
1.00
SNP effect on NCF2

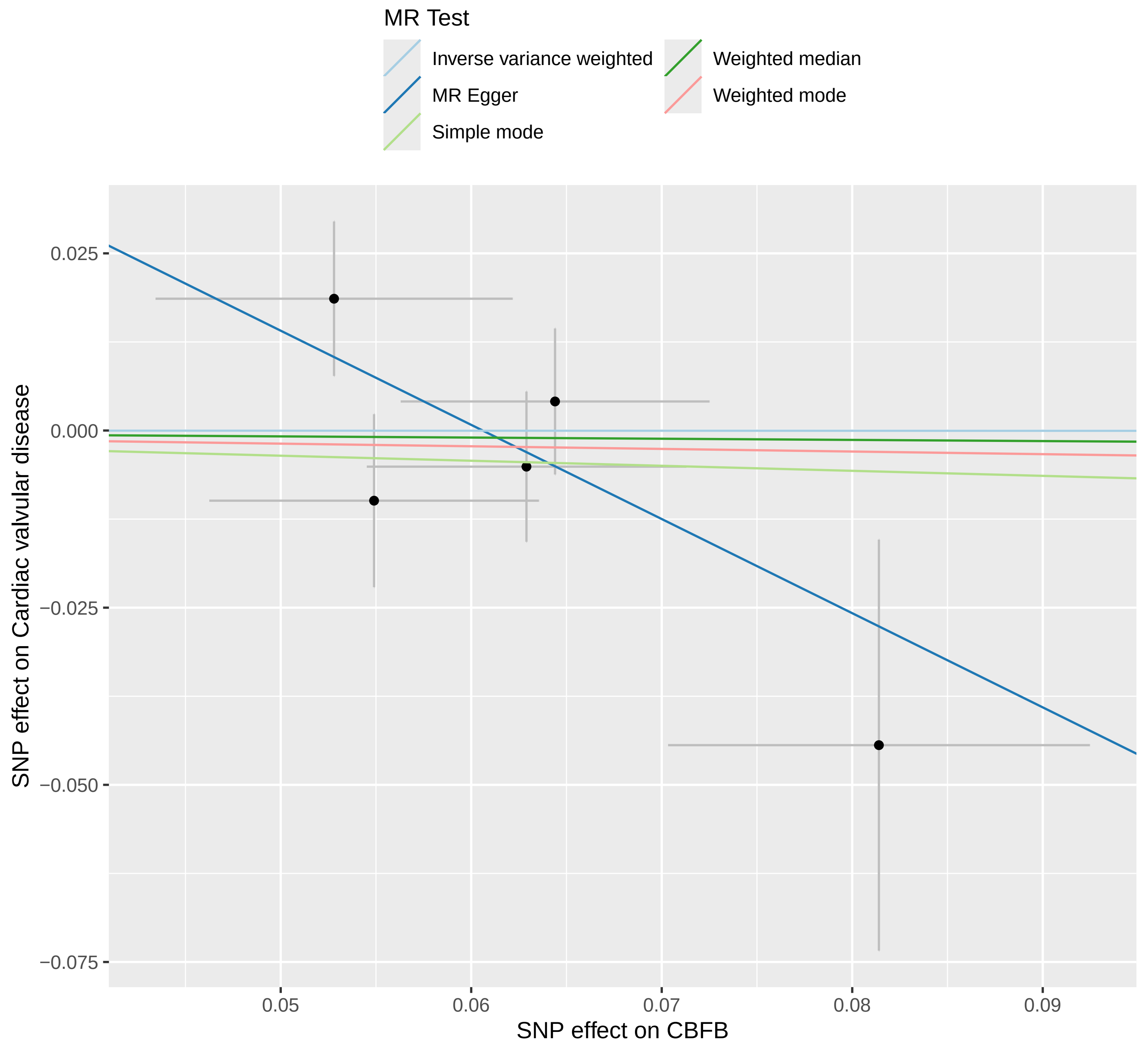

MR Test
Inverse variance weighted
MR Egger
Simple mode
Weighted median
Weighted mode
0.025
0.000
−0.025
−0.050
−0.075
SNP effect on Cardiac valvular disease
0.05
0.06
0.07
0.08
0.09
SNP effect on CBFB

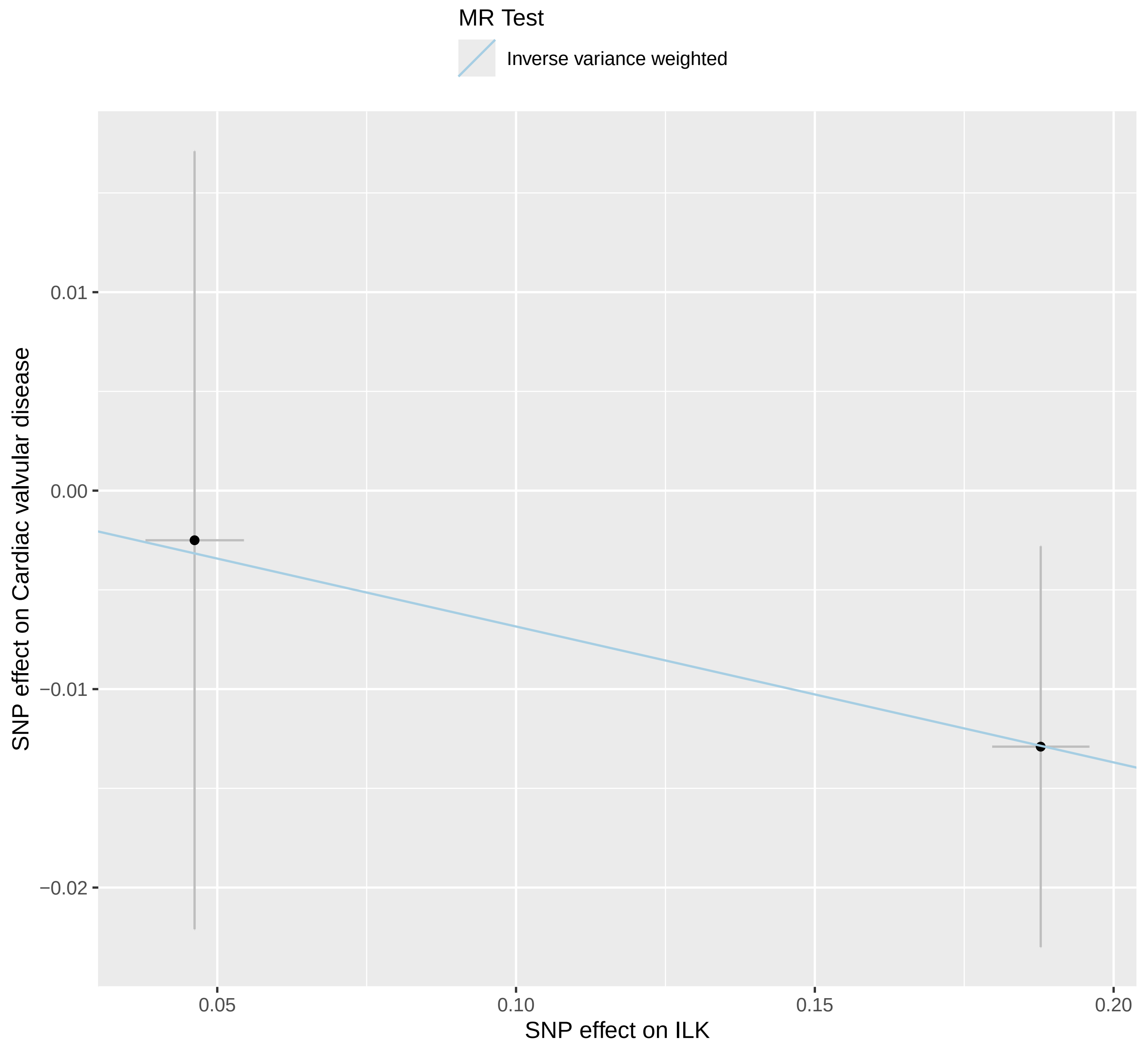

MR Test
Inverse variance weighted
0.01
0.00
−0.01
−0.02
0.05
0.10
0.15
0.20
SNP effect on ILK
SNP effect on Cardiac valvular disease

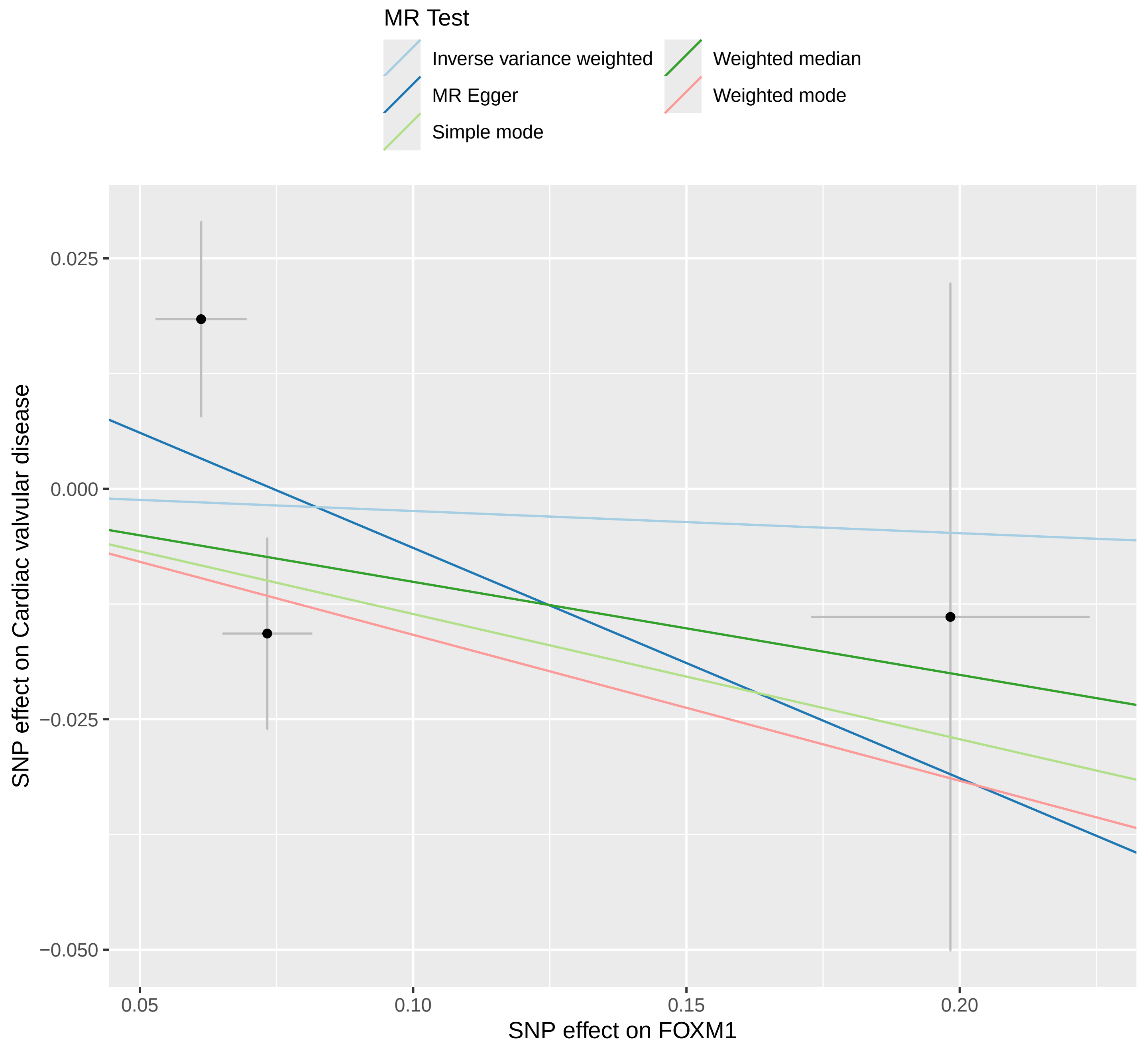

MR Test
Inverse variance weighted
MR Egger
Simple mode
Weighted median
Weighted mode
0.025
0.000
−0.025
−0.050
0.05
0.10
0.15
0.20
SNP effect on Cardiac valvular disease
SNP effect on FOXM1

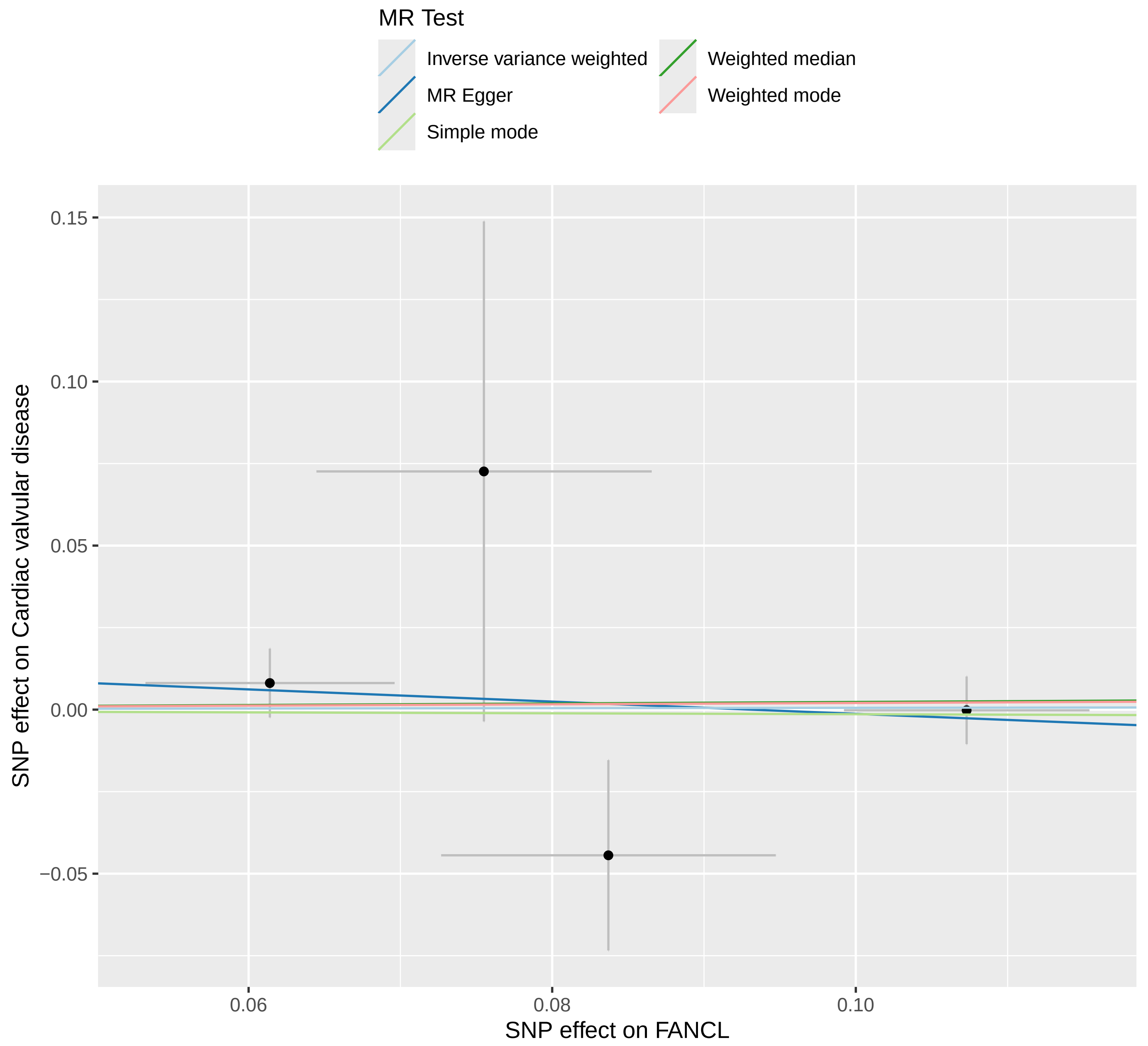

MR Test
Inverse variance weighted
MR Egger
Simple mode
Weighted median
Weighted mode
0.15
0.10
0.05
0.00
−0.05
0.06
0.08
0.10
SNP effect on Cardiac valvular disease
SNP effect on FANCL

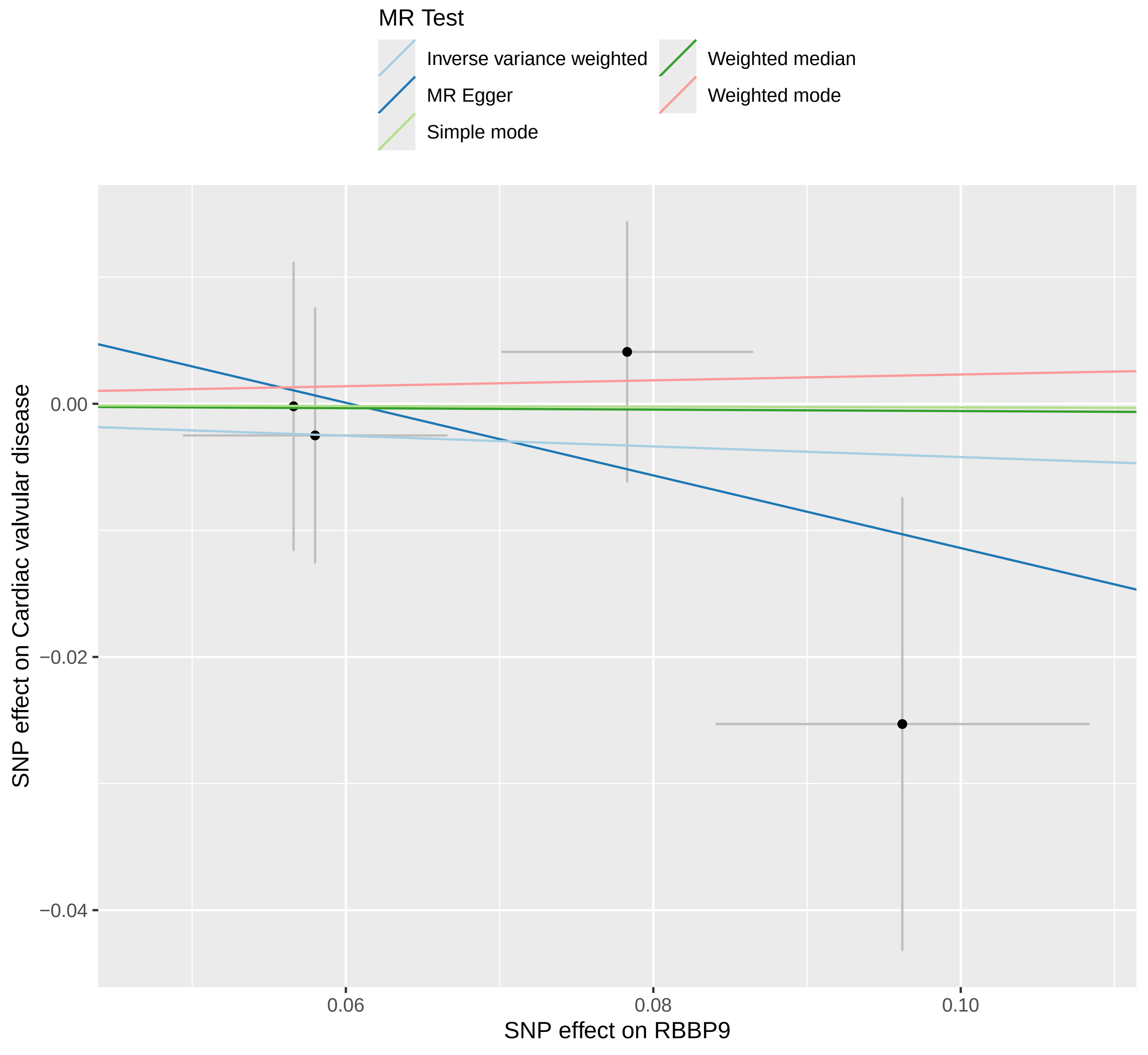
MR Test
Inverse variance weighted
MR Egger
Simple mode
Weighted median
Weighted mode
0.00
−0.02
−0.04
0.06
0.08
0.10
SNP effect on Cardiac valvular disease
SNP effect on RBBP9

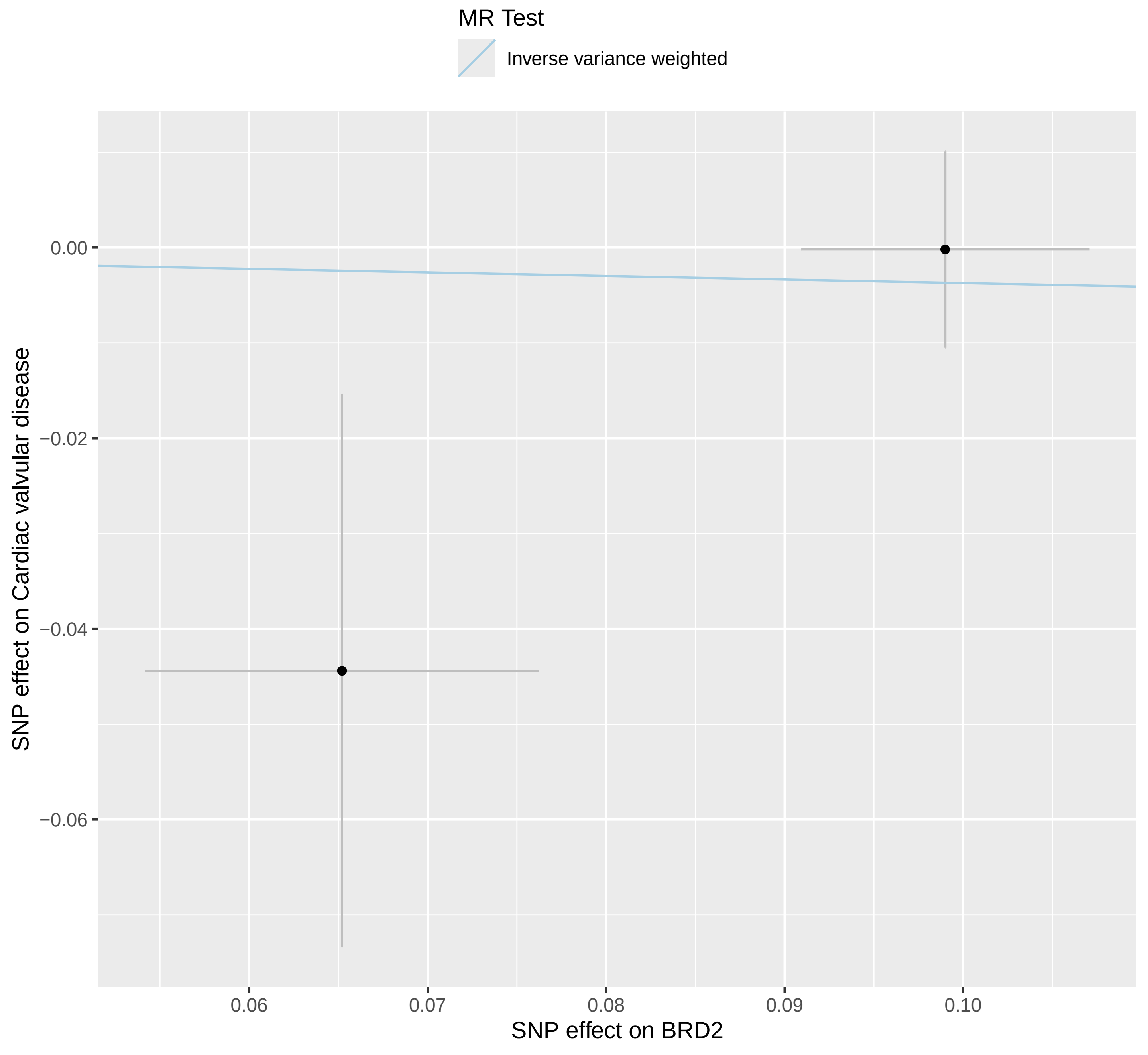

MR Test
Inverse variance weighted
0.00
−0.02
−0.04
−0.06
0.06
0.07
0.08
0.09
0.10
SNP effect on BRD2
SNP effect on Cardiac valvular disease

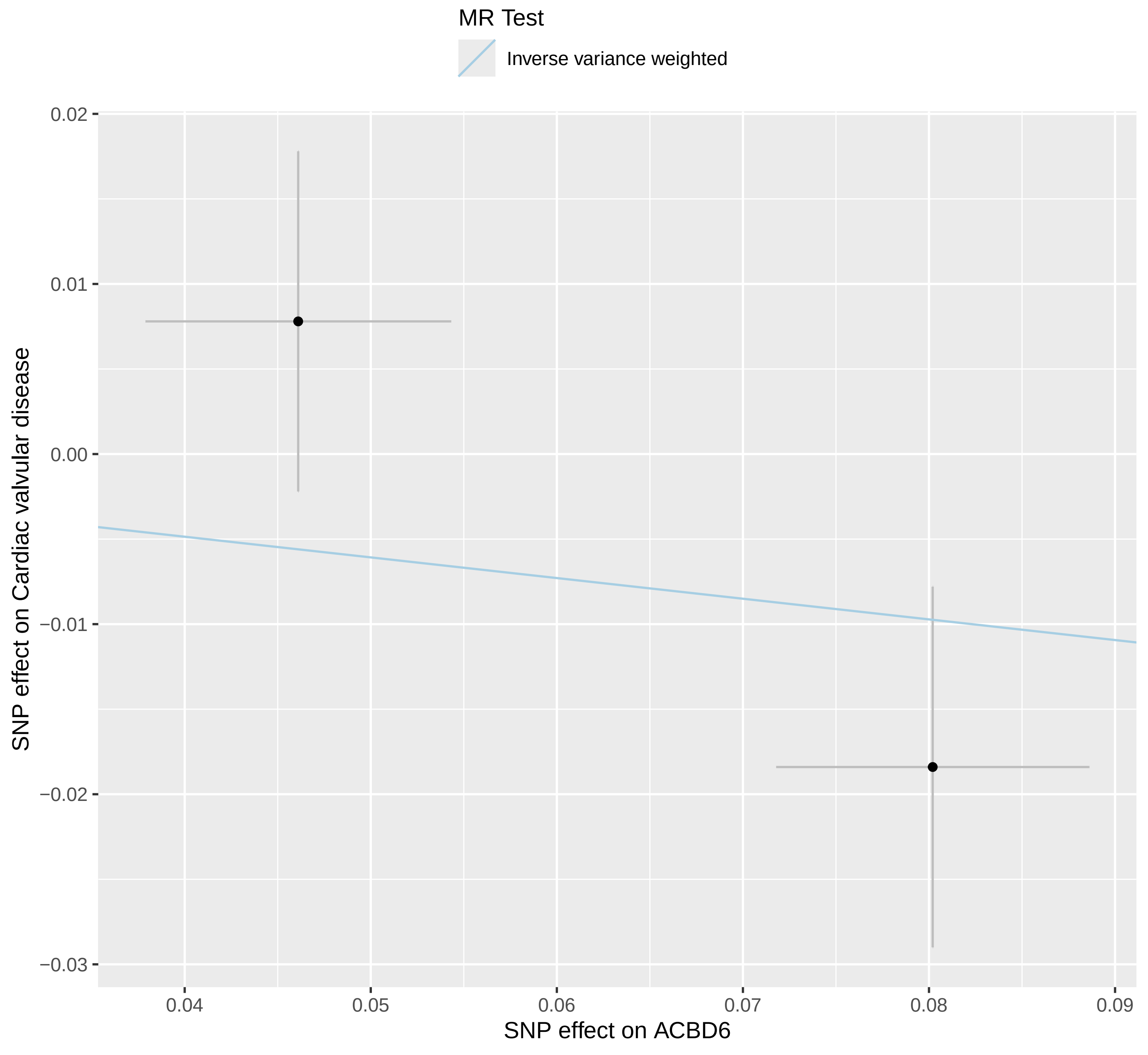

MR Test
Inverse variance weighted
0.02
0.01
0.00
−0.01
−0.02
−0.03
SNP effect on Cardiac valvular disease
0.04
0.05
0.06
0.07
0.08
0.09
SNP effect on ACBD6

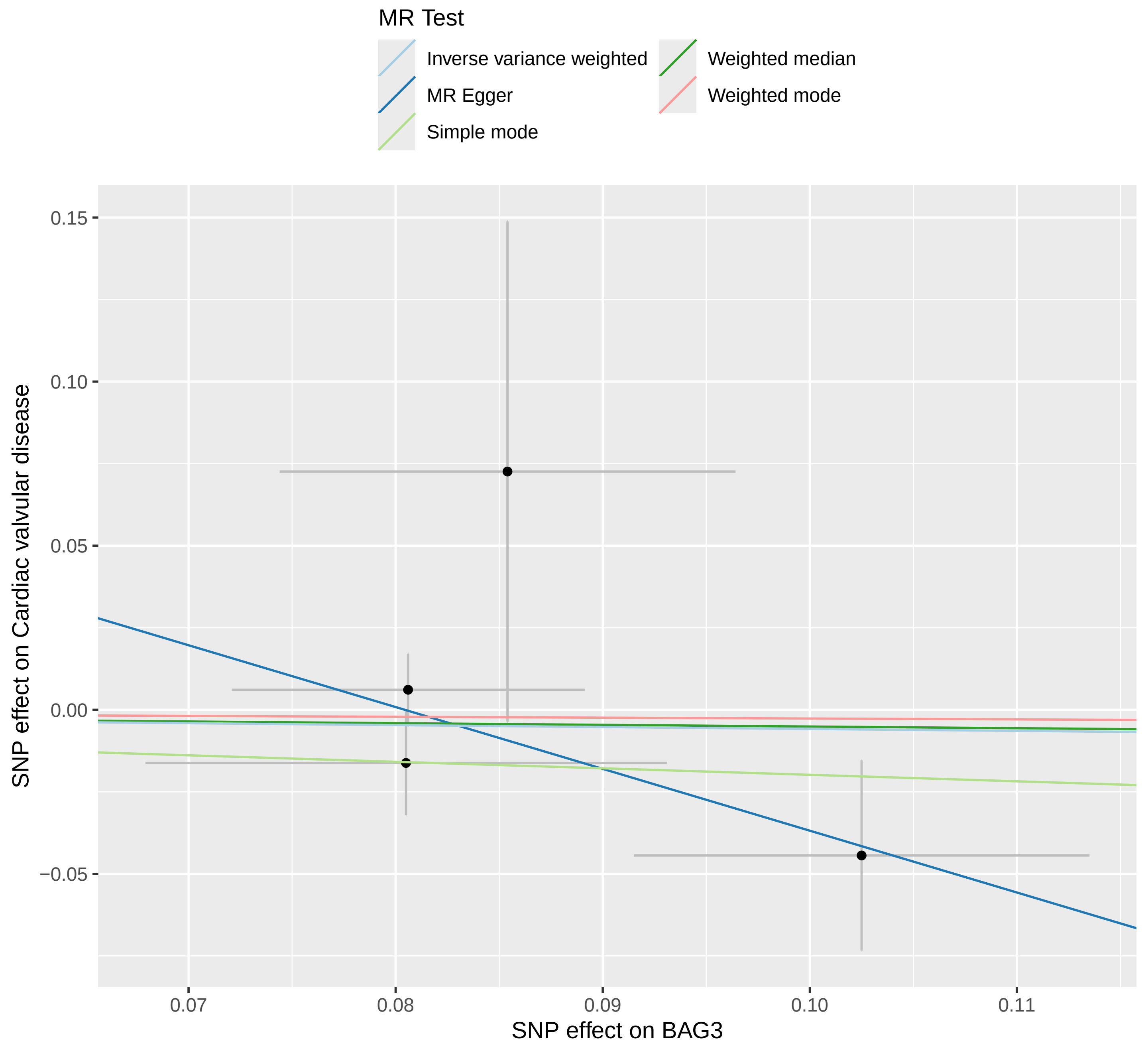
MR Test
Inverse variance weighted
MR Egger
Simple mode
Weighted median
Weighted mode
0.15
0.10
0.05
0.00
−0.05
SNP effect on Cardiac valvular disease
0.07
0.08
0.09
0.10
0.11
SNP effect on BAG3

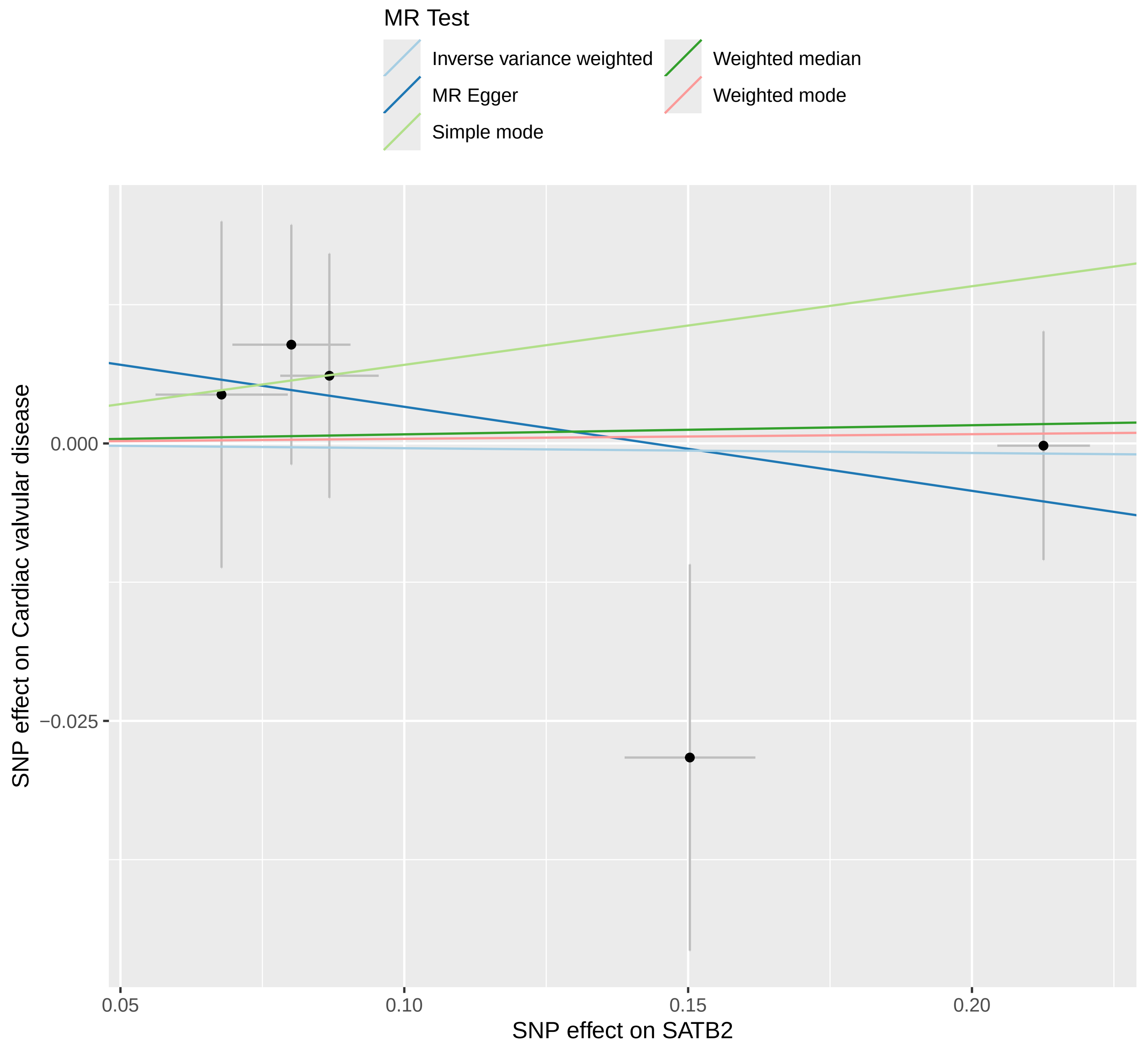
MR Test
Inverse variance weighted
MR Egger
Simple mode
Weighted median
Weighted mode
0.000
−0.025
0.05
0.10
0.15
0.20
SNP effect on SATB2
SNP effect on Cardiac valvular disease

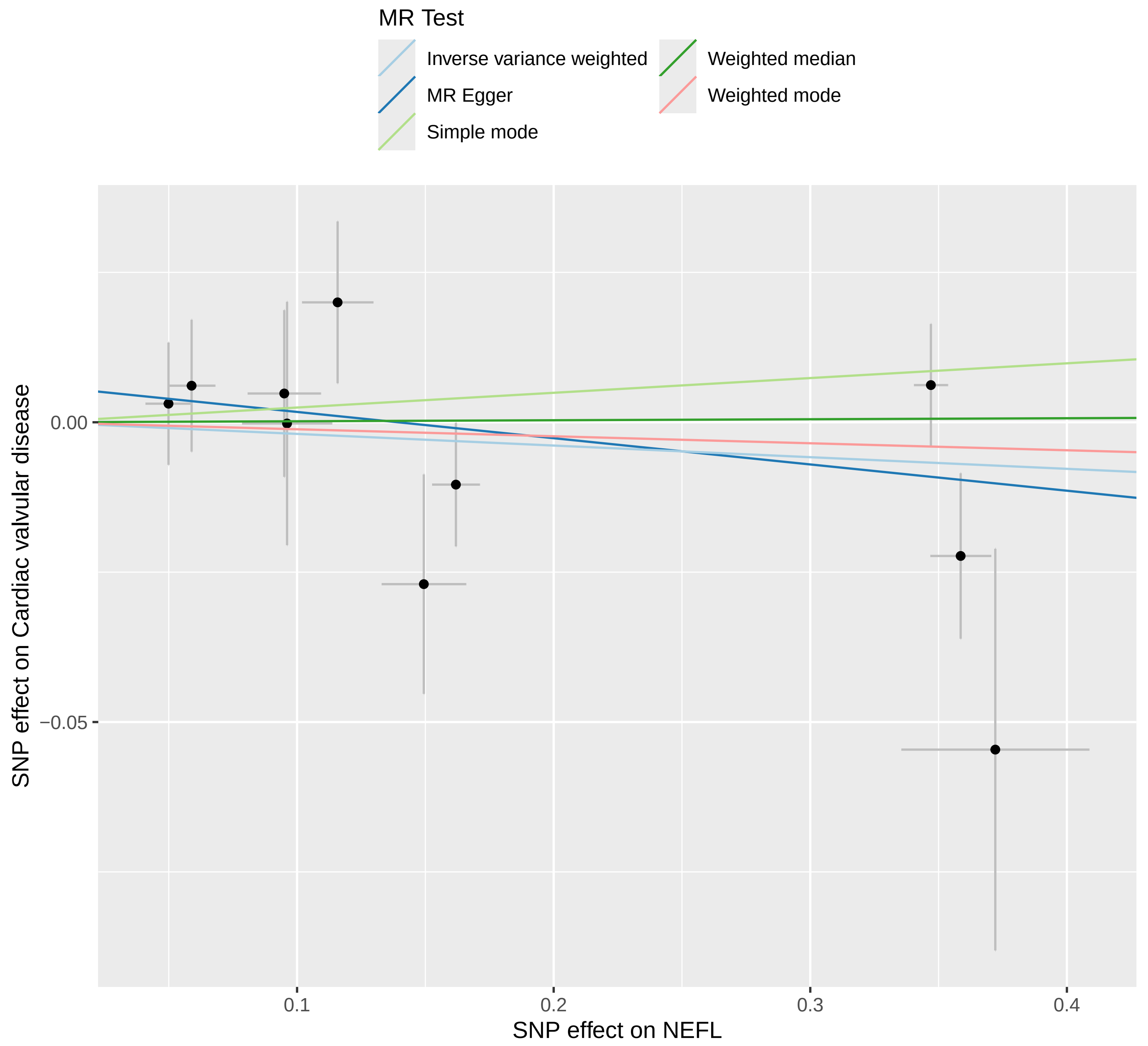
MR Test
Inverse variance weighted
MR Egger
Simple mode
Weighted median
Weighted mode
SNP effect on Cardiac valvular disease
0.00
−0.05
0.1
0.2
0.3
0.4
SNP effect on NEFL

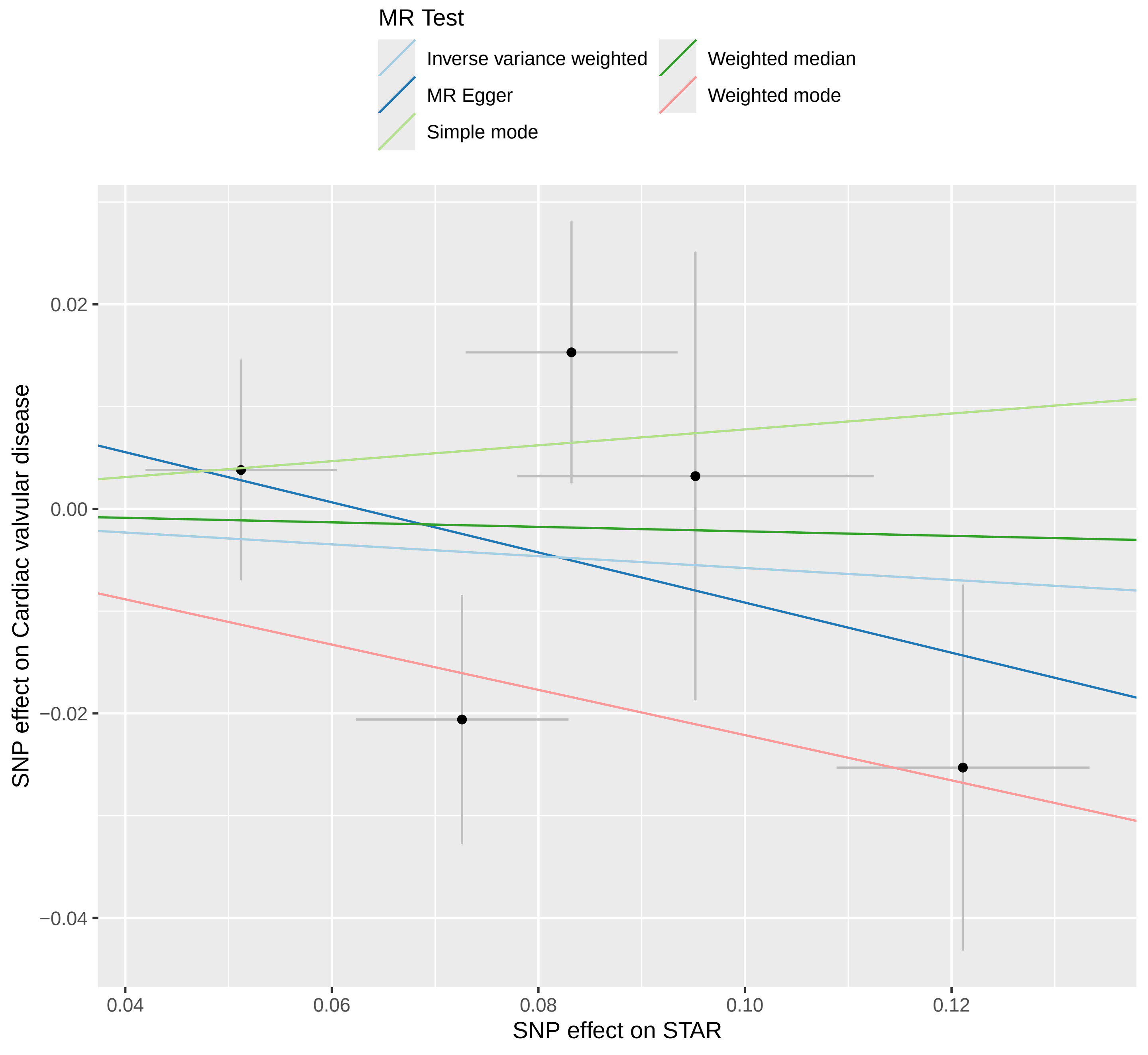

MR Test
Inverse variance weighted
MR Egger
Simple mode
Weighted median
Weighted mode
0.02
0.00
−0.02
−0.04
SNP effect on Cardiac valvular disease
0.04
0.06
0.08
0.10
0.12
SNP effect on STAR

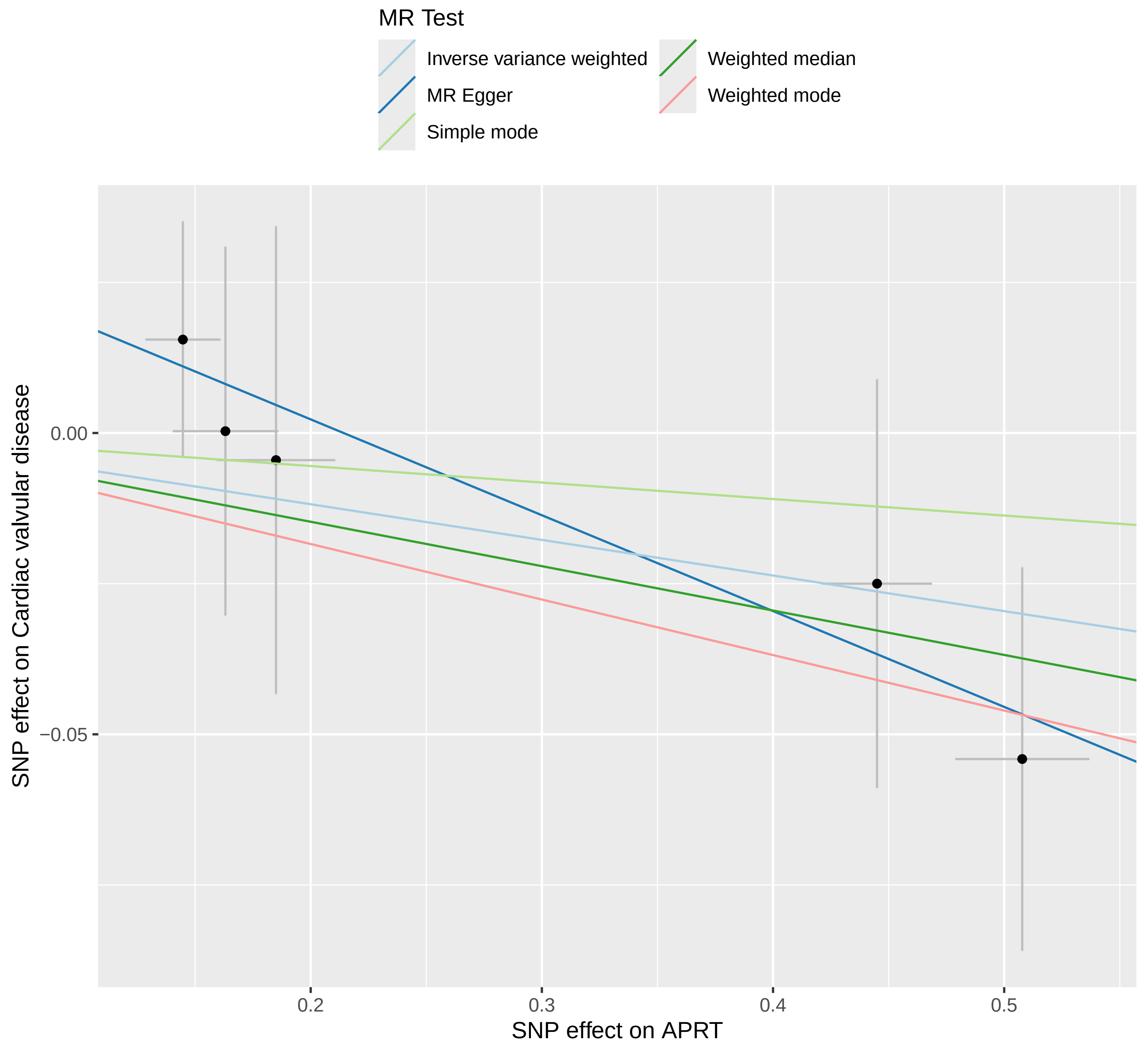
MR Test
Inverse variance weighted
MR Egger
Simple mode
Weighted median
Weighted mode
0.00
−0.05
0.2
0.3
0.4
0.5
SNP effect on APRT
SNP effect on Cardiac valvular disease

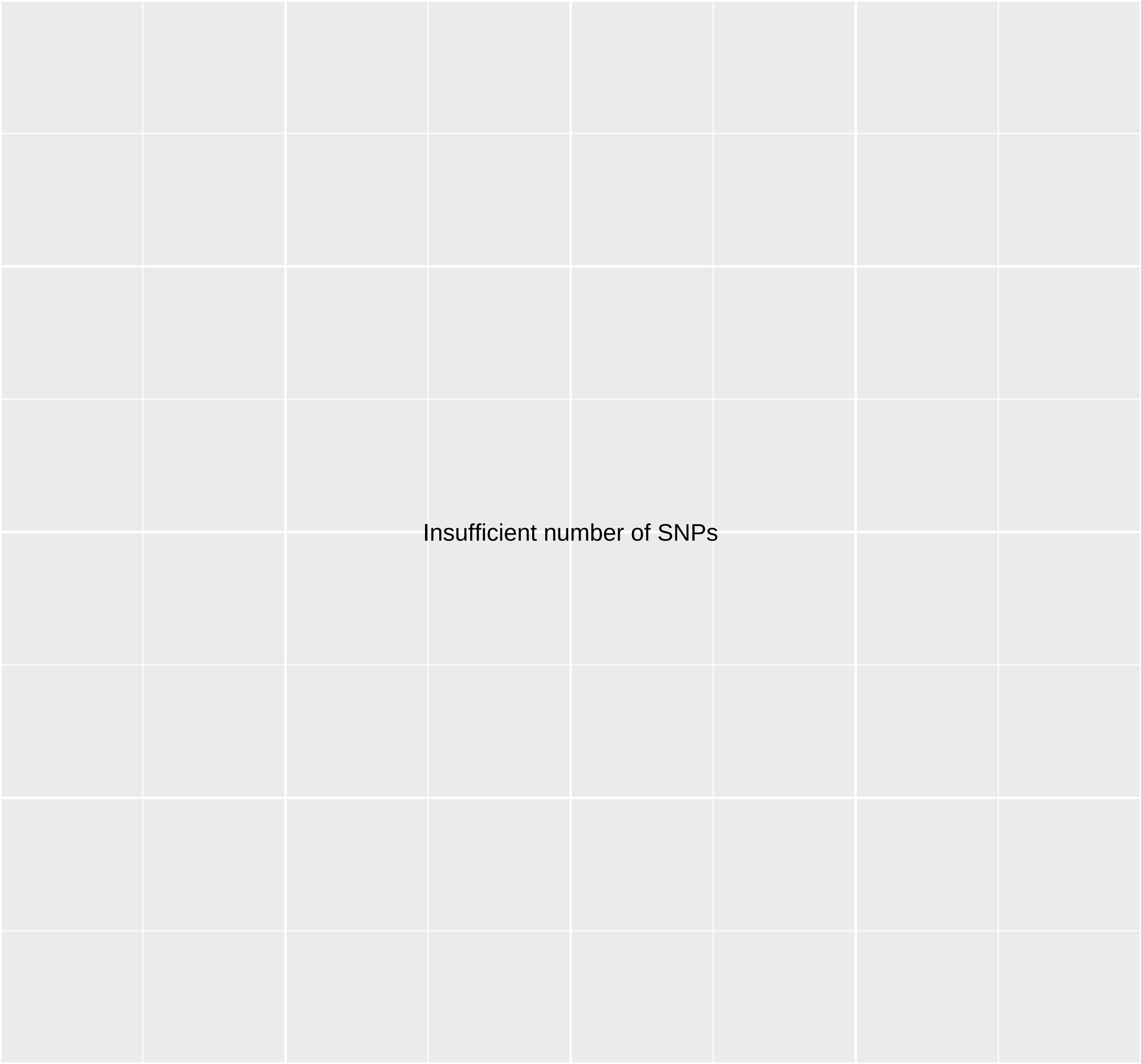
Insufficient number of SNPs

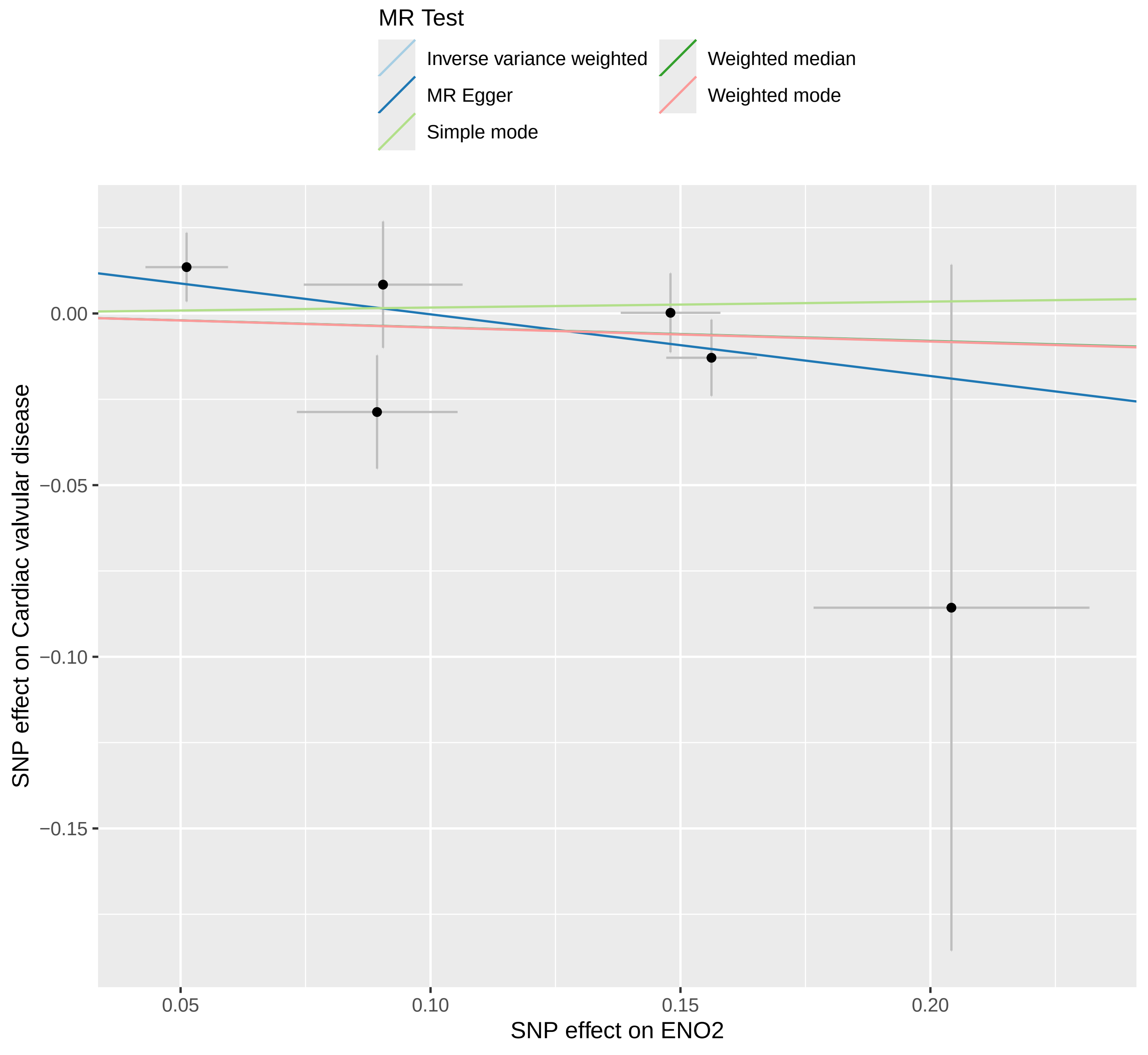

MR Test
Inverse variance weighted
MR Egger
Simple mode
Weighted median
Weighted mode
SNP effect on Cardiac valvular disease
0.00
−0.05
−0.10
−0.15
0.05
0.10
0.15
0.20
SNP effect on ENO2

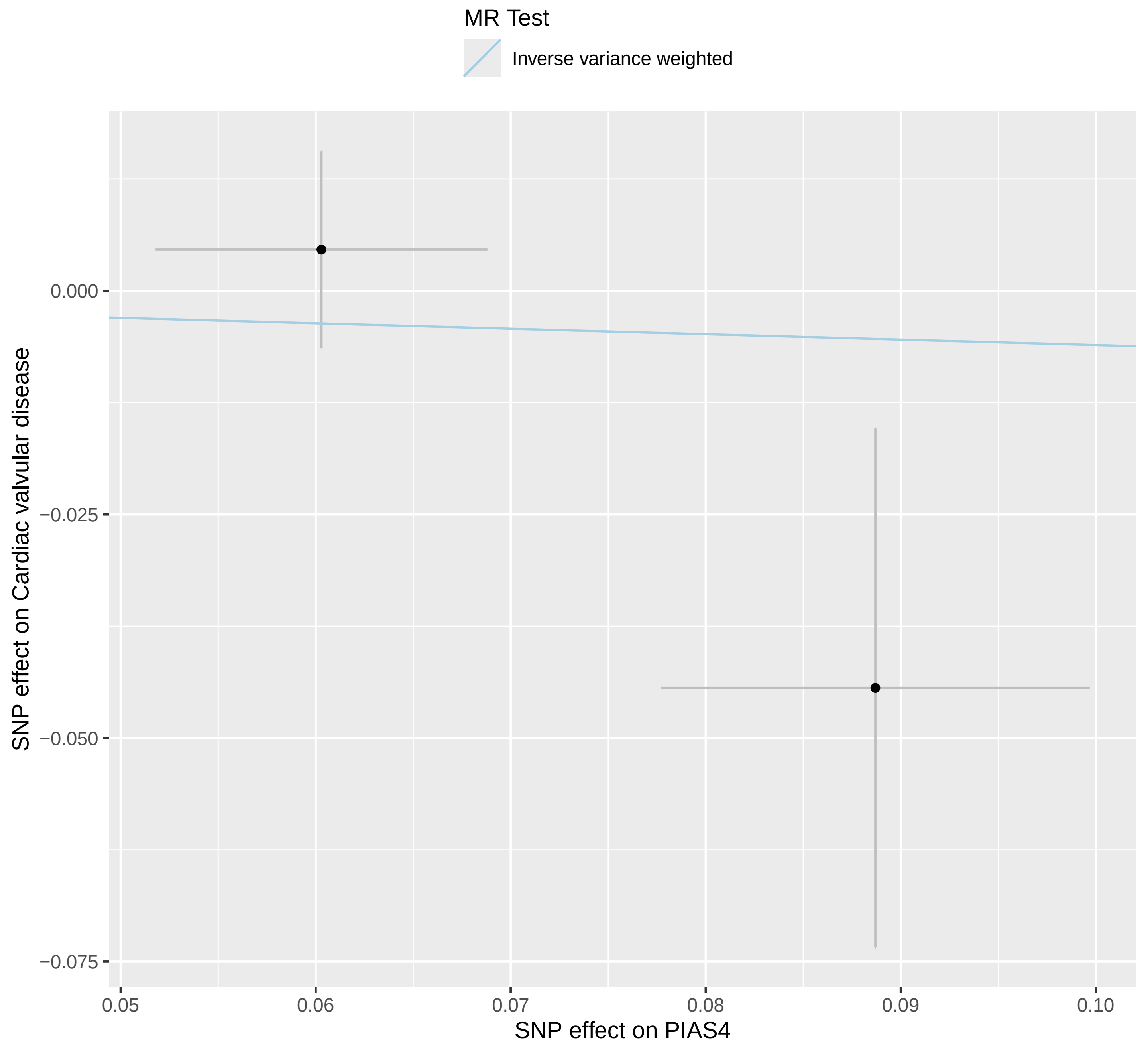

MR Test
Inverse variance weighted
0.000
−0.025
−0.050
−0.075
SNP effect on Cardiac valvular disease
0.05
0.06
0.07
0.08
0.09
0.10
SNP effect on PIAS4

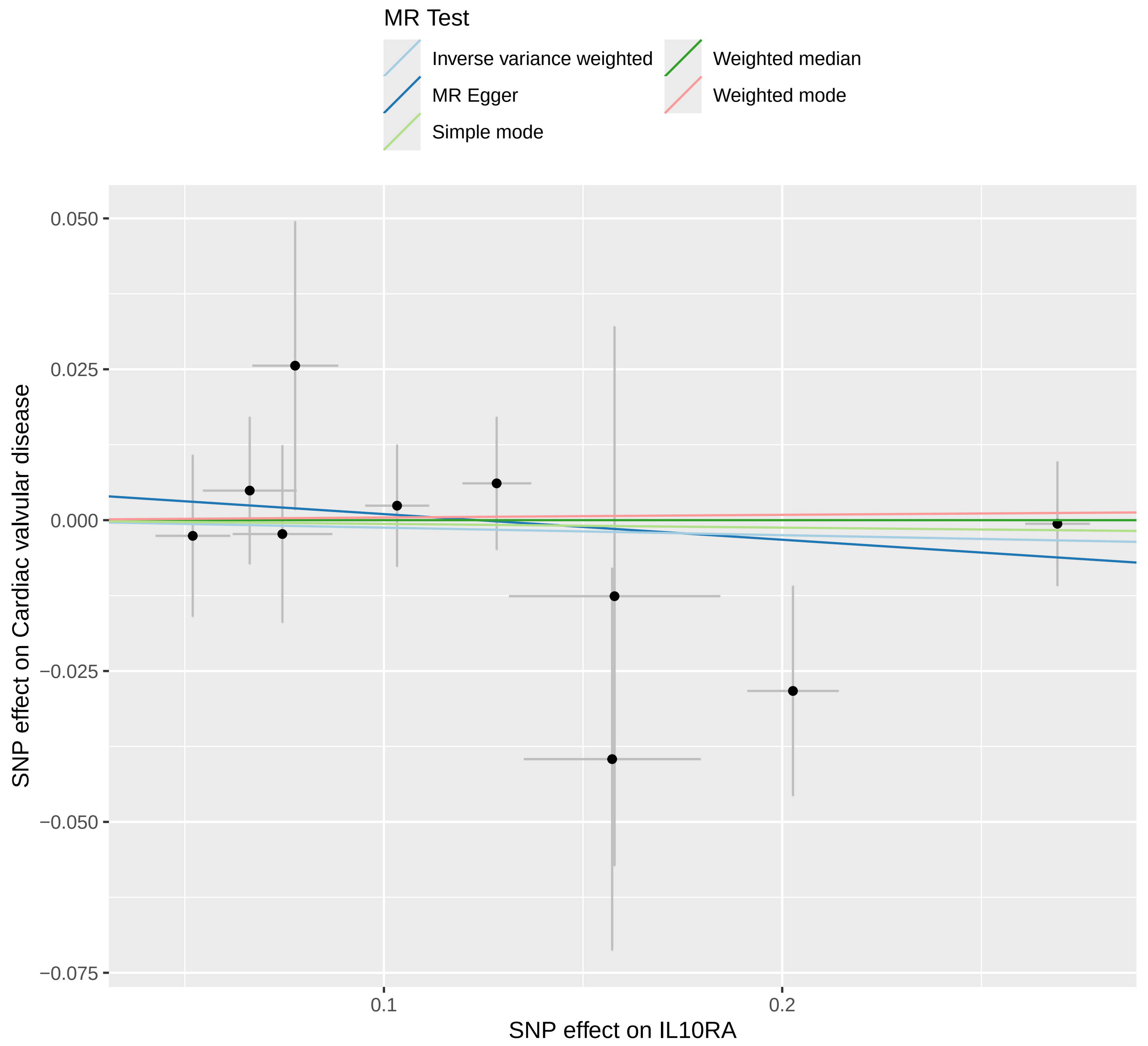

MR Test
Inverse variance weighted
MR Egger
Simple mode
Weighted median
Weighted mode
0.050
0.025
0.000
−0.025
−0.050
−0.075
0.1
0.2
SNP effect on Cardiac valvular disease
SNP effect on IL10RA

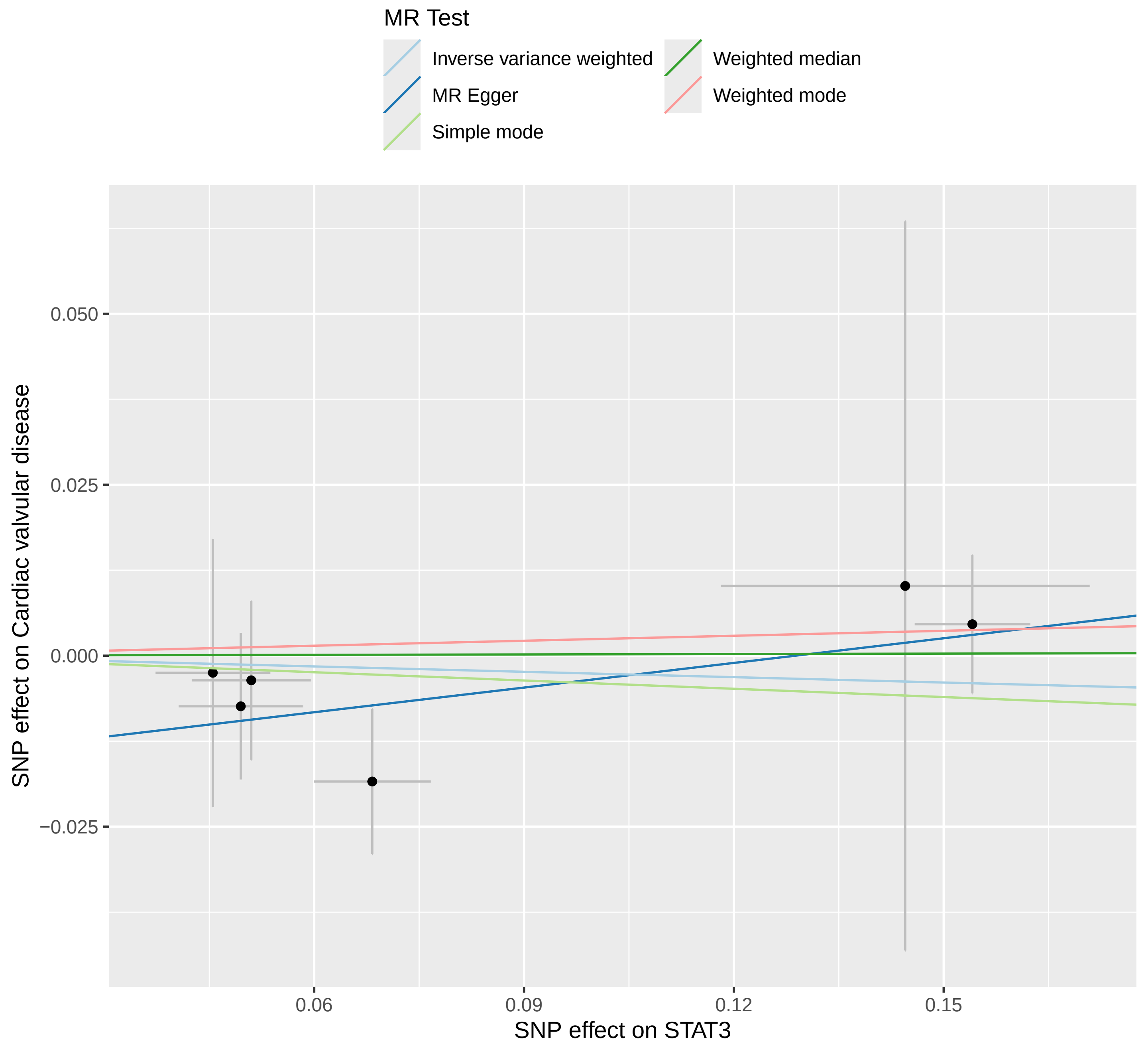
MR Test
Inverse variance weighted
MR Egger
Simple mode
Weighted median
Weighted mode
0.050
0.025
0.000
−0.025
0.06
0.09
0.12
0.15
SNP effect on Cardiac valvular disease
SNP effect on STAT3

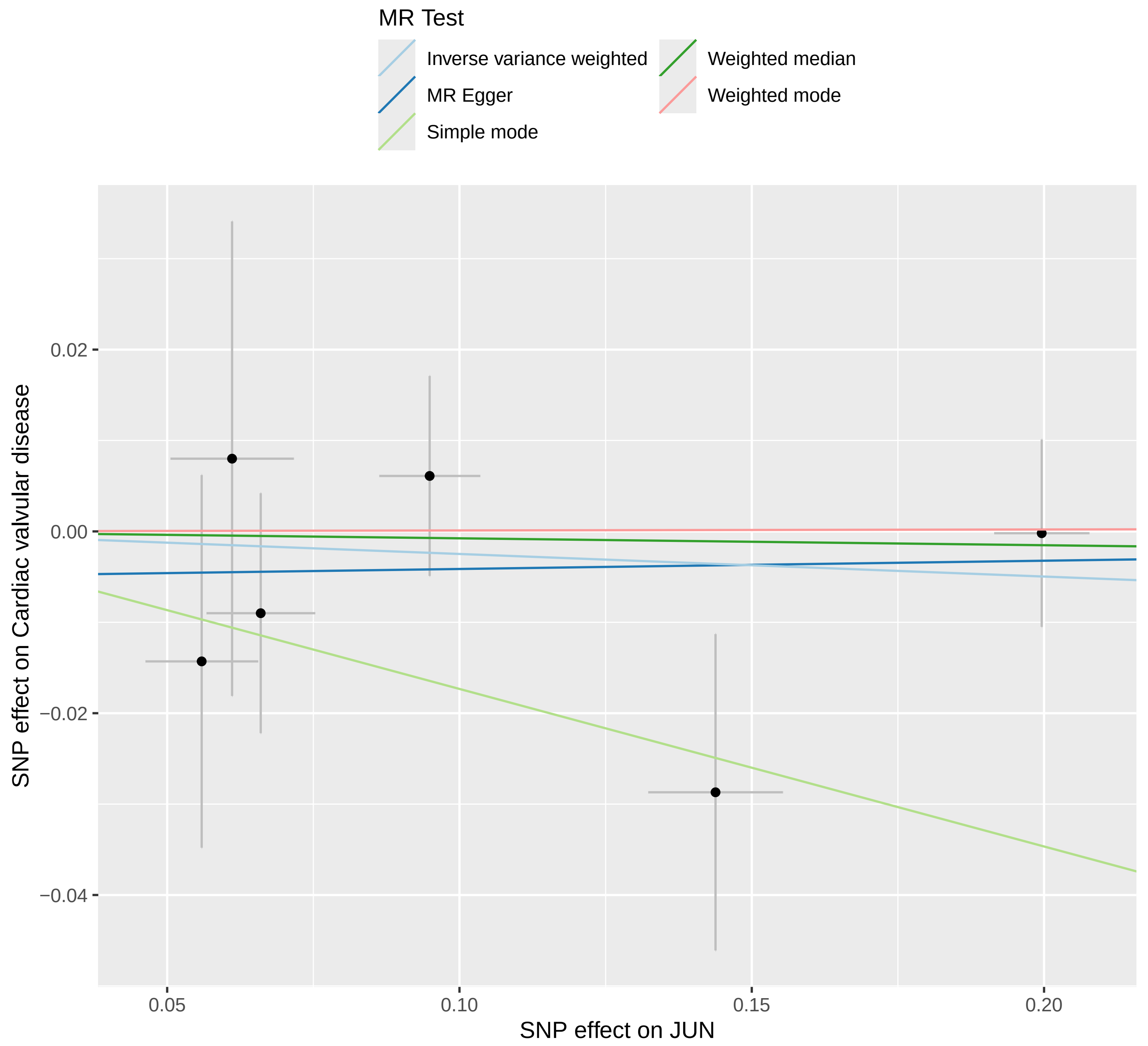

MR Test
Inverse variance weighted
MR Egger
Simple mode
Weighted median
Weighted mode
0.02
0.00
−0.02
−0.04
0.05
0.10
0.15
0.20
SNP effect on Cardiac valvular disease
SNP effect on JUN

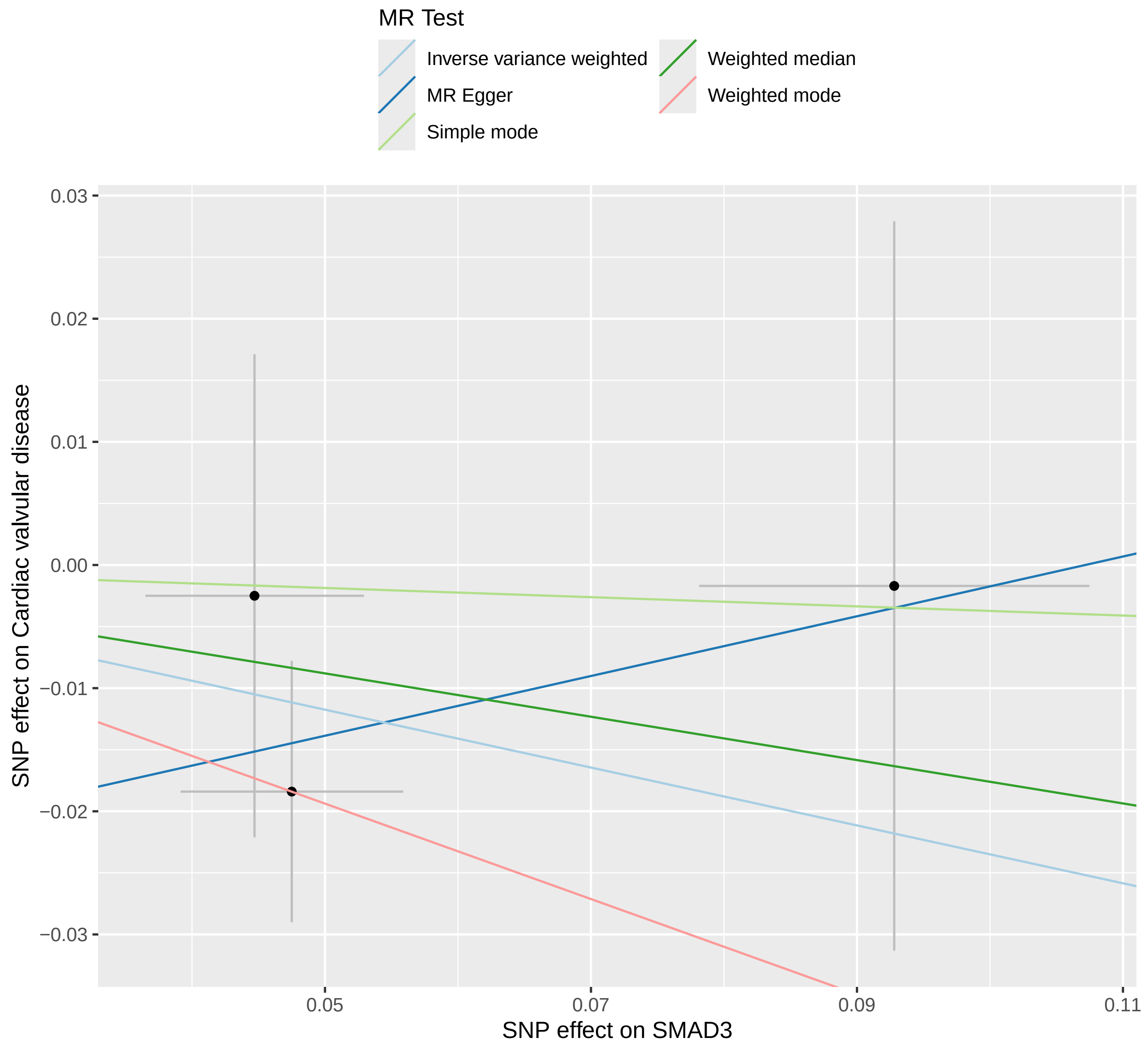
MR Test
Inverse variance weighted
MR Egger
Simple mode
Weighted median
Weighted mode
0.03
0.02
0.01
0.00
−0.01
−0.02
−0.03
SNP effect on Cardiac valvular disease
0.05
0.07
0.09
0.11
SNP effect on SMAD3

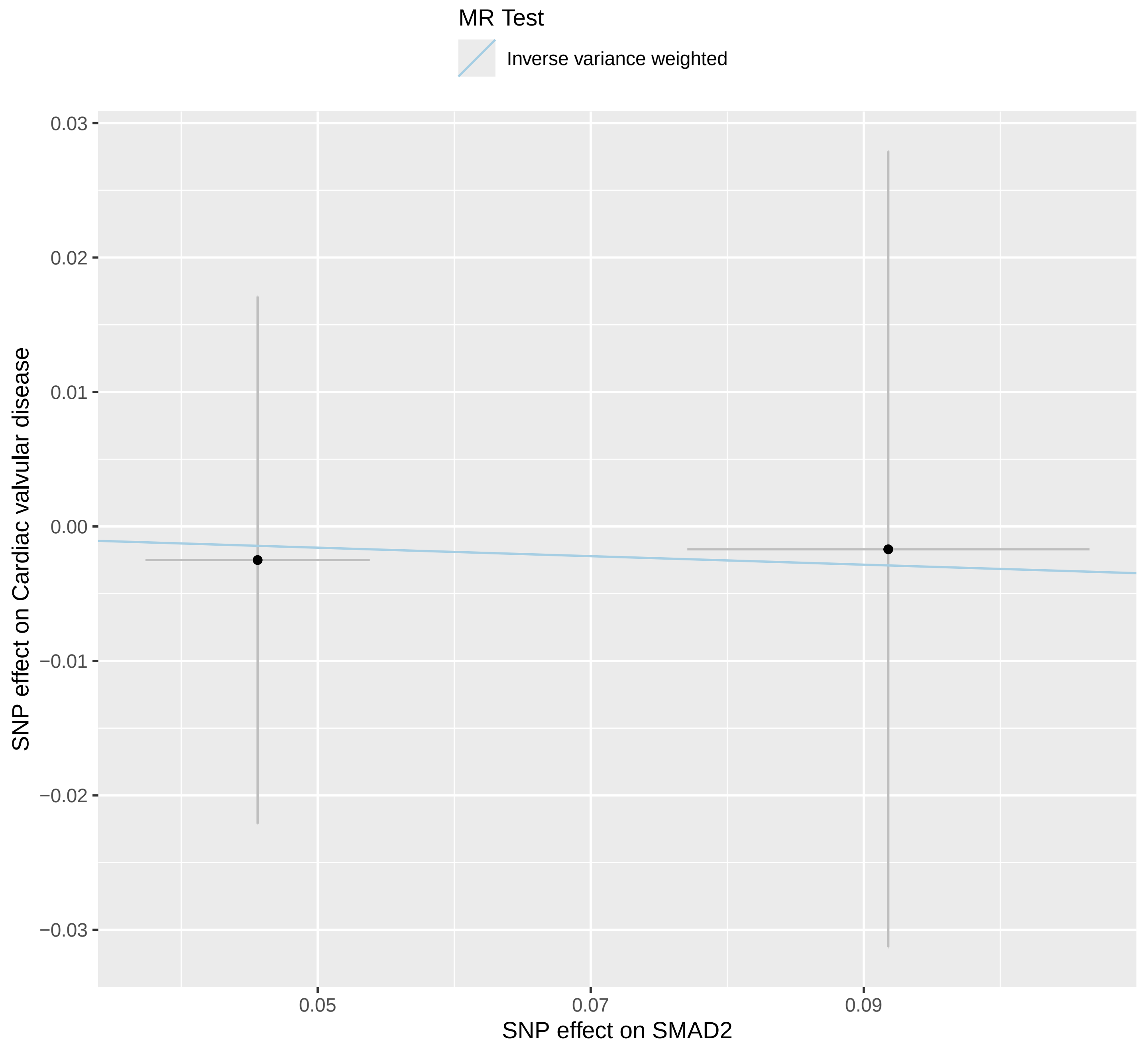

MR Test
Inverse variance weighted
0.03
0.02
0.01
0.00
−0.01
−0.02
−0.03
SNP effect on Cardiac valvular disease
0.05
0.07
0.09
SNP effect on SMAD2

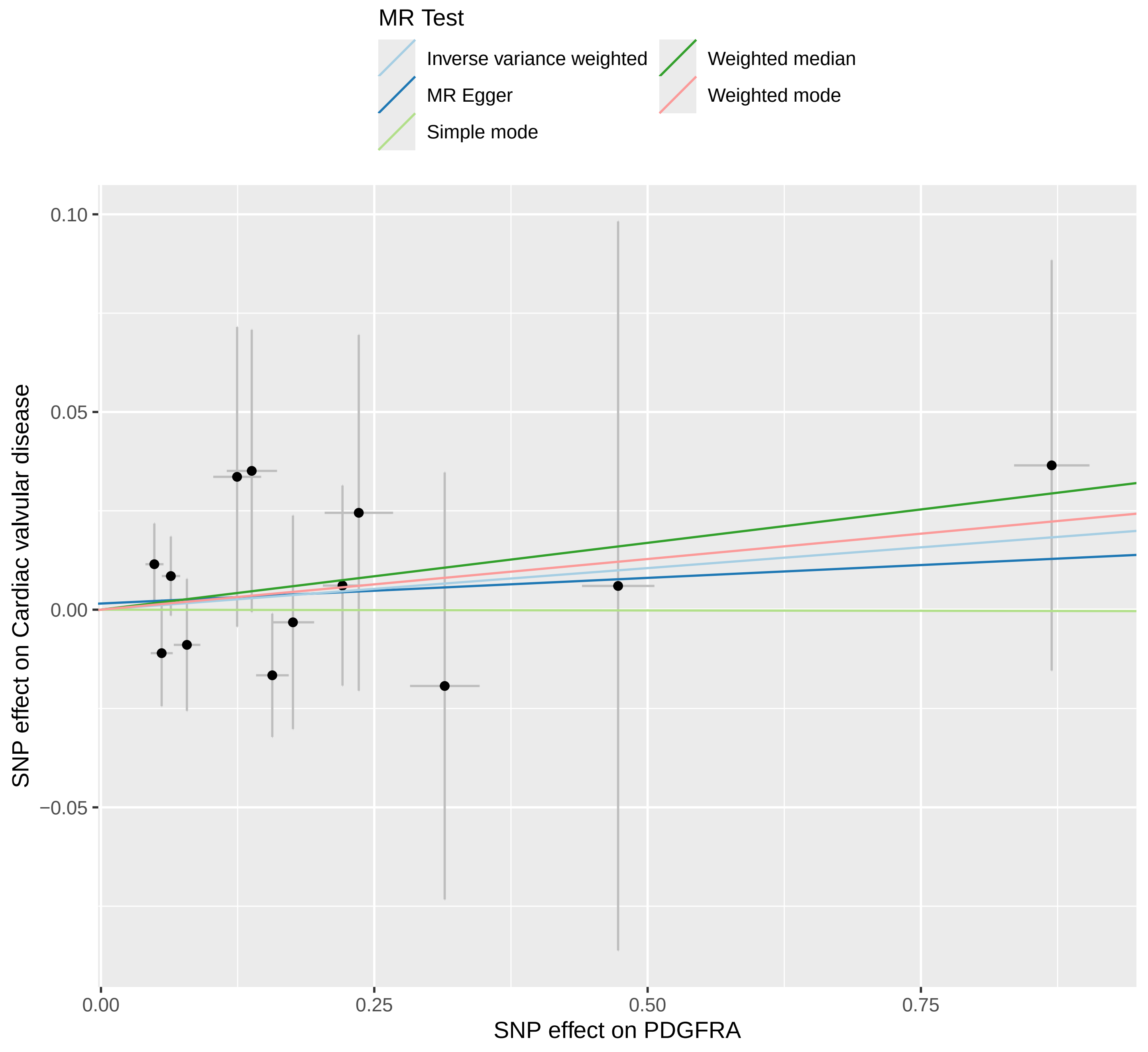
MR Test
Inverse variance weighted
MR Egger
Simple mode
Weighted median
Weighted mode
0.10
0.05
0.00
−0.05
SNP effect on Cardiac valvular disease
0.00
0.25
0.50
0.75
SNP effect on PDGFRA

Insufficient number of SNPs

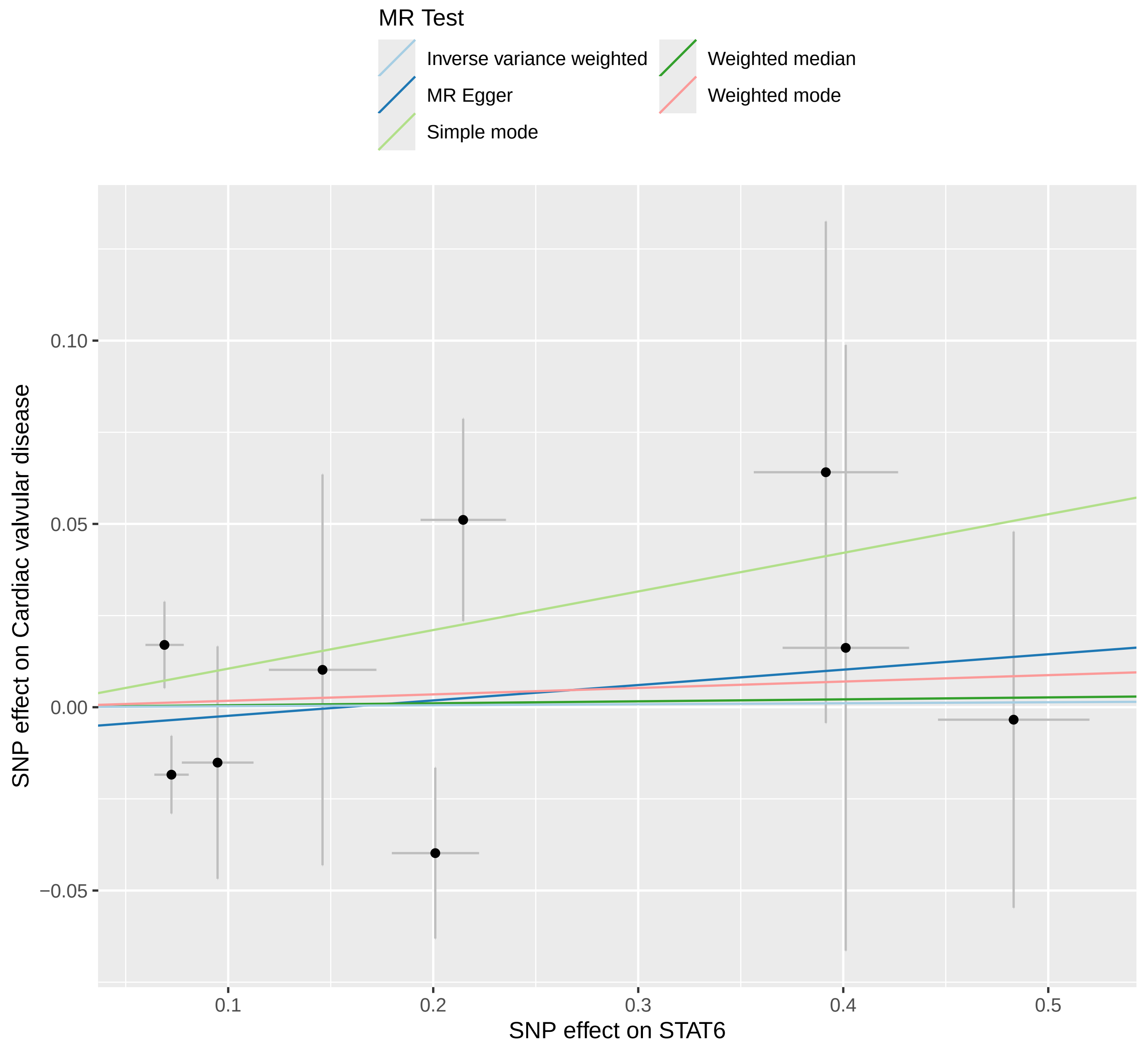
MR Test
Inverse variance weighted
MR Egger
Simple mode
Weighted median
Weighted mode
0.10
0.05
0.00
−0.05
0.1
0.2
0.3
0.4
0.5
SNP effect on Cardiac valvular disease
SNP effect on STAT6

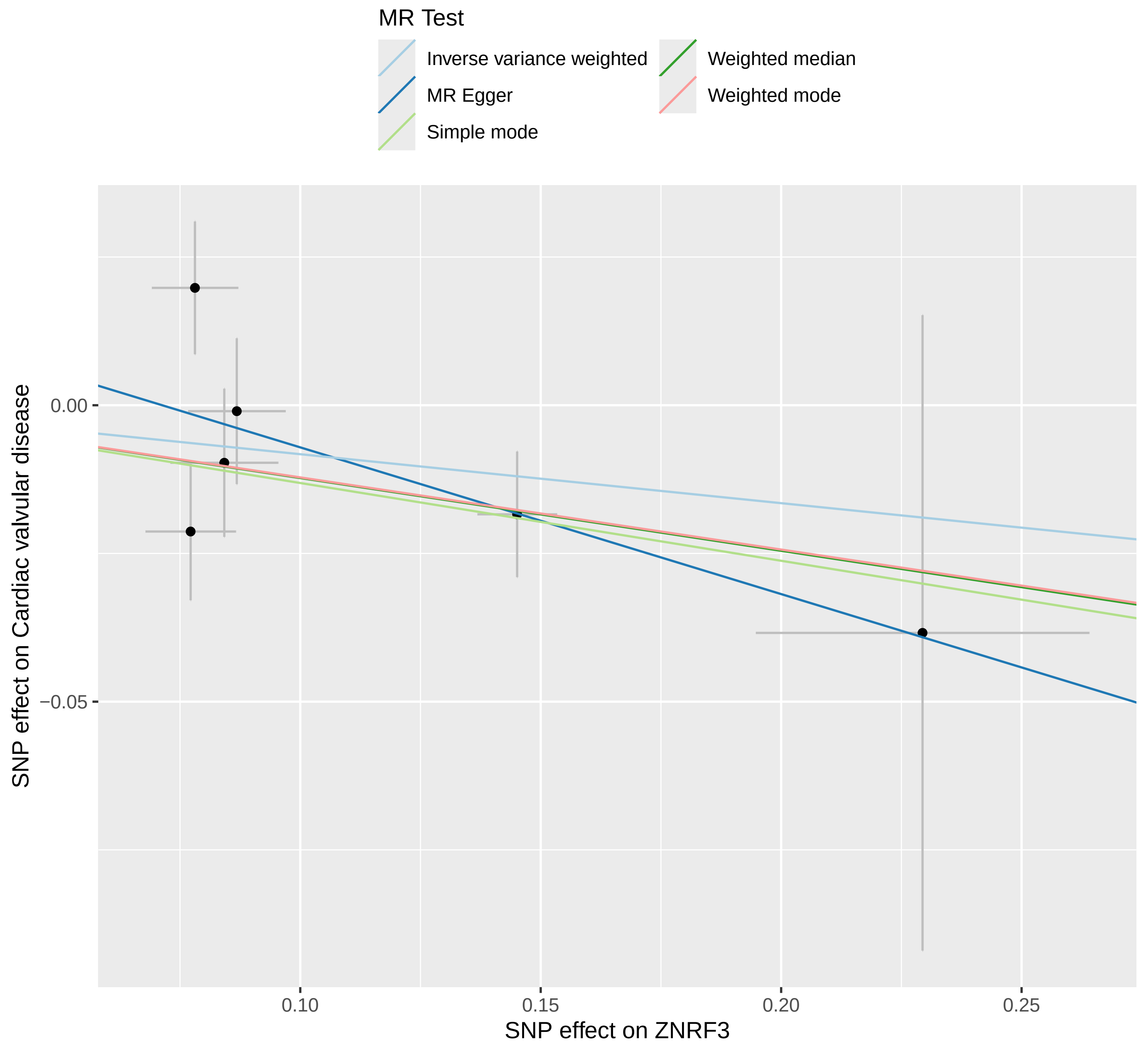
MR Test
Inverse variance weighted
MR Egger
Simple mode
Weighted median
Weighted mode
0.00
−0.05
0.10
0.15
0.20
0.25
SNP effect on Cardiac valvular disease
SNP effect on ZNRF3

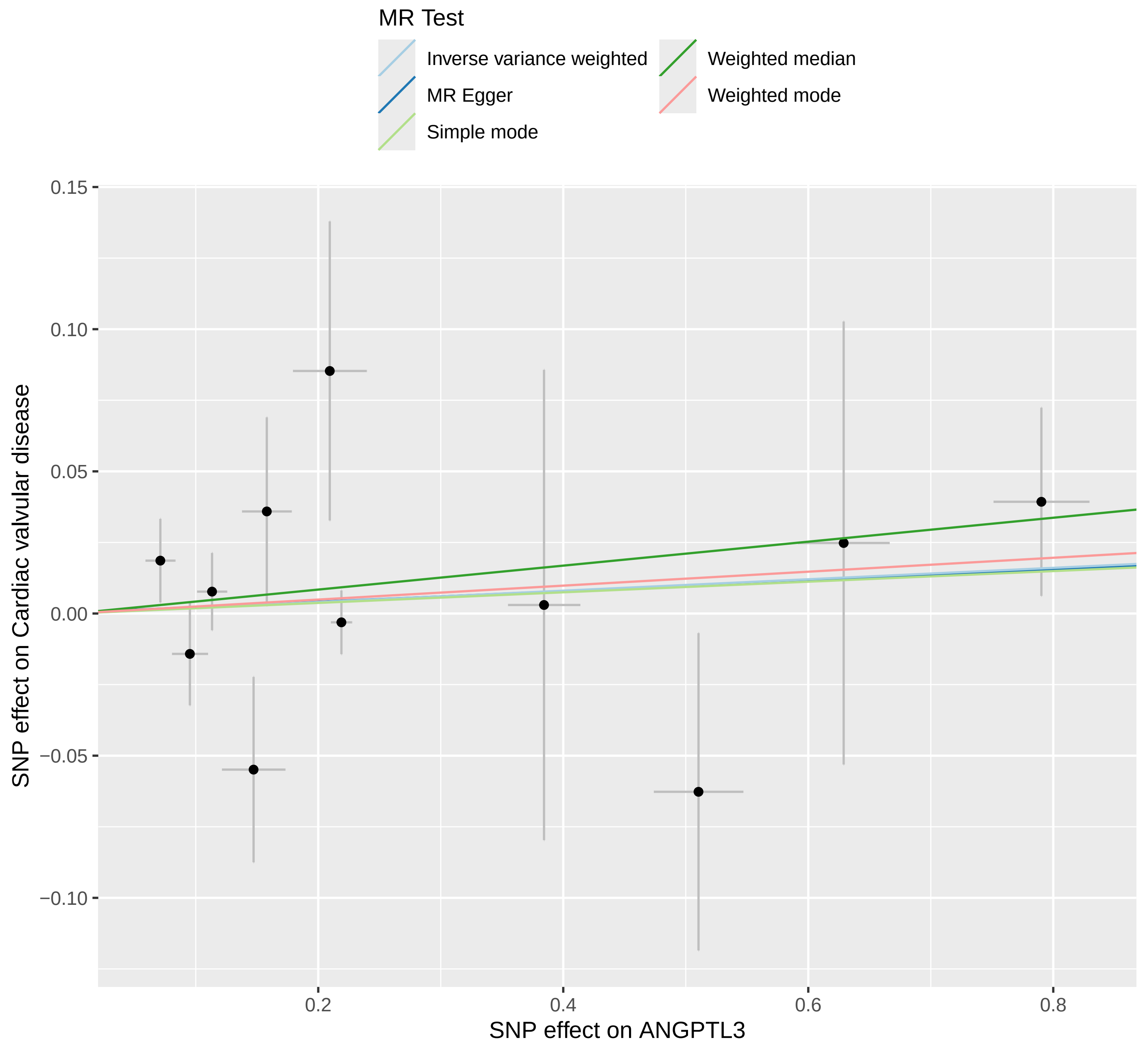
MR Test
Inverse variance weighted
MR Egger
Simple mode
Weighted median
Weighted mode
0.15
0.10
0.05
0.00
−0.05
−0.10
0.2
0.4
0.6
0.8
SNP effect on ANGPTL3
SNP effect on Cardiac valvular disease

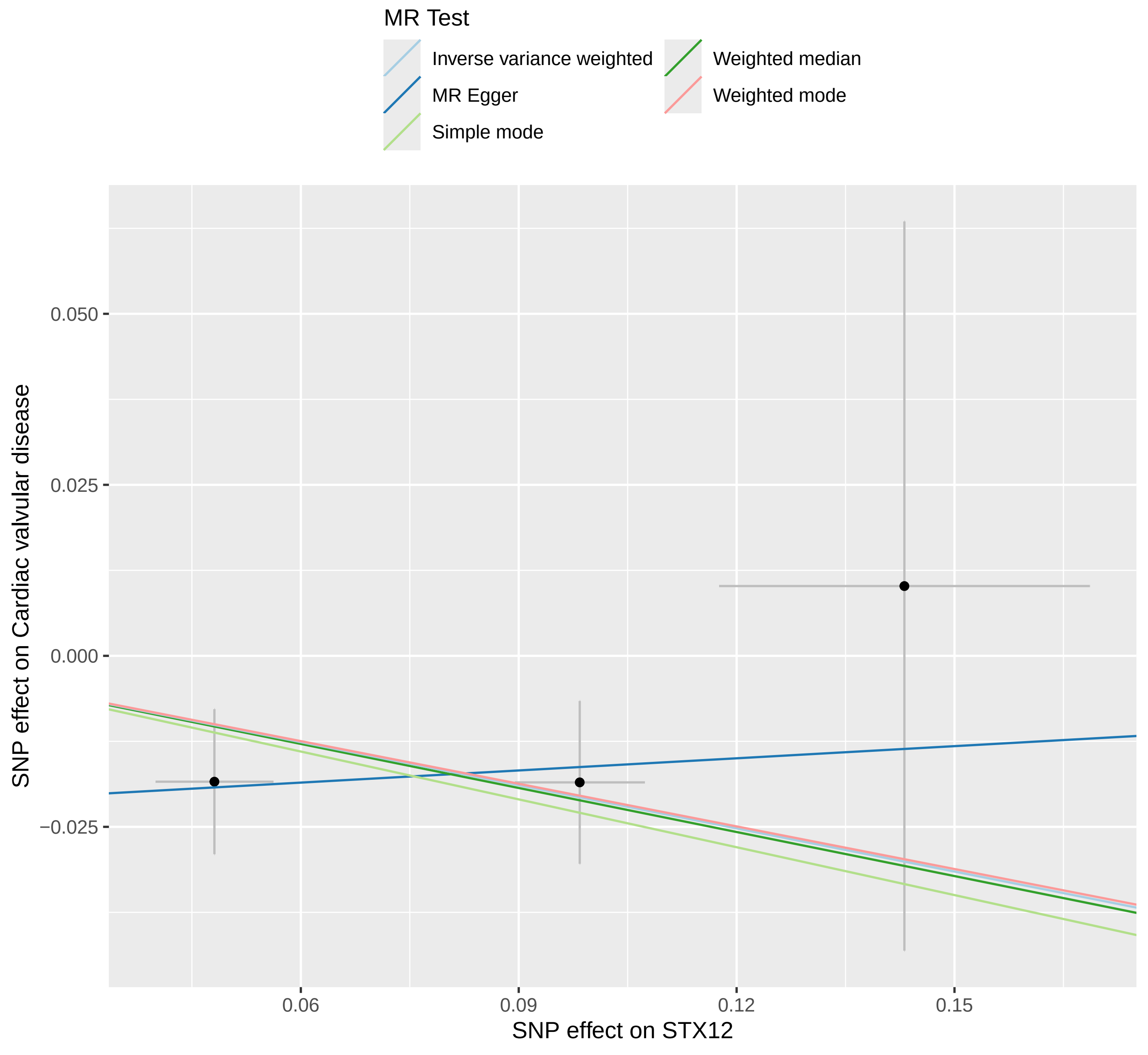
MR Test
Inverse variance weighted
MR Egger
Simple mode
Weighted median
Weighted mode
0.050
0.025
0.000
−0.025
SNP effect on Cardiac valvular disease
0.06
0.09
0.12
0.15
SNP effect on STX12

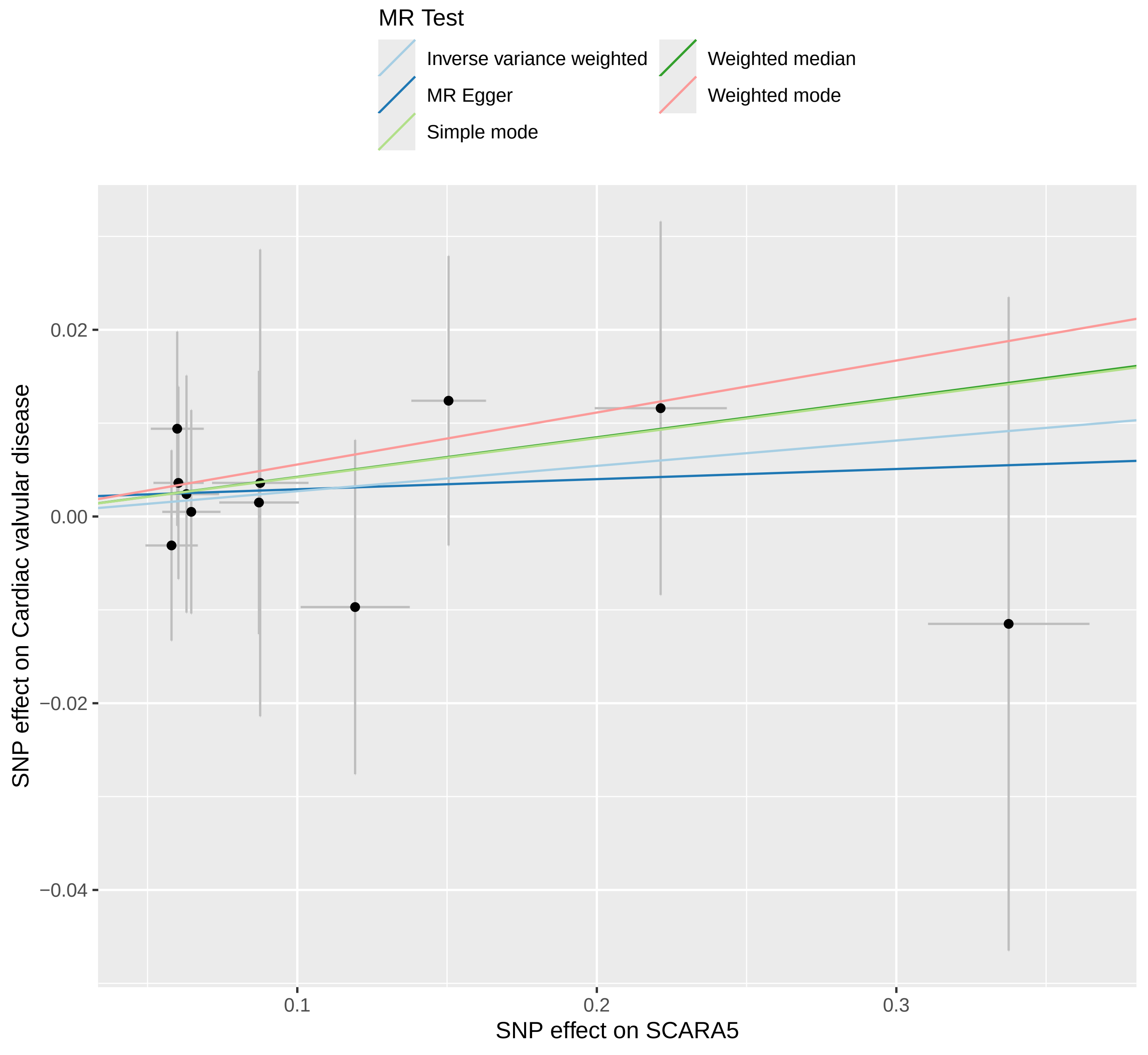

MR Test
Inverse variance weighted
MR Egger
Simple mode
Weighted median
Weighted mode
0.02
0.00
−0.02
−0.04
0.1
0.2
0.3
SNP effect on Cardiac valvular disease
SNP effect on SCARA5

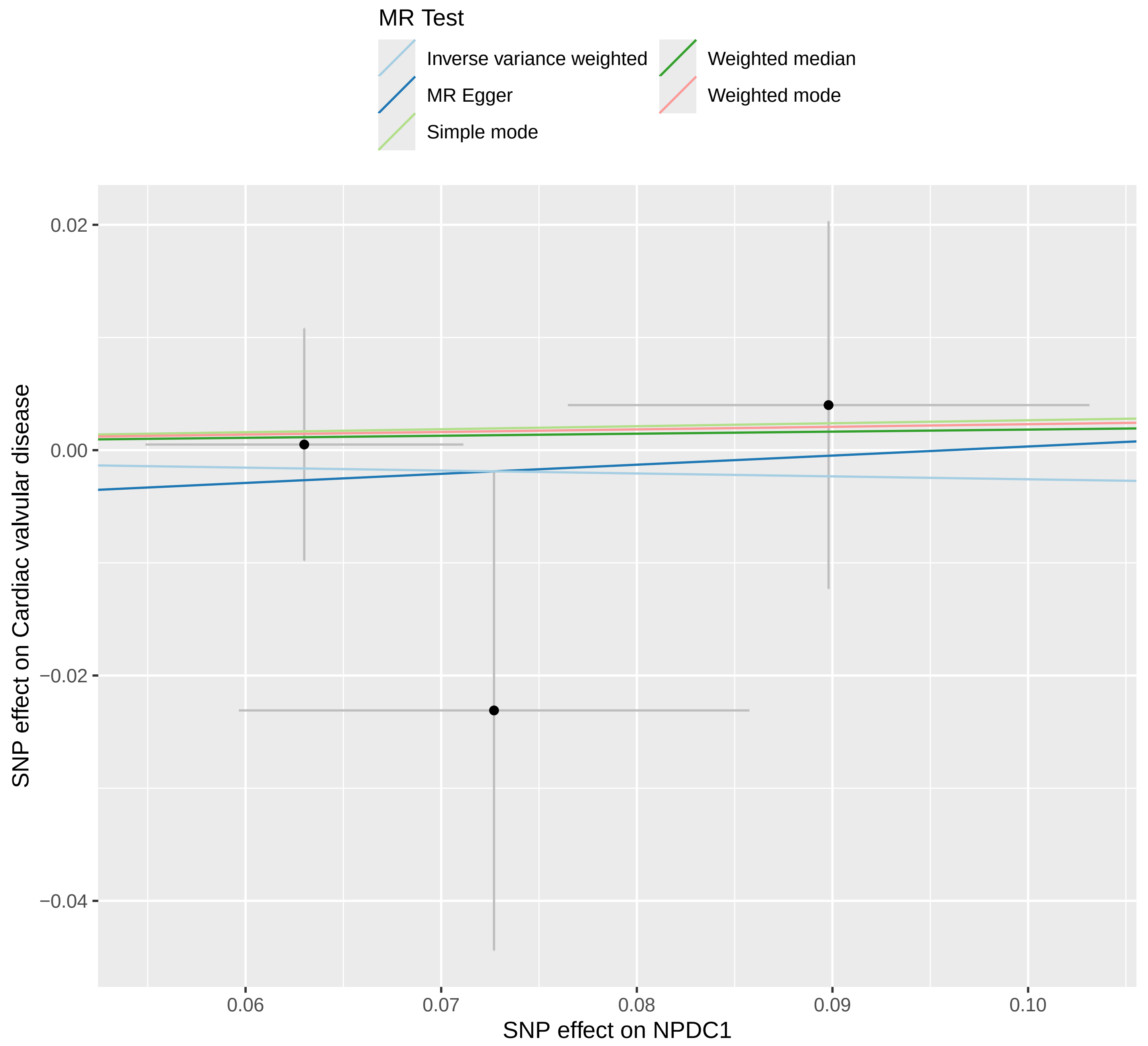

MR Test
Inverse variance weighted
MR Egger
Simple mode
Weighted median
Weighted mode
0.02
0.00
−0.02
−0.04
0.06
0.07
0.08
0.09
0.10
SNP effect on NPDC1
SNP effect on Cardiac valvular disease

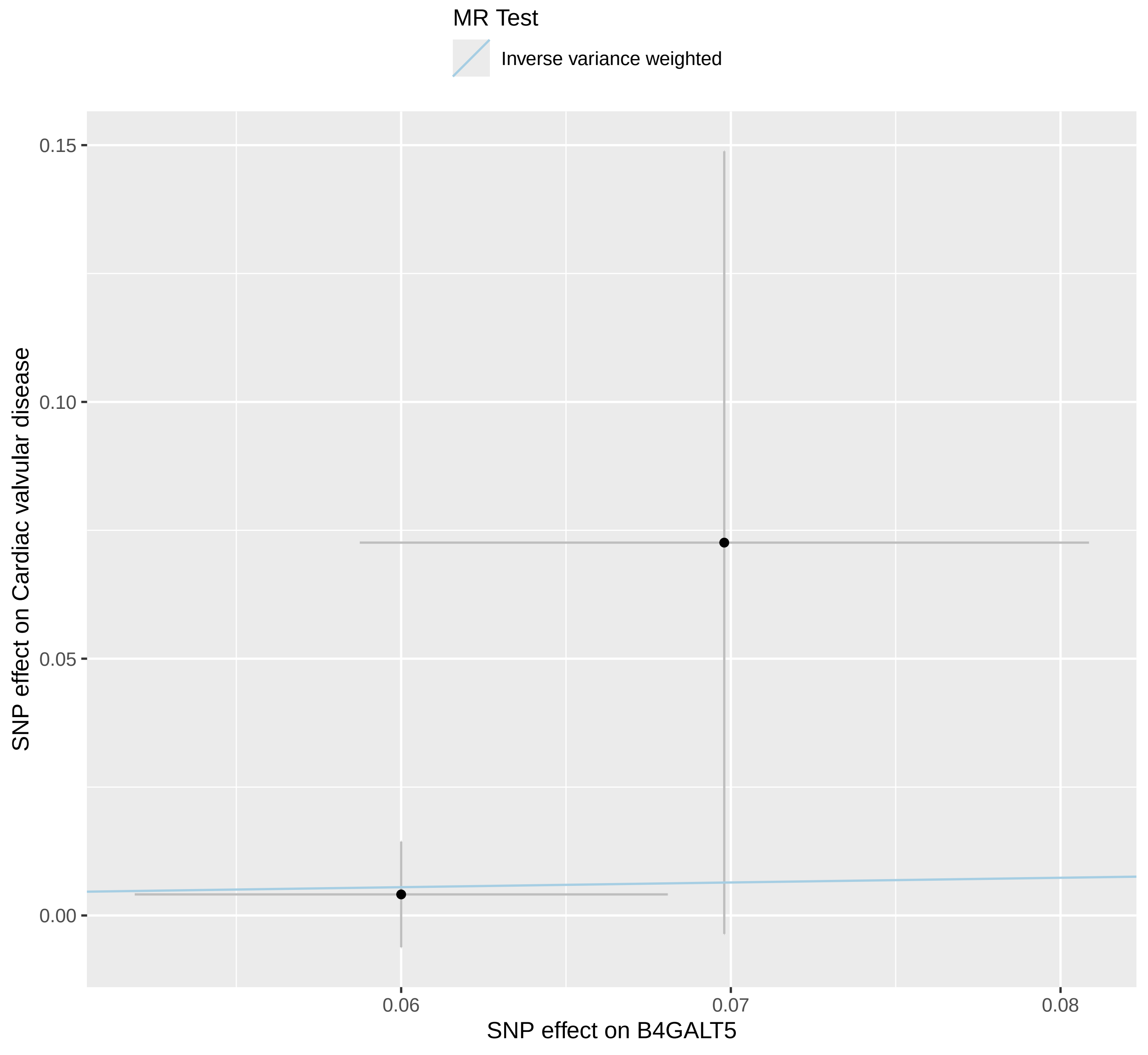

MR Test
Inverse variance weighted
0.15
0.10
0.05
0.00
0.06
0.07
0.08
SNP effect on Cardiac valvular disease
SNP effect on B4GALT5

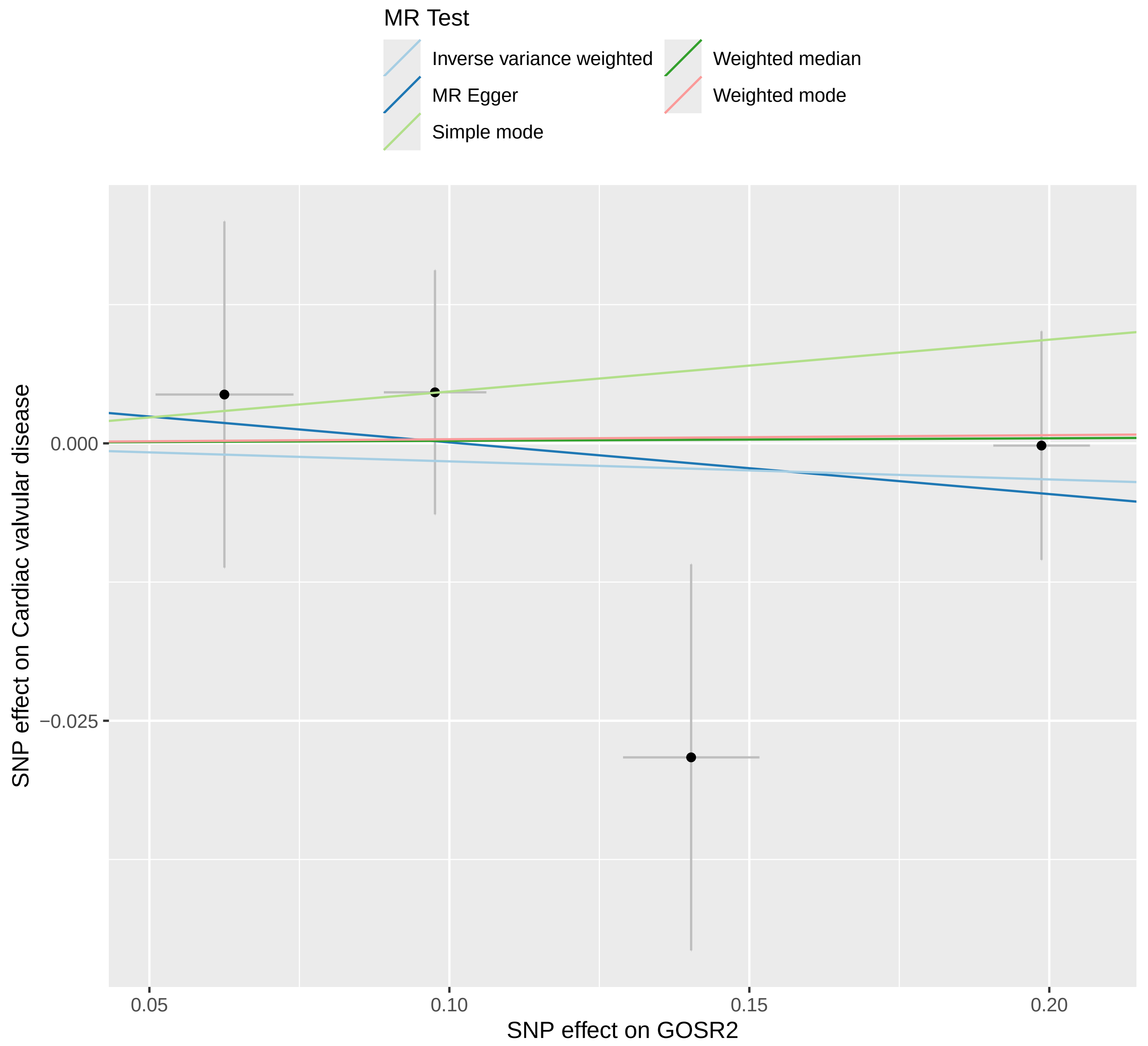

MR Test
Inverse variance weighted
MR Egger
Simple mode
Weighted median
Weighted mode
0.000
−0.025
0.05
0.10
0.15
0.20
SNP effect on GOSR2
SNP effect on Cardiac valvular disease

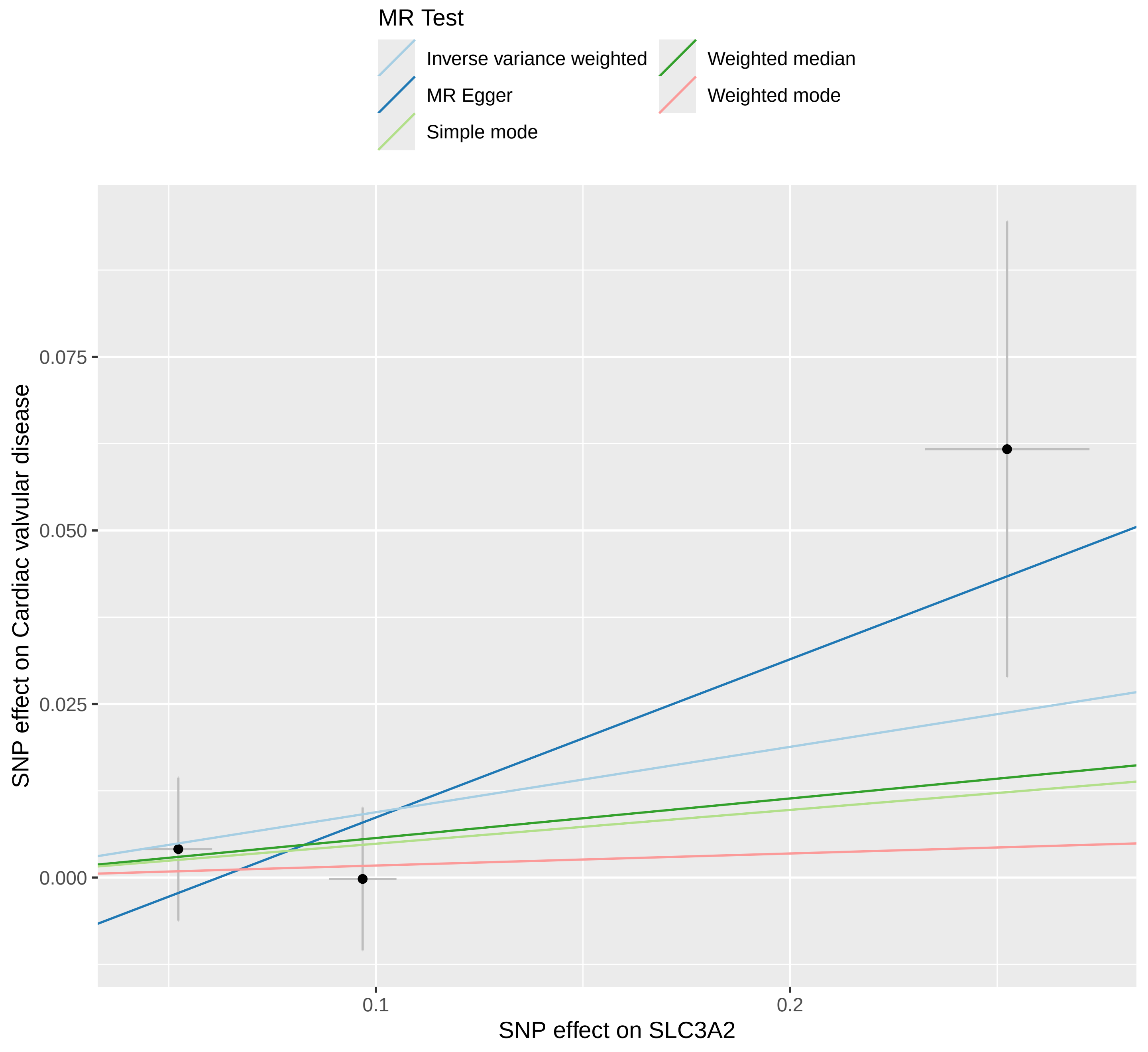
MR Test
Inverse variance weighted
MR Egger
Simple mode
Weighted median
Weighted mode
0.075
0.050
0.025
0.000
SNP effect on Cardiac valvular disease
0.1
0.2
SNP effect on SLC3A2

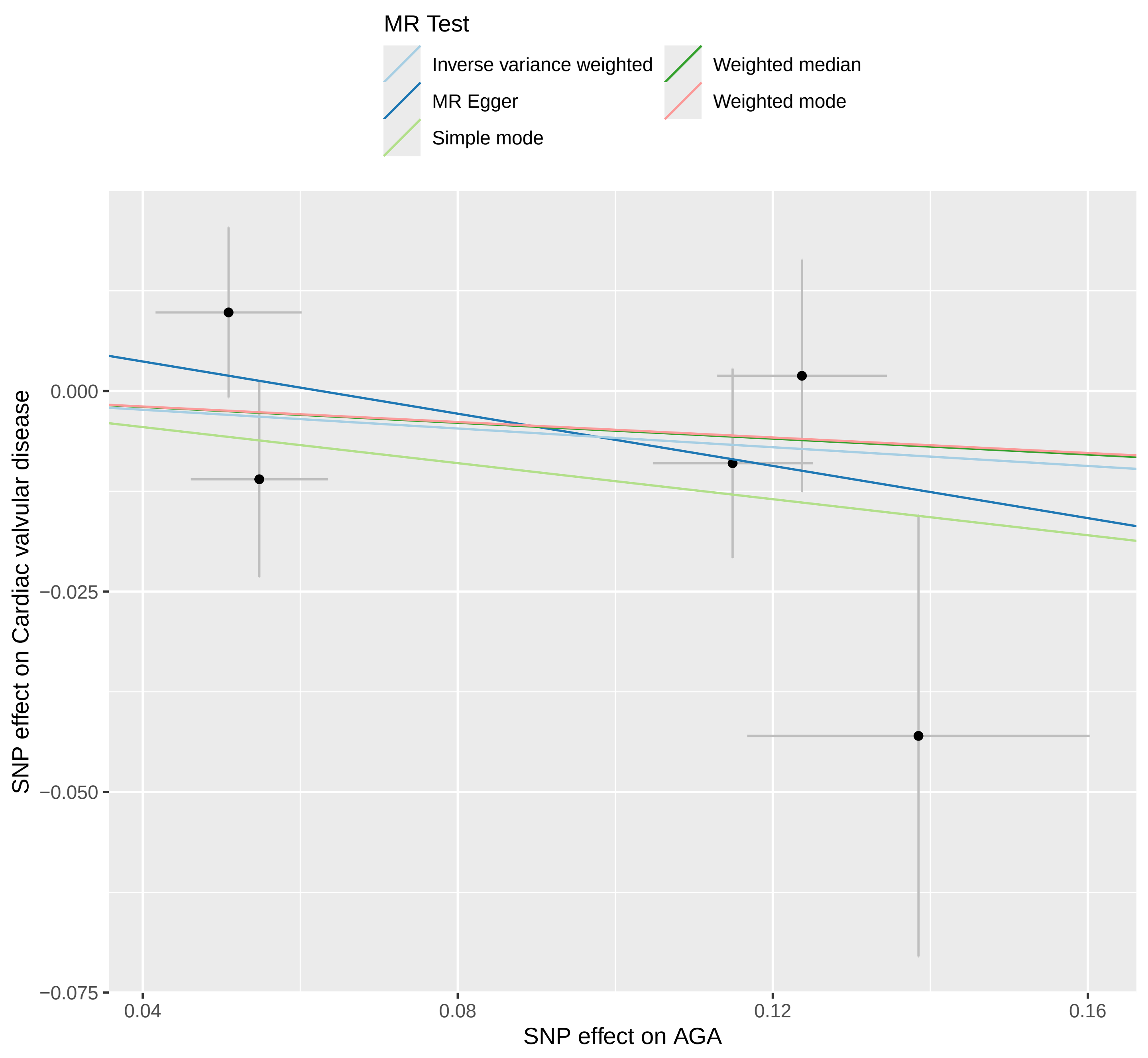

MR Test
Inverse variance weighted
MR Egger
Simple mode
Weighted median
Weighted mode
0.000
−0.025
−0.050
−0.075
0.04
0.08
0.12
0.16
SNP effect on Cardiac valvular disease
SNP effect on AGA

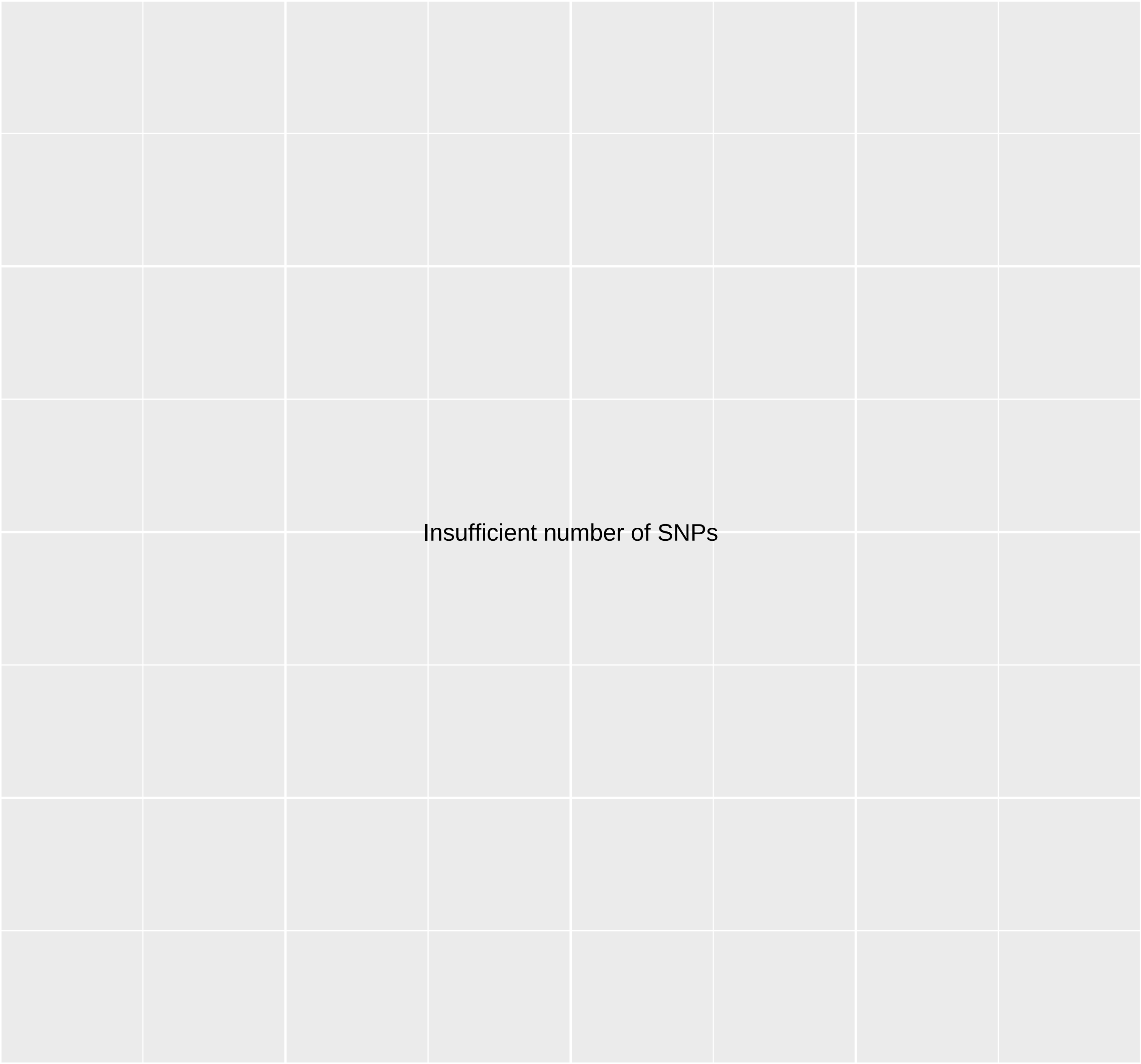
Insufficient number of SNPs

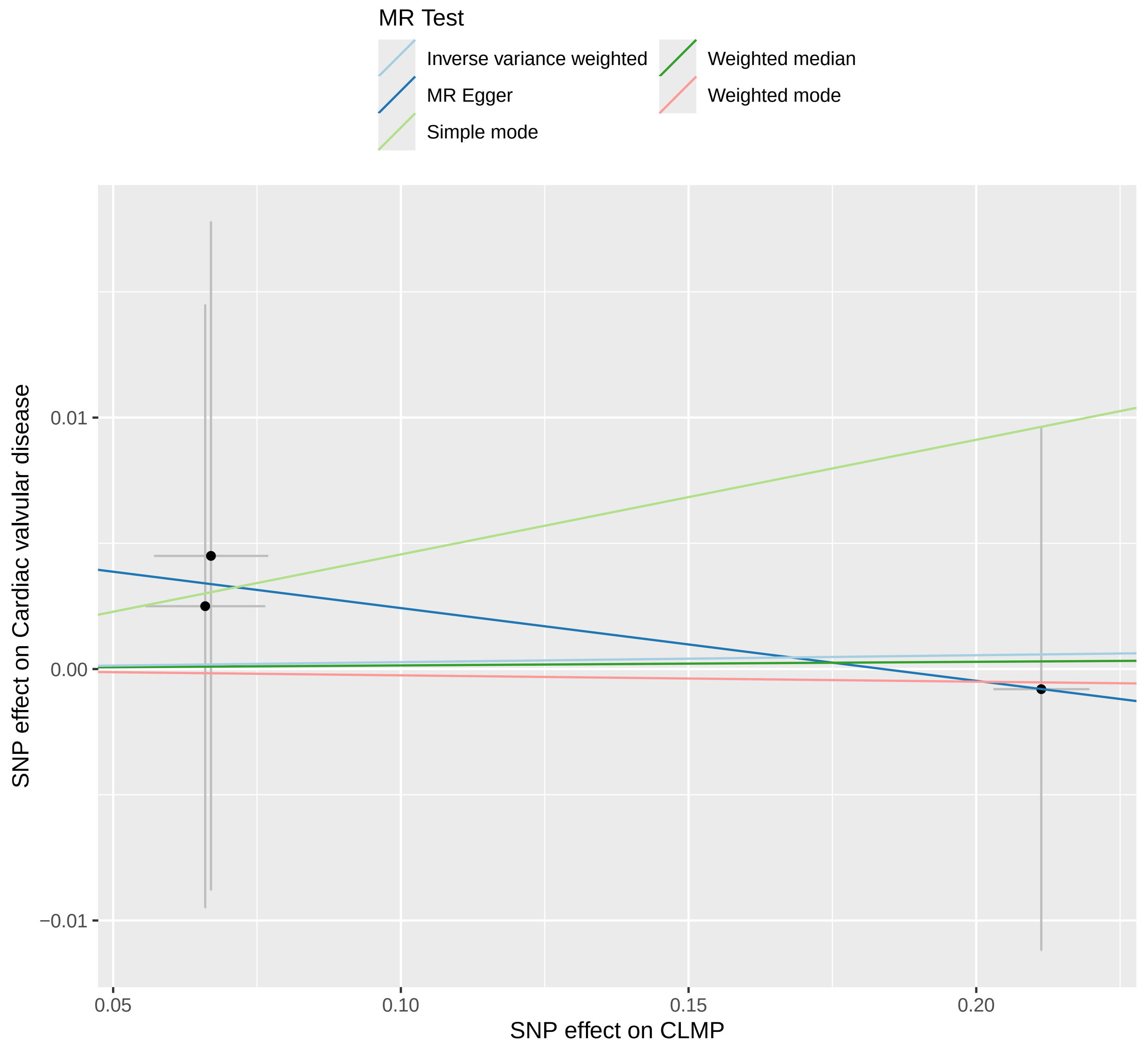
MR Test
Inverse variance weighted
MR Egger
Simple mode
Weighted median
Weighted mode
SNP effect on Cardiac valvular disease
0.01
0.00
−0.01
0.05
0.10
0.15
0.20
SNP effect on CLMP

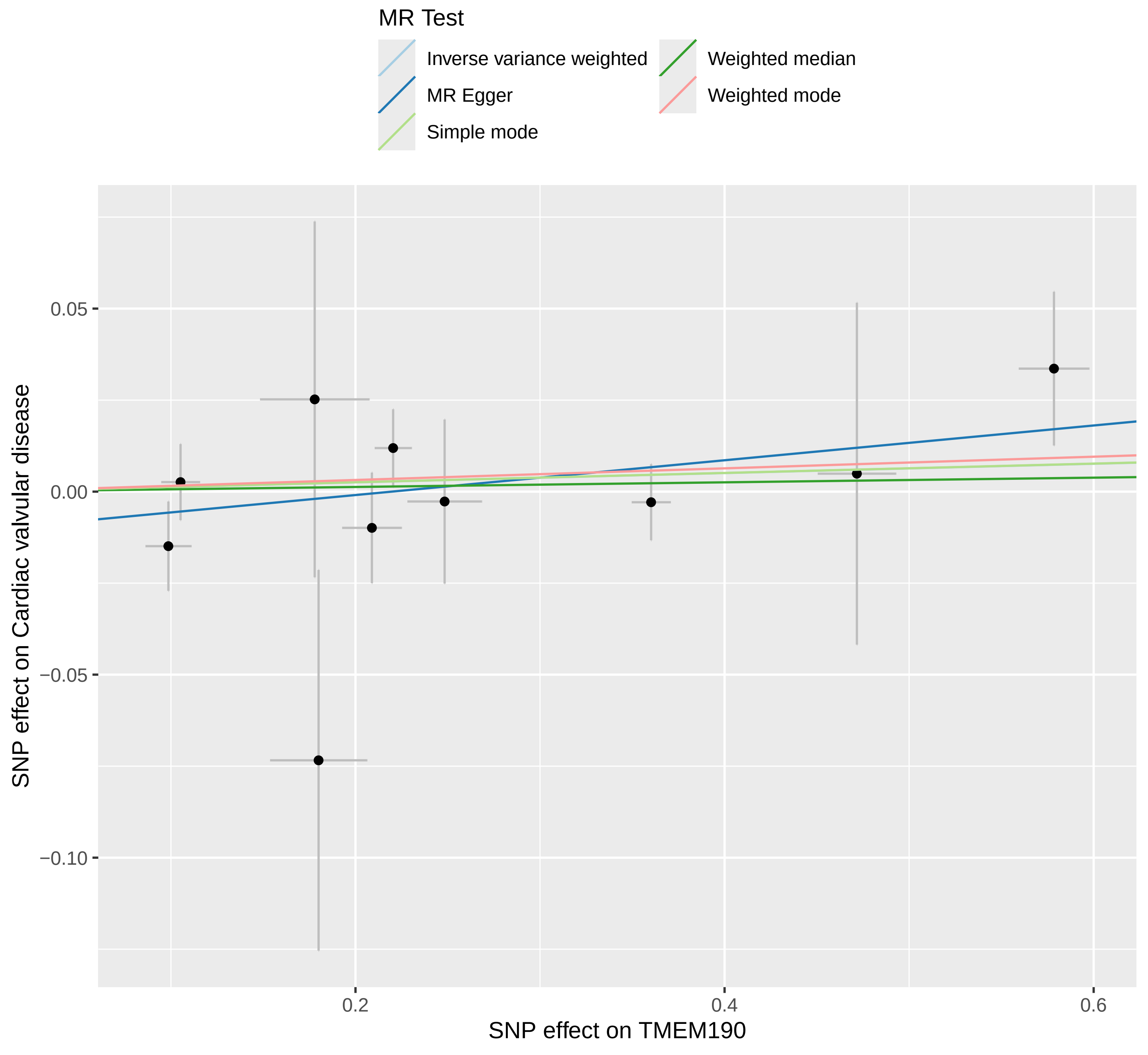
MR Test
Inverse variance weighted
MR Egger
Simple mode
Weighted median
Weighted mode
0.05
0.00
−0.05
−0.10
0.2
0.4
0.6
SNP effect on Cardiac valvular disease
SNP effect on TMEM190

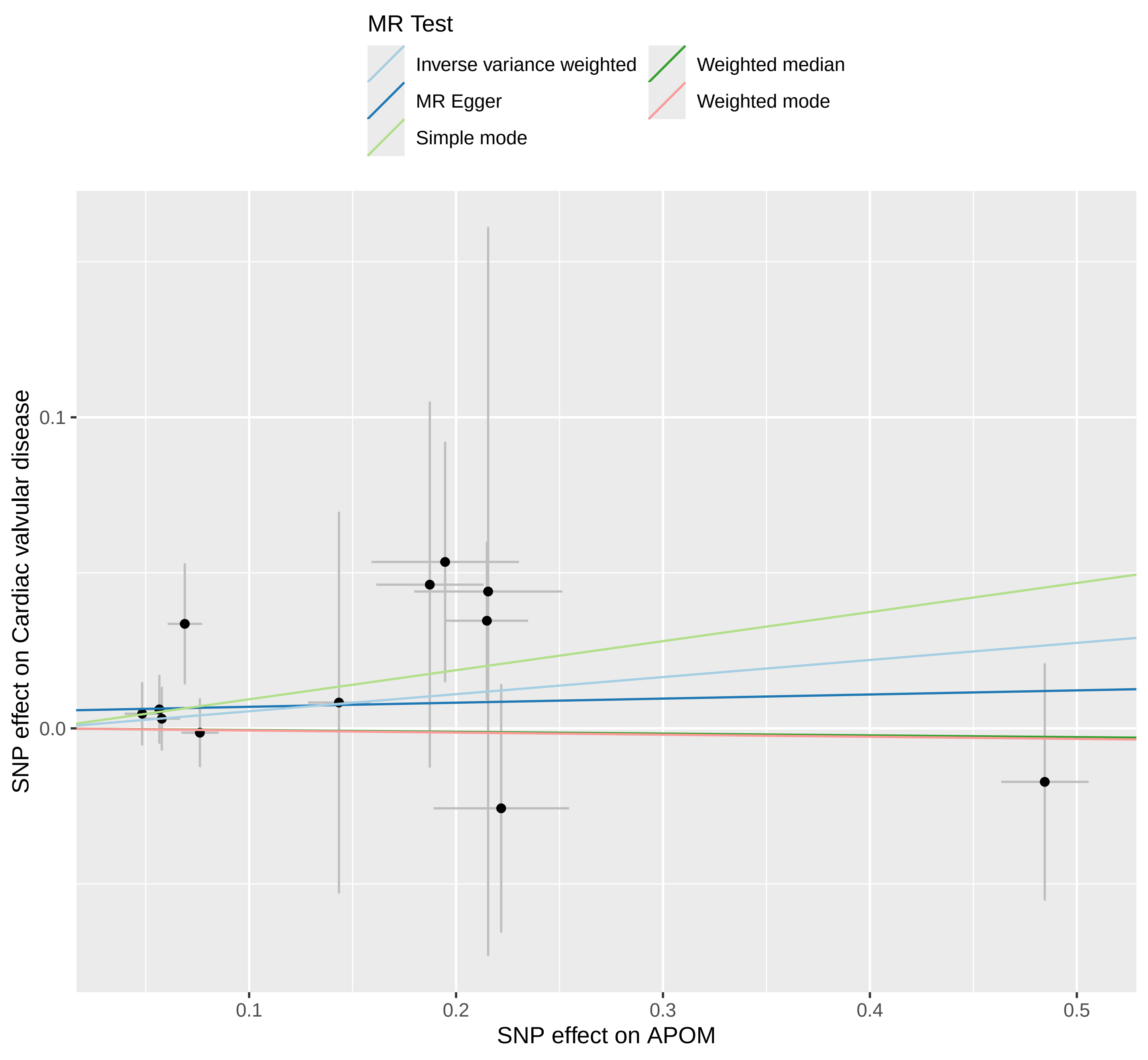
MR Test
Inverse variance weighted
MR Egger
Simple mode
Weighted median
Weighted mode
0.1
0.0
SNP effect on Cardiac valvular disease
0.1
0.2
0.3
0.4
0.5
SNP effect on APOM

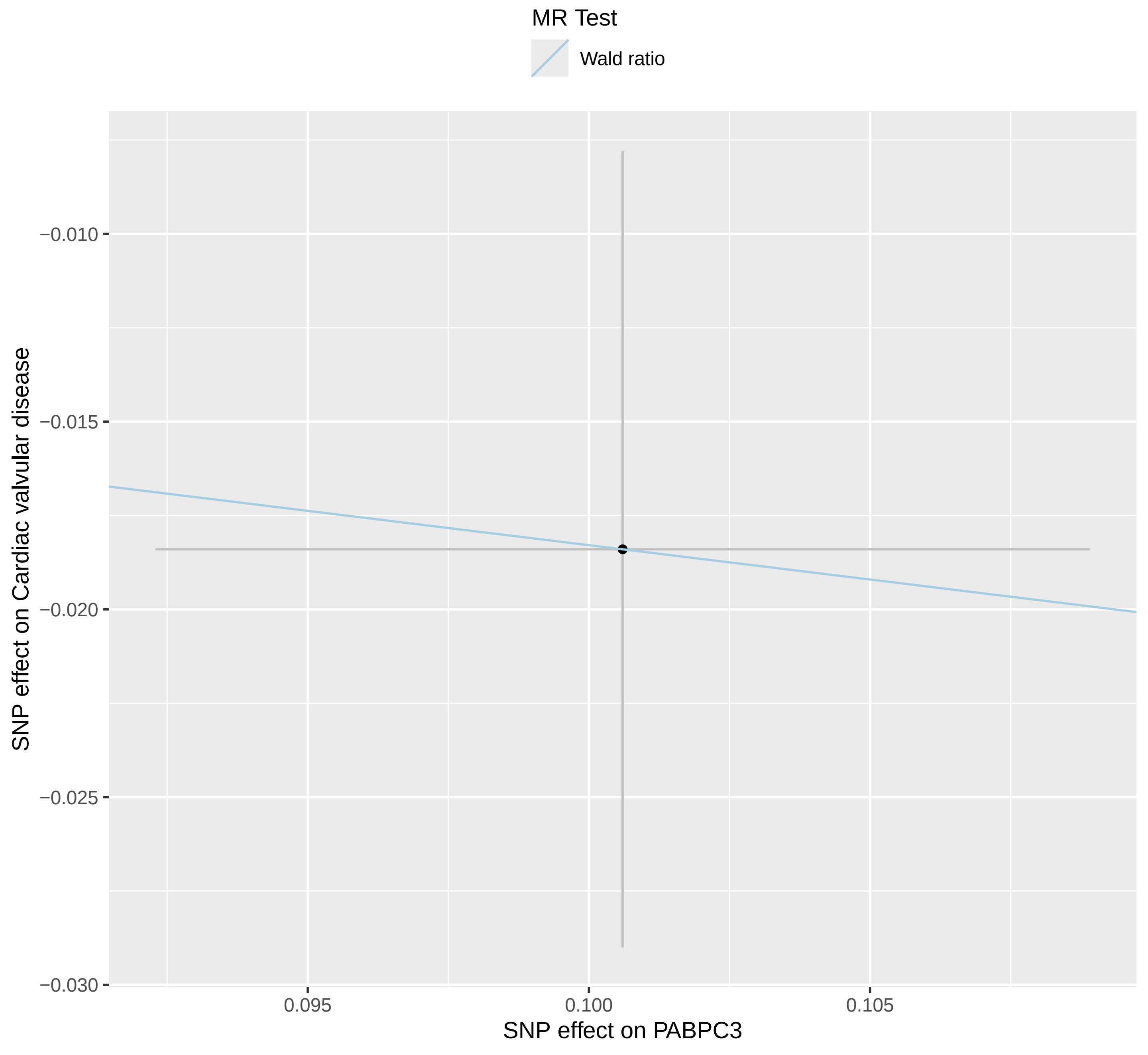

MR Test
Wald ratio
−0.010
−0.015
−0.020
−0.025
−0.030
0.095
0.100
0.105
SNP effect on Cardiac valvular disease
SNP effect on PABPC3

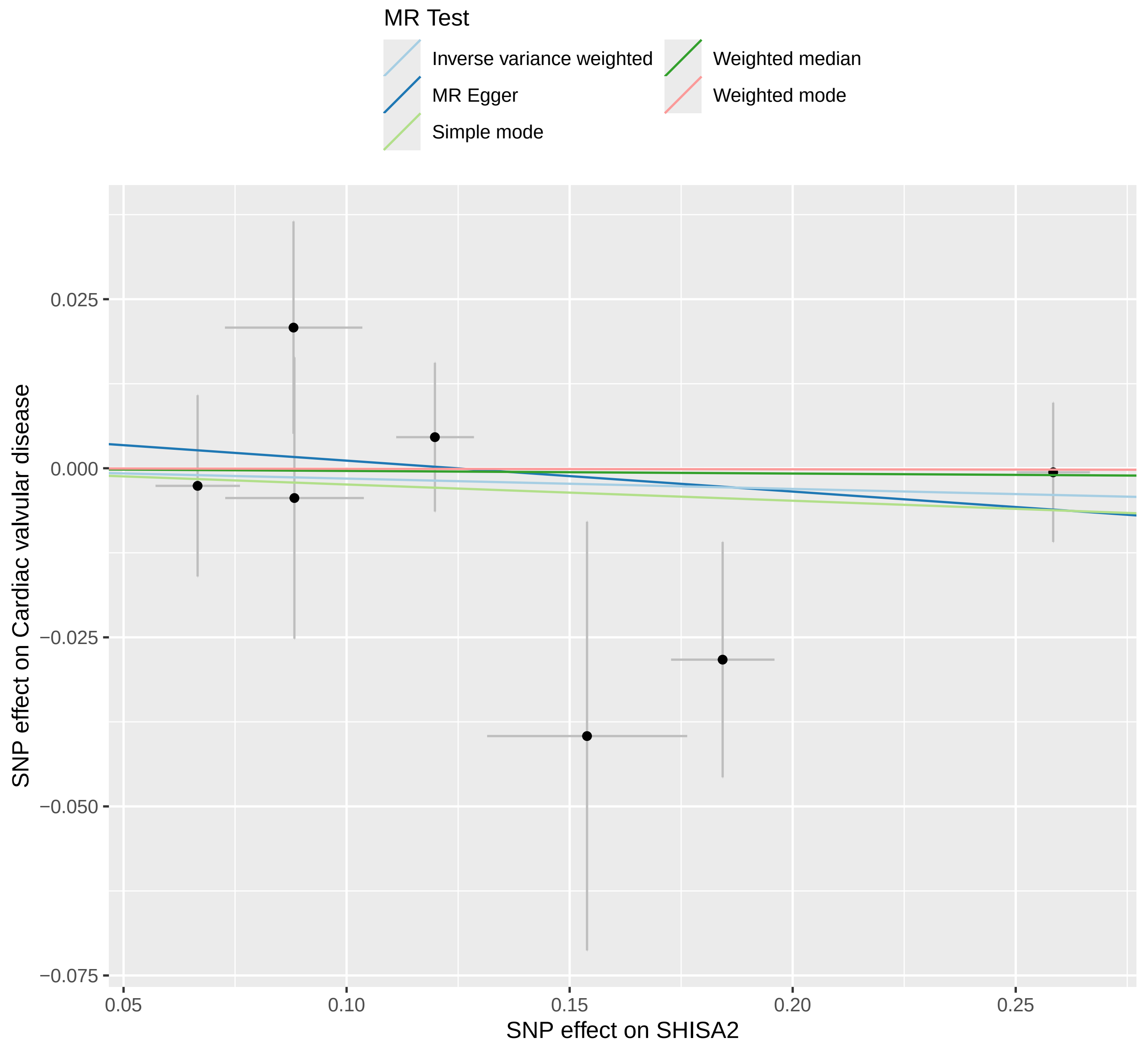

MR Test
Inverse variance weighted
MR Egger
Simple mode
Weighted median
Weighted mode
0.025
0.000
−0.025
−0.050
−0.075
0.05
0.10
0.15
0.20
0.25
SNP effect on Cardiac valvular disease
SNP effect on SHISA2

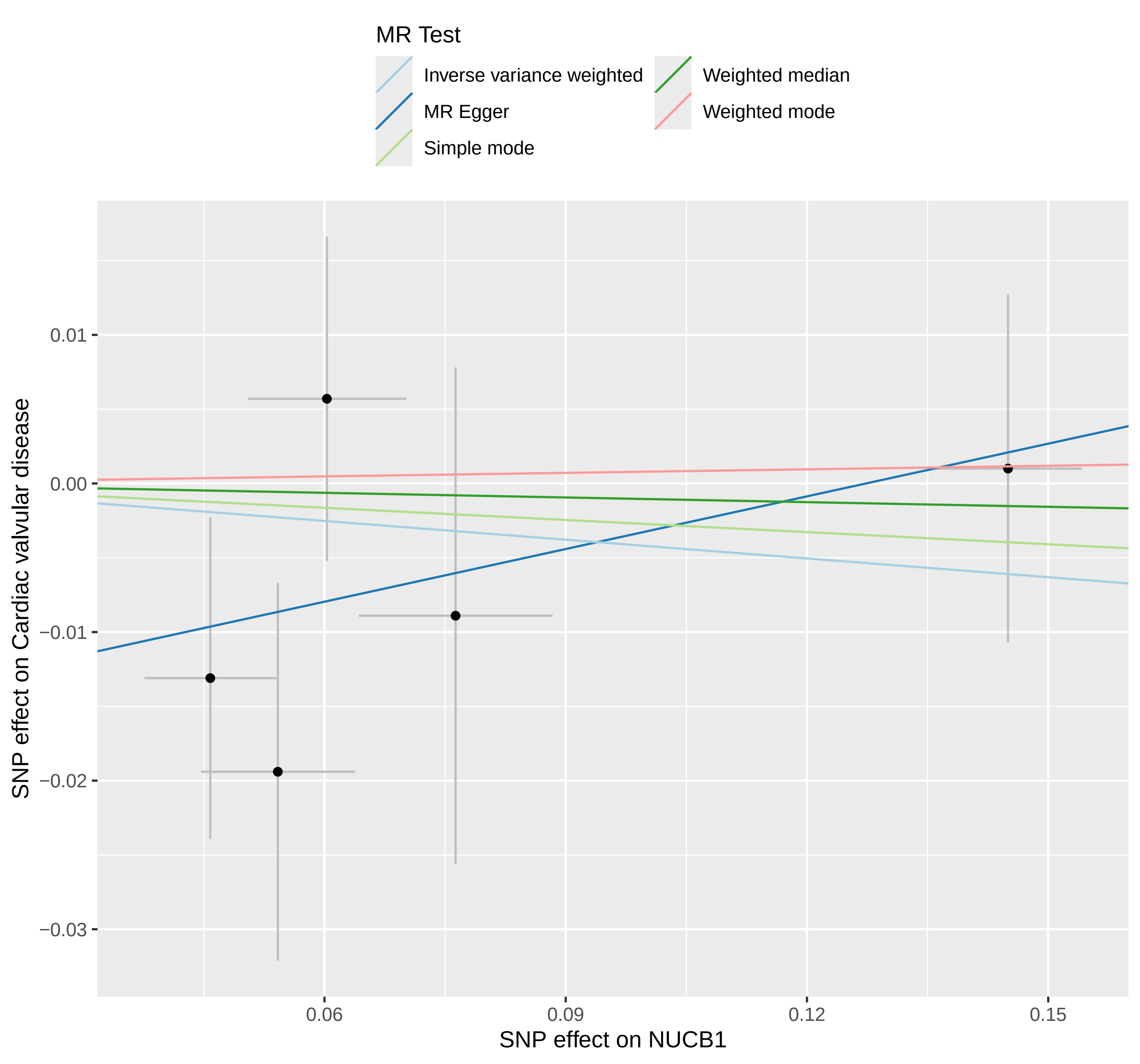
MR Test
Inverse variance weighted
MR Egger
Simple mode
Weighted median
Weighted mode
SNP effect on Cardiac valvular disease
0.01
0.00
−0.01
−0.02
−0.03
0.06
0.09
0.12
0.15
SNP effect on NUCB1

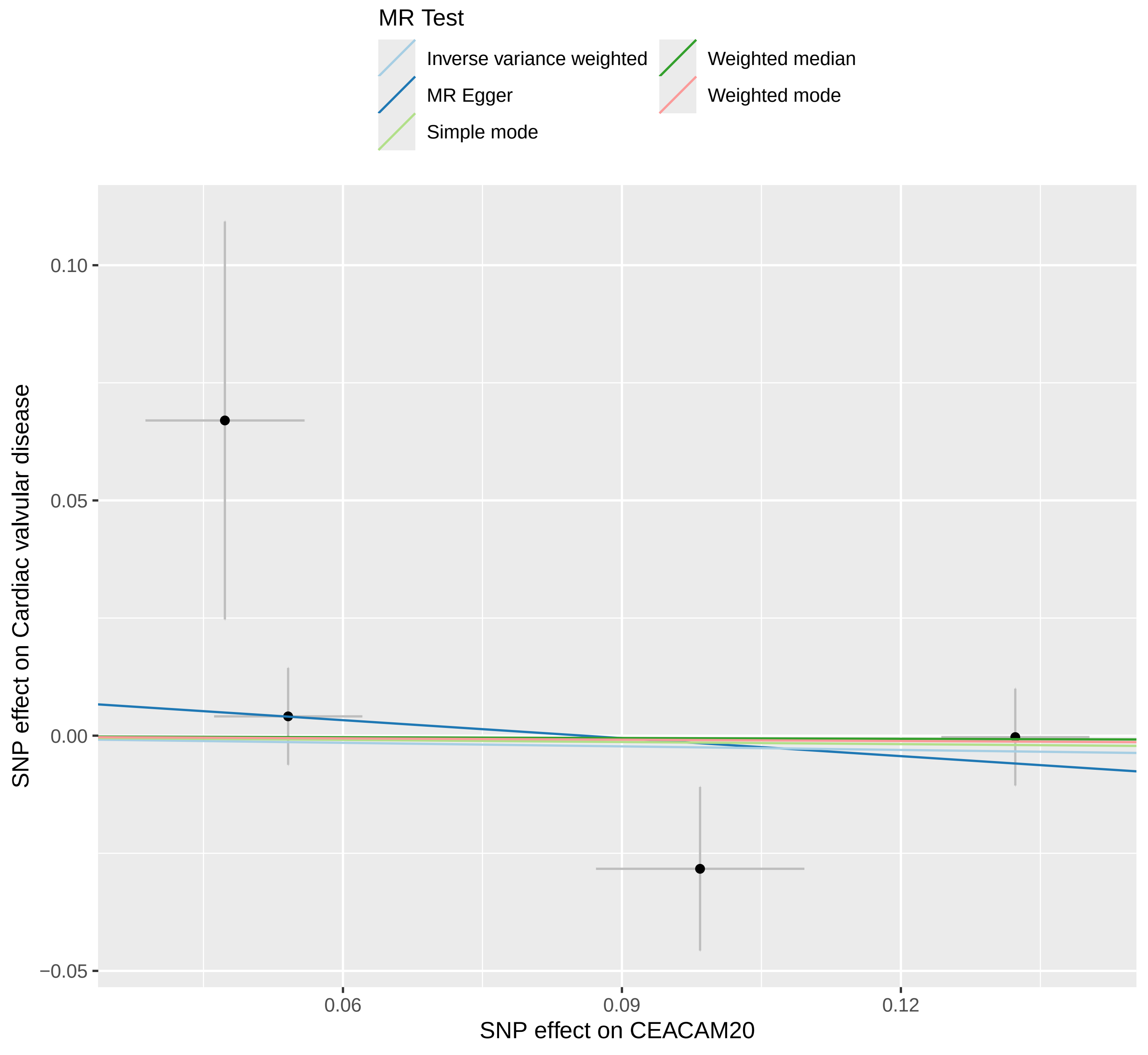

MR Test
Inverse variance weighted
MR Egger
Simple mode
Weighted median
Weighted mode
0.10
0.05
0.00
−0.05
0.06
0.09
0.12
SNP effect on Cardiac valvular disease
SNP effect on CEACAM20

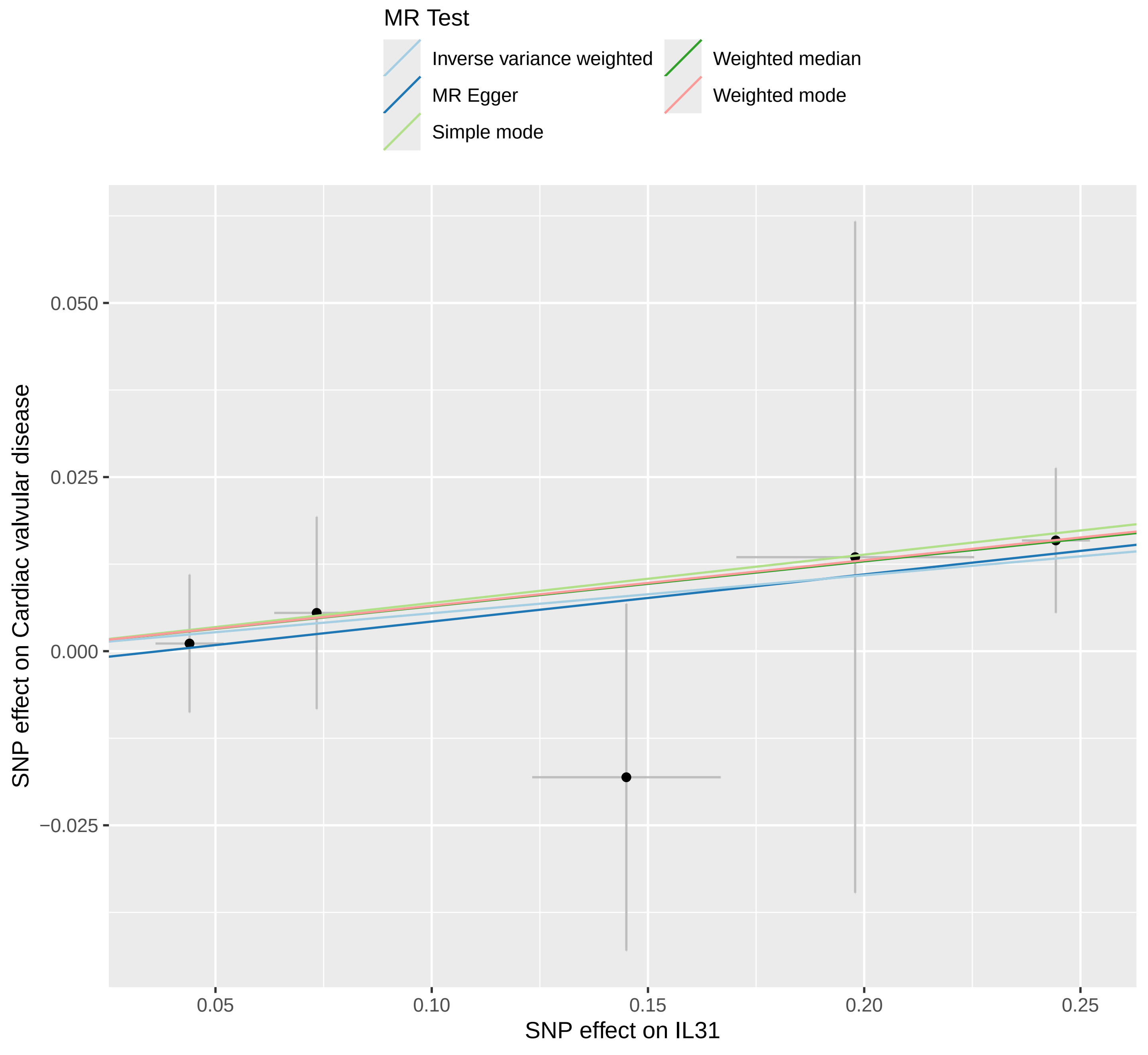
MR Test
Inverse variance weighted
MR Egger
Simple mode
Weighted median
Weighted mode
0.050
0.025
0.000
−0.025
SNP effect on Cardiac valvular disease
0.05
0.10
0.15
0.20
0.25
SNP effect on IL31

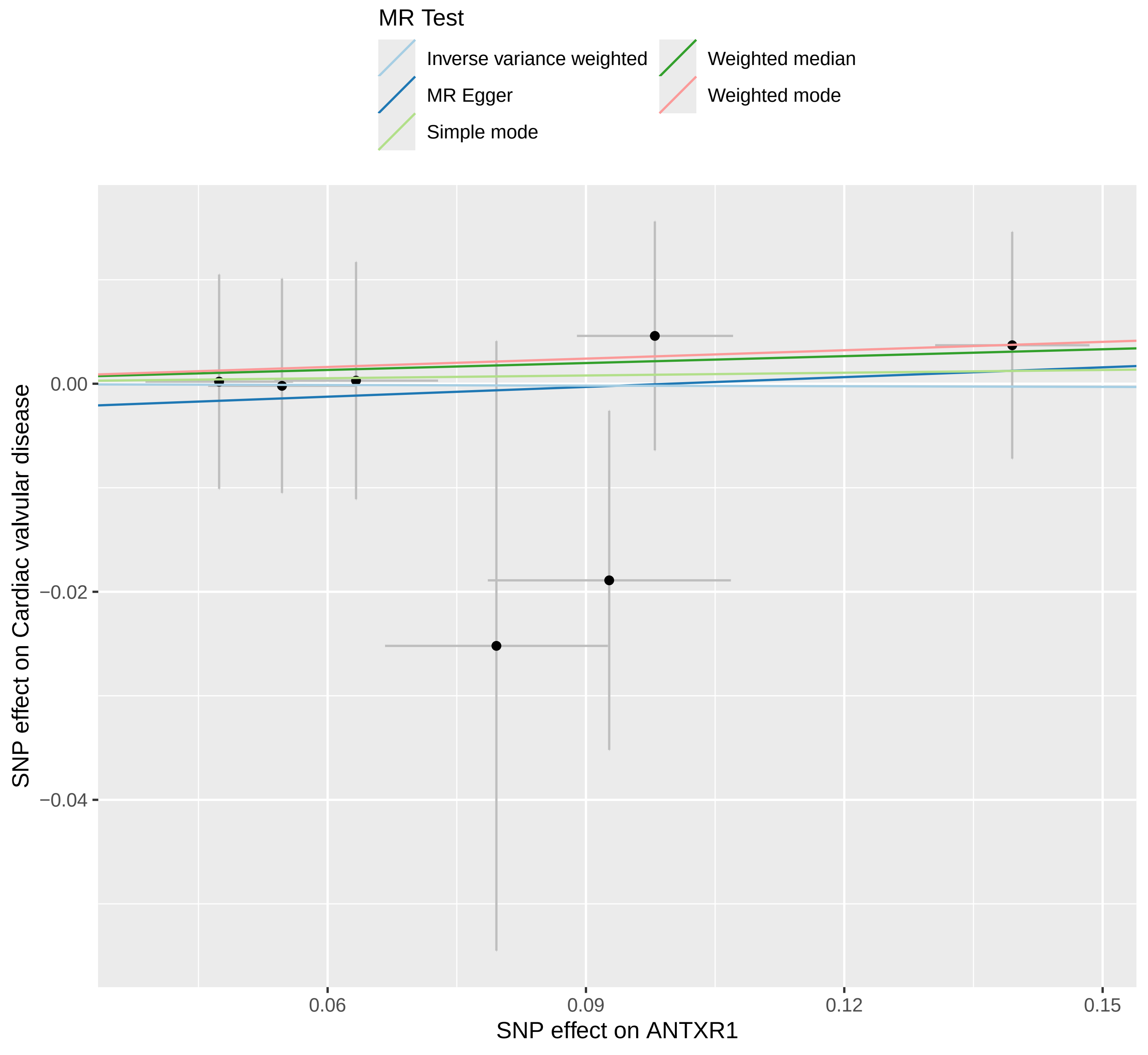
MR Test
Inverse variance weighted
MR Egger
Simple mode
Weighted median
Weighted mode
SNP effect on Cardiac valvular disease
0.00
−0.02
−0.04
0.06
0.09
0.12
0.15
SNP effect on ANTXR1

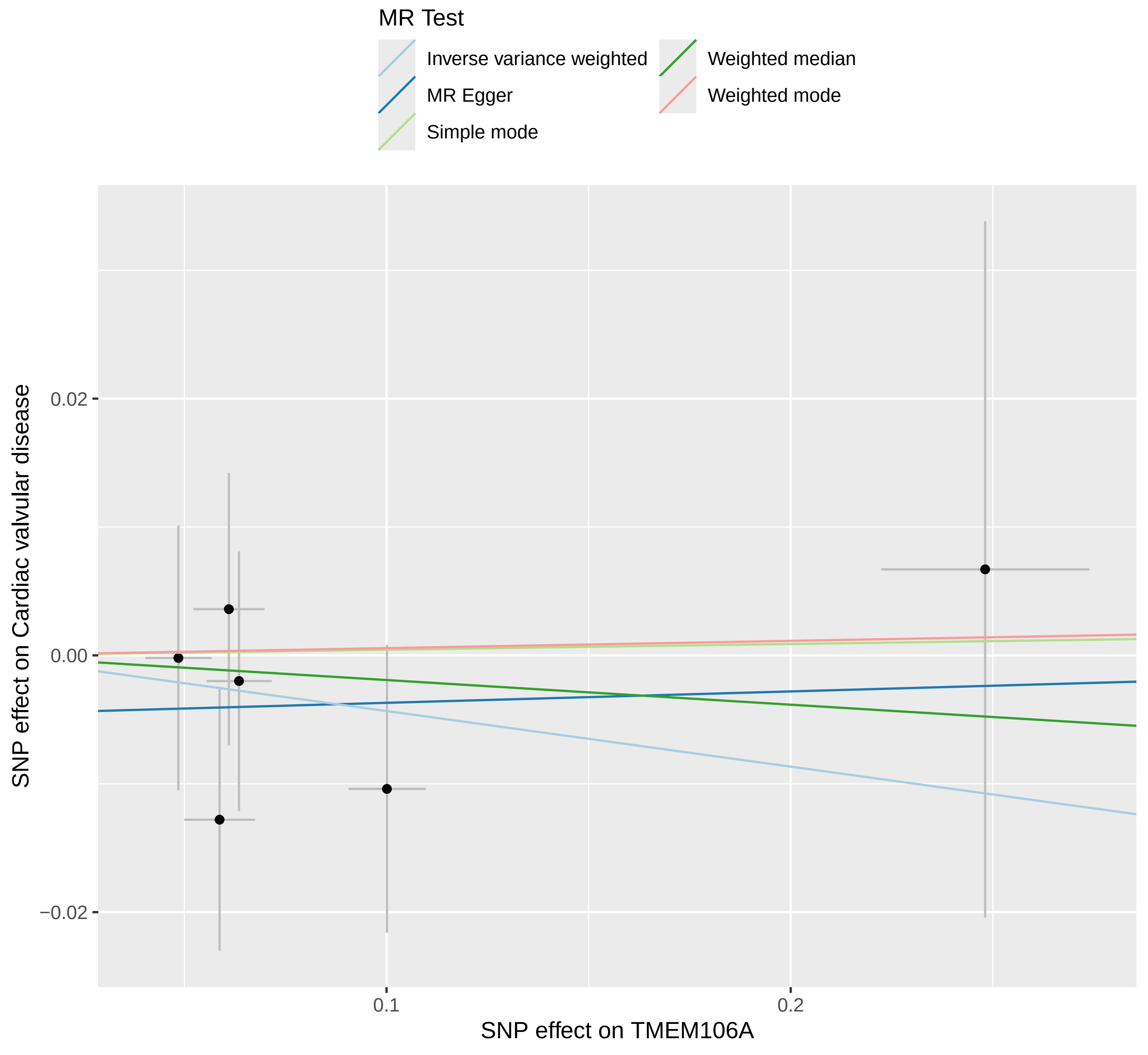
MR Test
Inverse variance weighted
MR Egger
Simple mode
Weighted median
Weighted mode
0.02
0.00
−0.02
0.1
0.2
SNP effect on Cardiac valvular disease
SNP effect on TMEM106A

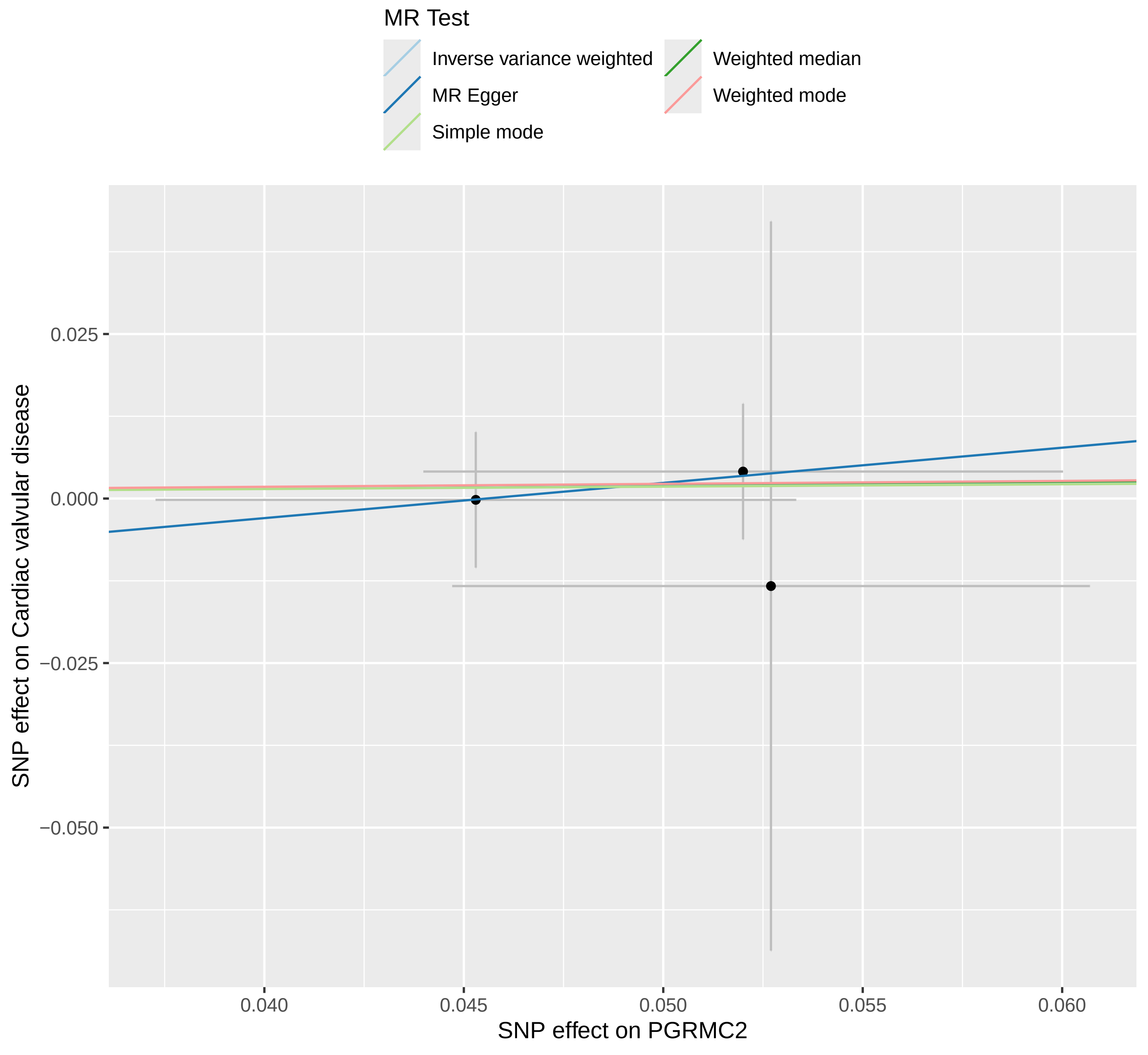

MR Test
Inverse variance weighted
MR Egger
Simple mode
Weighted median
Weighted mode
0.025
0.000
−0.025
−0.050
0.040
0.045
0.050
0.055
0.060
SNP effect on PGRMC2
SNP effect on Cardiac valvular disease

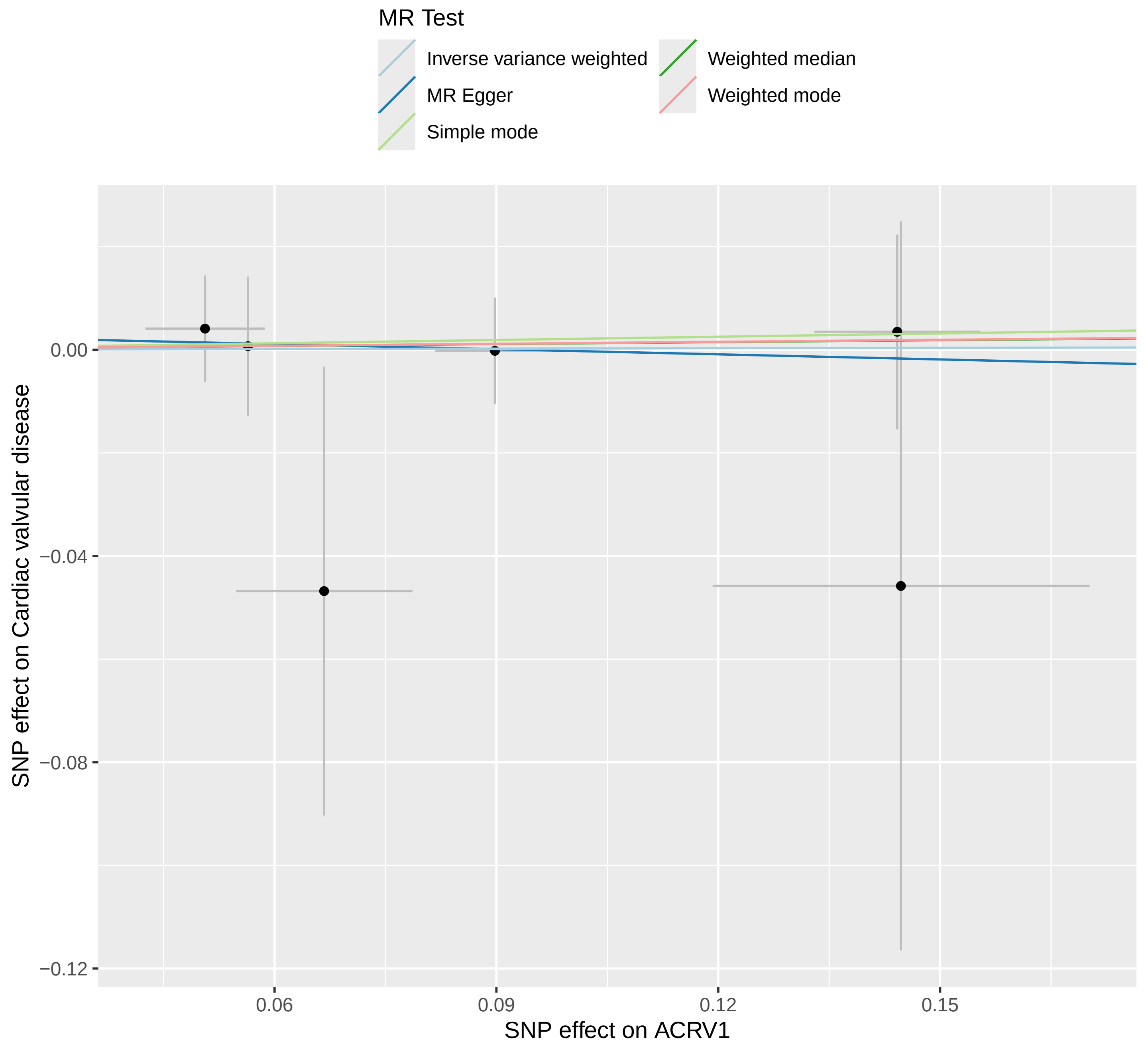
MR Test
Inverse variance weighted
MR Egger
Simple mode
Weighted median
Weighted mode
0.00
−0.04
−0.08
−0.12
SNP effect on Cardiac valvular disease
0.06
0.09
0.12
0.15
SNP effect on ACRV1

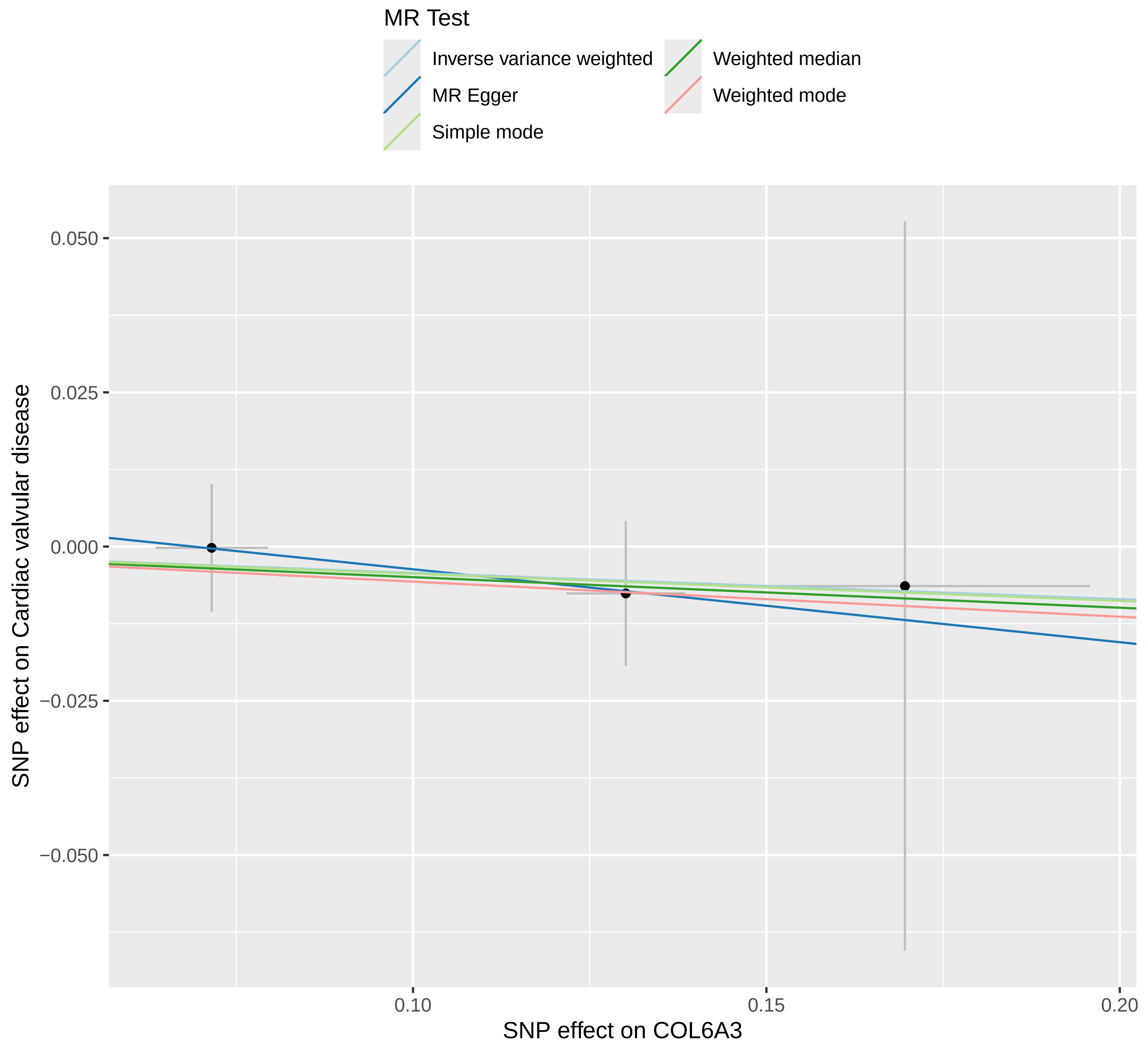
MR Test
Inverse variance weighted
MR Egger
Simple mode
Weighted median
Weighted mode
0.050
0.025
0.000
−0.025
−0.050
0.10
0.15
0.20
SNP effect on COL6A3
SNP effect on Cardiac valvular disease

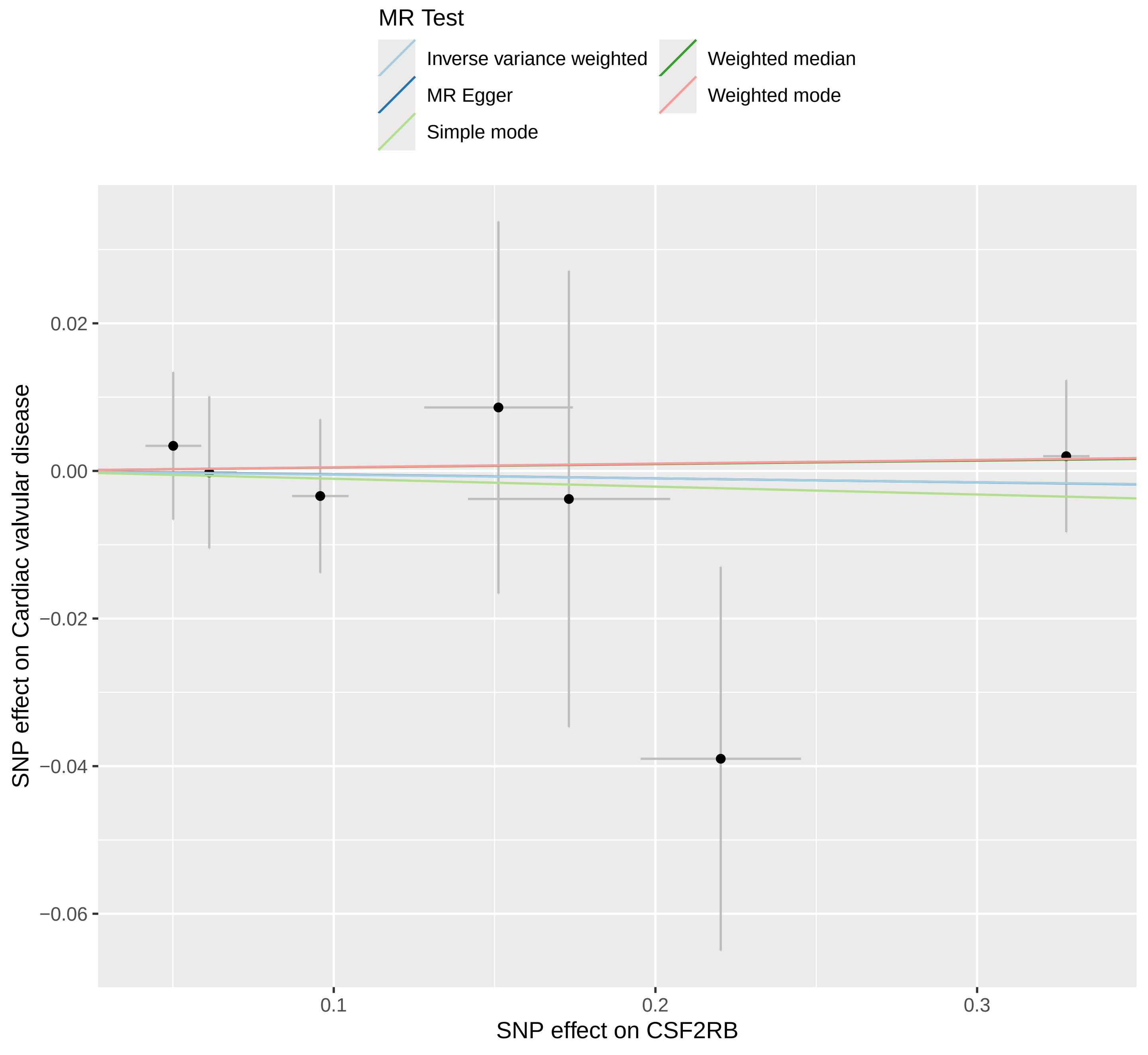
MR Test
Inverse variance weighted
MR Egger
Simple mode
Weighted median
Weighted mode
SNP effect on Cardiac valvular disease
0.02
0.00
−0.02
−0.04
−0.06
0.1
0.2
0.3
SNP effect on CSF2RB

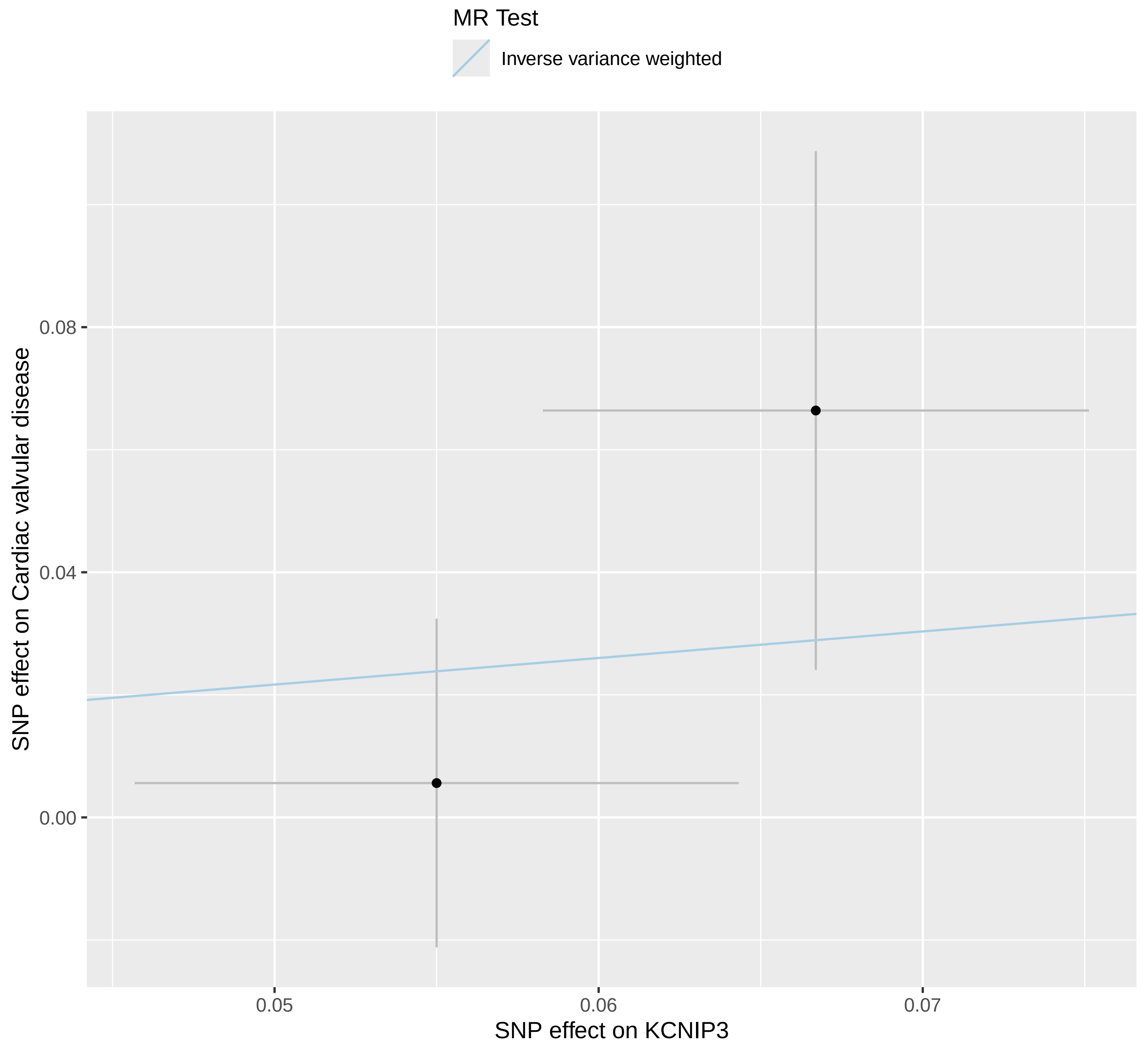

MR Test
Inverse variance weighted
0.08
0.04
0.00
0.05
0.06
0.07
SNP effect on Cardiac valvular disease
SNP effect on KCNIP3

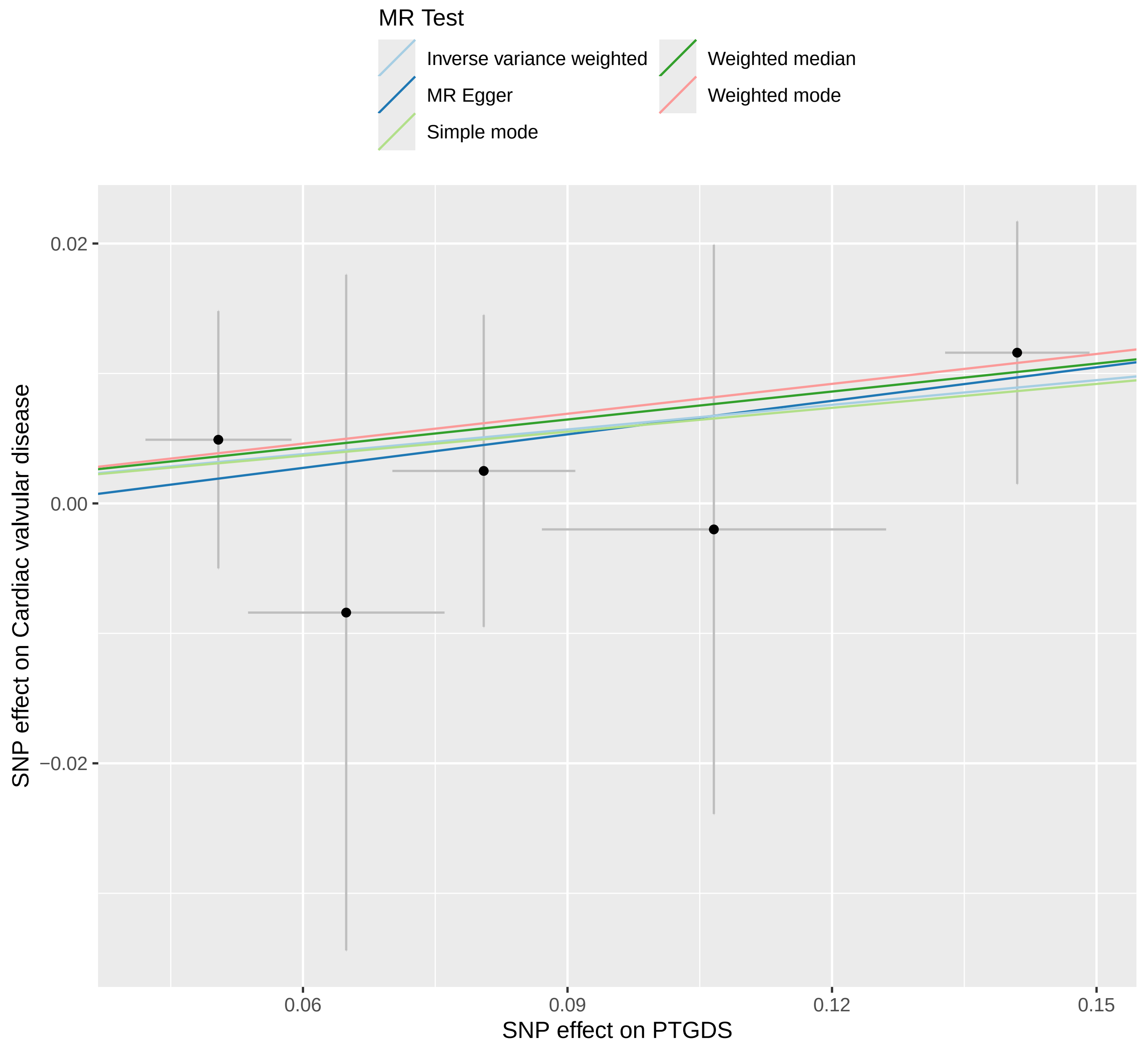

MR Test
Inverse variance weighted
MR Egger
Simple mode
Weighted median
Weighted mode
0.02
0.00
−0.02
0.06
0.09
0.12
0.15
SNP effect on PTGDS
SNP effect on Cardiac valvular disease

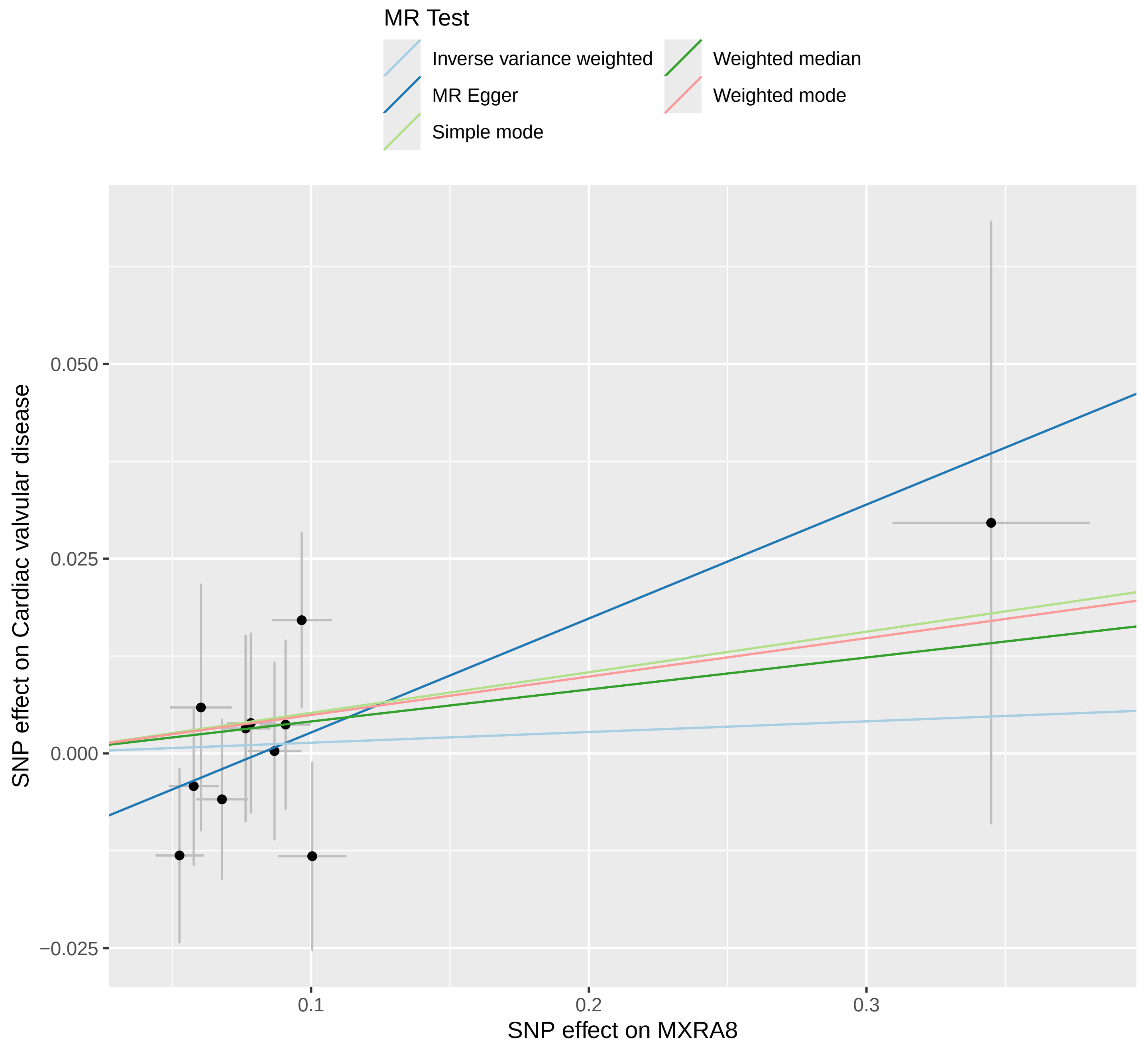
MR Test
Inverse variance weighted
MR Egger
Simple mode
Weighted median
Weighted mode
SNP effect on Cardiac valvular disease
0.050
0.025
0.000
−0.025
0.1
0.2
0.3
SNP effect on MXRA8

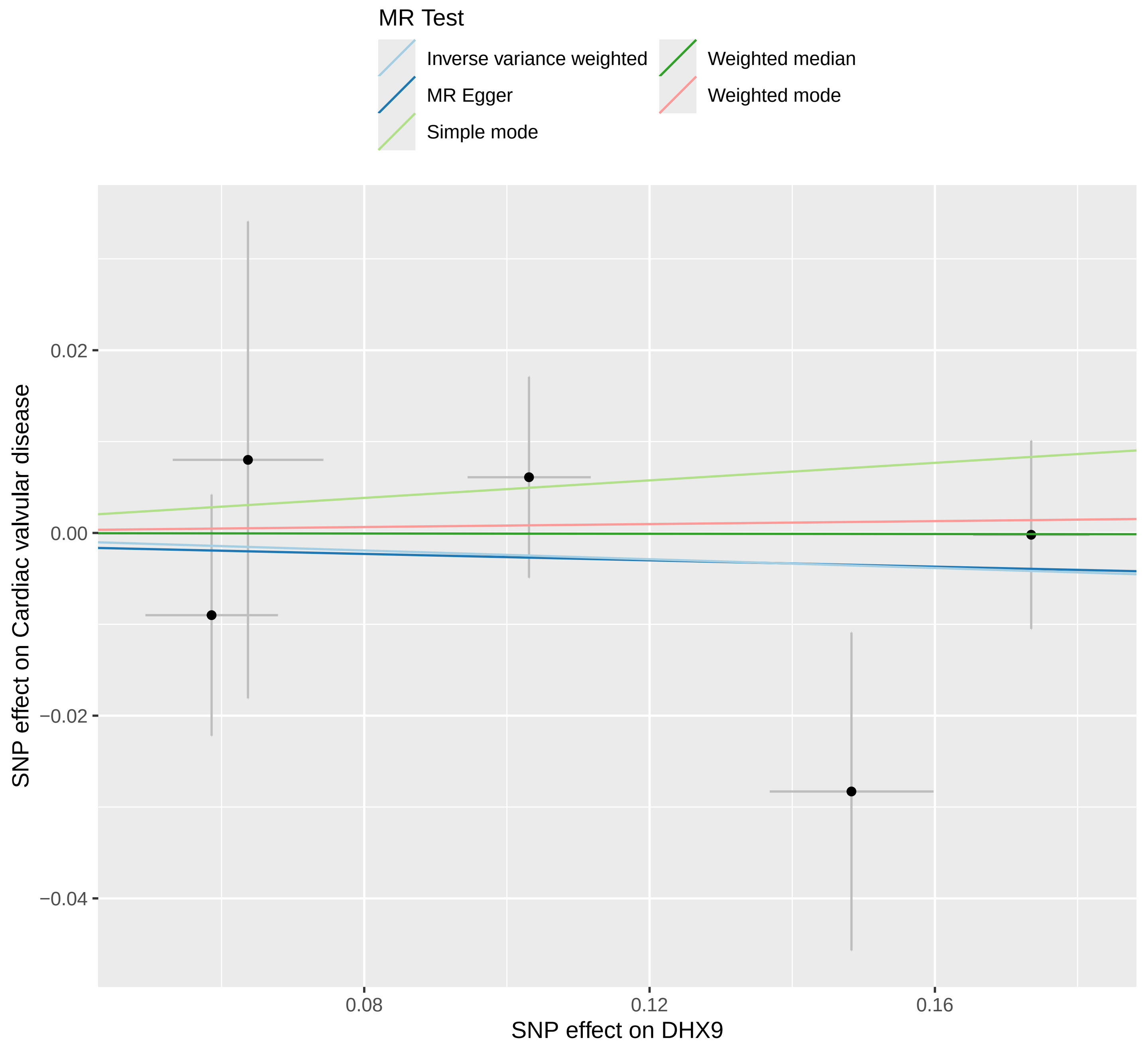

MR Test
Inverse variance weighted
MR Egger
Simple mode
Weighted median
Weighted mode
0.02
0.00
−0.02
−0.04
0.08
0.12
0.16
SNP effect on Cardiac valvular disease
SNP effect on DHX9

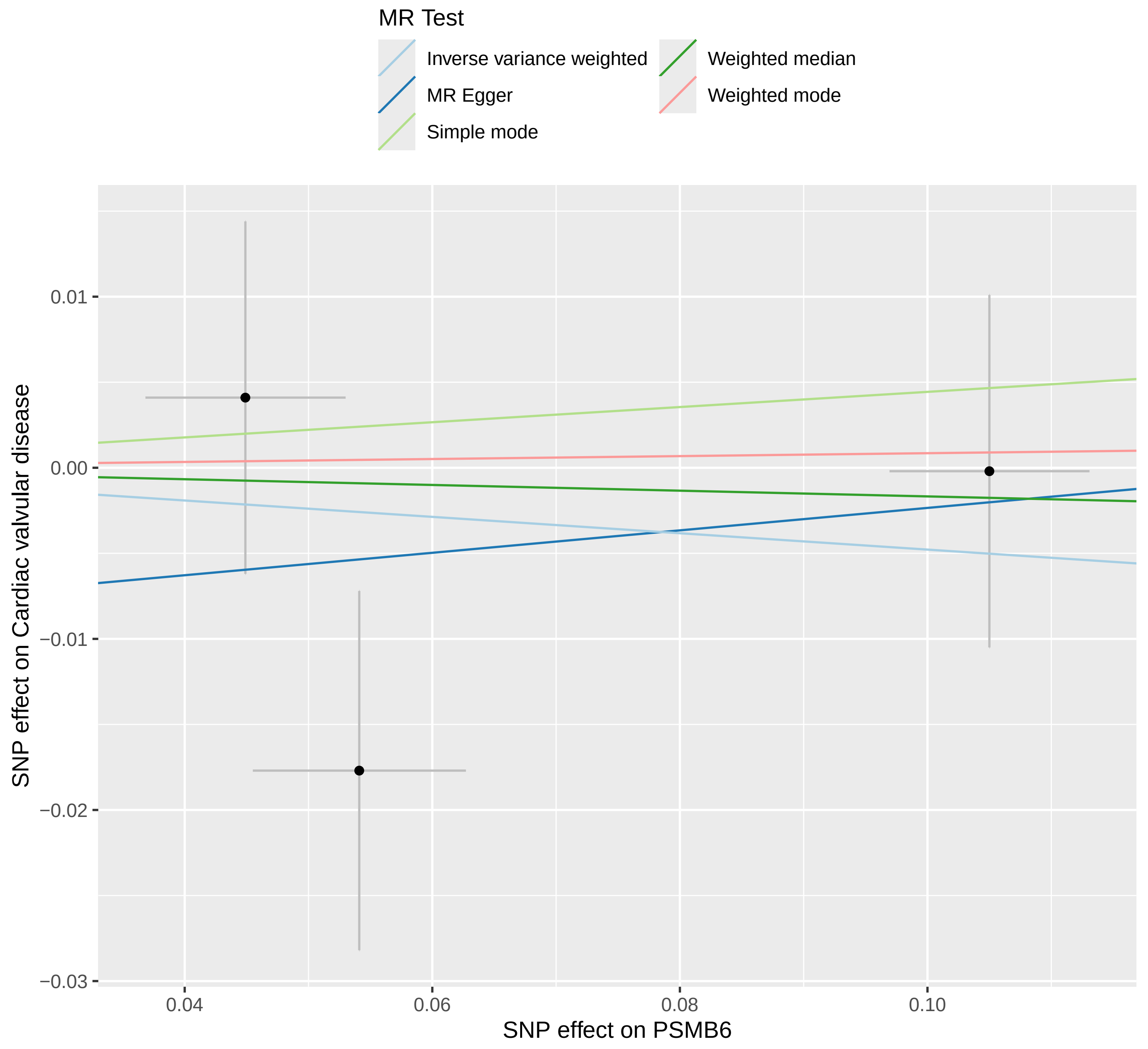

MR Test
Inverse variance weighted
MR Egger
Simple mode
Weighted median
Weighted mode
SNP effect on Cardiac valvular disease
0.01
0.00
−0.01
−0.02
−0.03
0.04
0.06
0.08
0.10
SNP effect on PSMB6

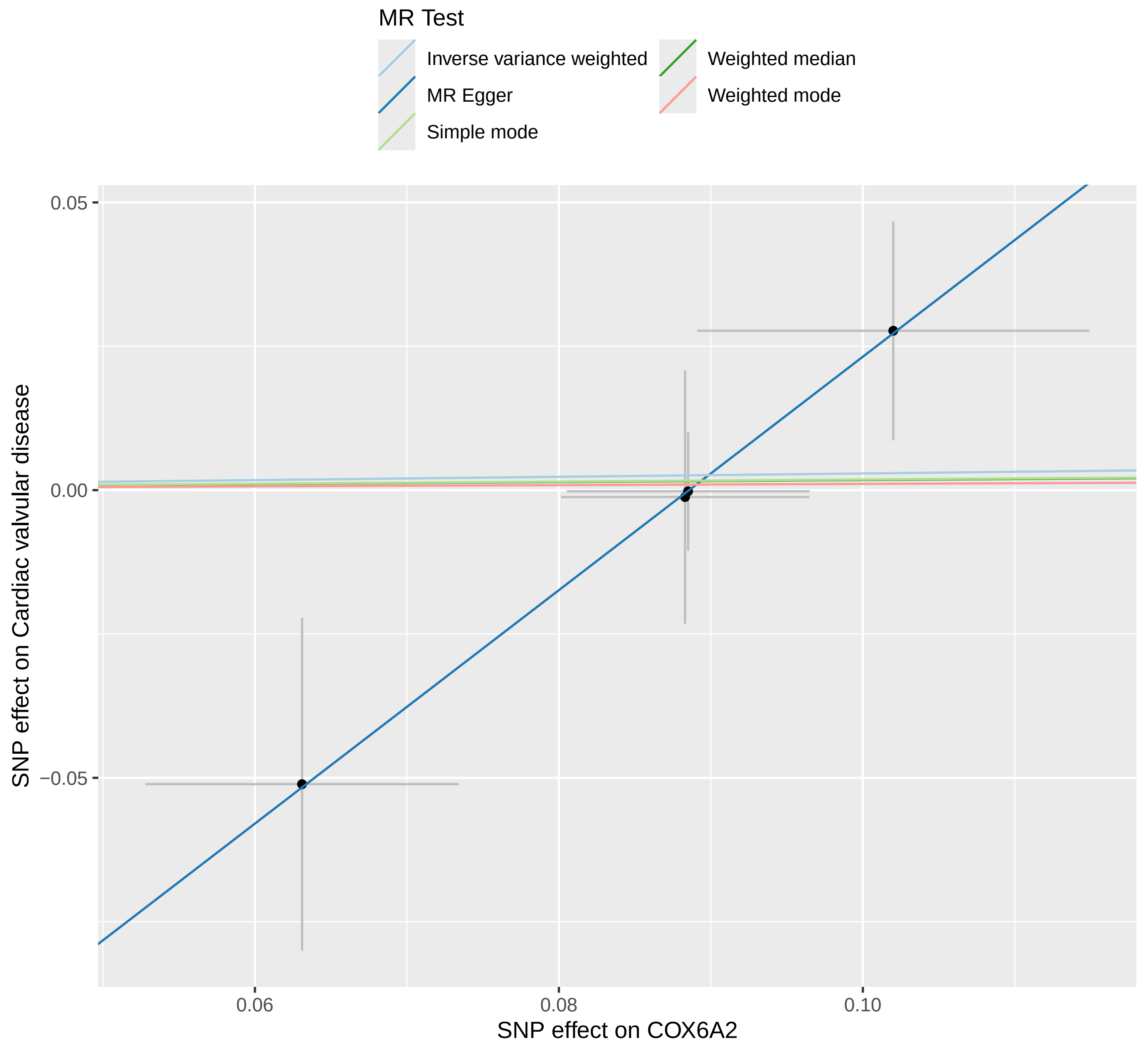

MR Test
Inverse variance weighted
MR Egger
Simple mode
Weighted median
Weighted mode
0.05
0.00
−0.05
SNP effect on Cardiac valvular disease
0.06
0.08
0.10
SNP effect on COX6A2

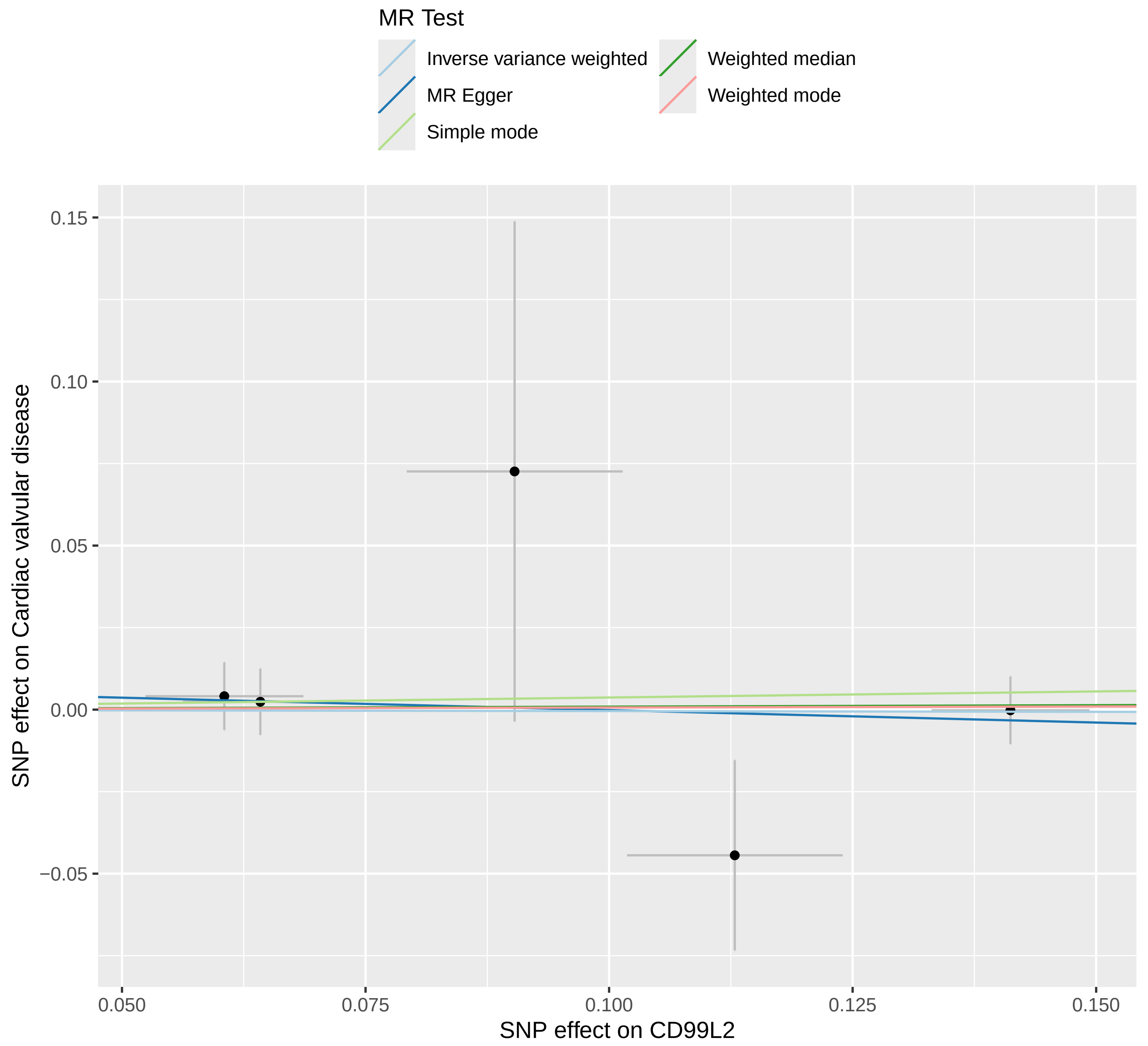

MR Test
Inverse variance weighted
MR Egger
Simple mode
Weighted median
Weighted mode
0.15
0.10
0.05
0.00
−0.05
0.050
0.075
0.100
0.125
0.150
SNP effect on CD99L2
SNP effect on Cardiac valvular disease

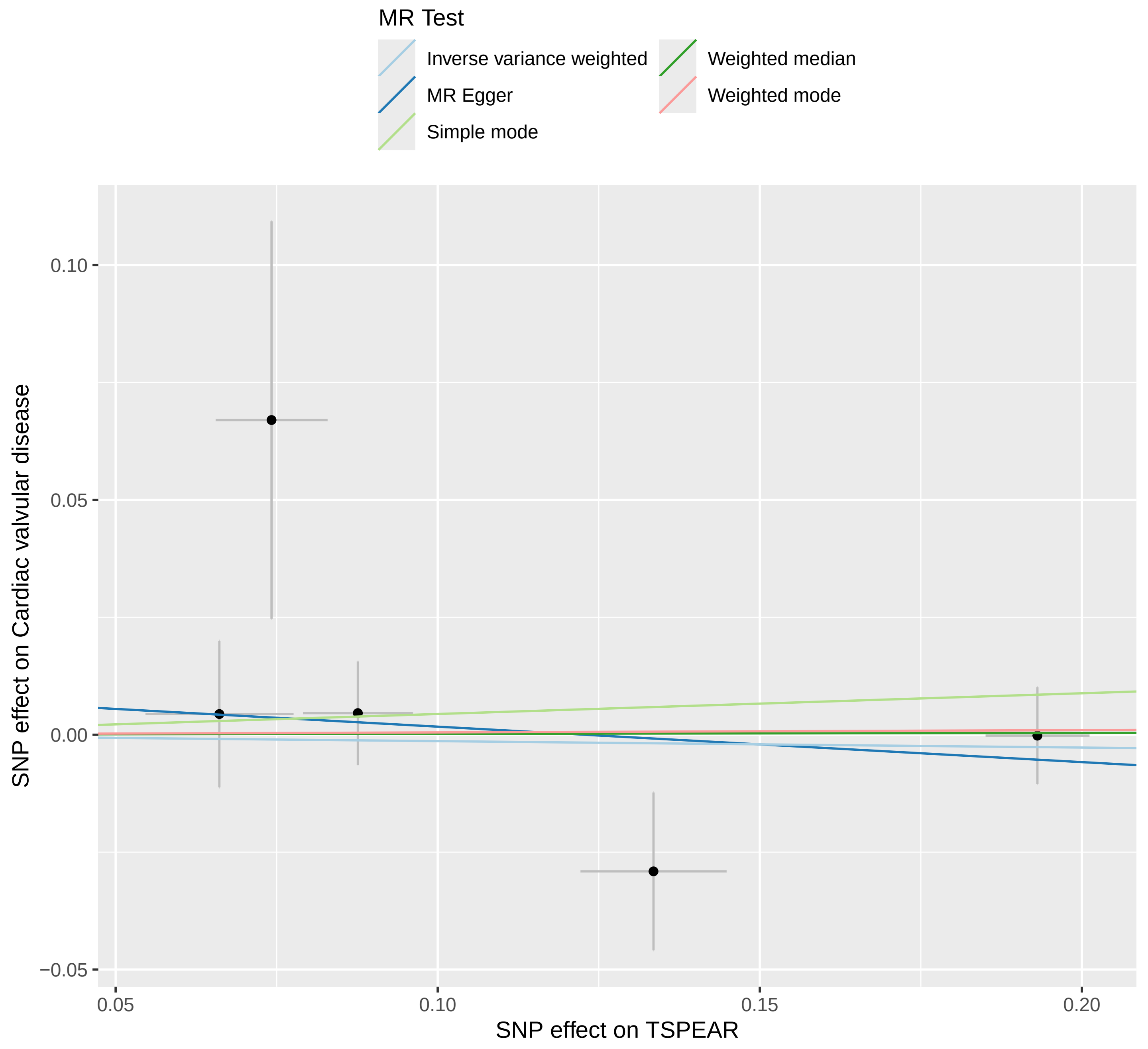
MR Test
Inverse variance weighted
MR Egger
Simple mode
Weighted median
Weighted mode
0.10
0.05
0.00
−0.05
0.05
0.10
0.15
0.20
SNP effect on Cardiac valvular disease
SNP effect on TSPEAR

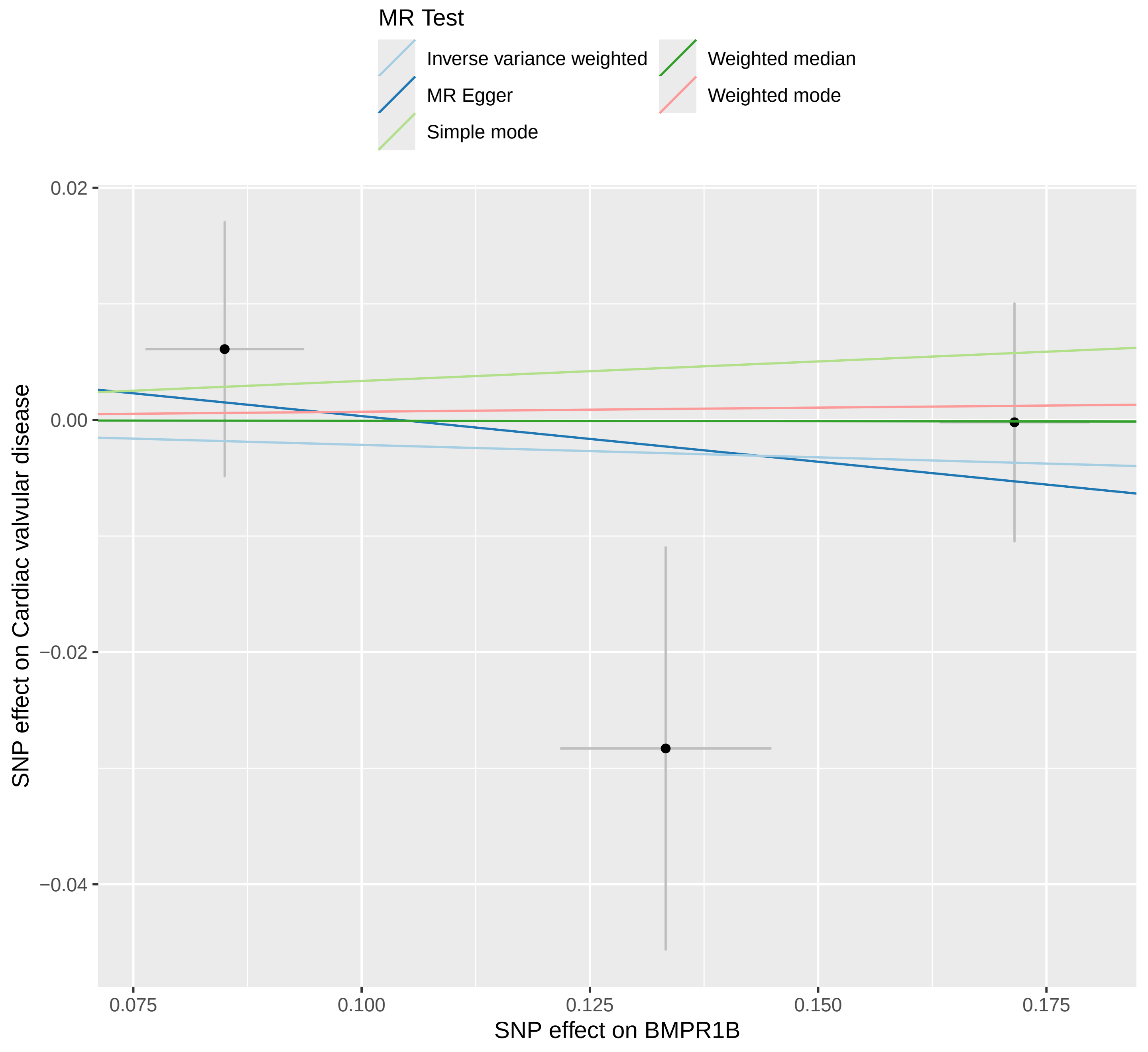

MR Test
Inverse variance weighted
MR Egger
Simple mode
Weighted median
Weighted mode
0.02
0.00
−0.02
−0.04
SNP effect on Cardiac valvular disease
0.075
0.100
0.125
0.150
0.175
SNP effect on BMPR1B

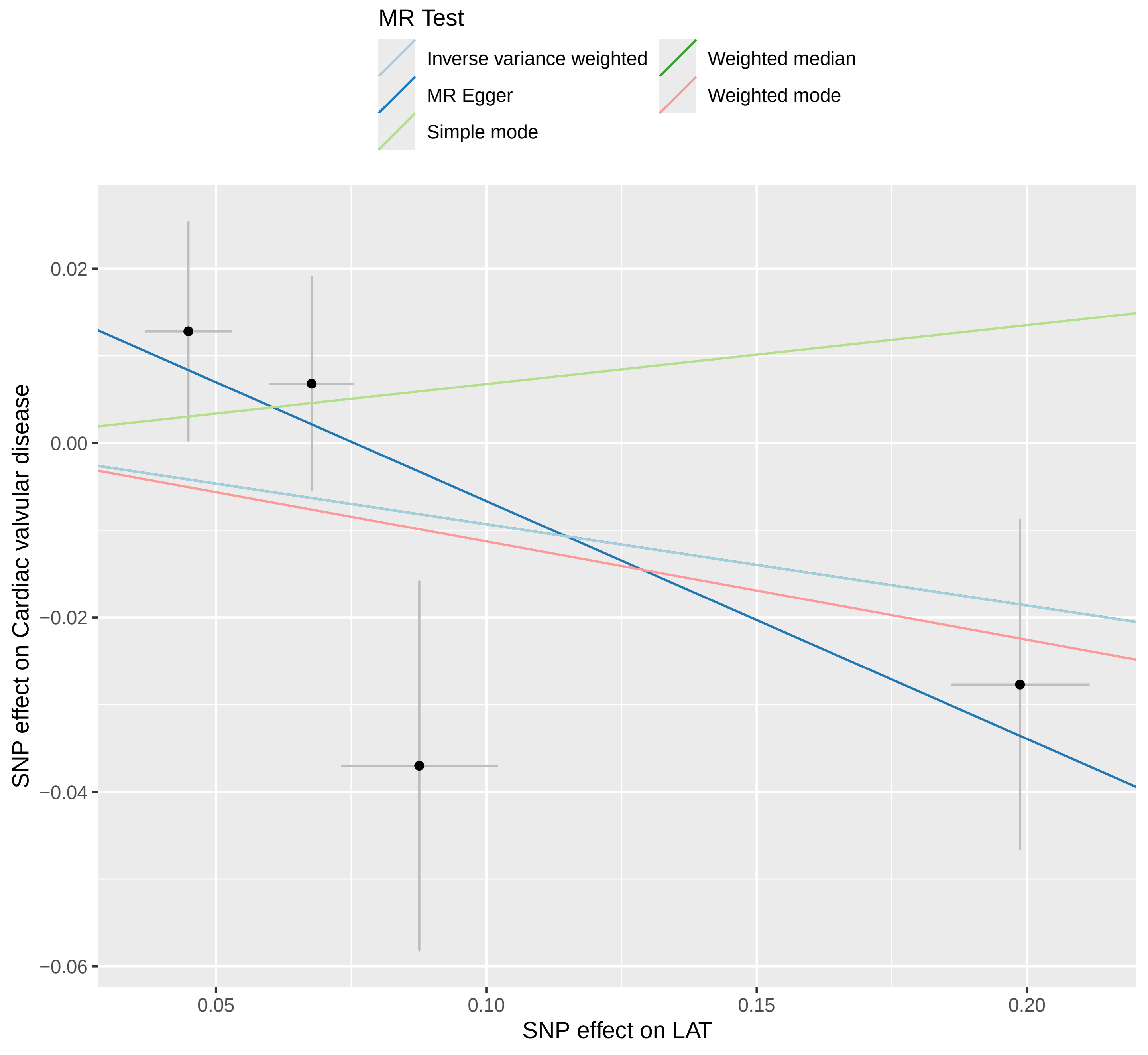
MR Test
Inverse variance weighted
MR Egger
Simple mode
Weighted median
Weighted mode
0.02
0.00
−0.02
−0.04
−0.06
0.05
0.10
0.15
0.20
SNP effect on Cardiac valvular disease
SNP effect on LAT

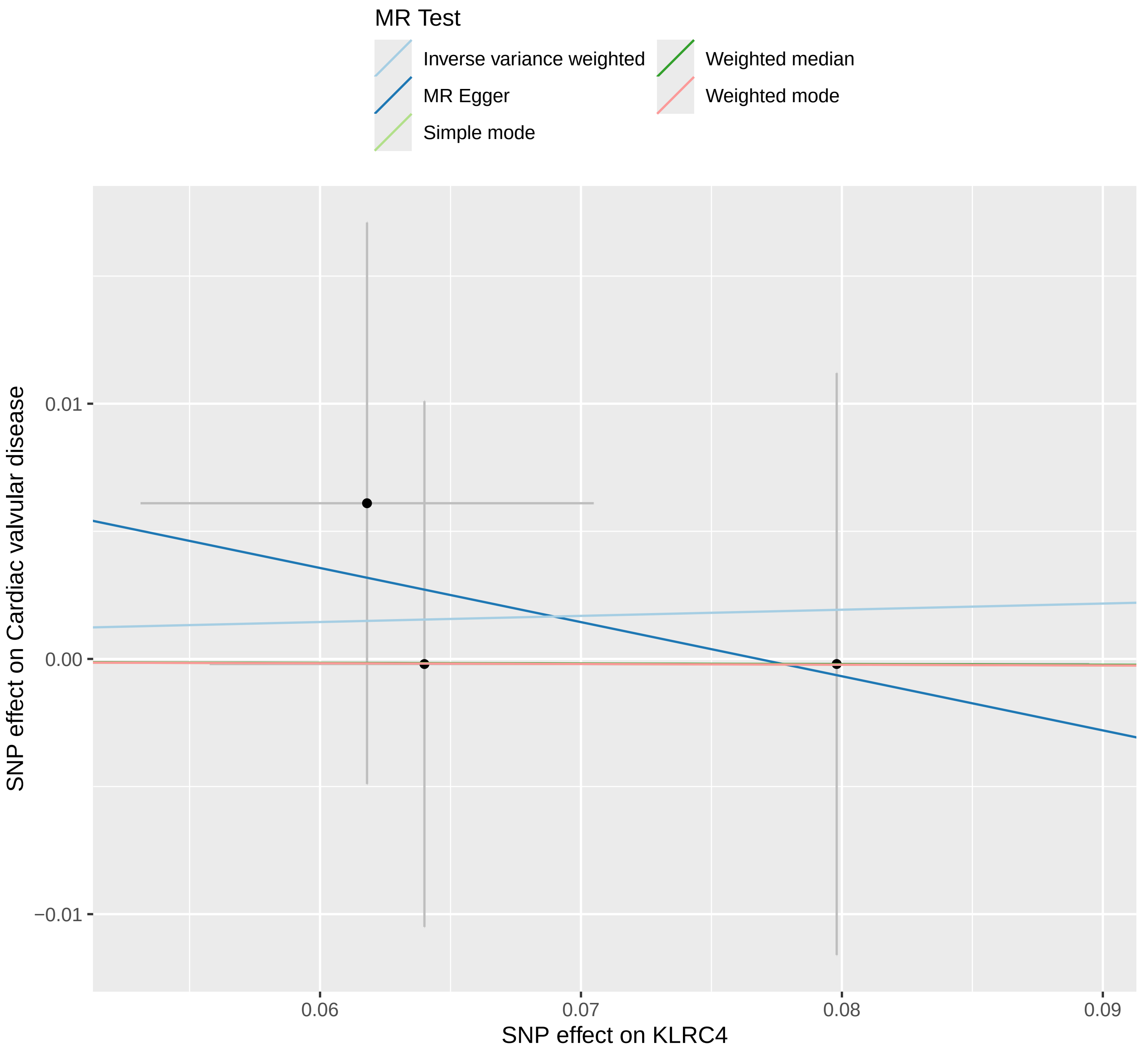
MR Test
Inverse variance weighted
MR Egger
Simple mode
Weighted median
Weighted mode
0.01
0.00
−0.01
0.06
0.07
0.08
0.09
SNP effect on KLRC4
SNP effect on Cardiac valvular disease

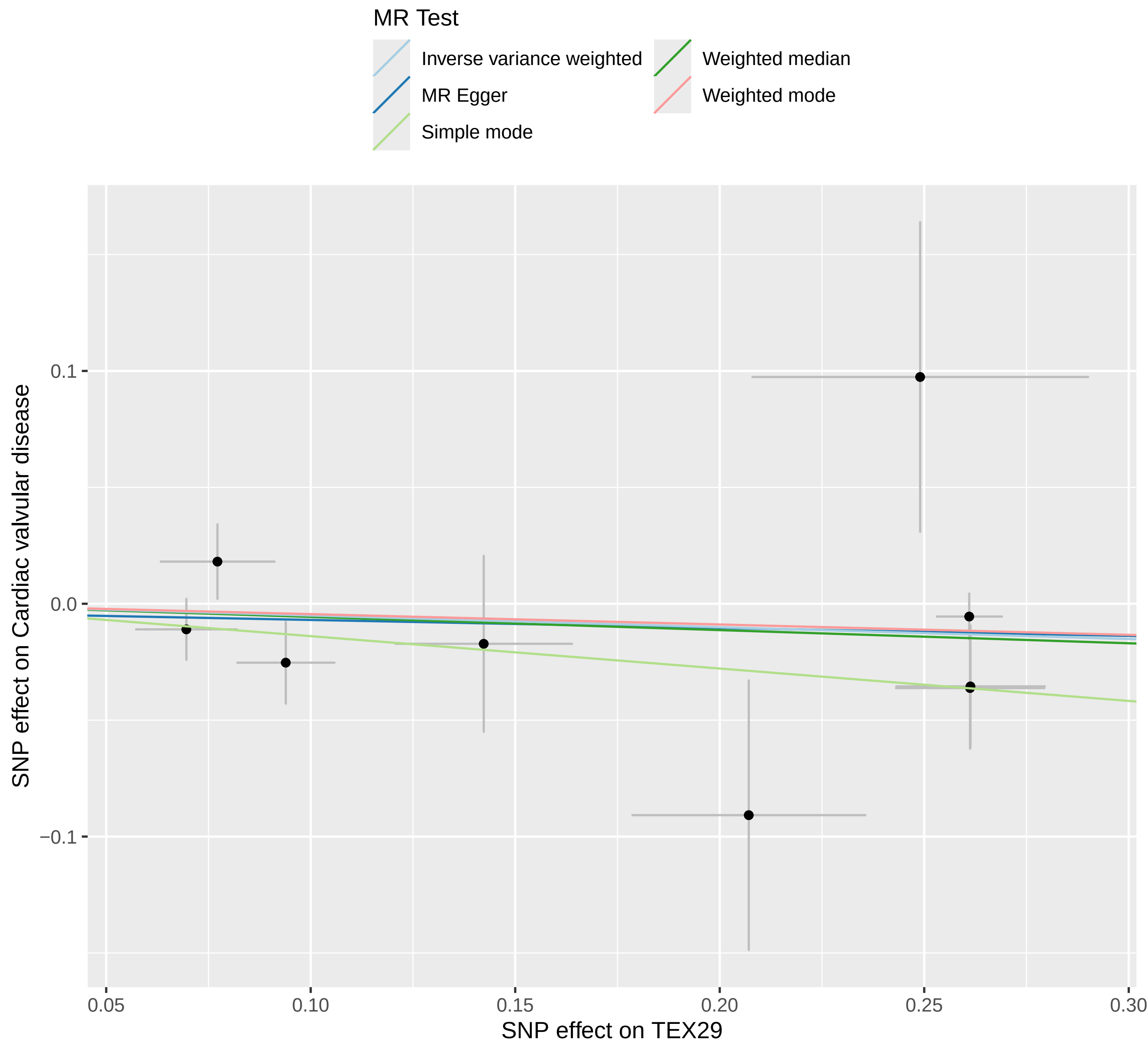

MR Test
Inverse variance weighted
MR Egger
Simple mode
Weighted median
Weighted mode
SNP effect on Cardiac valvular disease
0.1
0.0
−0.1
0.05
0.10
0.15
0.20
0.25
0.30
SNP effect on TEX29

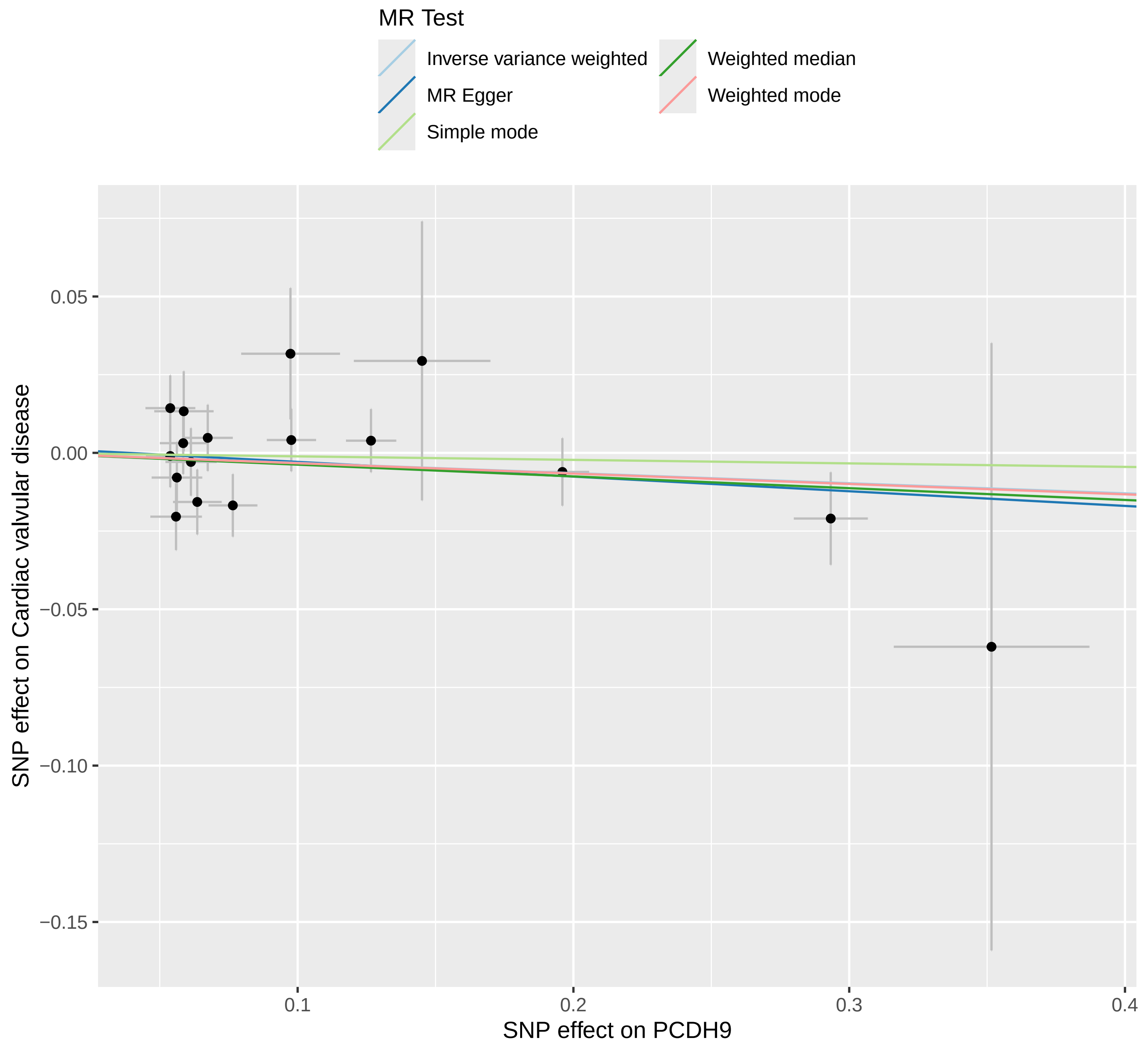

MR Test
Inverse variance weighted
MR Egger
Simple mode
Weighted median
Weighted mode
0.05
0.00
−0.05
−0.10
−0.15
SNP effect on Cardiac valvular disease
0.1
0.2
0.3
0.4
SNP effect on PCDH9

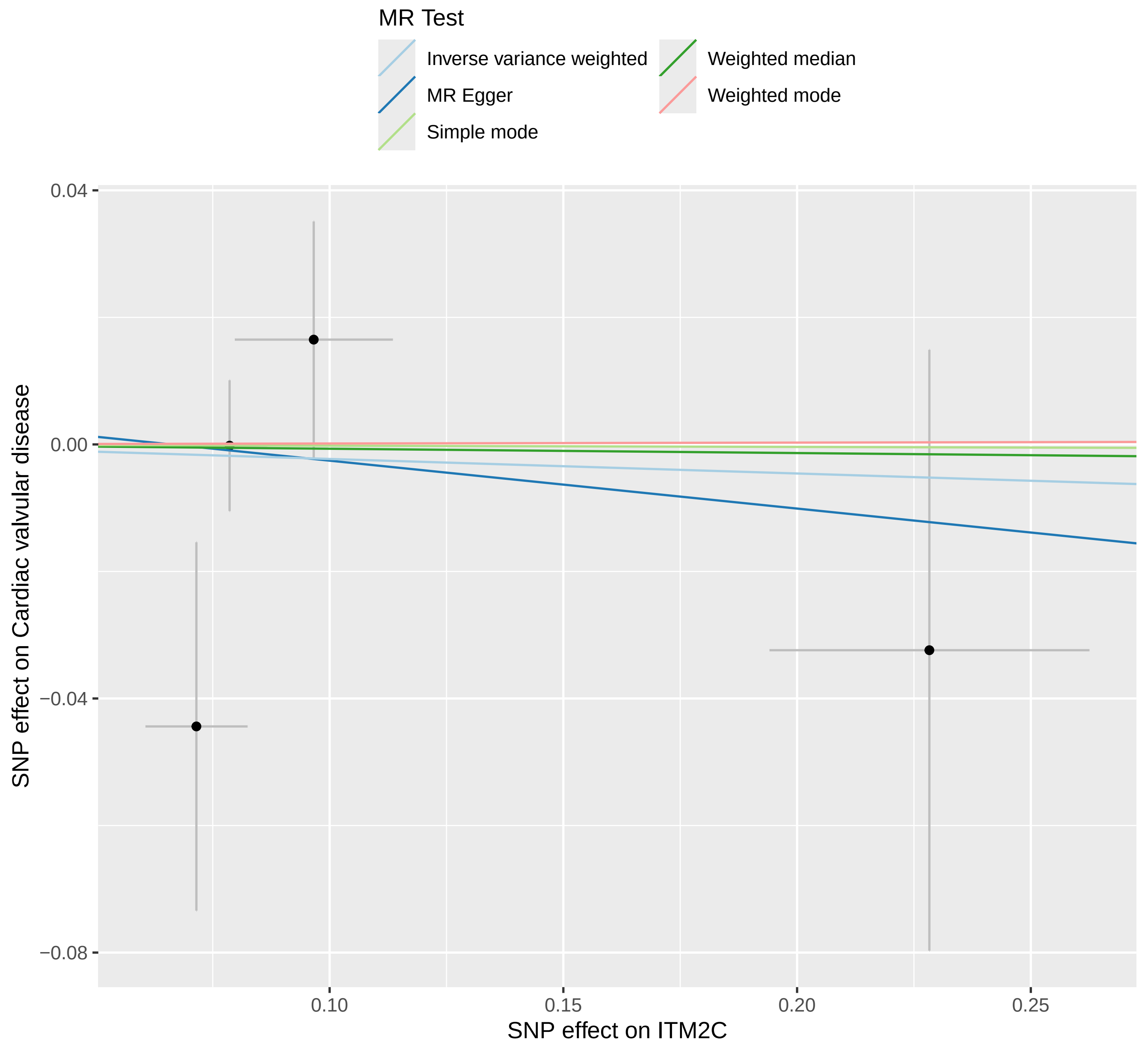

MR Test
Inverse variance weighted
MR Egger
Simple mode
Weighted median
Weighted mode
0.04
0.00
−0.04
−0.08
0.10
0.15
0.20
0.25
SNP effect on Cardiac valvular disease
SNP effect on ITM2C

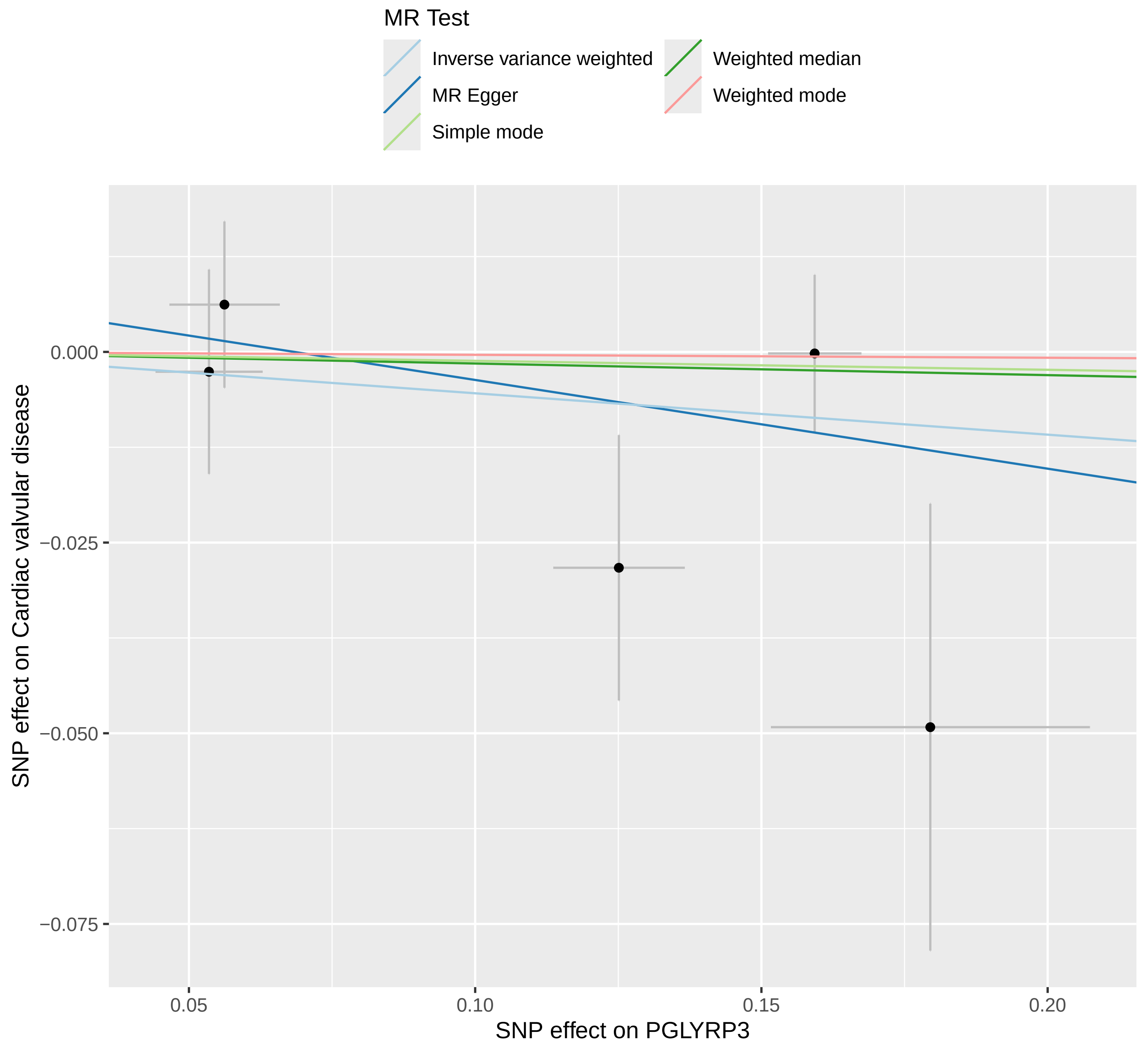

MR Test
Inverse variance weighted
MR Egger
Simple mode
Weighted median
Weighted mode
0.000
−0.025
−0.050
−0.075
0.05
0.10
0.15
0.20
SNP effect on Cardiac valvular disease
SNP effect on PGLYRP3

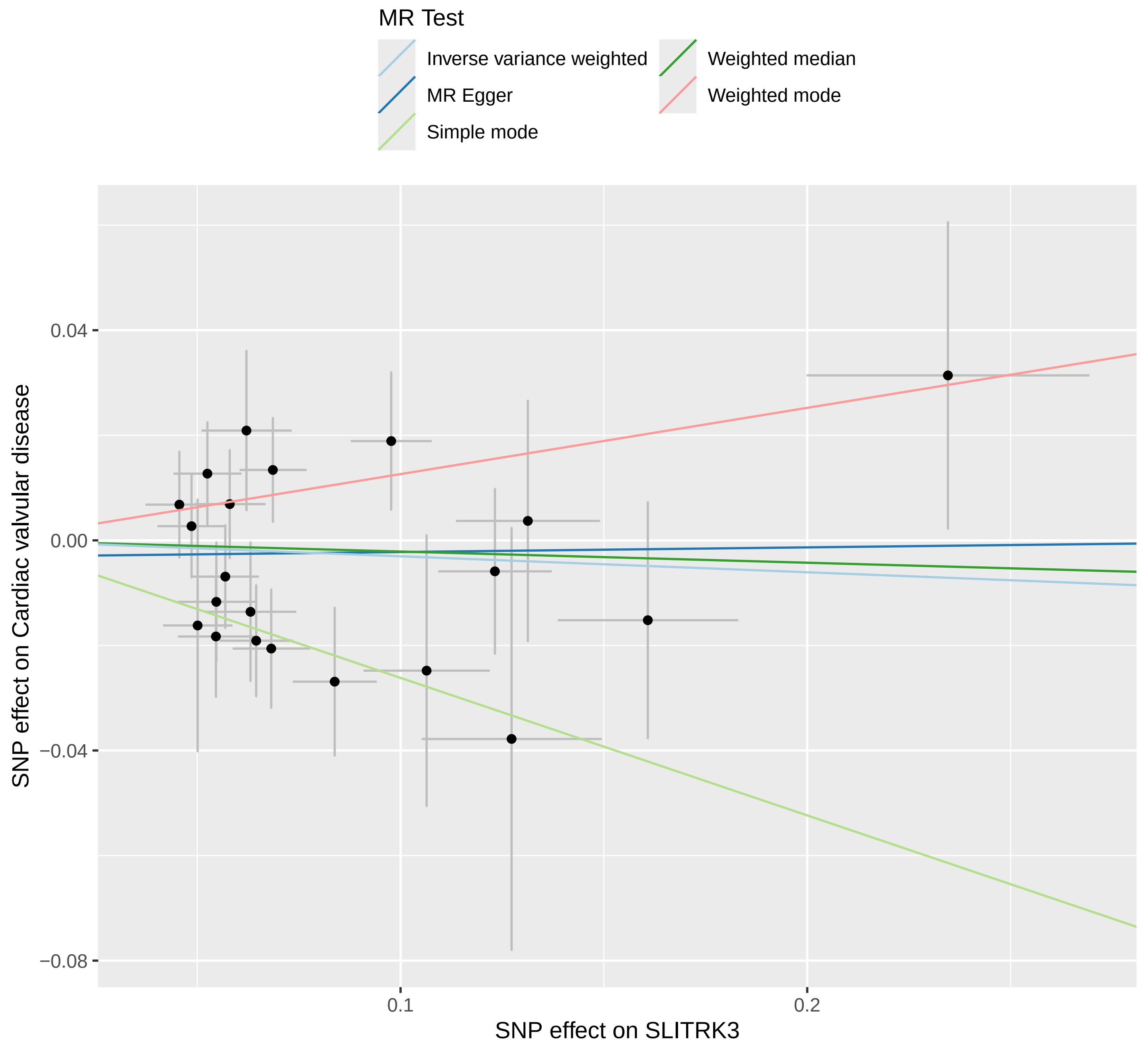
MR Test
Inverse variance weighted
MR Egger
Simple mode
Weighted median
Weighted mode
0.04
0.00
−0.04
−0.08
0.1
0.2
SNP effect on Cardiac valvular disease
SNP effect on SLITRK3

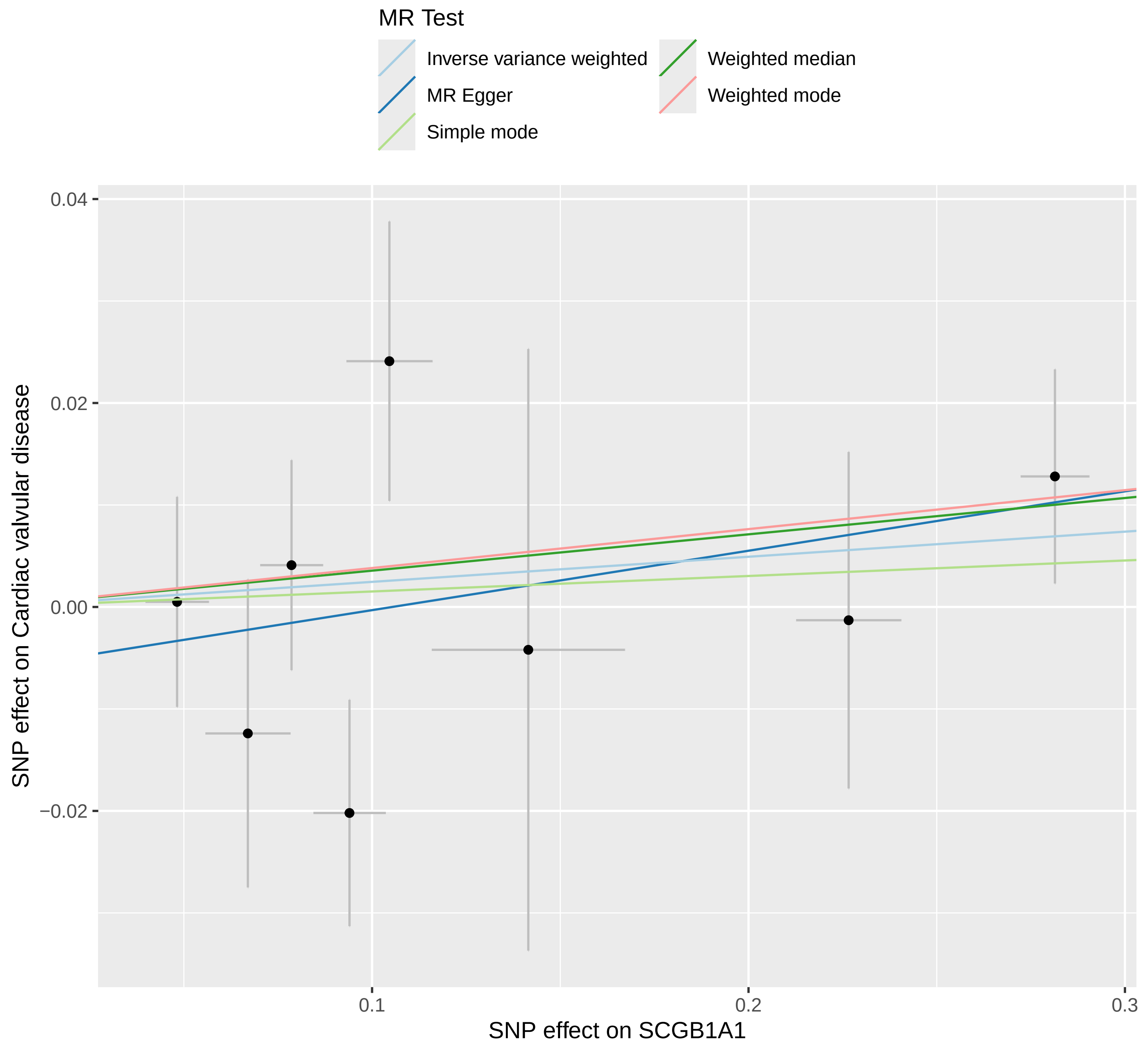

MR Test
Inverse variance weighted
MR Egger
Simple mode
Weighted median
Weighted mode
0.04
0.02
0.00
−0.02
0.1
0.2
0.3
SNP effect on Cardiac valvular disease
SNP effect on SCGB1A1

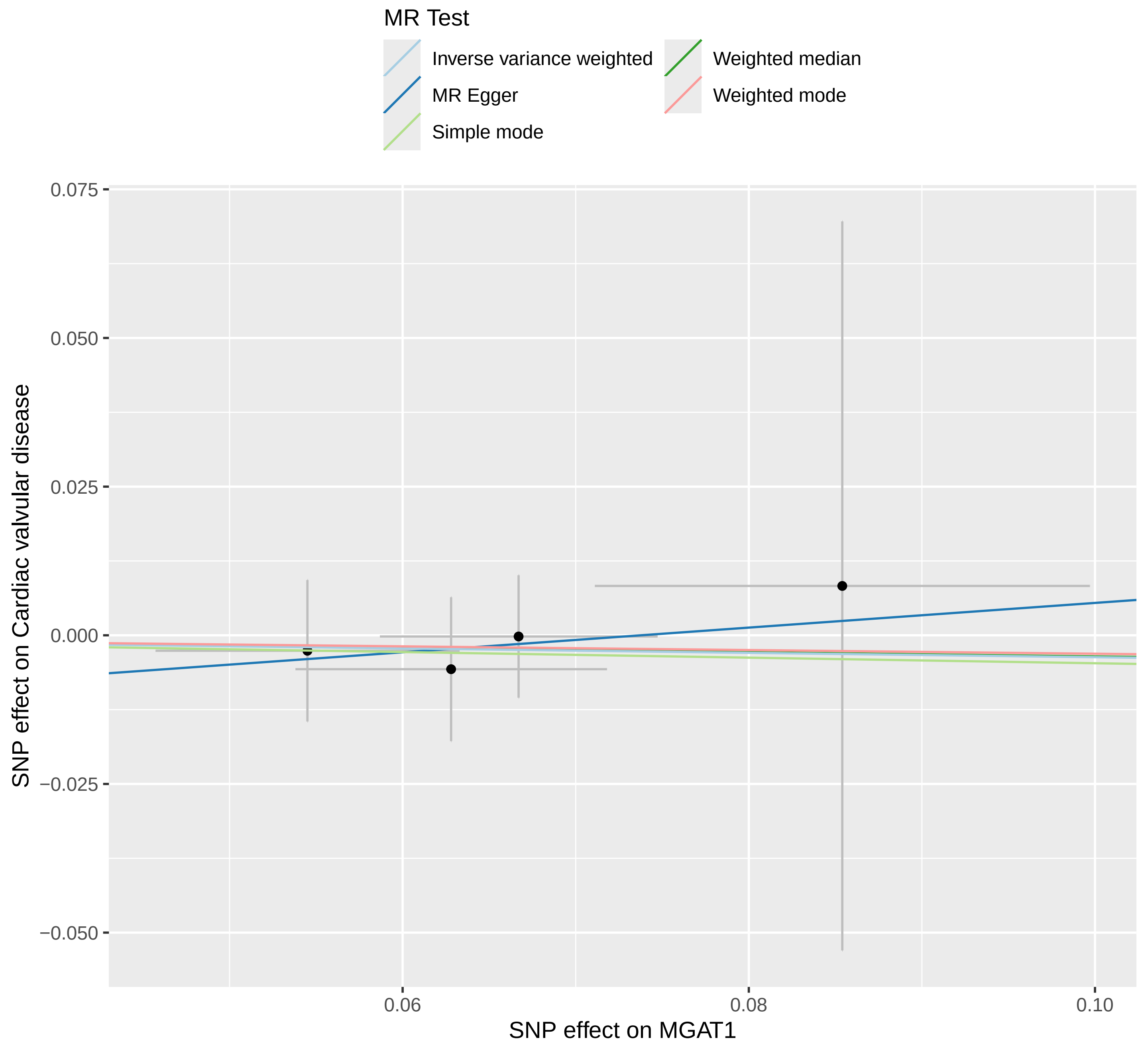

MR Test
Inverse variance weighted
MR Egger
Simple mode
Weighted median
Weighted mode
0.075
0.050
0.025
0.000
−0.025
−0.050
0.06
0.08
0.10
SNP effect on MGAT1
SNP effect on Cardiac valvular disease

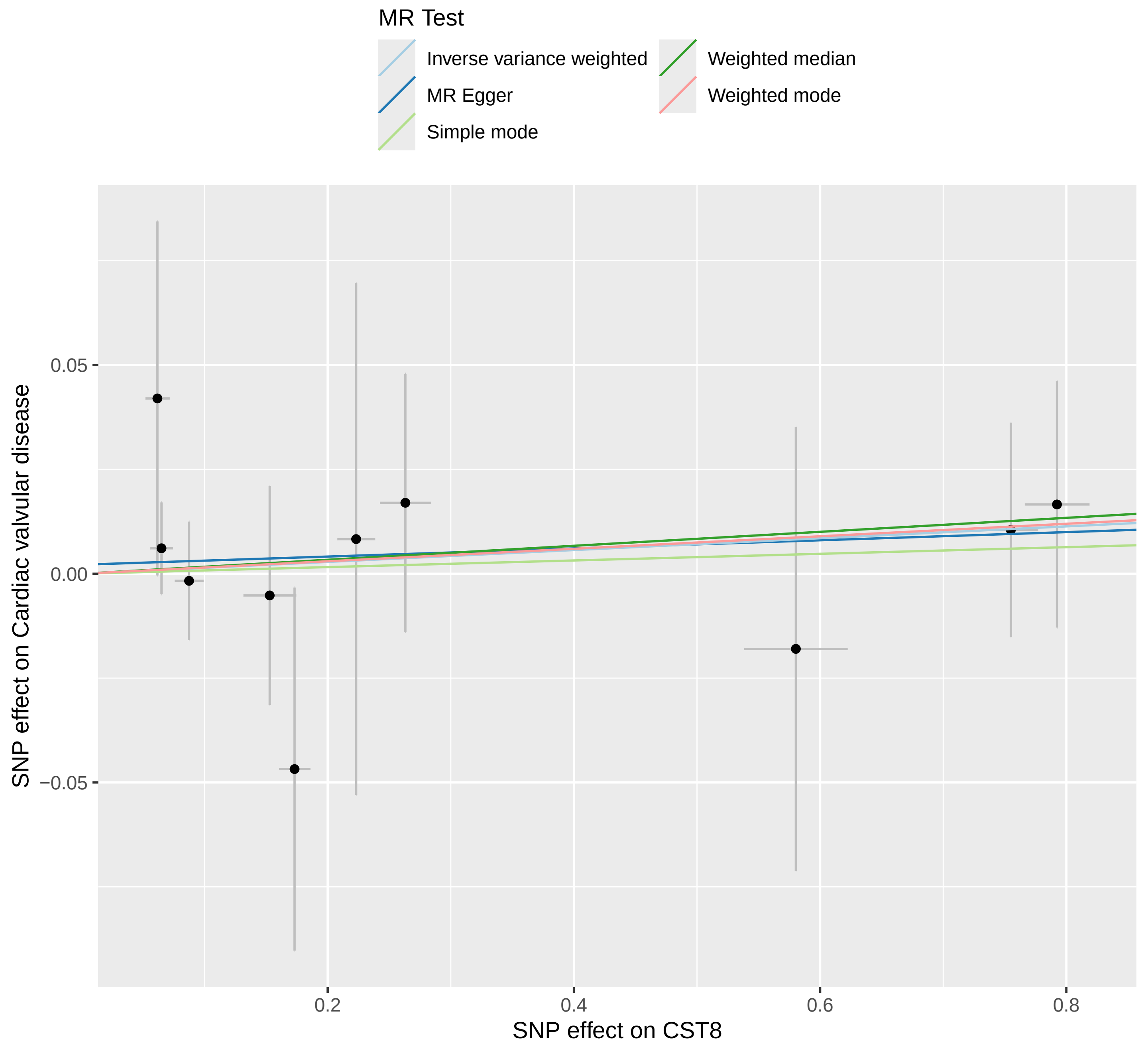
MR Test
Inverse variance weighted
MR Egger
Simple mode
Weighted median
Weighted mode
0.05
0.00
−0.05
SNP effect on Cardiac valvular disease
0.2
0.4
0.6
0.8
SNP effect on CST8

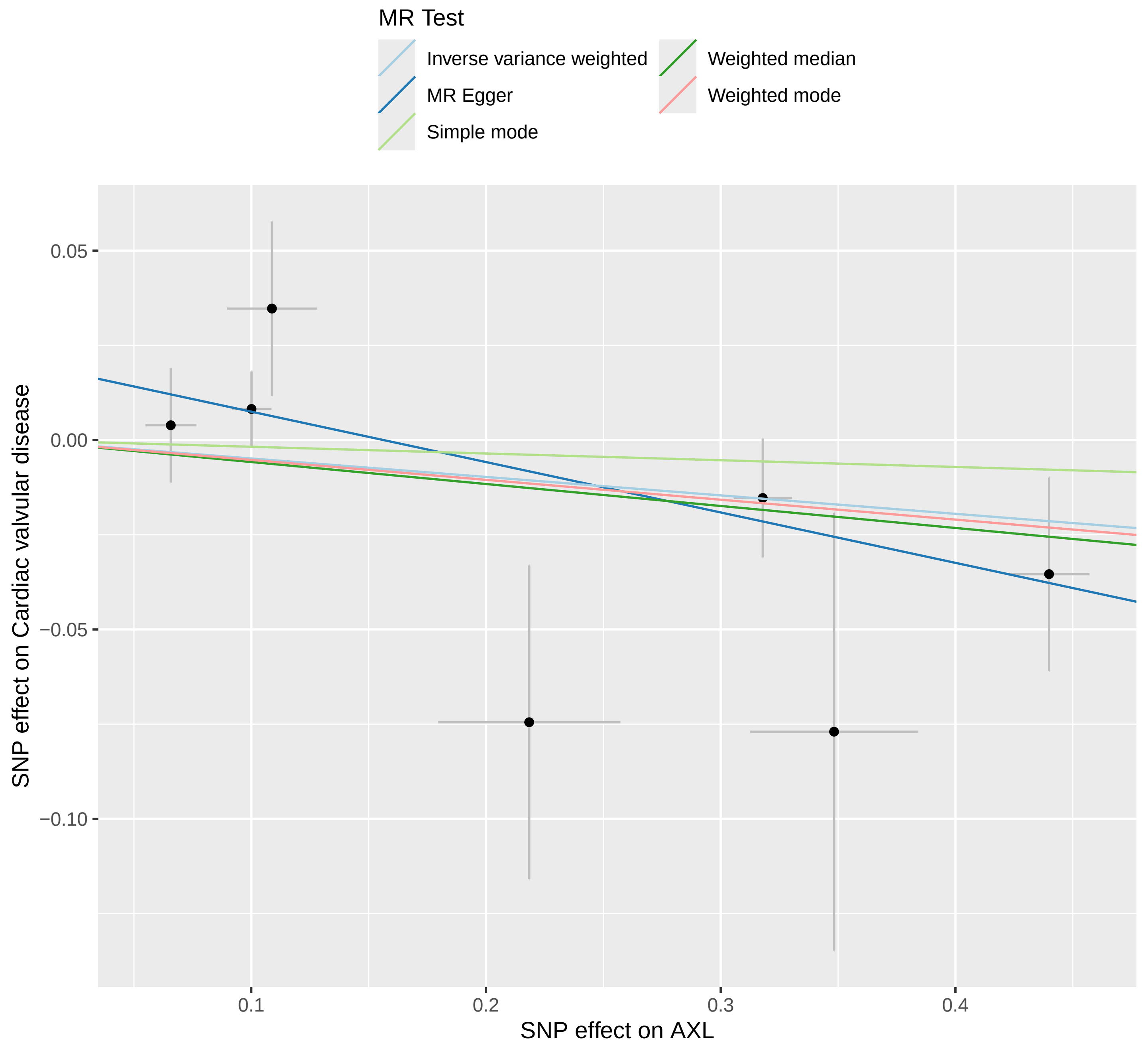

MR Test
Inverse variance weighted
MR Egger
Simple mode
Weighted median
Weighted mode
0.05
0.00
−0.05
−0.10
0.1
0.2
0.3
0.4
SNP effect on Cardiac valvular disease
SNP effect on AXL

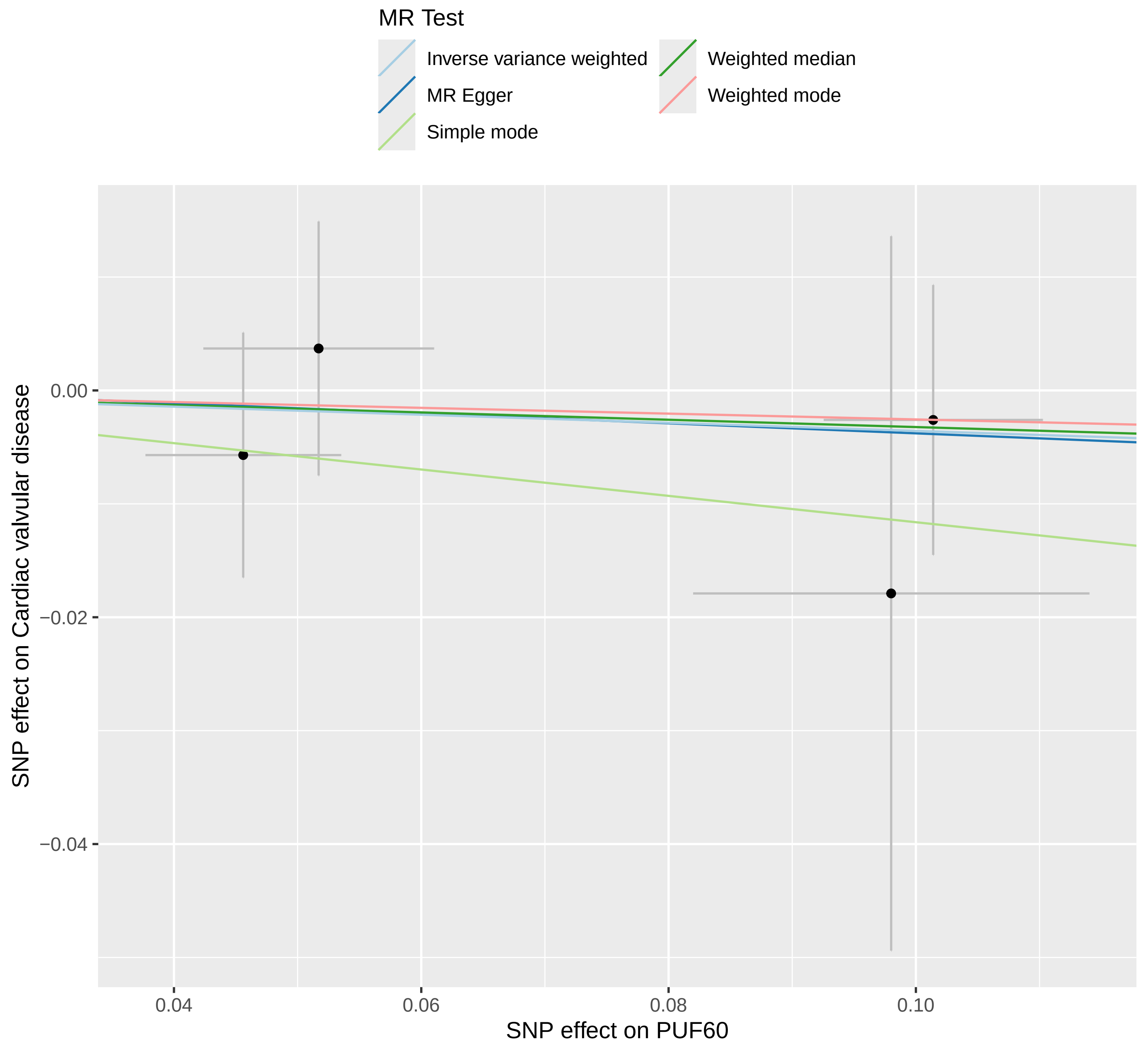
MR Test
Inverse variance weighted
MR Egger
Simple mode
Weighted median
Weighted mode
0.00
−0.02
−0.04
0.04
0.06
0.08
0.10
SNP effect on PUF60
SNP effect on Cardiac valvular disease

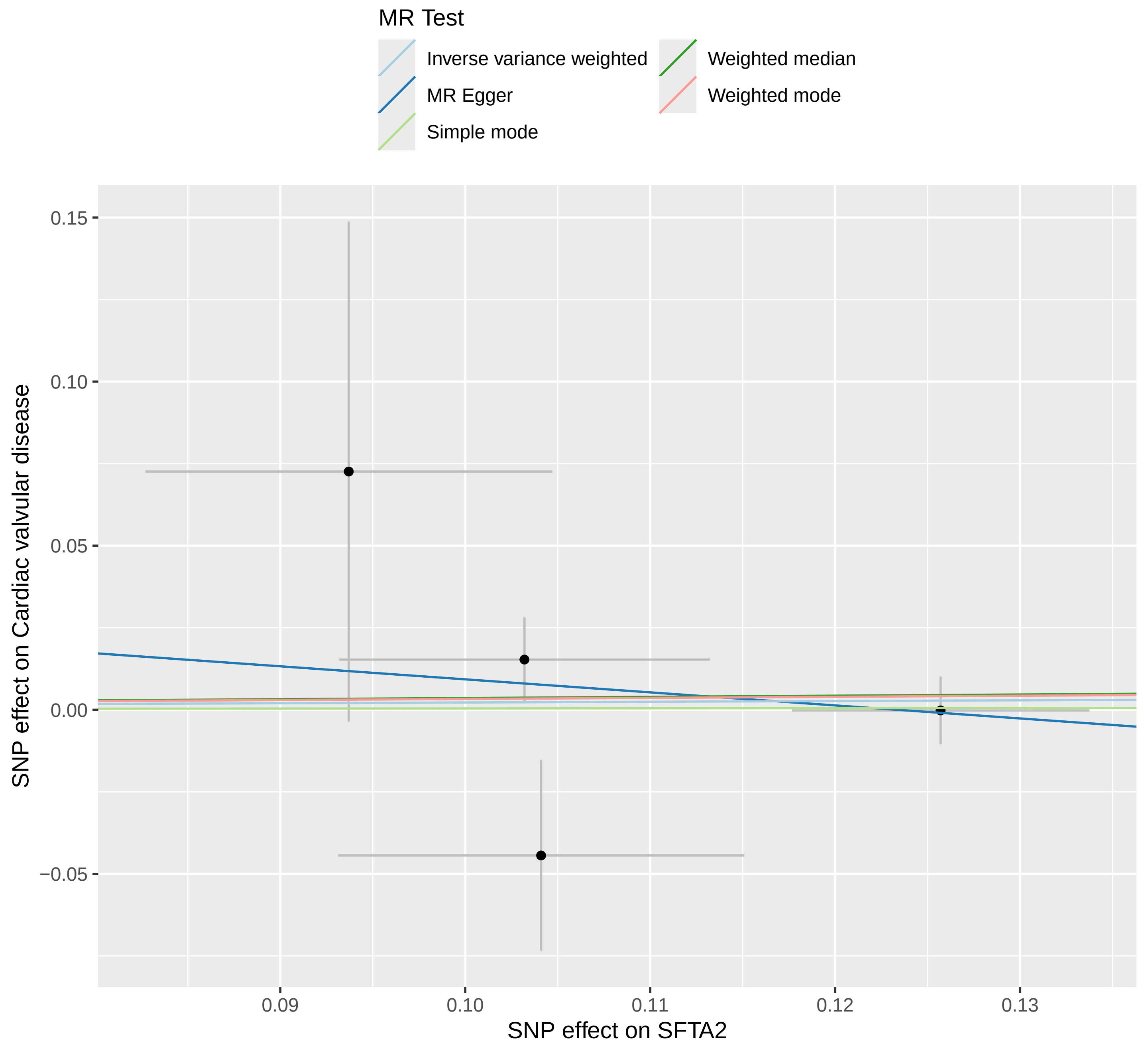

MR Test
Inverse variance weighted
MR Egger
Simple mode
Weighted median
Weighted mode
0.15
0.10
0.05
0.00
−0.05
0.09
0.10
0.11
0.12
0.13
SNP effect on Cardiac valvular disease
SNP effect on SFTA2

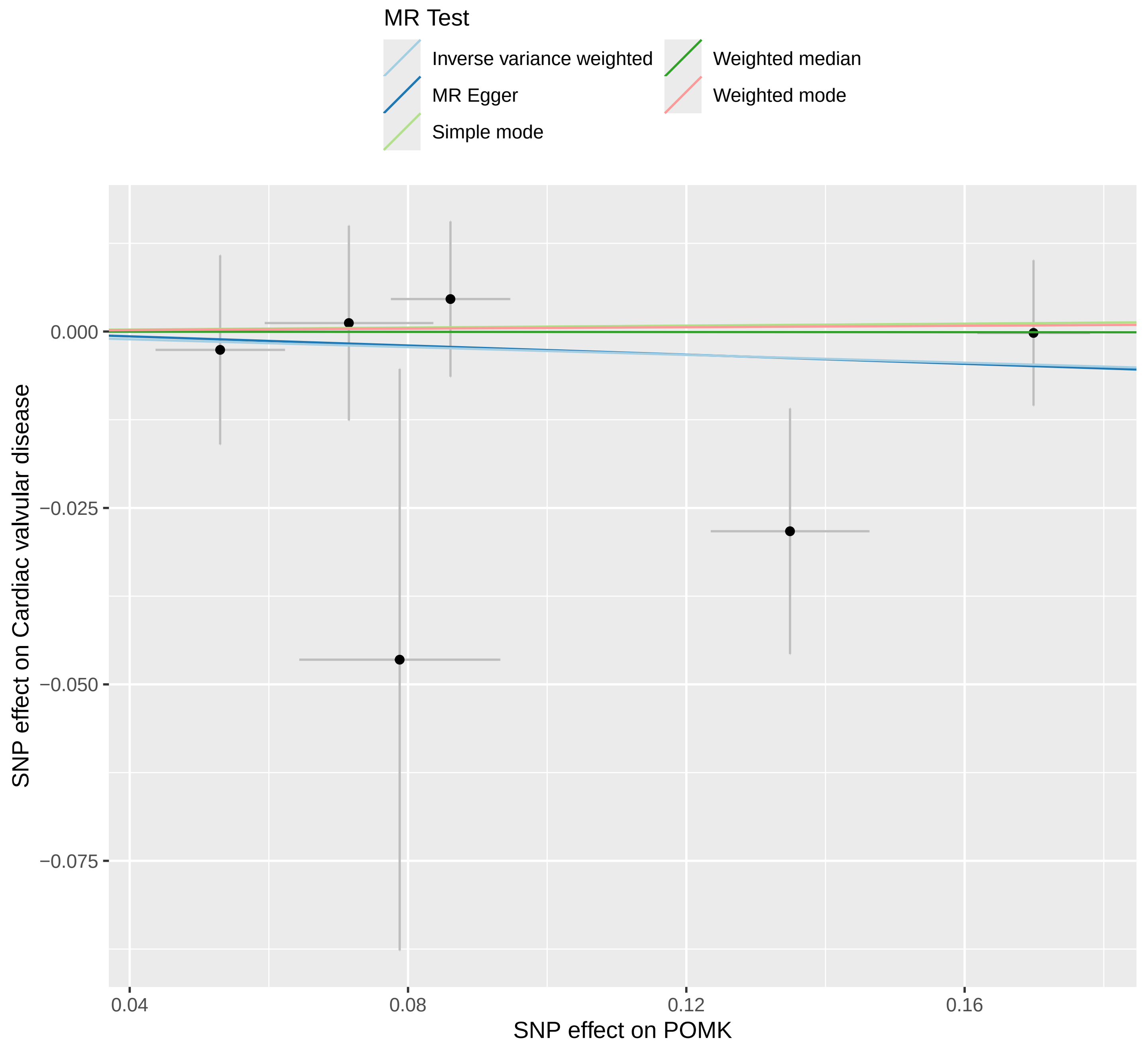

MR Test
Inverse variance weighted
MR Egger
Simple mode
Weighted median
Weighted mode
0.000
−0.025
−0.050
−0.075
SNP effect on Cardiac valvular disease
0.04
0.08
0.12
0.16
SNP effect on POMK

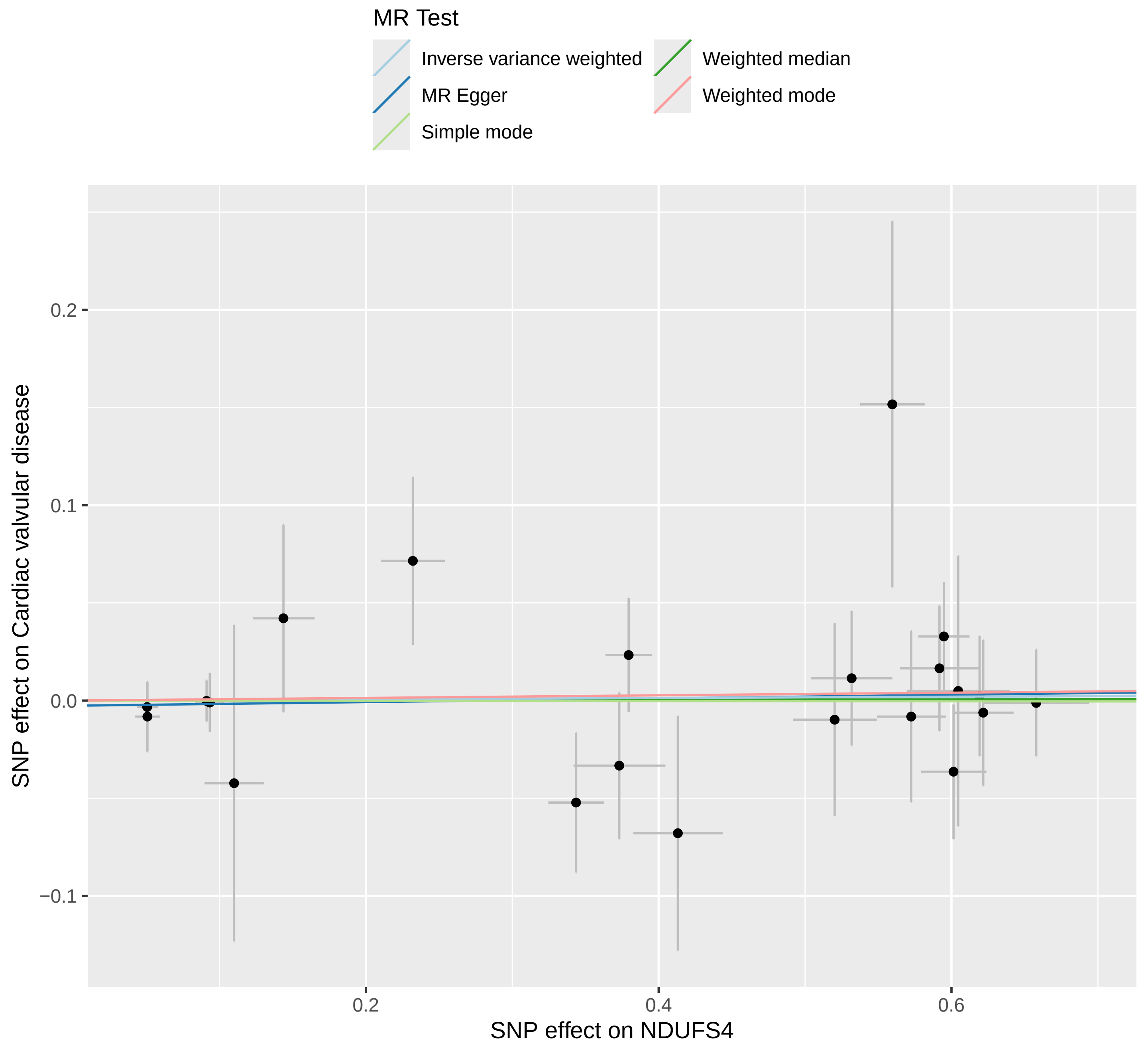
MR Test
Inverse variance weighted
MR Egger
Simple mode
Weighted median
Weighted mode
0.2
0.1
0.0
−0.1
0.2
0.4
0.6
SNP effect on Cardiac valvular disease
SNP effect on NDUFS4

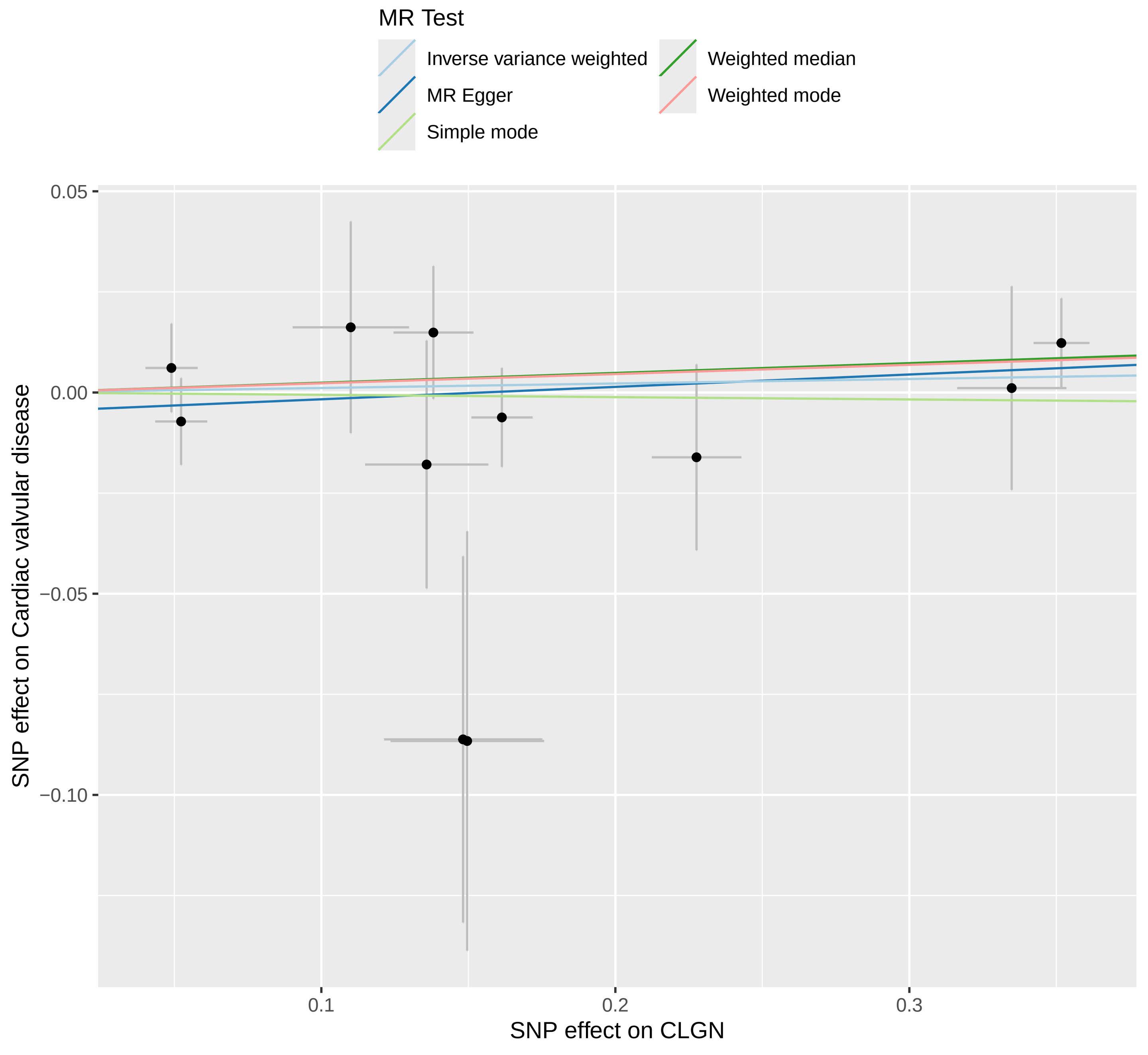
MR Test
Inverse variance weighted
MR Egger
Simple mode
Weighted median
Weighted mode
0.05
0.00
−0.05
−0.10
0.1
0.2
0.3
SNP effect on Cardiac valvular disease
SNP effect on CLGN

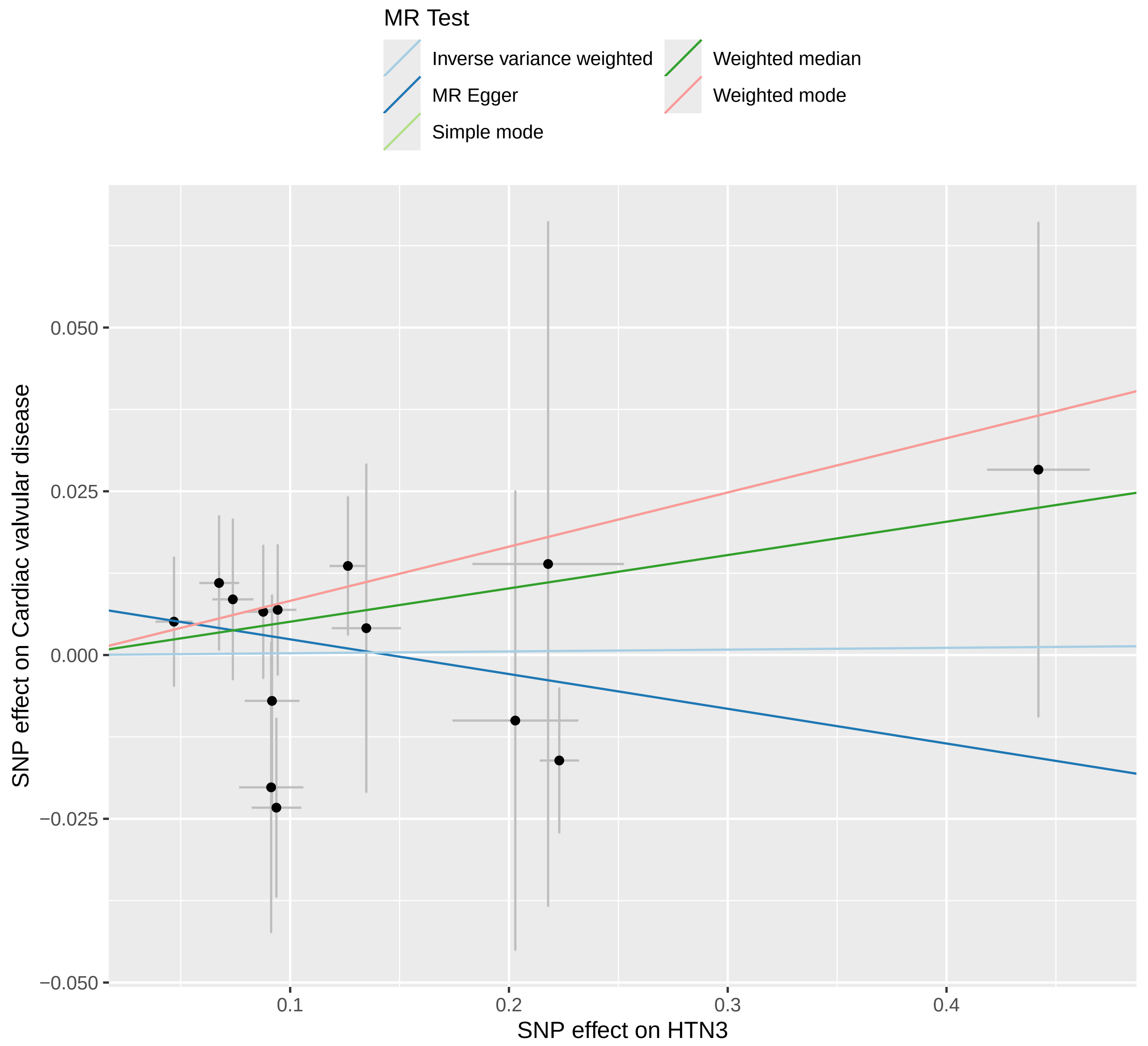

MR Test
Inverse variance weighted
MR Egger
Simple mode
Weighted median
Weighted mode
0.050
0.025
0.000
−0.025
−0.050
SNP effect on Cardiac valvular disease
0.1
0.2
0.3
0.4
SNP effect on HTN3

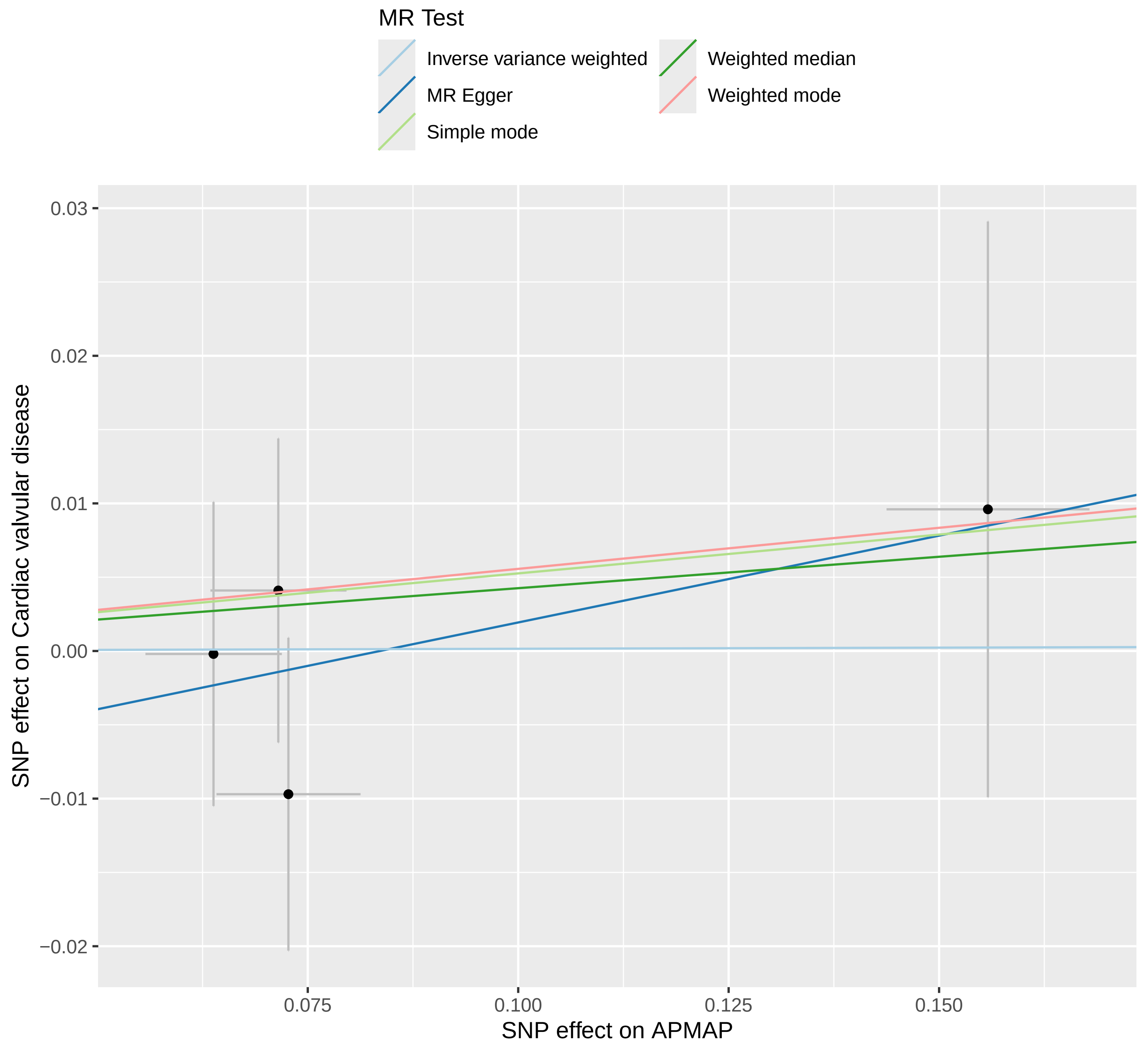

MR Test
Inverse variance weighted
MR Egger
Simple mode
Weighted median
Weighted mode
0.03
0.02
0.01
0.00
−0.01
−0.02
SNP effect on Cardiac valvular disease
0.075
0.100
0.125
0.150
SNP effect on APMAP

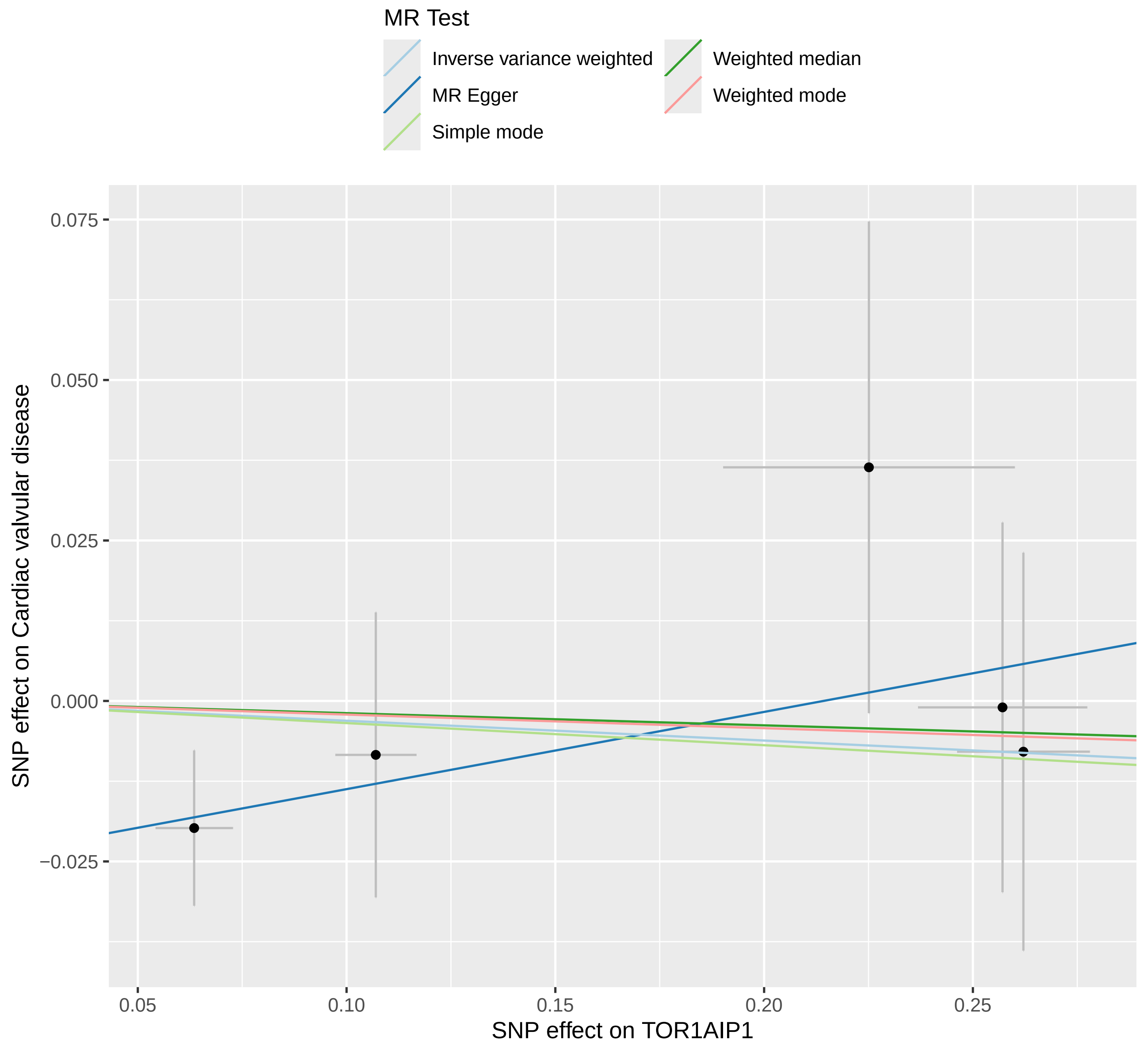

MR Test
Inverse variance weighted
MR Egger
Simple mode
Weighted median
Weighted mode
0.075
0.050
0.025
0.000
−0.025
SNP effect on Cardiac valvular disease
0.05
0.10
0.15
0.20
0.25
SNP effect on TOR1AIP1

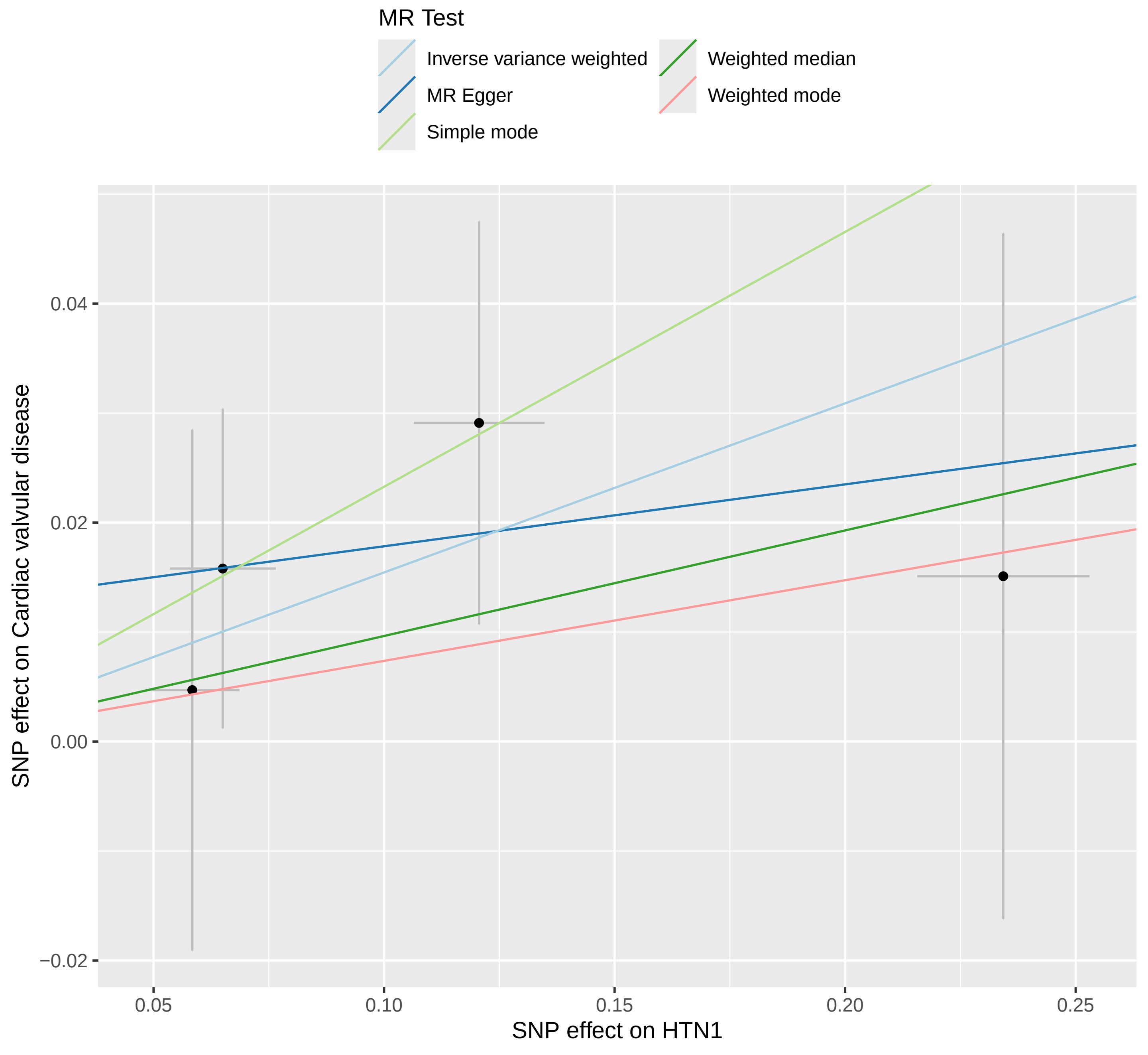
MR Test
Inverse variance weighted
MR Egger
Simple mode
Weighted median
Weighted mode
0.04
0.02
0.00
−0.02
0.05
0.10
0.15
0.20
0.25
SNP effect on Cardiac valvular disease
SNP effect on HTN1

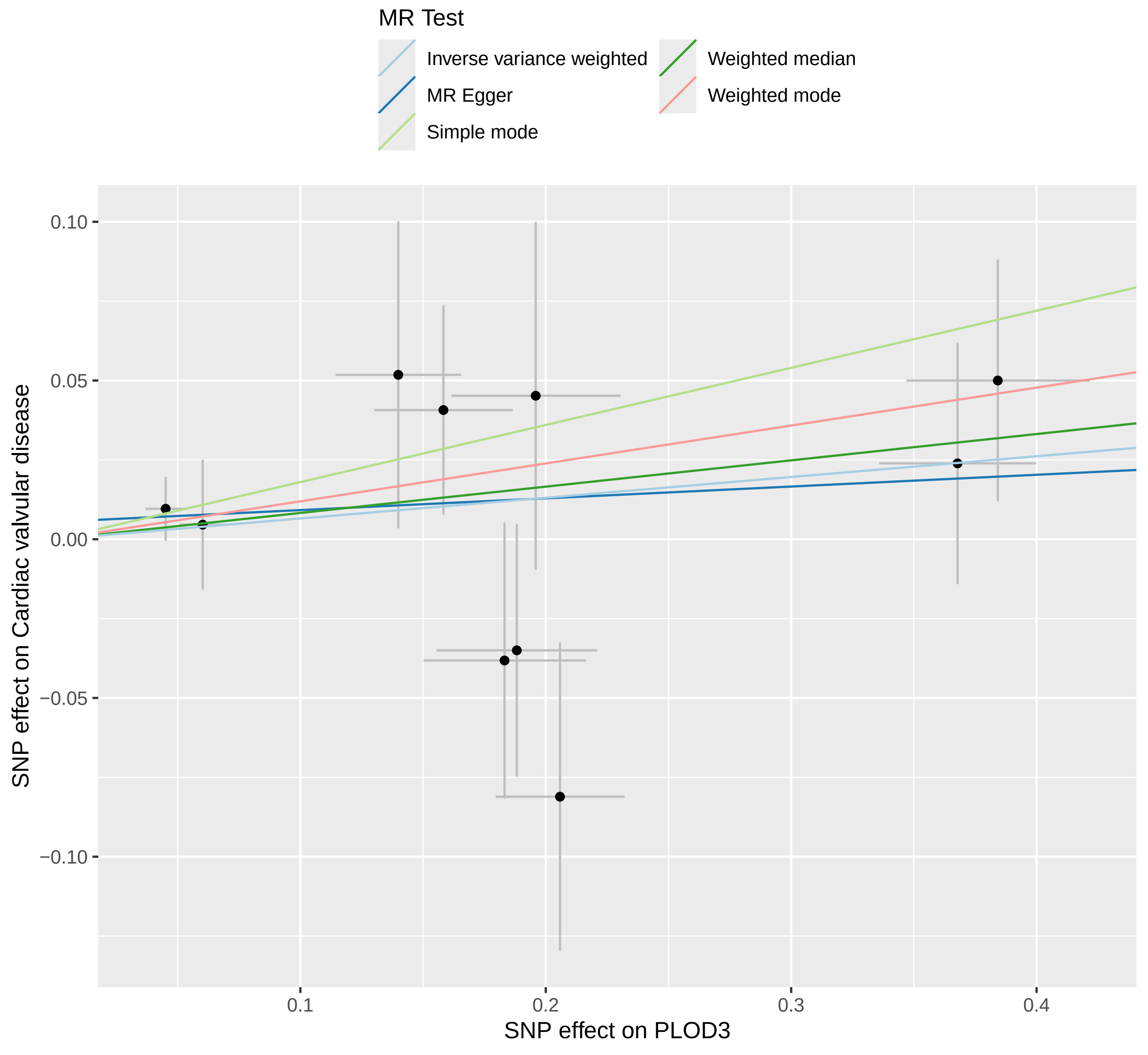
MR Test
Inverse variance weighted
MR Egger
Simple mode
Weighted median
Weighted mode
0.10
0.05
0.00
−0.05
−0.10
SNP effect on Cardiac valvular disease
0.1
0.2
0.3
0.4
SNP effect on PLOD3

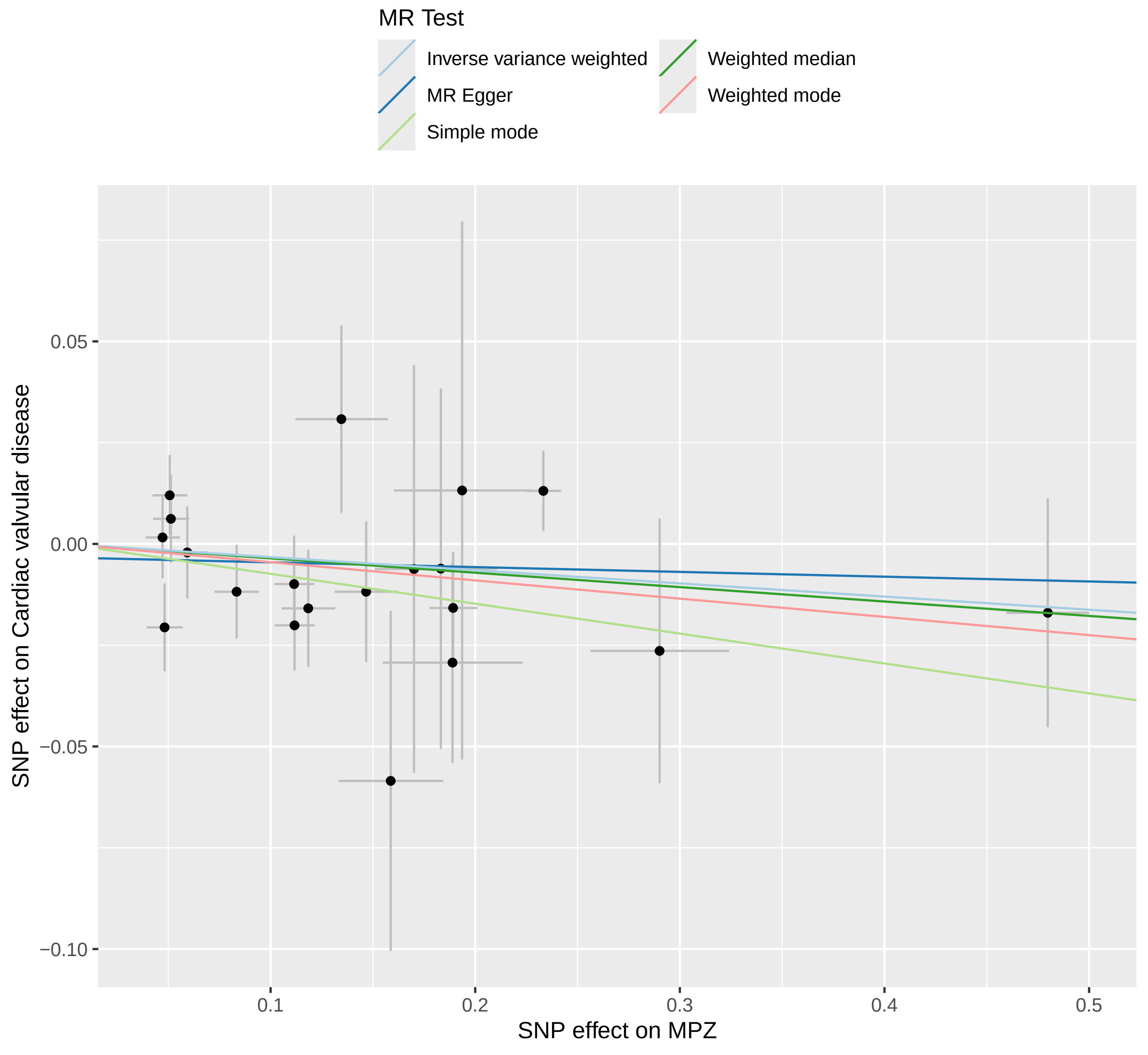

MR Test
Inverse variance weighted
MR Egger
Simple mode
Weighted median
Weighted mode
SNP effect on Cardiac valvular disease
0.05
0.00
−0.05
−0.10
0.1
0.2
0.3
0.4
0.5
SNP effect on MPZ

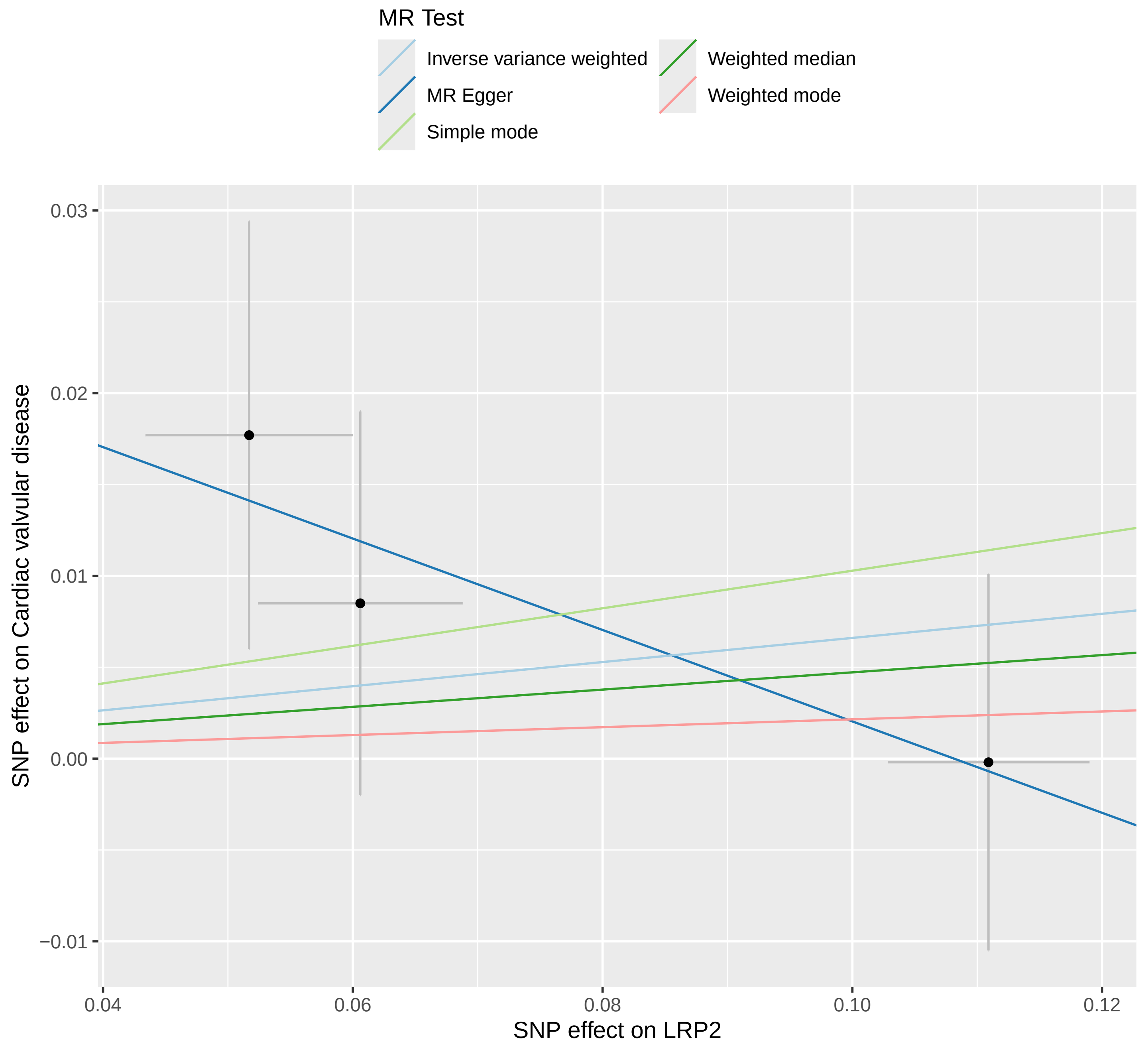
MR Test
Inverse variance weighted
MR Egger
Simple mode
Weighted median
Weighted mode
0.03
0.02
0.01
0.00
−0.01
0.04
0.06
0.08
0.10
0.12
SNP effect on Cardiac valvular disease
SNP effect on LRP2

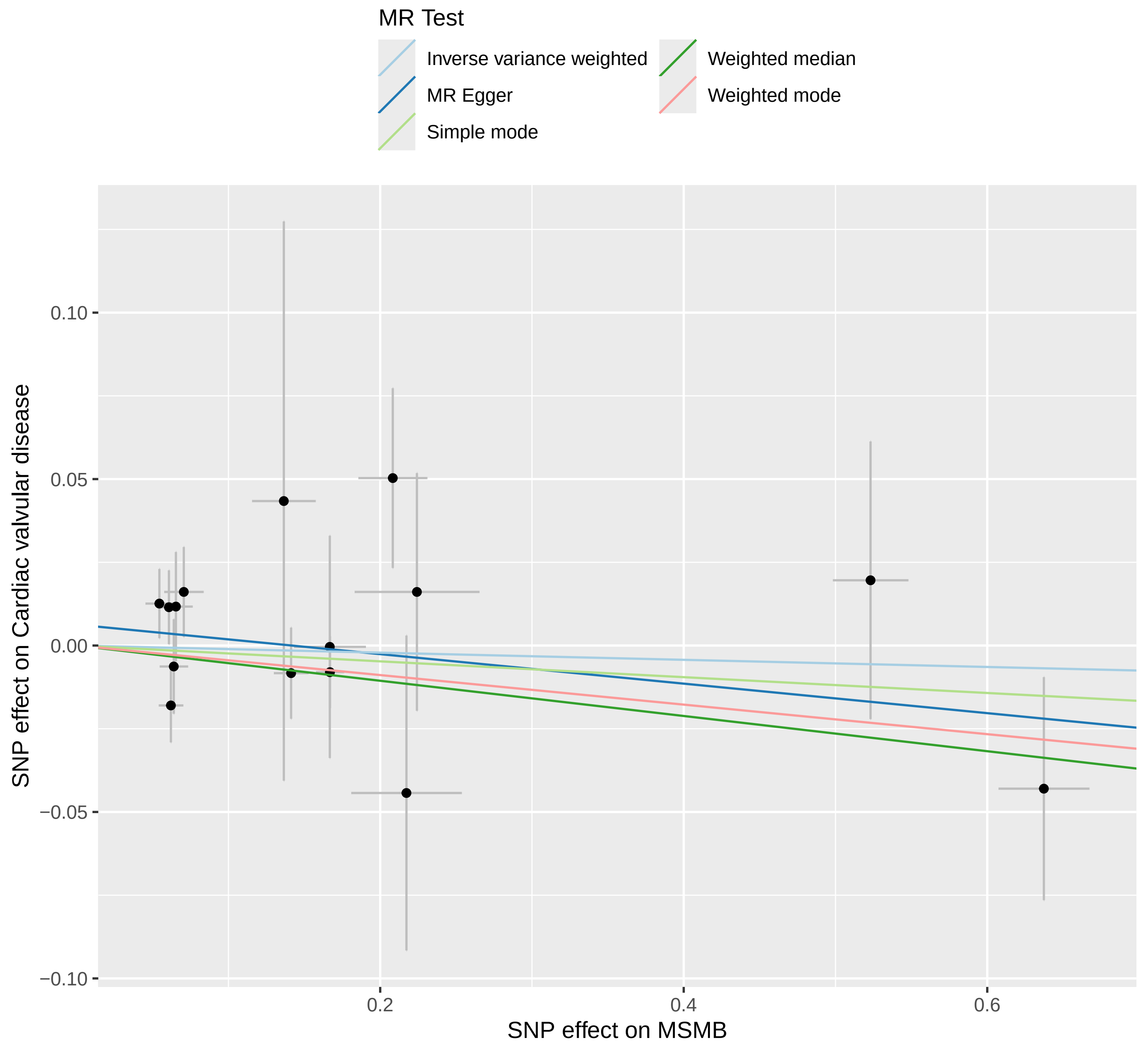
MR Test
Inverse variance weighted
MR Egger
Simple mode
Weighted median
Weighted mode
0.10
0.05
0.00
−0.05
−0.10
0.2
0.4
0.6
SNP effect on Cardiac valvular disease
SNP effect on MSMB

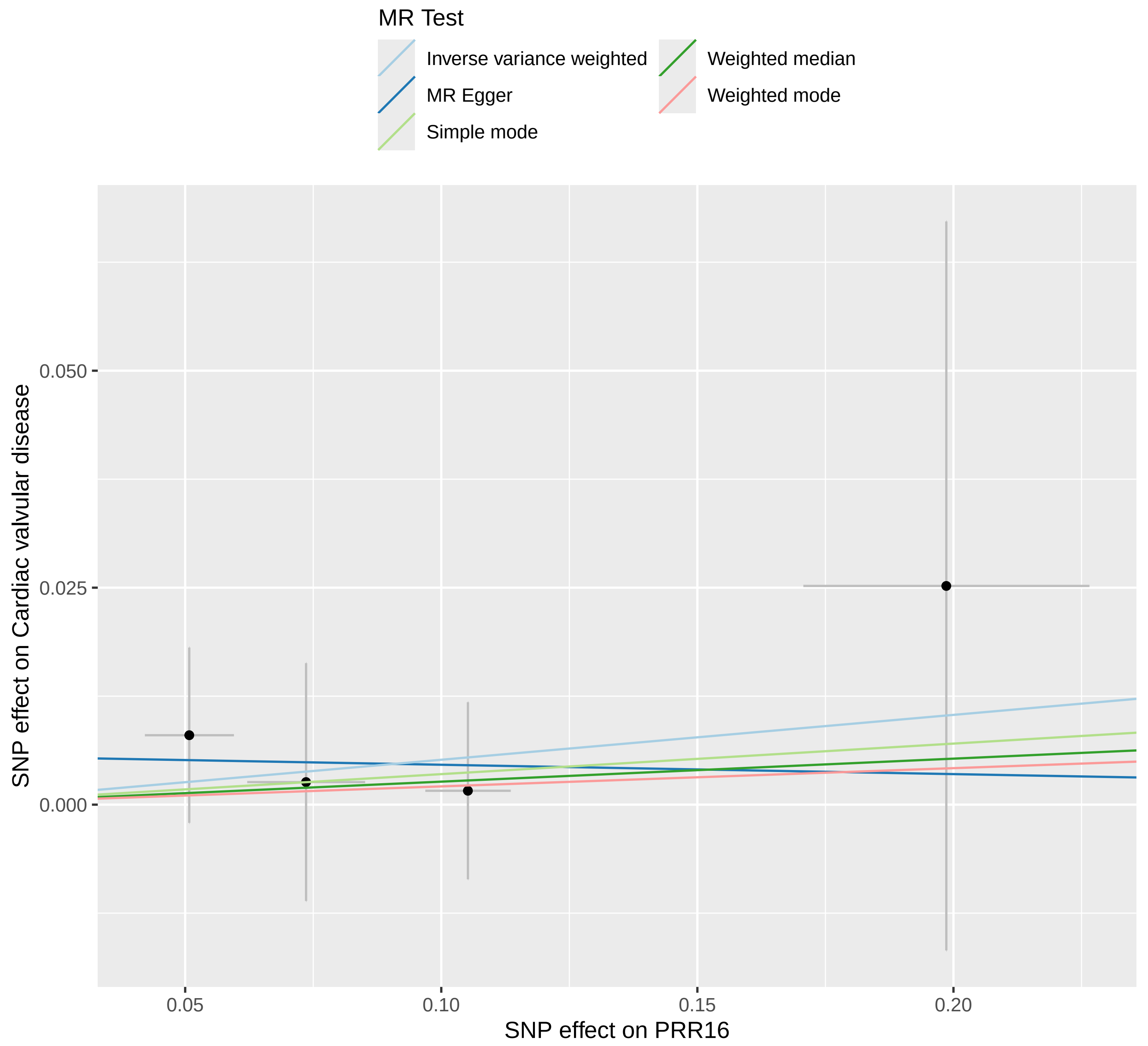

MR Test
Inverse variance weighted
MR Egger
Simple mode
Weighted median
Weighted mode
0.050
0.025
0.000
SNP effect on Cardiac valvular disease
0.05
0.10
0.15
0.20
SNP effect on PRR16

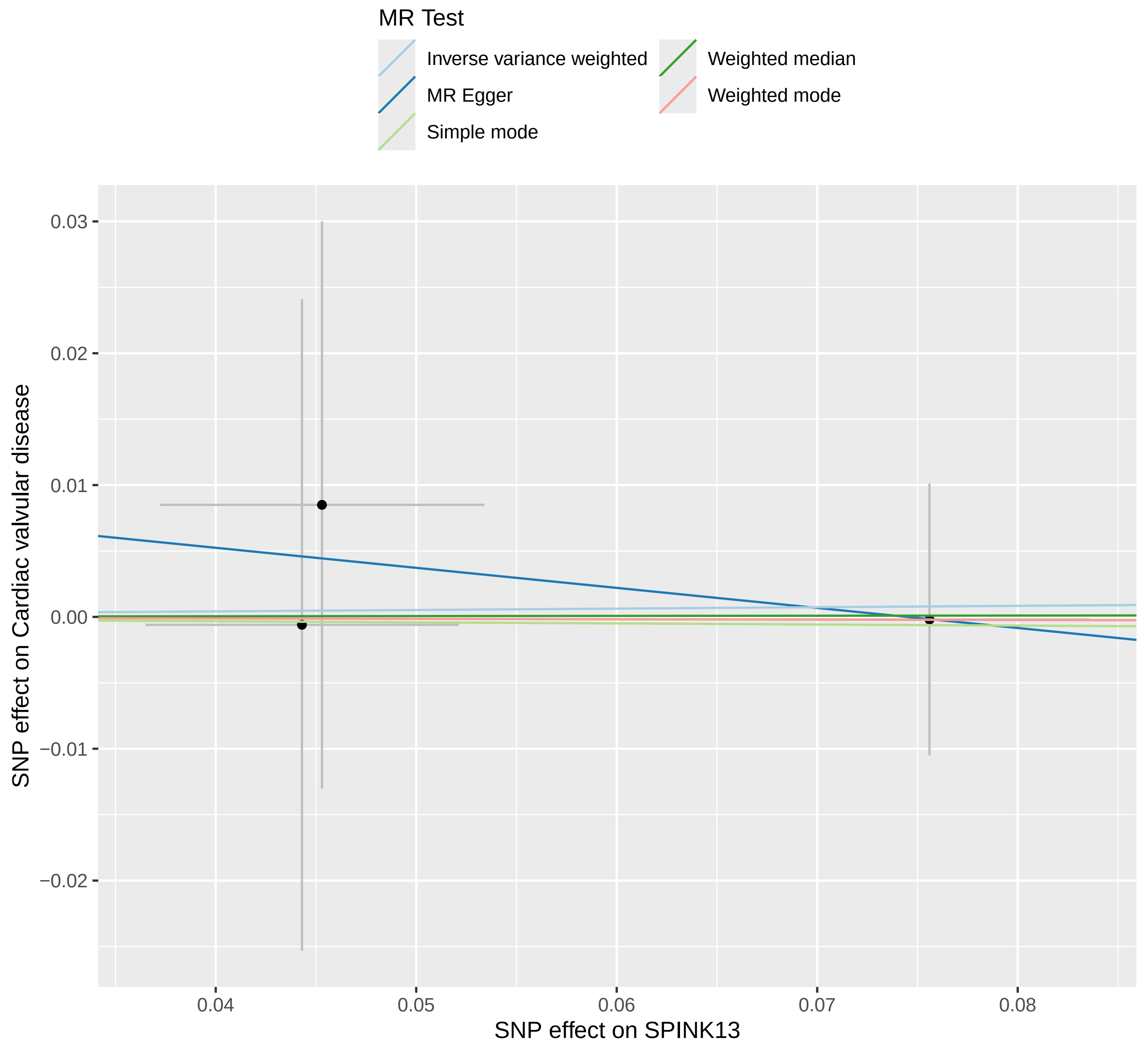

MR Test
Inverse variance weighted
MR Egger
Simple mode
Weighted median
Weighted mode
0.03
0.02
0.01
0.00
−0.01
−0.02
0.04
0.05
0.06
0.07
0.08
SNP effect on Cardiac valvular disease
SNP effect on SPINK13

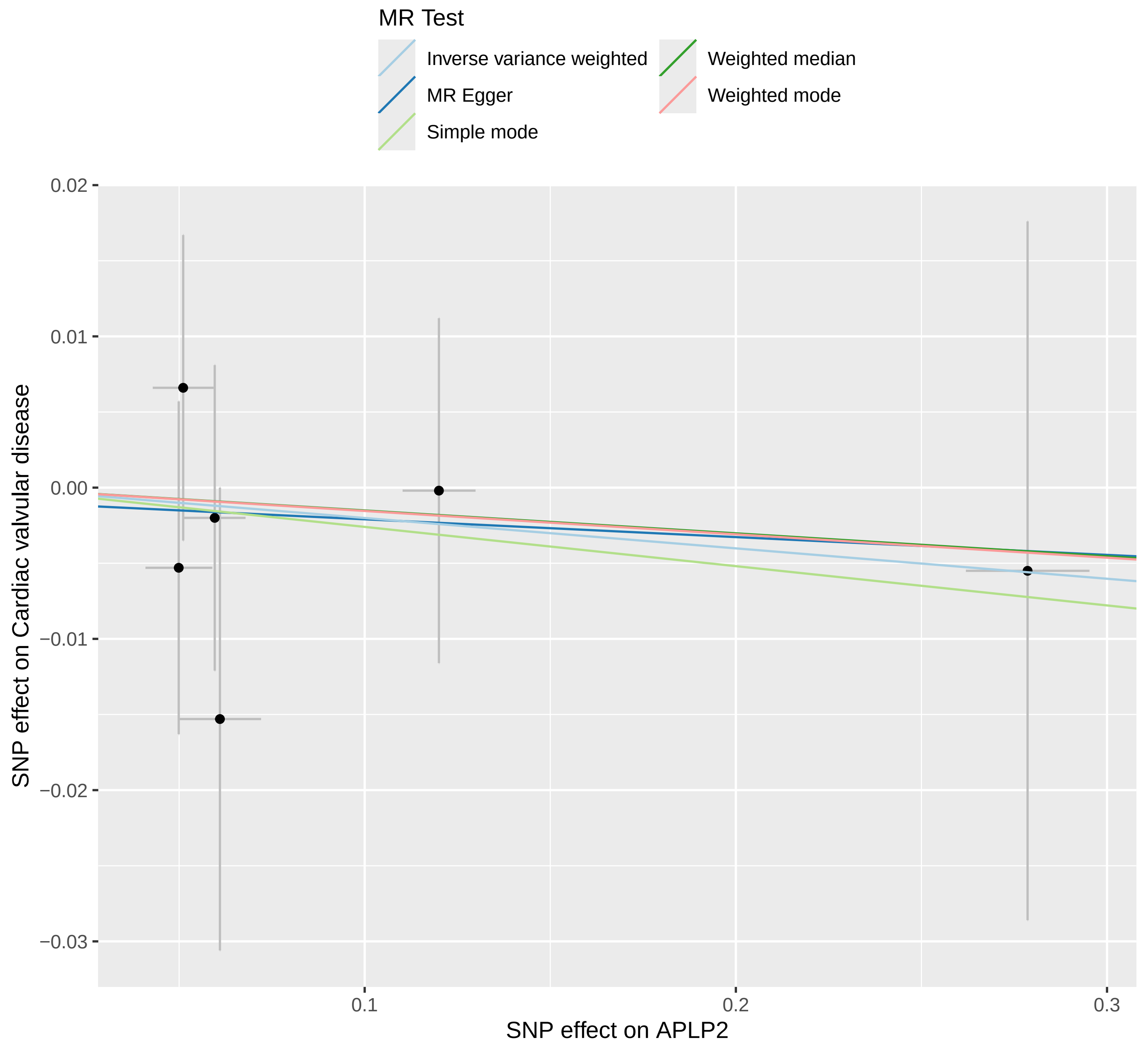

MR Test
Inverse variance weighted
MR Egger
Simple mode
Weighted median
Weighted mode
0.02
0.01
0.00
−0.01
−0.02
−0.03
SNP effect on Cardiac valvular disease
0.1
0.2
0.3
SNP effect on APLP2

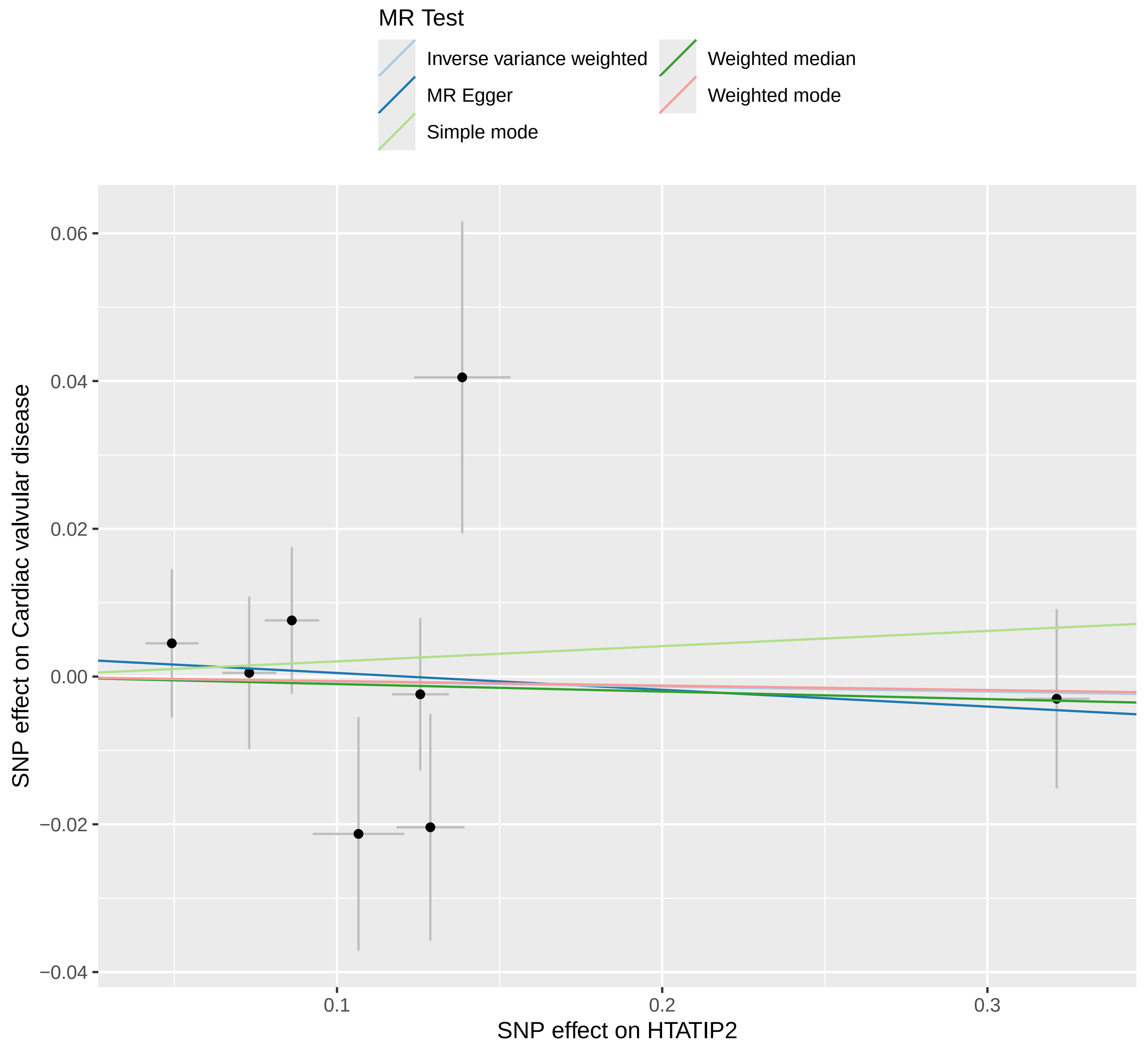

MR Test
Inverse variance weighted
MR Egger
Simple mode
Weighted median
Weighted mode
0.06
0.04
0.02
0.00
−0.02
−0.04
0.1
0.2
0.3
SNP effect on Cardiac valvular disease
SNP effect on HTATIP2

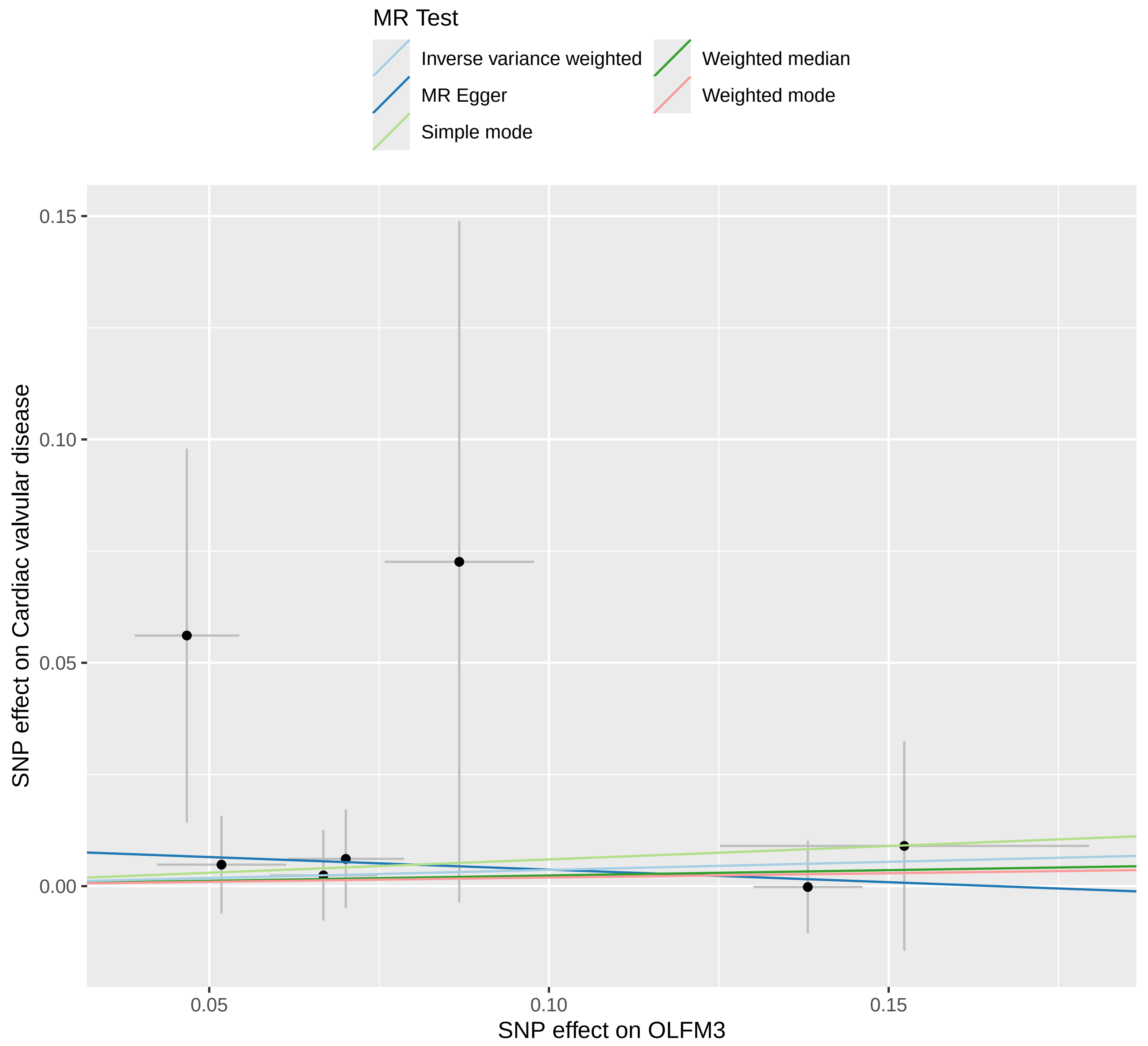

MR Test
Inverse variance weighted
MR Egger
Simple mode
Weighted median
Weighted mode
0.15
0.10
0.05
0.00
0.05
0.10
0.15
SNP effect on Cardiac valvular disease
SNP effect on OLFM3

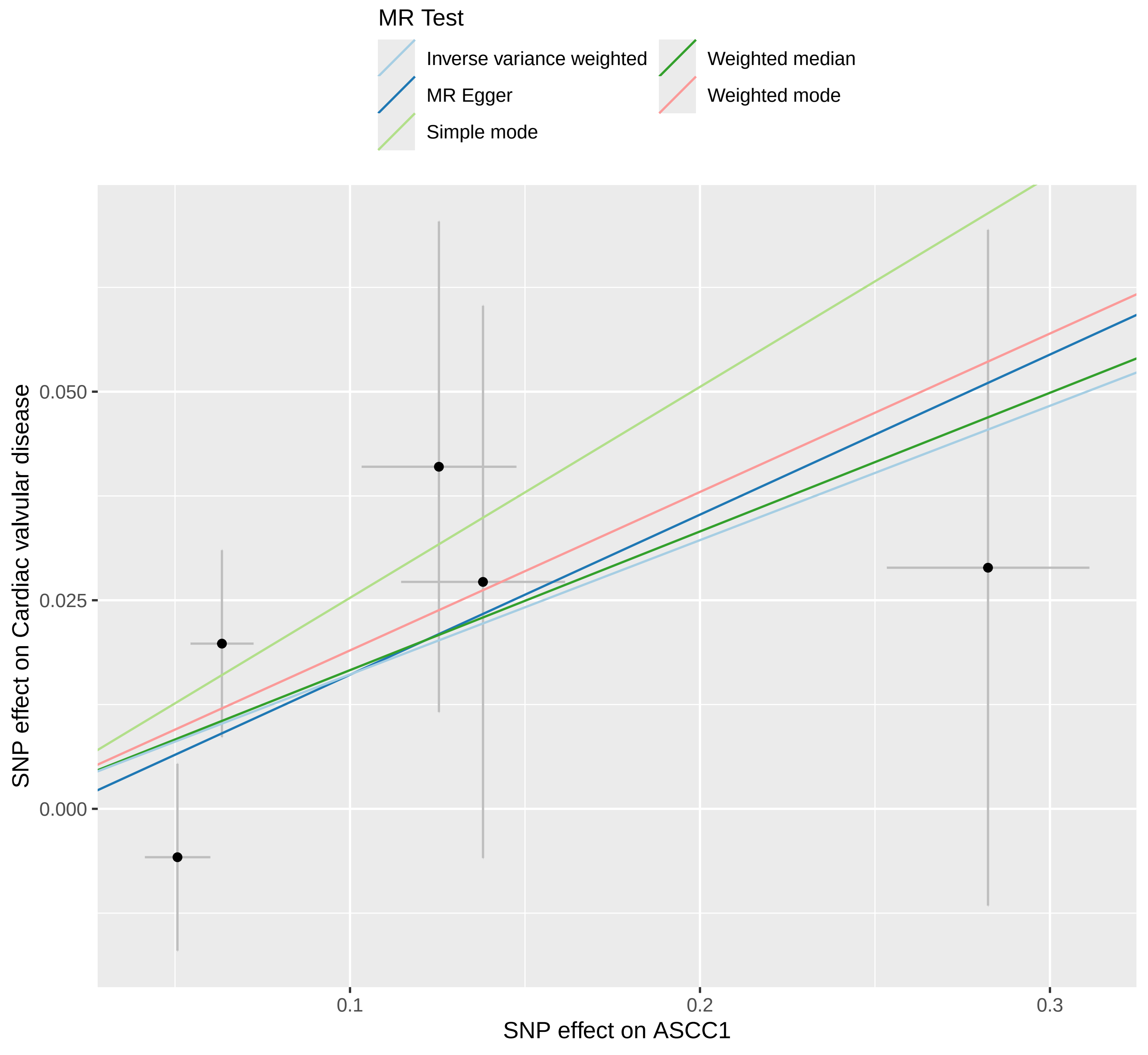
MR Test
Inverse variance weighted
MR Egger
Simple mode
Weighted median
Weighted mode
SNP effect on Cardiac valvular disease
0.050
0.025
0.000
0.1
0.2
0.3
SNP effect on ASCC1

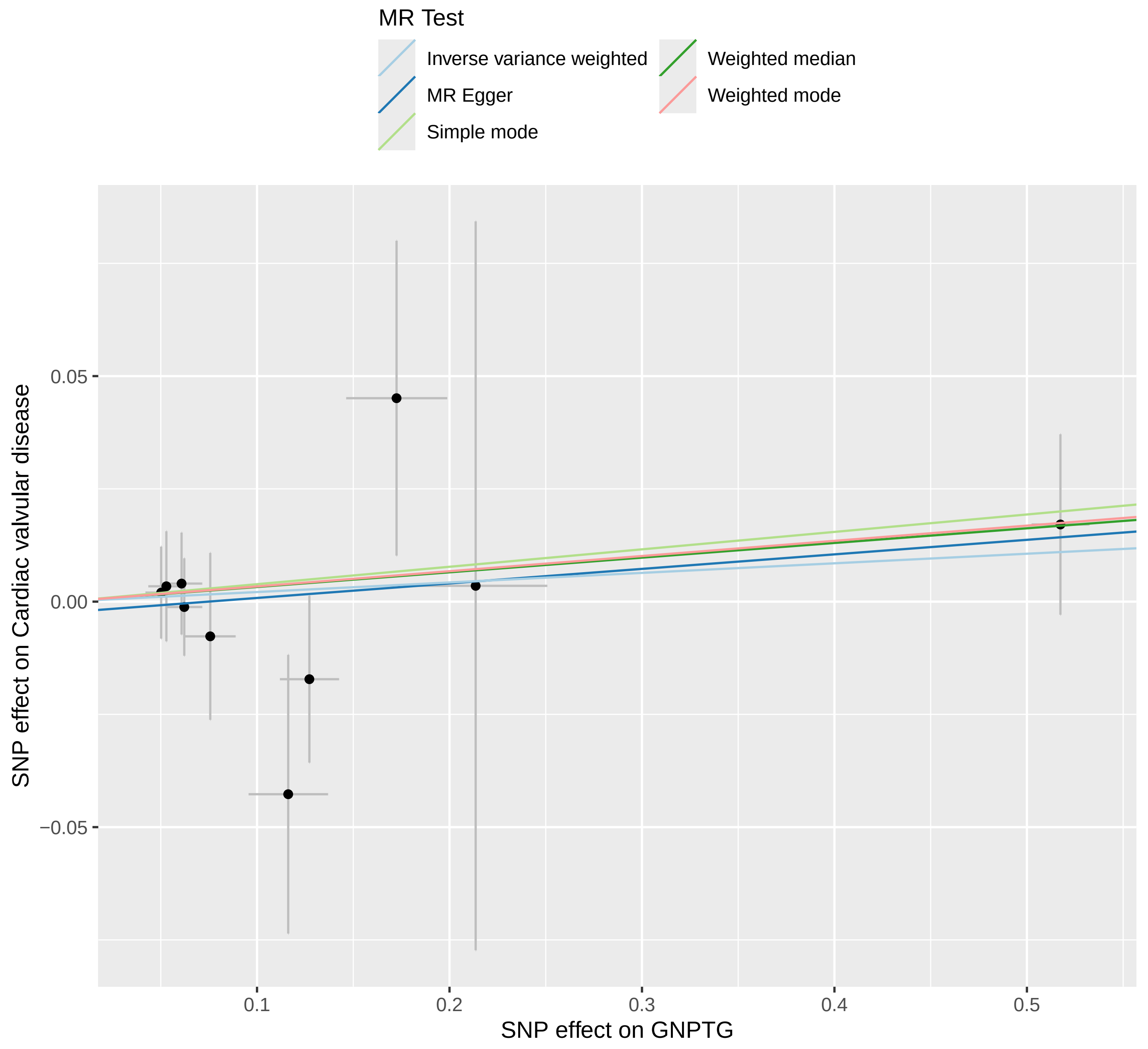
MR Test
Inverse variance weighted
MR Egger
Simple mode
Weighted median
Weighted mode
SNP effect on Cardiac valvular disease
0.05
0.00
−0.05
0.1
0.2
0.3
0.4
0.5
SNP effect on GNPTG

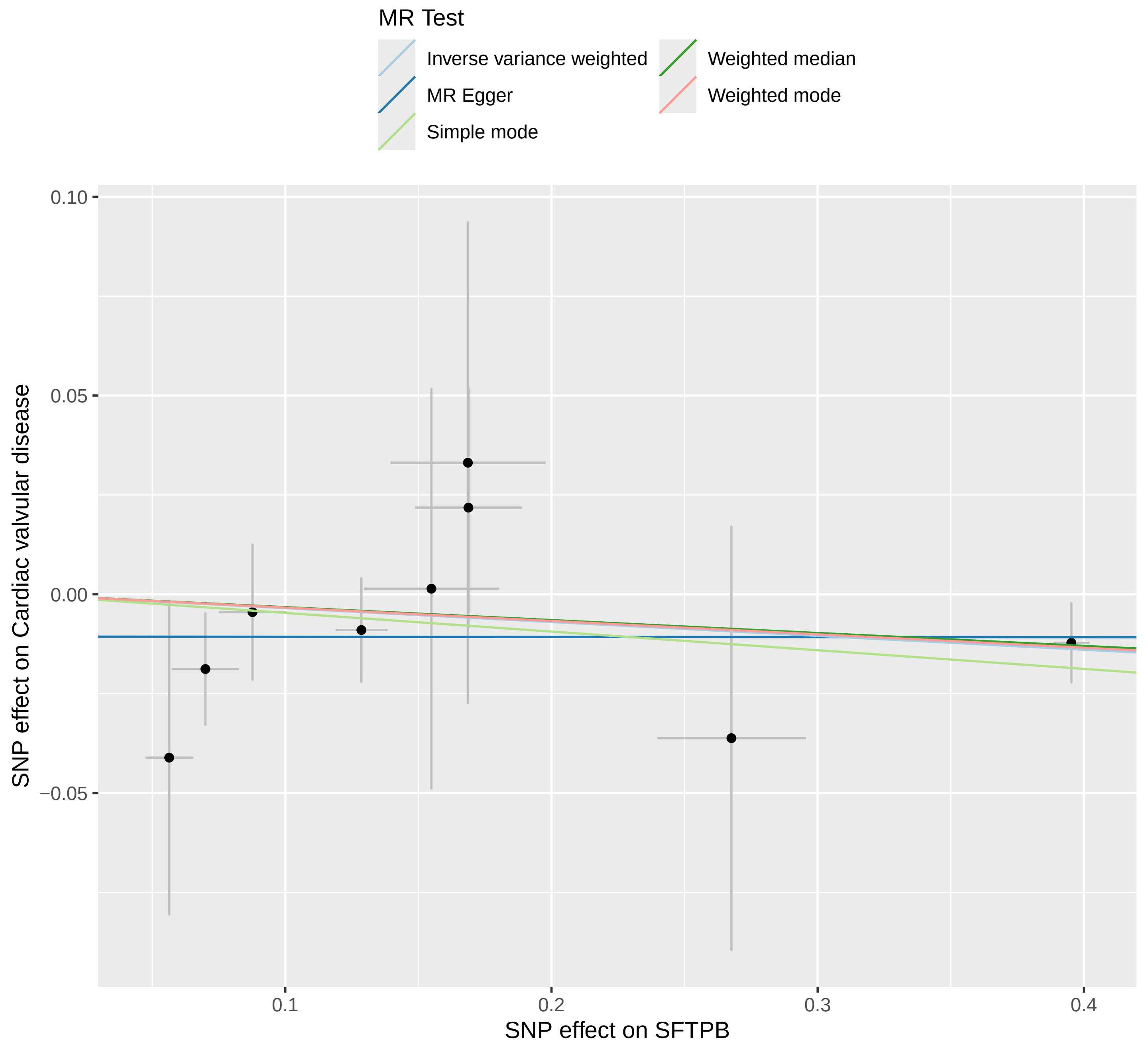

MR Test
Inverse variance weighted
MR Egger
Simple mode
Weighted median
Weighted mode
0.10
0.05
0.00
−0.05
SNP effect on Cardiac valvular disease
0.1
0.2
0.3
0.4
SNP effect on SFTPB

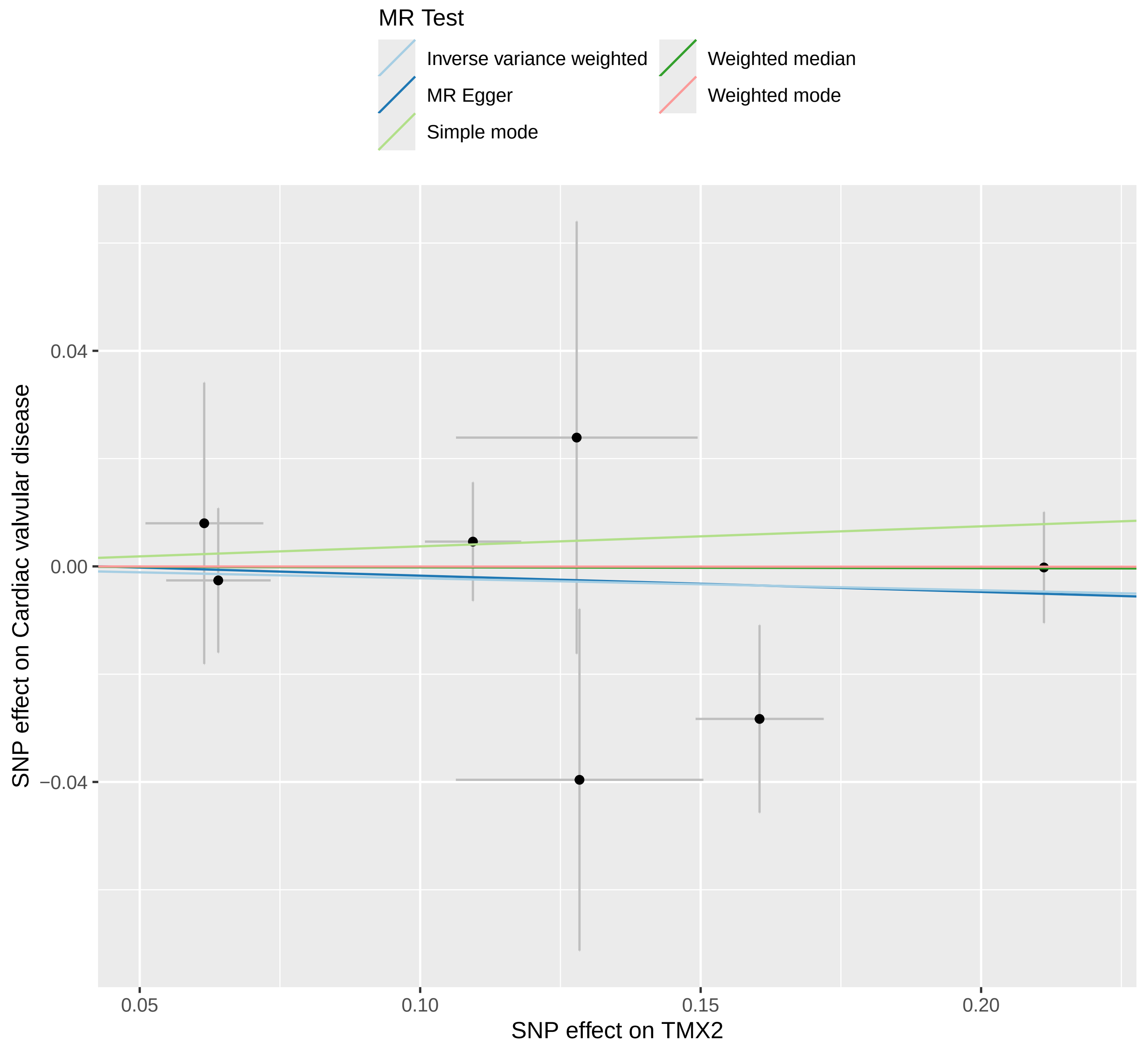
MR Test
Inverse variance weighted
MR Egger
Simple mode
Weighted median
Weighted mode
0.04
0.00
−0.04
SNP effect on Cardiac valvular disease
0.05
0.10
0.15
0.20
SNP effect on TMX2

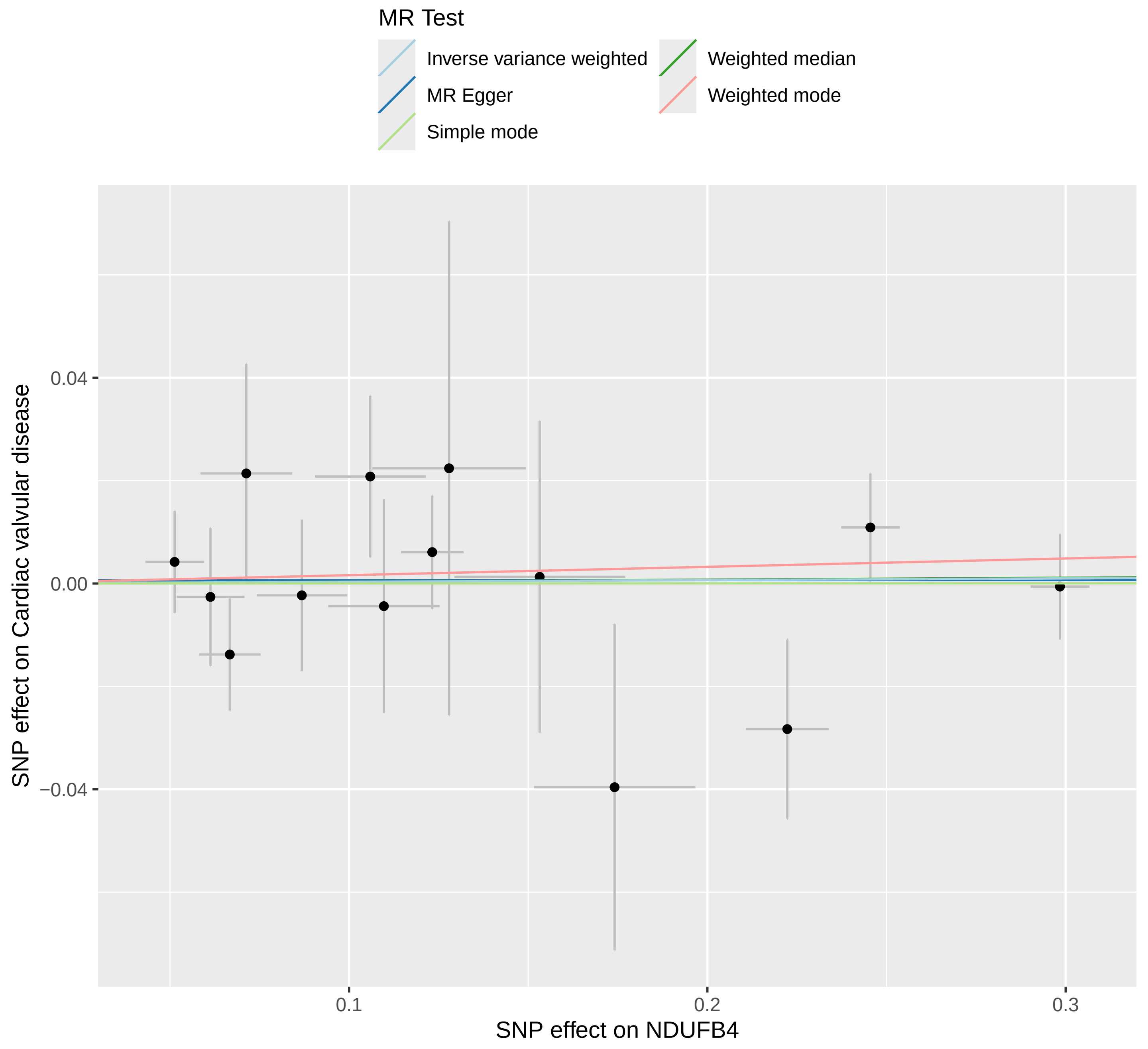

MR Test
Inverse variance weighted
MR Egger
Simple mode
Weighted median
Weighted mode
0.04
0.00
−0.04
0.1
0.2
0.3
SNP effect on Cardiac valvular disease
SNP effect on NDUFB4

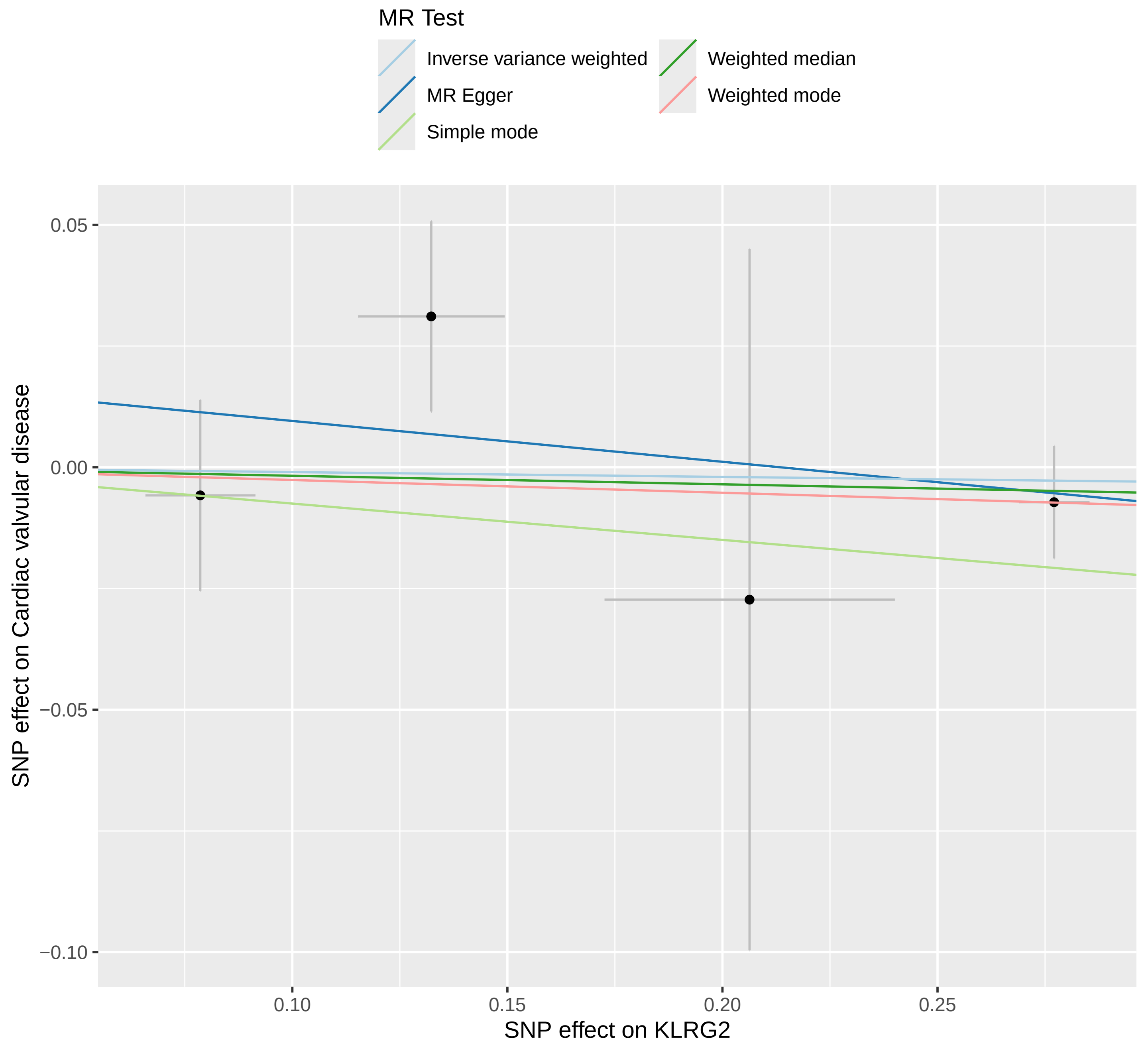
MR Test
Inverse variance weighted
MR Egger
Simple mode
Weighted median
Weighted mode
0.05
0.00
−0.05
−0.10
0.10
0.15
0.20
0.25
SNP effect on Cardiac valvular disease
SNP effect on KLRG2

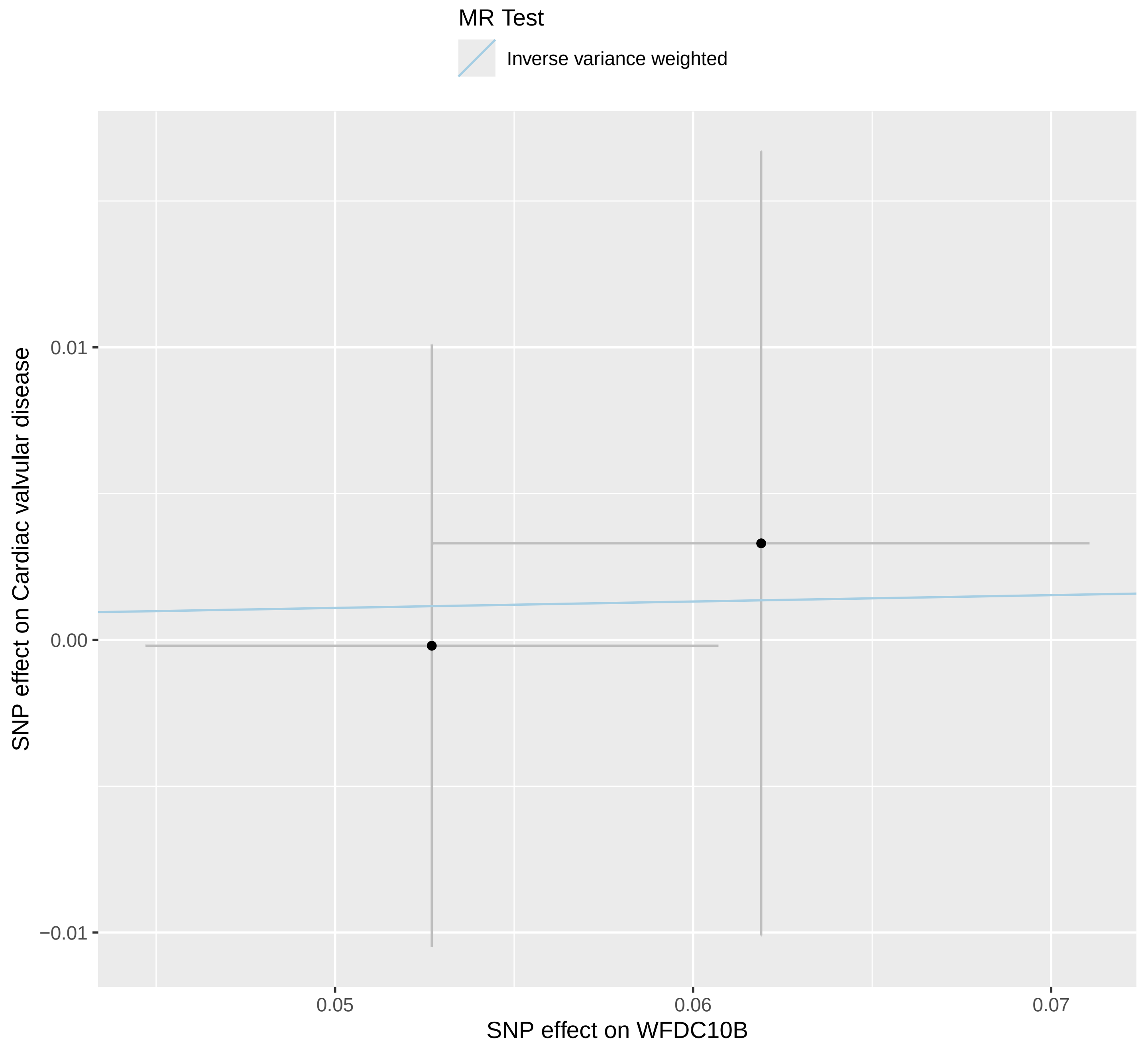

MR Test
Inverse variance weighted
SNP effect on Cardiac valvular disease
0.01
0.00
−0.01
0.05
0.06
0.07
SNP effect on WFDC10B

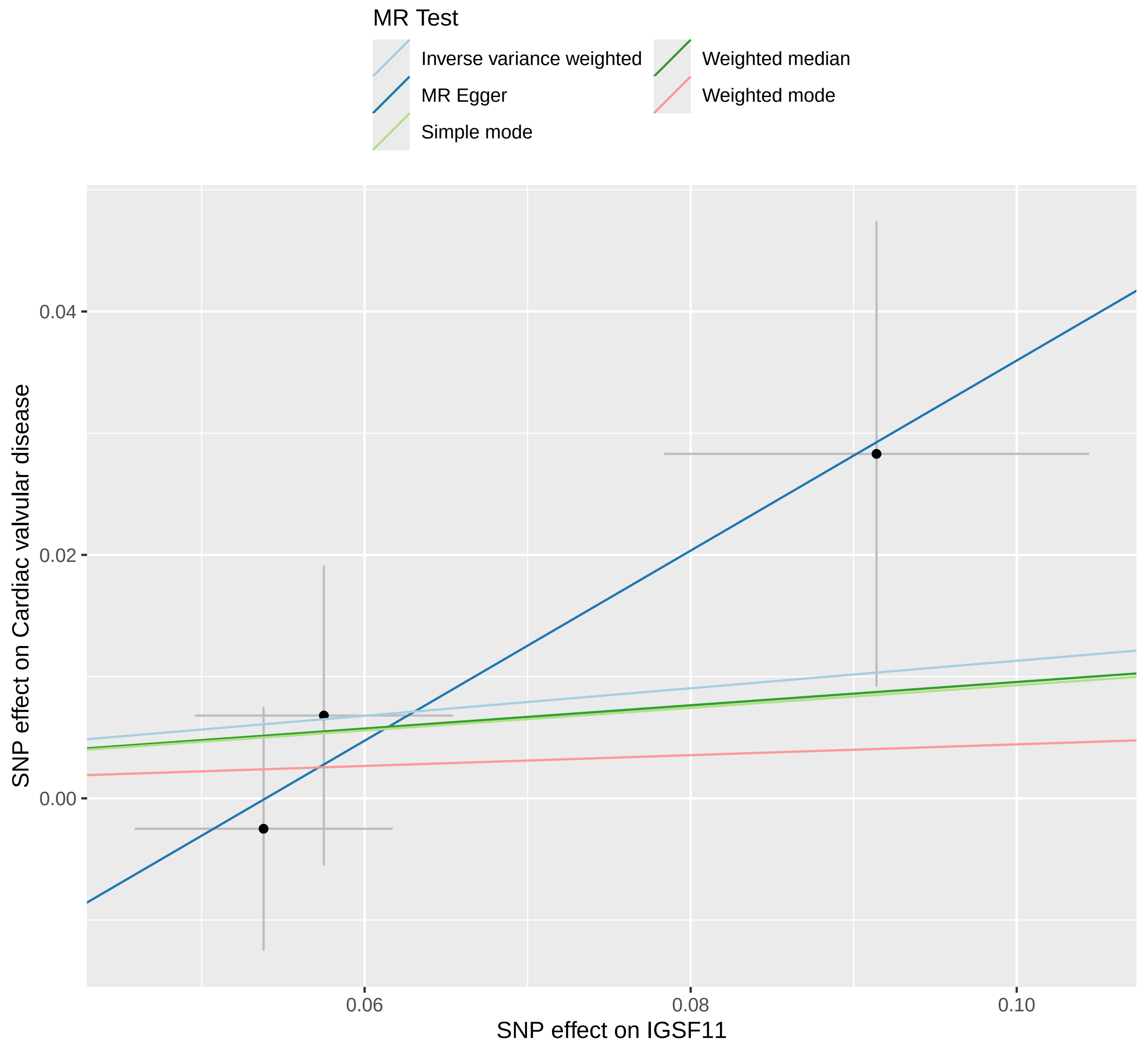

MR Test
Inverse variance weighted
MR Egger
Simple mode
Weighted median
Weighted mode
SNP effect on Cardiac valvular disease
0.04
0.02
0.00
0.06
0.08
0.10
SNP effect on IGSF11

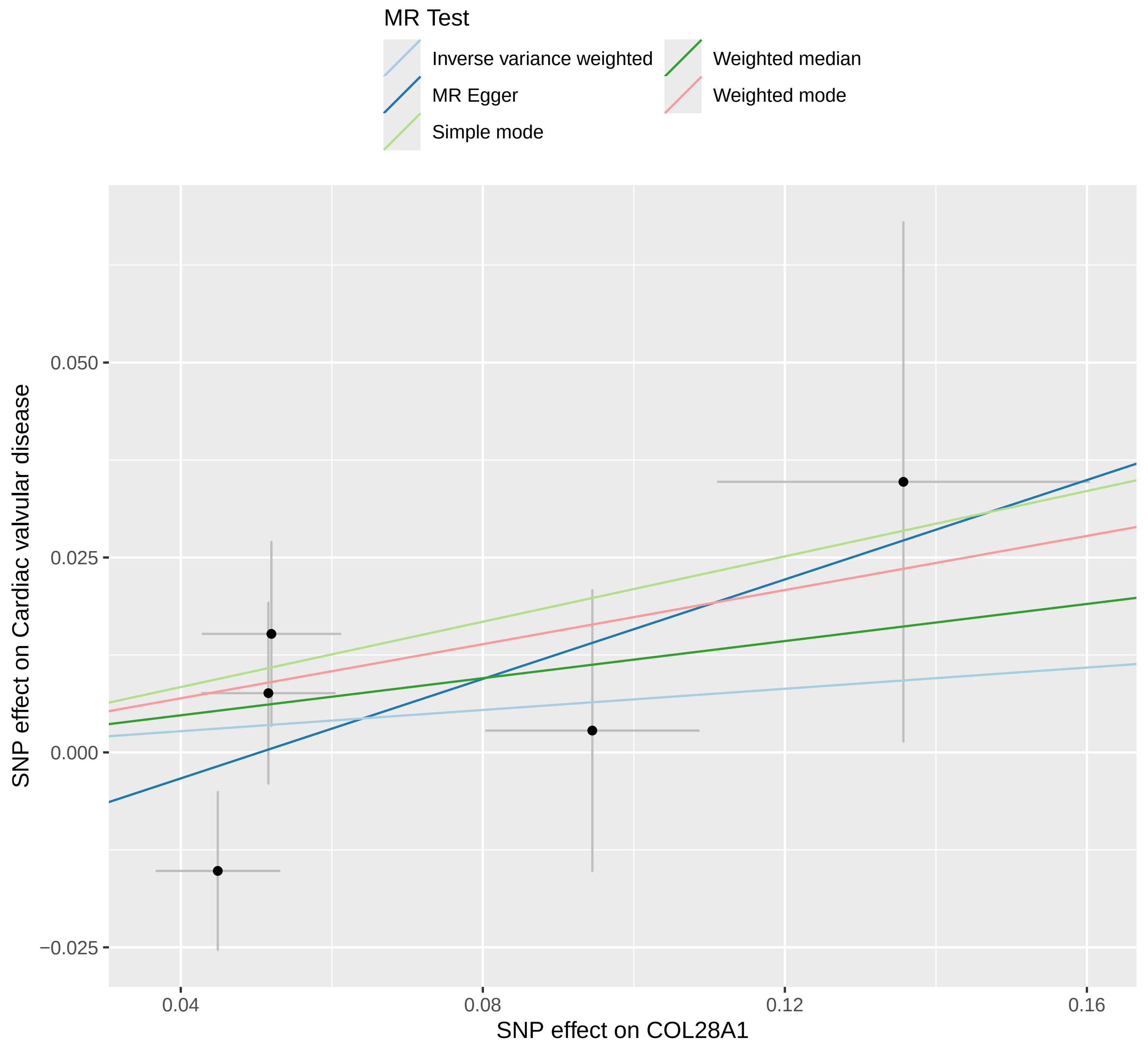

MR Test
Inverse variance weighted
MR Egger
Simple mode
Weighted median
Weighted mode
SNP effect on Cardiac valvular disease
0.050
0.025
0.000
−0.025
0.04
0.08
0.12
0.16
SNP effect on COL28A1

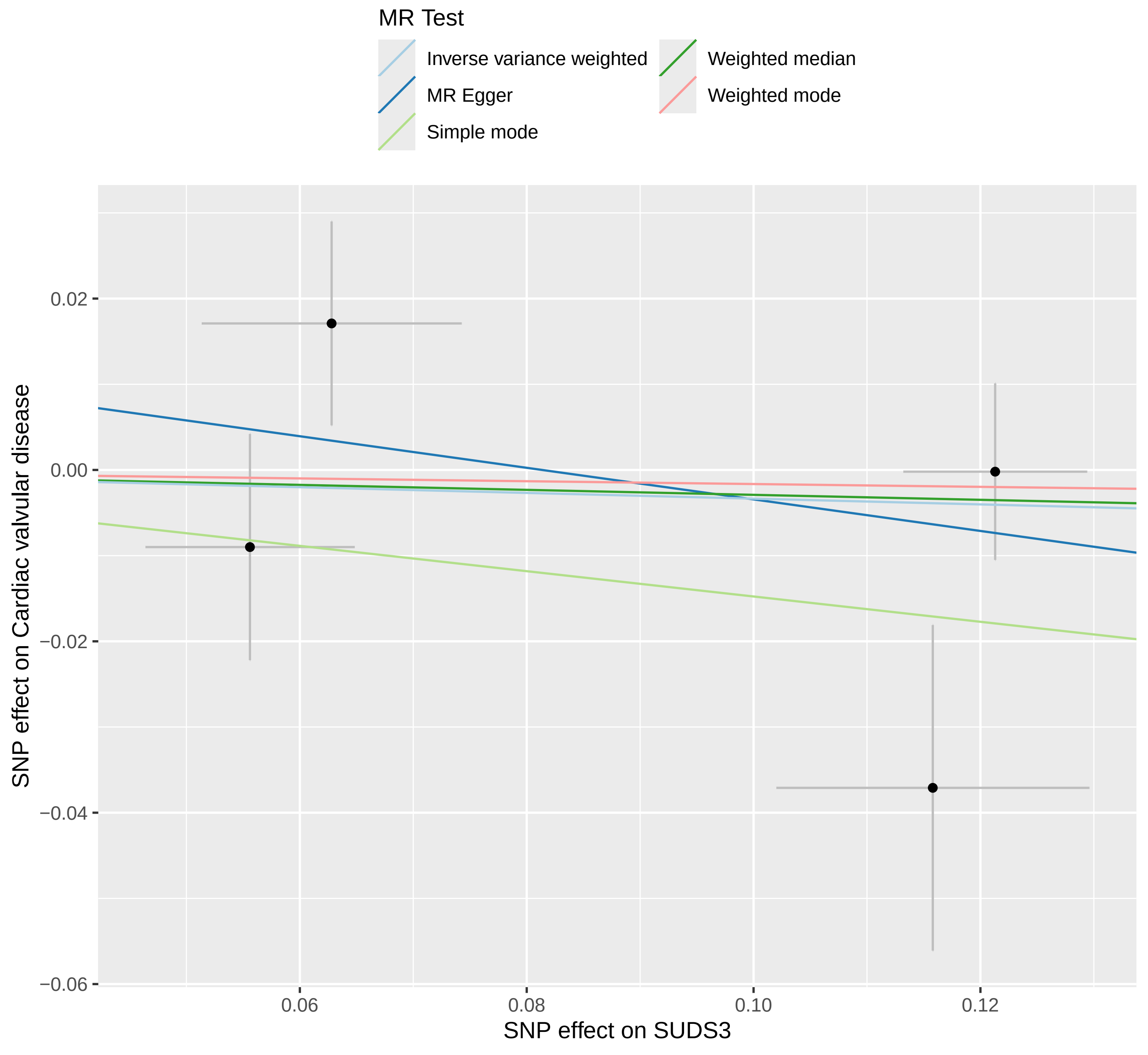

MR Test
Inverse variance weighted
MR Egger
Simple mode
Weighted median
Weighted mode
0.02
0.00
−0.02
−0.04
−0.06
0.06
0.08
0.10
0.12
SNP effect on Cardiac valvular disease
SNP effect on SUDS3

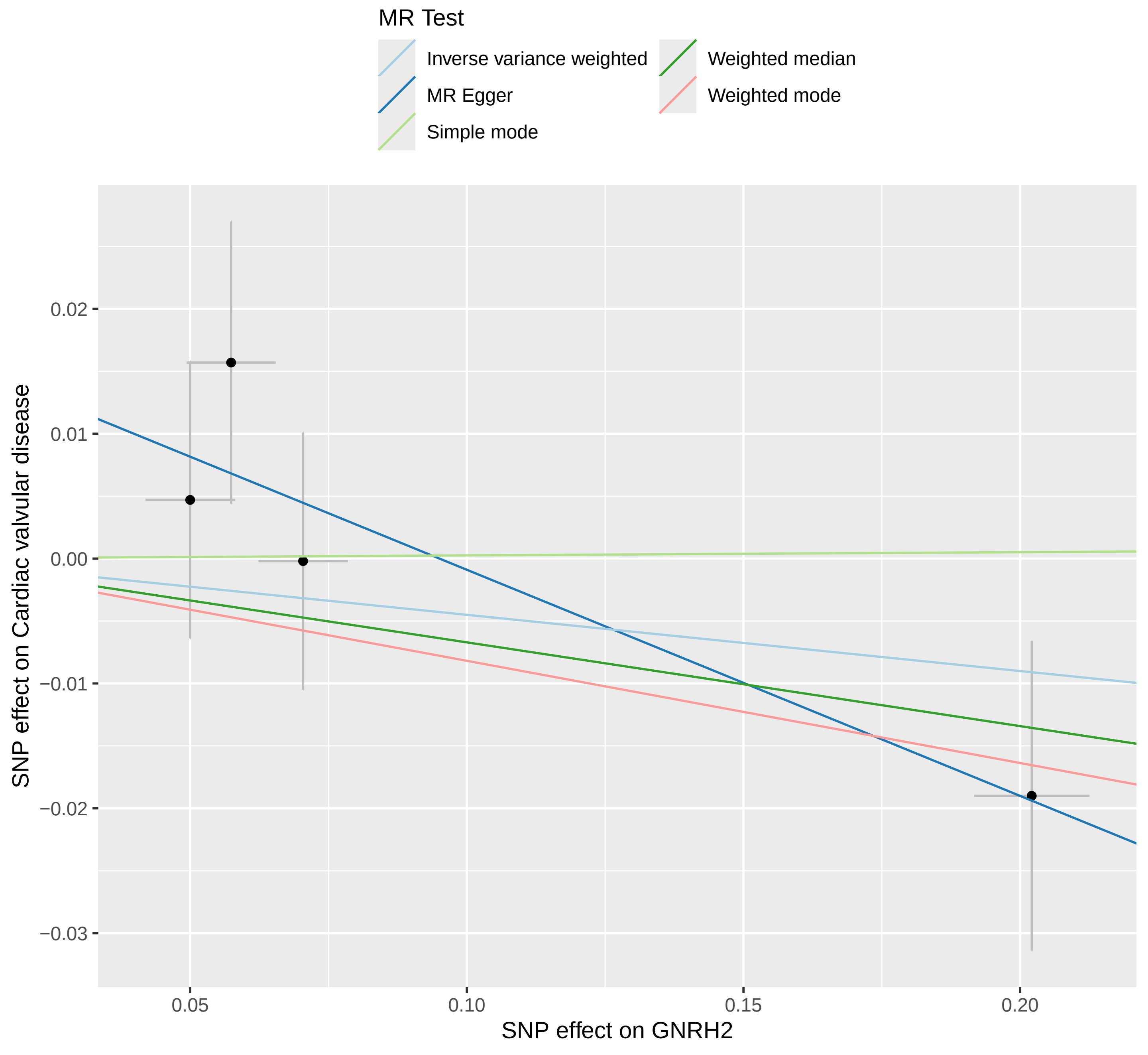
MR Test
Inverse variance weighted
MR Egger
Simple mode
Weighted median
Weighted mode
0.02
0.01
0.00
−0.01
−0.02
−0.03
SNP effect on Cardiac valvular disease
0.05
0.10
0.15
0.20
SNP effect on GNRH2

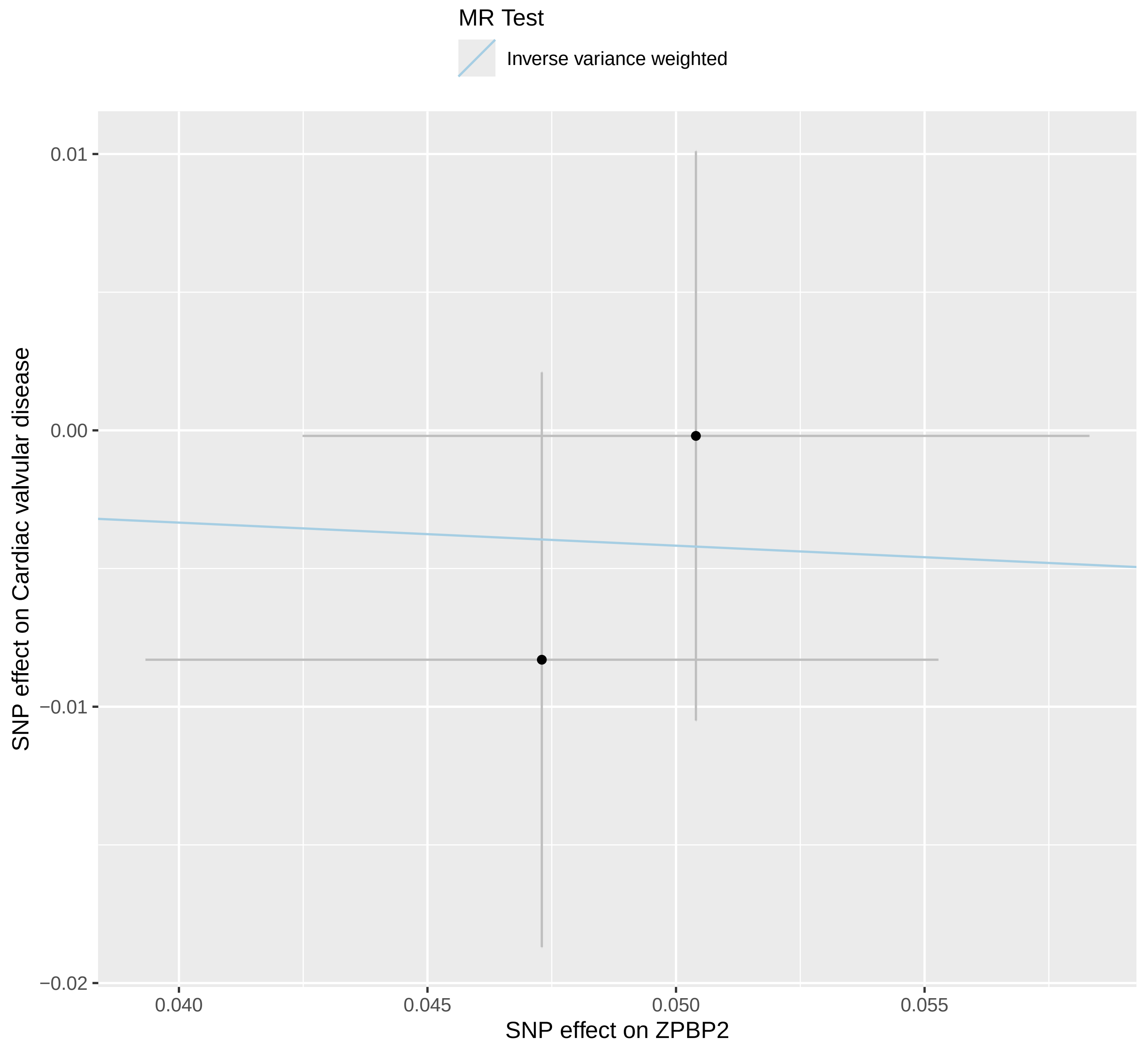

MR Test
Inverse variance weighted
0.01
0.00
−0.01
−0.02
0.040
0.045
0.050
0.055
SNP effect on ZPBP2
SNP effect on Cardiac valvular disease

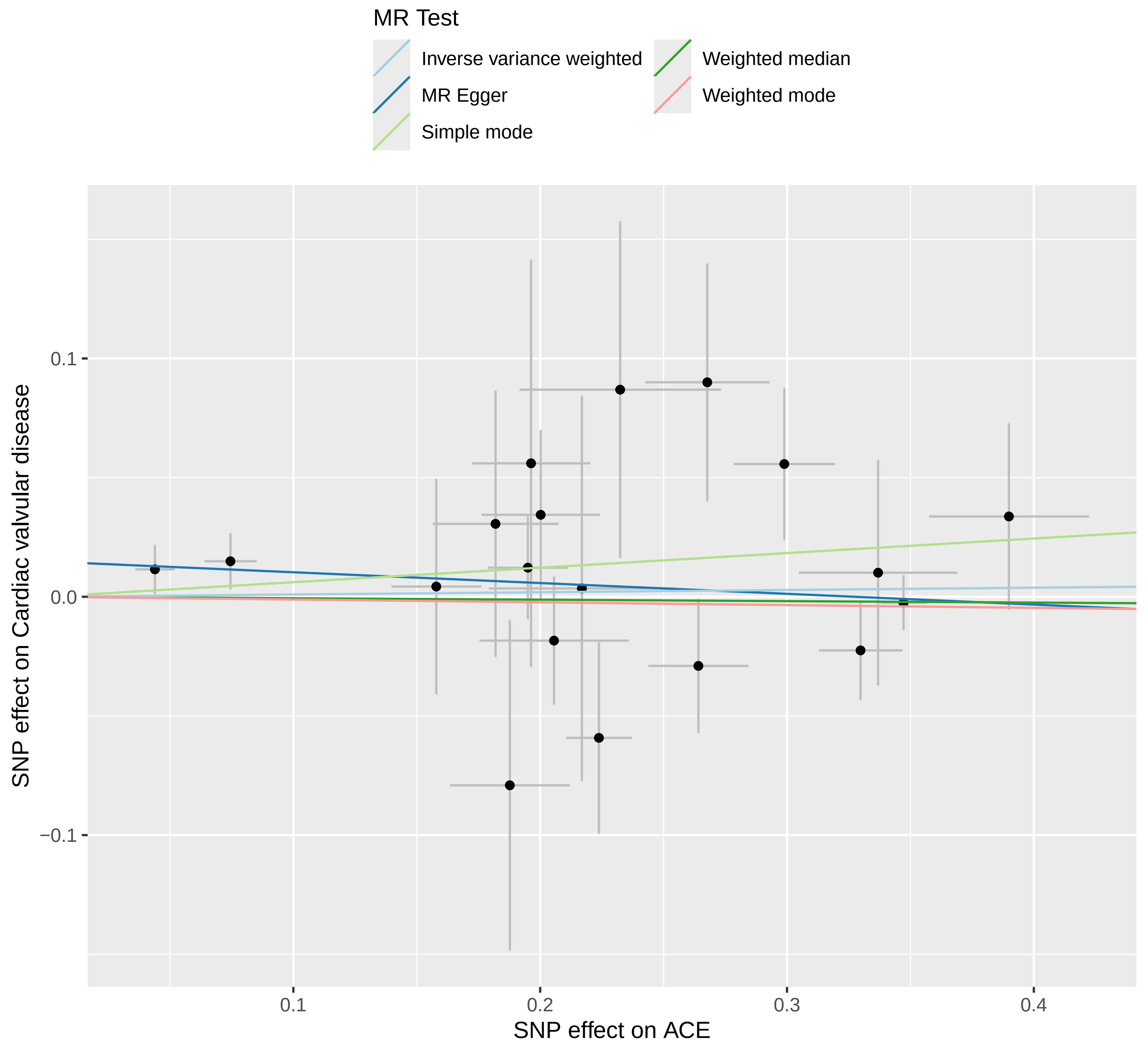

MR Test
Inverse variance weighted
MR Egger
Simple mode
Weighted median
Weighted mode
0.1
0.0
−0.1
SNP effect on Cardiac valvular disease
0.1
0.2
0.3
0.4
SNP effect on ACE

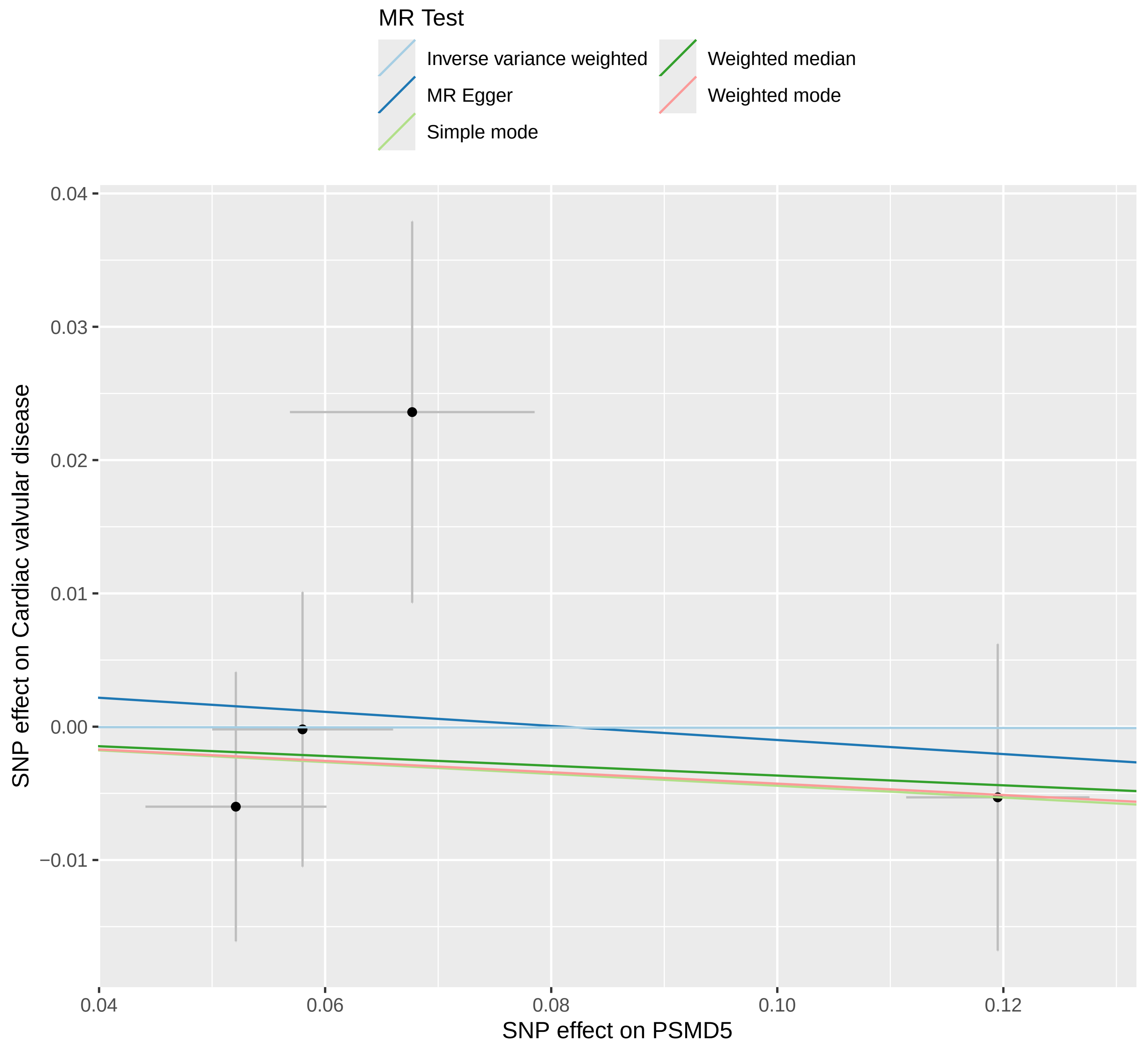

MR Test
Inverse variance weighted
MR Egger
Simple mode
Weighted median
Weighted mode
0.04
0.03
0.02
0.01
0.00
−0.01
SNP effect on Cardiac valvular disease
0.04
0.06
0.08
0.10
0.12
SNP effect on PSMD5

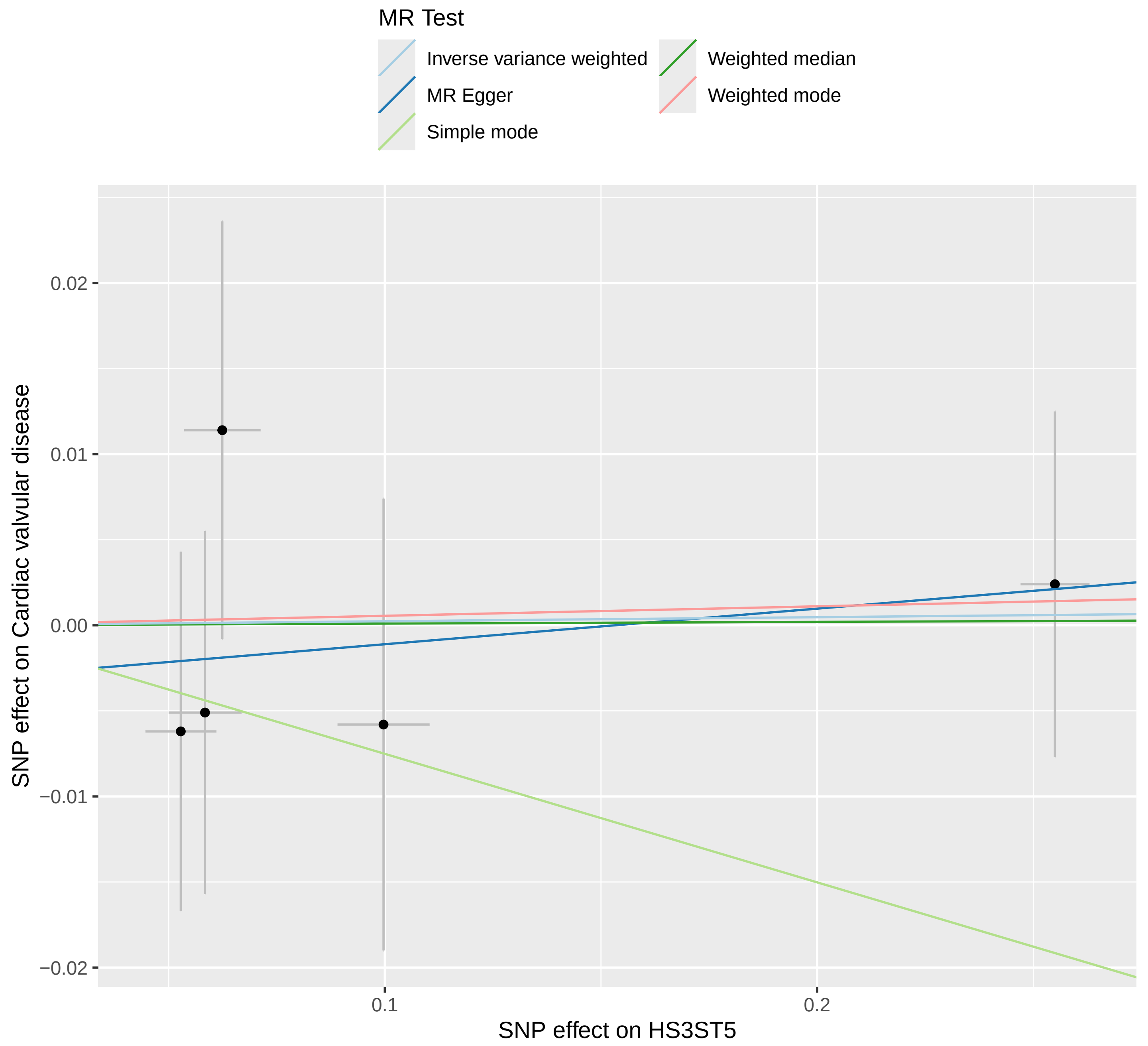

MR Test
Inverse variance weighted
MR Egger
Simple mode
Weighted median
Weighted mode
0.02
0.01
0.00
−0.01
−0.02
SNP effect on Cardiac valvular disease
0.1
0.2
SNP effect on HS3ST5

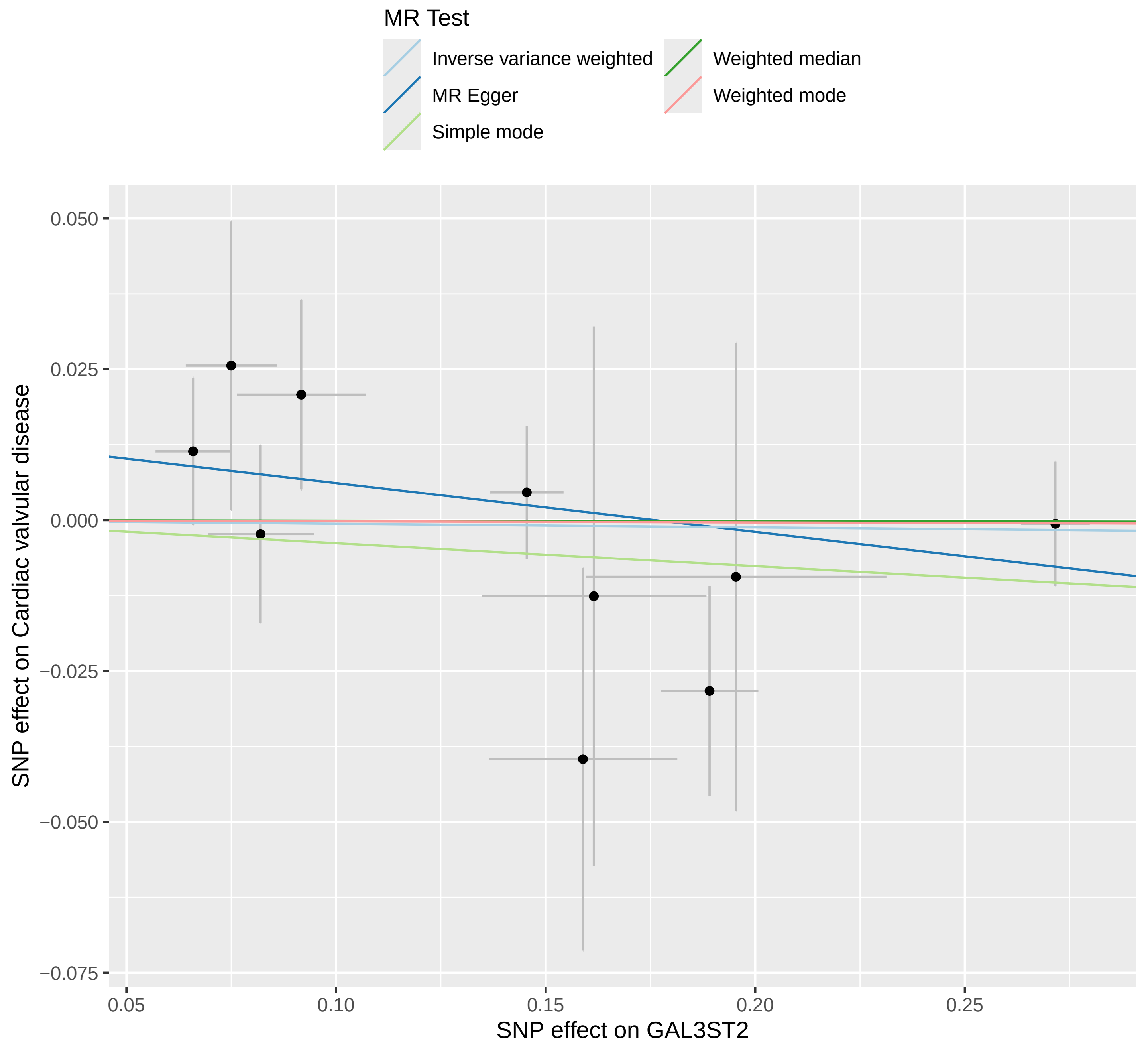

MR Test
Inverse variance weighted
MR Egger
Simple mode
Weighted median
Weighted mode
0.050
0.025
0.000
−0.025
−0.050
−0.075
SNP effect on Cardiac valvular disease
0.05
0.10
0.15
0.20
0.25
SNP effect on GAL3ST2

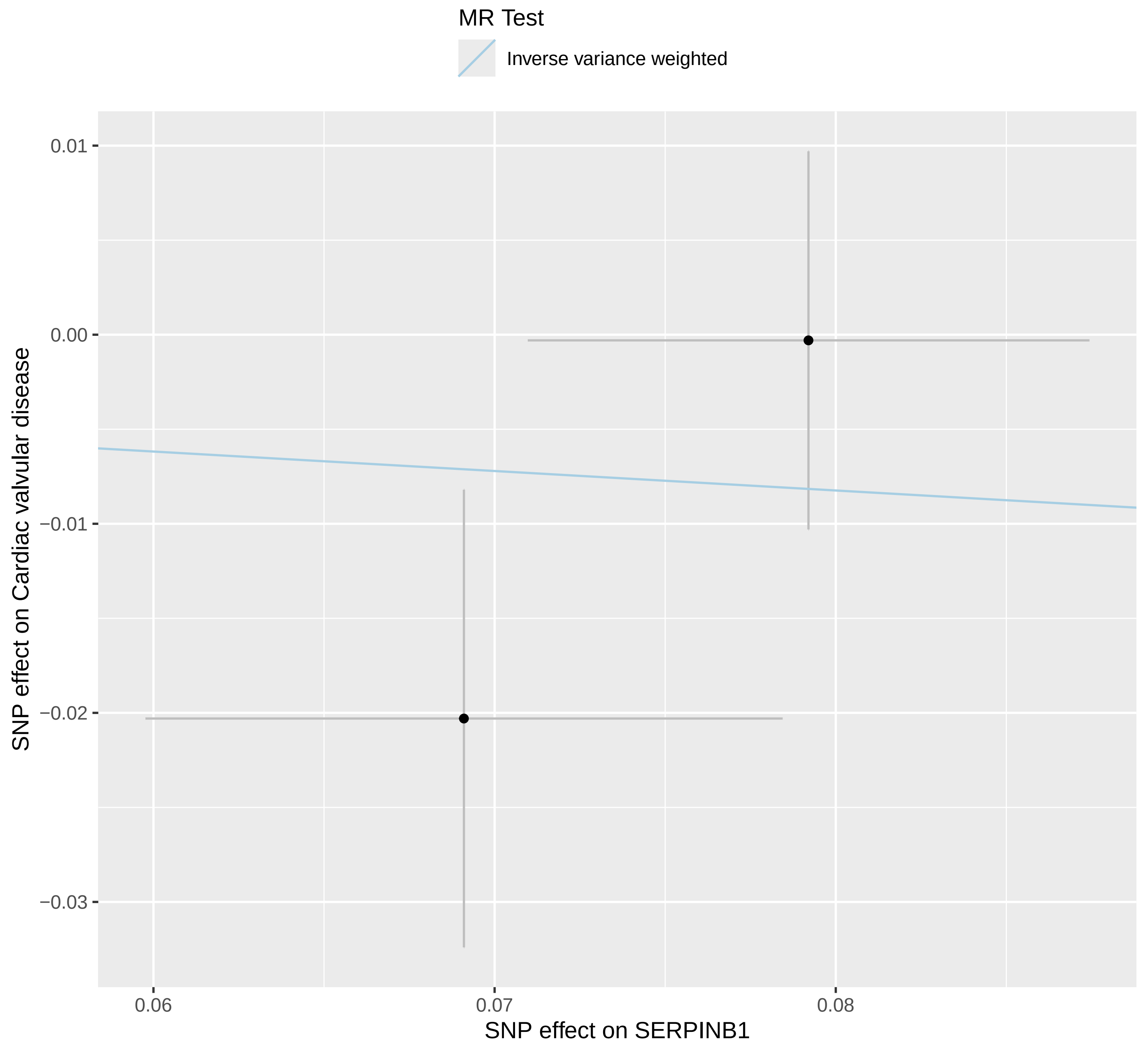

MR Test
Inverse variance weighted
0.01
0.00
−0.01
−0.02
−0.03
SNP effect on Cardiac valvular disease
0.06
0.07
0.08
SNP effect on SERPINB1

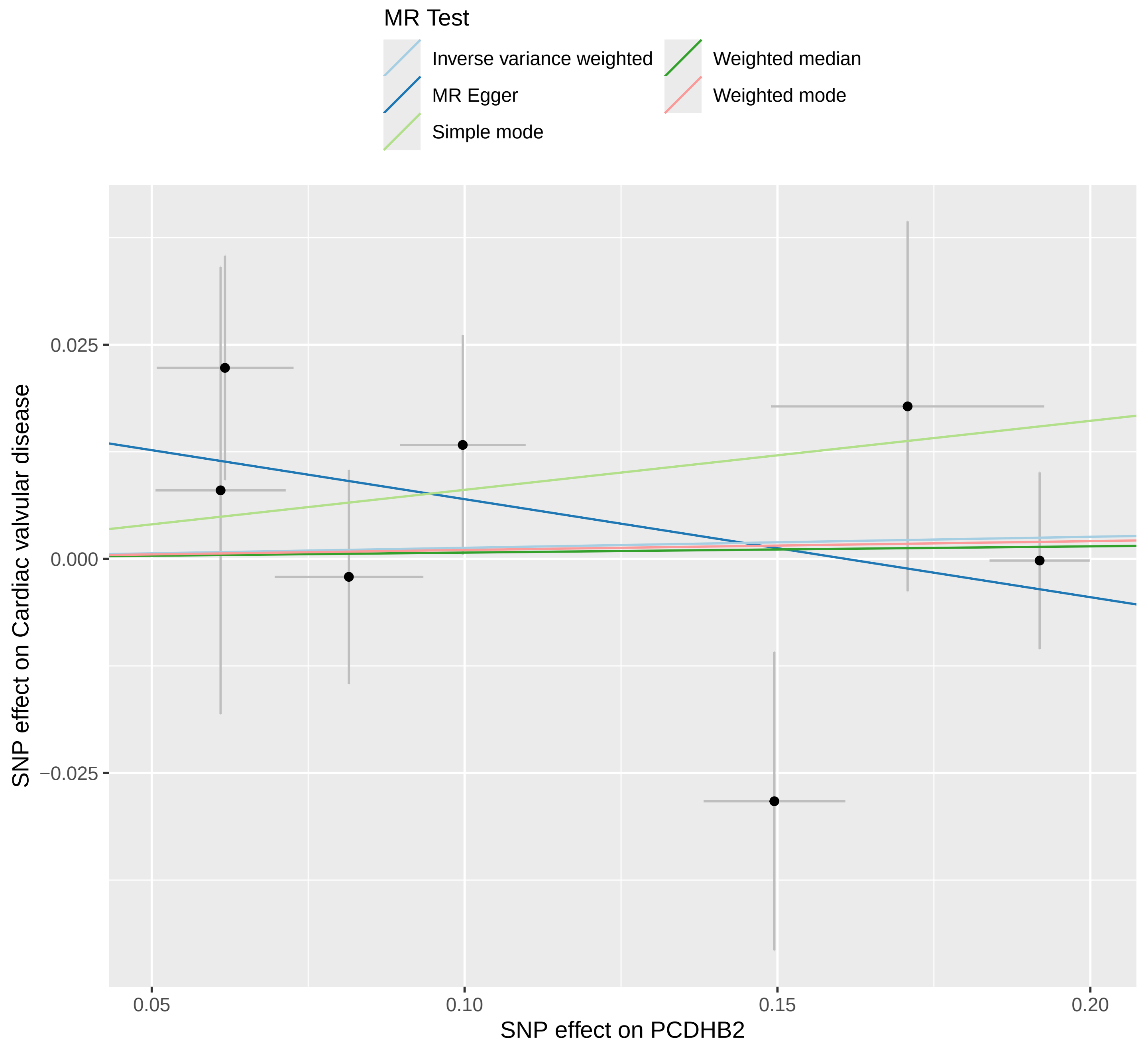

MR Test
Inverse variance weighted
MR Egger
Simple mode
Weighted median
Weighted mode
0.025
0.000
−0.025
0.05
0.10
0.15
0.20
SNP effect on Cardiac valvular disease
SNP effect on PCDHB2

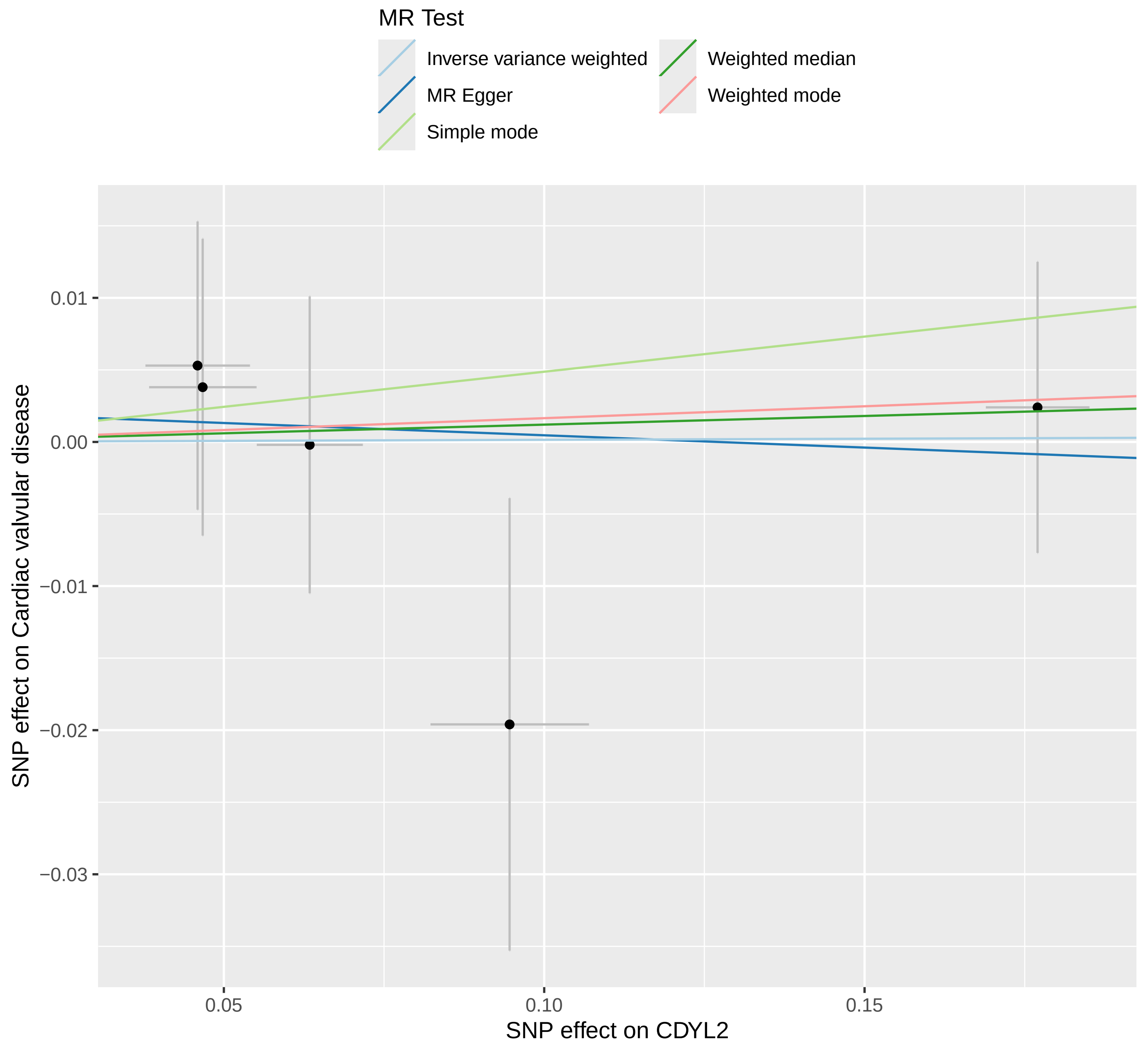
MR Test
Inverse variance weighted
MR Egger
Simple mode
Weighted median
Weighted mode
0.01
0.00
−0.01
−0.02
−0.03
SNP effect on Cardiac valvular disease
0.05
0.10
0.15
SNP effect on CDYL2

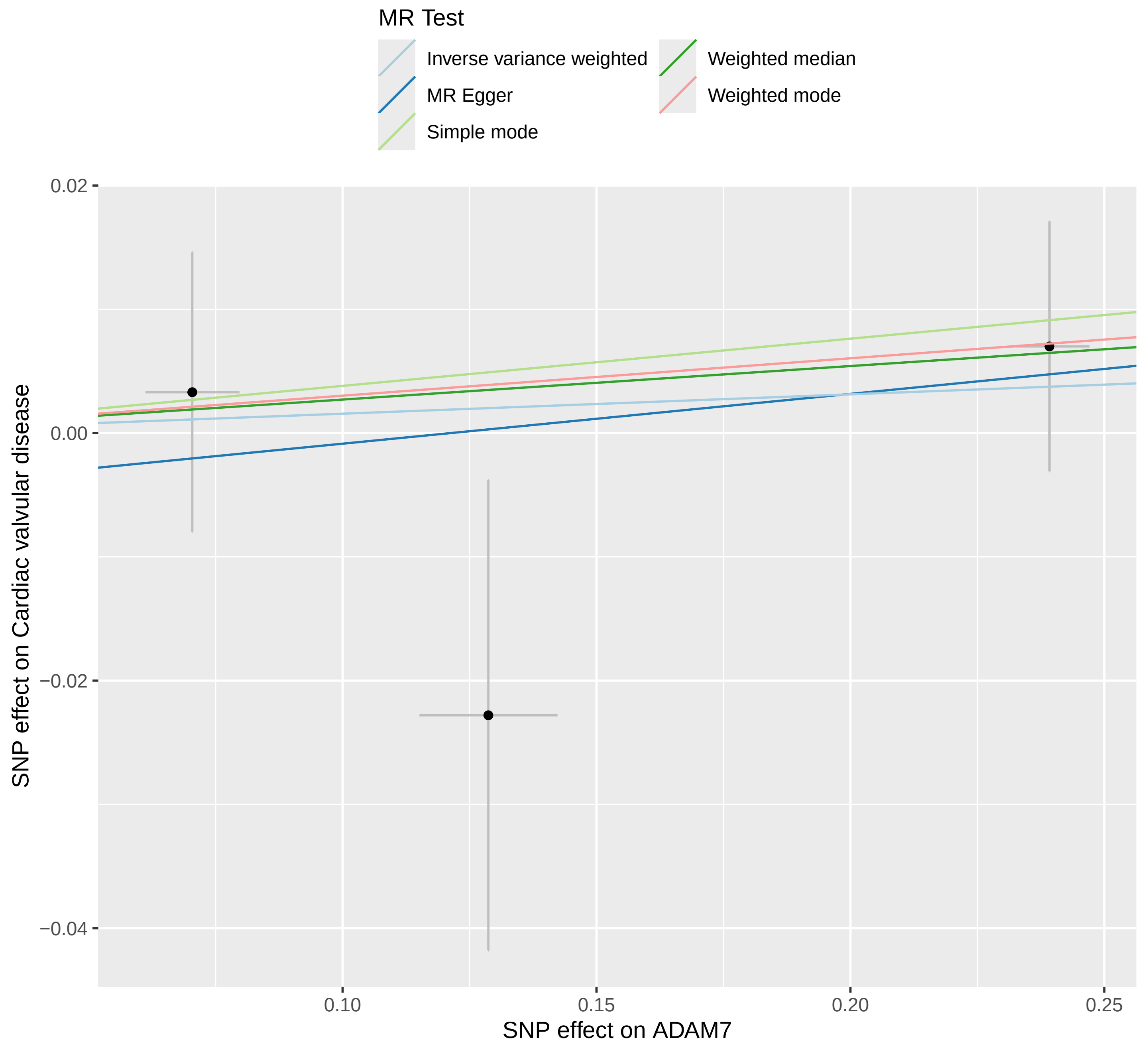

MR Test
Inverse variance weighted
MR Egger
Simple mode
Weighted median
Weighted mode
0.02
0.00
−0.02
−0.04
0.10
0.15
0.20
0.25
SNP effect on Cardiac valvular disease
SNP effect on ADAM7

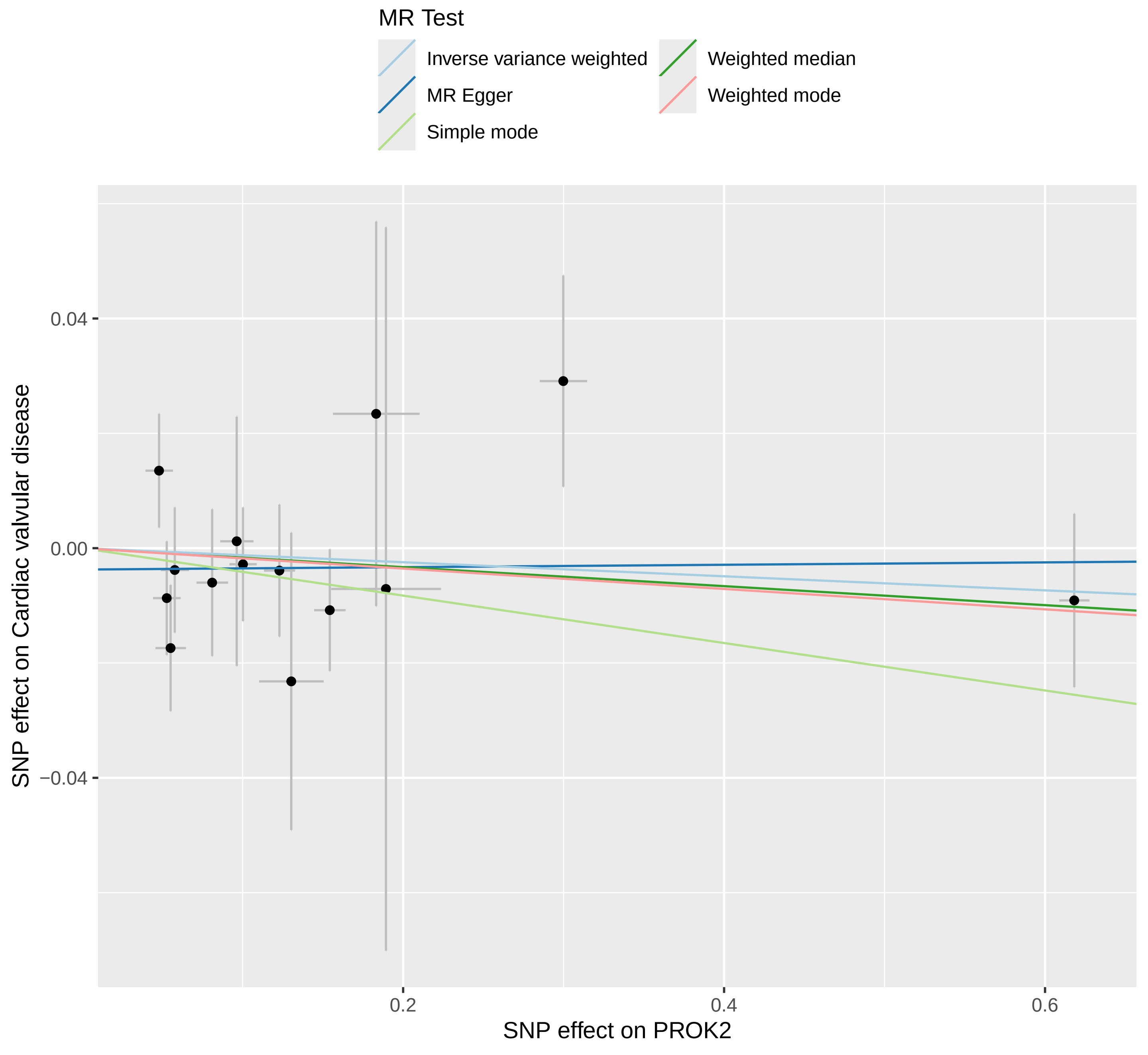
MR Test
Inverse variance weighted
MR Egger
Simple mode
Weighted median
Weighted mode
0.04
0.00
−0.04
0.2
0.4
0.6
SNP effect on Cardiac valvular disease
SNP effect on PROK2

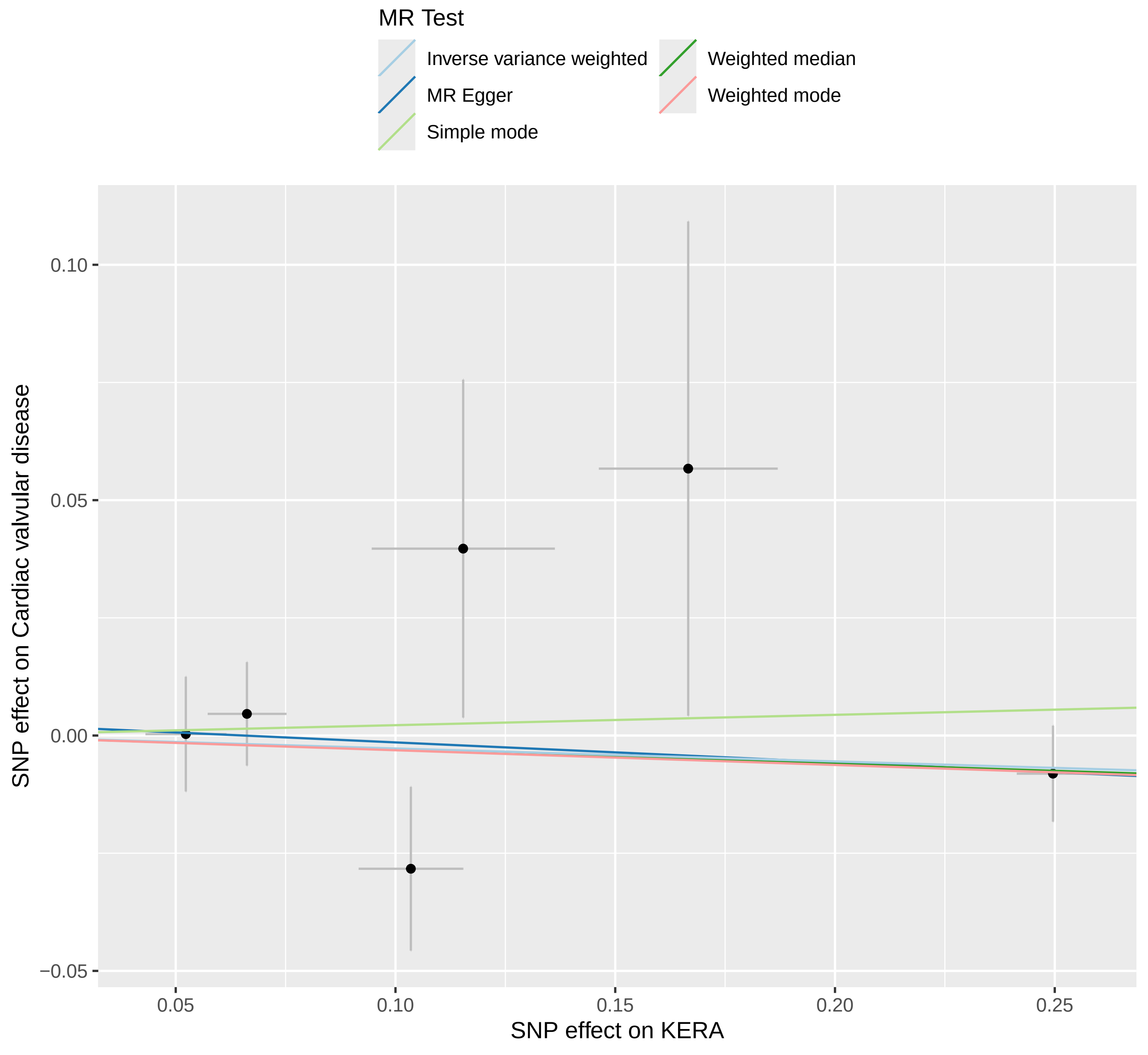
MR Test
Inverse variance weighted
MR Egger
Simple mode
Weighted median
Weighted mode
0.10
0.05
0.00
−0.05
0.05
0.10
0.15
0.20
0.25
SNP effect on KERA
SNP effect on Cardiac valvular disease

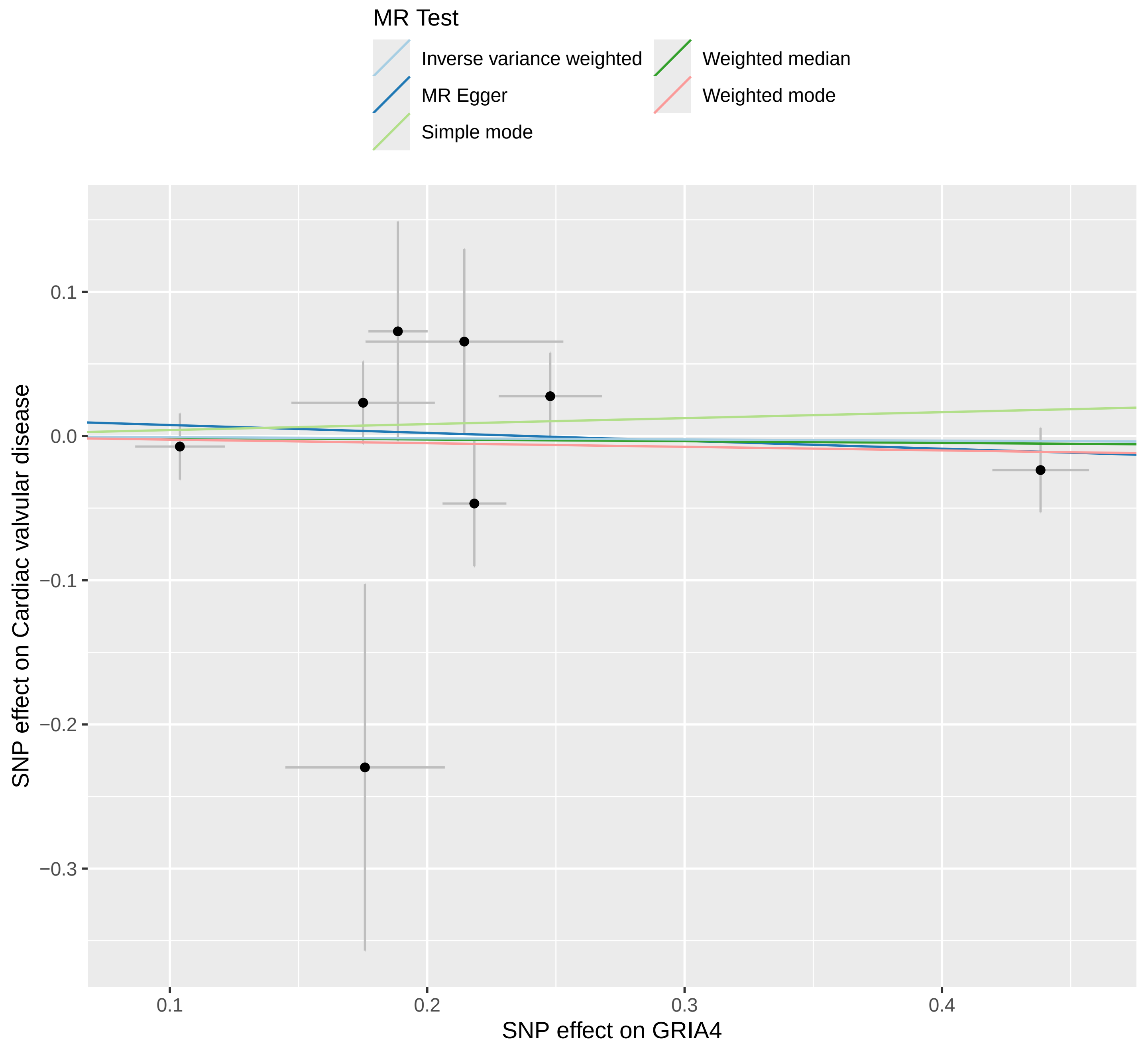
MR Test
Inverse variance weighted
MR Egger
Simple mode
Weighted median
Weighted mode
0.1
0.0
−0.1
−0.2
−0.3
SNP effect on Cardiac valvular disease
0.1
0.2
0.3
0.4
SNP effect on GRIA4

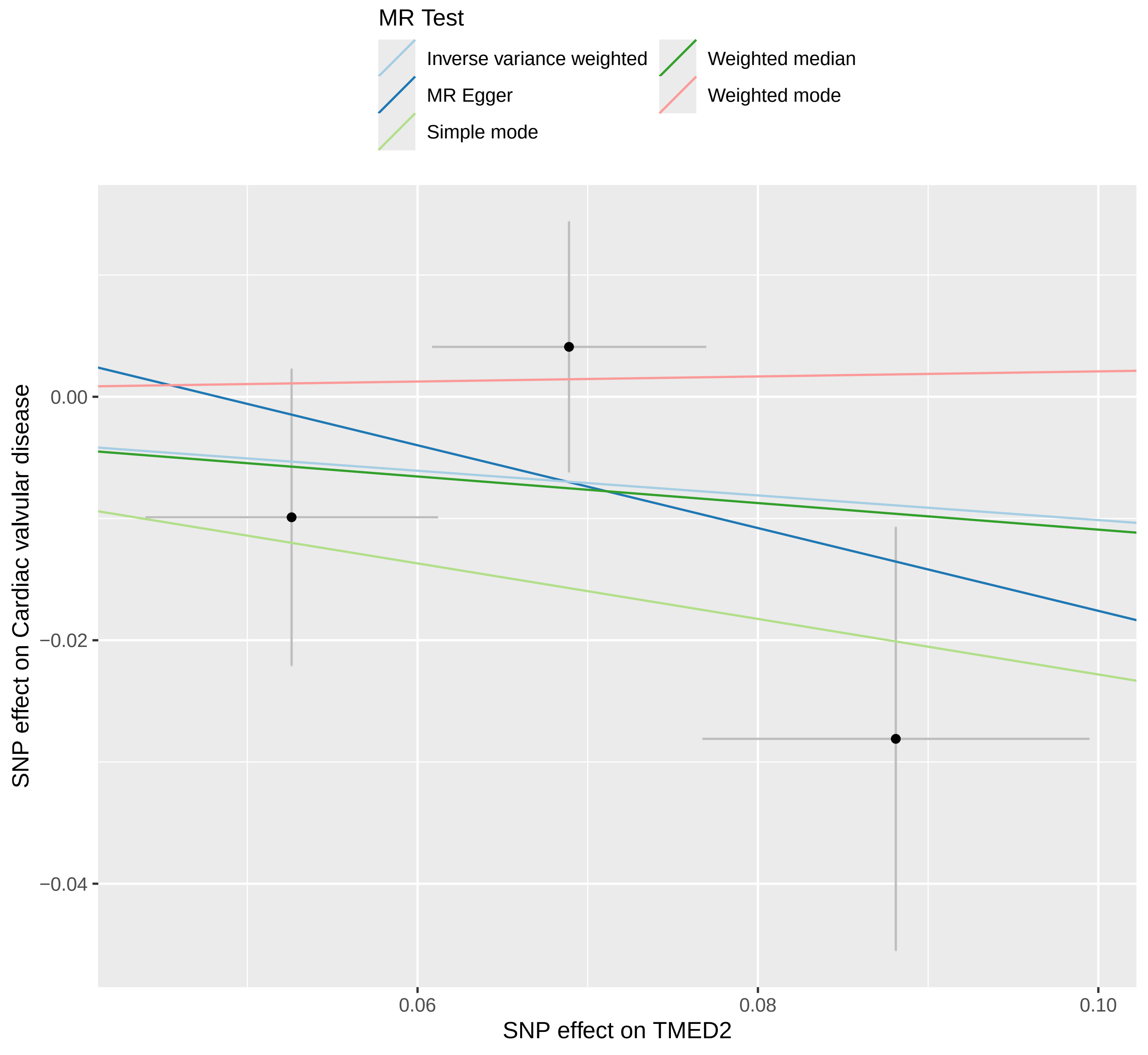

MR Test
Inverse variance weighted
MR Egger
Simple mode
Weighted median
Weighted mode
SNP effect on Cardiac valvular disease
0.00
−0.02
−0.04
0.06
0.08
0.10
SNP effect on TMED2

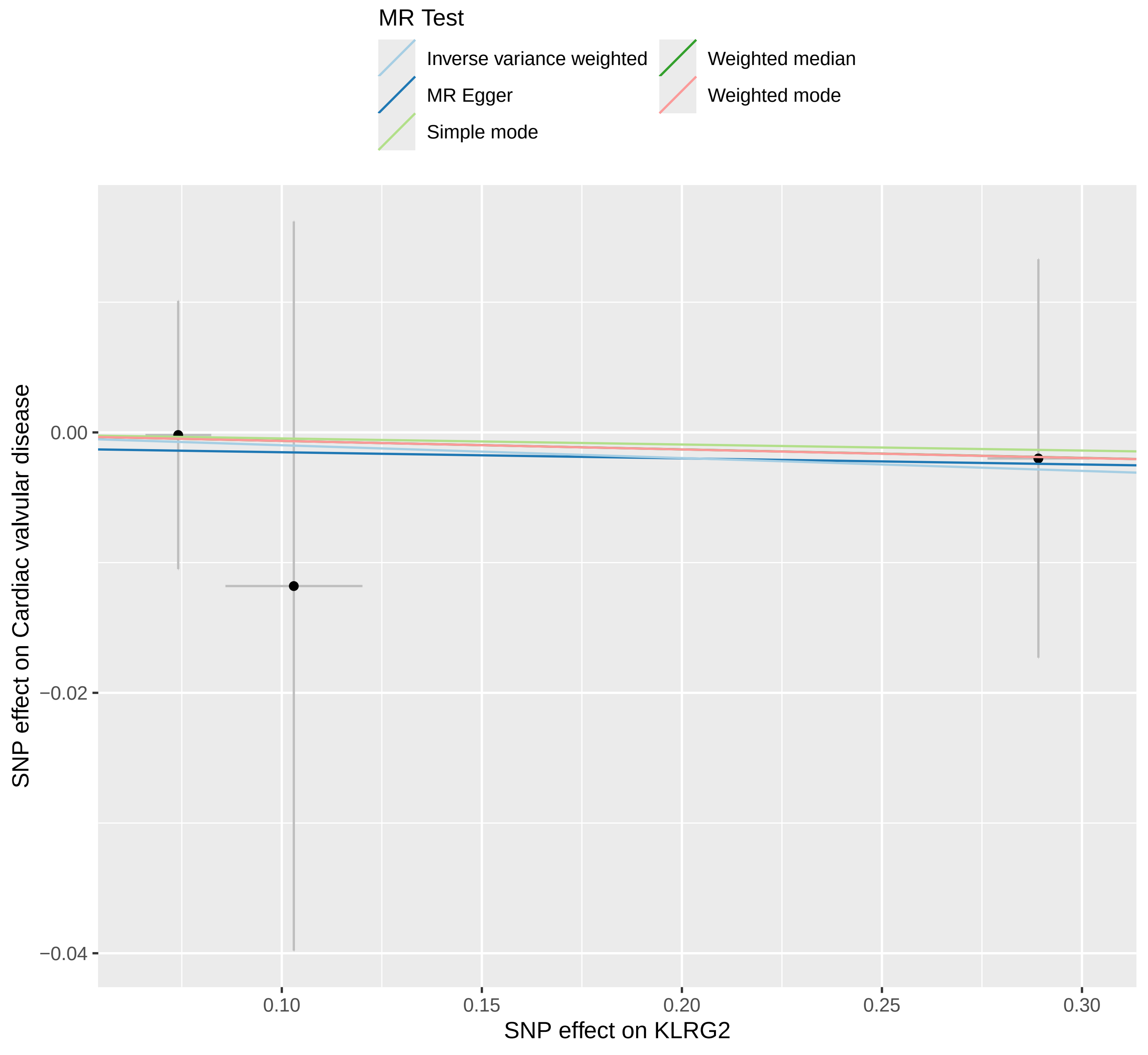

MR Test
Inverse variance weighted
MR Egger
Simple mode
Weighted median
Weighted mode
0.00
−0.02
−0.04
0.10
0.15
0.20
0.25
0.30
SNP effect on Cardiac valvular disease
SNP effect on KLRG2

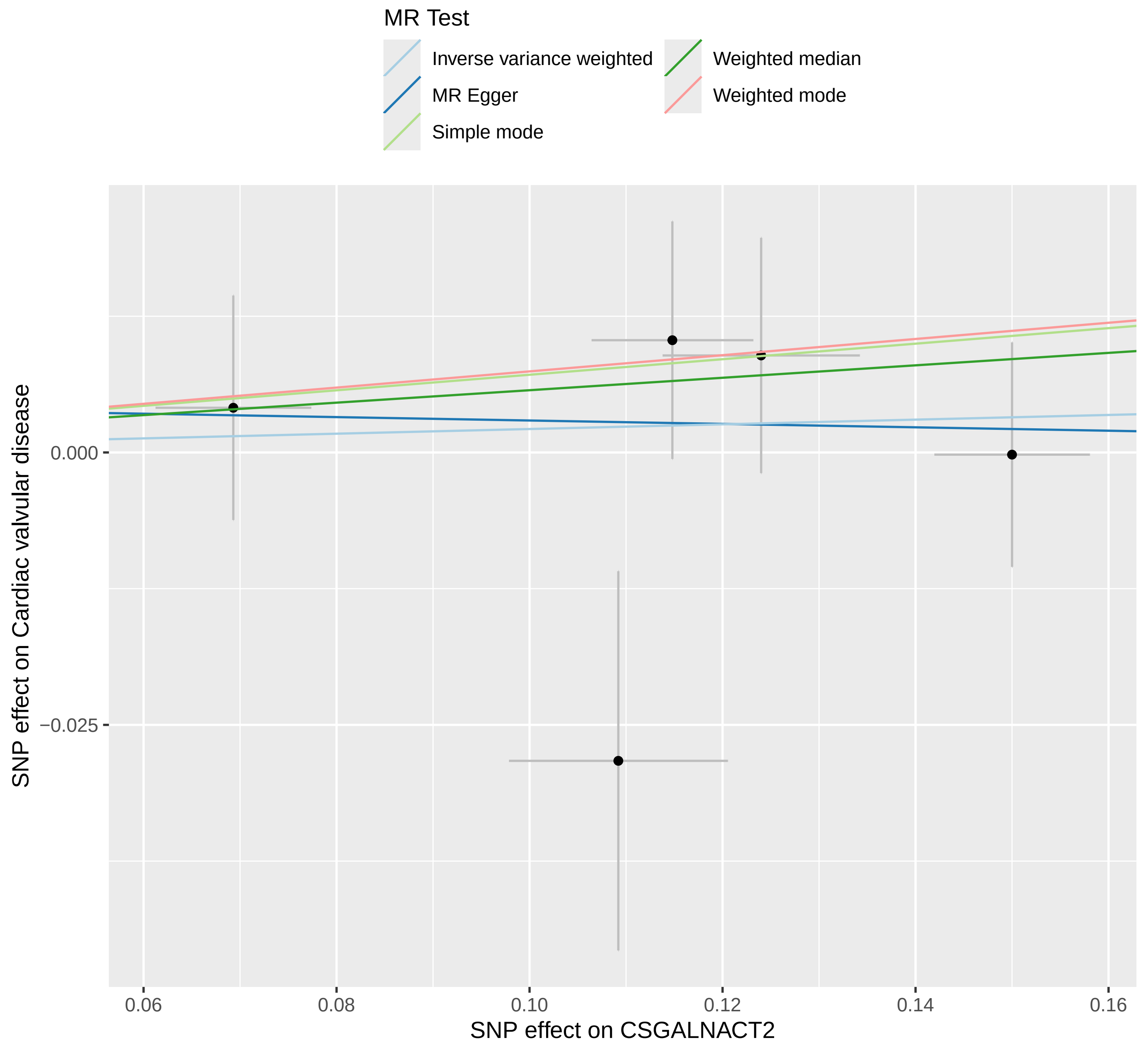

MR Test
Inverse variance weighted
MR Egger
Simple mode
Weighted median
Weighted mode
SNP effect on Cardiac valvular disease
0.000
−0.025
0.06
0.08
0.10
0.12
0.14
0.16
SNP effect on CSGALNACT2

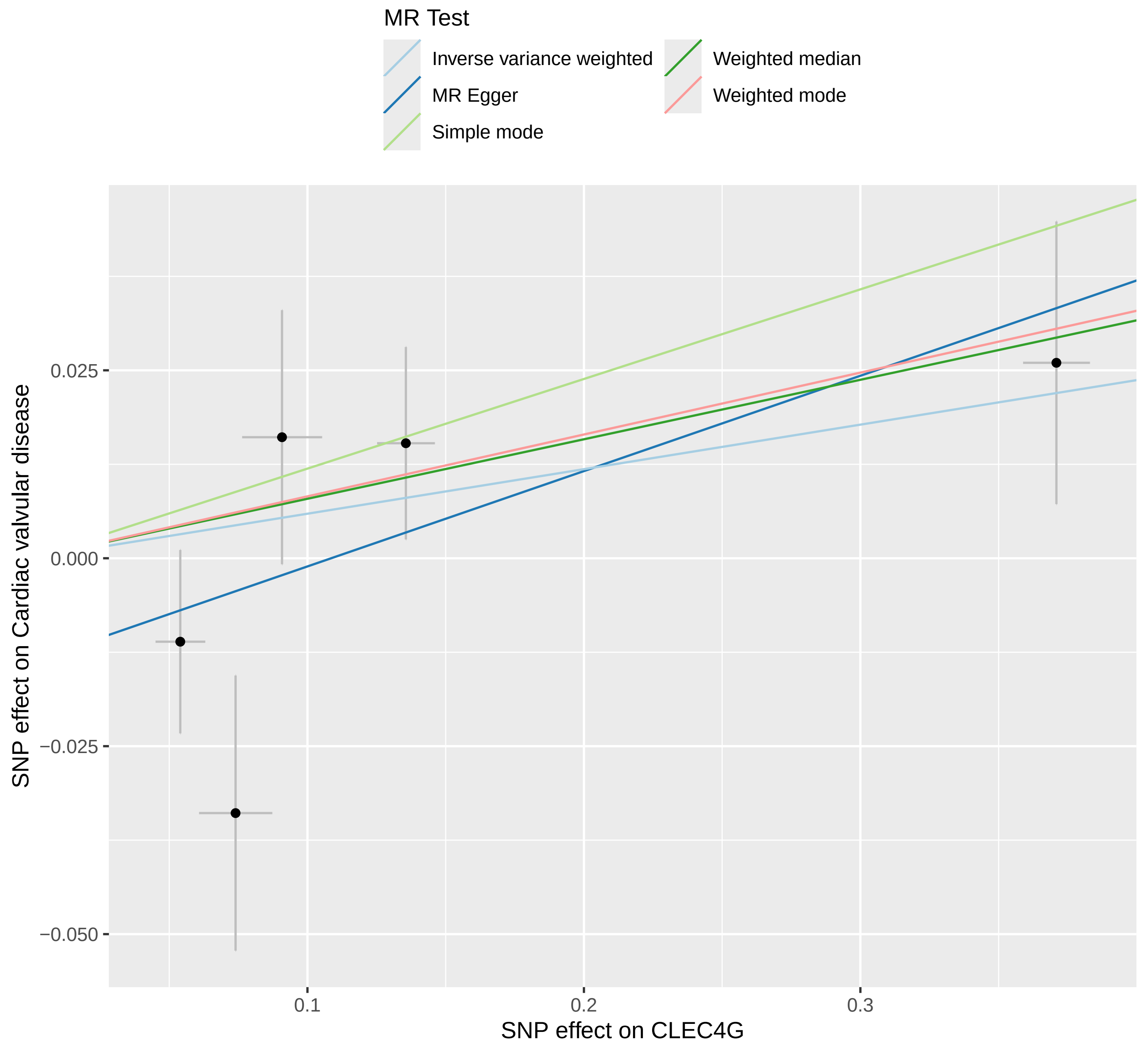
MR Test
Inverse variance weighted
MR Egger
Simple mode
Weighted median
Weighted mode
SNP effect on Cardiac valvular disease
0.025
0.000
−0.025
−0.050
0.1
0.2
0.3
SNP effect on CLEC4G

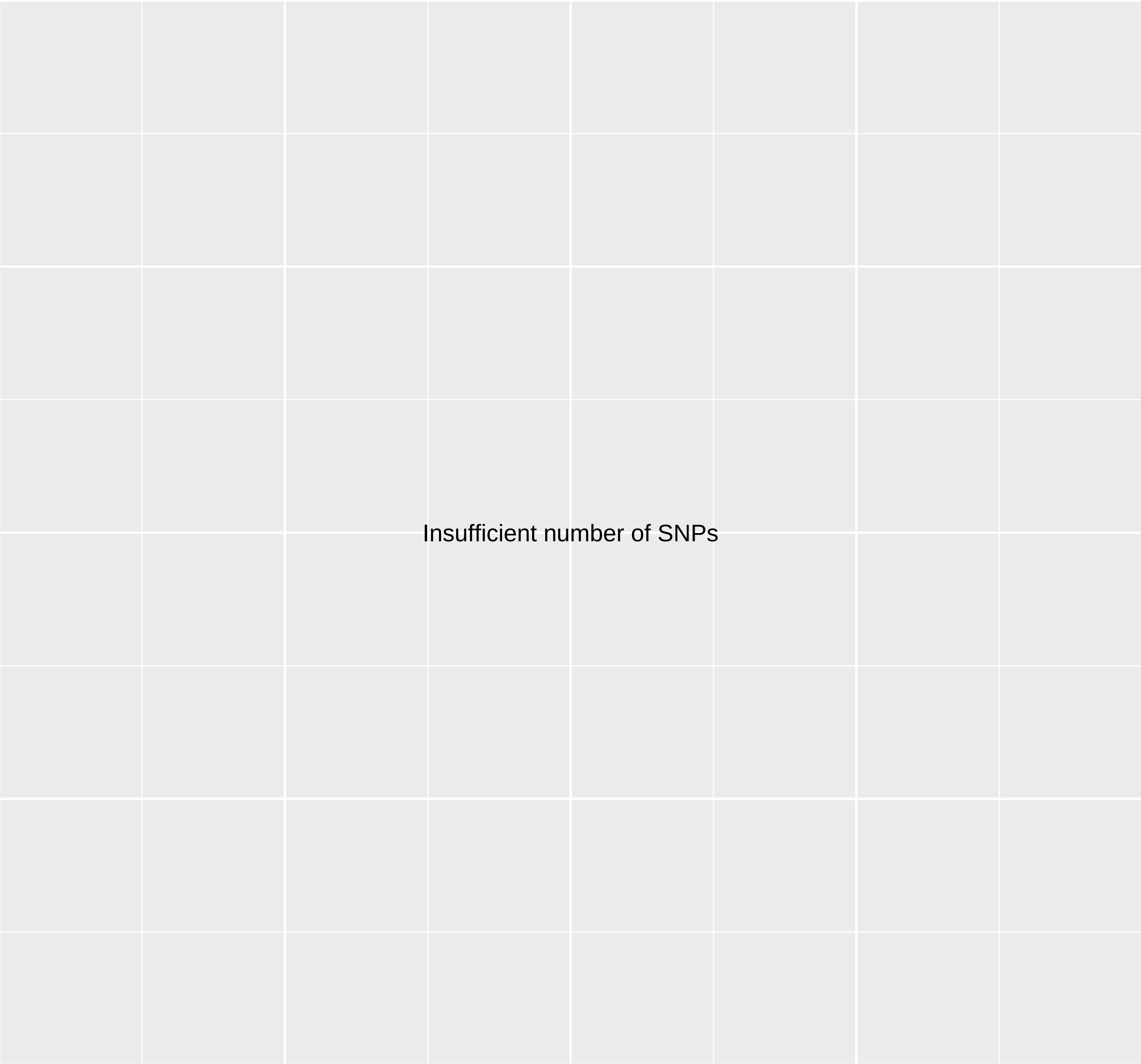
Insufficient number of SNPs

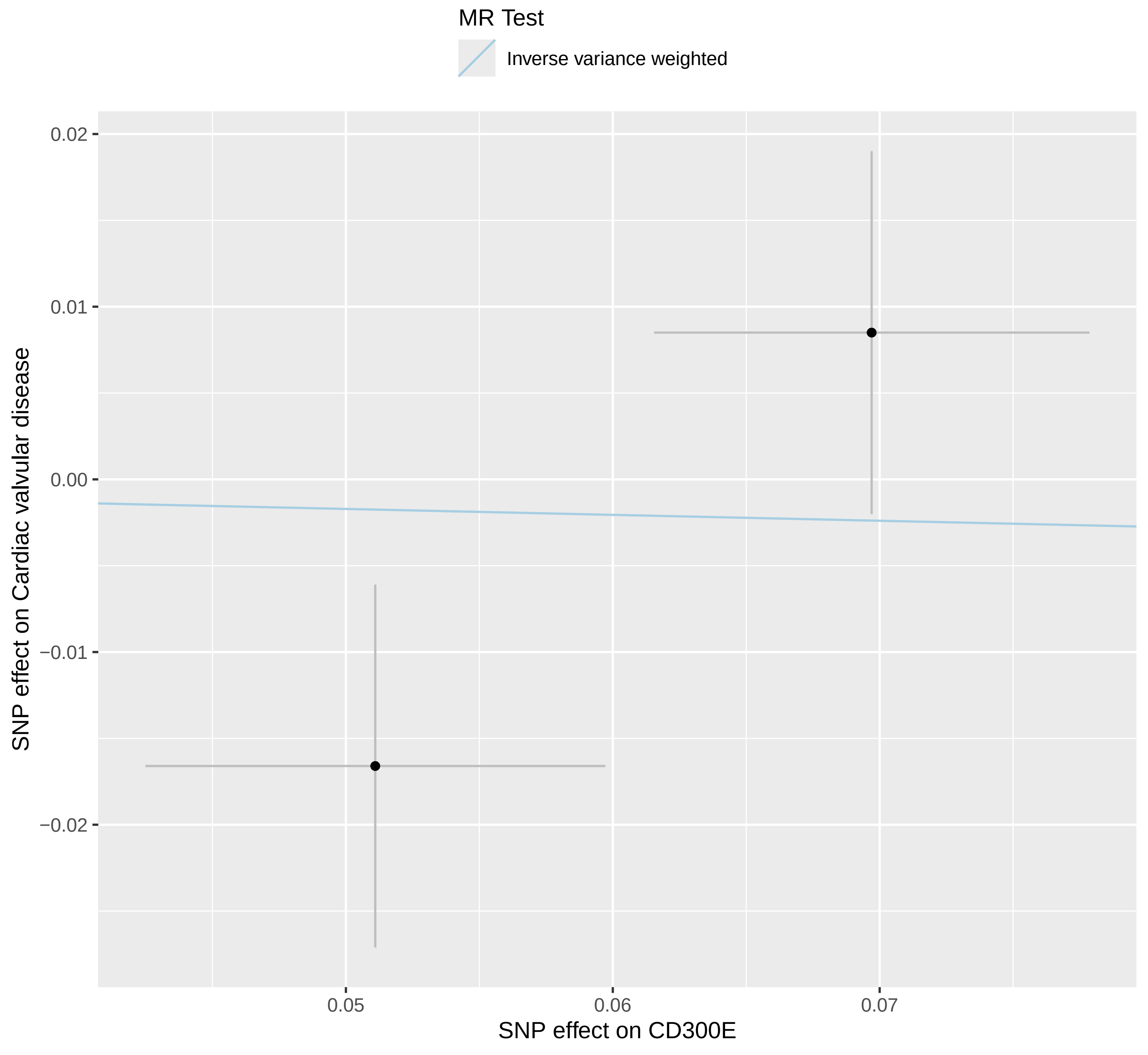
MR Test
Inverse variance weighted
0.02
0.01
0.00
−0.01
−0.02
0.05
0.06
0.07
SNP effect on CD300E
SNP effect on Cardiac valvular disease

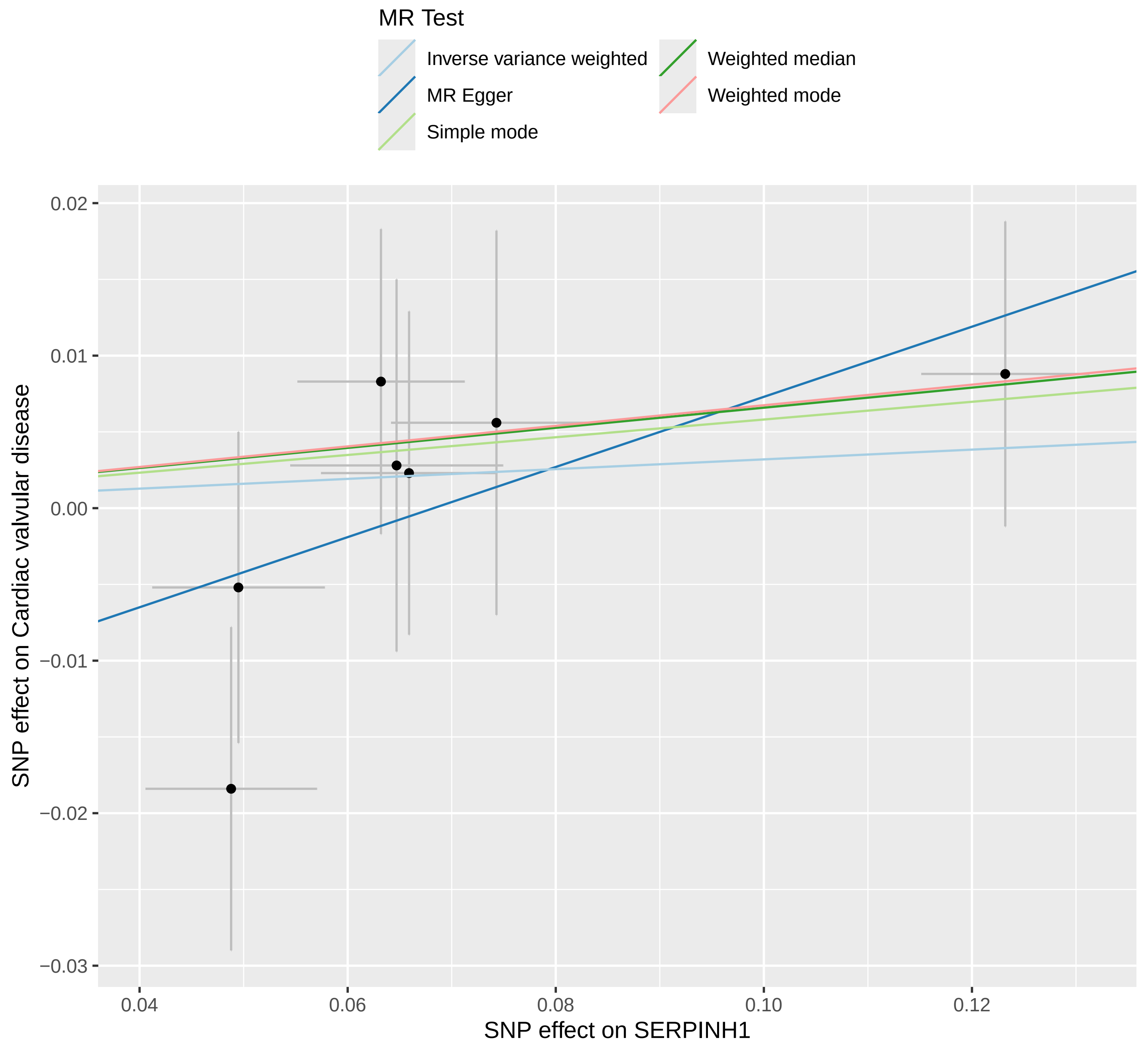

MR Test
Inverse variance weighted
MR Egger
Simple mode
Weighted median
Weighted mode
0.02
0.01
0.00
−0.01
−0.02
−0.03
SNP effect on Cardiac valvular disease
0.04
0.06
0.08
0.10
0.12
SNP effect on SERPINH1

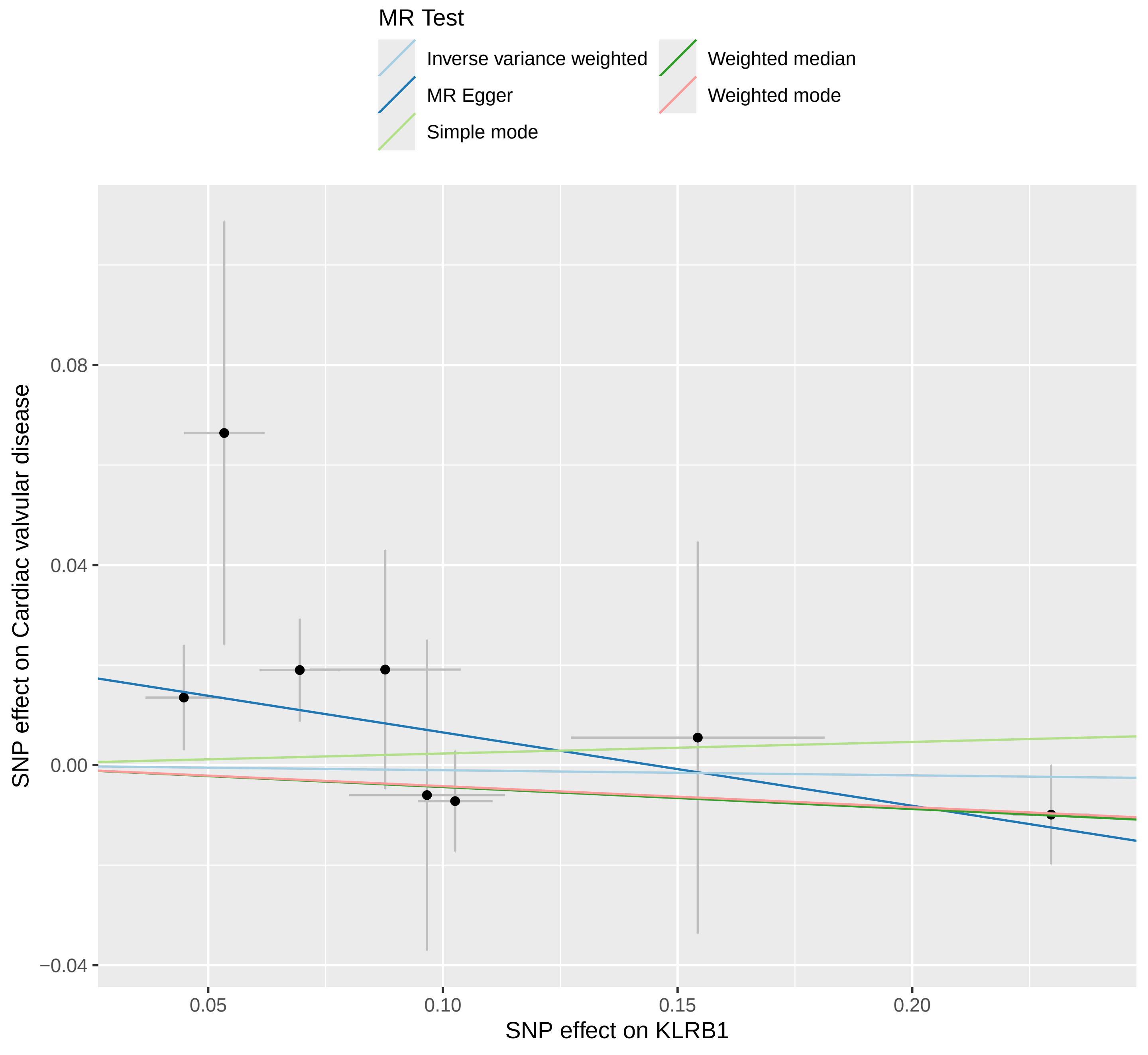
MR Test
Inverse variance weighted
MR Egger
Simple mode
Weighted median
Weighted mode
0.08
0.04
0.00
−0.04
0.05
0.10
0.15
0.20
SNP effect on Cardiac valvular disease
SNP effect on KLRB1

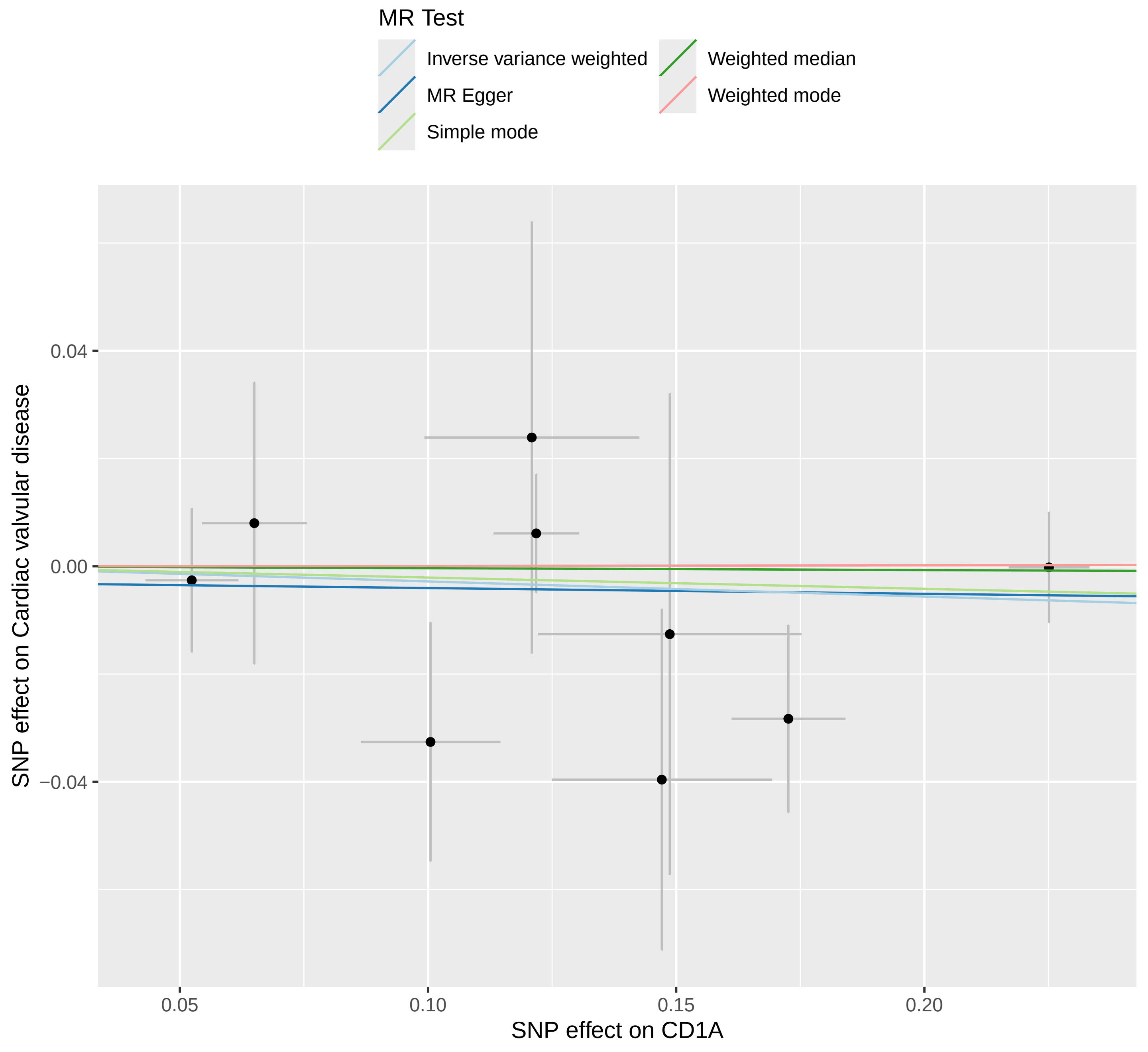

MR Test
Inverse variance weighted
MR Egger
Simple mode
Weighted median
Weighted mode
0.04
0.00
−0.04
SNP effect on Cardiac valvular disease
0.05
0.10
0.15
0.20
SNP effect on CD1A

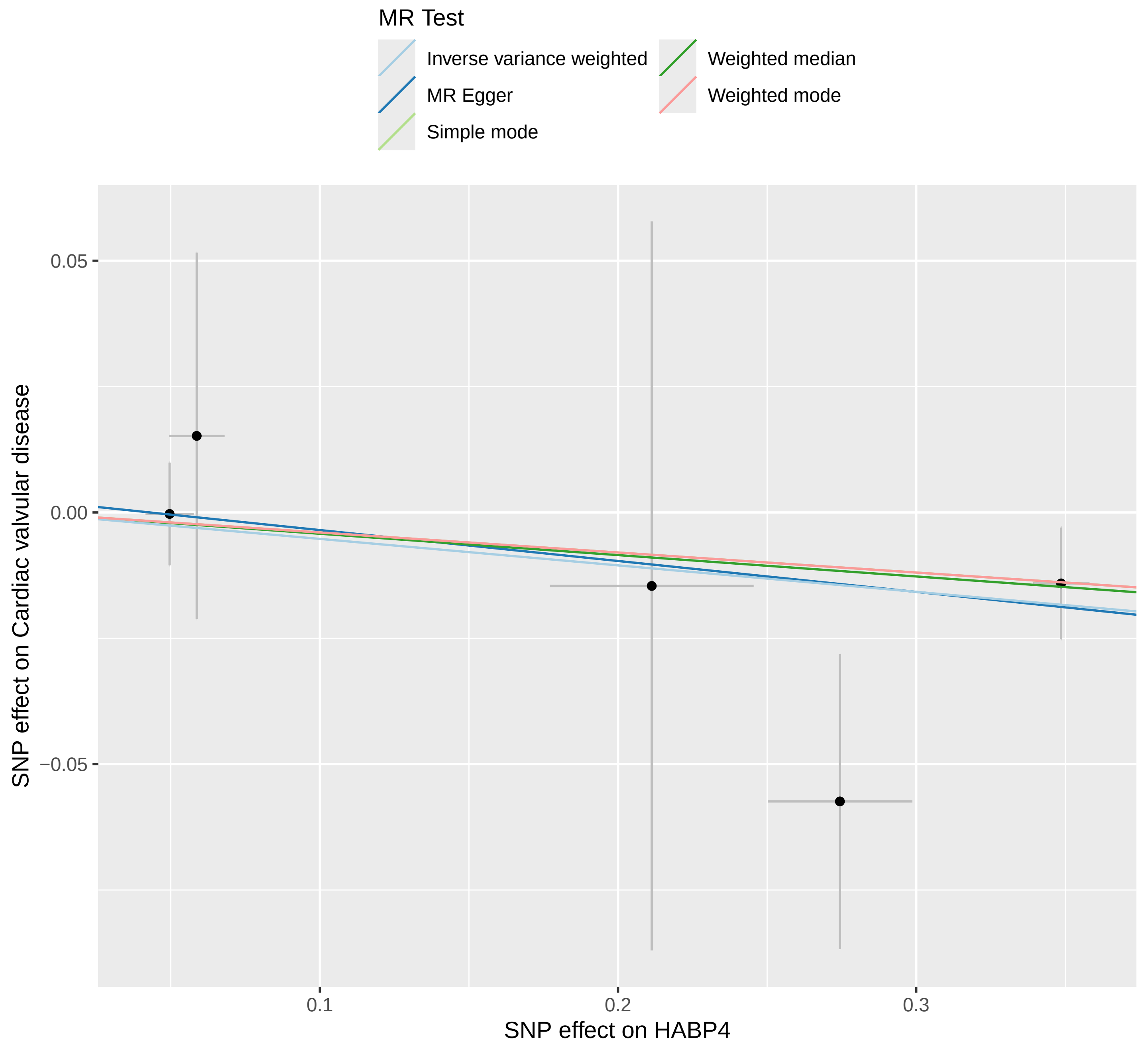
MR Test
Inverse variance weighted
MR Egger
Simple mode
Weighted median
Weighted mode
0.05
0.00
−0.05
0.1
0.2
0.3
SNP effect on Cardiac valvular disease
SNP effect on HABP4

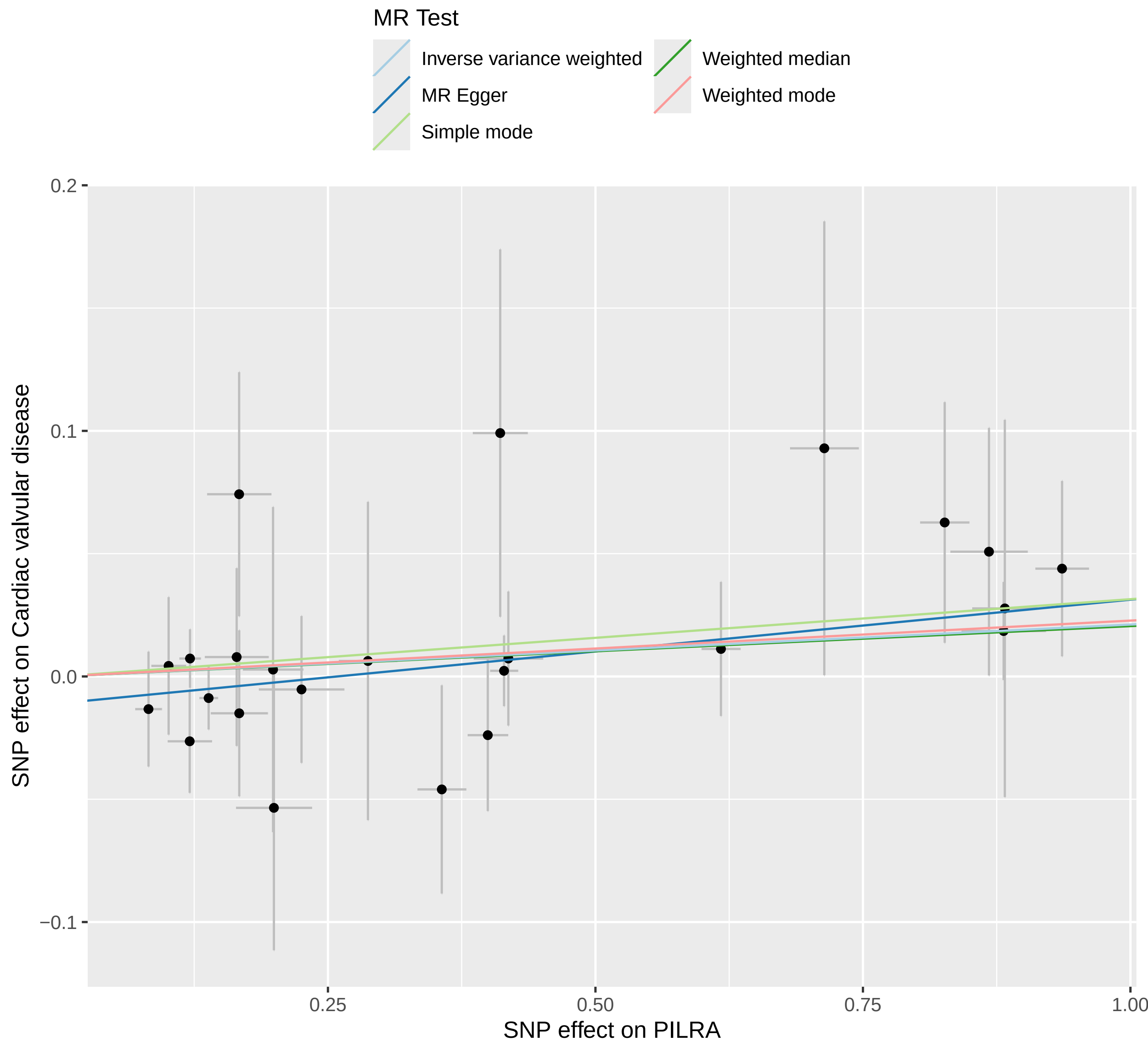

MR Test
Inverse variance weighted
MR Egger
Simple mode
Weighted median
Weighted mode
0.2
0.1
0.0
−0.1
0.25
0.50
0.75
1.00
SNP effect on PILRA
SNP effect on Cardiac valvular disease

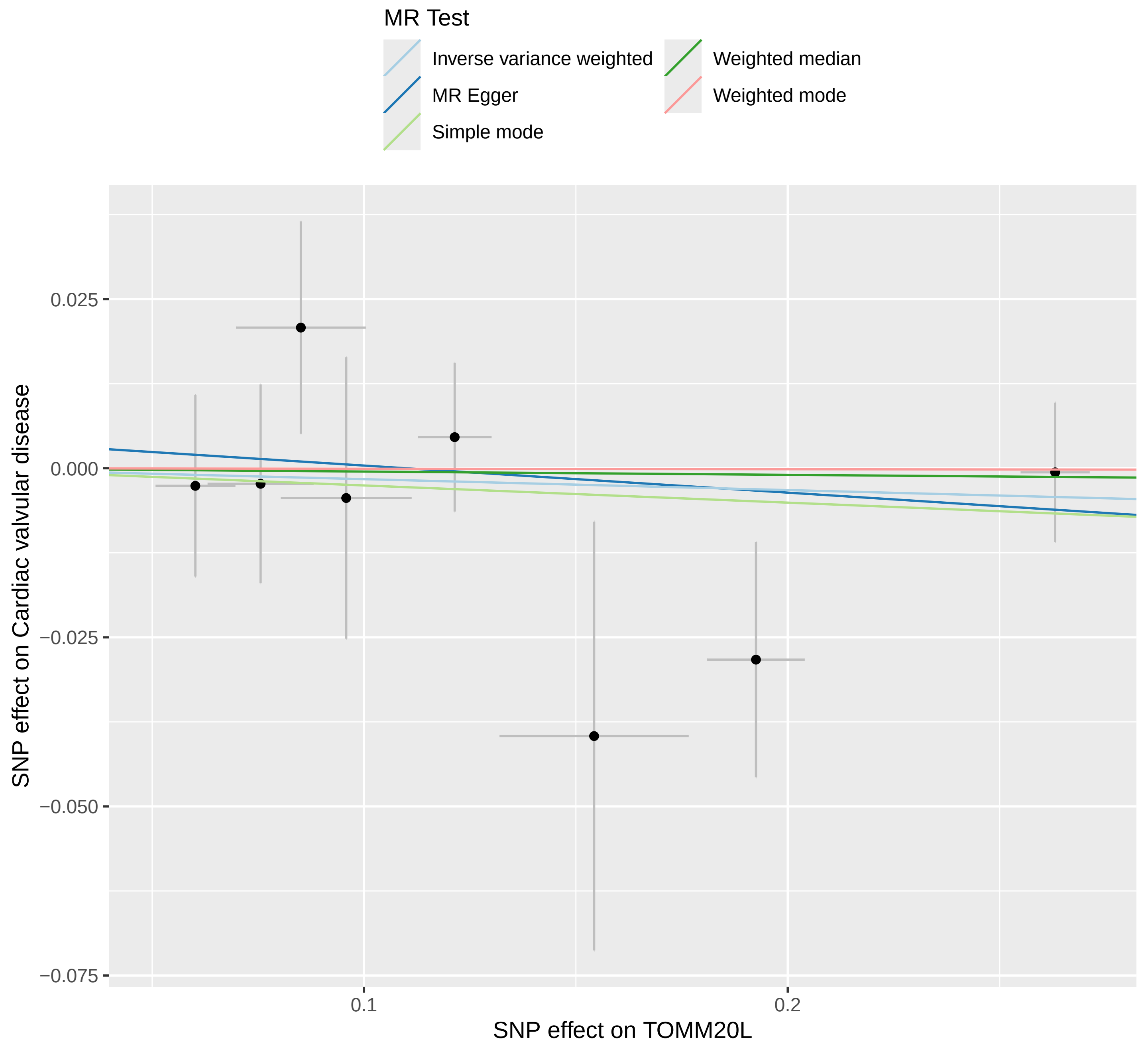

MR Test
Inverse variance weighted
MR Egger
Simple mode
Weighted median
Weighted mode
0.025
0.000
−0.025
−0.050
−0.075
0.1
0.2
SNP effect on Cardiac valvular disease
SNP effect on TOMM20L

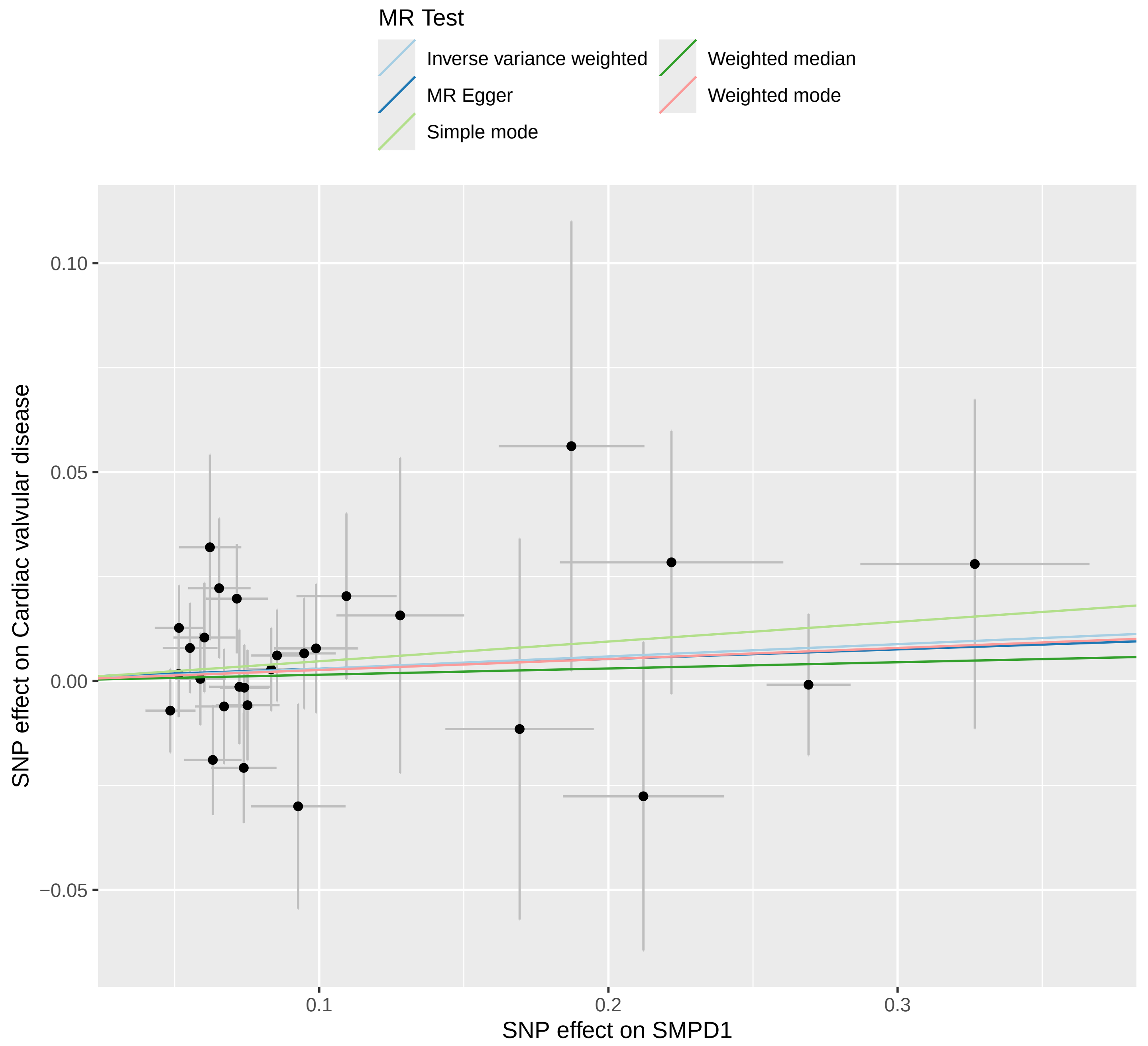

MR Test
Inverse variance weighted
MR Egger
Simple mode
Weighted median
Weighted mode
0.10
0.05
0.00
−0.05
0.1
0.2
0.3
SNP effect on Cardiac valvular disease
SNP effect on SMPD1

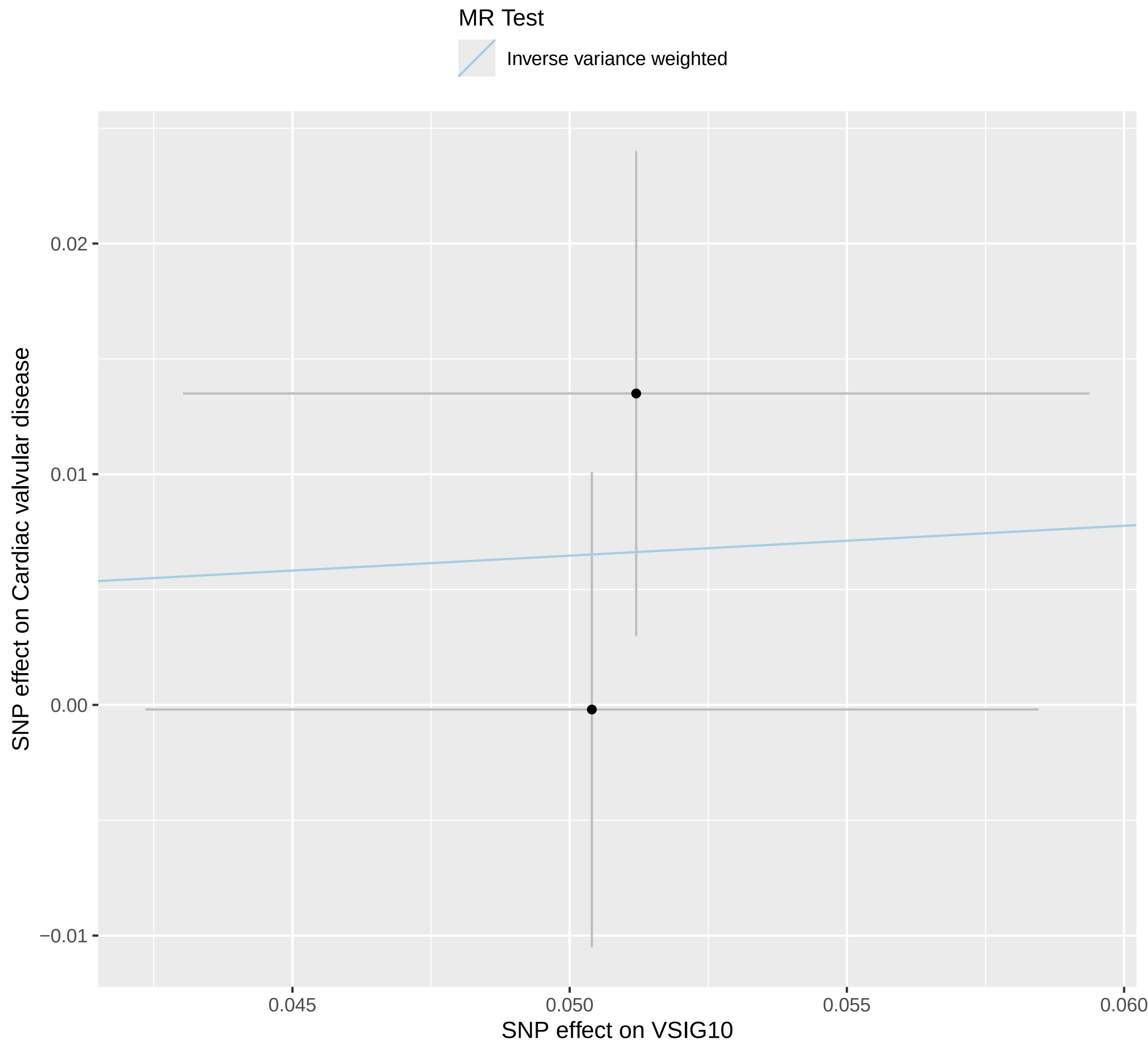

MR Test
Inverse variance weighted
SNP effect on Cardiac valvular disease
0.02
0.01
0.00
−0.01
0.045
0.050
0.055
0.060
SNP effect on VSIG10

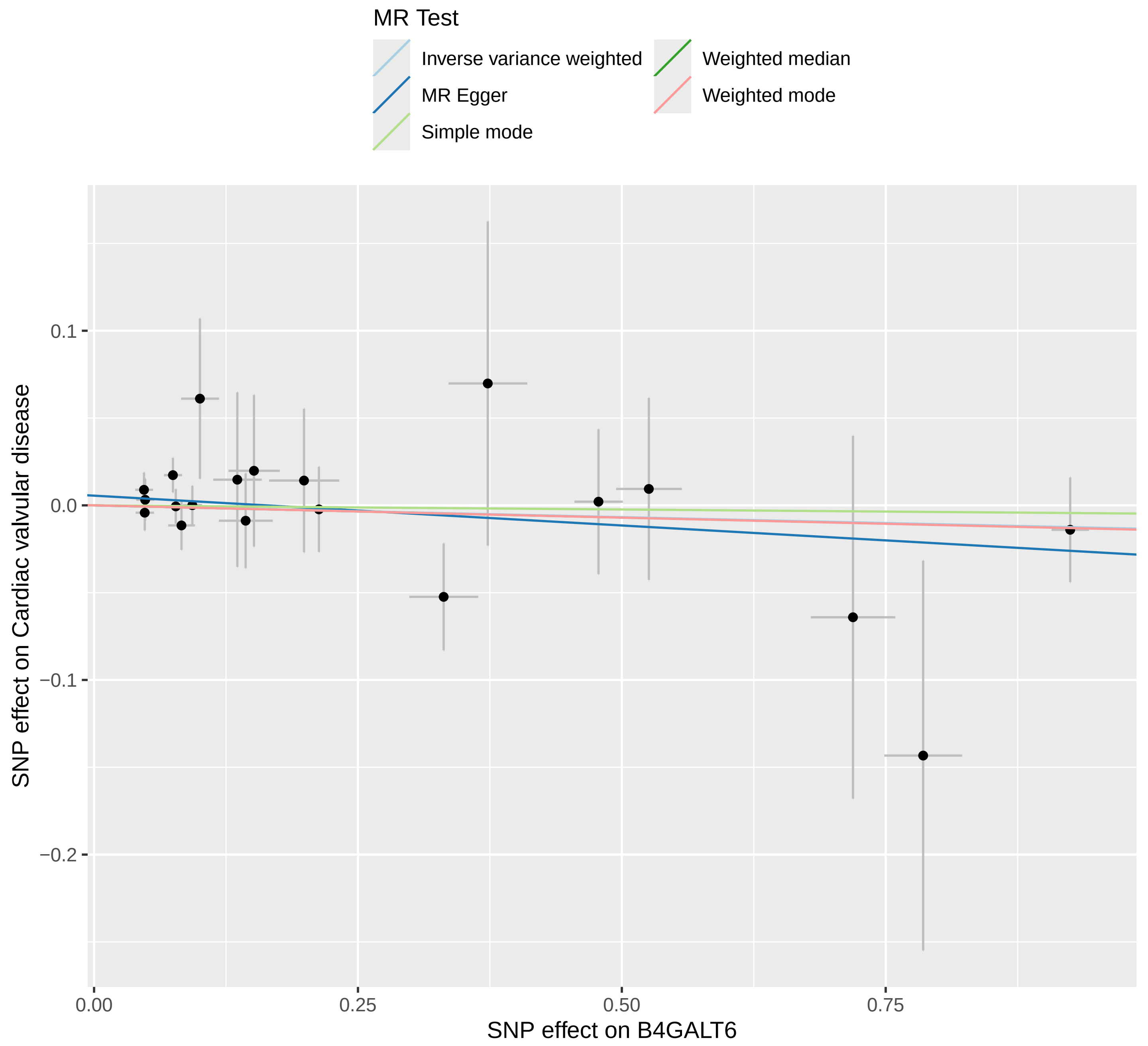
MR Test
Inverse variance weighted
MR Egger
Simple mode
Weighted median
Weighted mode
0.1
0.0
−0.1
−0.2
0.00
0.25
0.50
0.75
SNP effect on Cardiac valvular disease
SNP effect on B4GALT6

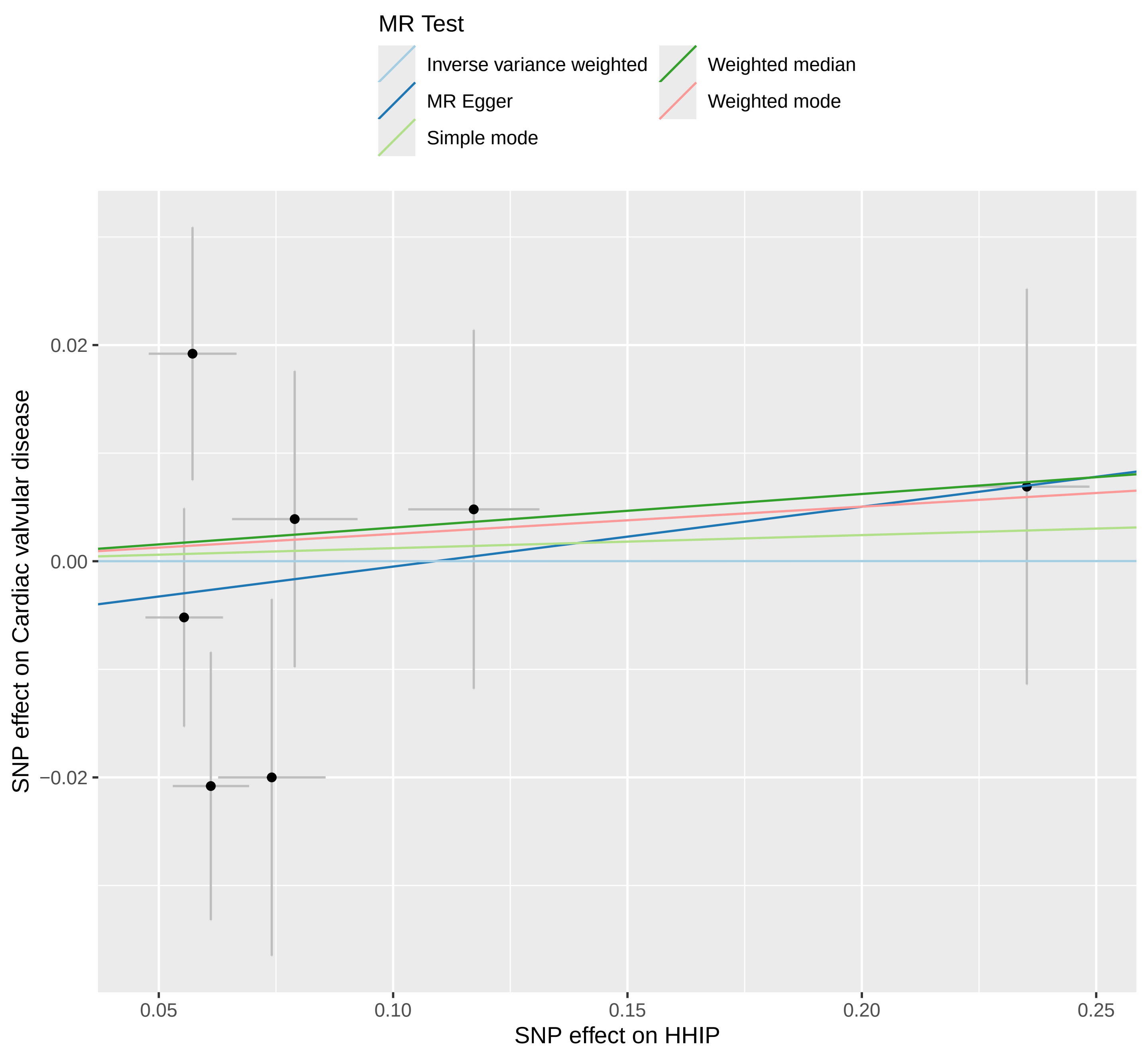

MR Test
Inverse variance weighted
MR Egger
Simple mode
Weighted median
Weighted mode
0.02
0.00
−0.02
0.05
0.10
0.15
0.20
0.25
SNP effect on Cardiac valvular disease
SNP effect on HHIP

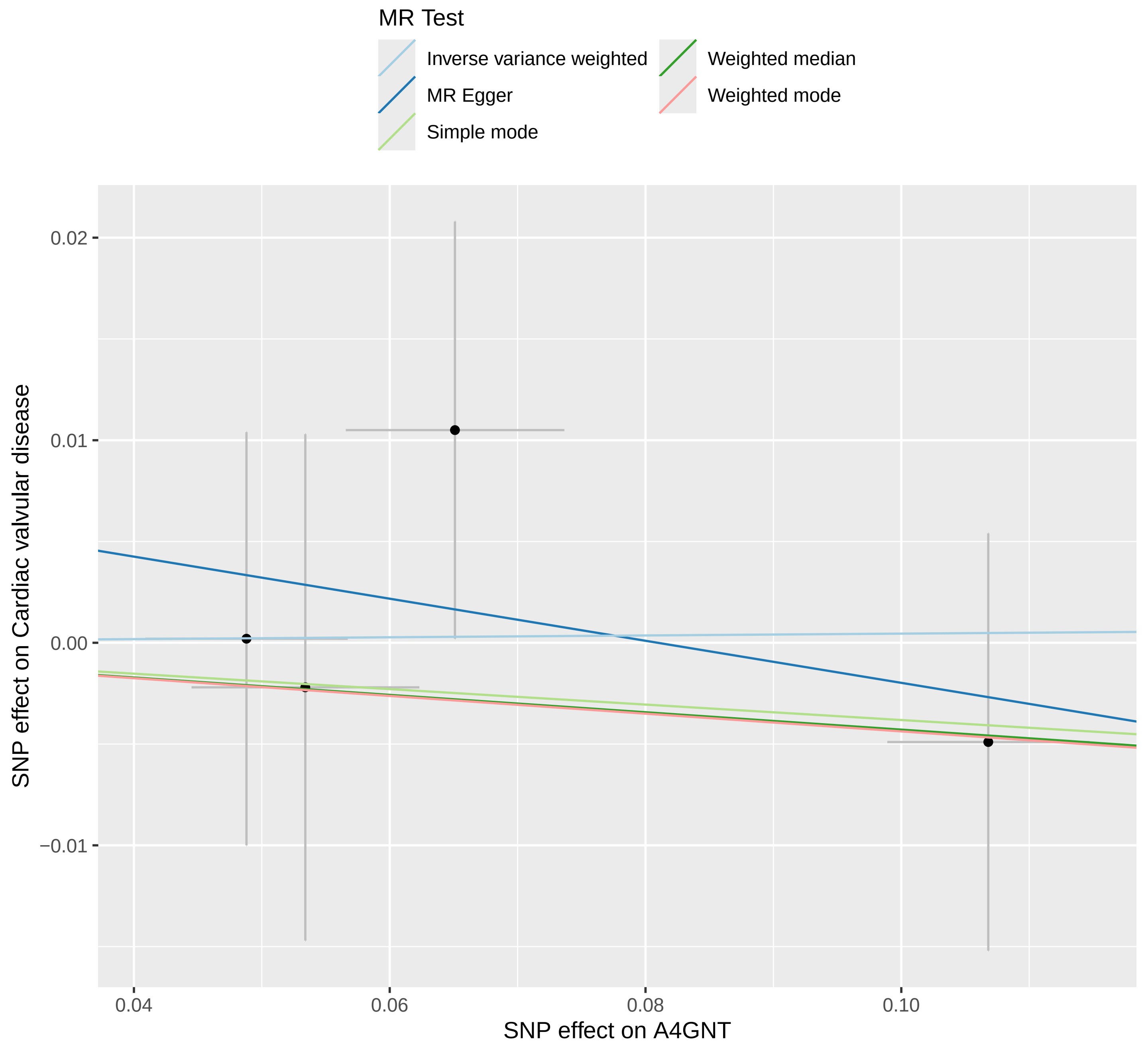

MR Test
Inverse variance weighted
MR Egger
Simple mode
Weighted median
Weighted mode
0.02
0.01
0.00
−0.01
0.04
0.06
0.08
0.10
SNP effect on Cardiac valvular disease
SNP effect on A4GNT

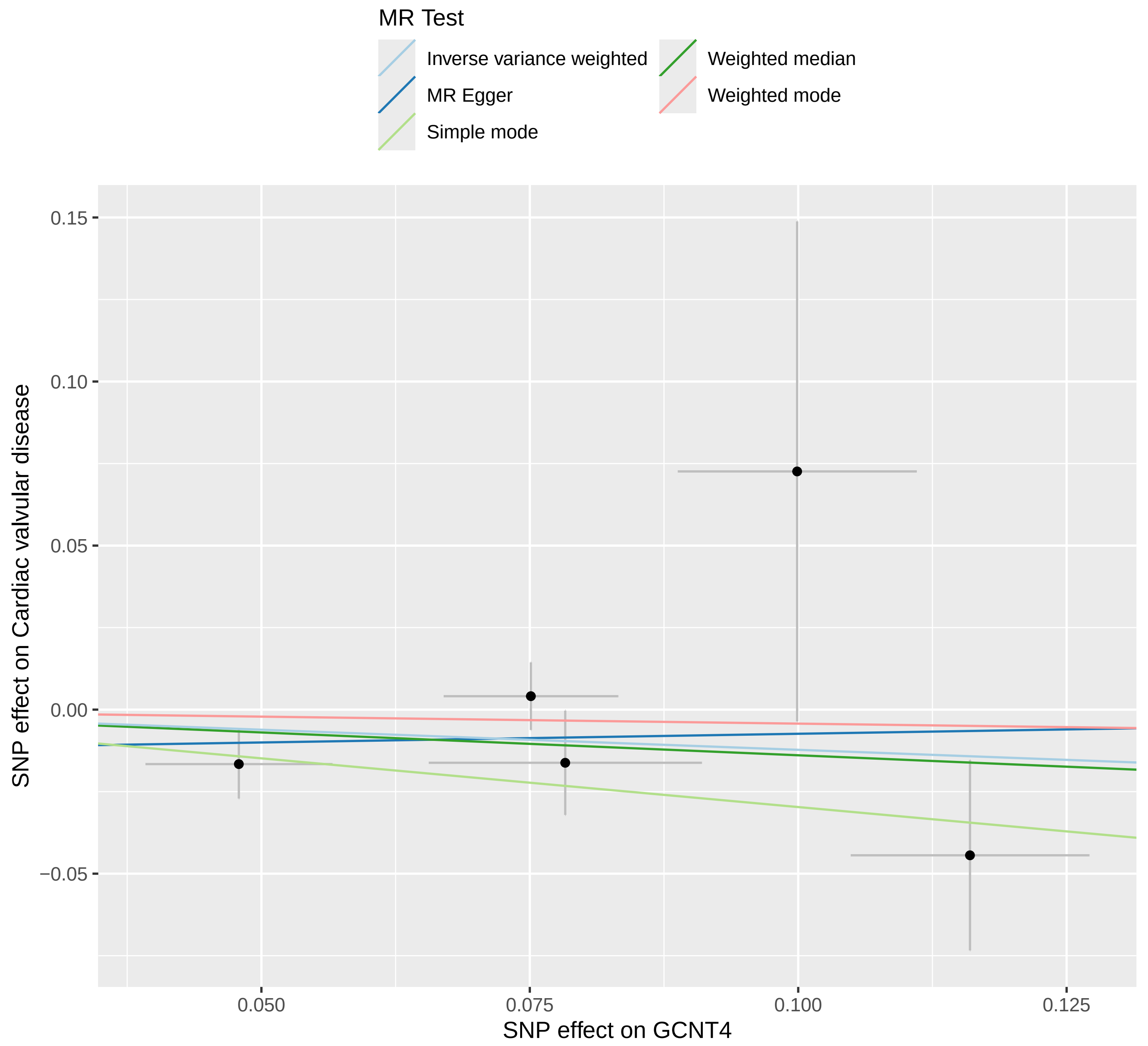

MR Test
Inverse variance weighted
MR Egger
Simple mode
Weighted median
Weighted mode
SNP effect on Cardiac valvular disease
0.15
0.10
0.05
0.00
−0.05
0.050
0.075
0.100
0.125
SNP effect on GCNT4

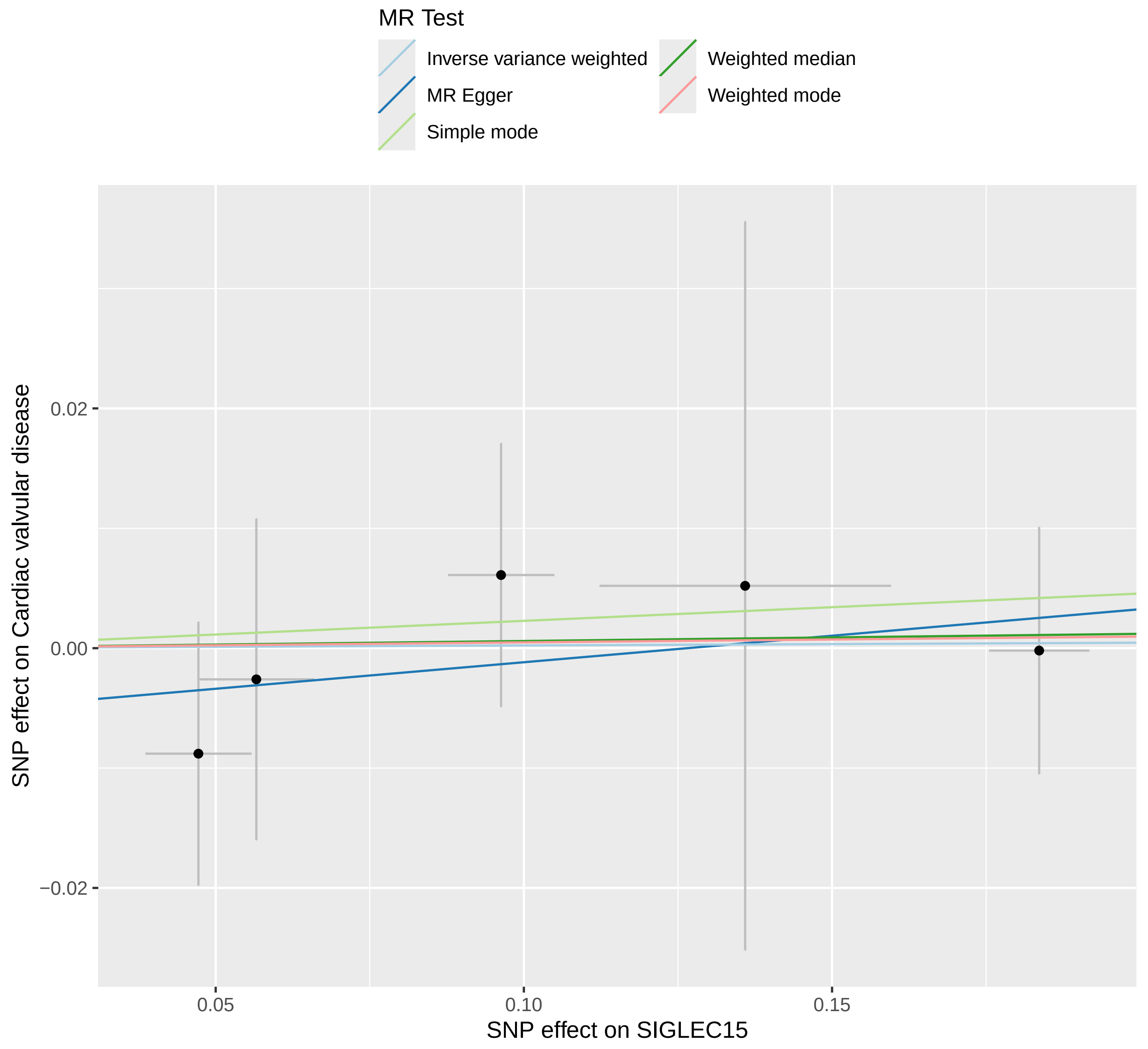

MR Test
Inverse variance weighted
MR Egger
Simple mode
Weighted median
Weighted mode
0.02
0.00
−0.02
0.05
0.10
0.15
SNP effect on Cardiac valvular disease
SNP effect on SIGLEC15

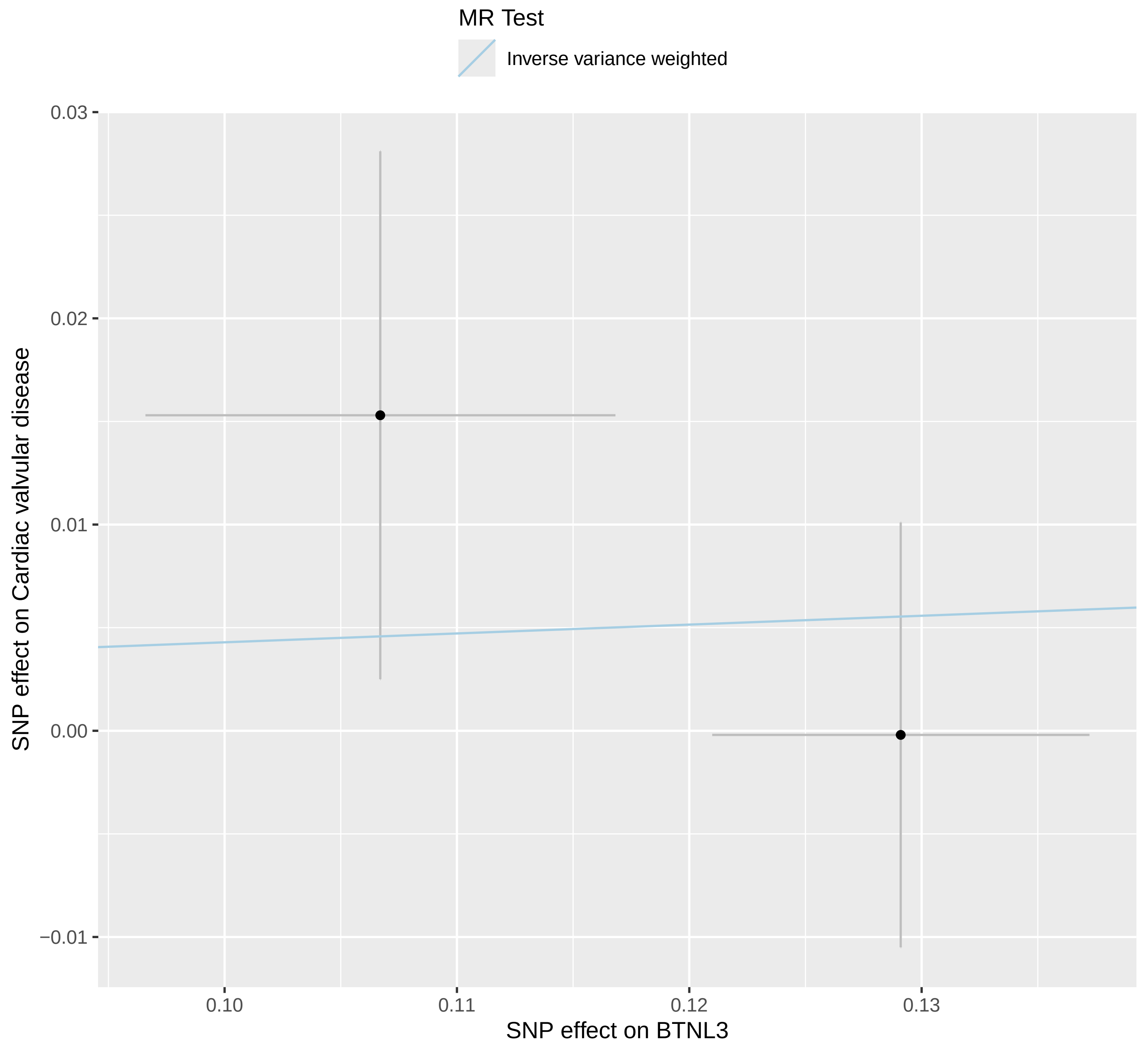

MR Test
Inverse variance weighted
0.03
0.02
0.01
0.00
−0.01
SNP effect on Cardiac valvular disease
0.10
0.11
0.12
0.13
SNP effect on BTNL3

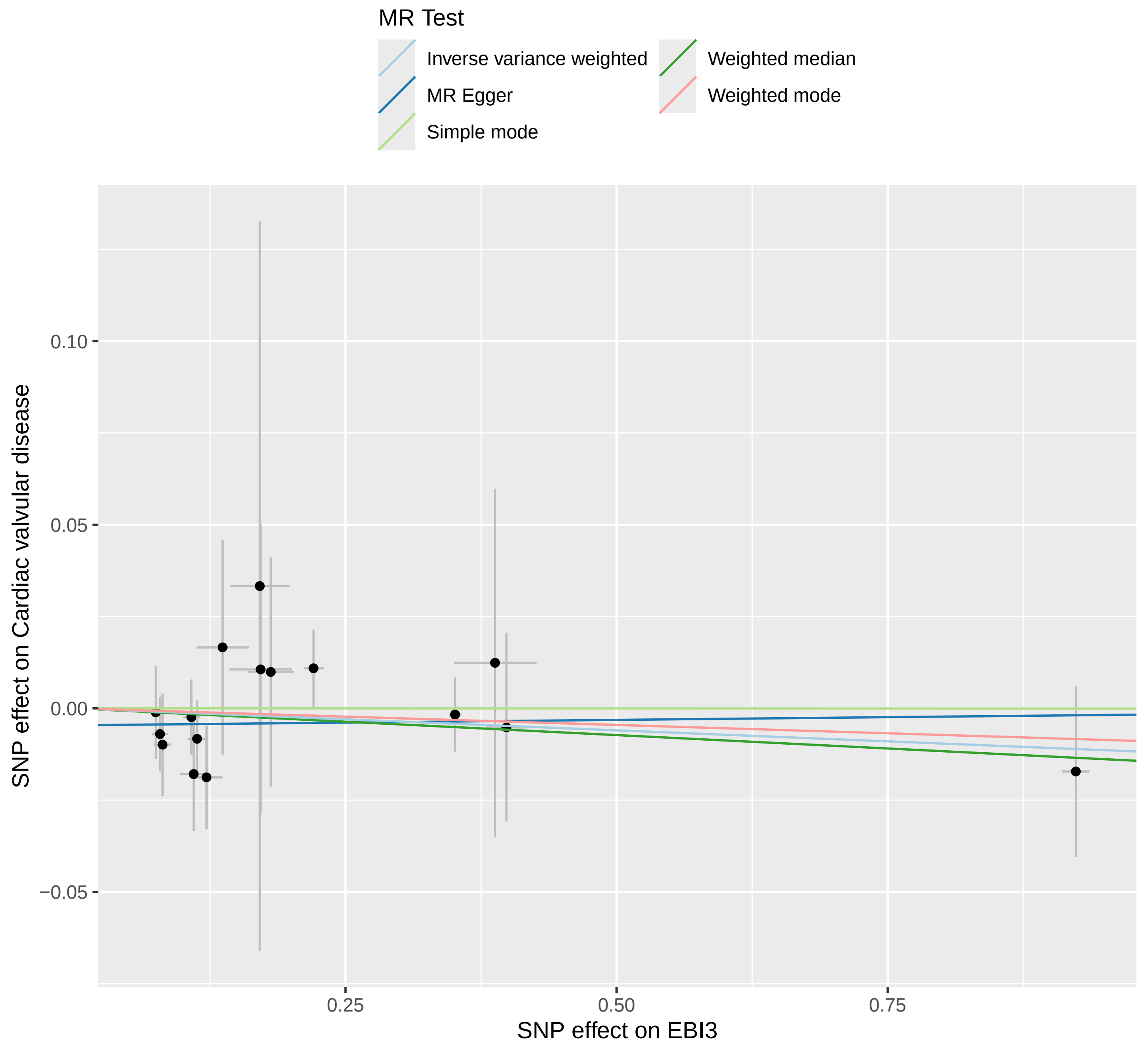

MR Test
Inverse variance weighted
MR Egger
Simple mode
Weighted median
Weighted mode
0.10
0.05
0.00
−0.05
SNP effect on Cardiac valvular disease
0.25
0.50
0.75
SNP effect on EBI3

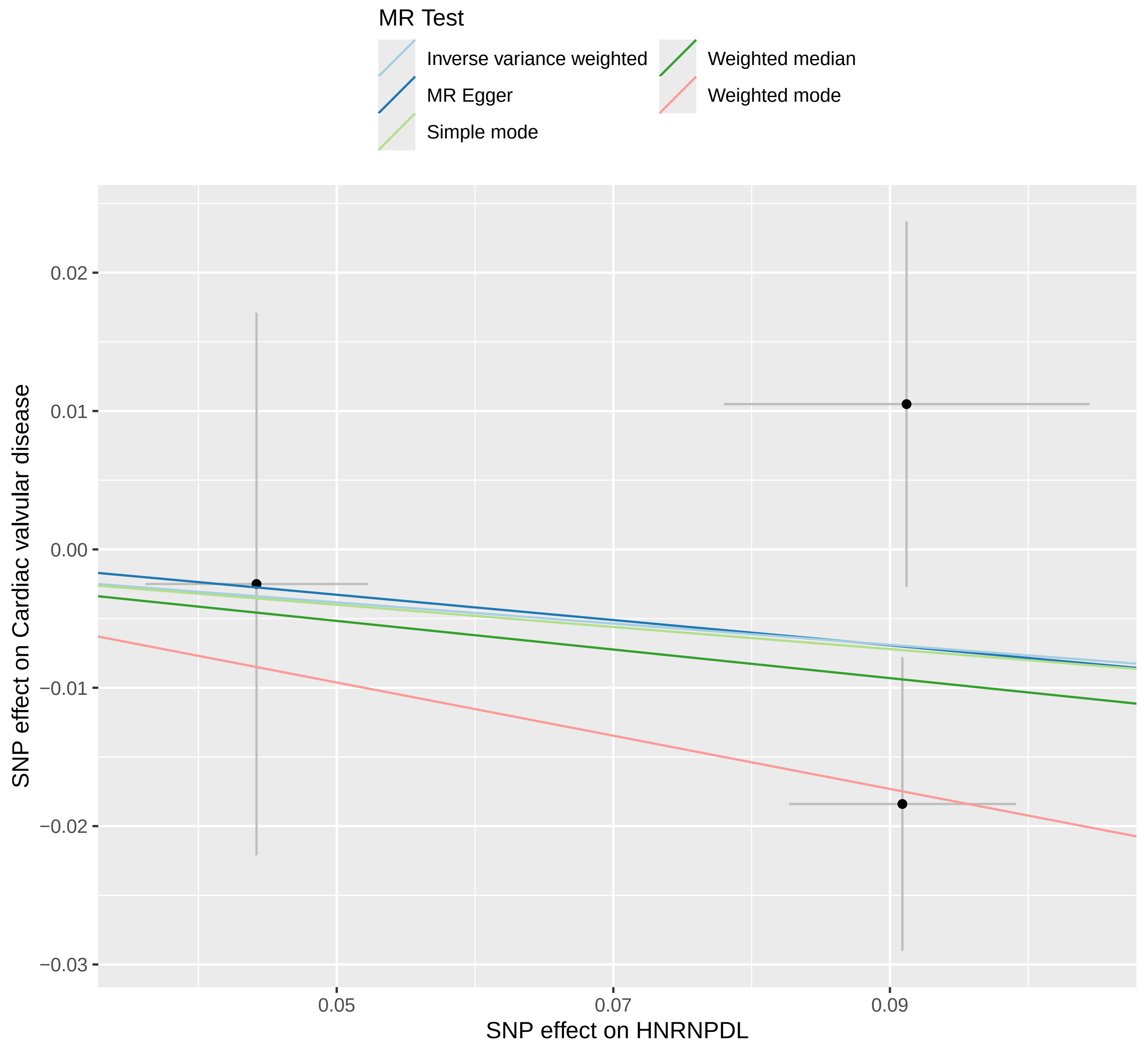

MR Test
Inverse variance weighted
MR Egger
Simple mode
Weighted median
Weighted mode
SNP effect on Cardiac valvular disease
0.02
0.01
0.00
−0.01
−0.02
−0.03
0.05
0.07
0.09
SNP effect on HNRNPDL

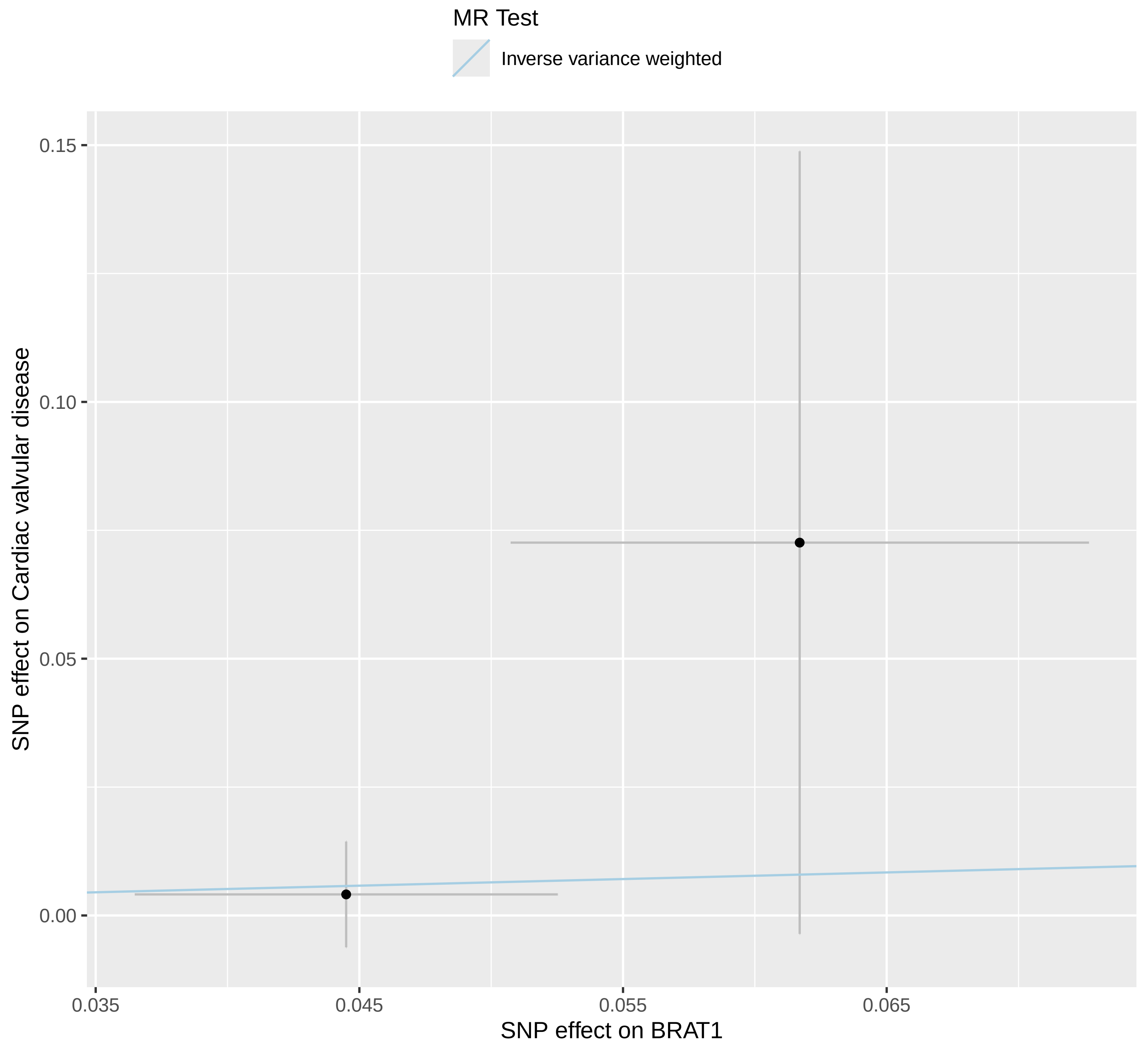
MR Test
Inverse variance weighted
0.15
0.10
0.05
0.00
SNP effect on Cardiac valvular disease
0.035
0.045
0.055
0.065
SNP effect on BRAT1

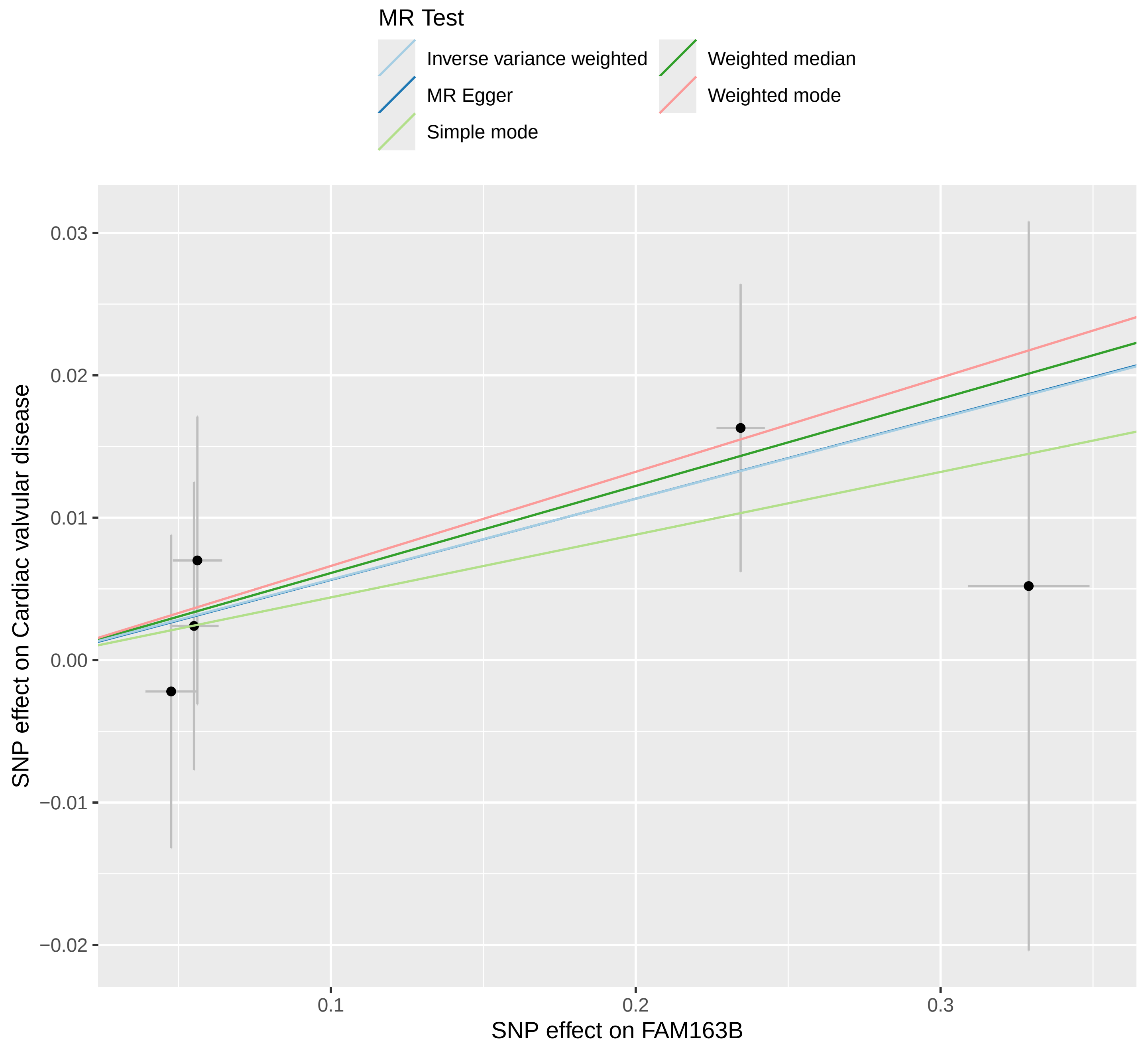

MR Test
Inverse variance weighted
MR Egger
Simple mode
Weighted median
Weighted mode
SNP effect on Cardiac valvular disease
0.03
0.02
0.01
0.00
−0.01
−0.02
0.1
0.2
0.3
SNP effect on FAM163B

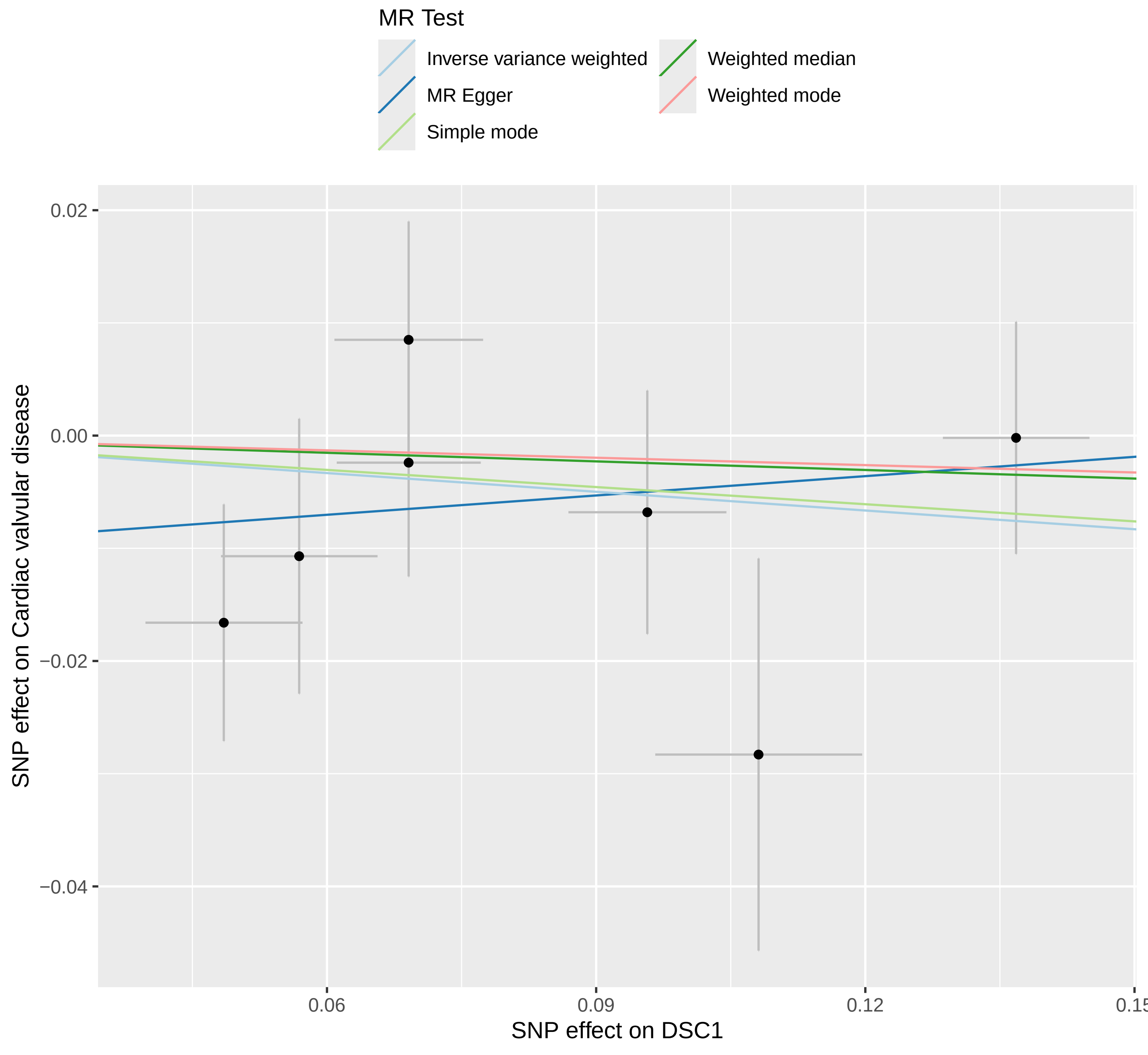
MR Test
Inverse variance weighted
MR Egger
Simple mode
Weighted median
Weighted mode
0.02
0.00
−0.02
−0.04
SNP effect on Cardiac valvular disease
0.06
0.09
0.12
0.15
SNP effect on DSC1

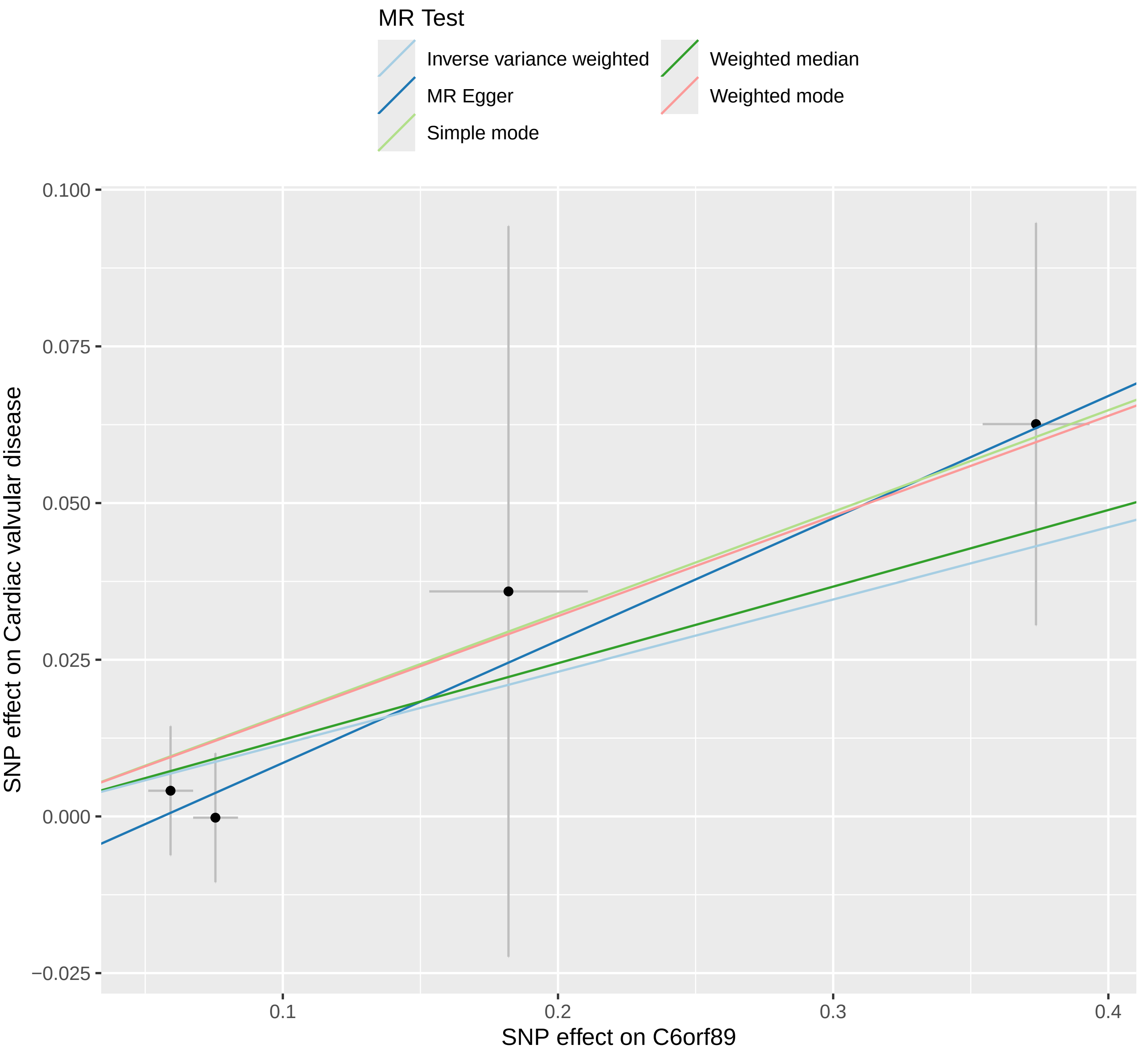

MR Test
Inverse variance weighted
MR Egger
Simple mode
Weighted median
Weighted mode
0.100
0.075
0.050
0.025
0.000
−0.025
SNP effect on Cardiac valvular disease
0.1
0.2
0.3
0.4
SNP effect on C6orf89

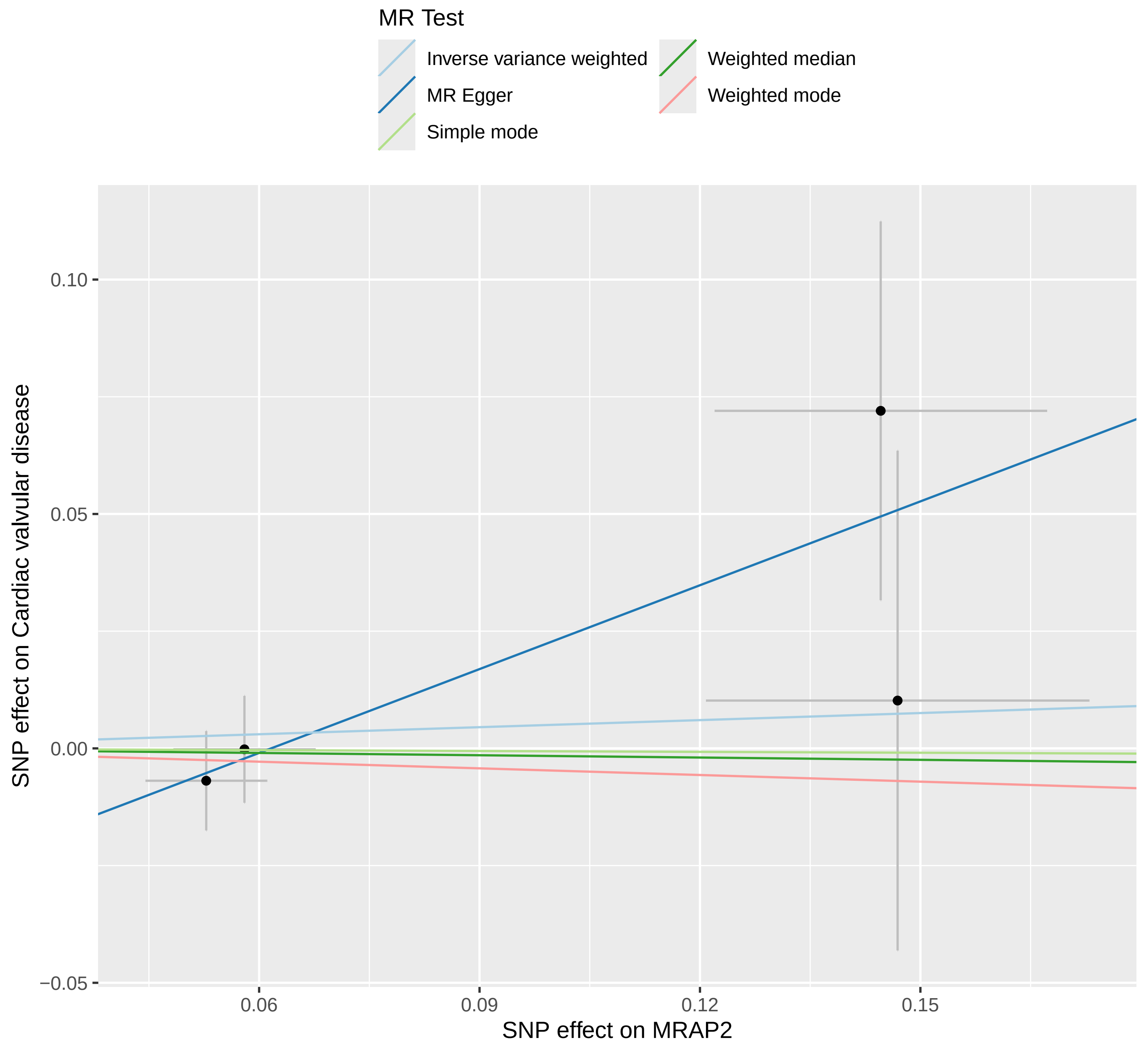

MR Test
Inverse variance weighted
MR Egger
Simple mode
Weighted median
Weighted mode
SNP effect on Cardiac valvular disease
0.10
0.05
0.00
−0.05
0.06
0.09
0.12
0.15
SNP effect on MRAP2

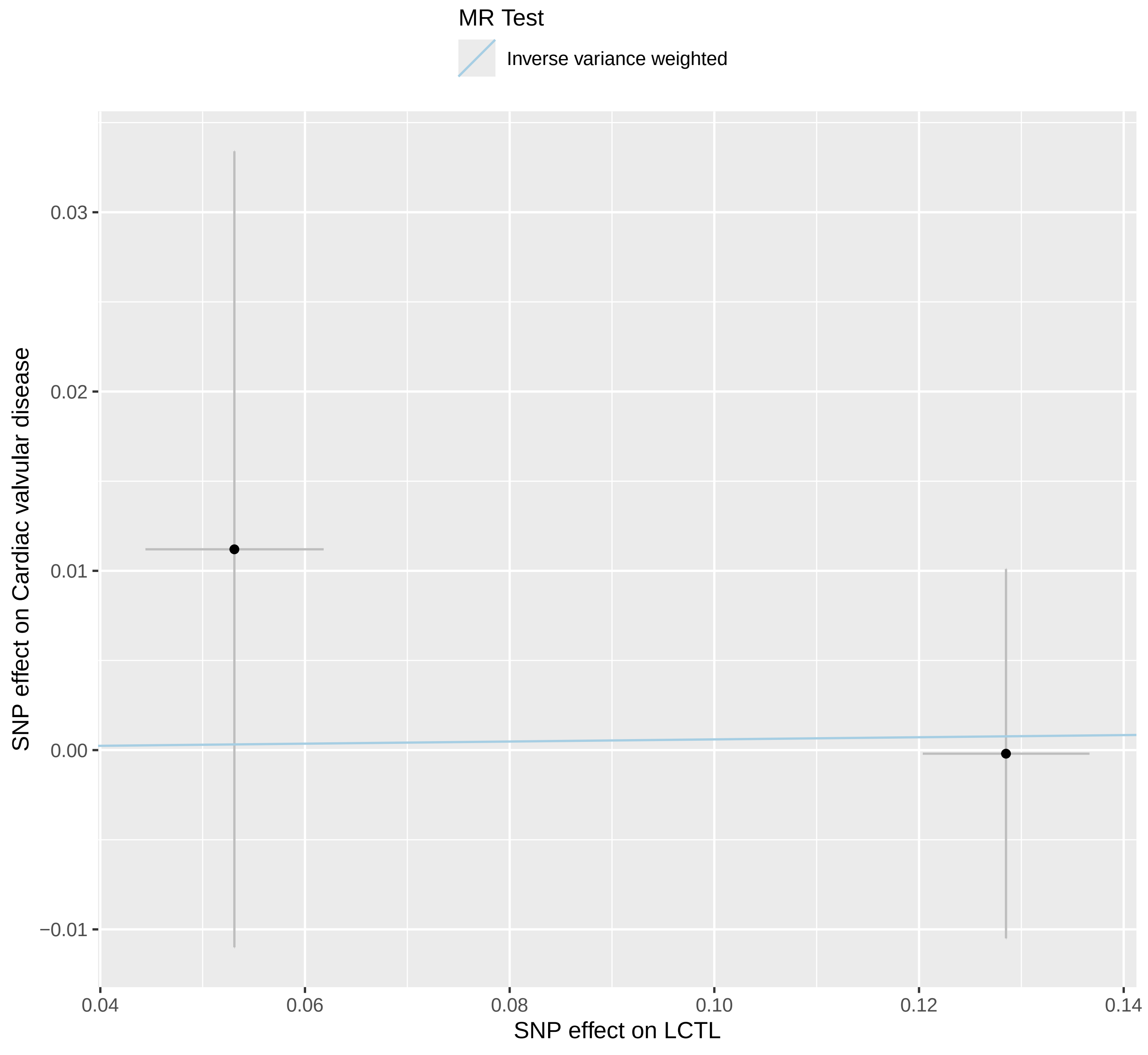

MR Test
Inverse variance weighted
0.03
0.02
0.01
0.00
−0.01
0.04
0.06
0.08
0.10
0.12
0.14
SNP effect on Cardiac valvular disease
SNP effect on LCTL

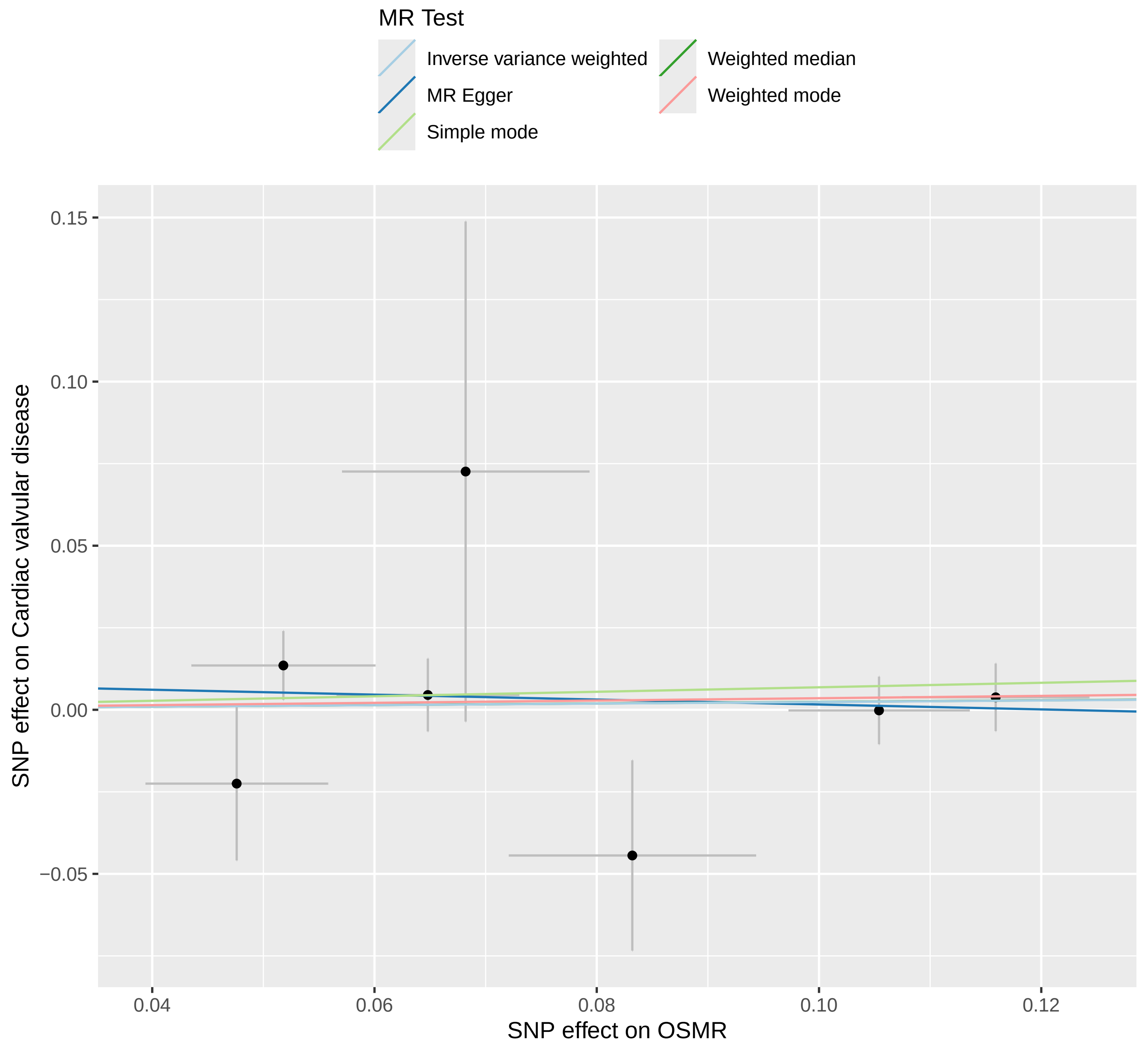

MR Test
Inverse variance weighted
MR Egger
Simple mode
Weighted median
Weighted mode
0.15
0.10
0.05
0.00
−0.05
0.04
0.06
0.08
0.10
0.12
SNP effect on Cardiac valvular disease
SNP effect on OSMR

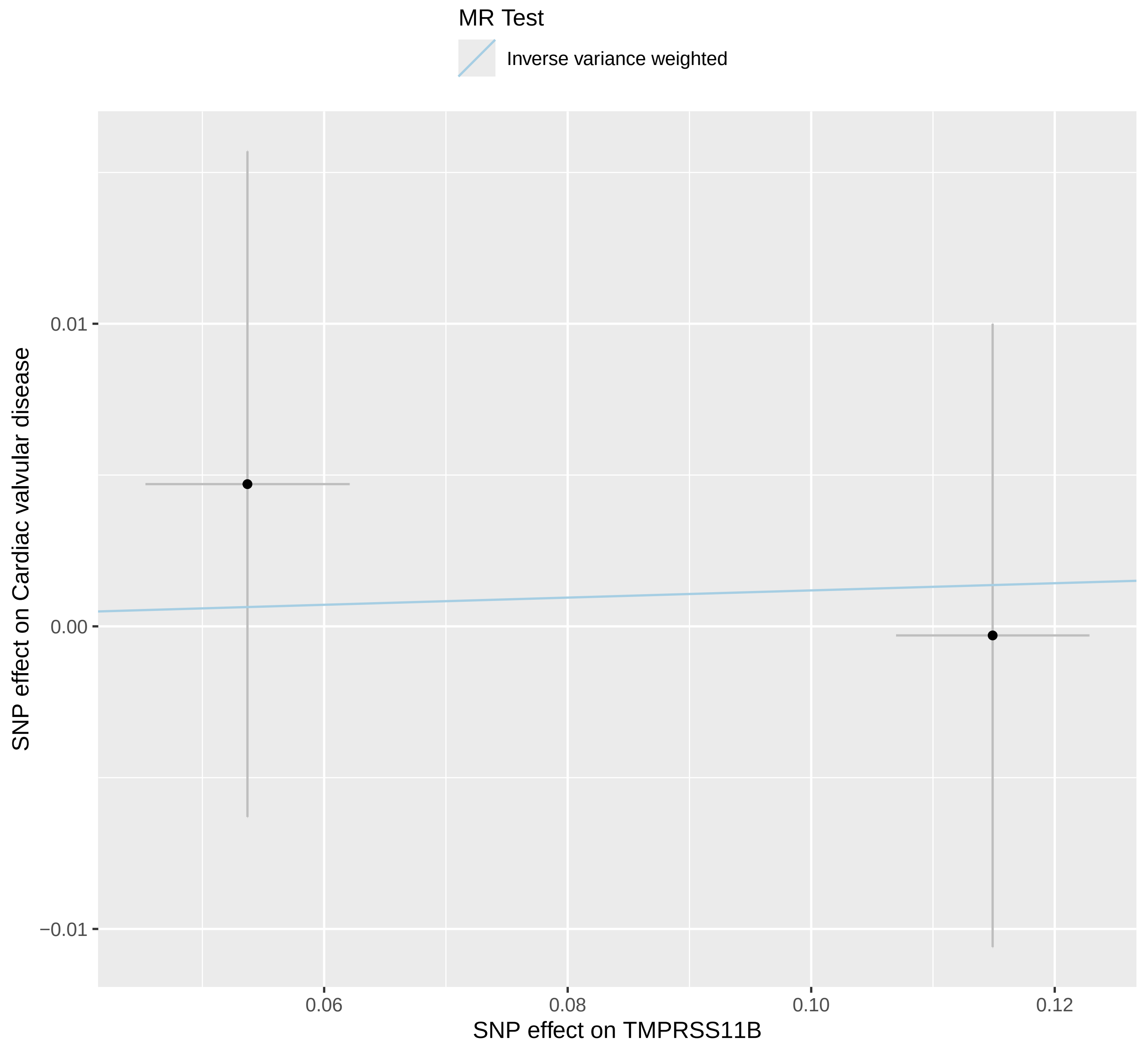

MR Test
Inverse variance weighted
0.01
0.00
−0.01
0.06
0.08
0.10
0.12
SNP effect on TMPRSS11B
SNP effect on Cardiac valvular disease

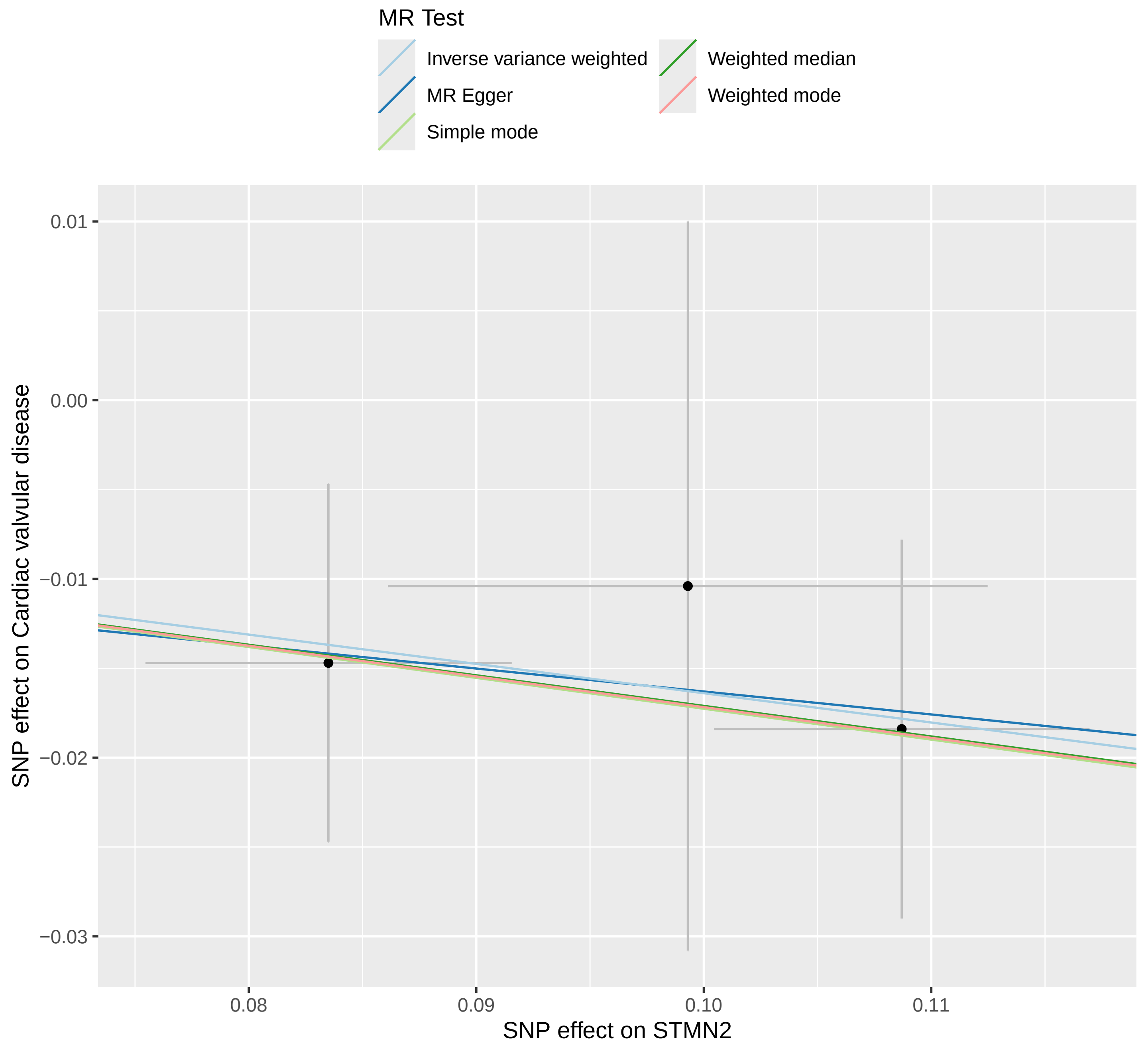

MR Test
Inverse variance weighted
MR Egger
Simple mode
Weighted median
Weighted mode
0.01
0.00
−0.01
−0.02
−0.03
SNP effect on Cardiac valvular disease
0.08
0.09
0.10
0.11
SNP effect on STMN2

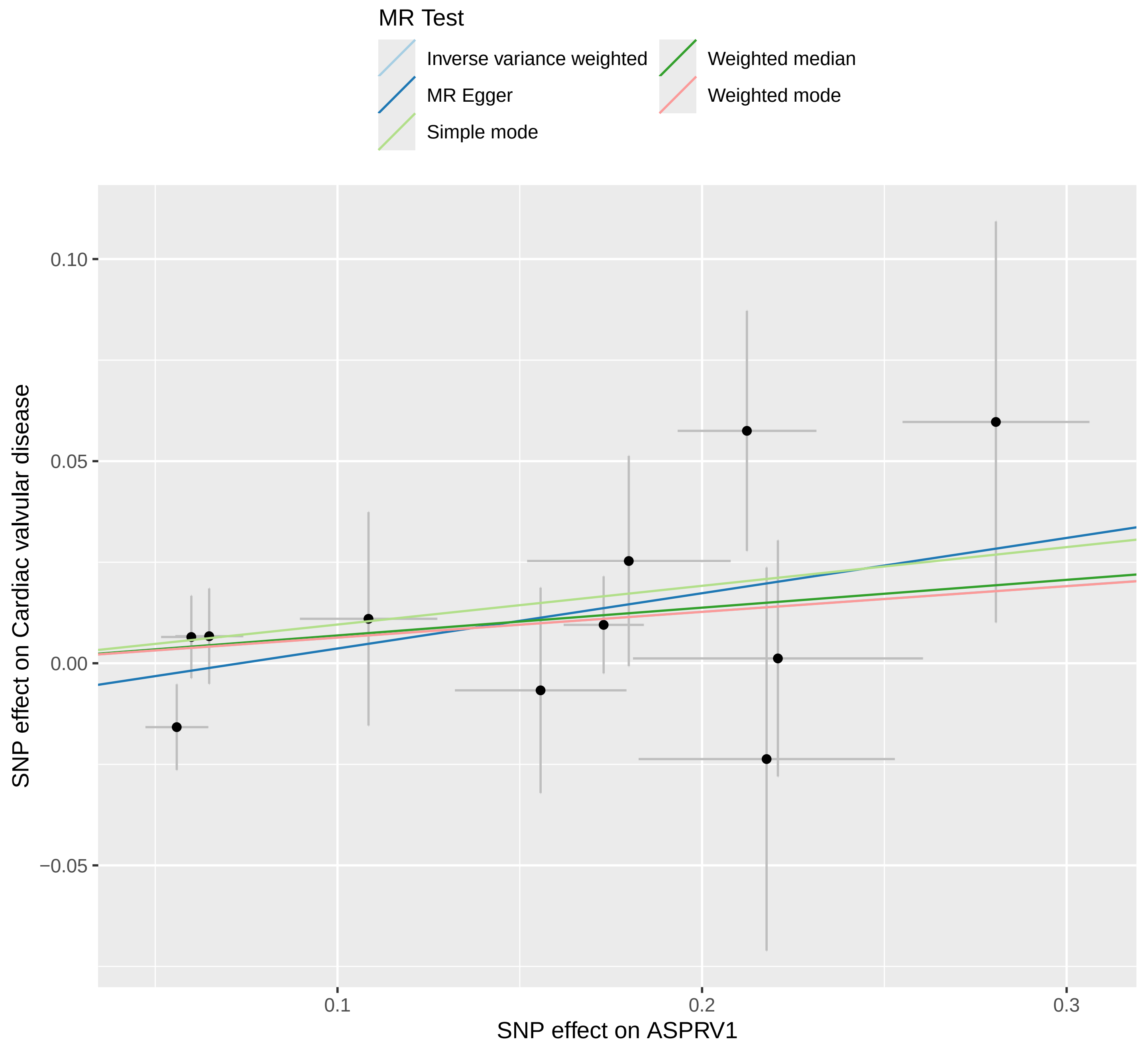

MR Test
Inverse variance weighted
MR Egger
Simple mode
Weighted median
Weighted mode
0.10
0.05
0.00
−0.05
0.1
0.2
0.3
SNP effect on ASPRV1
SNP effect on Cardiac valvular disease

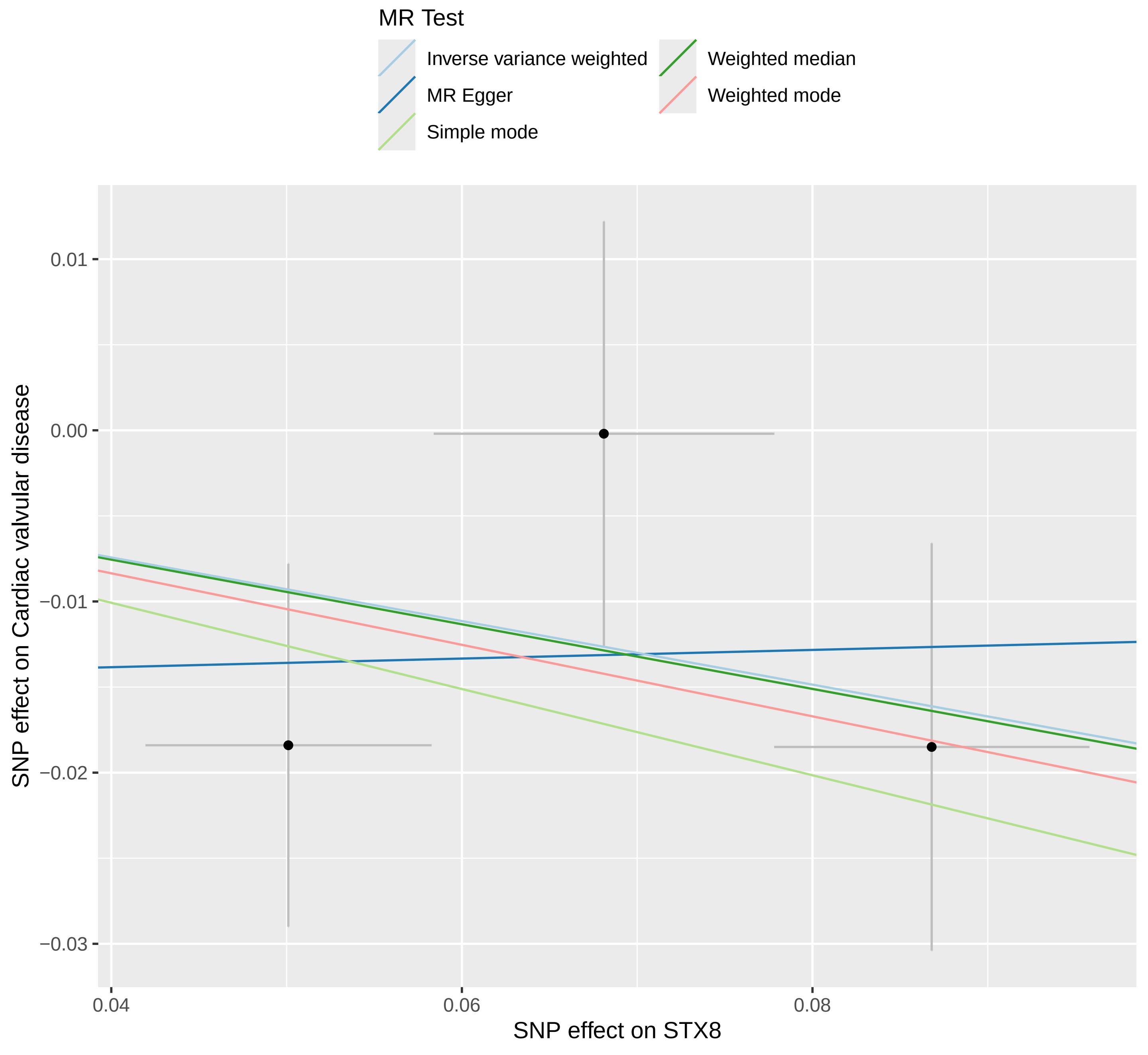
MR Test
Inverse variance weighted
MR Egger
Simple mode
Weighted median
Weighted mode
0.01
0.00
−0.01
−0.02
−0.03
0.04
0.06
0.08
SNP effect on STX8
SNP effect on Cardiac valvular disease

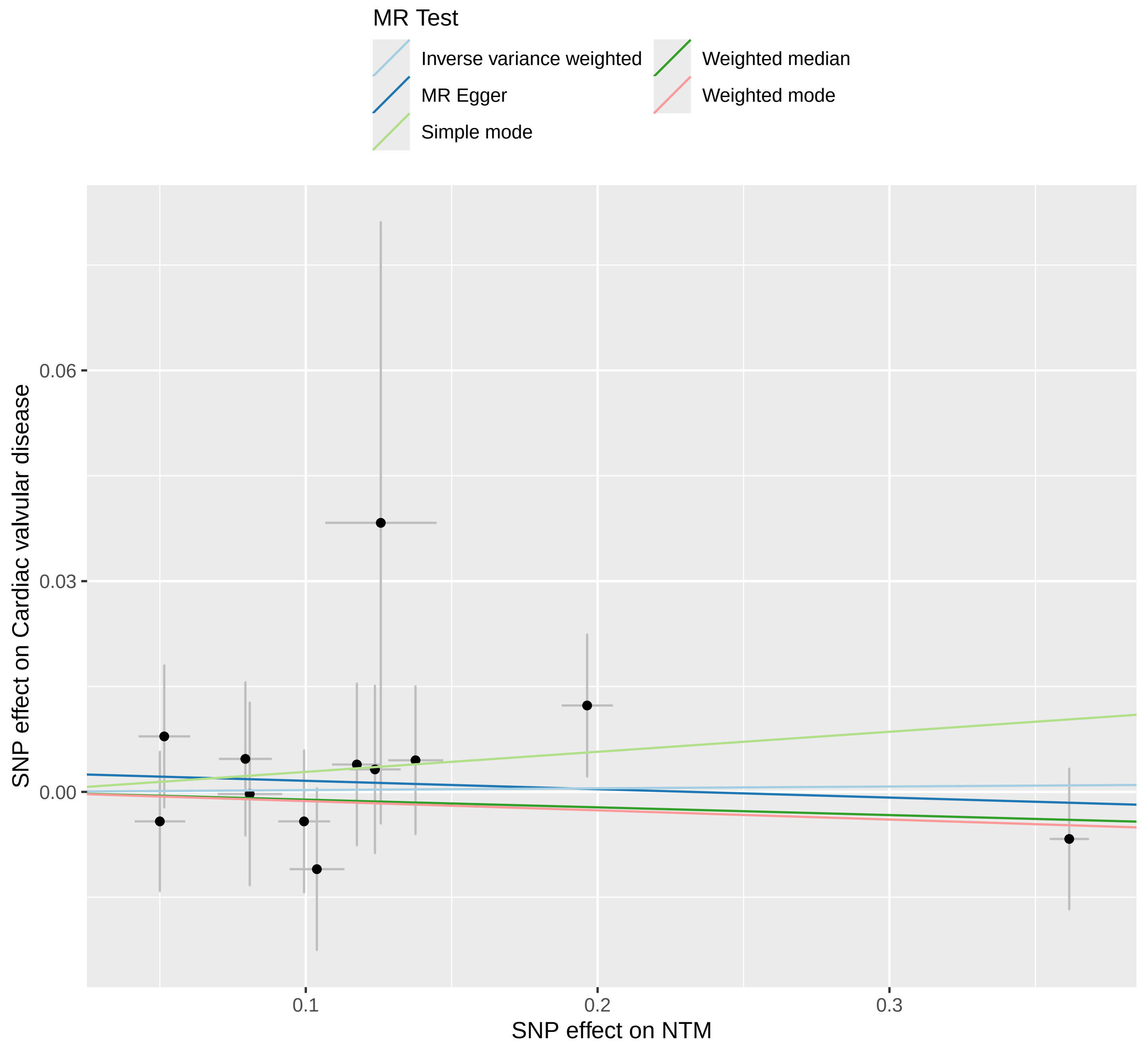
MR Test
Inverse variance weighted
MR Egger
Simple mode
Weighted median
Weighted mode
0.06
0.03
0.00
0.1
0.2
0.3
SNP effect on NTM
SNP effect on Cardiac valvular disease

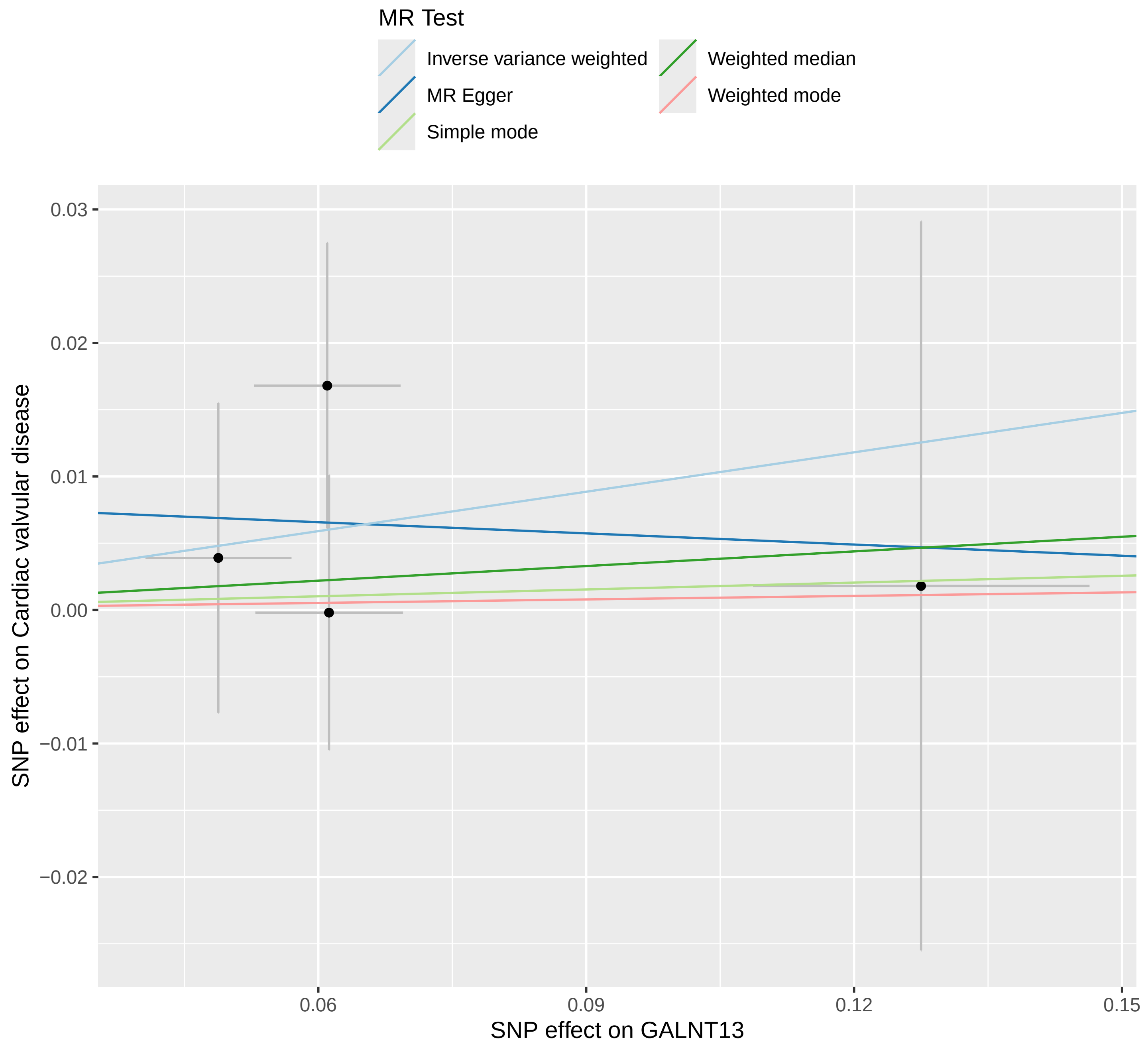

MR Test
Inverse variance weighted
MR Egger
Simple mode
Weighted median
Weighted mode
0.03
0.02
0.01
0.00
−0.01
−0.02
SNP effect on Cardiac valvular disease
0.06
0.09
0.12
0.15
SNP effect on GALNT13

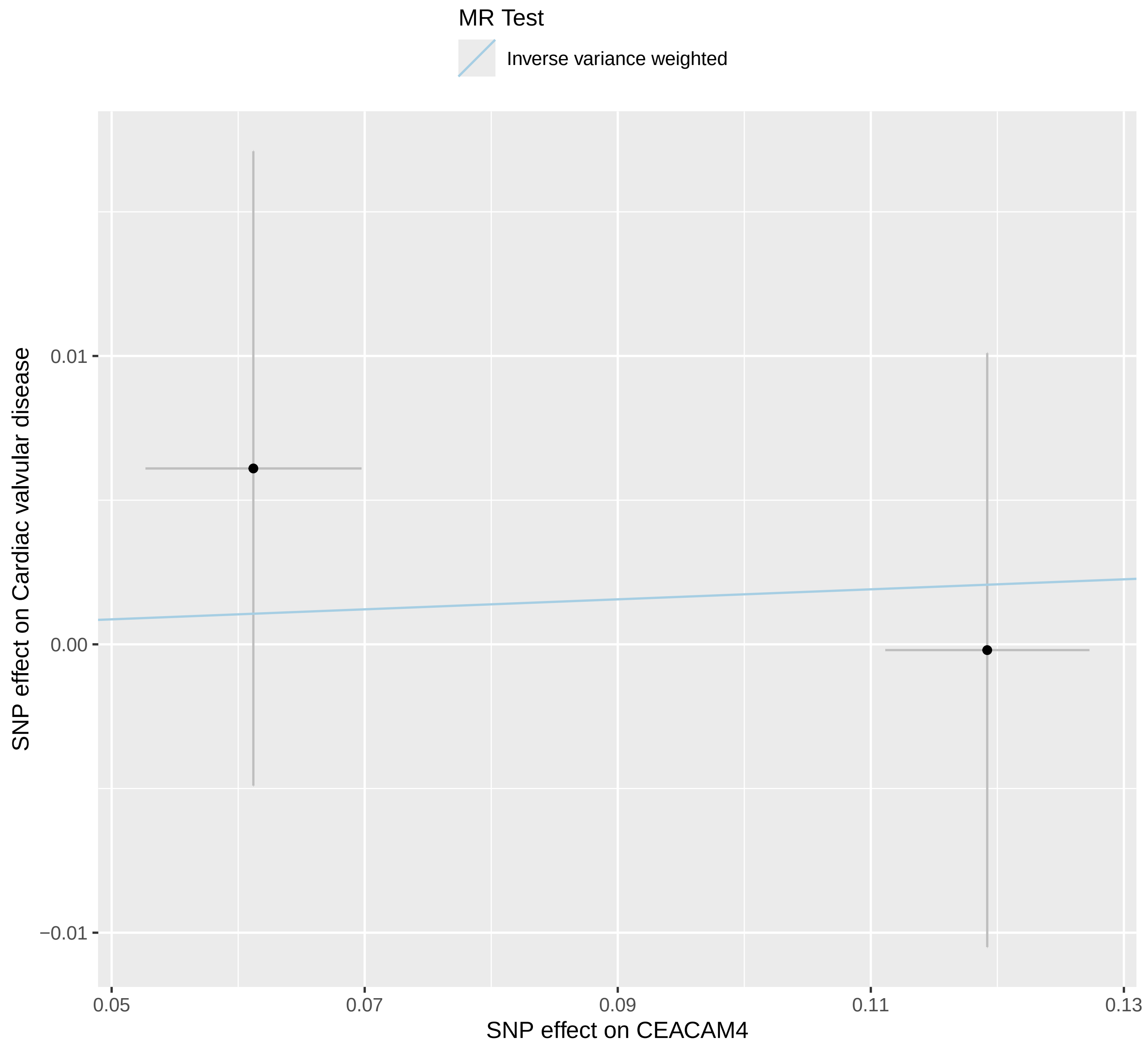

MR Test
Inverse variance weighted
SNP effect on Cardiac valvular disease
0.01
0.00
−0.01
0.05
0.07
0.09
0.11
0.13
SNP effect on CEACAM4

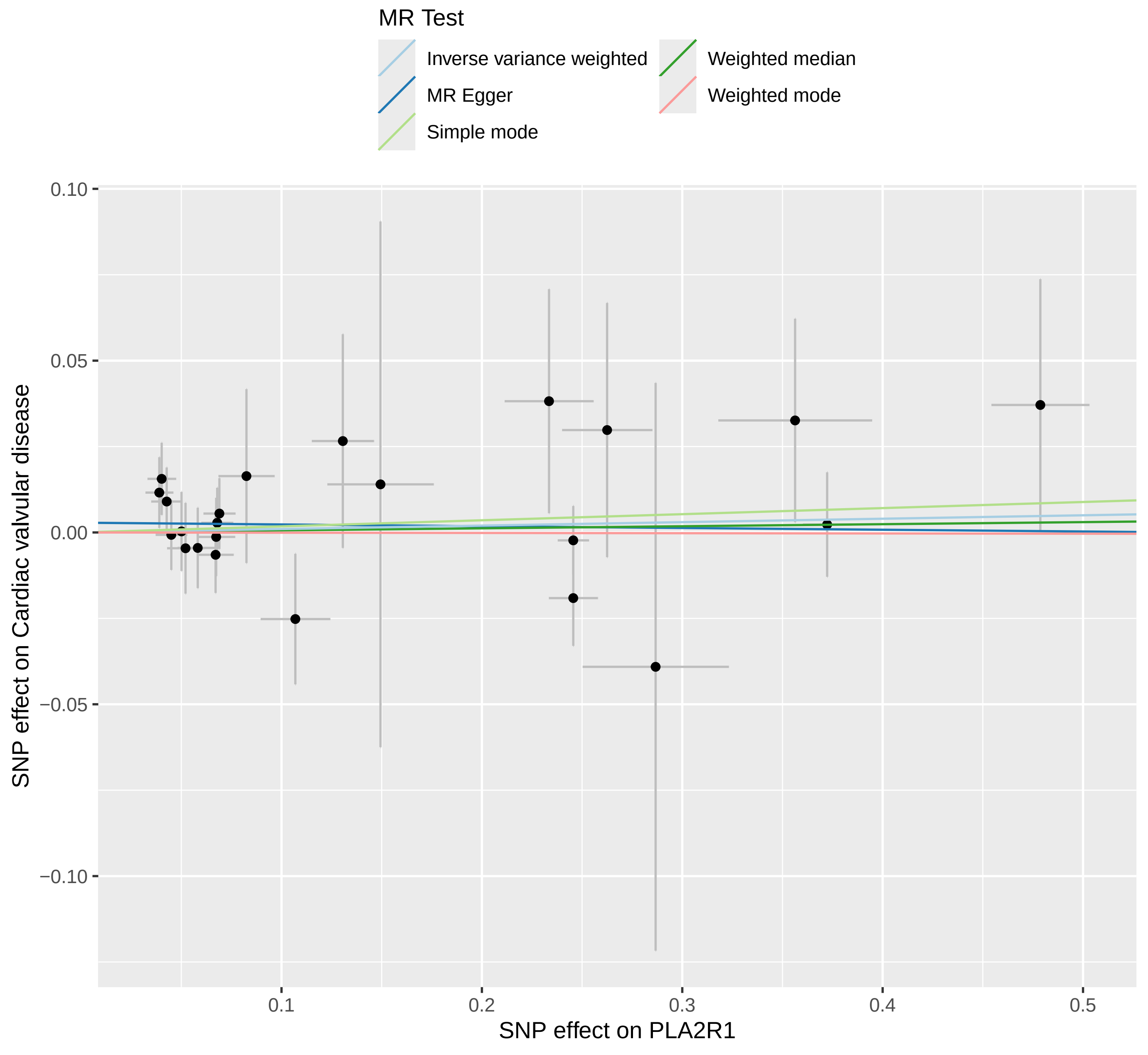

MR Test
Inverse variance weighted
MR Egger
Simple mode
Weighted median
Weighted mode
0.10
0.05
0.00
−0.05
−0.10
SNP effect on Cardiac valvular disease
0.1
0.2
0.3
0.4
0.5
SNP effect on PLA2R1

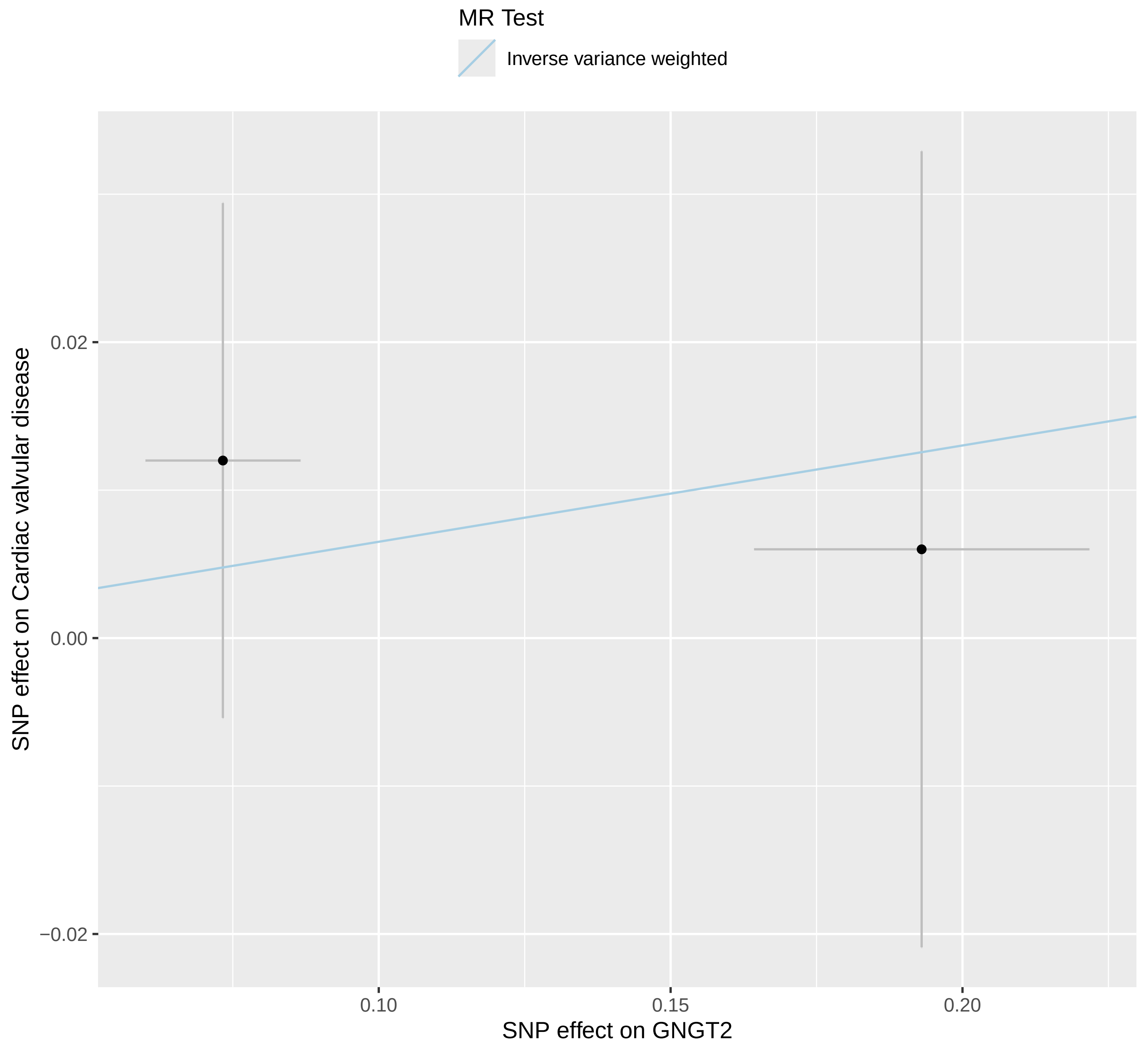

MR Test
Inverse variance weighted
0.02
0.00
−0.02
SNP effect on Cardiac valvular disease
0.10
0.15
0.20
SNP effect on GNGT2

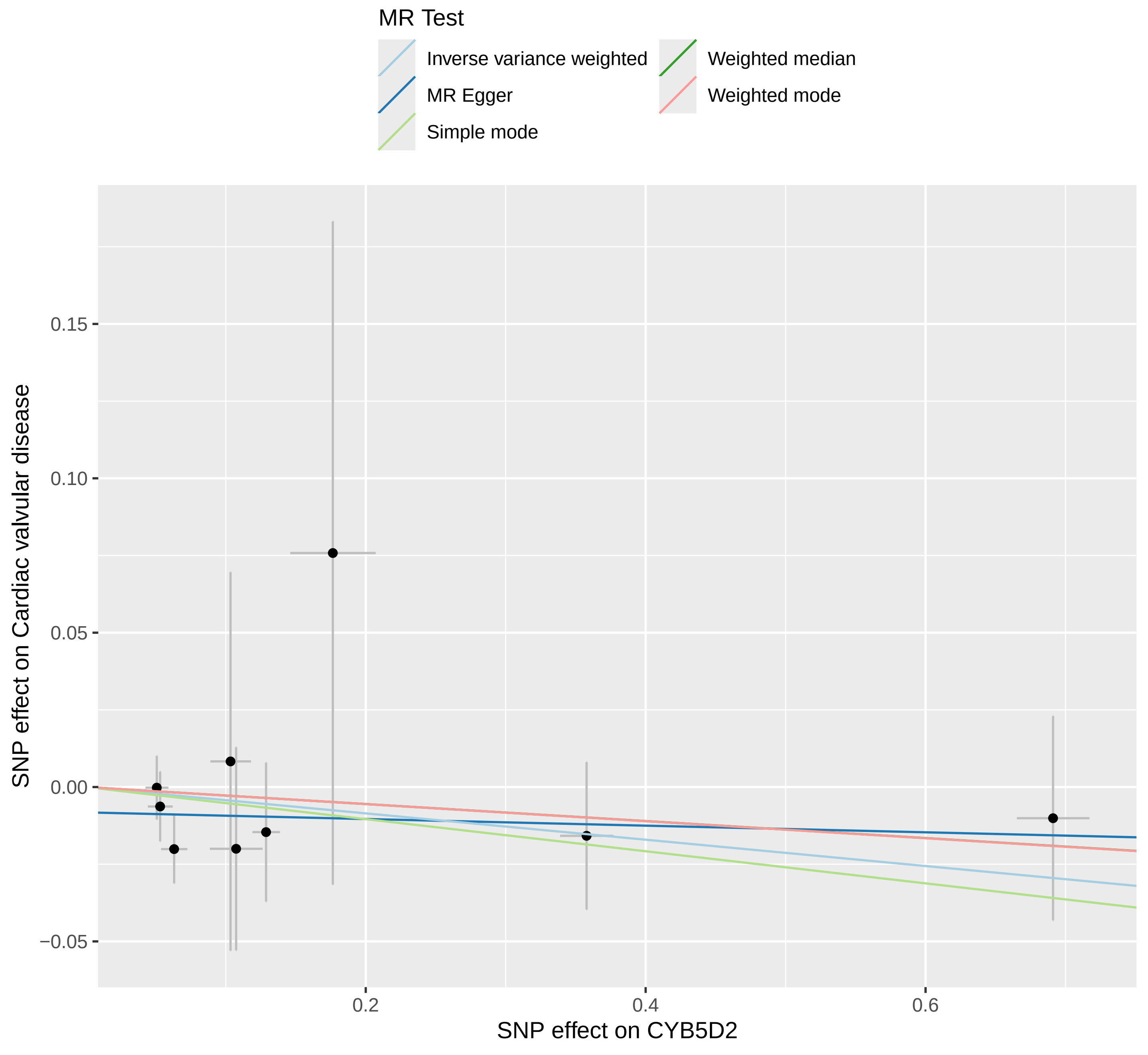

MR Test
Inverse variance weighted
MR Egger
Simple mode
Weighted median
Weighted mode
SNP effect on Cardiac valvular disease
0.15
0.10
0.05
0.00
−0.05
0.2
0.4
0.6
SNP effect on CYB5D2

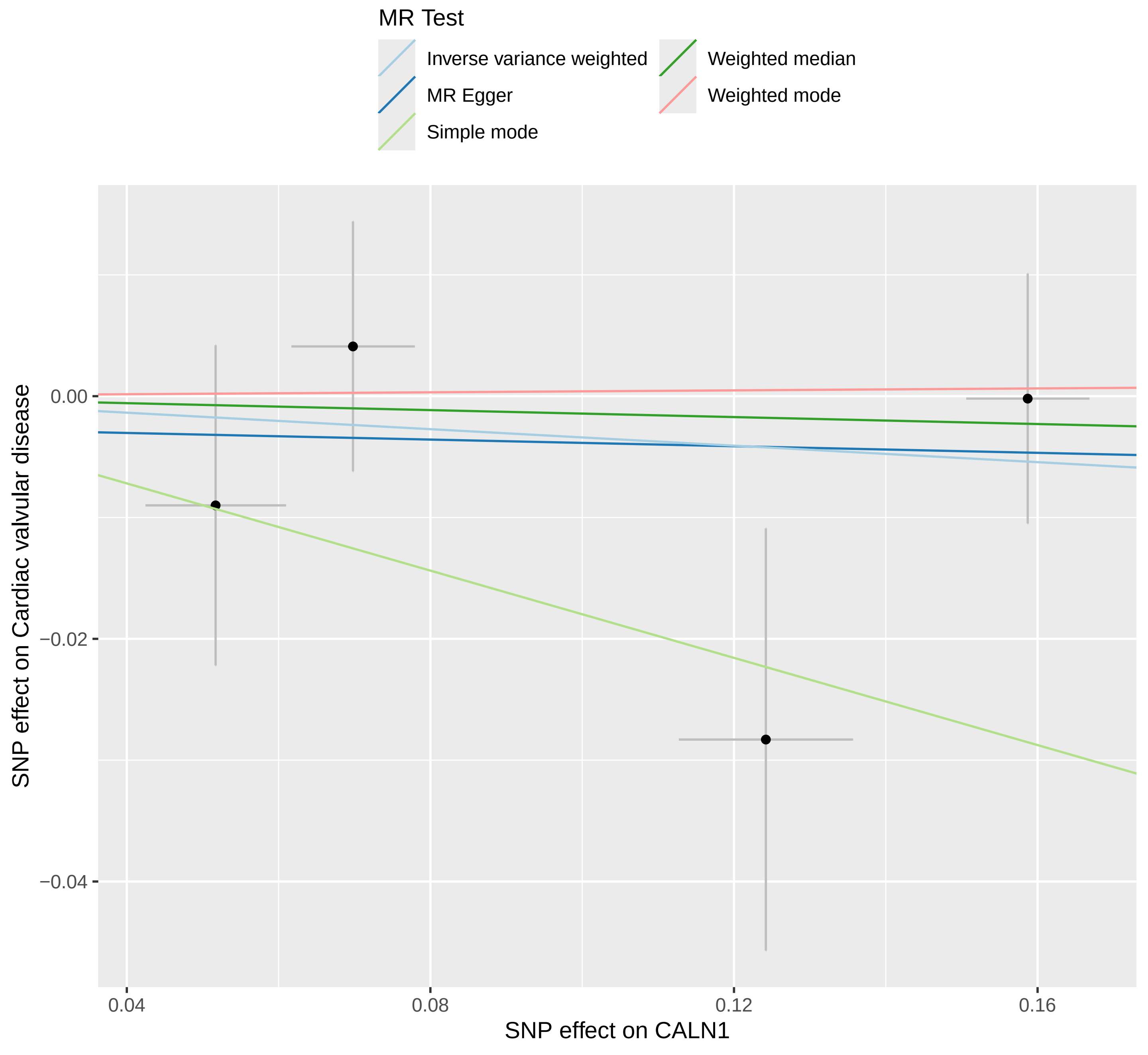

MR Test
Inverse variance weighted
MR Egger
Simple mode
Weighted median
Weighted mode
0.00
−0.02
−0.04
0.04
0.08
0.12
0.16
SNP effect on Cardiac valvular disease
SNP effect on CALN1

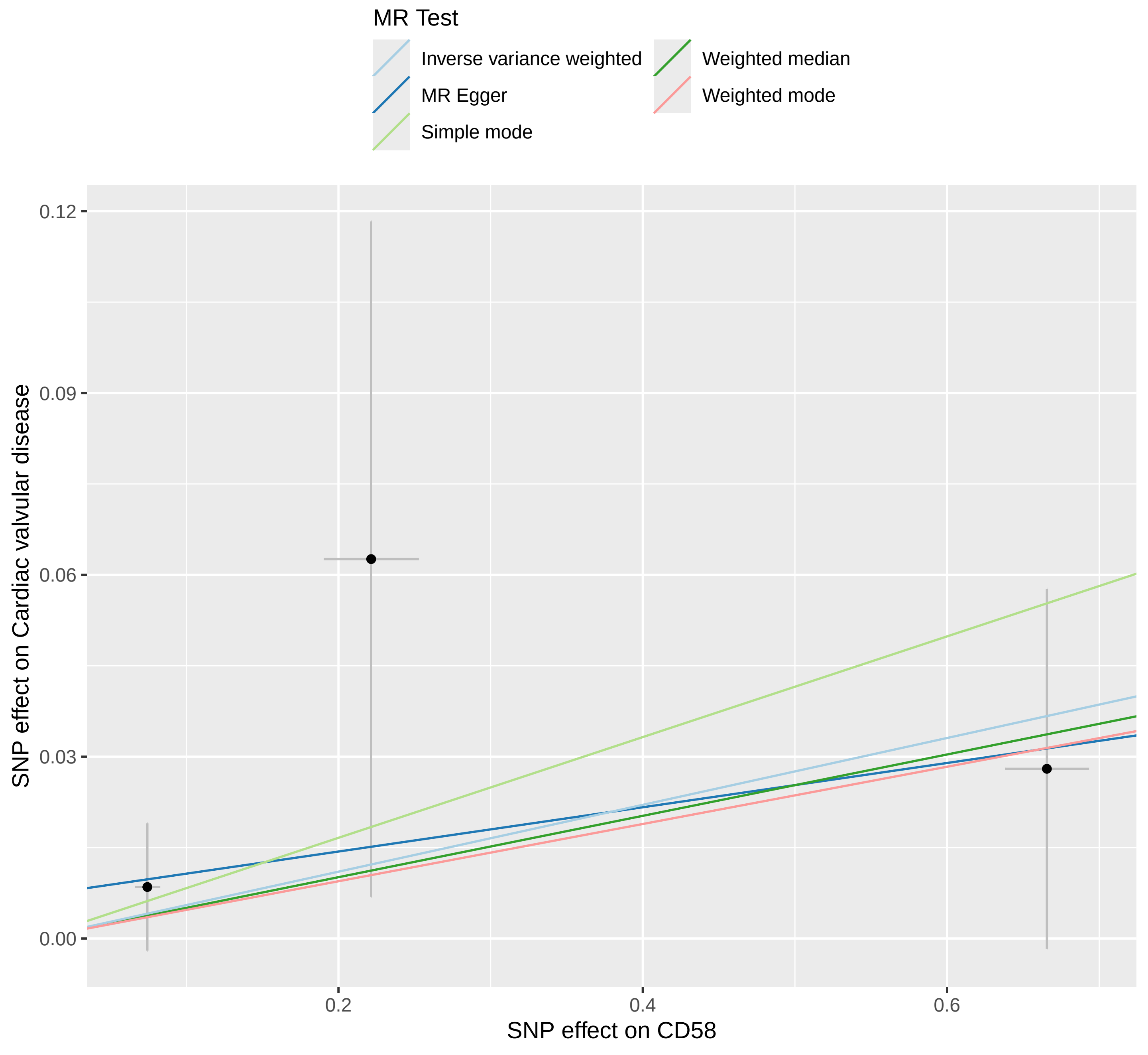
MR Test
Inverse variance weighted
MR Egger
Simple mode
Weighted median
Weighted mode
0.12
0.09
0.06
0.03
0.00
SNP effect on Cardiac valvular disease
0.2
0.4
0.6
SNP effect on CD58

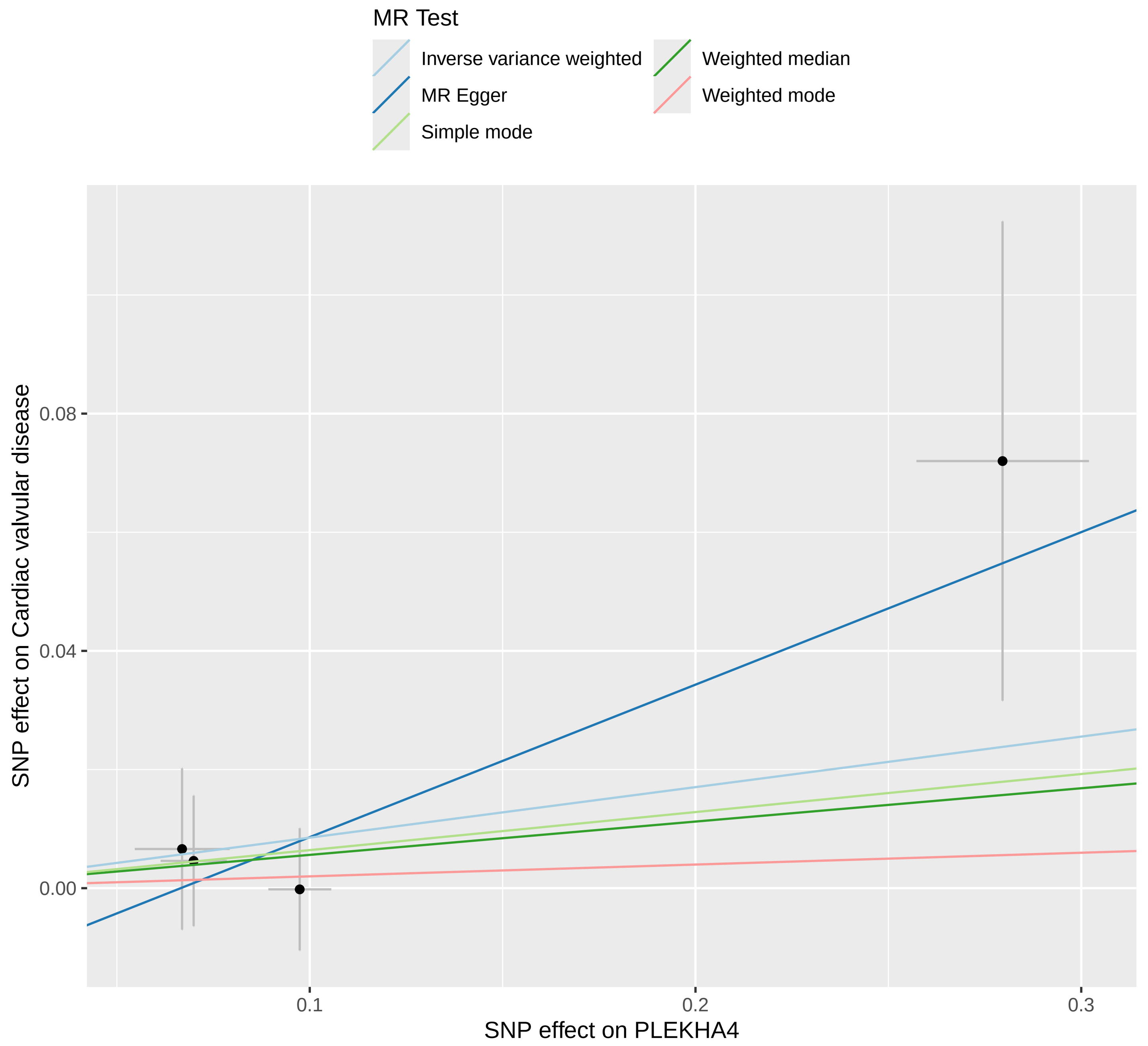

MR Test
Inverse variance weighted
MR Egger
Simple mode
Weighted median
Weighted mode
0.08
0.04
0.00
0.1
0.2
0.3
SNP effect on PLEKHA4
SNP effect on Cardiac valvular disease

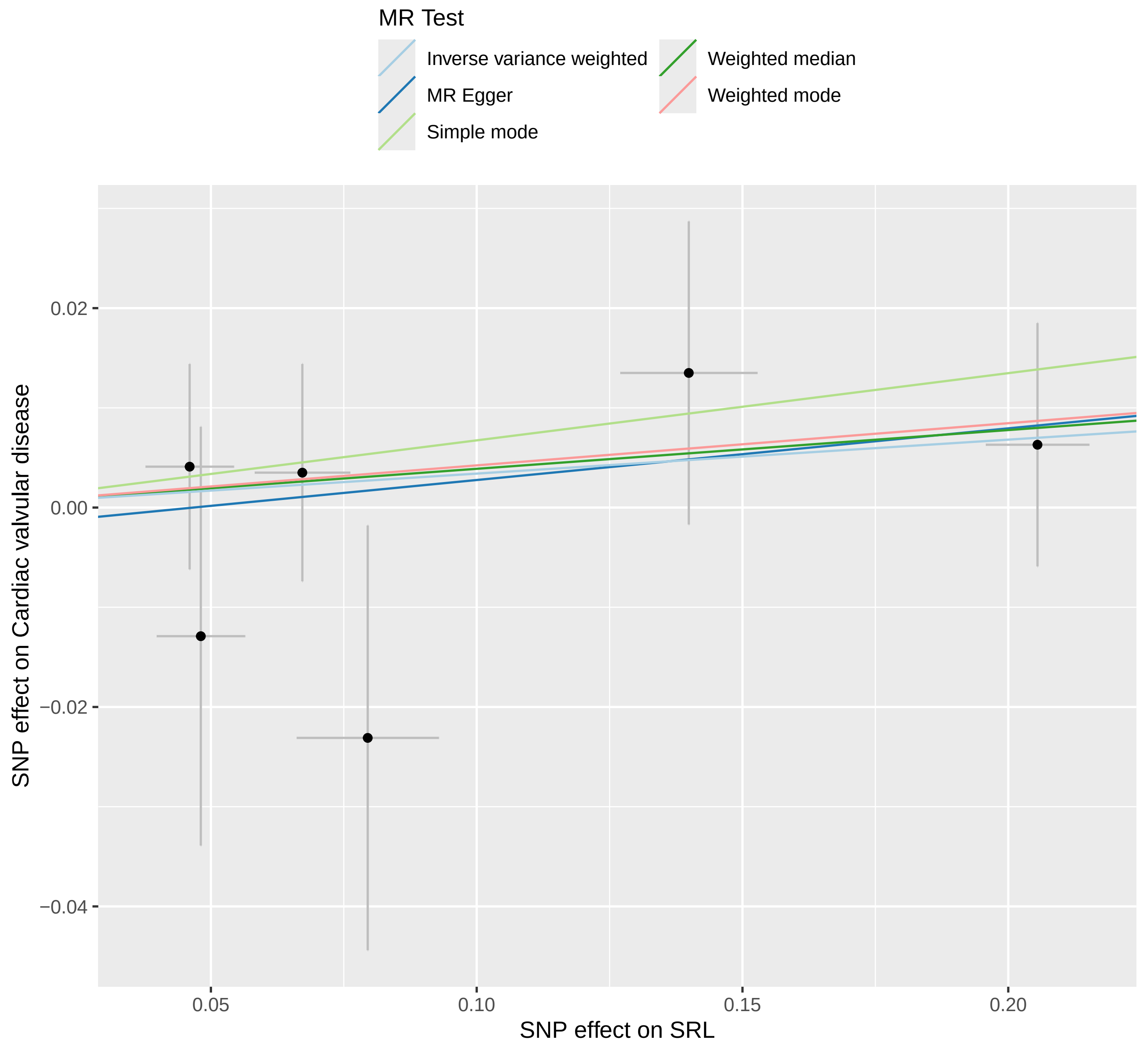

MR Test
Inverse variance weighted
MR Egger
Simple mode
Weighted median
Weighted mode
0.02
0.00
−0.02
−0.04
SNP effect on Cardiac valvular disease
0.05
0.10
0.15
0.20
SNP effect on SRL

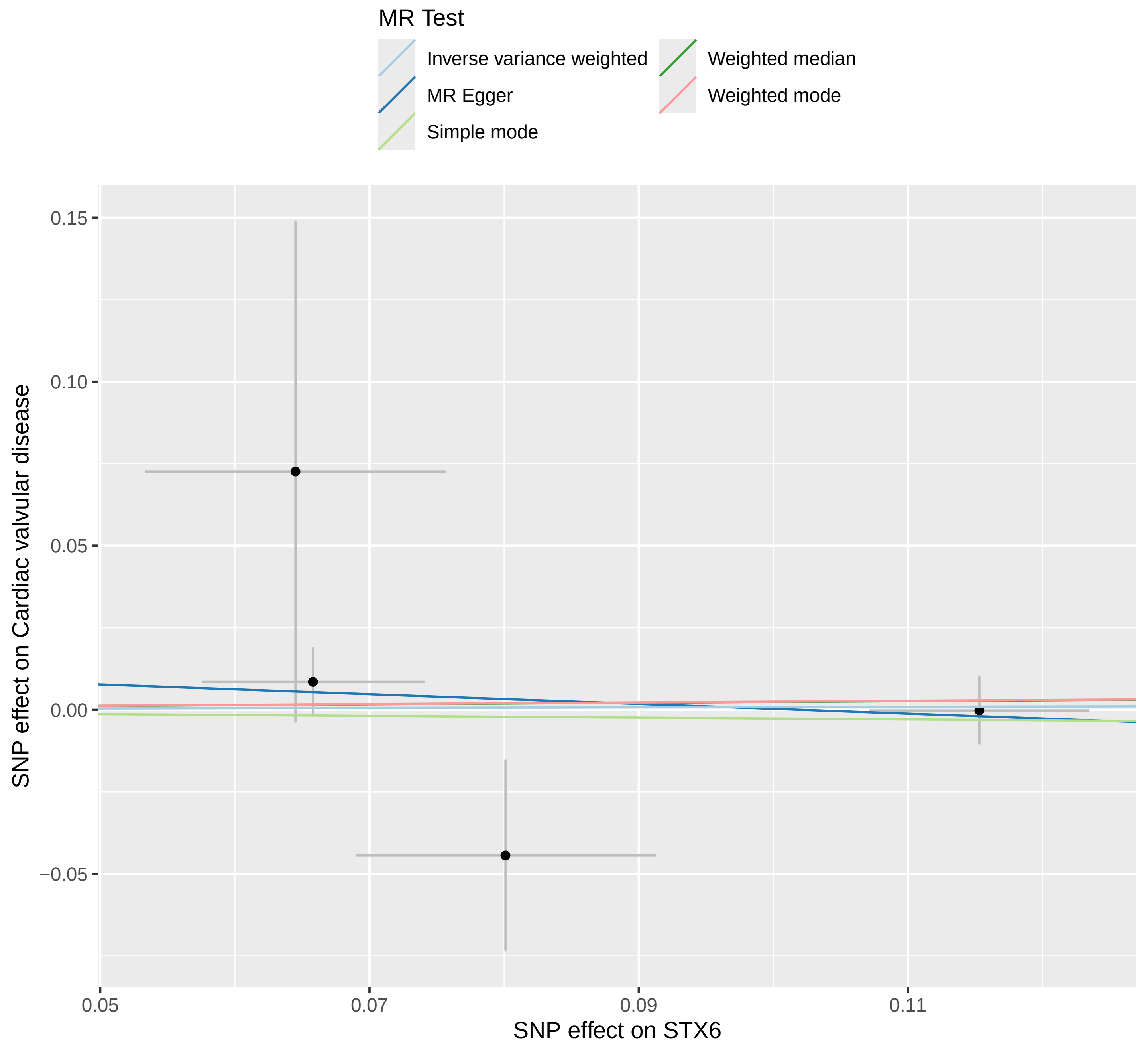
MR Test
Inverse variance weighted
MR Egger
Simple mode
Weighted median
Weighted mode
0.15
0.10
0.05
0.00
−0.05
0.05
0.07
0.09
0.11
SNP effect on Cardiac valvular disease
SNP effect on STX6

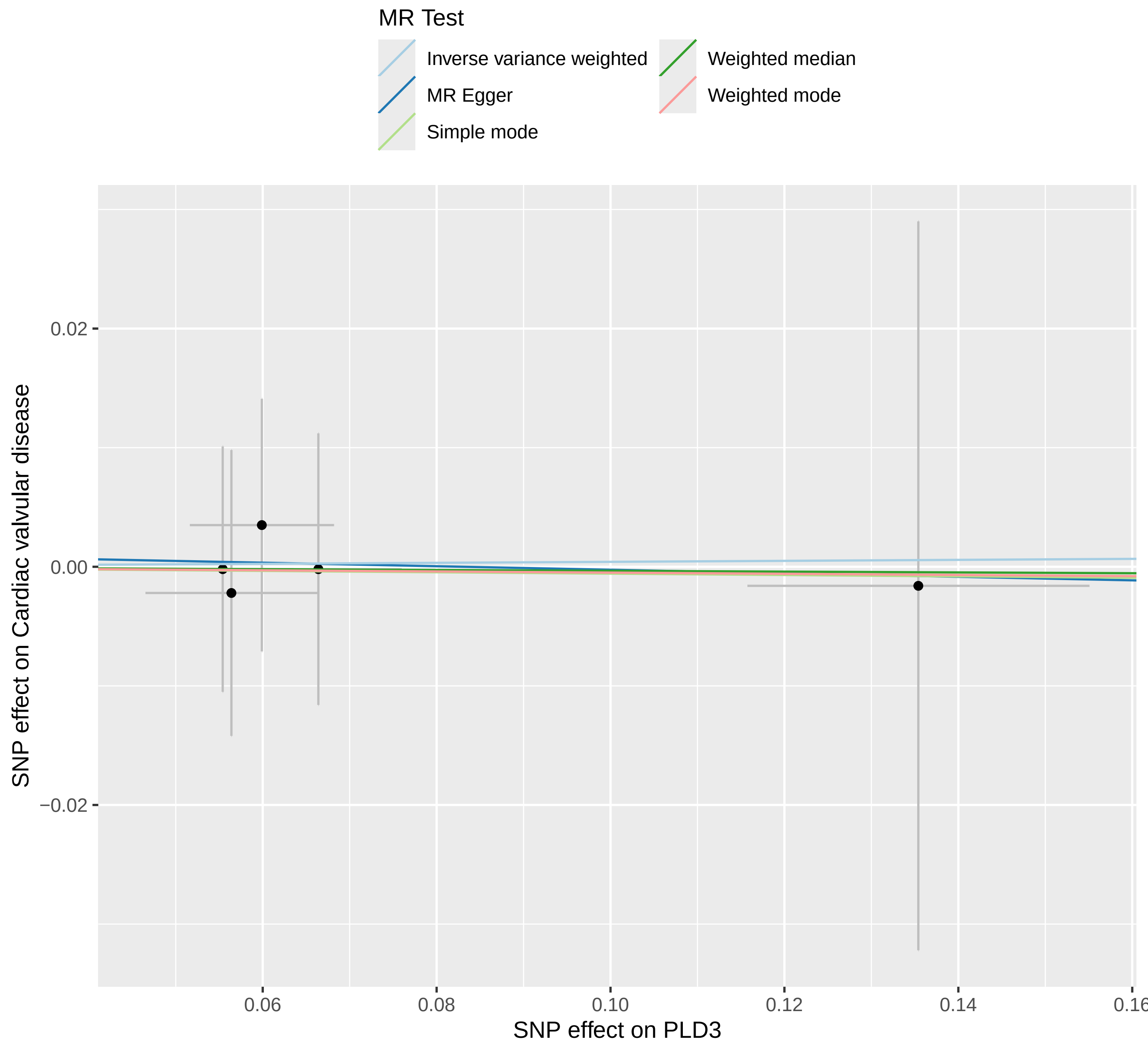

MR Test
Inverse variance weighted
MR Egger
Simple mode
Weighted median
Weighted mode
0.02
0.00
−0.02
0.06
0.08
0.10
0.12
0.14
SNP effect on PLD3
SNP effect on Cardiac valvular disease

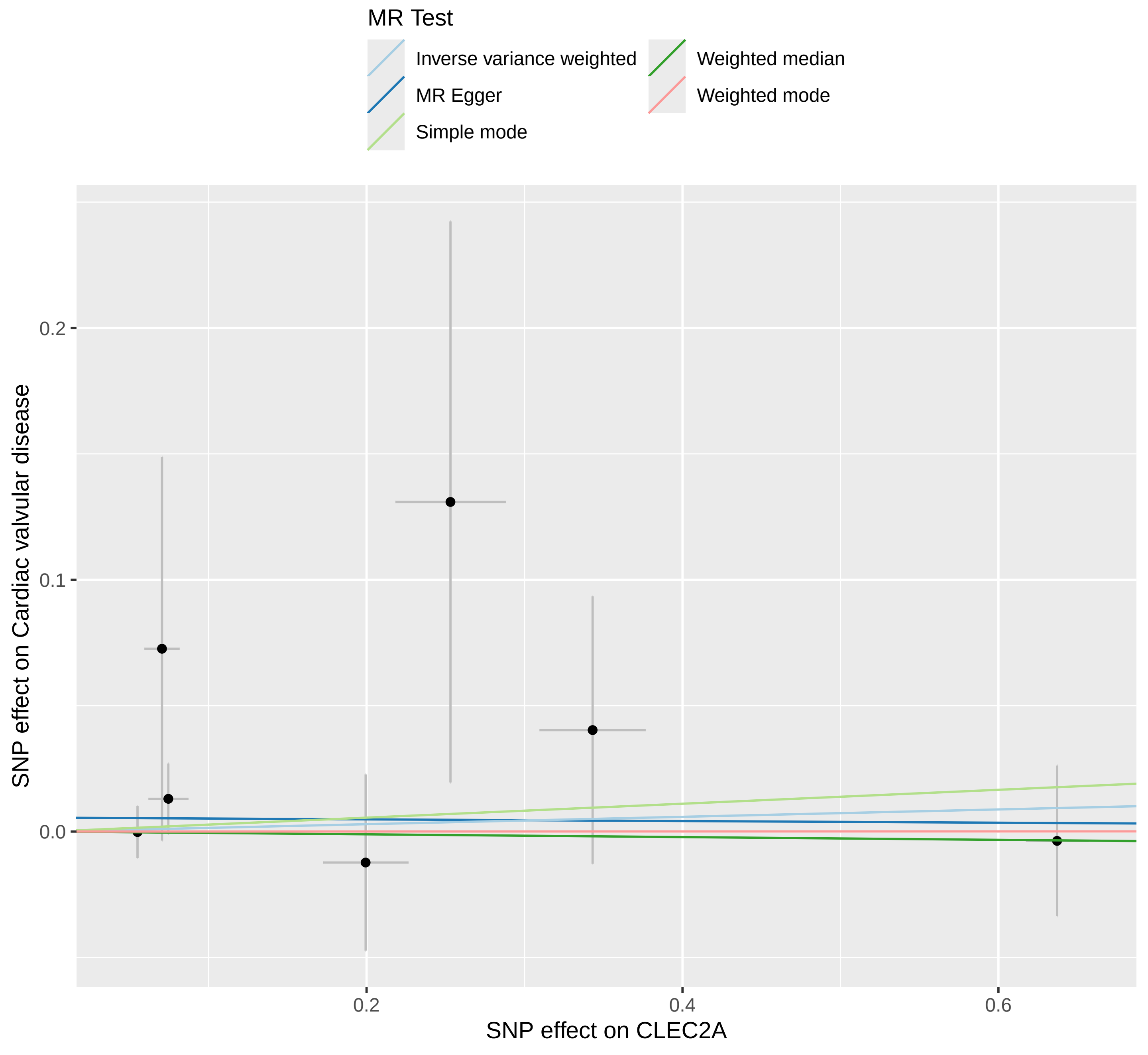

MR Test
Inverse variance weighted
MR Egger
Simple mode
Weighted median
Weighted mode
0.2
0.1
0.0
SNP effect on Cardiac valvular disease
0.2
0.4
0.6
SNP effect on CLEC2A

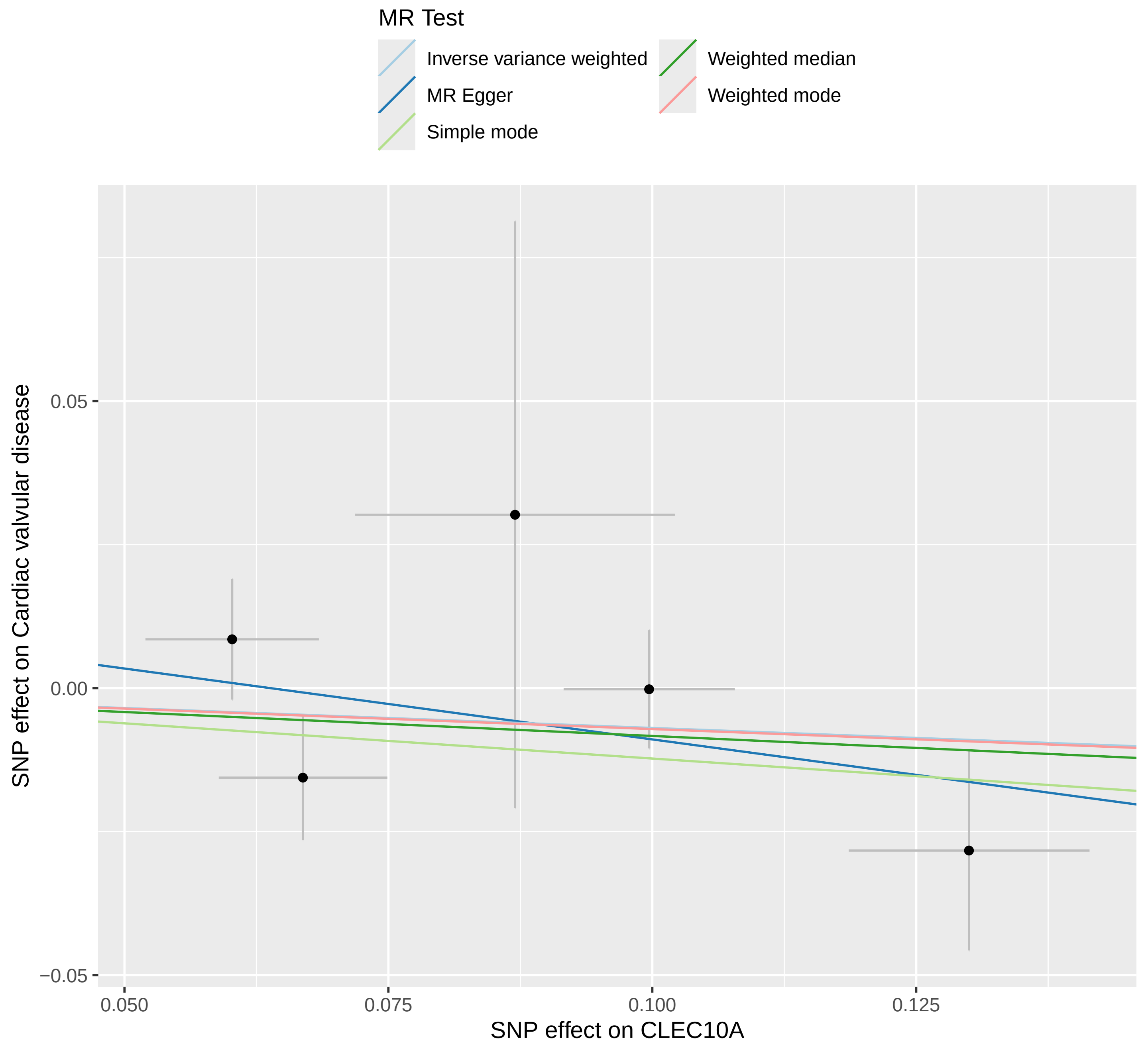
MR Test
Inverse variance weighted
MR Egger
Simple mode
Weighted median
Weighted mode
0.05
0.00
−0.05
0.050
0.075
0.100
0.125
SNP effect on Cardiac valvular disease
SNP effect on CLEC10A

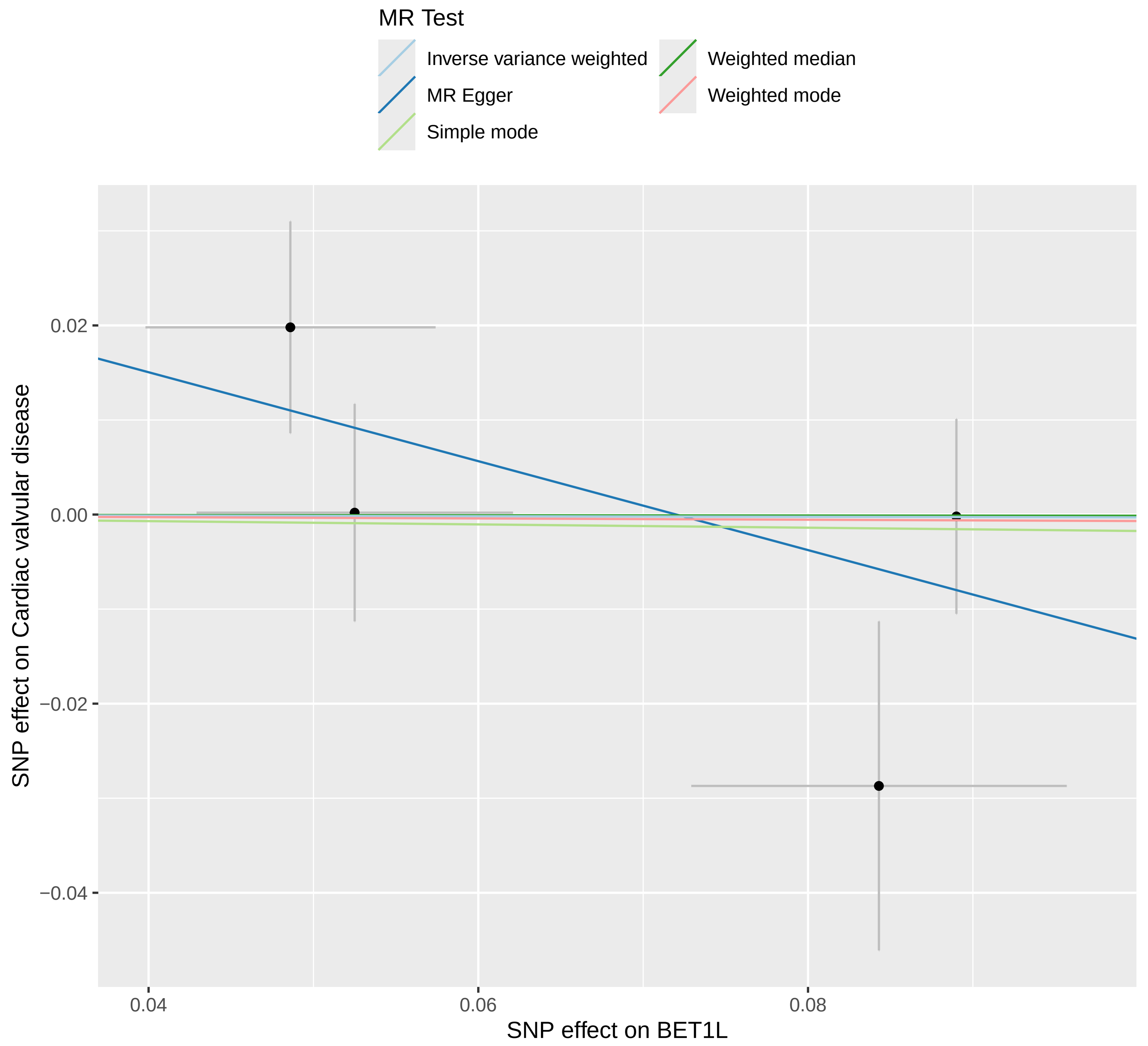

MR Test
Inverse variance weighted
MR Egger
Simple mode
Weighted median
Weighted mode
0.02
0.00
−0.02
−0.04
0.04
0.06
0.08
SNP effect on BET1L
SNP effect on Cardiac valvular disease

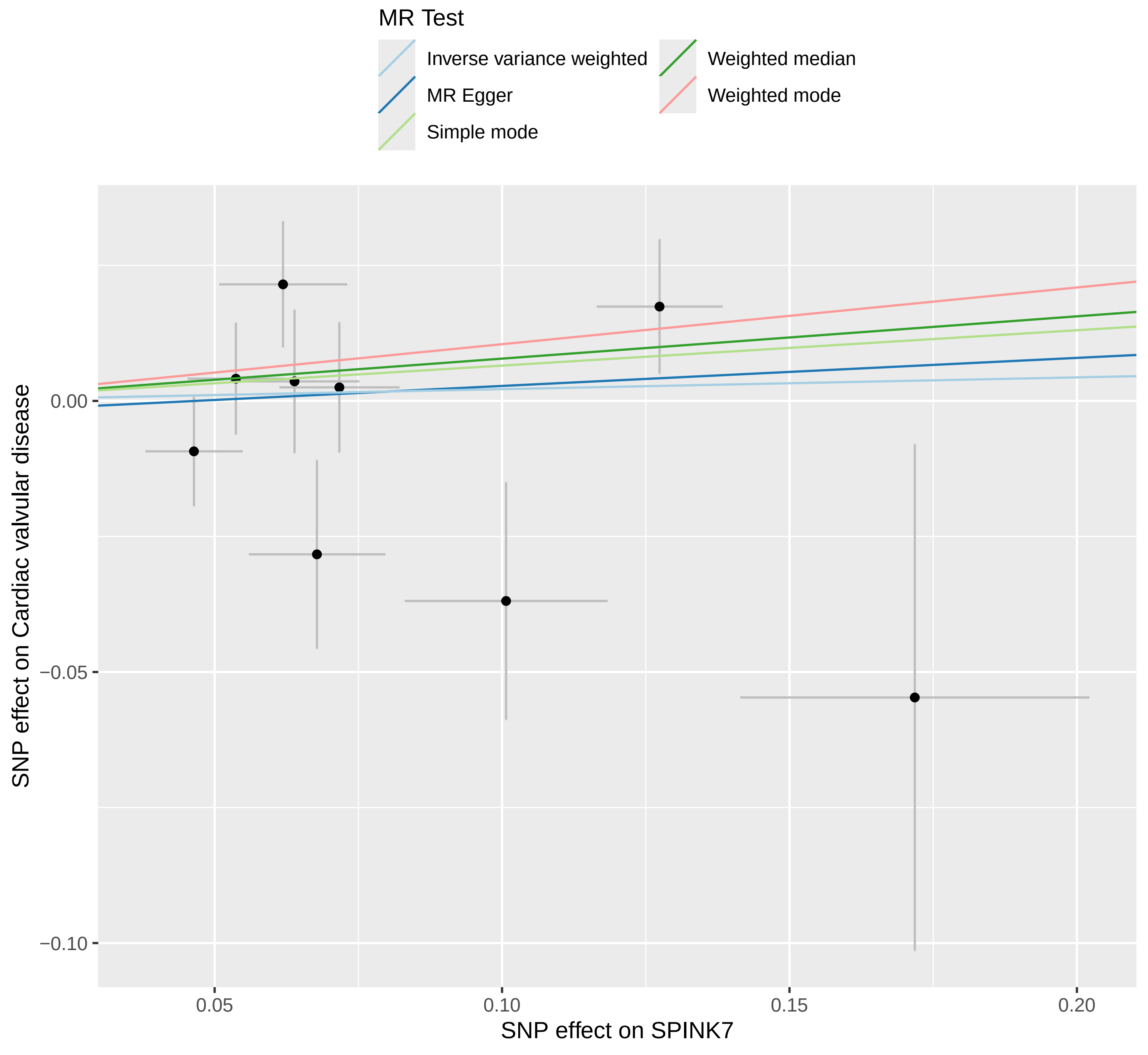

MR Test
Inverse variance weighted
MR Egger
Simple mode
Weighted median
Weighted mode
SNP effect on Cardiac valvular disease
0.00
−0.05
−0.10
0.05
0.10
0.15
0.20
SNP effect on SPINK7

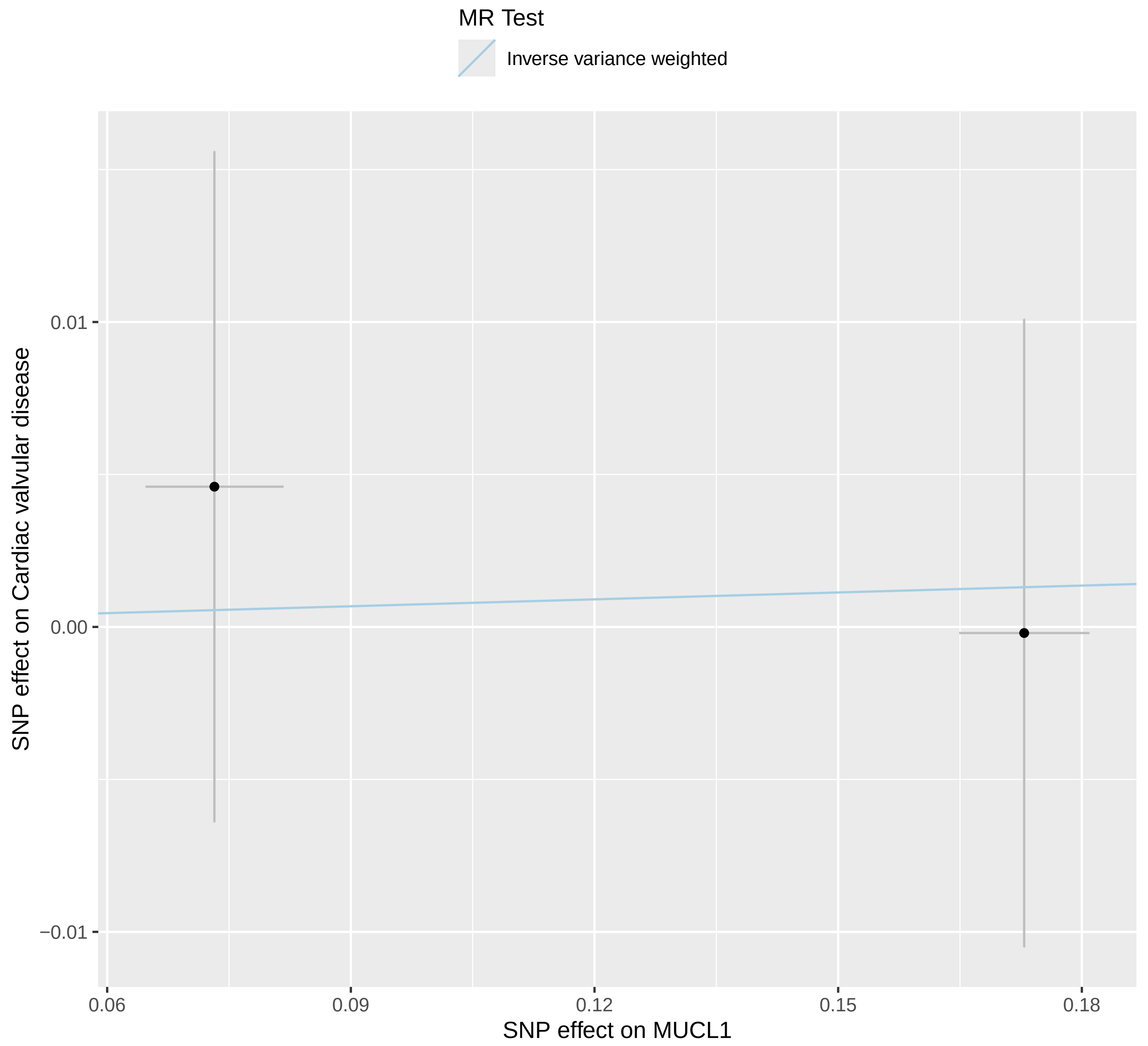
MR Test
Inverse variance weighted
SNP effect on Cardiac valvular disease
0.01
0.00
−0.01
0.06
0.09
0.12
0.15
0.18
SNP effect on MUCL1

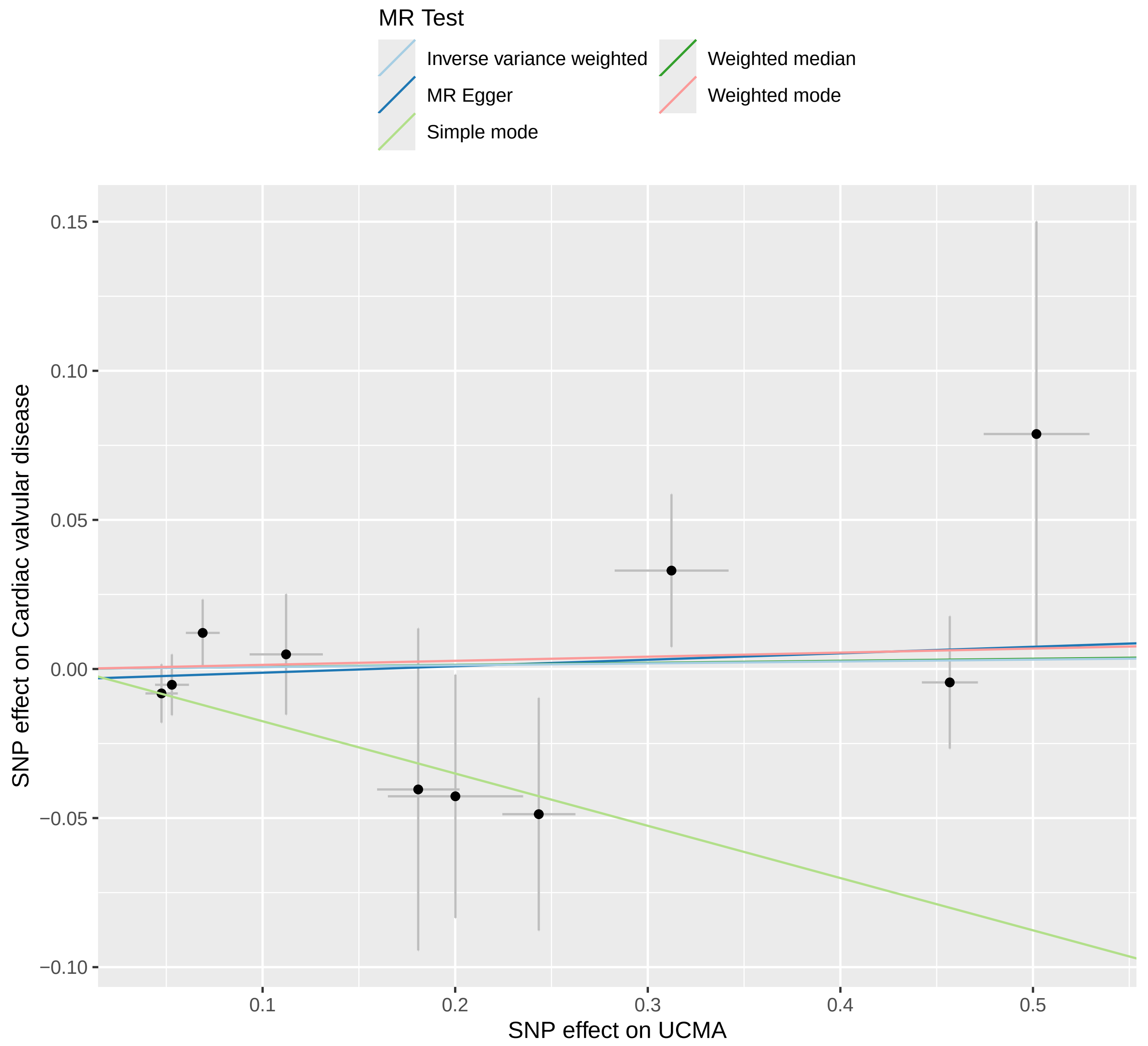
MR Test
Inverse variance weighted
MR Egger
Simple mode
Weighted median
Weighted mode
0.15
0.10
0.05
0.00
−0.05
−0.10
SNP effect on Cardiac valvular disease
0.1
0.2
0.3
0.4
0.5
SNP effect on UCMA

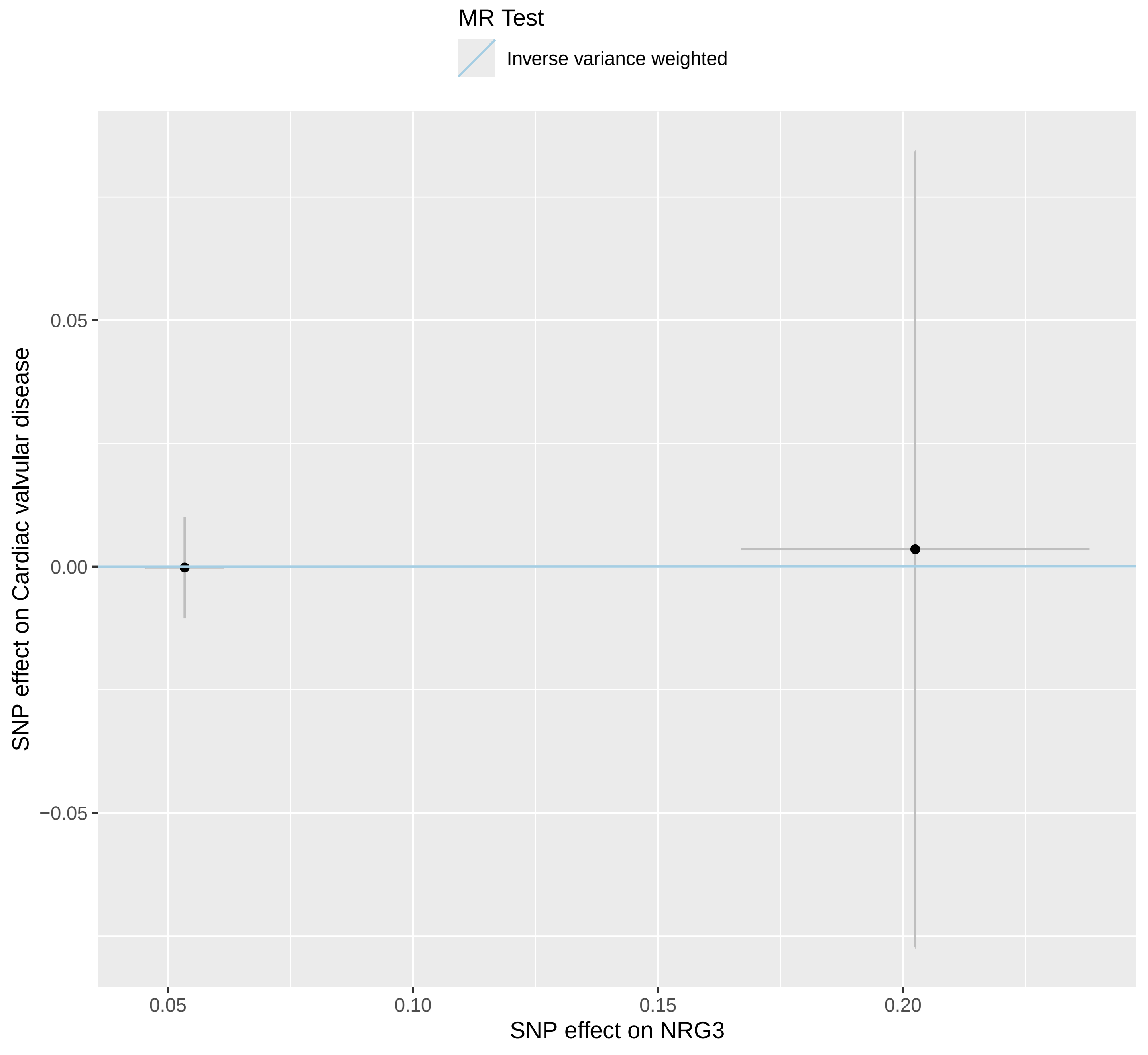

MR Test
Inverse variance weighted
SNP effect on Cardiac valvular disease
0.05
0.00
−0.05
0.05
0.10
0.15
0.20
SNP effect on NRG3

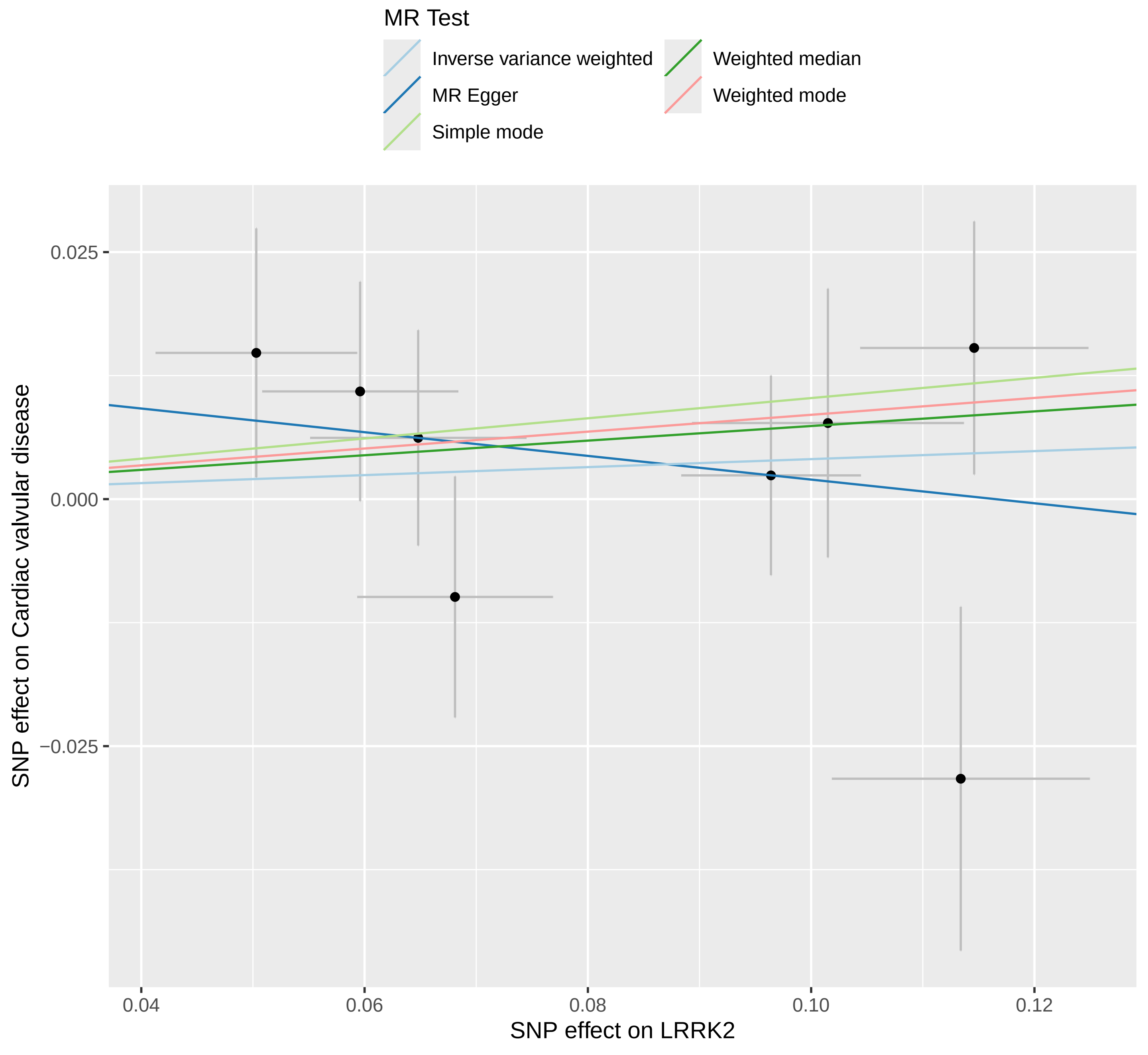

MR Test
Inverse variance weighted
MR Egger
Simple mode
Weighted median
Weighted mode
0.025
0.000
−0.025
0.04
0.06
0.08
0.10
0.12
SNP effect on LRRK2
SNP effect on Cardiac valvular disease

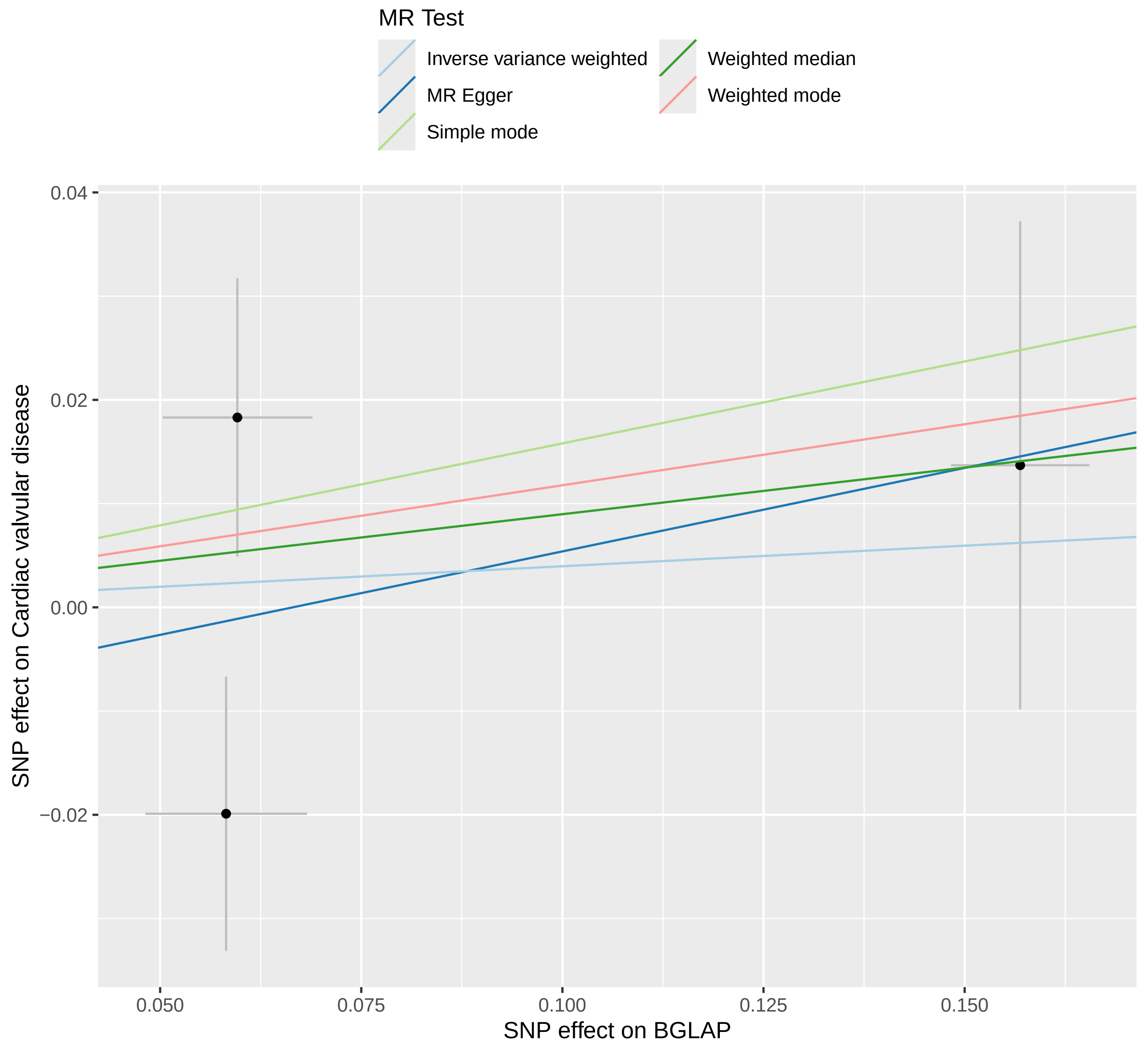

MR Test
Inverse variance weighted
MR Egger
Simple mode
Weighted median
Weighted mode
0.04
0.02
0.00
−0.02
SNP effect on Cardiac valvular disease
0.050
0.075
0.100
0.125
0.150
SNP effect on BGLAP

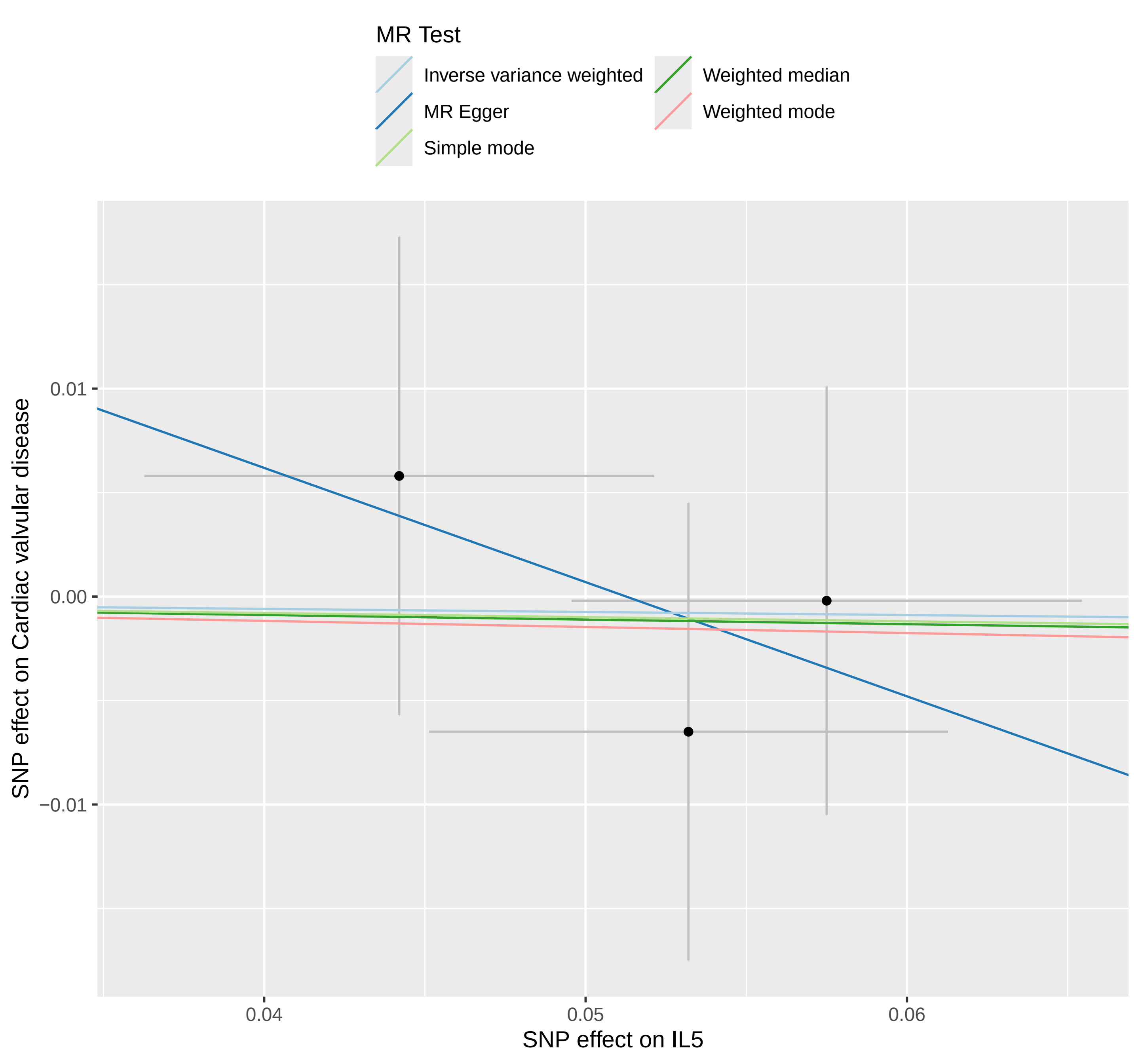
MR Test
Inverse variance weighted
MR Egger
Simple mode
Weighted median
Weighted mode
0.01
0.00
−0.01
SNP effect on Cardiac valvular disease
0.04
0.05
0.06
SNP effect on IL5

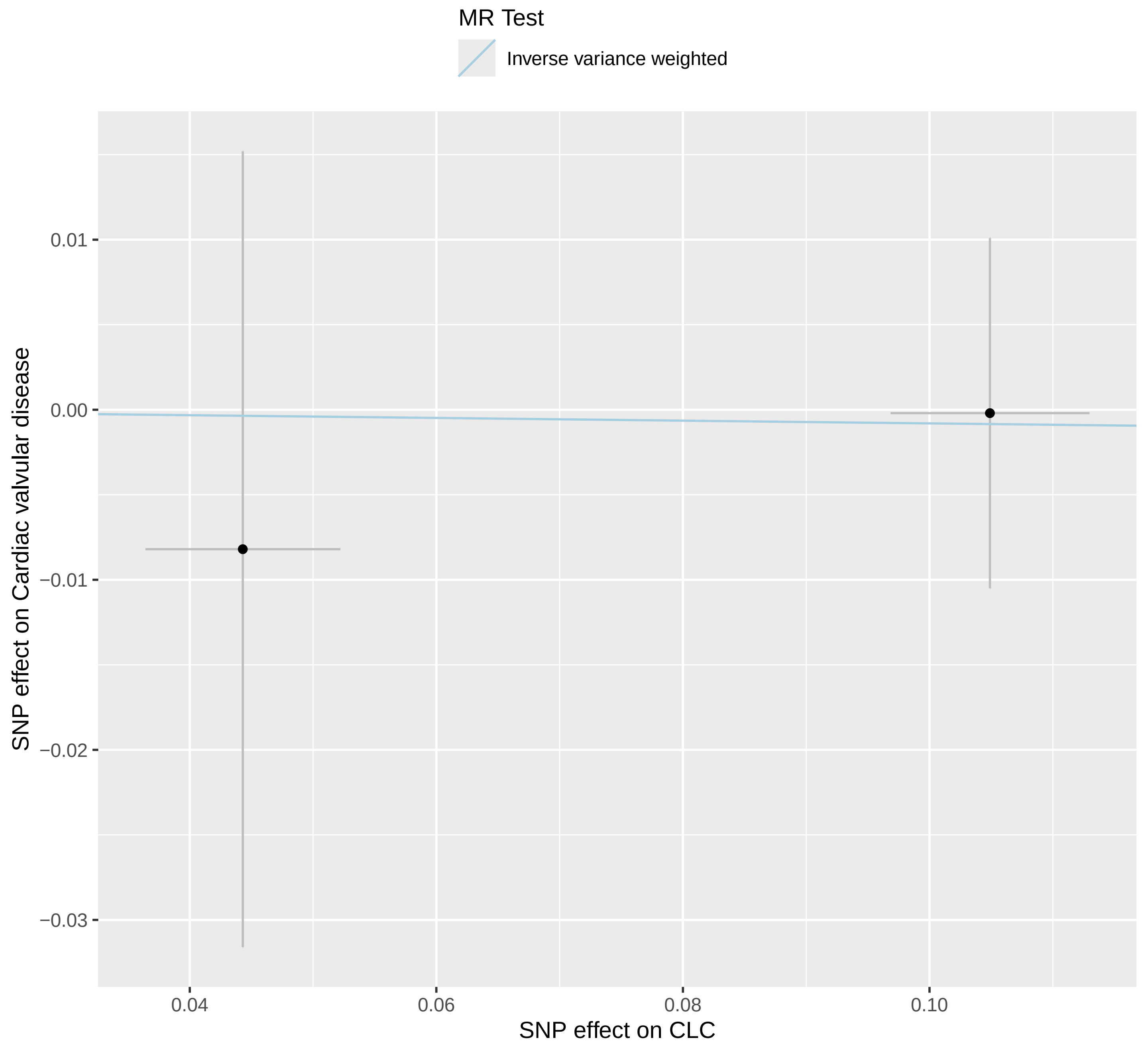
MR Test
Inverse variance weighted
0.01
0.00
−0.01
−0.02
−0.03
SNP effect on Cardiac valvular disease
0.04
0.06
0.08
0.10
SNP effect on CLC

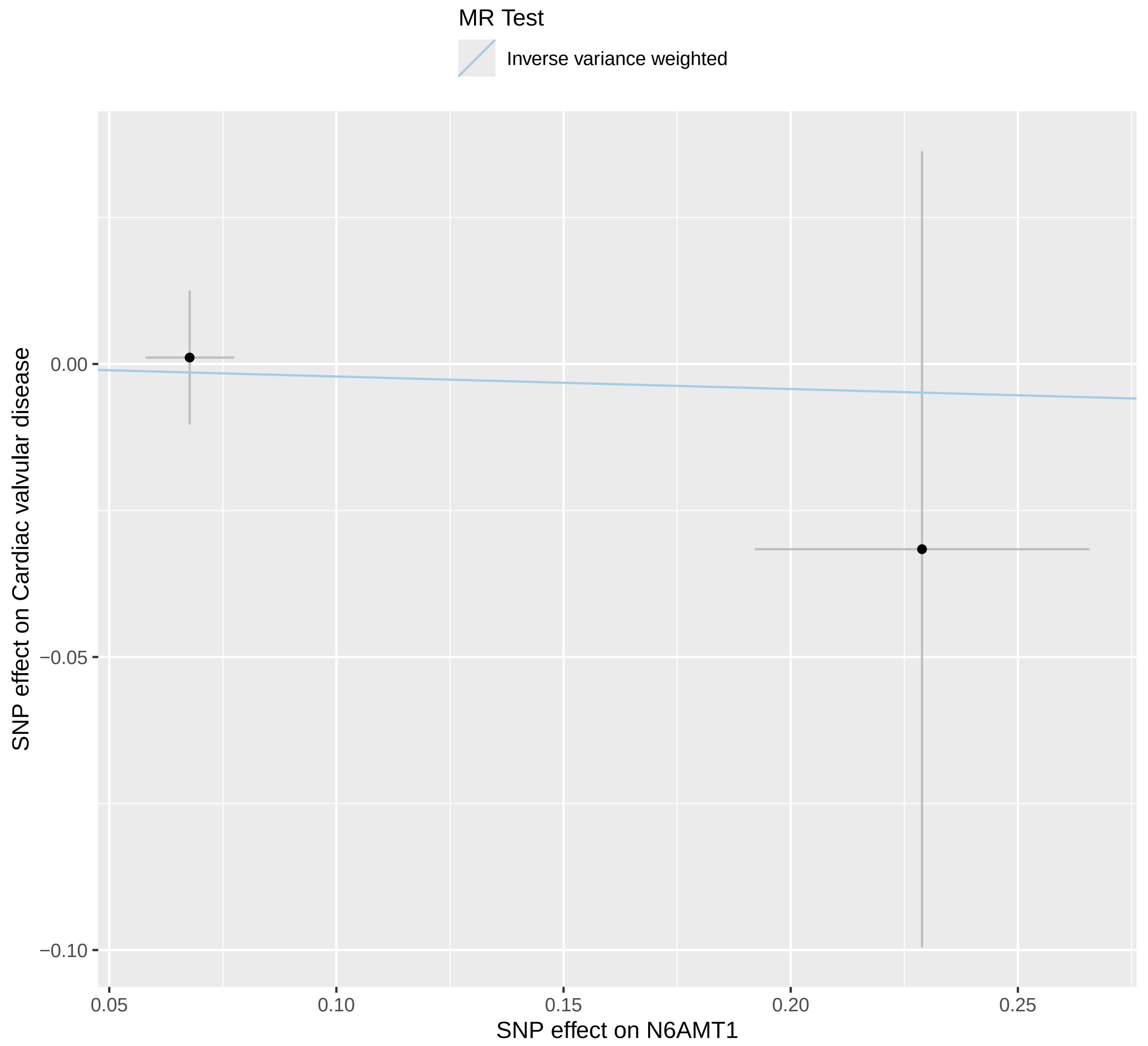
MR Test
Inverse variance weighted
SNP effect on Cardiac valvular disease
0.00
−0.05
−0.10
0.05
0.10
0.15
0.20
0.25
SNP effect on N6AMT1

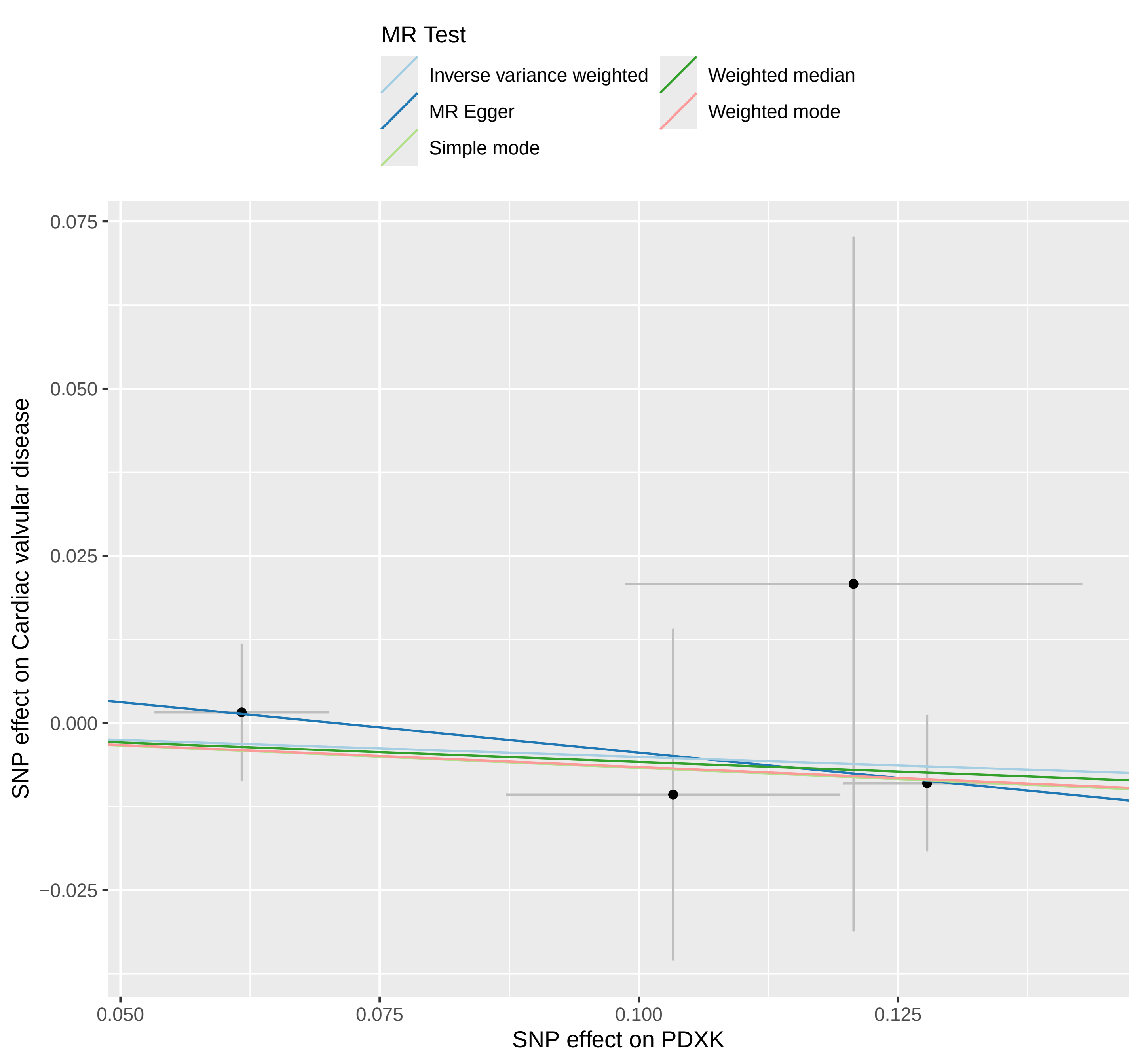
MR Test
Inverse variance weighted
MR Egger
Simple mode
Weighted median
Weighted mode
0.075
0.050
0.025
0.000
−0.025
SNP effect on Cardiac valvular disease
0.050
0.075
0.100
0.125
SNP effect on PDXK

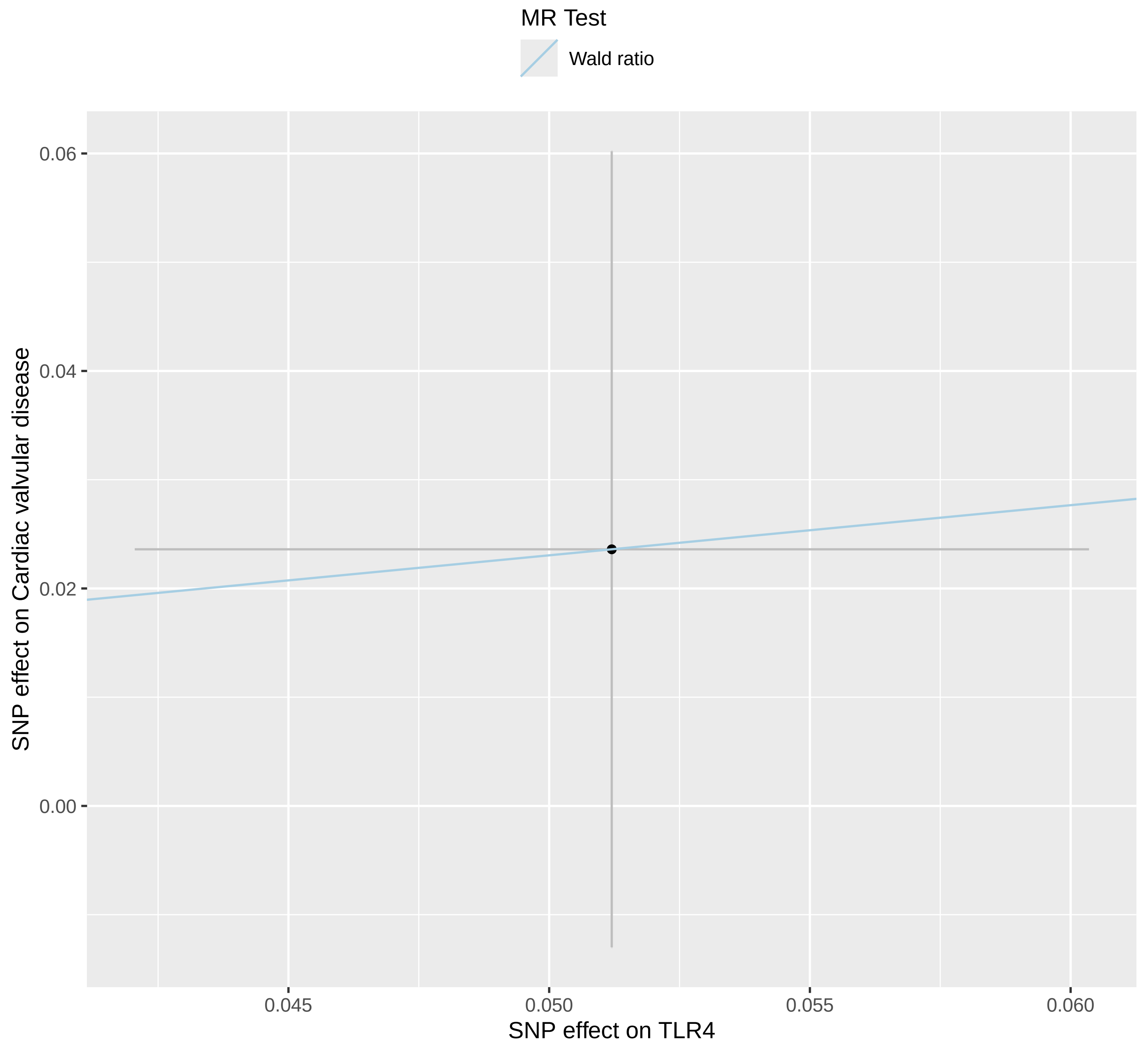
MR Test
Wald ratio
0.06
0.04
0.02
0.00
SNP effect on Cardiac valvular disease
0.045
0.050
0.055
0.060
SNP effect on TLR4

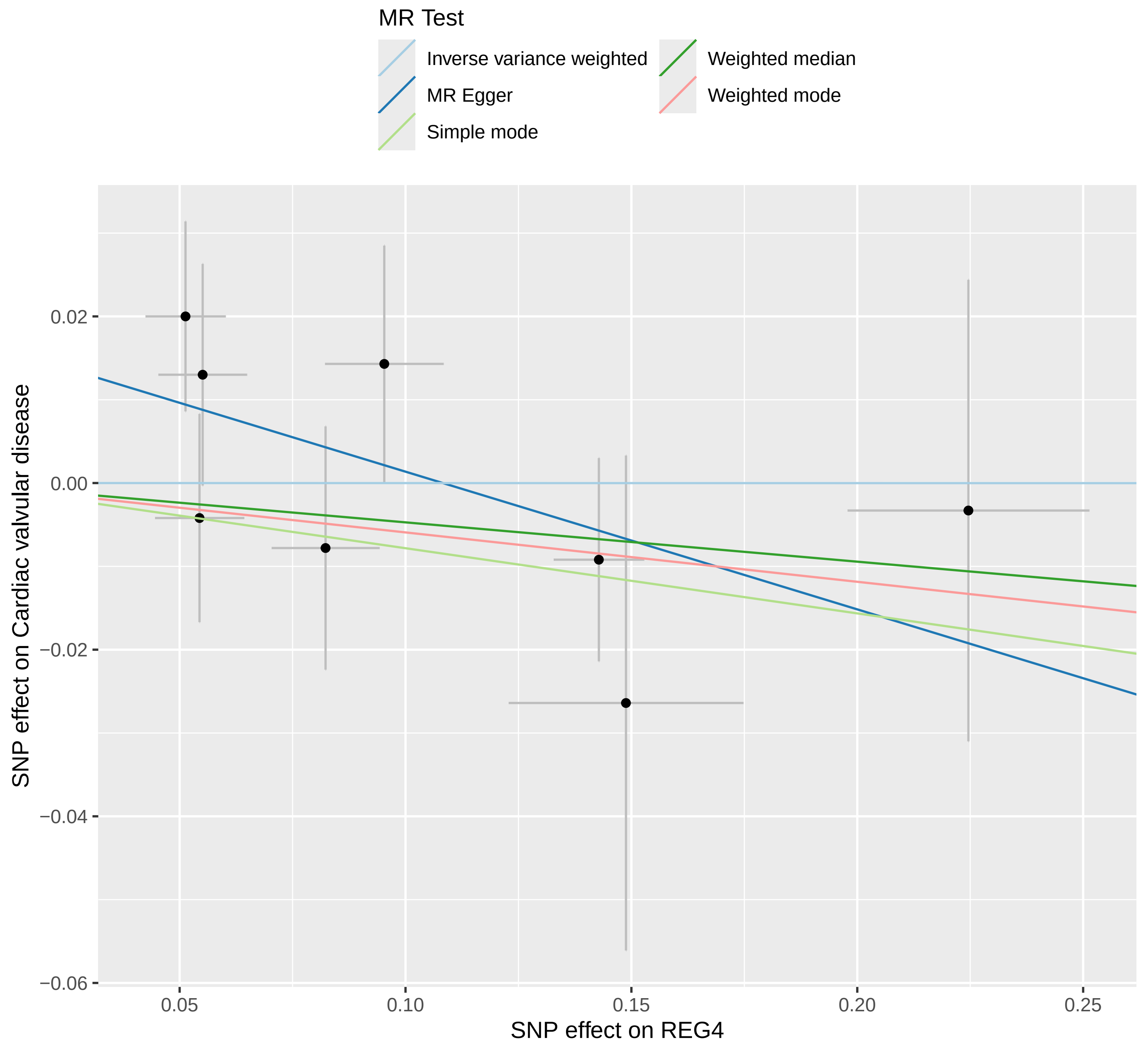

MR Test
Inverse variance weighted
MR Egger
Simple mode
Weighted median
Weighted mode
SNP effect on Cardiac valvular disease
0.02
0.00
−0.02
−0.04
−0.06
0.05
0.10
0.15
0.20
0.25
SNP effect on REG4

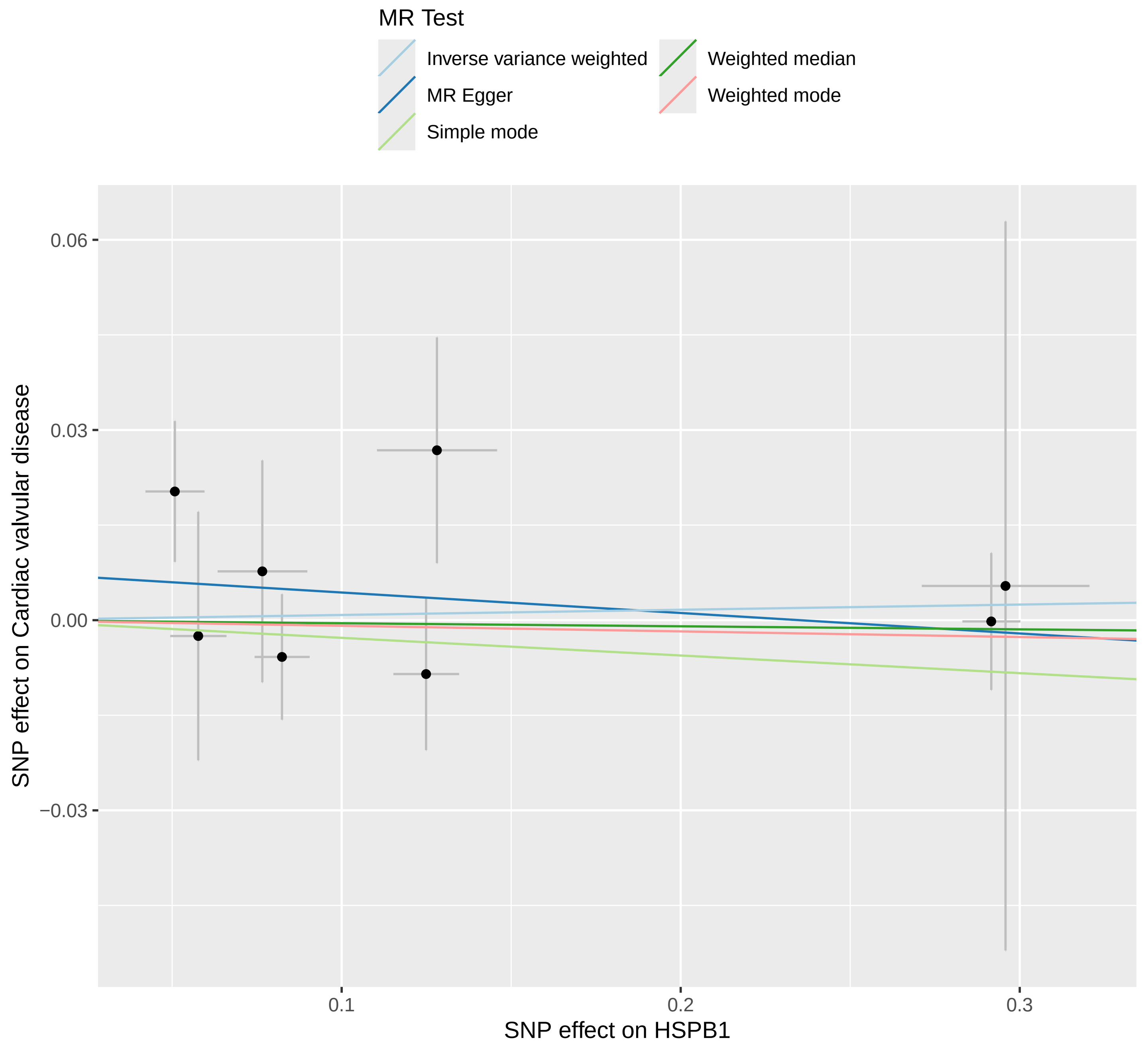

MR Test
Inverse variance weighted
MR Egger
Simple mode
Weighted median
Weighted mode
0.06
0.03
0.00
−0.03
0.1
0.2
0.3
SNP effect on Cardiac valvular disease
SNP effect on HSPB1

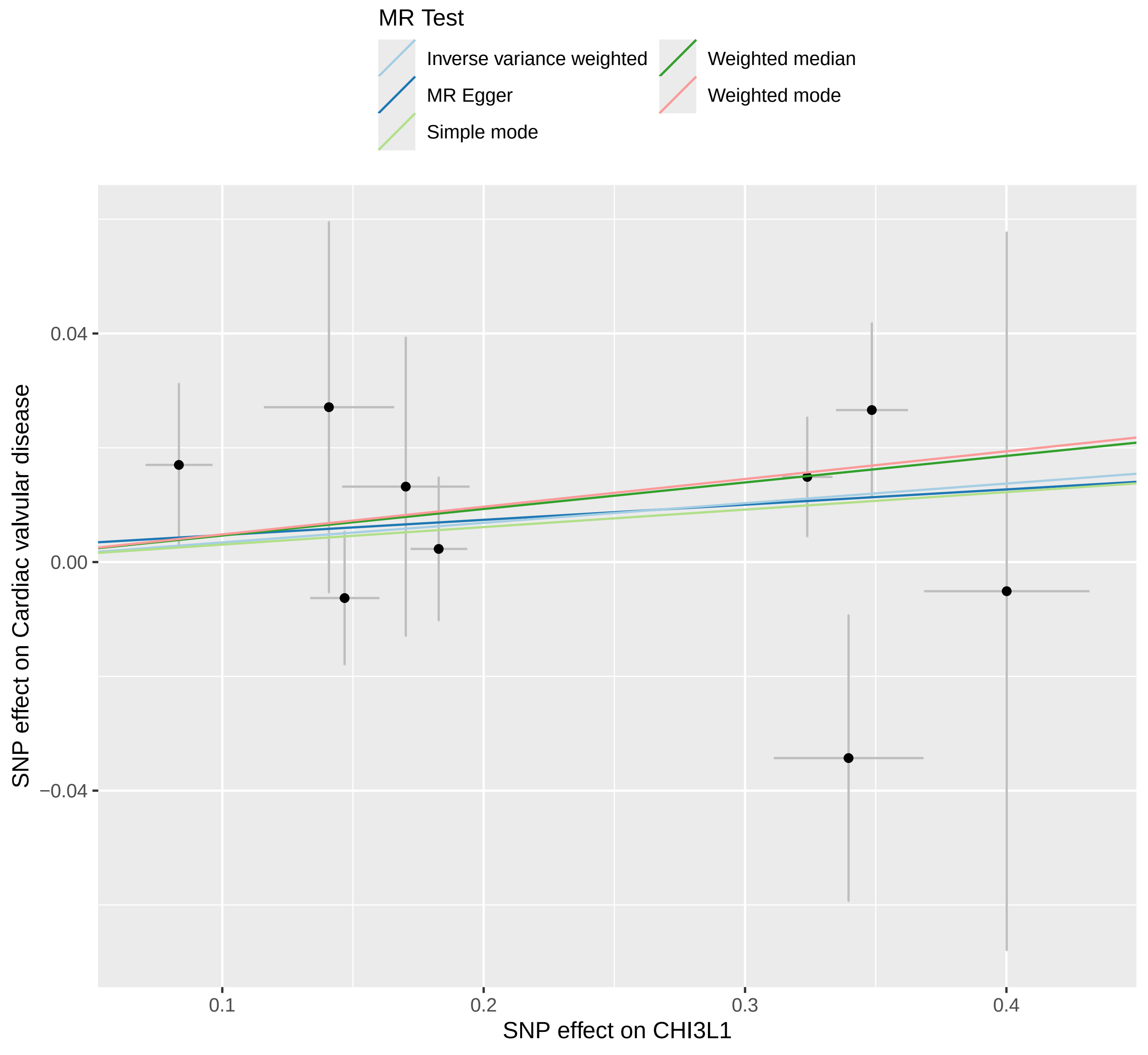

MR Test
Inverse variance weighted
MR Egger
Simple mode
Weighted median
Weighted mode
0.04
0.00
−0.04
0.1
0.2
0.3
0.4
SNP effect on Cardiac valvular disease
SNP effect on CHI3L1

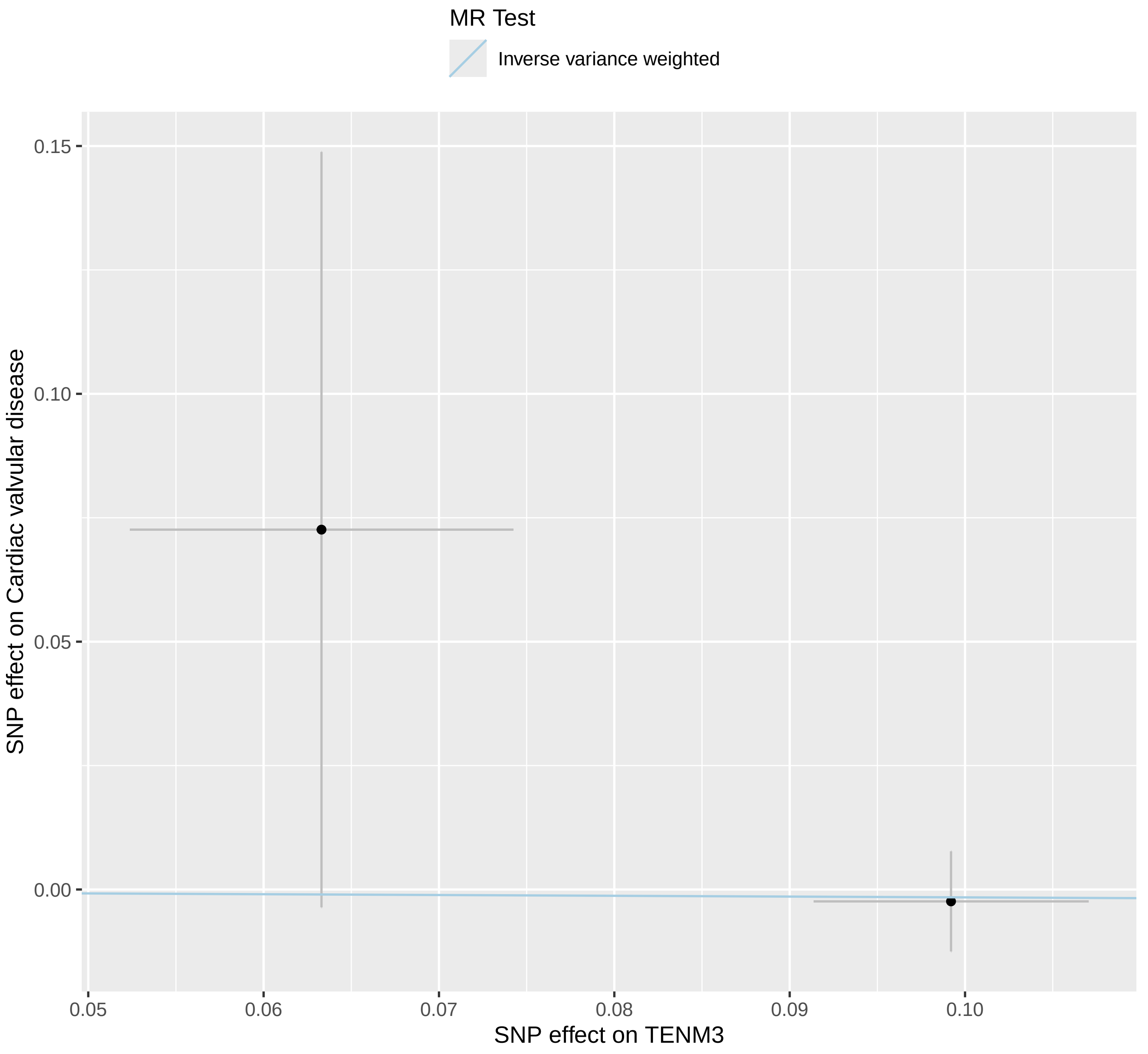
MR Test
Inverse variance weighted
0.15
0.10
0.05
0.00
SNP effect on Cardiac valvular disease
0.05
0.06
0.07
0.08
0.09
0.10
SNP effect on TENM3

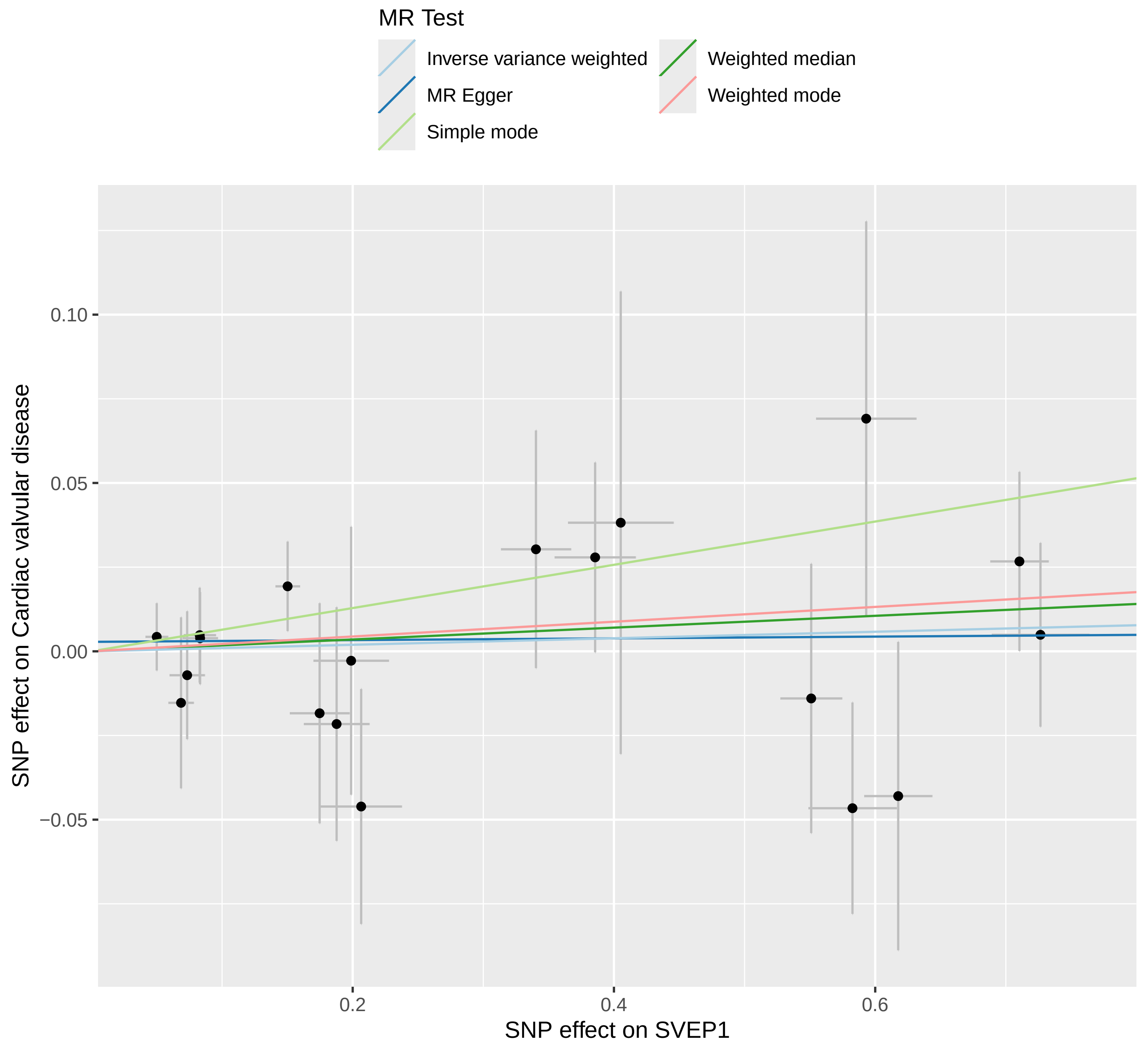
MR Test
Inverse variance weighted
MR Egger
Simple mode
Weighted median
Weighted mode
0.10
0.05
0.00
−0.05
SNP effect on Cardiac valvular disease
0.2
0.4
0.6
SNP effect on SVEP1

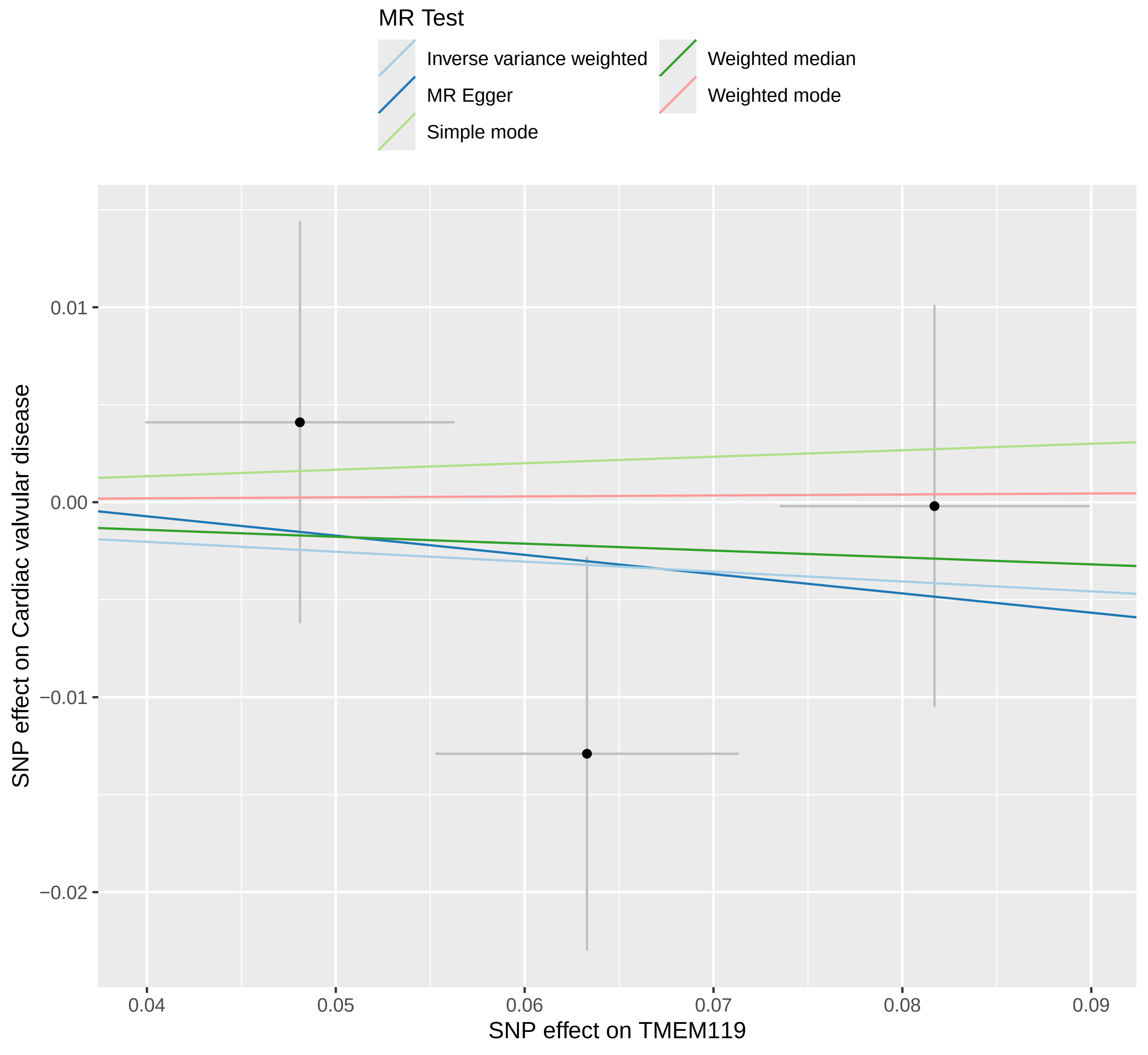
MR Test
Inverse variance weighted
MR Egger
Simple mode
Weighted median
Weighted mode
0.01
0.00
−0.01
−0.02
SNP effect on Cardiac valvular disease
0.04
0.05
0.06
0.07
0.08
0.09
SNP effect on TMEM119

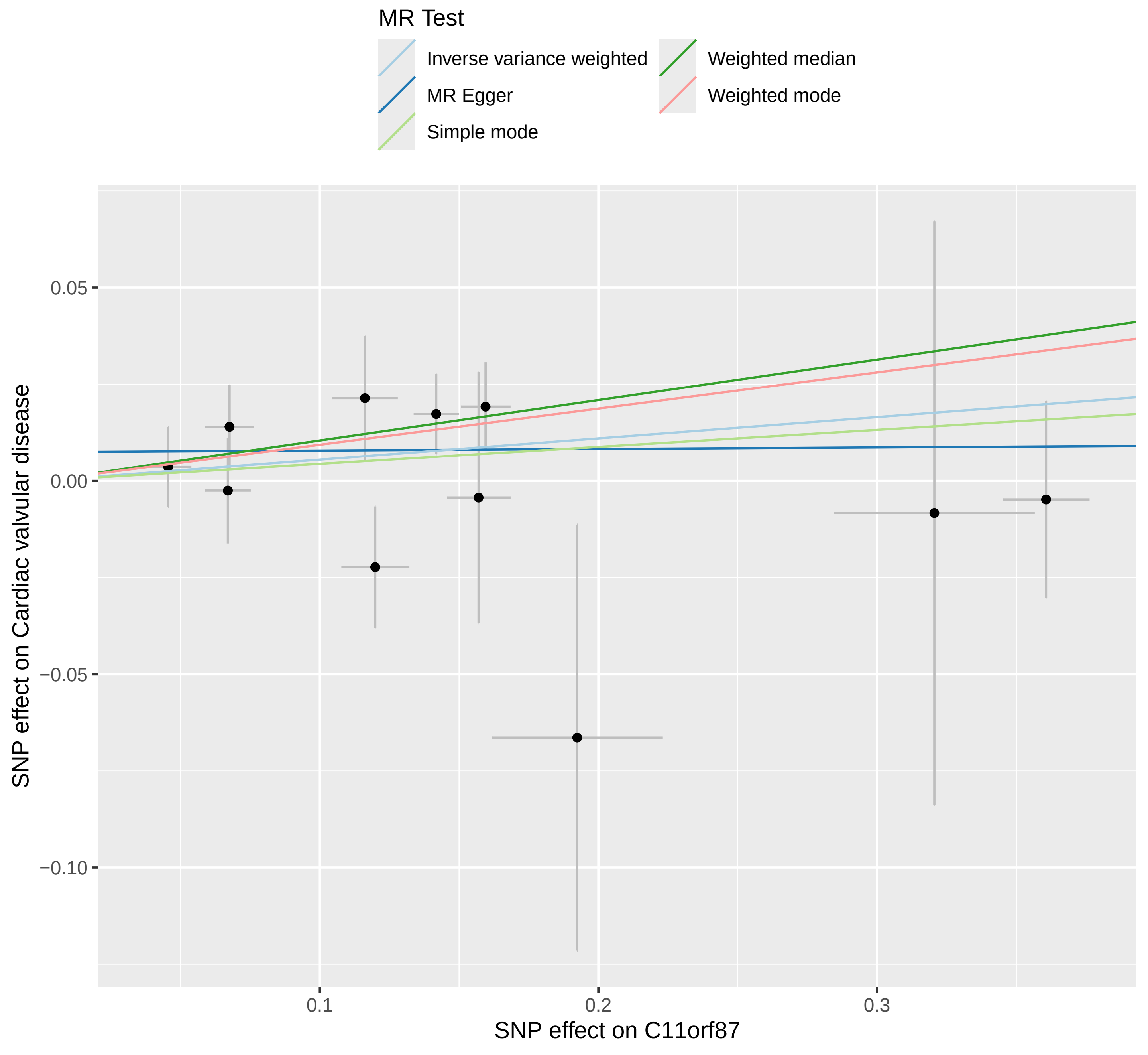

MR Test
Inverse variance weighted
MR Egger
Simple mode
Weighted median
Weighted mode
0.05
0.00
−0.05
−0.10
SNP effect on Cardiac valvular disease
0.1
0.2
0.3
SNP effect on C11orf87

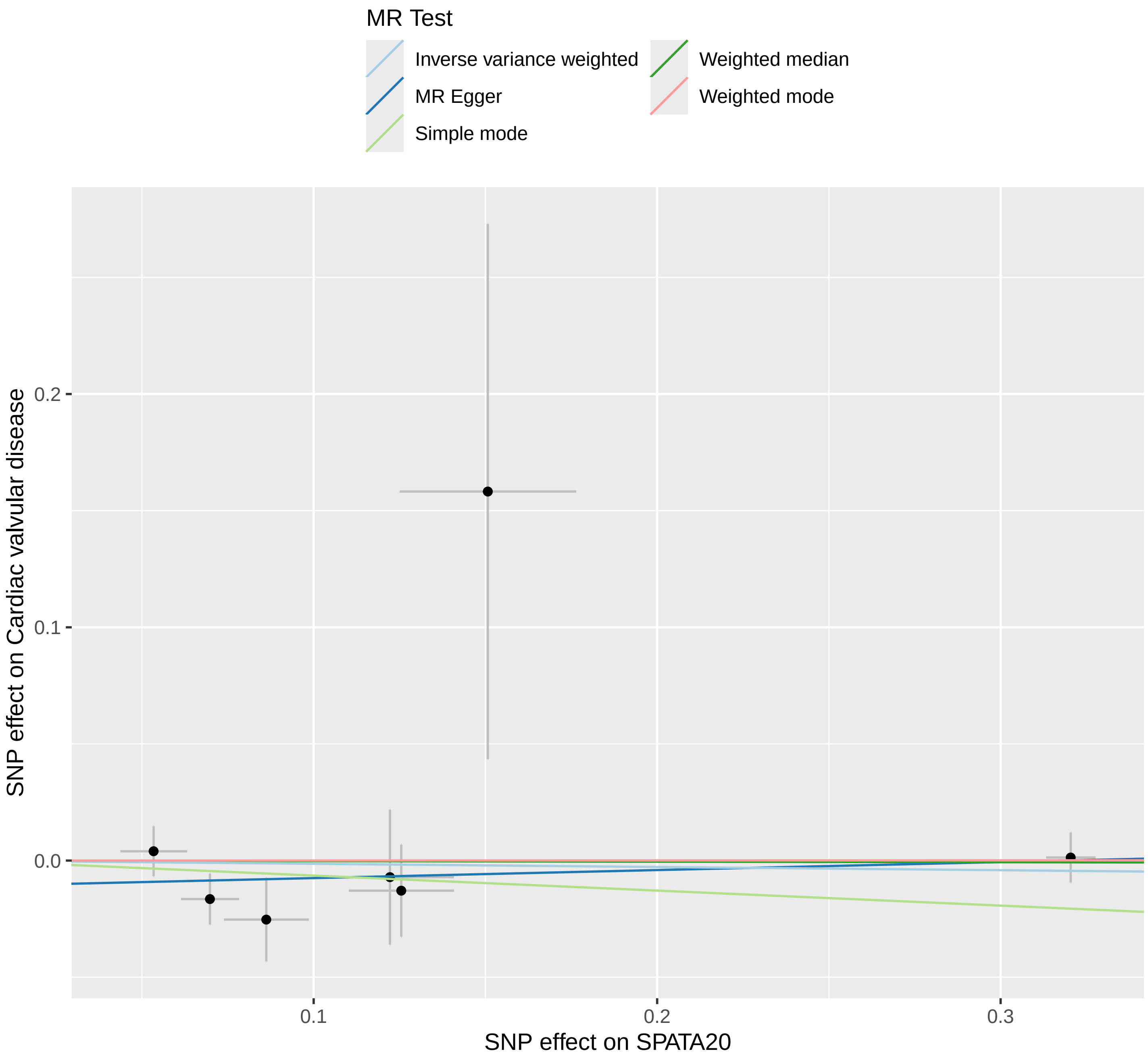

MR Test
Inverse variance weighted
MR Egger
Simple mode
Weighted median
Weighted mode
SNP effect on Cardiac valvular disease
0.2
0.1
0.0
0.1
0.2
0.3
SNP effect on SPATA20

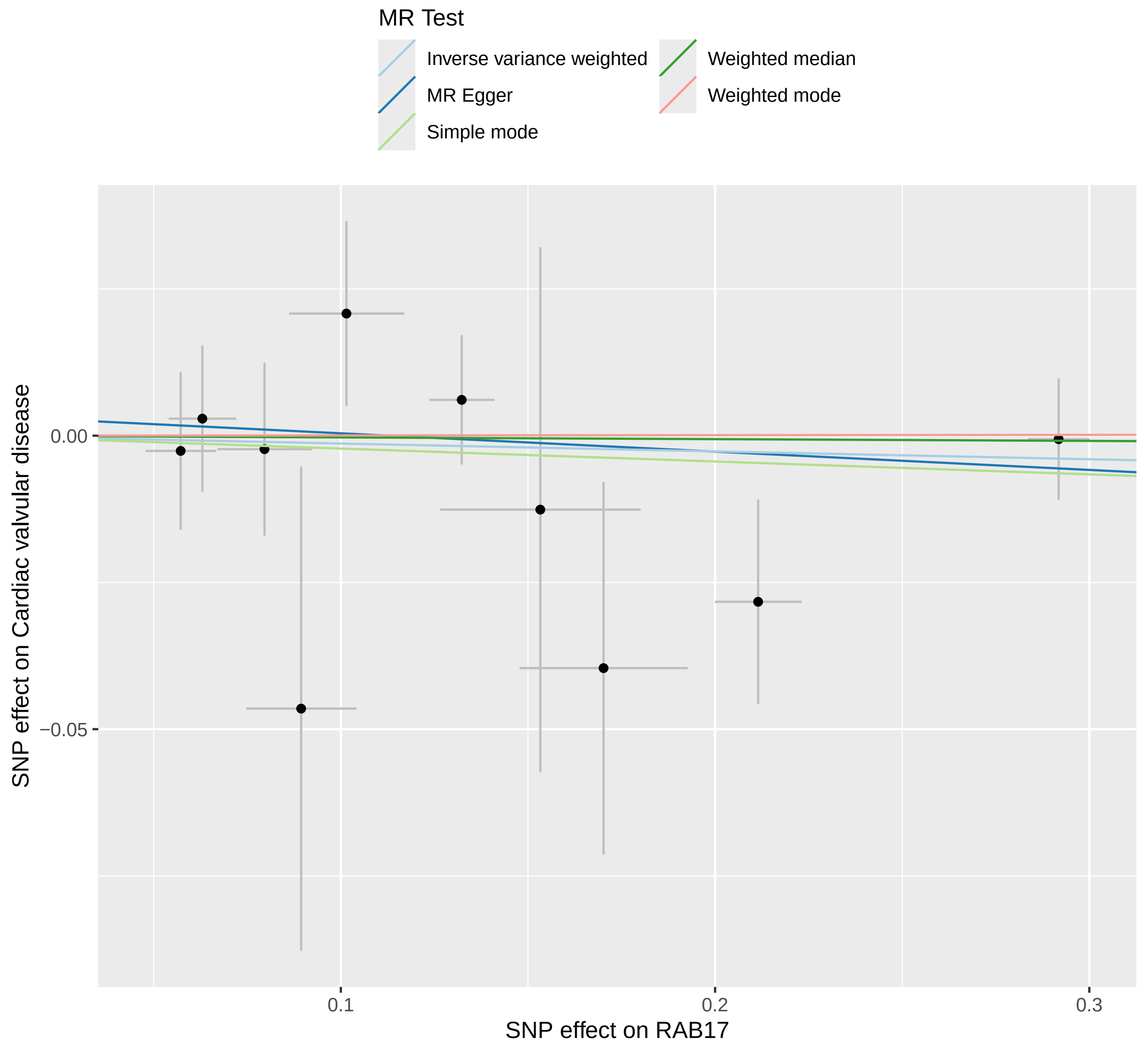

MR Test
Inverse variance weighted
MR Egger
Simple mode
Weighted median
Weighted mode
0.00
−0.05
0.1
0.2
0.3
SNP effect on RAB17
SNP effect on Cardiac valvular disease

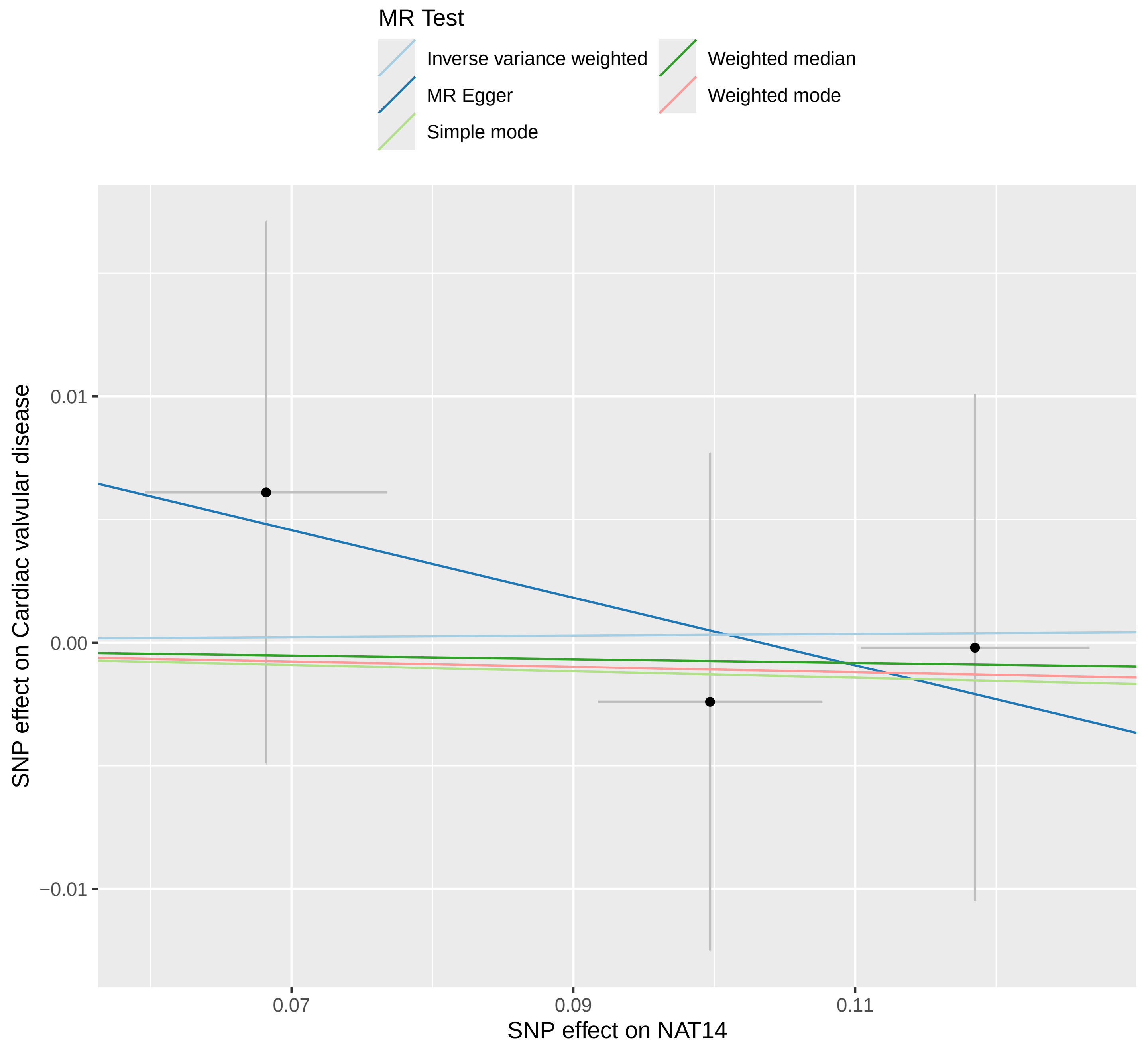
MR Test
Inverse variance weighted
MR Egger
Simple mode
Weighted median
Weighted mode
0.01
0.00
−0.01
SNP effect on Cardiac valvular disease
0.07
0.09
0.11
SNP effect on NAT14

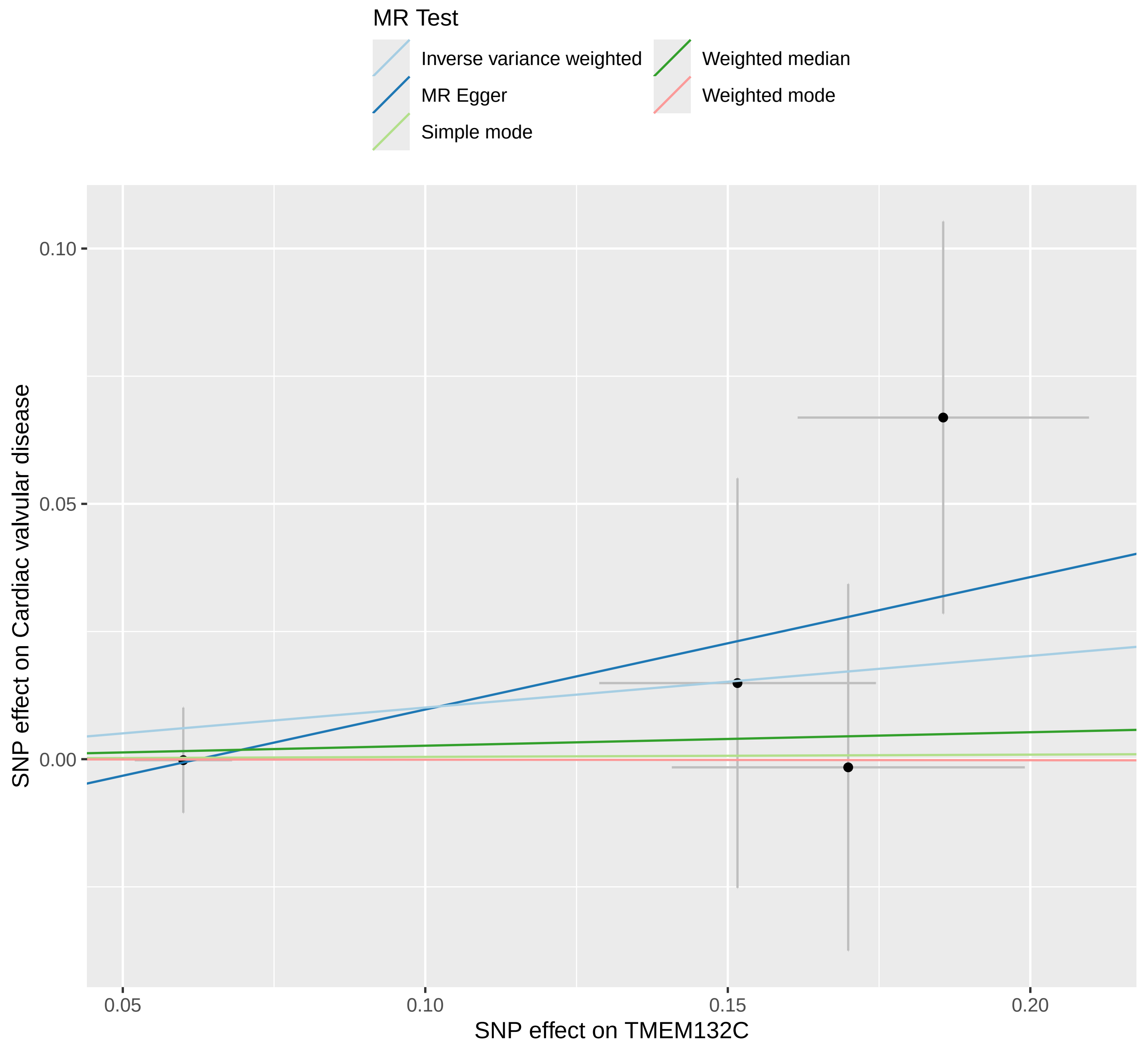

MR Test
Inverse variance weighted
MR Egger
Simple mode
Weighted median
Weighted mode
SNP effect on Cardiac valvular disease
0.10
0.05
0.00
0.05
0.10
0.15
0.20
SNP effect on TMEM132C

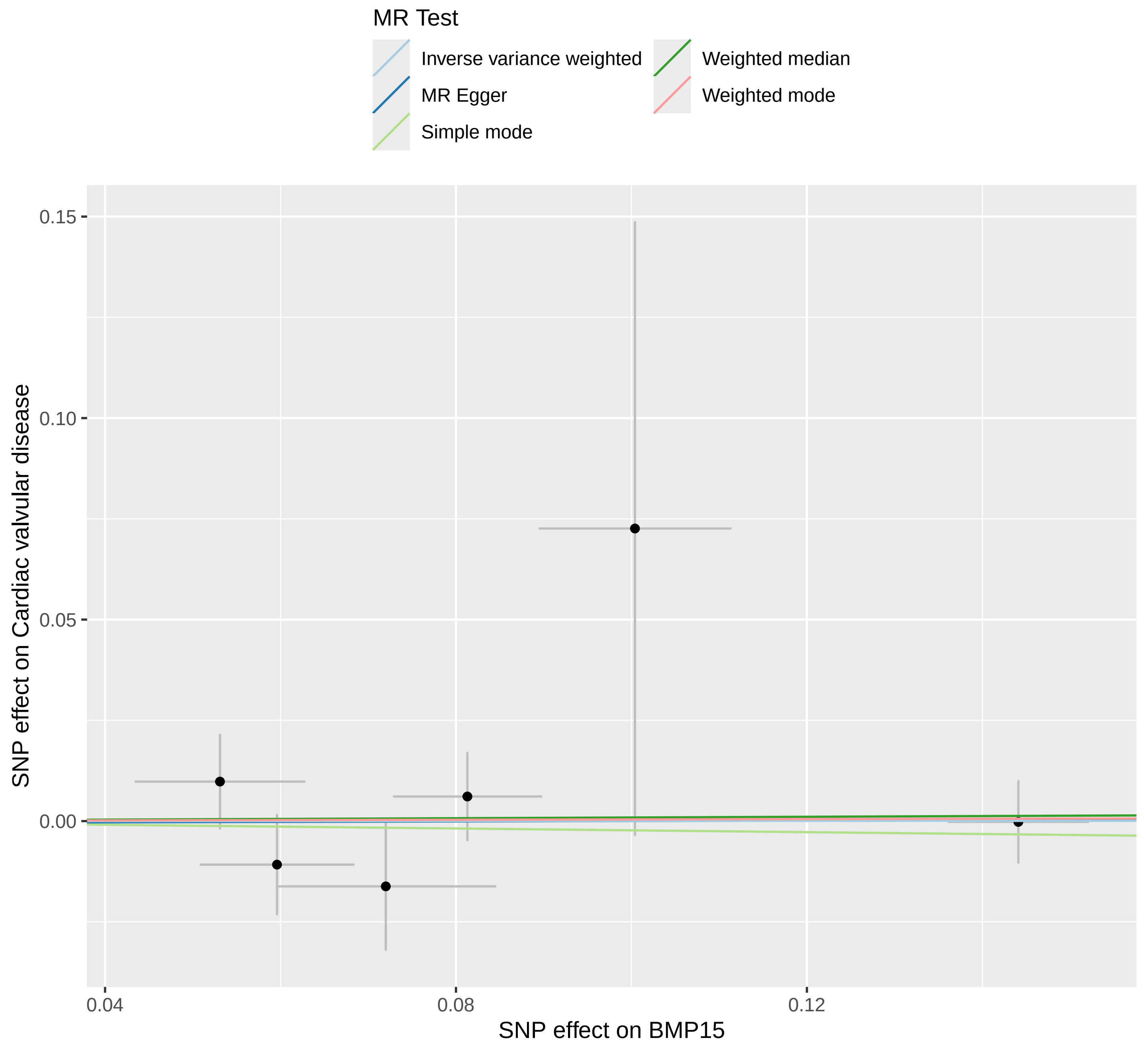

MR Test
Inverse variance weighted
MR Egger
Simple mode
Weighted median
Weighted mode
0.15
0.10
0.05
0.00
SNP effect on Cardiac valvular disease
0.04
0.08
0.12
SNP effect on BMP15

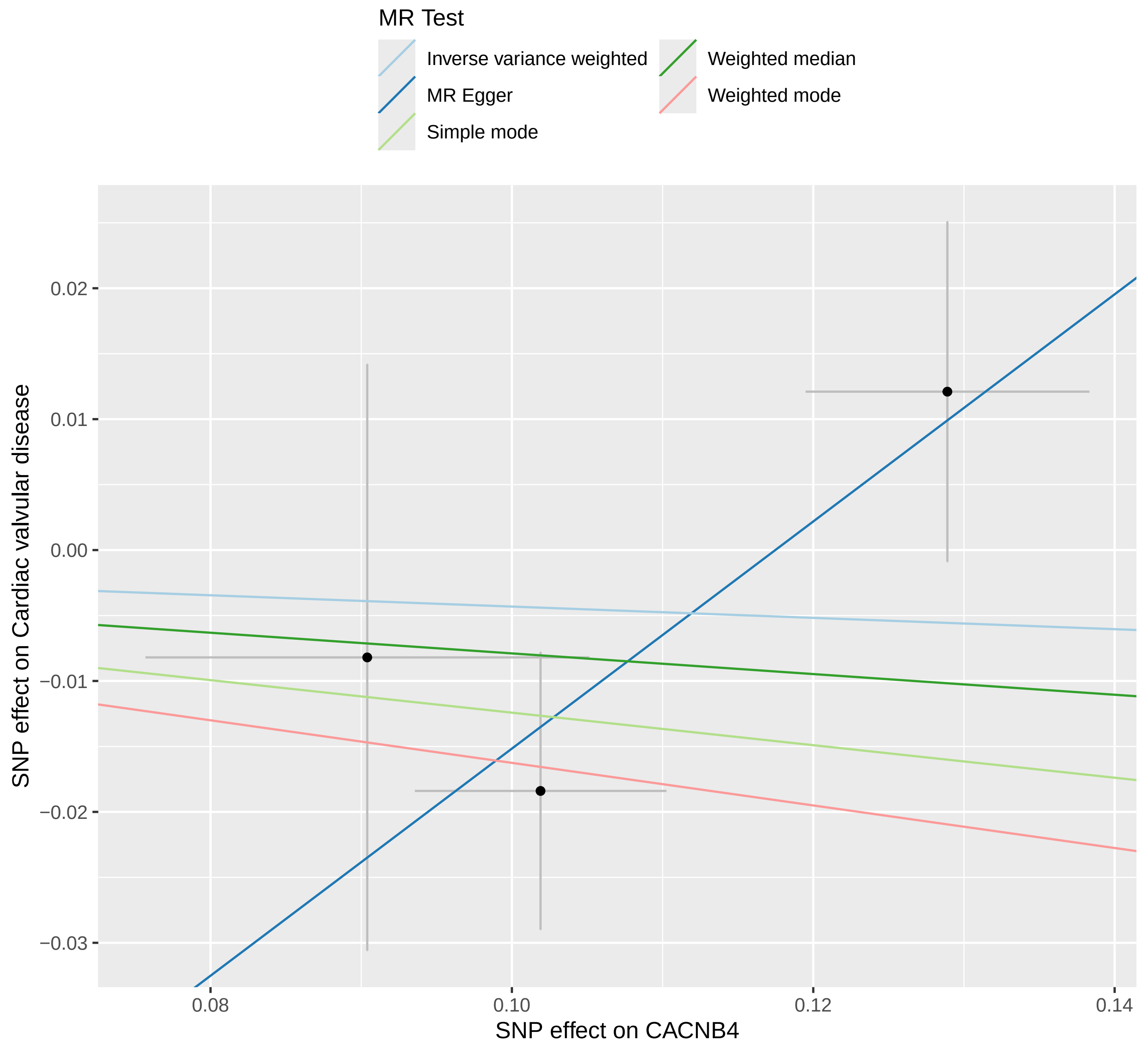

MR Test
Inverse variance weighted
MR Egger
Simple mode
Weighted median
Weighted mode
0.02
0.01
0.00
−0.01
−0.02
−0.03
SNP effect on Cardiac valvular disease
0.08
0.10
0.12
0.14
SNP effect on CACNB4

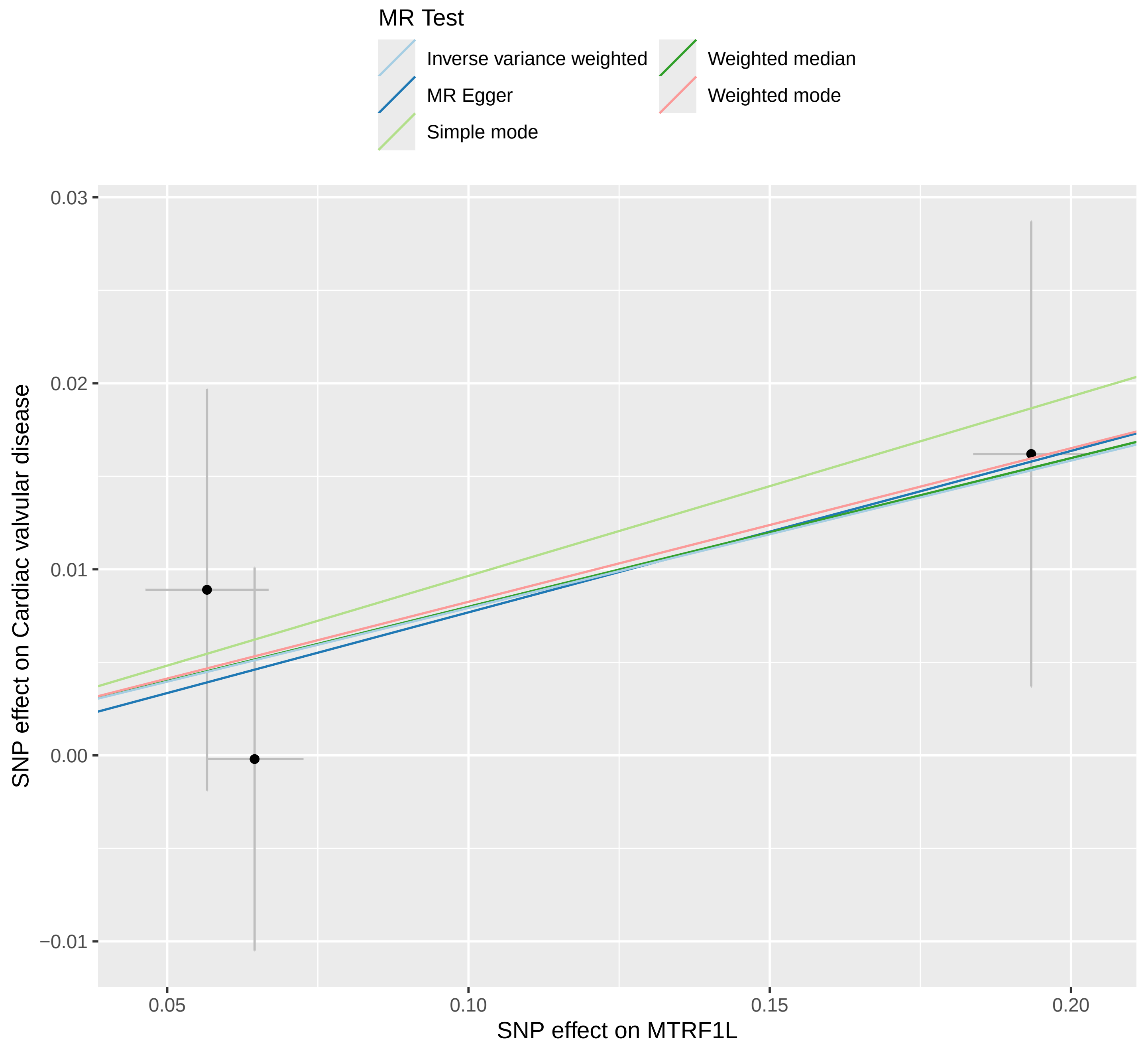
MR Test
Inverse variance weighted
MR Egger
Simple mode
Weighted median
Weighted mode
0.03
0.02
0.01
0.00
−0.01
0.05
0.10
0.15
0.20
SNP effect on Cardiac valvular disease
SNP effect on MTRF1L

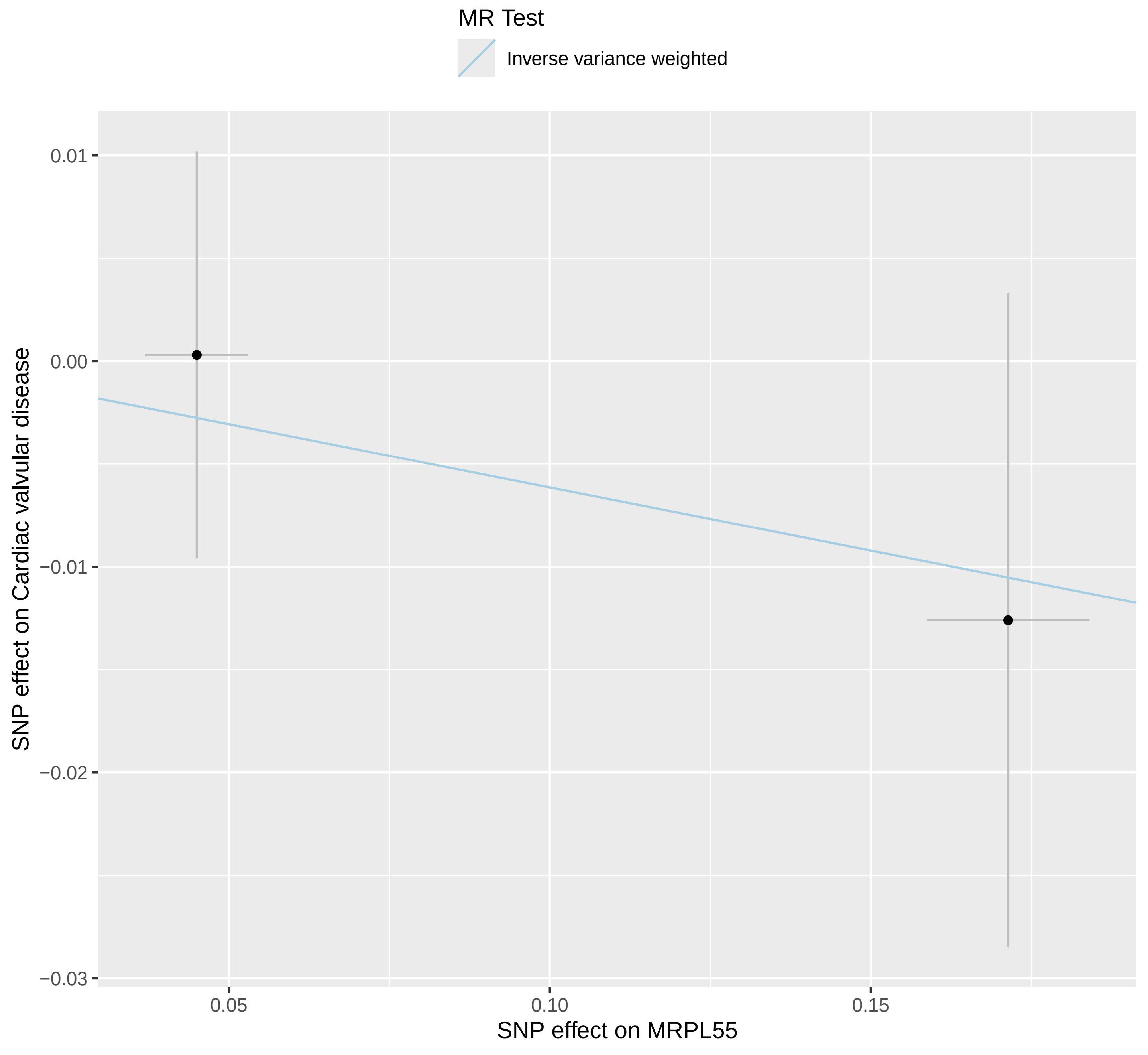

MR Test
Inverse variance weighted
0.01
0.00
−0.01
−0.02
−0.03
SNP effect on Cardiac valvular disease
0.05
0.10
0.15
SNP effect on MRPL55

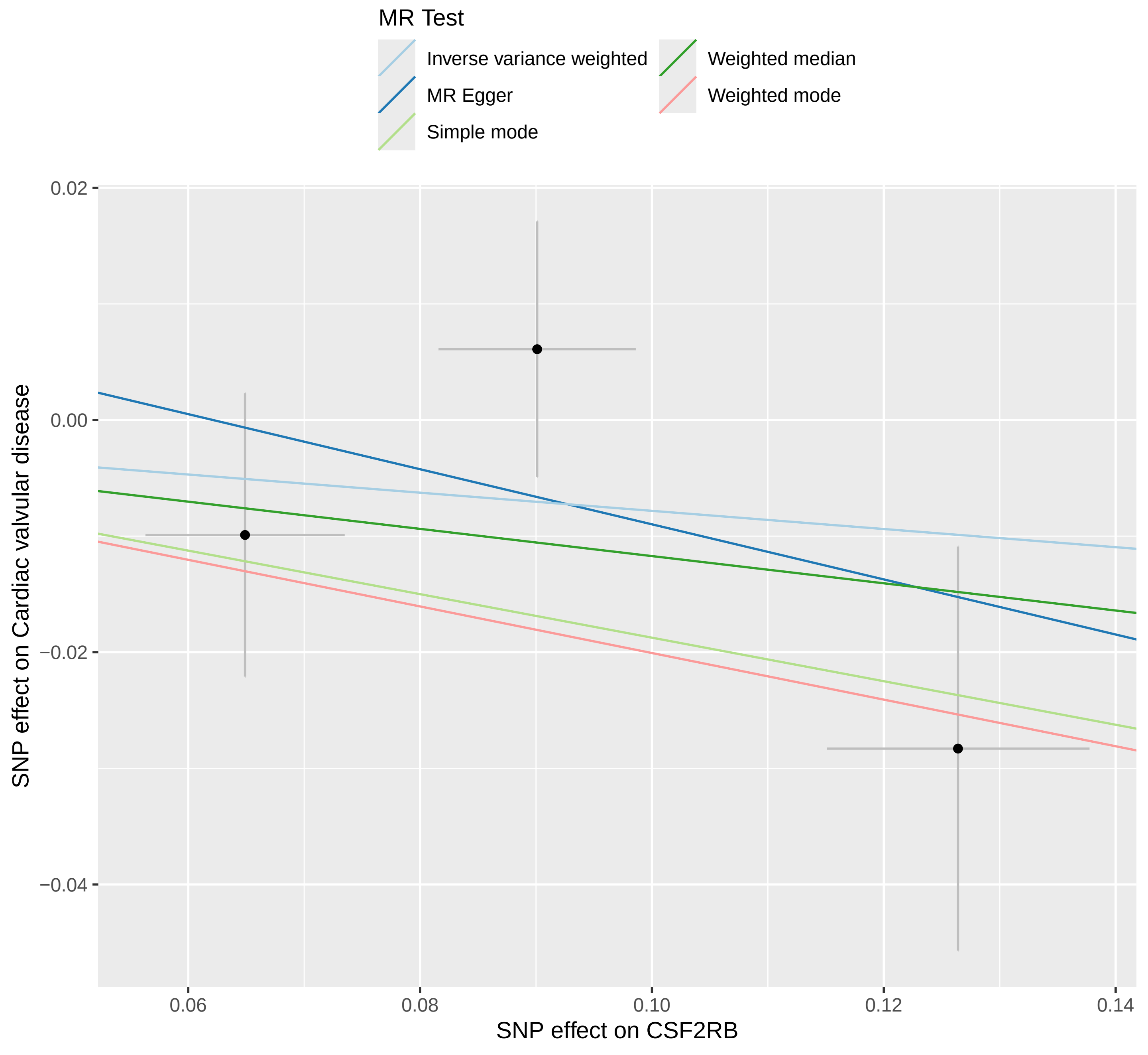

MR Test
Inverse variance weighted
MR Egger
Simple mode
Weighted median
Weighted mode
0.02
0.00
−0.02
−0.04
0.06
0.08
0.10
0.12
0.14
SNP effect on Cardiac valvular disease
SNP effect on CSF2RB

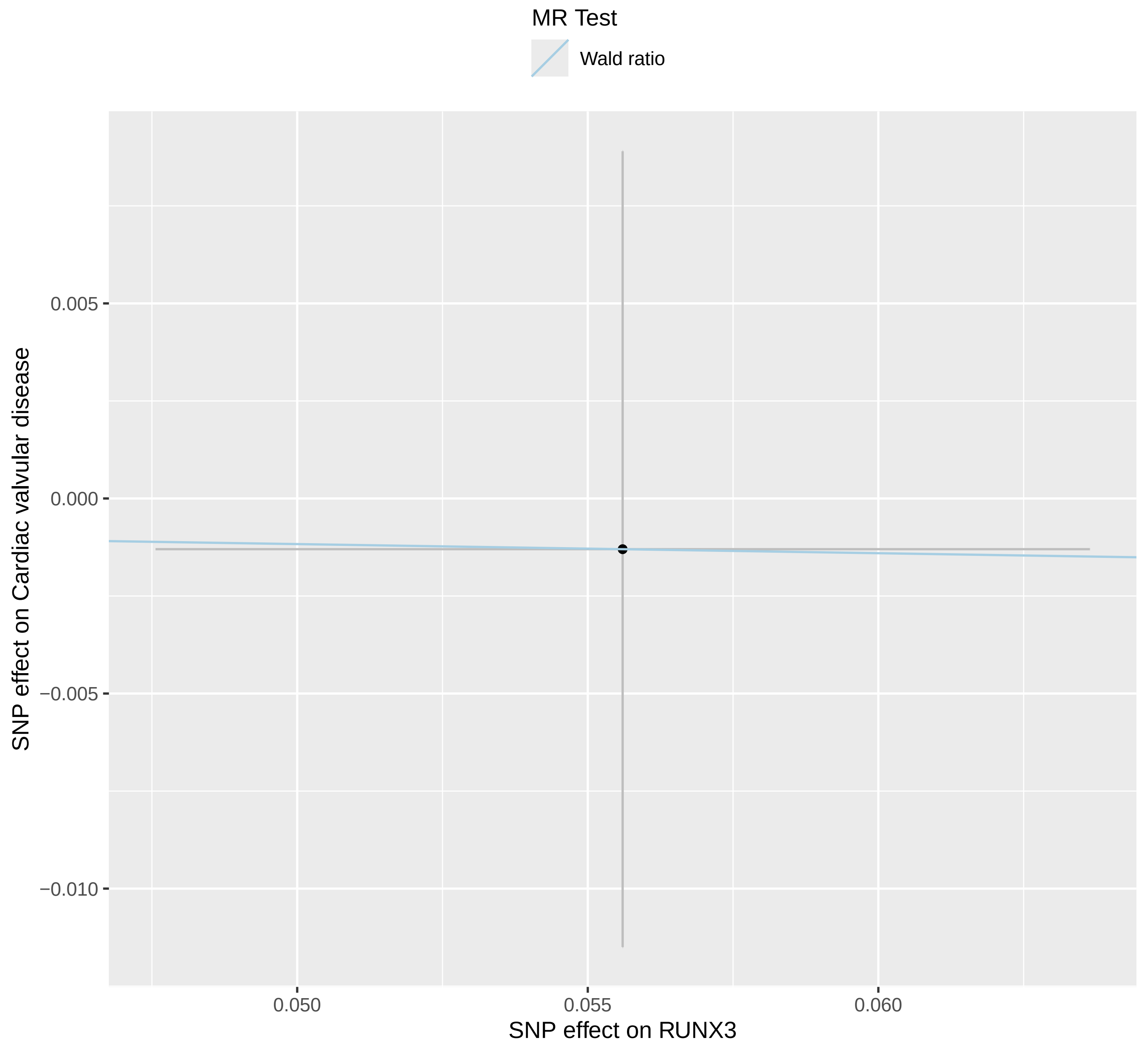

MR Test
Wald ratio
0.005
0.000
−0.005
−0.010
SNP effect on Cardiac valvular disease
0.050
0.055
0.060
SNP effect on RUNX3

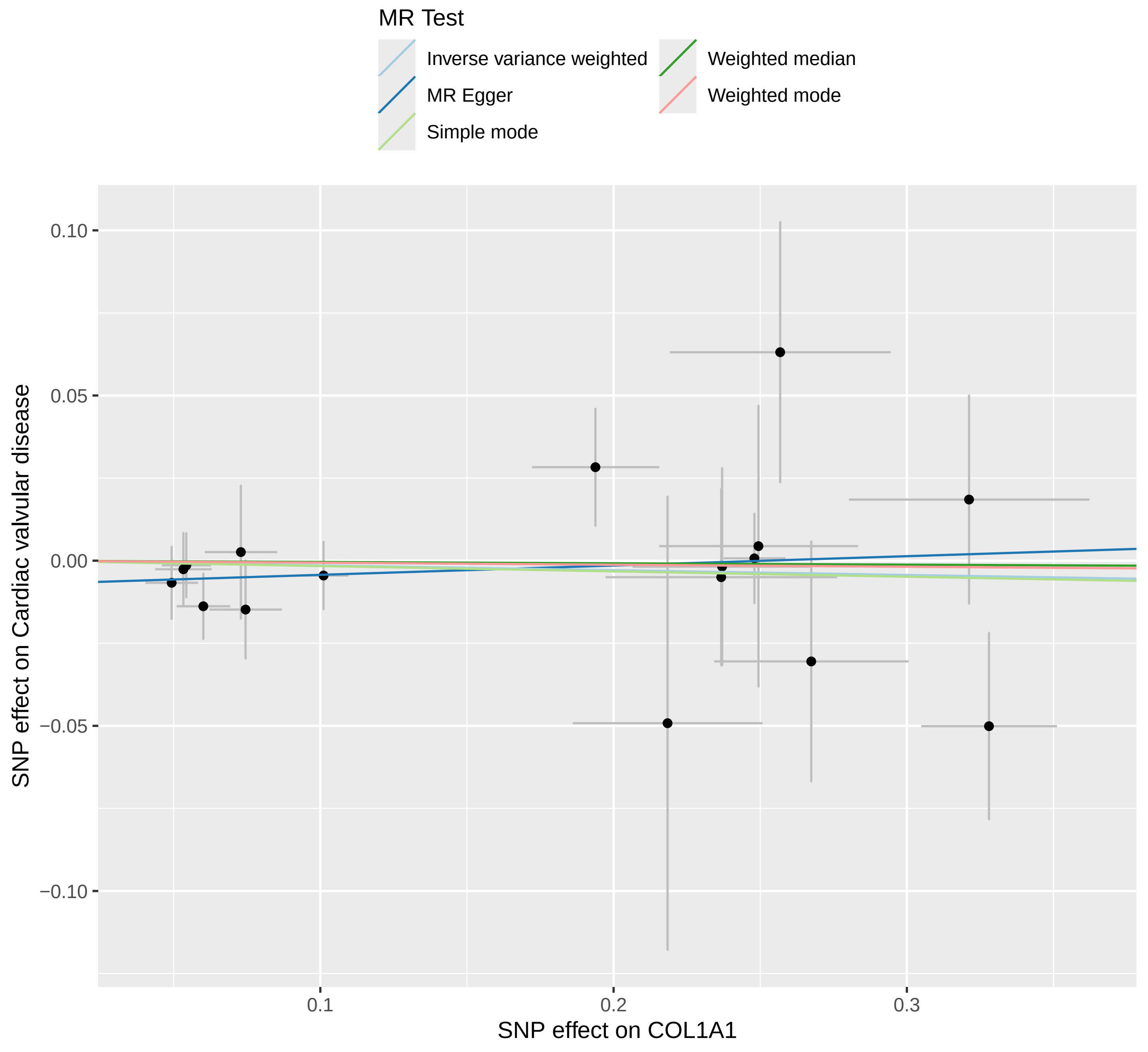

MR Test
Inverse variance weighted
MR Egger
Simple mode
Weighted median
Weighted mode
0.10
0.05
0.00
−0.05
−0.10
SNP effect on Cardiac valvular disease
0.1
0.2
0.3
SNP effect on COL1A1

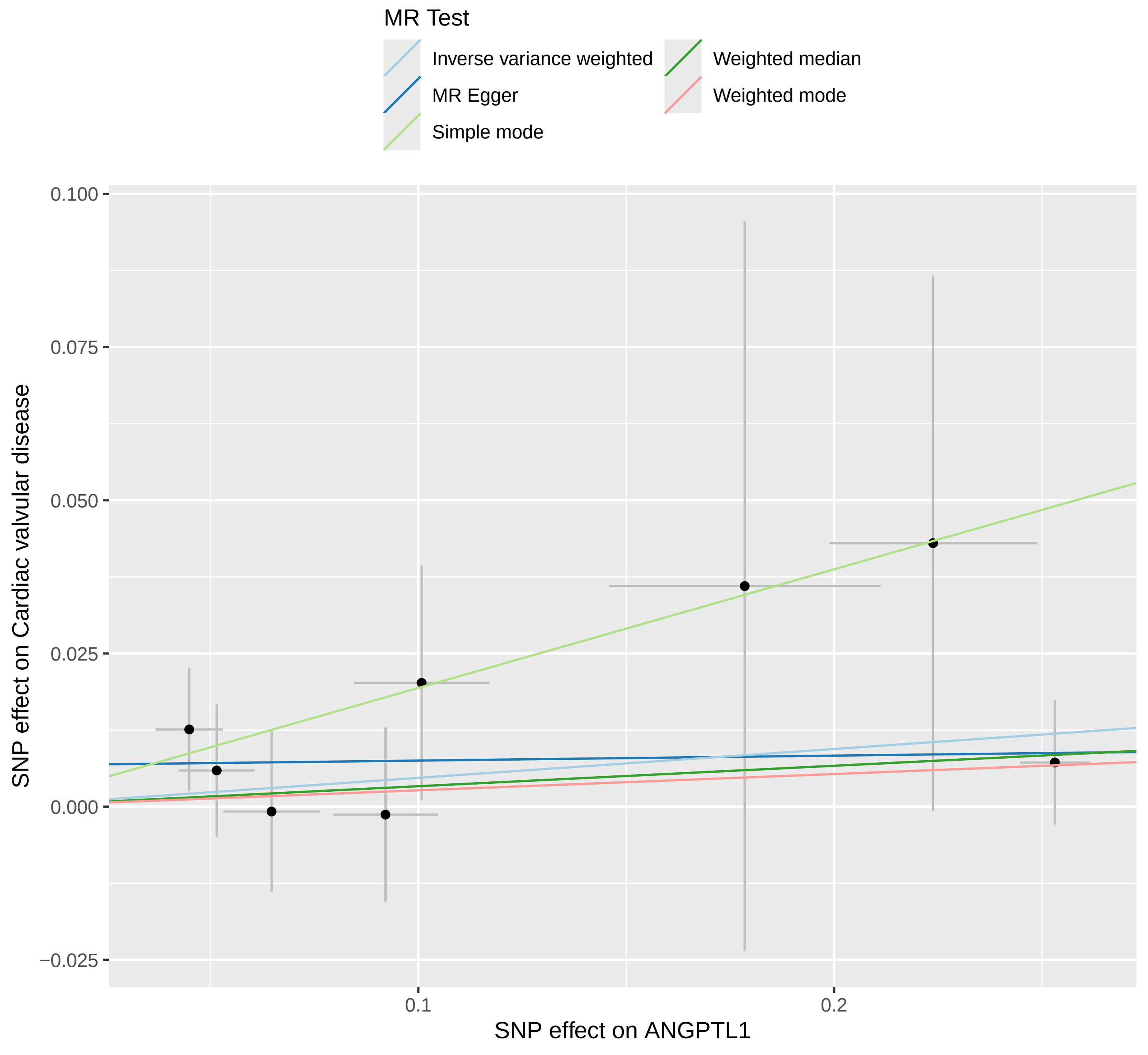

MR Test
Inverse variance weighted
MR Egger
Simple mode
Weighted median
Weighted mode
0.100
0.075
0.050
0.025
0.000
−0.025
0.1
0.2
SNP effect on ANGPTL1
SNP effect on Cardiac valvular disease

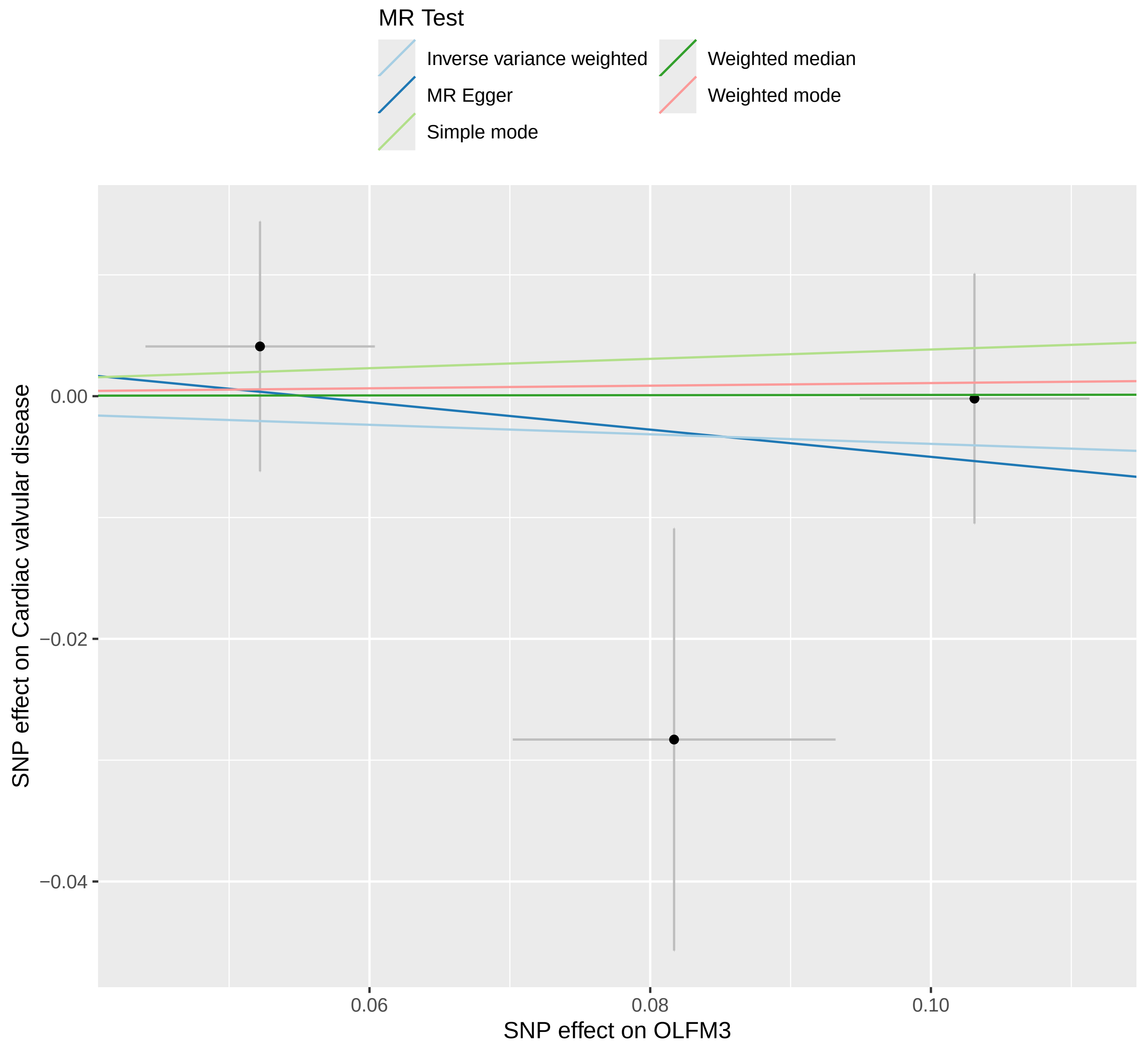
MR Test
Inverse variance weighted
MR Egger
Simple mode
Weighted median
Weighted mode
SNP effect on Cardiac valvular disease
0.00
−0.02
−0.04
0.06
0.08
0.10
SNP effect on OLFM3

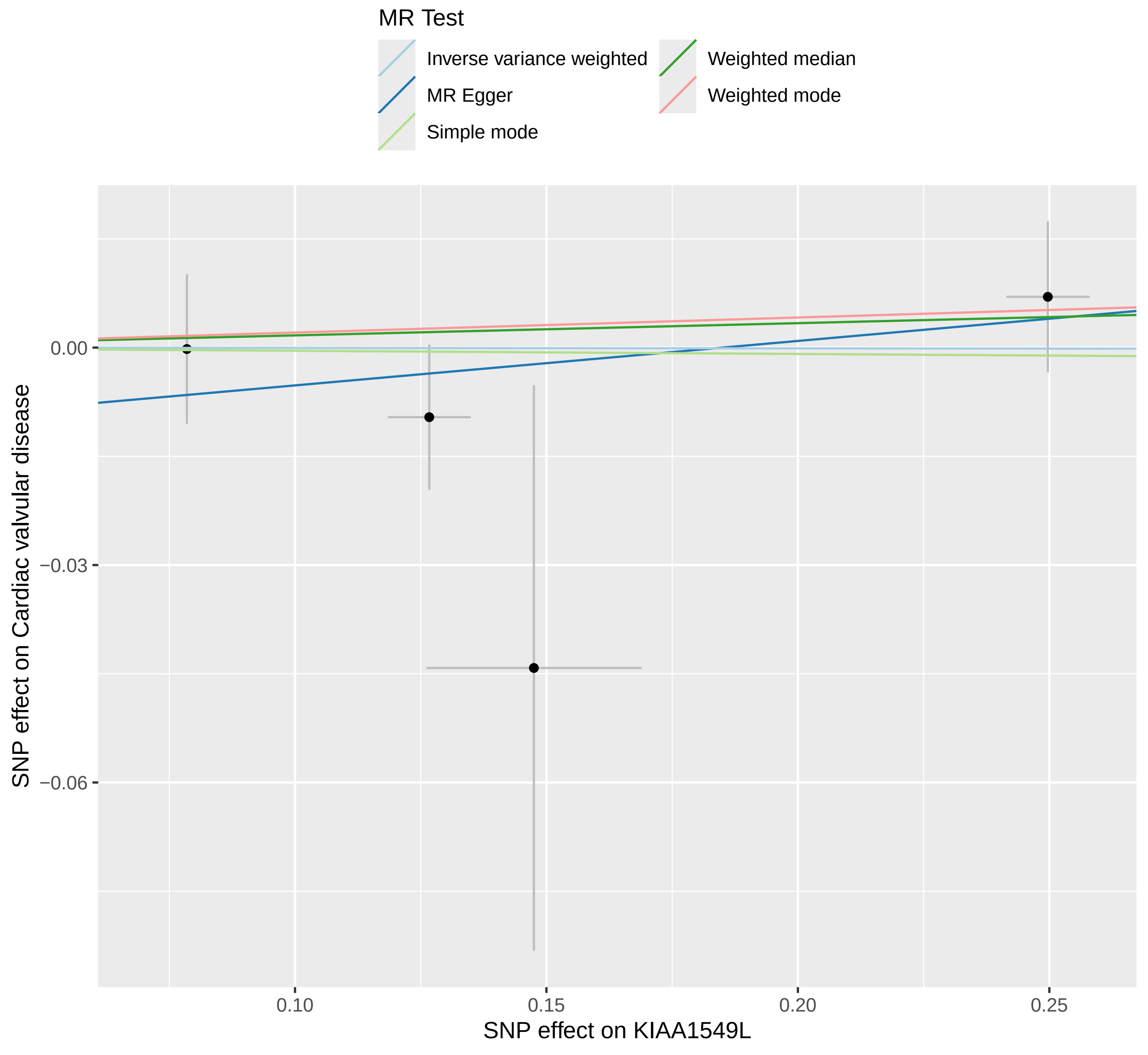

MR Test
Inverse variance weighted
MR Egger
Simple mode
Weighted median
Weighted mode
0.00
−0.03
−0.06
0.10
0.15
0.20
0.25
SNP effect on KIAA1549L
SNP effect on Cardiac valvular disease

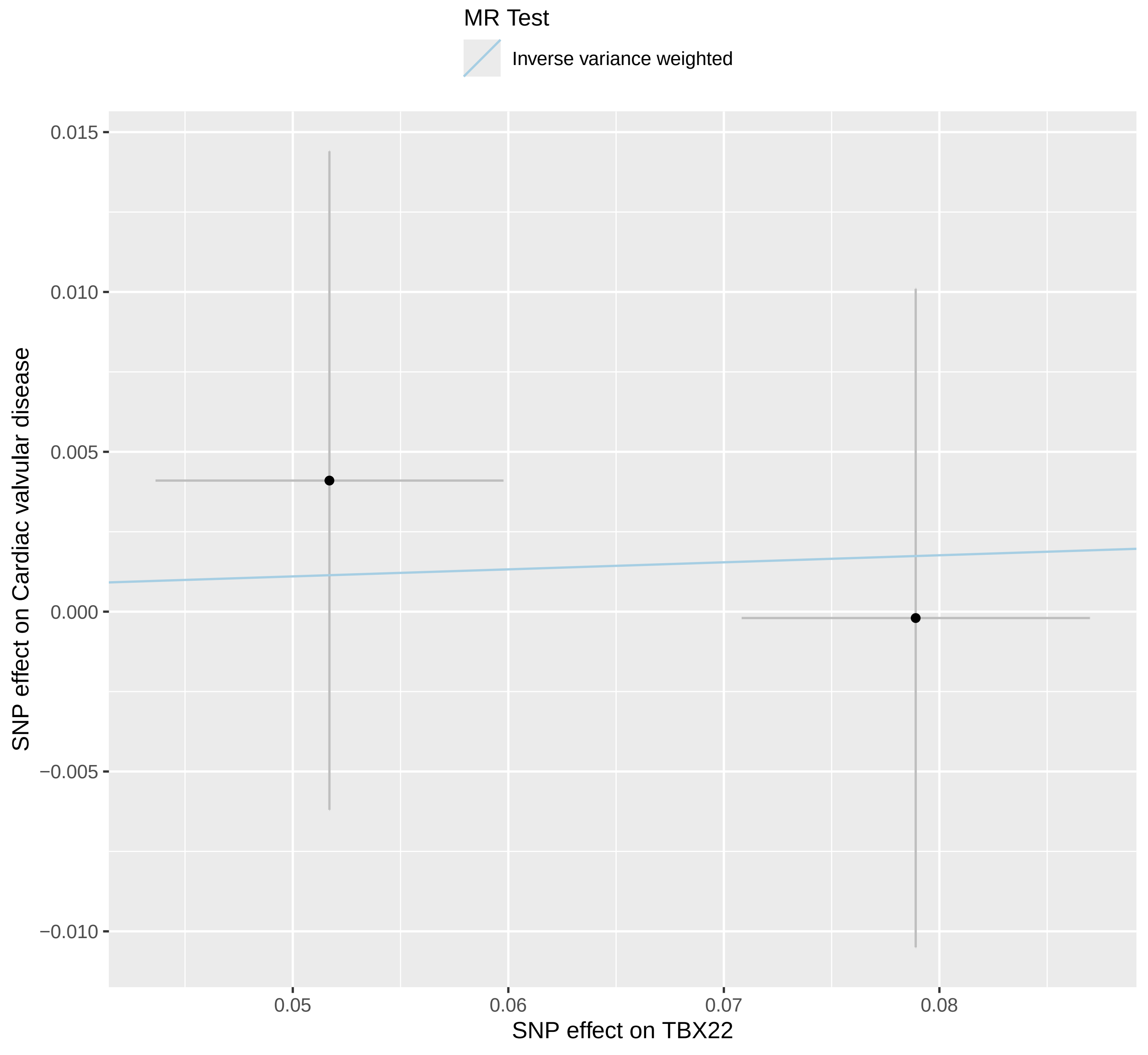
MR Test
Inverse variance weighted
0.015
0.010
0.005
0.000
−0.005
−0.010
SNP effect on Cardiac valvular disease
0.05
0.06
0.07
0.08
SNP effect on TBX22

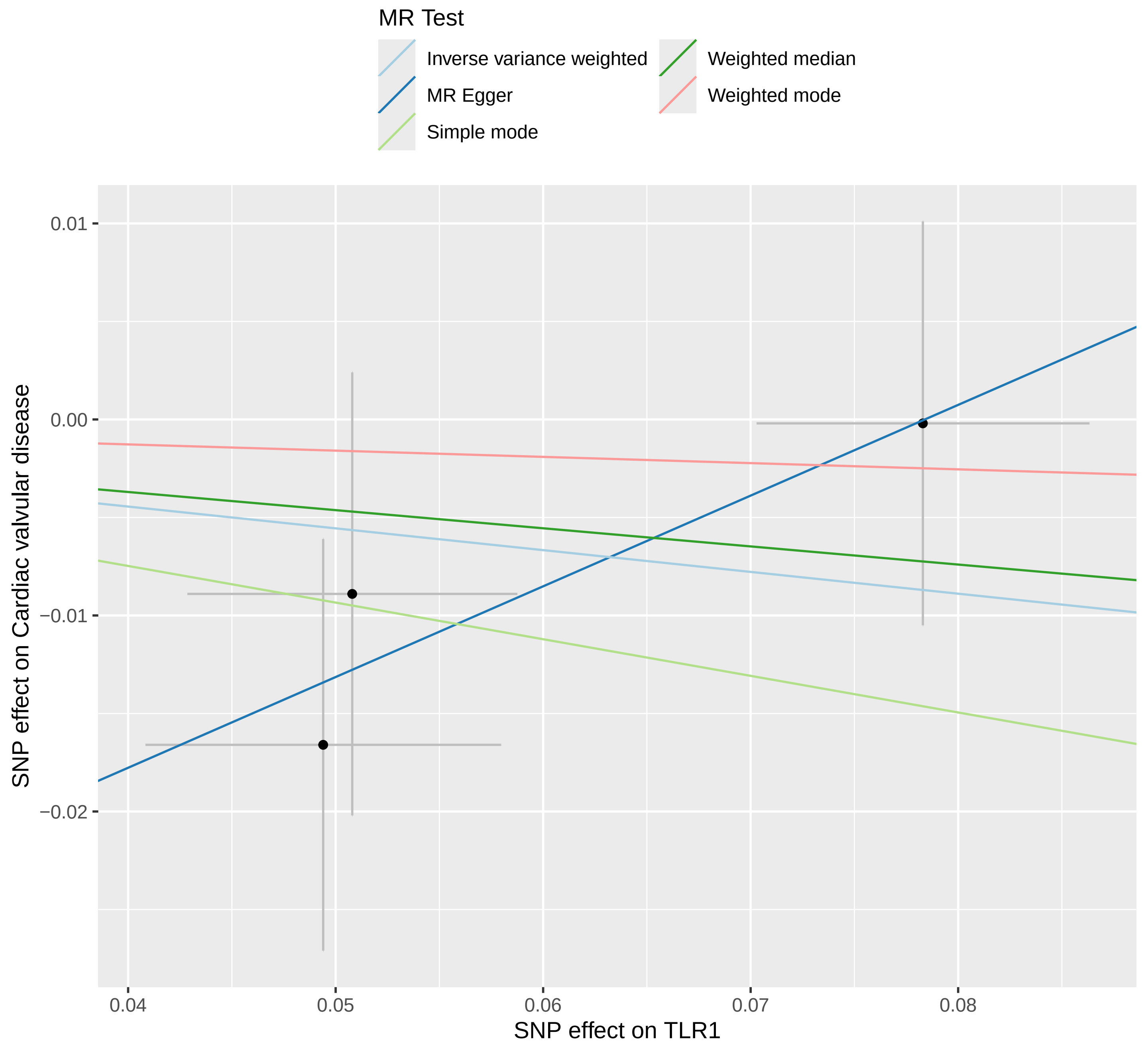
MR Test
Inverse variance weighted
MR Egger
Simple mode
Weighted median
Weighted mode
0.01
0.00
−0.01
−0.02
0.04
0.05
0.06
0.07
0.08
SNP effect on TLR1
SNP effect on Cardiac valvular disease

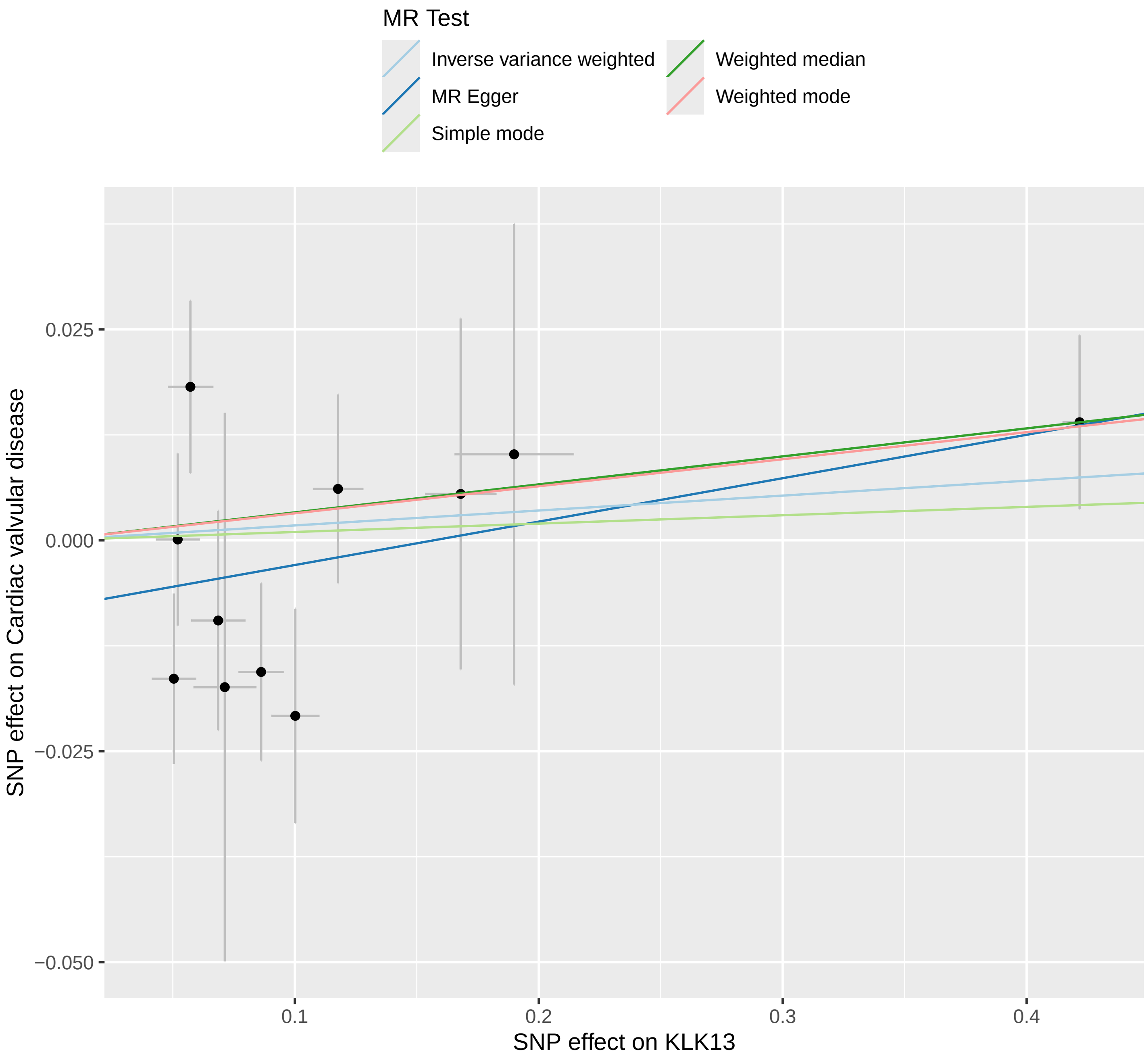
MR Test
Inverse variance weighted
MR Egger
Simple mode
Weighted median
Weighted mode
0.025
0.000
−0.025
−0.050
SNP effect on Cardiac valvular disease
0.1
0.2
0.3
0.4
SNP effect on KLK13

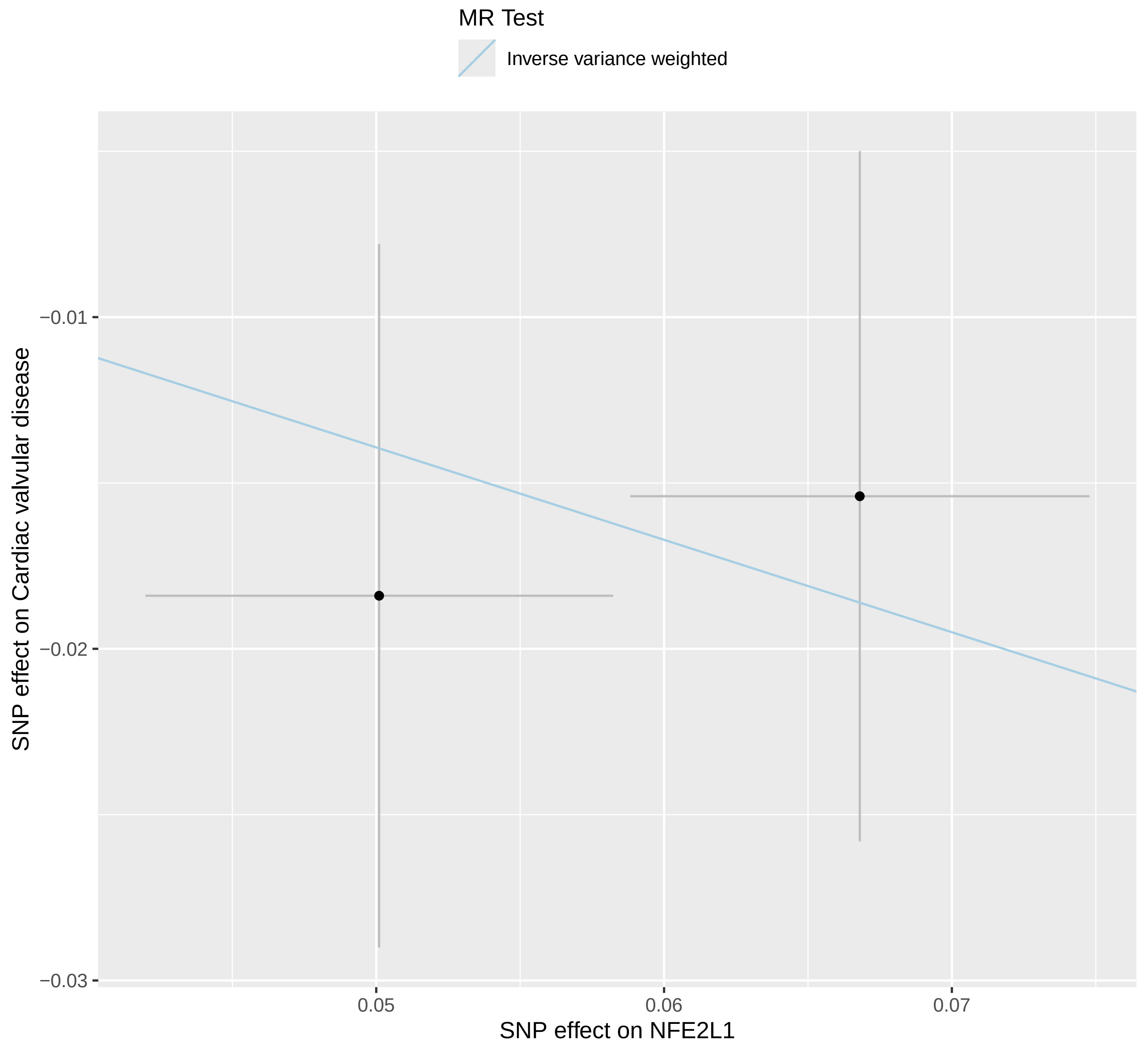
MR Test
Inverse variance weighted
−0.01
−0.02
−0.03
0.05
0.06
0.07
SNP effect on NFE2L1
SNP effect on Cardiac valvular disease

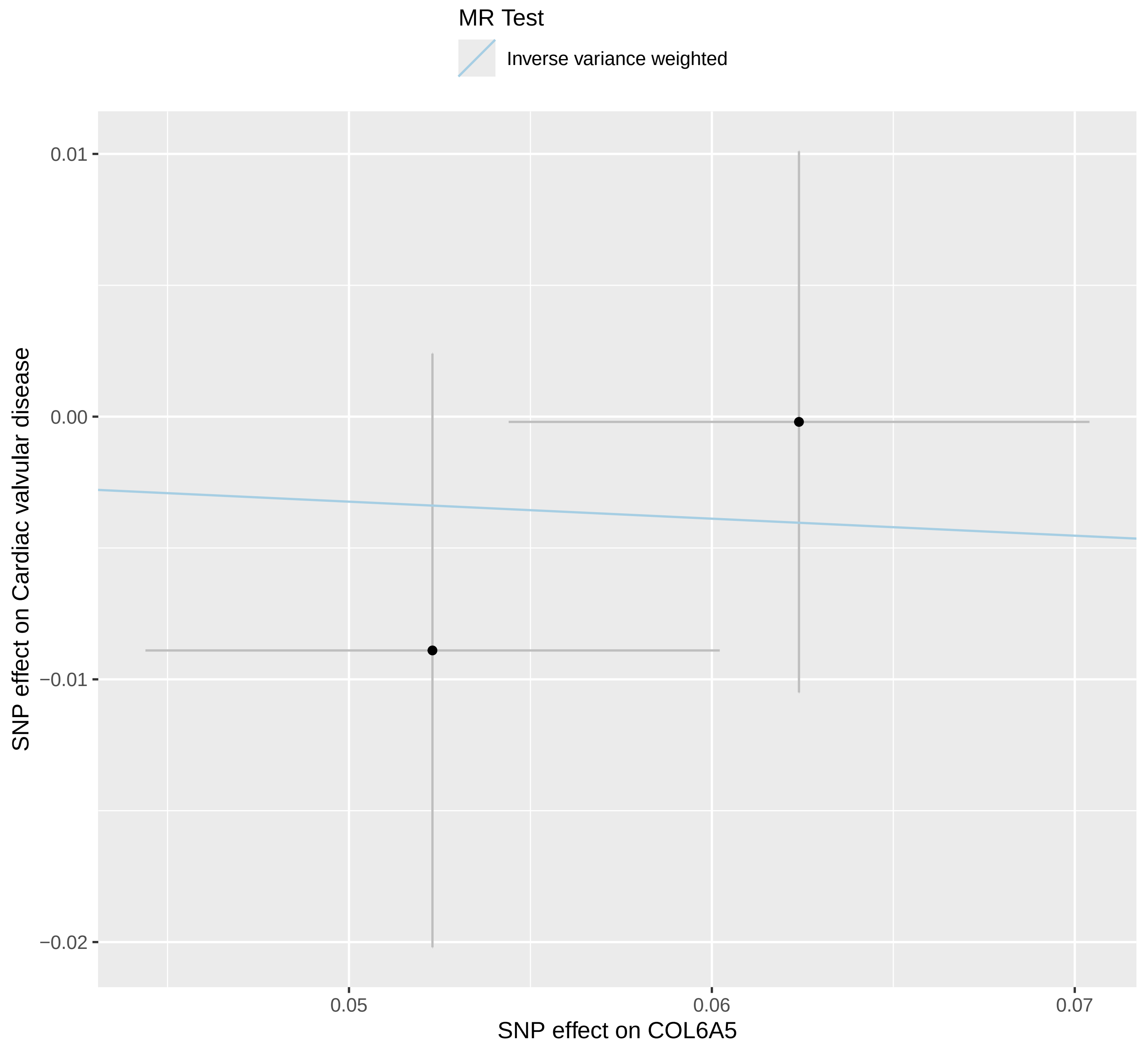
MR Test
Inverse variance weighted
0.01
0.00
−0.01
−0.02
0.05
0.06
0.07
SNP effect on Cardiac valvular disease
SNP effect on COL6A5

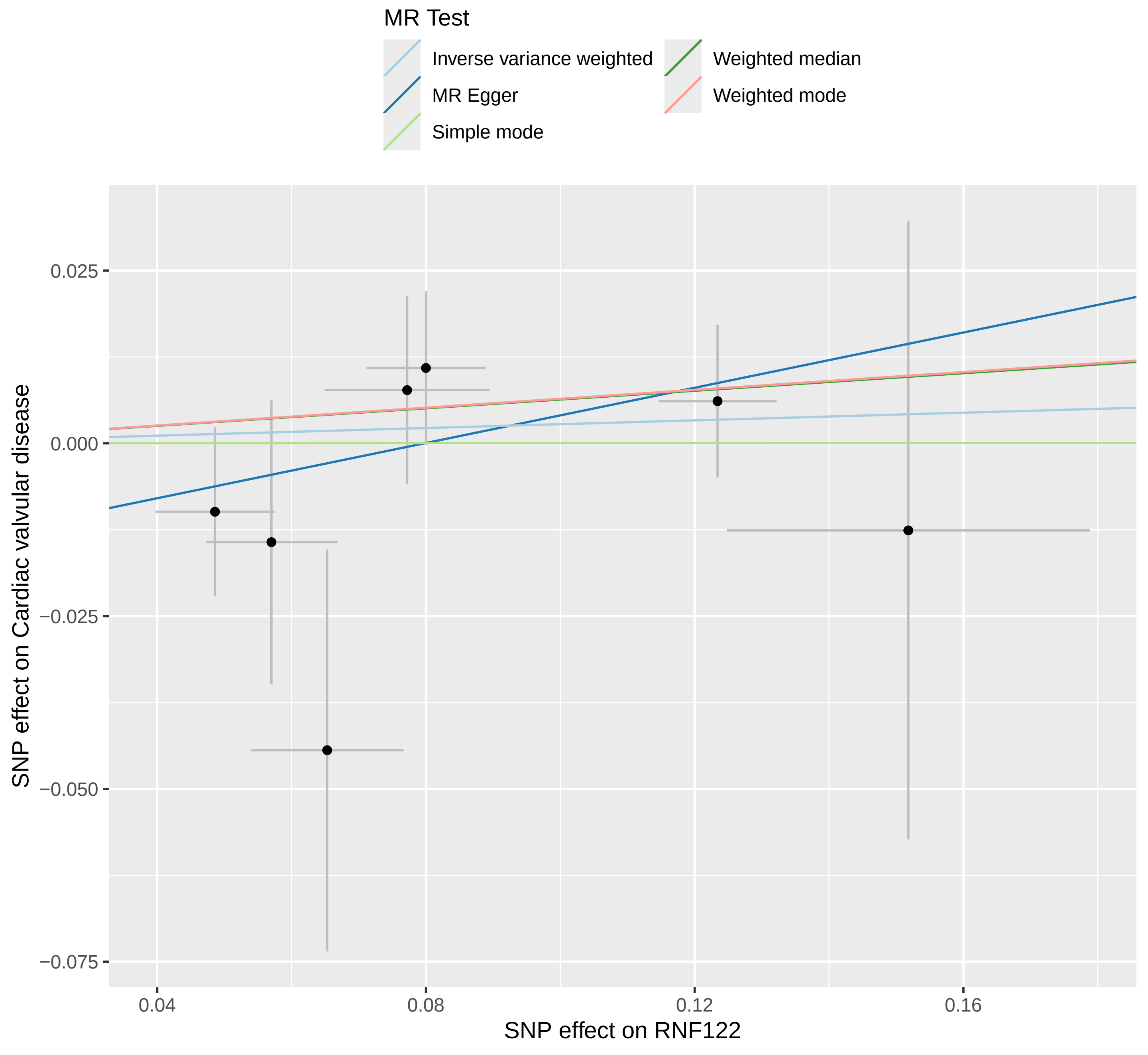
MR Test
Inverse variance weighted
MR Egger
Simple mode
Weighted median
Weighted mode
SNP effect on Cardiac valvular disease
0.025
0.000
−0.025
−0.050
−0.075
0.04
0.08
0.12
0.16
SNP effect on RNF122

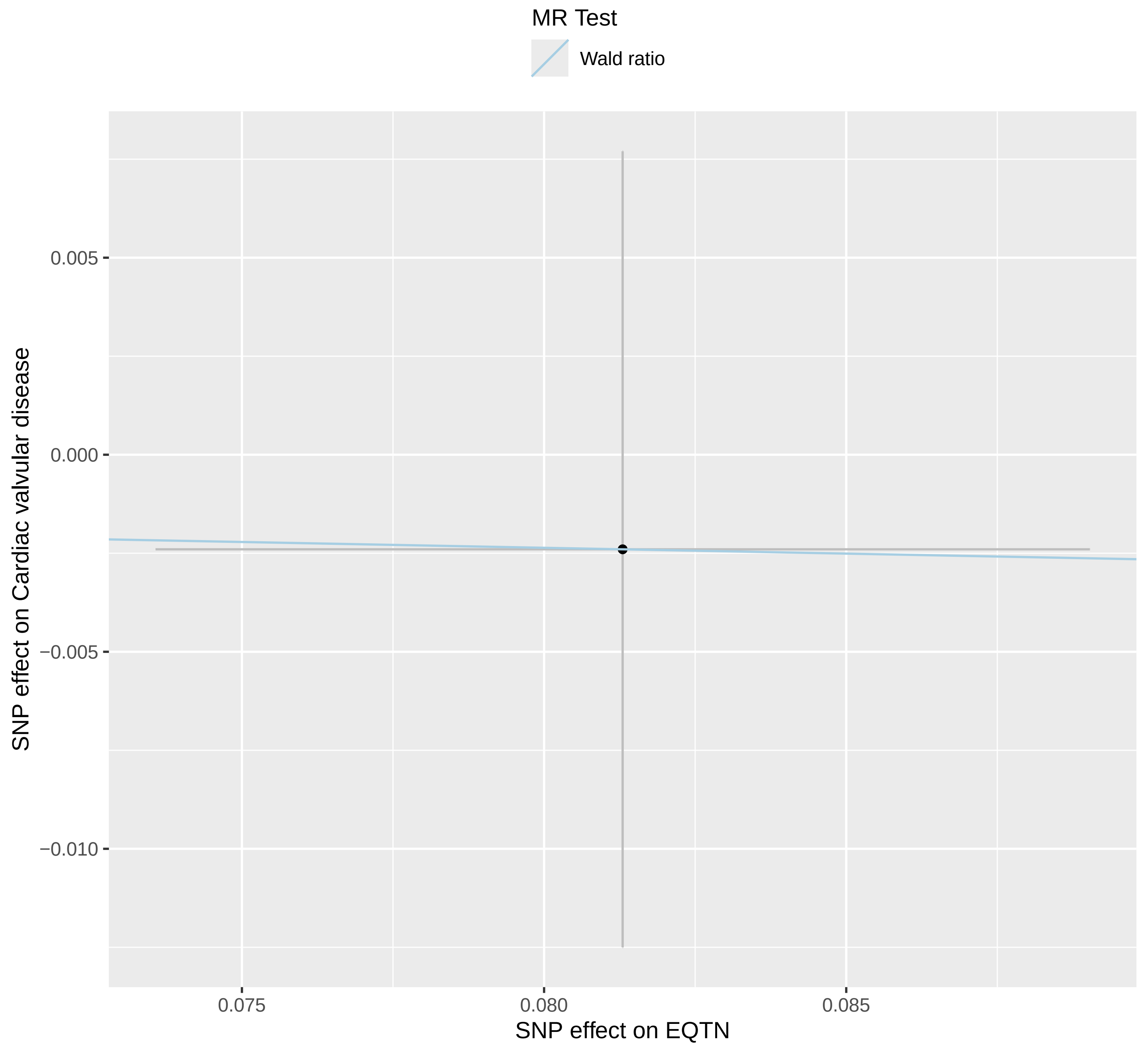
MR Test
Wald ratio
0.005
0.000
−0.005
−0.010
0.075
0.080
0.085
SNP effect on Cardiac valvular disease
SNP effect on EQTN

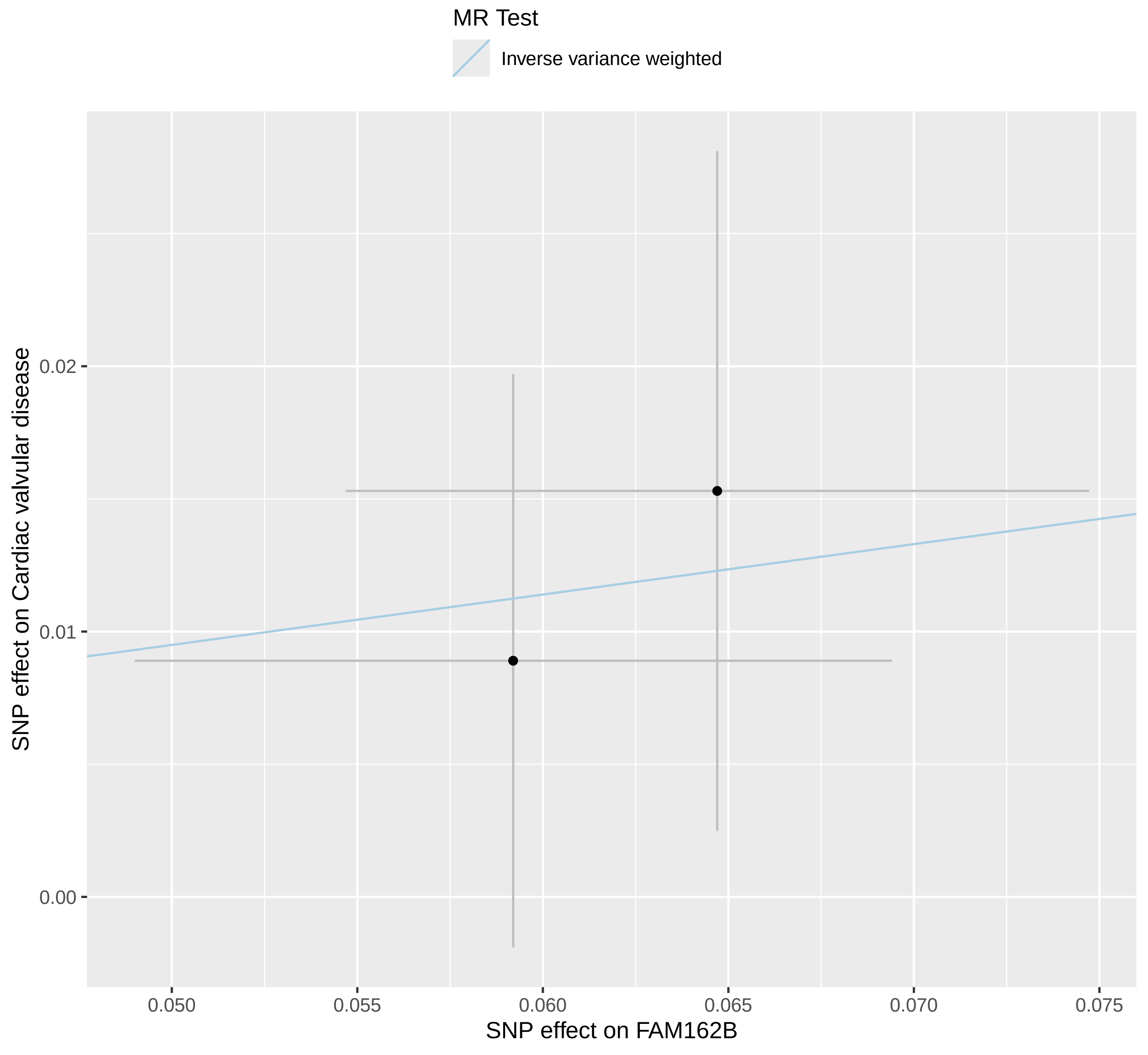
MR Test
Inverse variance weighted
0.02
0.01
0.00
0.050
0.055
0.060
0.065
0.070
0.075
SNP effect on FAM162B
SNP effect on Cardiac valvular disease

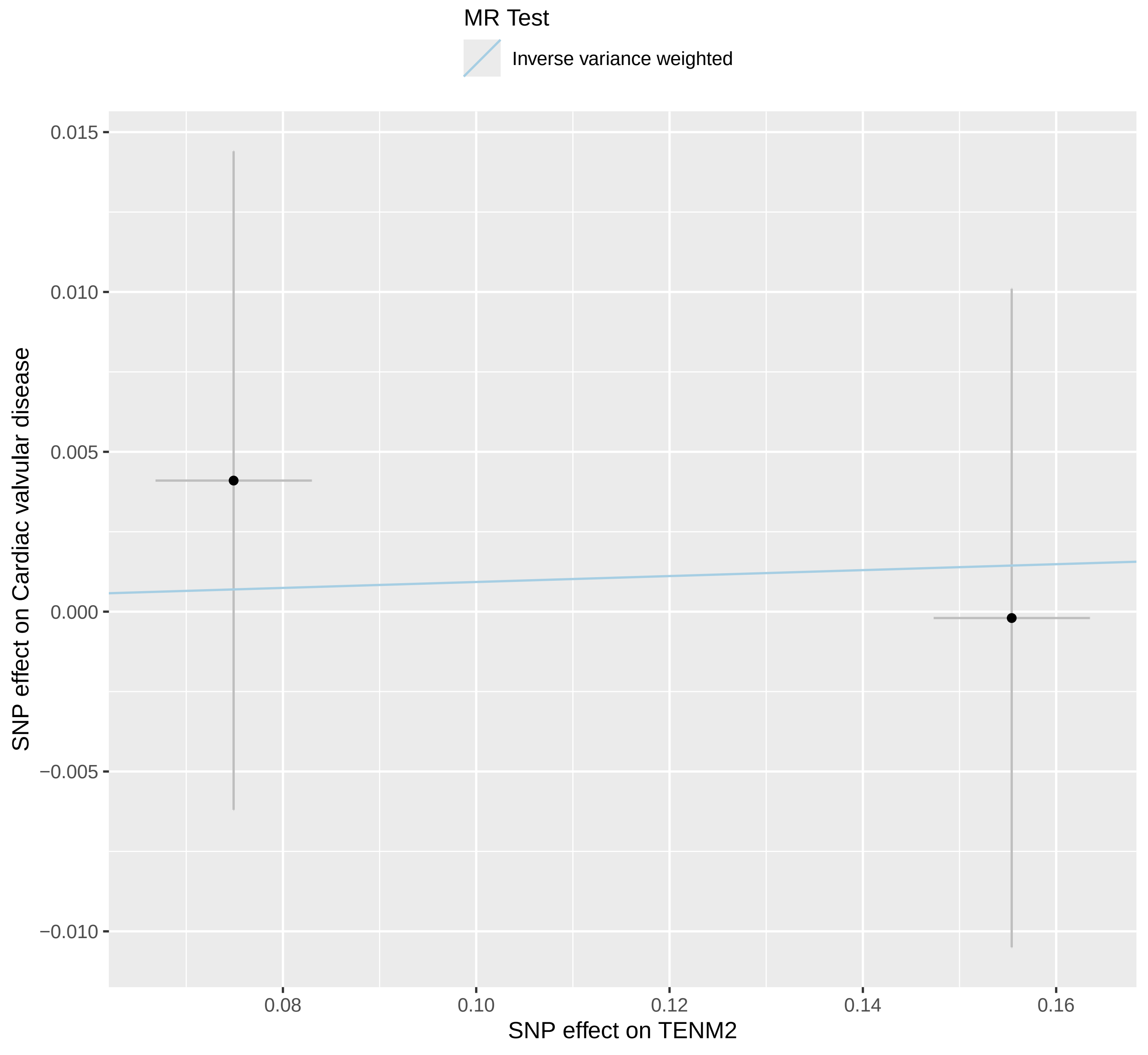
MR Test
Inverse variance weighted
SNP effect on Cardiac valvular disease
0.015
0.010
0.005
0.000
−0.005
−0.010
0.08
0.10
0.12
0.14
0.16
SNP effect on TENM2

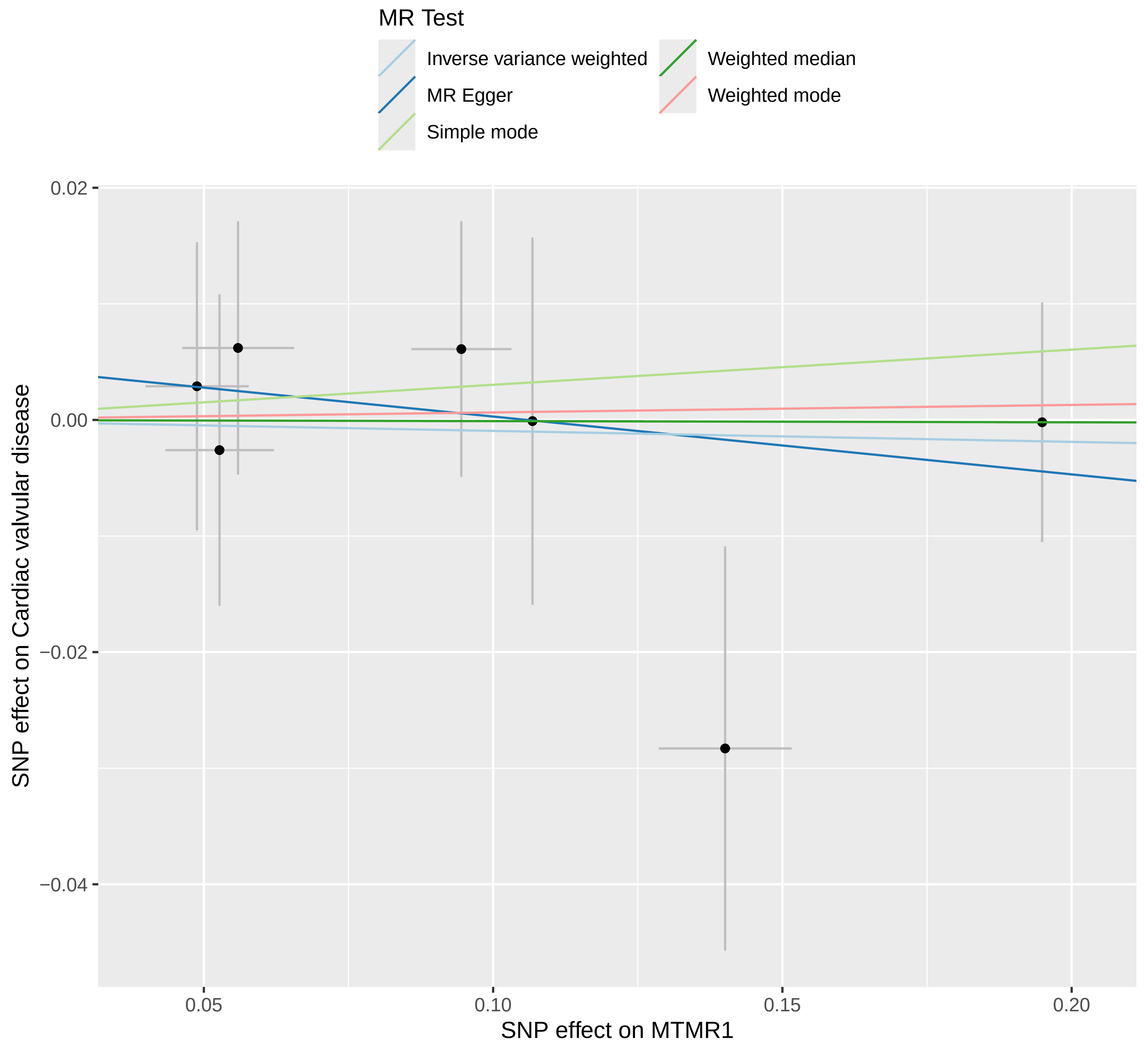

MR Test
Inverse variance weighted
MR Egger
Simple mode
Weighted median
Weighted mode
0.02
0.00
−0.02
−0.04
0.05
0.10
0.15
0.20
SNP effect on MTMR1
SNP effect on Cardiac valvular disease

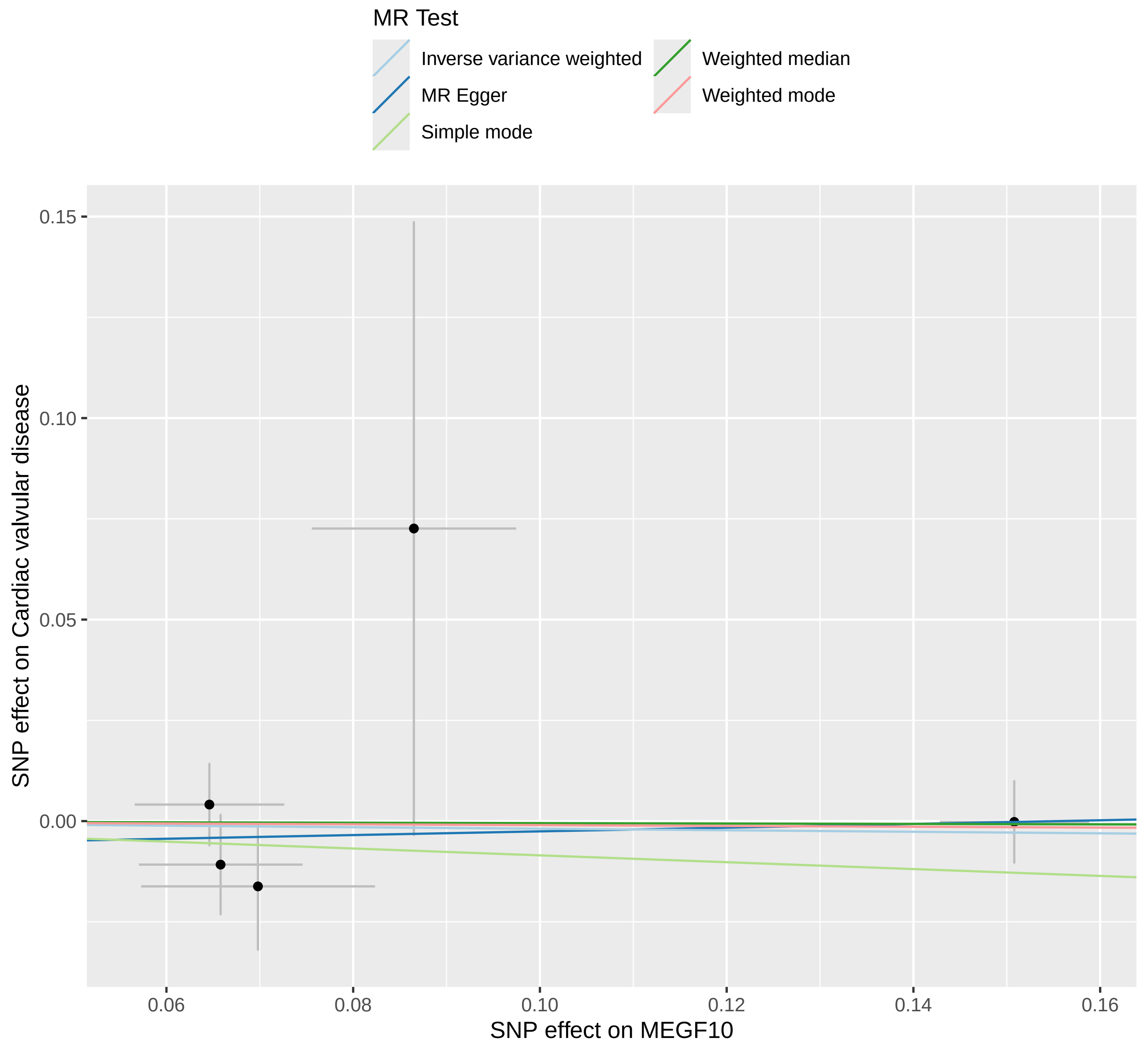

MR Test
Inverse variance weighted
MR Egger
Simple mode
Weighted median
Weighted mode
0.15
0.10
0.05
0.00
SNP effect on Cardiac valvular disease
0.06
0.08
0.10
0.12
0.14
0.16
SNP effect on MEGF10

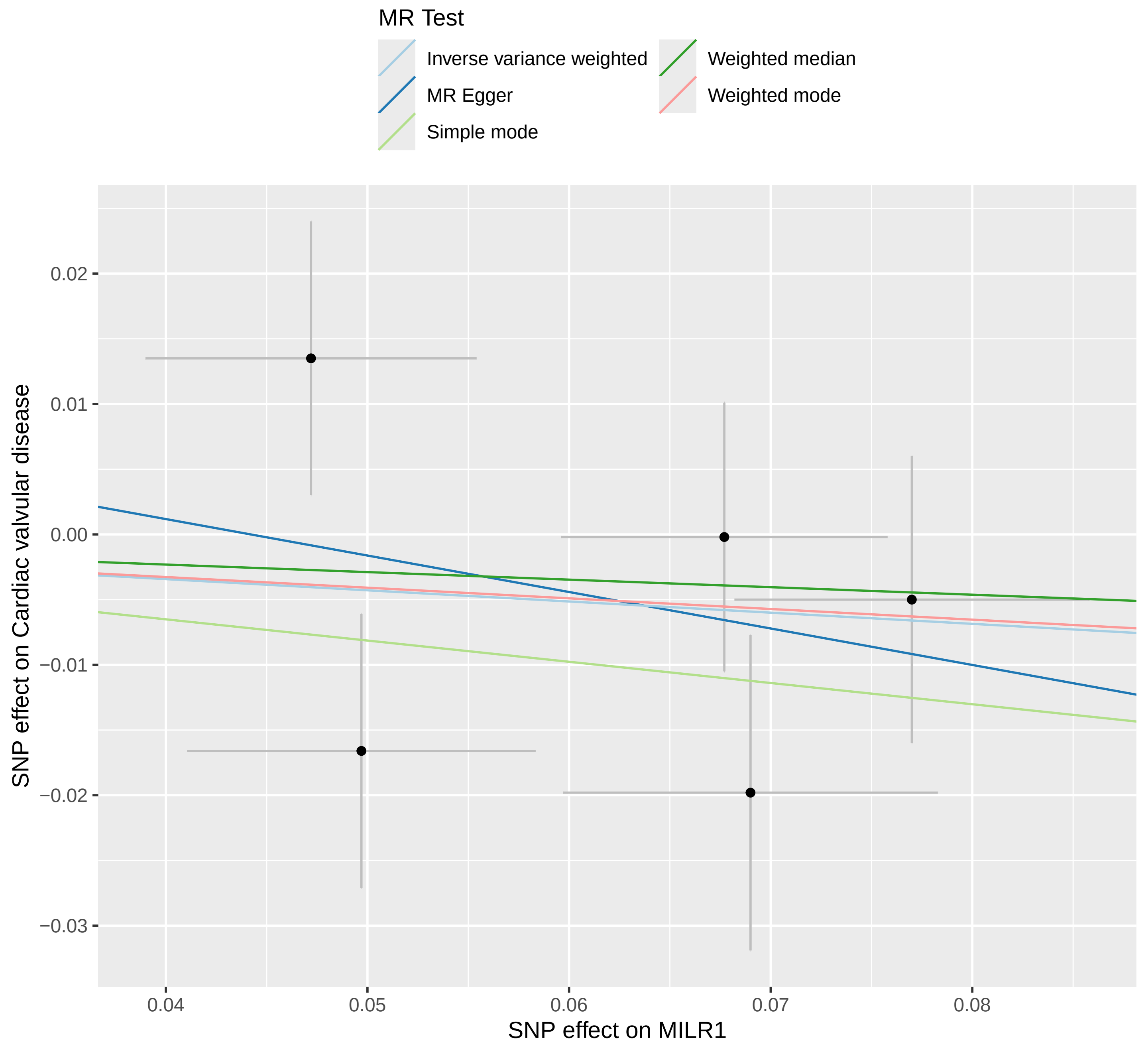

MR Test
Inverse variance weighted
MR Egger
Simple mode
Weighted median
Weighted mode
0.02
0.01
0.00
−0.01
−0.02
−0.03
SNP effect on Cardiac valvular disease
0.04
0.05
0.06
0.07
0.08
SNP effect on MILR1

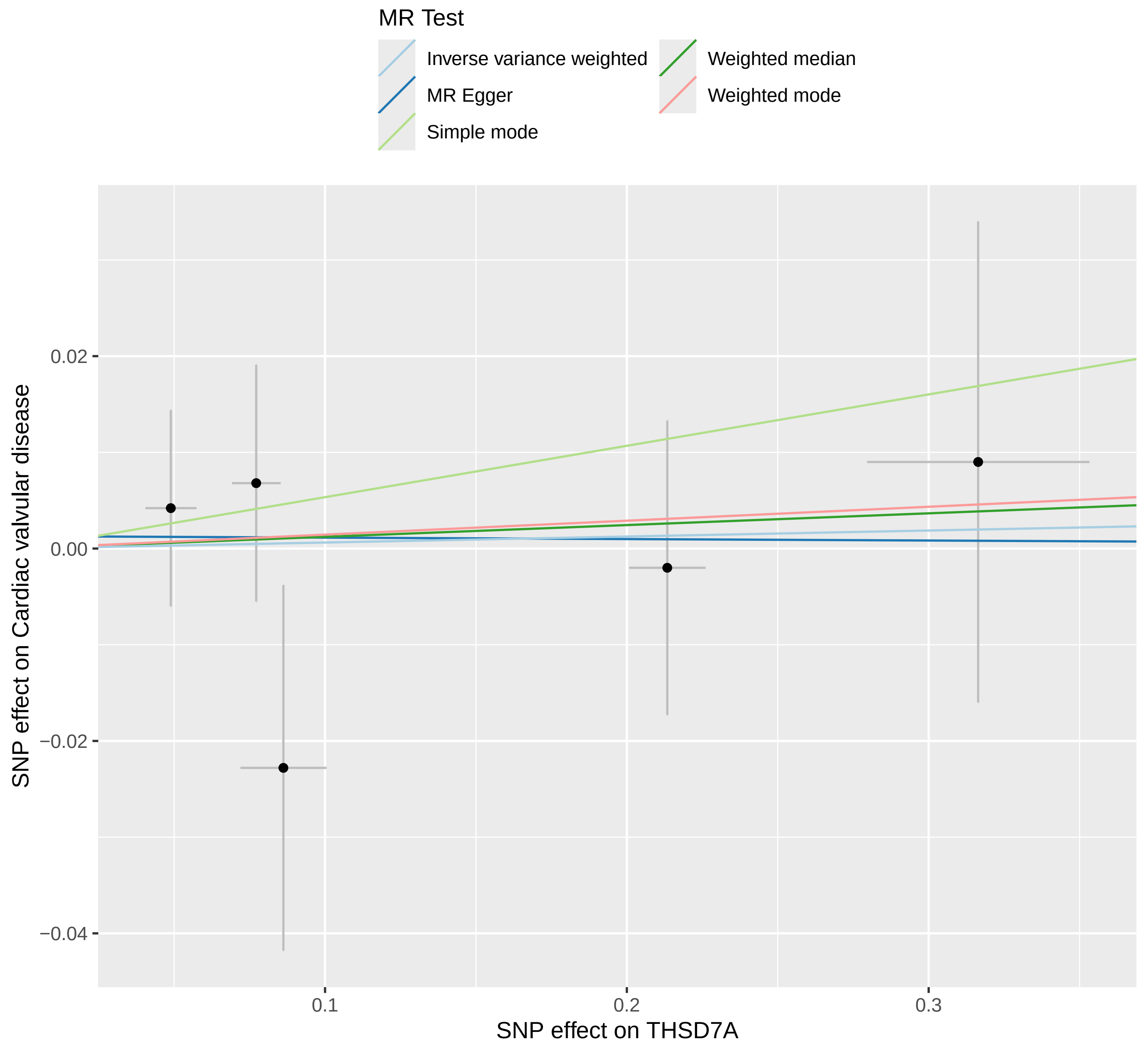
MR Test
Inverse variance weighted
MR Egger
Simple mode
Weighted median
Weighted mode
0.02
0.00
−0.02
−0.04
SNP effect on Cardiac valvular disease
0.1
0.2
0.3
SNP effect on THSD7A

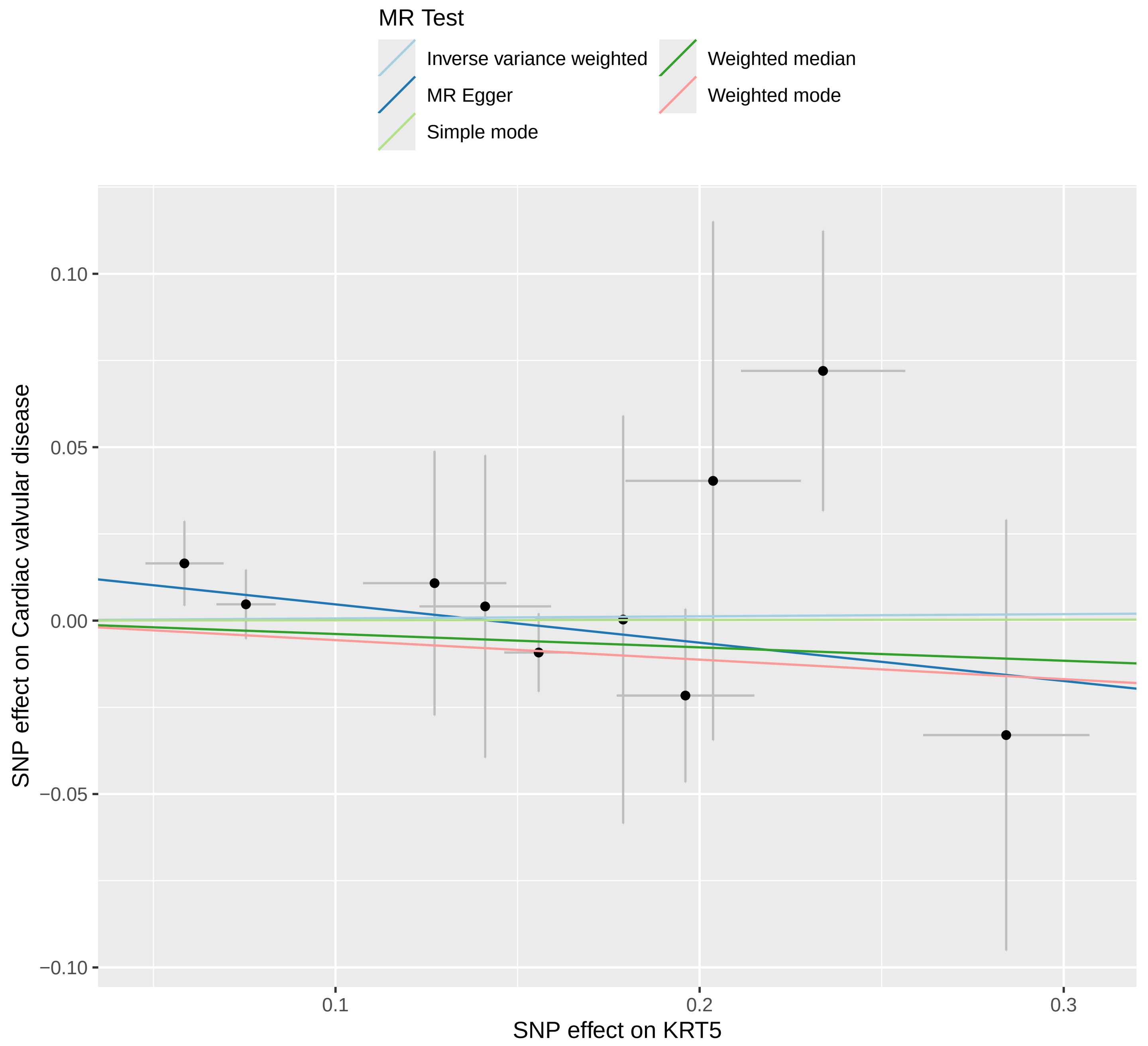
MR Test
Inverse variance weighted
MR Egger
Simple mode
Weighted median
Weighted mode
SNP effect on Cardiac valvular disease
0.10
0.05
0.00
−0.05
−0.10
SNP effect on KRT5
0.1
0.2
0.3

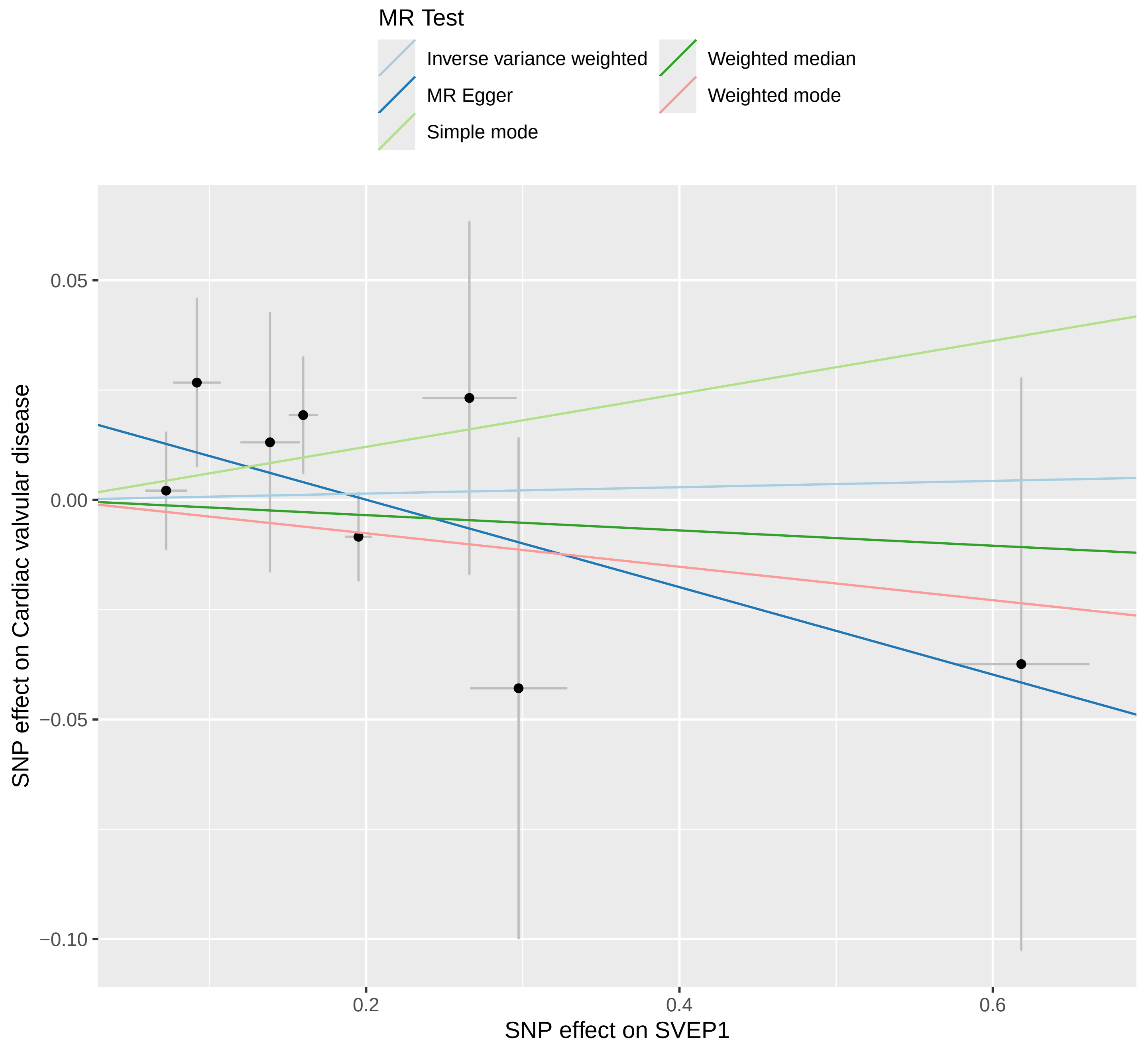
MR Test
Inverse variance weighted
MR Egger
Simple mode
Weighted median
Weighted mode
0.05
0.00
−0.05
−0.10
SNP effect on Cardiac valvular disease
0.2
0.4
0.6
SNP effect on SVEP1

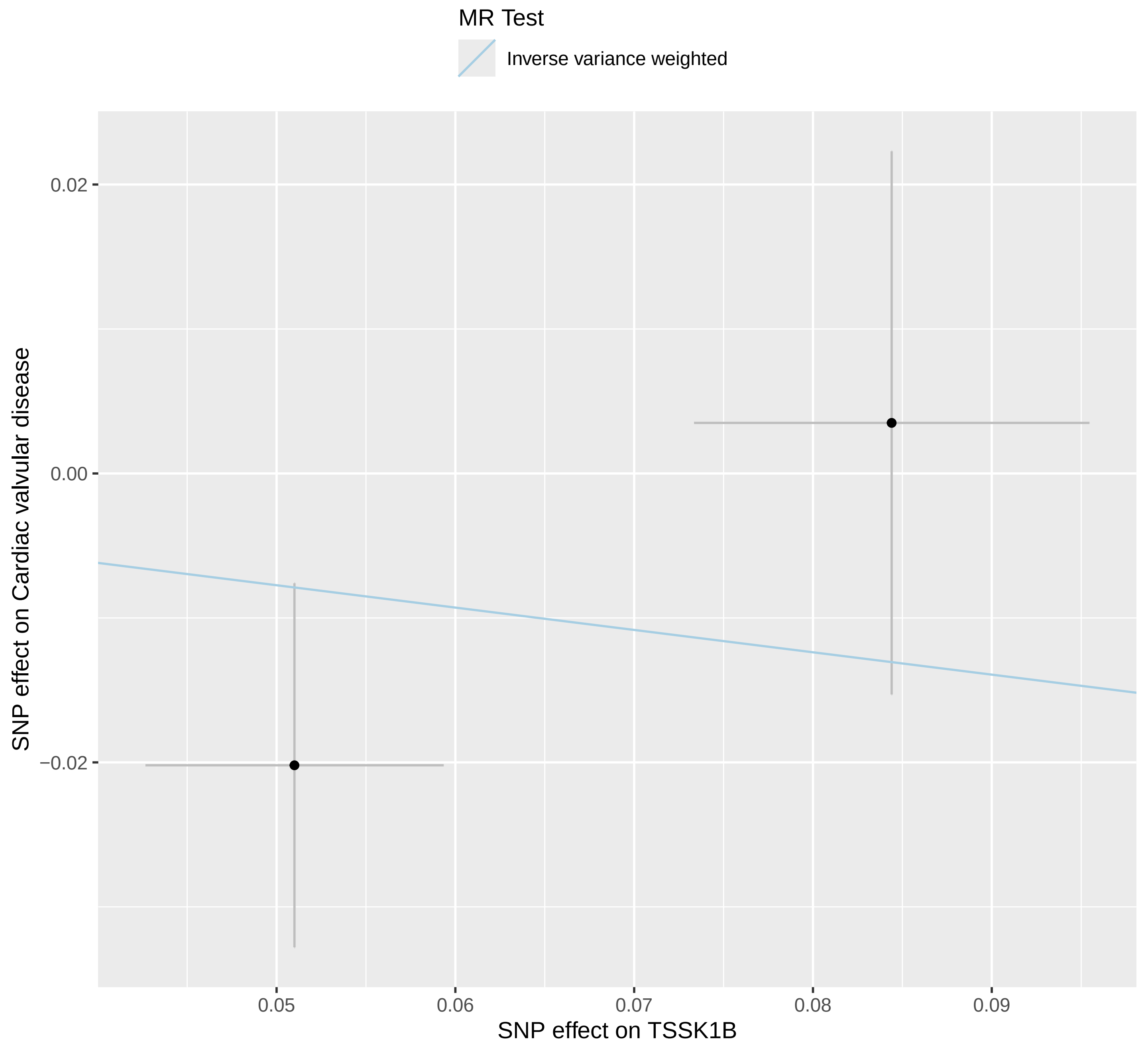

MR Test
Inverse variance weighted
0.02
0.00
−0.02
0.05
0.06
0.07
0.08
0.09
SNP effect on Cardiac valvular disease
SNP effect on TSSK1B

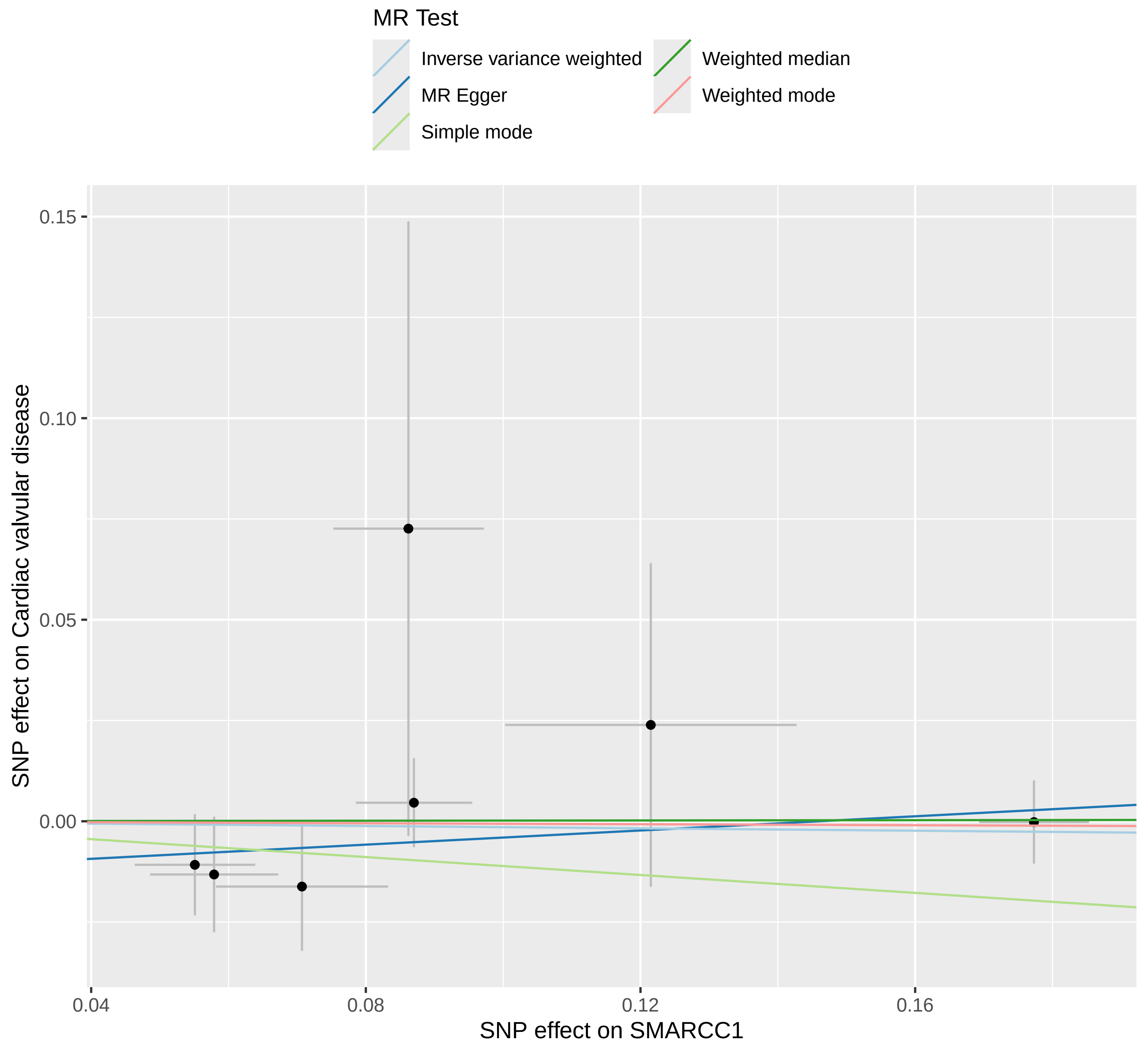

MR Test
Inverse variance weighted
MR Egger
Simple mode
Weighted median
Weighted mode
0.15
0.10
0.05
0.00
SNP effect on Cardiac valvular disease
0.04
0.08
0.12
0.16
SNP effect on SMARCC1

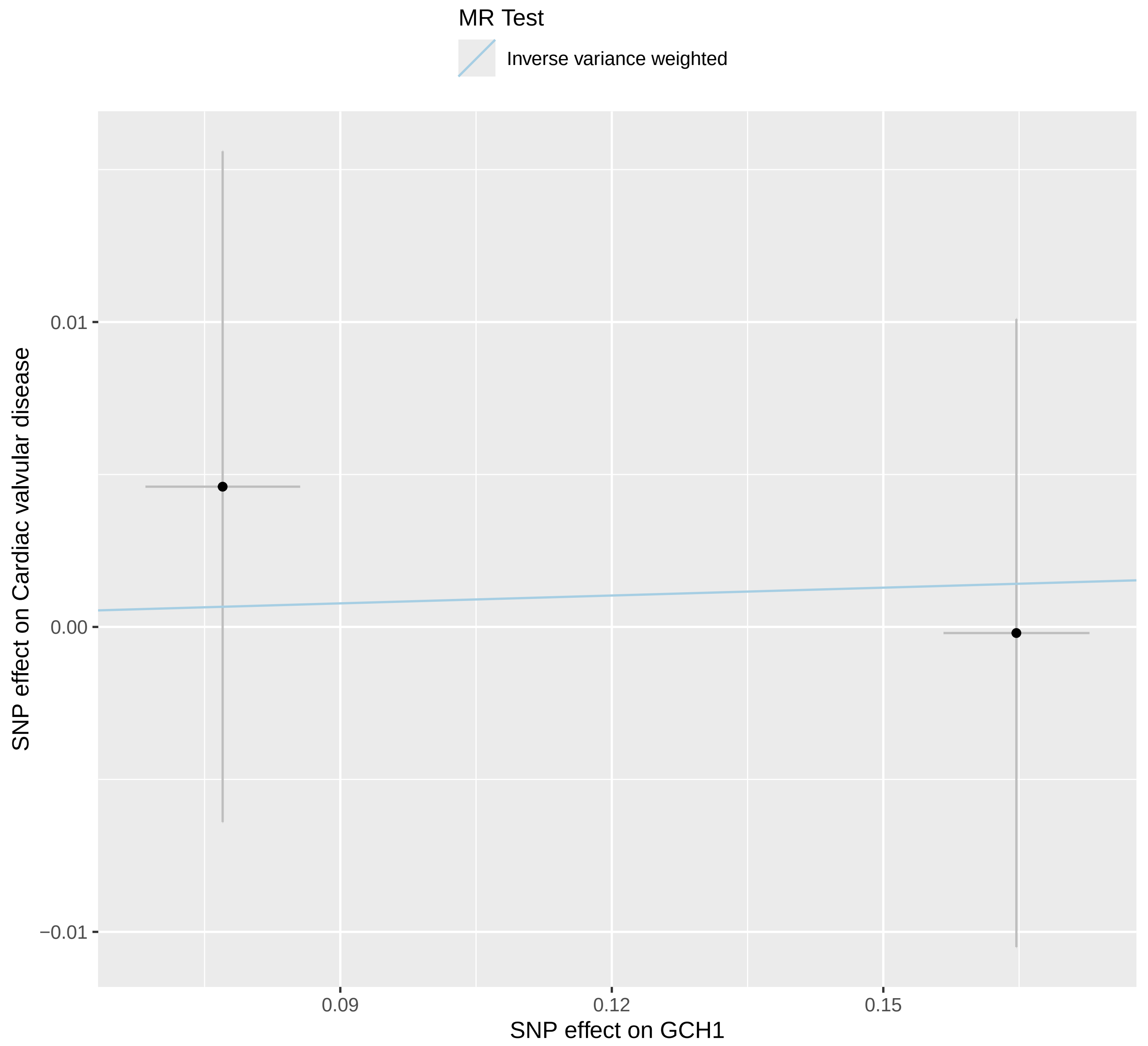
MR Test
Inverse variance weighted
0.01
0.00
−0.01
0.09
0.12
0.15
SNP effect on Cardiac valvular disease
SNP effect on GCH1

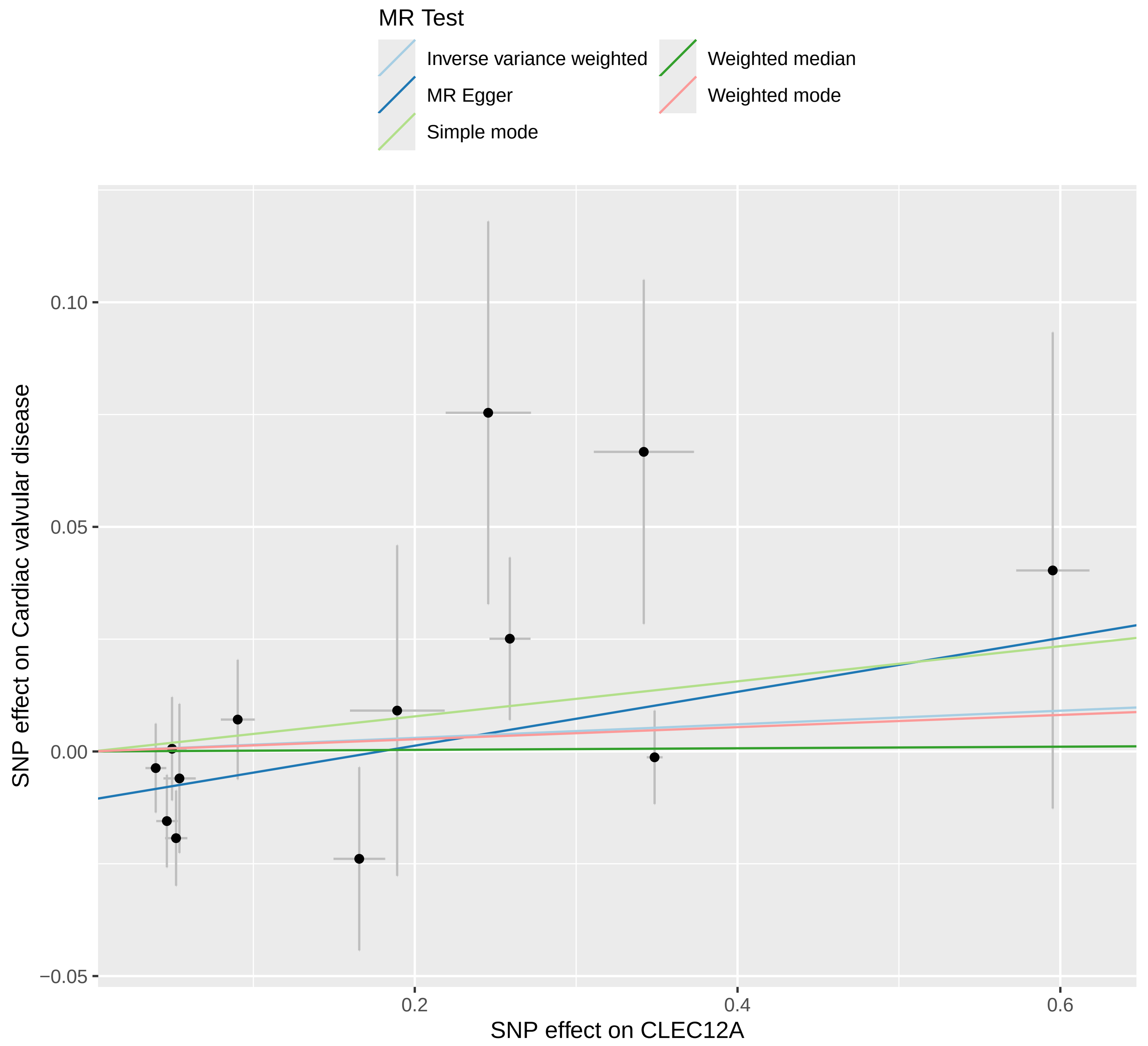
MR Test
Inverse variance weighted
MR Egger
Simple mode
Weighted median
Weighted mode
0.10
0.05
0.00
−0.05
0.2
0.4
0.6
SNP effect on Cardiac valvular disease
SNP effect on CLEC12A

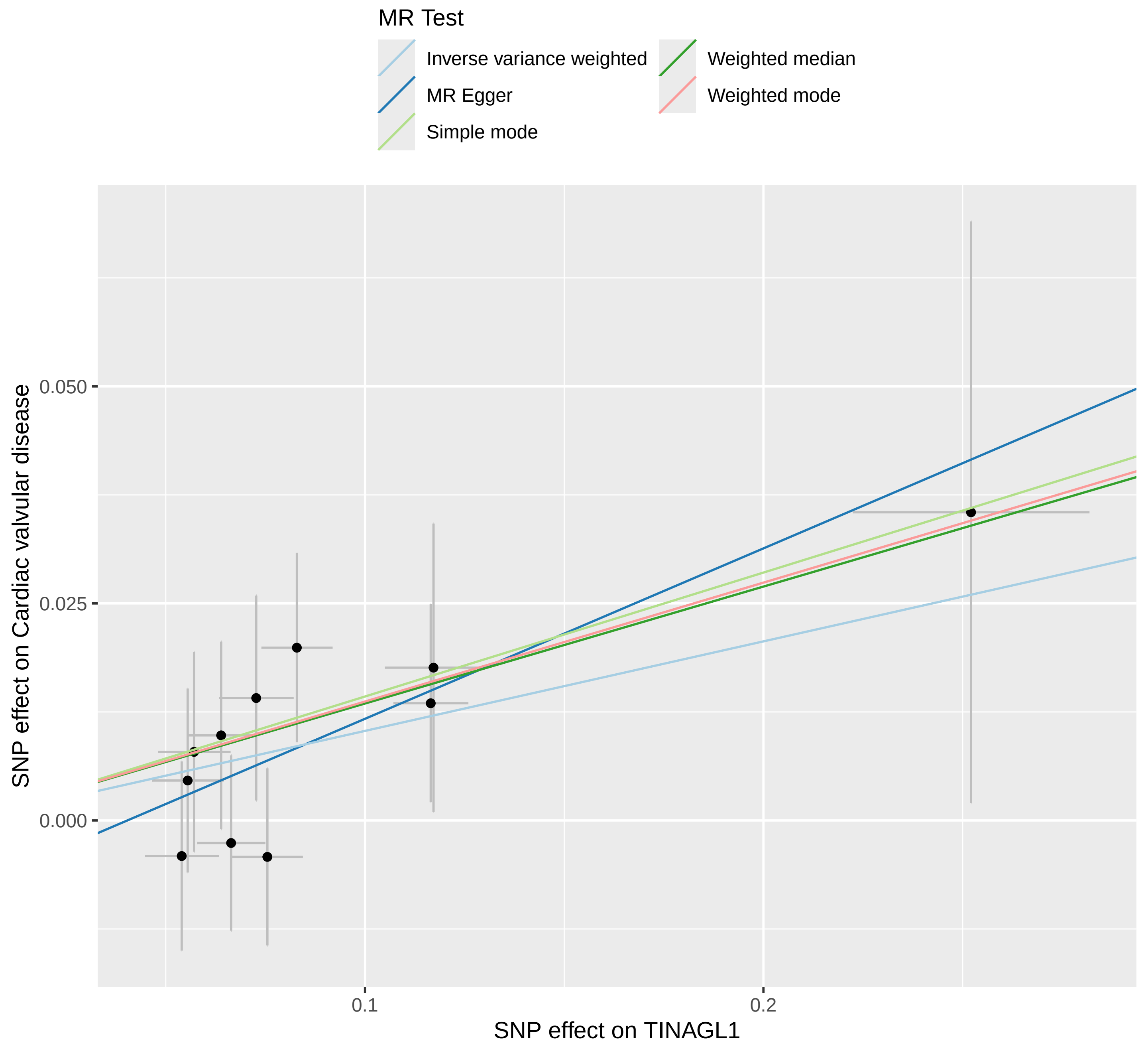
MR Test
Inverse variance weighted
MR Egger
Simple mode
Weighted median
Weighted mode
SNP effect on Cardiac valvular disease
0.050
0.025
0.000
0.1
0.2
SNP effect on TINAGL1

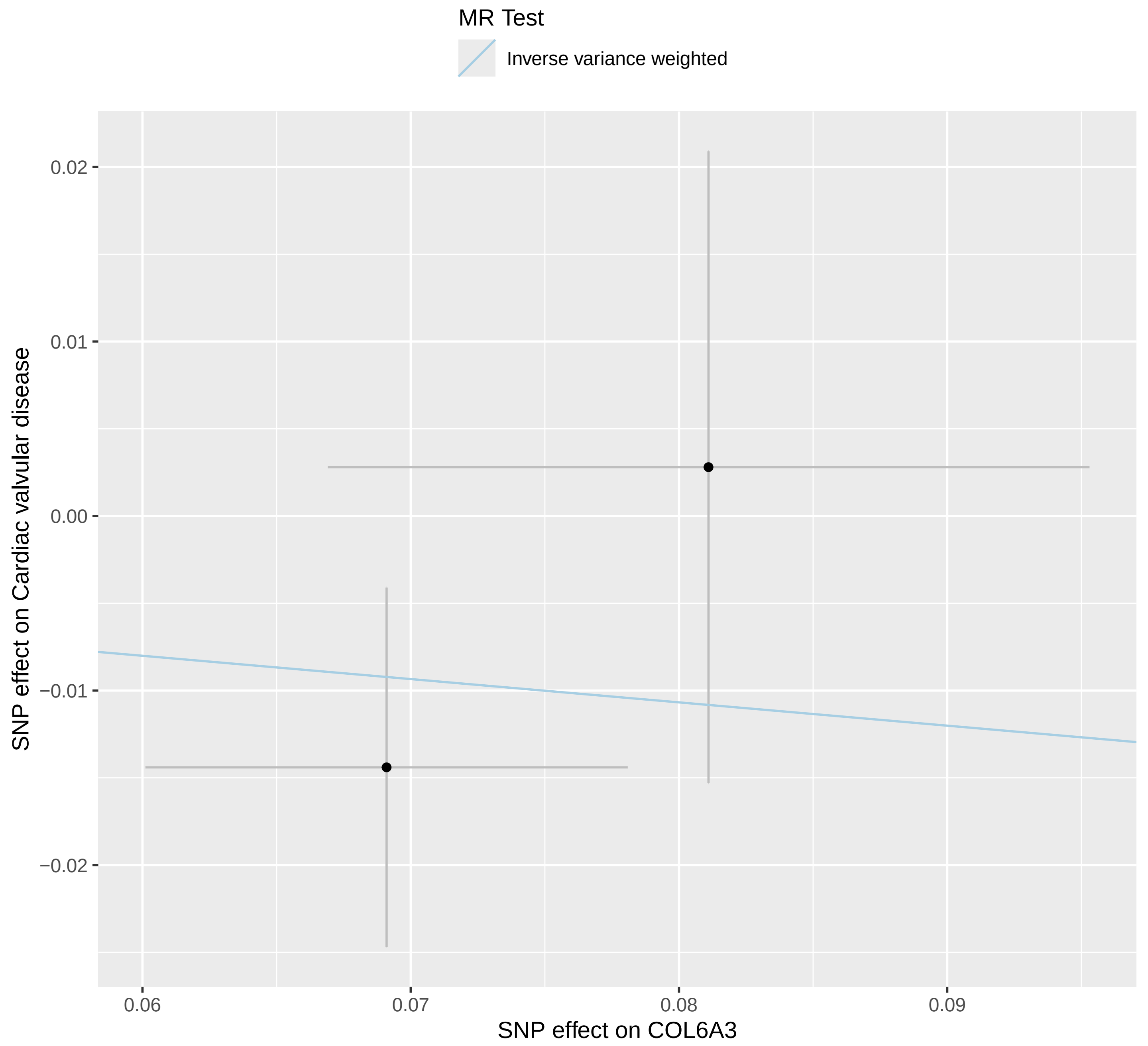
MR Test
Inverse variance weighted
0.02
0.01
0.00
−0.01
−0.02
0.06
0.07
0.08
0.09
SNP effect on COL6A3
SNP effect on Cardiac valvular disease

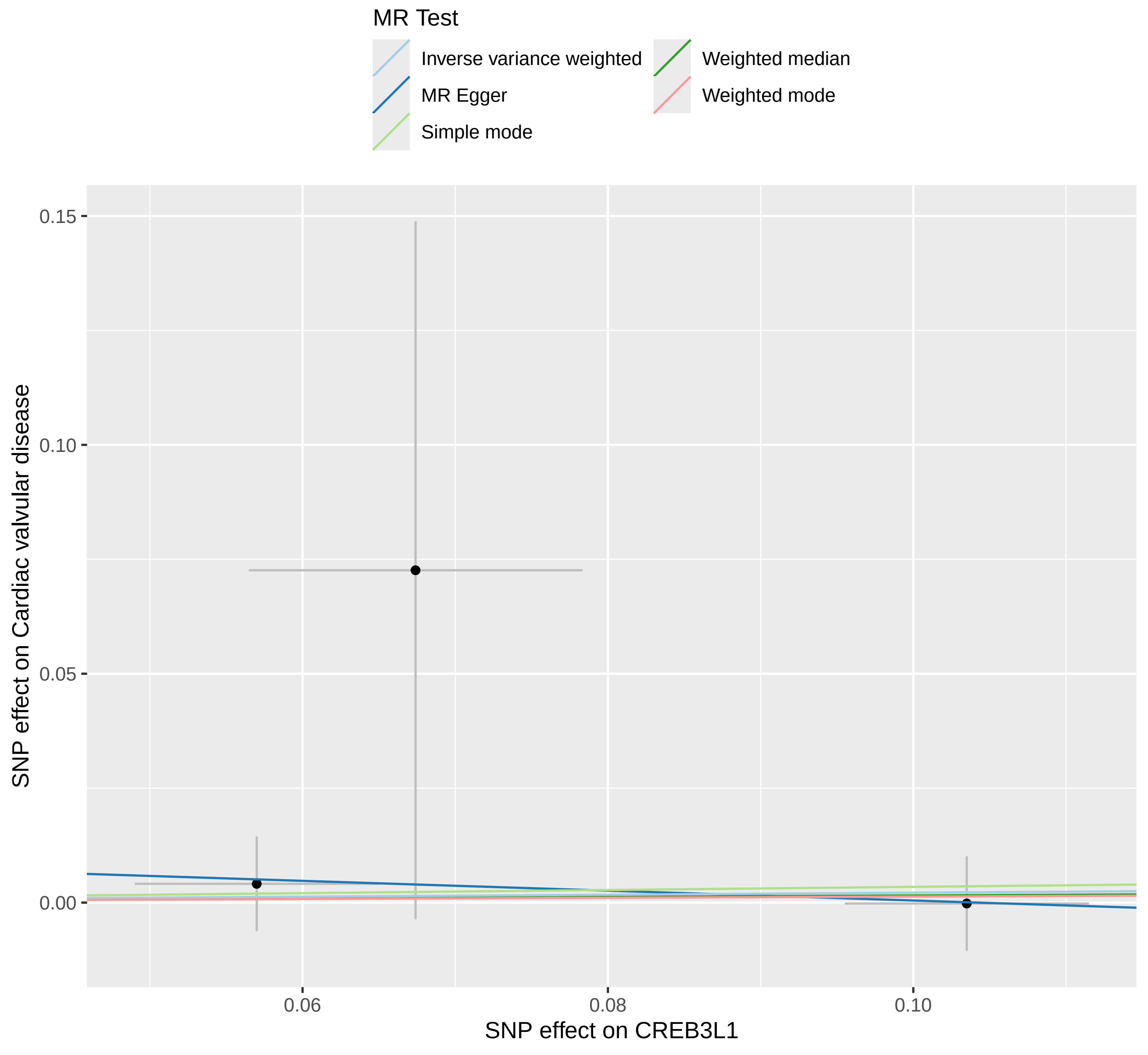

MR Test
Inverse variance weighted
MR Egger
Simple mode
Weighted median
Weighted mode
0.15
0.10
0.05
0.00
SNP effect on Cardiac valvular disease
0.06
0.08
0.10
SNP effect on CREB3L1

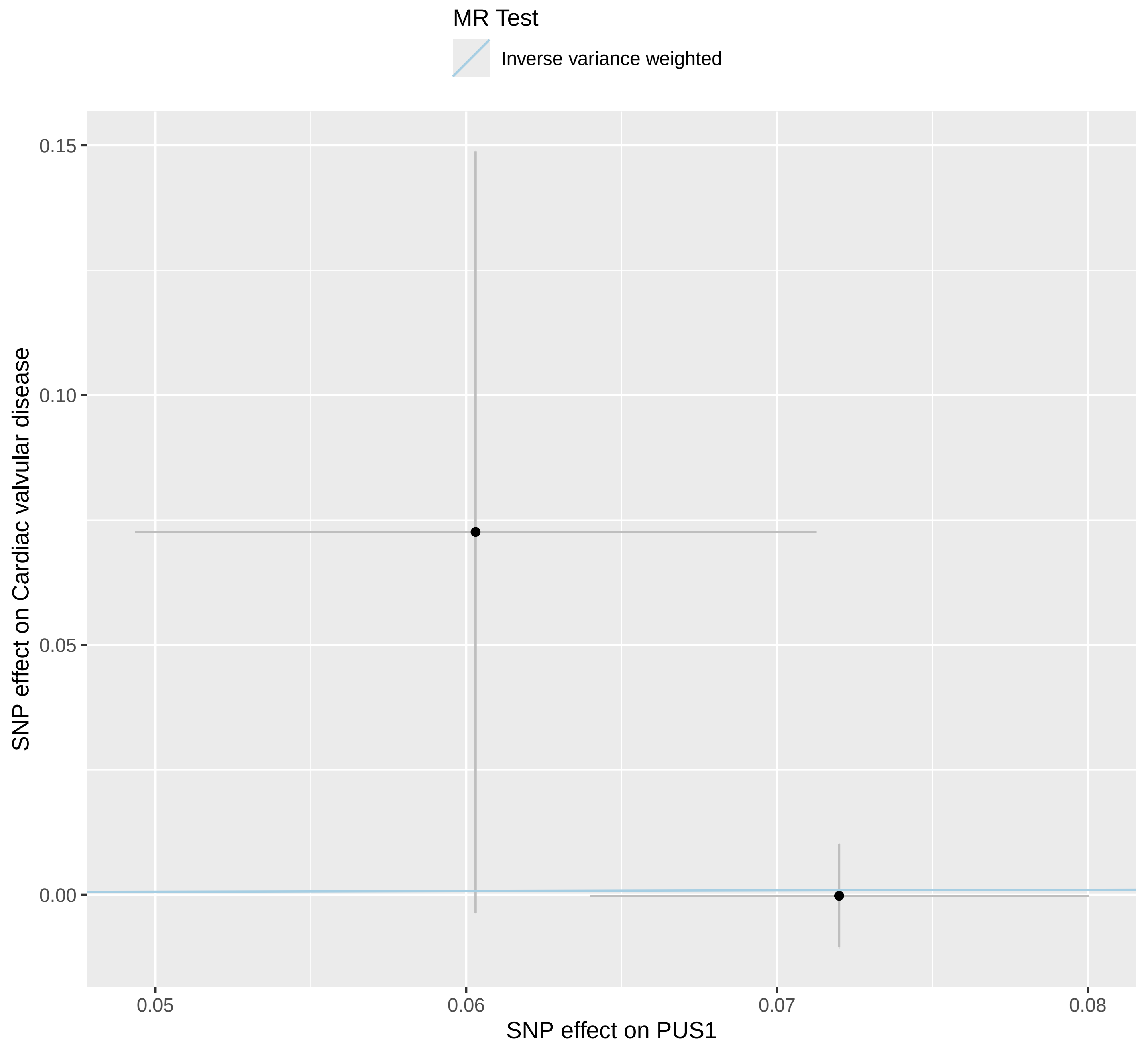

MR Test
Inverse variance weighted
0.15
0.10
0.05
0.00
0.05
0.06
0.07
0.08
SNP effect on Cardiac valvular disease
SNP effect on PUS1

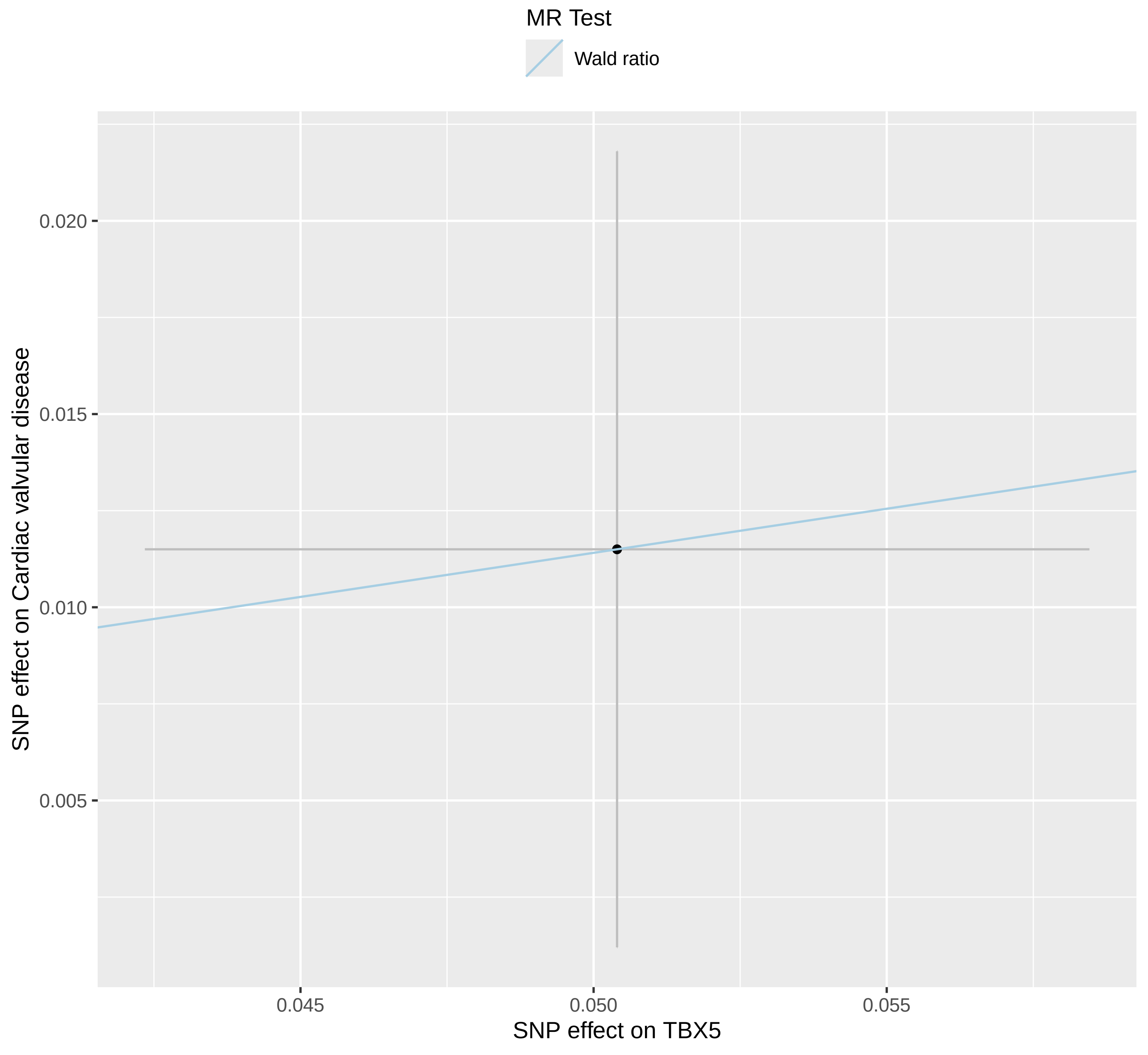
MR Test
Wald ratio
0.020
0.015
0.010
0.005
0.045
0.050
0.055
SNP effect on TBX5
SNP effect on Cardiac valvular disease

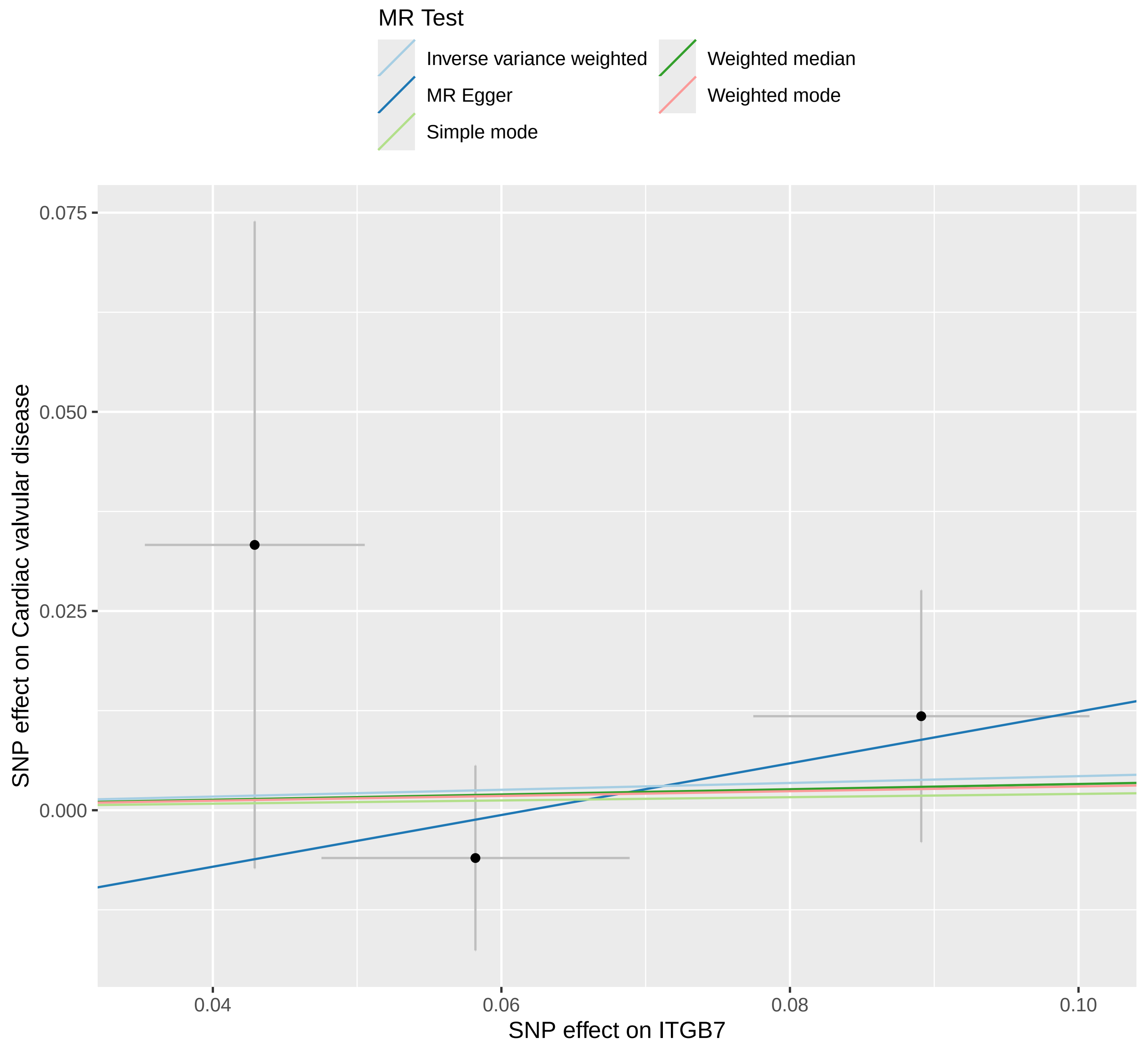

MR Test
Inverse variance weighted
MR Egger
Simple mode
Weighted median
Weighted mode
0.075
0.050
0.025
0.000
0.04
0.06
0.08
0.10
SNP effect on Cardiac valvular disease
SNP effect on ITGB7

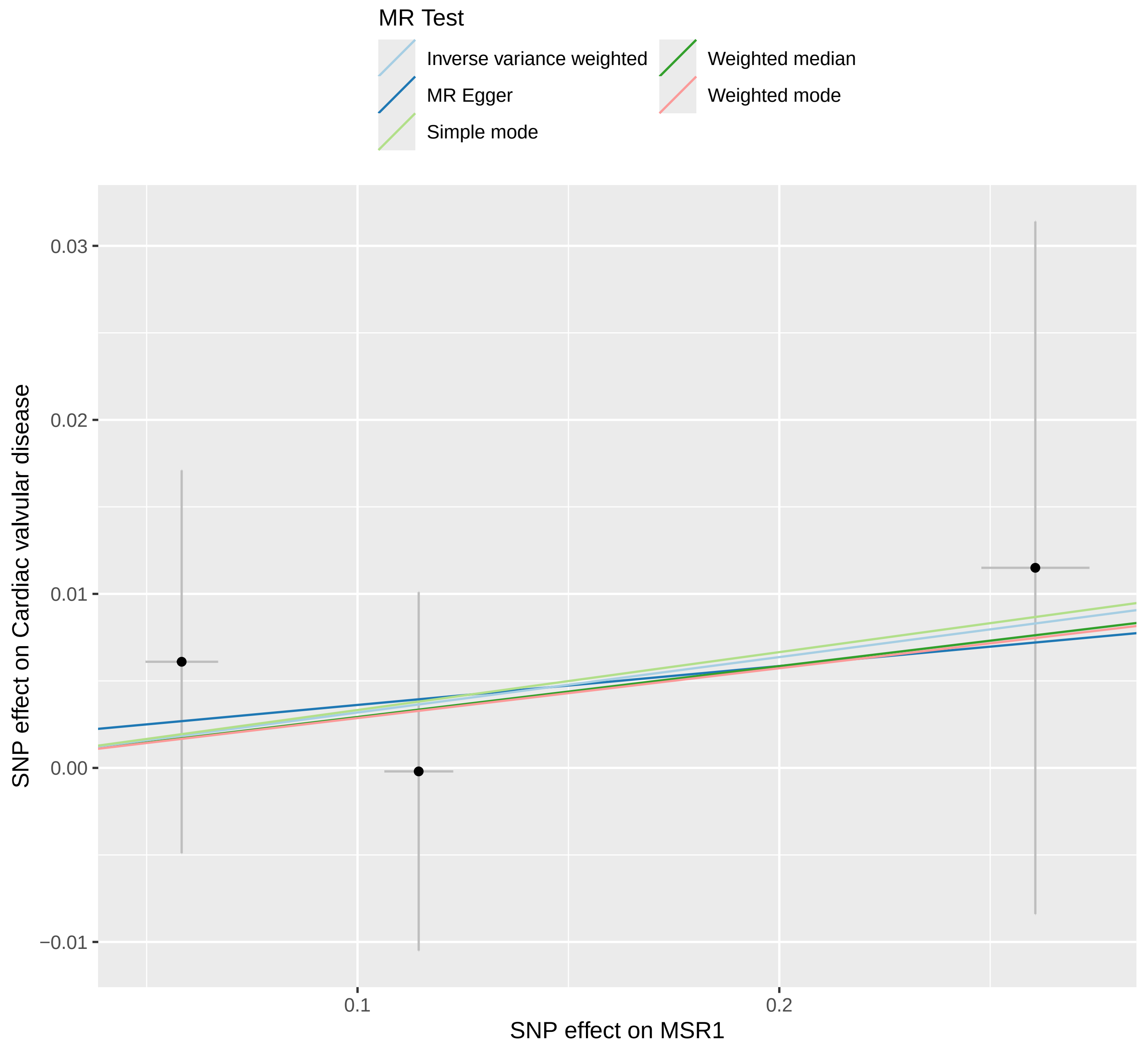
MR Test
Inverse variance weighted
MR Egger
Simple mode
Weighted median
Weighted mode
SNP effect on Cardiac valvular disease
0.03
0.02
0.01
0.00
−0.01
0.1
0.2
SNP effect on MSR1

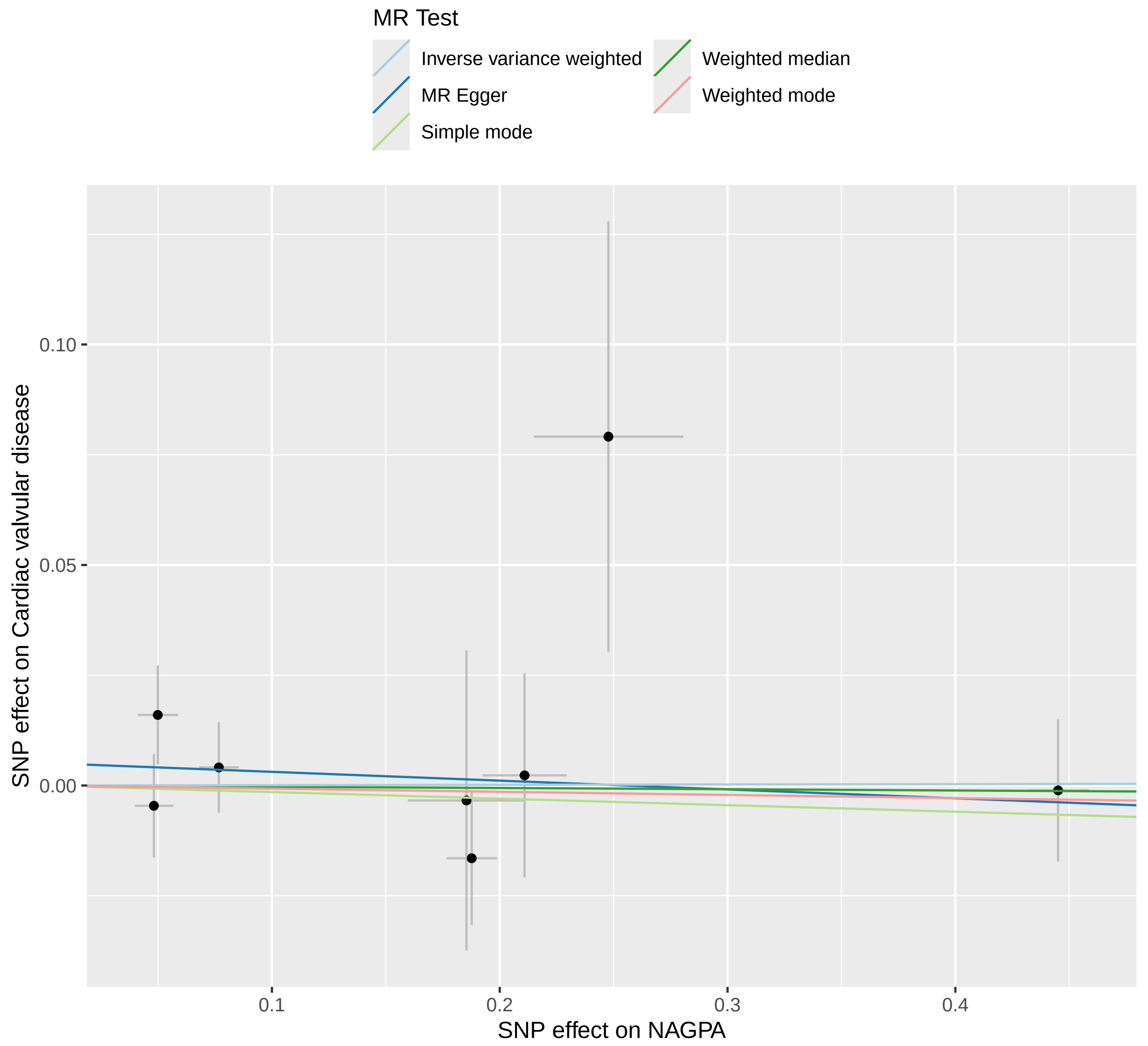
MR Test
Inverse variance weighted
MR Egger
Simple mode
Weighted median
Weighted mode
SNP effect on Cardiac valvular disease
0.10
0.05
0.00
0.1
0.2
0.3
0.4
SNP effect on NAGPA

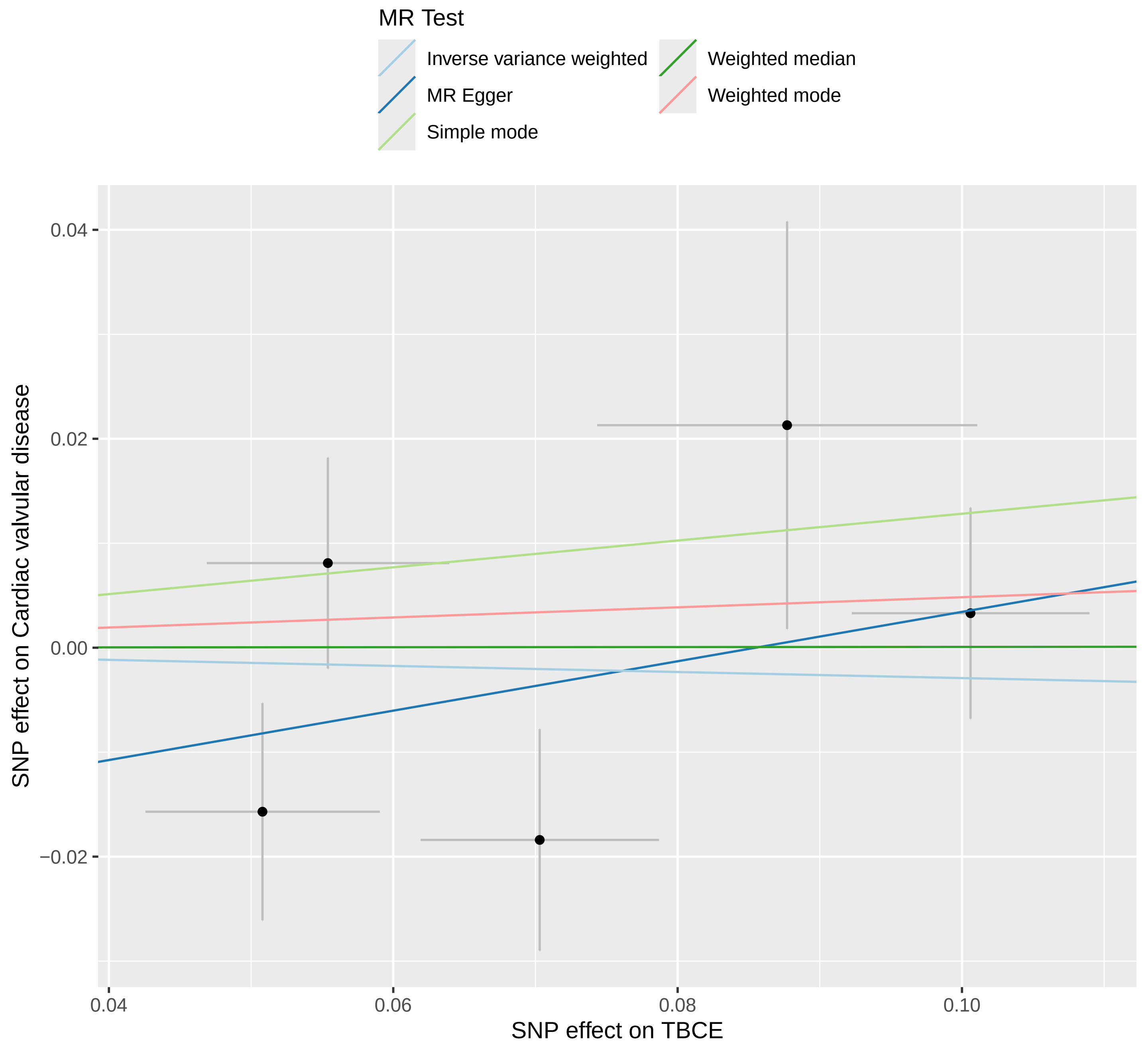

MR Test
Inverse variance weighted
MR Egger
Simple mode
Weighted median
Weighted mode
0.04
0.02
0.00
−0.02
SNP effect on Cardiac valvular disease
0.04
0.06
0.08
0.10
SNP effect on TBCE

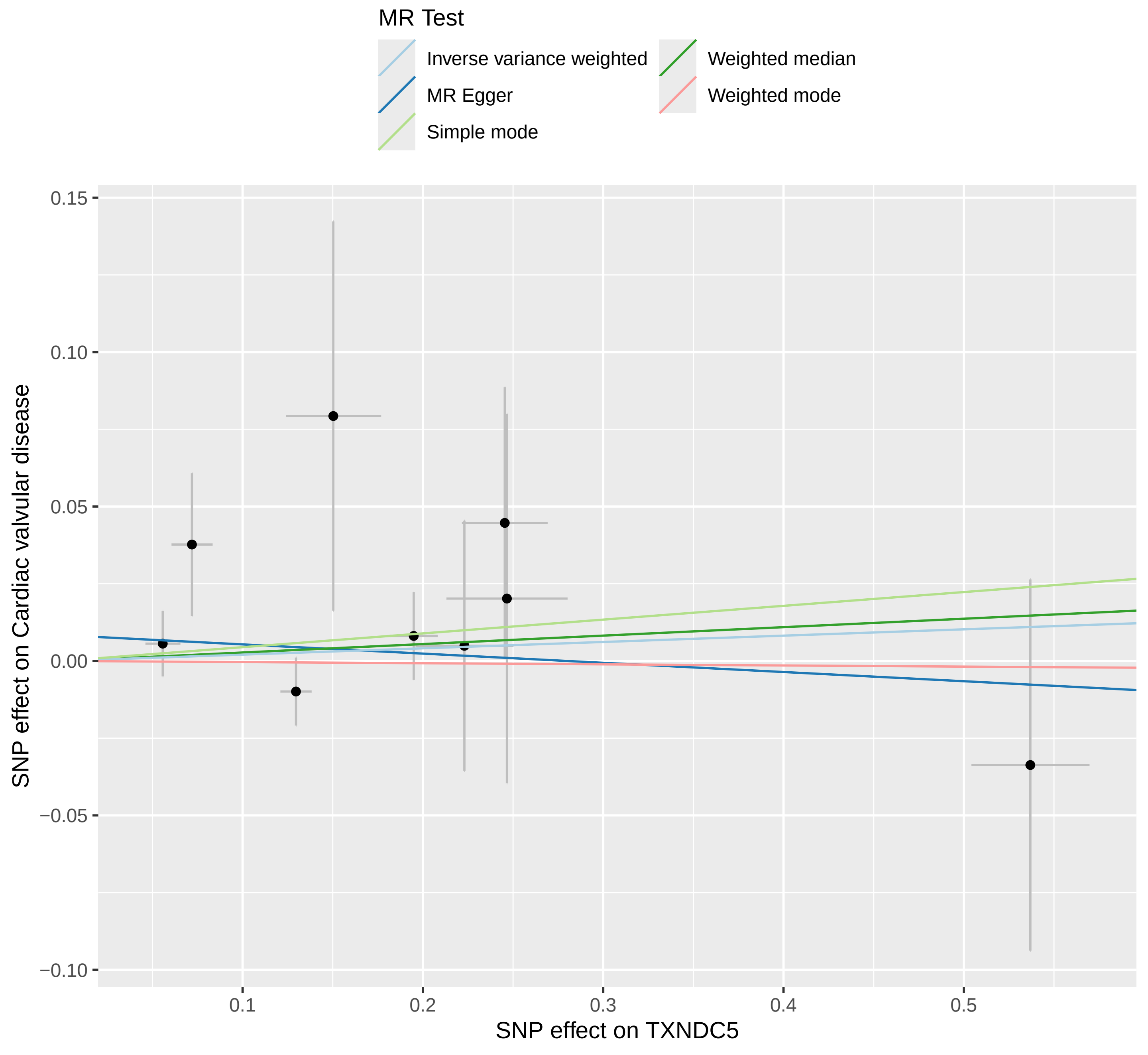
MR Test
Inverse variance weighted
MR Egger
Simple mode
Weighted median
Weighted mode
0.15
0.10
0.05
0.00
−0.05
−0.10
SNP effect on Cardiac valvular disease
0.1
0.2
0.3
0.4
0.5
SNP effect on TXNDC5

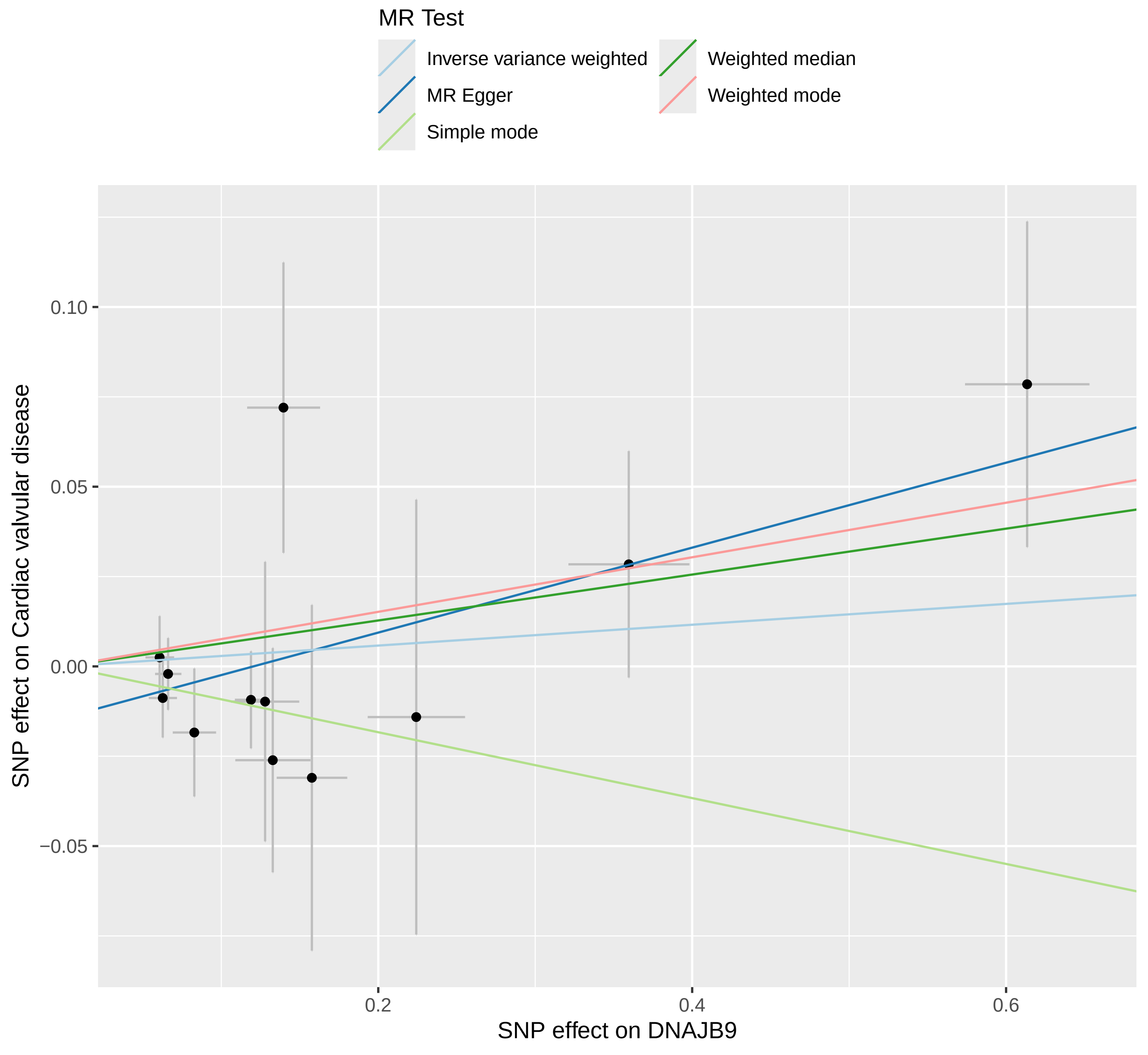
MR Test
Inverse variance weighted
MR Egger
Simple mode
Weighted median
Weighted mode
0.10
0.05
0.00
−0.05
0.2
0.4
0.6
SNP effect on Cardiac valvular disease
SNP effect on DNAJB9

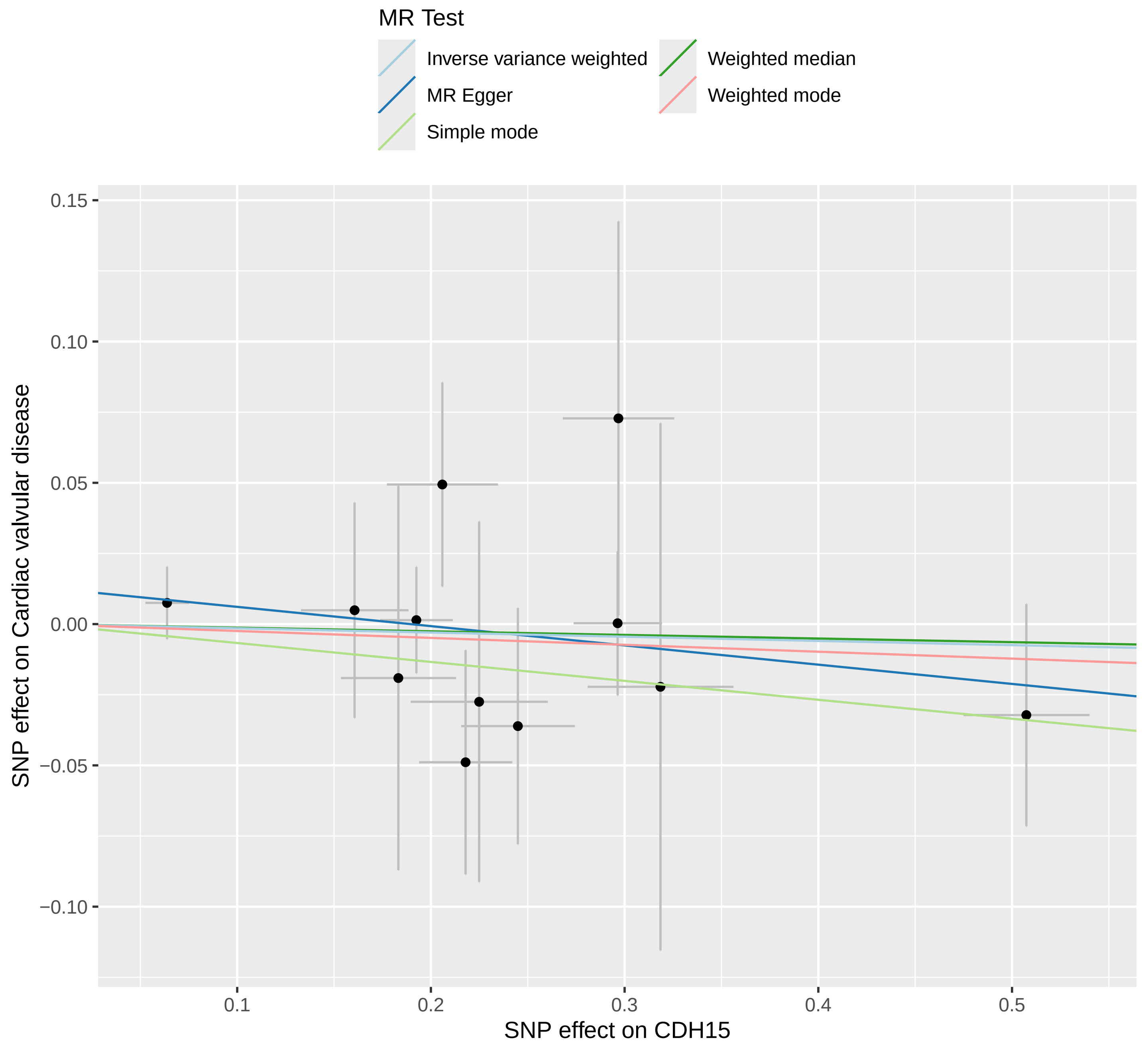
MR Test
Inverse variance weighted
MR Egger
Simple mode
Weighted median
Weighted mode
SNP effect on Cardiac valvular disease
0.15
0.10
0.05
0.00
−0.05
−0.10
0.1
0.2
0.3
0.4
0.5
SNP effect on CDH15

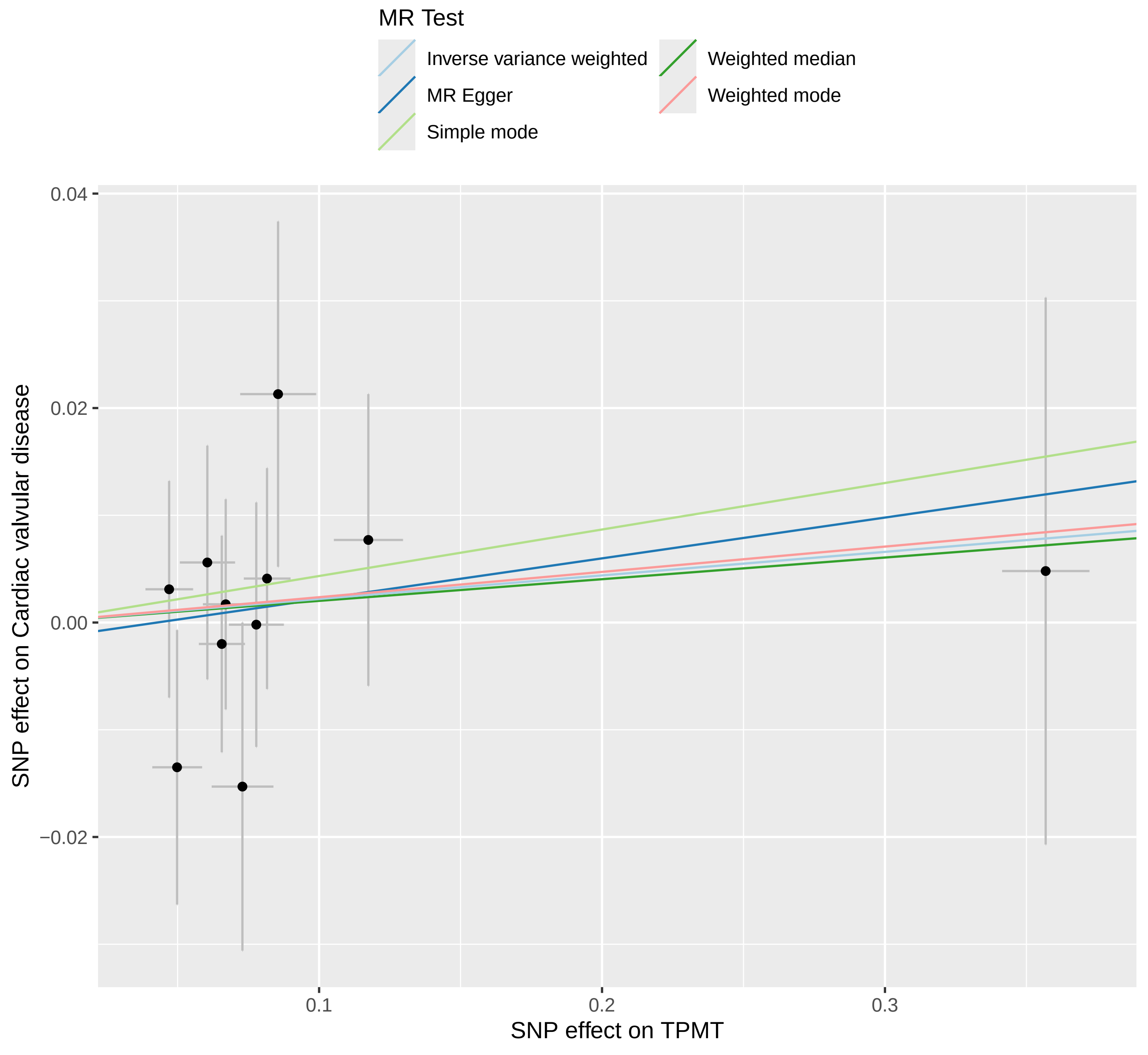

MR Test
Inverse variance weighted
MR Egger
Simple mode
Weighted median
Weighted mode
0.04
0.02
0.00
−0.02
0.1
0.2
0.3
SNP effect on Cardiac valvular disease
SNP effect on TPMT

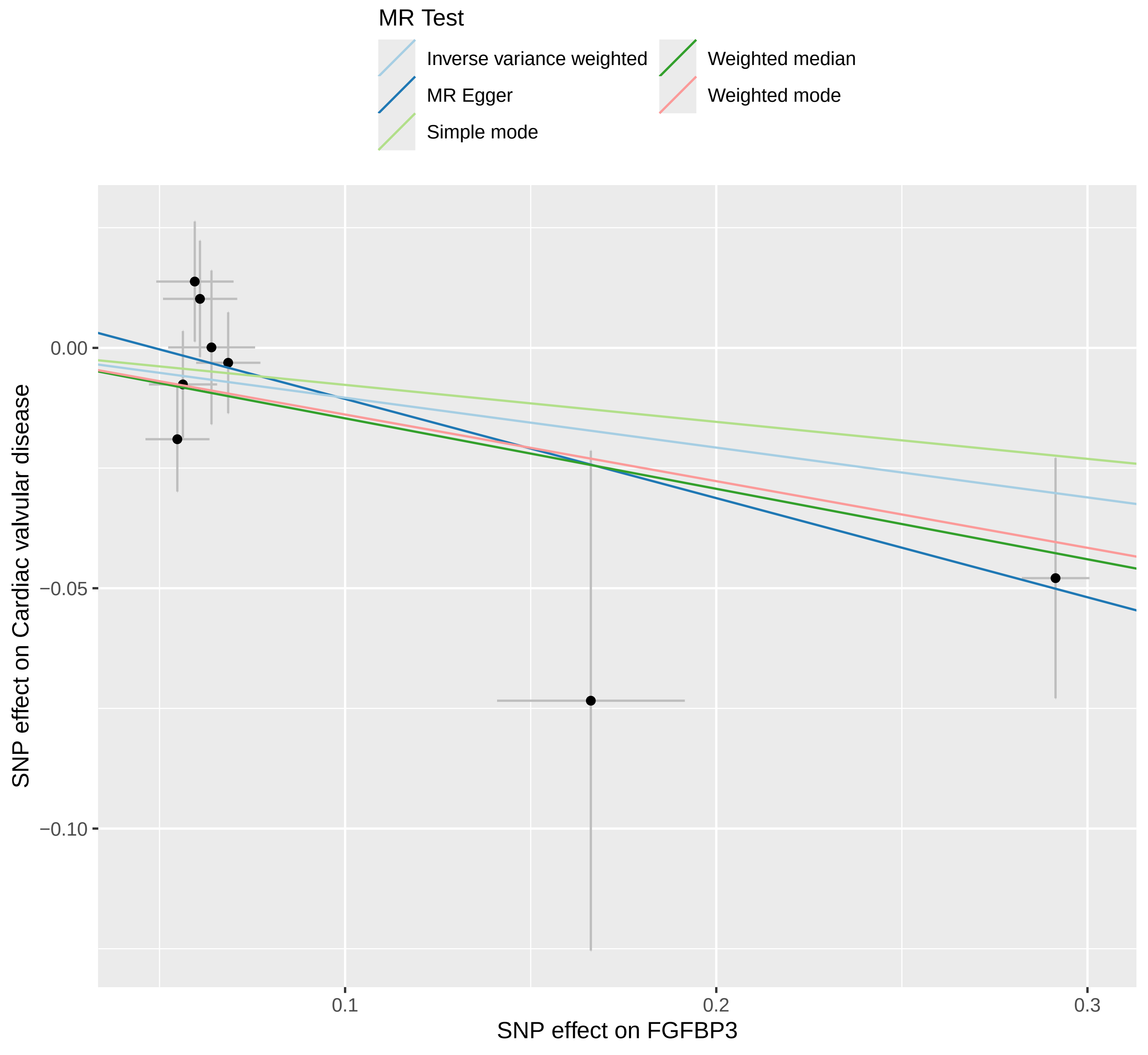

MR Test
Inverse variance weighted
MR Egger
Simple mode
Weighted median
Weighted mode
0.00
−0.05
−0.10
0.1
0.2
0.3
SNP effect on Cardiac valvular disease
SNP effect on FGFBP3

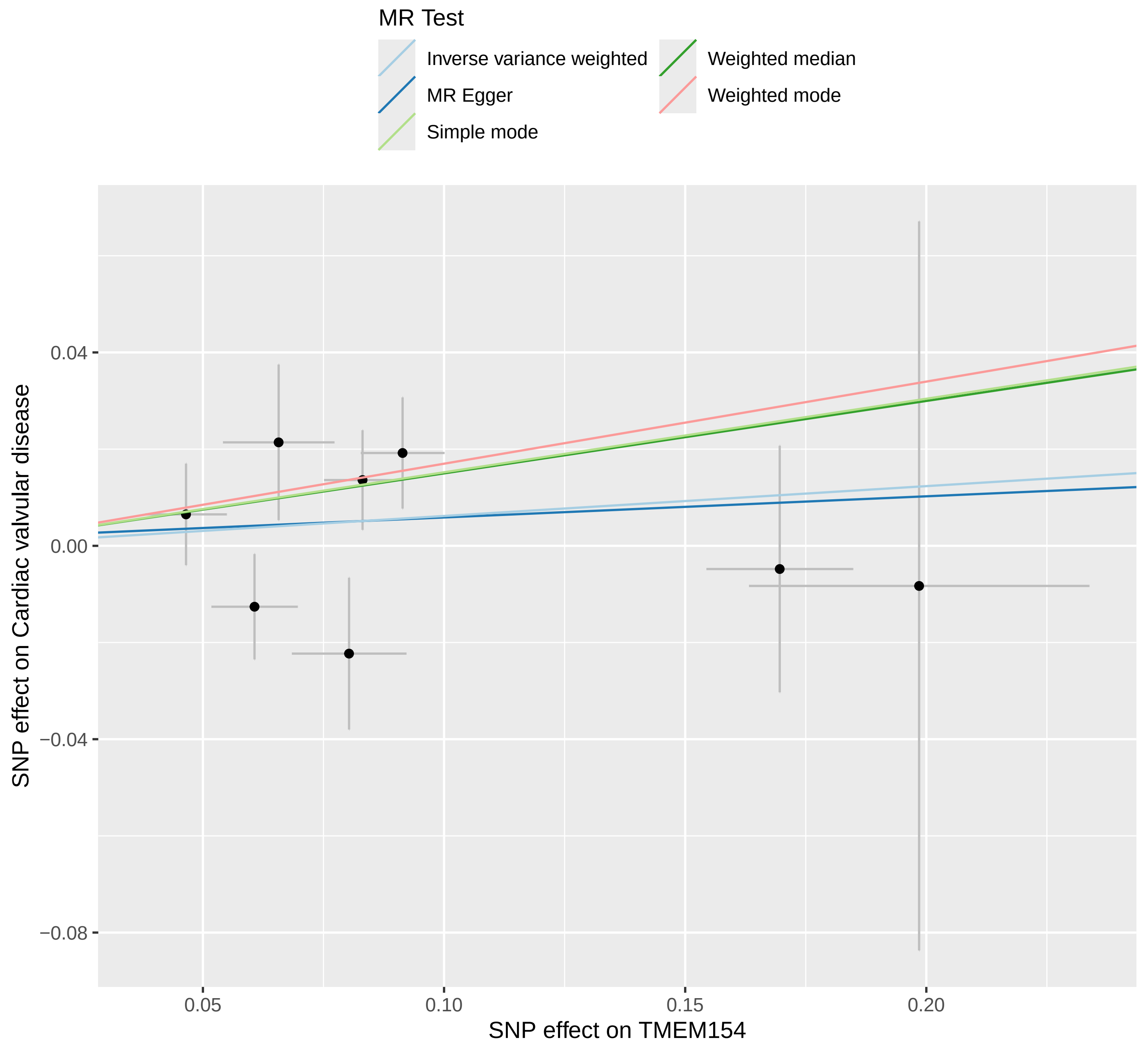
MR Test
Inverse variance weighted
MR Egger
Simple mode
Weighted median
Weighted mode
0.04
0.00
−0.04
−0.08
0.05
0.10
0.15
0.20
SNP effect on TMEM154
SNP effect on Cardiac valvular disease

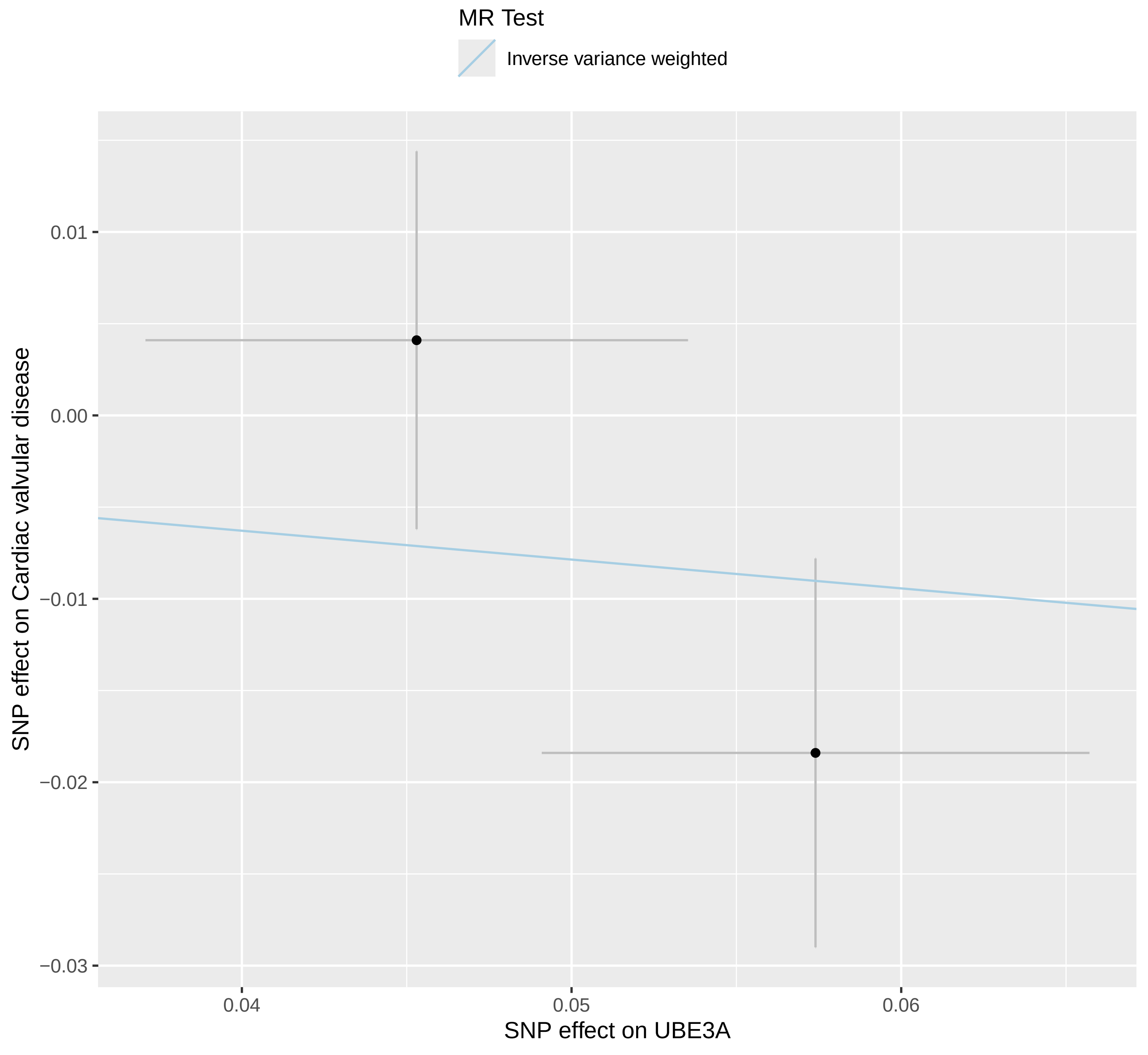

MR Test
Inverse variance weighted
0.01
0.00
−0.01
−0.02
−0.03
SNP effect on Cardiac valvular disease
0.04
0.05
0.06
SNP effect on UBE3A

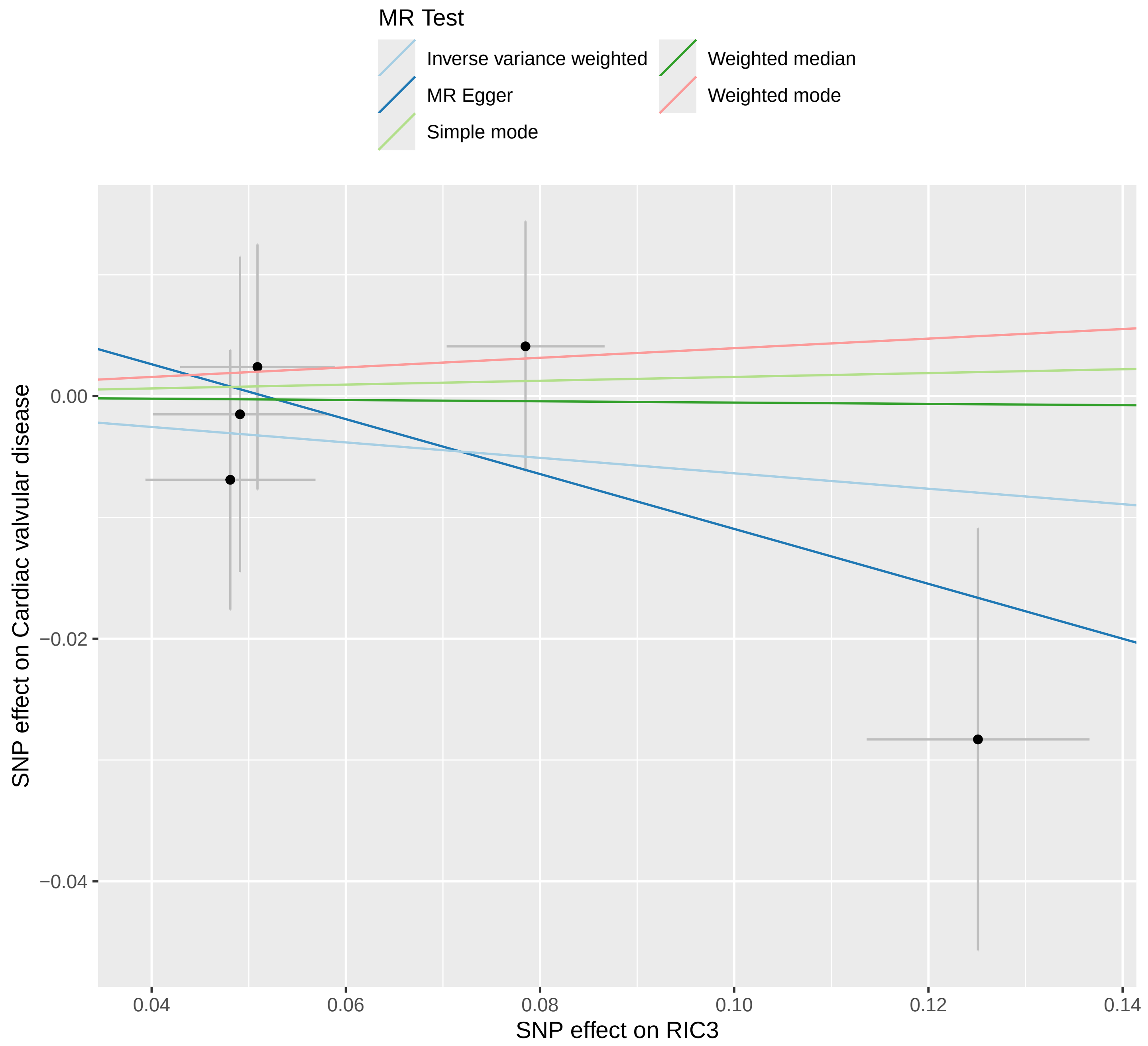

MR Test
Inverse variance weighted
MR Egger
Simple mode
Weighted median
Weighted mode
SNP effect on Cardiac valvular disease
0.00
−0.02
−0.04
0.04
0.06
0.08
0.10
0.12
0.14
SNP effect on RIC3

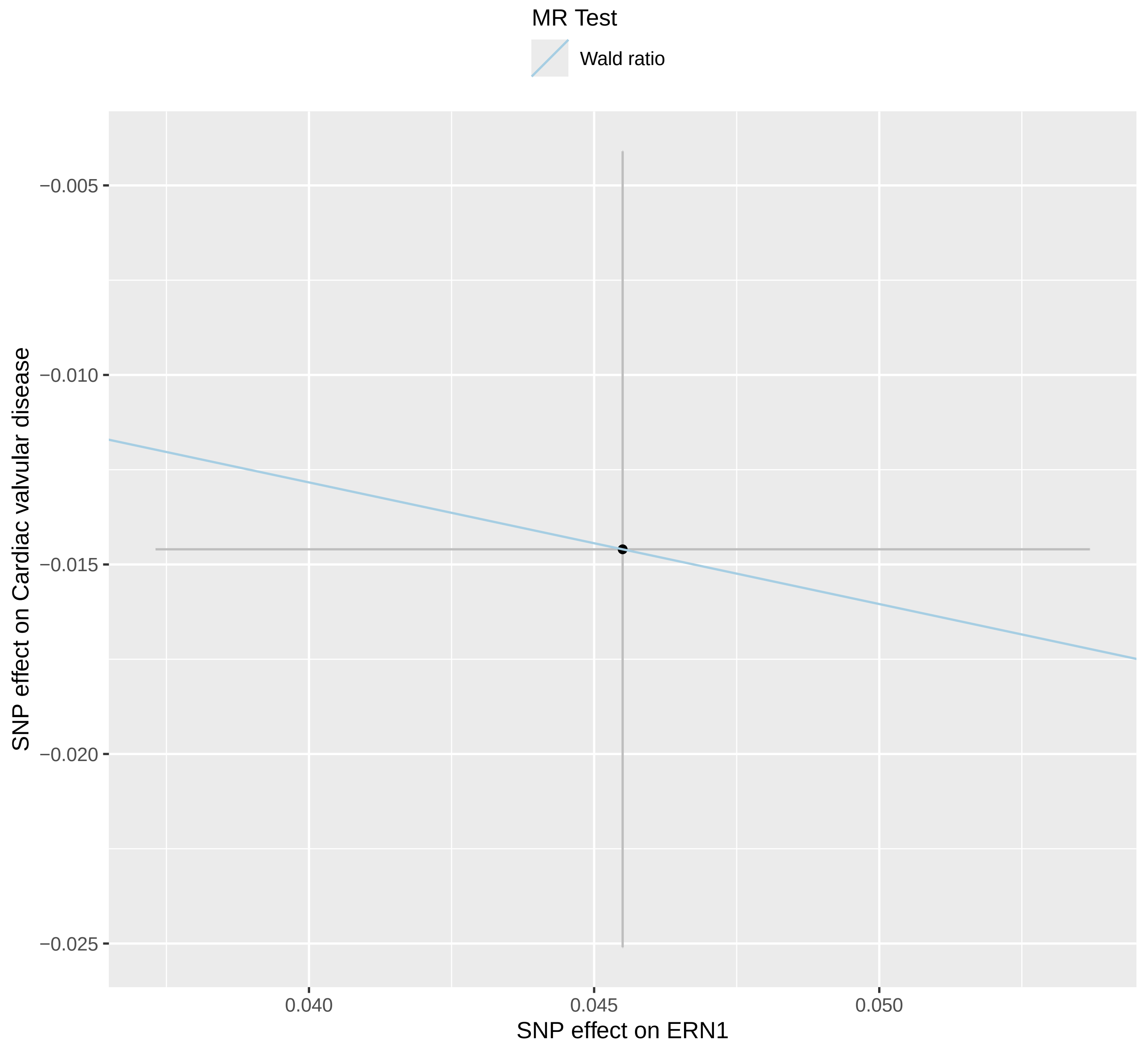
MR Test
Wald ratio
−0.005
−0.010
−0.015
−0.020
−0.025
SNP effect on Cardiac valvular disease
0.040
0.045
0.050
SNP effect on ERN1

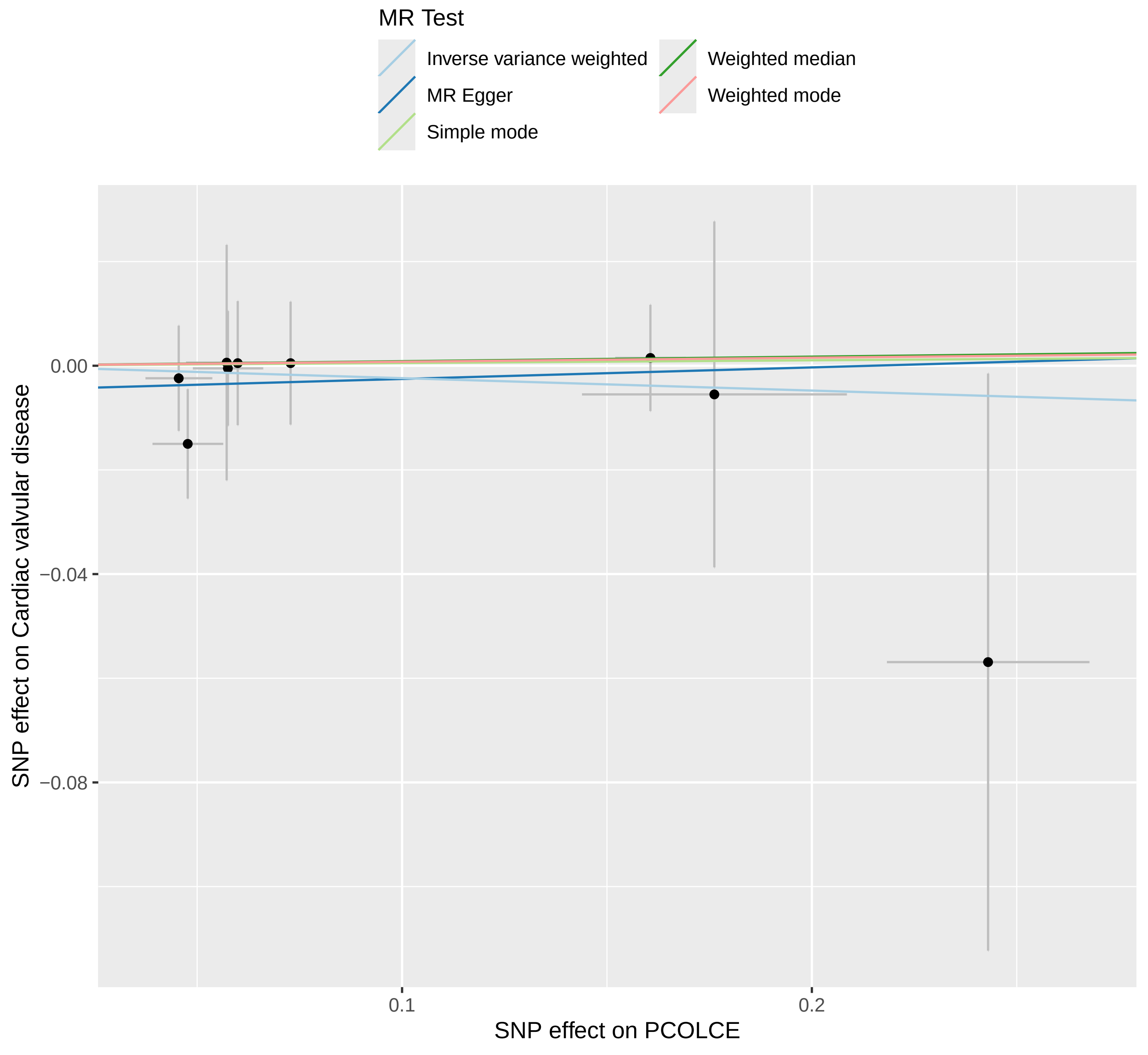

MR Test
Inverse variance weighted
MR Egger
Simple mode
Weighted median
Weighted mode
0.00
−0.04
−0.08
SNP effect on Cardiac valvular disease
0.1
0.2
SNP effect on PCOLCE

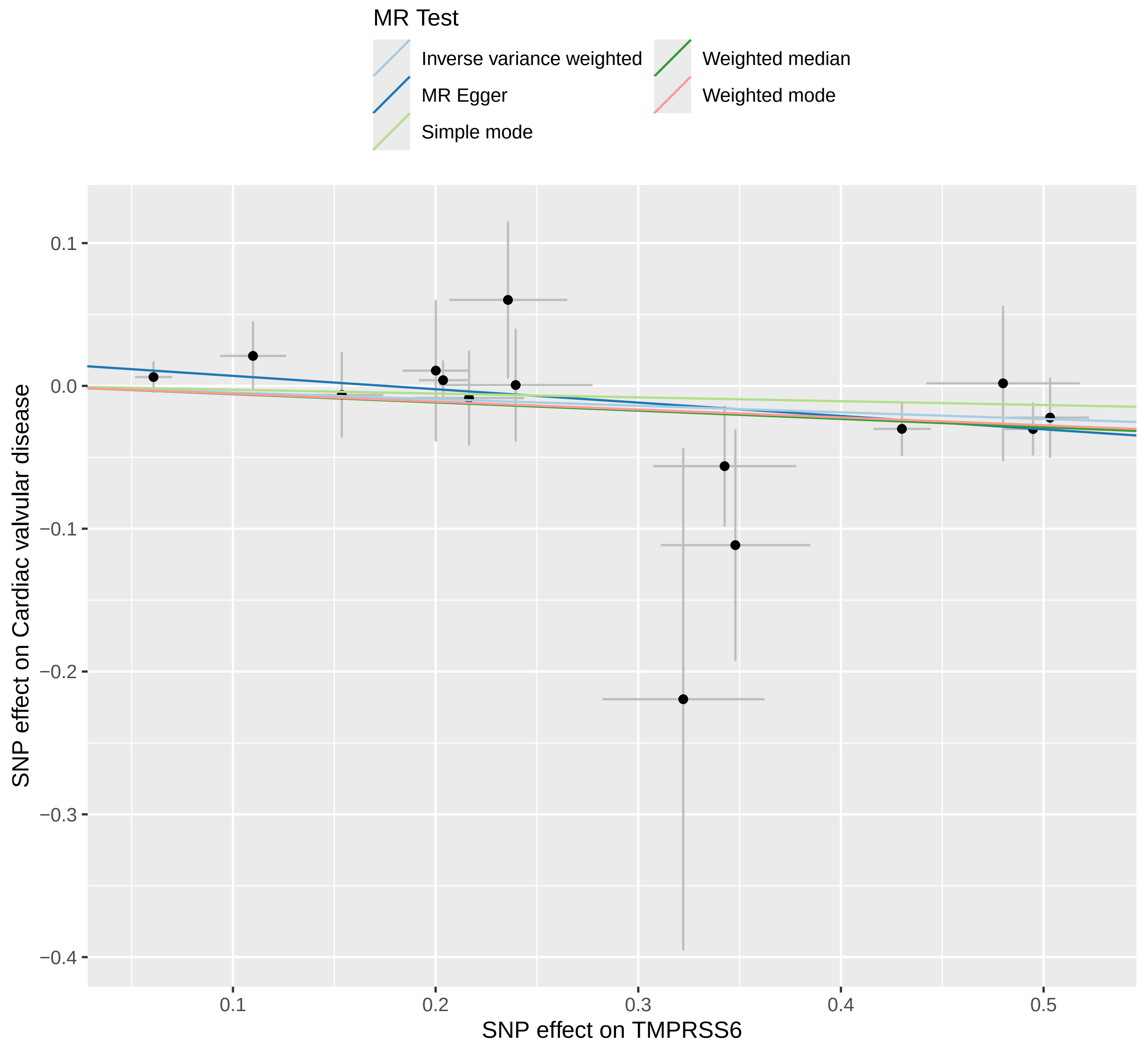
MR Test
Inverse variance weighted
MR Egger
Simple mode
Weighted median
Weighted mode
0.1
0.0
−0.1
−0.2
−0.3
−0.4
SNP effect on Cardiac valvular disease
0.1
0.2
0.3
0.4
0.5
SNP effect on TMPRSS6

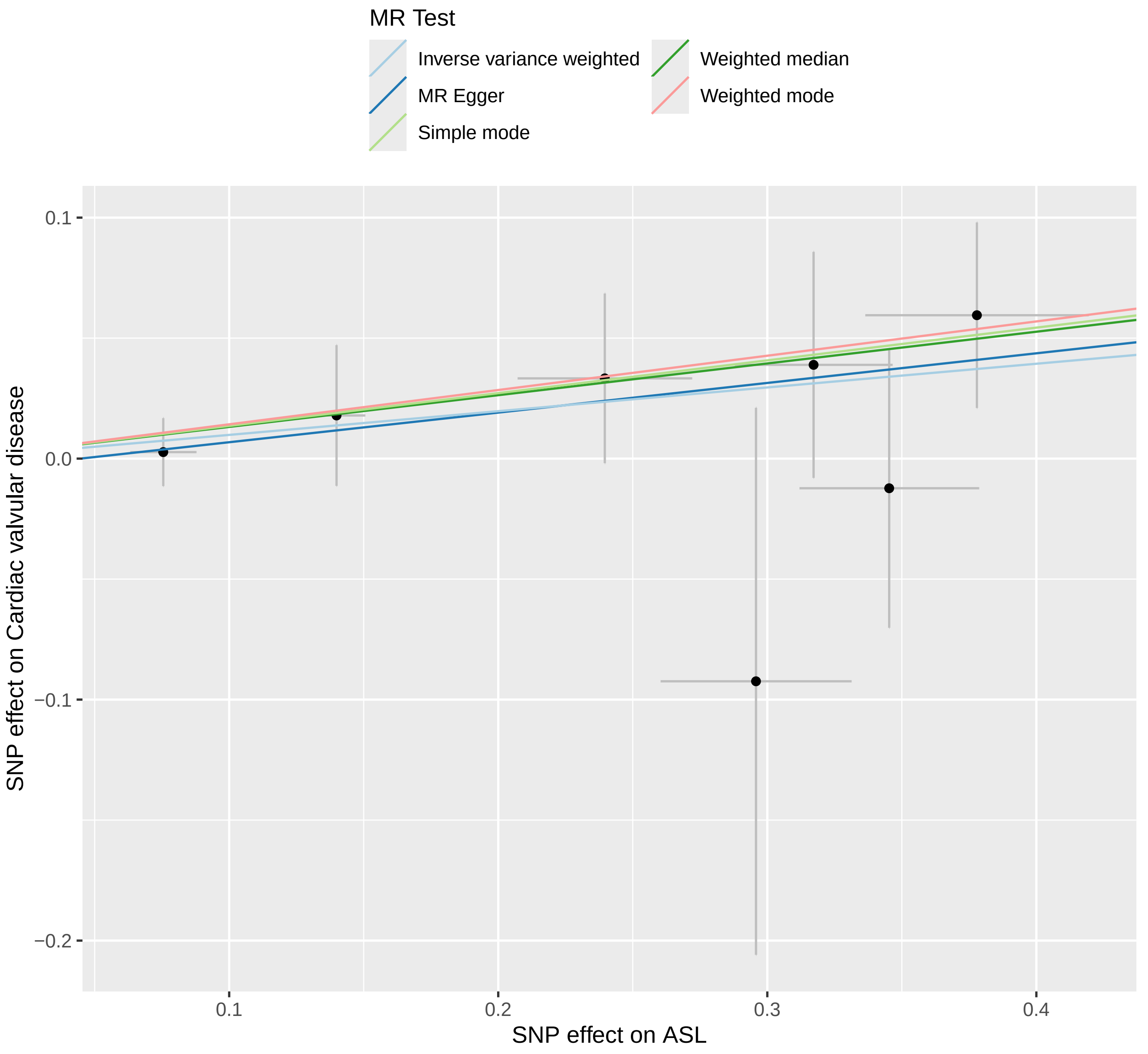
MR Test
Inverse variance weighted
MR Egger
Simple mode
Weighted median
Weighted mode
0.1
0.0
−0.1
−0.2
SNP effect on Cardiac valvular disease
0.1
0.2
0.3
0.4
SNP effect on ASL

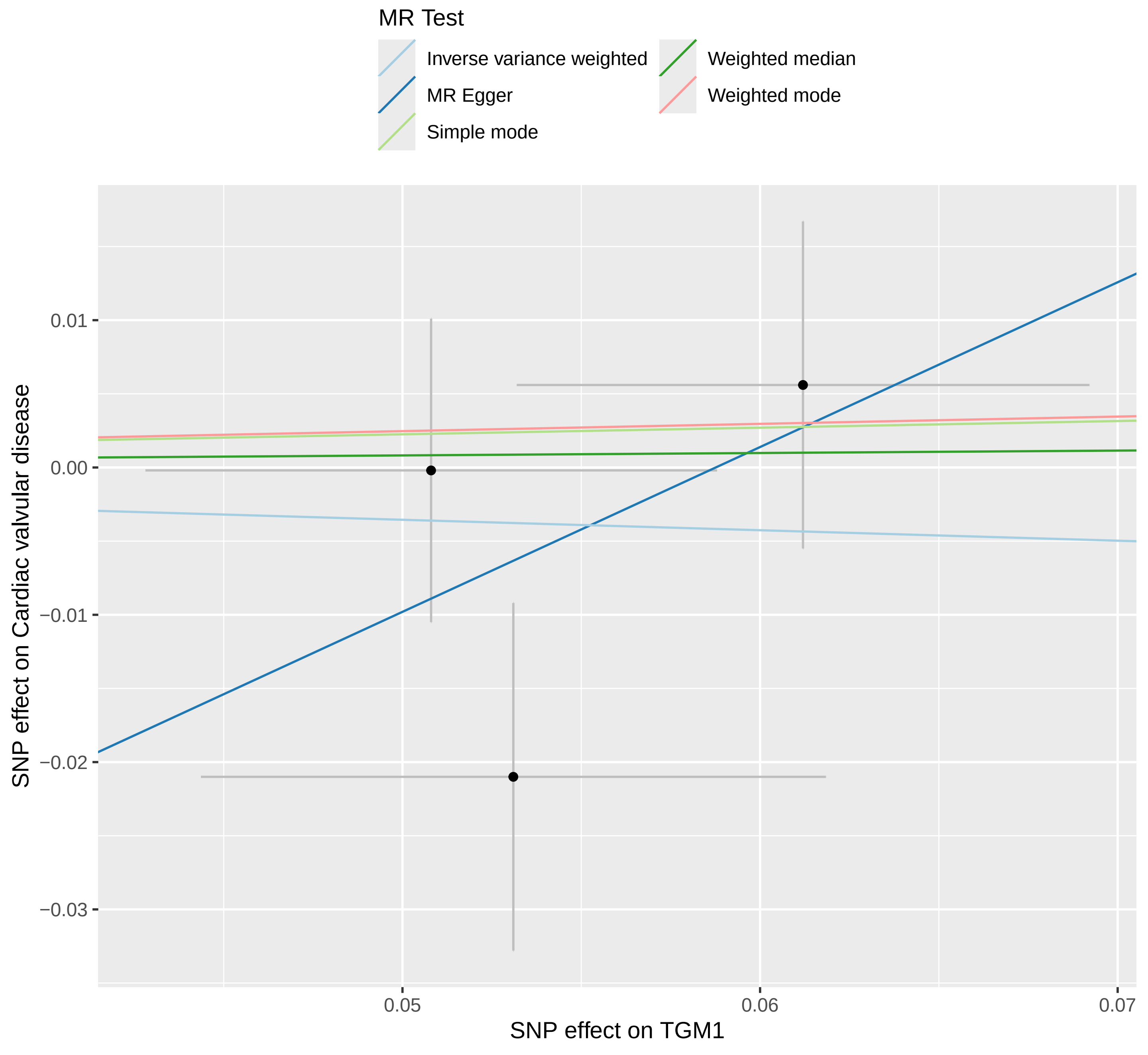

MR Test
Inverse variance weighted
MR Egger
Simple mode
Weighted median
Weighted mode
0.01
0.00
−0.01
−0.02
−0.03
SNP effect on Cardiac valvular disease
0.05
0.06
0.07
SNP effect on TGM1

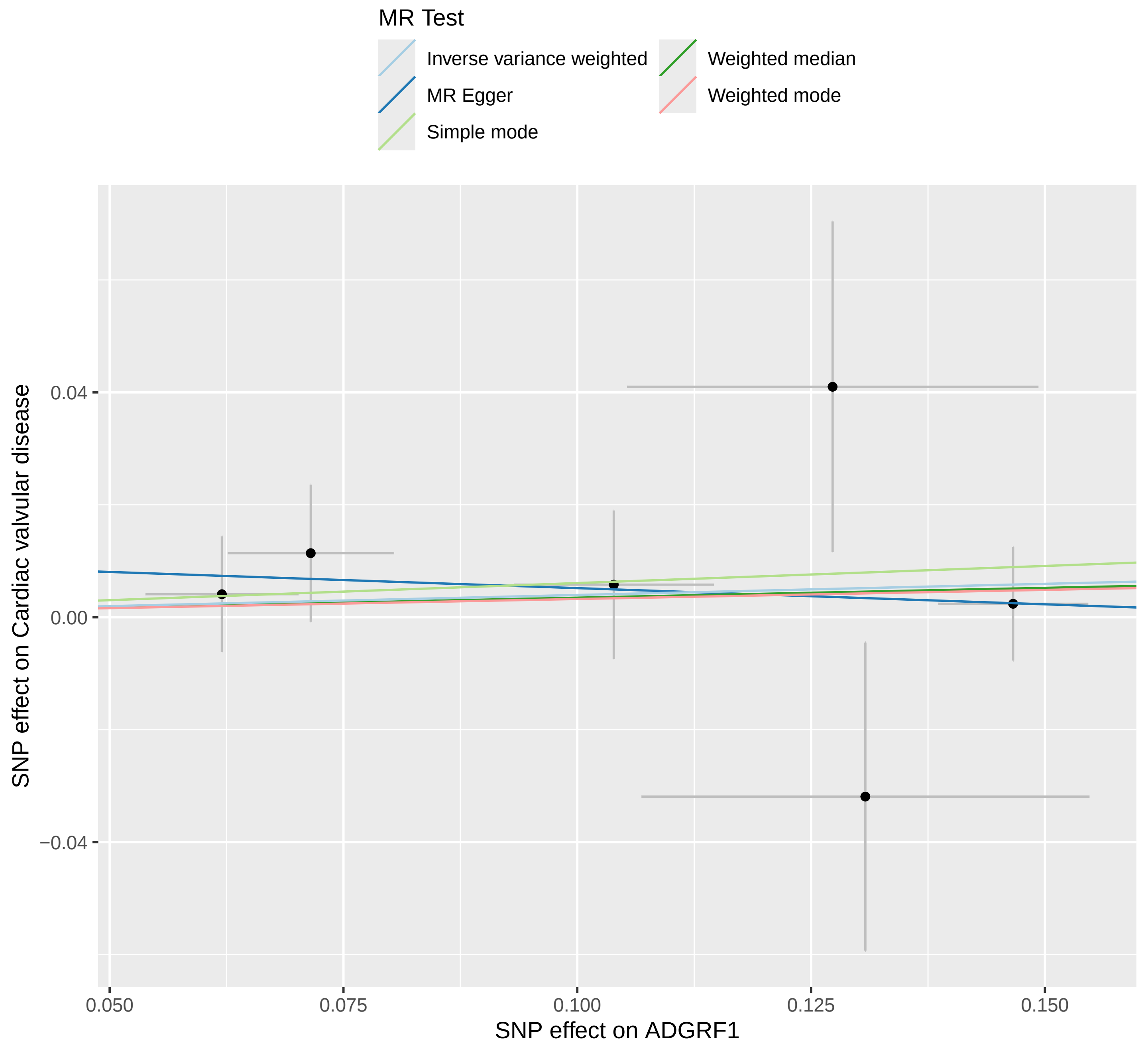
MR Test
Inverse variance weighted
MR Egger
Simple mode
Weighted median
Weighted mode
0.04
0.00
−0.04
0.050
0.075
0.100
0.125
0.150
SNP effect on ADGRF1
SNP effect on Cardiac valvular disease

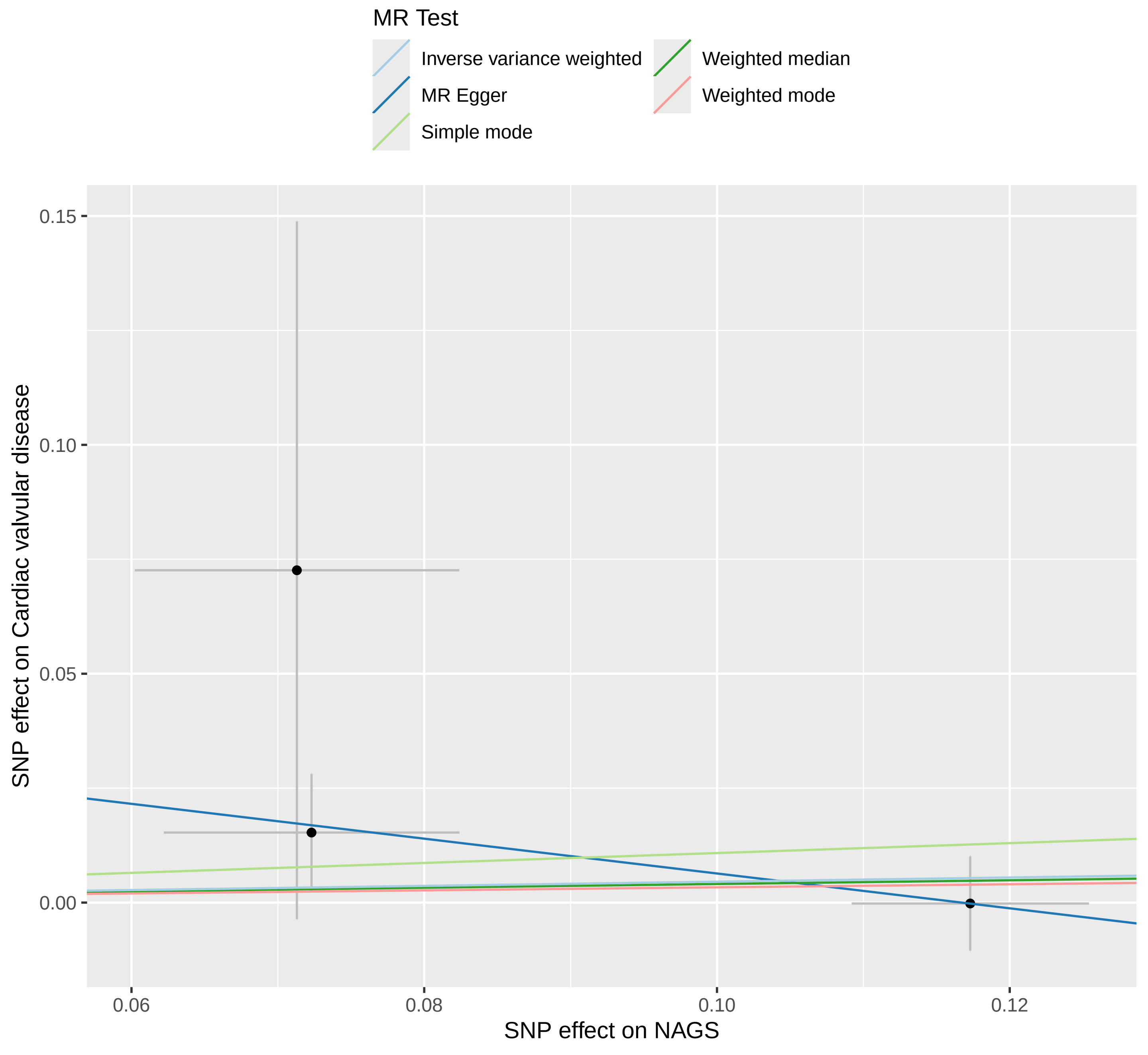

MR Test
Inverse variance weighted
MR Egger
Simple mode
Weighted median
Weighted mode
0.15
0.10
0.05
0.00
SNP effect on Cardiac valvular disease
0.06
0.08
0.10
0.12
SNP effect on NAGS

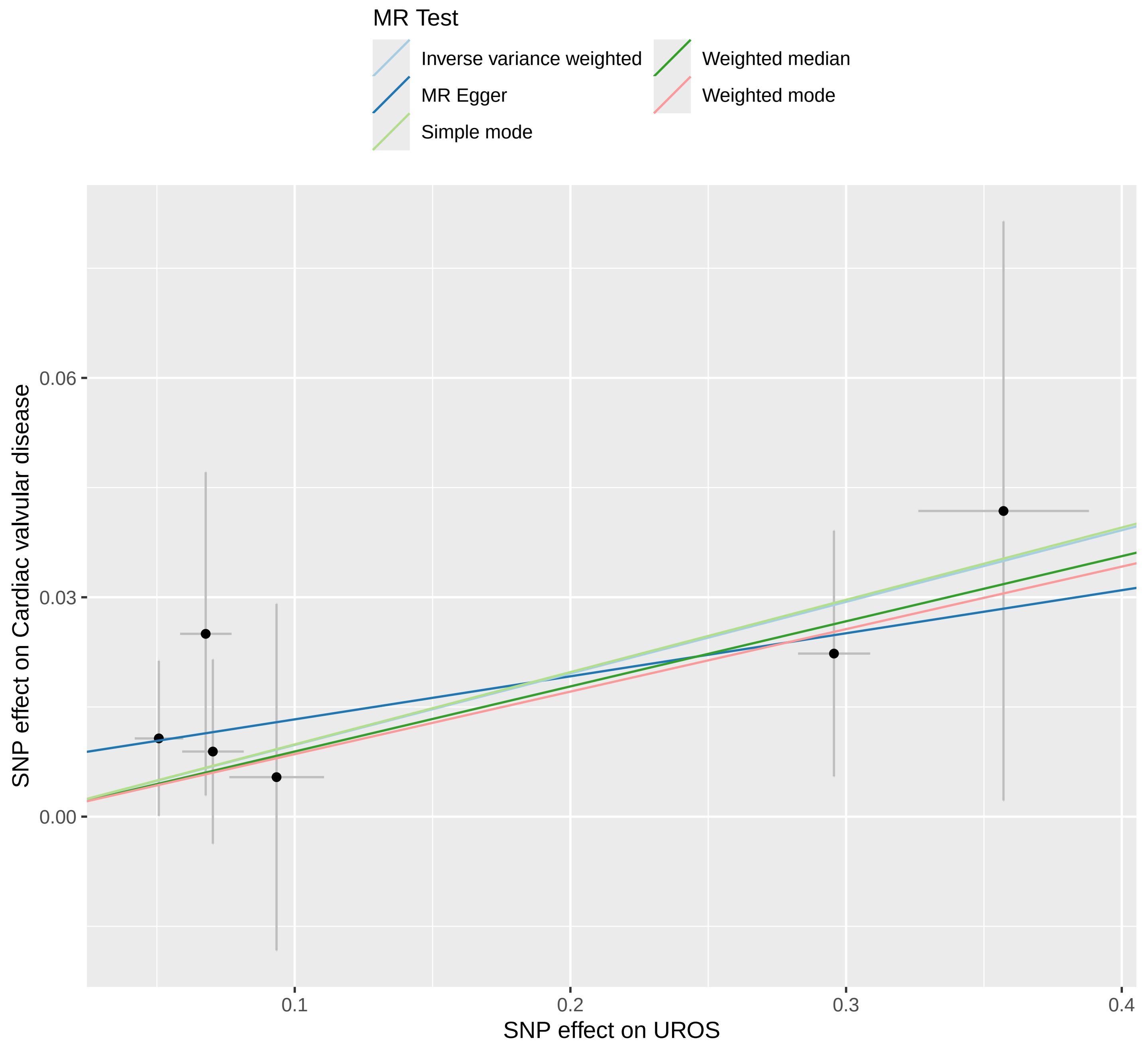

MR Test
Inverse variance weighted
MR Egger
Simple mode
Weighted median
Weighted mode
0.06
0.03
0.00
0.1
0.2
0.3
0.4
SNP effect on Cardiac valvular disease
SNP effect on UROS

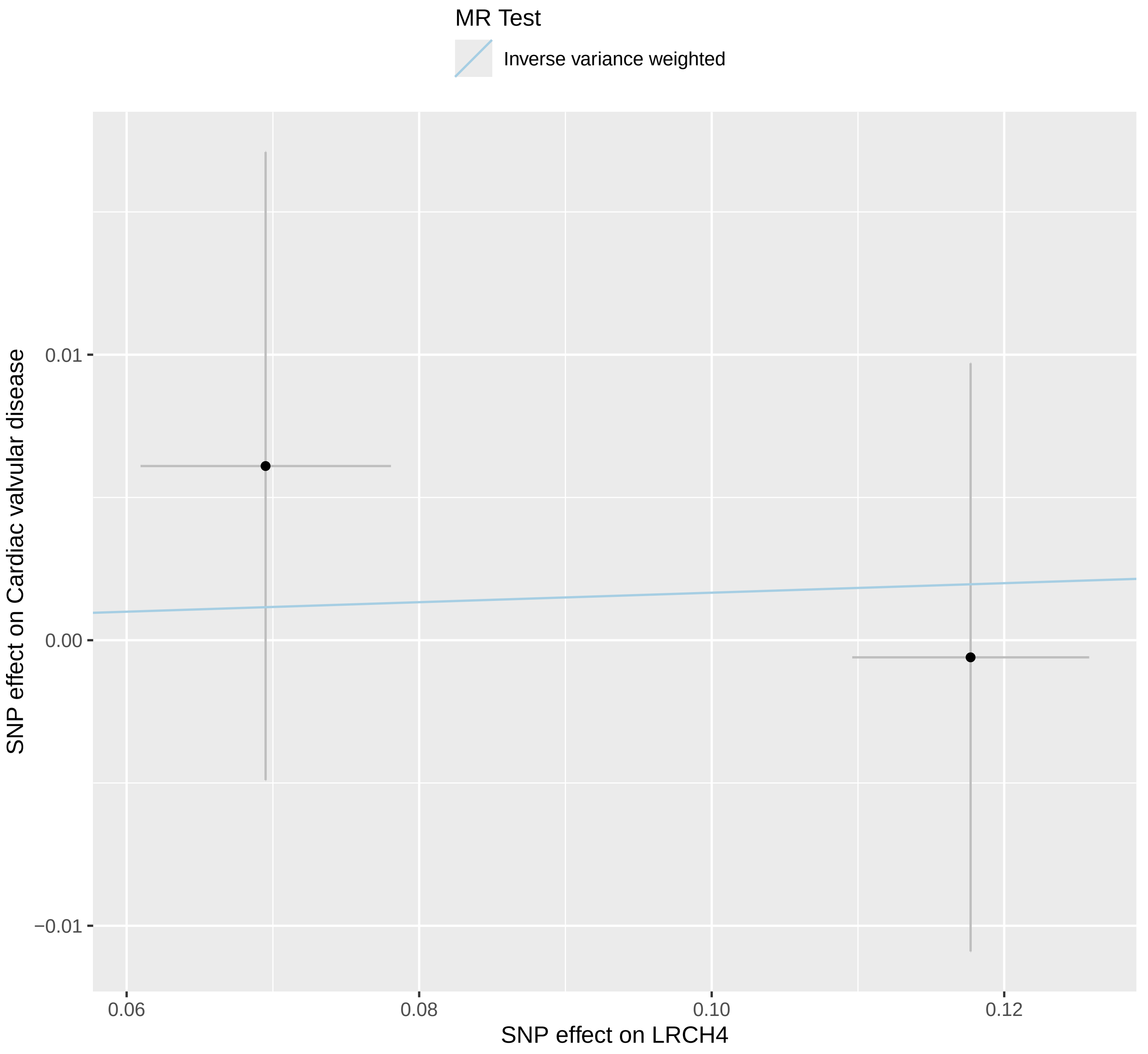

MR Test
Inverse variance weighted
SNP effect on Cardiac valvular disease
0.01
0.00
−0.01
0.06
0.08
0.10
0.12
SNP effect on LRCH4

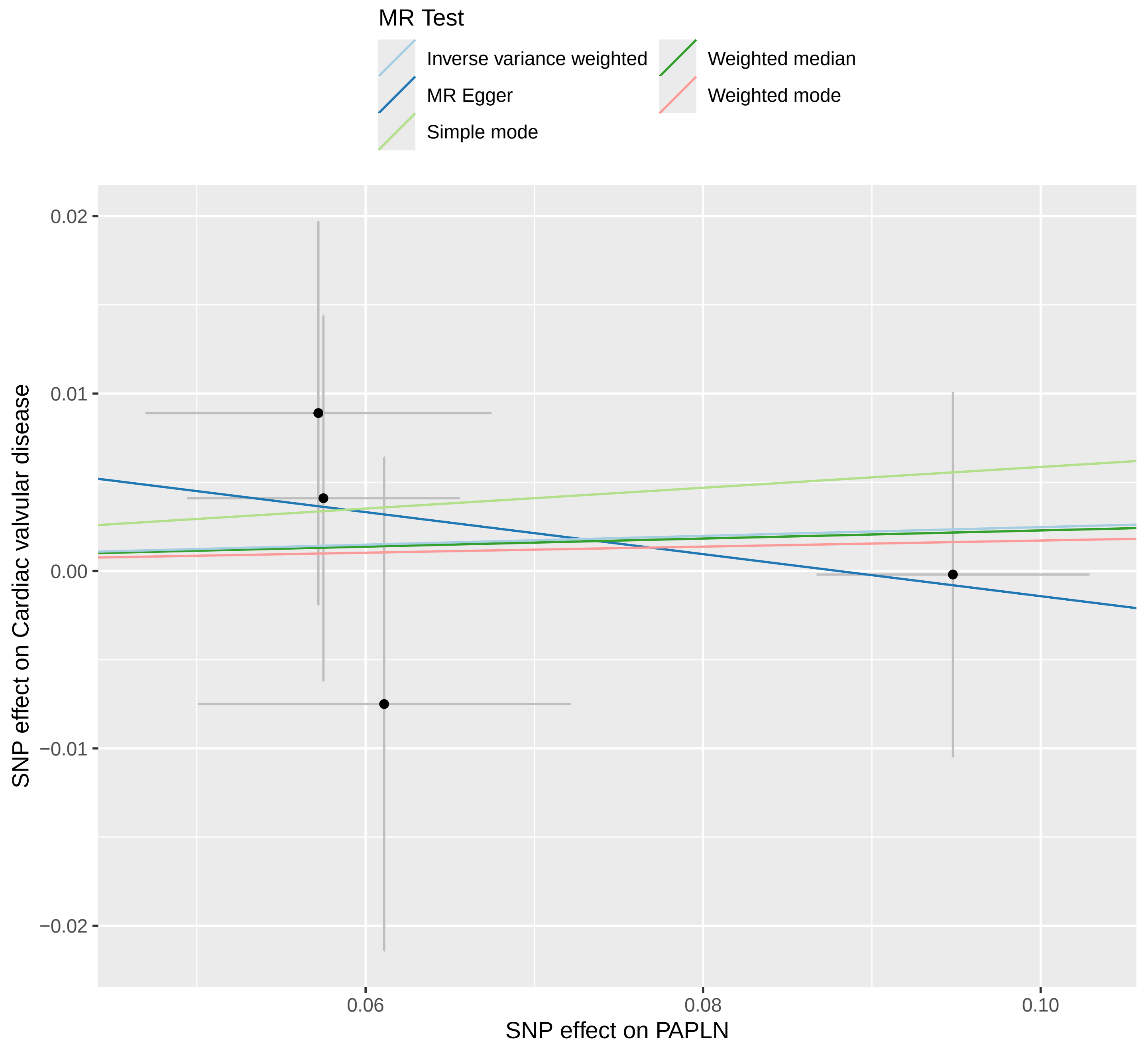

MR Test
Inverse variance weighted
MR Egger
Simple mode
Weighted median
Weighted mode
0.02
0.01
0.00
−0.01
−0.02
SNP effect on Cardiac valvular disease
0.06
0.08
0.10
SNP effect on PAPLN

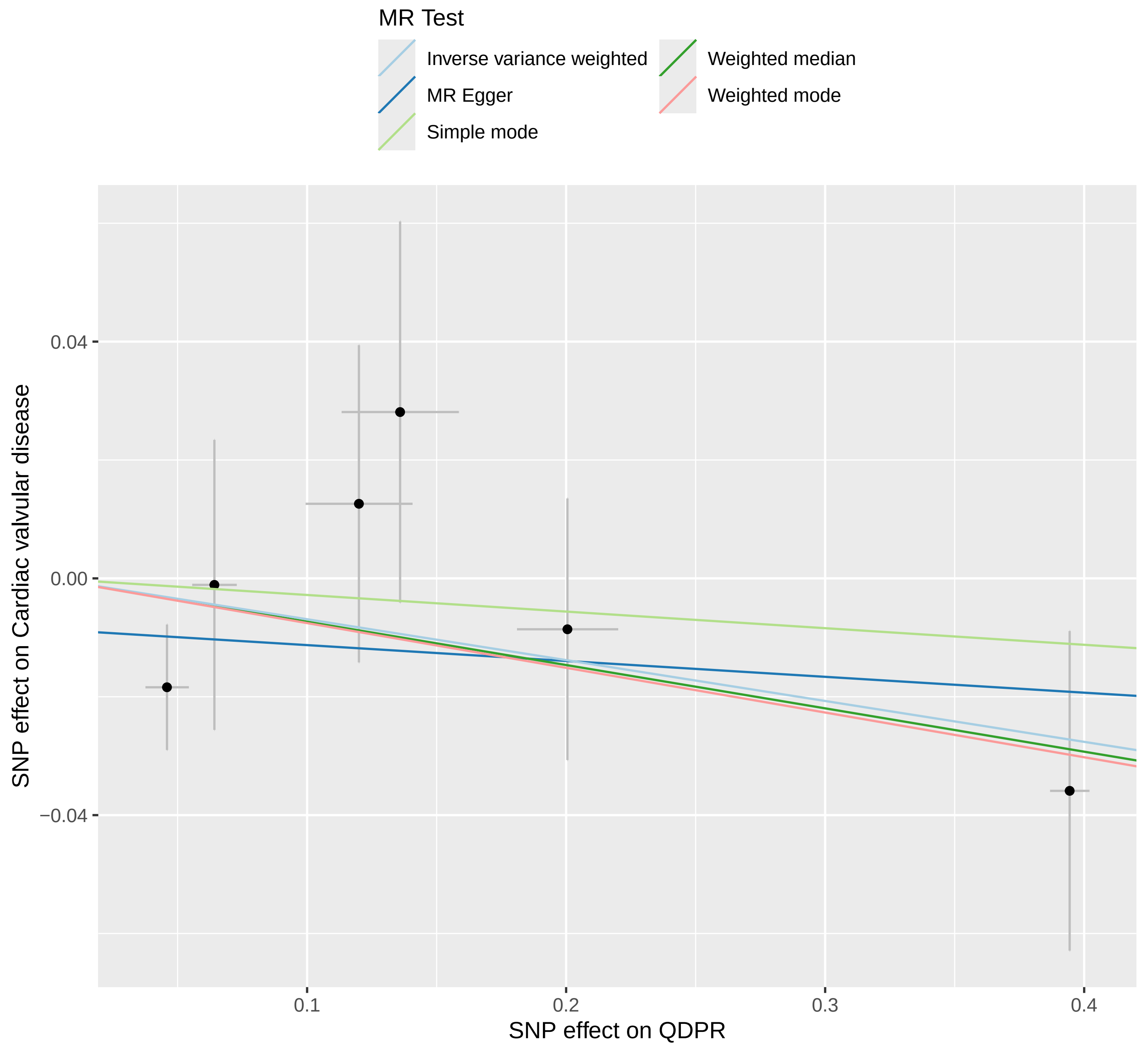

MR Test
Inverse variance weighted
MR Egger
Simple mode
Weighted median
Weighted mode
0.04
0.00
−0.04
SNP effect on Cardiac valvular disease
0.1
0.2
0.3
0.4
SNP effect on QDPR

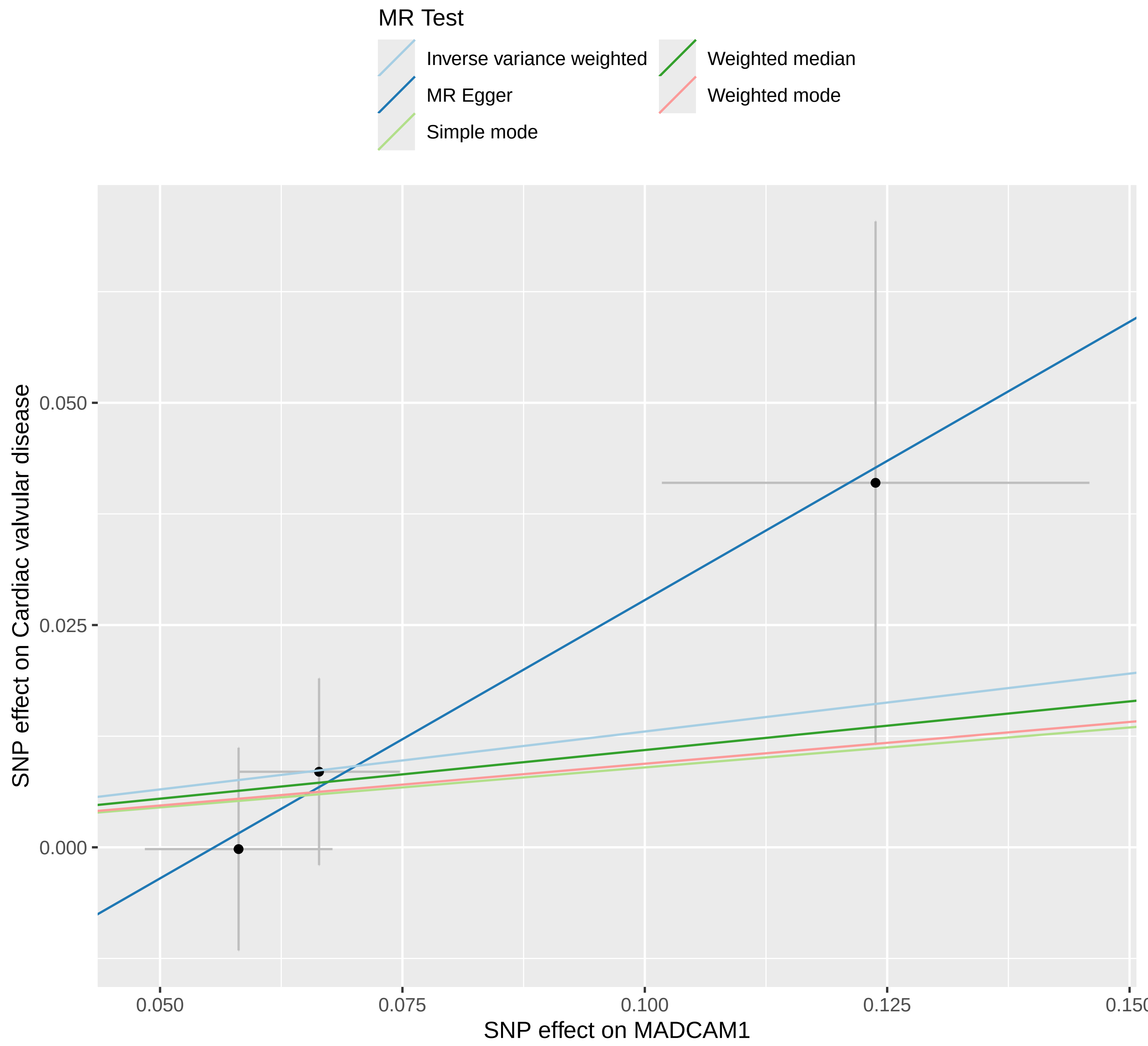

MR Test
Inverse variance weighted
MR Egger
Simple mode
Weighted median
Weighted mode
SNP effect on Cardiac valvular disease
0.050
0.025
0.000
0.050
0.075
0.100
0.125
0.150
SNP effect on MADCAM1

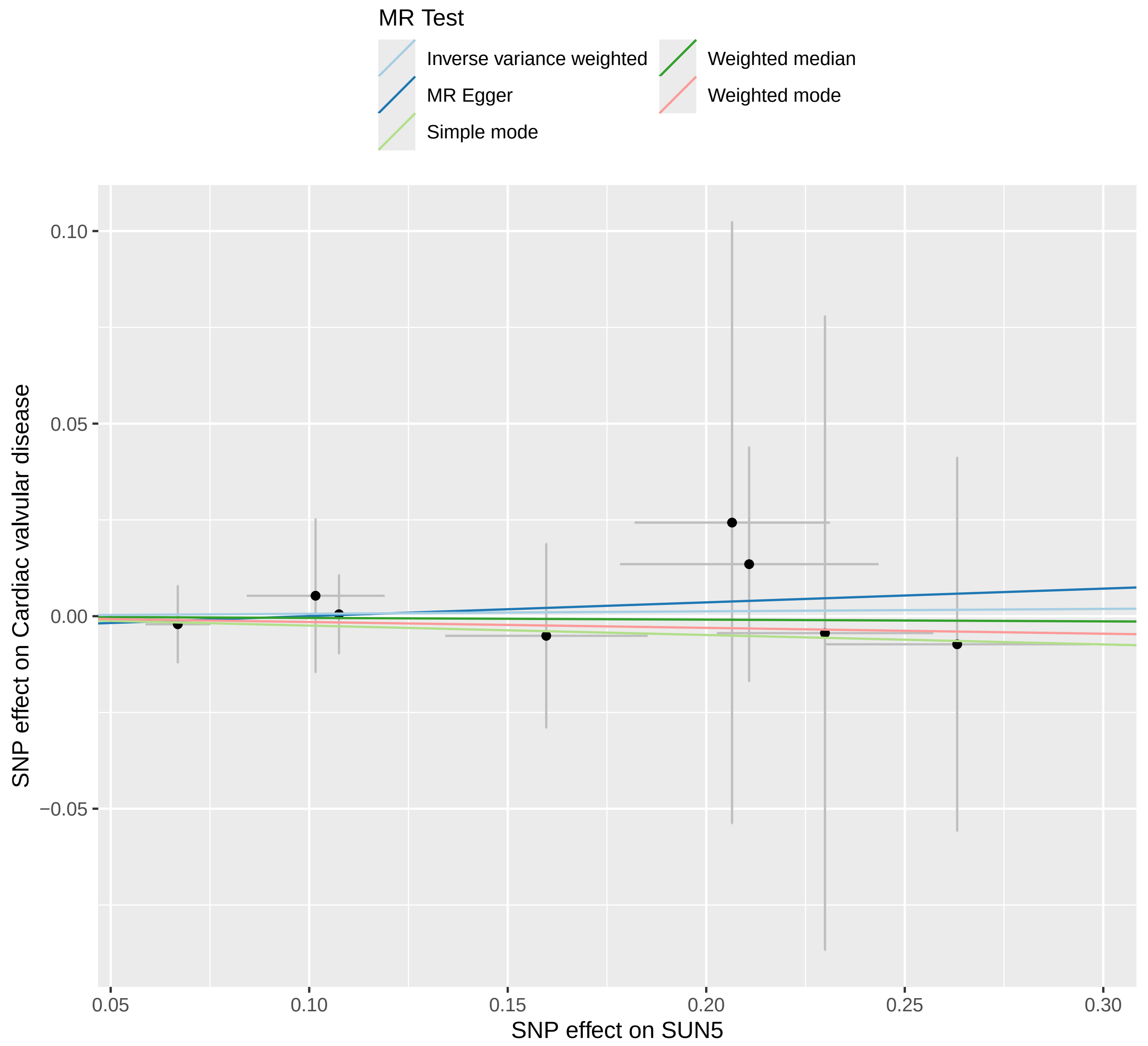
MR Test
Inverse variance weighted
MR Egger
Simple mode
Weighted median
Weighted mode
0.10
0.05
0.00
−0.05
SNP effect on Cardiac valvular disease
0.05
0.10
0.15
0.20
0.25
0.30
SNP effect on SUN5

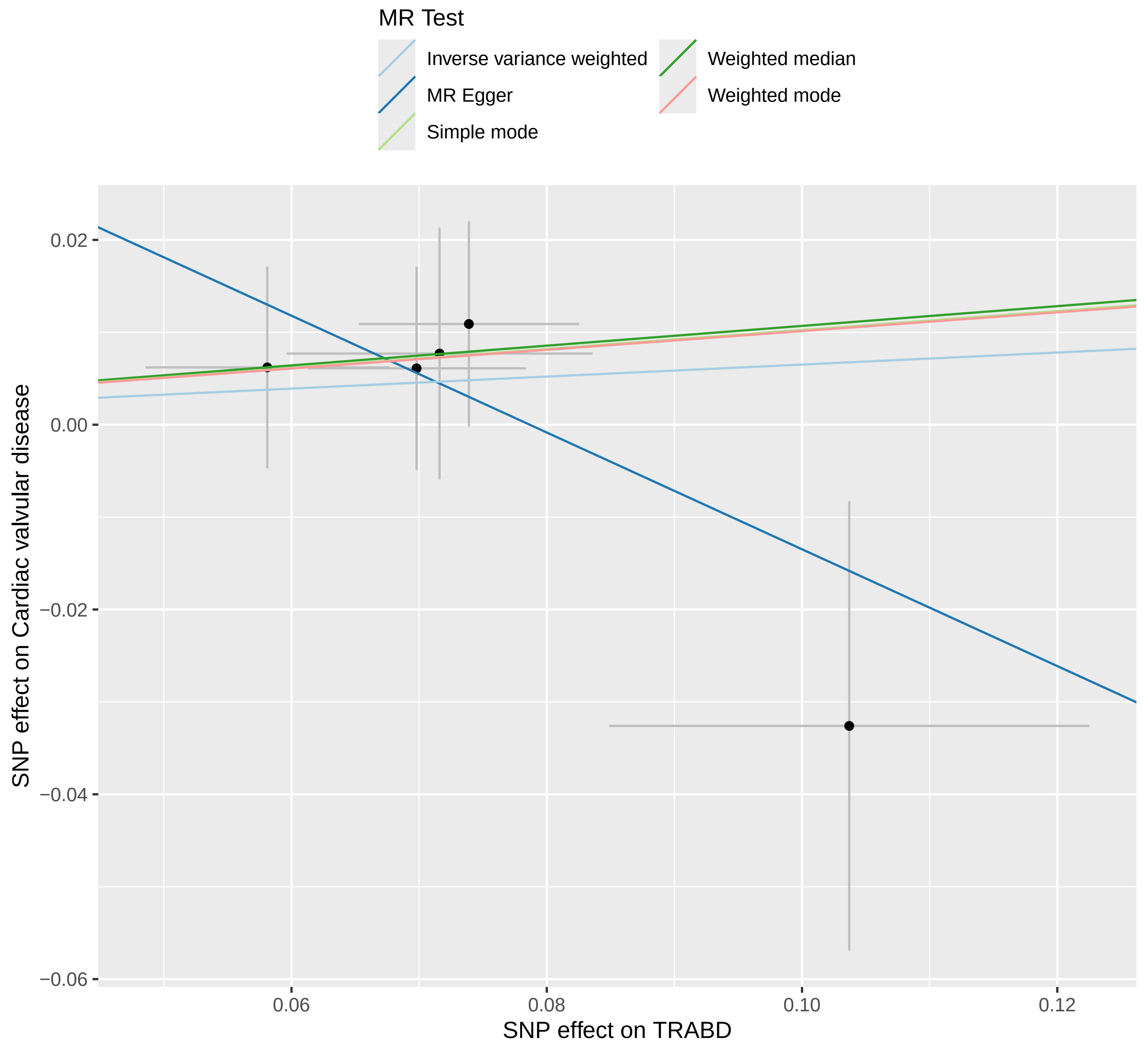

MR Test
Inverse variance weighted
MR Egger
Simple mode
Weighted median
Weighted mode
0.02
0.00
−0.02
−0.04
−0.06
0.06
0.08
0.10
0.12
SNP effect on TRABD
SNP effect on Cardiac valvular disease

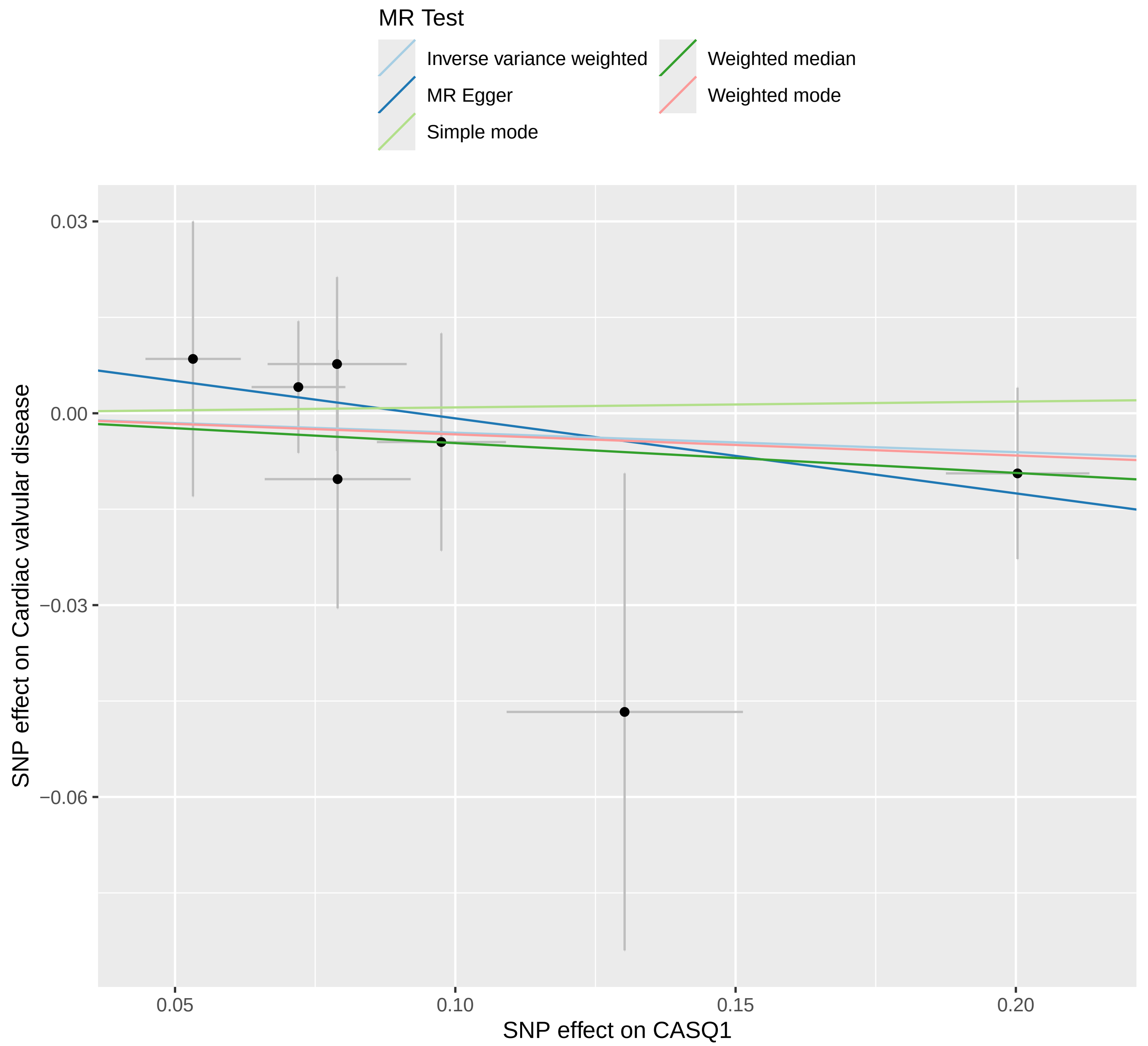

MR Test
Inverse variance weighted
MR Egger
Simple mode
Weighted median
Weighted mode
0.03
0.00
−0.03
−0.06
0.05
0.10
0.15
0.20
SNP effect on Cardiac valvular disease
SNP effect on CASQ1

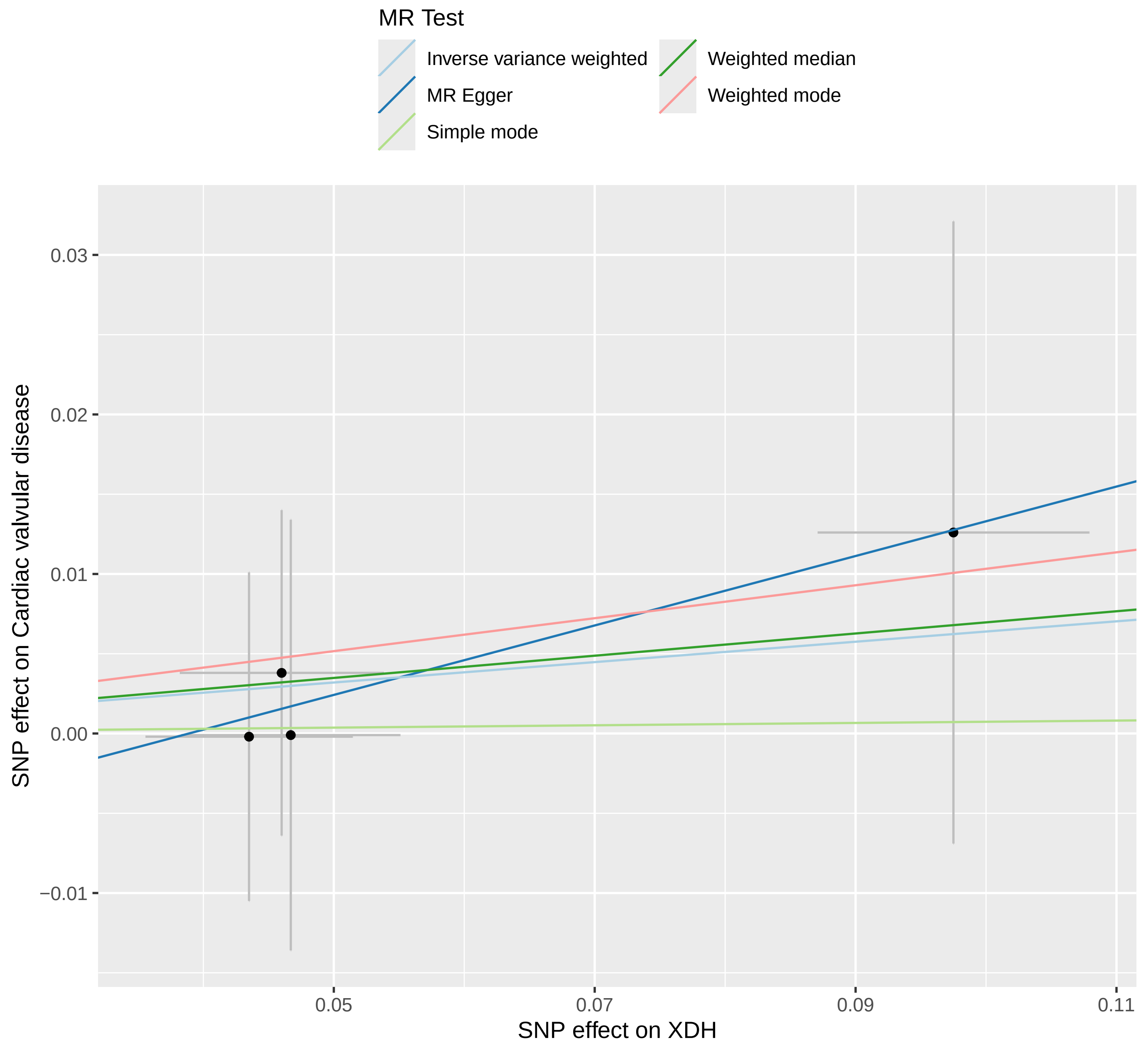

MR Test
Inverse variance weighted
MR Egger
Simple mode
Weighted median
Weighted mode
SNP effect on Cardiac valvular disease
0.03
0.02
0.01
0.00
−0.01
0.05
0.07
0.09
0.11
SNP effect on XDH

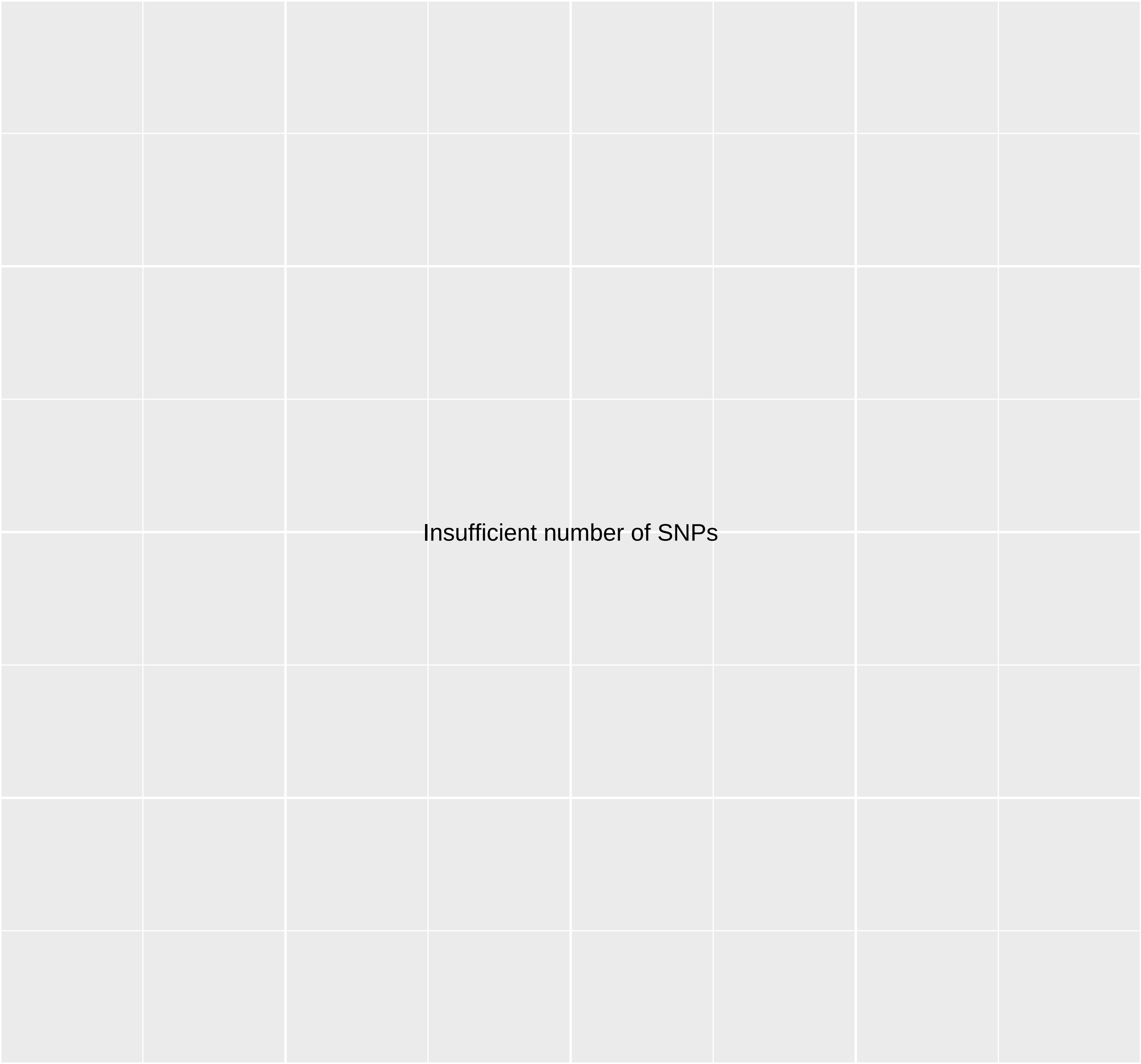
Insufficient number of SNPs

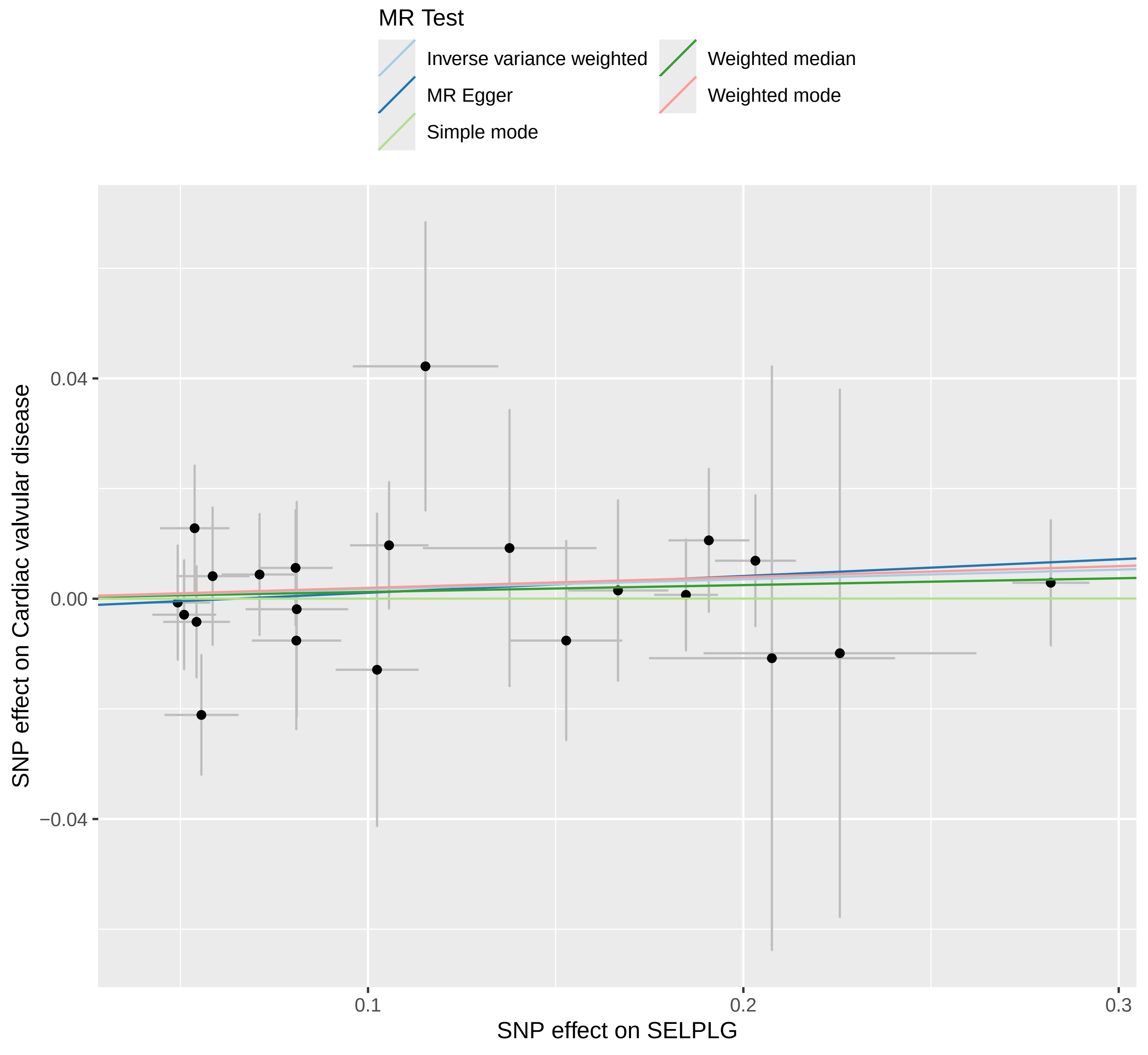
MR Test
Inverse variance weighted
MR Egger
Simple mode
Weighted median
Weighted mode
0.04
0.00
−0.04
0.1
0.2
0.3
SNP effect on Cardiac valvular disease
SNP effect on SELPLG

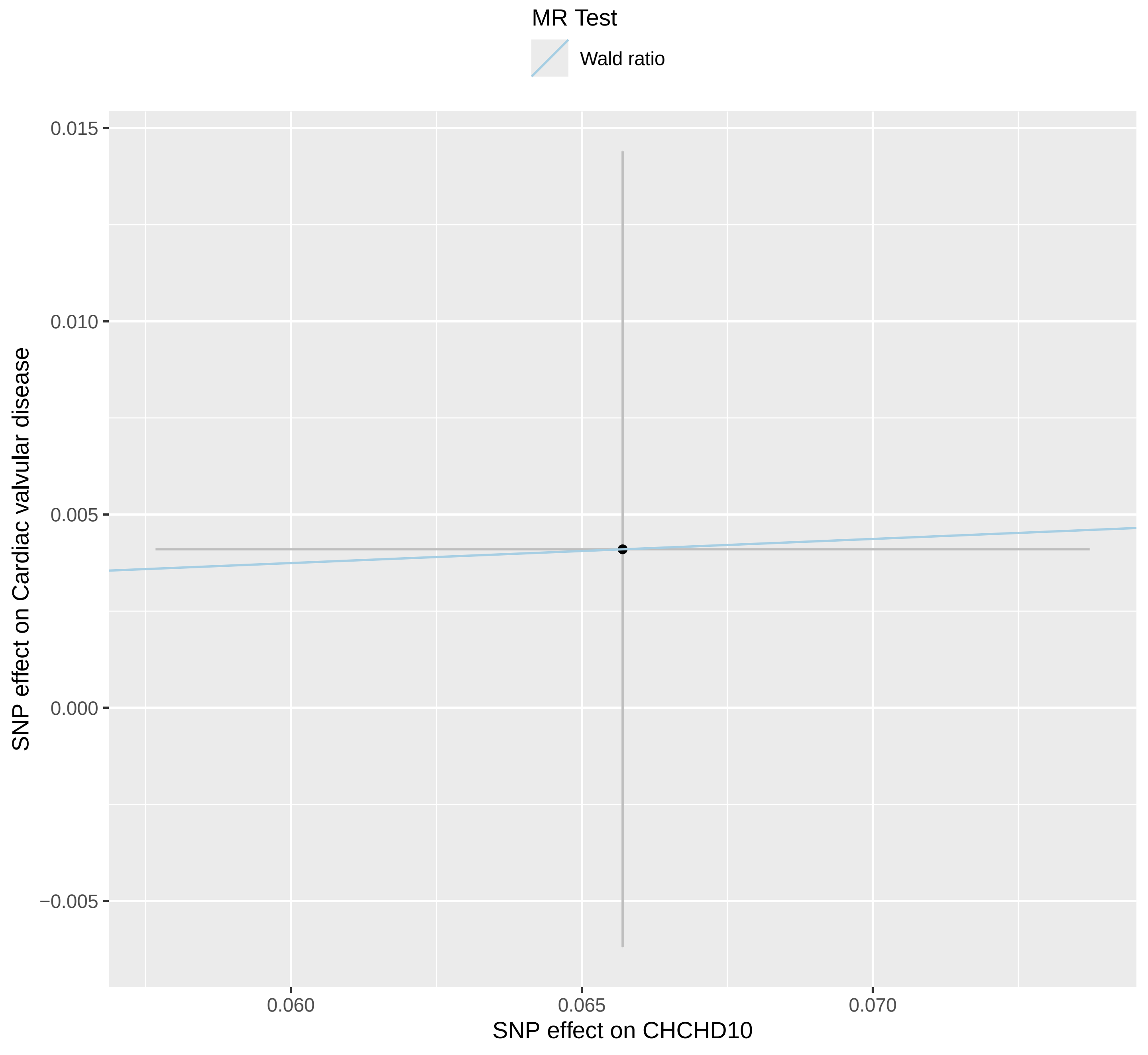

MR Test
Wald ratio
0.015
0.010
0.005
0.000
−0.005
0.060
0.065
0.070
SNP effect on Cardiac valvular disease
SNP effect on CHCHD10

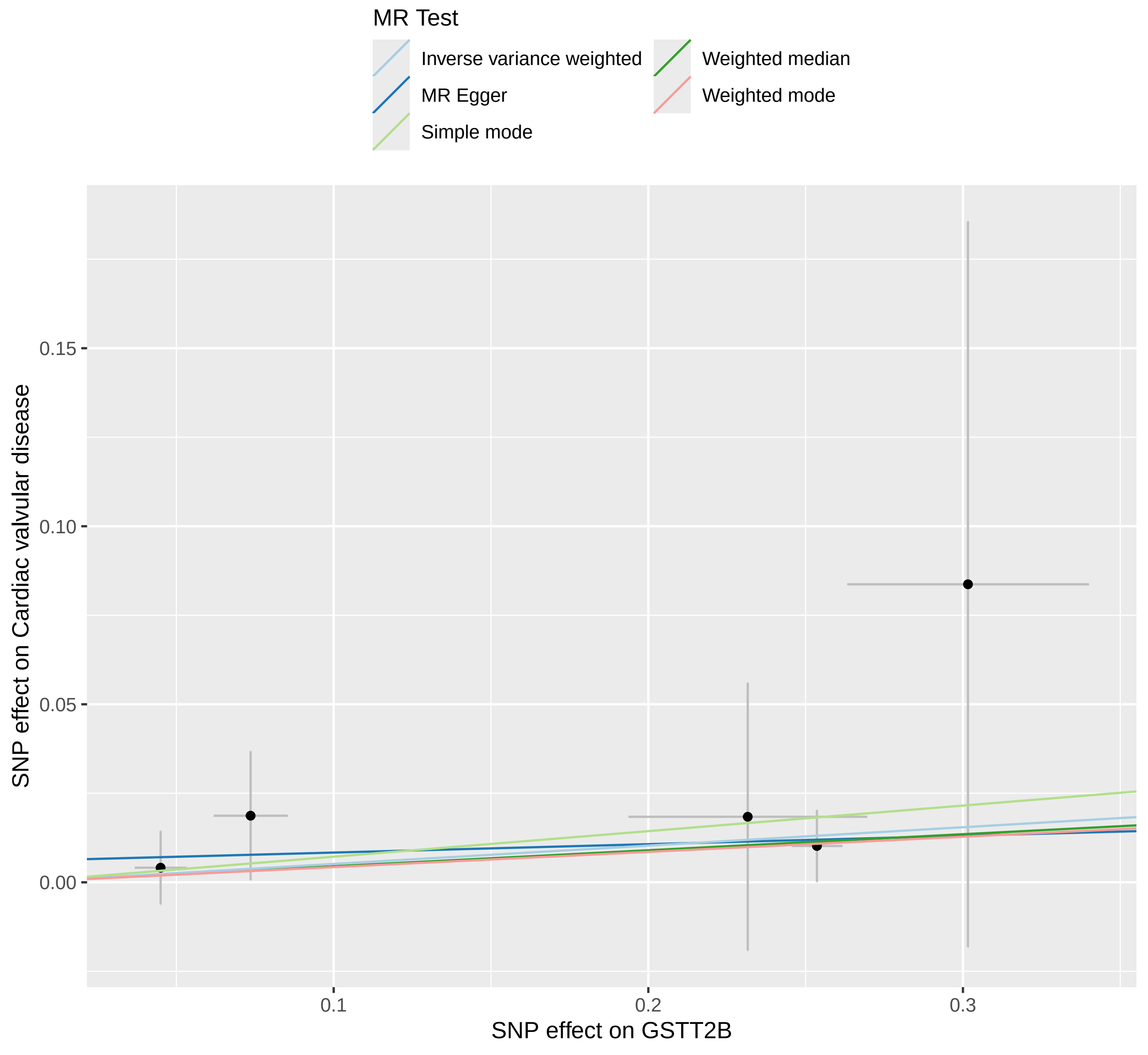
MR Test
Inverse variance weighted
MR Egger
Simple mode
Weighted median
Weighted mode
0.15
0.10
0.05
0.00
SNP effect on Cardiac valvular disease
0.1
0.2
0.3
SNP effect on GSTT2B

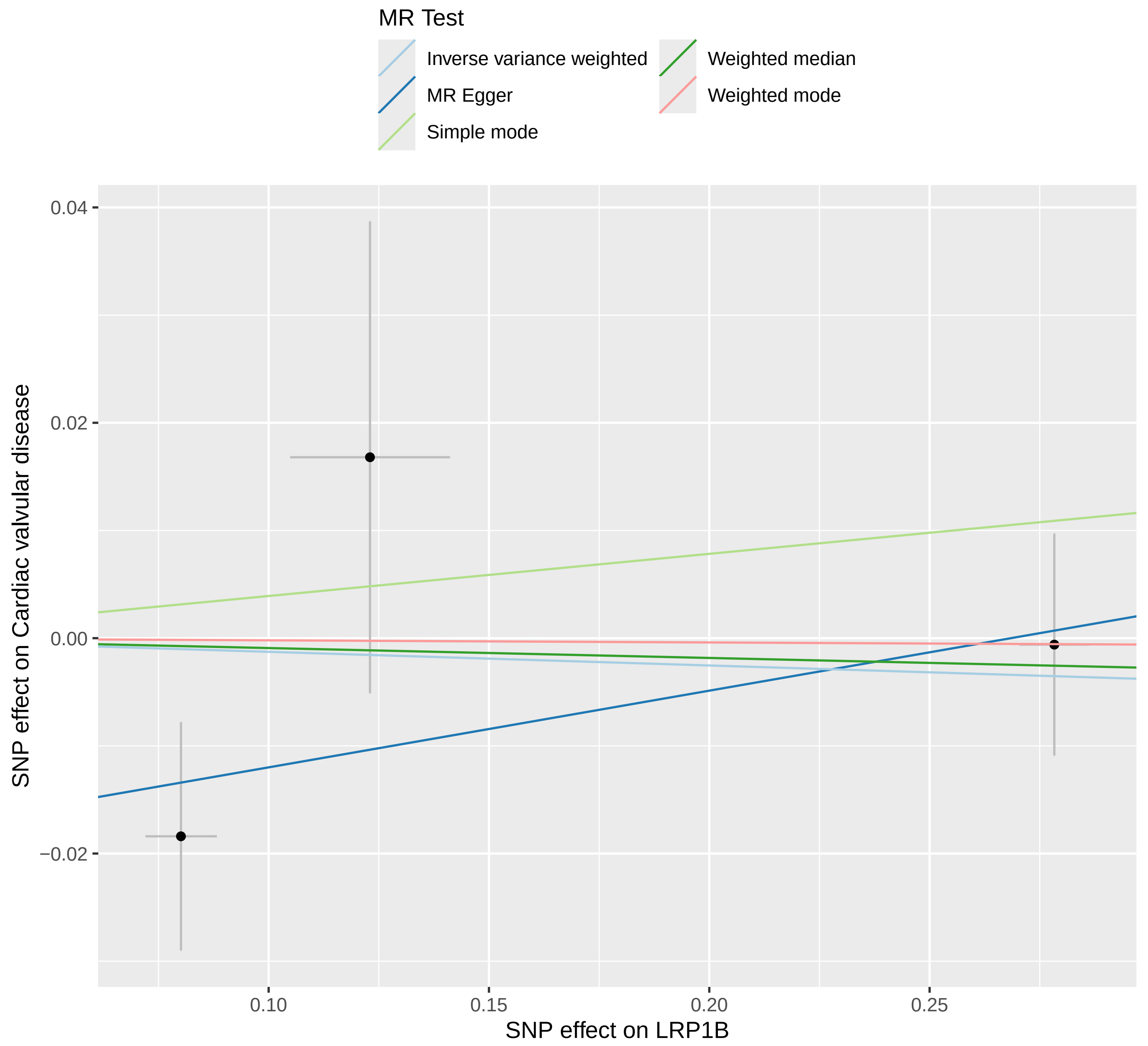
MR Test
Inverse variance weighted
MR Egger
Simple mode
Weighted median
Weighted mode
0.04
0.02
0.00
−0.02
0.10
0.15
0.20
0.25
SNP effect on Cardiac valvular disease
SNP effect on LRP1B

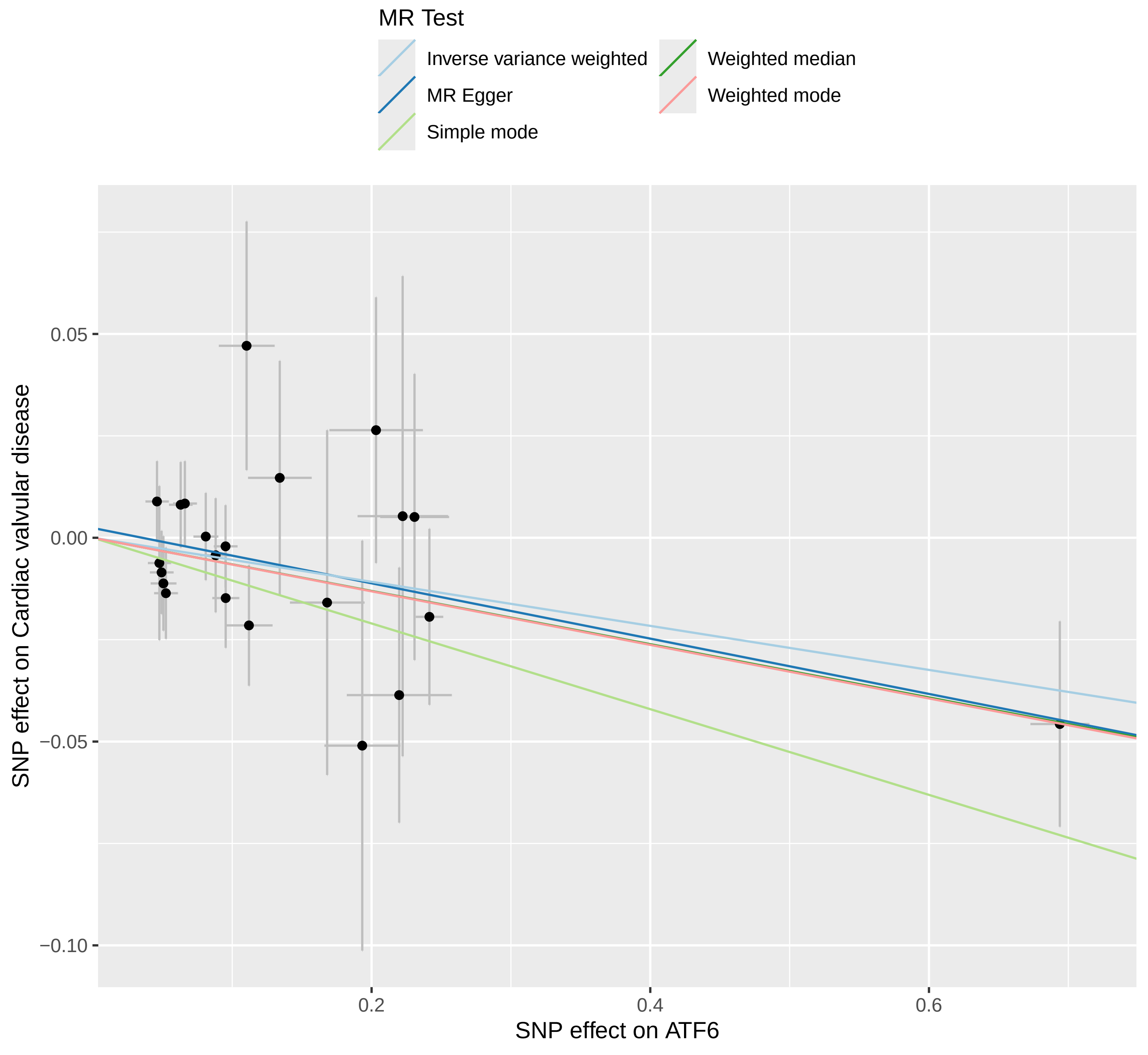
MR Test
Inverse variance weighted
MR Egger
Simple mode
Weighted median
Weighted mode
0.05
0.00
−0.05
−0.10
0.2
0.4
0.6
SNP effect on ATF6
SNP effect on Cardiac valvular disease

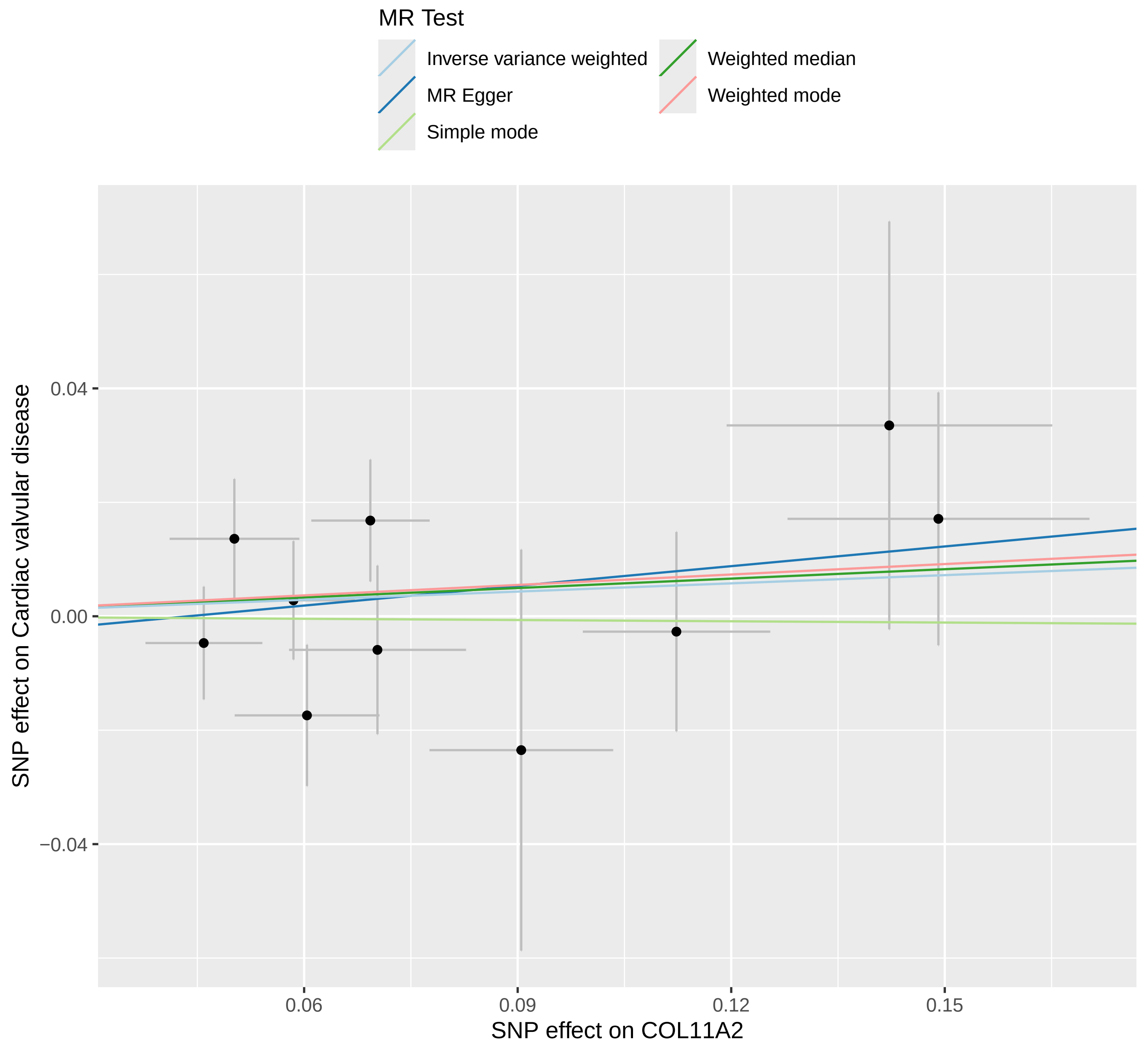
MR Test
Inverse variance weighted
MR Egger
Simple mode
Weighted median
Weighted mode
0.04
0.00
−0.04
0.06
0.09
0.12
0.15
SNP effect on Cardiac valvular disease
SNP effect on COL11A2

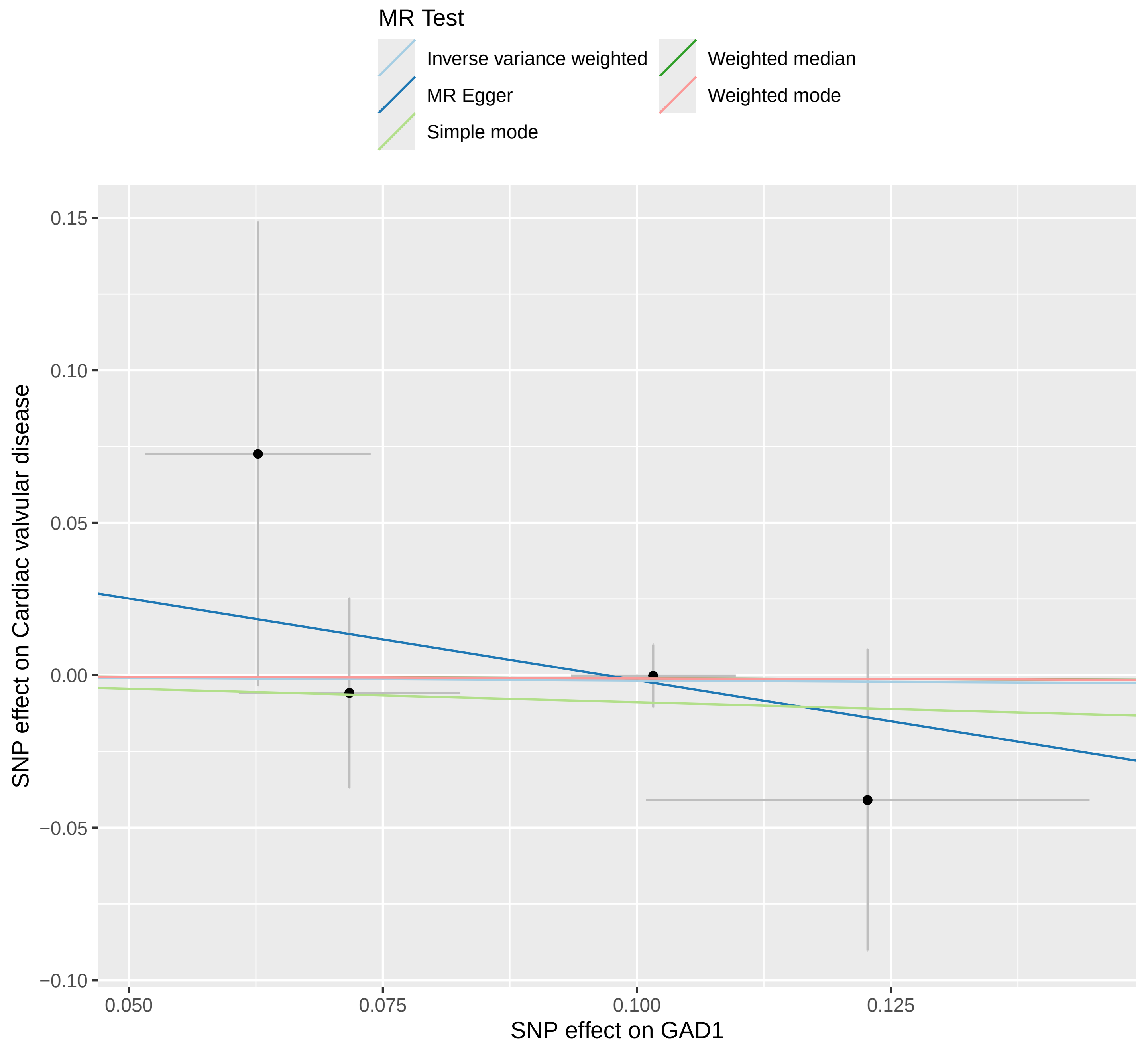
MR Test
Inverse variance weighted
MR Egger
Simple mode
Weighted median
Weighted mode
0.15
0.10
0.05
0.00
−0.05
−0.10
0.050
0.075
0.100
0.125
SNP effect on GAD1
SNP effect on Cardiac valvular disease

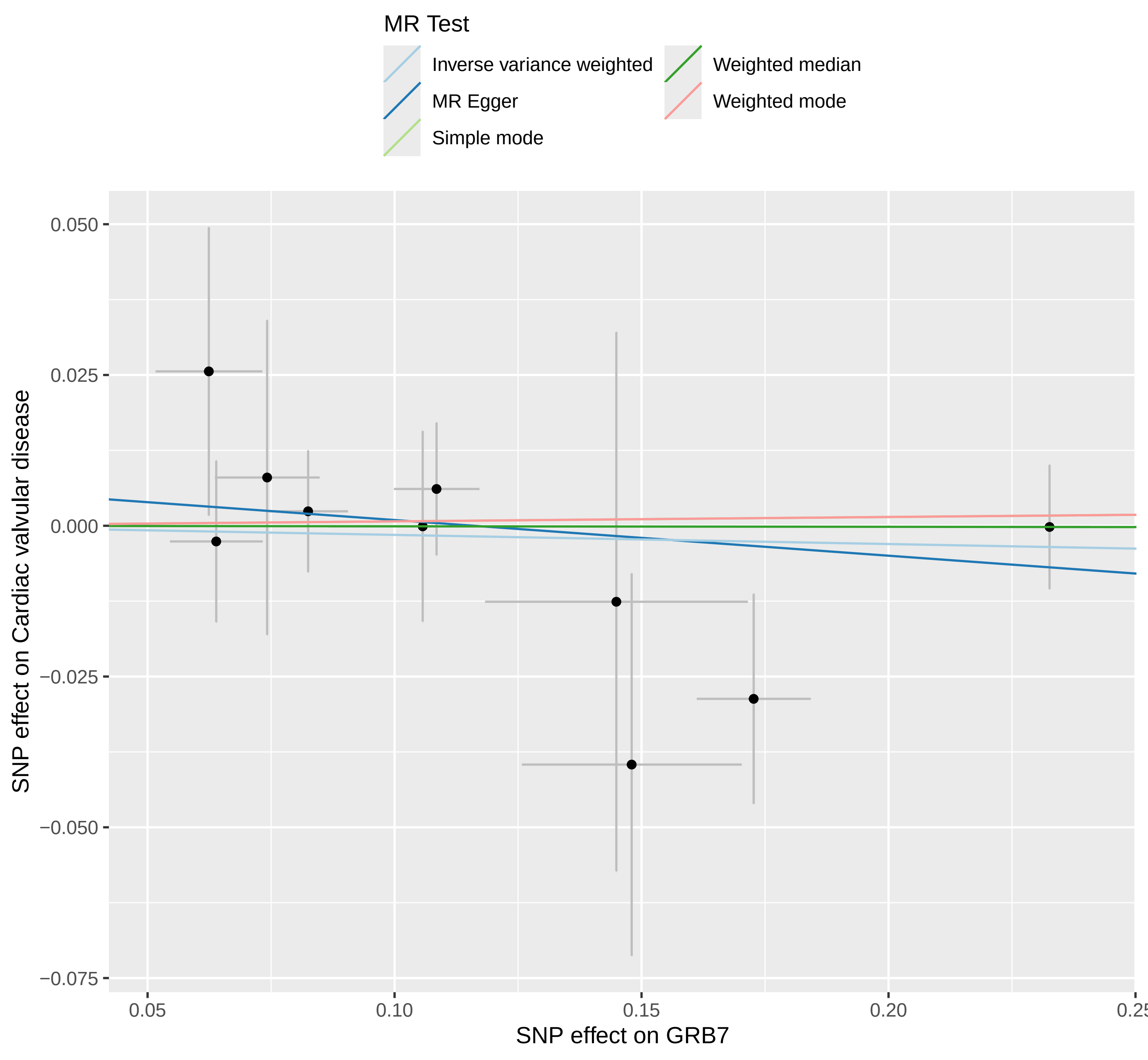
MR Test
Inverse variance weighted
MR Egger
Simple mode
Weighted median
Weighted mode
0.050
0.025
0.000
−0.025
−0.050
−0.075
SNP effect on Cardiac valvular disease
0.05
0.10
0.15
0.20
SNP effect on GRB7

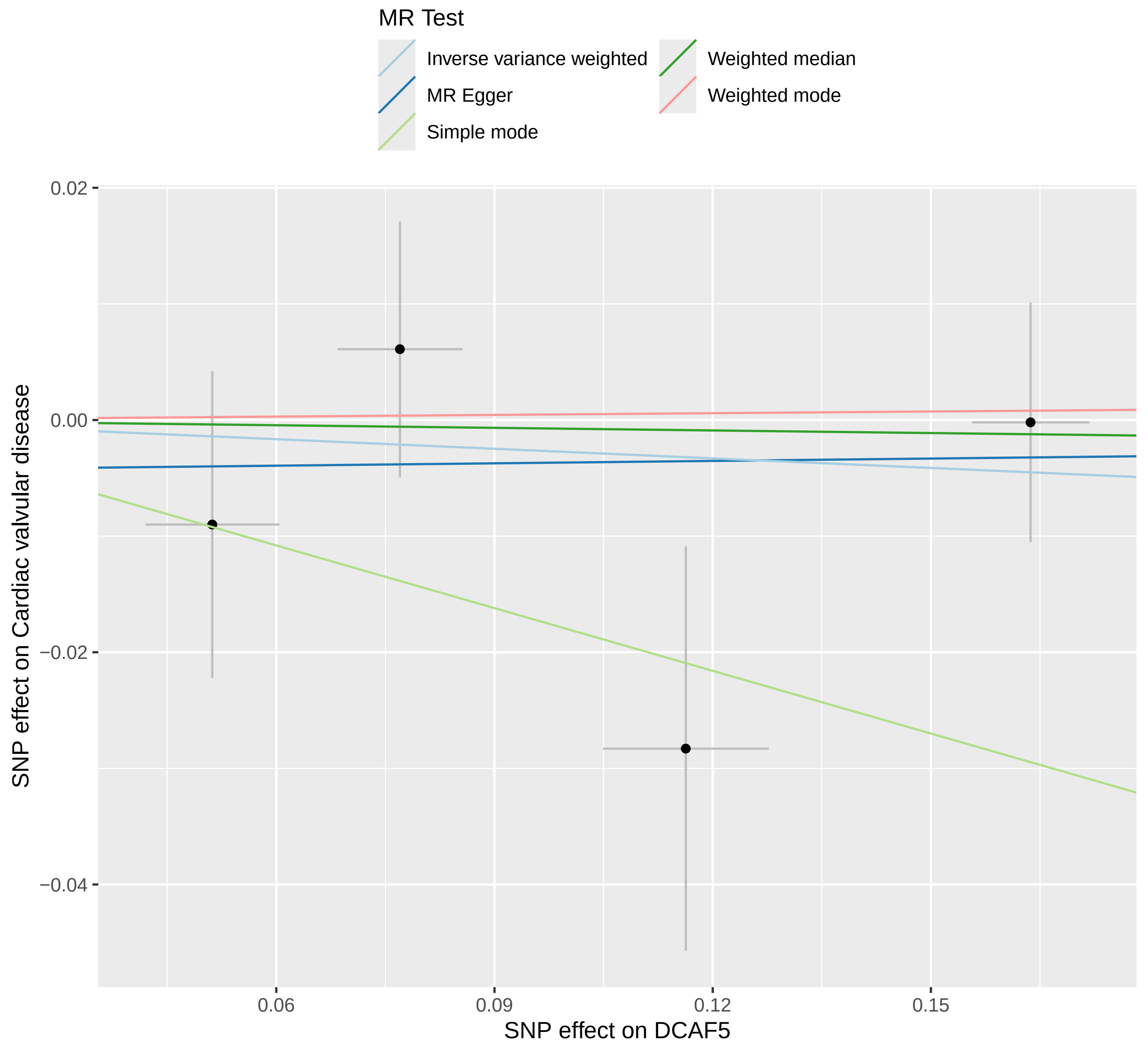

MR Test
Inverse variance weighted
MR Egger
Simple mode
Weighted median
Weighted mode
0.02
0.00
−0.02
−0.04
SNP effect on Cardiac valvular disease
0.06
0.09
0.12
0.15
SNP effect on DCAF5

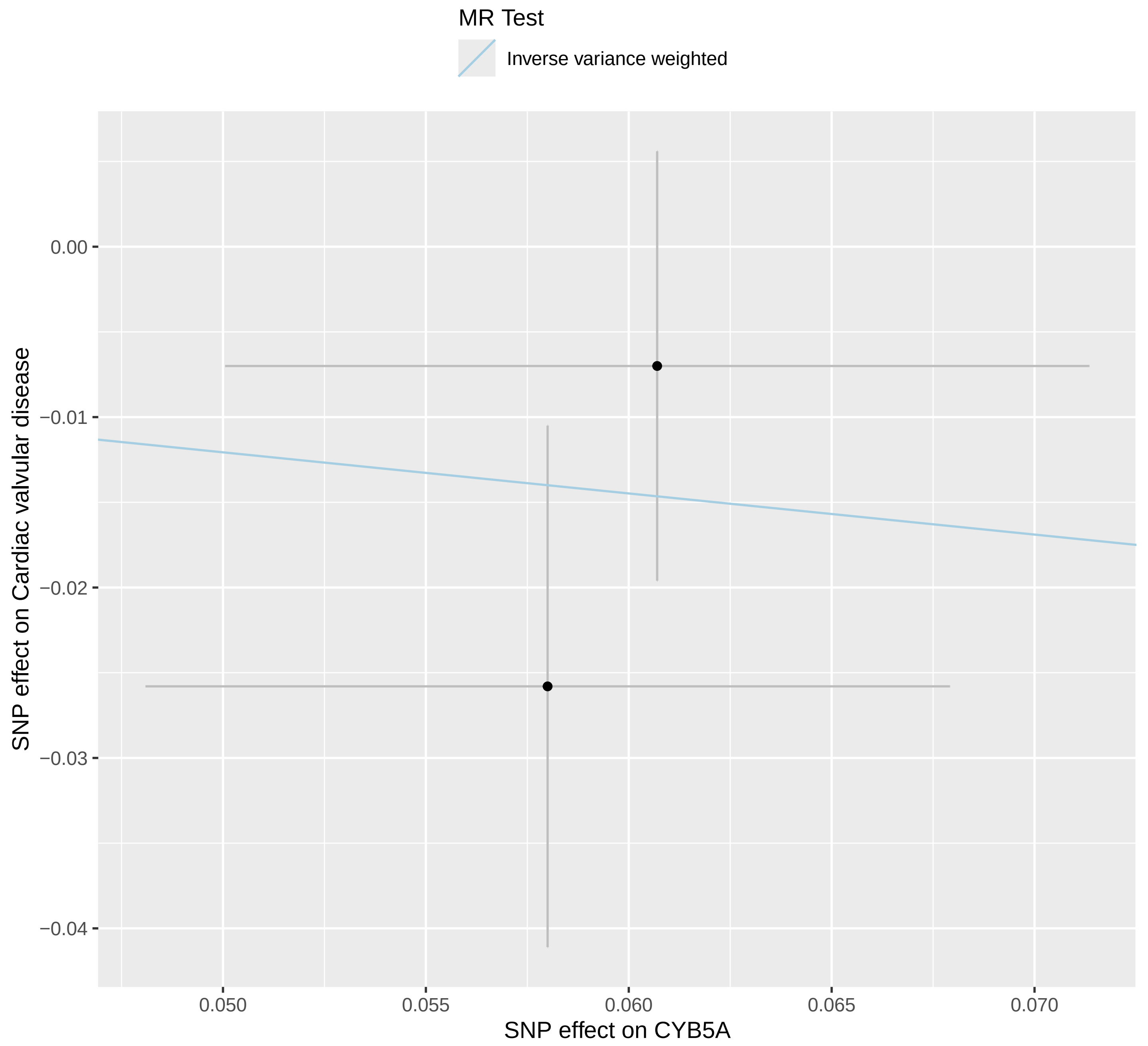

MR Test
Inverse variance weighted
0.00
−0.01
−0.02
−0.03
−0.04
0.050
0.055
0.060
0.065
0.070
SNP effect on Cardiac valvular disease
SNP effect on CYB5A

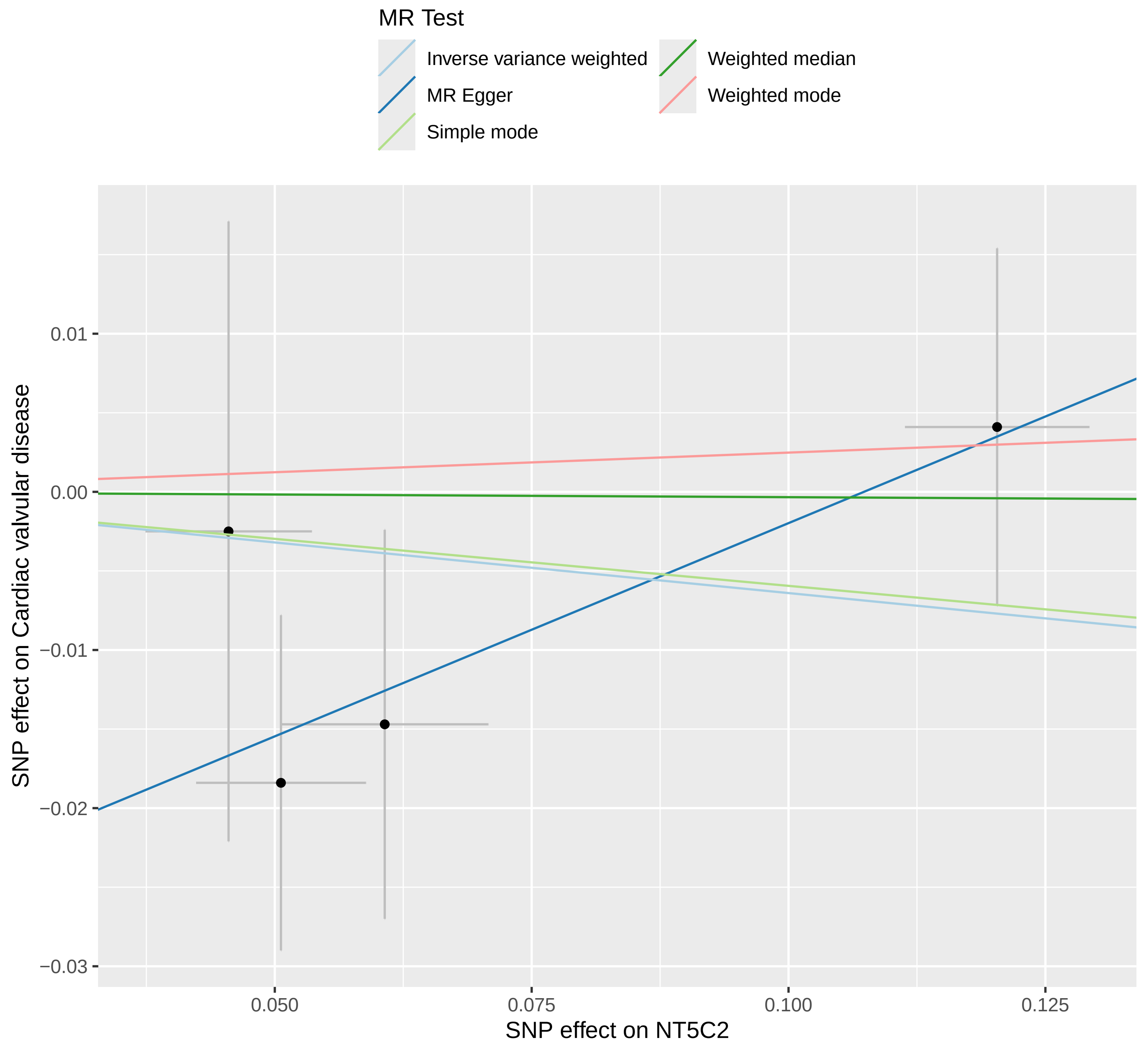

MR Test
Inverse variance weighted
MR Egger
Simple mode
Weighted median
Weighted mode
0.01
0.00
−0.01
−0.02
−0.03
0.050
0.075
0.100
0.125
SNP effect on NT5C2
SNP effect on Cardiac valvular disease

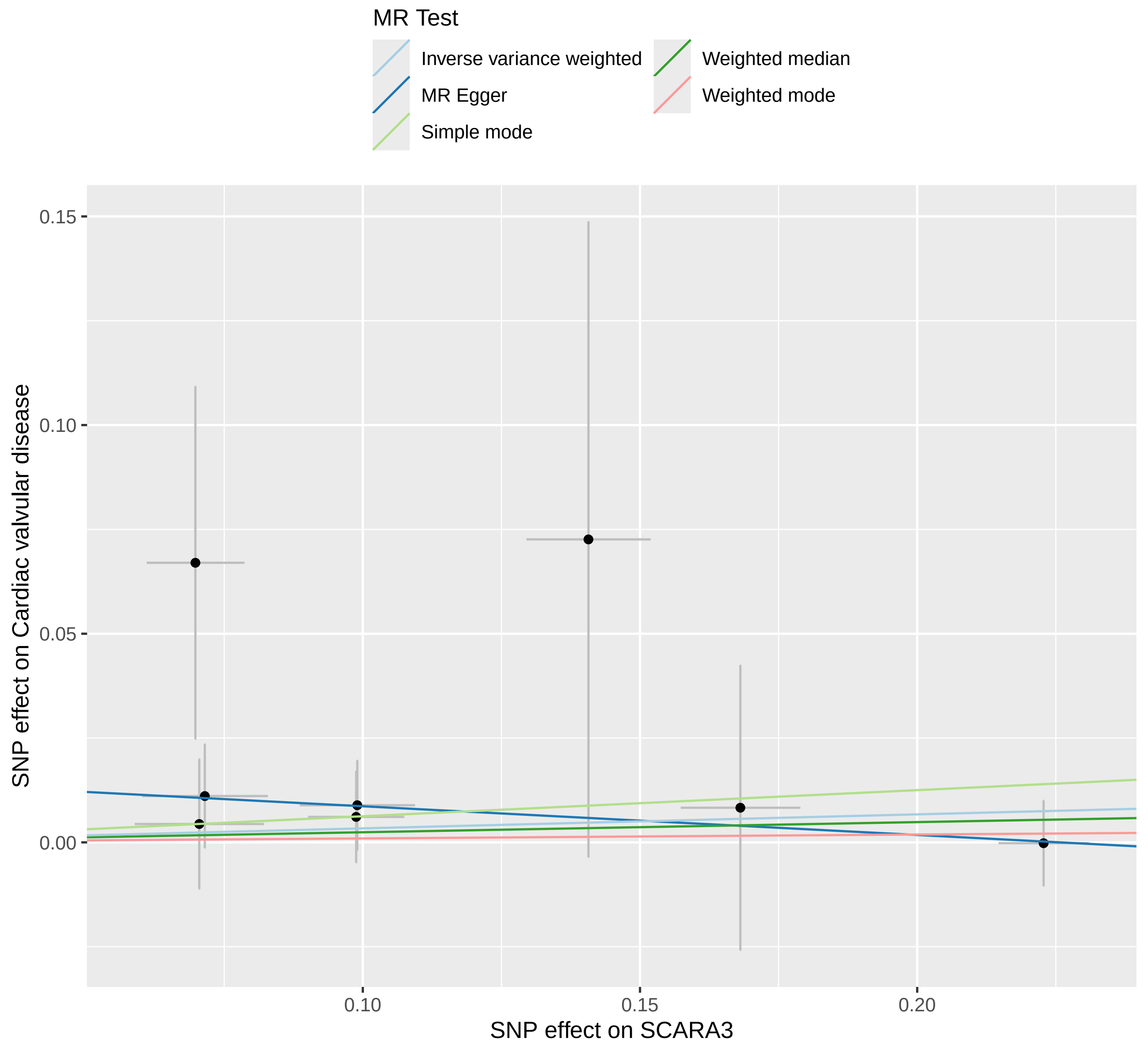
MR Test
Inverse variance weighted
MR Egger
Simple mode
Weighted median
Weighted mode
0.15
0.10
0.05
0.00
0.10
0.15
0.20
SNP effect on Cardiac valvular disease
SNP effect on SCARA3

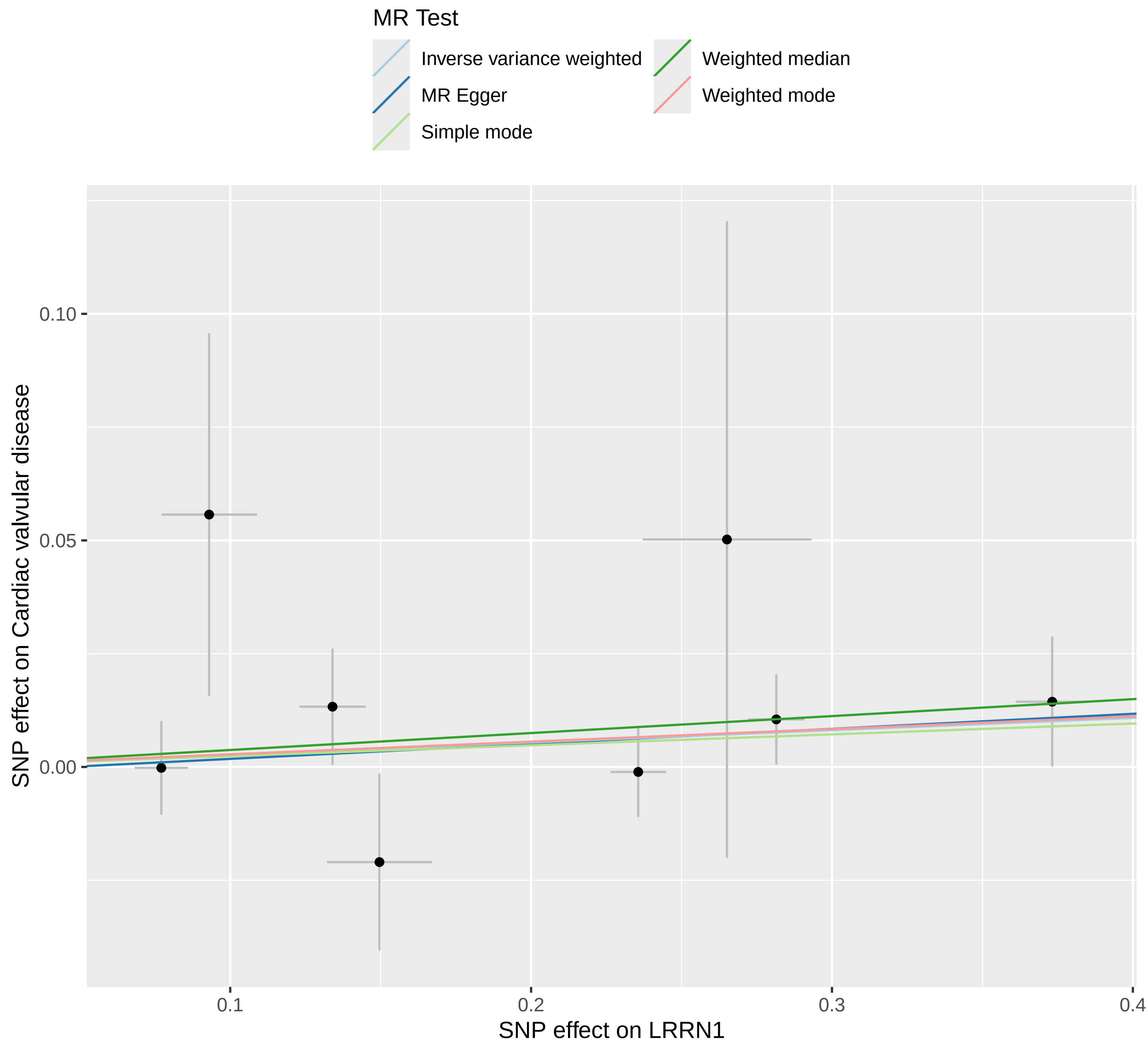
MR Test
Inverse variance weighted
MR Egger
Simple mode
Weighted median
Weighted mode
0.10
0.05
0.00
0.1
0.2
0.3
0.4
SNP effect on Cardiac valvular disease
SNP effect on LRRN1

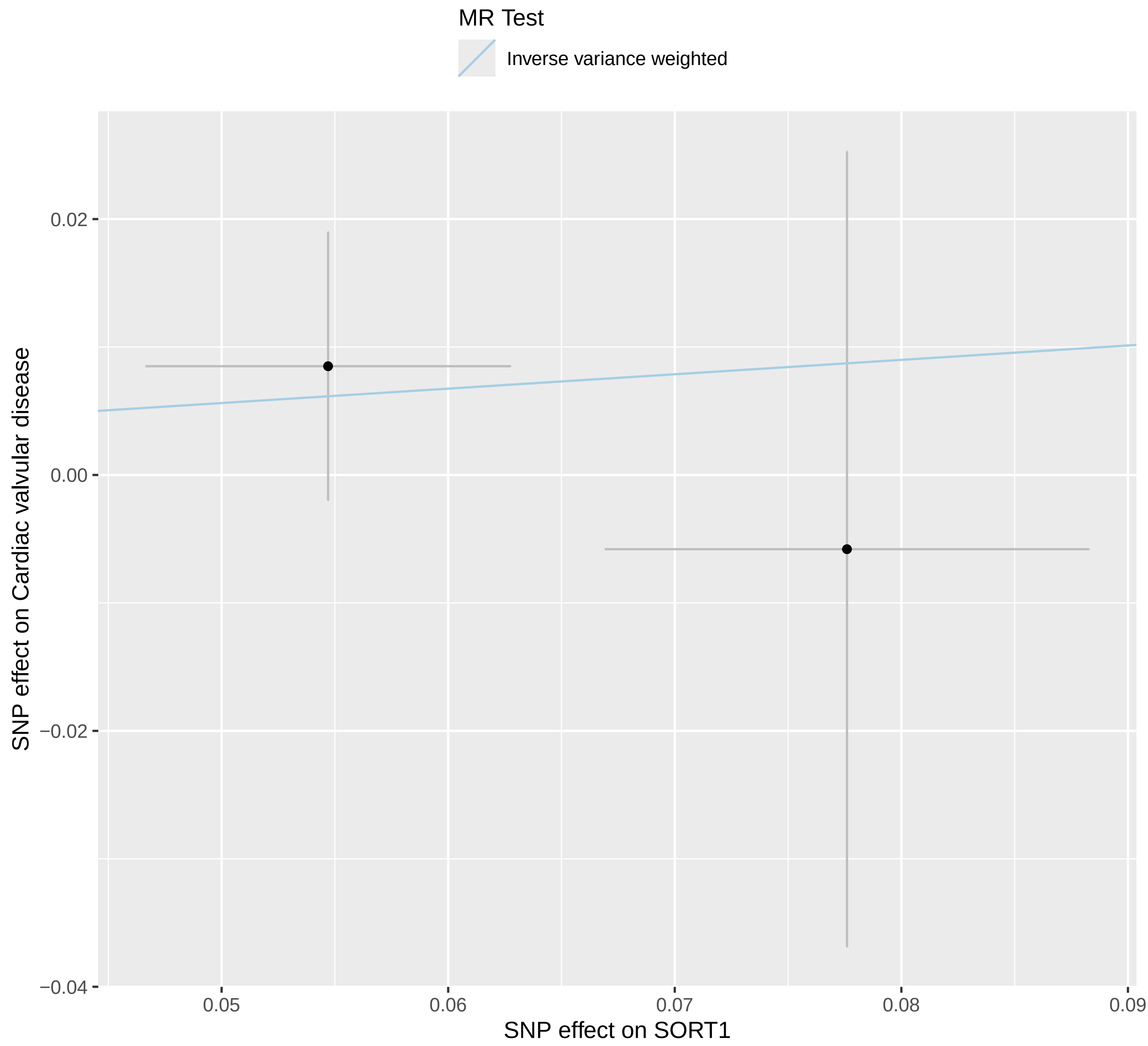
MR Test
Inverse variance weighted
0.02
0.00
−0.02
−0.04
0.05
0.06
0.07
0.08
0.09
SNP effect on Cardiac valvular disease
SNP effect on SORT1

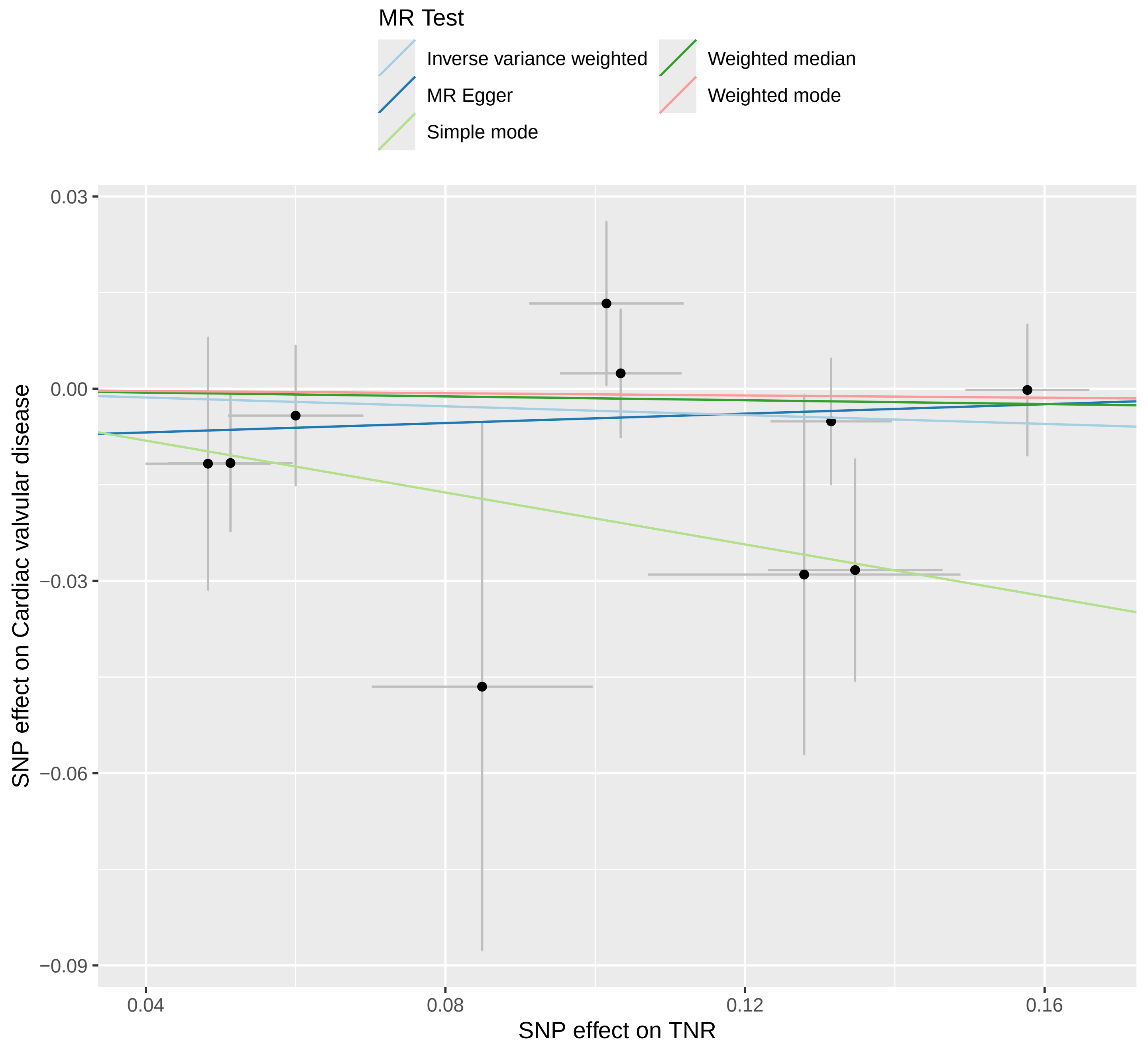

MR Test
Inverse variance weighted
MR Egger
Simple mode
Weighted median
Weighted mode
0.03
0.00
−0.03
−0.06
−0.09
0.04
0.08
0.12
0.16
SNP effect on TNR
SNP effect on Cardiac valvular disease

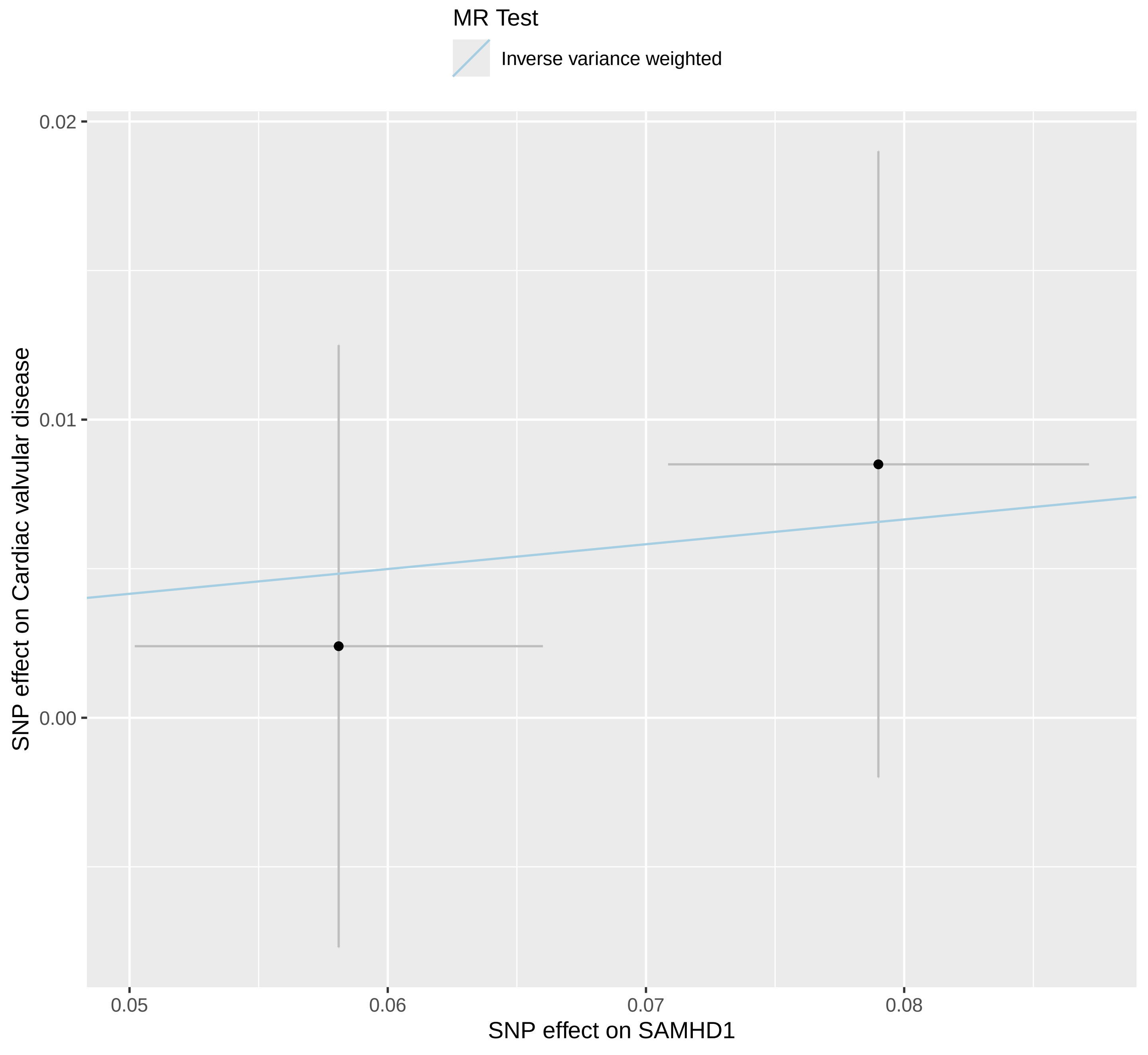

MR Test
Inverse variance weighted
0.02
0.01
0.00
0.05
0.06
0.07
0.08
SNP effect on SAMHD1
SNP effect on Cardiac valvular disease

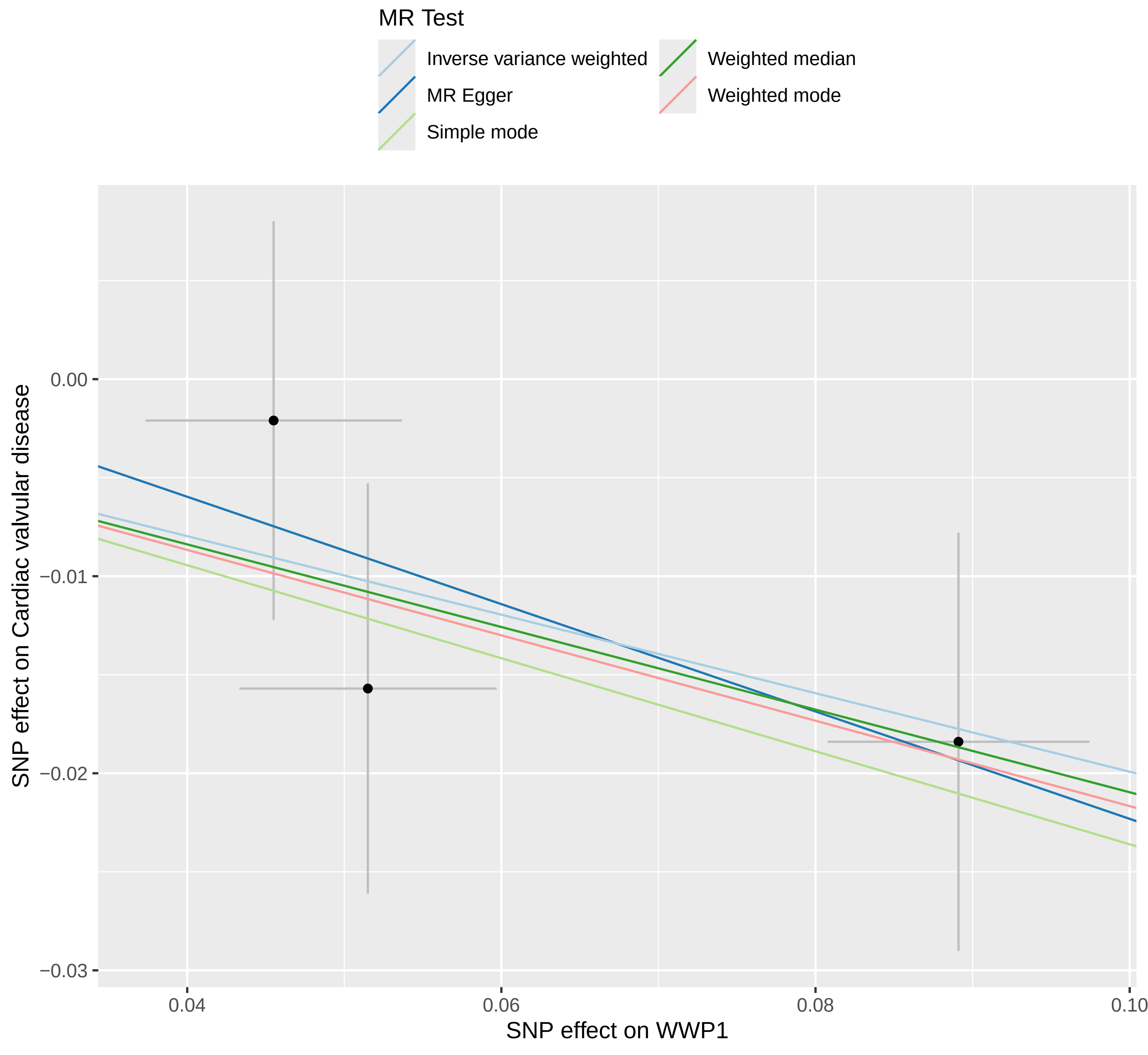

MR Test
Inverse variance weighted
MR Egger
Simple mode
Weighted median
Weighted mode
0.00
−0.01
−0.02
−0.03
0.04
0.06
0.08
0.10
SNP effect on Cardiac valvular disease
SNP effect on WWP1

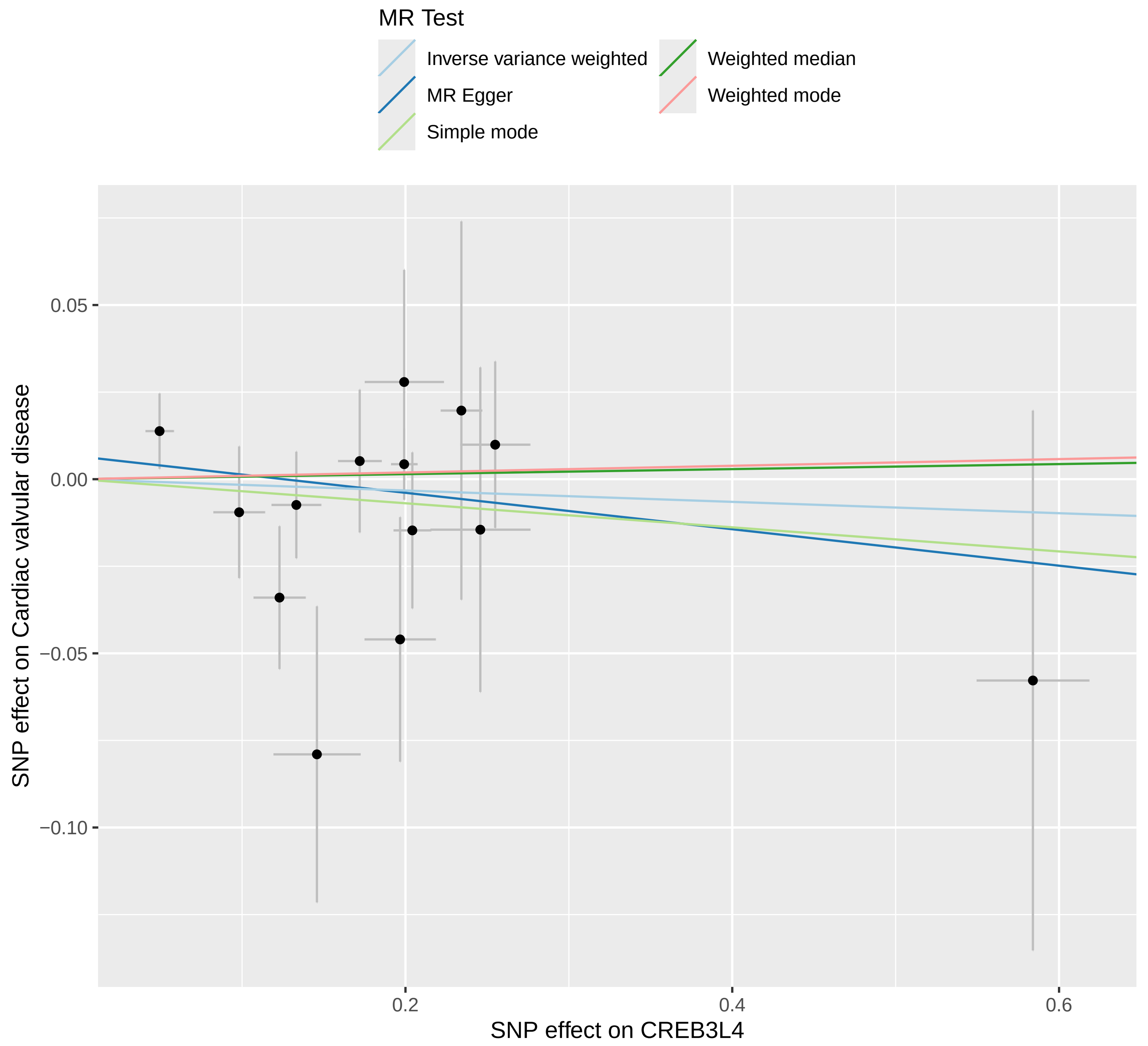
MR Test
Inverse variance weighted
MR Egger
Simple mode
Weighted median
Weighted mode
0.05
0.00
−0.05
−0.10
0.2
0.4
0.6
SNP effect on Cardiac valvular disease
SNP effect on CREB3L4

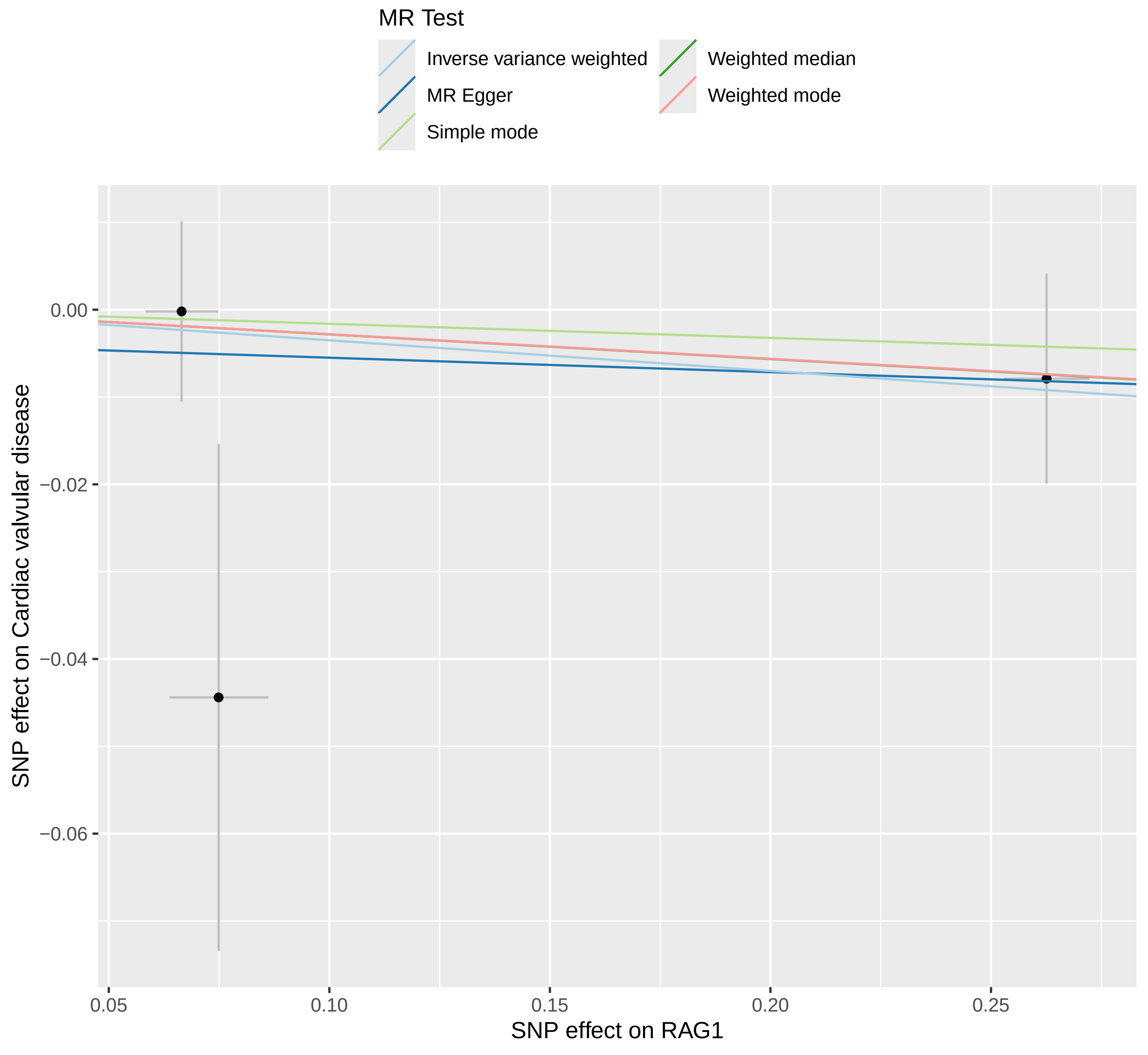

MR Test
Inverse variance weighted
MR Egger
Simple mode
Weighted median
Weighted mode
0.00
−0.02
−0.04
−0.06
0.05
0.10
0.15
0.20
0.25
SNP effect on Cardiac valvular disease
SNP effect on RAG1

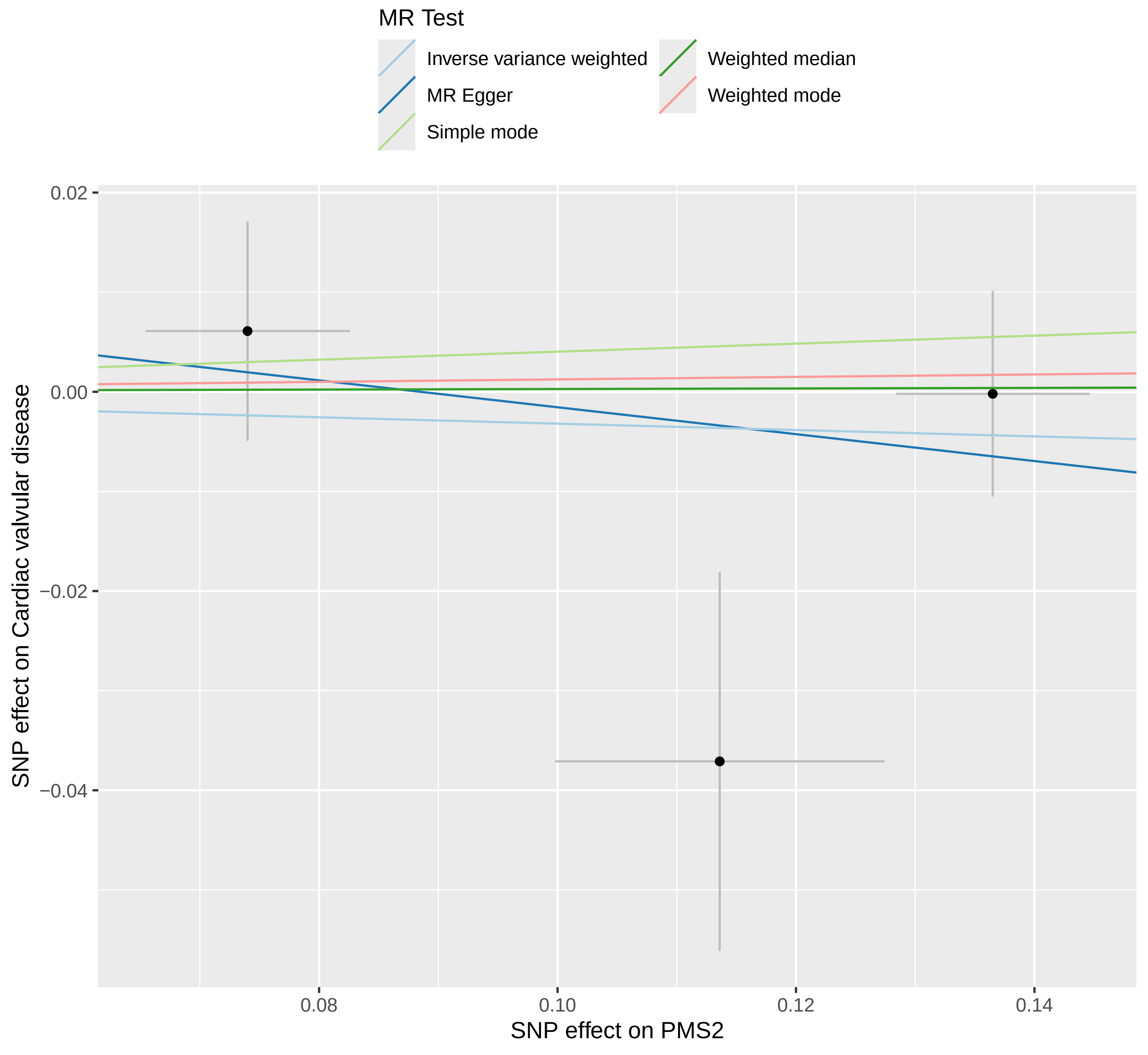

MR Test
Inverse variance weighted
MR Egger
Simple mode
Weighted median
Weighted mode
0.02
0.00
−0.02
−0.04
0.08
0.10
0.12
0.14
SNP effect on Cardiac valvular disease
SNP effect on PMS2

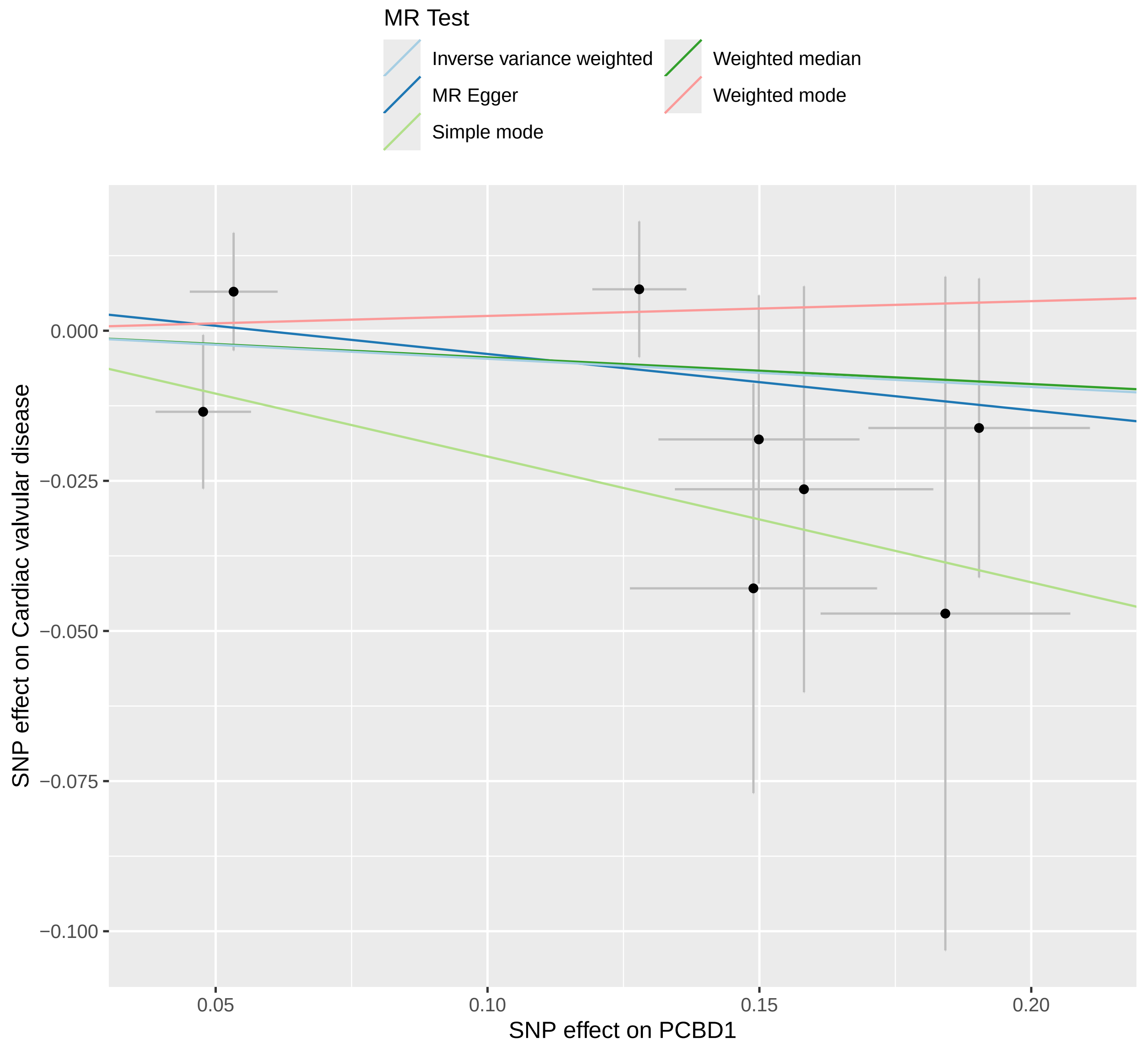
MR Test
Inverse variance weighted
MR Egger
Simple mode
Weighted median
Weighted mode
0.000
−0.025
−0.050
−0.075
−0.100
SNP effect on Cardiac valvular disease
0.05
0.10
0.15
0.20
SNP effect on PCBD1

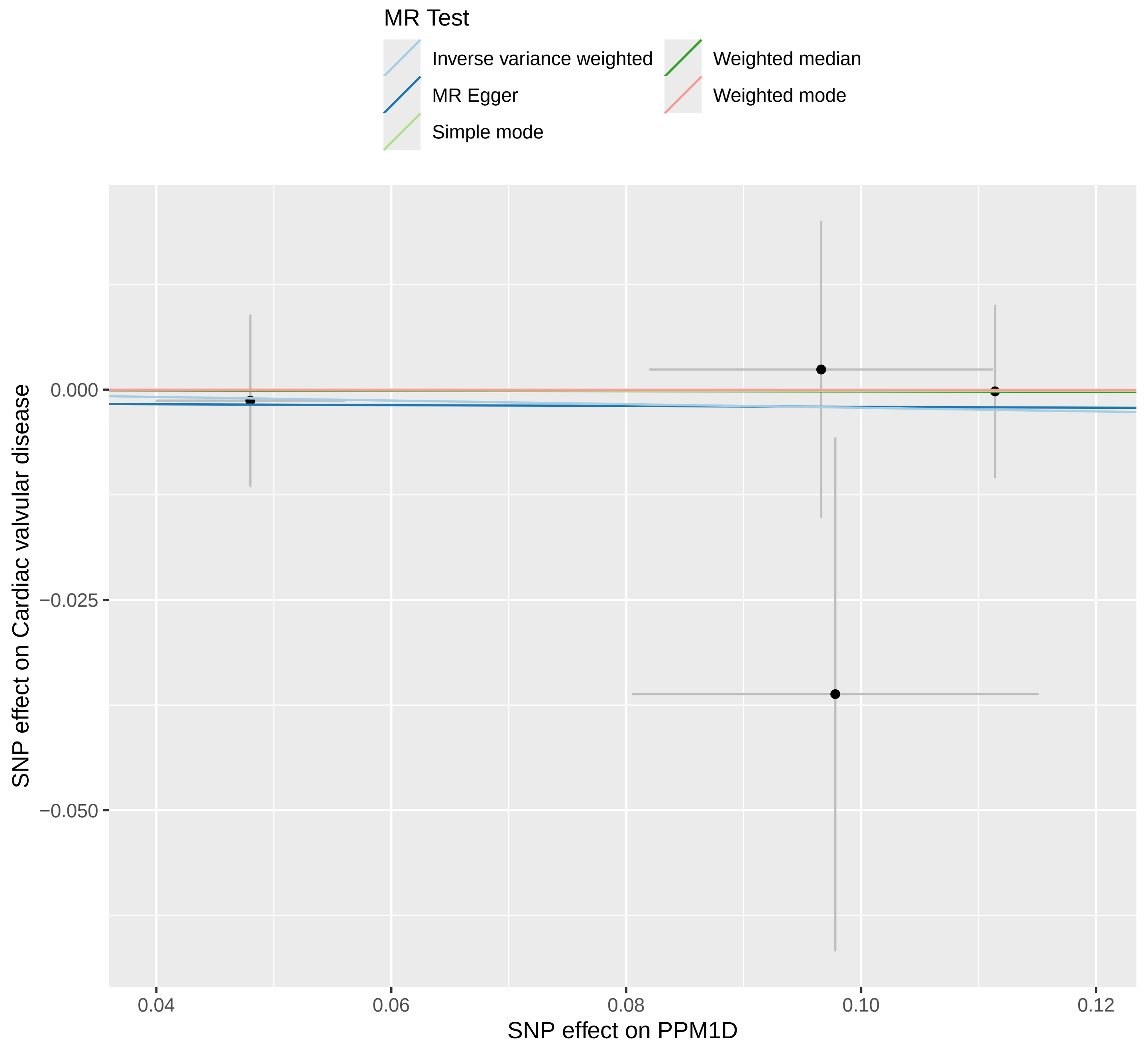
MR Test
Inverse variance weighted
MR Egger
Simple mode
Weighted median
Weighted mode
0.000
−0.025
−0.050
0.04
0.06
0.08
0.10
0.12
SNP effect on PPM1D
SNP effect on Cardiac valvular disease

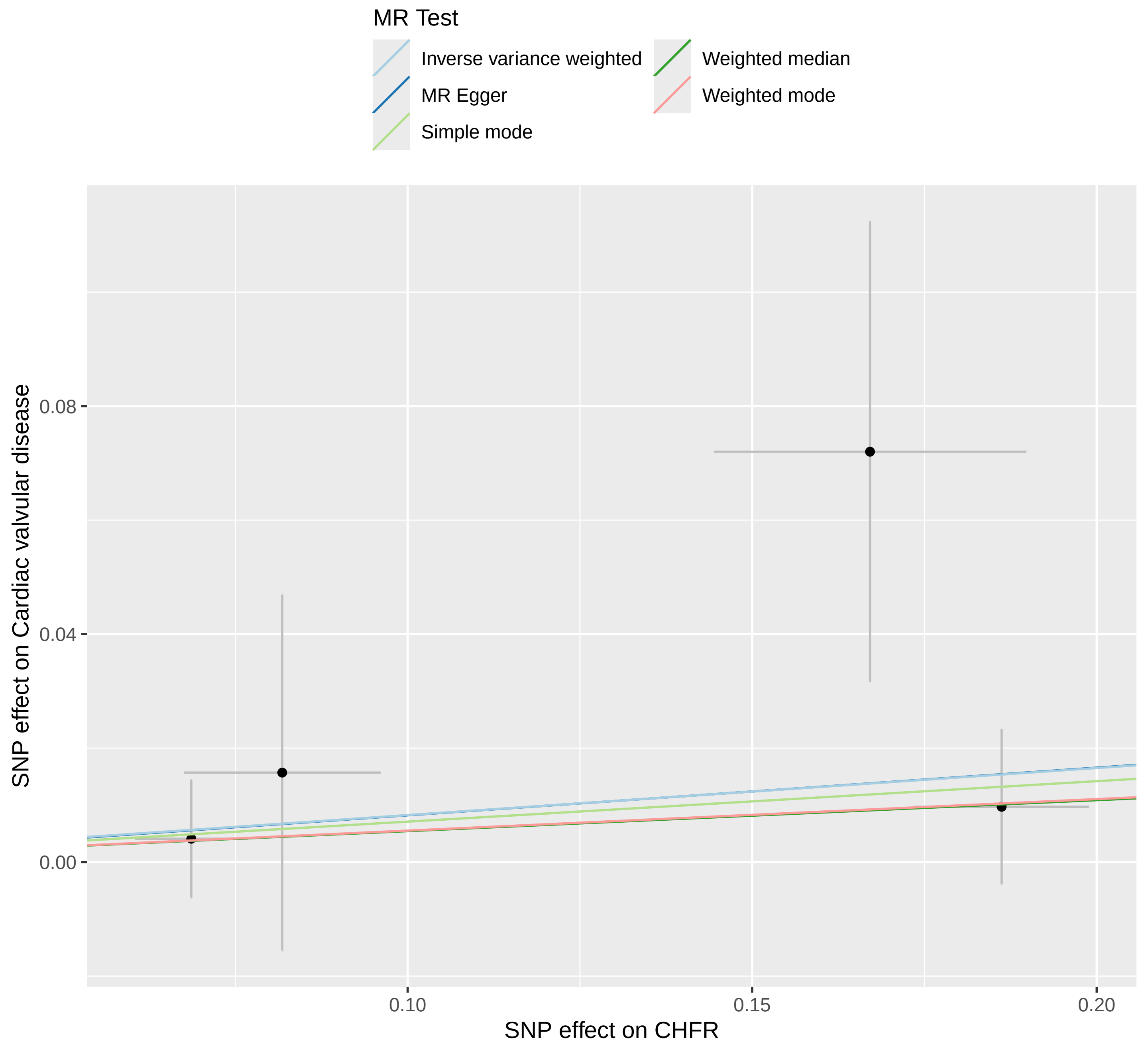
MR Test
Inverse variance weighted
MR Egger
Simple mode
Weighted median
Weighted mode
SNP effect on Cardiac valvular disease
0.08
0.04
0.00
0.10
0.15
0.20
SNP effect on CHFR

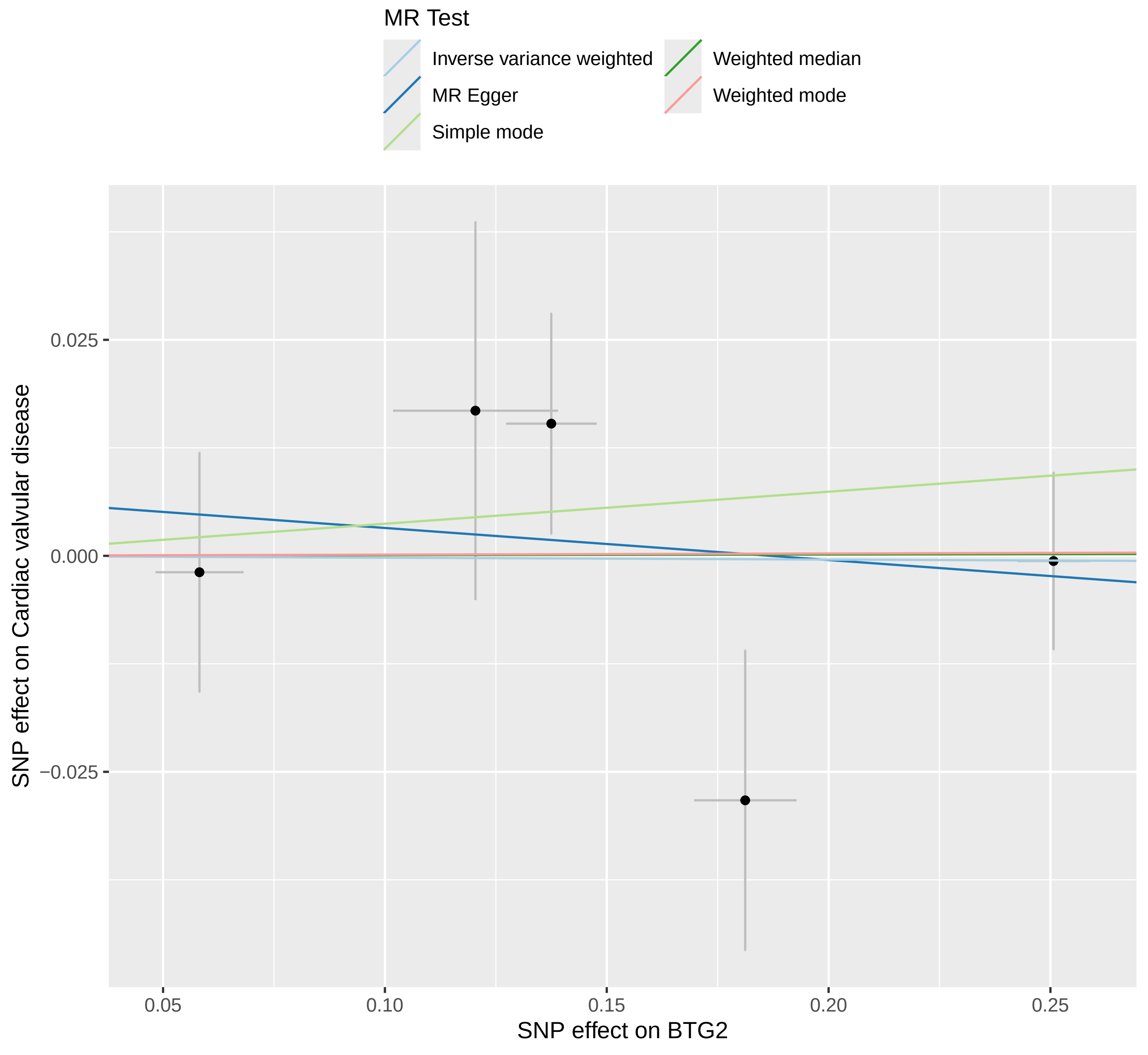
MR Test
Inverse variance weighted
MR Egger
Simple mode
Weighted median
Weighted mode
SNP effect on Cardiac valvular disease
0.025
0.000
−0.025
0.05
0.10
0.15
0.20
0.25
SNP effect on BTG2

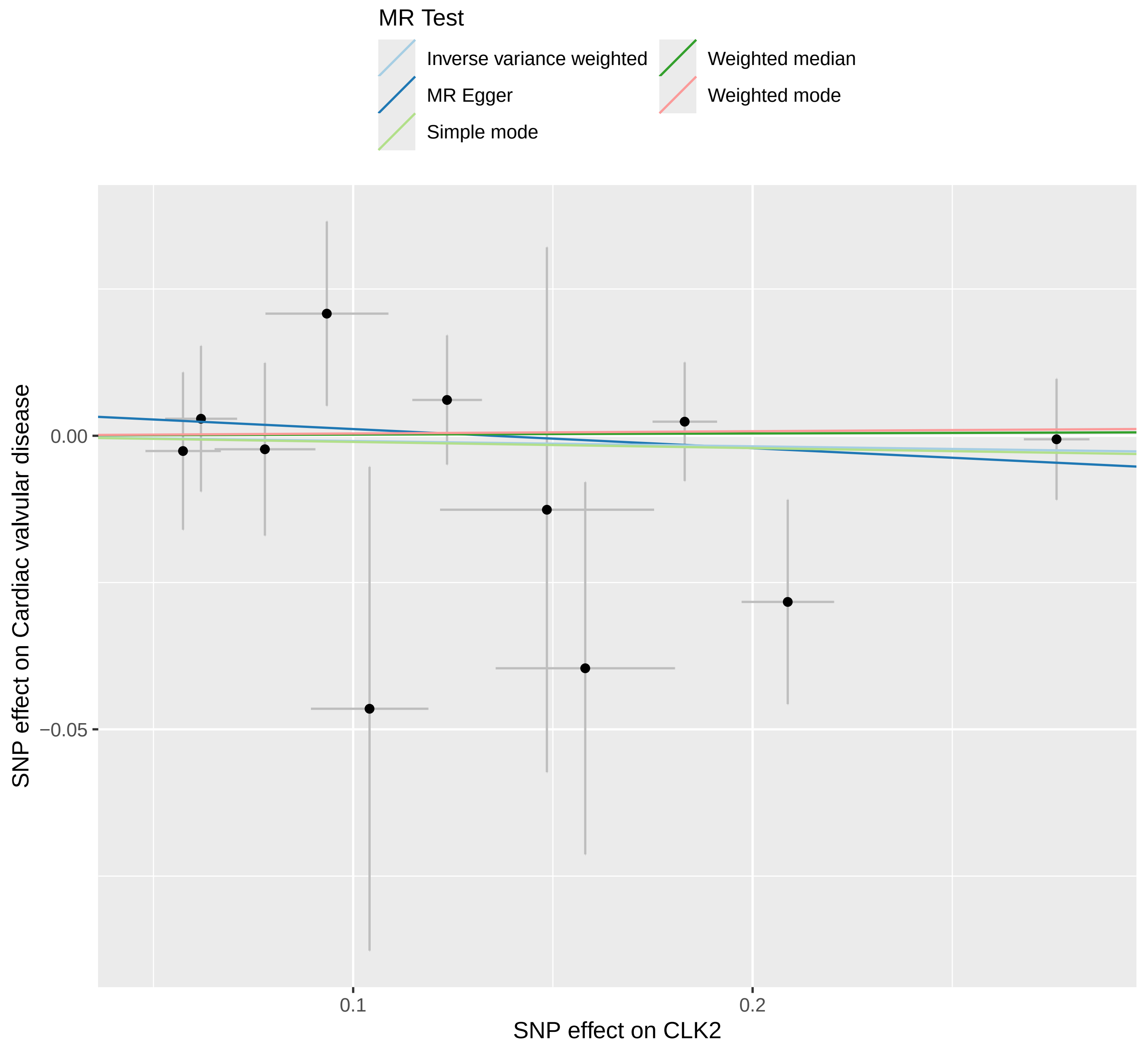
MR Test
Inverse variance weighted
MR Egger
Simple mode
Weighted median
Weighted mode
0.00
−0.05
0.1
0.2
SNP effect on Cardiac valvular disease
SNP effect on CLK2

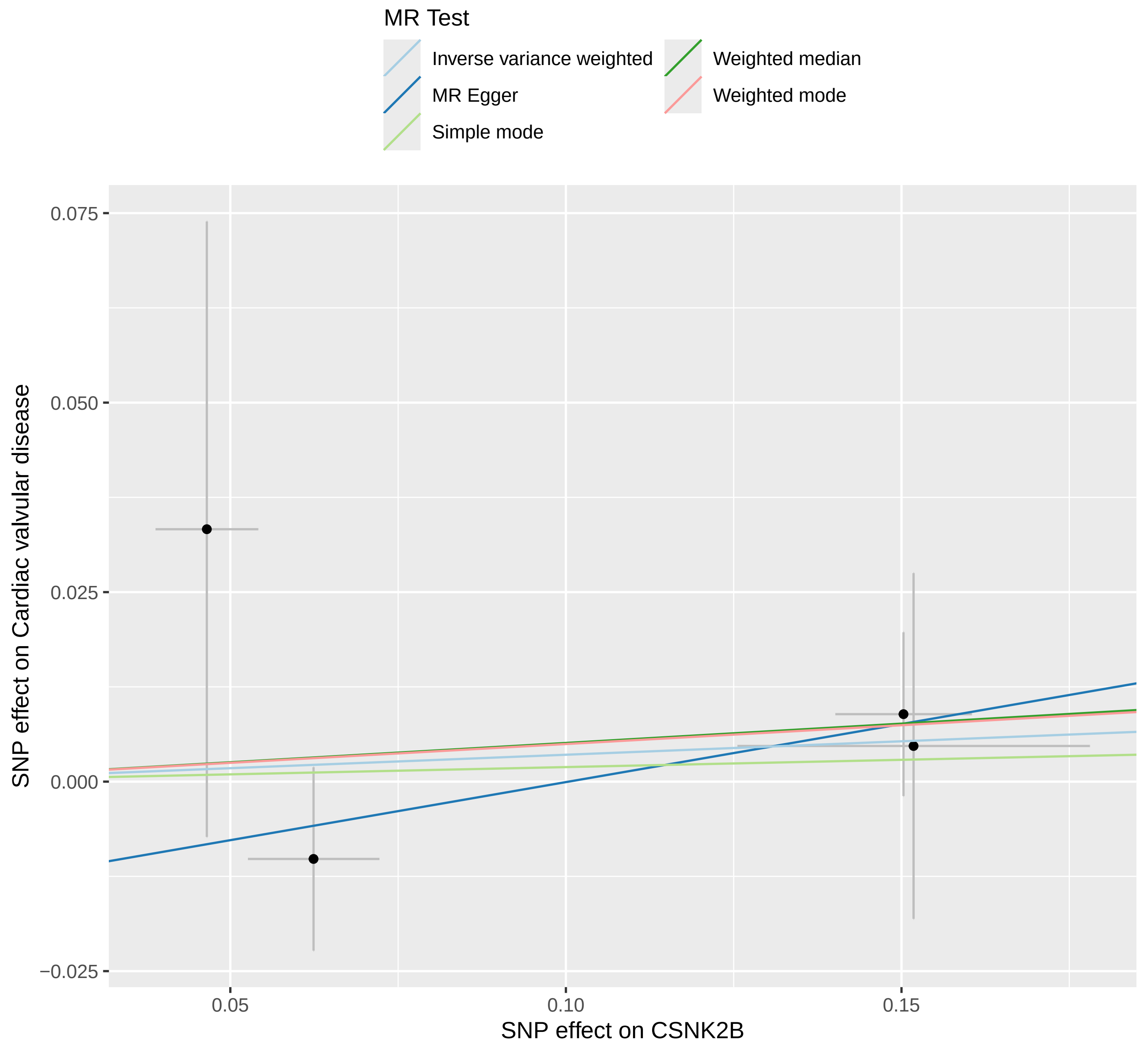

MR Test
Inverse variance weighted
MR Egger
Simple mode
Weighted median
Weighted mode
0.075
0.050
0.025
0.000
−0.025
0.05
0.10
0.15
SNP effect on Cardiac valvular disease
SNP effect on CSNK2B

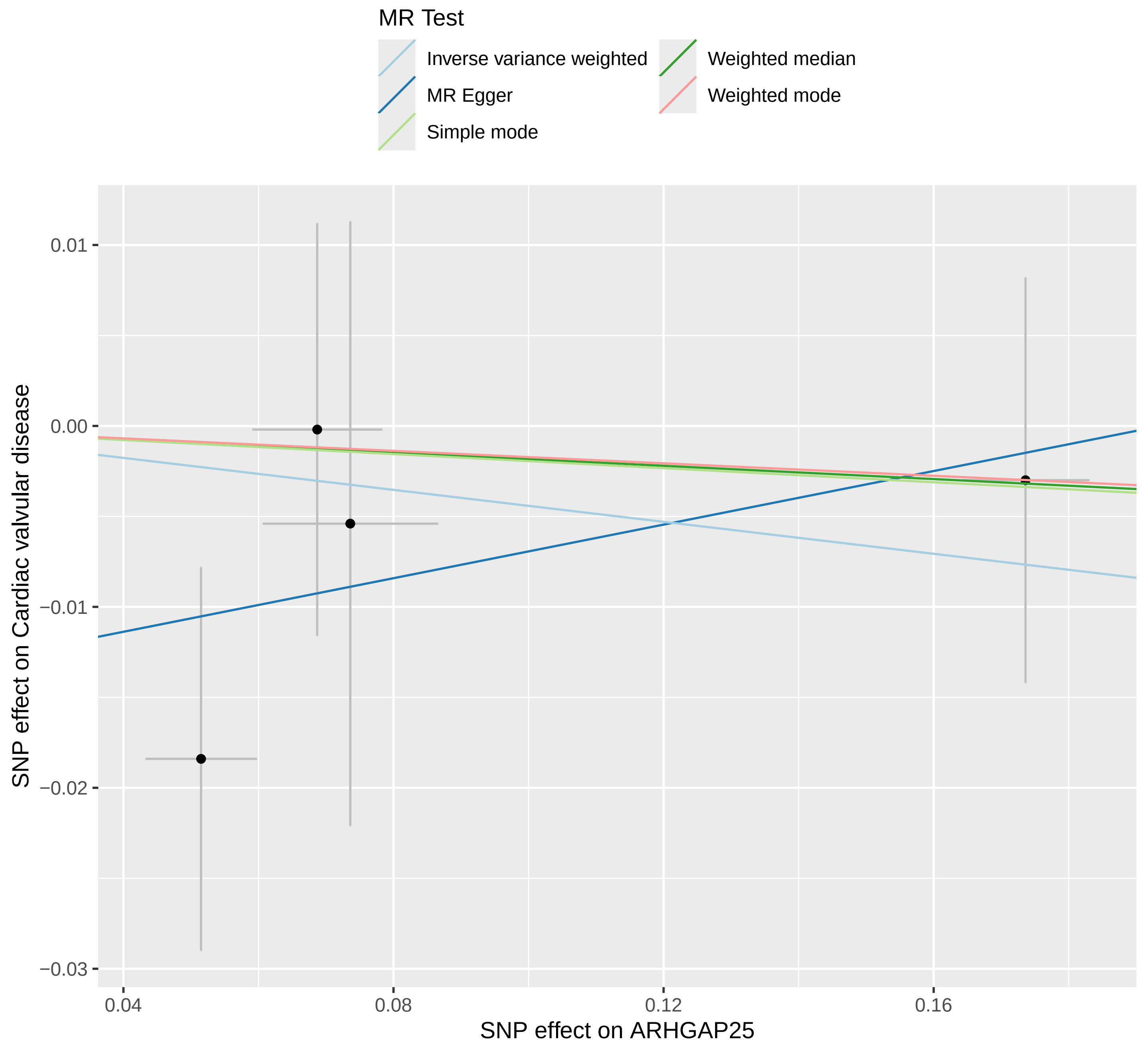

MR Test
Inverse variance weighted
MR Egger
Simple mode
Weighted median
Weighted mode
0.01
0.00
−0.01
−0.02
−0.03
0.04
0.08
0.12
0.16
SNP effect on Cardiac valvular disease
SNP effect on ARHGAP25

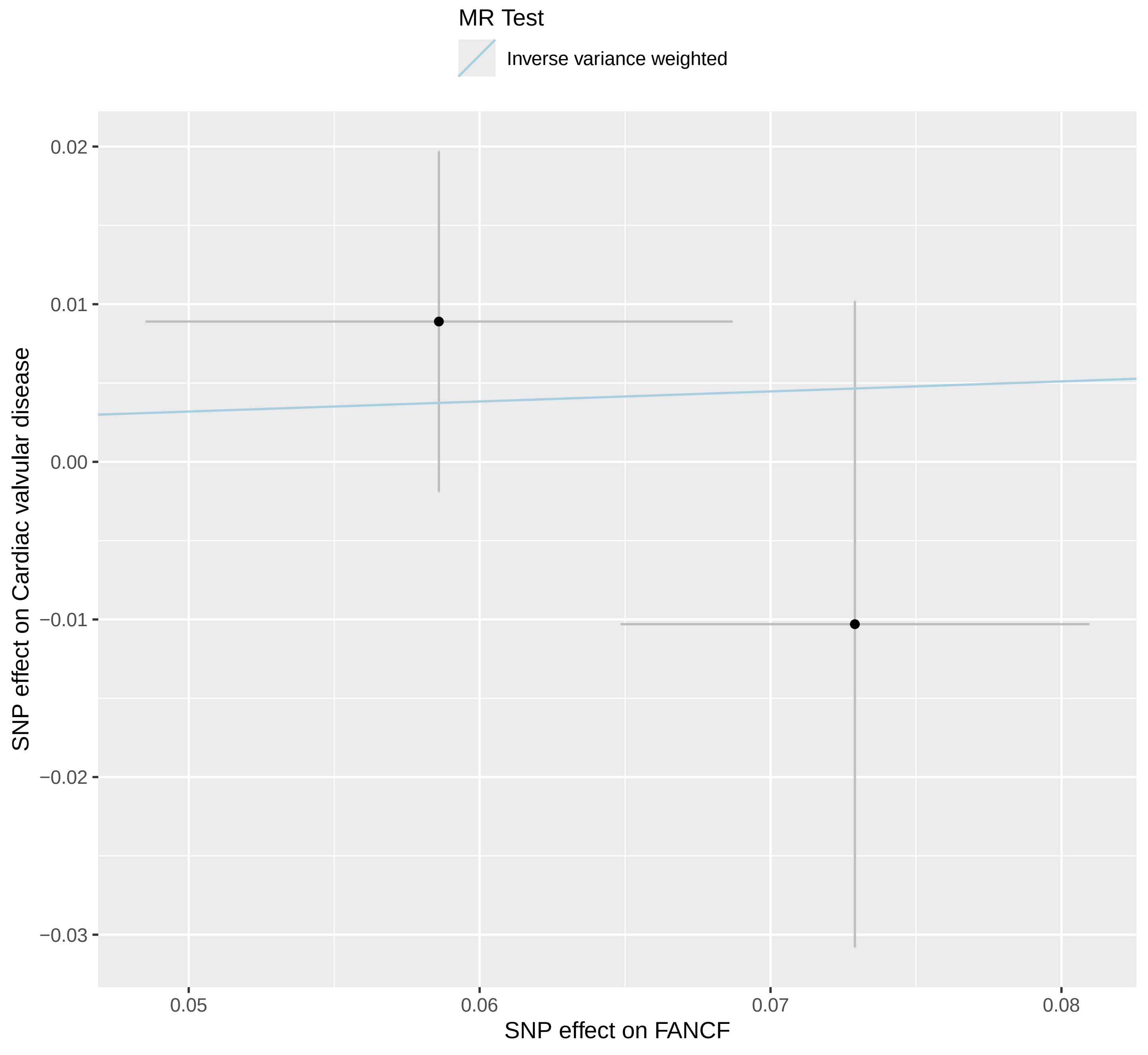

MR Test
Inverse variance weighted
0.02
0.01
0.00
−0.01
−0.02
−0.03
0.05
0.06
0.07
0.08
SNP effect on Cardiac valvular disease
SNP effect on FANCF

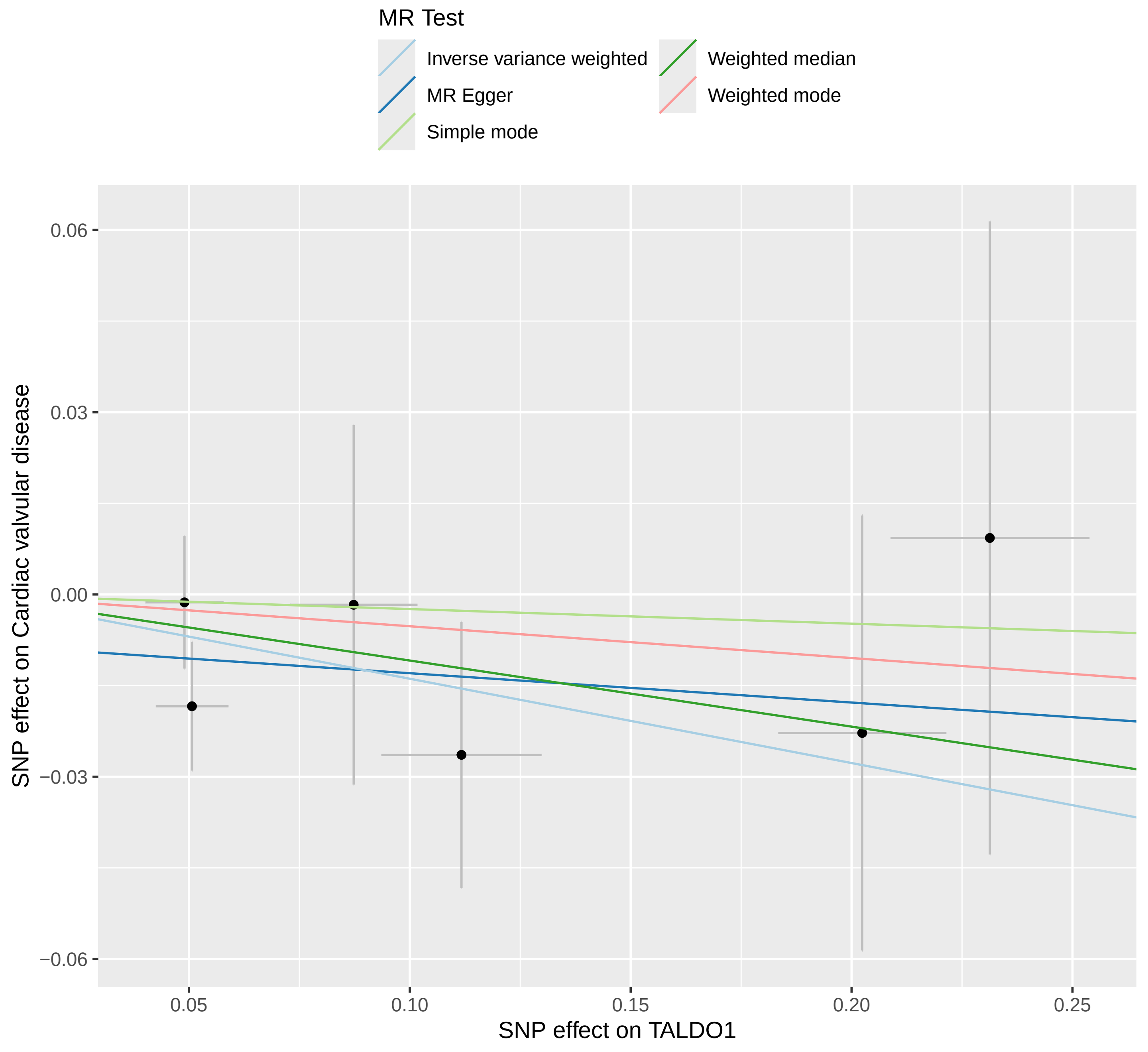

MR Test
Inverse variance weighted
MR Egger
Simple mode
Weighted median
Weighted mode
0.06
0.03
0.00
−0.03
−0.06
SNP effect on Cardiac valvular disease
0.05
0.10
0.15
0.20
0.25
SNP effect on TALDO1

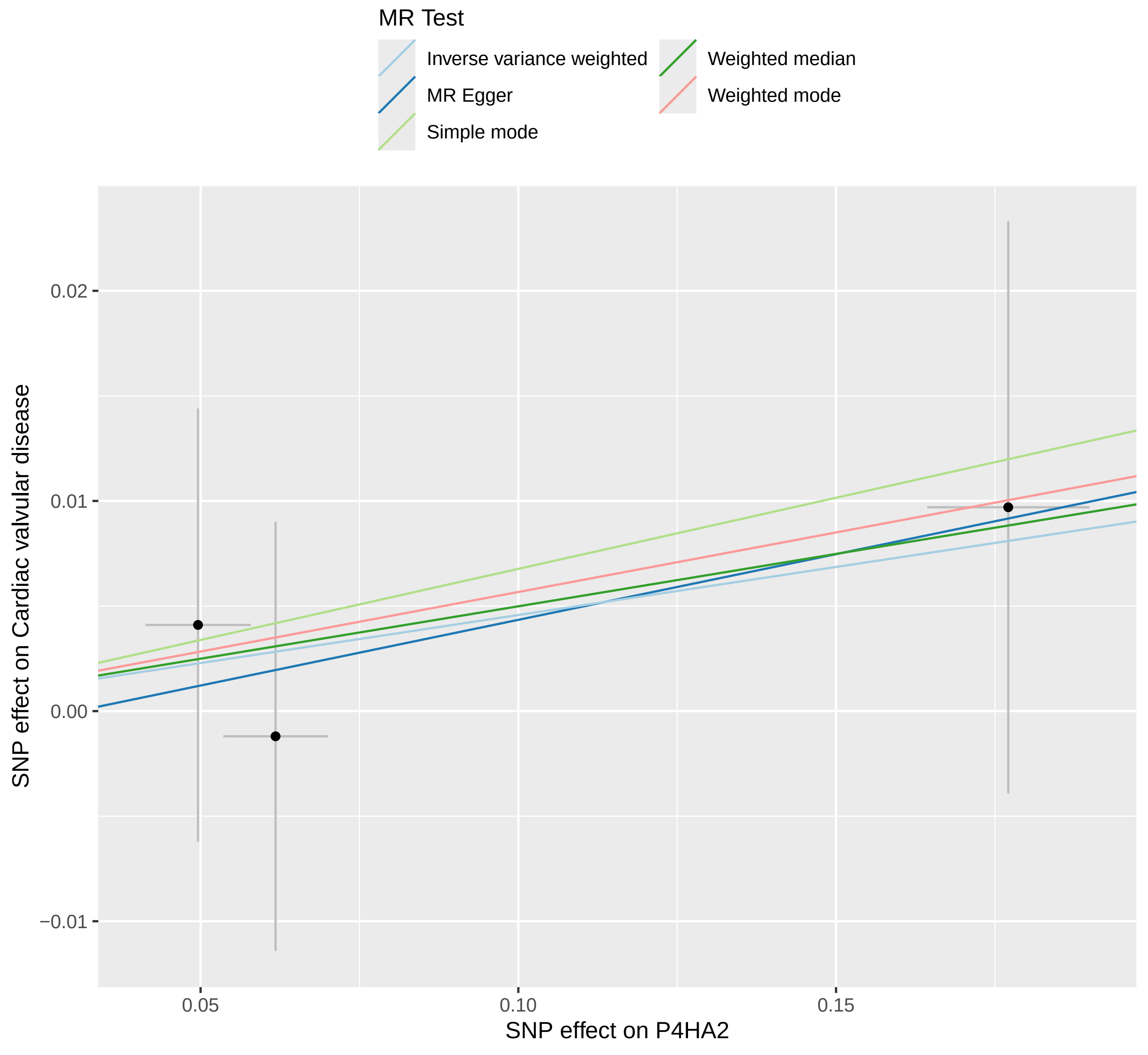

MR Test
Inverse variance weighted
MR Egger
Simple mode
Weighted median
Weighted mode
0.02
0.01
0.00
−0.01
0.05
0.10
0.15
SNP effect on Cardiac valvular disease
SNP effect on P4HA2

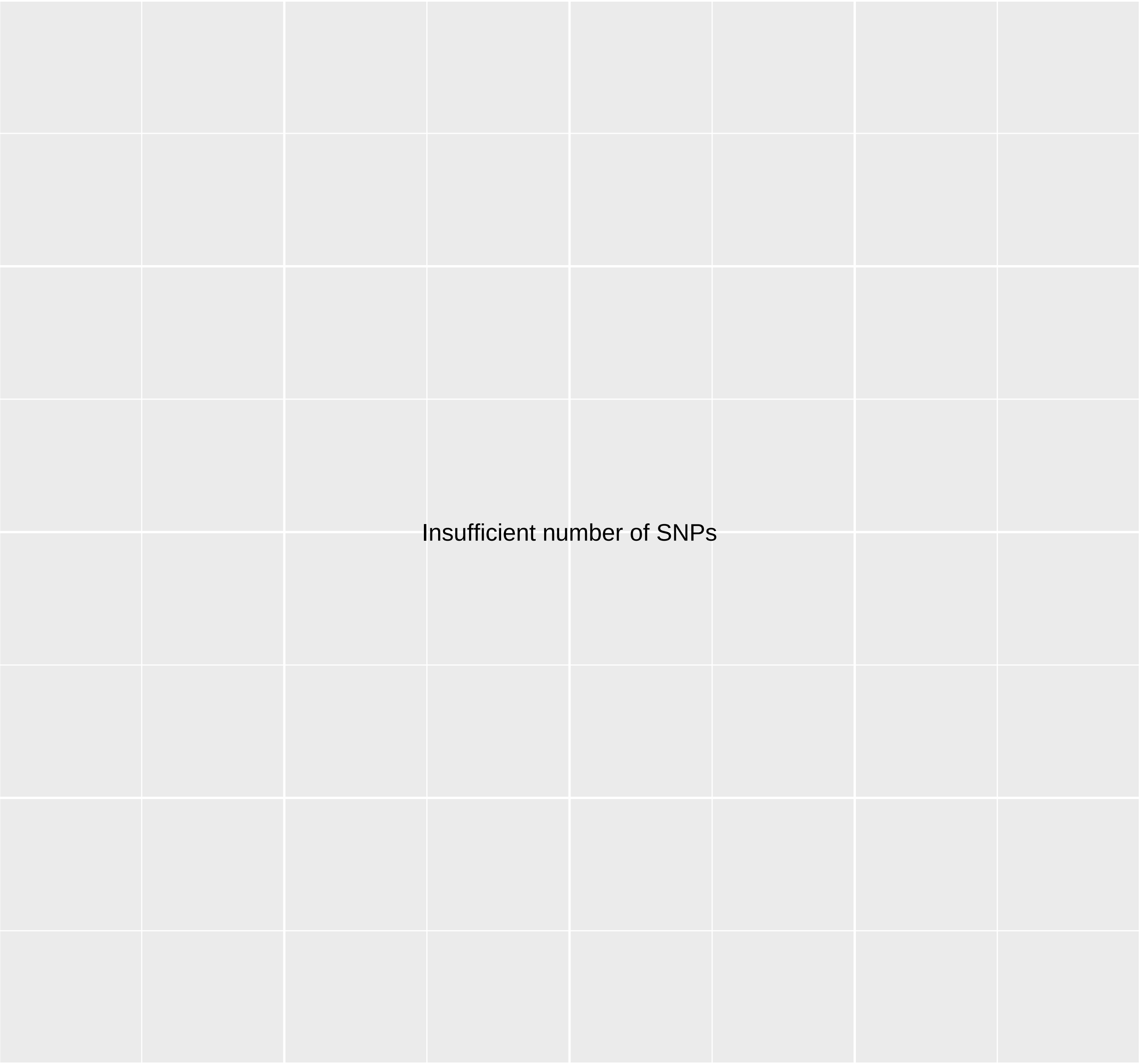
Insufficient number of SNPs

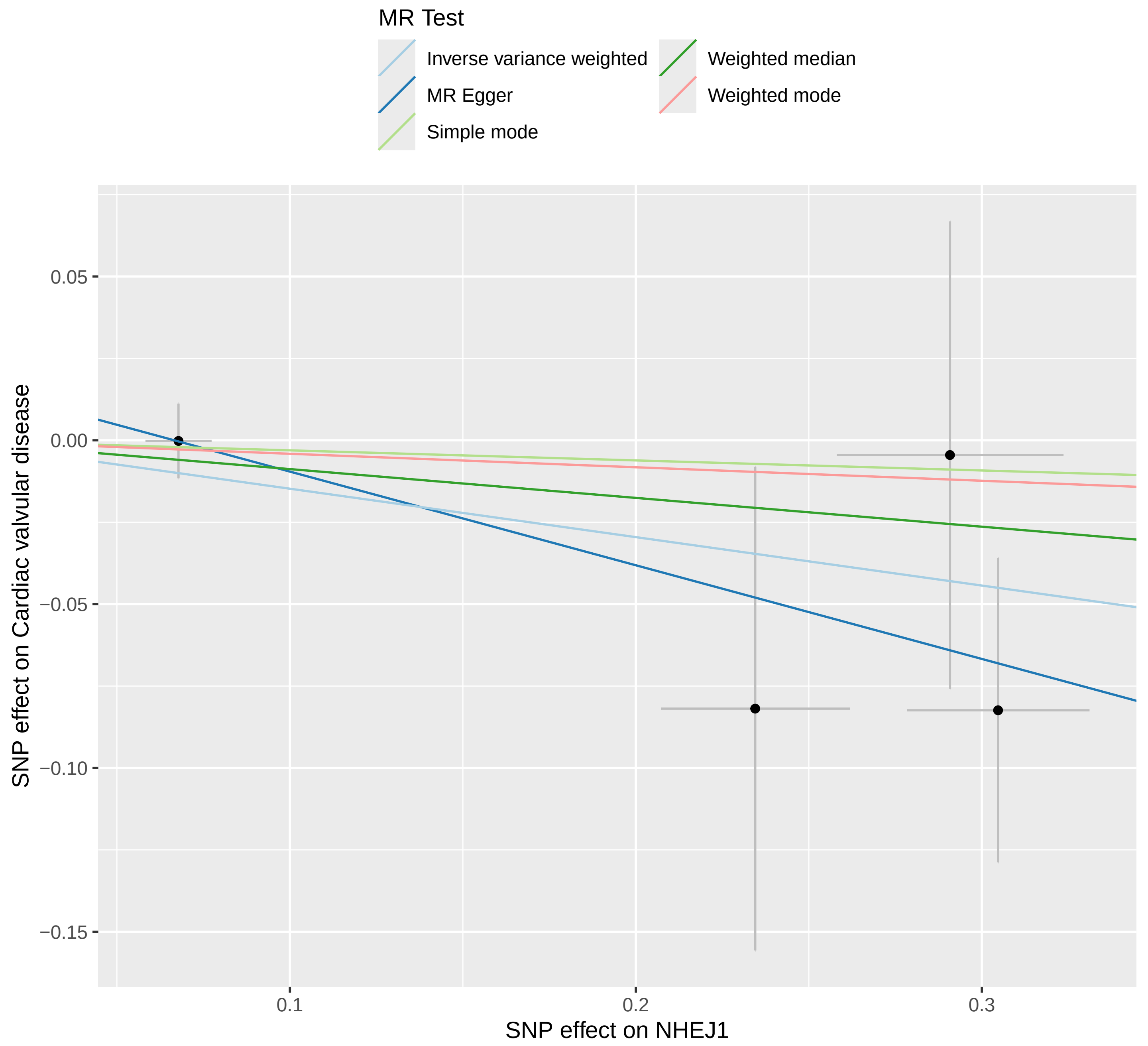
MR Test
Inverse variance weighted
MR Egger
Simple mode
Weighted median
Weighted mode
0.05
0.00
−0.05
−0.10
−0.15
SNP effect on Cardiac valvular disease
0.1
0.2
0.3
SNP effect on NHEJ1

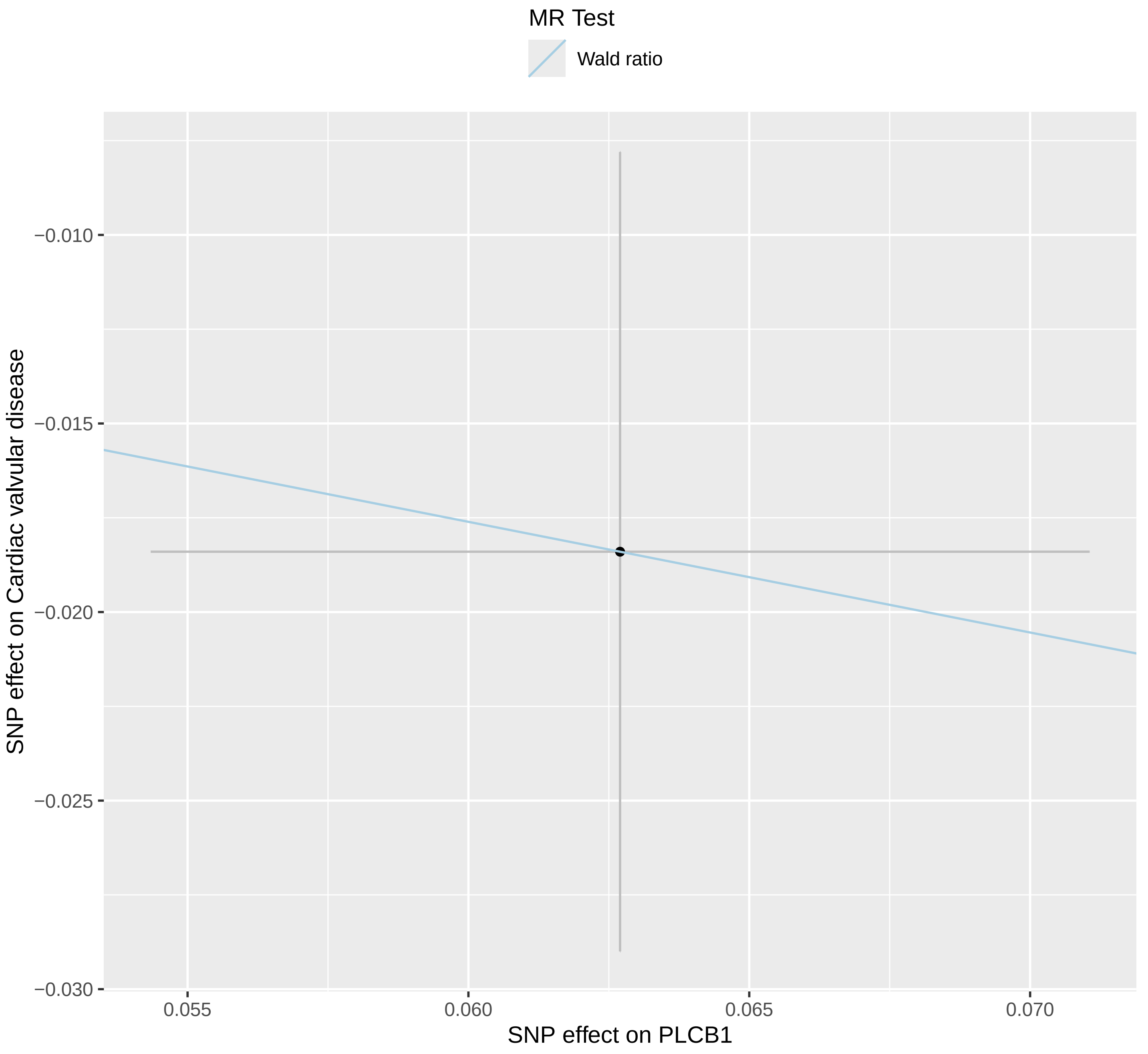

MR Test
Wald ratio
−0.010
−0.015
−0.020
−0.025
−0.030
SNP effect on Cardiac valvular disease
0.055
0.060
0.065
0.070
SNP effect on PLCB1

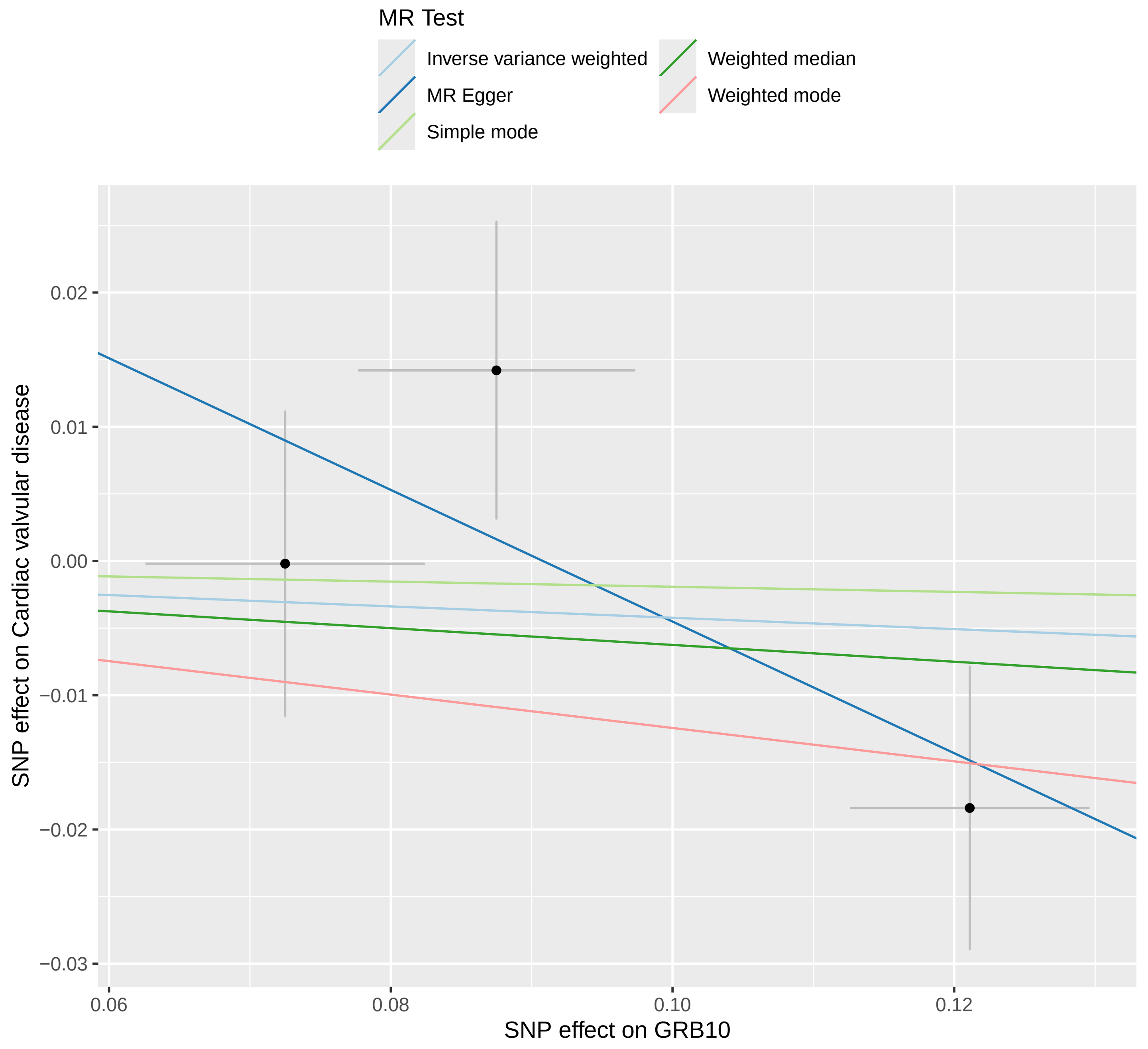
MR Test
Inverse variance weighted
MR Egger
Simple mode
Weighted median
Weighted mode
SNP effect on Cardiac valvular disease
0.02
0.01
0.00
−0.01
−0.02
−0.03
0.06
0.08
0.10
0.12
SNP effect on GRB10

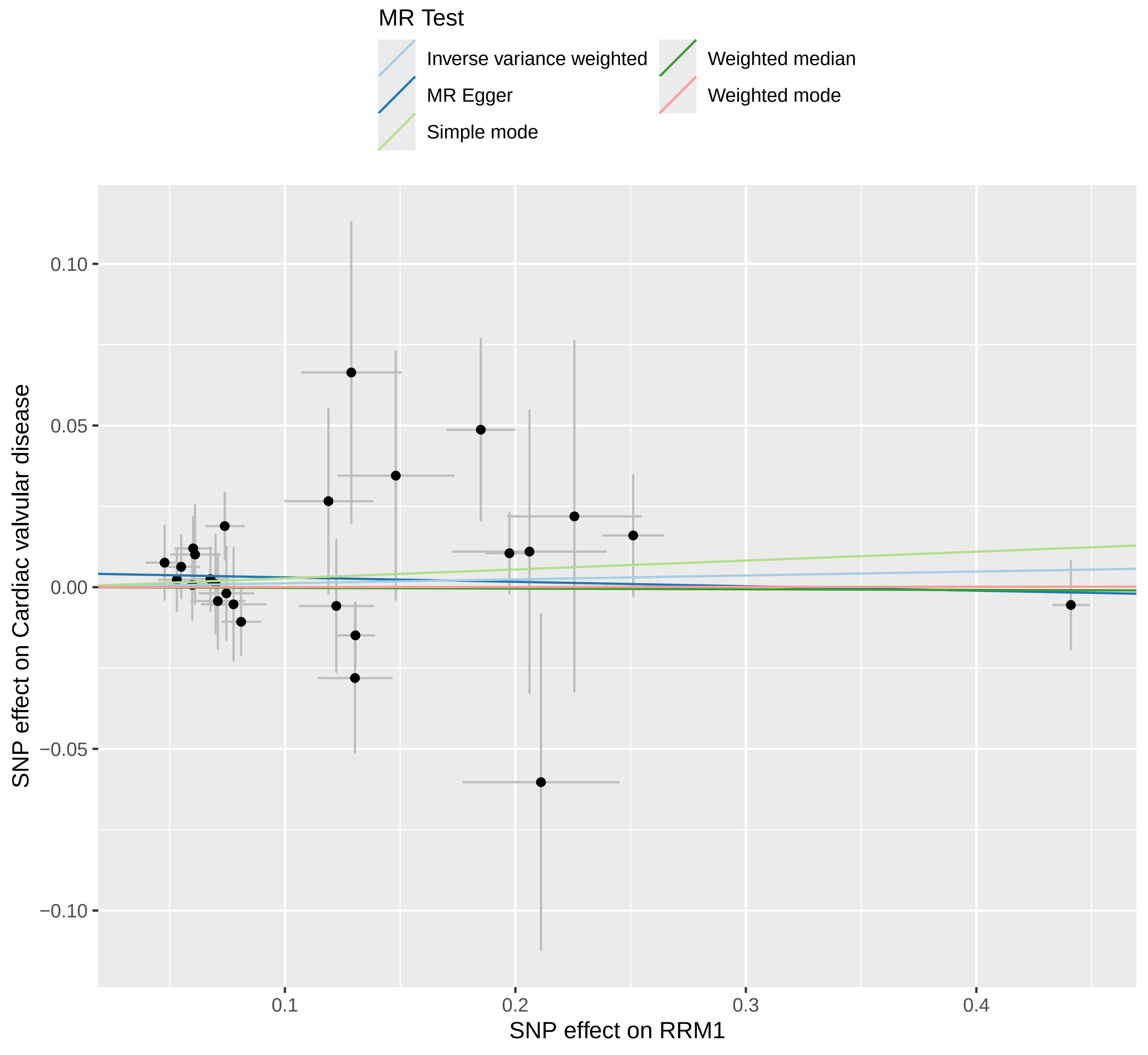

MR Test
Inverse variance weighted
MR Egger
Simple mode
Weighted median
Weighted mode
SNP effect on Cardiac valvular disease
0.10
0.05
0.00
−0.05
−0.10
0.1
0.2
0.3
0.4
SNP effect on RRM1

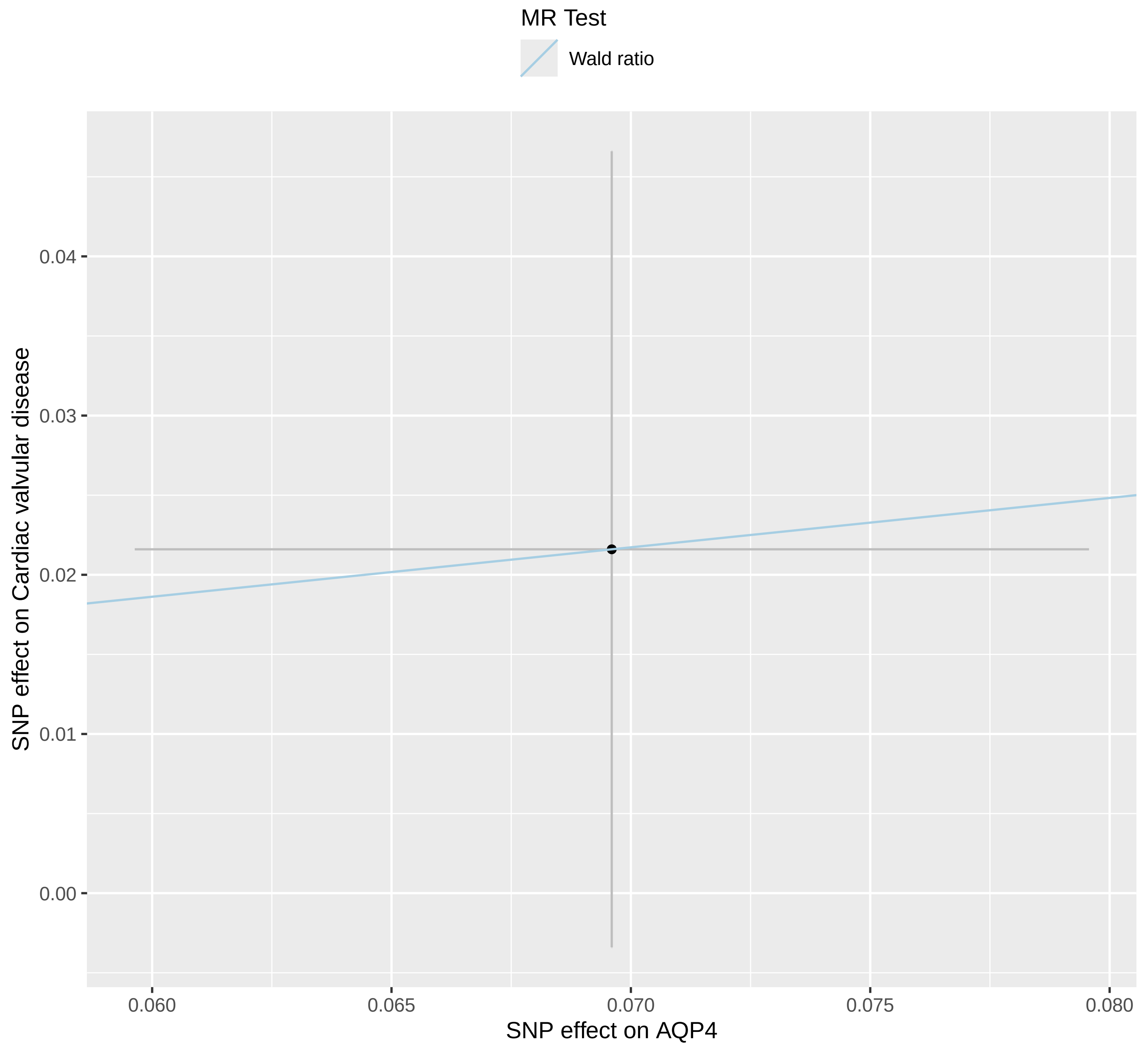

MR Test
Wald ratio
0.04
0.03
0.02
0.01
0.00
SNP effect on Cardiac valvular disease
0.060
0.065
0.070
0.075
0.080
SNP effect on AQP4

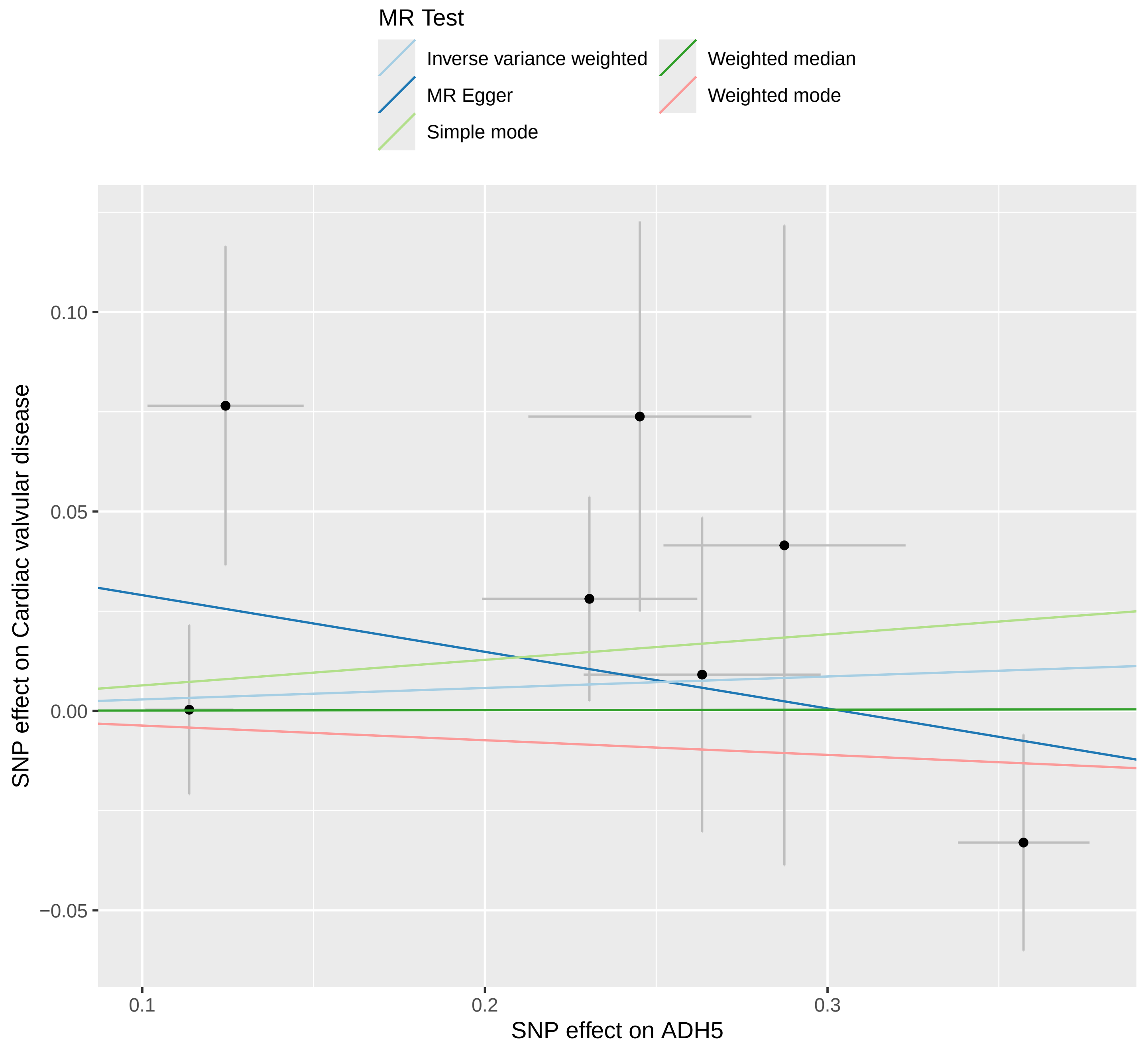
MR Test
Inverse variance weighted
MR Egger
Simple mode
Weighted median
Weighted mode
0.10
0.05
0.00
−0.05
SNP effect on Cardiac valvular disease
0.1
0.2
0.3
SNP effect on ADH5

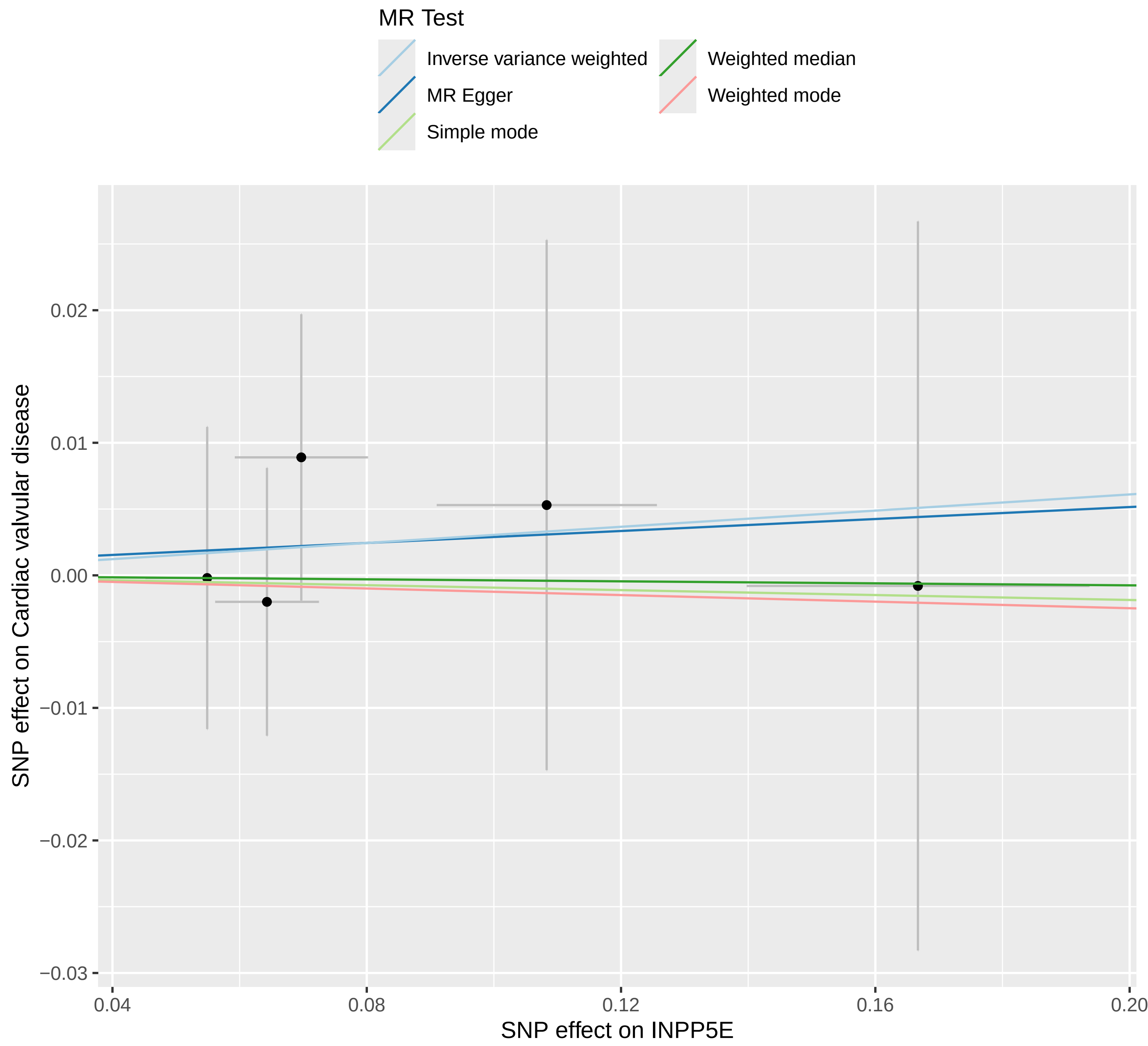
MR Test
Inverse variance weighted
MR Egger
Simple mode
Weighted median
Weighted mode
0.02
0.01
0.00
−0.01
−0.02
−0.03
SNP effect on Cardiac valvular disease
0.04
0.08
0.12
0.16
0.20
SNP effect on INPP5E

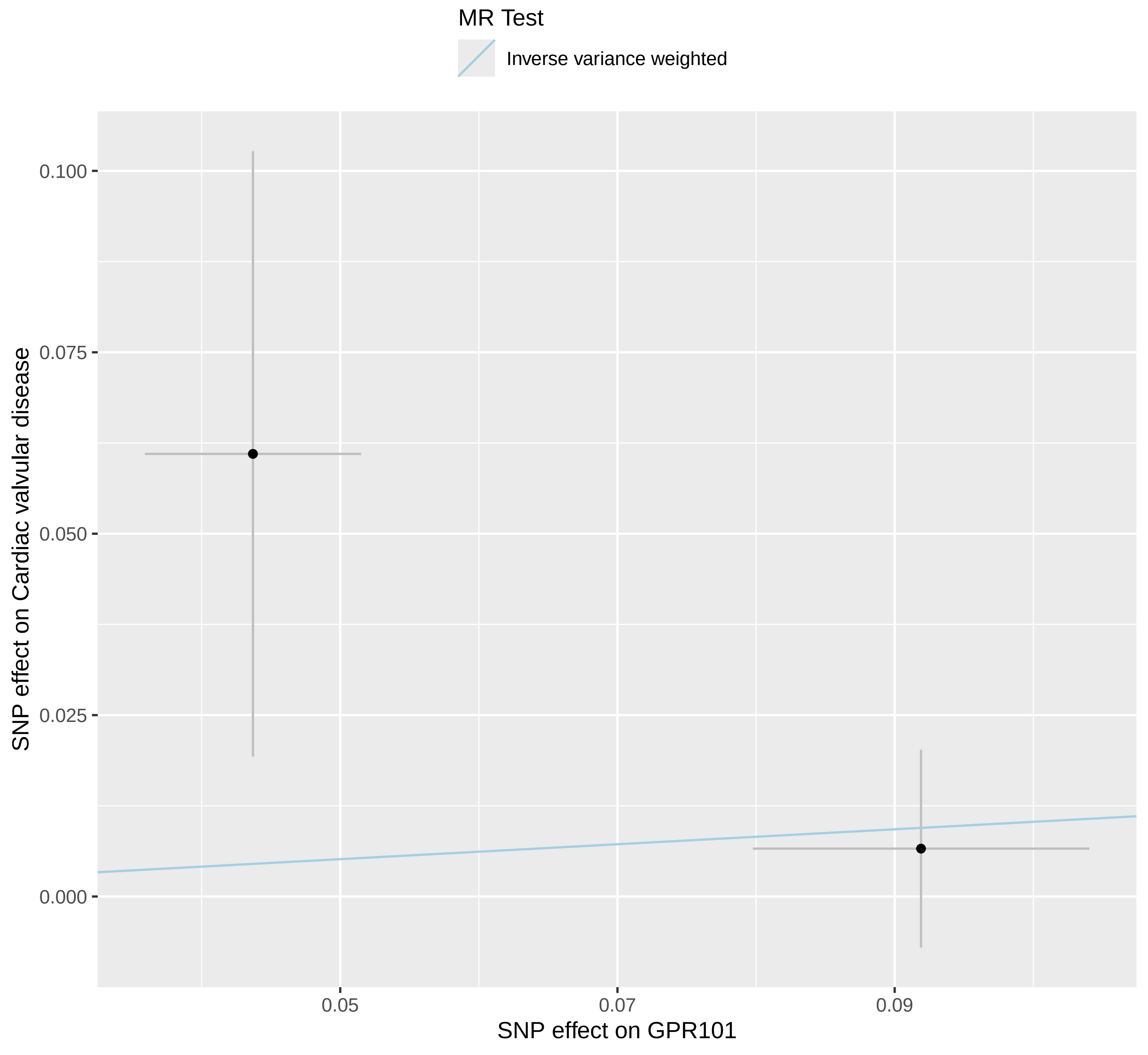
MR Test
Inverse variance weighted
SNP effect on Cardiac valvular disease
0.100
0.075
0.050
0.025
0.000
0.05
0.07
0.09
SNP effect on GPR101

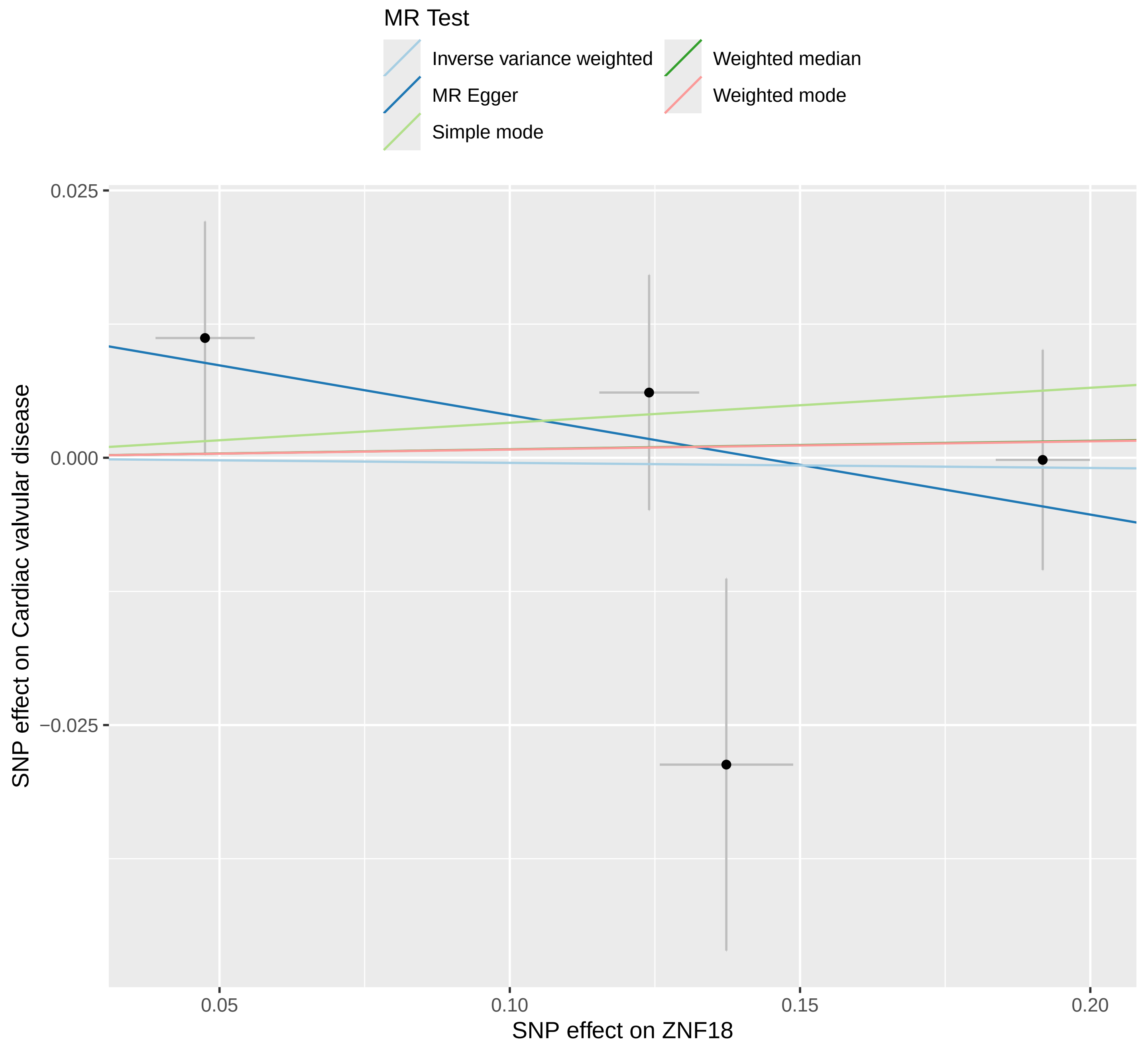
MR Test
Inverse variance weighted
MR Egger
Simple mode
Weighted median
Weighted mode
0.025
0.000
−0.025
0.05
0.10
0.15
0.20
SNP effect on Cardiac valvular disease
SNP effect on ZNF18

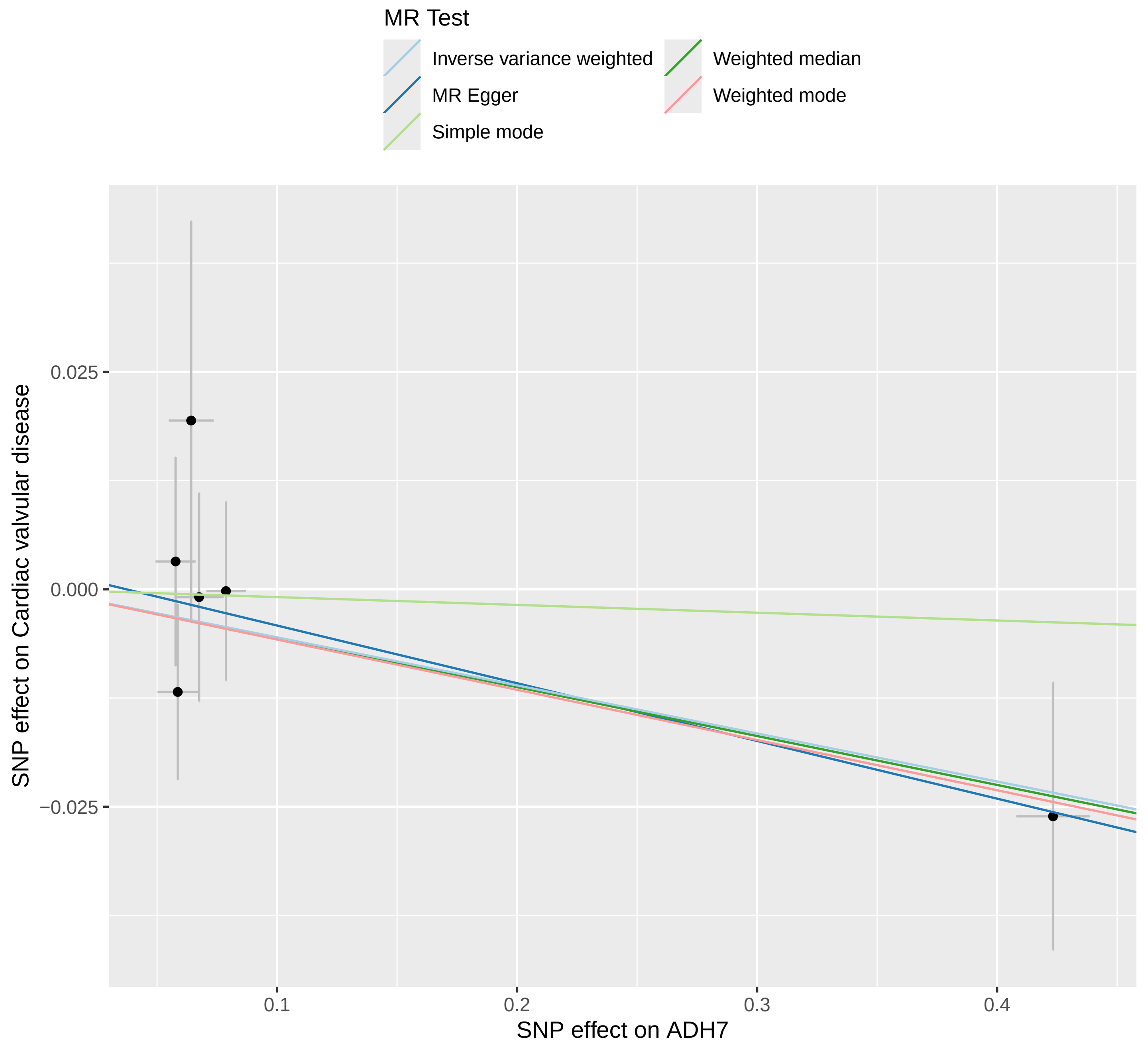
MR Test
Inverse variance weighted
MR Egger
Simple mode
Weighted median
Weighted mode
0.025
0.000
−0.025
0.1
0.2
0.3
0.4
SNP effect on Cardiac valvular disease
SNP effect on ADH7

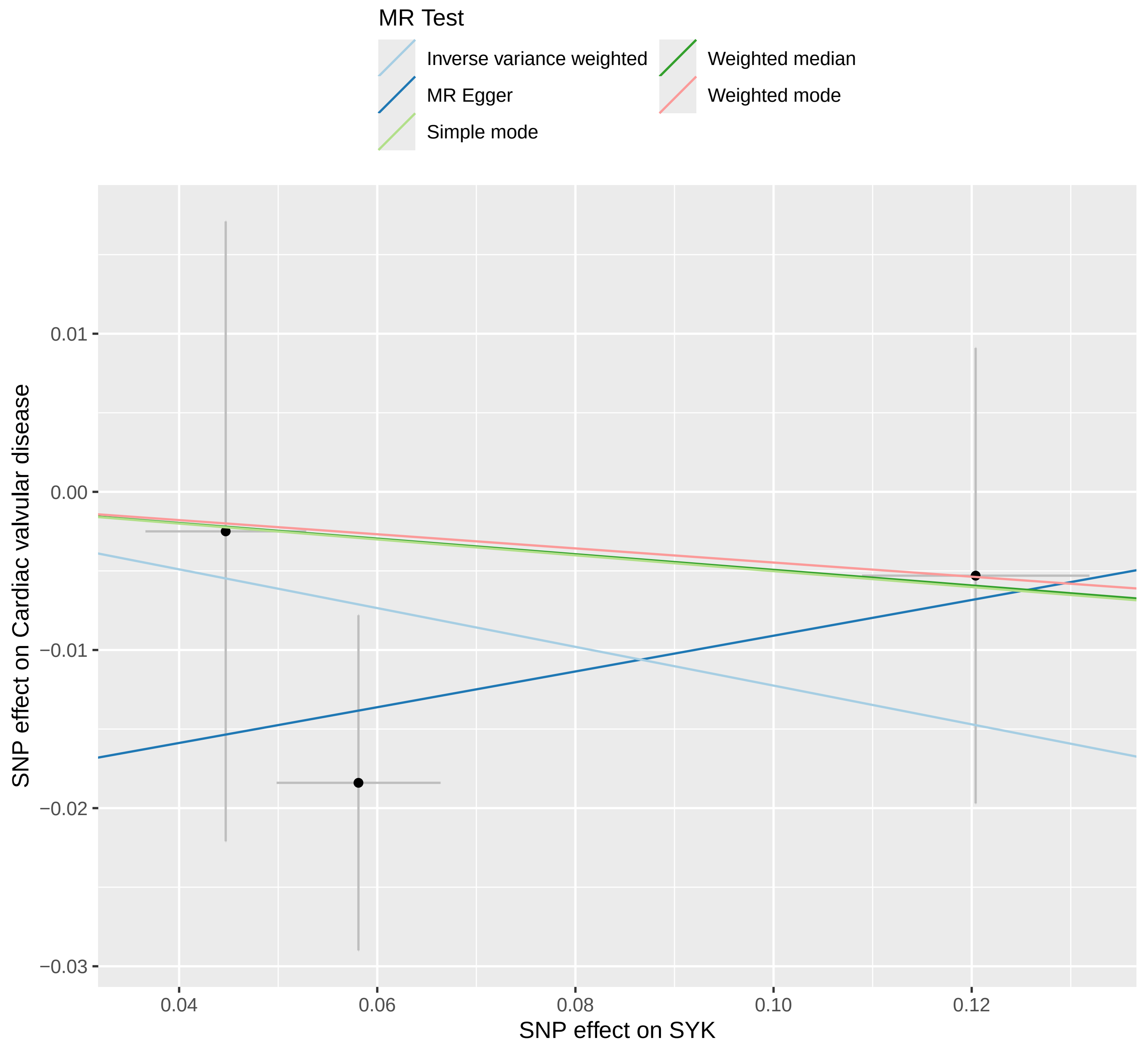
MR Test
Inverse variance weighted
MR Egger
Simple mode
Weighted median
Weighted mode
0.01
0.00
−0.01
−0.02
−0.03
SNP effect on Cardiac valvular disease
0.04
0.06
0.08
0.10
0.12
SNP effect on SYK

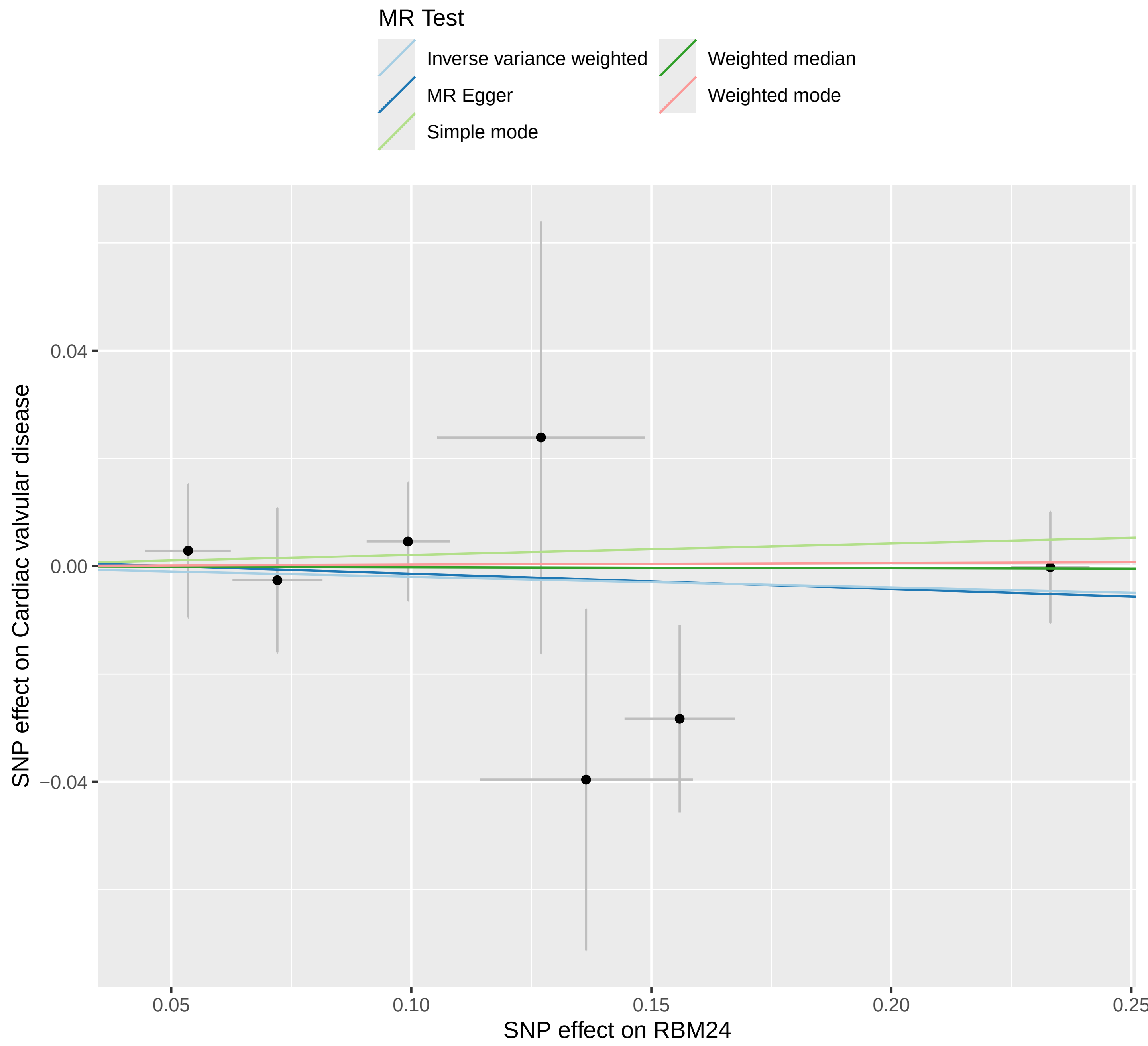

MR Test
Inverse variance weighted
MR Egger
Simple mode
Weighted median
Weighted mode
0.04
0.00
−0.04
0.05
0.10
0.15
0.20
0.25
SNP effect on Cardiac valvular disease
SNP effect on RBM24

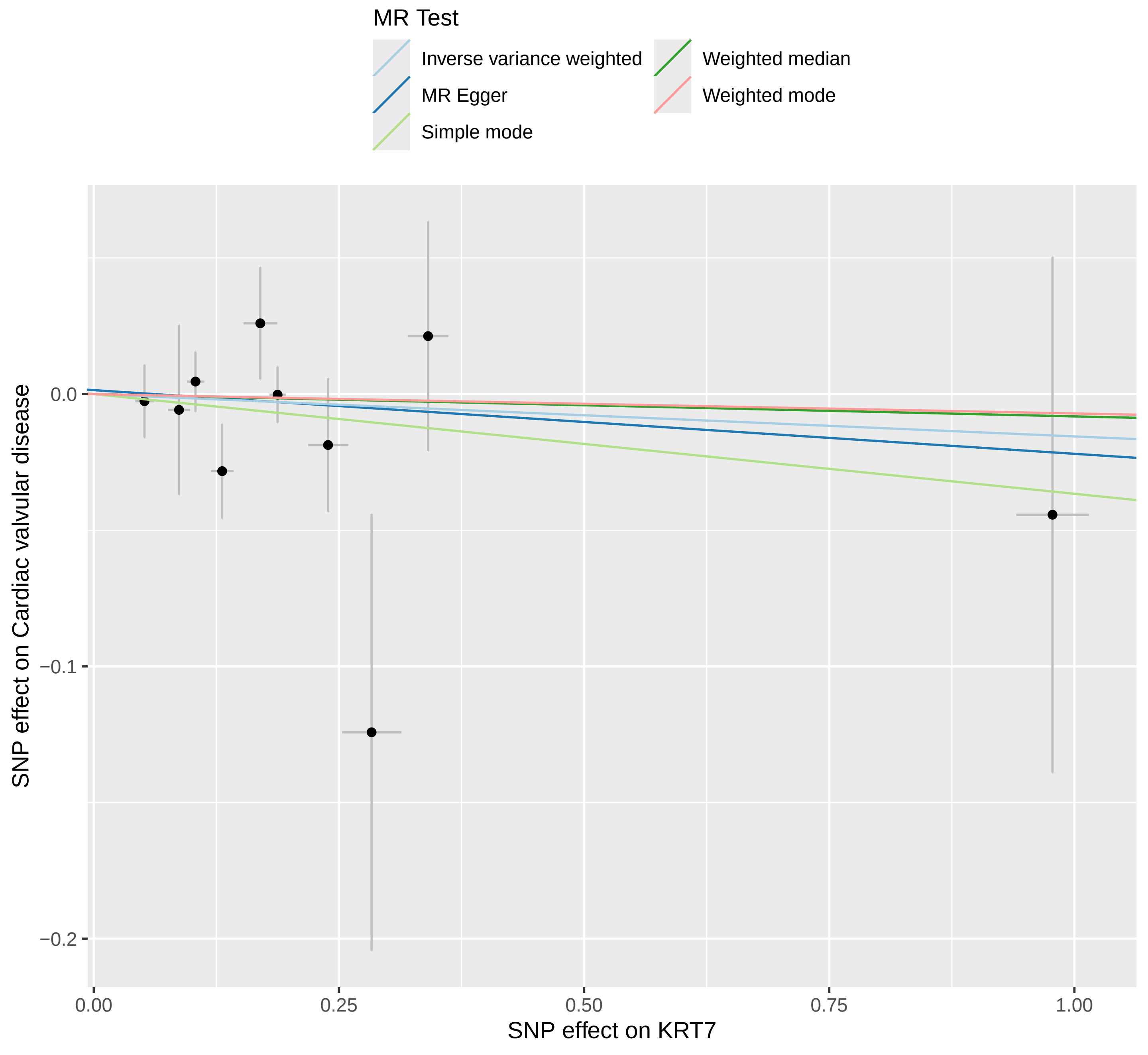

MR Test
Inverse variance weighted
MR Egger
Simple mode
Weighted median
Weighted mode
SNP effect on Cardiac valvular disease
0.0
−0.1
−0.2
0.00
0.25
0.50
0.75
1.00
SNP effect on KRT7

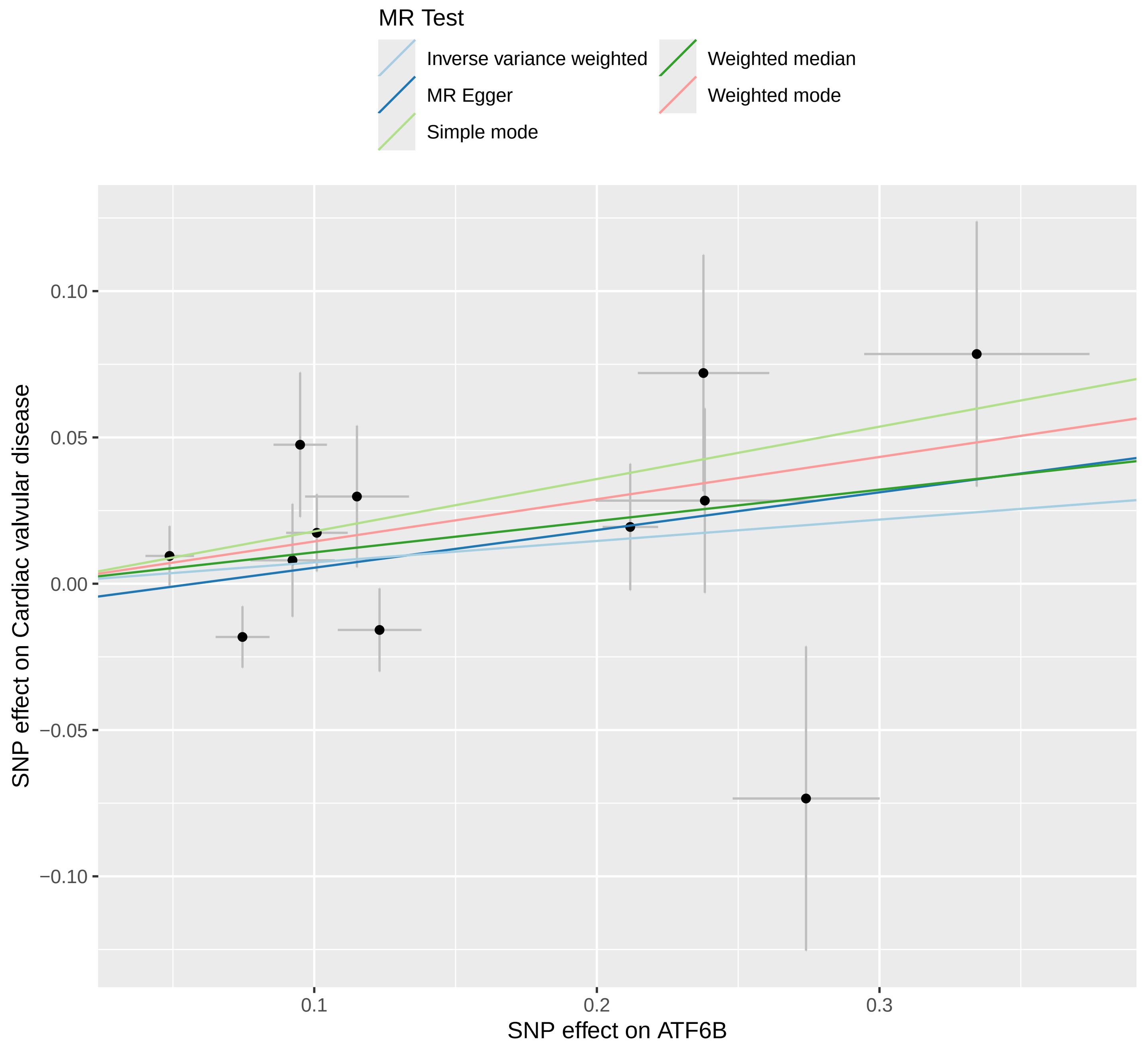

MR Test
Inverse variance weighted
MR Egger
Simple mode
Weighted median
Weighted mode
SNP effect on Cardiac valvular disease
0.10
0.05
0.00
−0.05
−0.10
0.1
0.2
0.3
SNP effect on ATF6B

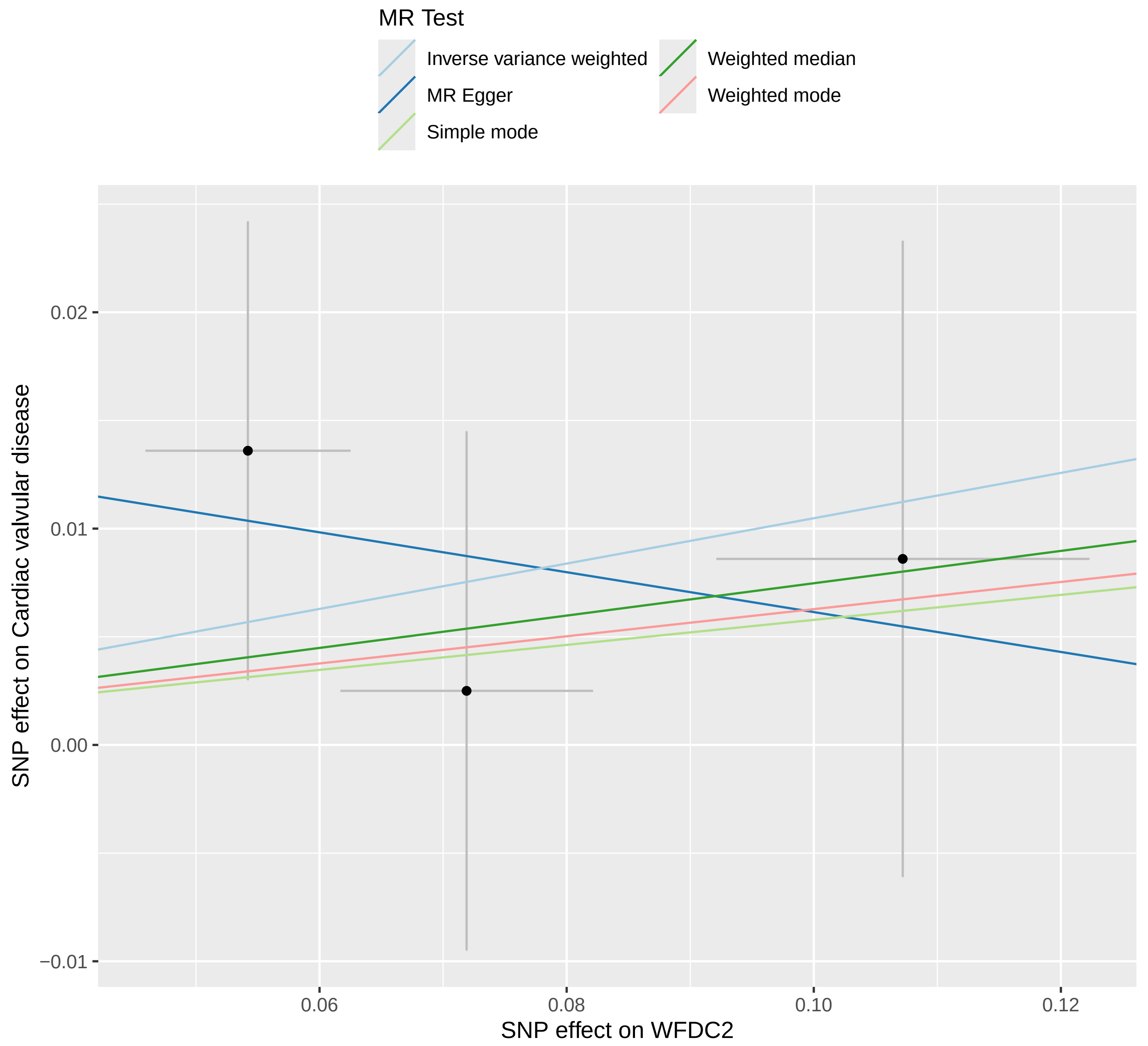
MR Test
Inverse variance weighted
MR Egger
Simple mode
Weighted median
Weighted mode
0.02
0.01
0.00
−0.01
SNP effect on Cardiac valvular disease
0.06
0.08
0.10
0.12
SNP effect on WFDC2

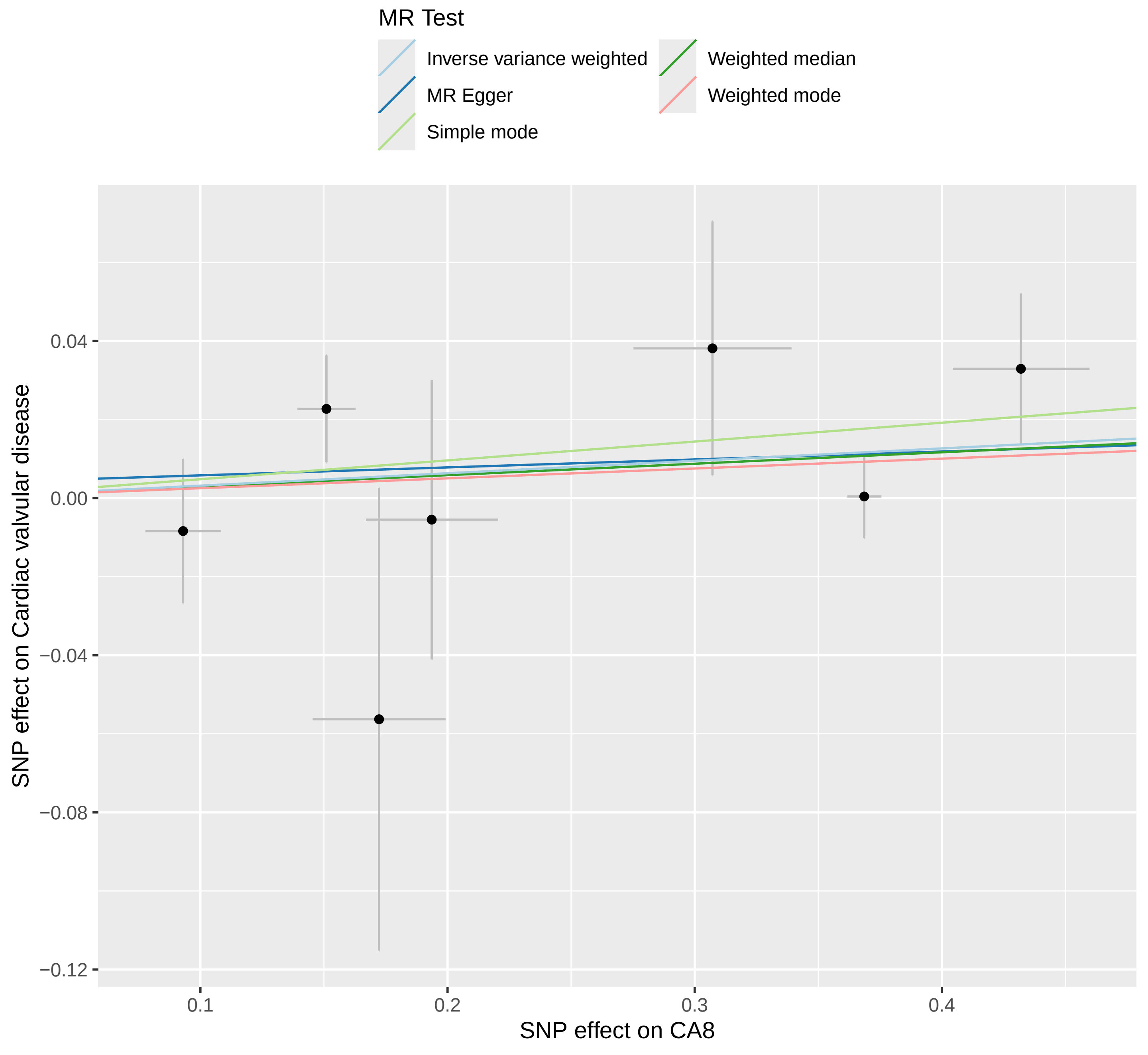

MR Test
Inverse variance weighted
MR Egger
Simple mode
Weighted median
Weighted mode
0.04
0.00
−0.04
−0.08
−0.12
SNP effect on Cardiac valvular disease
0.1
0.2
0.3
0.4
SNP effect on CA8

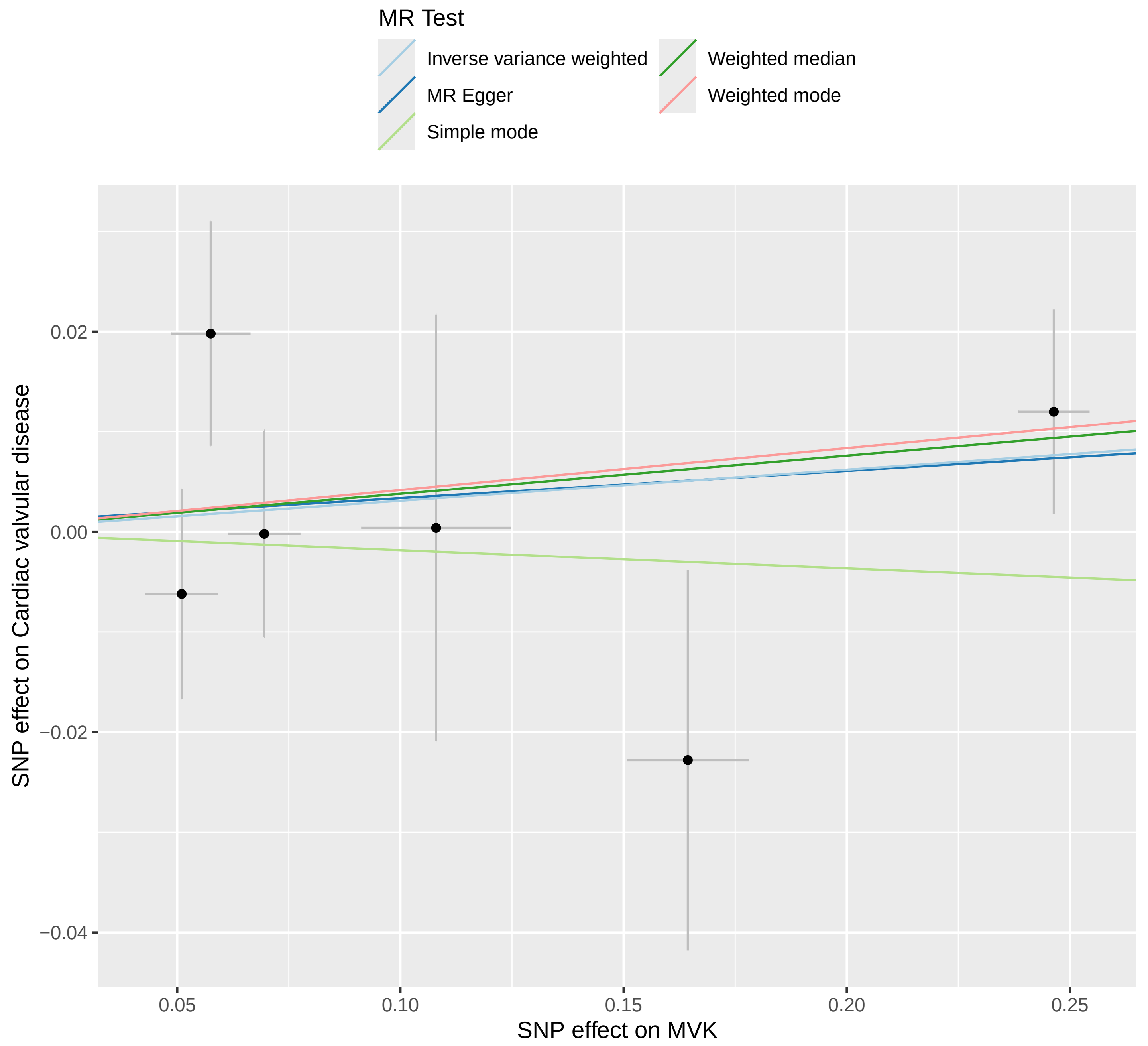
MR Test
Inverse variance weighted
MR Egger
Simple mode
Weighted median
Weighted mode
0.02
0.00
−0.02
−0.04
0.05
0.10
0.15
0.20
0.25
SNP effect on Cardiac valvular disease
SNP effect on MVK

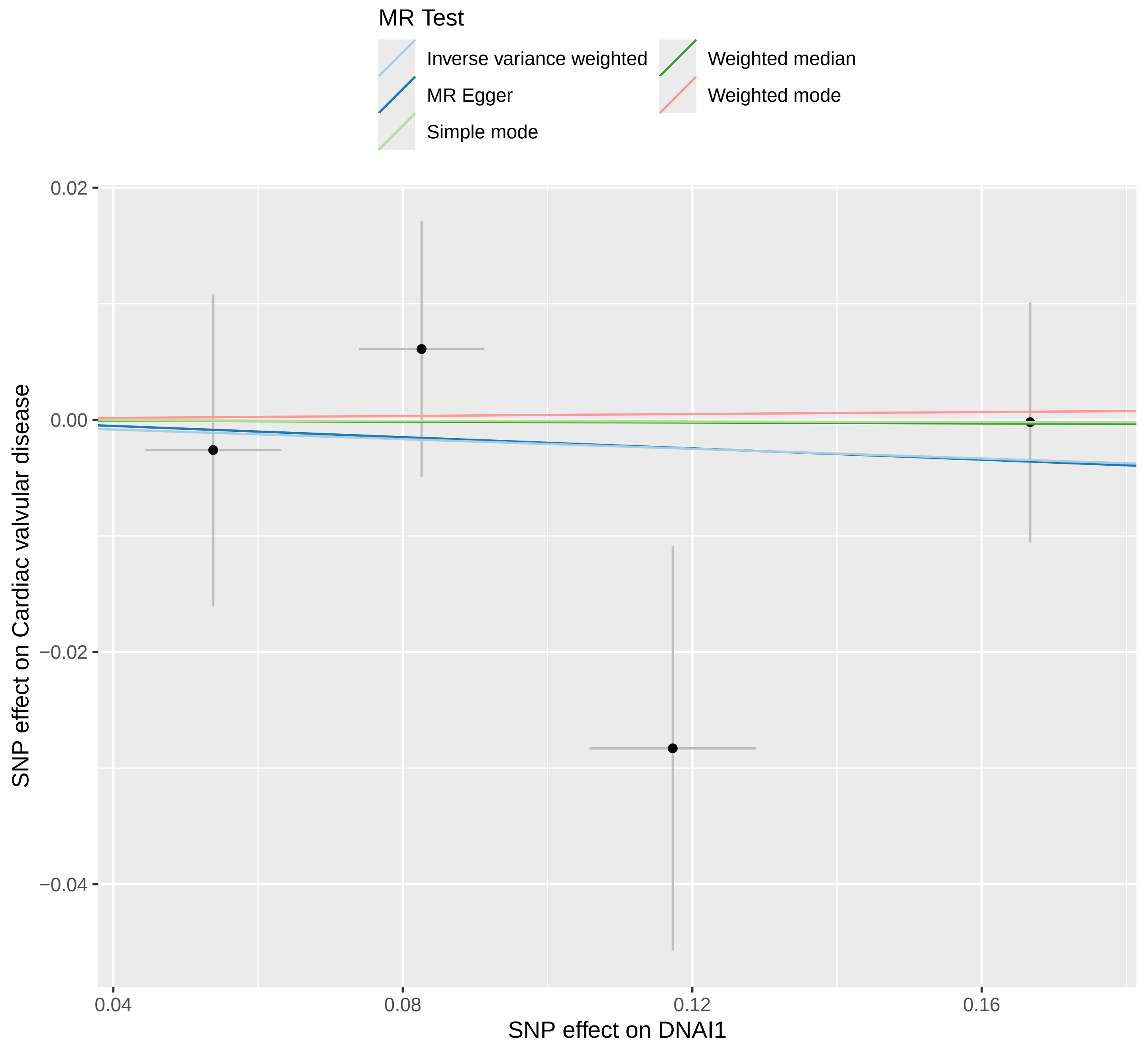
MR Test
Inverse variance weighted
MR Egger
Simple mode
Weighted median
Weighted mode
0.02
0.00
−0.02
−0.04
0.04
0.08
0.12
0.16
SNP effect on Cardiac valvular disease
SNP effect on DNAI1

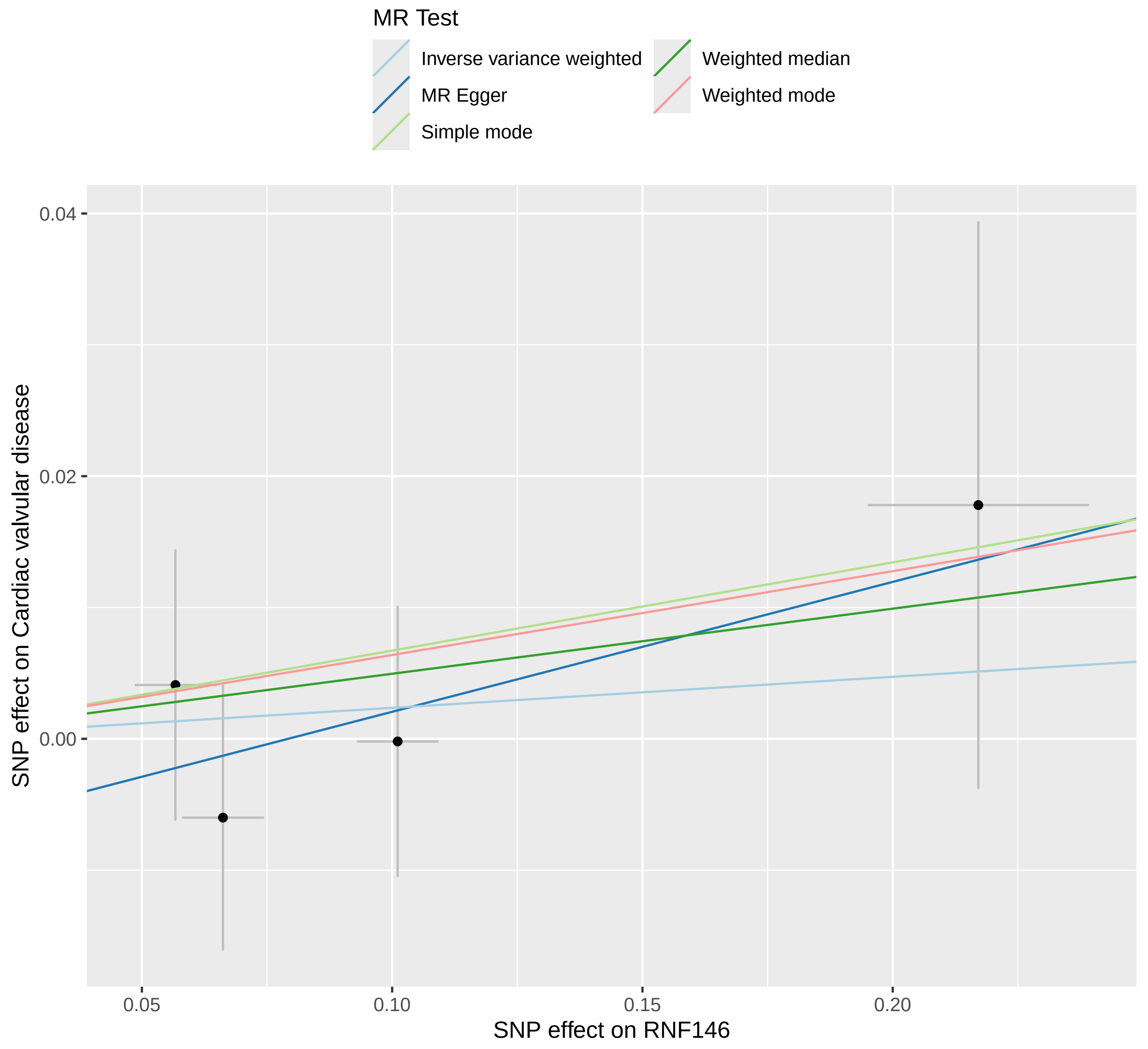
MR Test
Inverse variance weighted
MR Egger
Simple mode
Weighted median
Weighted mode
0.04
0.02
0.00
0.05
0.10
0.15
0.20
SNP effect on RNF146
SNP effect on Cardiac valvular disease

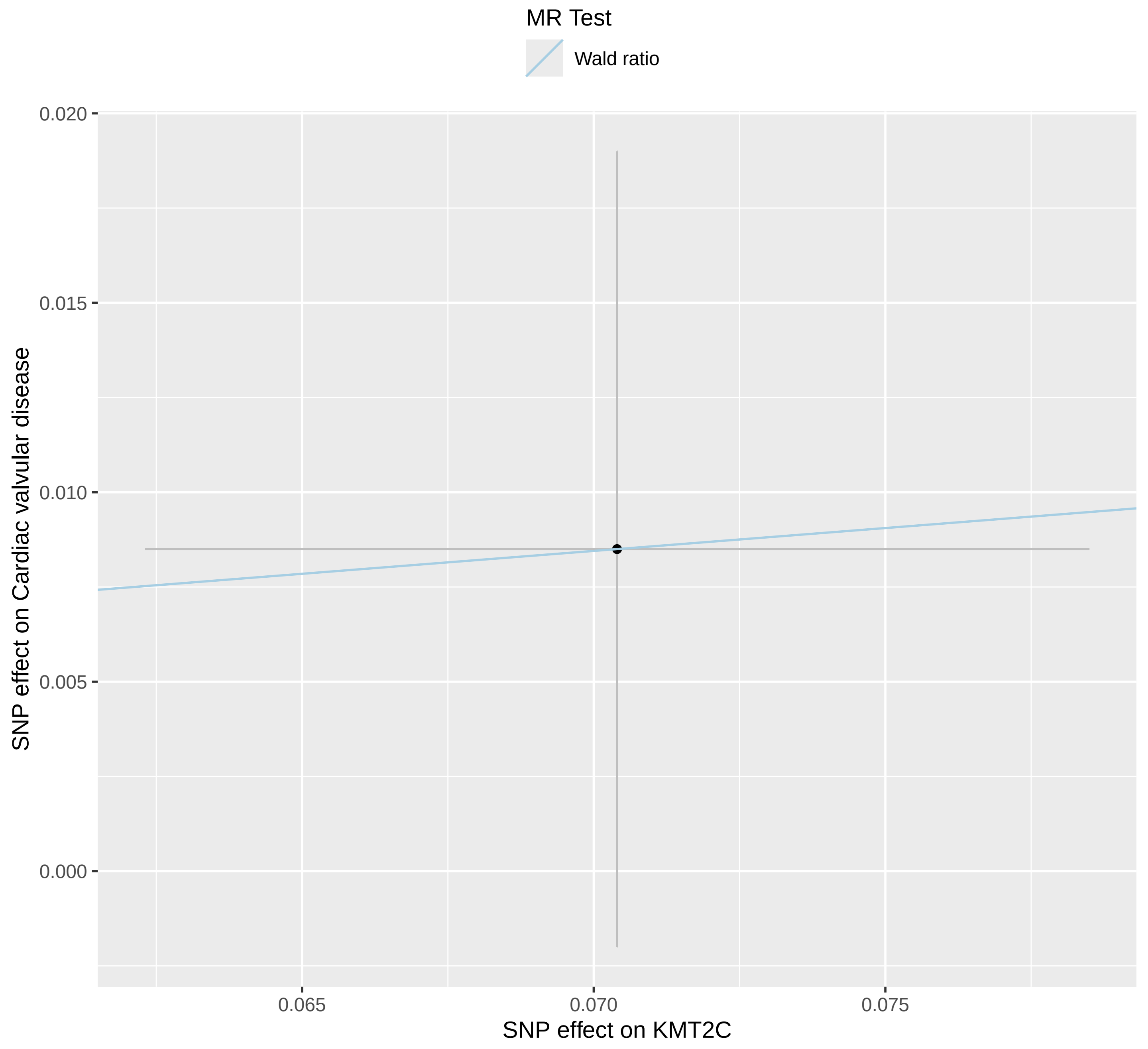

MR Test
Wald ratio
0.020
0.015
0.010
0.005
0.000
0.065
0.070
0.075
SNP effect on Cardiac valvular disease
SNP effect on KMT2C

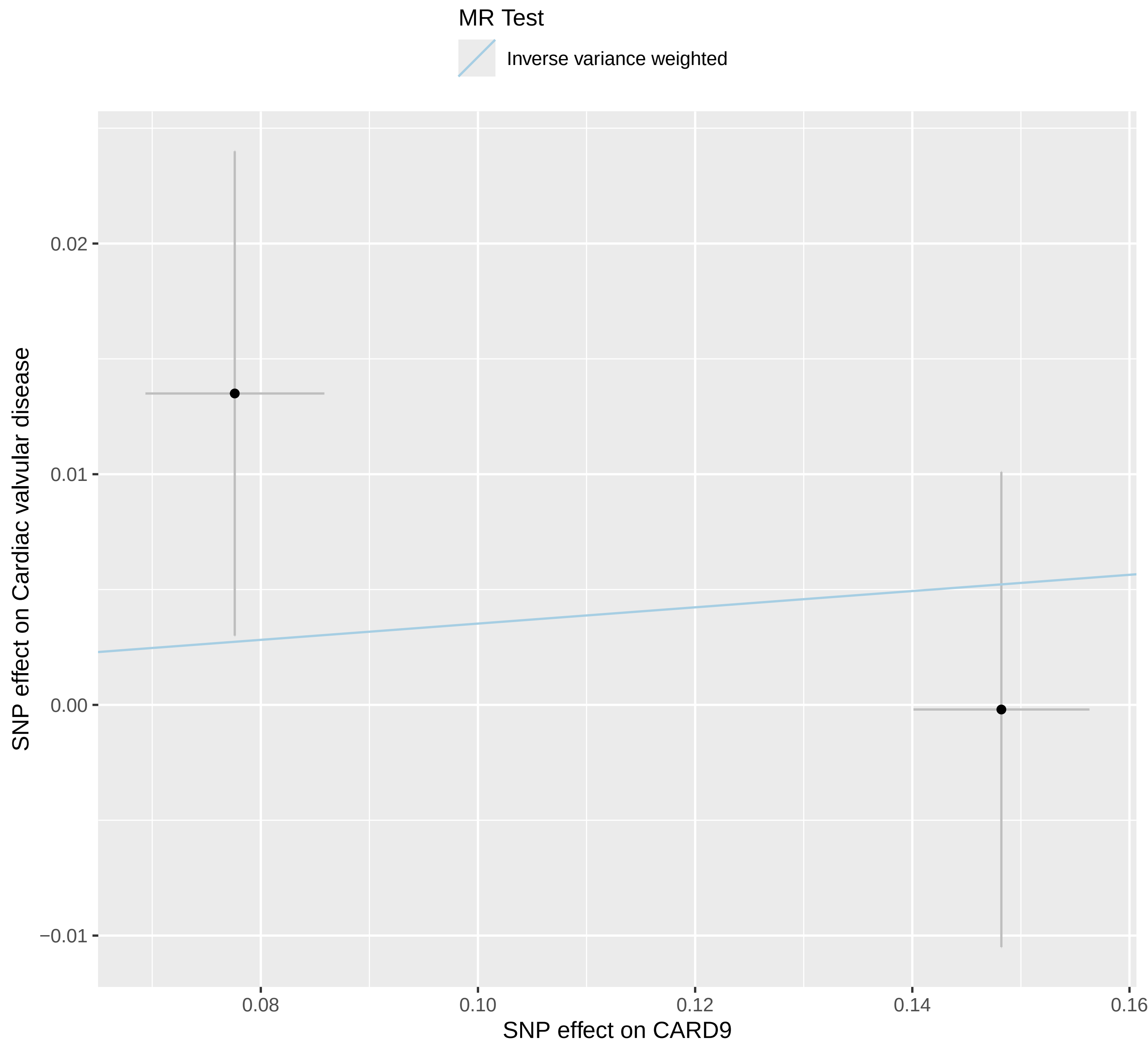

MR Test
Inverse variance weighted
0.02
0.01
0.00
−0.01
SNP effect on Cardiac valvular disease
0.08
0.10
0.12
0.14
0.16
SNP effect on CARD9

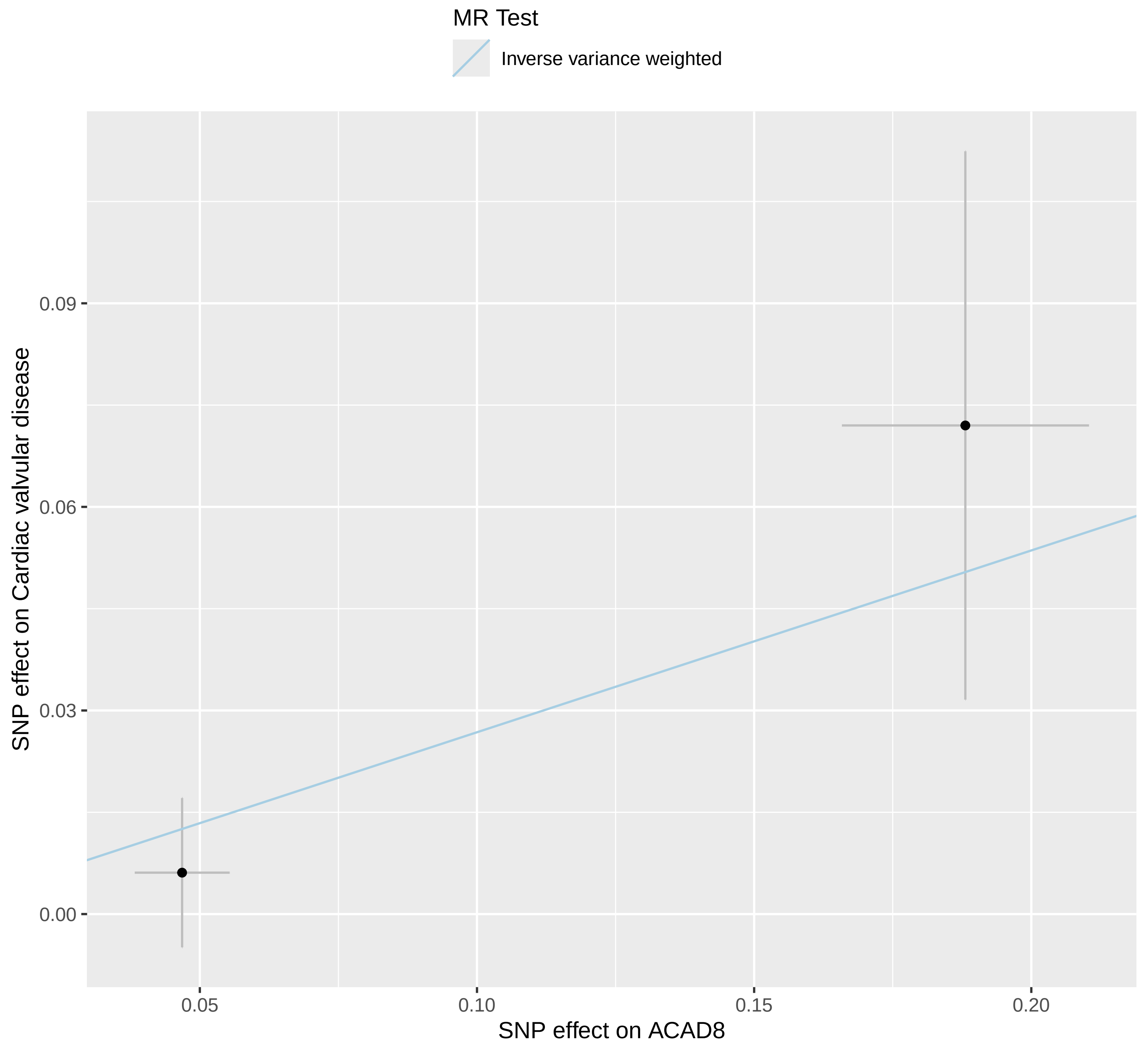

MR Test
Inverse variance weighted
0.09
0.06
0.03
0.00
SNP effect on Cardiac valvular disease
0.05
0.10
0.15
0.20
SNP effect on ACAD8

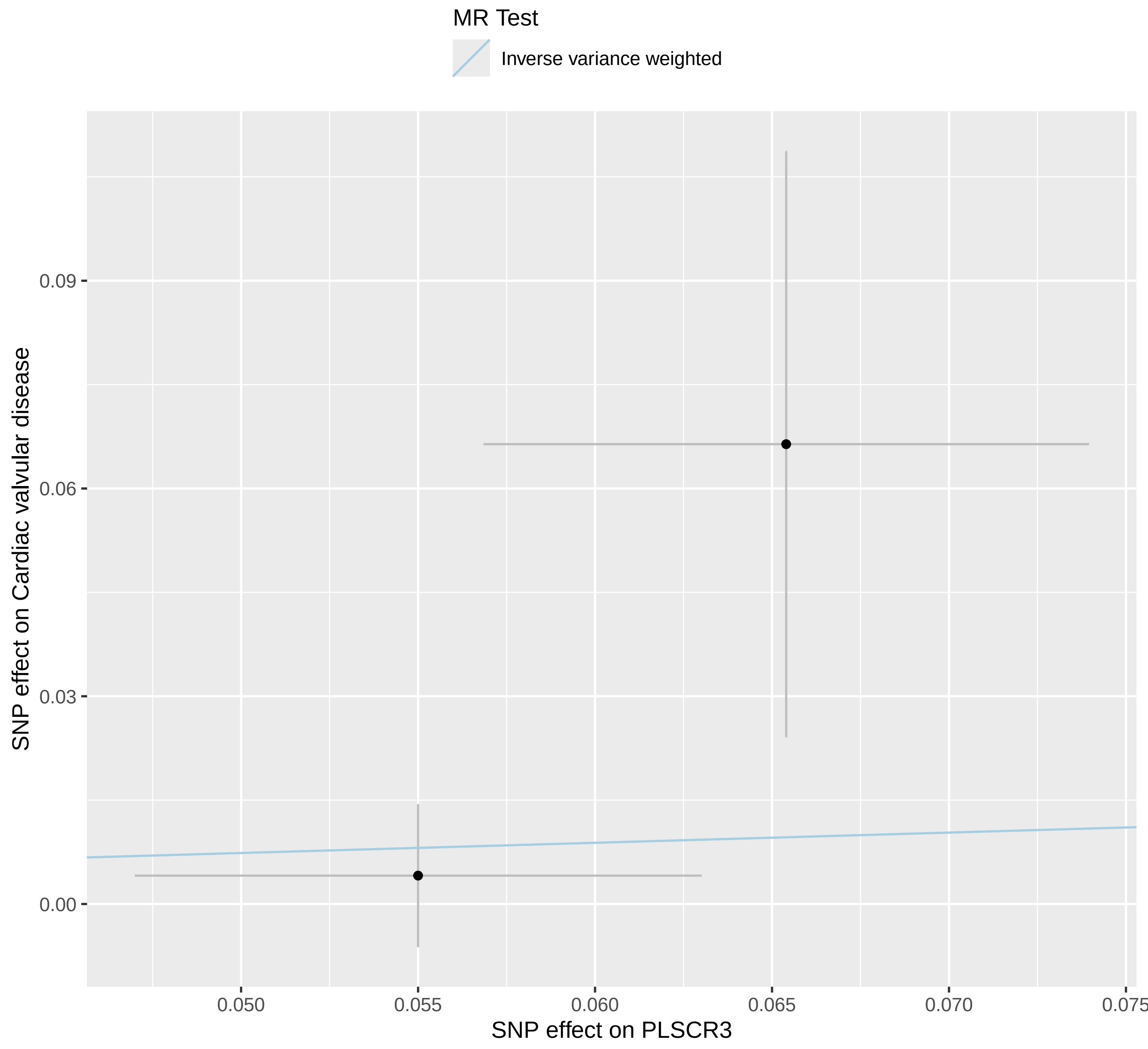

MR Test
Inverse variance weighted
0.09
0.06
0.03
0.00
SNP effect on Cardiac valvular disease
0.050
0.055
0.060
0.065
0.070
0.075
SNP effect on PLSCR3

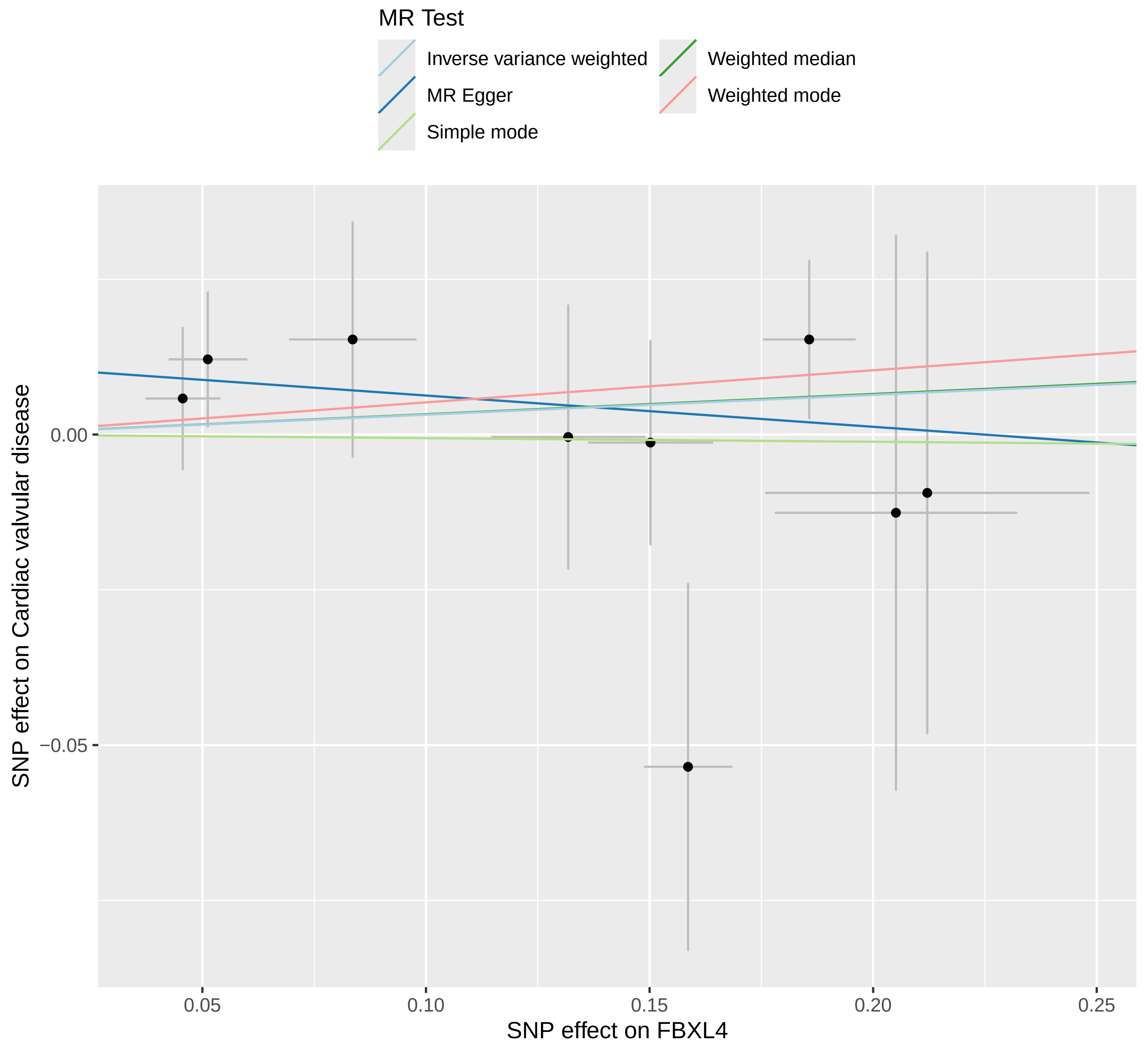

MR Test
Inverse variance weighted
MR Egger
Simple mode
Weighted median
Weighted mode
SNP effect on Cardiac valvular disease
0.00
−0.05
0.05
0.10
0.15
0.20
0.25
SNP effect on FBXL4

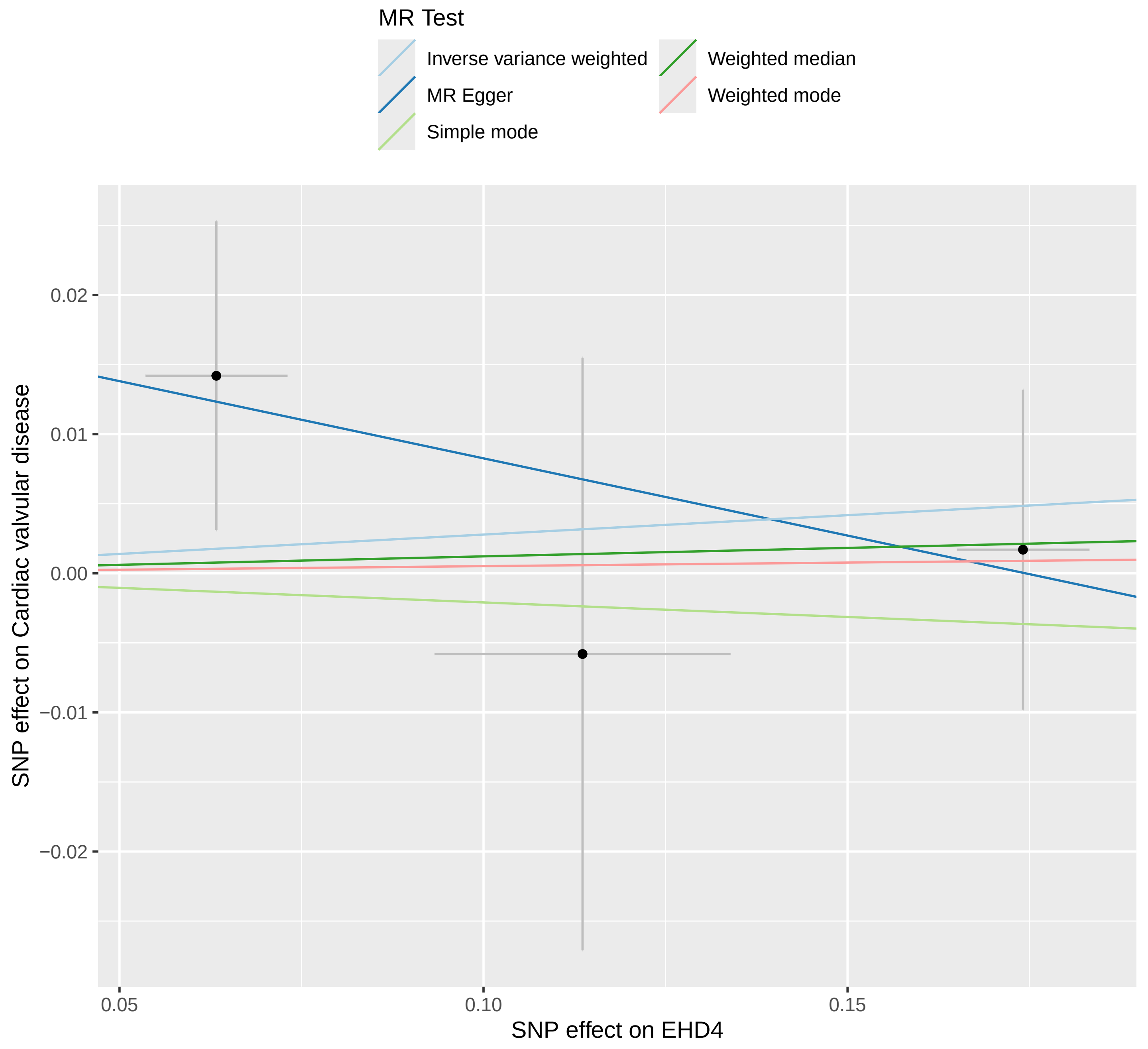

MR Test
Inverse variance weighted
MR Egger
Simple mode
Weighted median
Weighted mode
0.02
0.01
0.00
−0.01
−0.02
SNP effect on Cardiac valvular disease
0.05
0.10
0.15
SNP effect on EHD4

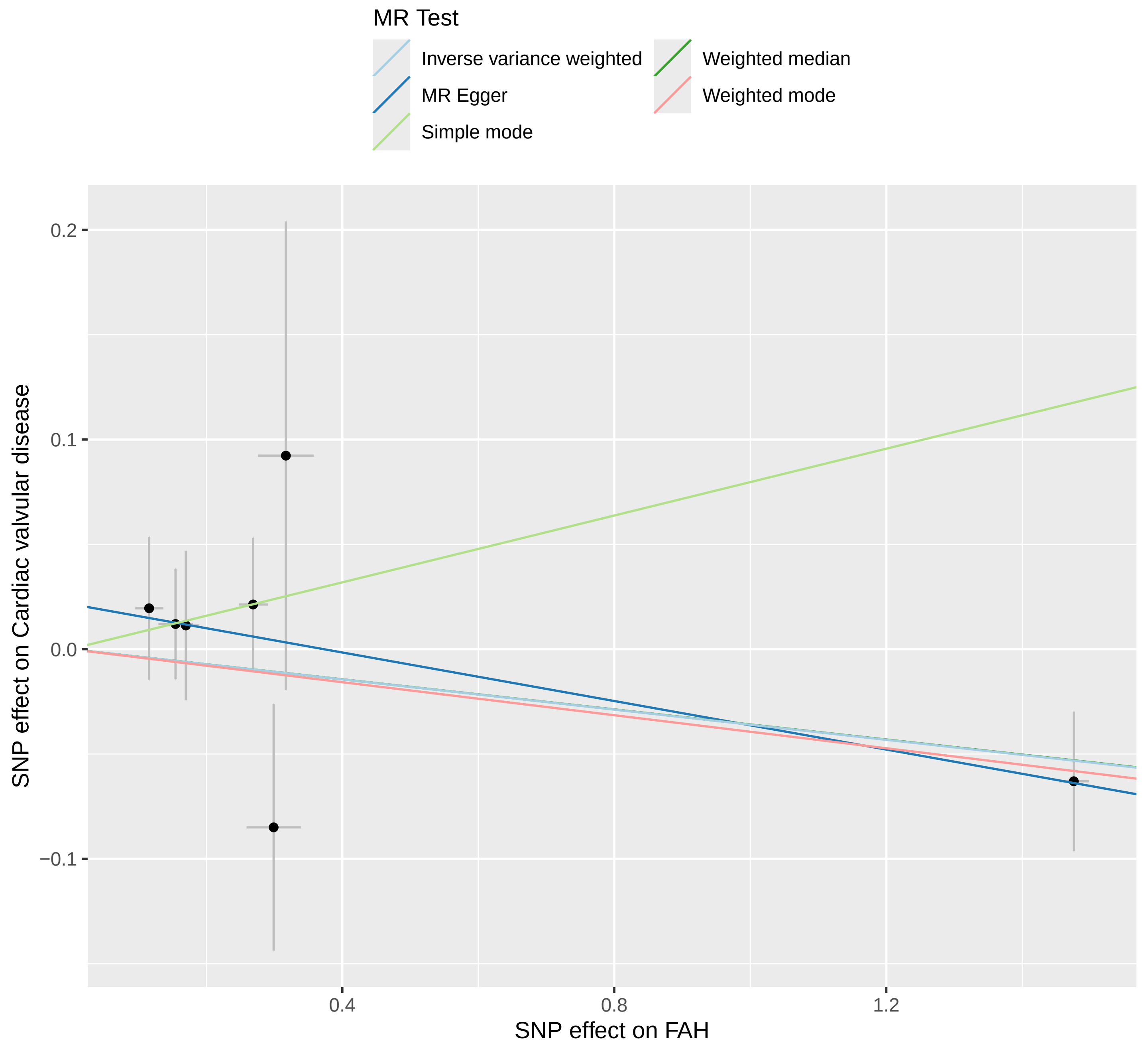

MR Test
Inverse variance weighted
MR Egger
Simple mode
Weighted median
Weighted mode
0.2
0.1
0.0
−0.1
SNP effect on Cardiac valvular disease
0.4
0.8
1.2
SNP effect on FAH

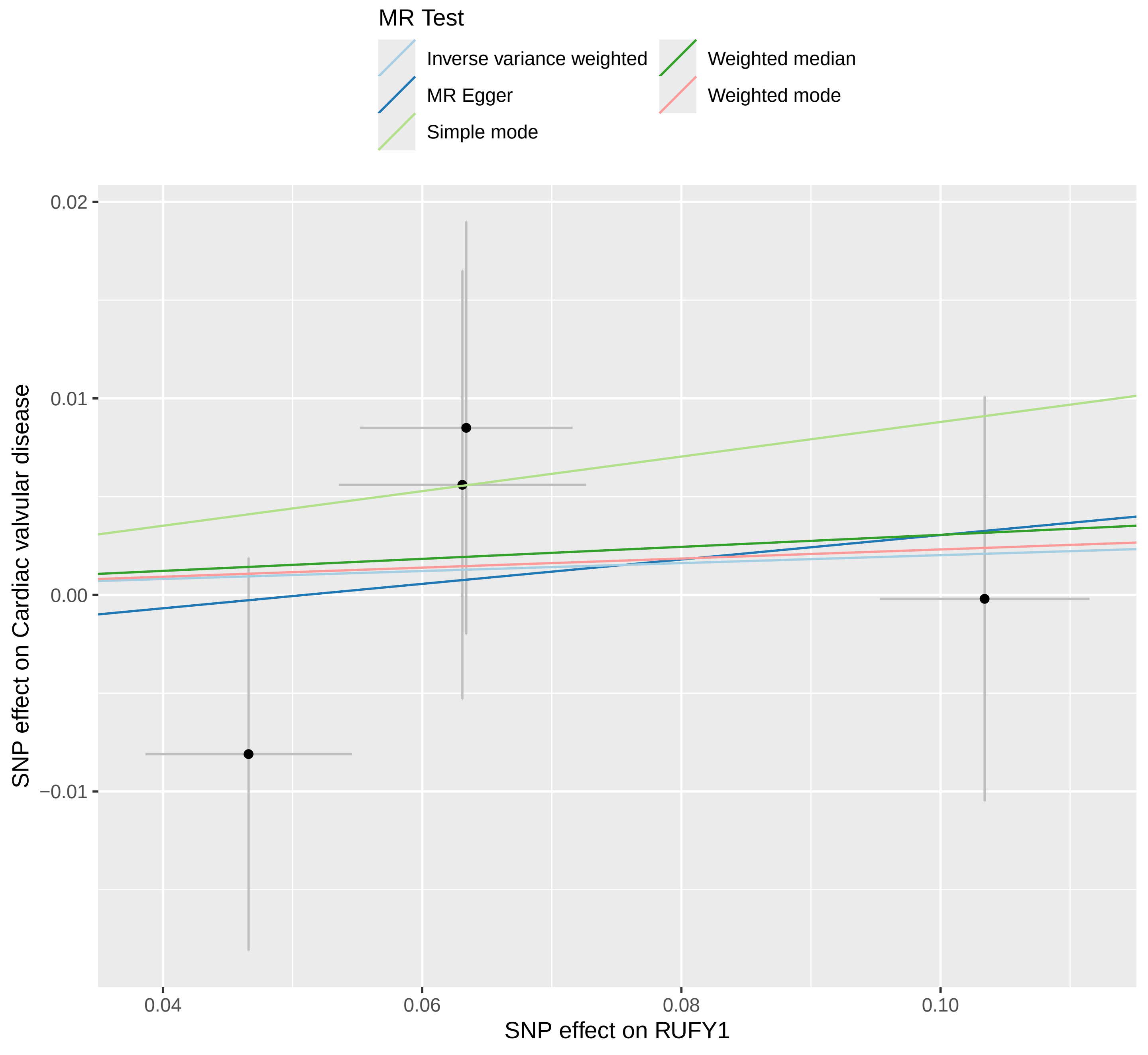

MR Test
Inverse variance weighted
MR Egger
Simple mode
Weighted median
Weighted mode
0.02
0.01
0.00
−0.01
SNP effect on Cardiac valvular disease
0.04
0.06
0.08
0.10
SNP effect on RUFY1

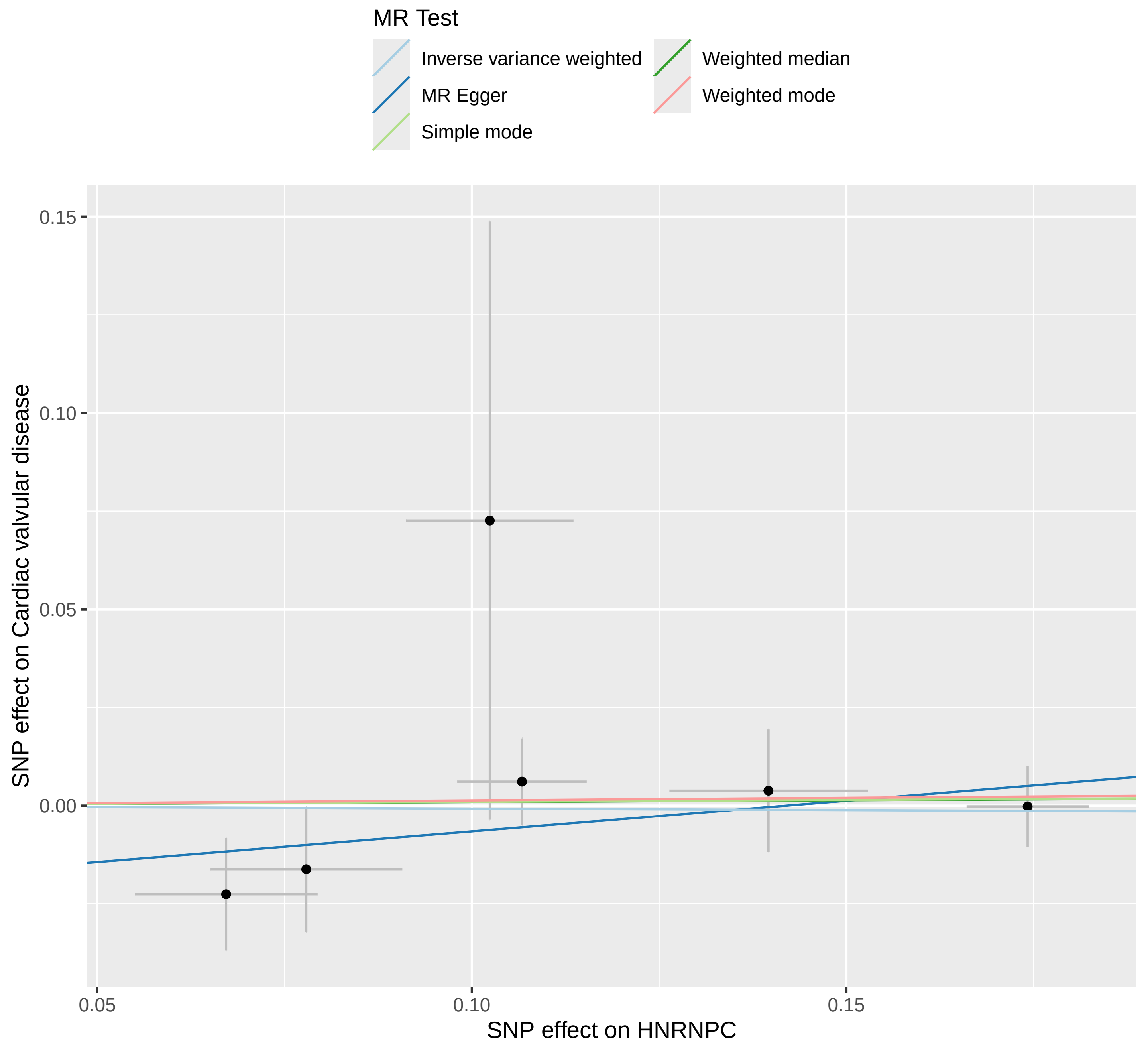
MR Test
Inverse variance weighted
MR Egger
Simple mode
Weighted median
Weighted mode
0.15
0.10
0.05
0.00
SNP effect on Cardiac valvular disease
0.05
0.10
0.15
SNP effect on HNRNPC

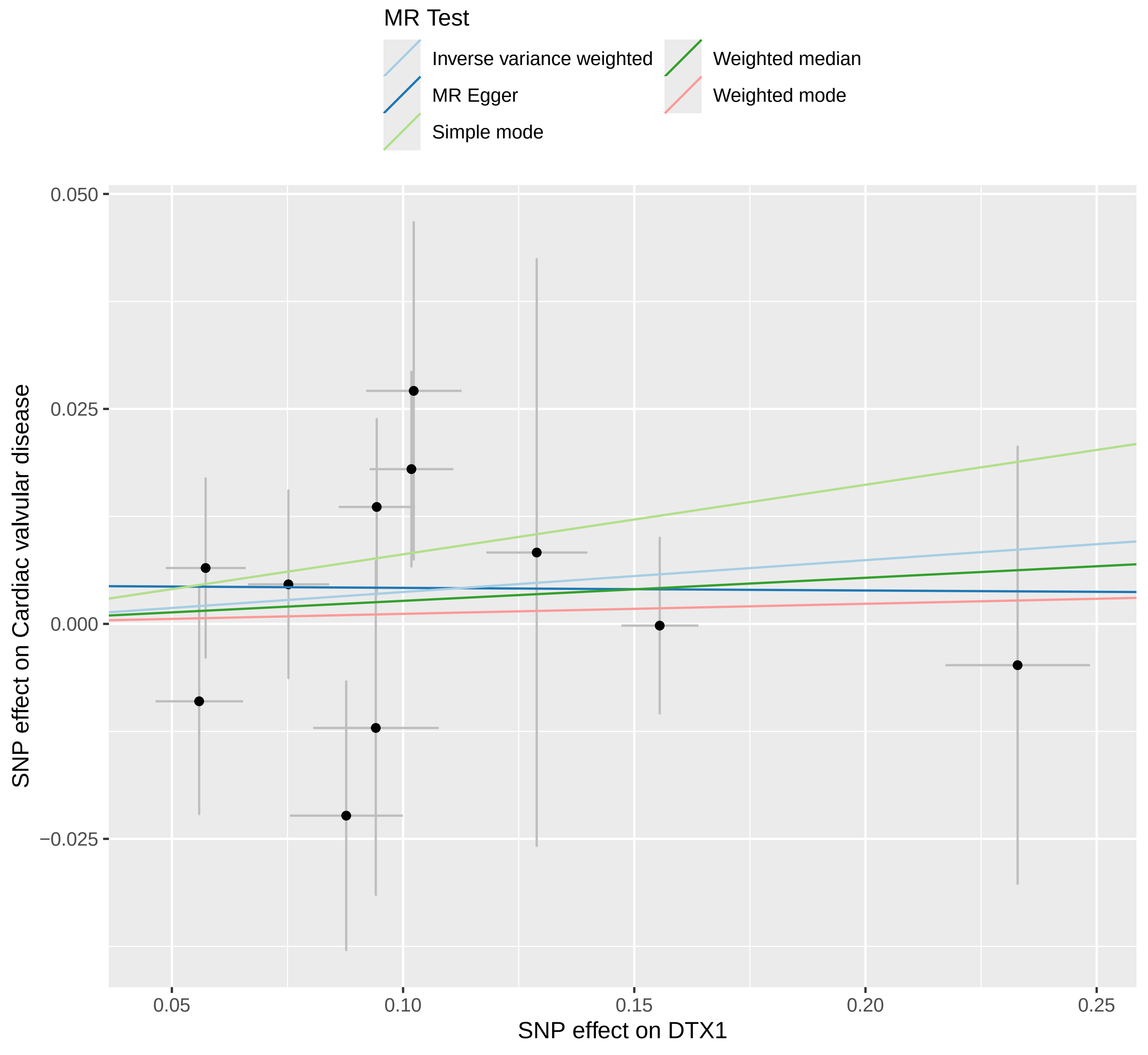
MR Test
Inverse variance weighted
MR Egger
Simple mode
Weighted median
Weighted mode
0.050
0.025
0.000
−0.025
SNP effect on Cardiac valvular disease
0.05
0.10
0.15
0.20
0.25
SNP effect on DTX1

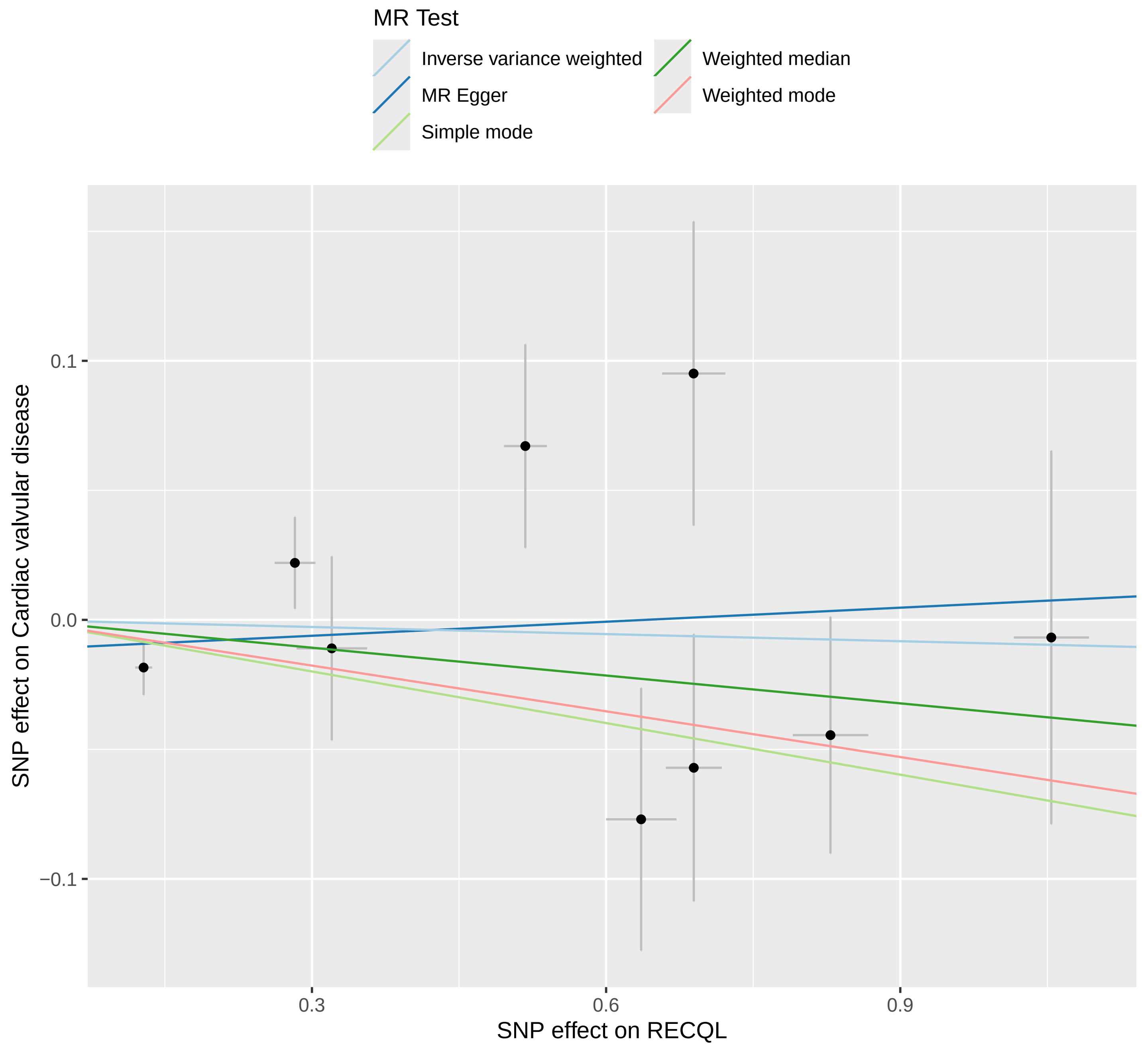
MR Test
Inverse variance weighted
MR Egger
Simple mode
Weighted median
Weighted mode
0.1
0.0
−0.1
0.3
0.6
0.9
SNP effect on Cardiac valvular disease
SNP effect on RECQL

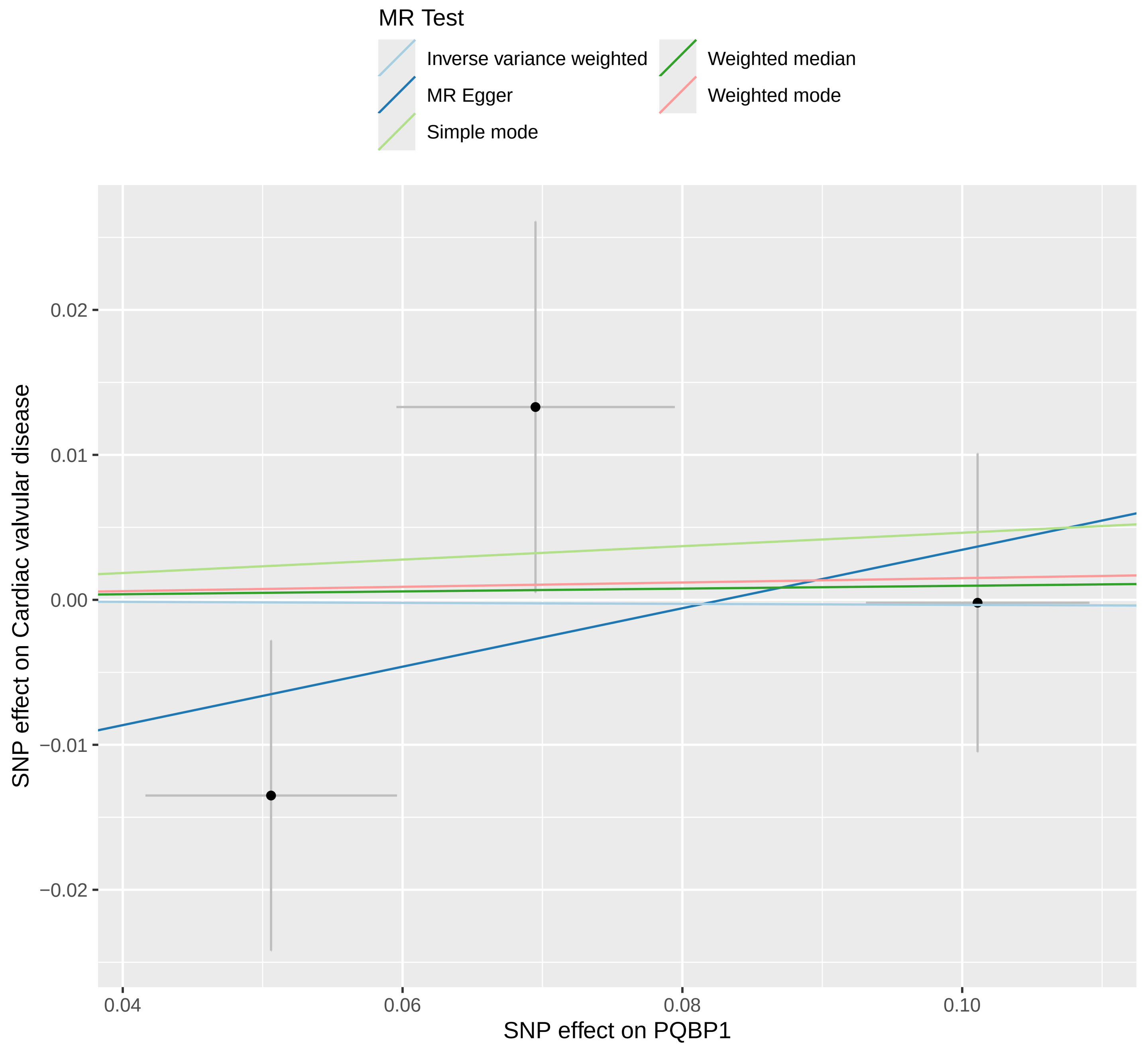
MR Test
Inverse variance weighted
MR Egger
Simple mode
Weighted median
Weighted mode
0.02
0.01
0.00
−0.01
−0.02
SNP effect on Cardiac valvular disease
0.04
0.06
0.08
0.10
SNP effect on PQBP1

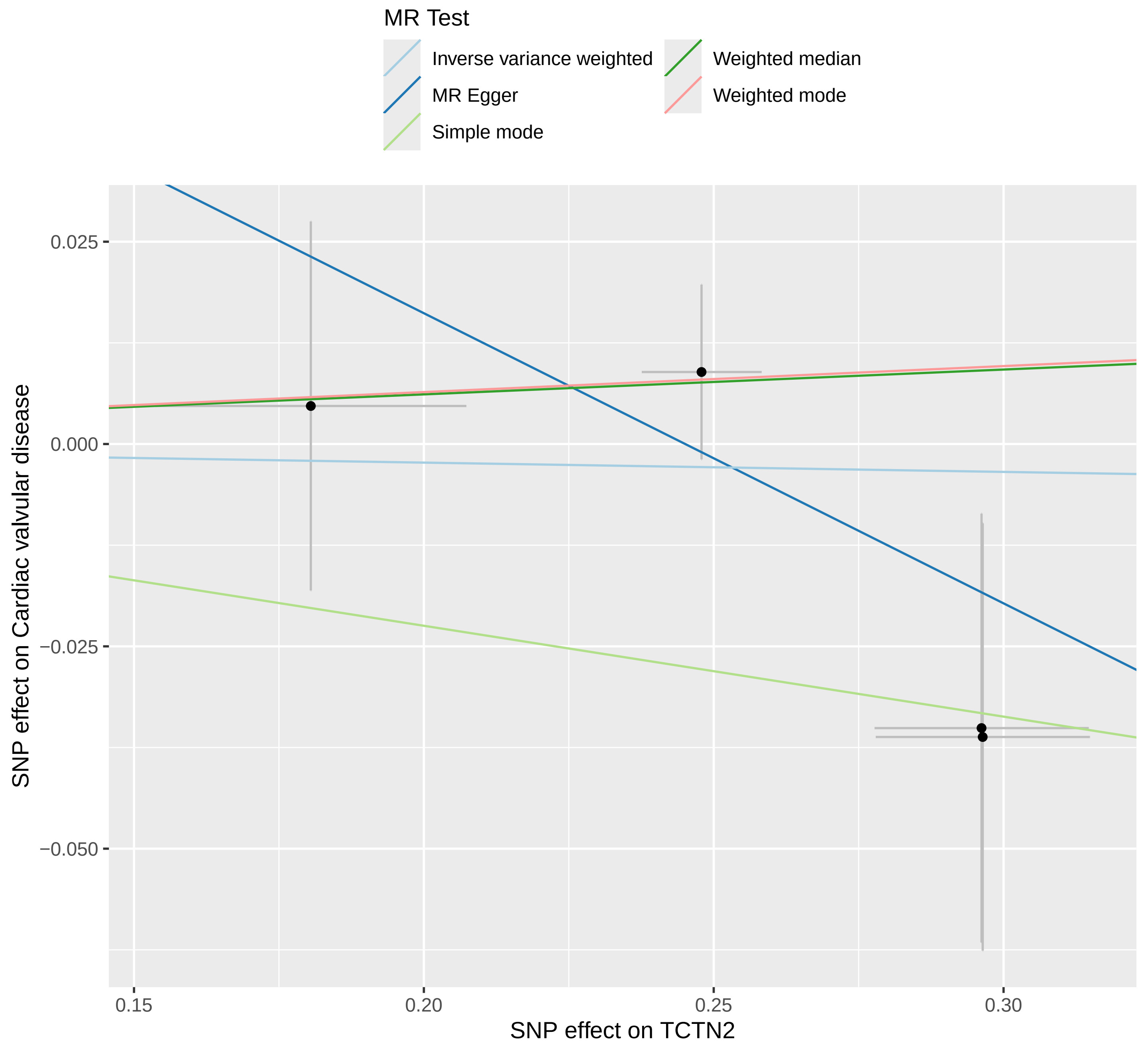

MR Test
Inverse variance weighted
MR Egger
Simple mode
Weighted median
Weighted mode
0.025
0.000
−0.025
−0.050
SNP effect on Cardiac valvular disease
0.15
0.20
0.25
0.30
SNP effect on TCTN2

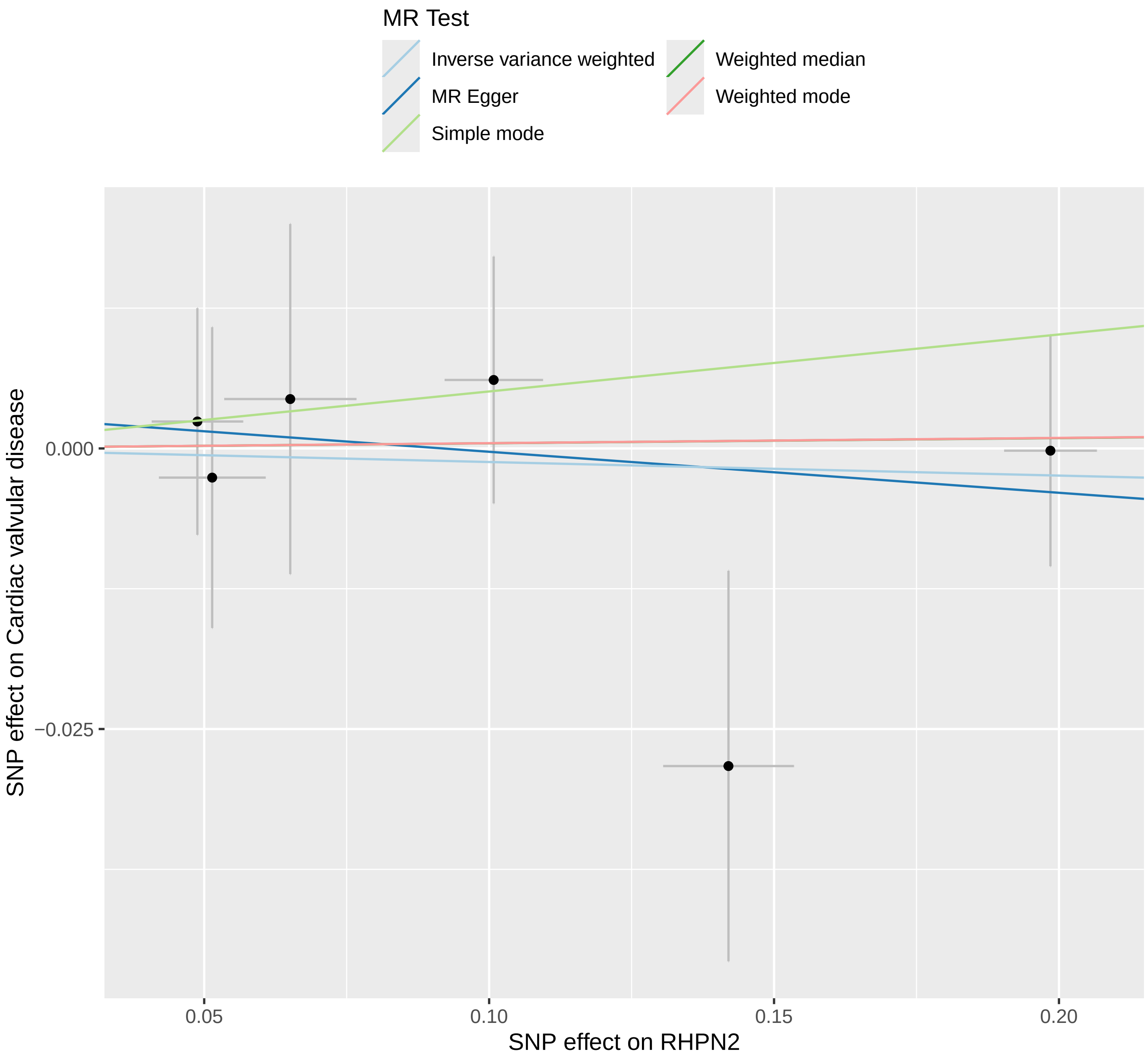

MR Test
Inverse variance weighted
MR Egger
Simple mode
Weighted median
Weighted mode
SNP effect on Cardiac valvular disease
0.000
−0.025
0.05
0.10
0.15
0.20
SNP effect on RHPN2

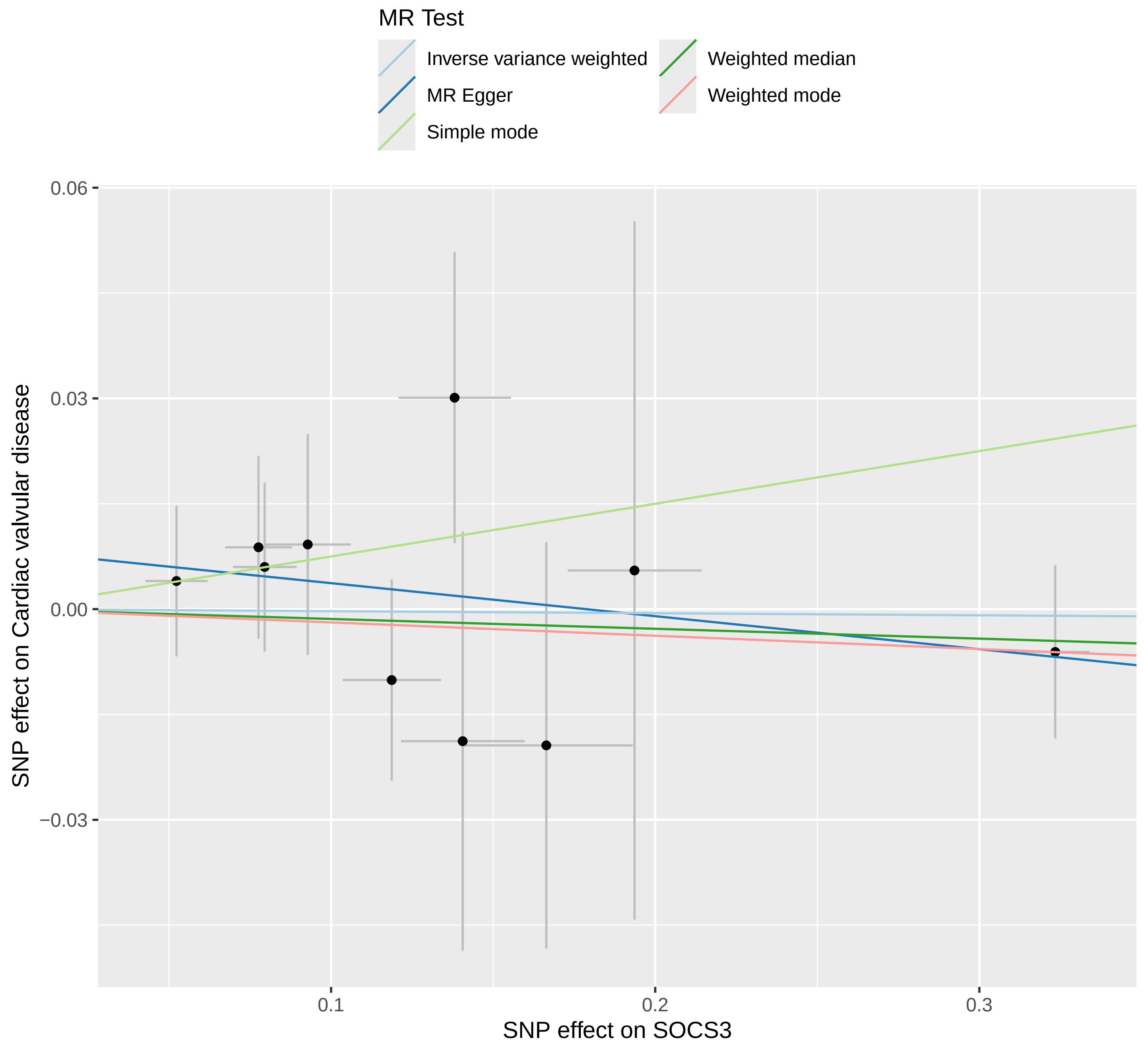

MR Test
Inverse variance weighted
MR Egger
Simple mode
Weighted median
Weighted mode
0.06
0.03
0.00
−0.03
0.1
0.2
0.3
SNP effect on Cardiac valvular disease
SNP effect on SOCS3

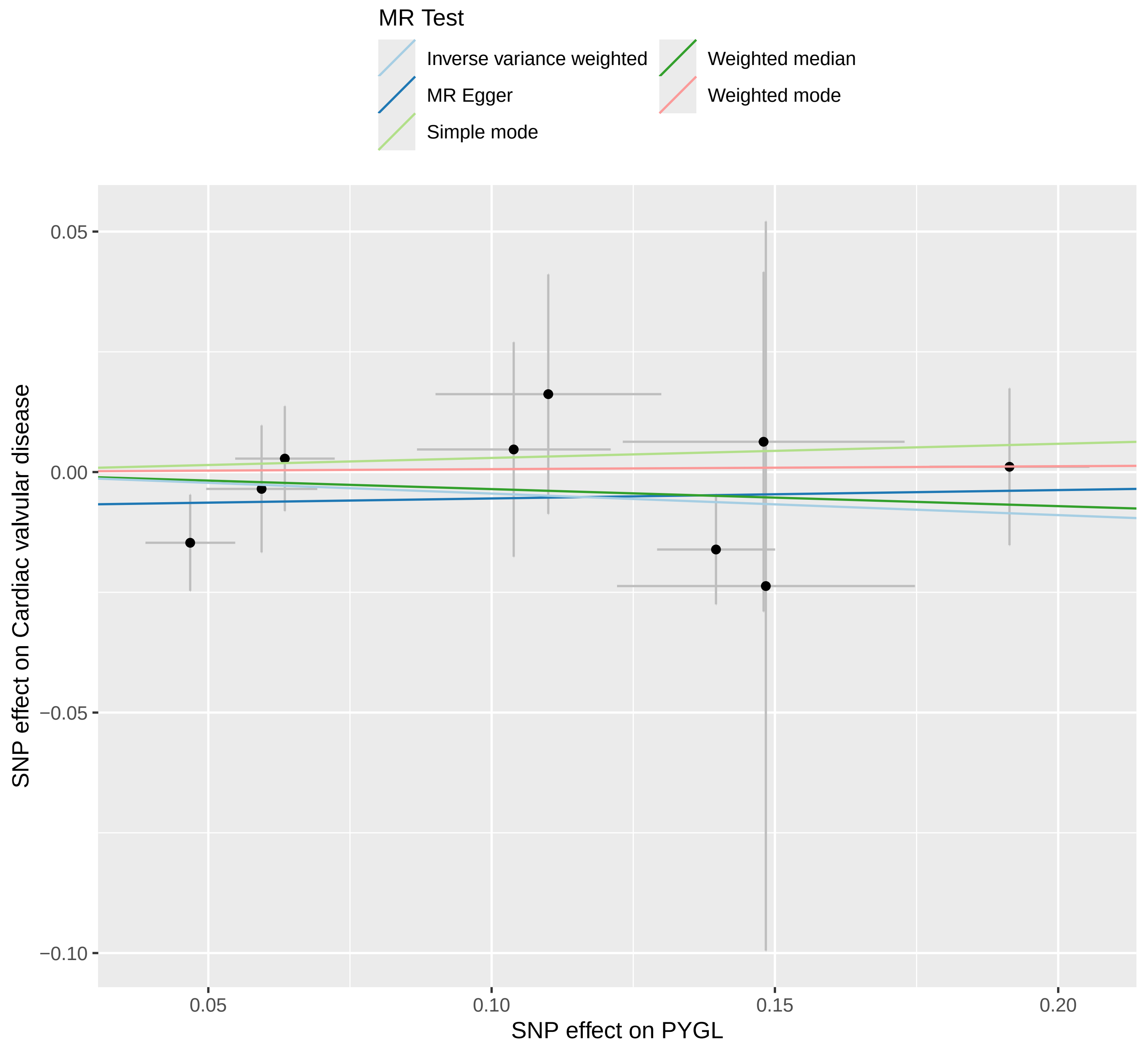

MR Test
Inverse variance weighted
MR Egger
Simple mode
Weighted median
Weighted mode
0.05
0.00
−0.05
−0.10
0.05
0.10
0.15
0.20
SNP effect on Cardiac valvular disease
SNP effect on PYGL

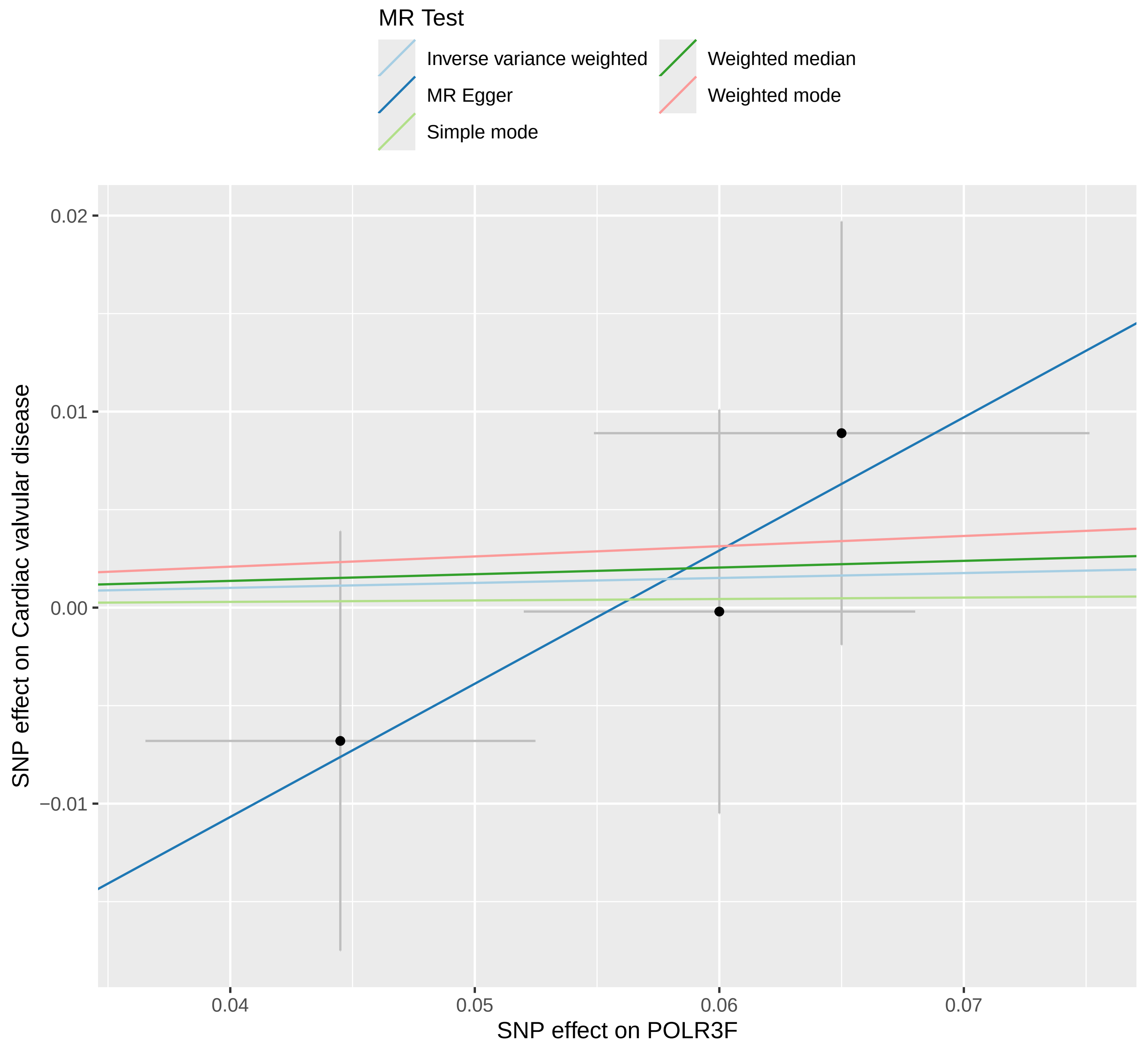

MR Test
Inverse variance weighted
MR Egger
Simple mode
Weighted median
Weighted mode
0.02
0.01
0.00
−0.01
0.04
0.05
0.06
0.07
SNP effect on Cardiac valvular disease
SNP effect on POLR3F

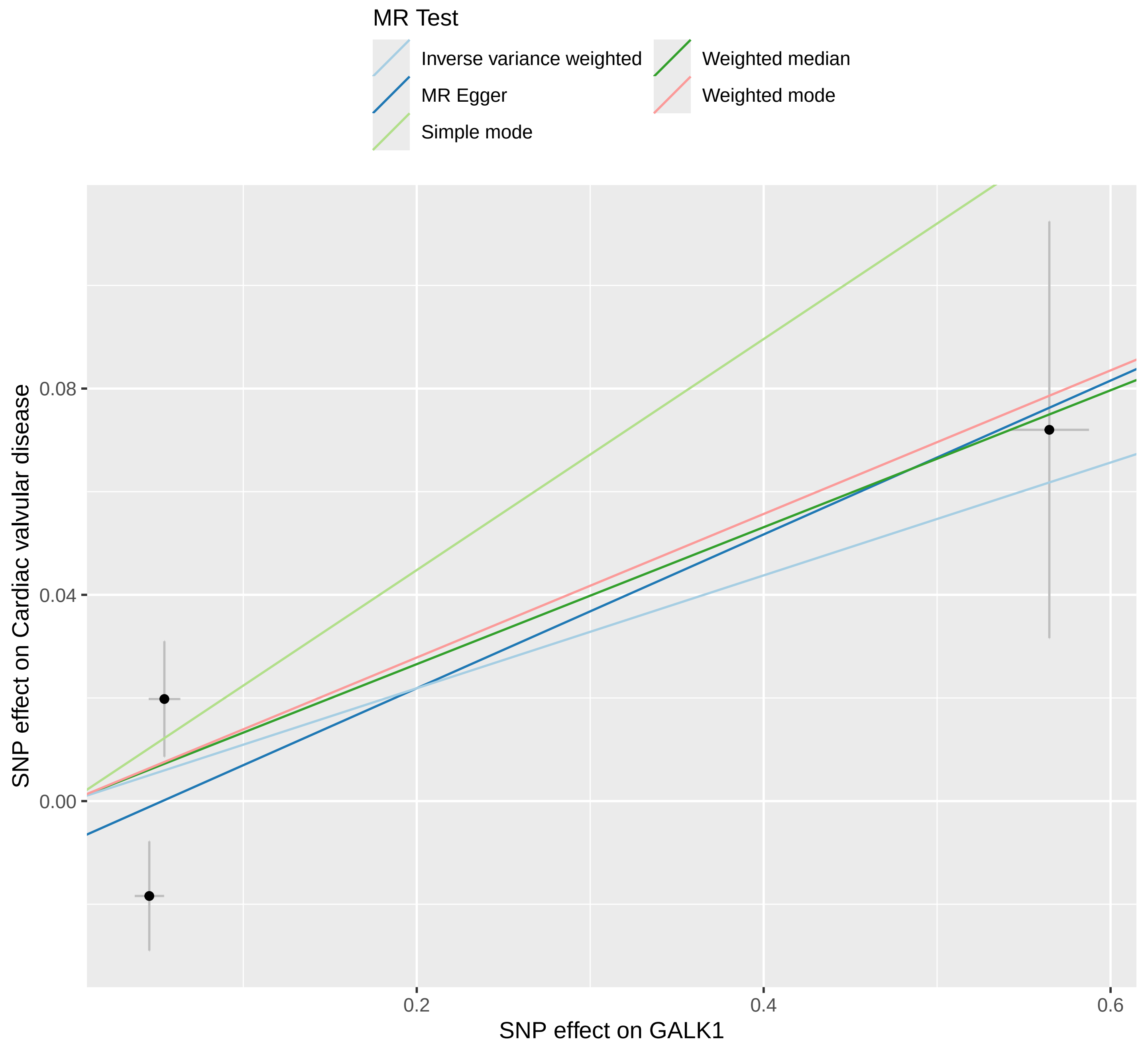
MR Test
Inverse variance weighted
MR Egger
Simple mode
Weighted median
Weighted mode
SNP effect on Cardiac valvular disease
0.08
0.04
0.00
0.2
0.4
0.6
SNP effect on GALK1

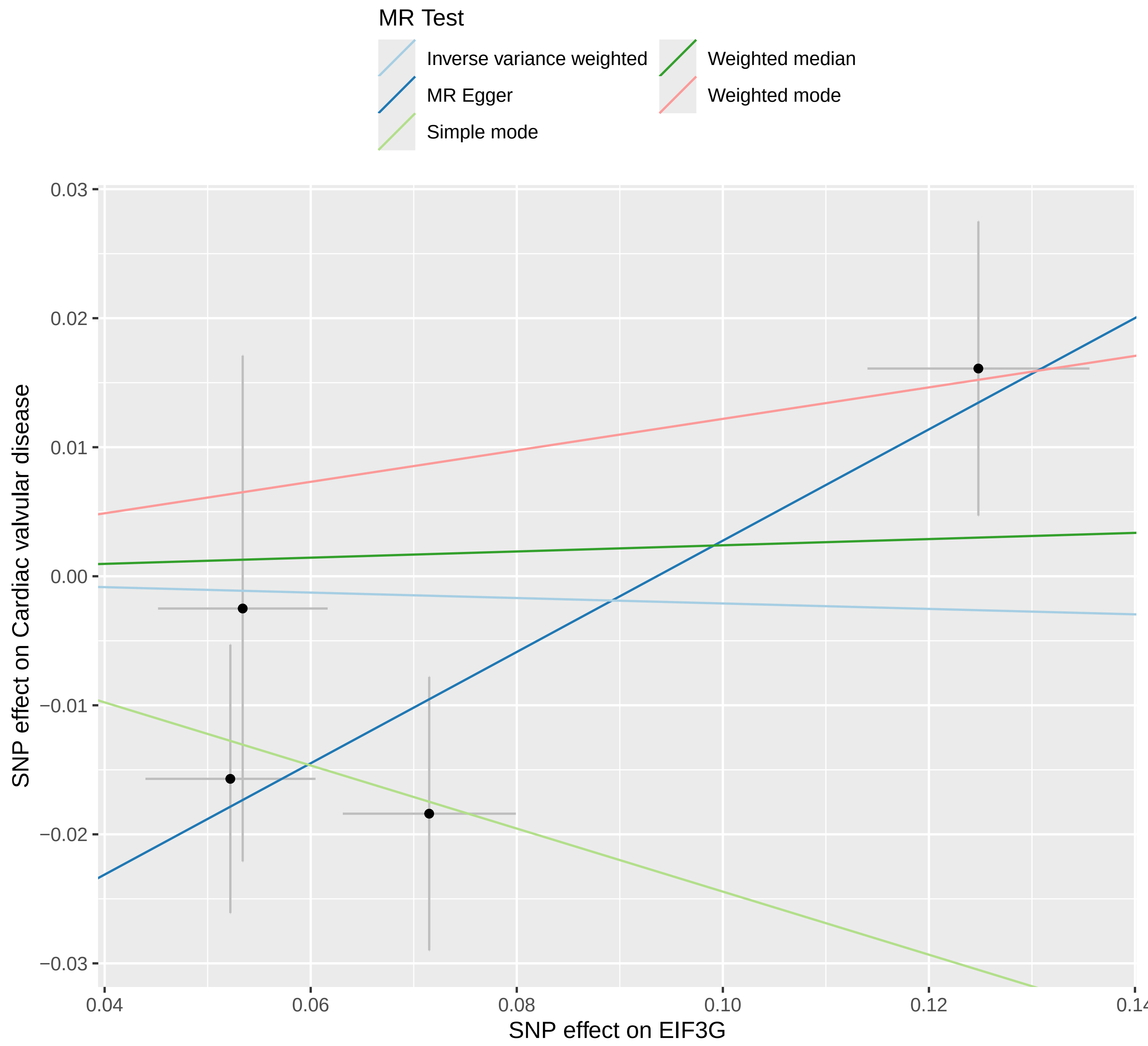

MR Test
Inverse variance weighted
MR Egger
Simple mode
Weighted median
Weighted mode
0.03
0.02
0.01
0.00
−0.01
−0.02
−0.03
SNP effect on Cardiac valvular disease
0.04
0.06
0.08
0.10
0.12
SNP effect on EIF3G

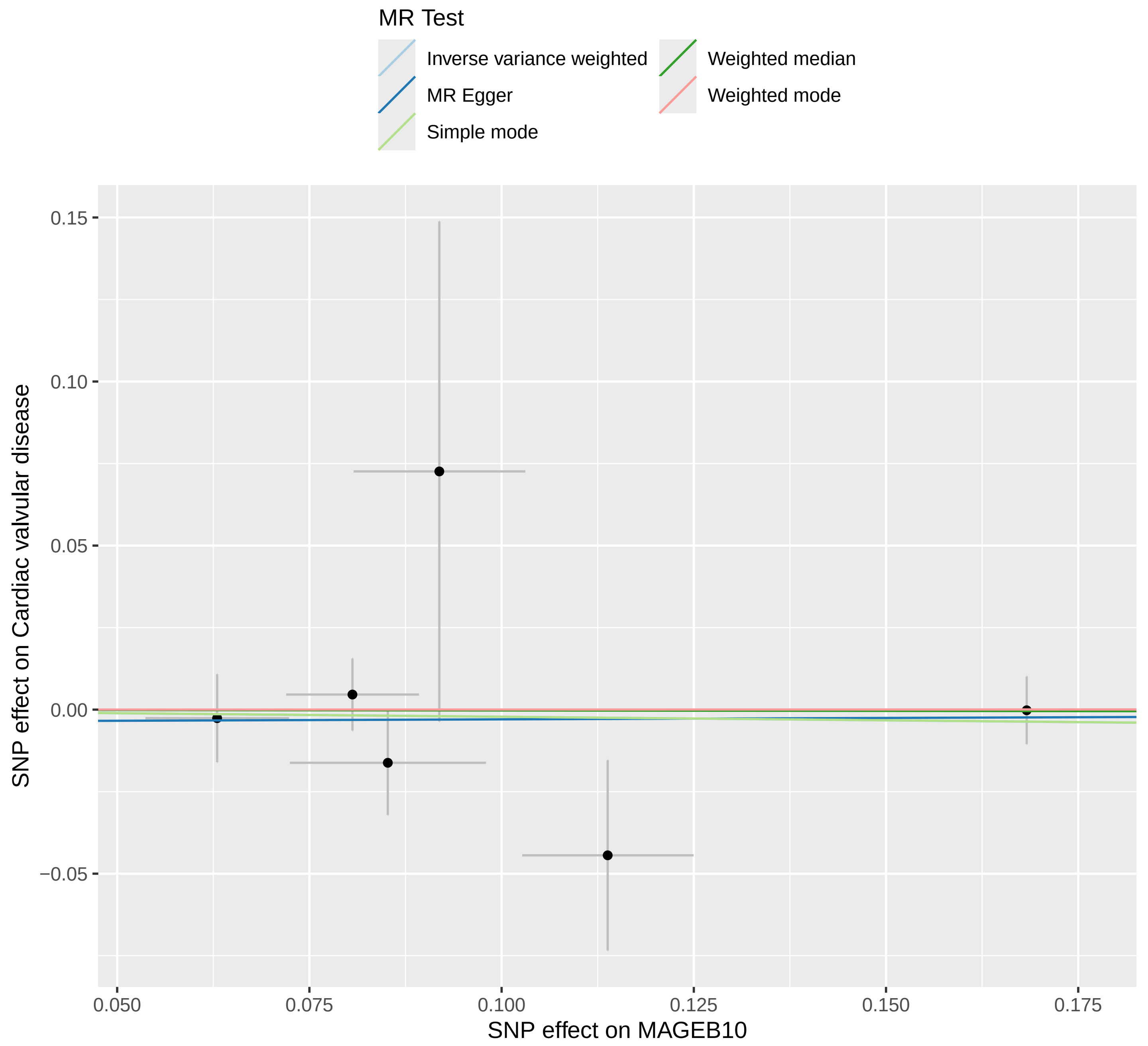

MR Test
Inverse variance weighted
MR Egger
Simple mode
Weighted median
Weighted mode
0.15
0.10
0.05
0.00
−0.05
SNP effect on Cardiac valvular disease
0.050
0.075
0.100
0.125
0.150
0.175
SNP effect on MAGEB10

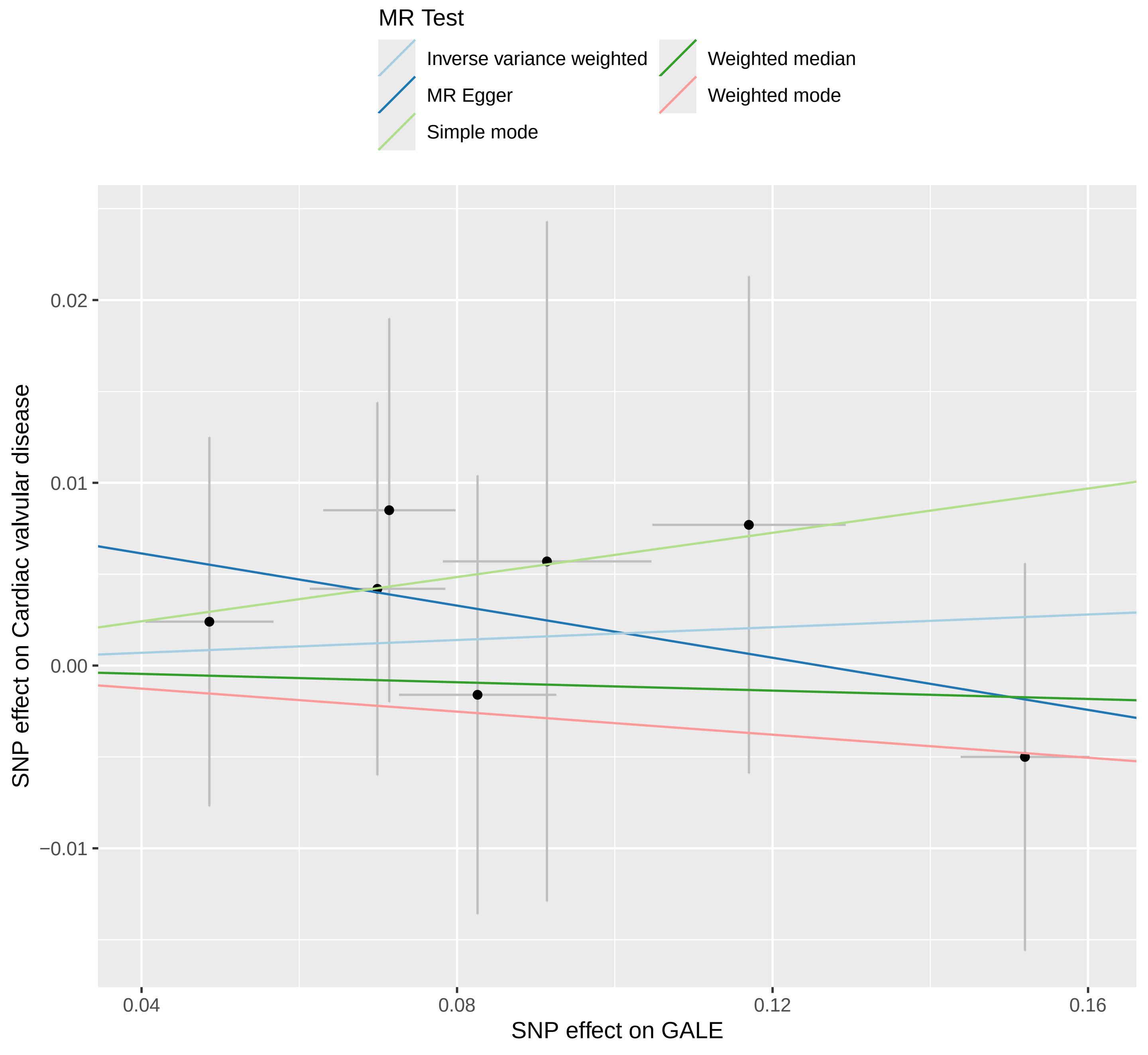

MR Test
Inverse variance weighted
MR Egger
Simple mode
Weighted median
Weighted mode
0.02
0.01
0.00
−0.01
0.04
0.08
0.12
0.16
SNP effect on Cardiac valvular disease
SNP effect on GALE

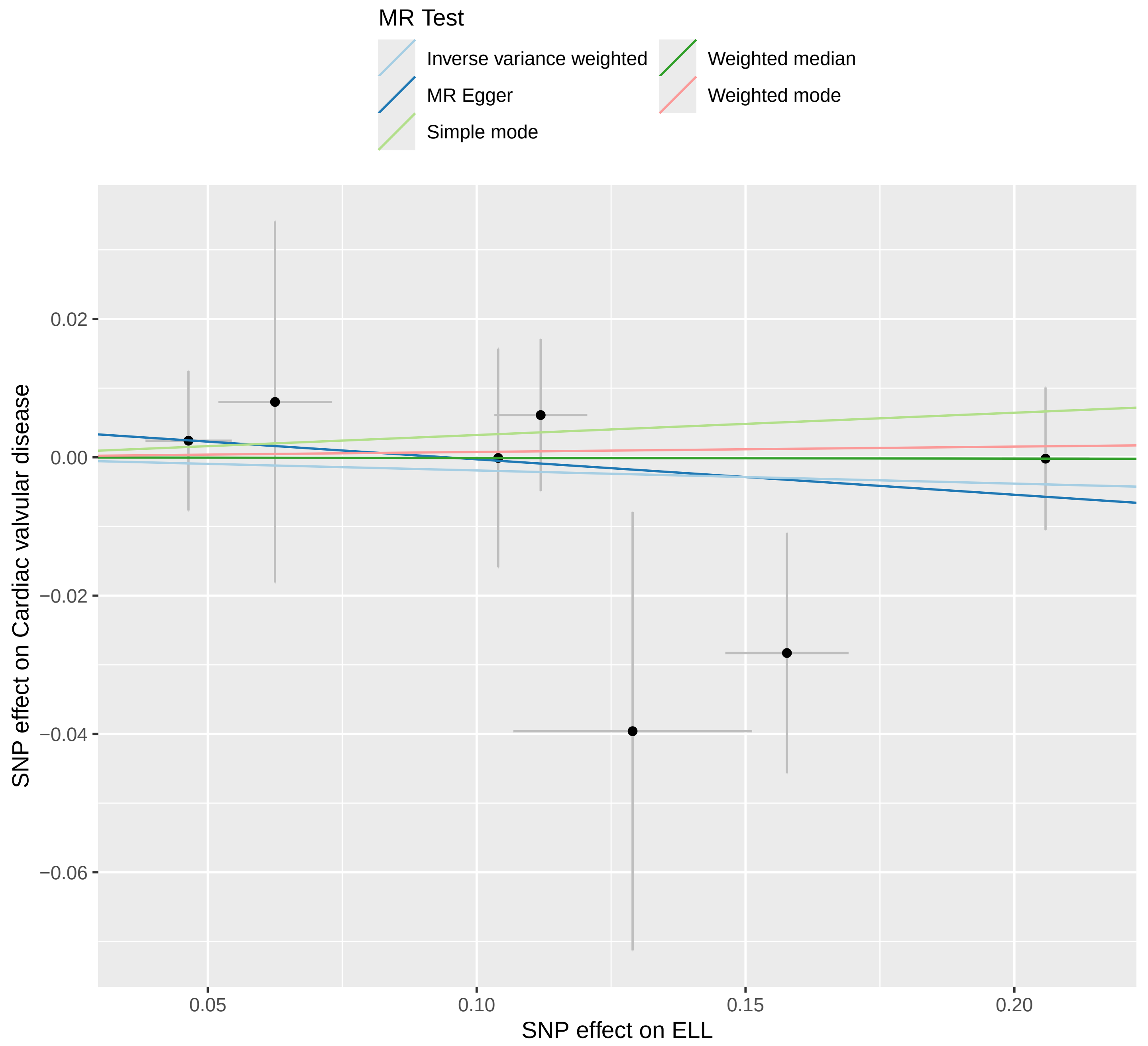

MR Test
Inverse variance weighted
MR Egger
Simple mode
Weighted median
Weighted mode
0.02
0.00
−0.02
−0.04
−0.06
0.05
0.10
0.15
0.20
SNP effect on Cardiac valvular disease
SNP effect on ELL

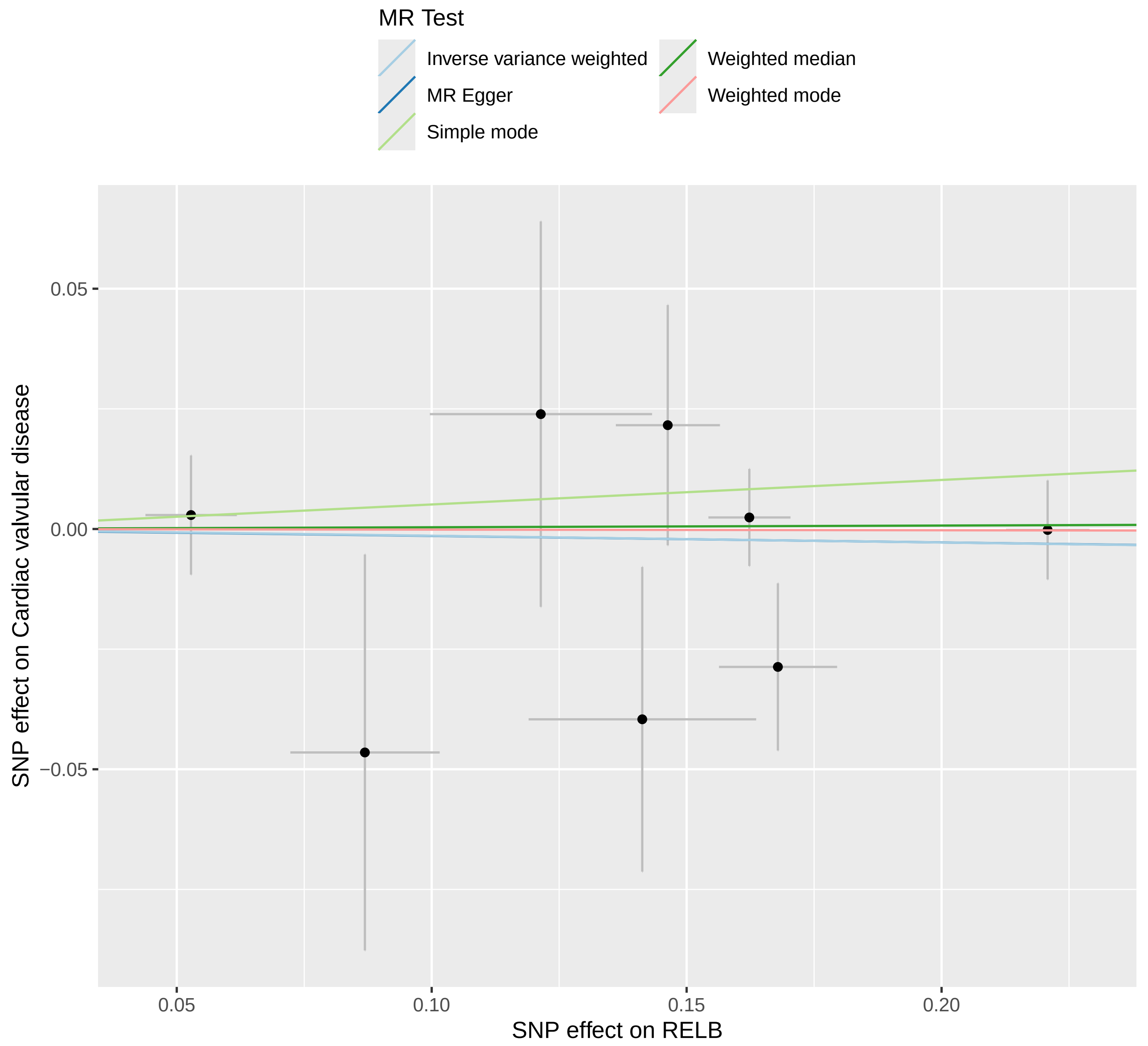
MR Test
Inverse variance weighted
MR Egger
Simple mode
Weighted median
Weighted mode
0.05
0.00
−0.05
0.05
0.10
0.15
0.20
SNP effect on Cardiac valvular disease
SNP effect on RELB

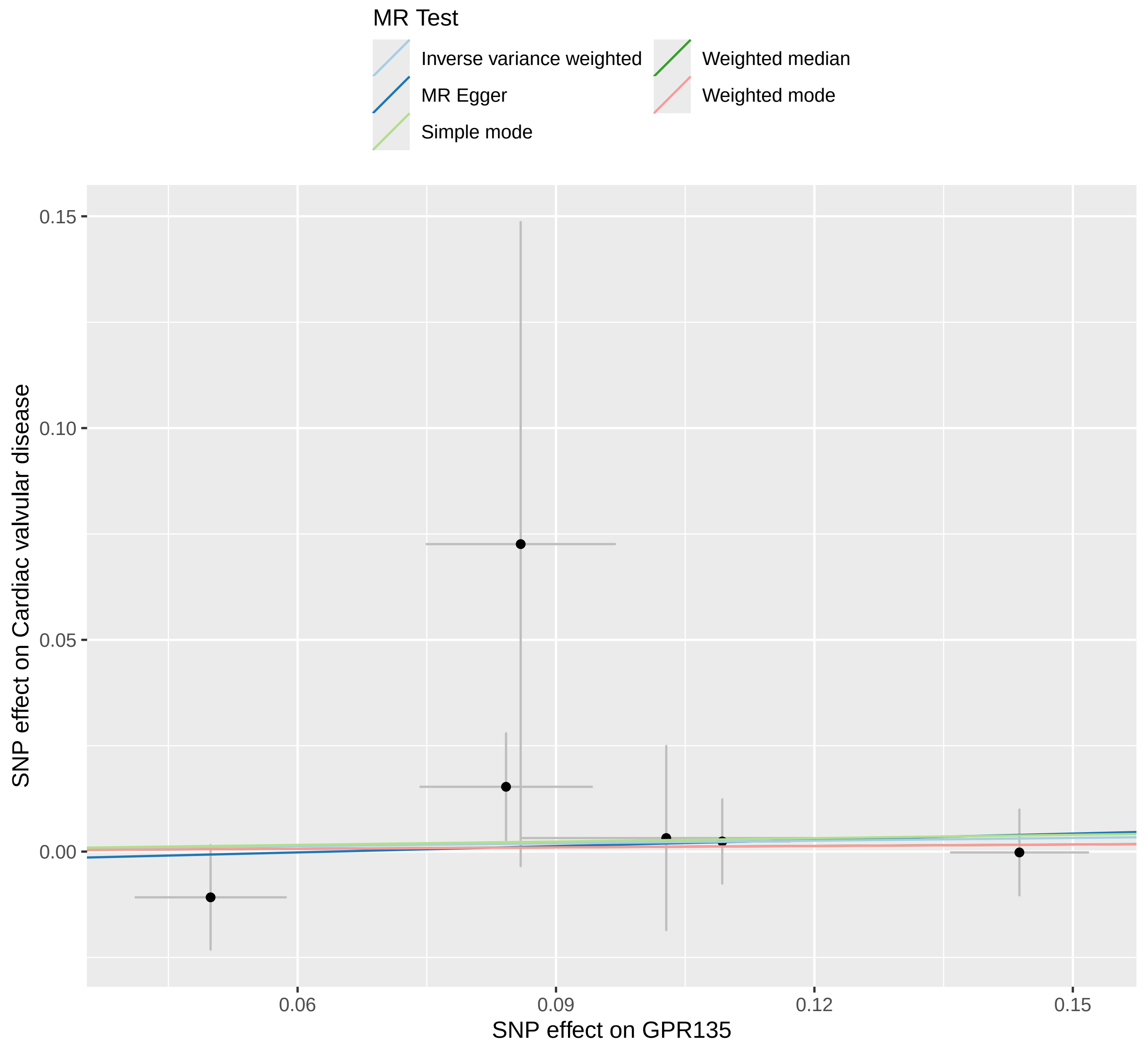

MR Test
Inverse variance weighted
MR Egger
Simple mode
Weighted median
Weighted mode
0.15
0.10
0.05
0.00
SNP effect on Cardiac valvular disease
0.06
0.09
0.12
0.15
SNP effect on GPR135

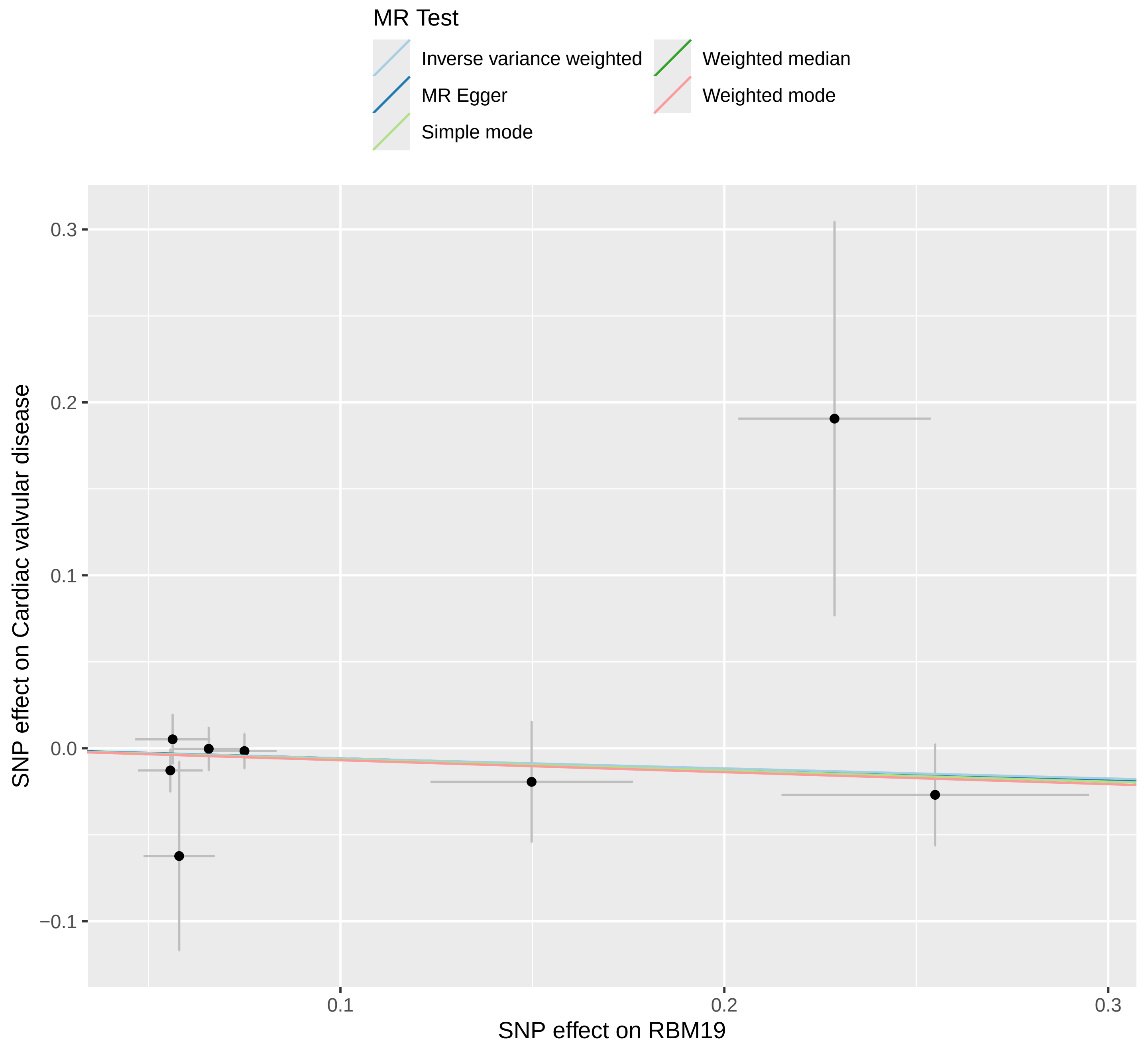

MR Test
Inverse variance weighted
MR Egger
Simple mode
Weighted median
Weighted mode
0.3
0.2
0.1
0.0
−0.1
SNP effect on Cardiac valvular disease
0.1
0.2
0.3
SNP effect on RBM19

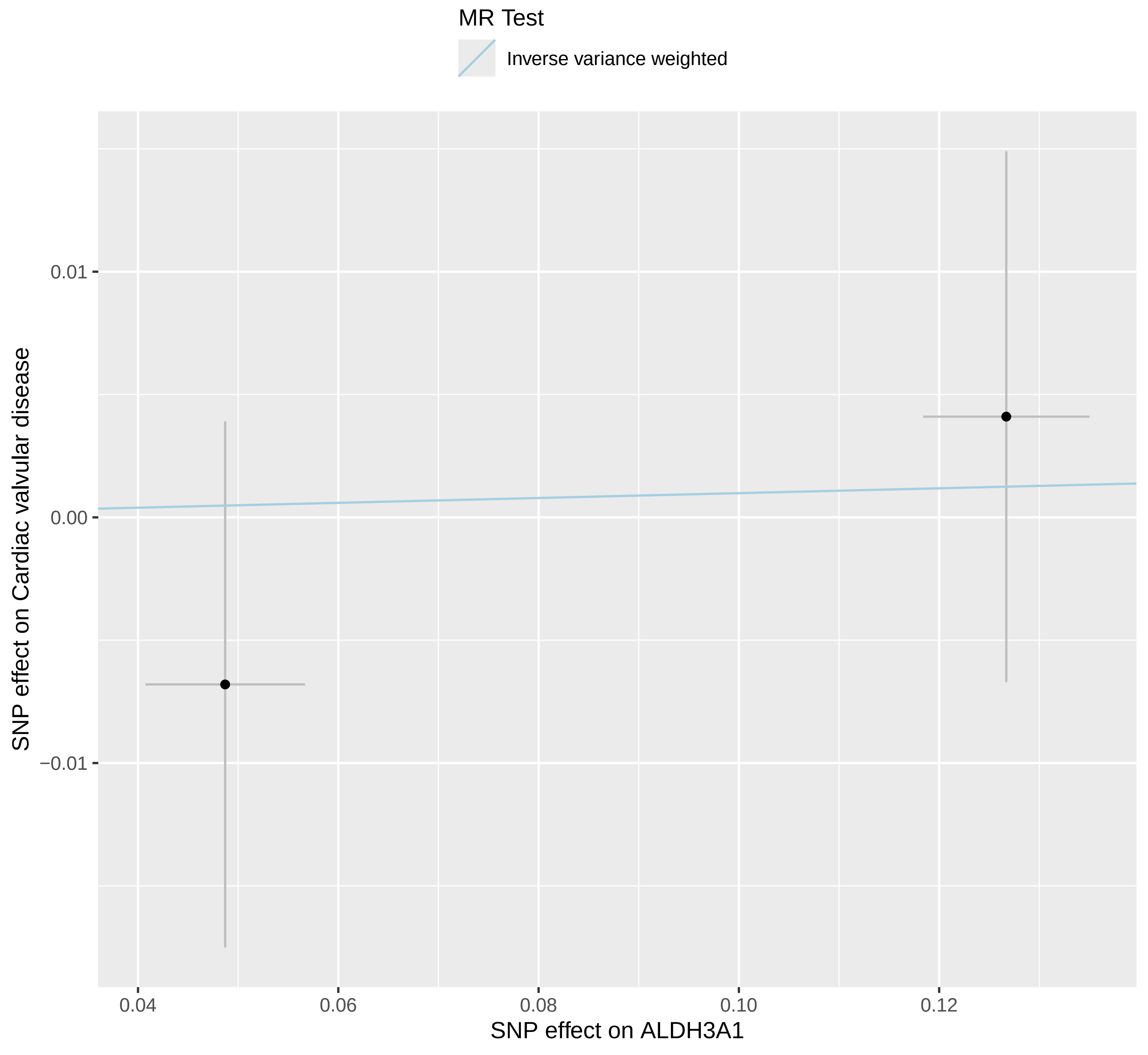

MR Test
Inverse variance weighted
0.01
0.00
−0.01
0.04
0.06
0.08
0.10
0.12
SNP effect on ALDH3A1
SNP effect on Cardiac valvular disease

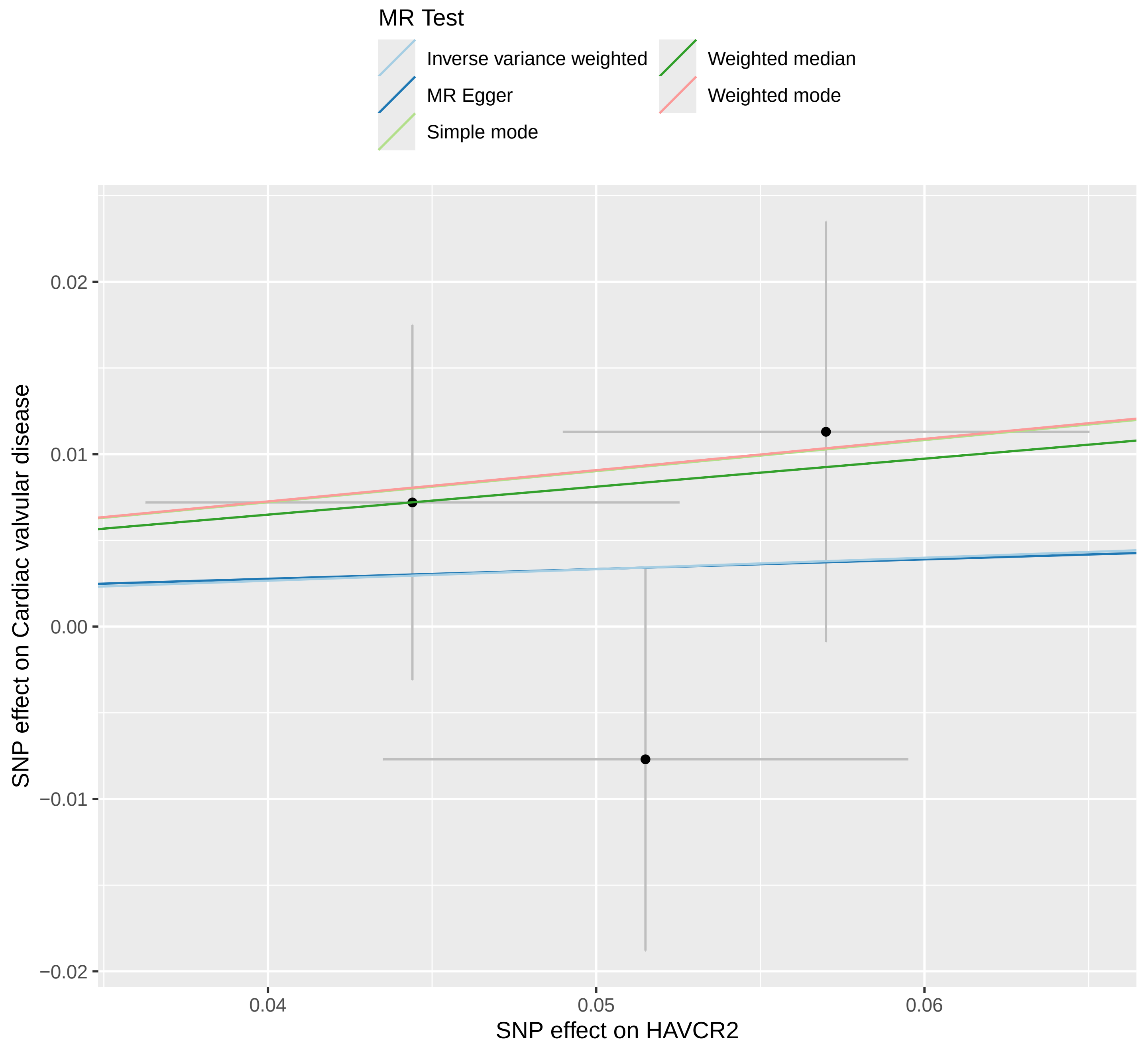
MR Test
Inverse variance weighted
MR Egger
Simple mode
Weighted median
Weighted mode
0.02
0.01
0.00
−0.01
−0.02
SNP effect on Cardiac valvular disease
0.04
0.05
0.06
SNP effect on HAVCR2

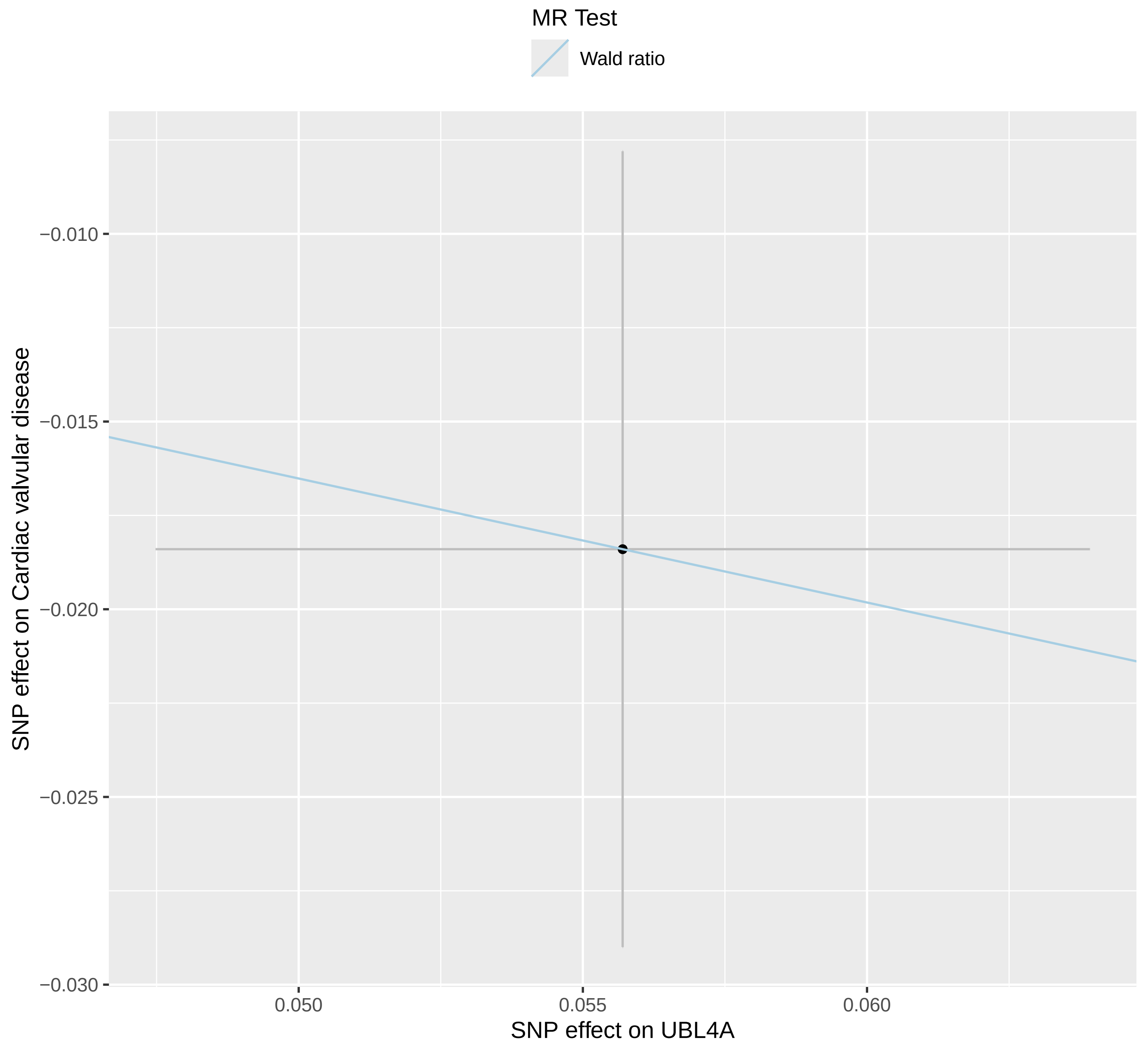
MR Test
Wald ratio
−0.010
−0.015
−0.020
−0.025
−0.030
SNP effect on Cardiac valvular disease
0.050
0.055
0.060
SNP effect on UBL4A

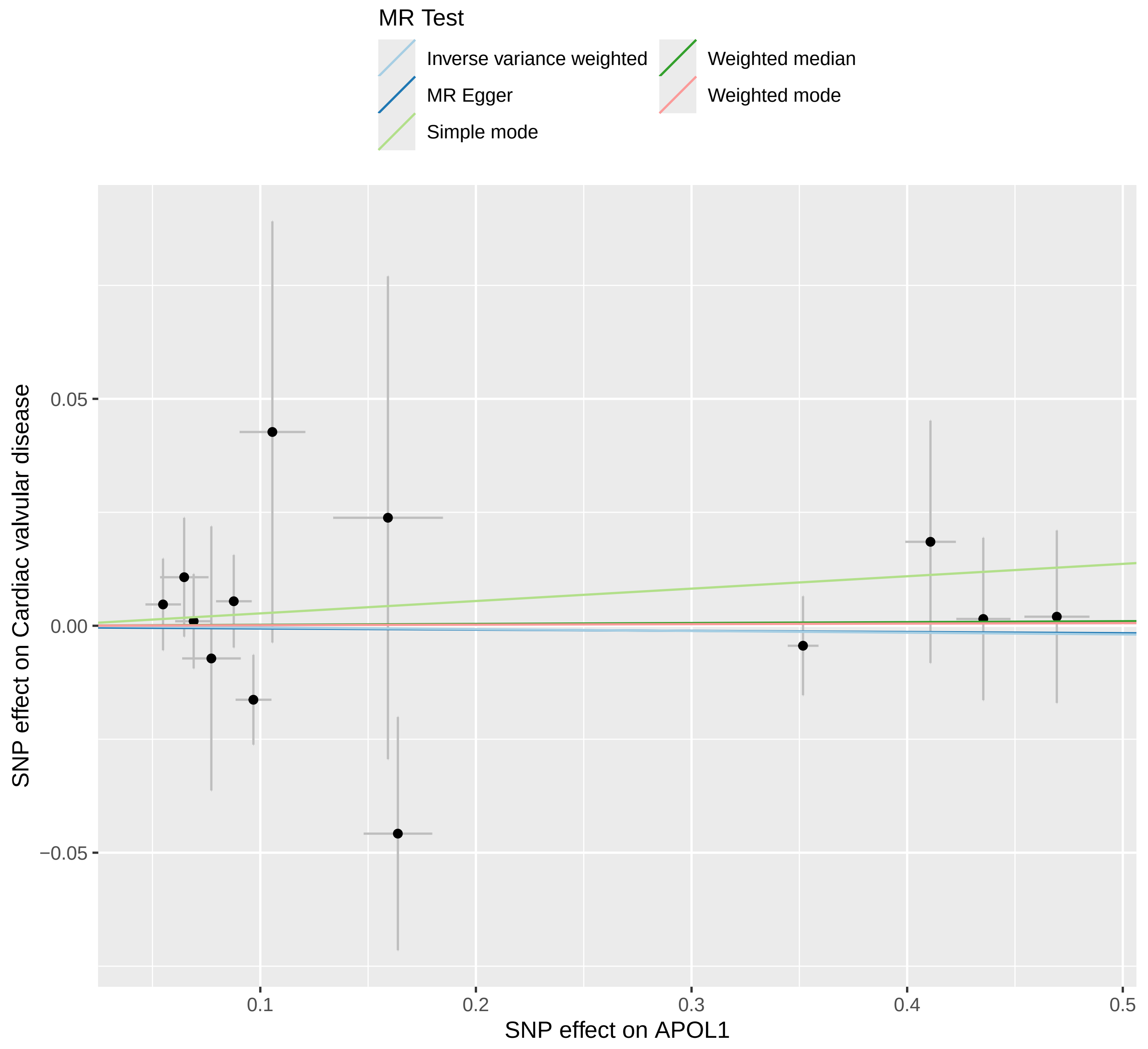

MR Test
Inverse variance weighted
MR Egger
Simple mode
Weighted median
Weighted mode
SNP effect on Cardiac valvular disease
0.05
0.00
−0.05
0.1
0.2
0.3
0.4
0.5
SNP effect on APOL1

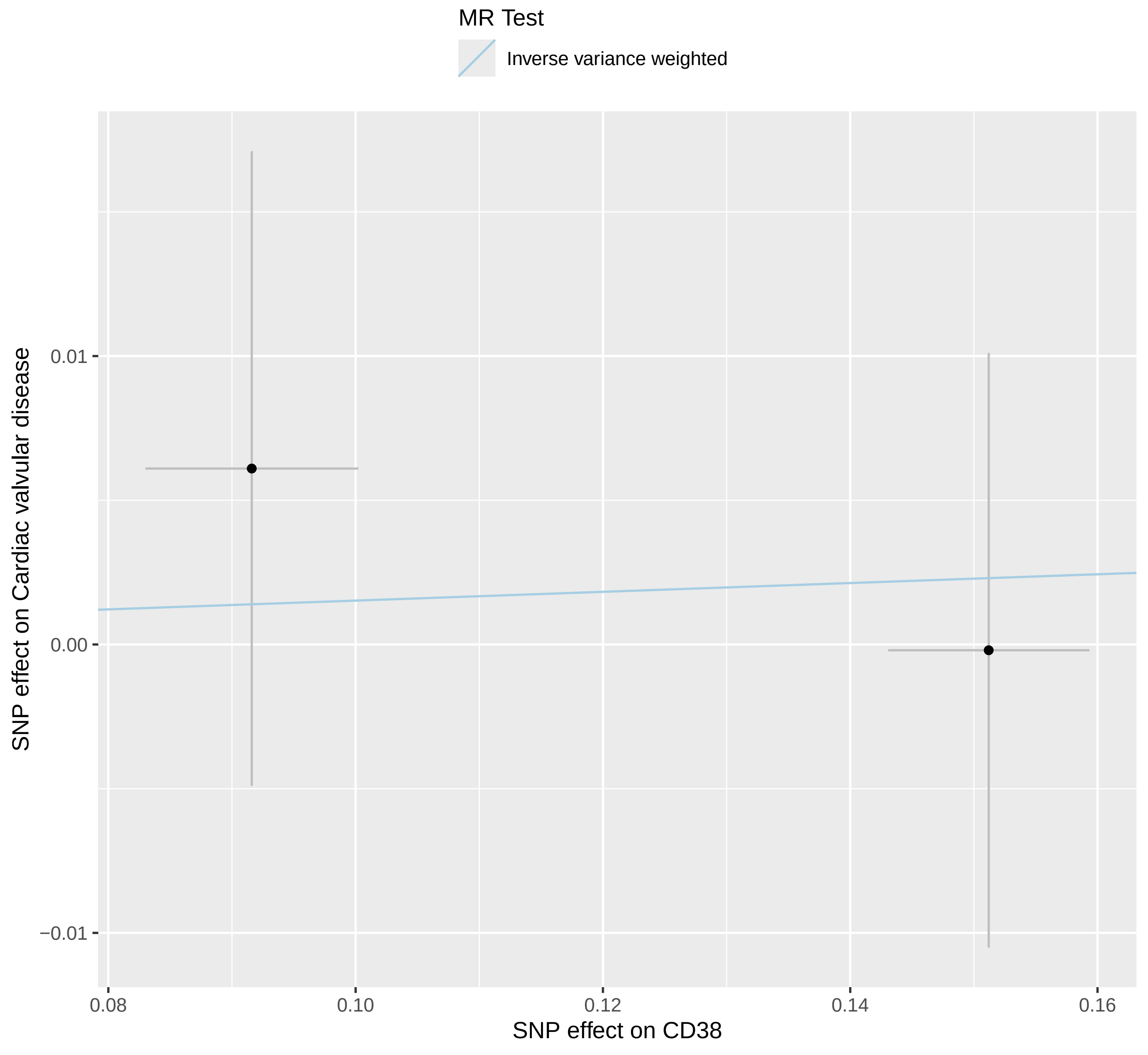

MR Test
Inverse variance weighted
SNP effect on Cardiac valvular disease
0.01
0.00
−0.01
0.08
0.10
0.12
0.14
0.16
SNP effect on CD38

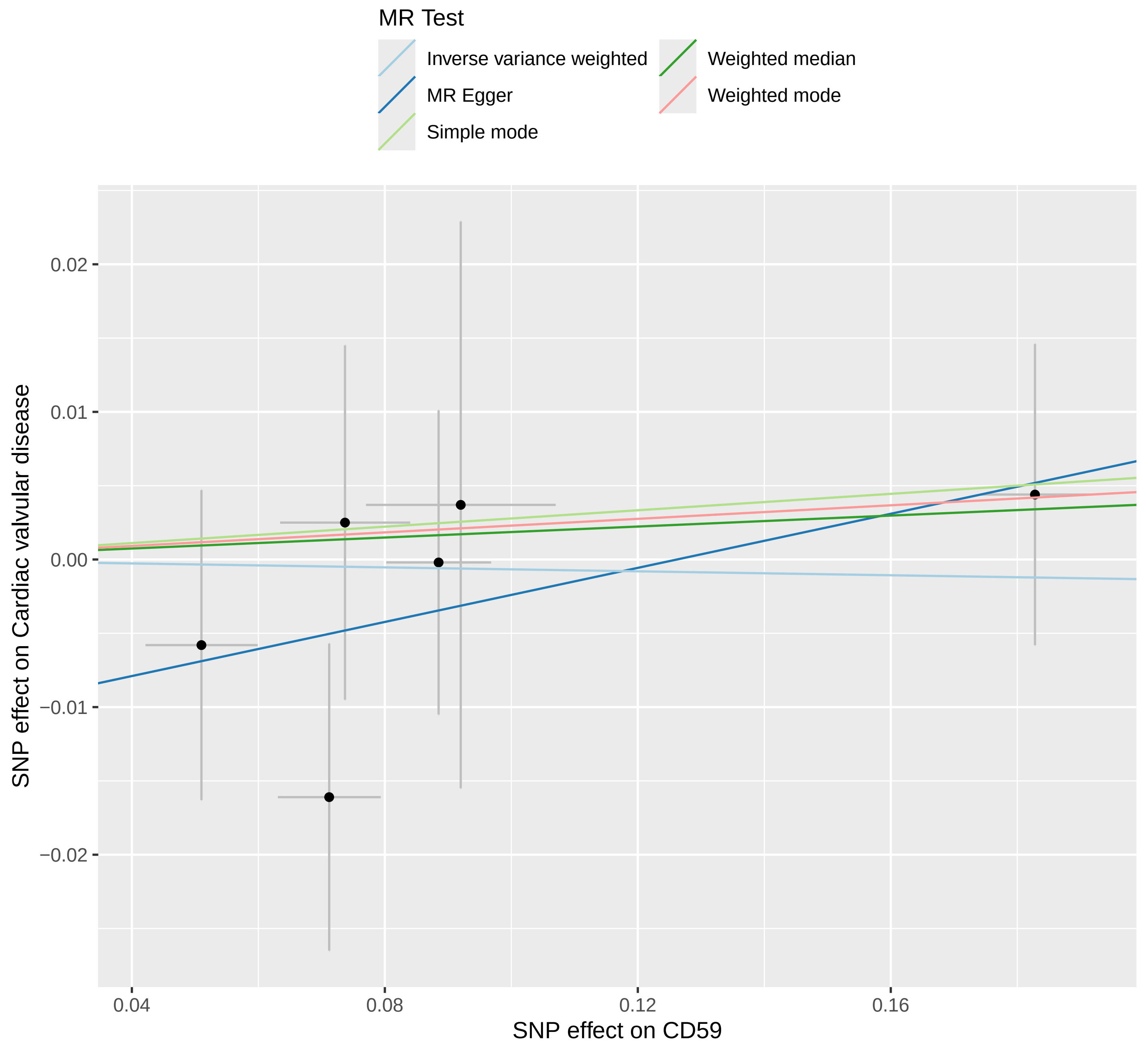

MR Test
Inverse variance weighted
MR Egger
Simple mode
Weighted median
Weighted mode
0.02
0.01
0.00
−0.01
−0.02
SNP effect on Cardiac valvular disease
0.04
0.08
0.12
0.16
SNP effect on CD59

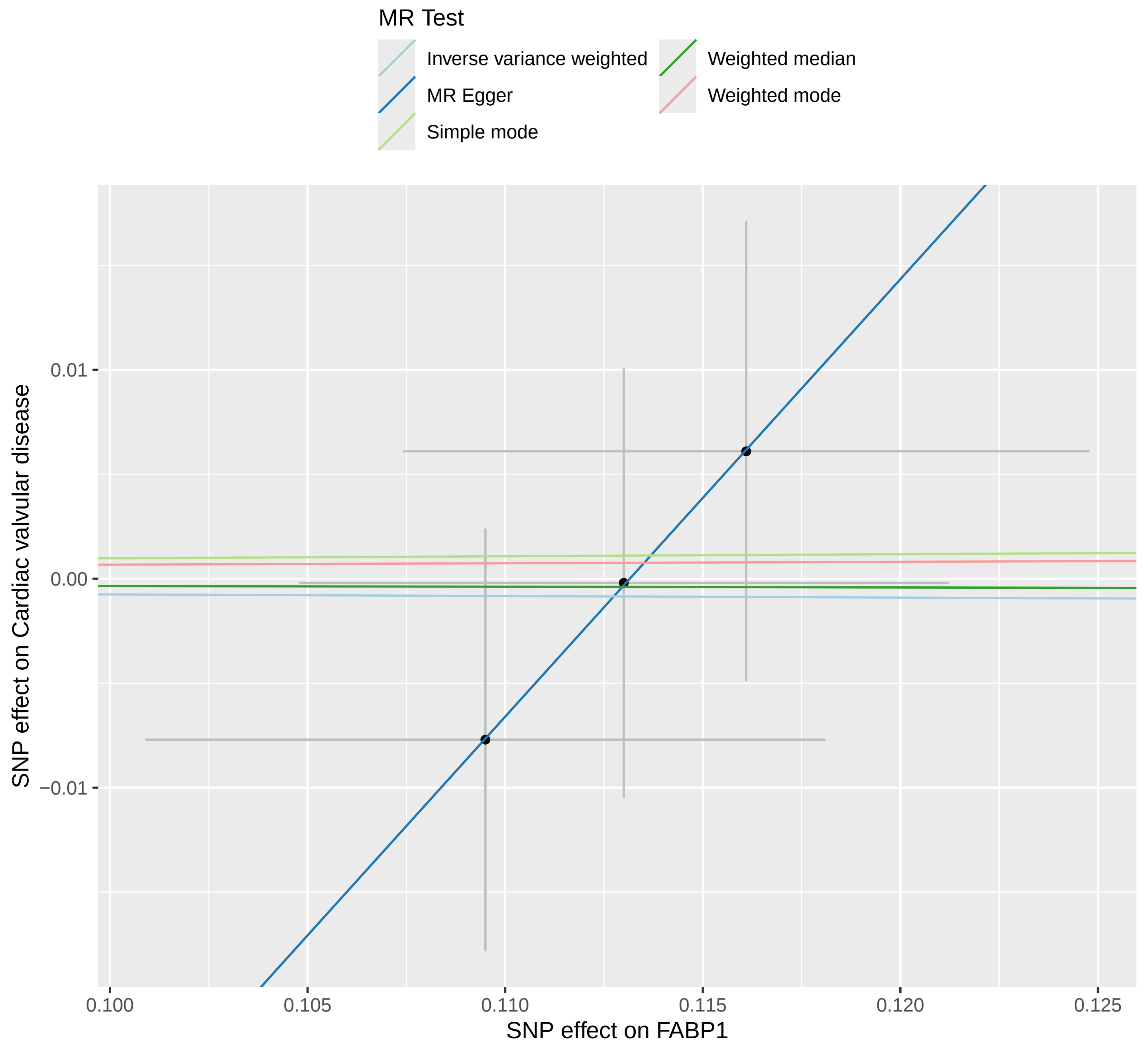
MR Test
Inverse variance weighted
MR Egger
Simple mode
Weighted median
Weighted mode
0.01
0.00
−0.01
0.100
0.105
0.110
0.115
0.120
0.125
SNP effect on Cardiac valvular disease
SNP effect on FABP1

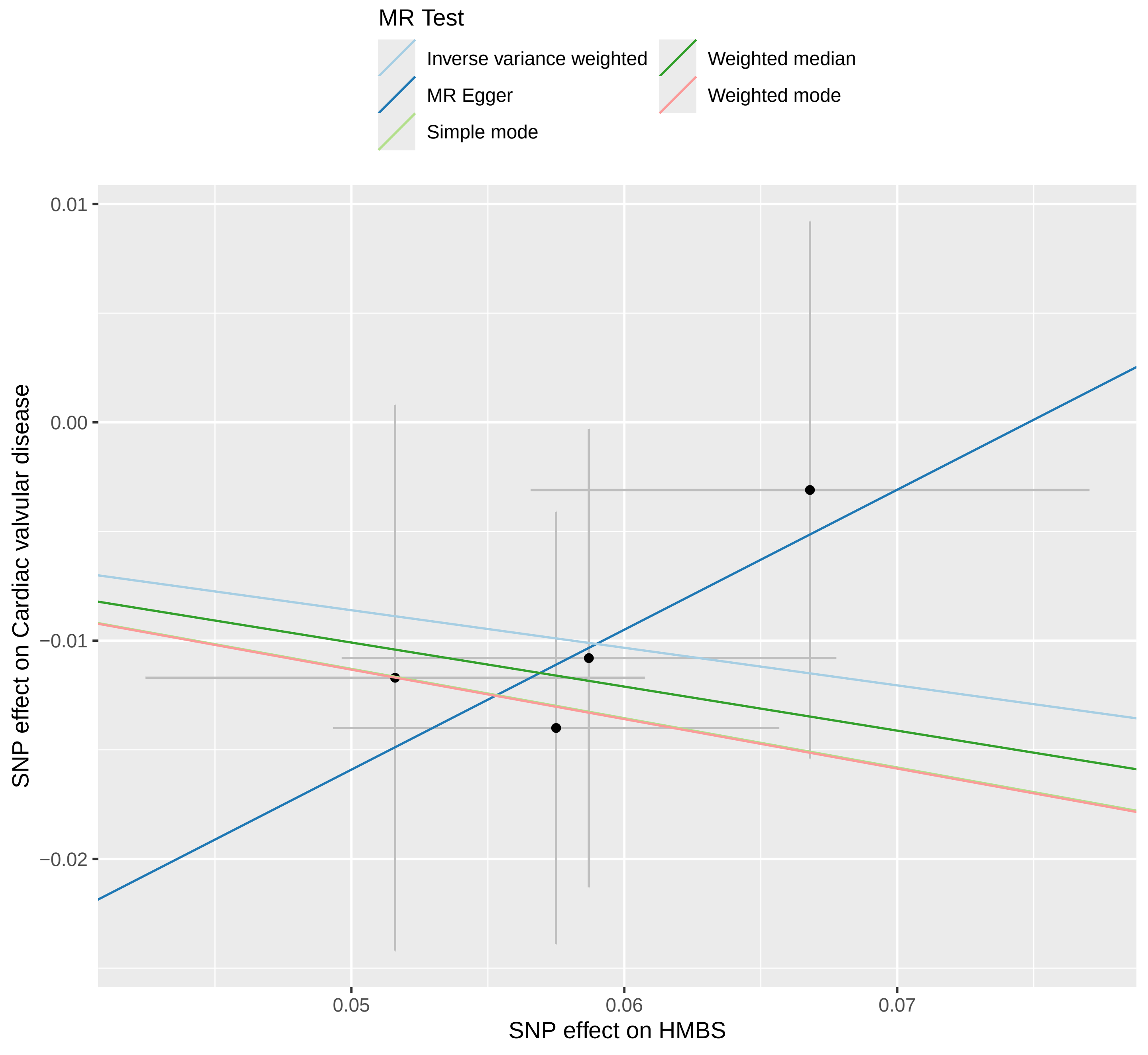

MR Test
Inverse variance weighted
MR Egger
Simple mode
Weighted median
Weighted mode
0.01
0.00
−0.01
−0.02
SNP effect on Cardiac valvular disease
0.05
0.06
0.07
SNP effect on HMBS

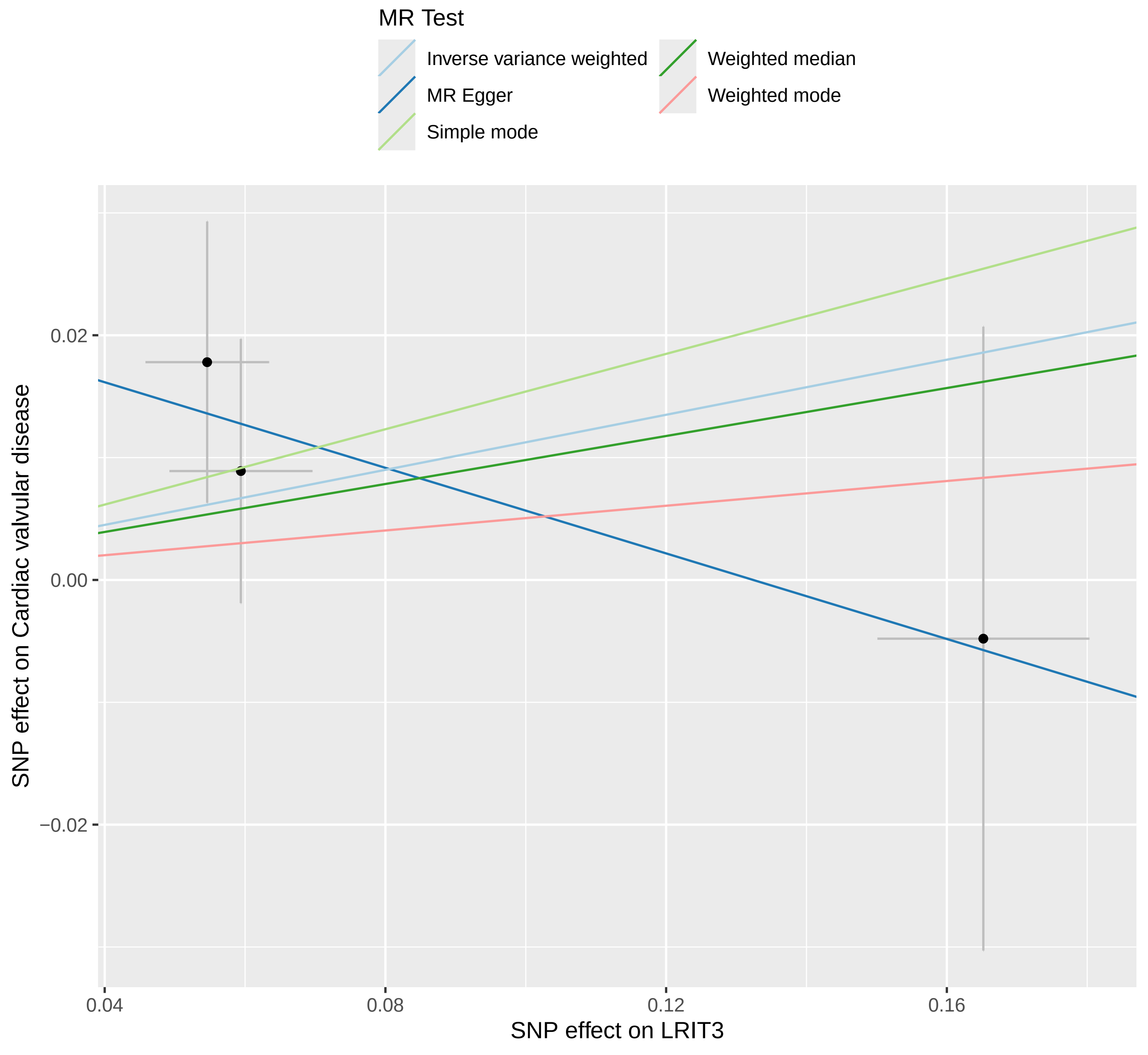
MR Test
Inverse variance weighted
MR Egger
Simple mode
Weighted median
Weighted mode
0.02
0.00
−0.02
0.04
0.08
0.12
0.16
SNP effect on Cardiac valvular disease
SNP effect on LRIT3

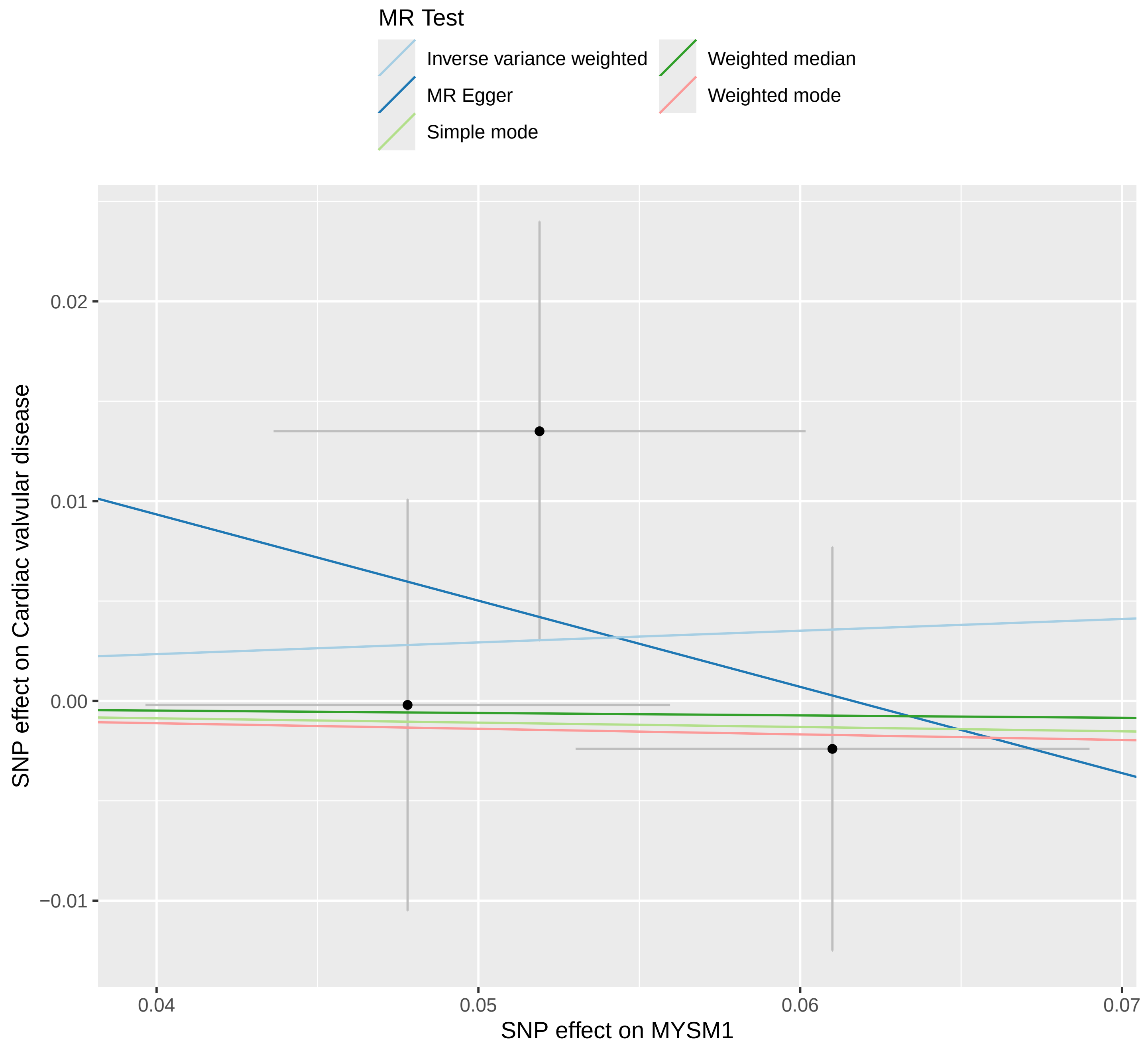
MR Test
Inverse variance weighted
MR Egger
Simple mode
Weighted median
Weighted mode
SNP effect on Cardiac valvular disease
0.02
0.01
0.00
−0.01
0.04
0.05
0.06
0.07
SNP effect on MYSM1

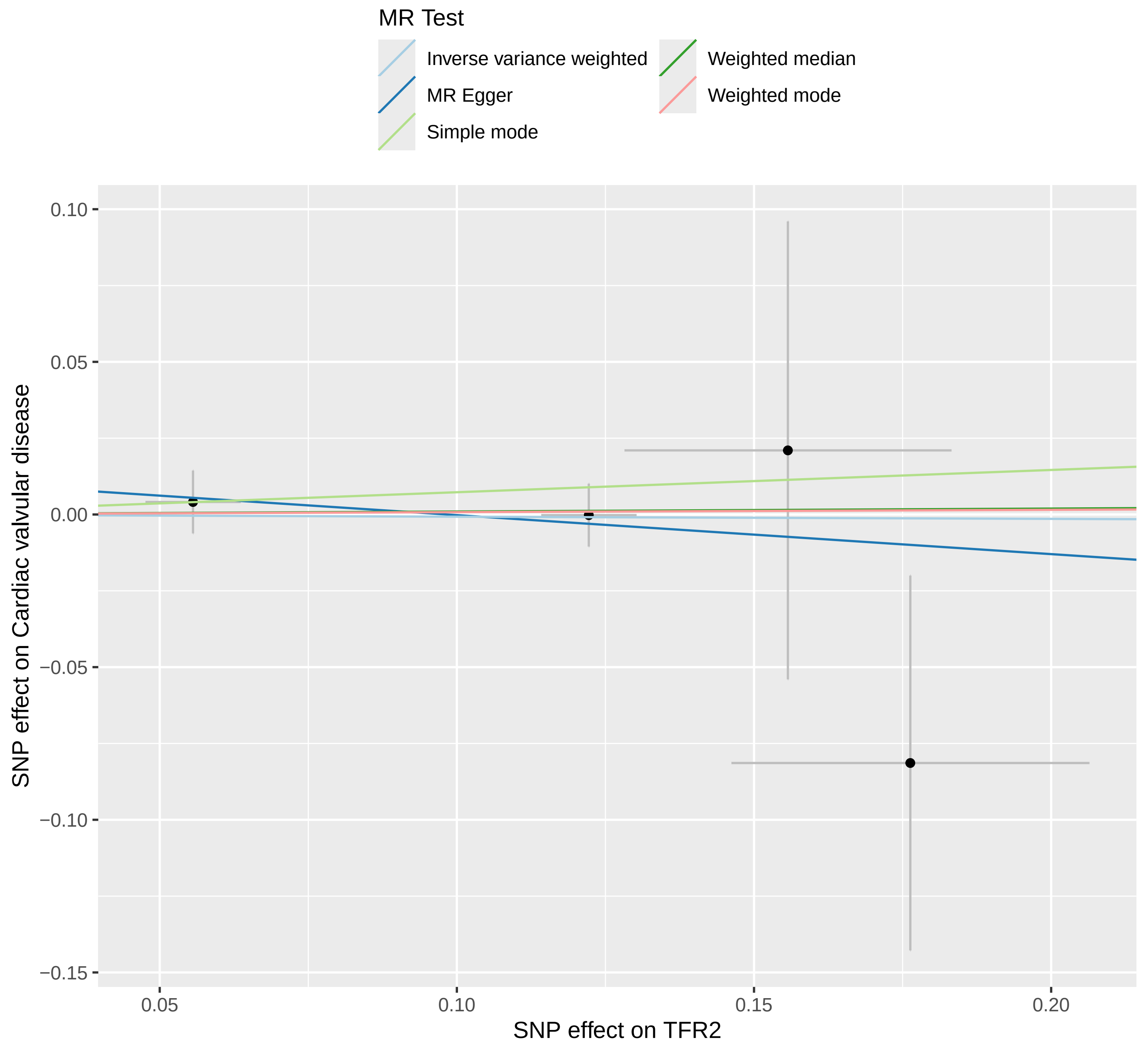

MR Test
Inverse variance weighted
MR Egger
Simple mode
Weighted median
Weighted mode
0.10
0.05
0.00
−0.05
−0.10
−0.15
0.05
0.10
0.15
0.20
SNP effect on Cardiac valvular disease
SNP effect on TFR2

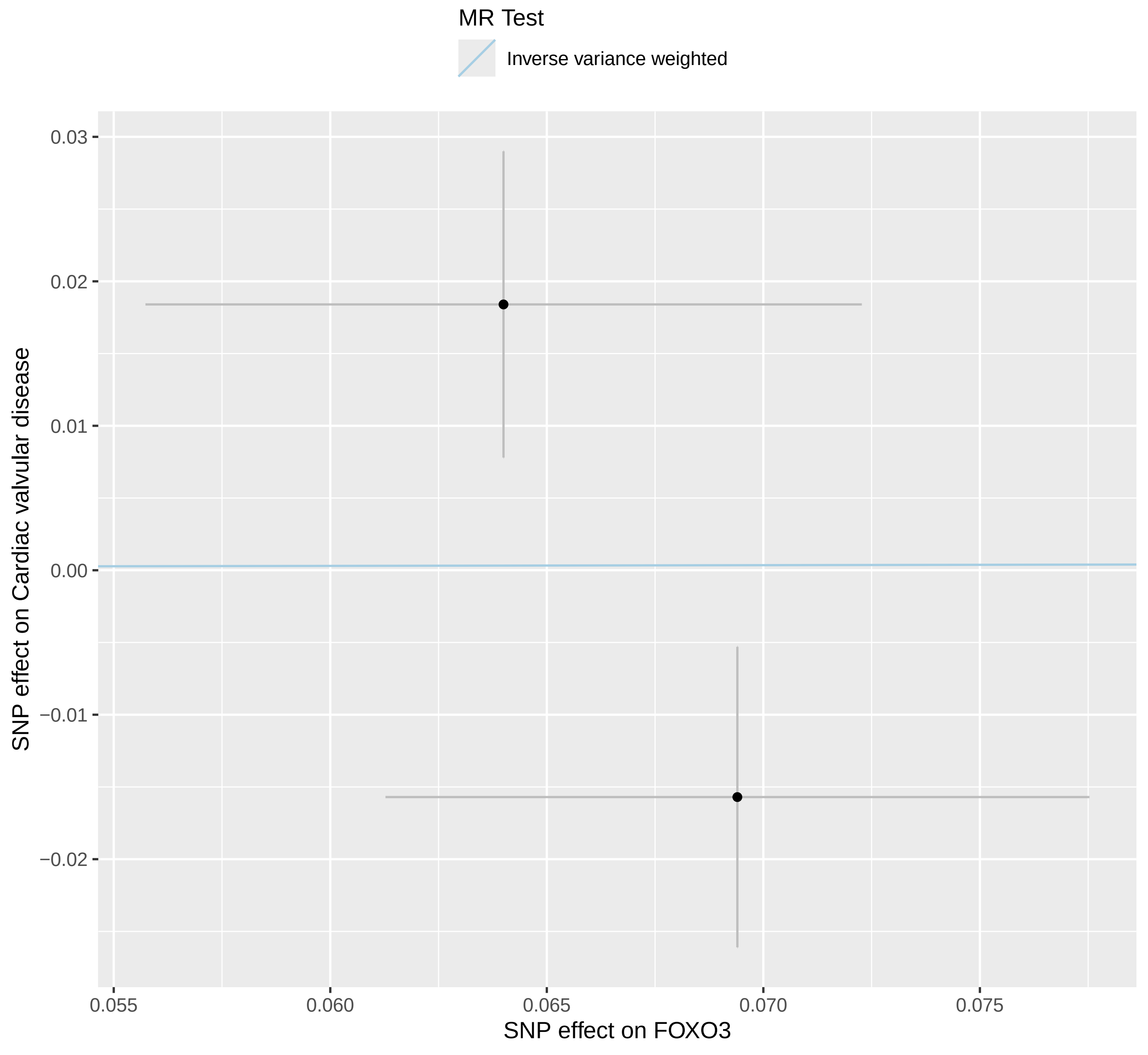
MR Test
Inverse variance weighted
0.03
0.02
0.01
0.00
−0.01
−0.02
SNP effect on Cardiac valvular disease
0.055
0.060
0.065
0.070
0.075
SNP effect on FOXO3

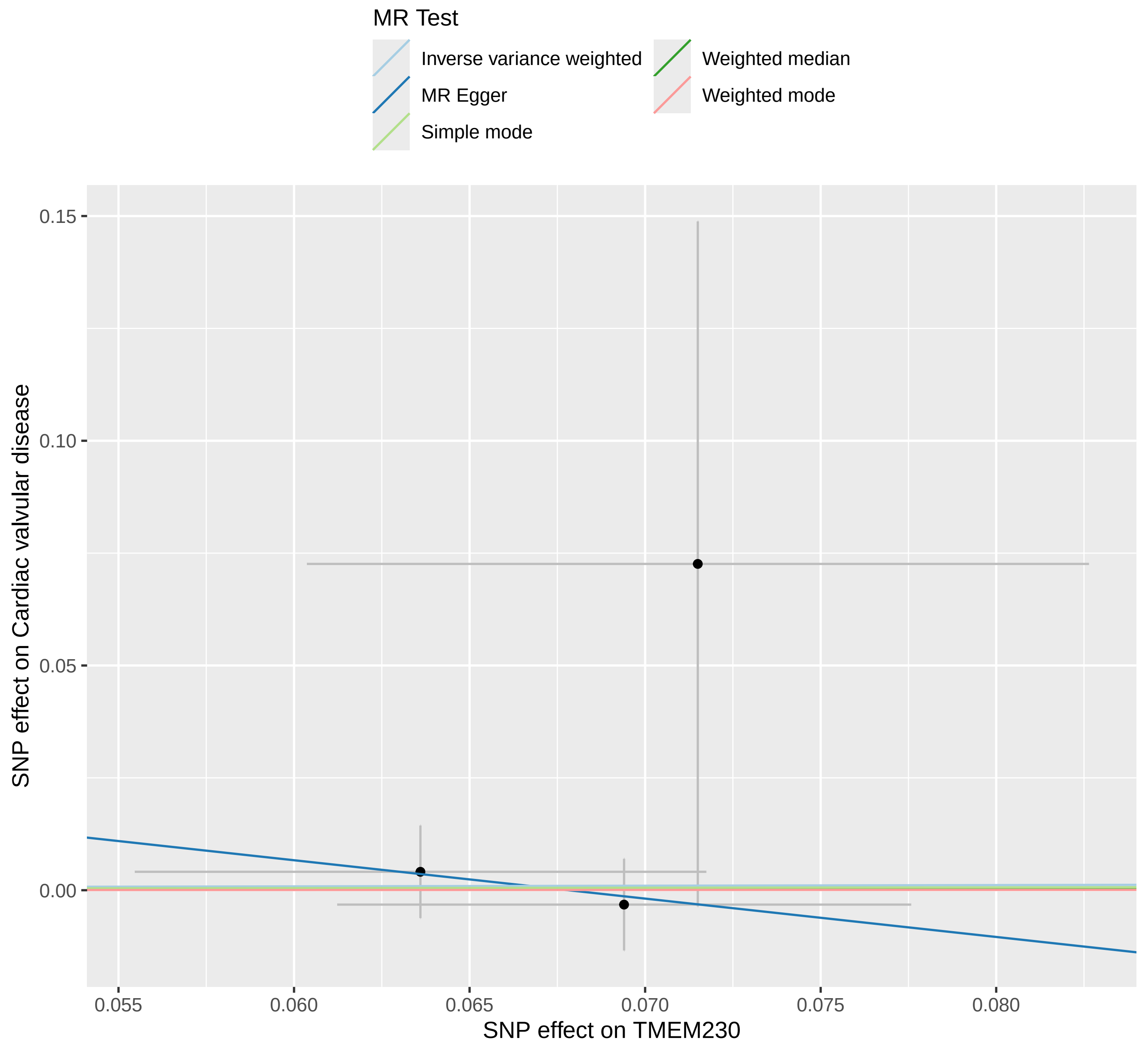

MR Test
Inverse variance weighted
MR Egger
Simple mode
Weighted median
Weighted mode
0.15
0.10
0.05
0.00
SNP effect on Cardiac valvular disease
0.055
0.060
0.065
0.070
0.075
0.080
SNP effect on TMEM230

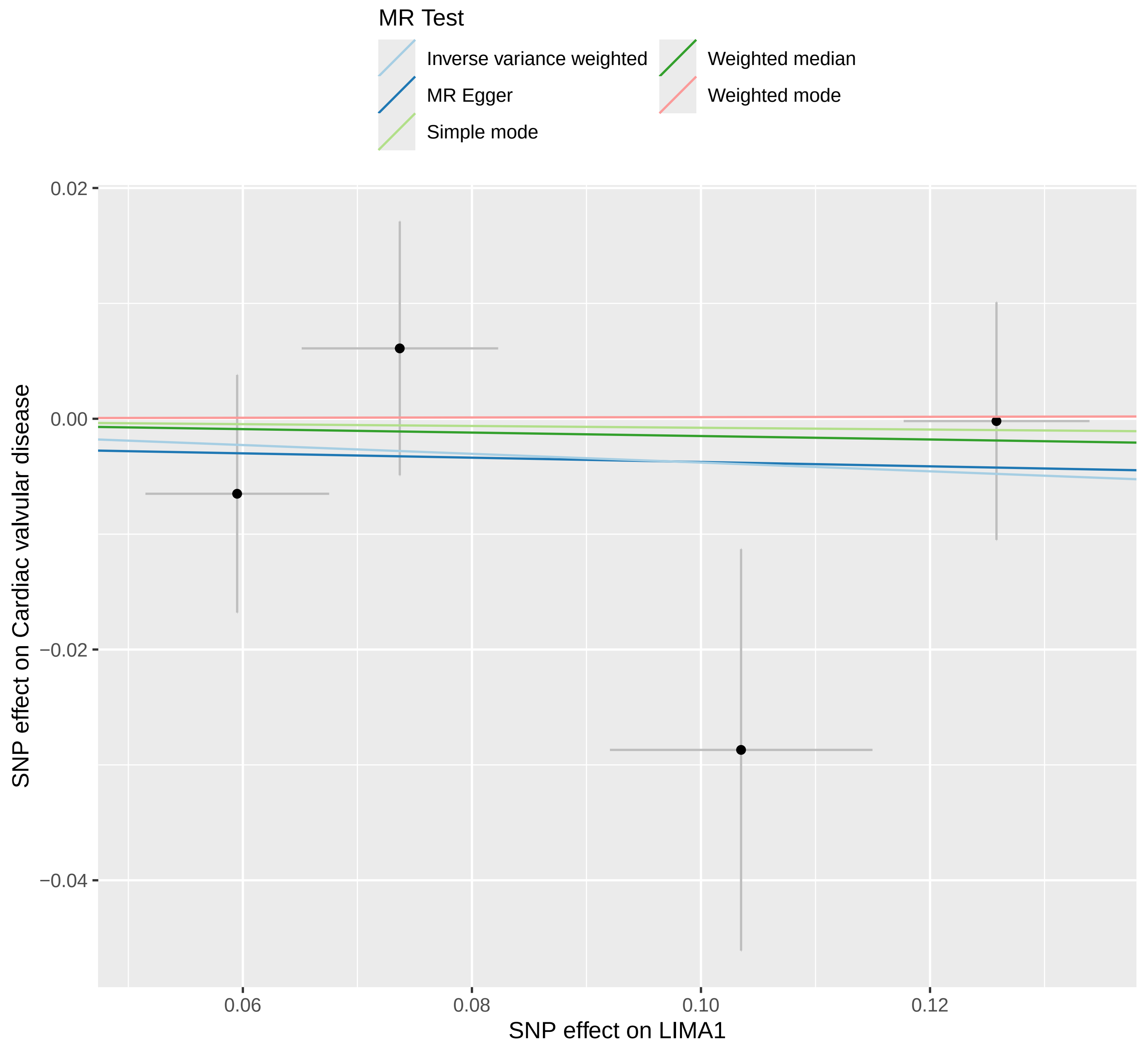

MR Test
Inverse variance weighted
MR Egger
Simple mode
Weighted median
Weighted mode
0.02
0.00
−0.02
−0.04
0.06
0.08
0.10
0.12
SNP effect on Cardiac valvular disease
SNP effect on LIMA1

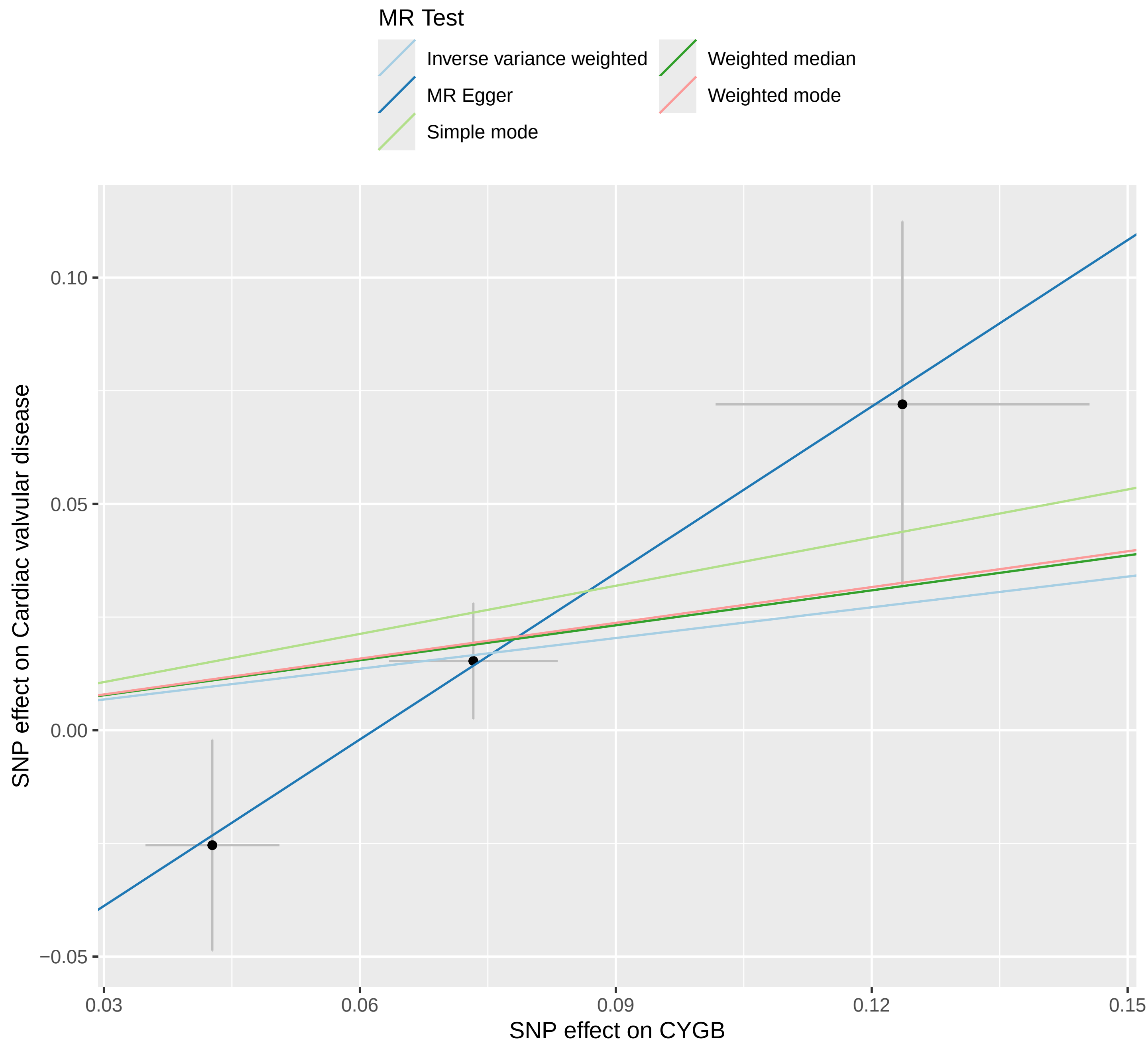

MR Test
Inverse variance weighted
MR Egger
Simple mode
Weighted median
Weighted mode
0.10
0.05
0.00
−0.05
SNP effect on Cardiac valvular disease
0.03
0.06
0.09
0.12
0.15
SNP effect on CYGB

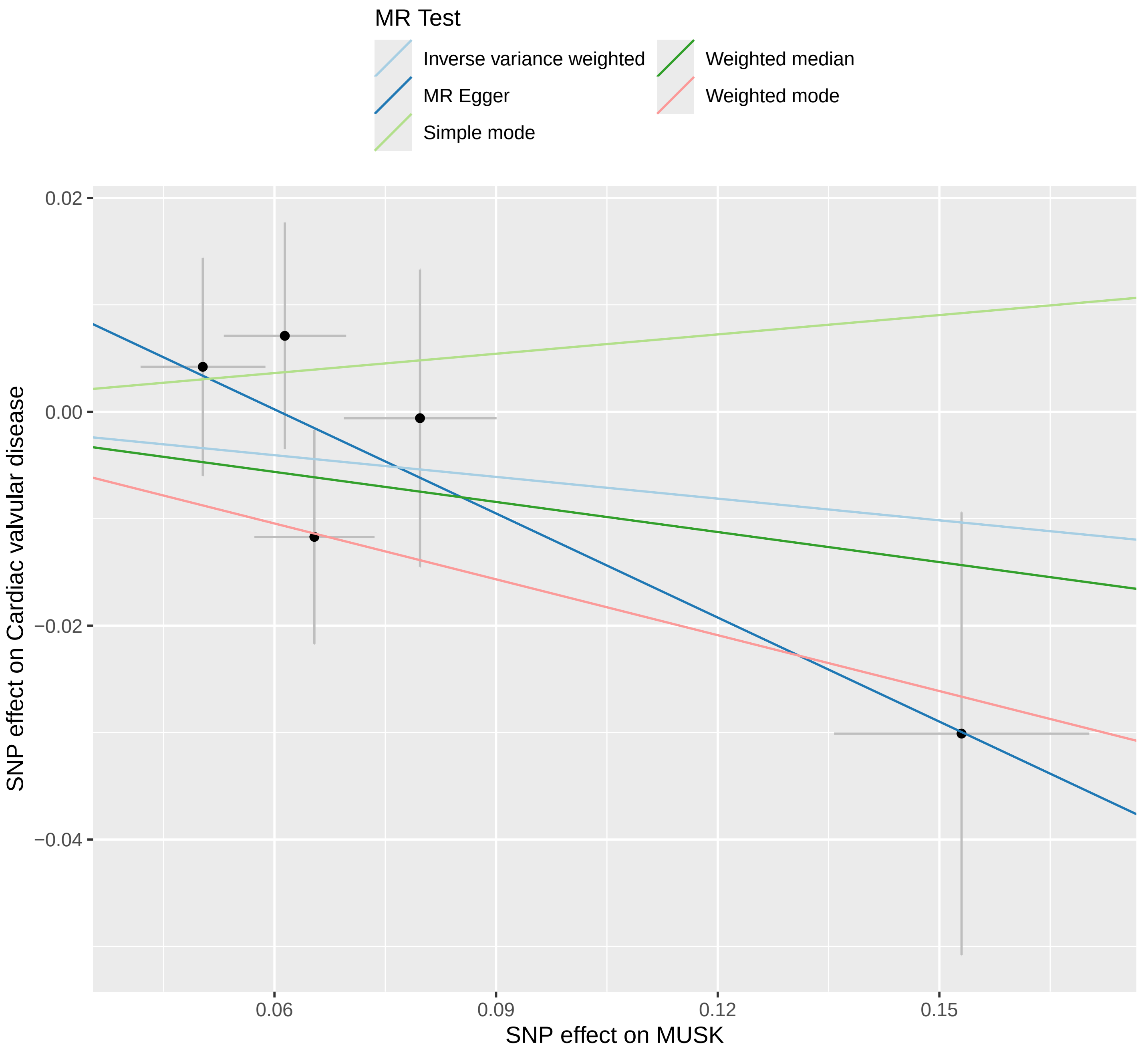

MR Test
Inverse variance weighted
MR Egger
Simple mode
Weighted median
Weighted mode
0.02
0.00
−0.02
−0.04
0.06
0.09
0.12
0.15
SNP effect on MUSK
SNP effect on Cardiac valvular disease

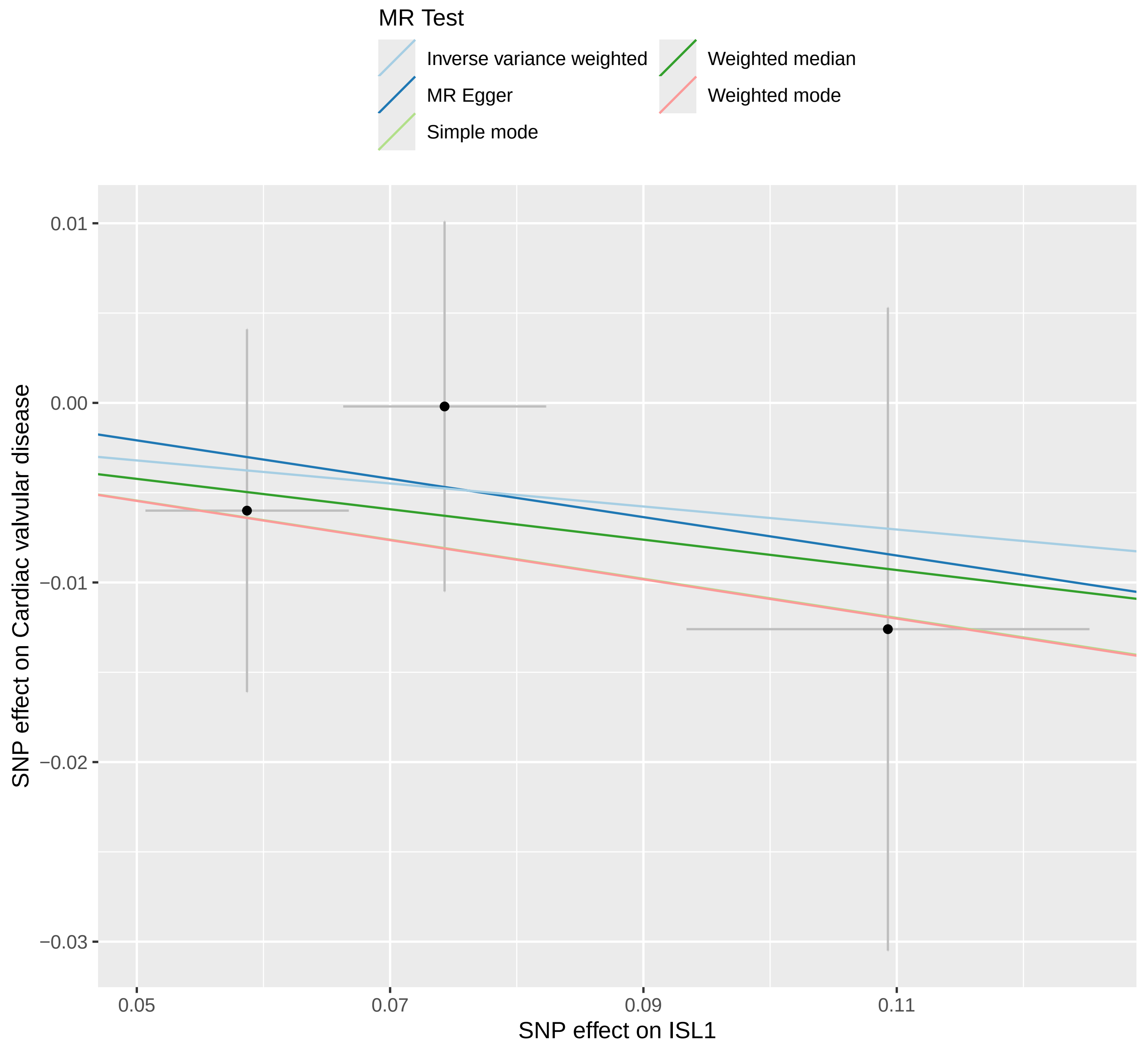
MR Test
Inverse variance weighted
MR Egger
Simple mode
Weighted median
Weighted mode
0.01
0.00
−0.01
−0.02
−0.03
0.05
0.07
0.09
0.11
SNP effect on ISL1
SNP effect on Cardiac valvular disease

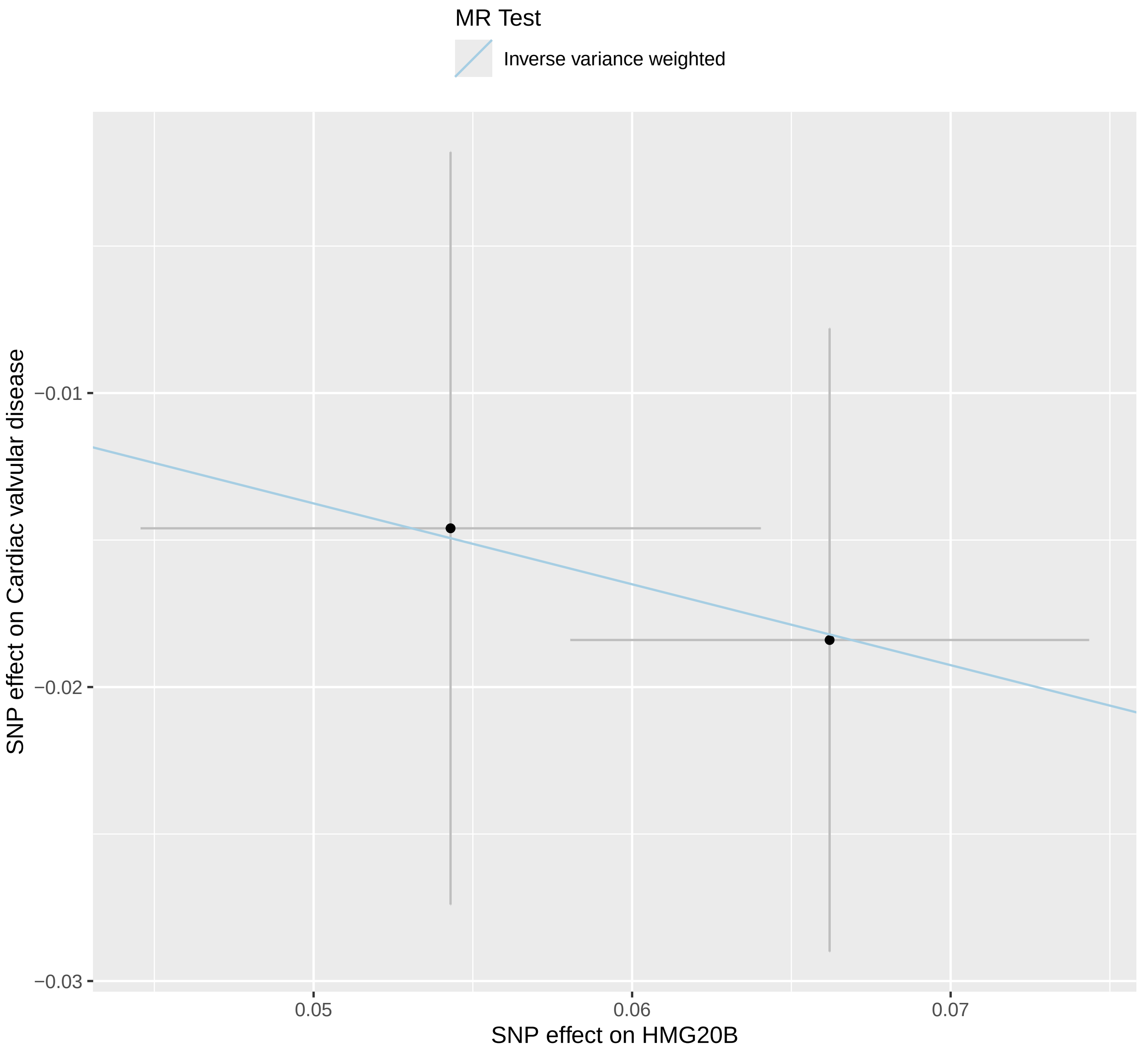

MR Test
Inverse variance weighted
SNP effect on Cardiac valvular disease
−0.01
−0.02
−0.03
0.05
0.06
0.07
SNP effect on HMG20B

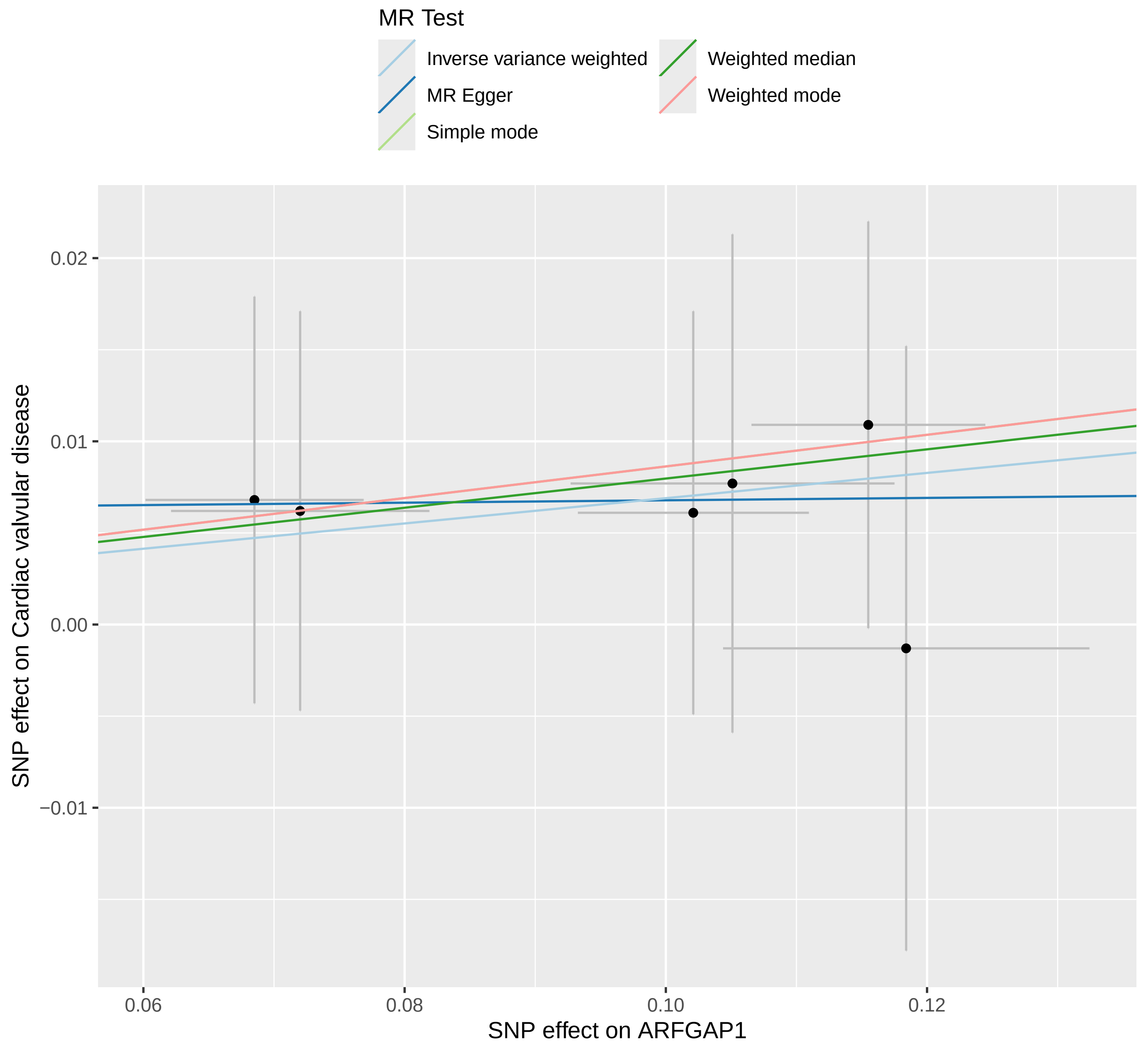
MR Test
Inverse variance weighted
MR Egger
Simple mode
Weighted median
Weighted mode
0.02
0.01
0.00
−0.01
0.06
0.08
0.10
0.12
SNP effect on Cardiac valvular disease
SNP effect on ARFGAP1

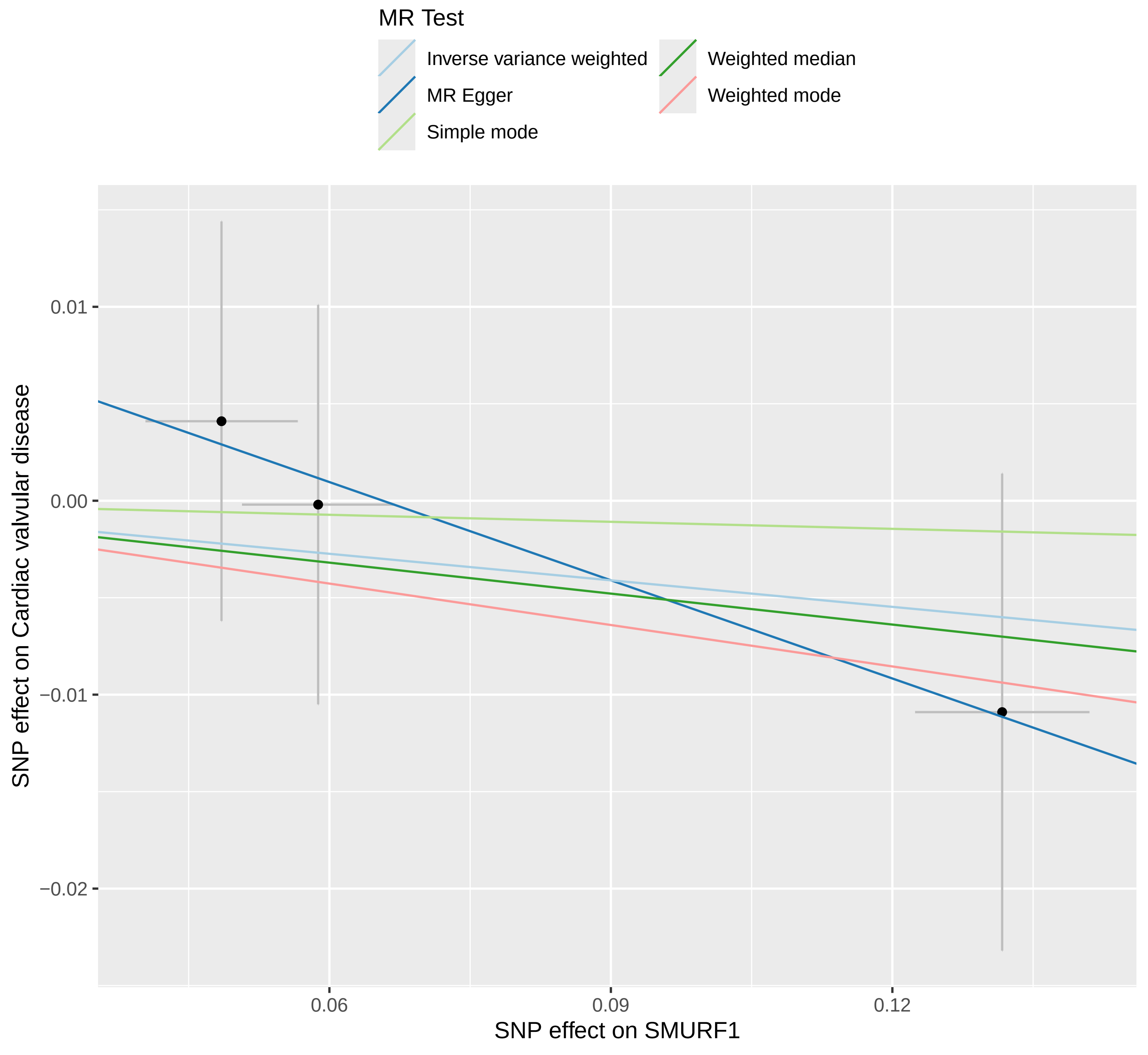

MR Test
Inverse variance weighted
MR Egger
Simple mode
Weighted median
Weighted mode
0.01
0.00
−0.01
−0.02
0.06
0.09
0.12
SNP effect on SMURF1
SNP effect on Cardiac valvular disease

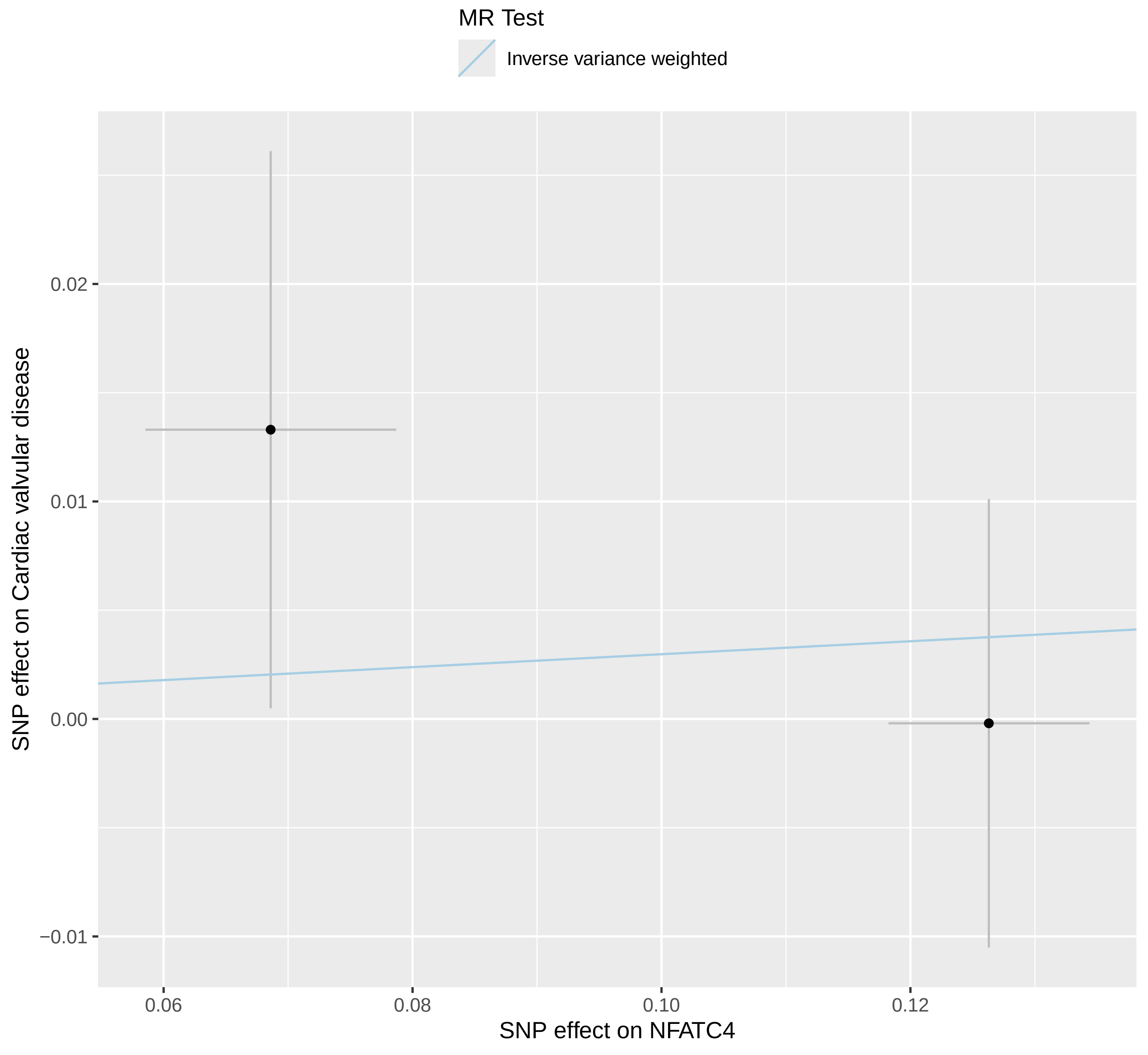
MR Test
Inverse variance weighted
SNP effect on Cardiac valvular disease
0.02
0.01
0.00
−0.01
0.06
0.08
0.10
0.12
SNP effect on NFATC4

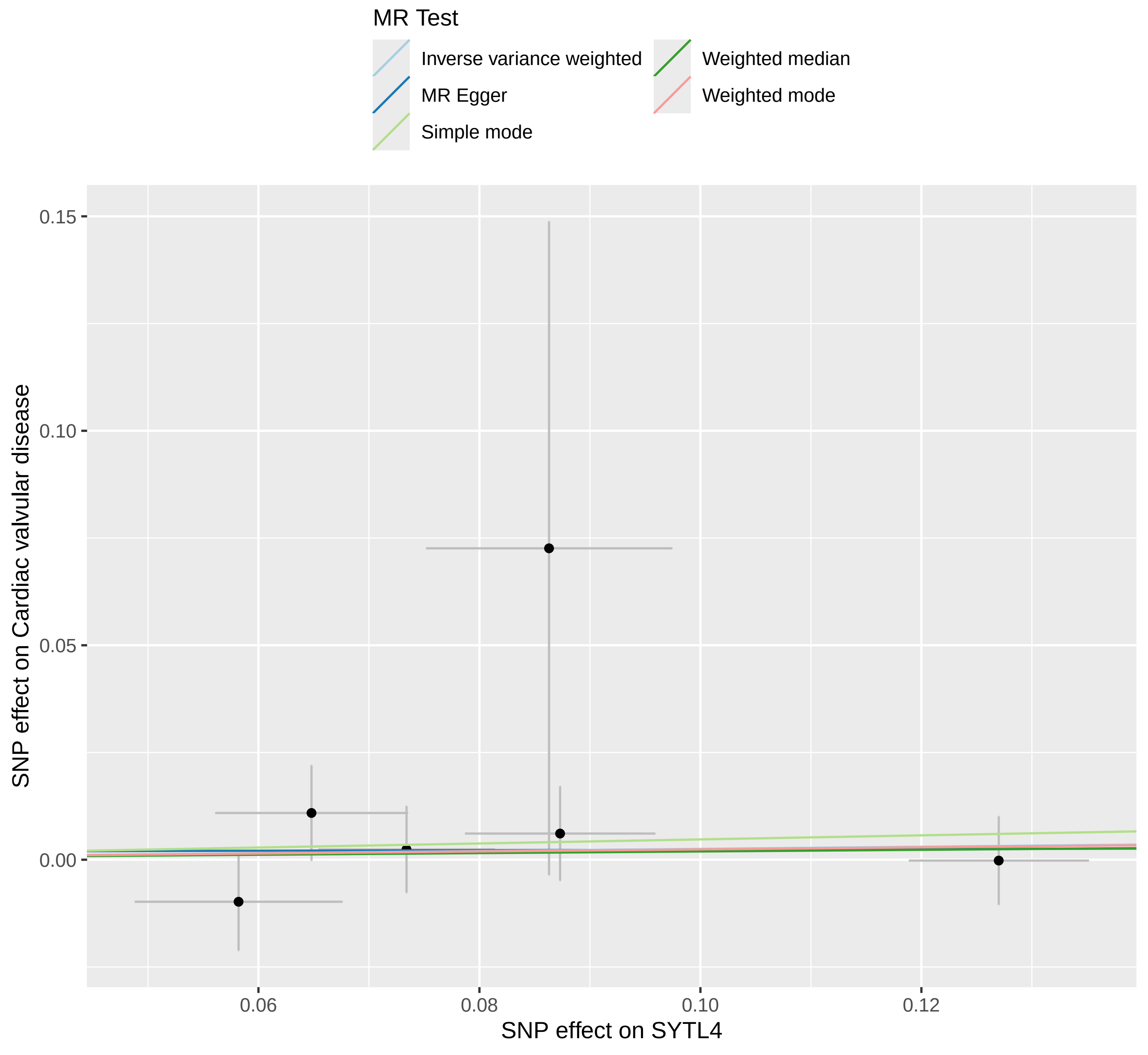

MR Test
Inverse variance weighted
MR Egger
Simple mode
Weighted median
Weighted mode
0.15
0.10
0.05
0.00
0.06
0.08
0.10
0.12
SNP effect on SYTL4
SNP effect on Cardiac valvular disease

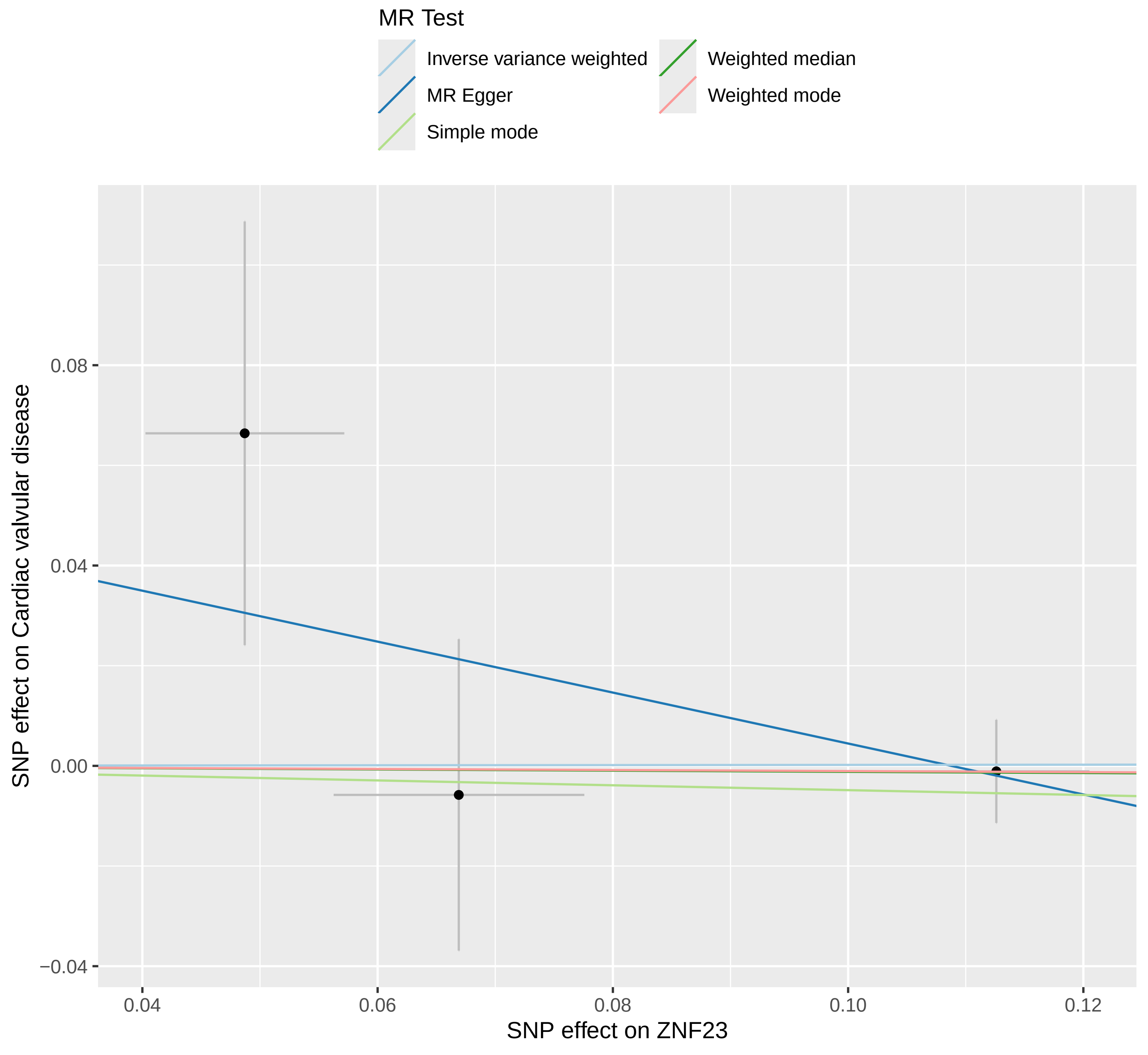

MR Test
Inverse variance weighted
MR Egger
Simple mode
Weighted median
Weighted mode
0.08
0.04
0.00
−0.04
SNP effect on Cardiac valvular disease
0.04
0.06
0.08
0.10
0.12
SNP effect on ZNF23

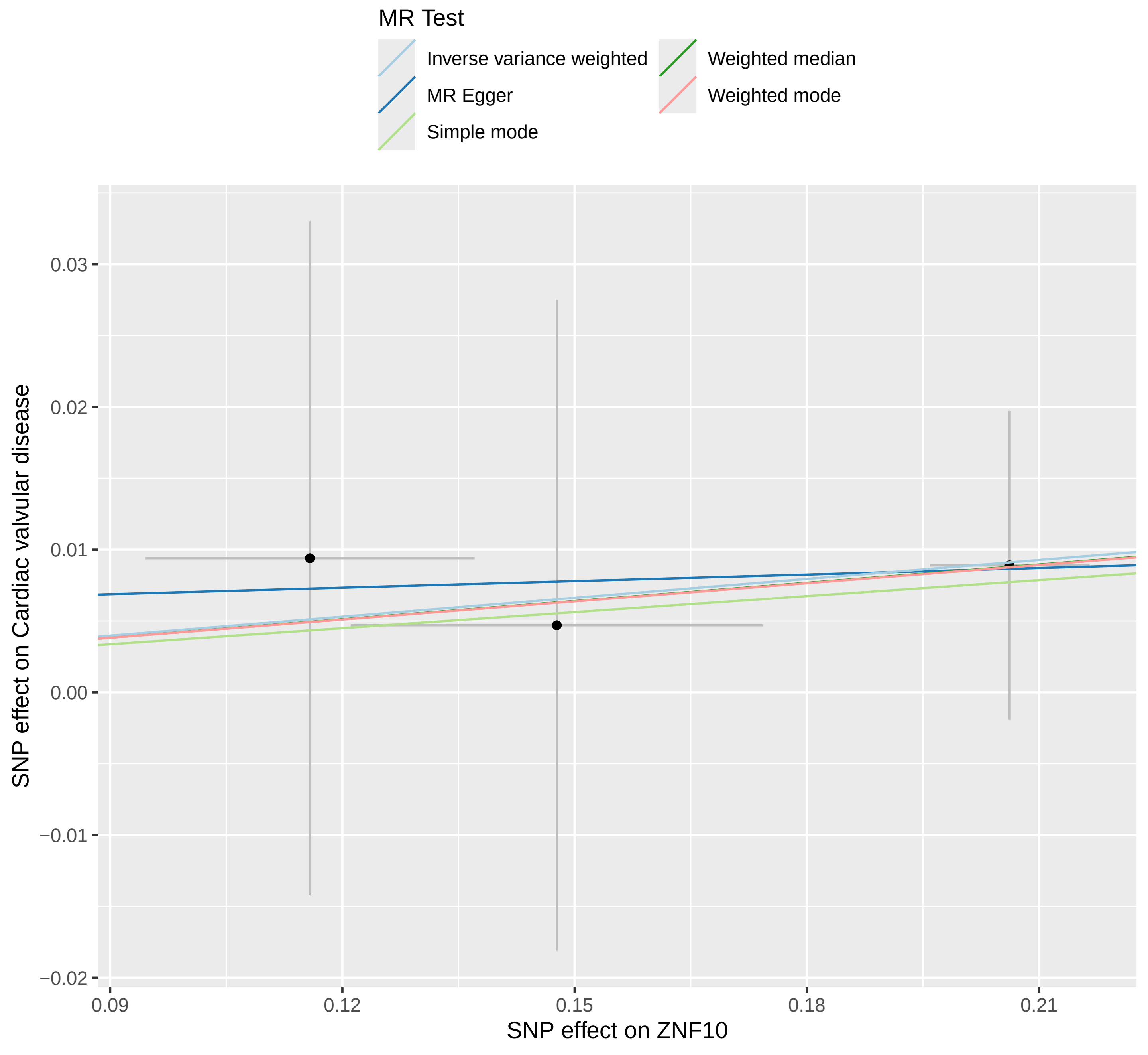

MR Test
Inverse variance weighted
MR Egger
Simple mode
Weighted median
Weighted mode
0.03
0.02
0.01
0.00
−0.01
−0.02
0.09
0.12
0.15
0.18
0.21
SNP effect on Cardiac valvular disease
SNP effect on ZNF10

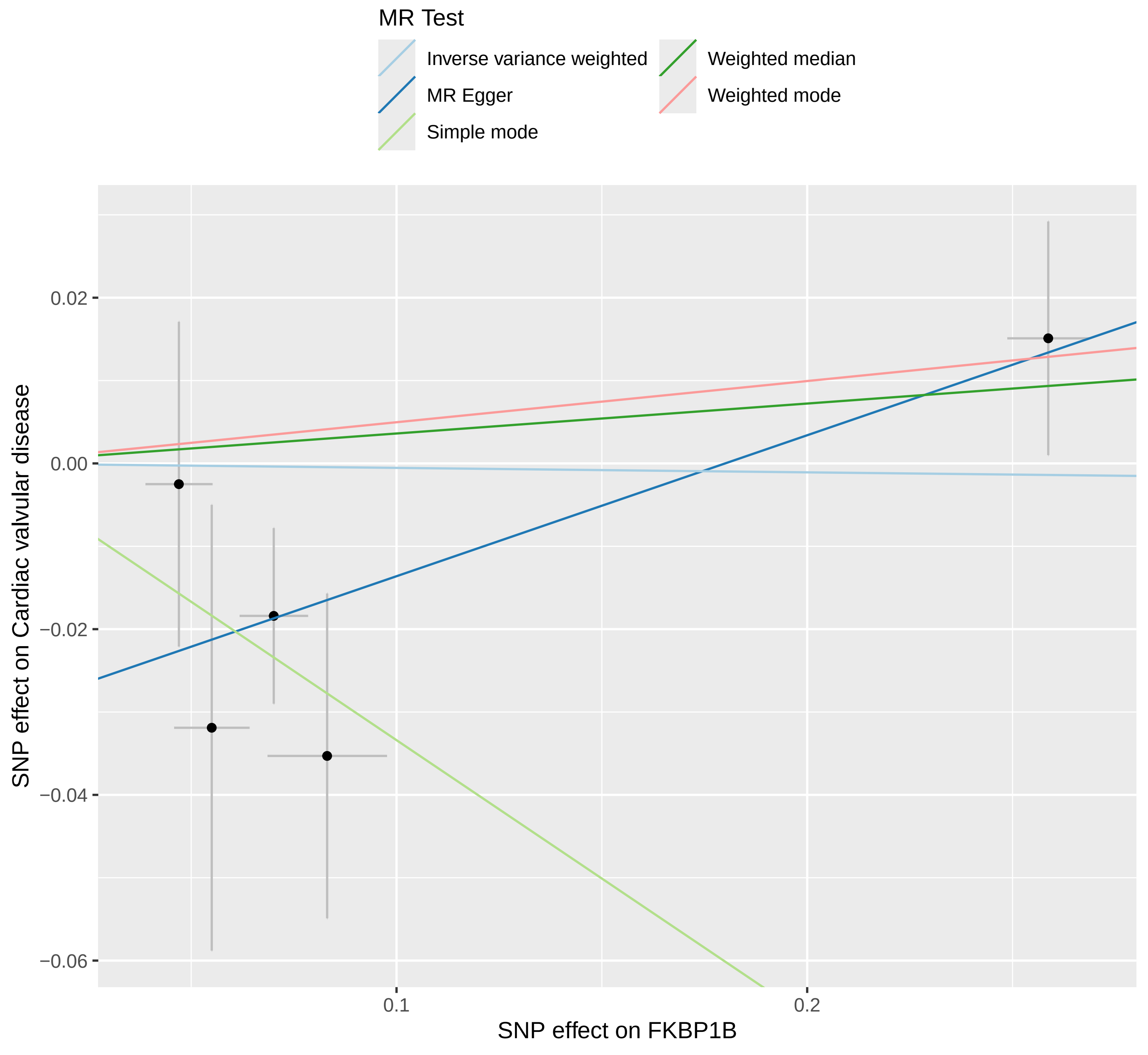

MR Test
Inverse variance weighted
MR Egger
Simple mode
Weighted median
Weighted mode
0.02
0.00
−0.02
−0.04
−0.06
0.1
0.2
SNP effect on Cardiac valvular disease
SNP effect on FKBP1B

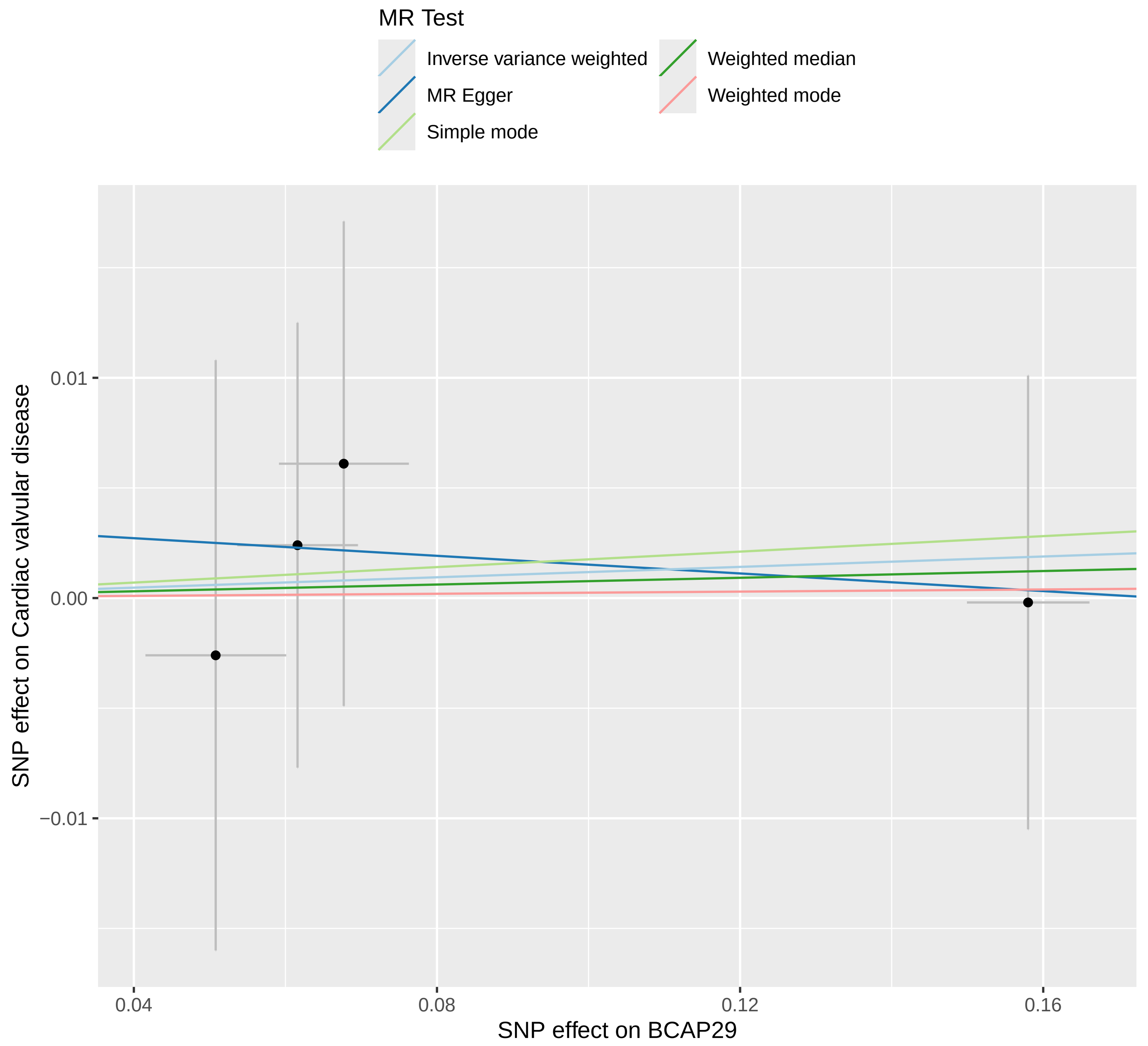
MR Test
Inverse variance weighted
MR Egger
Simple mode
Weighted median
Weighted mode
0.01
0.00
−0.01
0.04
0.08
0.12
0.16
SNP effect on Cardiac valvular disease
SNP effect on BCAP29

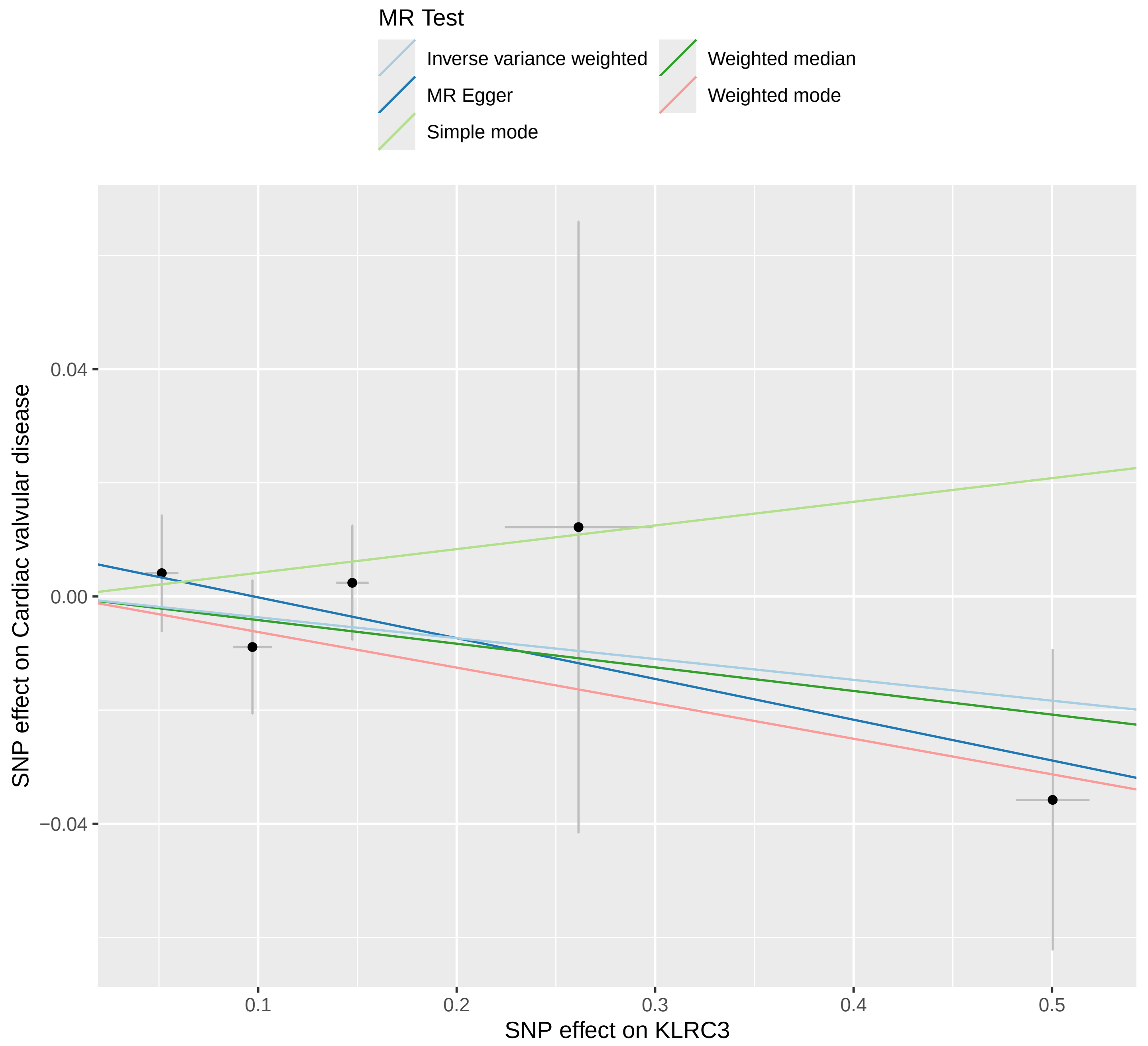

MR Test
Inverse variance weighted
MR Egger
Simple mode
Weighted median
Weighted mode
0.04
0.00
−0.04
0.1
0.2
0.3
0.4
0.5
SNP effect on Cardiac valvular disease
SNP effect on KLRC3

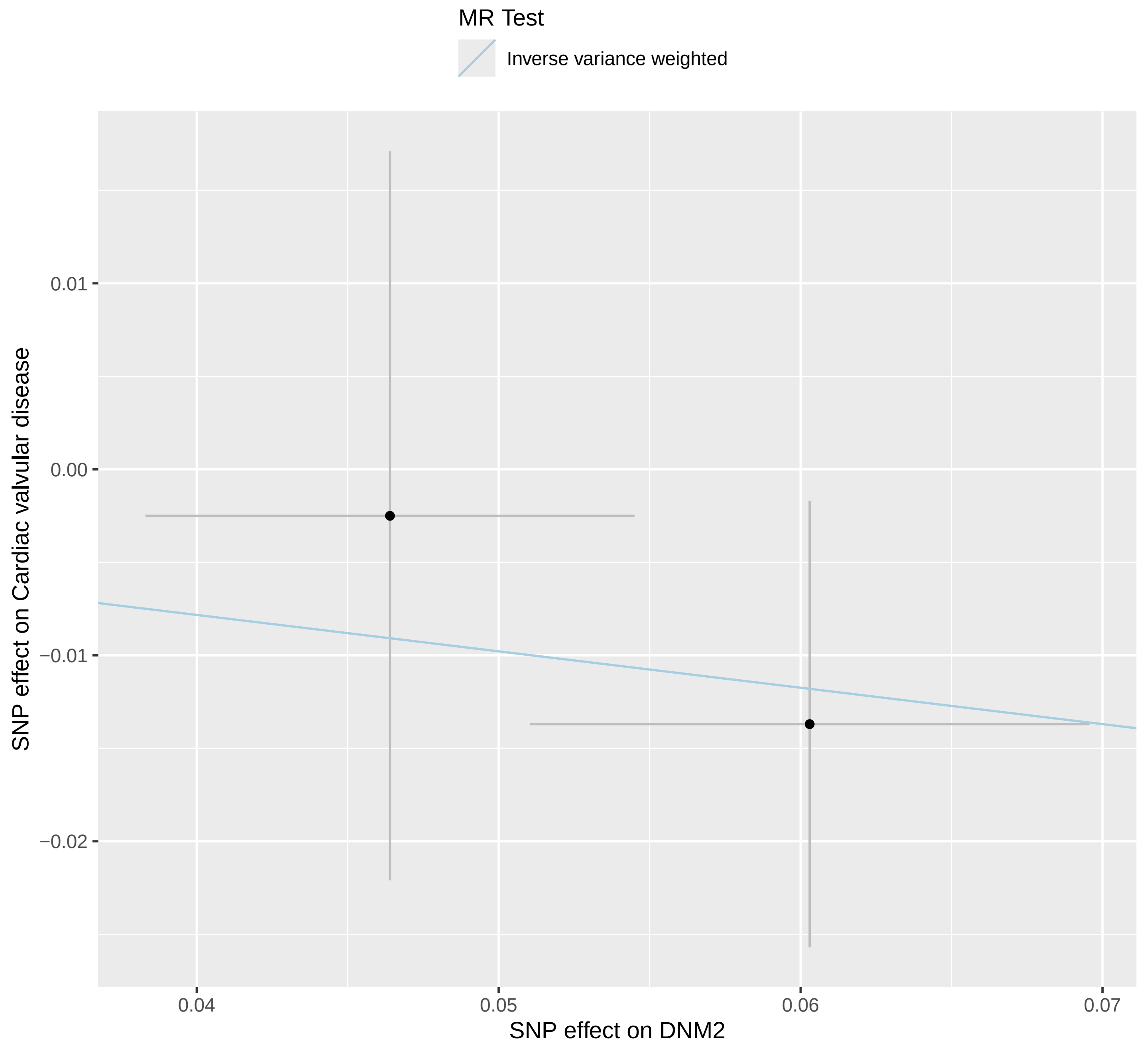
MR Test
Inverse variance weighted
0.01
0.00
−0.01
−0.02
SNP effect on Cardiac valvular disease
0.04
0.05
0.06
0.07
SNP effect on DNM2

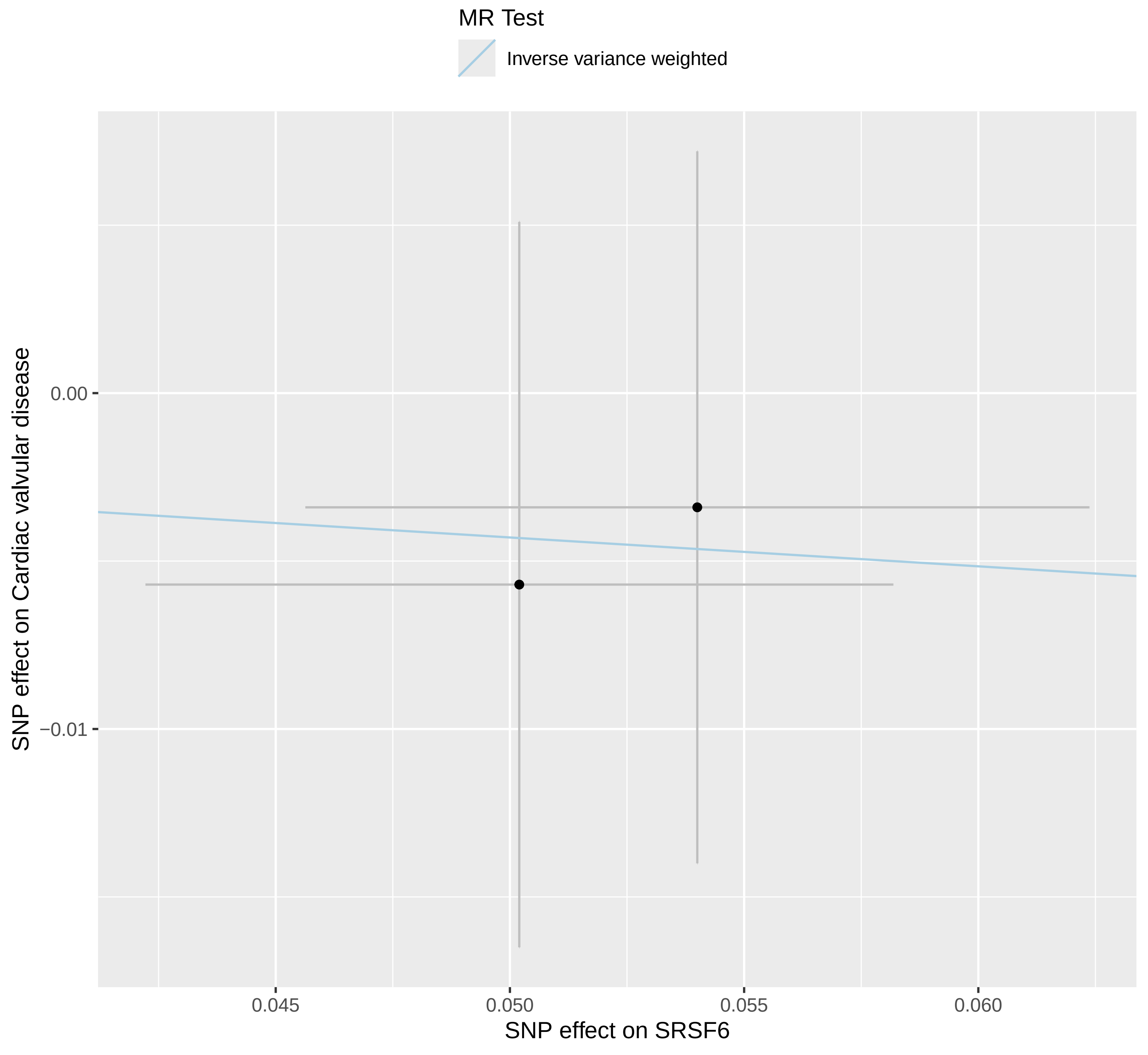

MR Test
Inverse variance weighted
SNP effect on Cardiac valvular disease
0.00
−0.01
0.045
0.050
0.055
0.060
SNP effect on SRSF6

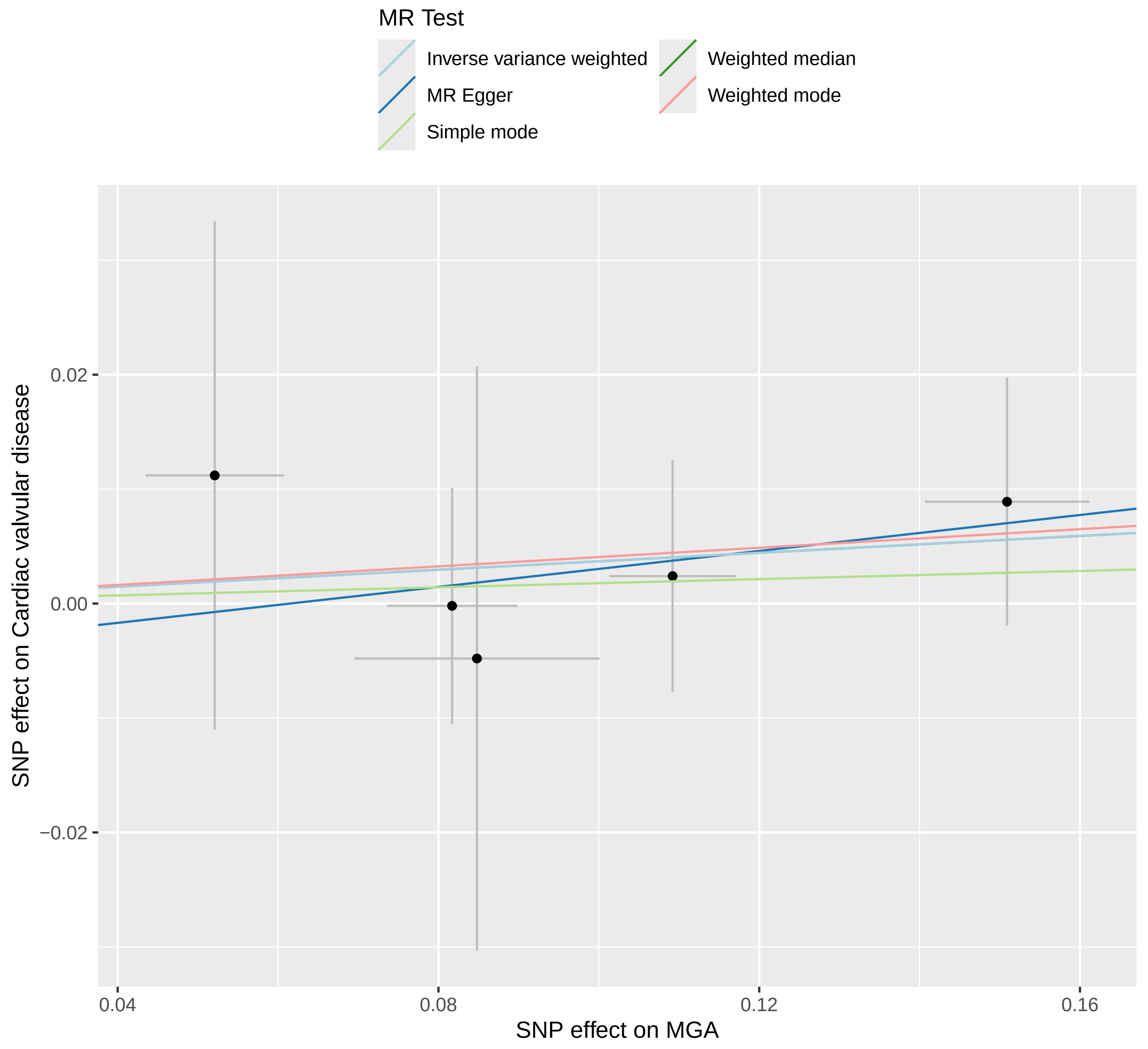

MR Test
Inverse variance weighted
MR Egger
Simple mode
Weighted median
Weighted mode
0.02
0.00
−0.02
0.04
0.08
0.12
0.16
SNP effect on Cardiac valvular disease
SNP effect on MGA

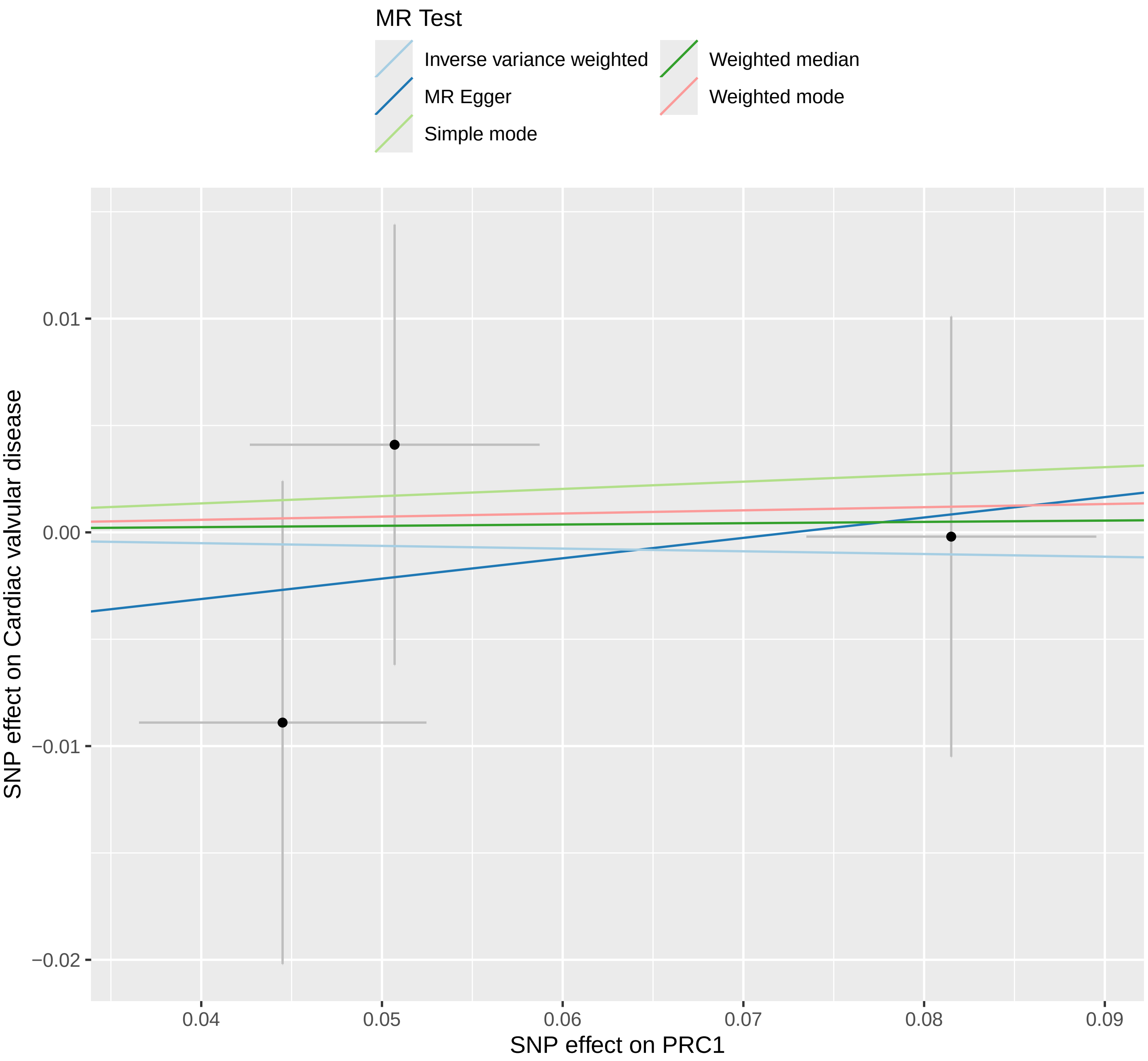
MR Test
Inverse variance weighted
MR Egger
Simple mode
Weighted median
Weighted mode
0.01
0.00
−0.01
−0.02
0.04
0.05
0.06
0.07
0.08
0.09
SNP effect on Cardiac valvular disease
SNP effect on PRC1

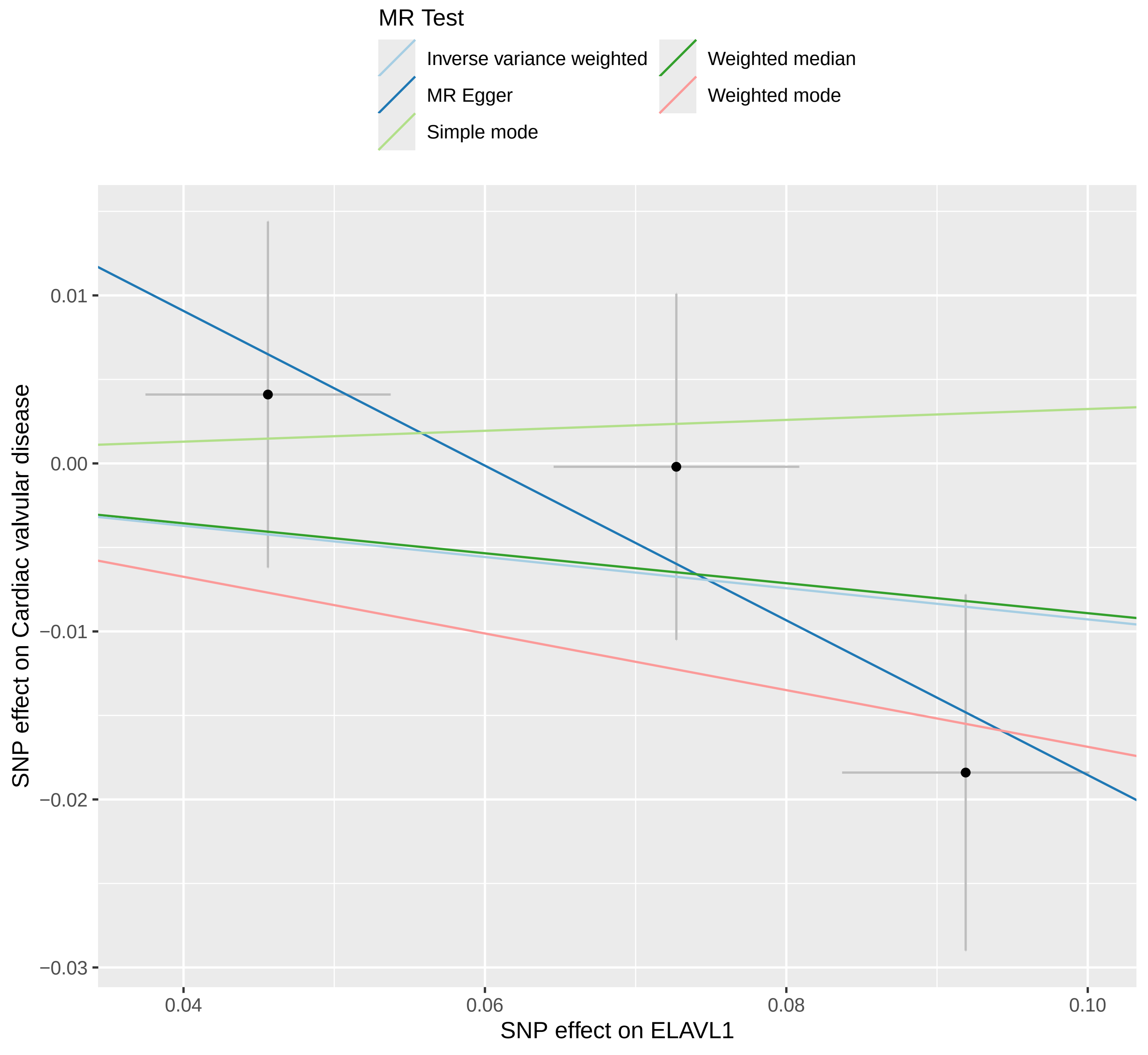
MR Test
Inverse variance weighted
MR Egger
Simple mode
Weighted median
Weighted mode
0.01
0.00
−0.01
−0.02
−0.03
SNP effect on Cardiac valvular disease
0.04
0.06
0.08
0.10
SNP effect on ELAVL1

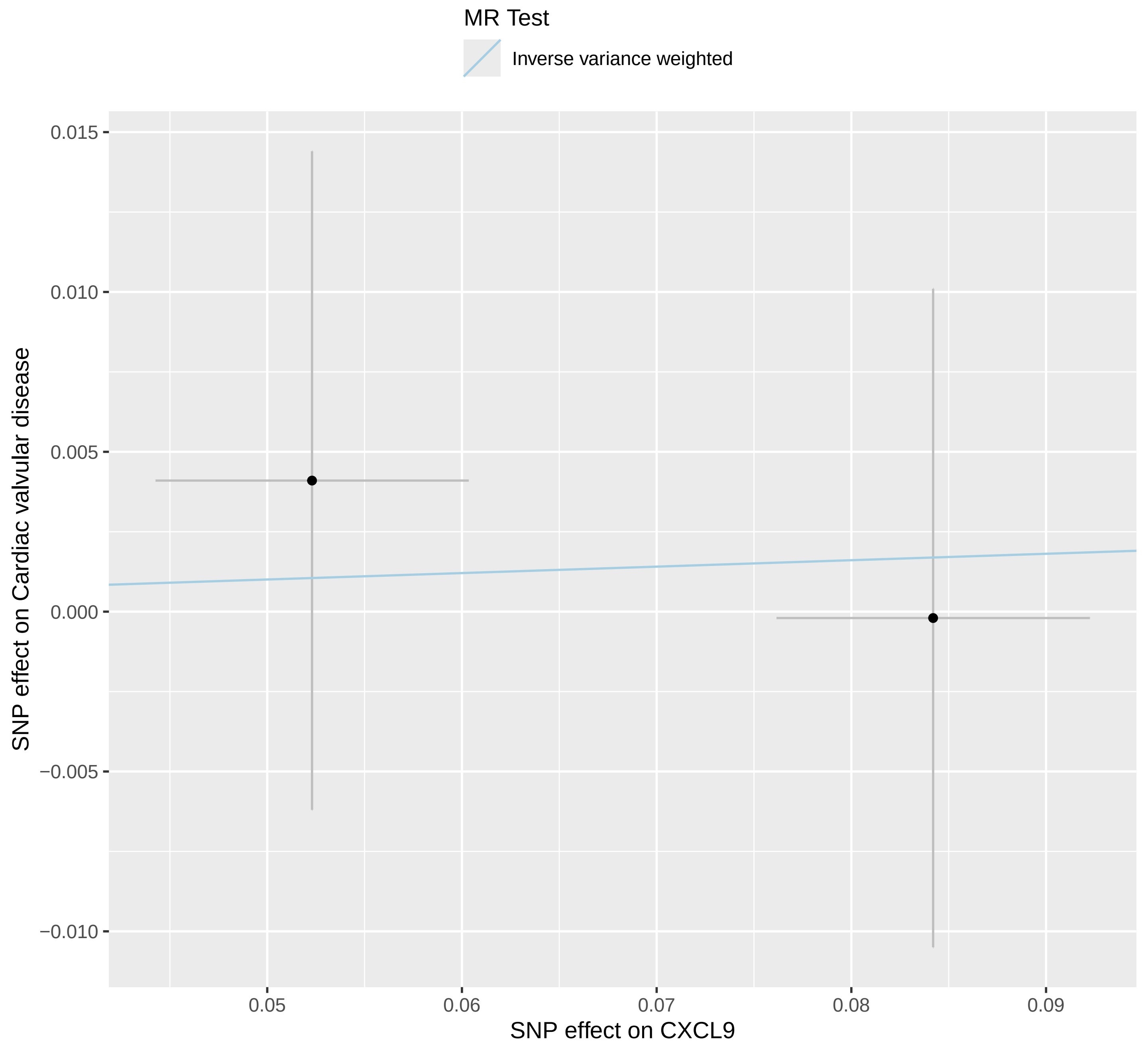

MR Test
Inverse variance weighted
0.015
0.010
0.005
0.000
−0.005
−0.010
SNP effect on Cardiac valvular disease
0.05
0.06
0.07
0.08
0.09
SNP effect on CXCL9

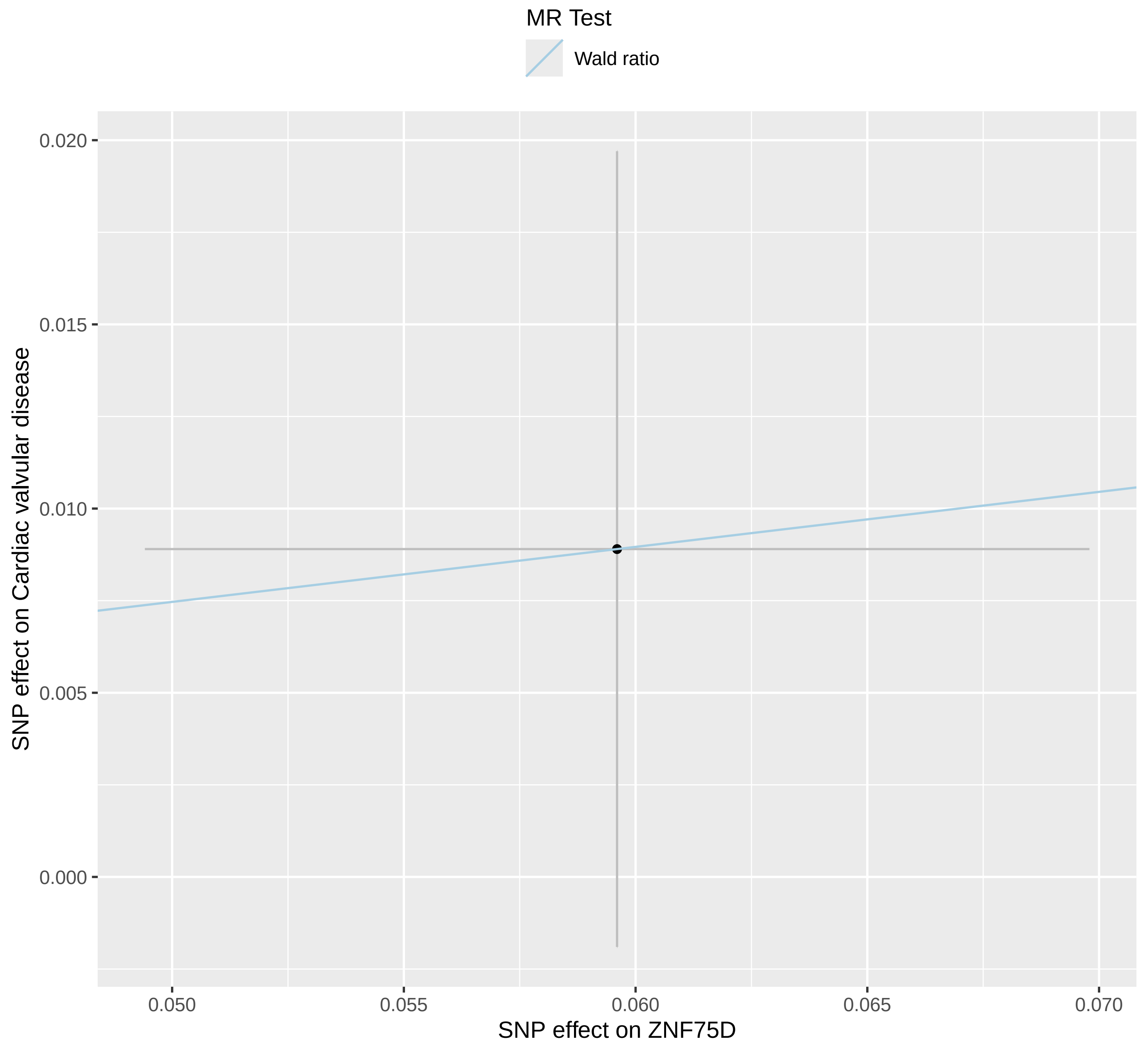

MR Test
Wald ratio
0.020
0.015
0.010
0.005
0.000
SNP effect on Cardiac valvular disease
0.050
0.055
0.060
0.065
0.070
SNP effect on ZNF75D

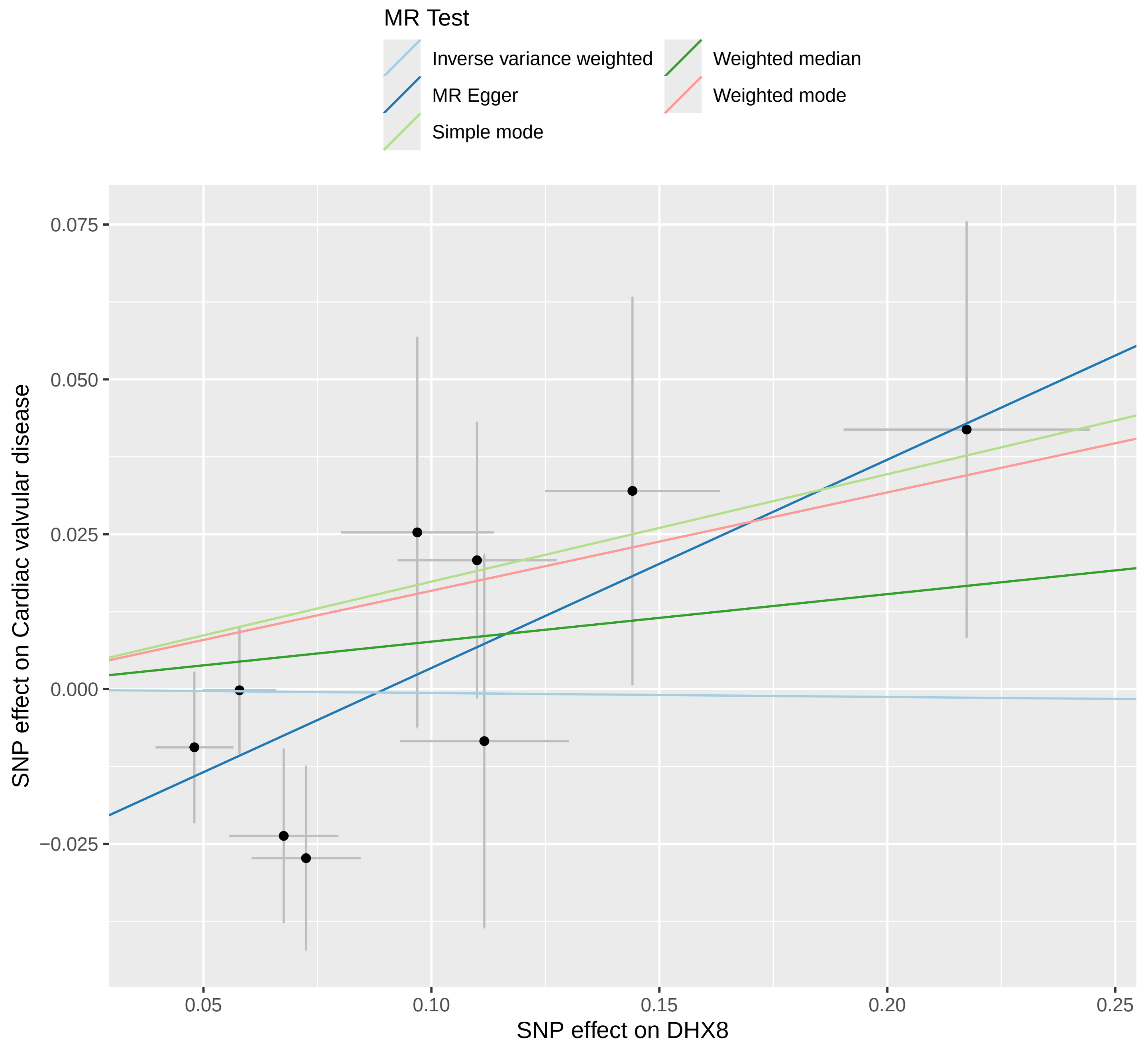
MR Test
Inverse variance weighted
MR Egger
Simple mode
Weighted median
Weighted mode
0.075
0.050
0.025
0.000
−0.025
SNP effect on Cardiac valvular disease
0.05
0.10
0.15
0.20
0.25
SNP effect on DHX8

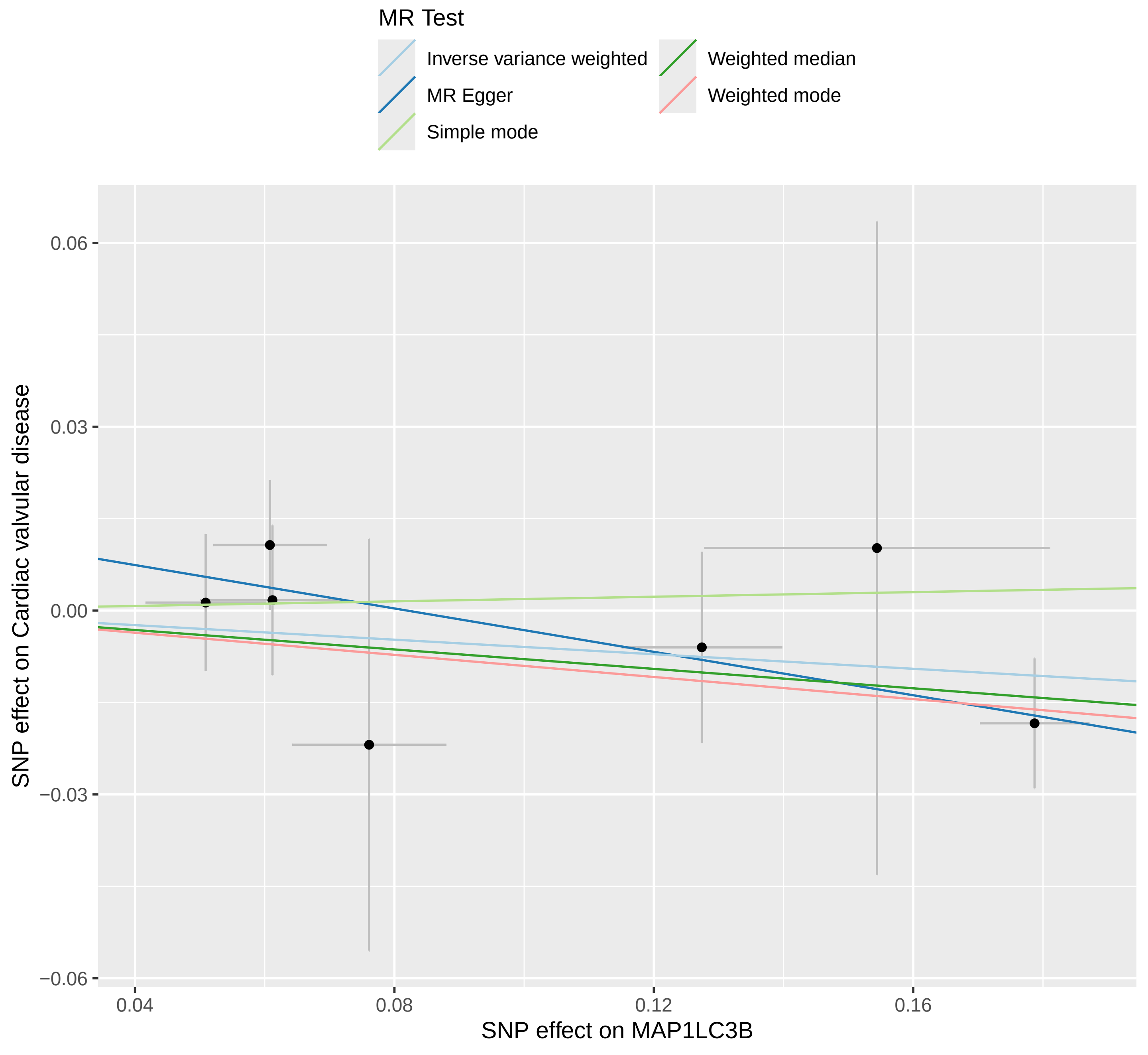
MR Test
Inverse variance weighted
MR Egger
Simple mode
Weighted median
Weighted mode
0.06
0.03
0.00
−0.03
−0.06
SNP effect on Cardiac valvular disease
0.04
0.08
0.12
0.16
SNP effect on MAP1LC3B

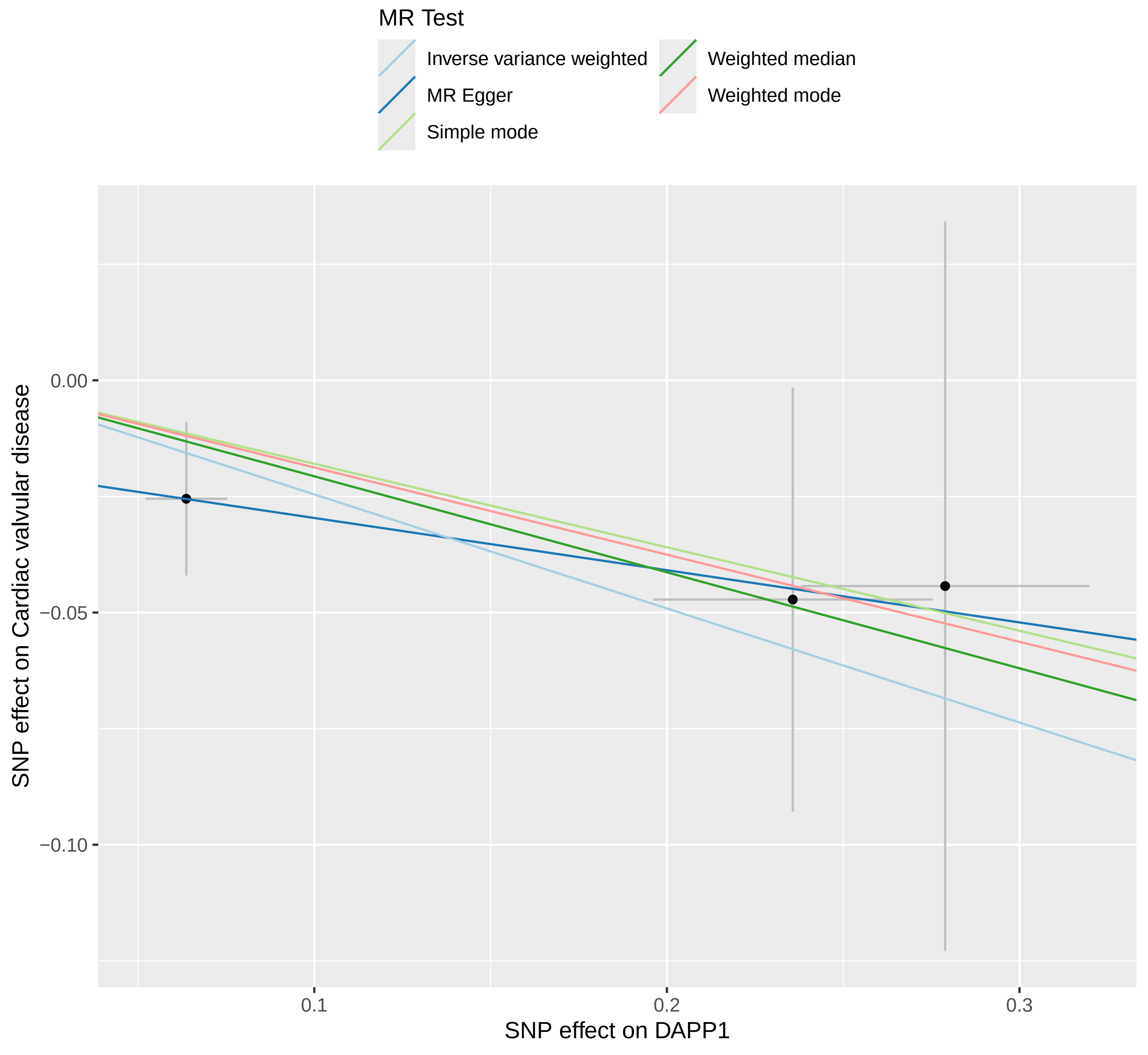

MR Test
Inverse variance weighted
MR Egger
Simple mode
Weighted median
Weighted mode
0.00
−0.05
−0.10
SNP effect on Cardiac valvular disease
0.1
0.2
0.3
SNP effect on DAPP1

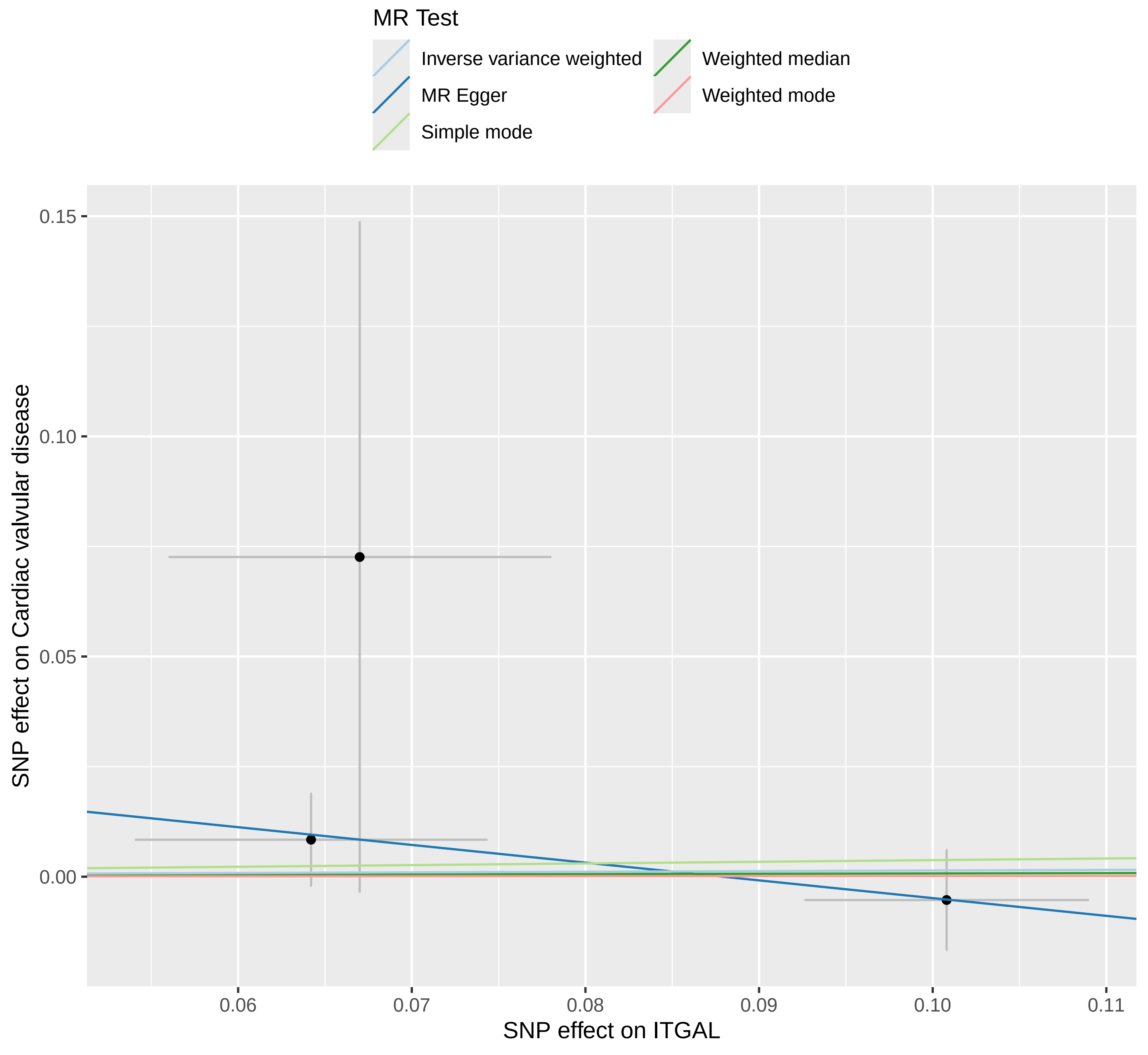
MR Test
Inverse variance weighted
MR Egger
Simple mode
Weighted median
Weighted mode
SNP effect on Cardiac valvular disease
0.15
0.10
0.05
0.00
0.06
0.07
0.08
0.09
0.10
0.11
SNP effect on ITGAL

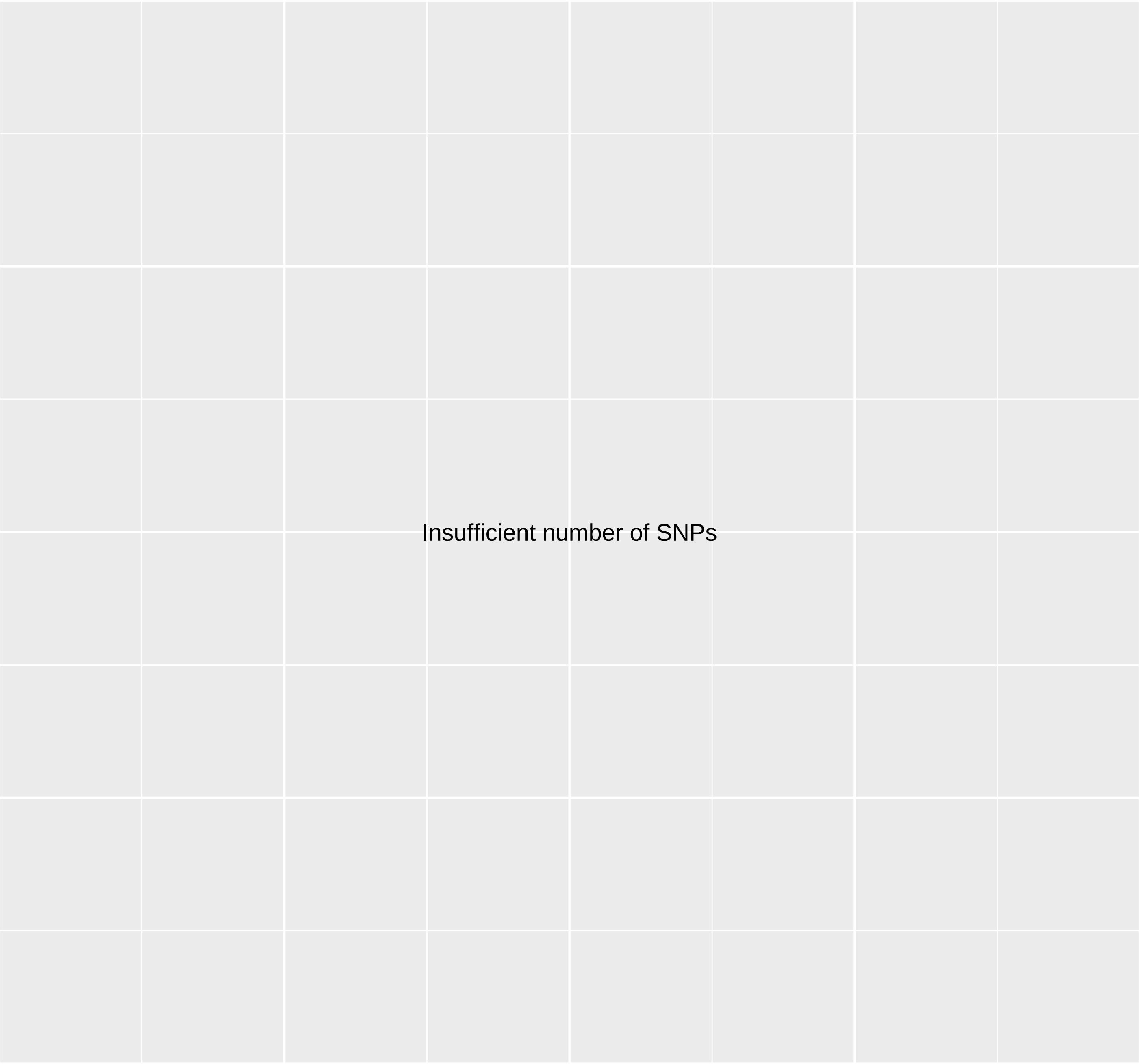
Insufficient number of SNPs

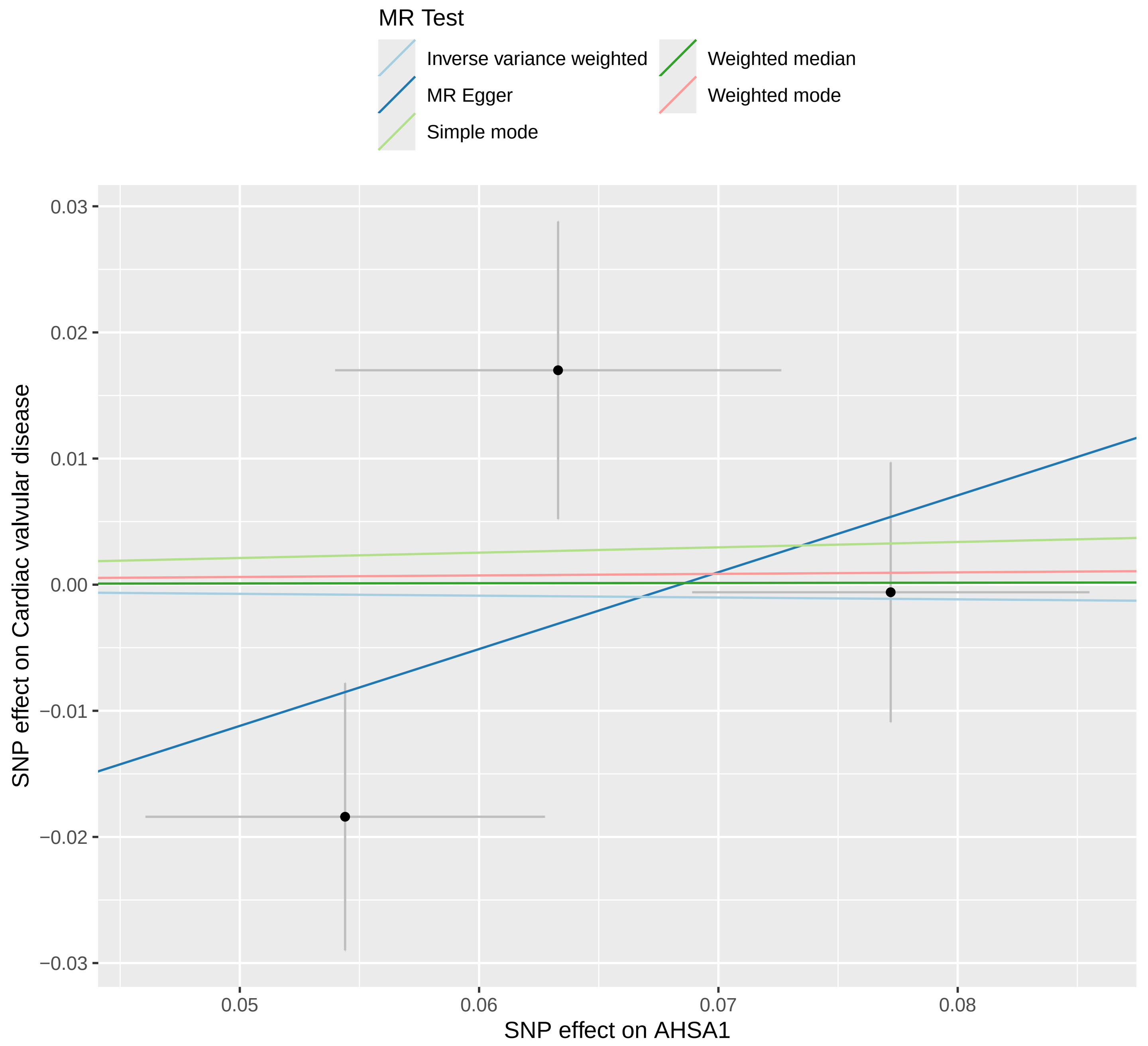

MR Test
Inverse variance weighted
MR Egger
Simple mode
Weighted median
Weighted mode
0.03
0.02
0.01
0.00
−0.01
−0.02
−0.03
SNP effect on Cardiac valvular disease
0.05
0.06
0.07
0.08
SNP effect on AHSA1

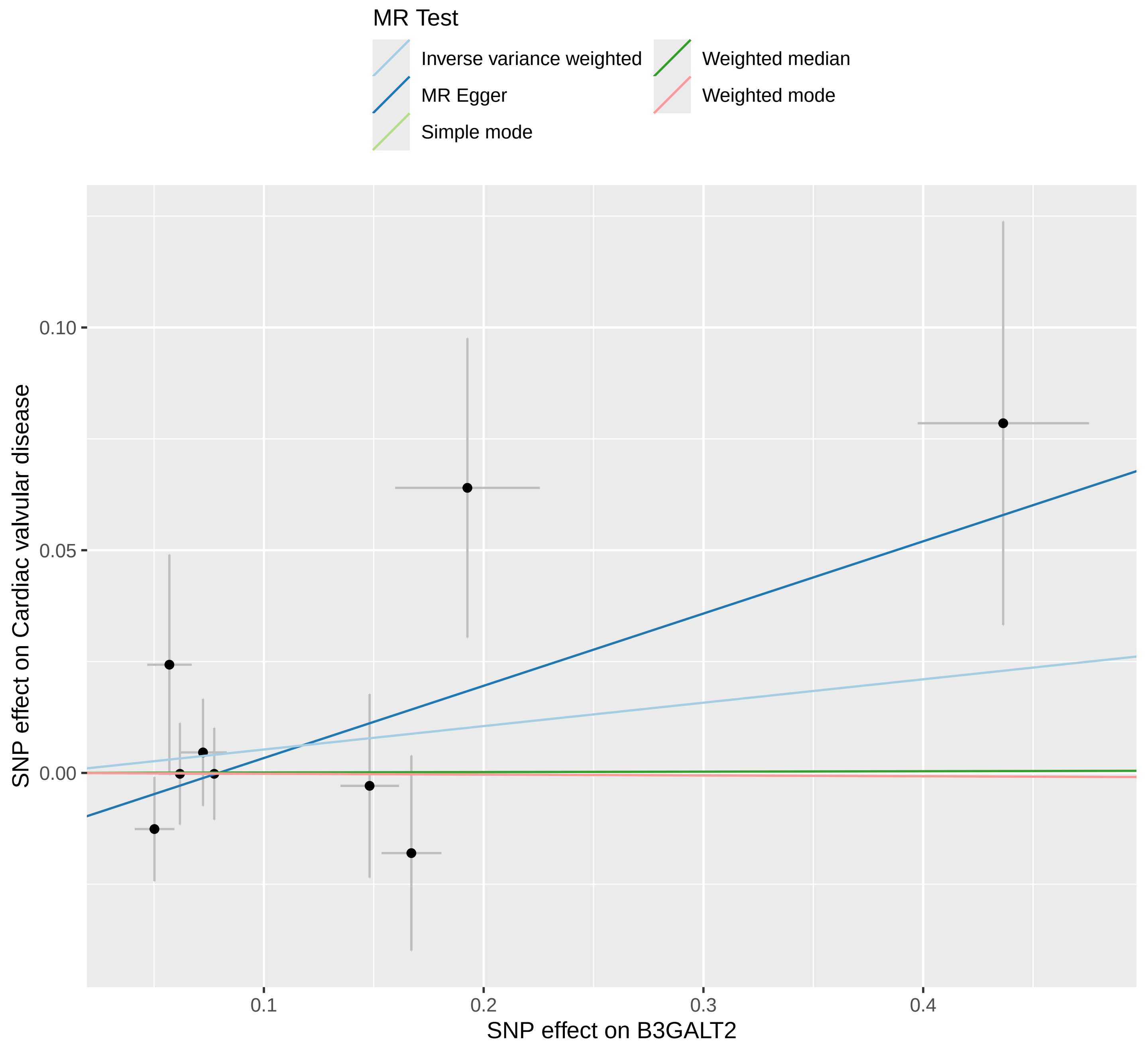

MR Test
Inverse variance weighted
MR Egger
Simple mode
Weighted median
Weighted mode
SNP effect on Cardiac valvular disease
0.10
0.05
0.00
0.1
0.2
0.3
0.4
SNP effect on B3GALT2

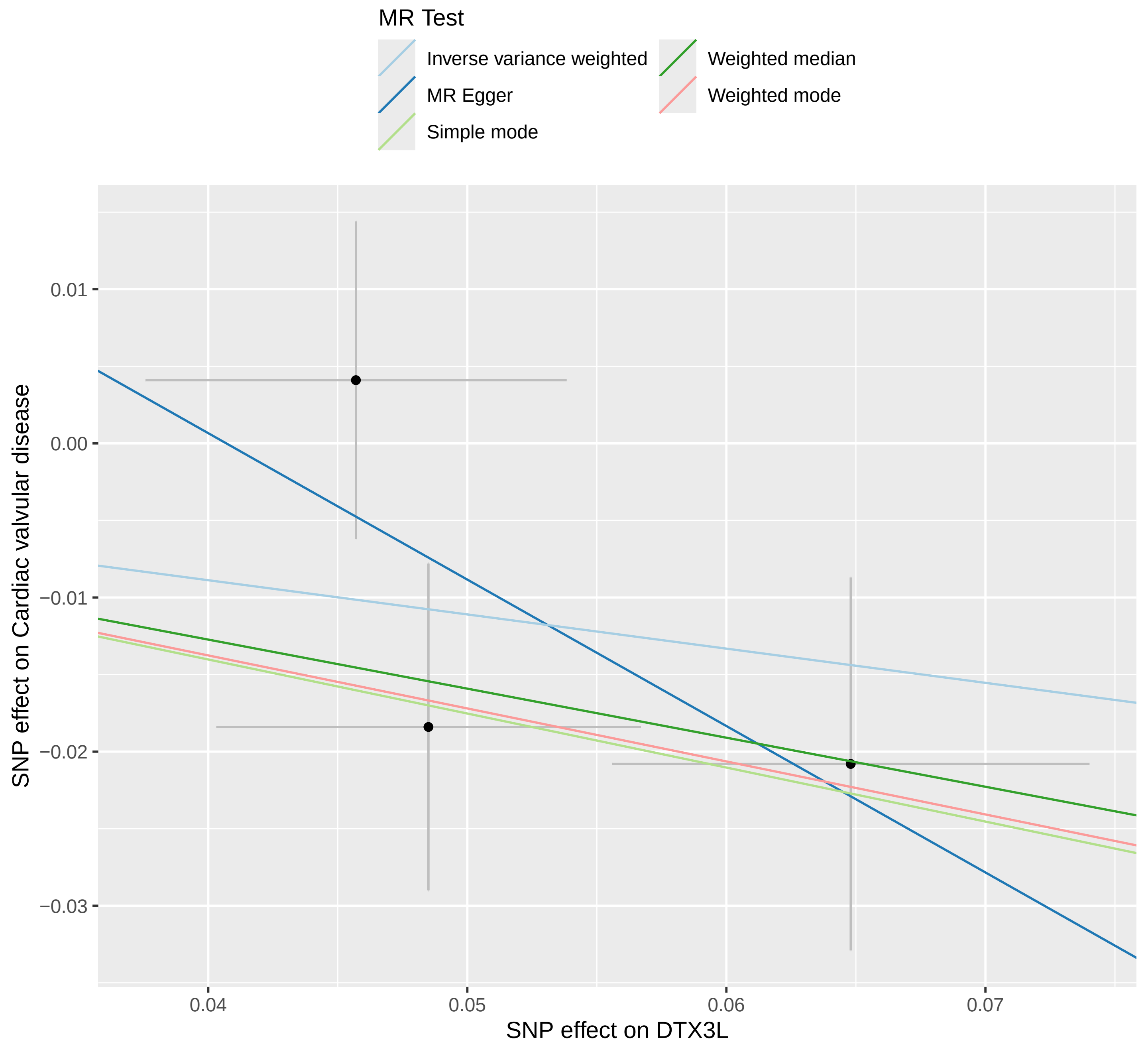
MR Test
Inverse variance weighted
MR Egger
Simple mode
Weighted median
Weighted mode
0.01
0.00
−0.01
−0.02
−0.03
SNP effect on Cardiac valvular disease
0.04
0.05
0.06
0.07
SNP effect on DTX3L

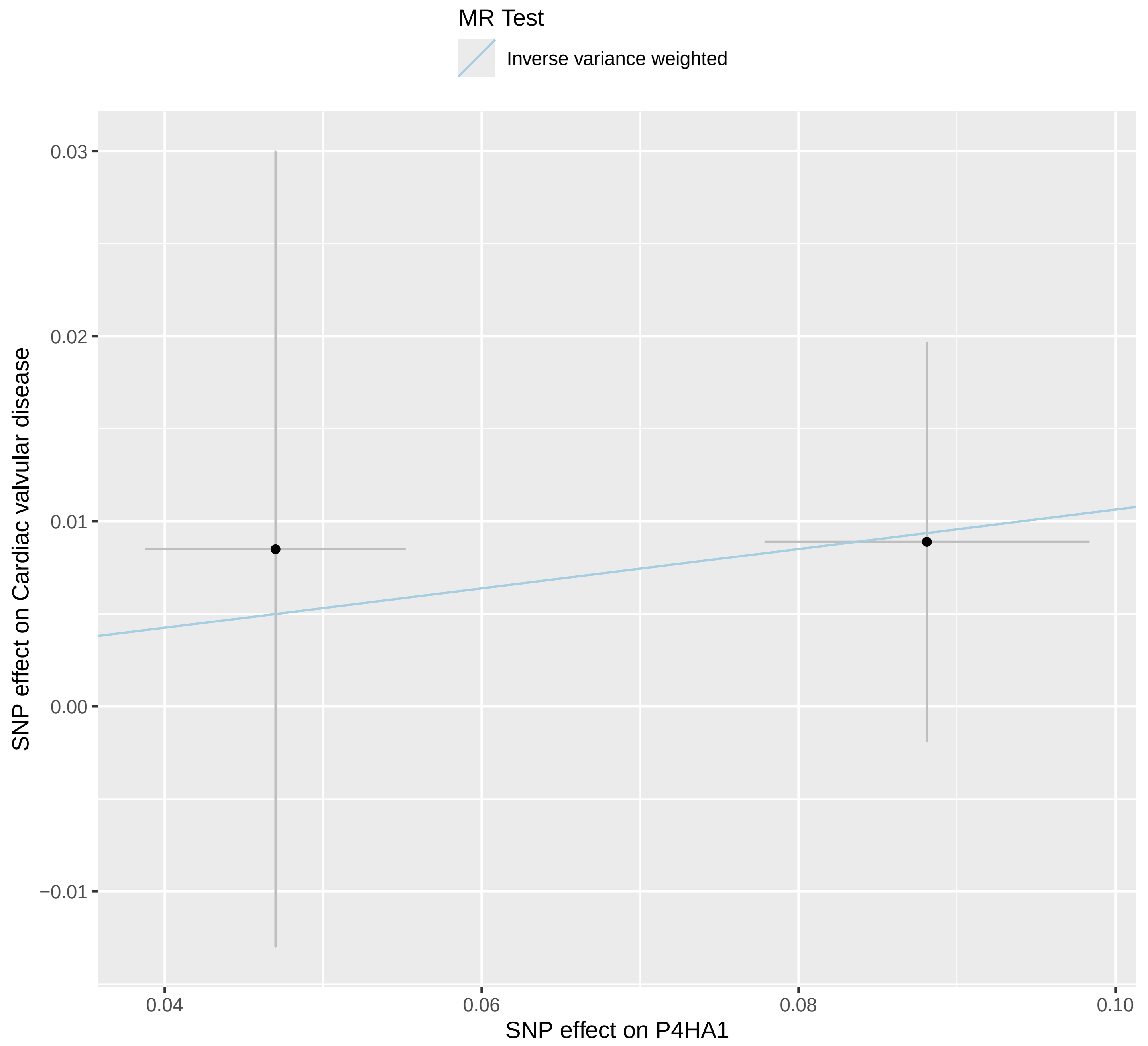

MR Test
Inverse variance weighted
0.03
0.02
0.01
0.00
−0.01
SNP effect on Cardiac valvular disease
0.04
0.06
0.08
0.10
SNP effect on P4HA1

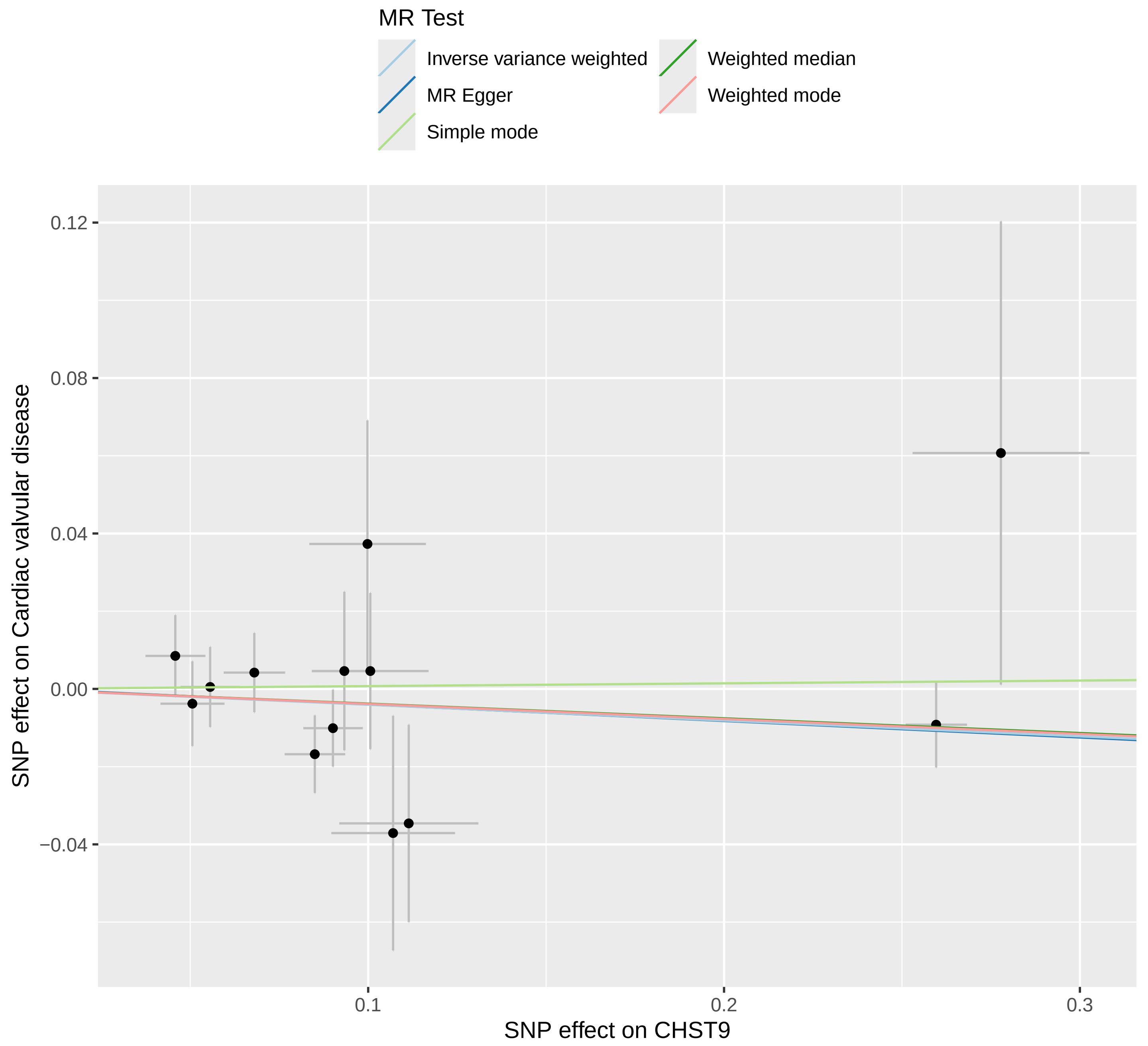

MR Test
Inverse variance weighted
MR Egger
Simple mode
Weighted median
Weighted mode
0.12
0.08
0.04
0.00
−0.04
0.1
0.2
0.3
SNP effect on Cardiac valvular disease
SNP effect on CHST9

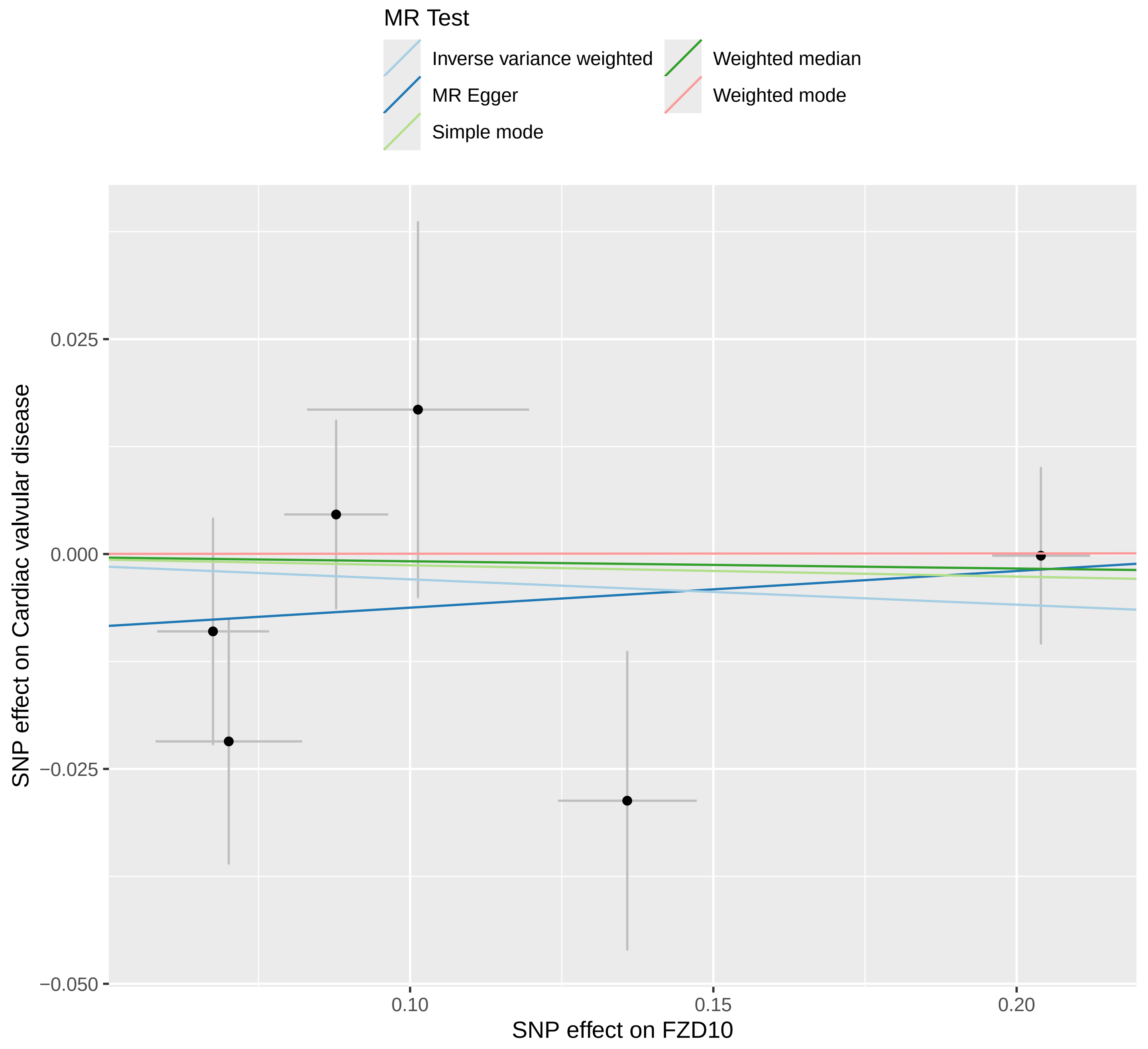
MR Test
Inverse variance weighted
MR Egger
Simple mode
Weighted median
Weighted mode
0.025
0.000
−0.025
−0.050
0.10
0.15
0.20
SNP effect on Cardiac valvular disease
SNP effect on FZD10

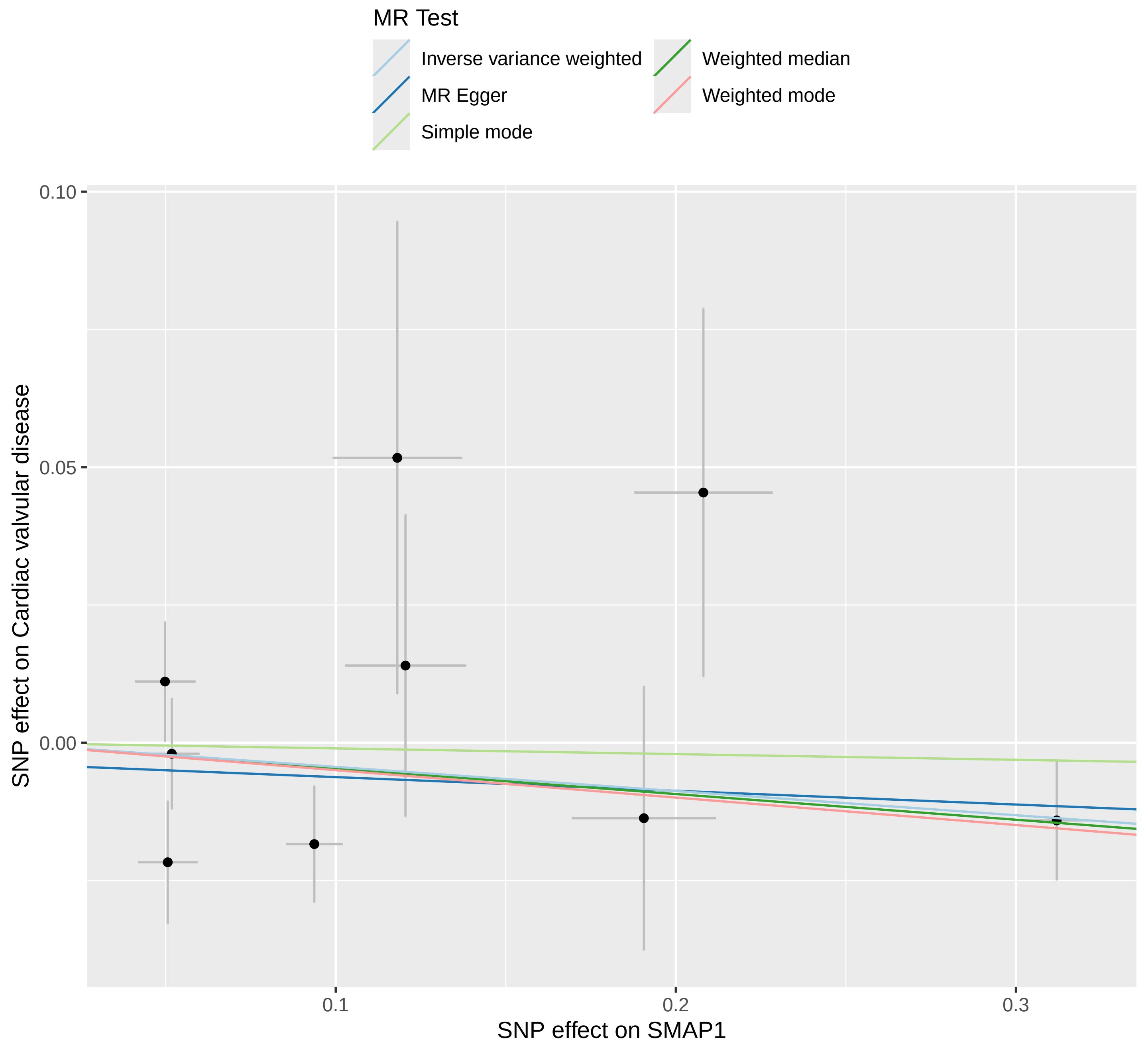

MR Test
Inverse variance weighted
MR Egger
Simple mode
Weighted median
Weighted mode
0.10
0.05
0.00
0.1
0.2
0.3
SNP effect on Cardiac valvular disease
SNP effect on SMAP1

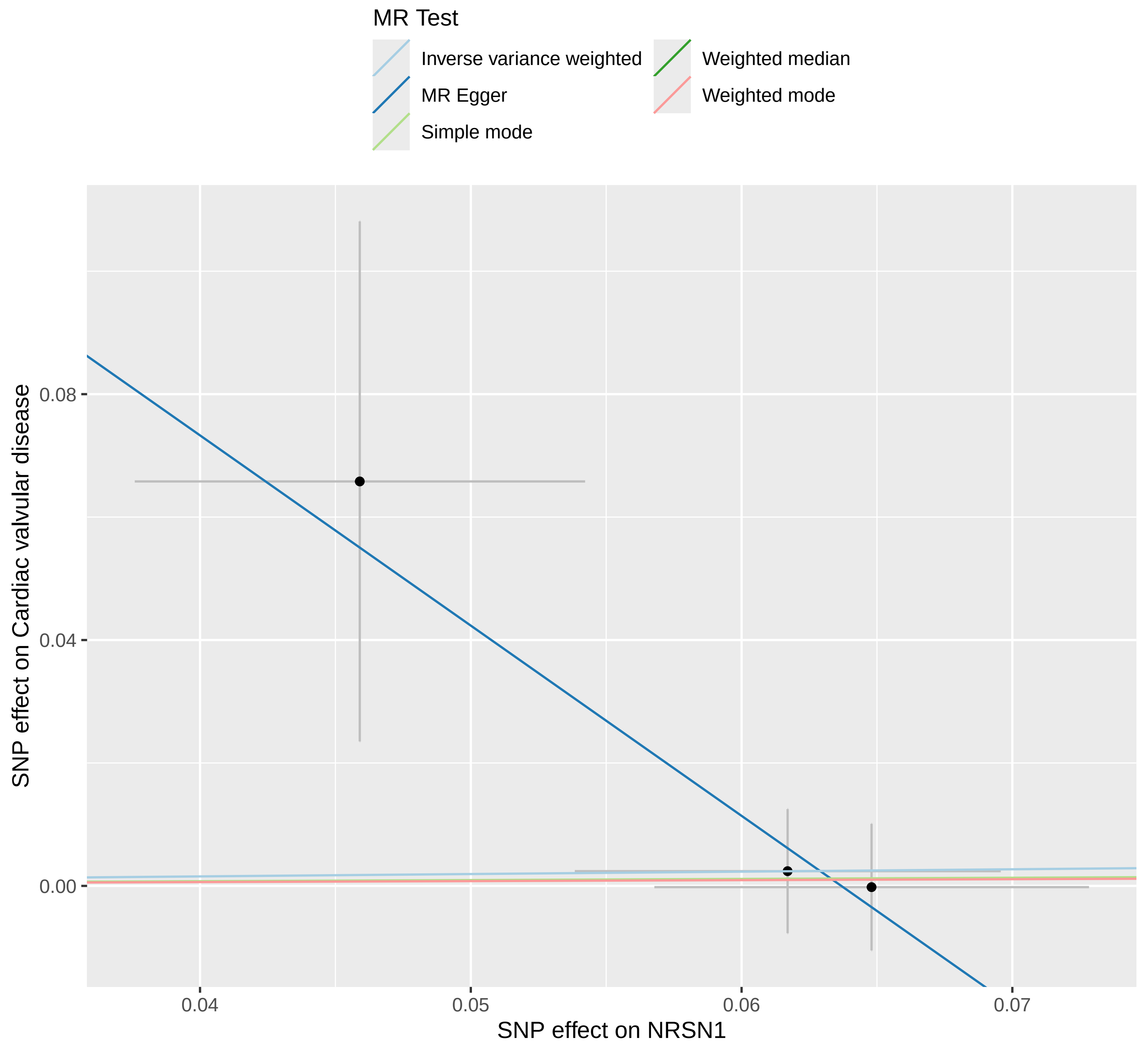
MR Test
Inverse variance weighted
MR Egger
Simple mode
Weighted median
Weighted mode
SNP effect on Cardiac valvular disease
0.08
0.04
0.00
0.04
0.05
0.06
0.07
SNP effect on NRSN1

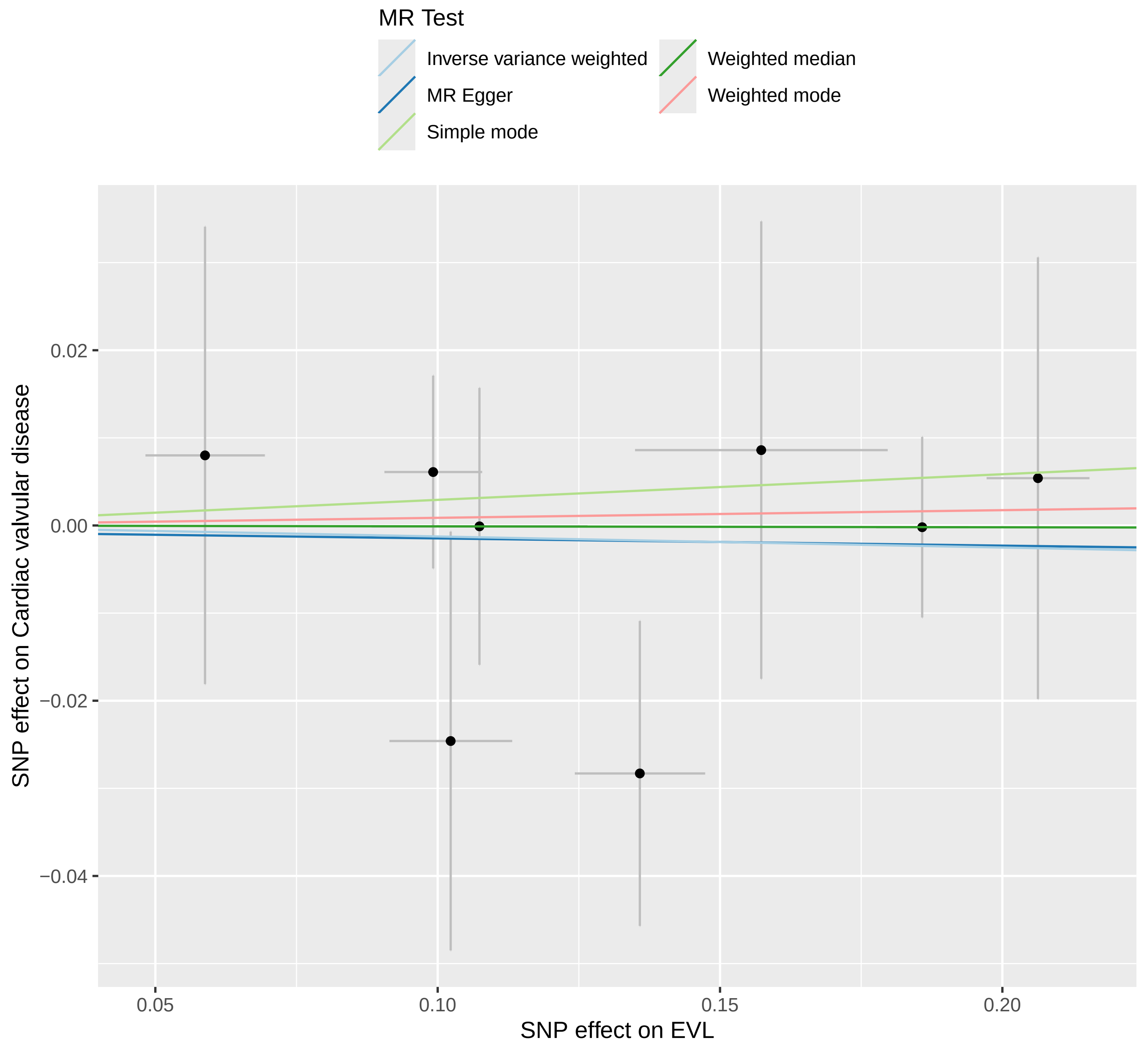
MR Test
Inverse variance weighted
MR Egger
Simple mode
Weighted median
Weighted mode
0.02
0.00
−0.02
−0.04
0.05
0.10
0.15
0.20
SNP effect on Cardiac valvular disease
SNP effect on EVL

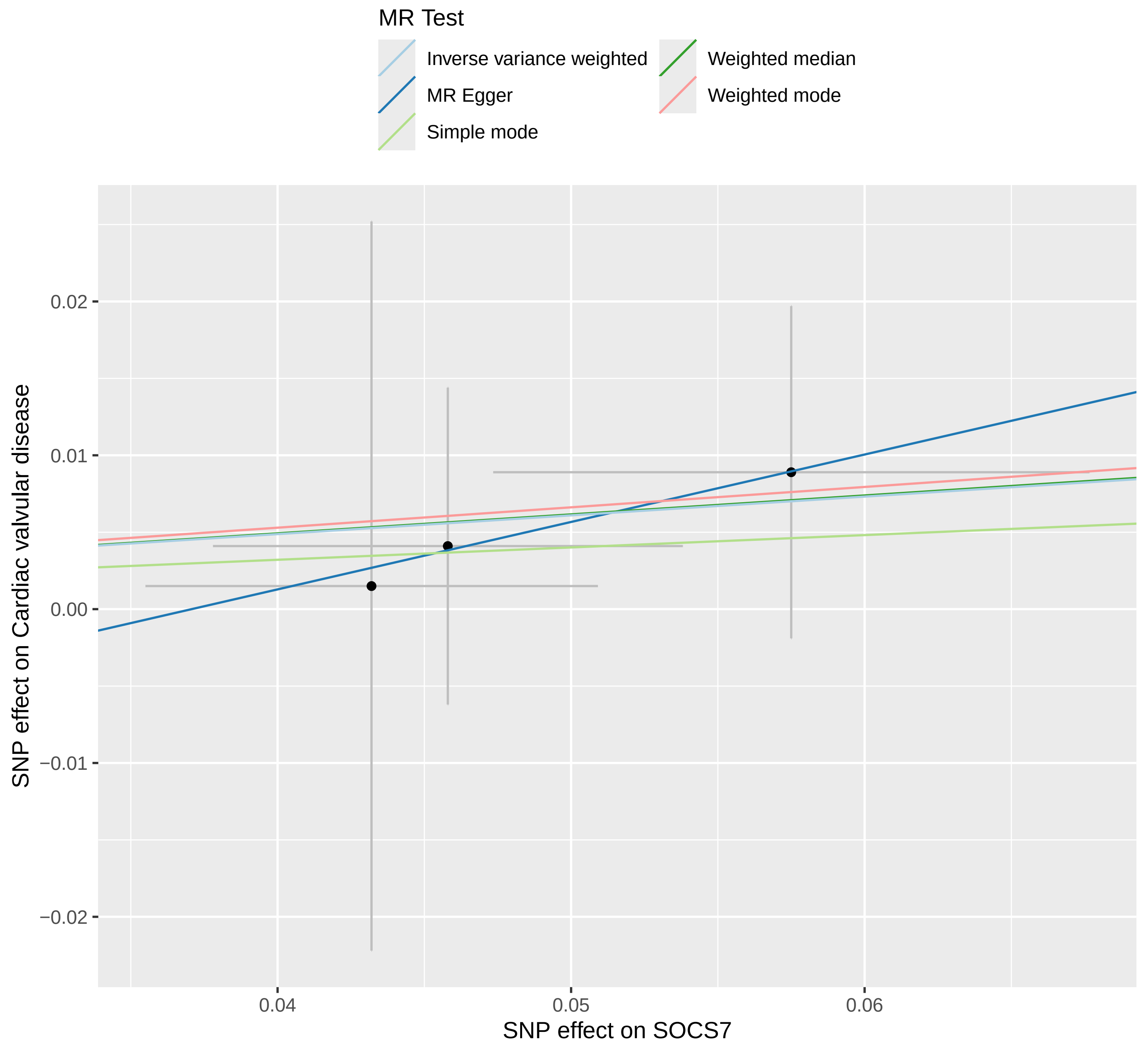
MR Test
Inverse variance weighted
MR Egger
Simple mode
Weighted median
Weighted mode
0.02
0.01
0.00
−0.01
−0.02
SNP effect on Cardiac valvular disease
0.04
0.05
0.06
SNP effect on SOCS7

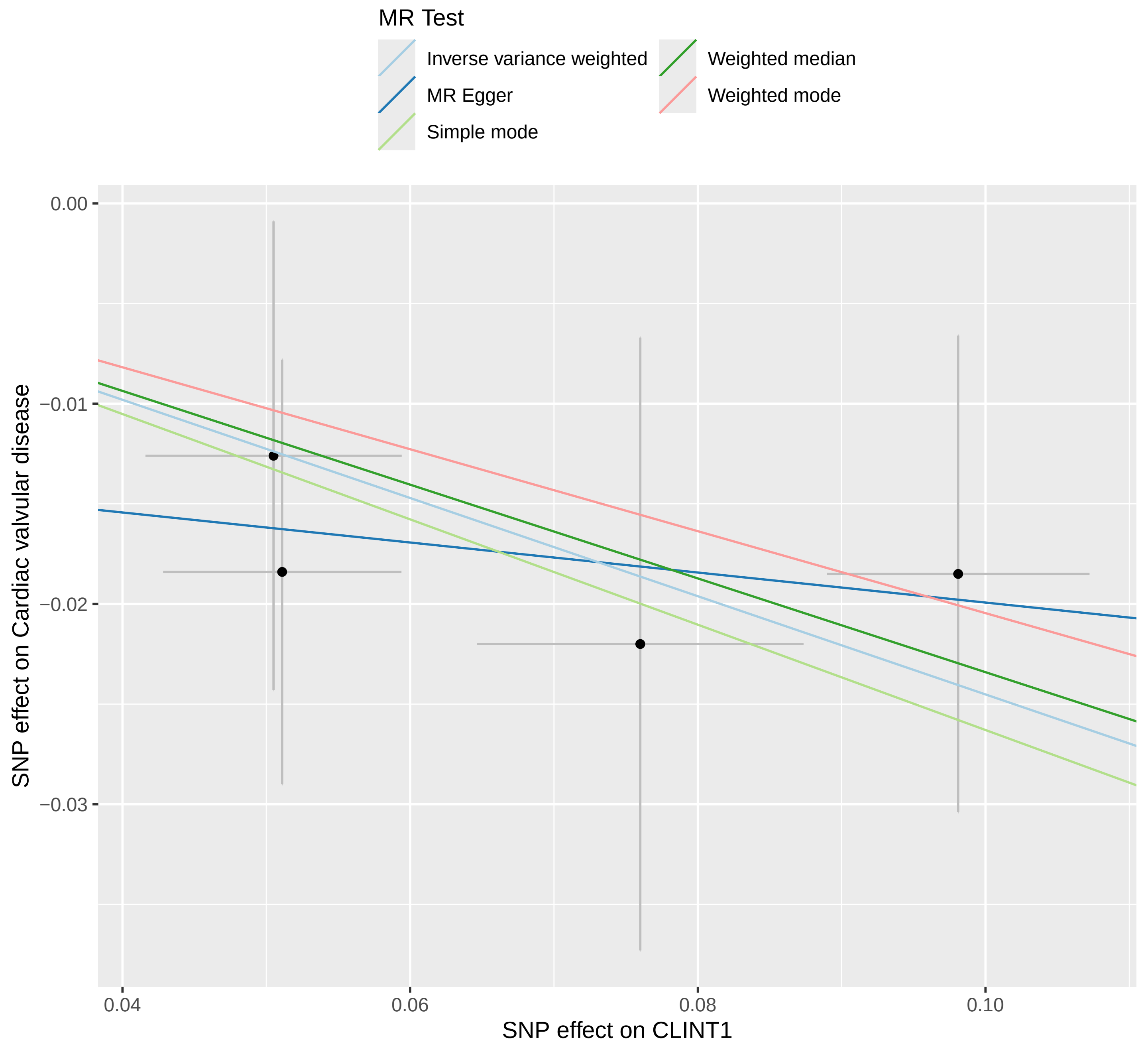
MR Test
Inverse variance weighted
MR Egger
Simple mode
Weighted median
Weighted mode
0.00
−0.01
−0.02
−0.03
0.04
0.06
0.08
0.10
SNP effect on Cardiac valvular disease
SNP effect on CLINT1

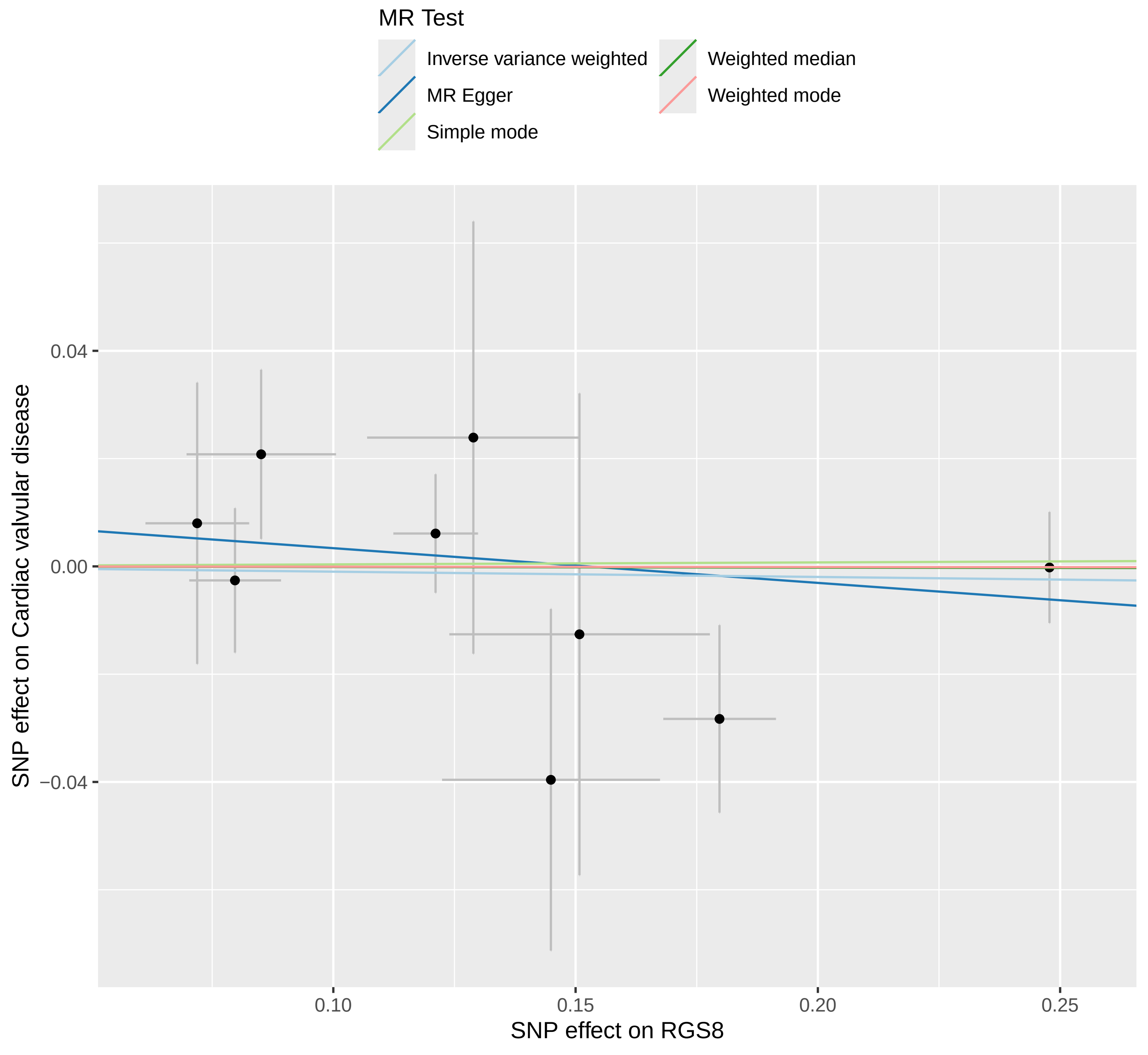
MR Test
Inverse variance weighted
MR Egger
Simple mode
Weighted median
Weighted mode
0.04
0.00
−0.04
SNP effect on Cardiac valvular disease
0.10
0.15
0.20
0.25
SNP effect on RGS8

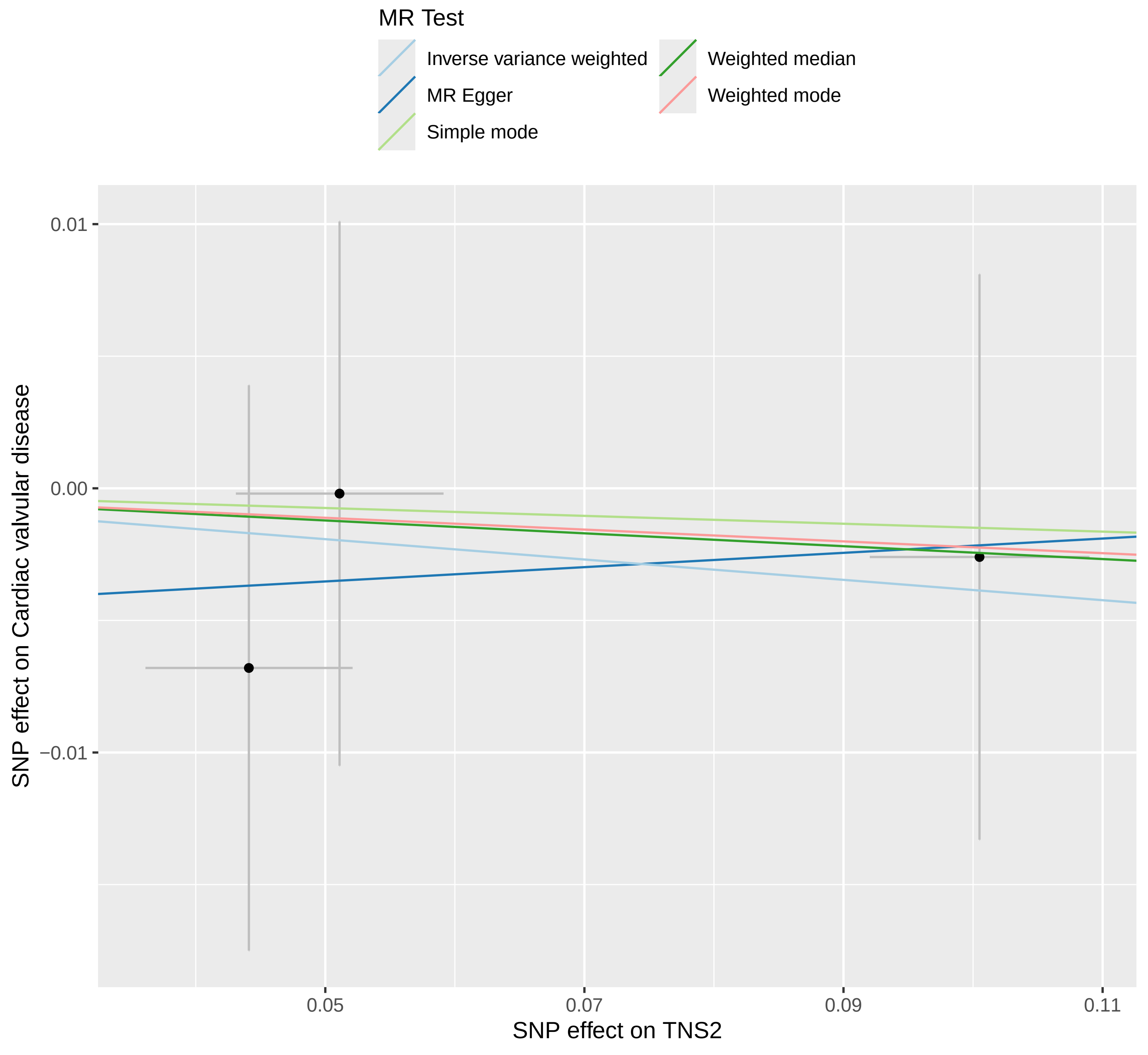
MR Test
Inverse variance weighted
MR Egger
Simple mode
Weighted median
Weighted mode
0.01
0.00
−0.01
0.05
0.07
0.09
0.11
SNP effect on Cardiac valvular disease
SNP effect on TNS2

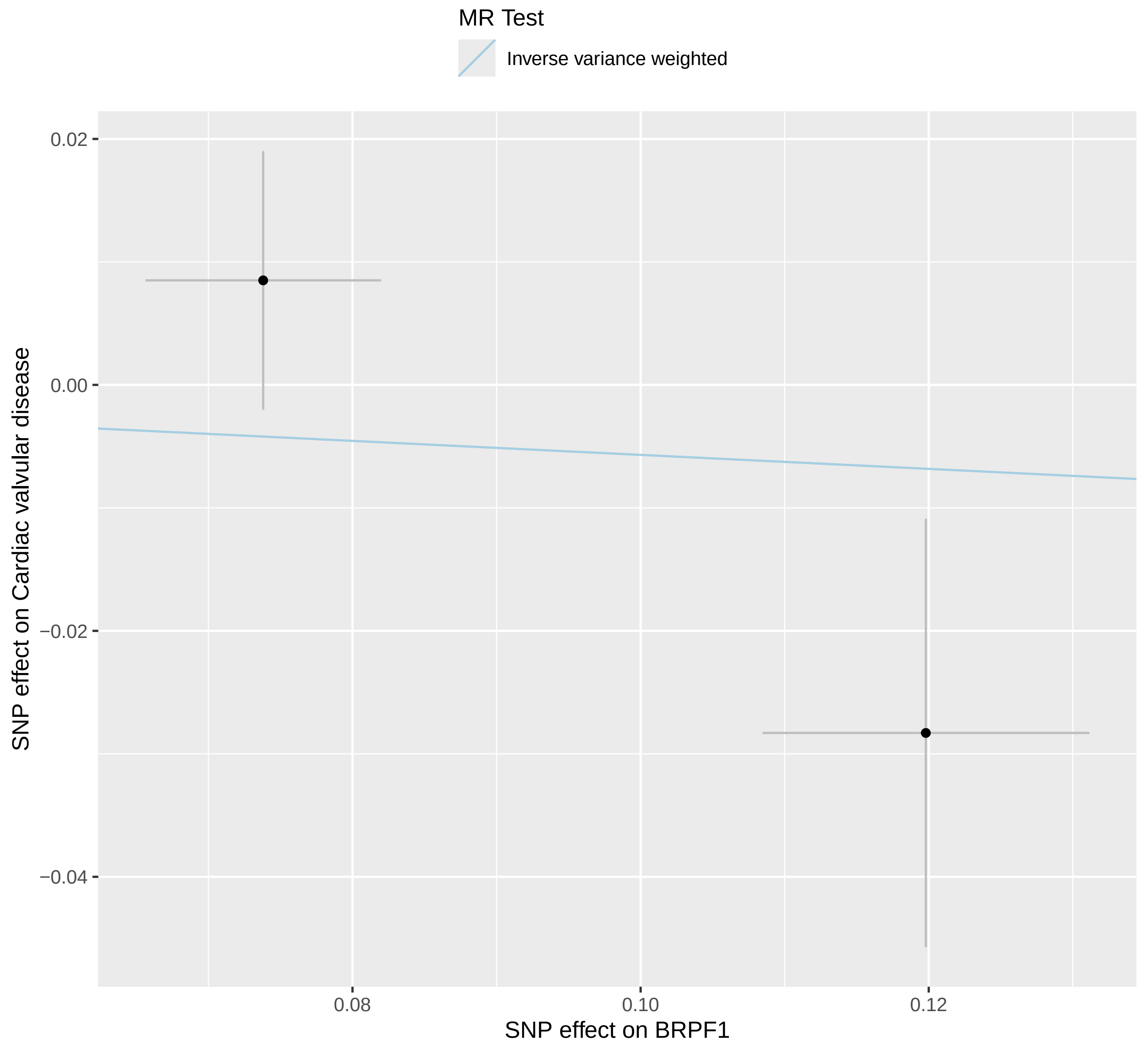

MR Test
Inverse variance weighted
0.02
0.00
−0.02
−0.04
SNP effect on Cardiac valvular disease
0.08
0.10
0.12
SNP effect on BRPF1

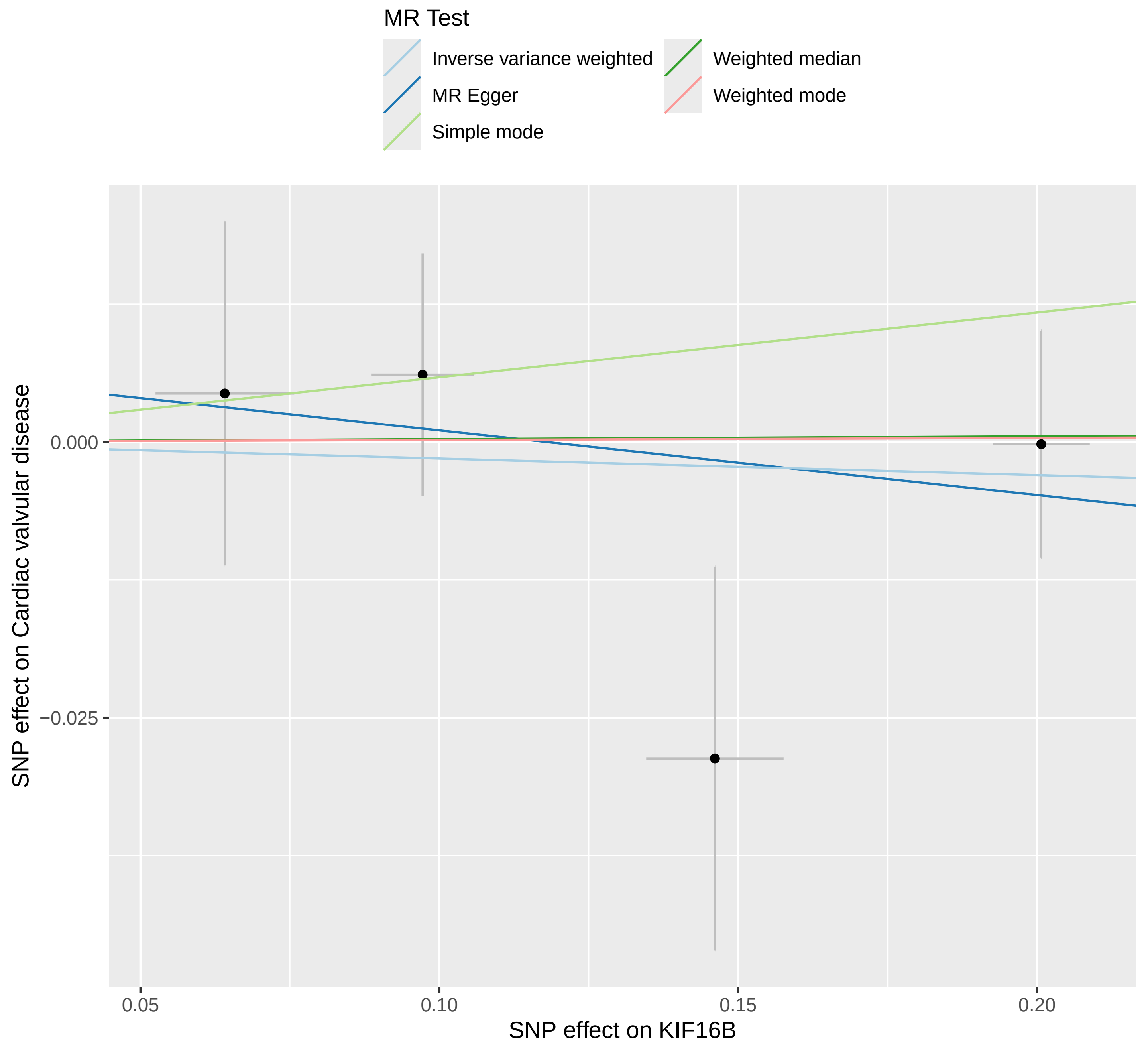

MR Test
Inverse variance weighted
MR Egger
Simple mode
Weighted median
Weighted mode
0.000
−0.025
0.05
0.10
0.15
0.20
SNP effect on KIF16B
SNP effect on Cardiac valvular disease

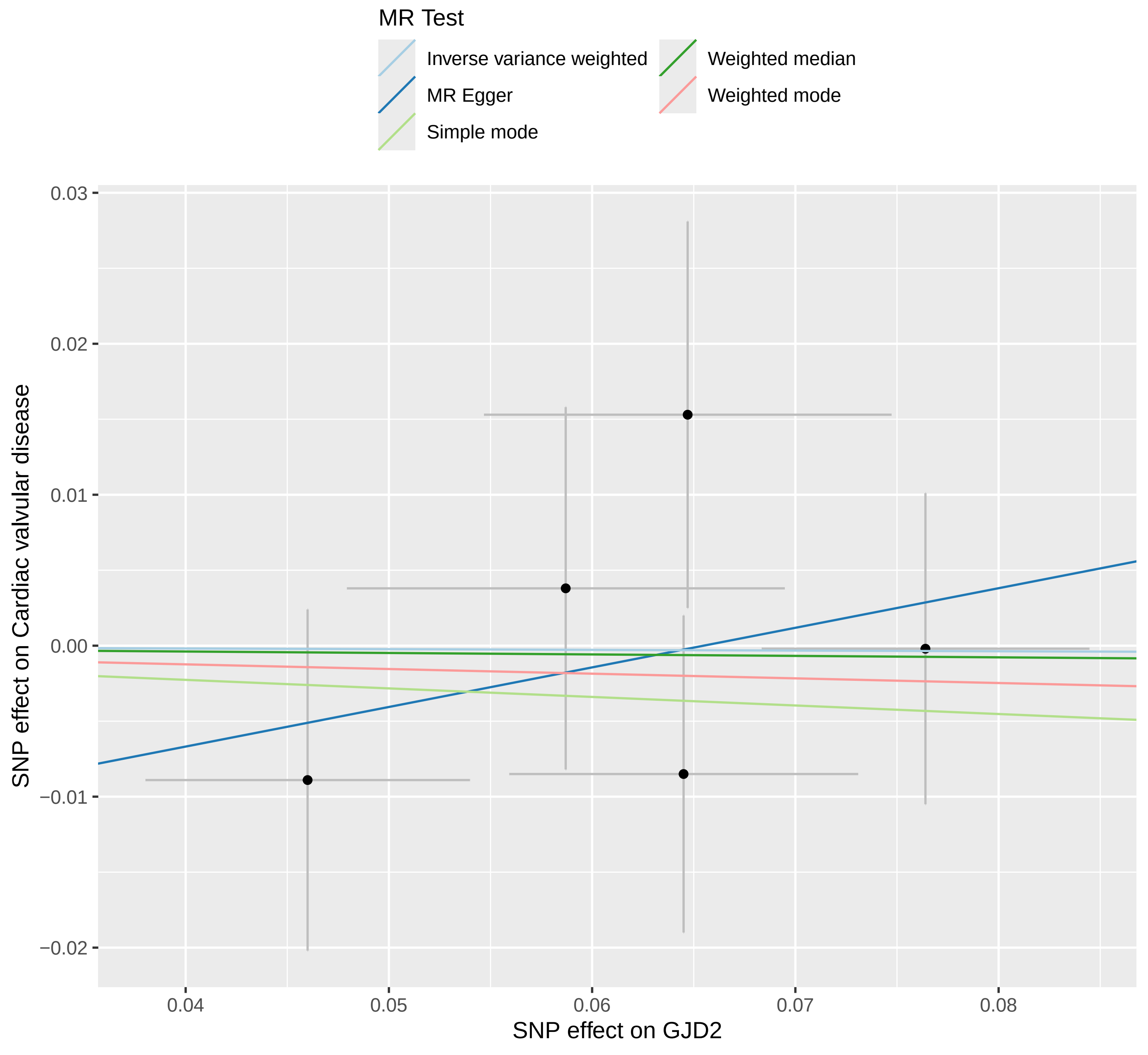

MR Test
Inverse variance weighted
MR Egger
Simple mode
Weighted median
Weighted mode
0.03
0.02
0.01
0.00
−0.01
−0.02
SNP effect on Cardiac valvular disease
0.04
0.05
0.06
0.07
0.08
SNP effect on GJD2

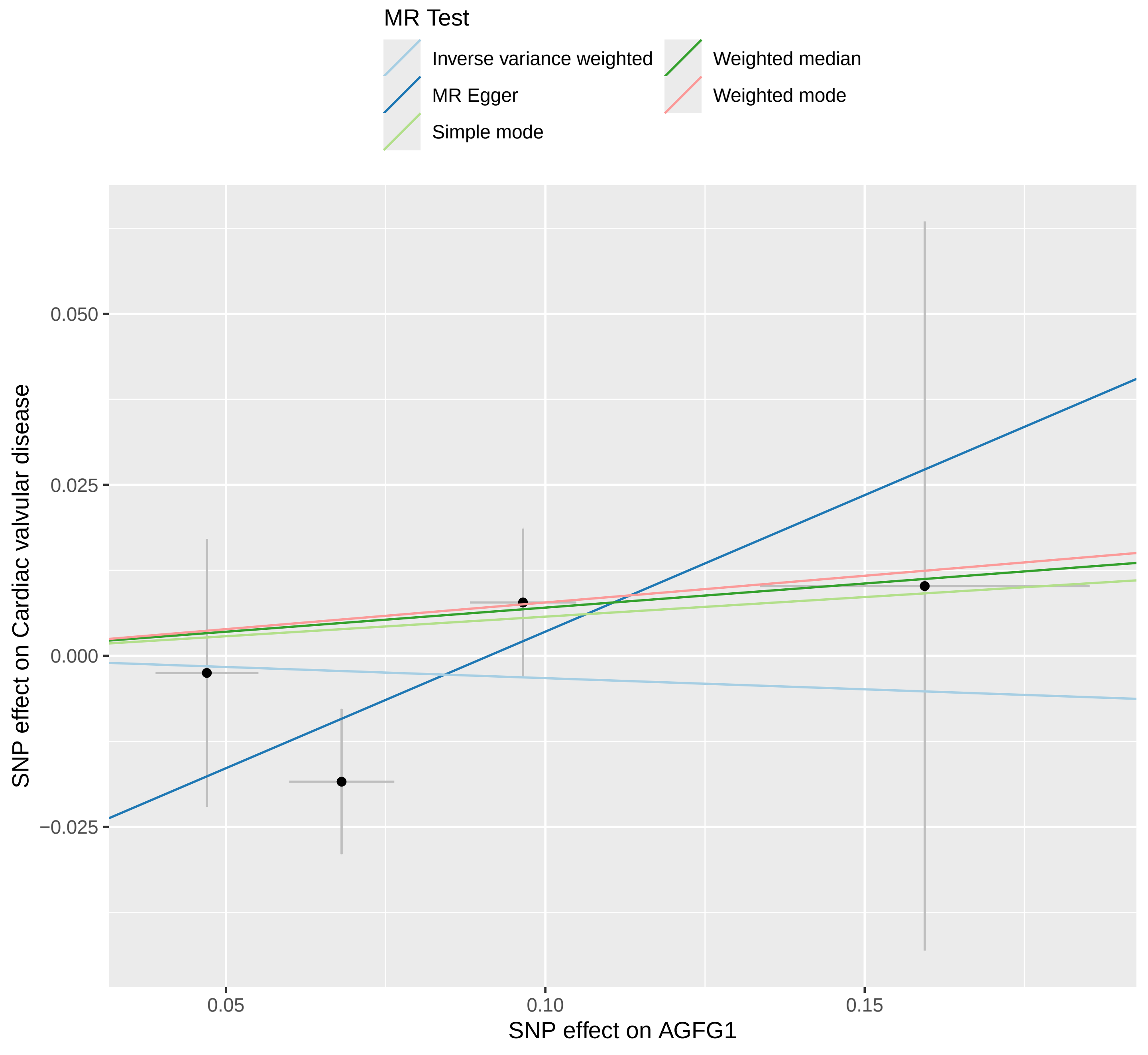

MR Test
Inverse variance weighted
MR Egger
Simple mode
Weighted median
Weighted mode
SNP effect on Cardiac valvular disease
0.050
0.025
0.000
−0.025
0.05
0.10
0.15
SNP effect on AGFG1

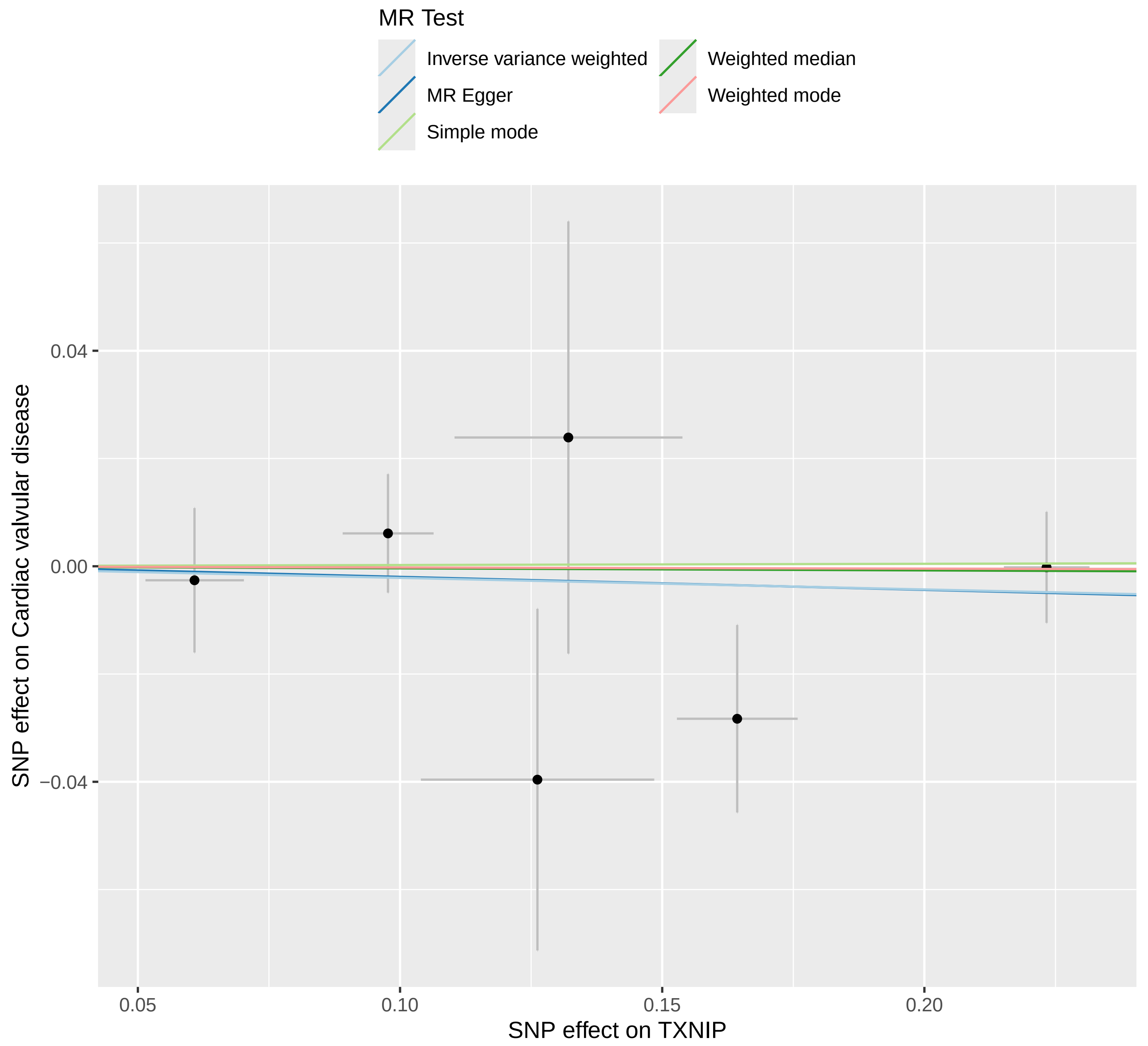
MR Test
Inverse variance weighted
MR Egger
Simple mode
Weighted median
Weighted mode
SNP effect on Cardiac valvular disease
0.04
0.00
−0.04
0.05
0.10
0.15
0.20
SNP effect on TXNIP

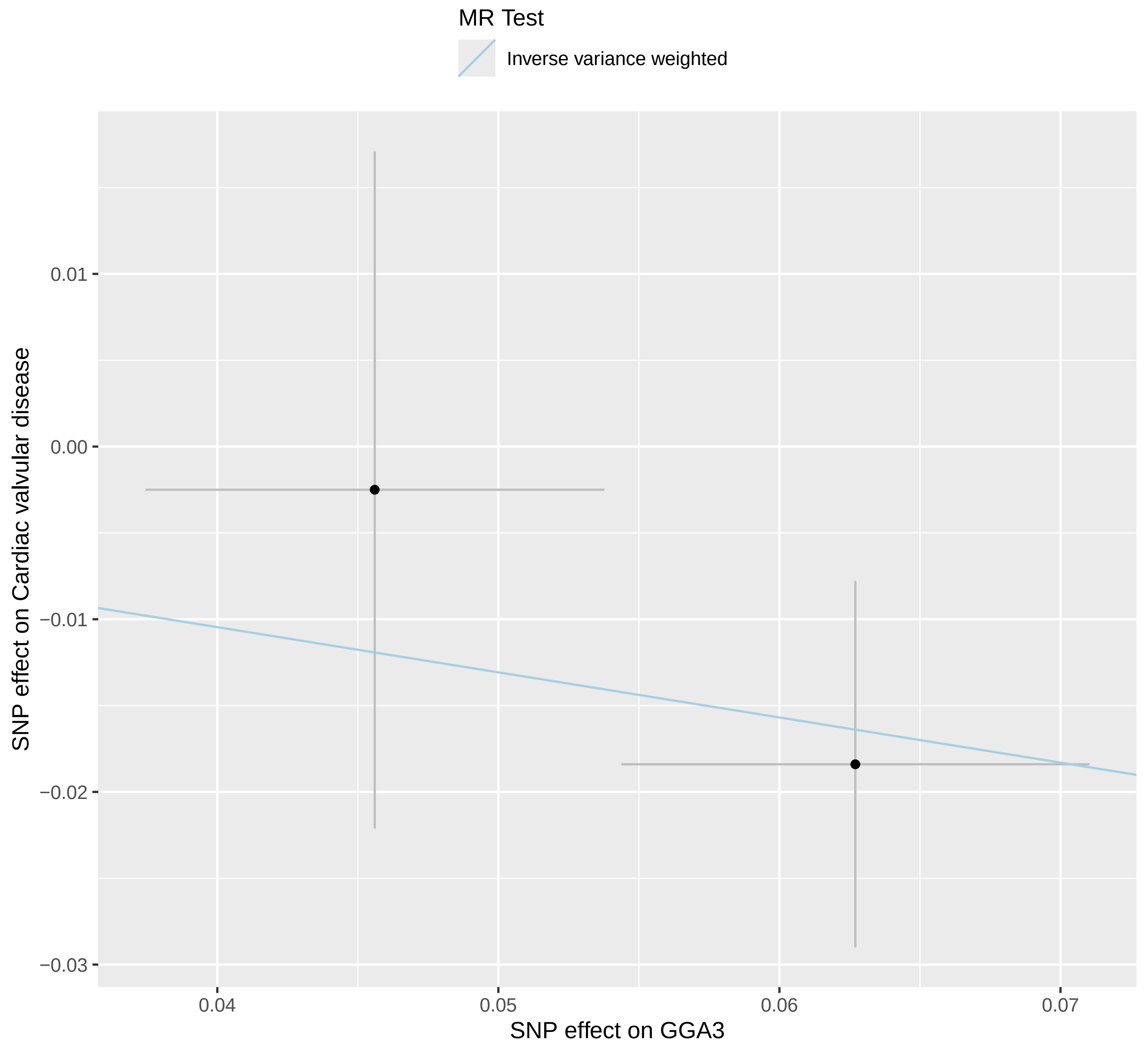

MR Test
Inverse variance weighted
SNP effect on Cardiac valvular disease
0.01
0.00
−0.01
−0.02
−0.03
0.04
0.05
0.06
0.07
SNP effect on GGA3

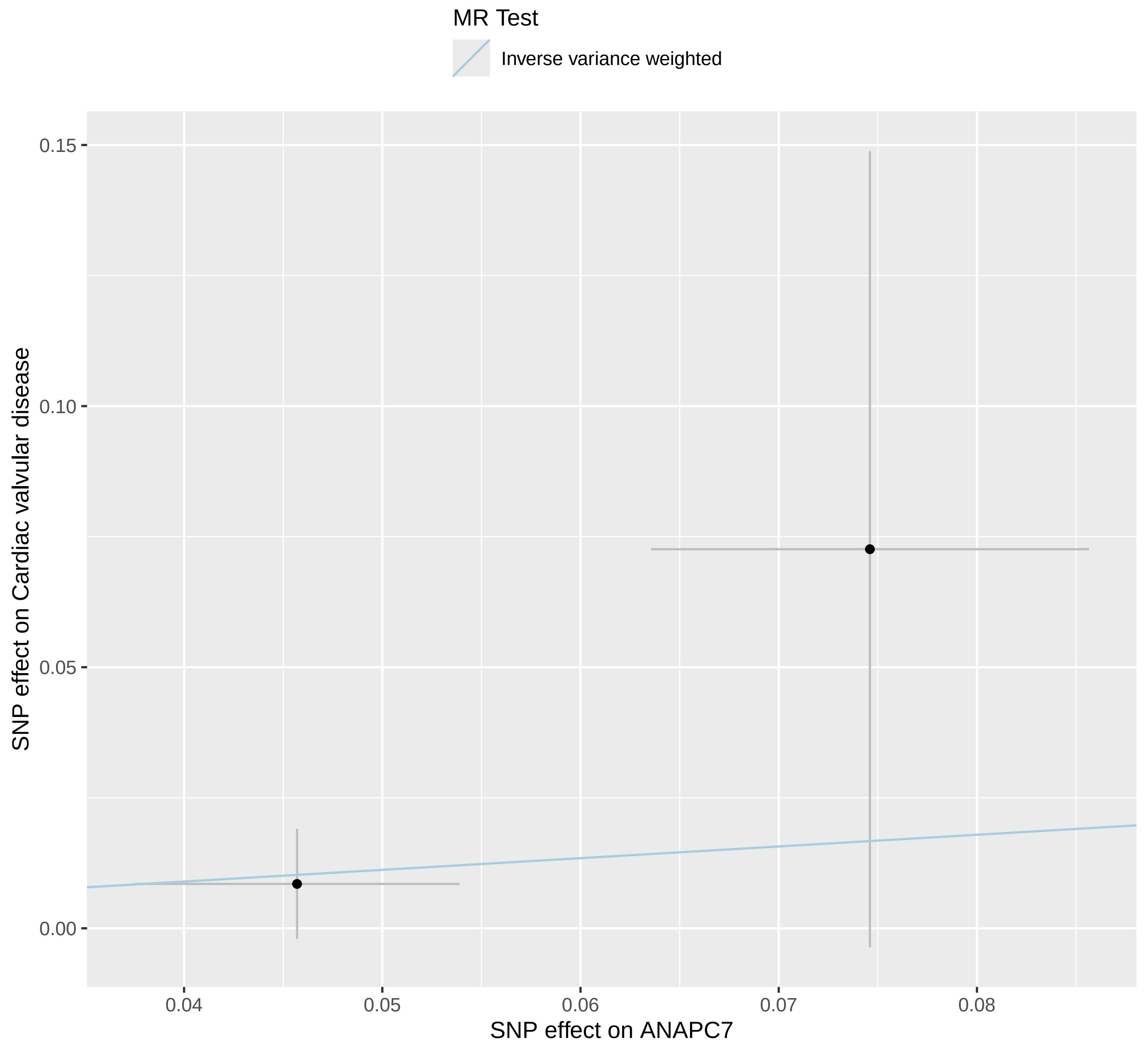
MR Test
Inverse variance weighted
0.15
0.10
0.05
0.00
SNP effect on Cardiac valvular disease
0.04
0.05
0.06
0.07
0.08
SNP effect on ANAPC7

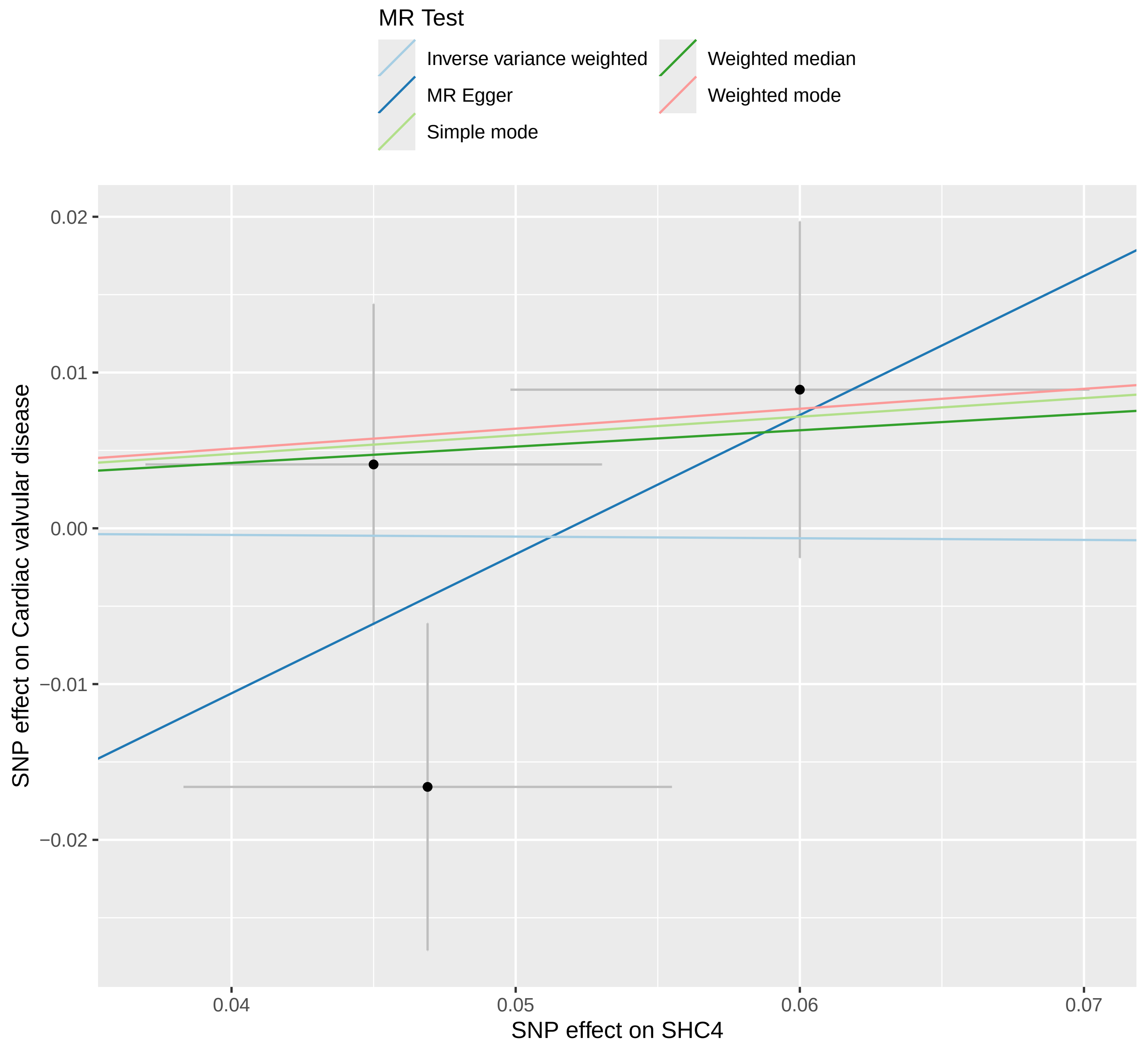
MR Test
Inverse variance weighted
MR Egger
Simple mode
Weighted median
Weighted mode
0.02
0.01
0.00
−0.01
−0.02
SNP effect on Cardiac valvular disease
0.04
0.05
0.06
0.07
SNP effect on SHC4

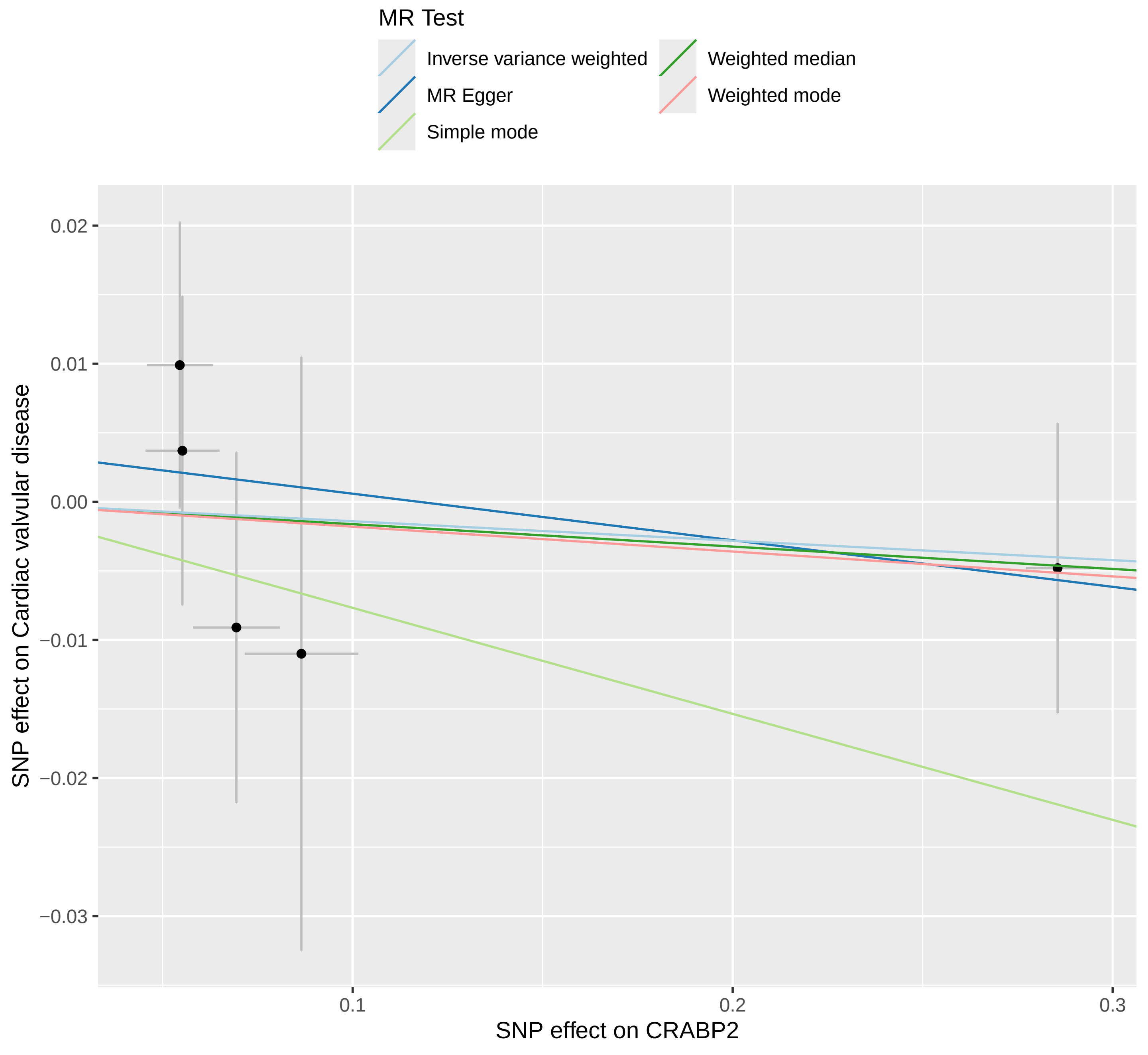
MR Test
Inverse variance weighted
MR Egger
Simple mode
Weighted median
Weighted mode
0.02
0.01
0.00
−0.01
−0.02
−0.03
SNP effect on Cardiac valvular disease
0.1
0.2
0.3
SNP effect on CRABP2

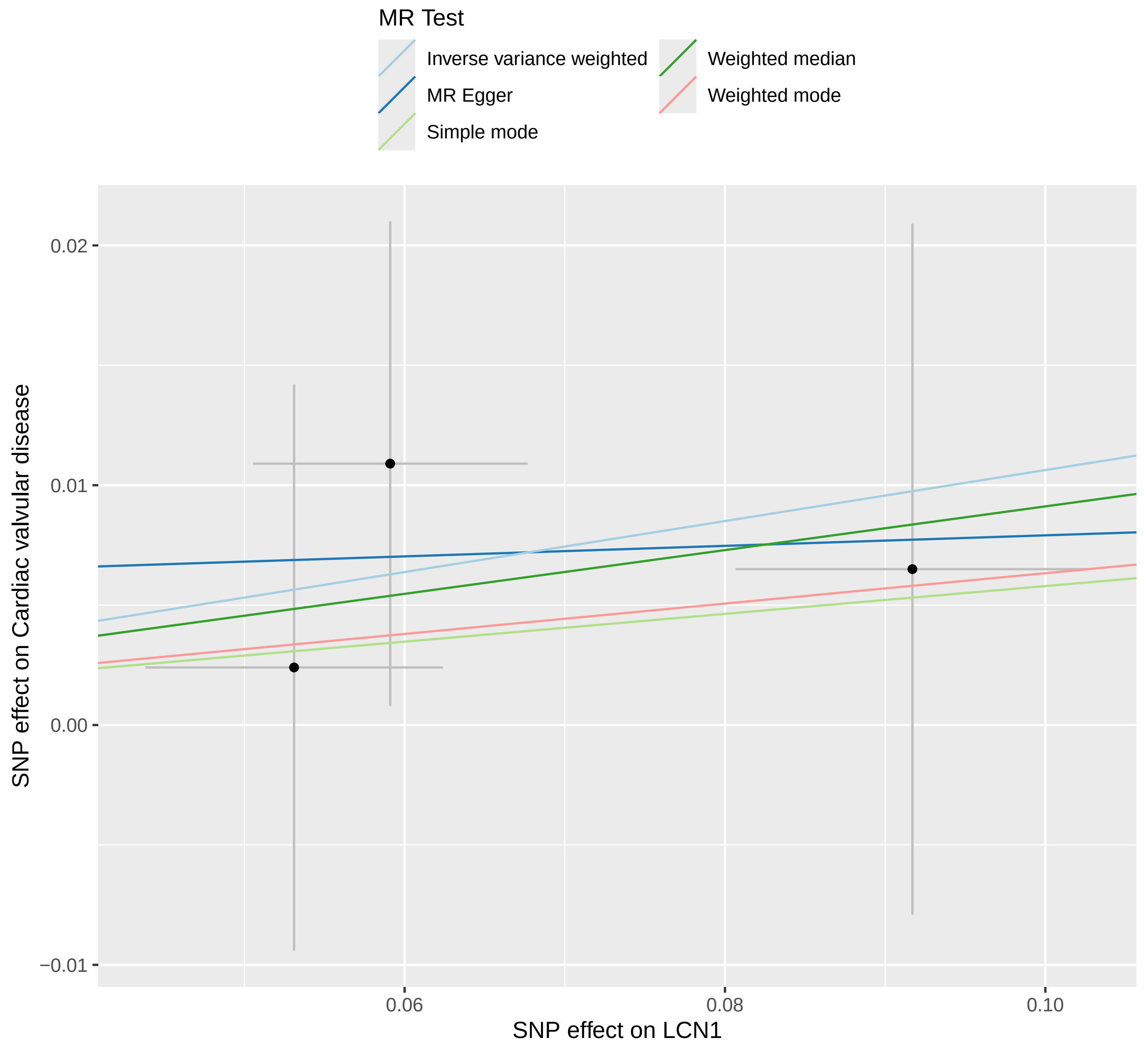
MR Test
Inverse variance weighted
MR Egger
Simple mode
Weighted median
Weighted mode
SNP effect on Cardiac valvular disease
0.02
0.01
0.00
−0.01
0.06
0.08
0.10
SNP effect on LCN1

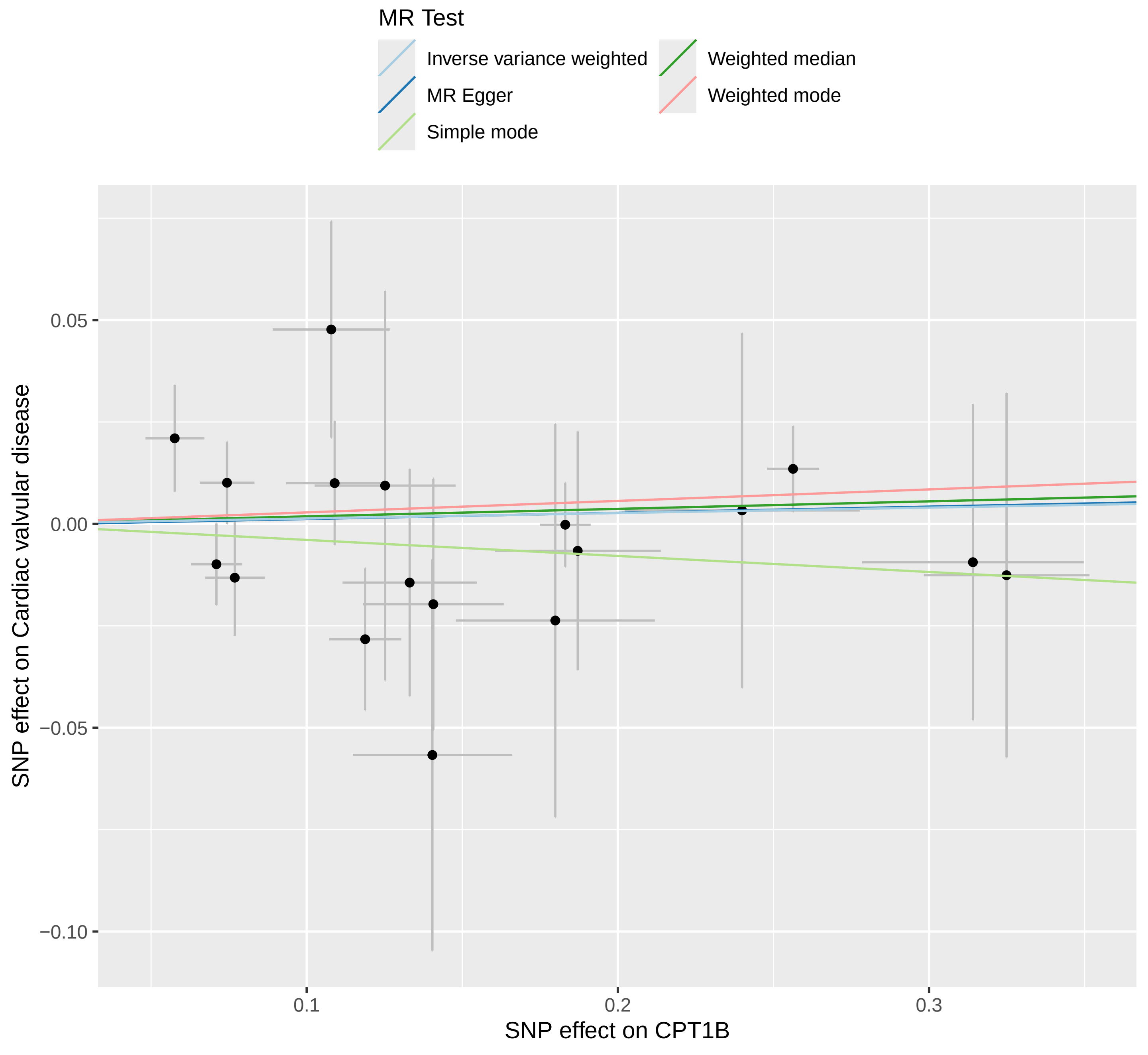
MR Test
Inverse variance weighted
MR Egger
Simple mode
Weighted median
Weighted mode
0.05
0.00
−0.05
−0.10
0.1
0.2
0.3
SNP effect on Cardiac valvular disease
SNP effect on CPT1B

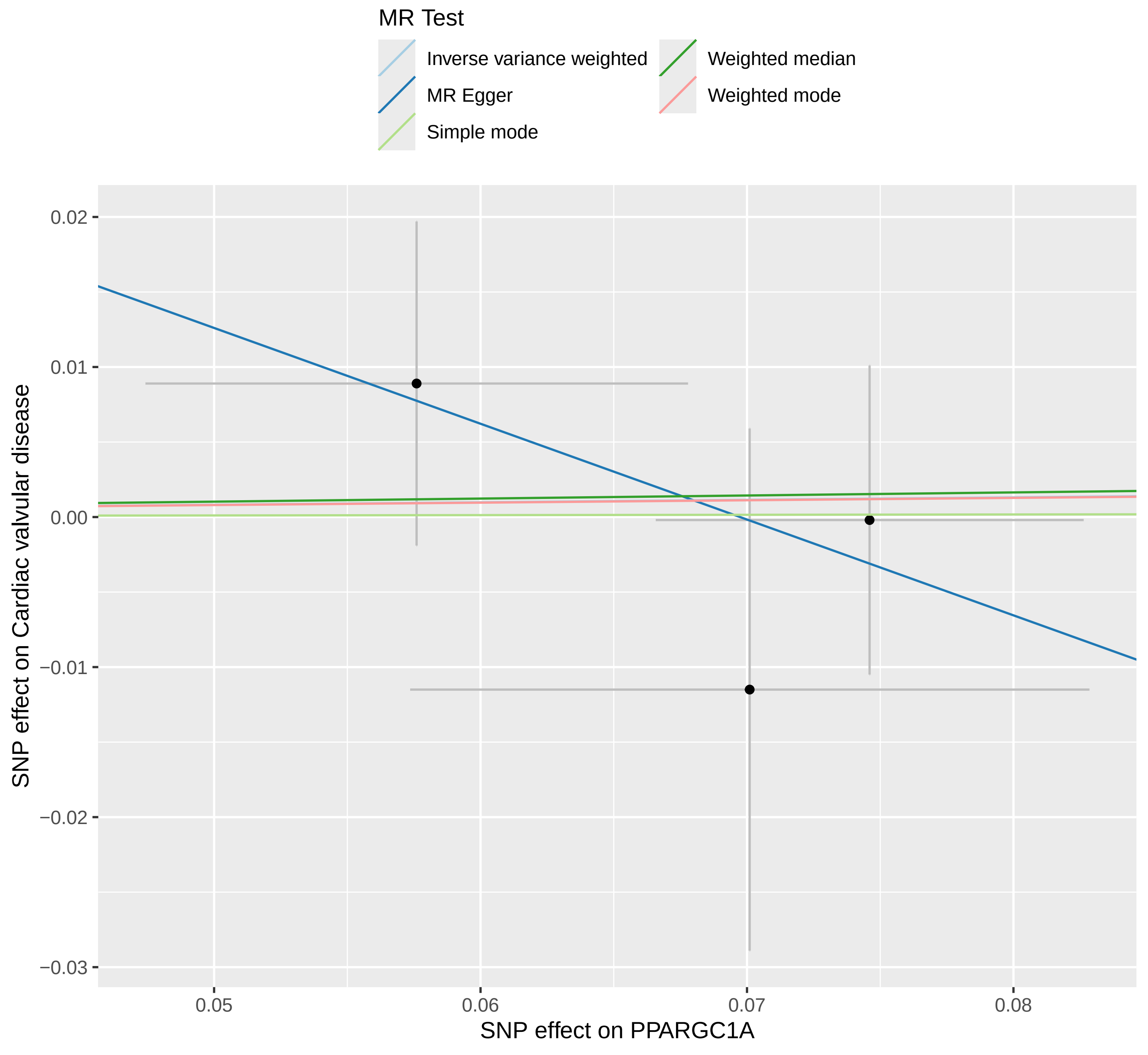

MR Test
Inverse variance weighted
MR Egger
Simple mode
Weighted median
Weighted mode
0.02
0.01
0.00
−0.01
−0.02
−0.03
0.05
0.06
0.07
0.08
SNP effect on PPARGC1A
SNP effect on Cardiac valvular disease

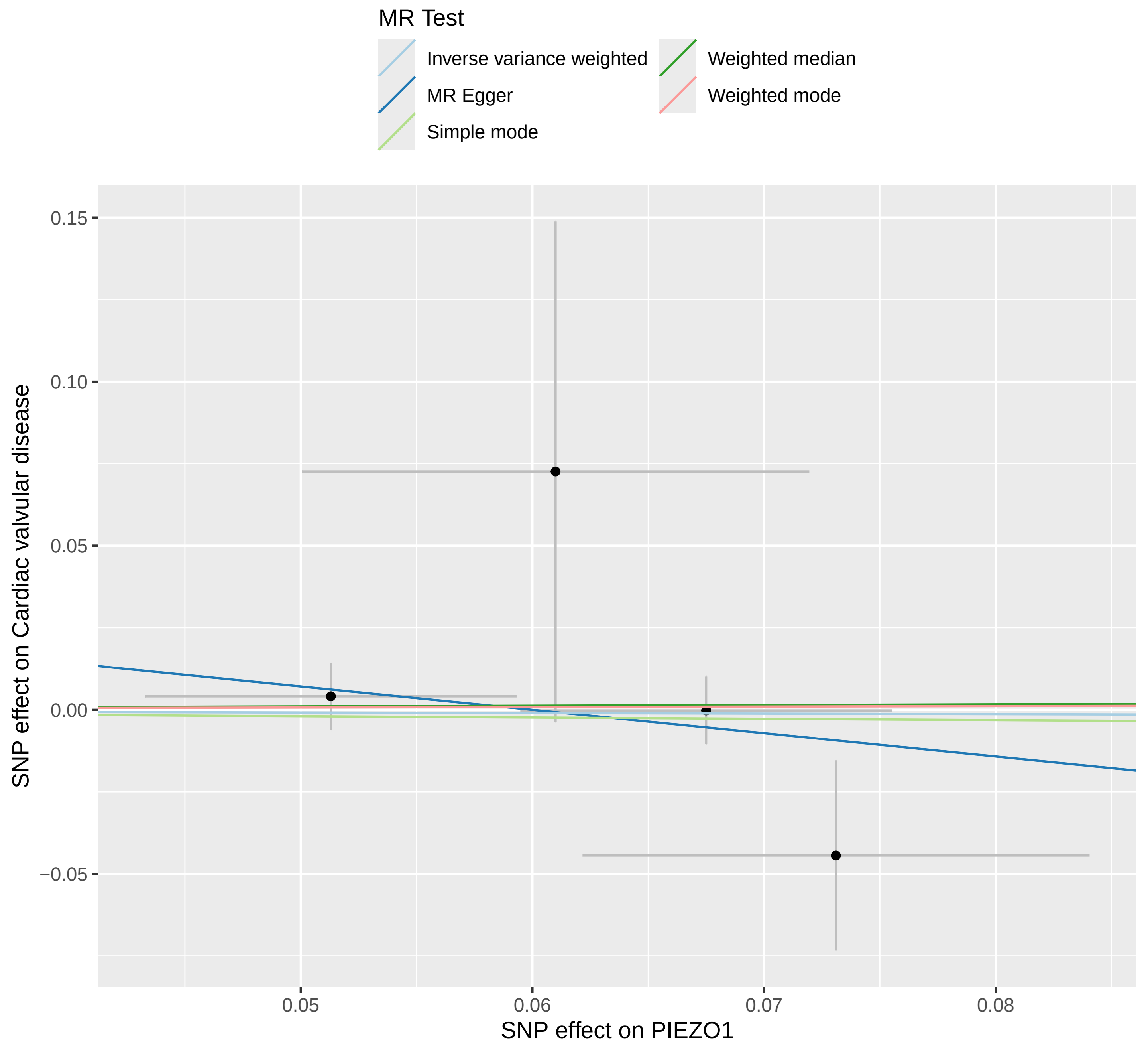
MR Test
Inverse variance weighted
MR Egger
Simple mode
Weighted median
Weighted mode
0.15
0.10
0.05
0.00
−0.05
0.05
0.06
0.07
0.08
SNP effect on PIEZO1
SNP effect on Cardiac valvular disease

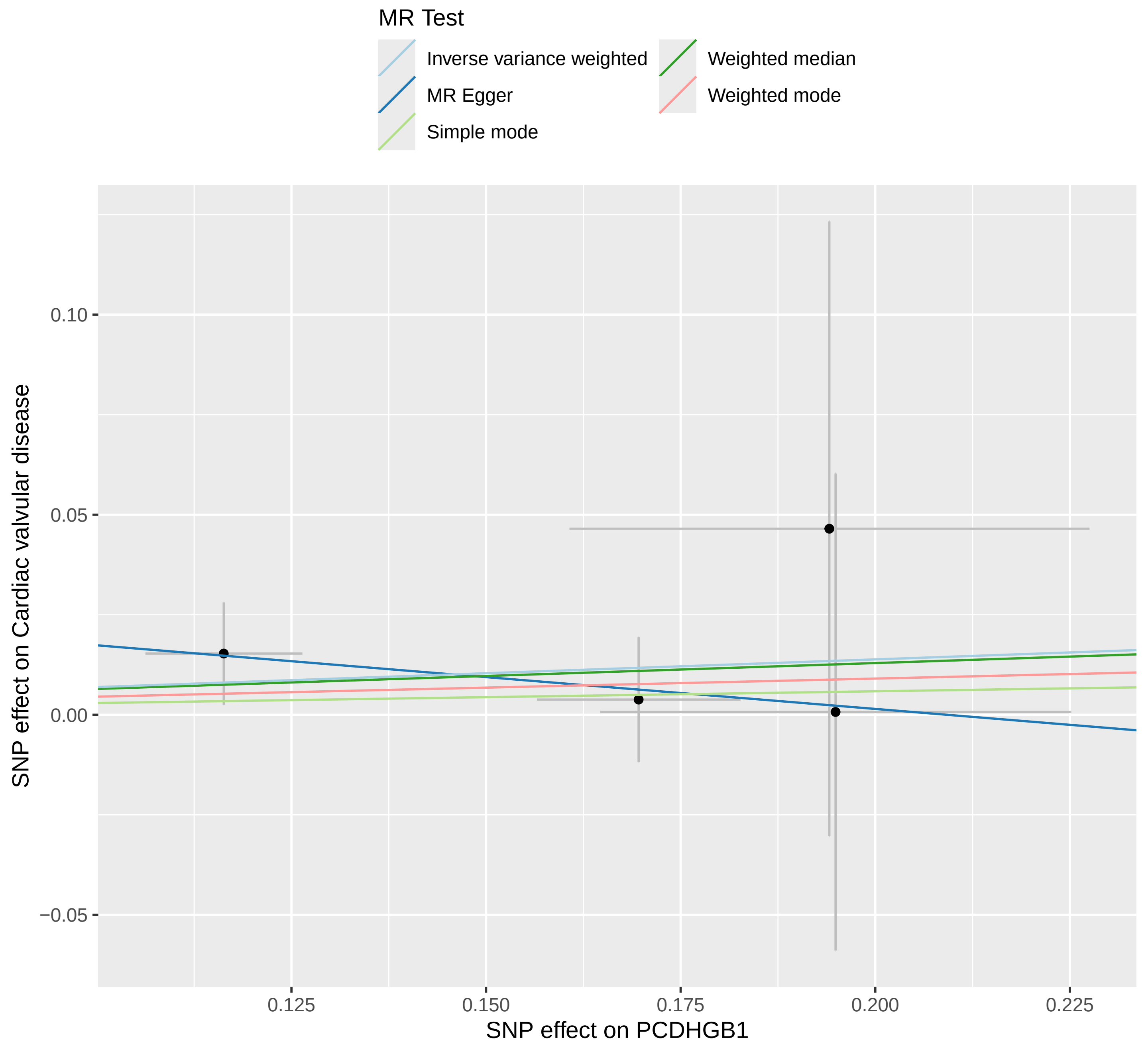
MR Test
Inverse variance weighted
MR Egger
Simple mode
Weighted median
Weighted mode
0.10
0.05
0.00
−0.05
0.125
0.150
0.175
0.200
0.225
SNP effect on PCDHGB1
SNP effect on Cardiac valvular disease

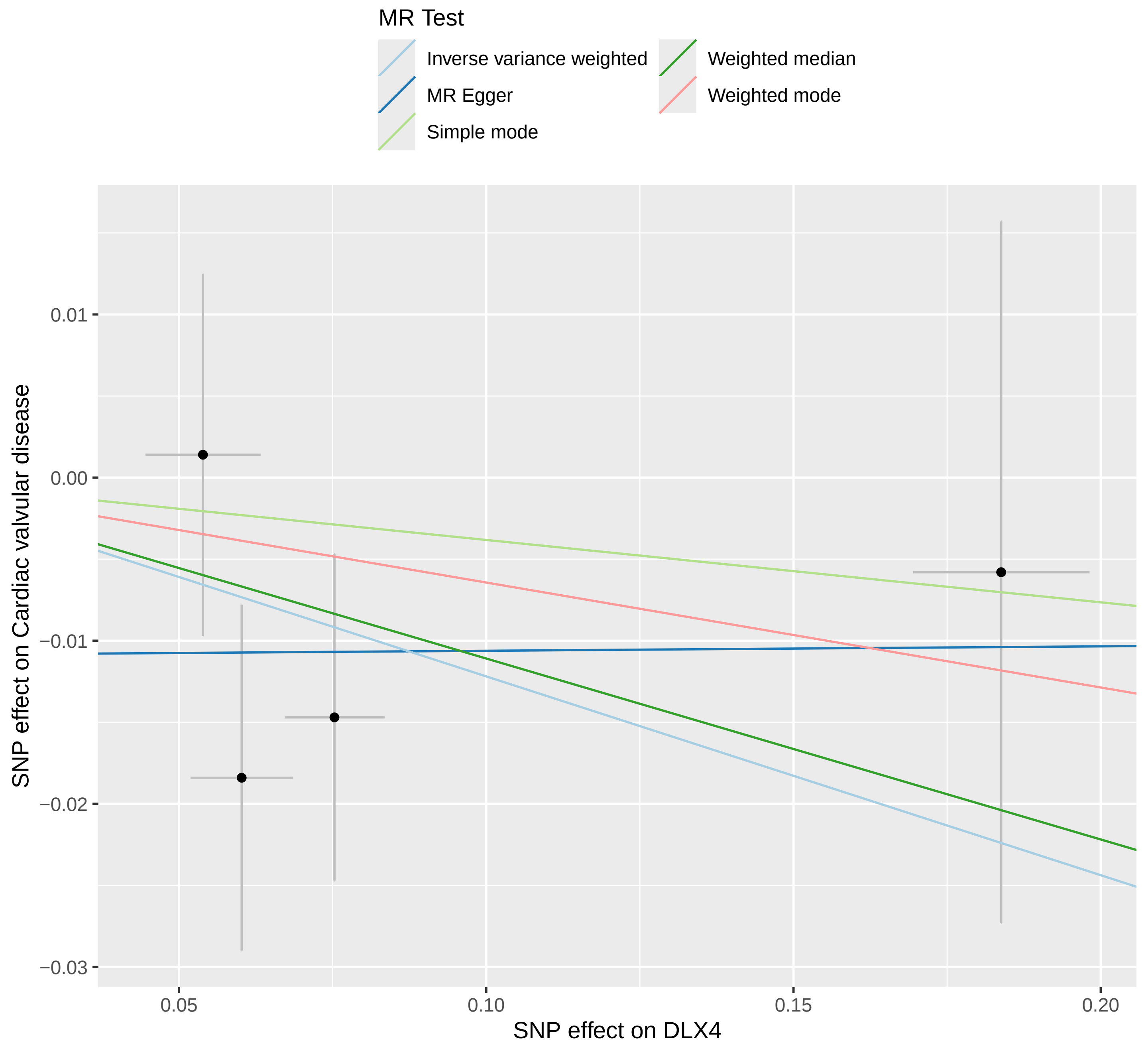

MR Test
Inverse variance weighted
MR Egger
Simple mode
Weighted median
Weighted mode
0.01
0.00
−0.01
−0.02
−0.03
SNP effect on Cardiac valvular disease
0.05
0.10
0.15
0.20
SNP effect on DLX4

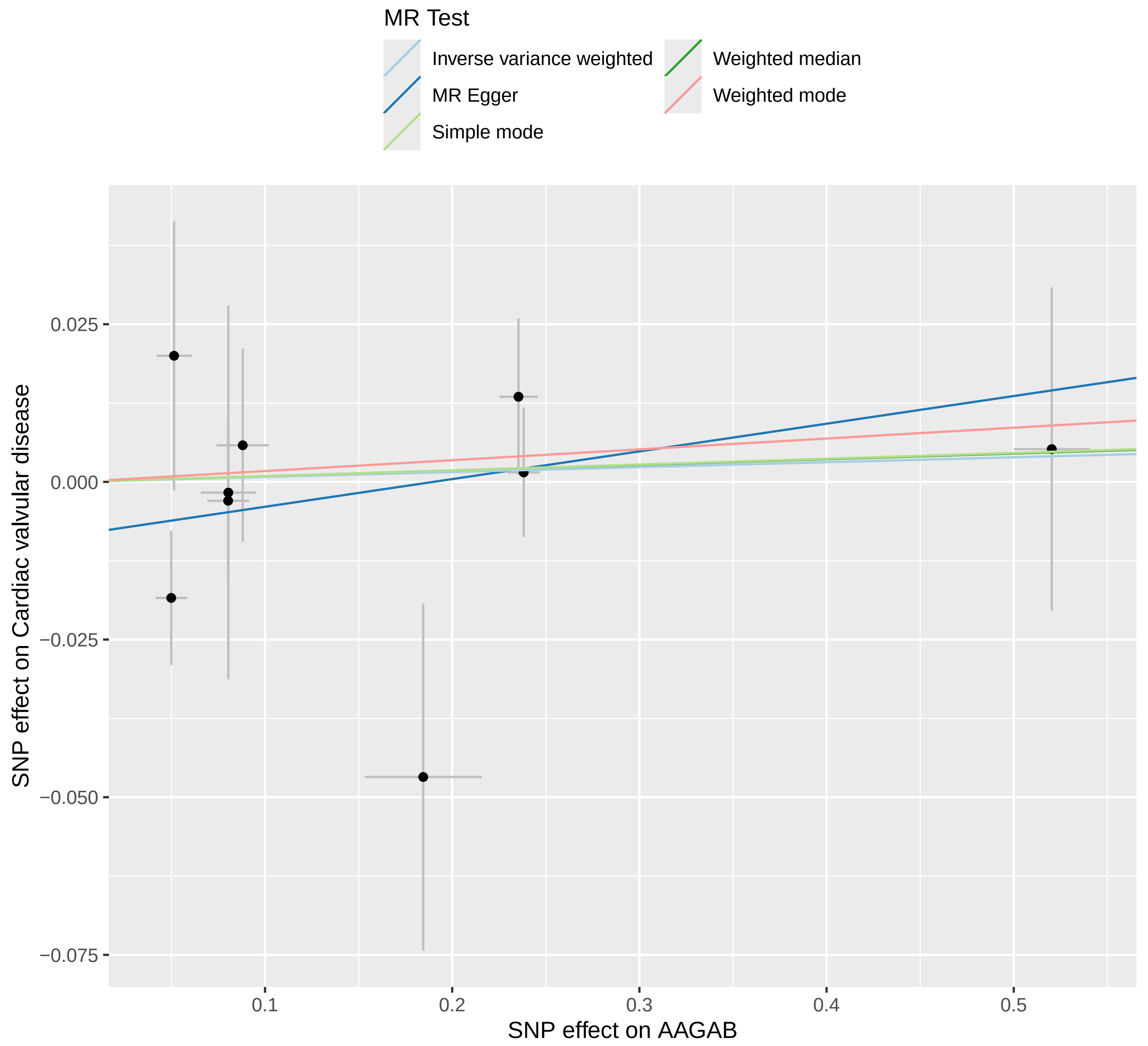

MR Test
Inverse variance weighted
MR Egger
Simple mode
Weighted median
Weighted mode
0.025
0.000
−0.025
−0.050
−0.075
SNP effect on Cardiac valvular disease
0.1
0.2
0.3
0.4
0.5
SNP effect on AAGAB

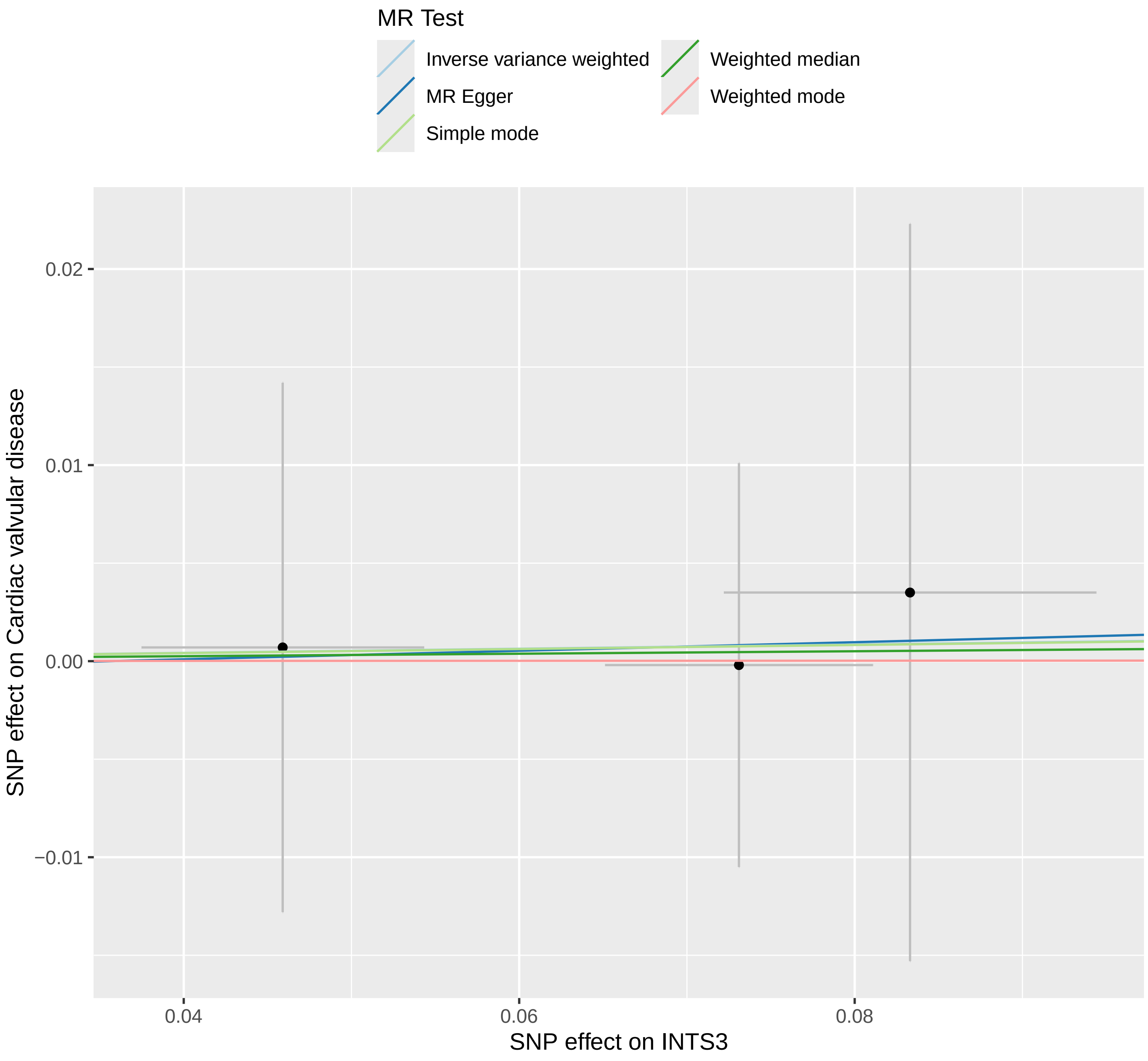

MR Test
Inverse variance weighted
MR Egger
Simple mode
Weighted median
Weighted mode
0.02
0.01
0.00
−0.01
0.04
0.06
0.08
SNP effect on INTS3
SNP effect on Cardiac valvular disease

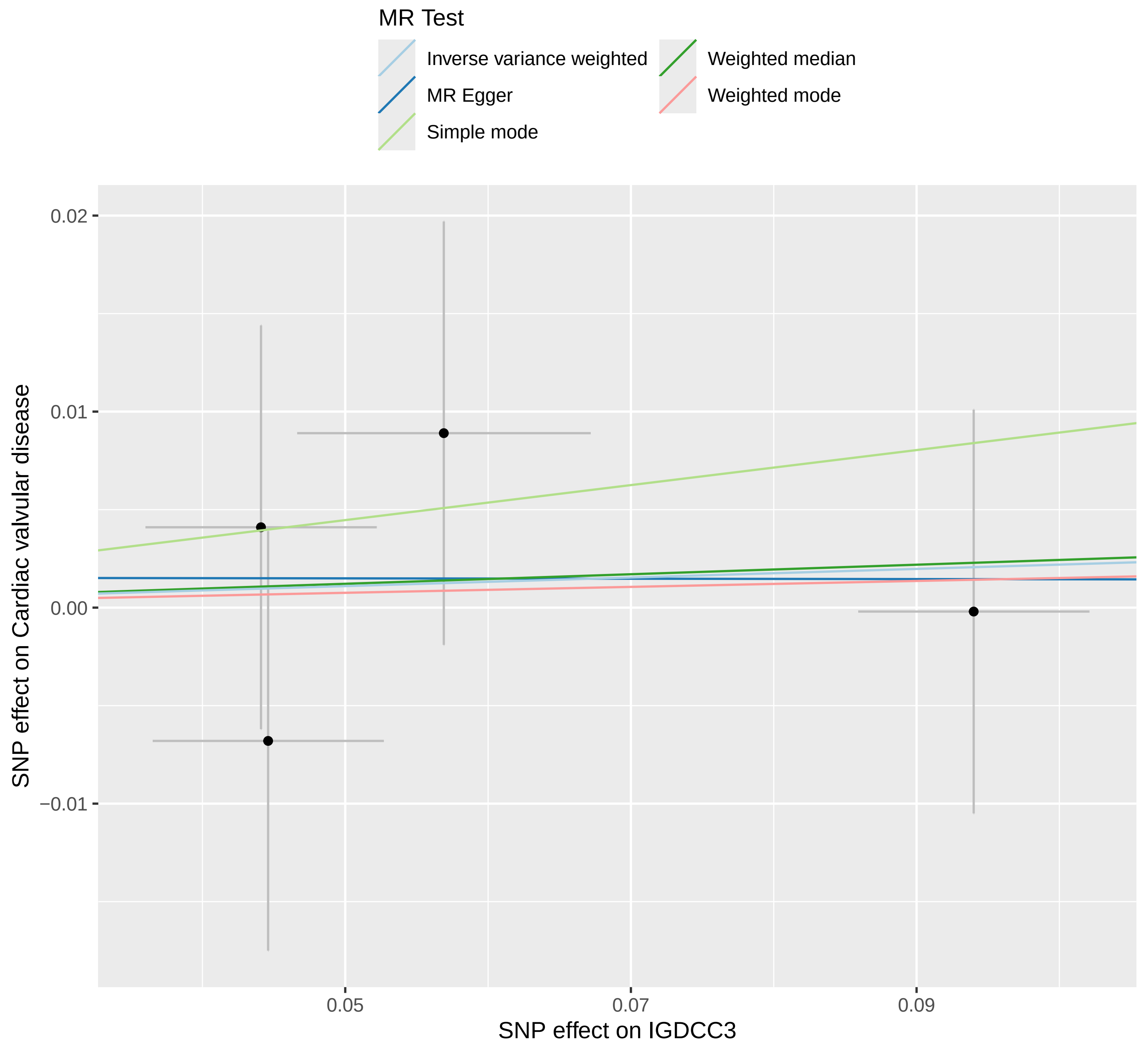

MR Test
Inverse variance weighted
MR Egger
Simple mode
Weighted median
Weighted mode
0.02
0.01
0.00
−0.01
0.05
0.07
0.09
SNP effect on Cardiac valvular disease
SNP effect on IGDCC3

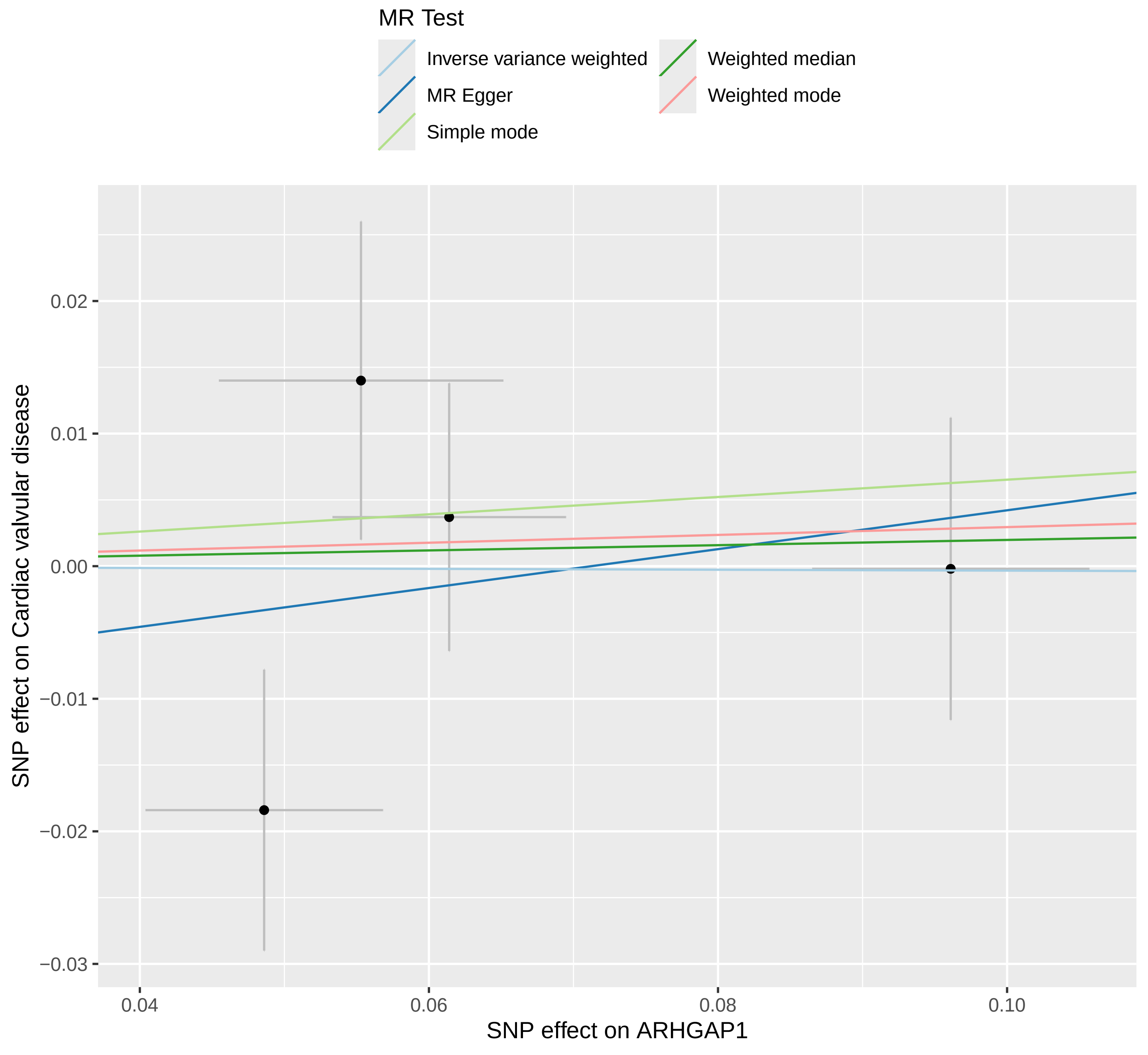
MR Test
Inverse variance weighted
MR Egger
Simple mode
Weighted median
Weighted mode
0.02
0.01
0.00
−0.01
−0.02
−0.03
SNP effect on Cardiac valvular disease
0.04
0.06
0.08
0.10
SNP effect on ARHGAP1

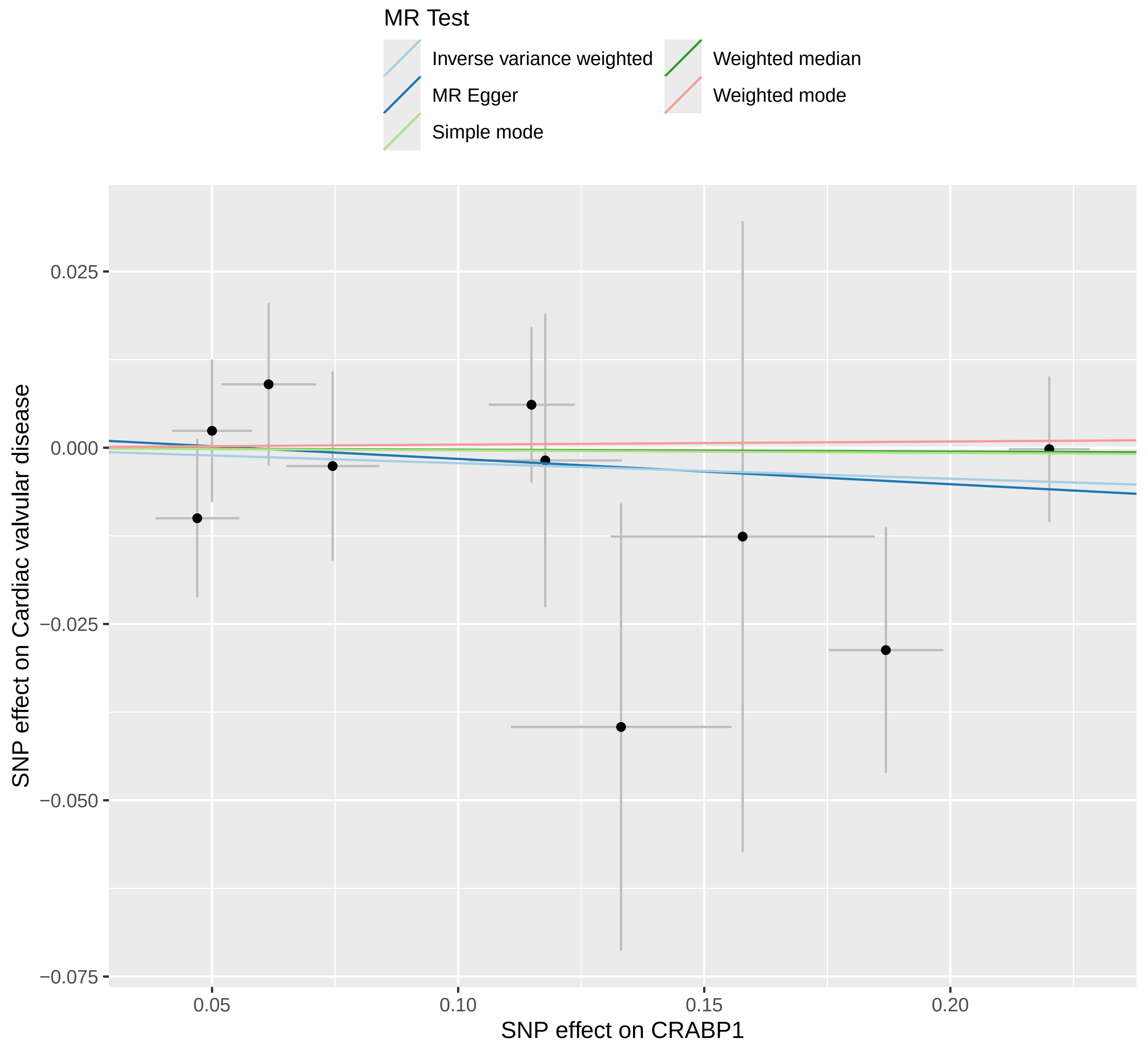
MR Test
Inverse variance weighted
MR Egger
Simple mode
Weighted median
Weighted mode
0.025
0.000
−0.025
−0.050
−0.075
SNP effect on Cardiac valvular disease
0.05
0.10
0.15
0.20
SNP effect on CRABP1

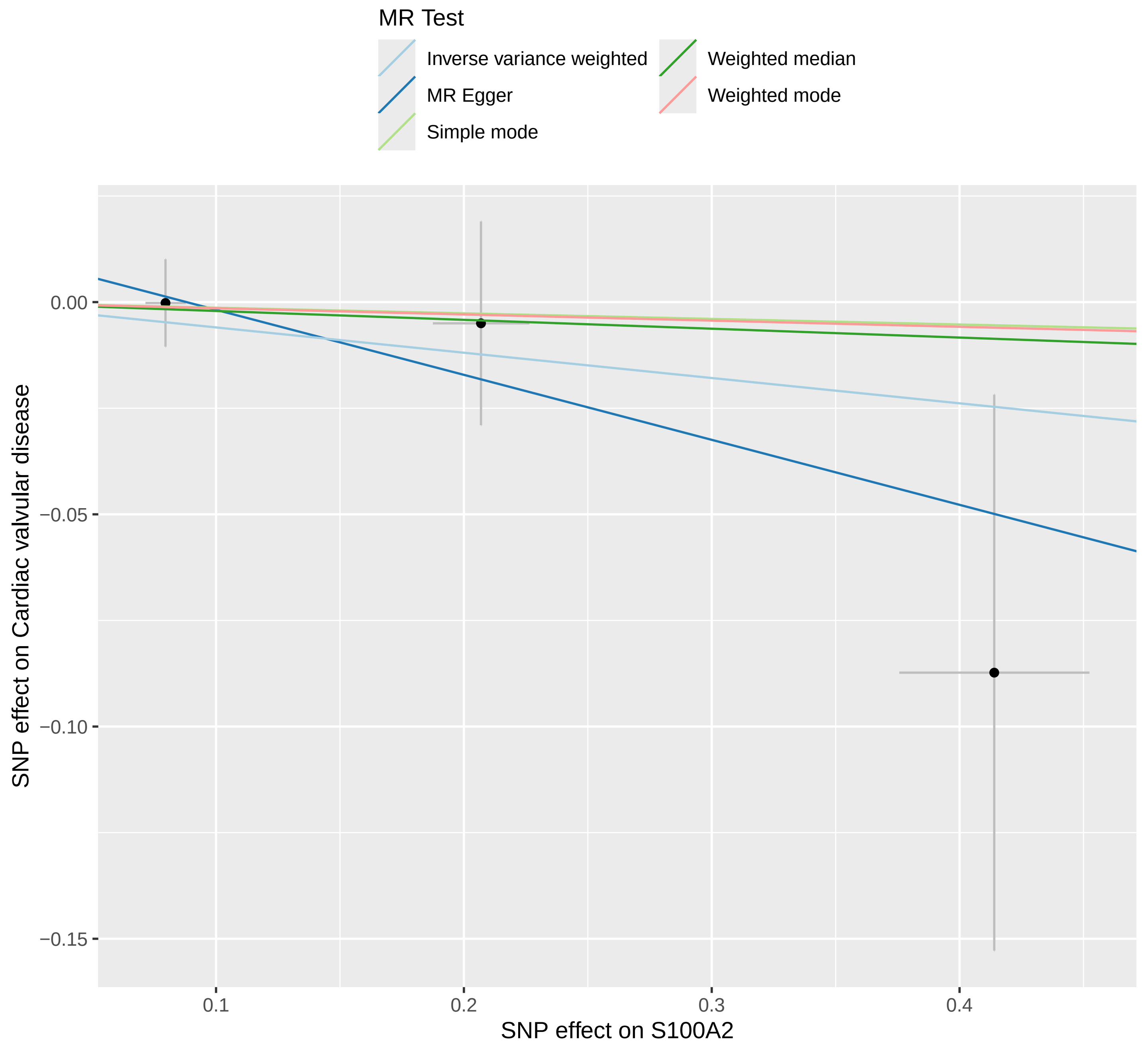
MR Test
Inverse variance weighted
MR Egger
Simple mode
Weighted median
Weighted mode
SNP effect on Cardiac valvular disease
0.00
−0.05
−0.10
−0.15
0.1
0.2
0.3
0.4
SNP effect on S100A2

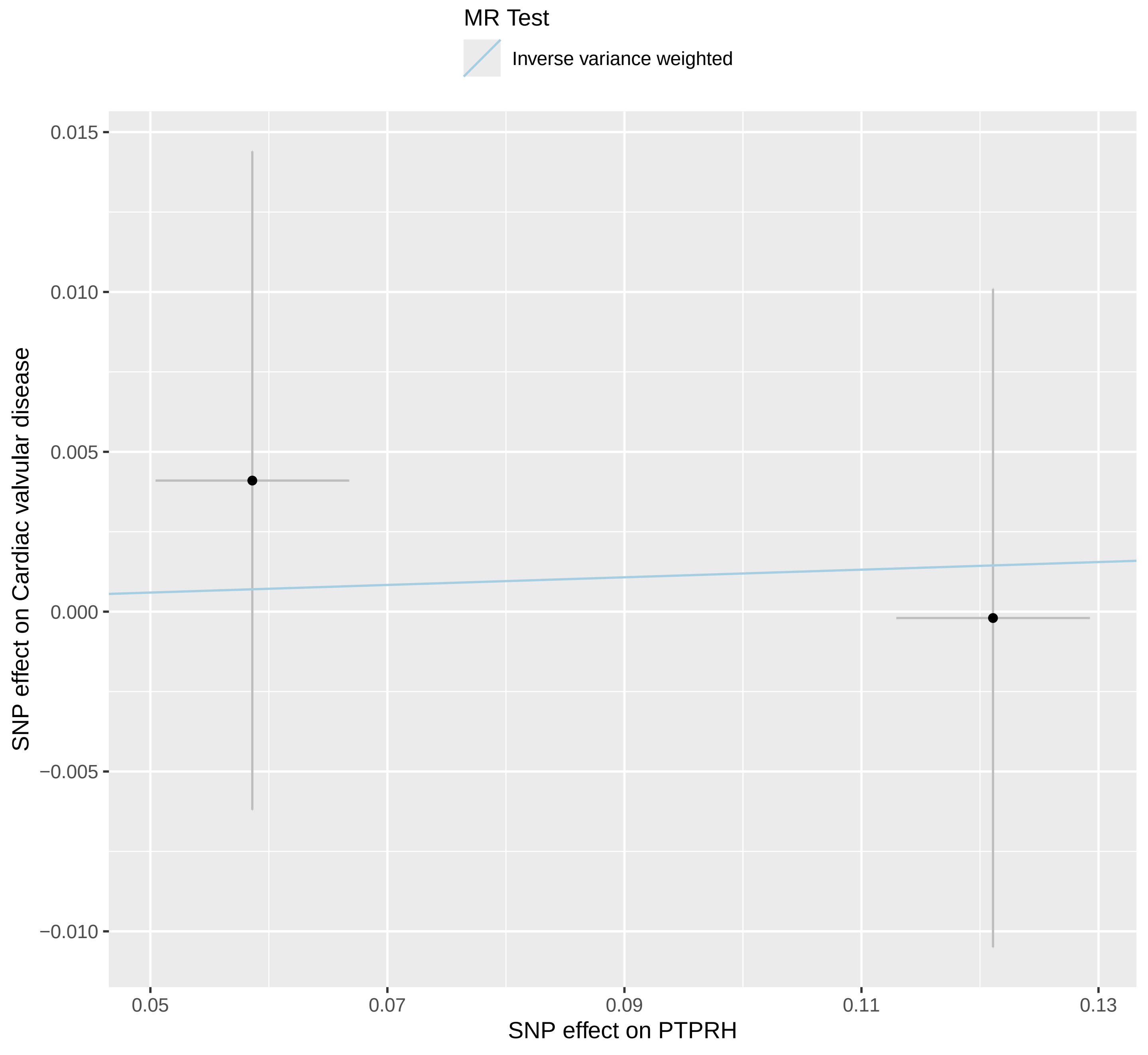

MR Test
Inverse variance weighted
0.015
0.010
0.005
0.000
−0.005
−0.010
SNP effect on Cardiac valvular disease
0.05
0.07
0.09
0.11
0.13
SNP effect on PTPRH

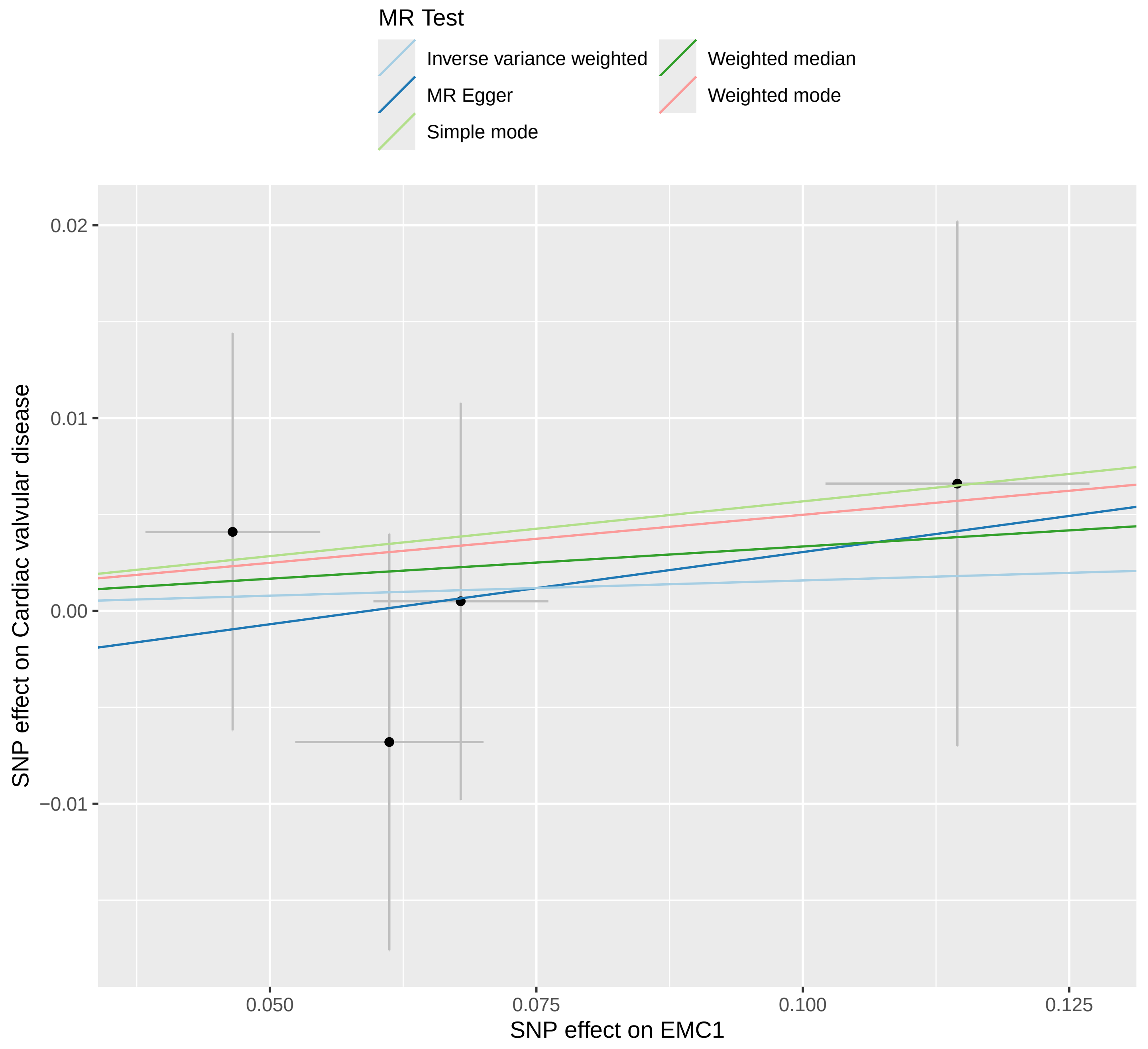
MR Test
Inverse variance weighted
MR Egger
Simple mode
Weighted median
Weighted mode
0.02
0.01
0.00
−0.01
0.050
0.075
0.100
0.125
SNP effect on Cardiac valvular disease
SNP effect on EMC1

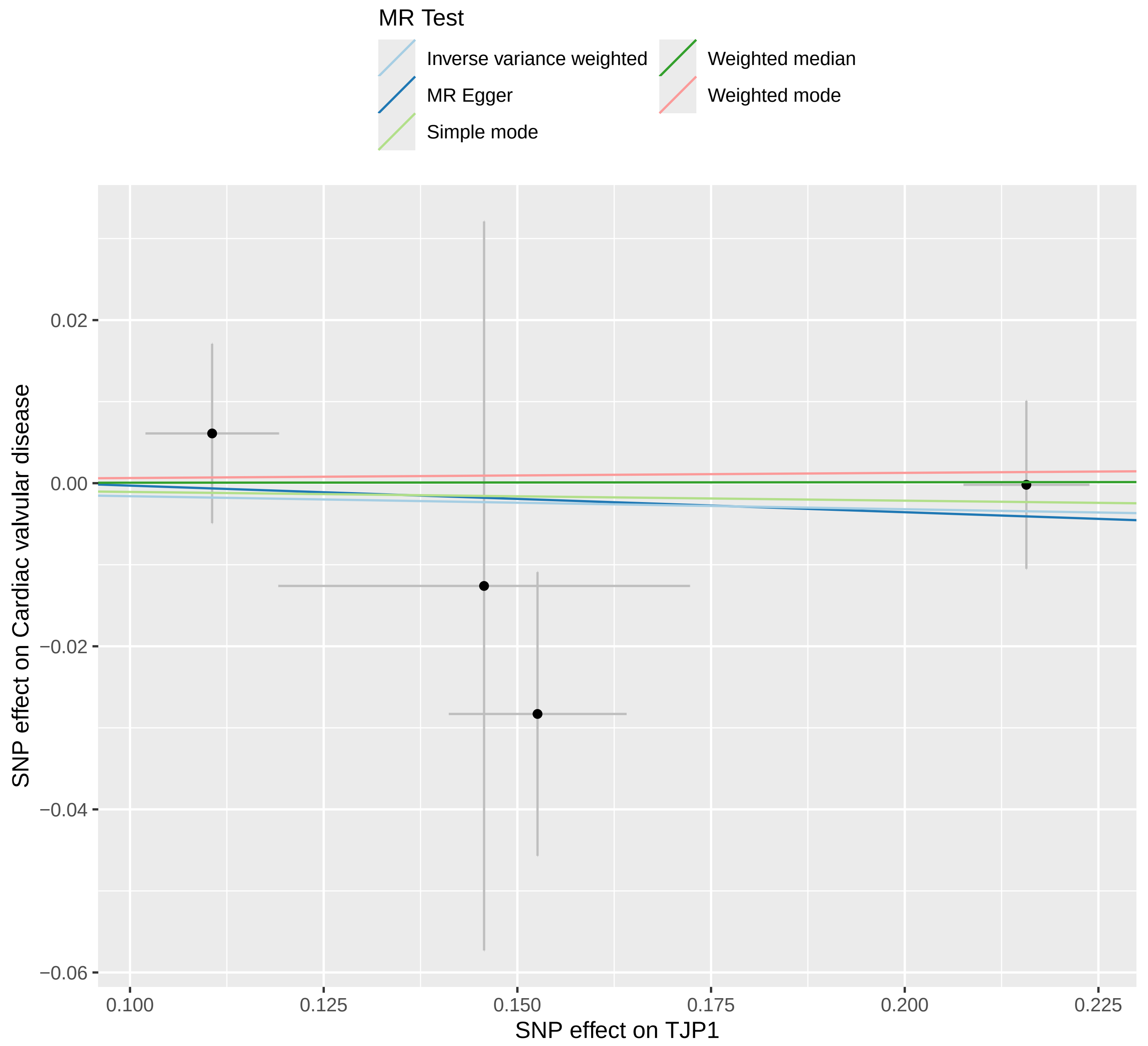

MR Test
Inverse variance weighted
MR Egger
Simple mode
Weighted median
Weighted mode
0.02
0.00
−0.02
−0.04
−0.06
SNP effect on Cardiac valvular disease
0.100
0.125
0.150
0.175
0.200
0.225
SNP effect on TJP1

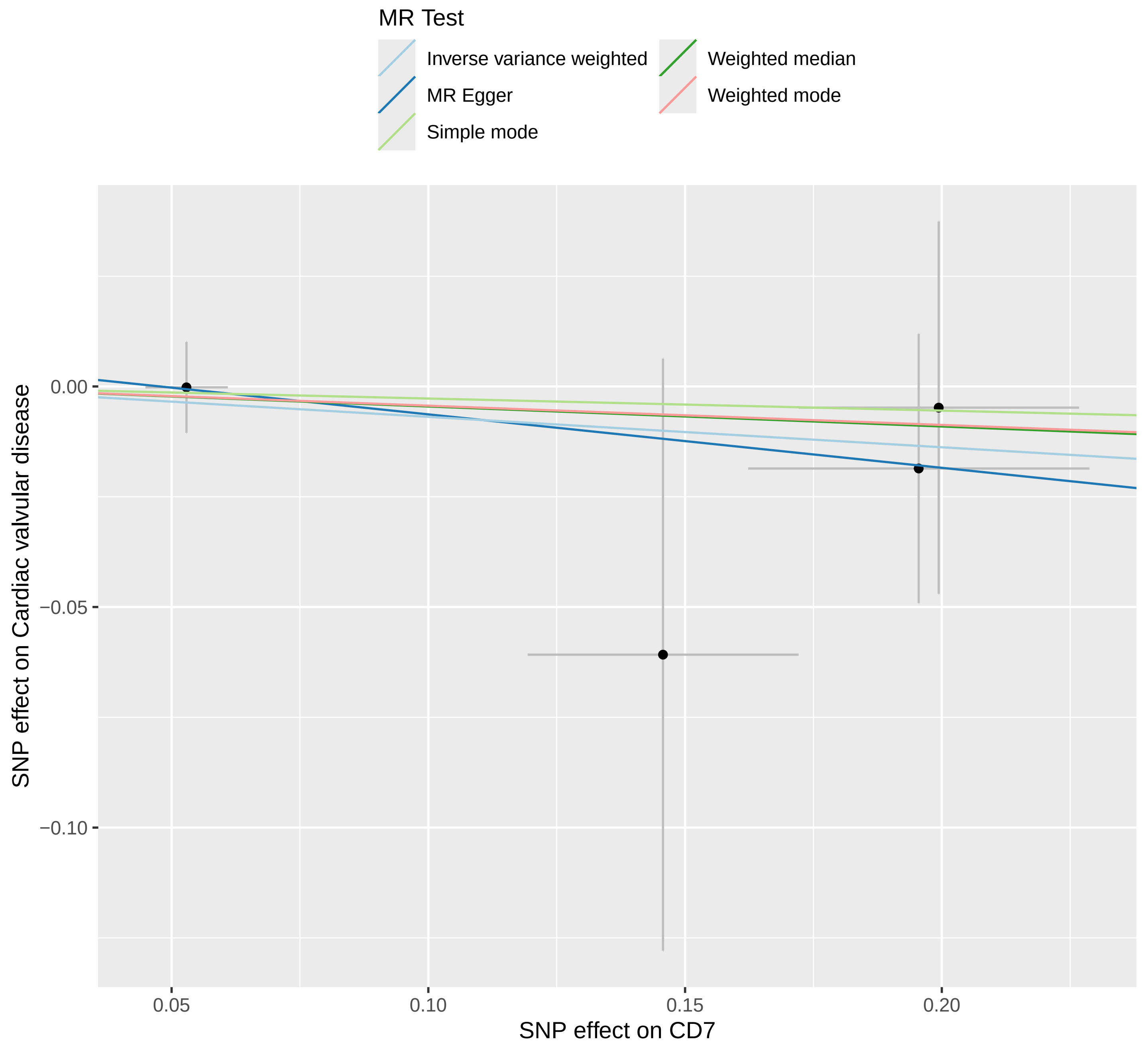
MR Test
Inverse variance weighted
MR Egger
Simple mode
Weighted median
Weighted mode
SNP effect on Cardiac valvular disease
0.00
−0.05
−0.10
0.05
0.10
0.15
0.20
SNP effect on CD7

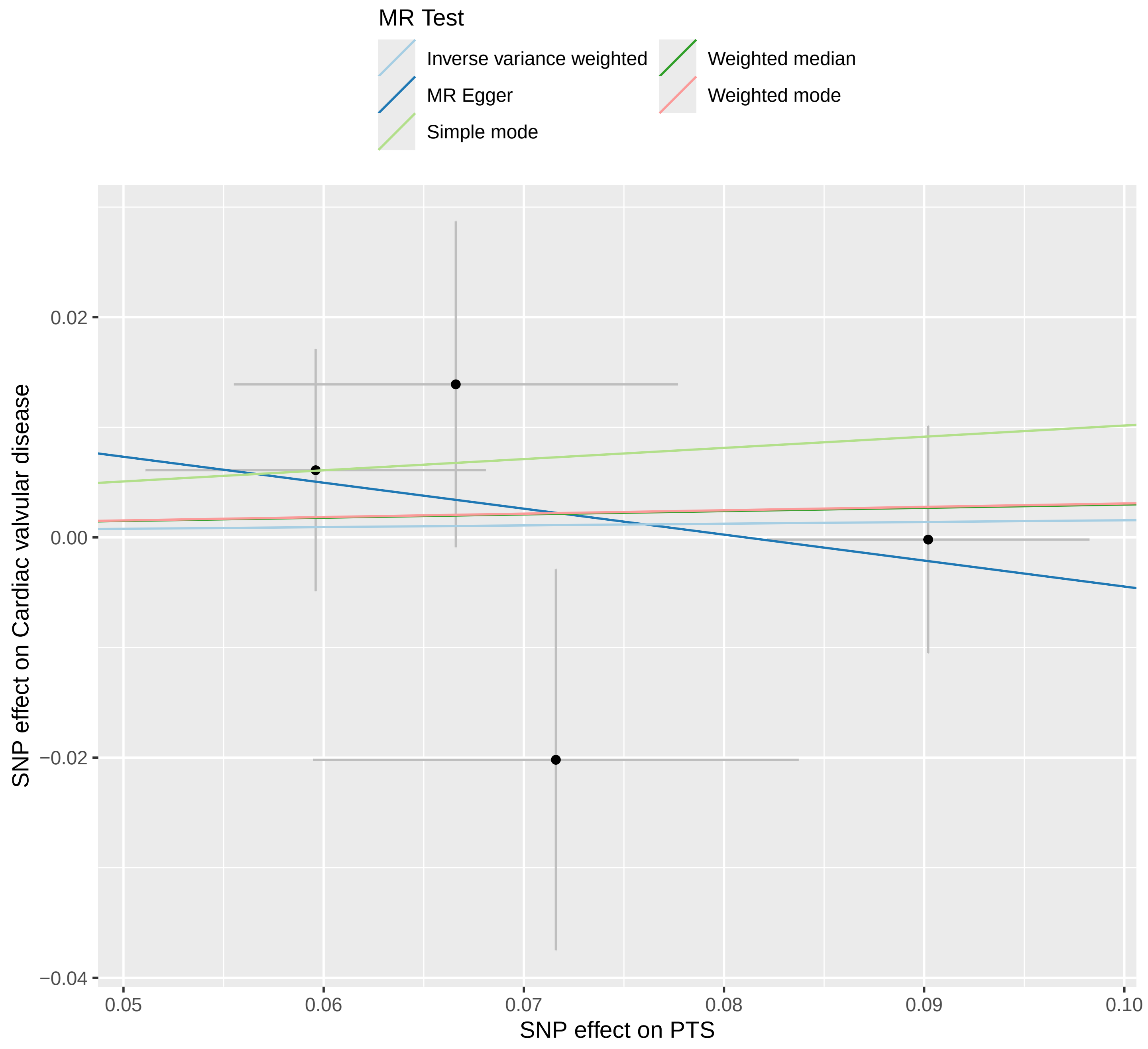
MR Test
Inverse variance weighted
MR Egger
Simple mode
Weighted median
Weighted mode
SNP effect on Cardiac valvular disease
0.02
0.00
−0.02
−0.04
0.05
0.06
0.07
0.08
0.09
0.10
SNP effect on PTS

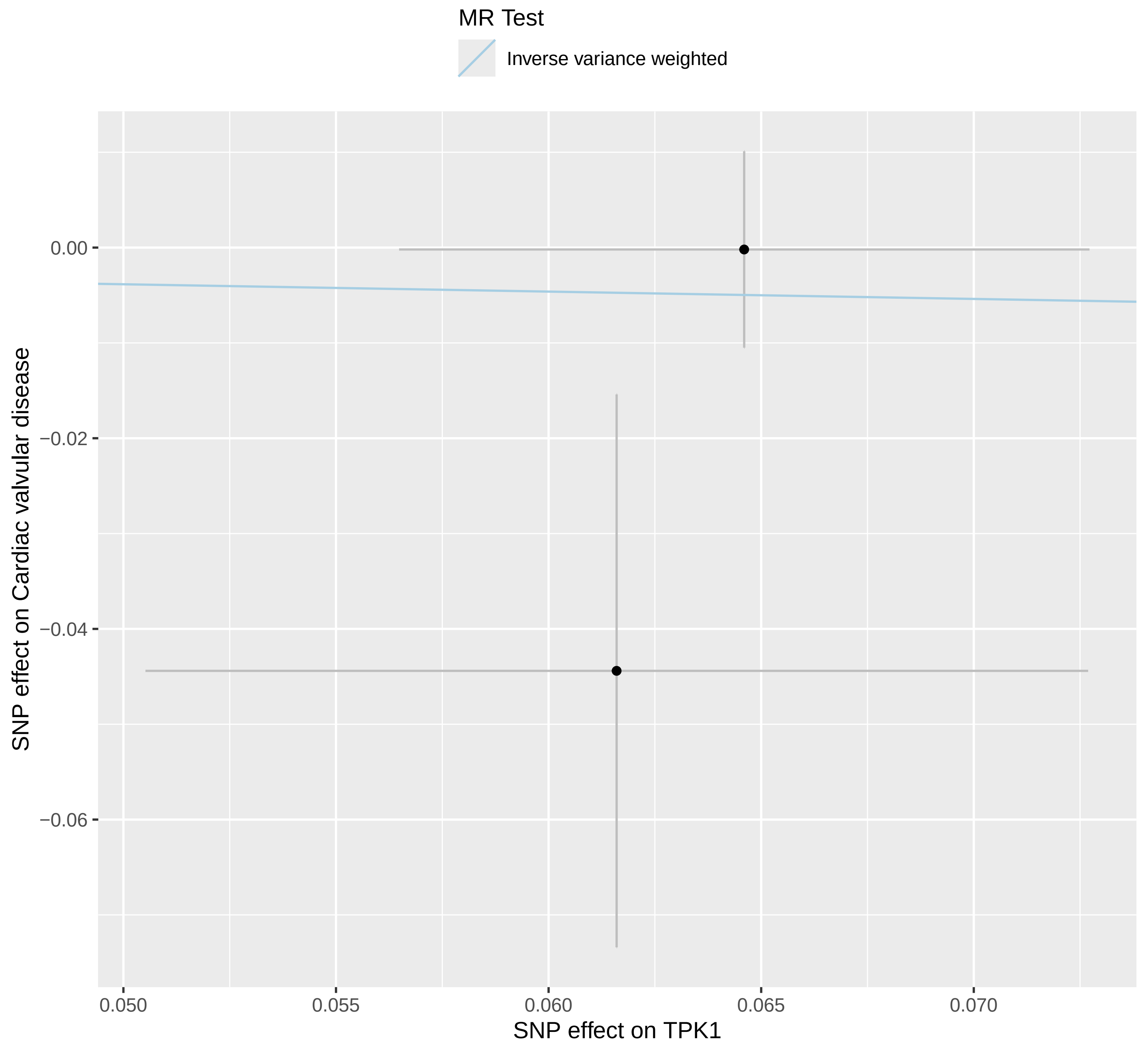
MR Test
Inverse variance weighted
0.00
−0.02
−0.04
−0.06
SNP effect on Cardiac valvular disease
0.050
0.055
0.060
0.065
0.070
SNP effect on TPK1

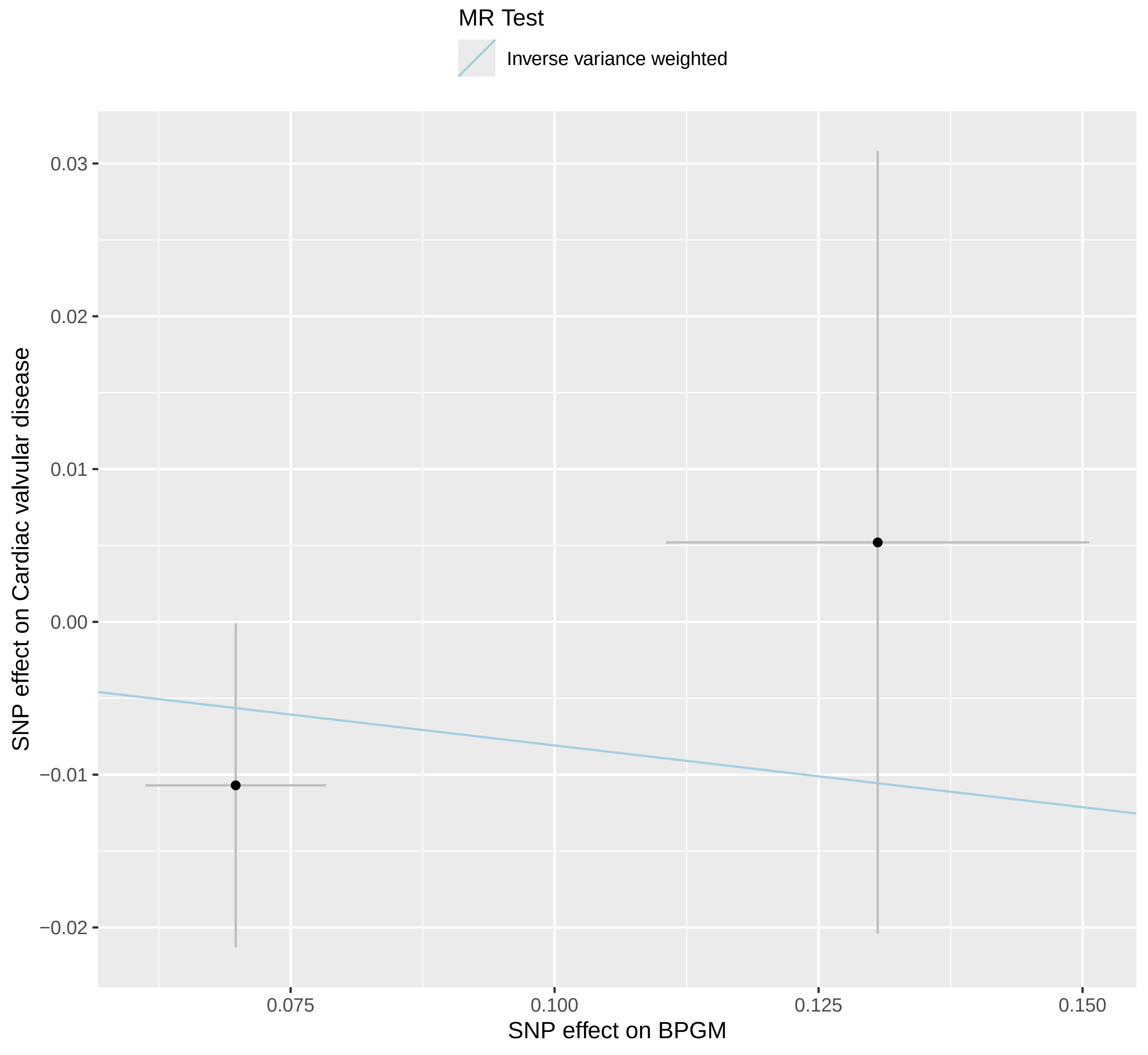

MR Test
Inverse variance weighted
0.03
0.02
0.01
0.00
−0.01
−0.02
0.075
0.100
0.125
0.150
SNP effect on BPGM
SNP effect on Cardiac valvular disease

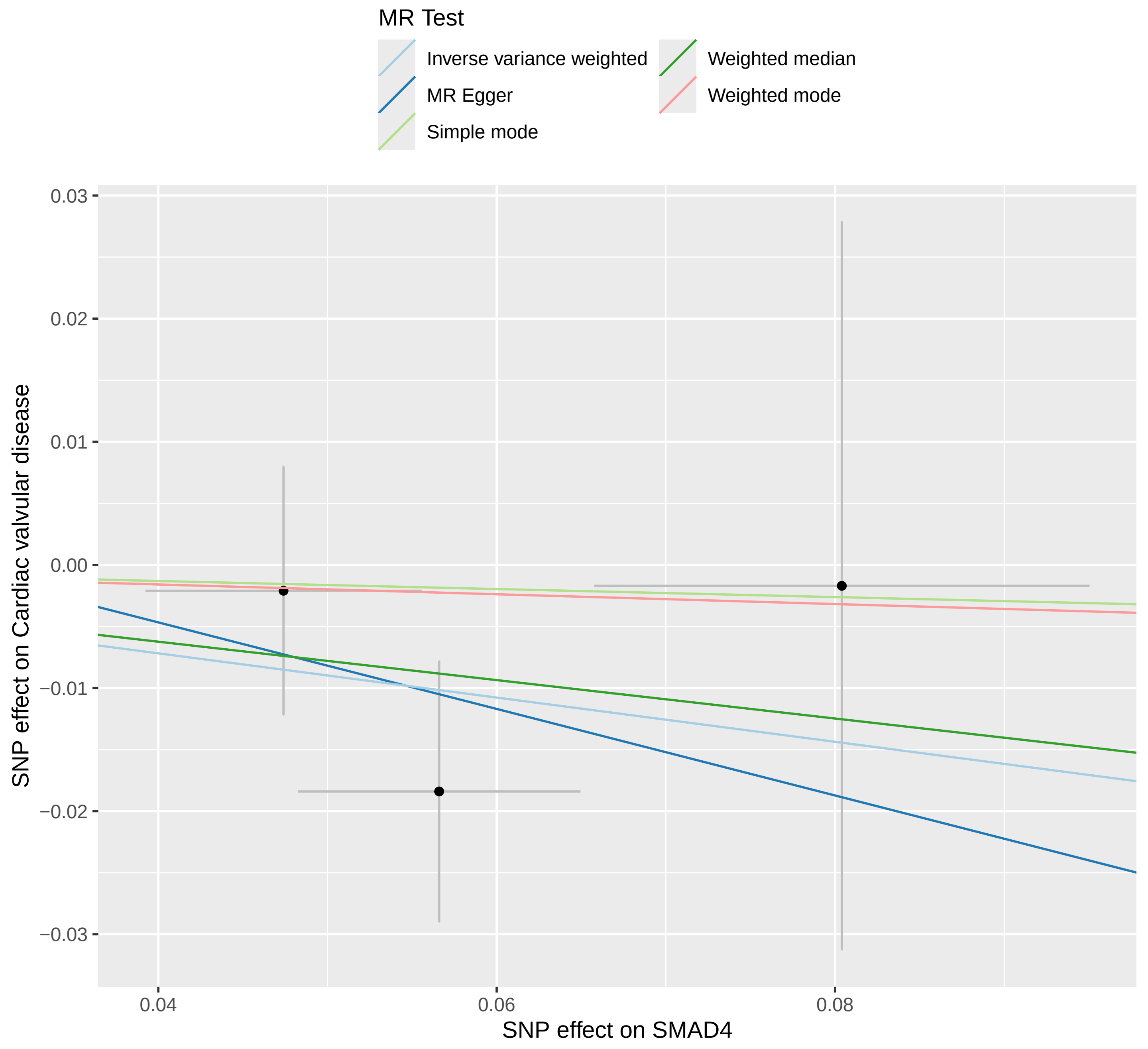

MR Test
Inverse variance weighted
MR Egger
Simple mode
Weighted median
Weighted mode
0.03
0.02
0.01
0.00
−0.01
−0.02
−0.03
0.04
0.06
0.08
SNP effect on Cardiac valvular disease
SNP effect on SMAD4

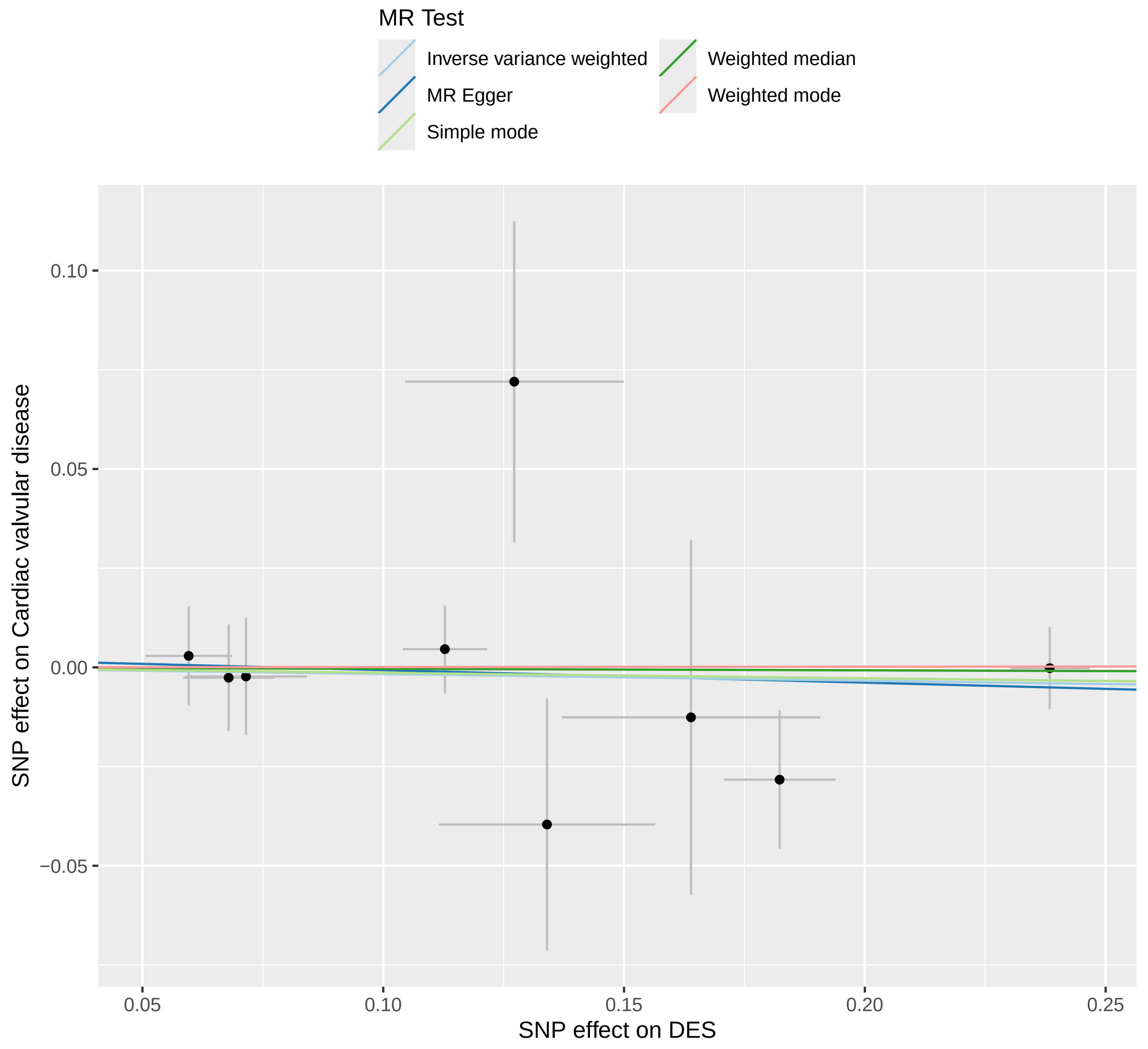
MR Test
Inverse variance weighted
MR Egger
Simple mode
Weighted median
Weighted mode
0.10
0.05
0.00
−0.05
0.05
0.10
0.15
0.20
0.25
SNP effect on DES
SNP effect on Cardiac valvular disease

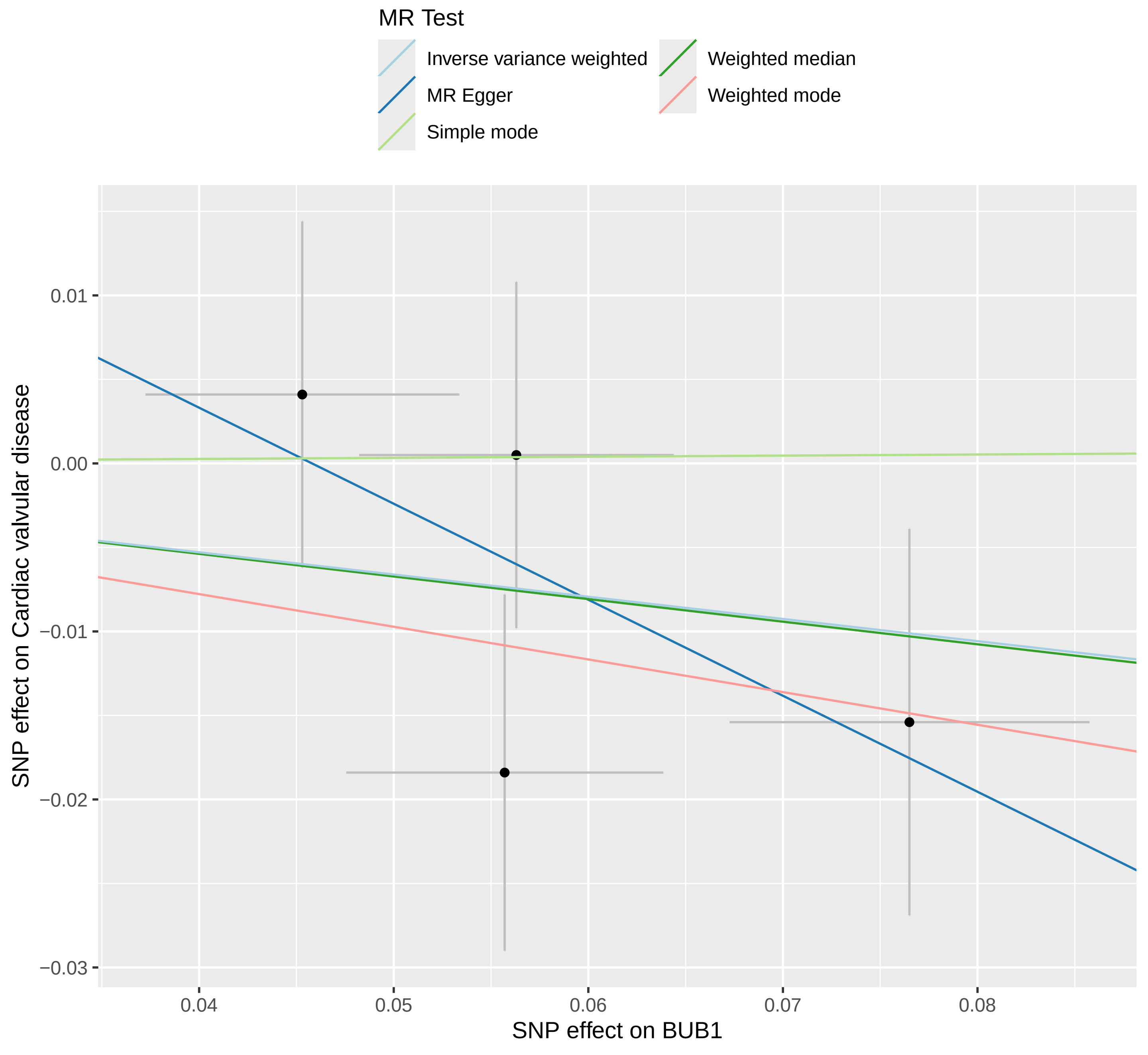

MR Test
Inverse variance weighted
MR Egger
Simple mode
Weighted median
Weighted mode
0.01
0.00
−0.01
−0.02
−0.03
0.04
0.05
0.06
0.07
0.08
SNP effect on Cardiac valvular disease
SNP effect on BUB1

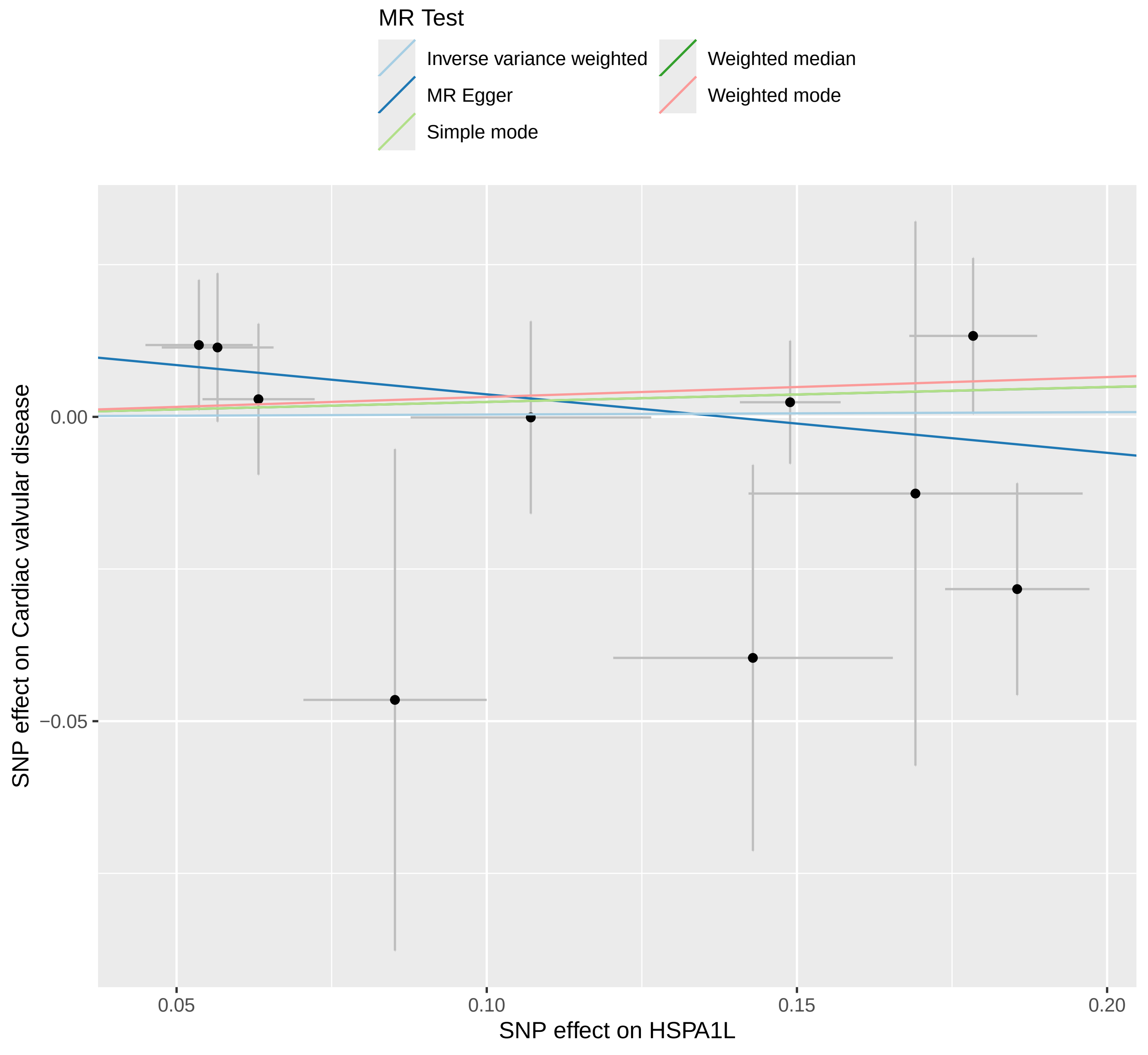

MR Test
Inverse variance weighted
MR Egger
Simple mode
Weighted median
Weighted mode
SNP effect on Cardiac valvular disease
0.00
−0.05
0.05
0.10
0.15
0.20
SNP effect on HSPA1L

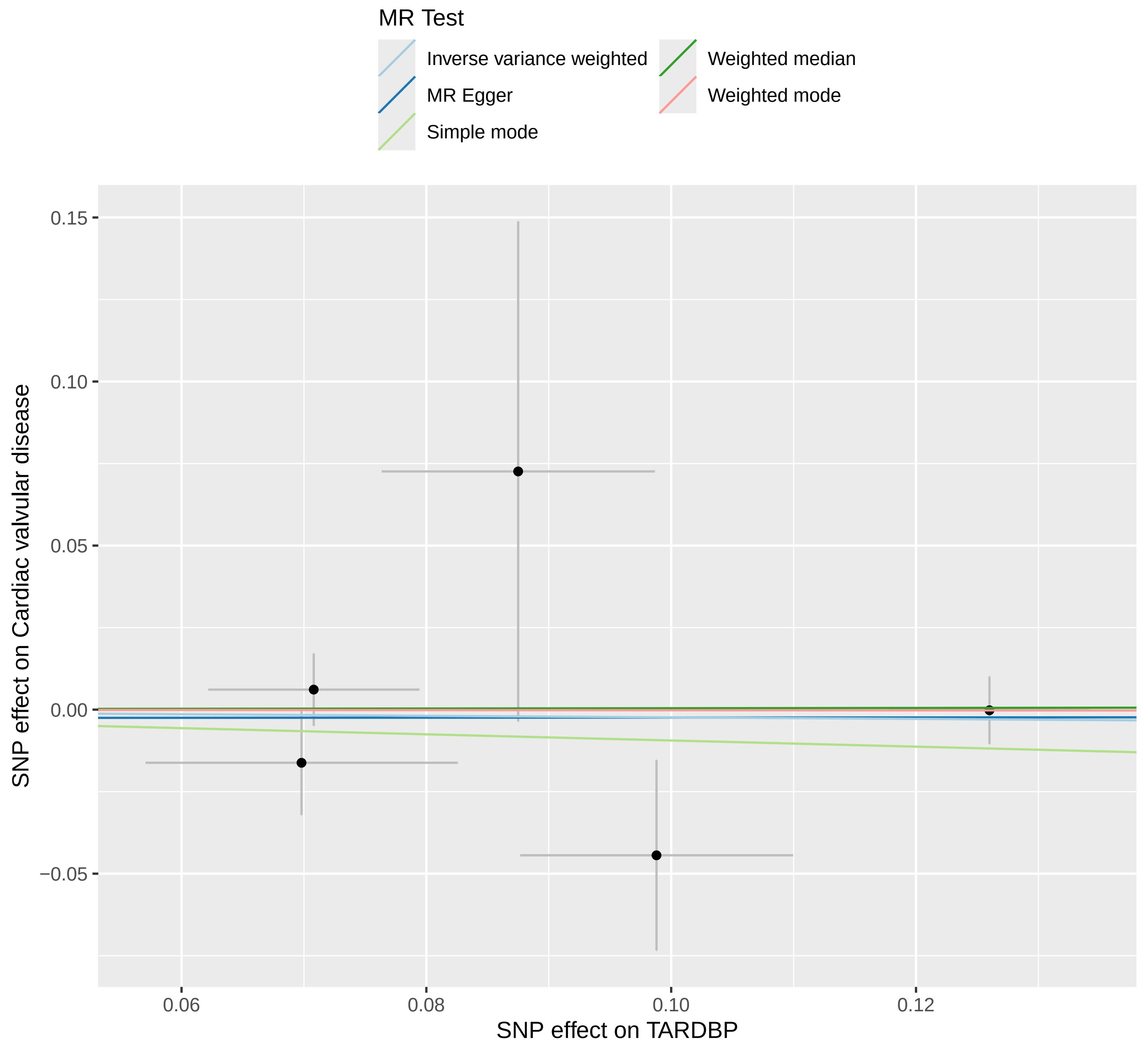

MR Test
Inverse variance weighted
MR Egger
Simple mode
Weighted median
Weighted mode
0.15
0.10
0.05
0.00
−0.05
0.06
0.08
0.10
0.12
SNP effect on Cardiac valvular disease
SNP effect on TARDBP

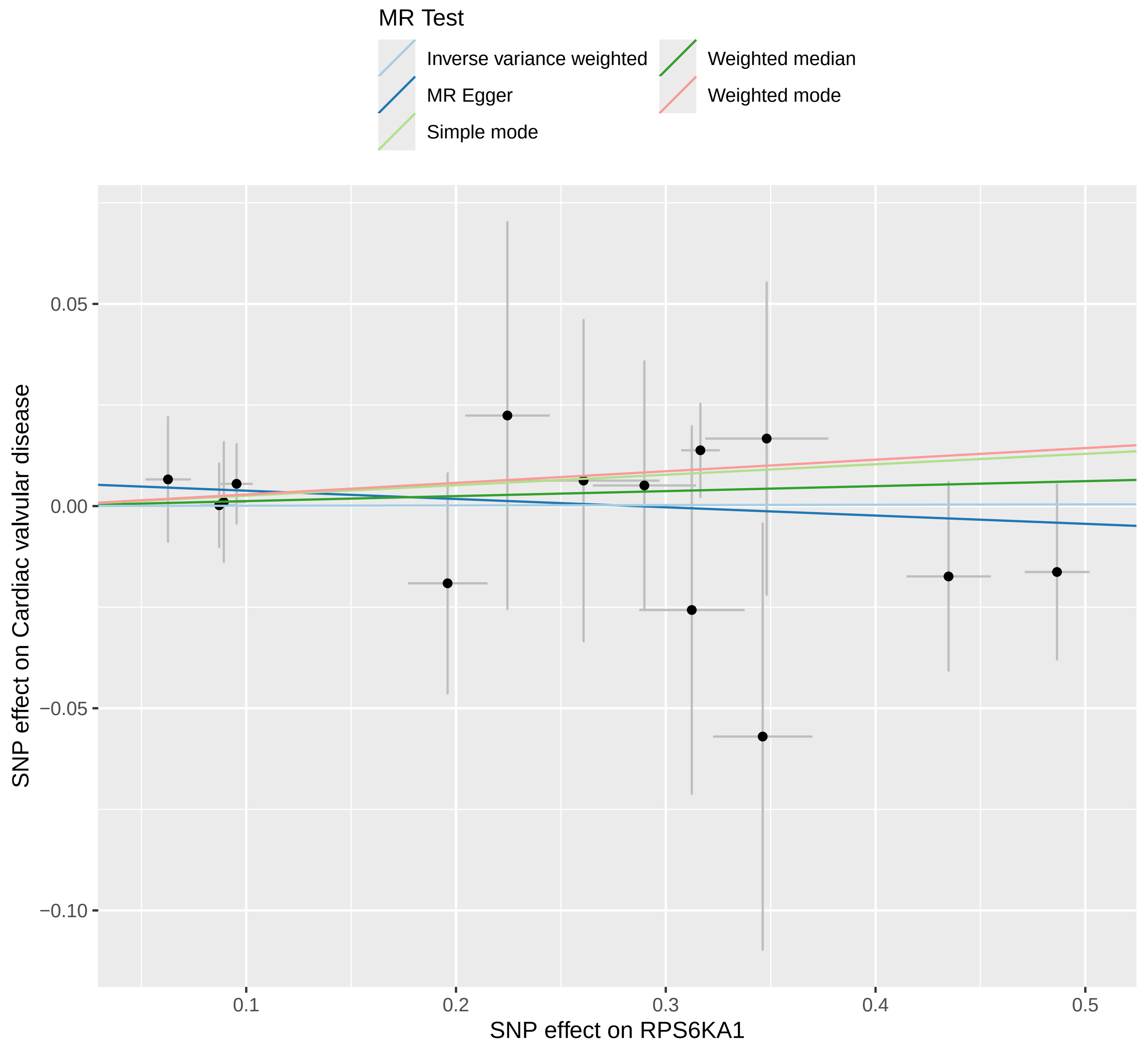

MR Test
Inverse variance weighted
MR Egger
Simple mode
Weighted median
Weighted mode
0.05
0.00
−0.05
−0.10
0.1
0.2
0.3
0.4
0.5
SNP effect on Cardiac valvular disease
SNP effect on RPS6KA1

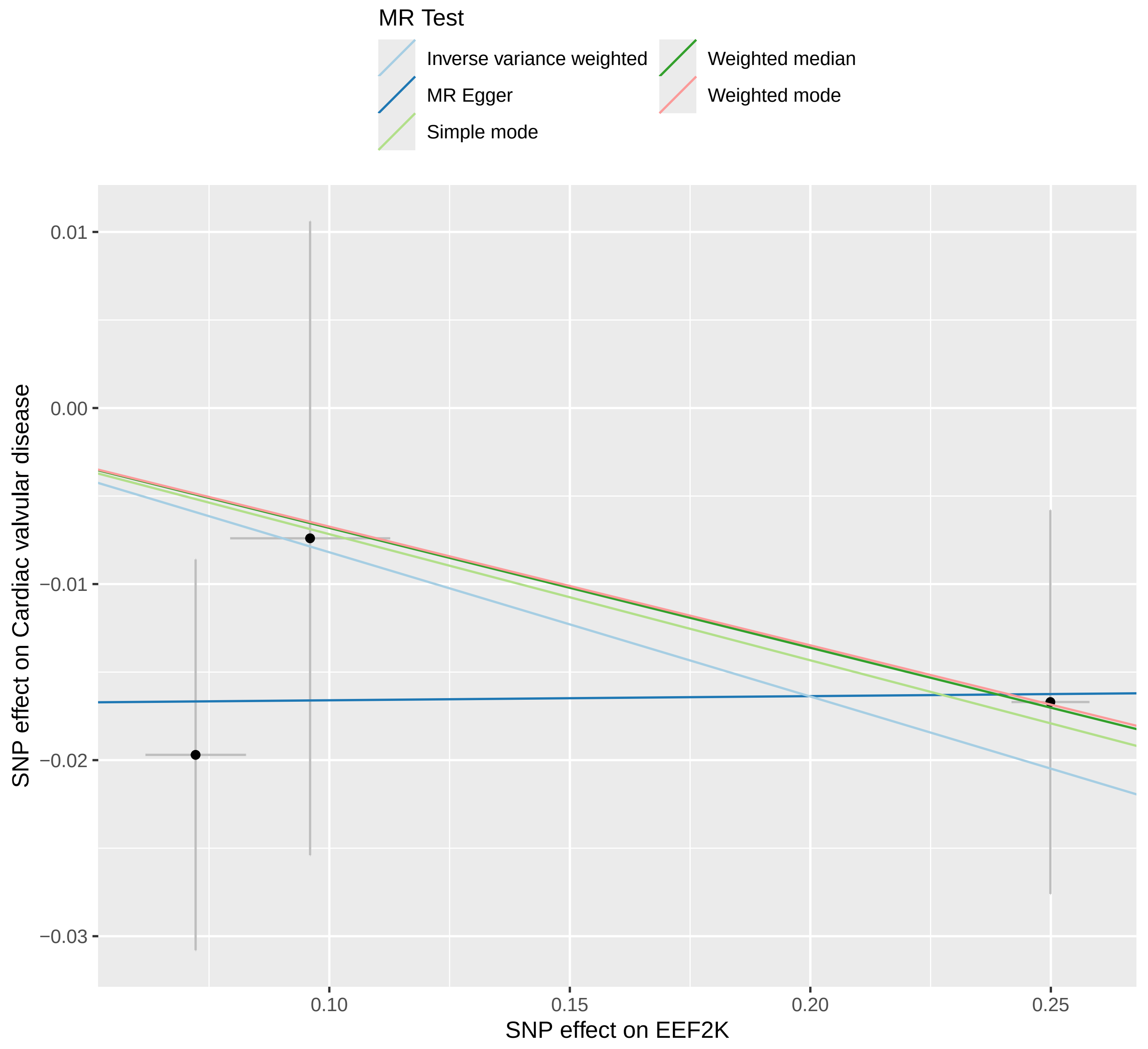
MR Test
Inverse variance weighted
MR Egger
Simple mode
Weighted median
Weighted mode
0.01
0.00
−0.01
−0.02
−0.03
SNP effect on Cardiac valvular disease
0.10
0.15
0.20
0.25
SNP effect on EEF2K

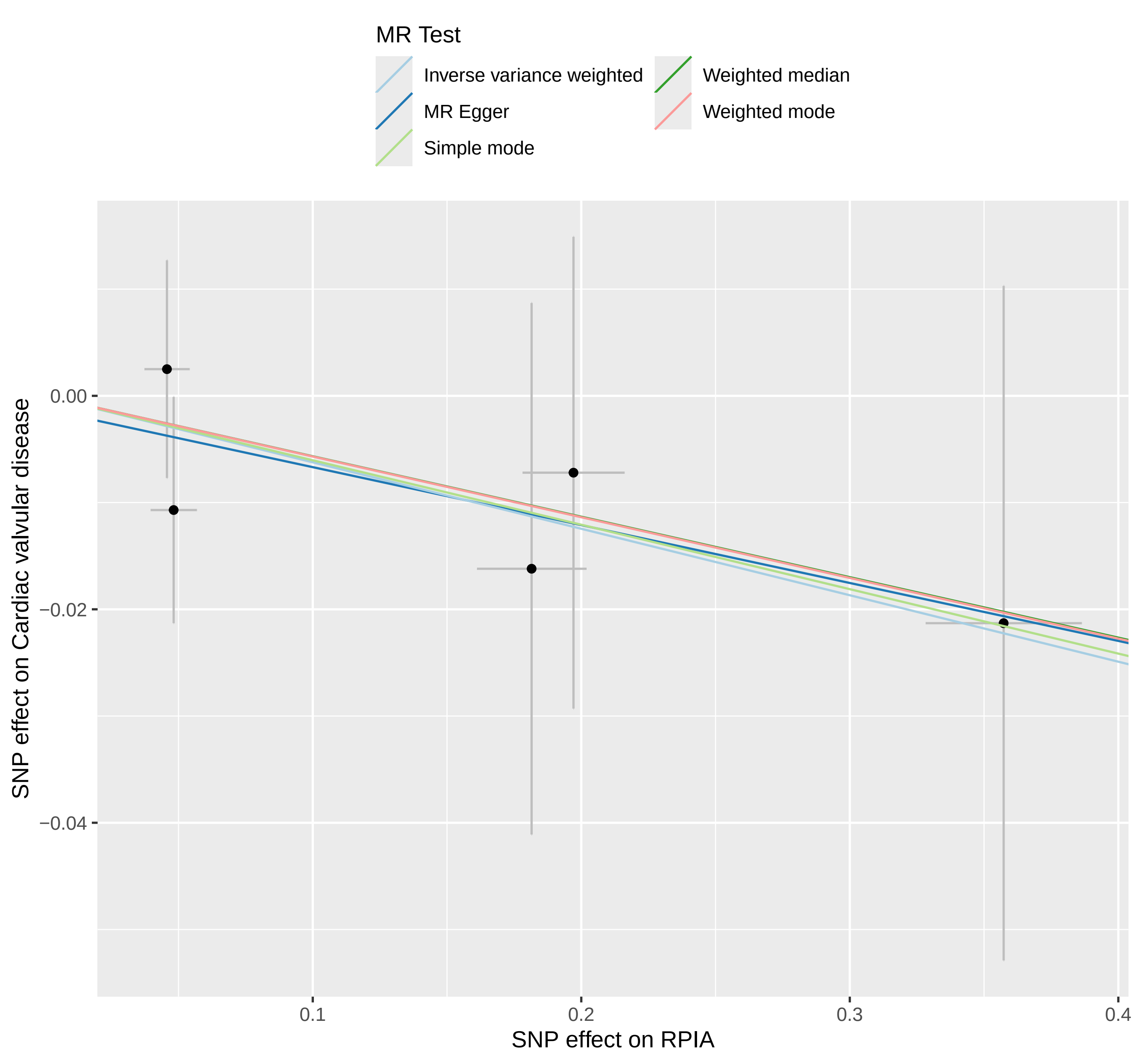

MR Test
Inverse variance weighted
MR Egger
Simple mode
Weighted median
Weighted mode
0.00
−0.02
−0.04
SNP effect on Cardiac valvular disease
0.1
0.2
0.3
0.4
SNP effect on RPIA

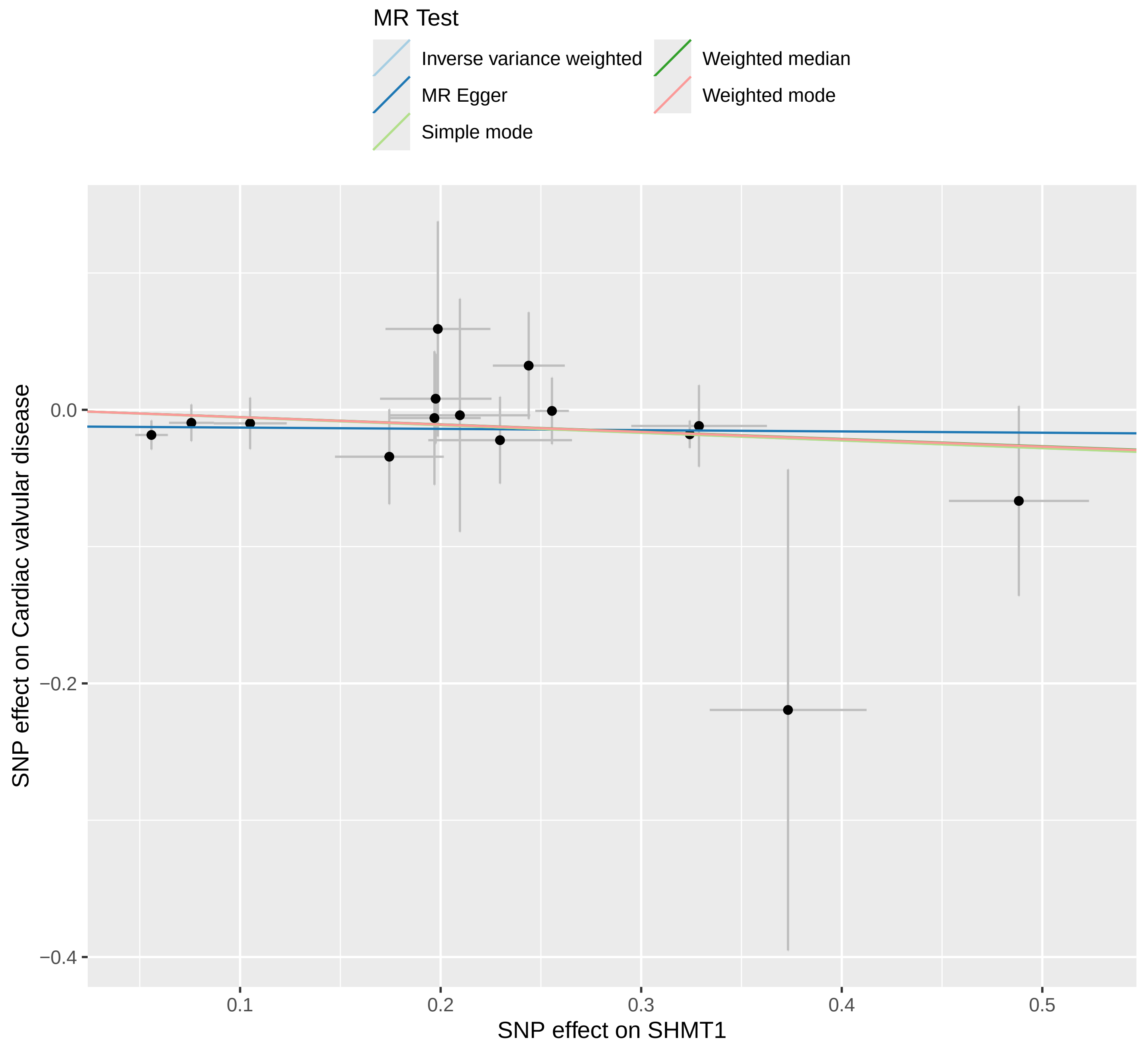

MR Test
Inverse variance weighted
MR Egger
Simple mode
Weighted median
Weighted mode
SNP effect on Cardiac valvular disease
0.0
−0.2
−0.4
0.1
0.2
0.3
0.4
0.5
SNP effect on SHMT1

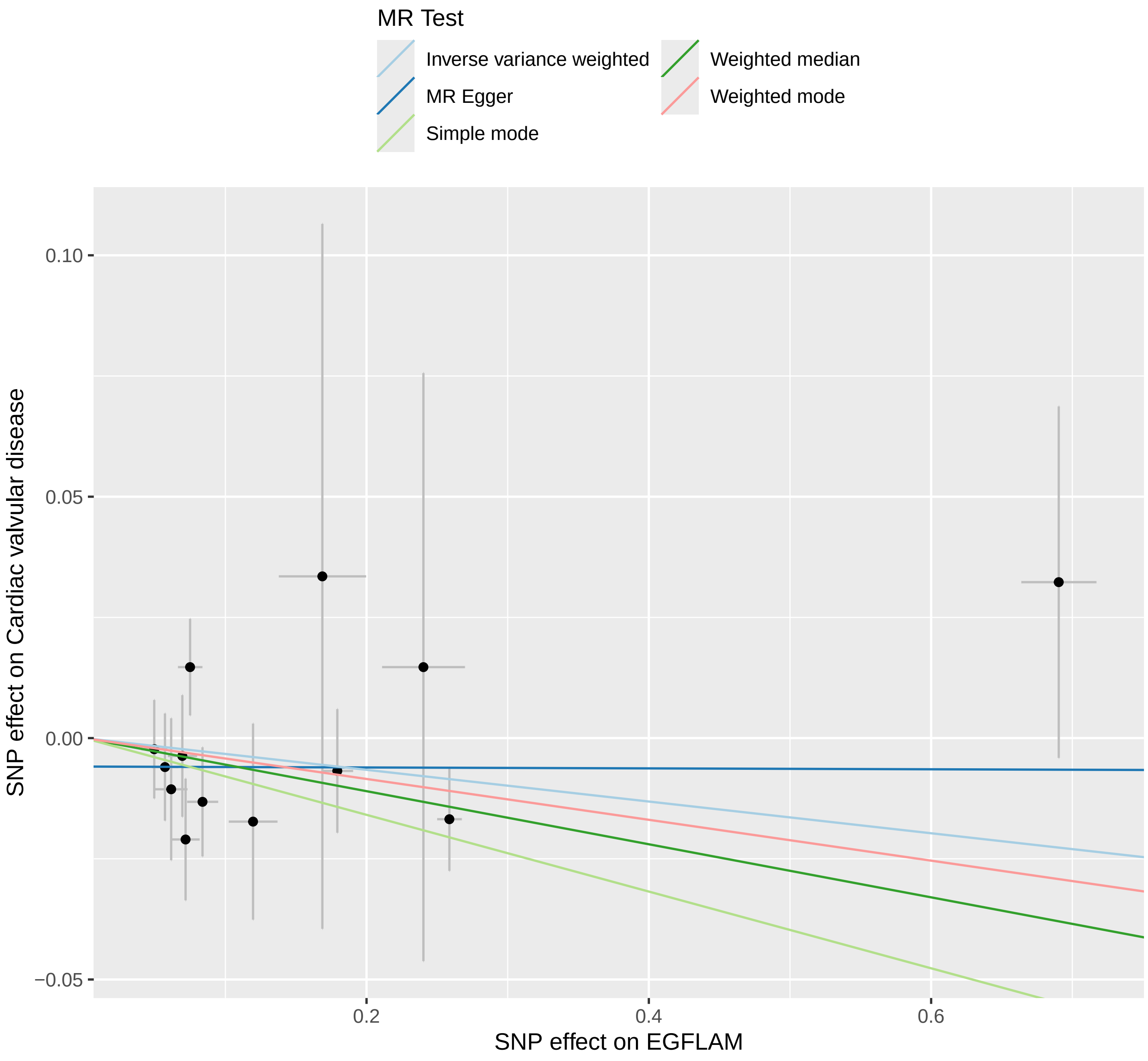
MR Test
Inverse variance weighted
MR Egger
Simple mode
Weighted median
Weighted mode
SNP effect on Cardiac valvular disease
0.10
0.05
0.00
−0.05
0.2
0.4
0.6
SNP effect on EGFLAM

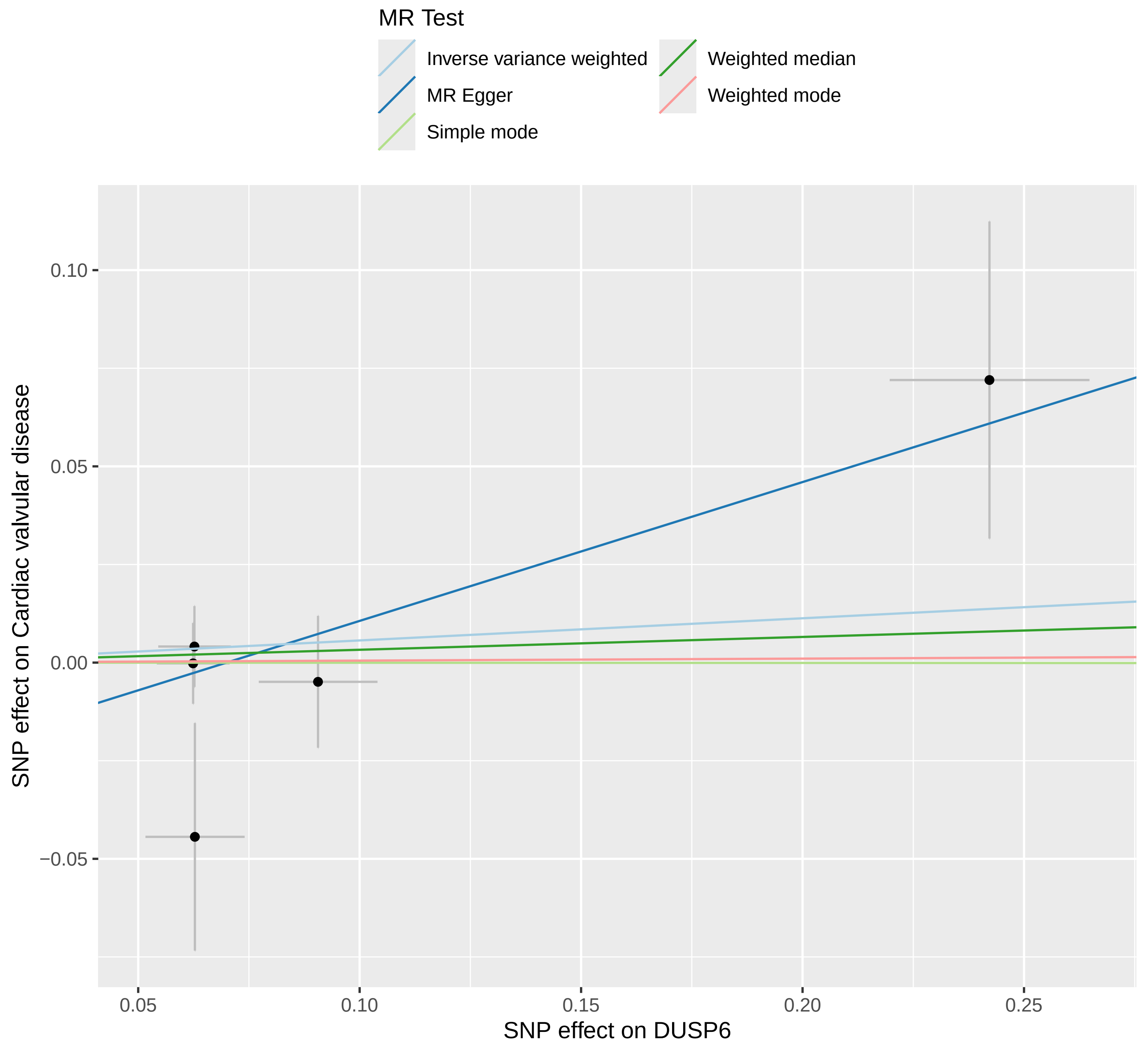
MR Test
Inverse variance weighted
MR Egger
Simple mode
Weighted median
Weighted mode
0.10
0.05
0.00
−0.05
0.05
0.10
0.15
0.20
0.25
SNP effect on Cardiac valvular disease
SNP effect on DUSP6

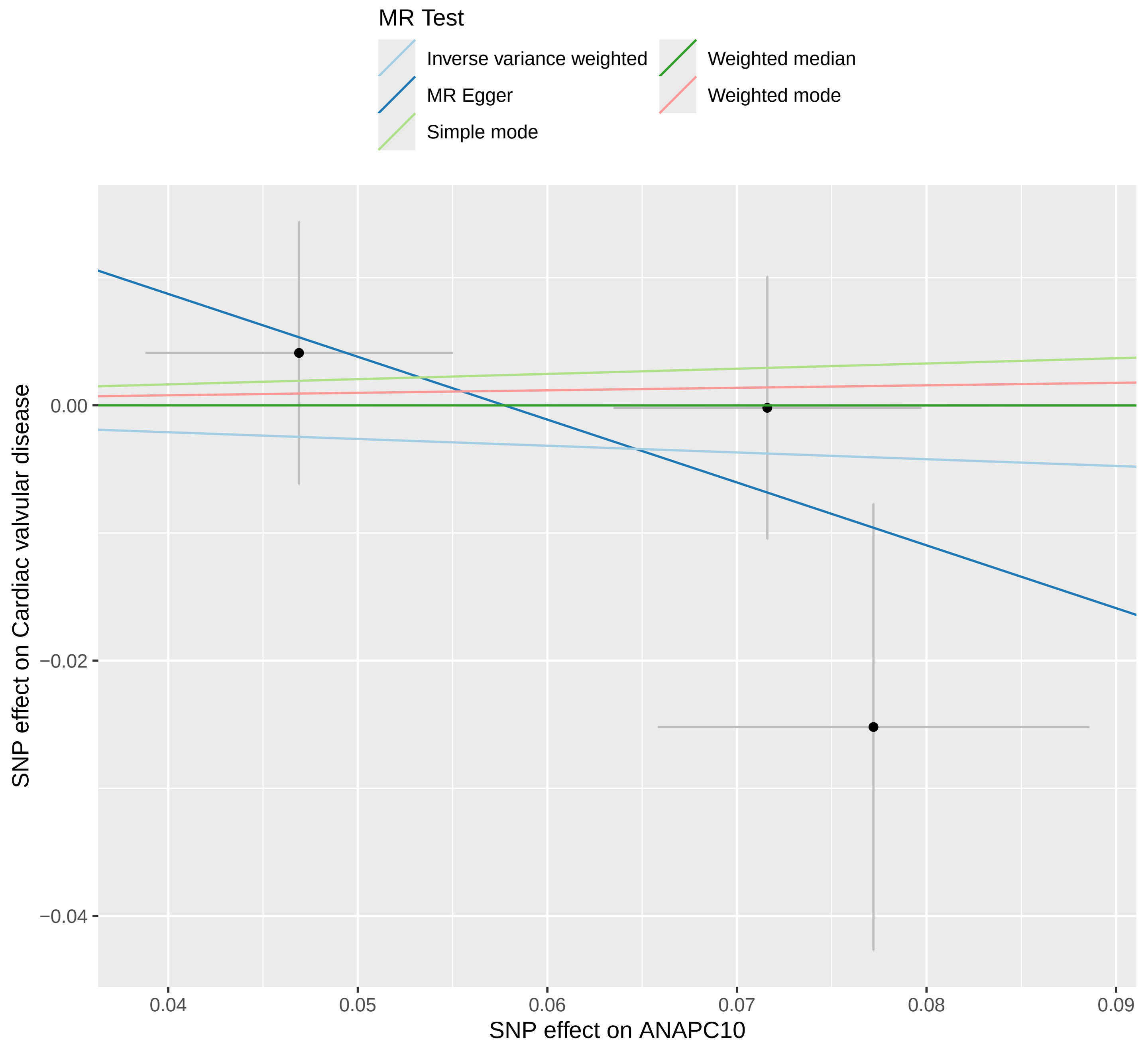
MR Test
Inverse variance weighted
MR Egger
Simple mode
Weighted median
Weighted mode
SNP effect on Cardiac valvular disease
0.00
−0.02
−0.04
0.04
0.05
0.06
0.07
0.08
0.09
SNP effect on ANAPC10

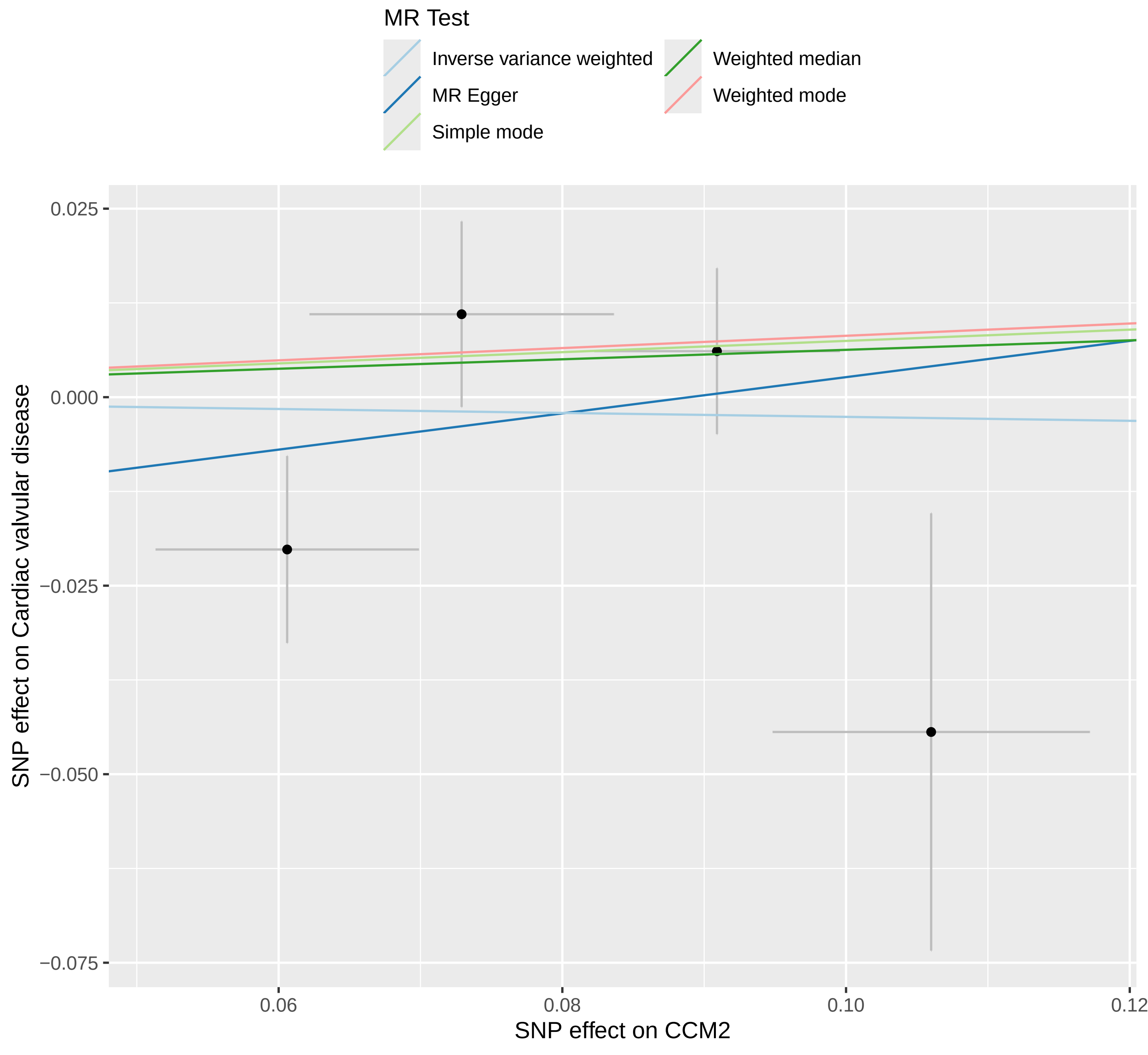
MR Test
Inverse variance weighted
MR Egger
Simple mode
Weighted median
Weighted mode
0.025
0.000
−0.025
−0.050
−0.075
SNP effect on Cardiac valvular disease
0.06
0.08
0.10
0.12
SNP effect on CCM2

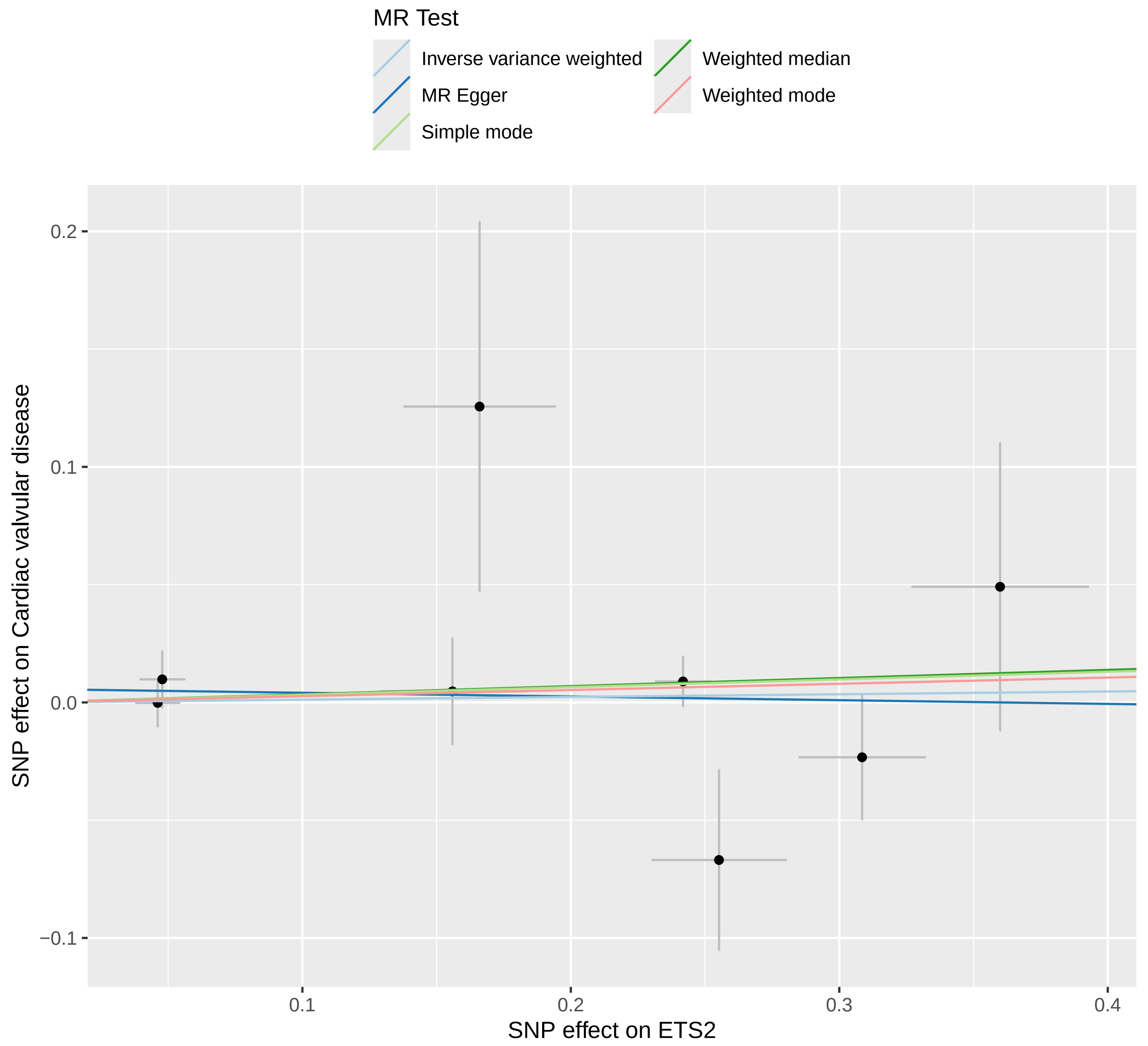
MR Test
Inverse variance weighted
MR Egger
Simple mode
Weighted median
Weighted mode
0.2
0.1
0.0
−0.1
0.1
0.2
0.3
0.4
SNP effect on Cardiac valvular disease
SNP effect on ETS2

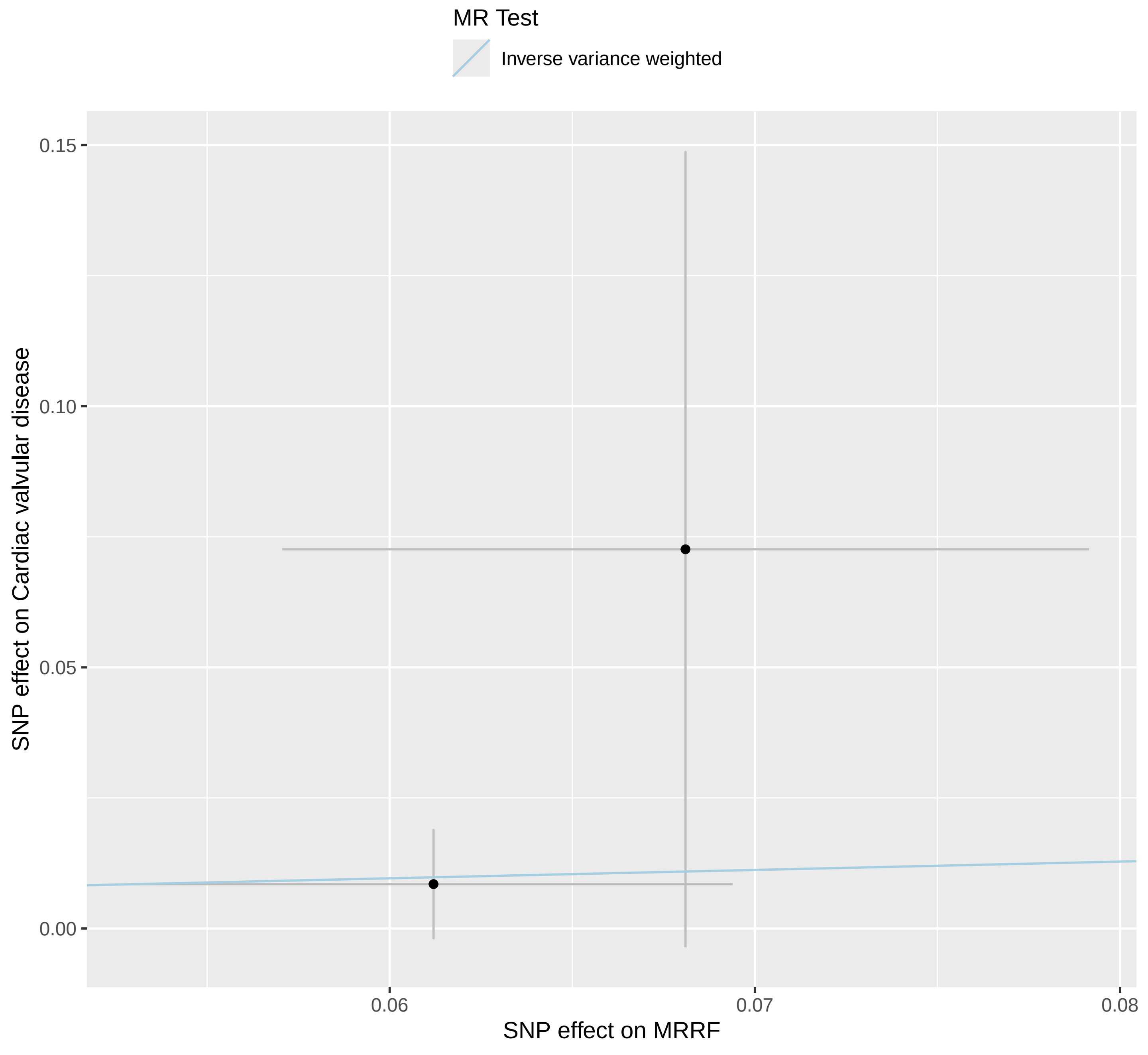

MR Test
Inverse variance weighted
SNP effect on Cardiac valvular disease
0.15
0.10
0.05
0.00
0.06
0.07
0.08
SNP effect on MRRF

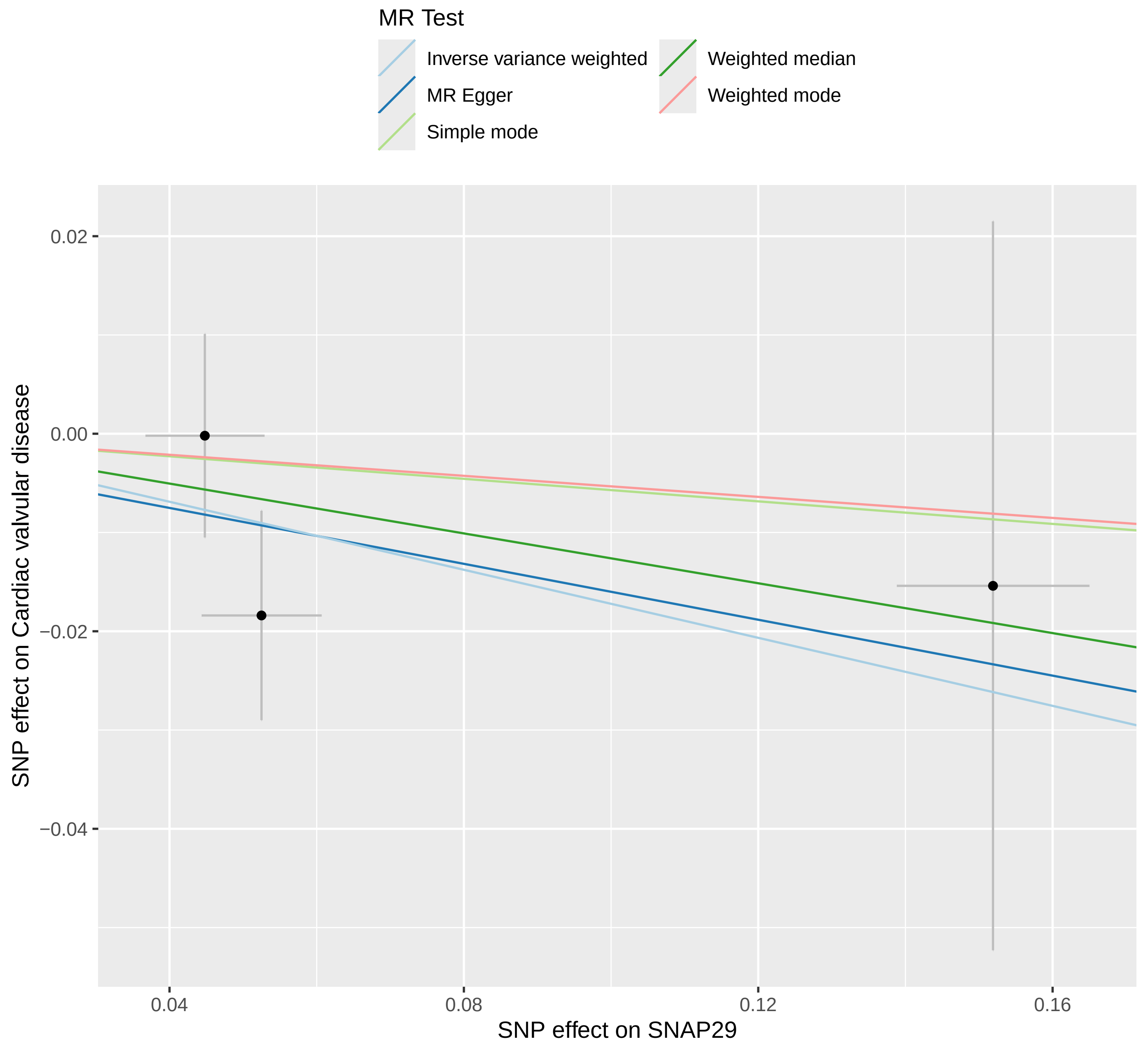
MR Test
Inverse variance weighted
MR Egger
Simple mode
Weighted median
Weighted mode
0.02
0.00
−0.02
−0.04
SNP effect on Cardiac valvular disease
0.04
0.08
0.12
0.16
SNP effect on SNAP29

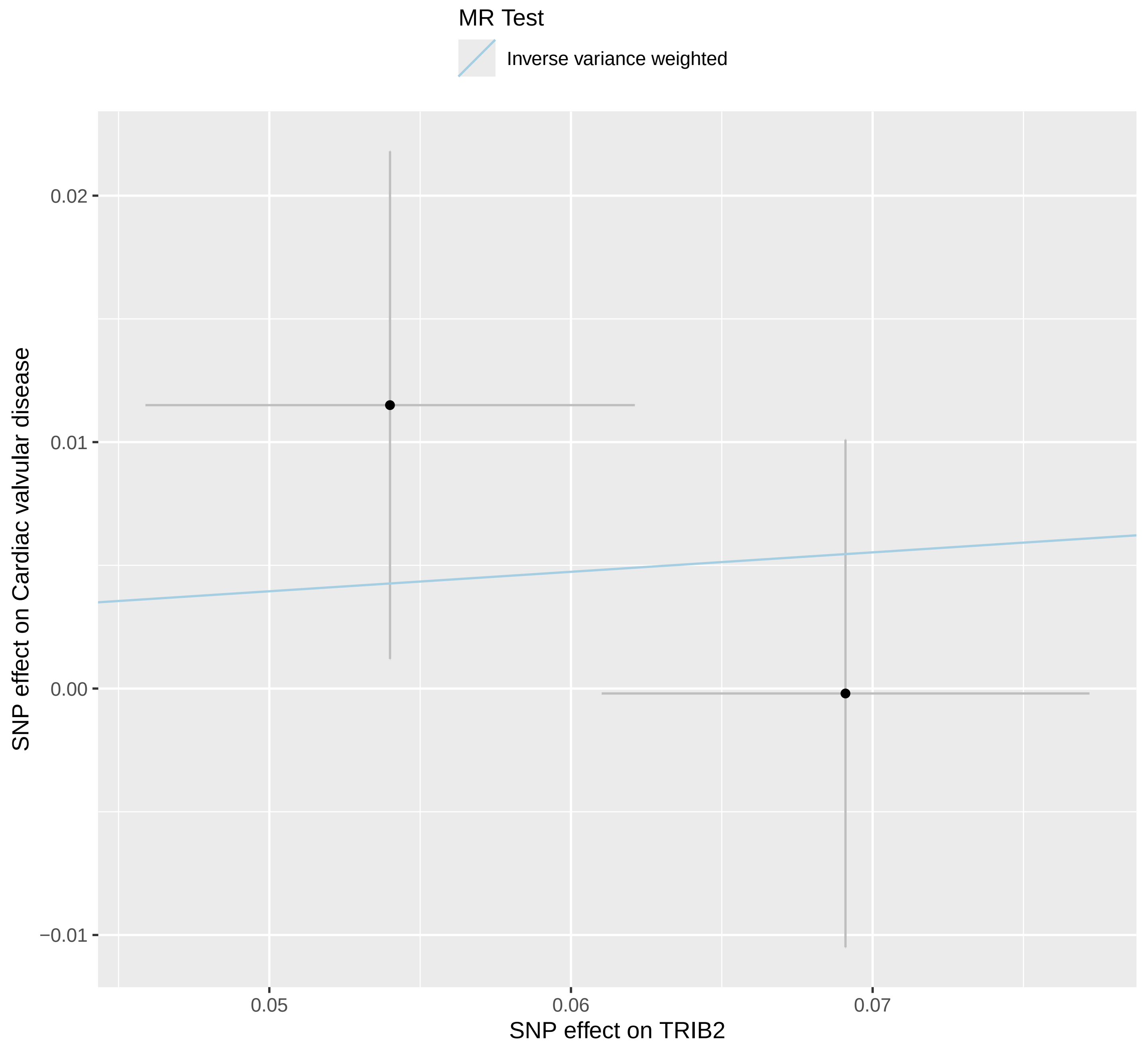
MR Test
Inverse variance weighted
0.02
0.01
0.00
−0.01
0.05
0.06
0.07
SNP effect on Cardiac valvular disease
SNP effect on TRIB2

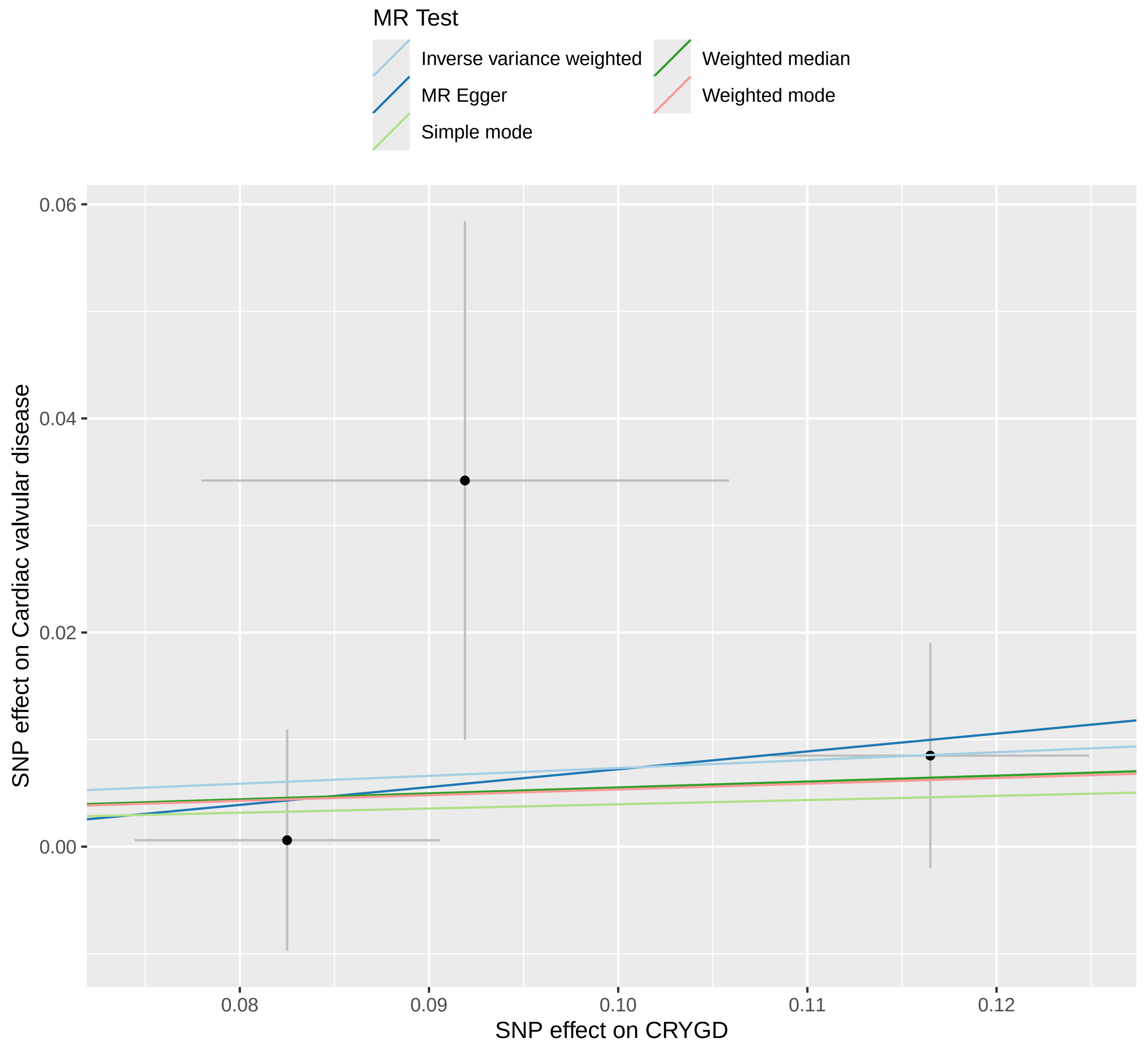
MR Test
Inverse variance weighted
MR Egger
Simple mode
Weighted median
Weighted mode
0.06
0.04
0.02
0.00
SNP effect on Cardiac valvular disease
0.08
0.09
0.10
0.11
0.12
SNP effect on CRYGD

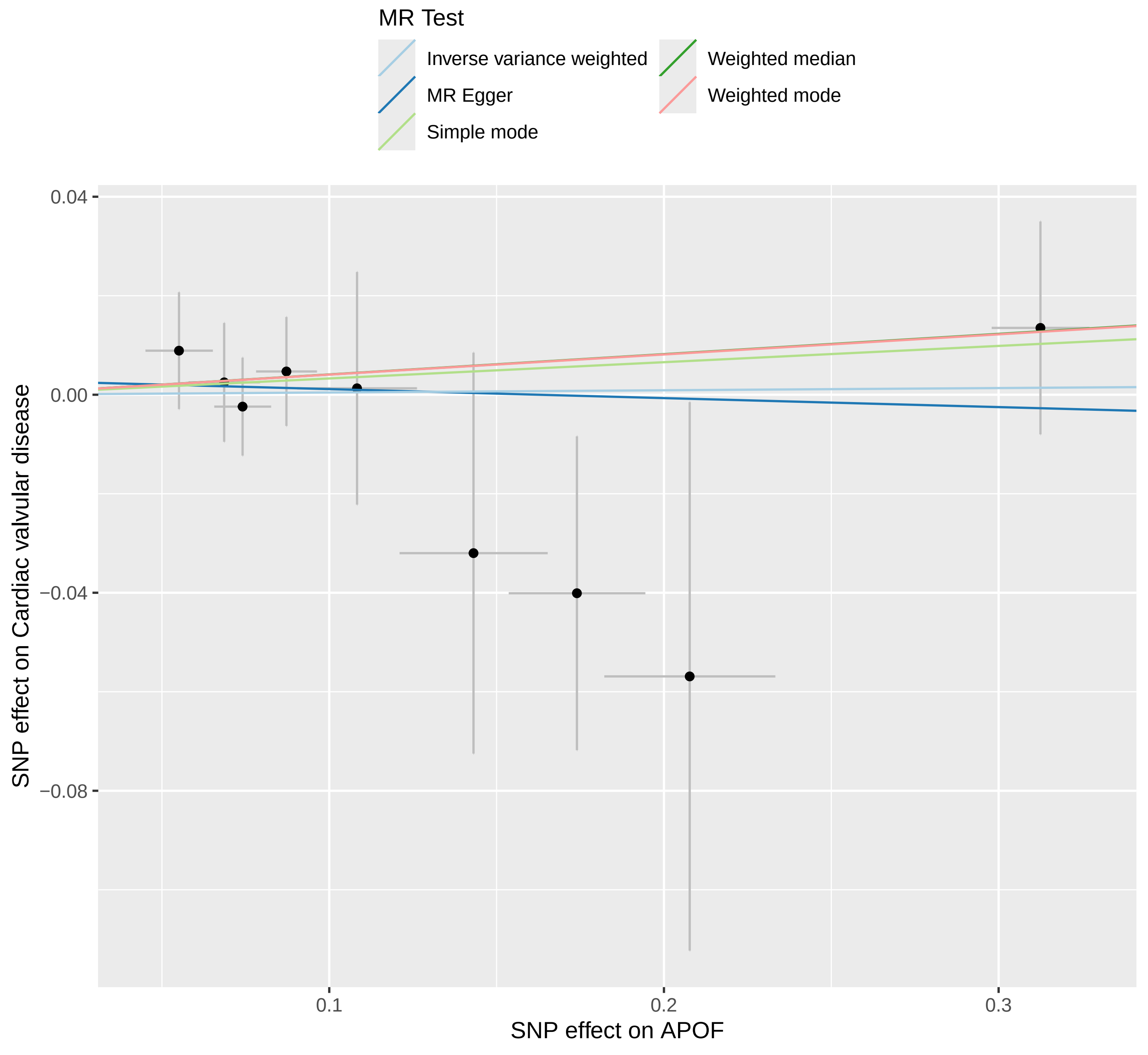
MR Test
Inverse variance weighted
MR Egger
Simple mode
Weighted median
Weighted mode
0.04
0.00
−0.04
−0.08
0.1
0.2
0.3
SNP effect on Cardiac valvular disease
SNP effect on APOF

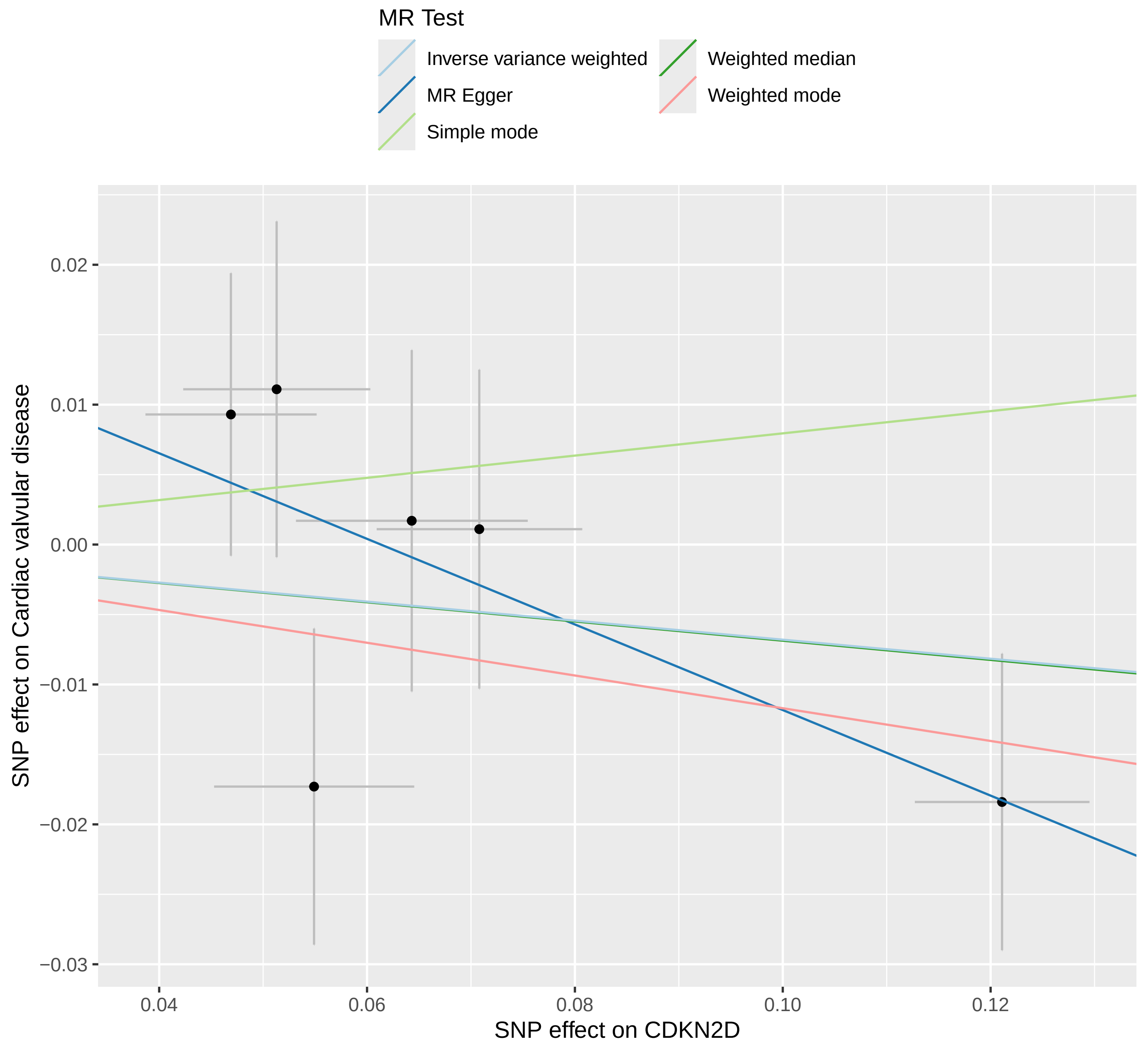

MR Test
Inverse variance weighted
MR Egger
Simple mode
Weighted median
Weighted mode
0.02
0.01
0.00
−0.01
−0.02
−0.03
0.04
0.06
0.08
0.10
0.12
SNP effect on Cardiac valvular disease
SNP effect on CDKN2D

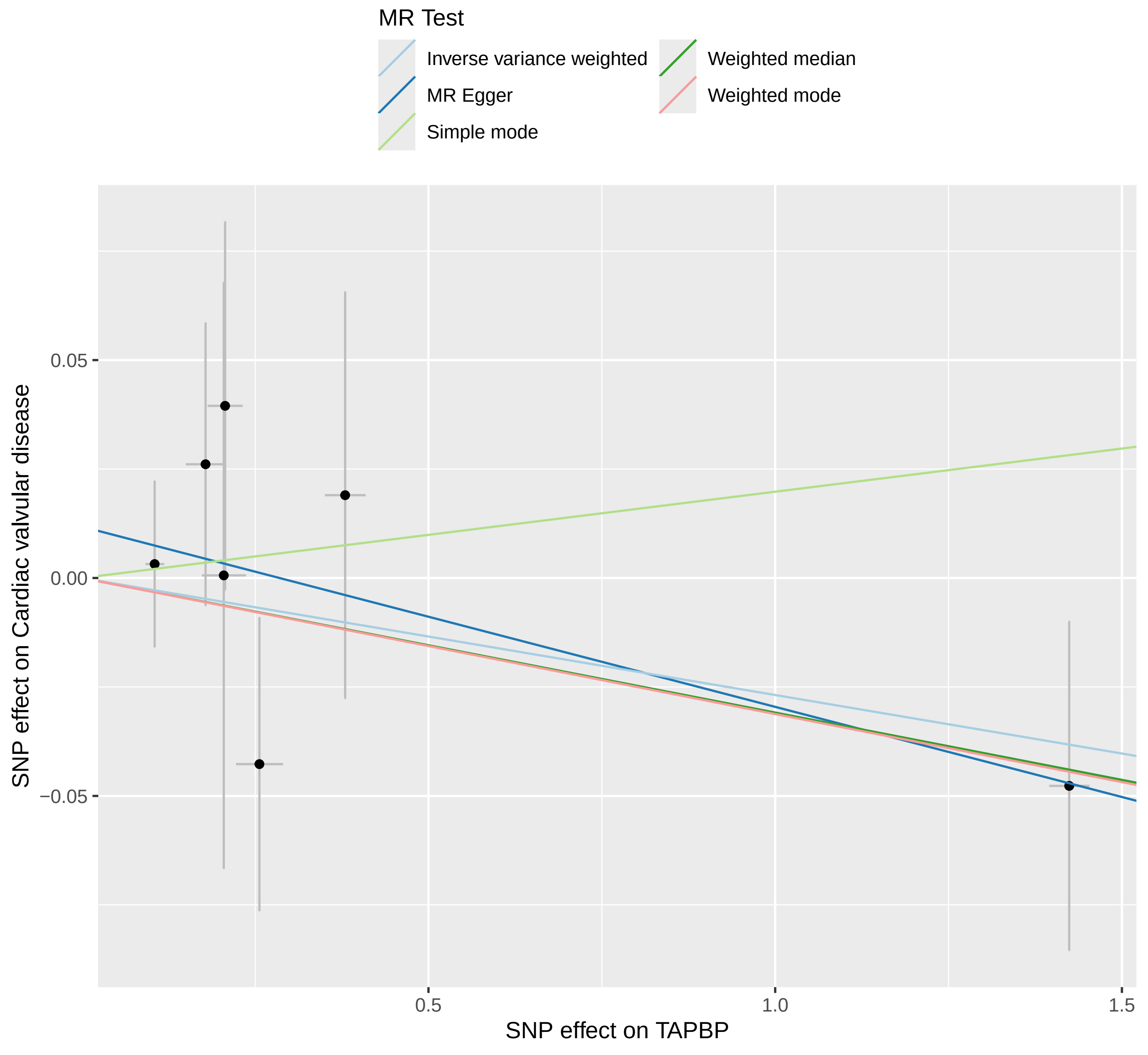

MR Test
Inverse variance weighted
MR Egger
Simple mode
Weighted median
Weighted mode
0.05
0.00
−0.05
SNP effect on Cardiac valvular disease
0.5
1.0
1.5
SNP effect on TAPBP

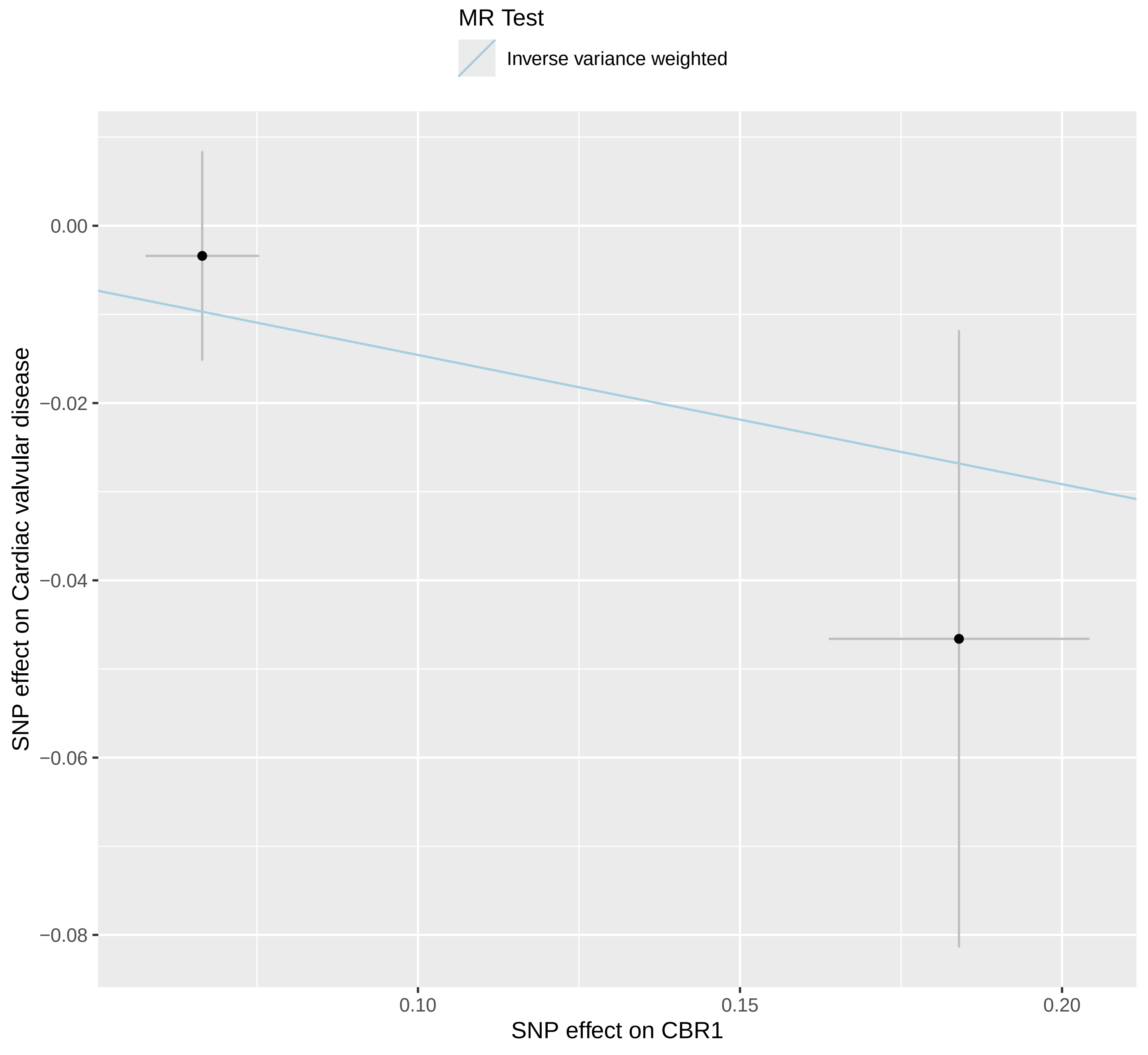

MR Test
Inverse variance weighted
0.00
−0.02
−0.04
−0.06
−0.08
0.10
0.15
0.20
SNP effect on CBR1
SNP effect on Cardiac valvular disease

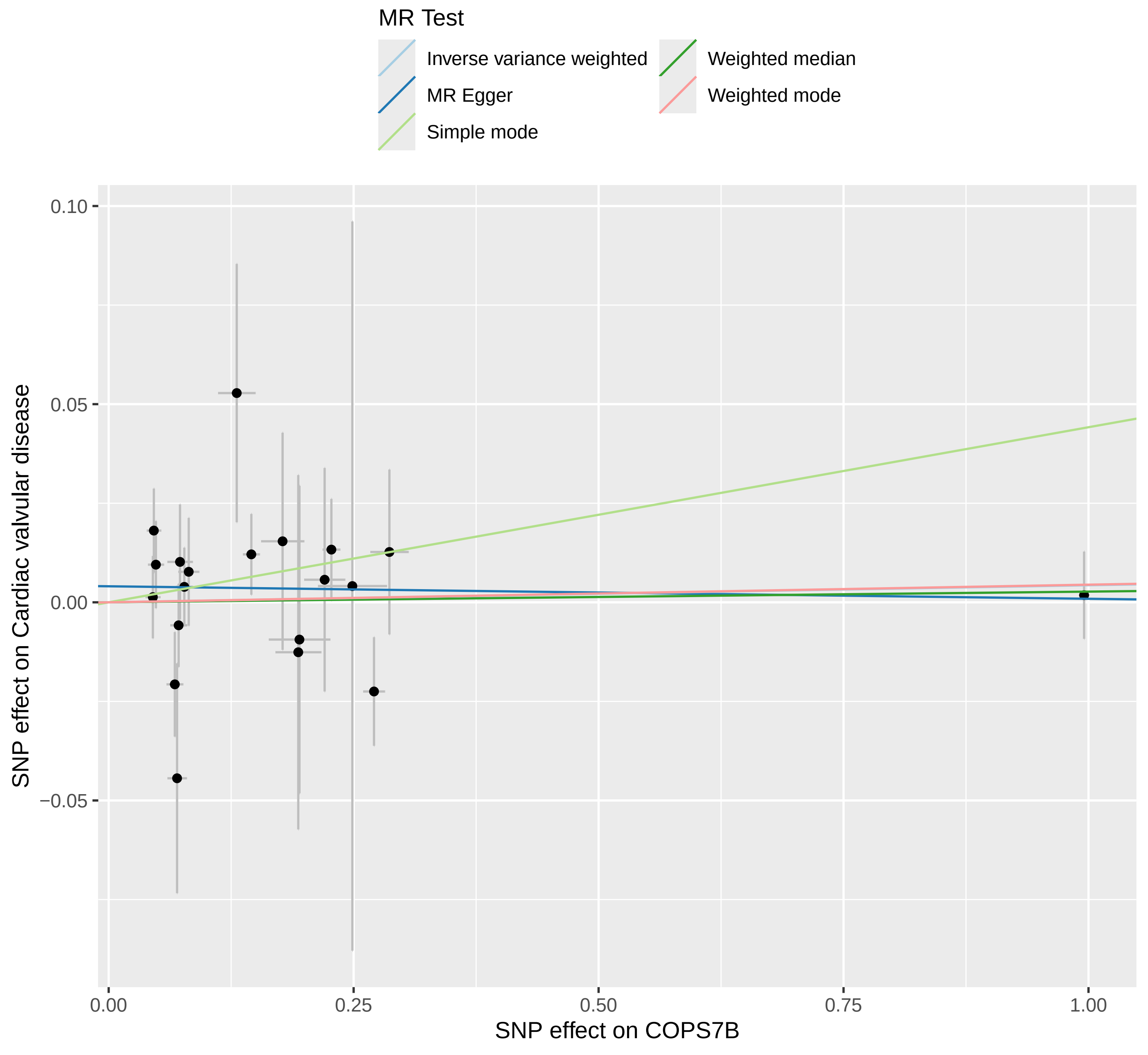

MR Test
Inverse variance weighted
MR Egger
Simple mode
Weighted median
Weighted mode
0.10
0.05
0.00
−0.05
0.00
0.25
0.50
0.75
1.00
SNP effect on Cardiac valvular disease
SNP effect on COPS7B

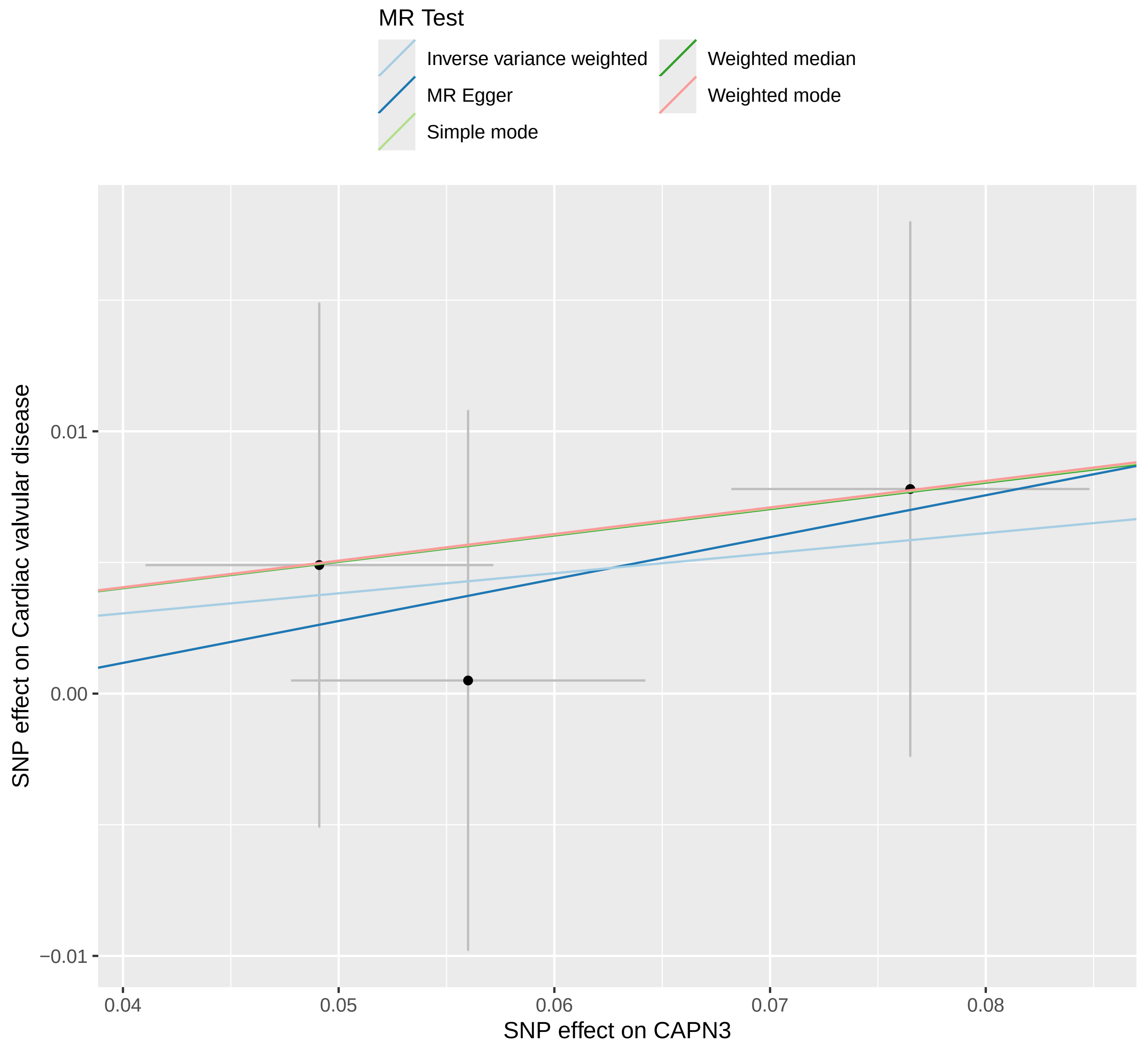

MR Test
Inverse variance weighted
MR Egger
Simple mode
Weighted median
Weighted mode
SNP effect on Cardiac valvular disease
0.01
0.00
−0.01
0.04
0.05
0.06
0.07
0.08
SNP effect on CAPN3

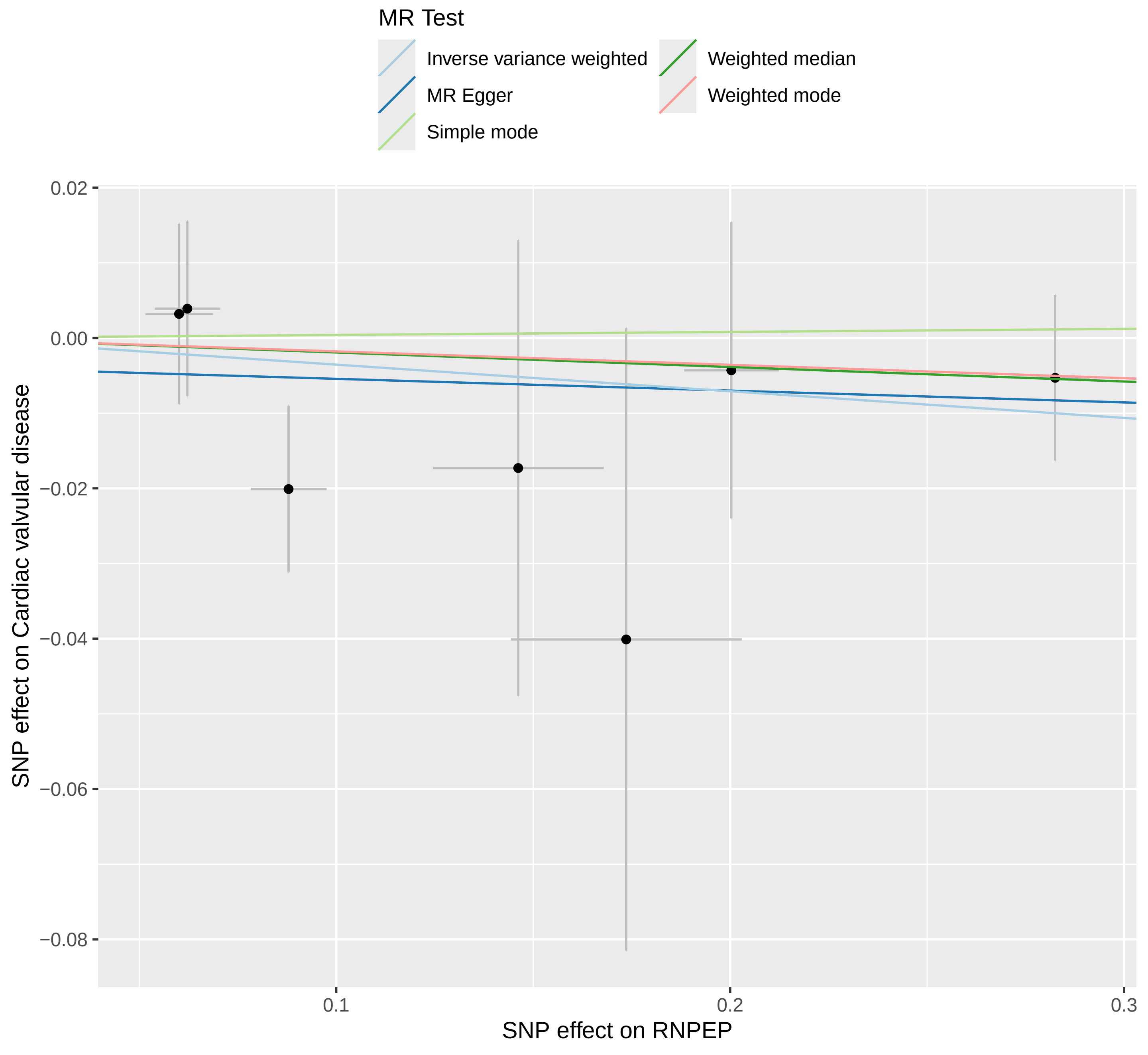
MR Test
Inverse variance weighted
MR Egger
Simple mode
Weighted median
Weighted mode
0.02
0.00
−0.02
−0.04
−0.06
−0.08
0.1
0.2
0.3
SNP effect on Cardiac valvular disease
SNP effect on RNPEP

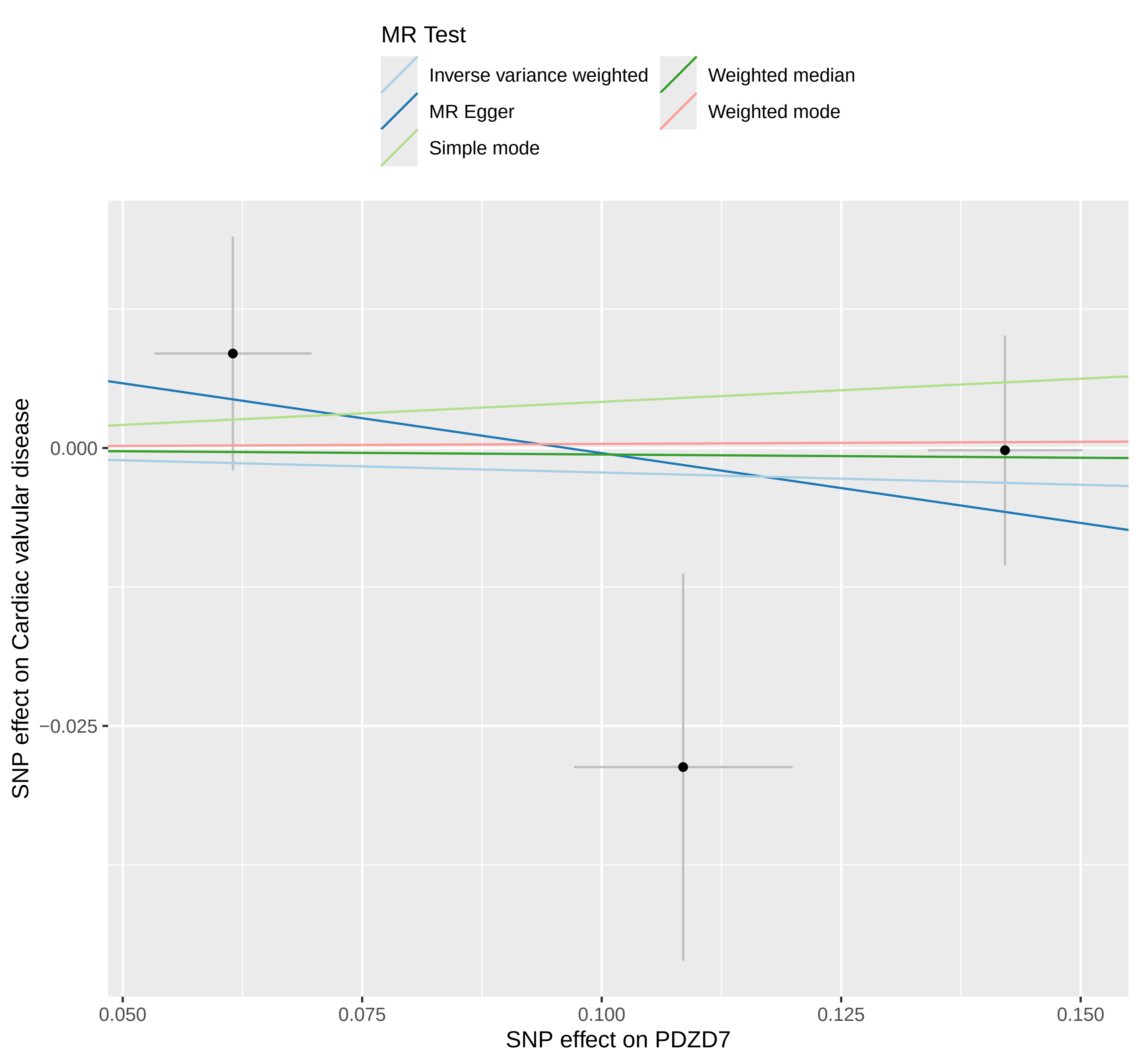
MR Test
Inverse variance weighted
MR Egger
Simple mode
Weighted median
Weighted mode
0.000
−0.025
SNP effect on Cardiac valvular disease
0.050
0.075
0.100
0.125
0.150
SNP effect on PDZD7

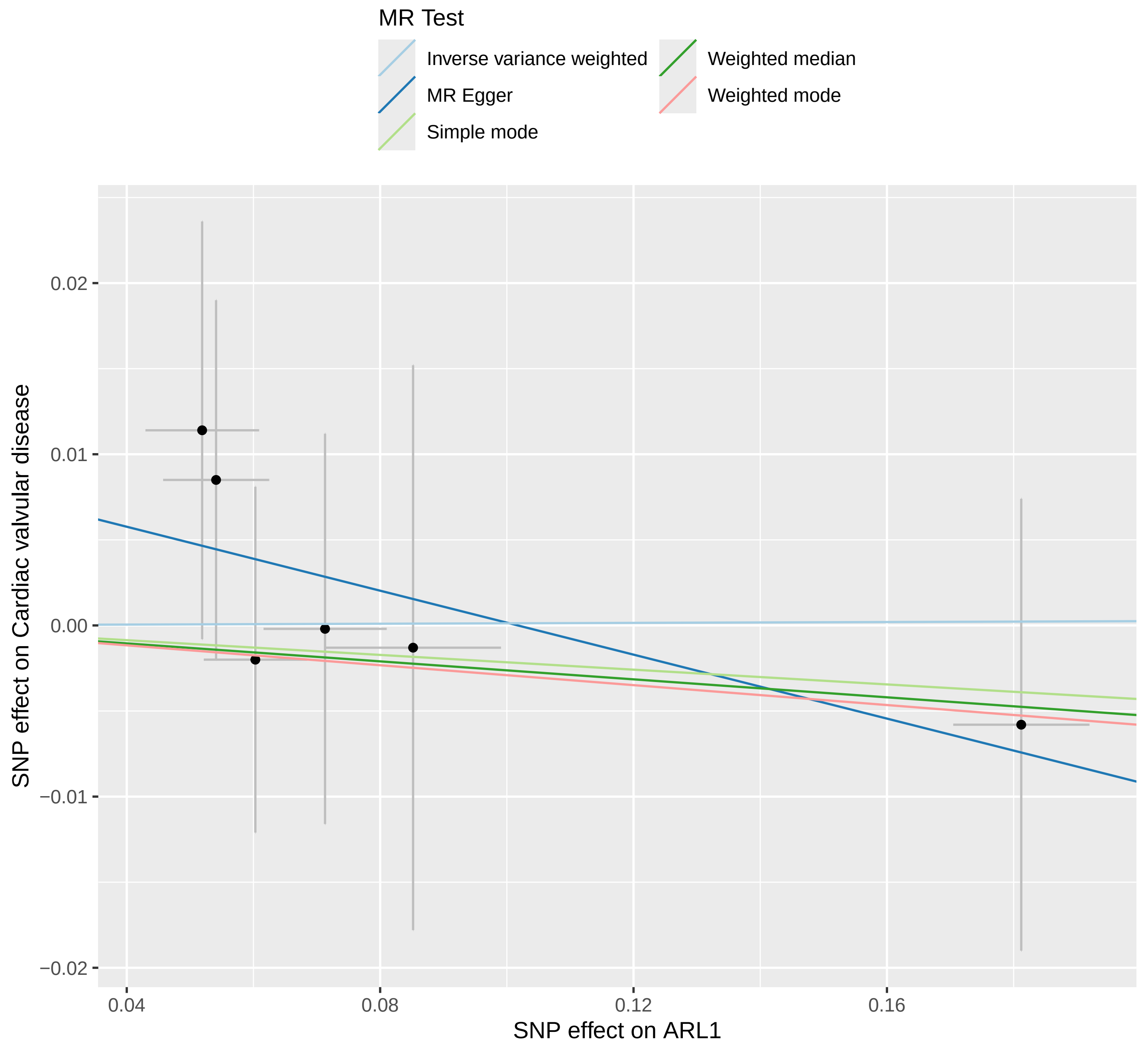
MR Test
Inverse variance weighted
MR Egger
Simple mode
Weighted median
Weighted mode
SNP effect on Cardiac valvular disease
0.02
0.01
0.00
−0.01
−0.02
0.04
0.08
0.12
0.16
SNP effect on ARL1

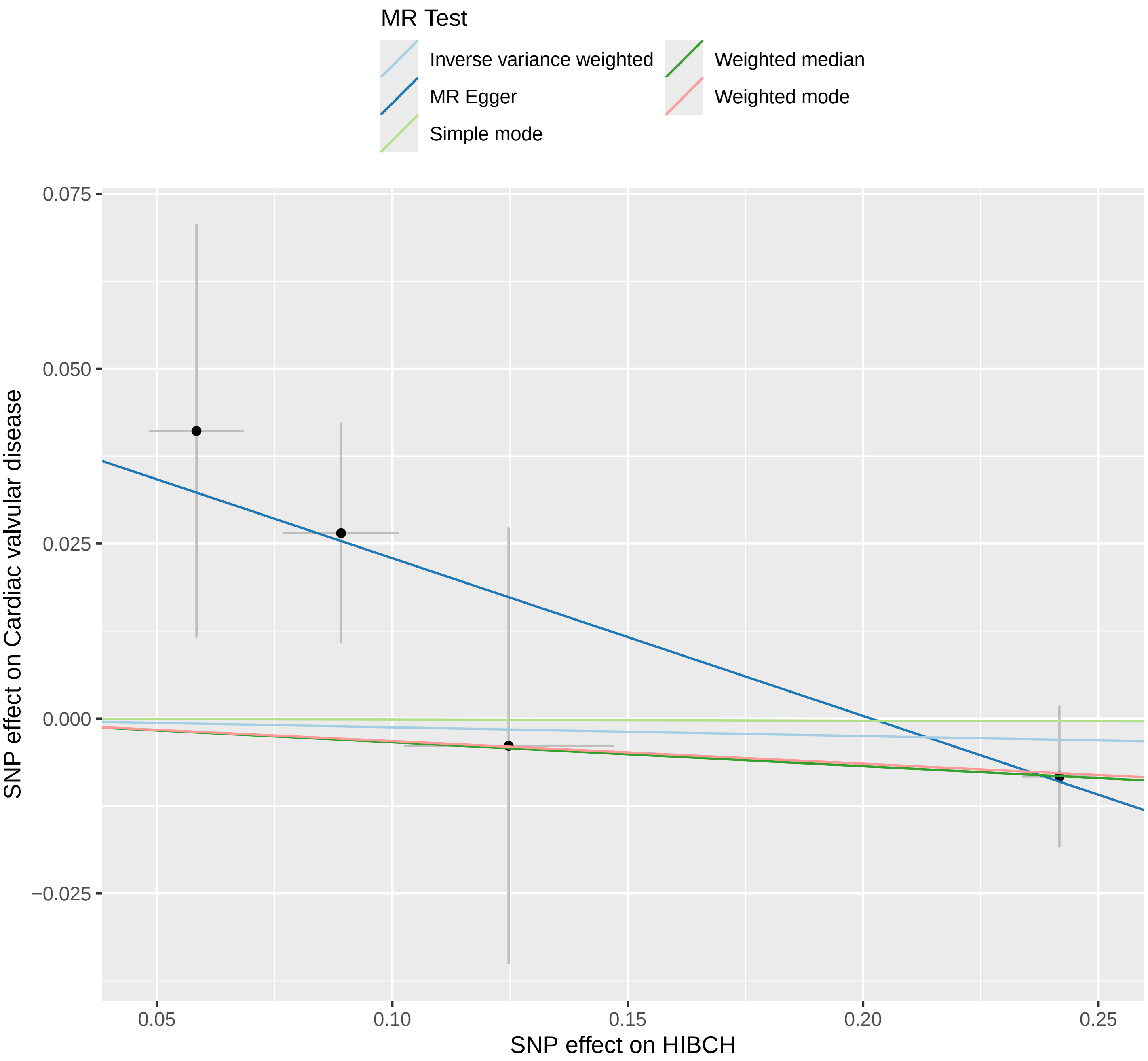
MR Test
Inverse variance weighted
MR Egger
Simple mode
Weighted median
Weighted mode
0.075
0.050
0.025
0.000
−0.025
SNP effect on Cardiac valvular disease
0.05
0.10
0.15
0.20
0.25
SNP effect on HIBCH

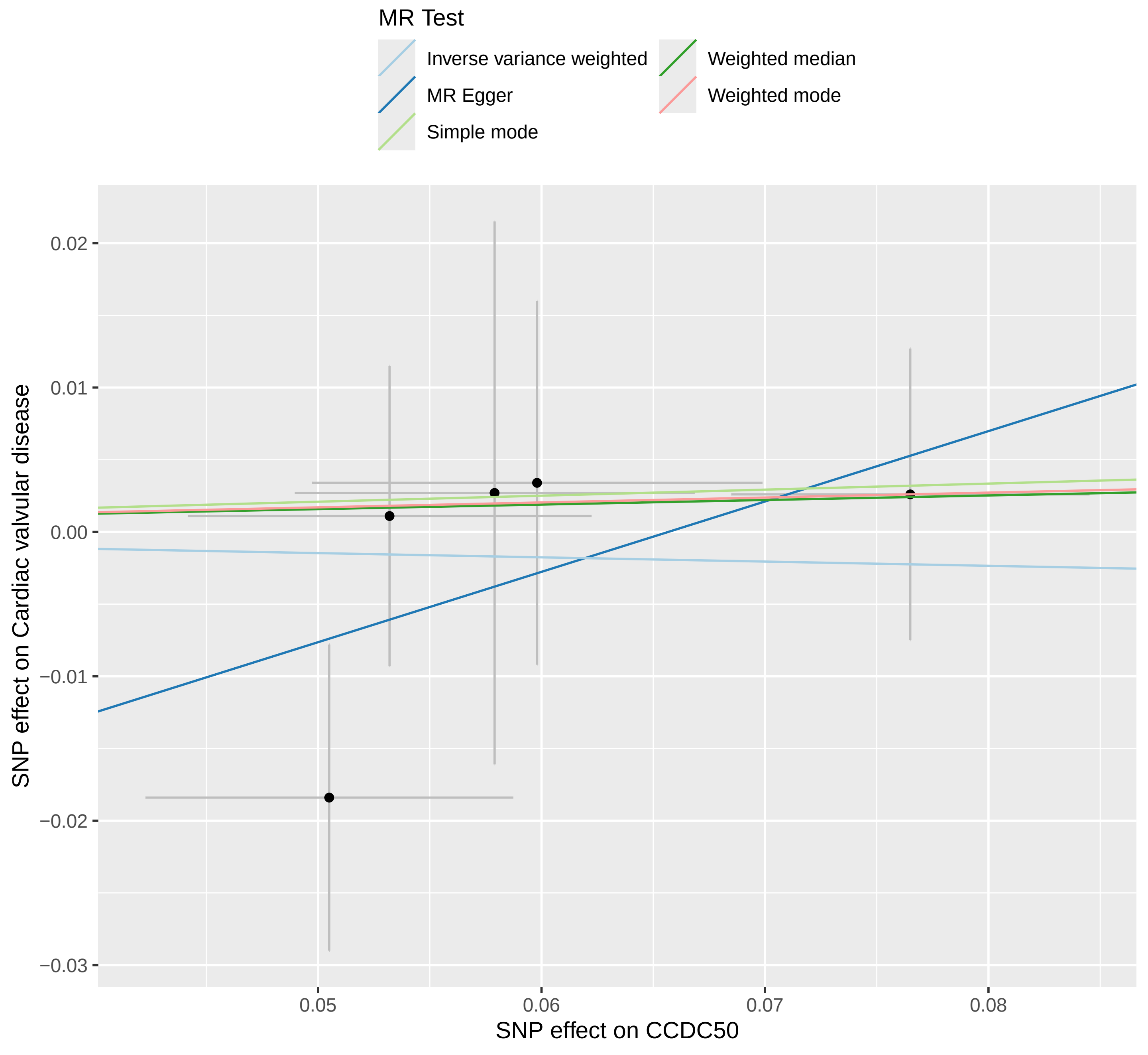

MR Test
Inverse variance weighted
MR Egger
Simple mode
Weighted median
Weighted mode
SNP effect on Cardiac valvular disease
0.02
0.01
0.00
−0.01
−0.02
−0.03
0.05
0.06
0.07
0.08
SNP effect on CCDC50

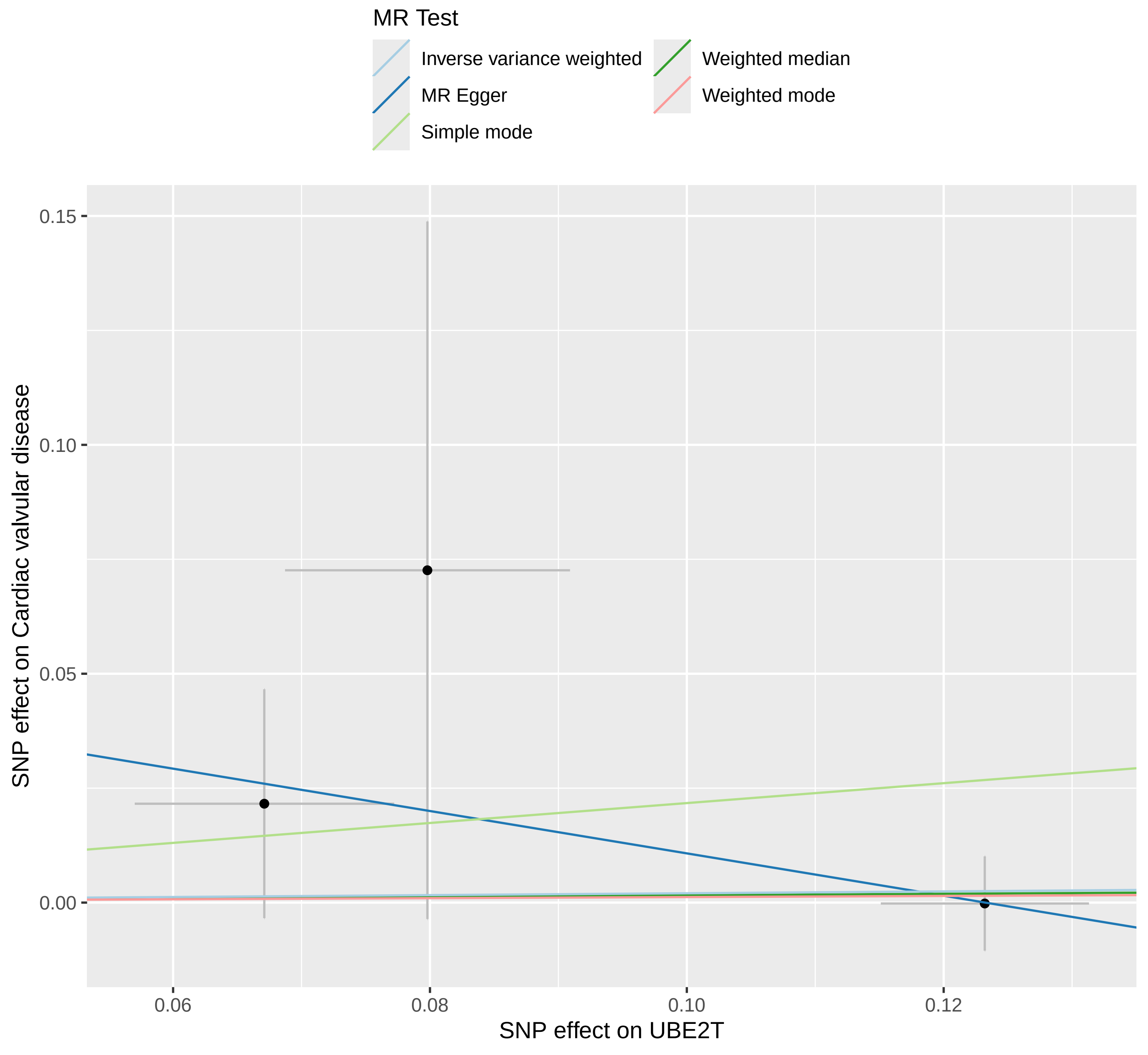

MR Test
Inverse variance weighted
MR Egger
Simple mode
Weighted median
Weighted mode
0.15
0.10
0.05
0.00
0.06
0.08
0.10
0.12
SNP effect on Cardiac valvular disease
SNP effect on UBE2T

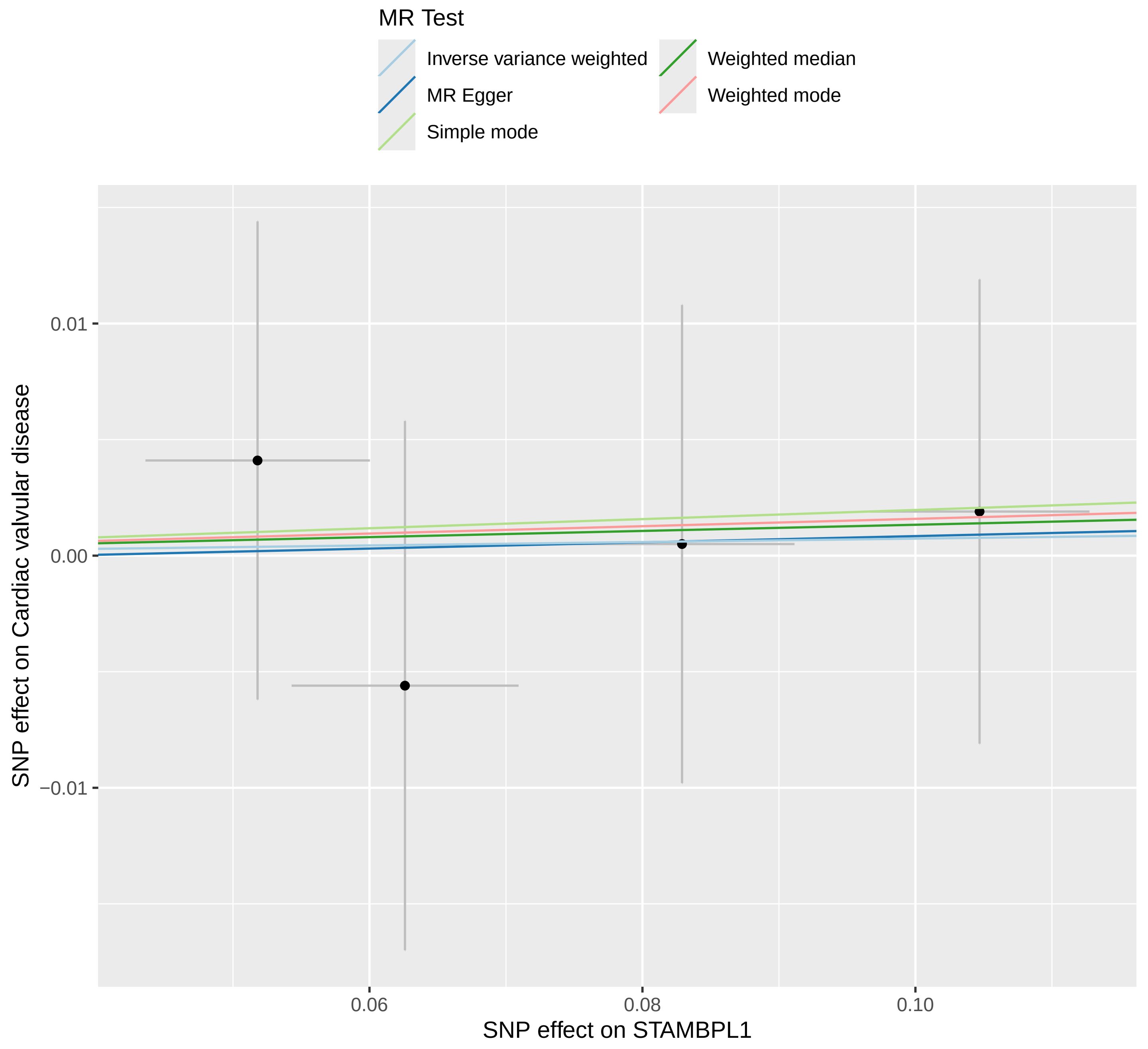

MR Test
Inverse variance weighted
MR Egger
Simple mode
Weighted median
Weighted mode
0.01
0.00
−0.01
SNP effect on Cardiac valvular disease
0.06
0.08
0.10
SNP effect on STAMBPL1

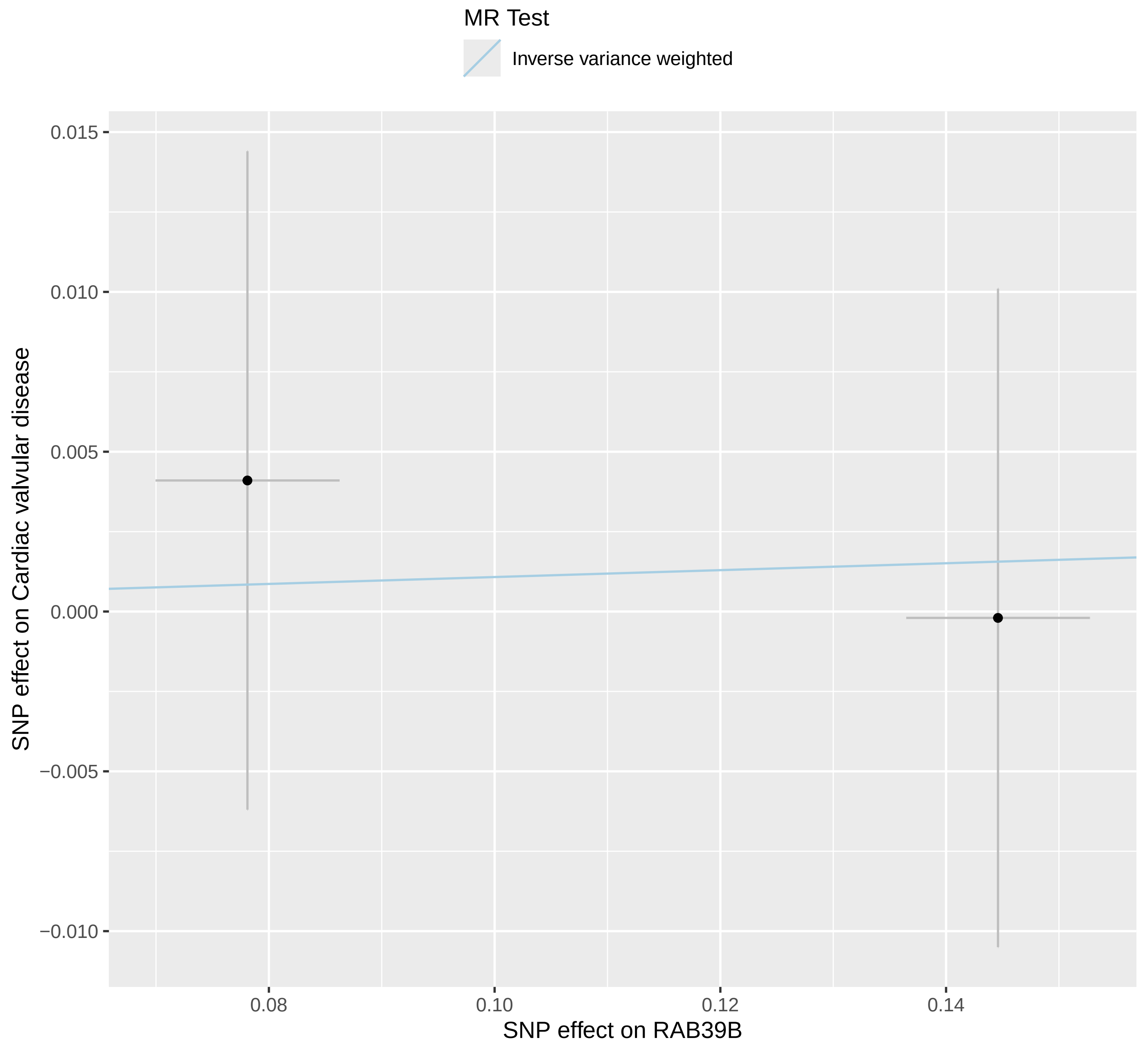
MR Test
Inverse variance weighted
0.015
0.010
0.005
0.000
−0.005
−0.010
SNP effect on Cardiac valvular disease
0.08
0.10
0.12
0.14
SNP effect on RAB39B

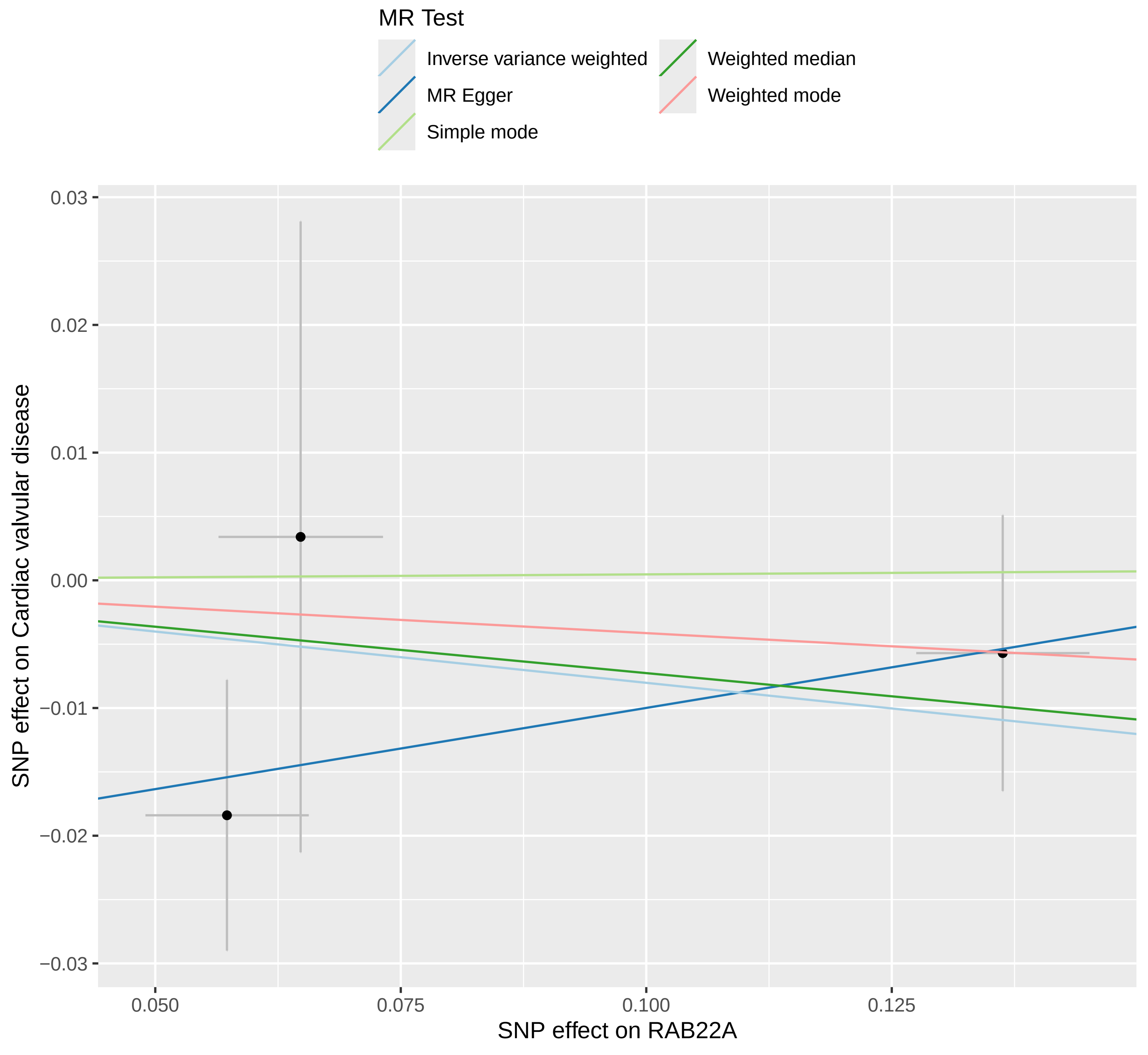

MR Test
Inverse variance weighted
MR Egger
Simple mode
Weighted median
Weighted mode
0.03
0.02
0.01
0.00
−0.01
−0.02
−0.03
SNP effect on Cardiac valvular disease
0.050
0.075
0.100
0.125
SNP effect on RAB22A

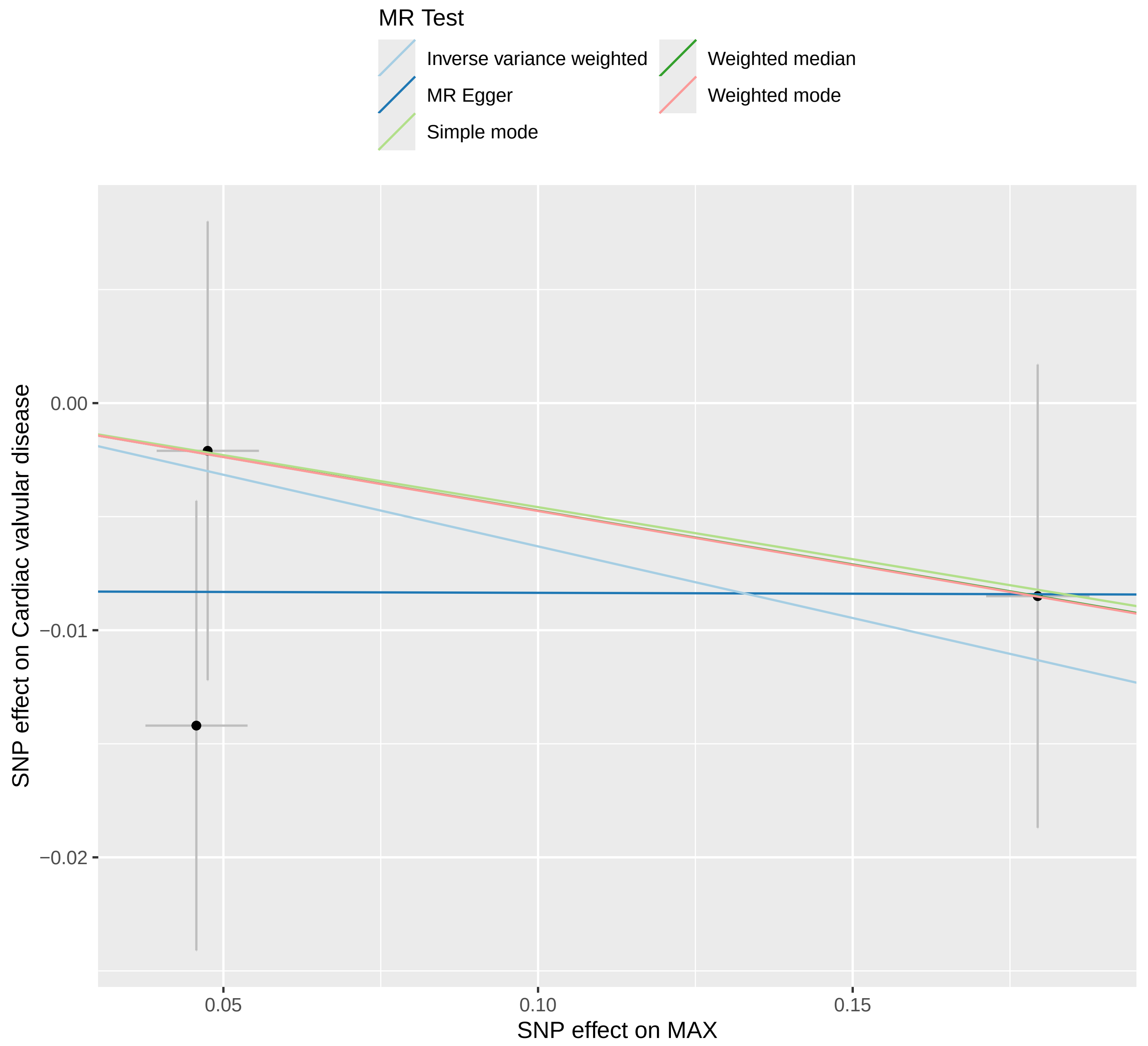
MR Test
Inverse variance weighted
MR Egger
Simple mode
Weighted median
Weighted mode
0.00
−0.01
−0.02
0.05
0.10
0.15
SNP effect on MAX
SNP effect on Cardiac valvular disease

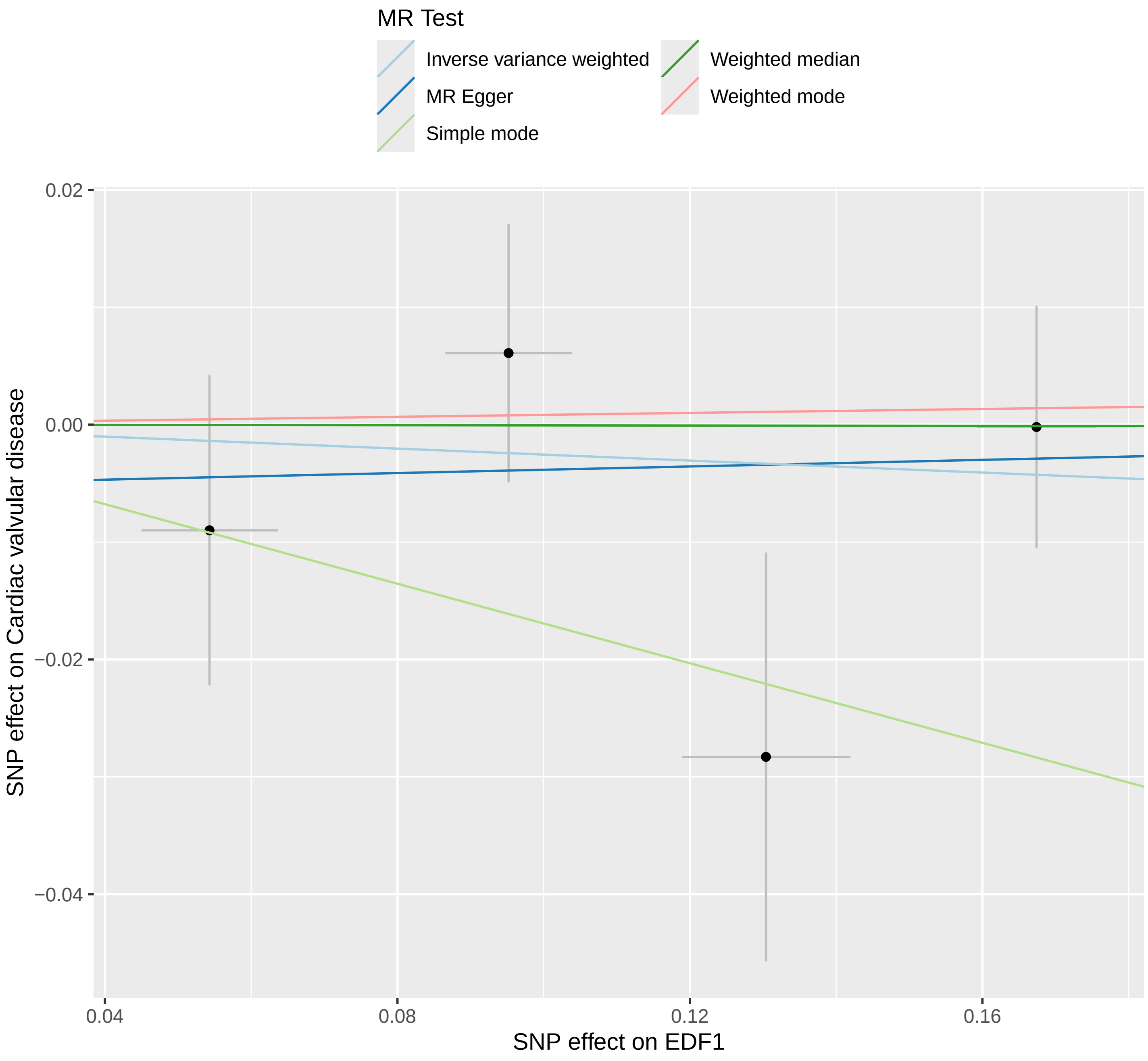
MR Test
Inverse variance weighted
MR Egger
Simple mode
Weighted median
Weighted mode
0.02
0.00
−0.02
−0.04
SNP effect on Cardiac valvular disease
0.04
0.08
0.12
0.16
SNP effect on EDF1

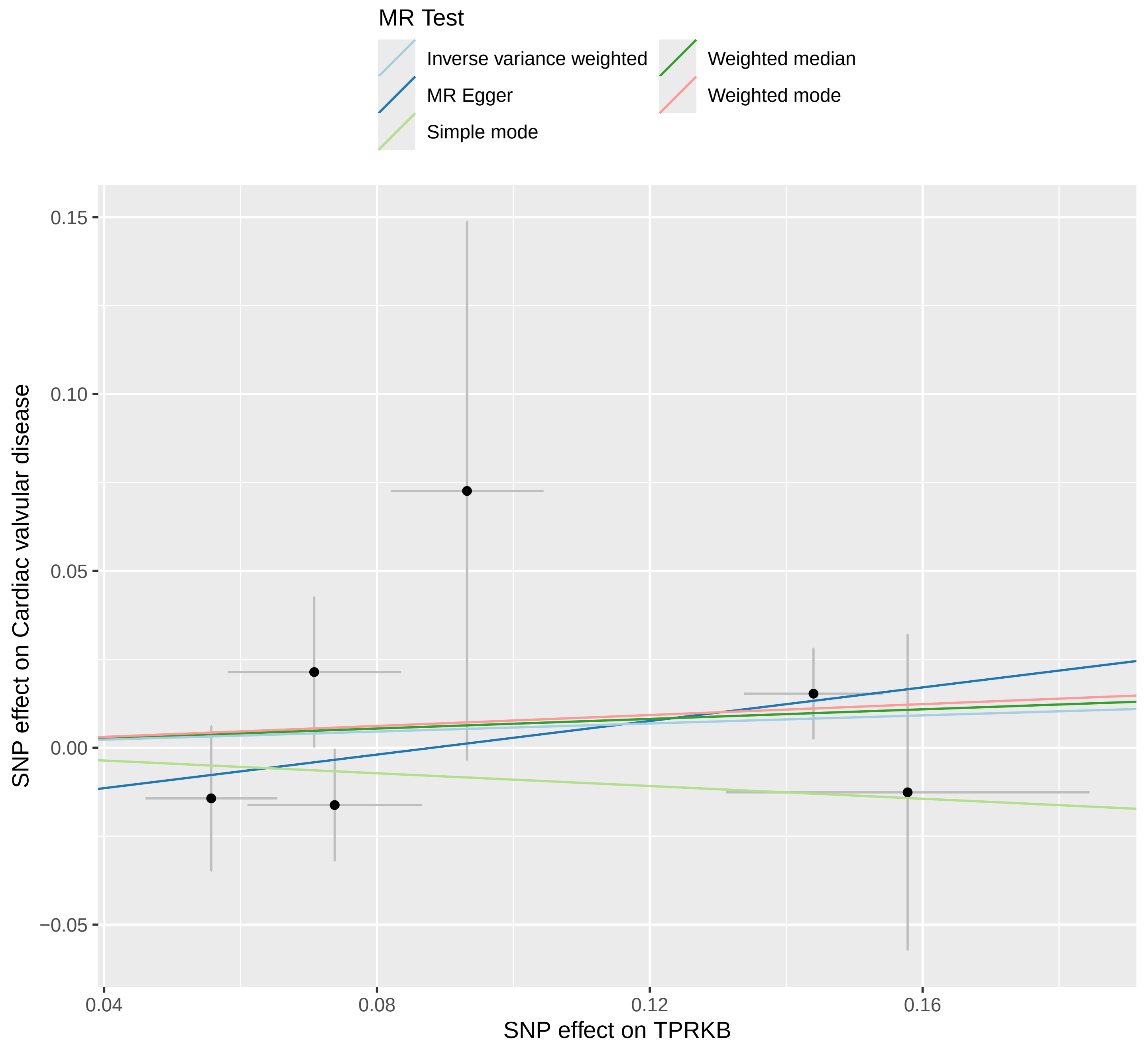
MR Test
Inverse variance weighted
MR Egger
Simple mode
Weighted median
Weighted mode
0.15
0.10
0.05
0.00
−0.05
0.04
0.08
0.12
0.16
SNP effect on Cardiac valvular disease
SNP effect on TPRKB

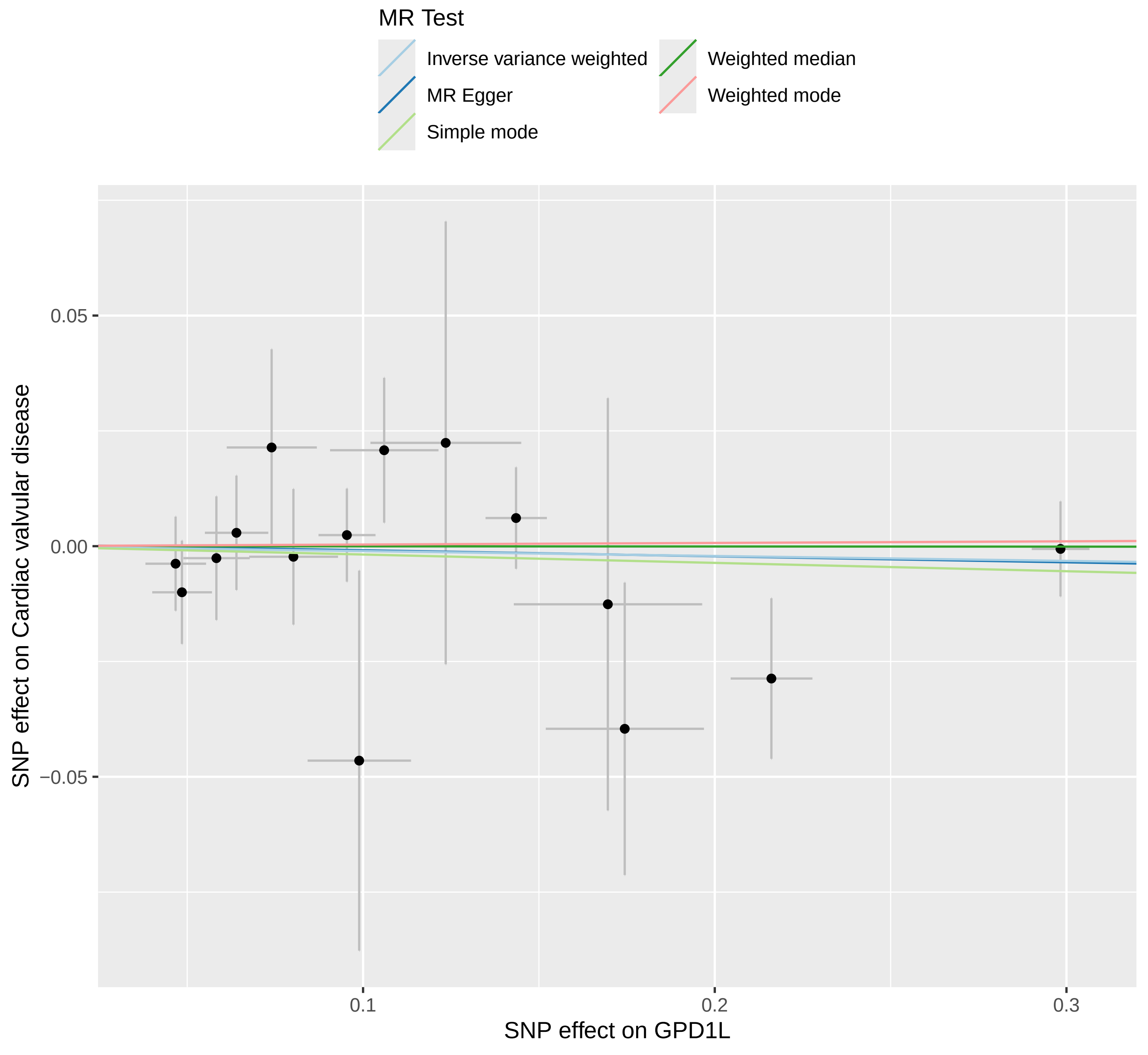

MR Test
Inverse variance weighted
MR Egger
Simple mode
Weighted median
Weighted mode
0.05
0.00
−0.05
0.1
0.2
0.3
SNP effect on GPD1L
SNP effect on Cardiac valvular disease

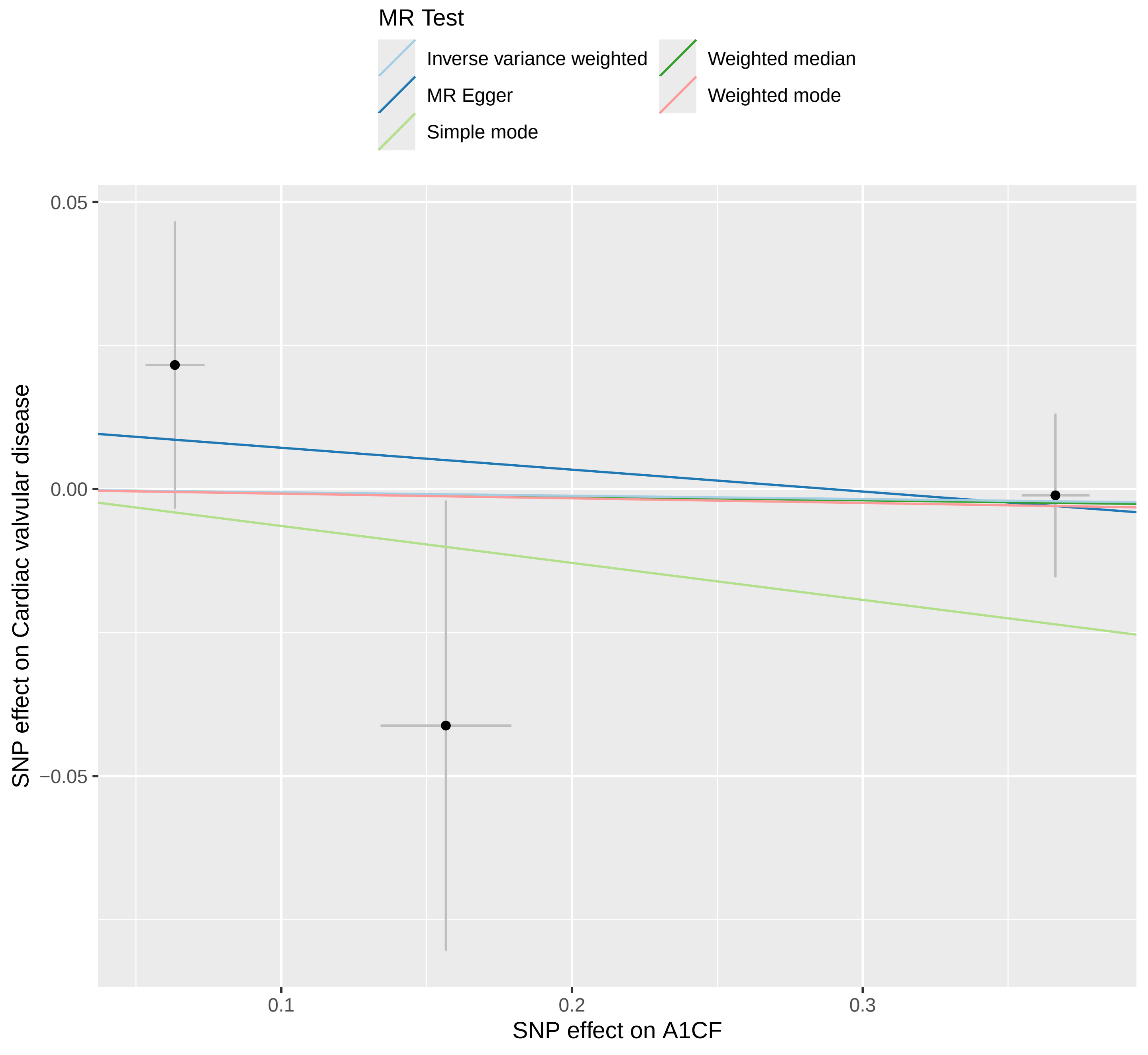

MR Test
Inverse variance weighted
MR Egger
Simple mode
Weighted median
Weighted mode
0.05
0.00
−0.05
0.1
0.2
0.3
SNP effect on Cardiac valvular disease
SNP effect on A1CF

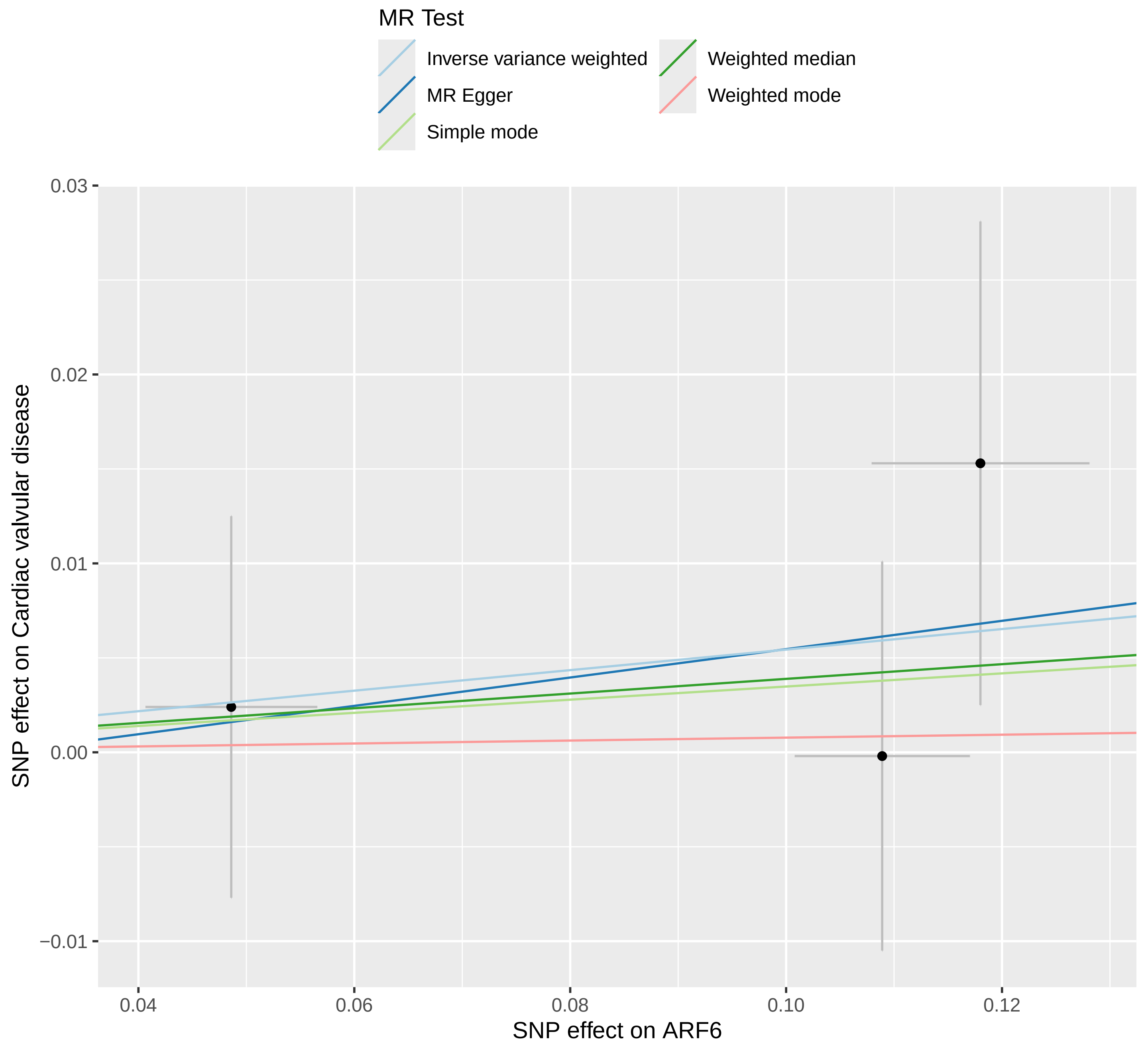

MR Test
Inverse variance weighted
MR Egger
Simple mode
Weighted median
Weighted mode
0.03
0.02
0.01
0.00
−0.01
SNP effect on Cardiac valvular disease
0.04
0.06
0.08
0.10
0.12
SNP effect on ARF6

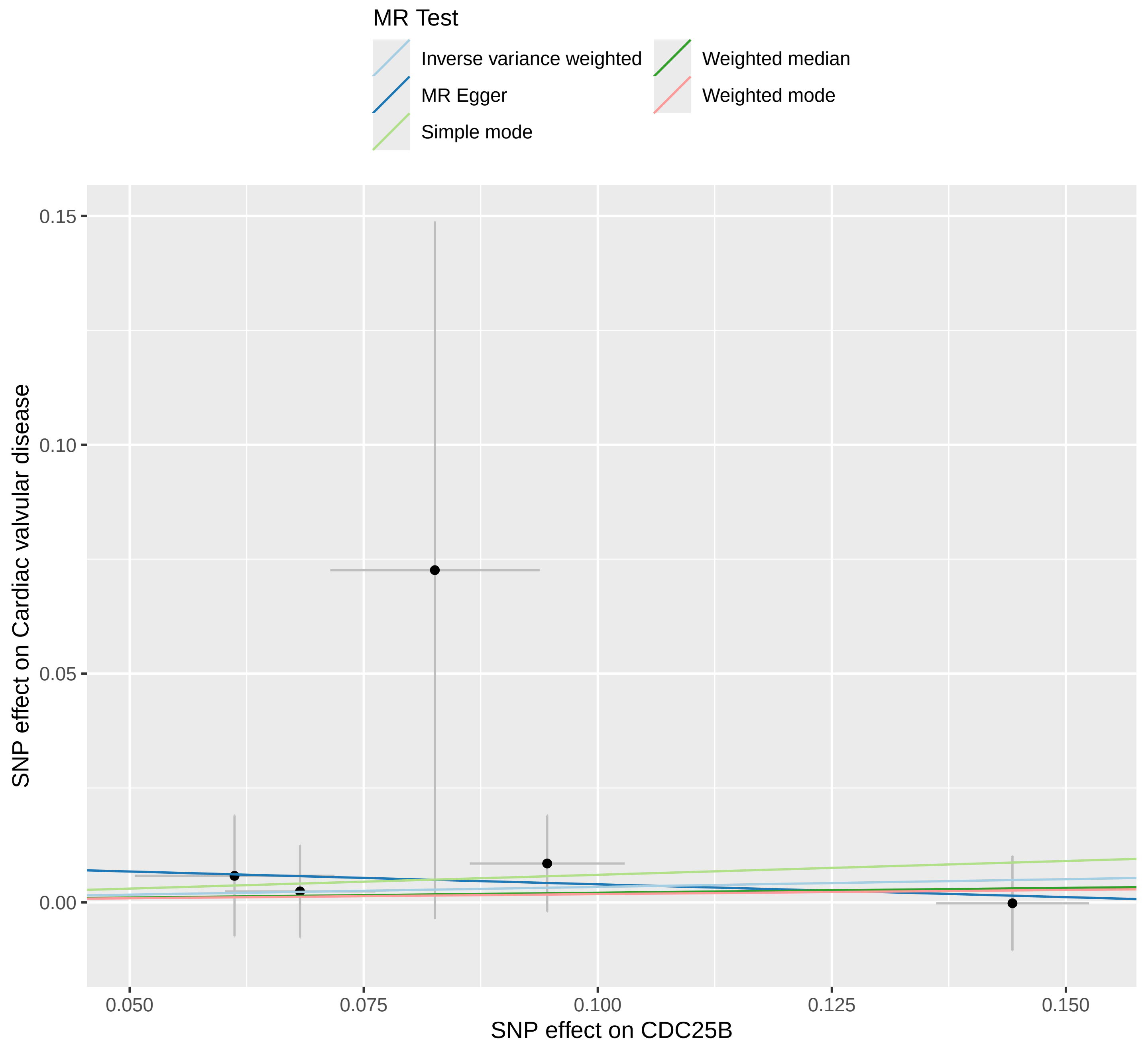

MR Test
Inverse variance weighted
MR Egger
Simple mode
Weighted median
Weighted mode
0.15
0.10
0.05
0.00
SNP effect on Cardiac valvular disease
0.050
0.075
0.100
0.125
0.150
SNP effect on CDC25B

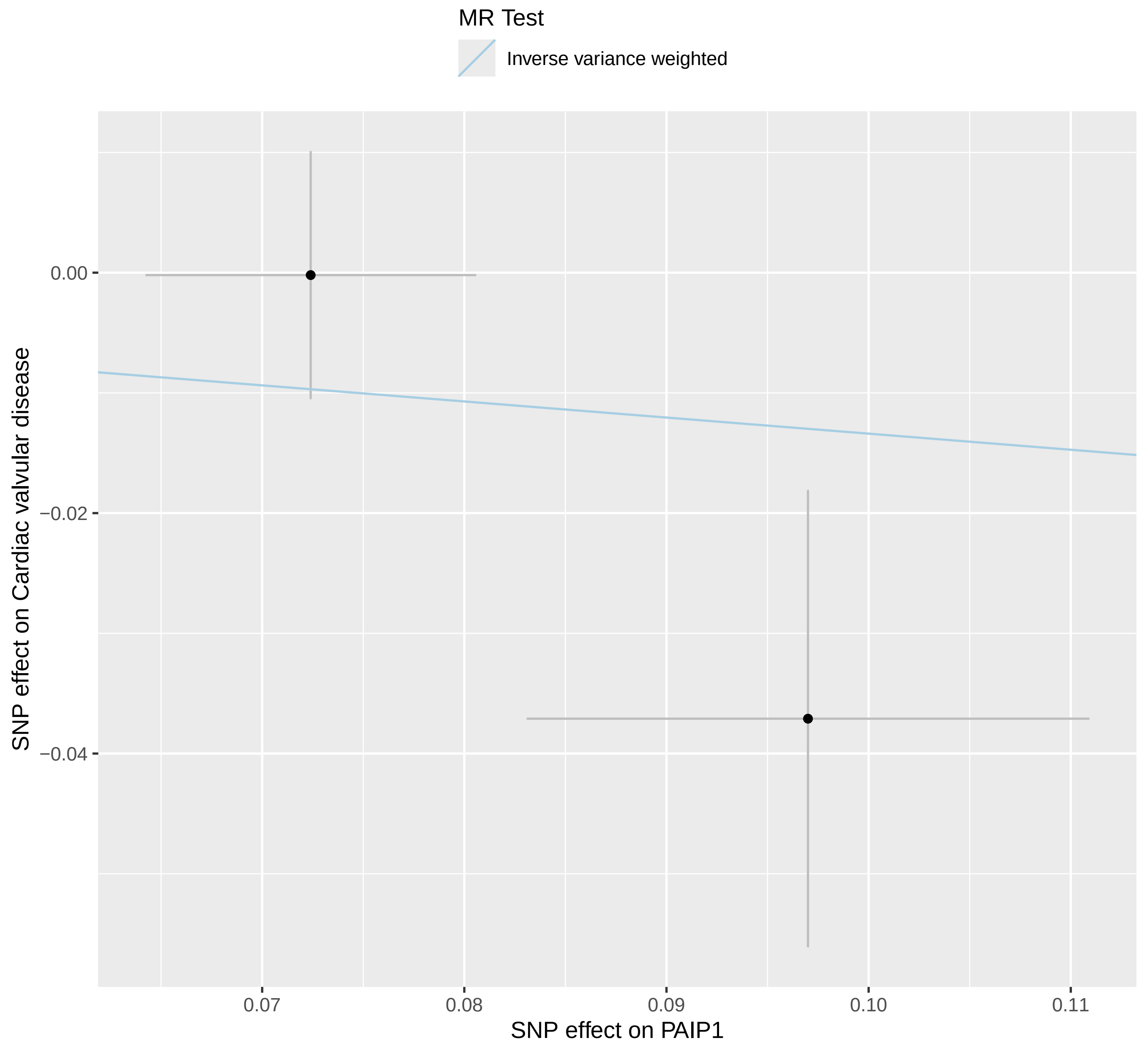

MR Test
Inverse variance weighted
0.00
−0.02
−0.04
0.07
0.08
0.09
0.10
0.11
SNP effect on PAIP1
SNP effect on Cardiac valvular disease

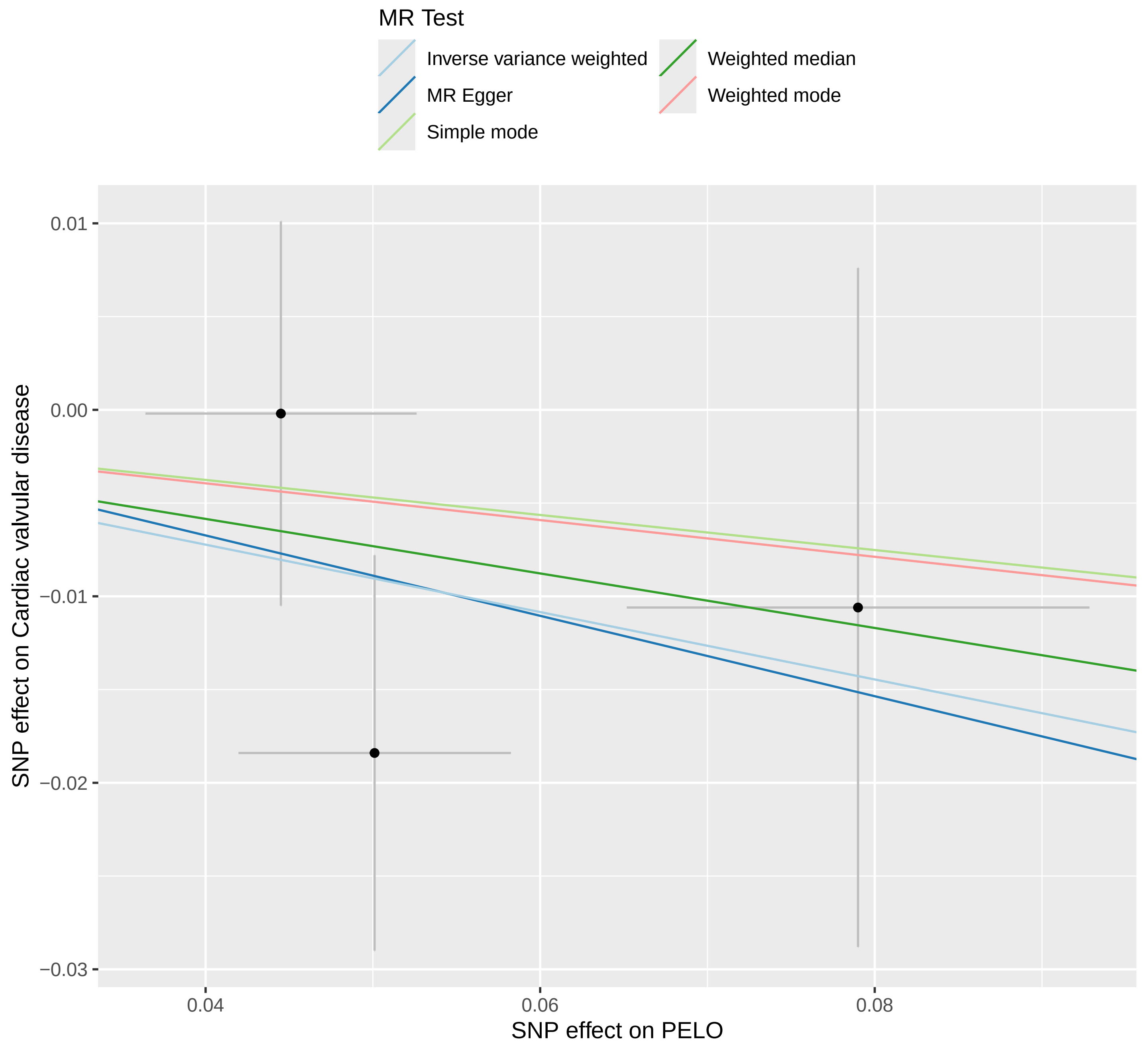
MR Test
Inverse variance weighted
MR Egger
Simple mode
Weighted median
Weighted mode
0.01
0.00
−0.01
−0.02
−0.03
0.04
0.06
0.08
SNP effect on PELO
SNP effect on Cardiac valvular disease

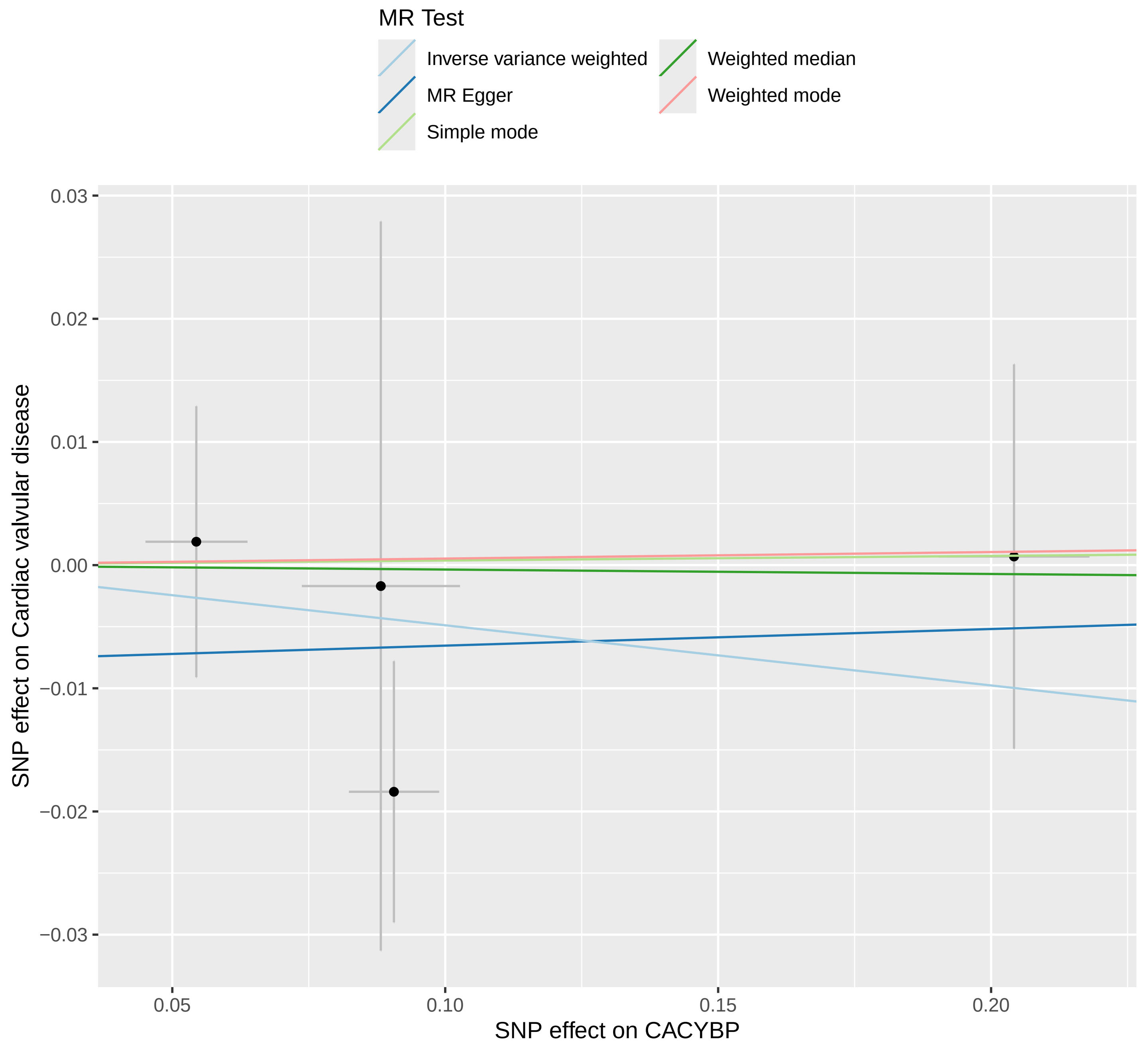

MR Test
Inverse variance weighted
MR Egger
Simple mode
Weighted median
Weighted mode
0.03
0.02
0.01
0.00
−0.01
−0.02
−0.03
SNP effect on Cardiac valvular disease
0.05
0.10
0.15
0.20
SNP effect on CACYBP

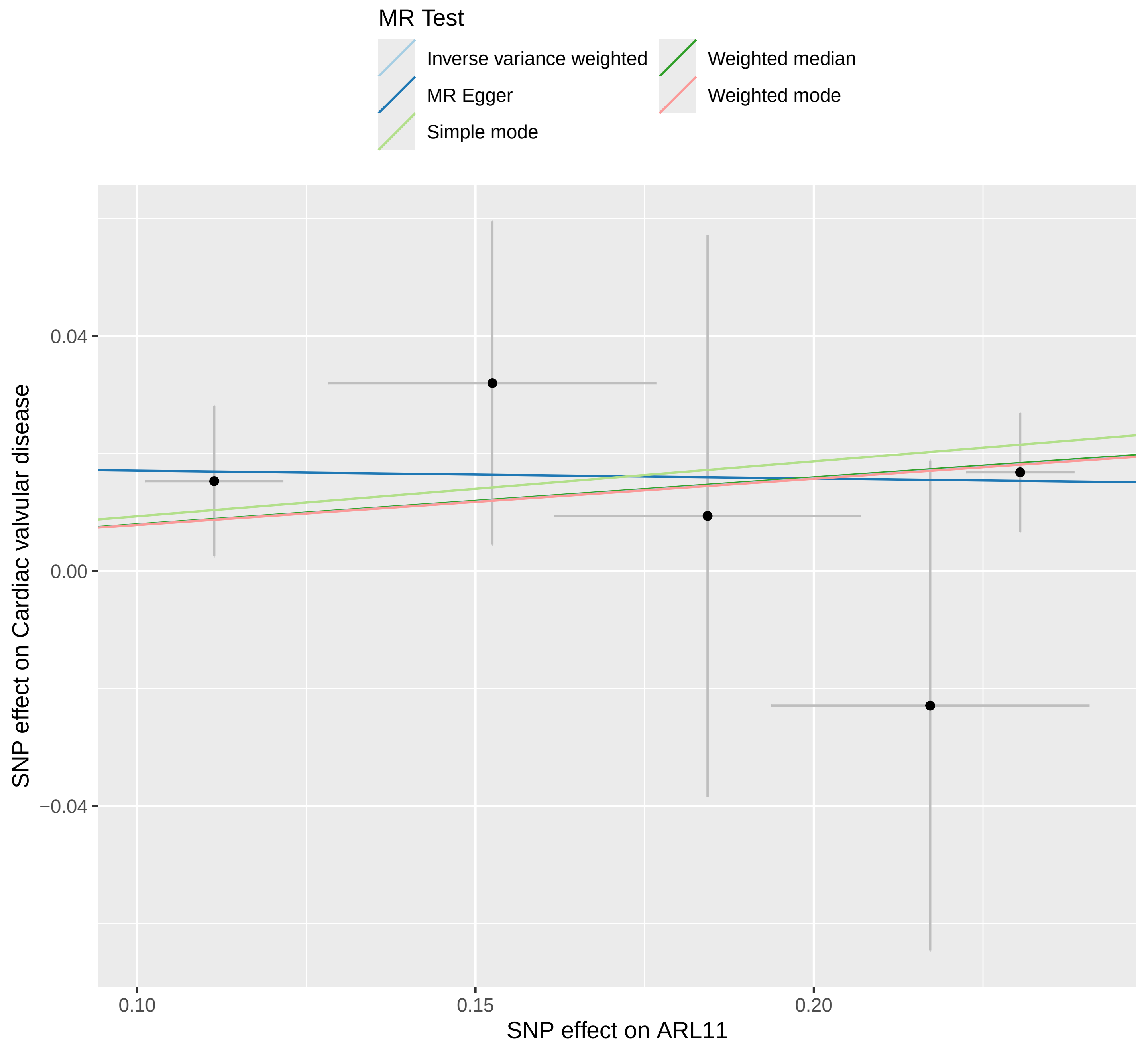

MR Test
Inverse variance weighted
MR Egger
Simple mode
Weighted median
Weighted mode
0.04
0.00
−0.04
0.10
0.15
0.20
SNP effect on Cardiac valvular disease
SNP effect on ARL11

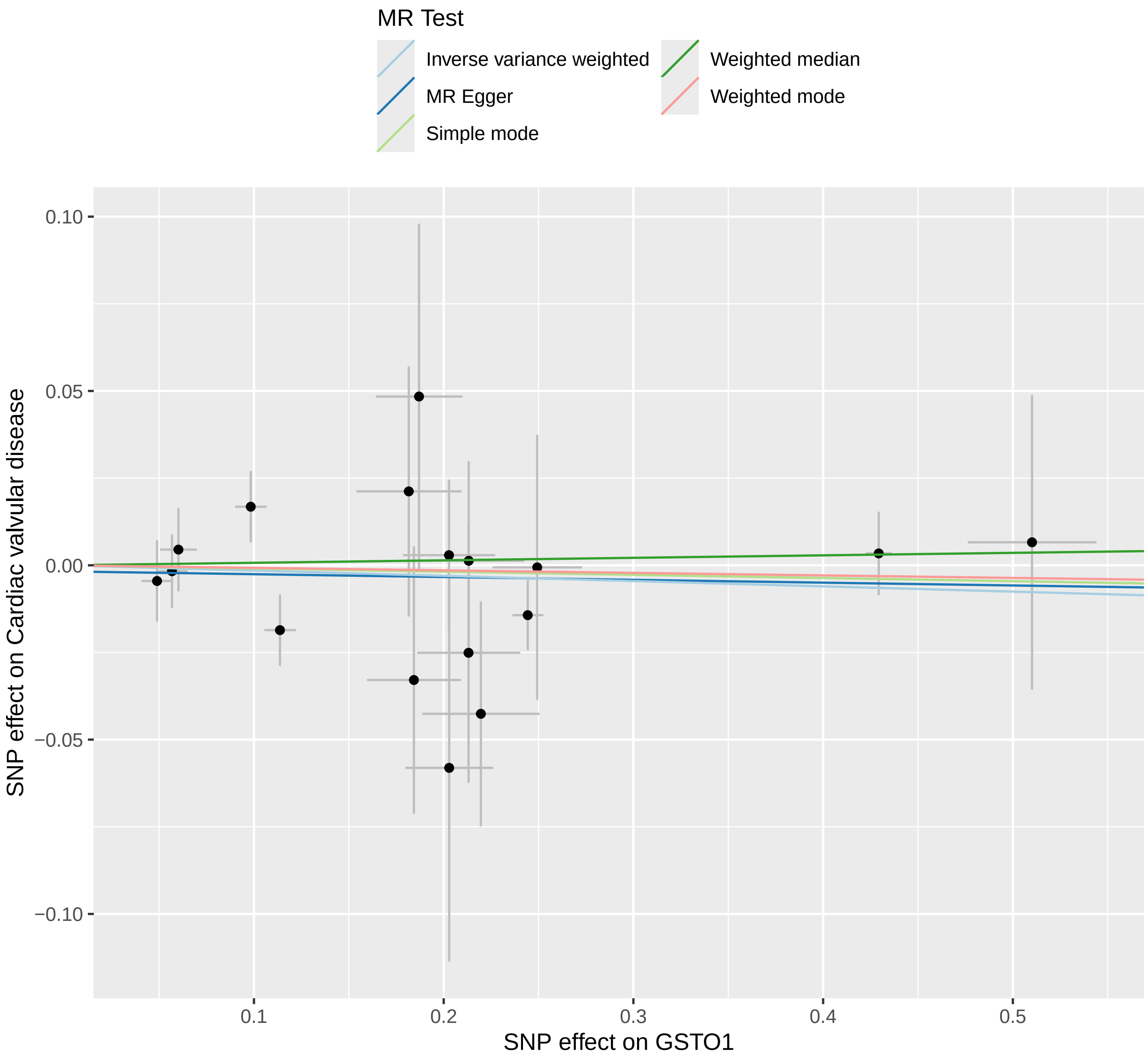

MR Test
Inverse variance weighted
MR Egger
Simple mode
Weighted median
Weighted mode
0.10
0.05
0.00
−0.05
−0.10
SNP effect on Cardiac valvular disease
0.1
0.2
0.3
0.4
0.5
SNP effect on GSTO1

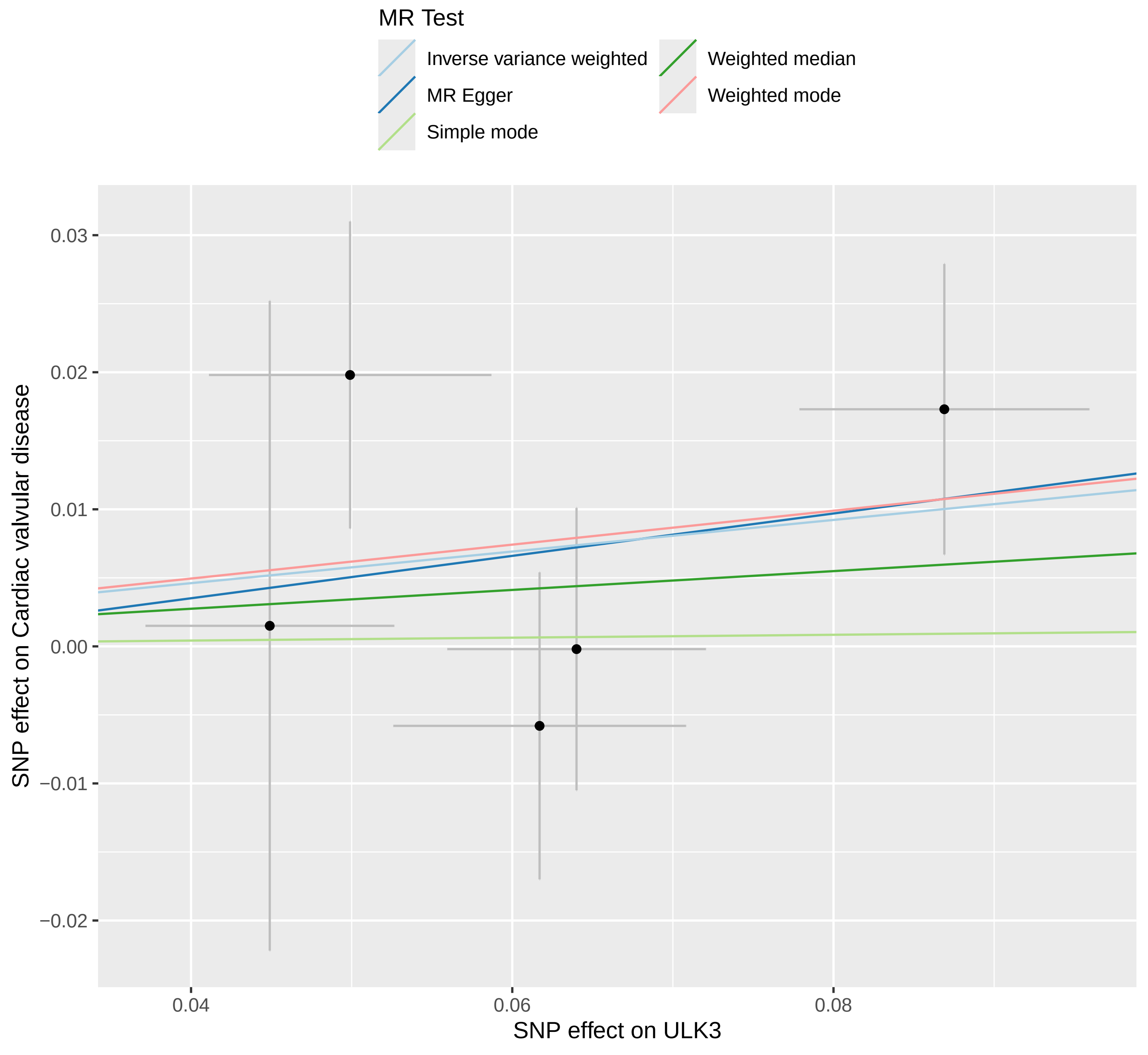

MR Test
Inverse variance weighted
MR Egger
Simple mode
Weighted median
Weighted mode
0.03
0.02
0.01
0.00
−0.01
−0.02
SNP effect on Cardiac valvular disease
0.04
0.06
0.08
SNP effect on ULK3

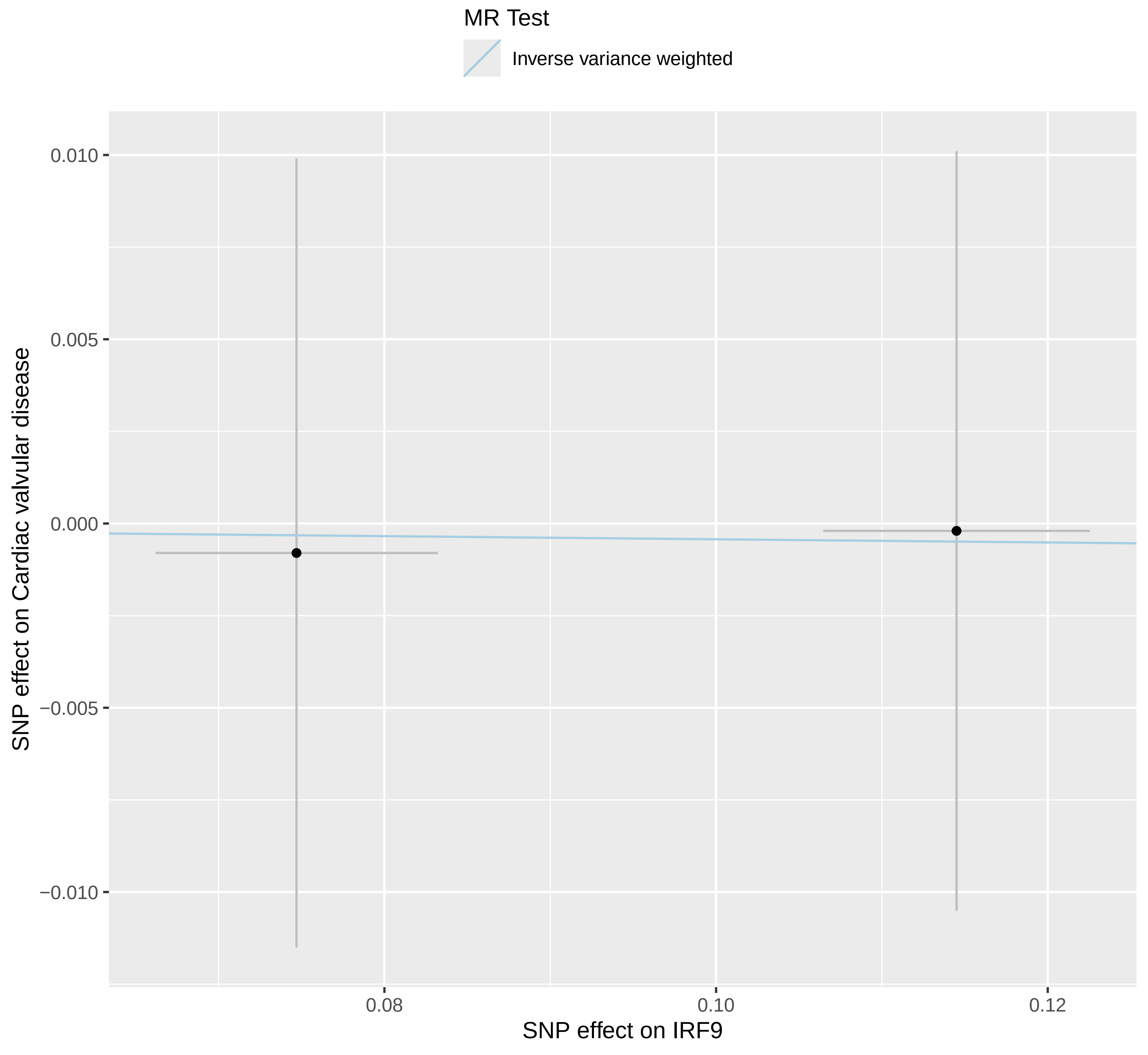

MR Test
Inverse variance weighted
0.010
0.005
0.000
−0.005
−0.010
0.08
0.10
0.12
SNP effect on IRF9
SNP effect on Cardiac valvular disease

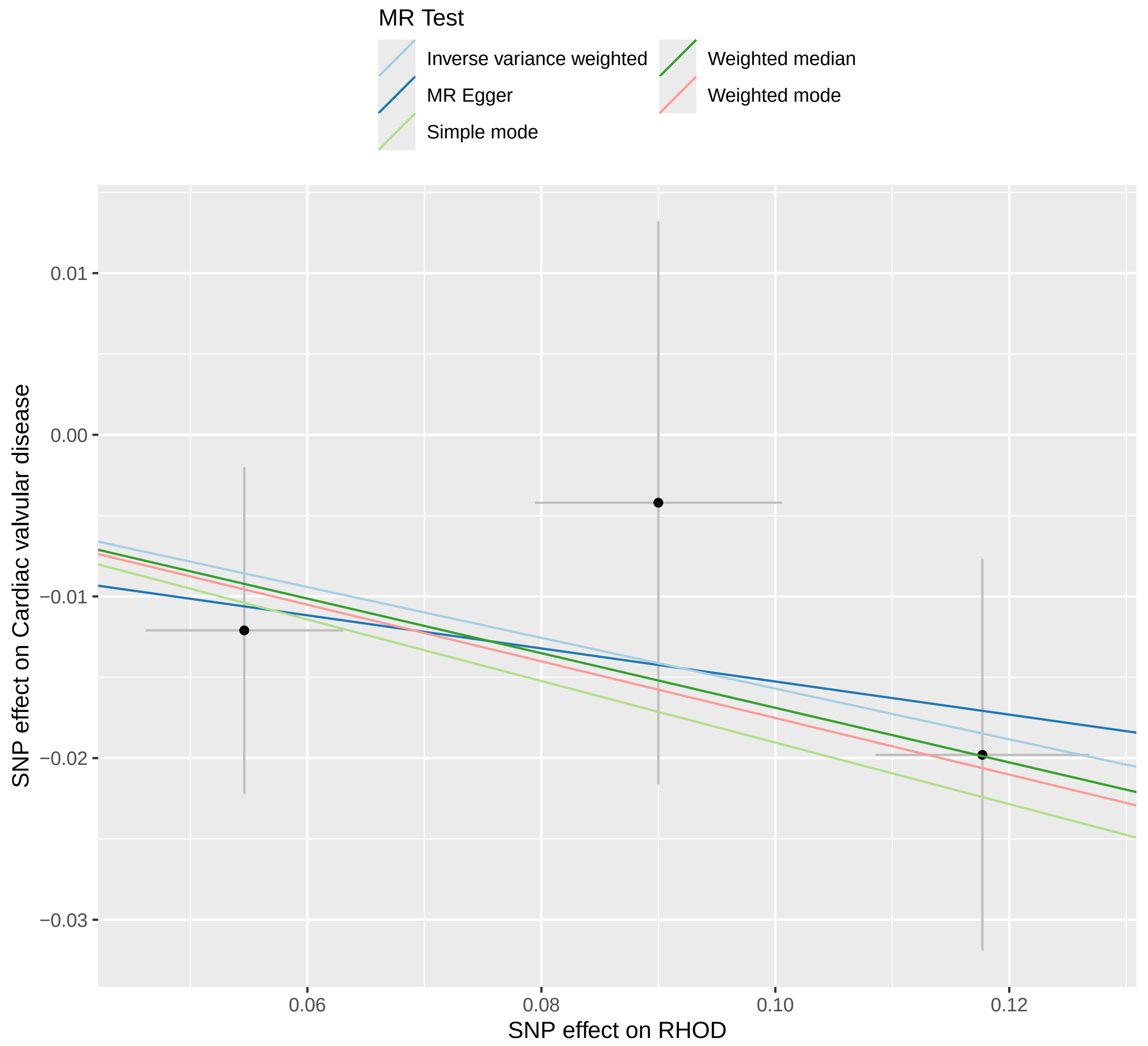
MR Test
Inverse variance weighted
MR Egger
Simple mode
Weighted median
Weighted mode
0.01
0.00
−0.01
−0.02
−0.03
0.06
0.08
0.10
0.12
SNP effect on Cardiac valvular disease
SNP effect on RHOD

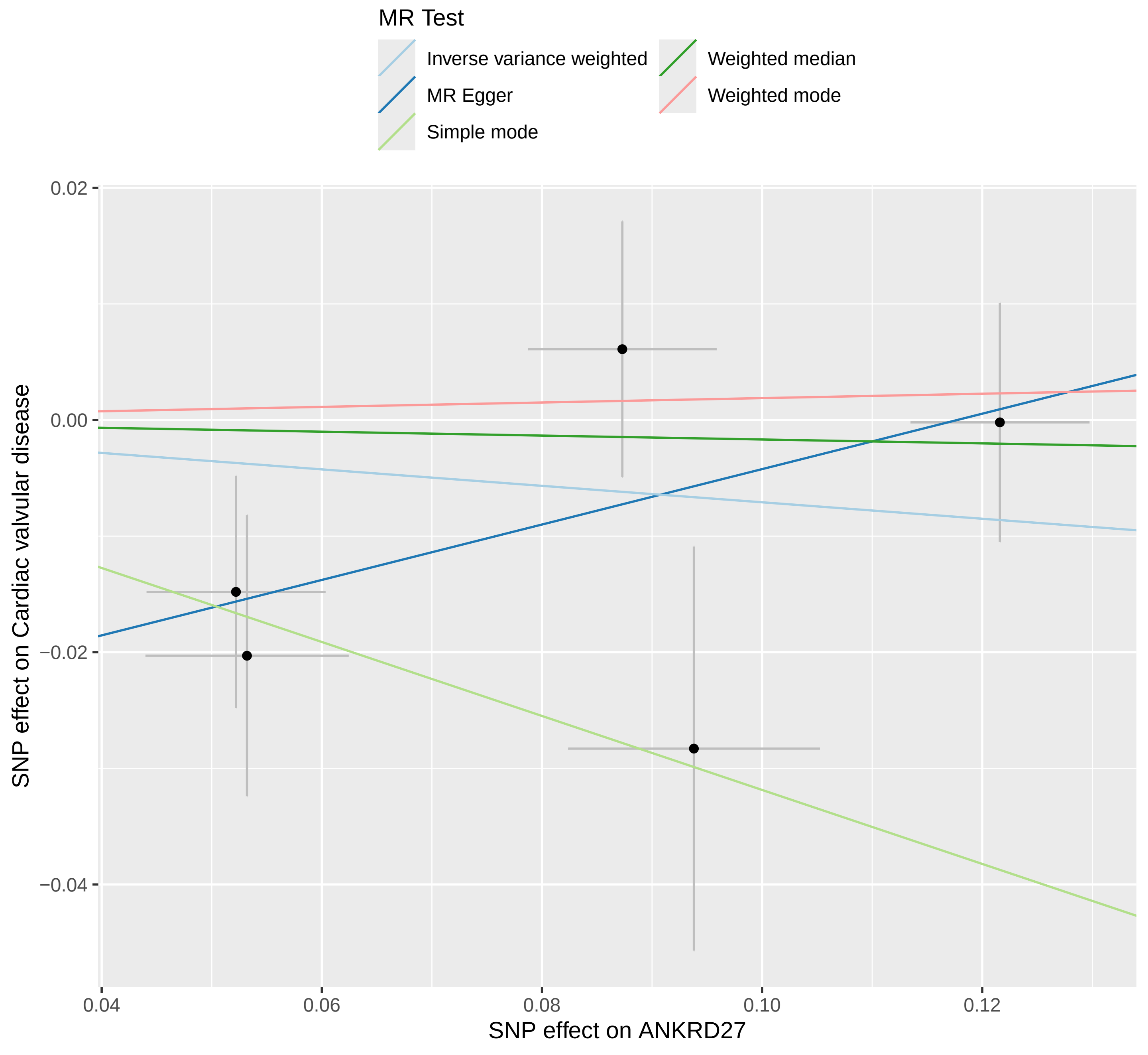

MR Test
Inverse variance weighted
MR Egger
Simple mode
Weighted median
Weighted mode
0.02
0.00
−0.02
−0.04
SNP effect on Cardiac valvular disease
0.04
0.06
0.08
0.10
0.12
SNP effect on ANKRD27

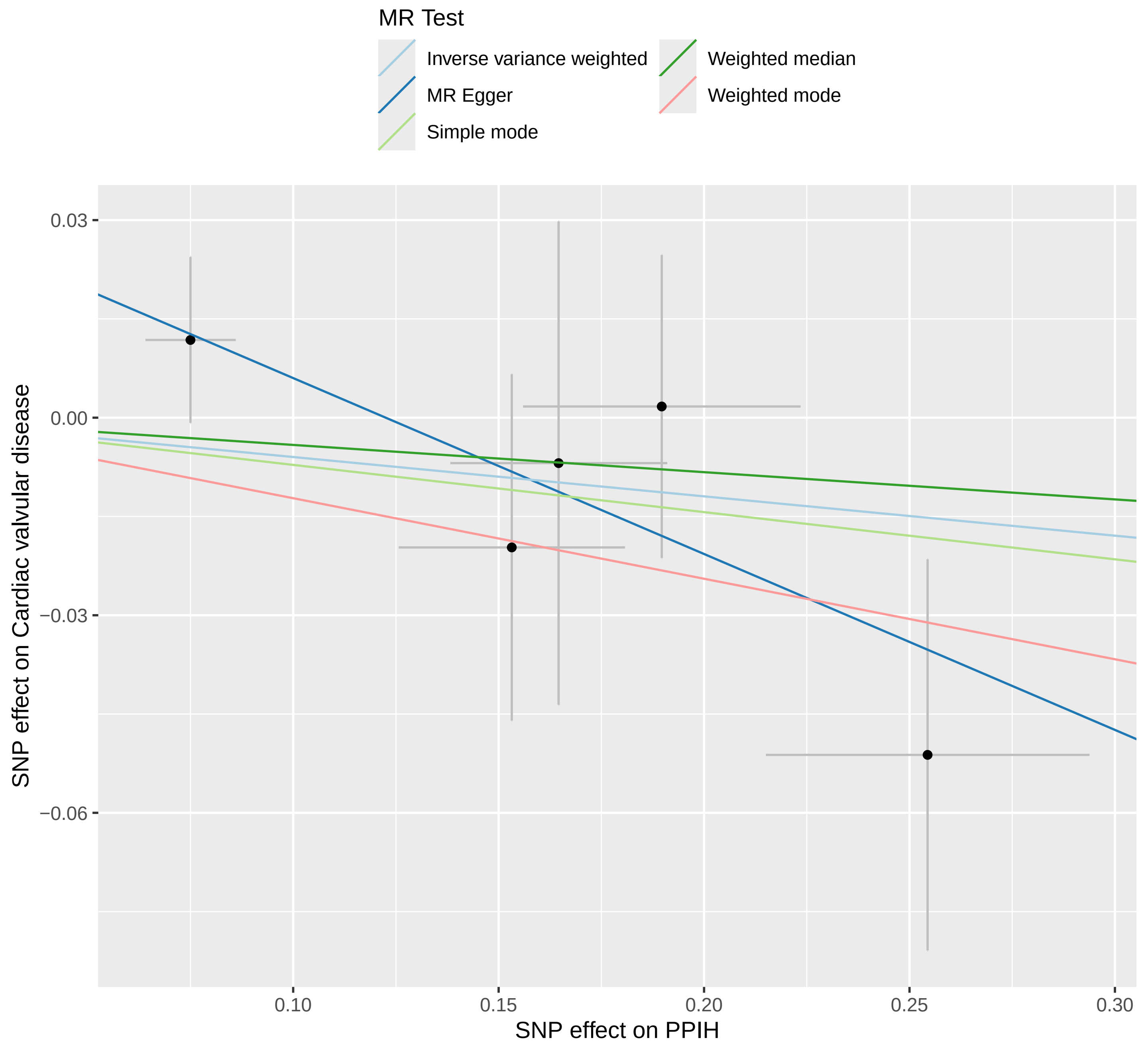
MR Test
Inverse variance weighted
MR Egger
Simple mode
Weighted median
Weighted mode
0.03
0.00
−0.03
−0.06
0.10
0.15
0.20
0.25
0.30
SNP effect on PPIH
SNP effect on Cardiac valvular disease

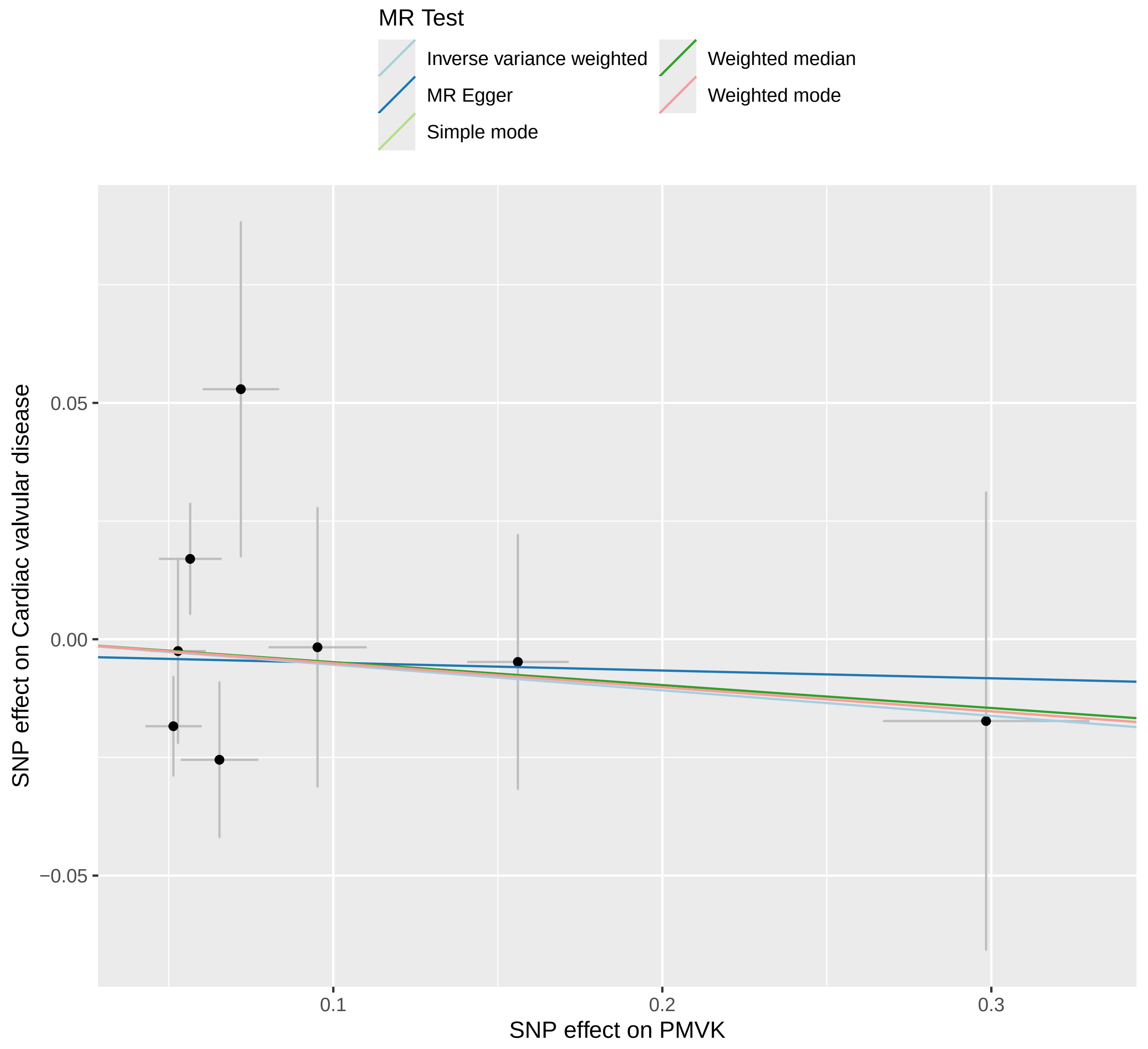
MR Test
Inverse variance weighted
MR Egger
Simple mode
Weighted median
Weighted mode
SNP effect on Cardiac valvular disease
0.05
0.00
−0.05
0.1
0.2
0.3
SNP effect on PMVK

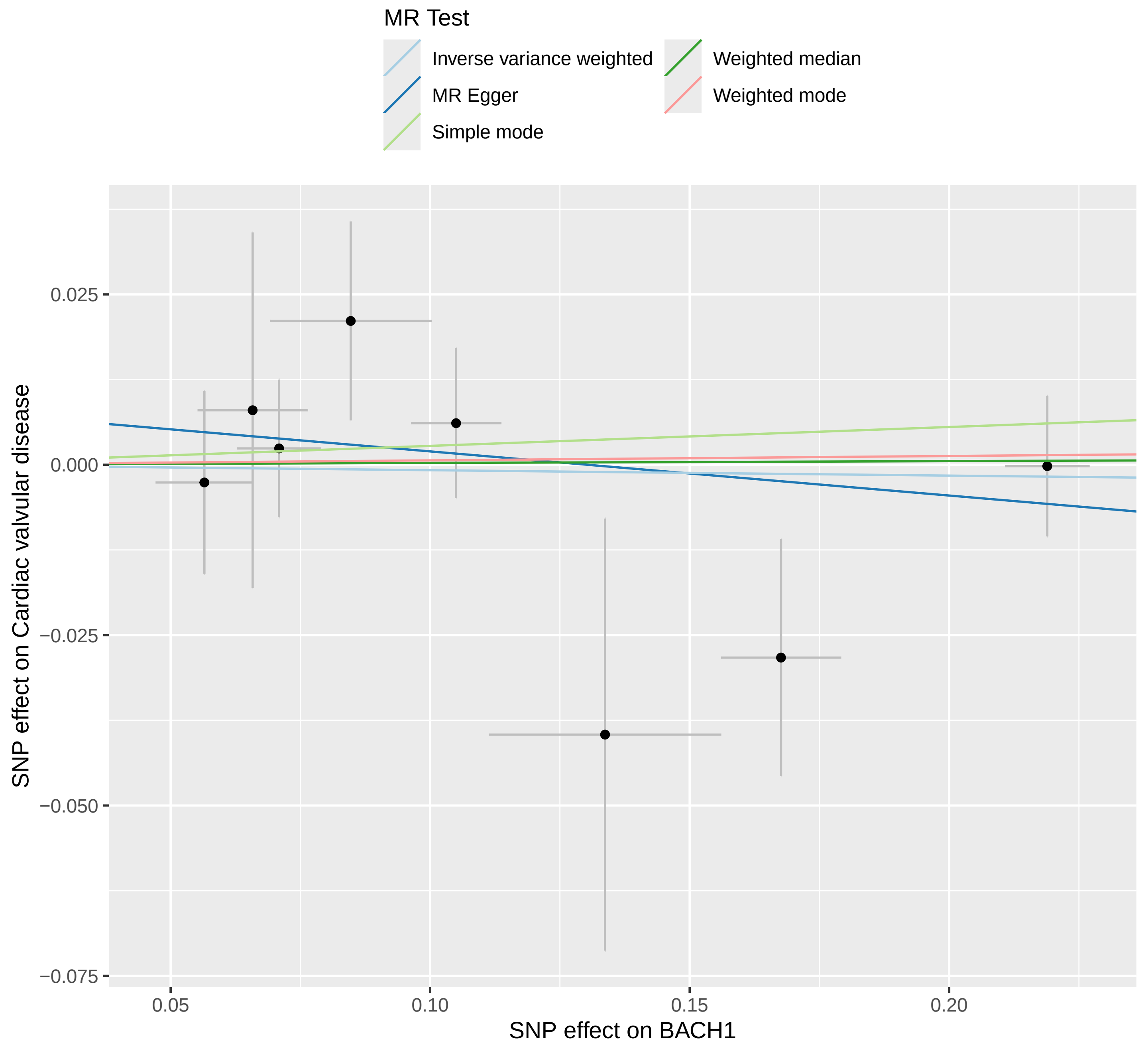

MR Test
Inverse variance weighted
MR Egger
Simple mode
Weighted median
Weighted mode
SNP effect on Cardiac valvular disease
0.025
0.000
−0.025
−0.050
−0.075
0.05
0.10
0.15
0.20
SNP effect on BACH1

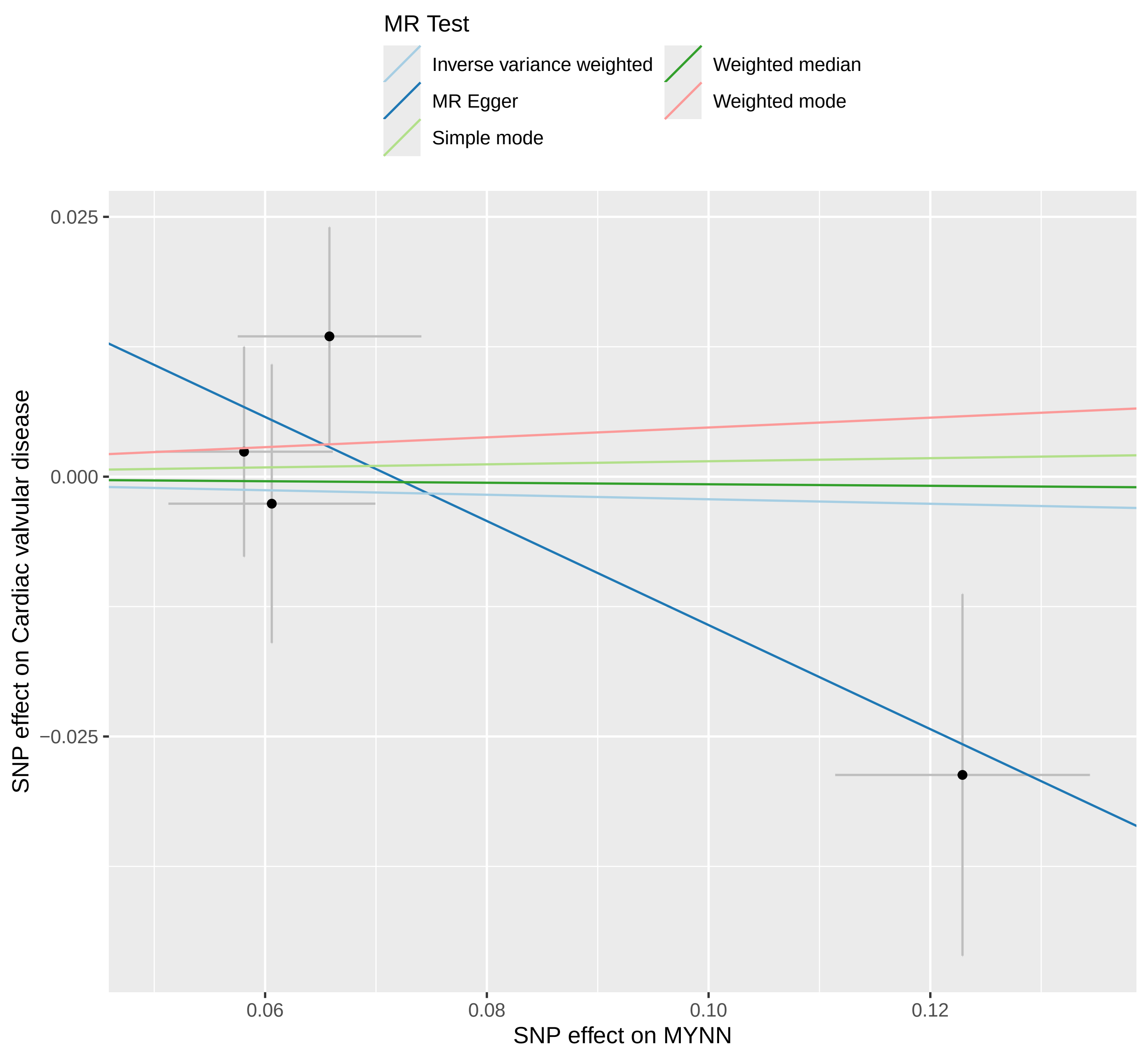
MR Test
Inverse variance weighted
MR Egger
Simple mode
Weighted median
Weighted mode
0.025
0.000
−0.025
0.06
0.08
0.10
0.12
SNP effect on MYNN
SNP effect on Cardiac valvular disease

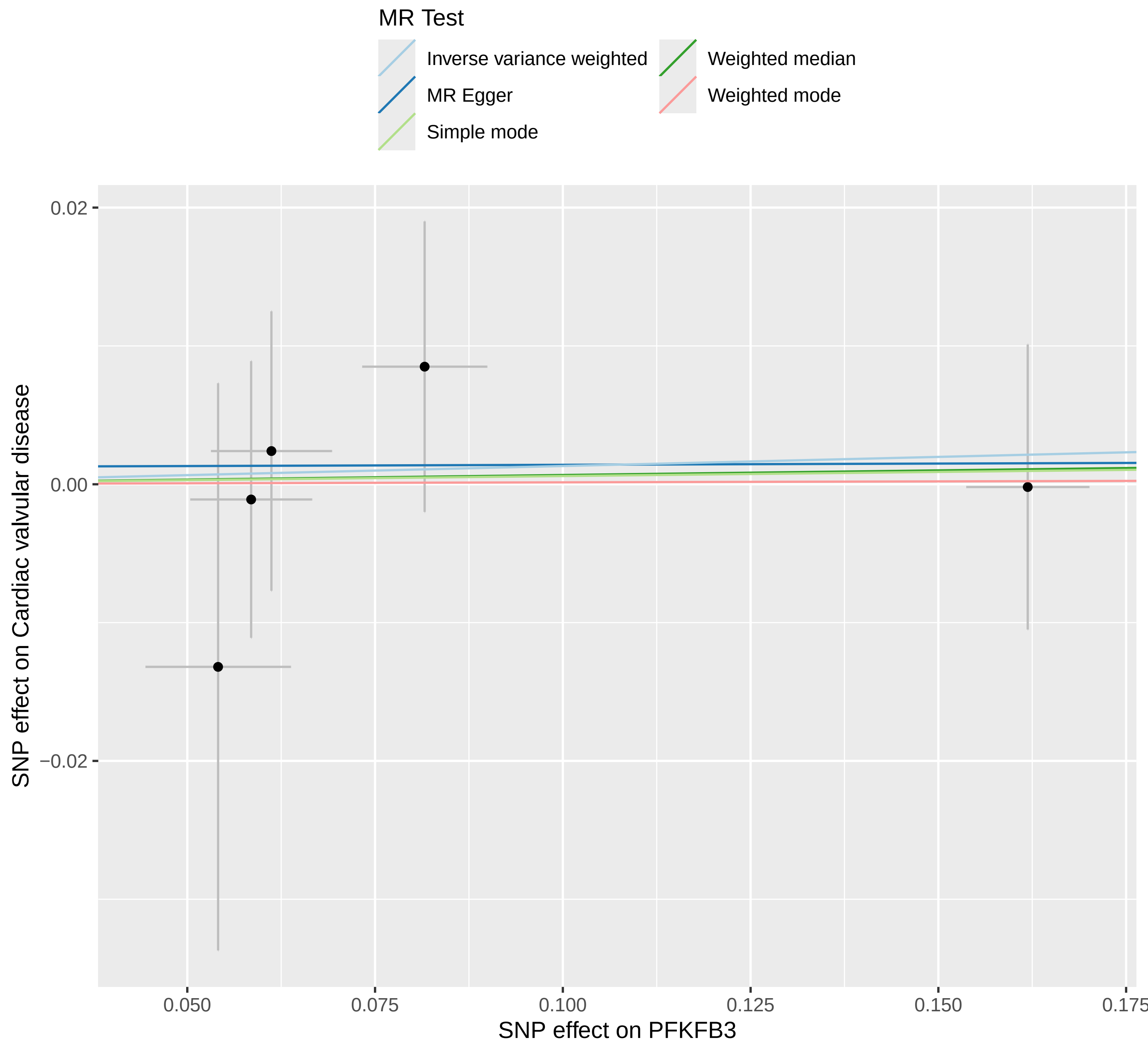
MR Test
Inverse variance weighted
MR Egger
Simple mode
Weighted median
Weighted mode
0.02
0.00
−0.02
0.050
0.075
0.100
0.125
0.150
0.175
SNP effect on Cardiac valvular disease
SNP effect on PFKFB3

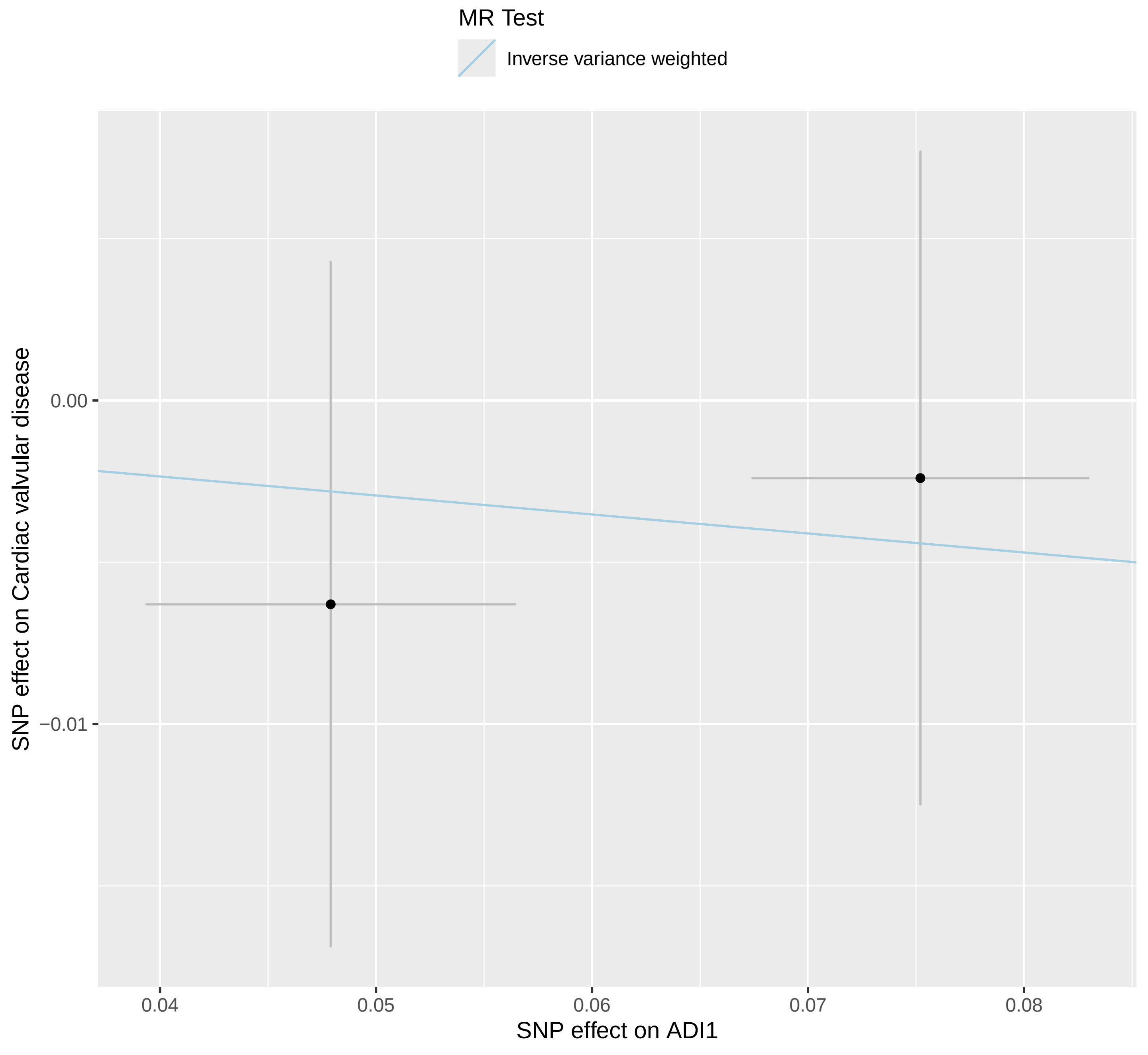

MR Test
Inverse variance weighted
0.00
−0.01
0.04
0.05
0.06
0.07
0.08
SNP effect on ADI1
SNP effect on Cardiac valvular disease

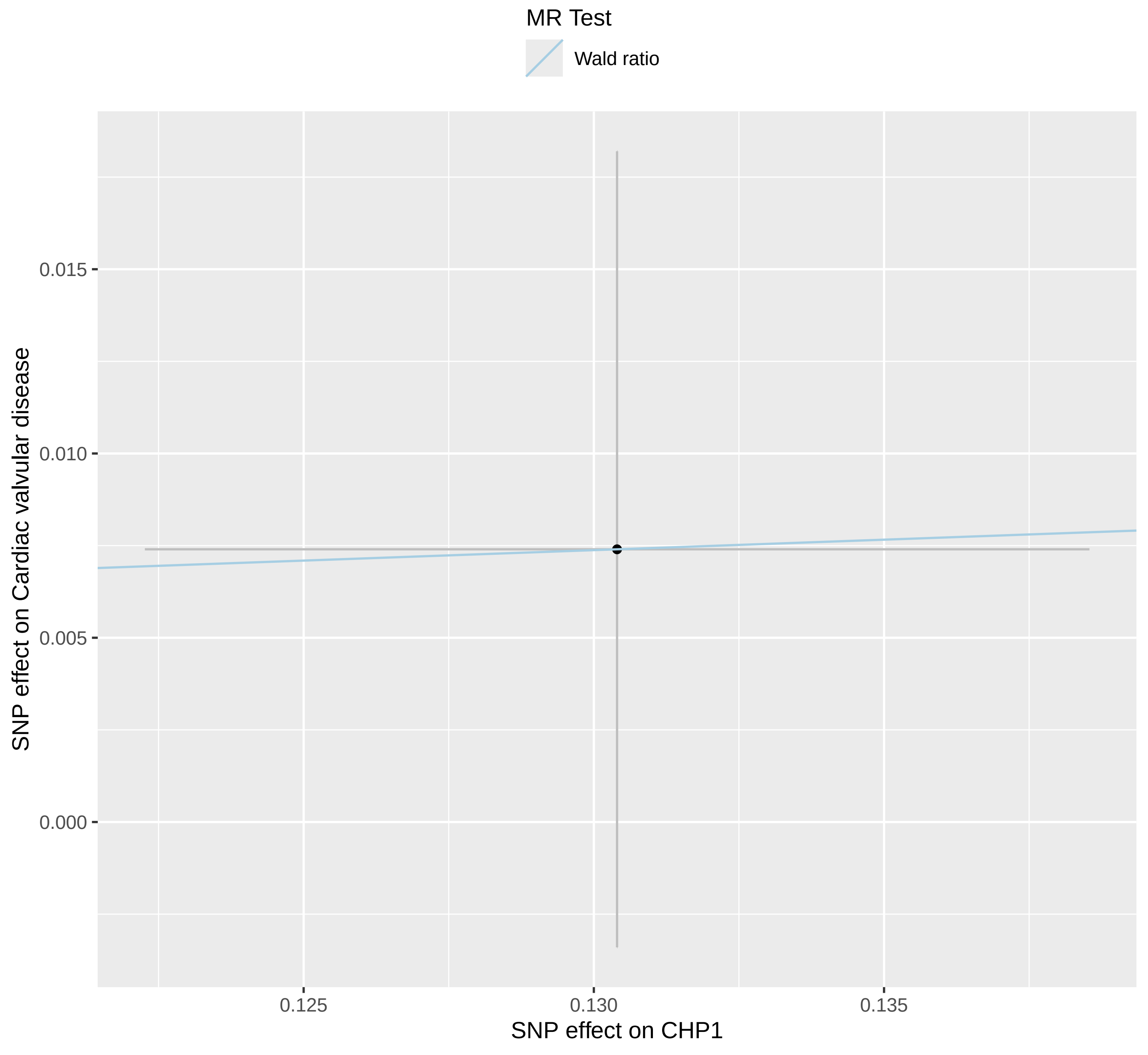
MR Test
Wald ratio
0.015
0.010
0.005
0.000
SNP effect on Cardiac valvular disease
0.125
0.130
0.135
SNP effect on CHP1

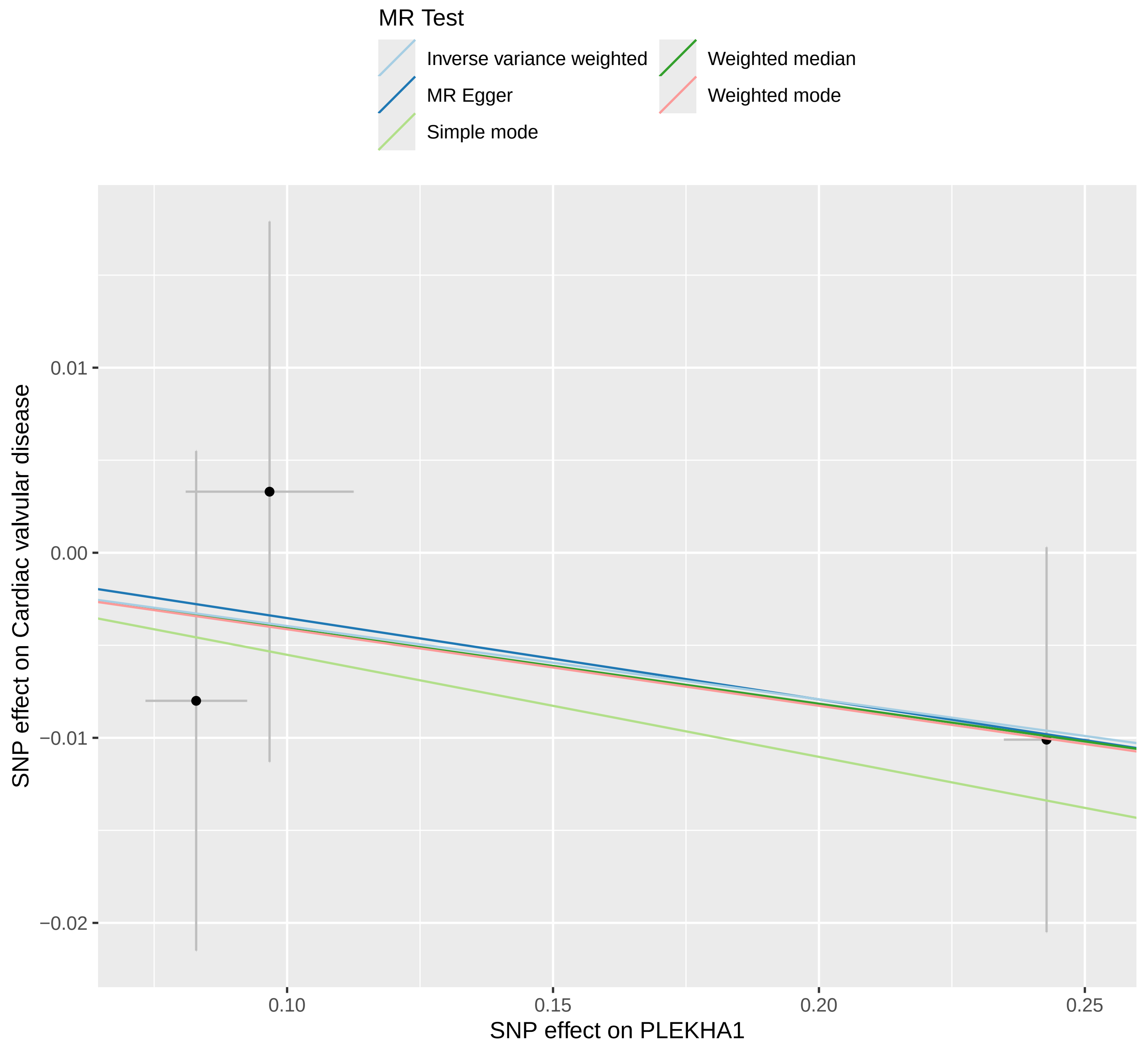
MR Test
Inverse variance weighted
MR Egger
Simple mode
Weighted median
Weighted mode
0.01
0.00
−0.01
−0.02
0.10
0.15
0.20
0.25
SNP effect on Cardiac valvular disease
SNP effect on PLEKHA1

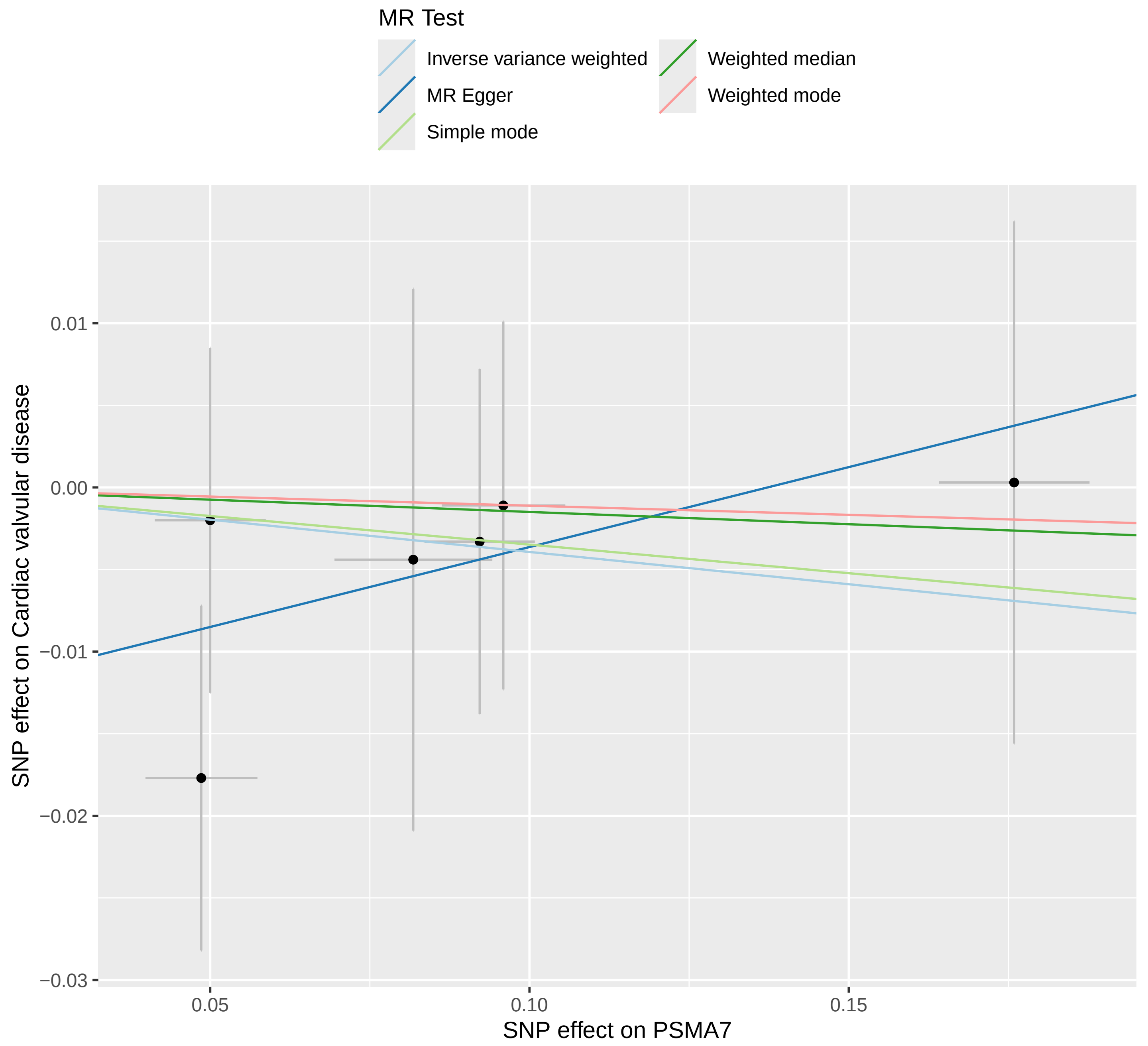

MR Test
Inverse variance weighted
MR Egger
Simple mode
Weighted median
Weighted mode
0.01
0.00
−0.01
−0.02
−0.03
0.05
0.10
0.15
SNP effect on PSMA7
SNP effect on Cardiac valvular disease

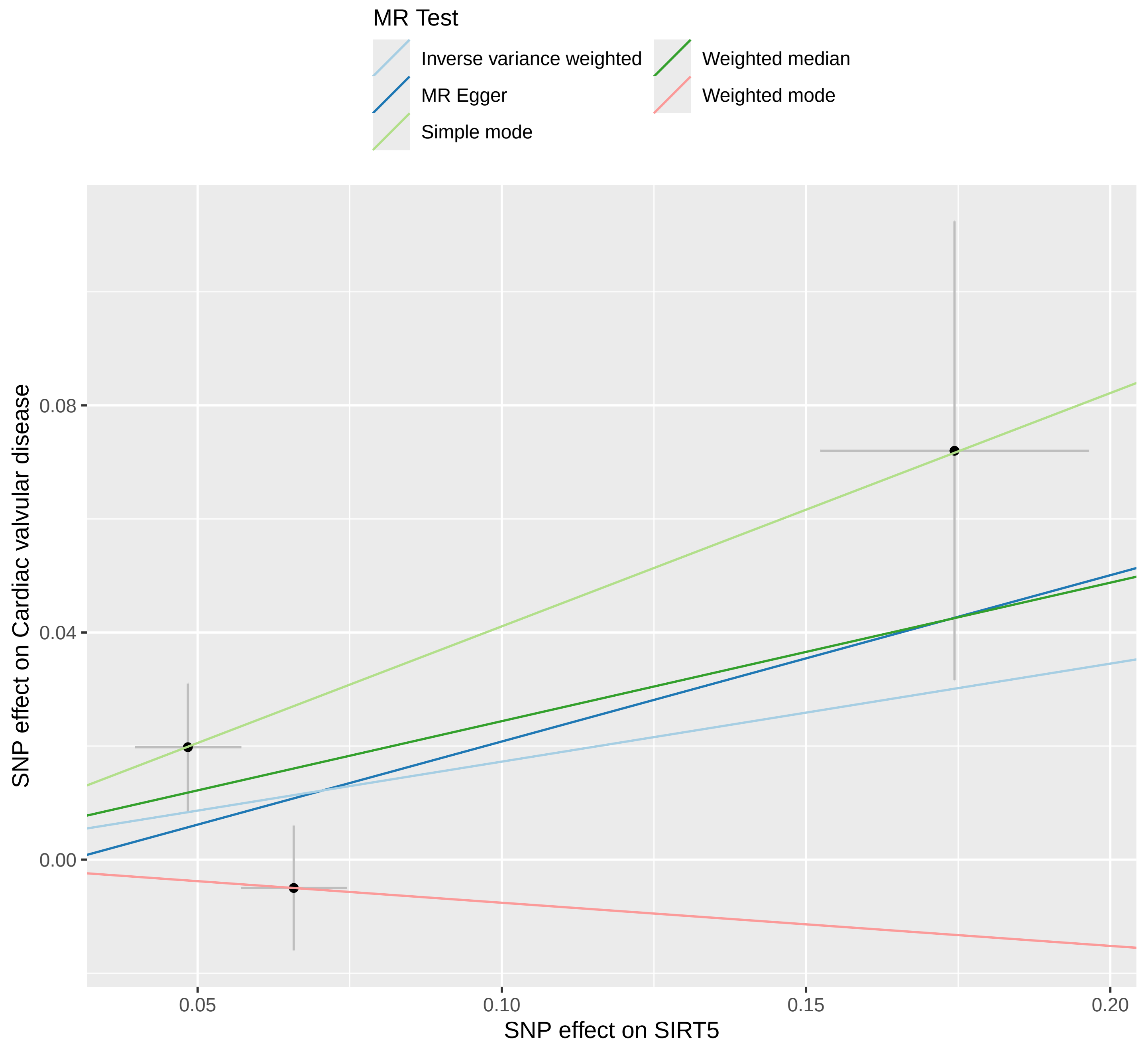

MR Test
Inverse variance weighted
MR Egger
Simple mode
Weighted median
Weighted mode
SNP effect on Cardiac valvular disease
0.08
0.04
0.00
0.05
0.10
0.15
0.20
SNP effect on SIRT5

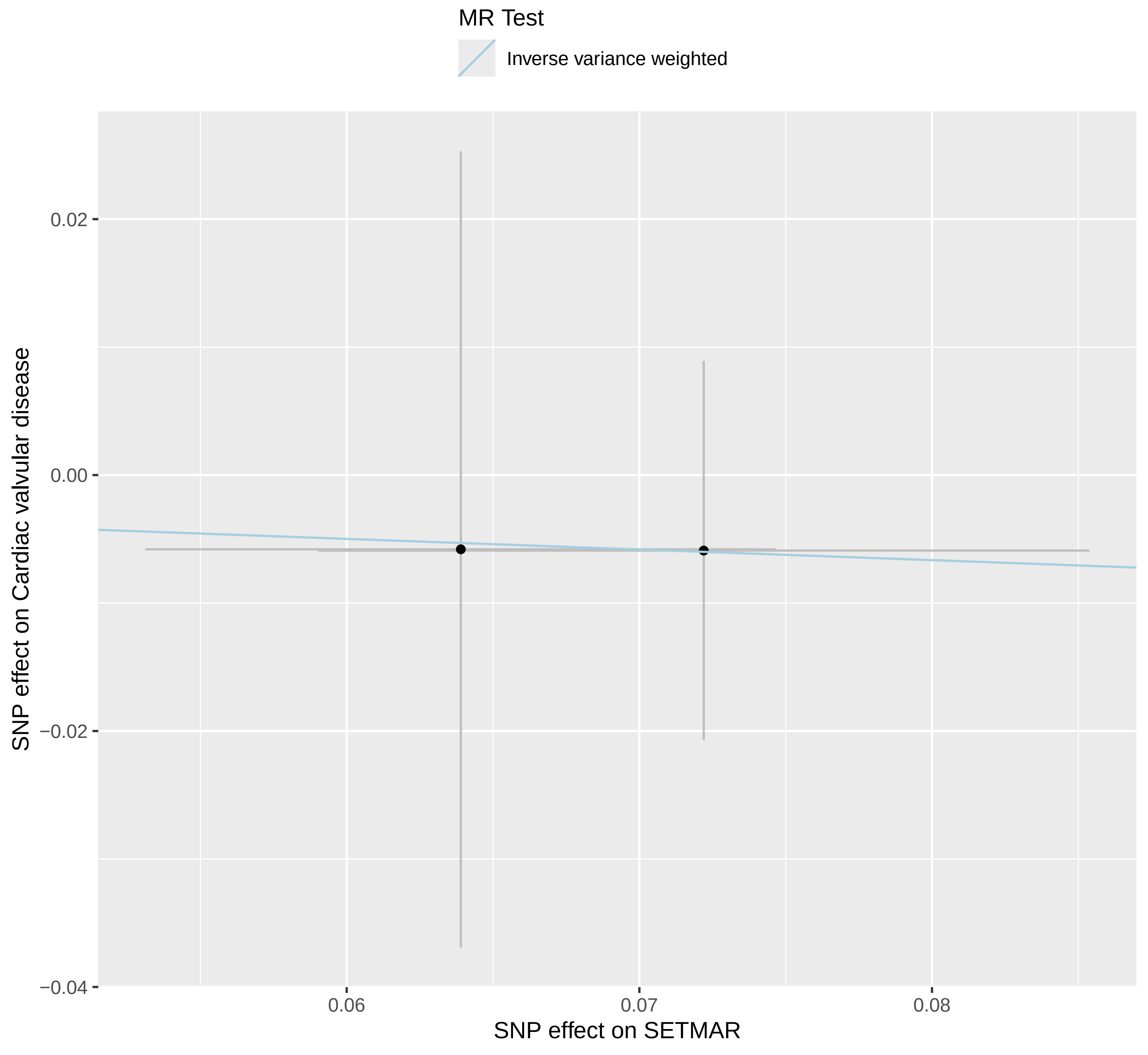
MR Test
Inverse variance weighted
SNP effect on Cardiac valvular disease
0.02
0.00
−0.02
−0.04
0.06
0.07
0.08
SNP effect on SETMAR

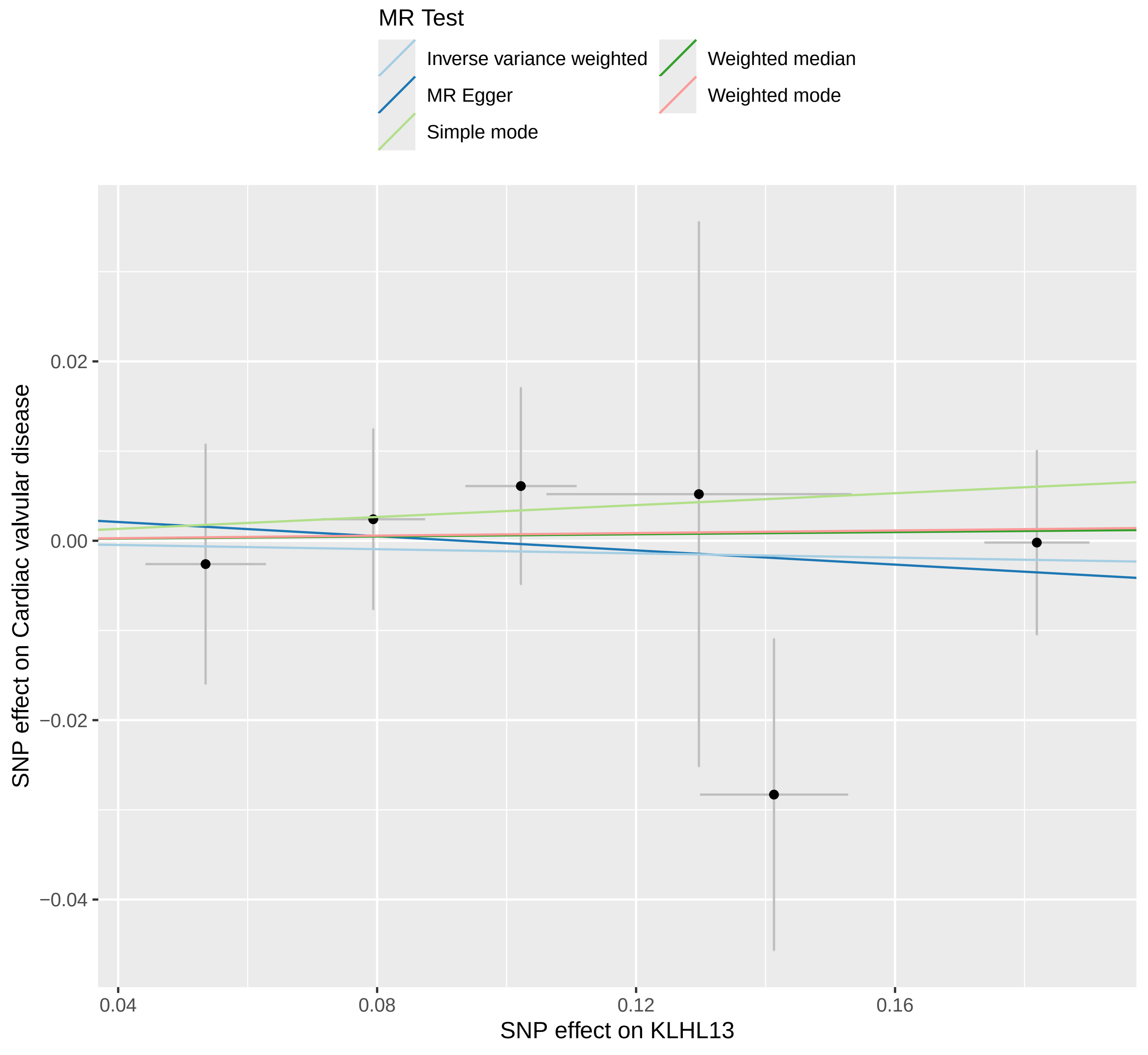
MR Test
Inverse variance weighted
MR Egger
Simple mode
Weighted median
Weighted mode
0.02
0.00
−0.02
−0.04
SNP effect on Cardiac valvular disease
0.04
0.08
0.12
0.16
SNP effect on KLHL13

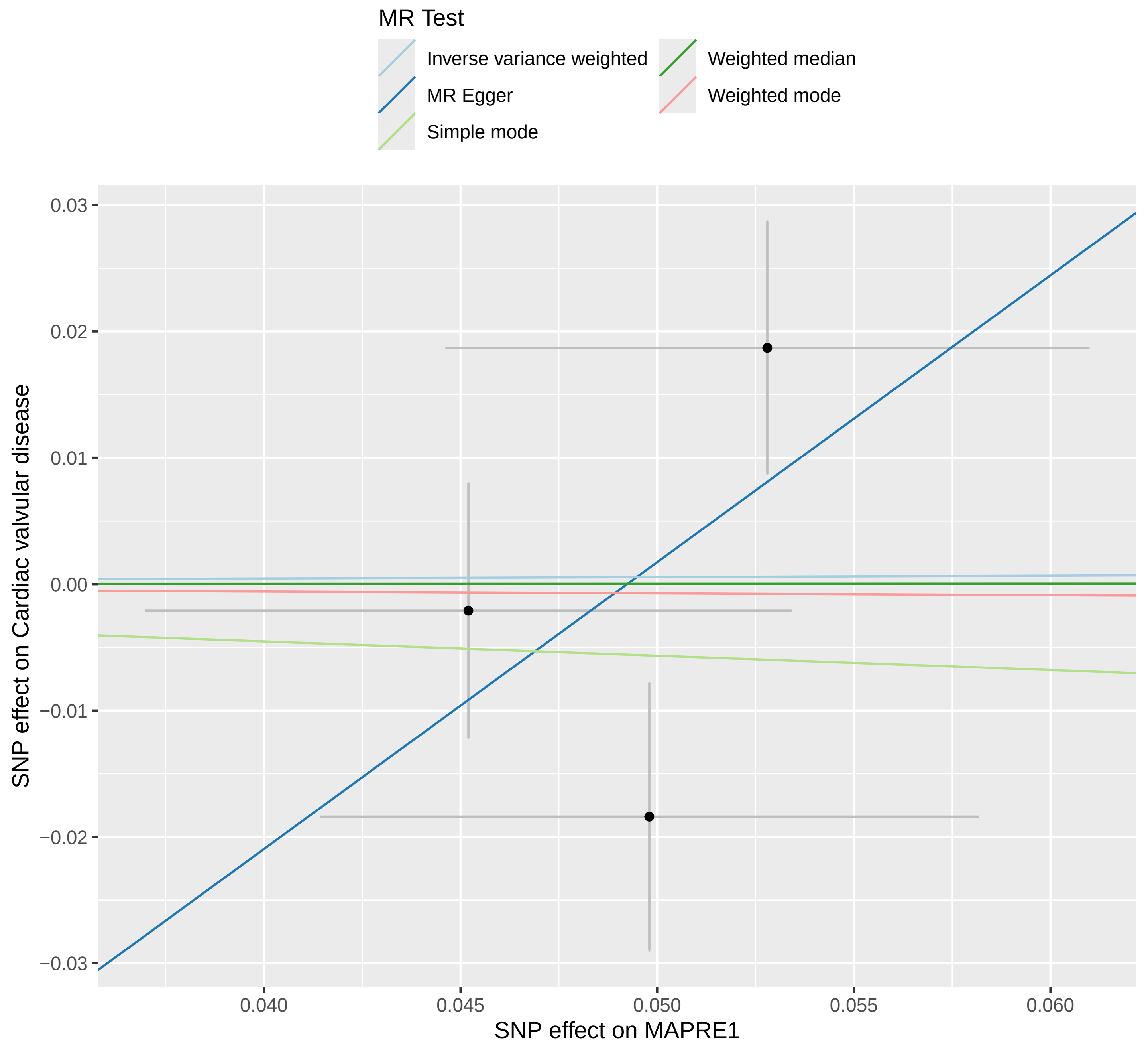

MR Test
Inverse variance weighted
MR Egger
Simple mode
Weighted median
Weighted mode
0.03
0.02
0.01
0.00
−0.01
−0.02
−0.03
SNP effect on Cardiac valvular disease
0.040
0.045
0.050
0.055
0.060
SNP effect on MAPRE1

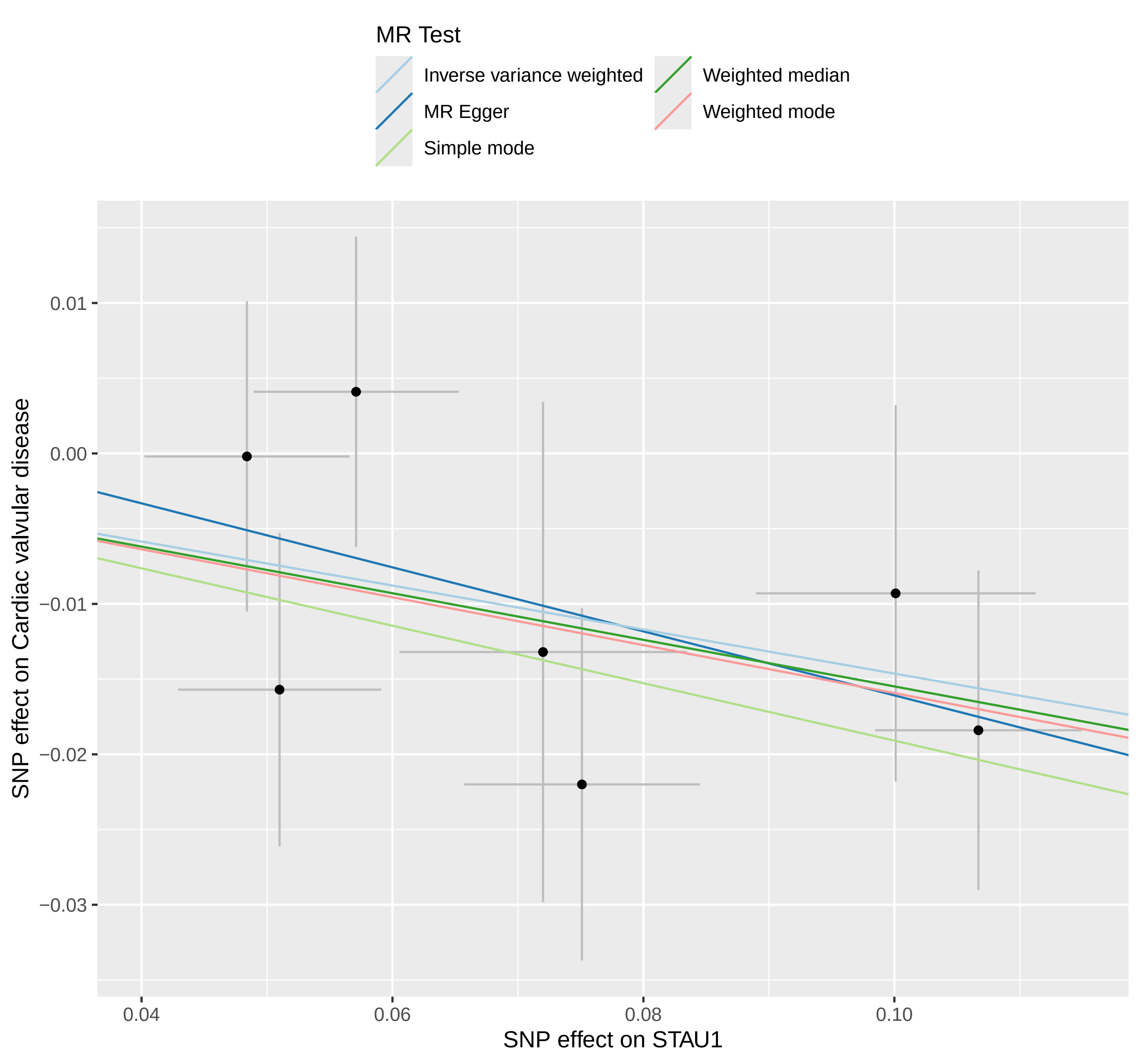
MR Test
Inverse variance weighted
MR Egger
Simple mode
Weighted median
Weighted mode
0.01
0.00
−0.01
−0.02
−0.03
SNP effect on Cardiac valvular disease
0.04
0.06
0.08
0.10
SNP effect on STAU1

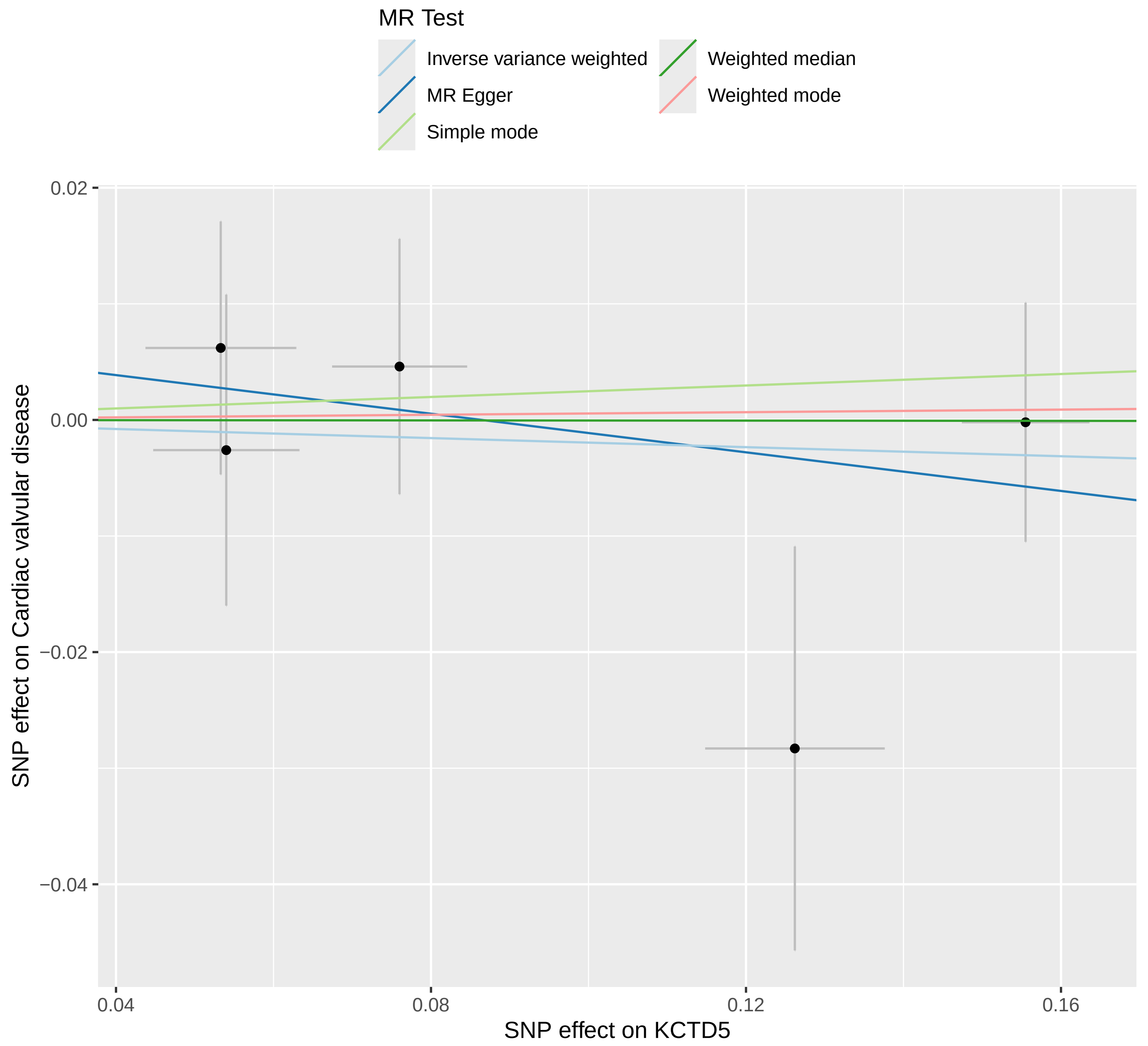

MR Test
Inverse variance weighted
MR Egger
Simple mode
Weighted median
Weighted mode
0.02
0.00
−0.02
−0.04
0.04
0.08
0.12
0.16
SNP effect on Cardiac valvular disease
SNP effect on KCTD5

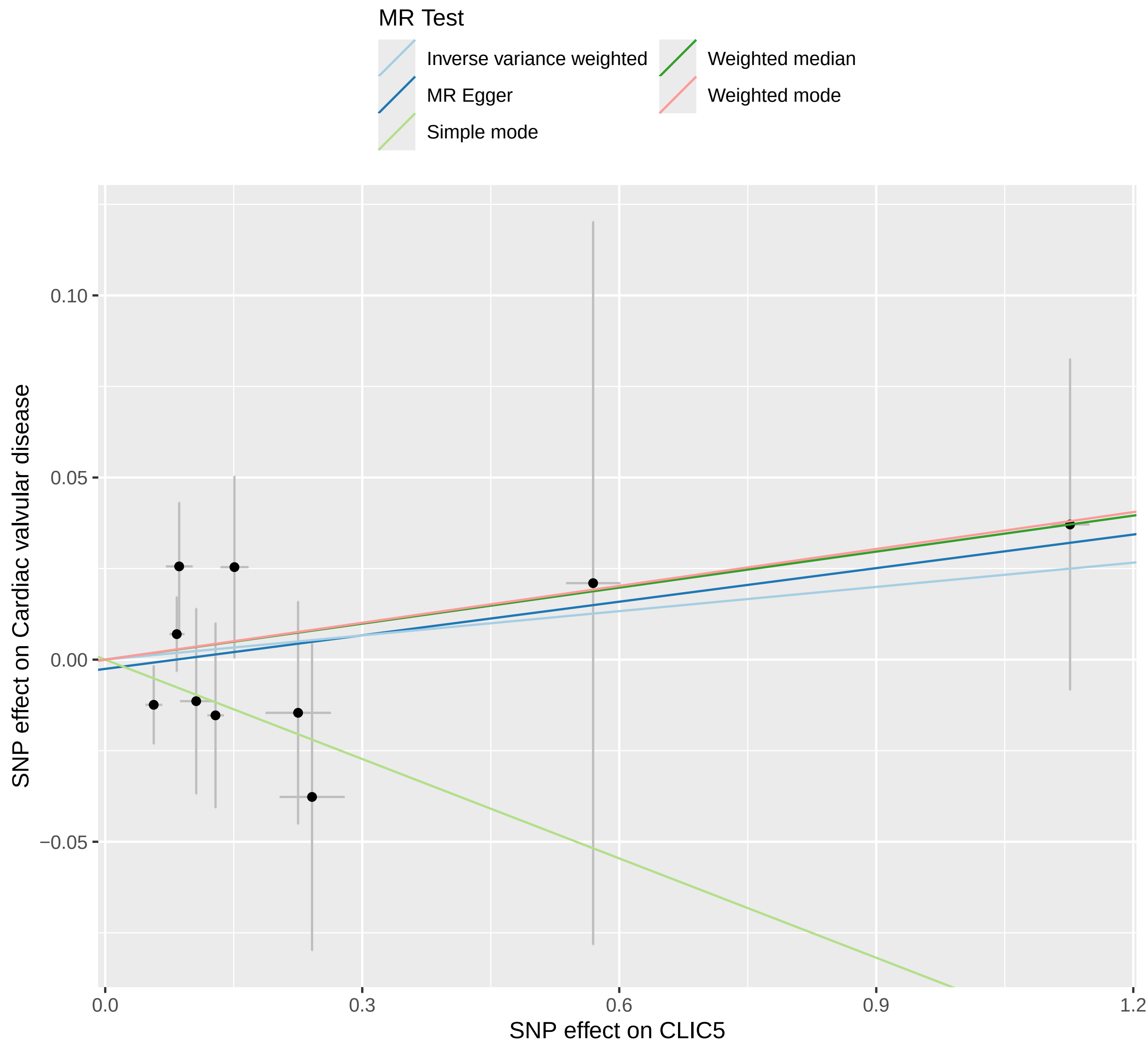
MR Test
Inverse variance weighted
MR Egger
Simple mode
Weighted median
Weighted mode
0.10
0.05
0.00
−0.05
0.0
0.3
0.6
0.9
1.2
SNP effect on CLIC5
SNP effect on Cardiac valvular disease

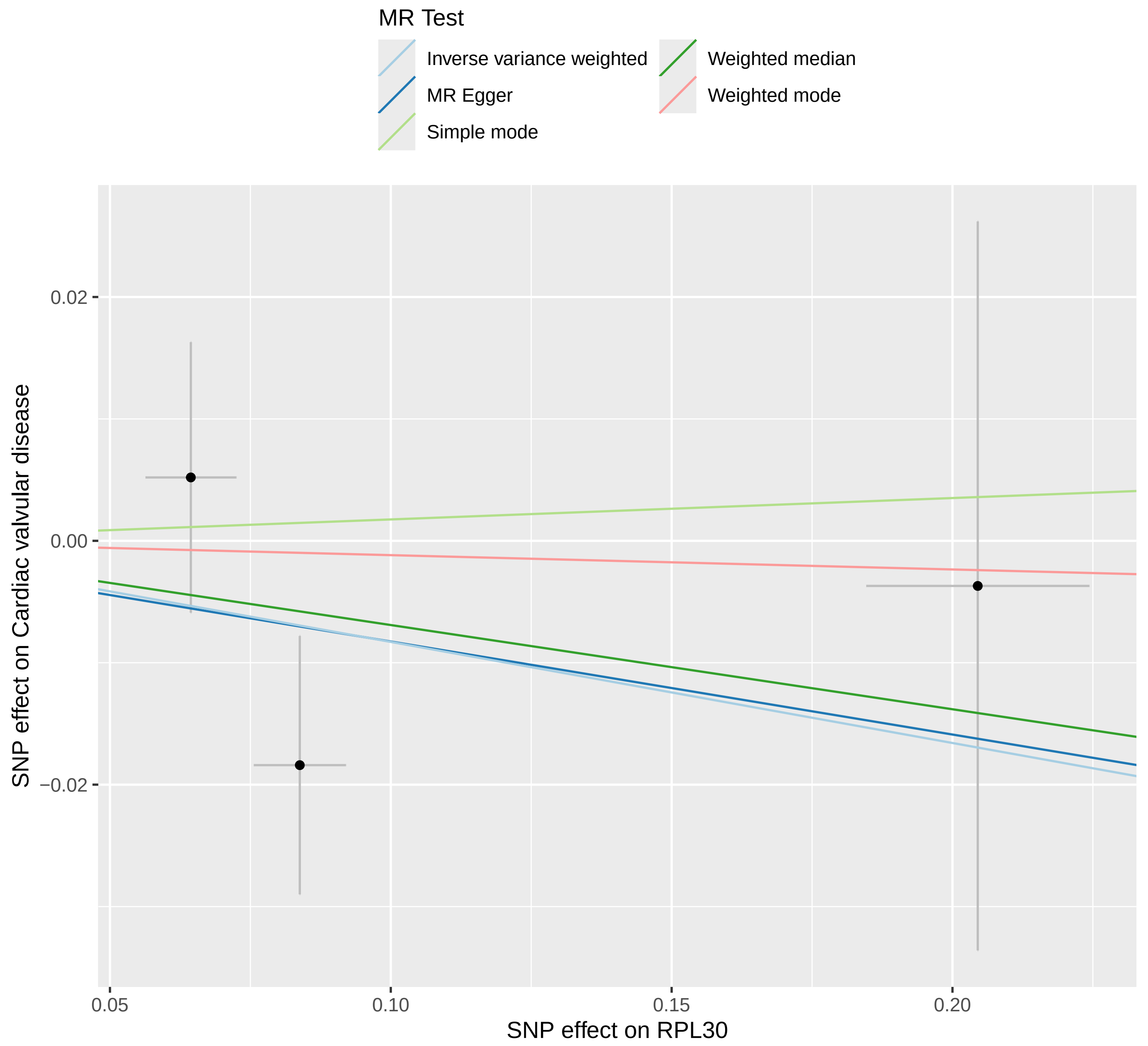

MR Test
Inverse variance weighted
MR Egger
Simple mode
Weighted median
Weighted mode
0.02
0.00
−0.02
0.05
0.10
0.15
0.20
SNP effect on Cardiac valvular disease
SNP effect on RPL30

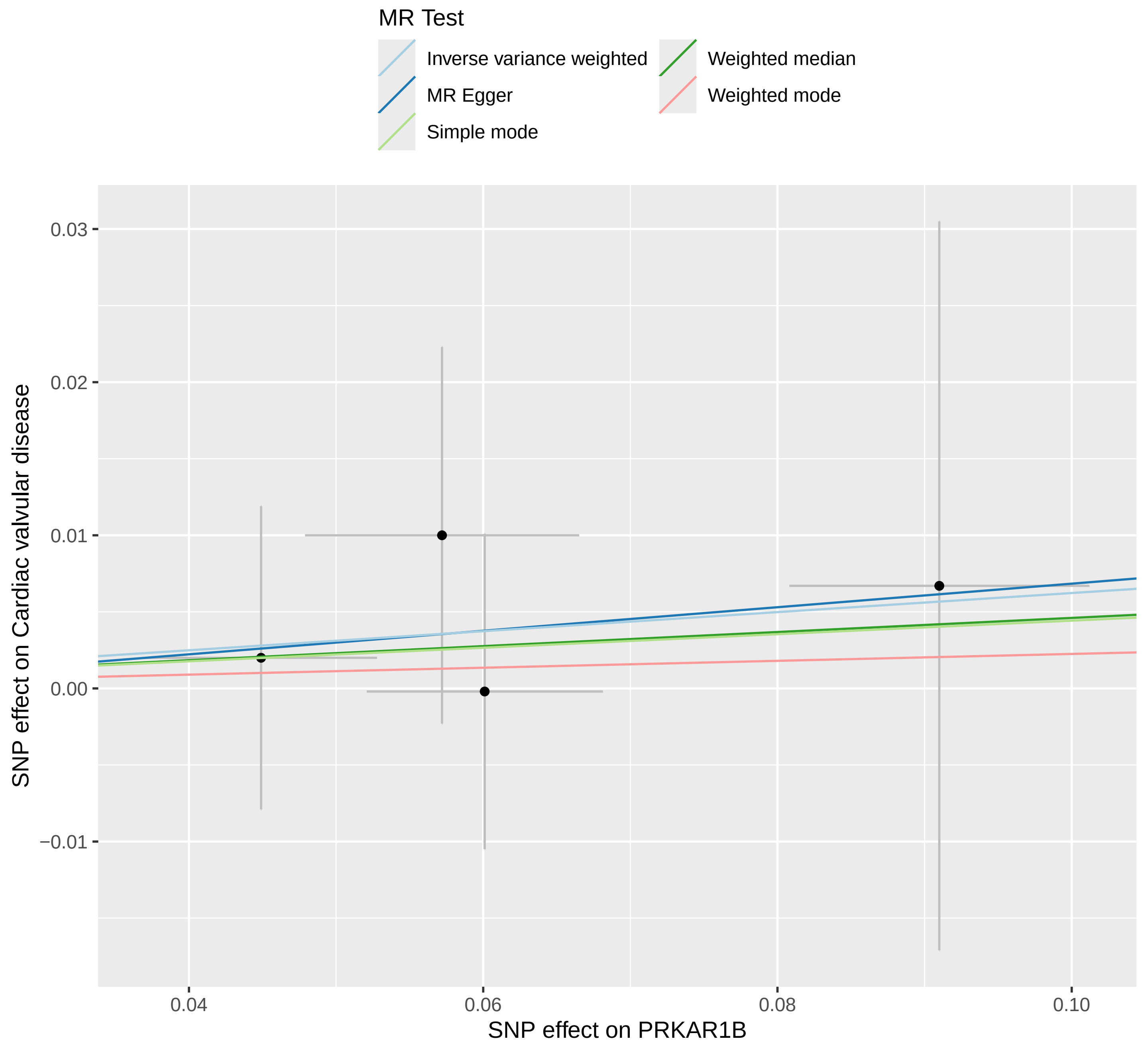

MR Test
Inverse variance weighted
MR Egger
Simple mode
Weighted median
Weighted mode
0.03
0.02
0.01
0.00
−0.01
SNP effect on Cardiac valvular disease
0.04
0.06
0.08
0.10
SNP effect on PRKAR1B

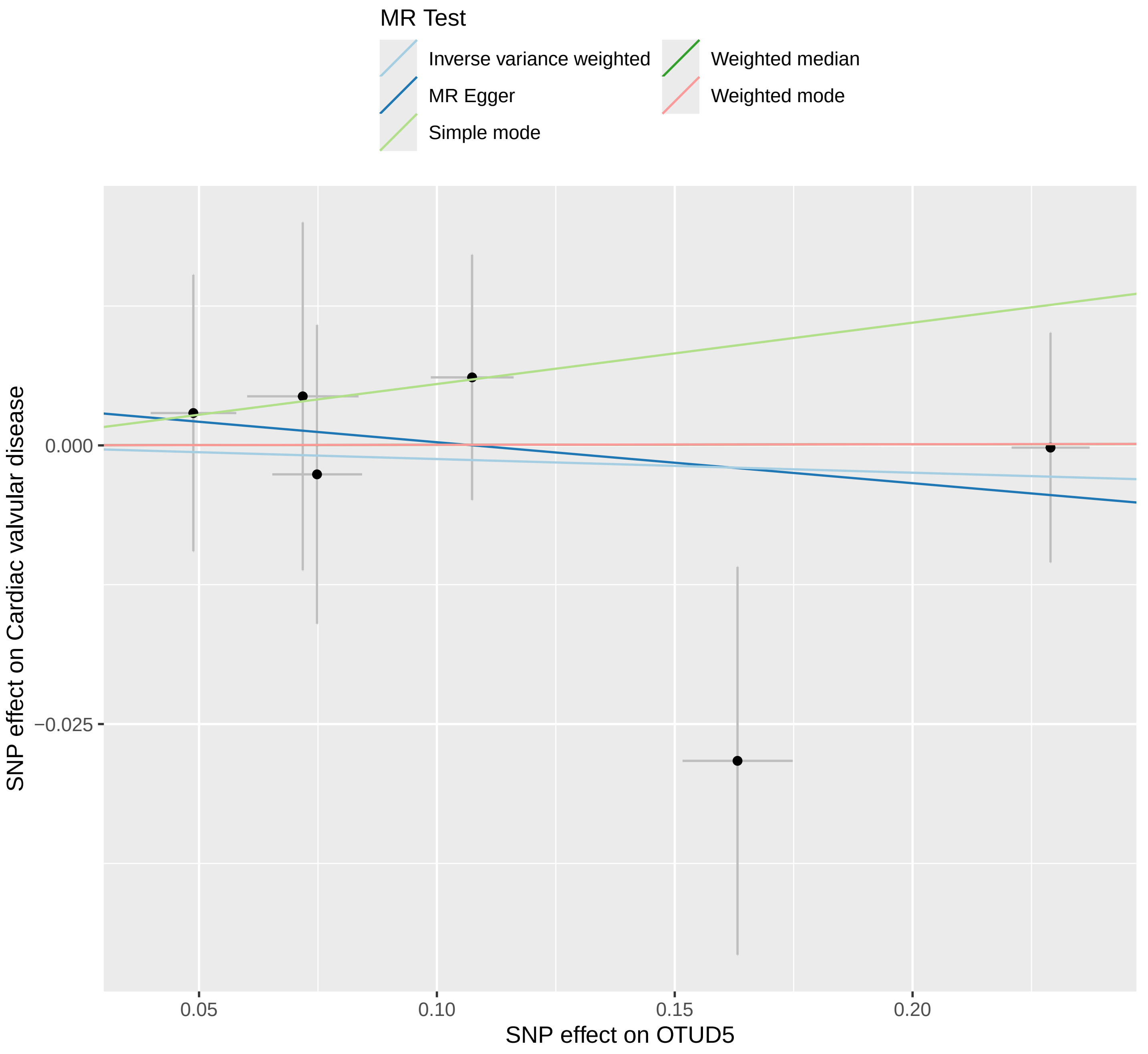
MR Test
Inverse variance weighted
MR Egger
Simple mode
Weighted median
Weighted mode
SNP effect on Cardiac valvular disease
0.000
−0.025
0.05
0.10
0.15
0.20
SNP effect on OTUD5

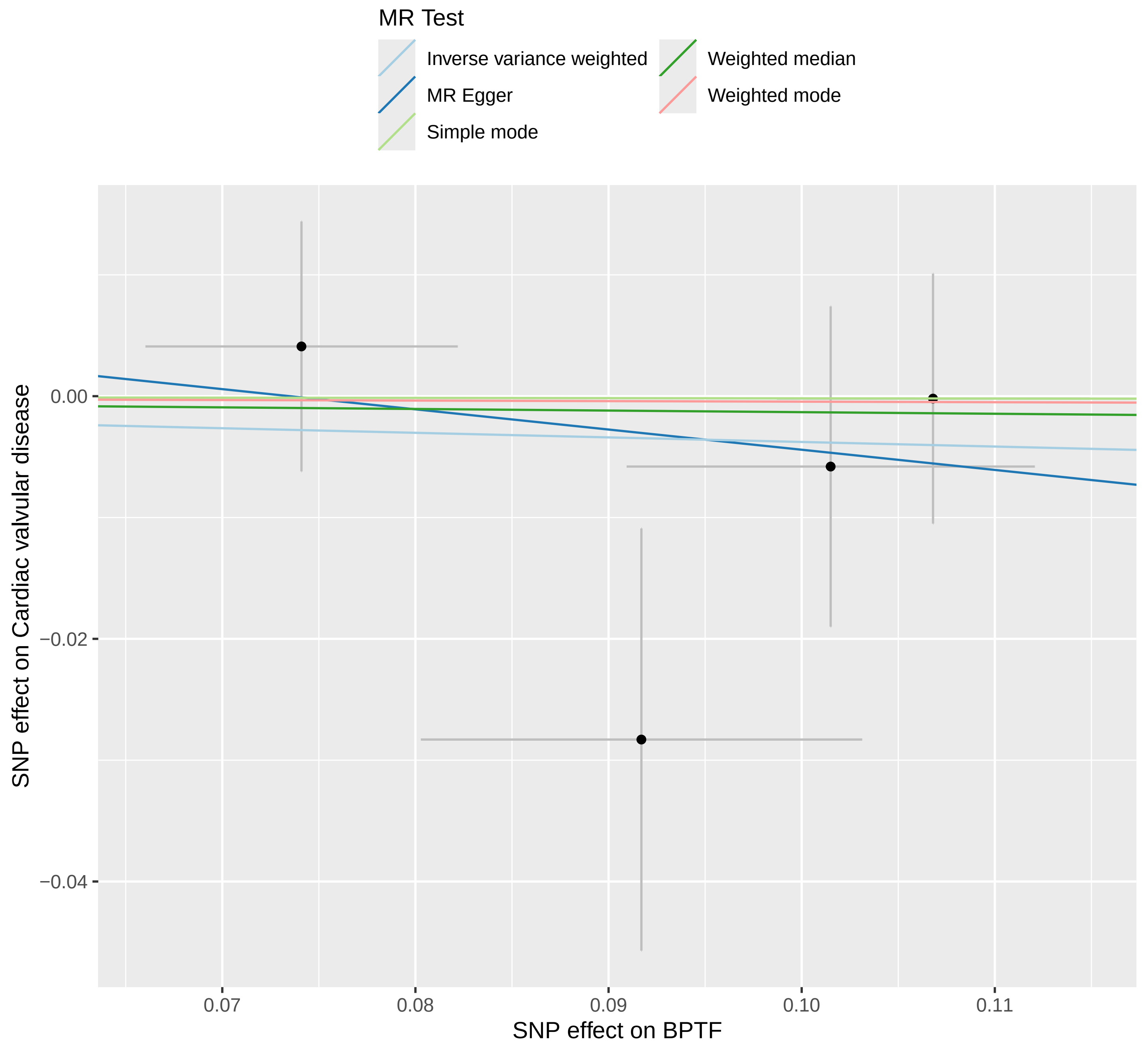

MR Test
Inverse variance weighted
MR Egger
Simple mode
Weighted median
Weighted mode
0.00
−0.02
−0.04
SNP effect on Cardiac valvular disease
0.07
0.08
0.09
0.10
0.11
SNP effect on BPTF

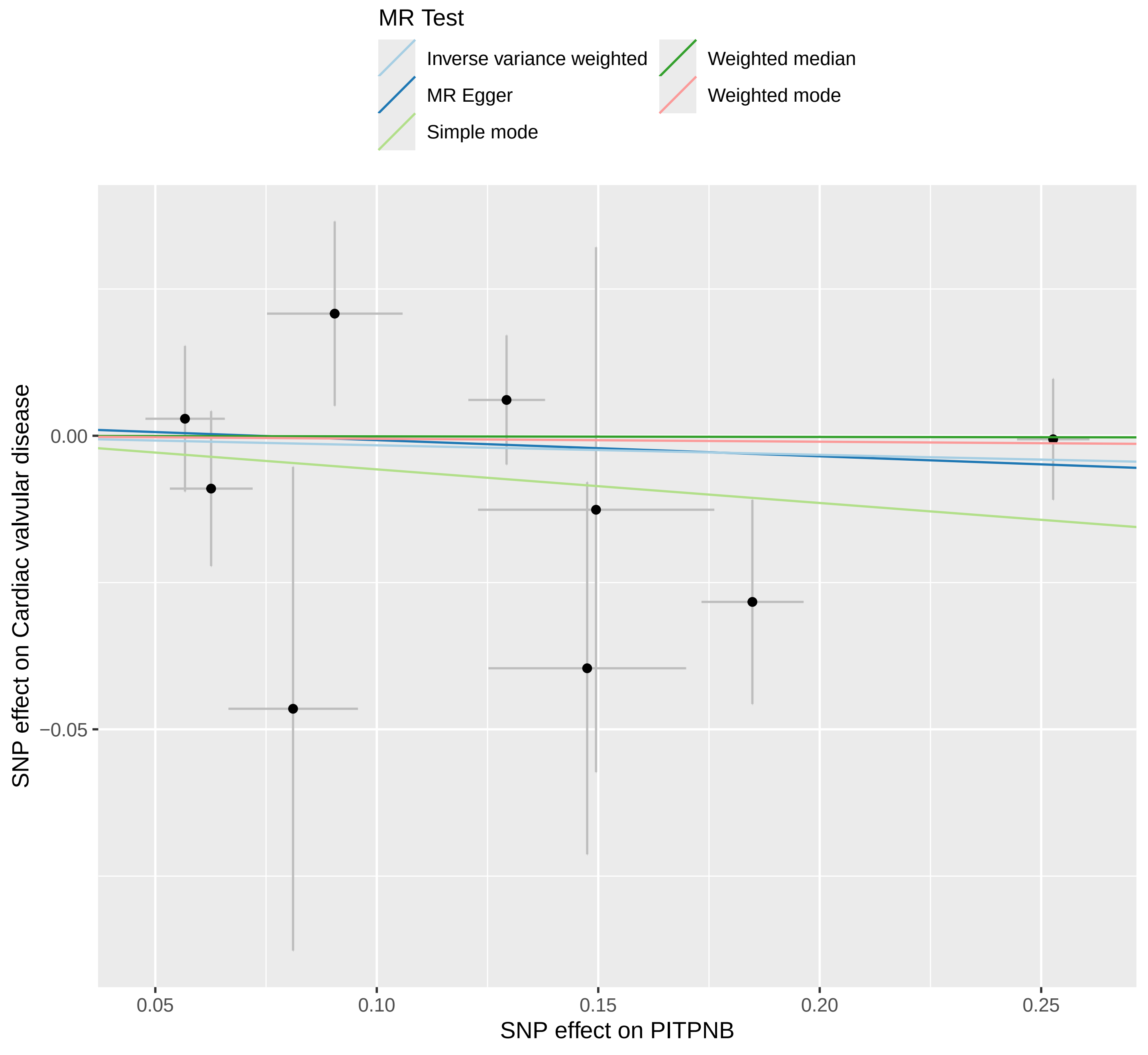
MR Test
Inverse variance weighted
MR Egger
Simple mode
Weighted median
Weighted mode
0.00
−0.05
0.05
0.10
0.15
0.20
0.25
SNP effect on PITPNB
SNP effect on Cardiac valvular disease

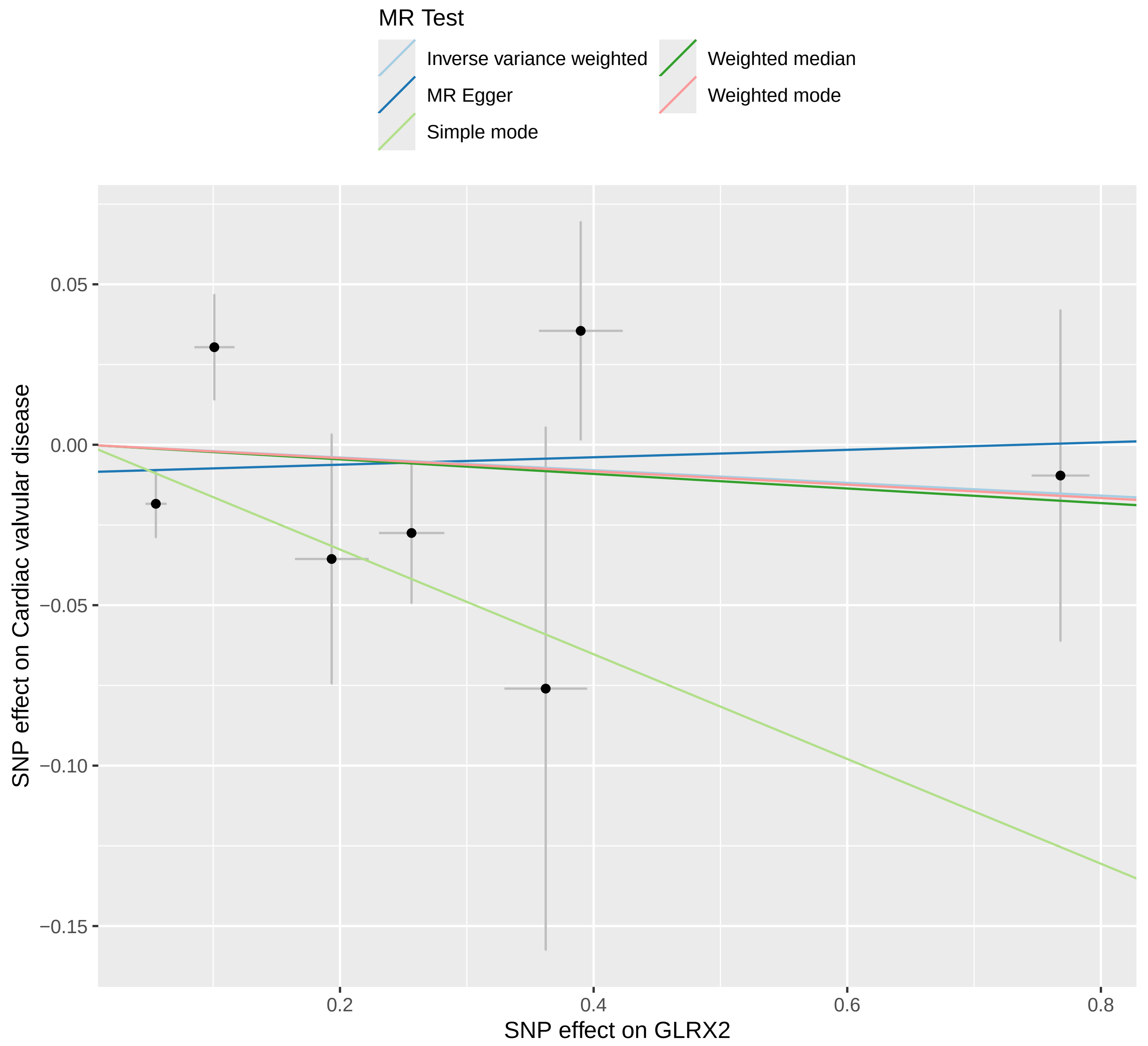

MR Test
Inverse variance weighted
MR Egger
Simple mode
Weighted median
Weighted mode
0.05
0.00
−0.05
−0.10
−0.15
0.2
0.4
0.6
0.8
SNP effect on Cardiac valvular disease
SNP effect on GLRX2

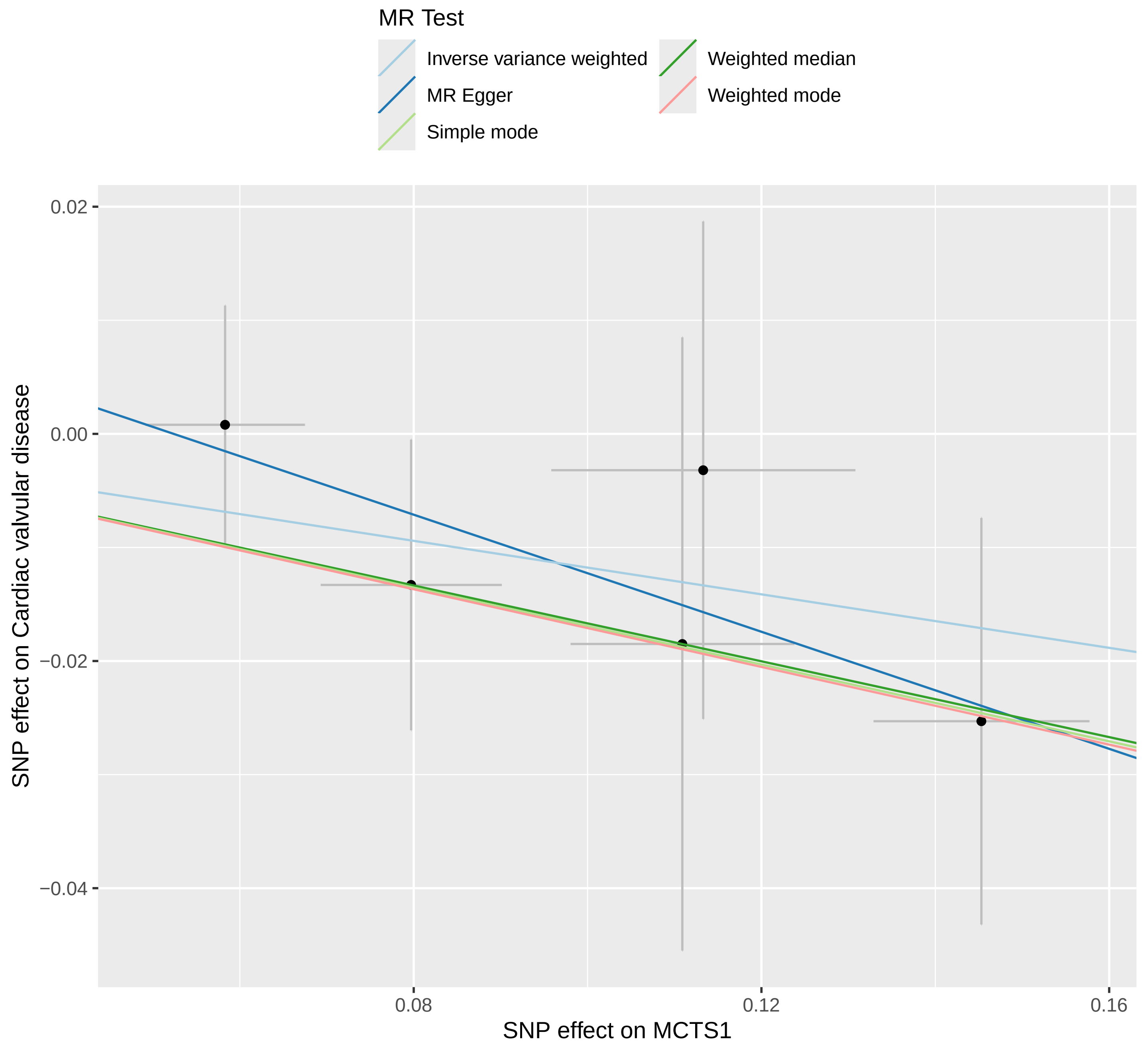
MR Test
Inverse variance weighted
MR Egger
Simple mode
Weighted median
Weighted mode
0.02
0.00
−0.02
−0.04
0.08
0.12
0.16
SNP effect on MCTS1
SNP effect on Cardiac valvular disease

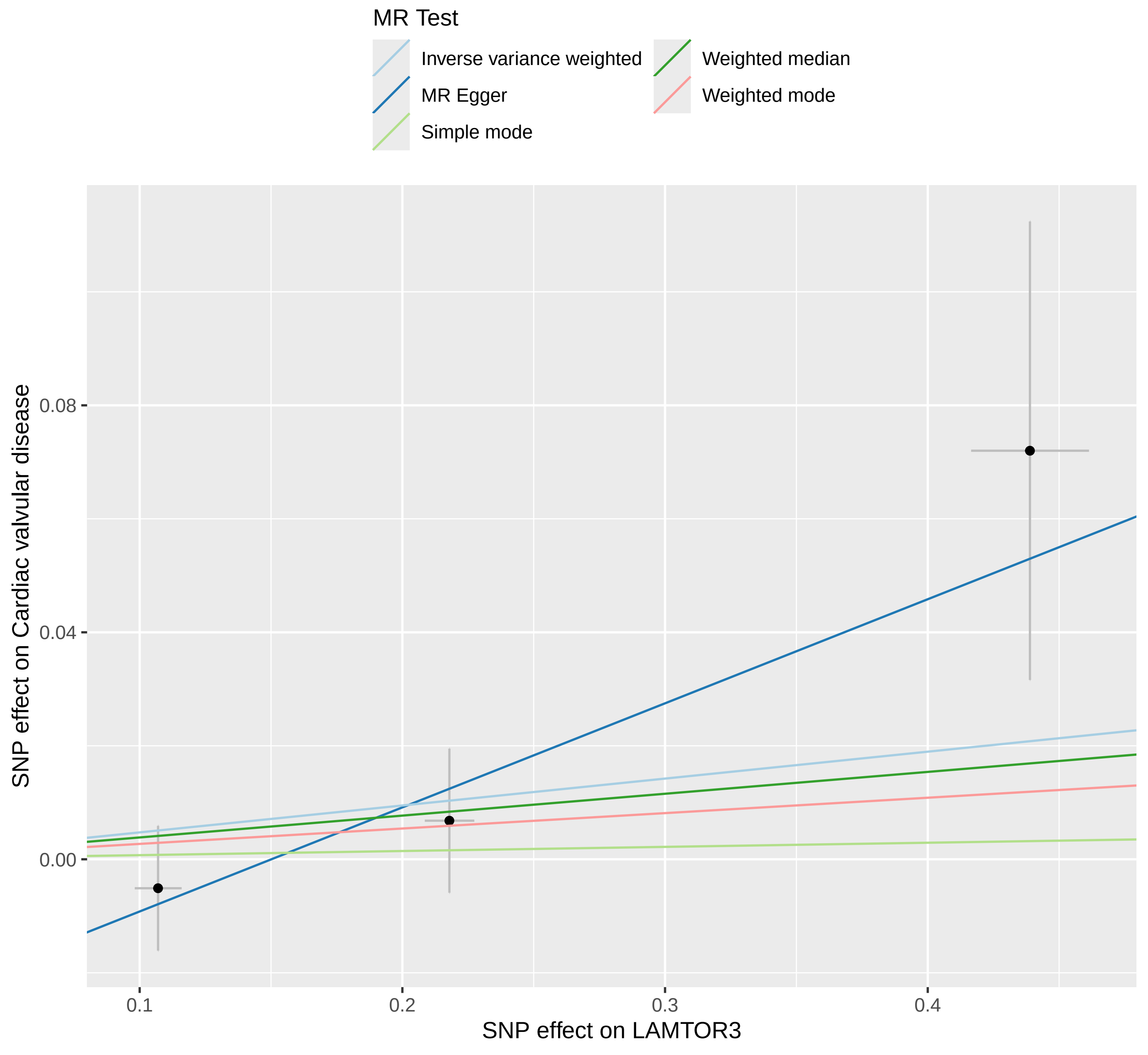
MR Test
Inverse variance weighted
MR Egger
Simple mode
Weighted median
Weighted mode
0.08
0.04
0.00
0.1
0.2
0.3
0.4
SNP effect on Cardiac valvular disease
SNP effect on LAMTOR3

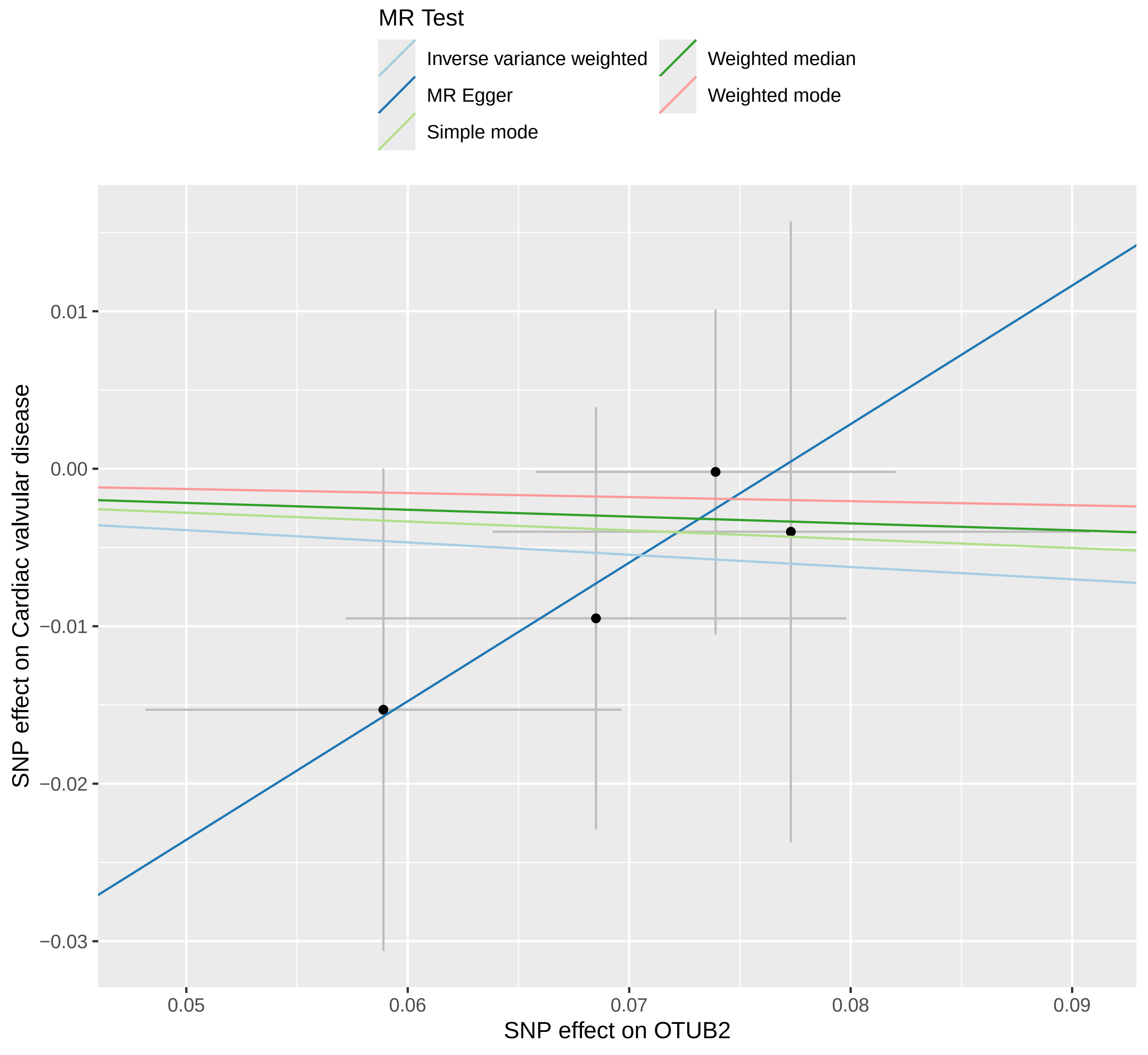

MR Test
Inverse variance weighted
MR Egger
Simple mode
Weighted median
Weighted mode
0.01
0.00
−0.01
−0.02
−0.03
SNP effect on Cardiac valvular disease
0.05
0.06
0.07
0.08
0.09
SNP effect on OTUB2

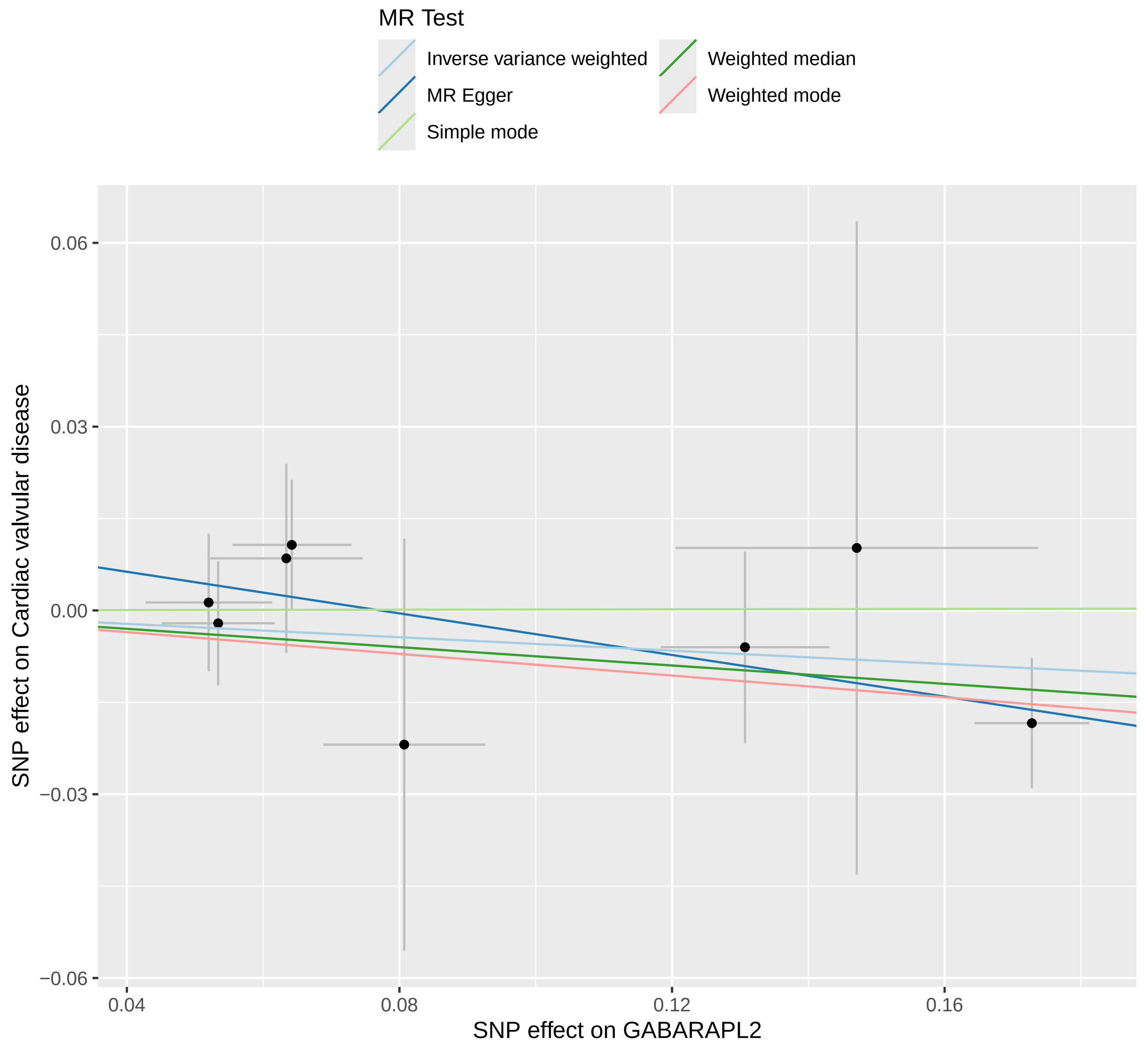
MR Test
Inverse variance weighted
MR Egger
Simple mode
Weighted median
Weighted mode
0.06
0.03
0.00
−0.03
−0.06
SNP effect on Cardiac valvular disease
0.04
0.08
0.12
0.16
SNP effect on GABARAPL2

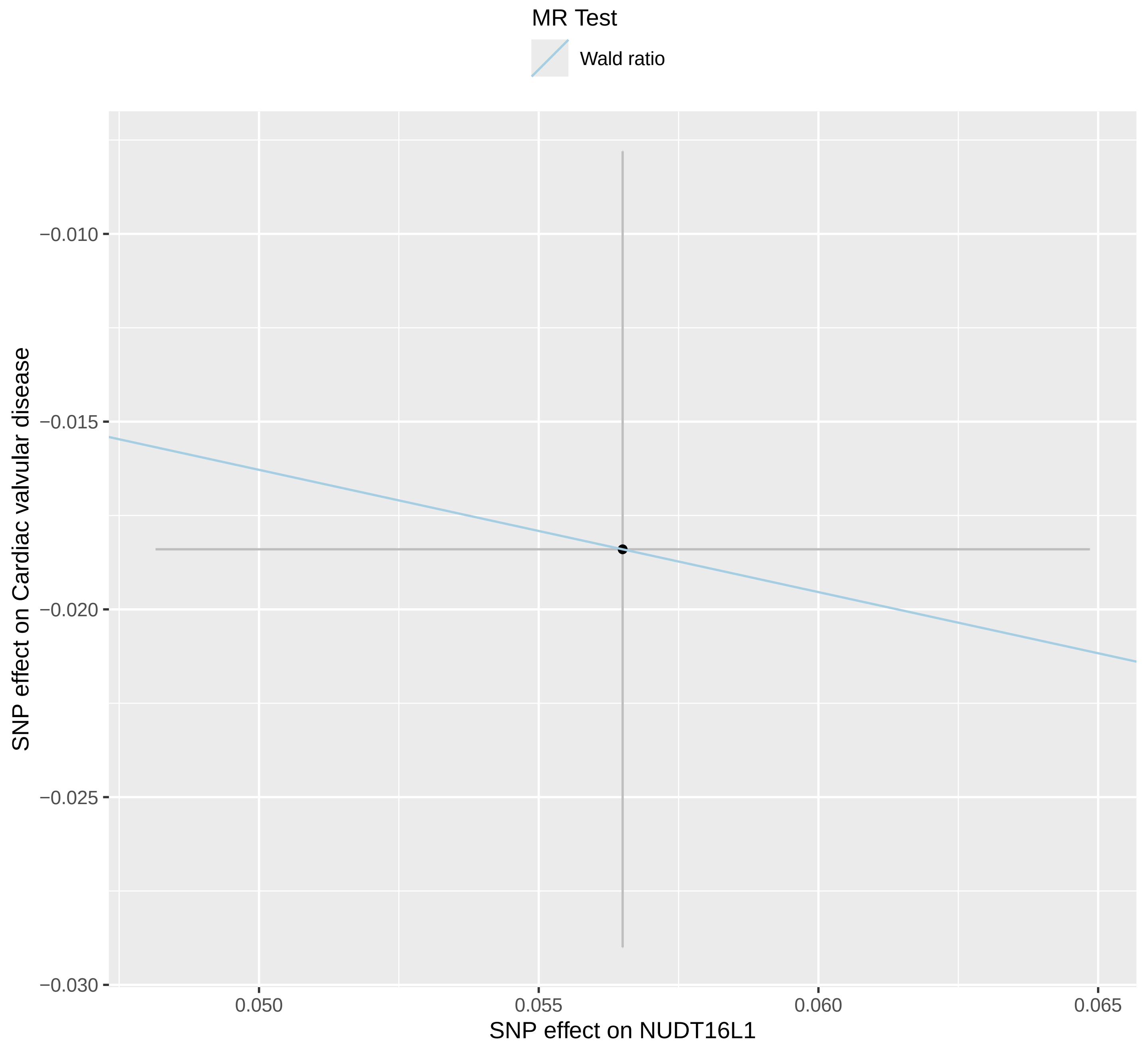
MR Test
Wald ratio
−0.010
−0.015
−0.020
−0.025
−0.030
0.050
0.055
0.060
0.065
SNP effect on Cardiac valvular disease
SNP effect on NUDT16L1

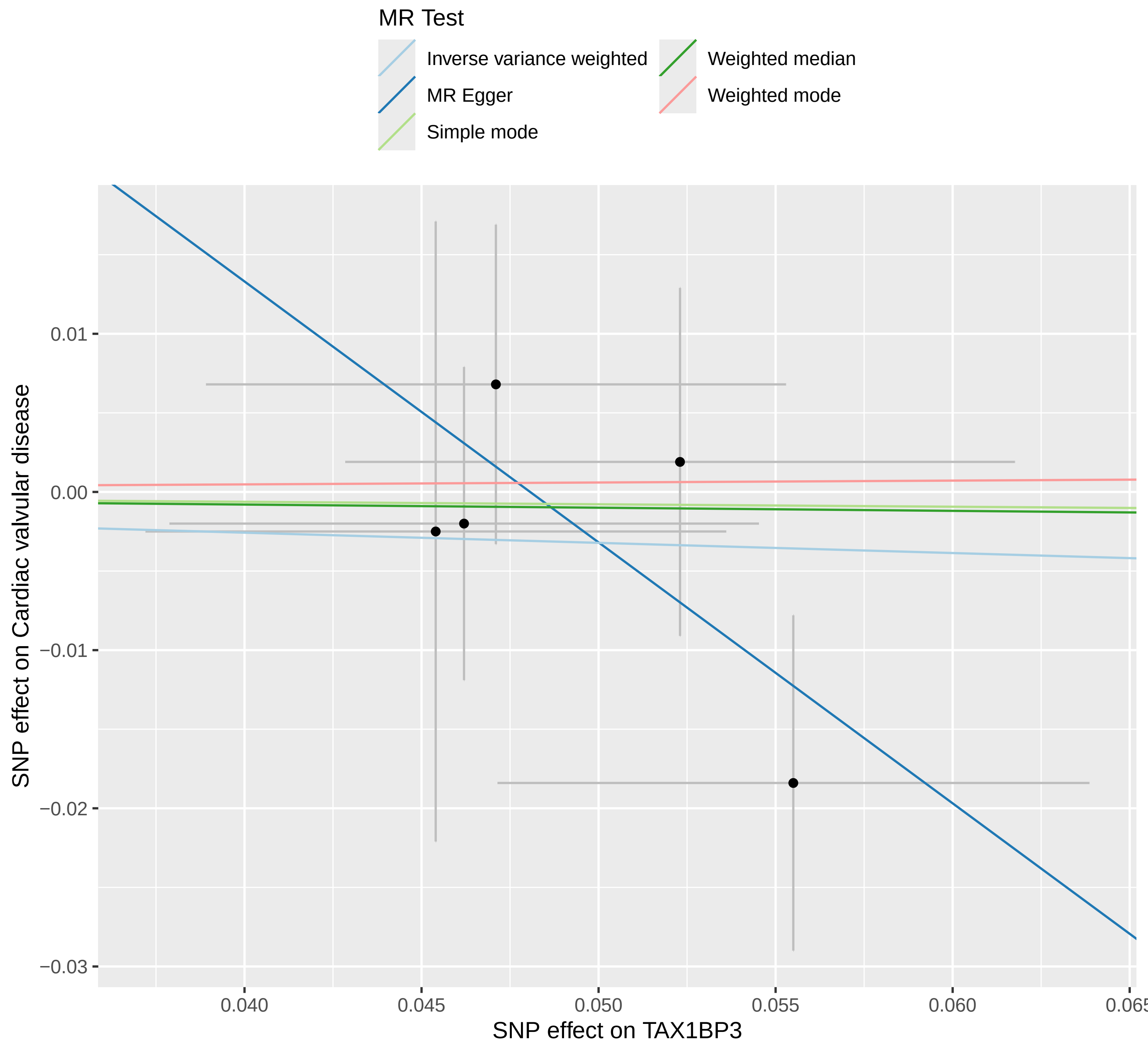

MR Test
Inverse variance weighted
MR Egger
Simple mode
Weighted median
Weighted mode
SNP effect on Cardiac valvular disease
0.01
0.00
−0.01
−0.02
−0.03
0.040
0.045
0.050
0.055
0.060
SNP effect on TAX1BP3

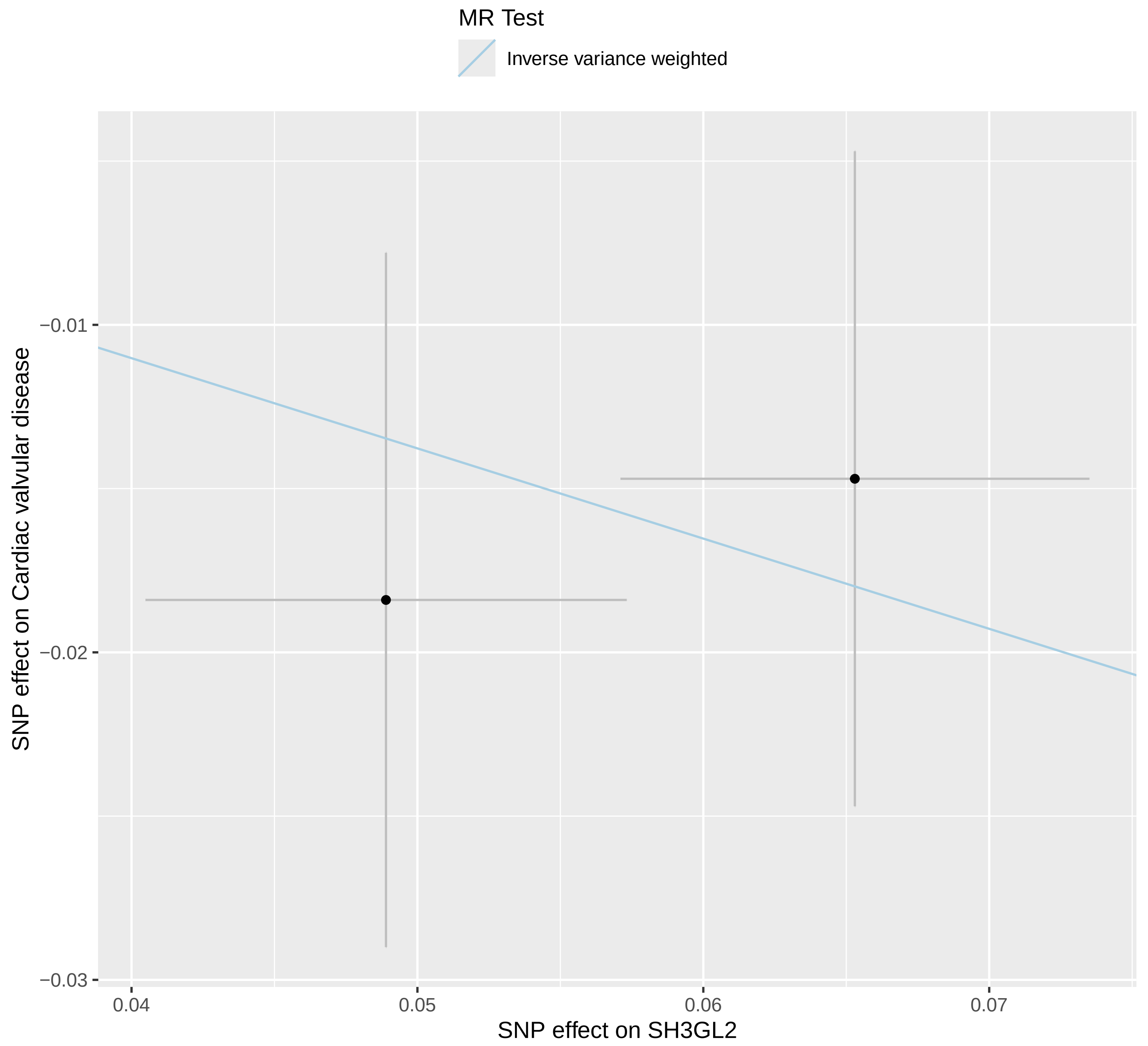

MR Test
Inverse variance weighted
−0.01
−0.02
−0.03
0.04
0.05
0.06
0.07
SNP effect on Cardiac valvular disease
SNP effect on SH3GL2

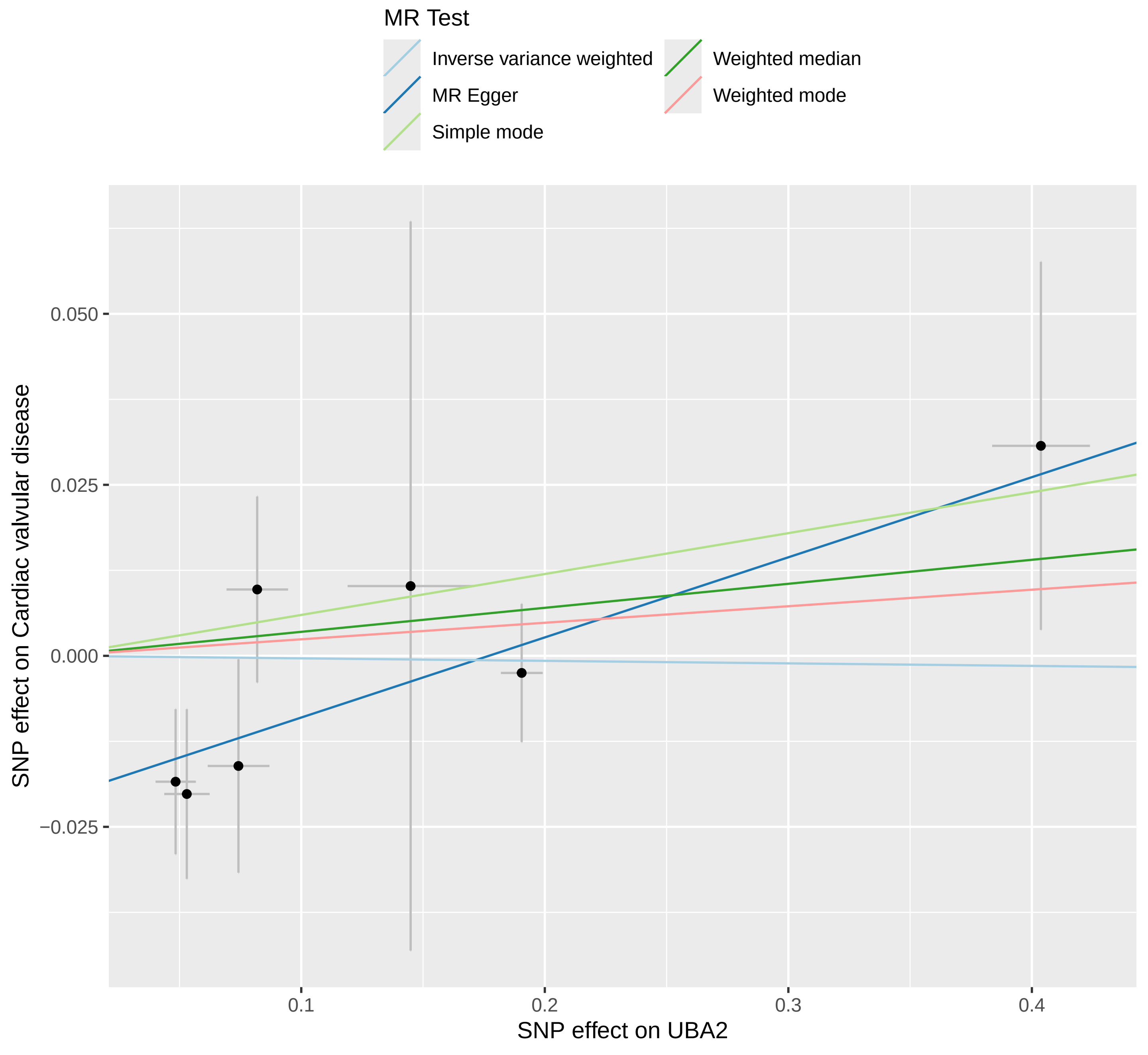
MR Test
Inverse variance weighted
MR Egger
Simple mode
Weighted median
Weighted mode
SNP effect on Cardiac valvular disease
0.050
0.025
0.000
−0.025
0.1
0.2
0.3
0.4
SNP effect on UBA2

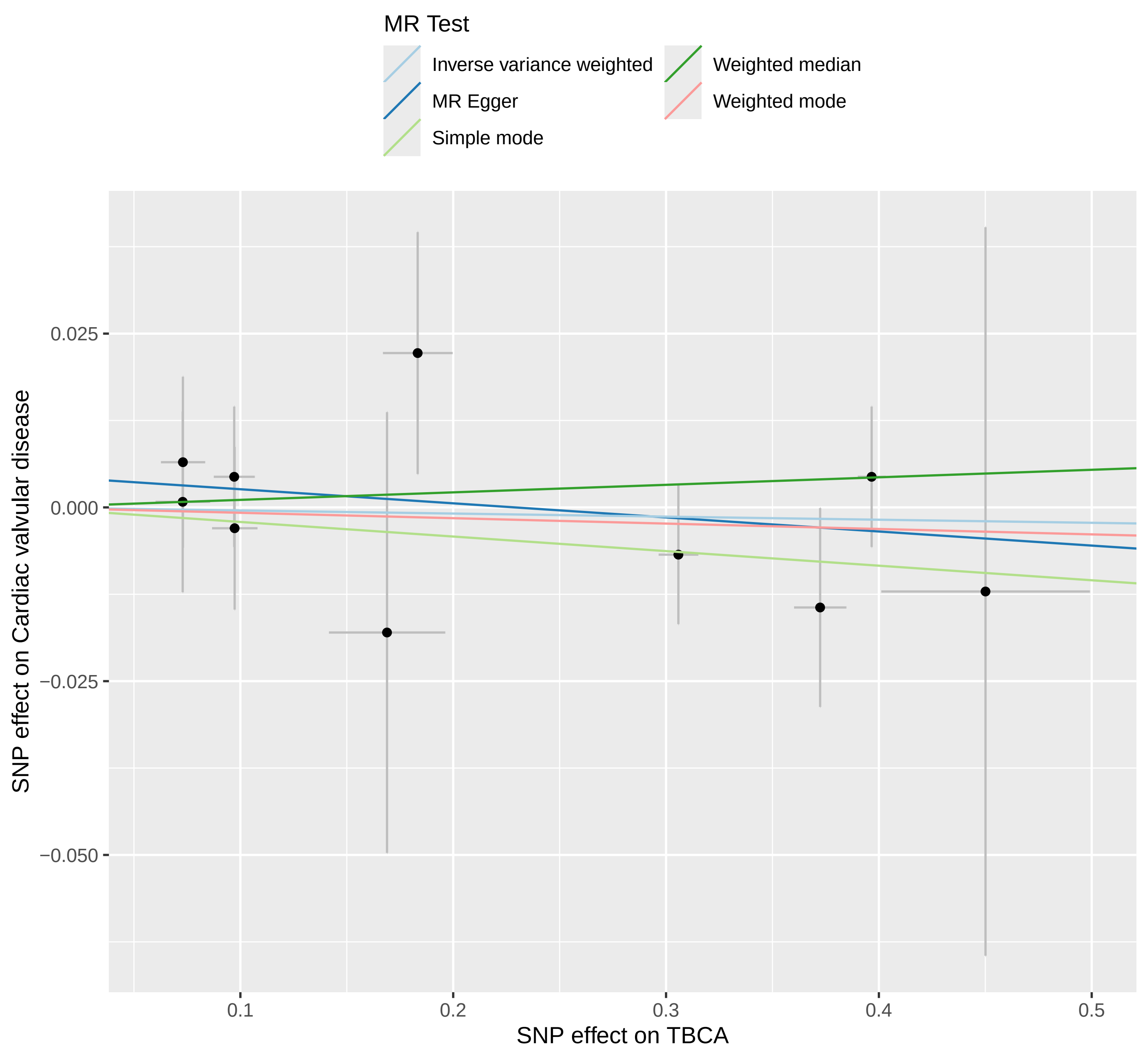
MR Test
Inverse variance weighted
MR Egger
Simple mode
Weighted median
Weighted mode
0.025
0.000
−0.025
−0.050
0.1
0.2
0.3
0.4
0.5
SNP effect on Cardiac valvular disease
SNP effect on TBCA

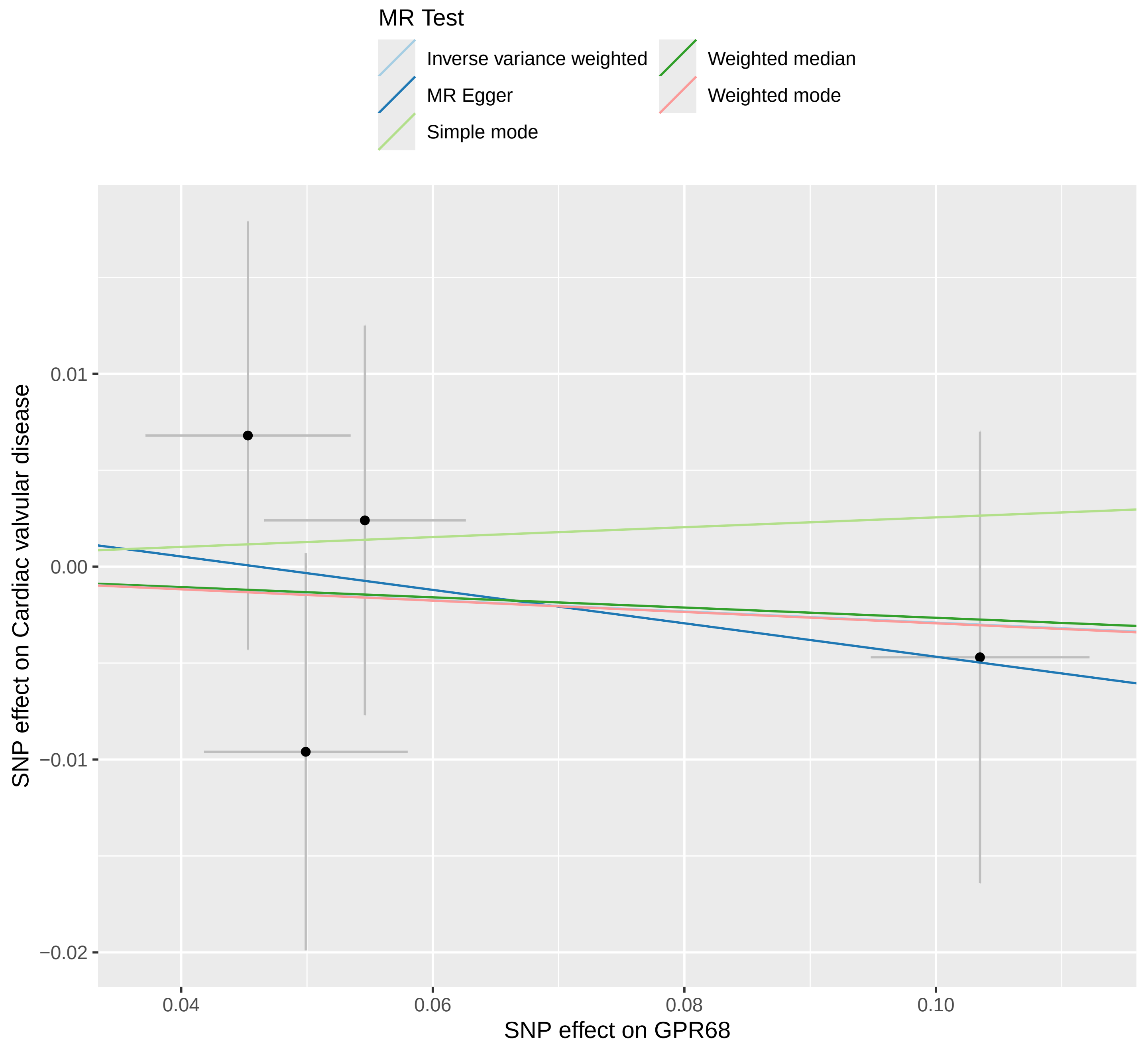
MR Test
Inverse variance weighted
MR Egger
Simple mode
Weighted median
Weighted mode
SNP effect on Cardiac valvular disease
0.01
0.00
−0.01
−0.02
0.04
0.06
0.08
0.10
SNP effect on GPR68

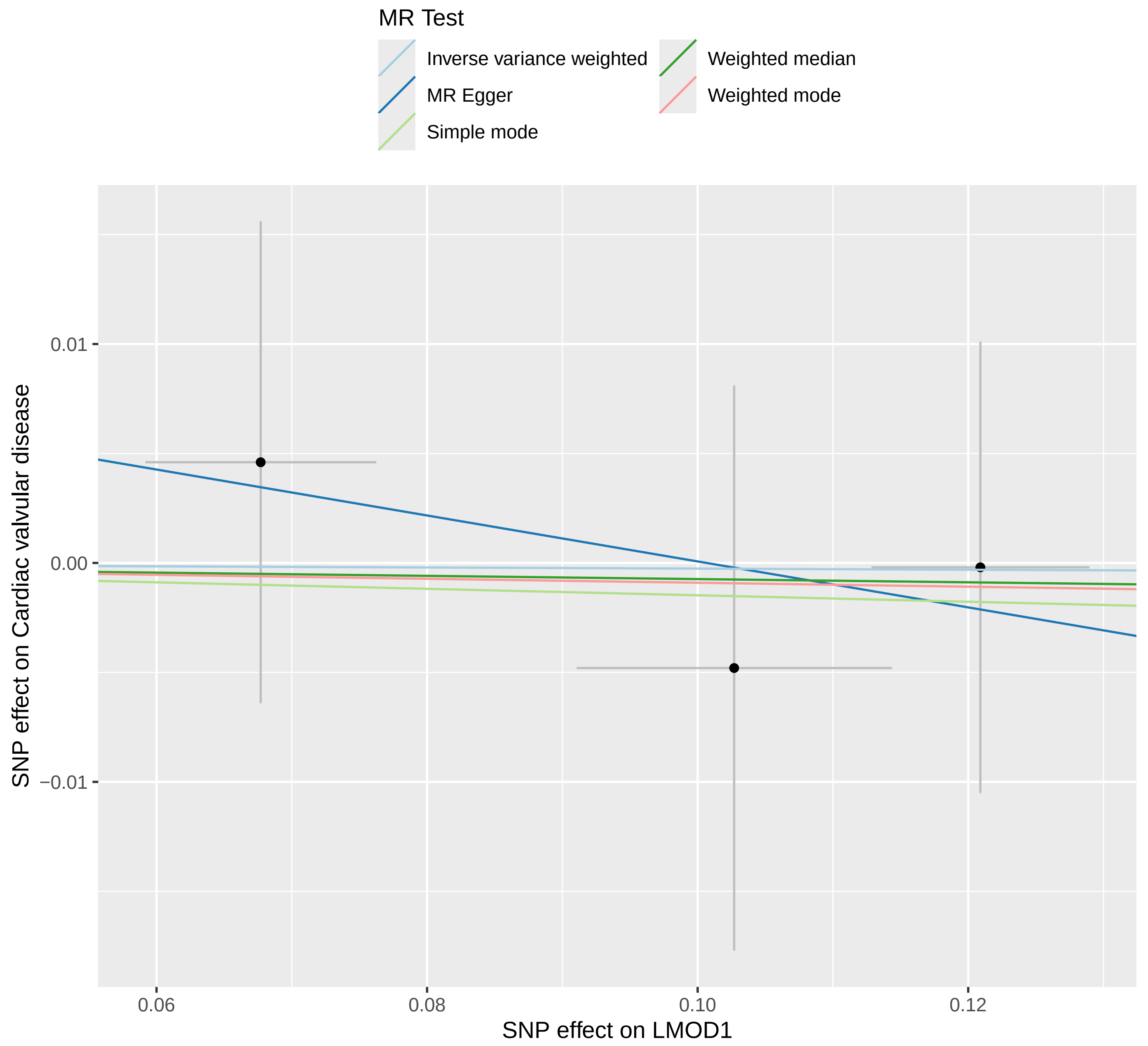
MR Test
Inverse variance weighted
MR Egger
Simple mode
Weighted median
Weighted mode
0.01
0.00
−0.01
SNP effect on Cardiac valvular disease
0.06
0.08
0.10
0.12
SNP effect on LMOD1

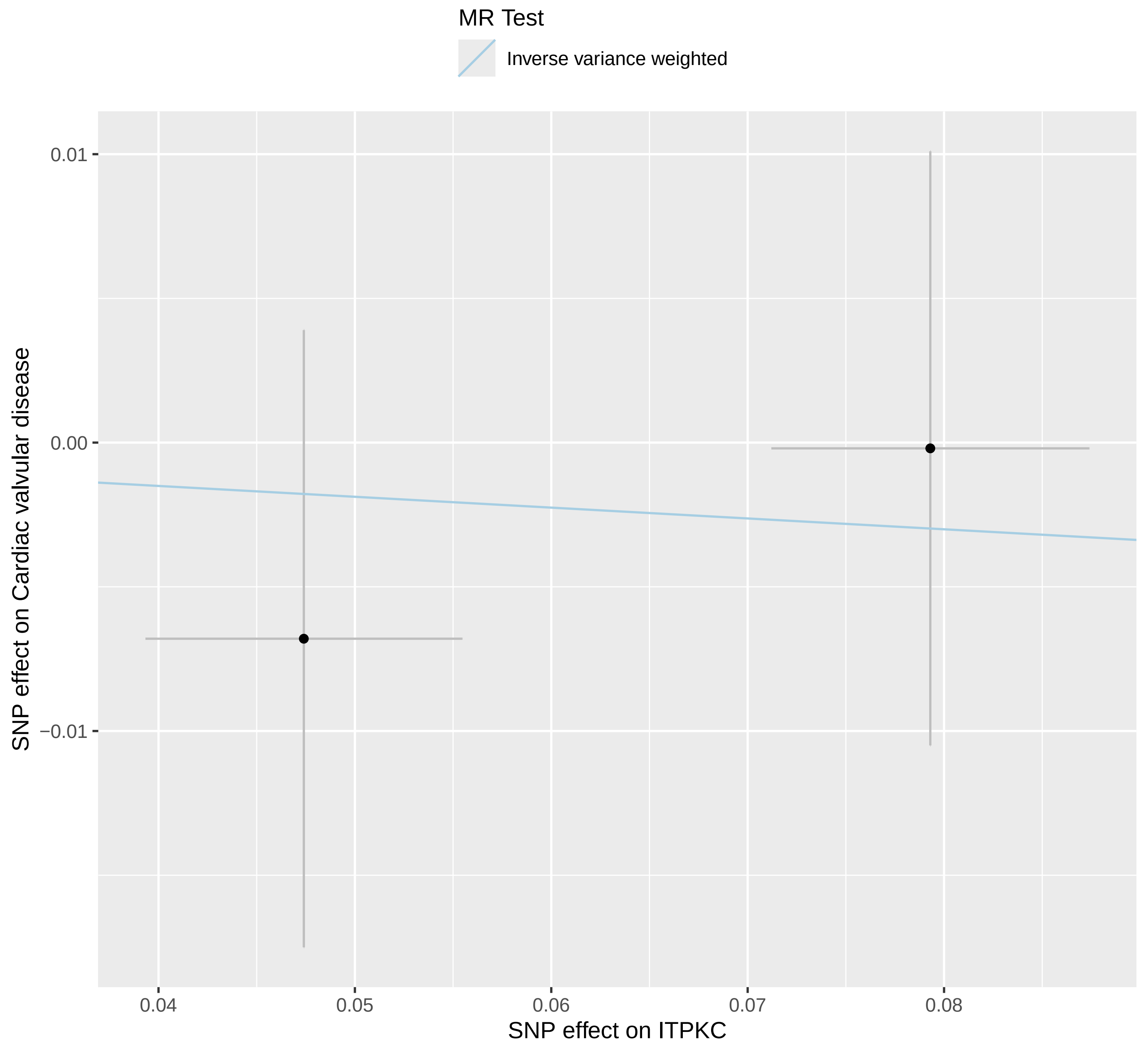

MR Test
Inverse variance weighted
0.01
0.00
−0.01
0.04
0.05
0.06
0.07
0.08
SNP effect on ITPKC
SNP effect on Cardiac valvular disease

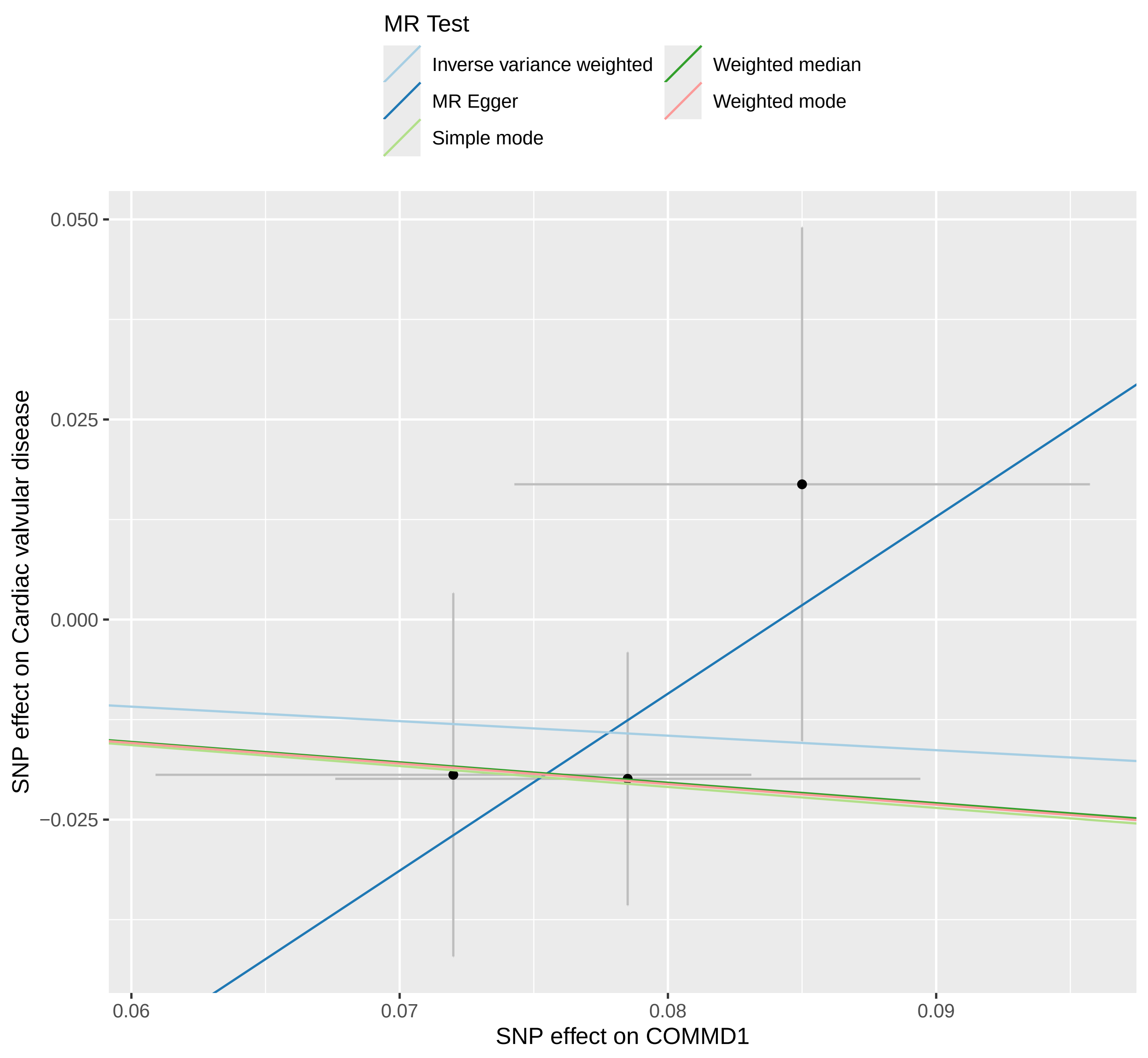
MR Test
Inverse variance weighted
MR Egger
Simple mode
Weighted median
Weighted mode
0.050
0.025
0.000
−0.025
0.06
0.07
0.08
0.09
SNP effect on Cardiac valvular disease
SNP effect on COMMD1

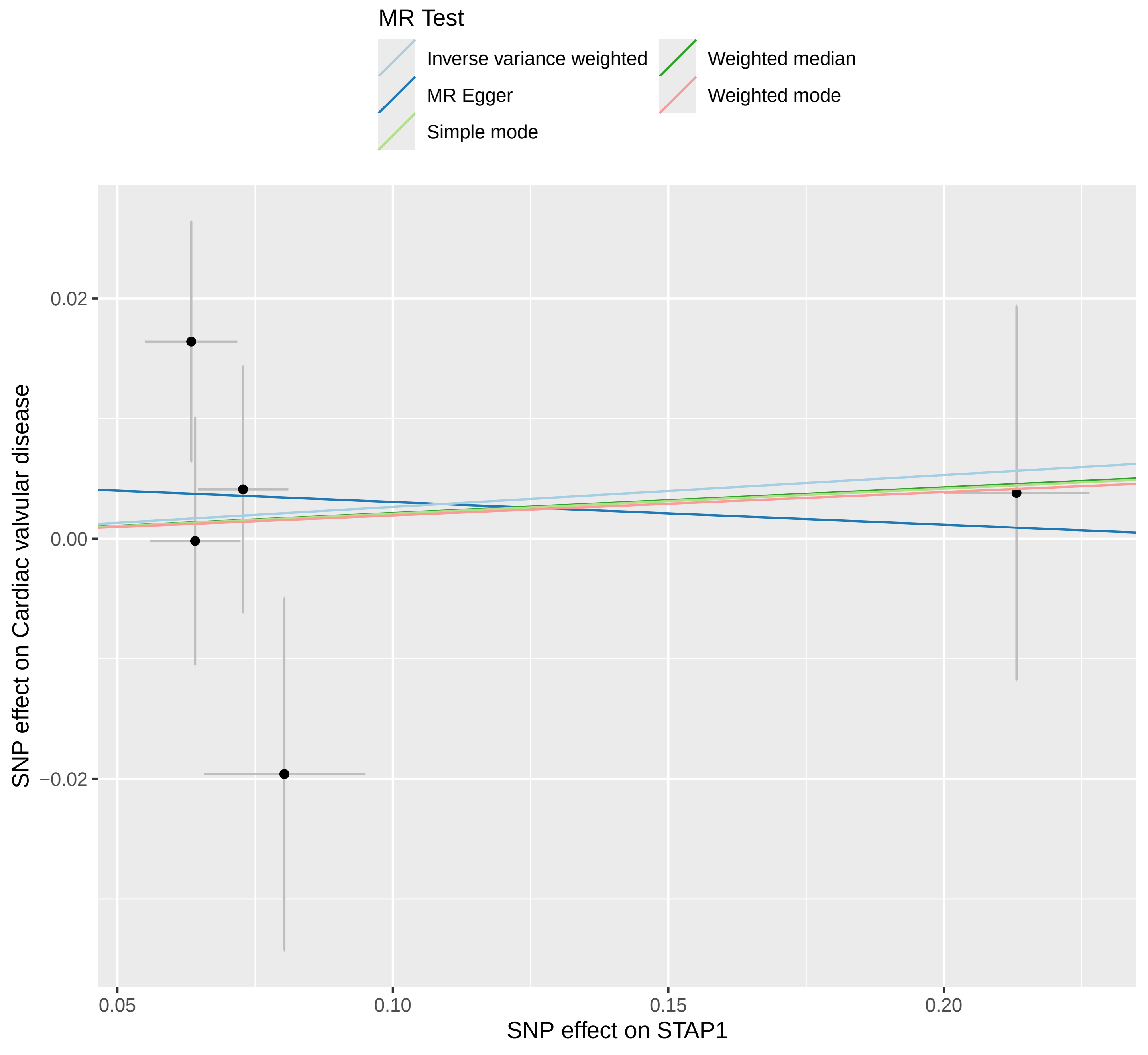

MR Test
Inverse variance weighted
MR Egger
Simple mode
Weighted median
Weighted mode
0.02
0.00
−0.02
0.05
0.10
0.15
0.20
SNP effect on STAP1
SNP effect on Cardiac valvular disease

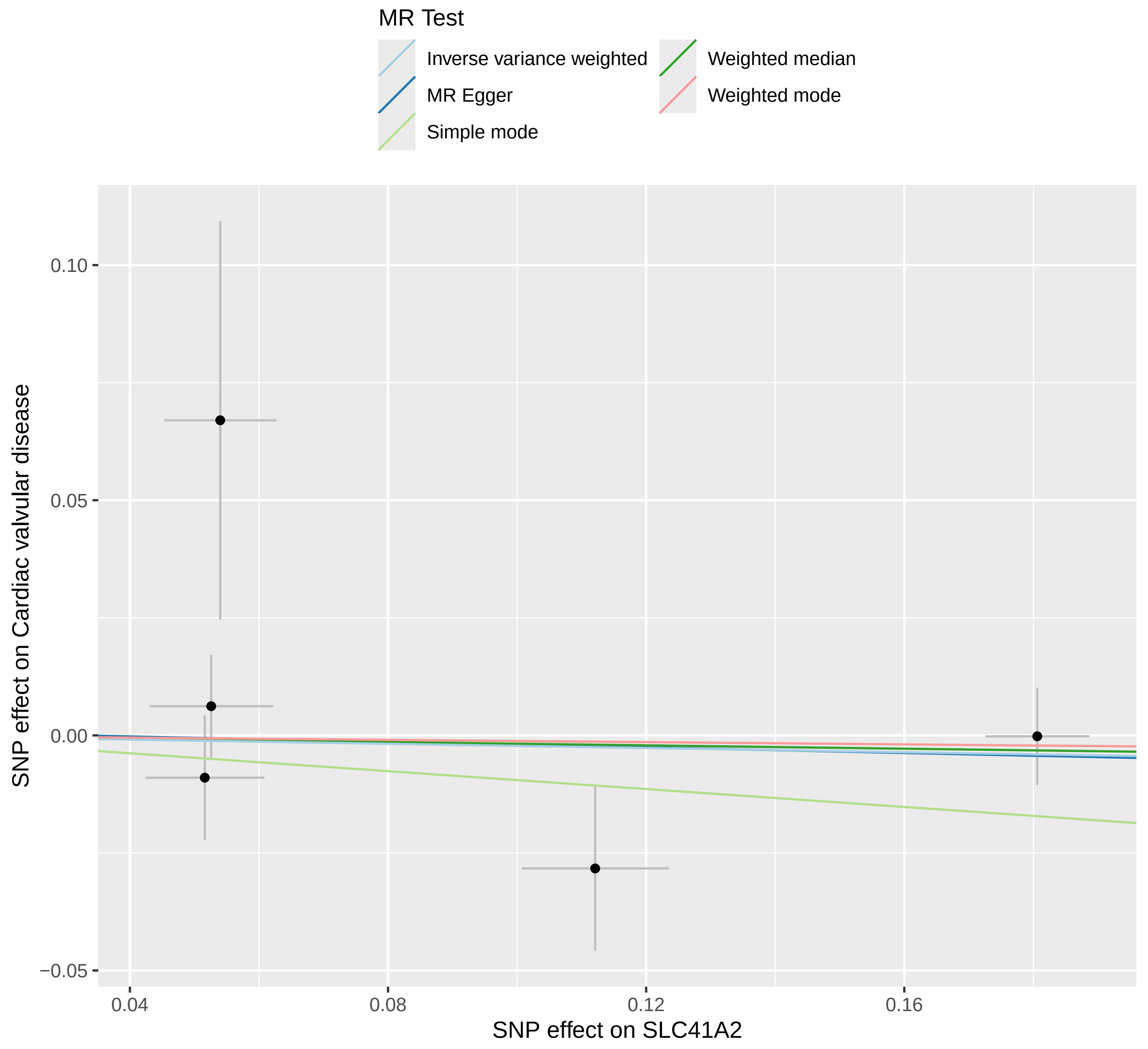

MR Test
Inverse variance weighted
MR Egger
Simple mode
Weighted median
Weighted mode
0.10
0.05
0.00
−0.05
0.04
0.08
0.12
0.16
SNP effect on Cardiac valvular disease
SNP effect on SLC41A2

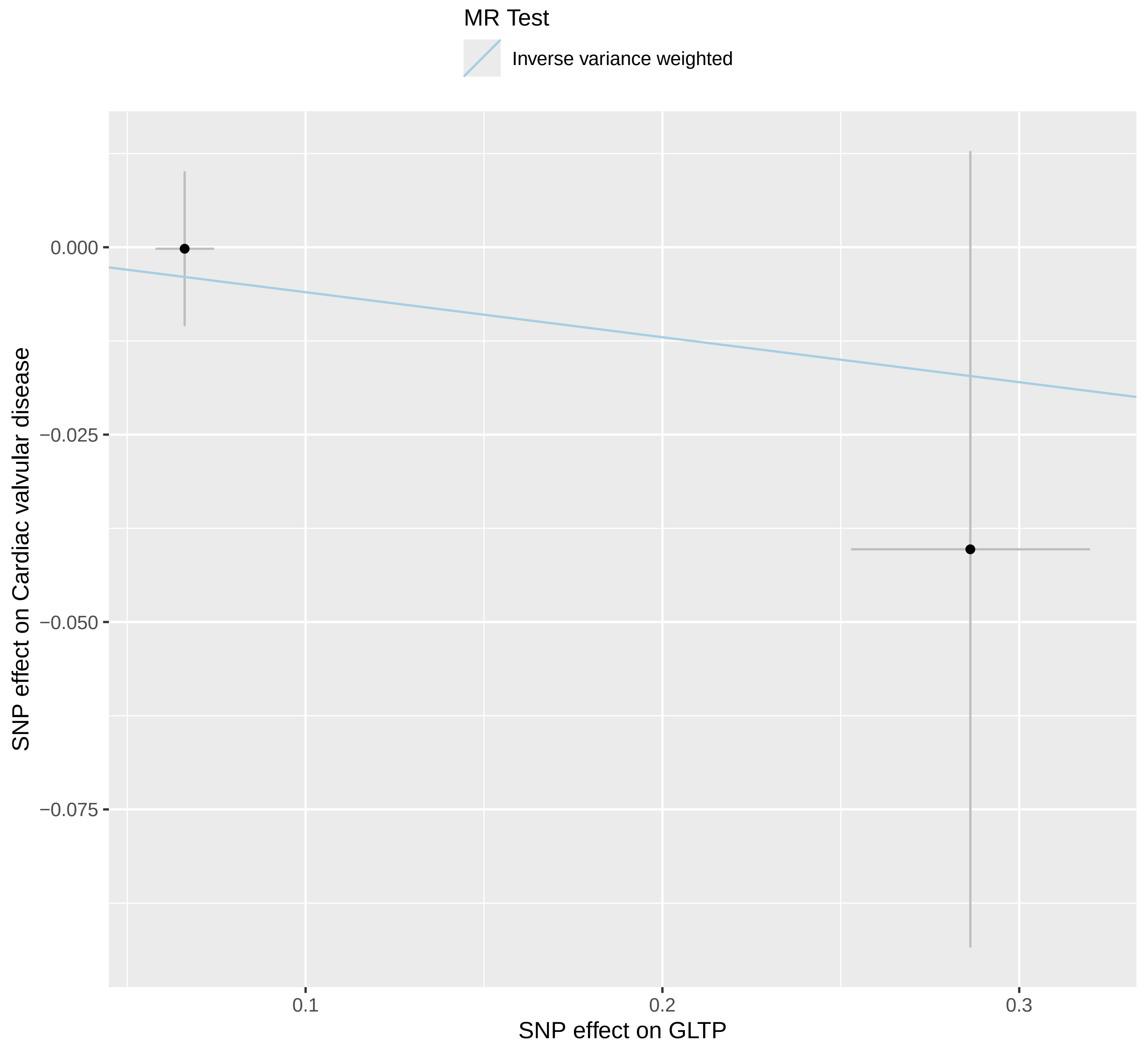
MR Test
Inverse variance weighted
0.000
−0.025
−0.050
−0.075
SNP effect on Cardiac valvular disease
0.1
0.2
0.3
SNP effect on GLTP

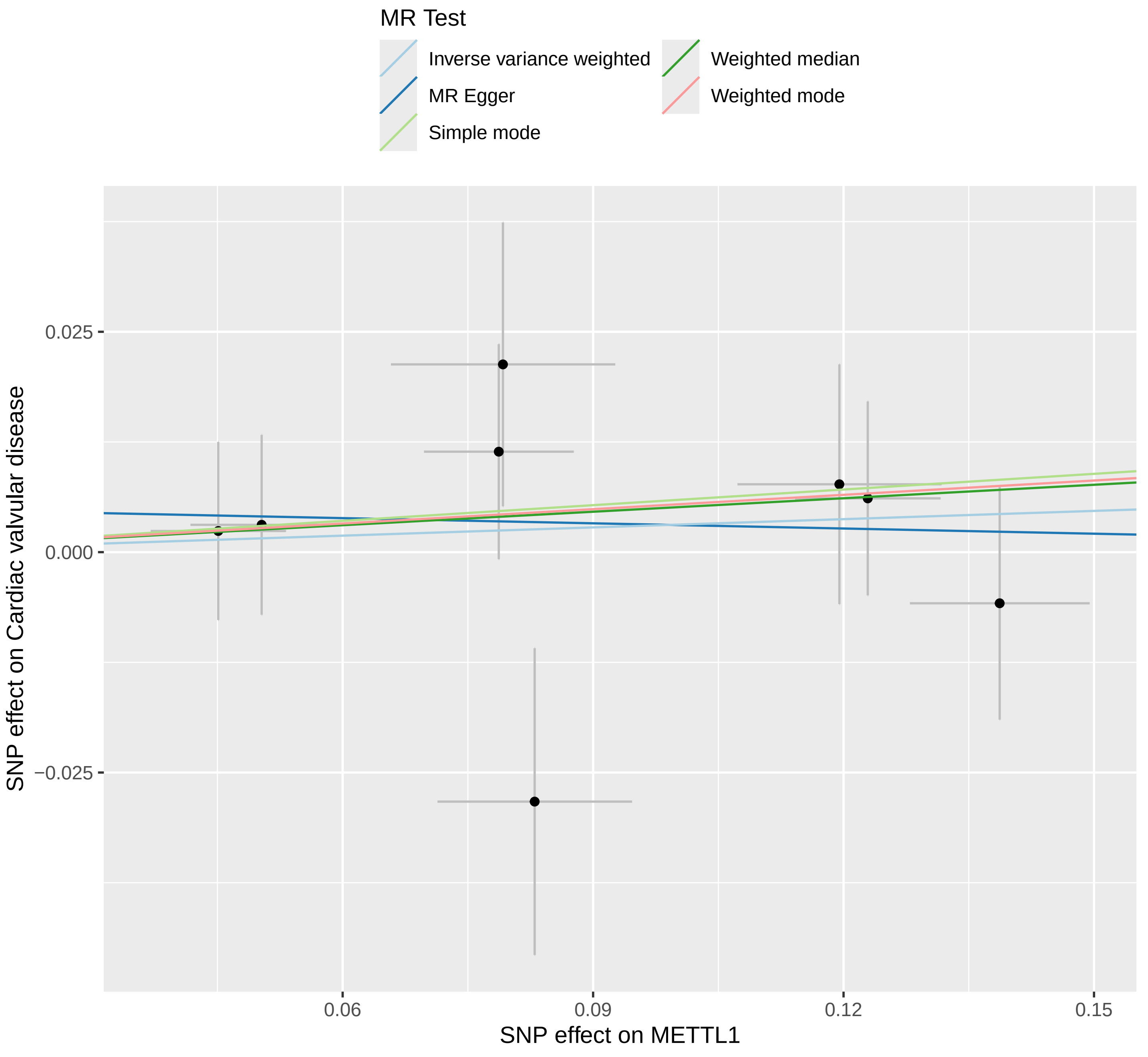

MR Test
Inverse variance weighted
MR Egger
Simple mode
Weighted median
Weighted mode
0.025
0.000
−0.025
0.06
0.09
0.12
0.15
SNP effect on METTL1
SNP effect on Cardiac valvular disease

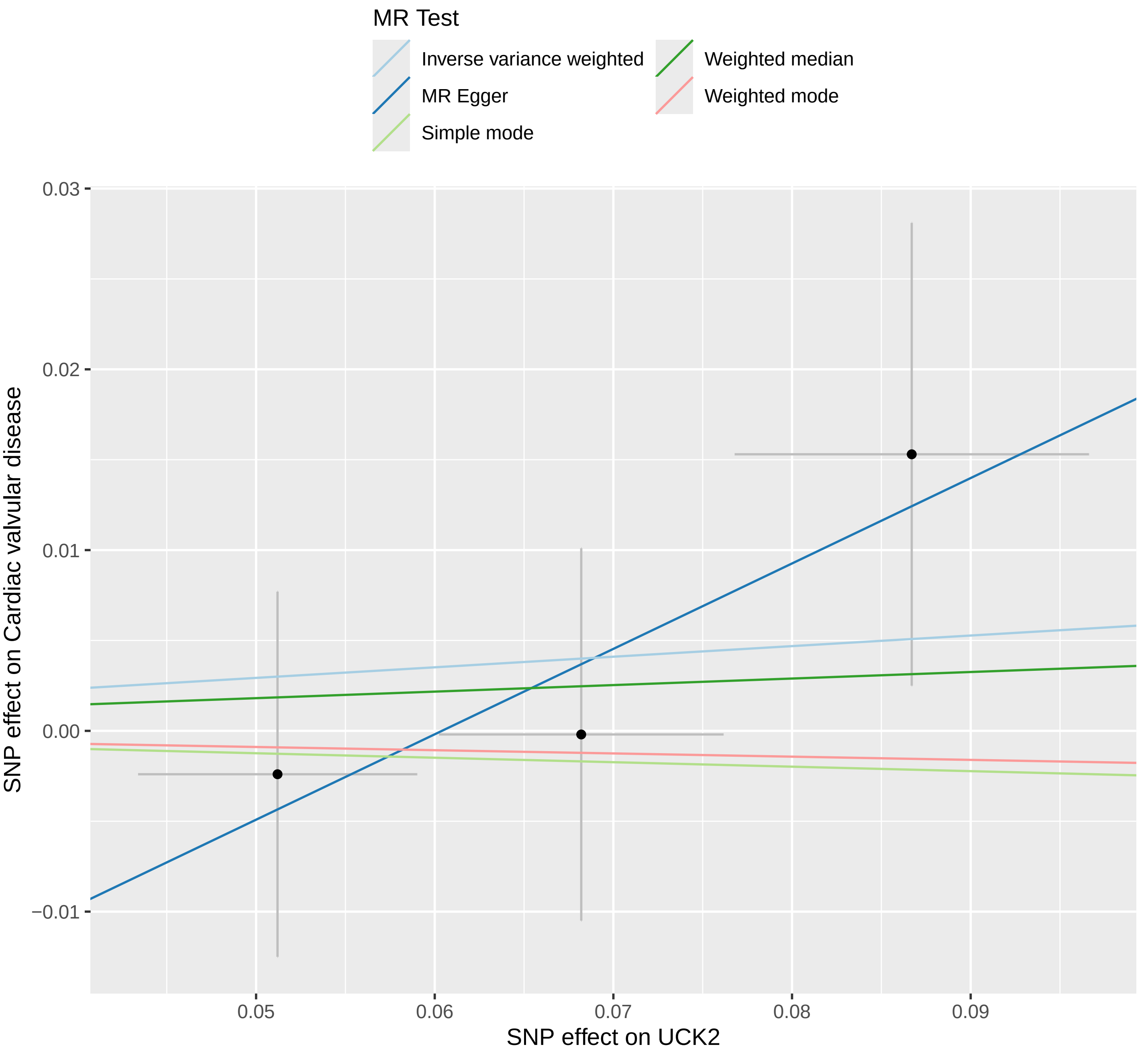
MR Test
Inverse variance weighted
MR Egger
Simple mode
Weighted median
Weighted mode
0.03
0.02
0.01
0.00
−0.01
0.05
0.06
0.07
0.08
0.09
SNP effect on Cardiac valvular disease
SNP effect on UCK2

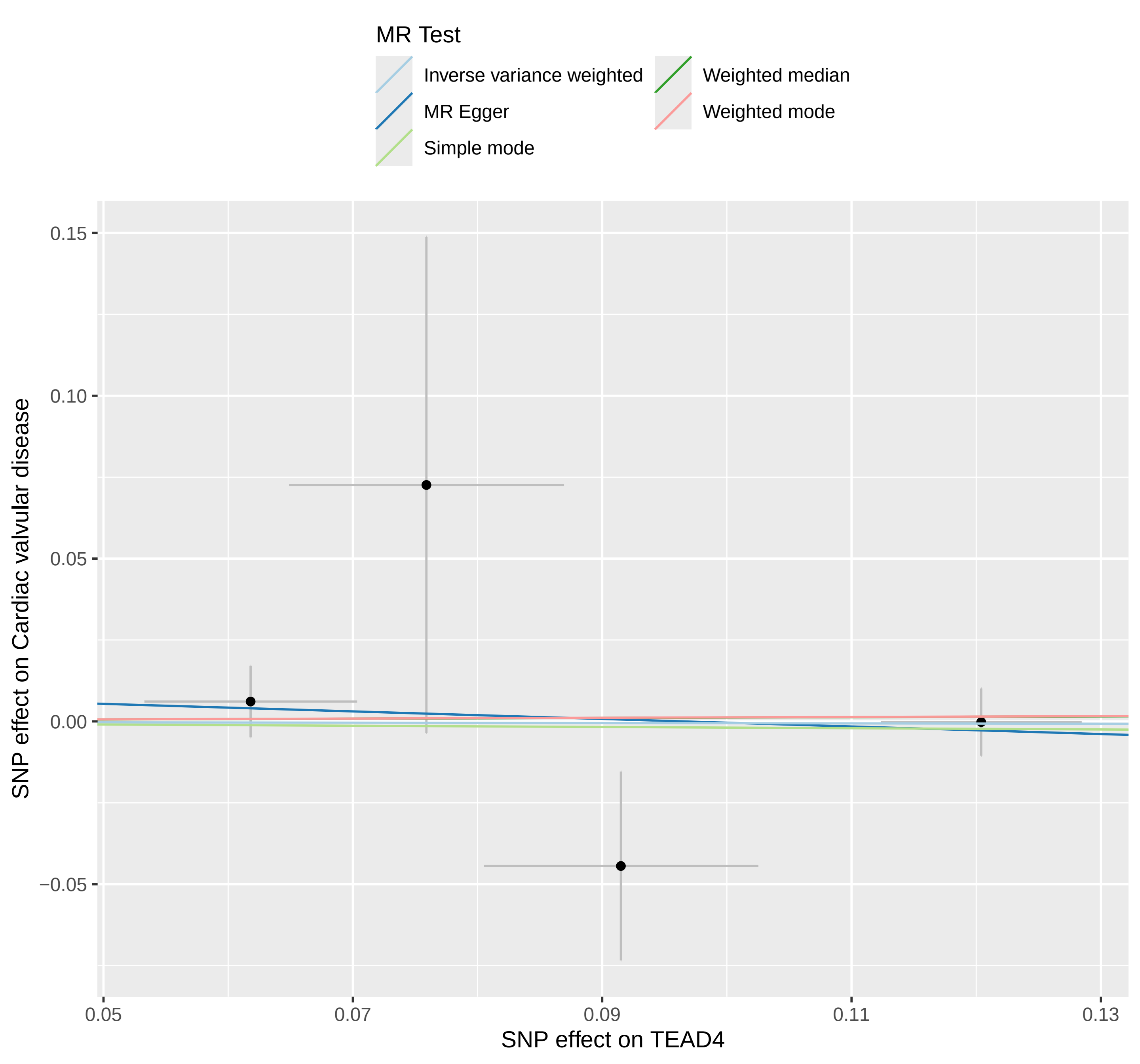

MR Test
Inverse variance weighted
MR Egger
Simple mode
Weighted median
Weighted mode
0.15
0.10
0.05
0.00
−0.05
0.05
0.07
0.09
0.11
0.13
SNP effect on TEAD4
SNP effect on Cardiac valvular disease

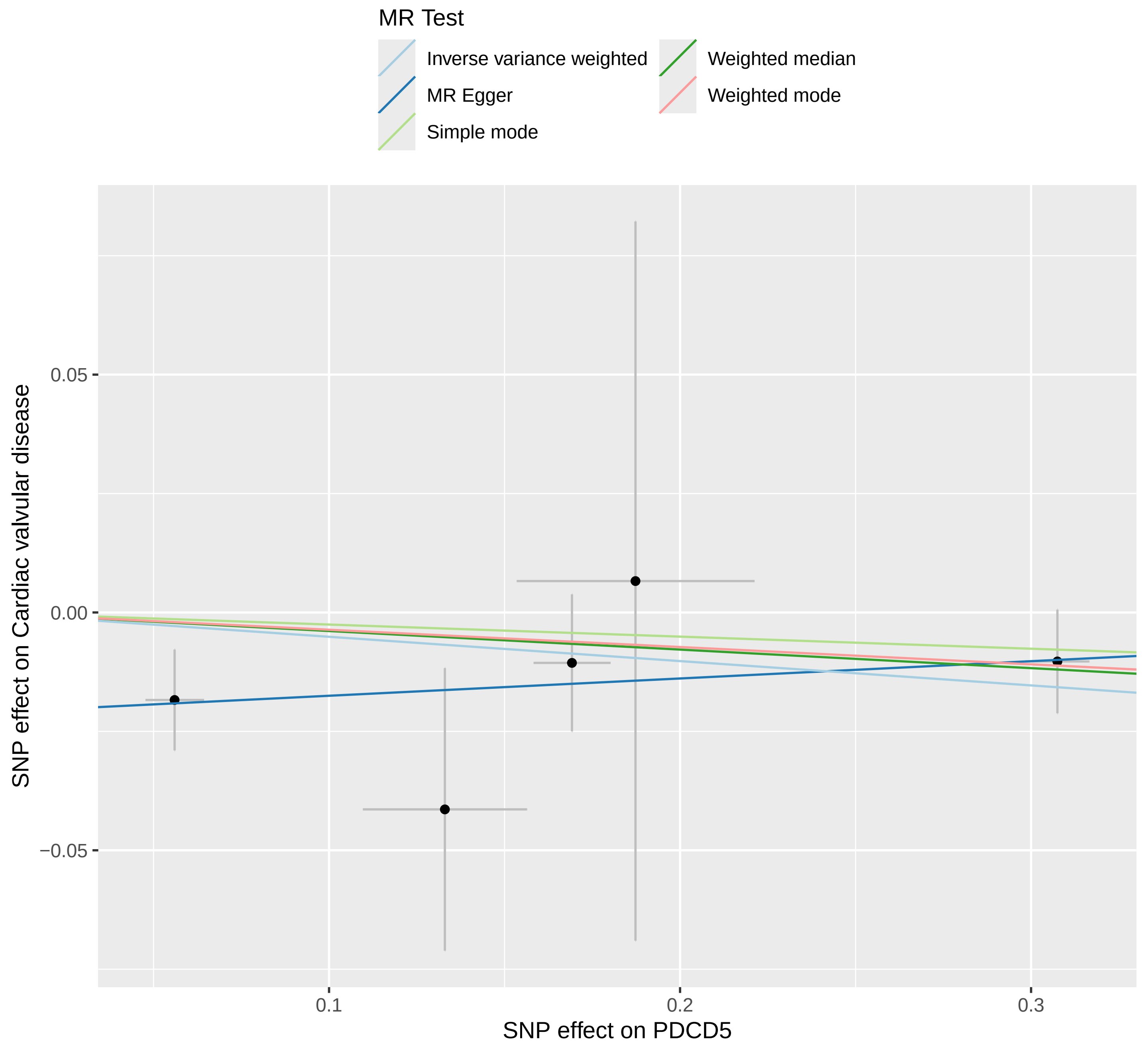
MR Test
Inverse variance weighted
MR Egger
Simple mode
Weighted median
Weighted mode
0.05
0.00
−0.05
0.1
0.2
0.3
SNP effect on Cardiac valvular disease
SNP effect on PDCD5

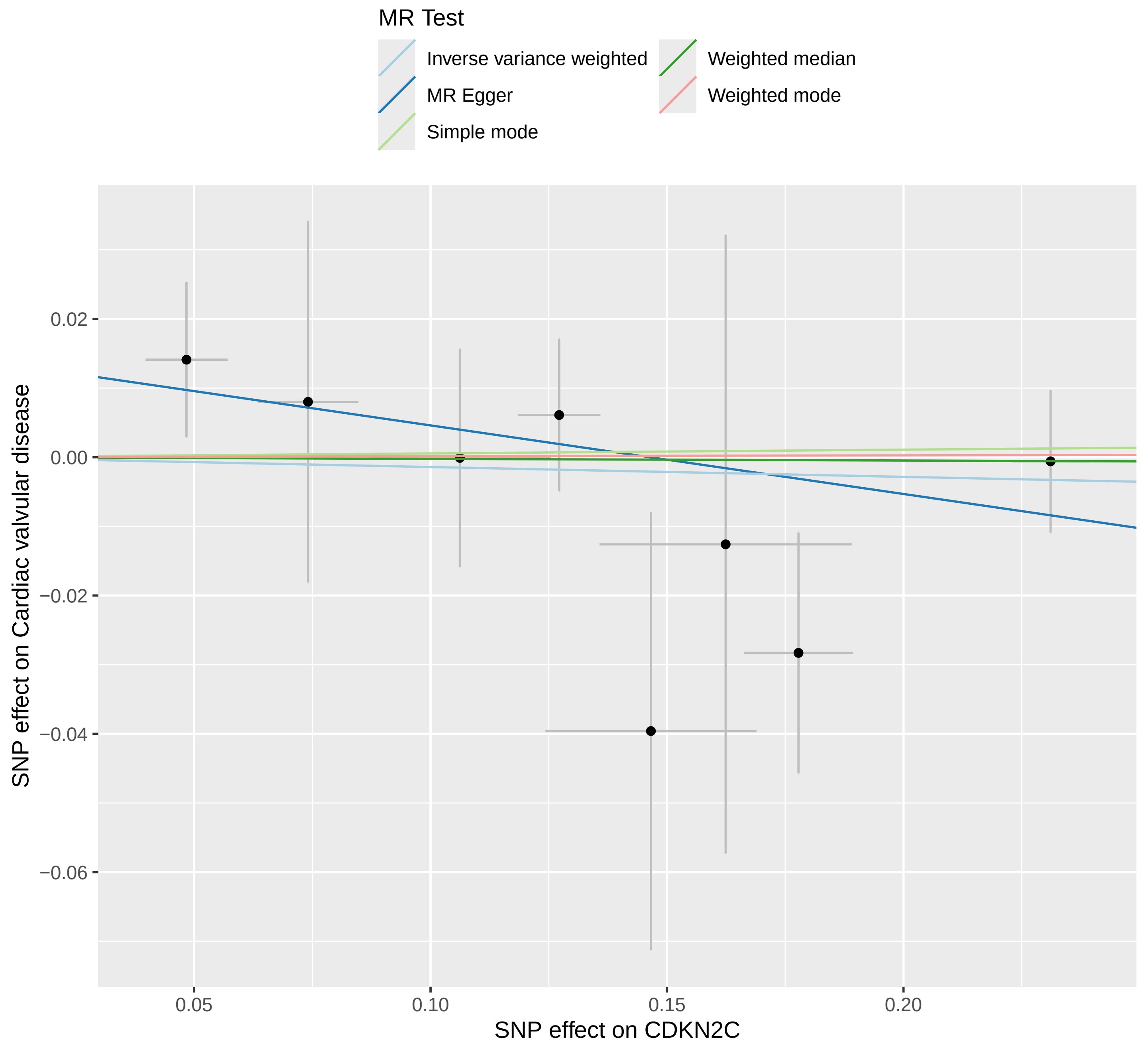

MR Test
Inverse variance weighted
MR Egger
Simple mode
Weighted median
Weighted mode
0.02
0.00
−0.02
−0.04
−0.06
0.05
0.10
0.15
0.20
SNP effect on Cardiac valvular disease
SNP effect on CDKN2C

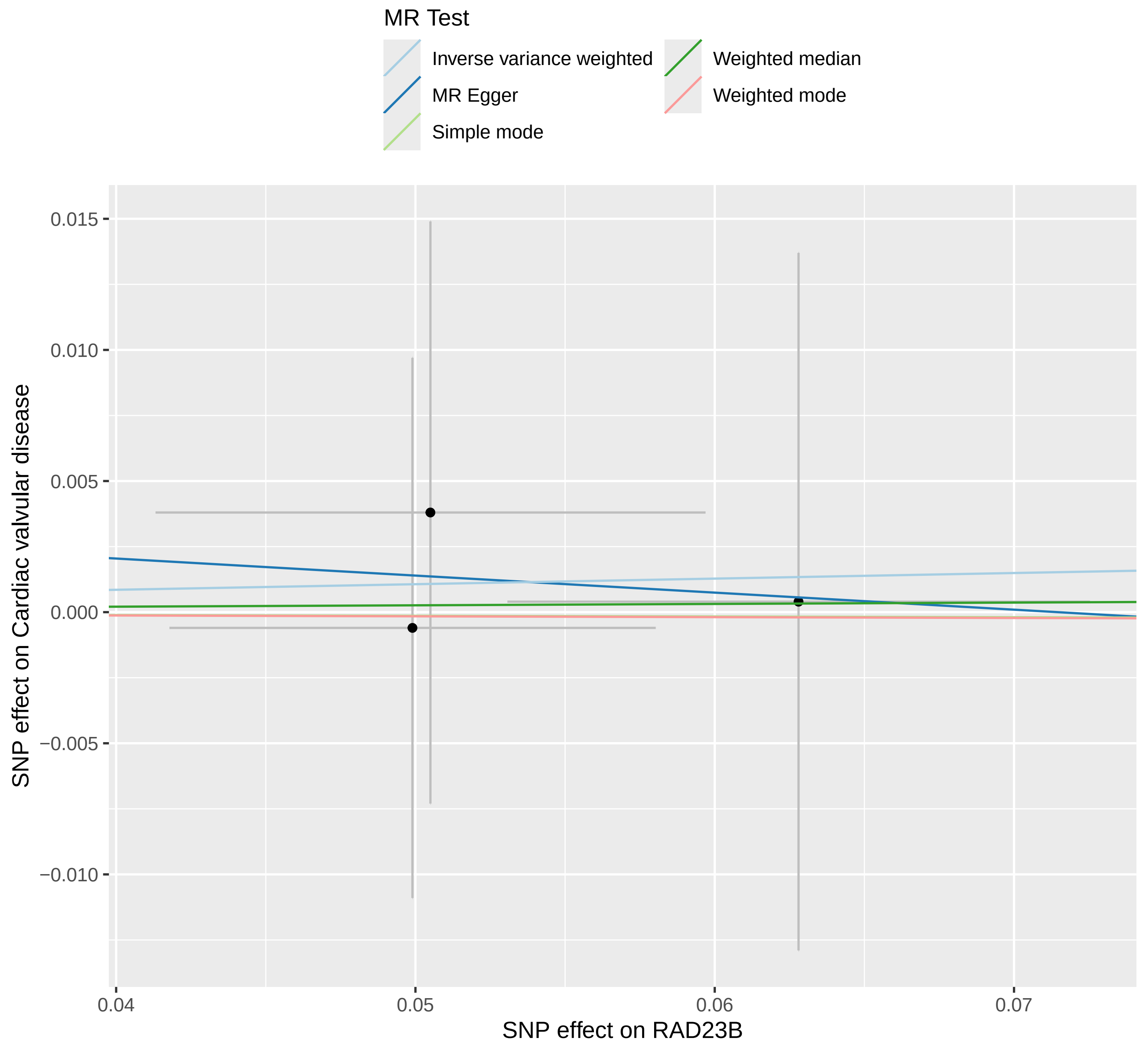

MR Test
Inverse variance weighted
MR Egger
Simple mode
Weighted median
Weighted mode
SNP effect on Cardiac valvular disease
0.015
0.010
0.005
0.000
−0.005
−0.010
0.04
0.05
0.06
0.07
SNP effect on RAD23B

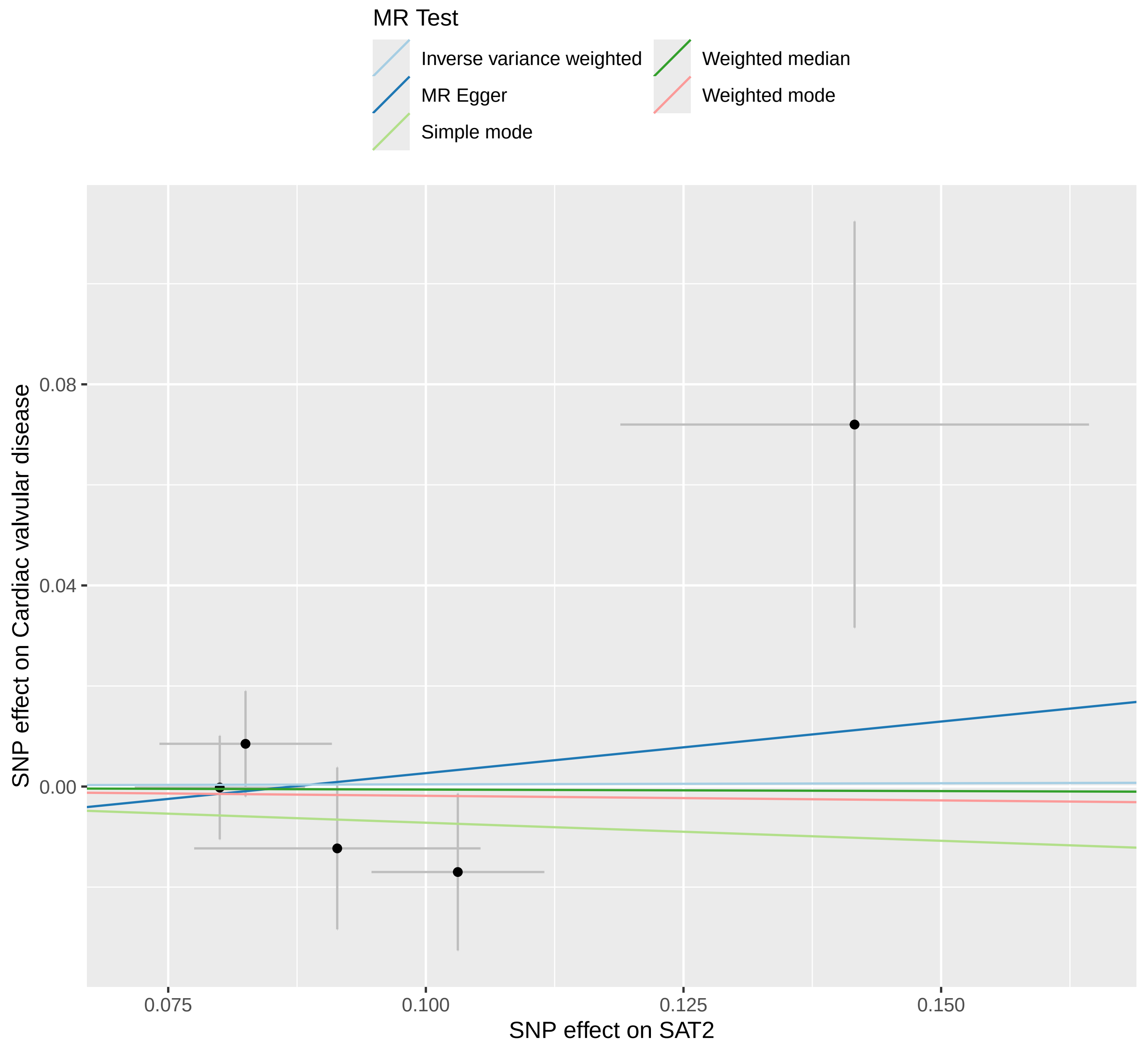

MR Test
Inverse variance weighted
MR Egger
Simple mode
Weighted median
Weighted mode
0.08
0.04
0.00
0.075
0.100
0.125
0.150
SNP effect on Cardiac valvular disease
SNP effect on SAT2

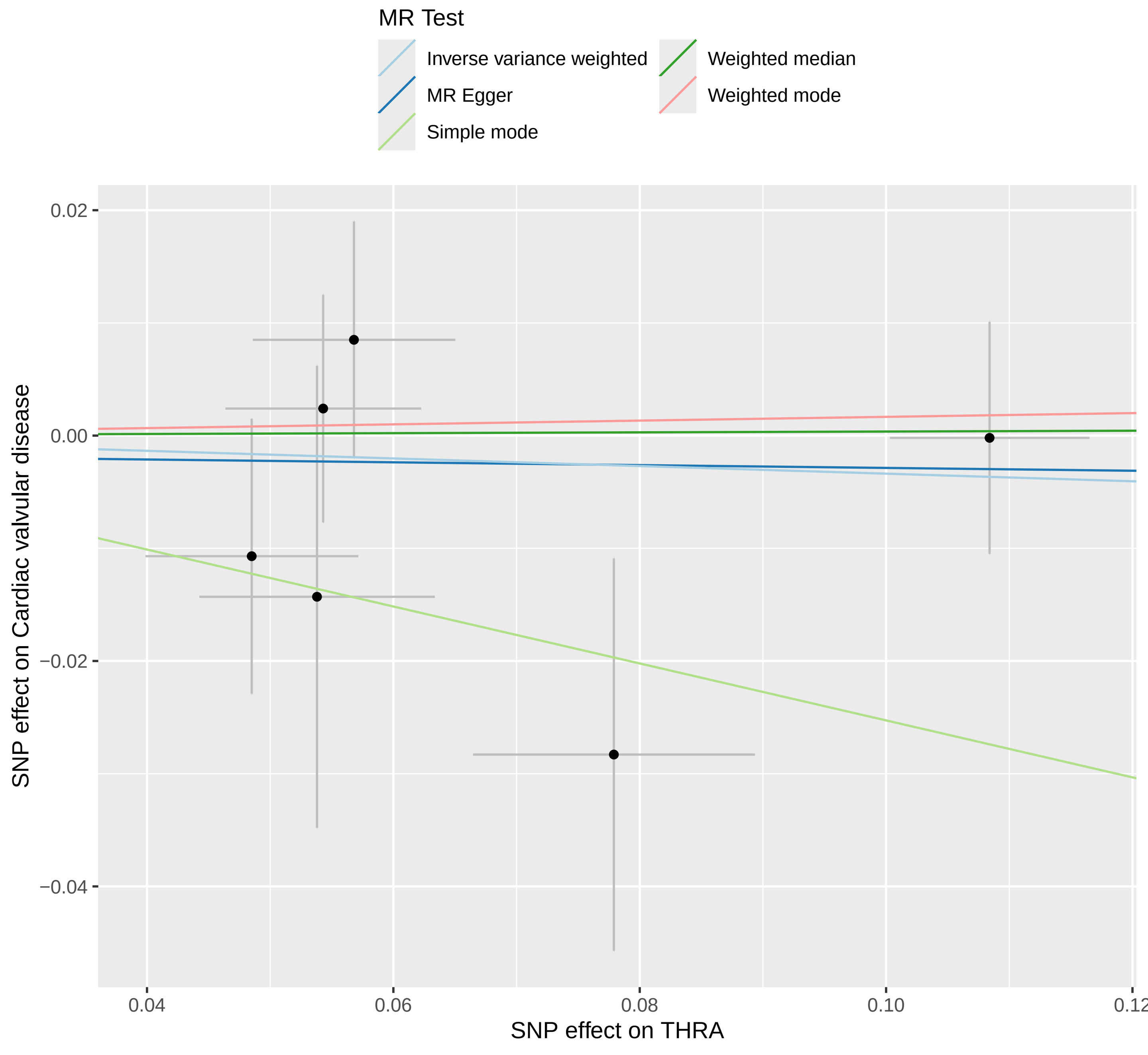
MR Test
Inverse variance weighted
MR Egger
Simple mode
Weighted median
Weighted mode
0.02
0.00
−0.02
−0.04
SNP effect on Cardiac valvular disease
0.04
0.06
0.08
0.10
0.12
SNP effect on THRA

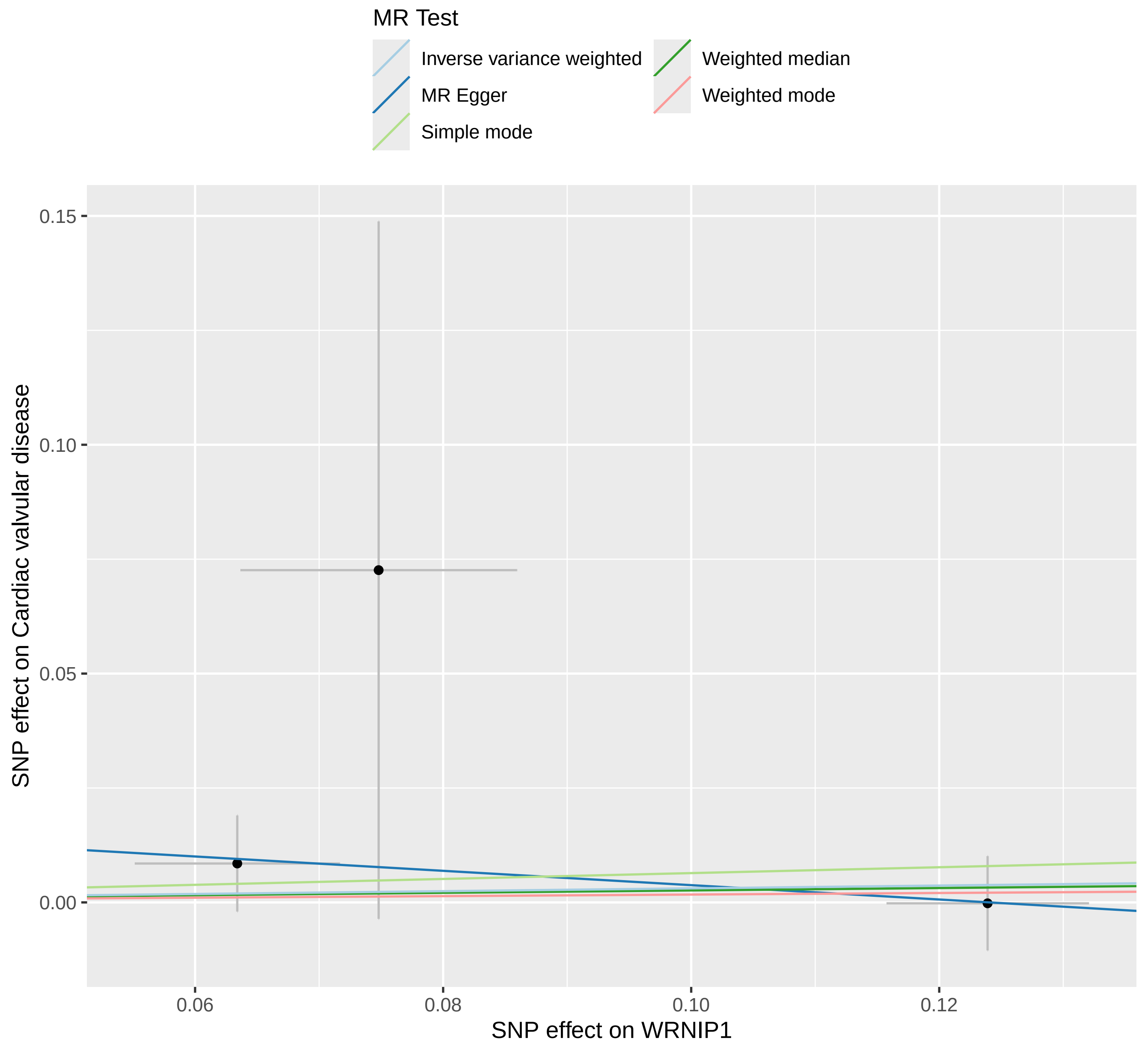
MR Test
Inverse variance weighted
MR Egger
Simple mode
Weighted median
Weighted mode
0.15
0.10
0.05
0.00
SNP effect on Cardiac valvular disease
0.06
0.08
0.10
0.12
SNP effect on WRNIP1

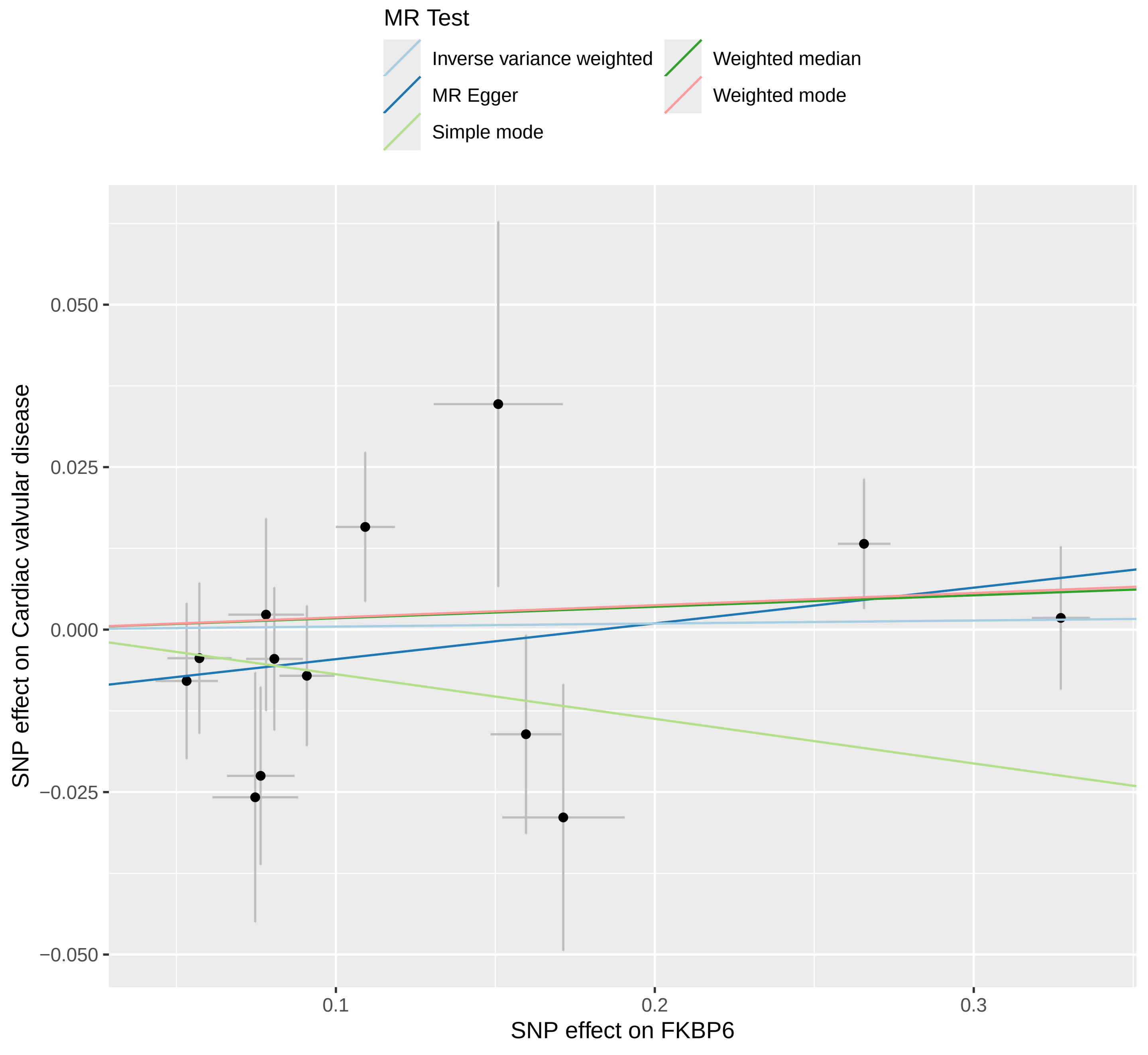

MR Test
Inverse variance weighted
MR Egger
Simple mode
Weighted median
Weighted mode
0.050
0.025
0.000
−0.025
−0.050
0.1
0.2
0.3
SNP effect on Cardiac valvular disease
SNP effect on FKBP6

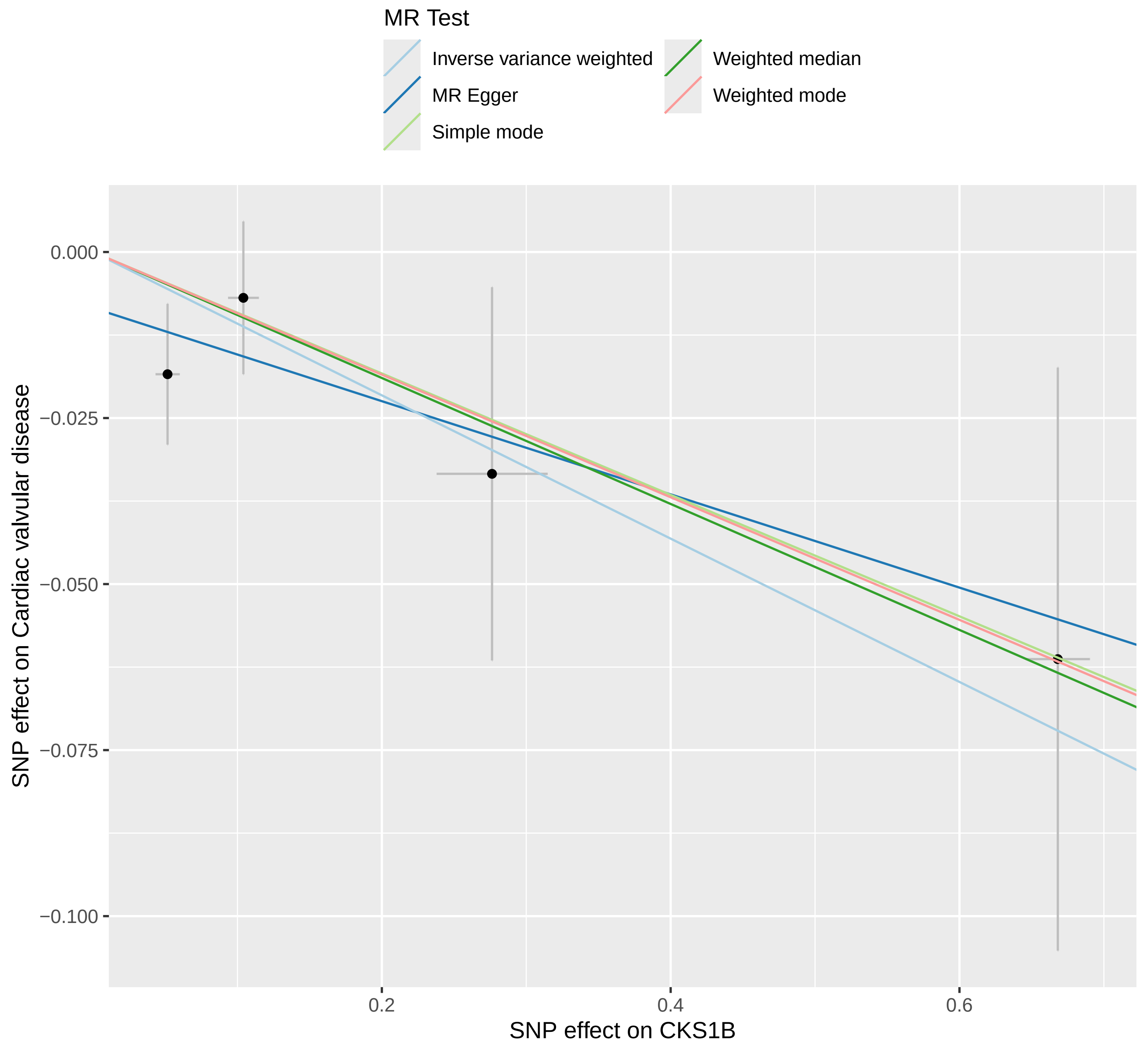

MR Test
Inverse variance weighted
MR Egger
Simple mode
Weighted median
Weighted mode
0.000
−0.025
−0.050
−0.075
−0.100
0.2
0.4
0.6
SNP effect on CKS1B
SNP effect on Cardiac valvular disease

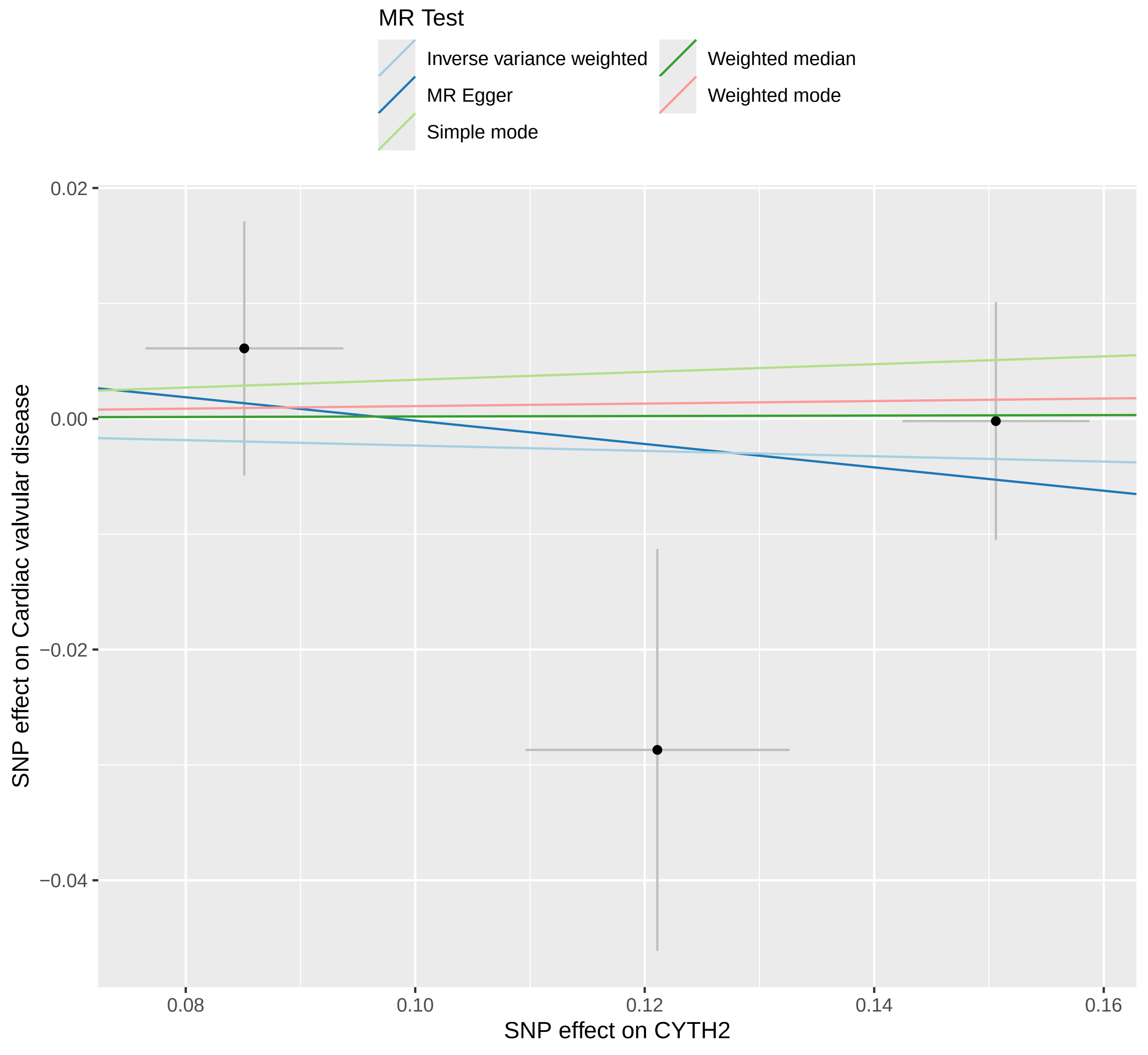
MR Test
Inverse variance weighted
MR Egger
Simple mode
Weighted median
Weighted mode
0.02
0.00
−0.02
−0.04
SNP effect on Cardiac valvular disease
0.08
0.10
0.12
0.14
0.16
SNP effect on CYTH2

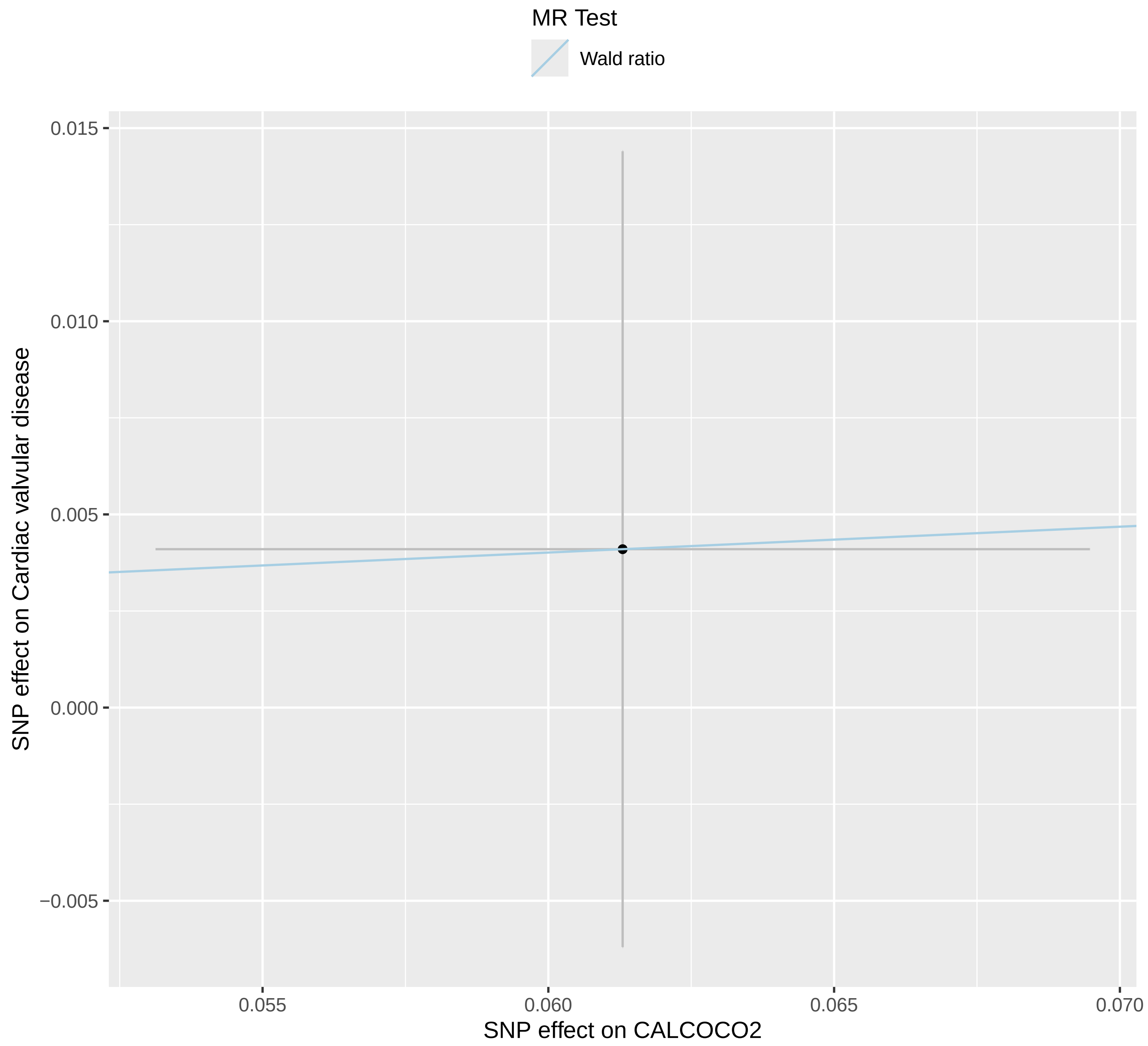
MR Test
Wald ratio
0.015
0.010
0.005
0.000
−0.005
SNP effect on Cardiac valvular disease
0.055
0.060
0.065
0.070
SNP effect on CALCOCO2

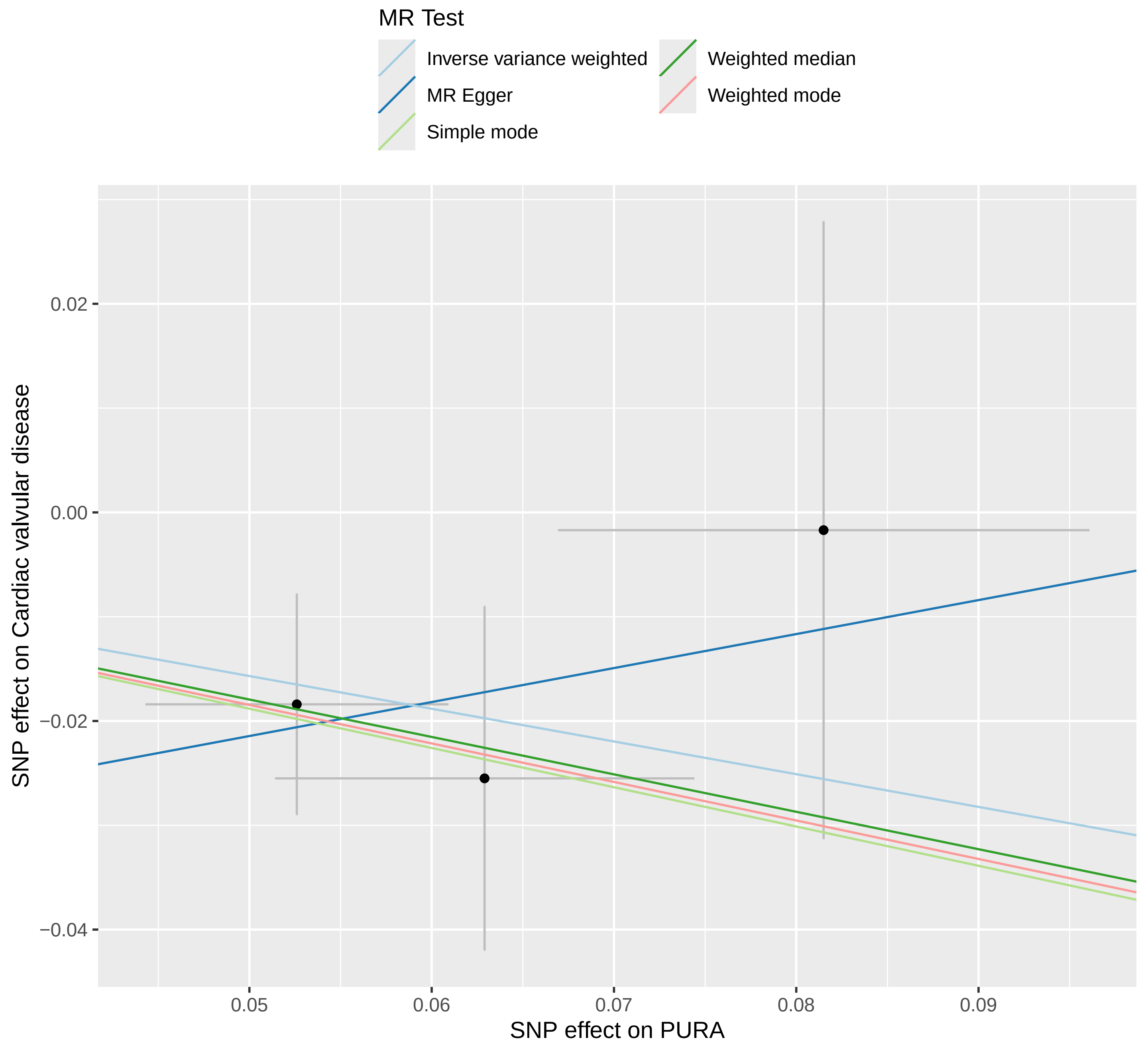

MR Test
Inverse variance weighted
MR Egger
Simple mode
Weighted median
Weighted mode
0.02
0.00
−0.02
−0.04
SNP effect on Cardiac valvular disease
0.05
0.06
0.07
0.08
0.09
SNP effect on PURA

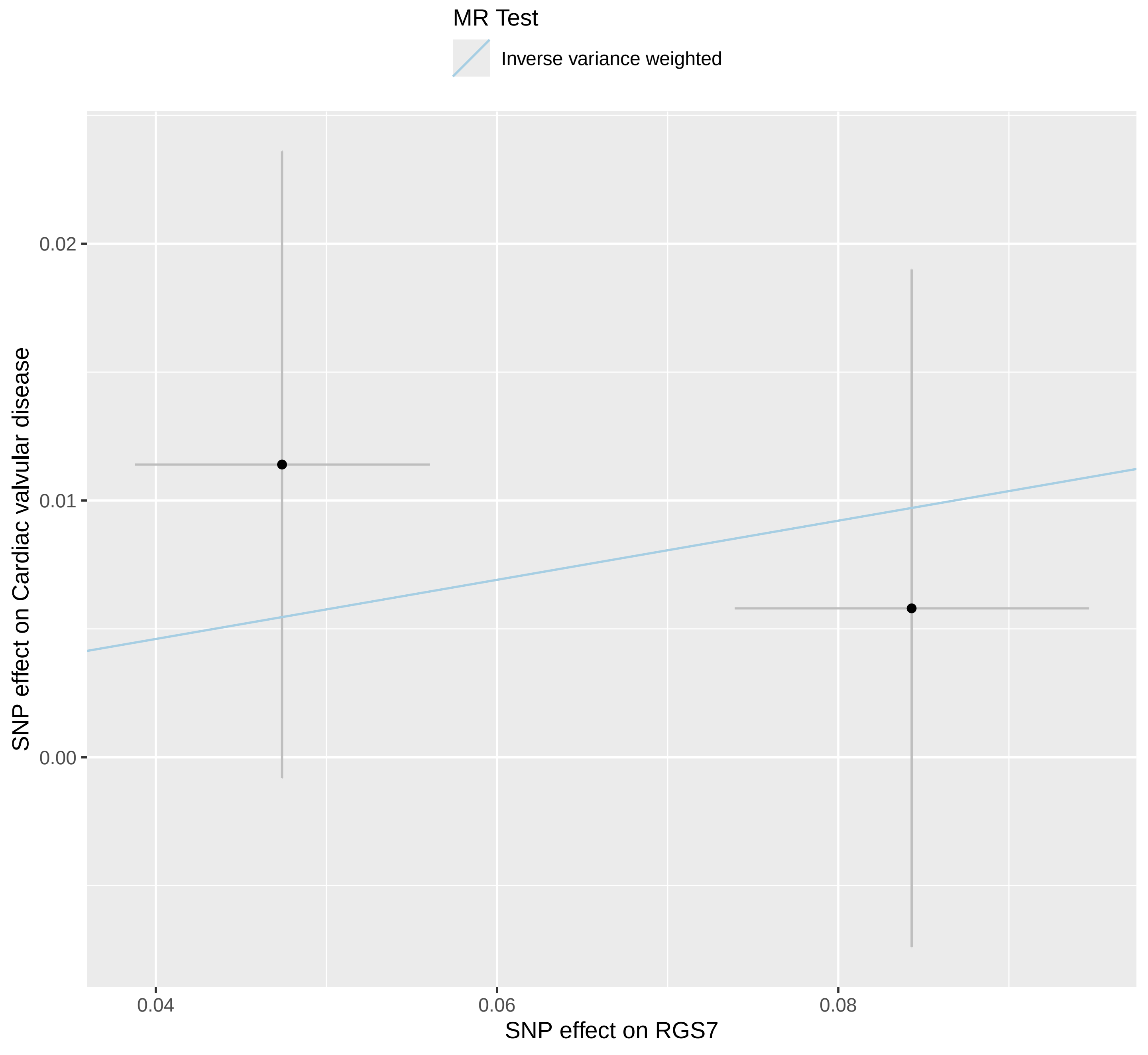
MR Test
Inverse variance weighted
0.02
0.01
0.00
SNP effect on Cardiac valvular disease
0.04
0.06
0.08
SNP effect on RGS7

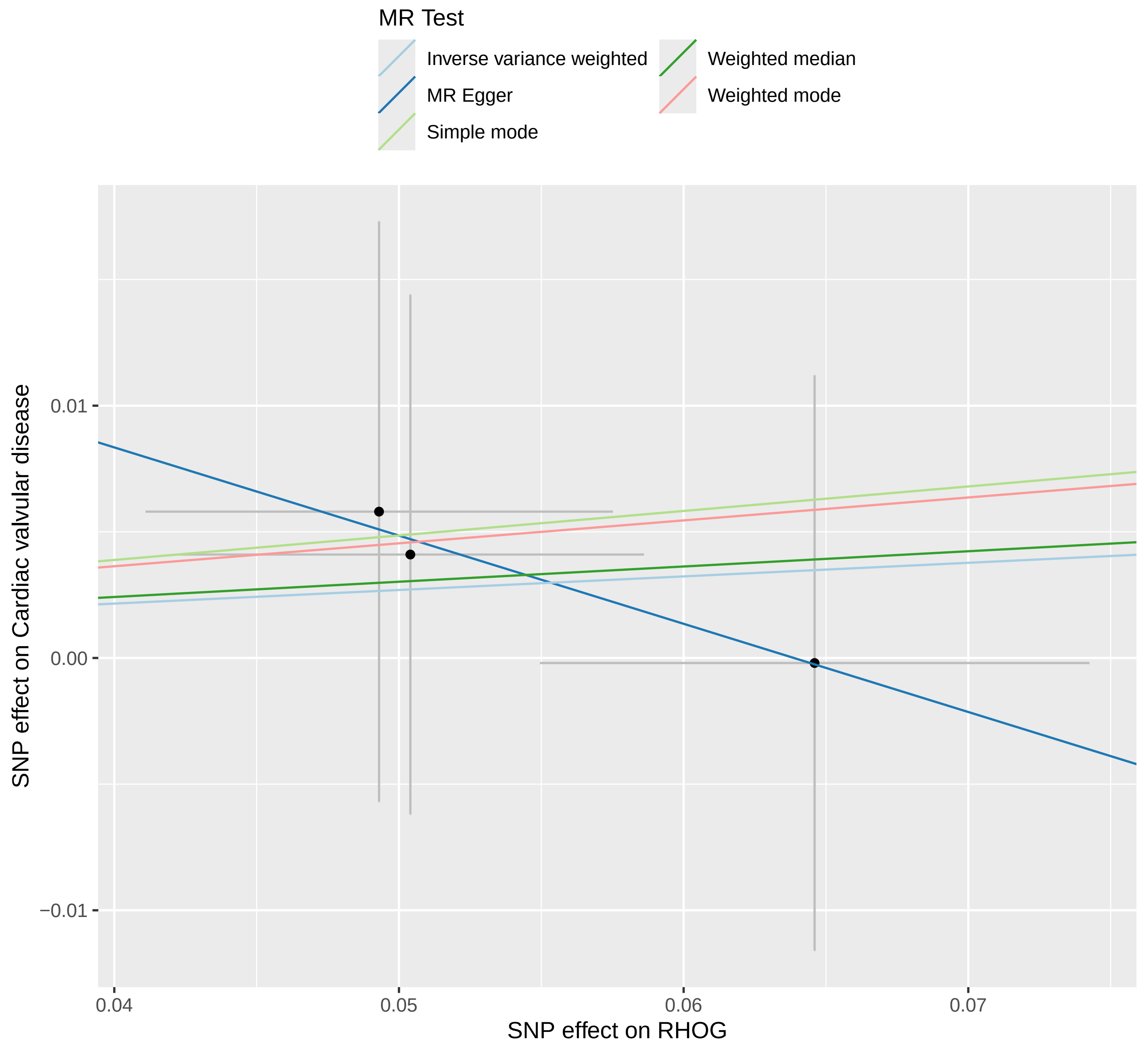
MR Test
Inverse variance weighted
MR Egger
Simple mode
Weighted median
Weighted mode
SNP effect on Cardiac valvular disease
0.01
0.00
−0.01
0.04
0.05
0.06
0.07
SNP effect on RHOG

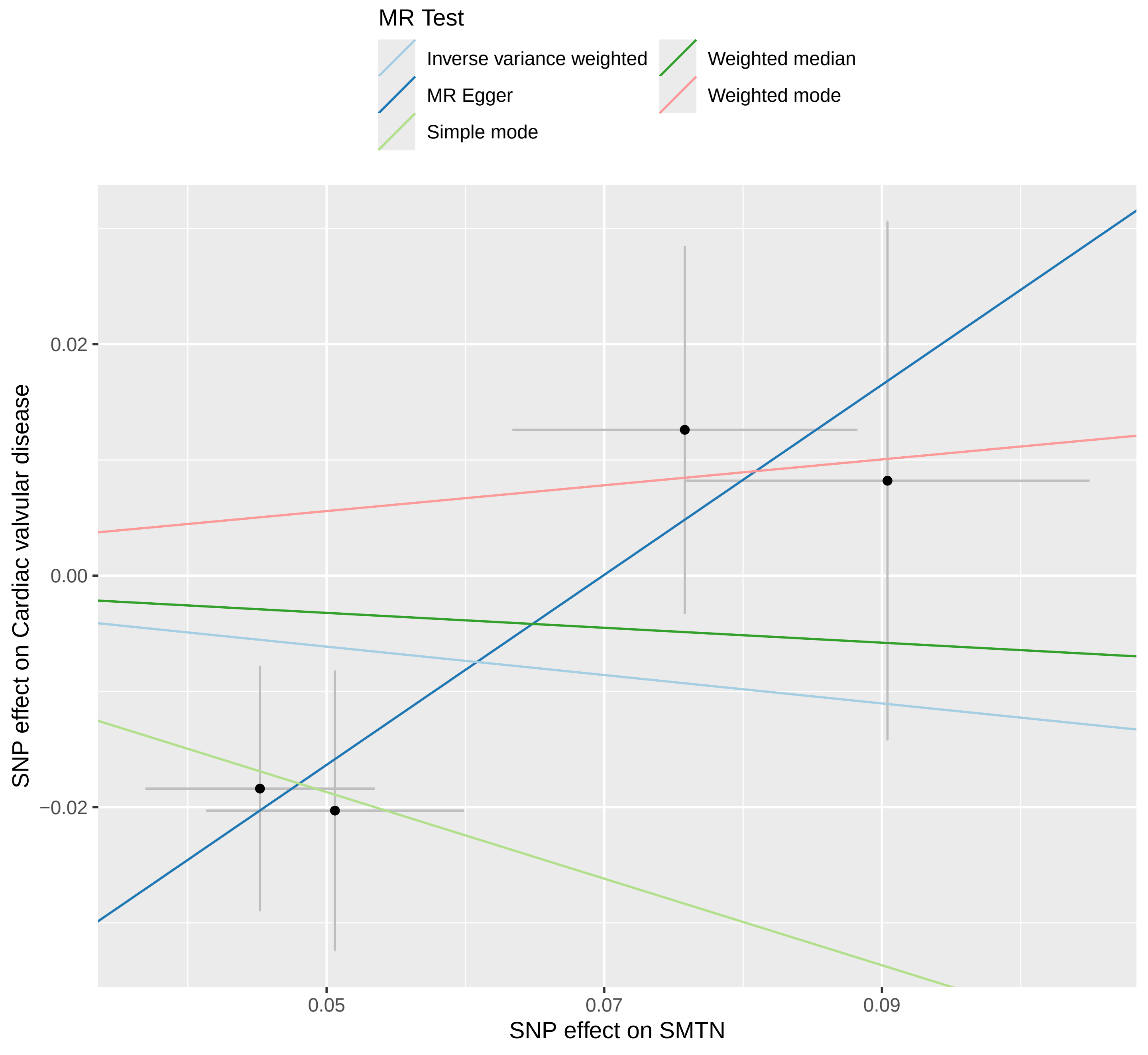

MR Test
Inverse variance weighted
MR Egger
Simple mode
Weighted median
Weighted mode
0.02
0.00
−0.02
0.05
0.07
0.09
SNP effect on Cardiac valvular disease
SNP effect on SMTN

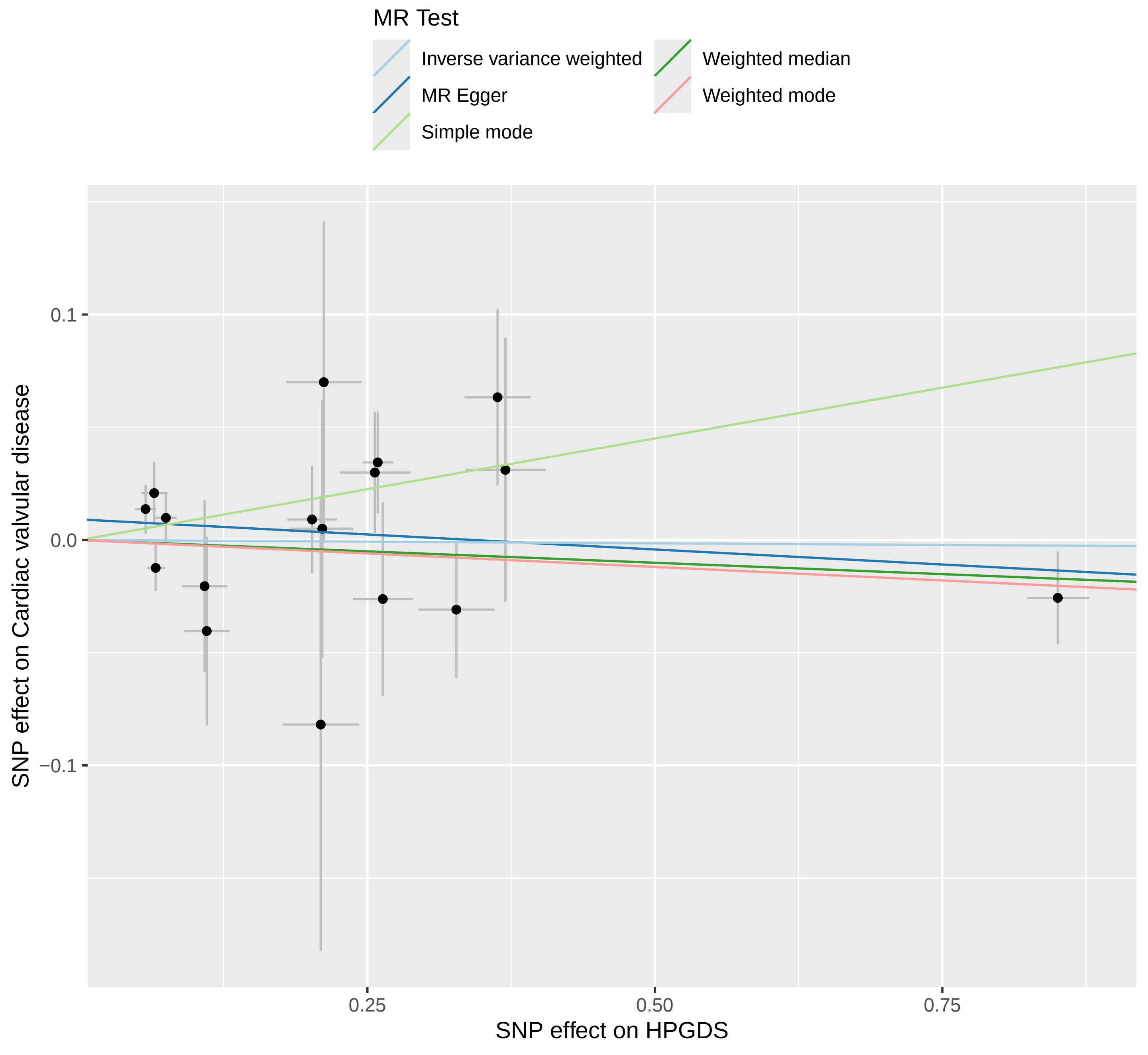

MR Test
Inverse variance weighted
MR Egger
Simple mode
Weighted median
Weighted mode
0.1
0.0
−0.1
0.25
0.50
0.75
SNP effect on HPGDS
SNP effect on Cardiac valvular disease

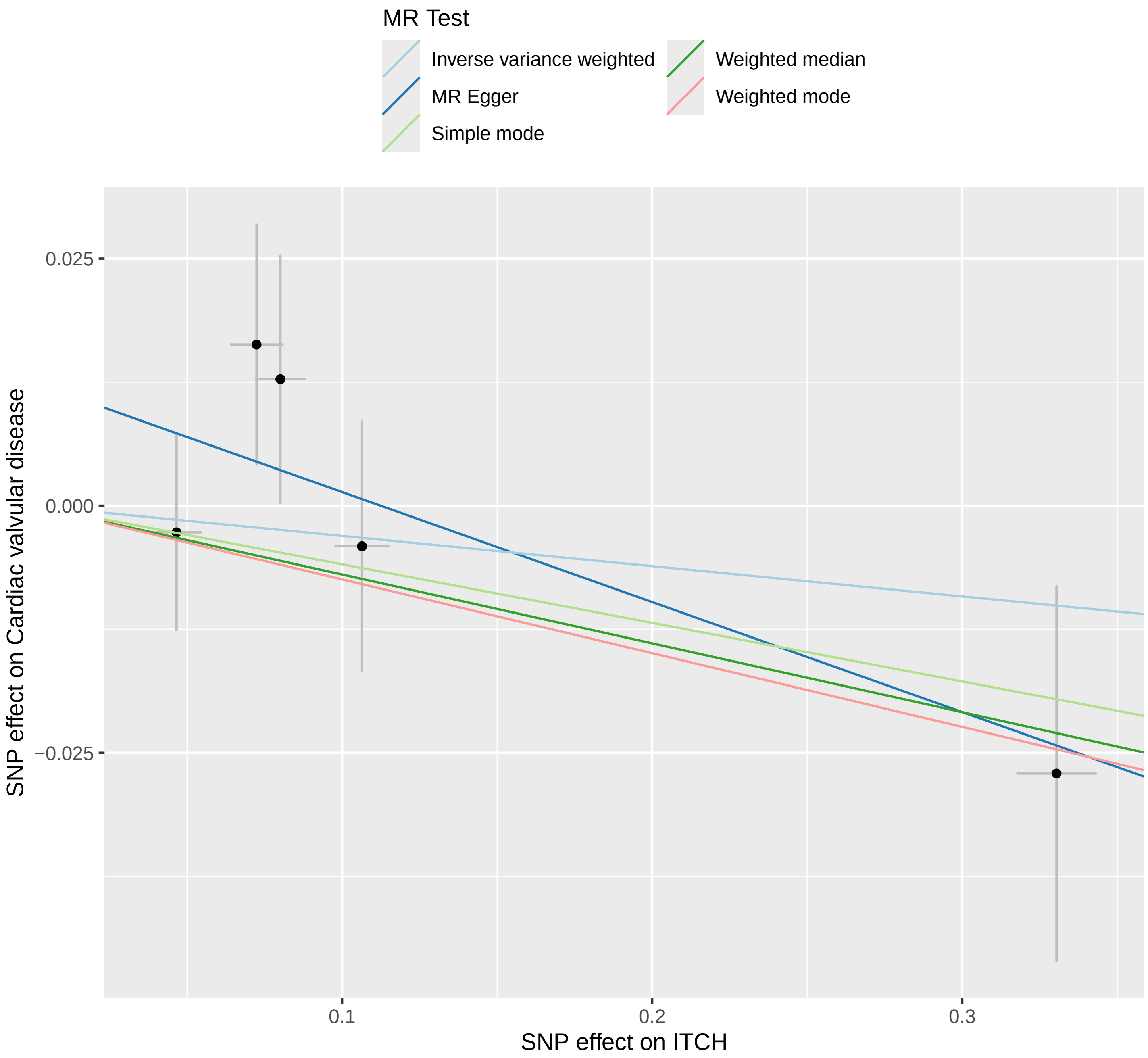
MR Test
Inverse variance weighted
MR Egger
Simple mode
Weighted median
Weighted mode
0.025
0.000
−0.025
SNP effect on Cardiac valvular disease
0.1
0.2
0.3
SNP effect on ITCH

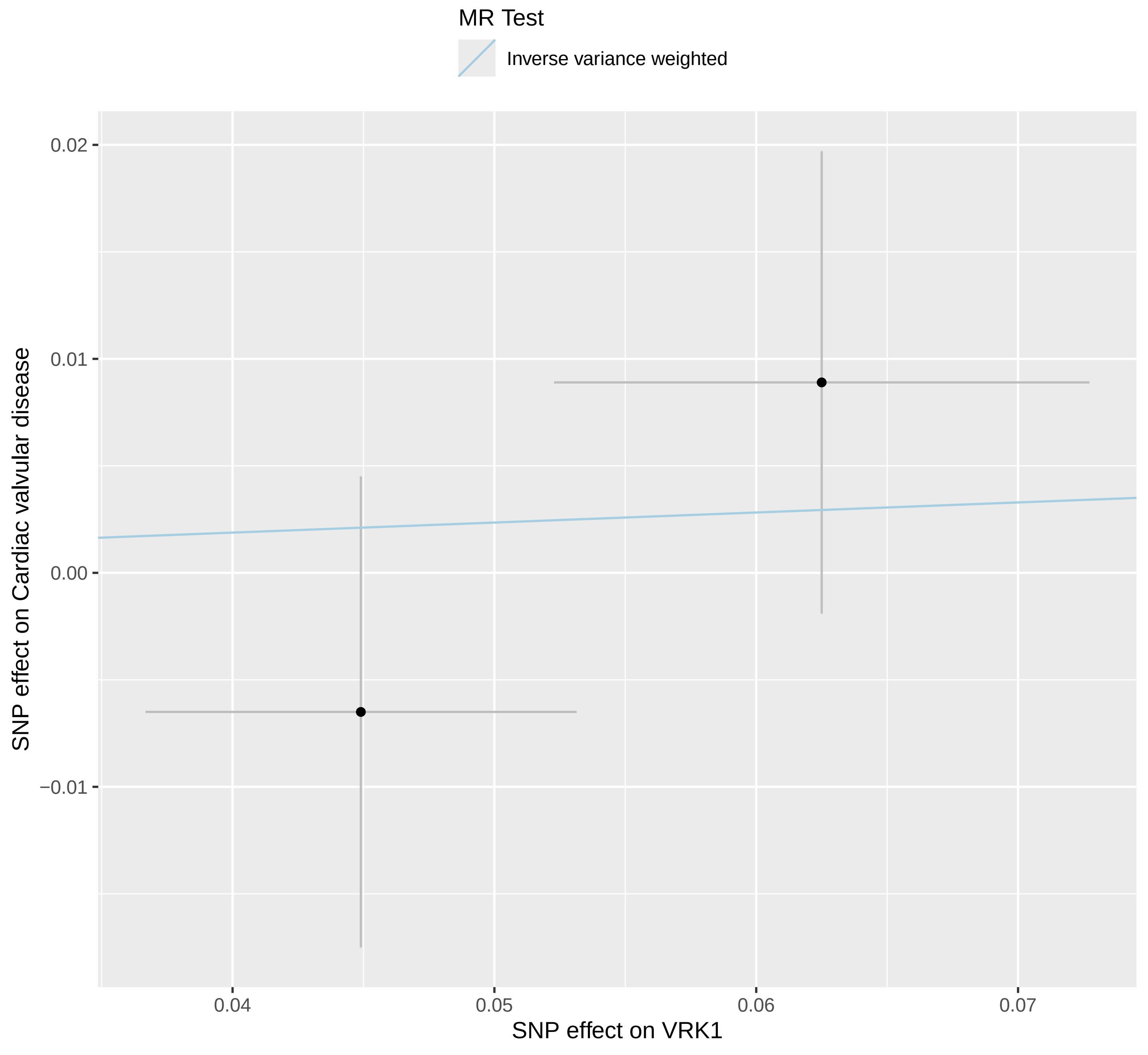
MR Test
Inverse variance weighted
0.02
0.01
0.00
−0.01
0.04
0.05
0.06
0.07
SNP effect on Cardiac valvular disease
SNP effect on VRK1

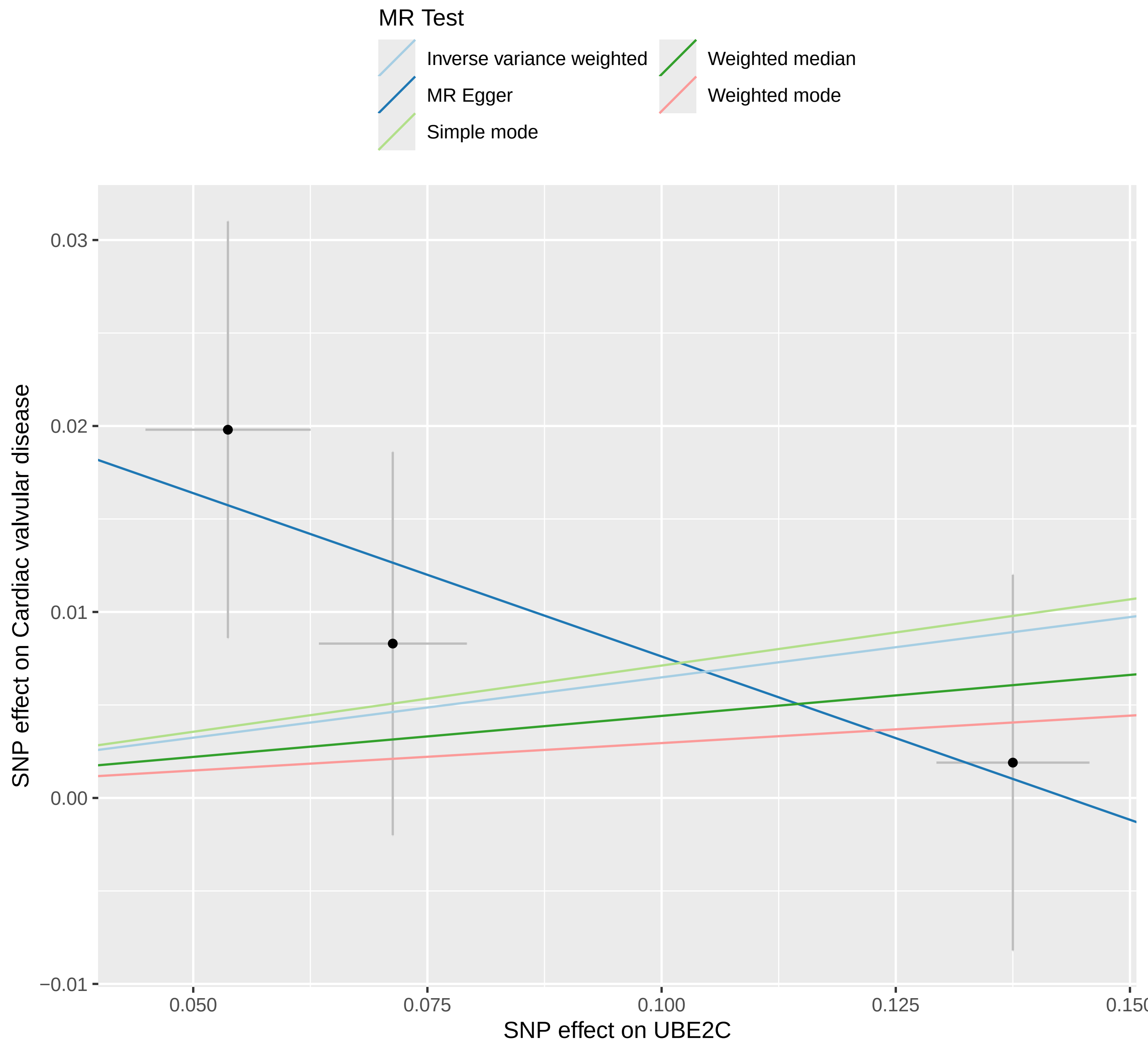
MR Test
Inverse variance weighted
MR Egger
Simple mode
Weighted median
Weighted mode
0.03
0.02
0.01
0.00
−0.01
0.050
0.075
0.100
0.125
0.150
SNP effect on UBE2C
SNP effect on Cardiac valvular disease

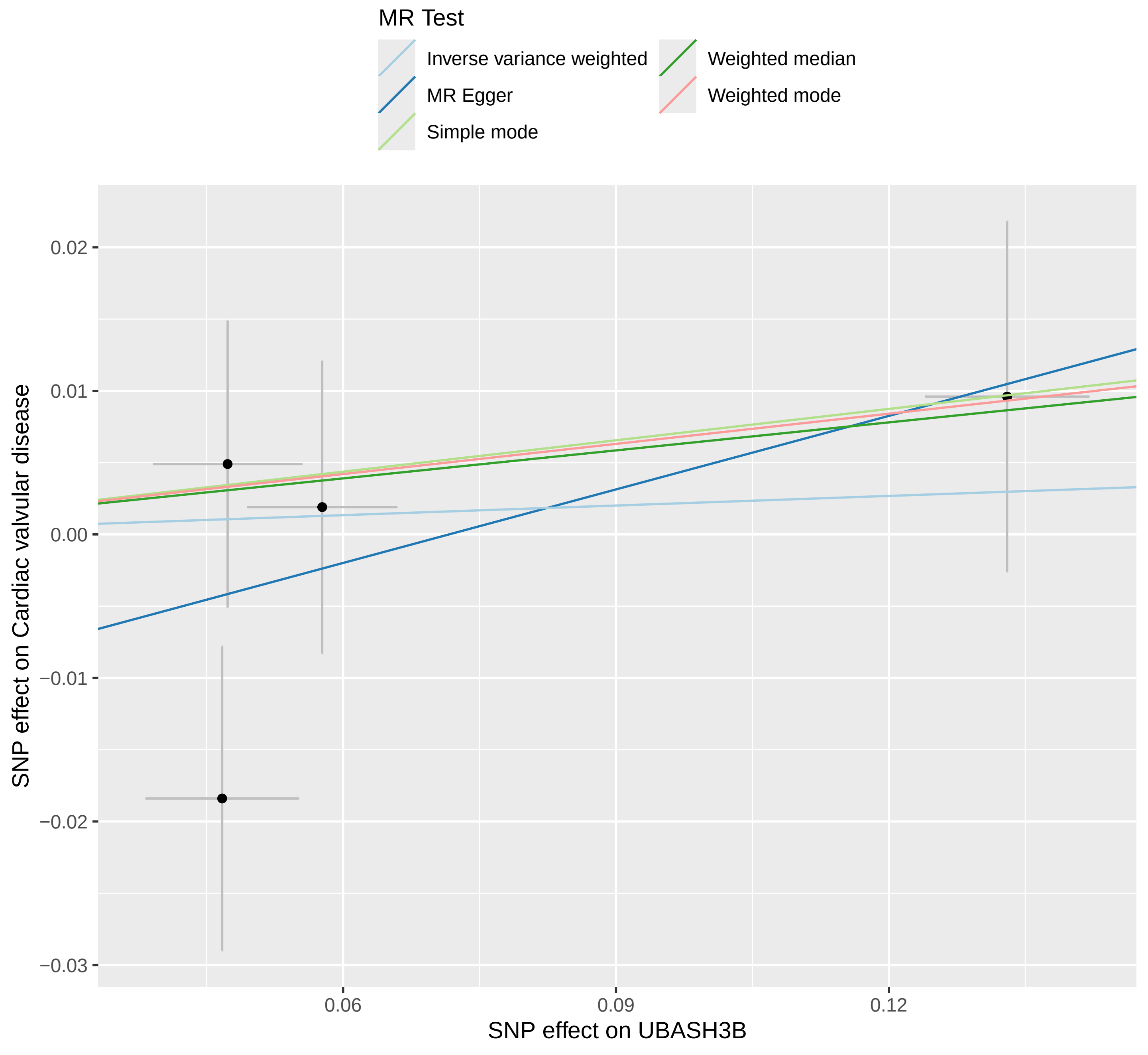

MR Test
Inverse variance weighted
MR Egger
Simple mode
Weighted median
Weighted mode
0.02
0.01
0.00
−0.01
−0.02
−0.03
SNP effect on Cardiac valvular disease
0.06
0.09
0.12
SNP effect on UBASH3B

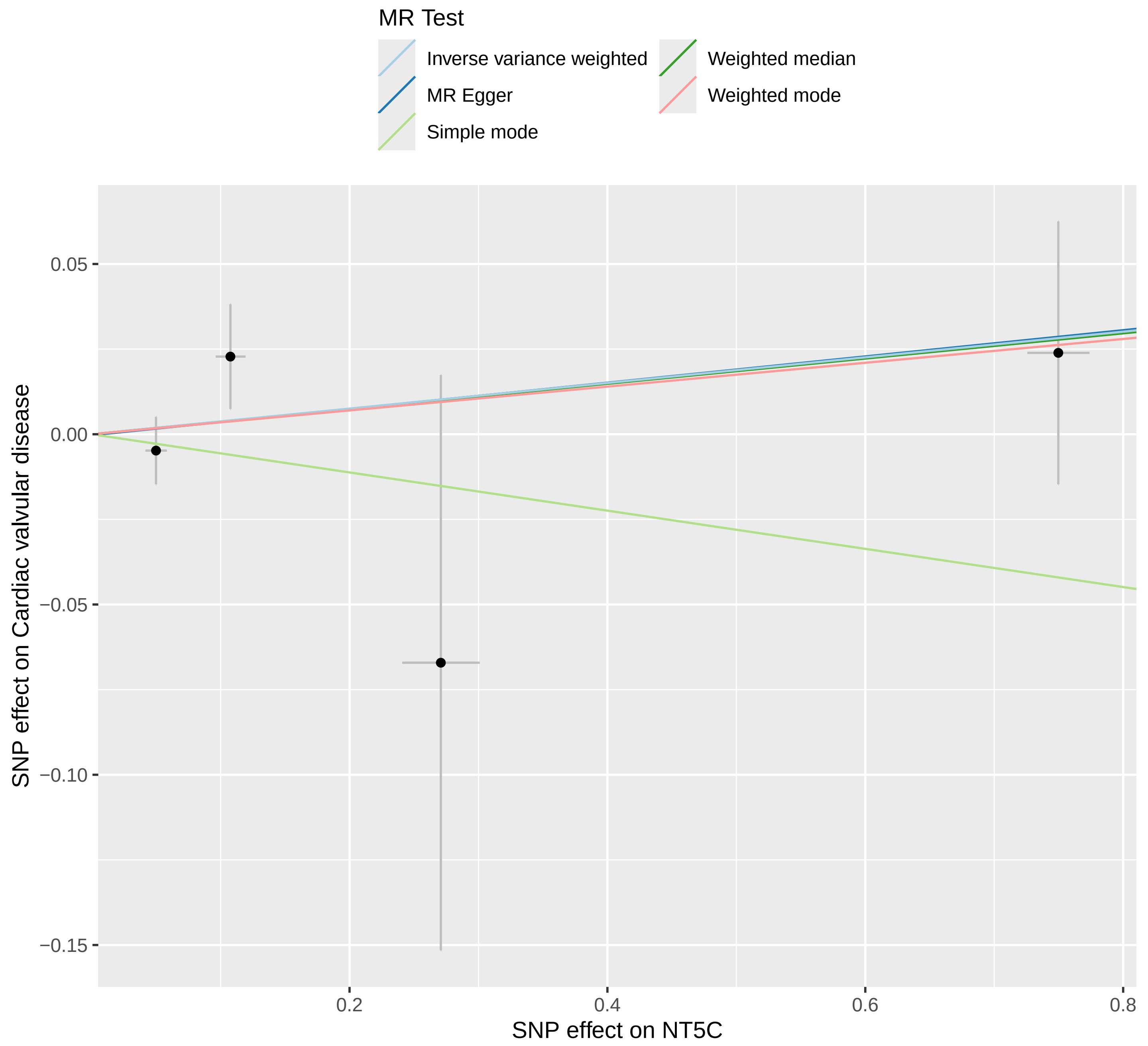

MR Test
Inverse variance weighted
MR Egger
Simple mode
Weighted median
Weighted mode
0.05
0.00
−0.05
−0.10
−0.15
0.2
0.4
0.6
0.8
SNP effect on Cardiac valvular disease
SNP effect on NT5C

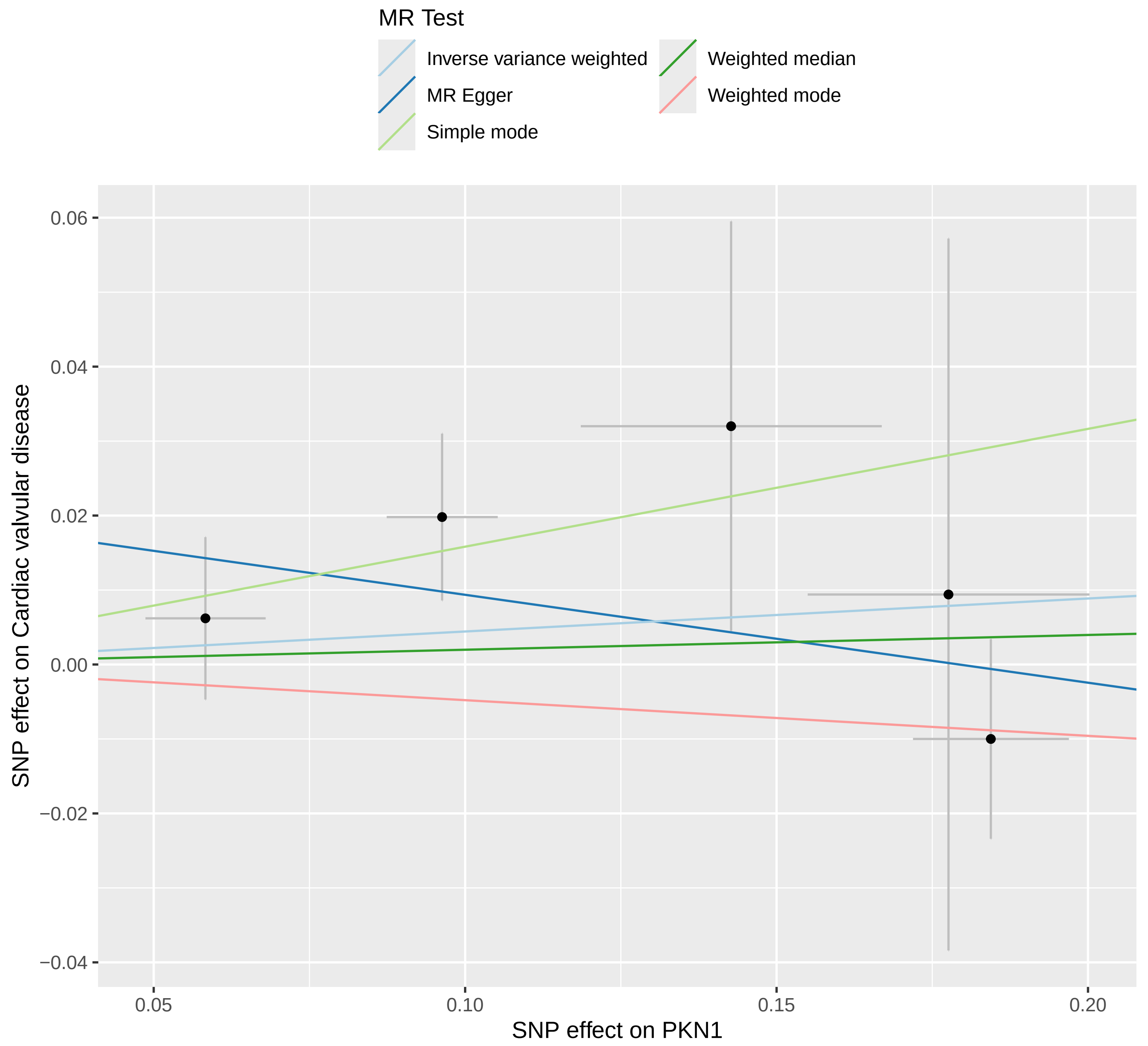
MR Test
Inverse variance weighted
MR Egger
Simple mode
Weighted median
Weighted mode
0.06
0.04
0.02
0.00
−0.02
−0.04
SNP effect on Cardiac valvular disease
0.05
0.10
0.15
0.20
SNP effect on PKN1

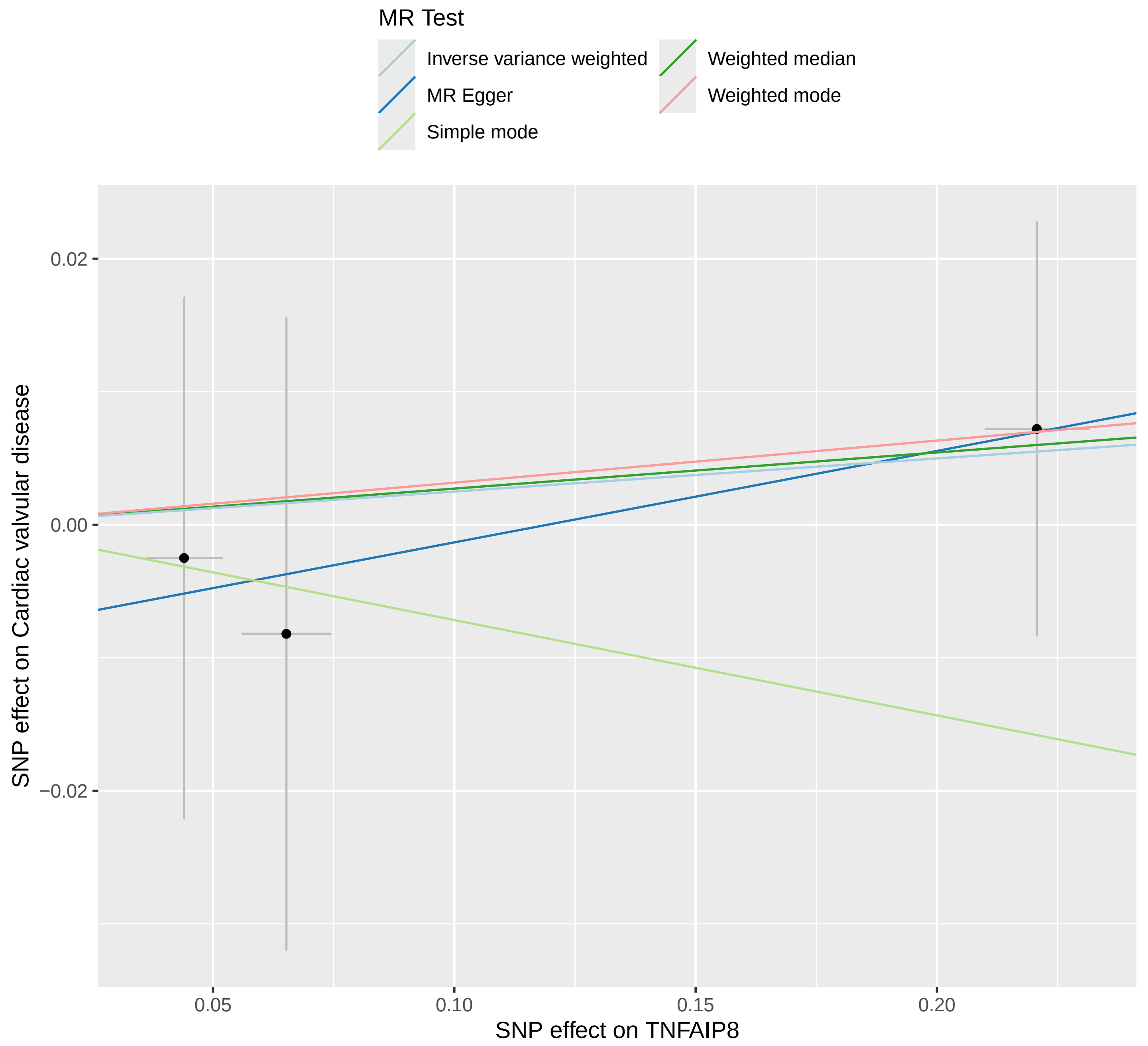

MR Test
Inverse variance weighted
MR Egger
Simple mode
Weighted median
Weighted mode
SNP effect on Cardiac valvular disease
0.02
0.00
−0.02
0.05
0.10
0.15
0.20
SNP effect on TNFAIP8

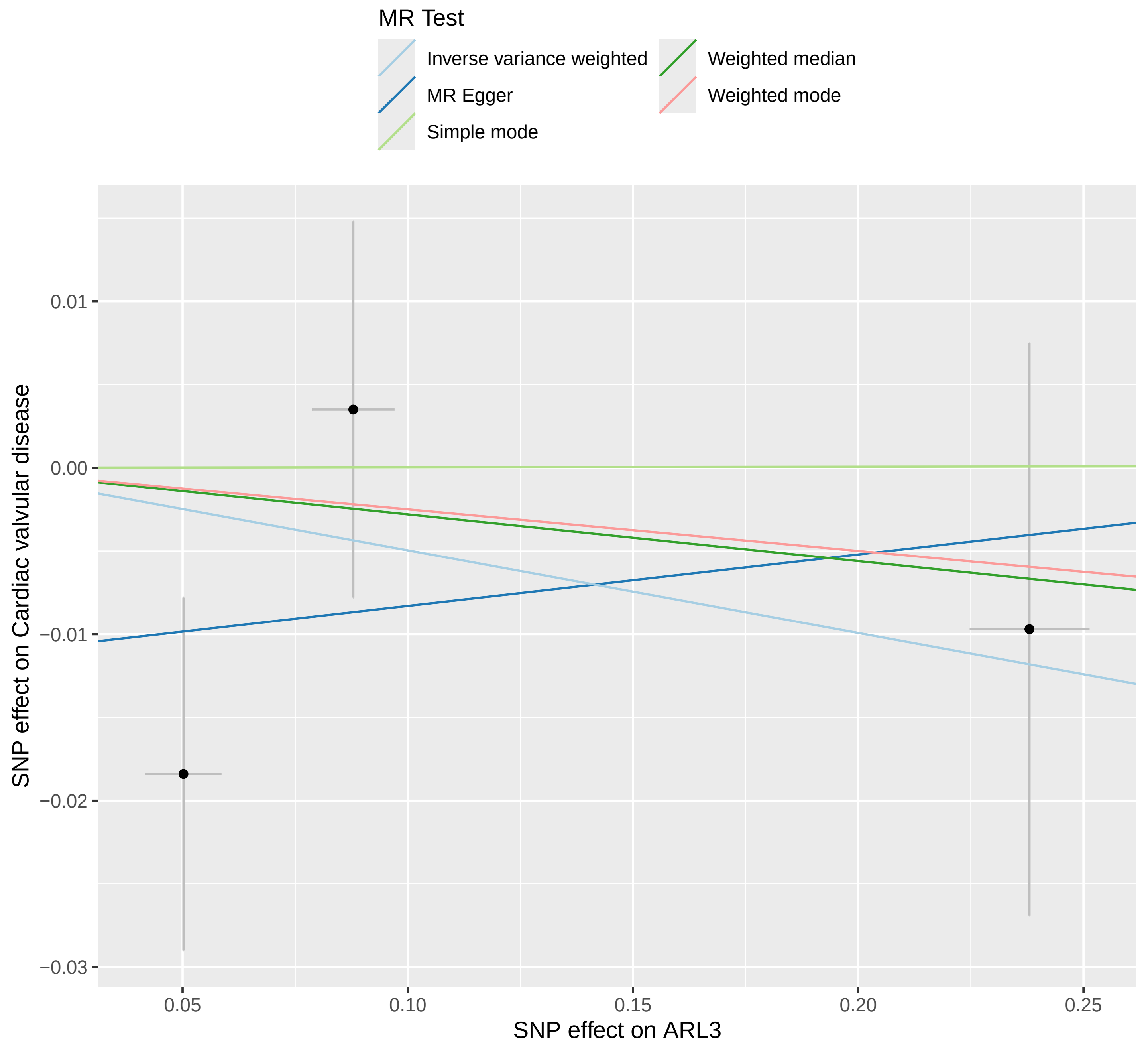

MR Test
Inverse variance weighted
MR Egger
Simple mode
Weighted median
Weighted mode
0.01
0.00
−0.01
−0.02
−0.03
SNP effect on Cardiac valvular disease
0.05
0.10
0.15
0.20
0.25
SNP effect on ARL3

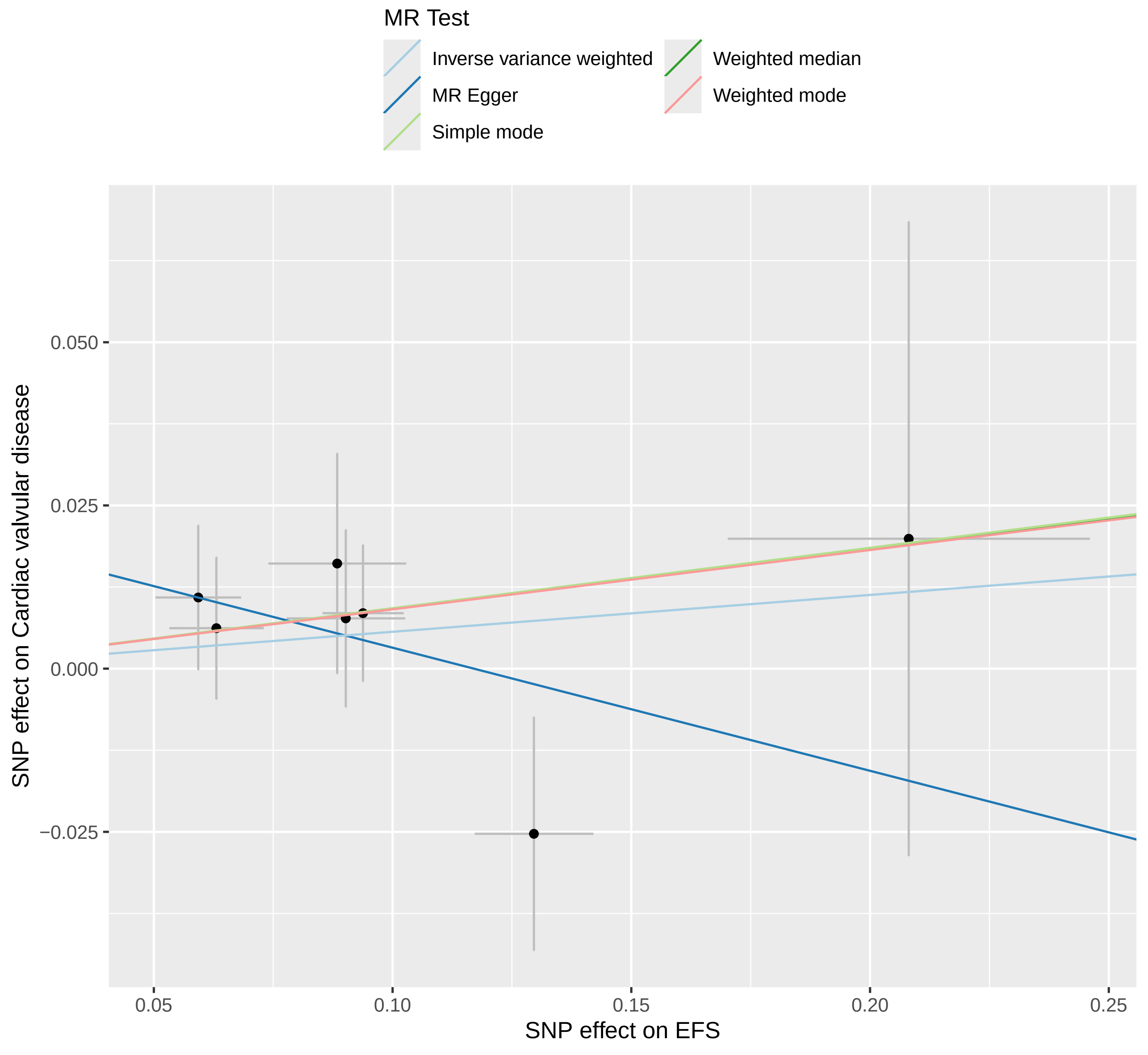

MR Test
Inverse variance weighted
MR Egger
Simple mode
Weighted median
Weighted mode
SNP effect on Cardiac valvular disease
0.050
0.025
0.000
−0.025
0.05
0.10
0.15
0.20
0.25
SNP effect on EFS

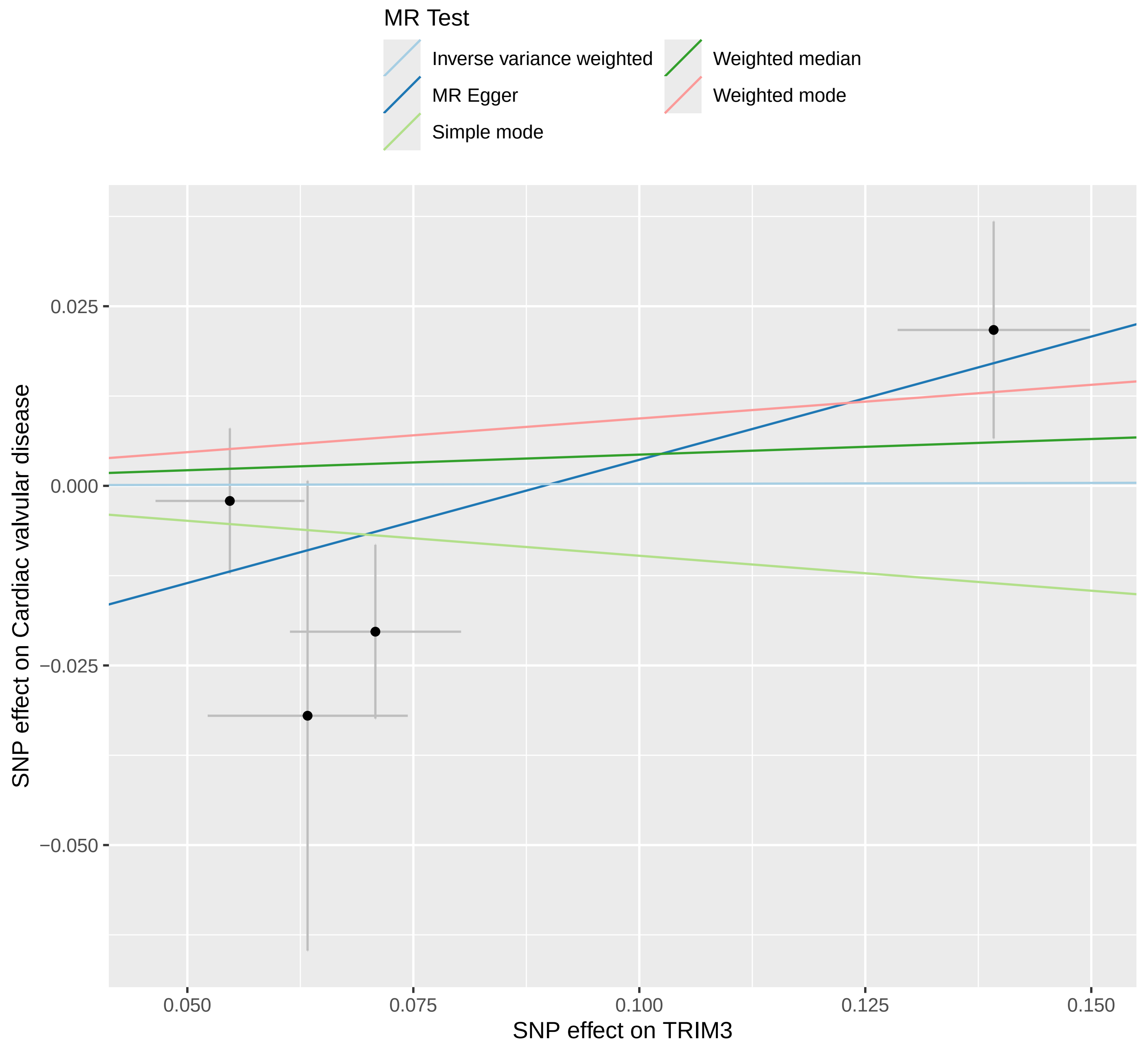

MR Test
Inverse variance weighted
MR Egger
Simple mode
Weighted median
Weighted mode
0.025
0.000
−0.025
−0.050
0.050
0.075
0.100
0.125
0.150
SNP effect on Cardiac valvular disease
SNP effect on TRIM3

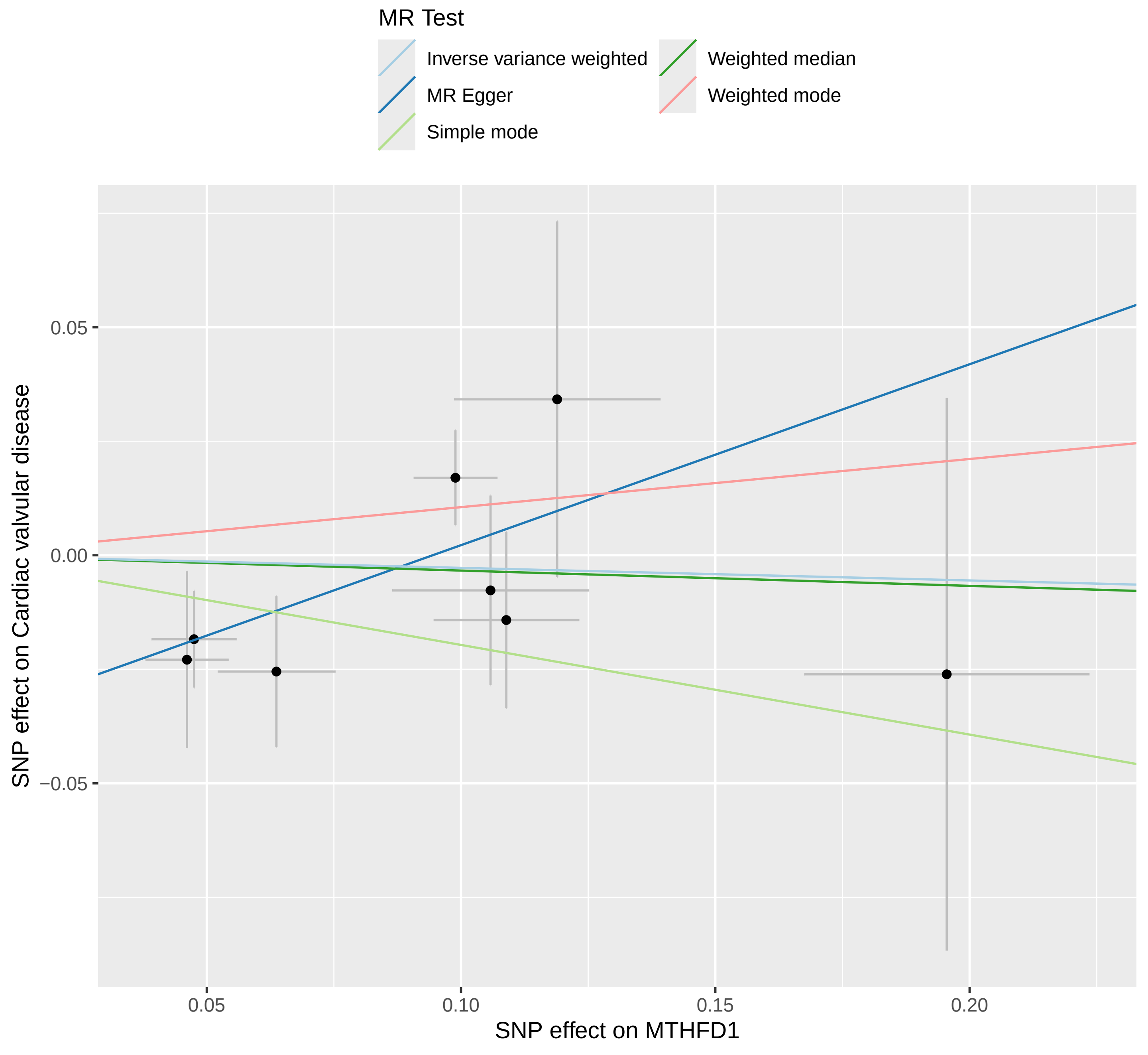
MR Test
Inverse variance weighted
MR Egger
Simple mode
Weighted median
Weighted mode
0.05
0.00
−0.05
0.05
0.10
0.15
0.20
SNP effect on Cardiac valvular disease
SNP effect on MTHFD1

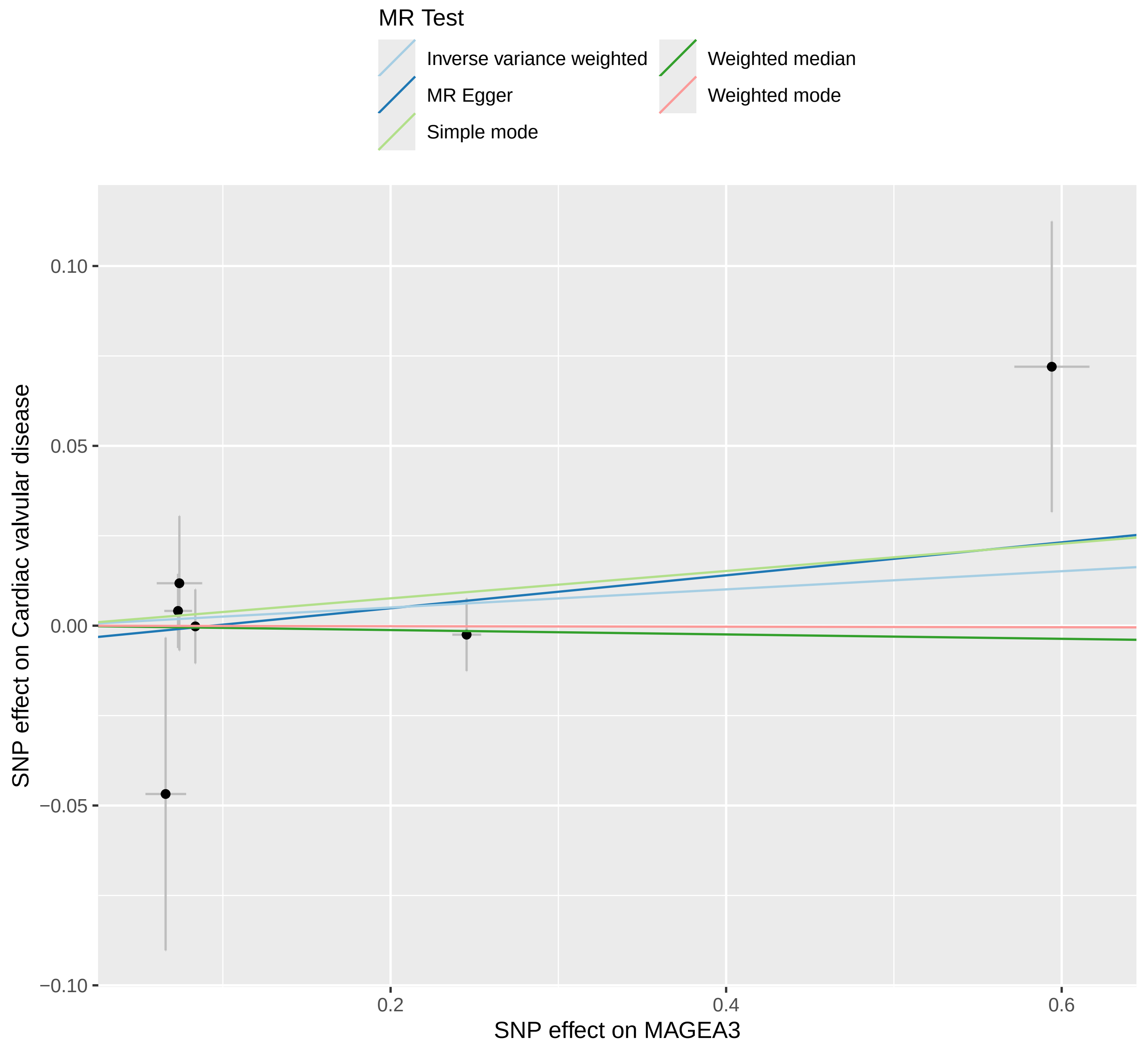
MR Test
Inverse variance weighted
MR Egger
Simple mode
Weighted median
Weighted mode
0.10
0.05
0.00
−0.05
−0.10
0.2
0.4
0.6
SNP effect on Cardiac valvular disease
SNP effect on MAGEA3

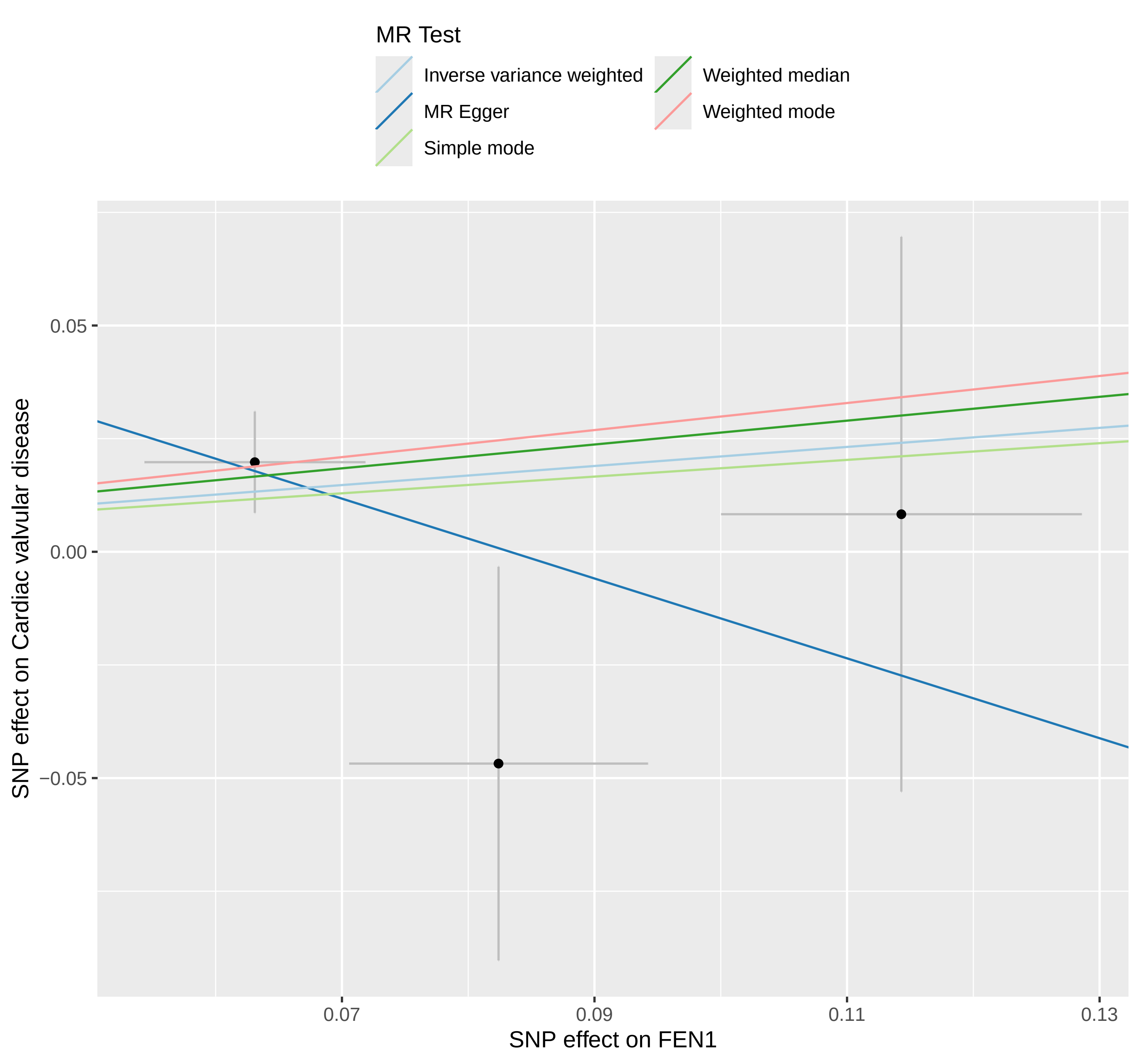
MR Test
Inverse variance weighted
MR Egger
Simple mode
Weighted median
Weighted mode
0.05
0.00
−0.05
0.07
0.09
0.11
0.13
SNP effect on Cardiac valvular disease
SNP effect on FEN1

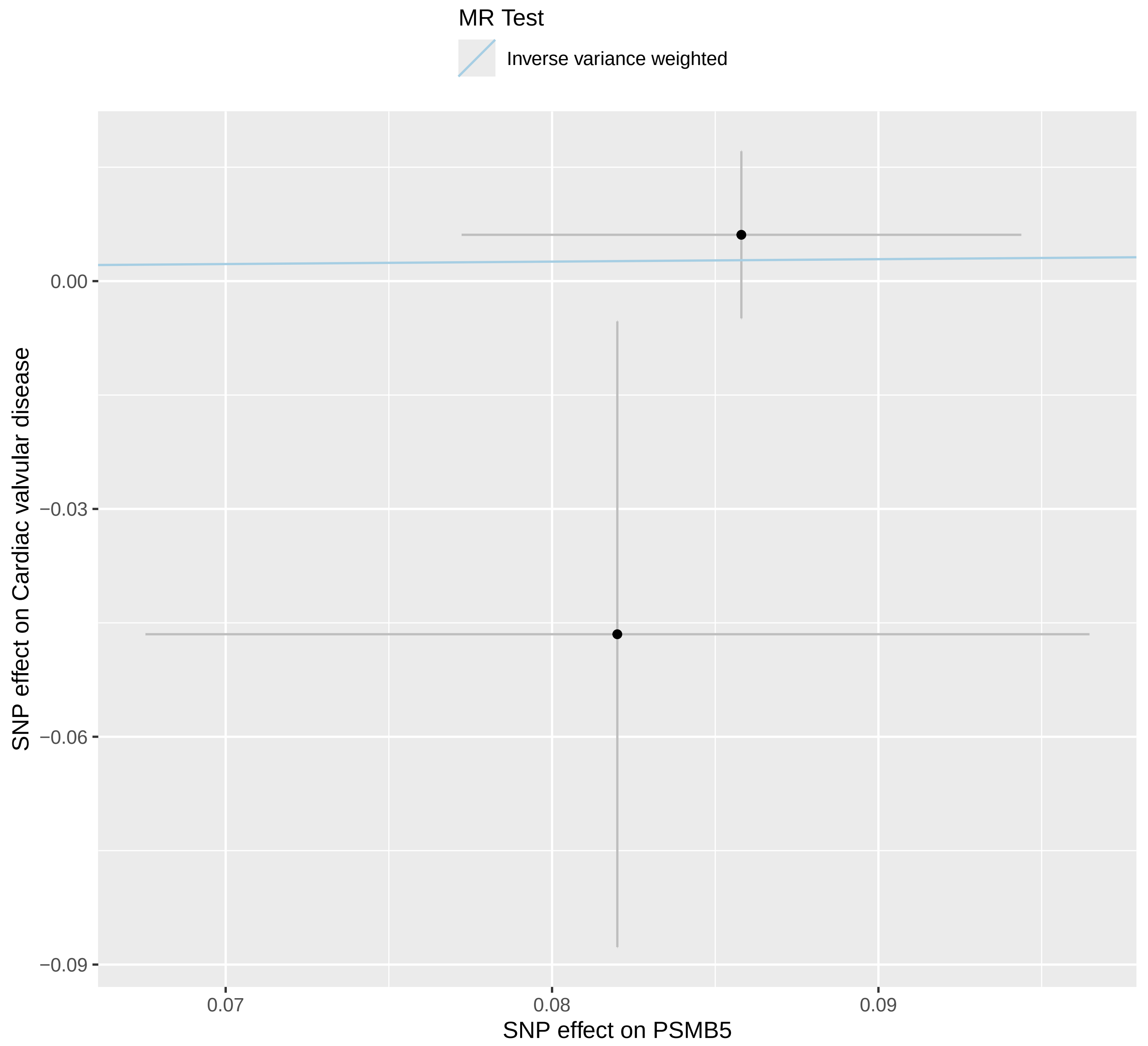

MR Test
Inverse variance weighted
SNP effect on Cardiac valvular disease
0.00
−0.03
−0.06
−0.09
0.07
0.08
0.09
SNP effect on PSMB5

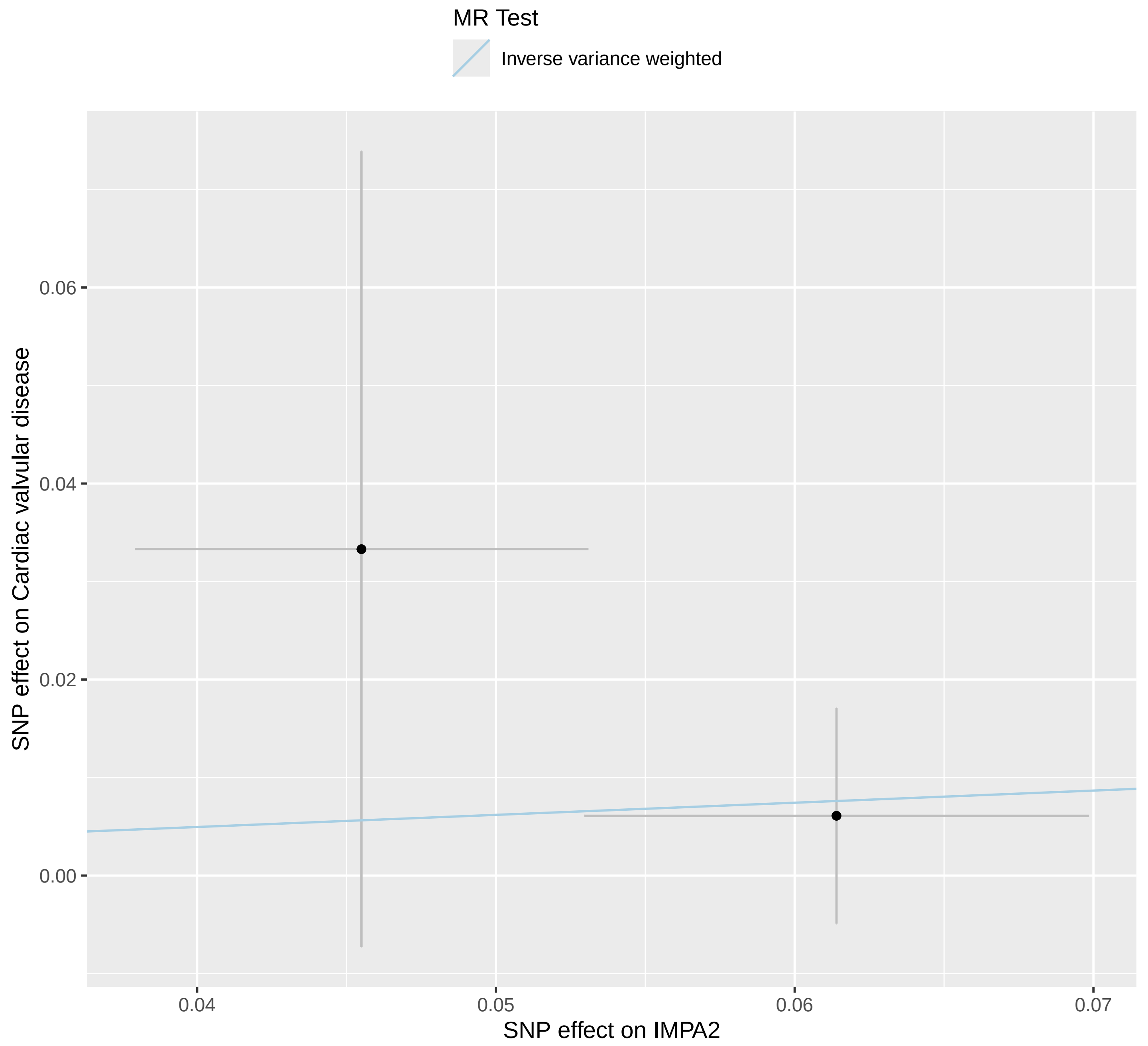
MR Test
Inverse variance weighted
SNP effect on Cardiac valvular disease
0.06
0.04
0.02
0.00
0.04
0.05
0.06
0.07
SNP effect on IMPA2

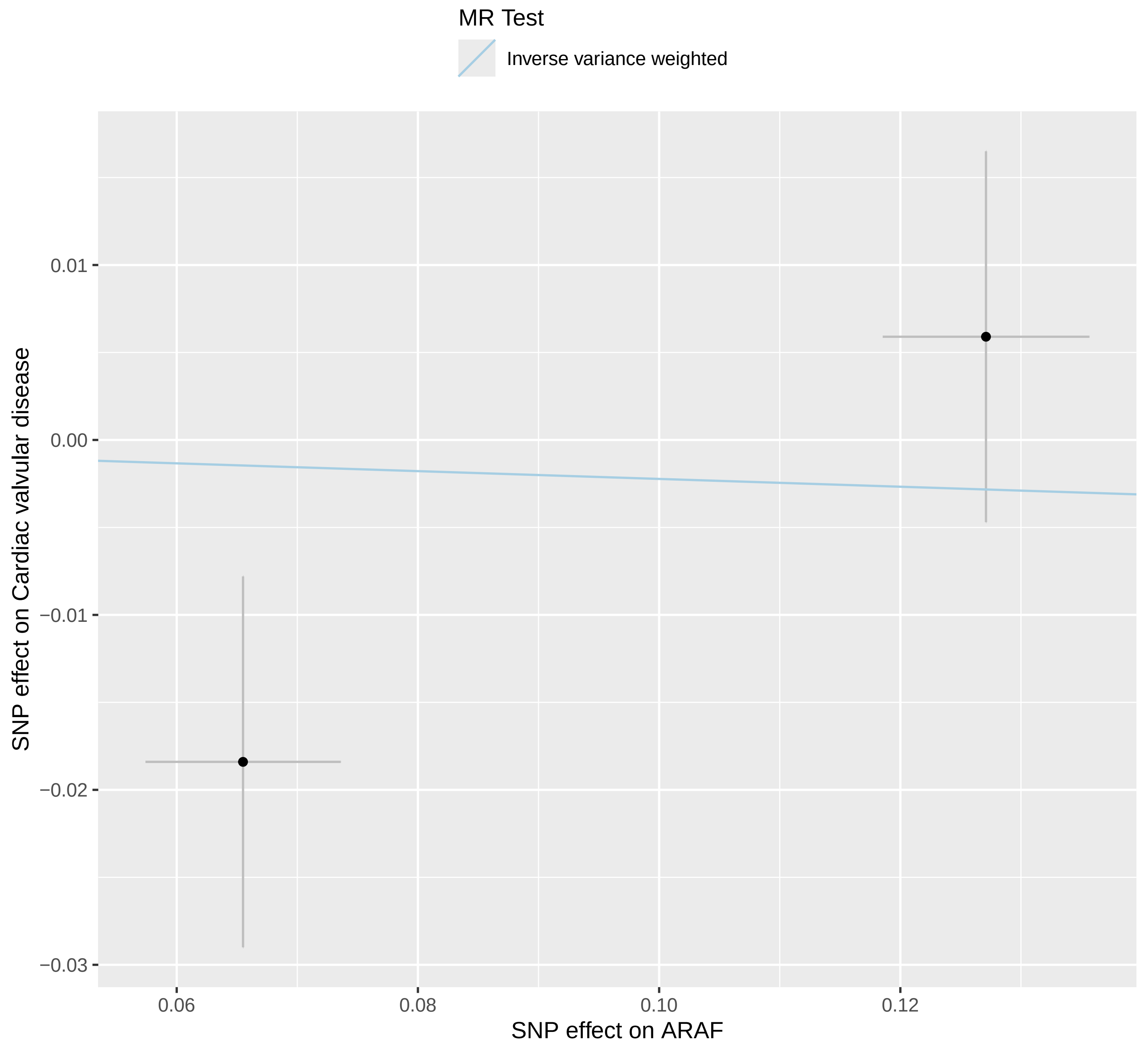

MR Test
Inverse variance weighted
0.01
0.00
−0.01
−0.02
−0.03
SNP effect on Cardiac valvular disease
0.06
0.08
0.10
0.12
SNP effect on ARAF

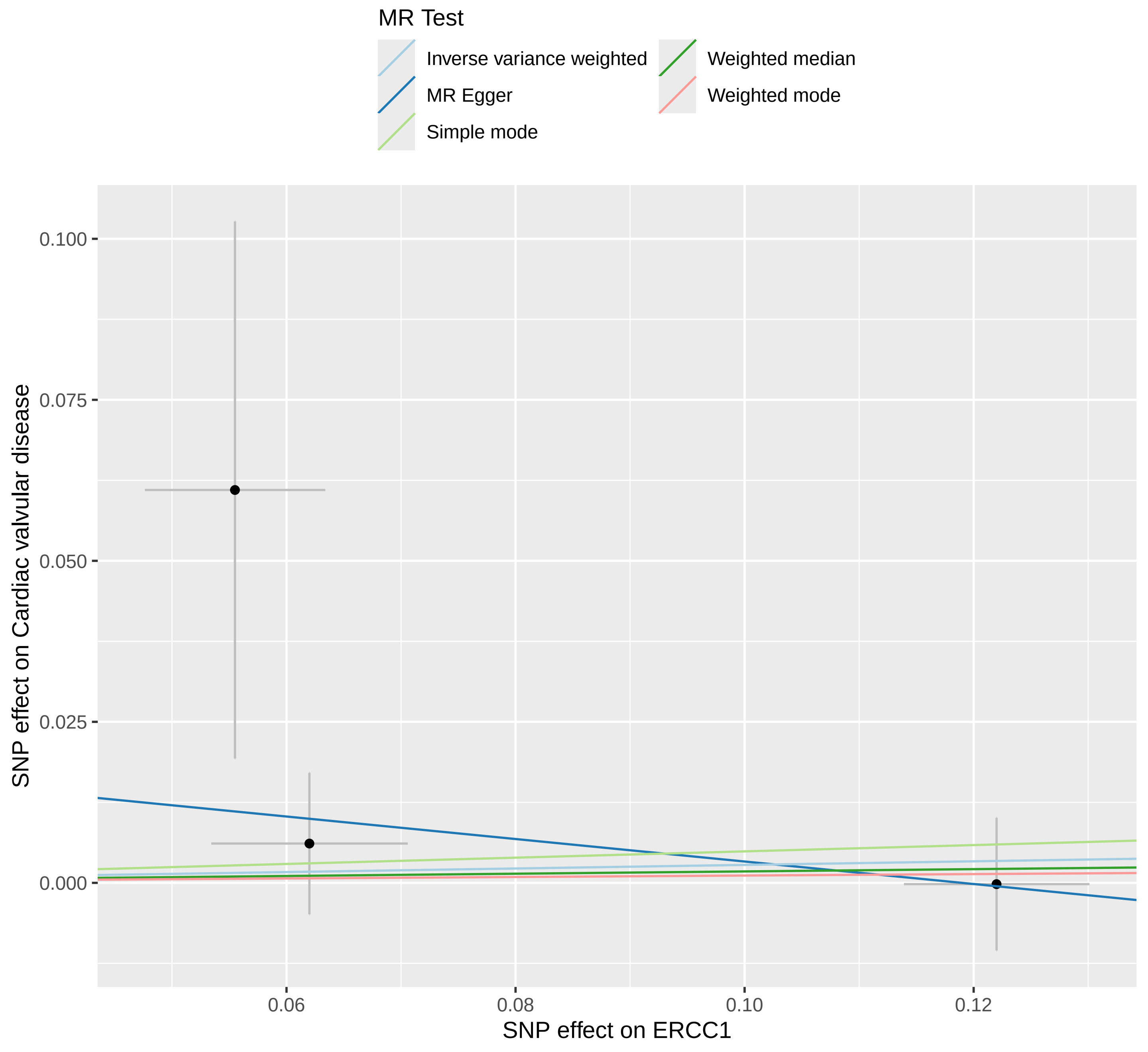
MR Test
Inverse variance weighted
MR Egger
Simple mode
Weighted median
Weighted mode
0.100
0.075
0.050
0.025
0.000
SNP effect on Cardiac valvular disease
0.06
0.08
0.10
0.12
SNP effect on ERCC1

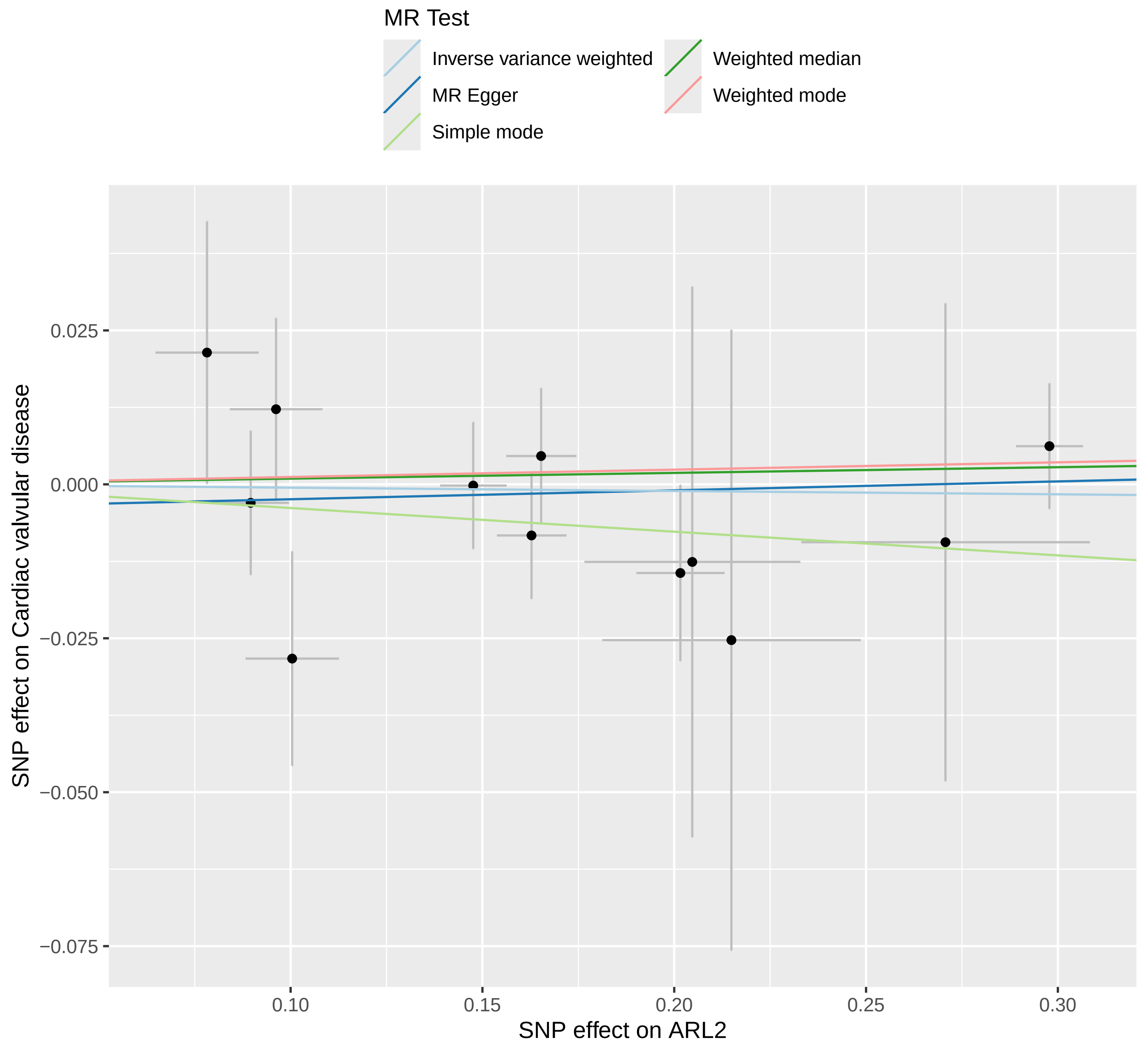

MR Test
Inverse variance weighted
MR Egger
Simple mode
Weighted median
Weighted mode
0.025
0.000
−0.025
−0.050
−0.075
SNP effect on Cardiac valvular disease
0.10
0.15
0.20
0.25
0.30
SNP effect on ARL2

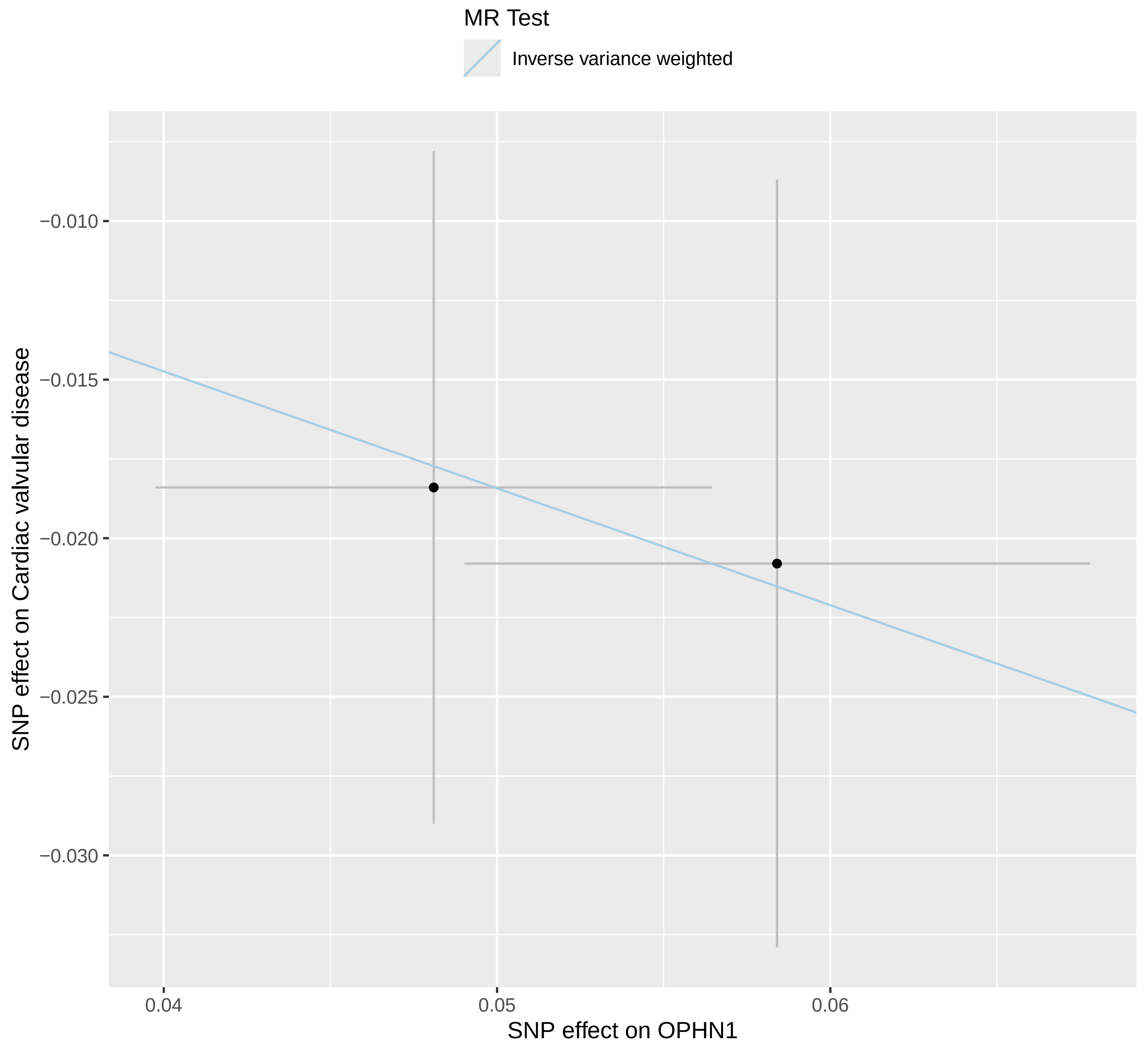
MR Test
Inverse variance weighted
SNP effect on Cardiac valvular disease
−0.010
−0.015
−0.020
−0.025
−0.030
0.04
0.05
0.06
SNP effect on OPHN1

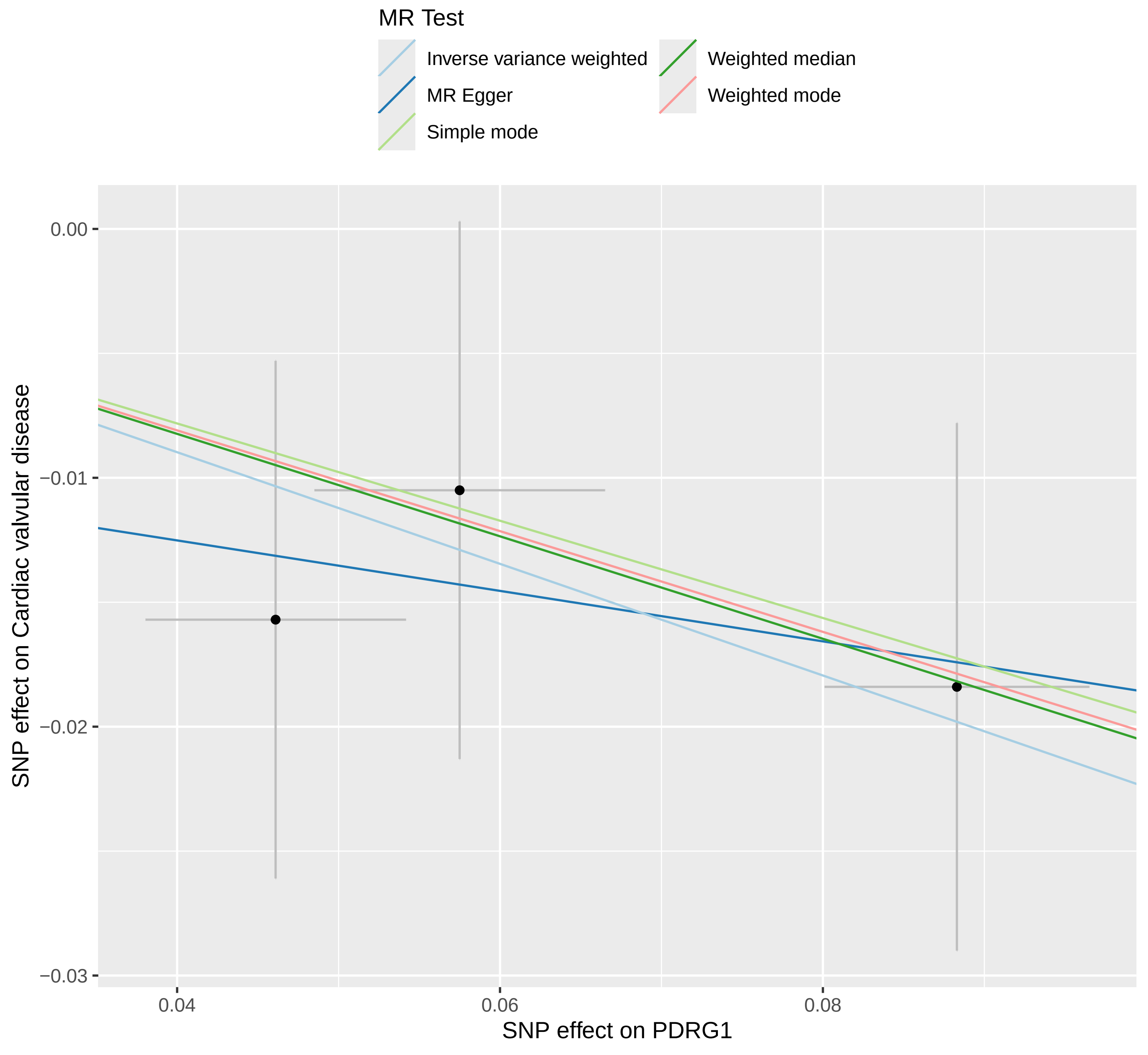
MR Test
Inverse variance weighted
MR Egger
Simple mode
Weighted median
Weighted mode
0.00
−0.01
−0.02
−0.03
0.04
0.06
0.08
SNP effect on Cardiac valvular disease
SNP effect on PDRG1

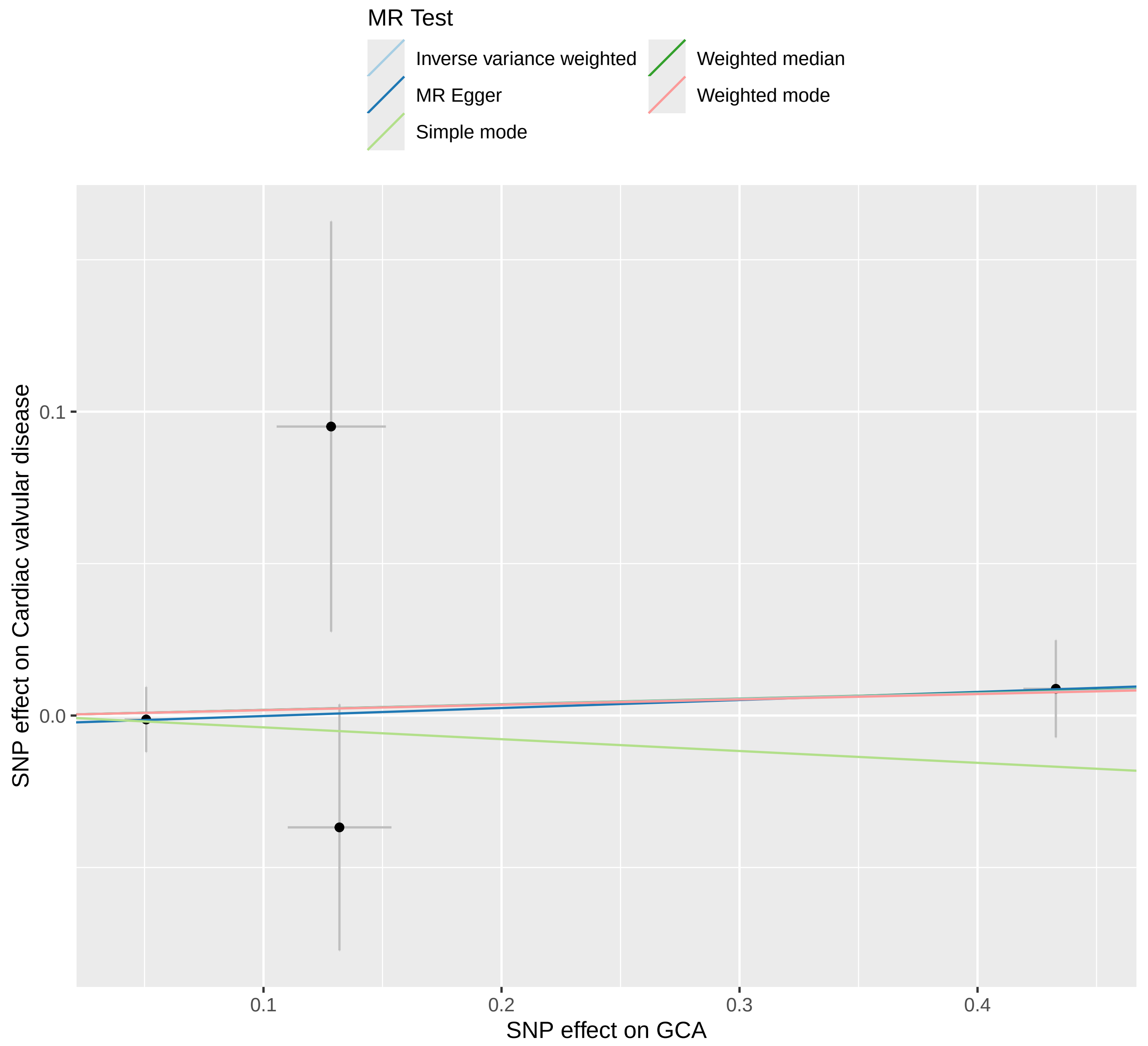

MR Test
Inverse variance weighted
MR Egger
Simple mode
Weighted median
Weighted mode
SNP effect on Cardiac valvular disease
0.1
0.0
0.1
0.2
0.3
0.4
SNP effect on GCA

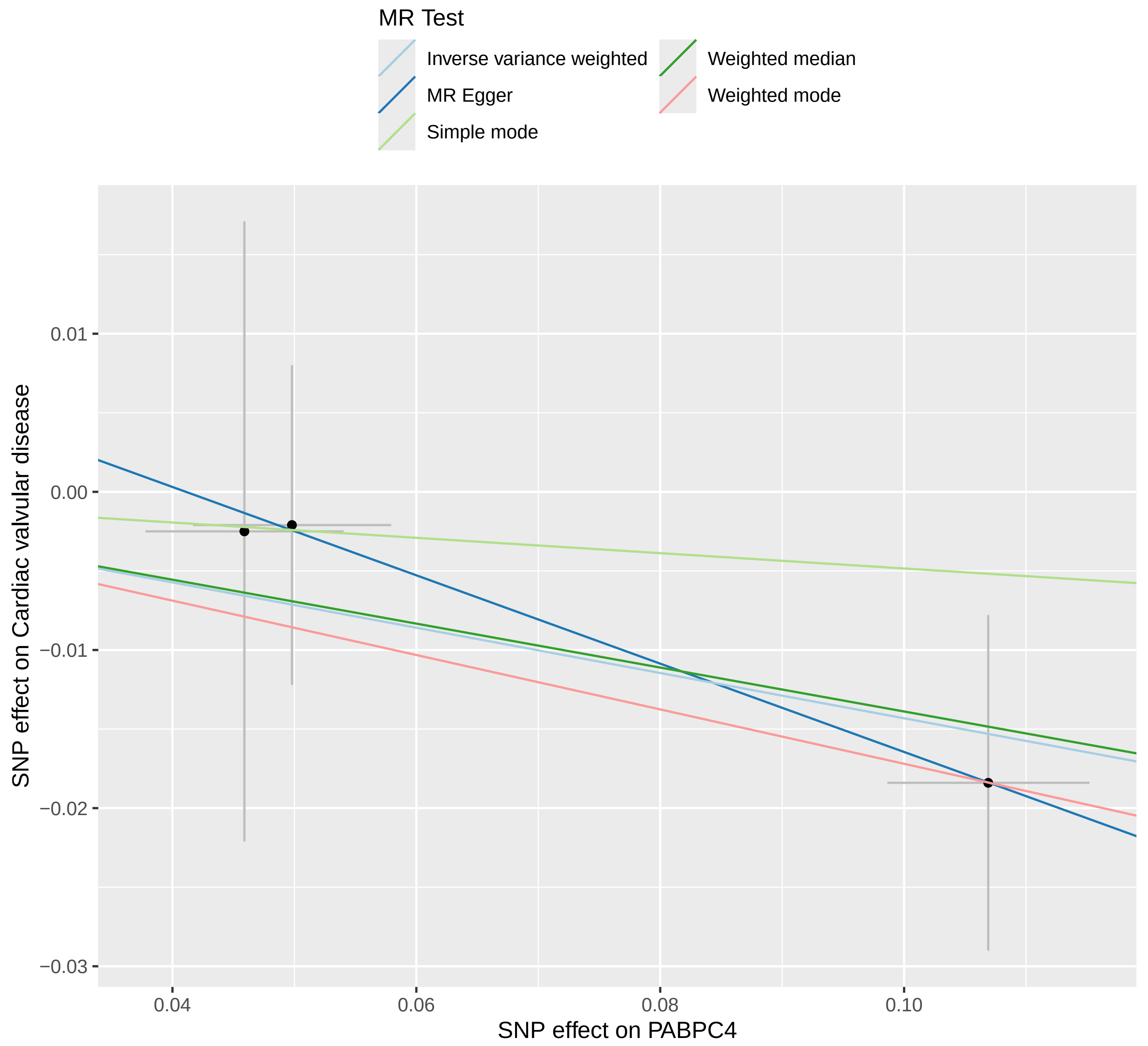
MR Test
Inverse variance weighted
MR Egger
Simple mode
Weighted median
Weighted mode
0.01
0.00
−0.01
−0.02
−0.03
SNP effect on Cardiac valvular disease
0.04
0.06
0.08
0.10
SNP effect on PABPC4

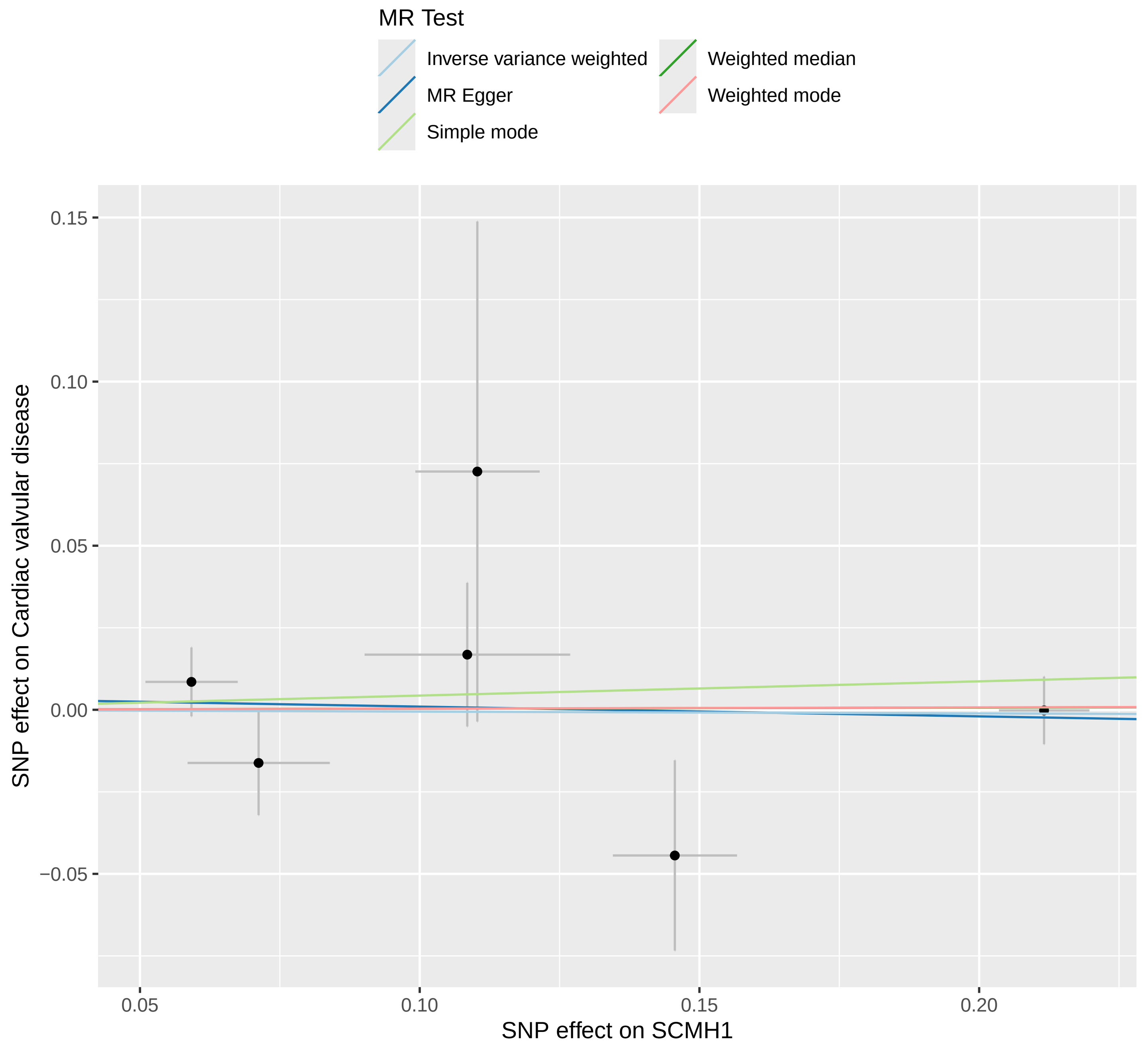

MR Test
Inverse variance weighted
MR Egger
Simple mode
Weighted median
Weighted mode
0.15
0.10
0.05
0.00
−0.05
0.05
0.10
0.15
0.20
SNP effect on Cardiac valvular disease
SNP effect on SCMH1

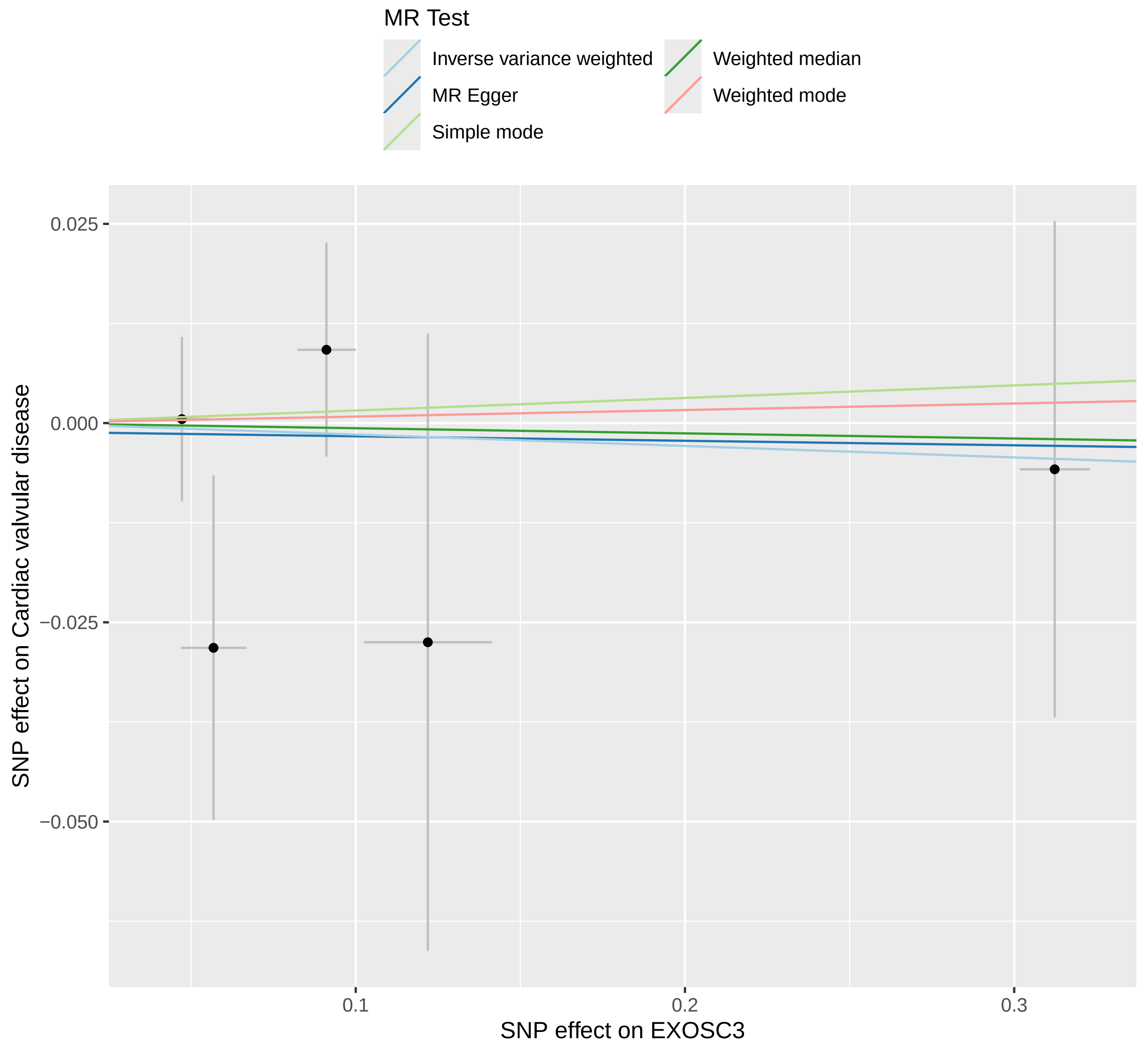
MR Test
Inverse variance weighted
MR Egger
Simple mode
Weighted median
Weighted mode
0.025
0.000
−0.025
−0.050
0.1
0.2
0.3
SNP effect on Cardiac valvular disease
SNP effect on EXOSC3

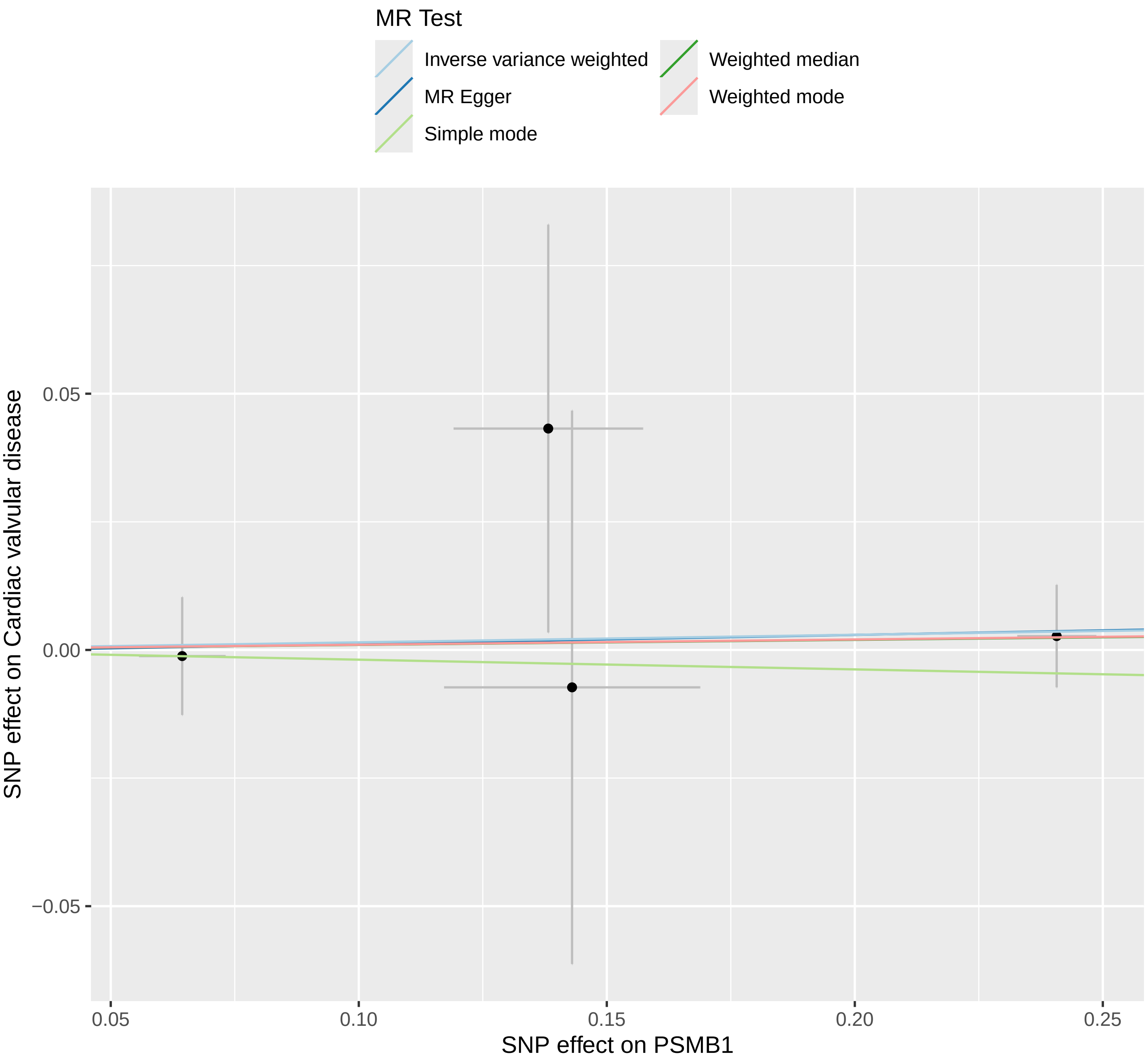
MR Test
Inverse variance weighted
MR Egger
Simple mode
Weighted median
Weighted mode
SNP effect on Cardiac valvular disease
0.05
0.00
−0.05
0.05
0.10
0.15
0.20
0.25
SNP effect on PSMB1

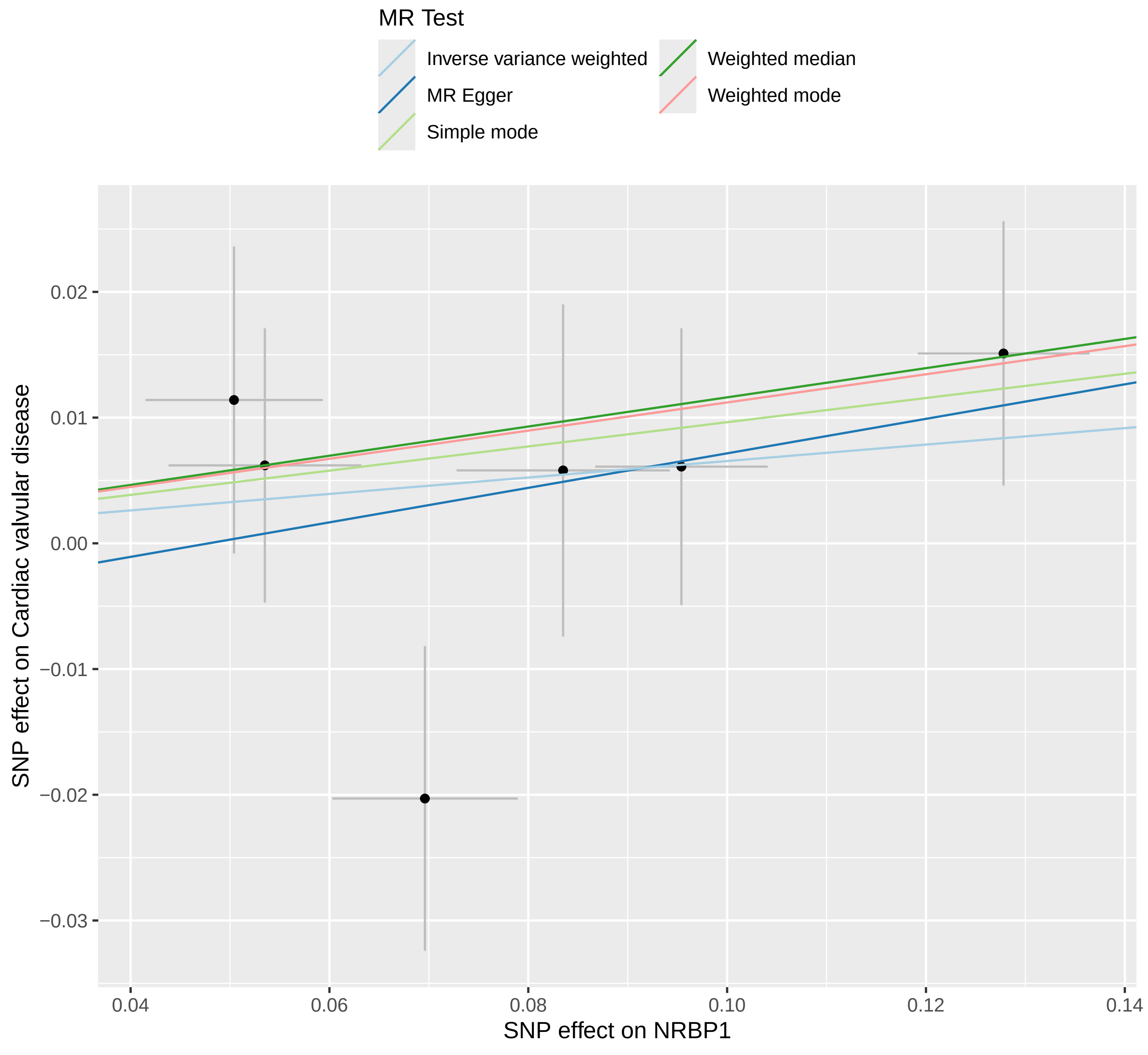
MR Test
Inverse variance weighted
MR Egger
Simple mode
Weighted median
Weighted mode
SNP effect on Cardiac valvular disease
0.02
0.01
0.00
−0.01
−0.02
−0.03
0.04
0.06
0.08
0.10
0.12
0.14
SNP effect on NRBP1

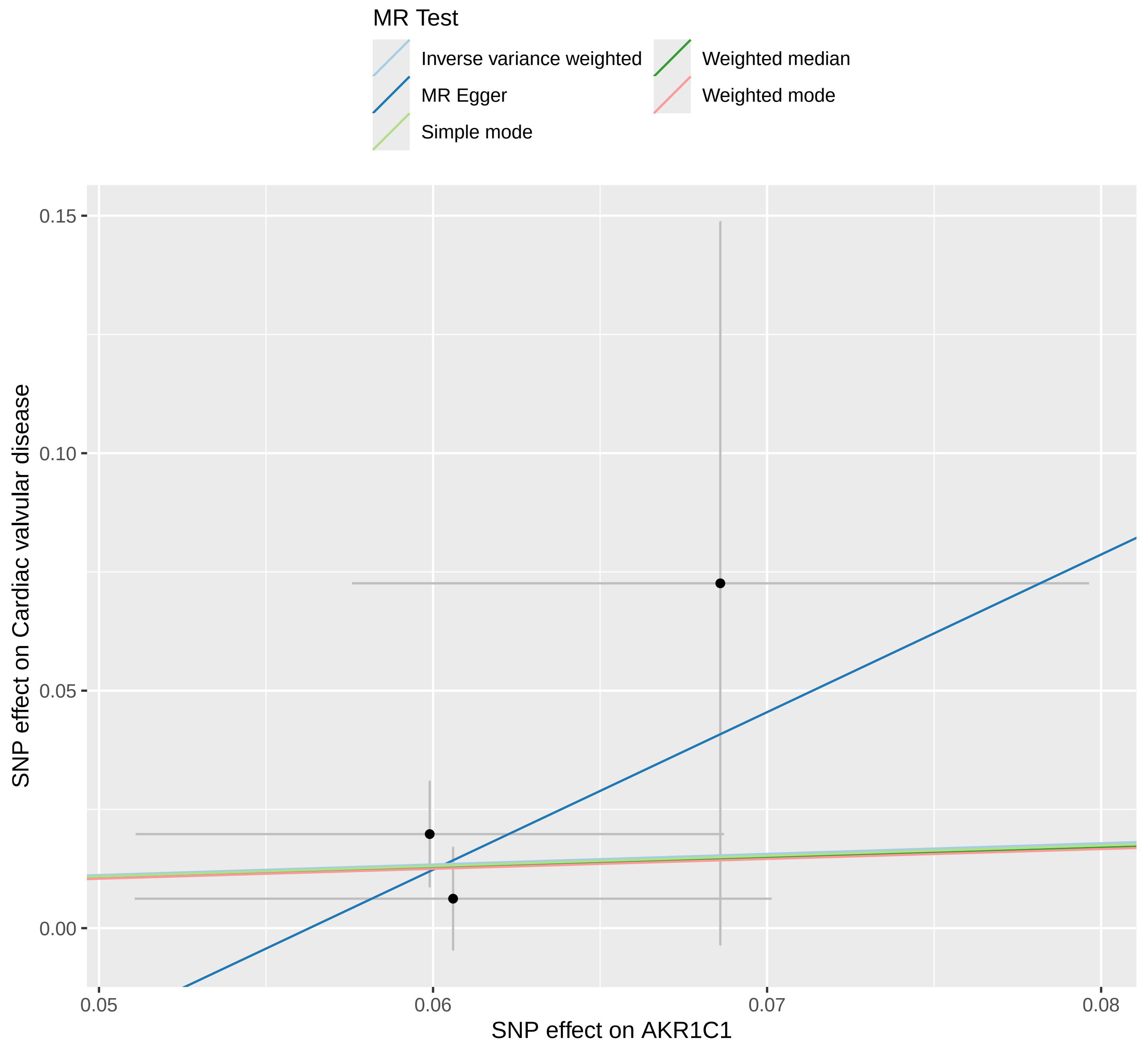

MR Test
Inverse variance weighted
MR Egger
Simple mode
Weighted median
Weighted mode
0.15
0.10
0.05
0.00
SNP effect on Cardiac valvular disease
0.05
0.06
0.07
0.08
SNP effect on AKR1C1

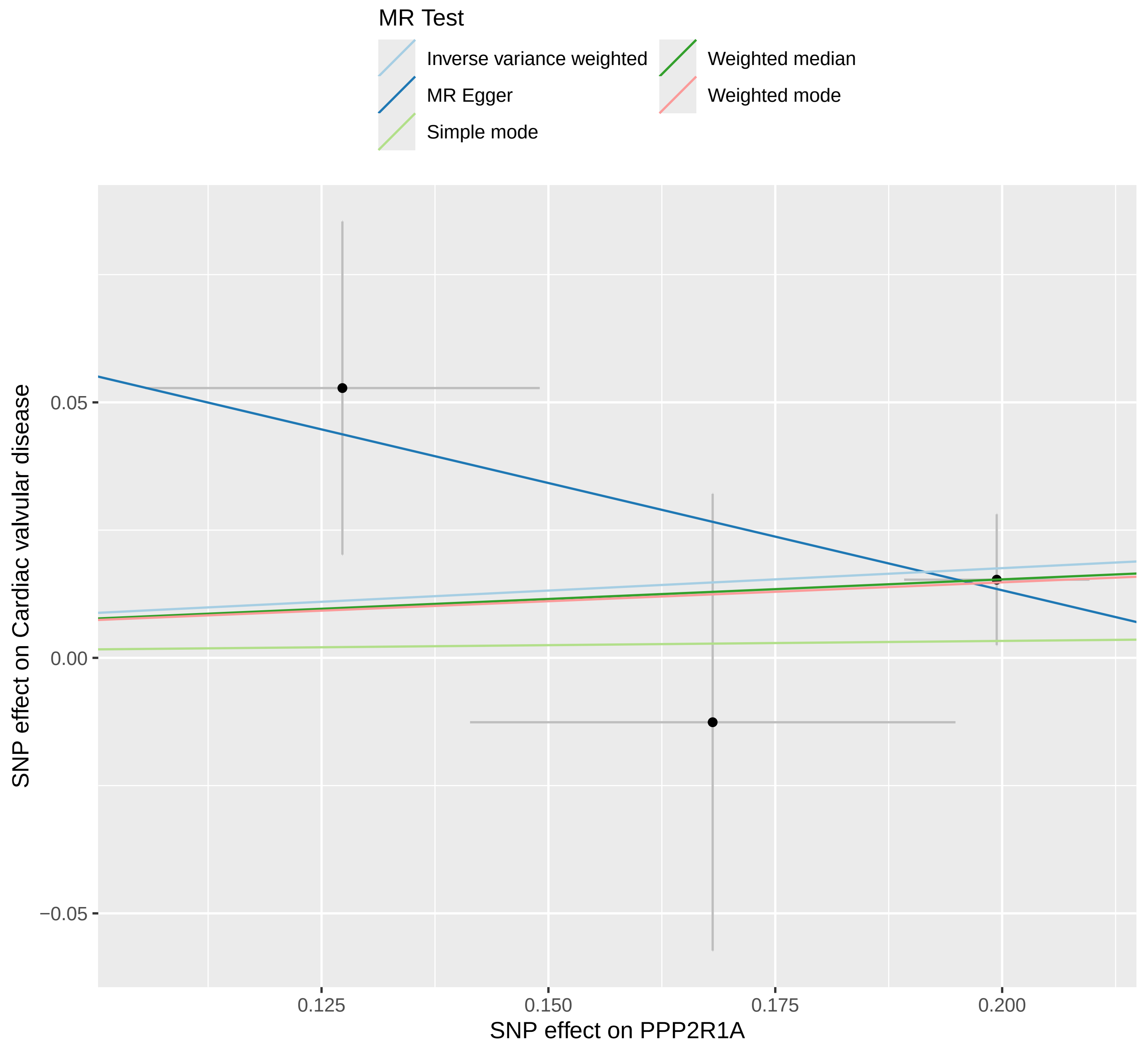

MR Test
Inverse variance weighted
MR Egger
Simple mode
Weighted median
Weighted mode
SNP effect on Cardiac valvular disease
0.05
0.00
−0.05
0.125
0.150
0.175
0.200
SNP effect on PPP2R1A

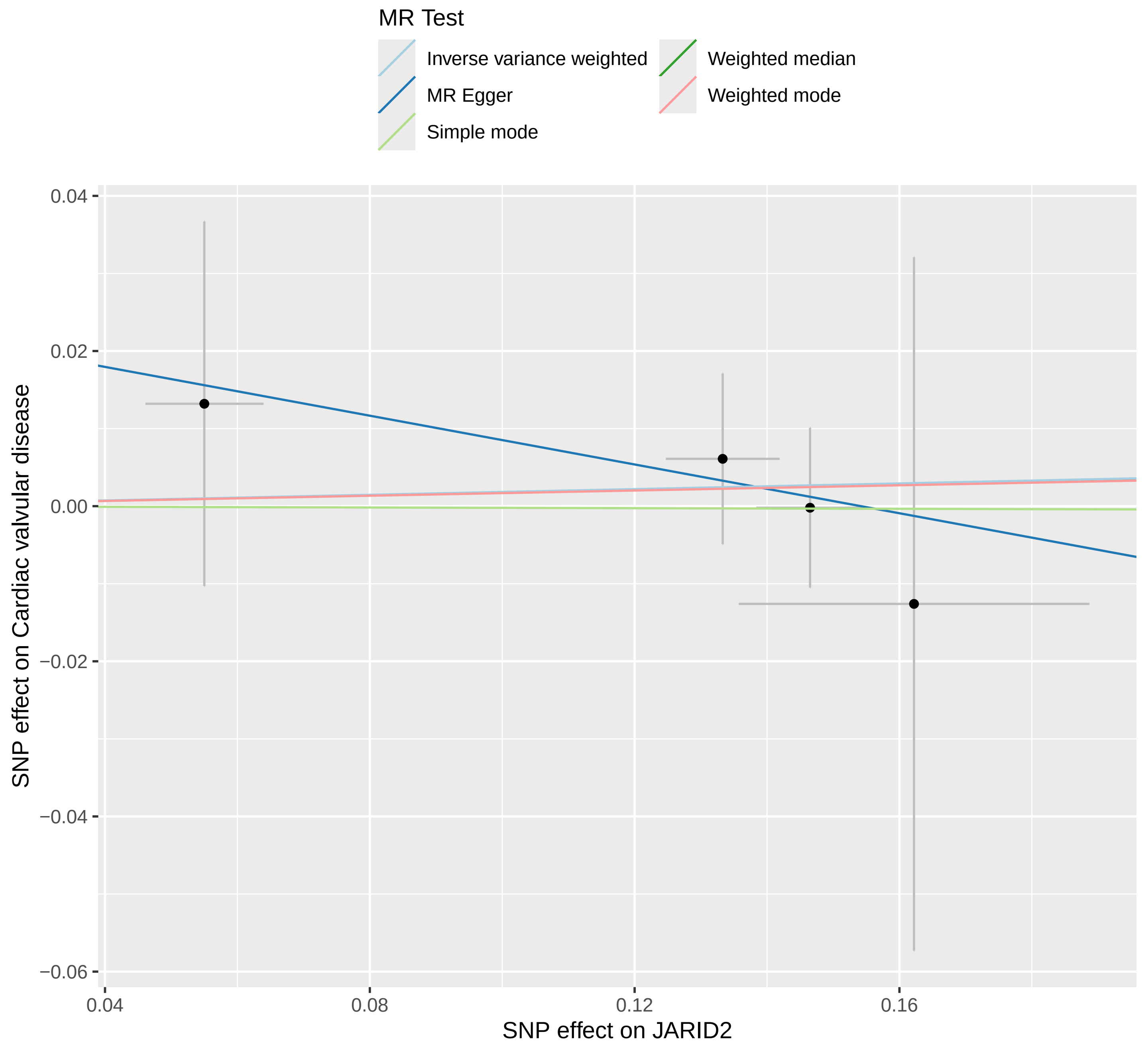

MR Test
Inverse variance weighted
MR Egger
Simple mode
Weighted median
Weighted mode
0.04
0.02
0.00
−0.02
−0.04
−0.06
SNP effect on Cardiac valvular disease
0.04
0.08
0.12
0.16
SNP effect on JARID2

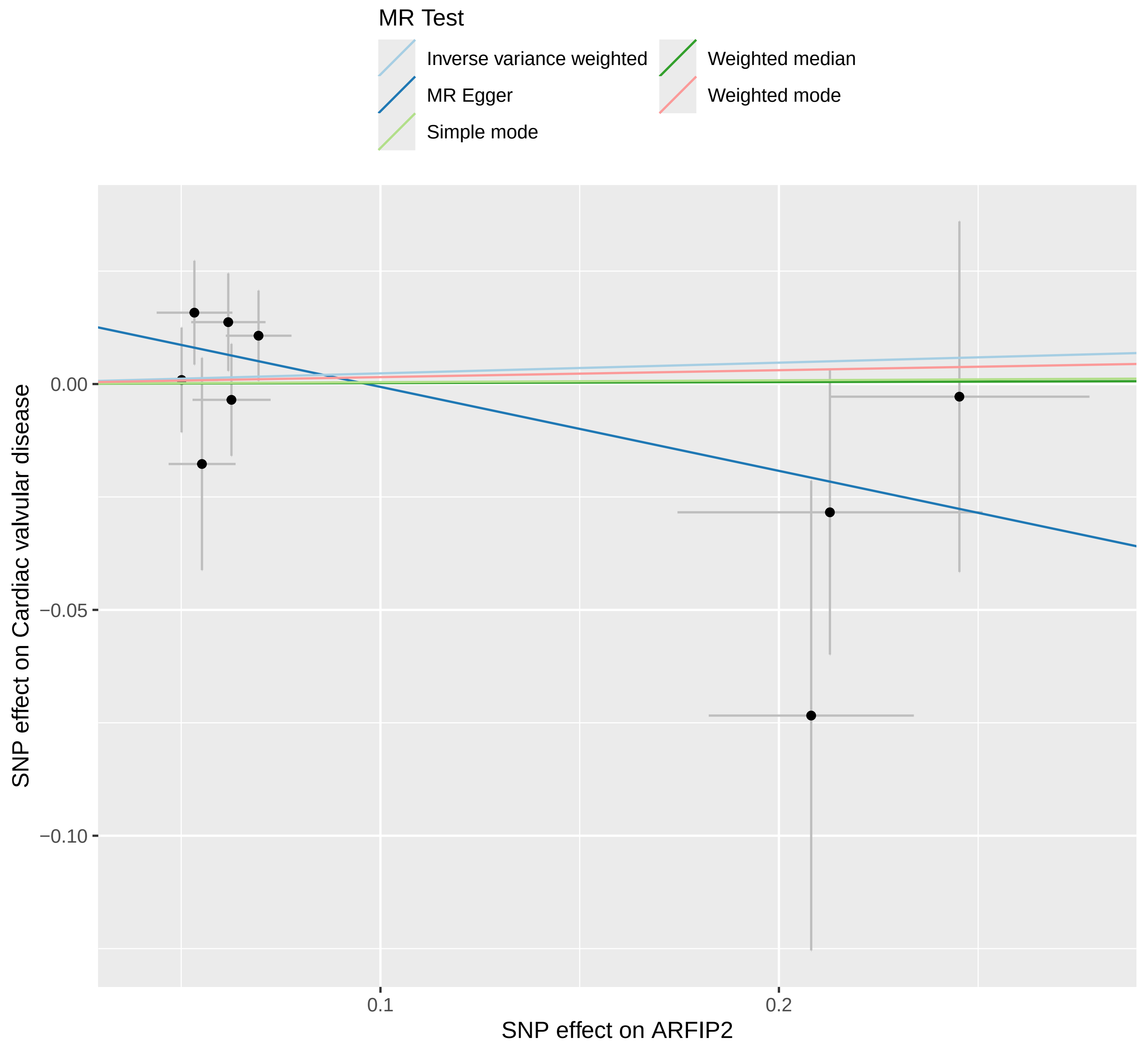

MR Test
Inverse variance weighted
MR Egger
Simple mode
Weighted median
Weighted mode
0.00
−0.05
−0.10
0.1
0.2
SNP effect on ARFIP2
SNP effect on Cardiac valvular disease

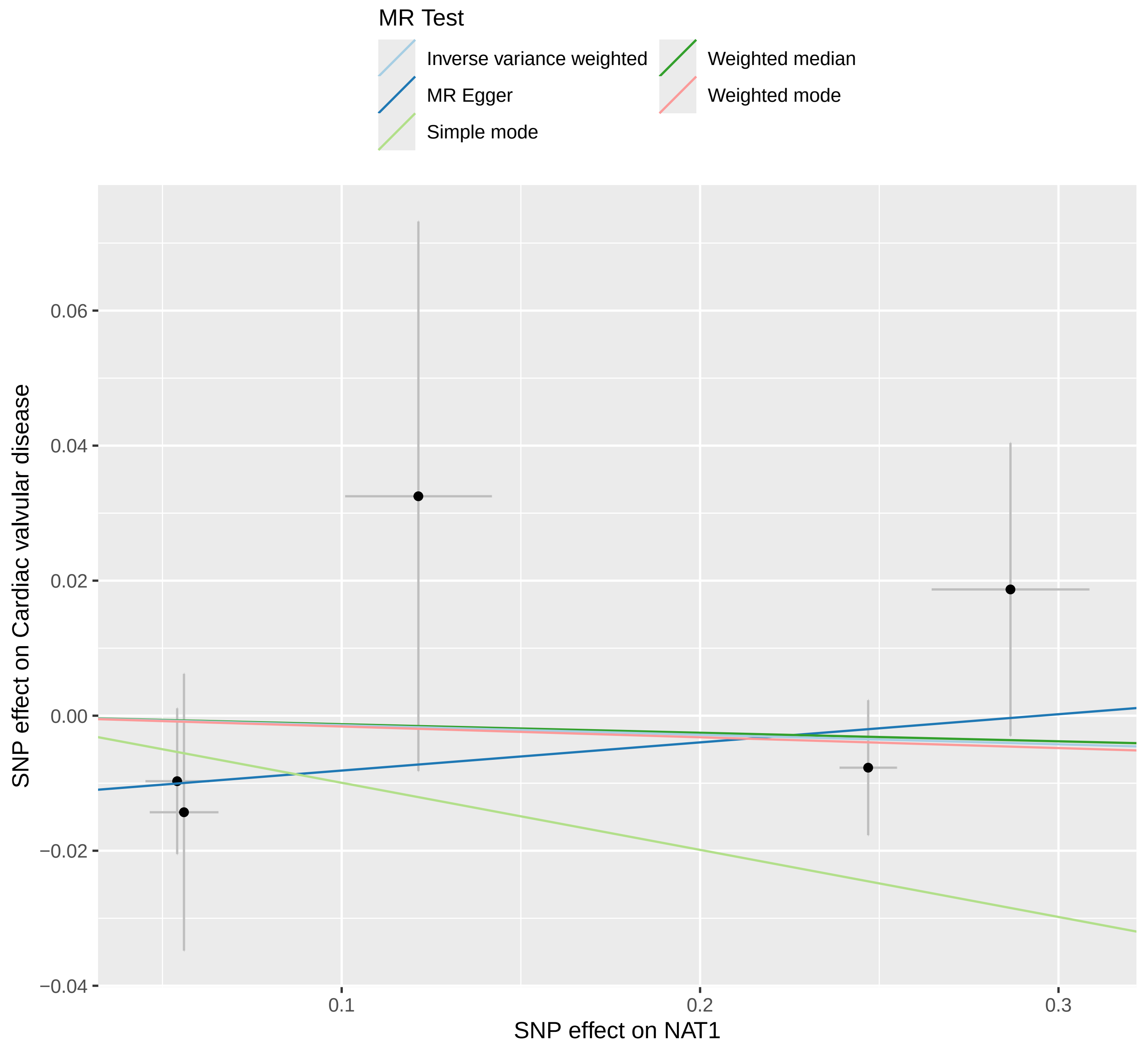

MR Test
Inverse variance weighted
MR Egger
Simple mode
Weighted median
Weighted mode
0.06
0.04
0.02
0.00
−0.02
−0.04
0.1
0.2
0.3
SNP effect on Cardiac valvular disease
SNP effect on NAT1

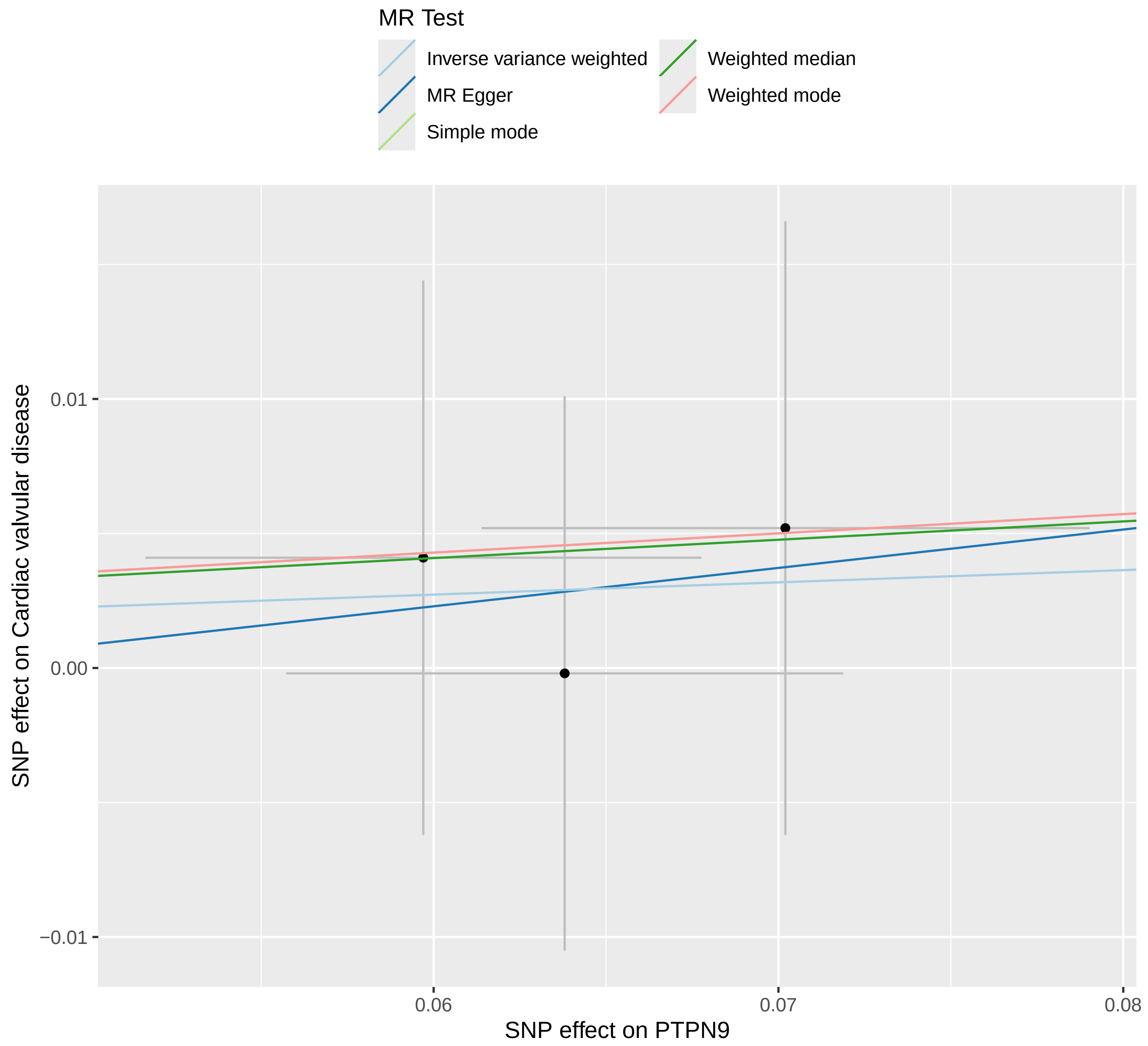

MR Test
Inverse variance weighted
MR Egger
Simple mode
Weighted median
Weighted mode
0.01
0.00
−0.01
0.06
0.07
0.08
SNP effect on PTPN9
SNP effect on Cardiac valvular disease

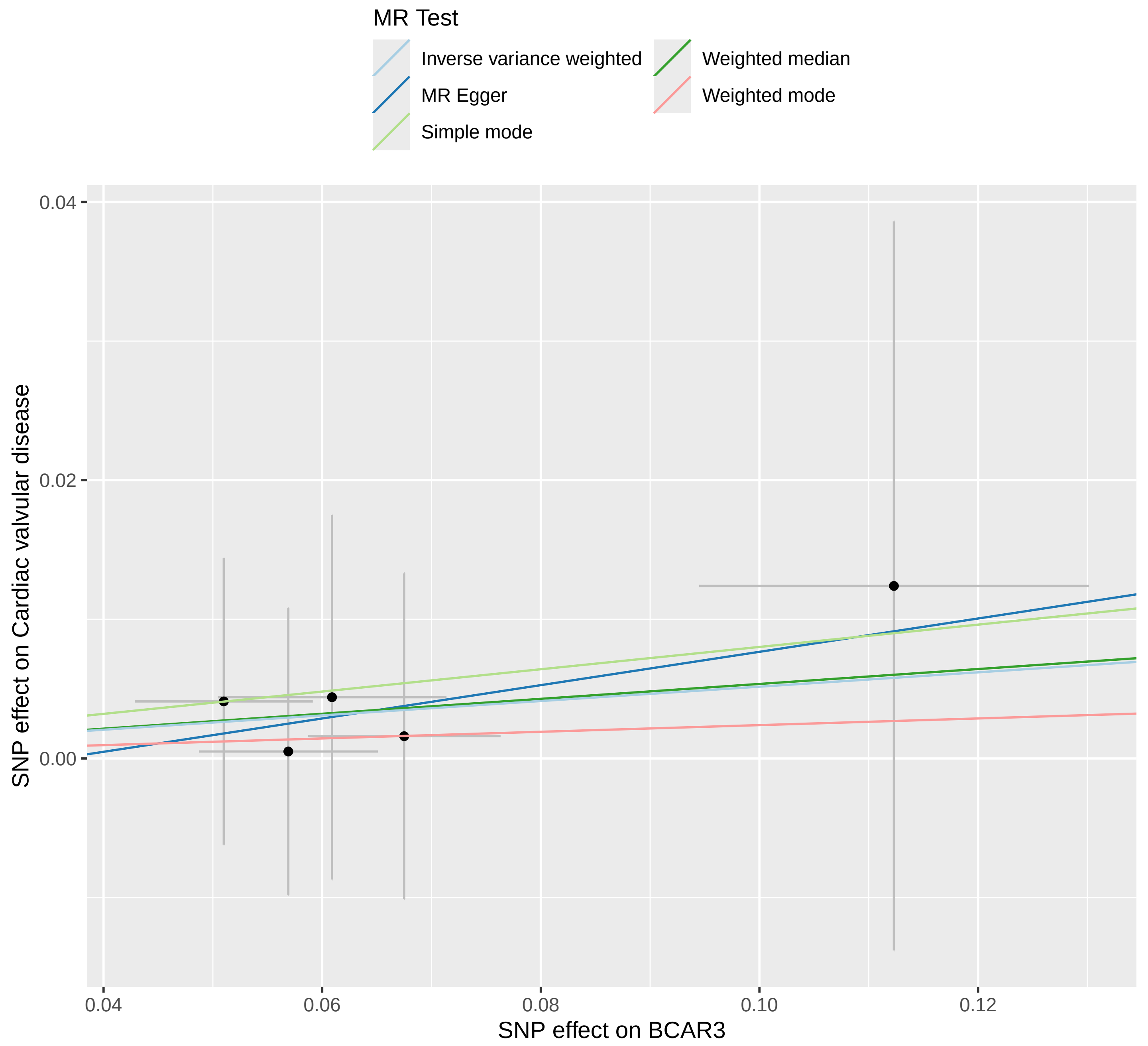
MR Test
Inverse variance weighted
MR Egger
Simple mode
Weighted median
Weighted mode
0.04
0.02
0.00
SNP effect on Cardiac valvular disease
0.04
0.06
0.08
0.10
0.12
SNP effect on BCAR3

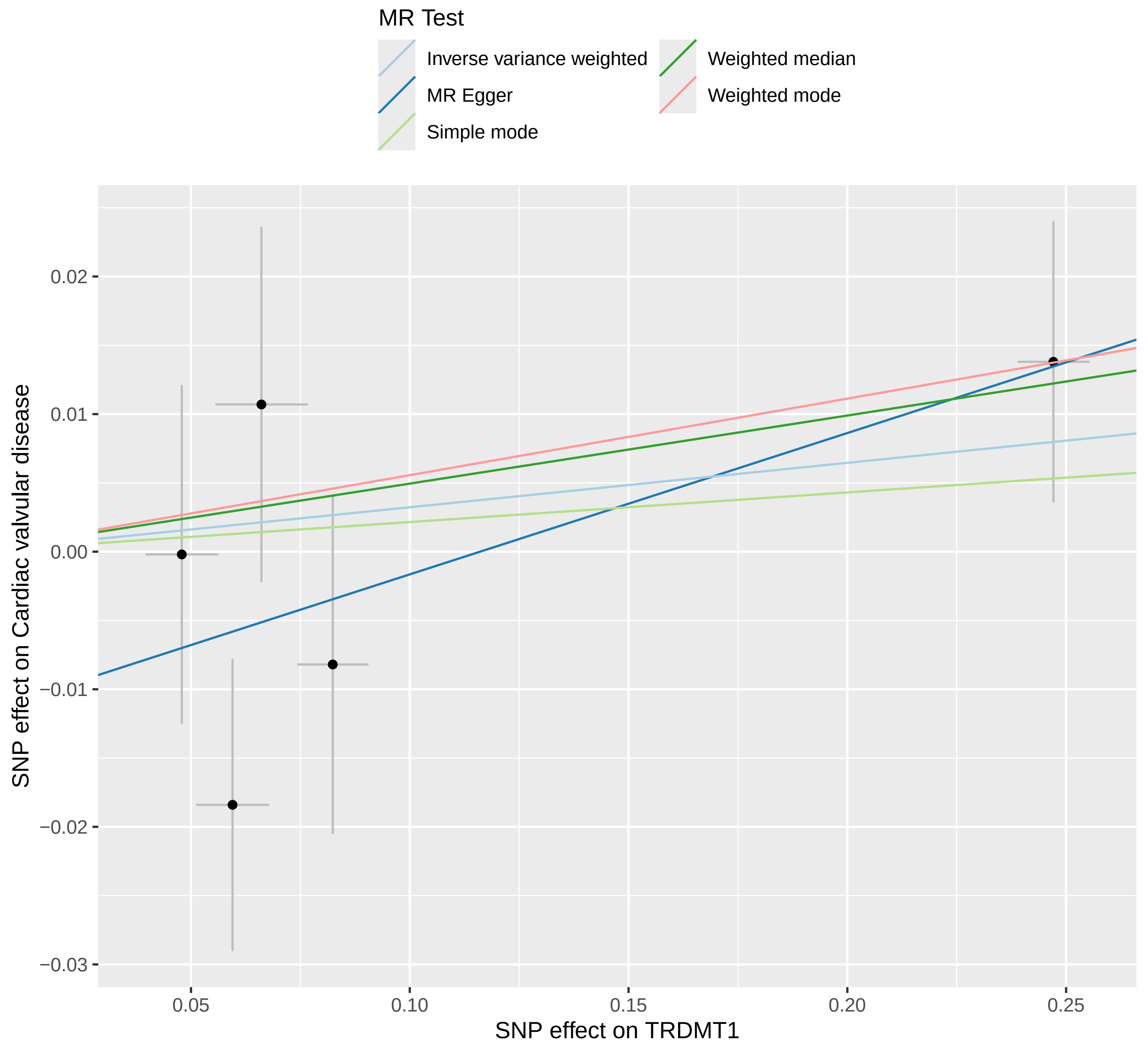

MR Test
Inverse variance weighted
MR Egger
Simple mode
Weighted median
Weighted mode
SNP effect on Cardiac valvular disease
0.02
0.01
0.00
−0.01
−0.02
−0.03
0.05
0.10
0.15
0.20
0.25
SNP effect on TRDMT1

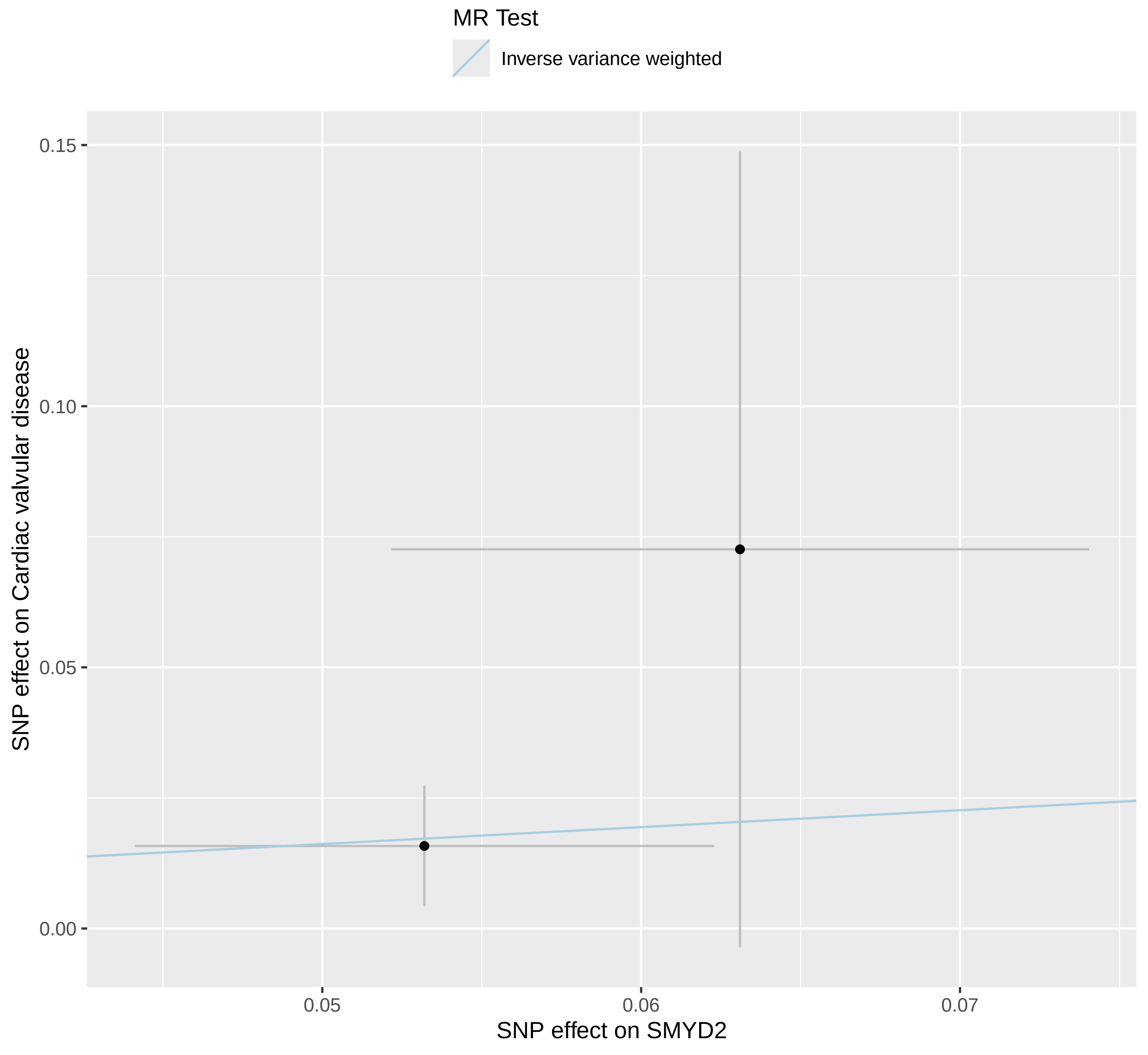

MR Test
Inverse variance weighted
0.15
0.10
0.05
0.00
0.05
0.06
0.07
SNP effect on Cardiac valvular disease
SNP effect on SMYD2

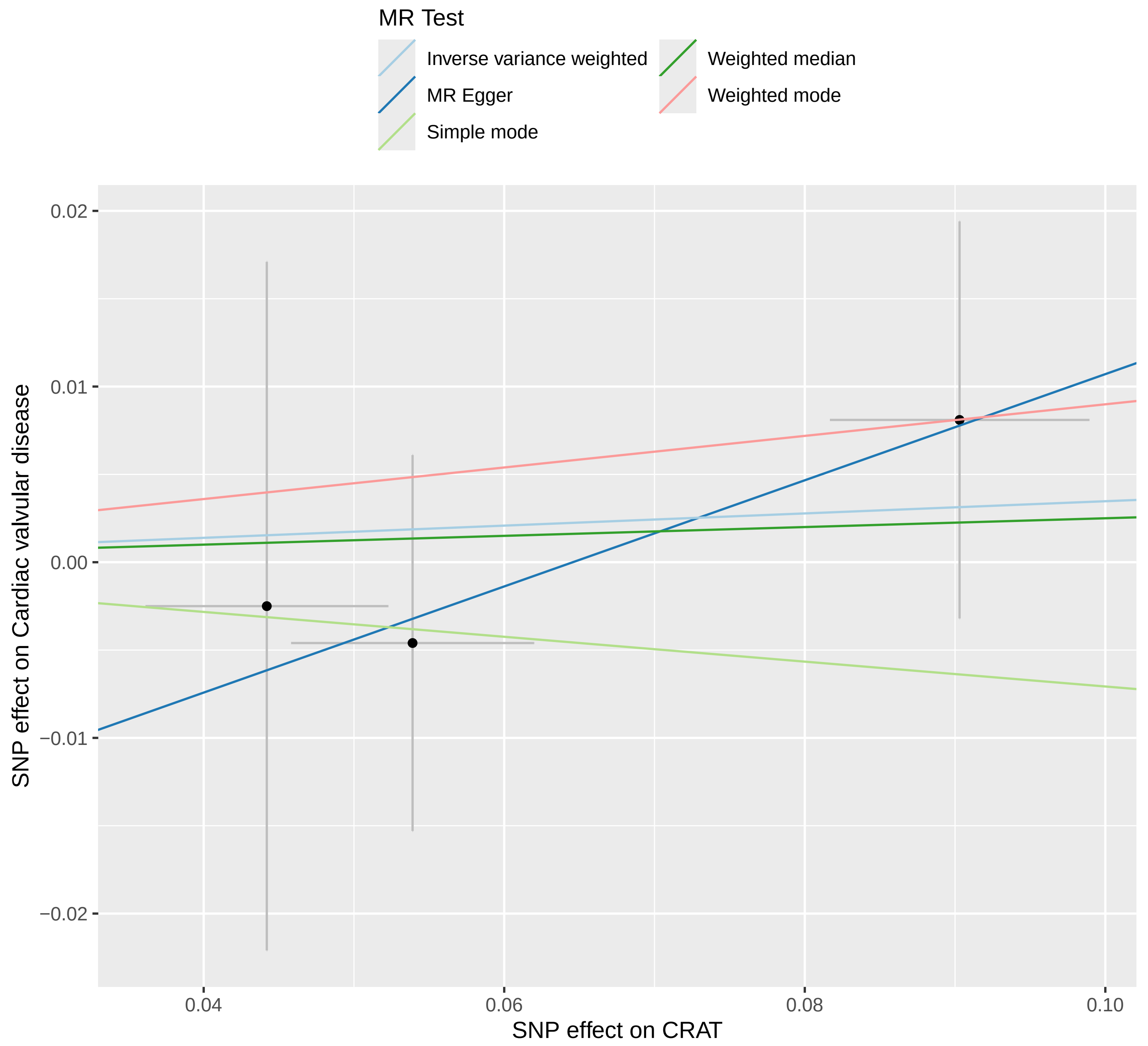

MR Test
Inverse variance weighted
MR Egger
Simple mode
Weighted median
Weighted mode
0.02
0.01
0.00
−0.01
−0.02
SNP effect on Cardiac valvular disease
0.04
0.06
0.08
0.10
SNP effect on CRAT

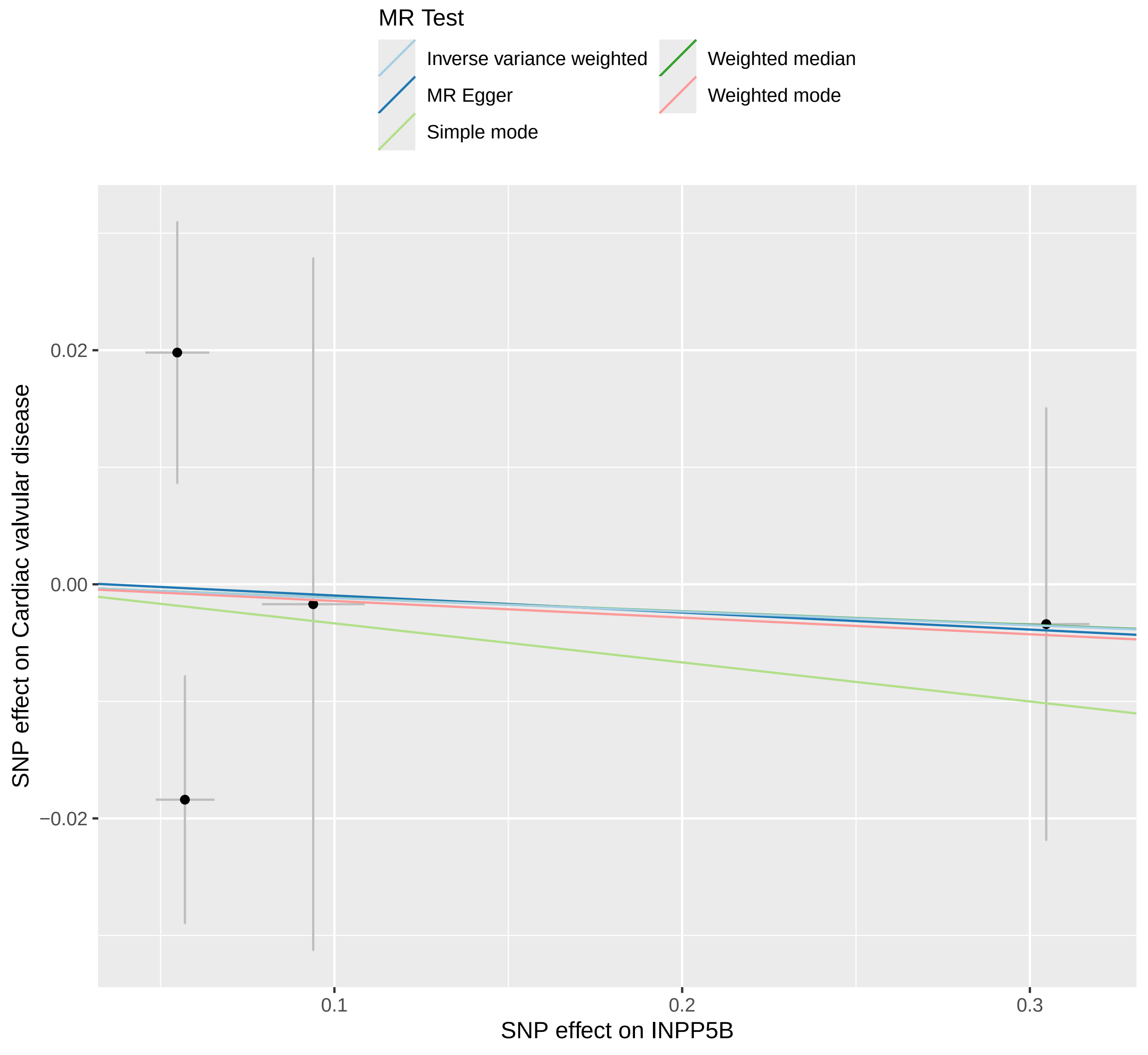
MR Test
Inverse variance weighted
MR Egger
Simple mode
Weighted median
Weighted mode
0.02
0.00
−0.02
0.1
0.2
0.3
SNP effect on Cardiac valvular disease
SNP effect on INPP5B

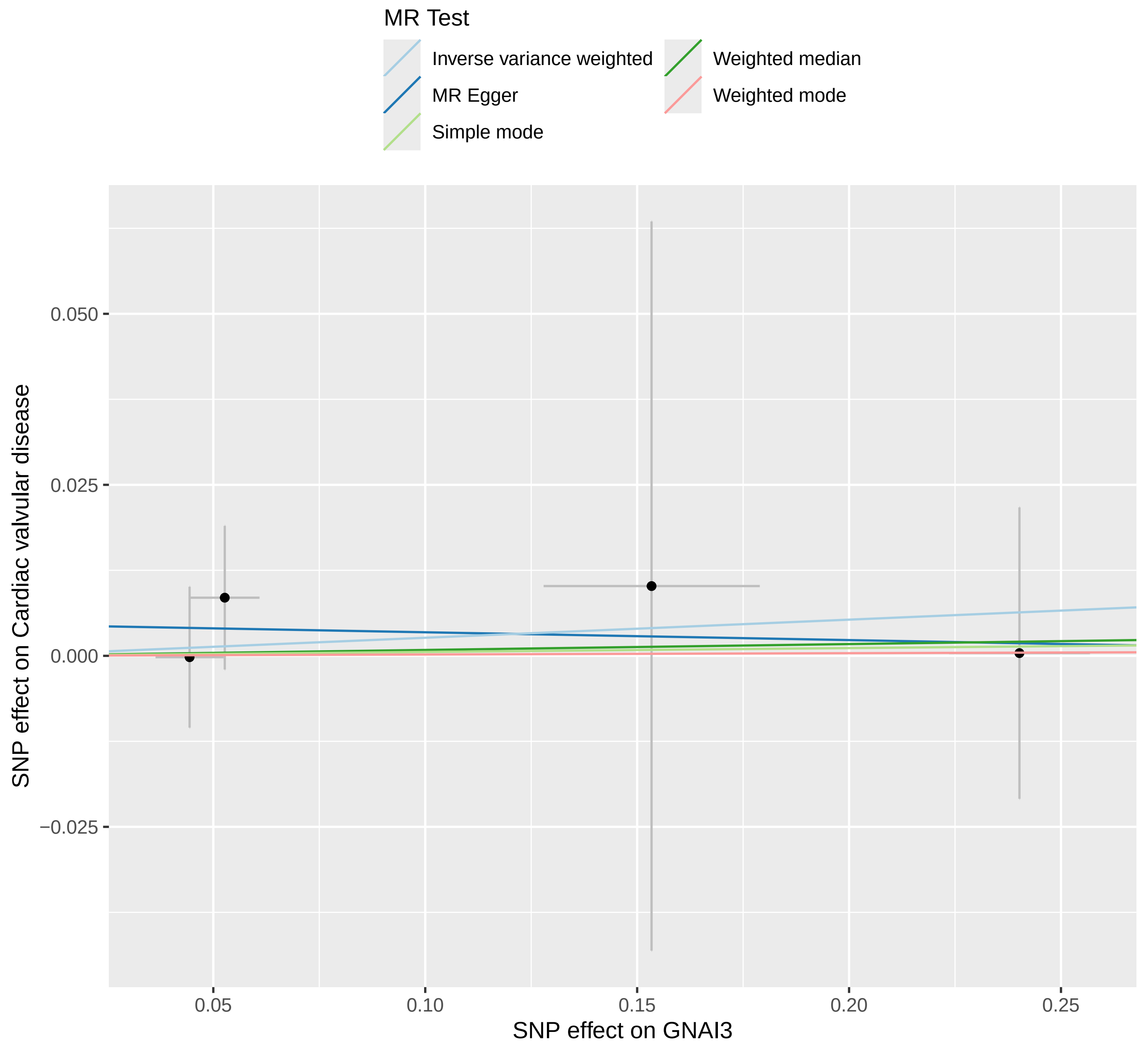
MR Test
Inverse variance weighted
MR Egger
Simple mode
Weighted median
Weighted mode
SNP effect on Cardiac valvular disease
0.050
0.025
0.000
−0.025
0.05
0.10
0.15
0.20
0.25
SNP effect on GNAI3

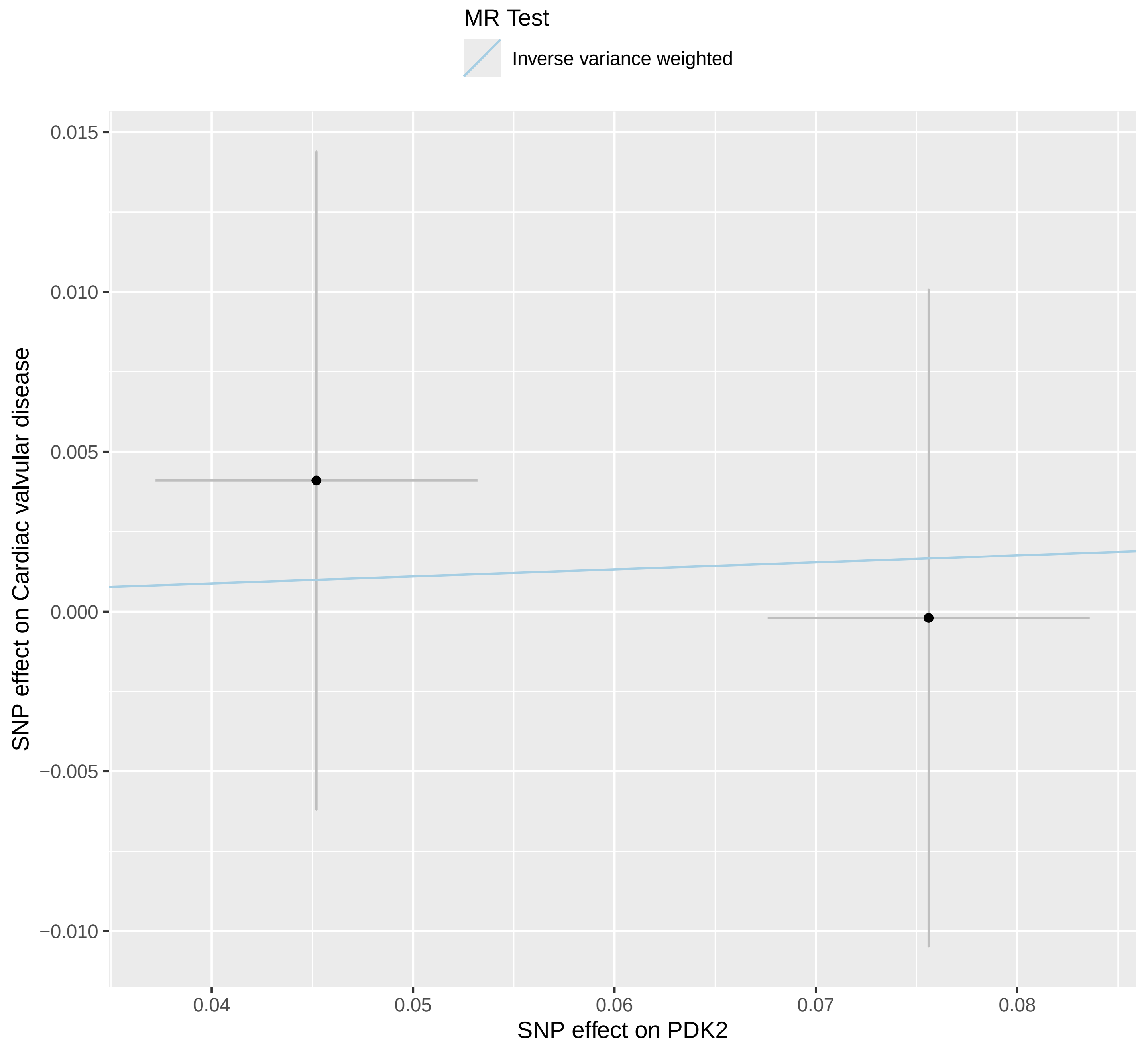
MR Test
Inverse variance weighted
0.015
0.010
0.005
0.000
−0.005
−0.010
SNP effect on Cardiac valvular disease
0.04
0.05
0.06
0.07
0.08
SNP effect on PDK2

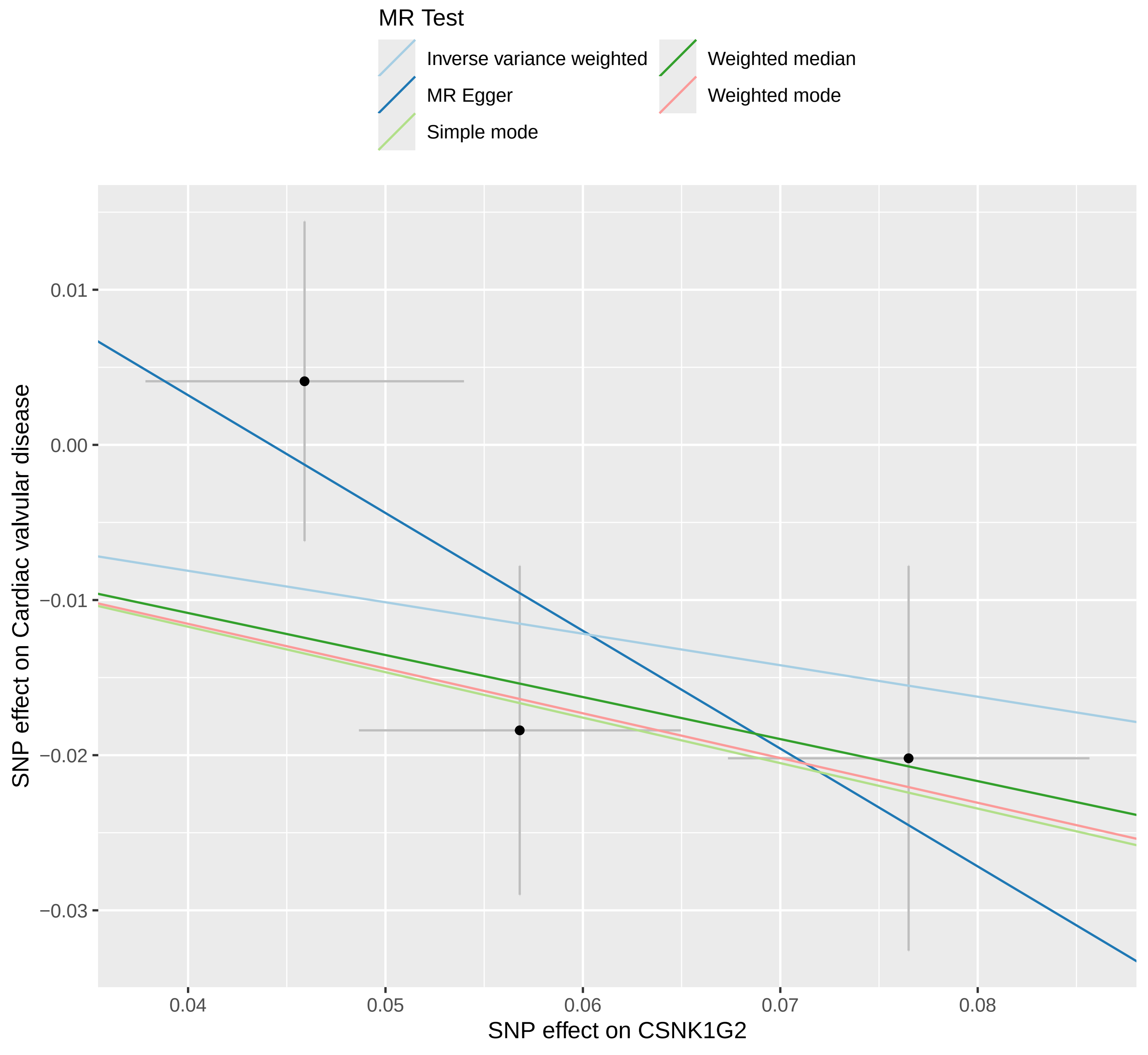

MR Test
Inverse variance weighted
MR Egger
Simple mode
Weighted median
Weighted mode
0.01
0.00
−0.01
−0.02
−0.03
SNP effect on Cardiac valvular disease
0.04
0.05
0.06
0.07
0.08
SNP effect on CSNK1G2

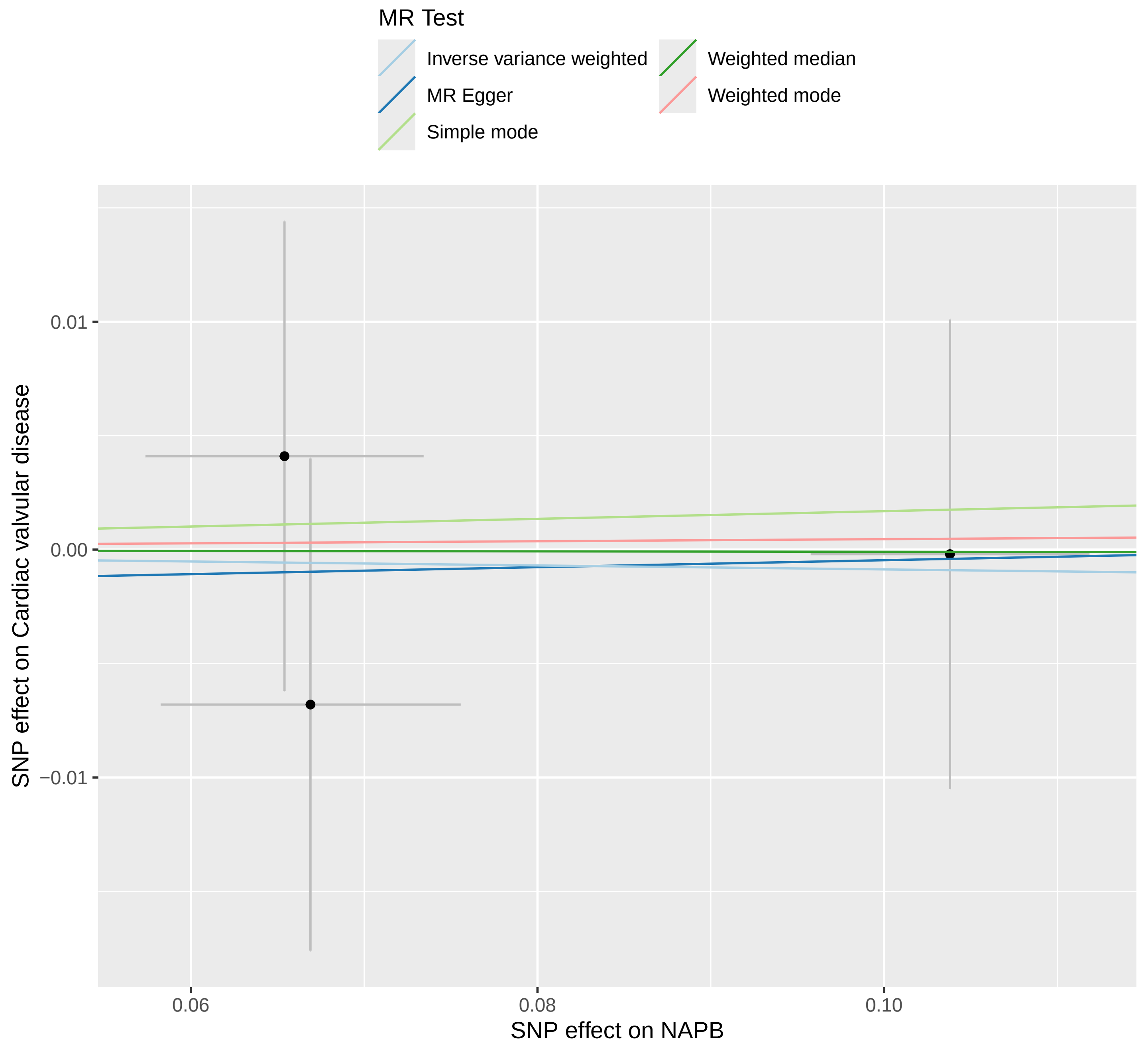
MR Test
Inverse variance weighted
MR Egger
Simple mode
Weighted median
Weighted mode
0.01
0.00
−0.01
SNP effect on Cardiac valvular disease
0.06
0.08
0.10
SNP effect on NAPB

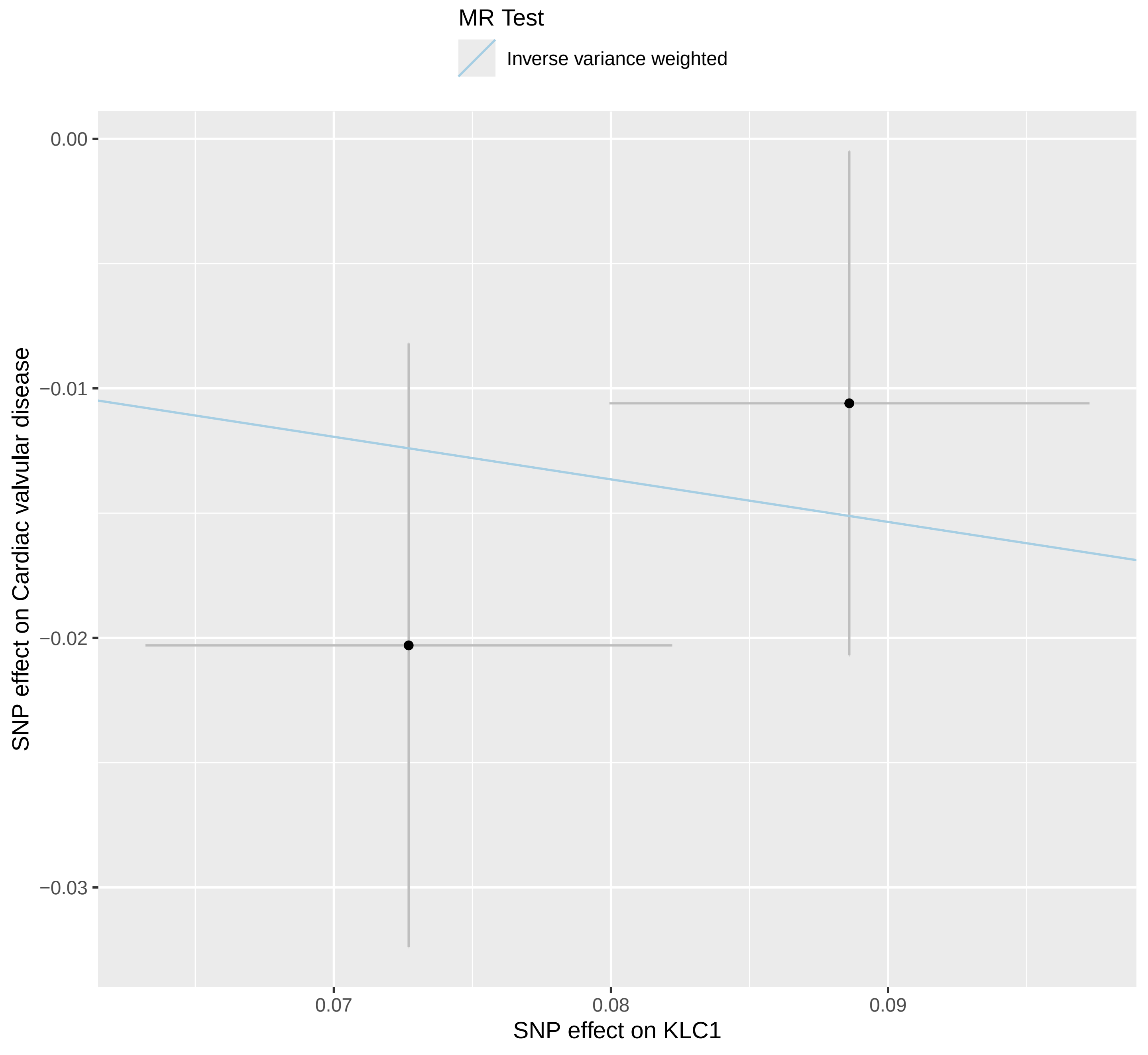

MR Test
Inverse variance weighted
0.00
−0.01
−0.02
−0.03
0.07
0.08
0.09
SNP effect on Cardiac valvular disease
SNP effect on KLC1

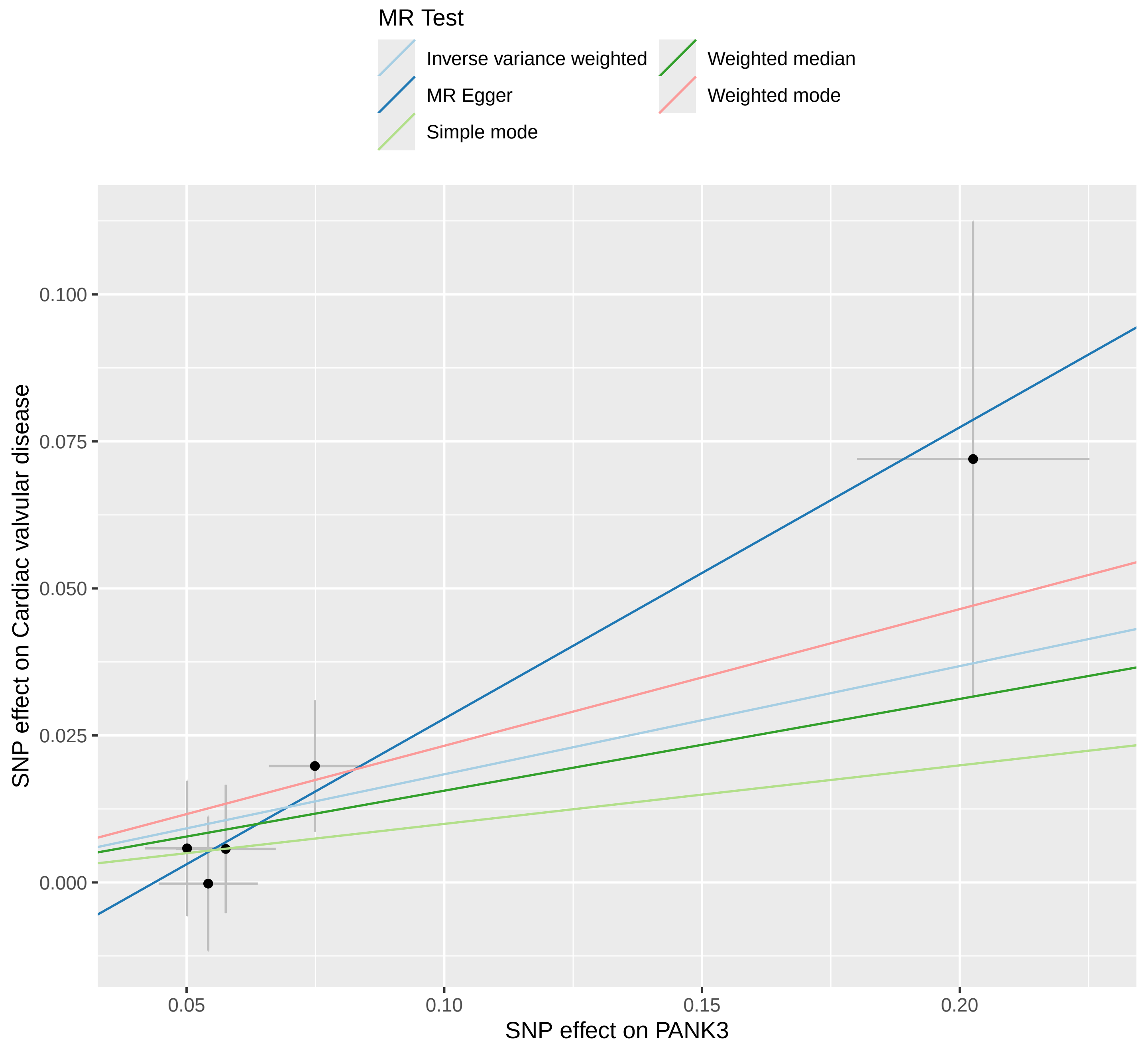
MR Test
Inverse variance weighted
MR Egger
Simple mode
Weighted median
Weighted mode
0.100
0.075
0.050
0.025
0.000
SNP effect on Cardiac valvular disease
0.05
0.10
0.15
0.20
SNP effect on PANK3

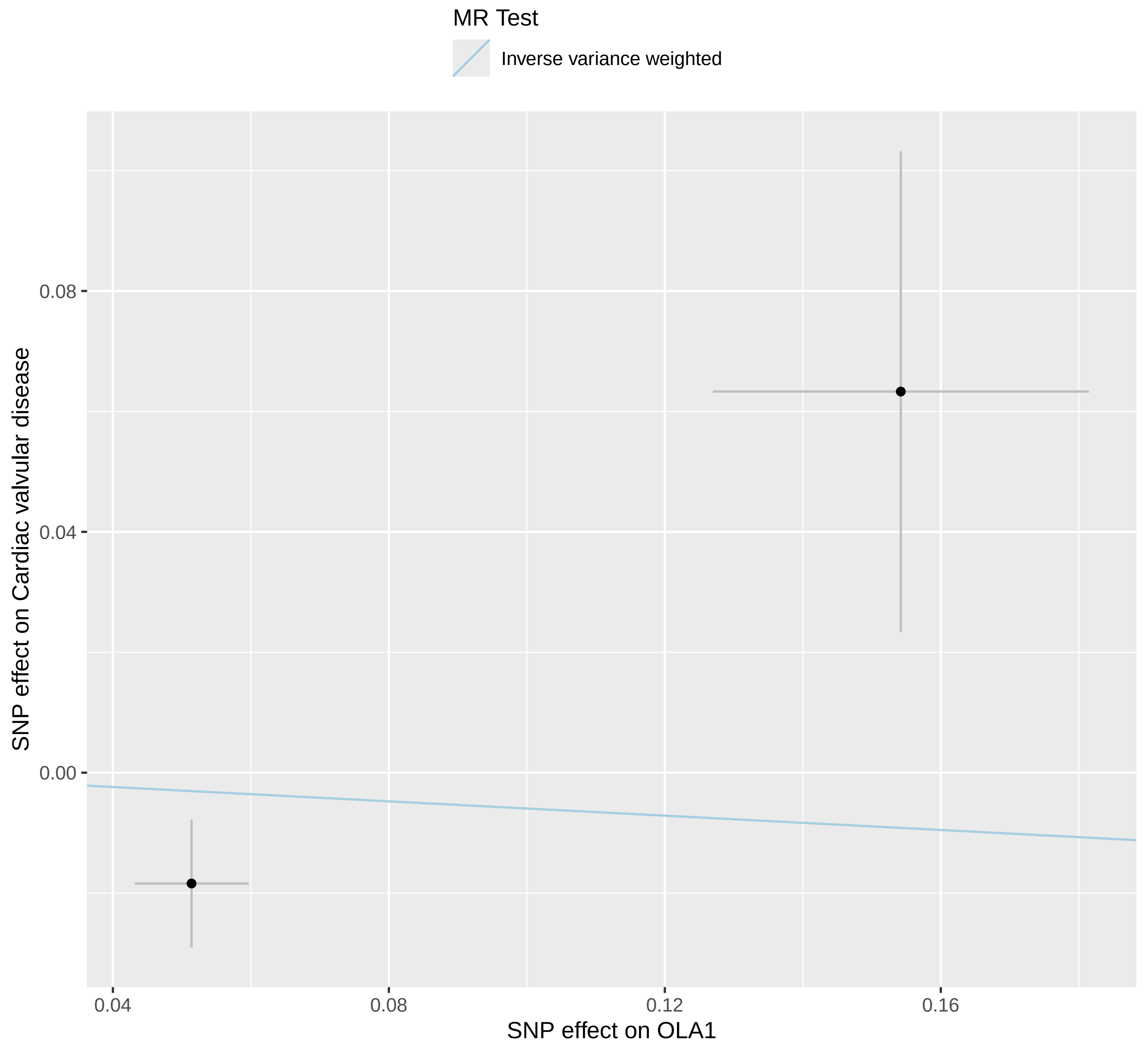
MR Test
Inverse variance weighted
SNP effect on Cardiac valvular disease
0.08
0.04
0.00
0.04
0.08
0.12
0.16
SNP effect on OLA1

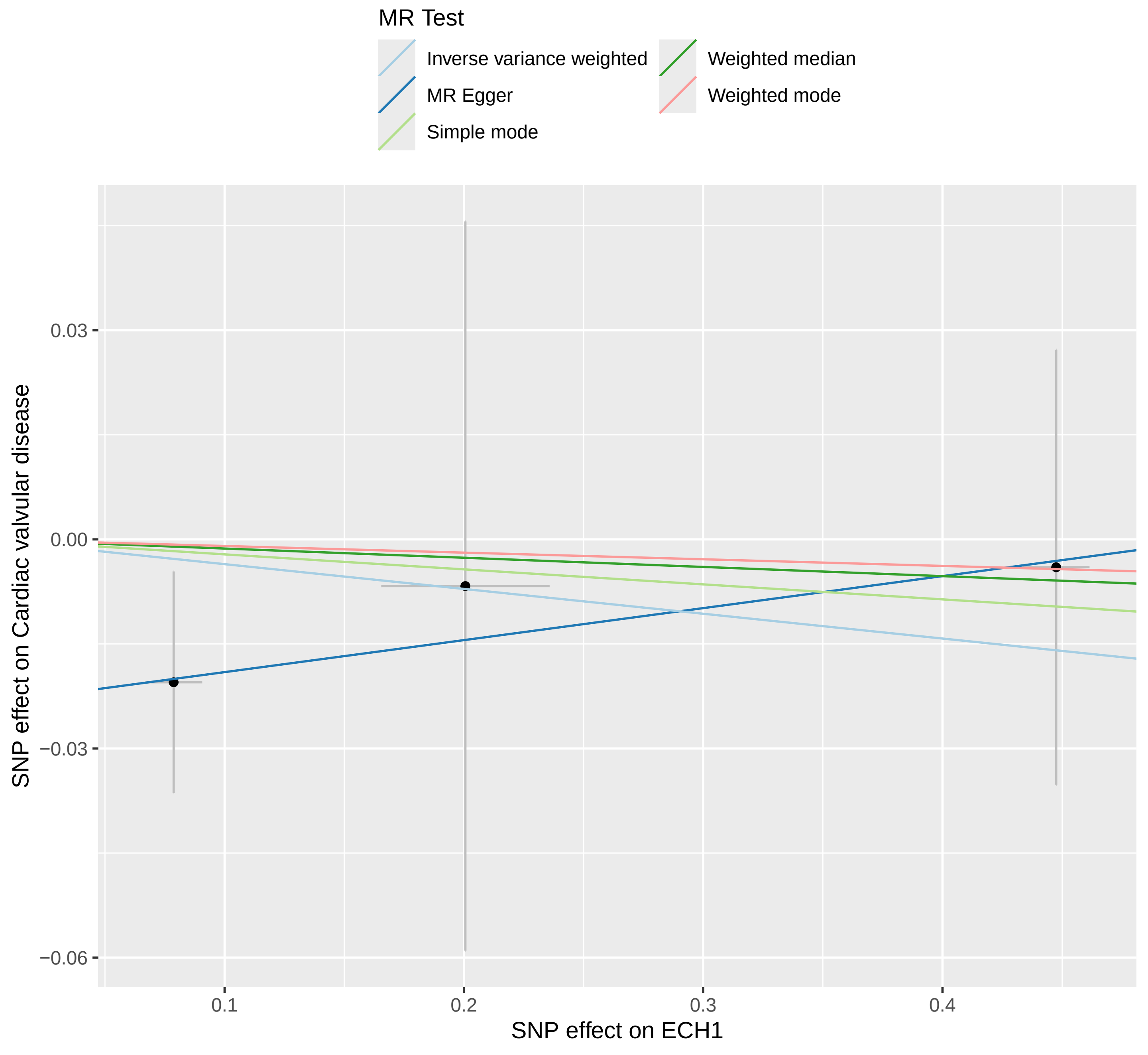

MR Test
Inverse variance weighted
MR Egger
Simple mode
Weighted median
Weighted mode
0.03
0.00
−0.03
−0.06
0.1
0.2
0.3
0.4
SNP effect on ECH1
SNP effect on Cardiac valvular disease

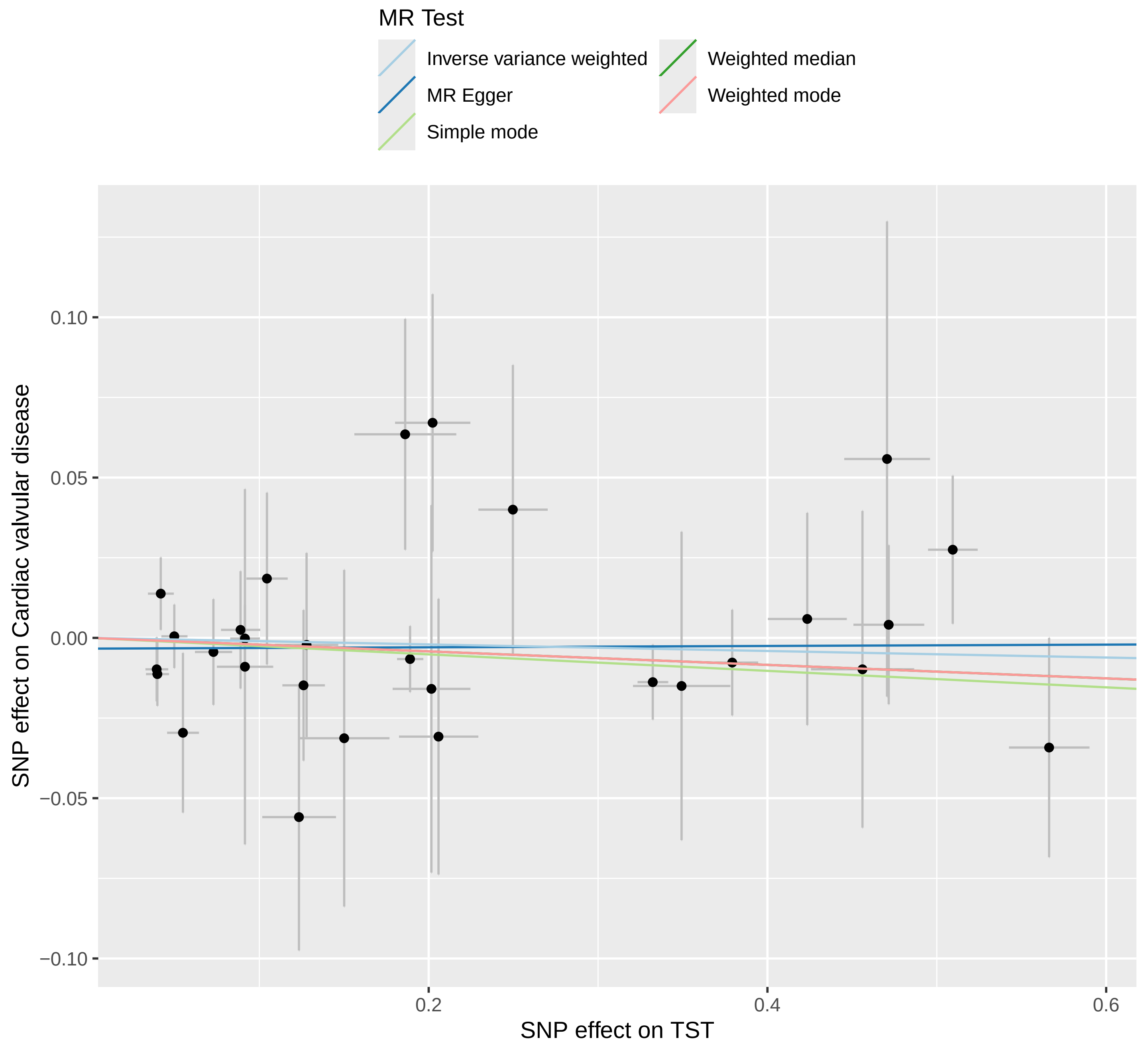

MR Test
Inverse variance weighted
MR Egger
Simple mode
Weighted median
Weighted mode
0.10
0.05
0.00
−0.05
−0.10
SNP effect on Cardiac valvular disease
0.2
0.4
0.6
SNP effect on TST

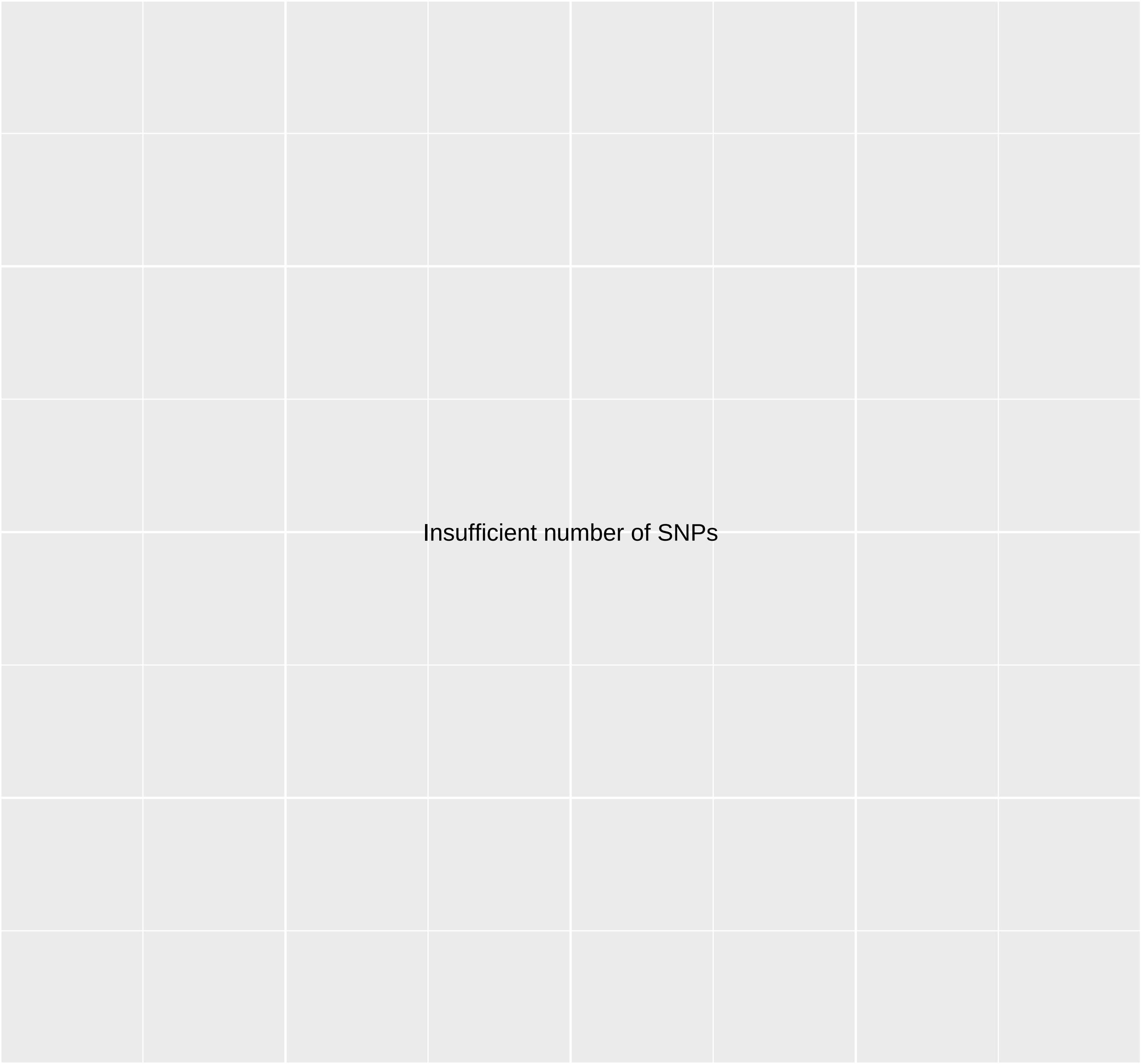
Insufficient number of SNPs

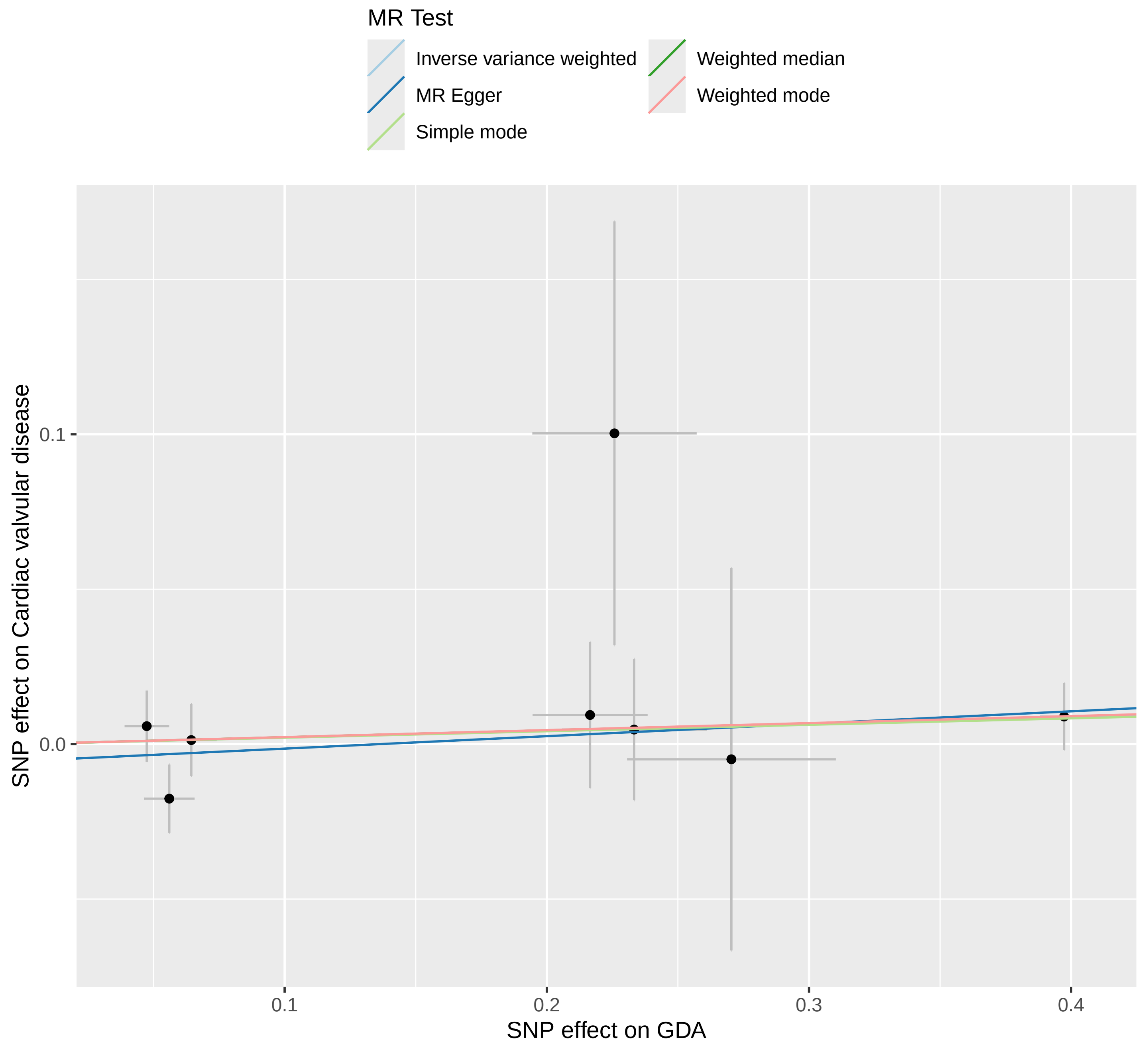

MR Test
Inverse variance weighted
MR Egger
Simple mode
Weighted median
Weighted mode
SNP effect on Cardiac valvular disease
0.1
0.0
0.1
0.2
0.3
0.4
SNP effect on GDA

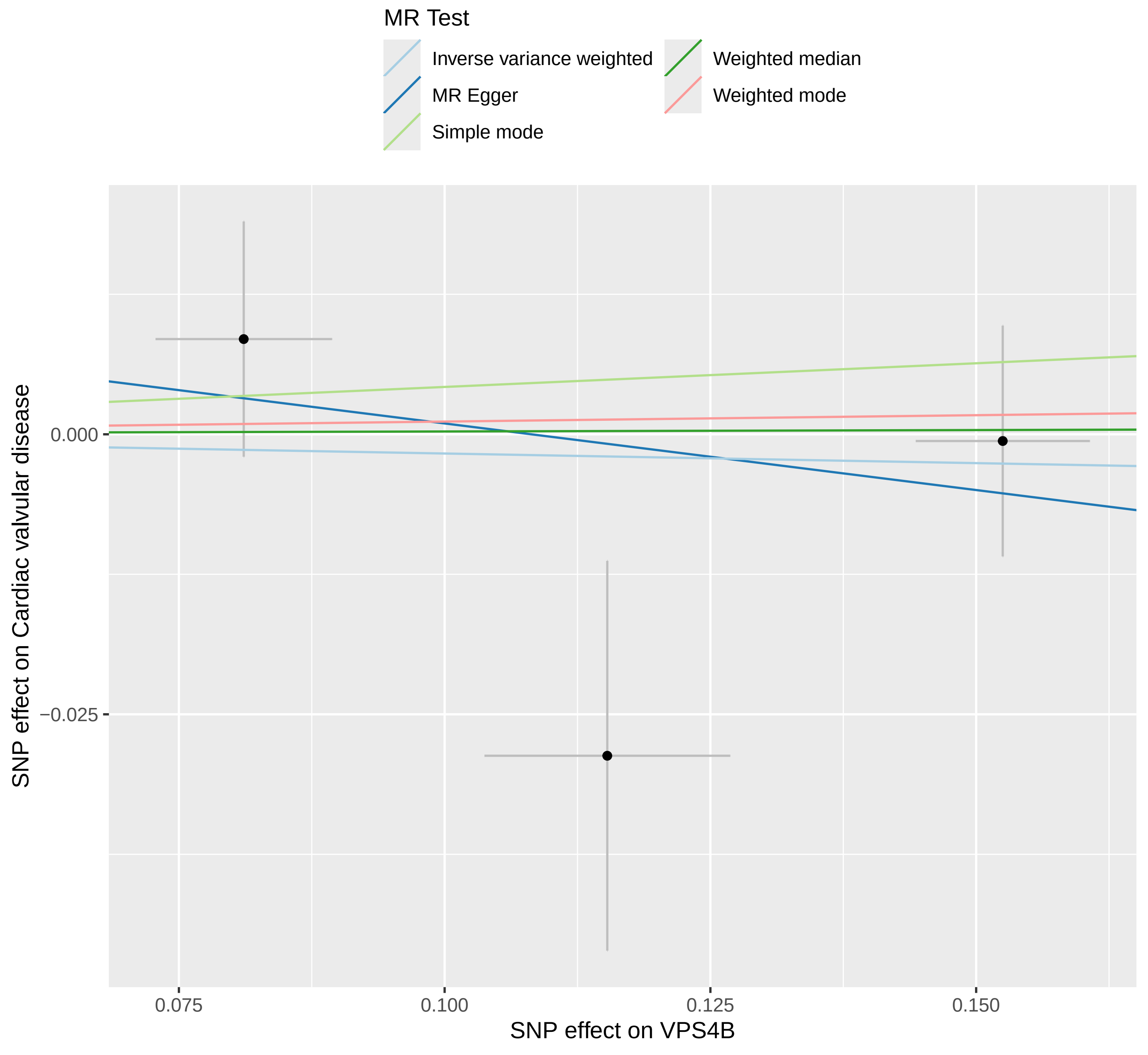
MR Test
Inverse variance weighted
MR Egger
Simple mode
Weighted median
Weighted mode
SNP effect on Cardiac valvular disease
0.000
−0.025
0.075
0.100
0.125
0.150
SNP effect on VPS4B

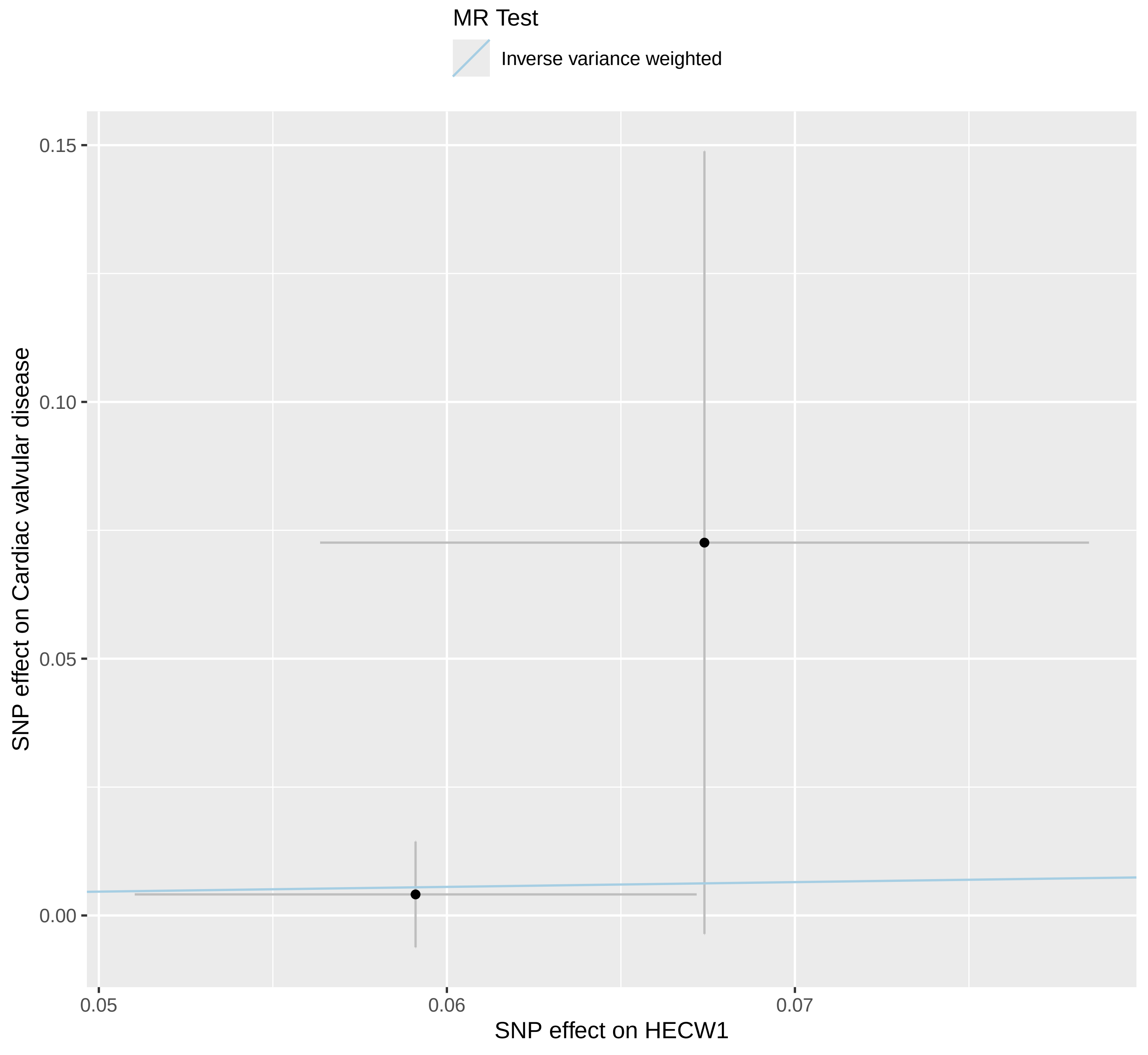

MR Test
Inverse variance weighted
0.15
0.10
0.05
0.00
0.05
0.06
0.07
SNP effect on Cardiac valvular disease
SNP effect on HECW1

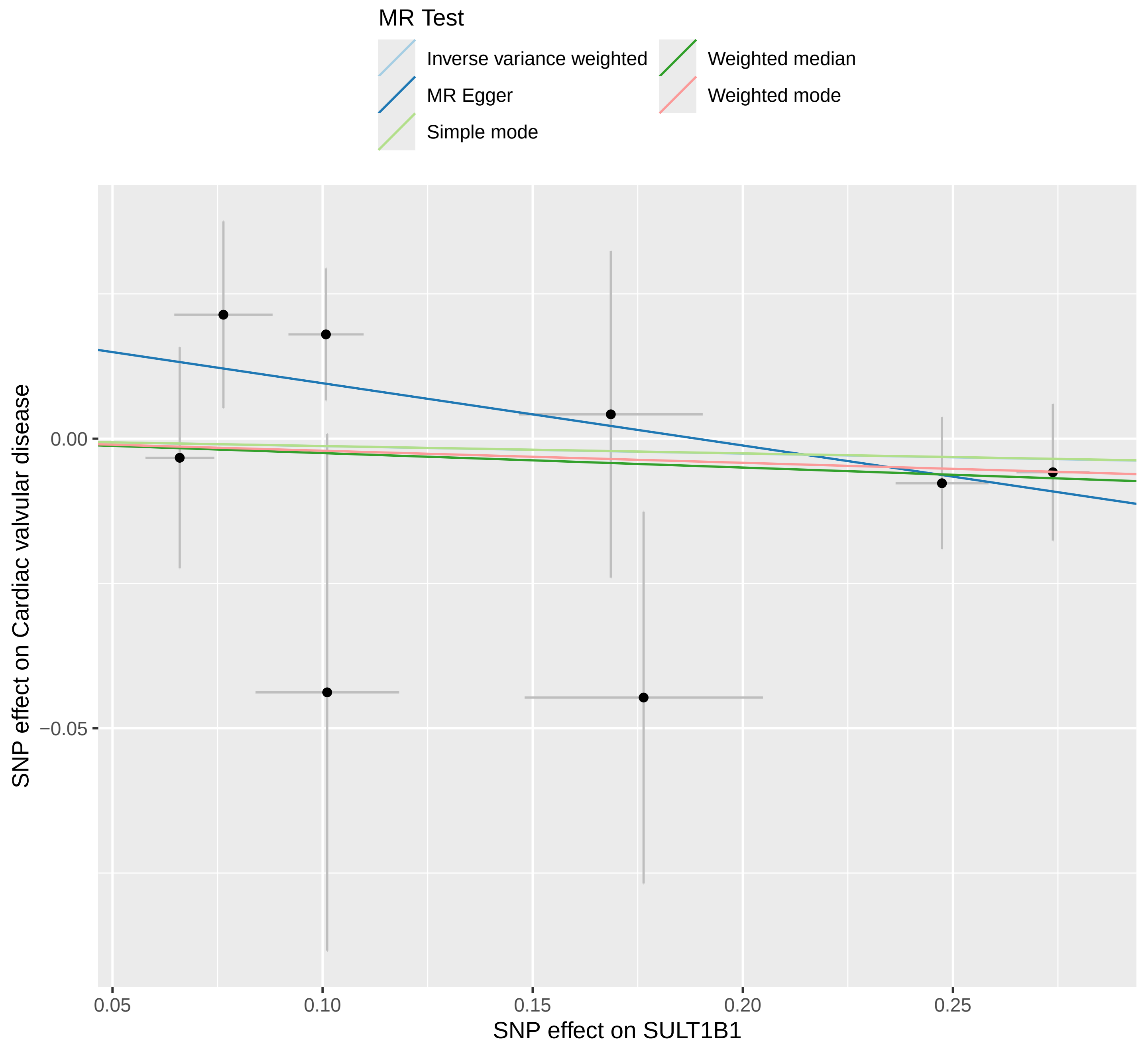

MR Test
Inverse variance weighted
MR Egger
Simple mode
Weighted median
Weighted mode
0.00
−0.05
0.05
0.10
0.15
0.20
0.25
SNP effect on Cardiac valvular disease
SNP effect on SULT1B1

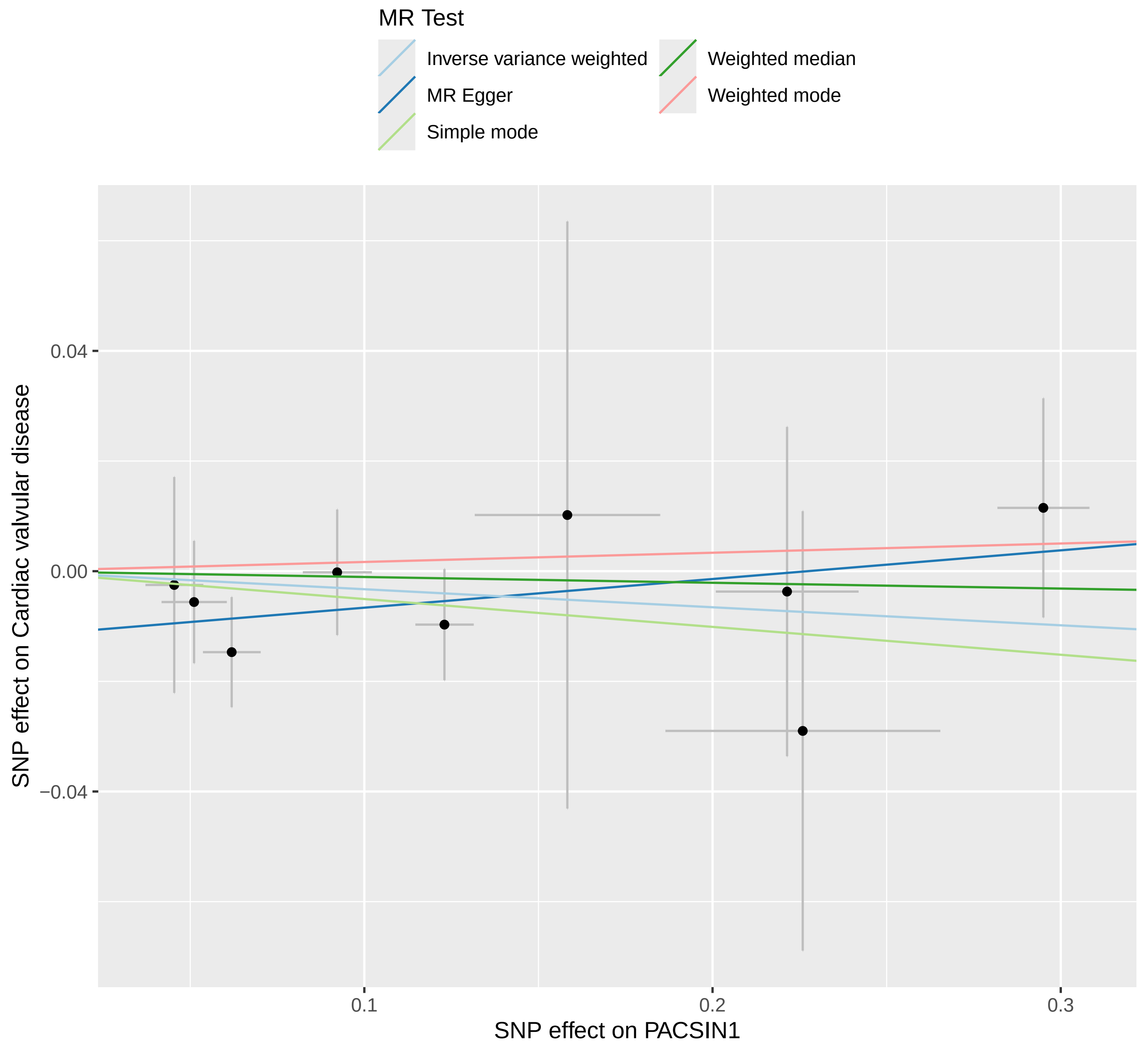

MR Test
Inverse variance weighted
MR Egger
Simple mode
Weighted median
Weighted mode
0.04
0.00
−0.04
0.1
0.2
0.3
SNP effect on Cardiac valvular disease
SNP effect on PACSIN1

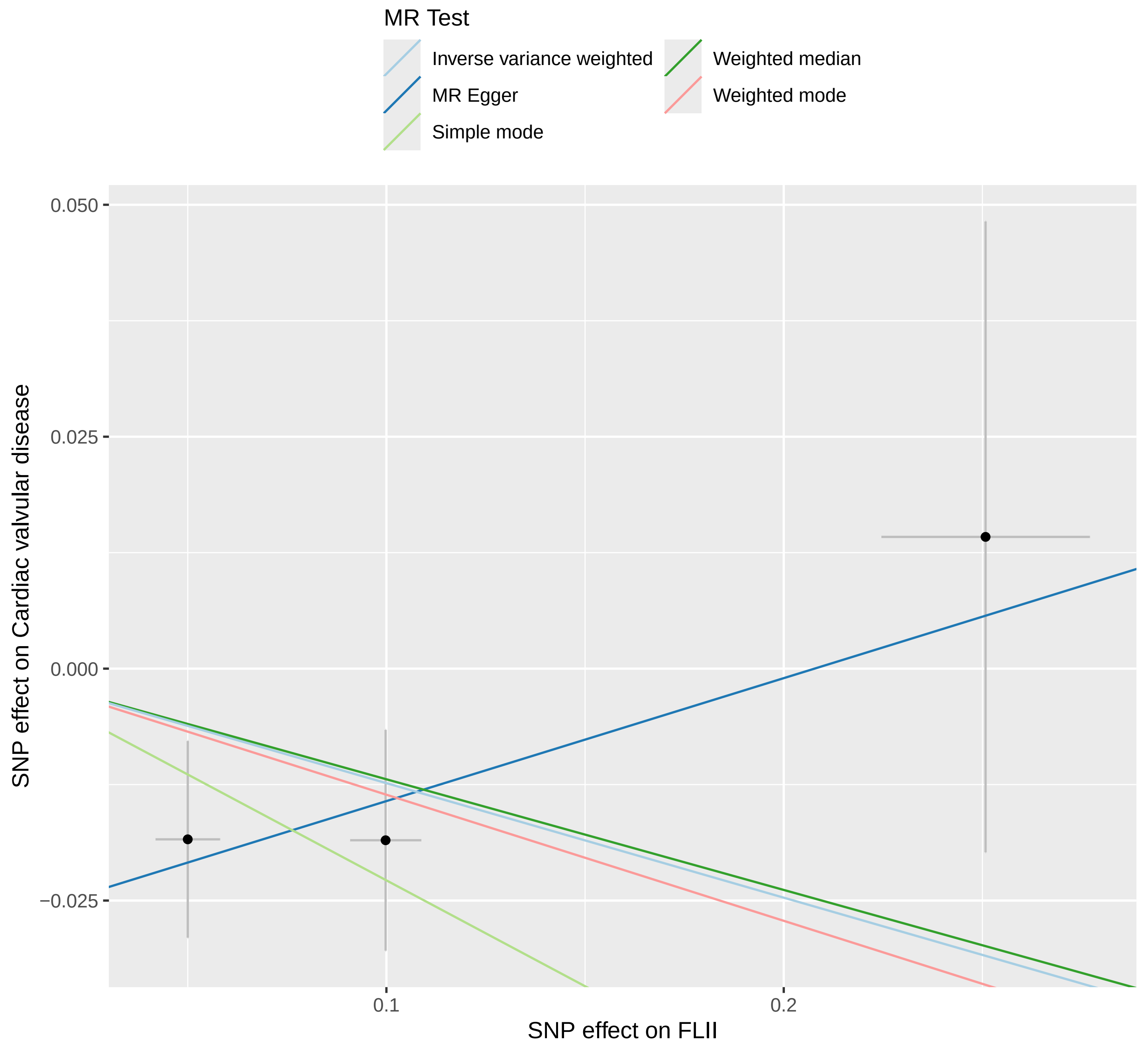
MR Test
Inverse variance weighted
MR Egger
Simple mode
Weighted median
Weighted mode
0.050
0.025
0.000
−0.025
0.1
0.2
SNP effect on Cardiac valvular disease
SNP effect on FLII

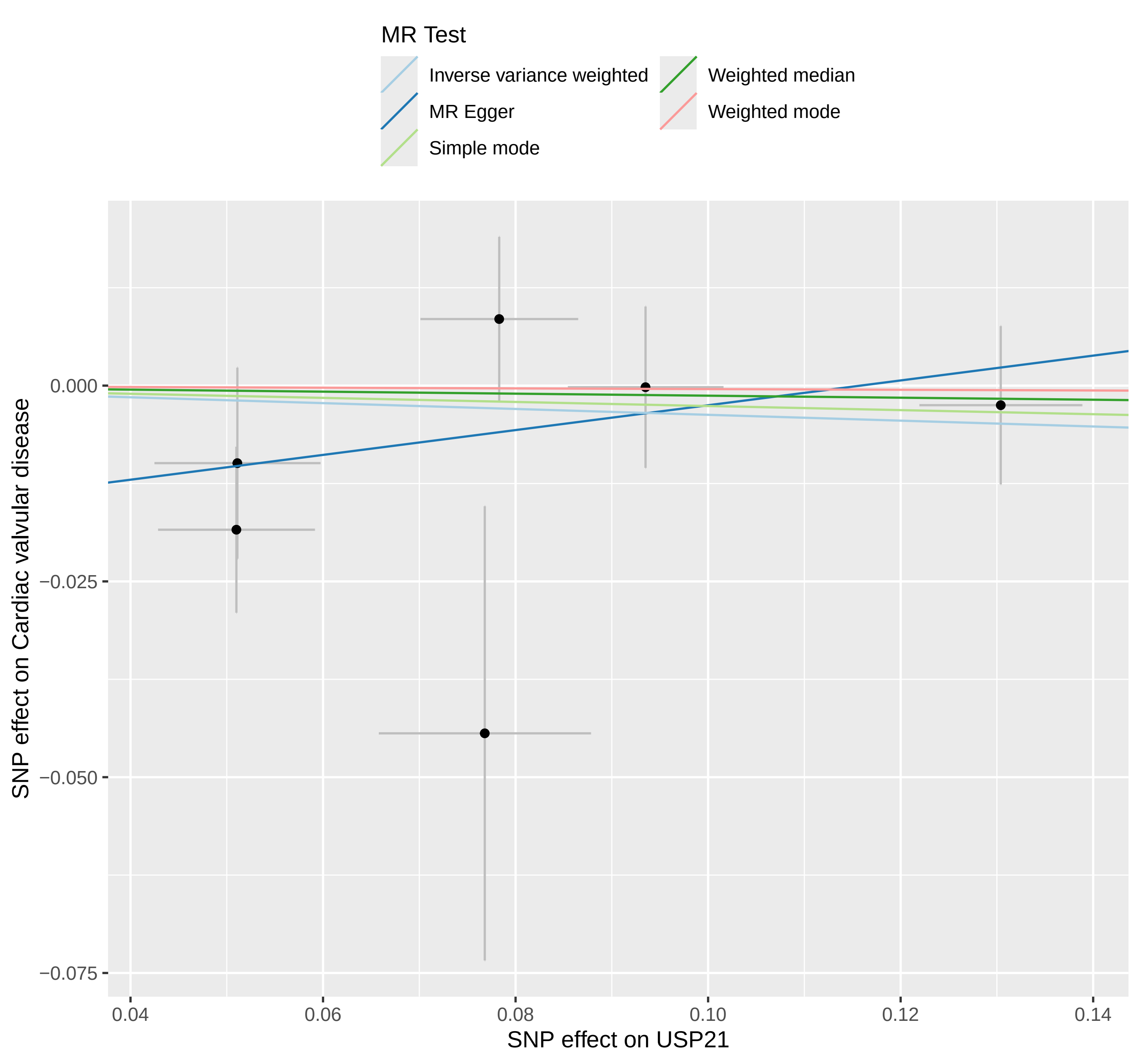

MR Test
Inverse variance weighted
MR Egger
Simple mode
Weighted median
Weighted mode
0.000
−0.025
−0.050
−0.075
SNP effect on Cardiac valvular disease
0.04
0.06
0.08
0.10
0.12
0.14
SNP effect on USP21

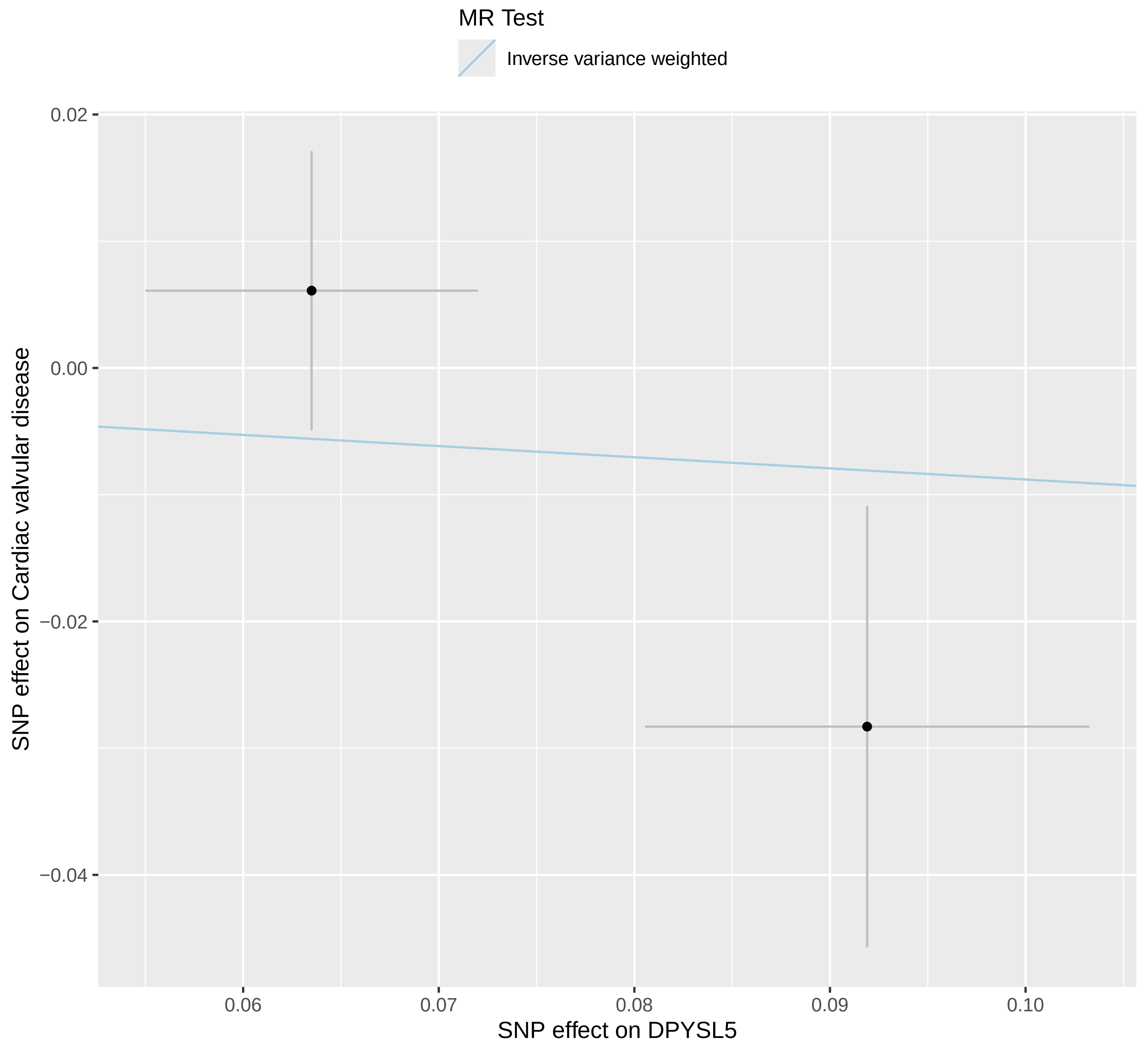
MR Test
Inverse variance weighted
0.02
0.00
−0.02
−0.04
SNP effect on Cardiac valvular disease
0.06
0.07
0.08
0.09
0.10
SNP effect on DPYSL5

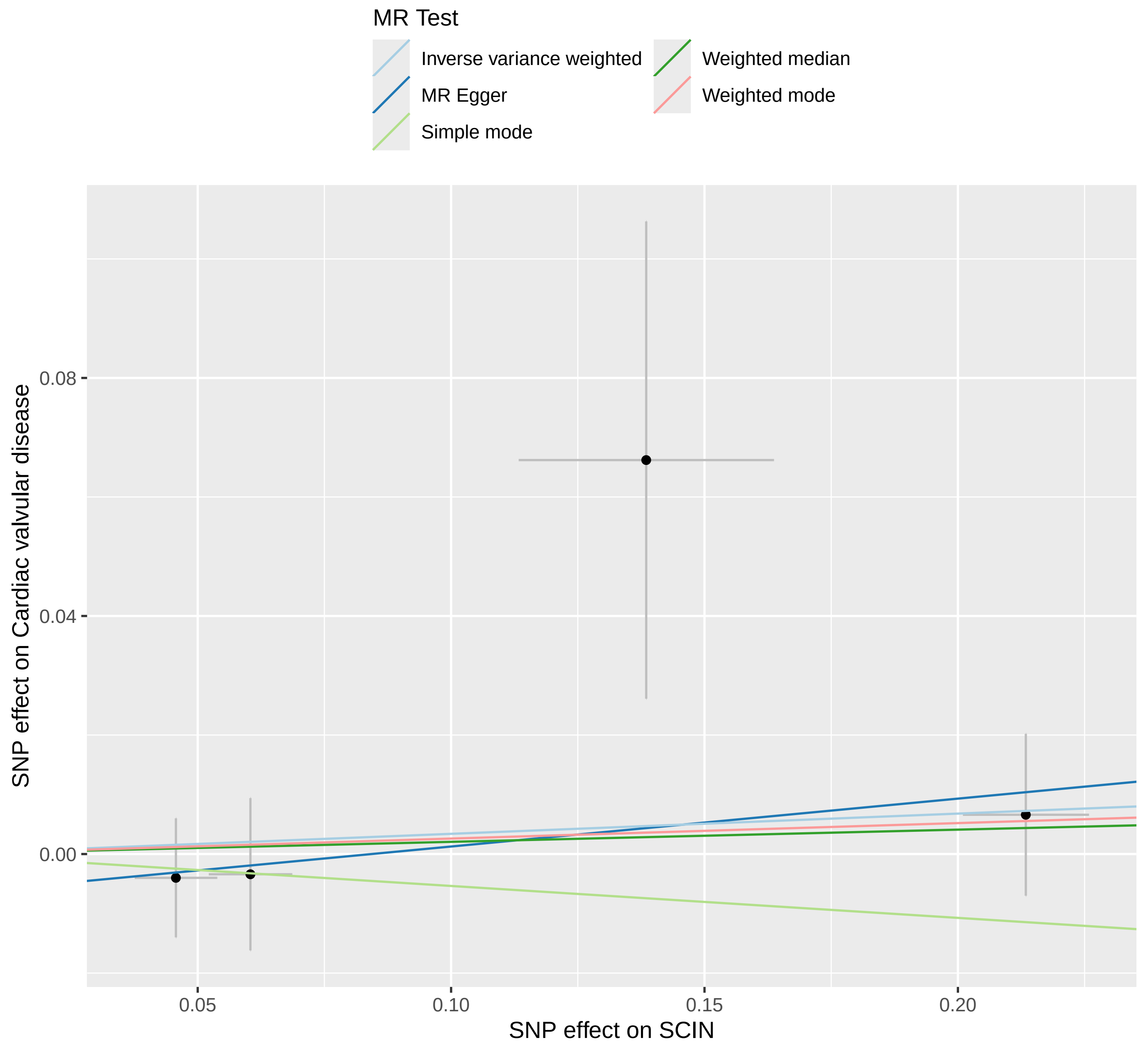
MR Test
Inverse variance weighted
MR Egger
Simple mode
Weighted median
Weighted mode
SNP effect on Cardiac valvular disease
0.08
0.04
0.00
0.05
0.10
0.15
0.20
SNP effect on SCIN

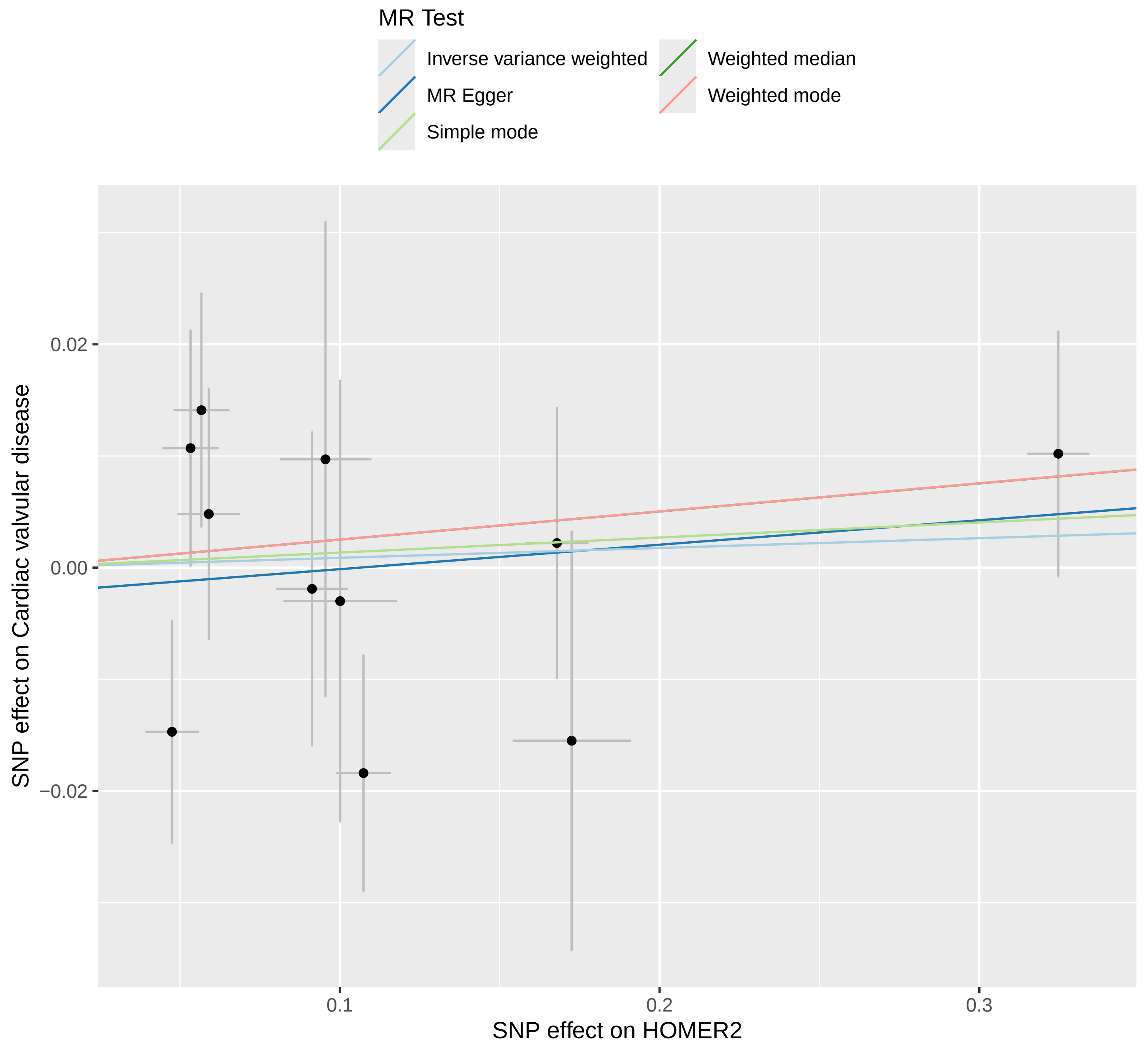
MR Test
Inverse variance weighted
MR Egger
Simple mode
Weighted median
Weighted mode
0.02
0.00
−0.02
0.1
0.2
0.3
SNP effect on Cardiac valvular disease
SNP effect on HOMER2

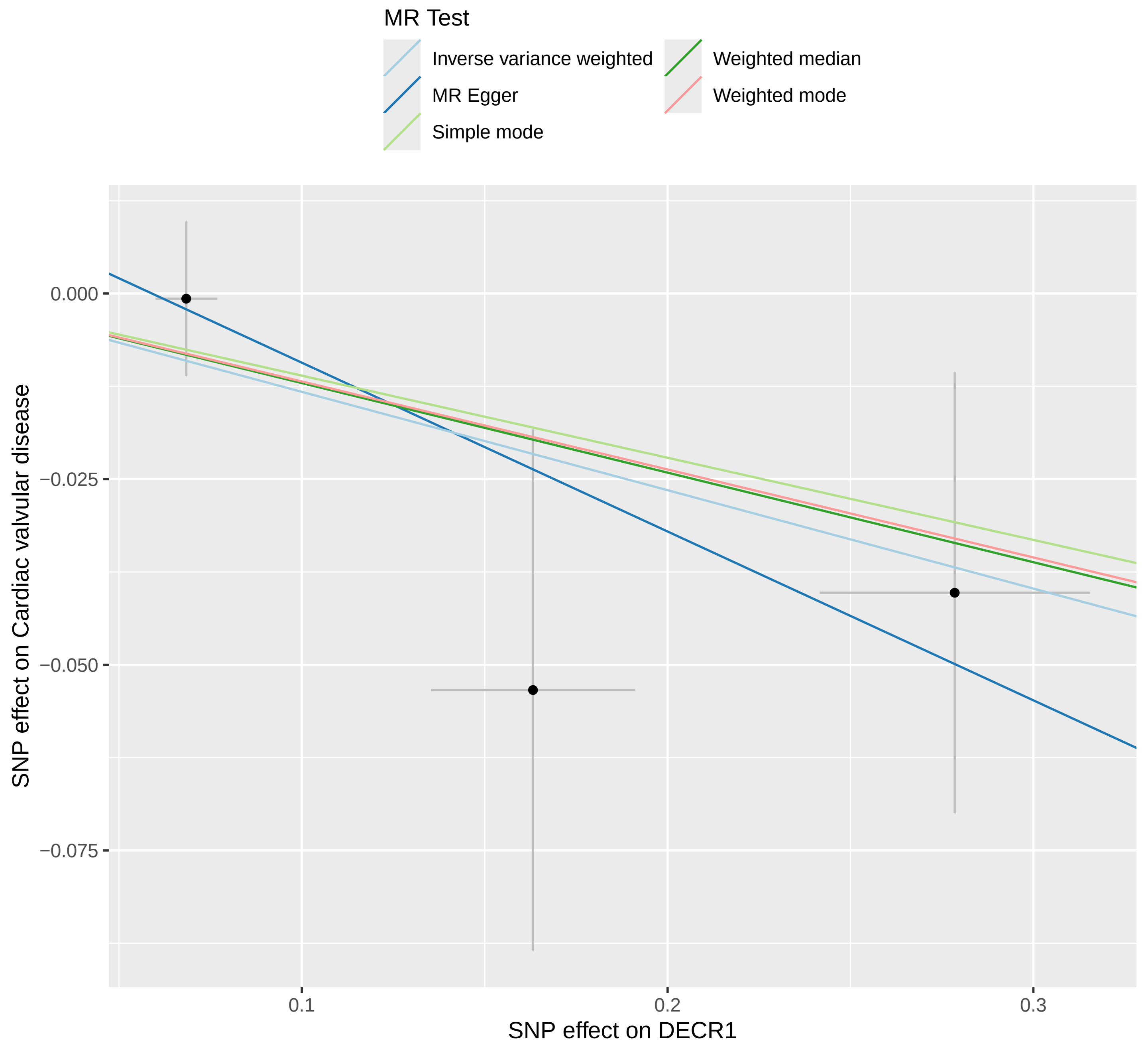

MR Test
Inverse variance weighted
MR Egger
Simple mode
Weighted median
Weighted mode
0.000
−0.025
−0.050
−0.075
SNP effect on Cardiac valvular disease
0.1
0.2
0.3
SNP effect on DECR1

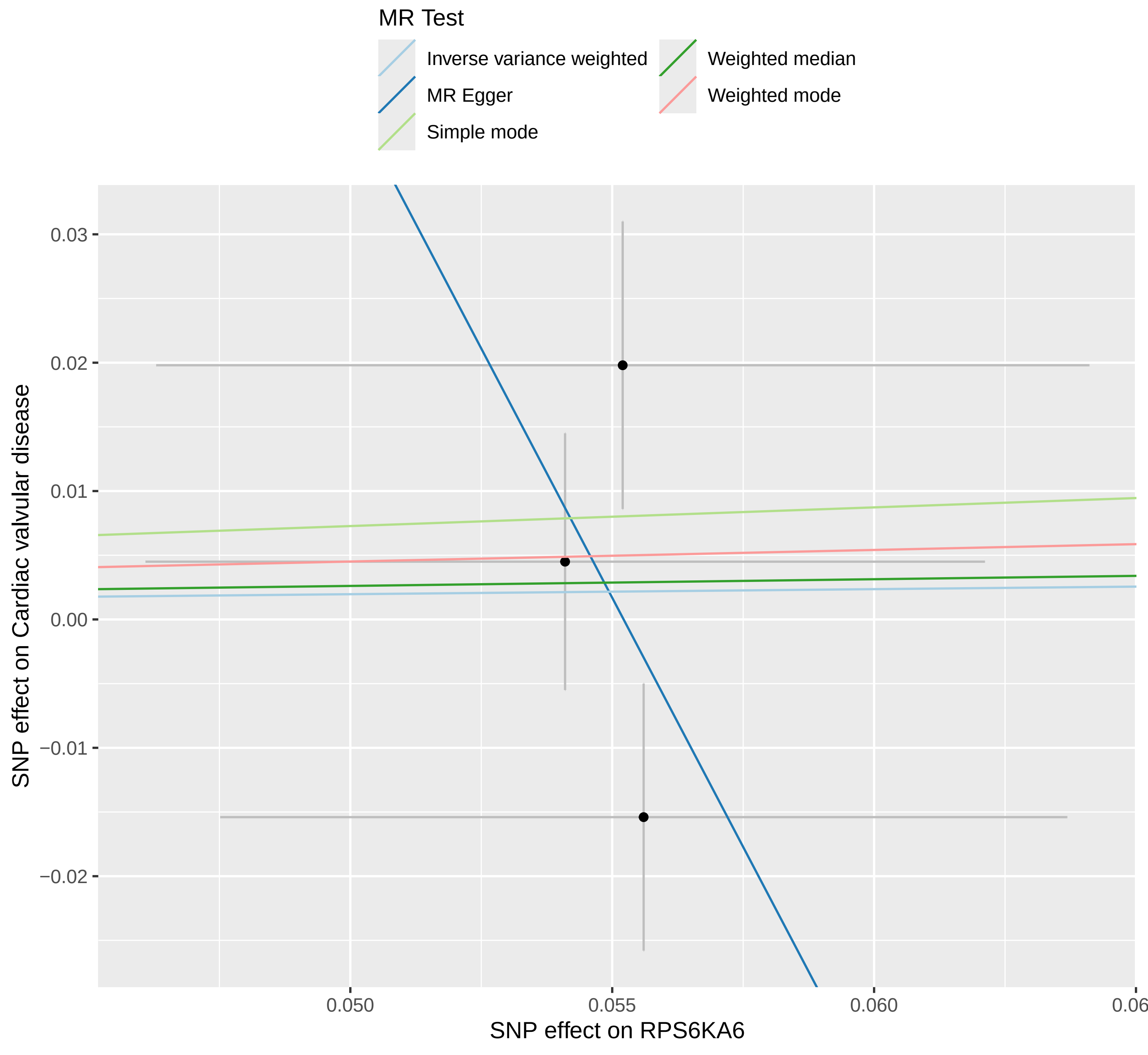

MR Test
Inverse variance weighted
MR Egger
Simple mode
Weighted median
Weighted mode
0.03
0.02
0.01
0.00
−0.01
−0.02
SNP effect on Cardiac valvular disease
0.050
0.055
0.060
SNP effect on RPS6KA6

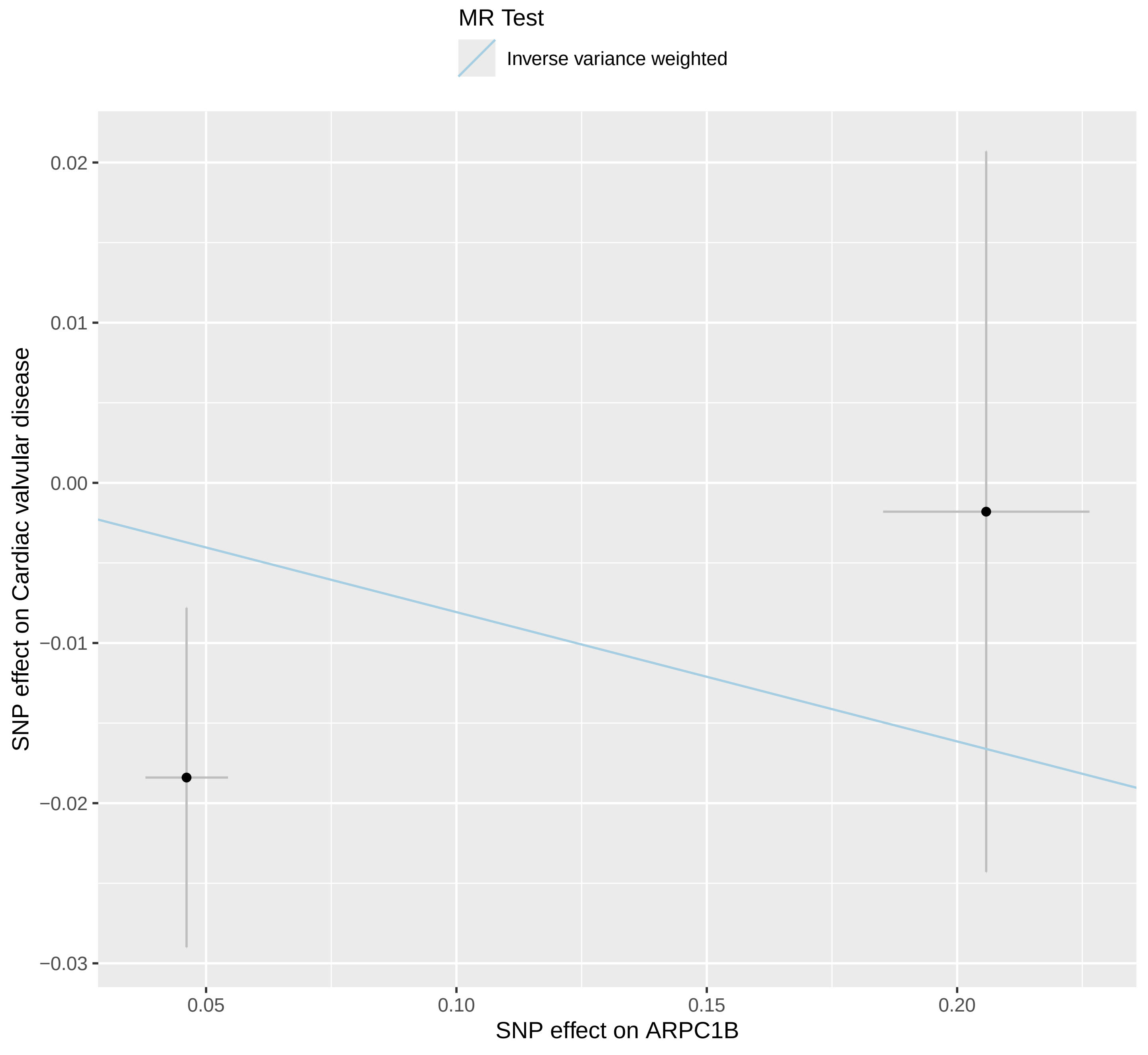
MR Test
Inverse variance weighted
0.02
0.01
0.00
−0.01
−0.02
−0.03
0.05
0.10
0.15
0.20
SNP effect on ARPC1B
SNP effect on Cardiac valvular disease

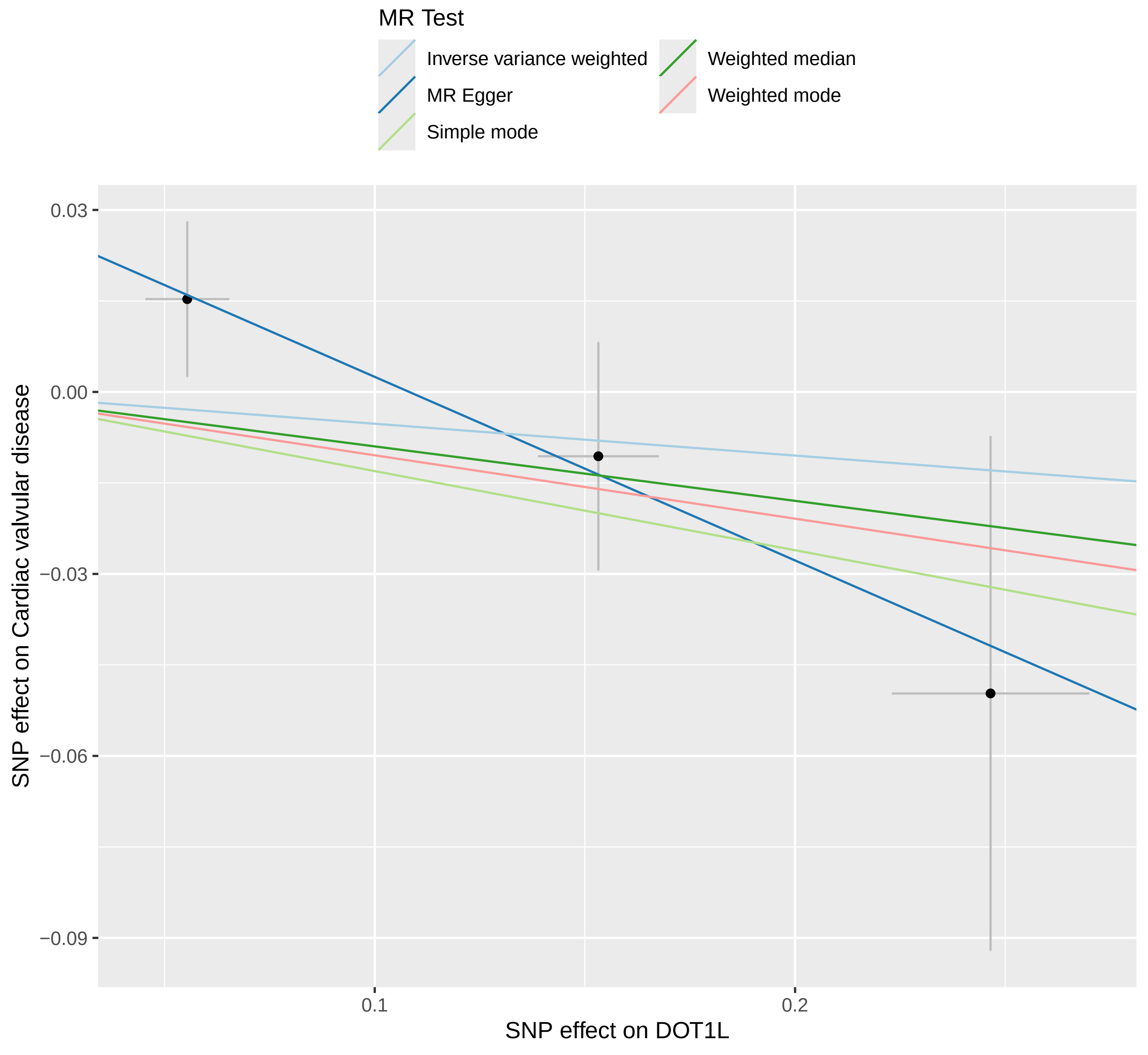

MR Test
Inverse variance weighted
MR Egger
Simple mode
Weighted median
Weighted mode
0.03
0.00
−0.03
−0.06
−0.09
0.1
0.2
SNP effect on Cardiac valvular disease
SNP effect on DOT1L

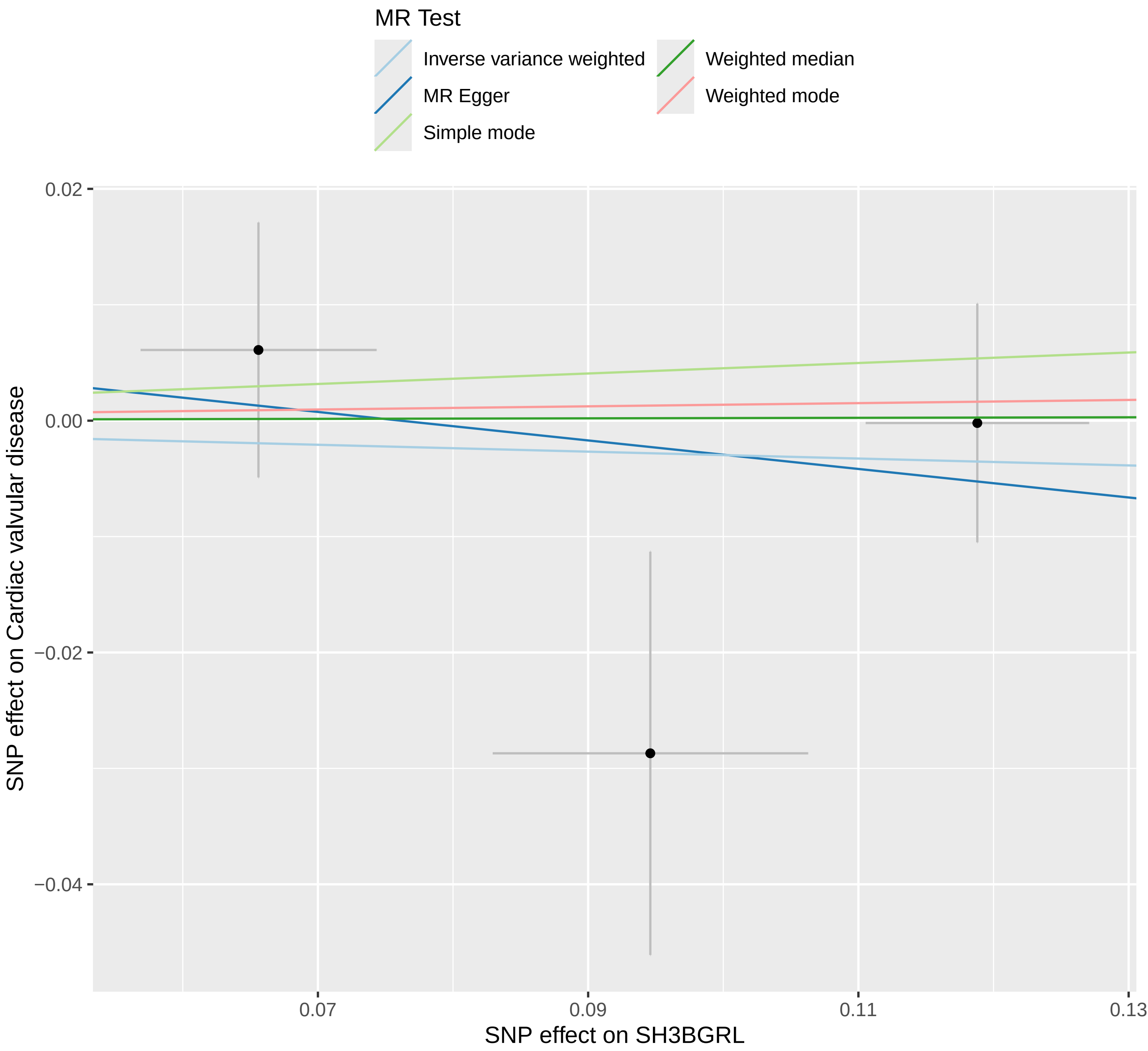

MR Test
Inverse variance weighted
MR Egger
Simple mode
Weighted median
Weighted mode
0.02
0.00
−0.02
−0.04
0.07
0.09
0.11
0.13
SNP effect on SH3BGRL
SNP effect on Cardiac valvular disease

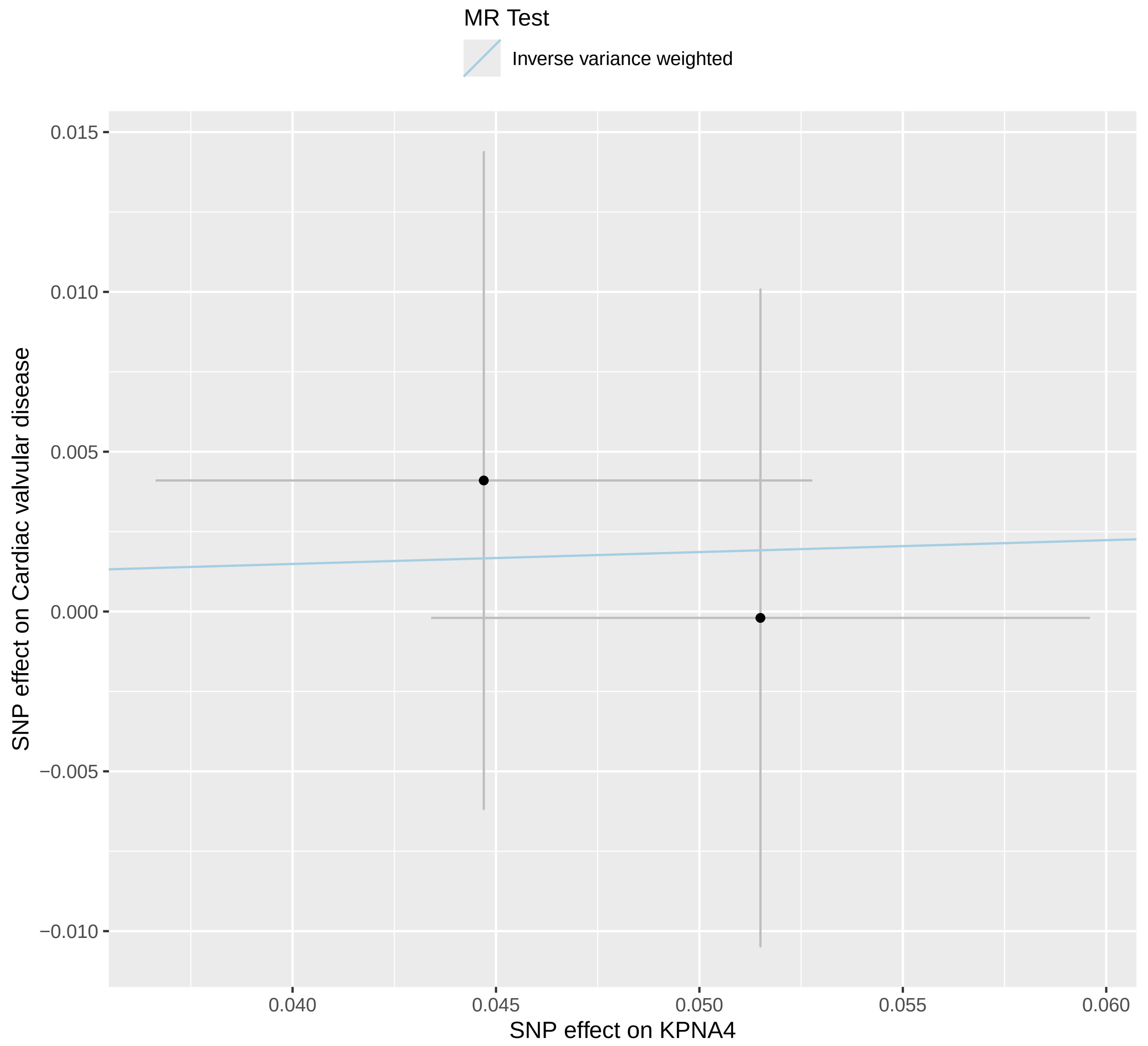
MR Test
Inverse variance weighted
0.015
0.010
0.005
0.000
−0.005
−0.010
SNP effect on Cardiac valvular disease
0.040
0.045
0.050
0.055
0.060
SNP effect on KPNA4

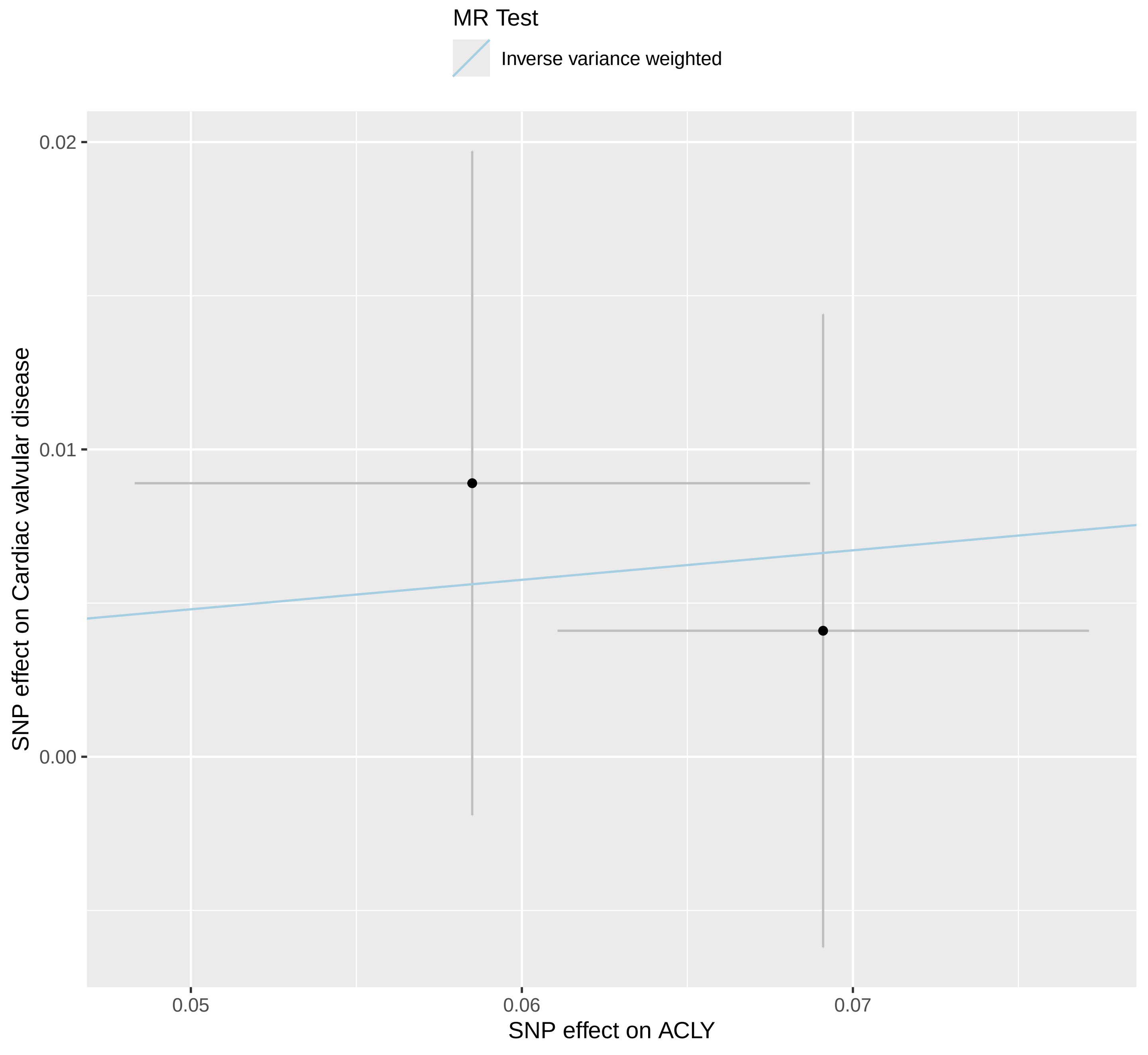

MR Test
Inverse variance weighted
0.02
0.01
0.00
0.05
0.06
0.07
SNP effect on ACLY
SNP effect on Cardiac valvular disease

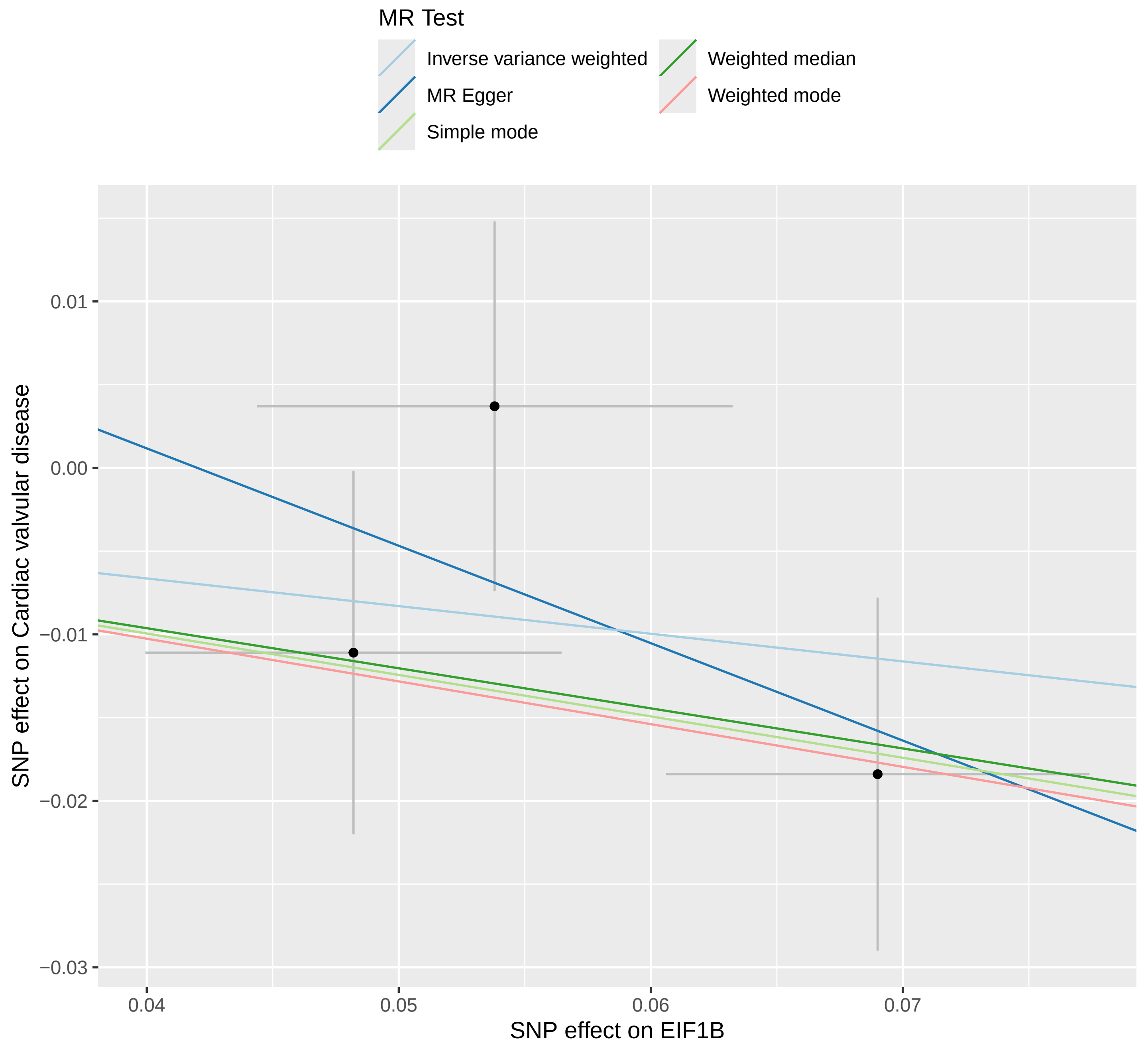
MR Test
Inverse variance weighted
MR Egger
Simple mode
Weighted median
Weighted mode
0.01
0.00
−0.01
−0.02
−0.03
0.04
0.05
0.06
0.07
SNP effect on Cardiac valvular disease
SNP effect on EIF1B

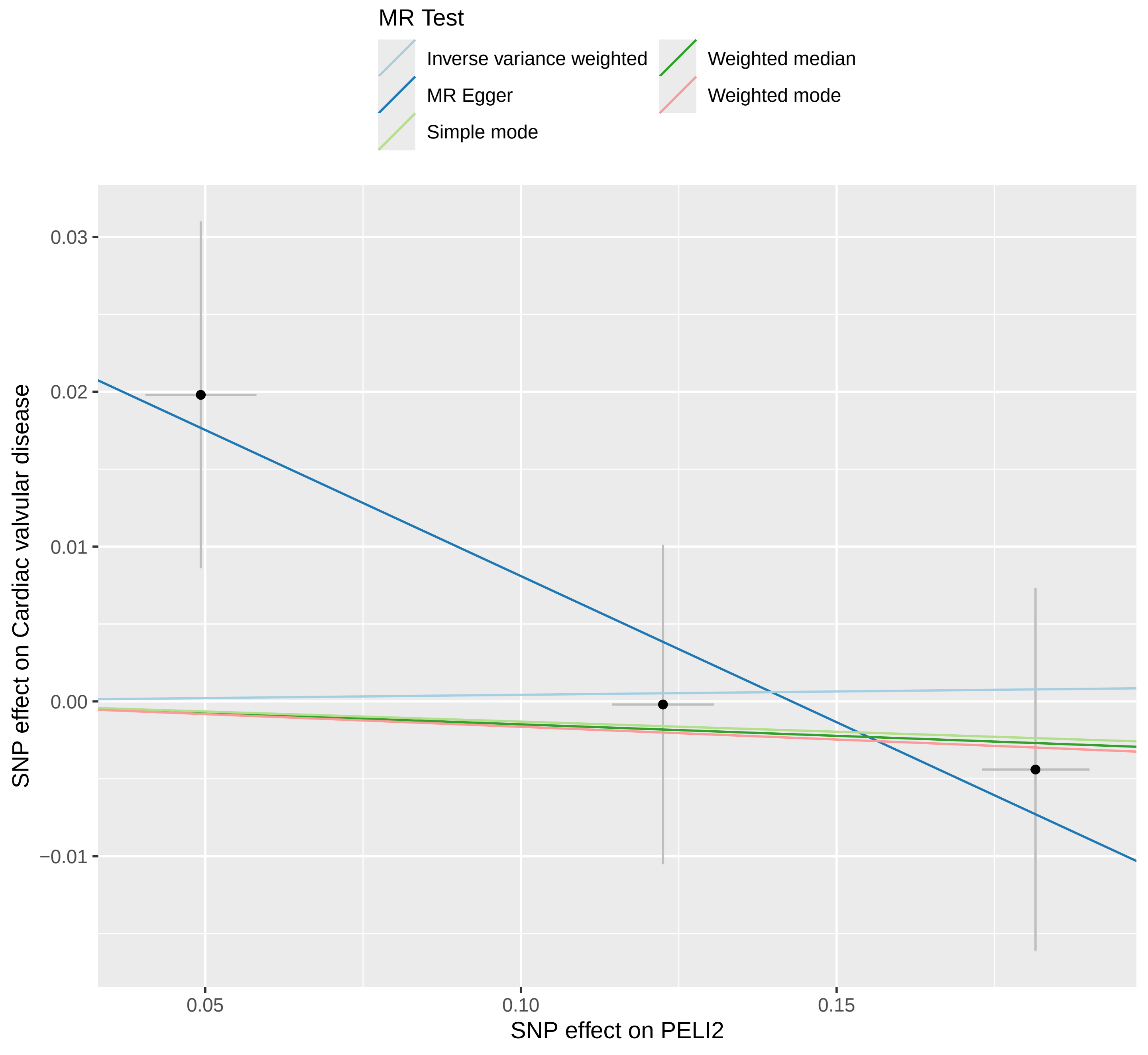

MR Test
Inverse variance weighted
MR Egger
Simple mode
Weighted median
Weighted mode
0.03
0.02
0.01
0.00
−0.01
0.05
0.10
0.15
SNP effect on Cardiac valvular disease
SNP effect on PELI2

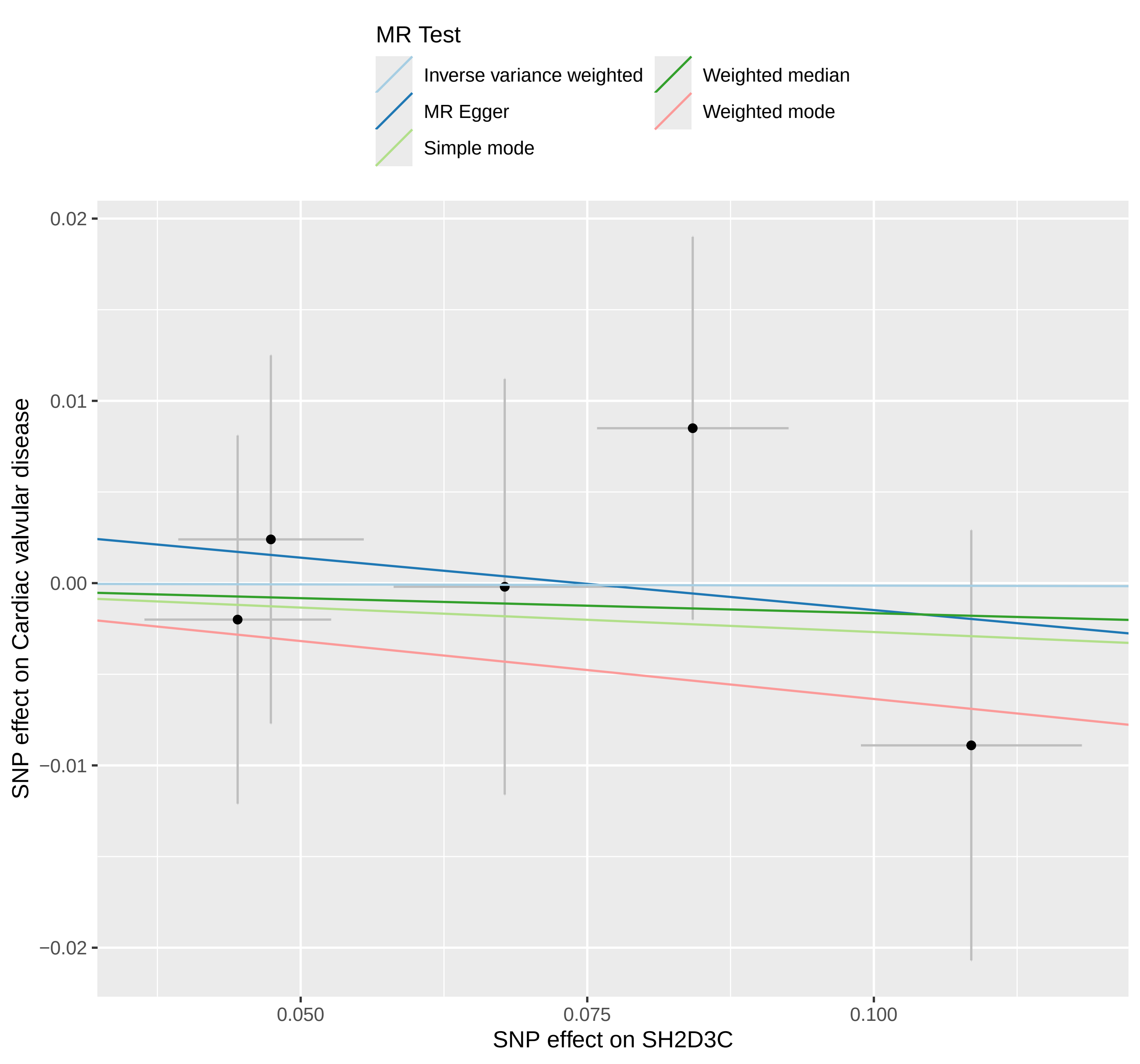

MR Test
Inverse variance weighted
MR Egger
Simple mode
Weighted median
Weighted mode
0.02
0.01
0.00
−0.01
−0.02
SNP effect on Cardiac valvular disease
0.050
0.075
0.100
SNP effect on SH2D3C

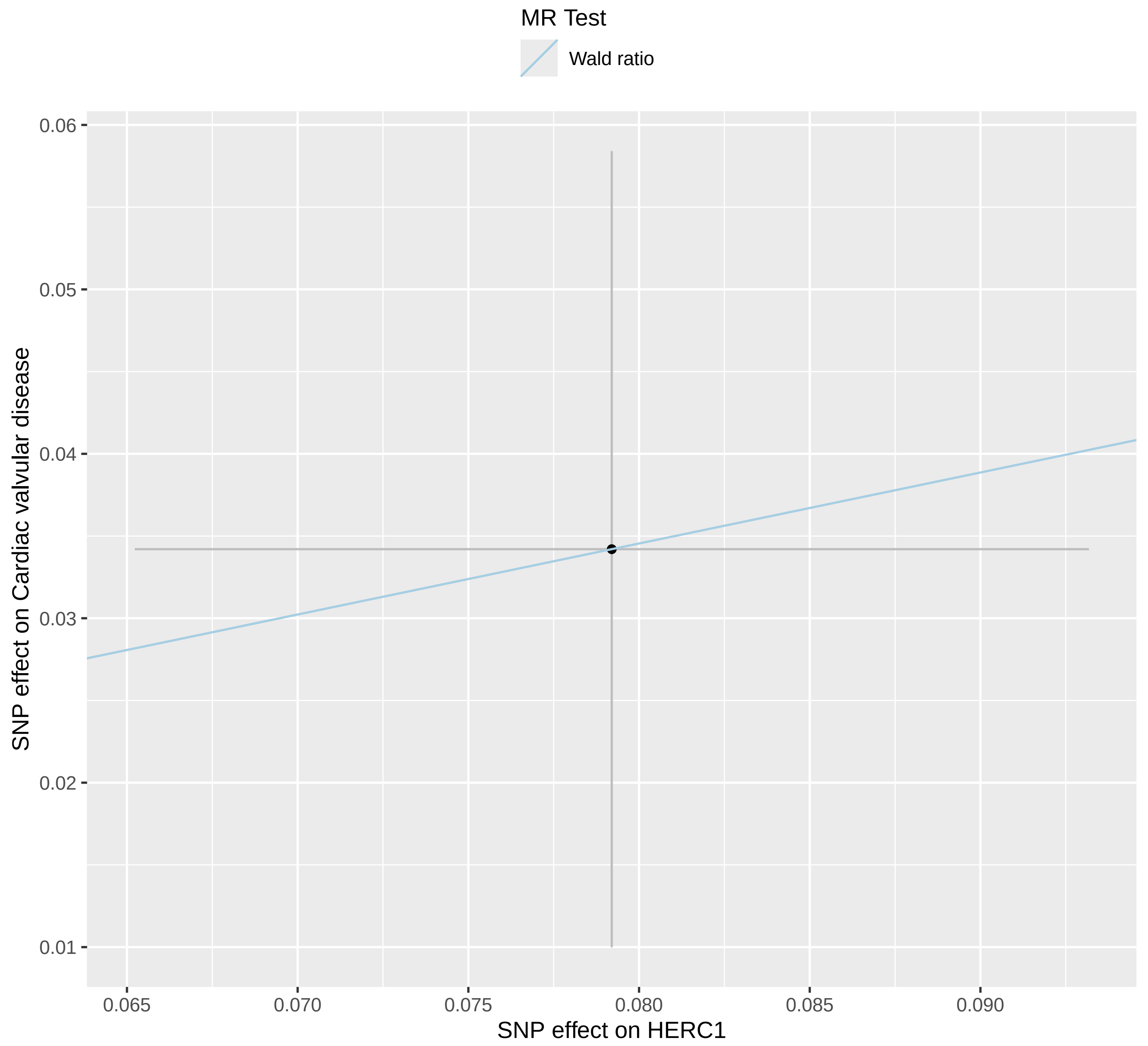
MR Test
Wald ratio
SNP effect on Cardiac valvular disease
0.06
0.05
0.04
0.03
0.02
0.01
0.065
0.070
0.075
0.080
0.085
0.090
SNP effect on HERC1

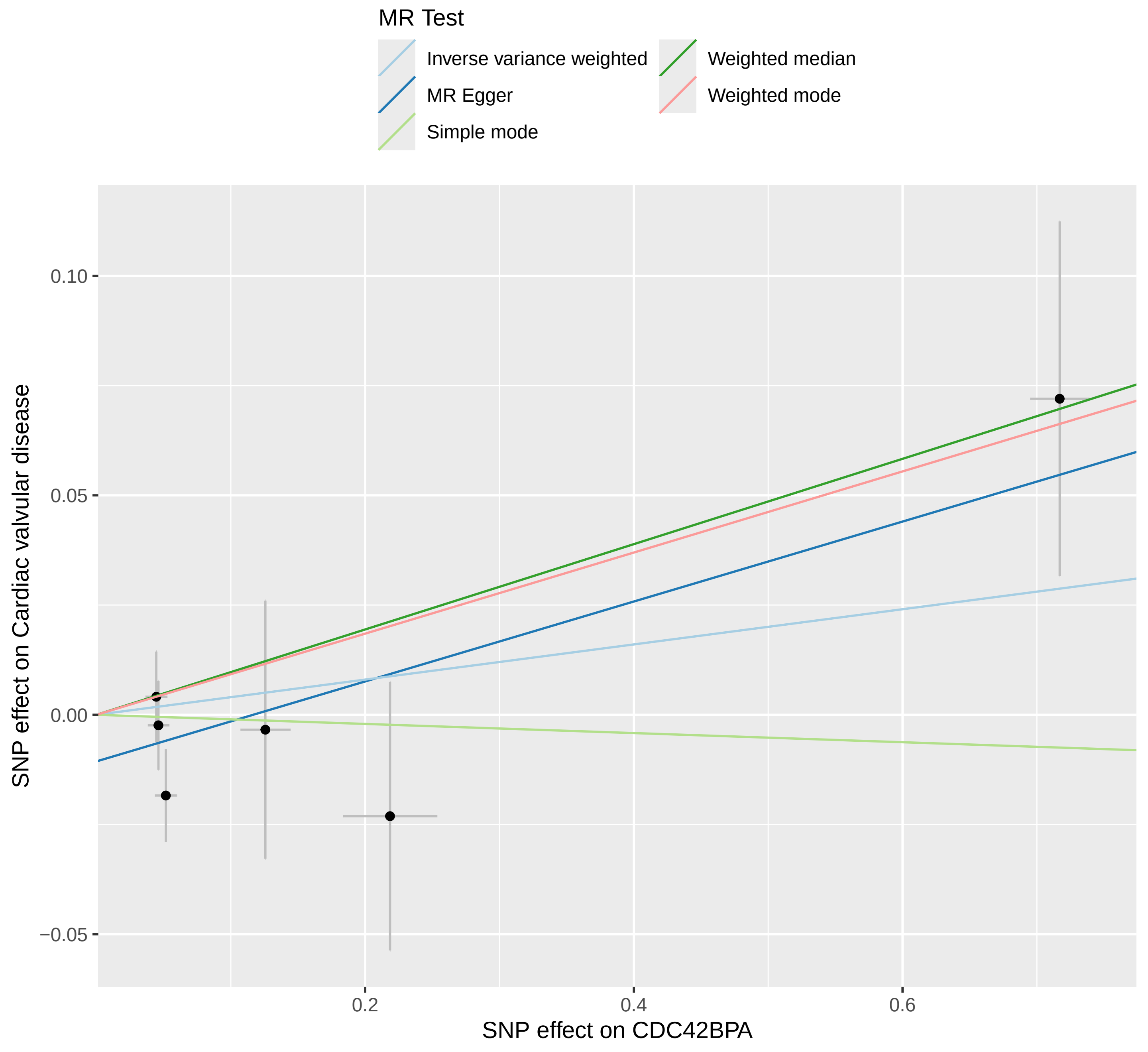
MR Test
Inverse variance weighted
MR Egger
Simple mode
Weighted median
Weighted mode
SNP effect on Cardiac valvular disease
0.10
0.05
0.00
−0.05
0.2
0.4
0.6
SNP effect on CDC42BPA

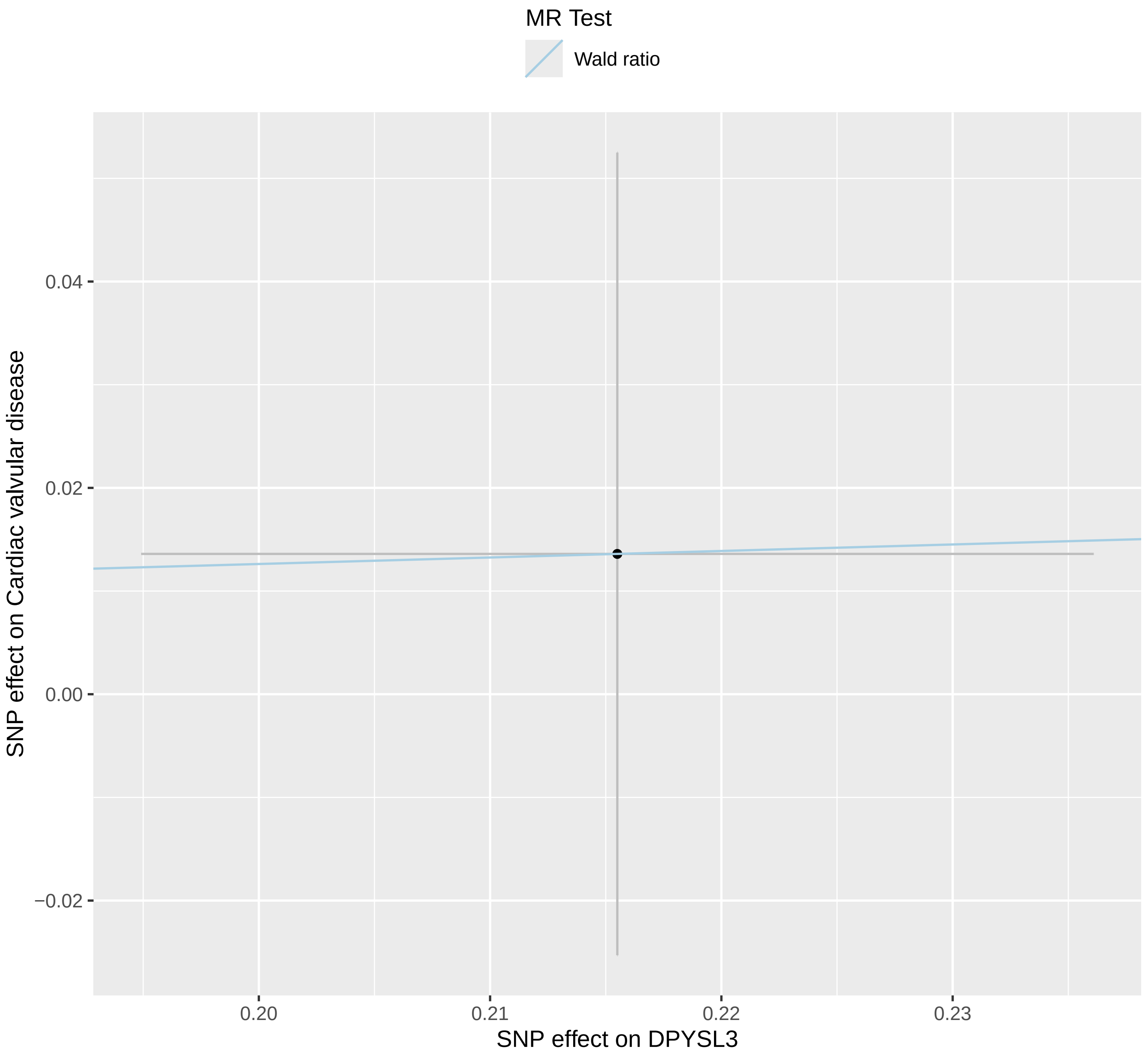

MR Test
Wald ratio
SNP effect on Cardiac valvular disease
0.04
0.02
0.00
−0.02
0.20
0.21
0.22
0.23
SNP effect on DPYSL3

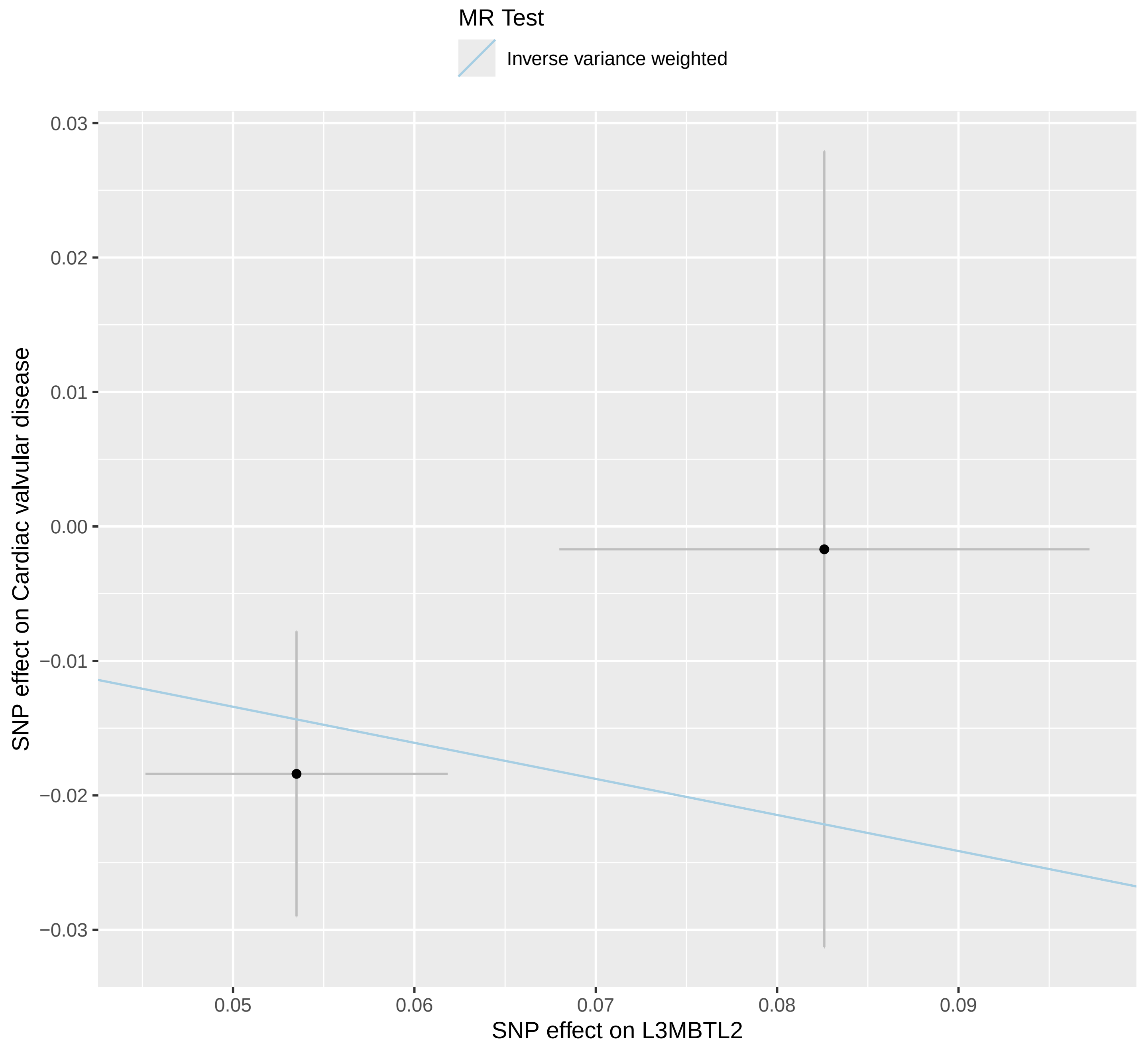
MR Test
Inverse variance weighted
0.03
0.02
0.01
0.00
−0.01
−0.02
−0.03
SNP effect on Cardiac valvular disease
0.05
0.06
0.07
0.08
0.09
SNP effect on L3MBTL2

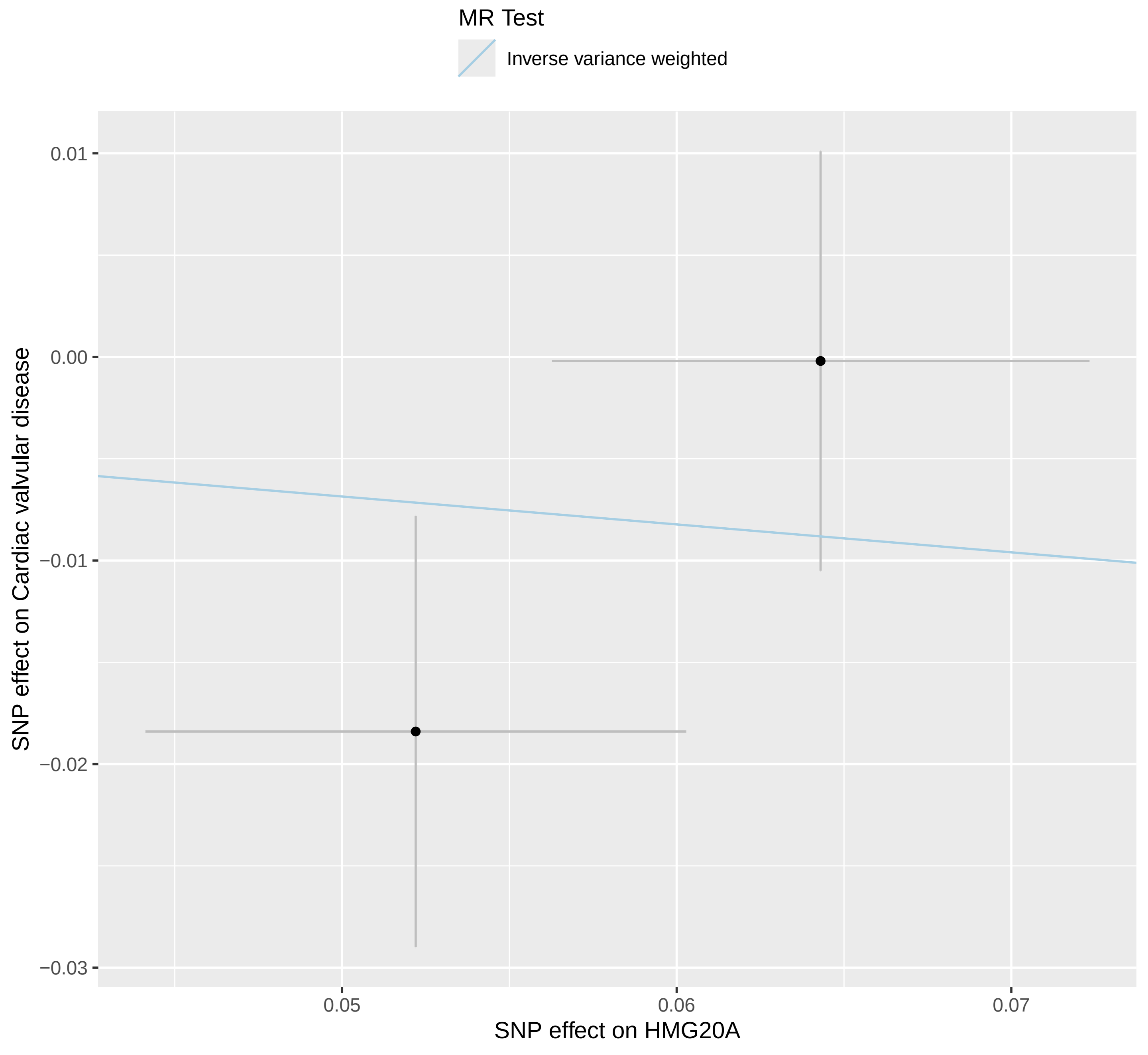

MR Test
Inverse variance weighted
0.01
0.00
−0.01
−0.02
−0.03
0.05
0.06
0.07
SNP effect on Cardiac valvular disease
SNP effect on HMG20A

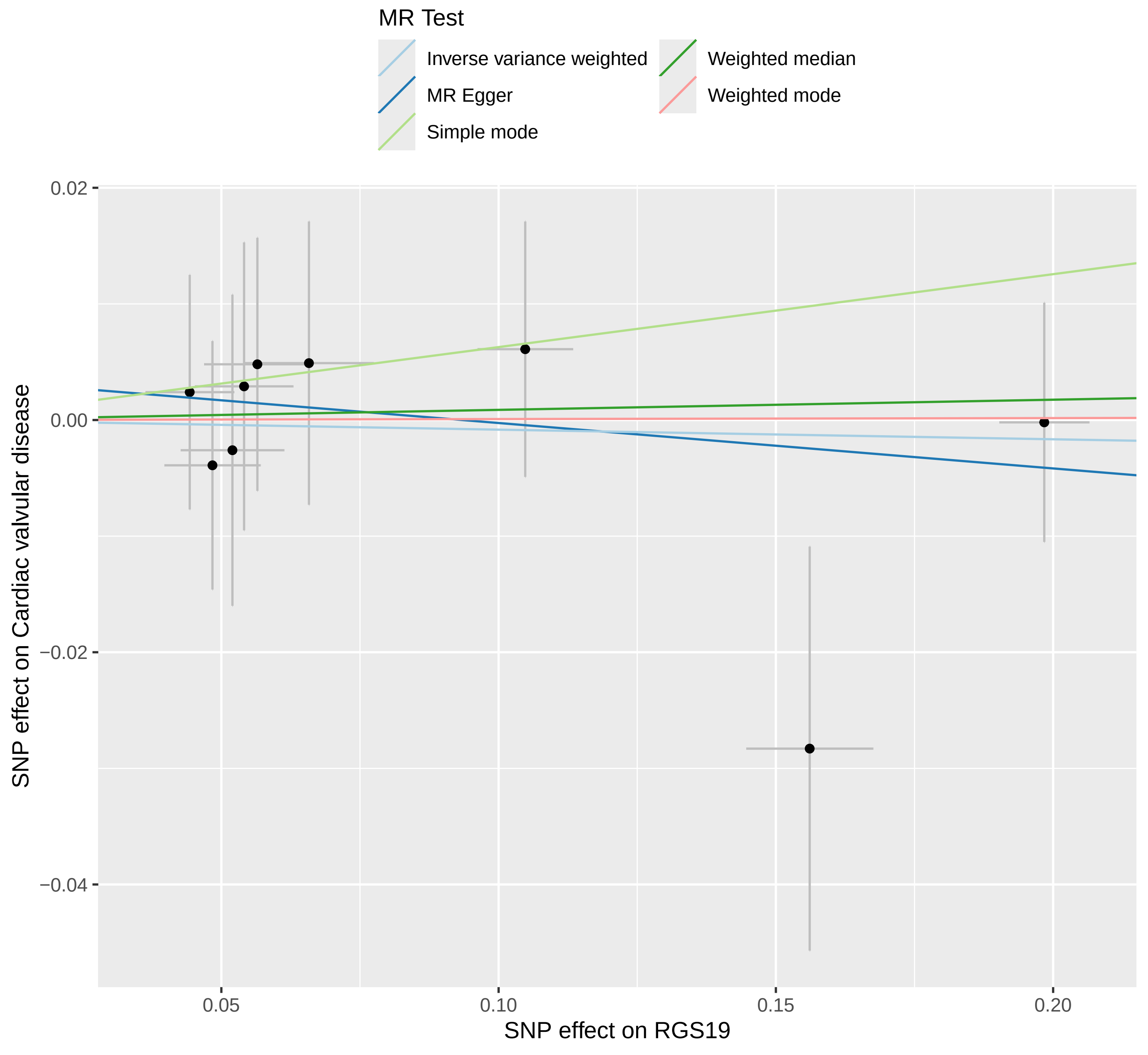
MR Test
Inverse variance weighted
MR Egger
Simple mode
Weighted median
Weighted mode
0.02
0.00
−0.02
−0.04
0.05
0.10
0.15
0.20
SNP effect on RGS19
SNP effect on Cardiac valvular disease

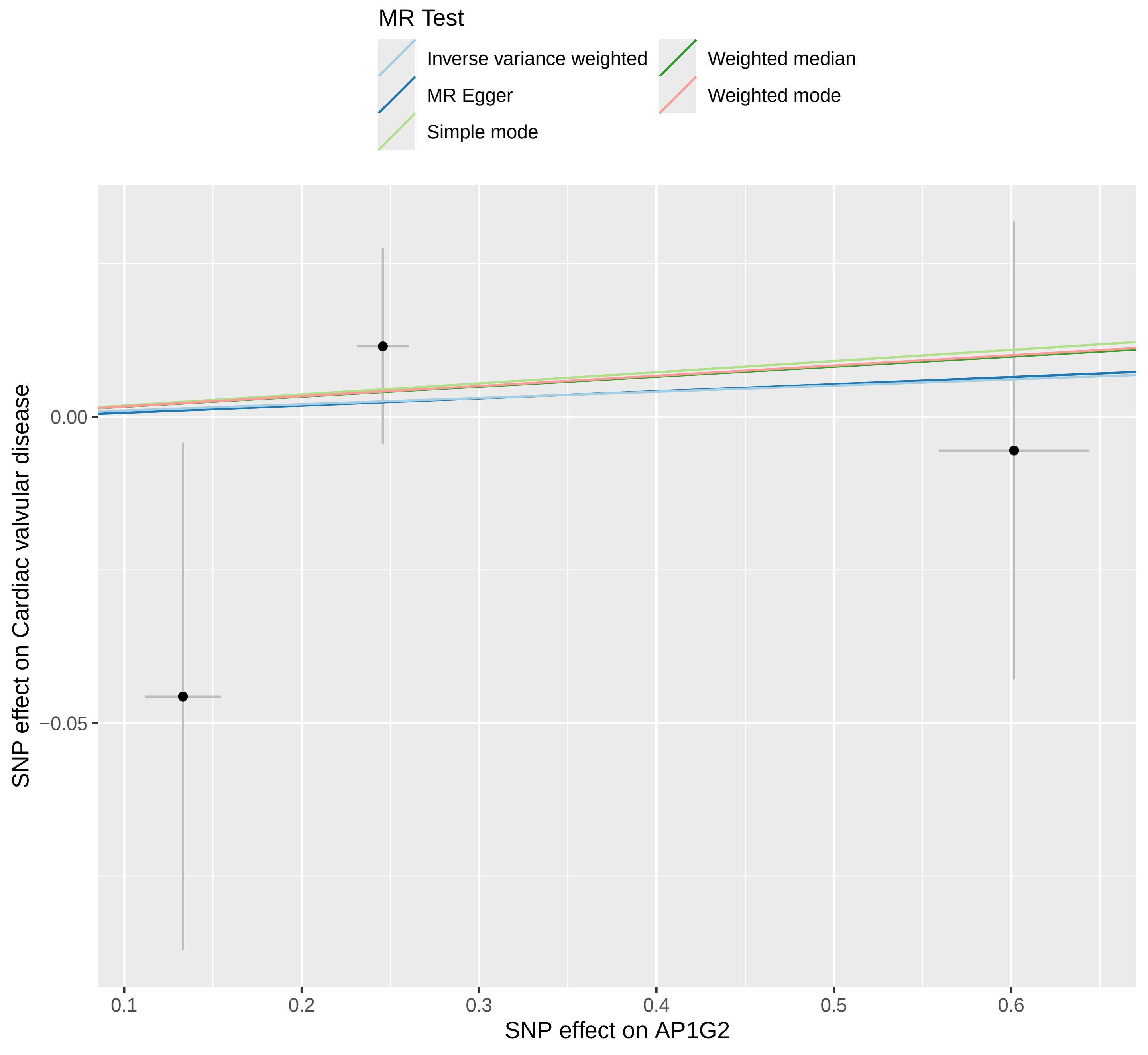
MR Test
Inverse variance weighted
MR Egger
Simple mode
Weighted median
Weighted mode
0.00
−0.05
0.1
0.2
0.3
0.4
0.5
0.6
SNP effect on AP1G2
SNP effect on Cardiac valvular disease

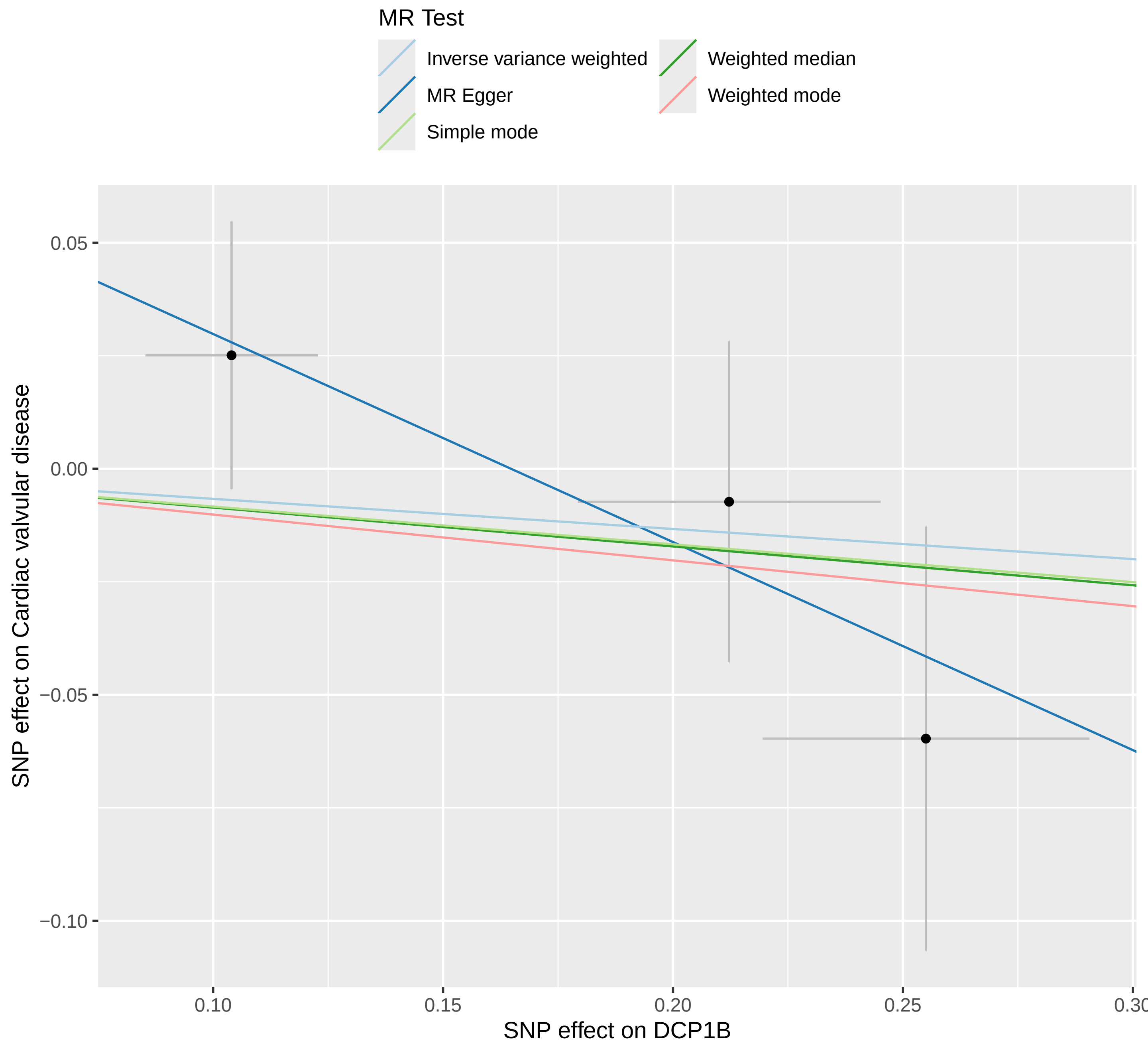
MR Test
Inverse variance weighted
MR Egger
Simple mode
Weighted median
Weighted mode
0.05
0.00
−0.05
−0.10
0.10
0.15
0.20
0.25
0.30
SNP effect on DCP1B
SNP effect on Cardiac valvular disease

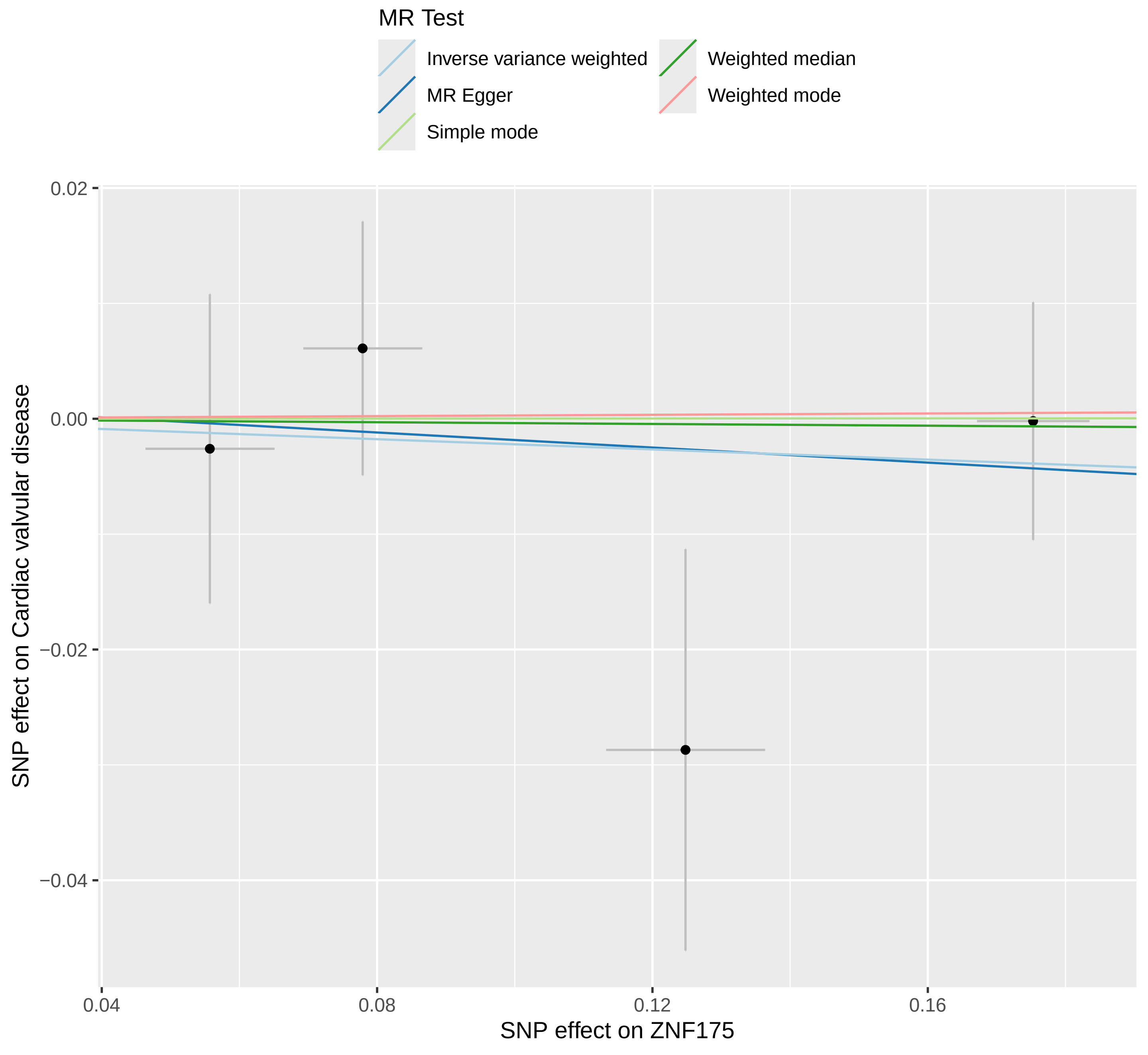
MR Test
Inverse variance weighted
MR Egger
Simple mode
Weighted median
Weighted mode
0.02
0.00
−0.02
−0.04
0.04
0.08
0.12
0.16
SNP effect on Cardiac valvular disease
SNP effect on ZNF175

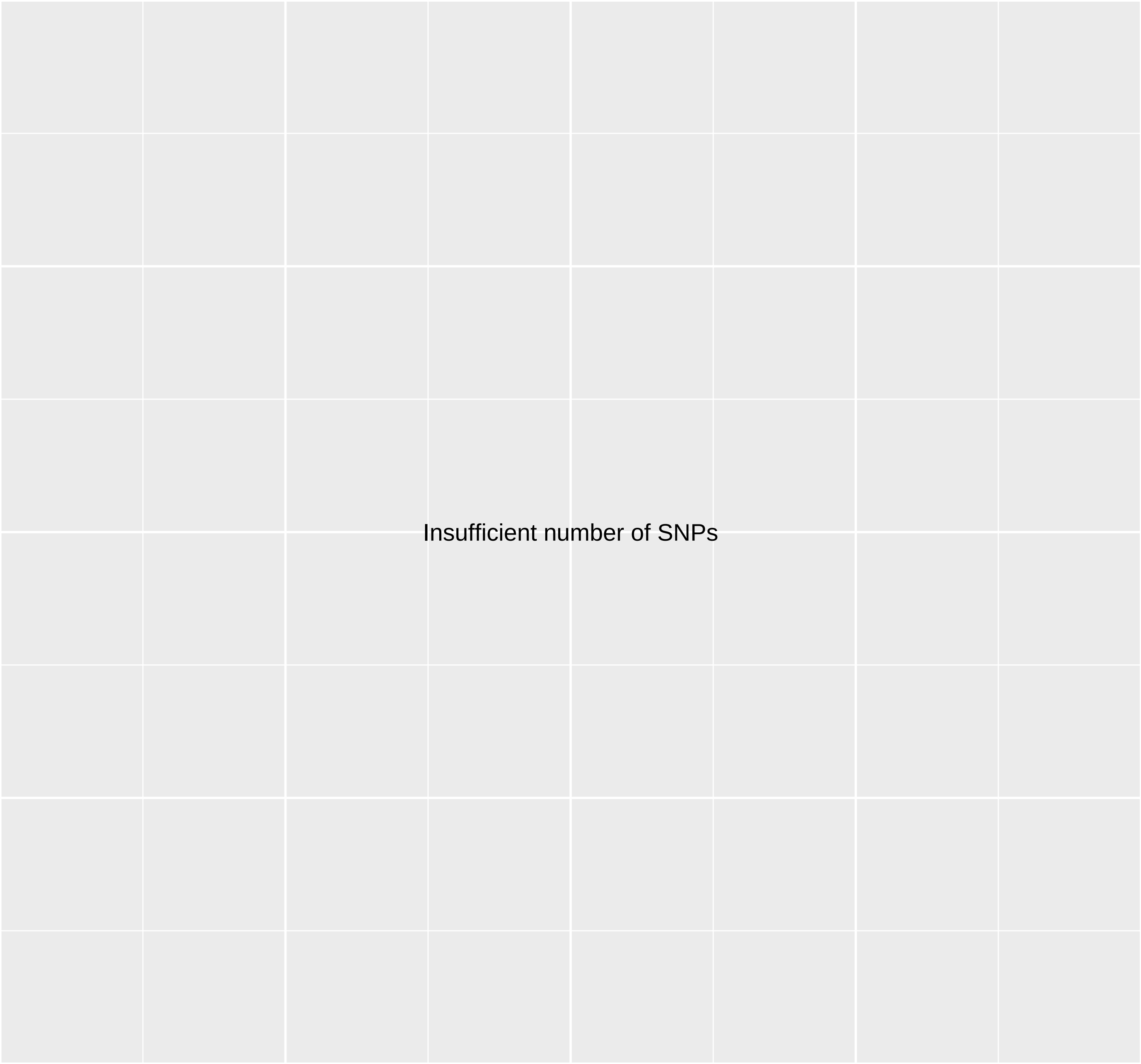
Insufficient number of SNPs

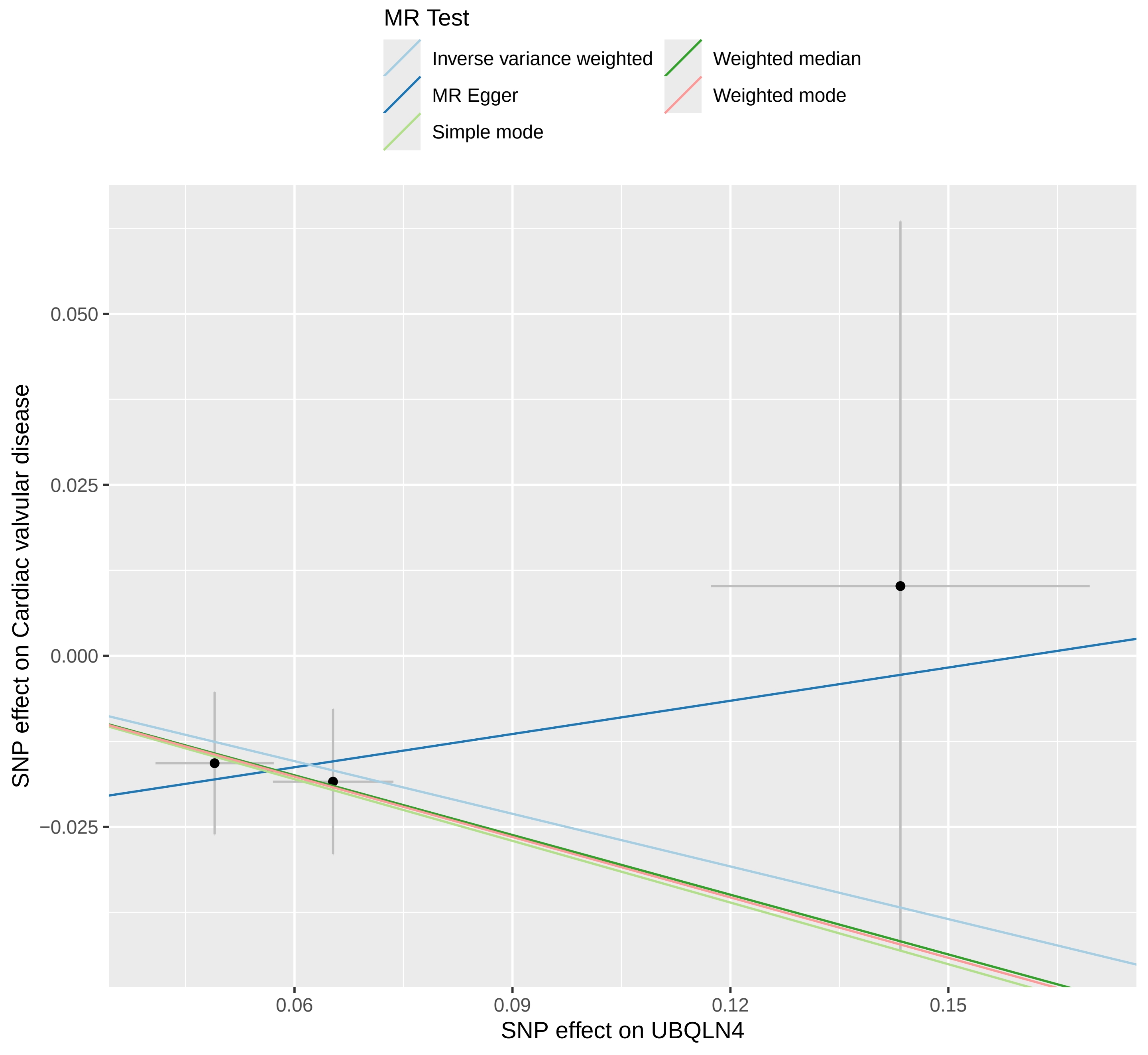

MR Test
Inverse variance weighted
MR Egger
Simple mode
Weighted median
Weighted mode
0.050
0.025
0.000
−0.025
SNP effect on Cardiac valvular disease
0.06
0.09
0.12
0.15
SNP effect on UBQLN4

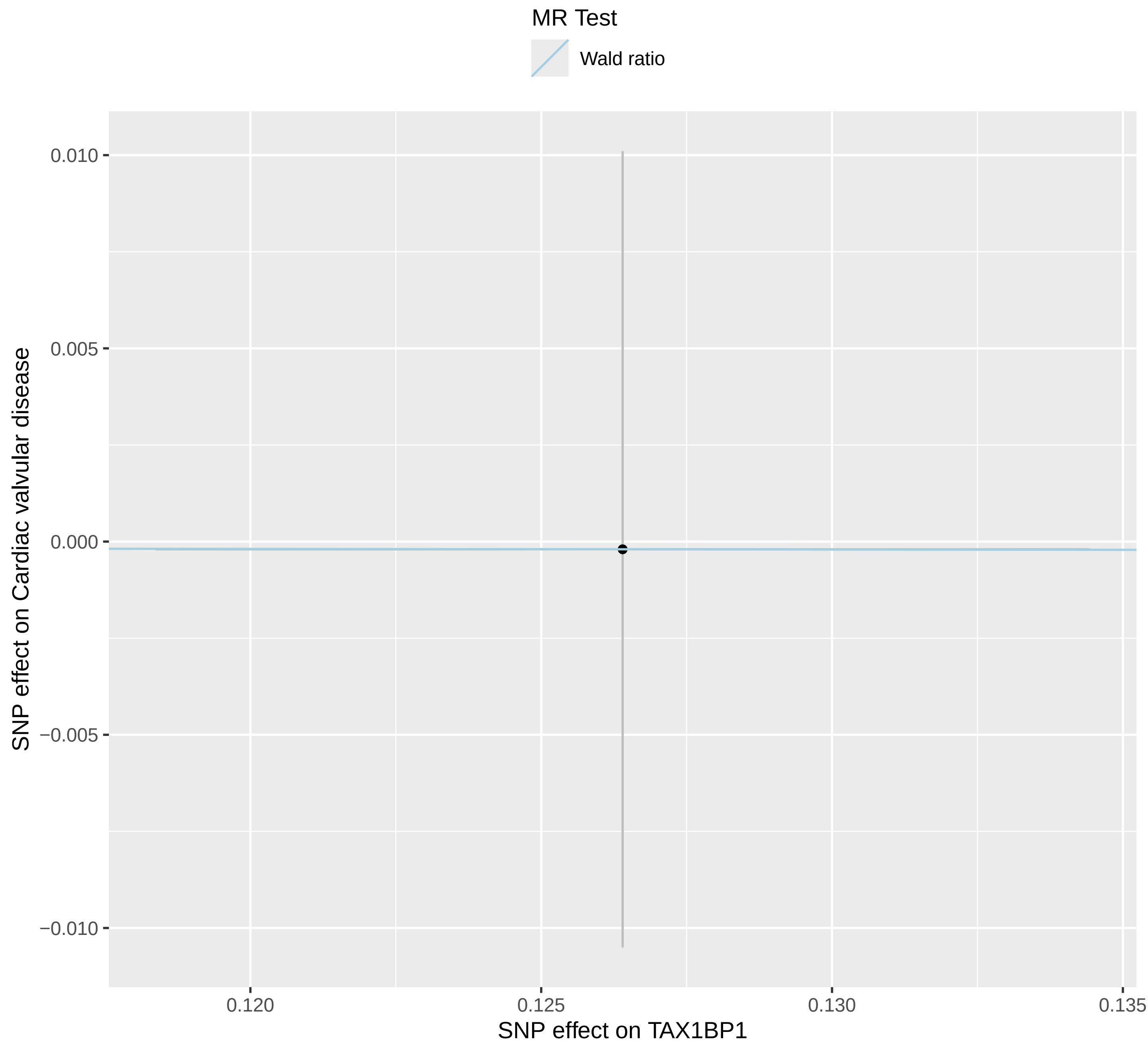
MR Test
Wald ratio
SNP effect on Cardiac valvular disease
0.010
0.005
0.000
−0.005
−0.010
0.120
0.125
0.130
0.135
SNP effect on TAX1BP1

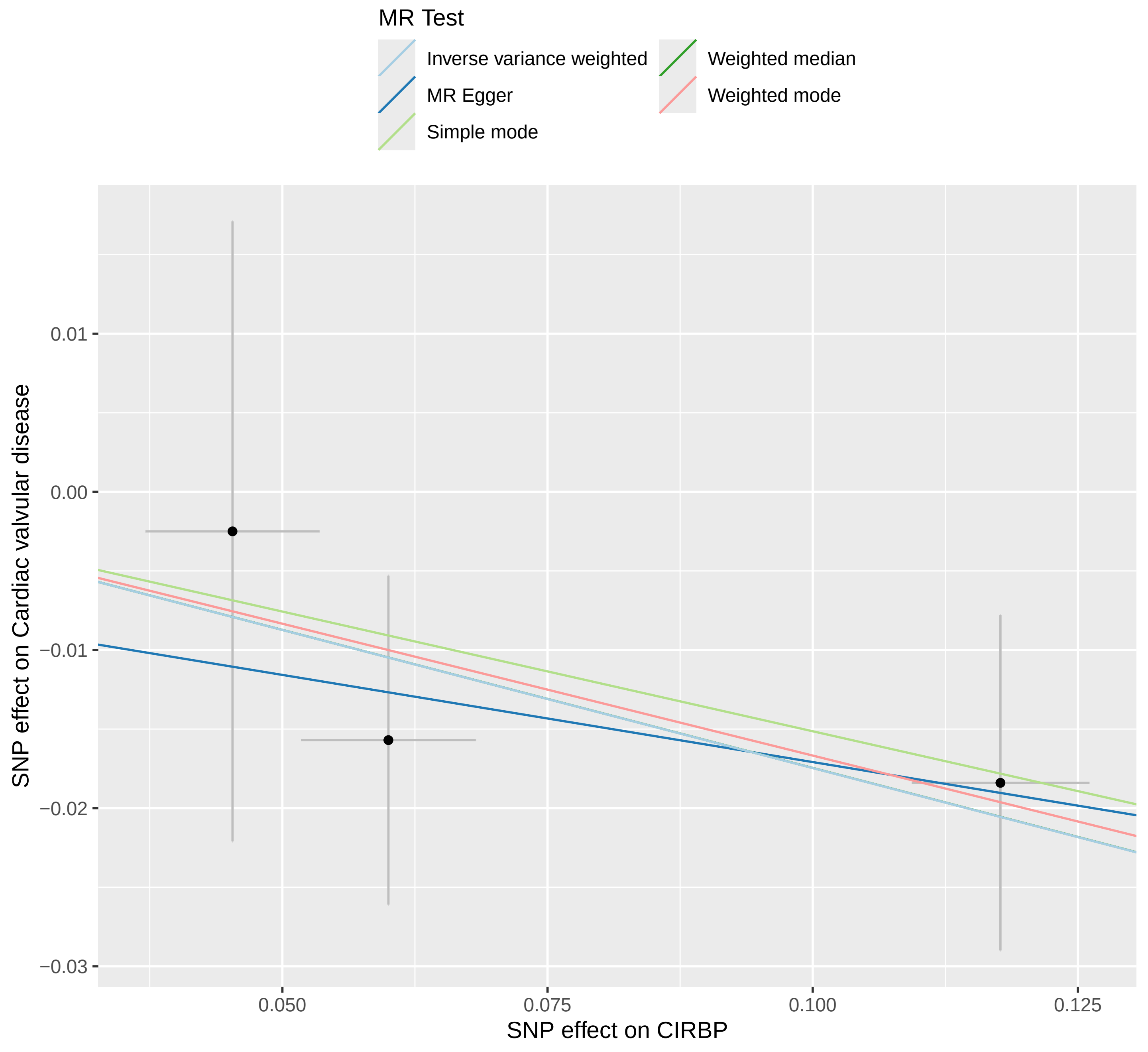

MR Test
Inverse variance weighted
MR Egger
Simple mode
Weighted median
Weighted mode
0.01
0.00
−0.01
−0.02
−0.03
0.050
0.075
0.100
0.125
SNP effect on Cardiac valvular disease
SNP effect on CIRBP

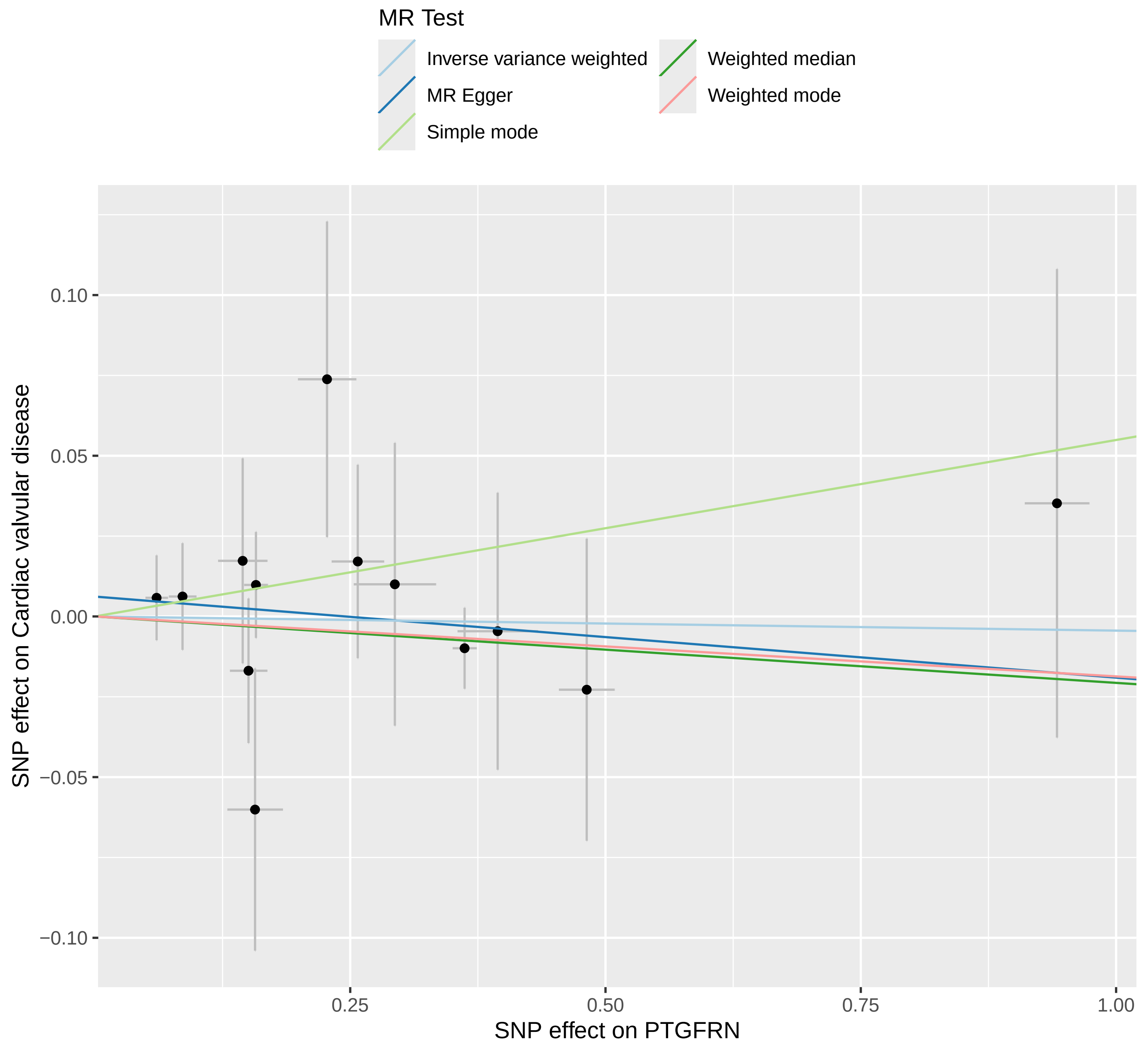
MR Test
Inverse variance weighted
MR Egger
Simple mode
Weighted median
Weighted mode
0.10
0.05
0.00
−0.05
−0.10
SNP effect on Cardiac valvular disease
0.25
0.50
0.75
1.00
SNP effect on PTGFRN

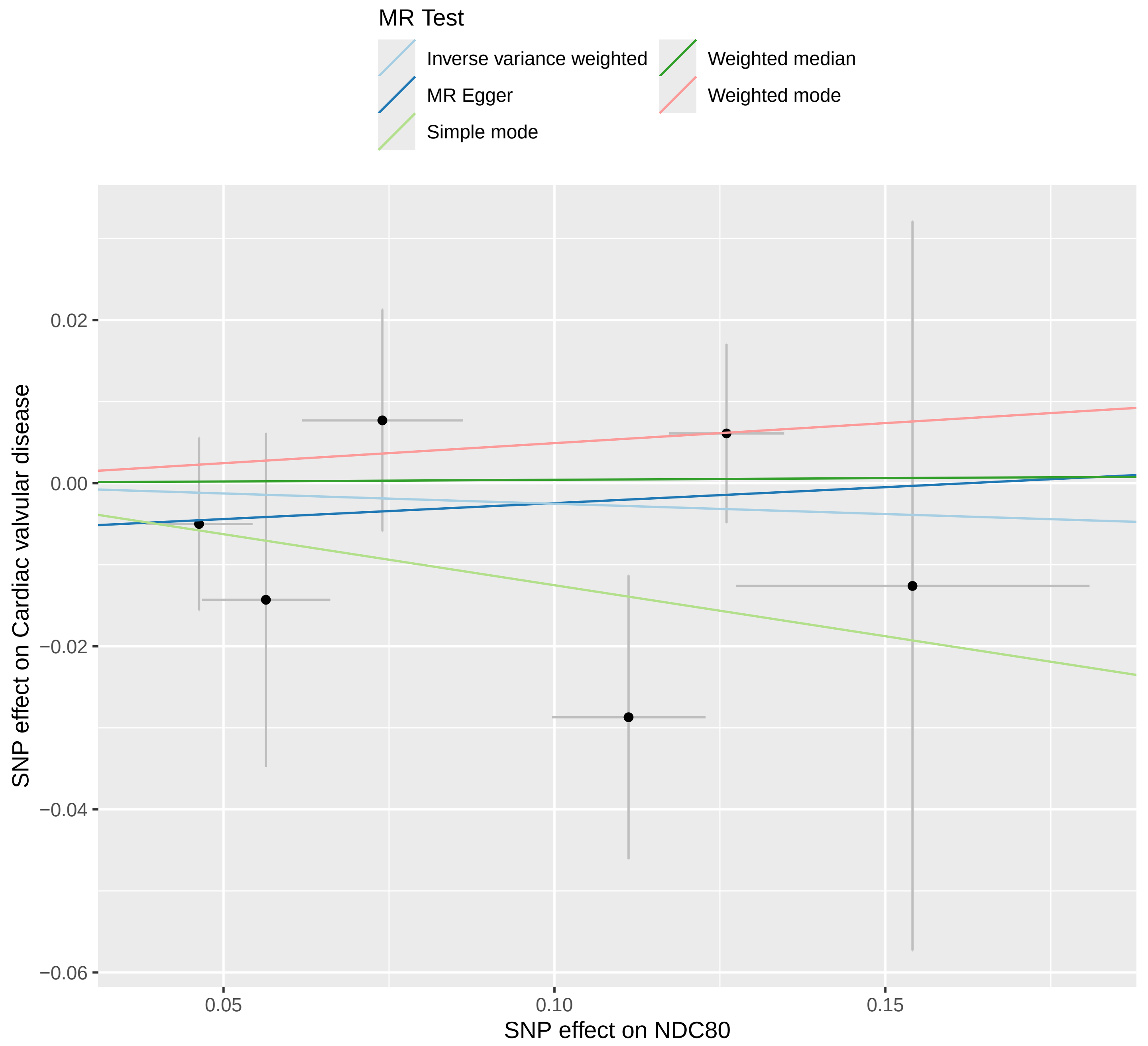
MR Test
Inverse variance weighted
MR Egger
Simple mode
Weighted median
Weighted mode
0.02
0.00
−0.02
−0.04
−0.06
SNP effect on Cardiac valvular disease
0.05
0.10
0.15
SNP effect on NDC80

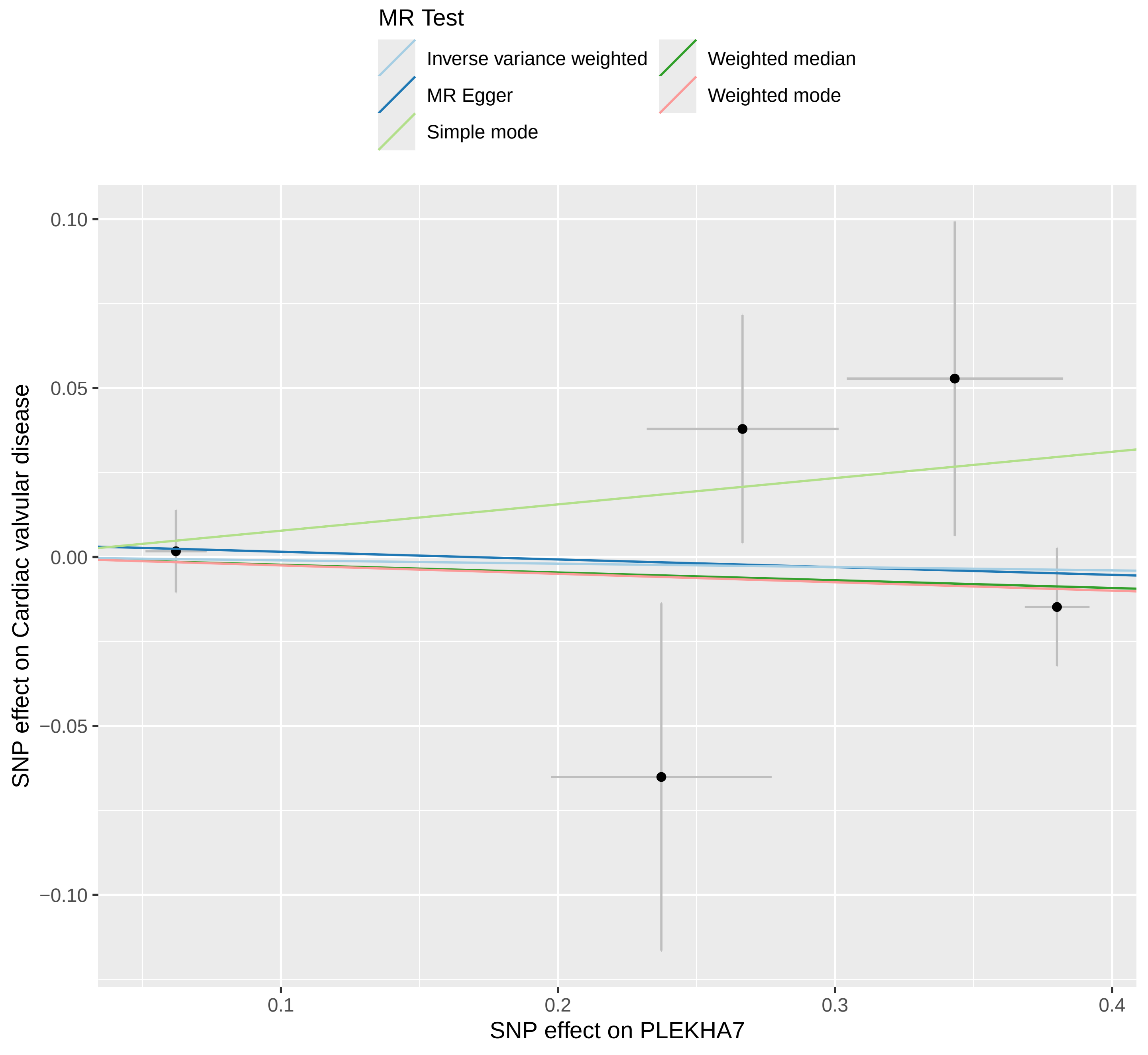
MR Test
Inverse variance weighted
MR Egger
Simple mode
Weighted median
Weighted mode
0.10
0.05
0.00
−0.05
−0.10
0.1
0.2
0.3
0.4
SNP effect on PLEKHA7
SNP effect on Cardiac valvular disease

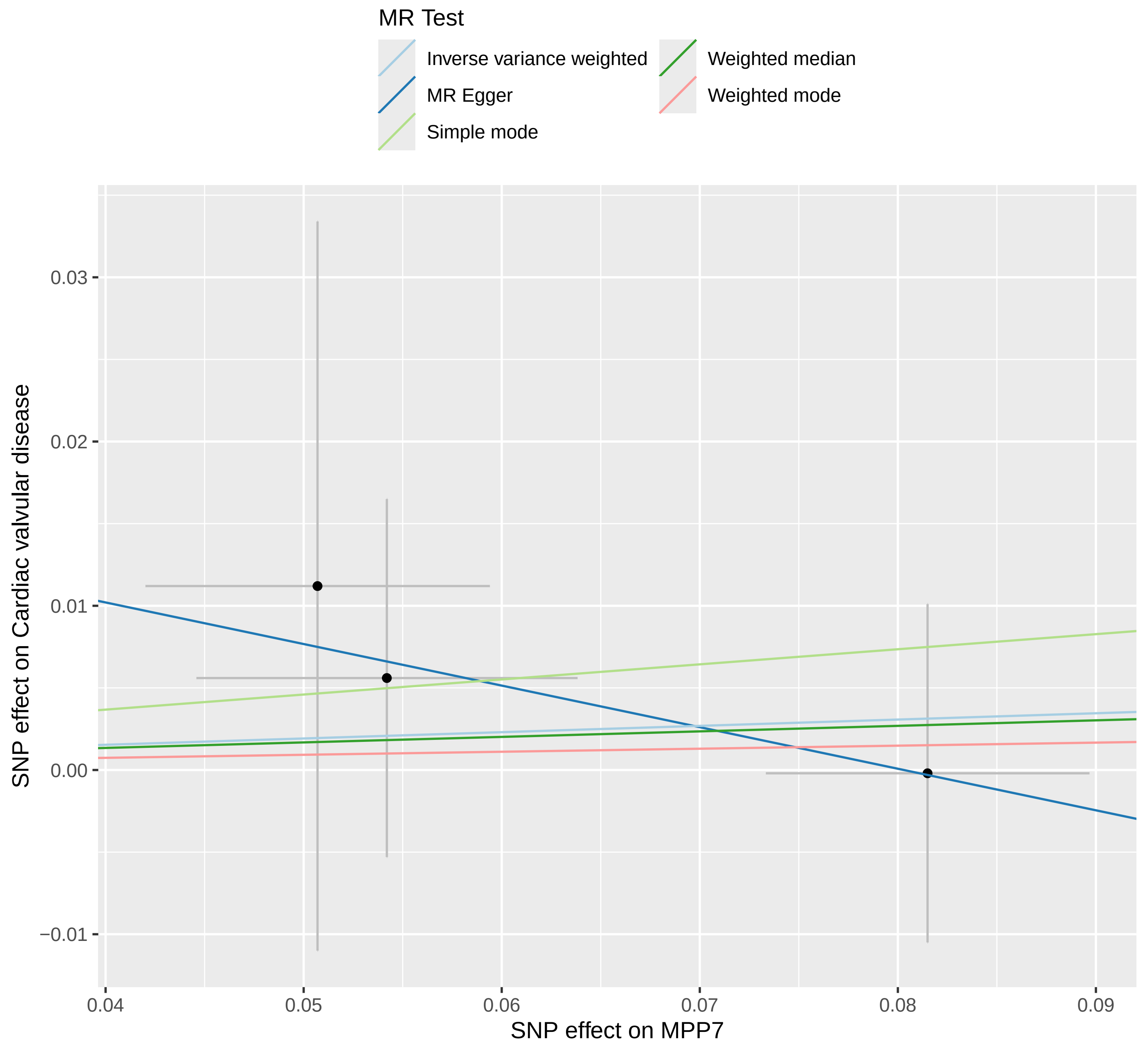

MR Test
Inverse variance weighted
MR Egger
Simple mode
Weighted median
Weighted mode
SNP effect on Cardiac valvular disease
0.03
0.02
0.01
0.00
−0.01
0.04
0.05
0.06
0.07
0.08
0.09
SNP effect on MPP7

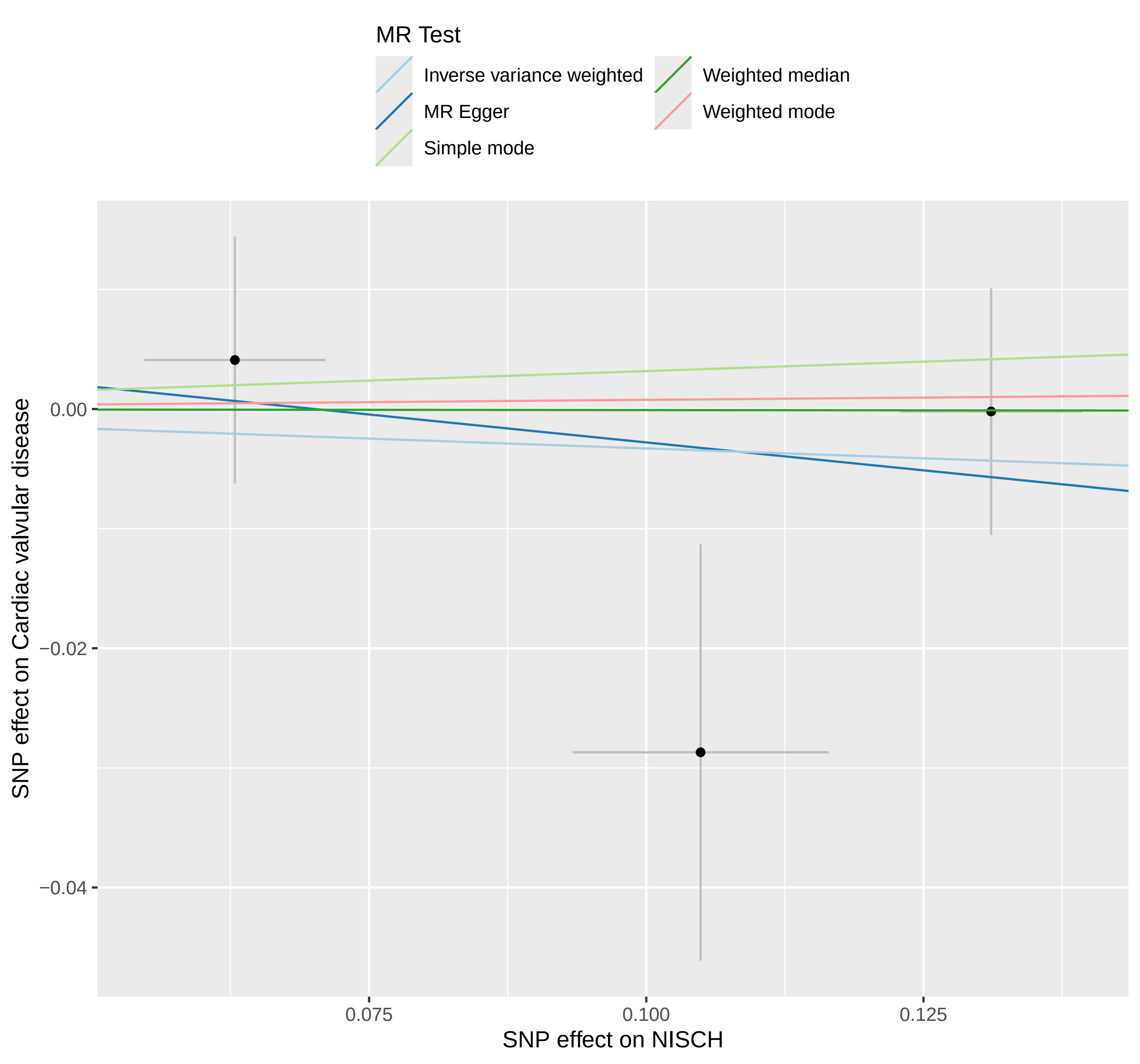
MR Test
Inverse variance weighted
MR Egger
Simple mode
Weighted median
Weighted mode
0.00
−0.02
−0.04
SNP effect on Cardiac valvular disease
0.075
0.100
0.125
SNP effect on NISCH

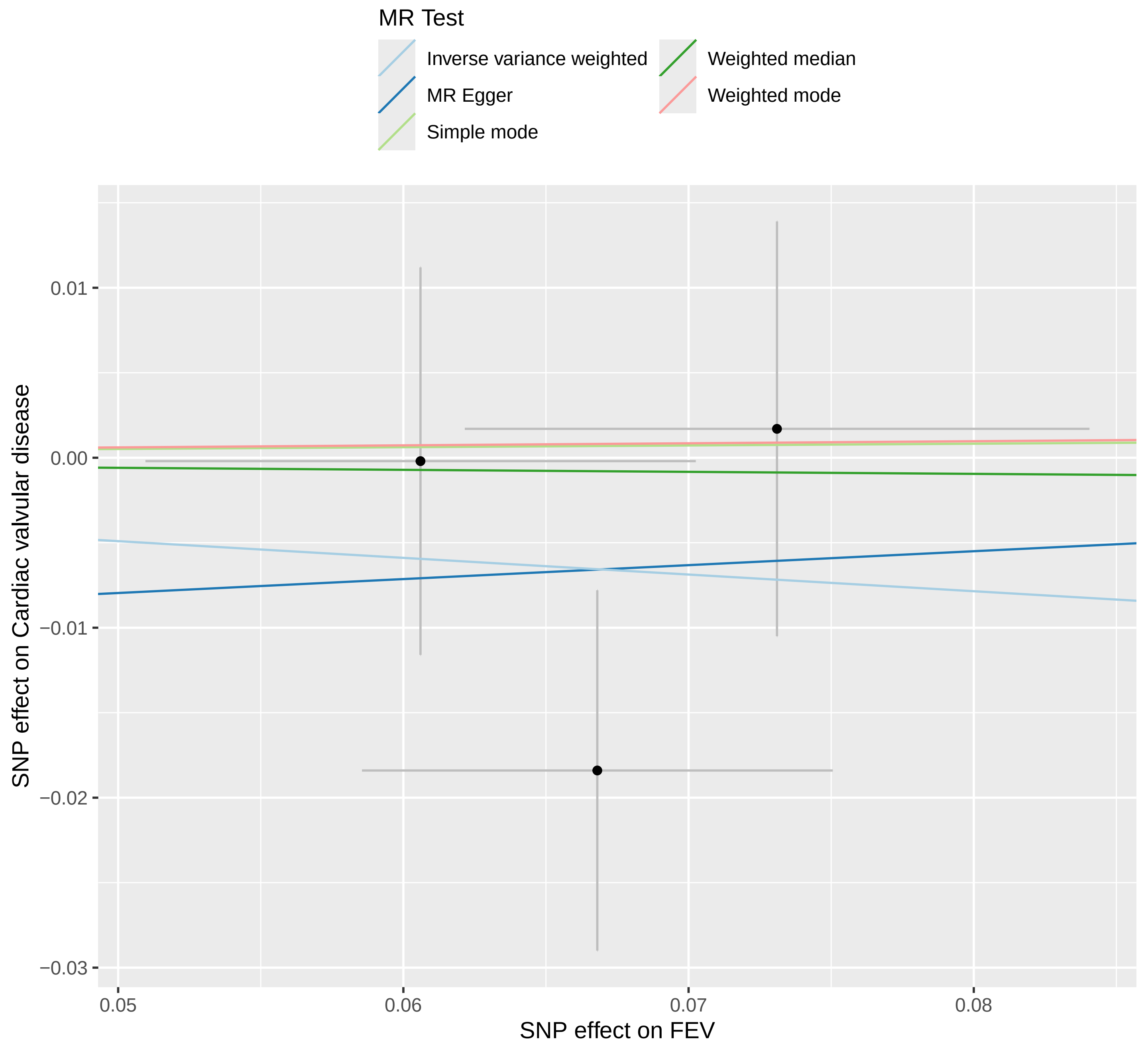
MR Test
Inverse variance weighted
MR Egger
Simple mode
Weighted median
Weighted mode
SNP effect on Cardiac valvular disease
0.01
0.00
−0.01
−0.02
−0.03
0.05
0.06
0.07
0.08
SNP effect on FEV

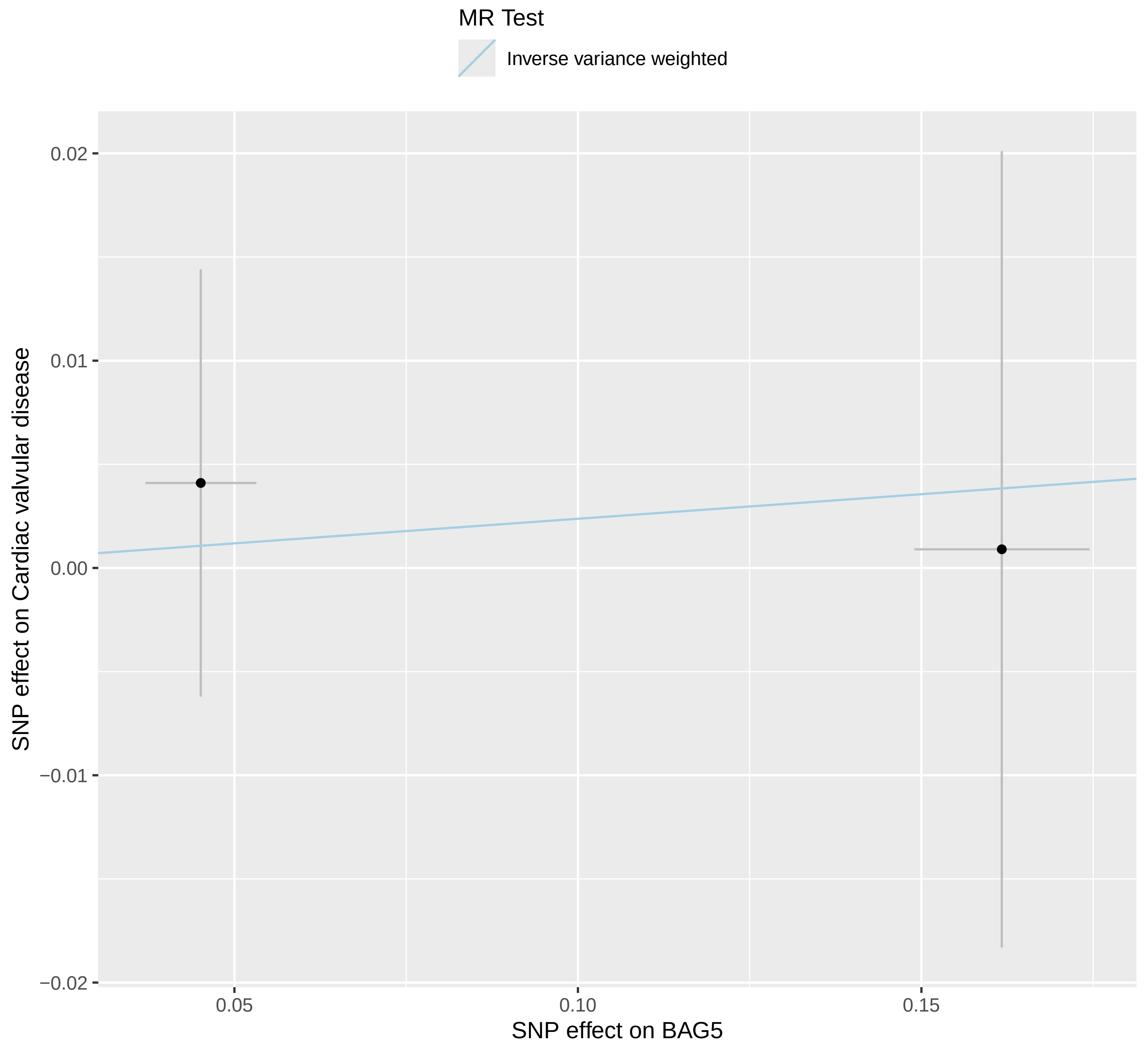

MR Test
Inverse variance weighted
0.02
0.01
0.00
−0.01
−0.02
SNP effect on Cardiac valvular disease
0.05
0.10
0.15
SNP effect on BAG5

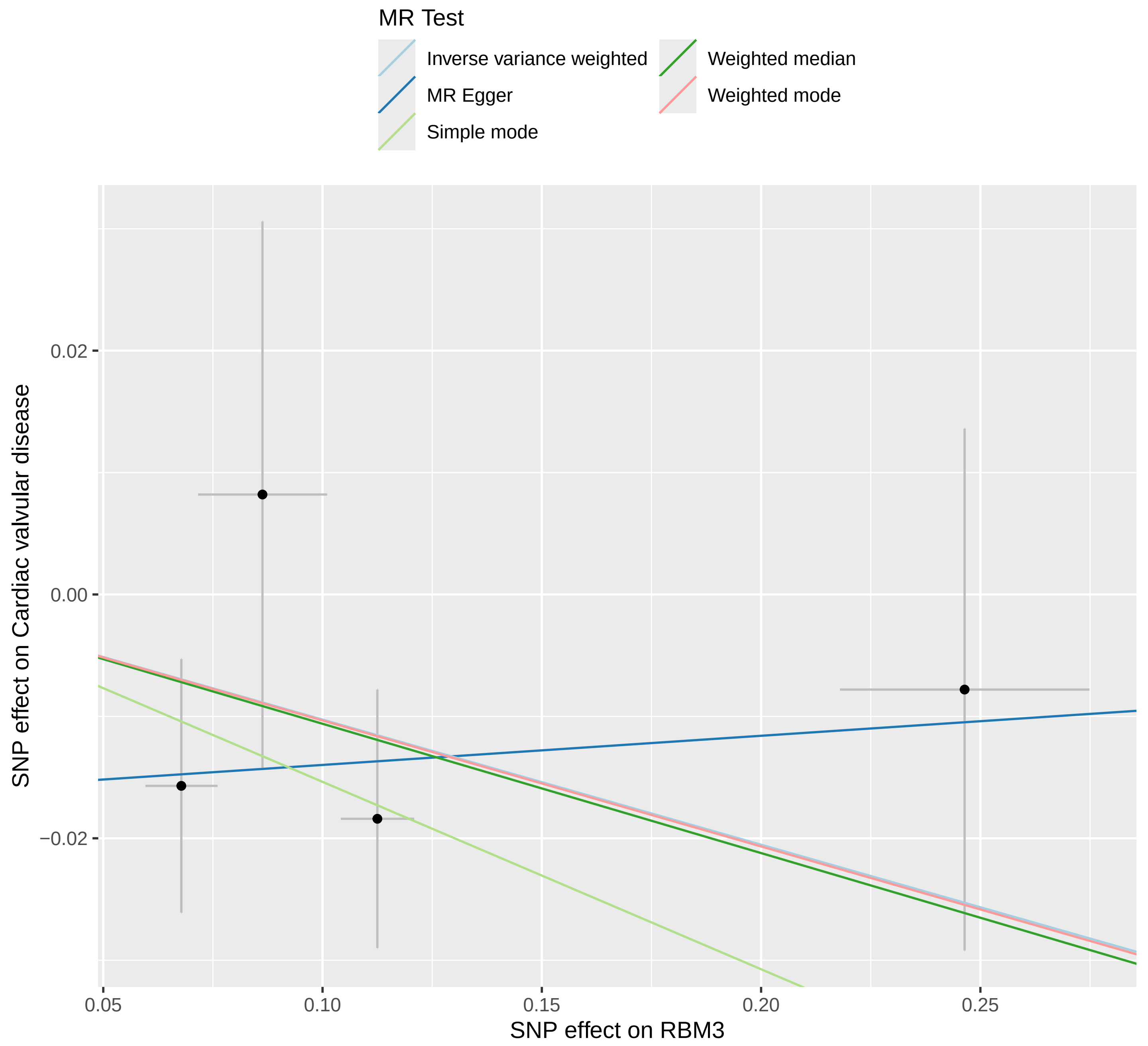
MR Test
Inverse variance weighted
MR Egger
Simple mode
Weighted median
Weighted mode
SNP effect on Cardiac valvular disease
0.02
0.00
−0.02
0.05
0.10
0.15
0.20
0.25
SNP effect on RBM3

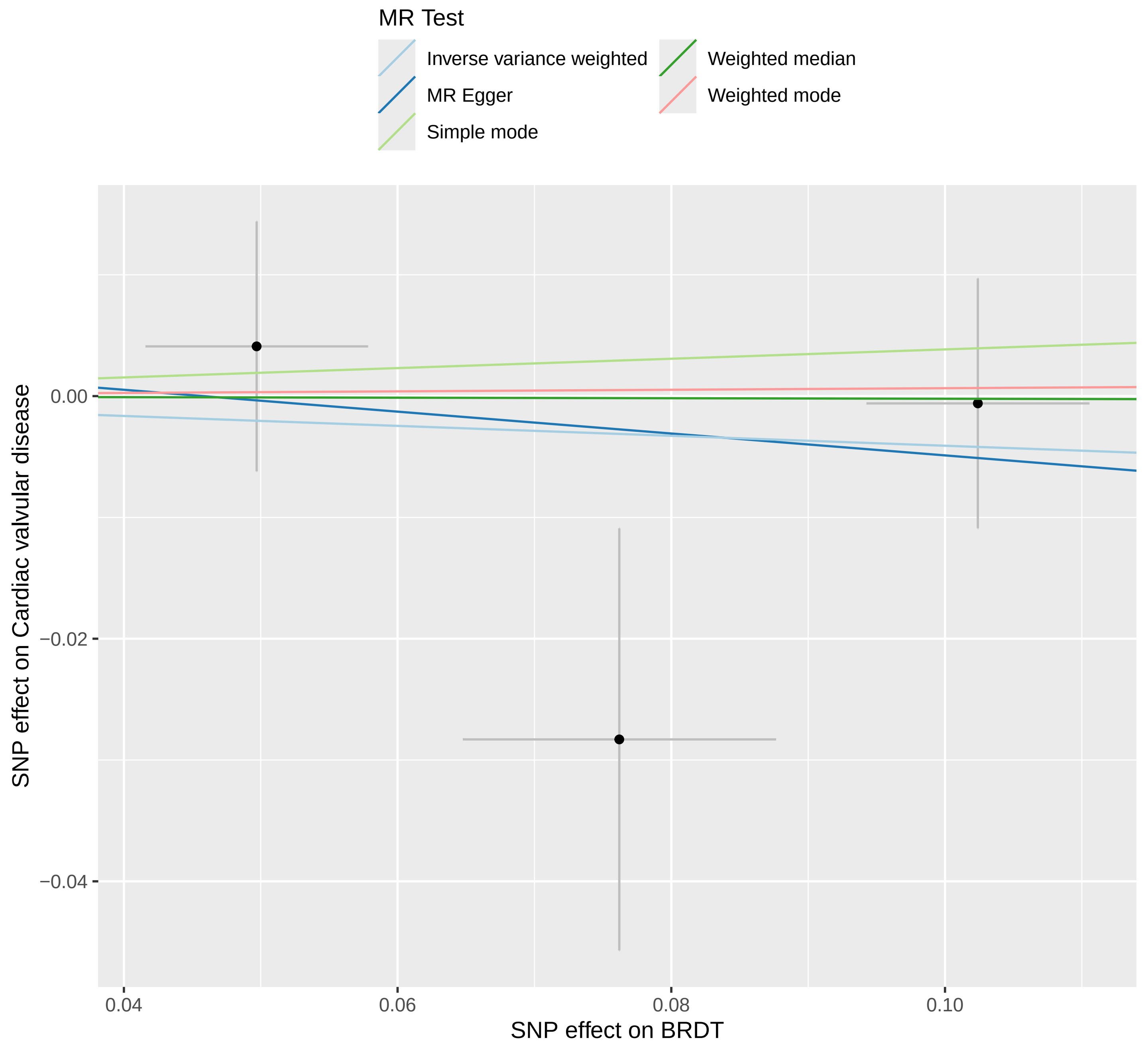
MR Test
Inverse variance weighted
MR Egger
Simple mode
Weighted median
Weighted mode
0.00
−0.02
−0.04
0.04
0.06
0.08
0.10
SNP effect on Cardiac valvular disease
SNP effect on BRDT

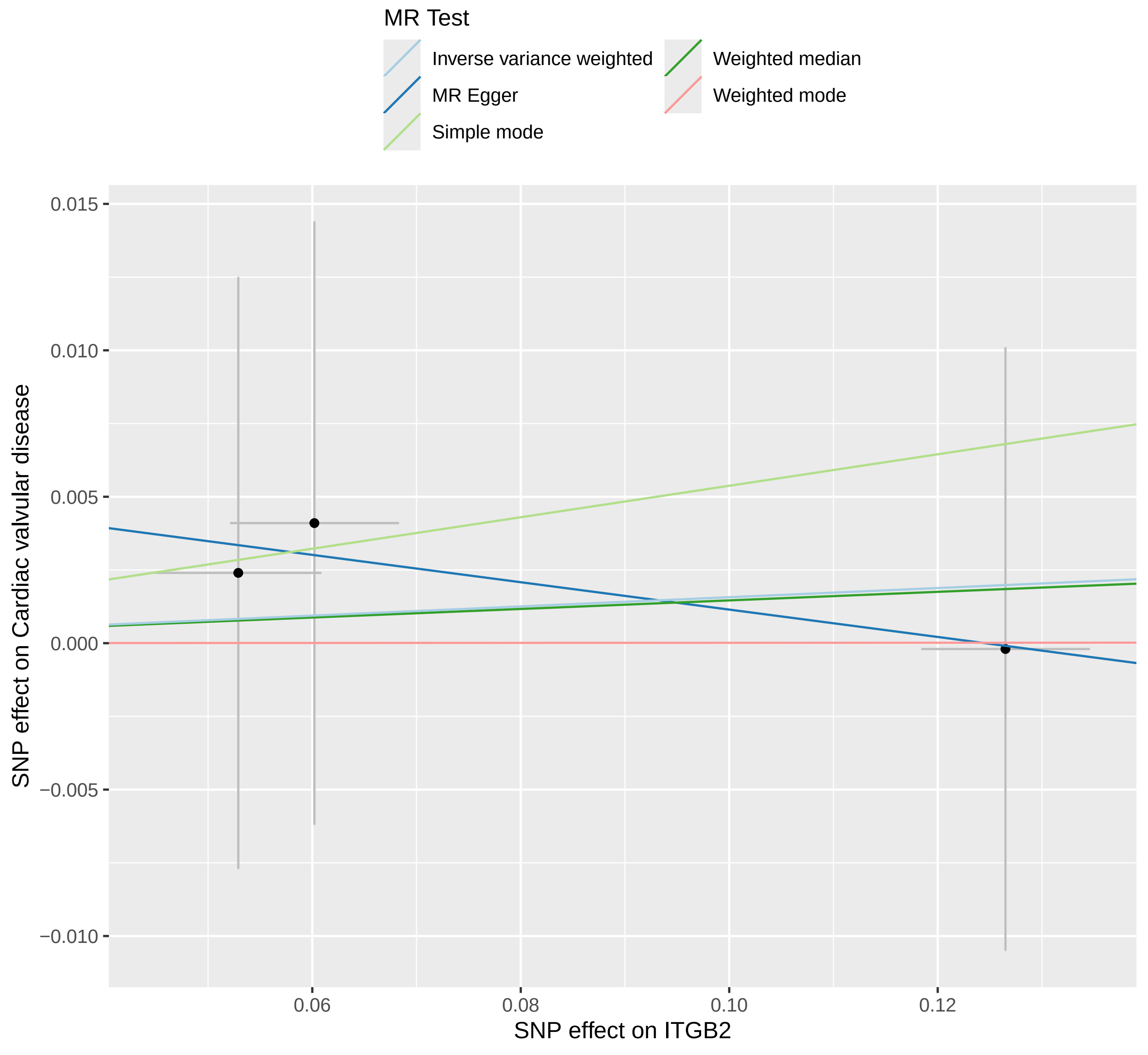

MR Test
Inverse variance weighted
MR Egger
Simple mode
Weighted median
Weighted mode
0.015
0.010
0.005
0.000
−0.005
−0.010
SNP effect on Cardiac valvular disease
0.06
0.08
0.10
0.12
SNP effect on ITGB2

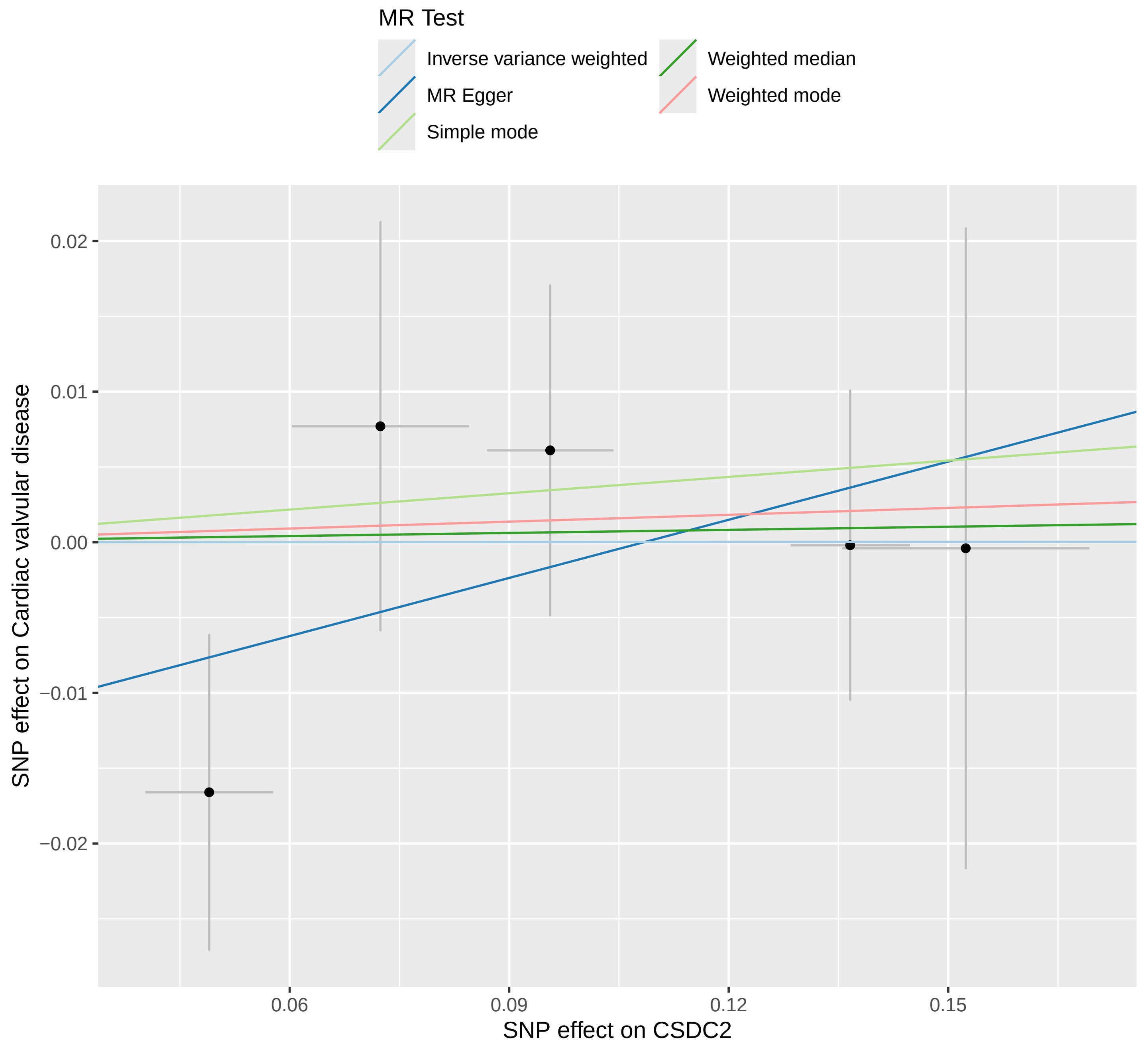

MR Test
Inverse variance weighted
MR Egger
Simple mode
Weighted median
Weighted mode
0.02
0.01
0.00
−0.01
−0.02
SNP effect on Cardiac valvular disease
0.06
0.09
0.12
0.15
SNP effect on CSDC2

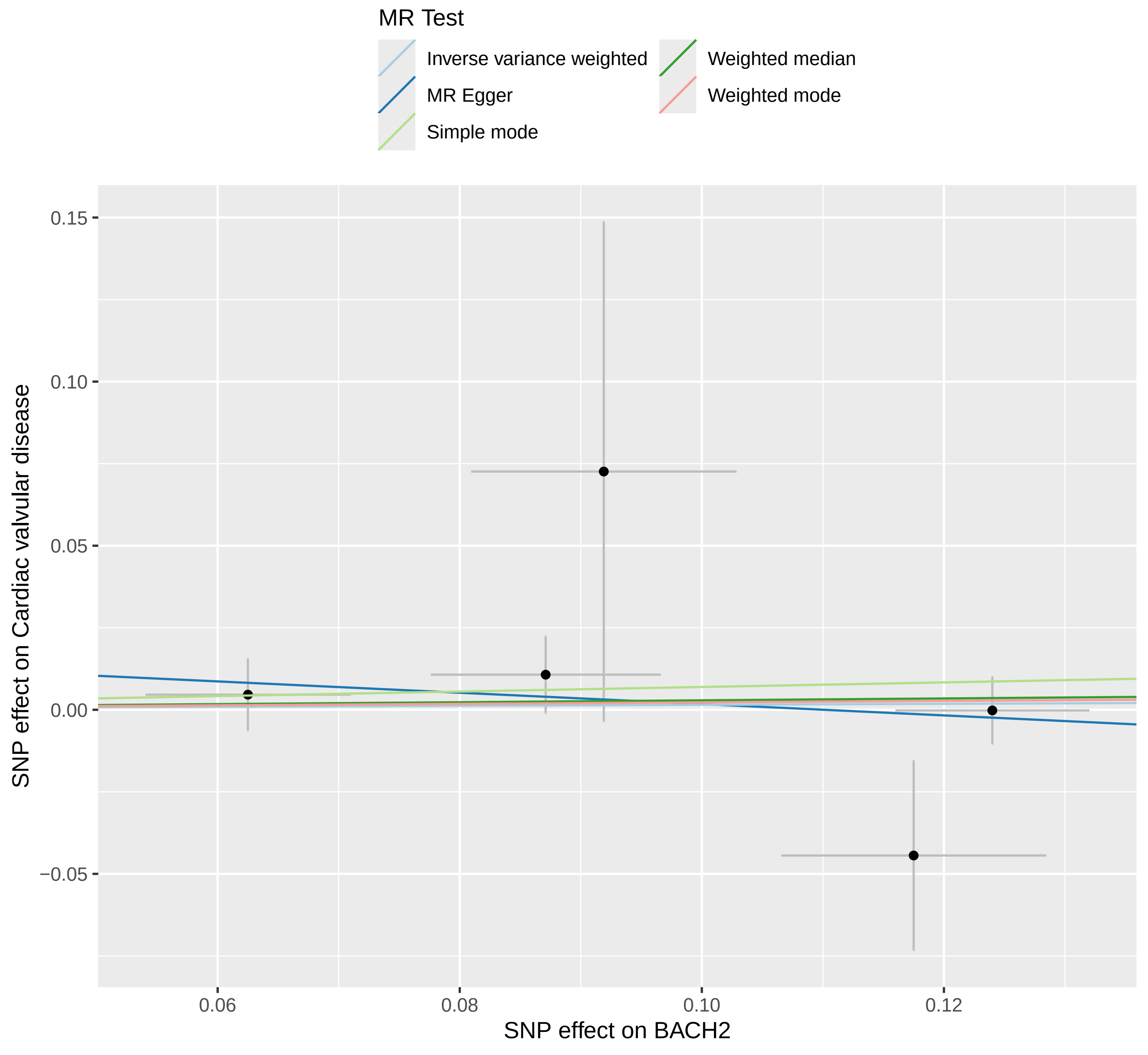
MR Test
Inverse variance weighted
MR Egger
Simple mode
Weighted median
Weighted mode
0.15
0.10
0.05
0.00
−0.05
0.06
0.08
0.10
0.12
SNP effect on Cardiac valvular disease
SNP effect on BACH2

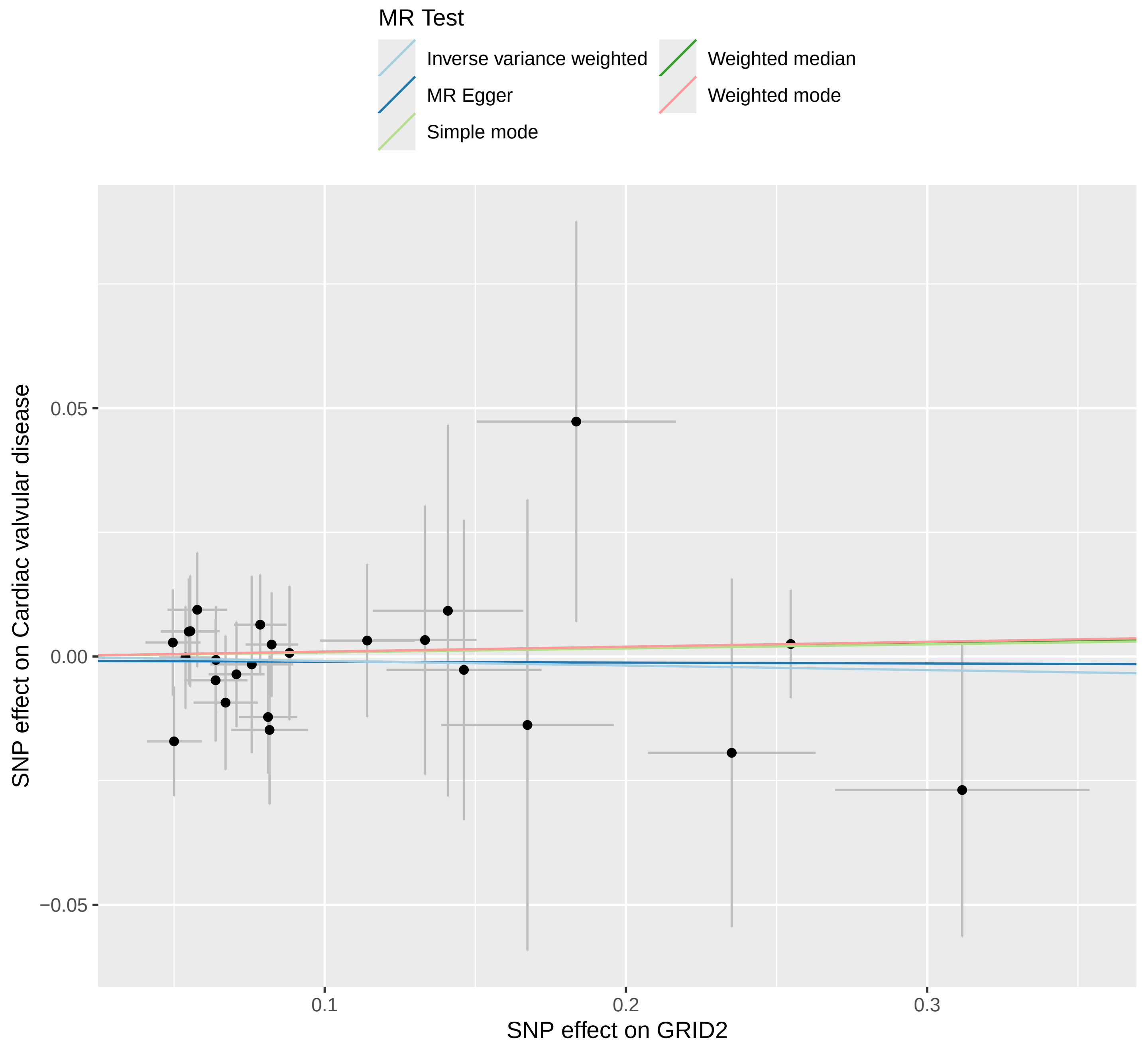
MR Test
Inverse variance weighted
MR Egger
Simple mode
Weighted median
Weighted mode
SNP effect on Cardiac valvular disease
0.05
0.00
−0.05
0.1
0.2
0.3
SNP effect on GRID2

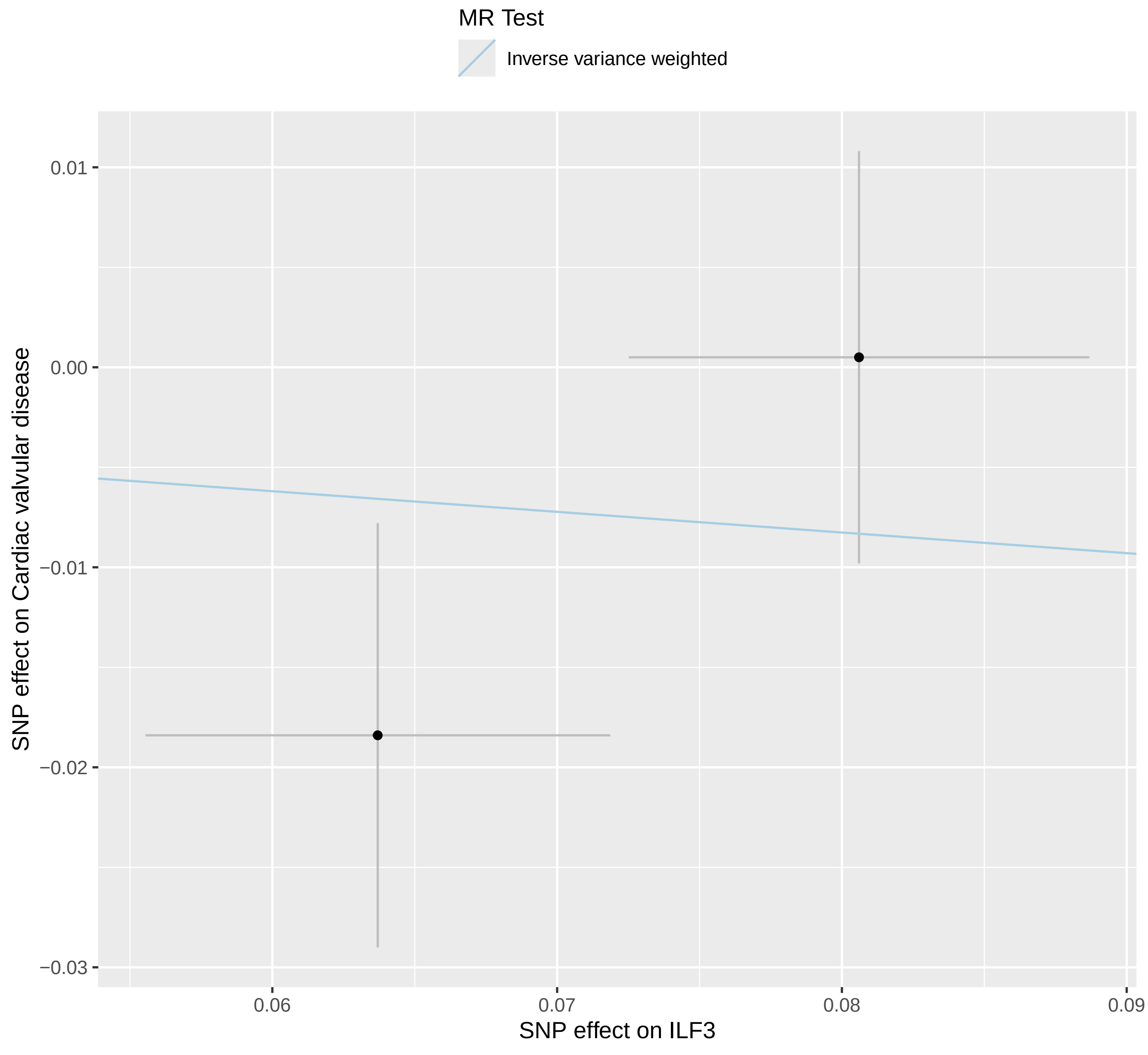

MR Test
Inverse variance weighted
SNP effect on Cardiac valvular disease
0.01
0.00
−0.01
−0.02
−0.03
0.06
0.07
0.08
0.09
SNP effect on ILF3

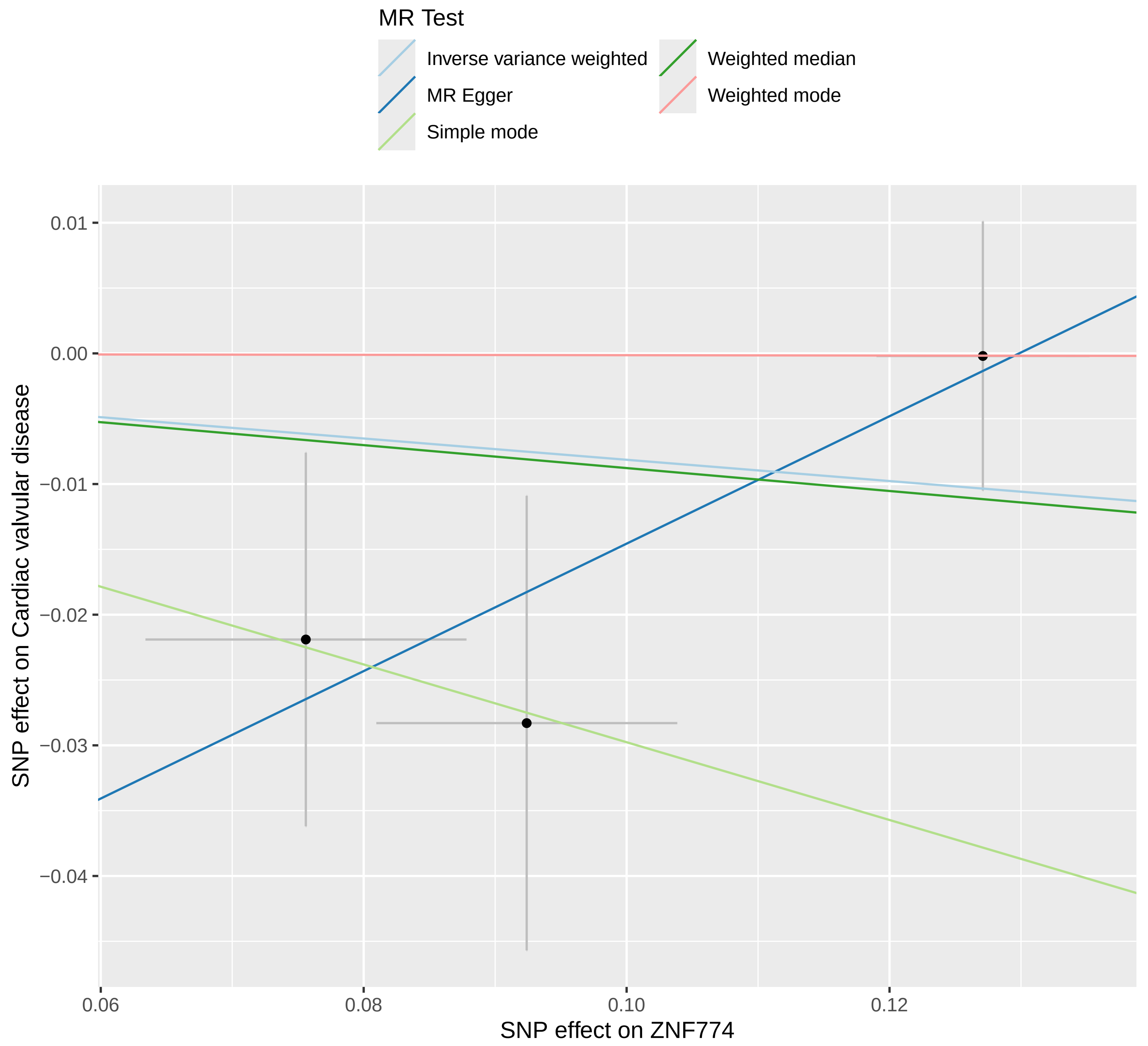

MR Test
Inverse variance weighted
MR Egger
Simple mode
Weighted median
Weighted mode
0.01
0.00
−0.01
−0.02
−0.03
−0.04
SNP effect on Cardiac valvular disease
0.06
0.08
0.10
0.12
SNP effect on ZNF774

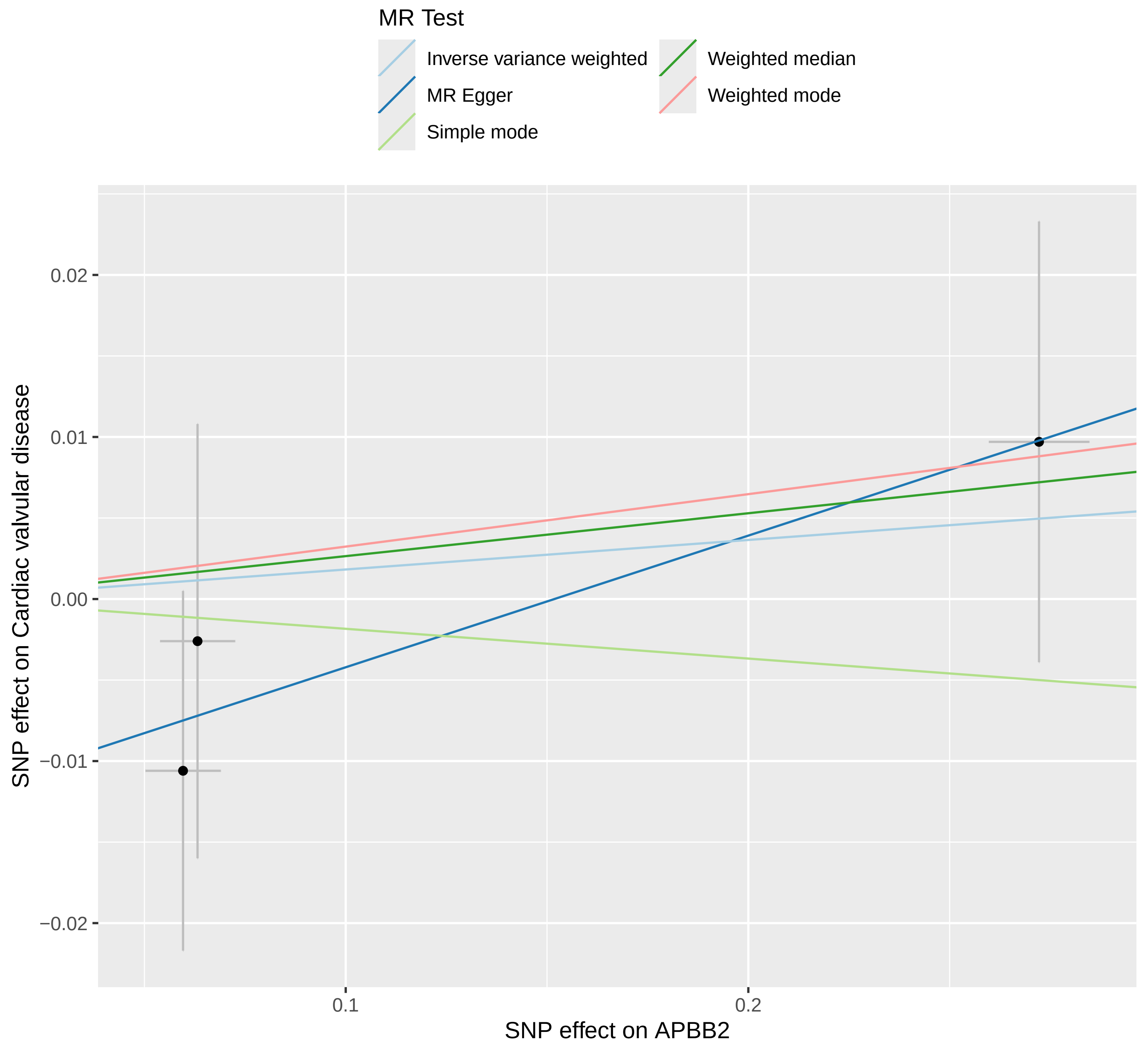

MR Test
Inverse variance weighted
MR Egger
Simple mode
Weighted median
Weighted mode
0.02
0.01
0.00
−0.01
−0.02
SNP effect on Cardiac valvular disease
0.1
0.2
SNP effect on APBB2

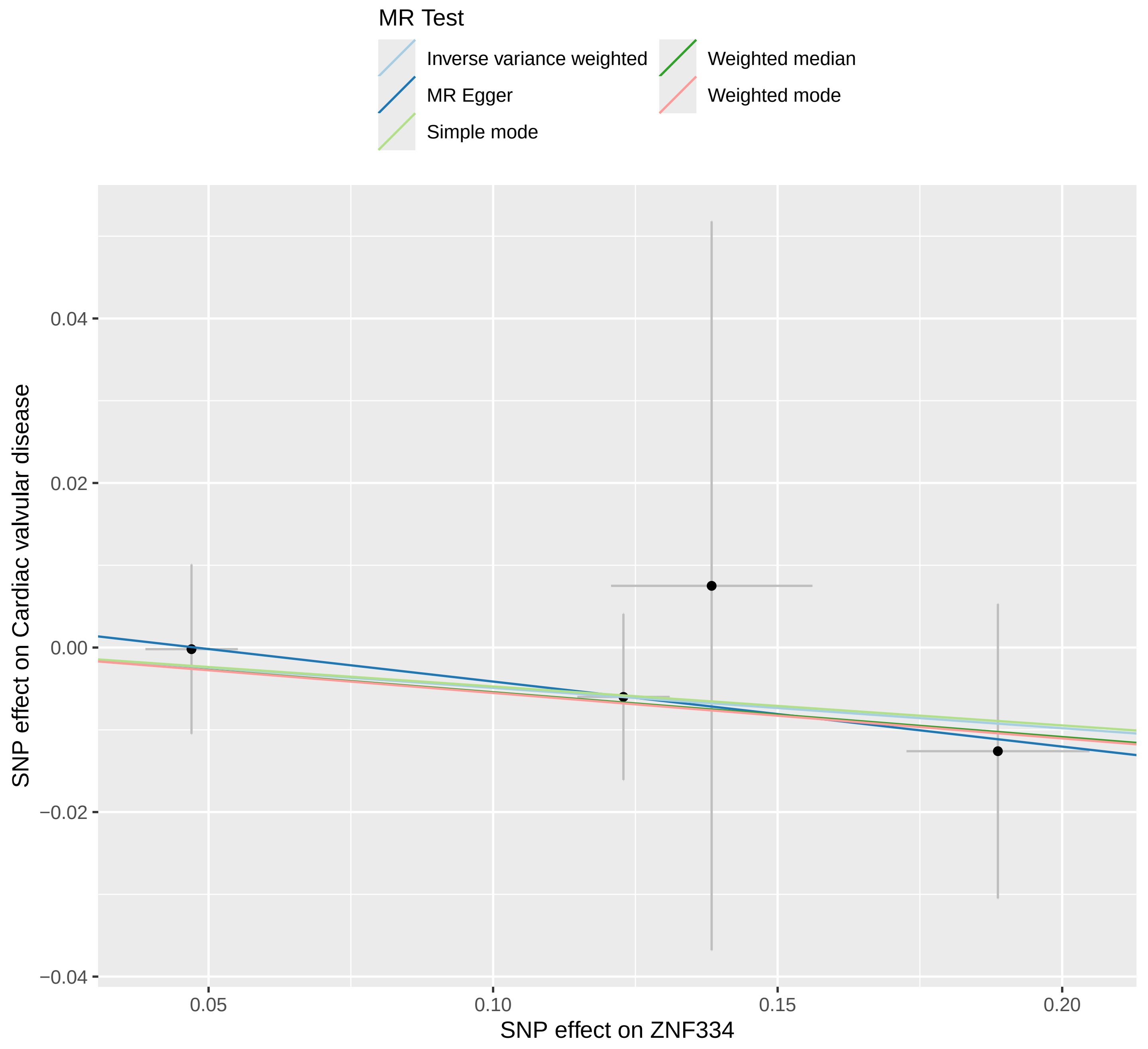

MR Test
Inverse variance weighted
MR Egger
Simple mode
Weighted median
Weighted mode
0.04
0.02
0.00
−0.02
−0.04
0.05
0.10
0.15
0.20
SNP effect on Cardiac valvular disease
SNP effect on ZNF334

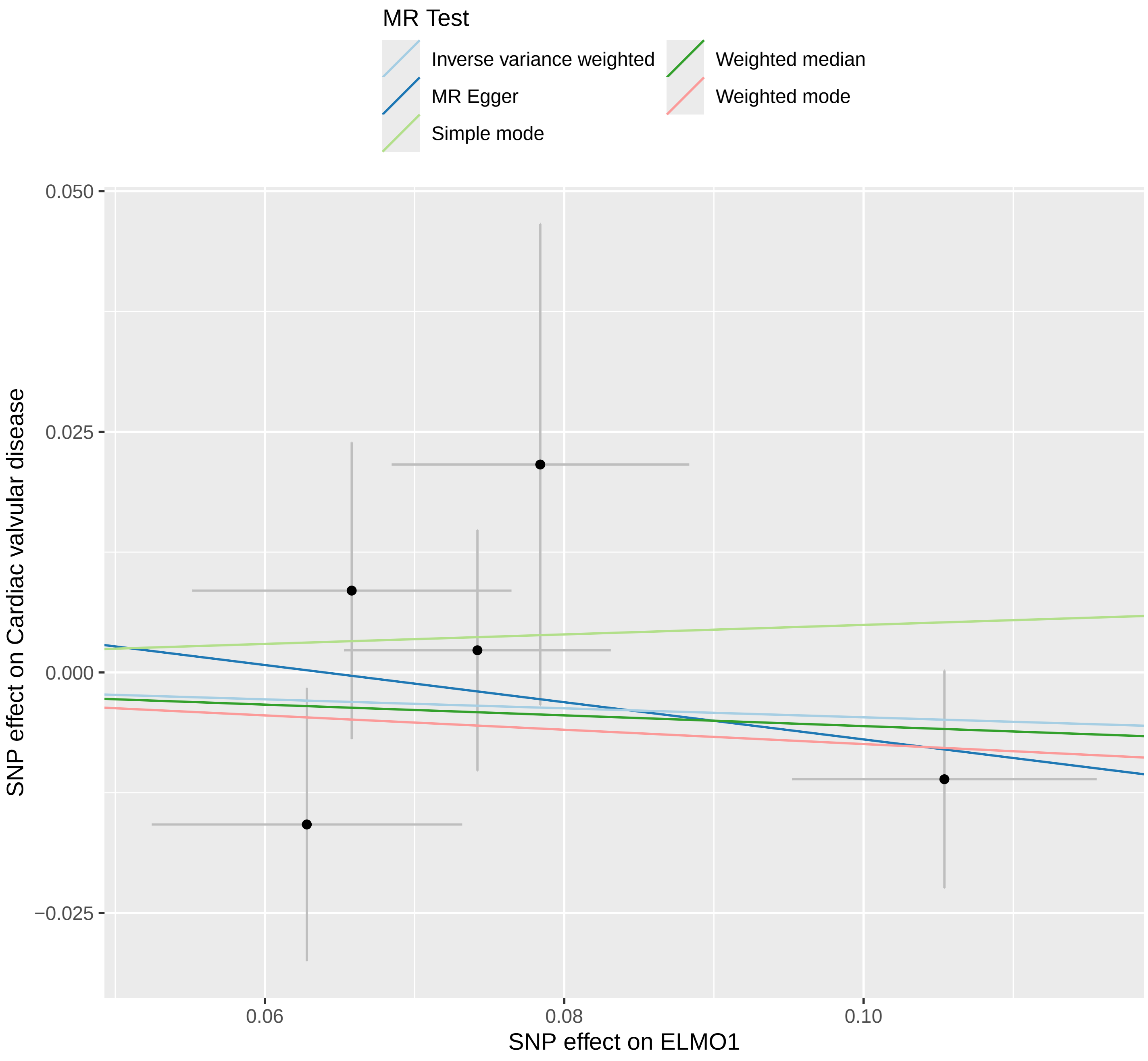

MR Test
Inverse variance weighted
MR Egger
Simple mode
Weighted median
Weighted mode
0.050
0.025
0.000
−0.025
SNP effect on Cardiac valvular disease
0.06
0.08
0.10
SNP effect on ELMO1

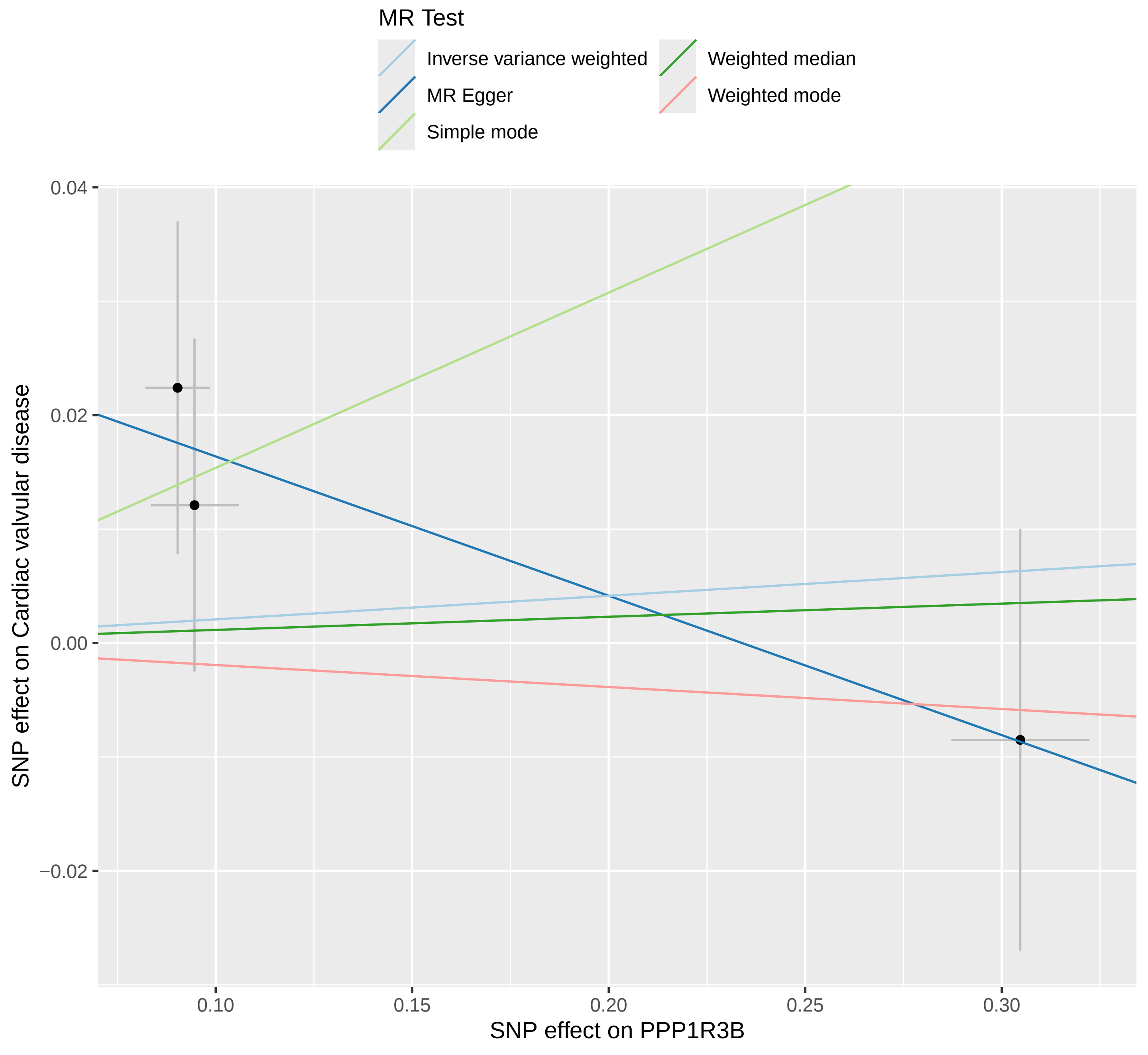
MR Test
Inverse variance weighted
MR Egger
Simple mode
Weighted median
Weighted mode
0.04
0.02
0.00
−0.02
SNP effect on Cardiac valvular disease
0.10
0.15
0.20
0.25
0.30
SNP effect on PPP1R3B

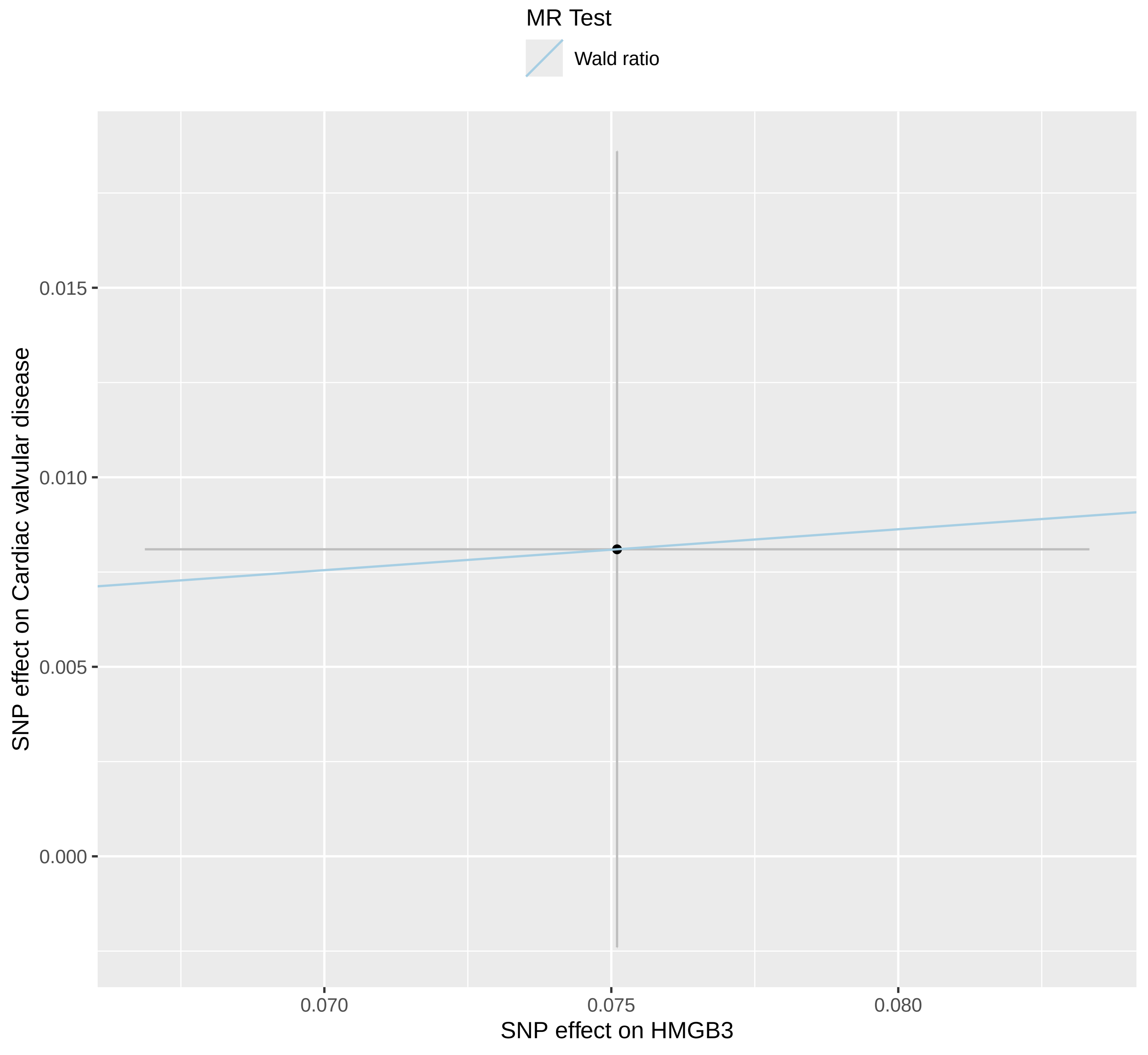

MR Test
Wald ratio
0.015
0.010
0.005
0.000
SNP effect on Cardiac valvular disease
0.070
0.075
0.080
SNP effect on HMGB3

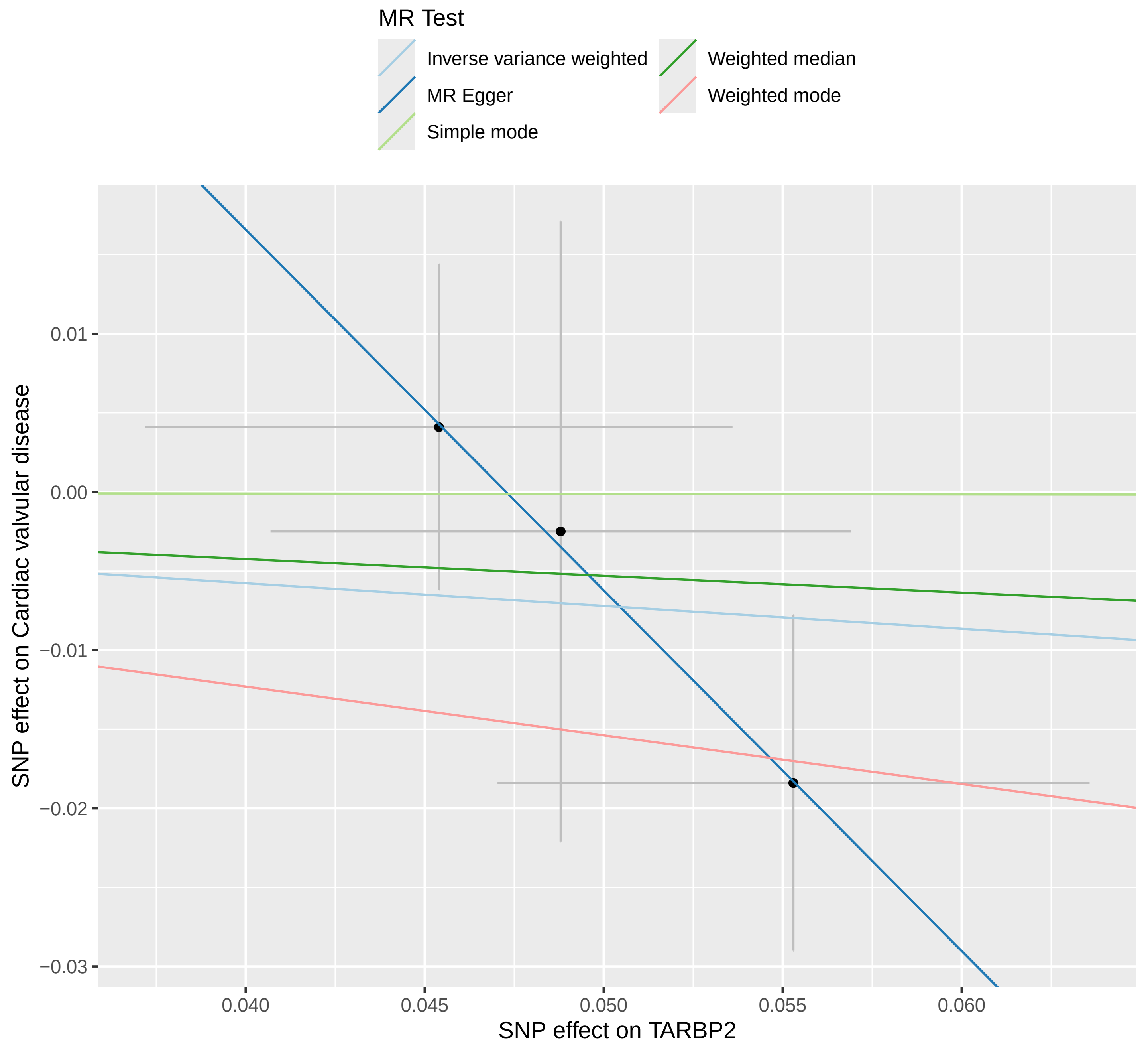
MR Test
Inverse variance weighted
MR Egger
Simple mode
Weighted median
Weighted mode
0.01
0.00
−0.01
−0.02
−0.03
SNP effect on Cardiac valvular disease
0.040
0.045
0.050
0.055
0.060
SNP effect on TARBP2

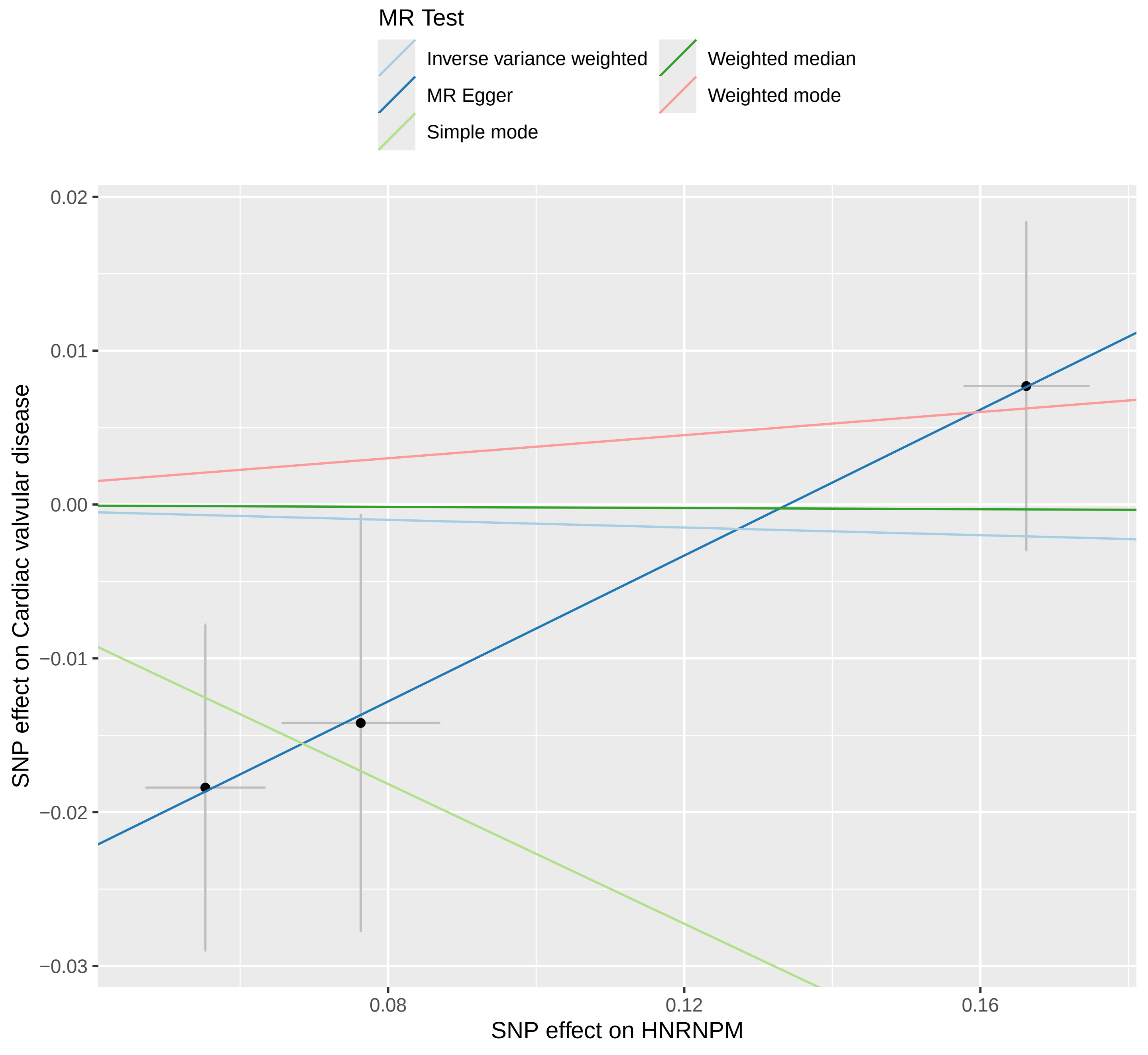
MR Test
Inverse variance weighted
MR Egger
Simple mode
Weighted median
Weighted mode
0.02
0.01
0.00
−0.01
−0.02
−0.03
SNP effect on Cardiac valvular disease
0.08
0.12
0.16
SNP effect on HNRNPM

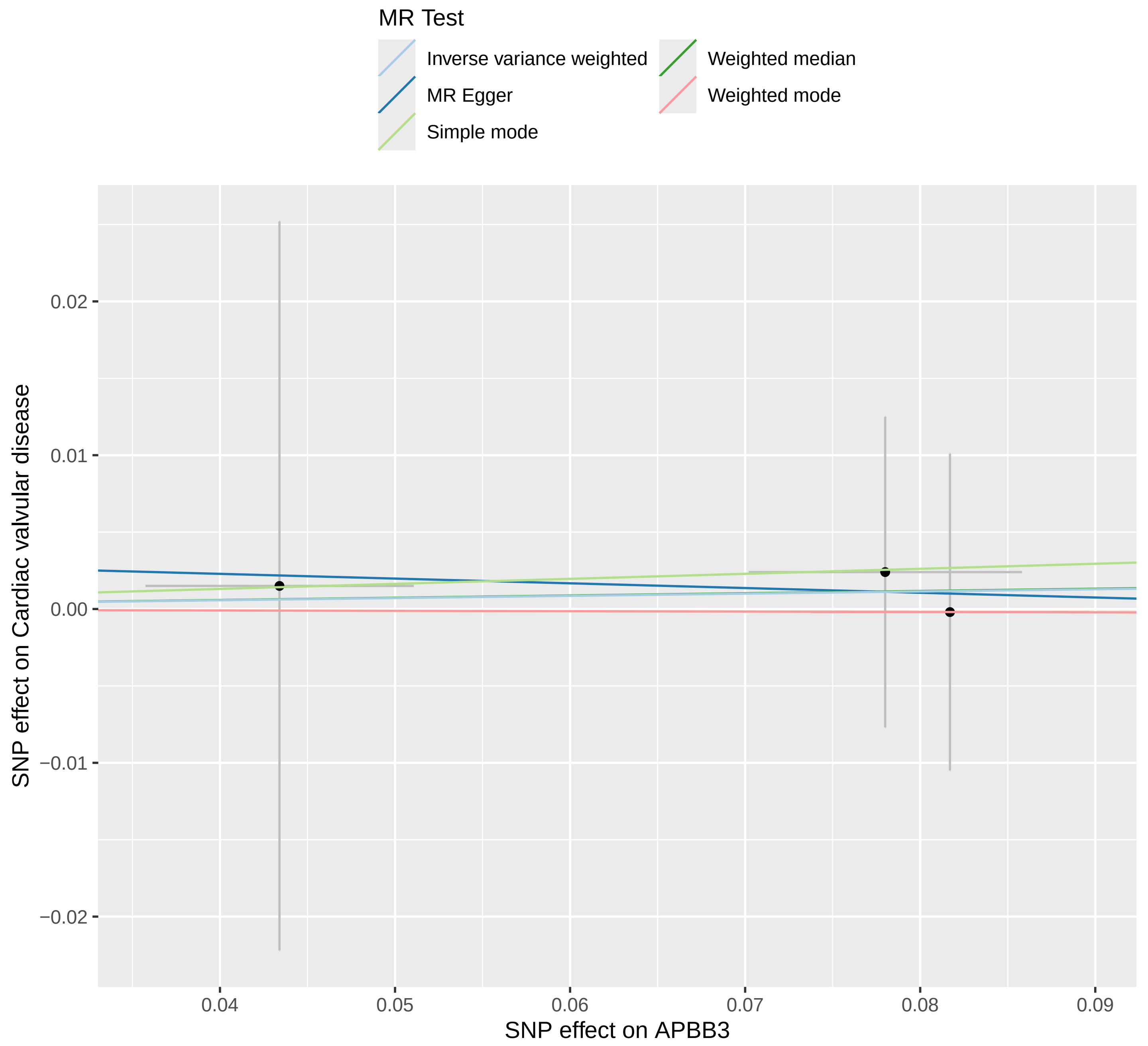
MR Test
Inverse variance weighted
MR Egger
Simple mode
Weighted median
Weighted mode
0.02
0.01
0.00
−0.01
−0.02
SNP effect on Cardiac valvular disease
0.04
0.05
0.06
0.07
0.08
0.09
SNP effect on APBB3

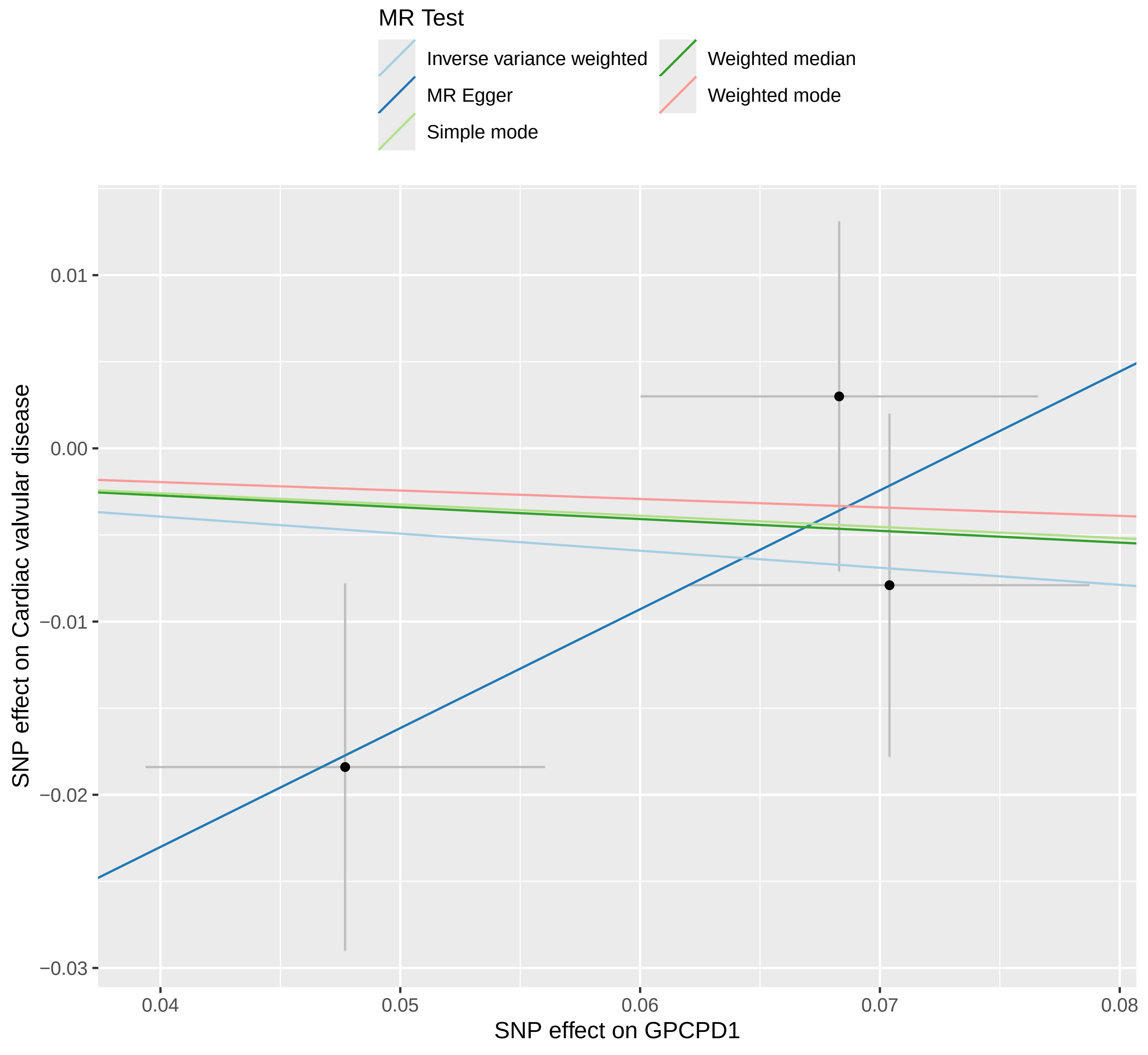

MR Test
Inverse variance weighted
MR Egger
Simple mode
Weighted median
Weighted mode
SNP effect on Cardiac valvular disease
0.01
0.00
−0.01
−0.02
−0.03
0.04
0.05
0.06
0.07
0.08
SNP effect on GPCPD1

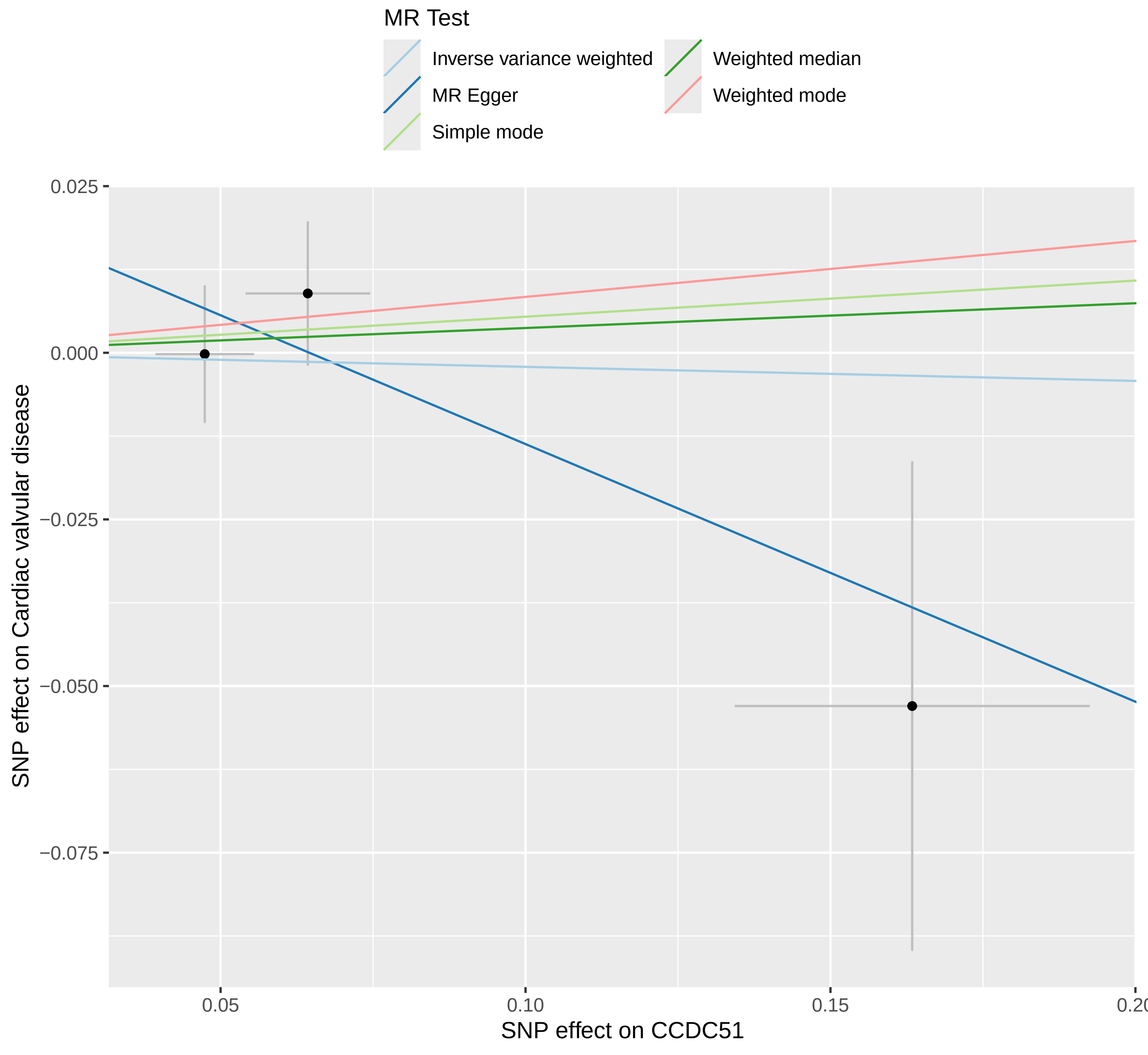

MR Test
Inverse variance weighted
MR Egger
Simple mode
Weighted median
Weighted mode
0.025
0.000
−0.025
−0.050
−0.075
0.05
0.10
0.15
0.20
SNP effect on CCDC51
SNP effect on Cardiac valvular disease

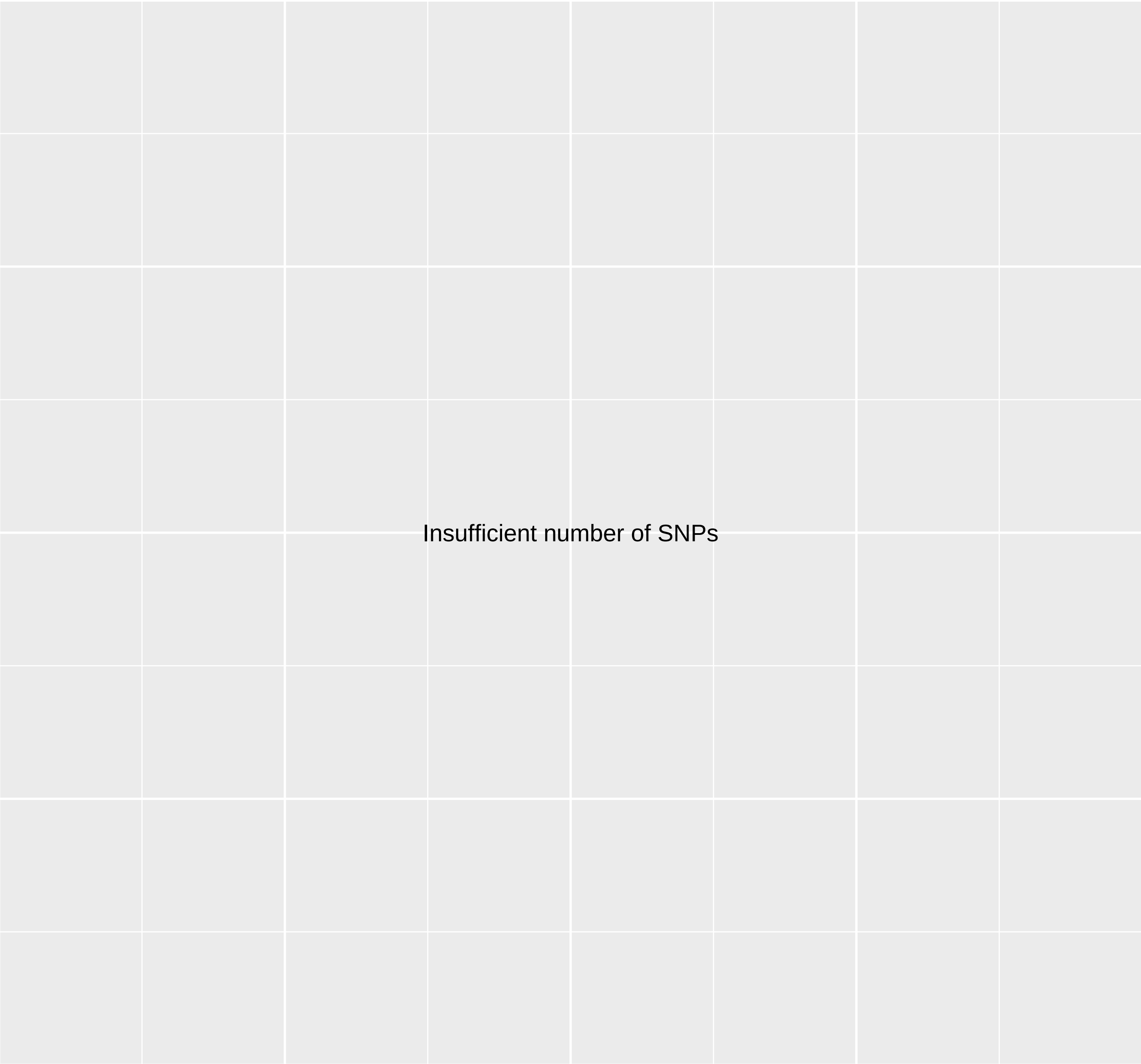
Insufficient number of SNPs

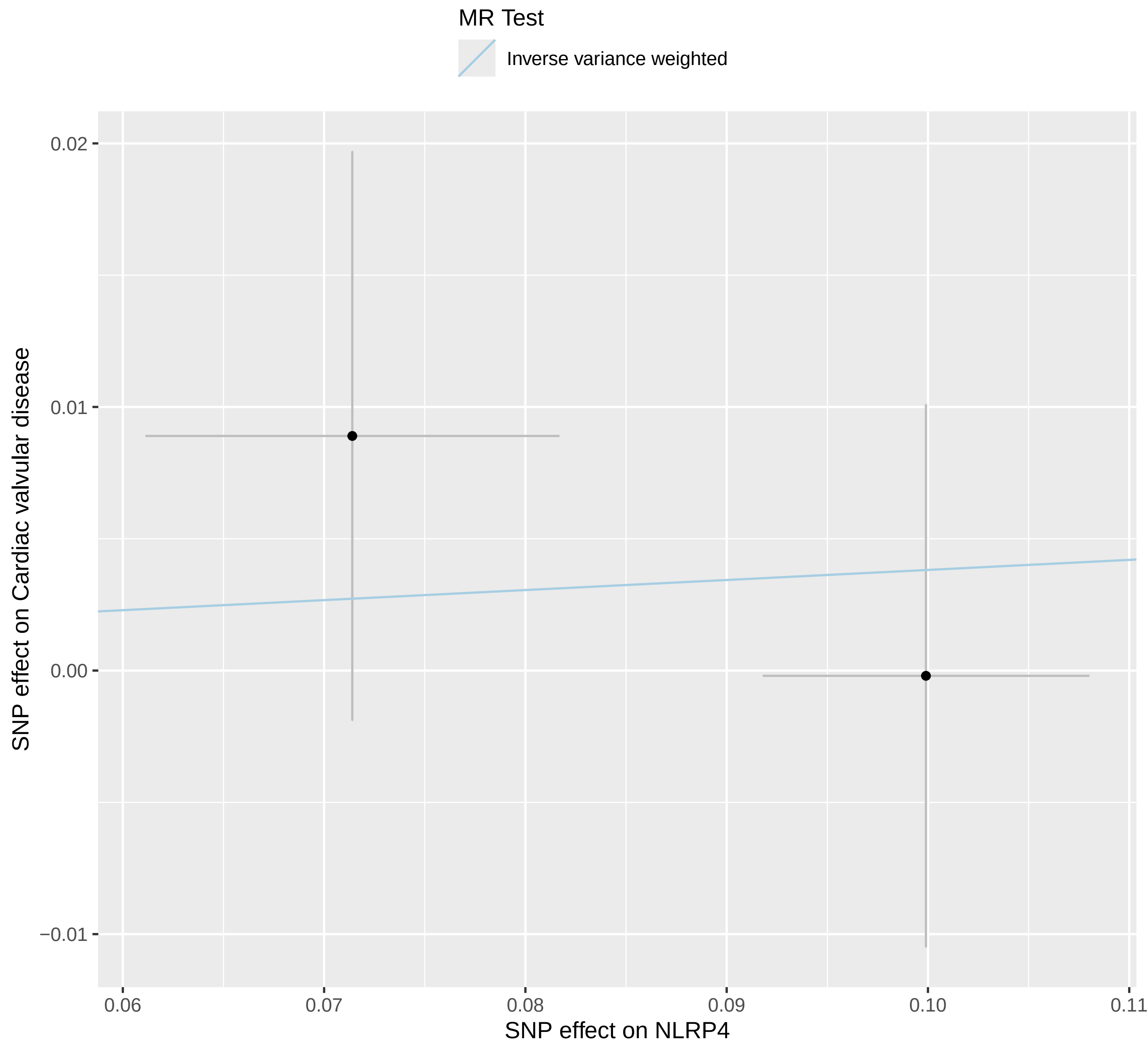
MR Test
Inverse variance weighted
0.02
0.01
0.00
−0.01
0.06
0.07
0.08
0.09
0.10
0.11
SNP effect on NLRP4
SNP effect on Cardiac valvular disease

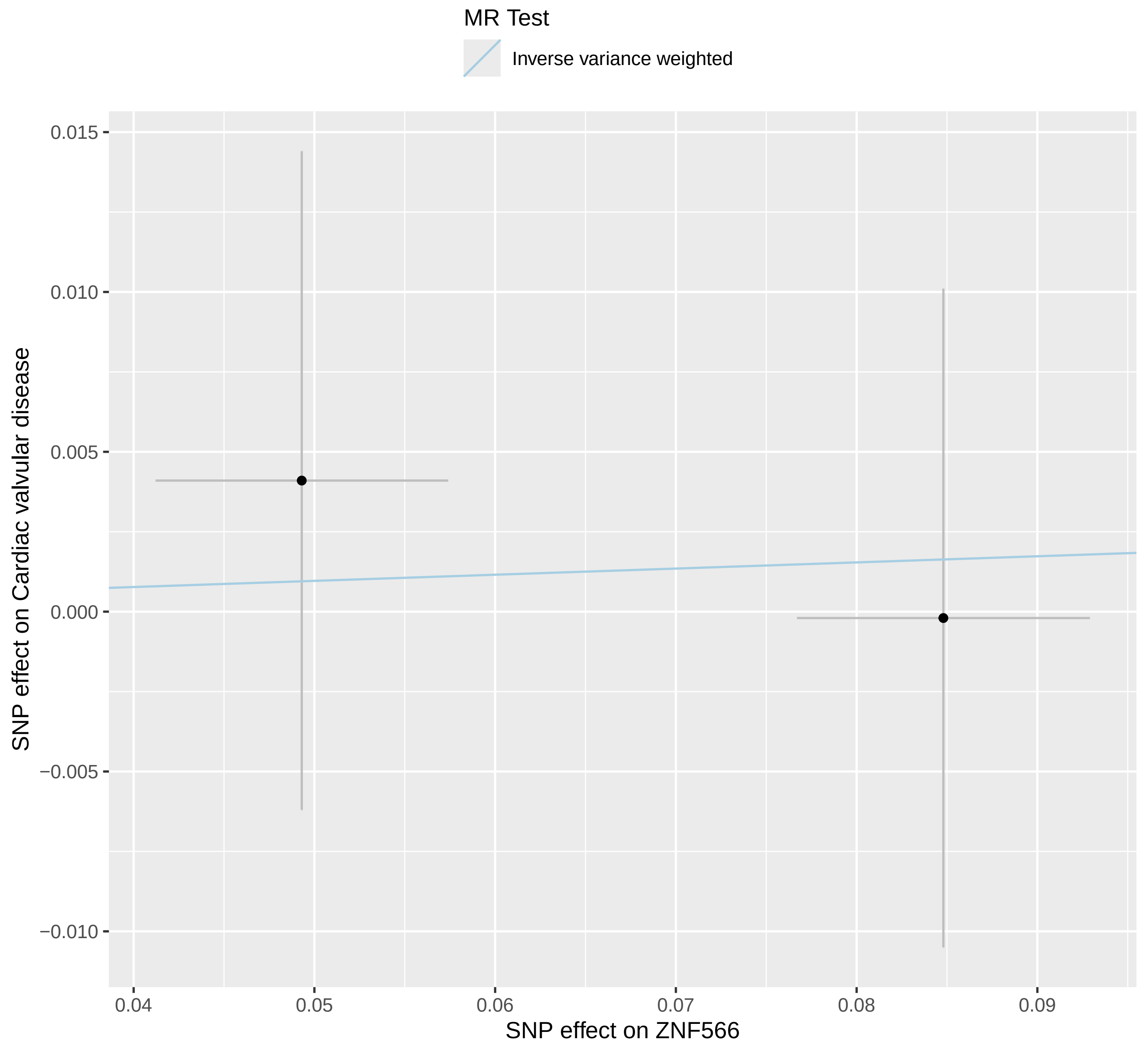
MR Test
Inverse variance weighted
0.015
0.010
0.005
0.000
−0.005
−0.010
0.04
0.05
0.06
0.07
0.08
0.09
SNP effect on ZNF566
SNP effect on Cardiac valvular disease

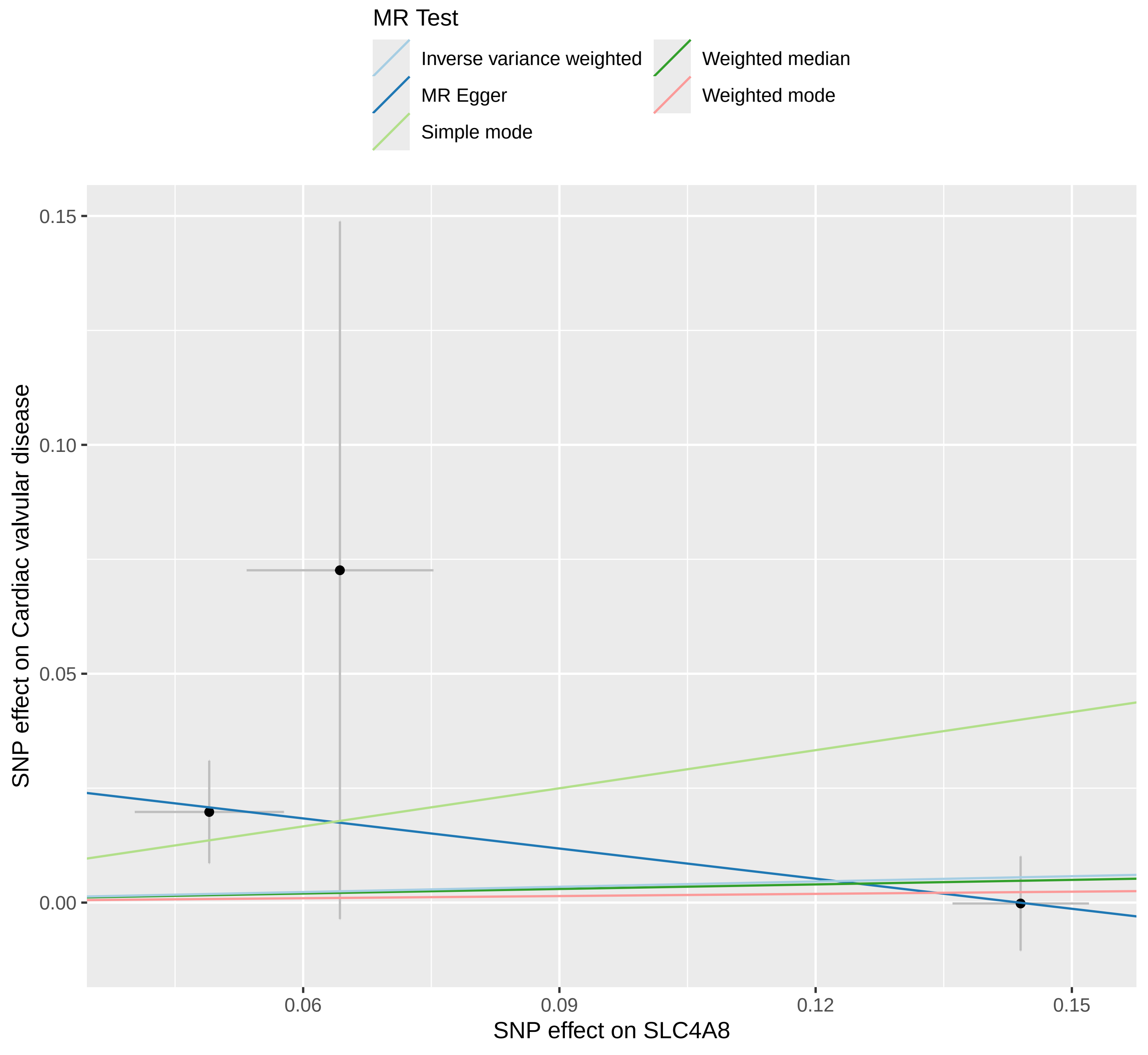

MR Test
Inverse variance weighted
MR Egger
Simple mode
Weighted median
Weighted mode
0.15
0.10
0.05
0.00
SNP effect on Cardiac valvular disease
0.06
0.09
0.12
0.15
SNP effect on SLC4A8

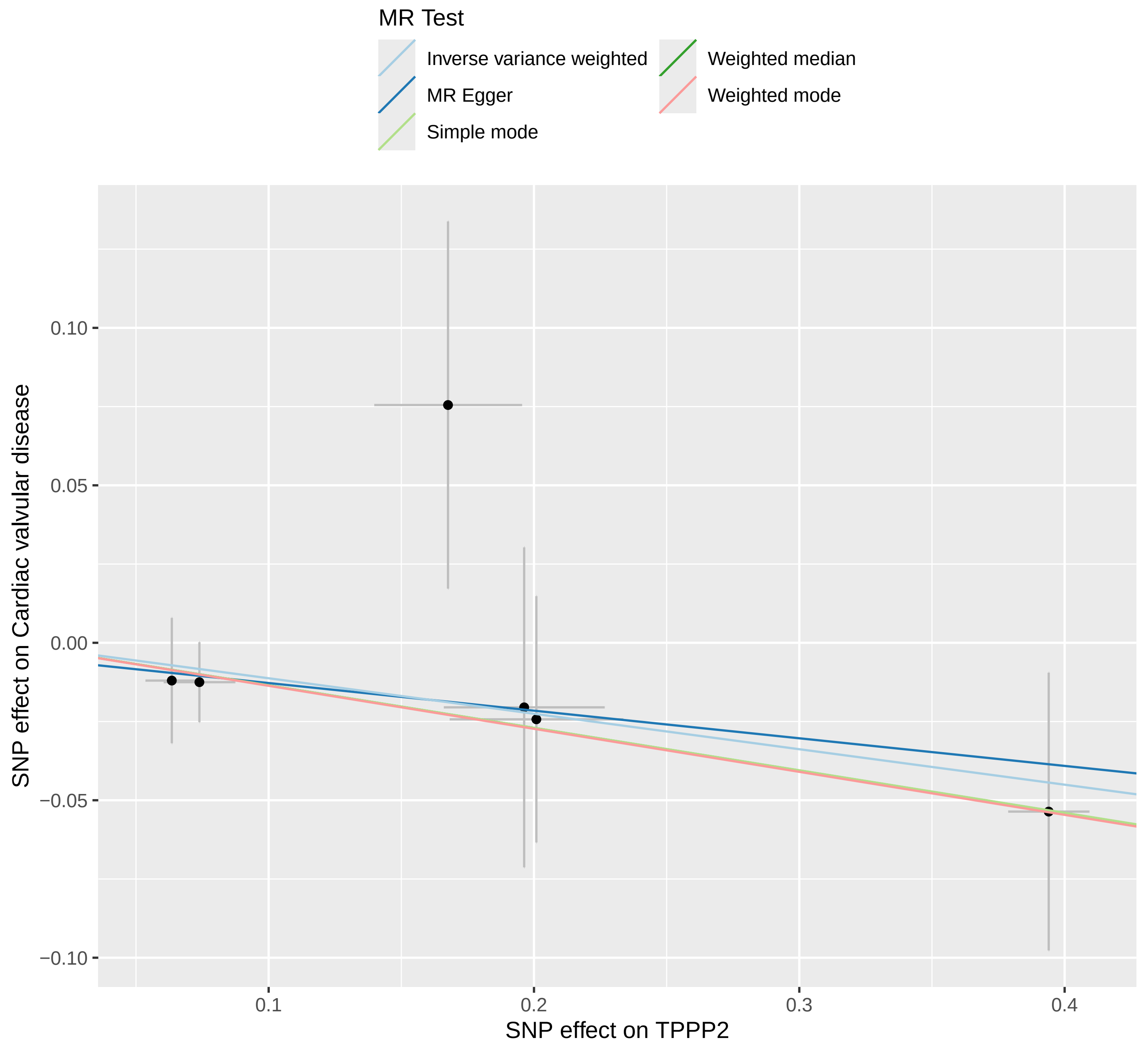
MR Test
Inverse variance weighted
MR Egger
Simple mode
Weighted median
Weighted mode
0.10
0.05
0.00
−0.05
−0.10
SNP effect on Cardiac valvular disease
0.1
0.2
0.3
0.4
SNP effect on TPPP2

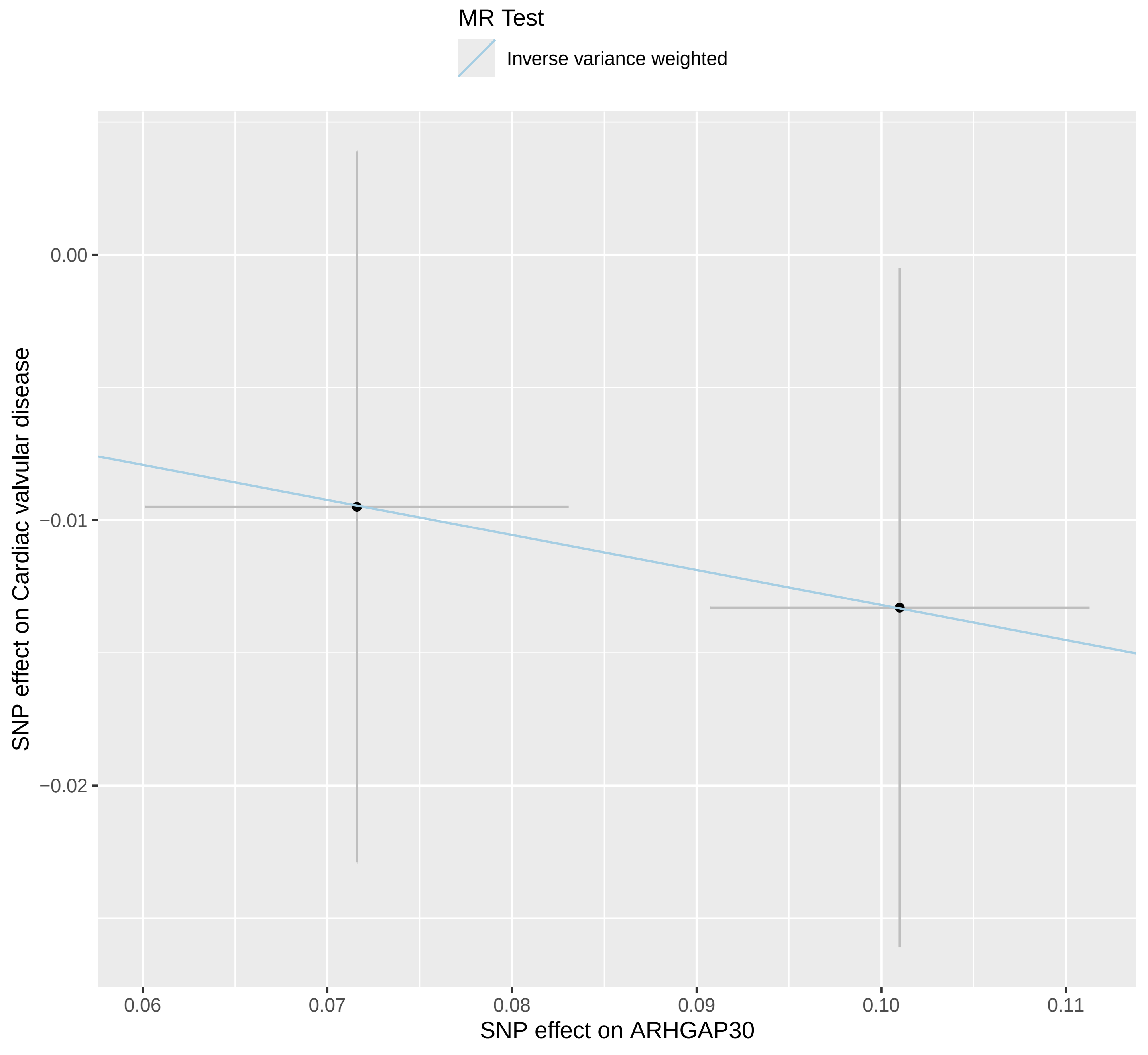
MR Test
Inverse variance weighted
0.00
−0.01
−0.02
SNP effect on Cardiac valvular disease
0.06
0.07
0.08
0.09
0.10
0.11
SNP effect on ARHGAP30

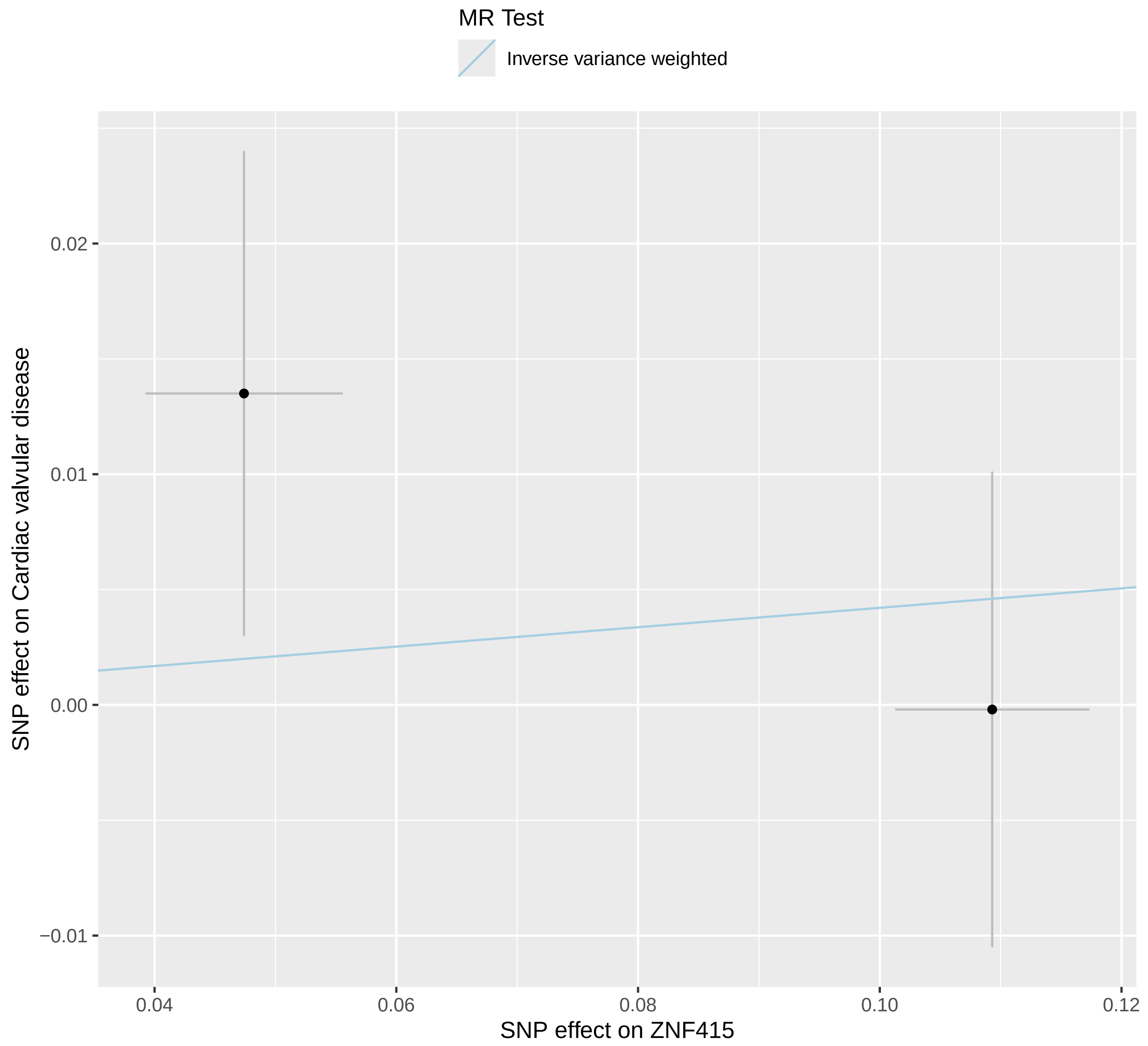
MR Test
Inverse variance weighted
0.02
0.01
0.00
−0.01
SNP effect on Cardiac valvular disease
0.04
0.06
0.08
0.10
0.12
SNP effect on ZNF415

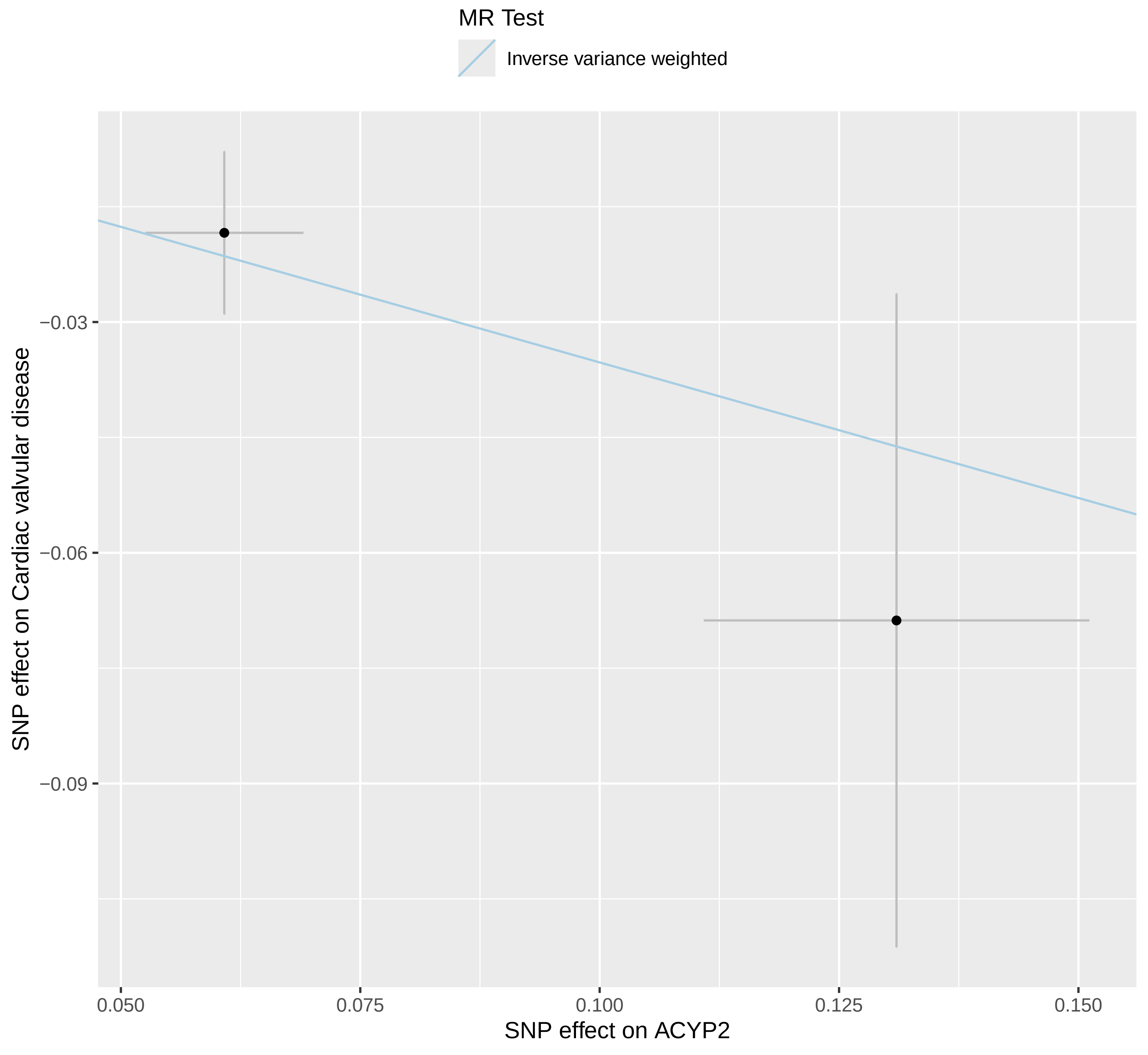

MR Test
Inverse variance weighted
−0.03
−0.06
−0.09
0.050
0.075
0.100
0.125
0.150
SNP effect on Cardiac valvular disease
SNP effect on ACYP2

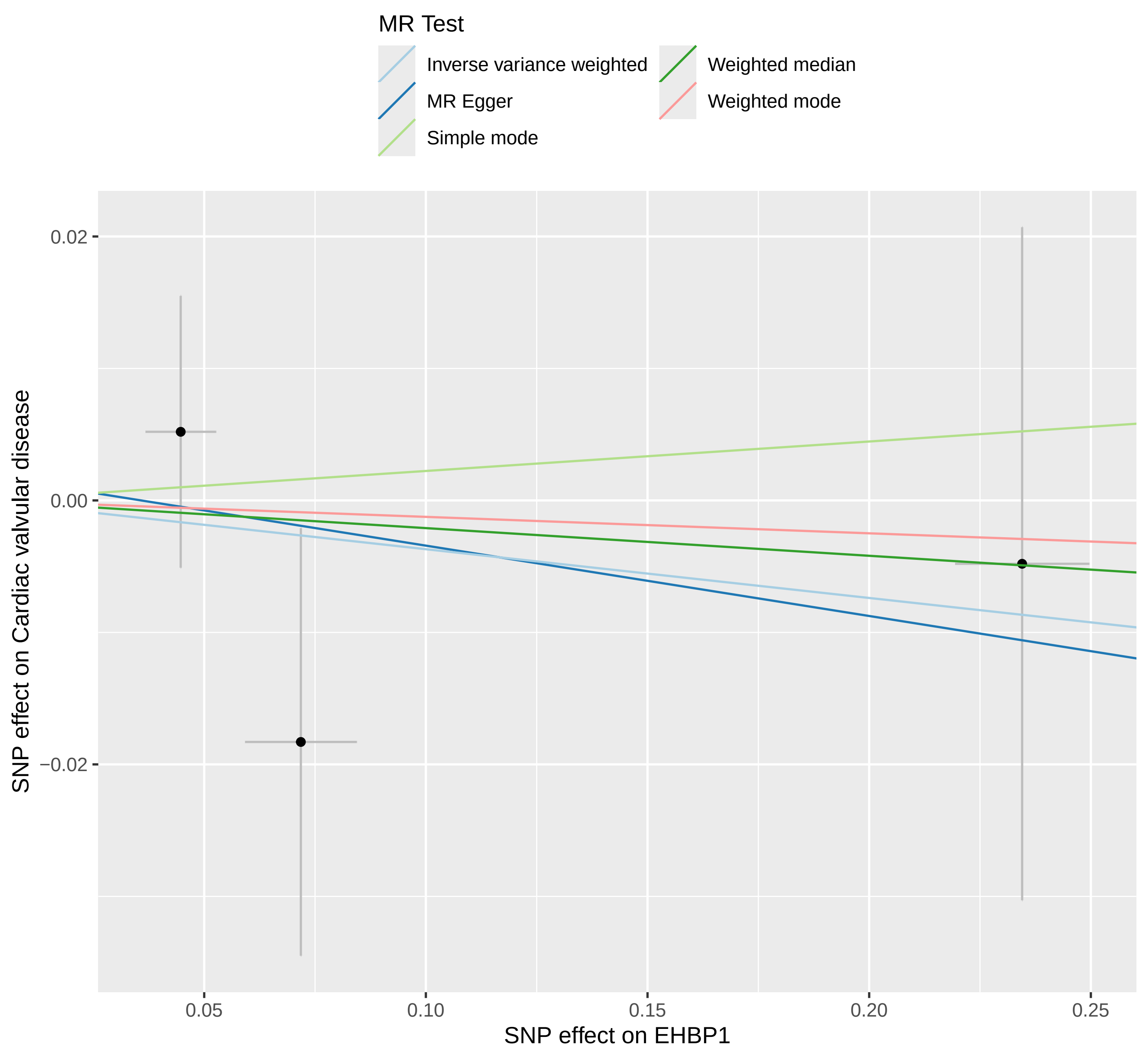

MR Test
Inverse variance weighted
MR Egger
Simple mode
Weighted median
Weighted mode
0.02
0.00
−0.02
0.05
0.10
0.15
0.20
0.25
SNP effect on EHBP1
SNP effect on Cardiac valvular disease

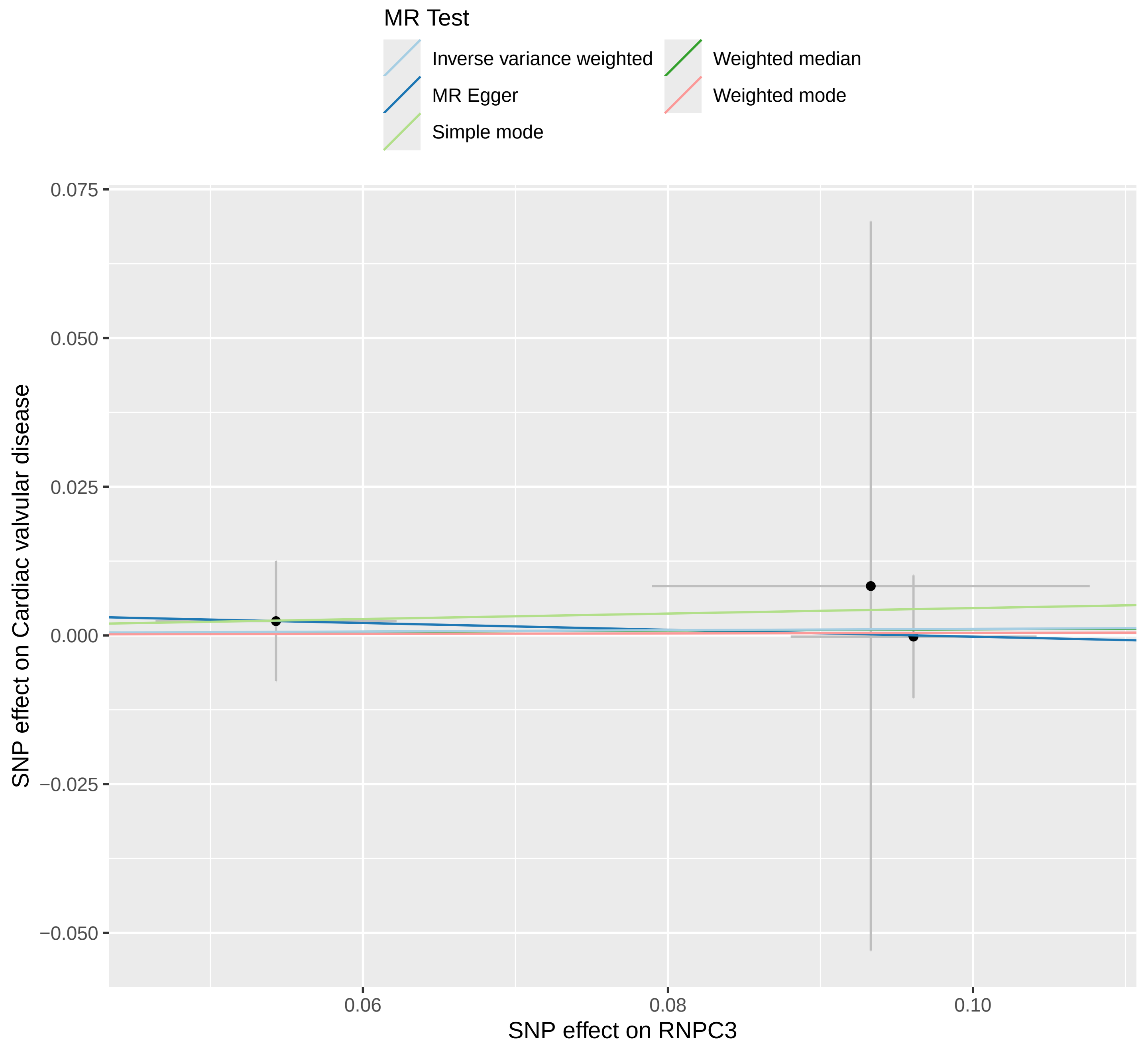

MR Test
Inverse variance weighted
MR Egger
Simple mode
Weighted median
Weighted mode
0.075
0.050
0.025
0.000
−0.025
−0.050
SNP effect on Cardiac valvular disease
0.06
0.08
0.10
SNP effect on RNPC3

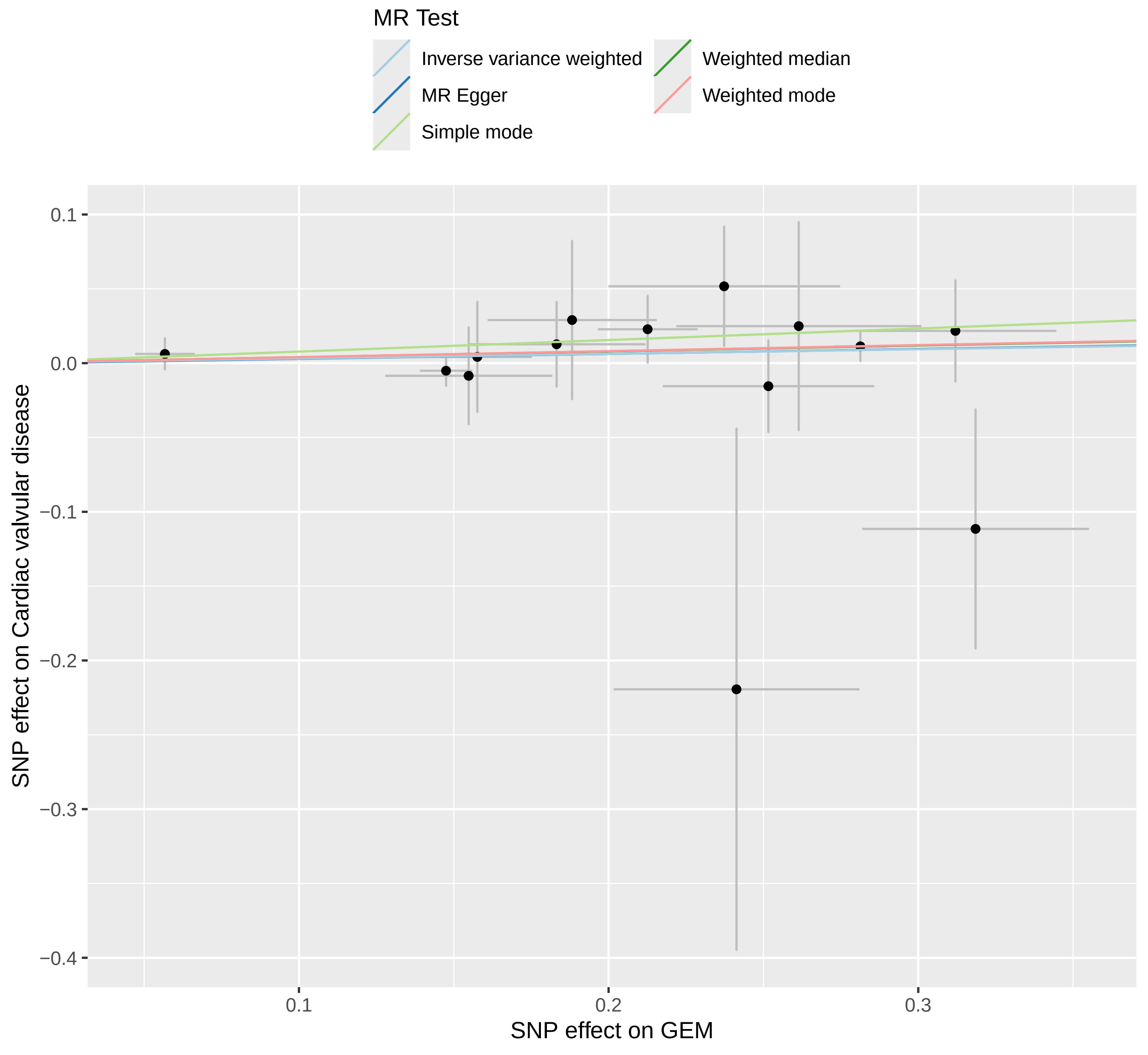

MR Test
Inverse variance weighted
MR Egger
Simple mode
Weighted median
Weighted mode
0.1
0.0
−0.1
−0.2
−0.3
−0.4
SNP effect on Cardiac valvular disease
0.1
0.2
0.3
SNP effect on GEM

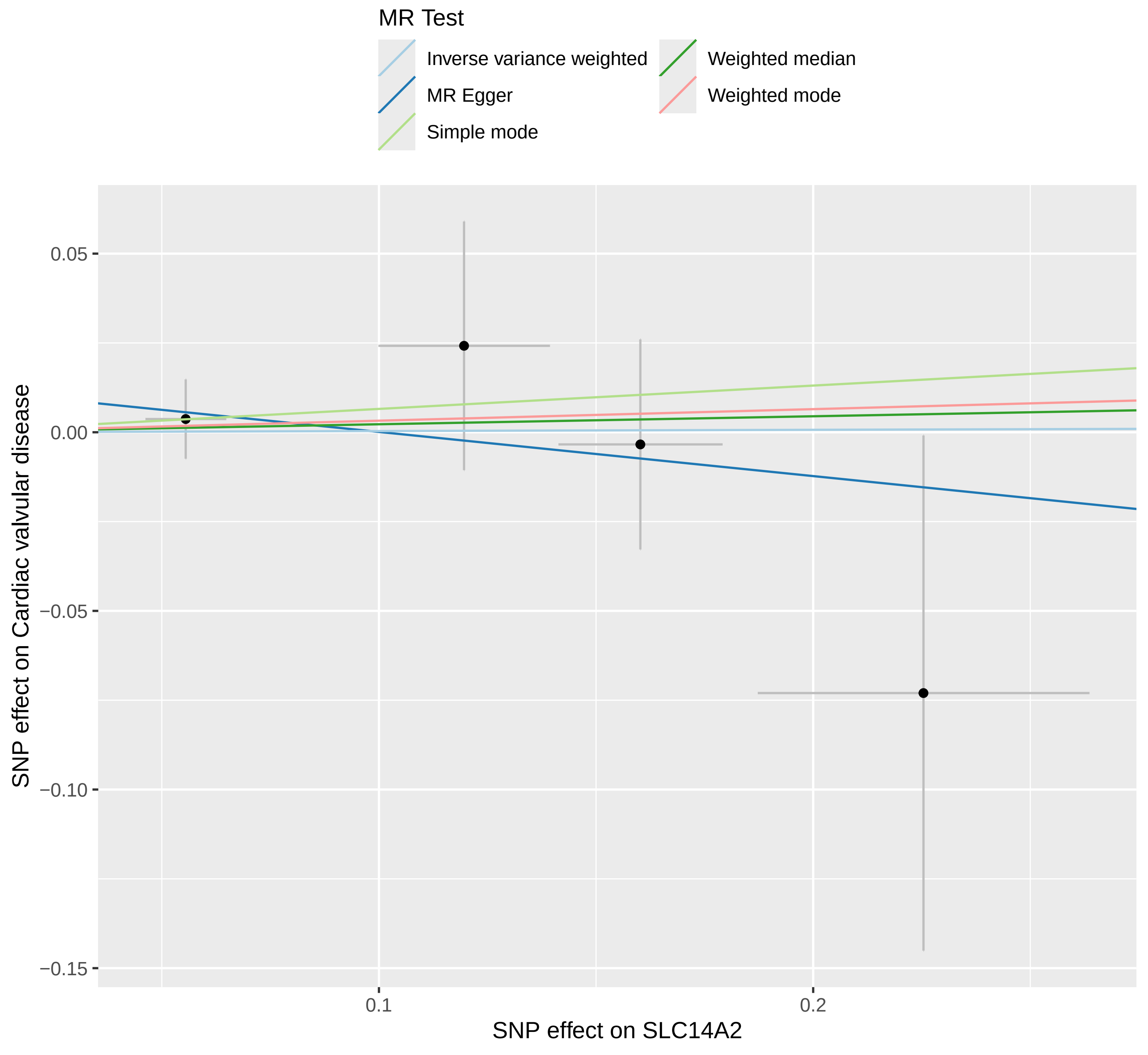
MR Test
Inverse variance weighted
MR Egger
Simple mode
Weighted median
Weighted mode
0.05
0.00
−0.05
−0.10
−0.15
0.1
0.2
SNP effect on Cardiac valvular disease
SNP effect on SLC14A2

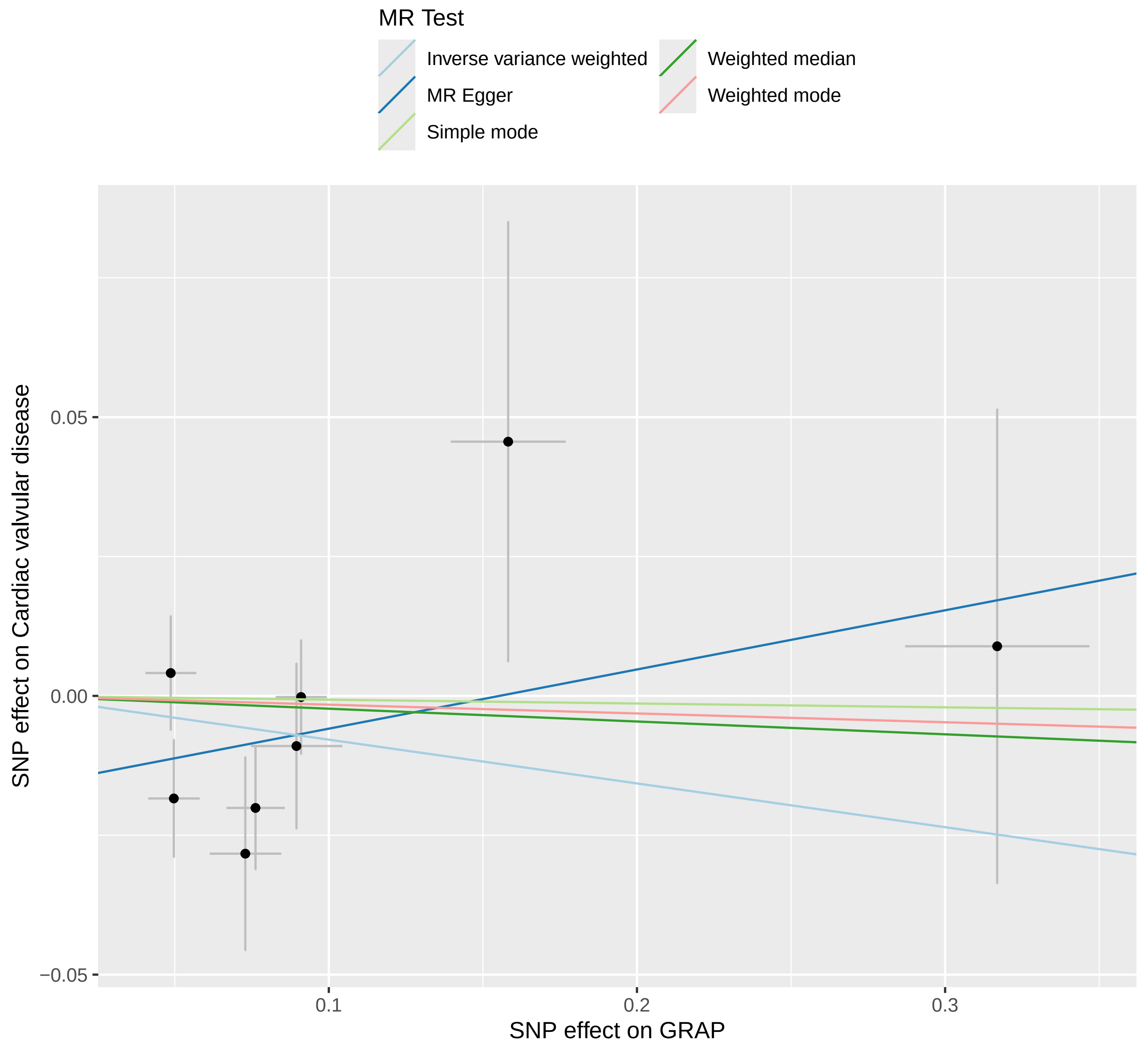

MR Test
Inverse variance weighted
MR Egger
Simple mode
Weighted median
Weighted mode
SNP effect on Cardiac valvular disease
0.05
0.00
−0.05
0.1
0.2
0.3
SNP effect on GRAP

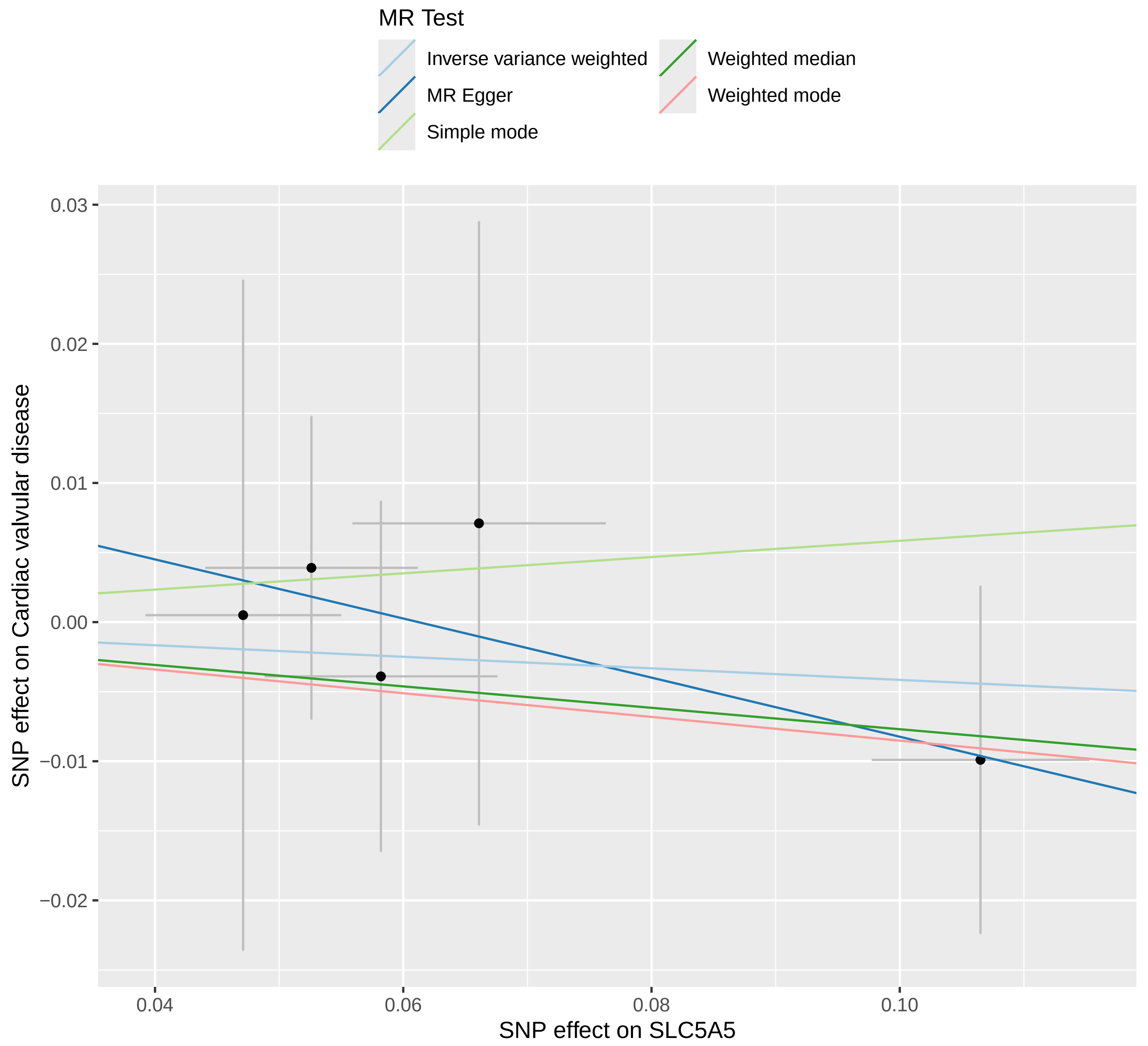
MR Test
Inverse variance weighted
MR Egger
Simple mode
Weighted median
Weighted mode
0.03
0.02
0.01
0.00
−0.01
−0.02
0.04
0.06
0.08
0.10
SNP effect on SLC5A5
SNP effect on Cardiac valvular disease

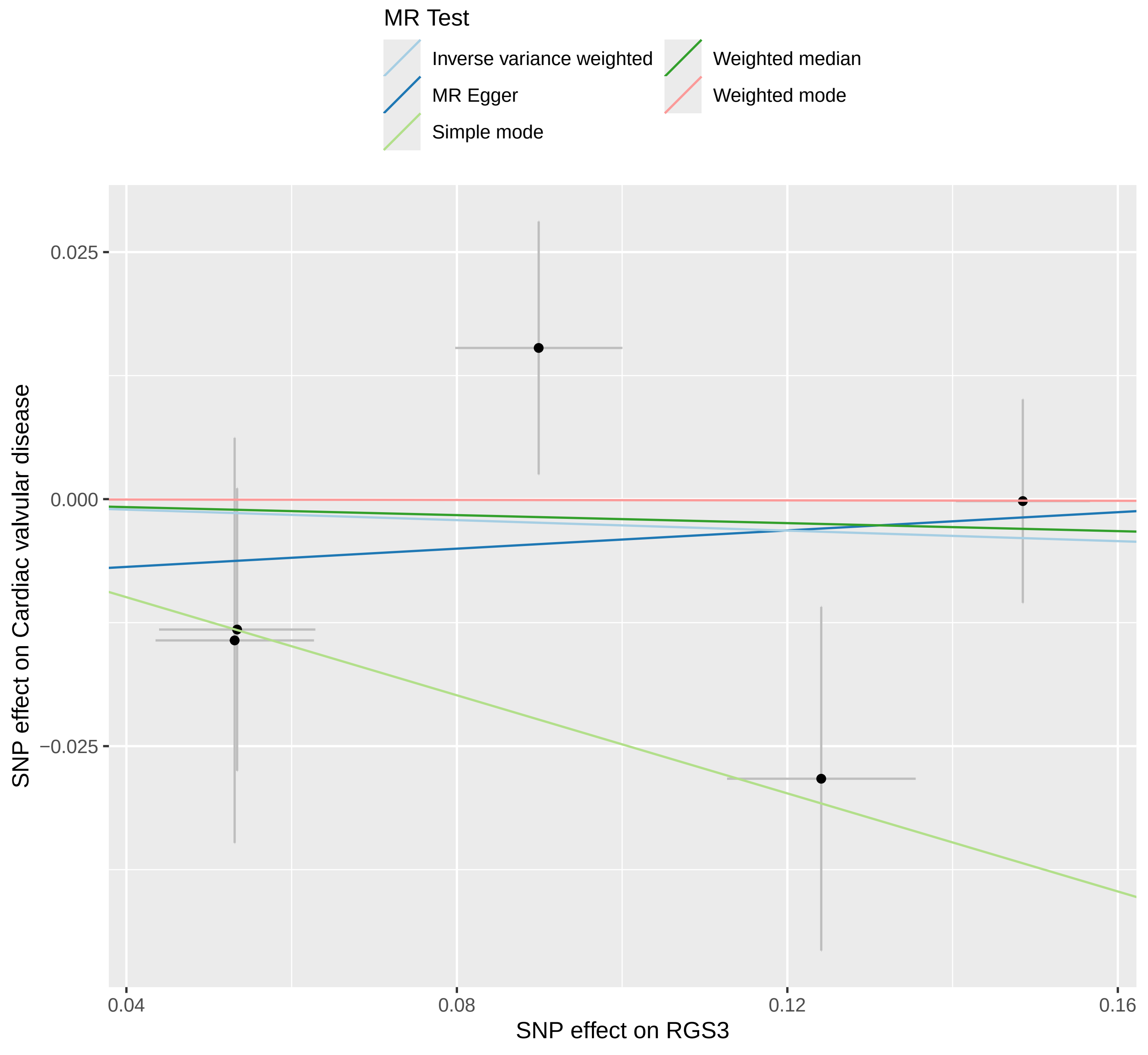

MR Test
Inverse variance weighted
MR Egger
Simple mode
Weighted median
Weighted mode
0.025
0.000
−0.025
0.04
0.08
0.12
0.16
SNP effect on RGS3
SNP effect on Cardiac valvular disease

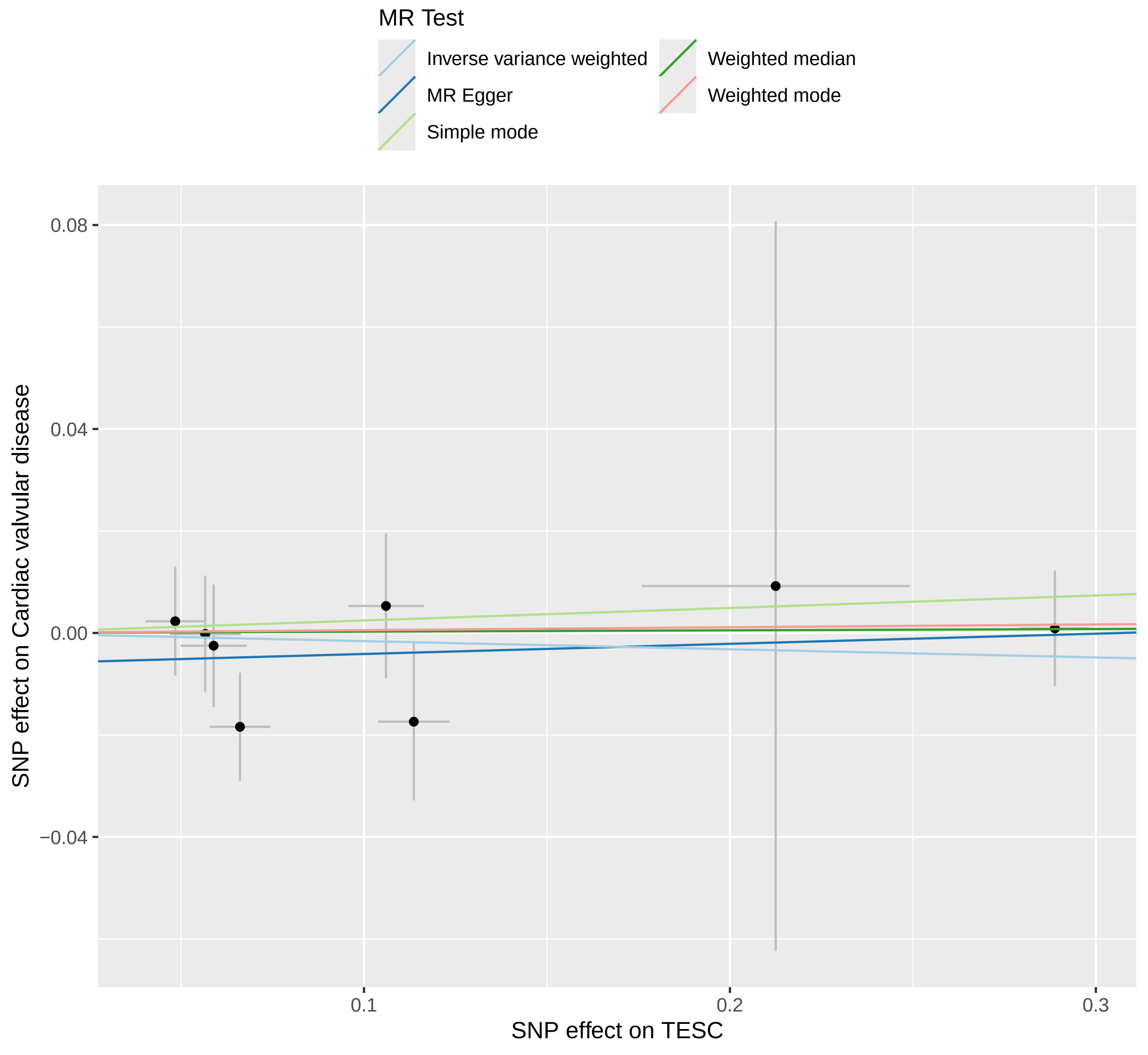

MR Test
Inverse variance weighted
MR Egger
Simple mode
Weighted median
Weighted mode
0.08
0.04
0.00
−0.04
0.1
0.2
0.3
SNP effect on TESC
SNP effect on Cardiac valvular disease

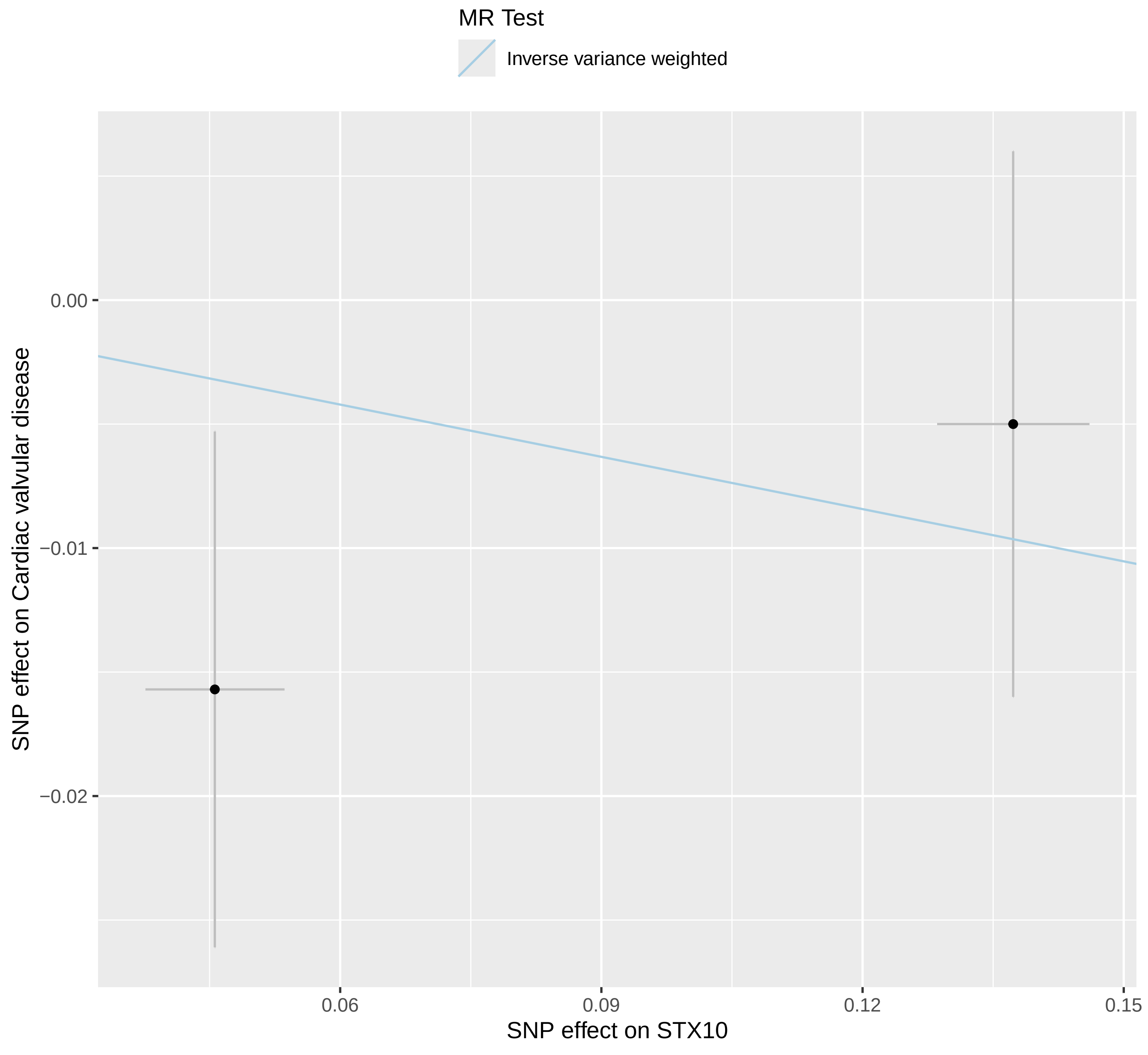
MR Test
Inverse variance weighted
0.00
−0.01
−0.02
SNP effect on Cardiac valvular disease
0.06
0.09
0.12
0.15
SNP effect on STX10

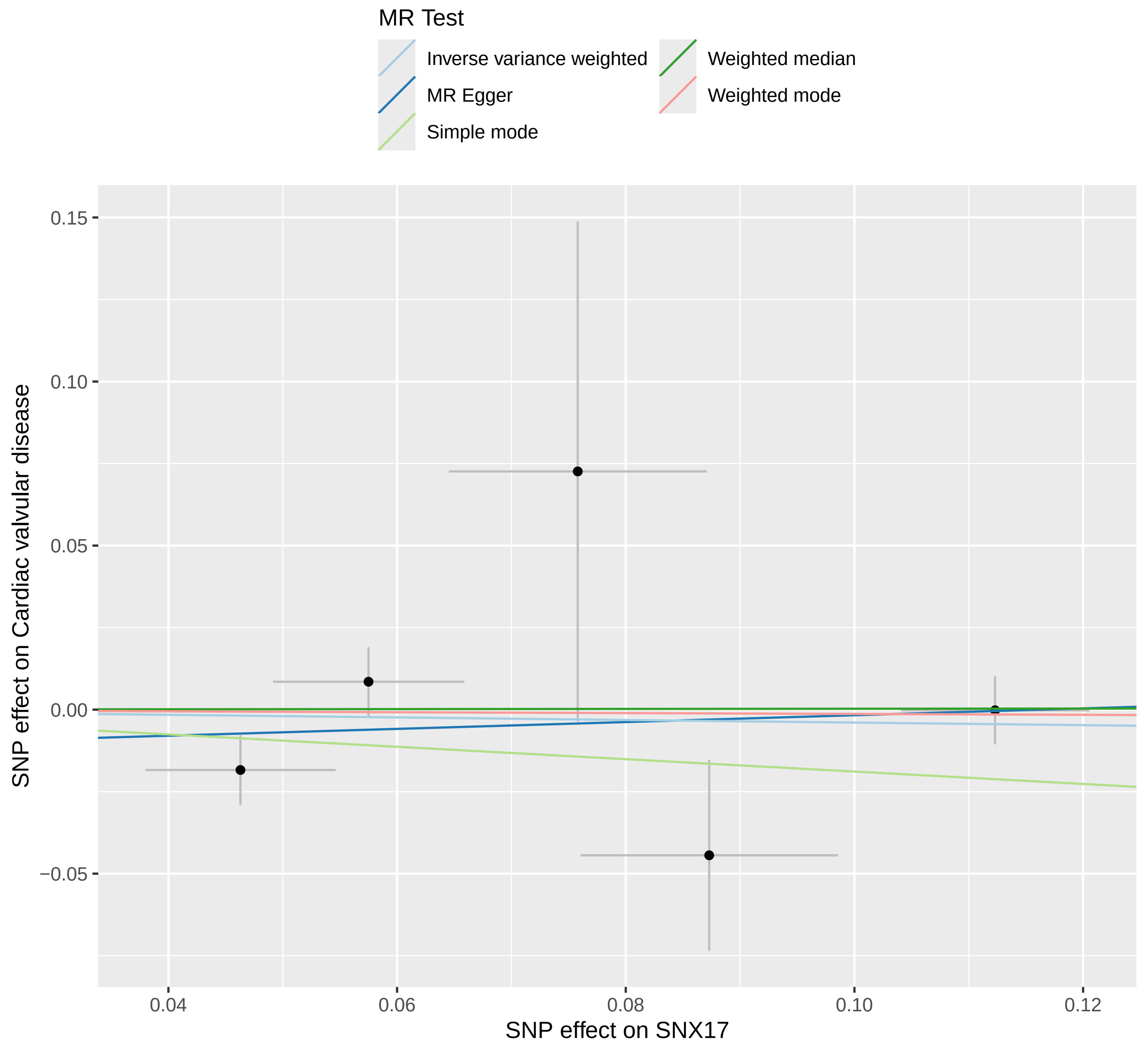
MR Test
Inverse variance weighted
MR Egger
Simple mode
Weighted median
Weighted mode
0.15
0.10
0.05
0.00
−0.05
0.04
0.06
0.08
0.10
0.12
SNP effect on Cardiac valvular disease
SNP effect on SNX17

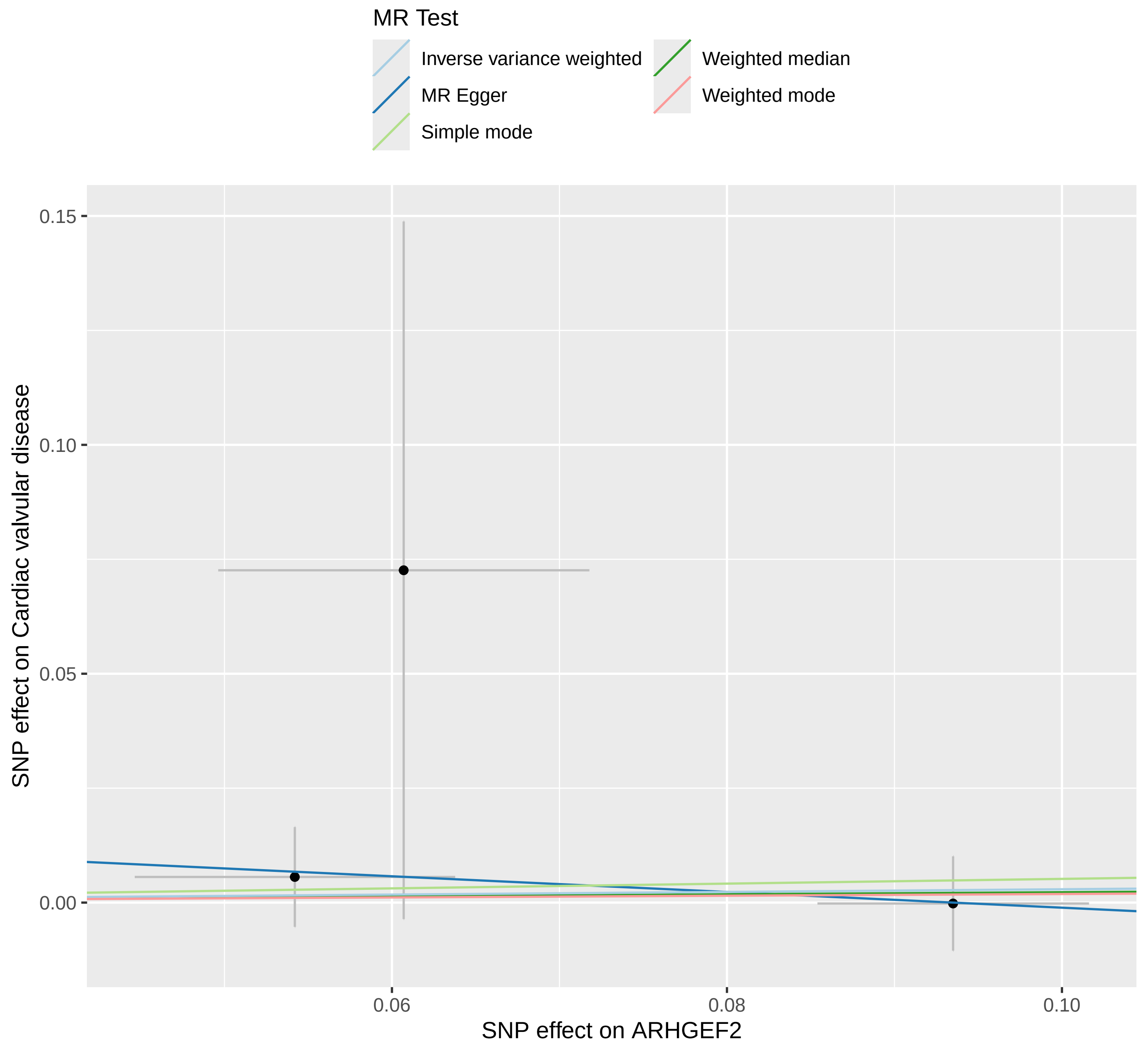

MR Test
Inverse variance weighted
MR Egger
Simple mode
Weighted median
Weighted mode
0.15
0.10
0.05
0.00
SNP effect on Cardiac valvular disease
0.06
0.08
0.10
SNP effect on ARHGEF2

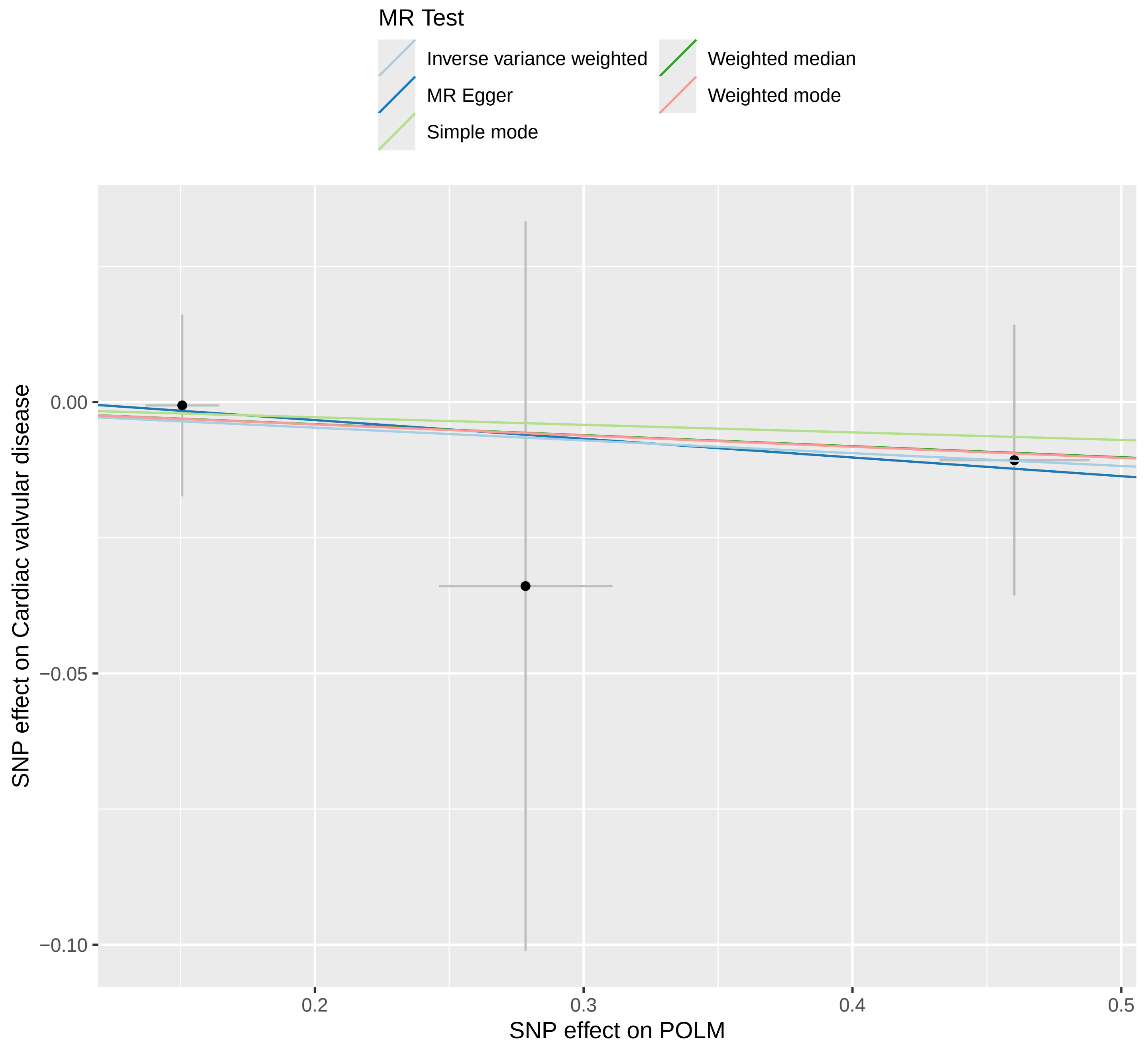
MR Test
Inverse variance weighted
MR Egger
Simple mode
Weighted median
Weighted mode
SNP effect on Cardiac valvular disease
0.00
−0.05
−0.10
0.2
0.3
0.4
0.5
SNP effect on POLM

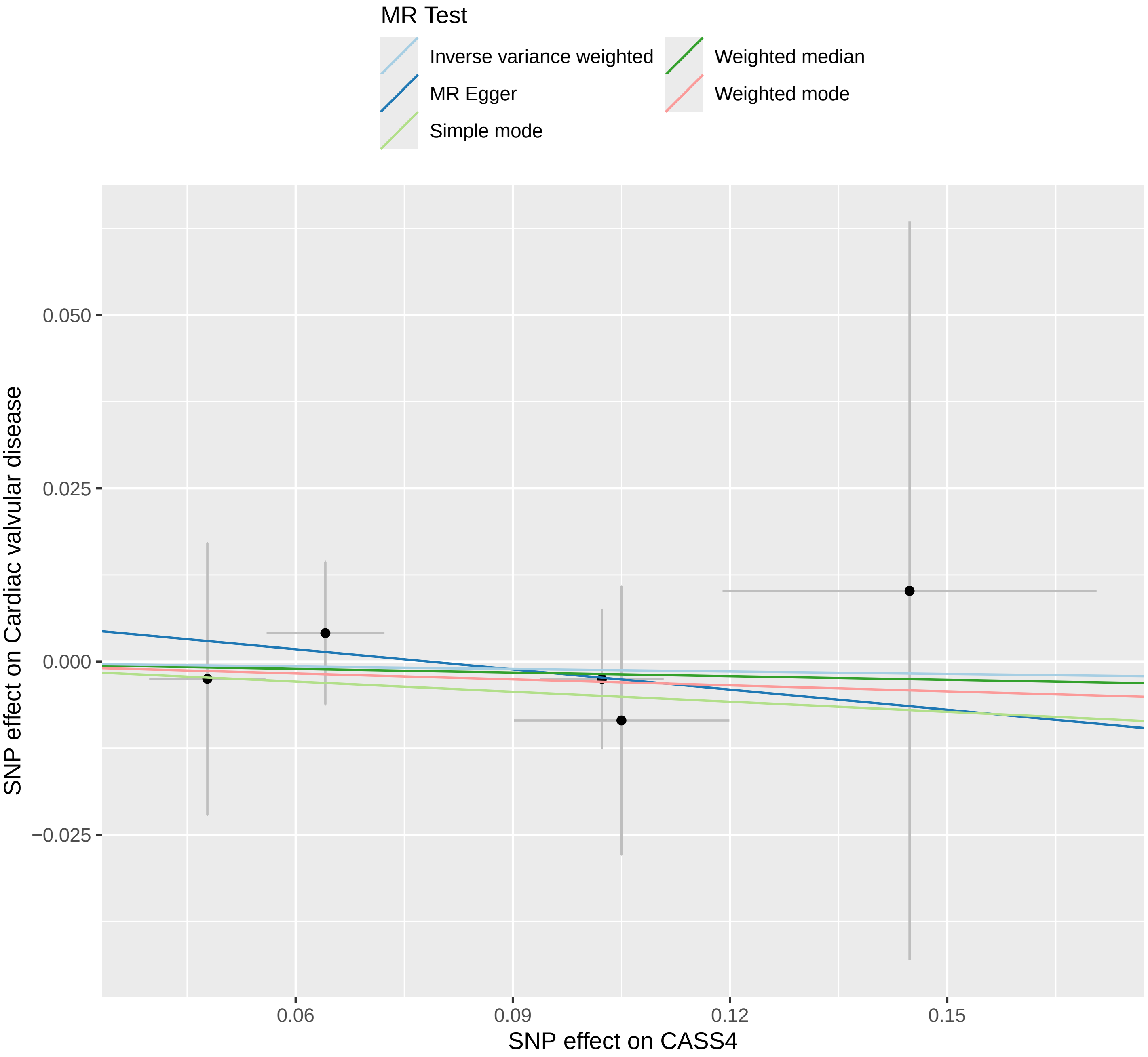

MR Test
Inverse variance weighted
MR Egger
Simple mode
Weighted median
Weighted mode
0.050
0.025
0.000
−0.025
SNP effect on Cardiac valvular disease
0.06
0.09
0.12
0.15
SNP effect on CASS4

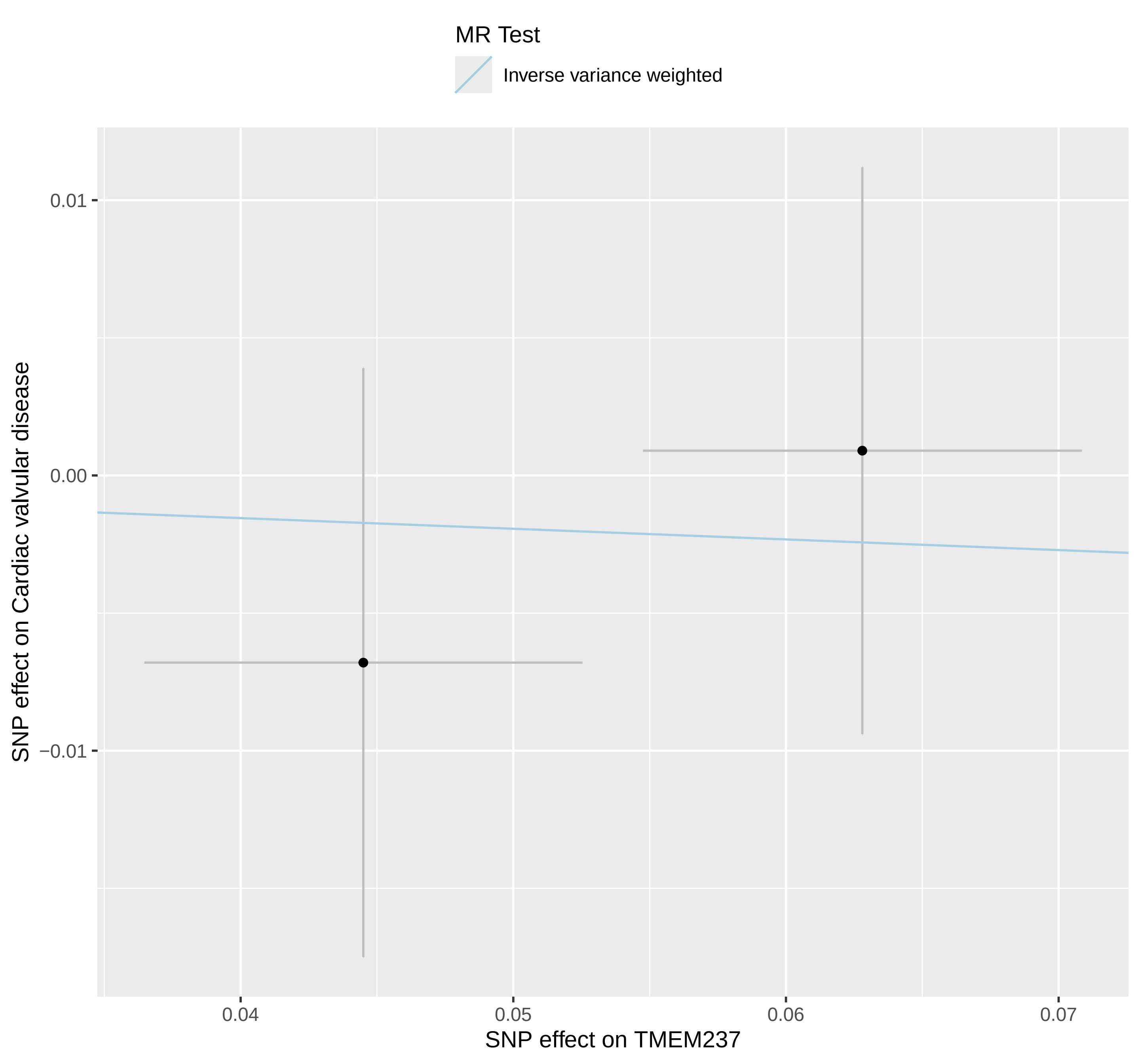
MR Test
Inverse variance weighted
0.01
0.00
−0.01
0.04
0.05
0.06
0.07
SNP effect on TMEM237
SNP effect on Cardiac valvular disease

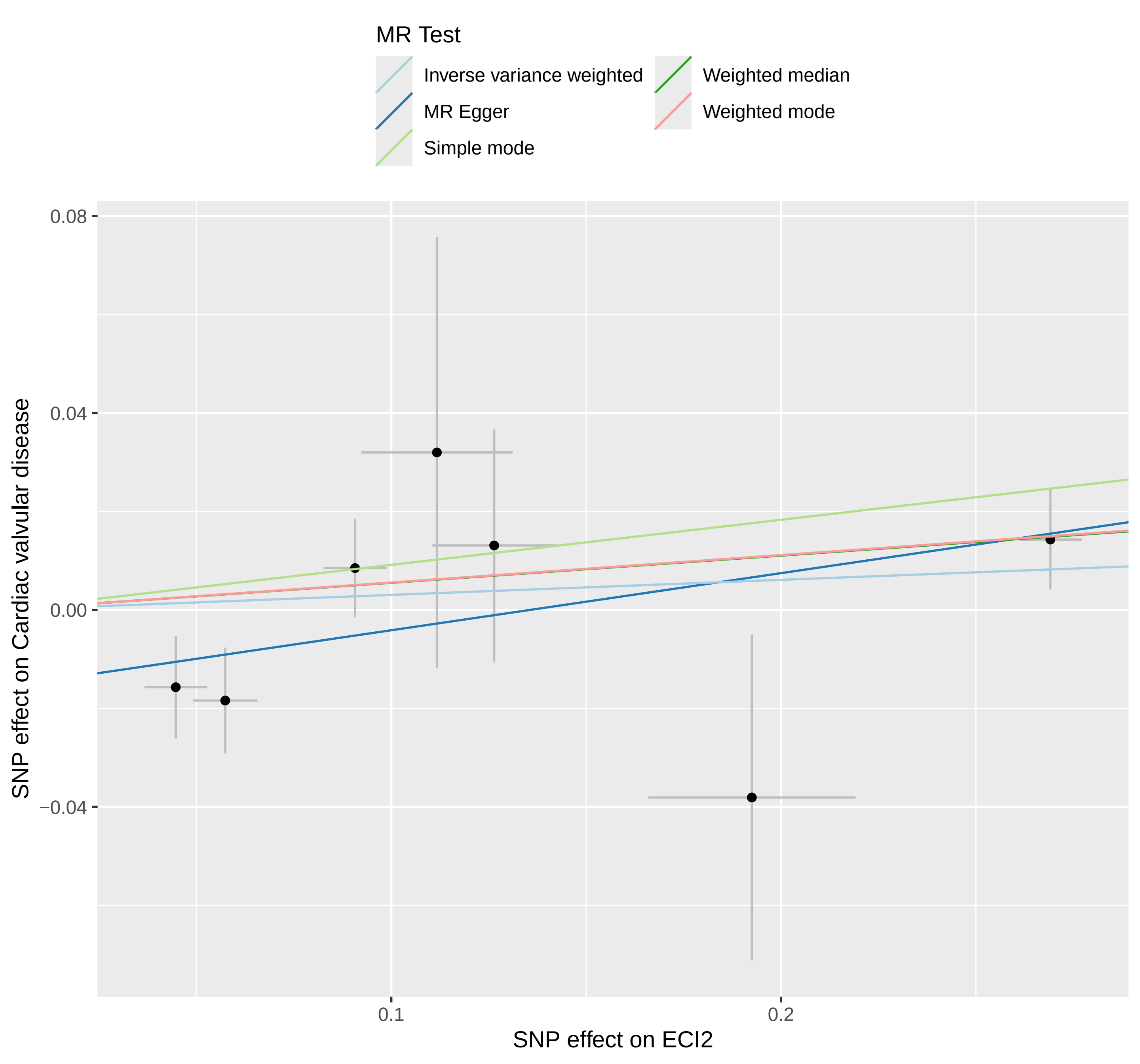
MR Test
Inverse variance weighted
MR Egger
Simple mode
Weighted median
Weighted mode
0.08
0.04
0.00
−0.04
0.1
0.2
SNP effect on Cardiac valvular disease
SNP effect on ECI2

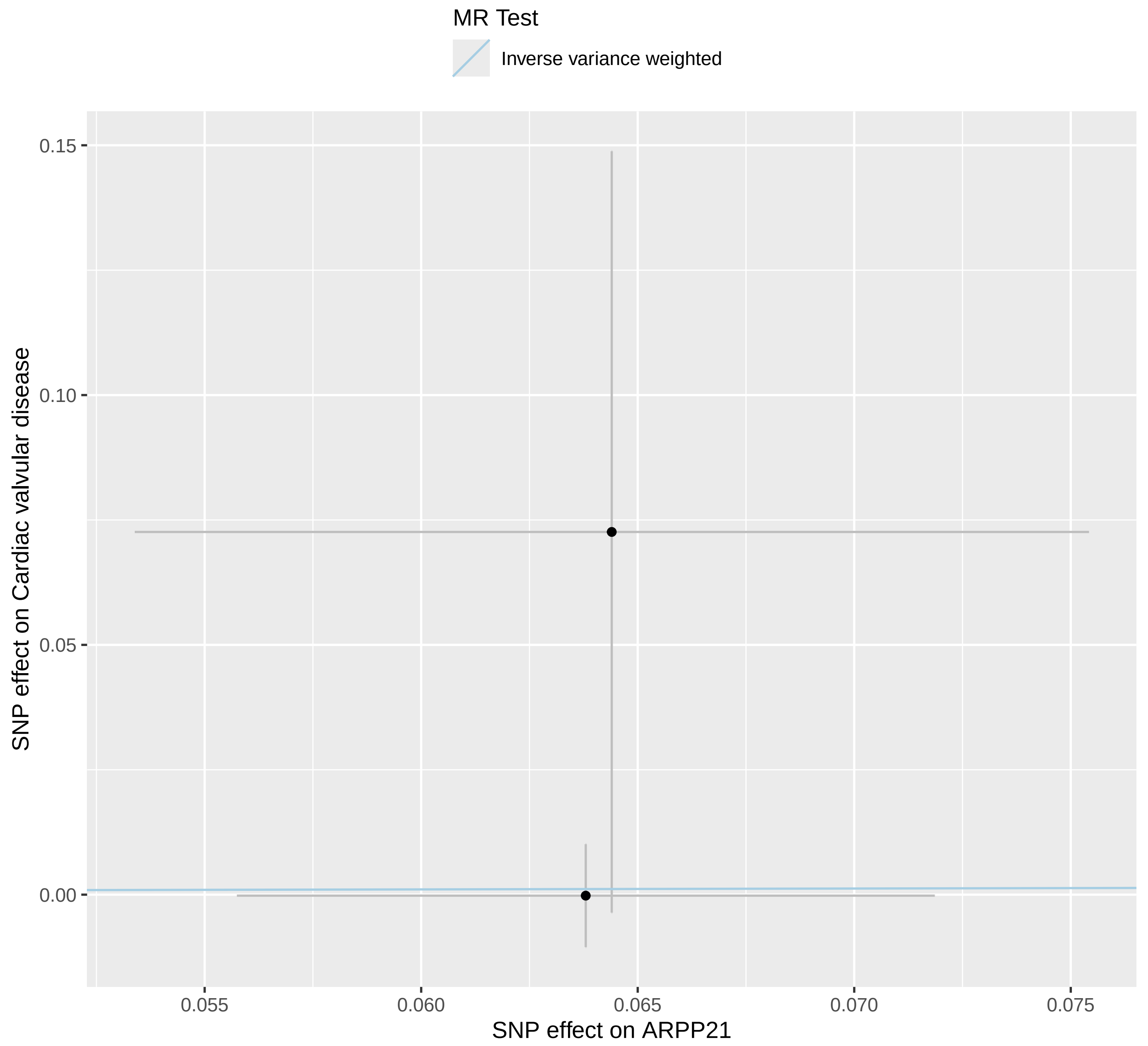
MR Test
Inverse variance weighted
0.15
0.10
0.05
0.00
SNP effect on Cardiac valvular disease
0.055
0.060
0.065
0.070
0.075
SNP effect on ARPP21

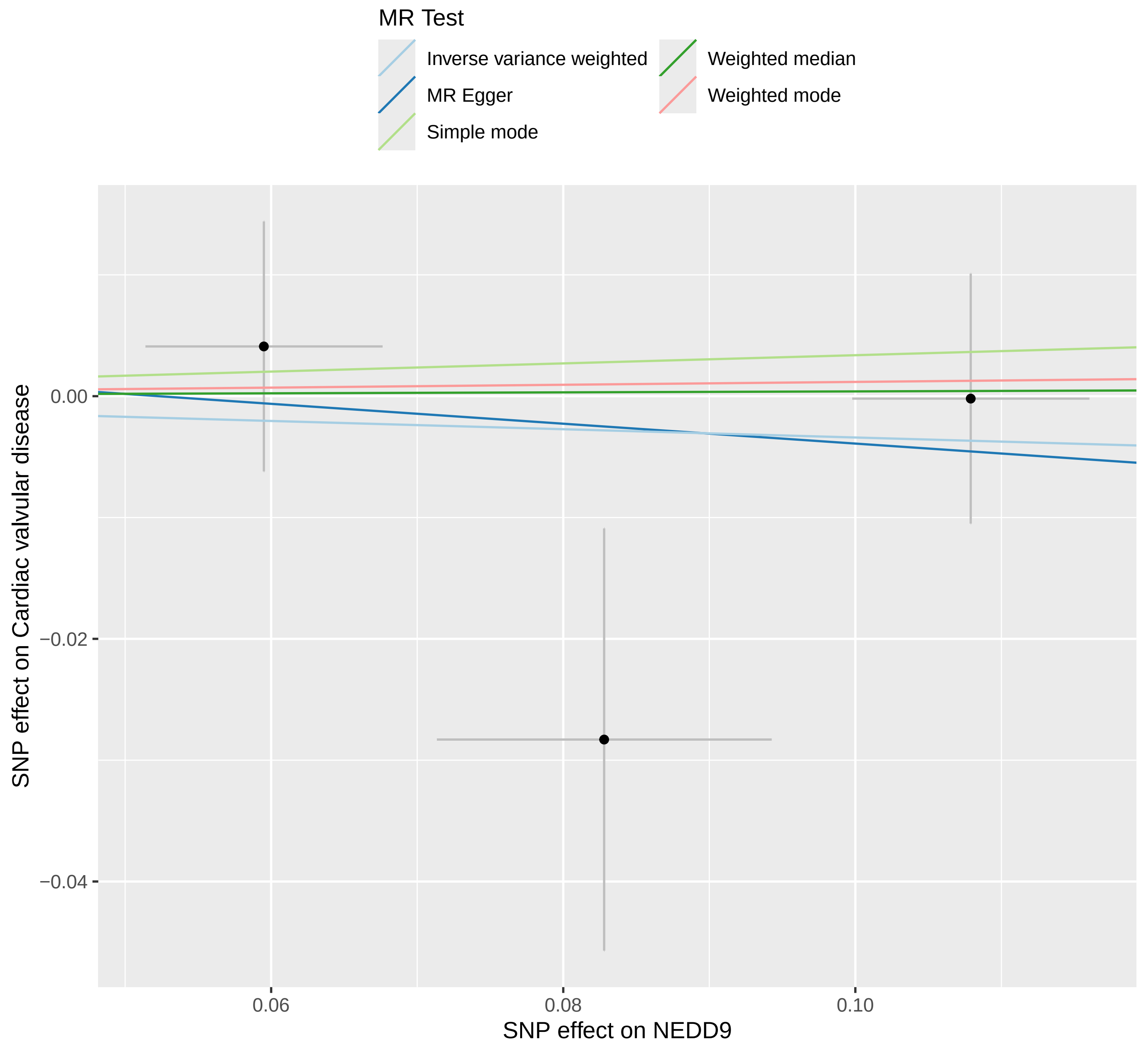
MR Test
Inverse variance weighted
MR Egger
Simple mode
Weighted median
Weighted mode
SNP effect on Cardiac valvular disease
0.00
−0.02
−0.04
0.06
0.08
0.10
SNP effect on NEDD9

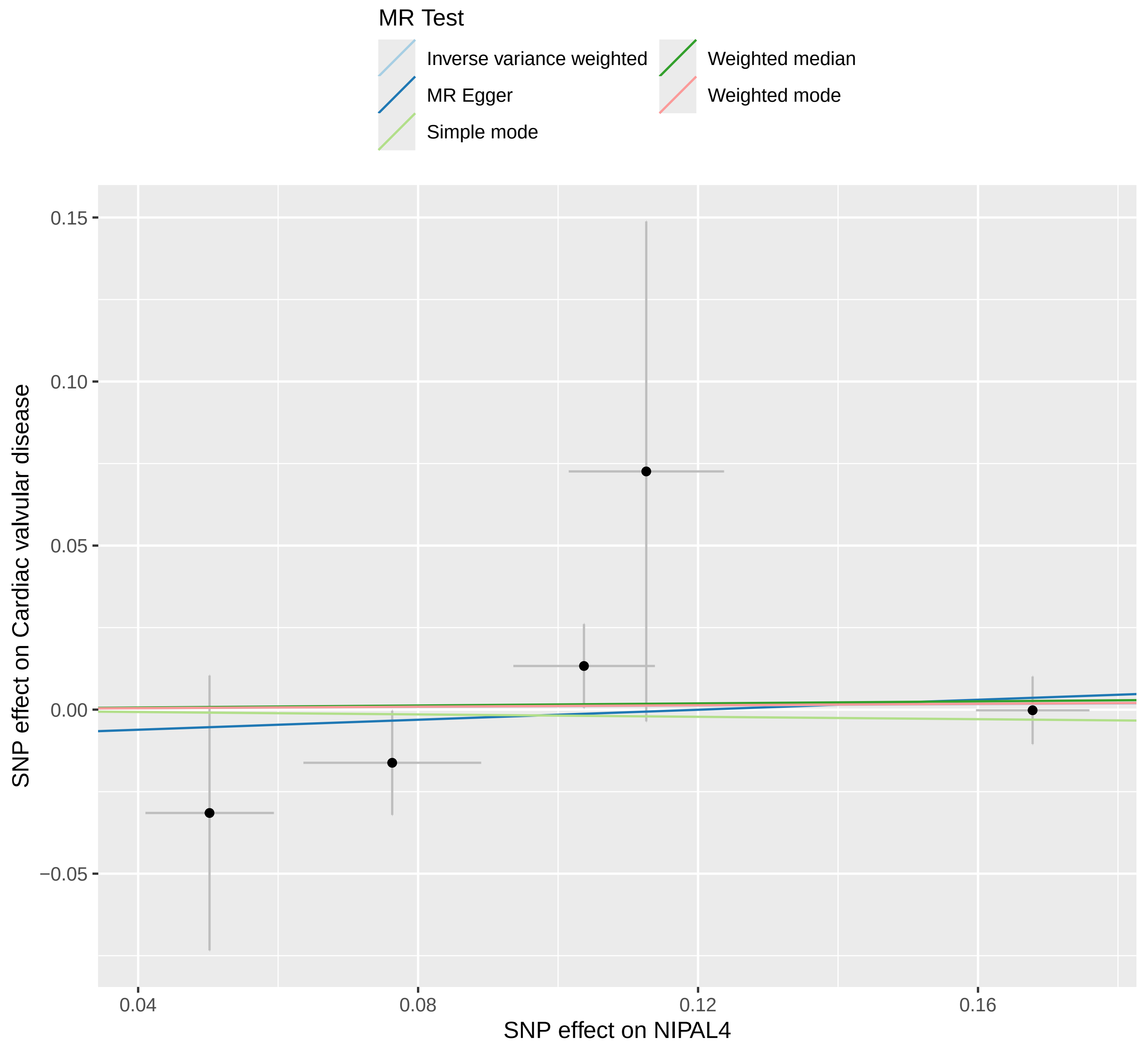
MR Test
Inverse variance weighted
MR Egger
Simple mode
Weighted median
Weighted mode
0.15
0.10
0.05
0.00
−0.05
SNP effect on Cardiac valvular disease
0.04
0.08
0.12
0.16
SNP effect on NIPAL4

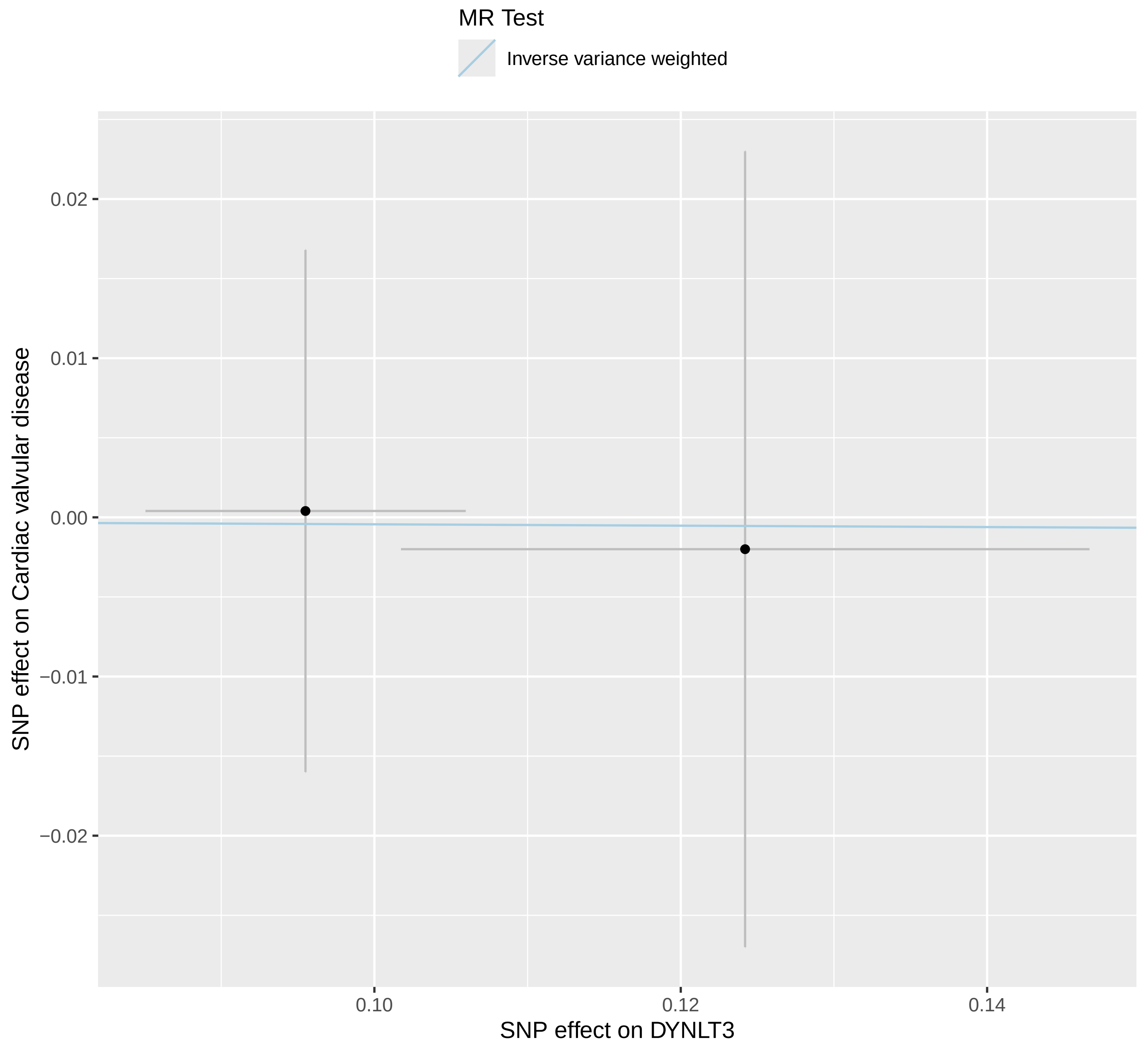

MR Test
Inverse variance weighted
0.02
0.01
0.00
−0.01
−0.02
SNP effect on Cardiac valvular disease
0.10
0.12
0.14
SNP effect on DYNLT3

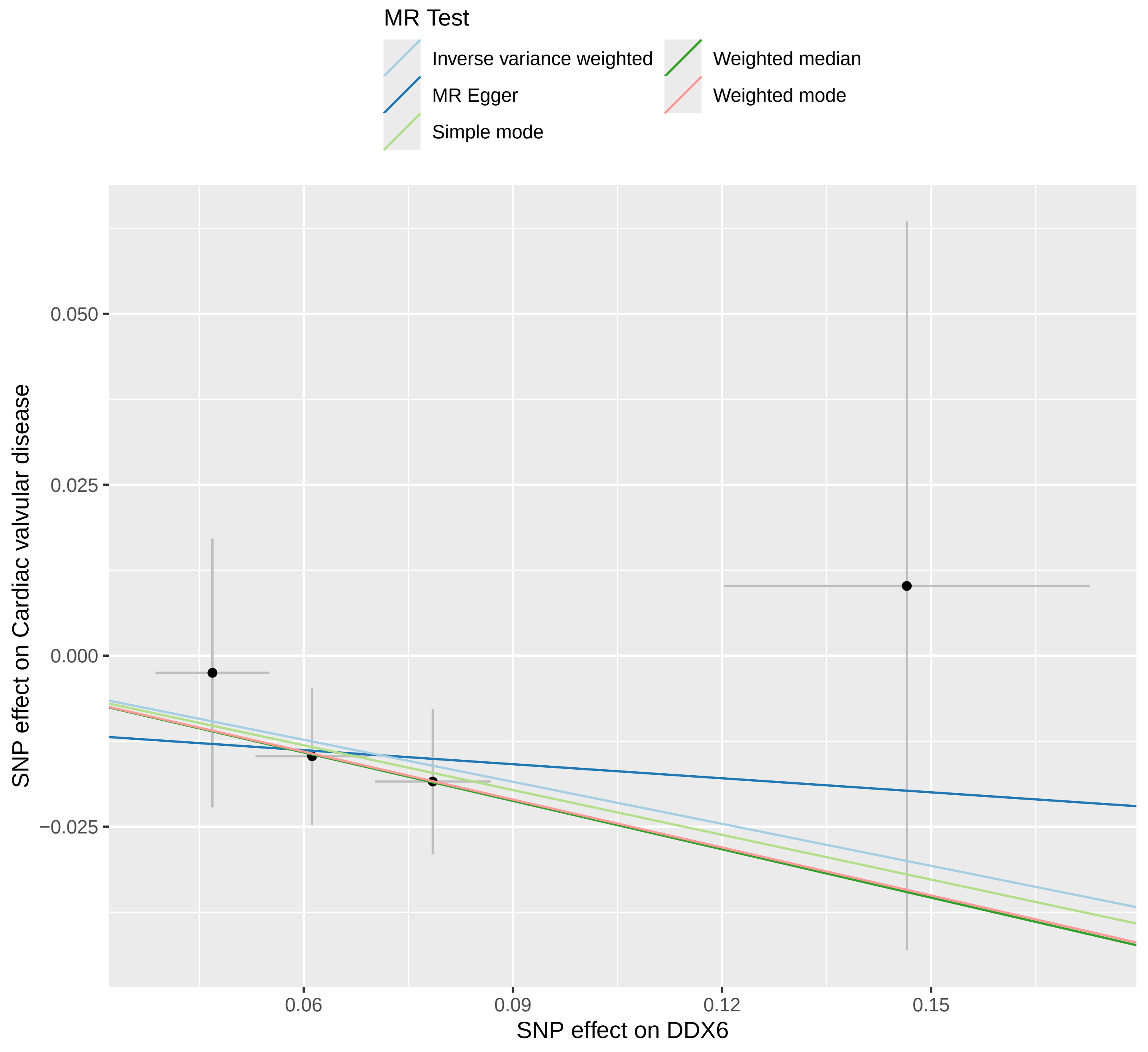
MR Test
Inverse variance weighted
MR Egger
Simple mode
Weighted median
Weighted mode
0.050
0.025
0.000
−0.025
SNP effect on Cardiac valvular disease
0.06
0.09
0.12
0.15
SNP effect on DDX6

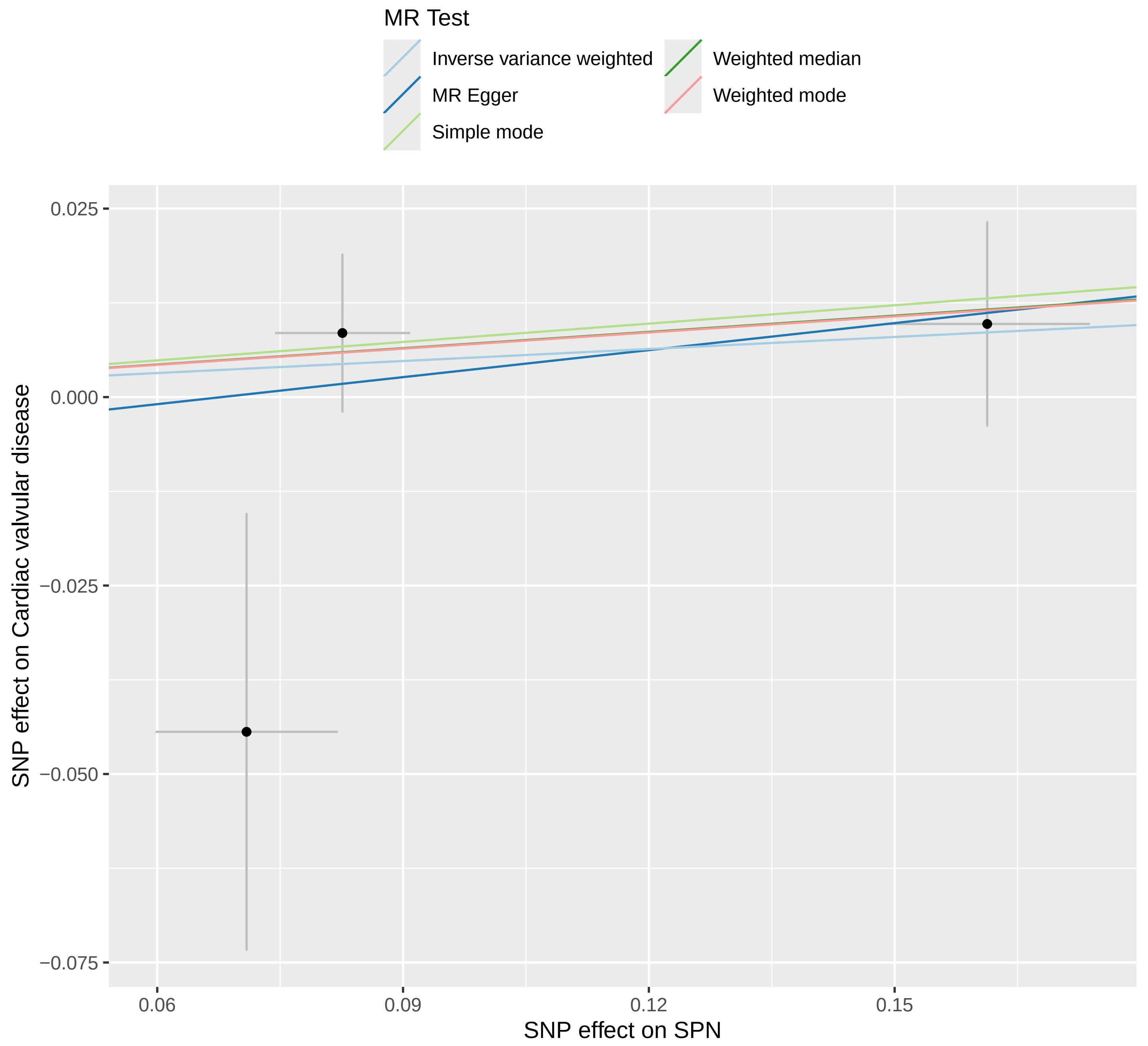

MR Test
Inverse variance weighted
MR Egger
Simple mode
Weighted median
Weighted mode
0.025
0.000
−0.025
−0.050
−0.075
SNP effect on Cardiac valvular disease
0.06
0.09
0.12
0.15
SNP effect on SPN

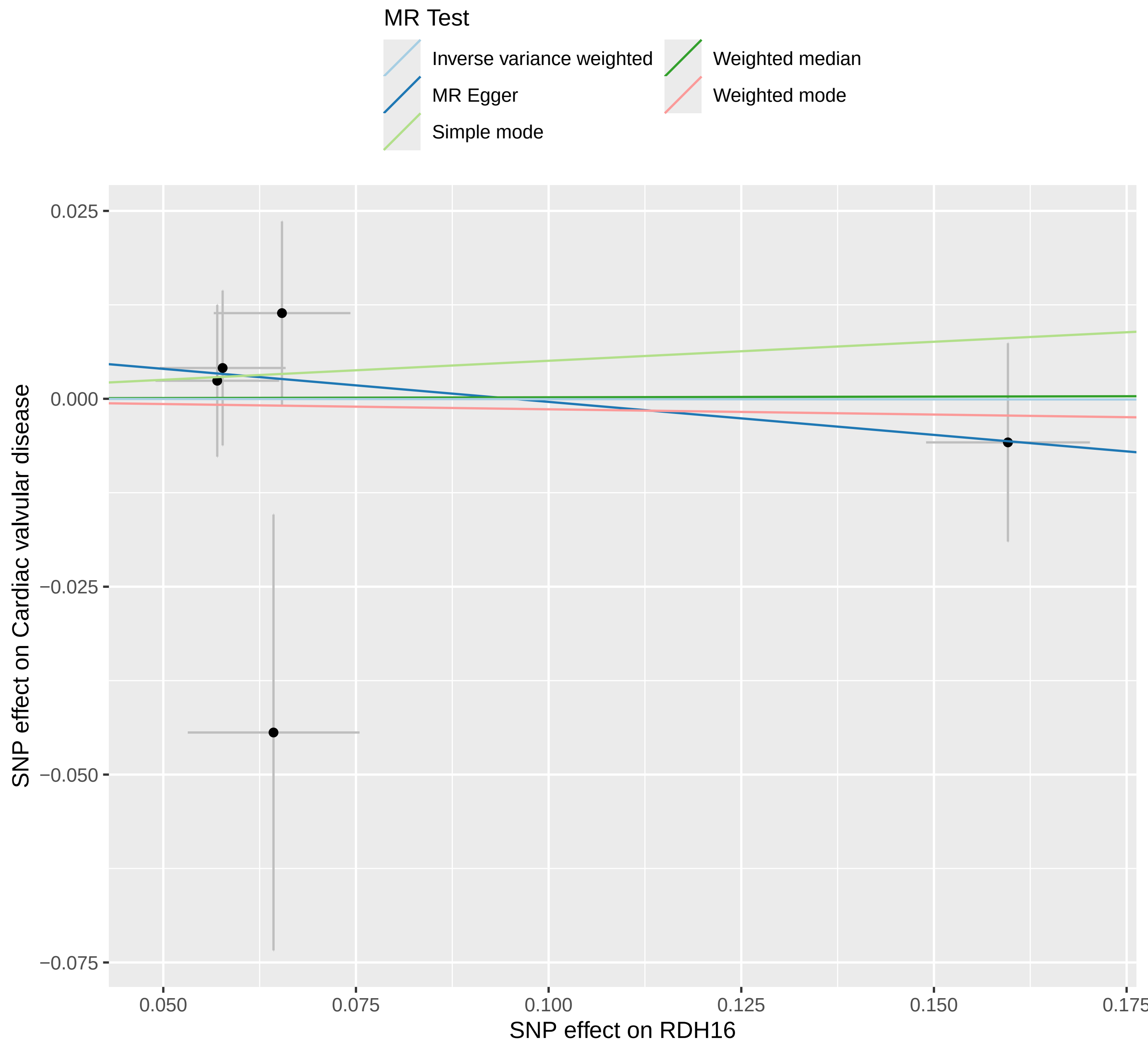
MR Test
Inverse variance weighted
MR Egger
Simple mode
Weighted median
Weighted mode
0.025
0.000
−0.025
−0.050
−0.075
SNP effect on Cardiac valvular disease
0.050
0.075
0.100
0.125
0.150
0.175
SNP effect on RDH16

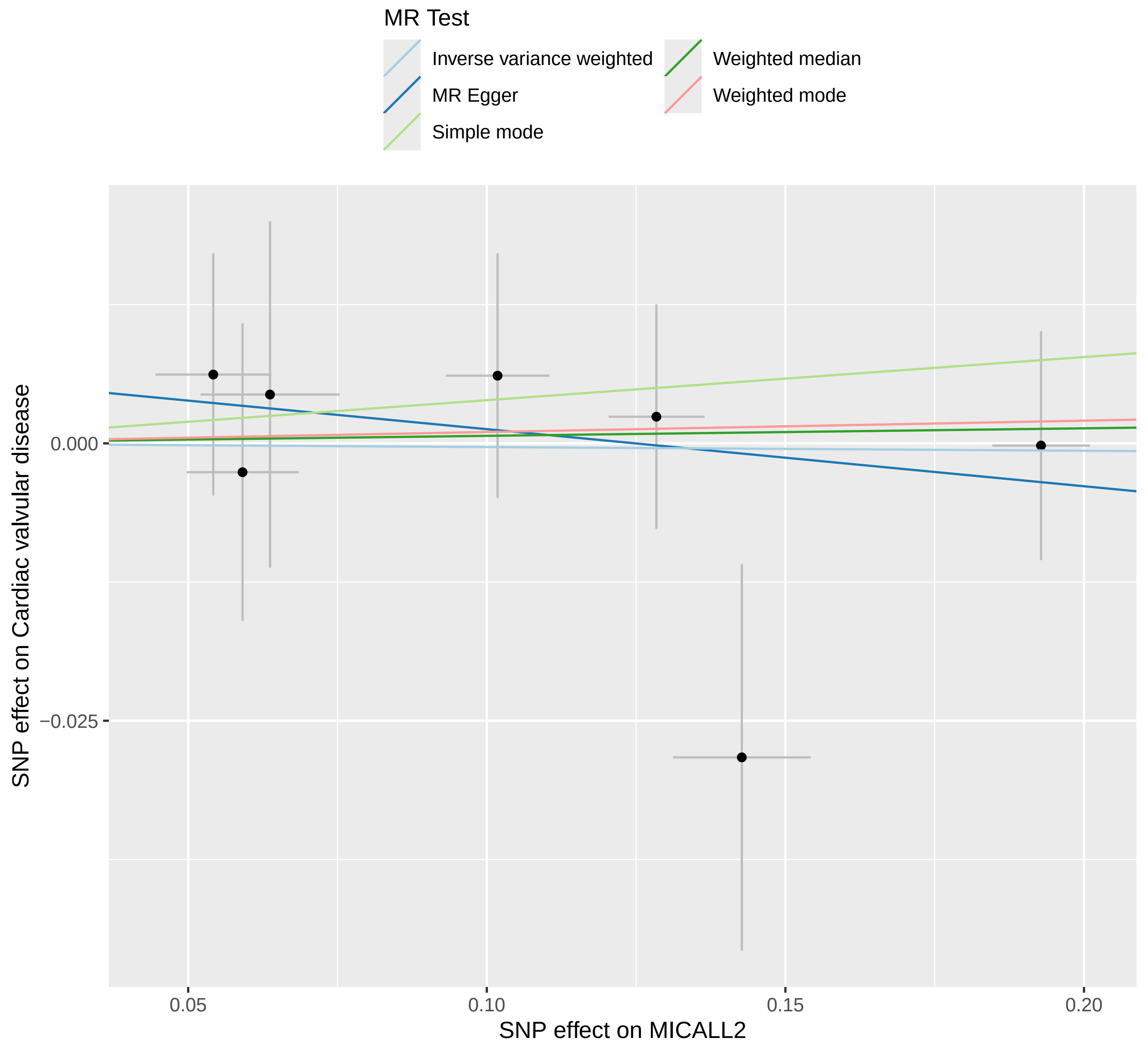
MR Test
Inverse variance weighted
MR Egger
Simple mode
Weighted median
Weighted mode
0.000
−0.025
0.05
0.10
0.15
0.20
SNP effect on MICALL2
SNP effect on Cardiac valvular disease

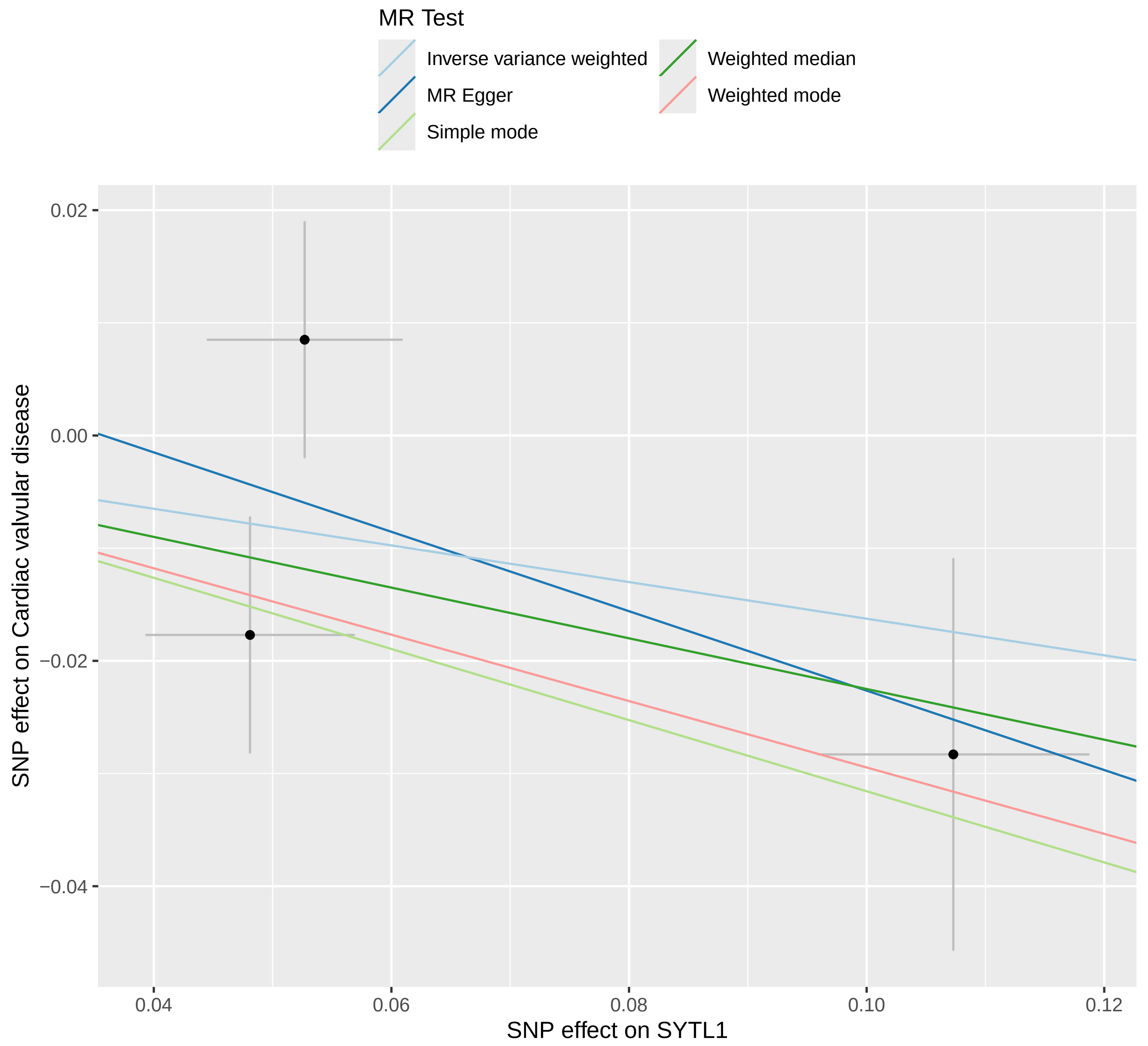

MR Test
Inverse variance weighted
MR Egger
Simple mode
Weighted median
Weighted mode
0.02
0.00
−0.02
−0.04
SNP effect on Cardiac valvular disease
0.04
0.06
0.08
0.10
0.12
SNP effect on SYTL1

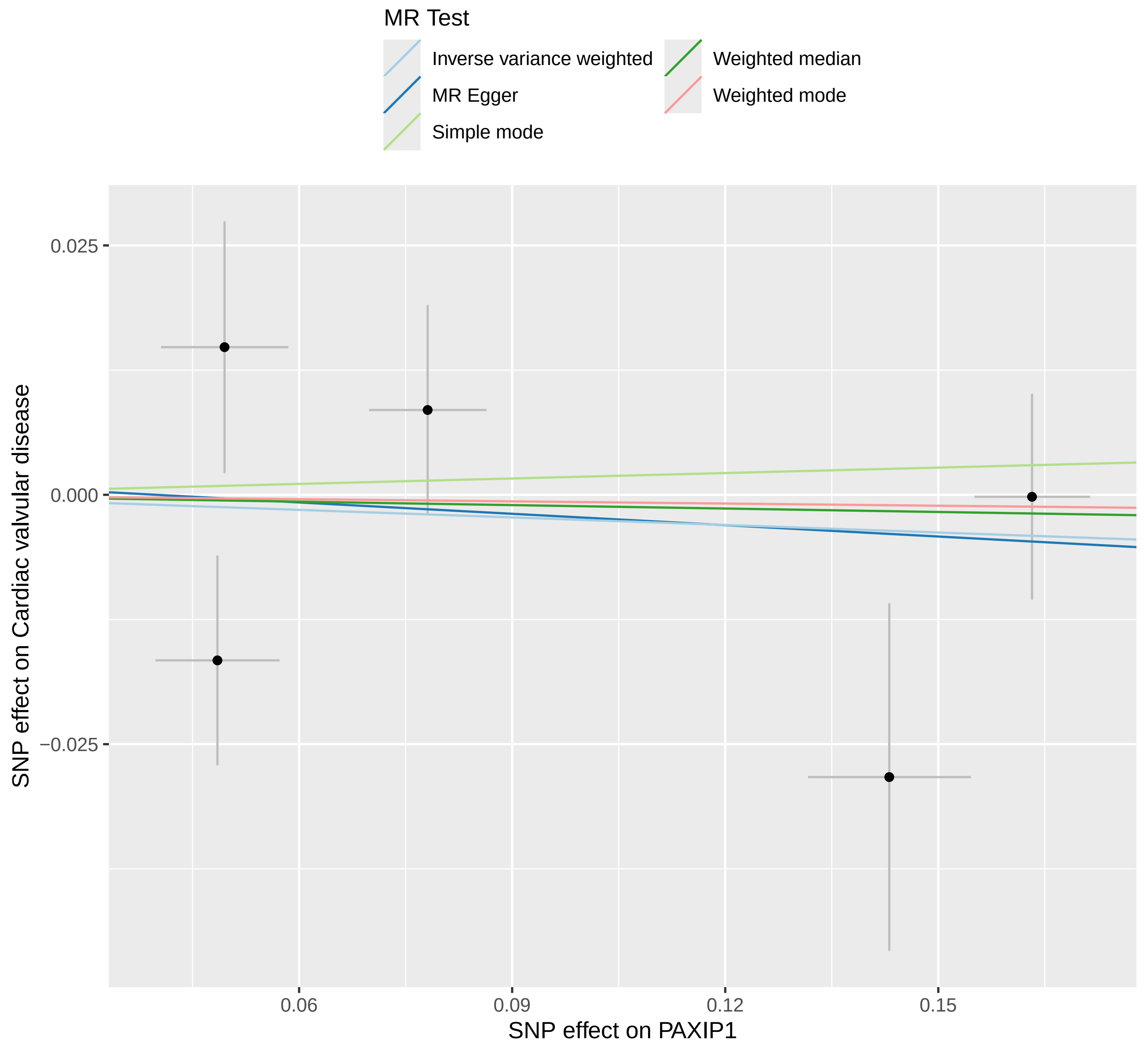
MR Test
Inverse variance weighted
MR Egger
Simple mode
Weighted median
Weighted mode
0.025
0.000
−0.025
0.06
0.09
0.12
0.15
SNP effect on PAXIP1
SNP effect on Cardiac valvular disease

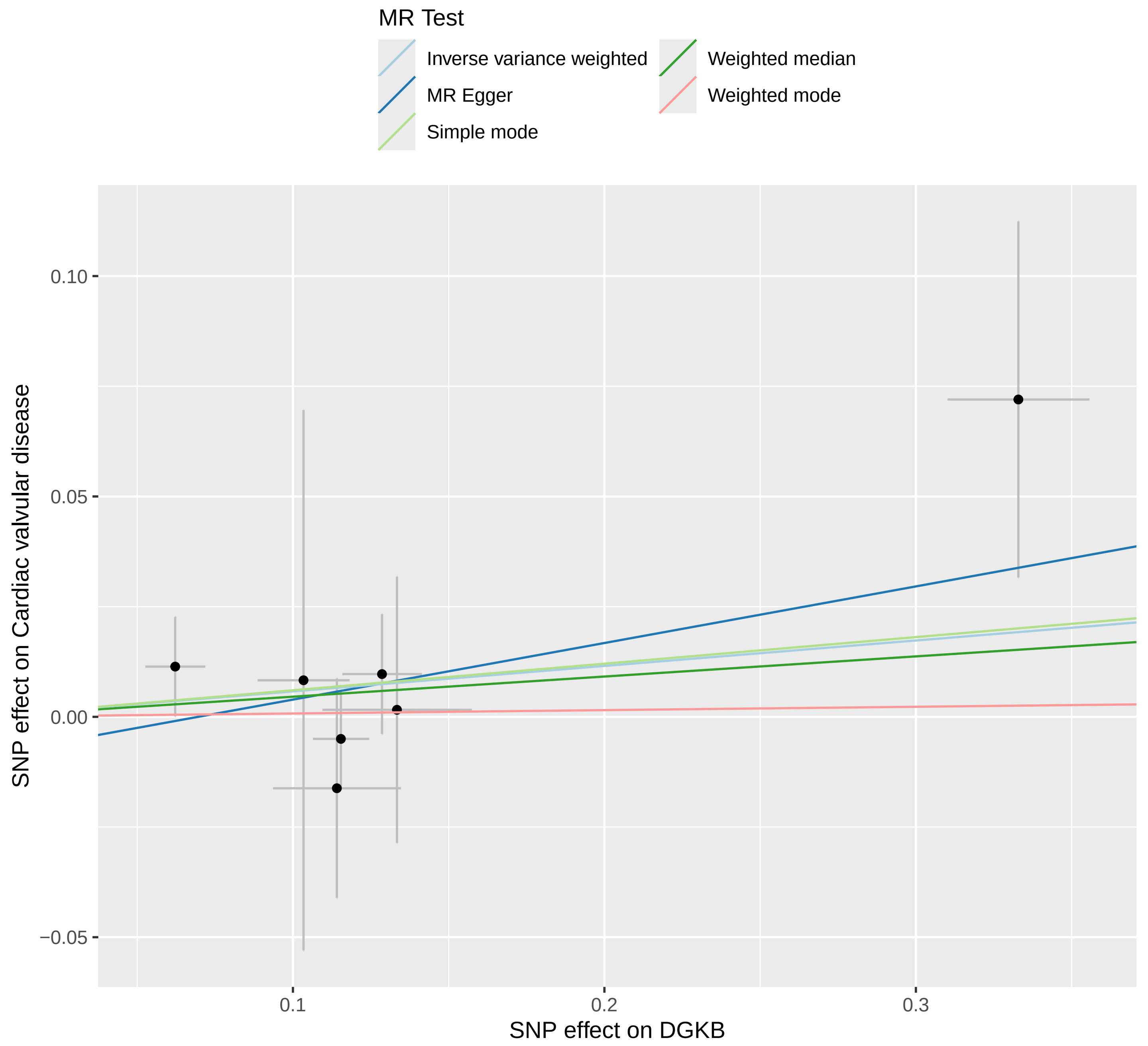
MR Test
Inverse variance weighted
MR Egger
Simple mode
Weighted median
Weighted mode
0.10
0.05
0.00
−0.05
SNP effect on Cardiac valvular disease
0.1
0.2
0.3
SNP effect on DGKB

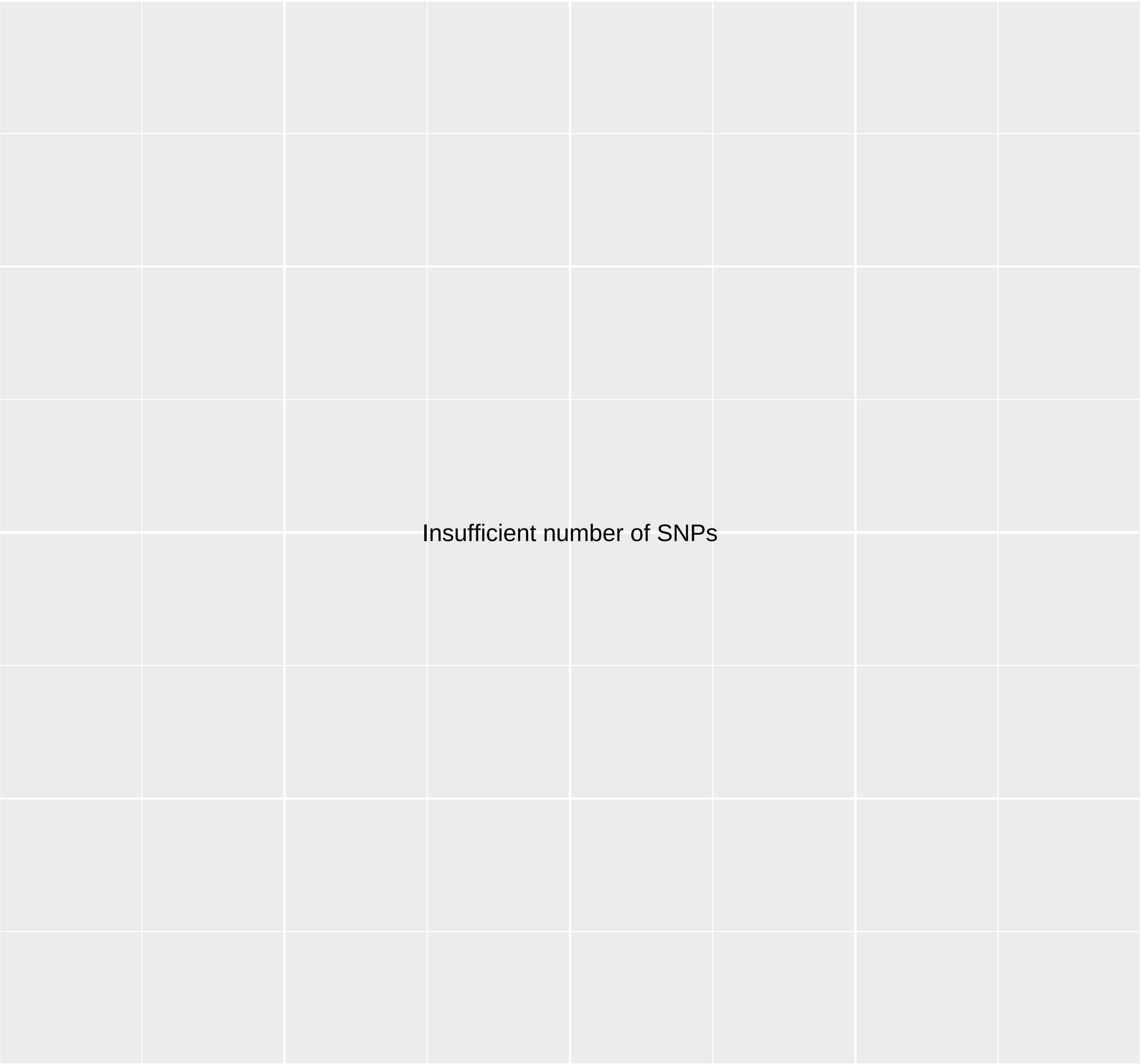
Insufficient number of SNPs

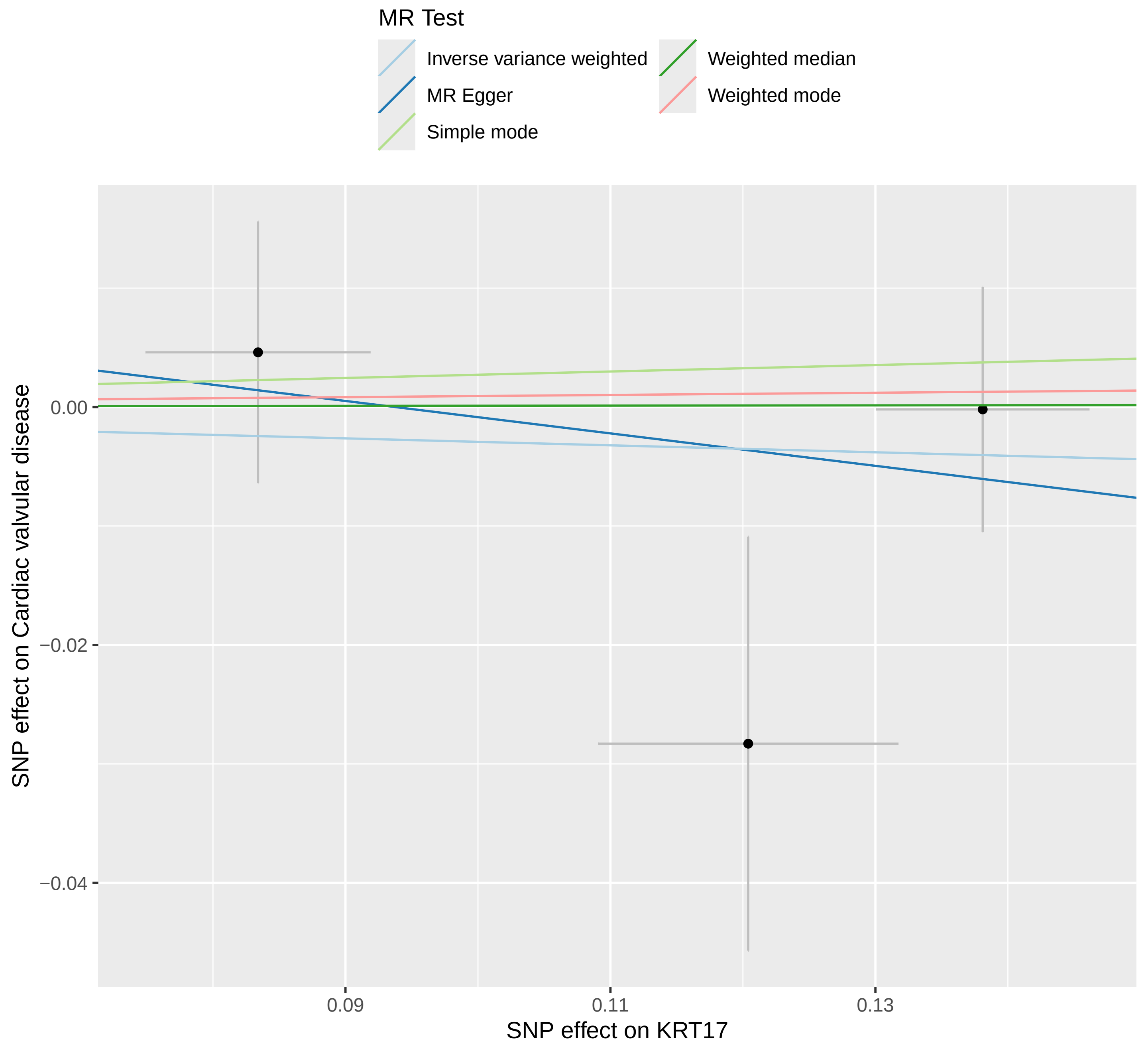
MR Test
Inverse variance weighted
MR Egger
Simple mode
Weighted median
Weighted mode
SNP effect on Cardiac valvular disease
0.00
−0.02
−0.04
0.09
0.11
0.13
SNP effect on KRT17

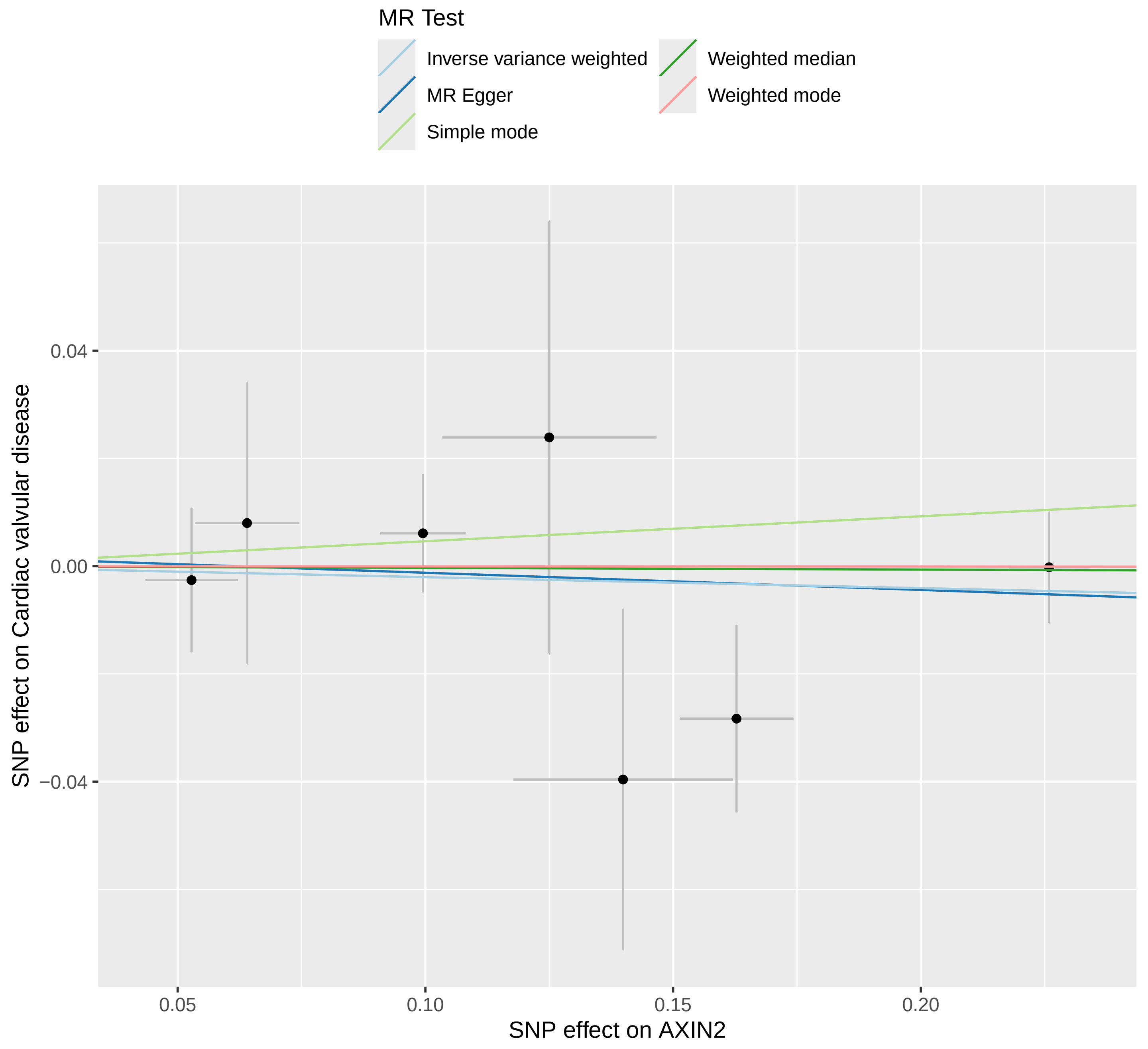
MR Test
Inverse variance weighted
MR Egger
Simple mode
Weighted median
Weighted mode
SNP effect on Cardiac valvular disease
0.04
0.00
−0.04
0.05
0.10
0.15
0.20
SNP effect on AXIN2

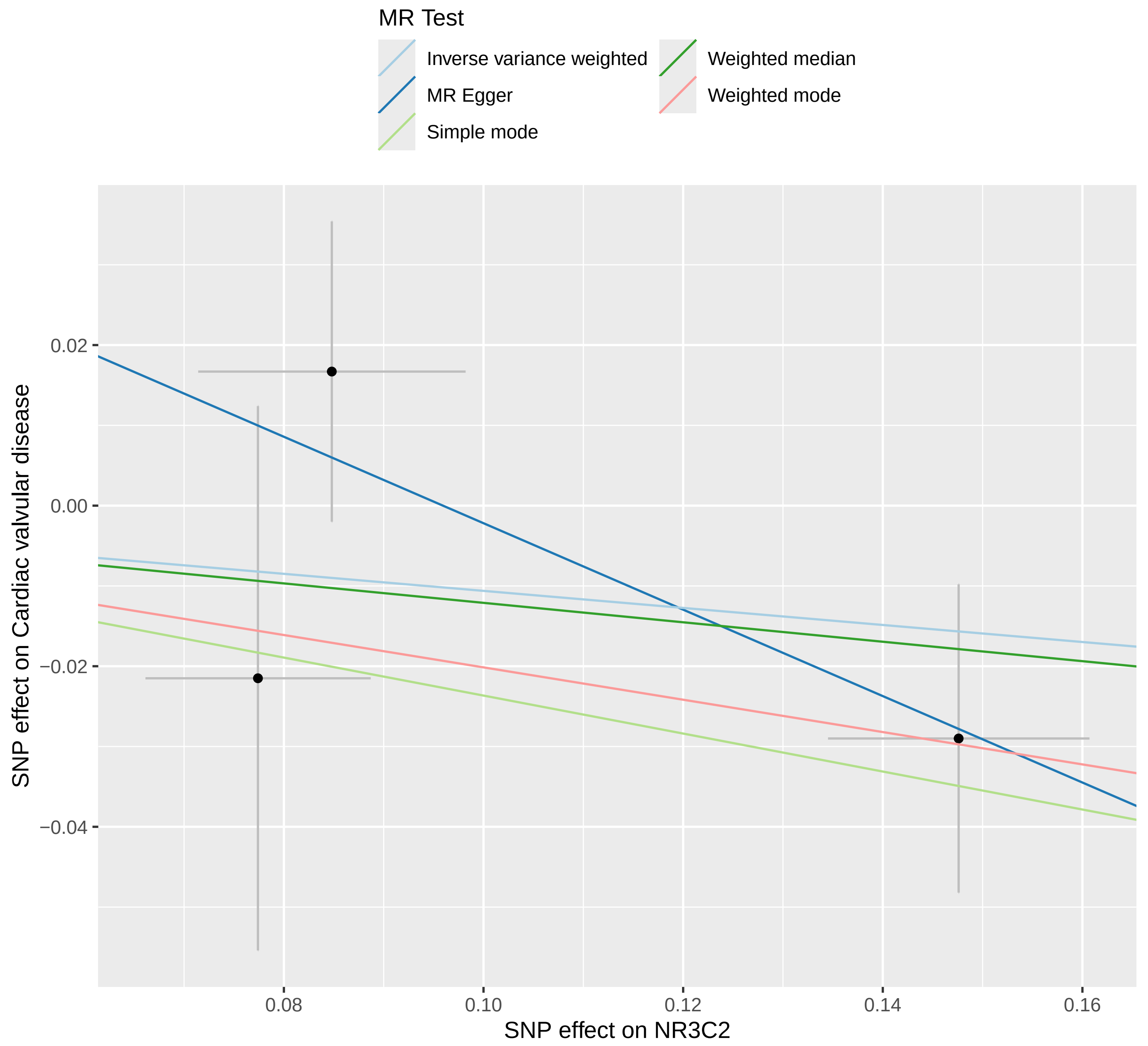

MR Test
Inverse variance weighted
MR Egger
Simple mode
Weighted median
Weighted mode
0.02
0.00
−0.02
−0.04
SNP effect on Cardiac valvular disease
0.08
0.10
0.12
0.14
0.16
SNP effect on NR3C2

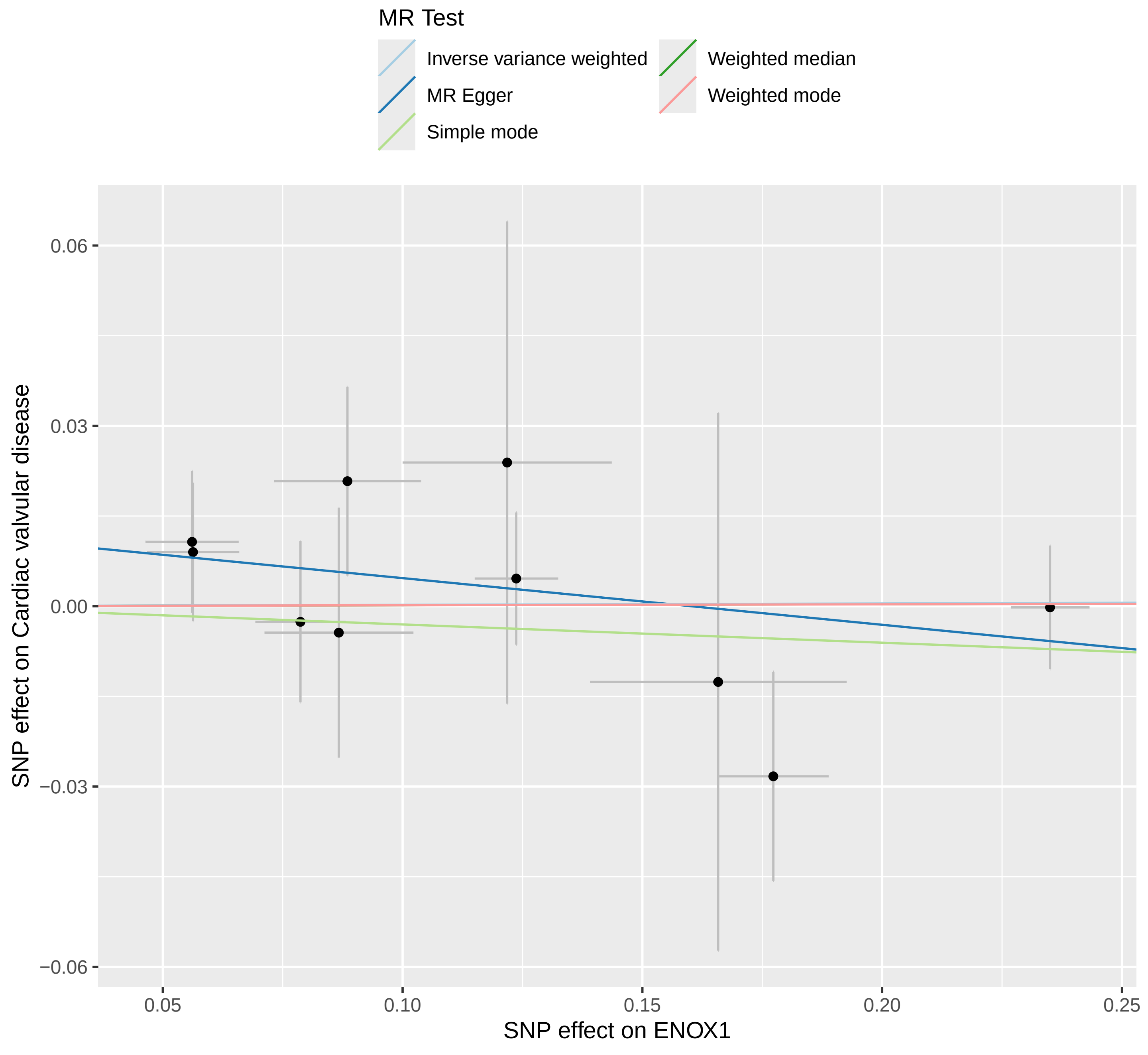

MR Test
Inverse variance weighted
MR Egger
Simple mode
Weighted median
Weighted mode
0.06
0.03
0.00
−0.03
−0.06
0.05
0.10
0.15
0.20
0.25
SNP effect on Cardiac valvular disease
SNP effect on ENOX1

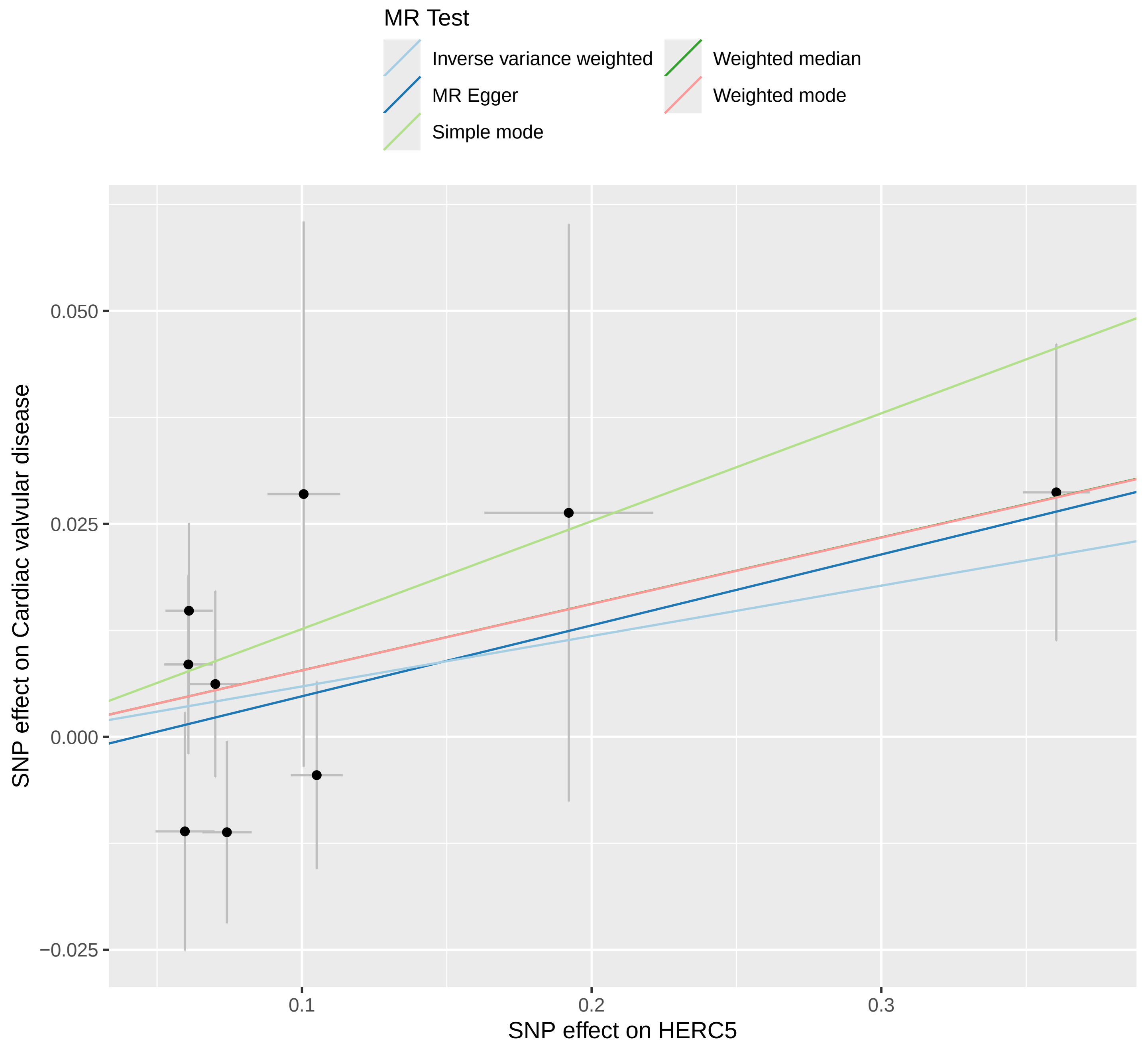
MR Test
Inverse variance weighted
MR Egger
Simple mode
Weighted median
Weighted mode
0.050
0.025
0.000
−0.025
0.1
0.2
0.3
SNP effect on Cardiac valvular disease
SNP effect on HERC5

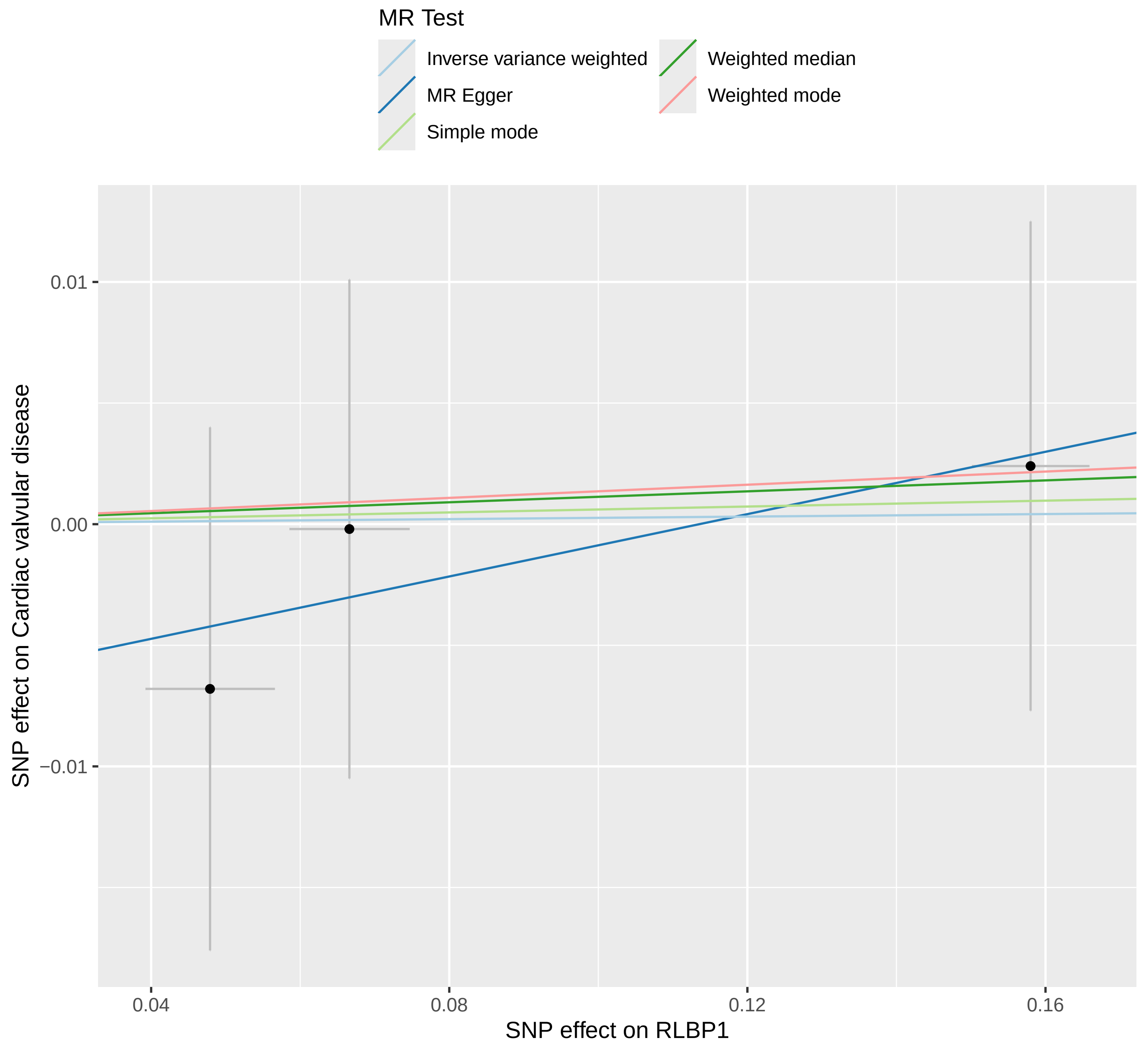
MR Test
Inverse variance weighted
MR Egger
Simple mode
Weighted median
Weighted mode
0.01
0.00
−0.01
SNP effect on Cardiac valvular disease
0.04
0.08
0.12
0.16
SNP effect on RLBP1

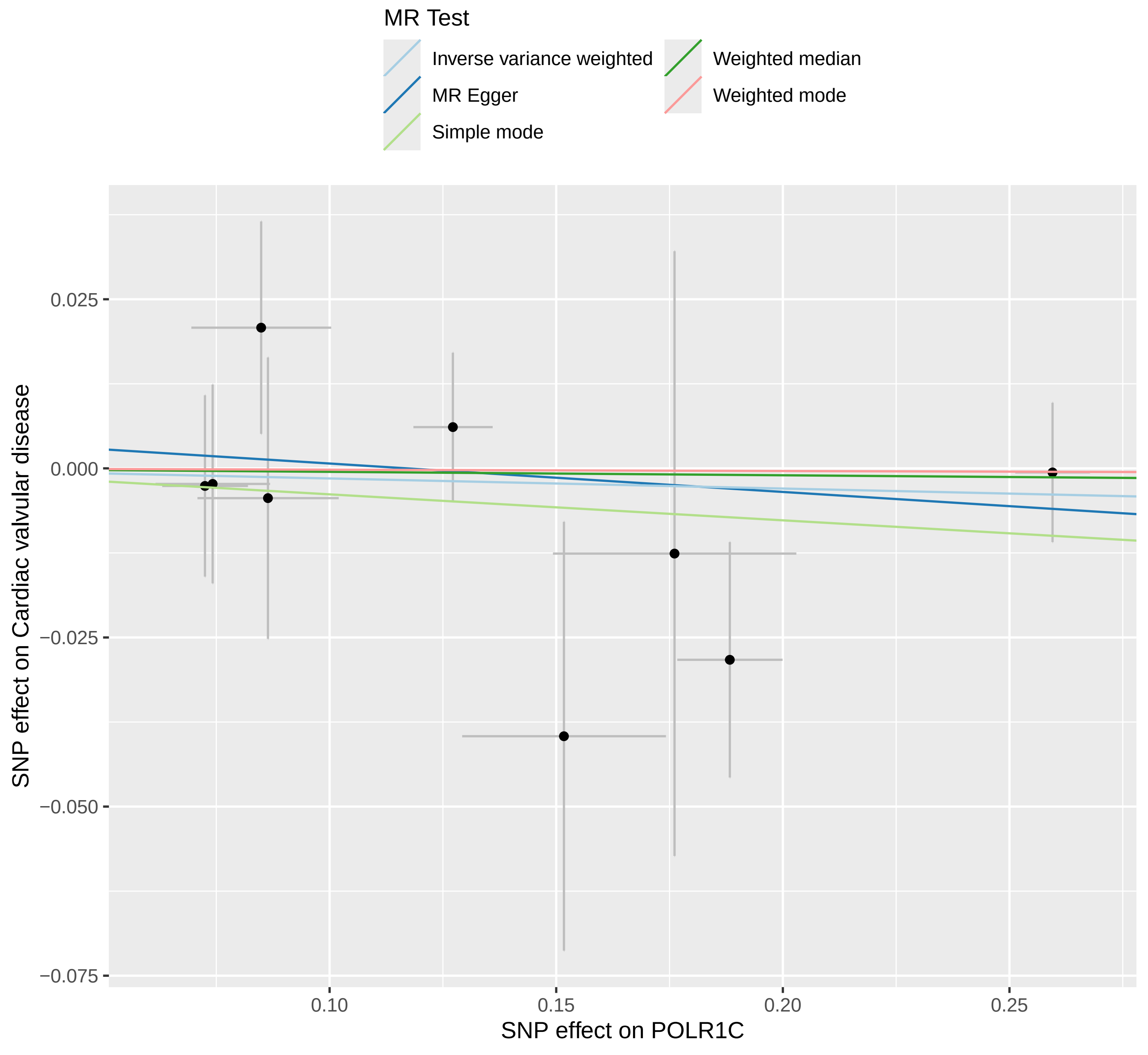
MR Test
Inverse variance weighted
MR Egger
Simple mode
Weighted median
Weighted mode
0.025
0.000
−0.025
−0.050
−0.075
0.10
0.15
0.20
0.25
SNP effect on Cardiac valvular disease
SNP effect on POLR1C

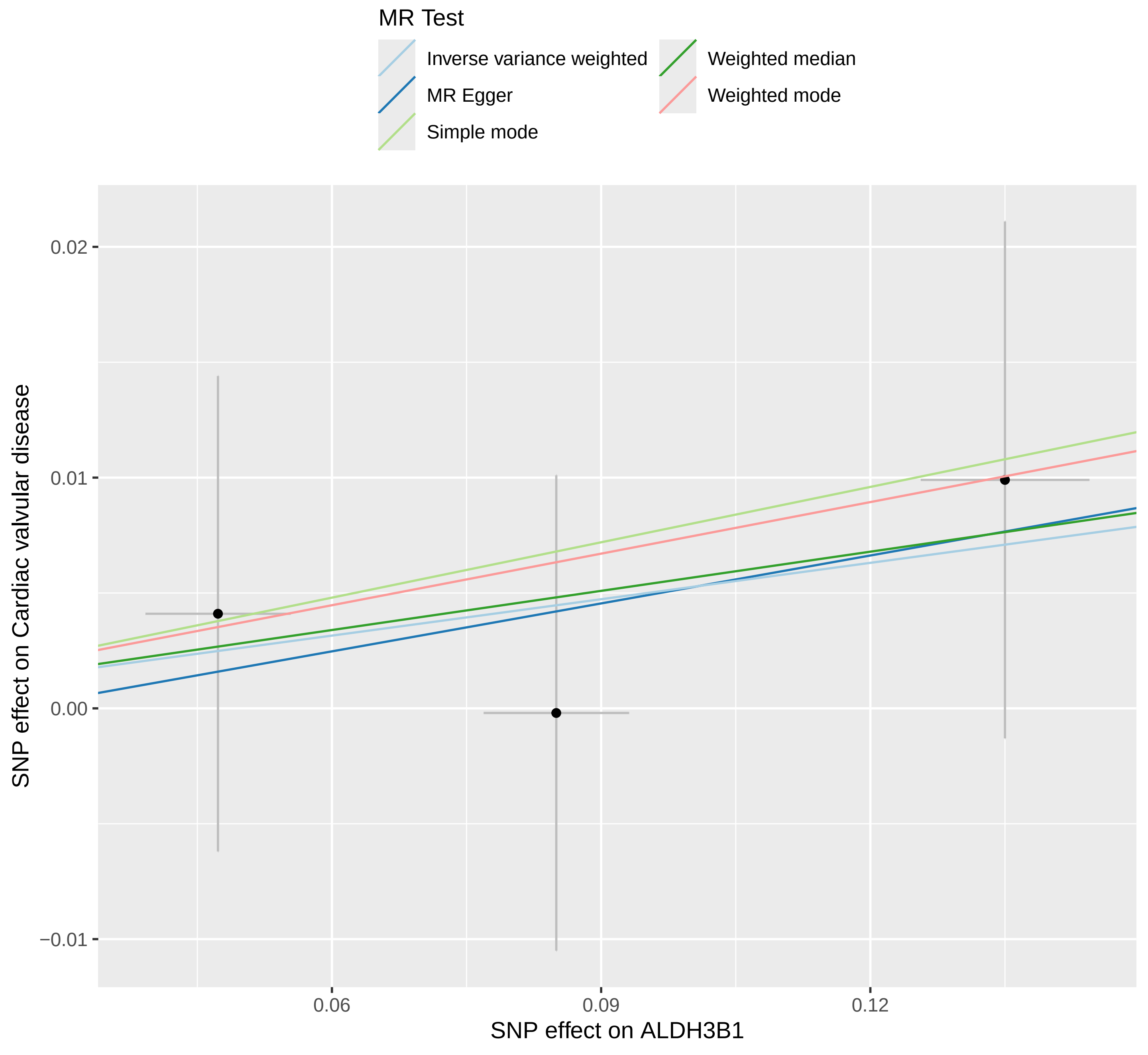
MR Test
Inverse variance weighted
MR Egger
Simple mode
Weighted median
Weighted mode
0.02
0.01
0.00
−0.01
0.06
0.09
0.12
SNP effect on Cardiac valvular disease
SNP effect on ALDH3B1

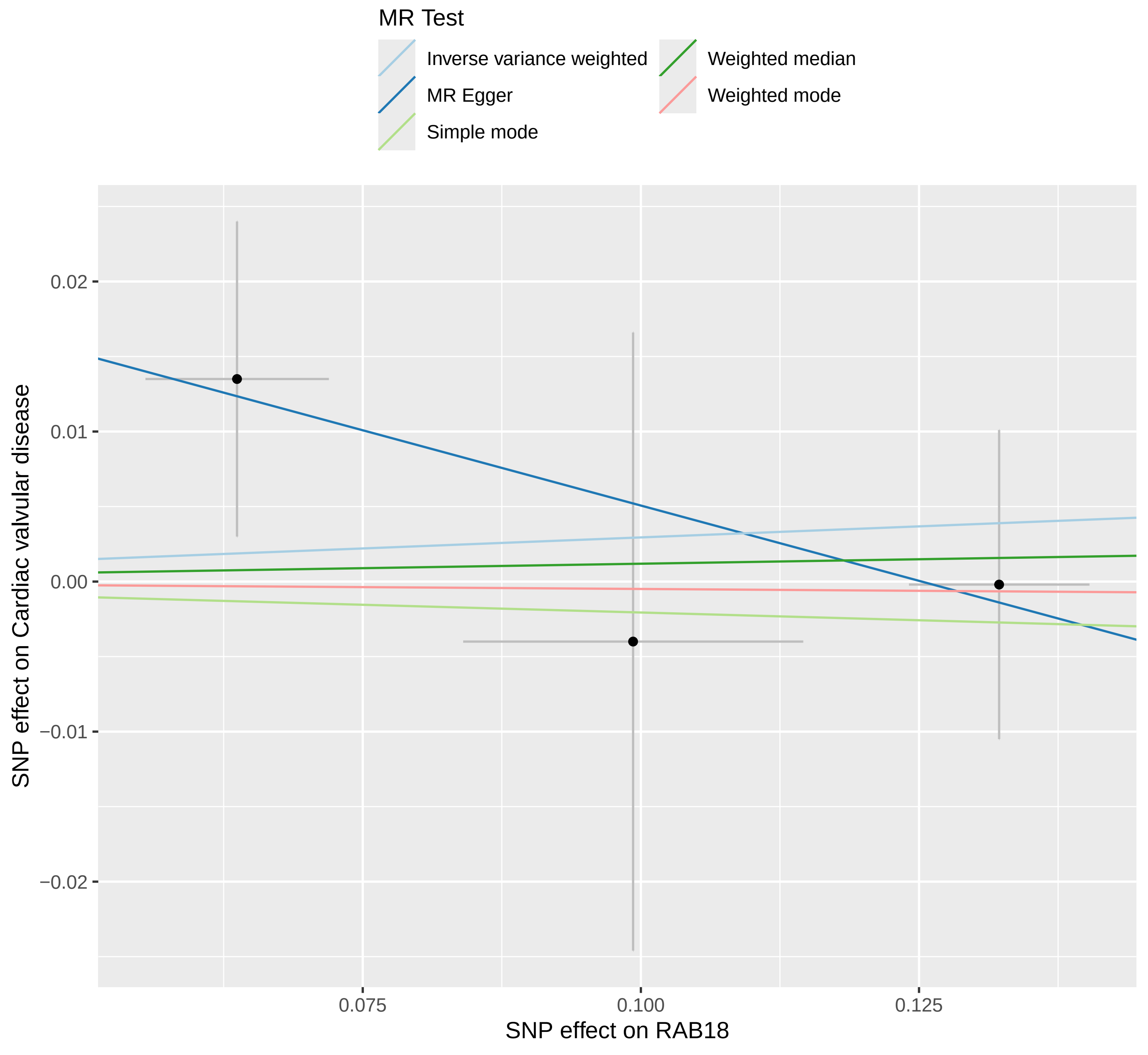

MR Test
Inverse variance weighted
MR Egger
Simple mode
Weighted median
Weighted mode
0.02
0.01
0.00
−0.01
−0.02
SNP effect on Cardiac valvular disease
0.075
0.100
0.125
SNP effect on RAB18

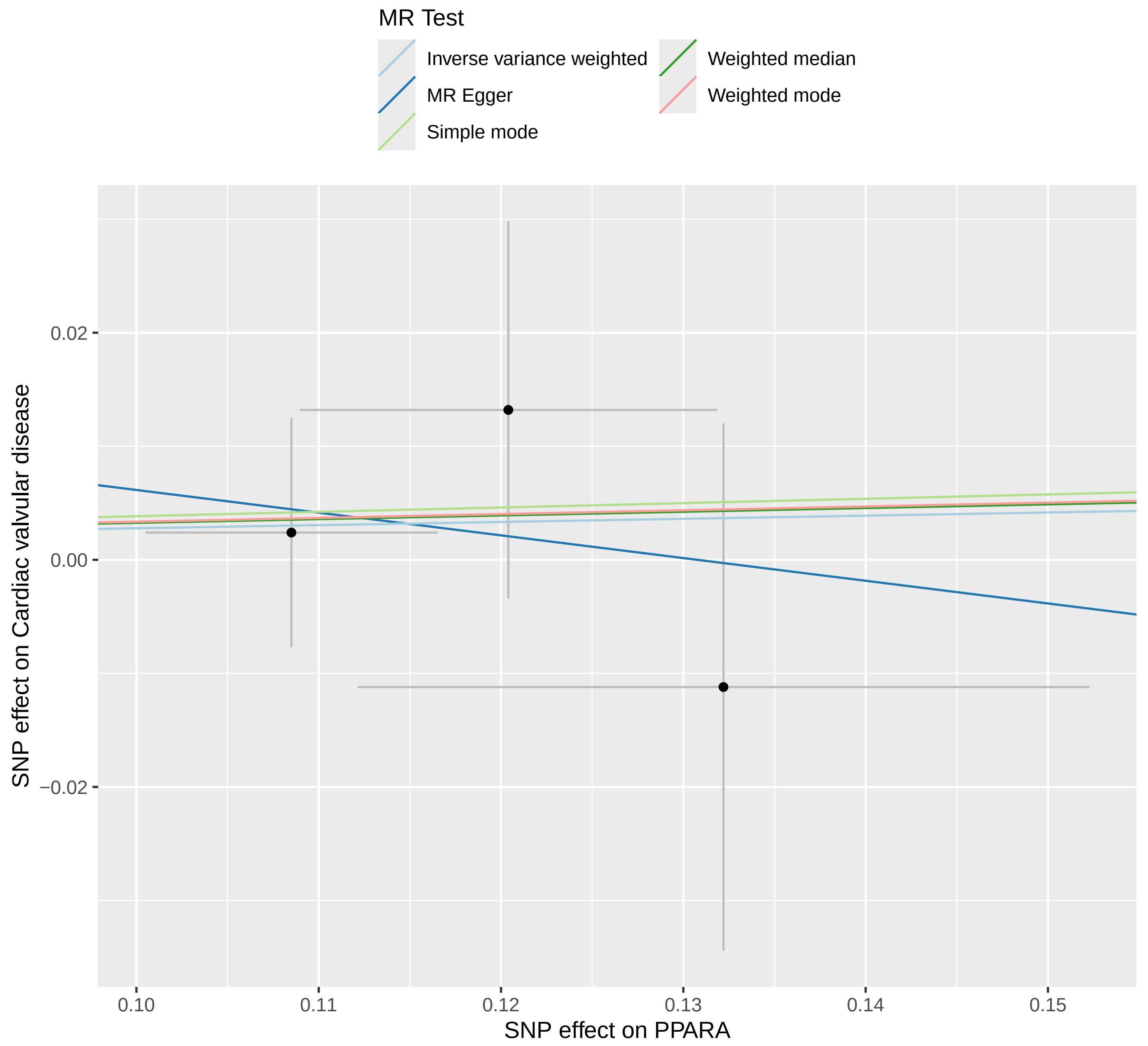
MR Test
Inverse variance weighted
MR Egger
Simple mode
Weighted median
Weighted mode
0.02
0.00
−0.02
0.10
0.11
0.12
0.13
0.14
0.15
SNP effect on PPARA
SNP effect on Cardiac valvular disease

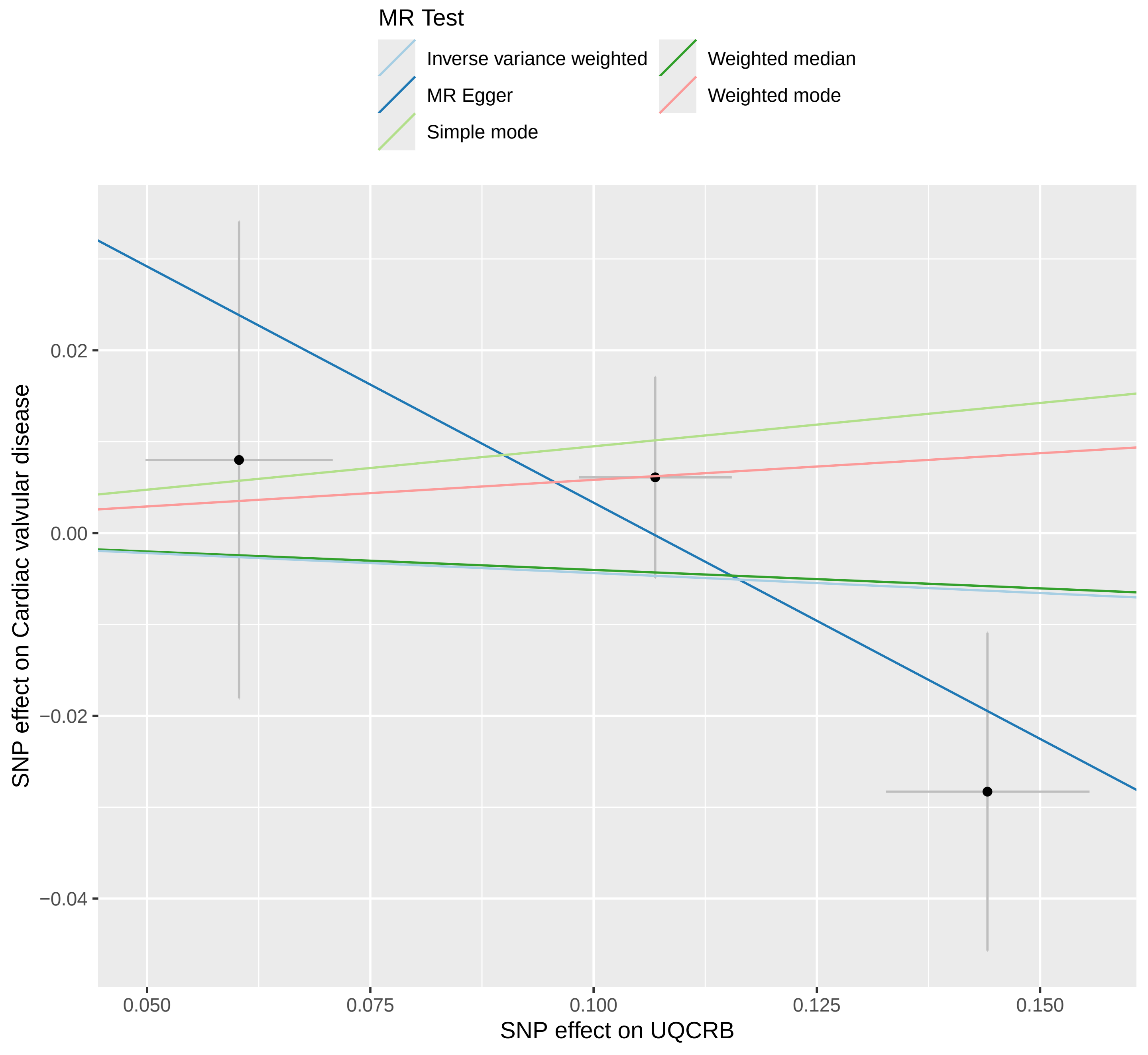
MR Test
Inverse variance weighted
MR Egger
Simple mode
Weighted median
Weighted mode
0.02
0.00
−0.02
−0.04
SNP effect on Cardiac valvular disease
0.050
0.075
0.100
0.125
0.150
SNP effect on UQCRB

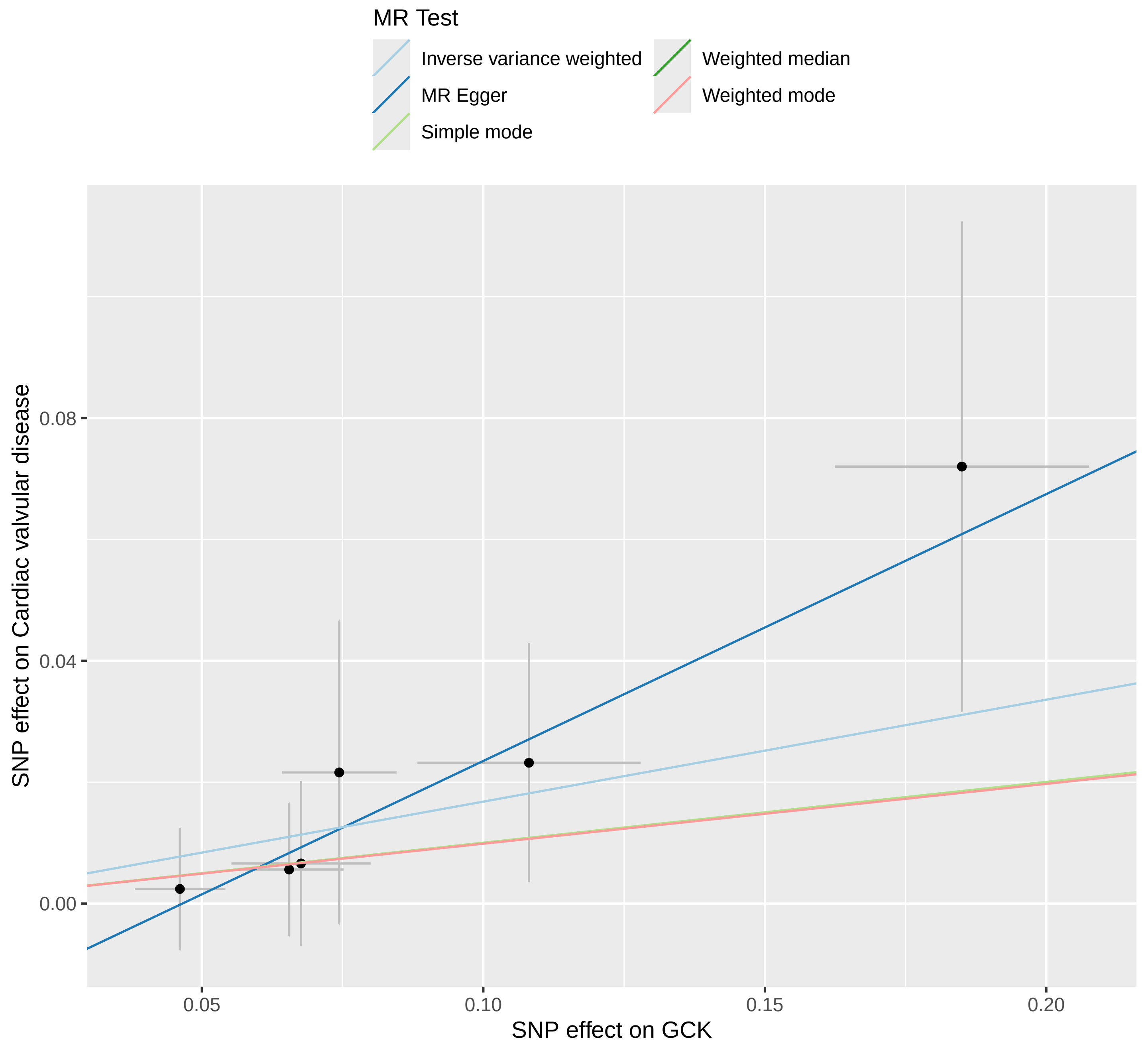
MR Test
Inverse variance weighted
MR Egger
Simple mode
Weighted median
Weighted mode
SNP effect on Cardiac valvular disease
0.08
0.04
0.00
0.05
0.10
0.15
0.20
SNP effect on GCK

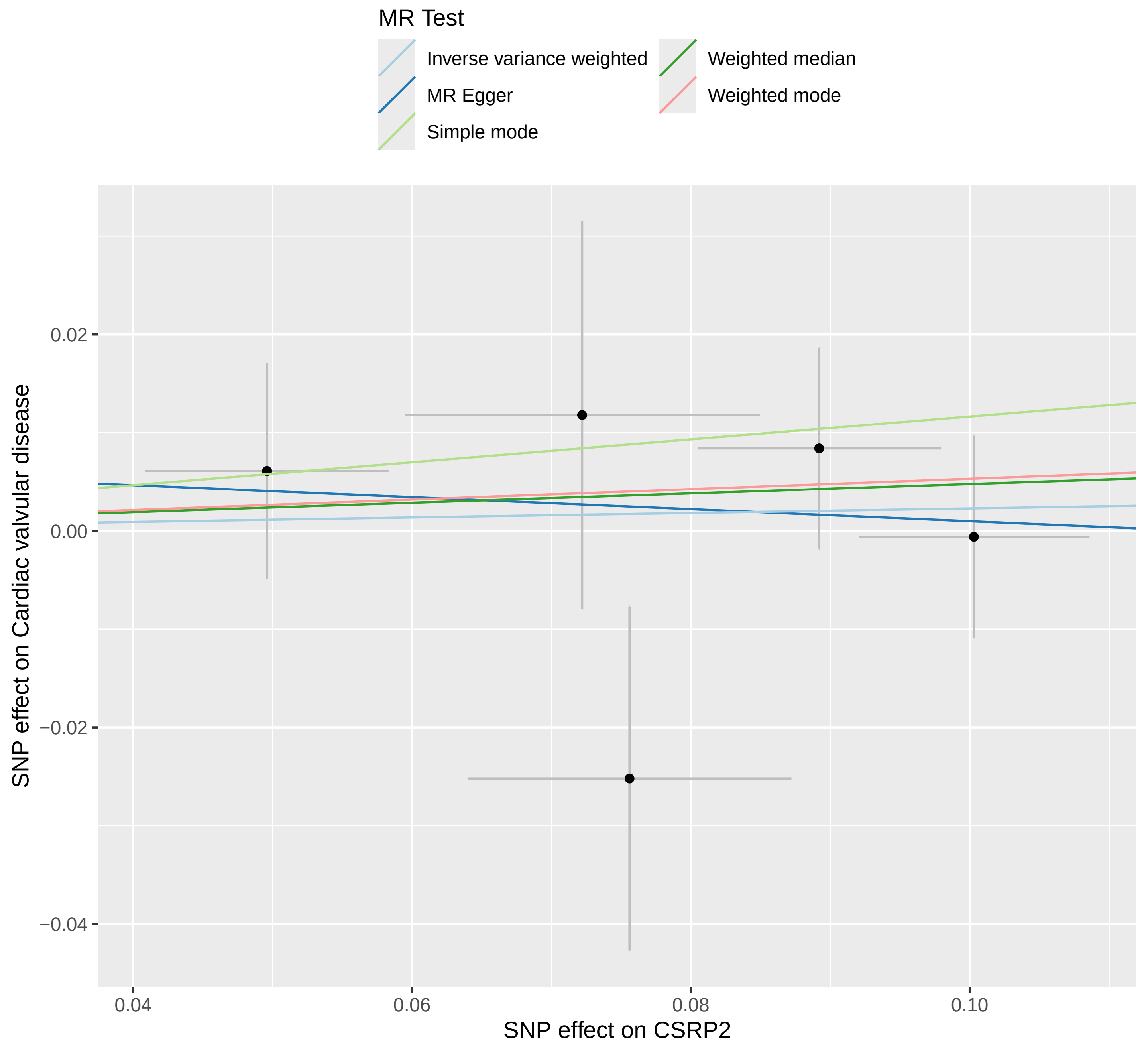

MR Test
Inverse variance weighted
MR Egger
Simple mode
Weighted median
Weighted mode
0.02
0.00
−0.02
−0.04
0.04
0.06
0.08
0.10
SNP effect on CSRP2
SNP effect on Cardiac valvular disease

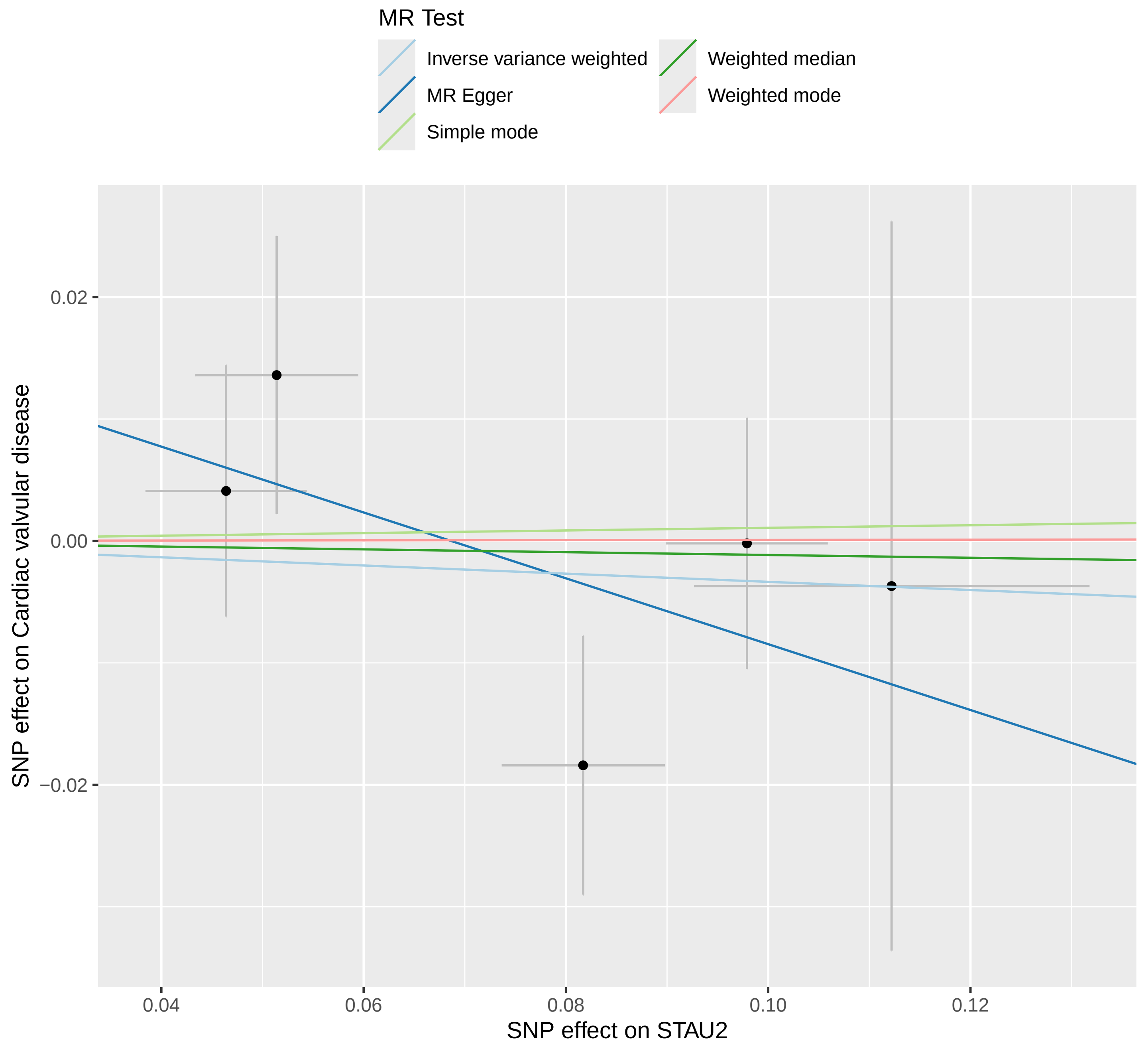
MR Test
Inverse variance weighted
MR Egger
Simple mode
Weighted median
Weighted mode
0.02
0.00
−0.02
SNP effect on Cardiac valvular disease
0.04
0.06
0.08
0.10
0.12
SNP effect on STAU2

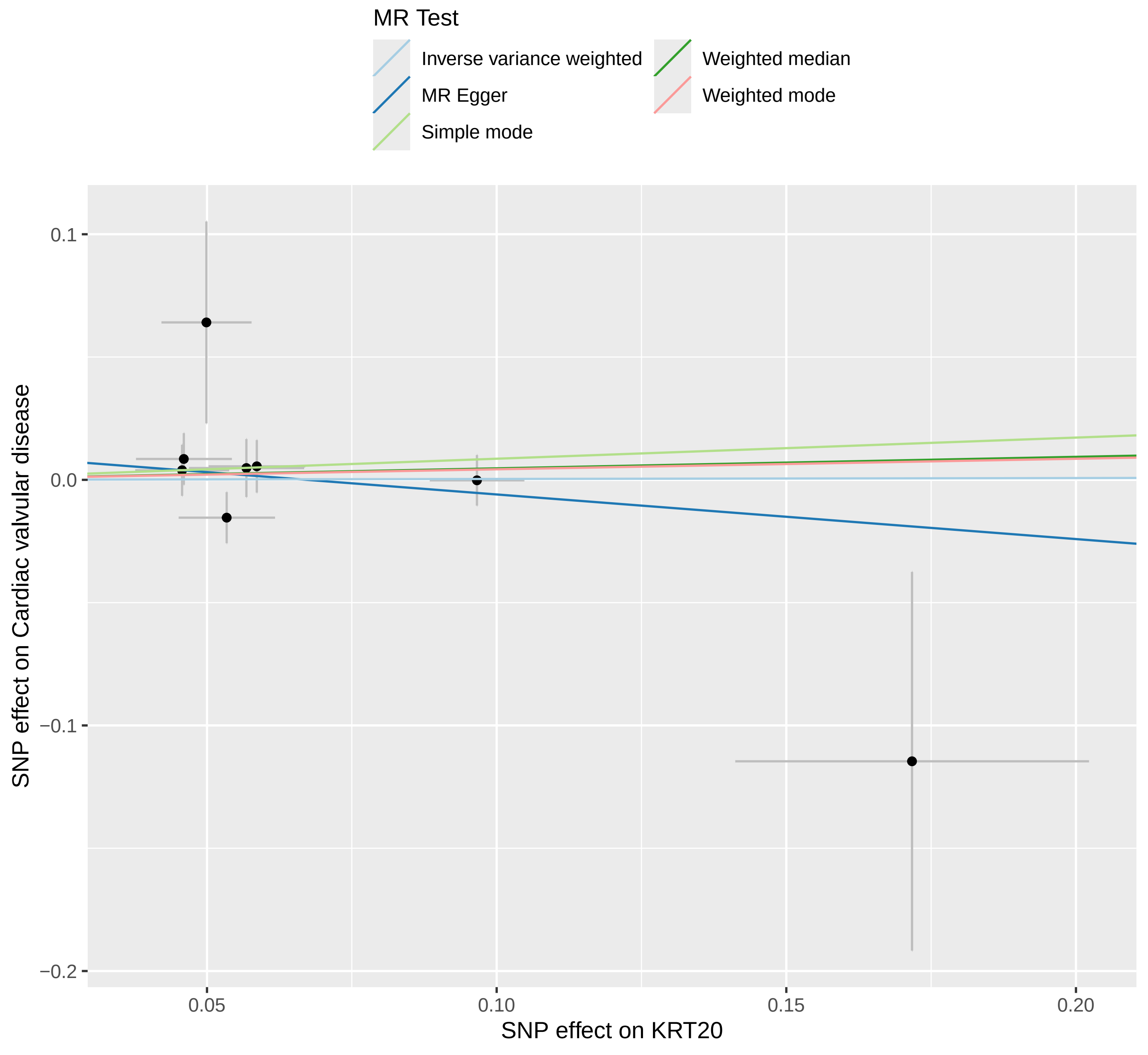

MR Test
Inverse variance weighted
MR Egger
Simple mode
Weighted median
Weighted mode
0.1
0.0
−0.1
−0.2
0.05
0.10
0.15
0.20
SNP effect on KRT20
SNP effect on Cardiac valvular disease

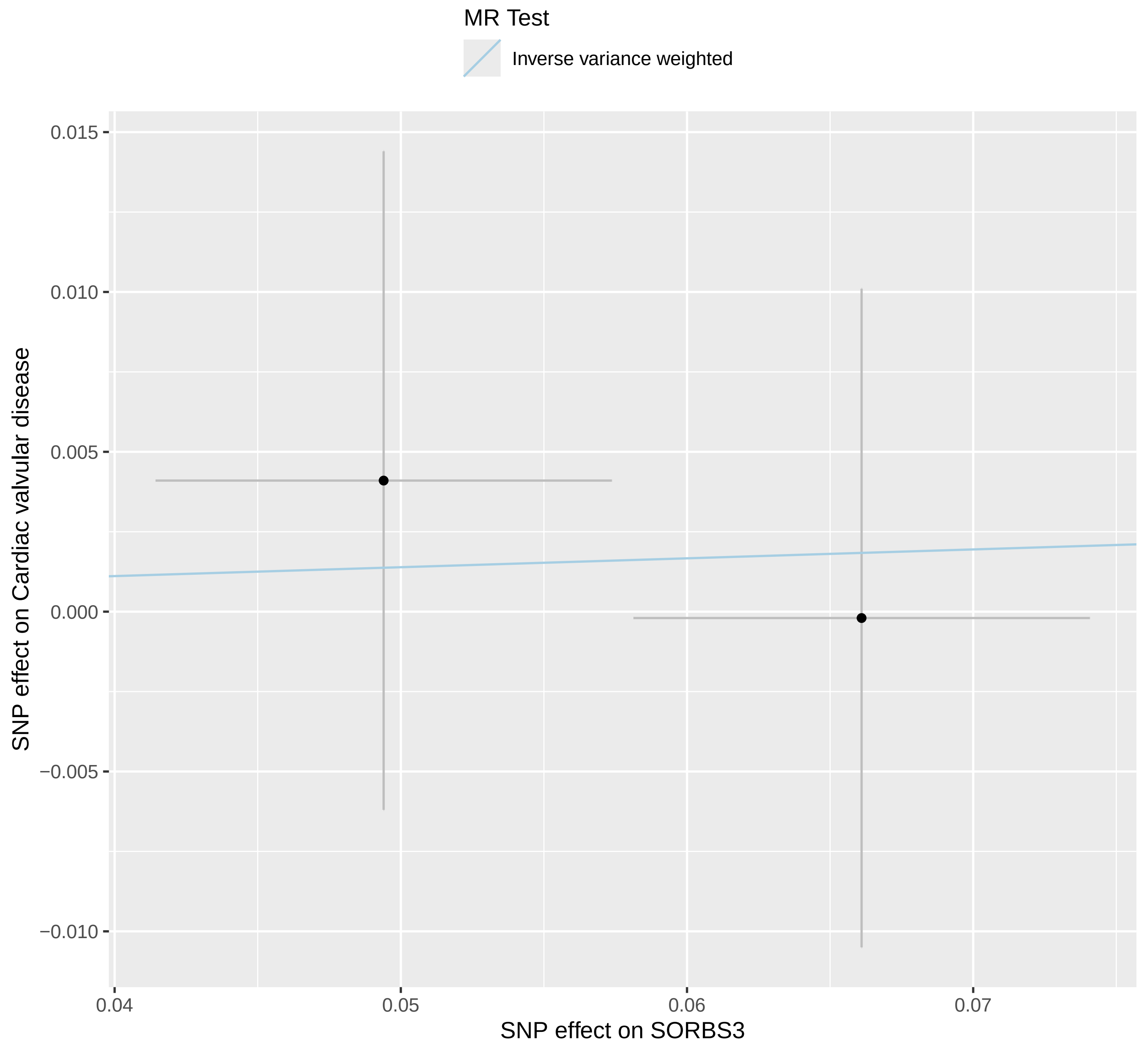
MR Test
Inverse variance weighted
0.015
0.010
0.005
0.000
−0.005
−0.010
SNP effect on Cardiac valvular disease
0.04
0.05
0.06
0.07
SNP effect on SORBS3

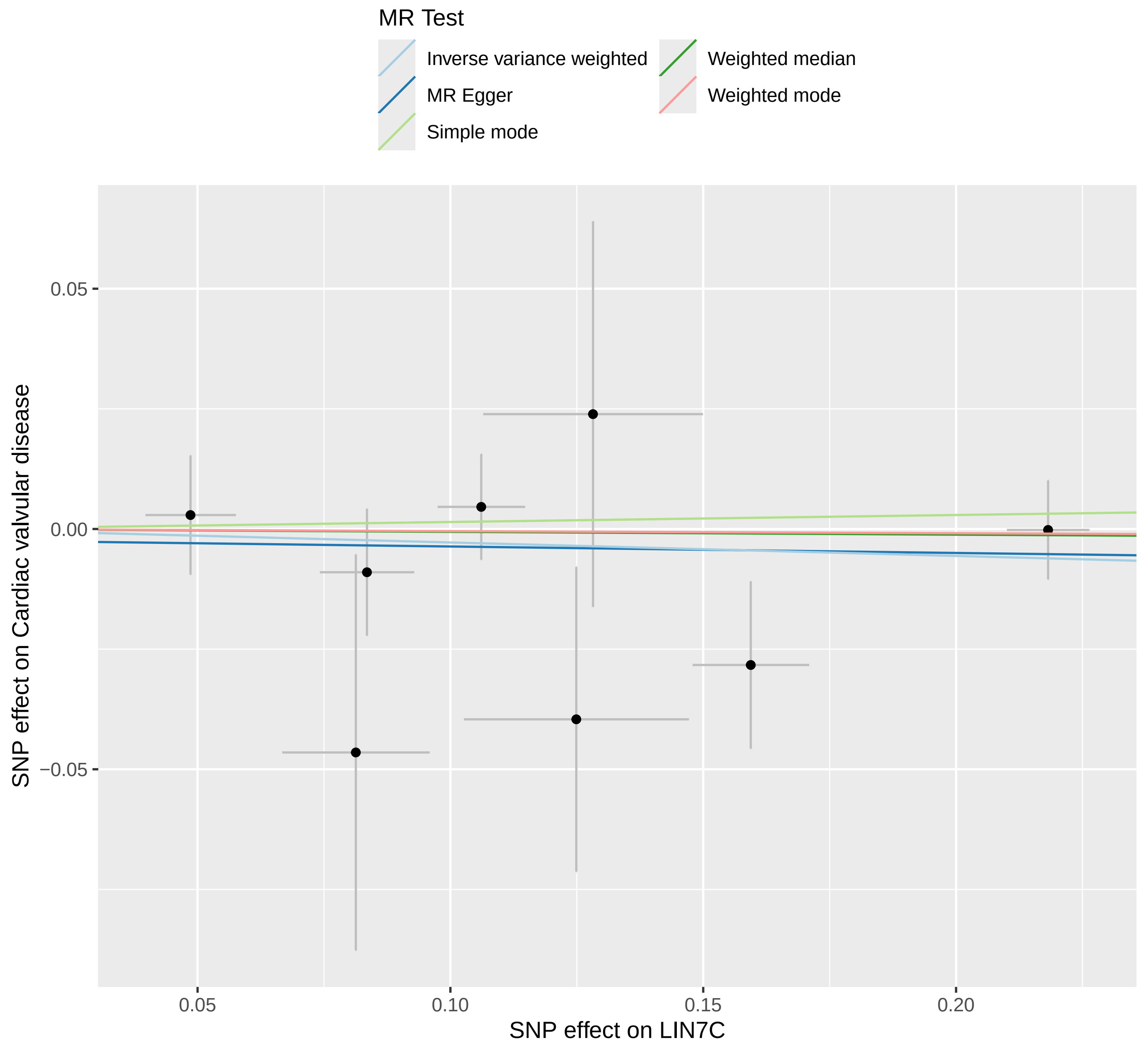
MR Test
Inverse variance weighted
MR Egger
Simple mode
Weighted median
Weighted mode
0.05
0.00
−0.05
0.05
0.10
0.15
0.20
SNP effect on LIN7C
SNP effect on Cardiac valvular disease

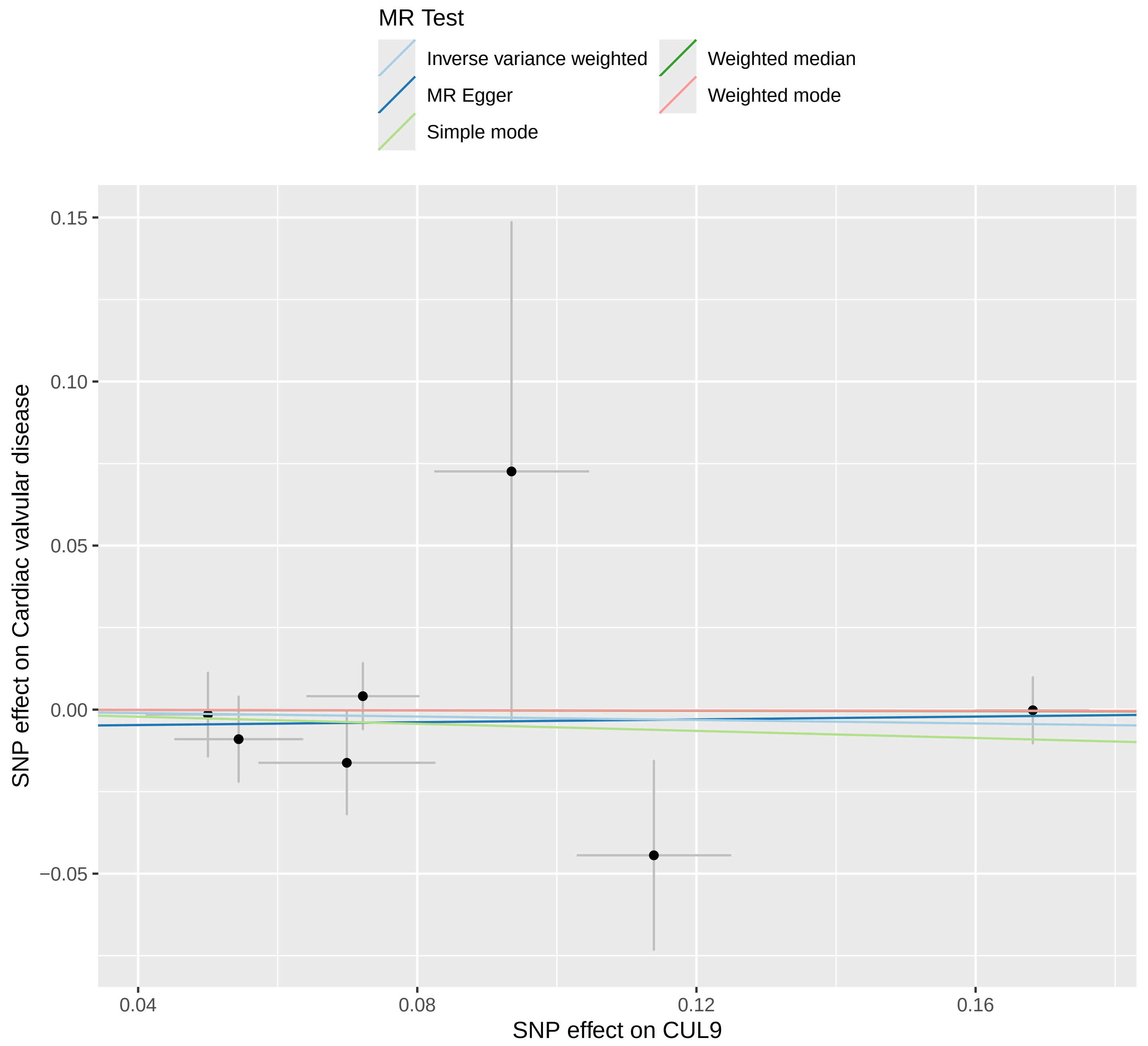

MR Test
Inverse variance weighted
MR Egger
Simple mode
Weighted median
Weighted mode
0.15
0.10
0.05
0.00
−0.05
SNP effect on Cardiac valvular disease
0.04
0.08
0.12
0.16
SNP effect on CUL9

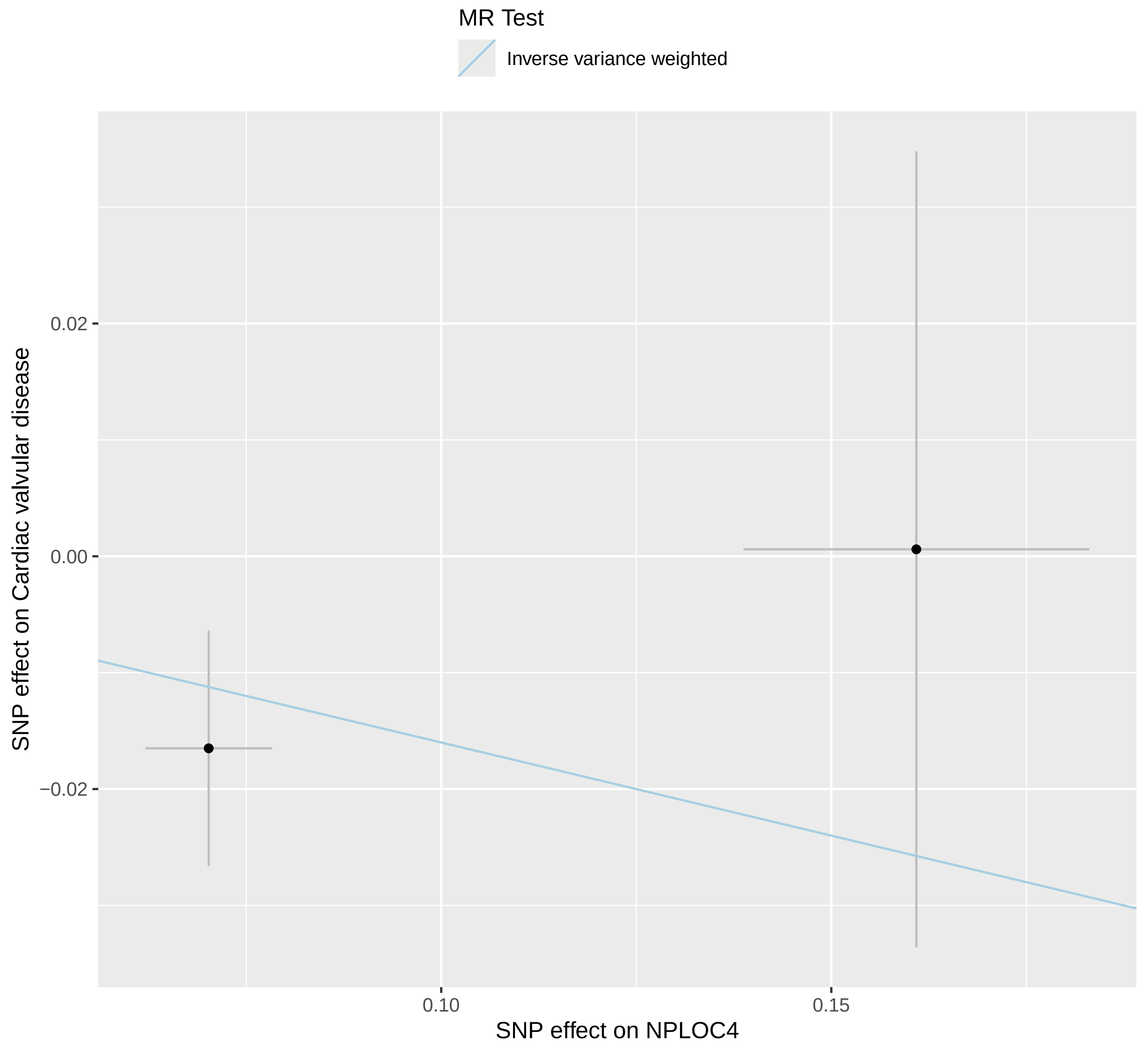
MR Test
Inverse variance weighted
SNP effect on Cardiac valvular disease
0.02
0.00
−0.02
0.10
0.15
SNP effect on NPLOC4

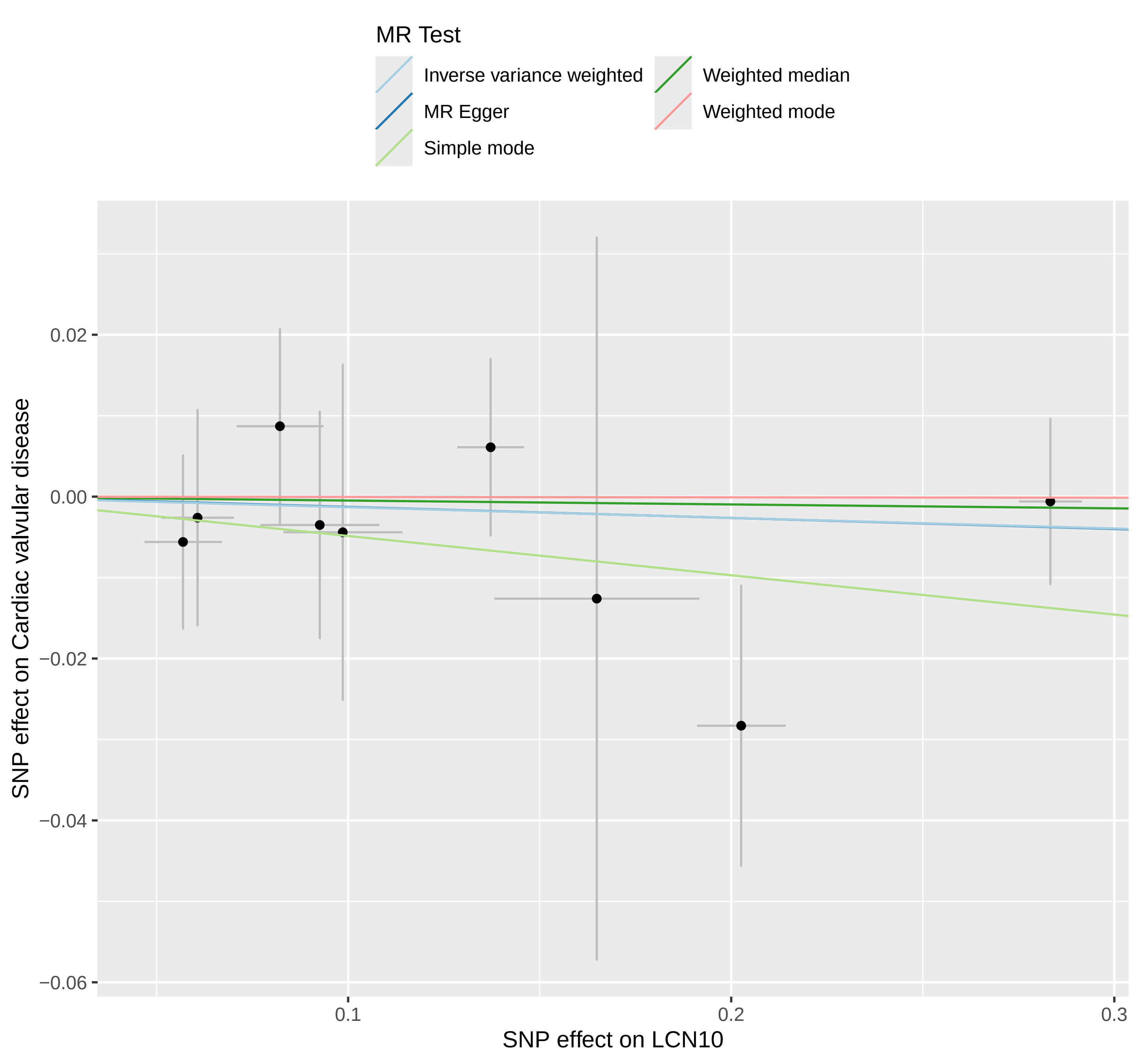
MR Test
Inverse variance weighted
MR Egger
Simple mode
Weighted median
Weighted mode
SNP effect on Cardiac valvular disease
0.02
0.00
−0.02
−0.04
−0.06
0.1
0.2
0.3
SNP effect on LCN10

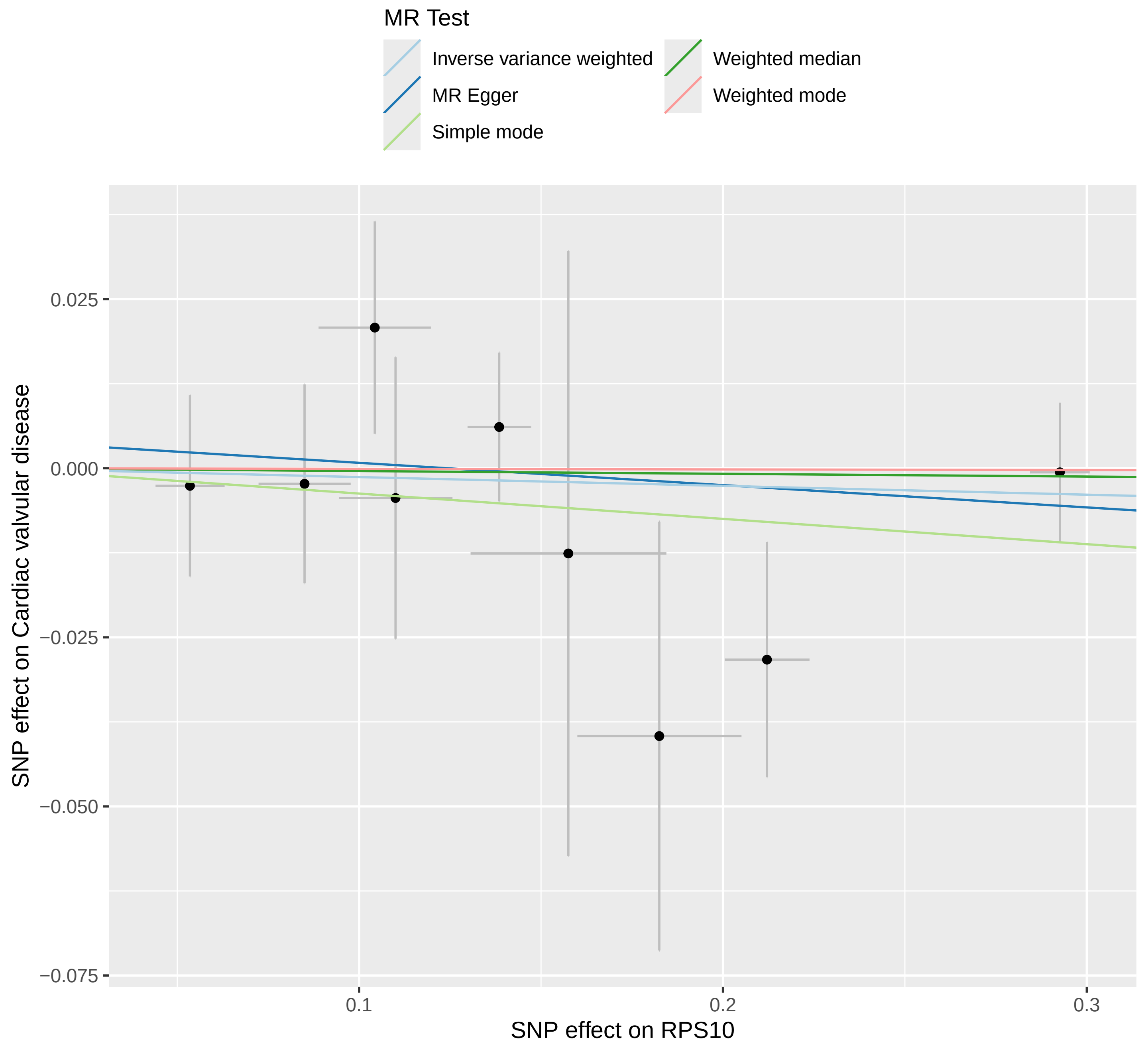
MR Test
Inverse variance weighted
MR Egger
Simple mode
Weighted median
Weighted mode
0.025
0.000
−0.025
−0.050
−0.075
0.1
0.2
0.3
SNP effect on Cardiac valvular disease
SNP effect on RPS10

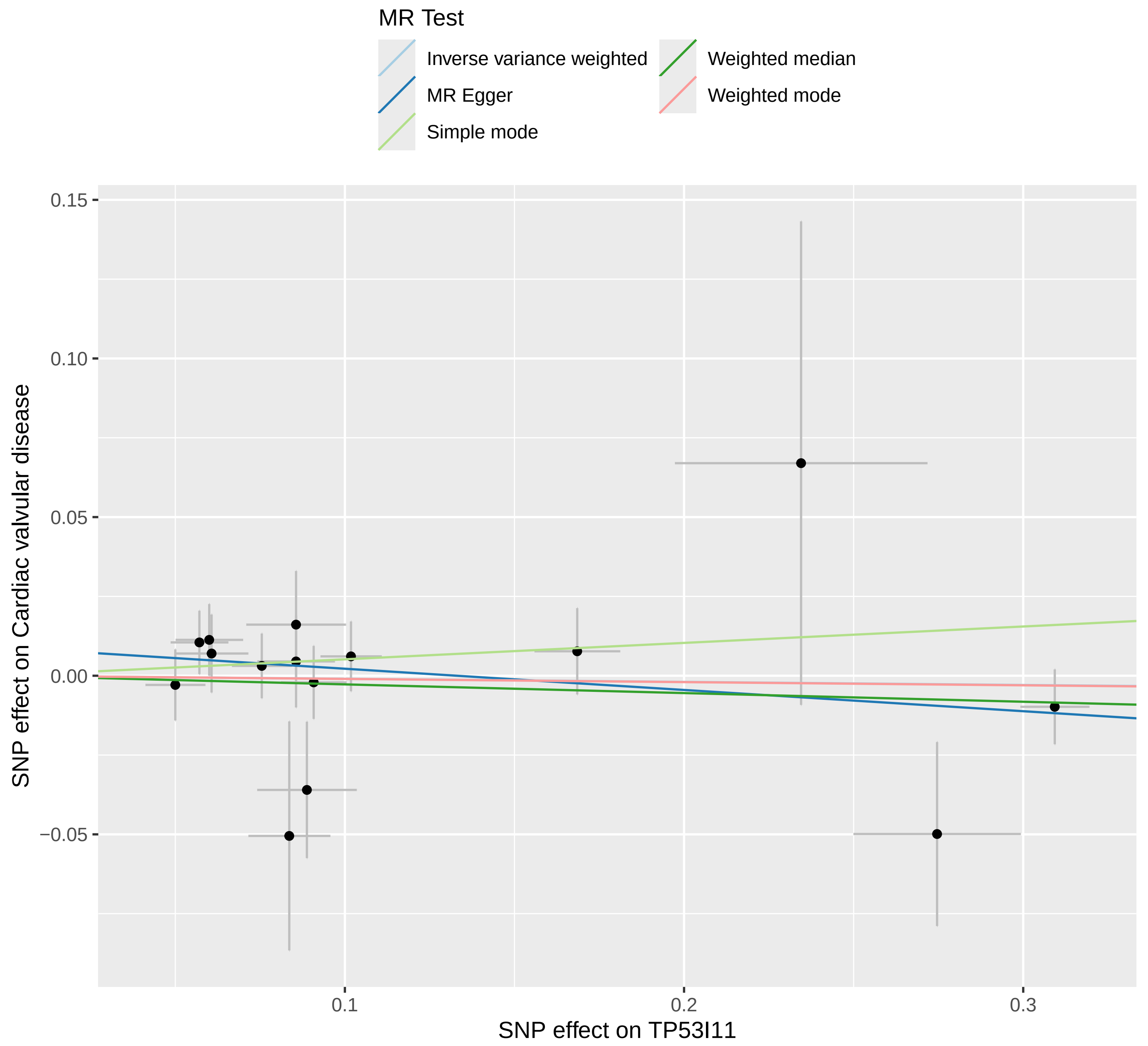
MR Test
Inverse variance weighted
MR Egger
Simple mode
Weighted median
Weighted mode
0.15
0.10
0.05
0.00
−0.05
0.1
0.2
0.3
SNP effect on Cardiac valvular disease
SNP effect on TP53I11

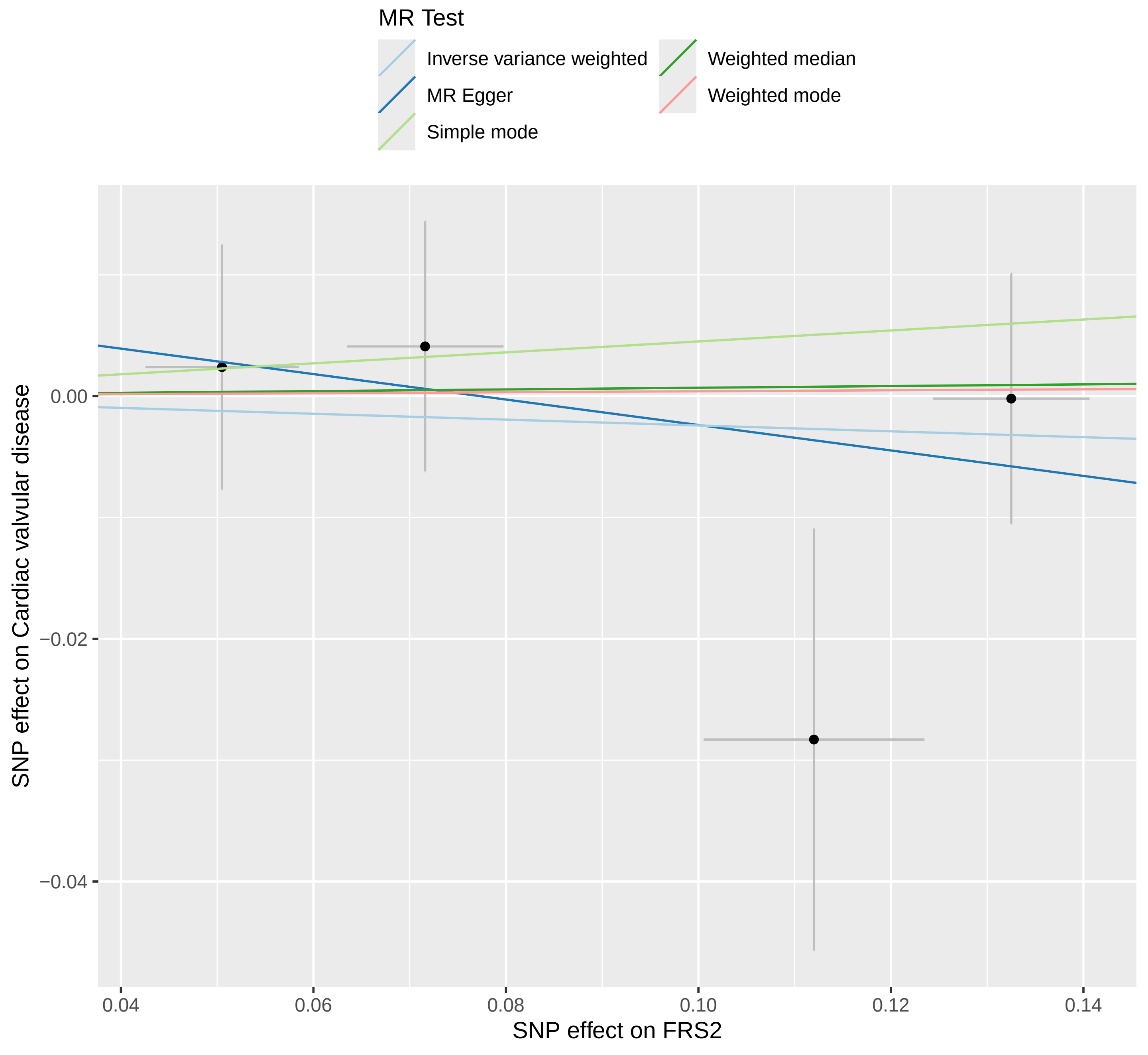
MR Test
Inverse variance weighted
MR Egger
Simple mode
Weighted median
Weighted mode
SNP effect on Cardiac valvular disease
0.00
−0.02
−0.04
0.04
0.06
0.08
0.10
0.12
0.14
SNP effect on FRS2

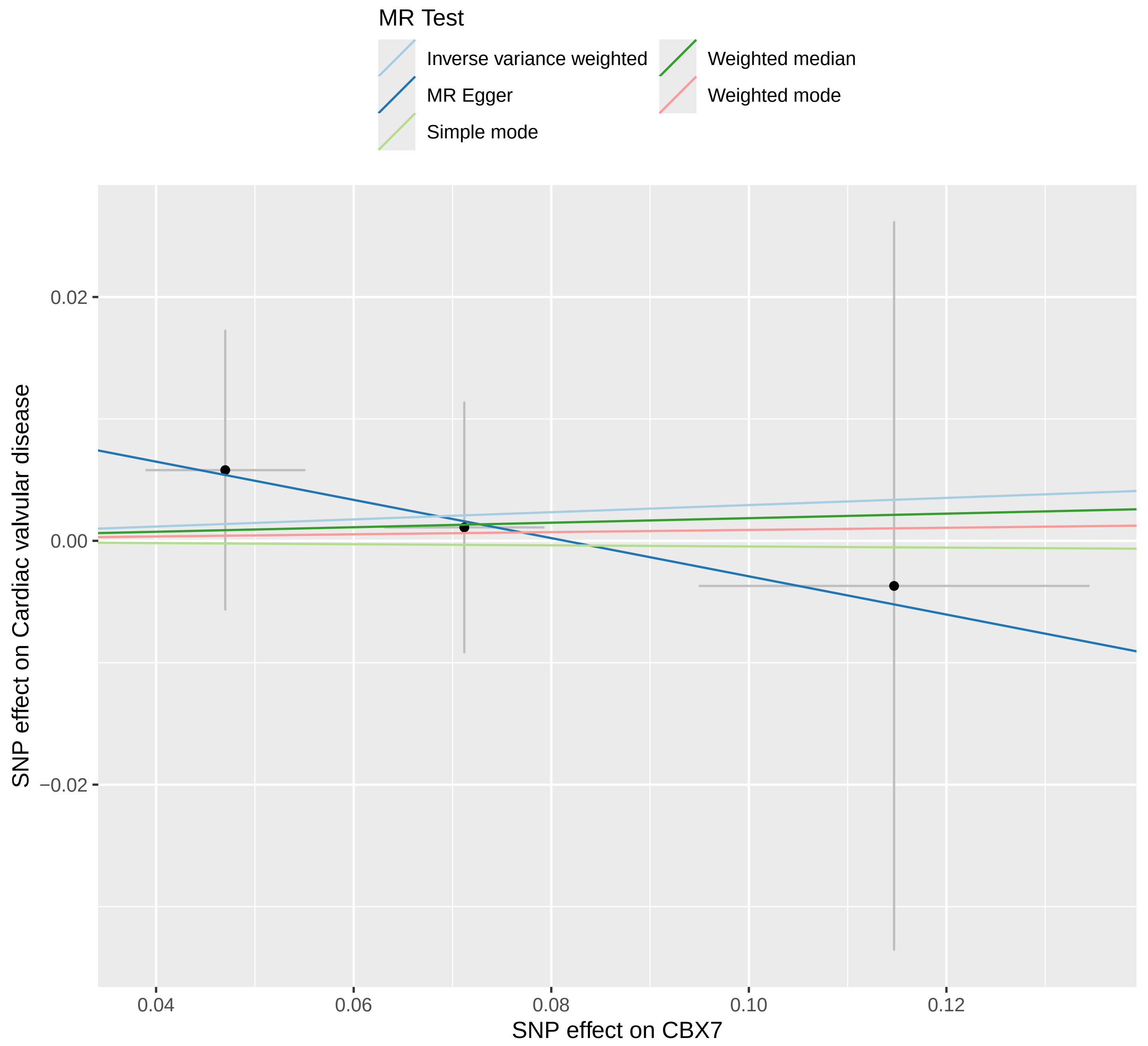
MR Test
Inverse variance weighted
MR Egger
Simple mode
Weighted median
Weighted mode
0.02
0.00
−0.02
0.04
0.06
0.08
0.10
0.12
SNP effect on CBX7
SNP effect on Cardiac valvular disease

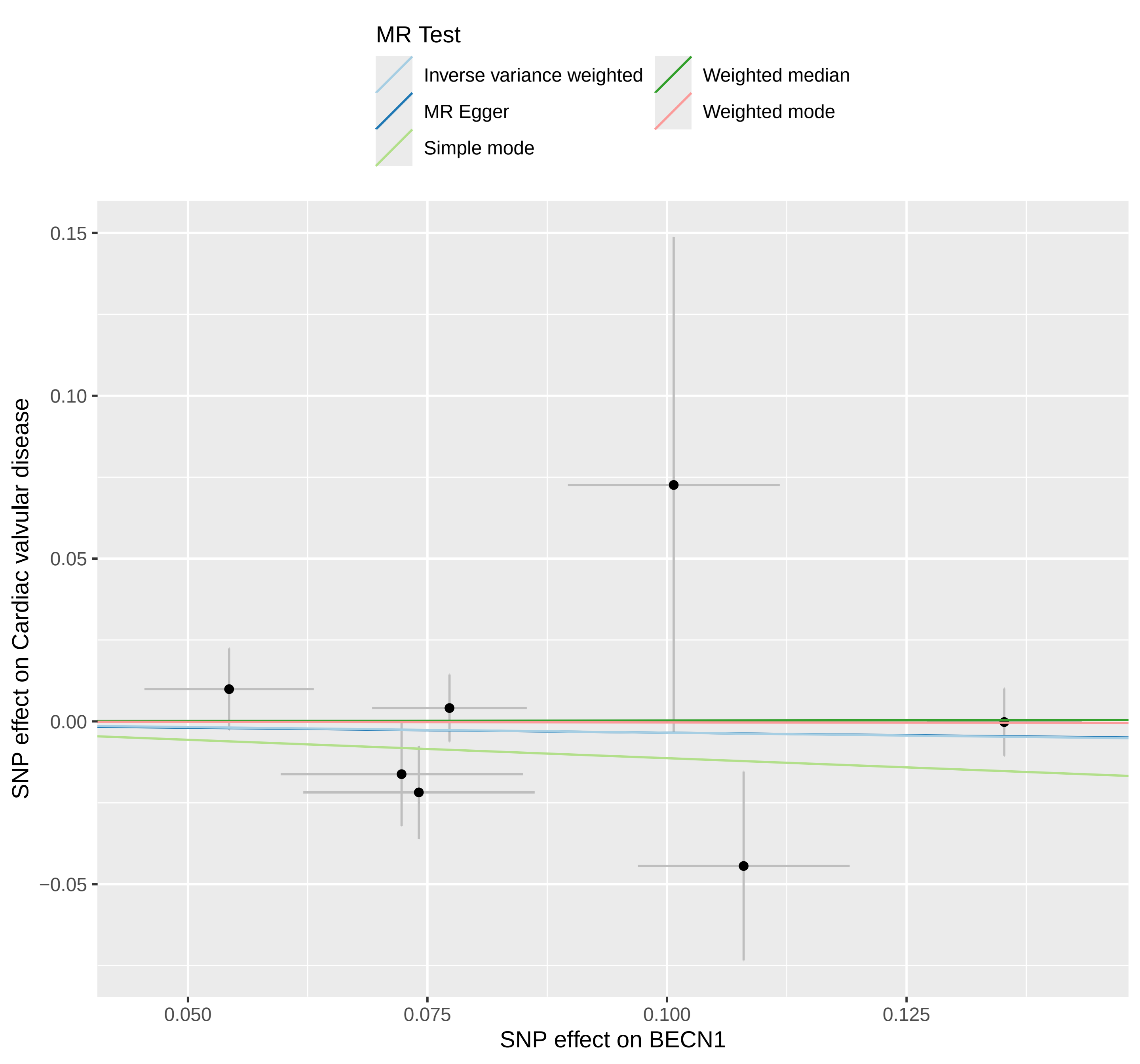

MR Test
Inverse variance weighted
MR Egger
Simple mode
Weighted median
Weighted mode
SNP effect on Cardiac valvular disease
0.15
0.10
0.05
0.00
−0.05
0.050
0.075
0.100
0.125
SNP effect on BECN1

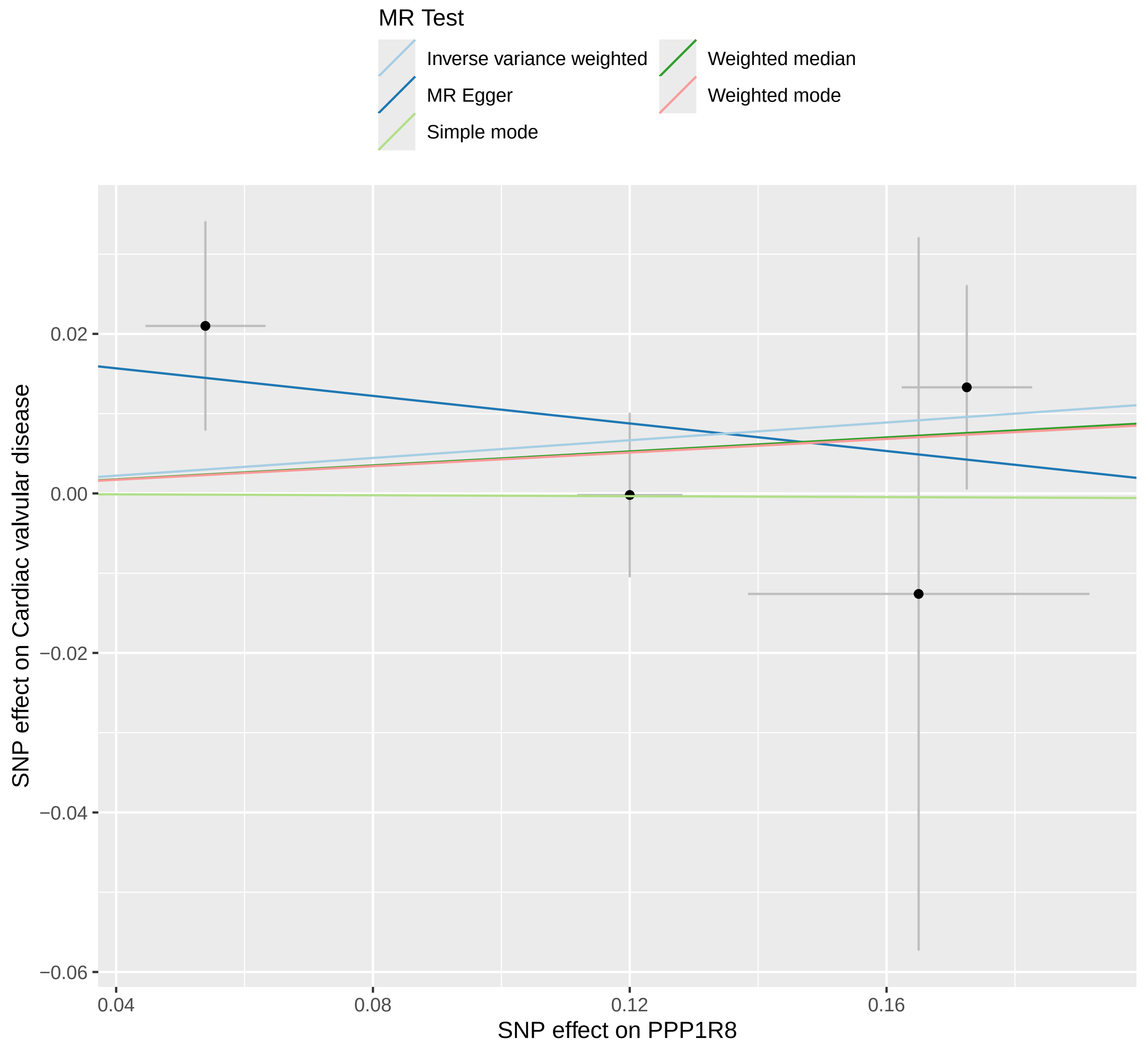
MR Test
Inverse variance weighted
MR Egger
Simple mode
Weighted median
Weighted mode
SNP effect on Cardiac valvular disease
0.02
0.00
−0.02
−0.04
−0.06
0.04
0.08
0.12
0.16
SNP effect on PPP1R8

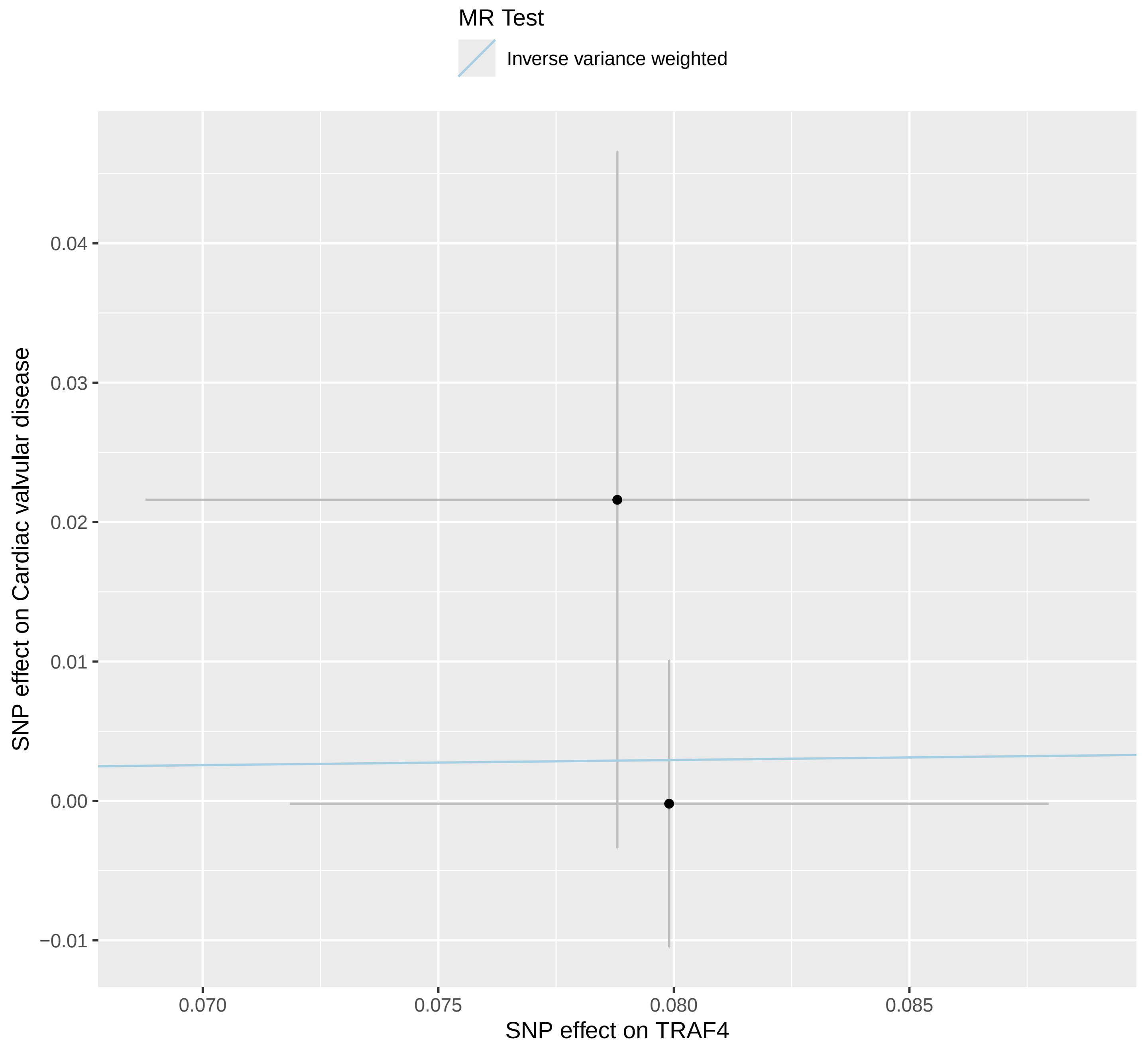
MR Test
Inverse variance weighted
0.04
0.03
0.02
0.01
0.00
−0.01
0.070
0.075
0.080
0.085
SNP effect on TRAF4
SNP effect on Cardiac valvular disease

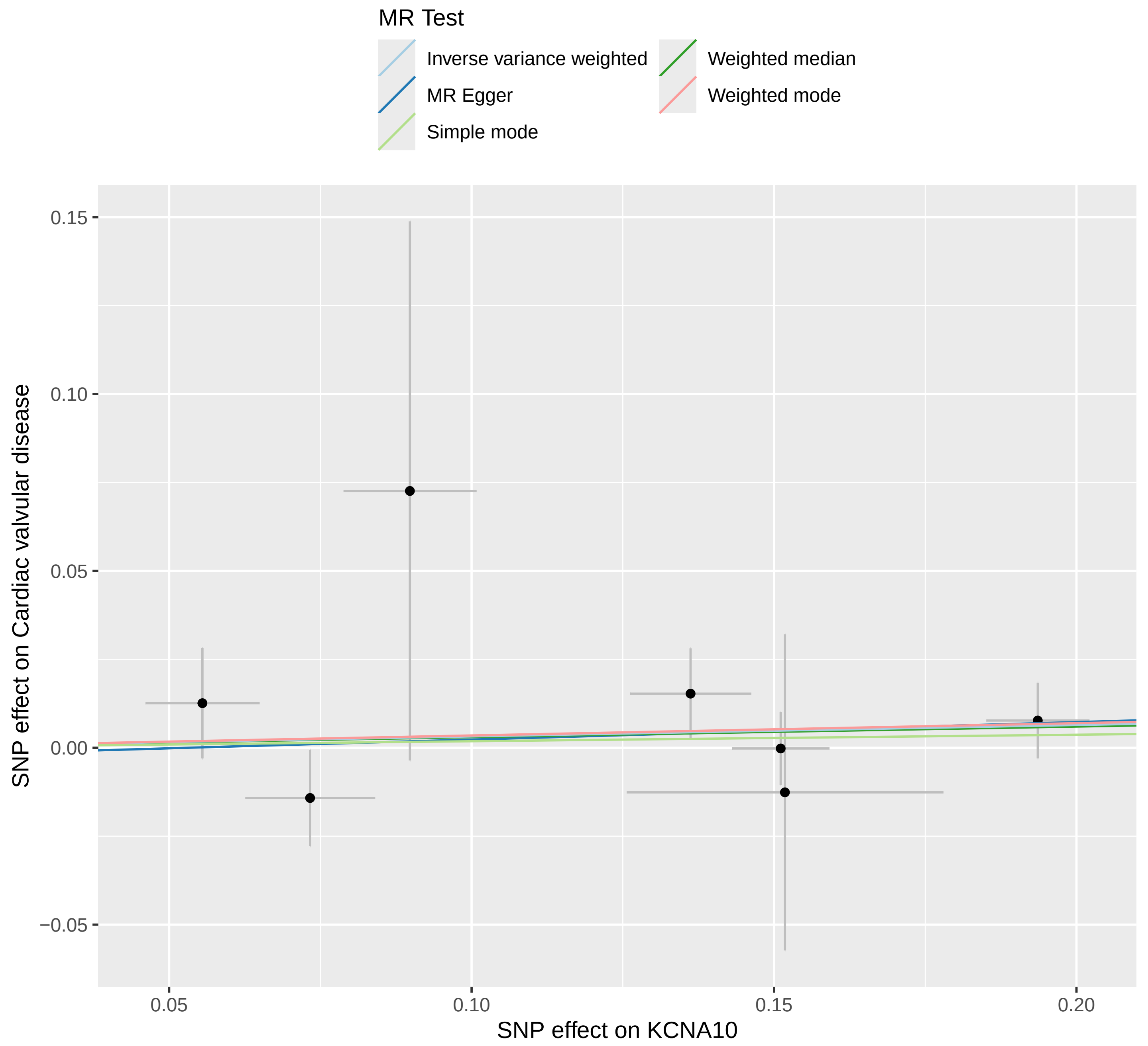
MR Test
Inverse variance weighted
MR Egger
Simple mode
Weighted median
Weighted mode
0.15
0.10
0.05
0.00
−0.05
SNP effect on Cardiac valvular disease
0.05
0.10
0.15
0.20
SNP effect on KCNA10

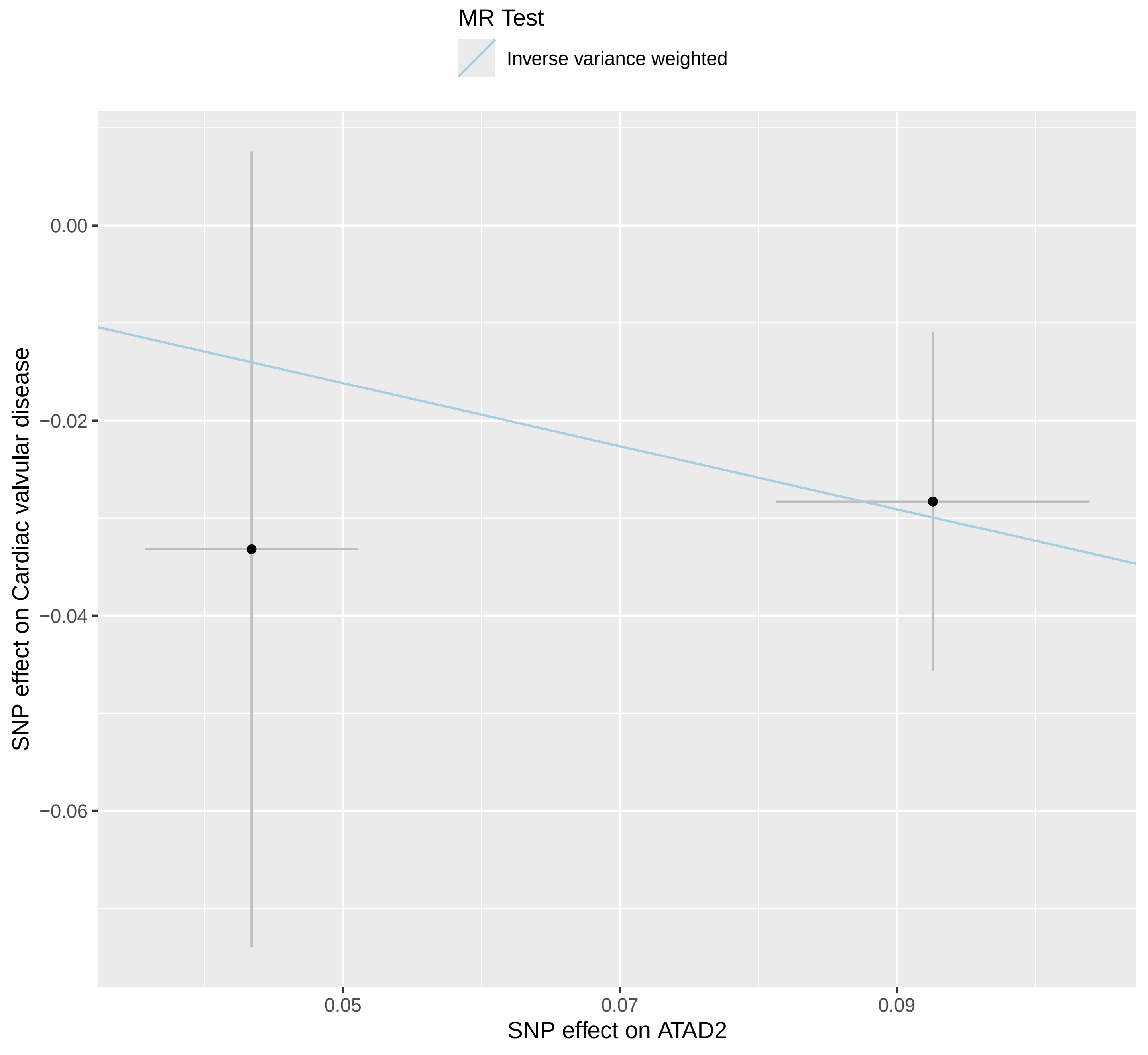
MR Test
Inverse variance weighted
0.00
−0.02
−0.04
−0.06
SNP effect on Cardiac valvular disease
0.05
0.07
0.09
SNP effect on ATAD2

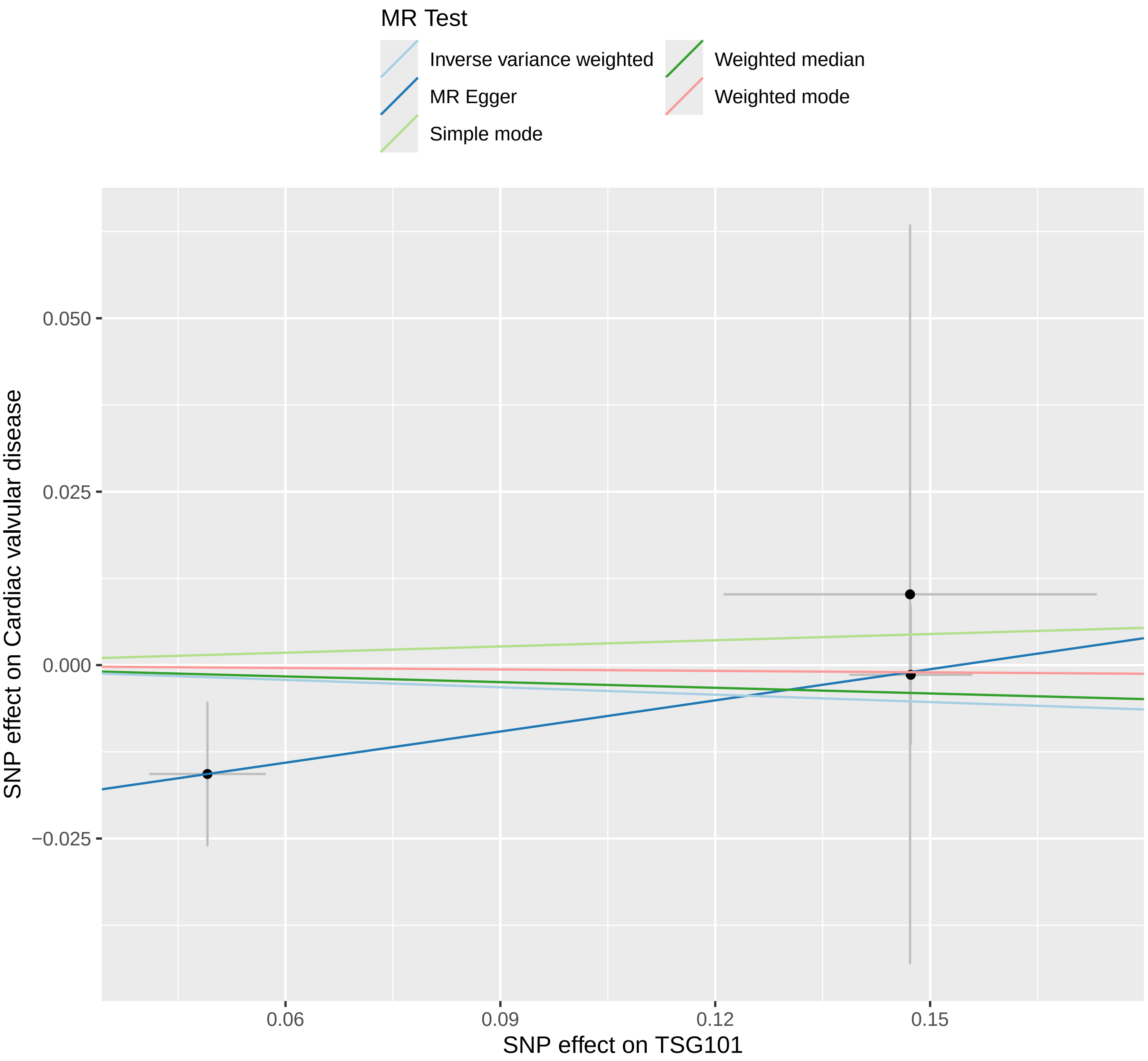
MR Test
Inverse variance weighted
MR Egger
Simple mode
Weighted median
Weighted mode
0.050
0.025
0.000
−0.025
SNP effect on Cardiac valvular disease
0.06
0.09
0.12
0.15
SNP effect on TSG101

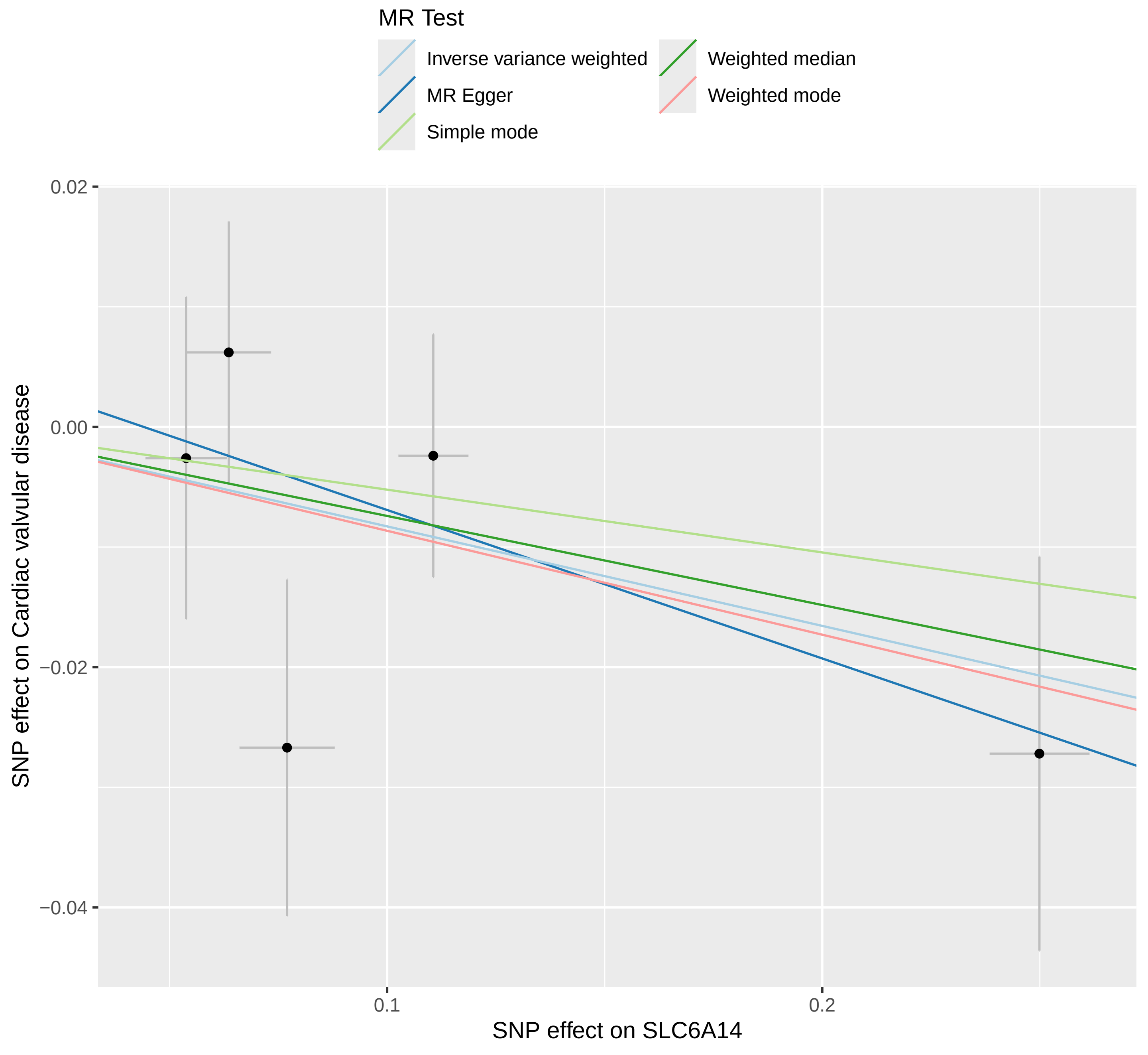
MR Test
Inverse variance weighted
MR Egger
Simple mode
Weighted median
Weighted mode
0.02
0.00
−0.02
−0.04
0.1
0.2
SNP effect on SLC6A14
SNP effect on Cardiac valvular disease

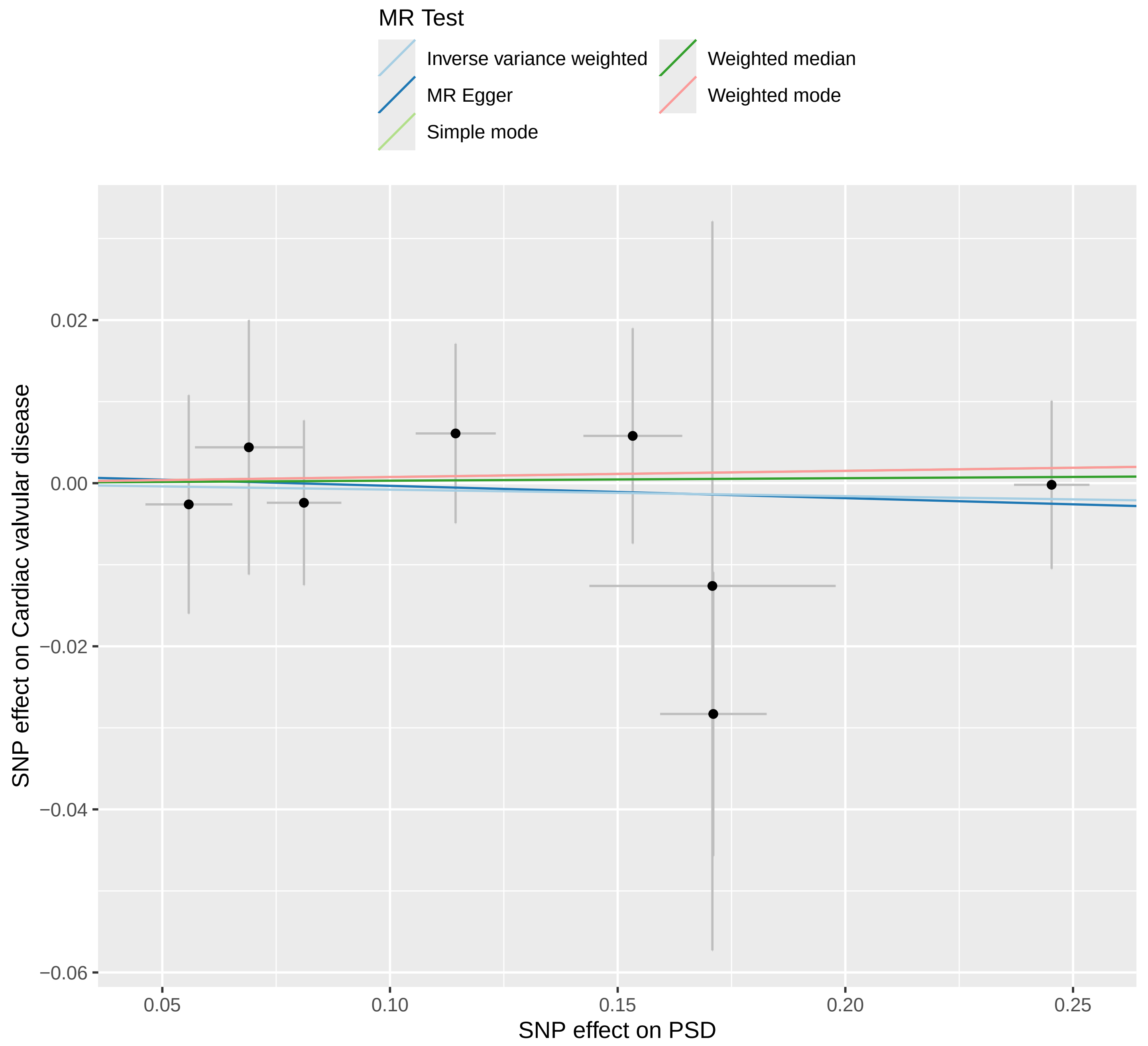

MR Test
Inverse variance weighted
MR Egger
Simple mode
Weighted median
Weighted mode
0.02
0.00
−0.02
−0.04
−0.06
0.05
0.10
0.15
0.20
0.25
SNP effect on Cardiac valvular disease
SNP effect on PSD

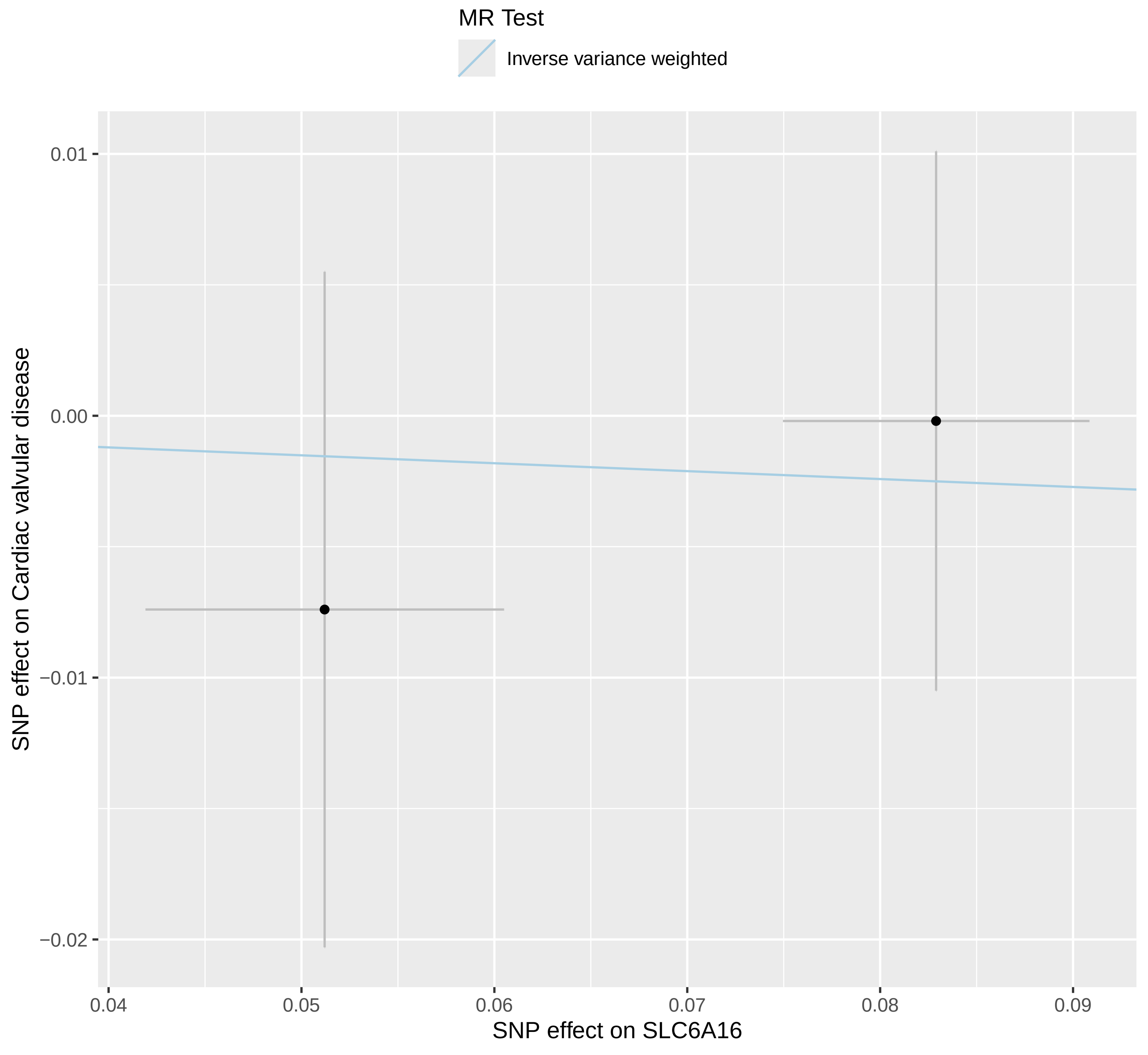
MR Test
Inverse variance weighted
0.01
0.00
−0.01
−0.02
SNP effect on Cardiac valvular disease
0.04
0.05
0.06
0.07
0.08
0.09
SNP effect on SLC6A16

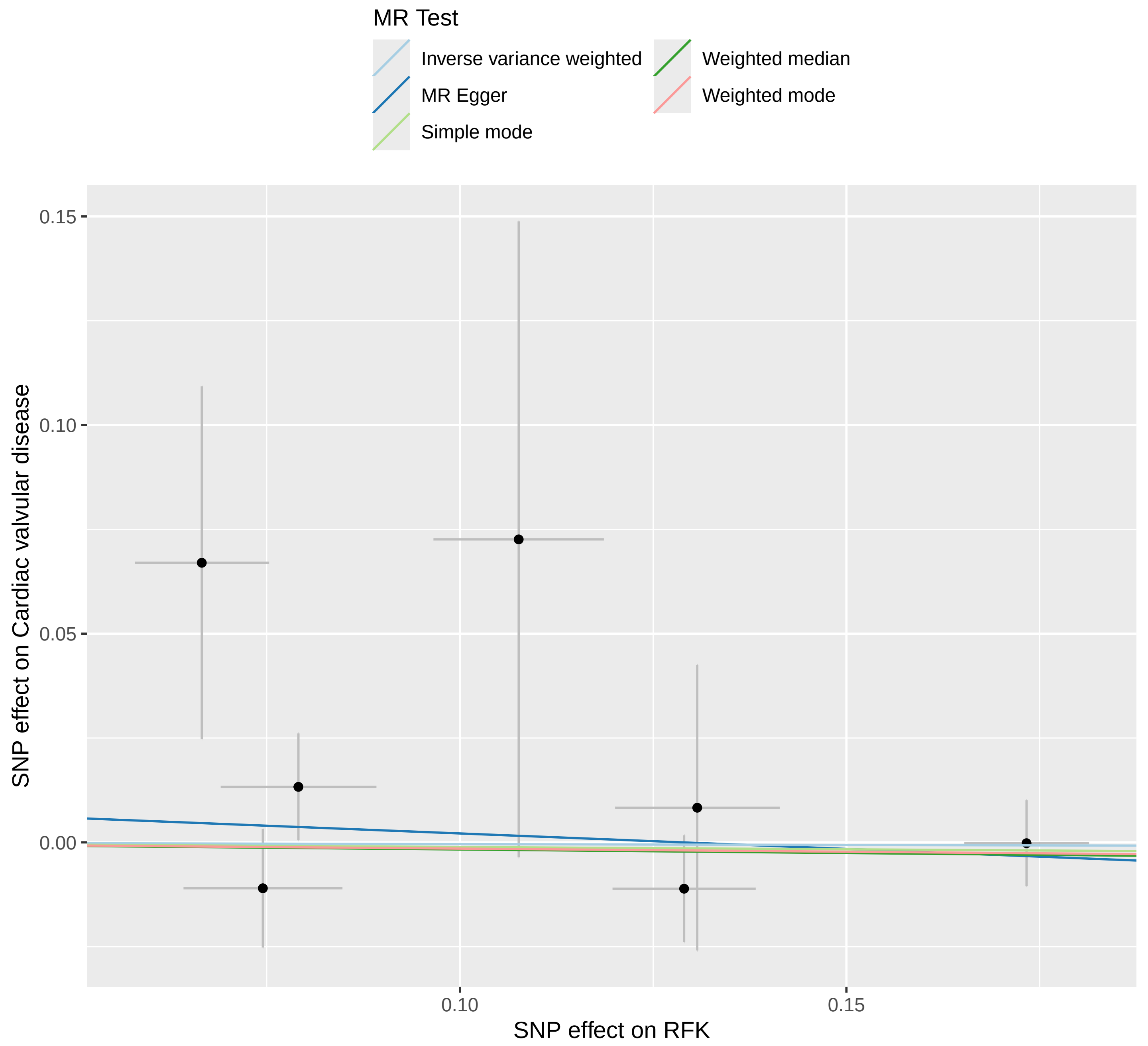
MR Test
Inverse variance weighted
MR Egger
Simple mode
Weighted median
Weighted mode
0.15
0.10
0.05
0.00
SNP effect on Cardiac valvular disease
0.10
0.15
SNP effect on RFK

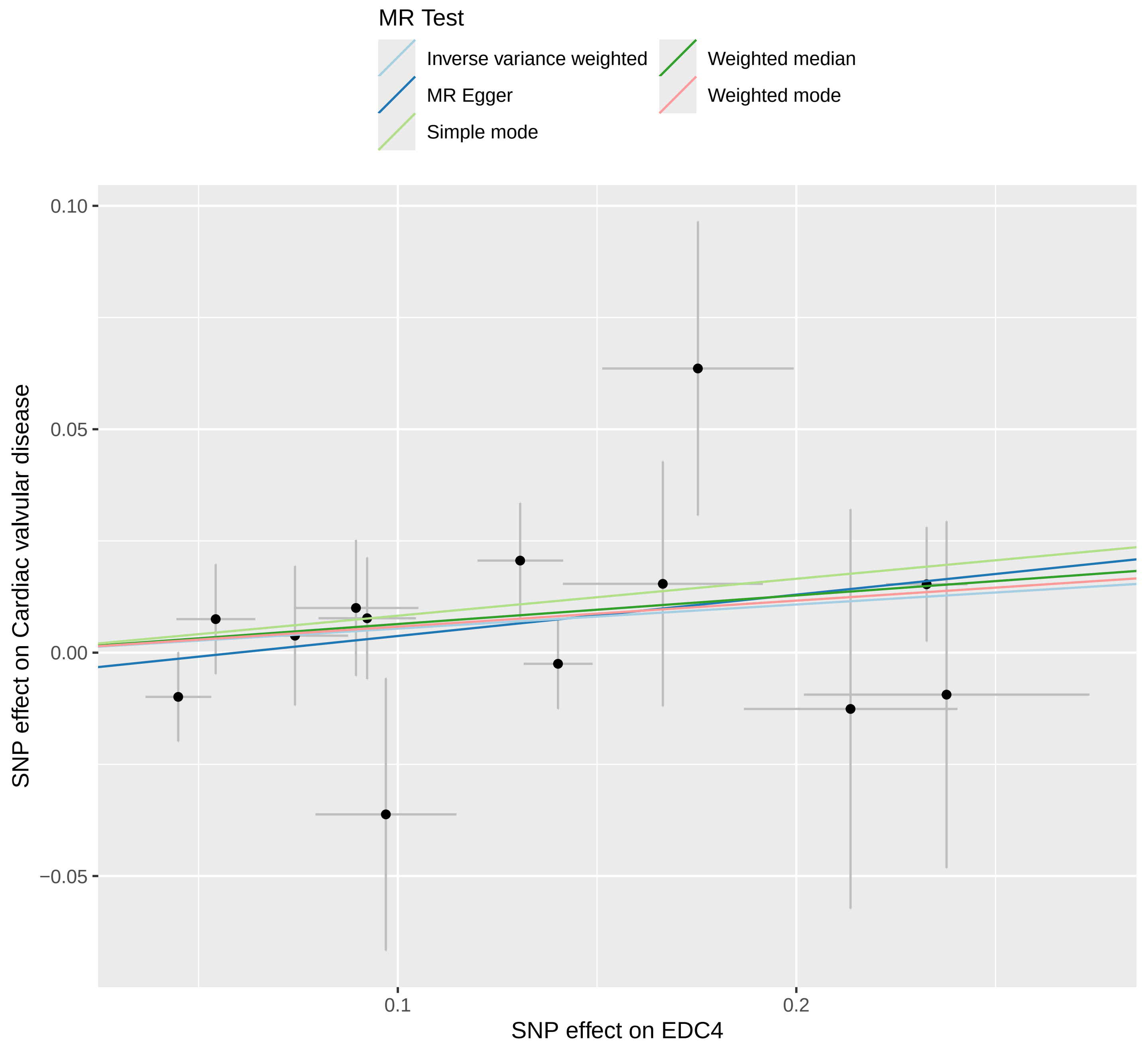
MR Test
Inverse variance weighted
MR Egger
Simple mode
Weighted median
Weighted mode
0.10
0.05
0.00
−0.05
0.1
0.2
SNP effect on Cardiac valvular disease
SNP effect on EDC4

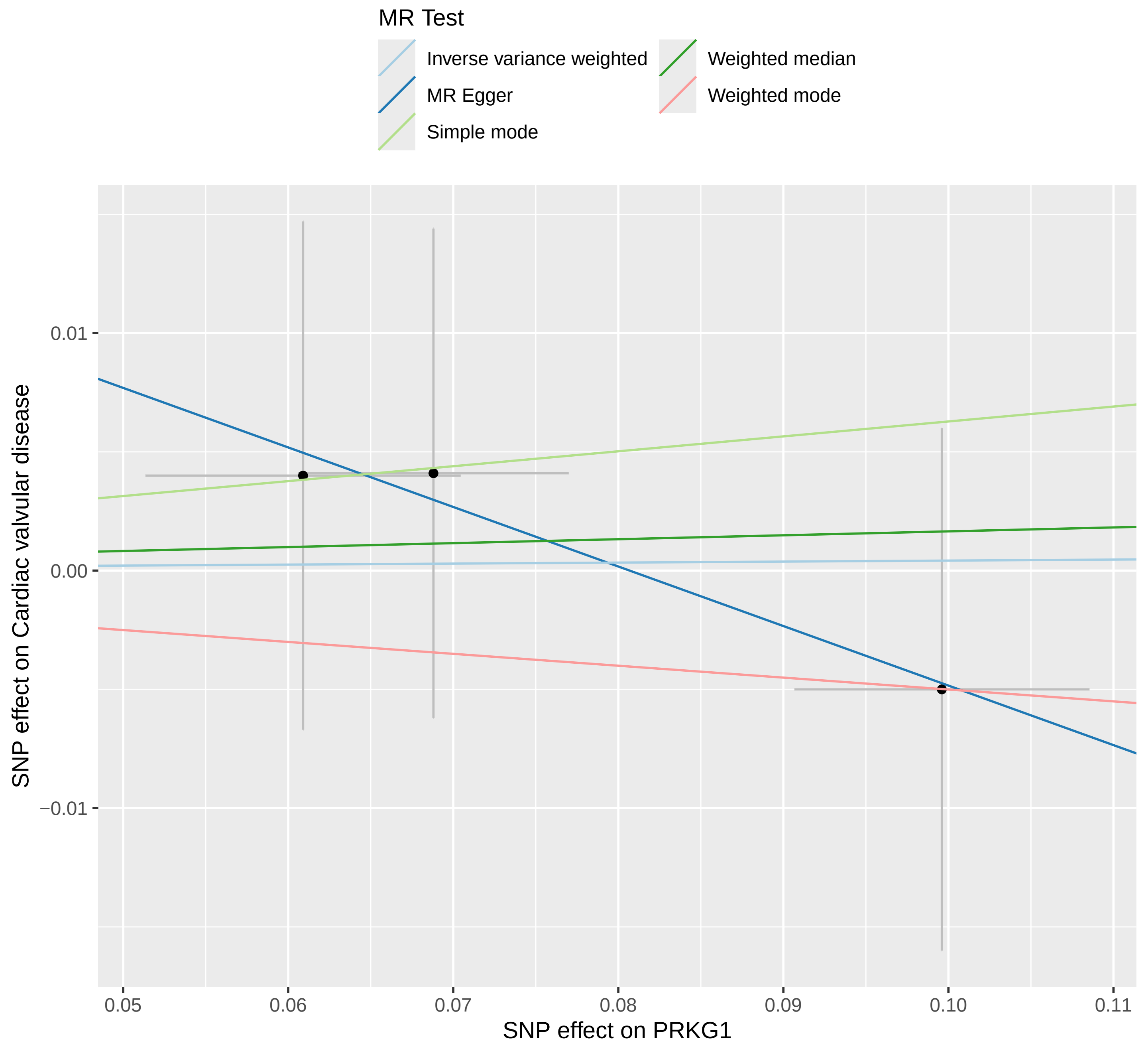
MR Test
Inverse variance weighted
MR Egger
Simple mode
Weighted median
Weighted mode
0.01
0.00
−0.01
0.05
0.06
0.07
0.08
0.09
0.10
0.11
SNP effect on PRKG1
SNP effect on Cardiac valvular disease

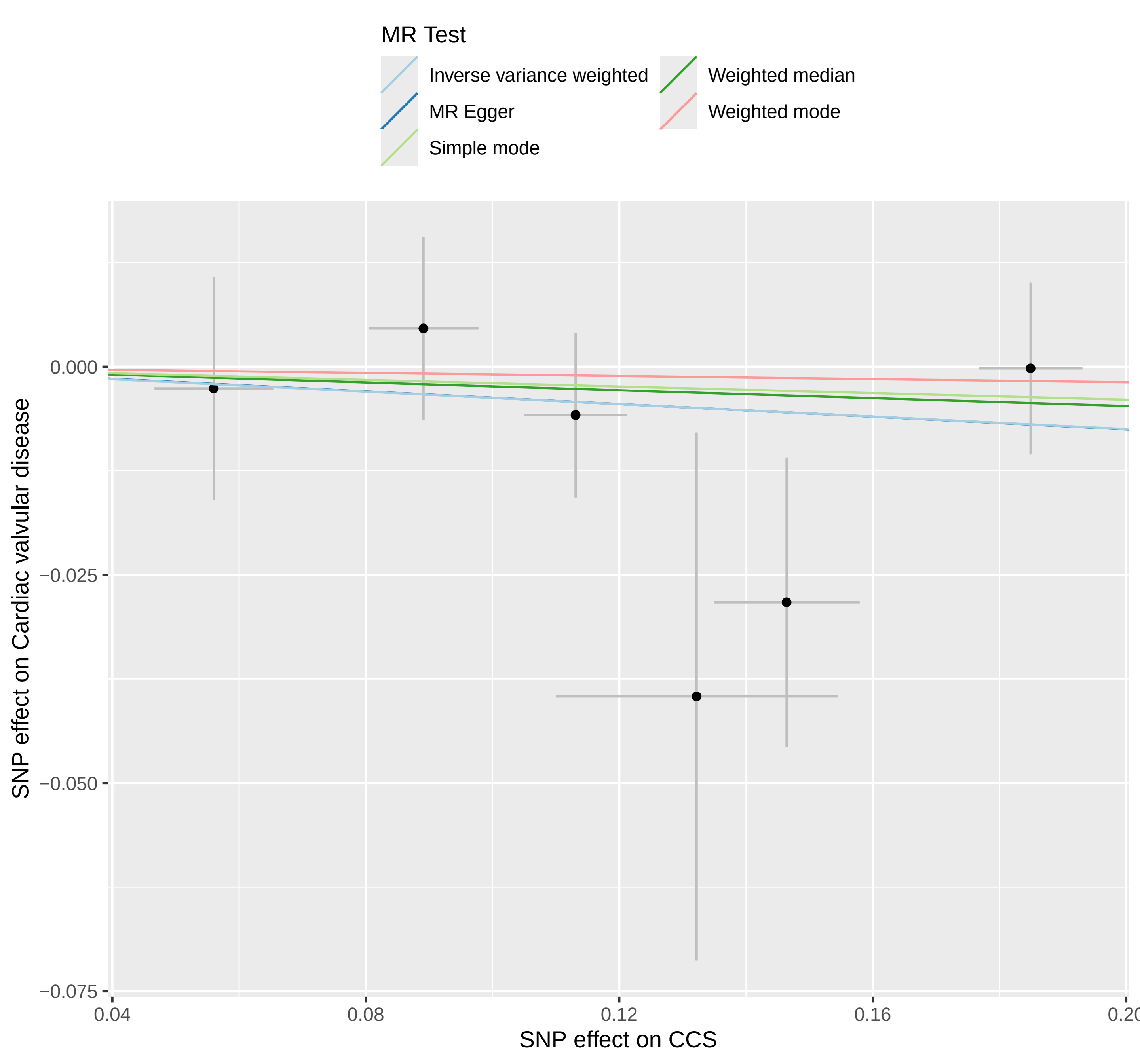
MR Test
Inverse variance weighted
MR Egger
Simple mode
Weighted median
Weighted mode
0.000
−0.025
−0.050
−0.075
SNP effect on Cardiac valvular disease
0.04
0.08
0.12
0.16
0.20
SNP effect on CCS

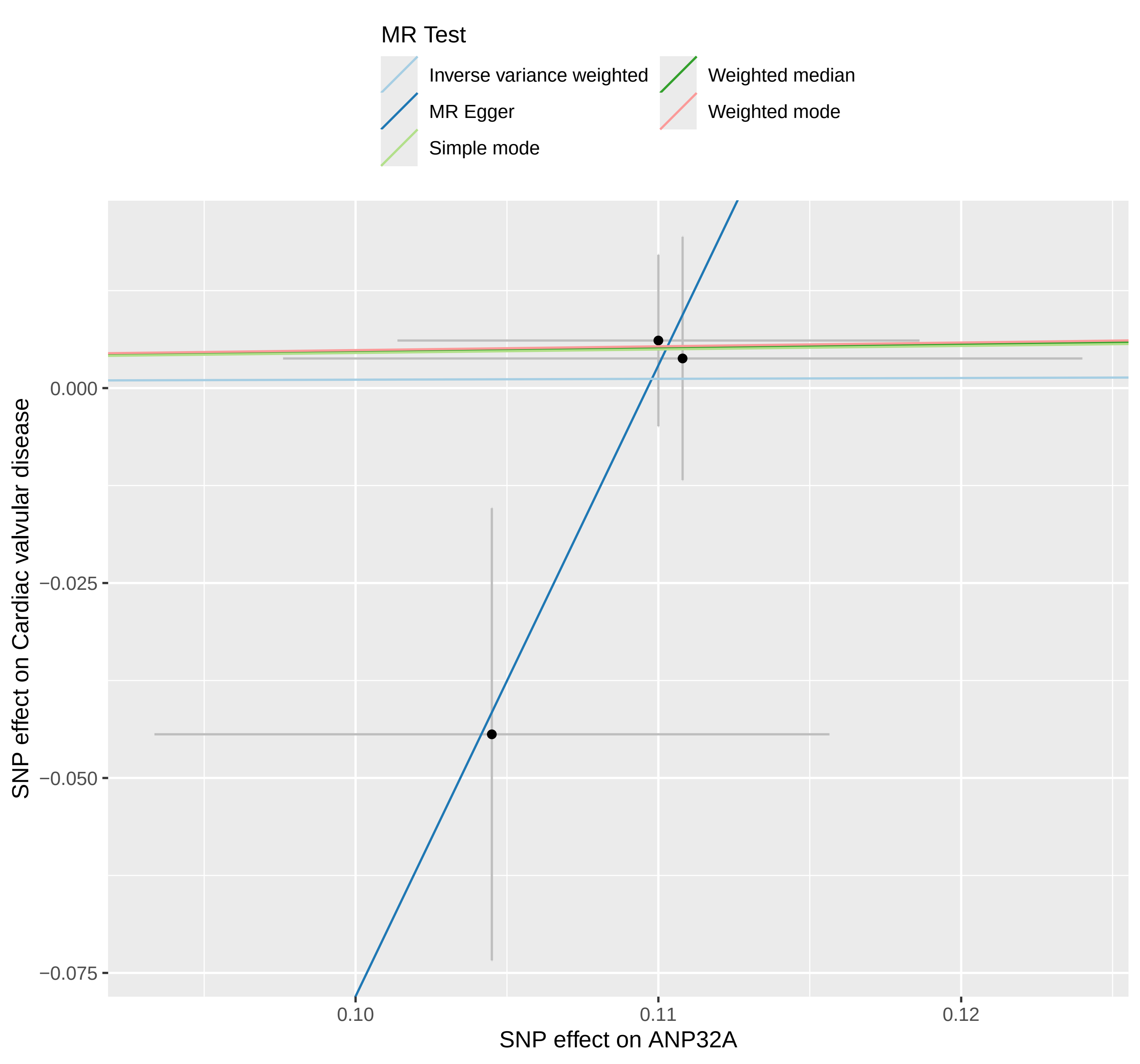
MR Test
Inverse variance weighted
MR Egger
Simple mode
Weighted median
Weighted mode
0.000
−0.025
−0.050
−0.075
0.10
0.11
0.12
SNP effect on Cardiac valvular disease
SNP effect on ANP32A

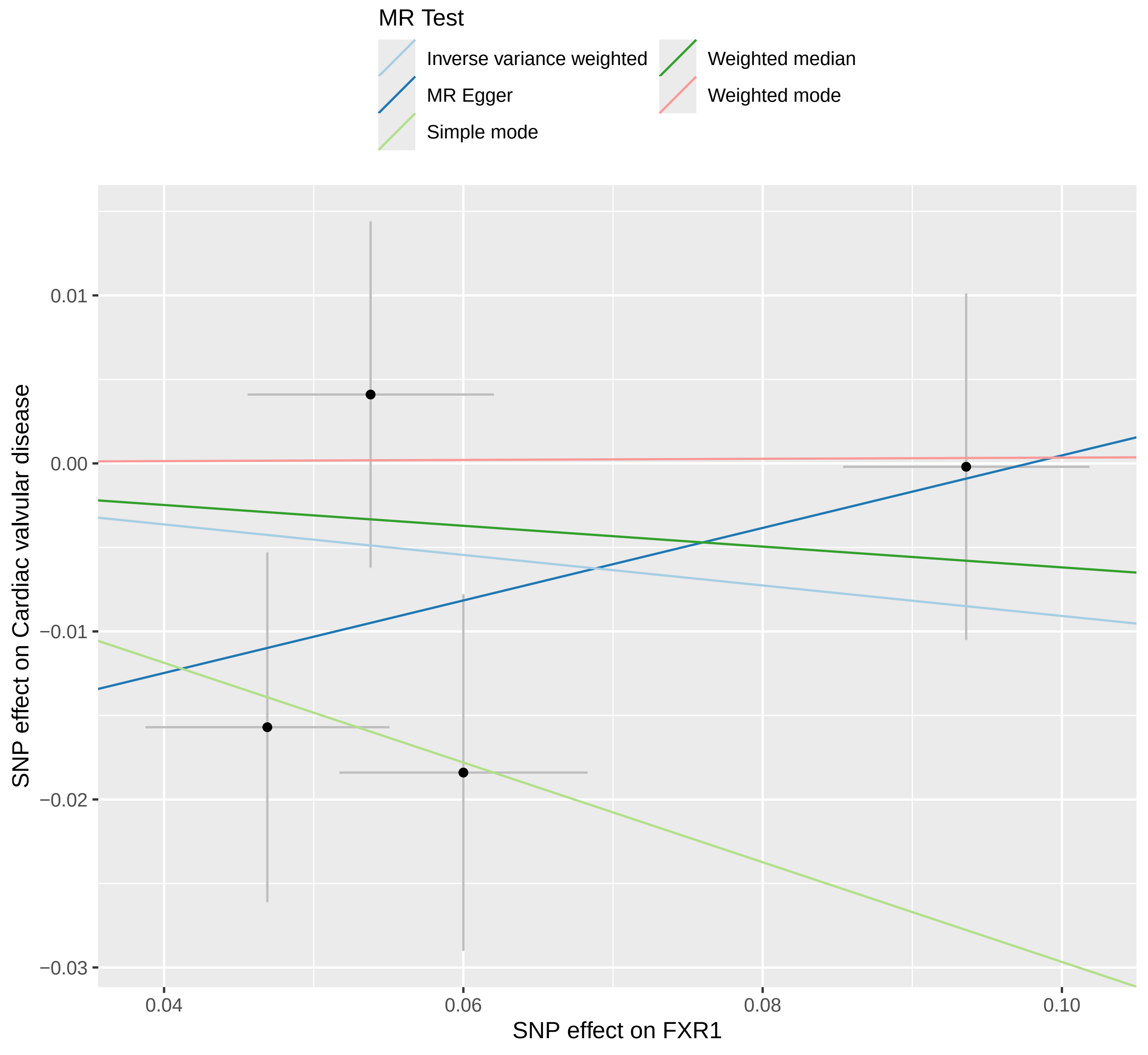
MR Test
Inverse variance weighted
MR Egger
Simple mode
Weighted median
Weighted mode
0.01
0.00
−0.01
−0.02
−0.03
0.04
0.06
0.08
0.10
SNP effect on Cardiac valvular disease
SNP effect on FXR1

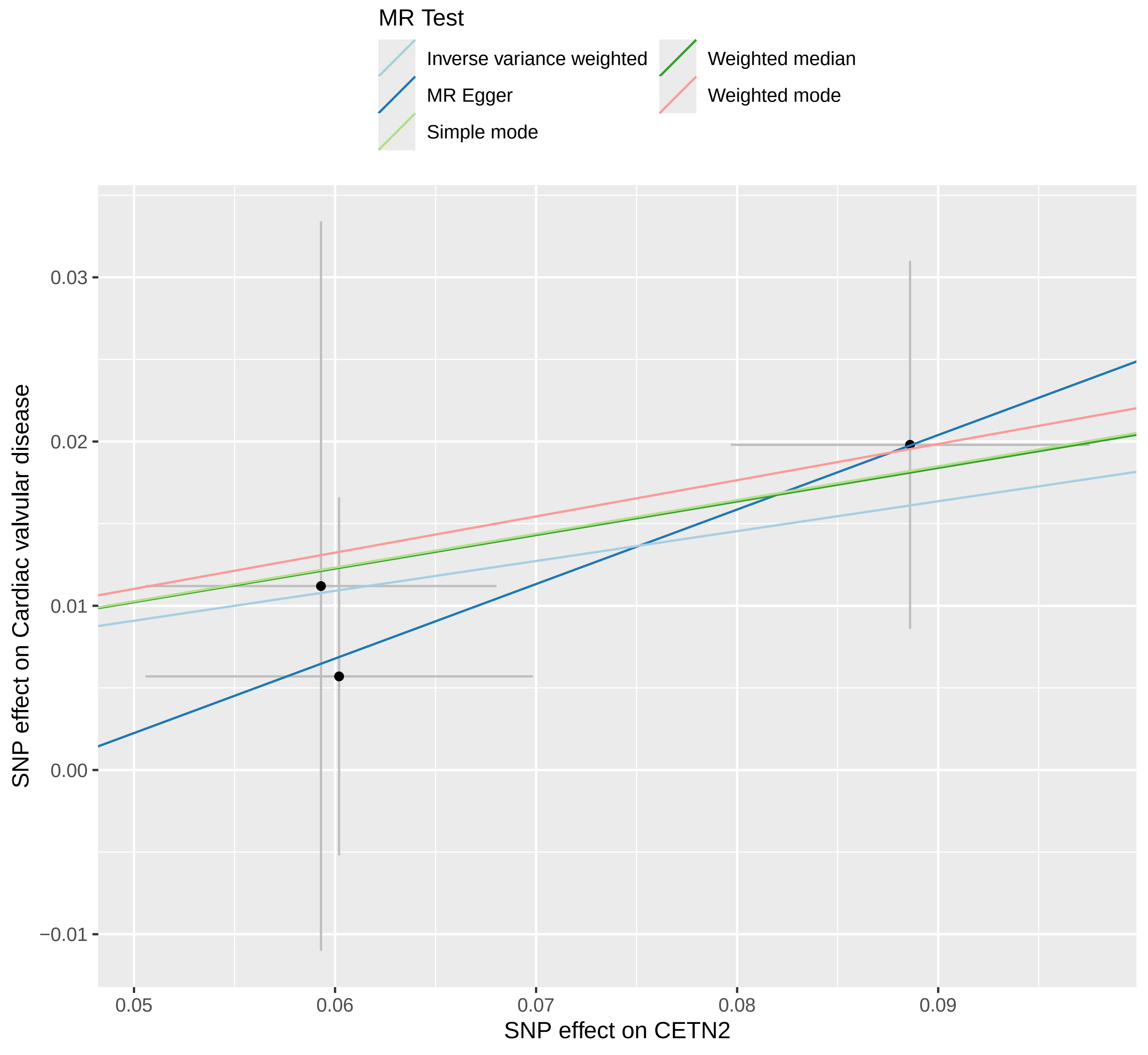
MR Test
Inverse variance weighted
MR Egger
Simple mode
Weighted median
Weighted mode
0.03
0.02
0.01
0.00
−0.01
SNP effect on Cardiac valvular disease
0.05
0.06
0.07
0.08
0.09
SNP effect on CETN2

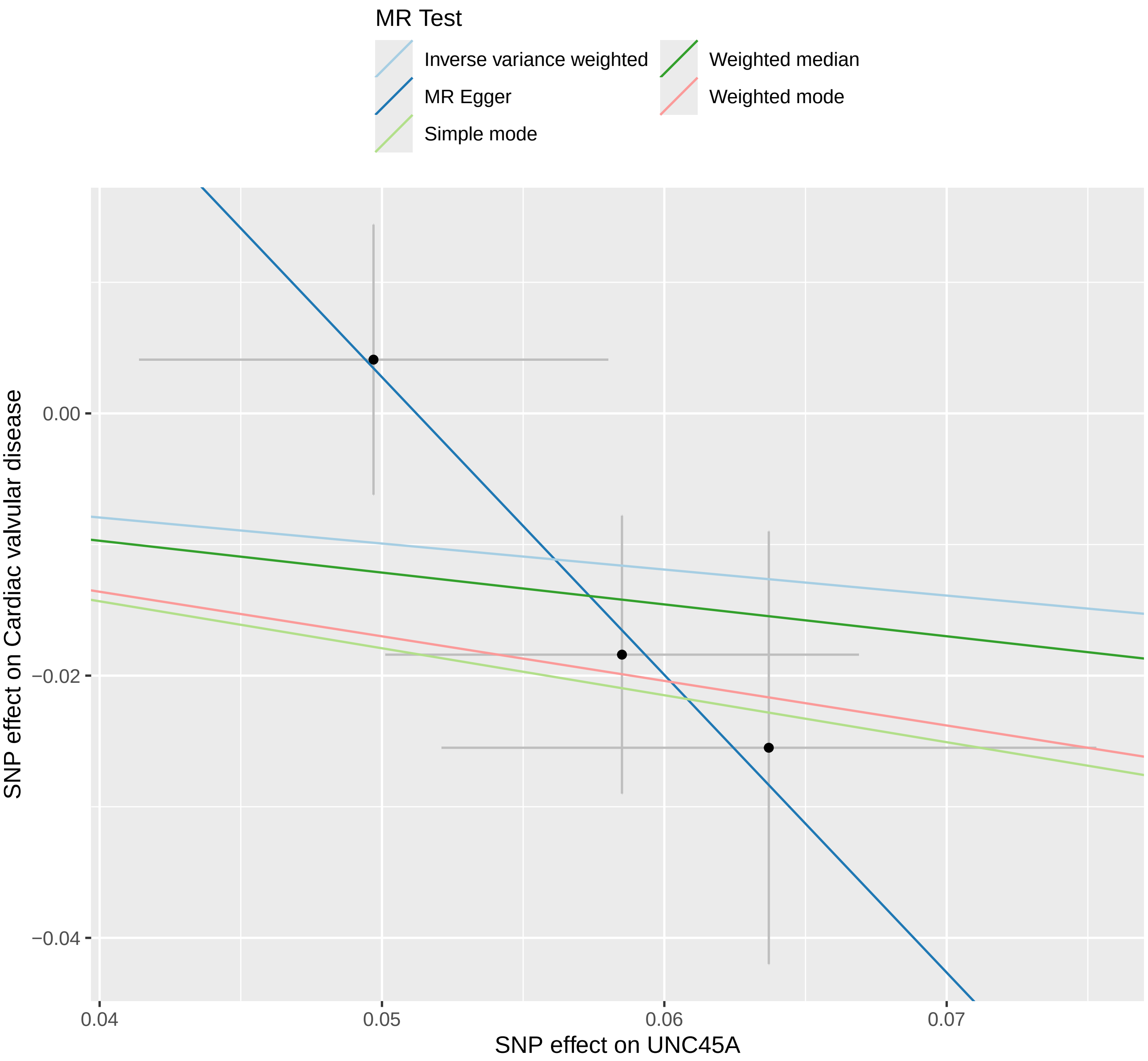
MR Test
Inverse variance weighted
MR Egger
Simple mode
Weighted median
Weighted mode
SNP effect on Cardiac valvular disease
0.00
−0.02
−0.04
0.04
0.05
0.06
0.07
SNP effect on UNC45A

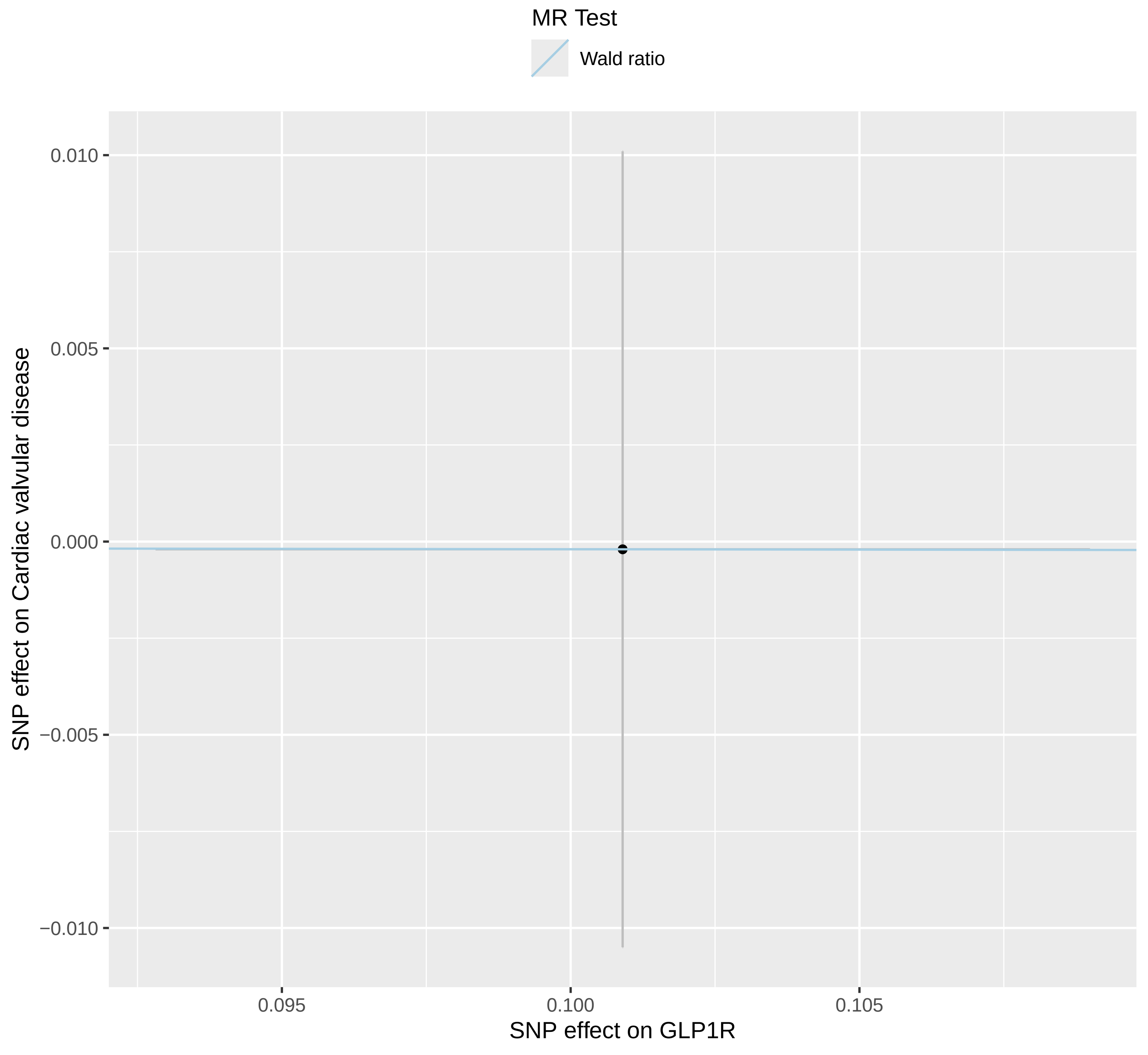
MR Test
Wald ratio
0.010
0.005
0.000
−0.005
−0.010
SNP effect on Cardiac valvular disease
0.095
0.100
0.105
SNP effect on GLP1R

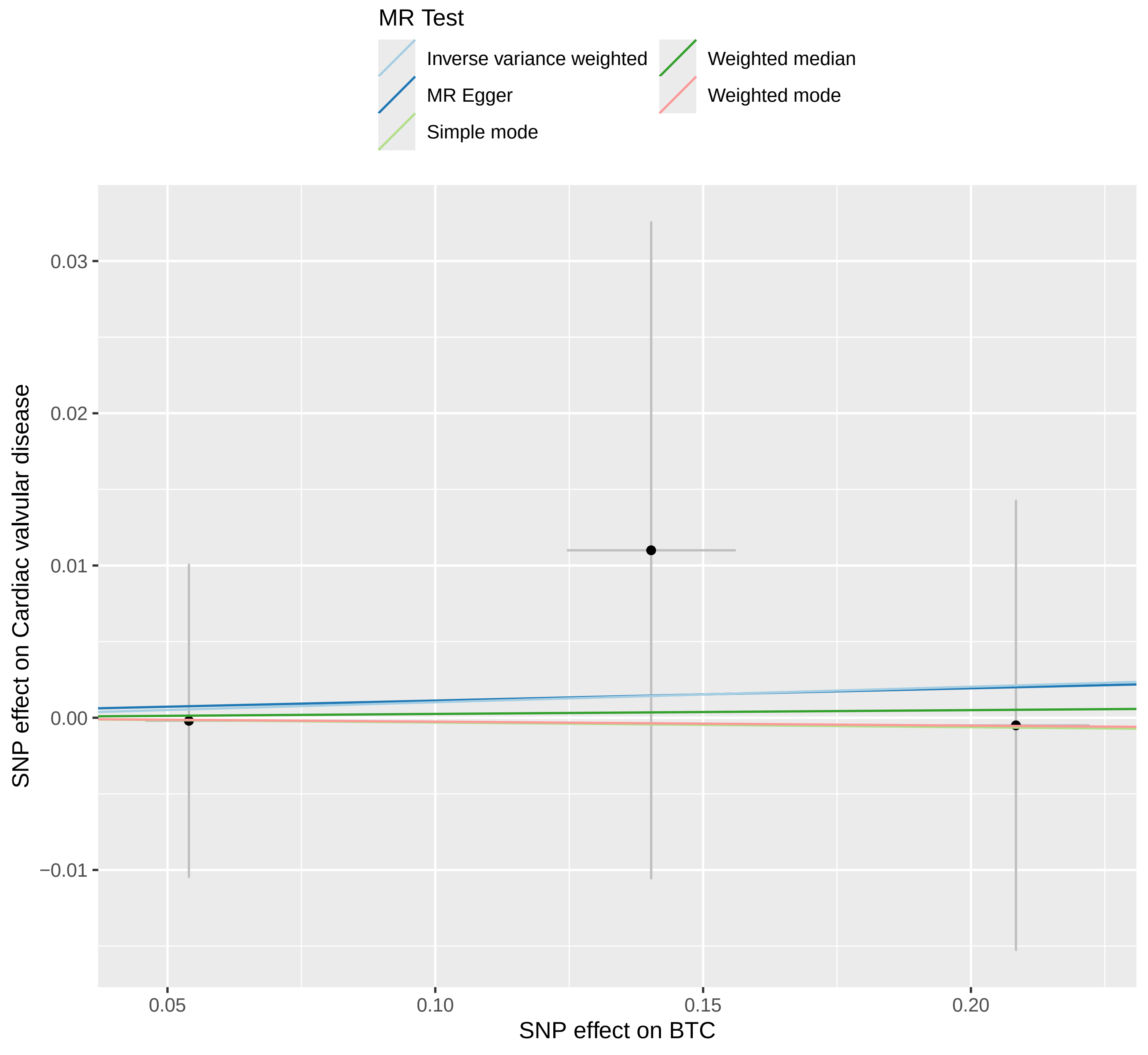

MR Test
Inverse variance weighted
MR Egger
Simple mode
Weighted median
Weighted mode
SNP effect on Cardiac valvular disease
0.03
0.02
0.01
0.00
−0.01
0.05
0.10
0.15
0.20
SNP effect on BTC

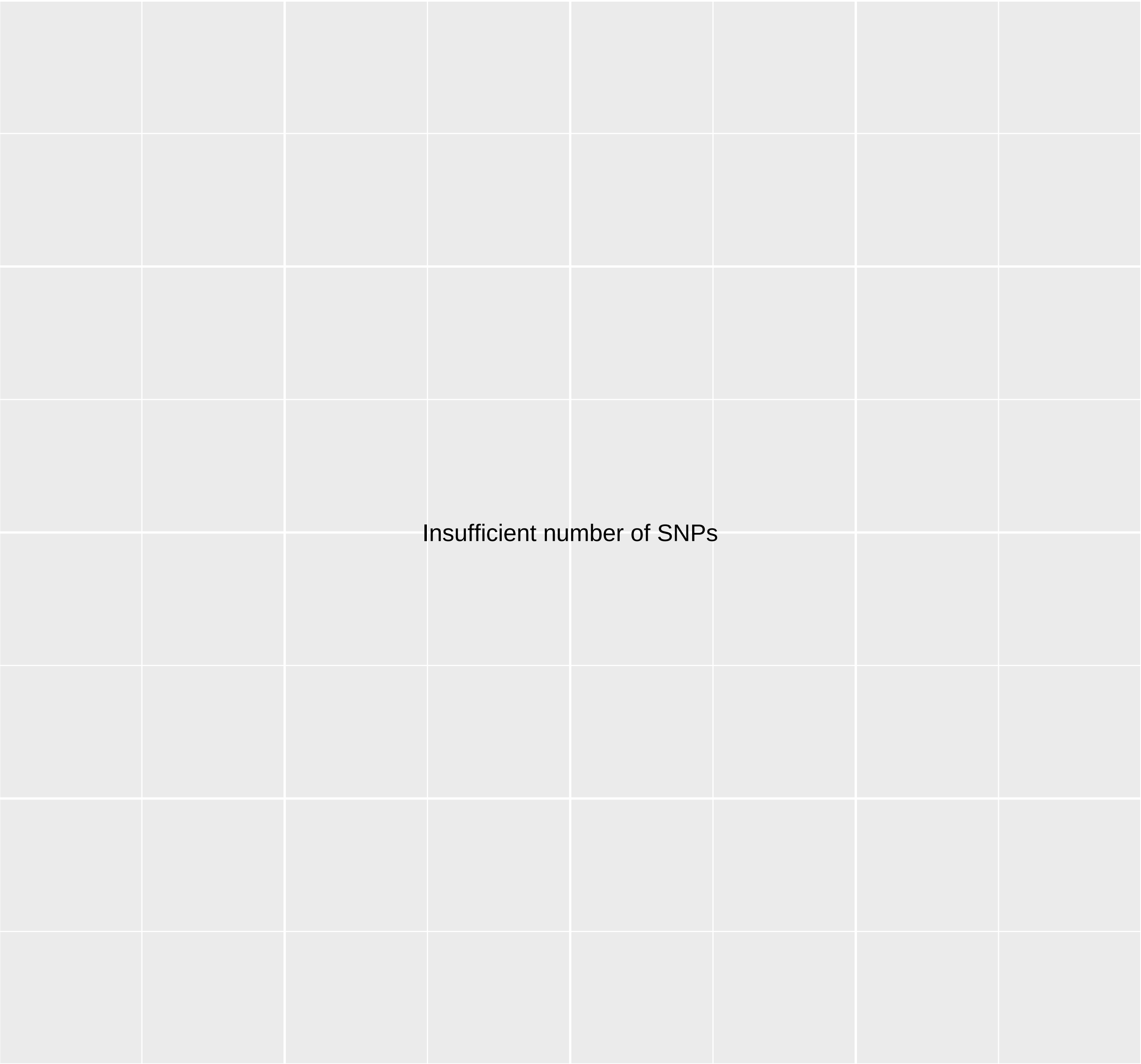
Insufficient number of SNPs

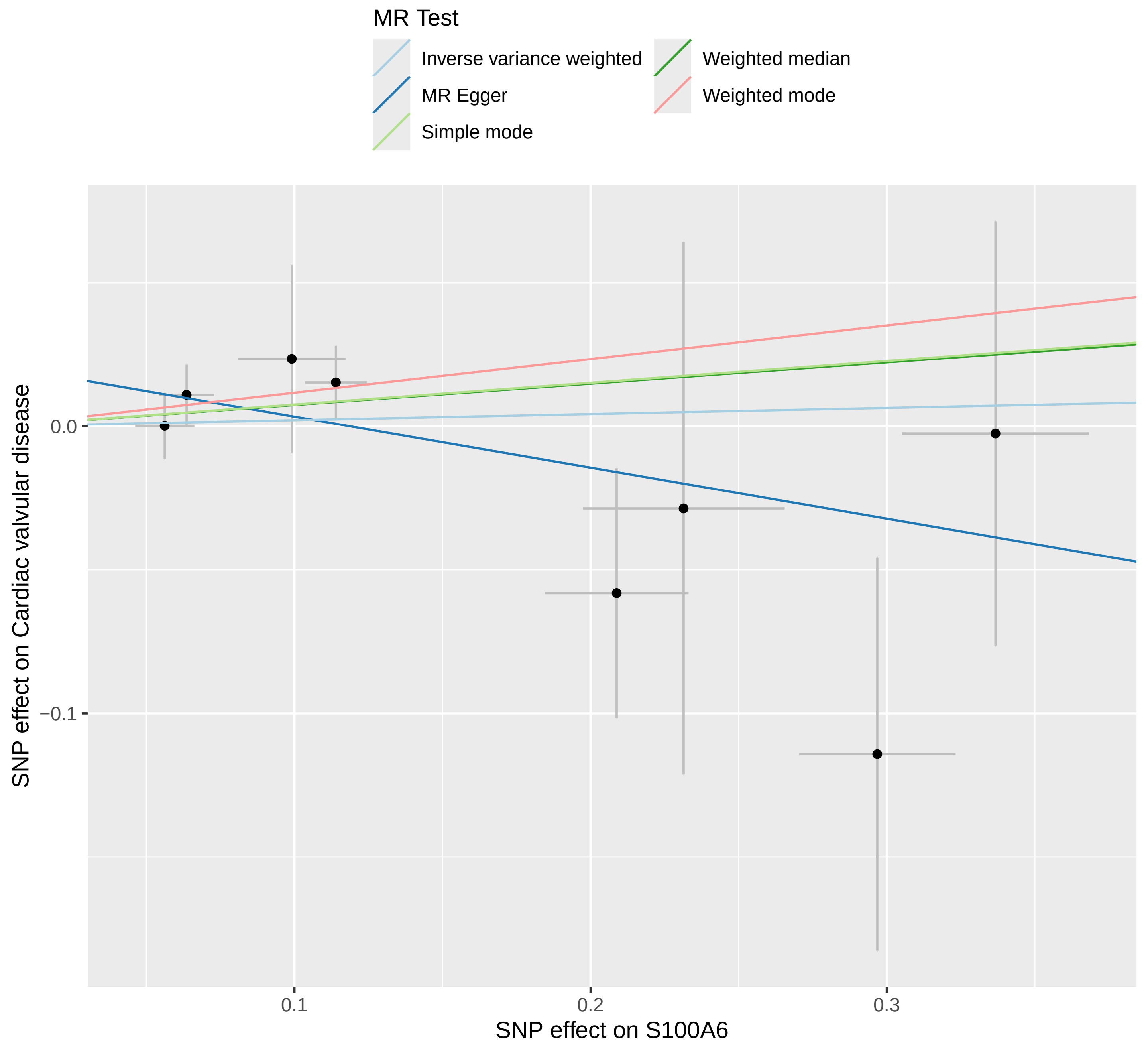

MR Test
Inverse variance weighted
MR Egger
Simple mode
Weighted median
Weighted mode
0.0
−0.1
0.1
0.2
0.3
SNP effect on Cardiac valvular disease
SNP effect on S100A6

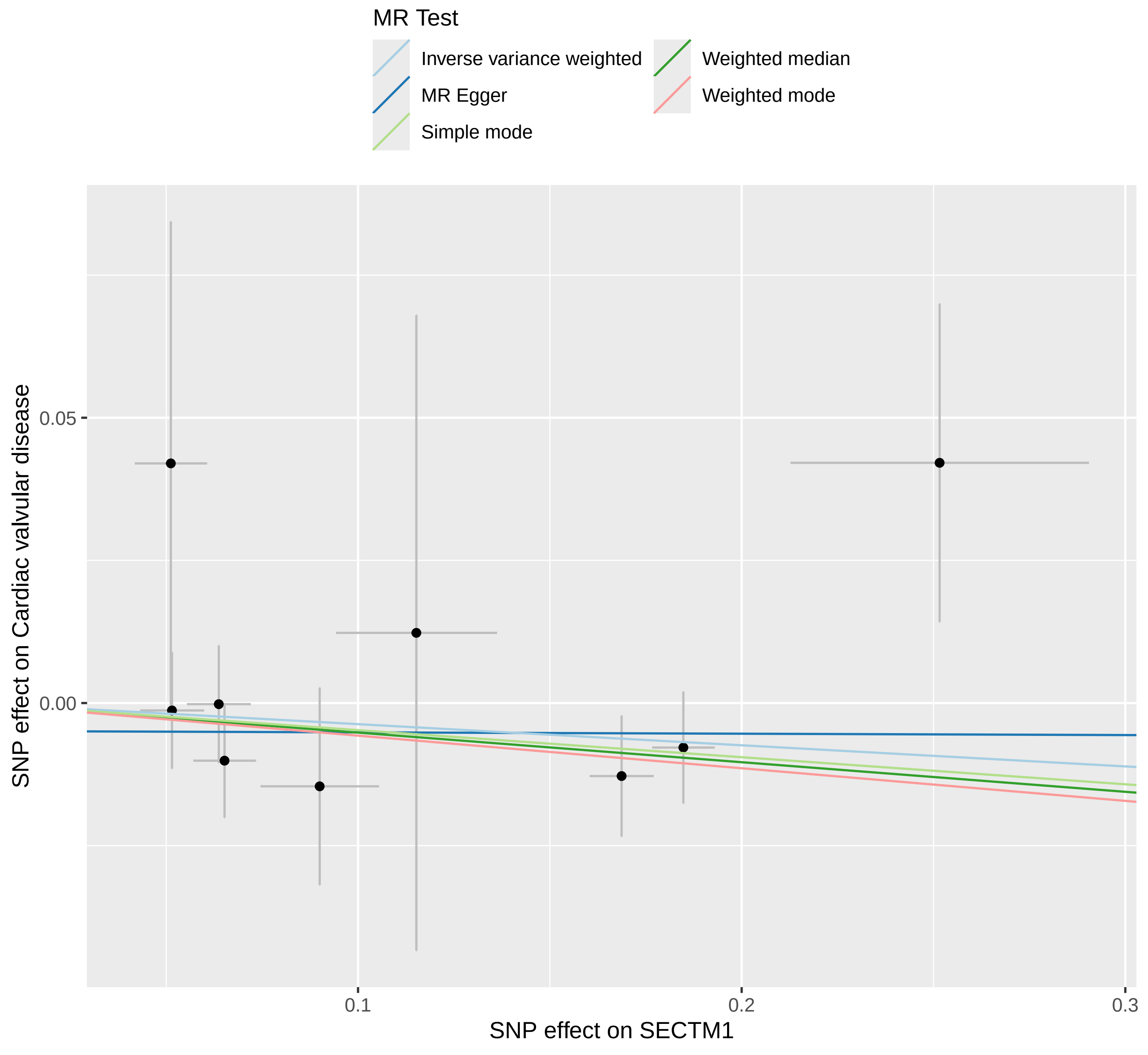

MR Test
Inverse variance weighted
MR Egger
Simple mode
Weighted median
Weighted mode
SNP effect on Cardiac valvular disease
0.05
0.00
0.1
0.2
0.3
SNP effect on SECTM1

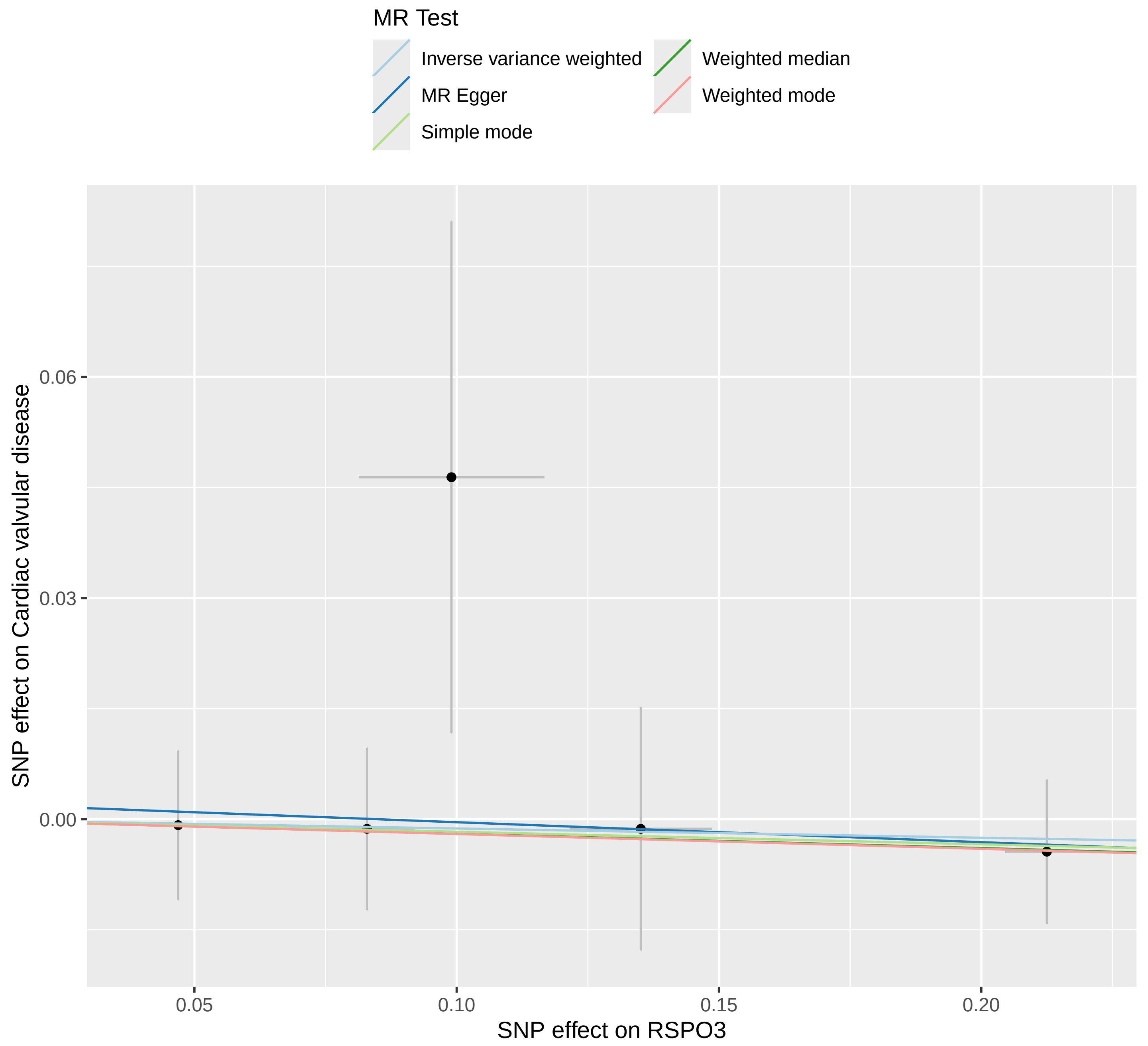

MR Test
Inverse variance weighted
MR Egger
Simple mode
Weighted median
Weighted mode
0.06
0.03
0.00
SNP effect on Cardiac valvular disease
0.05
0.10
0.15
0.20
SNP effect on RSPO3

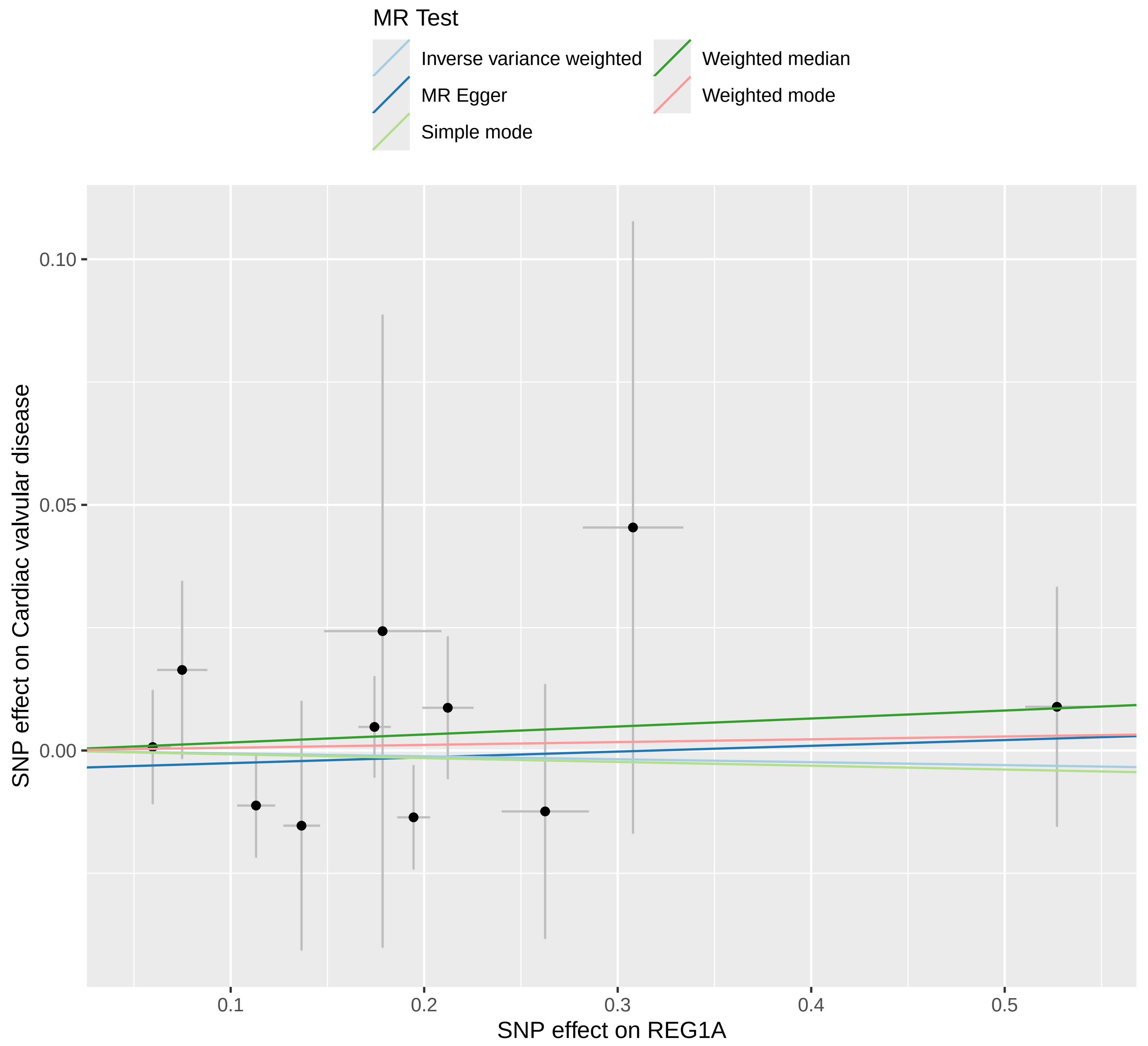

MR Test
Inverse variance weighted
MR Egger
Simple mode
Weighted median
Weighted mode
0.10
0.05
0.00
SNP effect on Cardiac valvular disease
0.1
0.2
0.3
0.4
0.5
SNP effect on REG1A

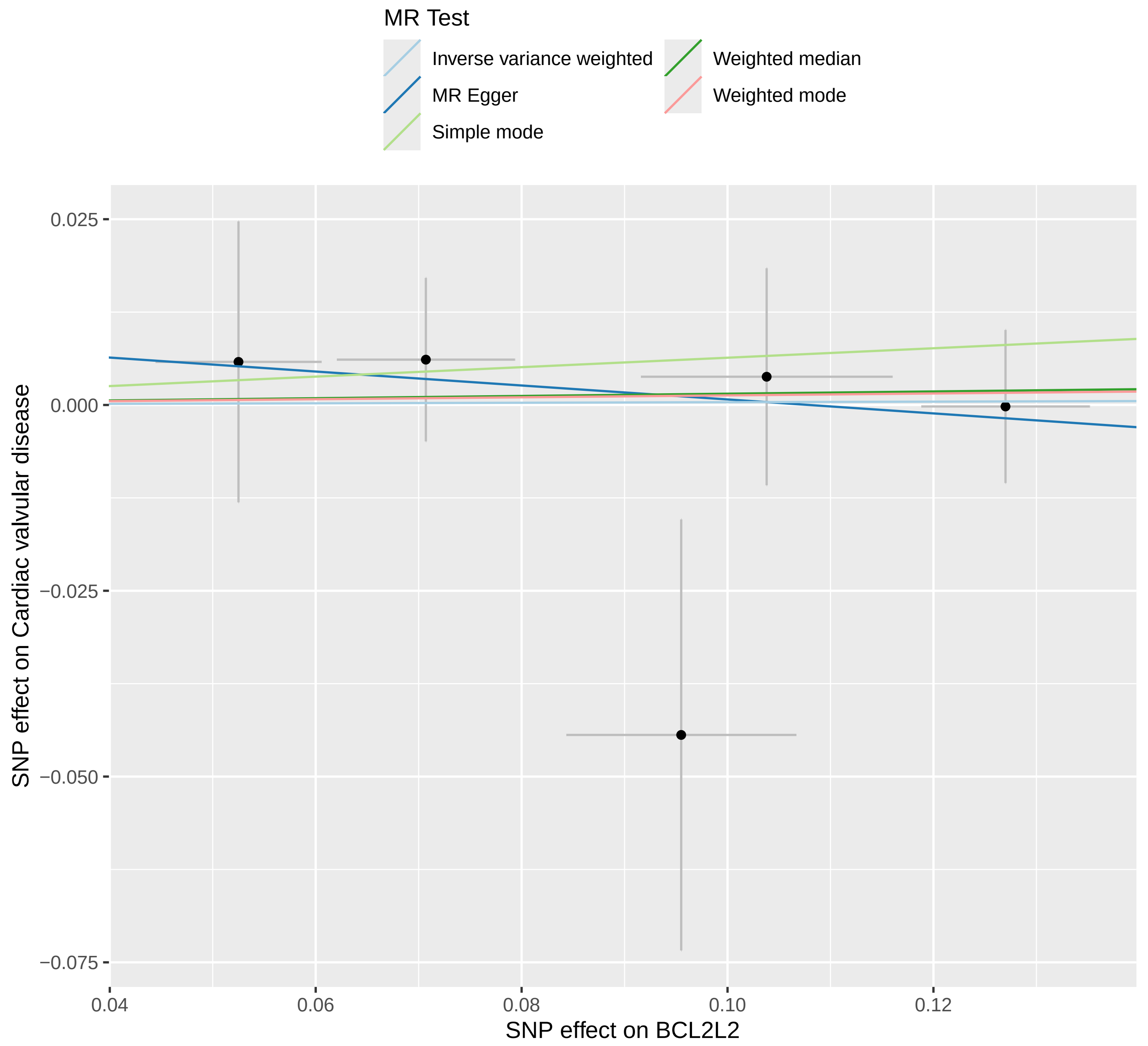
MR Test
Inverse variance weighted
MR Egger
Simple mode
Weighted median
Weighted mode
0.025
0.000
−0.025
−0.050
−0.075
SNP effect on Cardiac valvular disease
0.04
0.06
0.08
0.10
0.12
SNP effect on BCL2L2

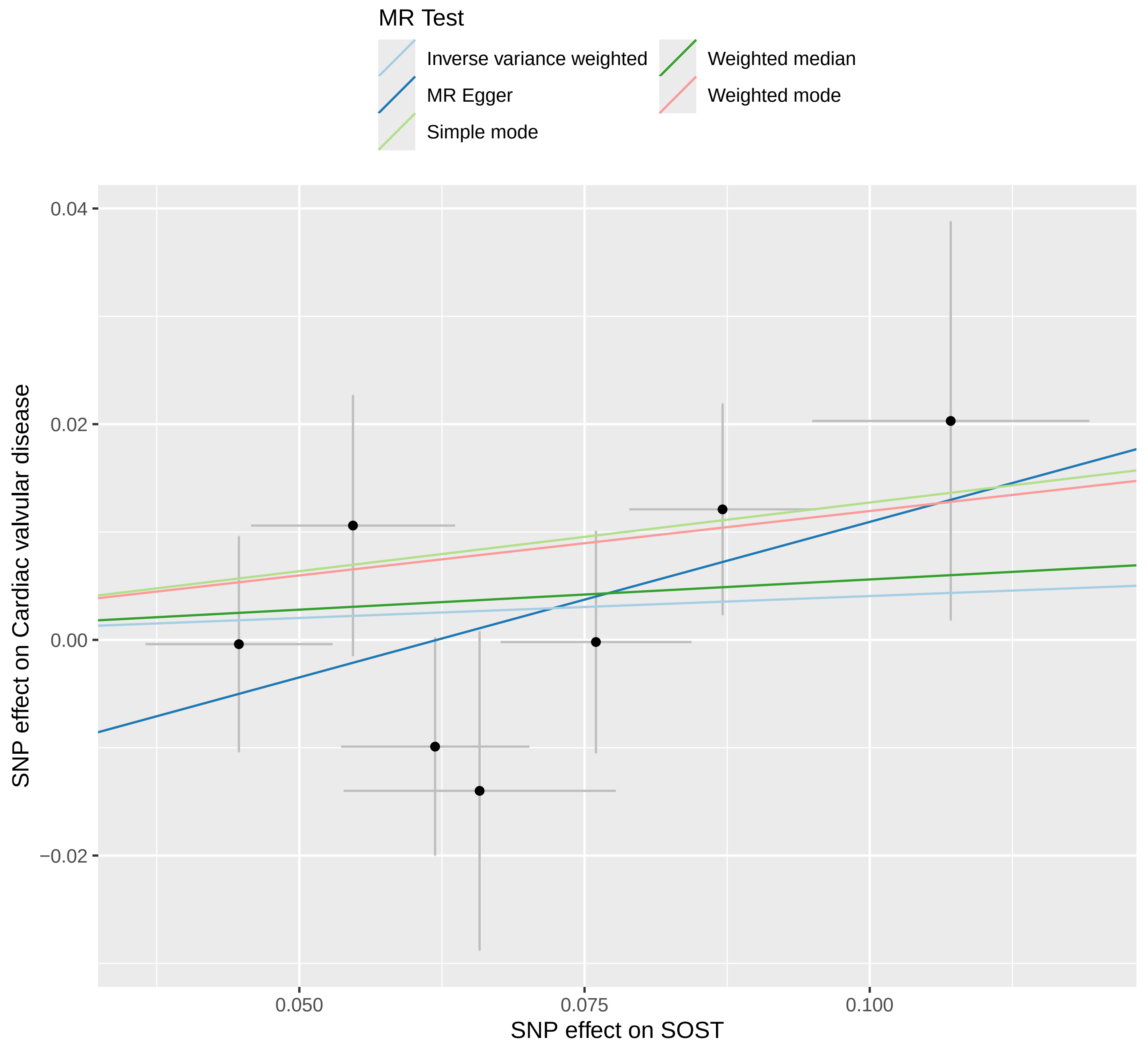
MR Test
Inverse variance weighted
MR Egger
Simple mode
Weighted median
Weighted mode
0.04
0.02
0.00
−0.02
SNP effect on Cardiac valvular disease
0.050
0.075
0.100
SNP effect on SOST

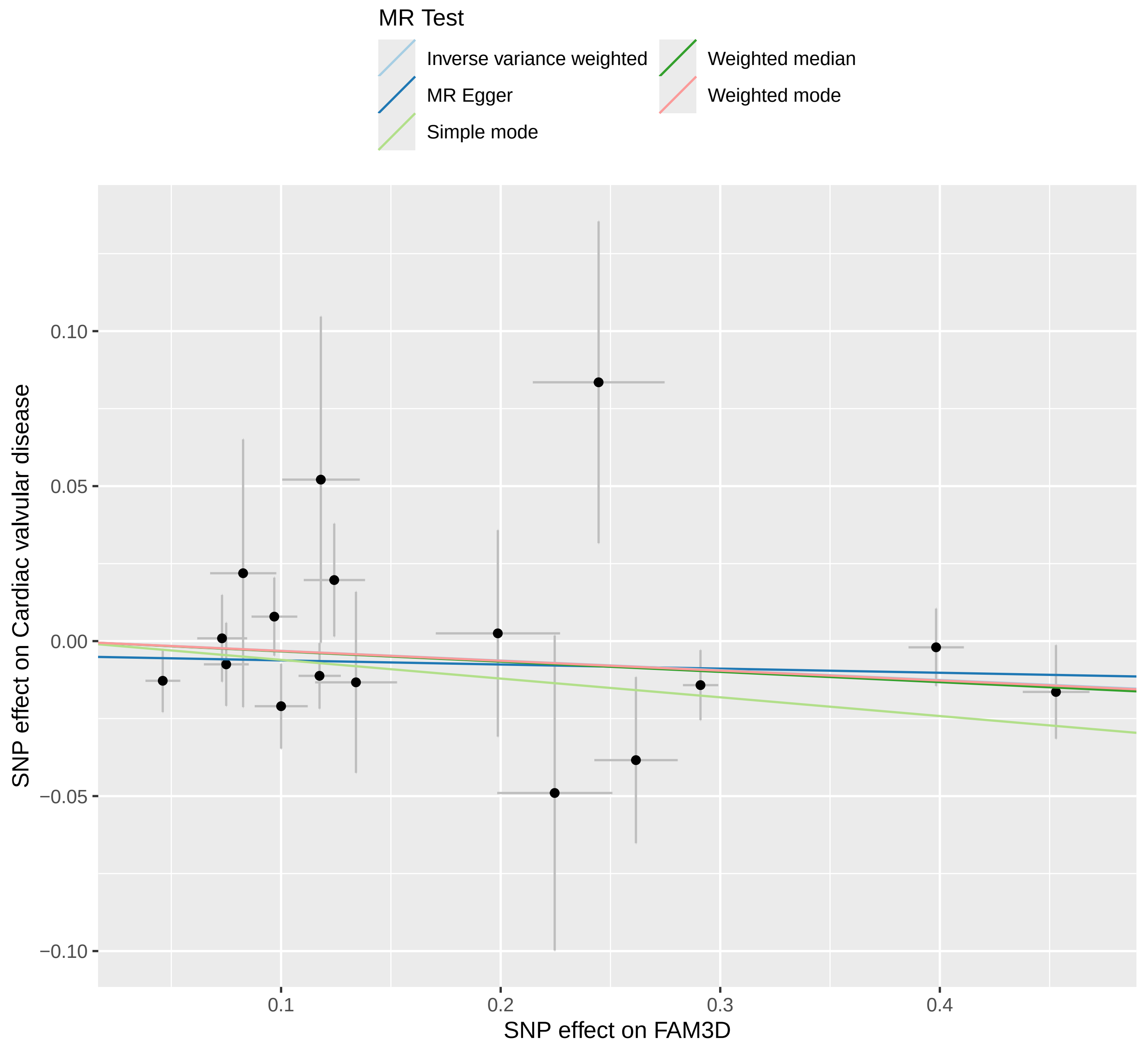
MR Test
Inverse variance weighted
MR Egger
Simple mode
Weighted median
Weighted mode
0.10
0.05
0.00
−0.05
−0.10
SNP effect on Cardiac valvular disease
0.1
0.2
0.3
0.4
SNP effect on FAM3D

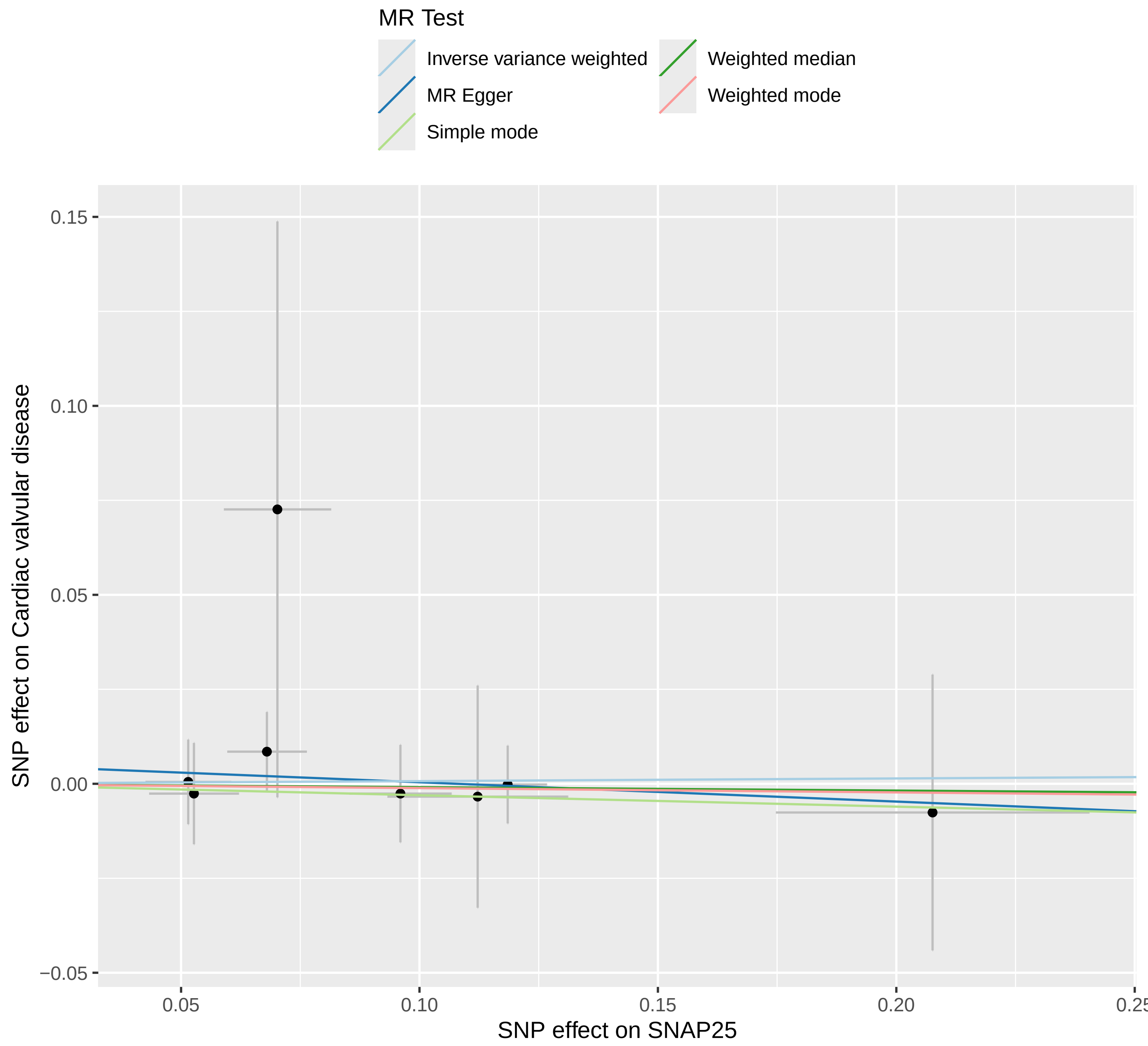
MR Test
Inverse variance weighted
MR Egger
Simple mode
Weighted median
Weighted mode
SNP effect on Cardiac valvular disease
0.15
0.10
0.05
0.00
−0.05
0.05
0.10
0.15
0.20
0.25
SNP effect on SNAP25

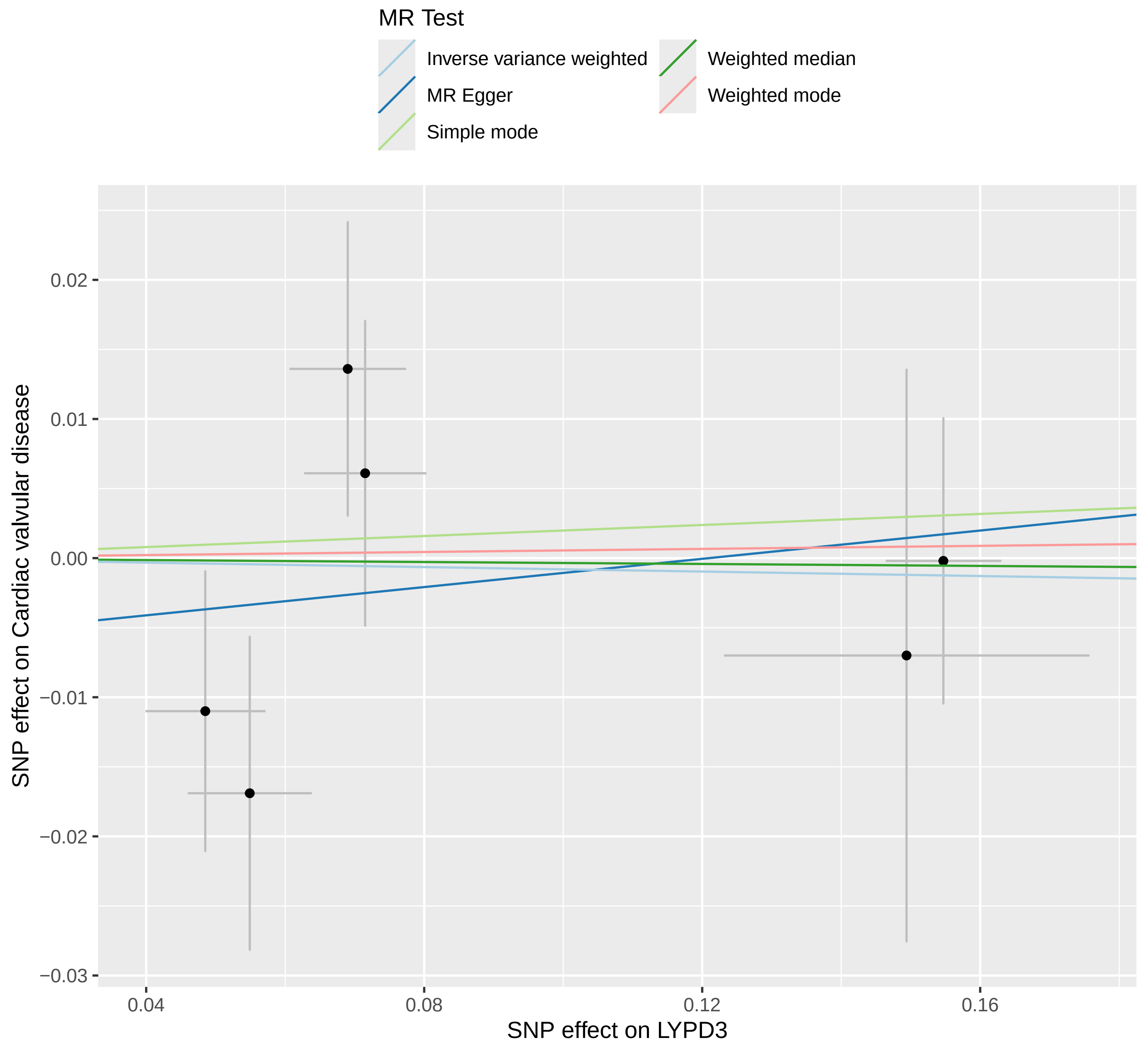

MR Test
Inverse variance weighted
MR Egger
Simple mode
Weighted median
Weighted mode
0.02
0.01
0.00
−0.01
−0.02
−0.03
SNP effect on Cardiac valvular disease
0.04
0.08
0.12
0.16
SNP effect on LYPD3

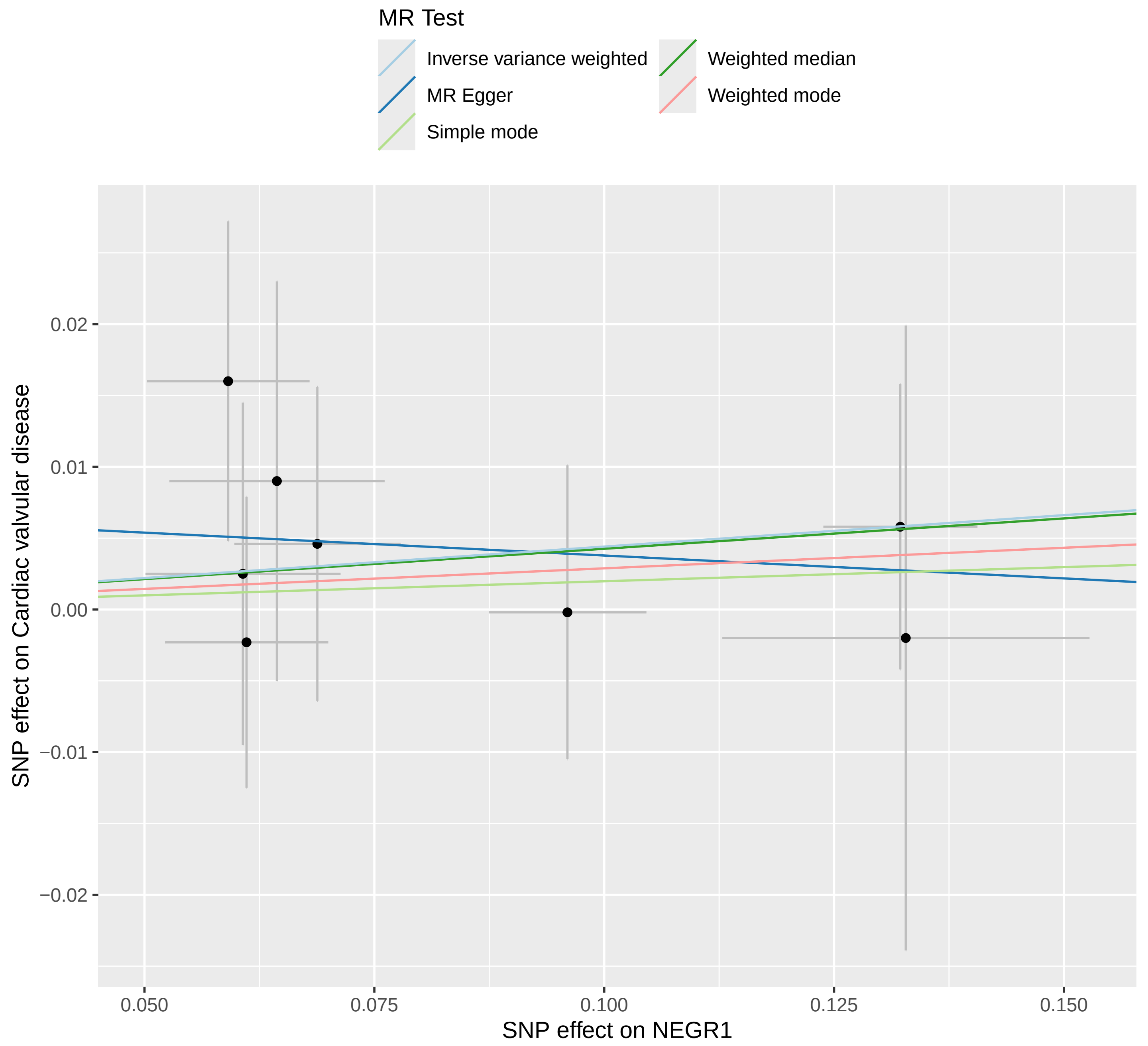

MR Test
Inverse variance weighted
MR Egger
Simple mode
Weighted median
Weighted mode
SNP effect on Cardiac valvular disease
0.02
0.01
0.00
−0.01
−0.02
0.050
0.075
0.100
0.125
0.150
SNP effect on NEGR1

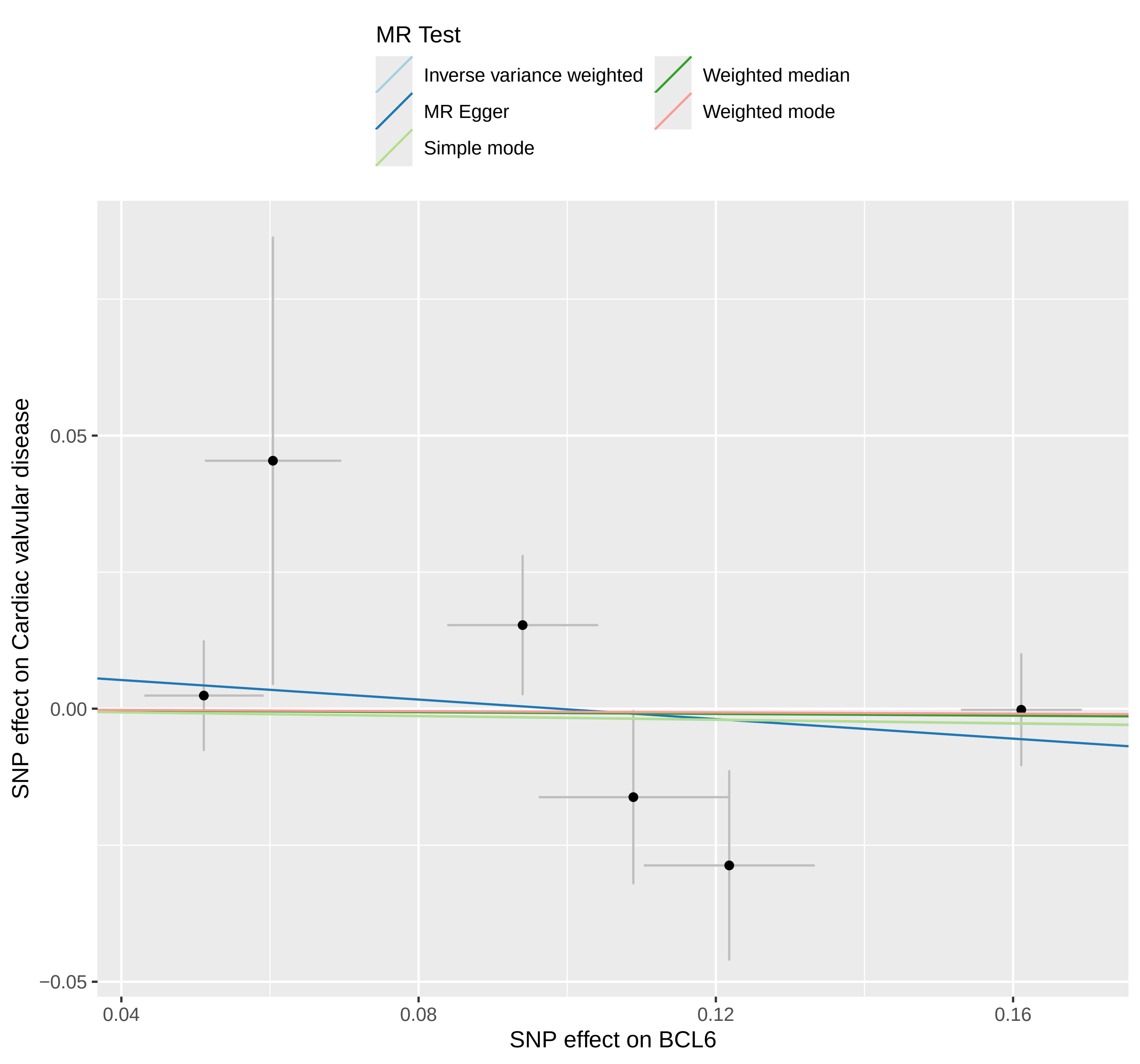
MR Test
Inverse variance weighted
MR Egger
Simple mode
Weighted median
Weighted mode
0.05
0.00
−0.05
0.04
0.08
0.12
0.16
SNP effect on BCL6
SNP effect on Cardiac valvular disease

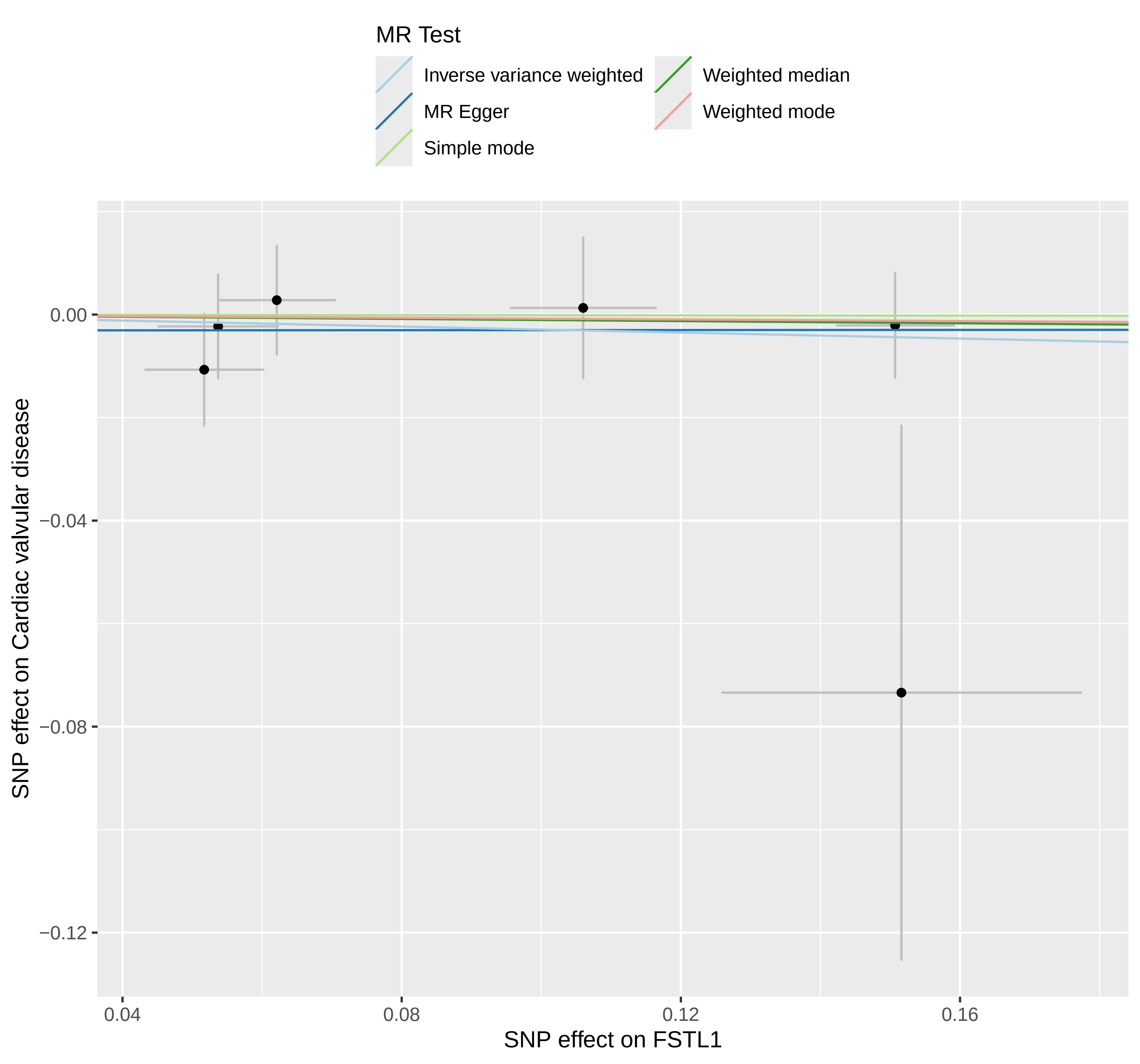
MR Test
Inverse variance weighted
MR Egger
Simple mode
Weighted median
Weighted mode
SNP effect on Cardiac valvular disease
0.00
−0.04
−0.08
−0.12
0.04
0.08
0.12
0.16
SNP effect on FSTL1

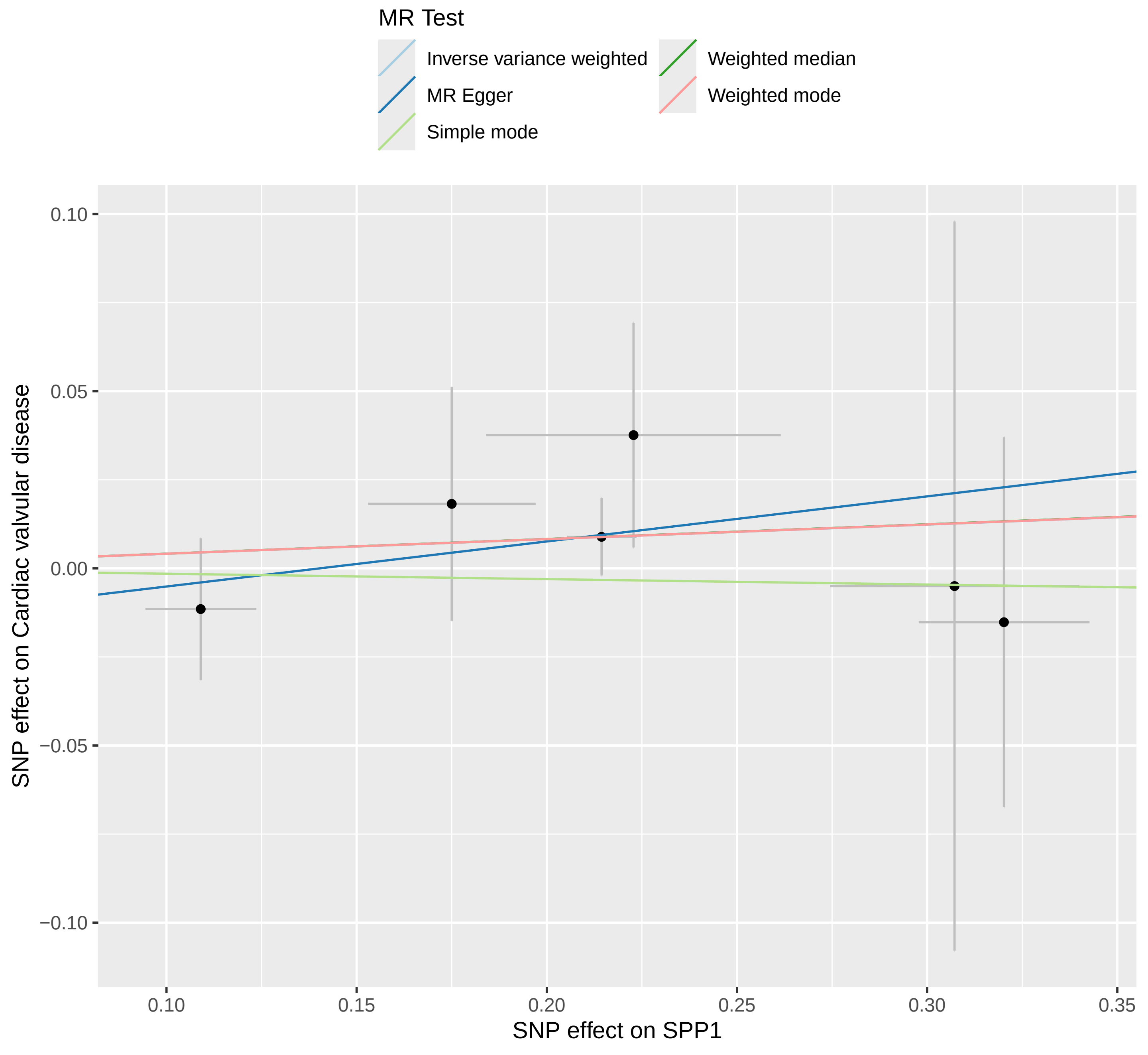

MR Test
Inverse variance weighted
MR Egger
Simple mode
Weighted median
Weighted mode
0.10
0.05
0.00
−0.05
−0.10
0.10
0.15
0.20
0.25
0.30
0.35
SNP effect on Cardiac valvular disease
SNP effect on SPP1

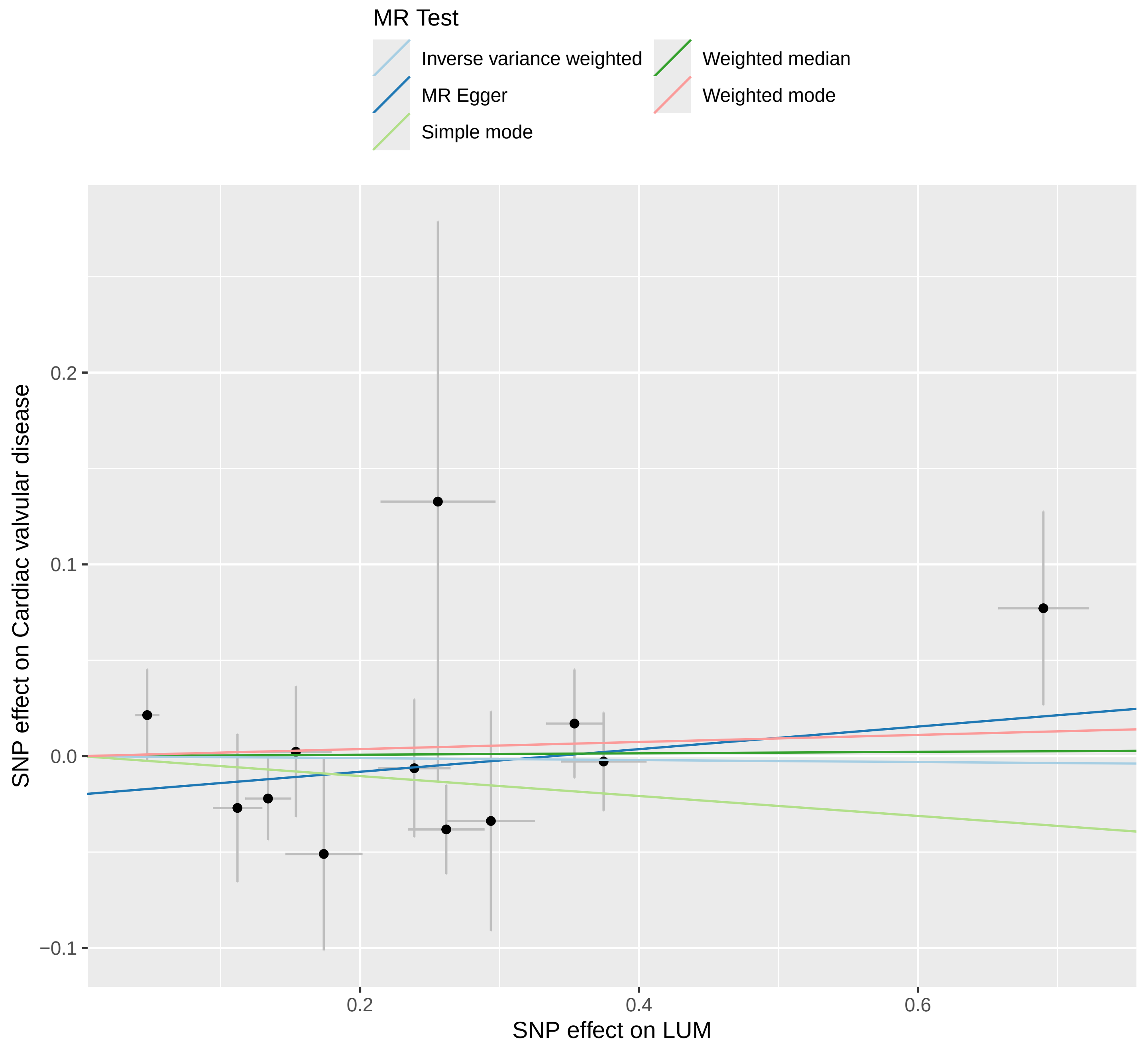

MR Test
Inverse variance weighted
MR Egger
Simple mode
Weighted median
Weighted mode
0.2
0.1
0.0
−0.1
SNP effect on Cardiac valvular disease
0.2
0.4
0.6
SNP effect on LUM

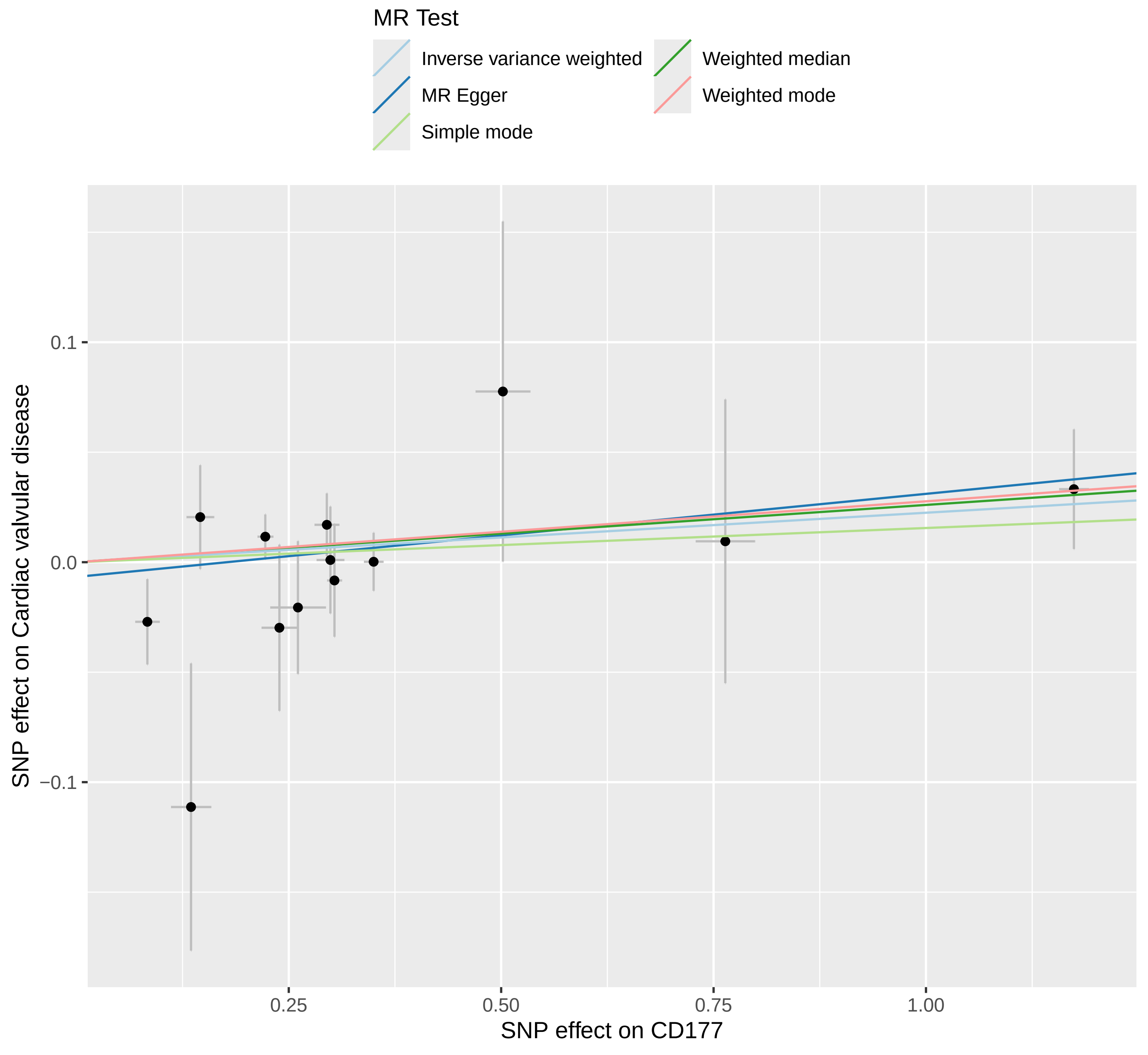

MR Test
Inverse variance weighted
MR Egger
Simple mode
Weighted median
Weighted mode
0.1
0.0
−0.1
SNP effect on Cardiac valvular disease
0.25
0.50
0.75
1.00
SNP effect on CD177

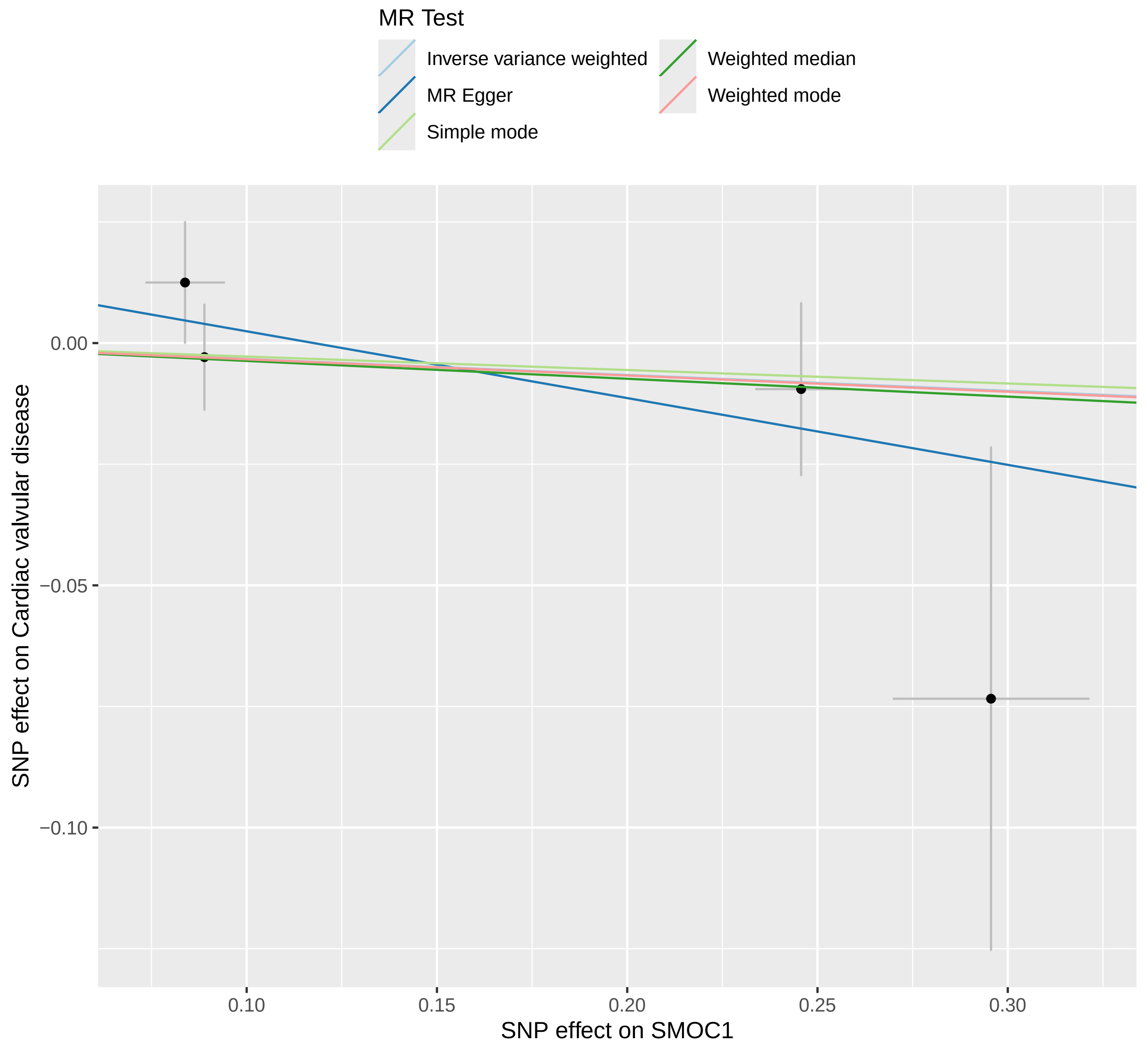
MR Test
Inverse variance weighted
MR Egger
Simple mode
Weighted median
Weighted mode
0.00
−0.05
−0.10
0.10
0.15
0.20
0.25
0.30
SNP effect on Cardiac valvular disease
SNP effect on SMOC1

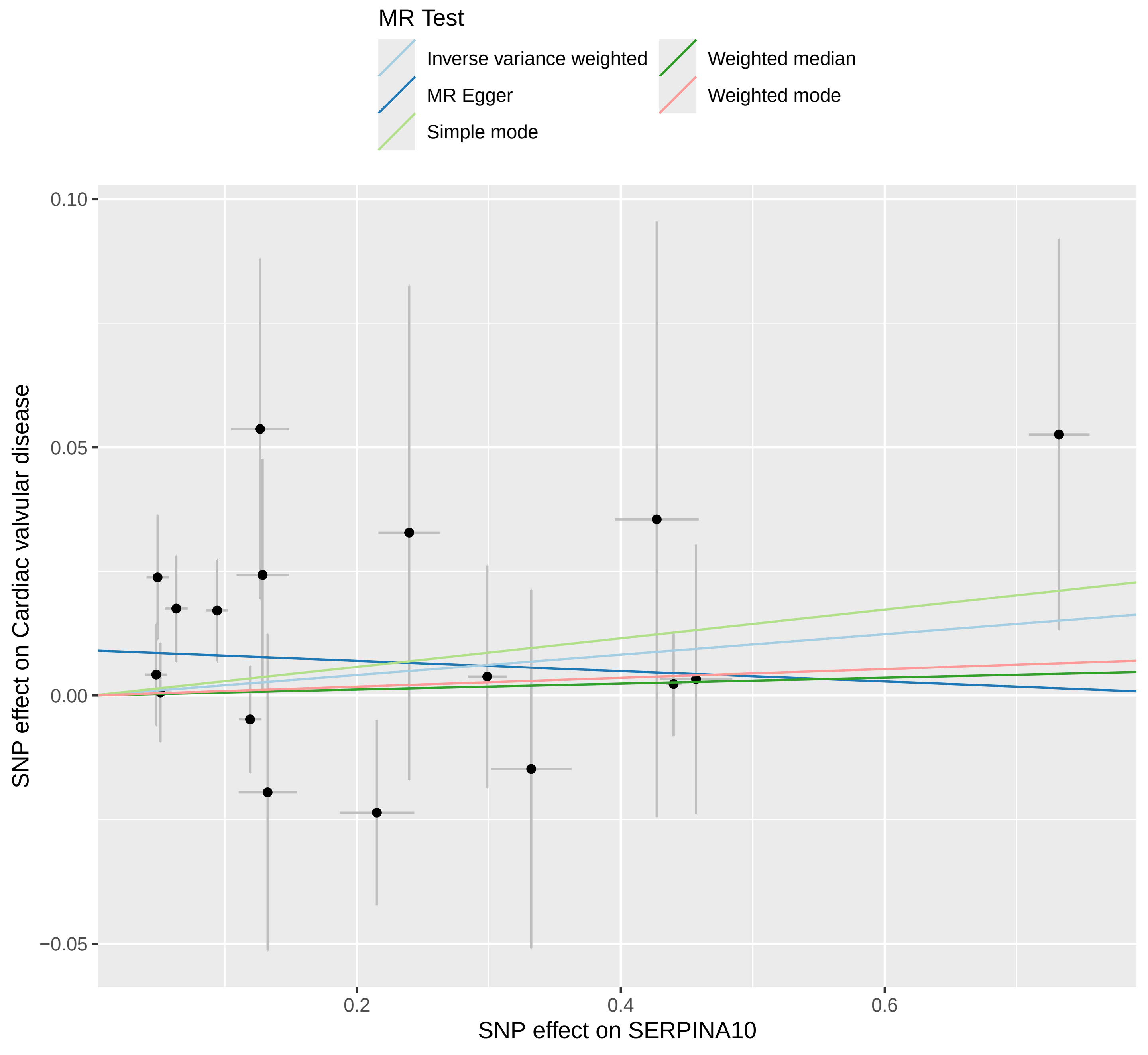

MR Test
Inverse variance weighted
MR Egger
Simple mode
Weighted median
Weighted mode
0.10
0.05
0.00
−0.05
SNP effect on Cardiac valvular disease
0.2
0.4
0.6
SNP effect on SERPINA10

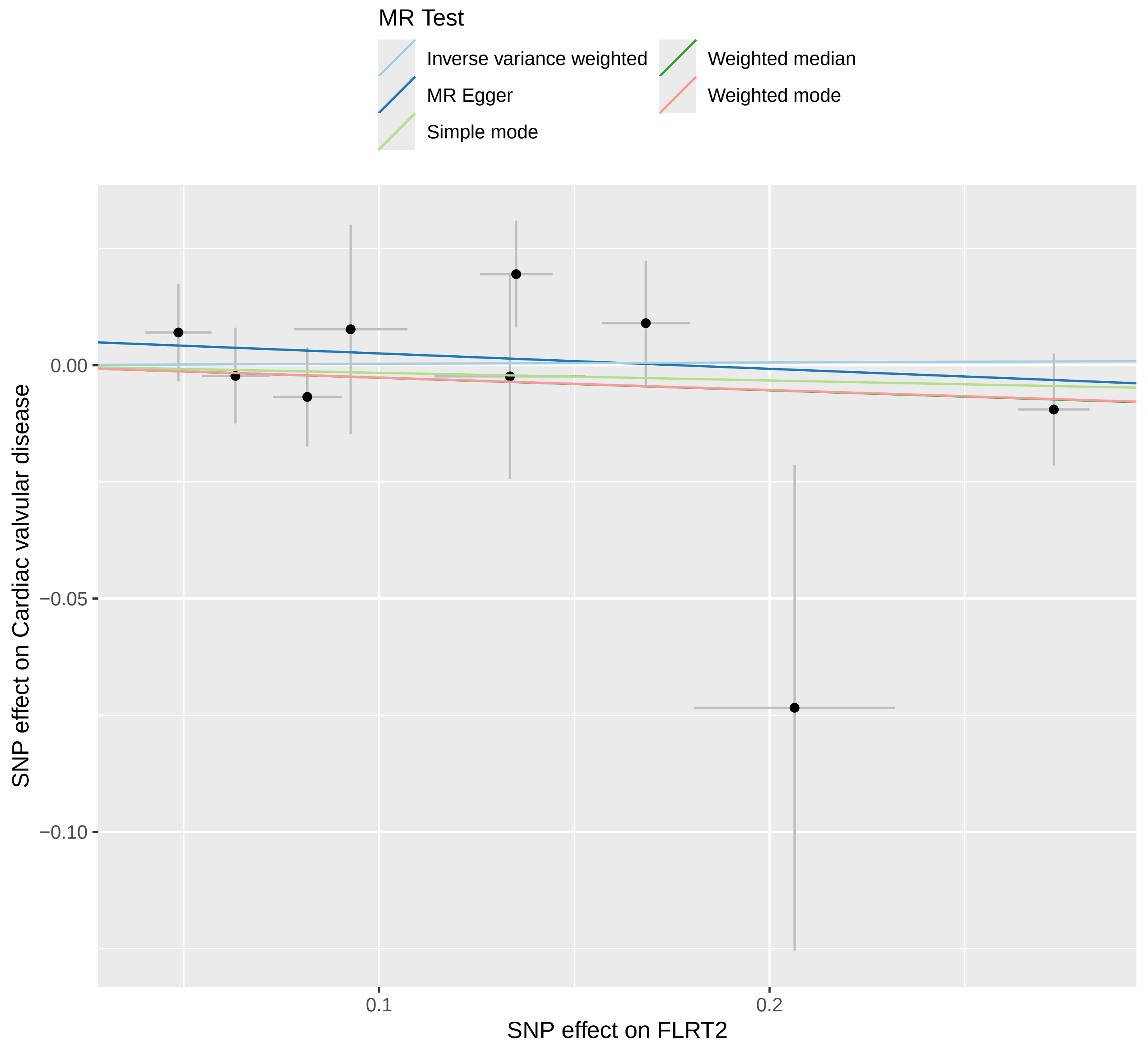
MR Test
Inverse variance weighted
MR Egger
Simple mode
Weighted median
Weighted mode
SNP effect on Cardiac valvular disease
0.00
−0.05
−0.10
0.1
0.2
SNP effect on FLRT2

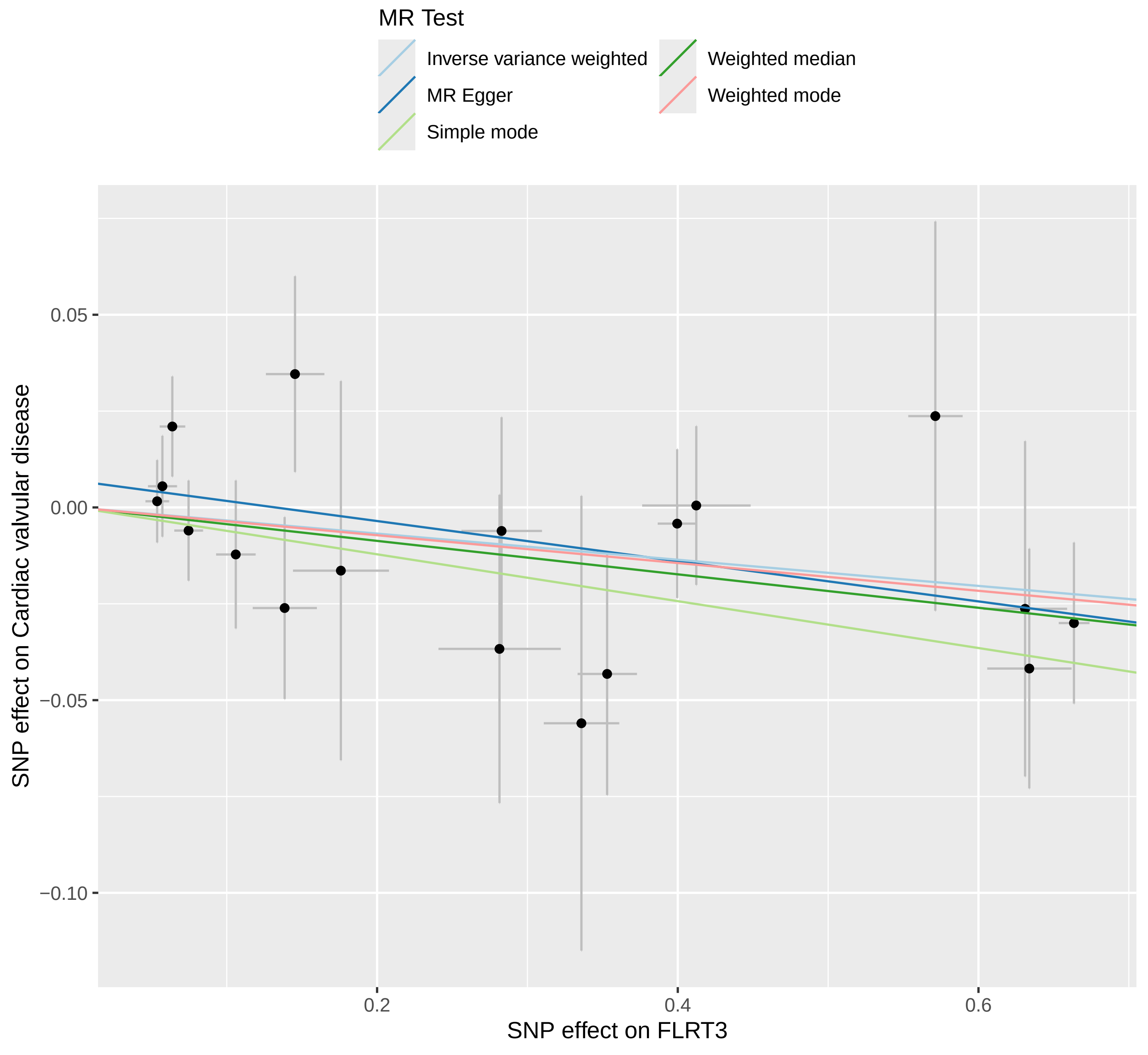

MR Test
Inverse variance weighted
MR Egger
Simple mode
Weighted median
Weighted mode
0.05
0.00
−0.05
−0.10
0.2
0.4
0.6
SNP effect on Cardiac valvular disease
SNP effect on FLRT3

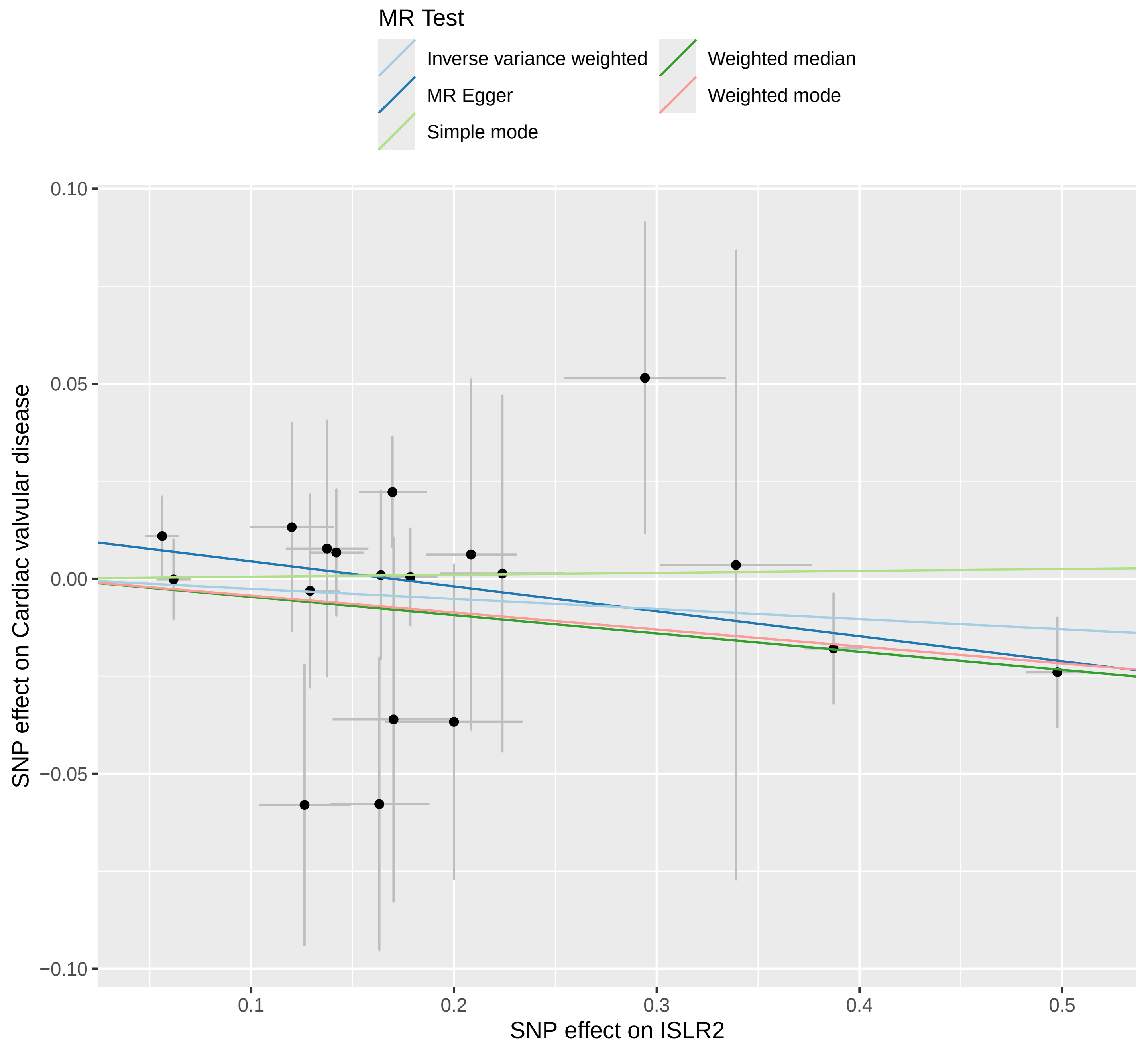

MR Test
Inverse variance weighted
MR Egger
Simple mode
Weighted median
Weighted mode
0.10
0.05
0.00
−0.05
−0.10
SNP effect on Cardiac valvular disease
0.1
0.2
0.3
0.4
0.5
SNP effect on ISLR2

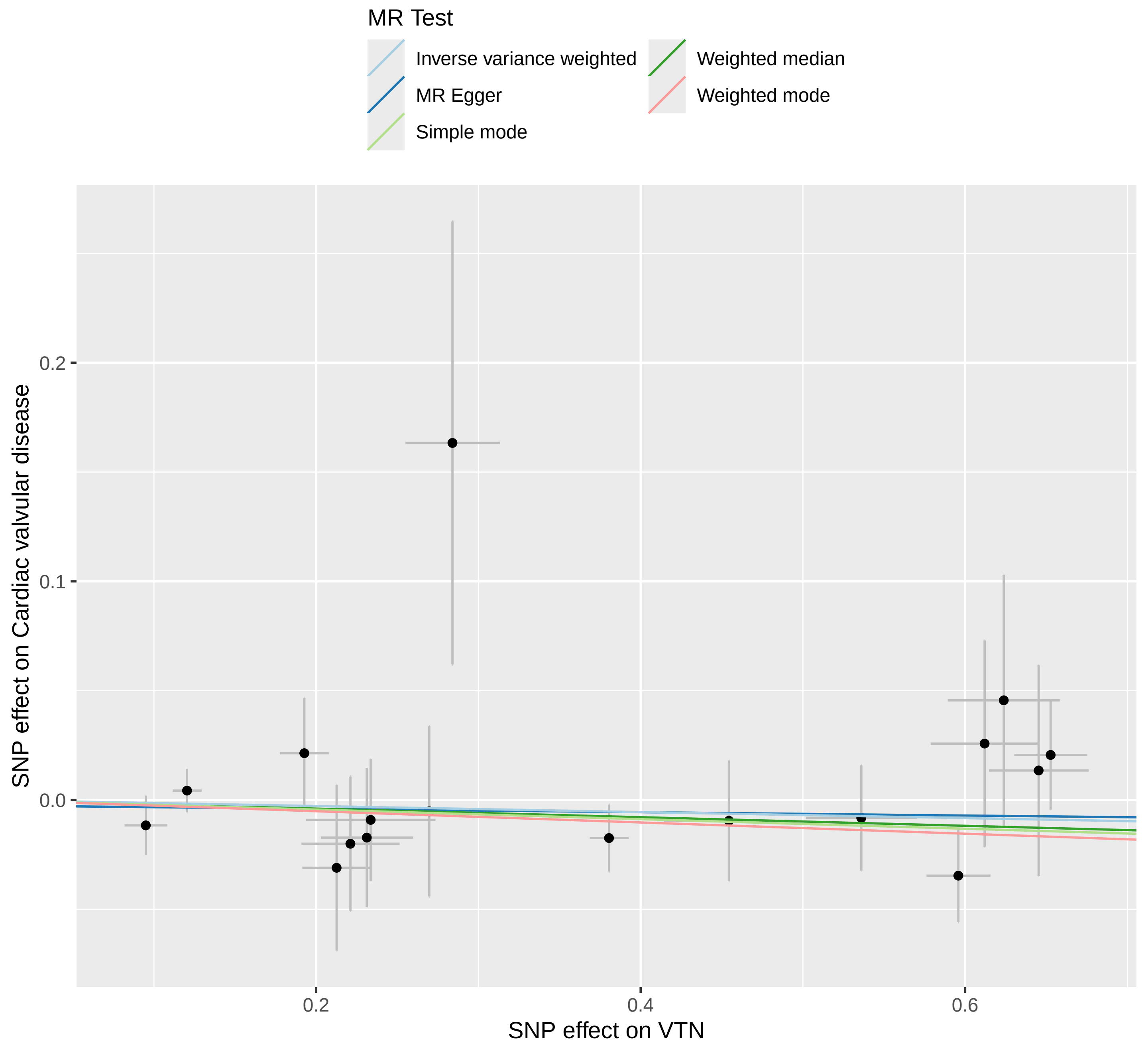

MR Test
Inverse variance weighted
MR Egger
Simple mode
Weighted median
Weighted mode
0.2
0.1
0.0
SNP effect on Cardiac valvular disease
0.2
0.4
0.6
SNP effect on VTN

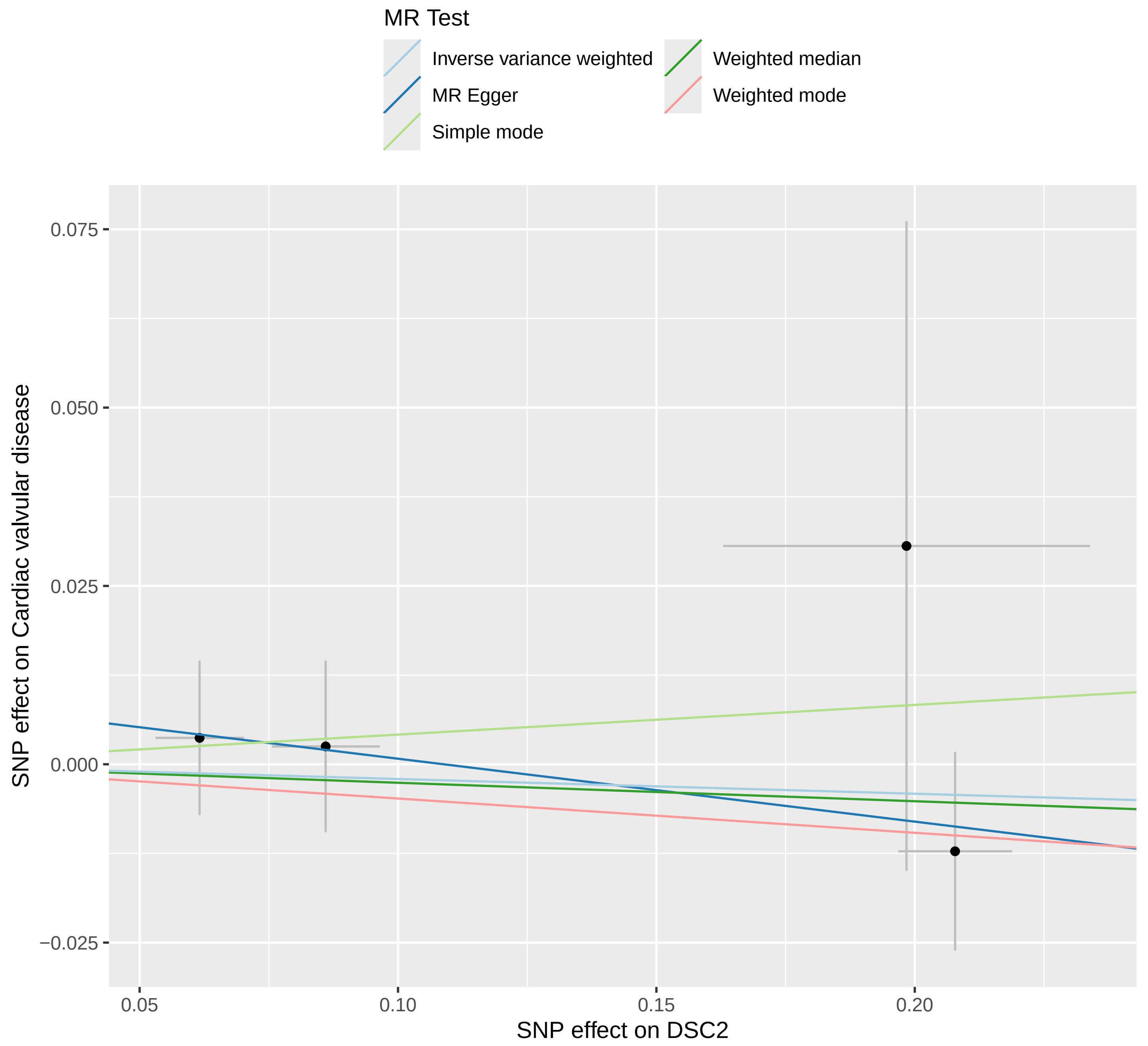

MR Test
Inverse variance weighted
MR Egger
Simple mode
Weighted median
Weighted mode
0.075
0.050
0.025
0.000
−0.025
SNP effect on Cardiac valvular disease
0.05
0.10
0.15
0.20
SNP effect on DSC2

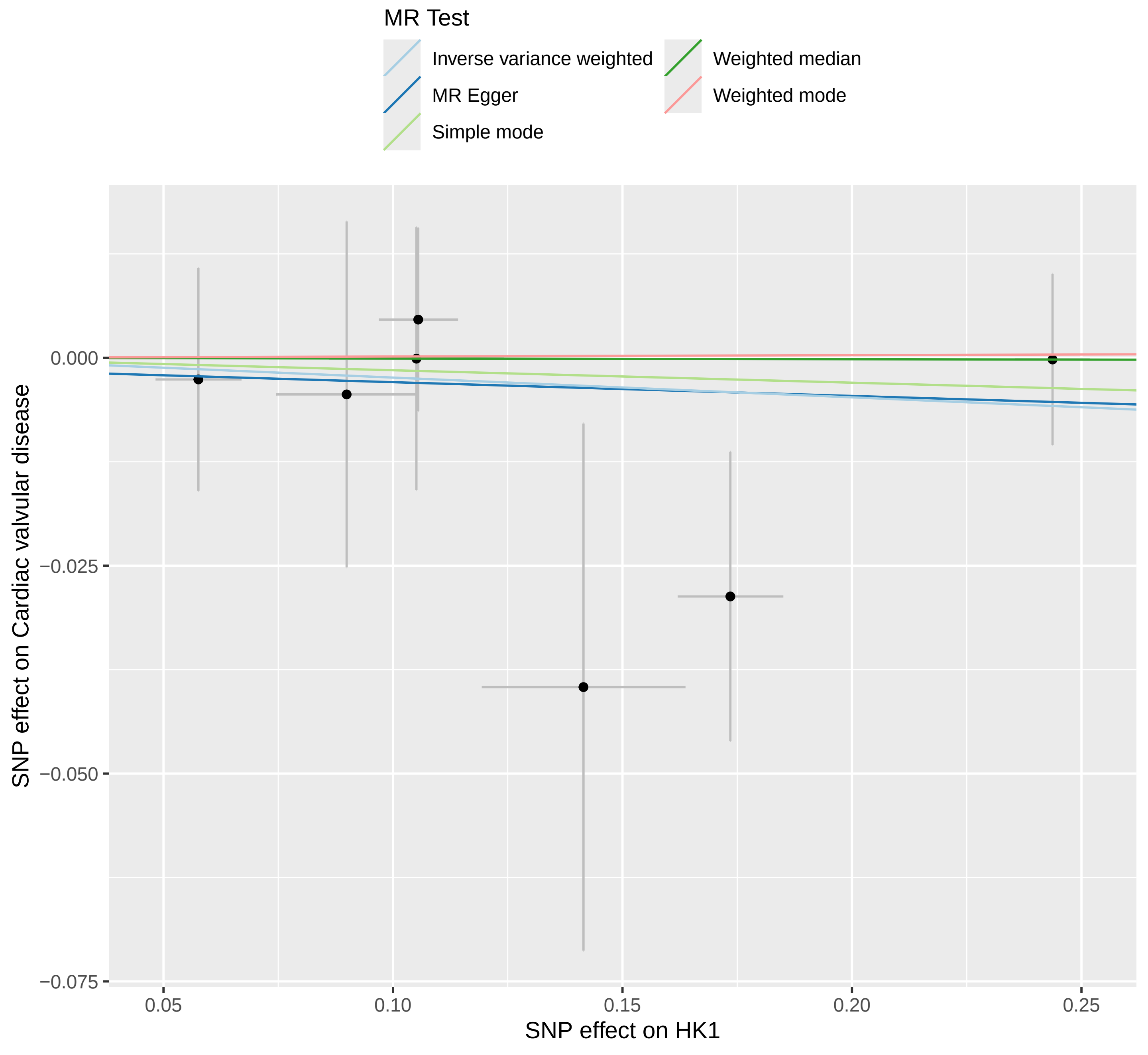

MR Test
Inverse variance weighted
MR Egger
Simple mode
Weighted median
Weighted mode
0.000
−0.025
−0.050
−0.075
0.05
0.10
0.15
0.20
0.25
SNP effect on Cardiac valvular disease
SNP effect on HK1

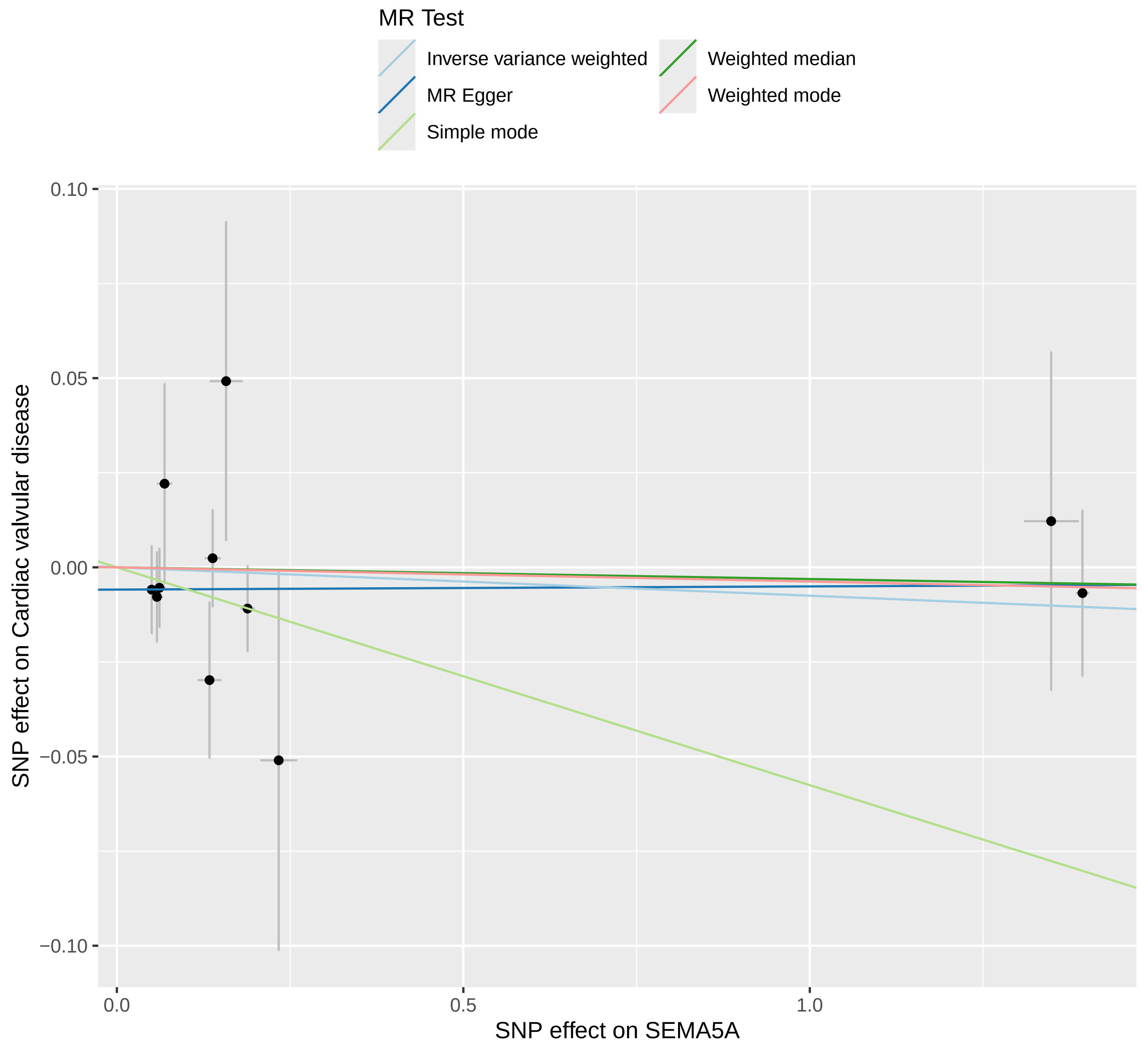
MR Test
Inverse variance weighted
MR Egger
Simple mode
Weighted median
Weighted mode
0.10
0.05
0.00
−0.05
−0.10
0.0
0.5
1.0
SNP effect on Cardiac valvular disease
SNP effect on SEMA5A

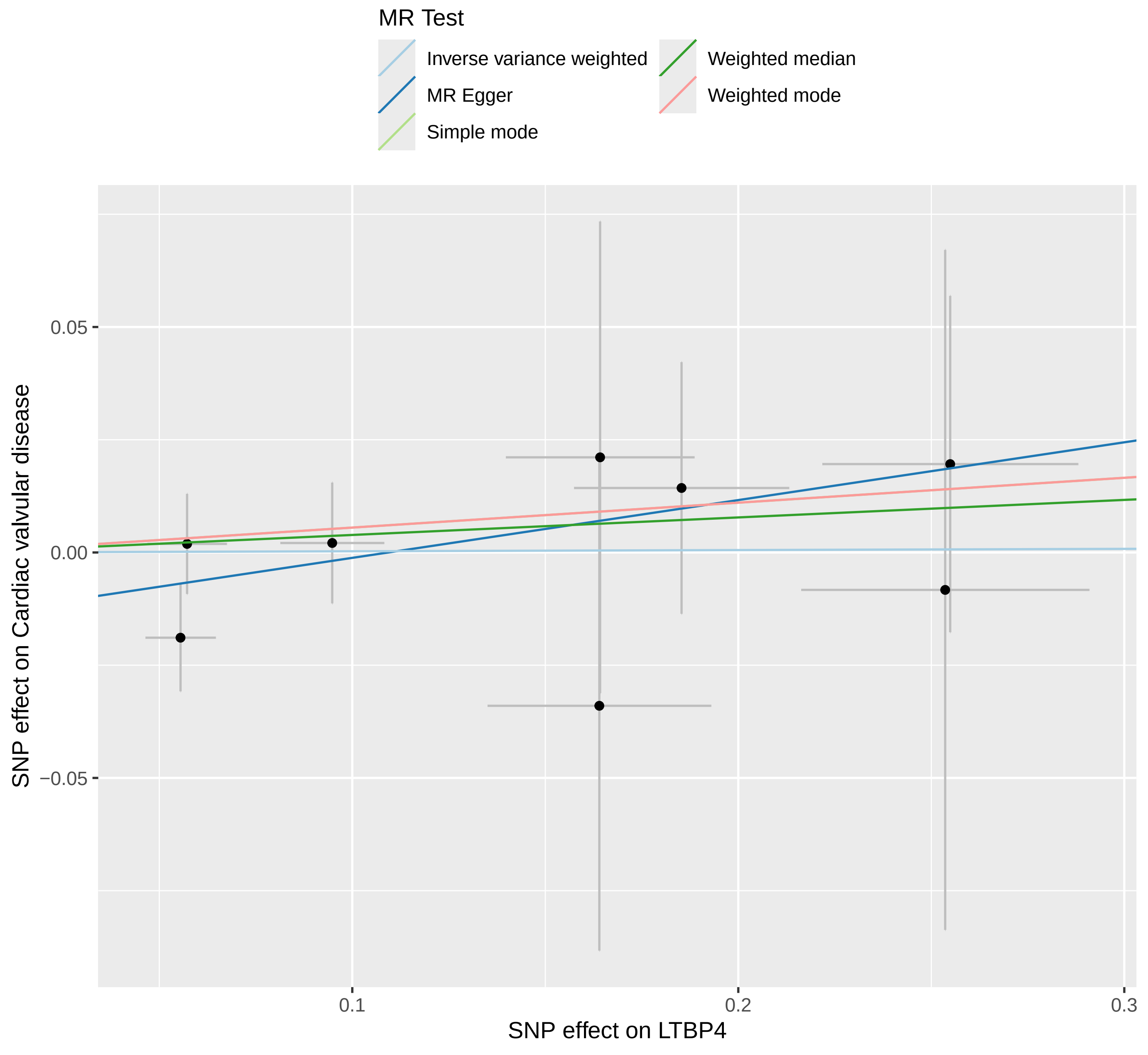

MR Test
Inverse variance weighted
MR Egger
Simple mode
Weighted median
Weighted mode
0.05
0.00
−0.05
0.1
0.2
0.3
SNP effect on LTBP4
SNP effect on Cardiac valvular disease

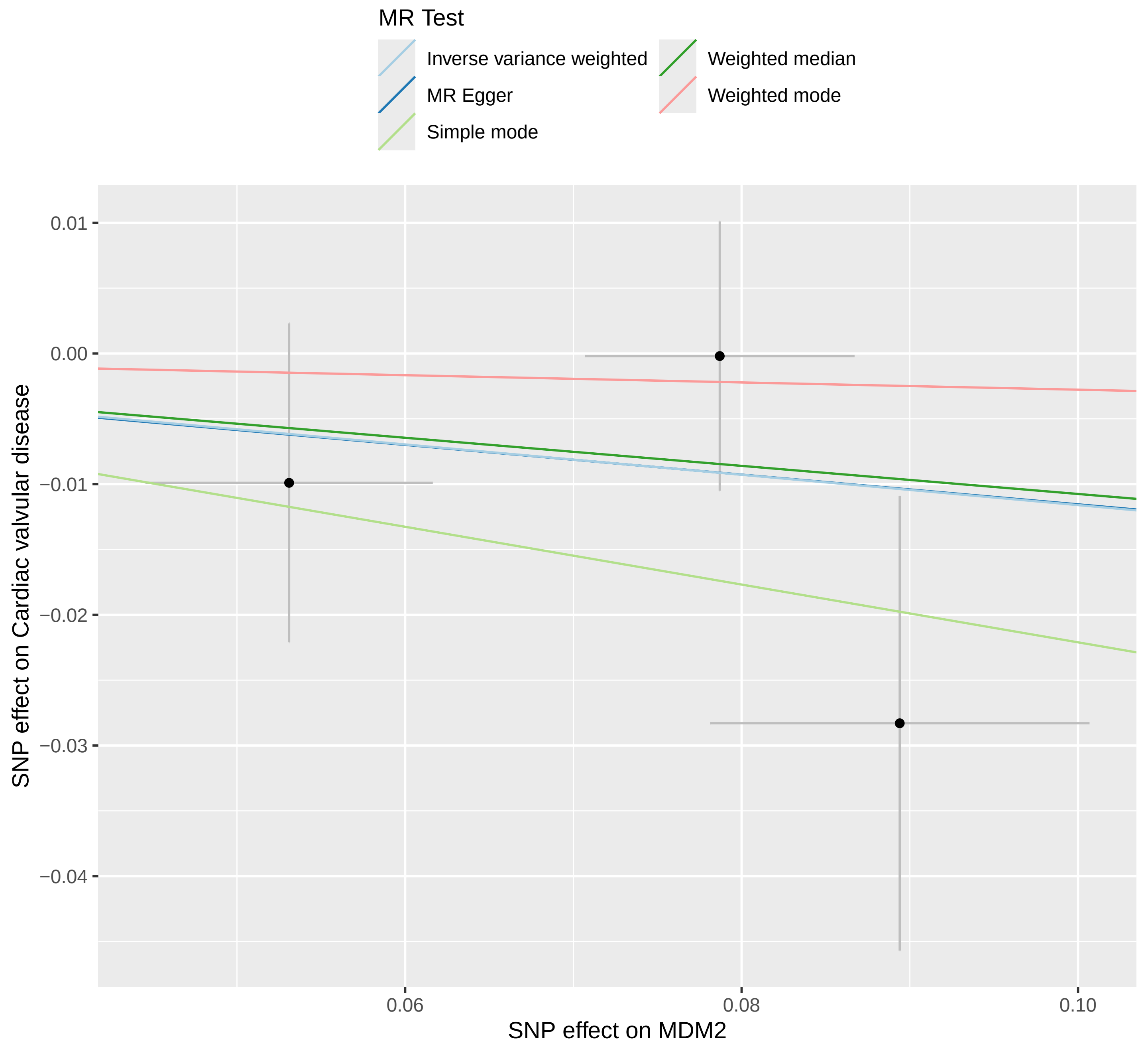

MR Test
Inverse variance weighted
MR Egger
Simple mode
Weighted median
Weighted mode
0.01
0.00
−0.01
−0.02
−0.03
−0.04
SNP effect on Cardiac valvular disease
0.06
0.08
0.10
SNP effect on MDM2

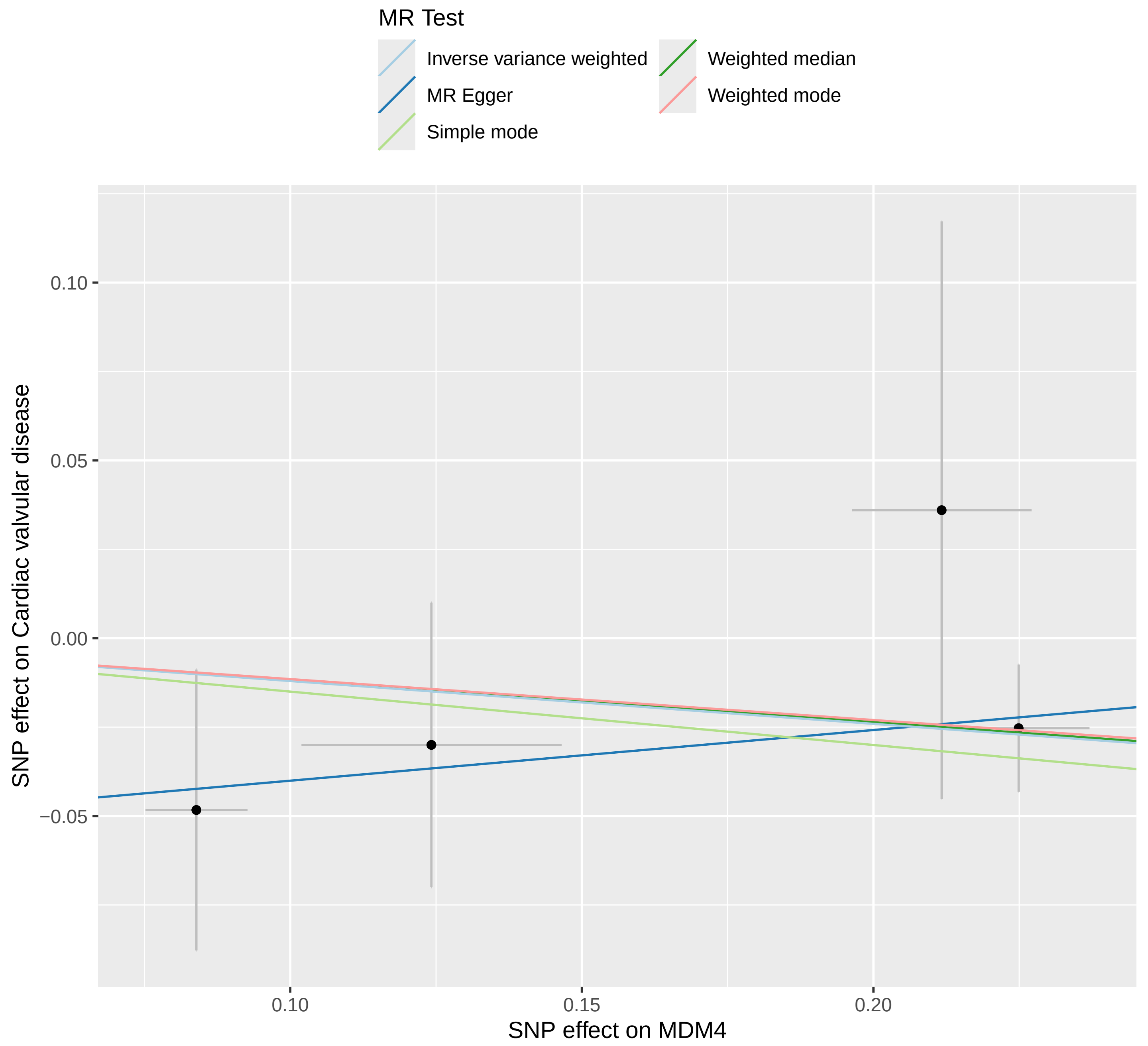
MR Test
Inverse variance weighted
MR Egger
Simple mode
Weighted median
Weighted mode
0.10
0.05
0.00
−0.05
SNP effect on Cardiac valvular disease
0.10
0.15
0.20
SNP effect on MDM4

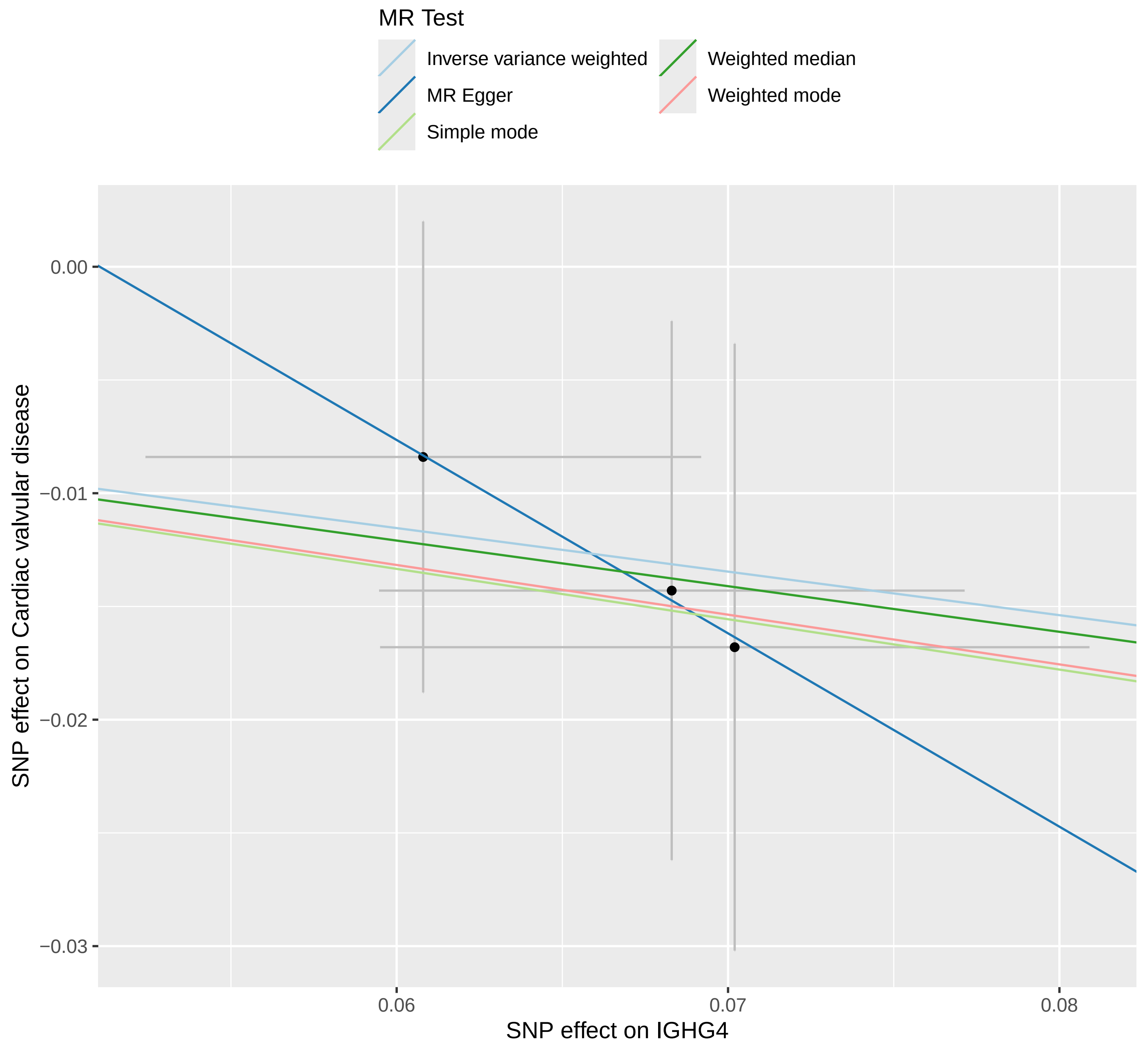
MR Test
Inverse variance weighted
MR Egger
Simple mode
Weighted median
Weighted mode
0.00
−0.01
−0.02
−0.03
SNP effect on Cardiac valvular disease
0.06
0.07
0.08
SNP effect on IGHG4

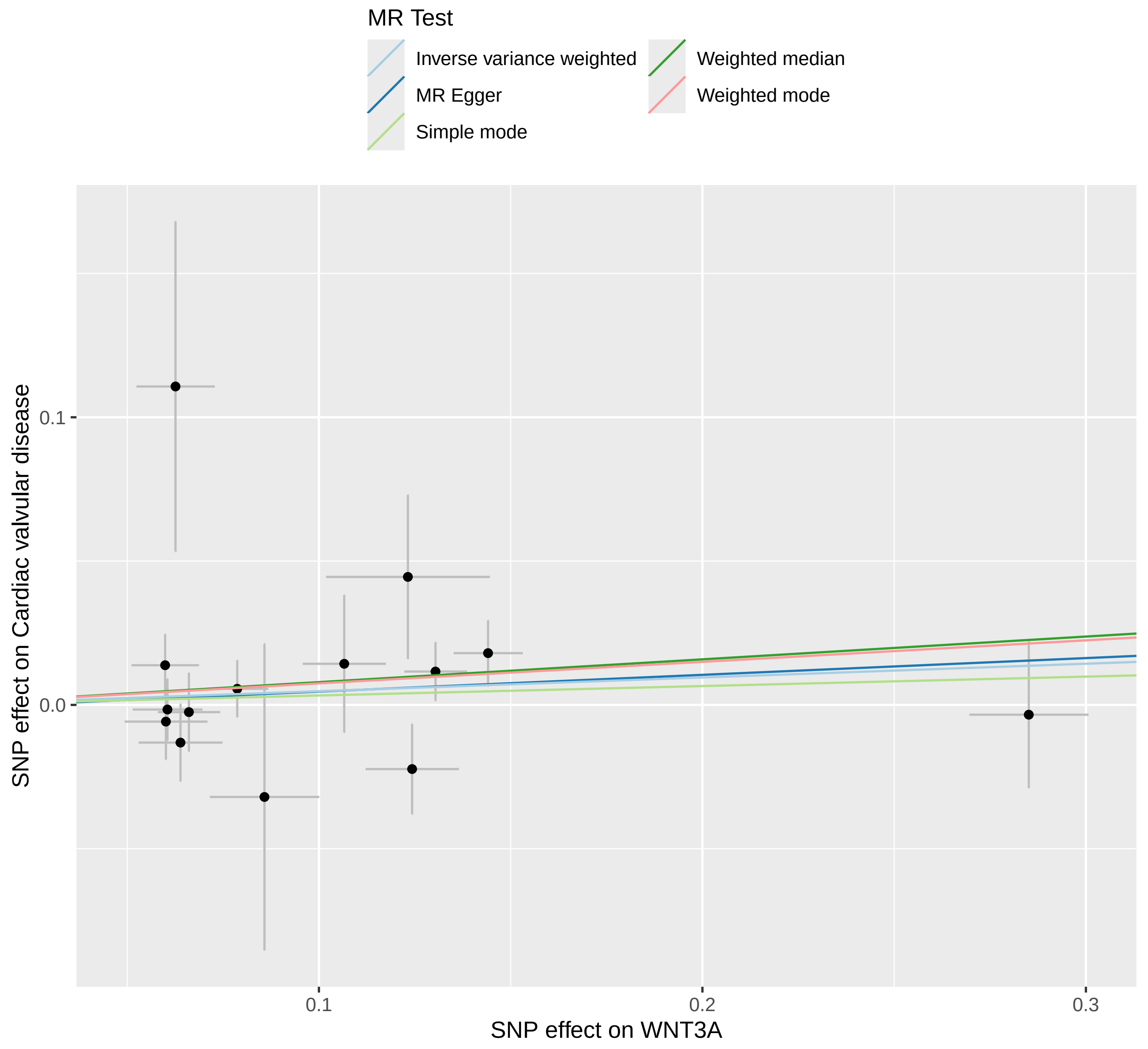

MR Test
Inverse variance weighted
MR Egger
Simple mode
Weighted median
Weighted mode
SNP effect on Cardiac valvular disease
0.1
0.0
0.1
0.2
0.3
SNP effect on WNT3A

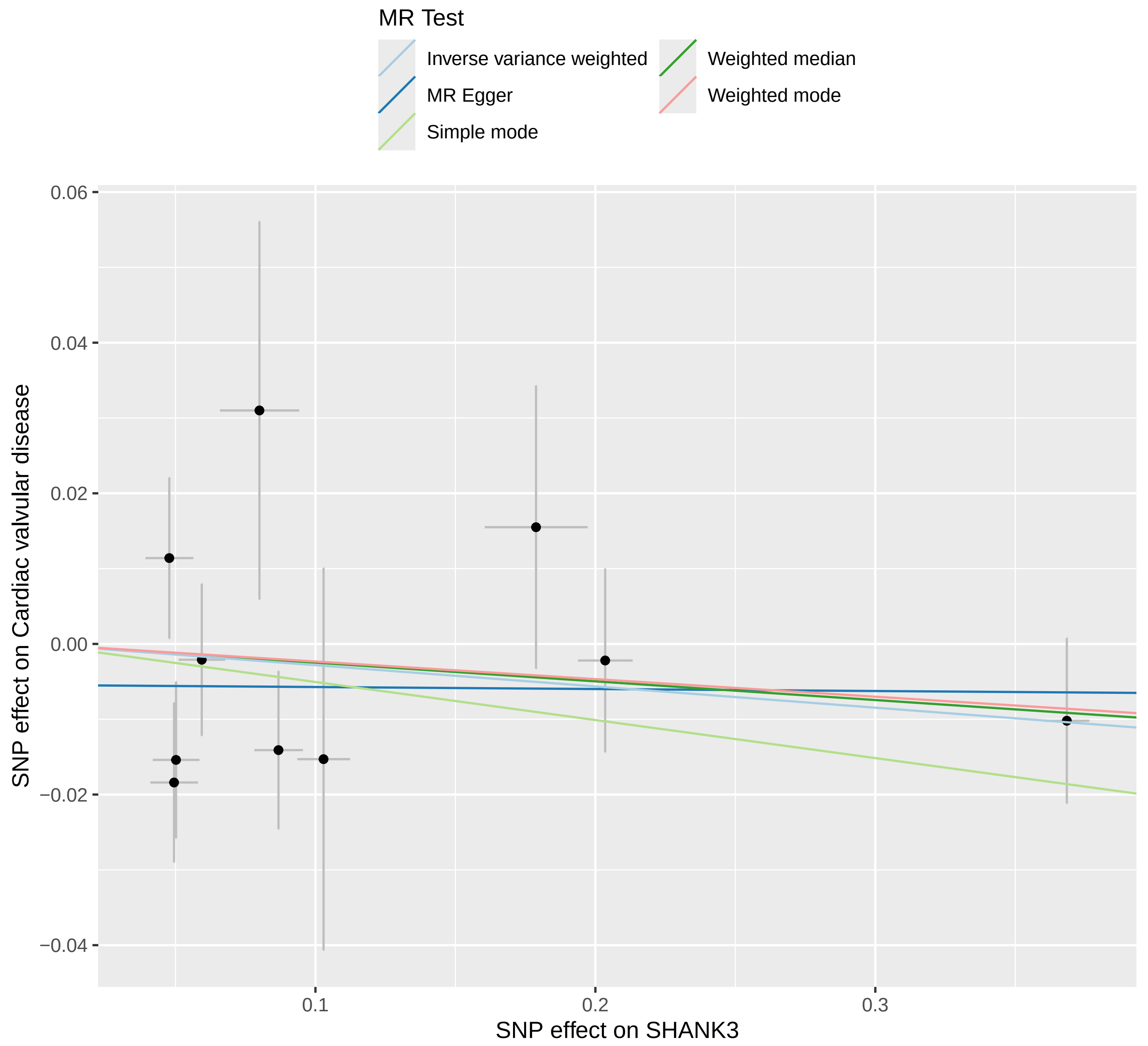
MR Test
Inverse variance weighted
MR Egger
Simple mode
Weighted median
Weighted mode
0.06
0.04
0.02
0.00
−0.02
−0.04
SNP effect on Cardiac valvular disease
0.1
0.2
0.3
SNP effect on SHANK3

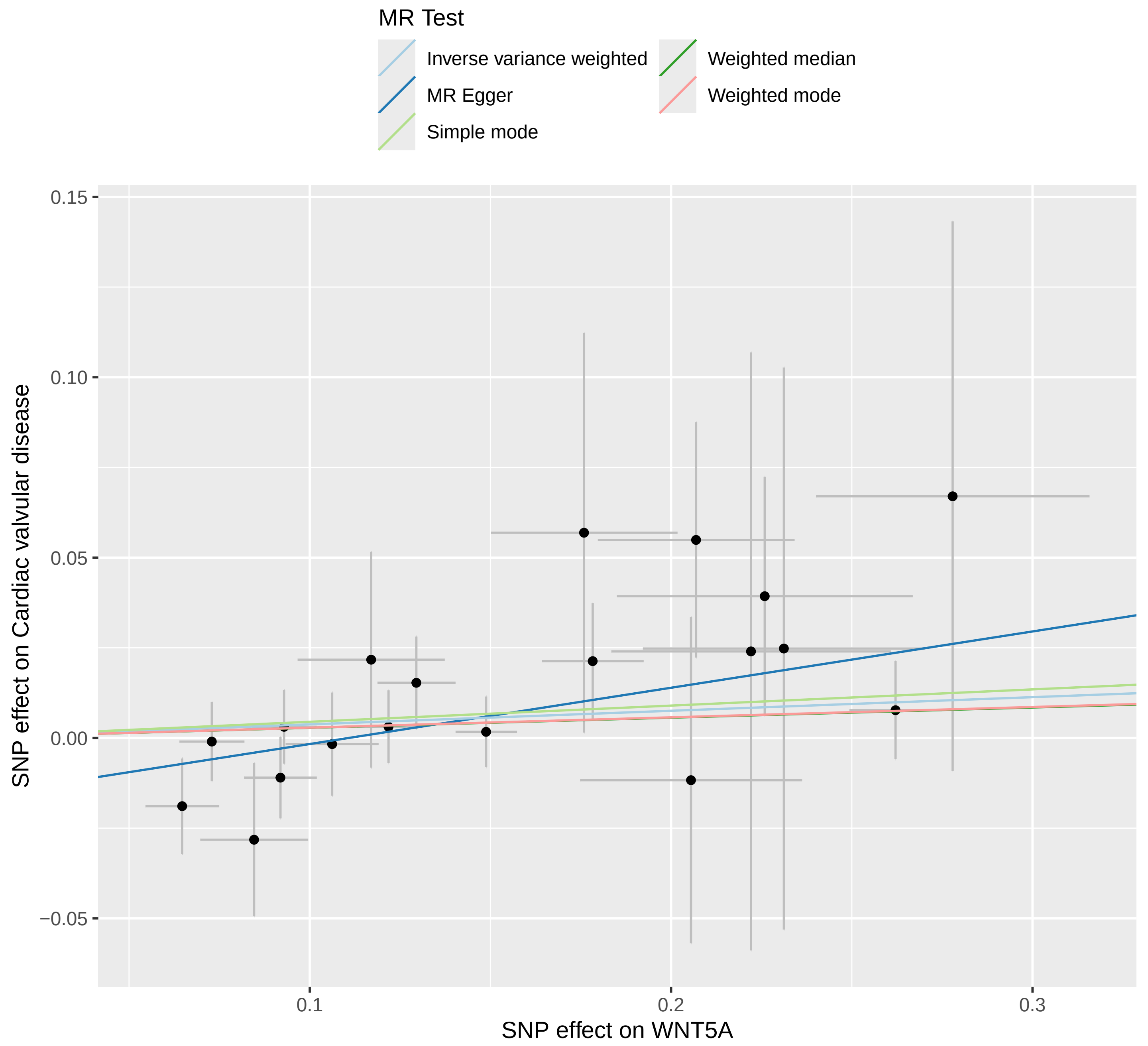

MR Test
Inverse variance weighted
MR Egger
Simple mode
Weighted median
Weighted mode
0.15
0.10
0.05
0.00
−0.05
0.1
0.2
0.3
SNP effect on Cardiac valvular disease
SNP effect on WNT5A

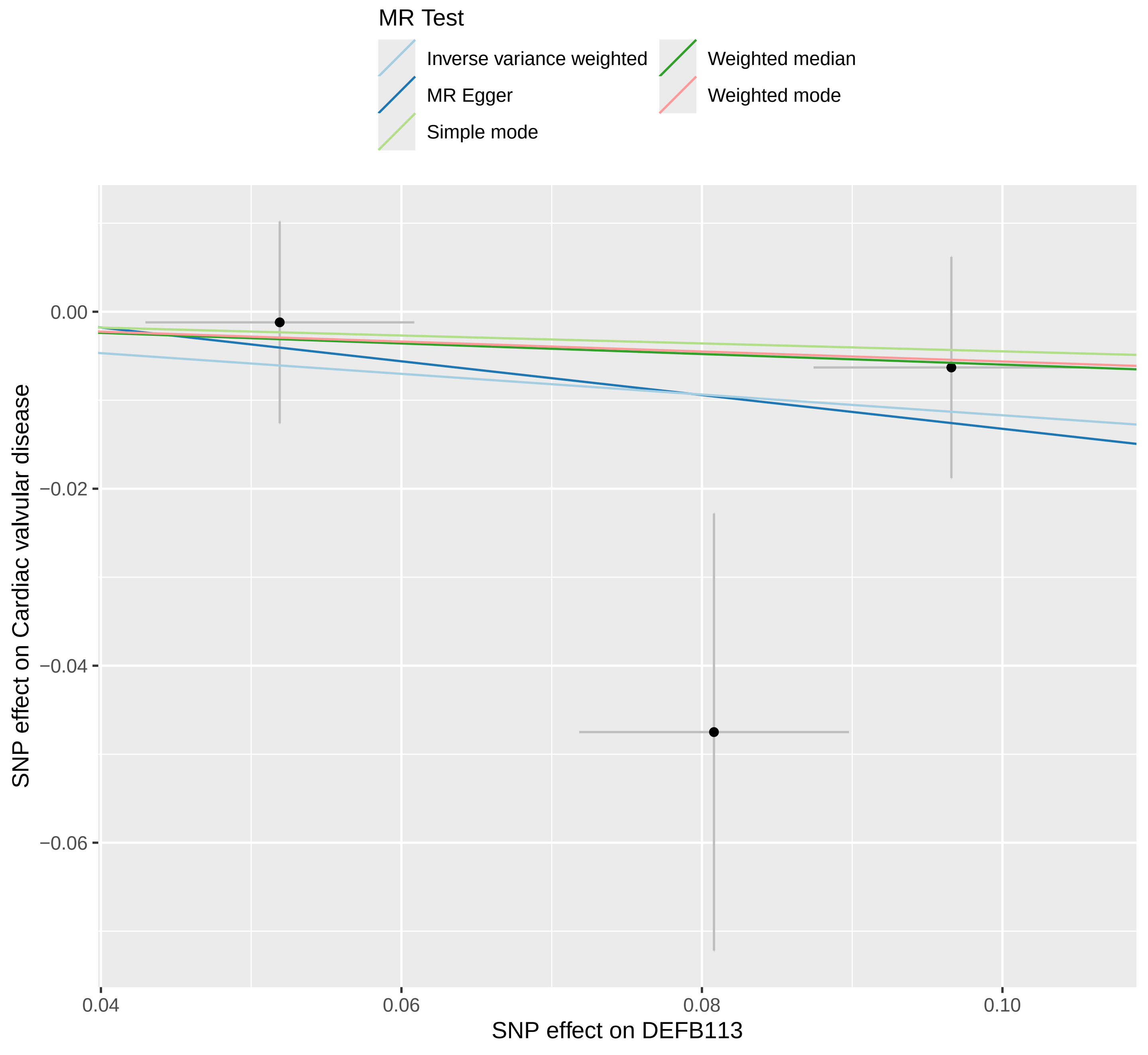
MR Test
Inverse variance weighted
MR Egger
Simple mode
Weighted median
Weighted mode
0.00
−0.02
−0.04
−0.06
SNP effect on Cardiac valvular disease
0.04
0.06
0.08
0.10
SNP effect on DEFB113

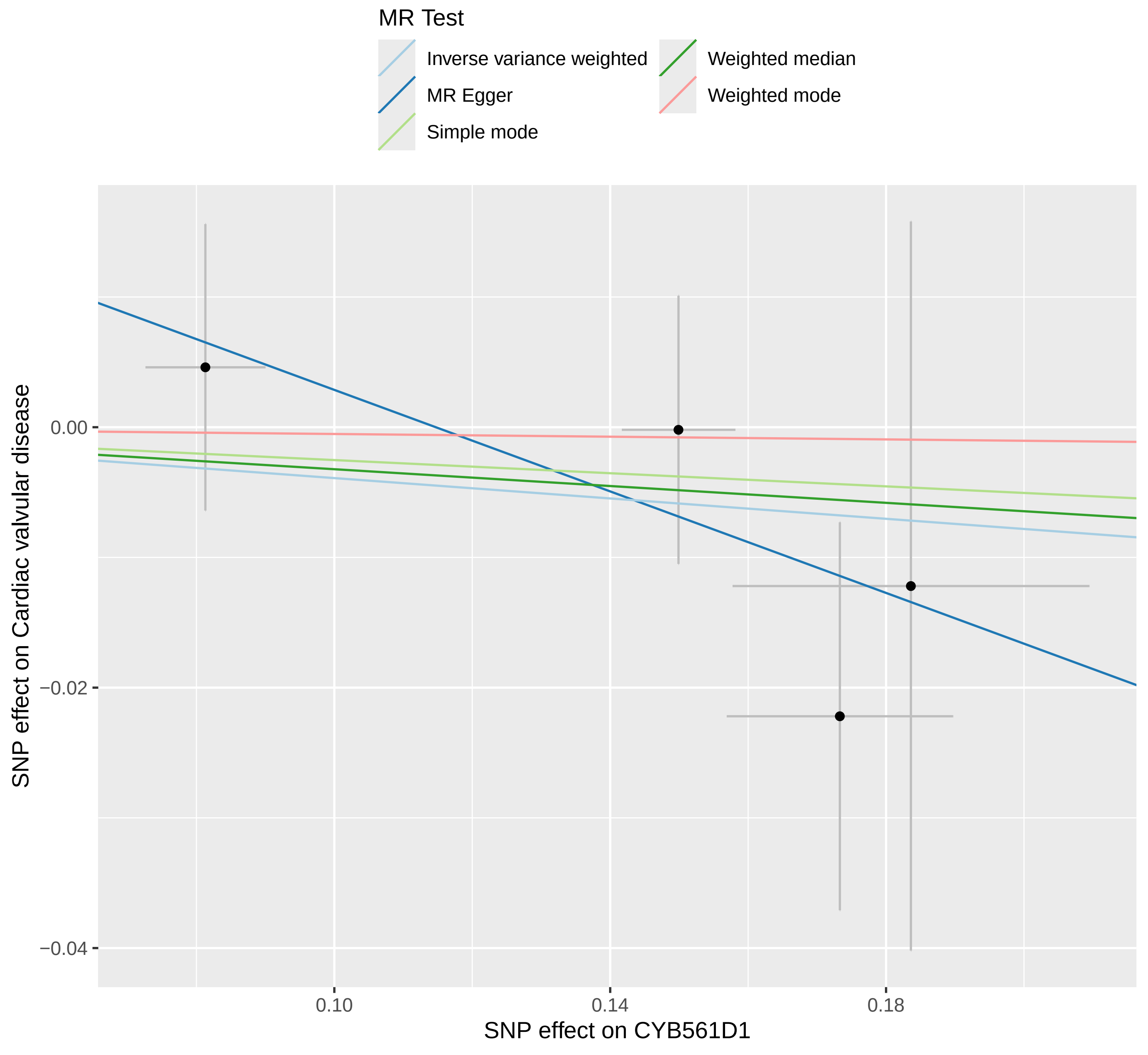

MR Test
Inverse variance weighted
MR Egger
Simple mode
Weighted median
Weighted mode
SNP effect on Cardiac valvular disease
0.00
−0.02
−0.04
0.10
0.14
0.18
SNP effect on CYB561D1

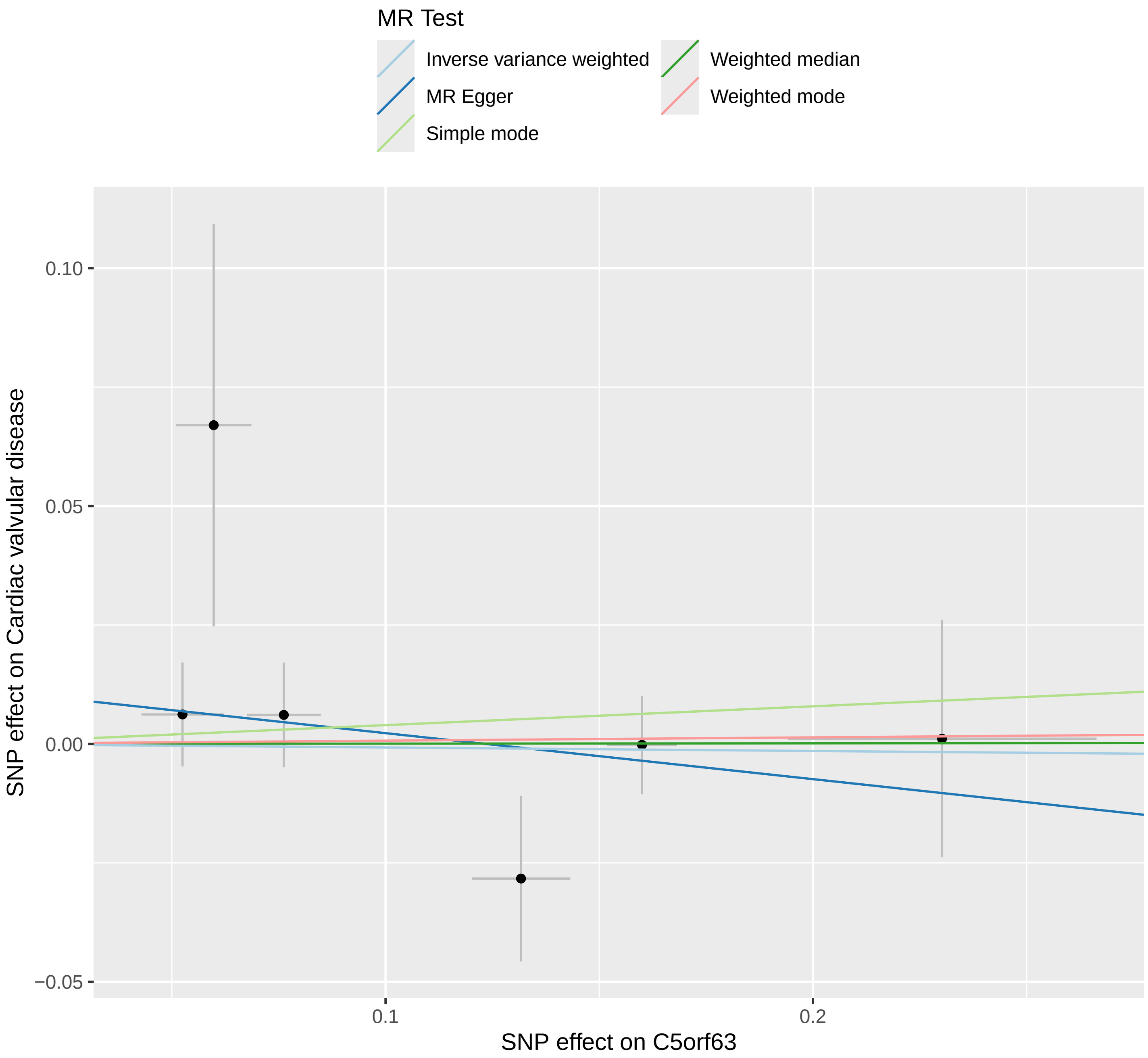
MR Test
Inverse variance weighted
MR Egger
Simple mode
Weighted median
Weighted mode
0.10
0.05
0.00
−0.05
0.1
0.2
SNP effect on C5orf63
SNP effect on Cardiac valvular disease

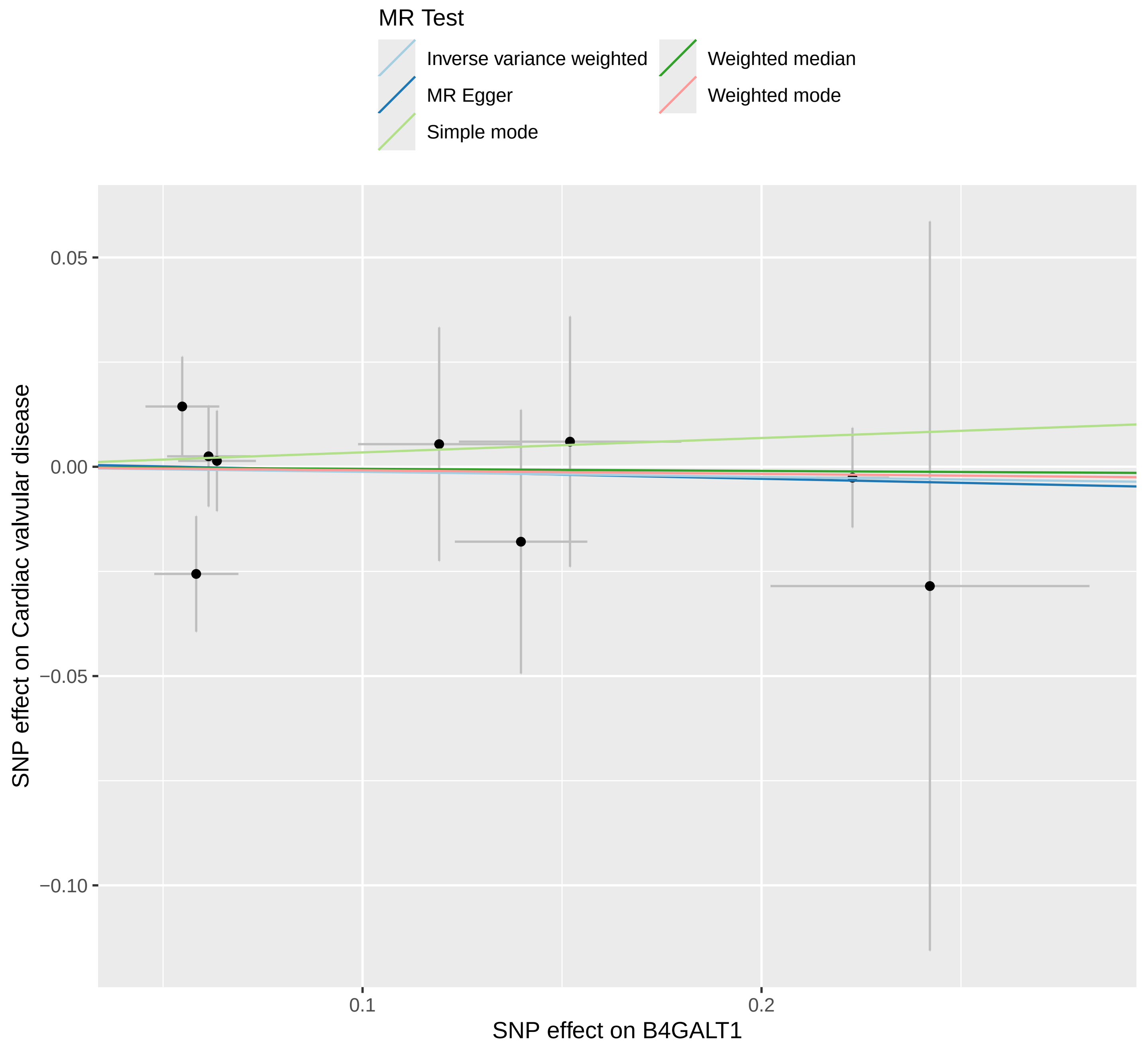
MR Test
Inverse variance weighted
MR Egger
Simple mode
Weighted median
Weighted mode
0.05
0.00
−0.05
−0.10
SNP effect on Cardiac valvular disease
0.1
0.2
SNP effect on B4GALT1

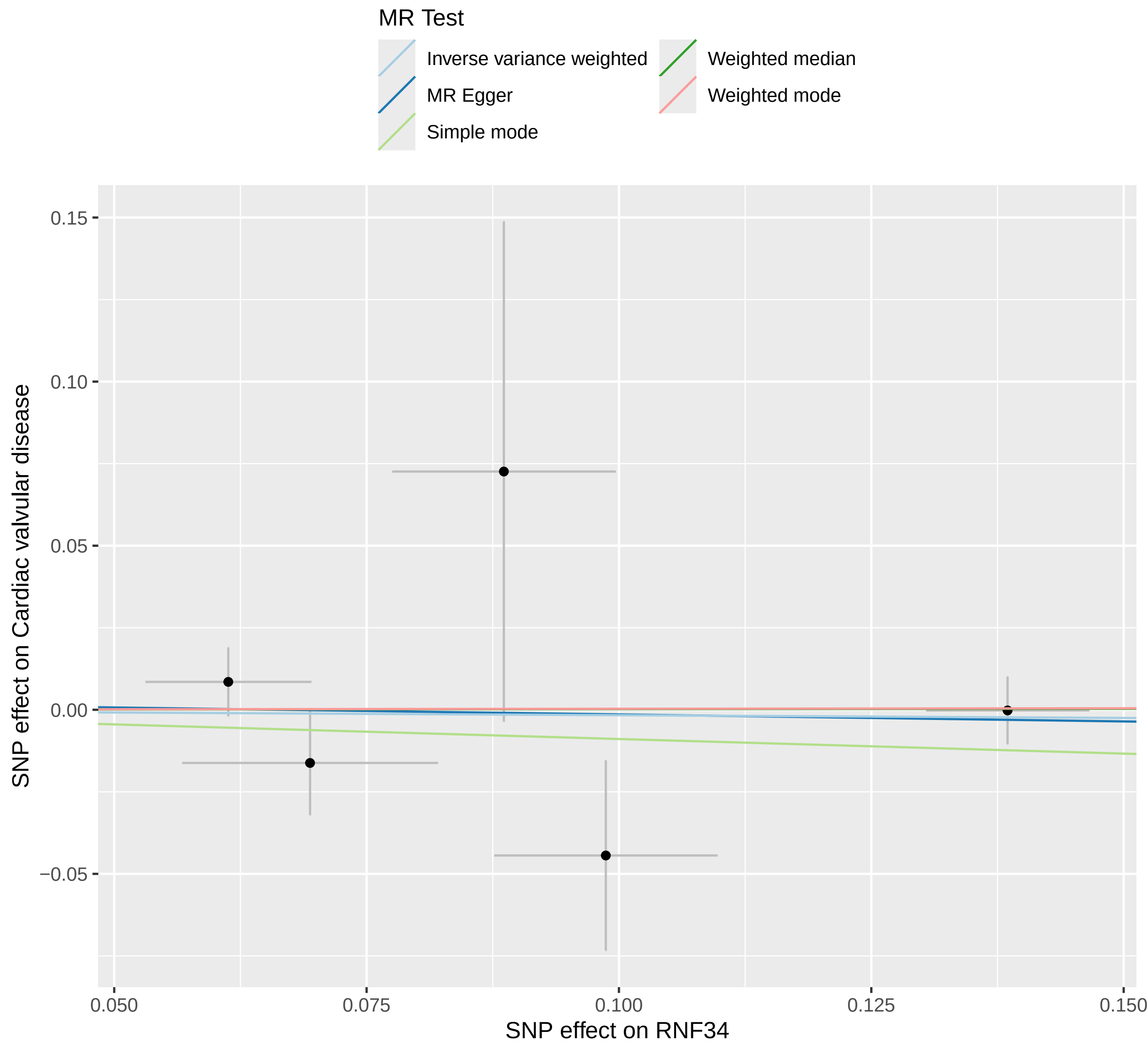

MR Test
Inverse variance weighted
MR Egger
Simple mode
Weighted median
Weighted mode
0.15
0.10
0.05
0.00
−0.05
0.050
0.075
0.100
0.125
0.150
SNP effect on RNF34
SNP effect on Cardiac valvular disease

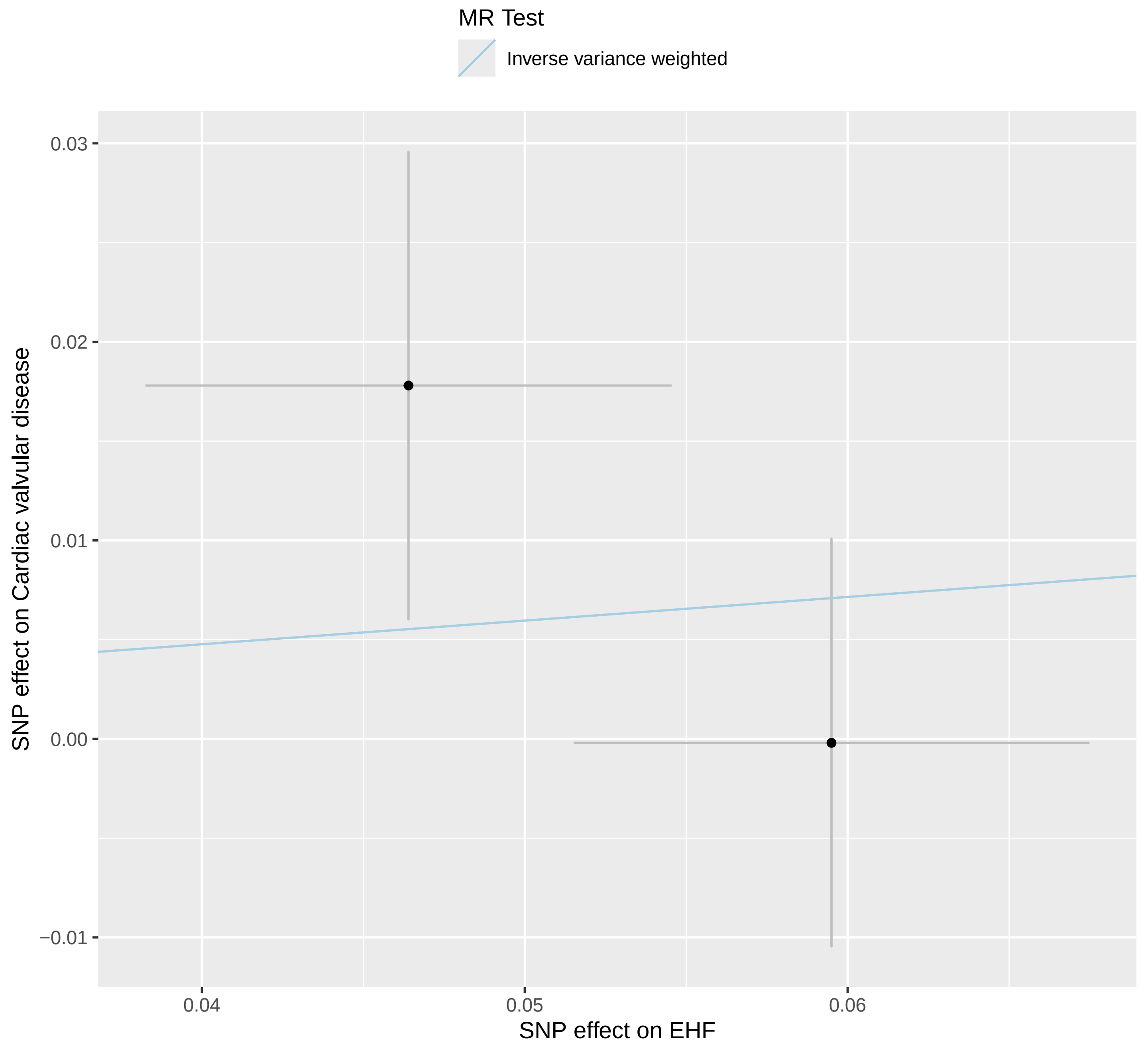

MR Test
Inverse variance weighted
0.03
0.02
0.01
0.00
−0.01
0.04
0.05
0.06
SNP effect on Cardiac valvular disease
SNP effect on EHF

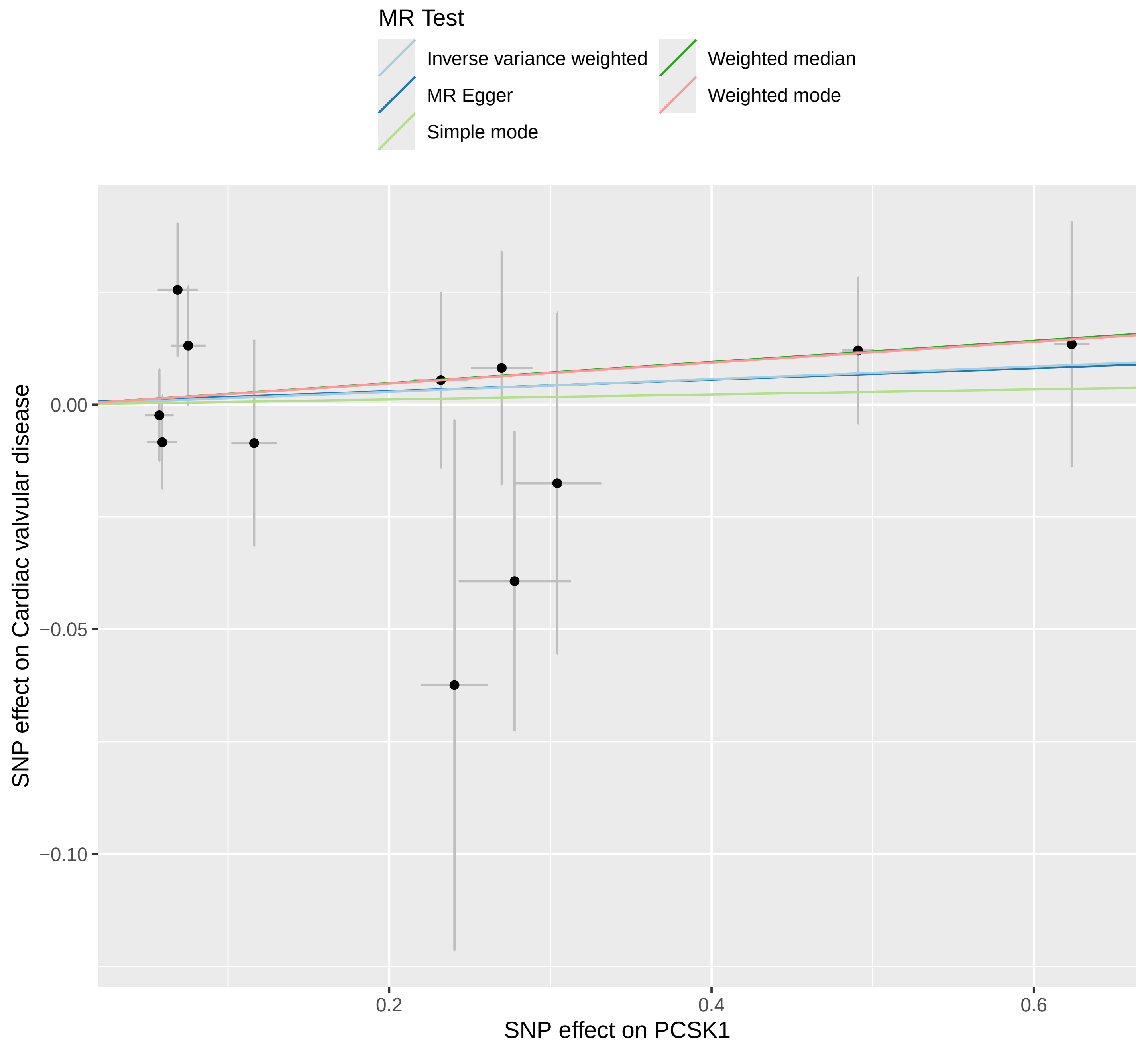

MR Test
Inverse variance weighted
MR Egger
Simple mode
Weighted median
Weighted mode
SNP effect on Cardiac valvular disease
0.00
−0.05
−0.10
0.2
0.4
0.6
SNP effect on PCSK1

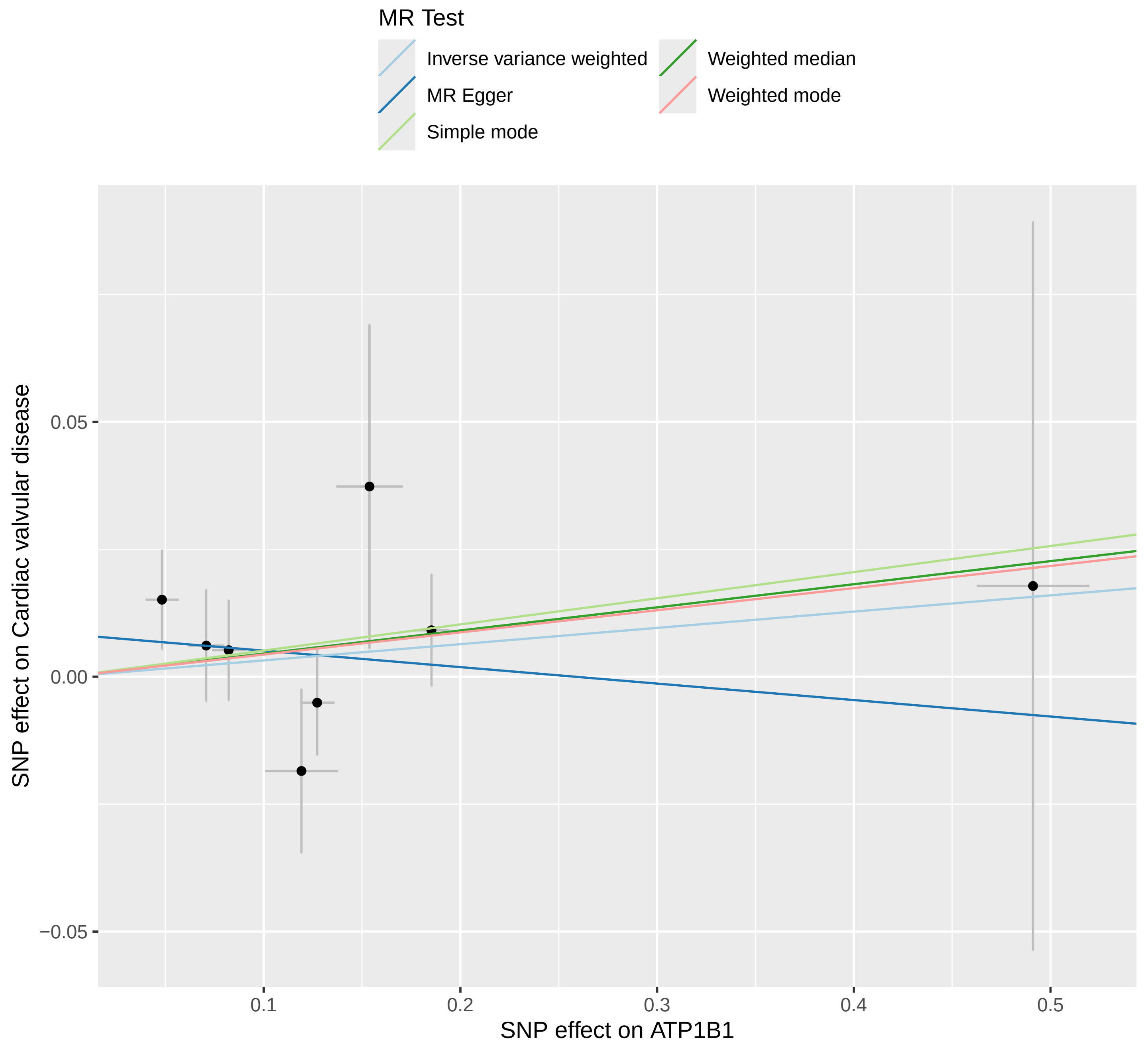
MR Test
Inverse variance weighted
MR Egger
Simple mode
Weighted median
Weighted mode
0.05
0.00
−0.05
0.1
0.2
0.3
0.4
0.5
SNP effect on ATP1B1
SNP effect on Cardiac valvular disease

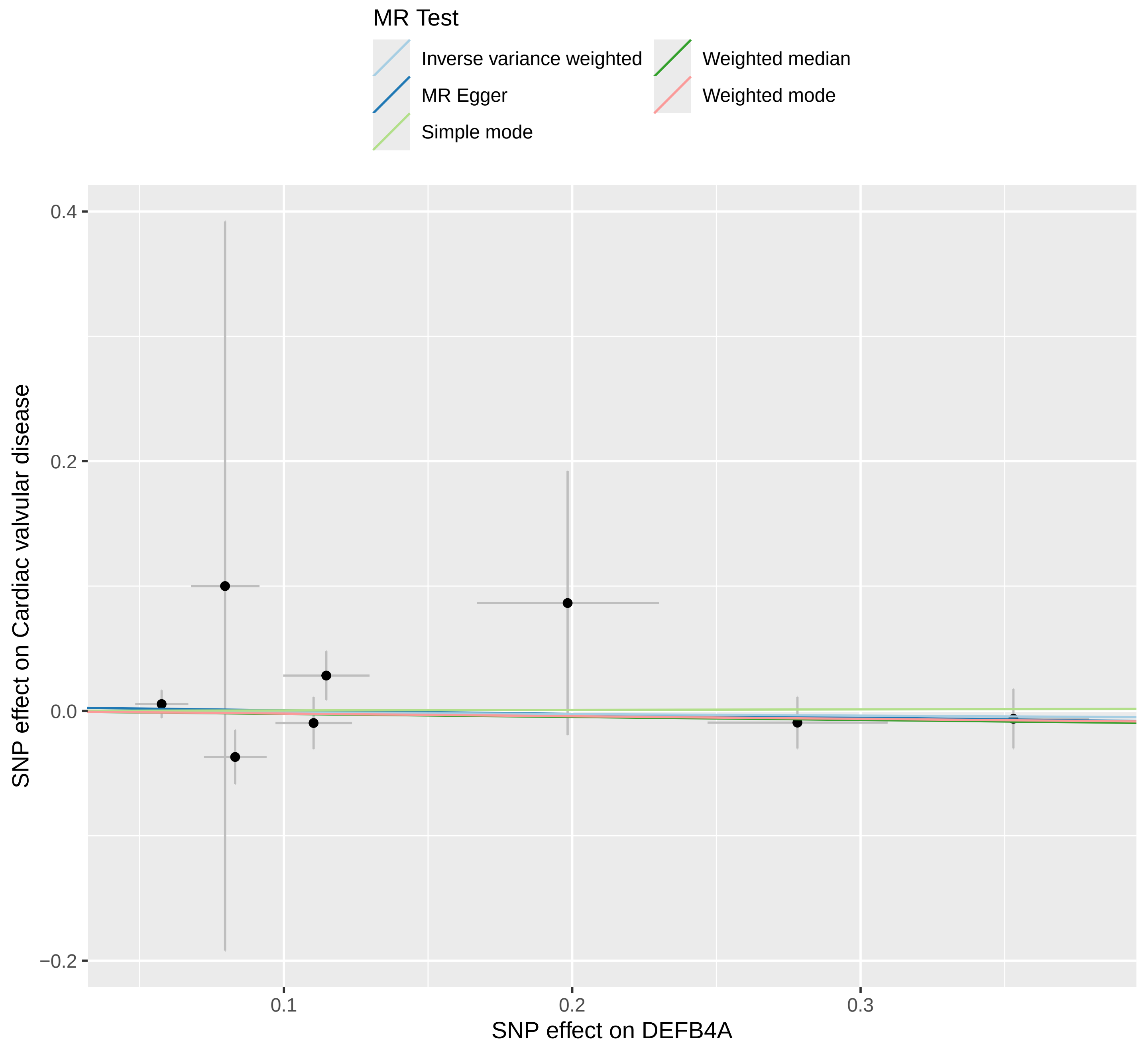
MR Test
Inverse variance weighted
MR Egger
Simple mode
Weighted median
Weighted mode
0.4
0.2
0.0
−0.2
SNP effect on Cardiac valvular disease
0.1
0.2
0.3
SNP effect on DEFB4A

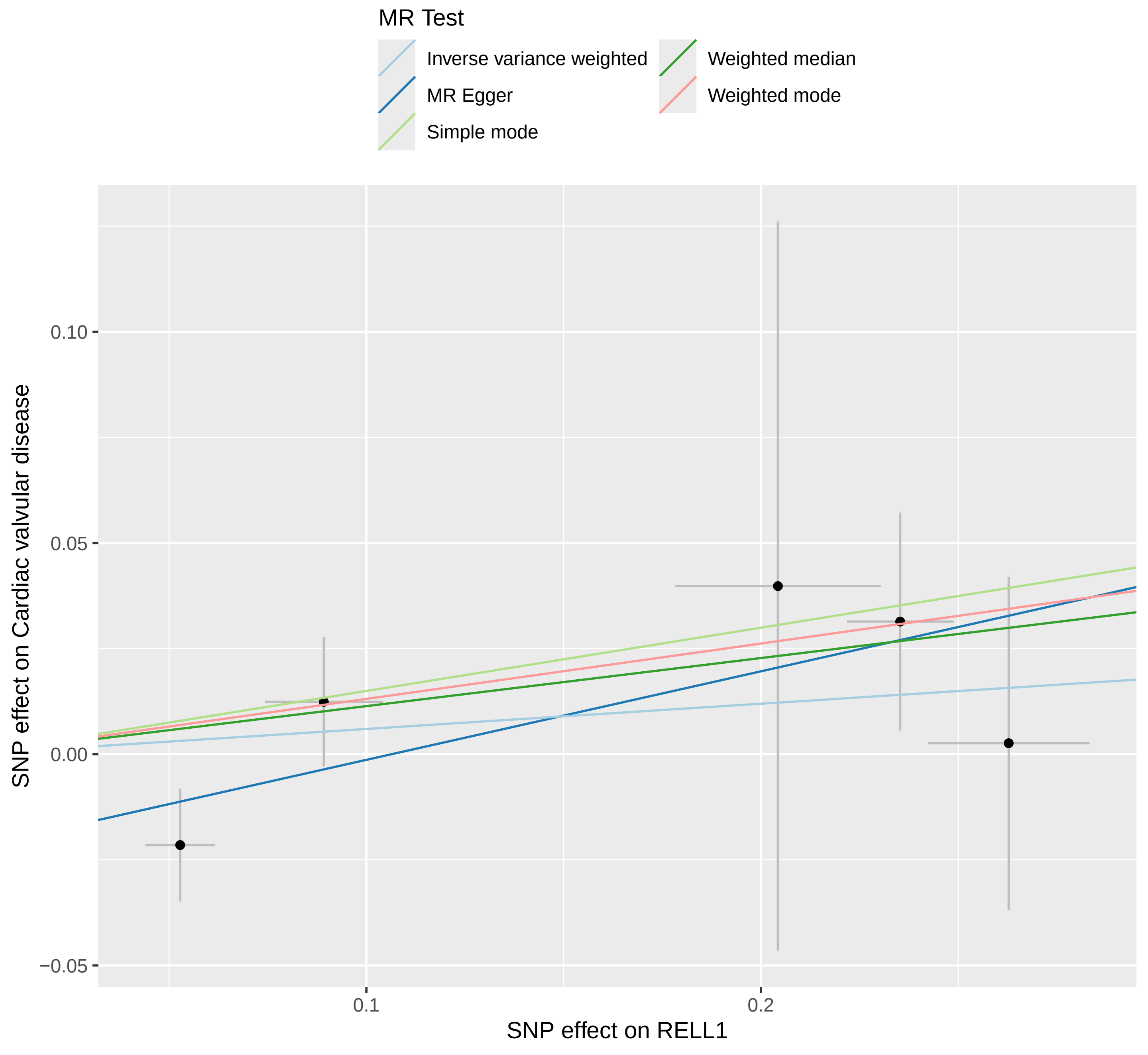

MR Test
Inverse variance weighted
MR Egger
Simple mode
Weighted median
Weighted mode
0.10
0.05
0.00
−0.05
0.1
0.2
SNP effect on Cardiac valvular disease
SNP effect on RELL1

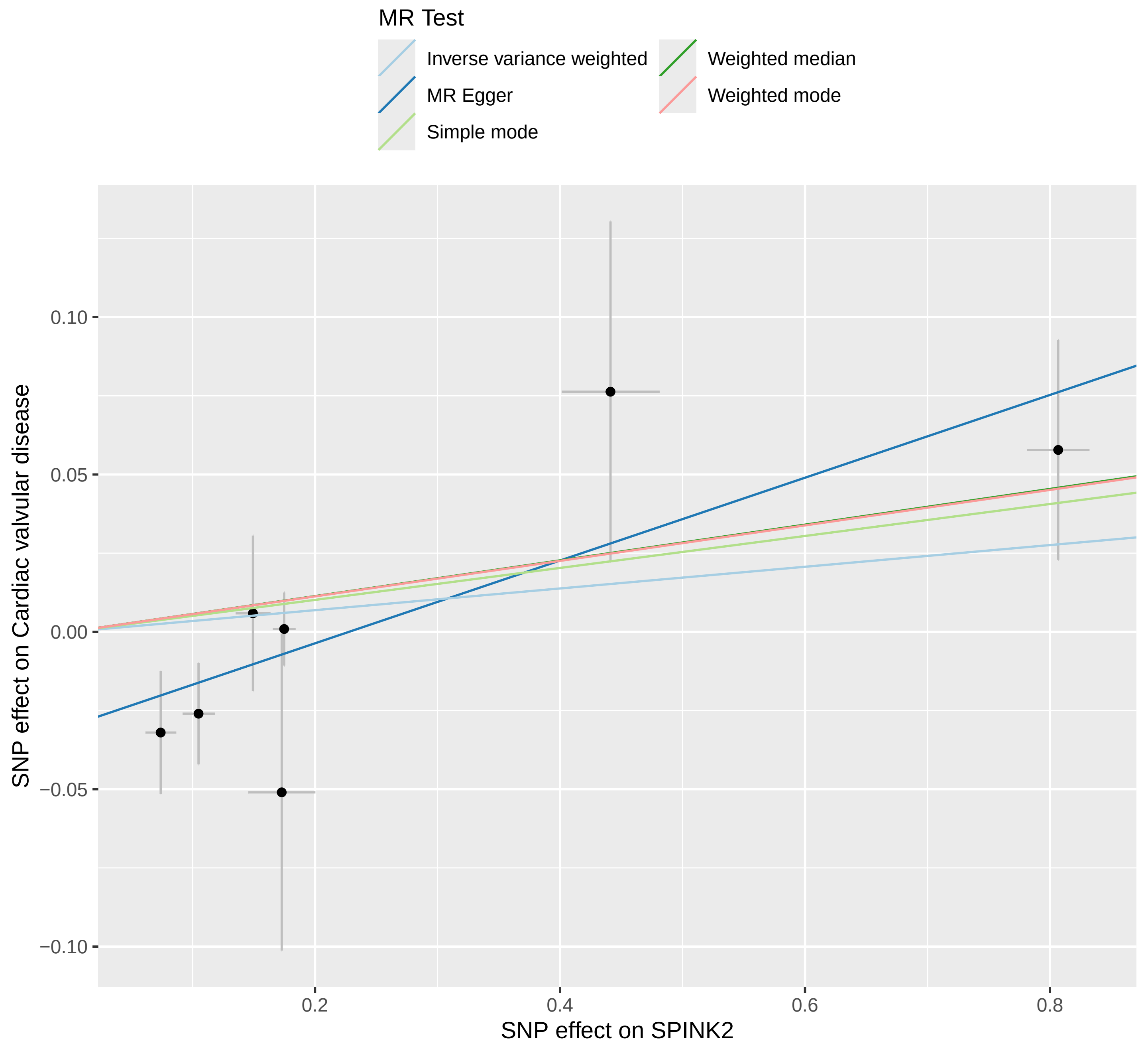

MR Test
Inverse variance weighted
MR Egger
Simple mode
Weighted median
Weighted mode
SNP effect on Cardiac valvular disease
0.10
0.05
0.00
−0.05
−0.10
0.2
0.4
0.6
0.8
SNP effect on SPINK2

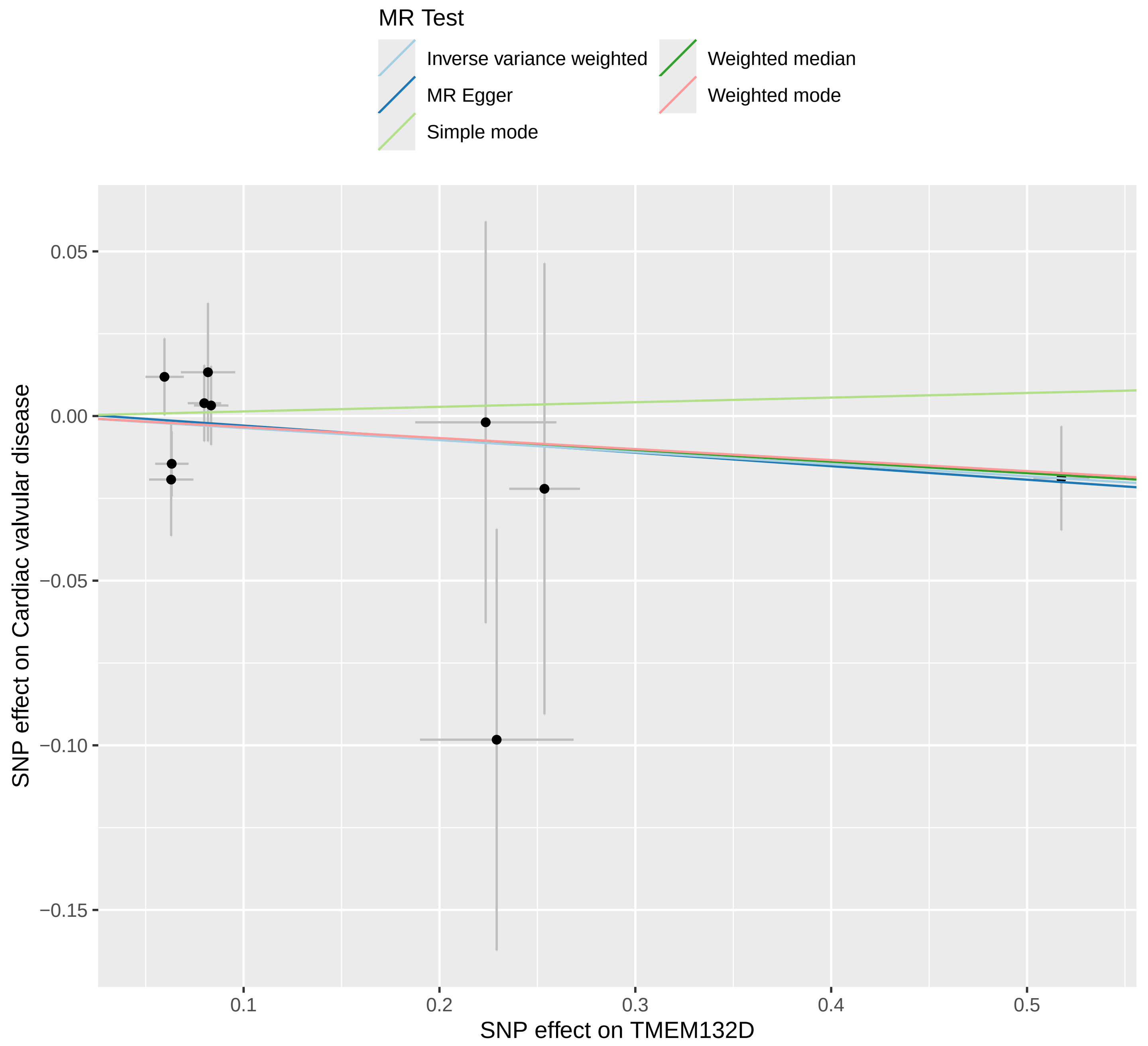
MR Test
Inverse variance weighted
MR Egger
Simple mode
Weighted median
Weighted mode
0.05
0.00
−0.05
−0.10
−0.15
SNP effect on Cardiac valvular disease
0.1
0.2
0.3
0.4
0.5
SNP effect on TMEM132D

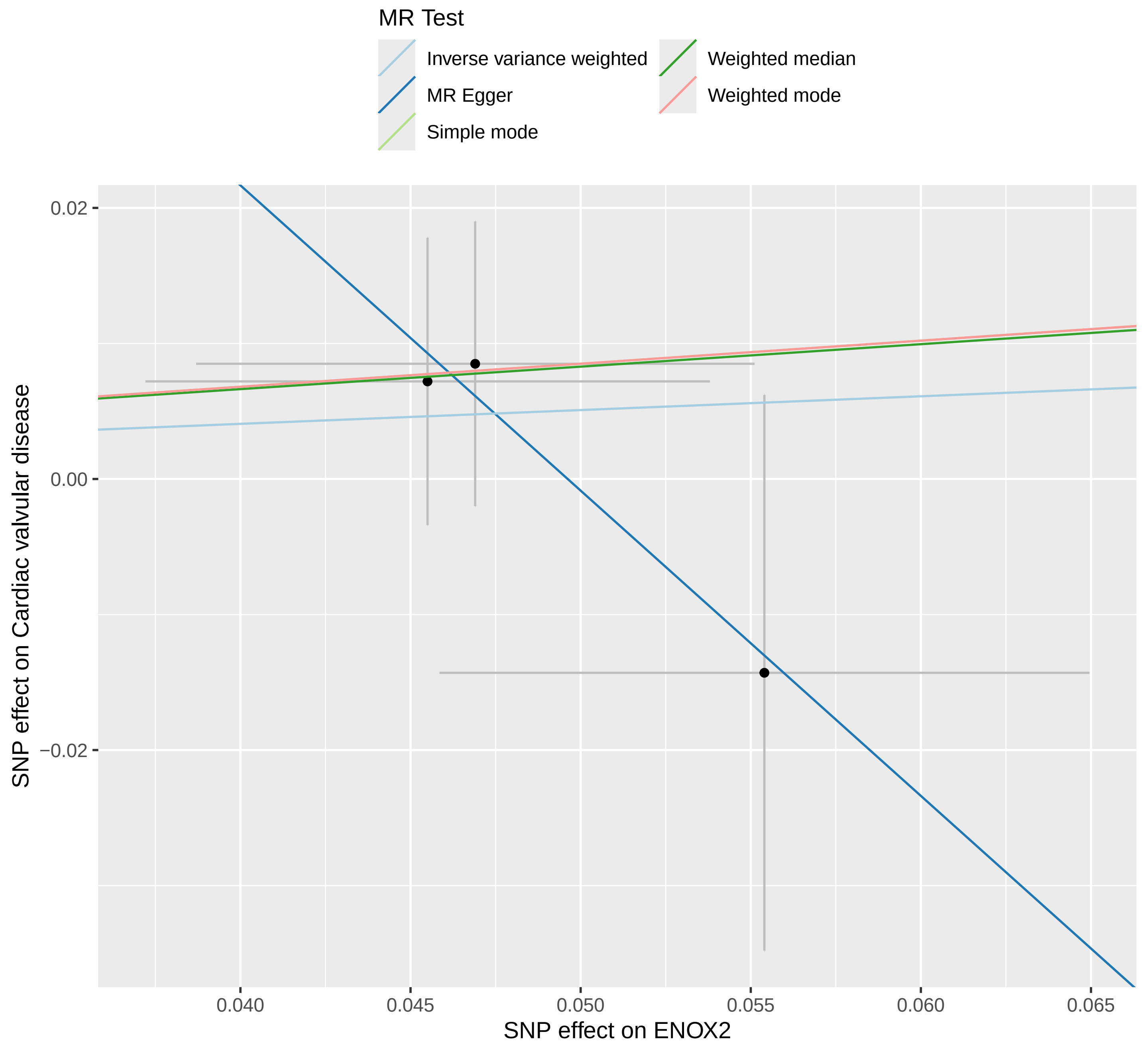

MR Test
Inverse variance weighted
MR Egger
Simple mode
Weighted median
Weighted mode
0.02
0.00
−0.02
0.040
0.045
0.050
0.055
0.060
0.065
SNP effect on ENOX2
SNP effect on Cardiac valvular disease

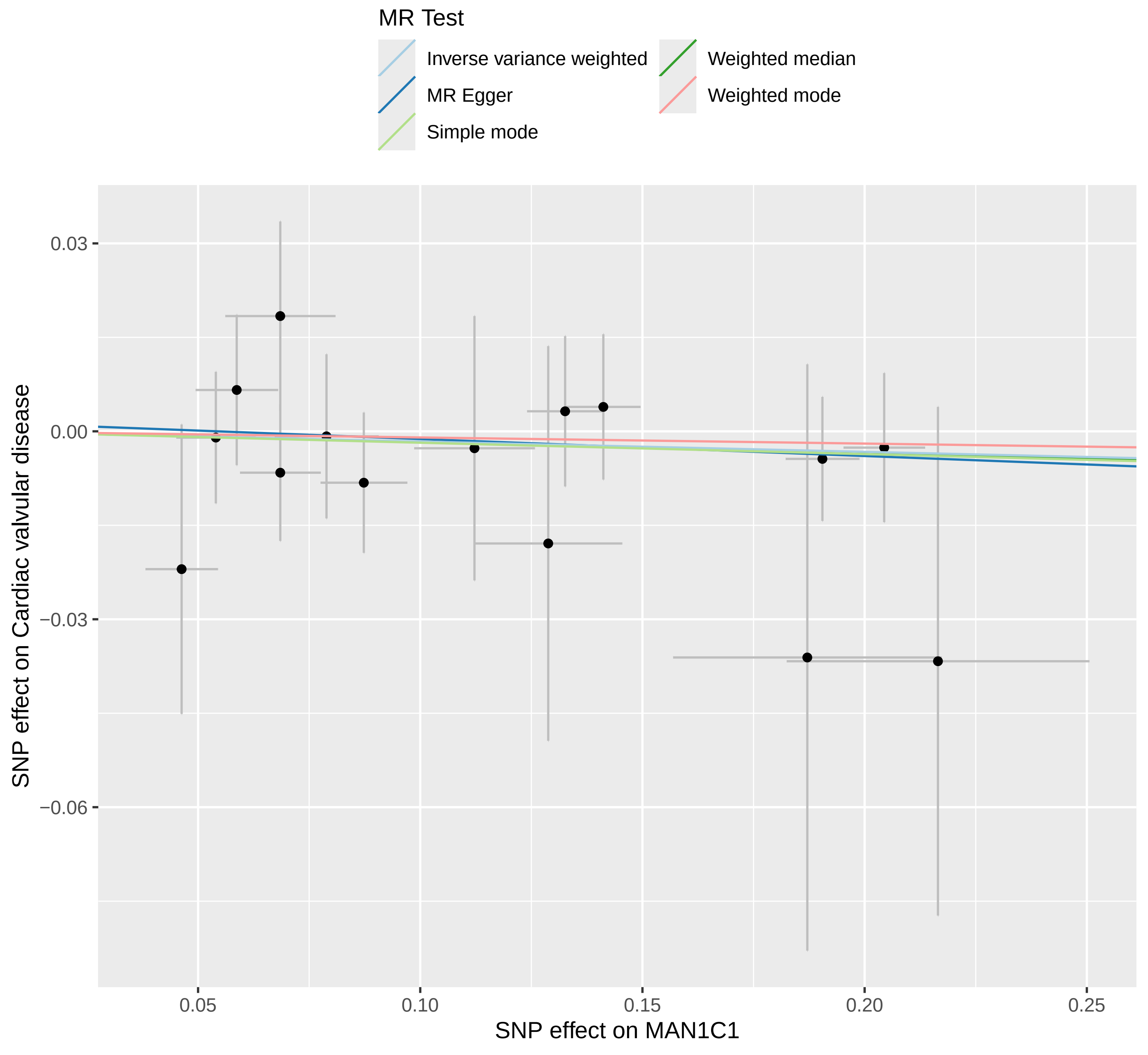

MR Test
Inverse variance weighted
MR Egger
Simple mode
Weighted median
Weighted mode
0.03
0.00
−0.03
−0.06
SNP effect on Cardiac valvular disease
0.05
0.10
0.15
0.20
0.25
SNP effect on MAN1C1

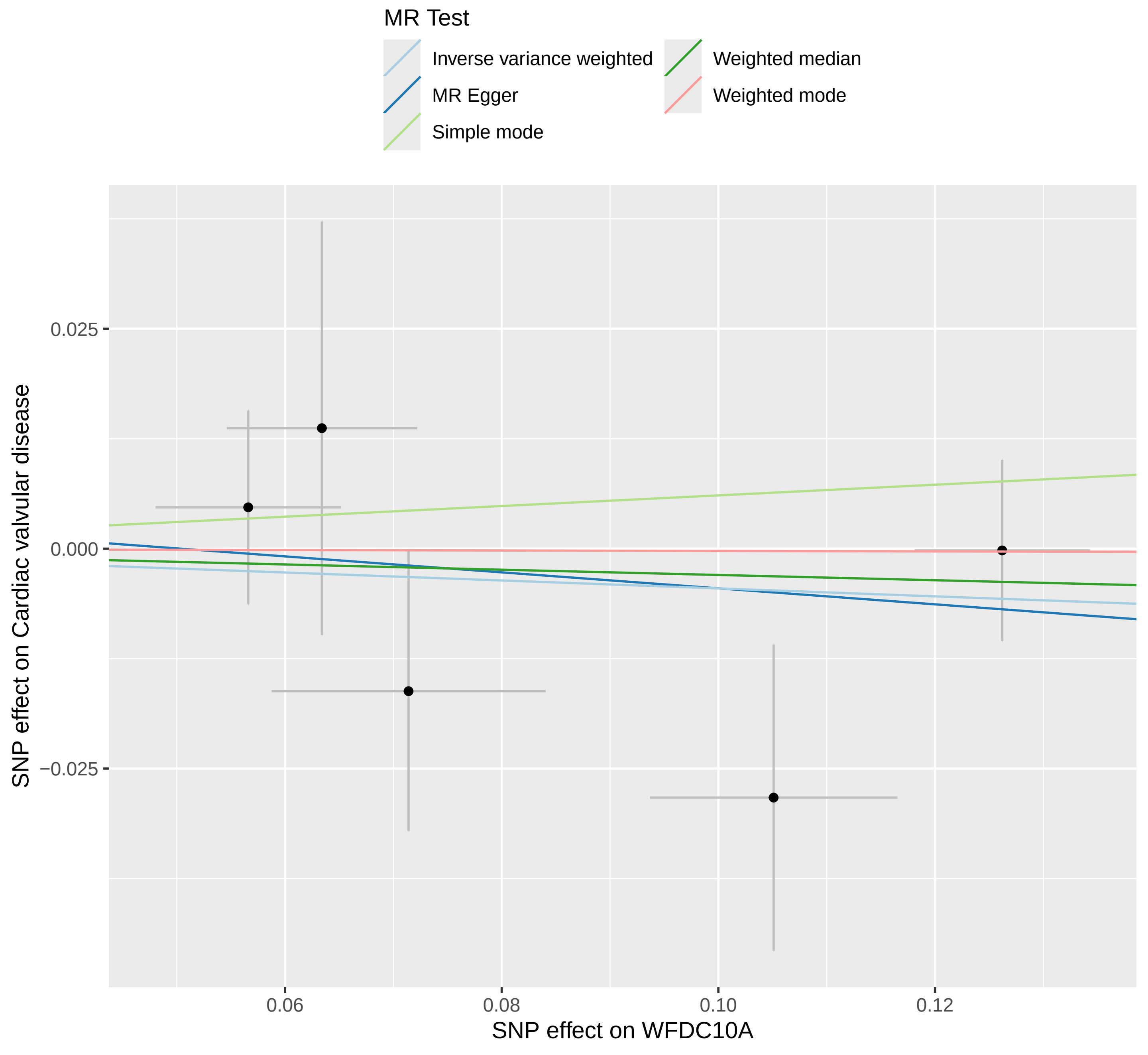

MR Test
Inverse variance weighted
MR Egger
Simple mode
Weighted median
Weighted mode
0.025
0.000
−0.025
SNP effect on Cardiac valvular disease
0.06
0.08
0.10
0.12
SNP effect on WFDC10A

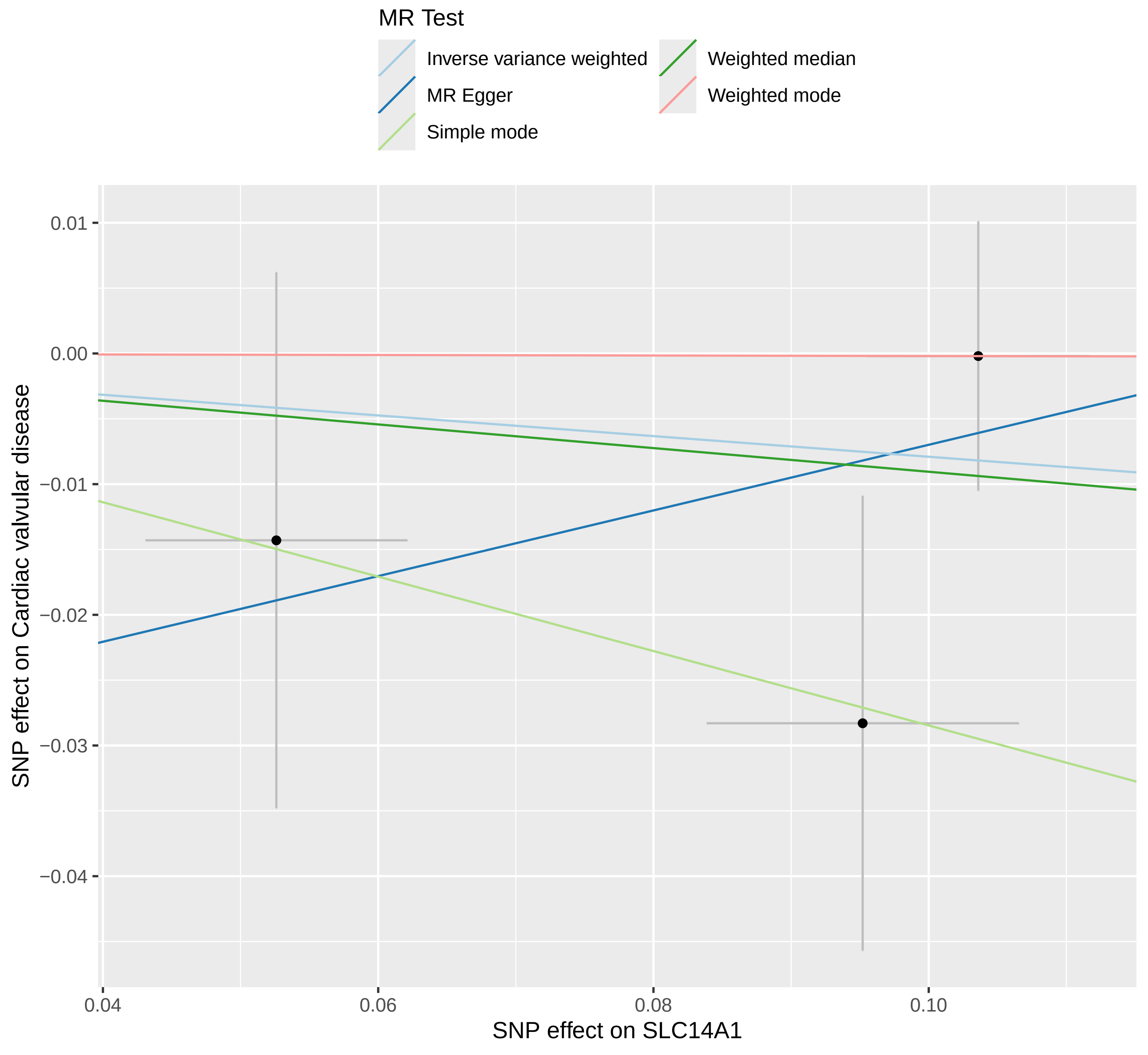
MR Test
Inverse variance weighted
MR Egger
Simple mode
Weighted median
Weighted mode
0.01
0.00
−0.01
−0.02
−0.03
−0.04
0.04
0.06
0.08
0.10
SNP effect on Cardiac valvular disease
SNP effect on SLC14A1

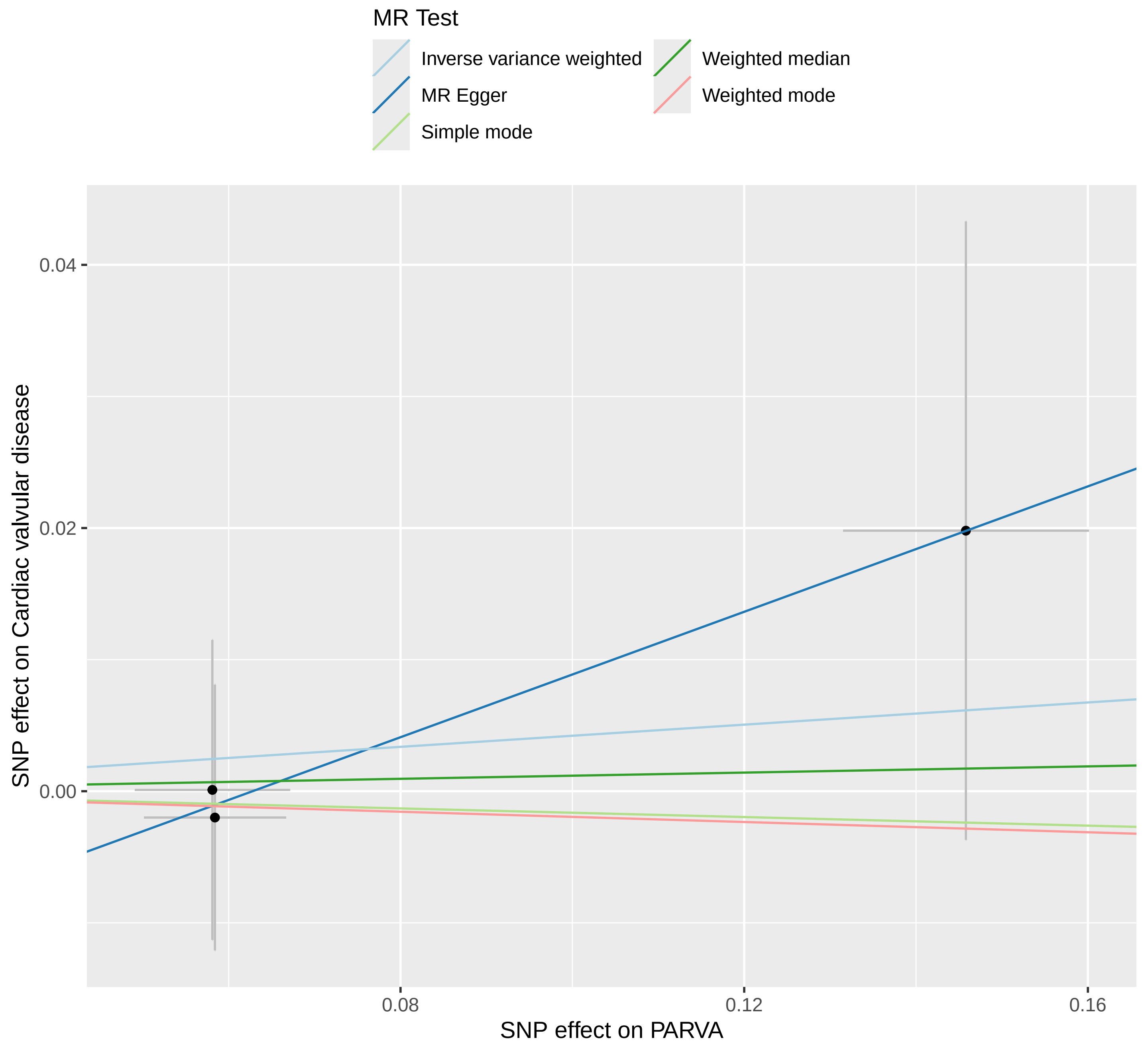
MR Test
Inverse variance weighted
MR Egger
Simple mode
Weighted median
Weighted mode
0.04
0.02
0.00
0.08
0.12
0.16
SNP effect on Cardiac valvular disease
SNP effect on PARVA

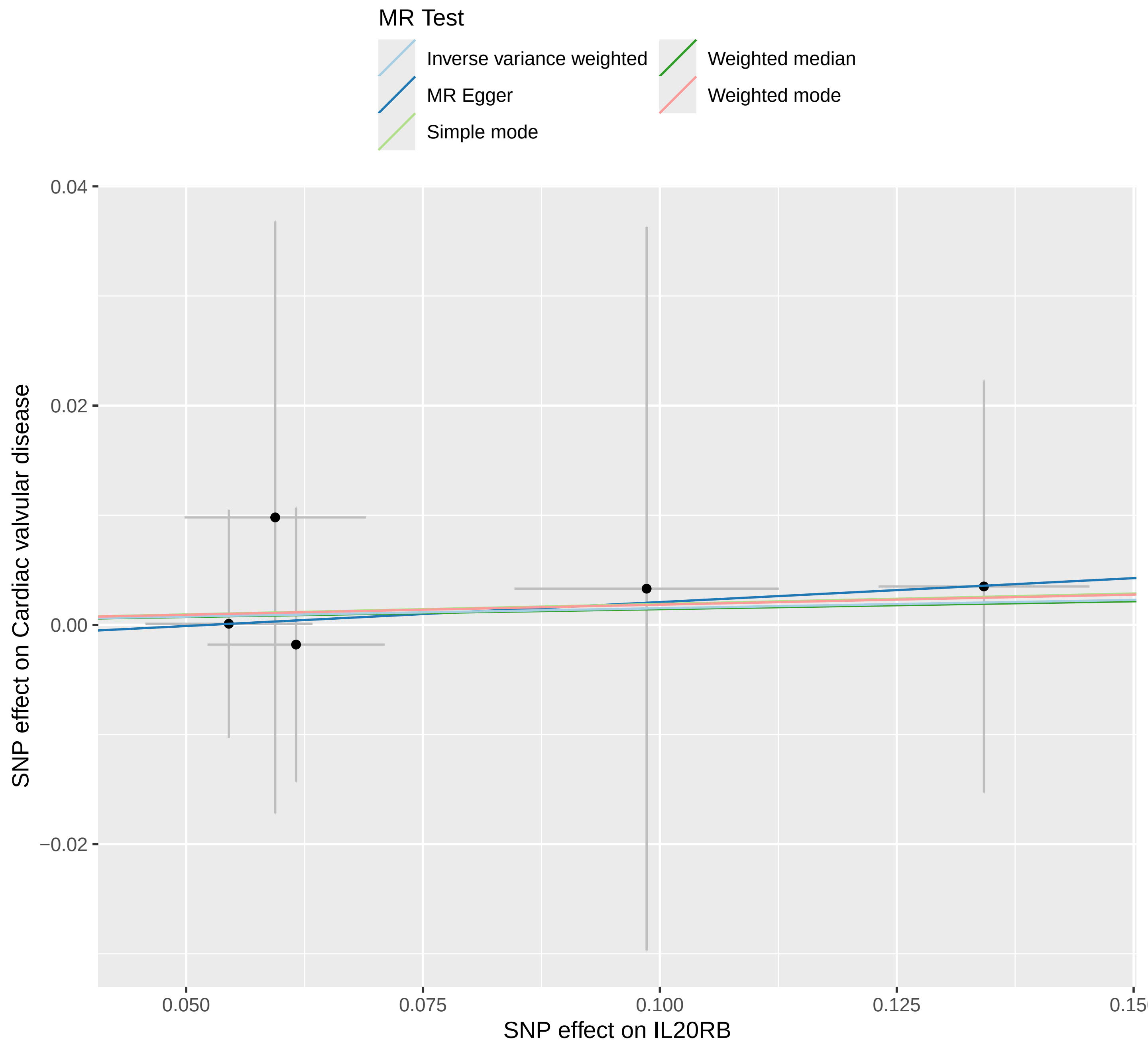

MR Test
Inverse variance weighted
MR Egger
Simple mode
Weighted median
Weighted mode
0.04
0.02
0.00
−0.02
SNP effect on Cardiac valvular disease
0.050
0.075
0.100
0.125
0.150
SNP effect on IL20RB

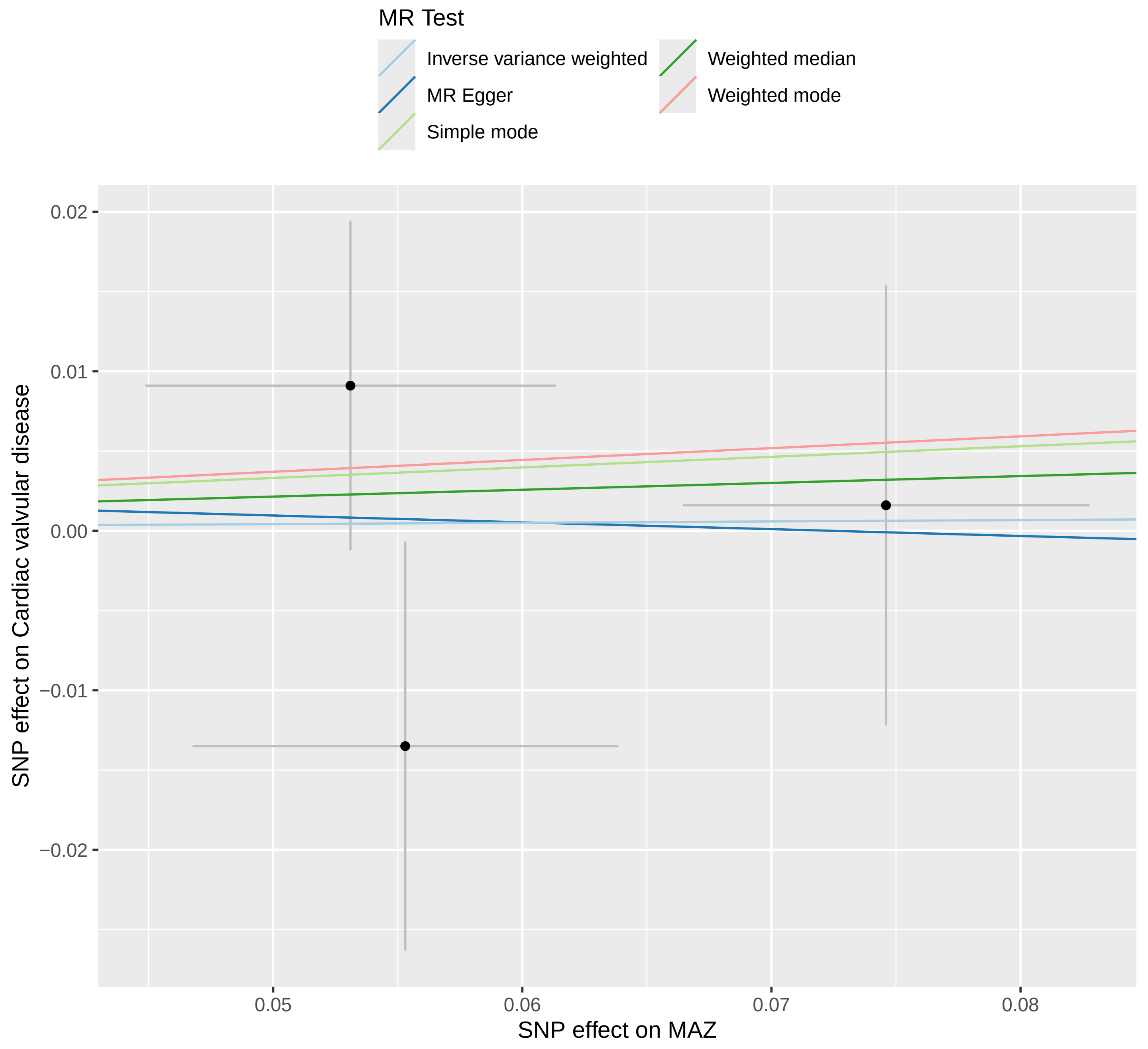
MR Test
Inverse variance weighted
MR Egger
Simple mode
Weighted median
Weighted mode
0.02
0.01
0.00
−0.01
−0.02
SNP effect on Cardiac valvular disease
0.05
0.06
0.07
0.08
SNP effect on MAZ

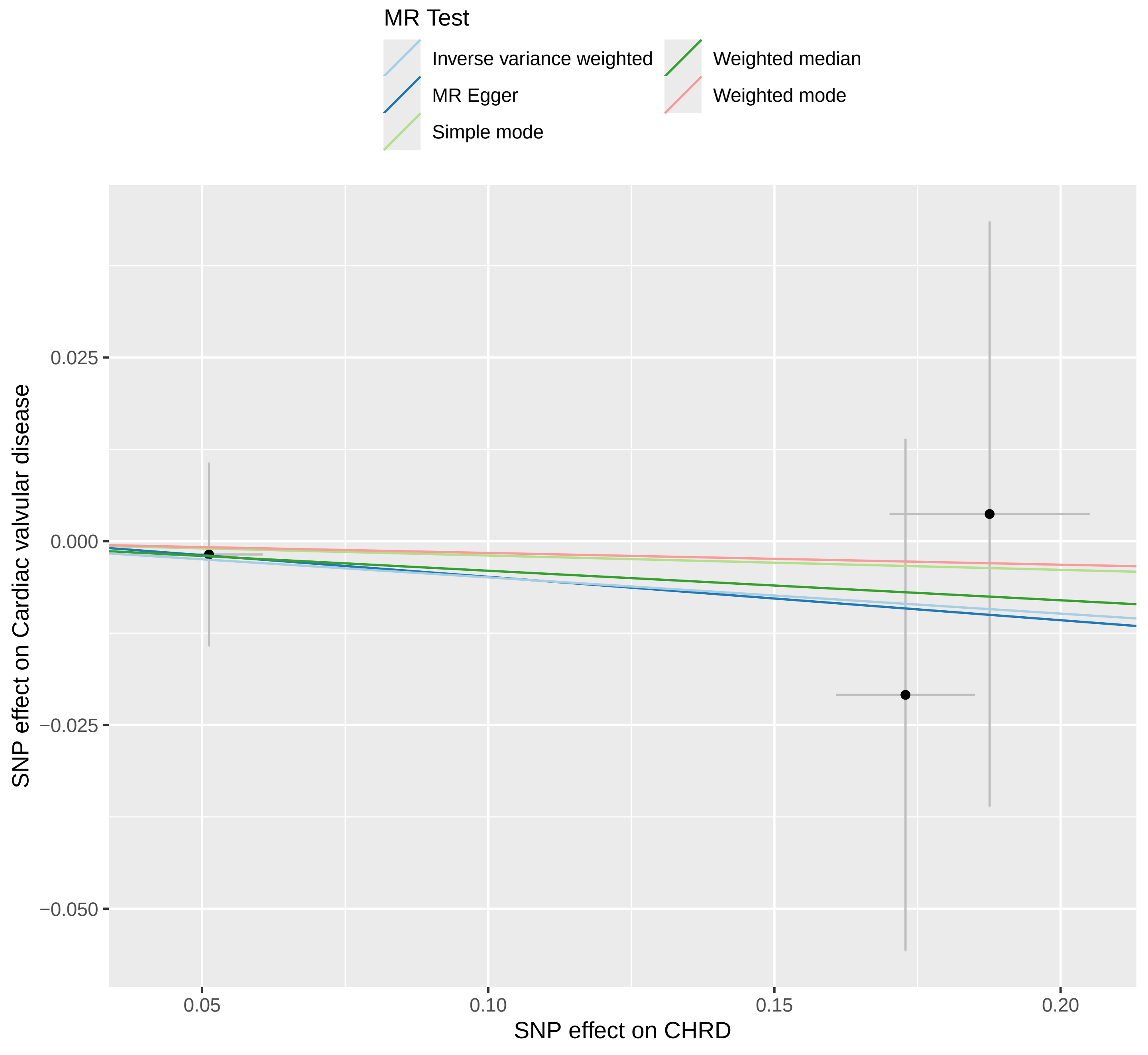
MR Test
Inverse variance weighted
MR Egger
Simple mode
Weighted median
Weighted mode
0.025
0.000
−0.025
−0.050
0.05
0.10
0.15
0.20
SNP effect on CHRD
SNP effect on Cardiac valvular disease

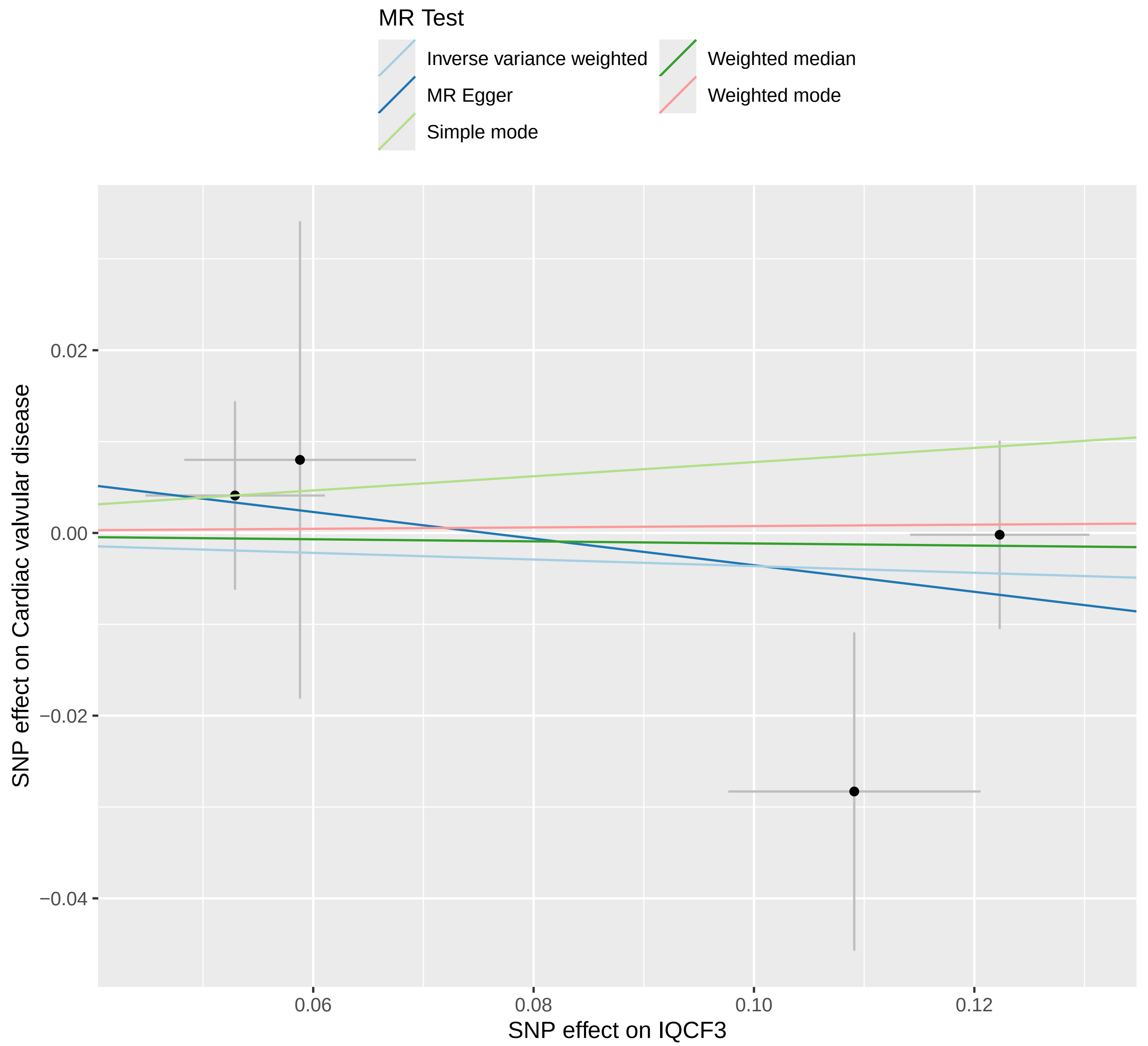

MR Test
Inverse variance weighted
MR Egger
Simple mode
Weighted median
Weighted mode
SNP effect on Cardiac valvular disease
0.02
0.00
−0.02
−0.04
0.06
0.08
0.10
0.12
SNP effect on IQCF3

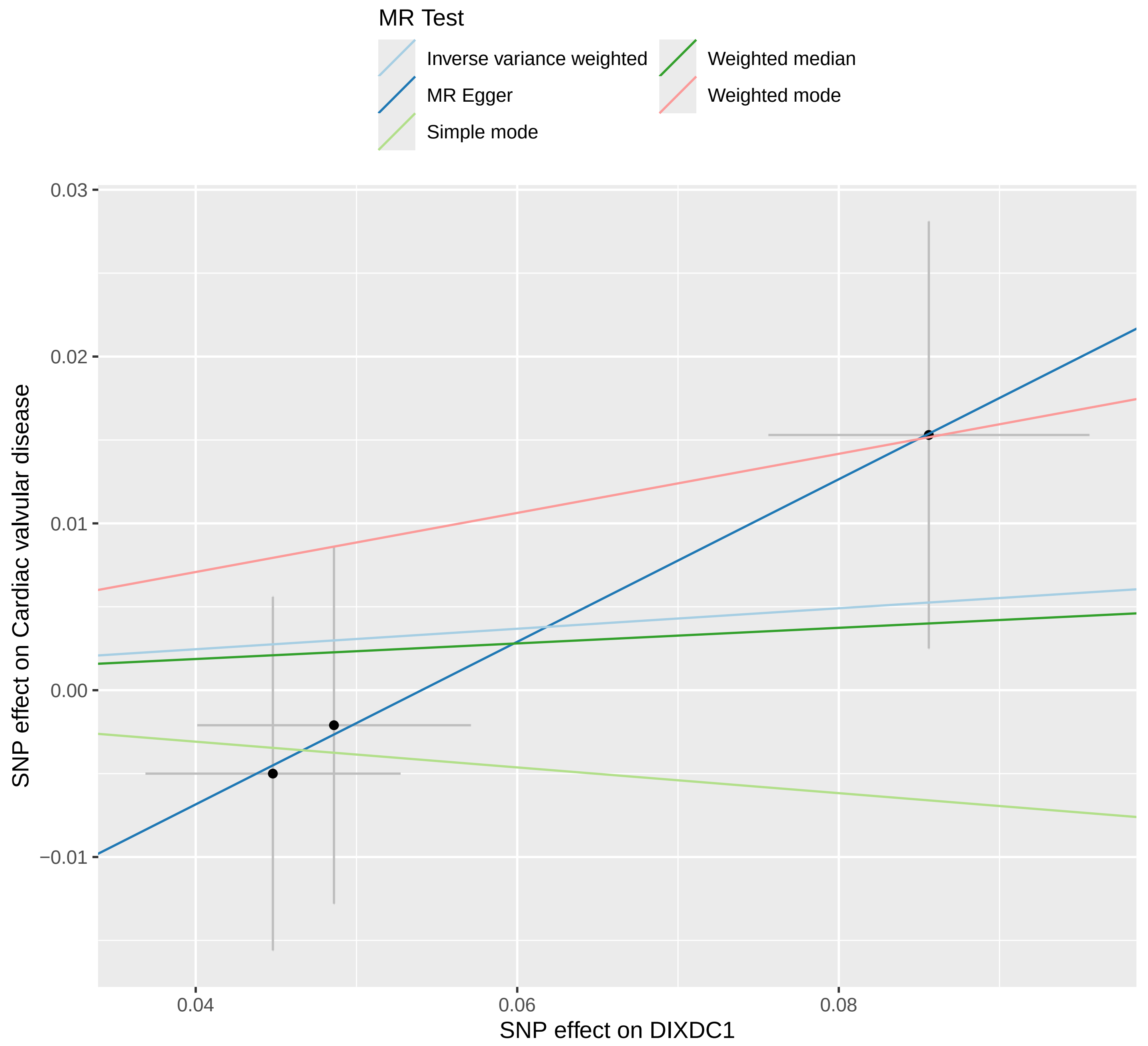
MR Test
Inverse variance weighted
MR Egger
Simple mode
Weighted median
Weighted mode
0.03
0.02
0.01
0.00
−0.01
0.04
0.06
0.08
SNP effect on Cardiac valvular disease
SNP effect on DIXDC1

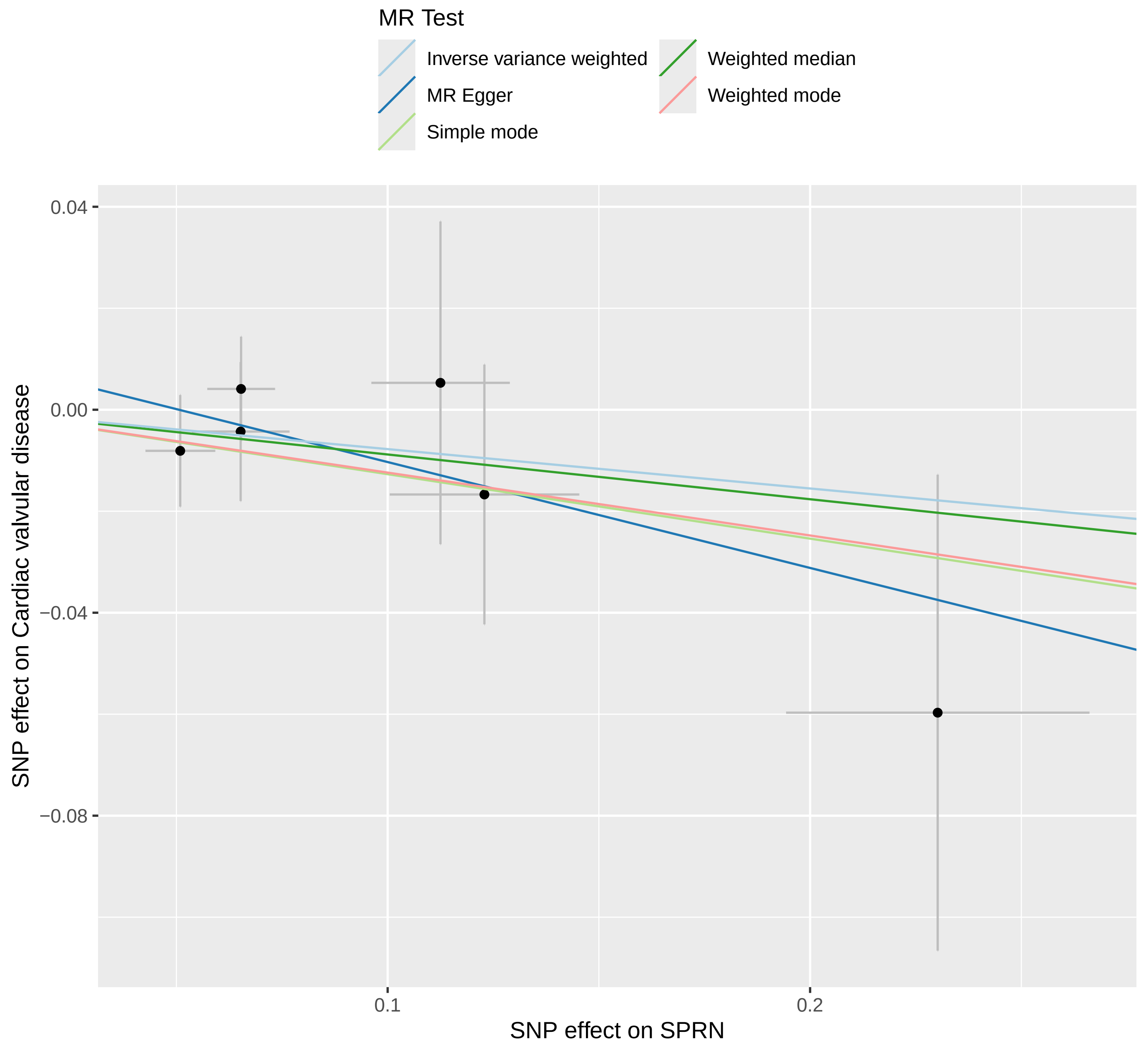

MR Test
Inverse variance weighted
MR Egger
Simple mode
Weighted median
Weighted mode
0.04
0.00
−0.04
−0.08
0.1
0.2
SNP effect on Cardiac valvular disease
SNP effect on SPRN

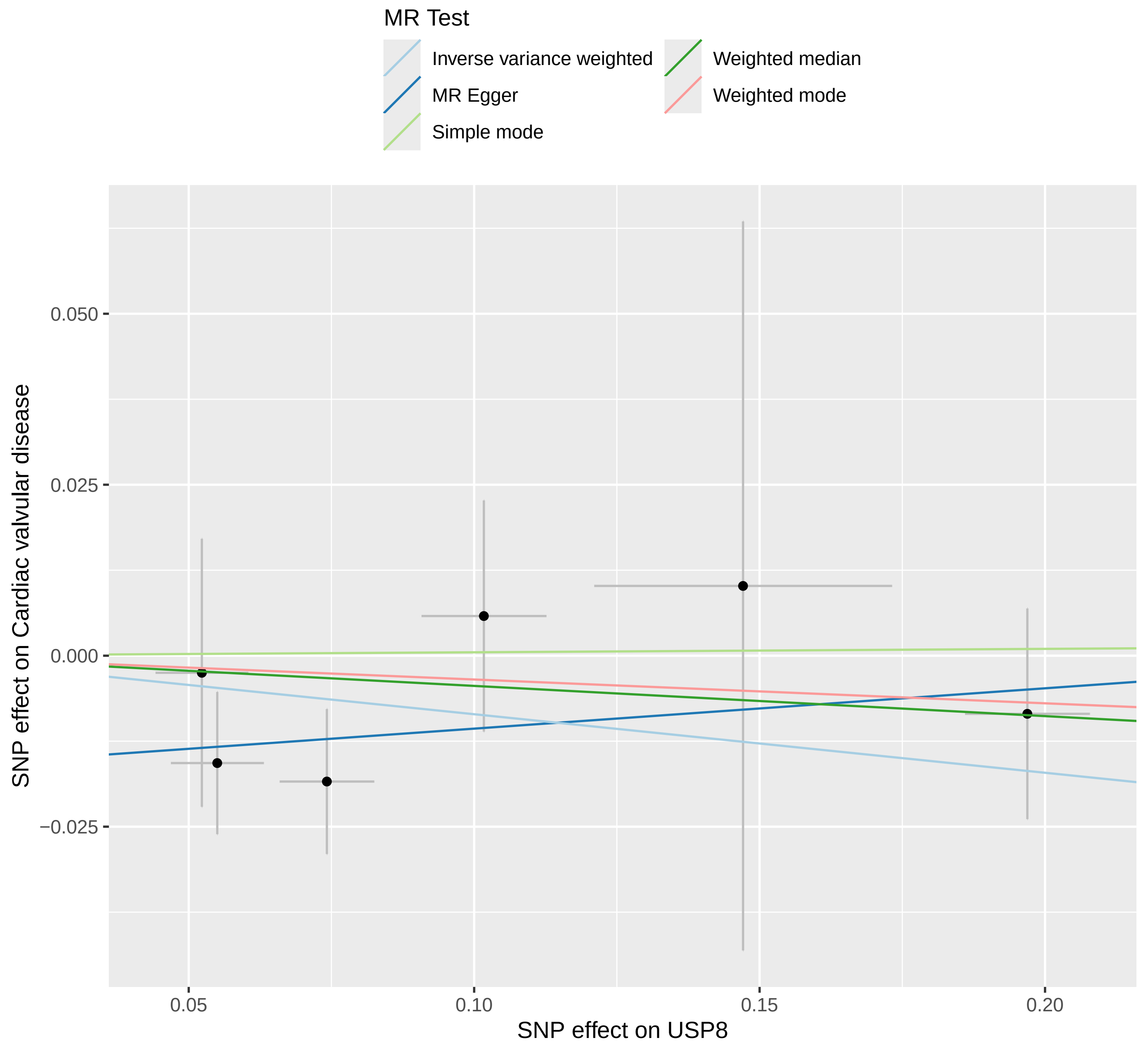
MR Test
Inverse variance weighted
MR Egger
Simple mode
Weighted median
Weighted mode
0.050
0.025
0.000
−0.025
0.05
0.10
0.15
0.20
SNP effect on Cardiac valvular disease
SNP effect on USP8

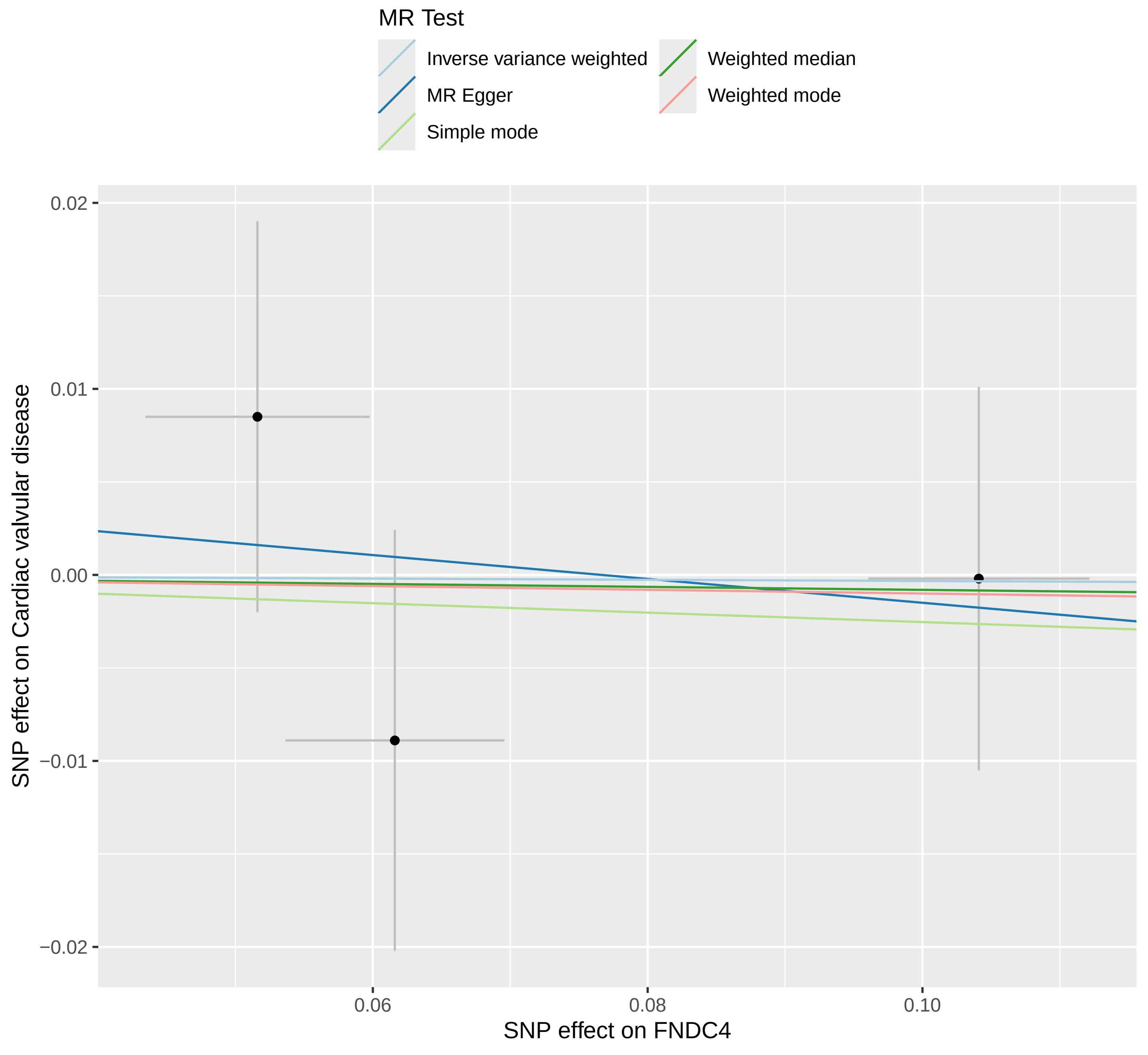

MR Test
Inverse variance weighted
MR Egger
Simple mode
Weighted median
Weighted mode
0.02
0.01
0.00
−0.01
−0.02
SNP effect on Cardiac valvular disease
0.06
0.08
0.10
SNP effect on FNDC4

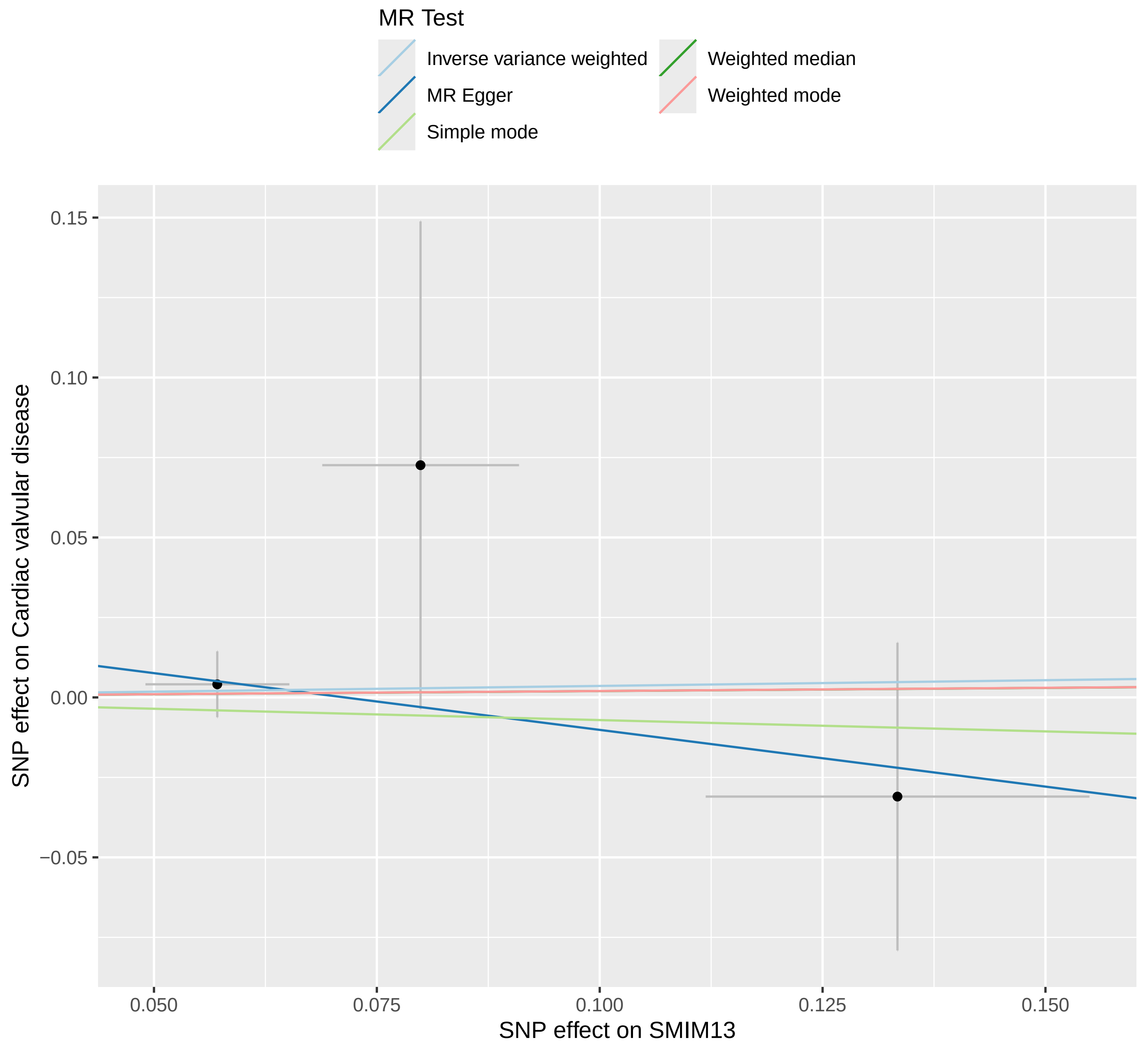
MR Test
Inverse variance weighted
MR Egger
Simple mode
Weighted median
Weighted mode
0.15
0.10
0.05
0.00
−0.05
0.050
0.075
0.100
0.125
0.150
SNP effect on SMIM13
SNP effect on Cardiac valvular disease

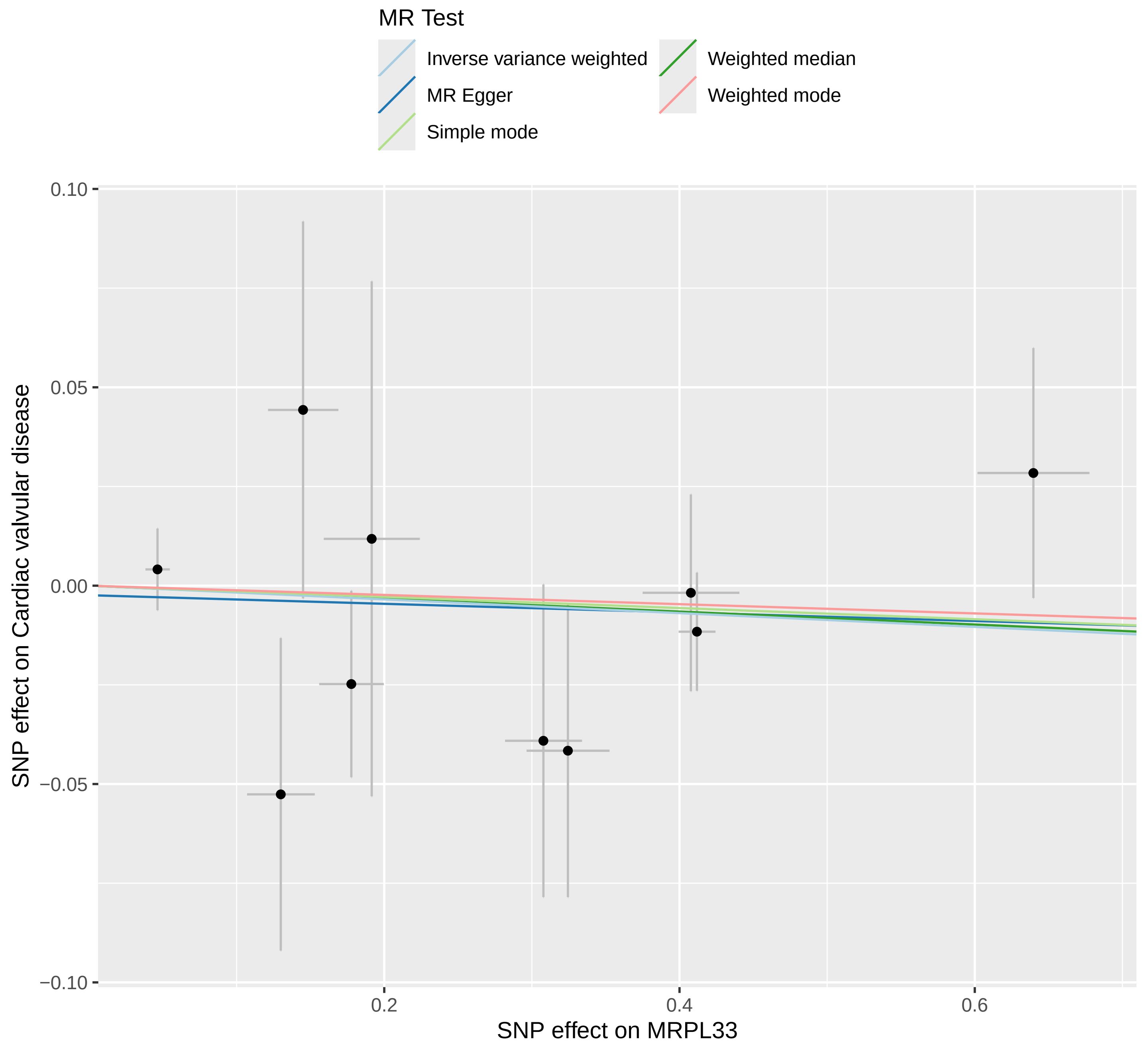
MR Test
Inverse variance weighted
MR Egger
Simple mode
Weighted median
Weighted mode
0.10
0.05
0.00
−0.05
−0.10
0.2
0.4
0.6
SNP effect on Cardiac valvular disease
SNP effect on MRPL33

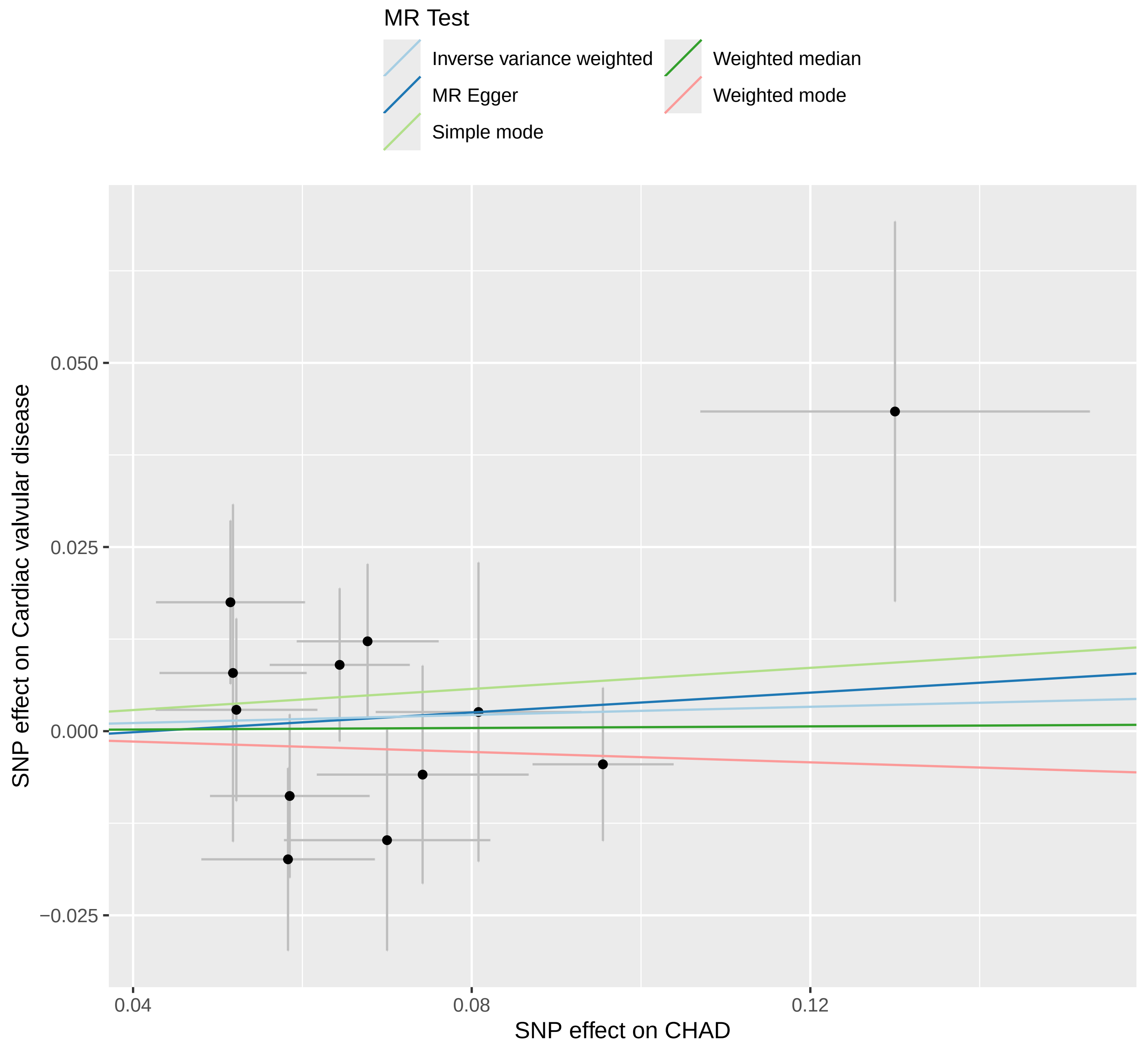

MR Test
Inverse variance weighted
MR Egger
Simple mode
Weighted median
Weighted mode
0.050
0.025
0.000
−0.025
0.04
0.08
0.12
SNP effect on Cardiac valvular disease
SNP effect on CHAD

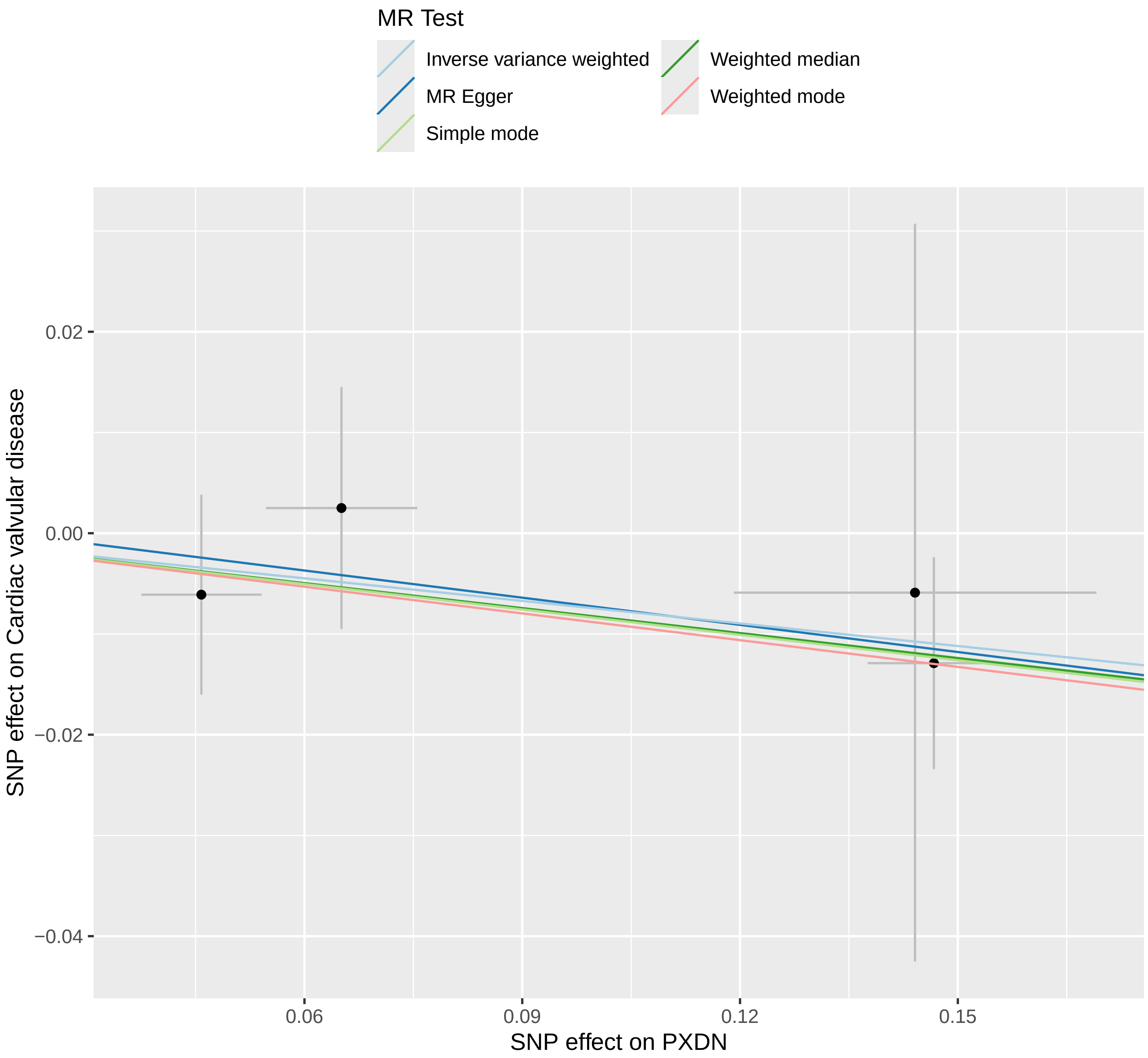
MR Test
Inverse variance weighted
MR Egger
Simple mode
Weighted median
Weighted mode
0.02
0.00
−0.02
−0.04
SNP effect on Cardiac valvular disease
0.06
0.09
0.12
0.15
SNP effect on PXDN

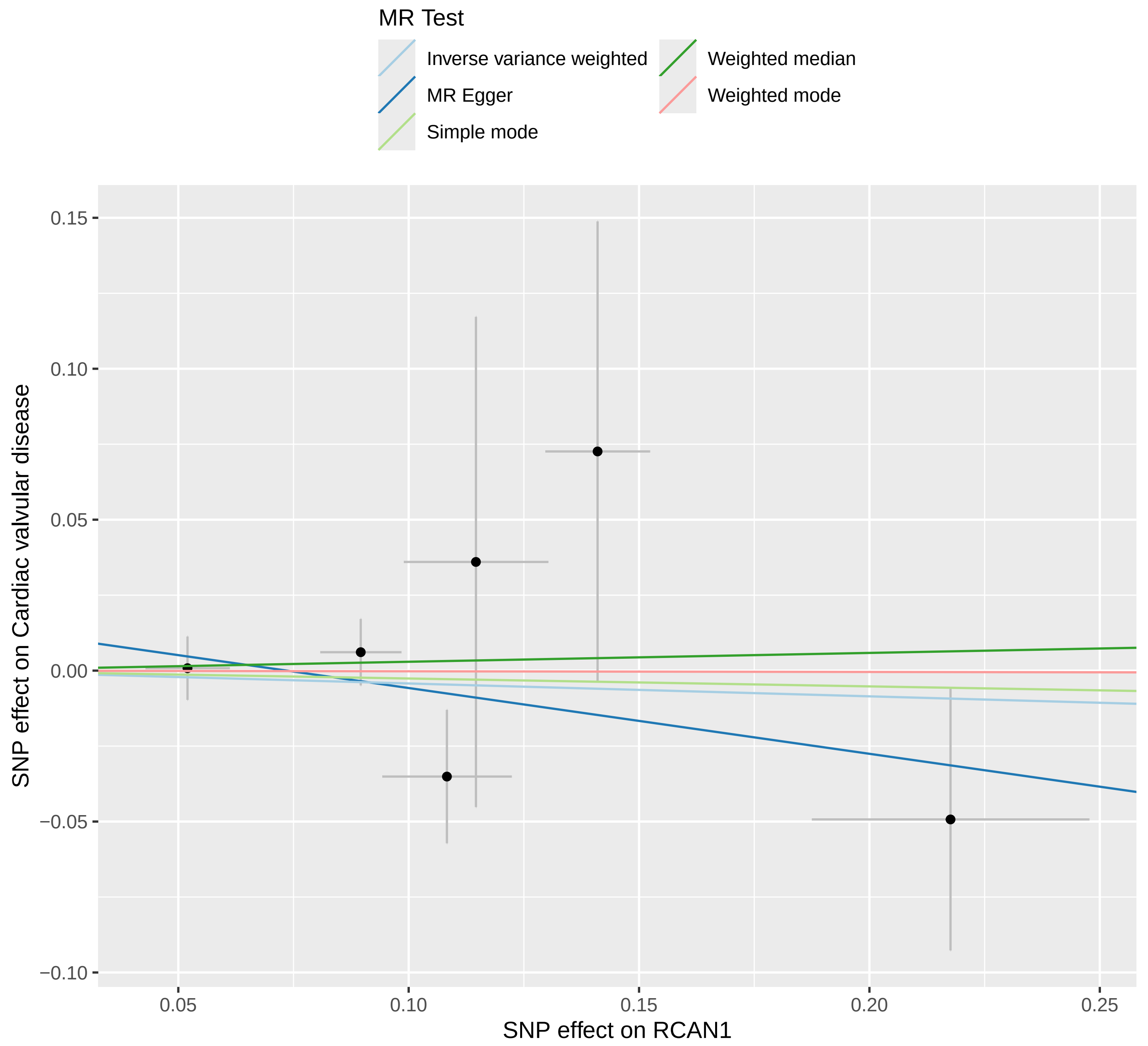
MR Test
Inverse variance weighted
MR Egger
Simple mode
Weighted median
Weighted mode
SNP effect on Cardiac valvular disease
0.15
0.10
0.05
0.00
−0.05
−0.10
0.05
0.10
0.15
0.20
0.25
SNP effect on RCAN1

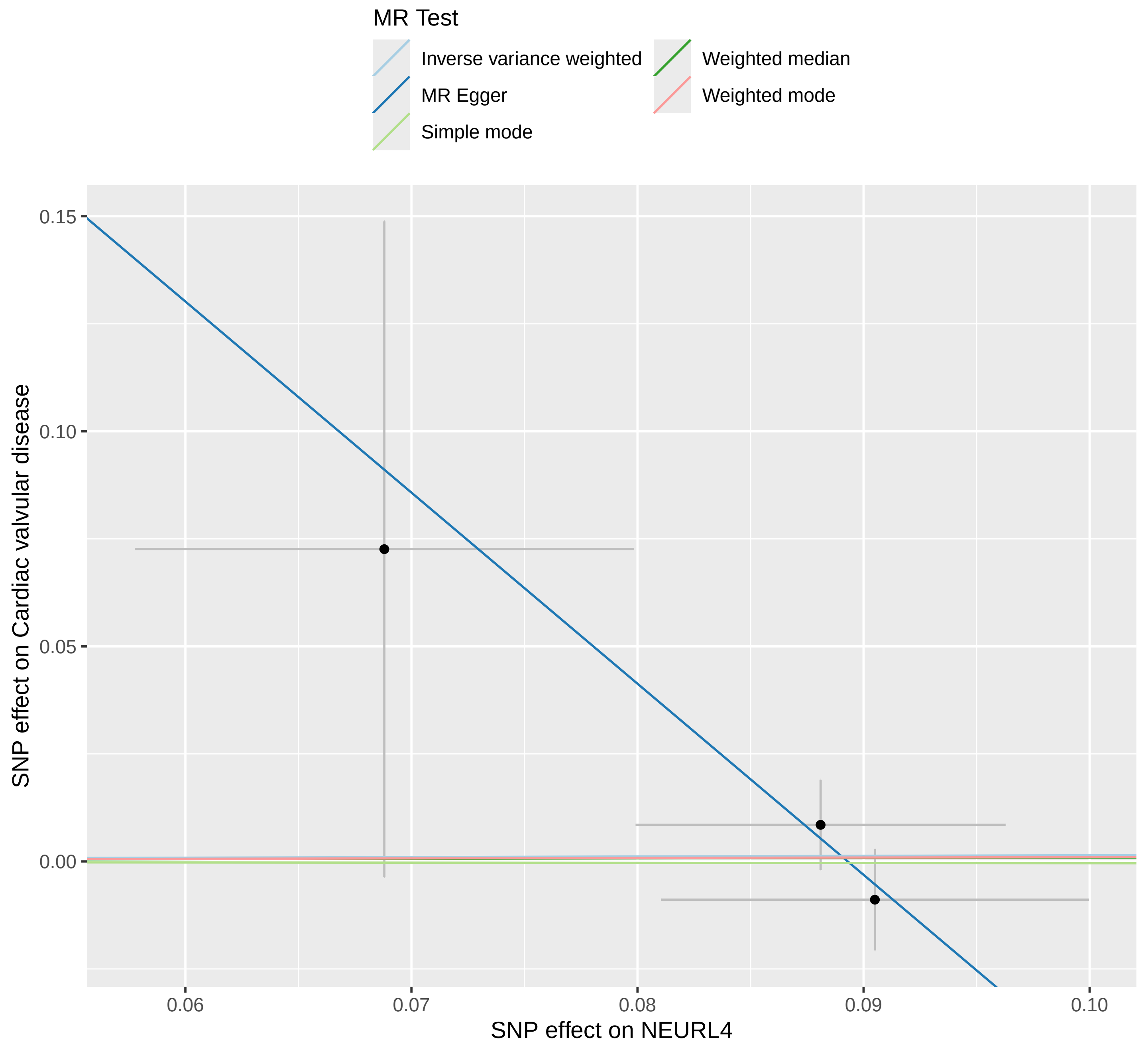
MR Test
Inverse variance weighted
MR Egger
Simple mode
Weighted median
Weighted mode
0.15
0.10
0.05
0.00
SNP effect on Cardiac valvular disease
0.06
0.07
0.08
0.09
0.10
SNP effect on NEURL4

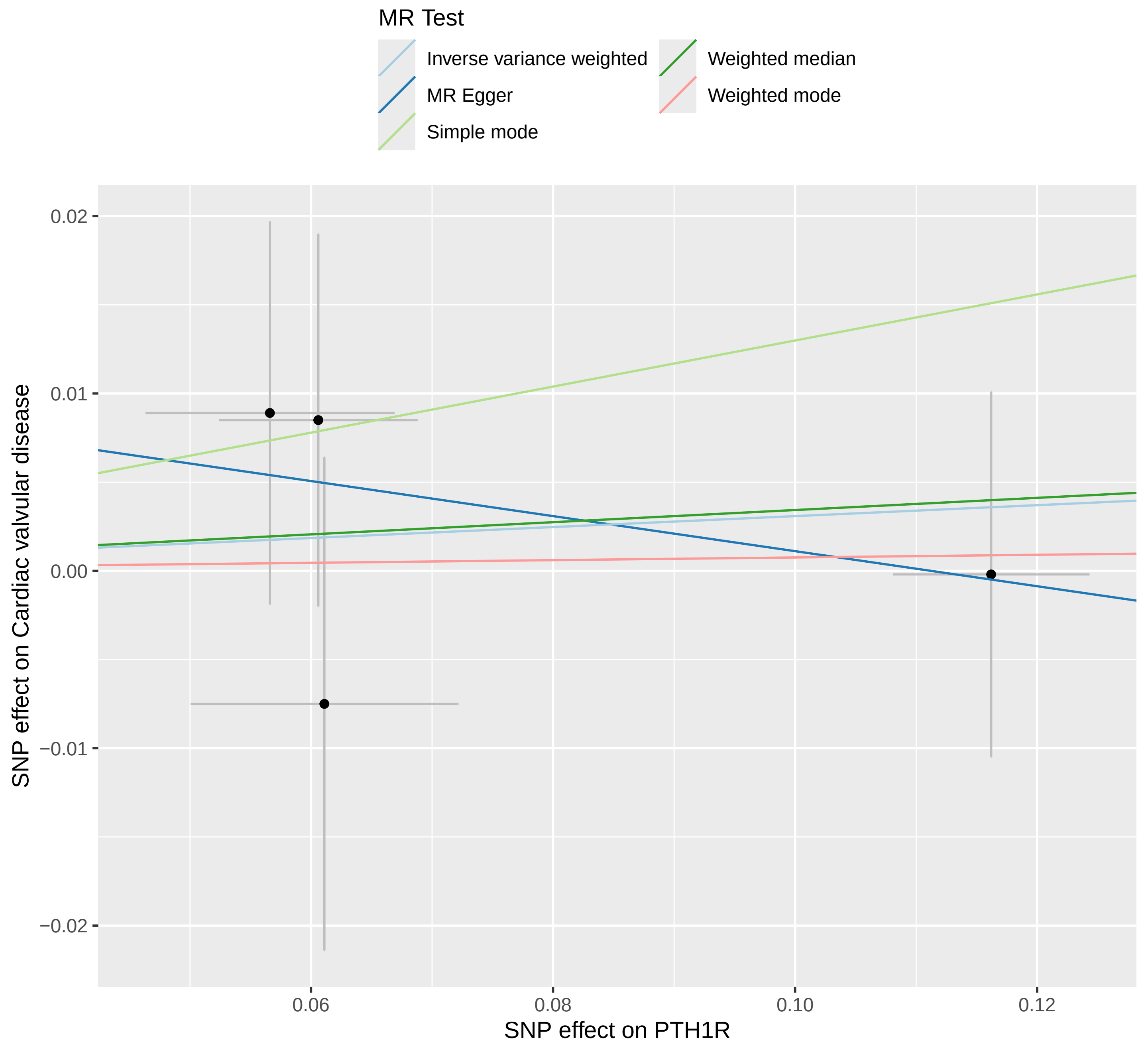

MR Test
Inverse variance weighted
MR Egger
Simple mode
Weighted median
Weighted mode
0.02
0.01
0.00
−0.01
−0.02
0.06
0.08
0.10
0.12
SNP effect on Cardiac valvular disease
SNP effect on PTH1R

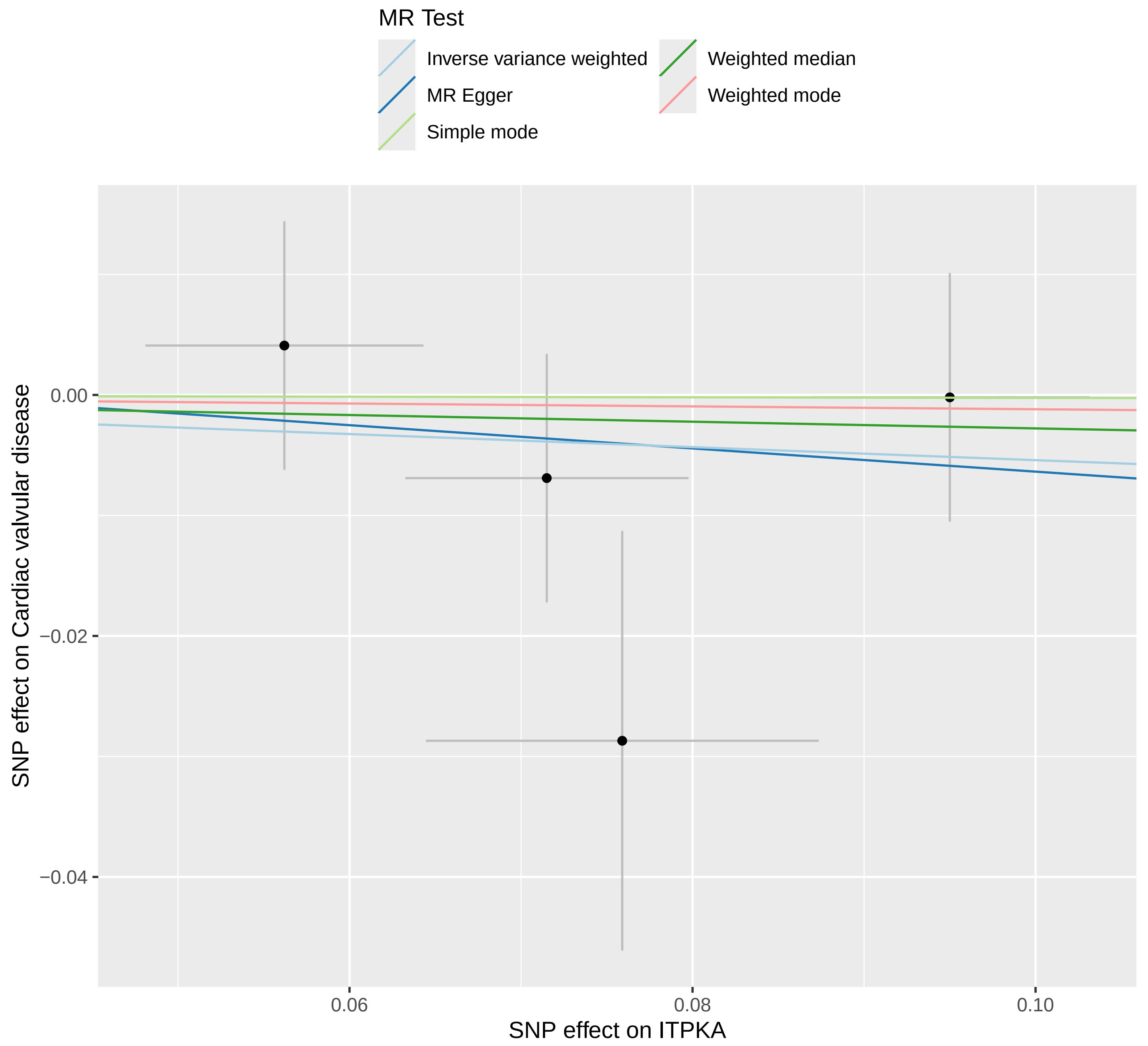
MR Test
Inverse variance weighted
MR Egger
Simple mode
Weighted median
Weighted mode
0.00
−0.02
−0.04
0.06
0.08
0.10
SNP effect on Cardiac valvular disease
SNP effect on ITPKA

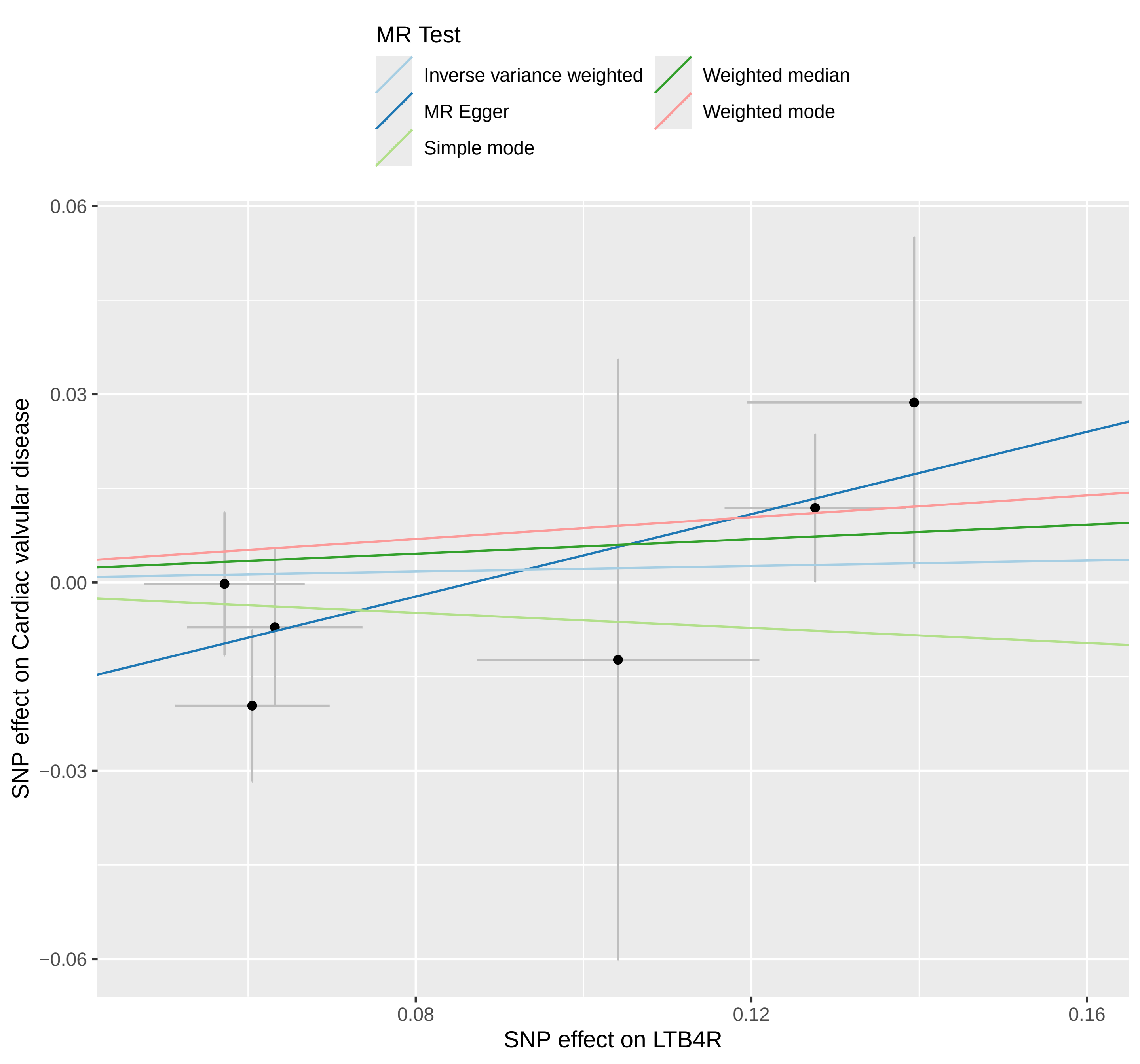

MR Test
Inverse variance weighted
MR Egger
Simple mode
Weighted median
Weighted mode
0.06
0.03
0.00
−0.03
−0.06
0.08
0.12
0.16
SNP effect on Cardiac valvular disease
SNP effect on LTB4R

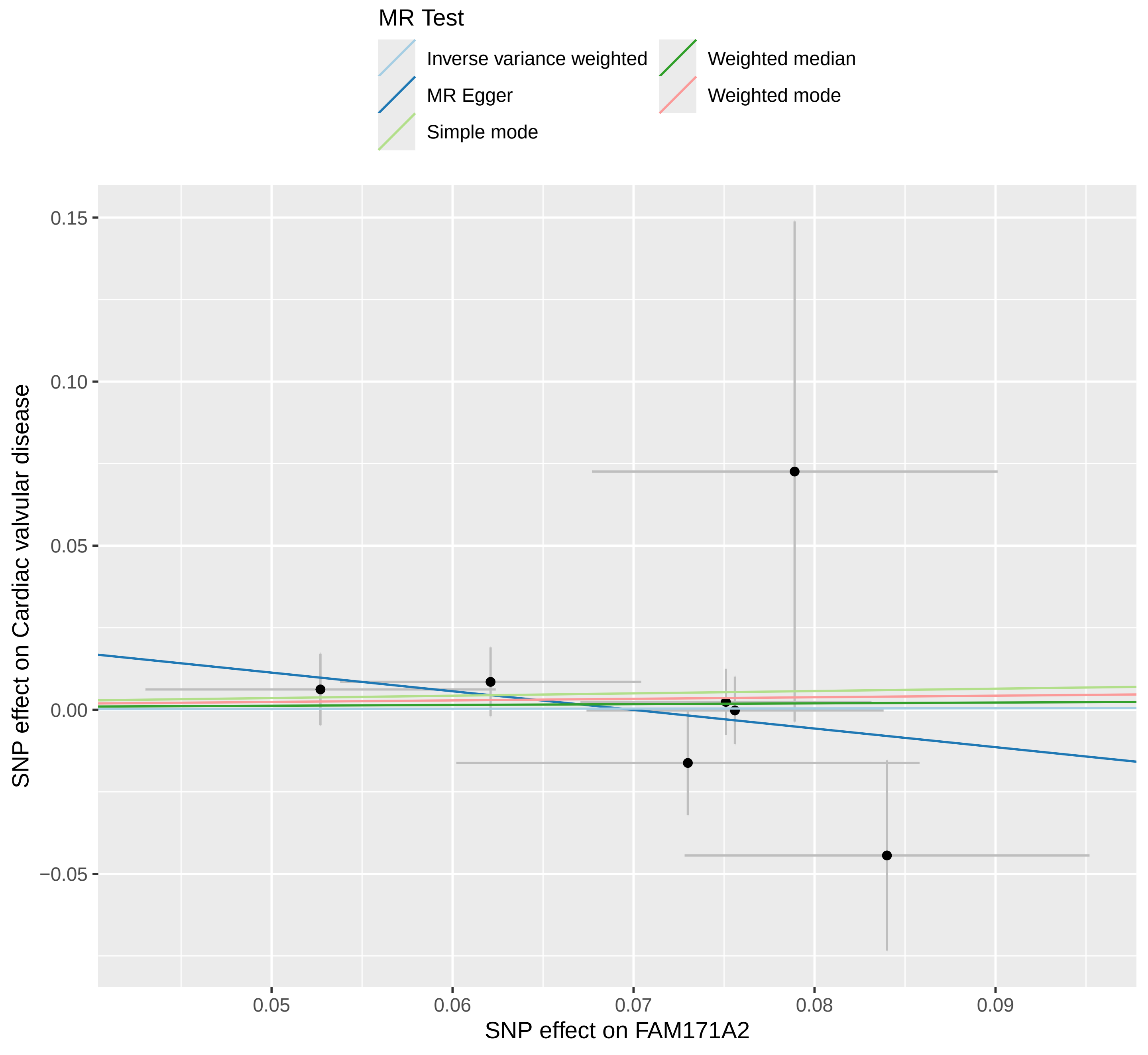
MR Test
Inverse variance weighted
MR Egger
Simple mode
Weighted median
Weighted mode
0.15
0.10
0.05
0.00
−0.05
0.05
0.06
0.07
0.08
0.09
SNP effect on Cardiac valvular disease
SNP effect on FAM171A2

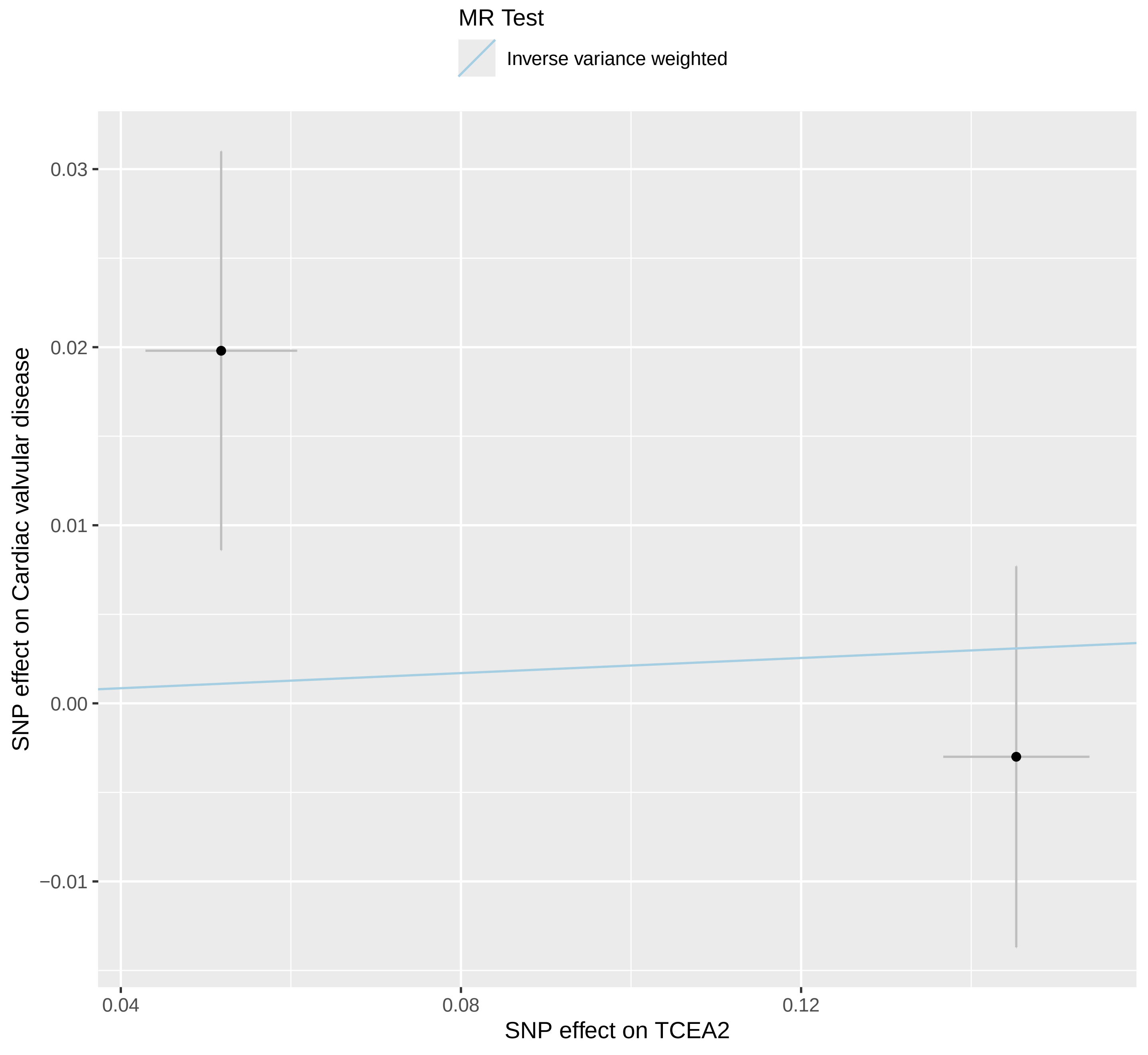

MR Test
Inverse variance weighted
0.03
0.02
0.01
0.00
−0.01
0.04
0.08
0.12
SNP effect on TCEA2
SNP effect on Cardiac valvular disease

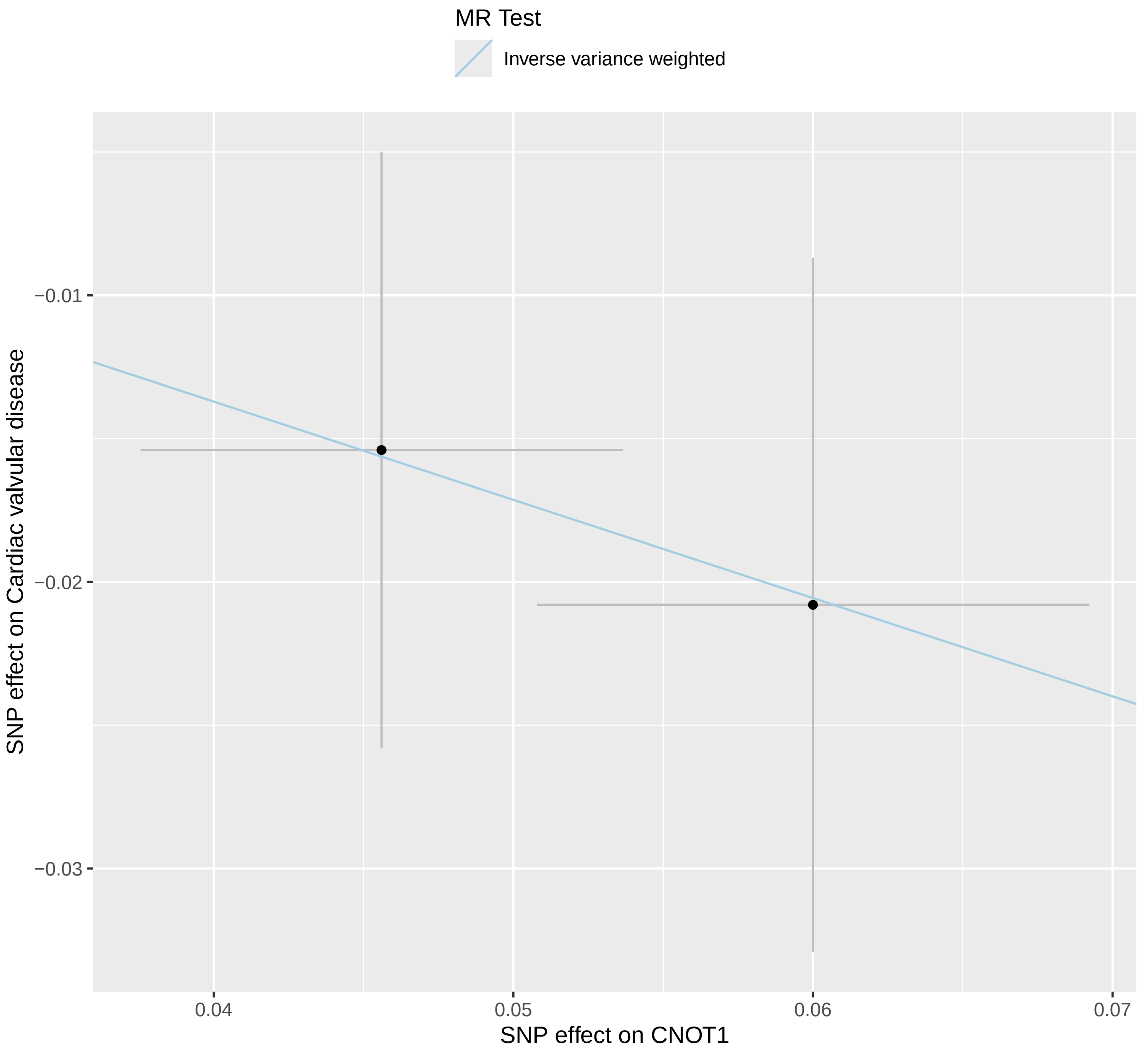

MR Test
Inverse variance weighted
−0.01
−0.02
−0.03
SNP effect on Cardiac valvular disease
0.04
0.05
0.06
0.07
SNP effect on CNOT1

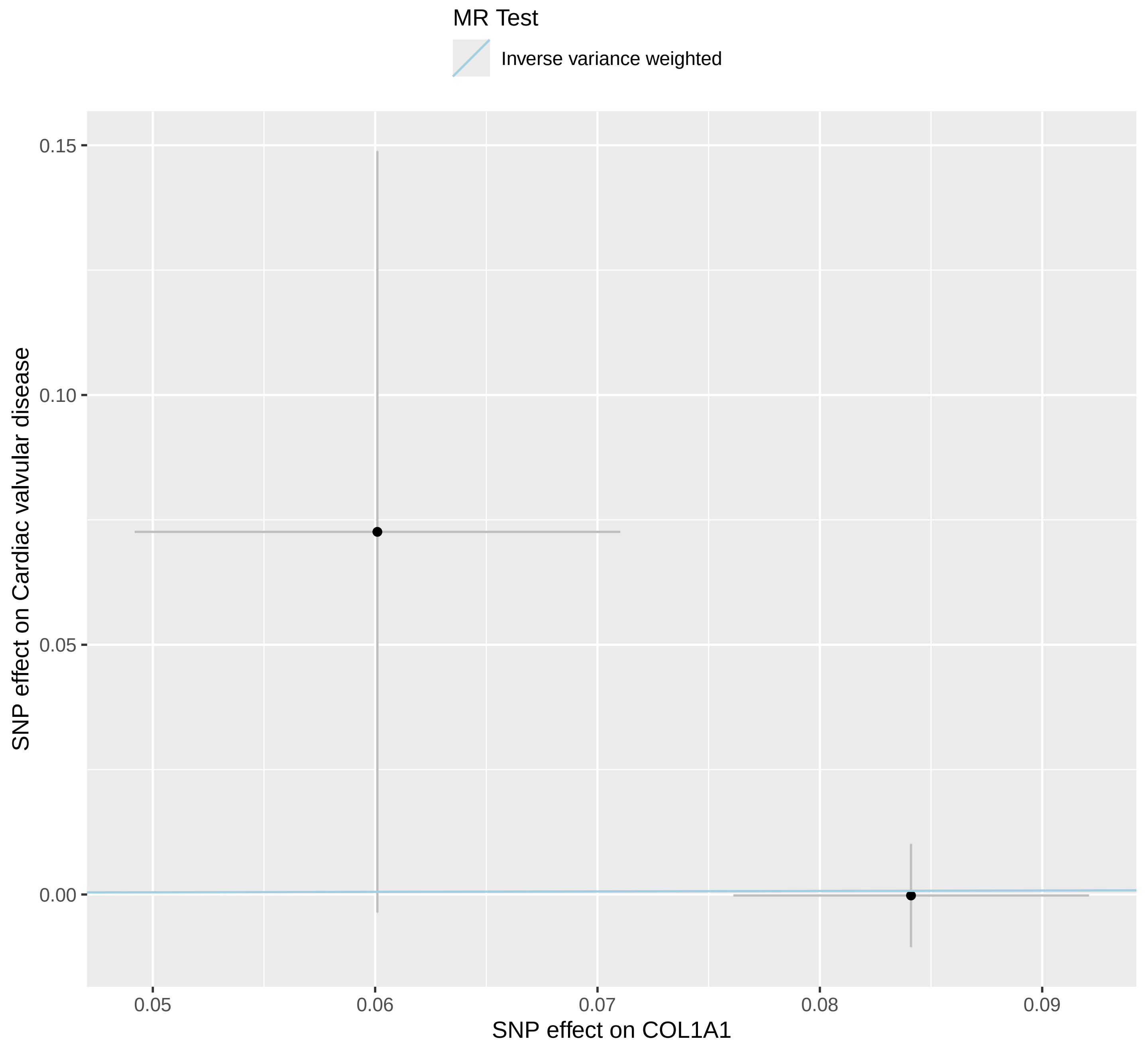
MR Test
Inverse variance weighted
SNP effect on Cardiac valvular disease
0.15
0.10
0.05
0.00
0.05
0.06
0.07
0.08
0.09
SNP effect on COL1A1

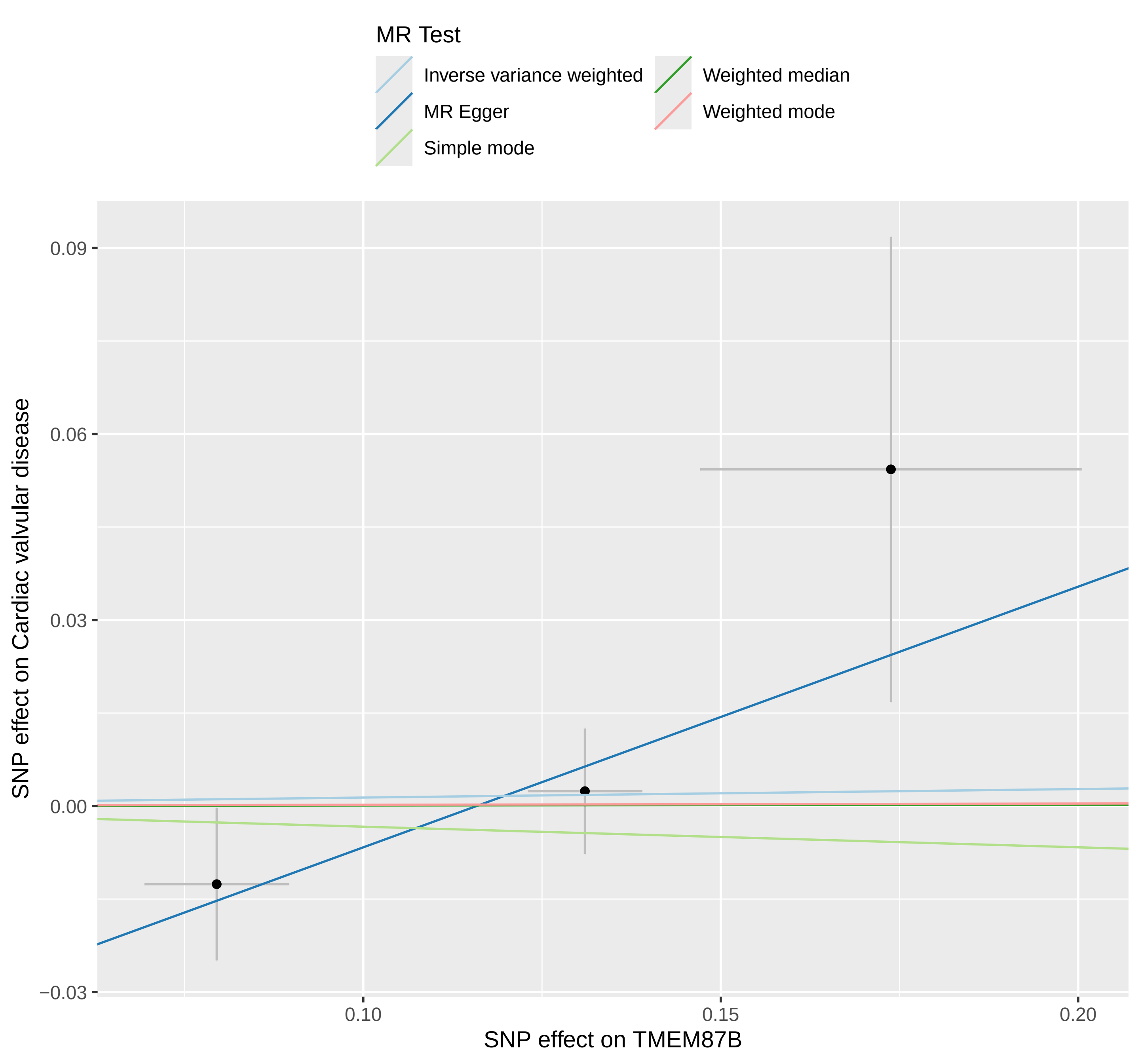

MR Test
Inverse variance weighted
MR Egger
Simple mode
Weighted median
Weighted mode
0.09
0.06
0.03
0.00
−0.03
0.10
0.15
0.20
SNP effect on Cardiac valvular disease
SNP effect on TMEM87B

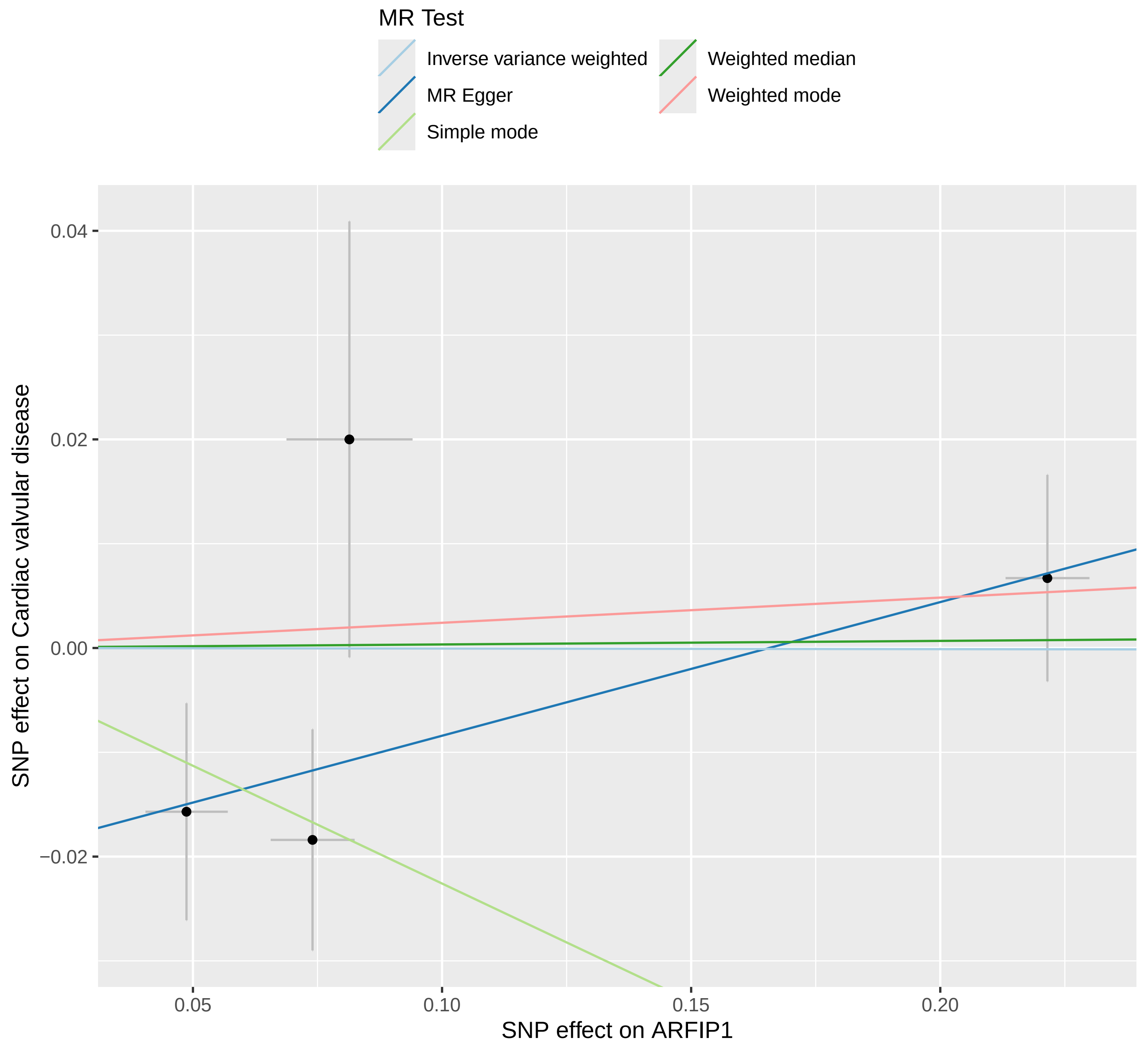
MR Test
Inverse variance weighted
MR Egger
Simple mode
Weighted median
Weighted mode
0.04
0.02
0.00
−0.02
SNP effect on Cardiac valvular disease
0.05
0.10
0.15
0.20
SNP effect on ARFIP1

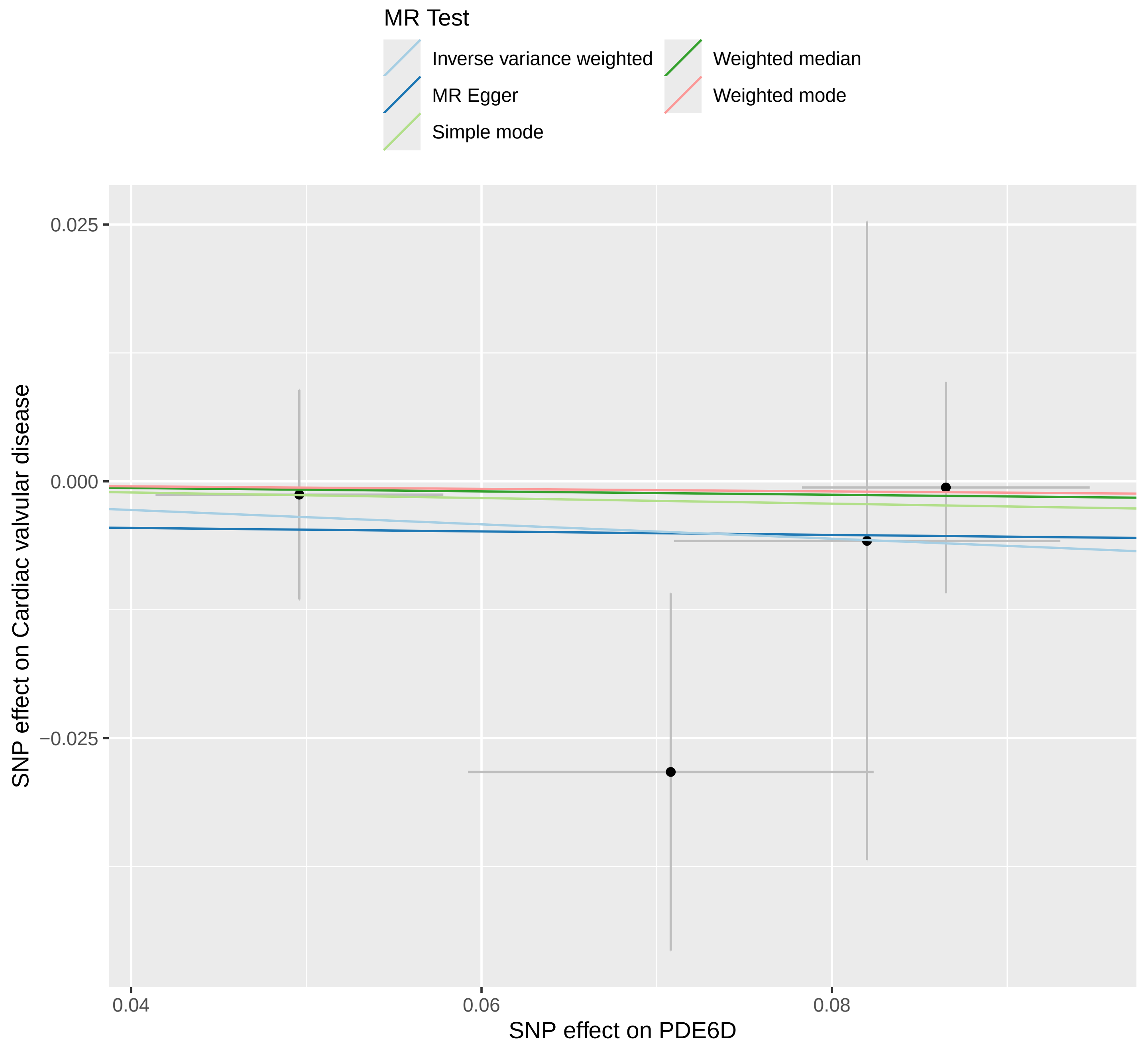

MR Test
Inverse variance weighted
MR Egger
Simple mode
Weighted median
Weighted mode
0.025
0.000
−0.025
0.04
0.06
0.08
SNP effect on PDE6D
SNP effect on Cardiac valvular disease

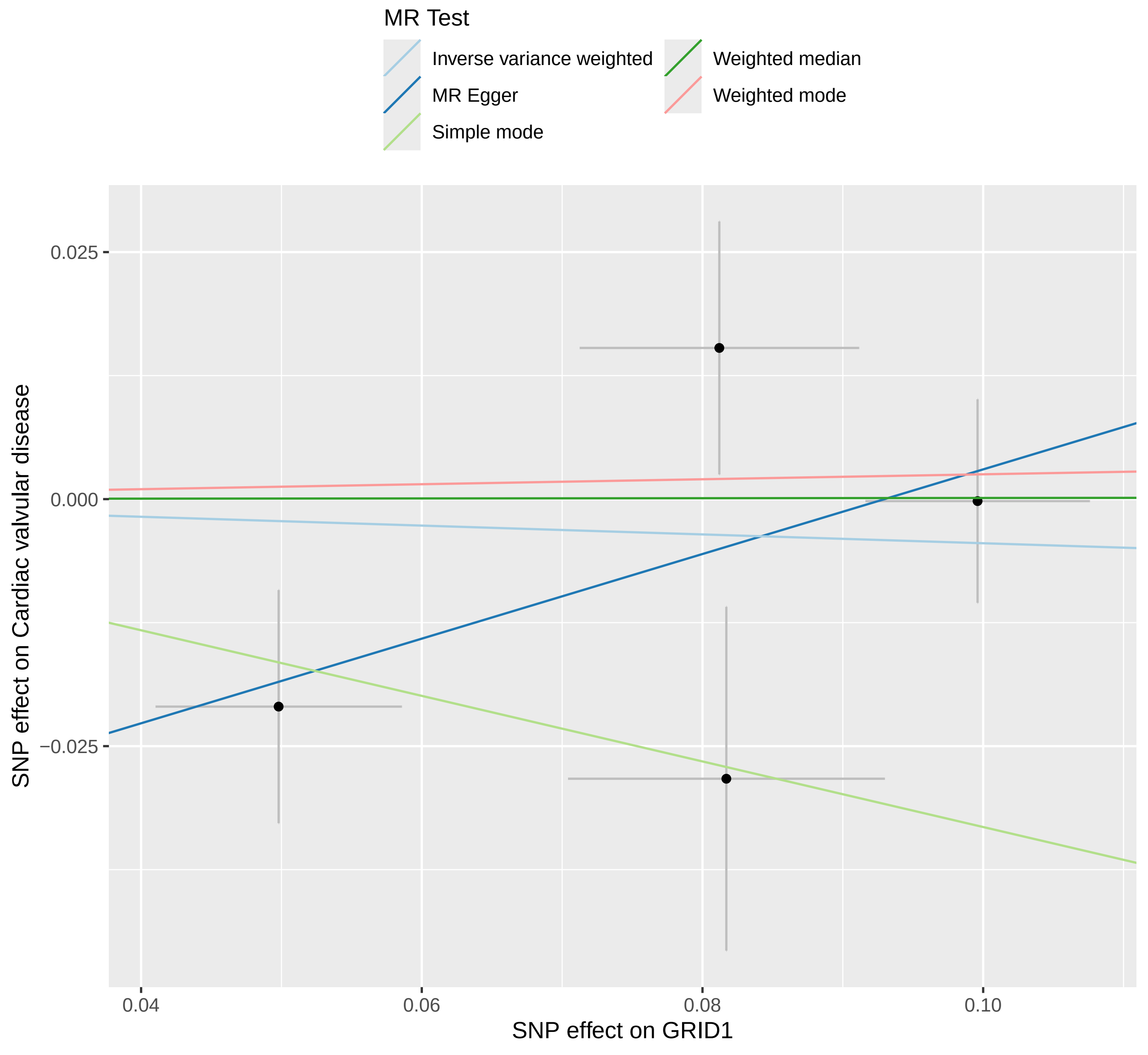
MR Test
Inverse variance weighted
MR Egger
Simple mode
Weighted median
Weighted mode
0.025
0.000
−0.025
SNP effect on Cardiac valvular disease
0.04
0.06
0.08
0.10
SNP effect on GRID1

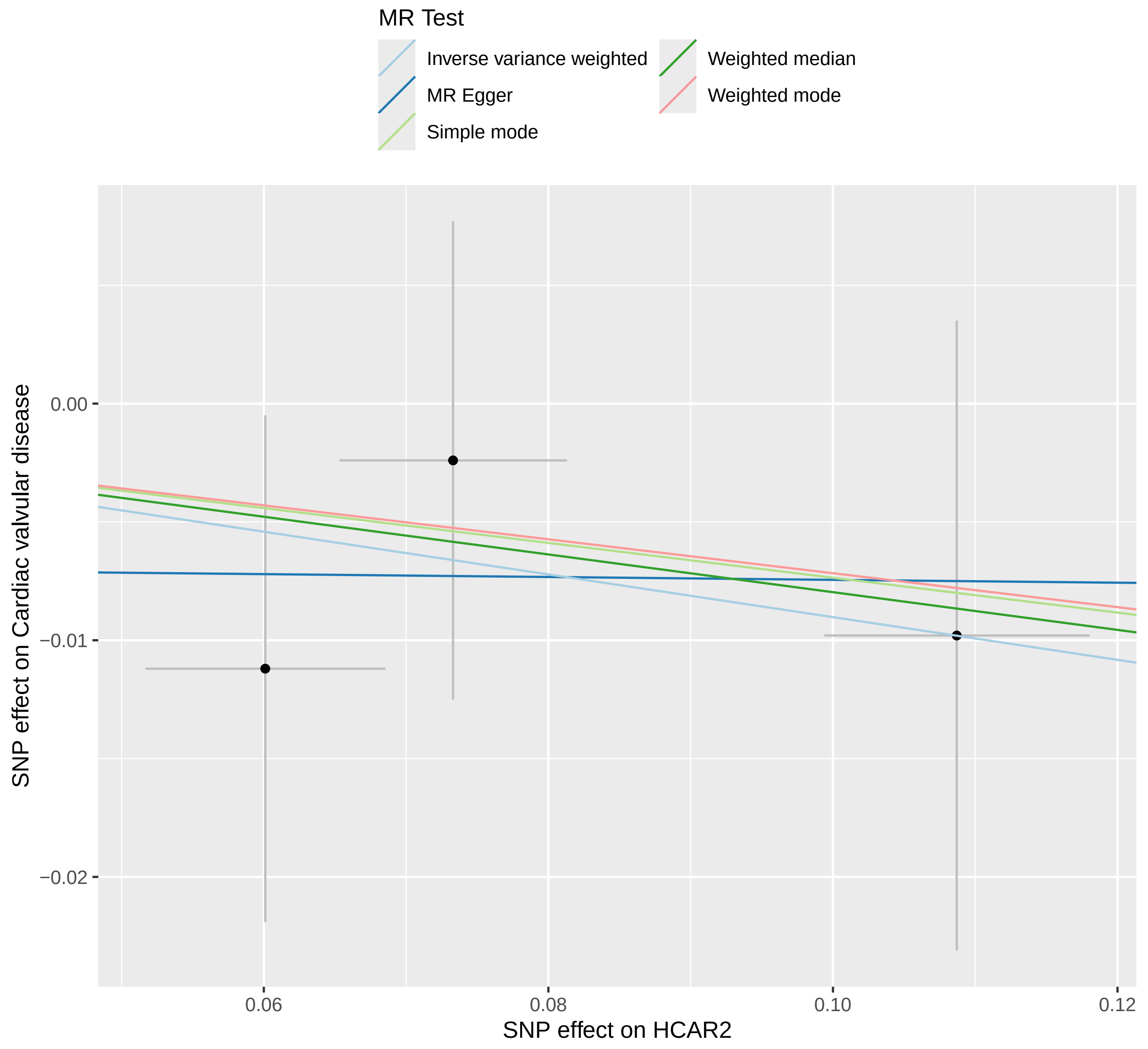
MR Test
Inverse variance weighted
MR Egger
Simple mode
Weighted median
Weighted mode
0.00
−0.01
−0.02
SNP effect on Cardiac valvular disease
0.06
0.08
0.10
0.12
SNP effect on HCAR2

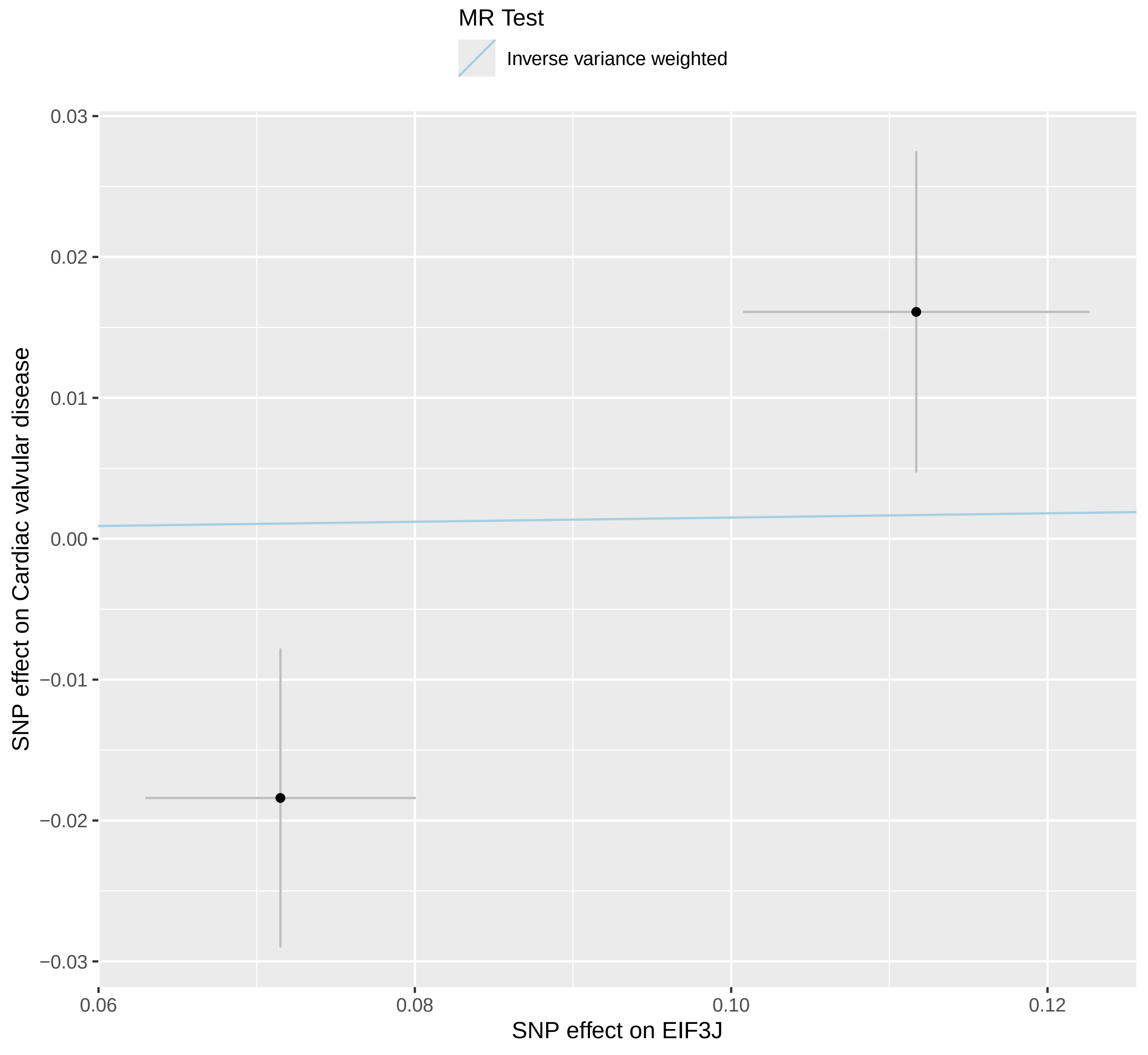
MR Test
Inverse variance weighted
0.03
0.02
0.01
0.00
−0.01
−0.02
−0.03
SNP effect on Cardiac valvular disease
0.06
0.08
0.10
0.12
SNP effect on EIF3J

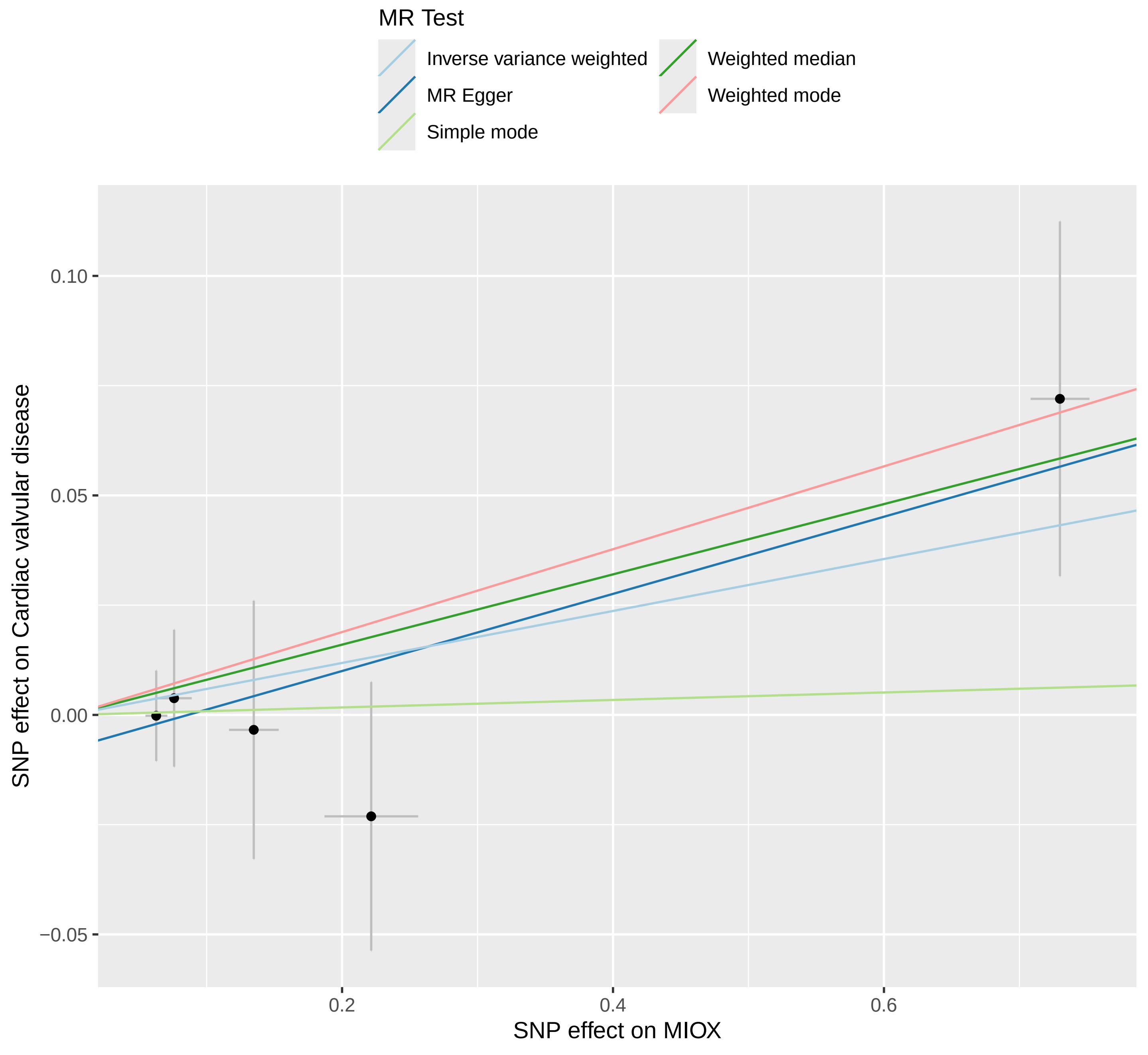
MR Test
Inverse variance weighted
MR Egger
Simple mode
Weighted median
Weighted mode
0.10
0.05
0.00
−0.05
0.2
0.4
0.6
SNP effect on Cardiac valvular disease
SNP effect on MIOX

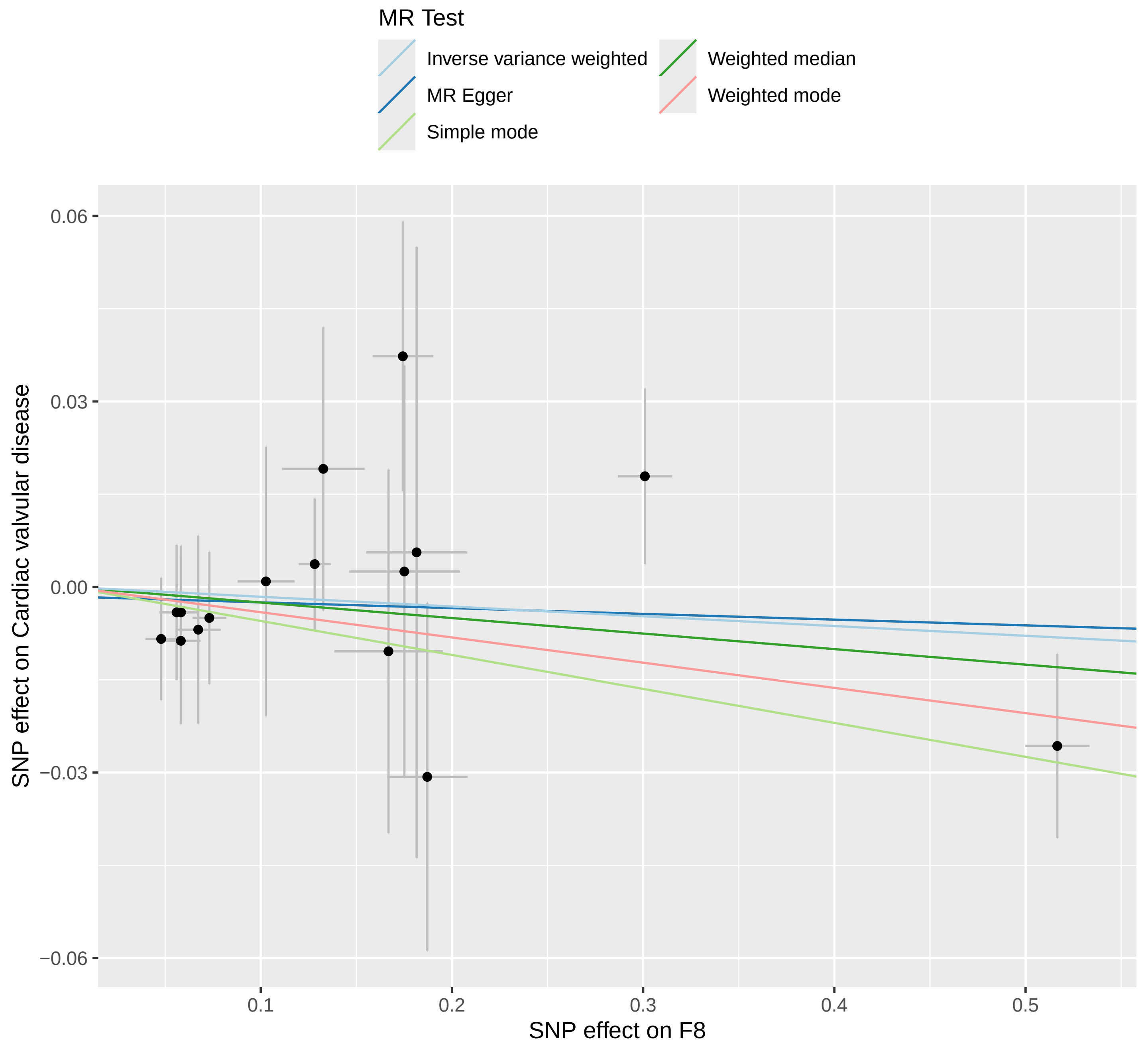
MR Test
Inverse variance weighted
MR Egger
Simple mode
Weighted median
Weighted mode
0.06
0.03
0.00
−0.03
−0.06
SNP effect on Cardiac valvular disease
0.1
0.2
0.3
0.4
0.5
SNP effect on F8

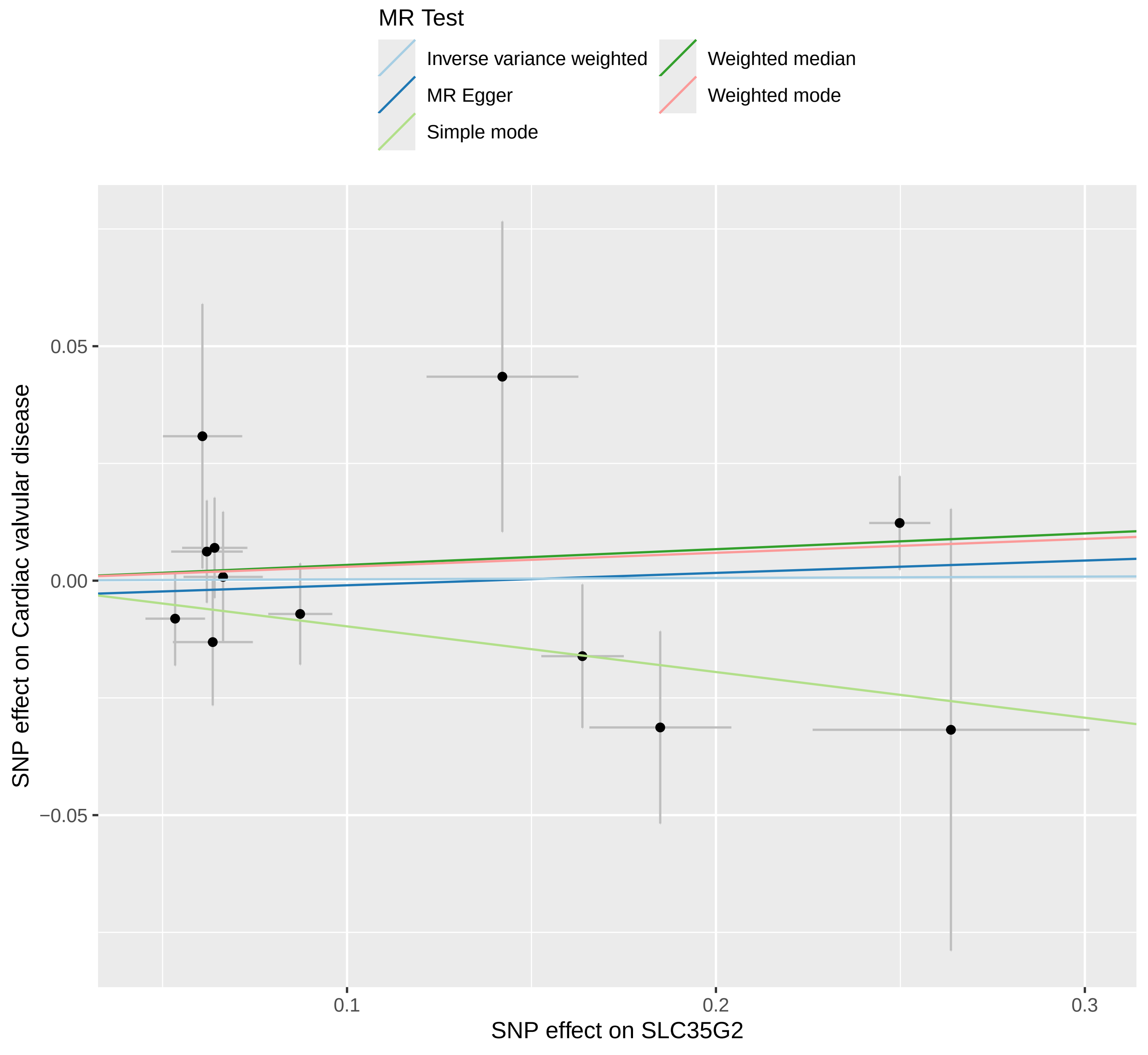
MR Test
Inverse variance weighted
MR Egger
Simple mode
Weighted median
Weighted mode
SNP effect on Cardiac valvular disease
0.05
0.00
−0.05
0.1
0.2
0.3
SNP effect on SLC35G2

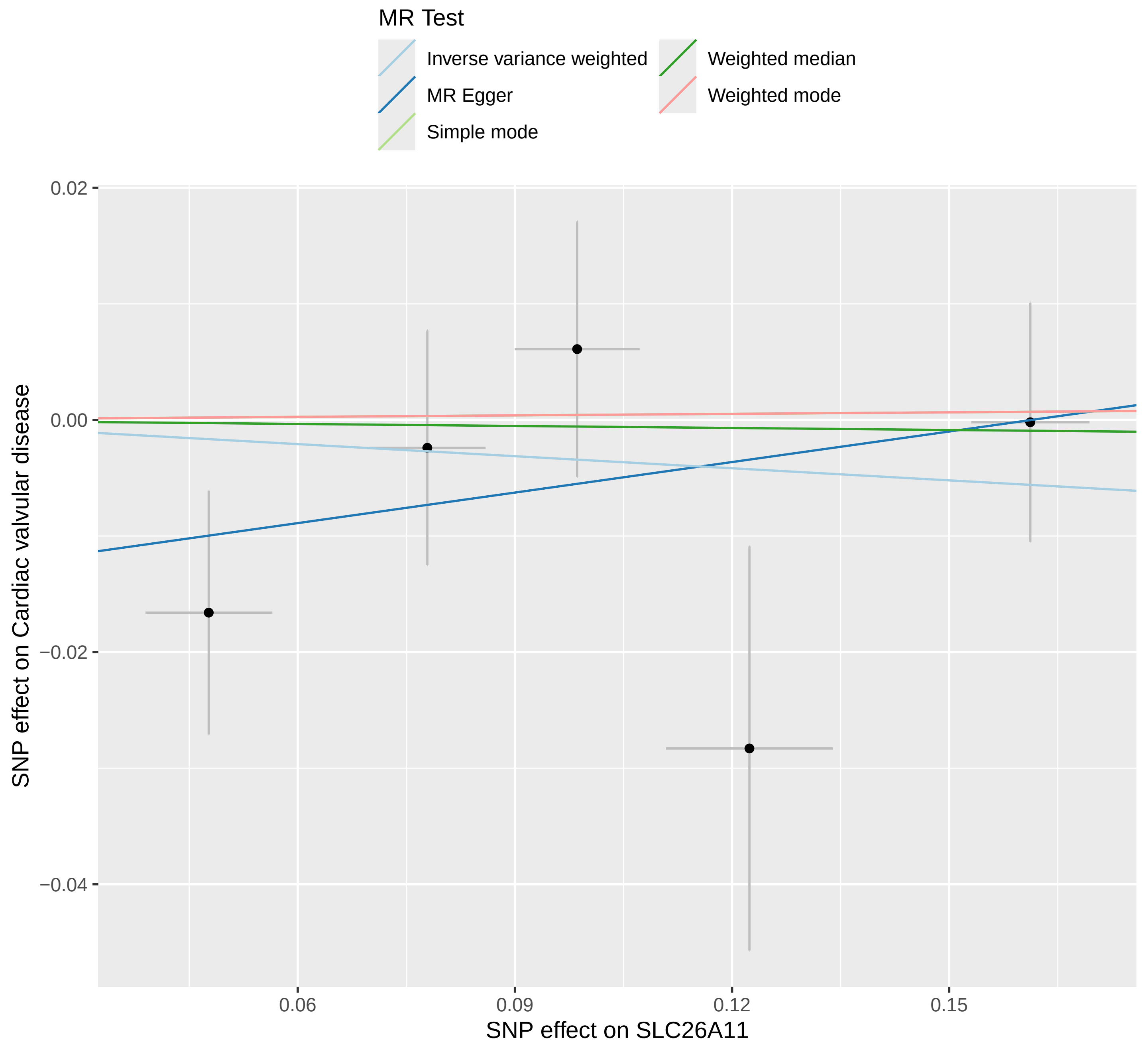

MR Test
Inverse variance weighted
MR Egger
Simple mode
Weighted median
Weighted mode
0.02
0.00
−0.02
−0.04
0.06
0.09
0.12
0.15
SNP effect on Cardiac valvular disease
SNP effect on SLC26A11

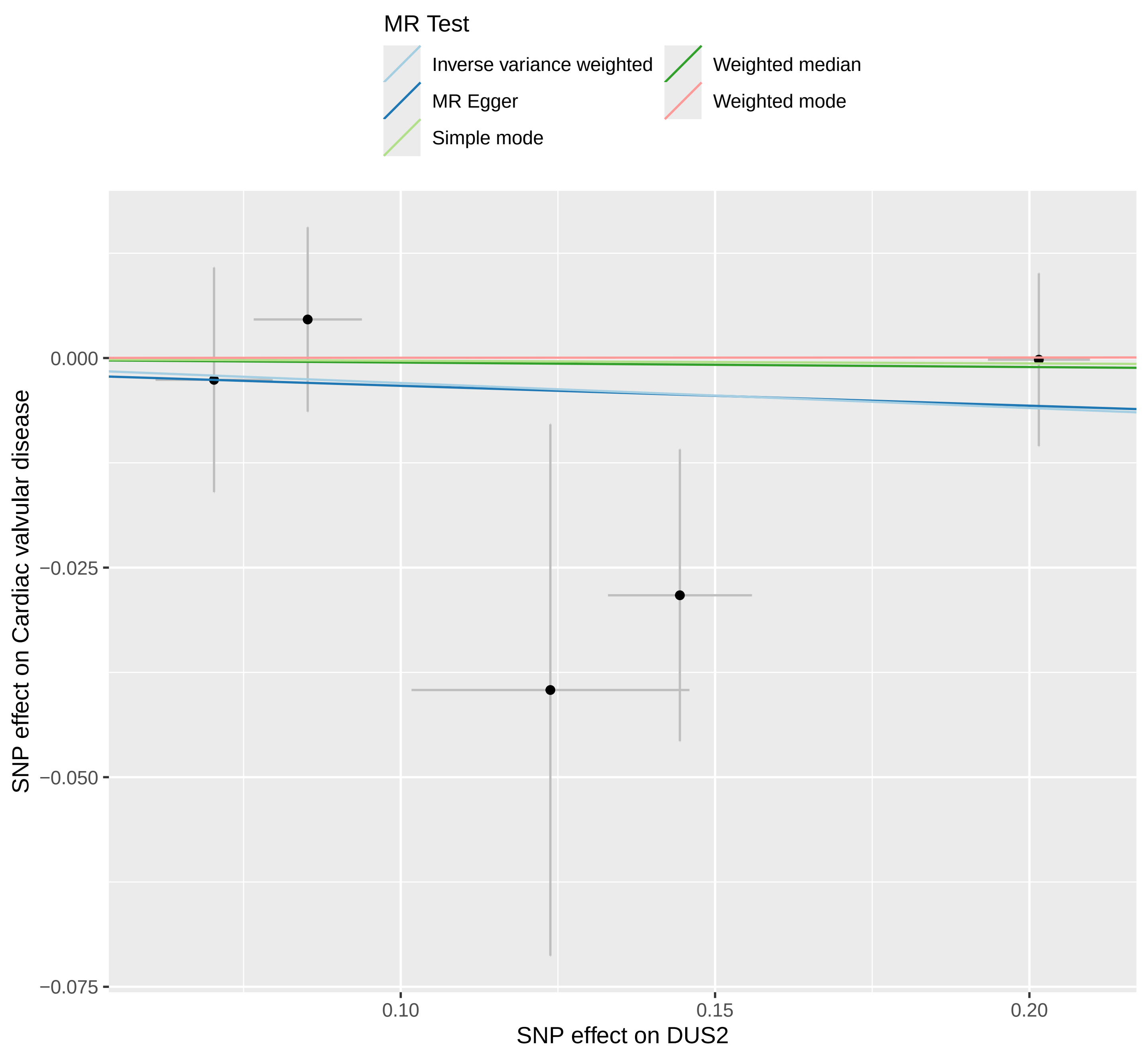

MR Test
Inverse variance weighted
MR Egger
Simple mode
Weighted median
Weighted mode
0.000
−0.025
−0.050
−0.075
0.10
0.15
0.20
SNP effect on DUS2
SNP effect on Cardiac valvular disease

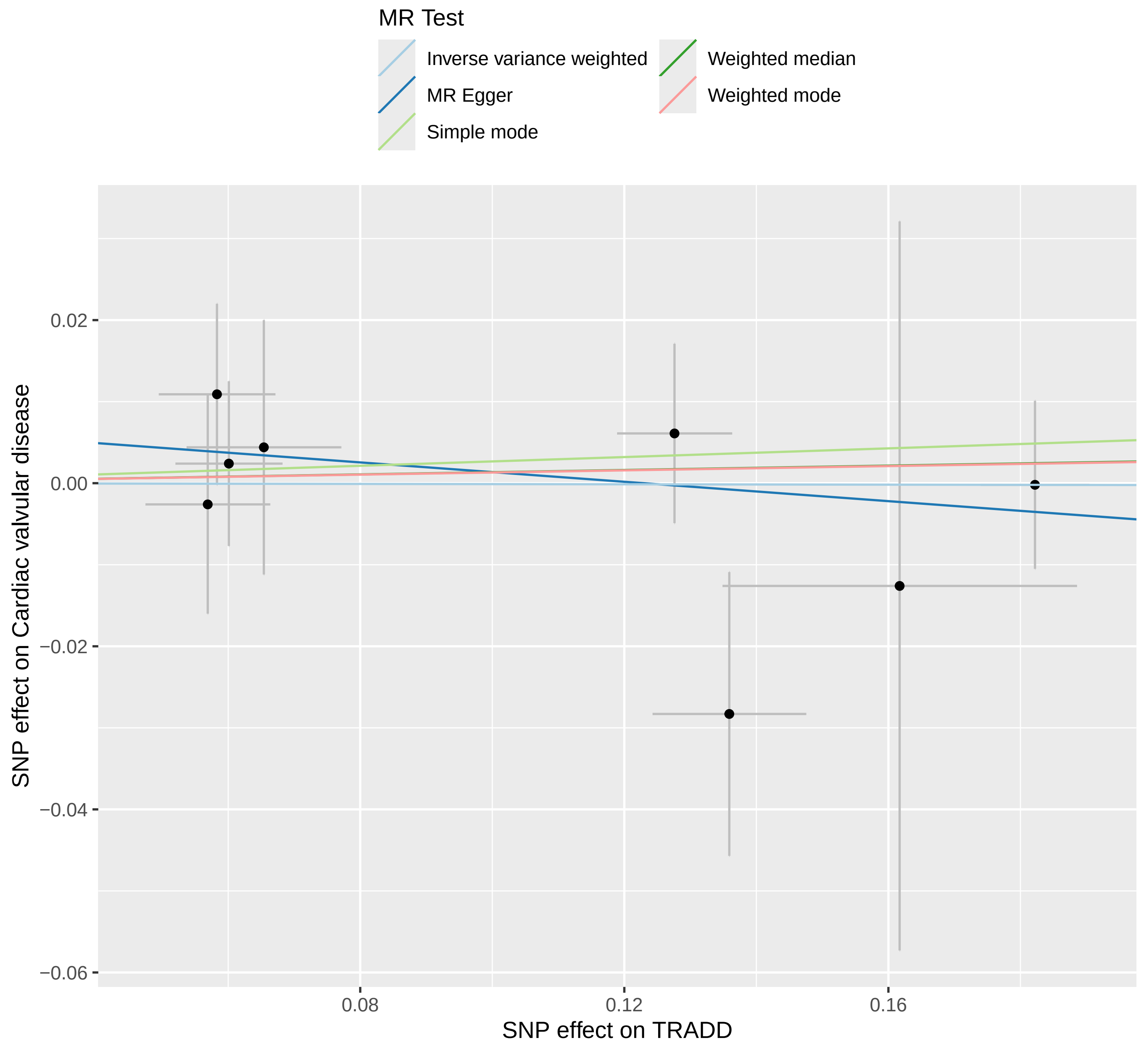
MR Test
Inverse variance weighted
MR Egger
Simple mode
Weighted median
Weighted mode
SNP effect on Cardiac valvular disease
0.02
0.00
−0.02
−0.04
−0.06
0.08
0.12
0.16
SNP effect on TRADD

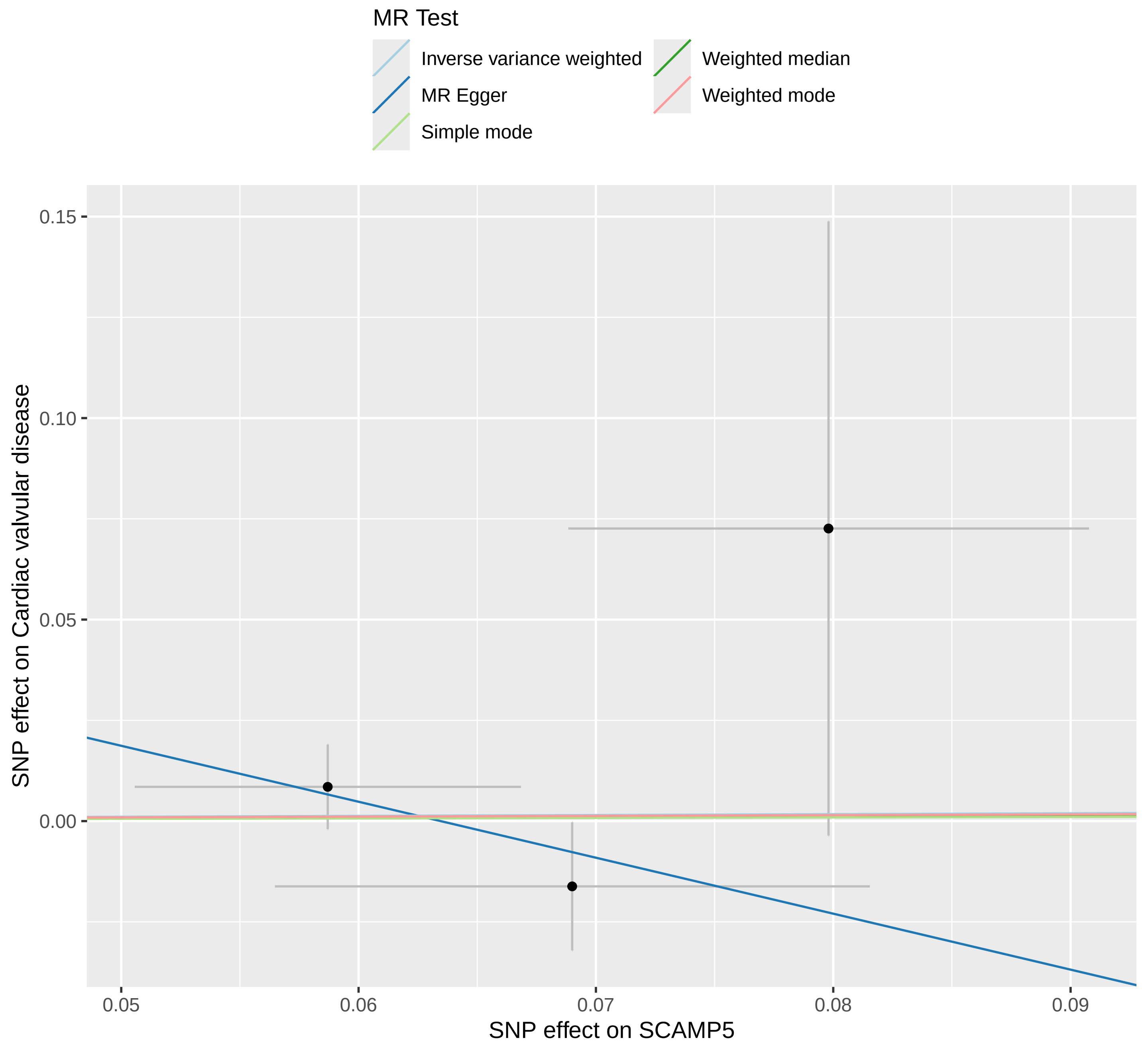
MR Test
Inverse variance weighted
MR Egger
Simple mode
Weighted median
Weighted mode
0.15
0.10
0.05
0.00
SNP effect on Cardiac valvular disease
0.05
0.06
0.07
0.08
0.09
SNP effect on SCAMP5

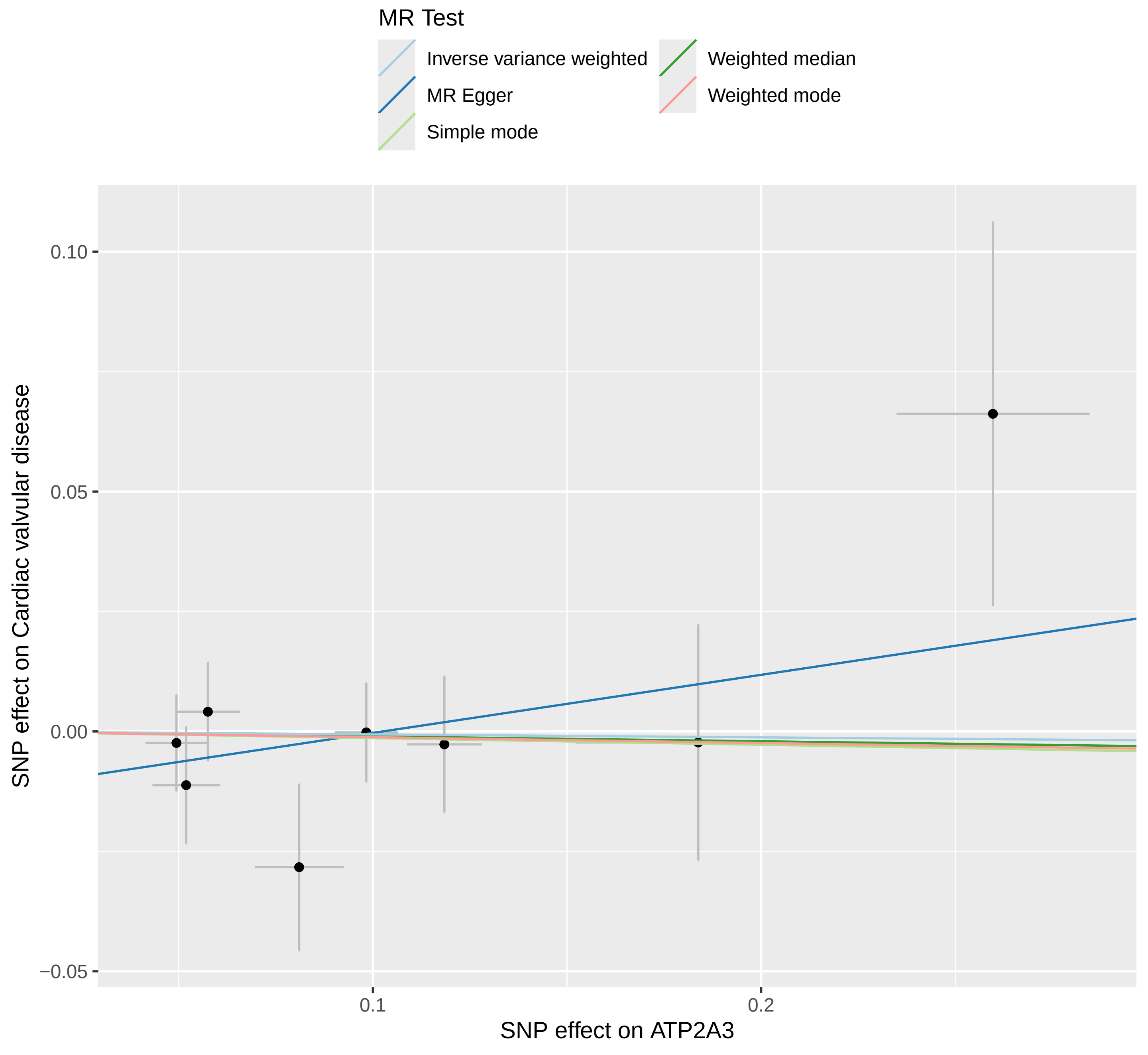

MR Test
Inverse variance weighted
MR Egger
Simple mode
Weighted median
Weighted mode
0.10
0.05
0.00
−0.05
SNP effect on Cardiac valvular disease
0.1
0.2
SNP effect on ATP2A3

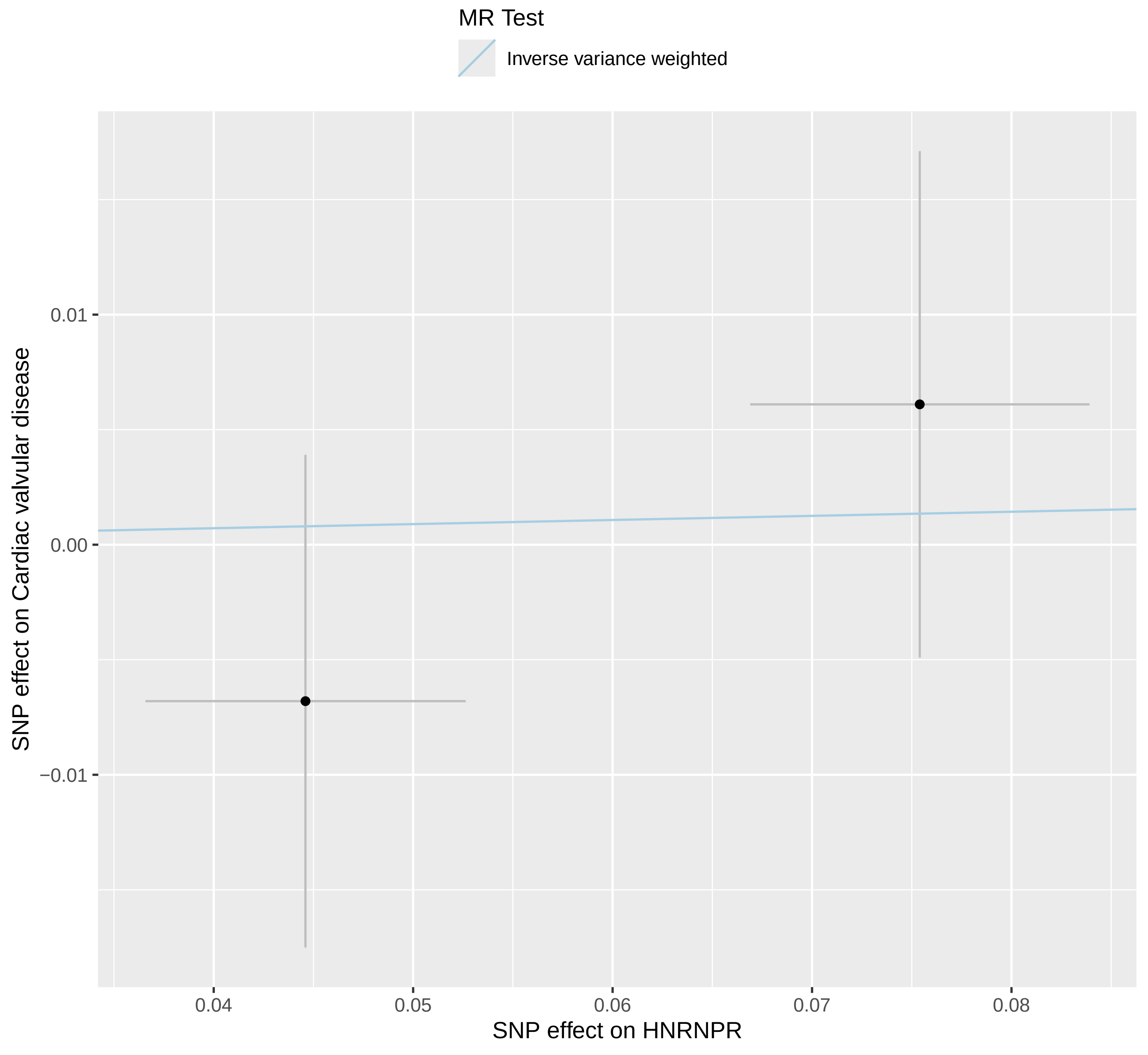

MR Test
Inverse variance weighted
SNP effect on Cardiac valvular disease
0.01
0.00
−0.01
0.04
0.05
0.06
0.07
0.08
SNP effect on HNRNPR

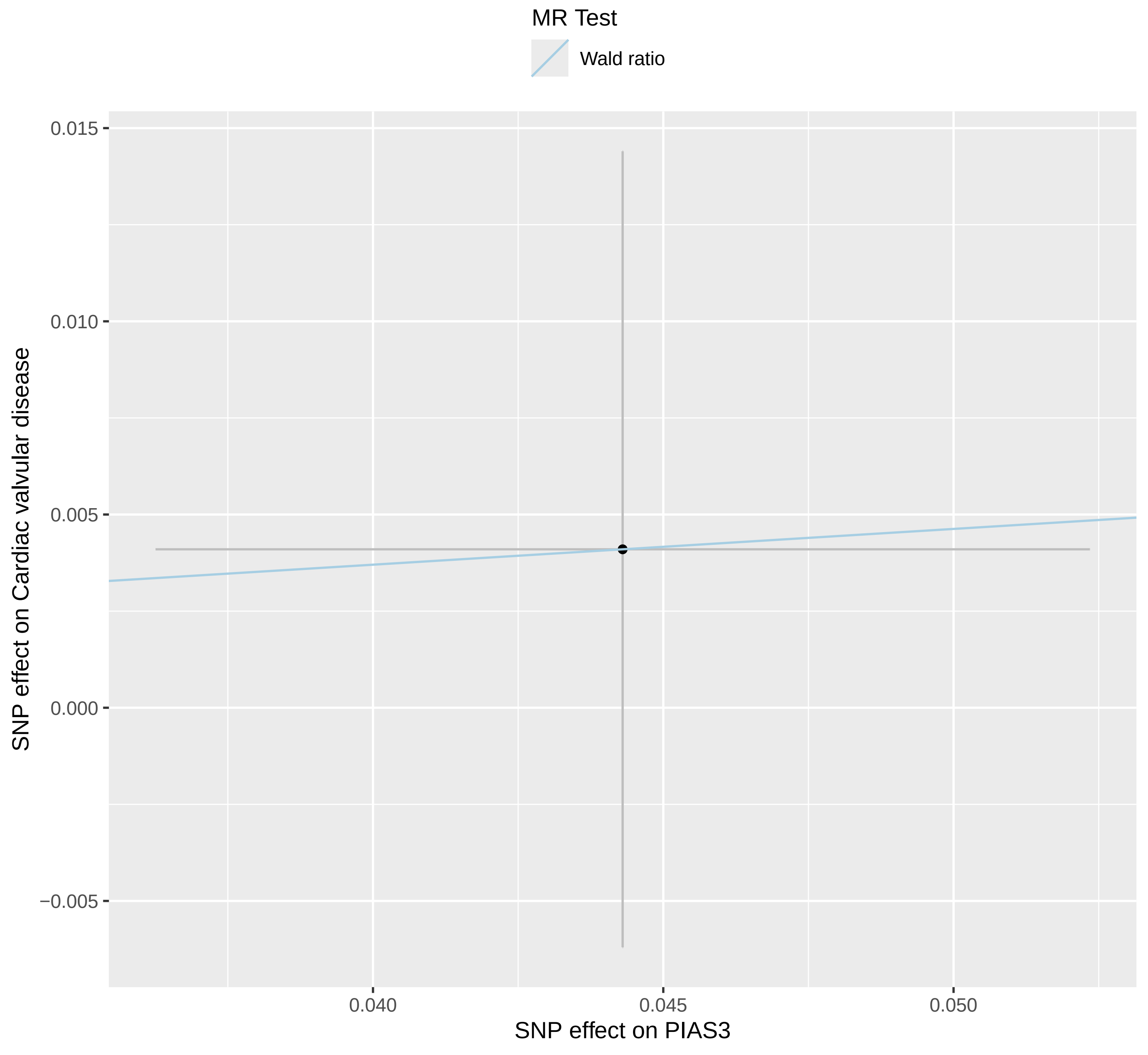

MR Test
Wald ratio
0.015
0.010
0.005
0.000
−0.005
SNP effect on Cardiac valvular disease
0.040
0.045
0.050
SNP effect on PIAS3

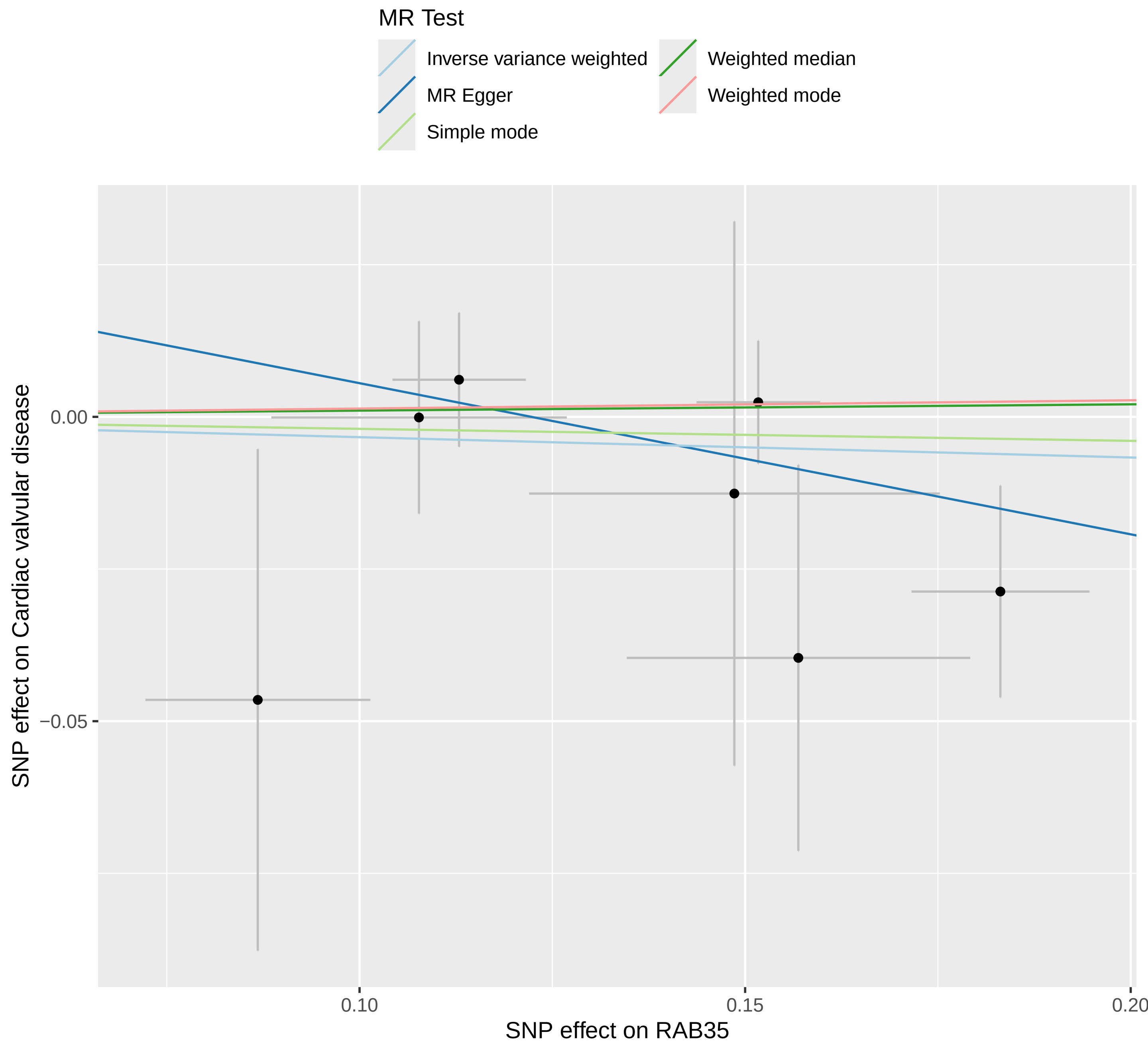
MR Test
Inverse variance weighted
MR Egger
Simple mode
Weighted median
Weighted mode
0.00
−0.05
0.10
0.15
0.20
SNP effect on RAB35
SNP effect on Cardiac valvular disease

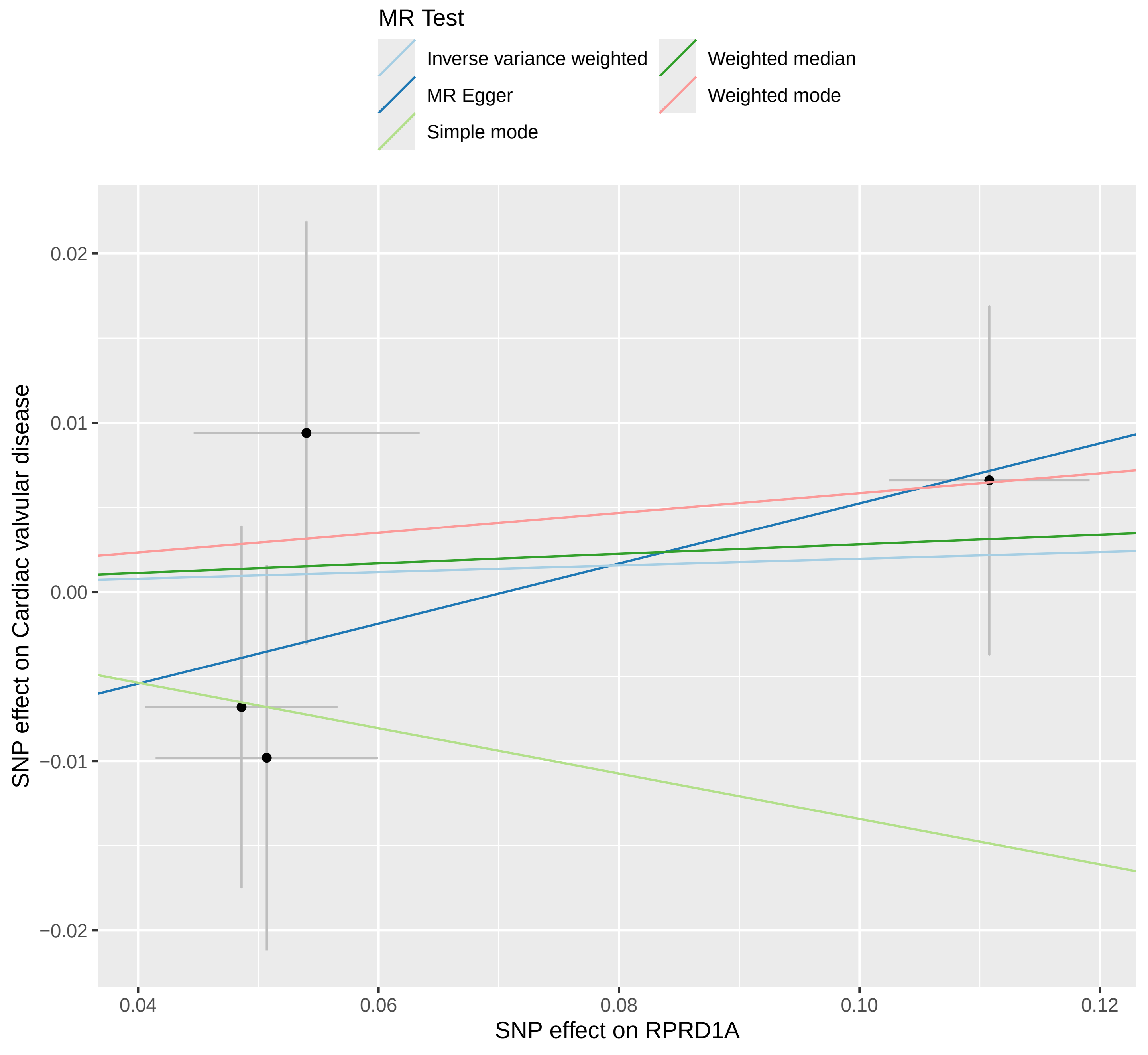
MR Test
Inverse variance weighted
MR Egger
Simple mode
Weighted median
Weighted mode
0.02
0.01
0.00
−0.01
−0.02
0.04
0.06
0.08
0.10
0.12
SNP effect on RPRD1A
SNP effect on Cardiac valvular disease

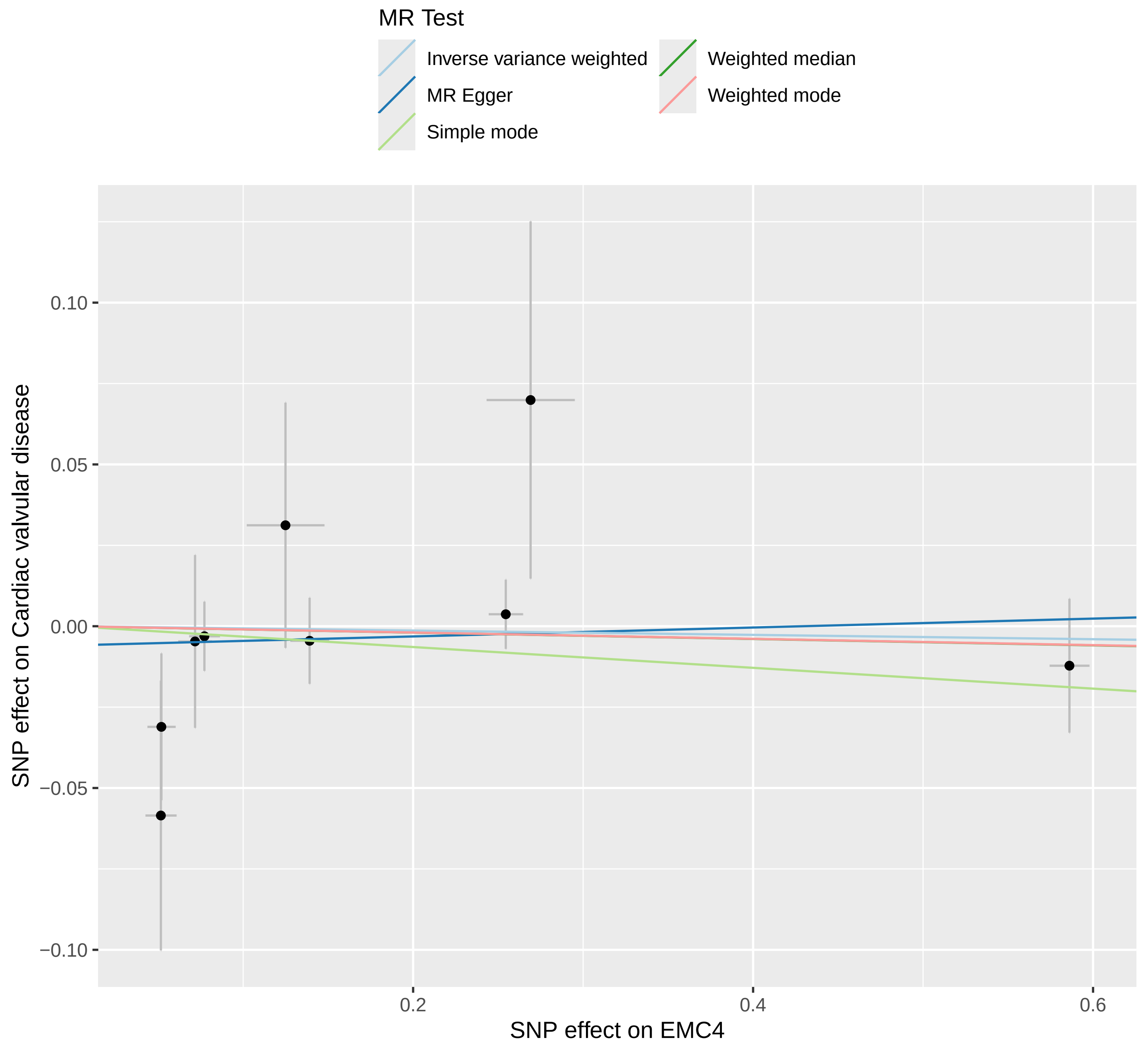
MR Test
Inverse variance weighted
MR Egger
Simple mode
Weighted median
Weighted mode
SNP effect on Cardiac valvular disease
0.10
0.05
0.00
−0.05
−0.10
0.2
0.4
0.6
SNP effect on EMC4

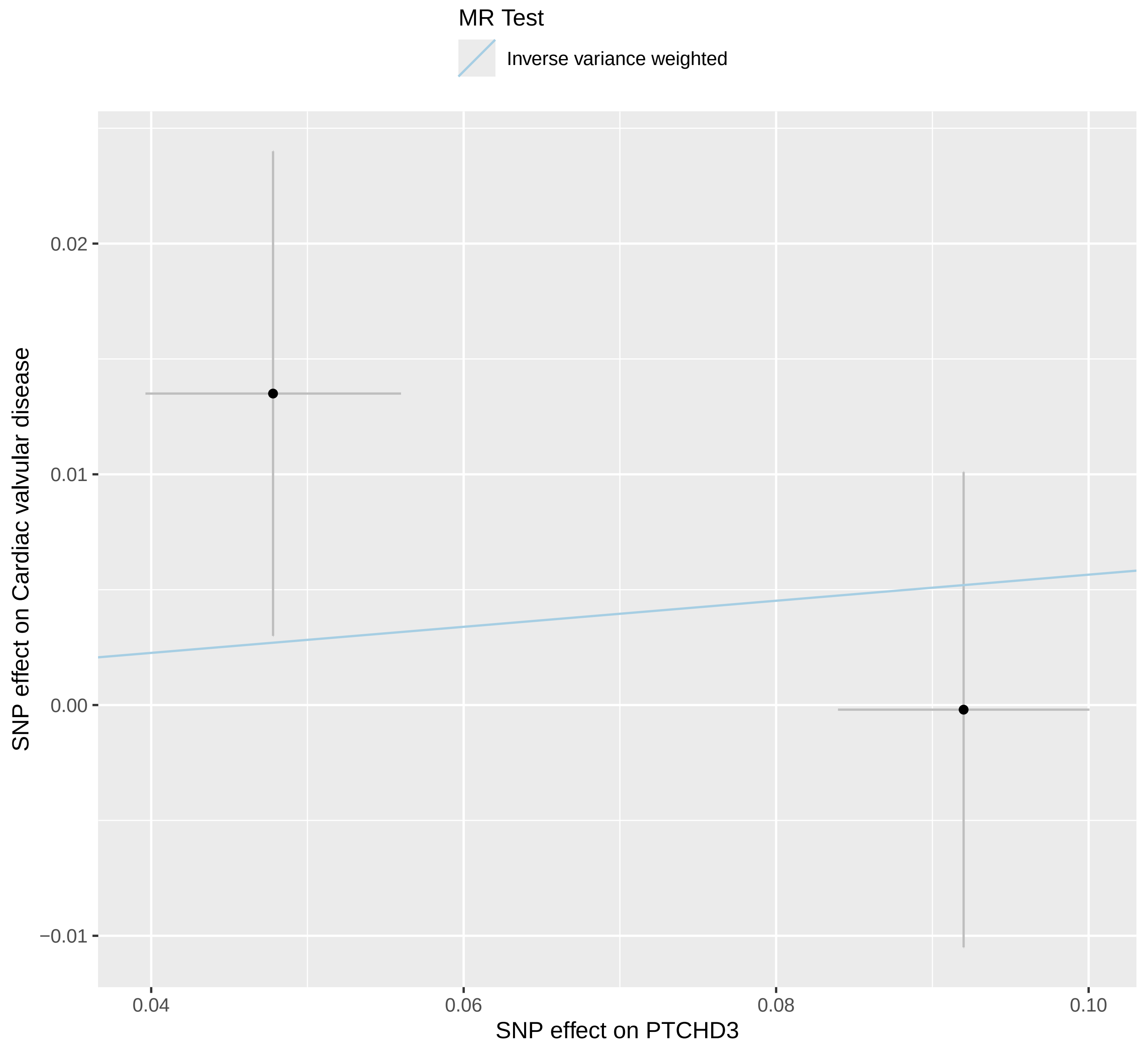
MR Test
Inverse variance weighted
0.02
0.01
0.00
−0.01
SNP effect on Cardiac valvular disease
0.04
0.06
0.08
0.10
SNP effect on PTCHD3

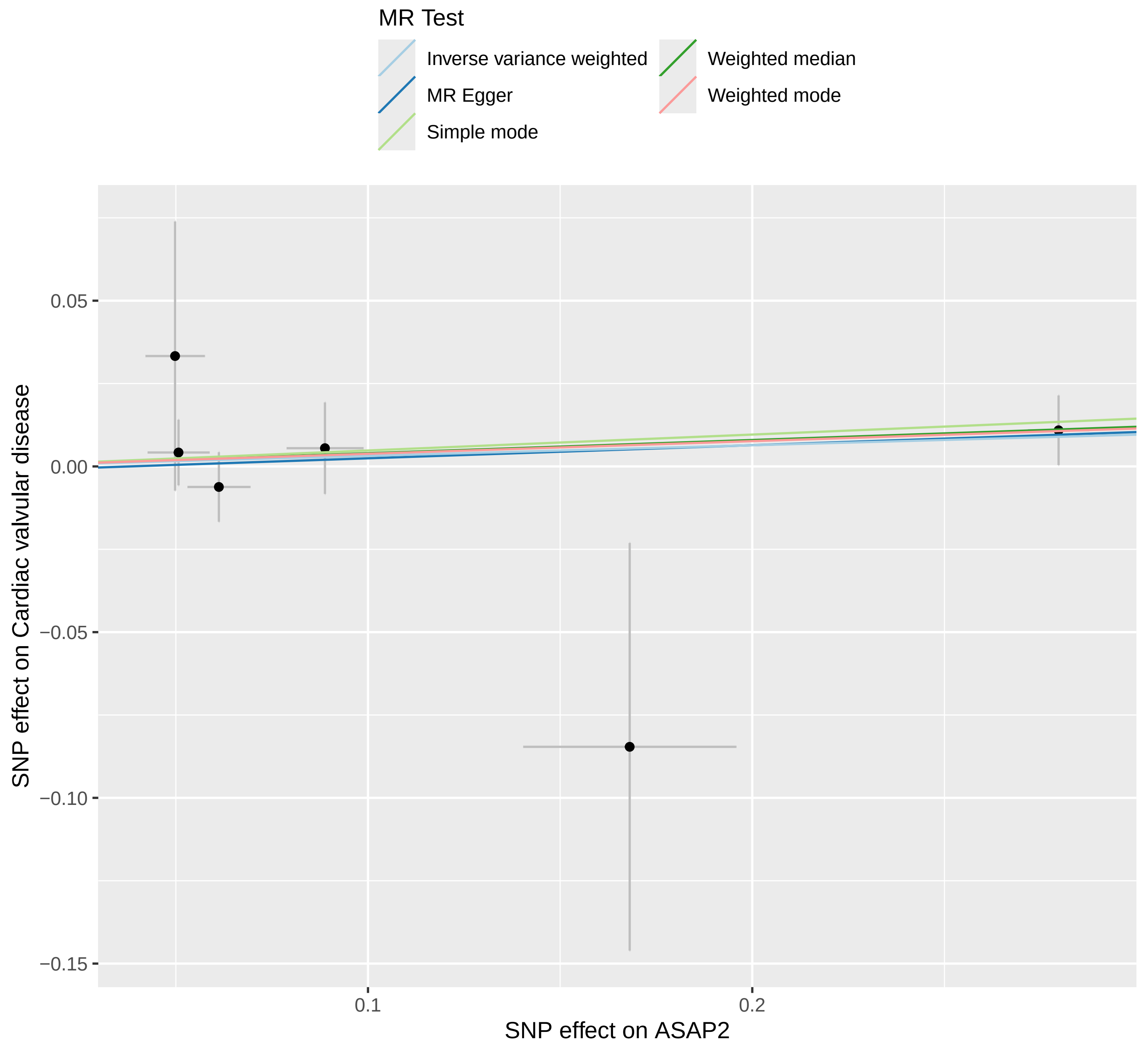
MR Test
Inverse variance weighted
MR Egger
Simple mode
Weighted median
Weighted mode
0.05
0.00
−0.05
−0.10
−0.15
SNP effect on Cardiac valvular disease
0.1
0.2
SNP effect on ASAP2

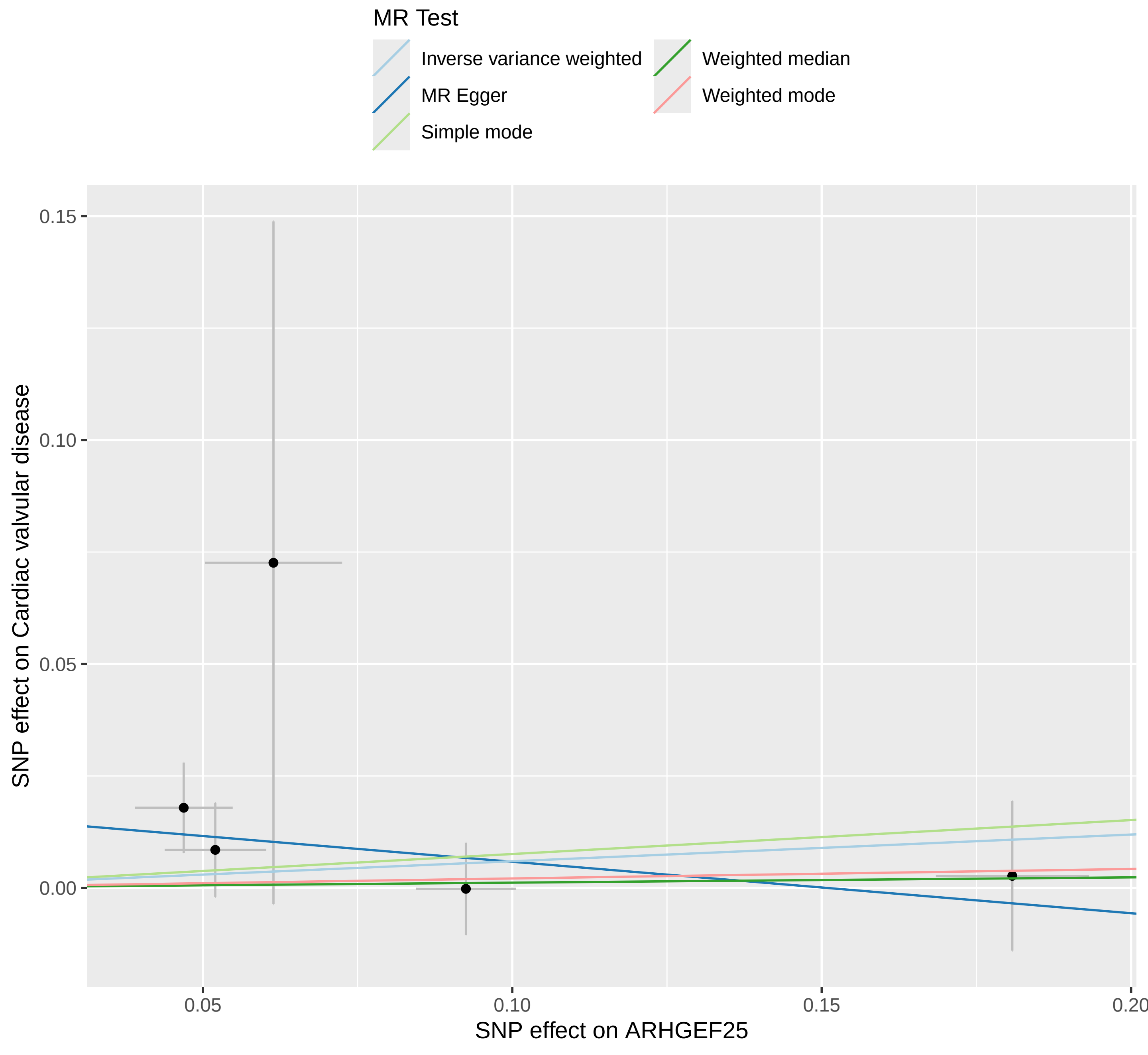

MR Test
Inverse variance weighted
MR Egger
Simple mode
Weighted median
Weighted mode
0.15
0.10
0.05
0.00
SNP effect on Cardiac valvular disease
0.05
0.10
0.15
0.20
SNP effect on ARHGEF25

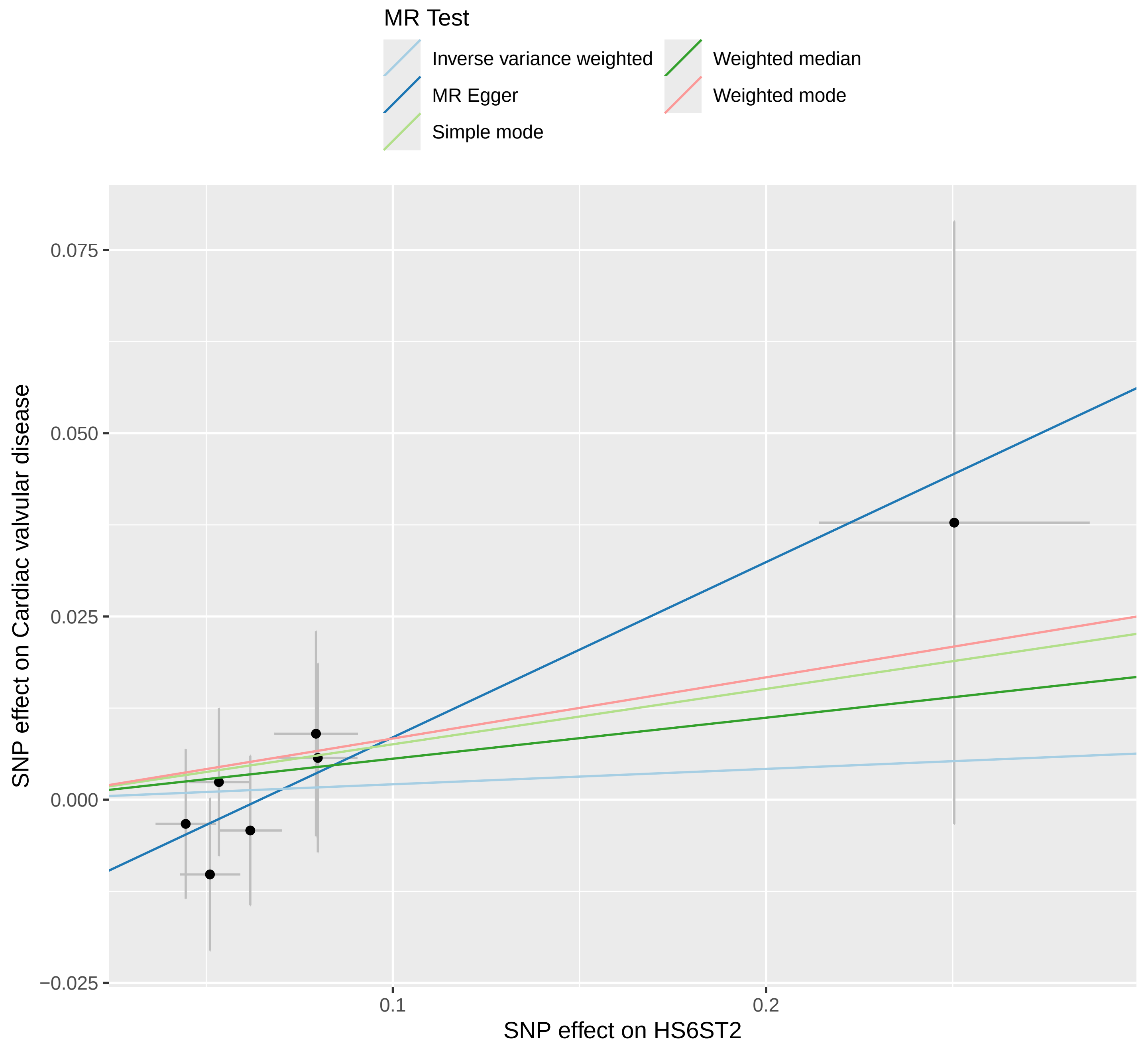

MR Test
Inverse variance weighted
MR Egger
Simple mode
Weighted median
Weighted mode
SNP effect on Cardiac valvular disease
0.075
0.050
0.025
0.000
−0.025
0.1
0.2
SNP effect on HS6ST2

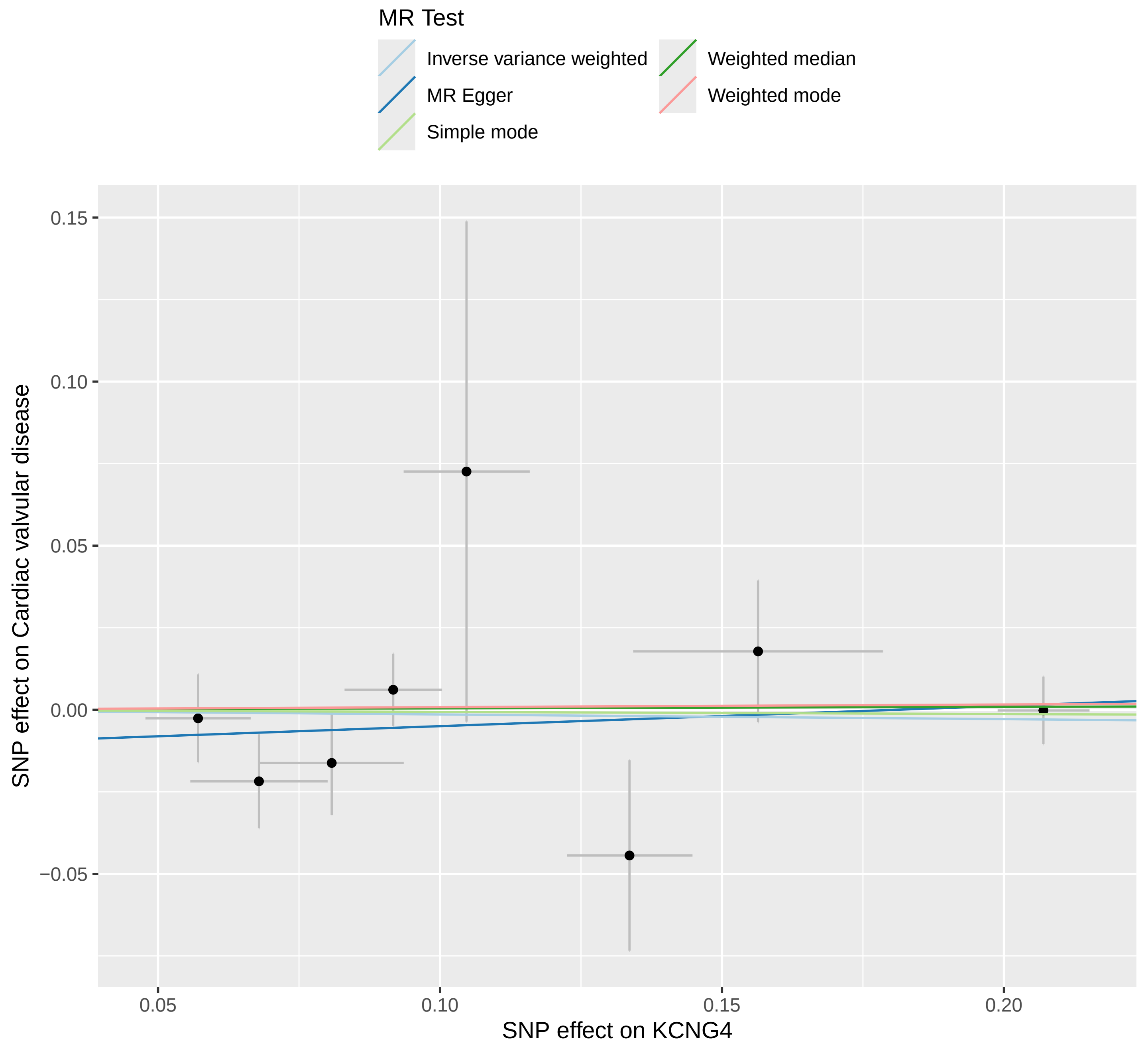

MR Test
Inverse variance weighted
MR Egger
Simple mode
Weighted median
Weighted mode
0.15
0.10
0.05
0.00
−0.05
0.05
0.10
0.15
0.20
SNP effect on Cardiac valvular disease
SNP effect on KCNG4

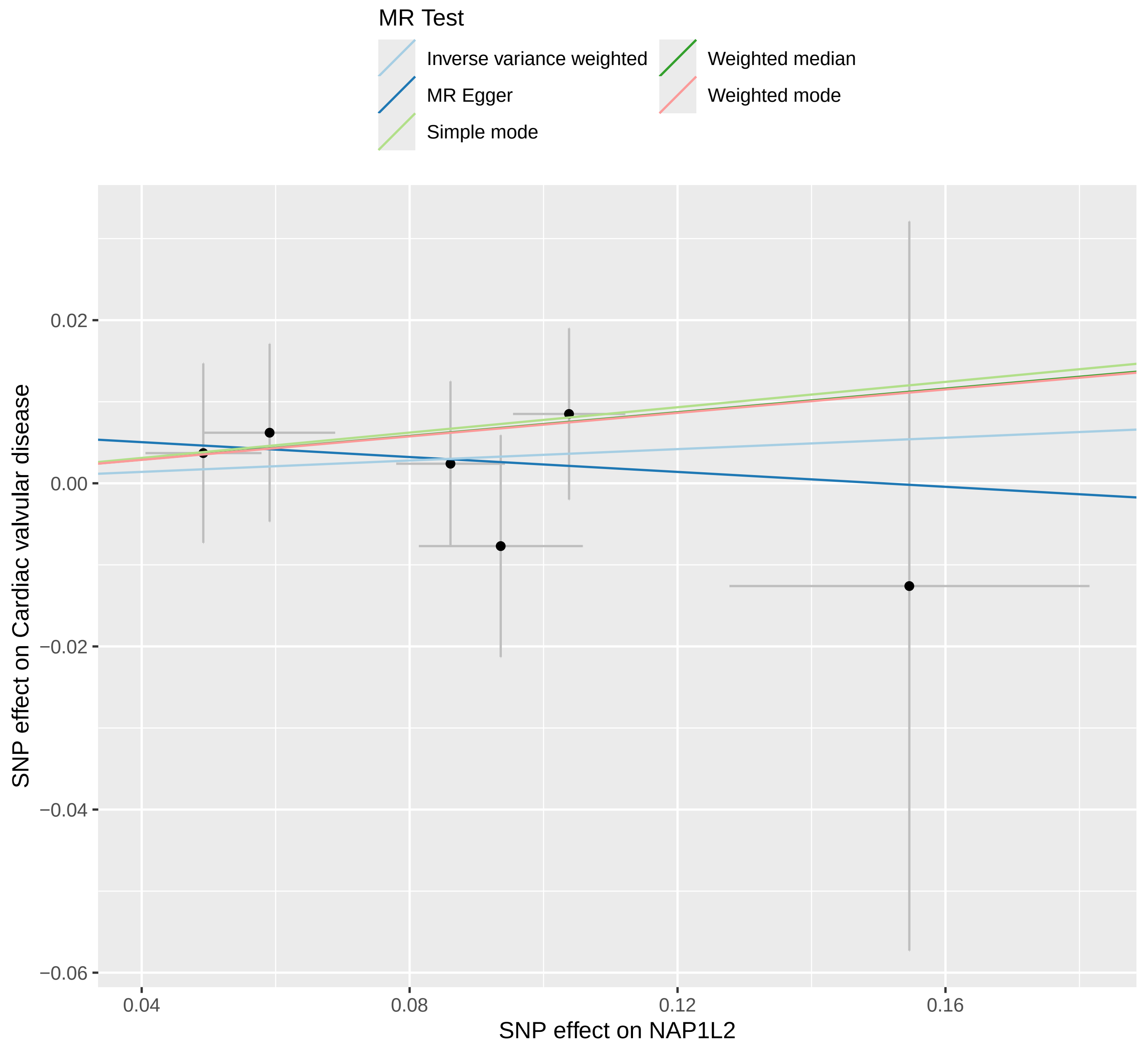
MR Test
Inverse variance weighted
MR Egger
Simple mode
Weighted median
Weighted mode
0.02
0.00
−0.02
−0.04
−0.06
0.04
0.08
0.12
0.16
SNP effect on Cardiac valvular disease
SNP effect on NAP1L2

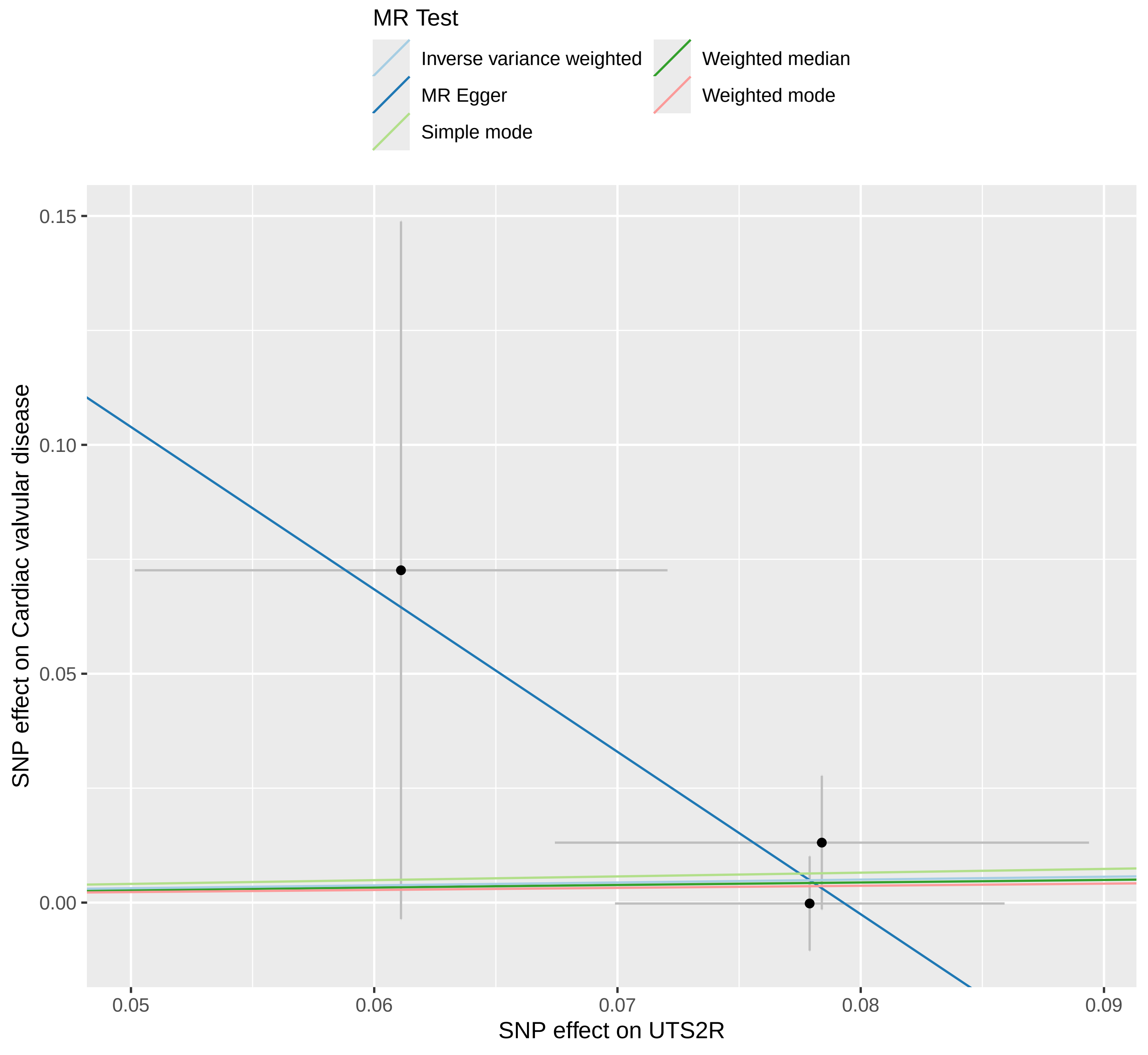

MR Test
Inverse variance weighted
MR Egger
Simple mode
Weighted median
Weighted mode
SNP effect on Cardiac valvular disease
0.15
0.10
0.05
0.00
0.05
0.06
0.07
0.08
0.09
SNP effect on UTS2R

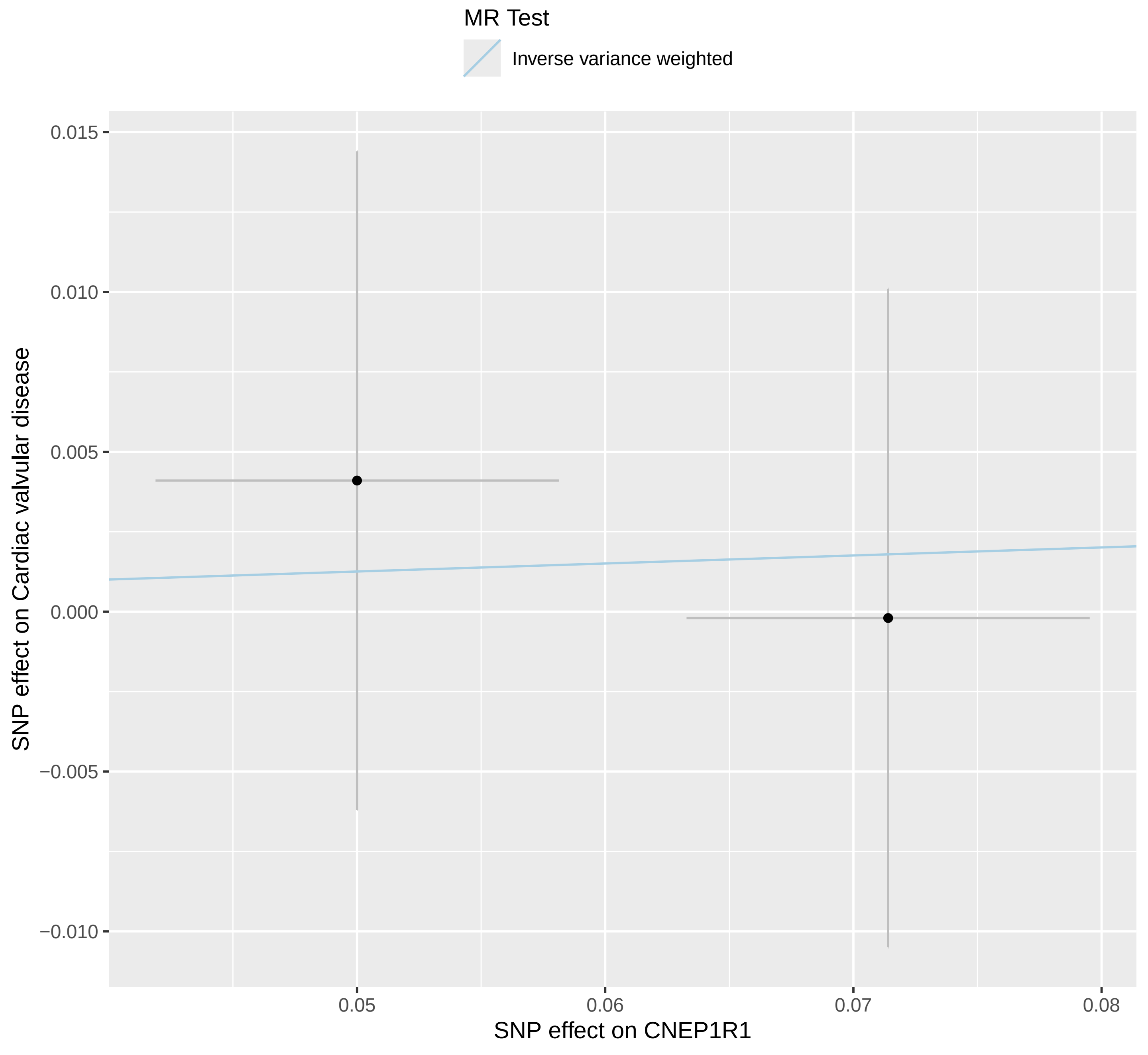
MR Test
Inverse variance weighted
0.015
0.010
0.005
0.000
−0.005
−0.010
SNP effect on Cardiac valvular disease
0.05
0.06
0.07
0.08
SNP effect on CNEP1R1

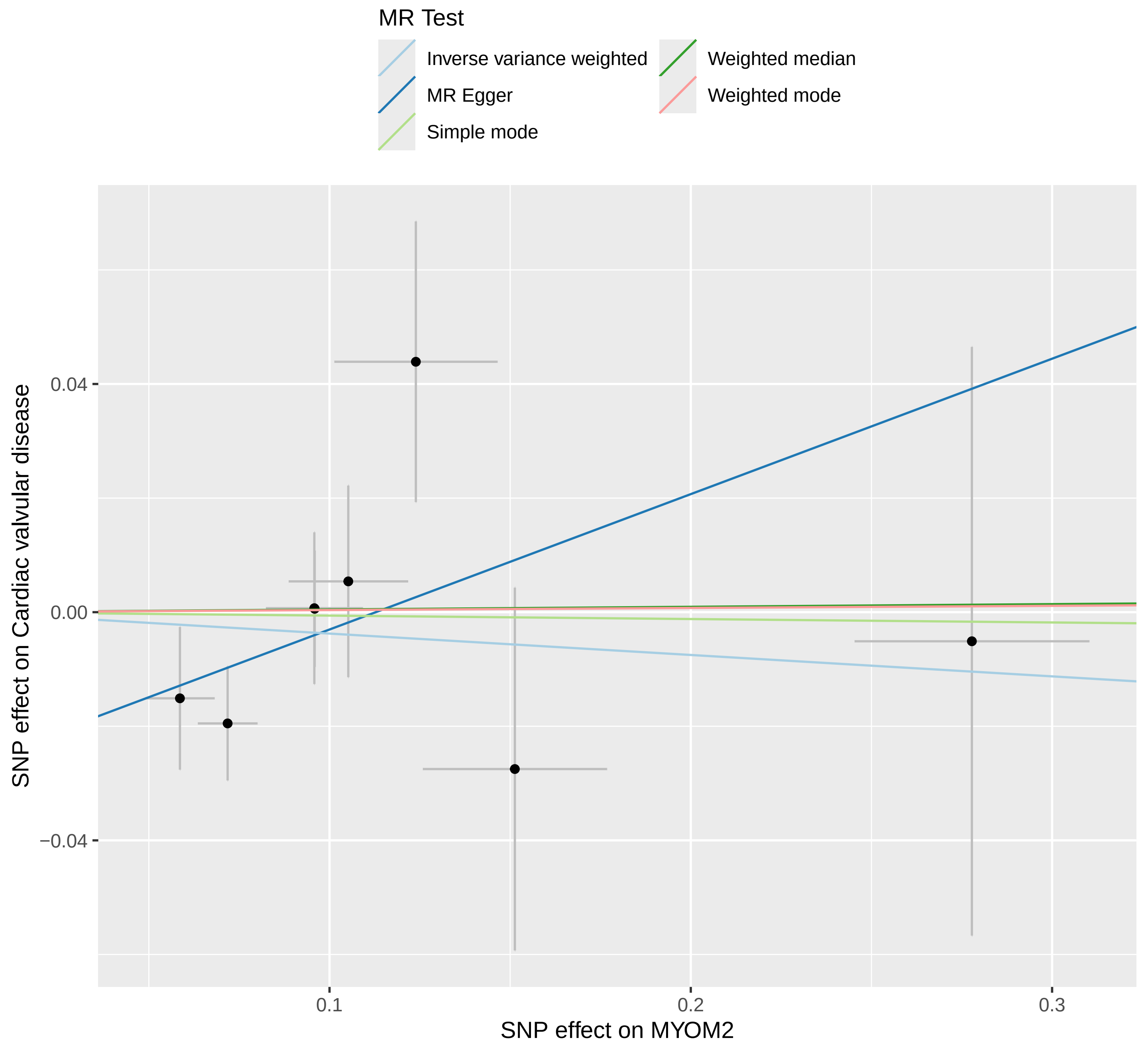

MR Test
Inverse variance weighted
MR Egger
Simple mode
Weighted median
Weighted mode
0.04
0.00
−0.04
0.1
0.2
0.3
SNP effect on Cardiac valvular disease
SNP effect on MYOM2

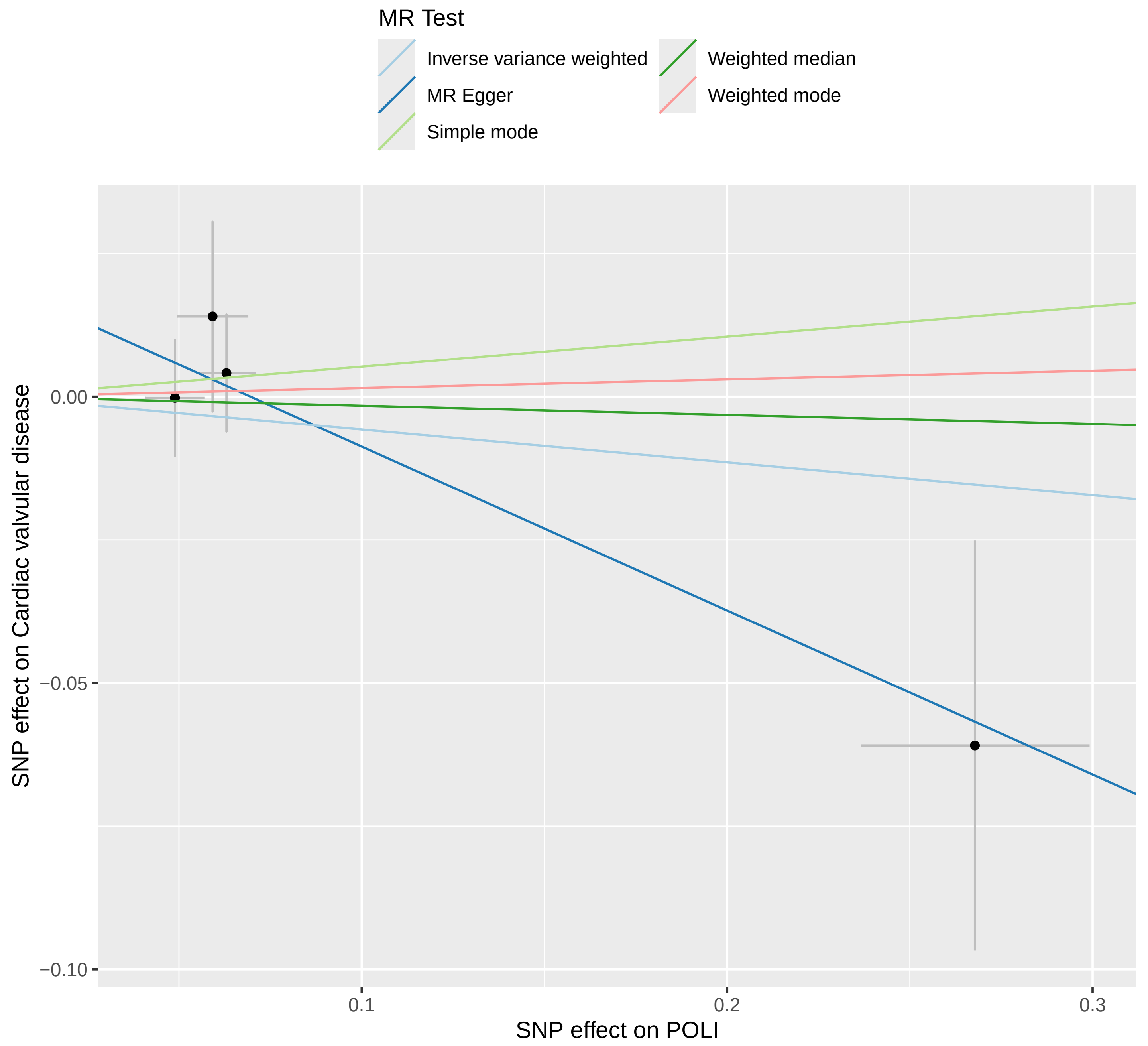

MR Test
Inverse variance weighted
MR Egger
Simple mode
Weighted median
Weighted mode
SNP effect on Cardiac valvular disease
0.00
−0.05
−0.10
0.1
0.2
0.3
SNP effect on POLI

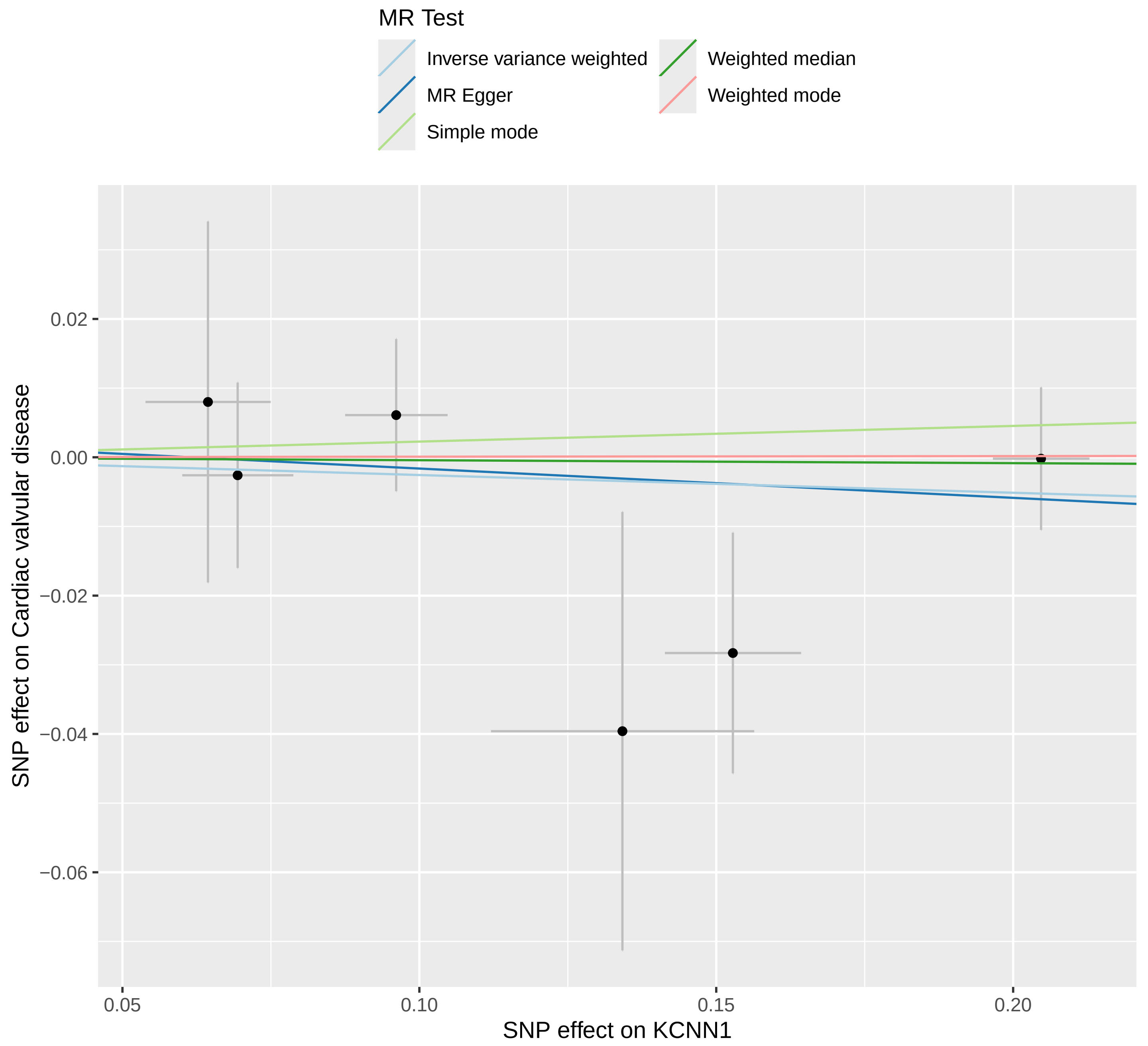
MR Test
Inverse variance weighted
MR Egger
Simple mode
Weighted median
Weighted mode
0.02
0.00
−0.02
−0.04
−0.06
SNP effect on Cardiac valvular disease
0.05
0.10
0.15
0.20
SNP effect on KCNN1

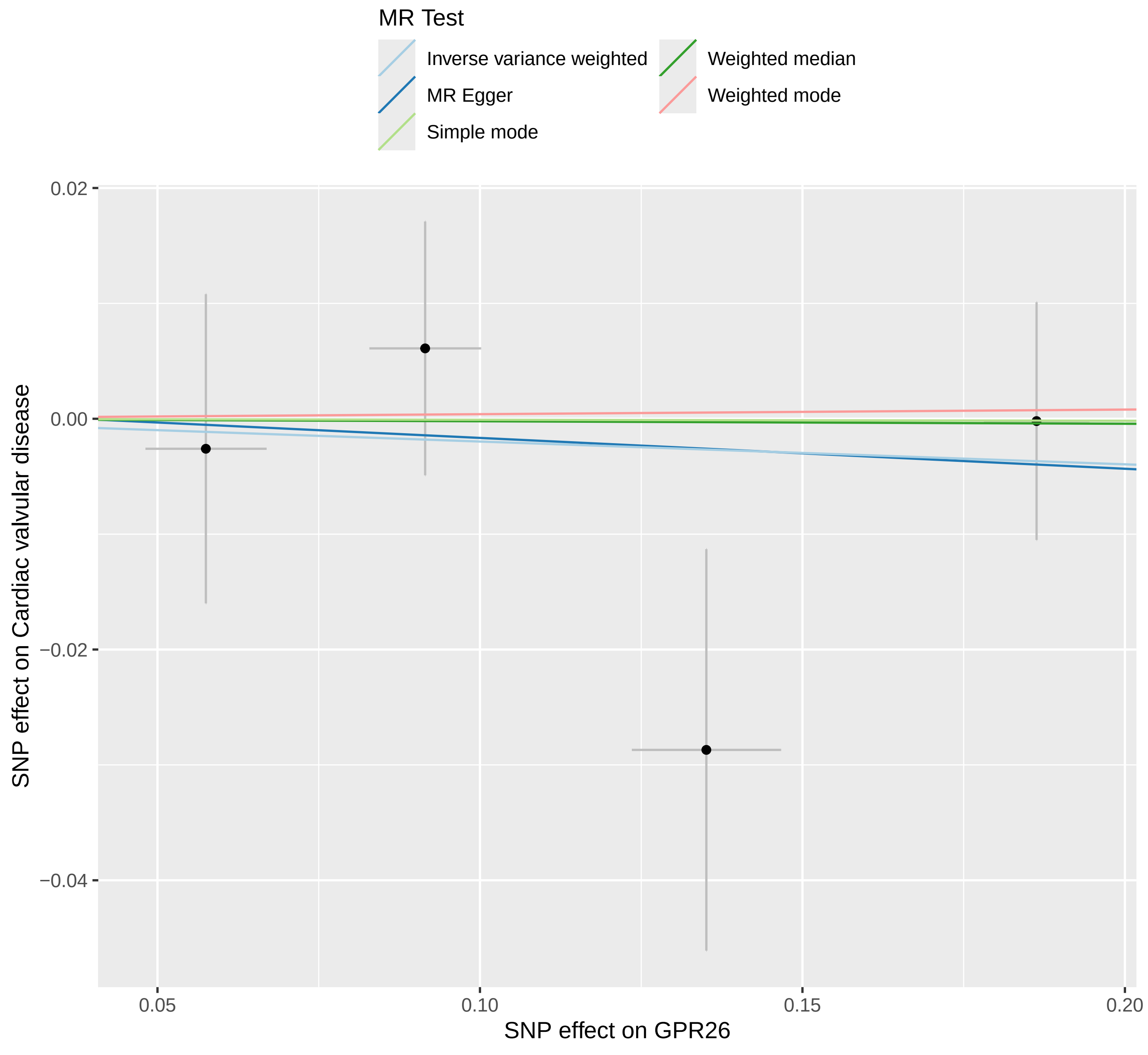
MR Test
Inverse variance weighted
MR Egger
Simple mode
Weighted median
Weighted mode
0.02
0.00
−0.02
−0.04
SNP effect on Cardiac valvular disease
0.05
0.10
0.15
0.20
SNP effect on GPR26

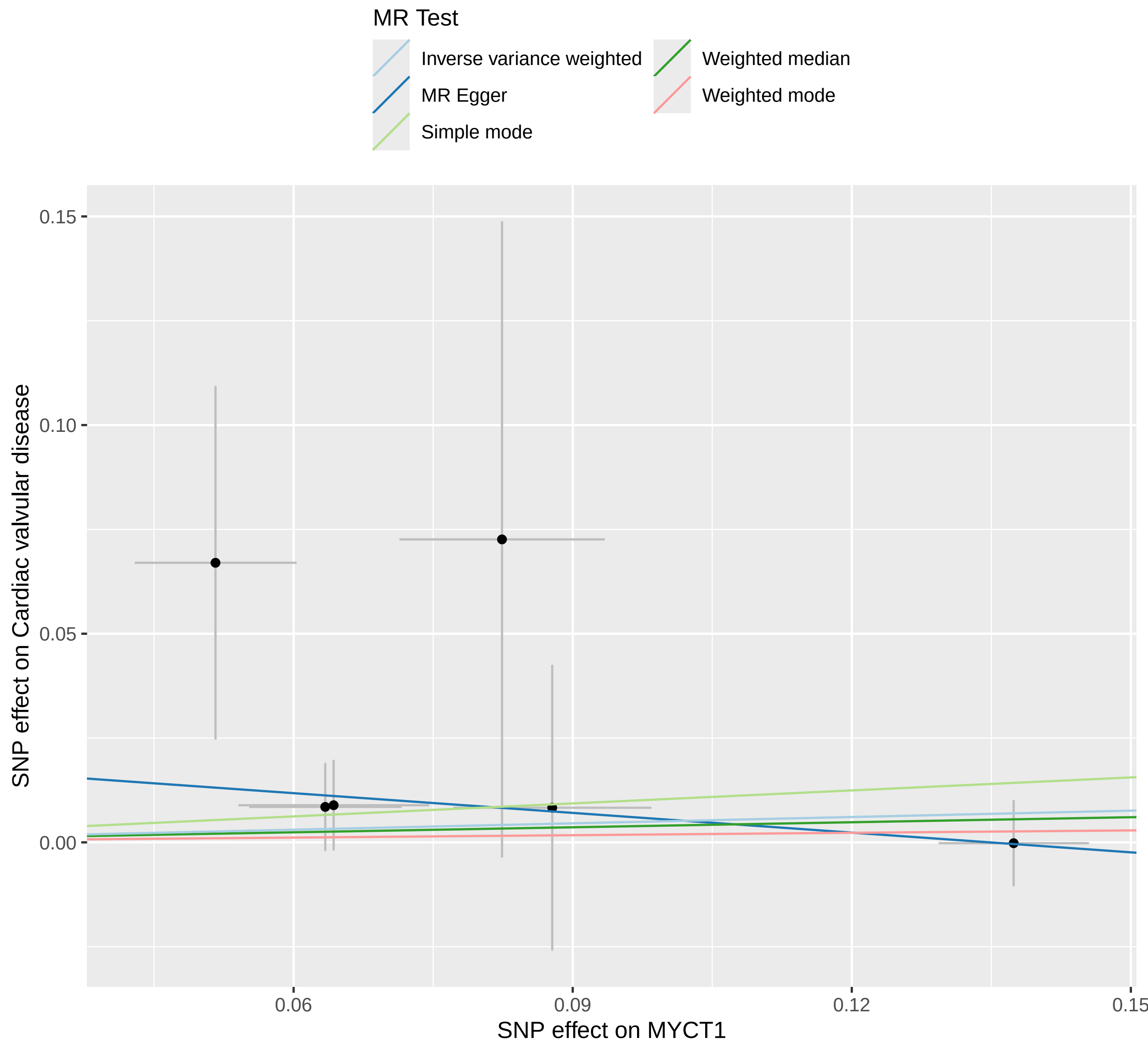

MR Test
Inverse variance weighted
MR Egger
Simple mode
Weighted median
Weighted mode
0.15
0.10
0.05
0.00
SNP effect on Cardiac valvular disease
0.06
0.09
0.12
0.15
SNP effect on MYCT1

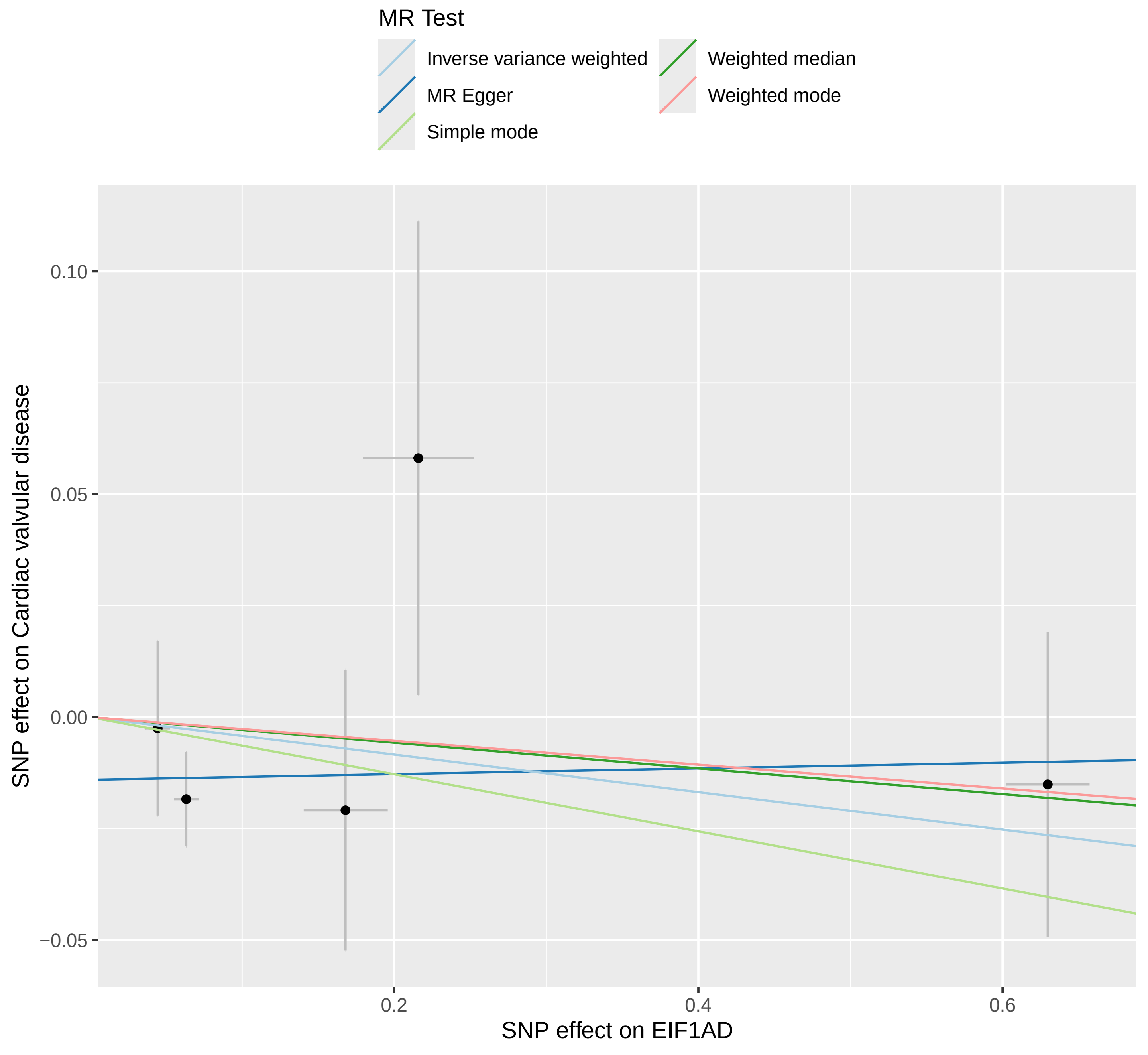
MR Test
Inverse variance weighted
MR Egger
Simple mode
Weighted median
Weighted mode
0.10
0.05
0.00
−0.05
SNP effect on Cardiac valvular disease
0.2
0.4
0.6
SNP effect on EIF1AD

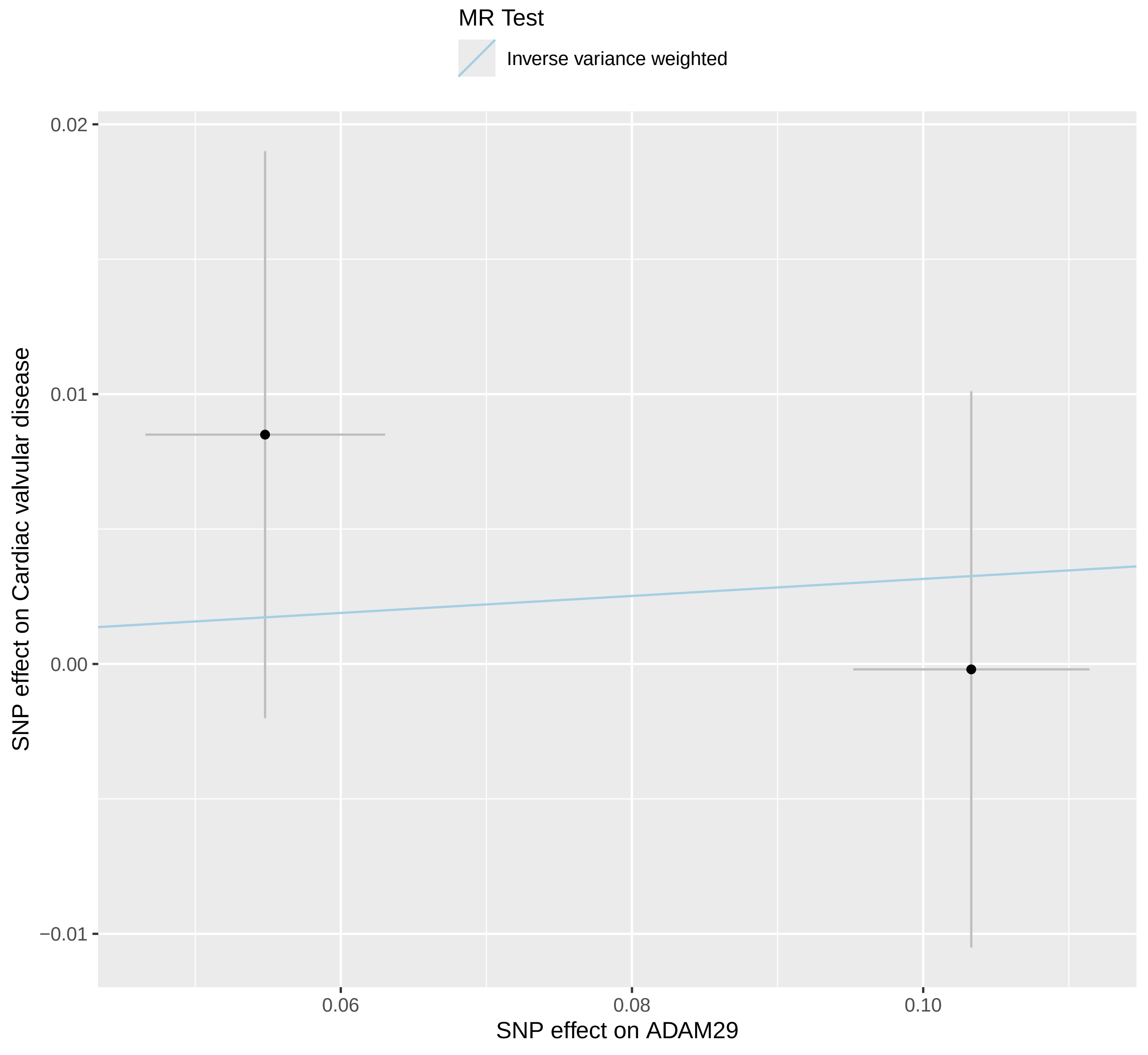

MR Test
Inverse variance weighted
0.02
0.01
0.00
−0.01
SNP effect on Cardiac valvular disease
0.06
0.08
0.10
SNP effect on ADAM29

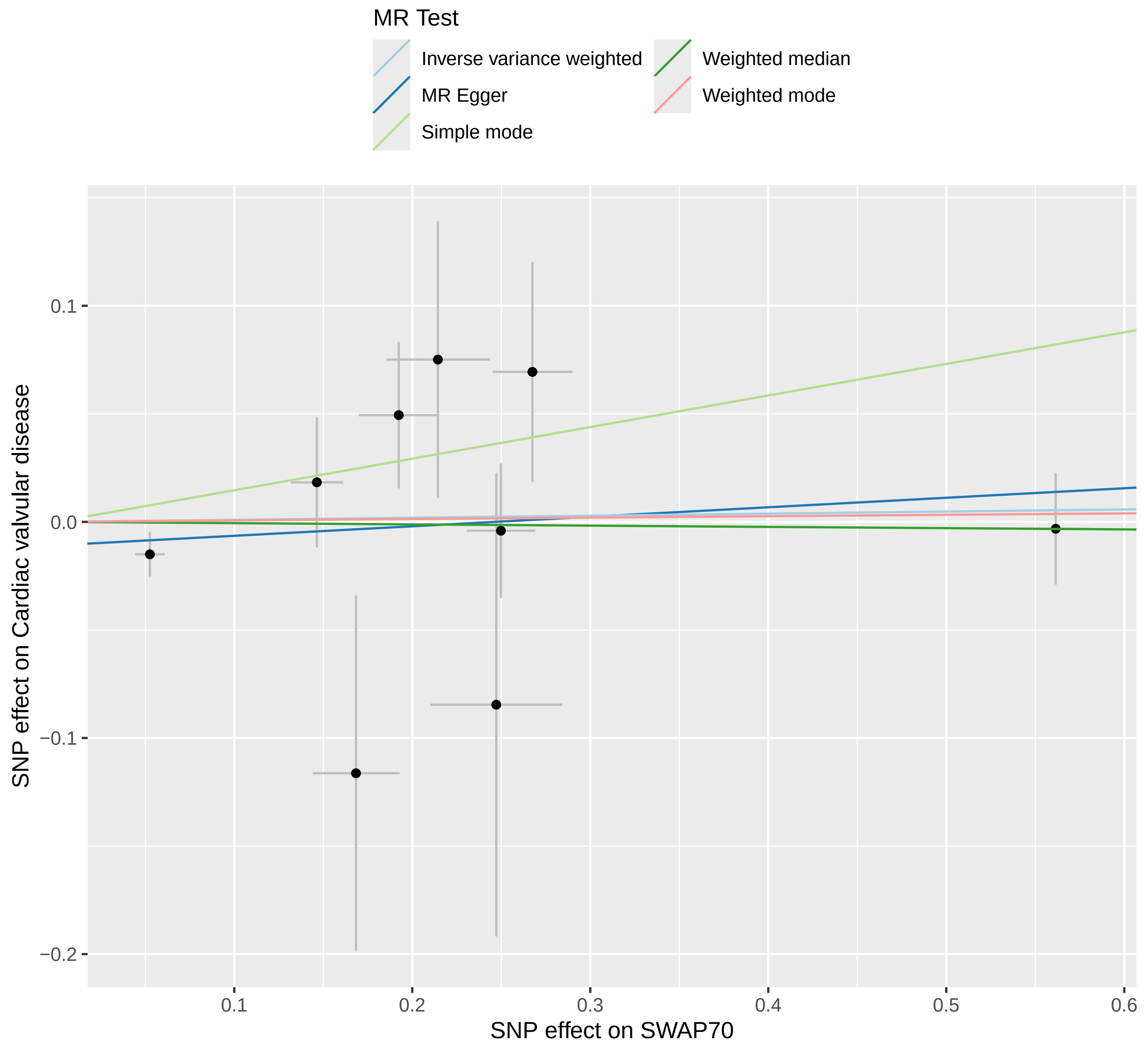
MR Test
Inverse variance weighted
MR Egger
Simple mode
Weighted median
Weighted mode
0.1
0.0
−0.1
−0.2
SNP effect on Cardiac valvular disease
0.1
0.2
0.3
0.4
0.5
0.6
SNP effect on SWAP70

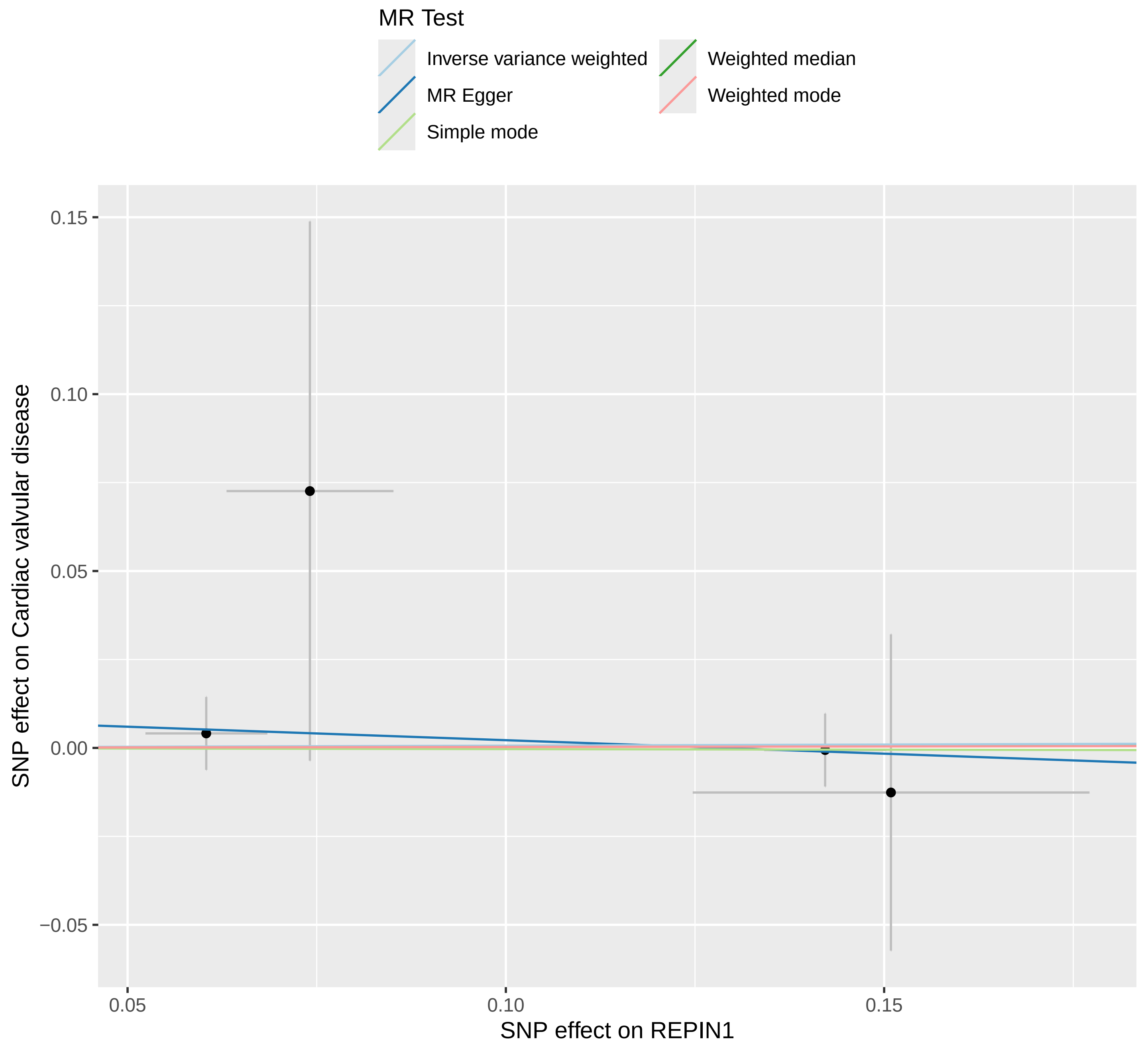
MR Test
Inverse variance weighted
MR Egger
Simple mode
Weighted median
Weighted mode
0.15
0.10
0.05
0.00
−0.05
SNP effect on Cardiac valvular disease
0.05
0.10
0.15
SNP effect on REPIN1

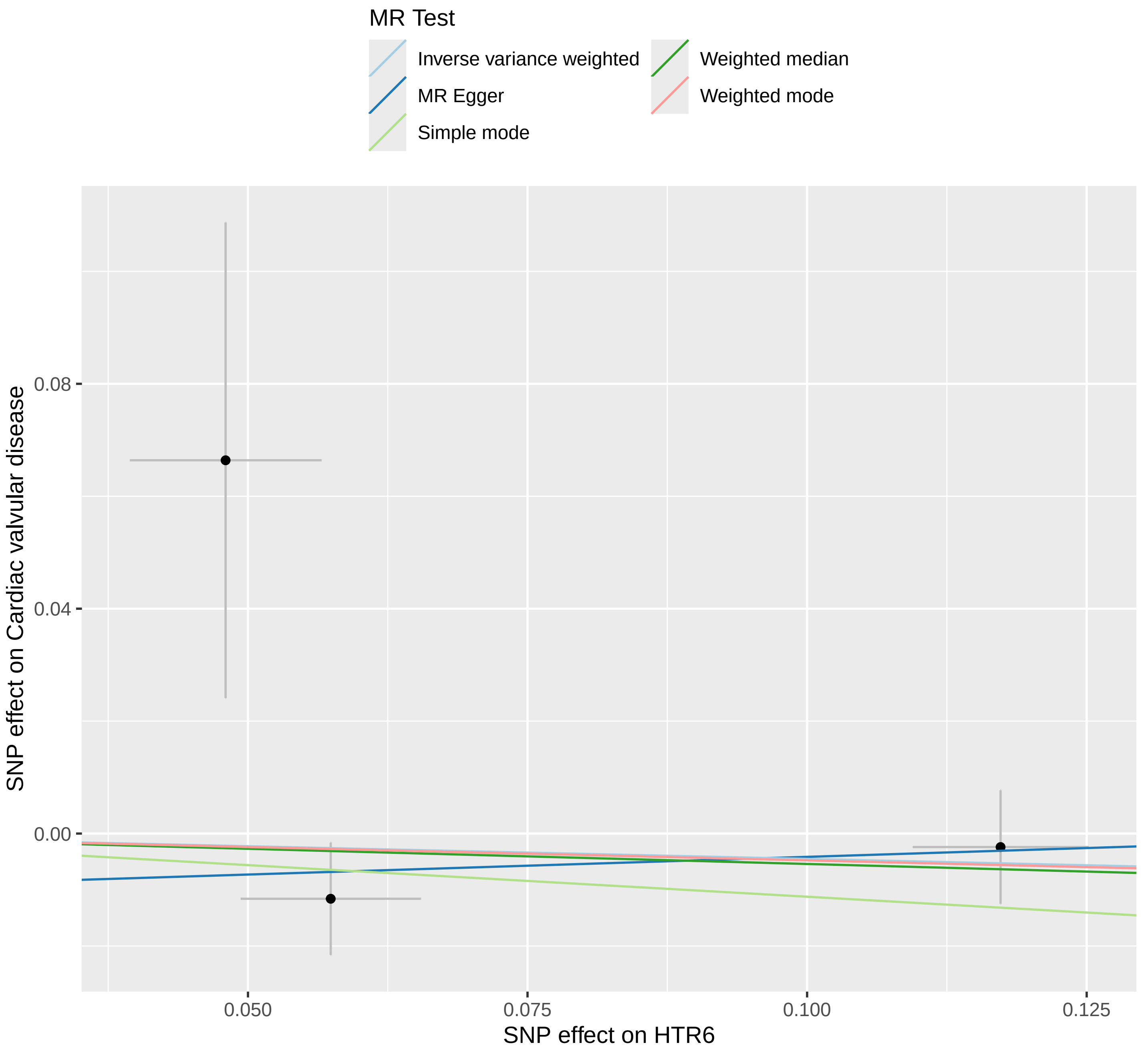

MR Test
Inverse variance weighted
MR Egger
Simple mode
Weighted median
Weighted mode
0.08
0.04
0.00
SNP effect on Cardiac valvular disease
0.050
0.075
0.100
0.125
SNP effect on HTR6

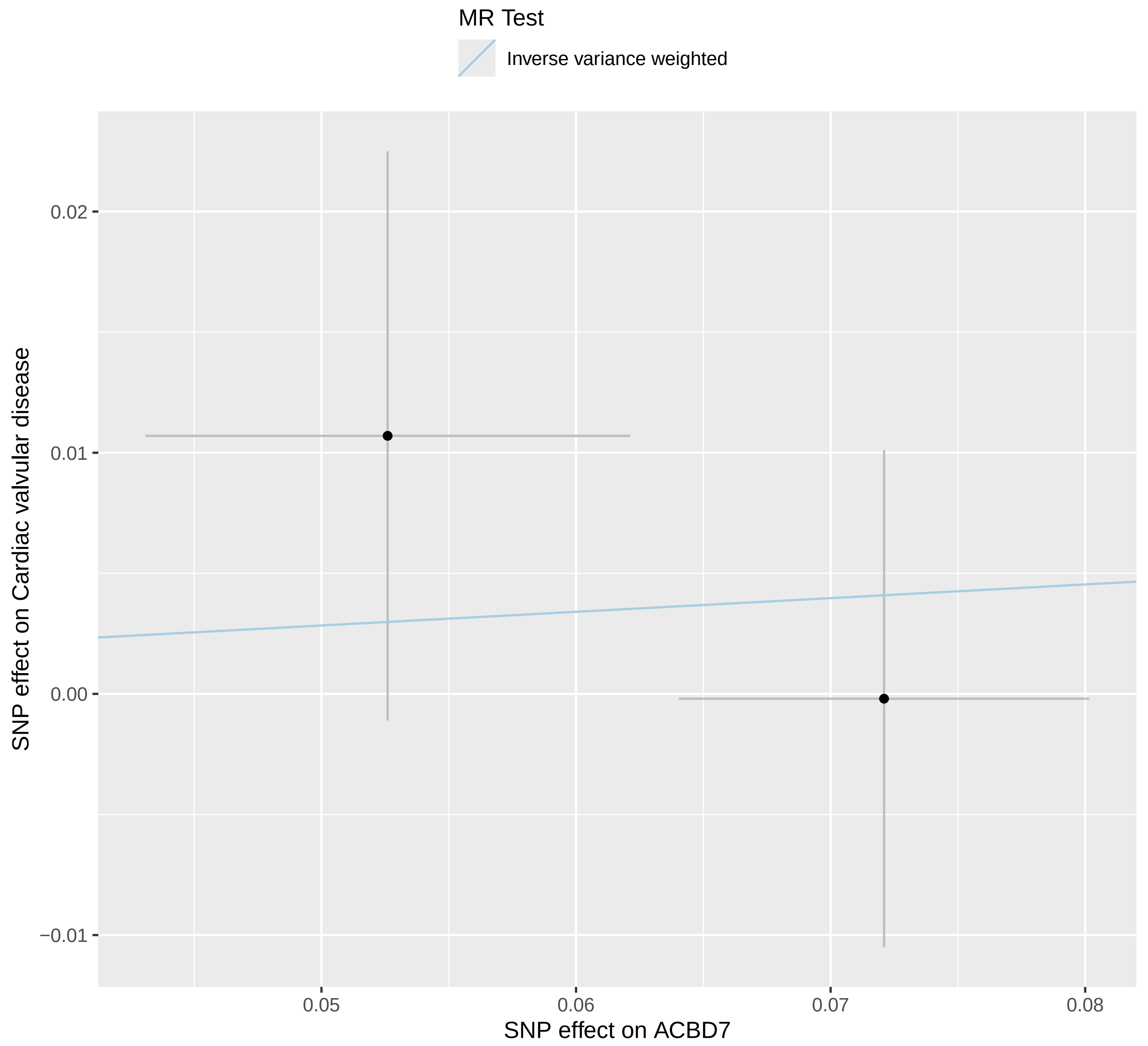

MR Test
Inverse variance weighted
0.02
0.01
0.00
−0.01
SNP effect on Cardiac valvular disease
0.05
0.06
0.07
0.08
SNP effect on ACBD7

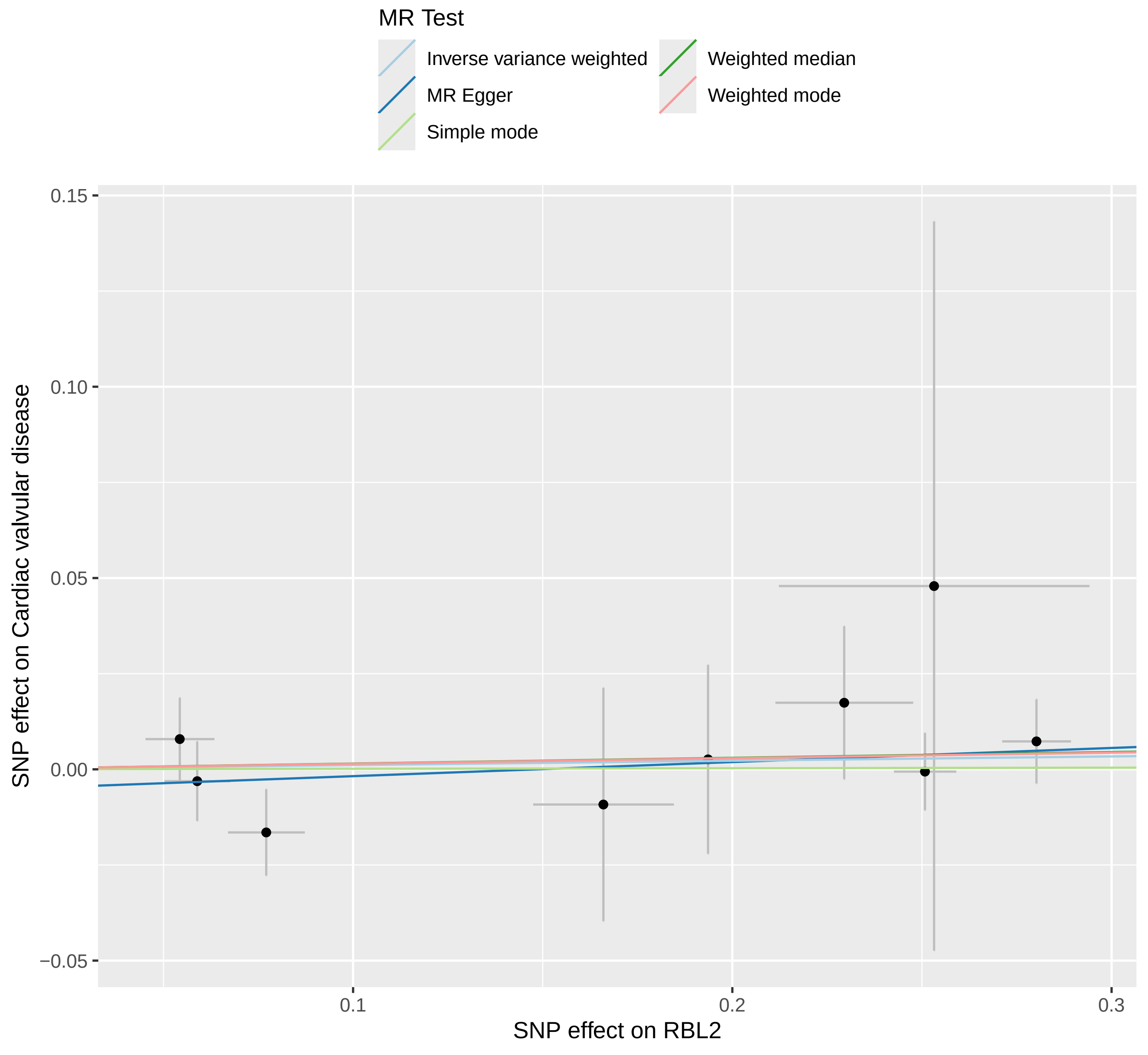

MR Test
Inverse variance weighted
MR Egger
Simple mode
Weighted median
Weighted mode
0.15
0.10
0.05
0.00
−0.05
SNP effect on Cardiac valvular disease
0.1
0.2
0.3
SNP effect on RBL2

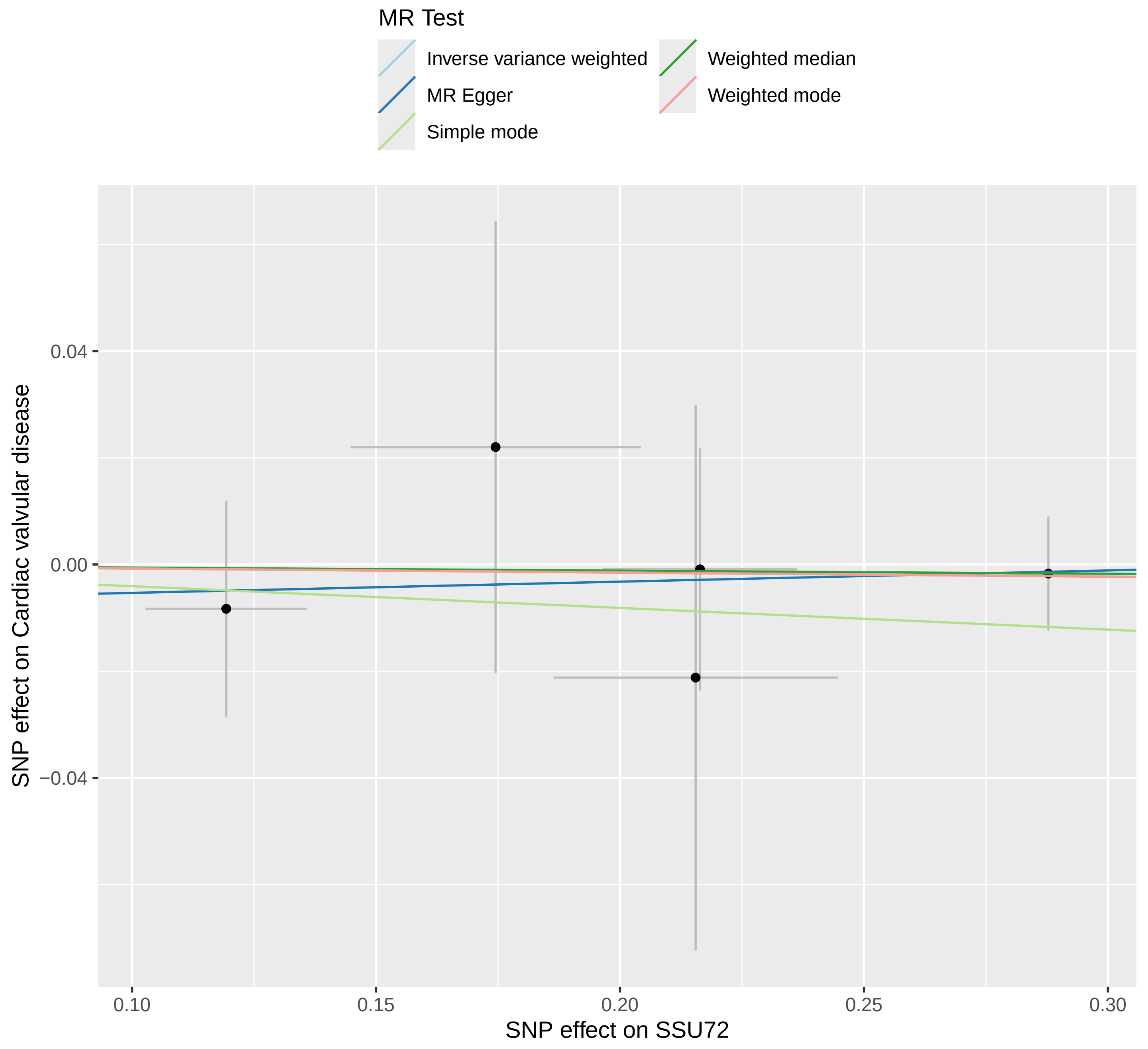

MR Test
Inverse variance weighted
MR Egger
Simple mode
Weighted median
Weighted mode
0.04
0.00
−0.04
0.10
0.15
0.20
0.25
0.30
SNP effect on Cardiac valvular disease
SNP effect on SSU72

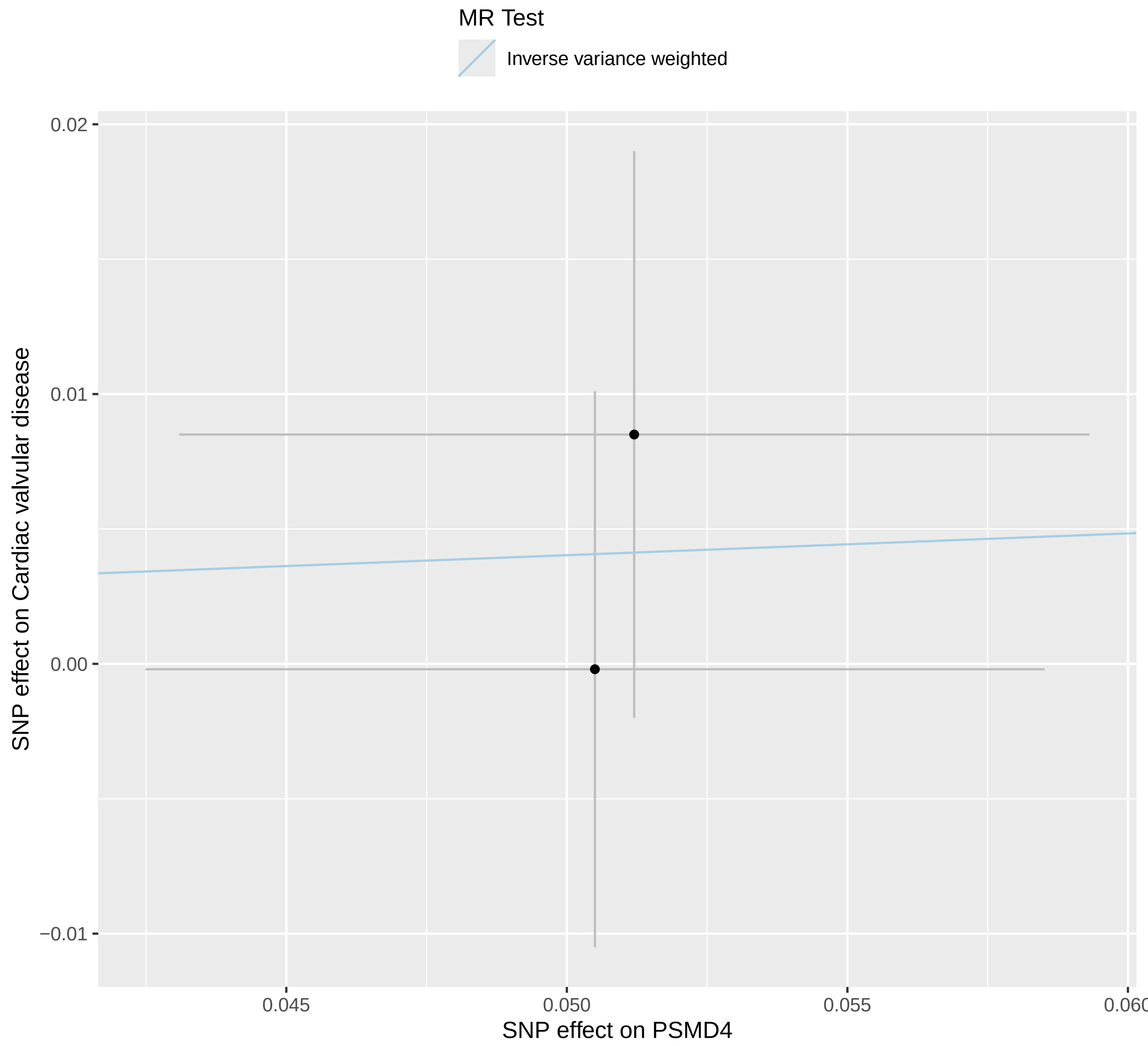
MR Test
Inverse variance weighted
0.02
0.01
0.00
−0.01
0.045
0.050
0.055
0.060
SNP effect on PSMD4
SNP effect on Cardiac valvular disease

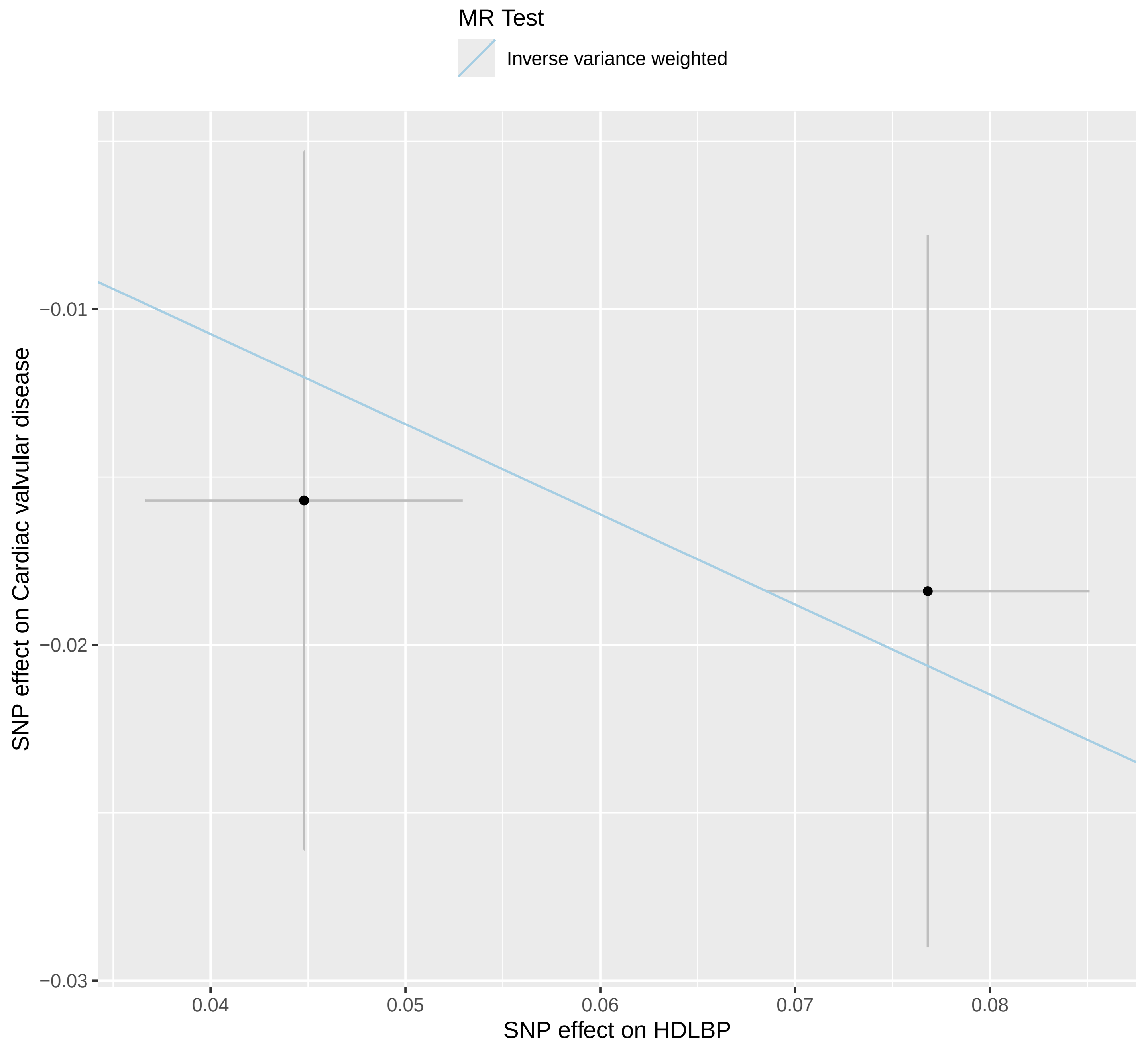
MR Test
Inverse variance weighted
SNP effect on Cardiac valvular disease
−0.01
−0.02
−0.03
0.04
0.05
0.06
0.07
0.08
SNP effect on HDLBP

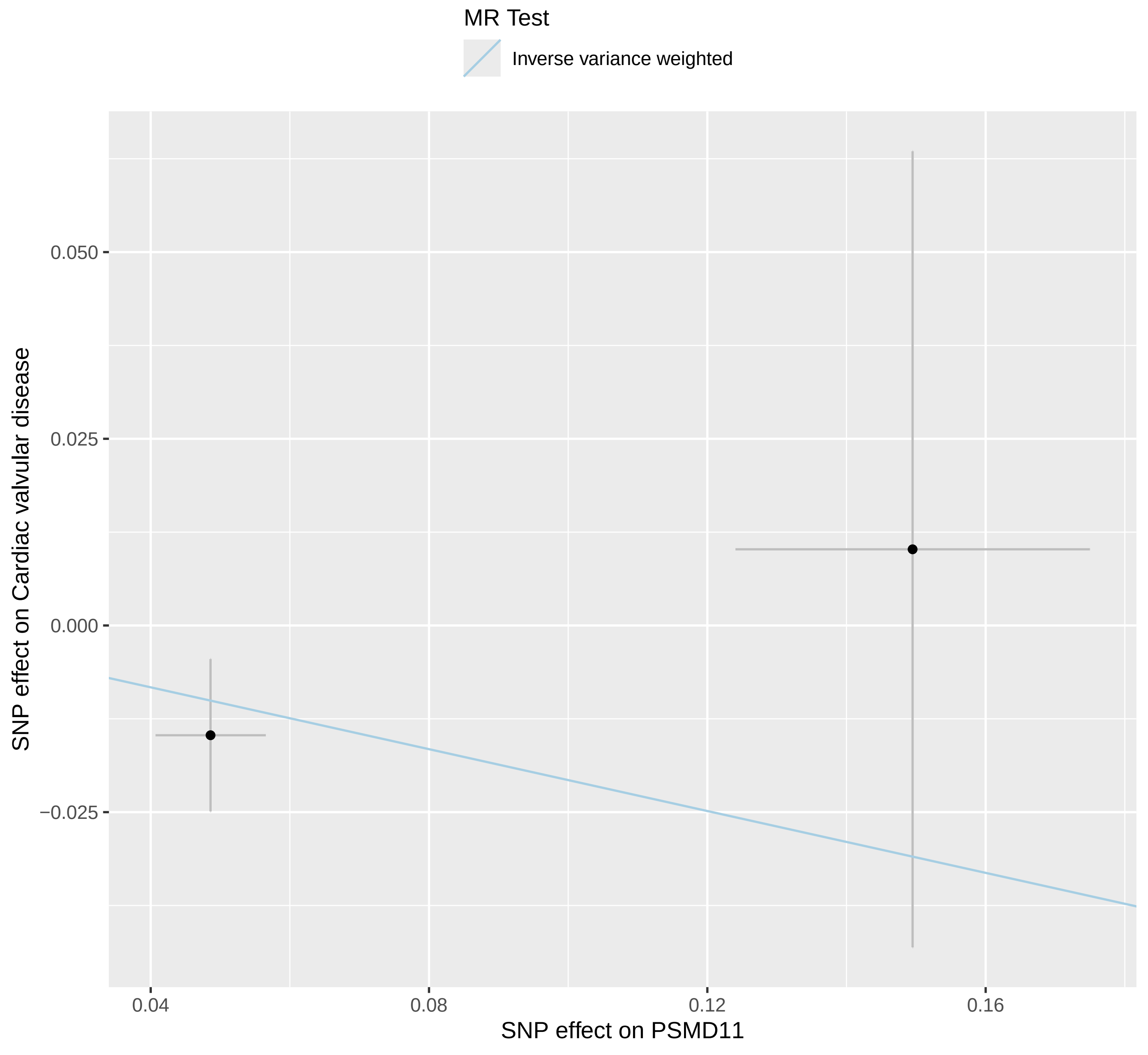

MR Test
Inverse variance weighted
0.050
0.025
0.000
−0.025
0.04
0.08
0.12
0.16
SNP effect on PSMD11
SNP effect on Cardiac valvular disease

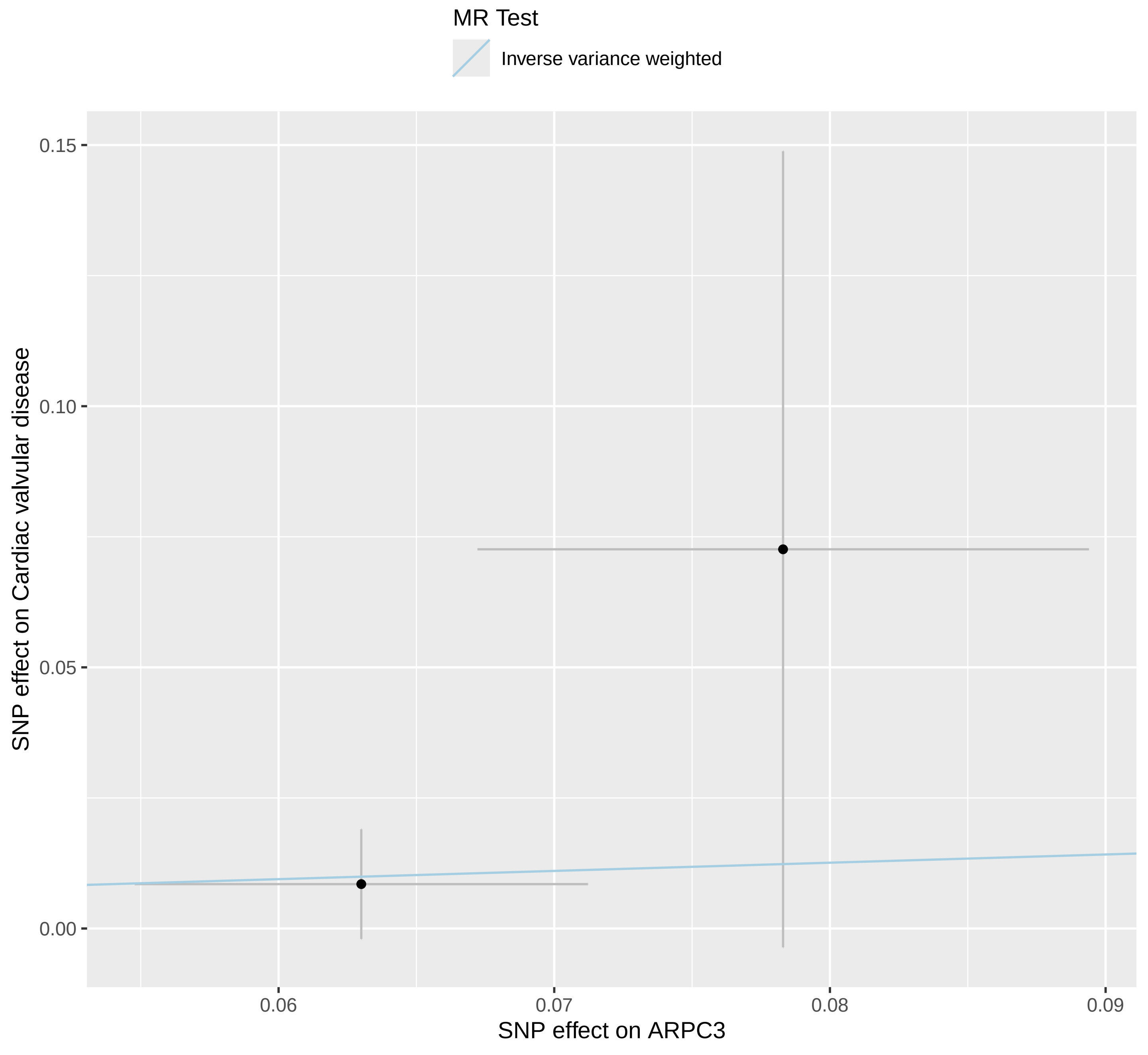
MR Test
Inverse variance weighted
0.15
0.10
0.05
0.00
SNP effect on Cardiac valvular disease
0.06
0.07
0.08
0.09
SNP effect on ARPC3

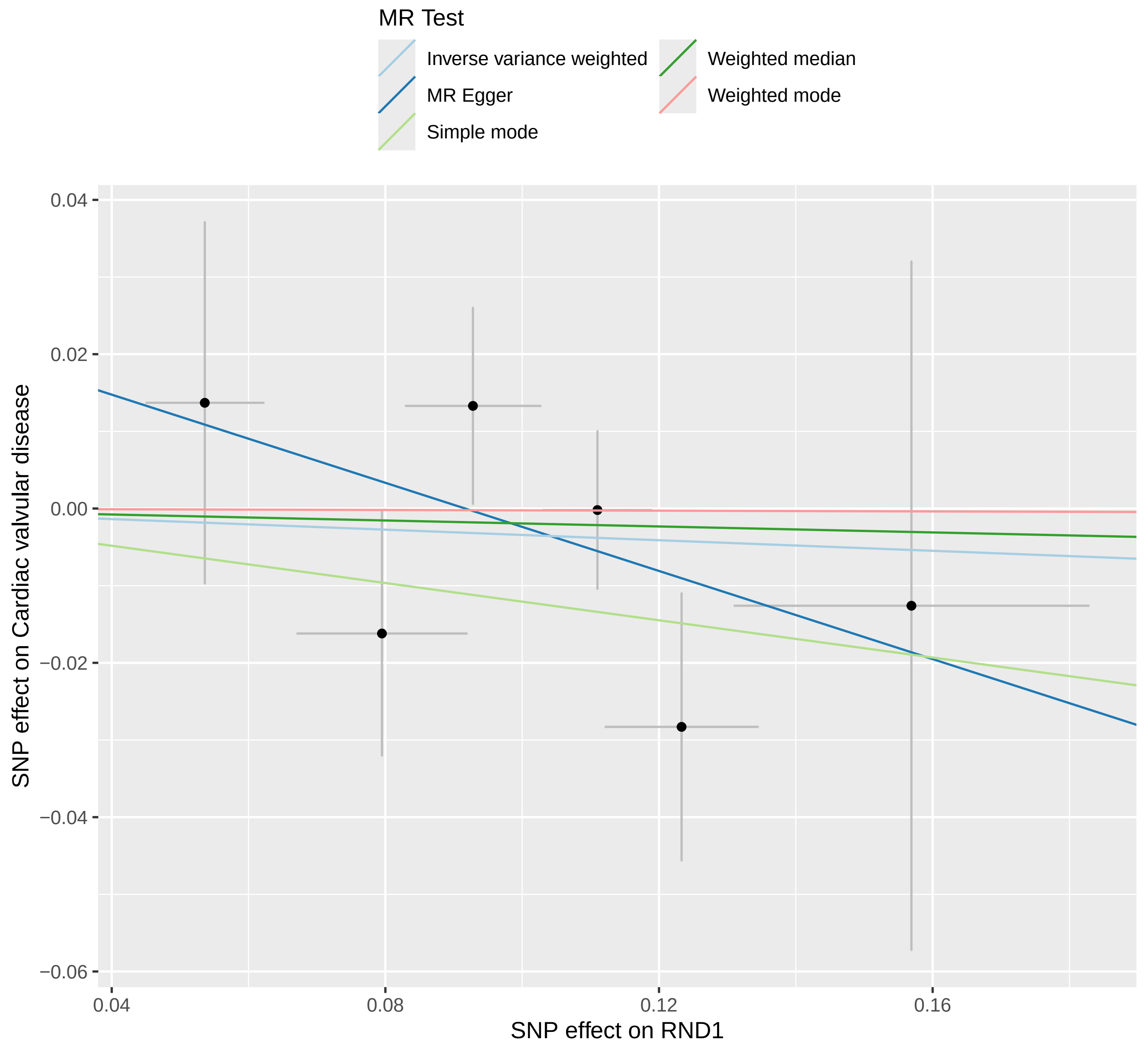
MR Test
Inverse variance weighted
MR Egger
Simple mode
Weighted median
Weighted mode
0.04
0.02
0.00
−0.02
−0.04
−0.06
SNP effect on Cardiac valvular disease
0.04
0.08
0.12
0.16
SNP effect on RND1

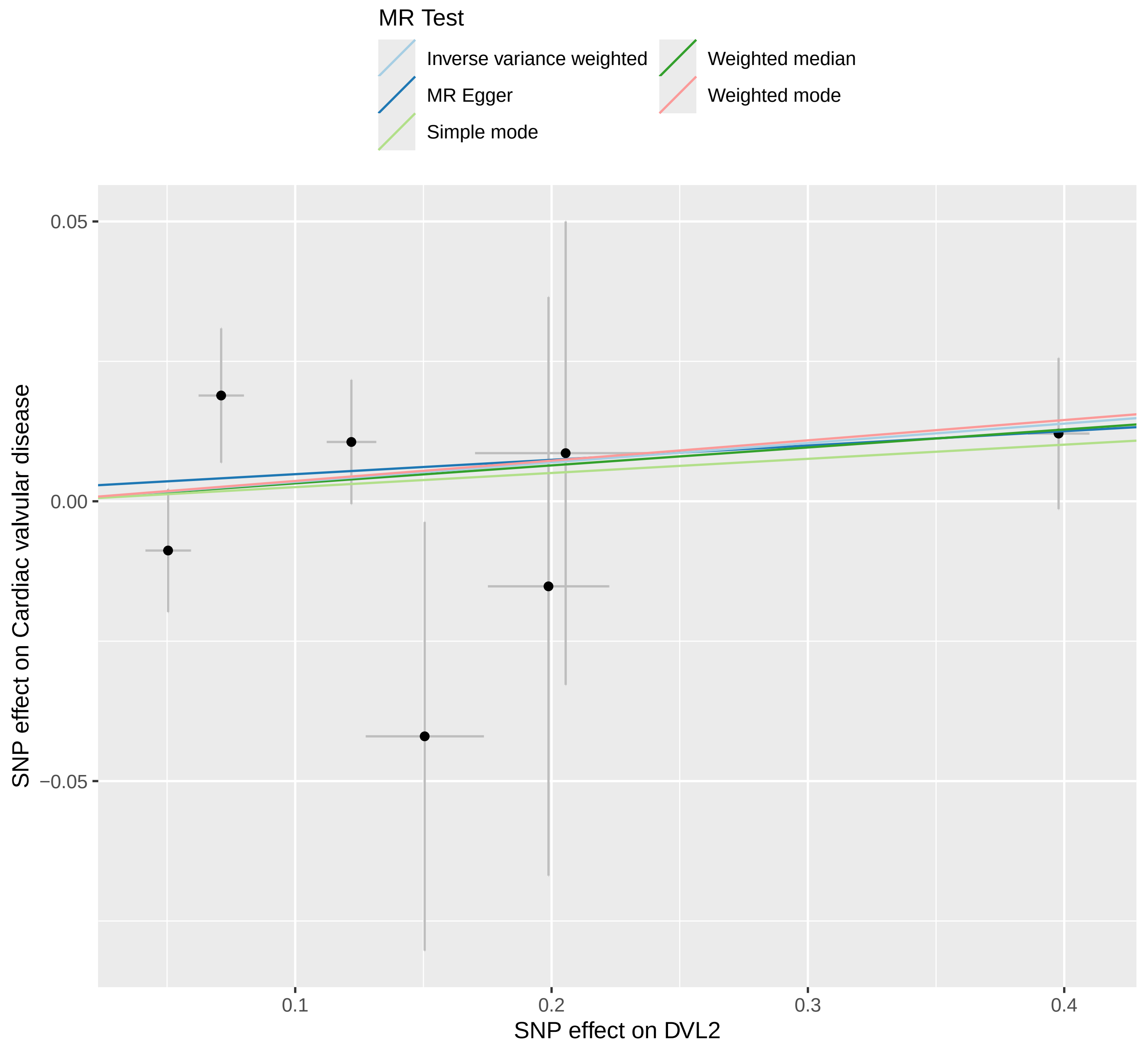
MR Test
Inverse variance weighted
MR Egger
Simple mode
Weighted median
Weighted mode
0.05
0.00
−0.05
SNP effect on Cardiac valvular disease
0.1
0.2
0.3
0.4
SNP effect on DVL2

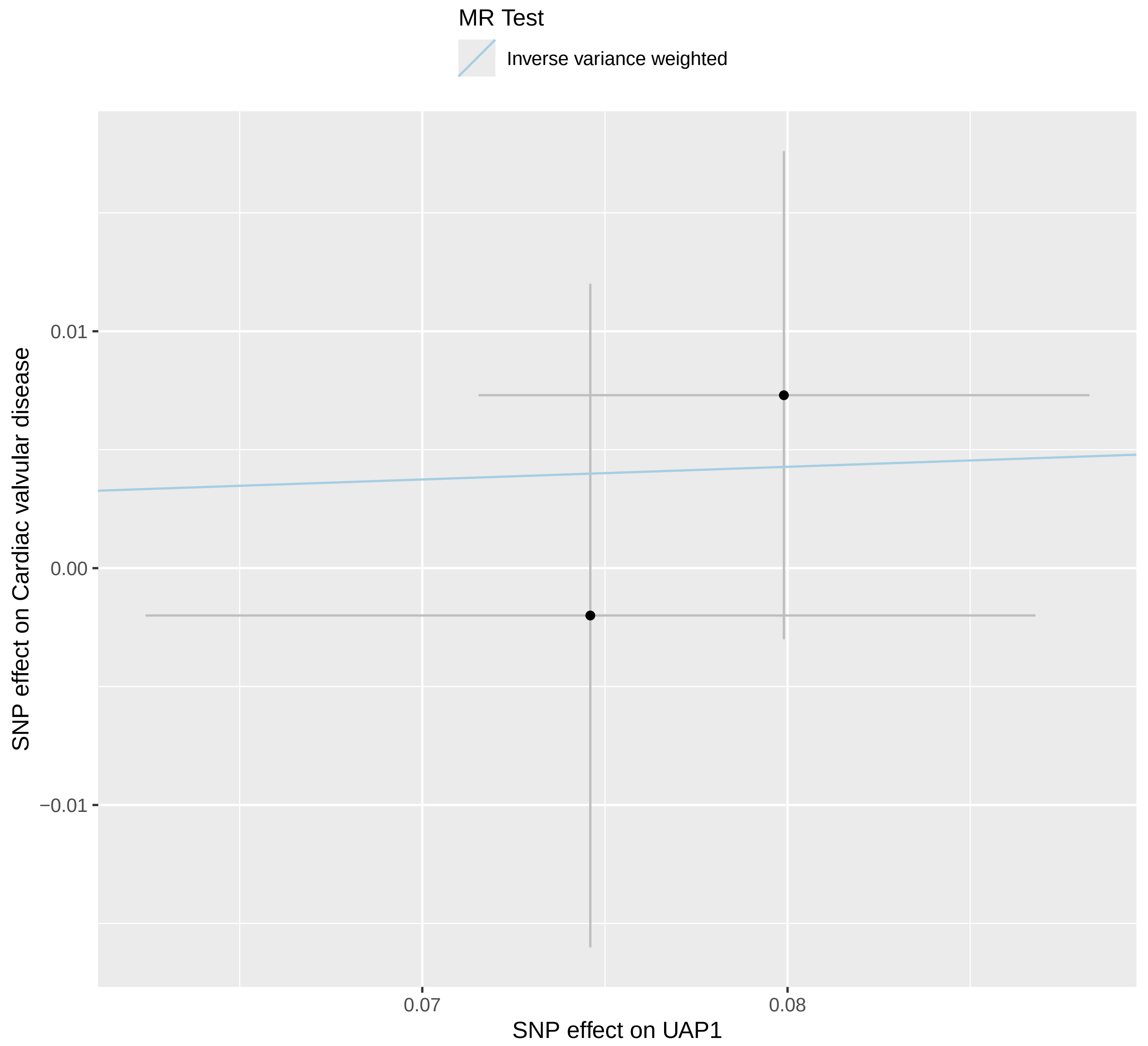

MR Test
Inverse variance weighted
0.01
0.00
−0.01
SNP effect on Cardiac valvular disease
0.07
0.08
SNP effect on UAP1

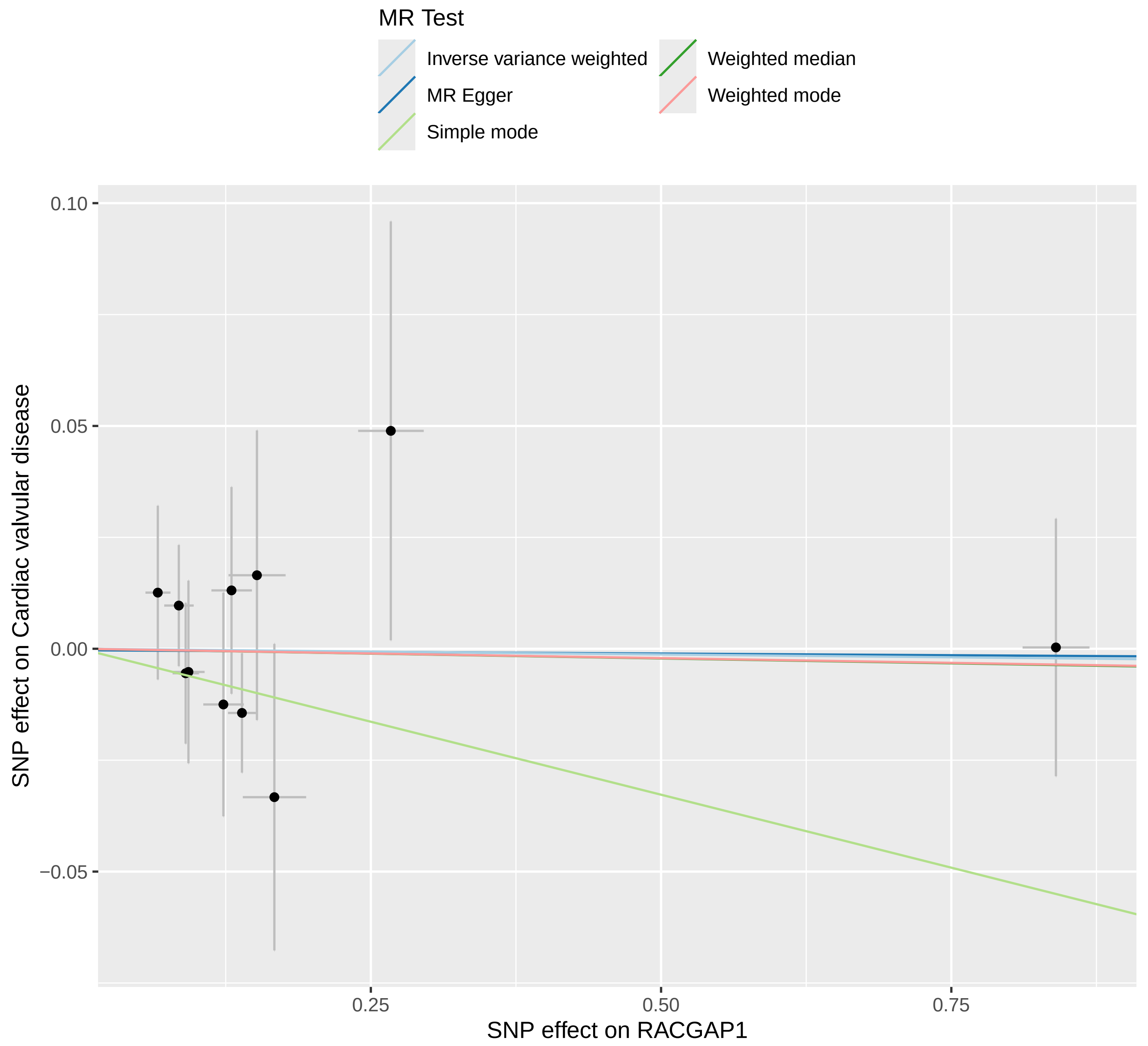

MR Test
Inverse variance weighted
MR Egger
Simple mode
Weighted median
Weighted mode
0.10
0.05
0.00
−0.05
0.25
0.50
0.75
SNP effect on Cardiac valvular disease
SNP effect on RACGAP1

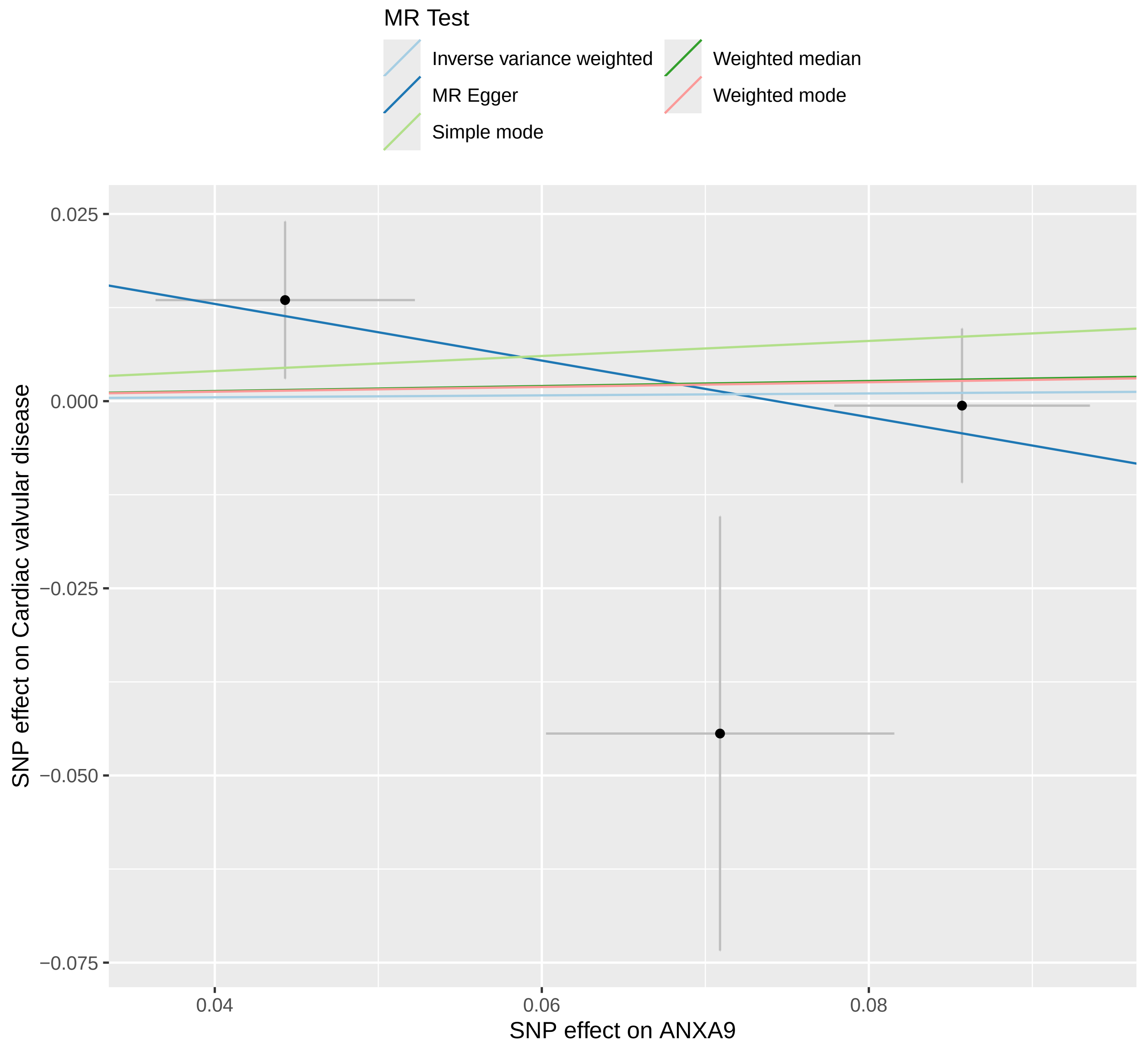
MR Test
Inverse variance weighted
MR Egger
Simple mode
Weighted median
Weighted mode
0.025
0.000
−0.025
−0.050
−0.075
SNP effect on Cardiac valvular disease
0.04
0.06
0.08
SNP effect on ANXA9

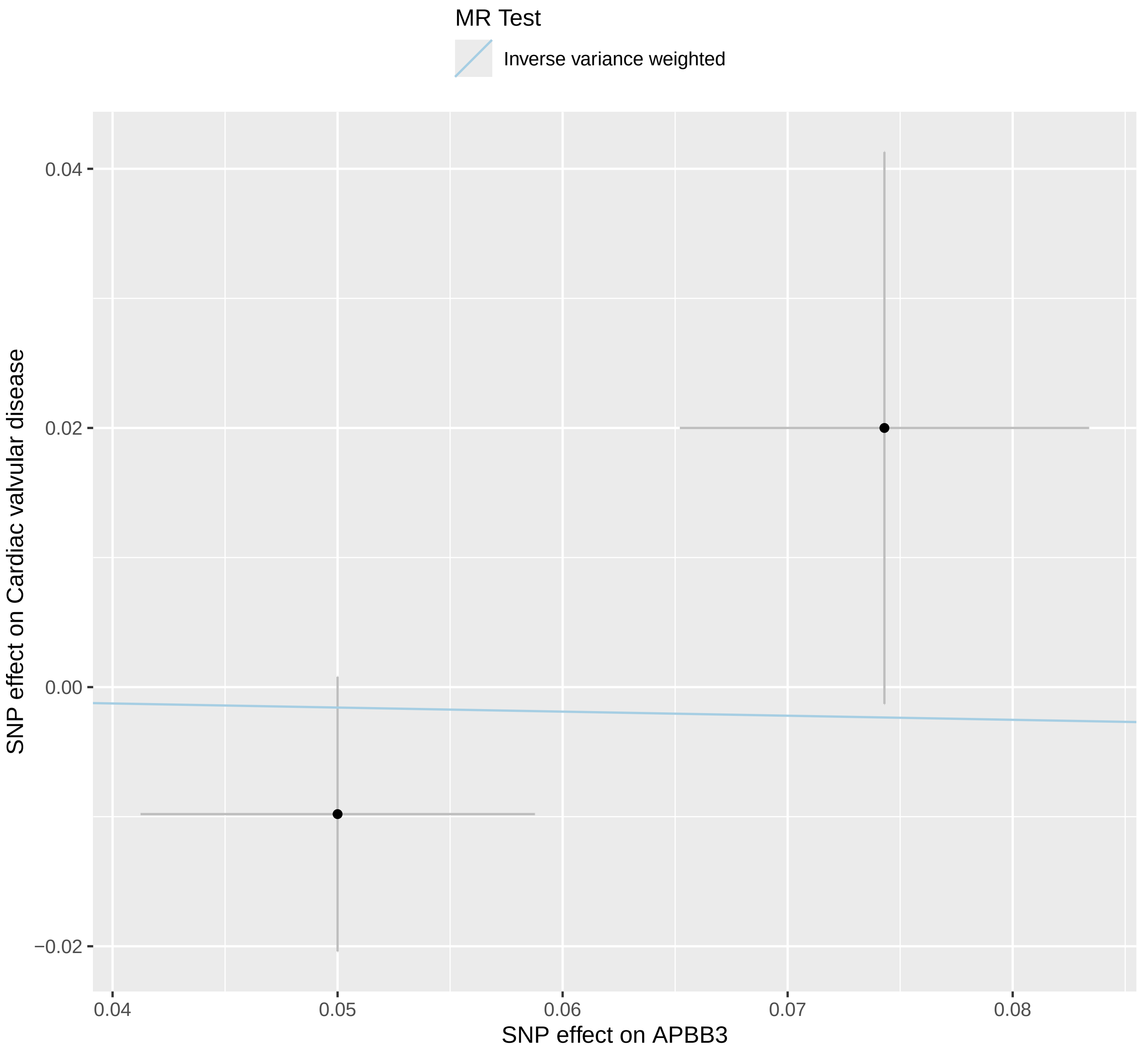
MR Test
Inverse variance weighted
0.04
0.02
0.00
−0.02
SNP effect on Cardiac valvular disease
0.04
0.05
0.06
0.07
0.08
SNP effect on APBB3

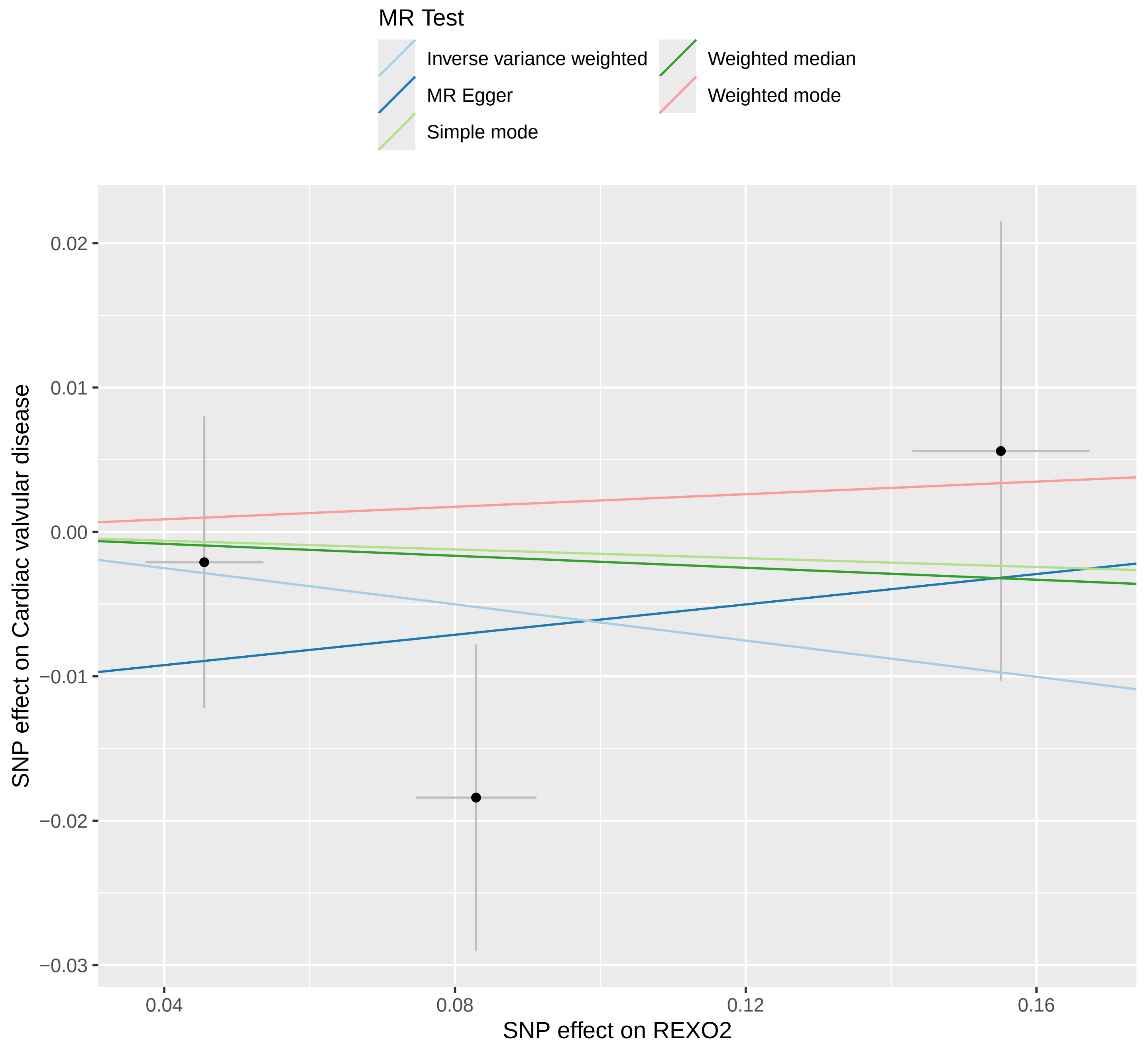
MR Test
Inverse variance weighted
MR Egger
Simple mode
Weighted median
Weighted mode
0.02
0.01
0.00
−0.01
−0.02
−0.03
SNP effect on Cardiac valvular disease
0.04
0.08
0.12
0.16
SNP effect on REXO2

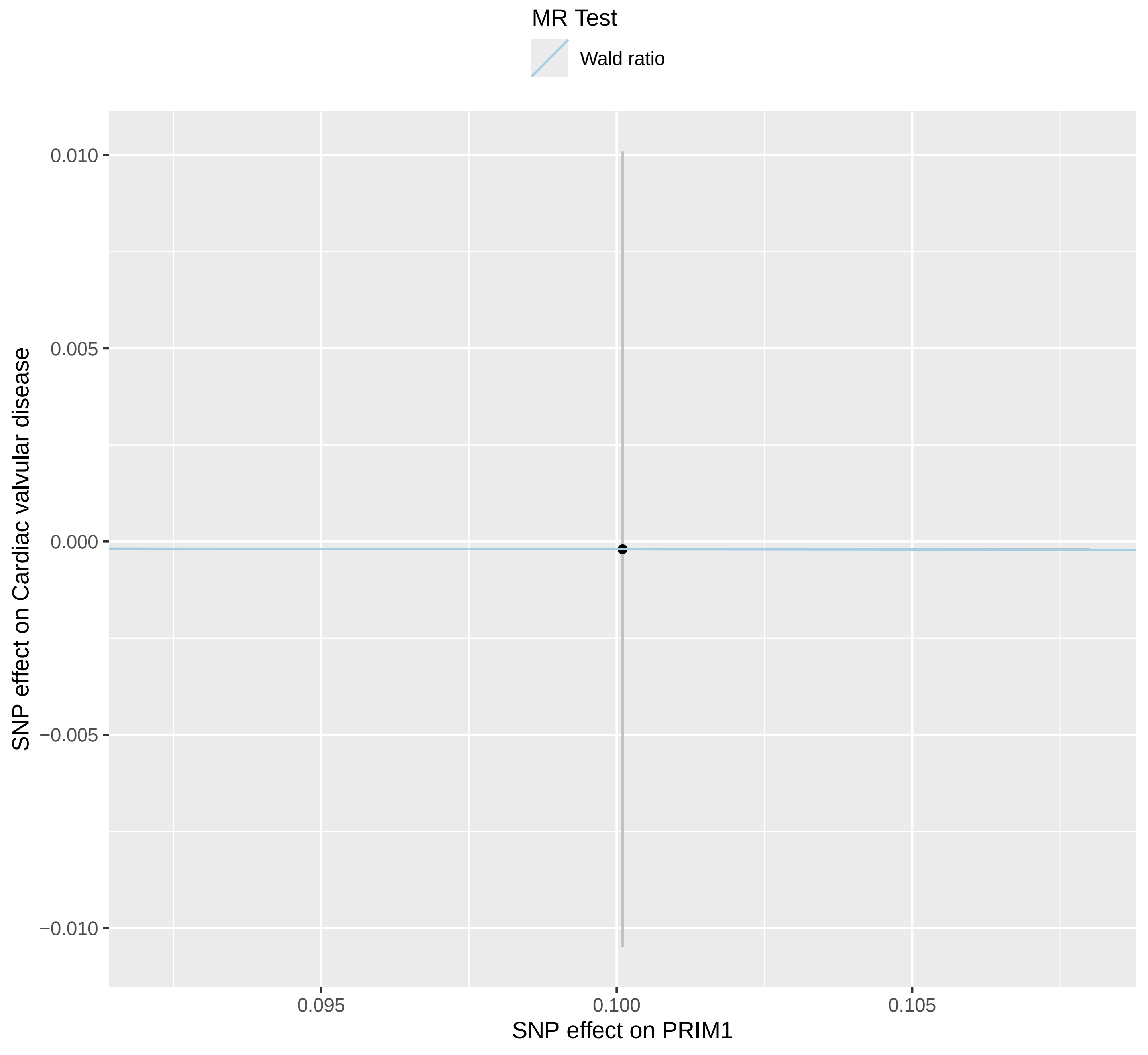

MR Test
Wald ratio
0.010
0.005
0.000
−0.005
−0.010
0.095
0.100
0.105
SNP effect on PRIM1
SNP effect on Cardiac valvular disease

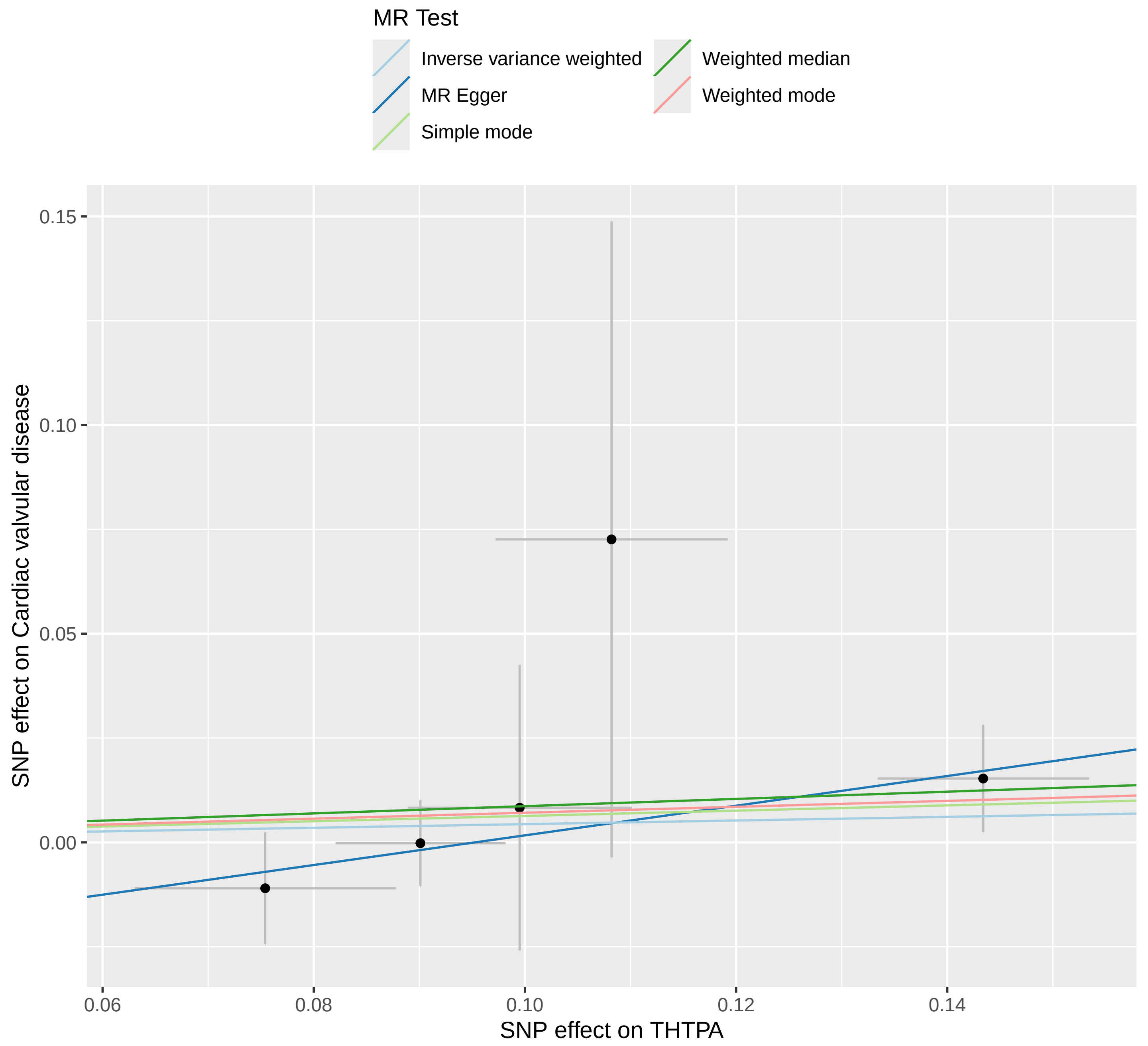

MR Test
Inverse variance weighted
MR Egger
Simple mode
Weighted median
Weighted mode
0.15
0.10
0.05
0.00
SNP effect on Cardiac valvular disease
0.06
0.08
0.10
0.12
0.14
SNP effect on THTPA

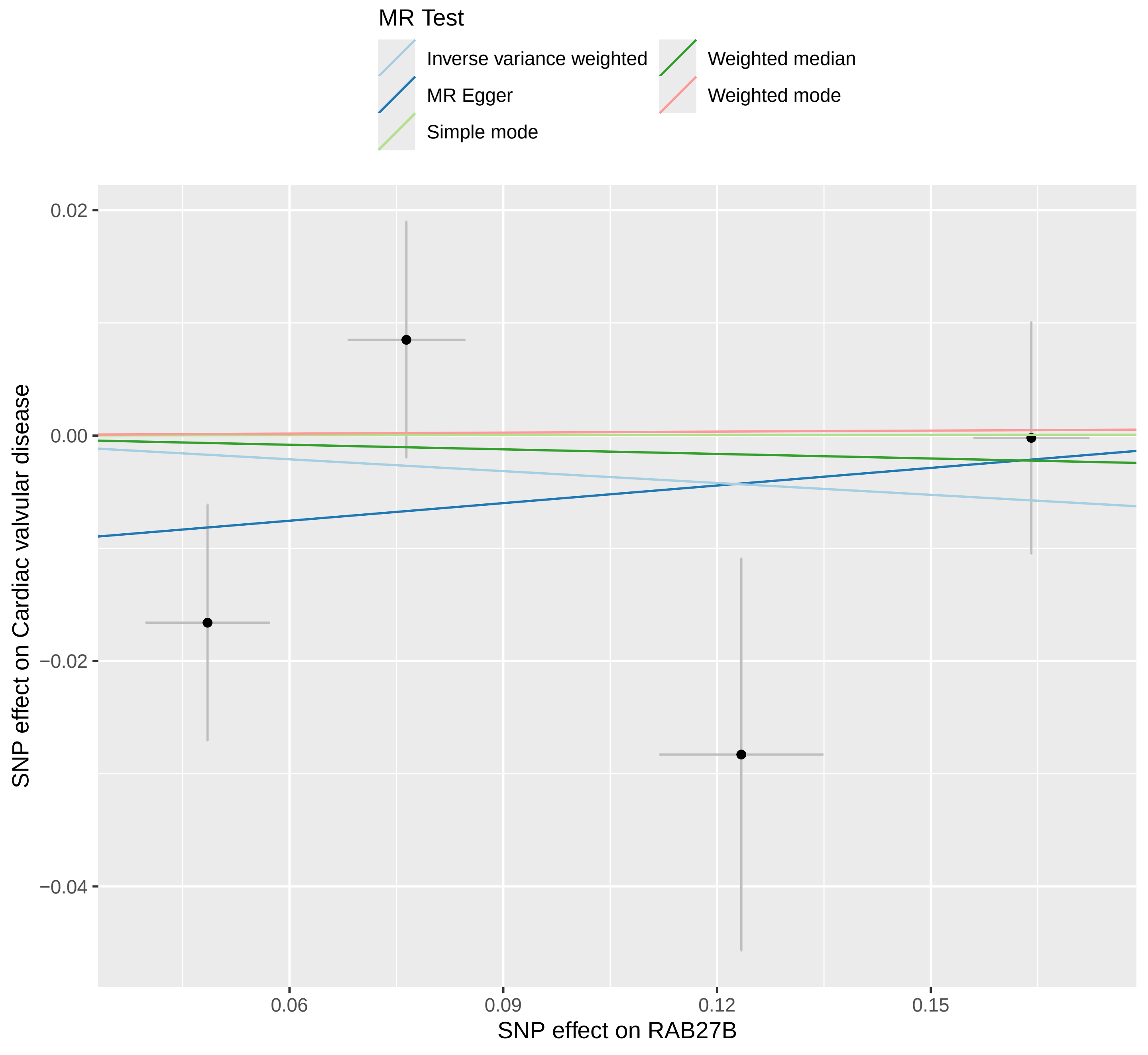
MR Test
Inverse variance weighted
MR Egger
Simple mode
Weighted median
Weighted mode
0.02
0.00
−0.02
−0.04
0.06
0.09
0.12
0.15
SNP effect on Cardiac valvular disease
SNP effect on RAB27B

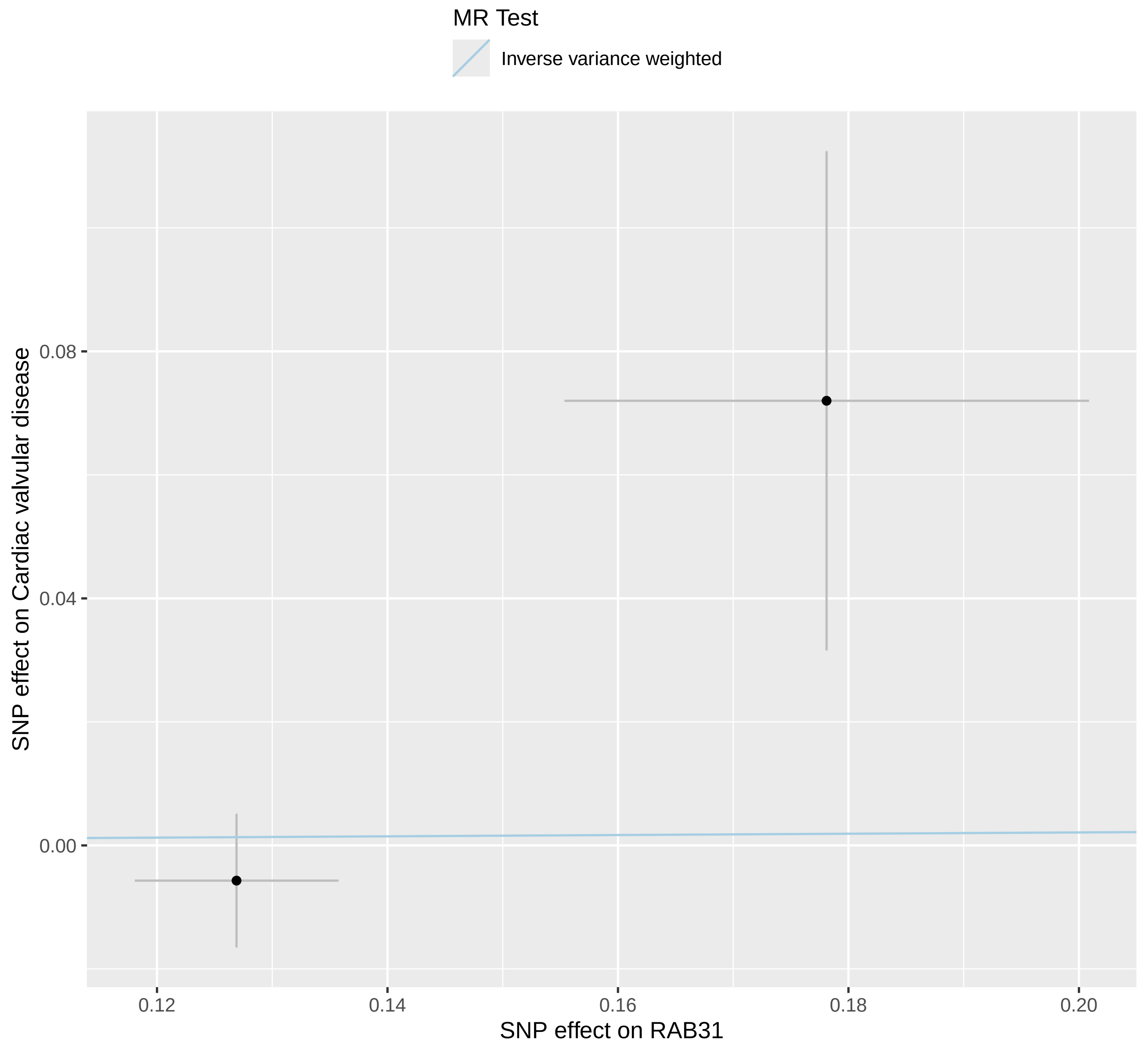

MR Test
Inverse variance weighted
SNP effect on Cardiac valvular disease
0.08
0.04
0.00
0.12
0.14
0.16
0.18
0.20
SNP effect on RAB31

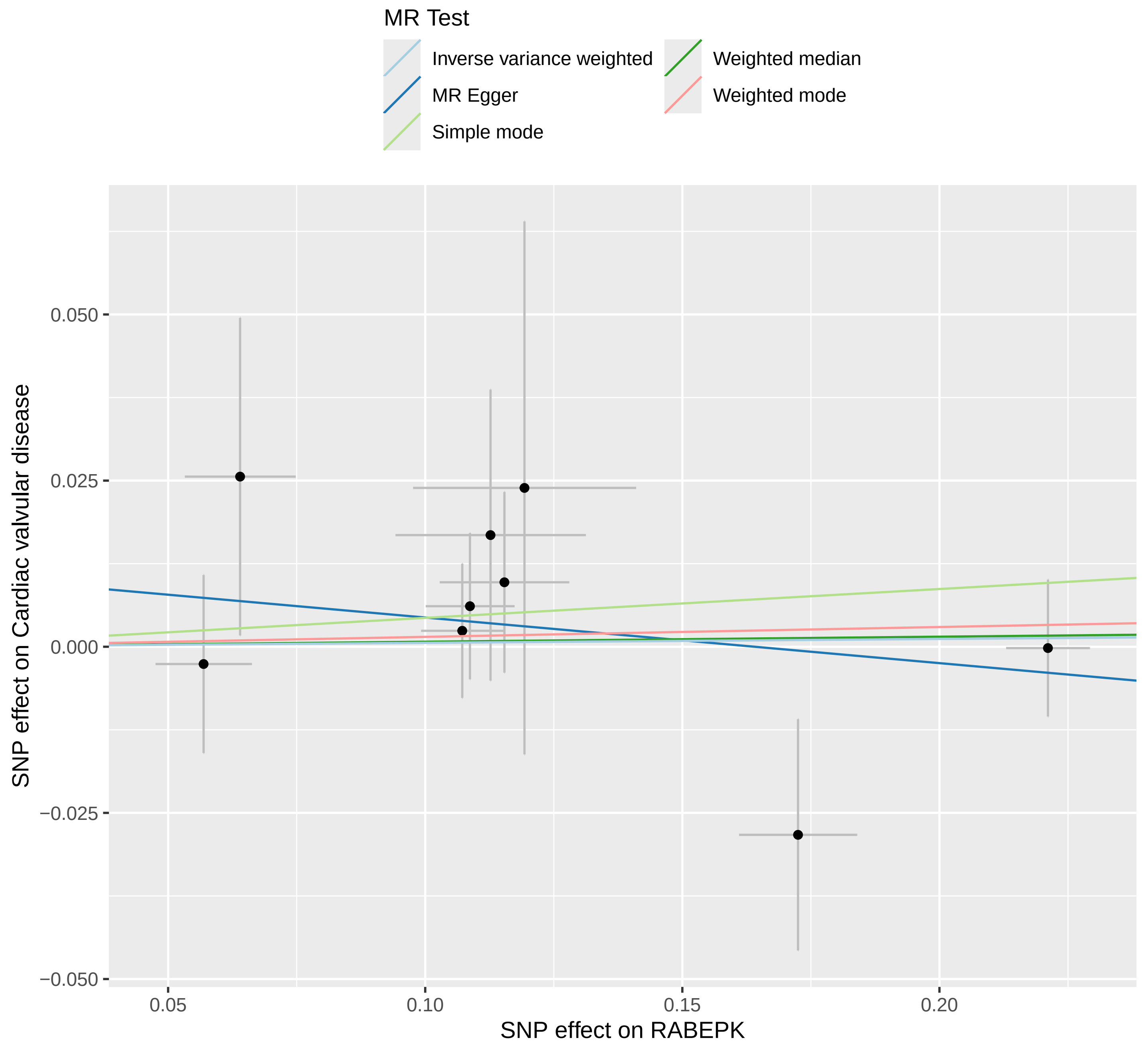

MR Test
Inverse variance weighted
MR Egger
Simple mode
Weighted median
Weighted mode
0.050
0.025
0.000
−0.025
−0.050
SNP effect on Cardiac valvular disease
0.05
0.10
0.15
0.20
SNP effect on RABEPK

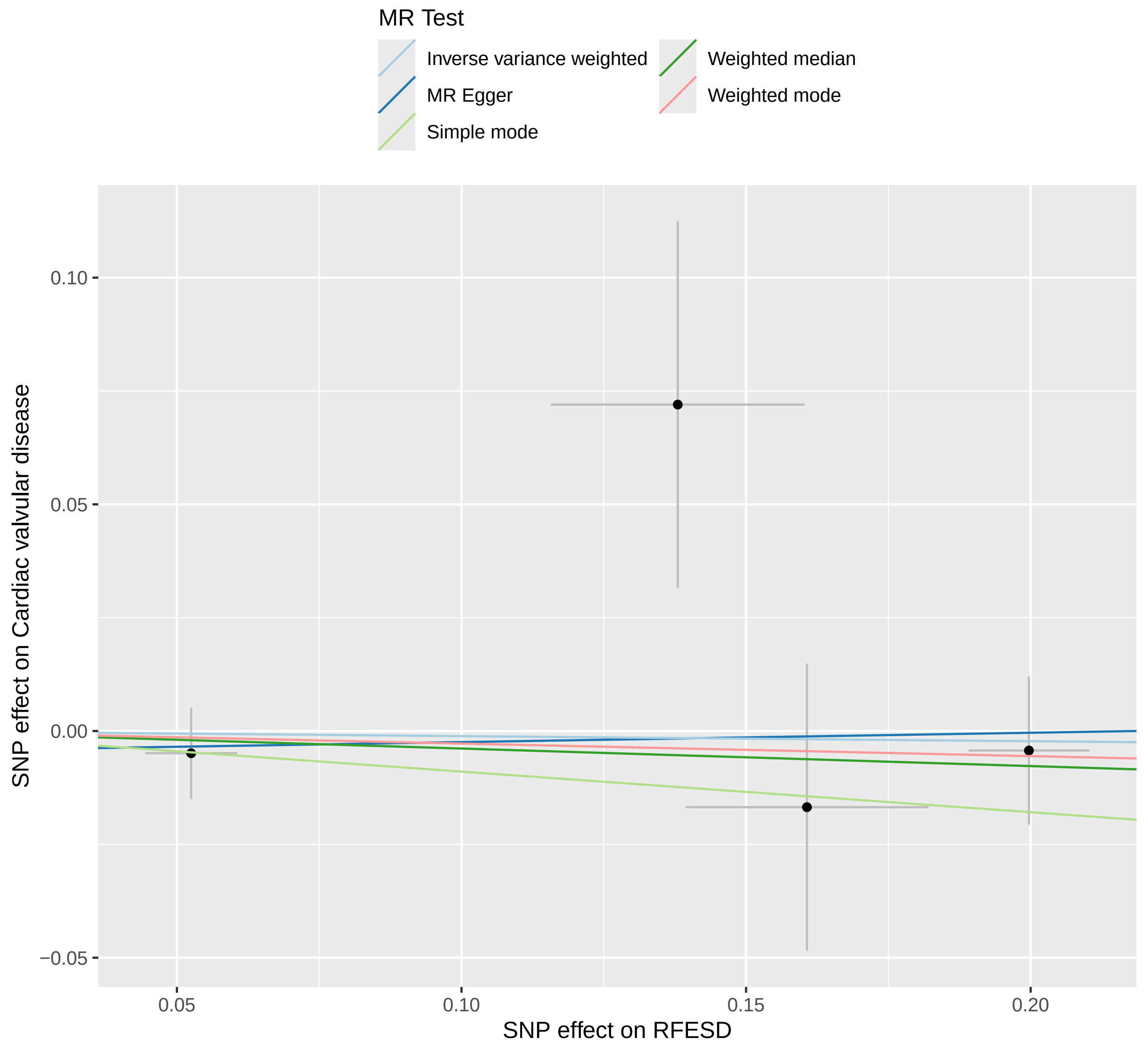
MR Test
Inverse variance weighted
MR Egger
Simple mode
Weighted median
Weighted mode
0.10
0.05
0.00
−0.05
SNP effect on Cardiac valvular disease
0.05
0.10
0.15
0.20
SNP effect on RFESD

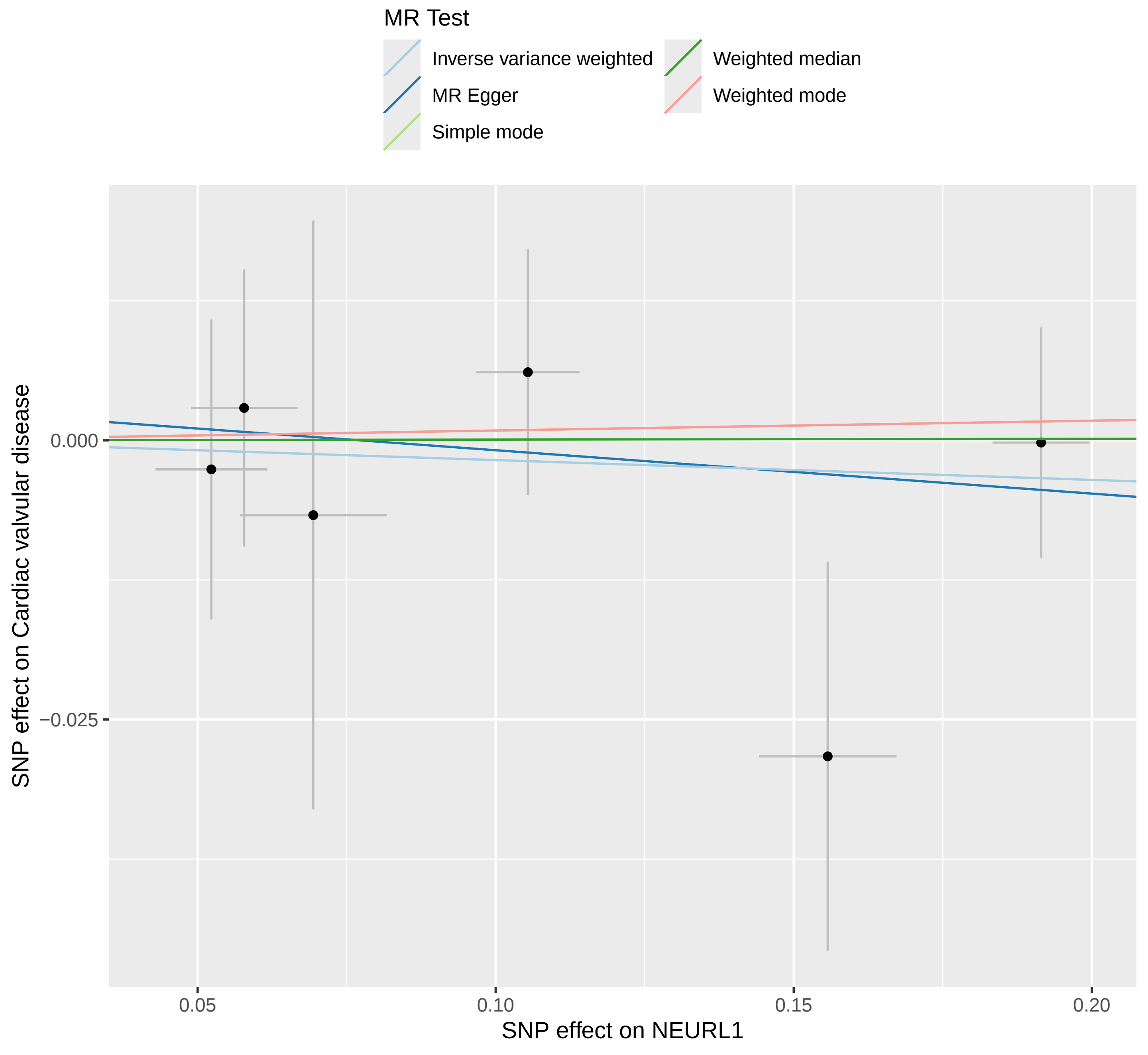
MR Test
Inverse variance weighted
MR Egger
Simple mode
Weighted median
Weighted mode
0.000
−0.025
0.05
0.10
0.15
0.20
SNP effect on NEURL1
SNP effect on Cardiac valvular disease

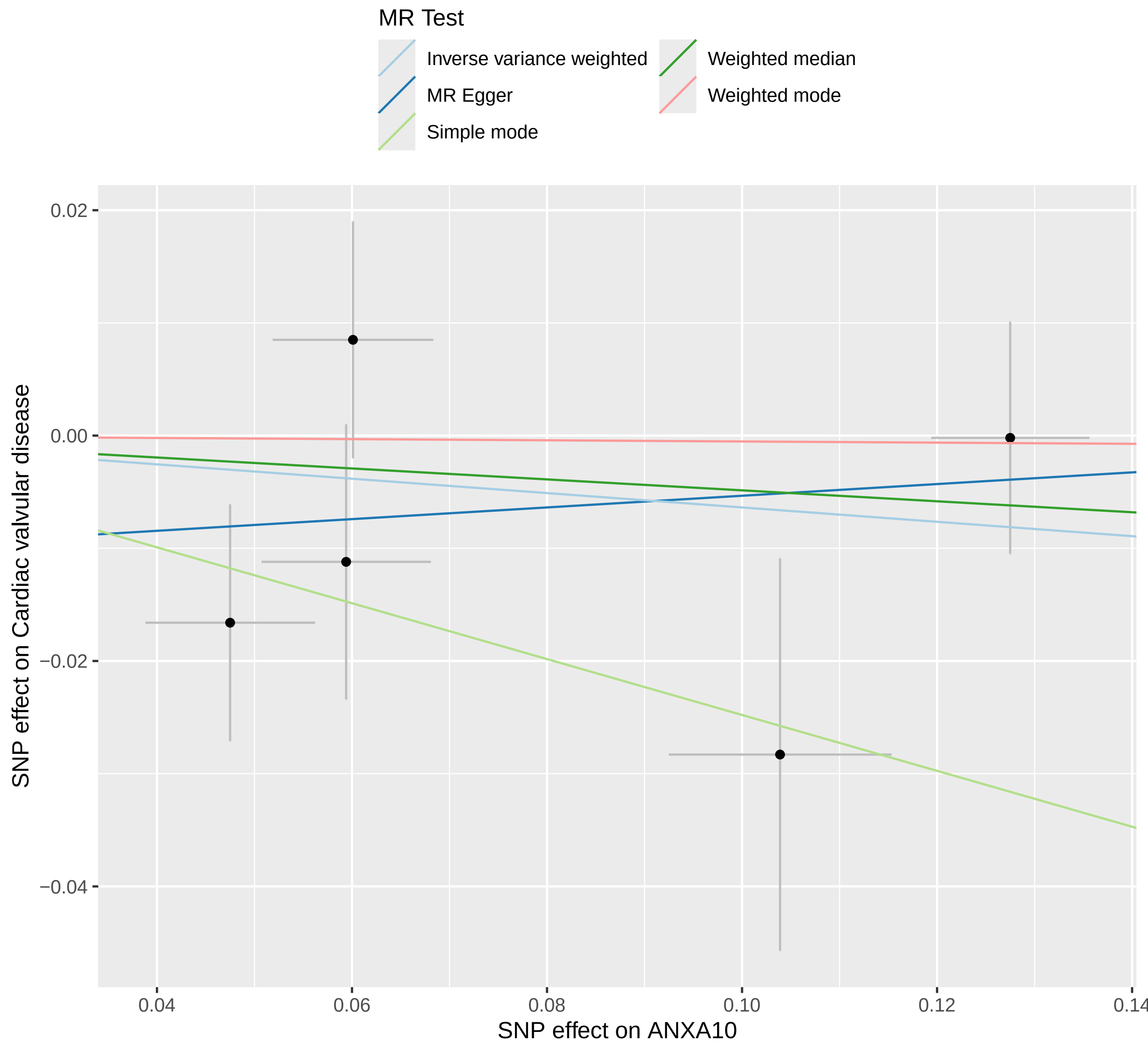
MR Test
Inverse variance weighted
MR Egger
Simple mode
Weighted median
Weighted mode
0.02
0.00
−0.02
−0.04
SNP effect on Cardiac valvular disease
0.04
0.06
0.08
0.10
0.12
0.14
SNP effect on ANXA10

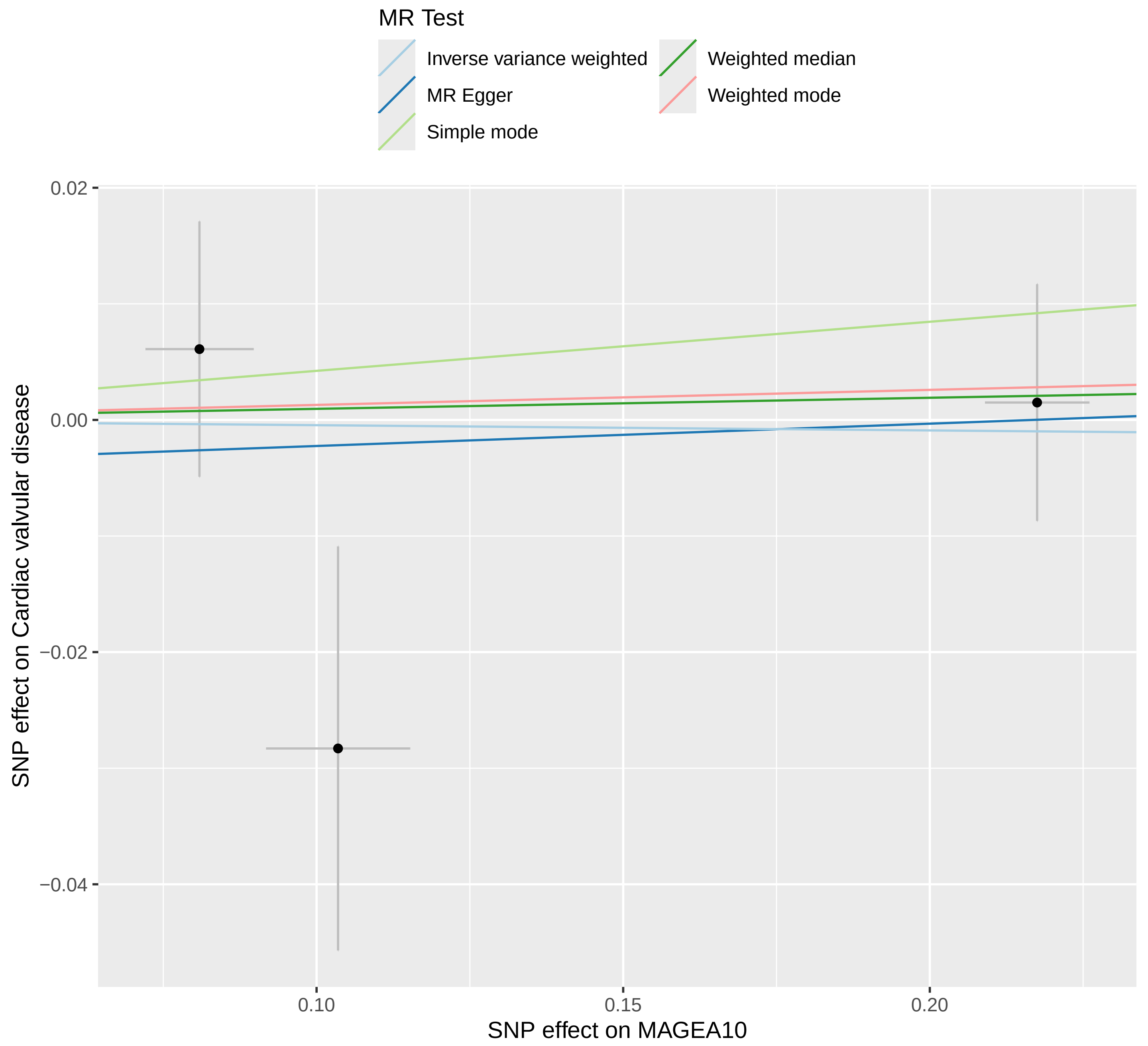

MR Test
Inverse variance weighted
MR Egger
Simple mode
Weighted median
Weighted mode
0.02
0.00
−0.02
−0.04
0.10
0.15
0.20
SNP effect on Cardiac valvular disease
SNP effect on MAGEA10

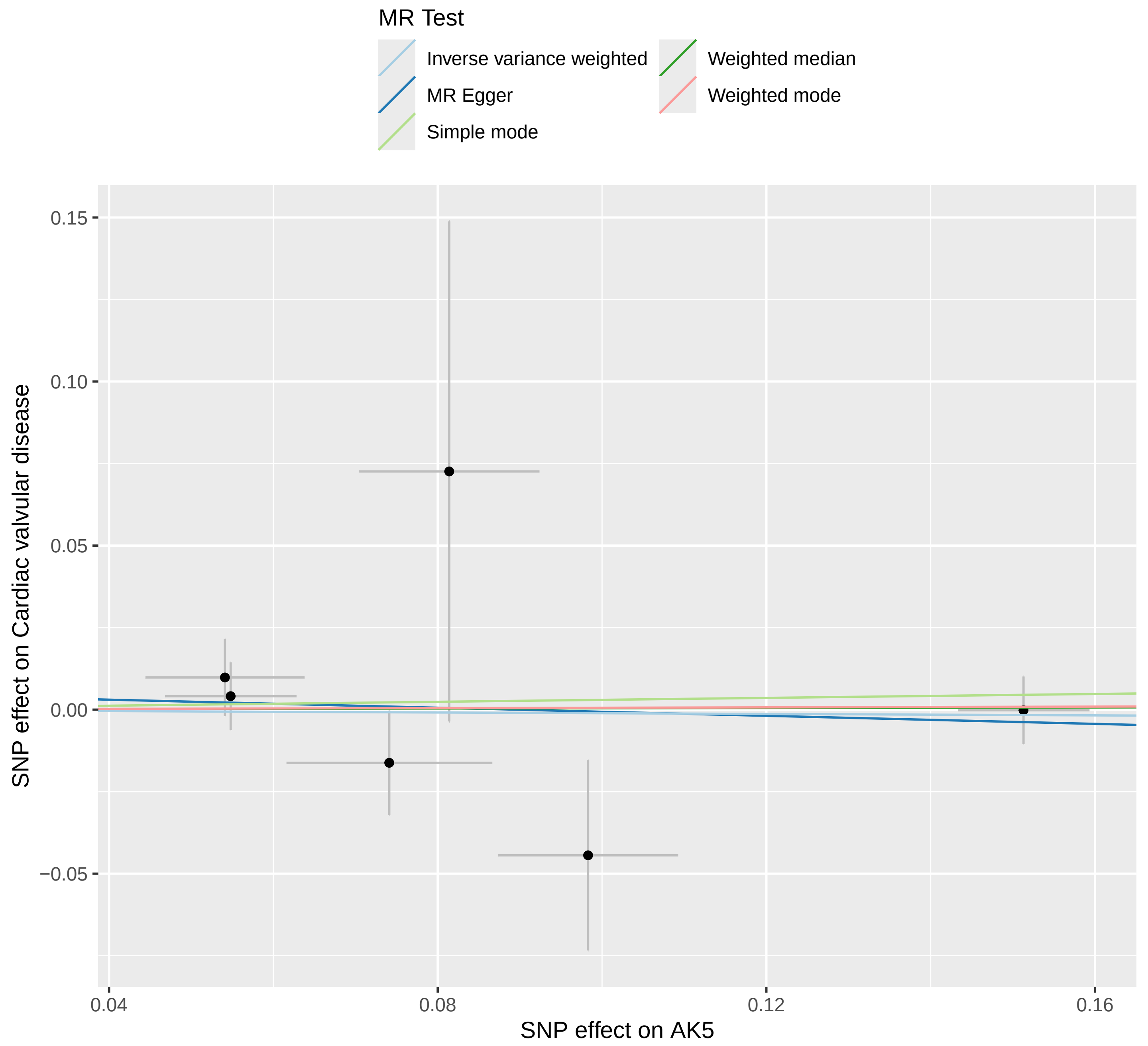
MR Test
Inverse variance weighted
MR Egger
Simple mode
Weighted median
Weighted mode
0.15
0.10
0.05
0.00
−0.05
0.04
0.08
0.12
0.16
SNP effect on Cardiac valvular disease
SNP effect on AK5

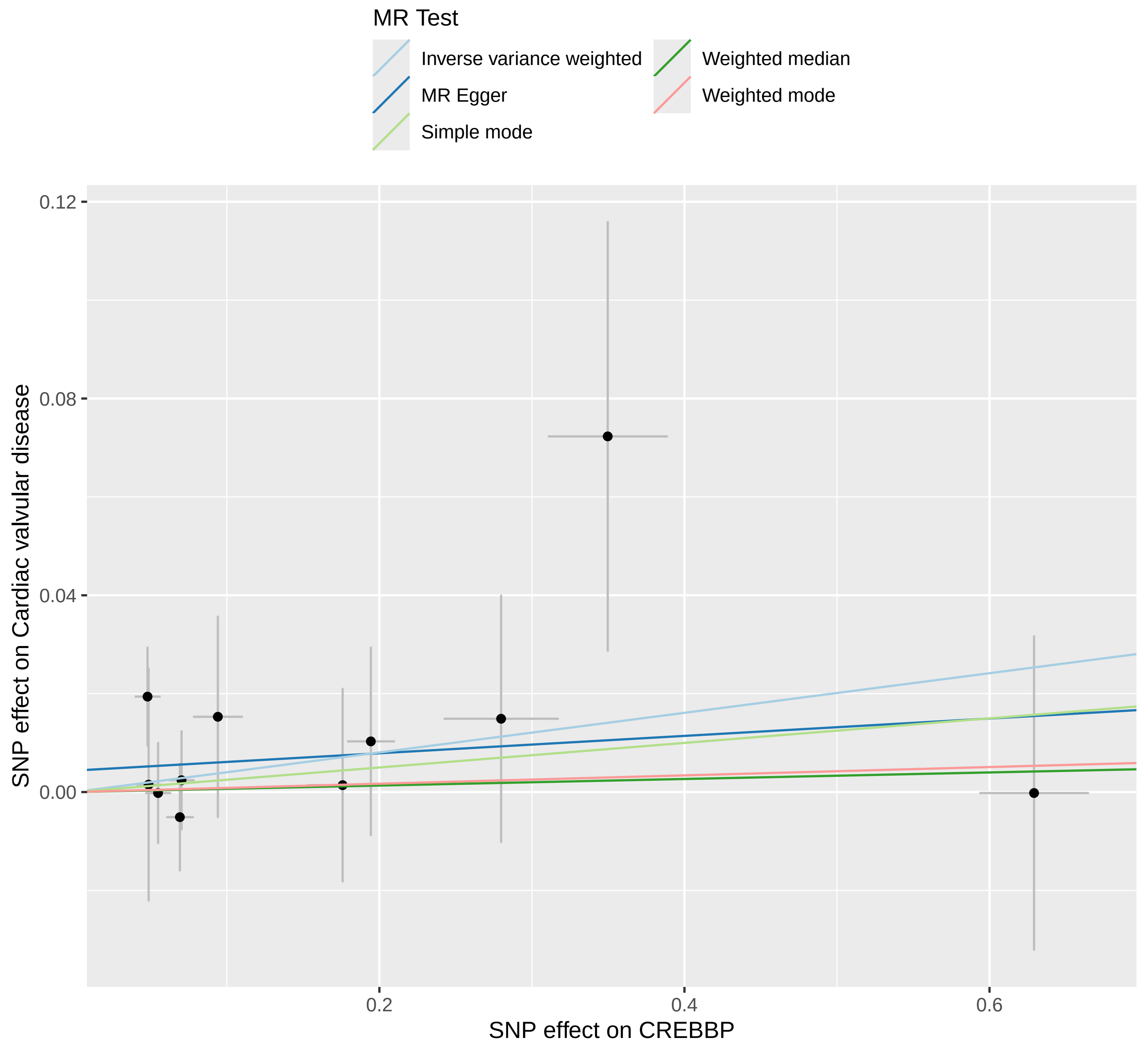
MR Test
Inverse variance weighted
MR Egger
Simple mode
Weighted median
Weighted mode
0.12
0.08
0.04
0.00
0.2
0.4
0.6
SNP effect on CREBBP
SNP effect on Cardiac valvular disease

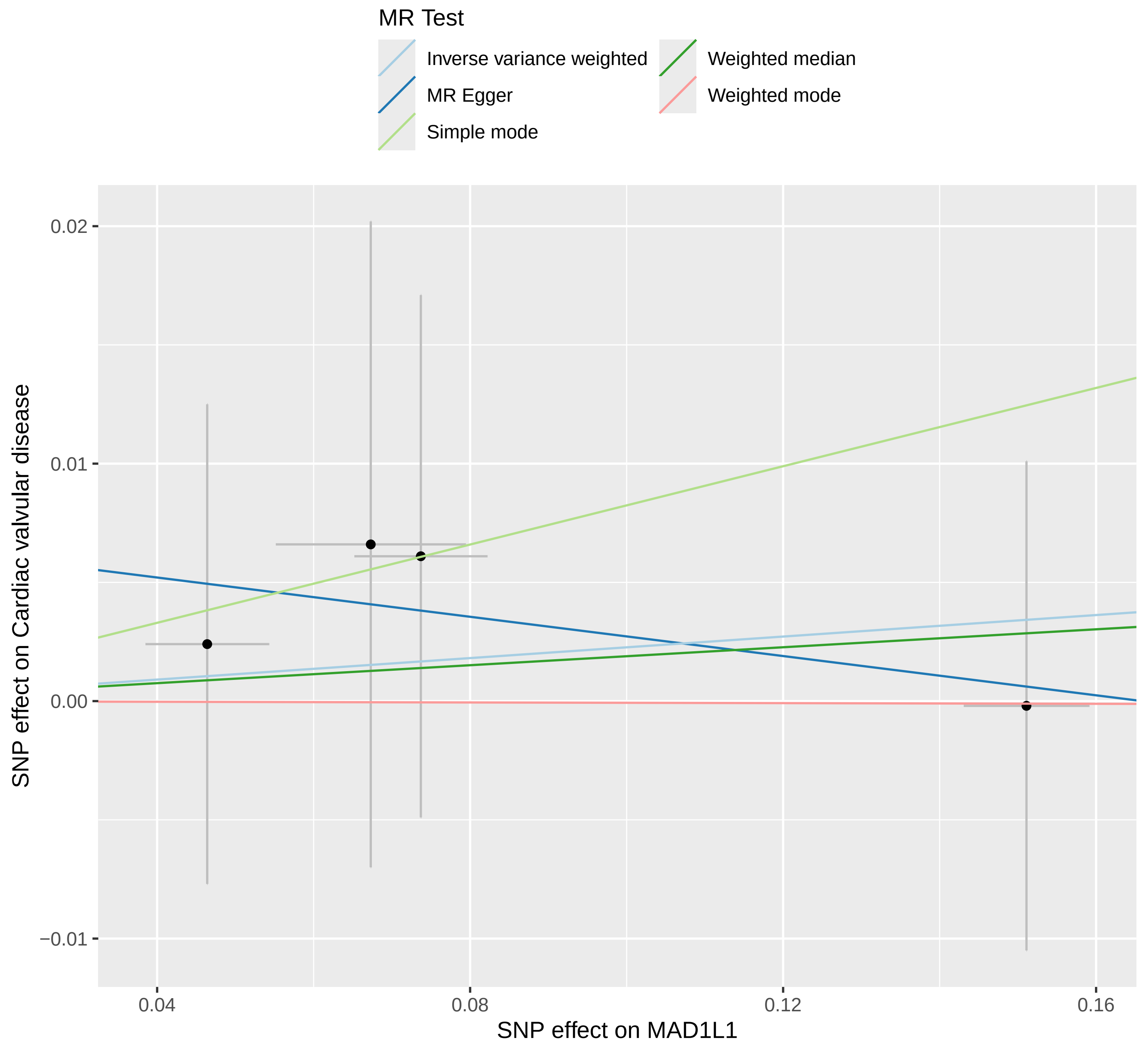
MR Test
Inverse variance weighted
MR Egger
Simple mode
Weighted median
Weighted mode
0.02
0.01
0.00
−0.01
0.04
0.08
0.12
0.16
SNP effect on Cardiac valvular disease
SNP effect on MAD1L1

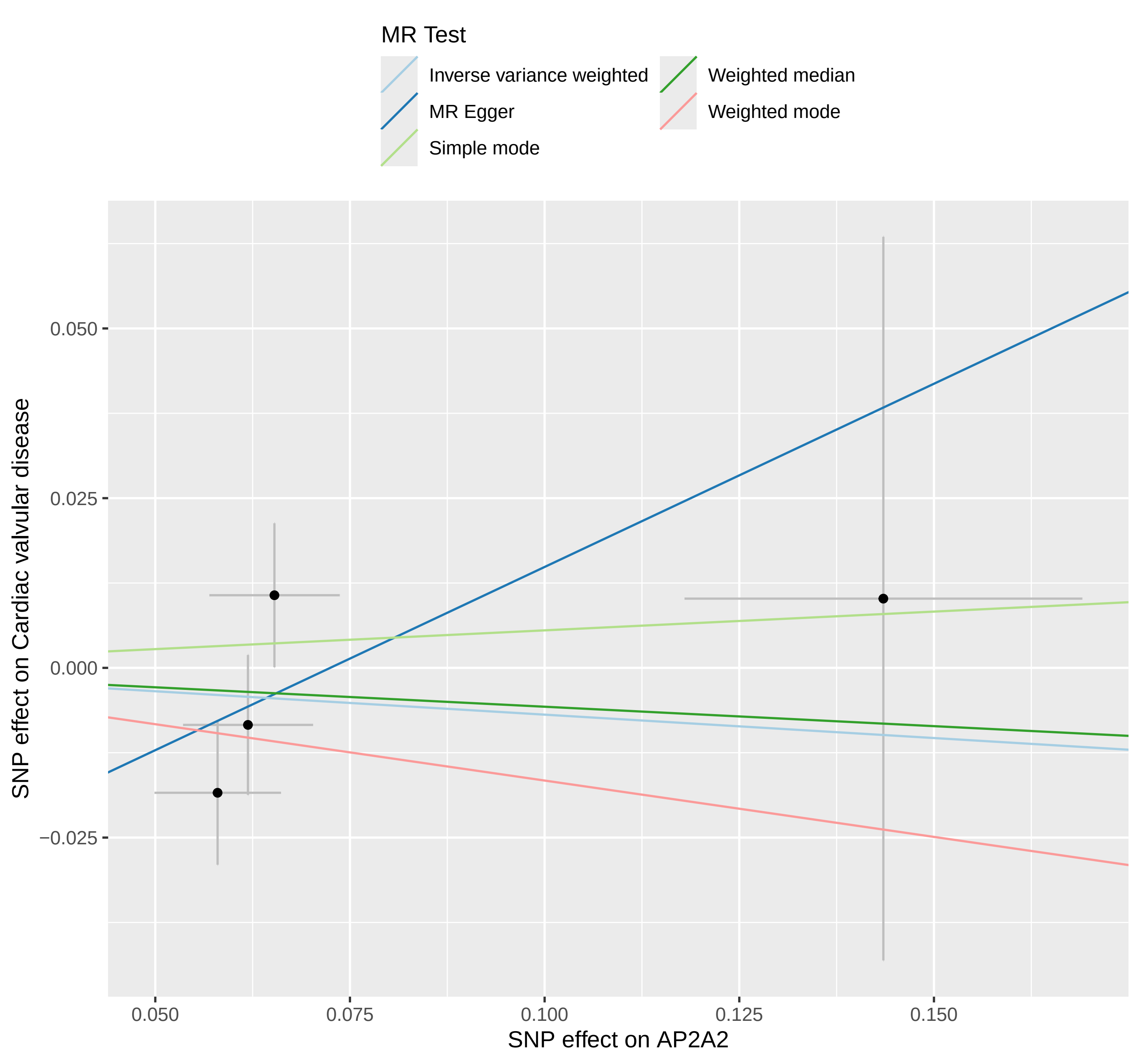

MR Test
Inverse variance weighted
MR Egger
Simple mode
Weighted median
Weighted mode
0.050
0.025
0.000
−0.025
0.050
0.075
0.100
0.125
0.150
SNP effect on AP2A2
SNP effect on Cardiac valvular disease

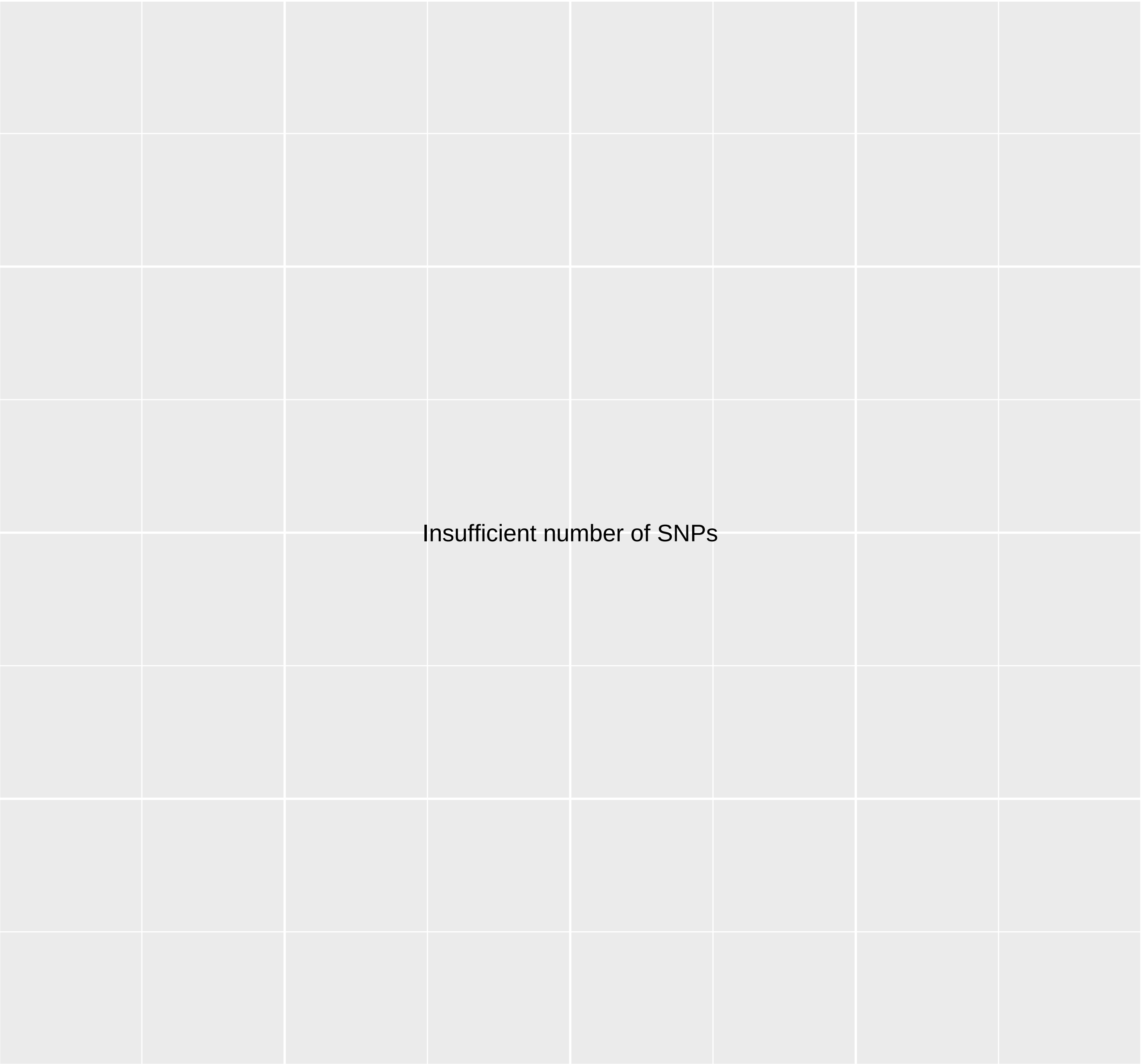
Insufficient number of SNPs

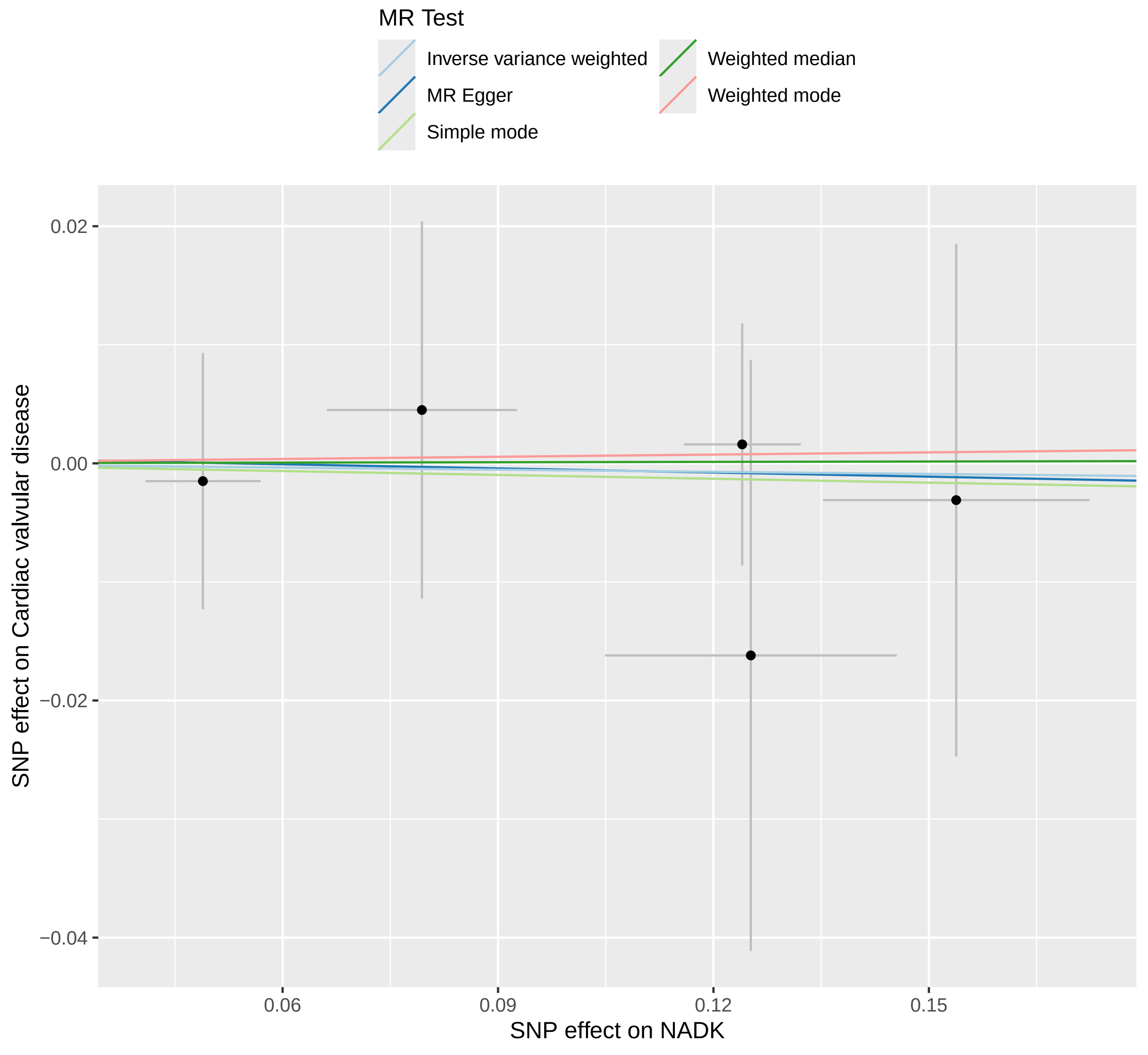

MR Test
Inverse variance weighted
MR Egger
Simple mode
Weighted median
Weighted mode
0.02
0.00
−0.02
−0.04
0.06
0.09
0.12
0.15
SNP effect on NADK
SNP effect on Cardiac valvular disease

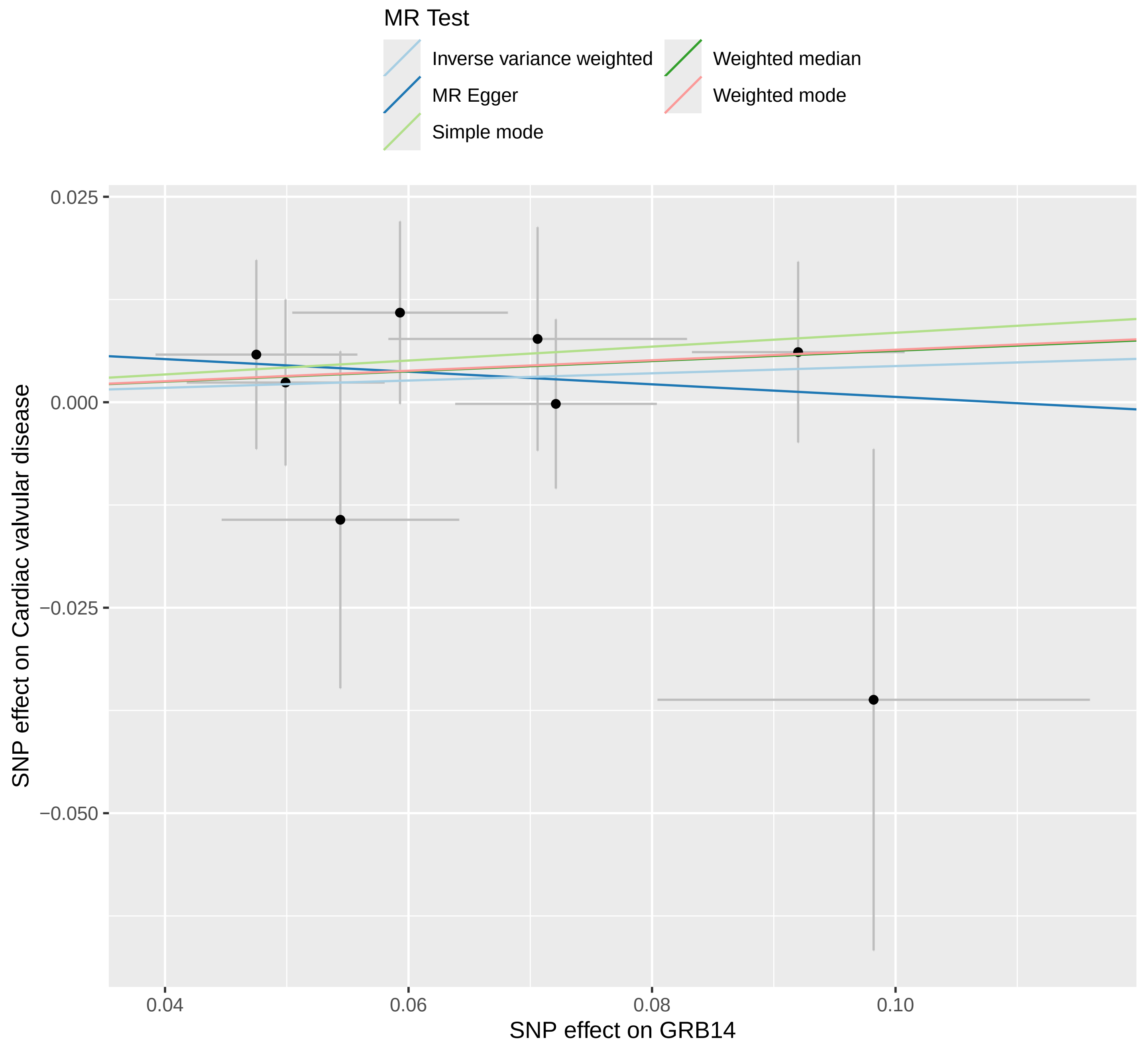
MR Test
Inverse variance weighted
MR Egger
Simple mode
Weighted median
Weighted mode
0.025
0.000
−0.025
−0.050
0.04
0.06
0.08
0.10
SNP effect on GRB14
SNP effect on Cardiac valvular disease

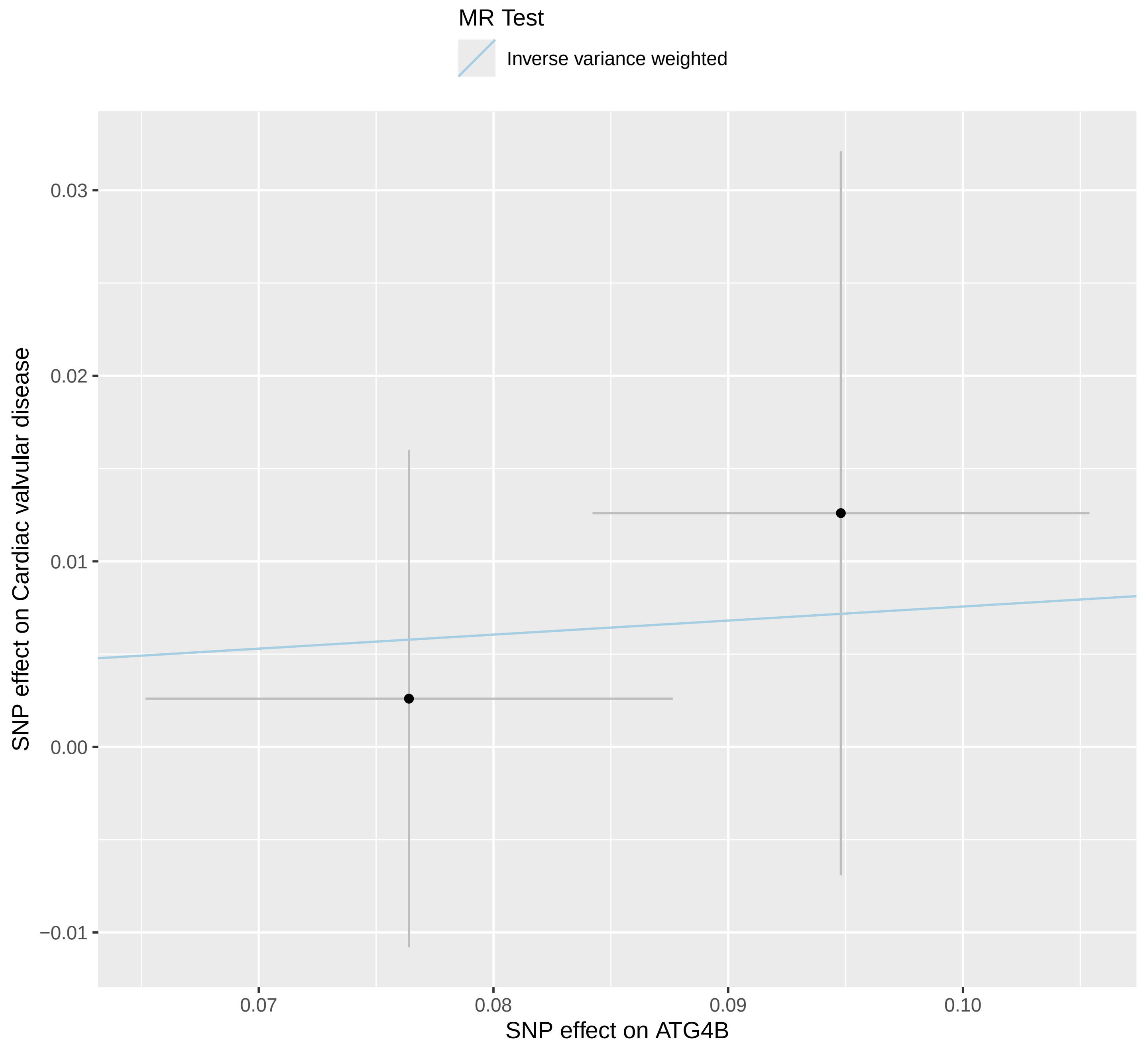
MR Test
Inverse variance weighted
0.03
0.02
0.01
0.00
−0.01
0.07
0.08
0.09
0.10
SNP effect on Cardiac valvular disease
SNP effect on ATG4B

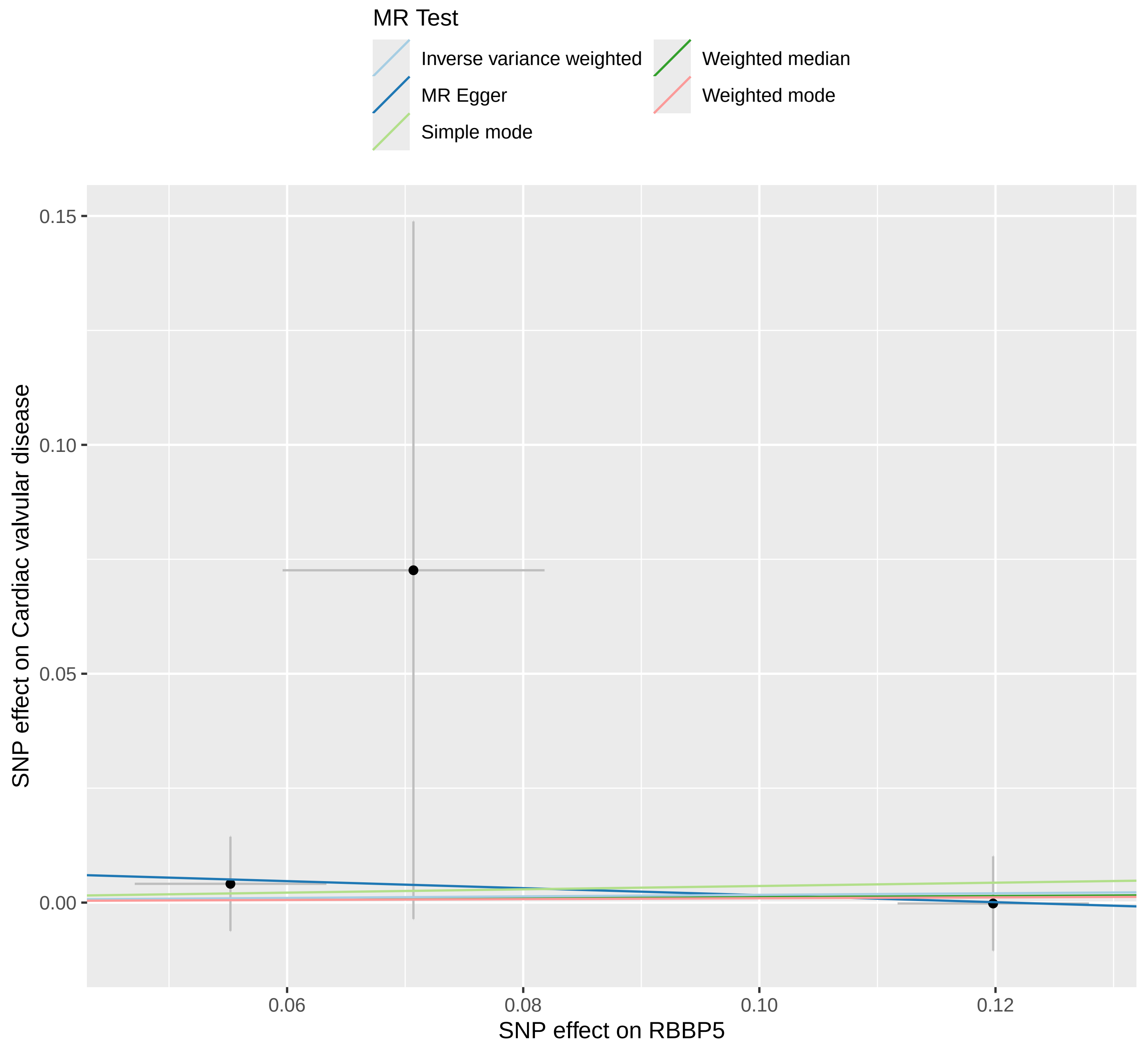

MR Test
Inverse variance weighted
MR Egger
Simple mode
Weighted median
Weighted mode
0.15
0.10
0.05
0.00
0.06
0.08
0.10
0.12
SNP effect on Cardiac valvular disease
SNP effect on RBBP5

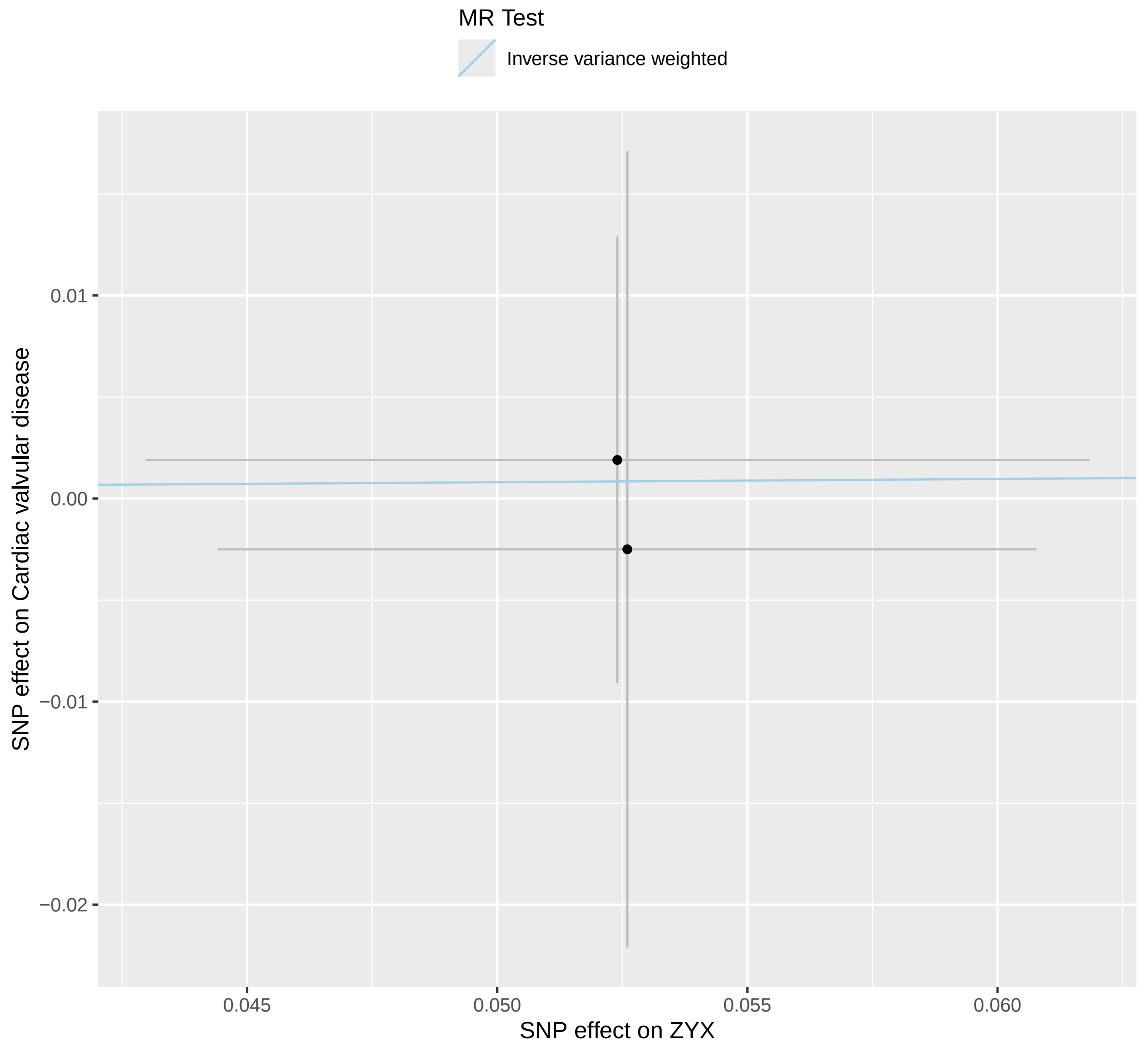

MR Test
Inverse variance weighted
0.01
0.00
−0.01
−0.02
0.045
0.050
0.055
0.060
SNP effect on ZYX
SNP effect on Cardiac valvular disease

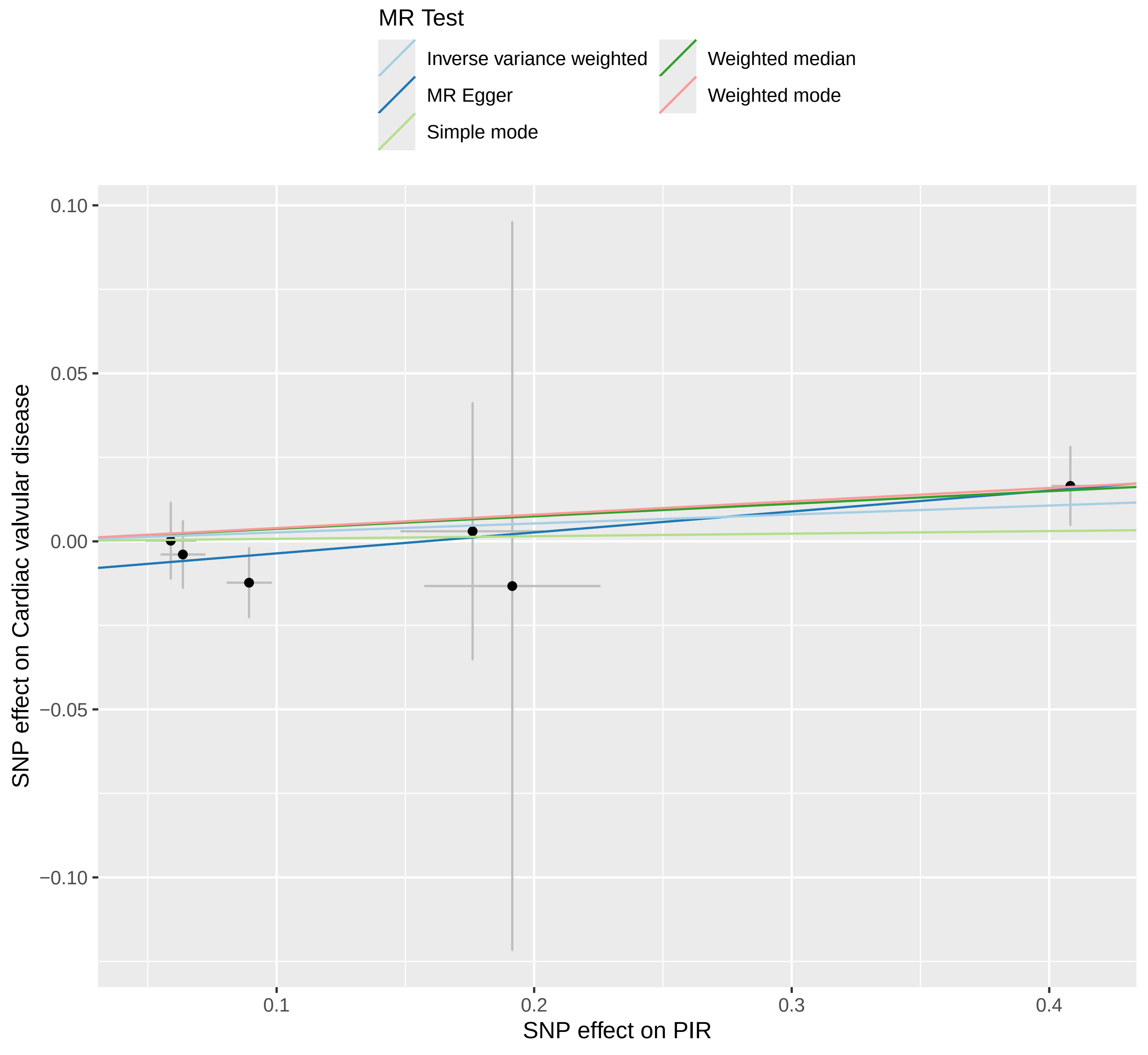
MR Test
Inverse variance weighted
MR Egger
Simple mode
Weighted median
Weighted mode
0.10
0.05
0.00
−0.05
−0.10
SNP effect on Cardiac valvular disease
0.1
0.2
0.3
0.4
SNP effect on PIR

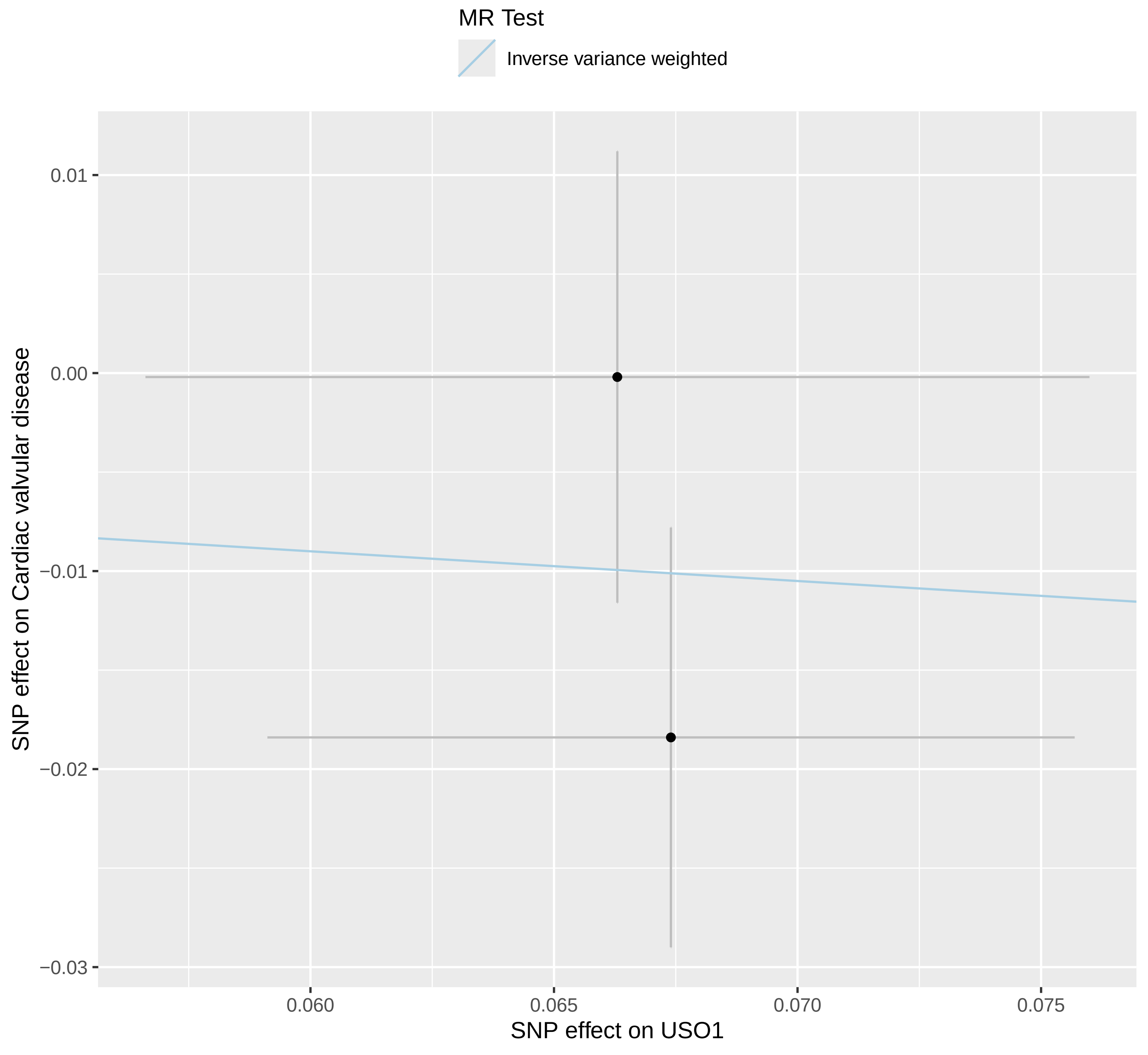
MR Test
Inverse variance weighted
0.01
0.00
−0.01
−0.02
−0.03
SNP effect on Cardiac valvular disease
0.060
0.065
0.070
0.075
SNP effect on USO1

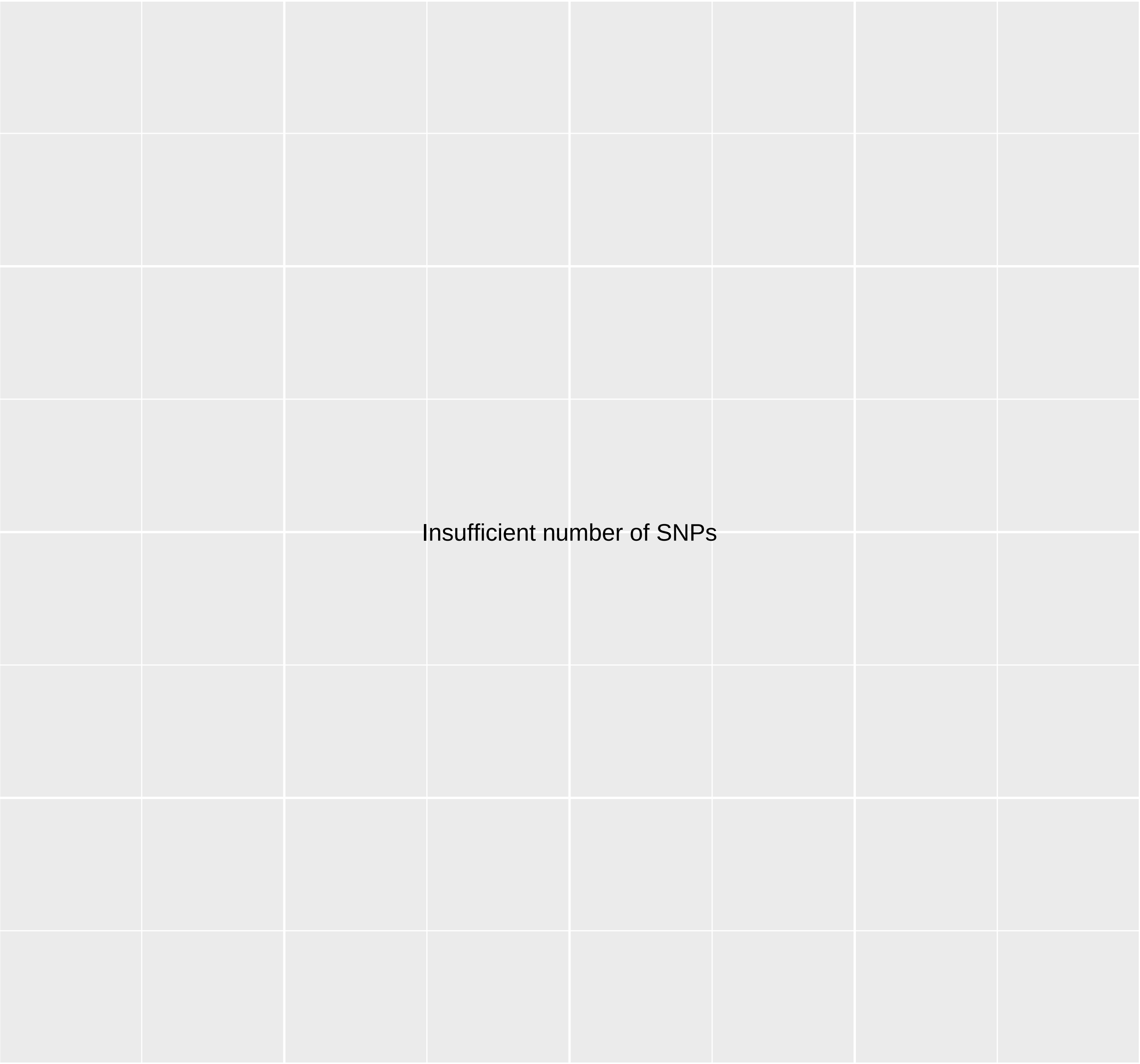
Insufficient number of SNPs

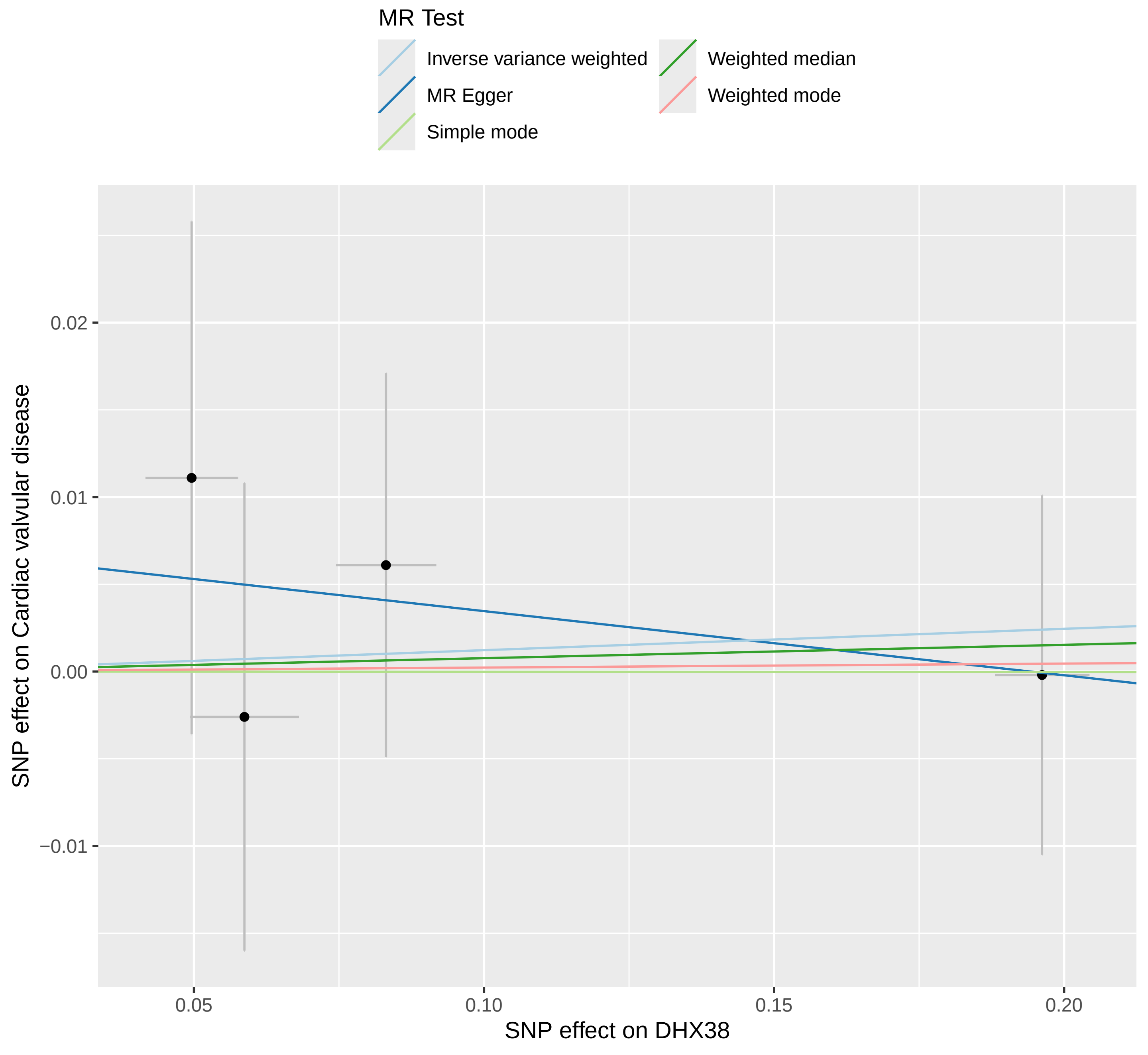
MR Test
Inverse variance weighted
MR Egger
Simple mode
Weighted median
Weighted mode
0.02
0.01
0.00
−0.01
0.05
0.10
0.15
0.20
SNP effect on DHX38
SNP effect on Cardiac valvular disease

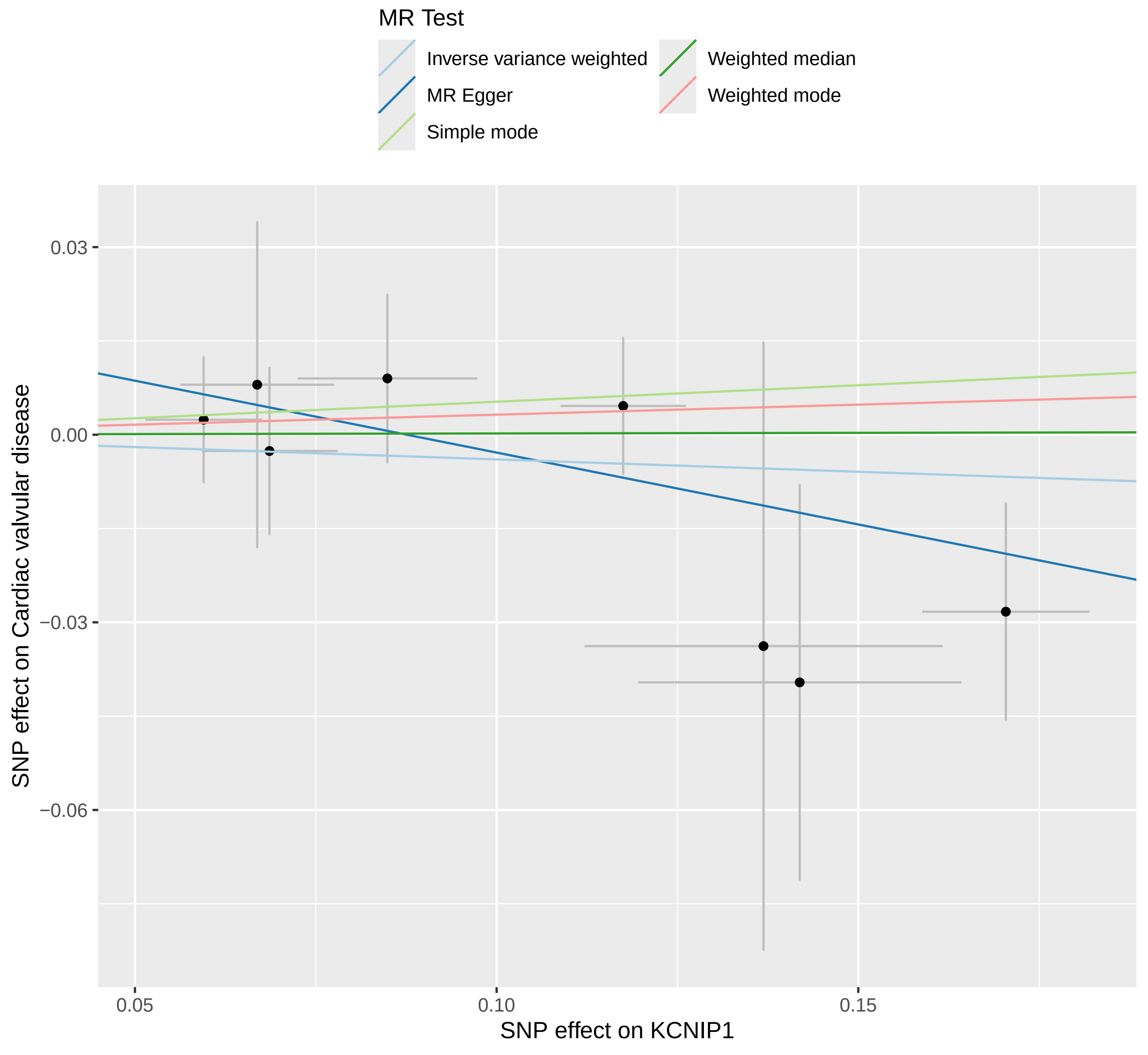

MR Test
Inverse variance weighted
MR Egger
Simple mode
Weighted median
Weighted mode
0.03
0.00
−0.03
−0.06
0.05
0.10
0.15
SNP effect on KCNIP1
SNP effect on Cardiac valvular disease

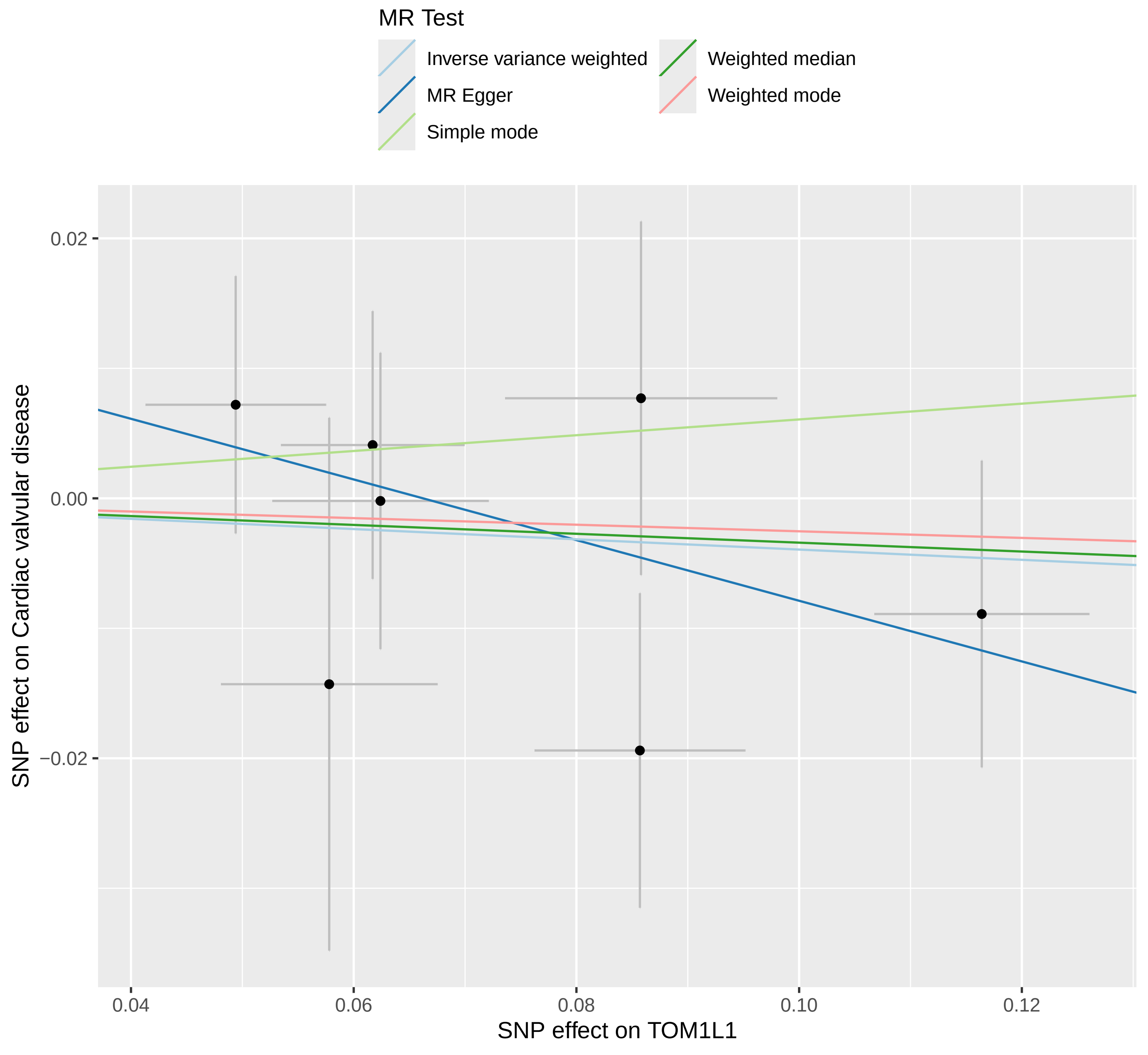
MR Test
Inverse variance weighted
MR Egger
Simple mode
Weighted median
Weighted mode
0.02
0.00
−0.02
0.04
0.06
0.08
0.10
0.12
SNP effect on Cardiac valvular disease
SNP effect on TOM1L1

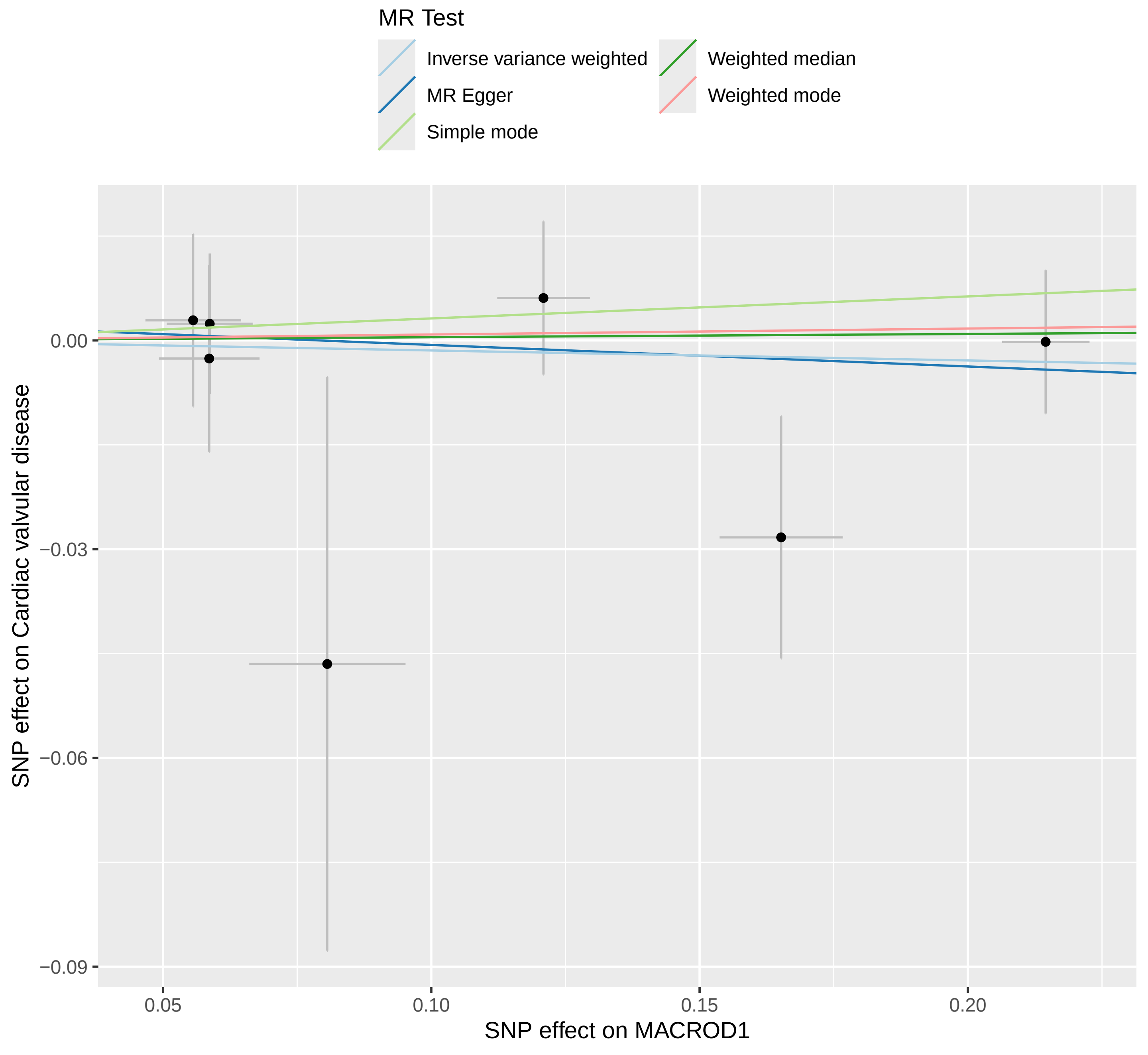
MR Test
Inverse variance weighted
MR Egger
Simple mode
Weighted median
Weighted mode
0.00
−0.03
−0.06
−0.09
SNP effect on Cardiac valvular disease
0.05
0.10
0.15
0.20
SNP effect on MACROD1

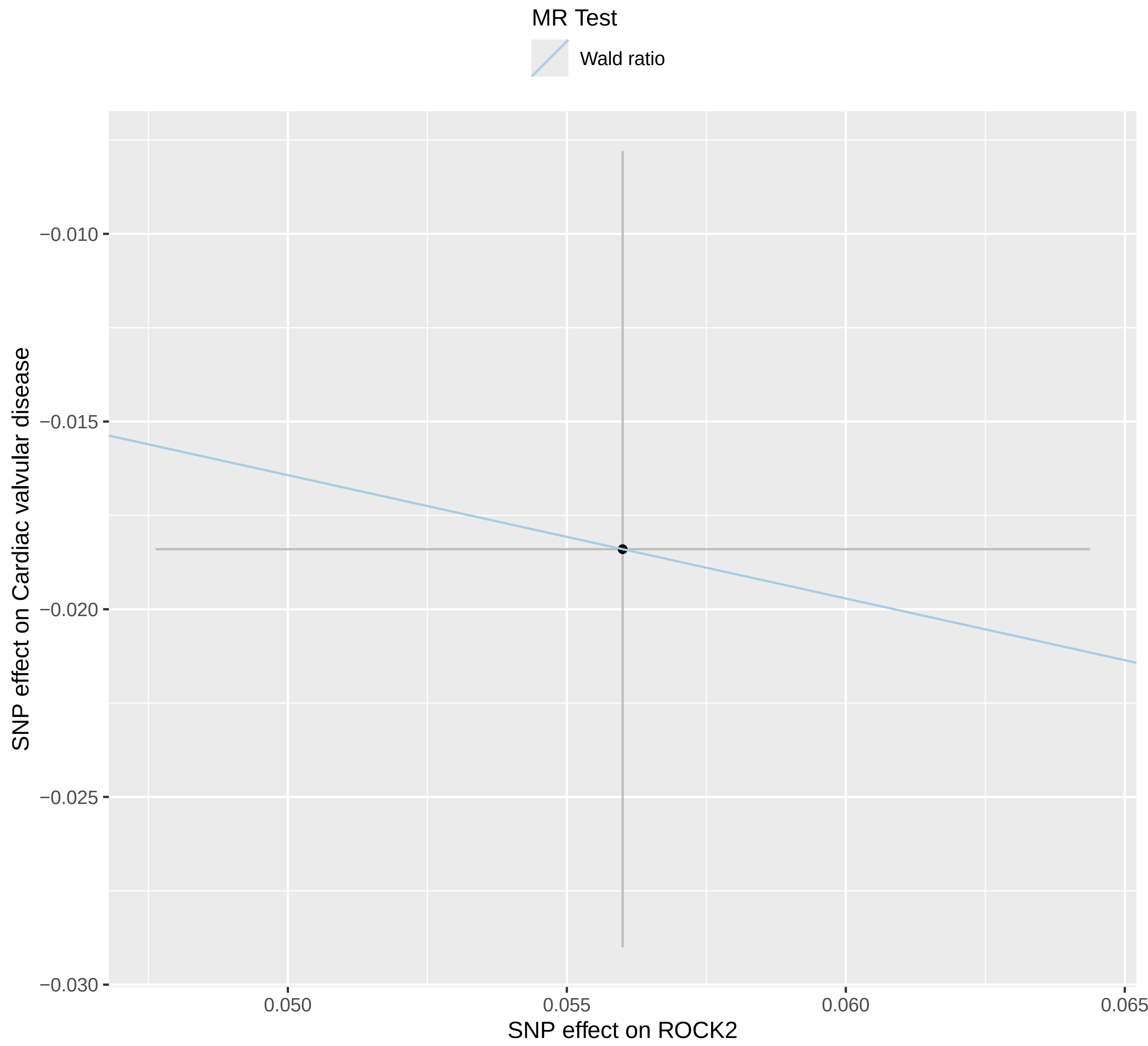

MR Test
Wald ratio
SNP effect on Cardiac valvular disease
−0.010
−0.015
−0.020
−0.025
−0.030
0.050
0.055
0.060
0.065
SNP effect on ROCK2

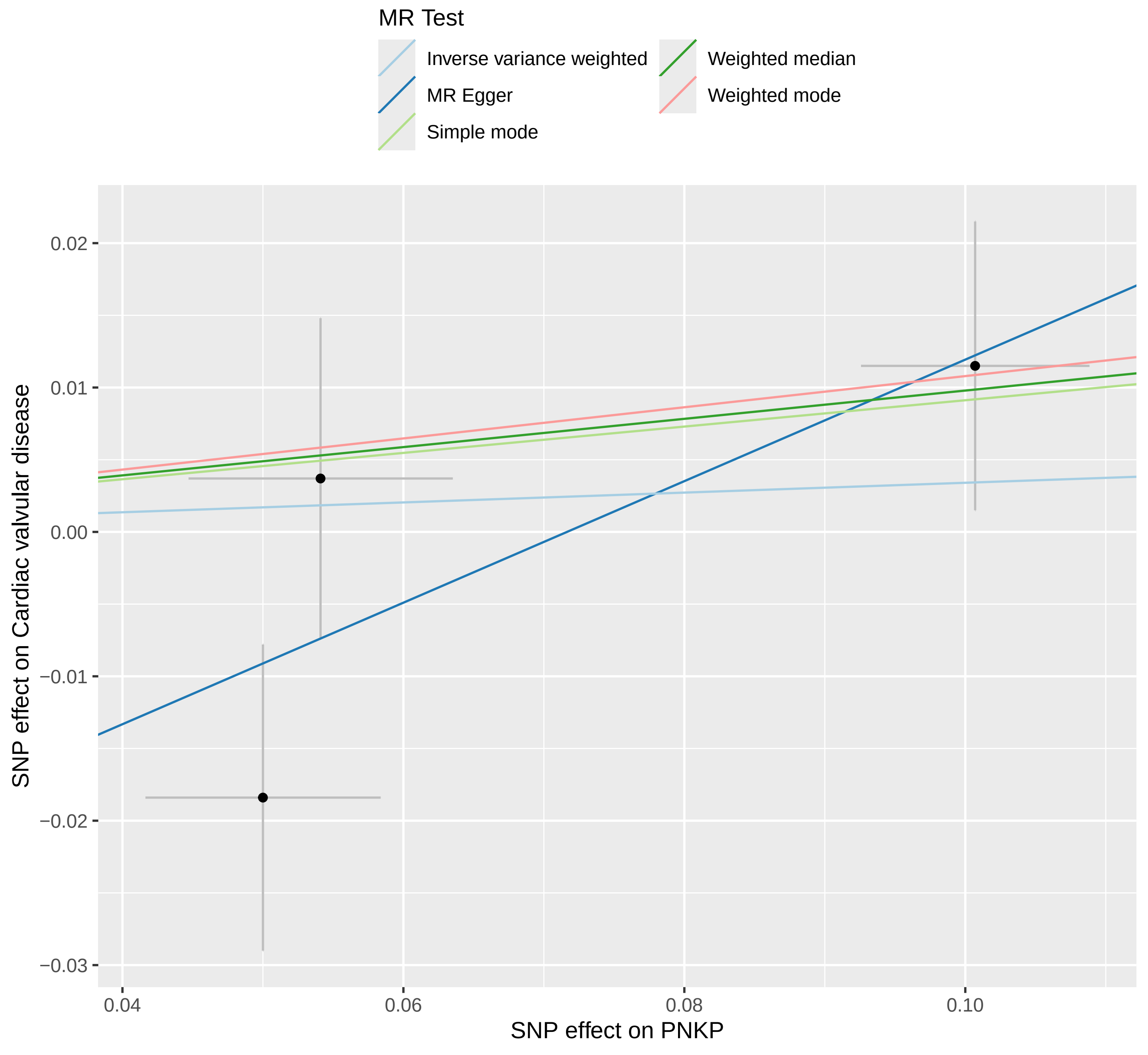

MR Test
Inverse variance weighted
MR Egger
Simple mode
Weighted median
Weighted mode
0.02
0.01
0.00
−0.01
−0.02
−0.03
SNP effect on Cardiac valvular disease
0.04
0.06
0.08
0.10
SNP effect on PNKP

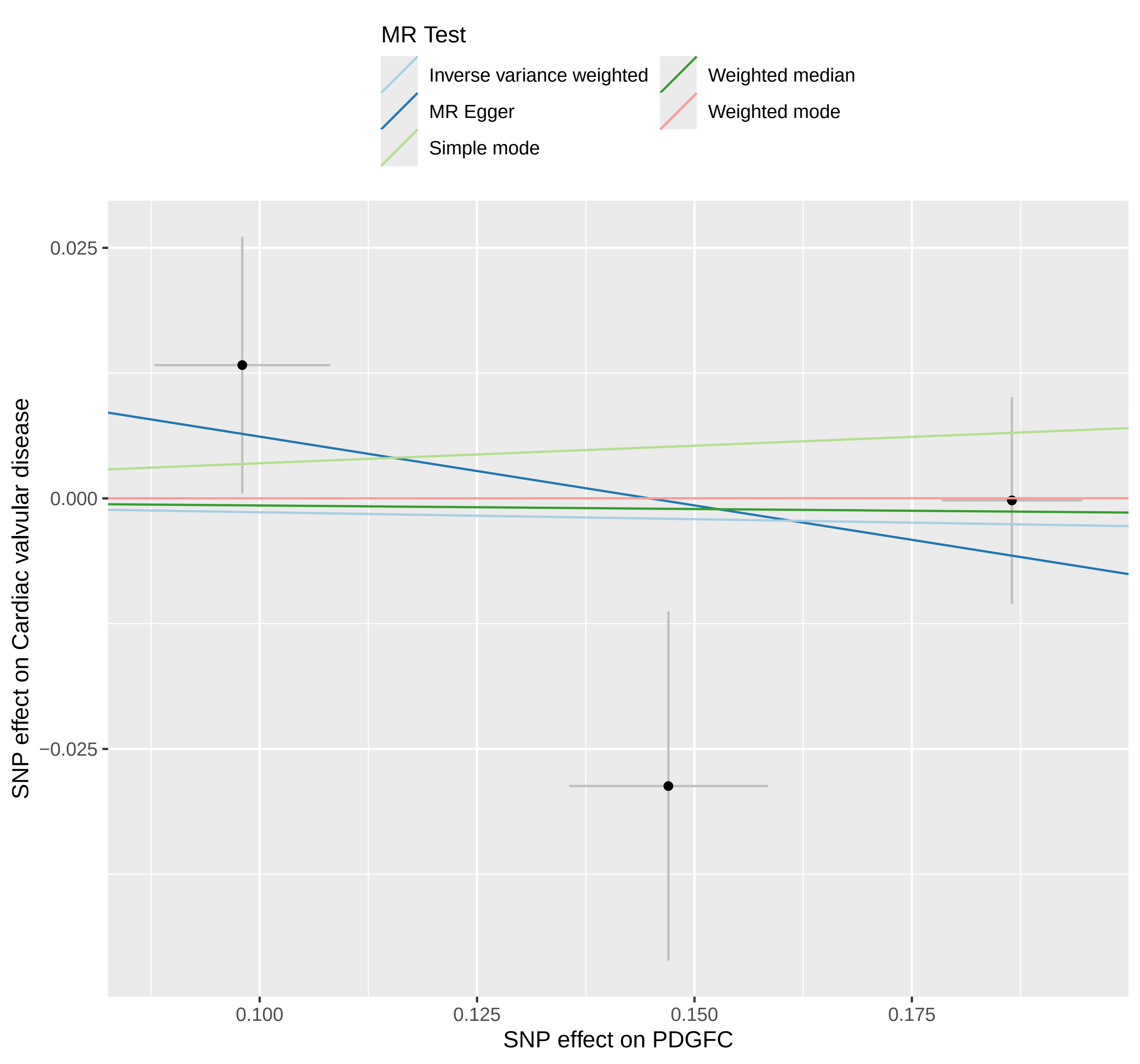
MR Test
Inverse variance weighted
MR Egger
Simple mode
Weighted median
Weighted mode
0.025
0.000
−0.025
0.100
0.125
0.150
0.175
SNP effect on PDGFC
SNP effect on Cardiac valvular disease

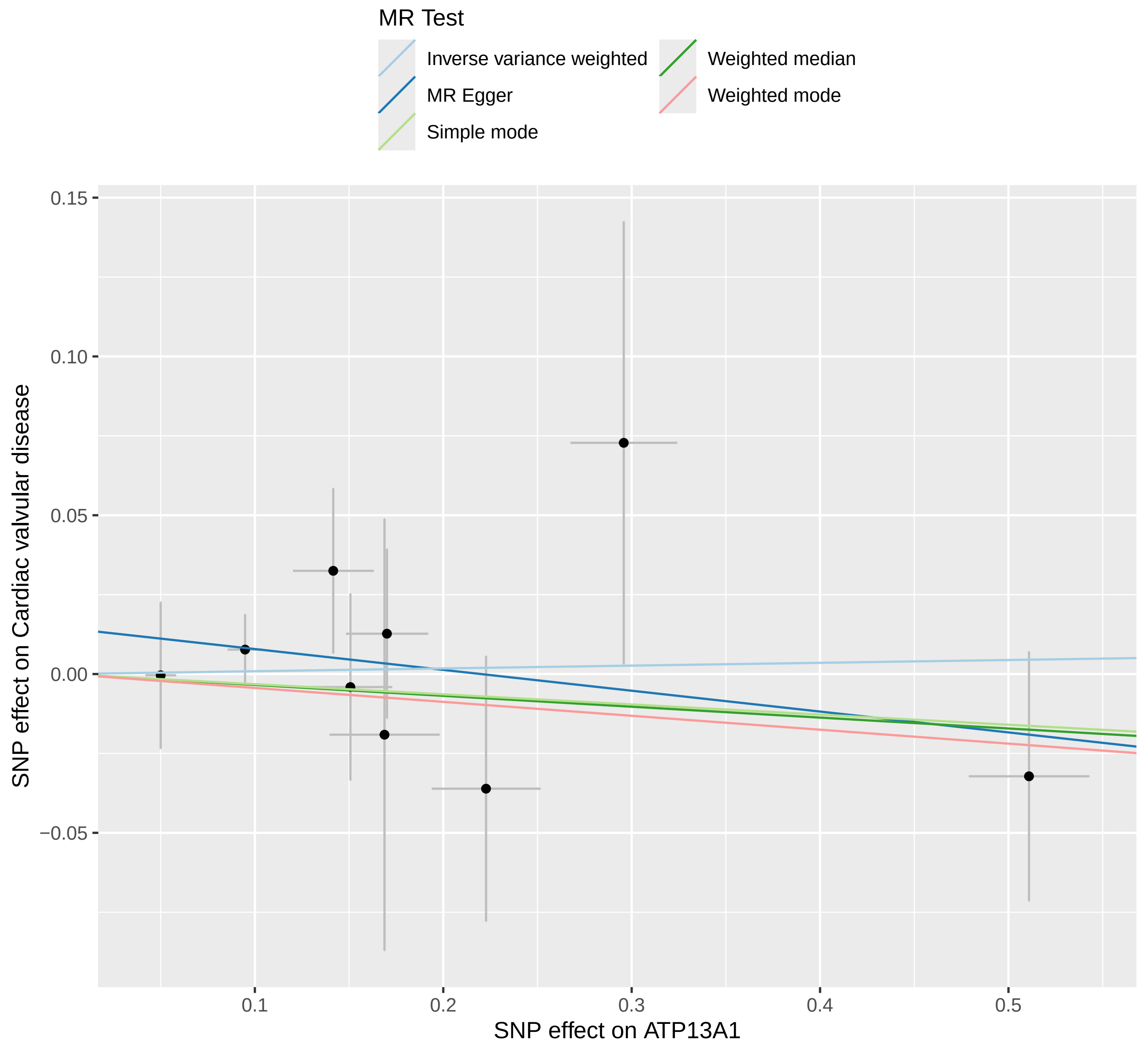

MR Test
Inverse variance weighted
MR Egger
Simple mode
Weighted median
Weighted mode
0.15
0.10
0.05
0.00
−0.05
SNP effect on Cardiac valvular disease
0.1
0.2
0.3
0.4
0.5
SNP effect on ATP13A1

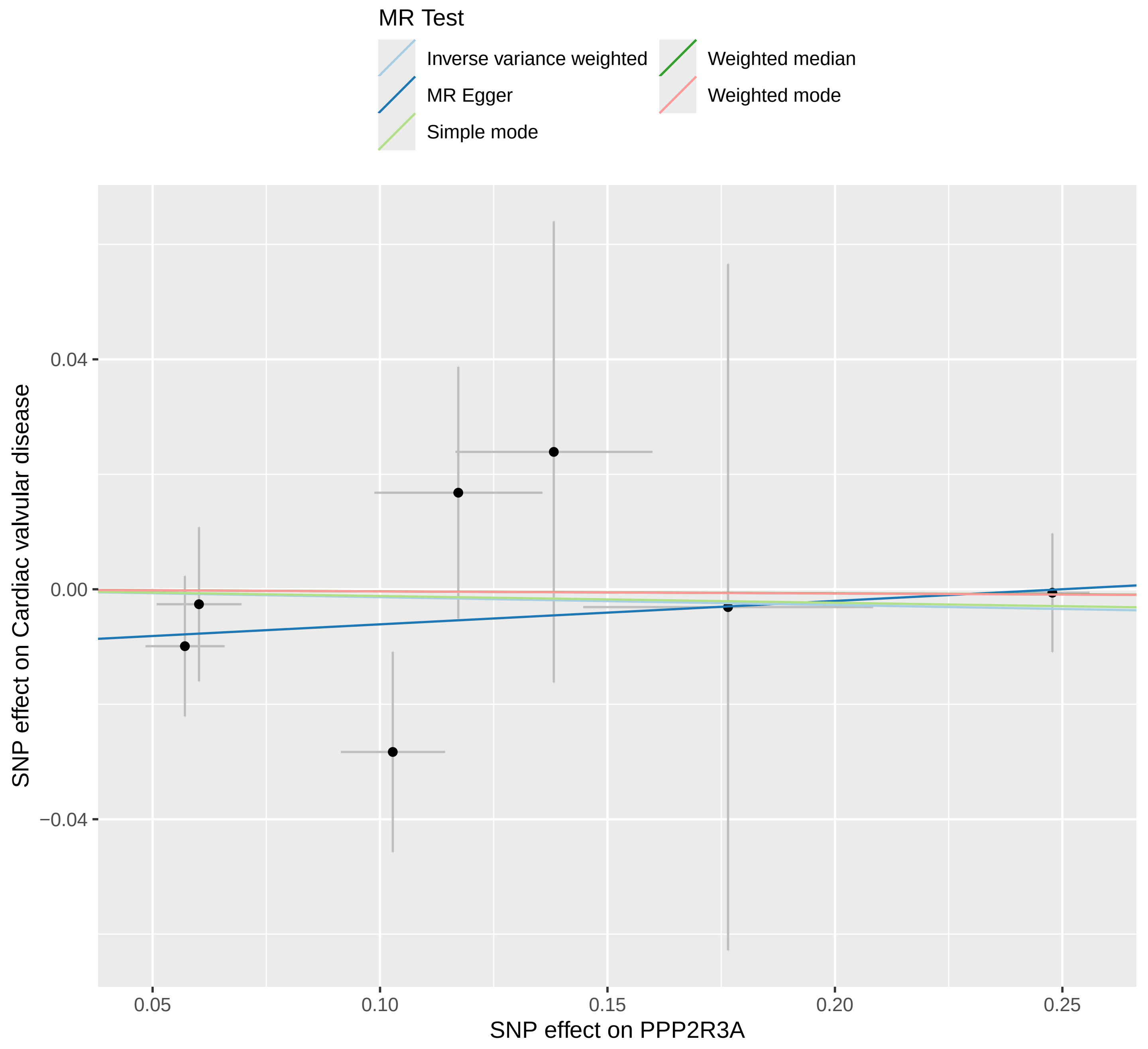

MR Test
Inverse variance weighted
MR Egger
Simple mode
Weighted median
Weighted mode
0.04
0.00
−0.04
SNP effect on Cardiac valvular disease
0.05
0.10
0.15
0.20
0.25
SNP effect on PPP2R3A

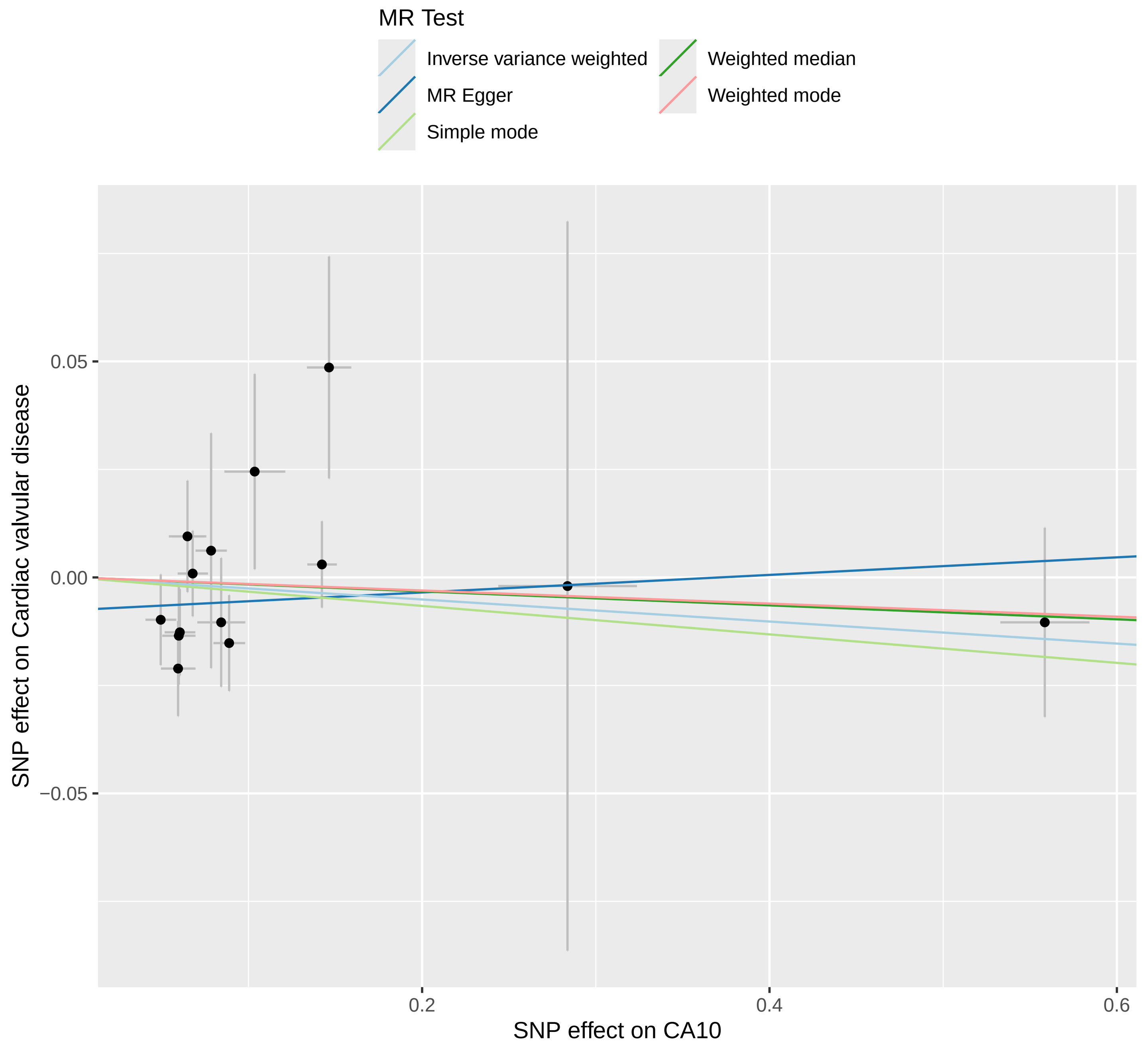

MR Test
Inverse variance weighted
MR Egger
Simple mode
Weighted median
Weighted mode
0.05
0.00
−0.05
SNP effect on Cardiac valvular disease
0.2
0.4
0.6
SNP effect on CA10

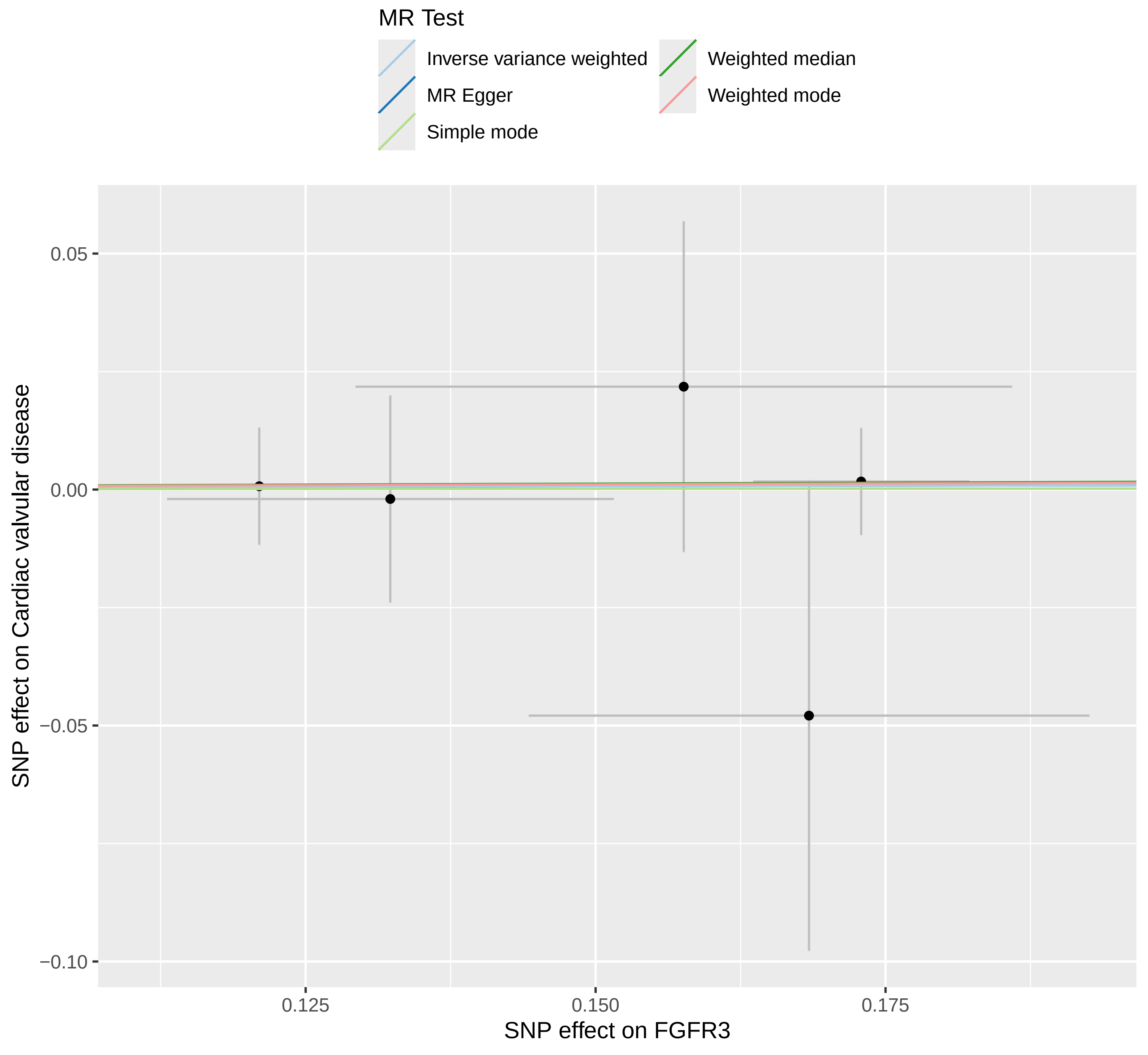
MR Test
Inverse variance weighted
MR Egger
Simple mode
Weighted median
Weighted mode
0.05
0.00
−0.05
−0.10
SNP effect on Cardiac valvular disease
0.125
0.150
0.175
SNP effect on FGFR3

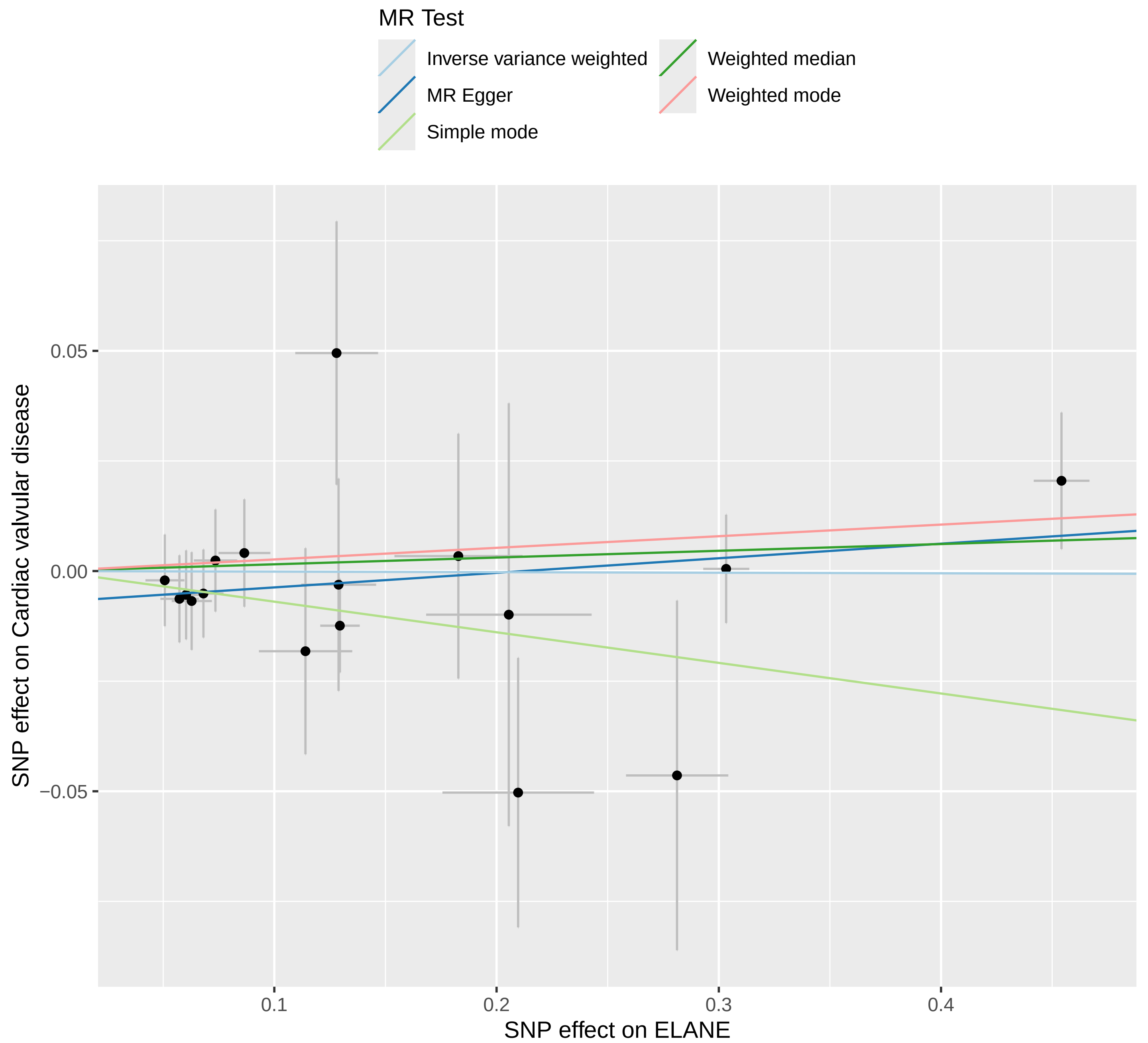

MR Test
Inverse variance weighted
MR Egger
Simple mode
Weighted median
Weighted mode
0.05
0.00
−0.05
SNP effect on Cardiac valvular disease
0.1
0.2
0.3
0.4
SNP effect on ELANE

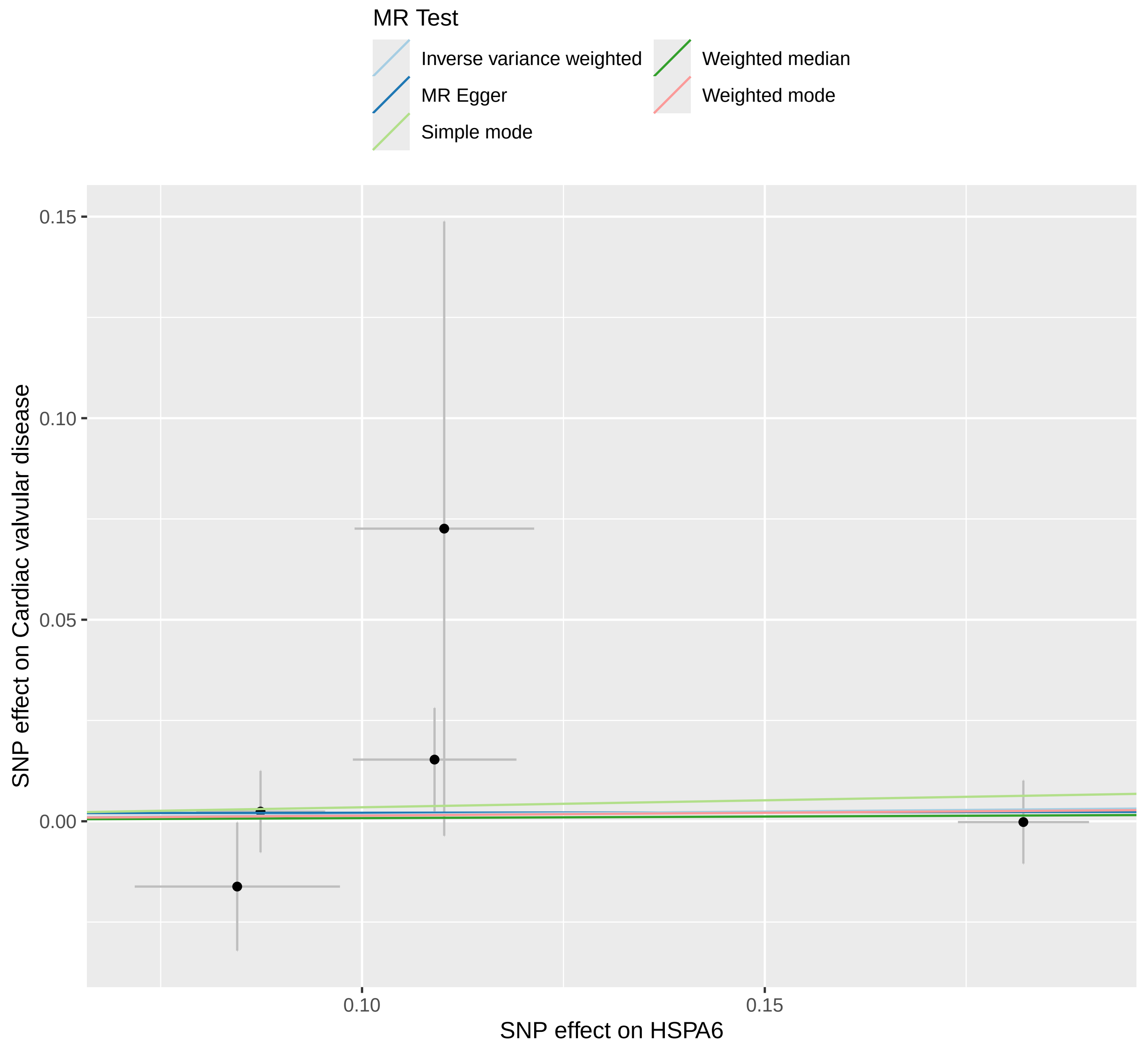

MR Test
Inverse variance weighted
MR Egger
Simple mode
Weighted median
Weighted mode
0.15
0.10
0.05
0.00
SNP effect on Cardiac valvular disease
0.10
0.15
SNP effect on HSPA6

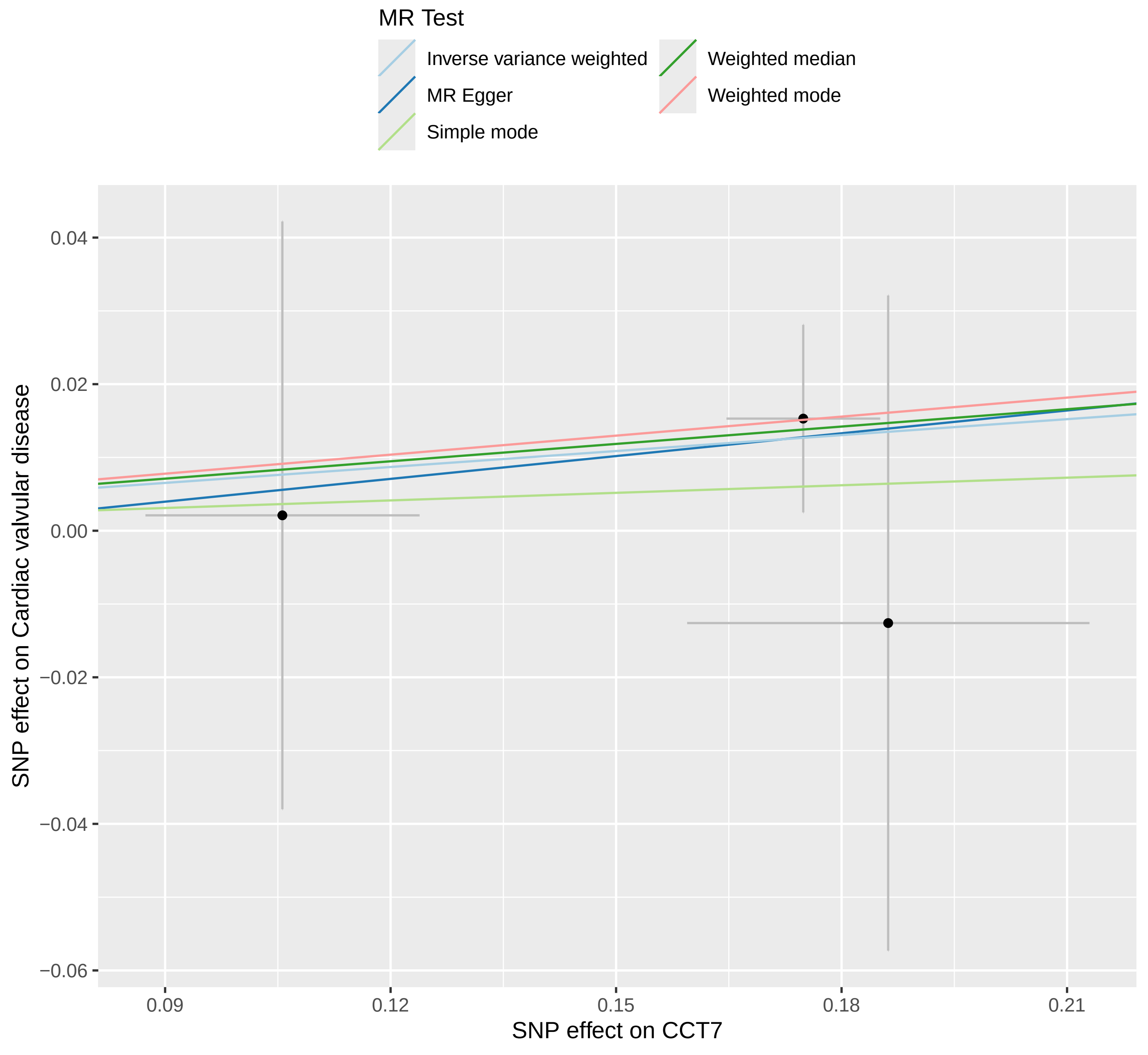

MR Test
Inverse variance weighted
MR Egger
Simple mode
Weighted median
Weighted mode
SNP effect on Cardiac valvular disease
0.04
0.02
0.00
−0.02
−0.04
−0.06
0.09
0.12
0.15
0.18
0.21
SNP effect on CCT7

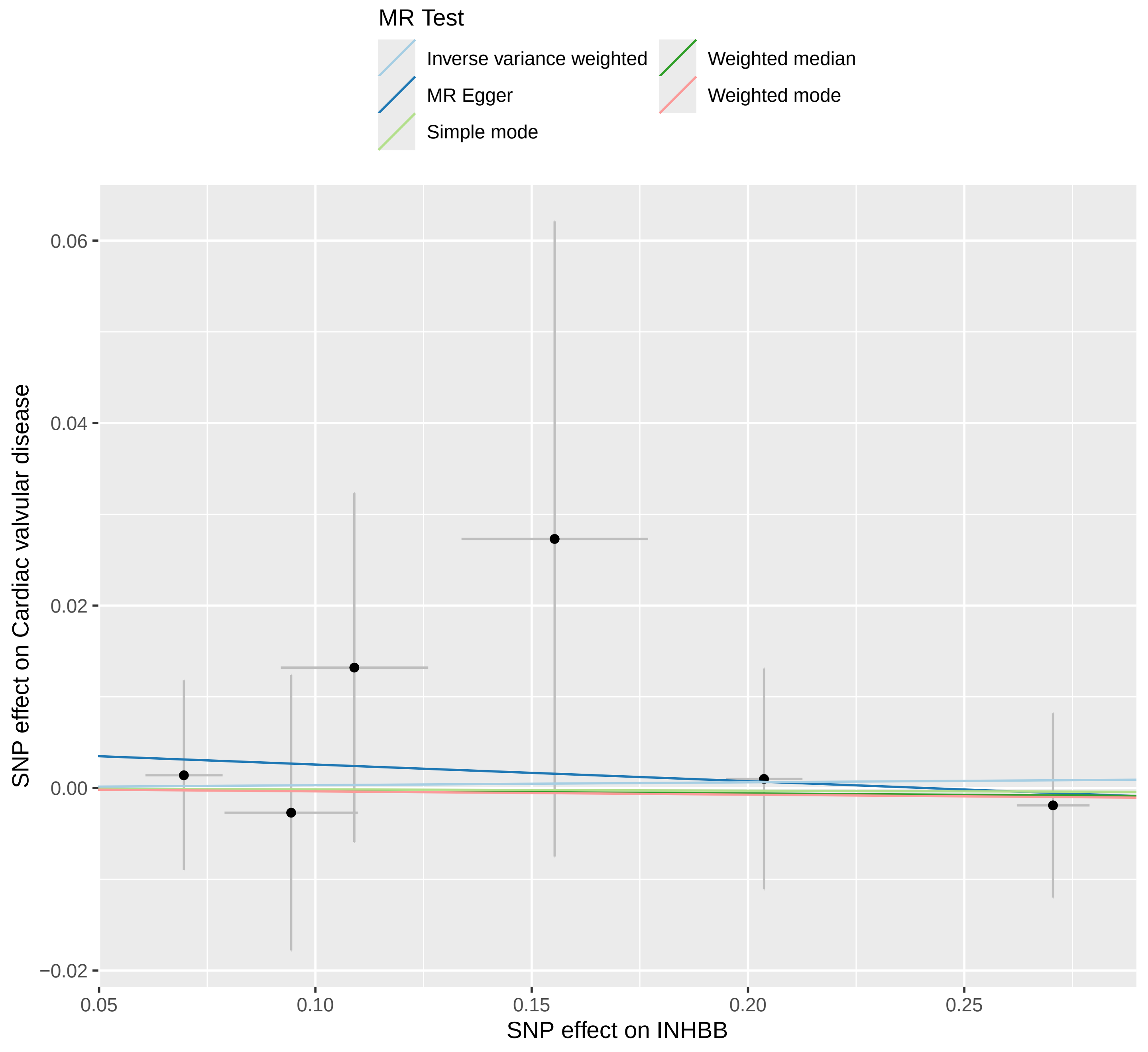
MR Test
Inverse variance weighted
MR Egger
Simple mode
Weighted median
Weighted mode
0.06
0.04
0.02
0.00
−0.02
SNP effect on Cardiac valvular disease
0.05
0.10
0.15
0.20
0.25
SNP effect on INHBB

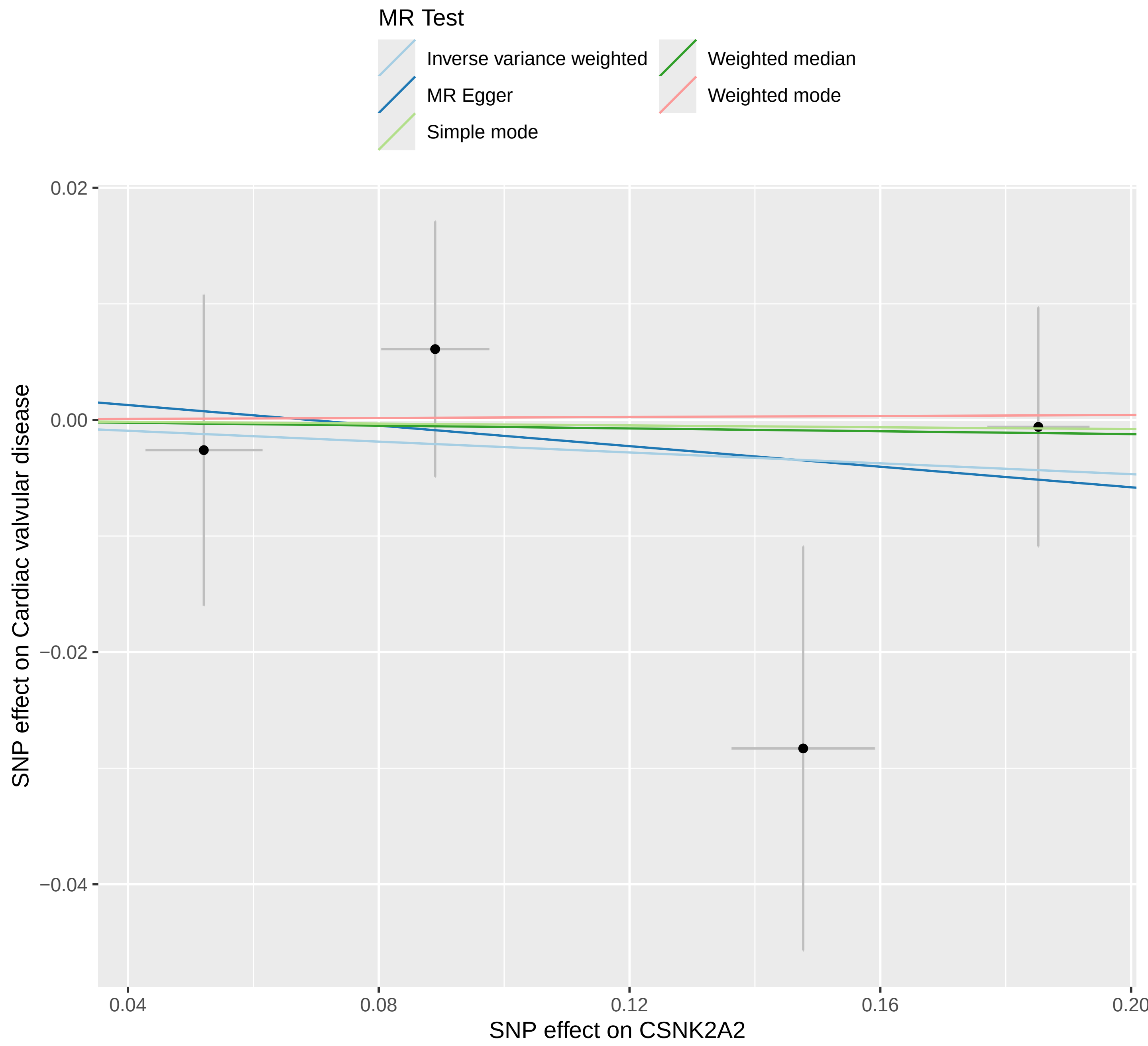

MR Test
Inverse variance weighted
MR Egger
Simple mode
Weighted median
Weighted mode
0.02
0.00
−0.02
−0.04
0.04
0.08
0.12
0.16
0.20
SNP effect on CSNK2A2
SNP effect on Cardiac valvular disease

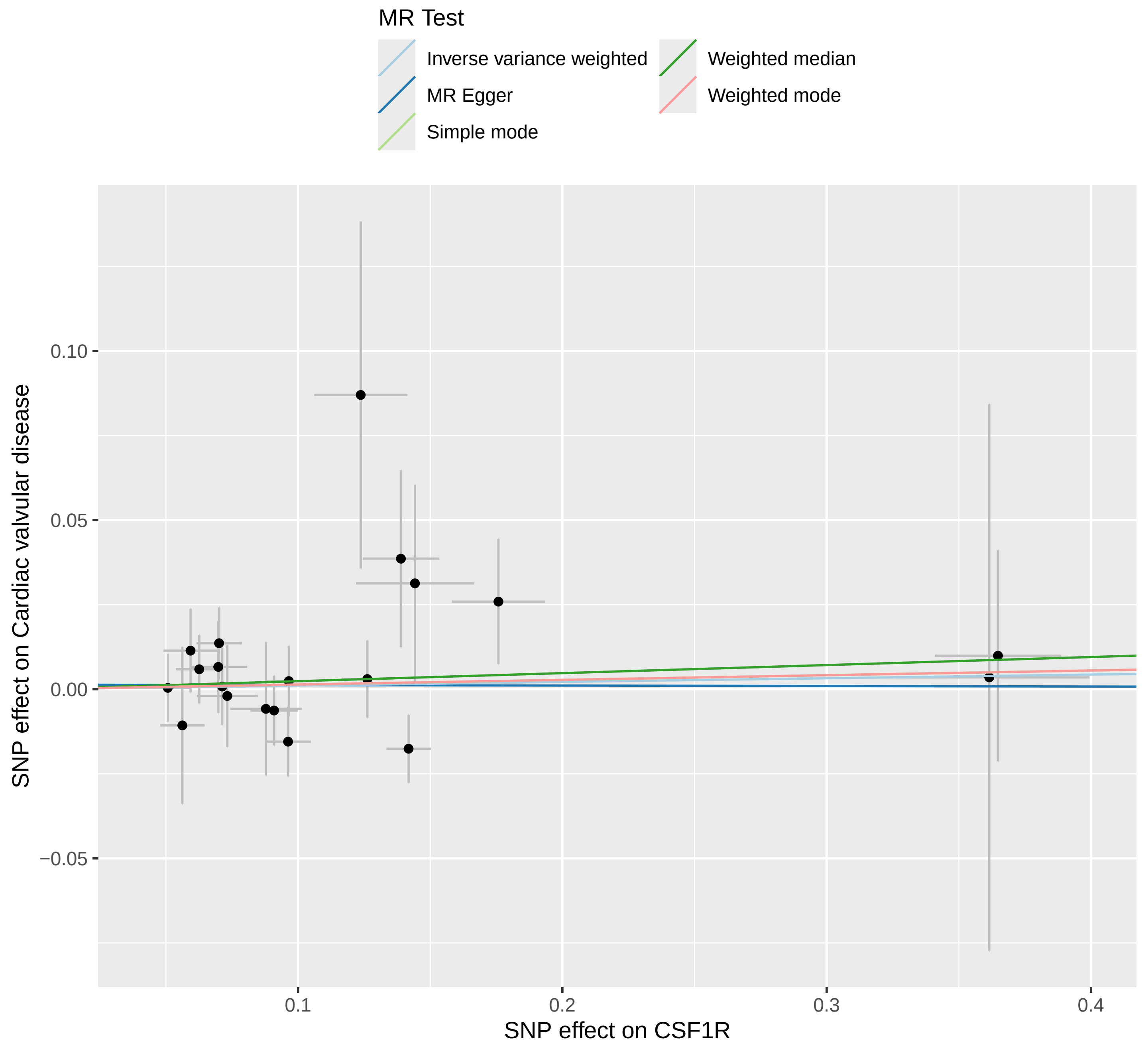

MR Test
Inverse variance weighted
MR Egger
Simple mode
Weighted median
Weighted mode
SNP effect on Cardiac valvular disease
0.10
0.05
0.00
−0.05
0.1
0.2
0.3
0.4
SNP effect on CSF1R

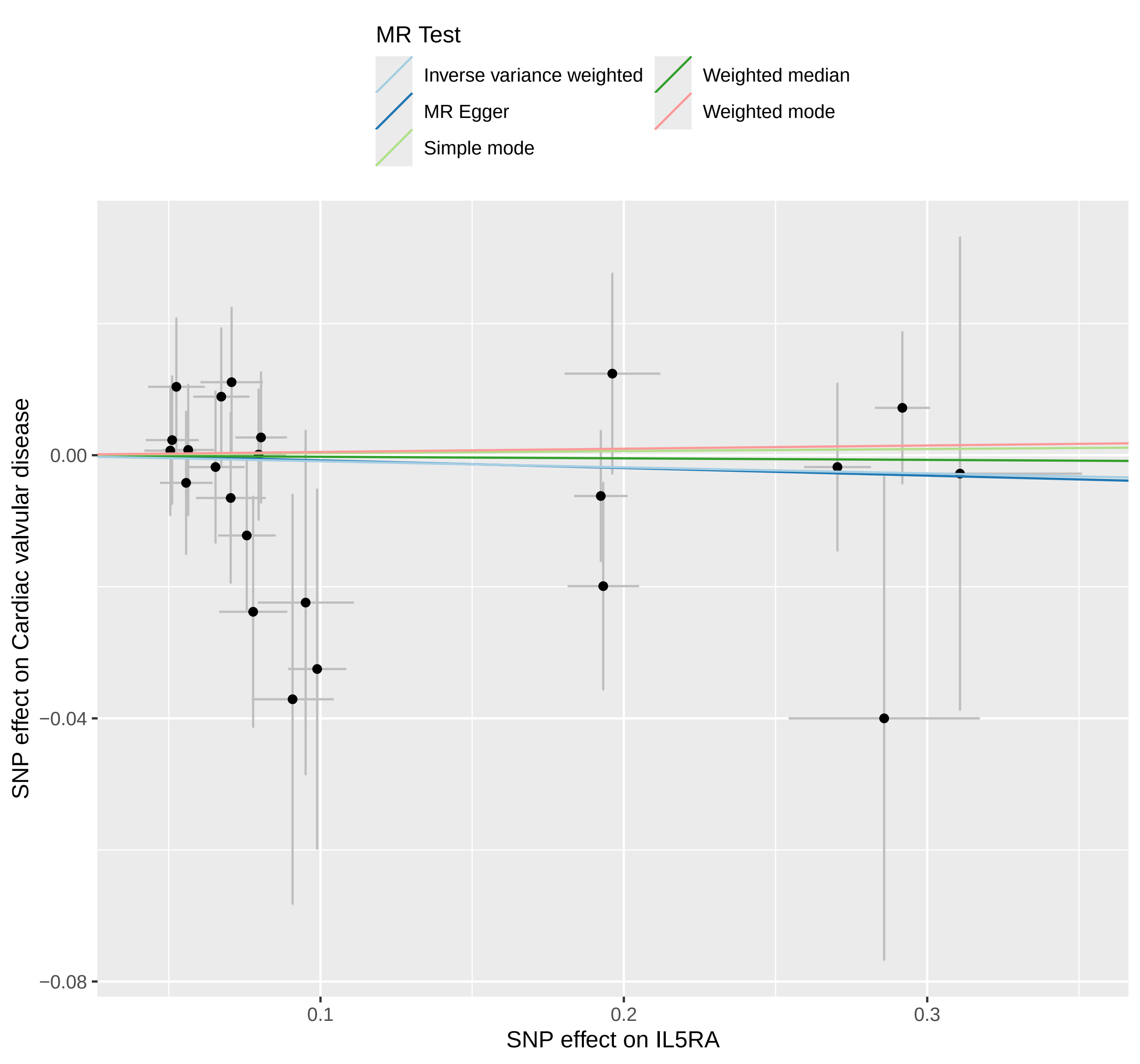
MR Test
Inverse variance weighted
MR Egger
Simple mode
Weighted median
Weighted mode
0.00
−0.04
−0.08
0.1
0.2
0.3
SNP effect on IL5RA
SNP effect on Cardiac valvular disease

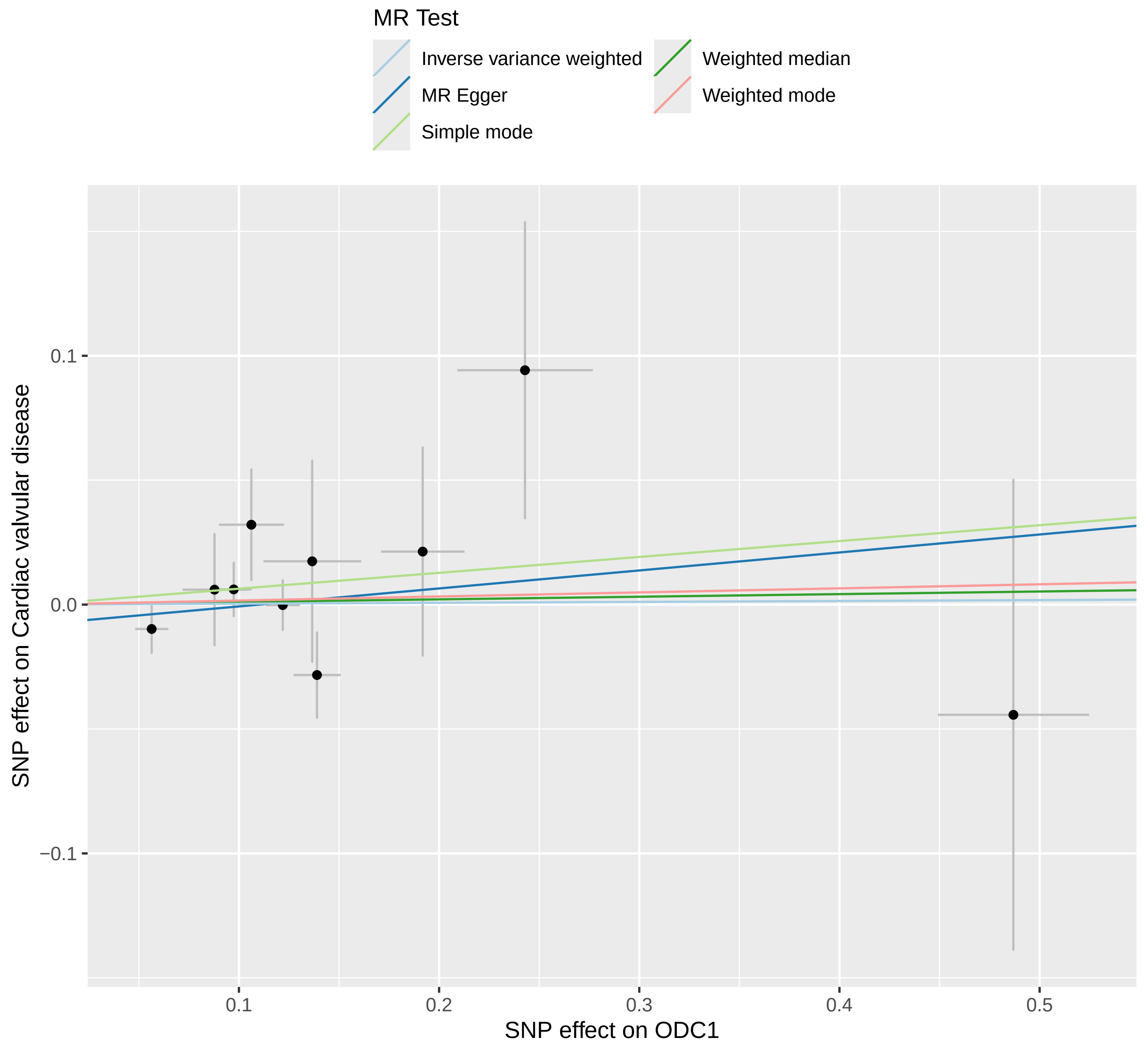

MR Test
Inverse variance weighted
MR Egger
Simple mode
Weighted median
Weighted mode
0.1
0.0
−0.1
SNP effect on Cardiac valvular disease
0.1
0.2
0.3
0.4
0.5
SNP effect on ODC1

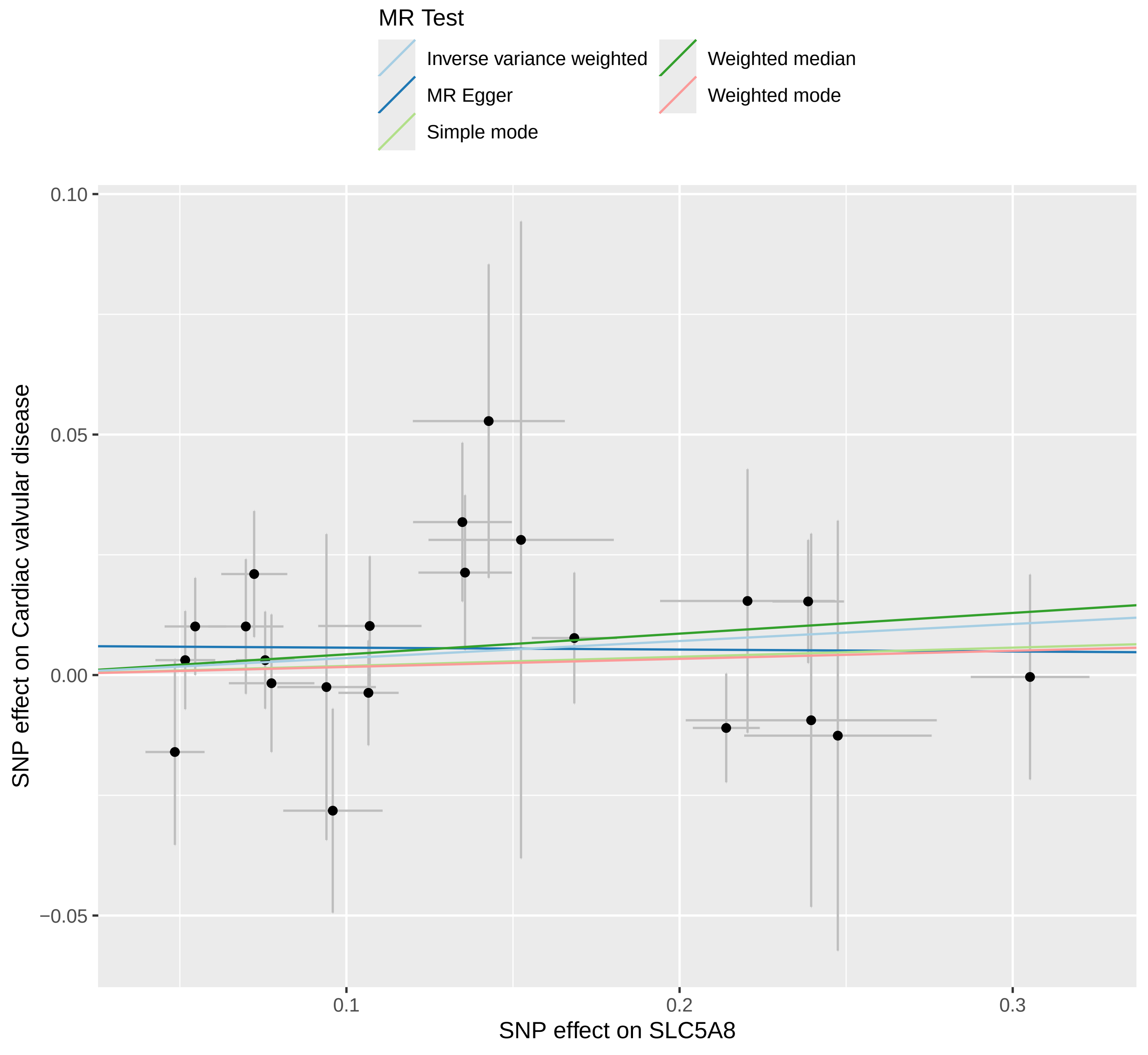

MR Test
Inverse variance weighted
MR Egger
Simple mode
Weighted median
Weighted mode
0.10
0.05
0.00
−0.05
SNP effect on Cardiac valvular disease
0.1
0.2
0.3
SNP effect on SLC5A8

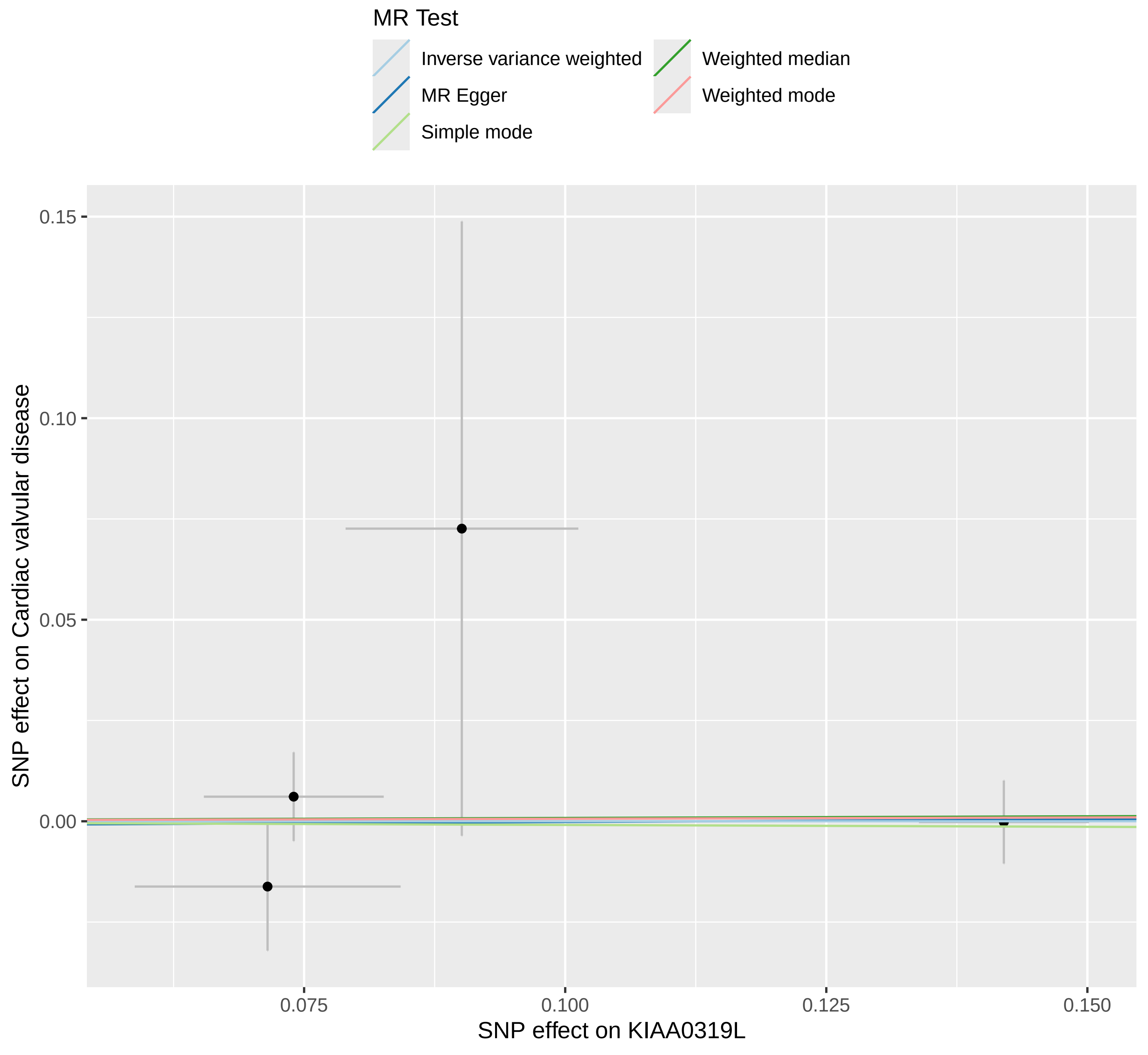

MR Test
Inverse variance weighted
MR Egger
Simple mode
Weighted median
Weighted mode
0.15
0.10
0.05
0.00
SNP effect on Cardiac valvular disease
0.075
0.100
0.125
0.150
SNP effect on KIAA0319L

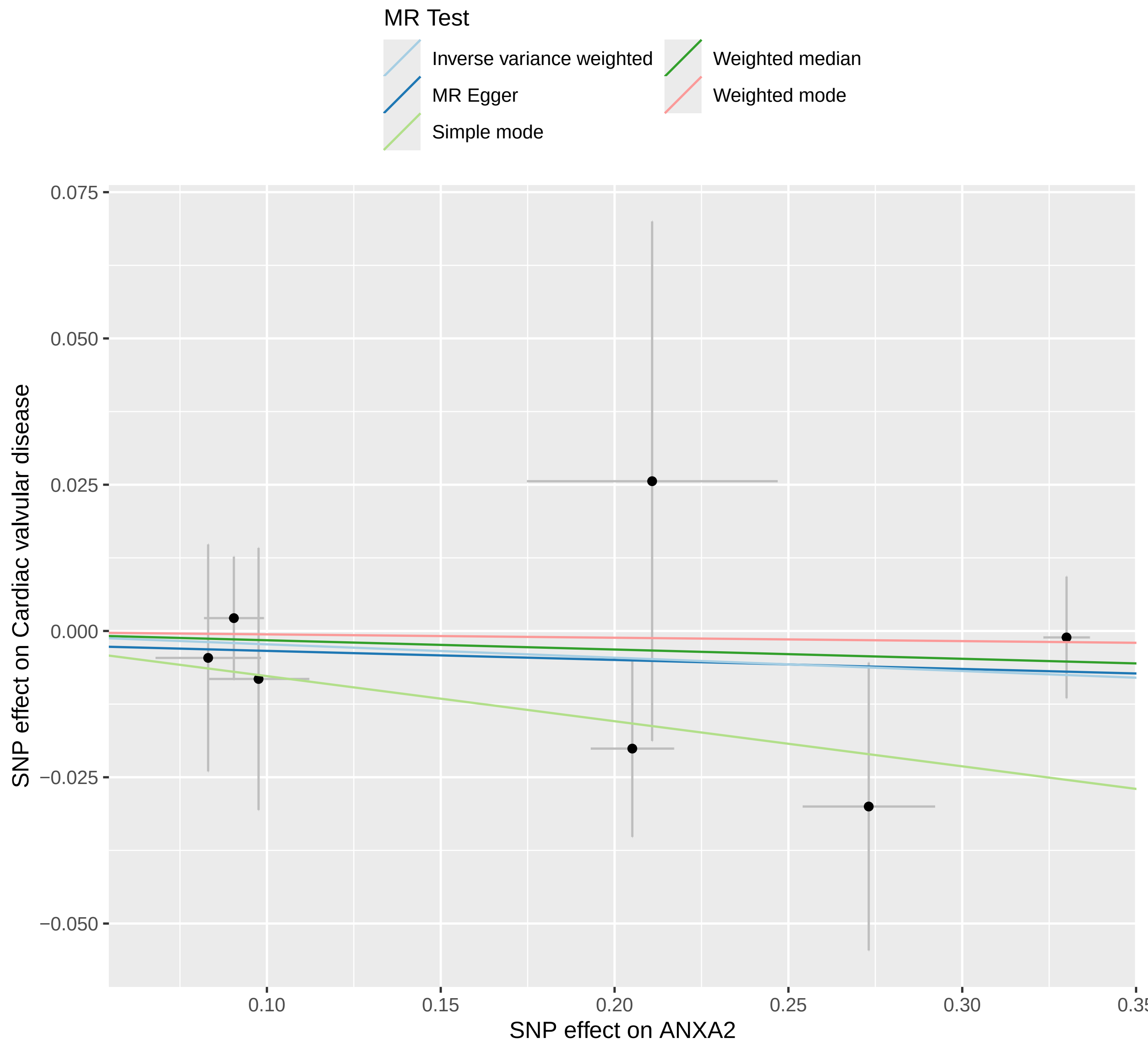

MR Test
Inverse variance weighted
MR Egger
Simple mode
Weighted median
Weighted mode
0.075
0.050
0.025
0.000
−0.025
−0.050
0.10
0.15
0.20
0.25
0.30
SNP effect on Cardiac valvular disease
SNP effect on ANXA2

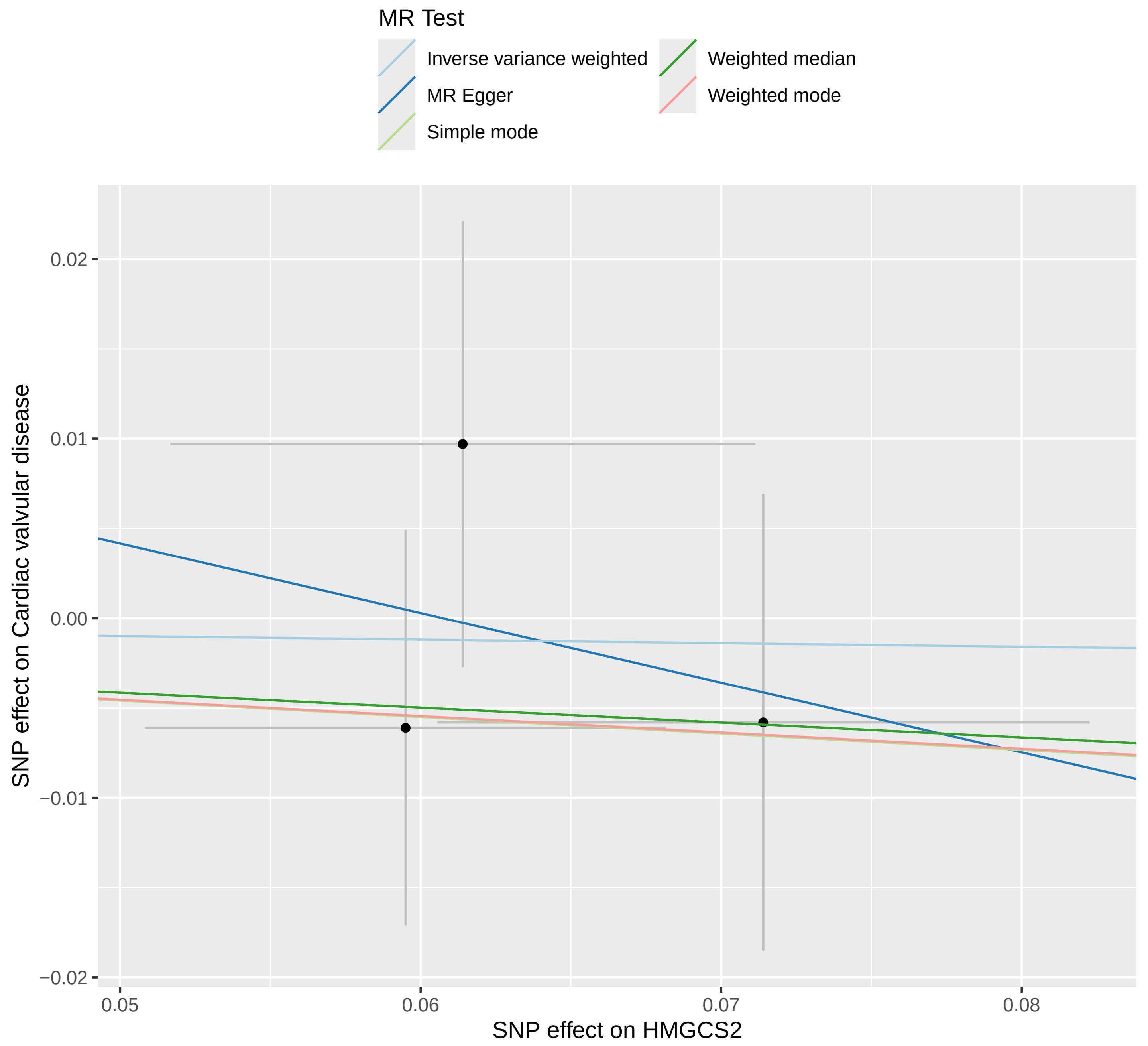
MR Test
Inverse variance weighted
MR Egger
Simple mode
Weighted median
Weighted mode
SNP effect on Cardiac valvular disease
0.02
0.01
0.00
−0.01
−0.02
0.05
0.06
0.07
0.08
SNP effect on HMGCS2

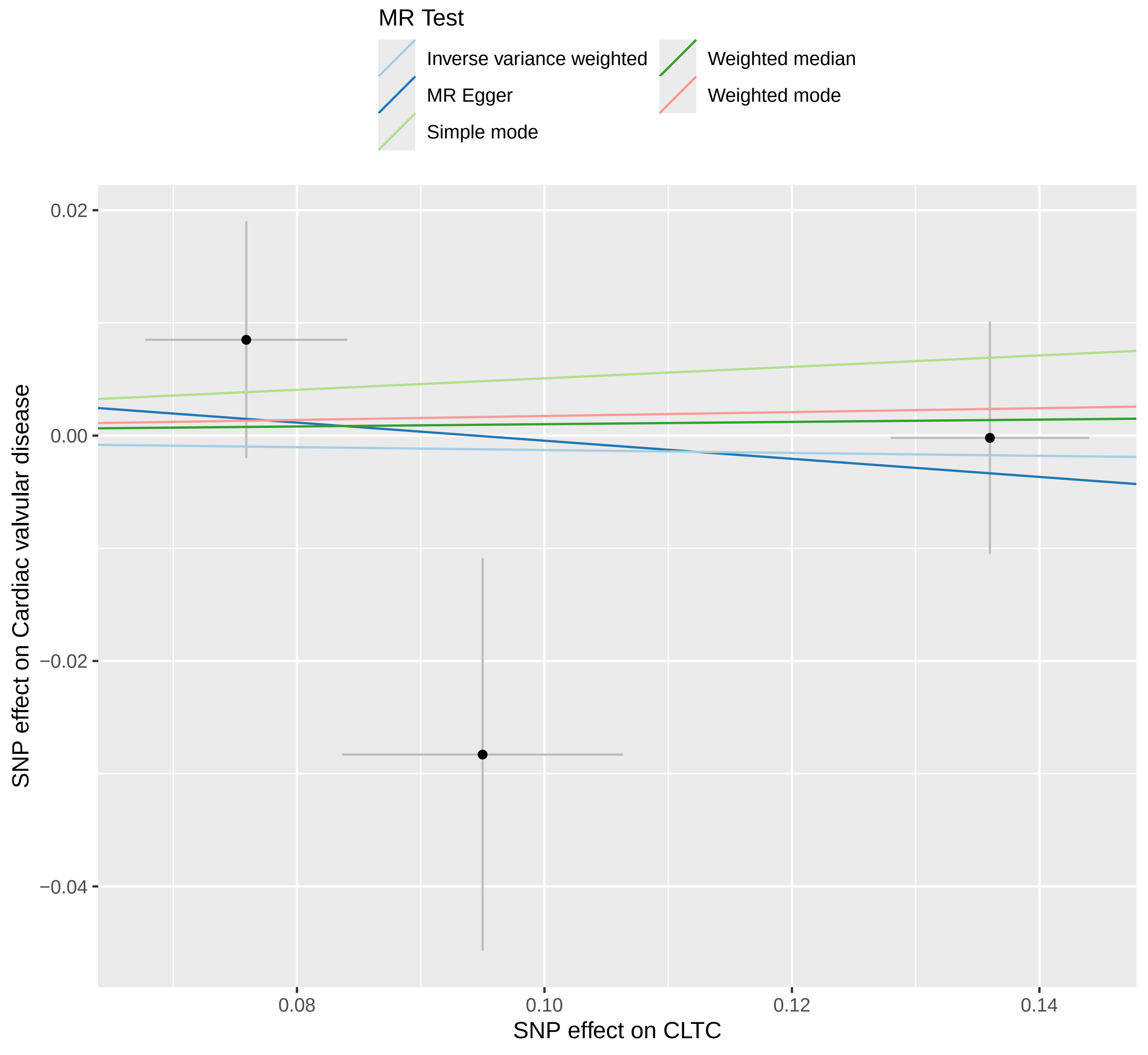
MR Test
Inverse variance weighted
MR Egger
Simple mode
Weighted median
Weighted mode
SNP effect on Cardiac valvular disease
0.02
0.00
−0.02
−0.04
0.08
0.10
0.12
0.14
SNP effect on CLTC

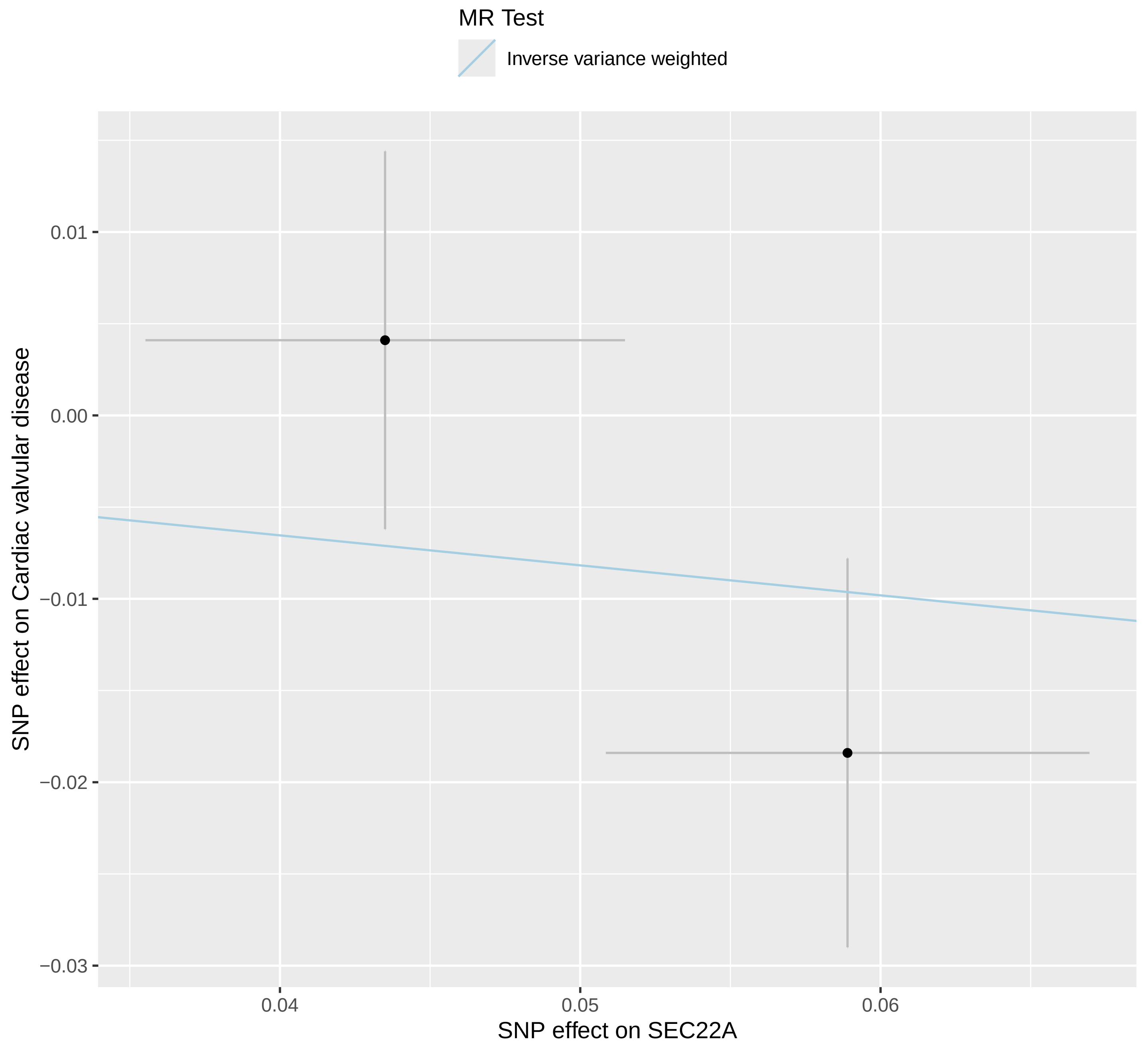

MR Test
Inverse variance weighted
0.01
0.00
−0.01
−0.02
−0.03
SNP effect on Cardiac valvular disease
0.04
0.05
0.06
SNP effect on SEC22A

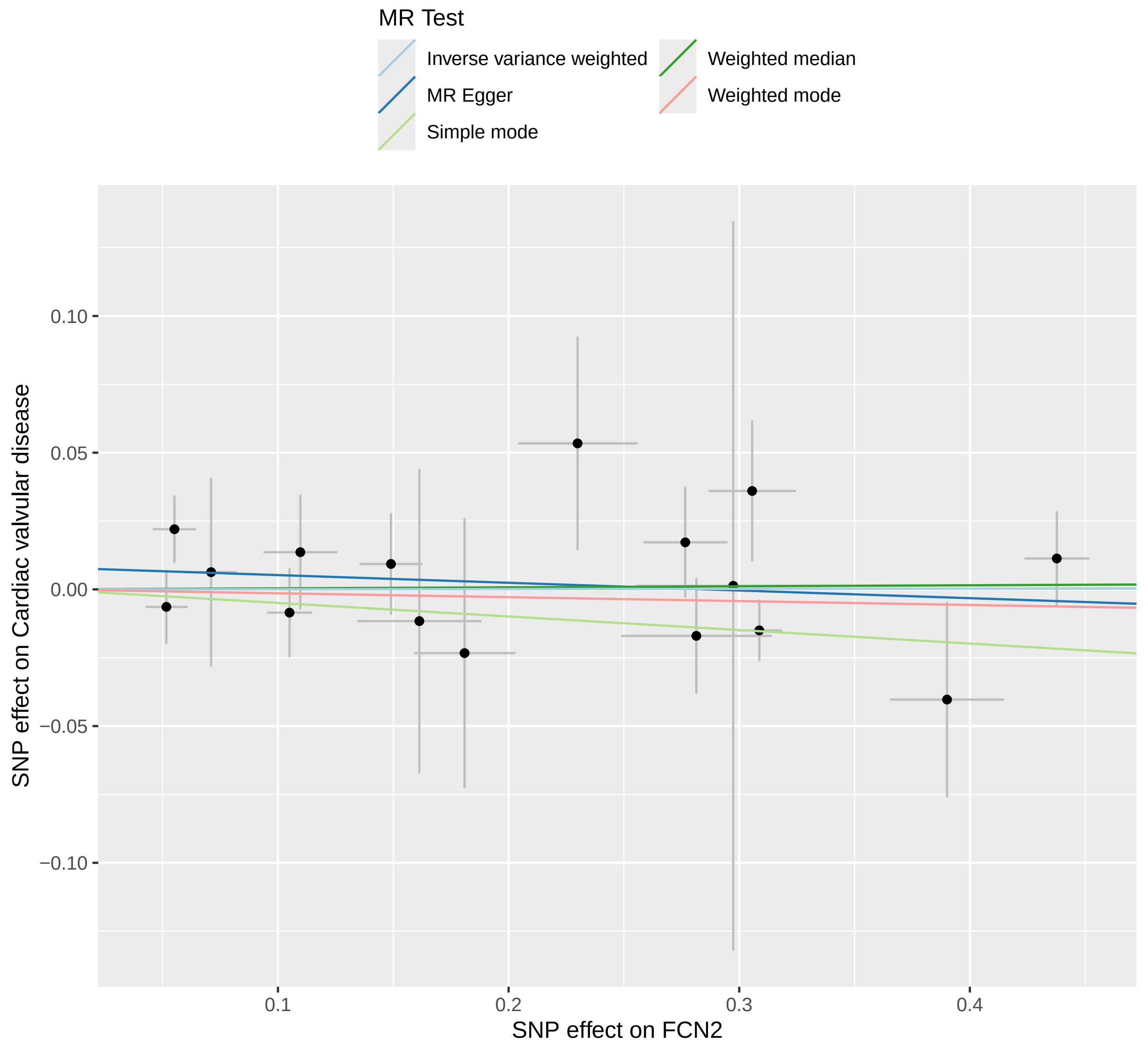

MR Test
Inverse variance weighted
MR Egger
Simple mode
Weighted median
Weighted mode
0.10
0.05
0.00
−0.05
−0.10
SNP effect on Cardiac valvular disease
0.1
0.2
0.3
0.4
SNP effect on FCN2

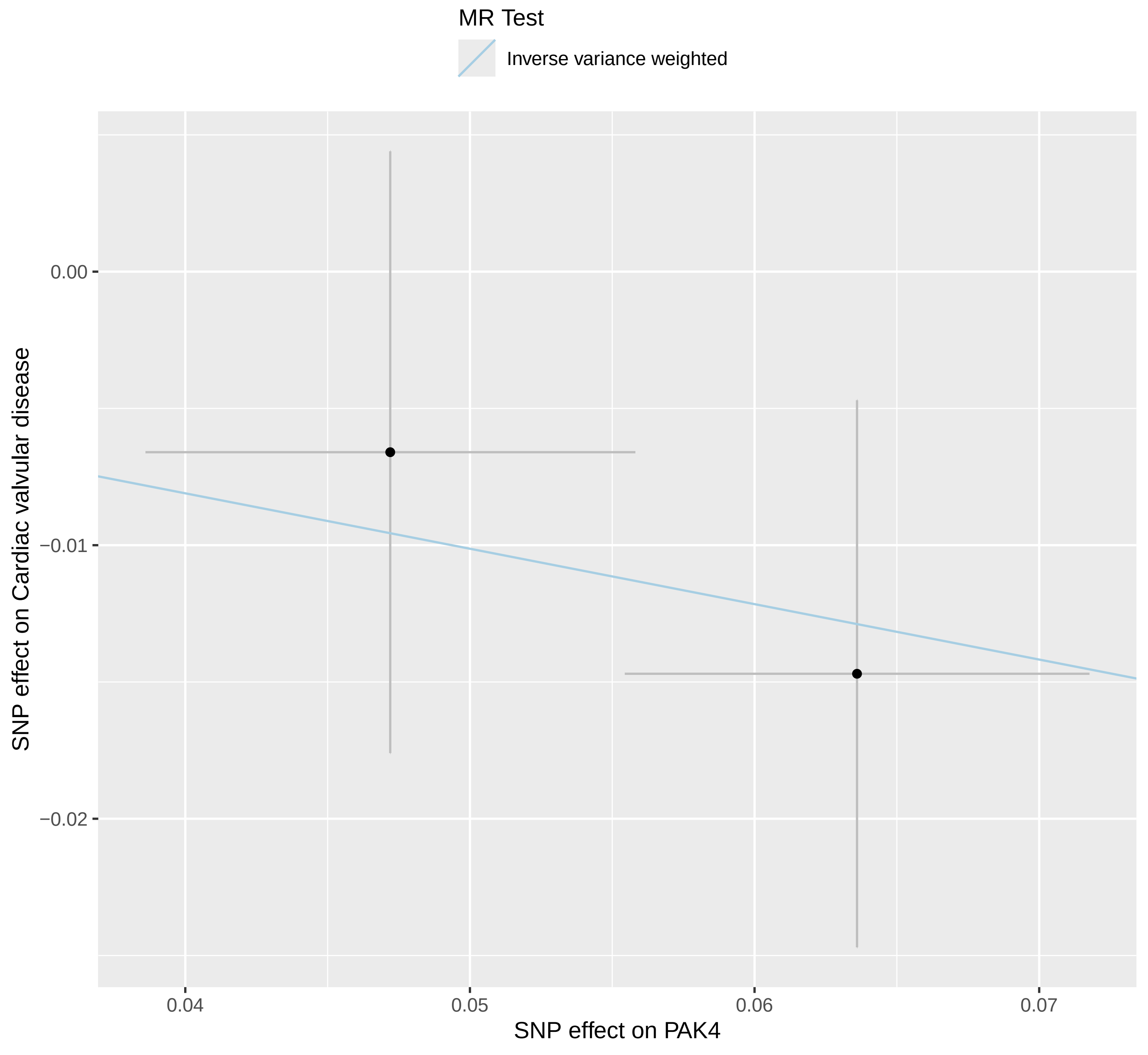
MR Test
Inverse variance weighted
0.00
−0.01
−0.02
SNP effect on Cardiac valvular disease
0.04
0.05
0.06
0.07
SNP effect on PAK4

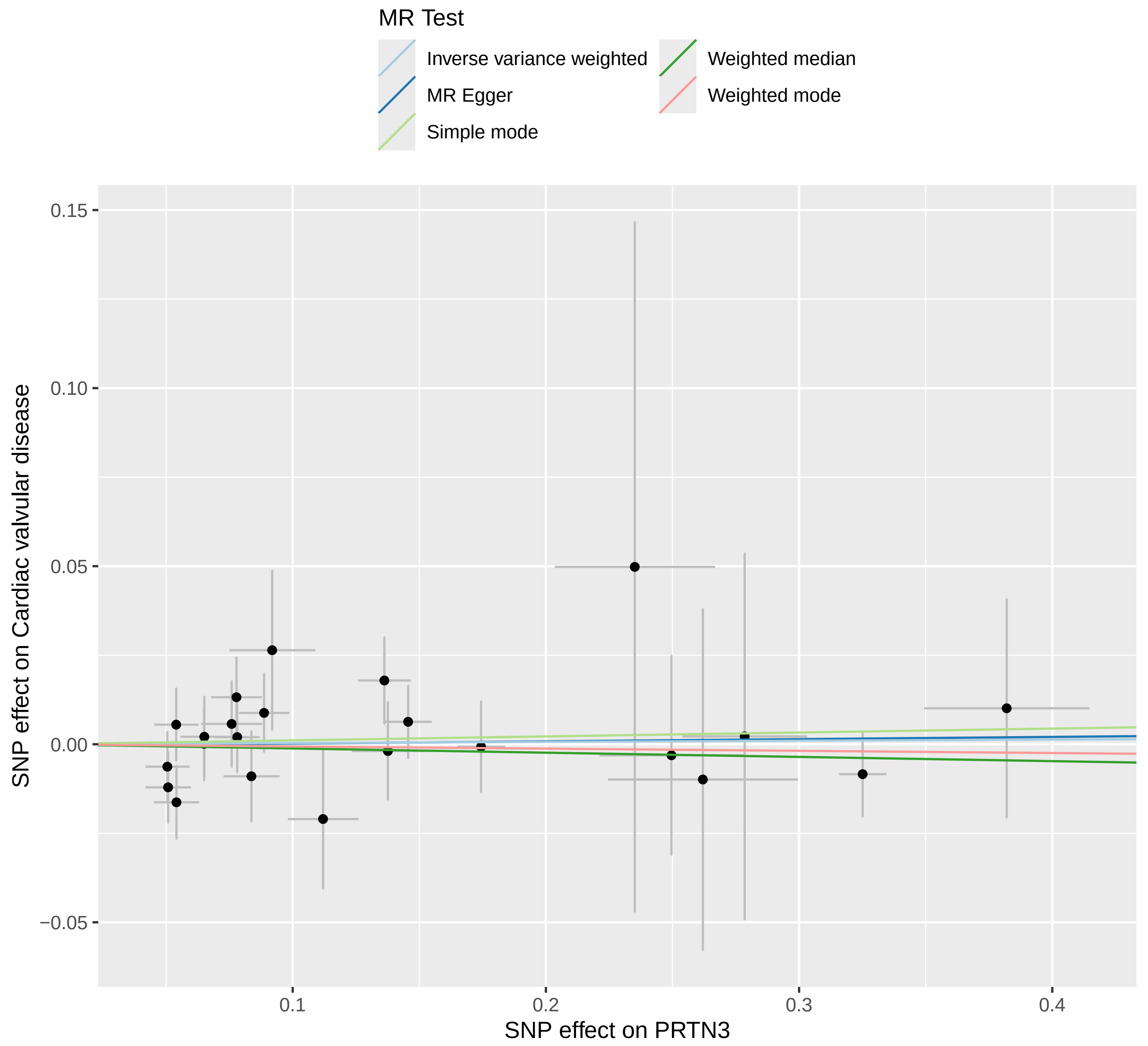
MR Test
Inverse variance weighted
MR Egger
Simple mode
Weighted median
Weighted mode
0.15
0.10
0.05
0.00
−0.05
0.1
0.2
0.3
0.4
SNP effect on Cardiac valvular disease
SNP effect on PRTN3

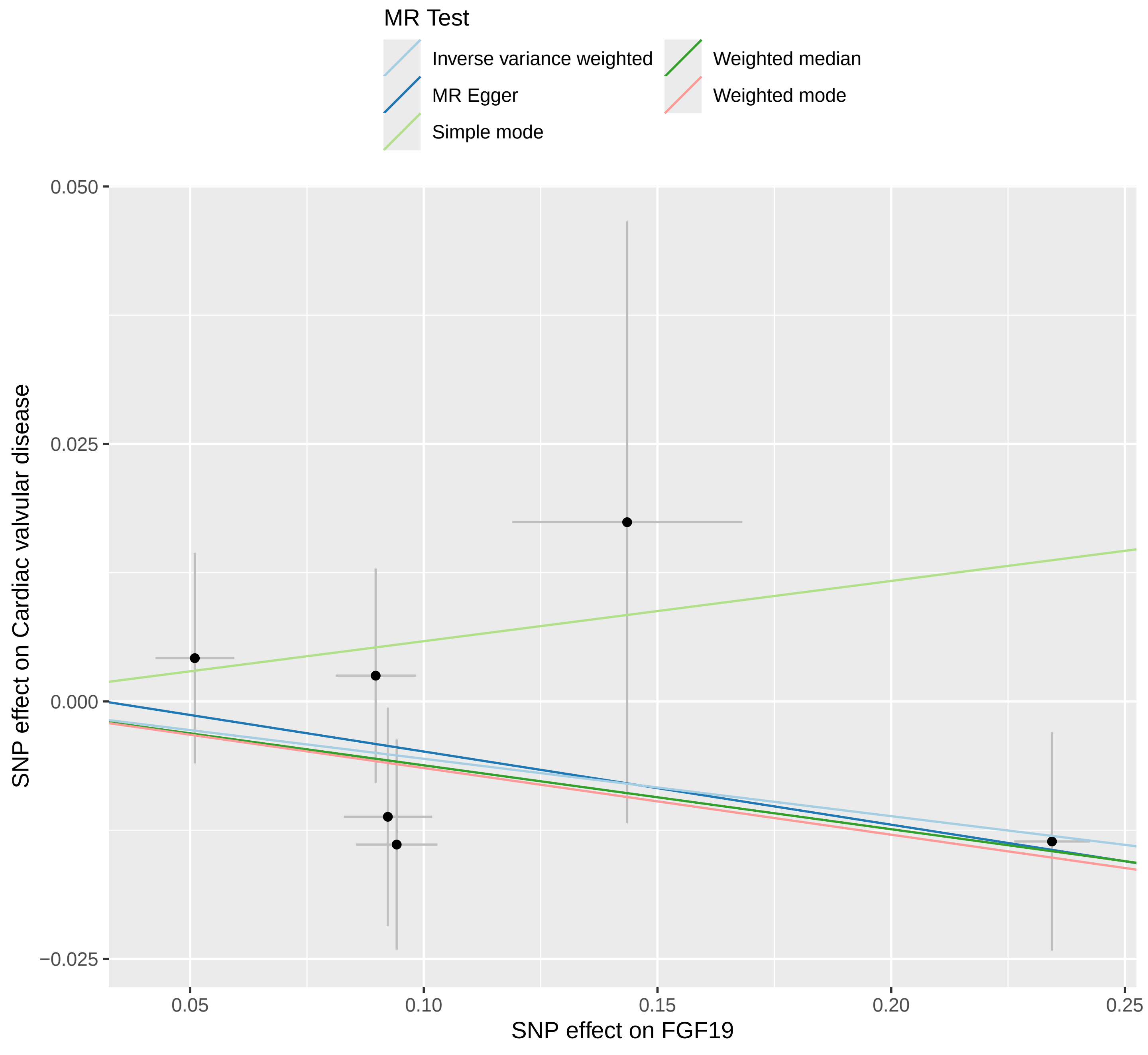
MR Test
Inverse variance weighted
MR Egger
Simple mode
Weighted median
Weighted mode
0.050
0.025
0.000
−0.025
0.05
0.10
0.15
0.20
0.25
SNP effect on Cardiac valvular disease
SNP effect on FGF19

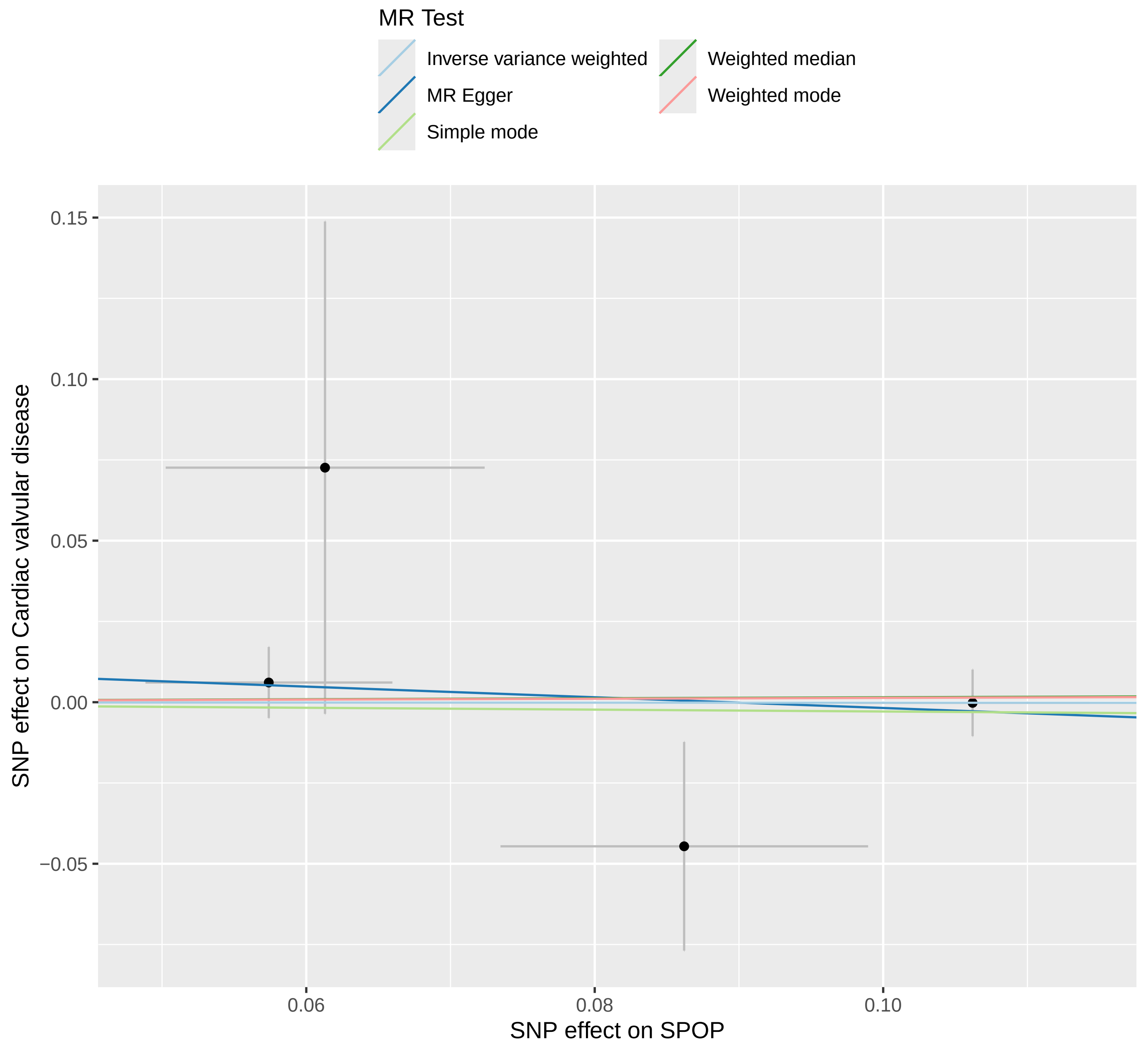
MR Test
Inverse variance weighted
MR Egger
Simple mode
Weighted median
Weighted mode
0.15
0.10
0.05
0.00
−0.05
0.06
0.08
0.10
SNP effect on Cardiac valvular disease
SNP effect on SPOP

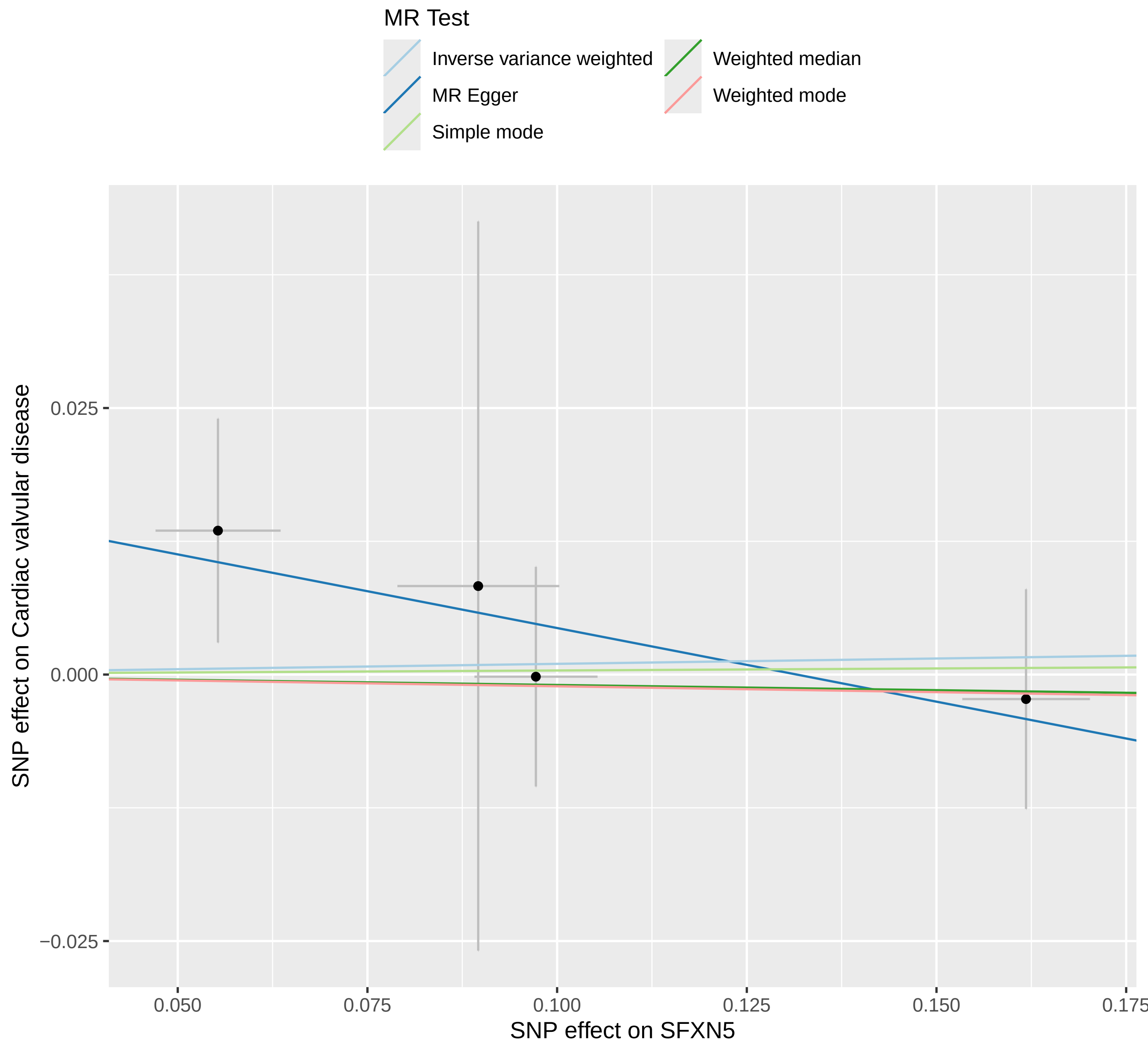
MR Test
Inverse variance weighted
MR Egger
Simple mode
Weighted median
Weighted mode
0.025
0.000
−0.025
0.050
0.075
0.100
0.125
0.150
0.175
SNP effect on Cardiac valvular disease
SNP effect on SFXN5

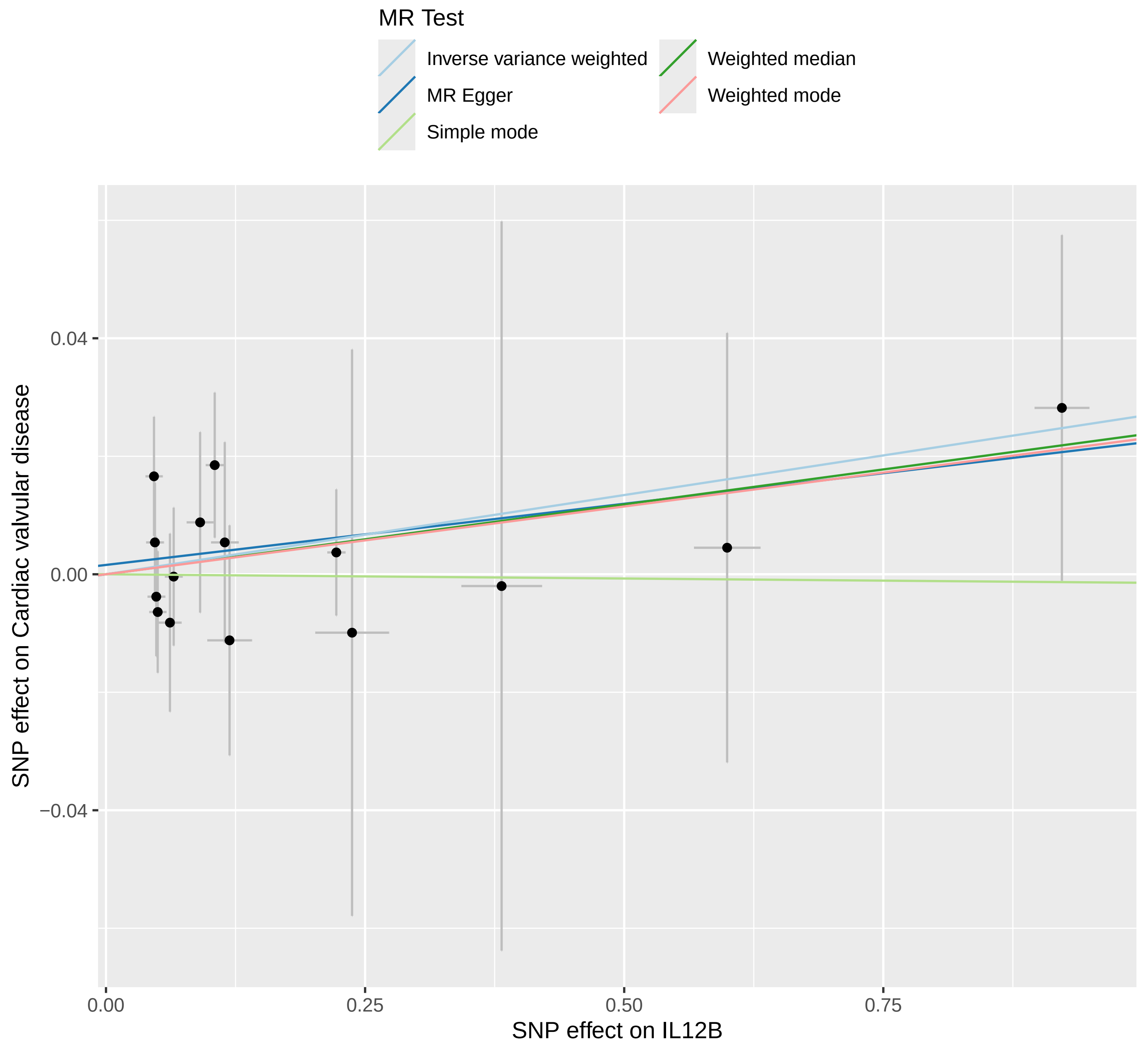

MR Test
Inverse variance weighted
MR Egger
Simple mode
Weighted median
Weighted mode
0.04
0.00
−0.04
SNP effect on Cardiac valvular disease
0.00
0.25
0.50
0.75
SNP effect on IL12B

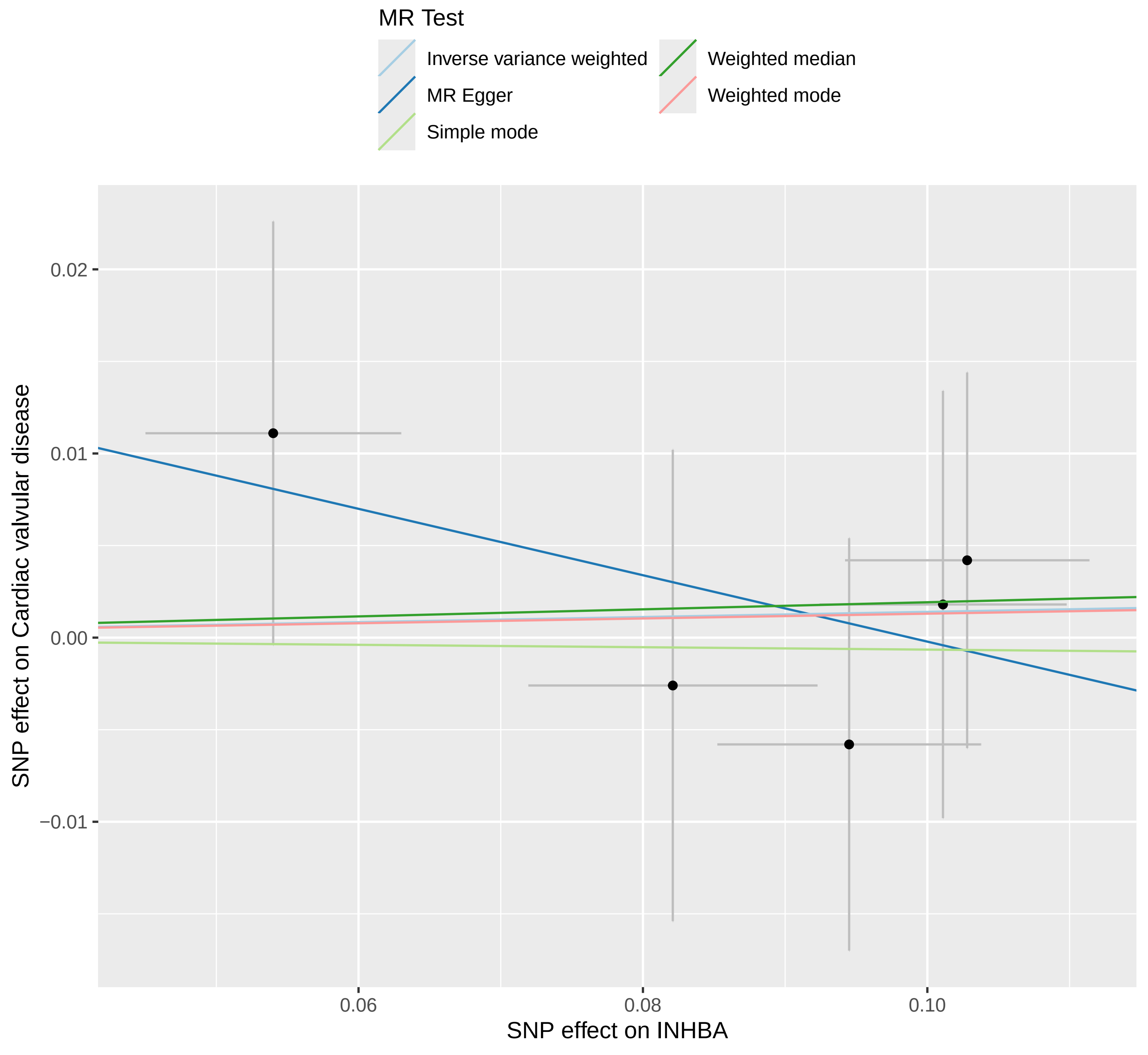
MR Test
Inverse variance weighted
MR Egger
Simple mode
Weighted median
Weighted mode
0.02
0.01
0.00
−0.01
SNP effect on Cardiac valvular disease
0.06
0.08
0.10
SNP effect on INHBA

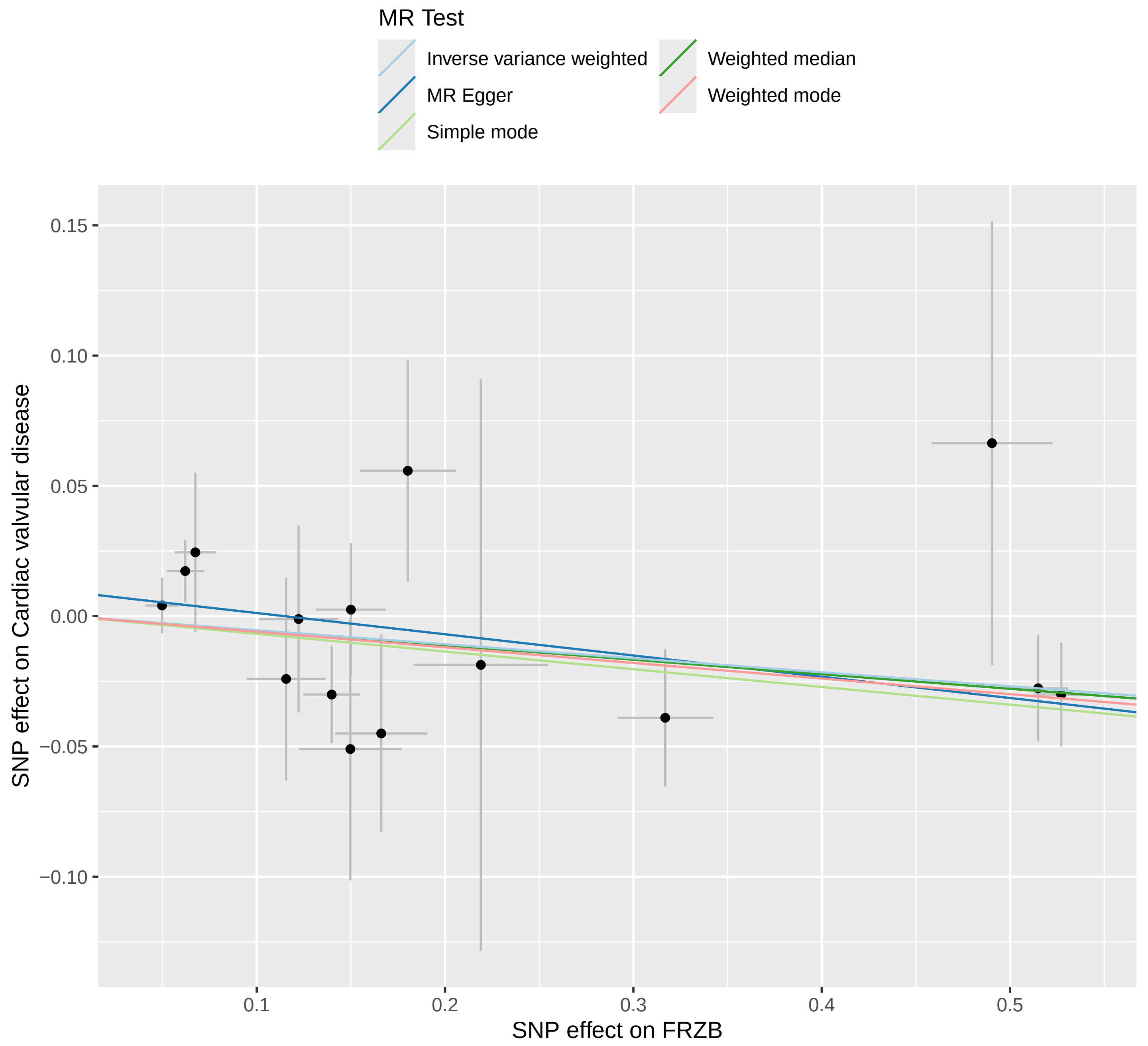
MR Test
Inverse variance weighted
MR Egger
Simple mode
Weighted median
Weighted mode
0.15
0.10
0.05
0.00
−0.05
−0.10
SNP effect on Cardiac valvular disease
0.1
0.2
0.3
0.4
0.5
SNP effect on FRZB

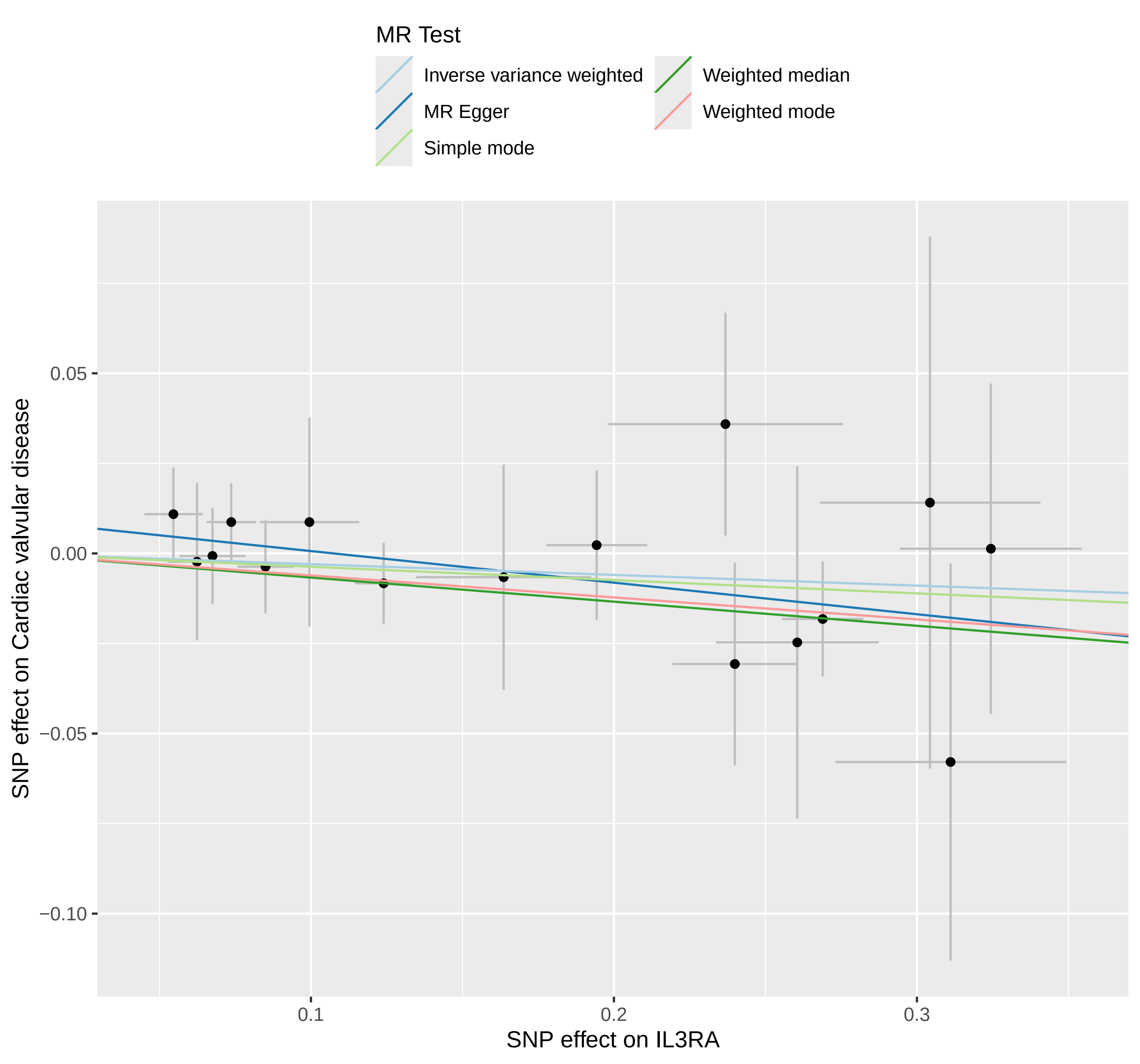

MR Test
Inverse variance weighted
MR Egger
Simple mode
Weighted median
Weighted mode
0.05
0.00
−0.05
−0.10
0.1
0.2
0.3
SNP effect on Cardiac valvular disease
SNP effect on IL3RA

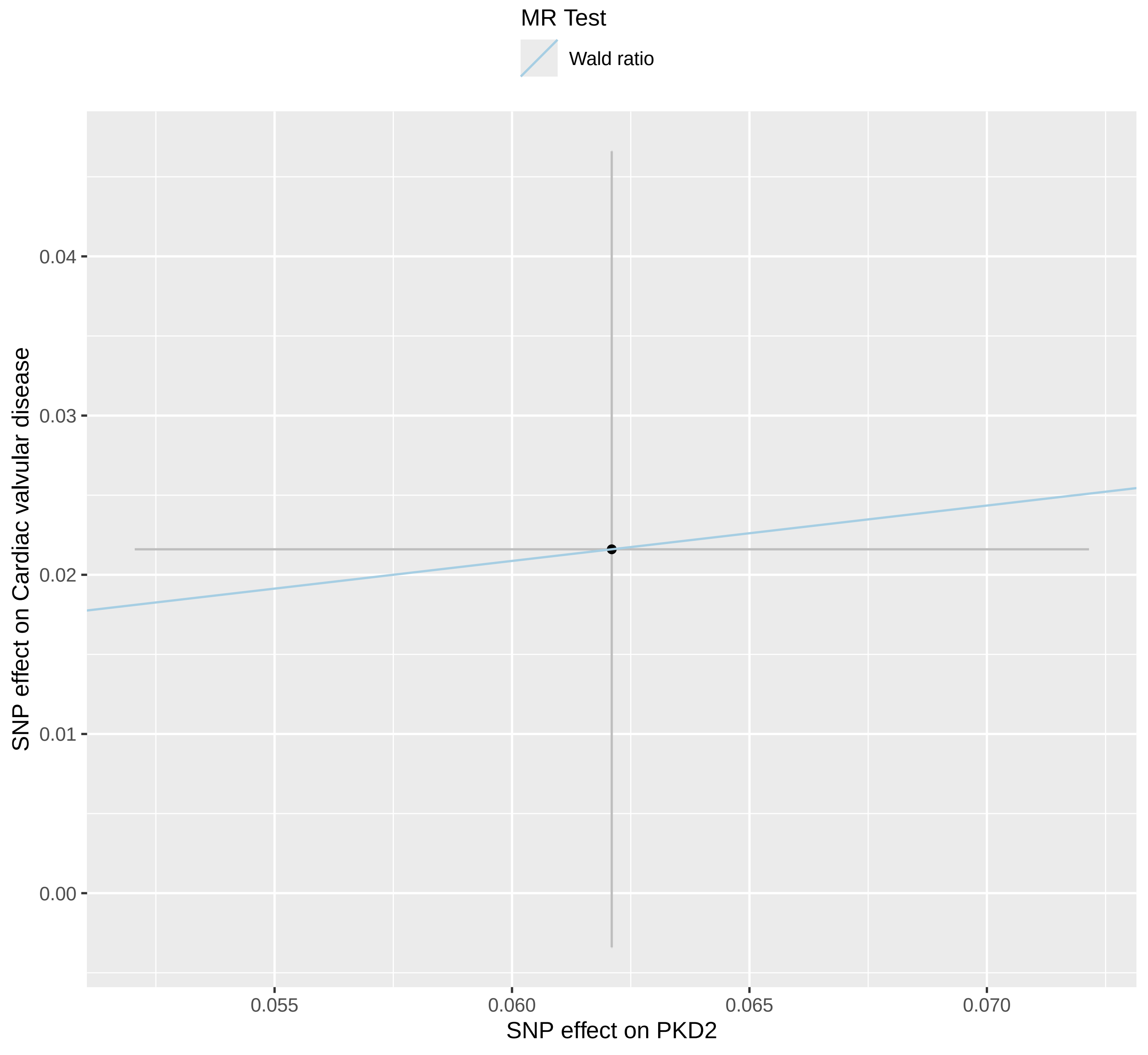

MR Test
Wald ratio
0.04
0.03
0.02
0.01
0.00
0.055
0.060
0.065
0.070
SNP effect on PKD2
SNP effect on Cardiac valvular disease

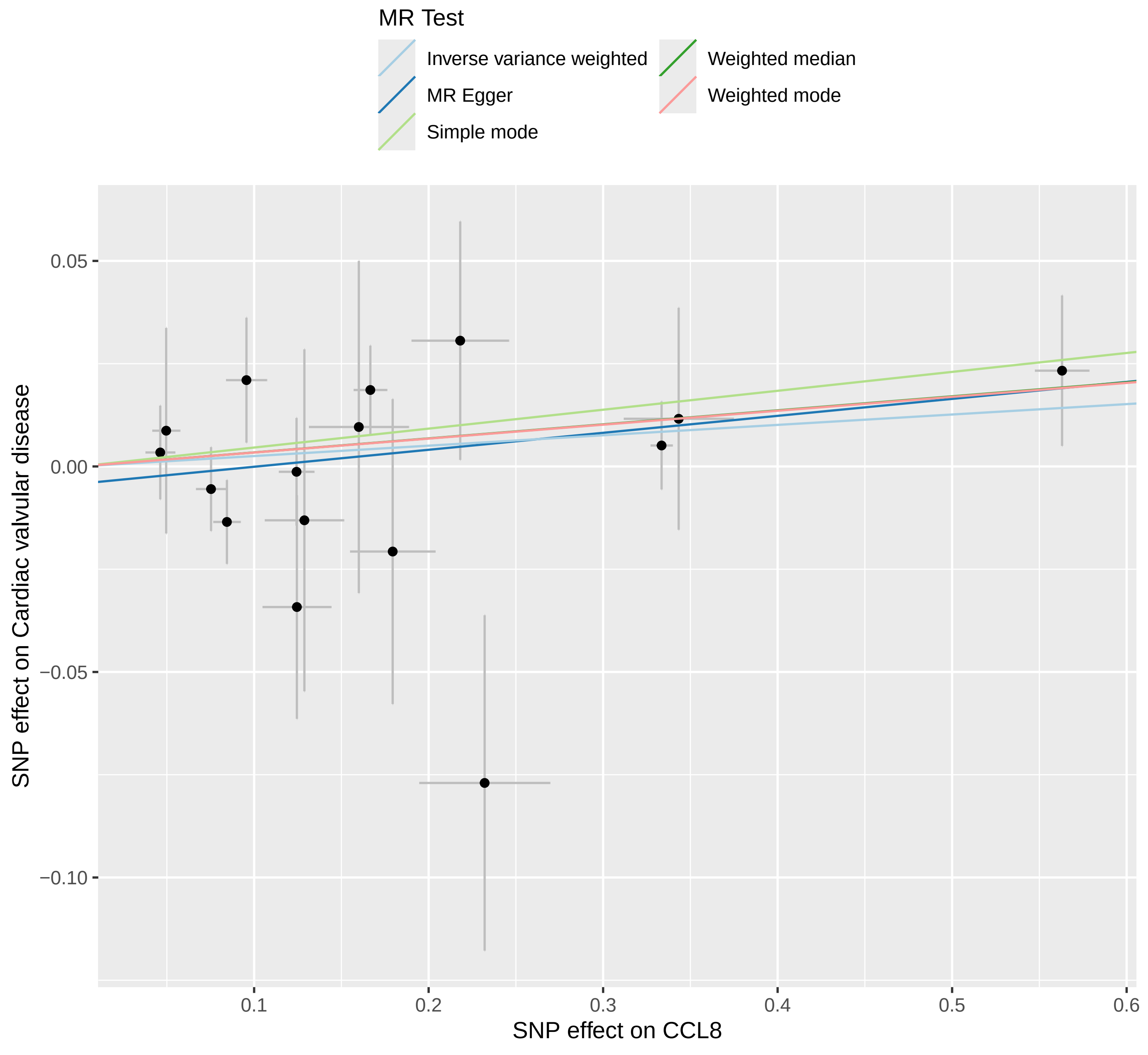

MR Test
Inverse variance weighted
MR Egger
Simple mode
Weighted median
Weighted mode
0.05
0.00
−0.05
−0.10
SNP effect on Cardiac valvular disease
0.1
0.2
0.3
0.4
0.5
0.6
SNP effect on CCL8

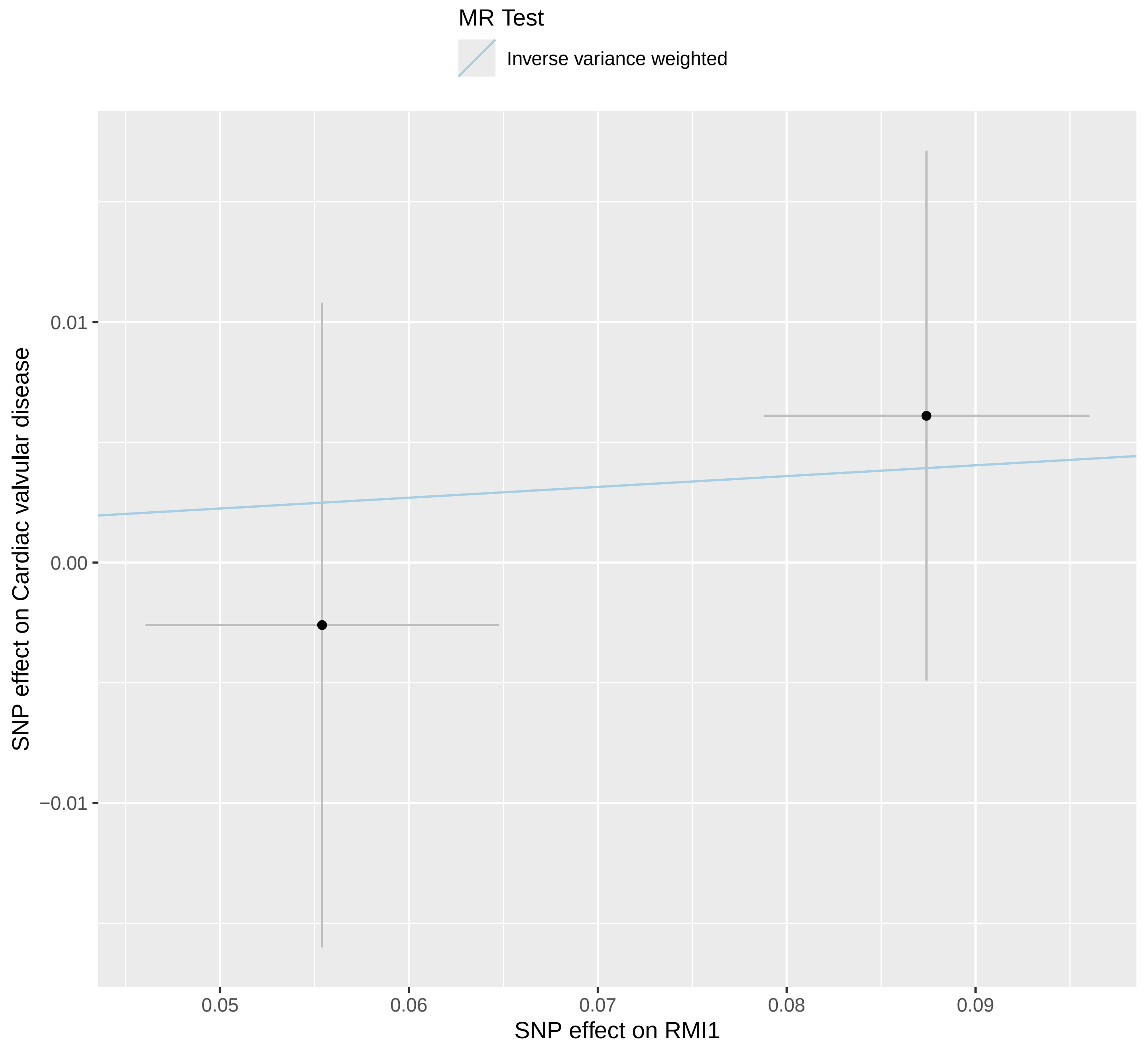

MR Test
Inverse variance weighted
0.01
0.00
−0.01
SNP effect on Cardiac valvular disease
0.05
0.06
0.07
0.08
0.09
SNP effect on RMI1

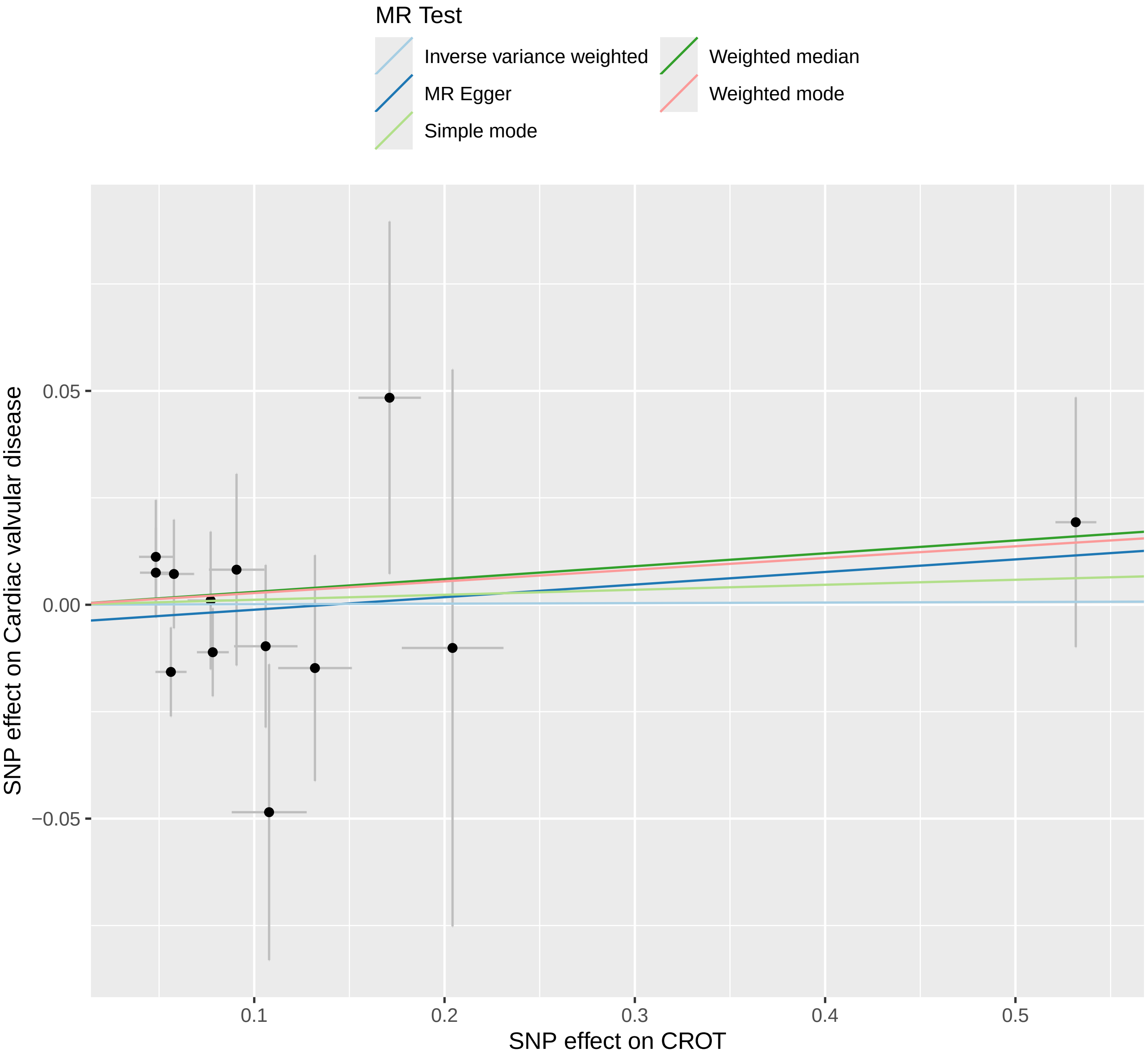
MR Test
Inverse variance weighted
MR Egger
Simple mode
Weighted median
Weighted mode
0.05
0.00
−0.05
SNP effect on Cardiac valvular disease
0.1
0.2
0.3
0.4
0.5
SNP effect on CROT

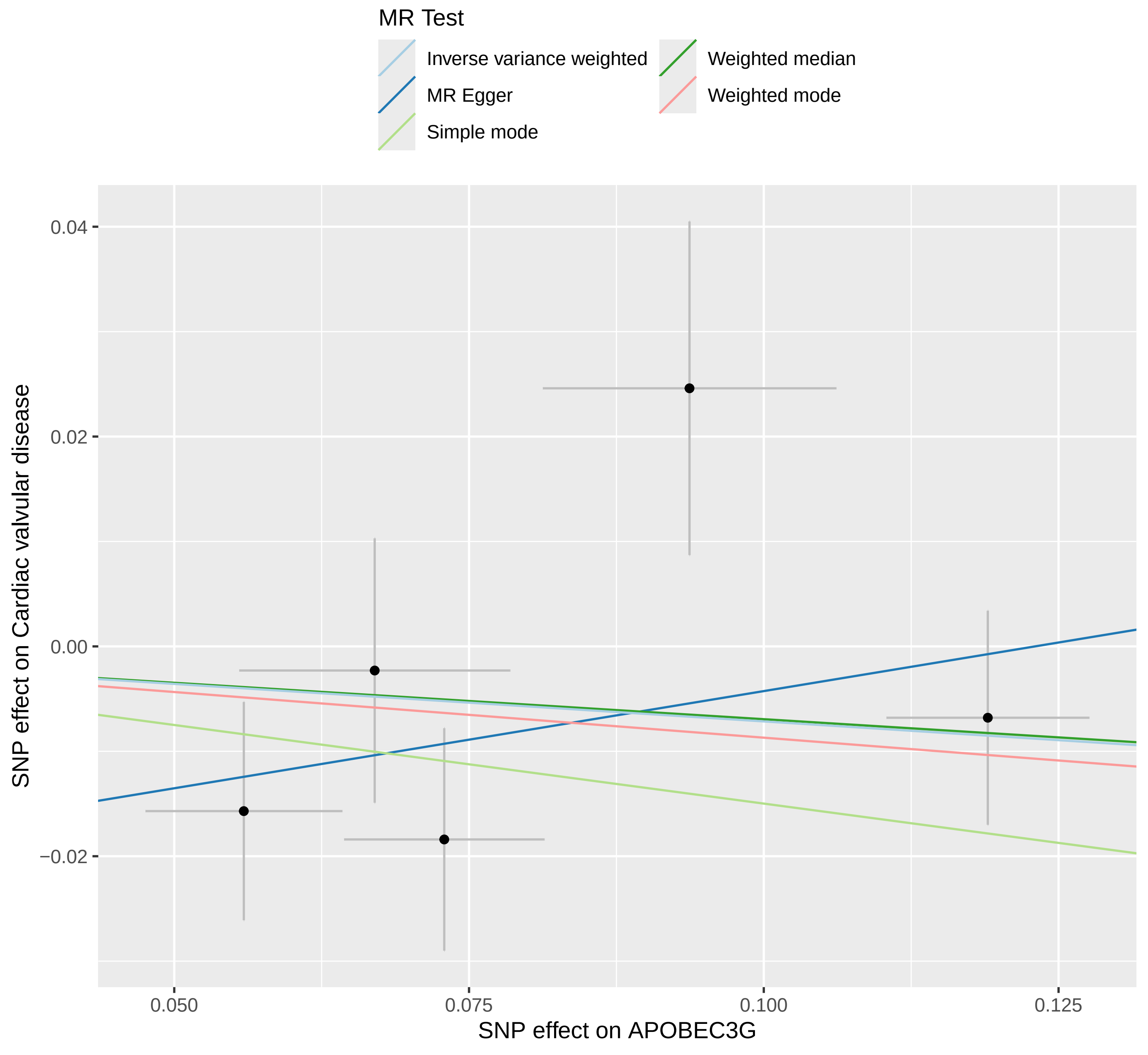
MR Test
Inverse variance weighted
MR Egger
Simple mode
Weighted median
Weighted mode
0.04
0.02
0.00
−0.02
0.050
0.075
0.100
0.125
SNP effect on Cardiac valvular disease
SNP effect on APOBEC3G

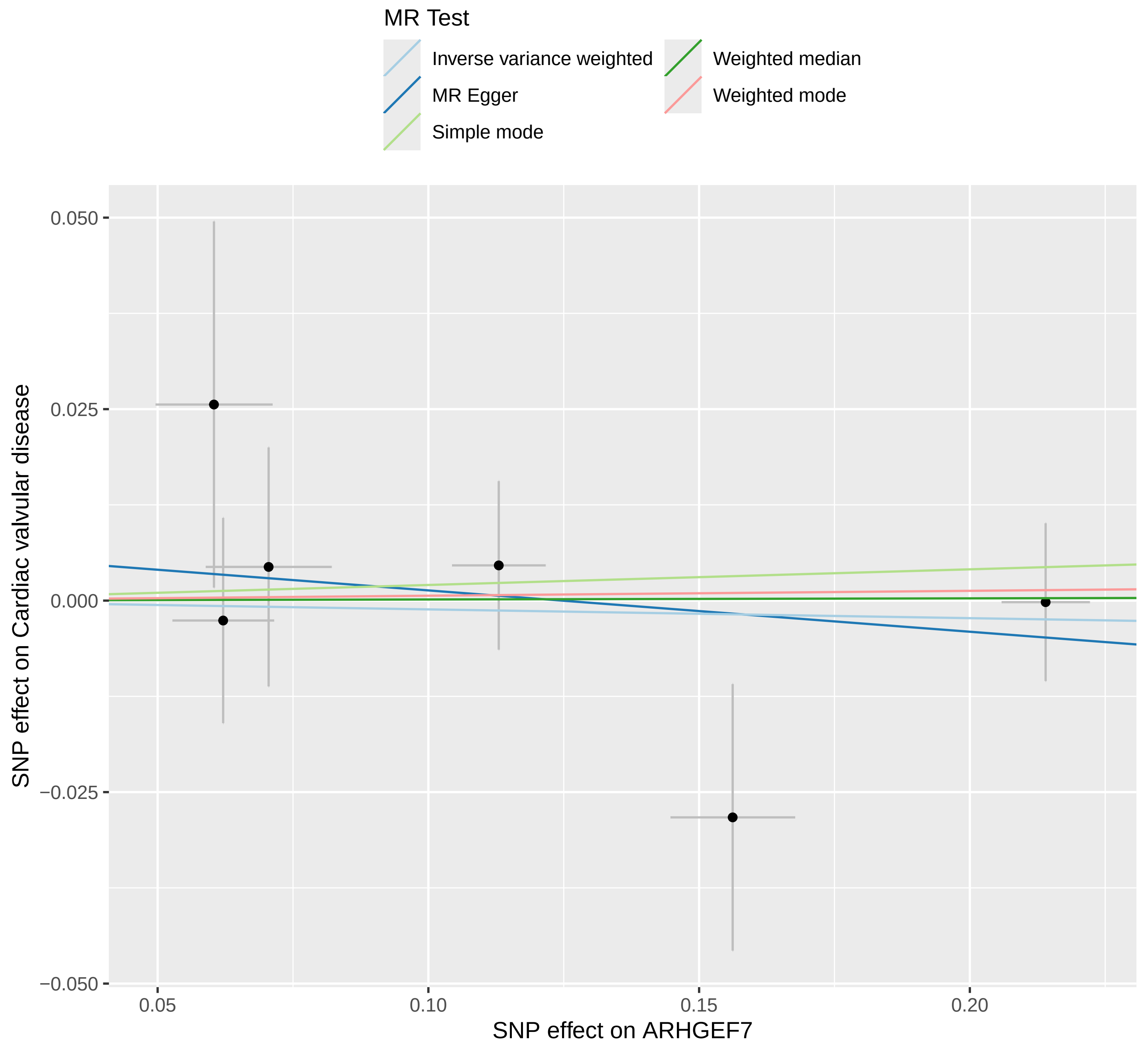
MR Test
Inverse variance weighted
MR Egger
Simple mode
Weighted median
Weighted mode
0.050
0.025
0.000
−0.025
−0.050
SNP effect on Cardiac valvular disease
0.05
0.10
0.15
0.20
SNP effect on ARHGEF7

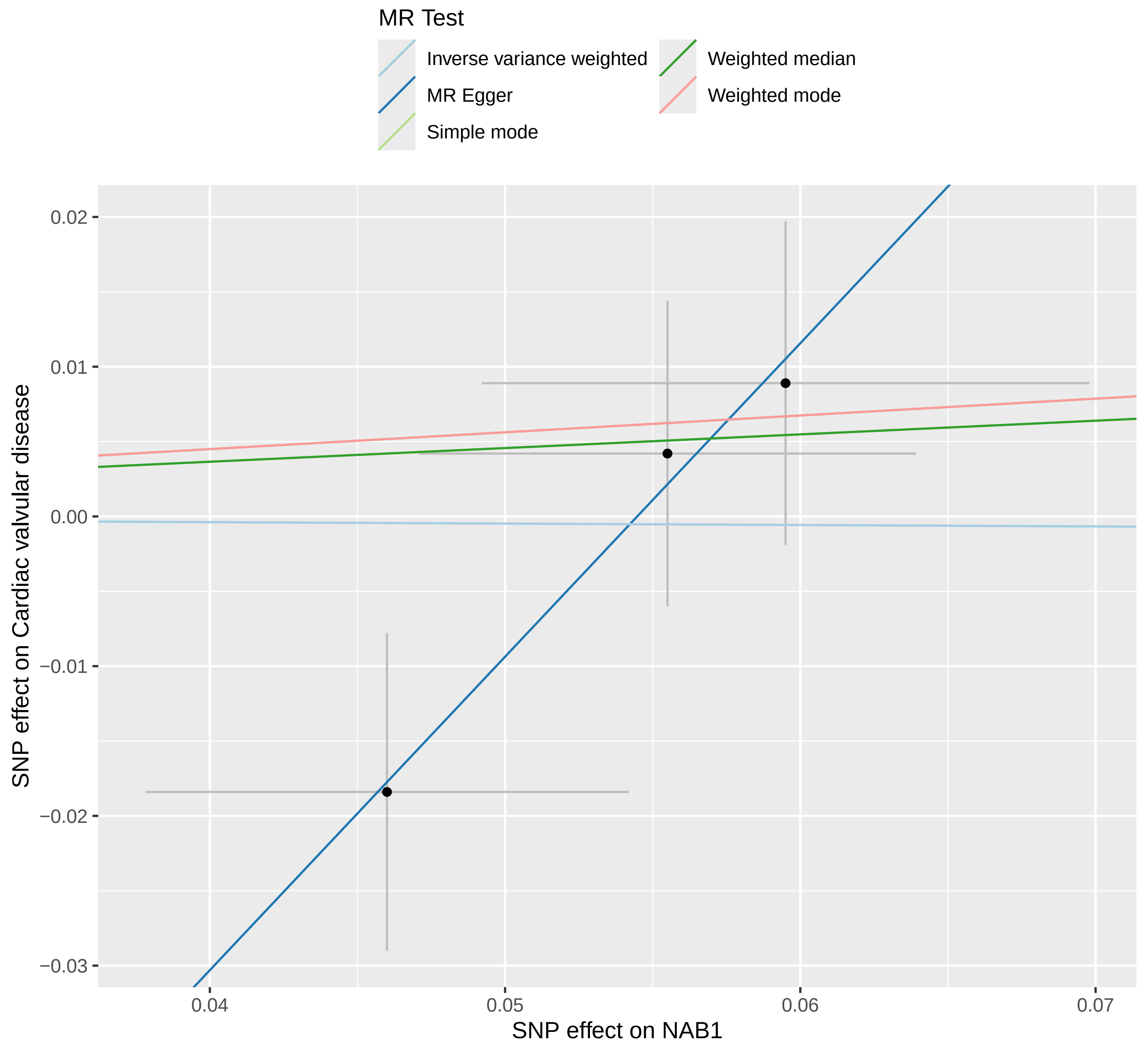

MR Test
Inverse variance weighted
MR Egger
Simple mode
Weighted median
Weighted mode
0.02
0.01
0.00
−0.01
−0.02
−0.03
0.04
0.05
0.06
0.07
SNP effect on NAB1
SNP effect on Cardiac valvular disease

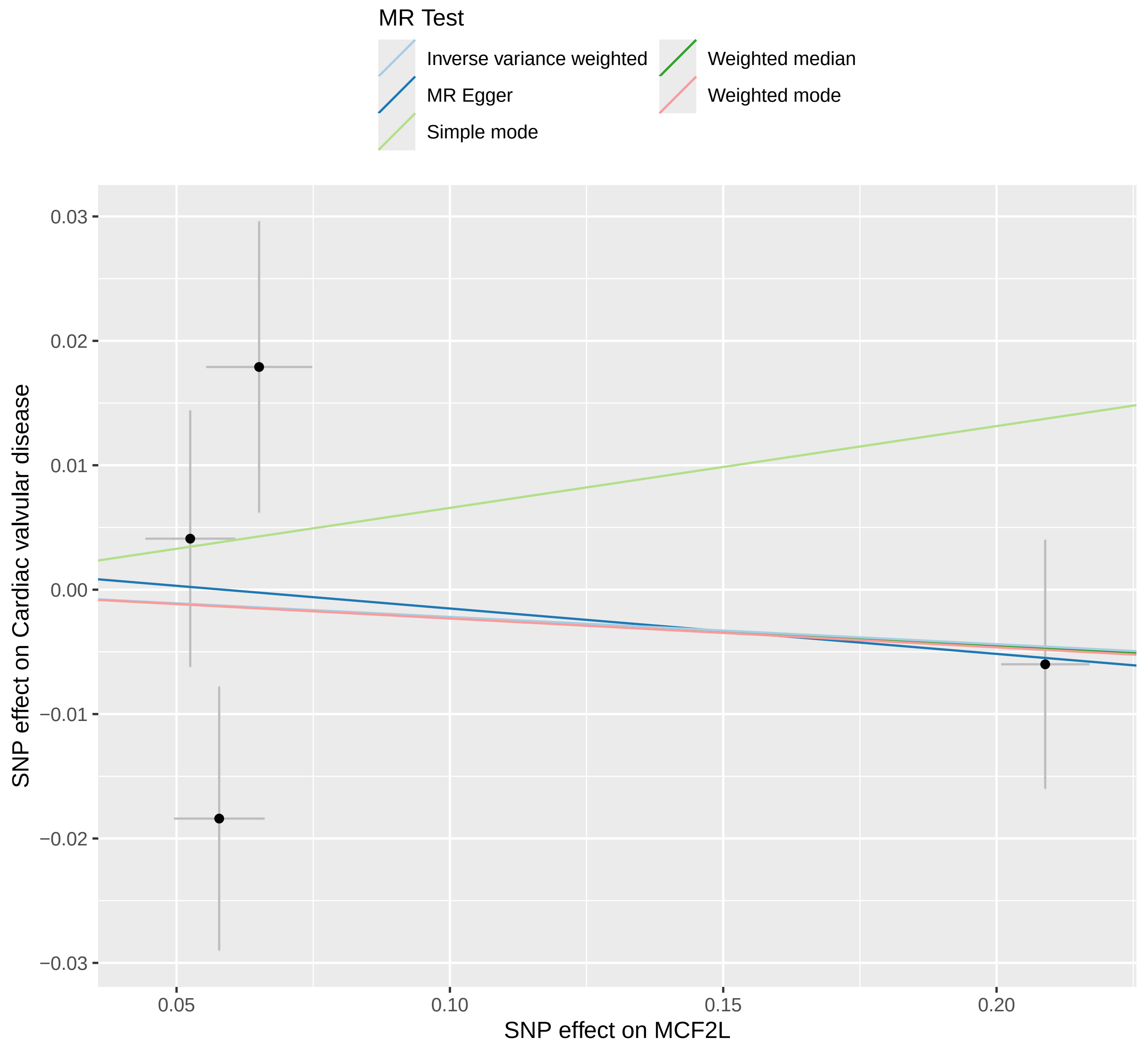
MR Test
Inverse variance weighted
MR Egger
Simple mode
Weighted median
Weighted mode
0.03
0.02
0.01
0.00
−0.01
−0.02
−0.03
0.05
0.10
0.15
0.20
SNP effect on Cardiac valvular disease
SNP effect on MCF2L

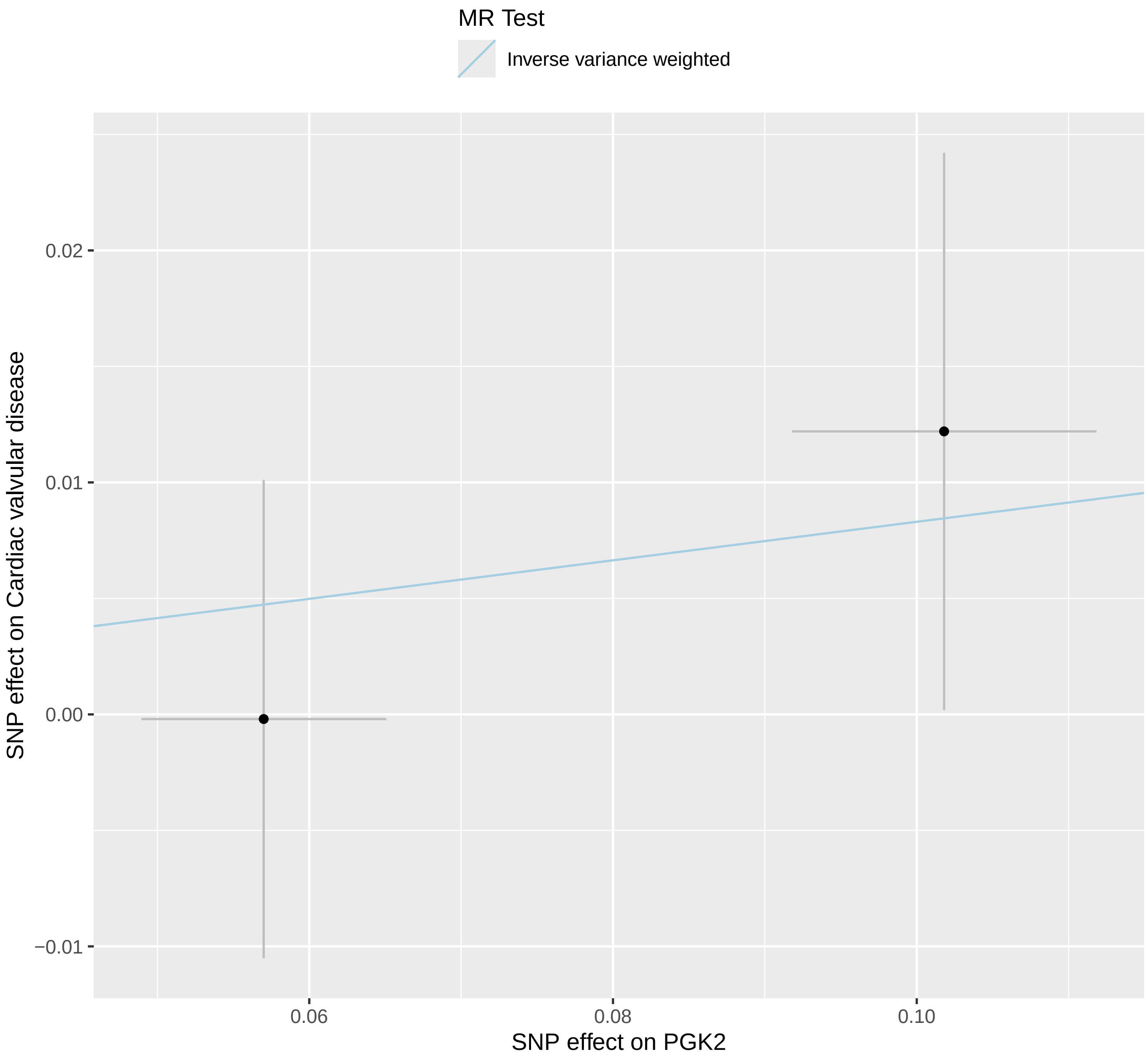
MR Test
Inverse variance weighted
0.02
0.01
0.00
−0.01
SNP effect on Cardiac valvular disease
0.06
0.08
0.10
SNP effect on PGK2

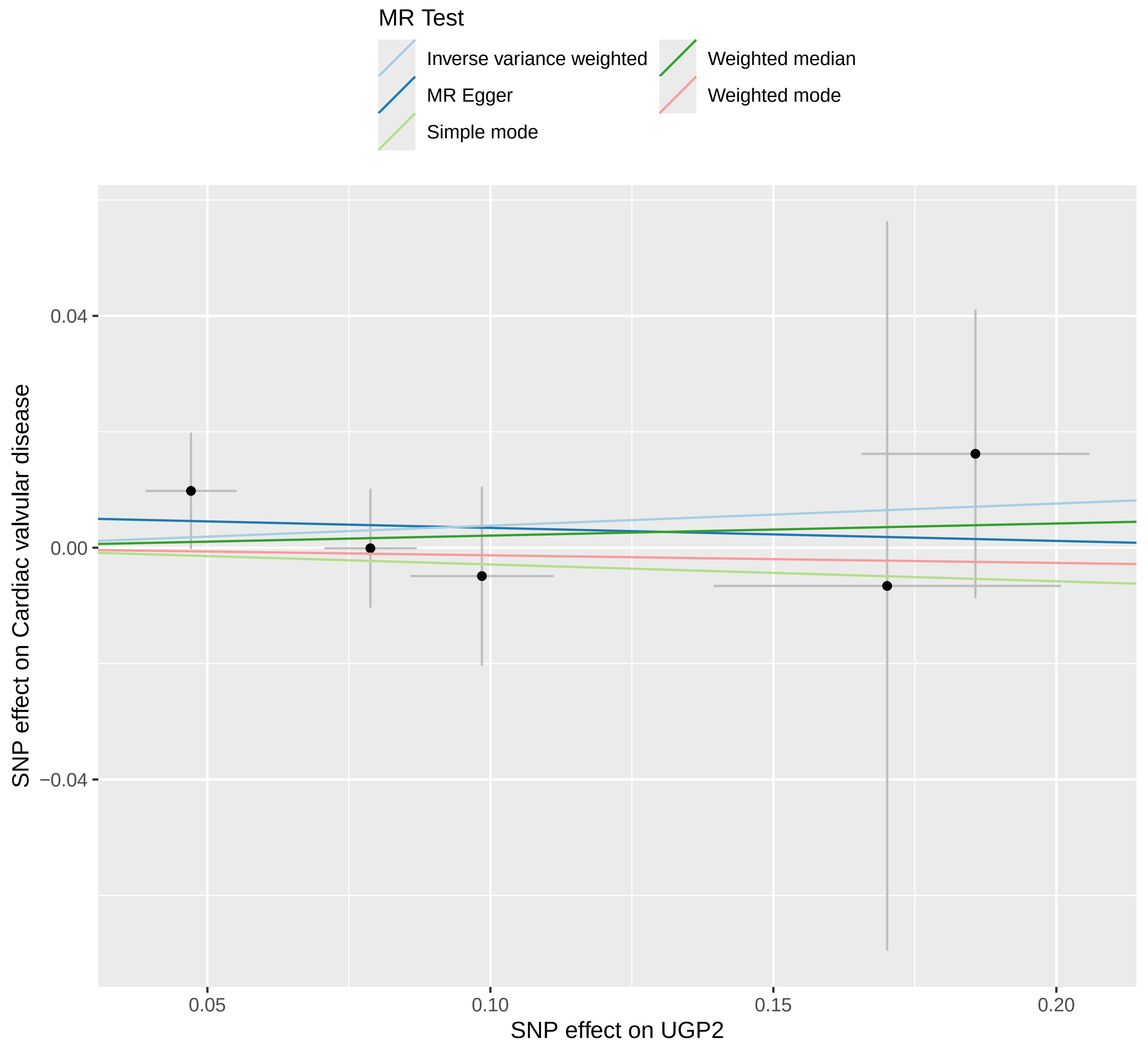
MR Test
Inverse variance weighted
MR Egger
Simple mode
Weighted median
Weighted mode
0.04
0.00
−0.04
0.05
0.10
0.15
0.20
SNP effect on Cardiac valvular disease
SNP effect on UGP2

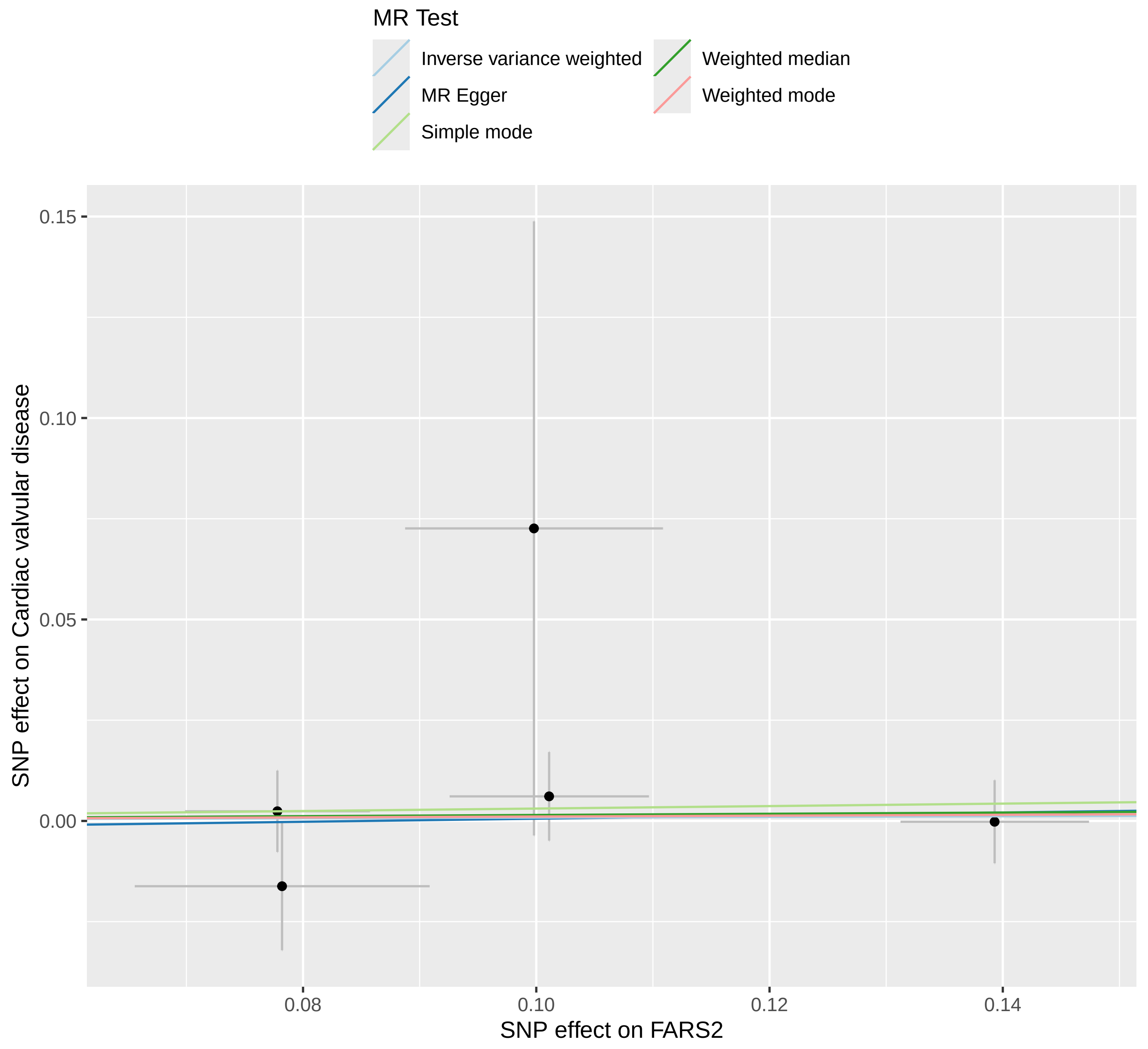

MR Test
Inverse variance weighted
MR Egger
Simple mode
Weighted median
Weighted mode
0.15
0.10
0.05
0.00
0.08
0.10
0.12
0.14
SNP effect on Cardiac valvular disease
SNP effect on FARS2

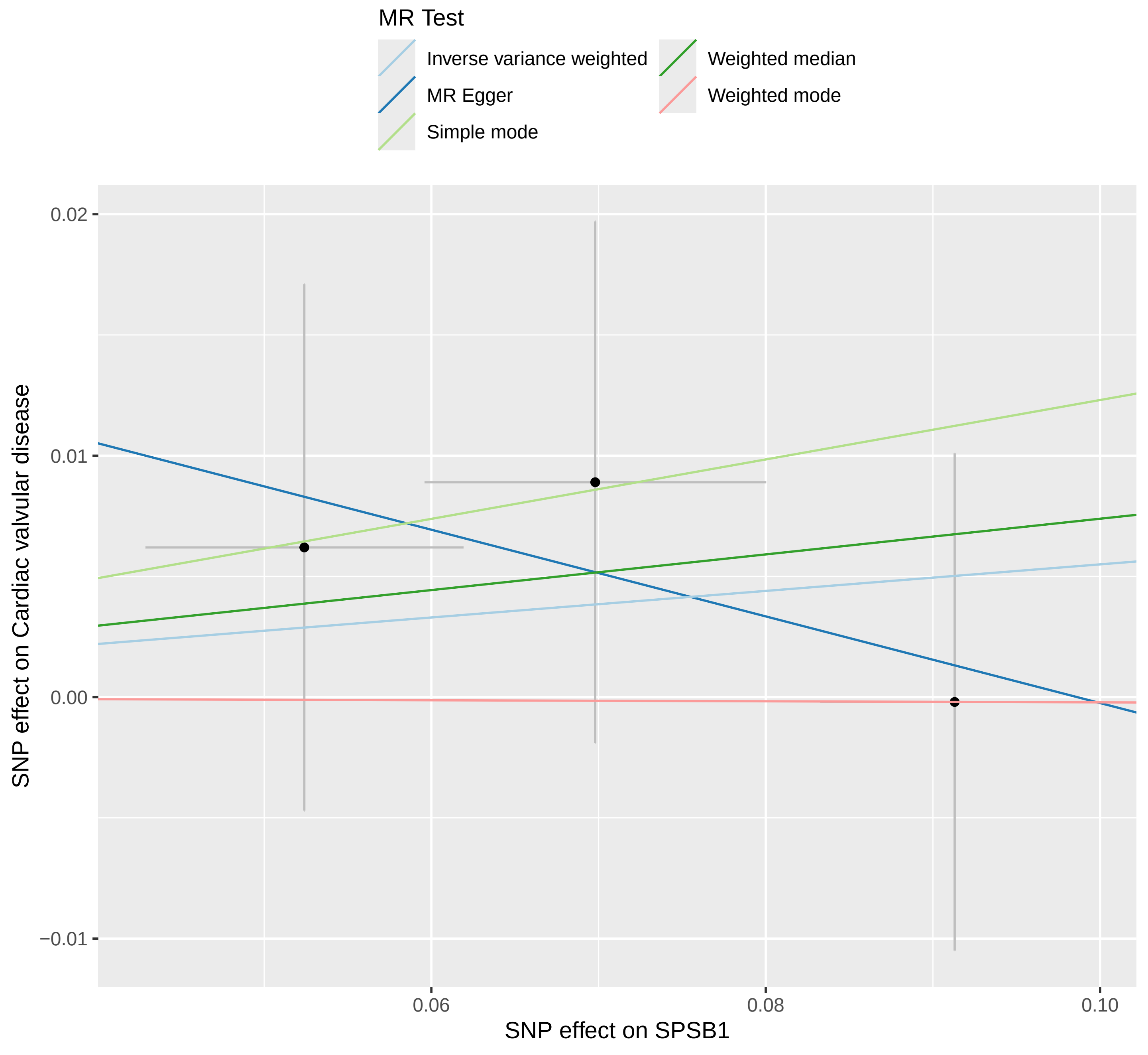

MR Test
Inverse variance weighted
MR Egger
Simple mode
Weighted median
Weighted mode
0.02
0.01
0.00
−0.01
SNP effect on Cardiac valvular disease
0.06
0.08
0.10
SNP effect on SPSB1

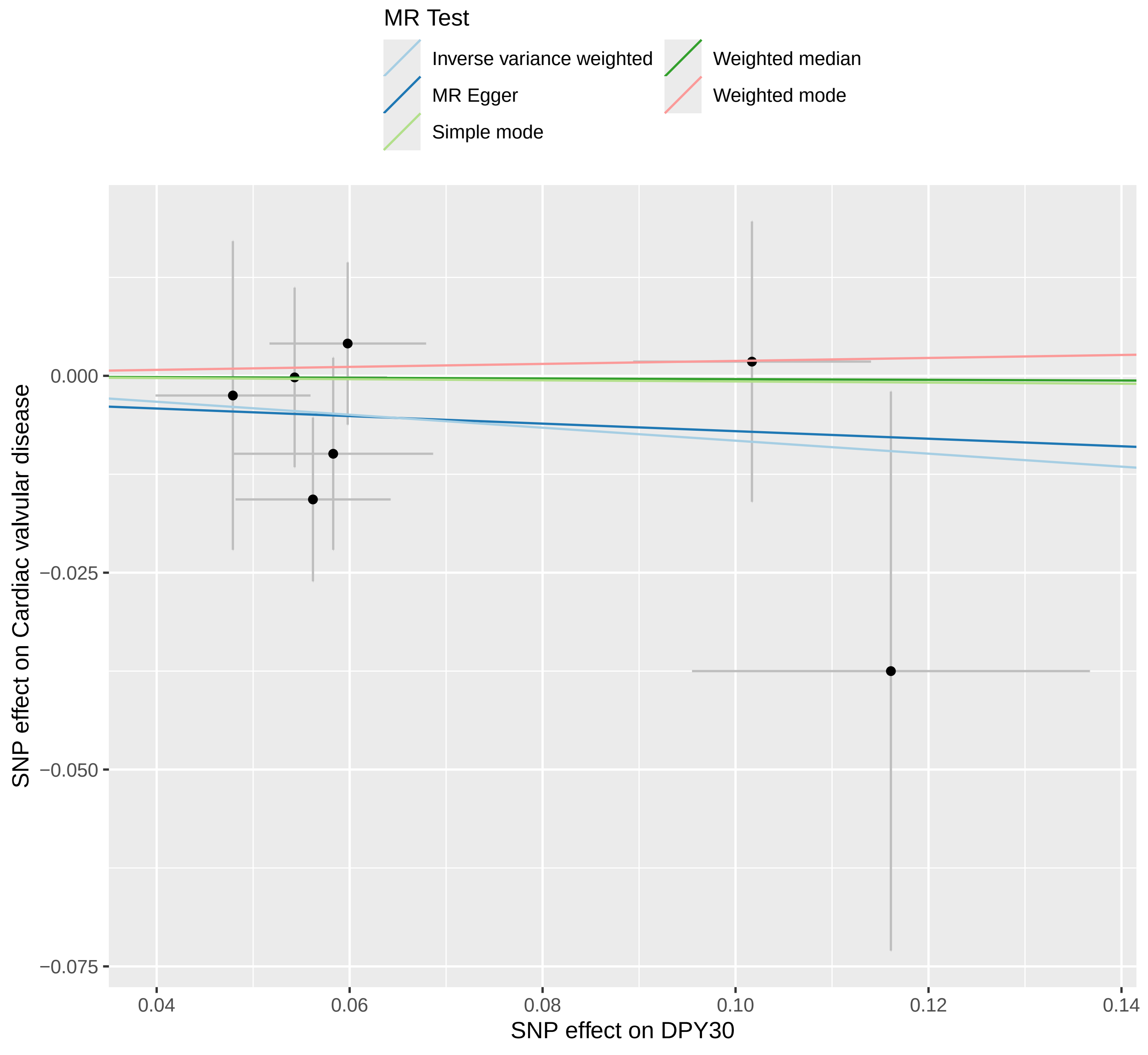

MR Test
Inverse variance weighted
MR Egger
Simple mode
Weighted median
Weighted mode
0.000
−0.025
−0.050
−0.075
SNP effect on Cardiac valvular disease
0.04
0.06
0.08
0.10
0.12
0.14
SNP effect on DPY30

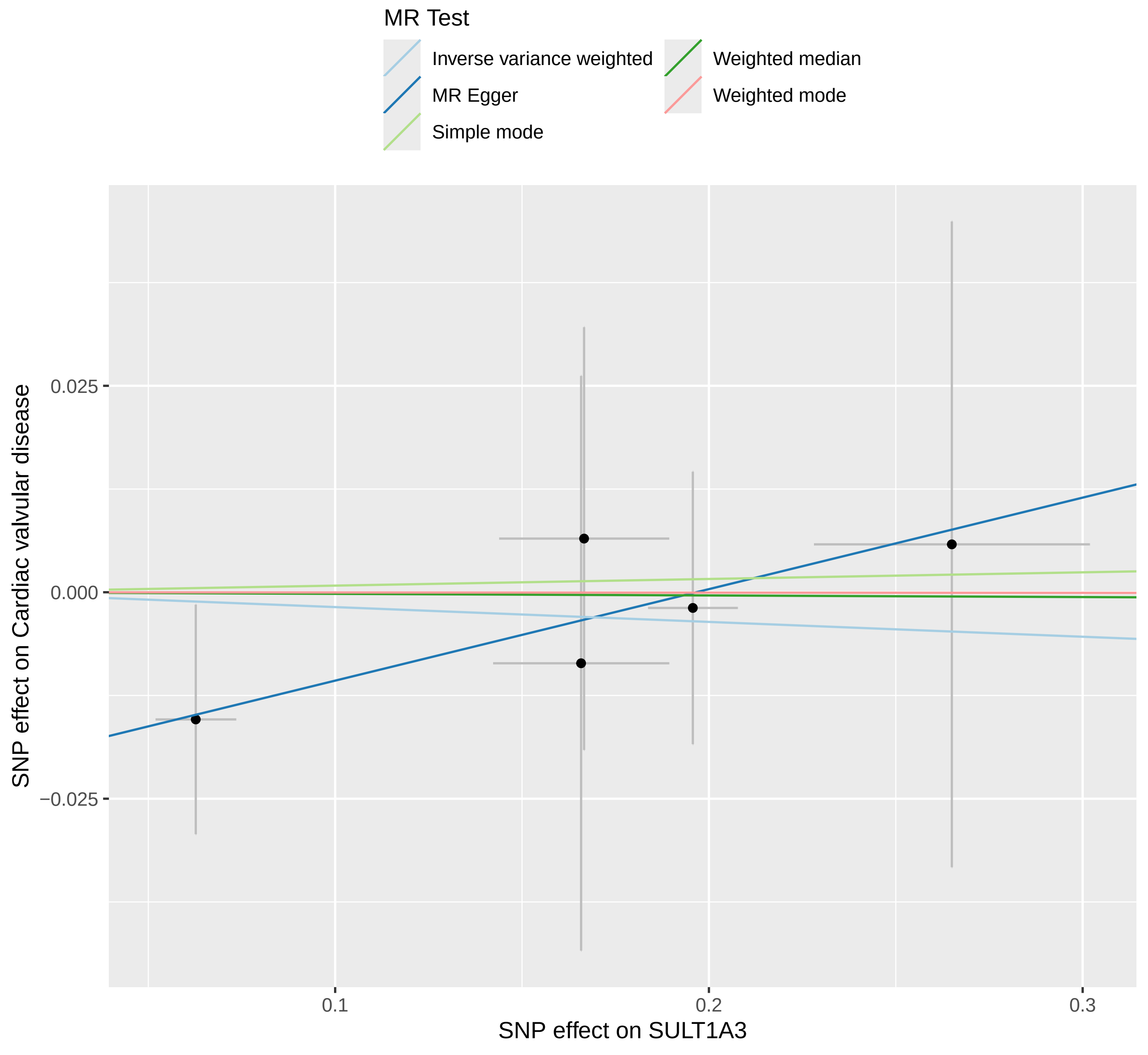

MR Test
Inverse variance weighted
MR Egger
Simple mode
Weighted median
Weighted mode
0.025
0.000
−0.025
SNP effect on Cardiac valvular disease
0.1
0.2
0.3
SNP effect on SULT1A3

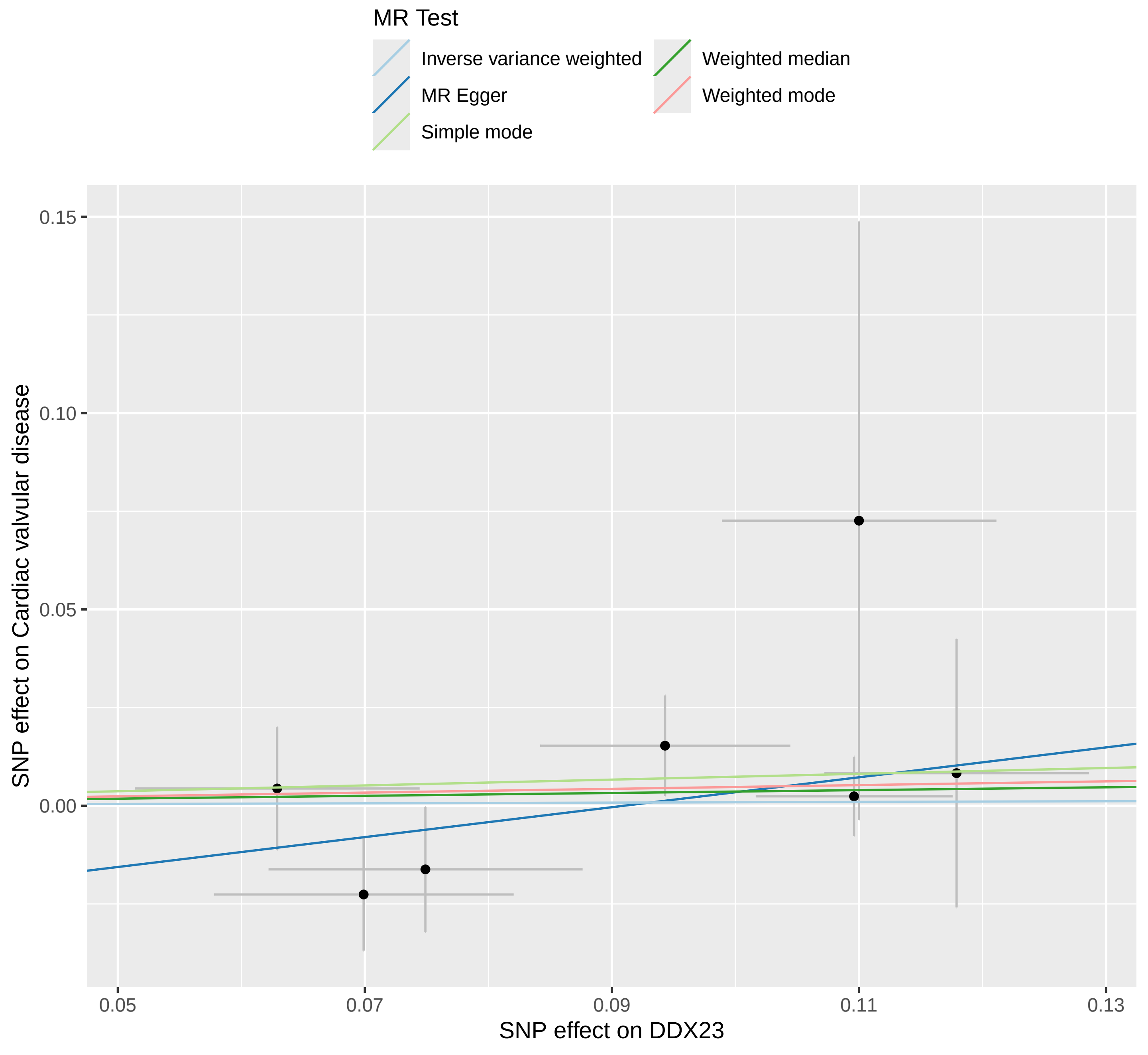
MR Test
Inverse variance weighted
MR Egger
Simple mode
Weighted median
Weighted mode
0.15
0.10
0.05
0.00
SNP effect on Cardiac valvular disease
0.05
0.07
0.09
0.11
0.13
SNP effect on DDX23

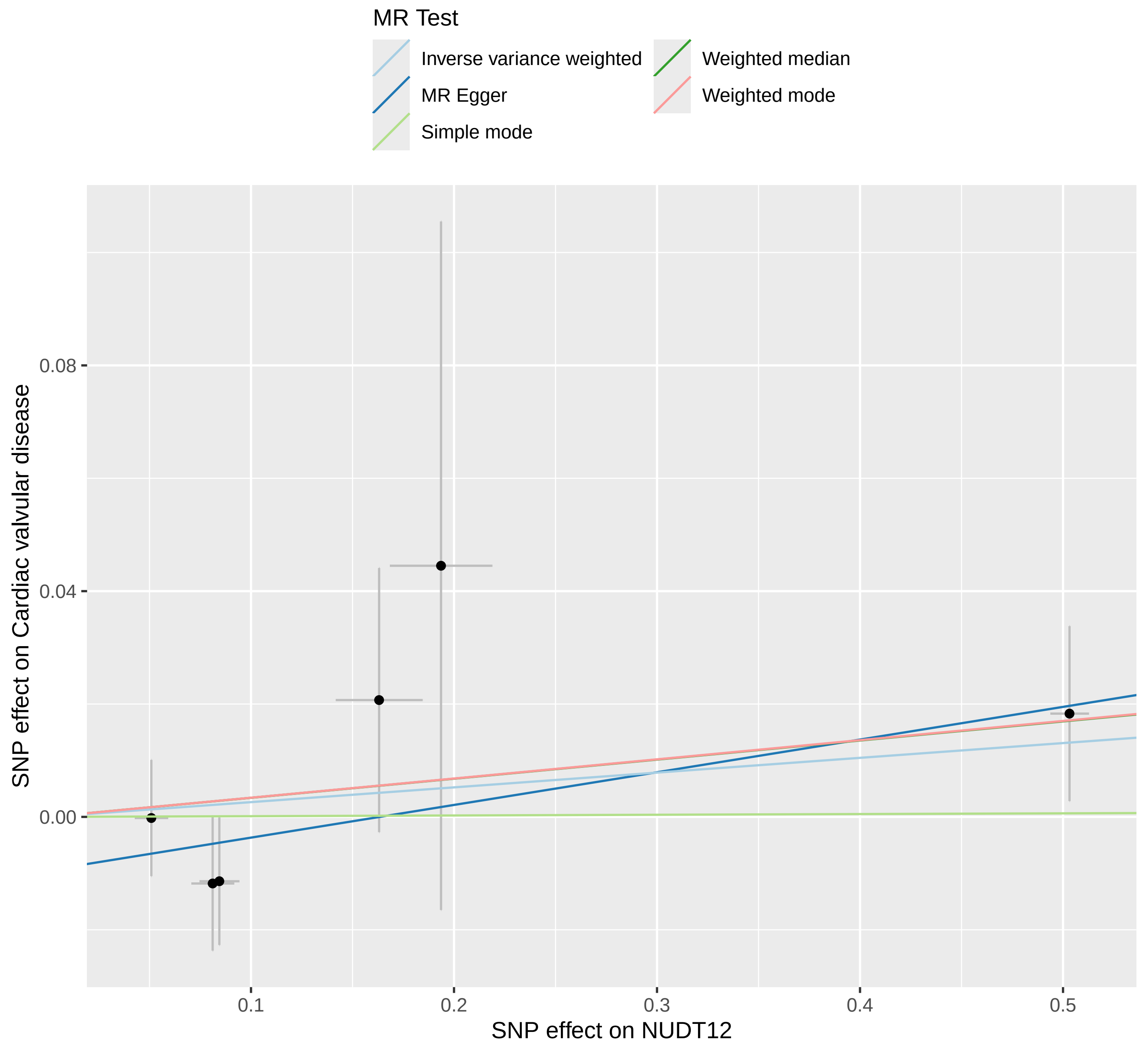

MR Test
Inverse variance weighted
MR Egger
Simple mode
Weighted median
Weighted mode
0.08
0.04
0.00
0.1
0.2
0.3
0.4
0.5
SNP effect on NUDT12
SNP effect on Cardiac valvular disease

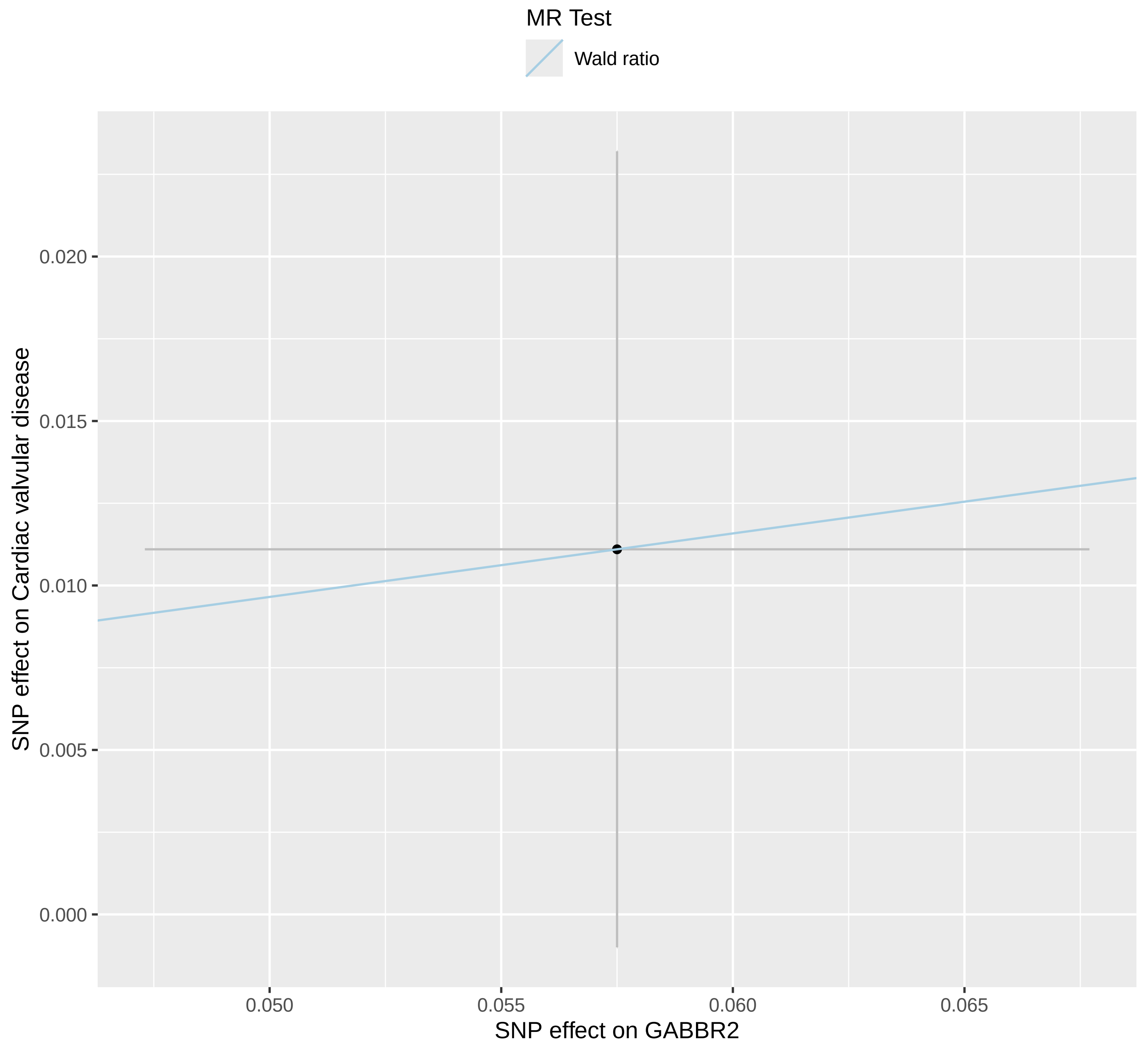

MR Test
Wald ratio
0.020
0.015
0.010
0.005
0.000
SNP effect on Cardiac valvular disease
0.050
0.055
0.060
0.065
SNP effect on GABBR2

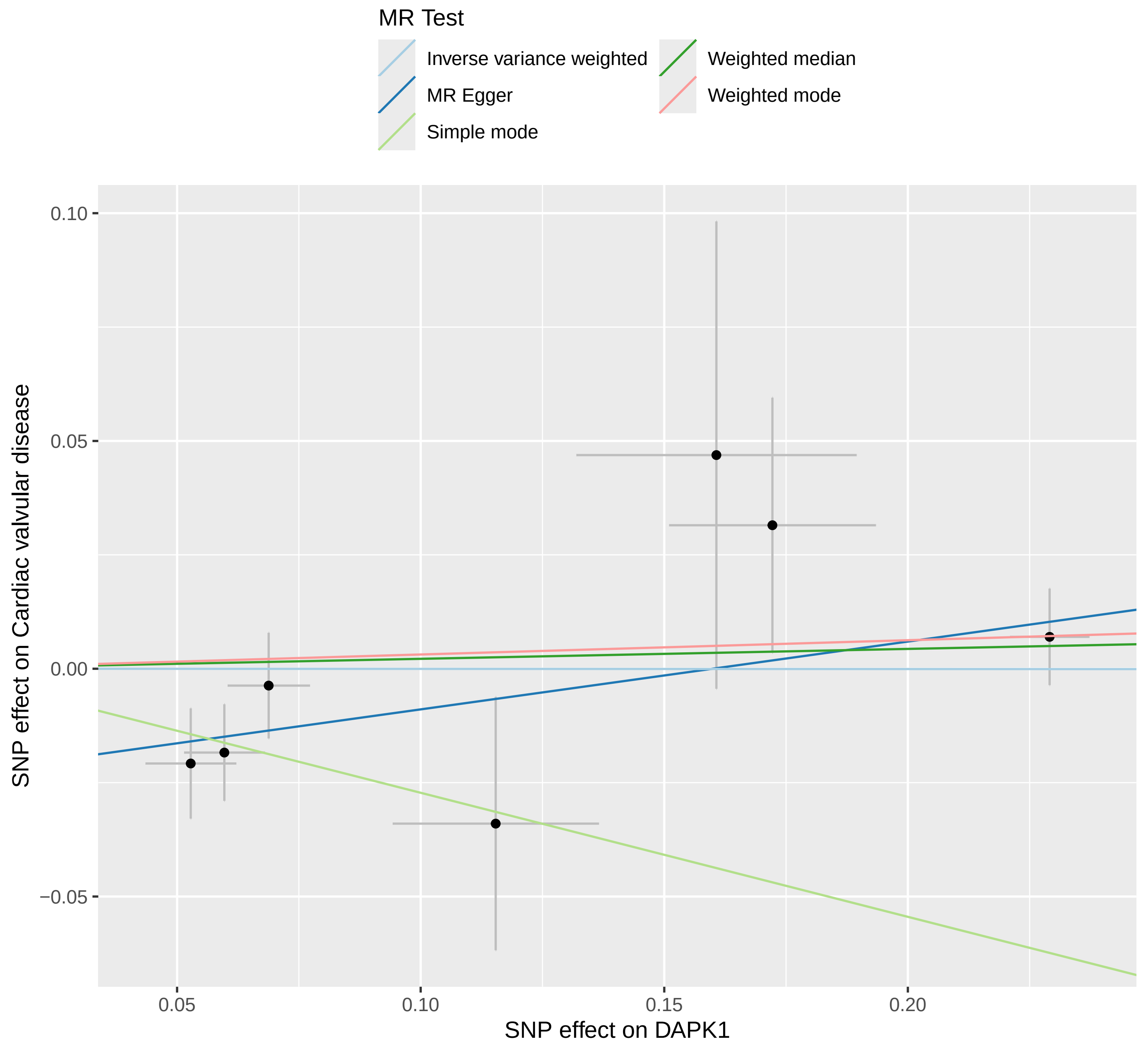

MR Test
Inverse variance weighted
MR Egger
Simple mode
Weighted median
Weighted mode
0.10
0.05
0.00
−0.05
0.05
0.10
0.15
0.20
SNP effect on Cardiac valvular disease
SNP effect on DAPK1

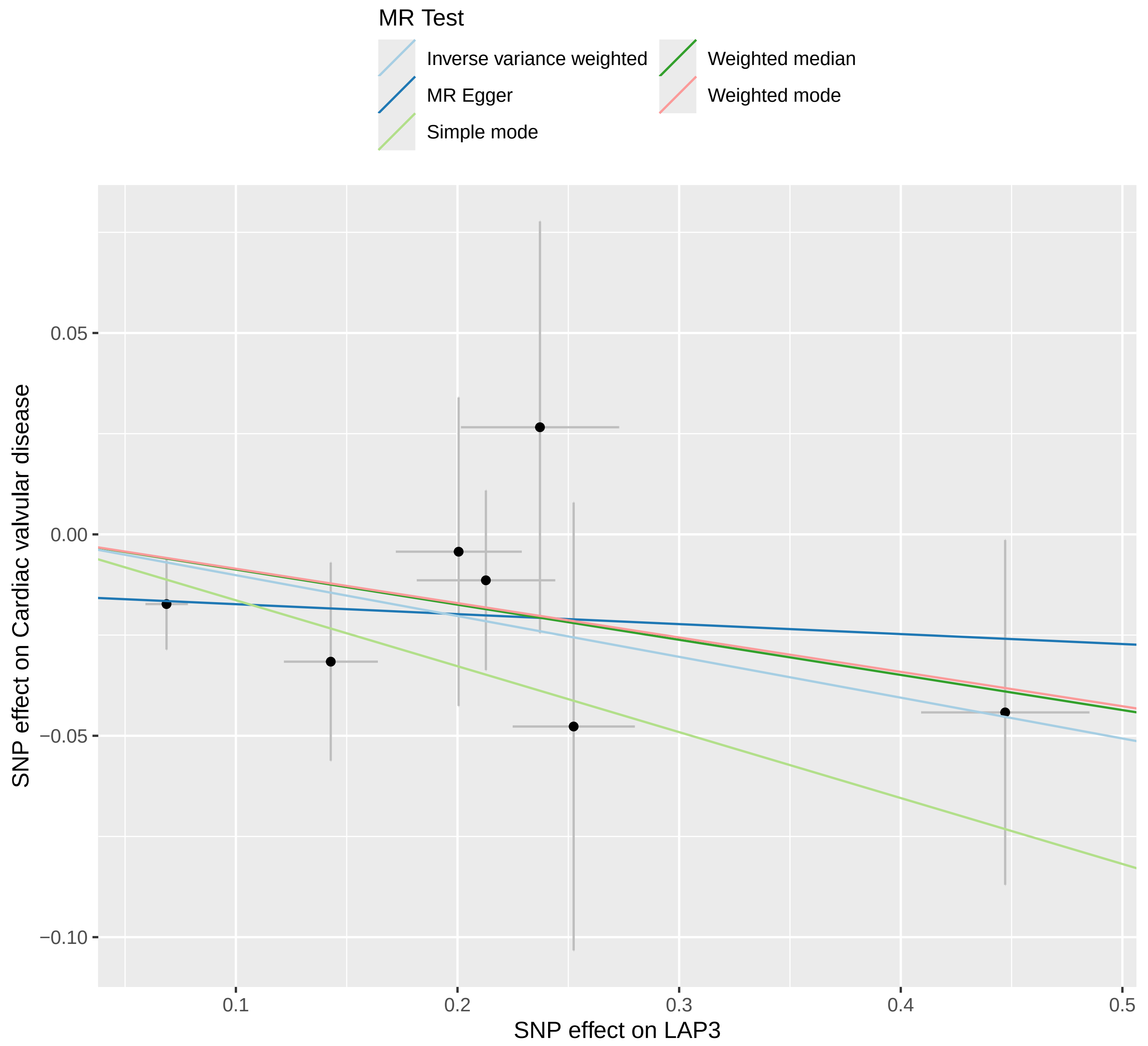
MR Test
Inverse variance weighted
MR Egger
Simple mode
Weighted median
Weighted mode
0.05
0.00
−0.05
−0.10
0.1
0.2
0.3
0.4
0.5
SNP effect on Cardiac valvular disease
SNP effect on LAP3

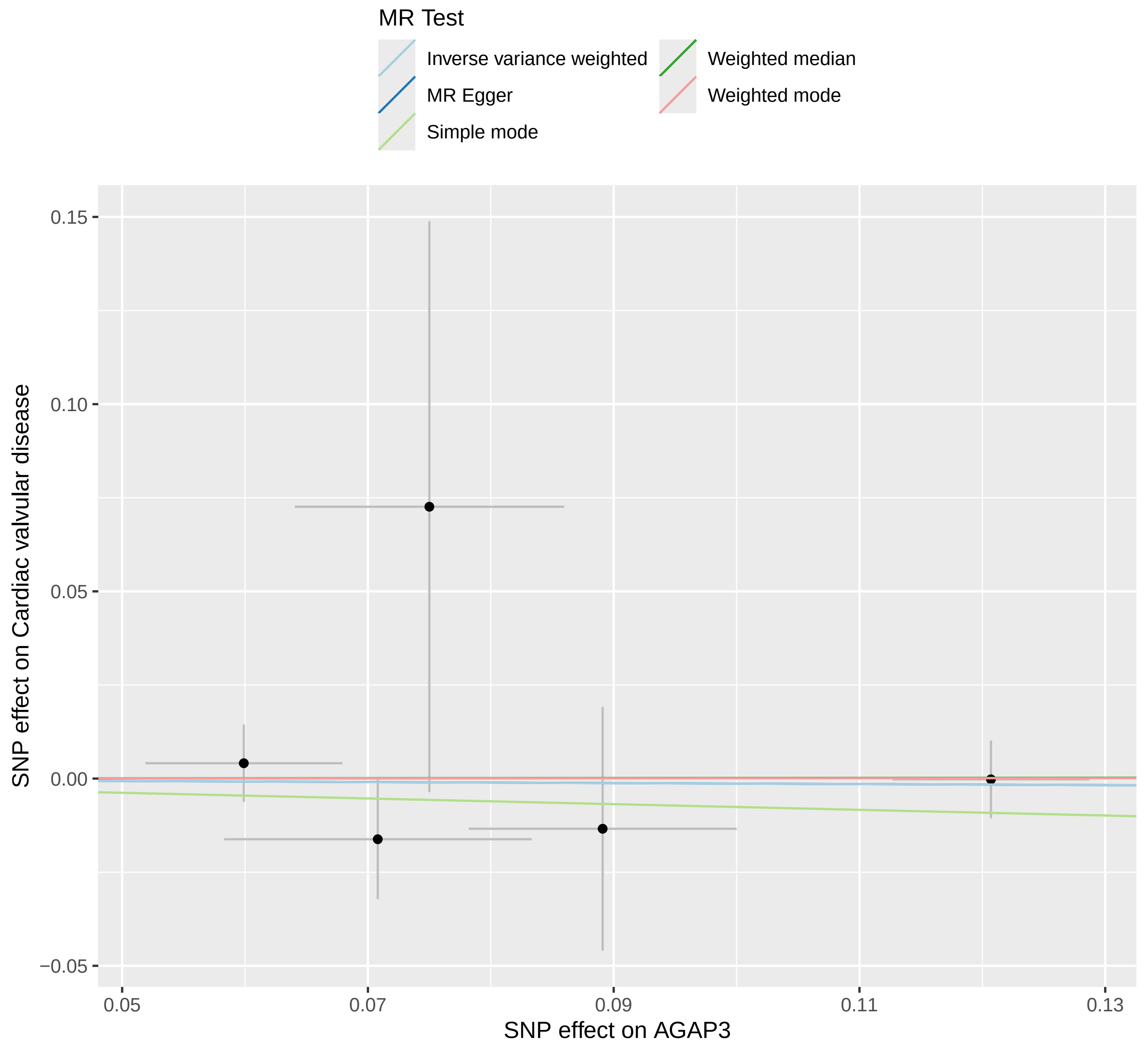

MR Test
Inverse variance weighted
MR Egger
Simple mode
Weighted median
Weighted mode
0.15
0.10
0.05
0.00
−0.05
SNP effect on Cardiac valvular disease
0.05
0.07
0.09
0.11
0.13
SNP effect on AGAP3

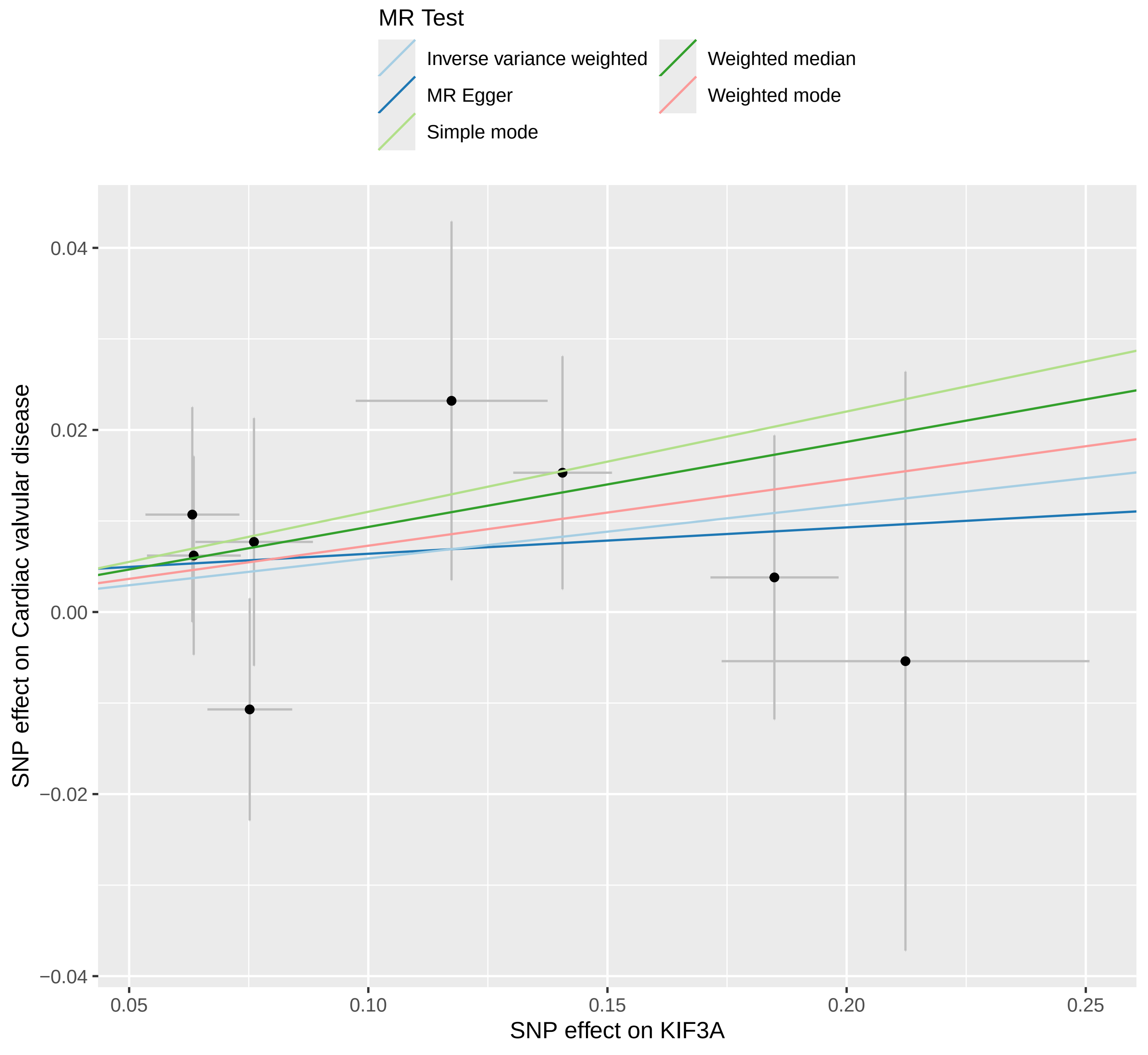
MR Test
Inverse variance weighted
MR Egger
Simple mode
Weighted median
Weighted mode
SNP effect on Cardiac valvular disease
0.04
0.02
0.00
−0.02
−0.04
0.05
0.10
0.15
0.20
0.25
SNP effect on KIF3A

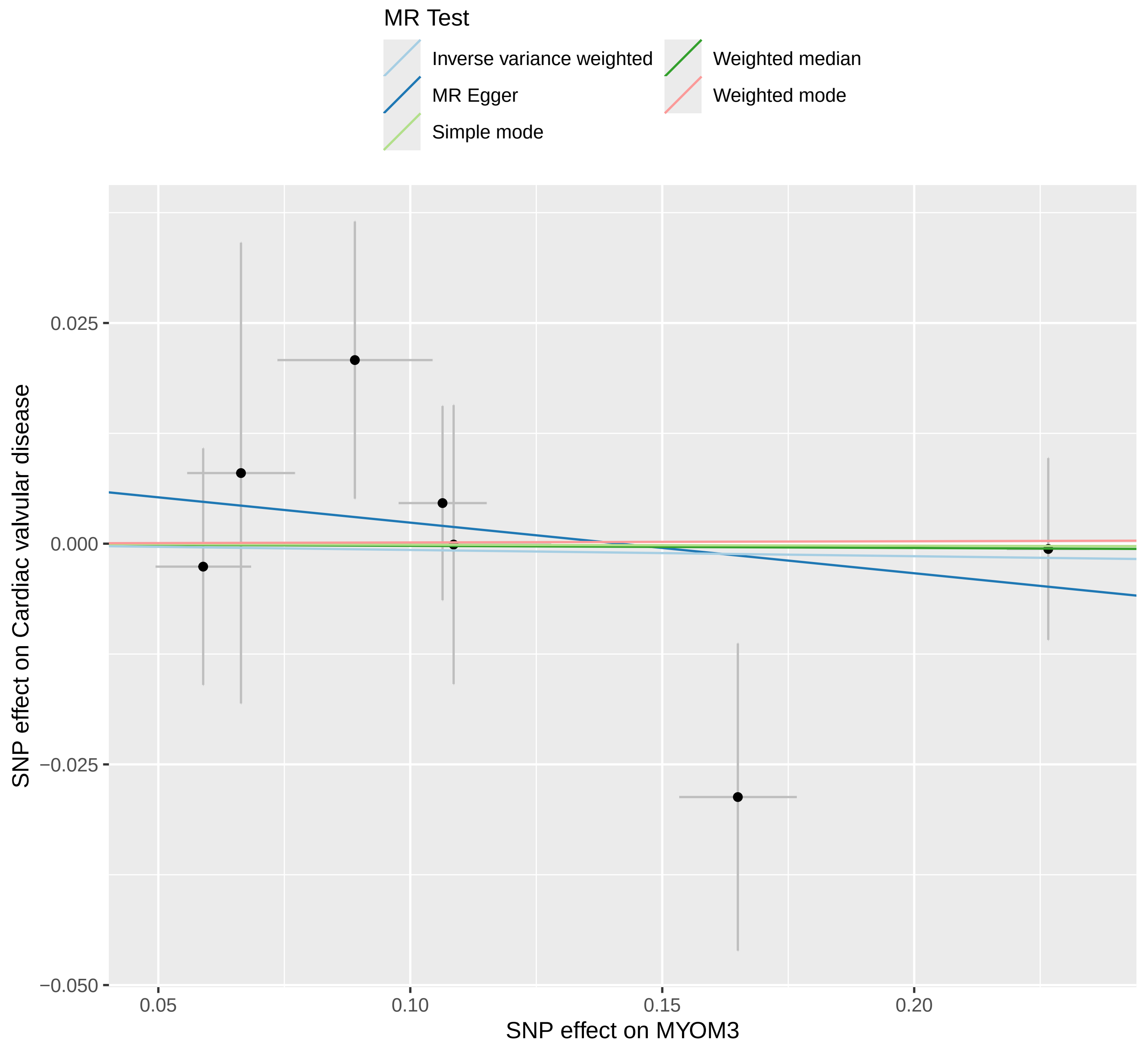

MR Test
Inverse variance weighted
MR Egger
Simple mode
Weighted median
Weighted mode
0.025
0.000
−0.025
−0.050
0.05
0.10
0.15
0.20
SNP effect on Cardiac valvular disease
SNP effect on MYOM3

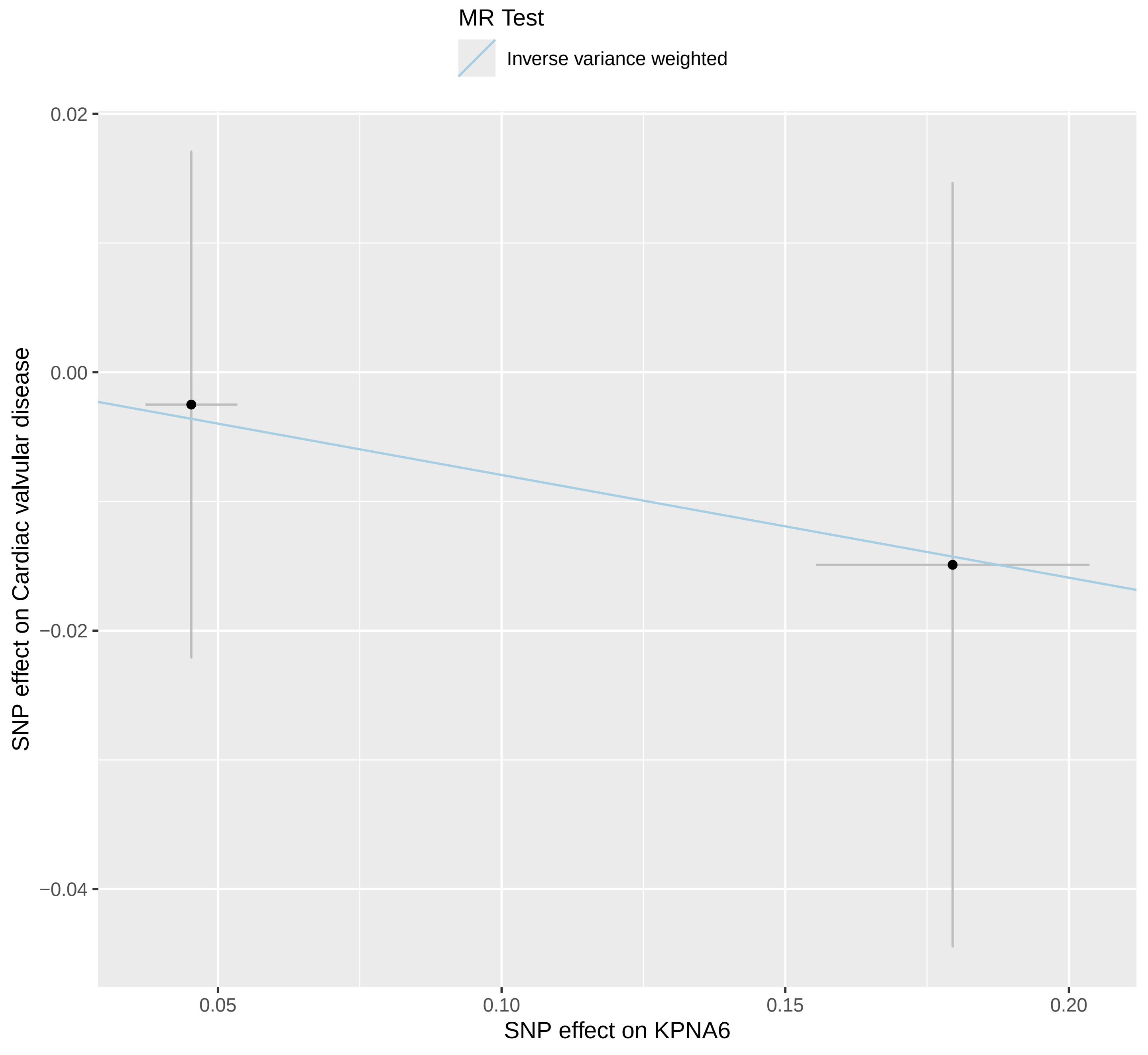

MR Test
Inverse variance weighted
0.02
0.00
−0.02
−0.04
SNP effect on Cardiac valvular disease
0.05
0.10
0.15
0.20
SNP effect on KPNA6

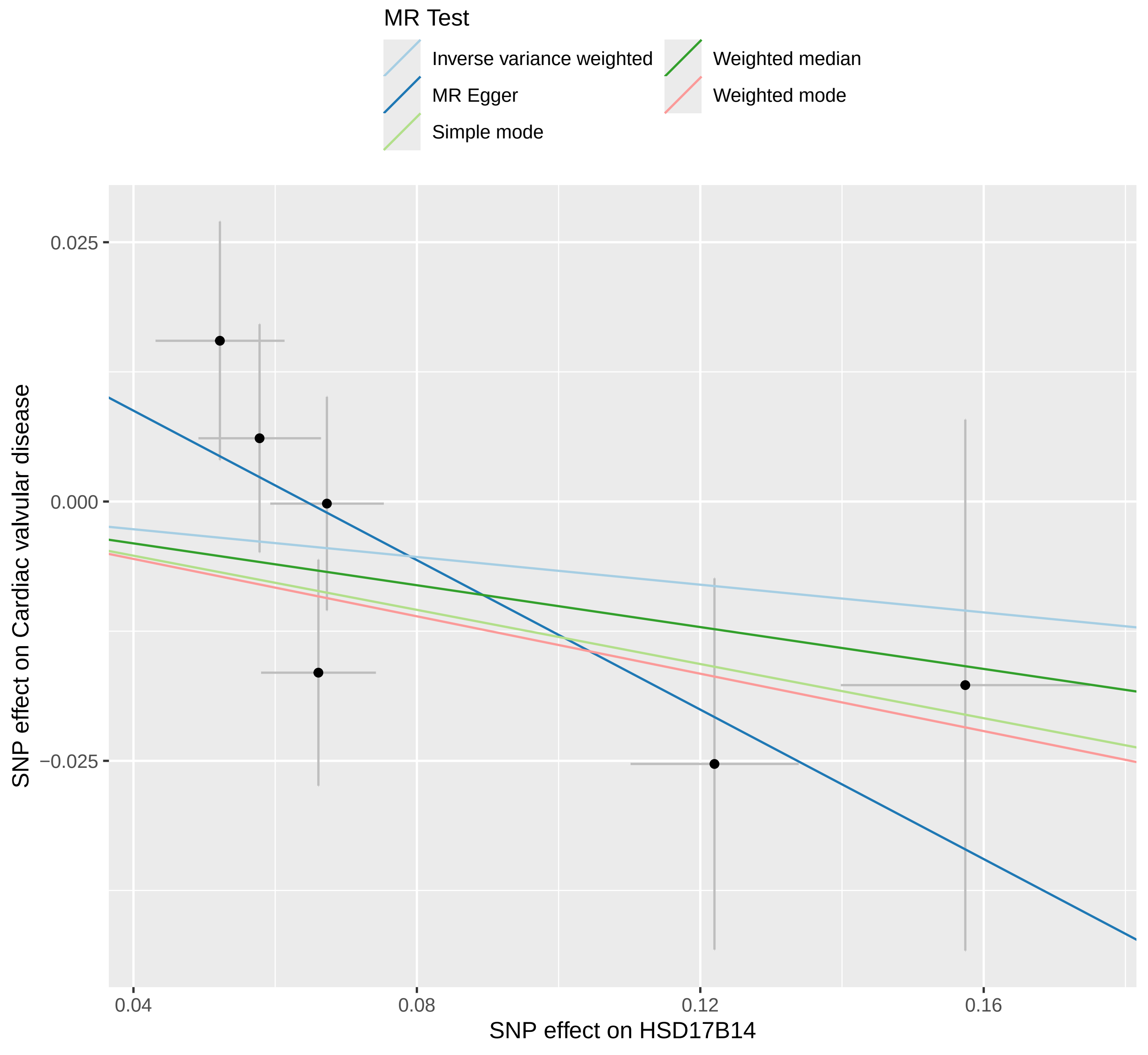
MR Test
Inverse variance weighted
MR Egger
Simple mode
Weighted median
Weighted mode
0.025
0.000
−0.025
0.04
0.08
0.12
0.16
SNP effect on Cardiac valvular disease
SNP effect on HSD17B14

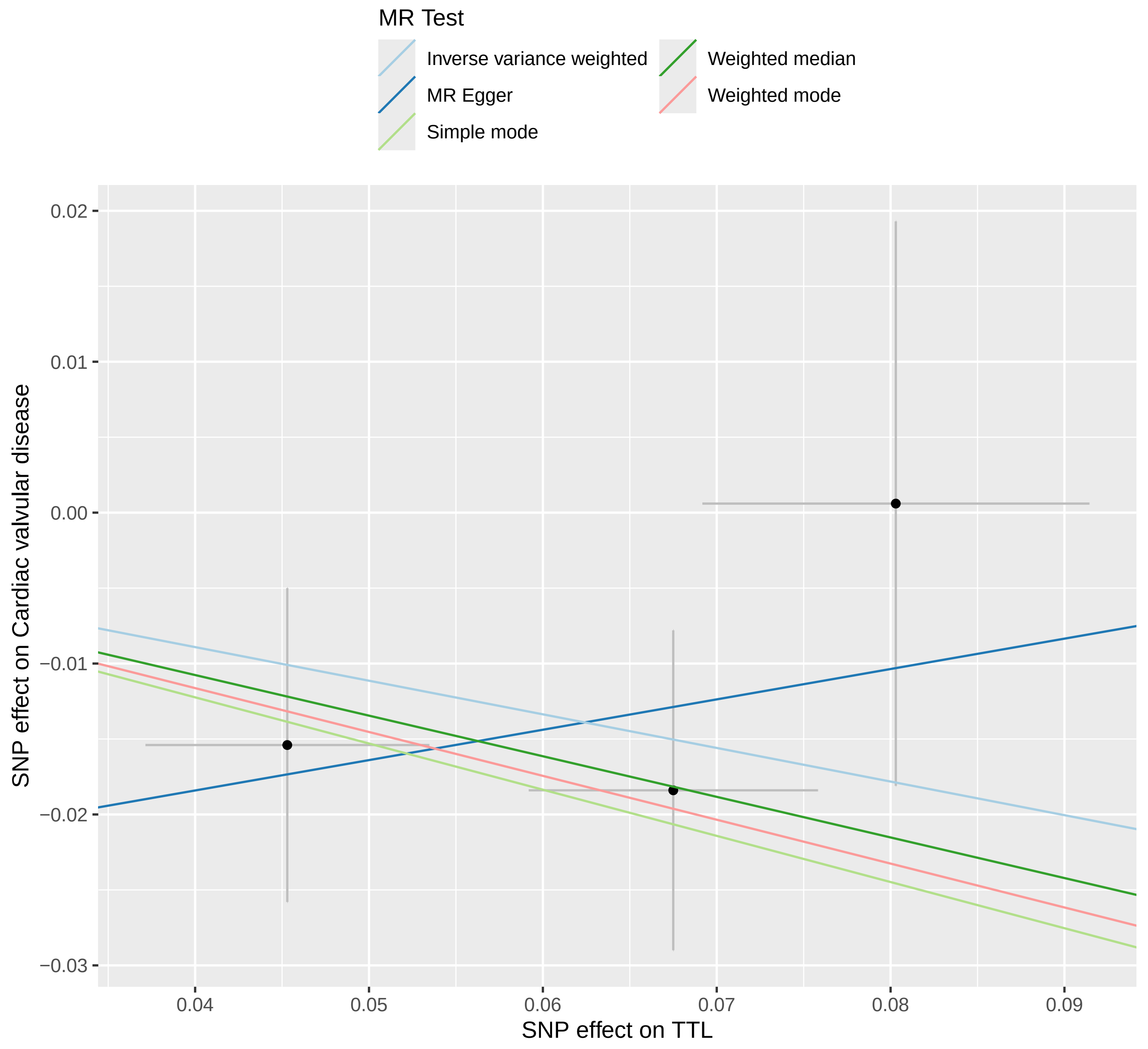

MR Test
Inverse variance weighted
MR Egger
Simple mode
Weighted median
Weighted mode
0.02
0.01
0.00
−0.01
−0.02
−0.03
SNP effect on Cardiac valvular disease
0.04
0.05
0.06
0.07
0.08
0.09
SNP effect on TTL

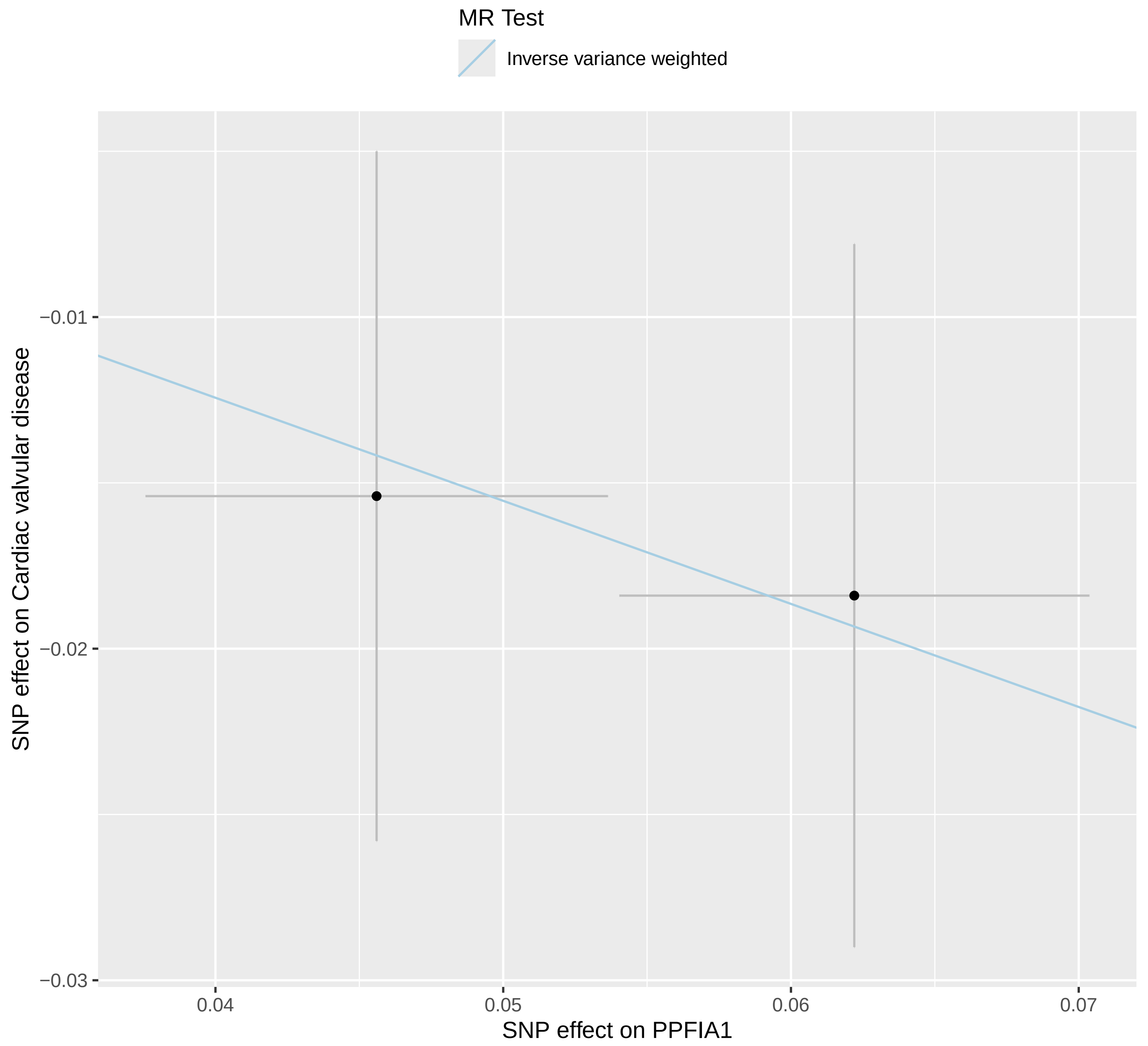

MR Test
Inverse variance weighted
SNP effect on Cardiac valvular disease
−0.01
−0.02
−0.03
0.04
0.05
0.06
0.07
SNP effect on PPFIA1

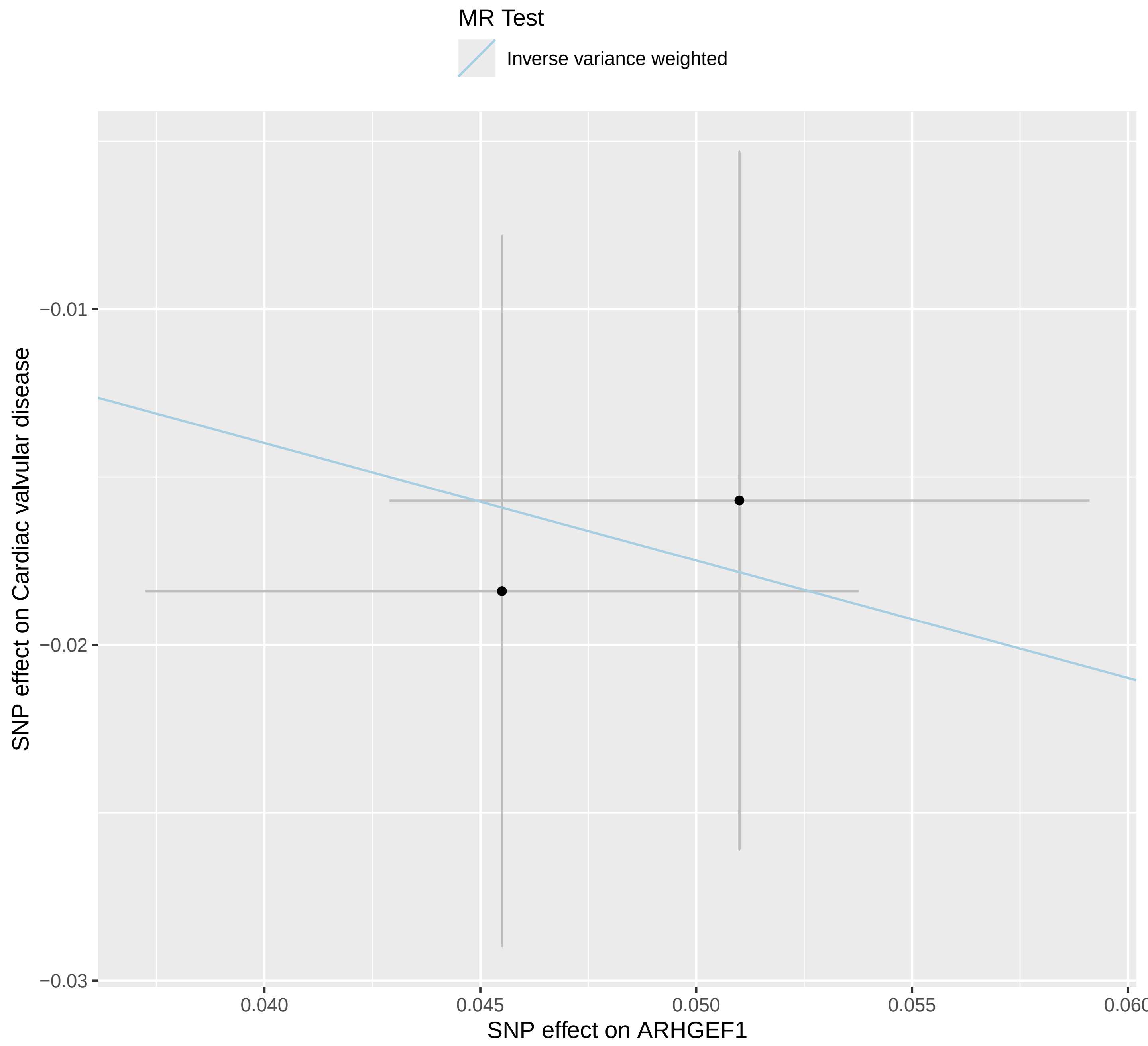

MR Test
Inverse variance weighted
−0.01
−0.02
−0.03
0.040
0.045
0.050
0.055
0.060
SNP effect on ARHGEF1
SNP effect on Cardiac valvular disease

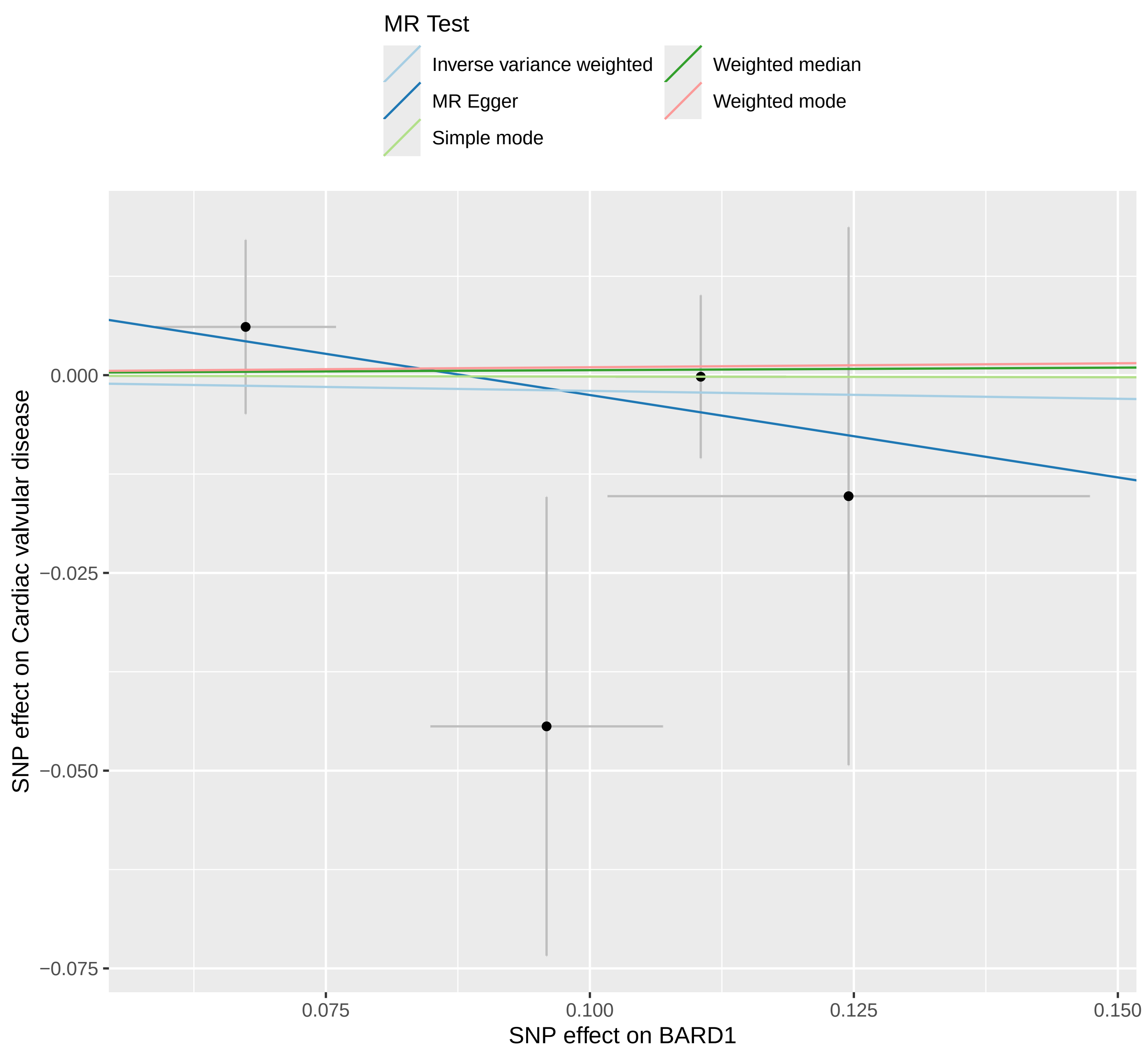
MR Test
Inverse variance weighted
MR Egger
Simple mode
Weighted median
Weighted mode
SNP effect on Cardiac valvular disease
0.000
−0.025
−0.050
−0.075
0.075
0.100
0.125
0.150
SNP effect on BARD1

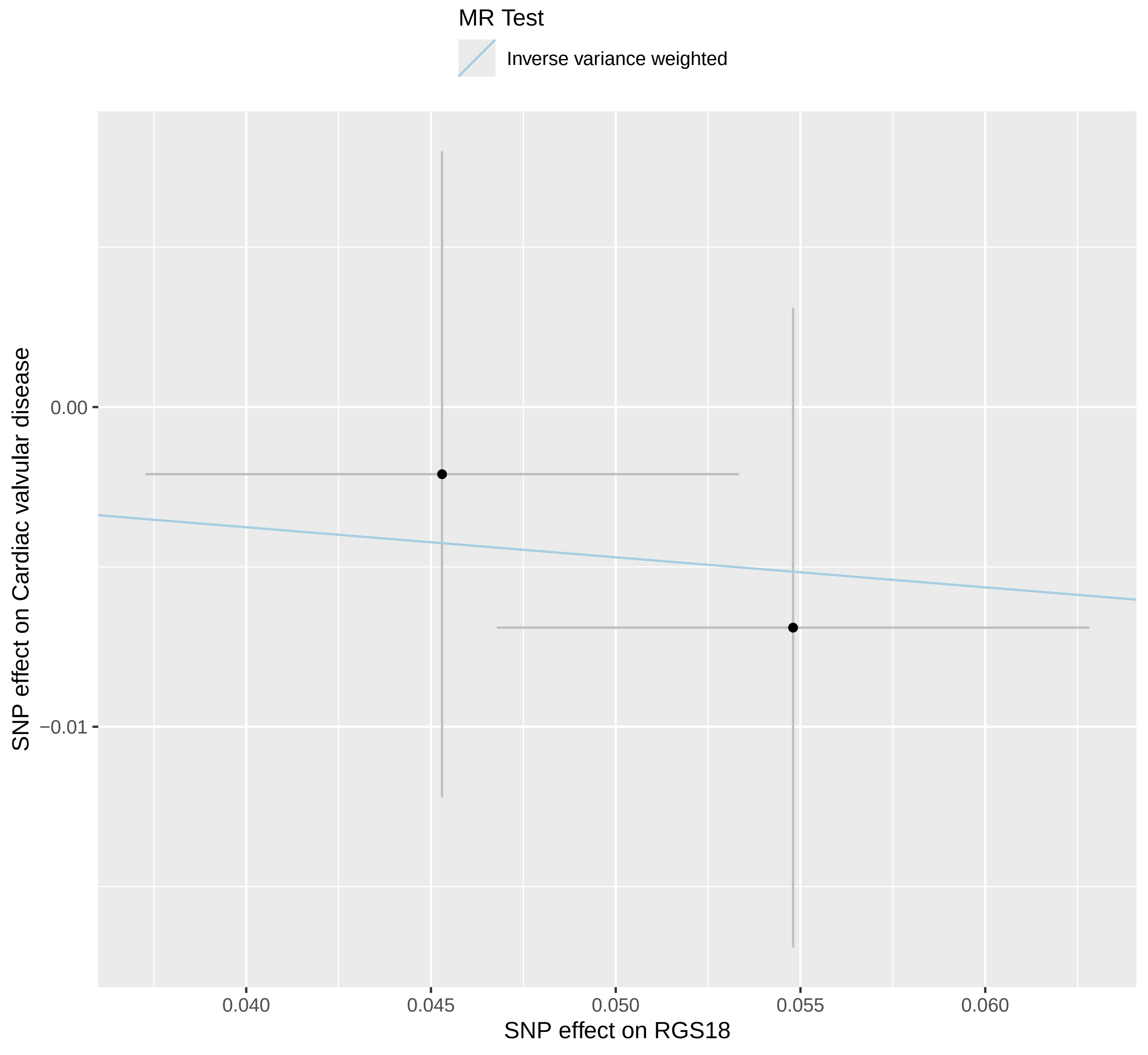

MR Test
Inverse variance weighted
0.00
−0.01
0.040
0.045
0.050
0.055
0.060
SNP effect on RGS18
SNP effect on Cardiac valvular disease

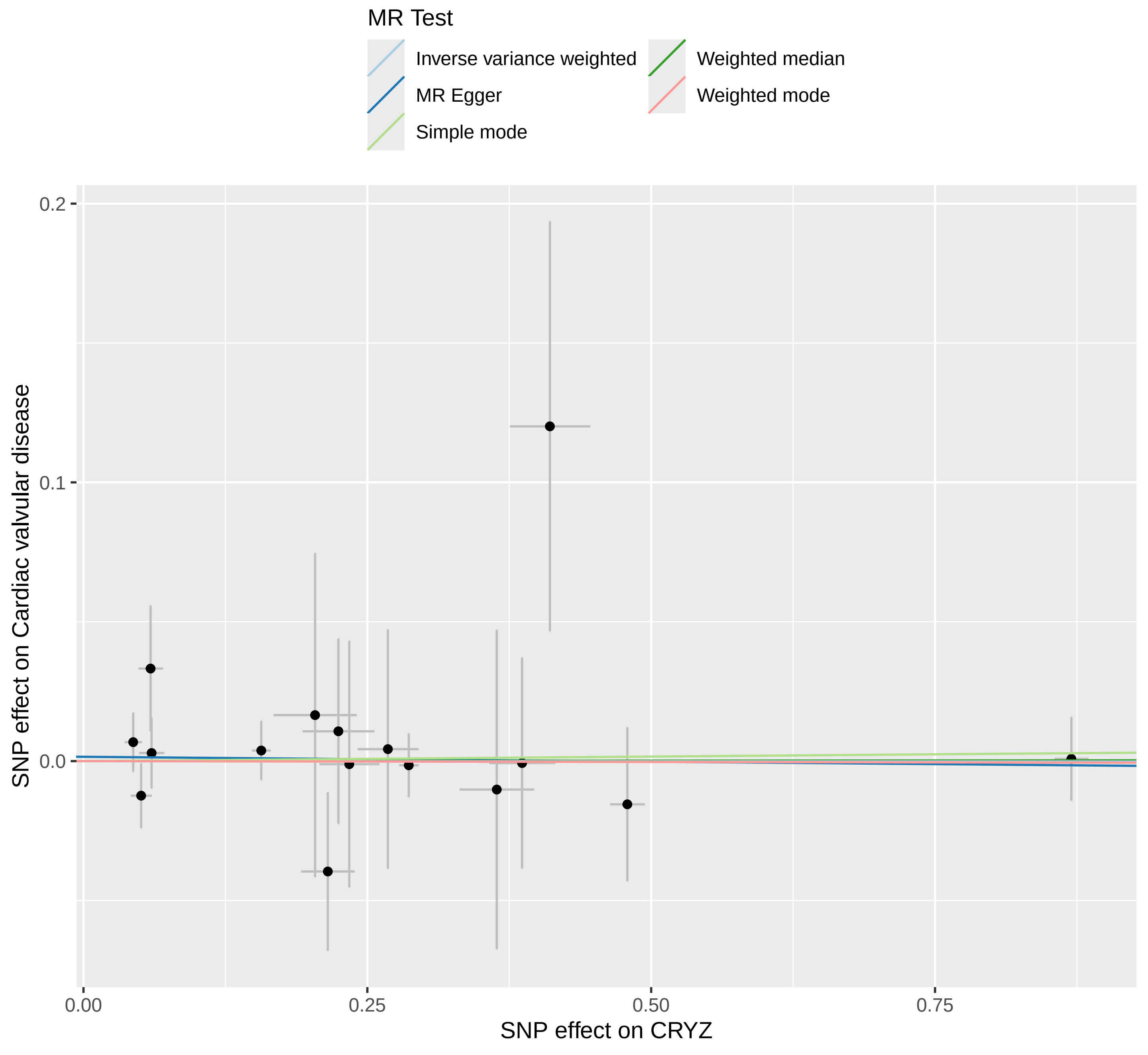

MR Test
Inverse variance weighted
MR Egger
Simple mode
Weighted median
Weighted mode
0.2
0.1
0.0
SNP effect on Cardiac valvular disease
0.00
0.25
0.50
0.75
SNP effect on CRYZ

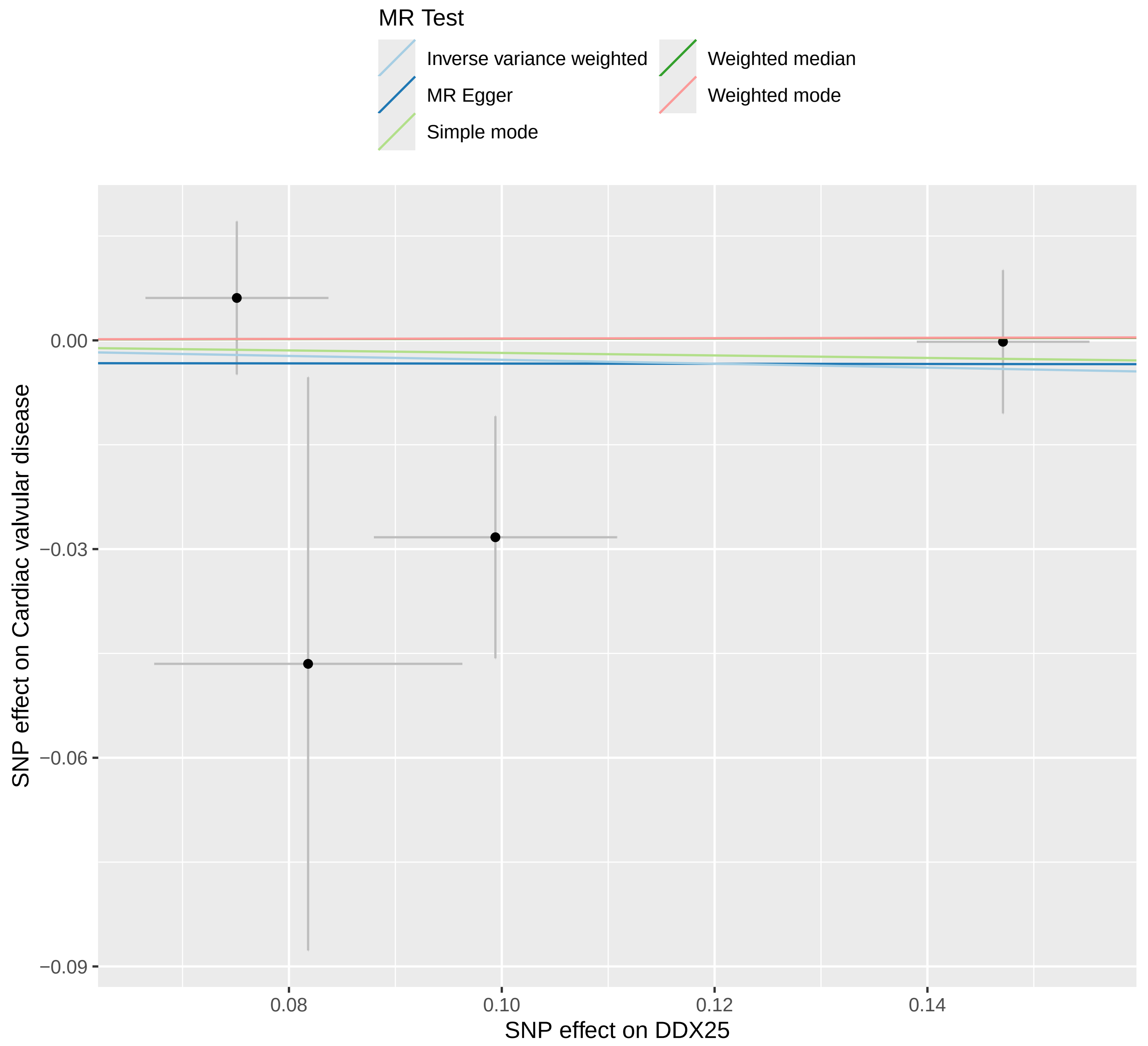

MR Test
Inverse variance weighted
MR Egger
Simple mode
Weighted median
Weighted mode
SNP effect on Cardiac valvular disease
0.00
−0.03
−0.06
−0.09
0.08
0.10
0.12
0.14
SNP effect on DDX25

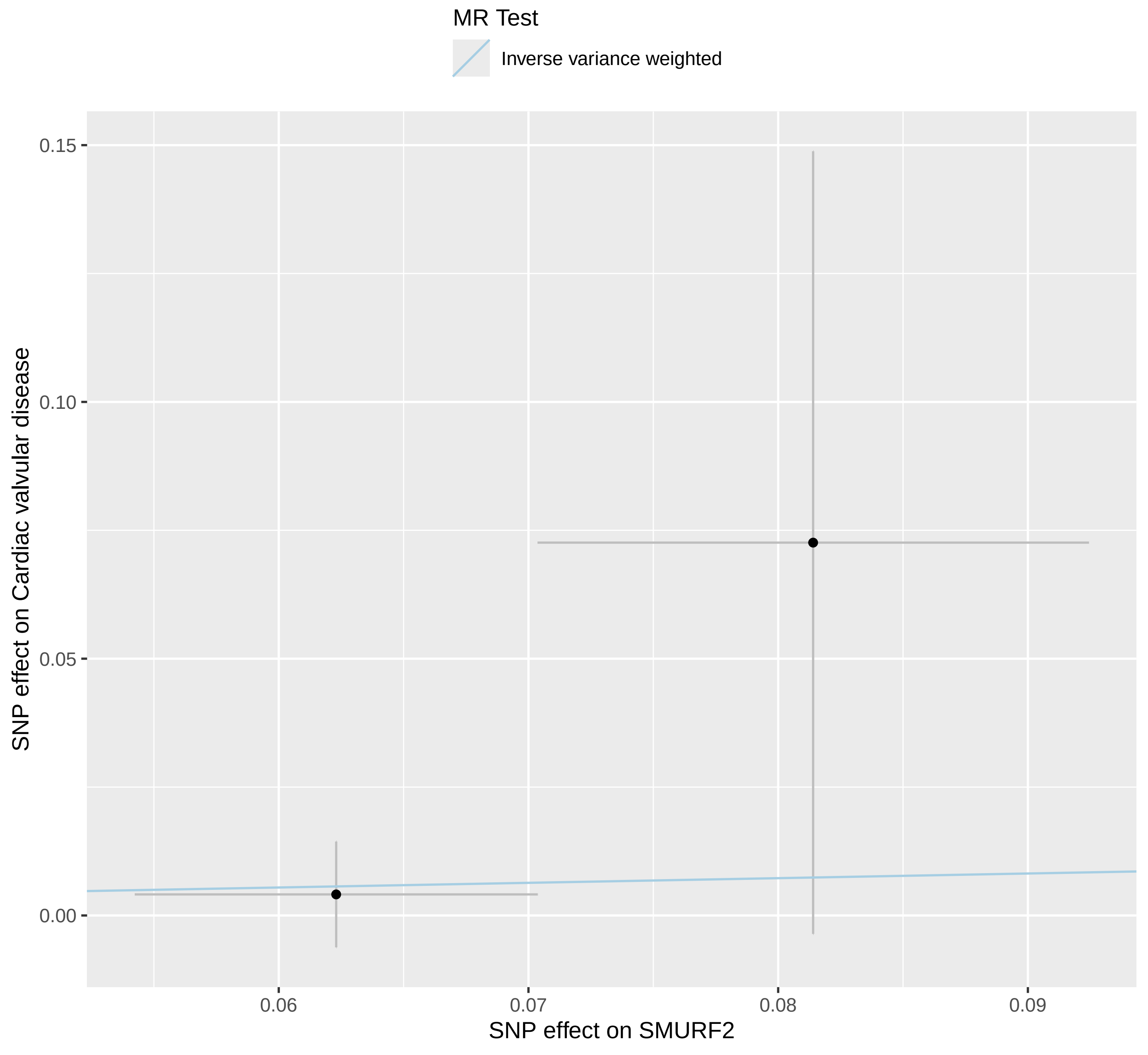
MR Test
Inverse variance weighted
SNP effect on Cardiac valvular disease
0.15
0.10
0.05
0.00
0.06
0.07
0.08
0.09
SNP effect on SMURF2

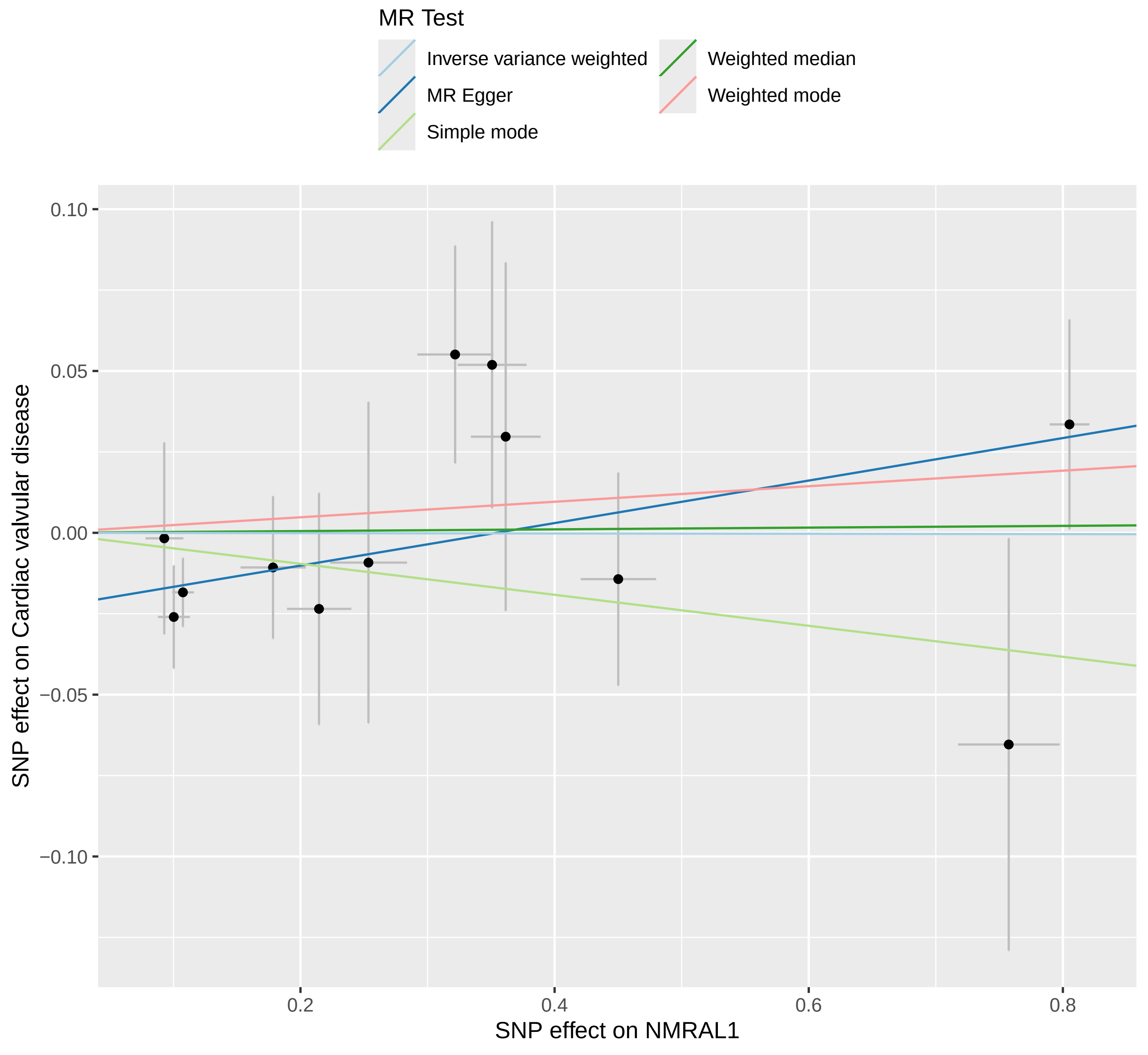
MR Test
Inverse variance weighted
MR Egger
Simple mode
Weighted median
Weighted mode
0.10
0.05
0.00
−0.05
−0.10
SNP effect on Cardiac valvular disease
0.2
0.4
0.6
0.8
SNP effect on NMRAL1

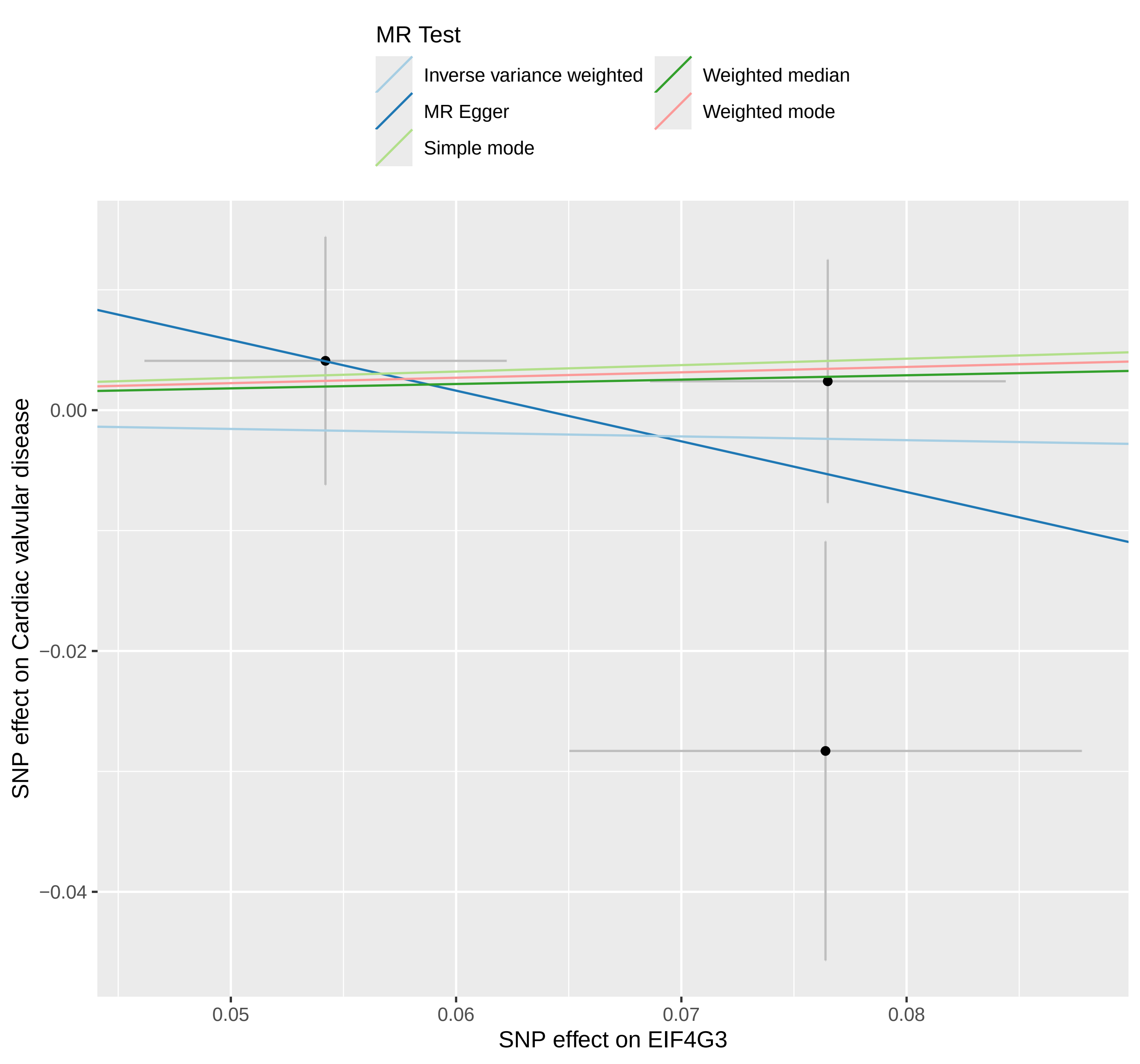
MR Test
Inverse variance weighted
MR Egger
Simple mode
Weighted median
Weighted mode
SNP effect on Cardiac valvular disease
0.00
−0.02
−0.04
0.05
0.06
0.07
0.08
SNP effect on EIF4G3

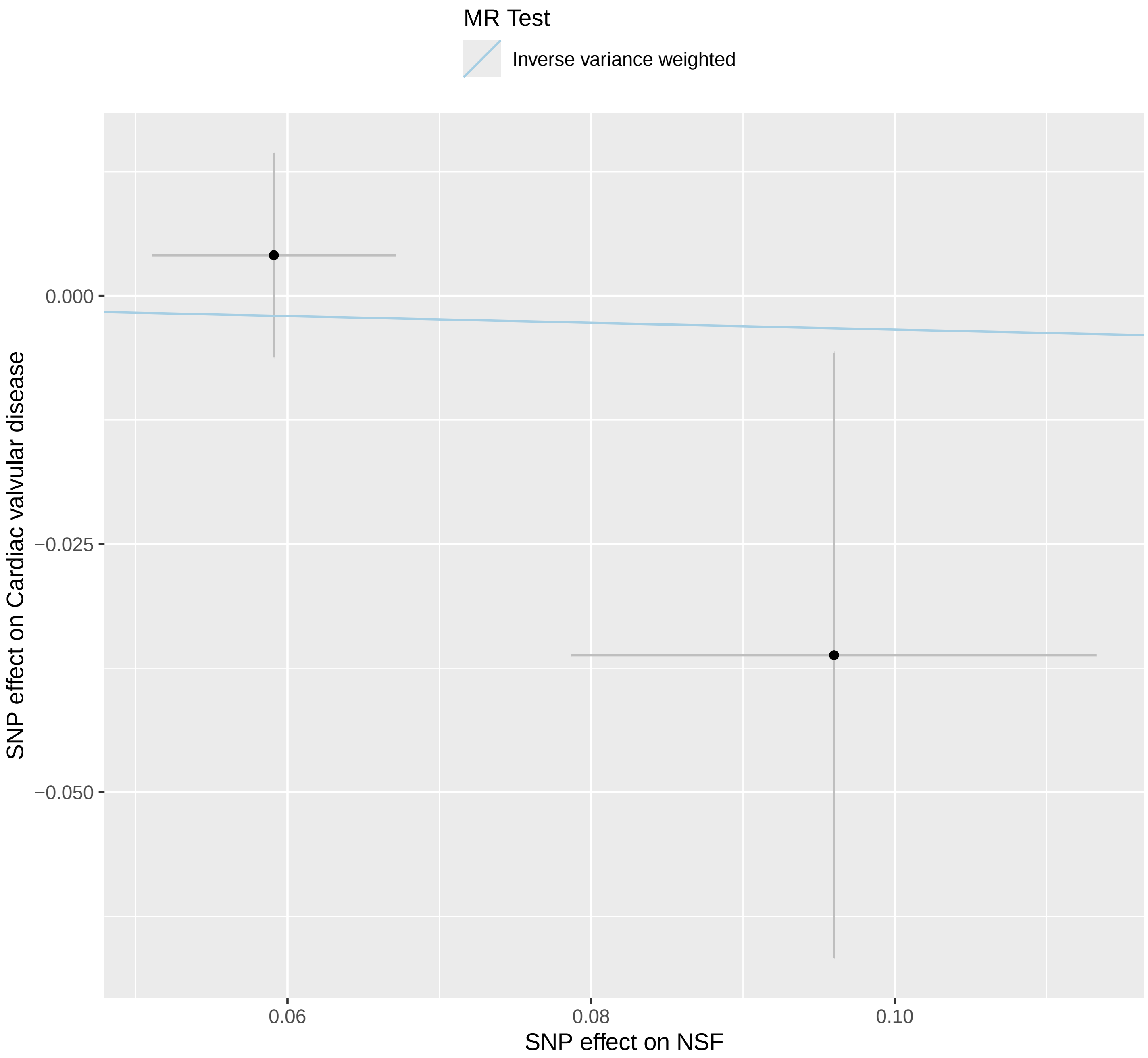
MR Test
Inverse variance weighted
0.000
−0.025
−0.050
0.06
0.08
0.10
SNP effect on NSF
SNP effect on Cardiac valvular disease

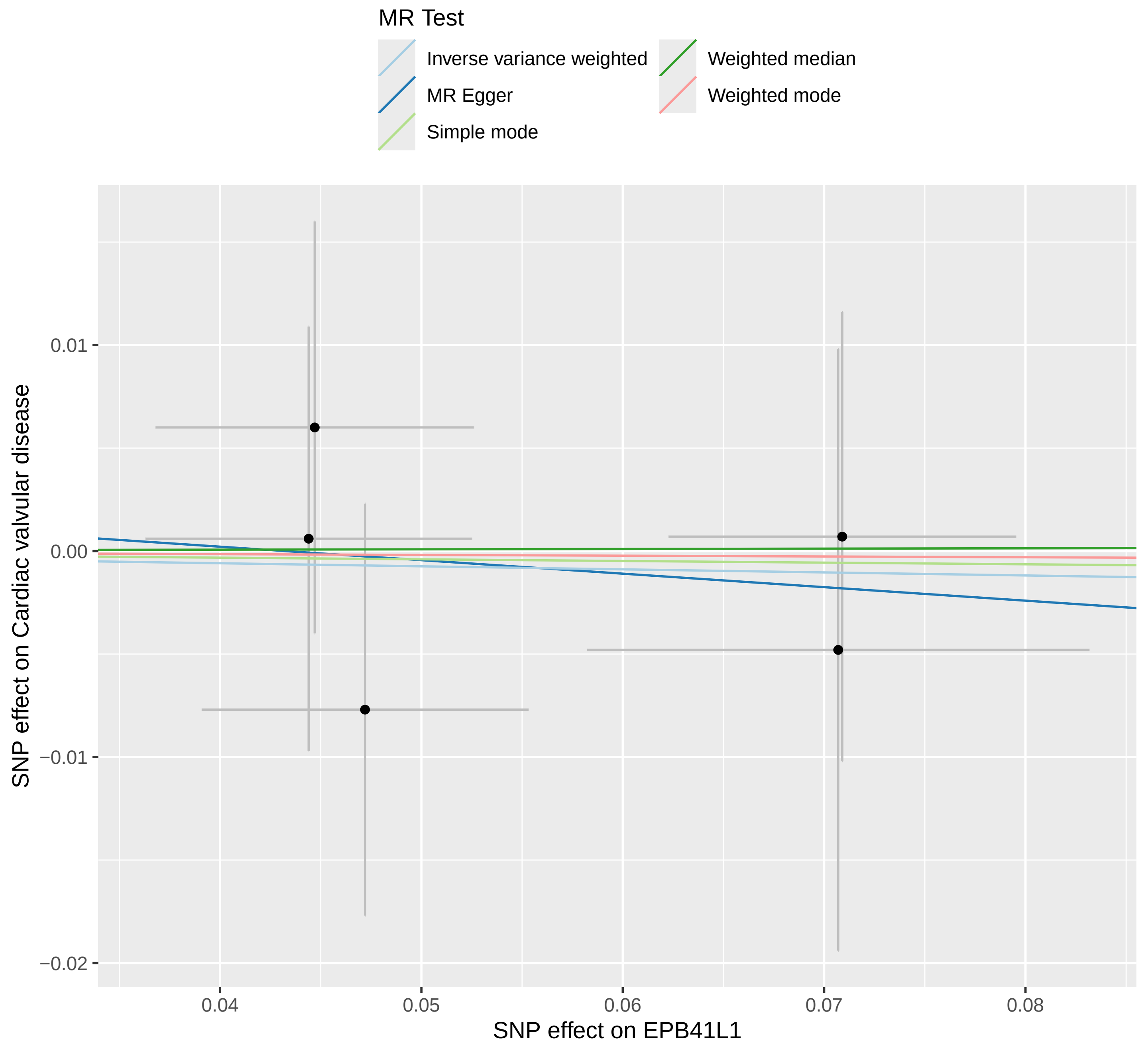
MR Test
Inverse variance weighted
MR Egger
Simple mode
Weighted median
Weighted mode
0.01
0.00
−0.01
−0.02
SNP effect on Cardiac valvular disease
0.04
0.05
0.06
0.07
0.08
SNP effect on EPB41L1

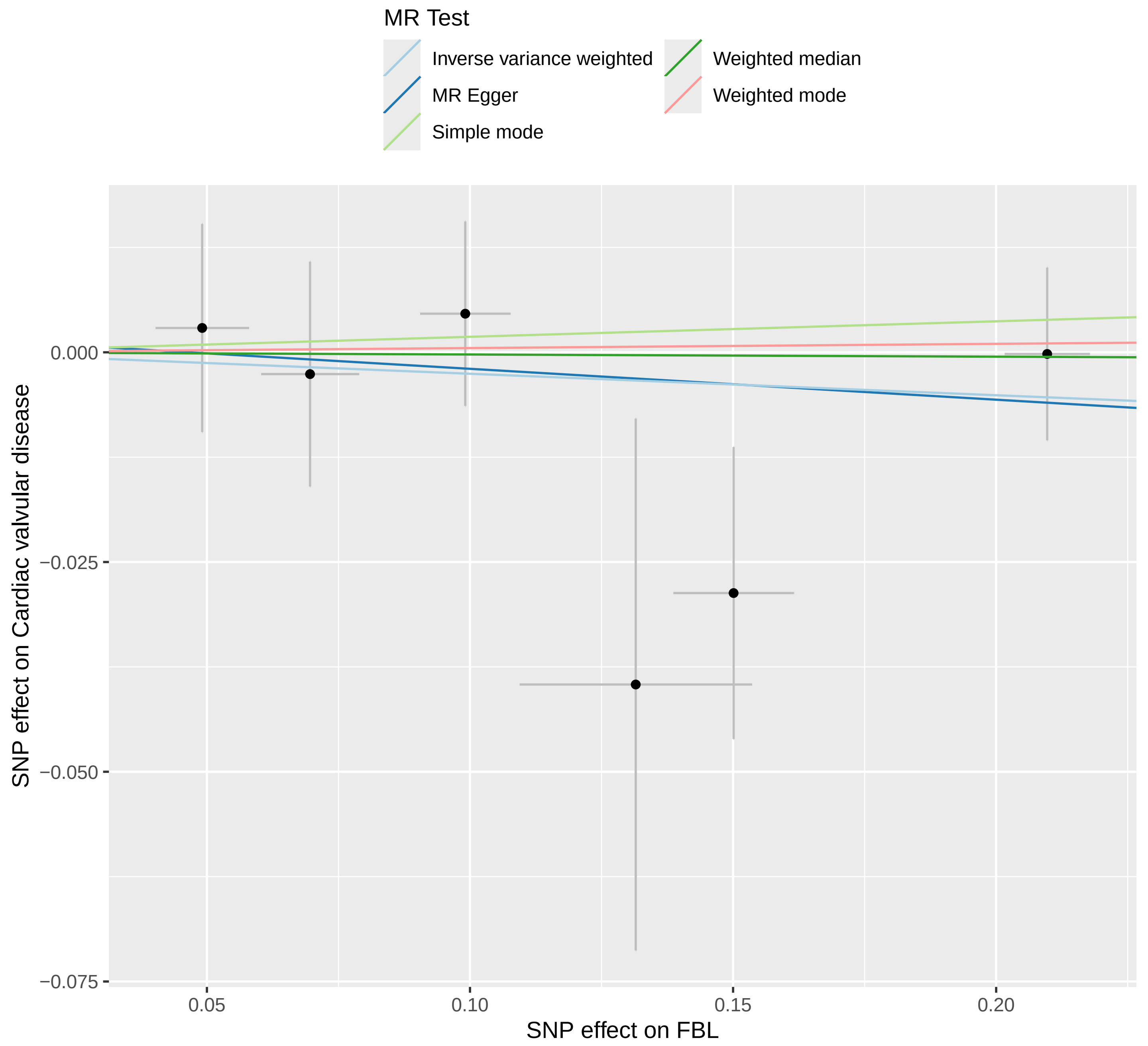
MR Test
Inverse variance weighted
MR Egger
Simple mode
Weighted median
Weighted mode
0.000
−0.025
−0.050
−0.075
0.05
0.10
0.15
0.20
SNP effect on FBL
SNP effect on Cardiac valvular disease

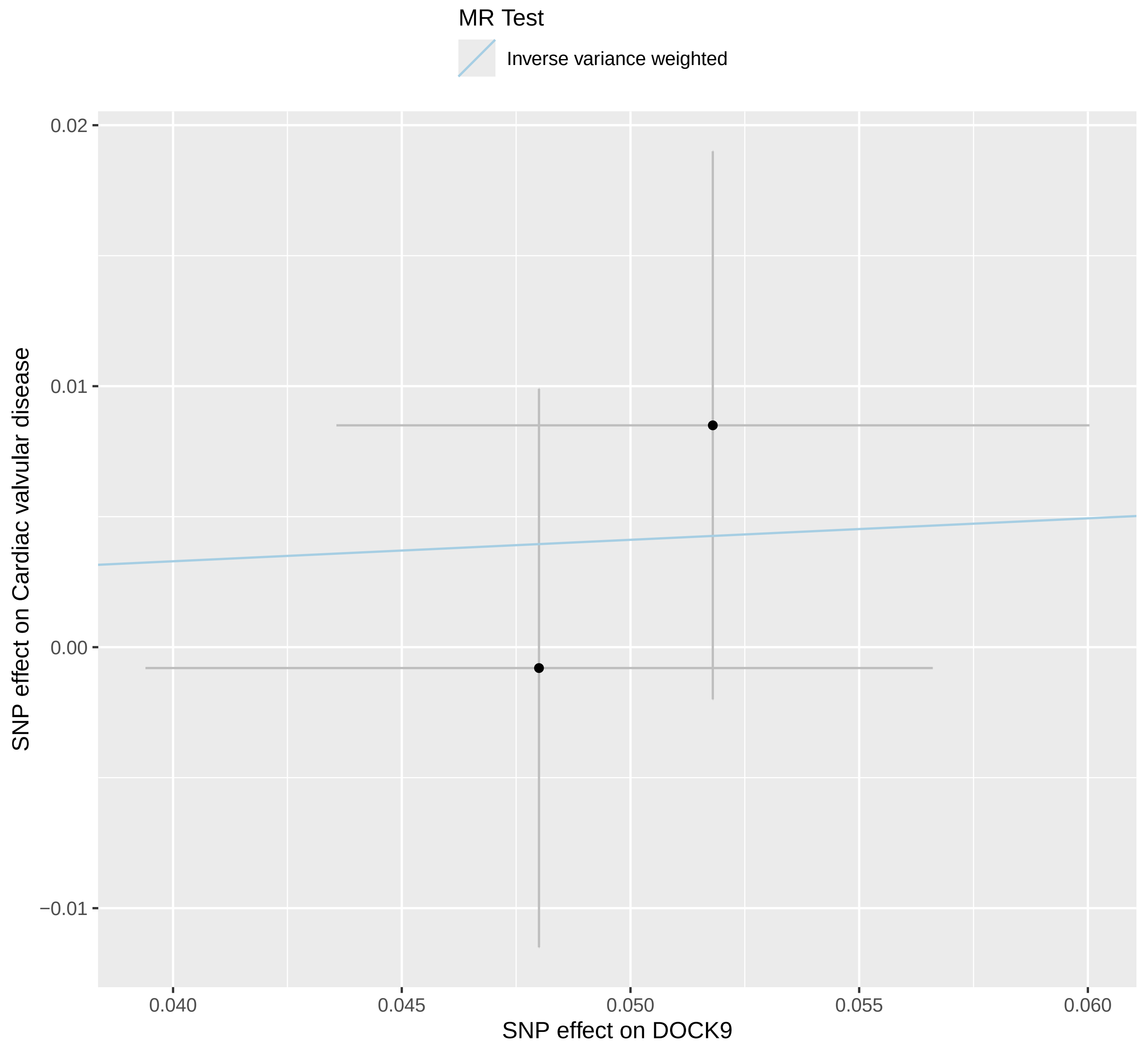

MR Test
Inverse variance weighted
0.02
0.01
0.00
−0.01
SNP effect on Cardiac valvular disease
0.040
0.045
0.050
0.055
0.060
SNP effect on DOCK9

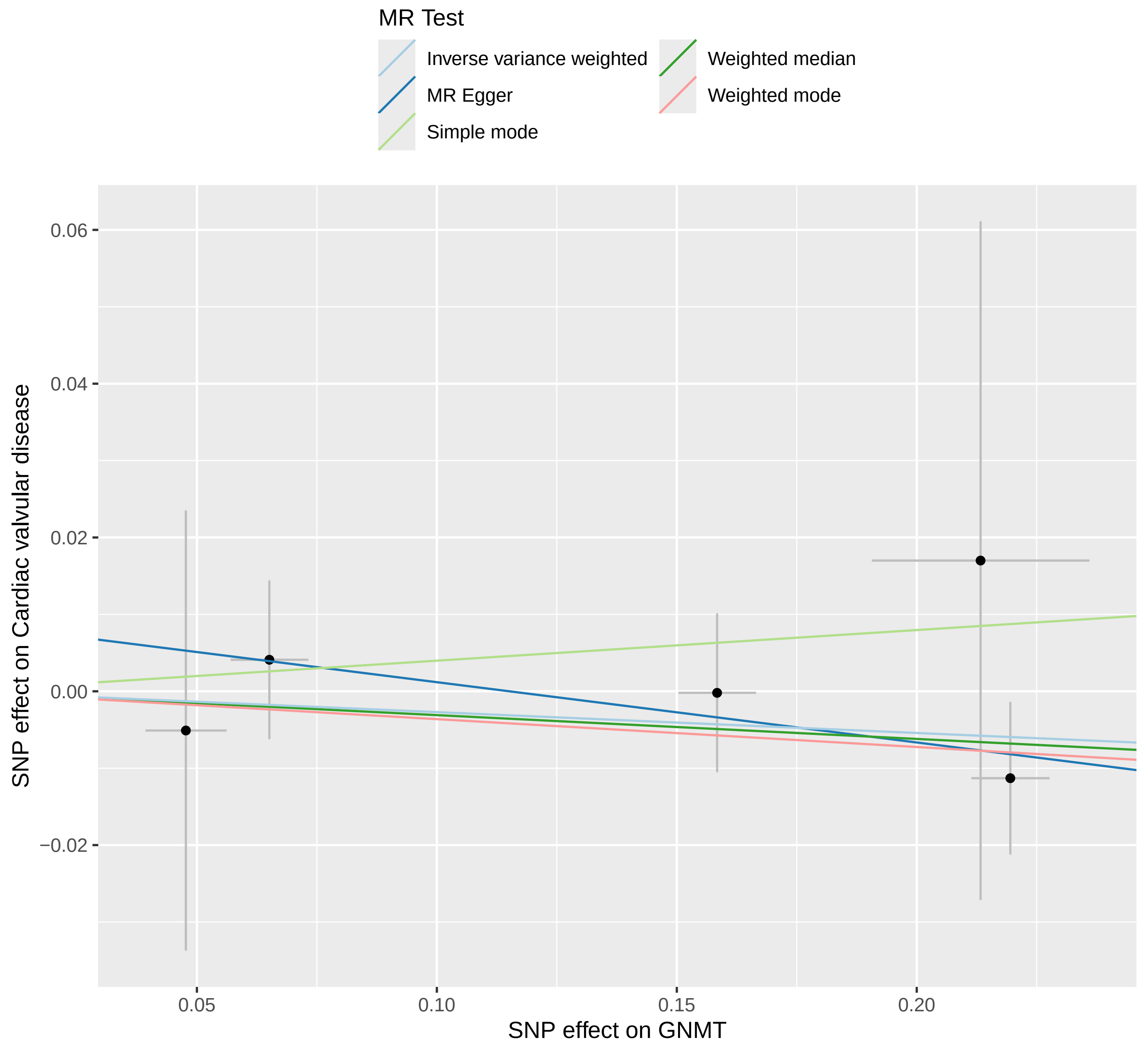

MR Test
Inverse variance weighted
MR Egger
Simple mode
Weighted median
Weighted mode
0.06
0.04
0.02
0.00
−0.02
0.05
0.10
0.15
0.20
SNP effect on Cardiac valvular disease
SNP effect on GNMT

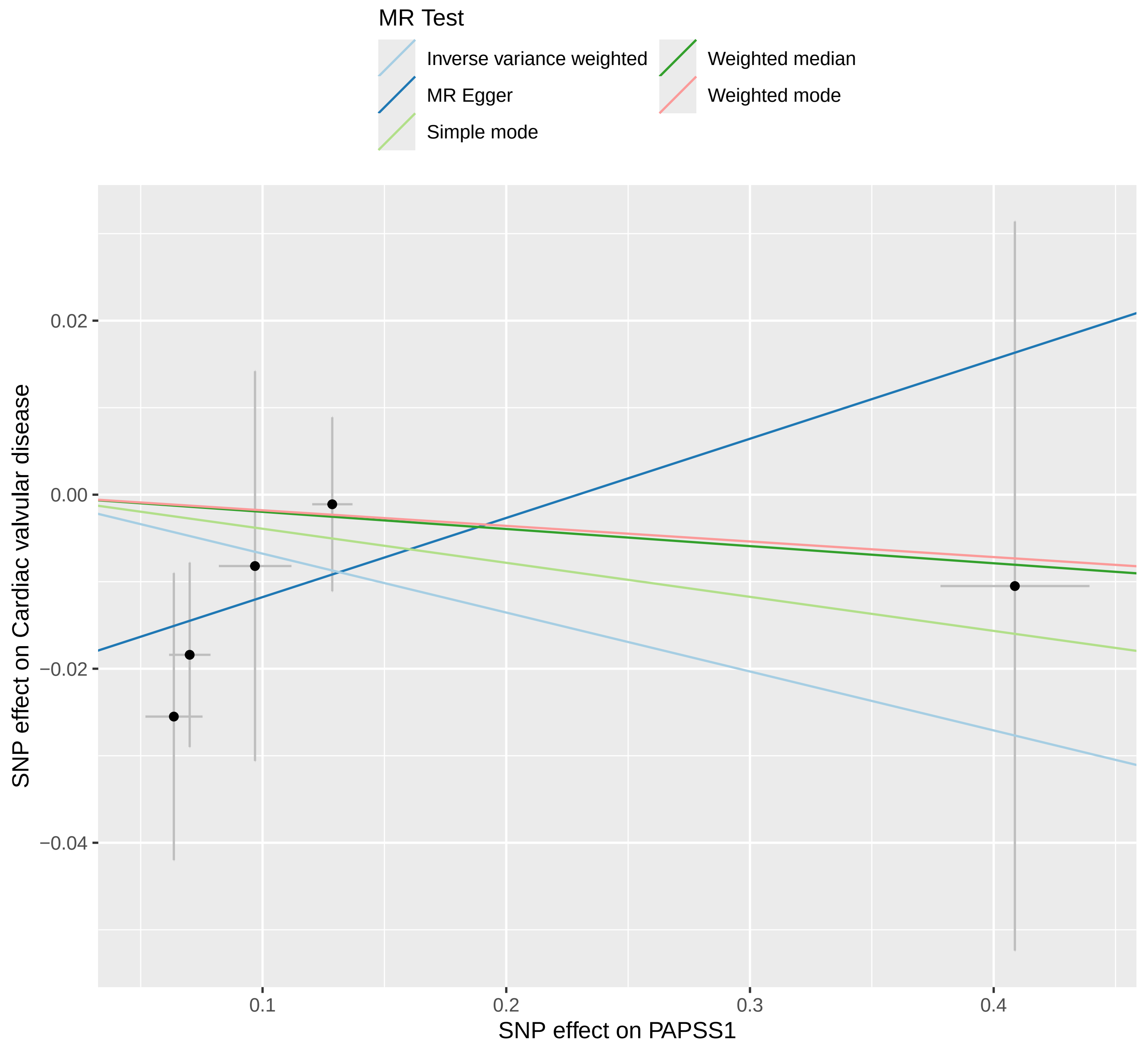

MR Test
Inverse variance weighted
MR Egger
Simple mode
Weighted median
Weighted mode
0.02
0.00
−0.02
−0.04
SNP effect on Cardiac valvular disease
0.1
0.2
0.3
0.4
SNP effect on PAPSS1

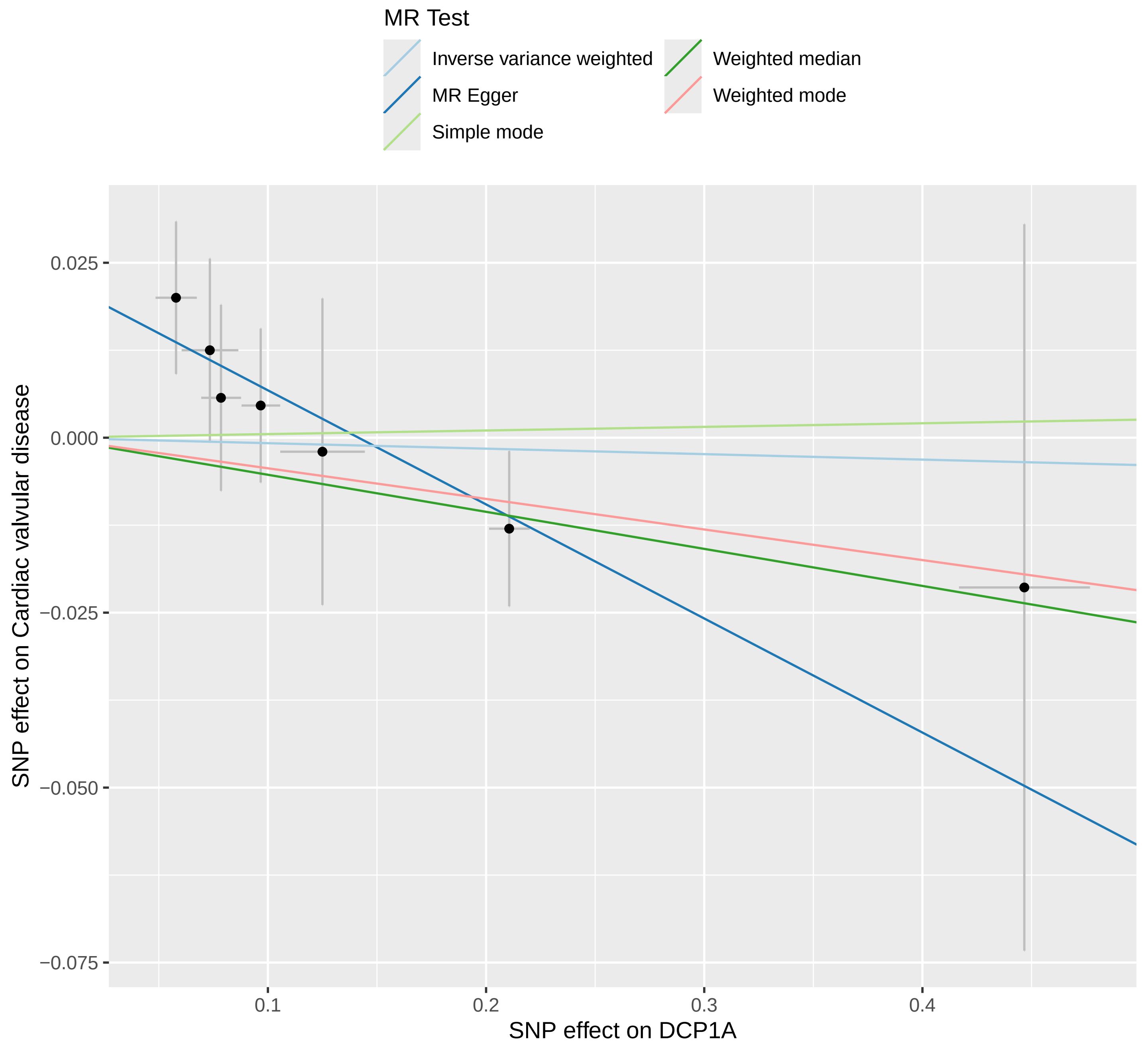
MR Test
Inverse variance weighted
MR Egger
Simple mode
Weighted median
Weighted mode
0.025
0.000
−0.025
−0.050
−0.075
SNP effect on Cardiac valvular disease
0.1
0.2
0.3
0.4
SNP effect on DCP1A

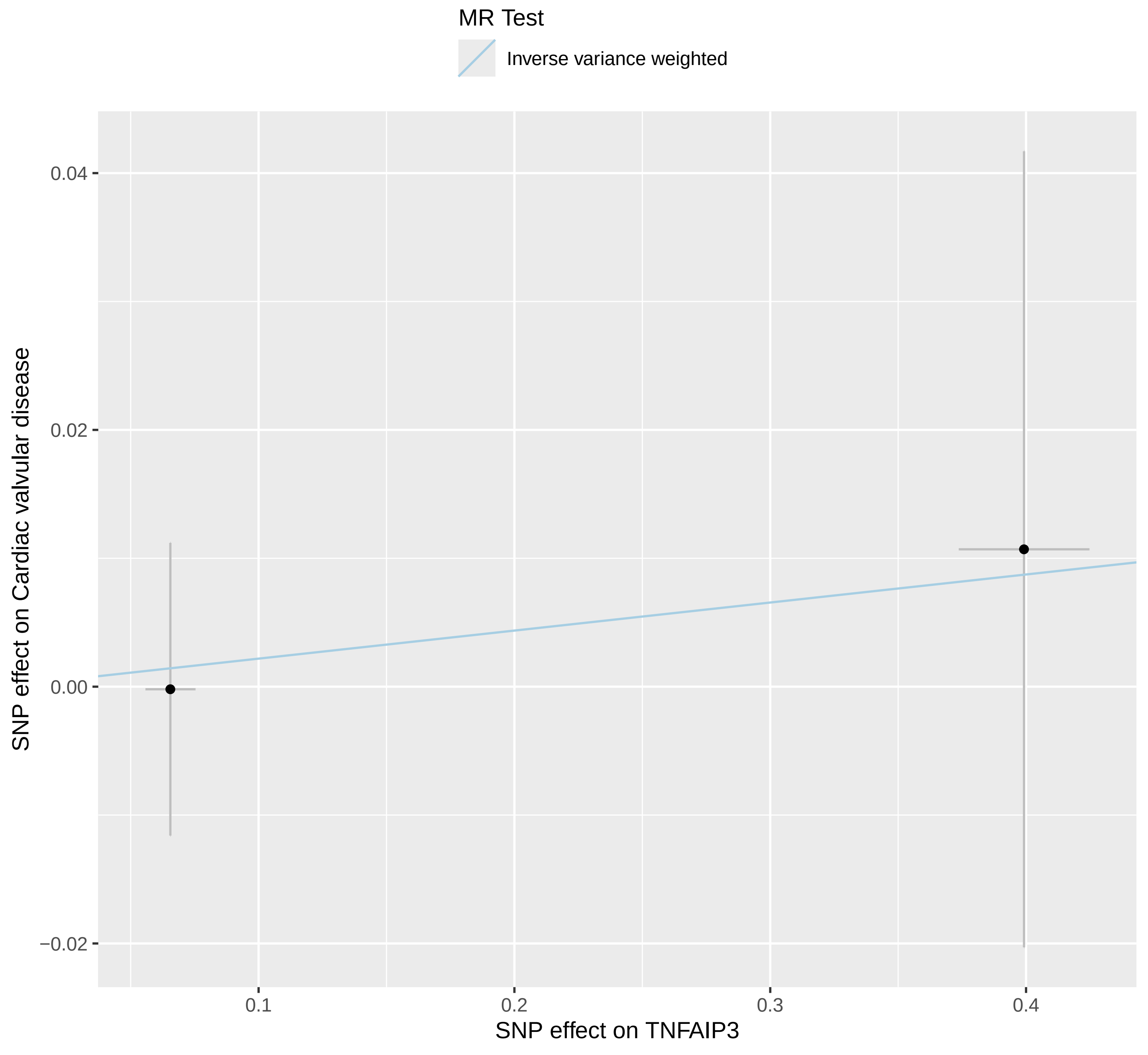

MR Test
Inverse variance weighted
0.04
0.02
0.00
−0.02
0.1
0.2
0.3
0.4
SNP effect on Cardiac valvular disease
SNP effect on TNFAIP3

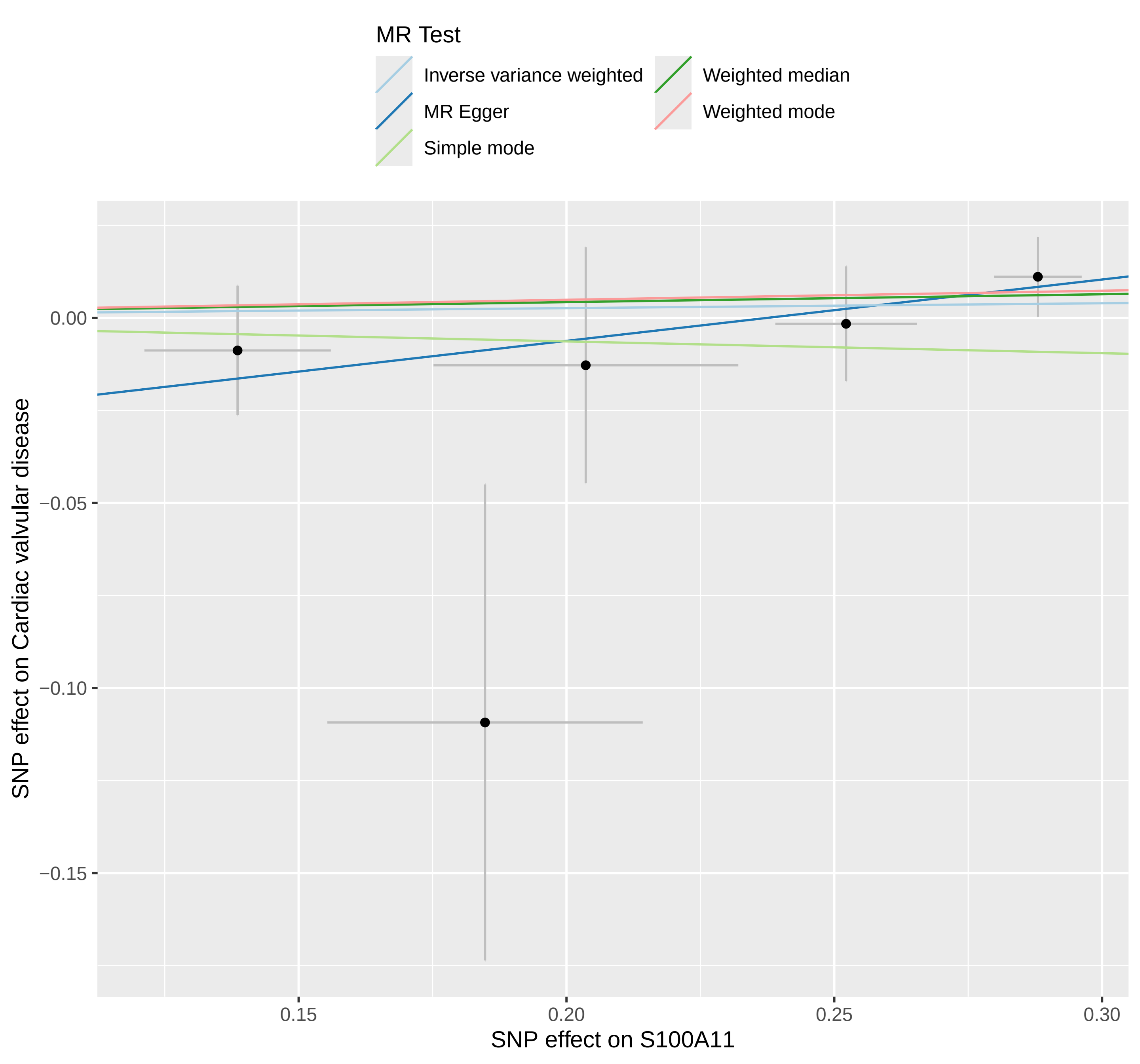

MR Test
Inverse variance weighted
MR Egger
Simple mode
Weighted median
Weighted mode
0.00
−0.05
−0.10
−0.15
SNP effect on Cardiac valvular disease
0.15
0.20
0.25
0.30
SNP effect on S100A11

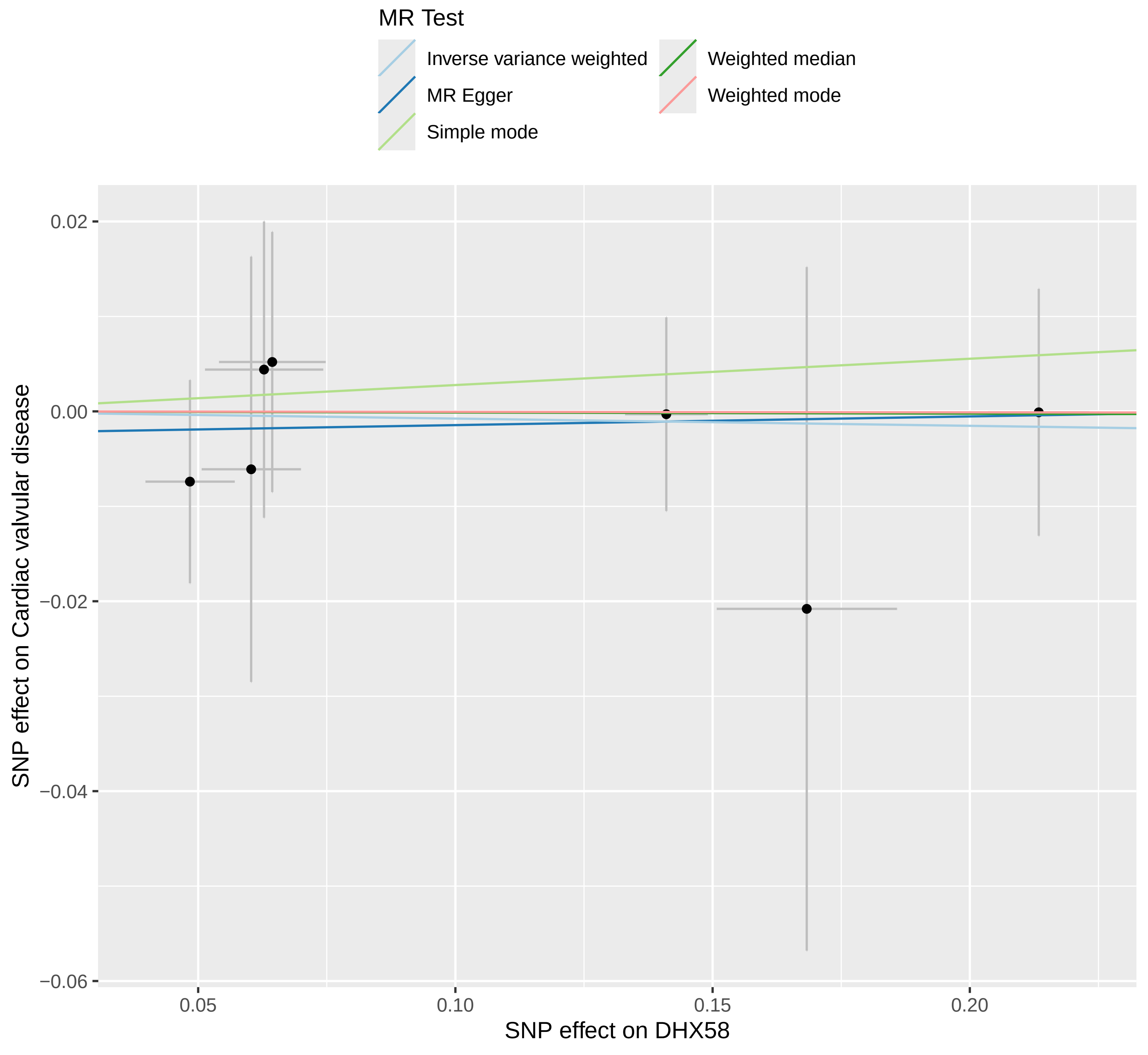

MR Test
Inverse variance weighted
MR Egger
Simple mode
Weighted median
Weighted mode
0.02
0.00
−0.02
−0.04
−0.06
SNP effect on Cardiac valvular disease
0.05
0.10
0.15
0.20
SNP effect on DHX58

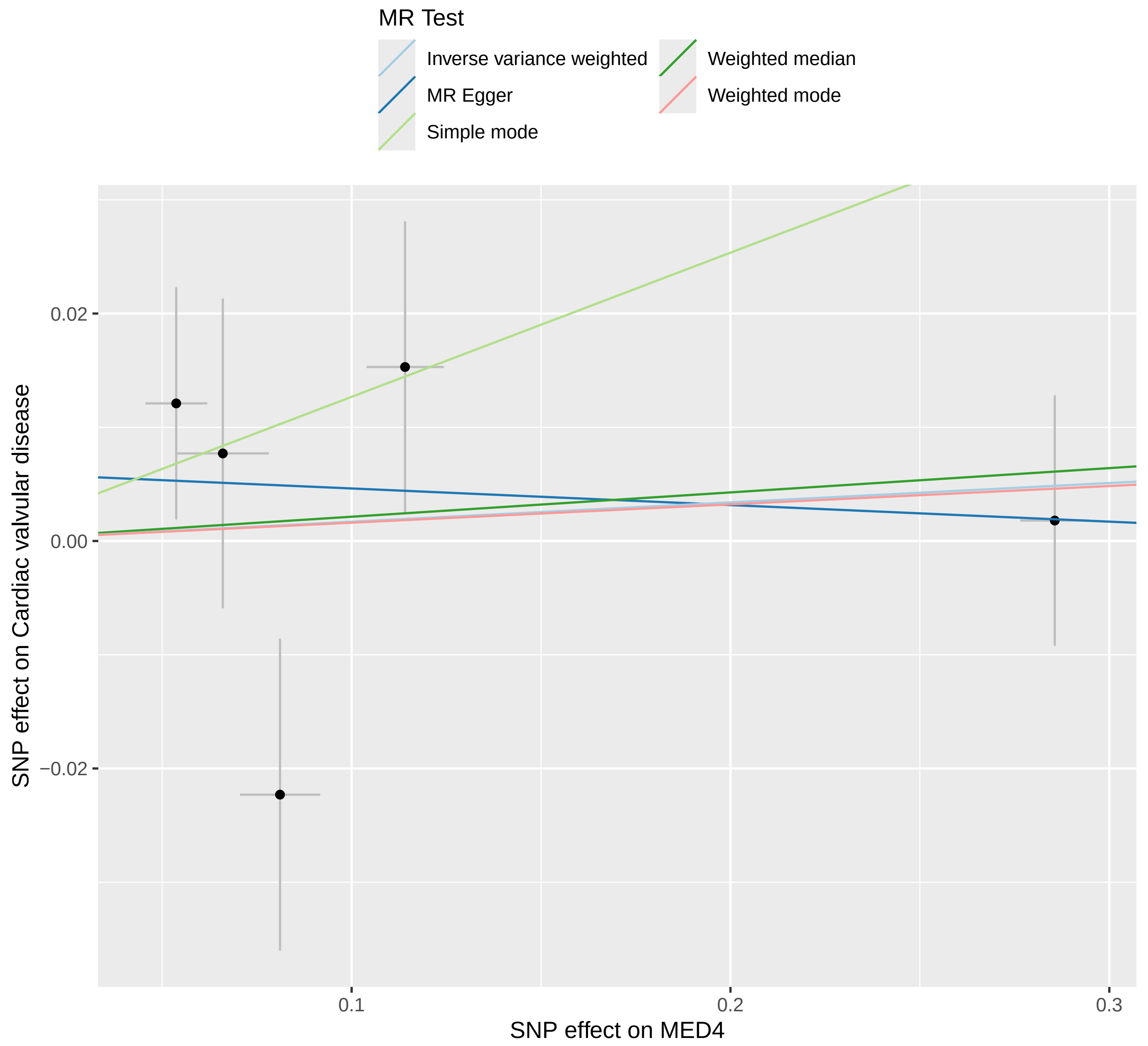

MR Test
Inverse variance weighted
MR Egger
Simple mode
Weighted median
Weighted mode
0.02
0.00
−0.02
0.1
0.2
0.3
SNP effect on Cardiac valvular disease
SNP effect on MED4

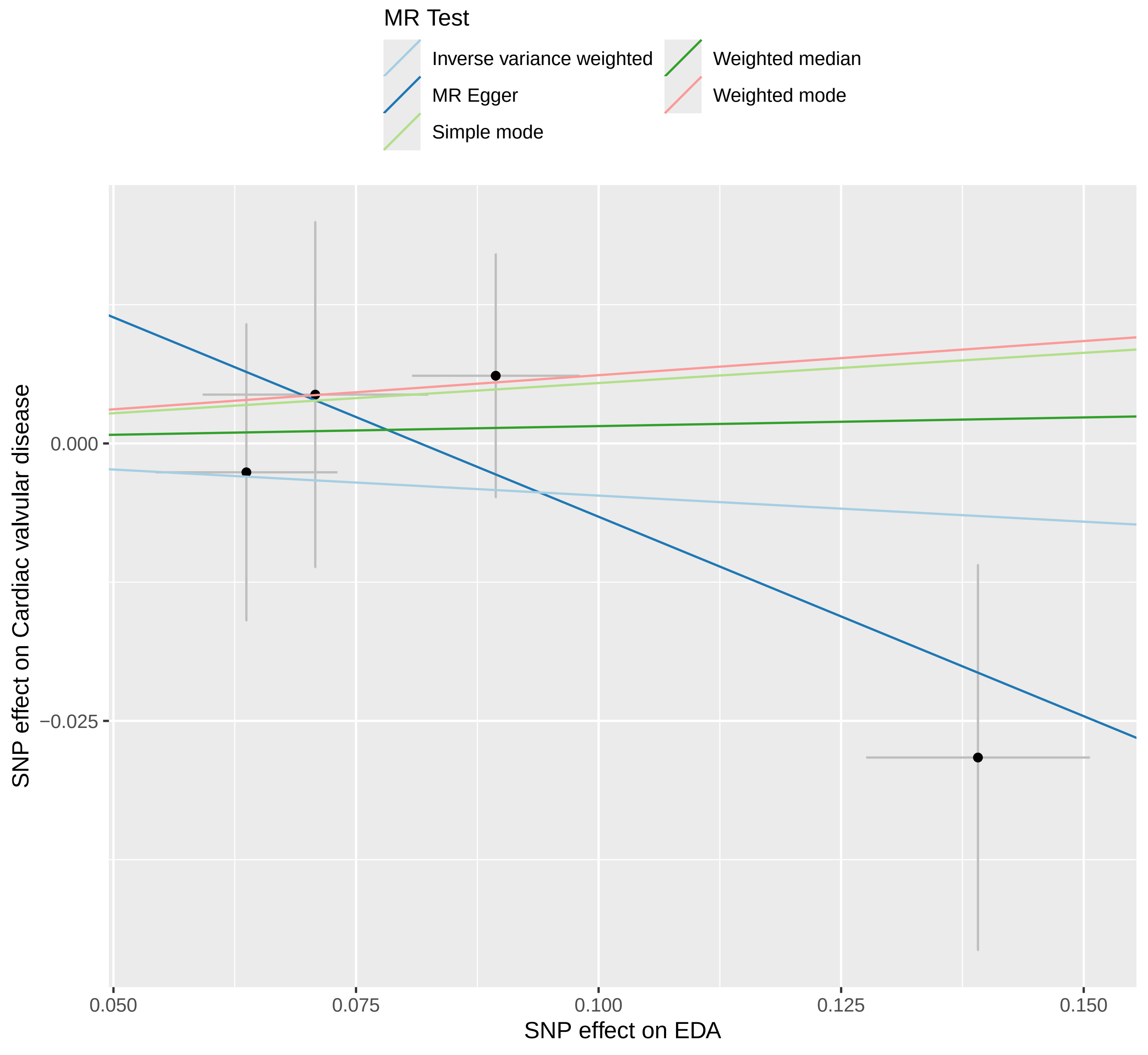
MR Test
Inverse variance weighted
MR Egger
Simple mode
Weighted median
Weighted mode
0.000
−0.025
0.050
0.075
0.100
0.125
0.150
SNP effect on EDA
SNP effect on Cardiac valvular disease

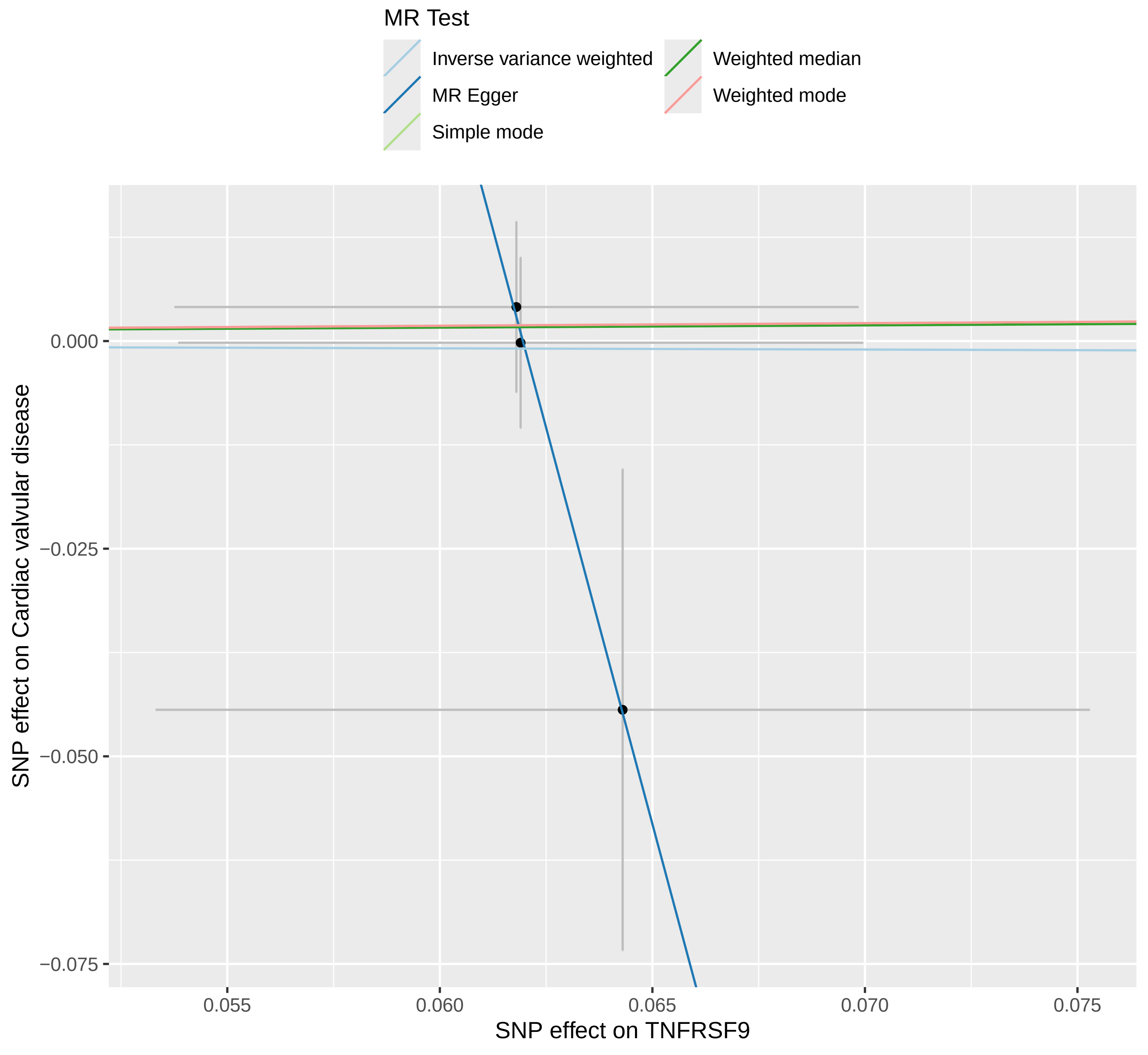

MR Test
Inverse variance weighted
MR Egger
Simple mode
Weighted median
Weighted mode
0.000
−0.025
−0.050
−0.075
SNP effect on Cardiac valvular disease
0.055
0.060
0.065
0.070
0.075
SNP effect on TNFRSF9

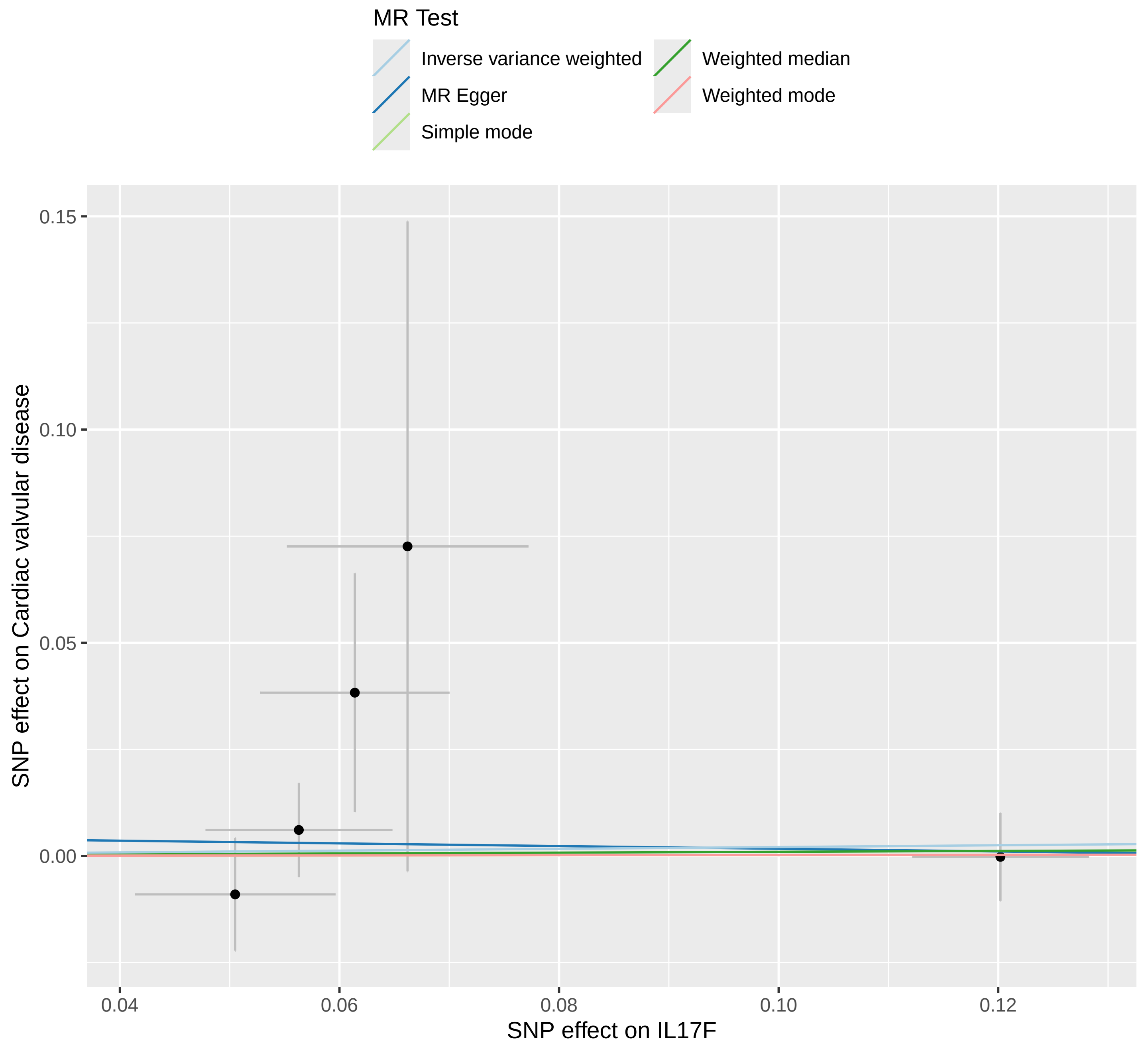
MR Test
Inverse variance weighted
MR Egger
Simple mode
Weighted median
Weighted mode
0.15
0.10
0.05
0.00
0.04
0.06
0.08
0.10
0.12
SNP effect on Cardiac valvular disease
SNP effect on IL17F

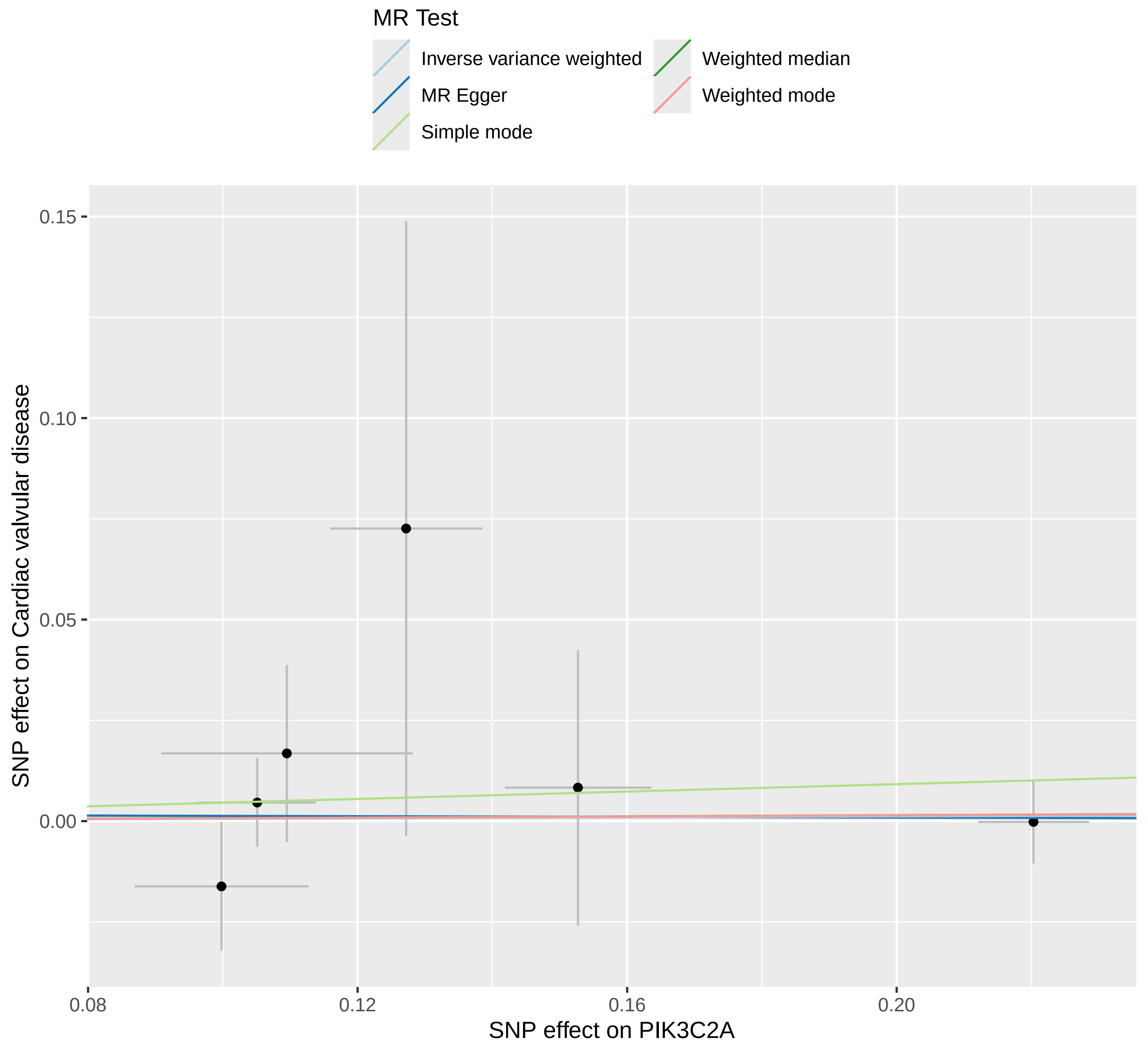
MR Test
Inverse variance weighted
MR Egger
Simple mode
Weighted median
Weighted mode
0.15
0.10
0.05
0.00
SNP effect on Cardiac valvular disease
0.08
0.12
0.16
0.20
SNP effect on PIK3C2A

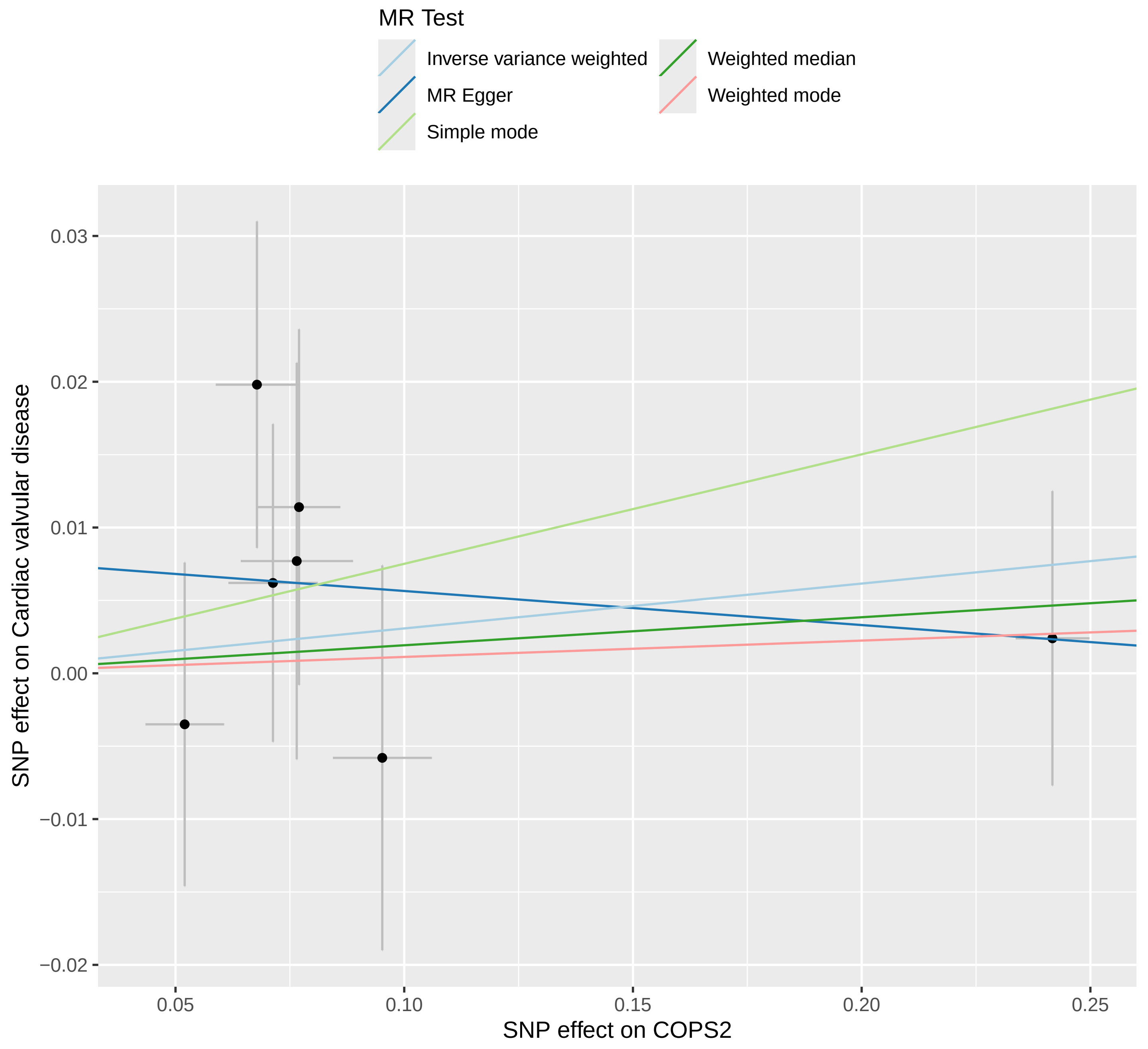

MR Test
Inverse variance weighted
MR Egger
Simple mode
Weighted median
Weighted mode
0.03
0.02
0.01
0.00
−0.01
−0.02
0.05
0.10
0.15
0.20
0.25
SNP effect on Cardiac valvular disease
SNP effect on COPS2

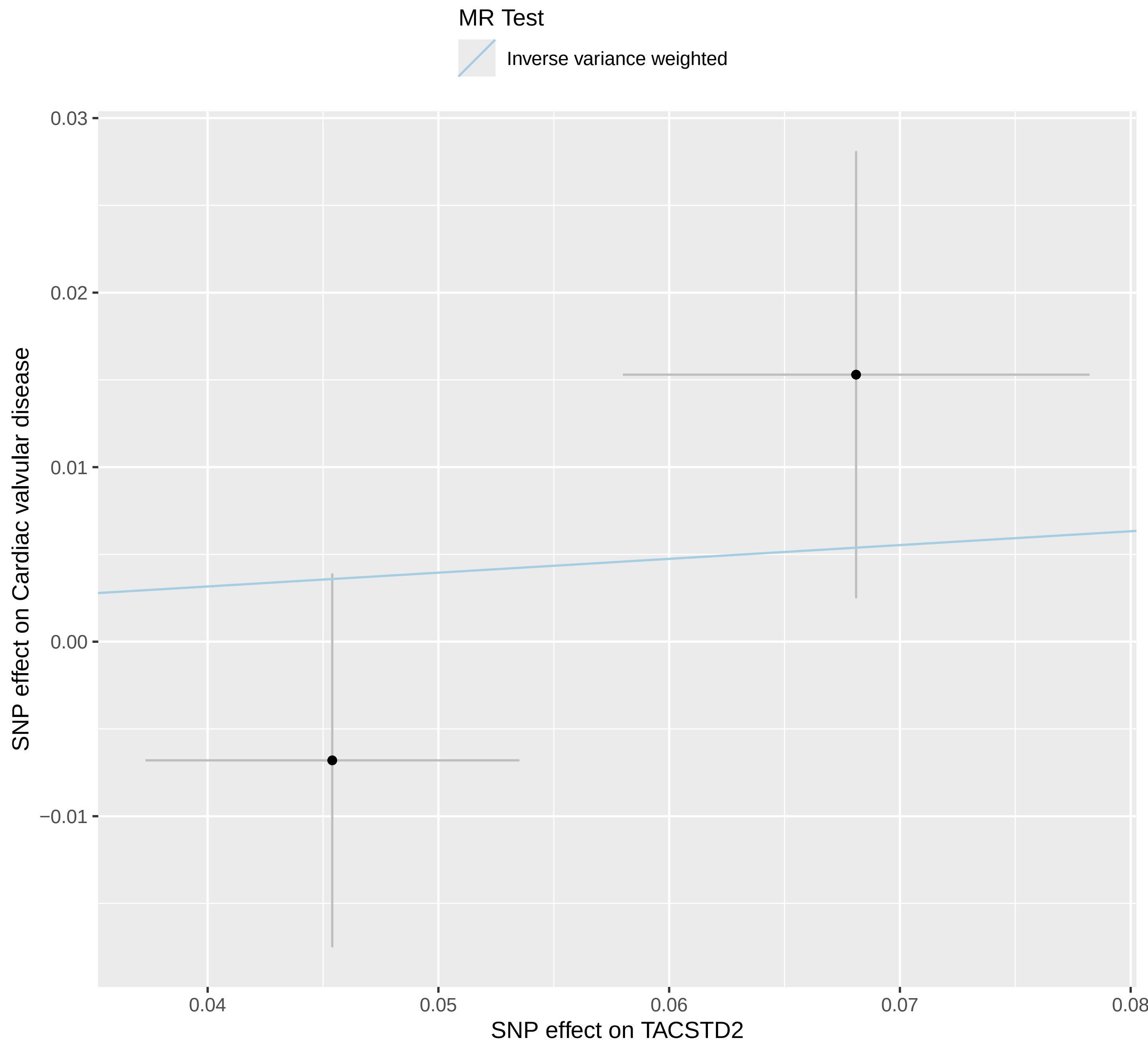
MR Test
Inverse variance weighted
0.03
0.02
0.01
0.00
−0.01
0.04
0.05
0.06
0.07
0.08
SNP effect on Cardiac valvular disease
SNP effect on TACSTD2

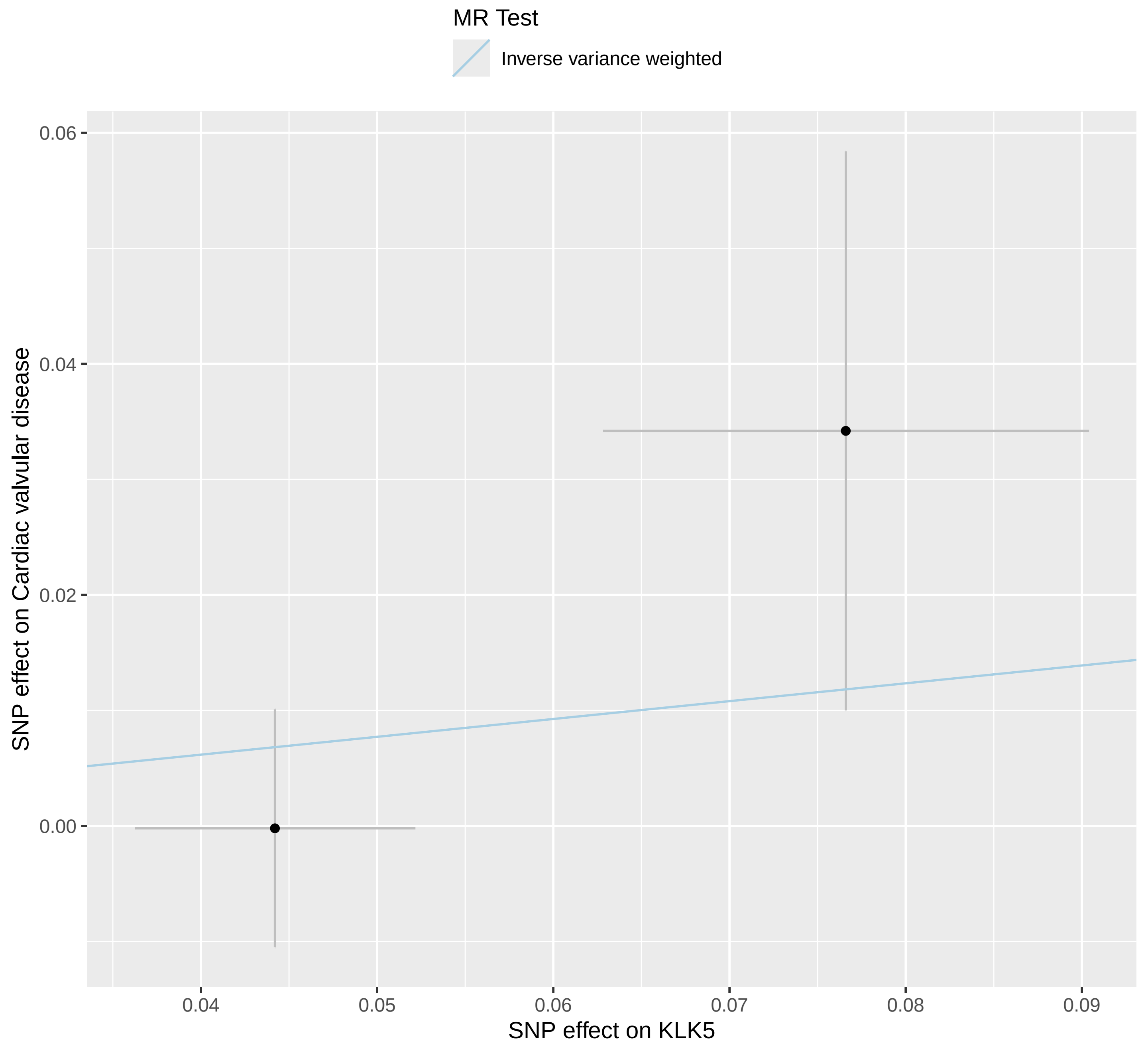

MR Test
Inverse variance weighted
SNP effect on Cardiac valvular disease
0.06
0.04
0.02
0.00
0.04
0.05
0.06
0.07
0.08
0.09
SNP effect on KLK5

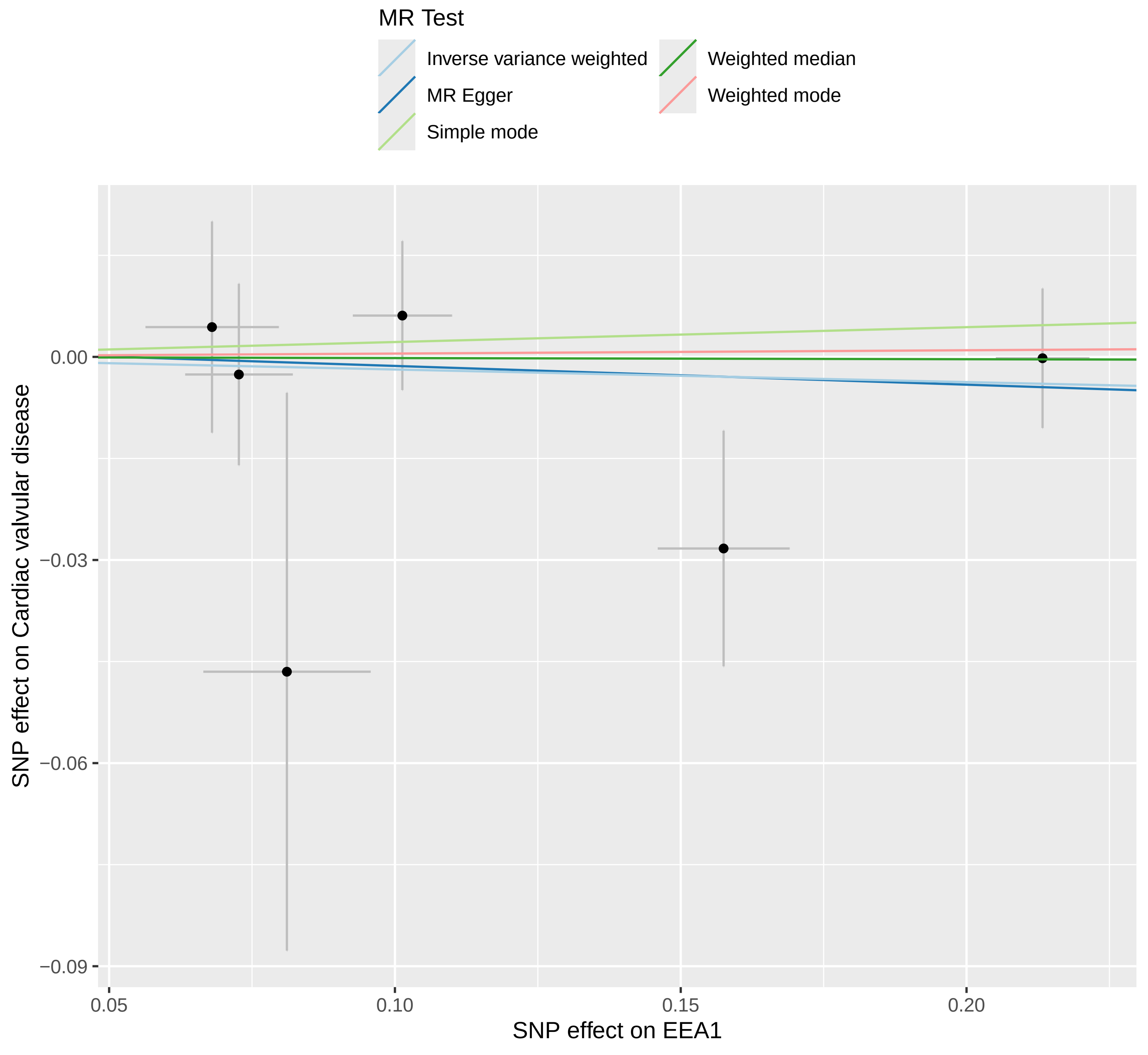

MR Test
Inverse variance weighted
MR Egger
Simple mode
Weighted median
Weighted mode
0.00
−0.03
−0.06
−0.09
0.05
0.10
0.15
0.20
SNP effect on EEA1
SNP effect on Cardiac valvular disease

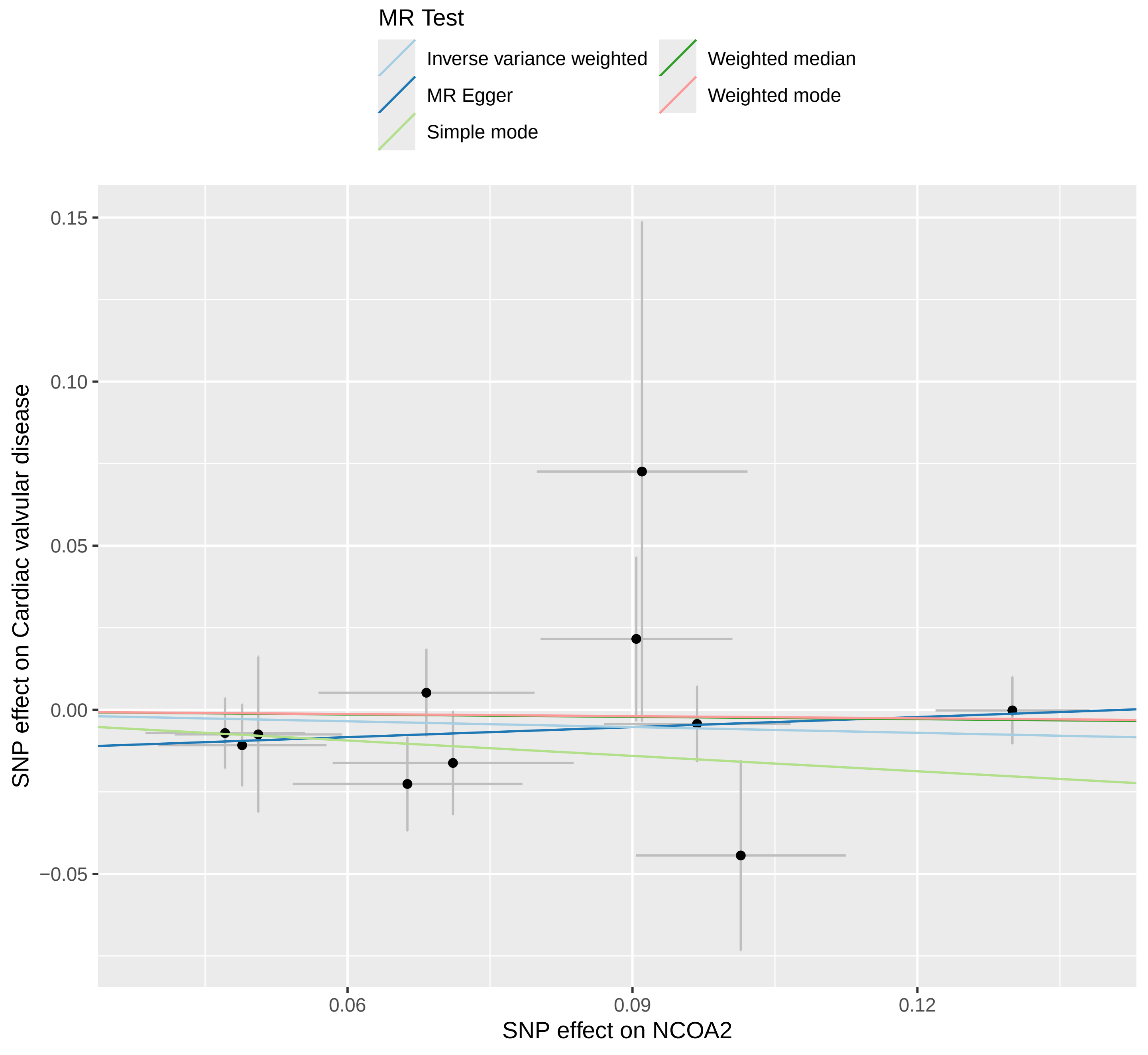
MR Test
Inverse variance weighted
MR Egger
Simple mode
Weighted median
Weighted mode
0.15
0.10
0.05
0.00
−0.05
0.06
0.09
0.12
SNP effect on Cardiac valvular disease
SNP effect on NCOA2

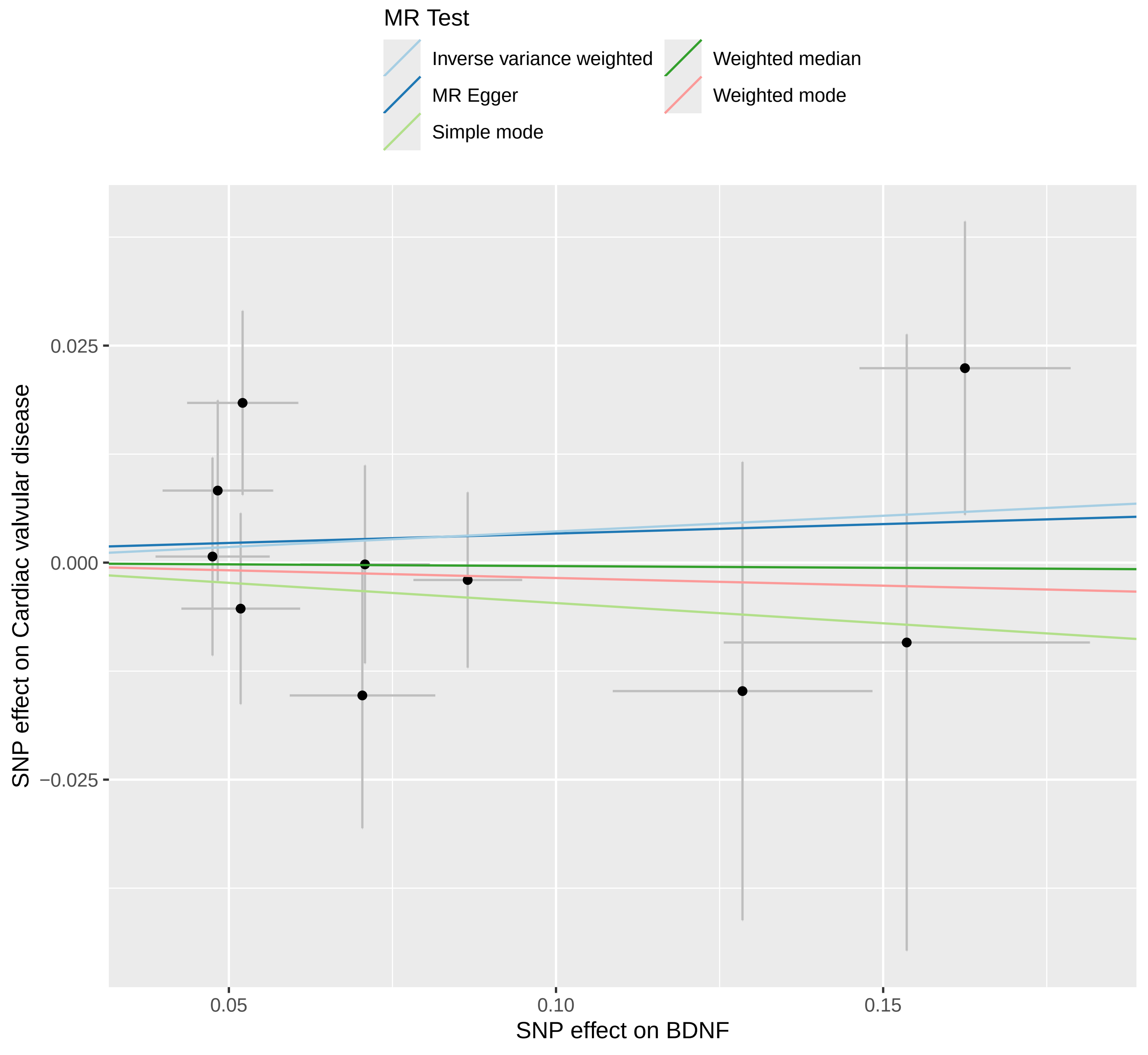

MR Test
Inverse variance weighted
MR Egger
Simple mode
Weighted median
Weighted mode
0.025
0.000
−0.025
SNP effect on Cardiac valvular disease
0.05
0.10
0.15
SNP effect on BDNF

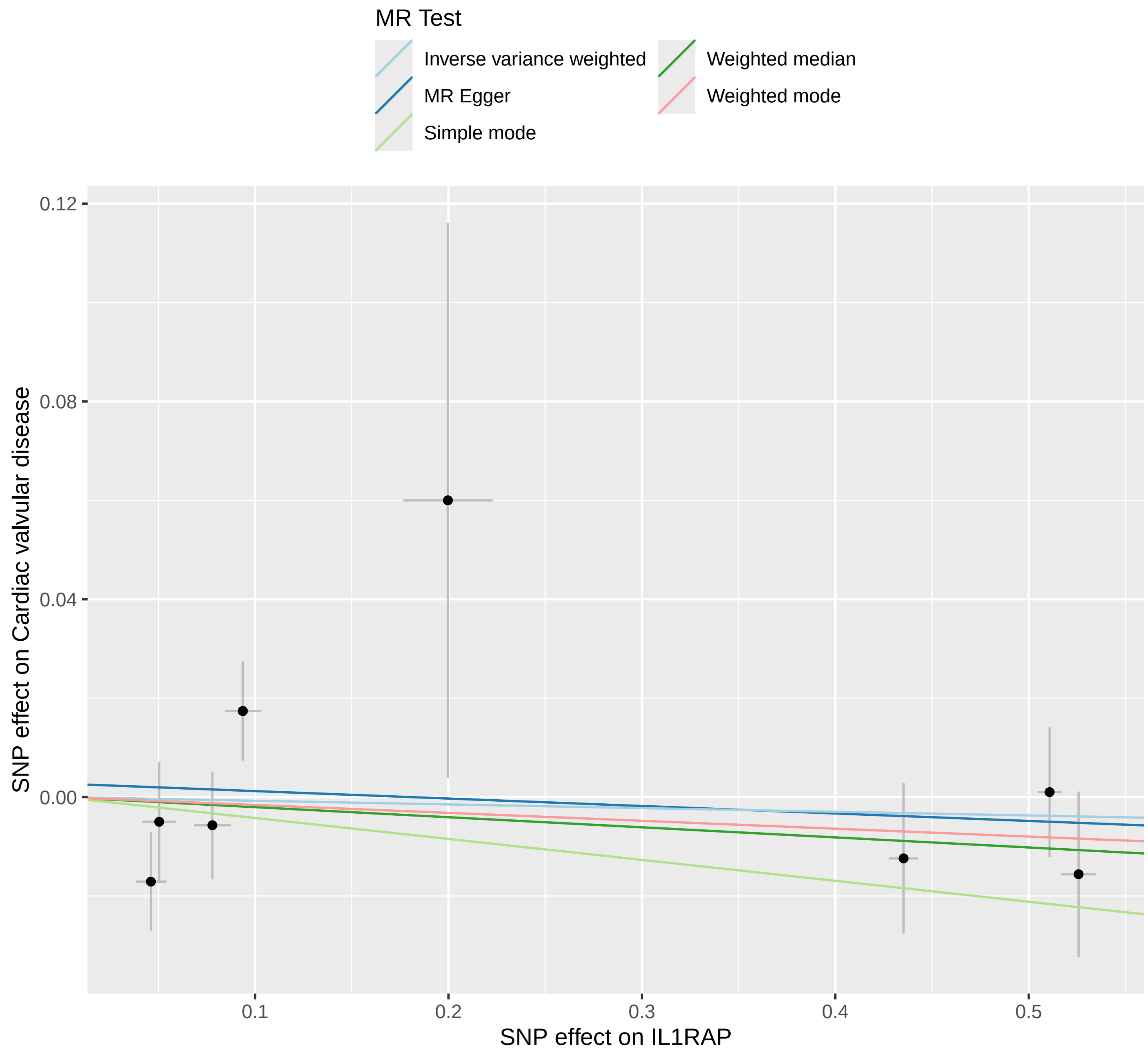

MR Test
Inverse variance weighted
MR Egger
Simple mode
Weighted median
Weighted mode
0.12
0.08
0.04
0.00
SNP effect on Cardiac valvular disease
0.1
0.2
0.3
0.4
0.5
SNP effect on IL1RAP

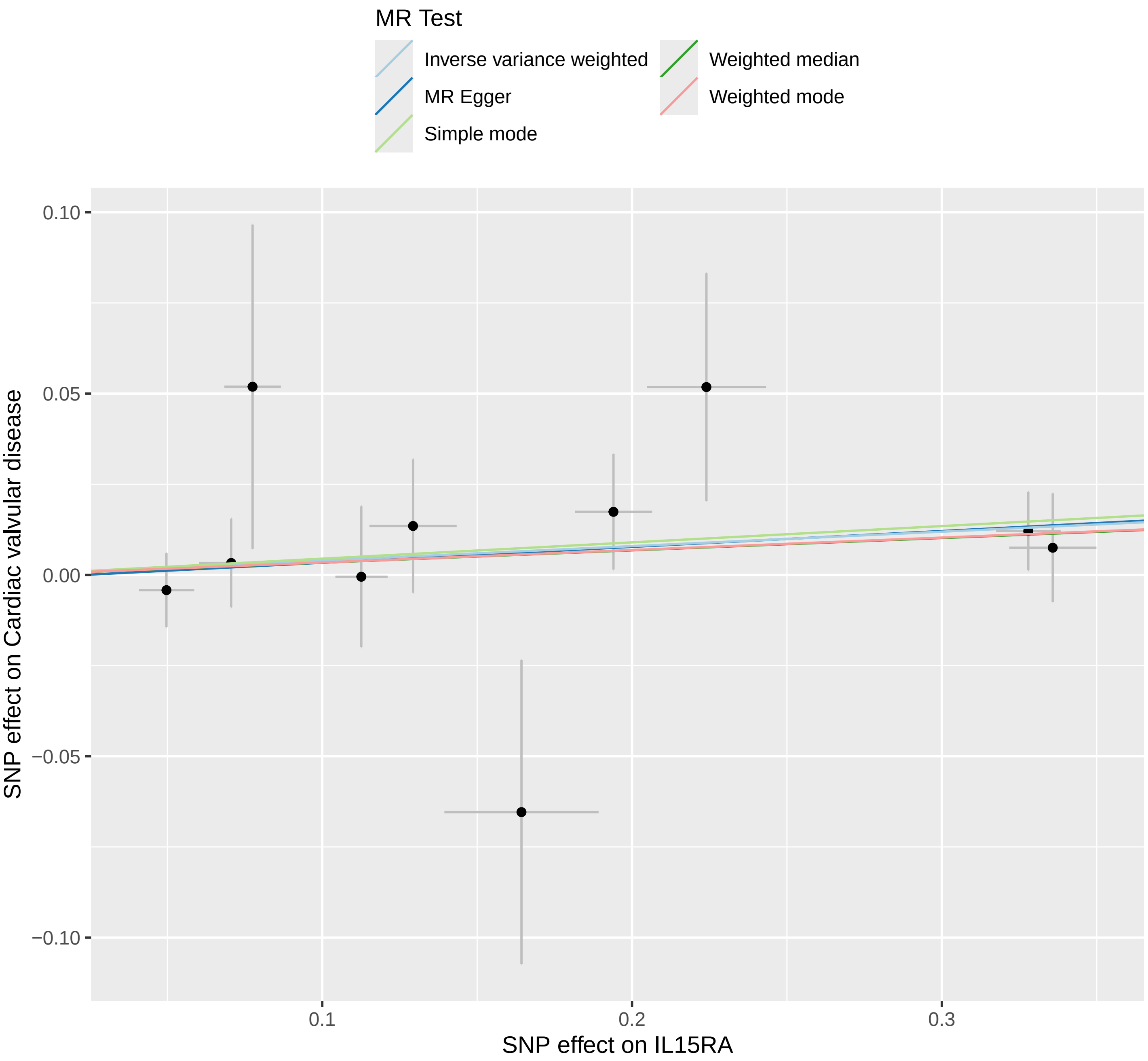

MR Test
Inverse variance weighted
MR Egger
Simple mode
Weighted median
Weighted mode
0.10
0.05
0.00
−0.05
−0.10
0.1
0.2
0.3
SNP effect on Cardiac valvular disease
SNP effect on IL15RA

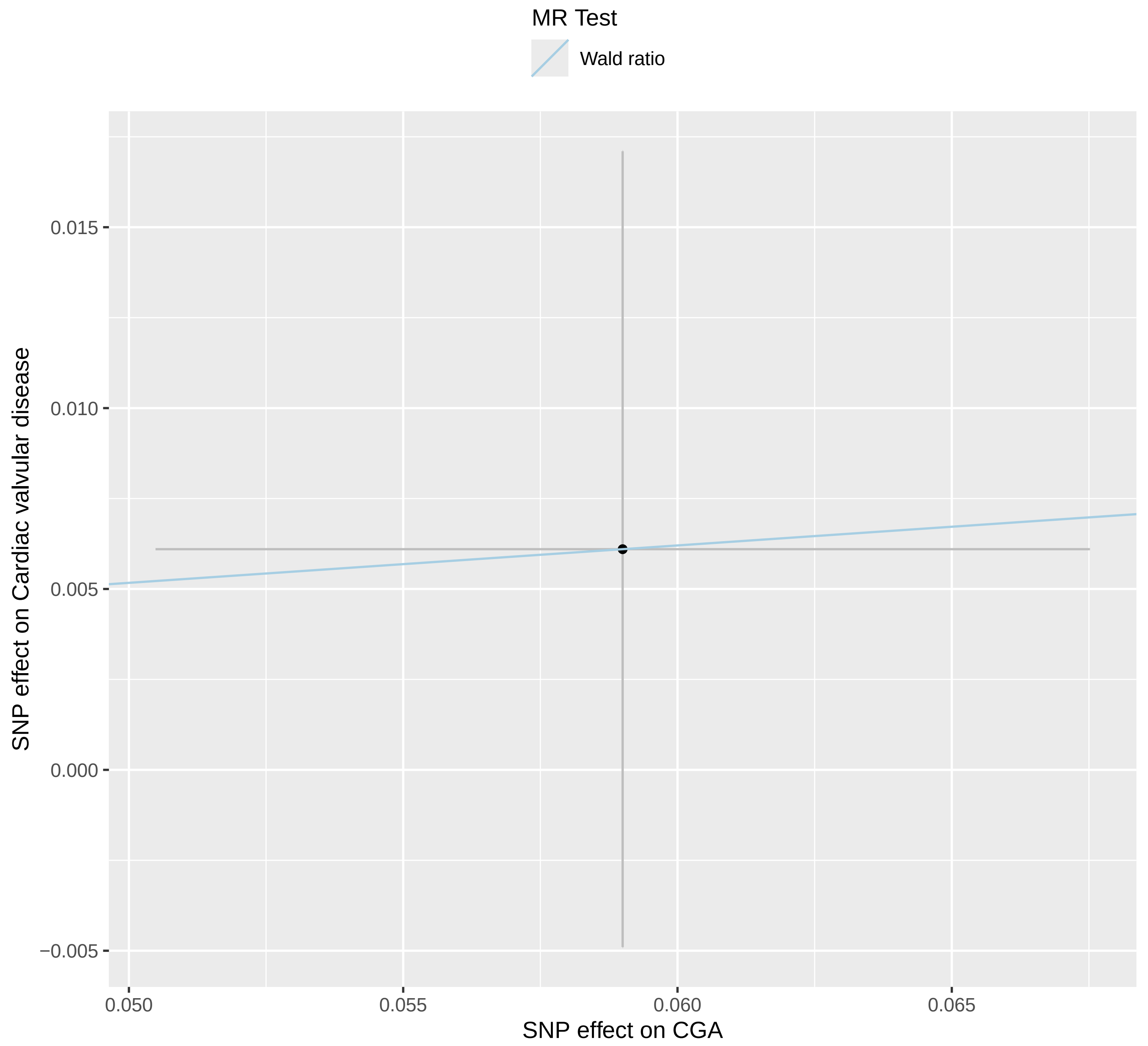
MR Test
Wald ratio
SNP effect on Cardiac valvular disease
0.015
0.010
0.005
0.000
−0.005
0.050
0.055
0.060
0.065
SNP effect on CGA

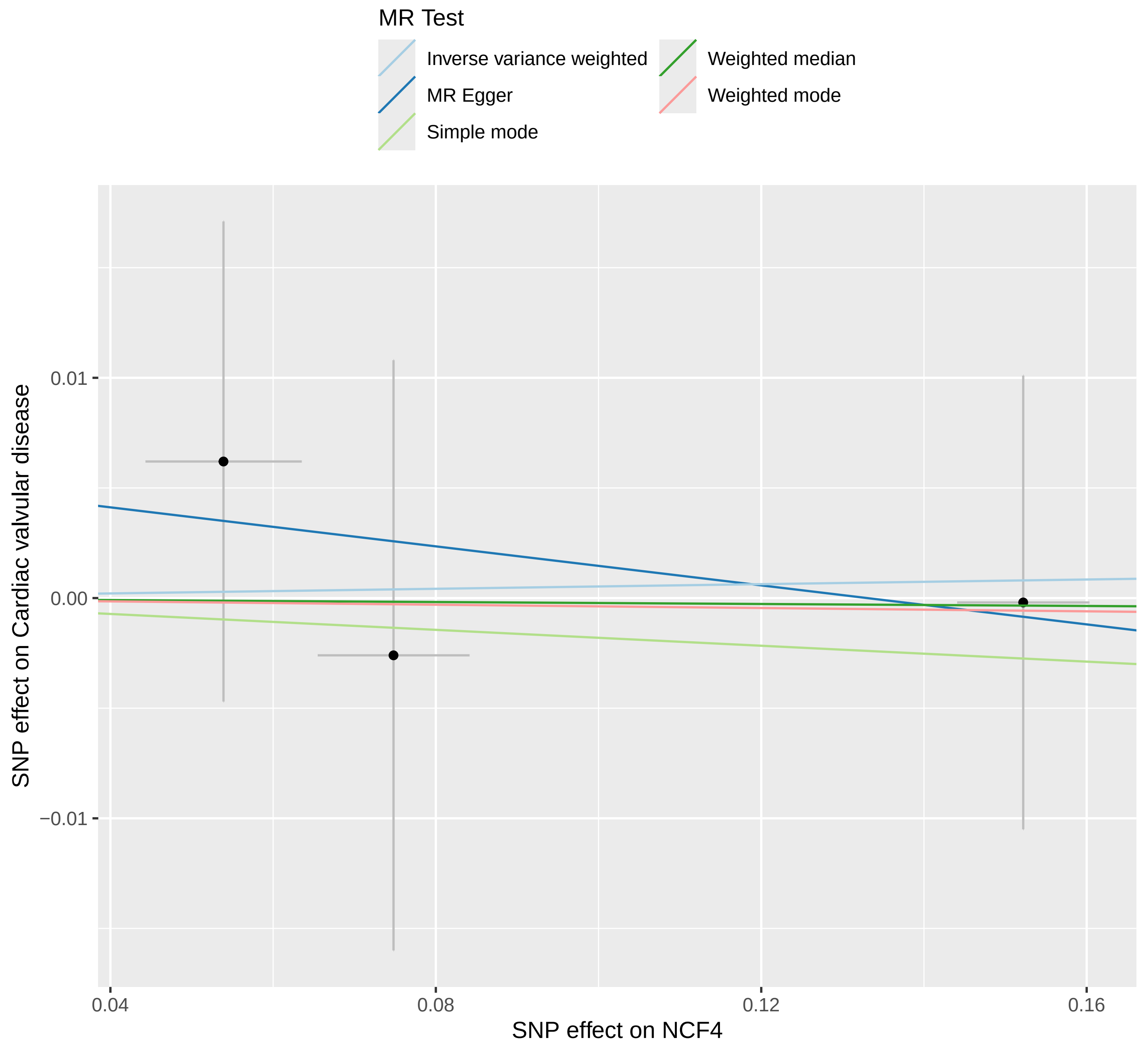
MR Test
Inverse variance weighted
MR Egger
Simple mode
Weighted median
Weighted mode
0.01
0.00
−0.01
SNP effect on Cardiac valvular disease
0.04
0.08
0.12
0.16
SNP effect on NCF4

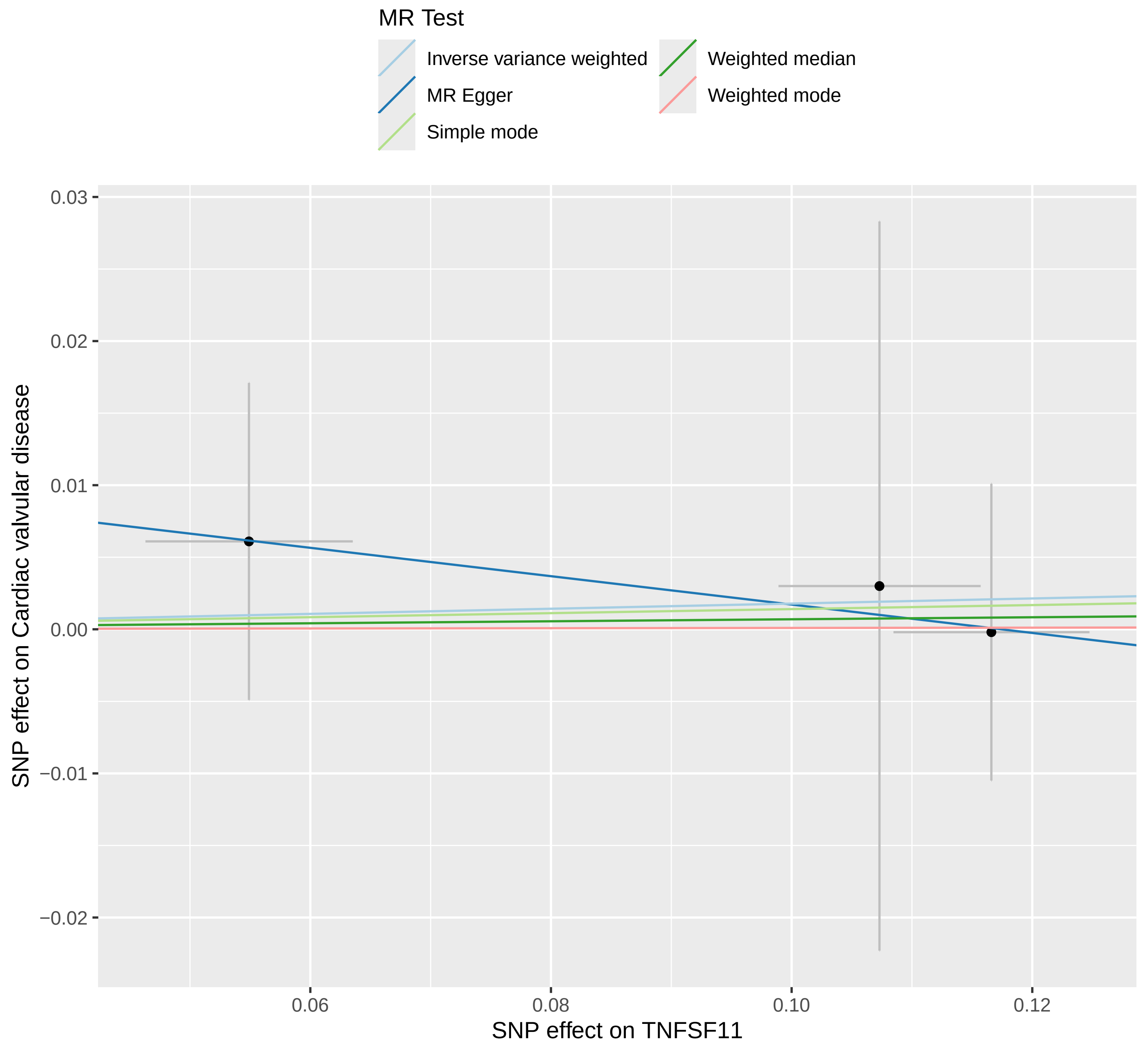

MR Test
Inverse variance weighted
MR Egger
Simple mode
Weighted median
Weighted mode
0.03
0.02
0.01
0.00
−0.01
−0.02
SNP effect on Cardiac valvular disease
0.06
0.08
0.10
0.12
SNP effect on TNFSF11

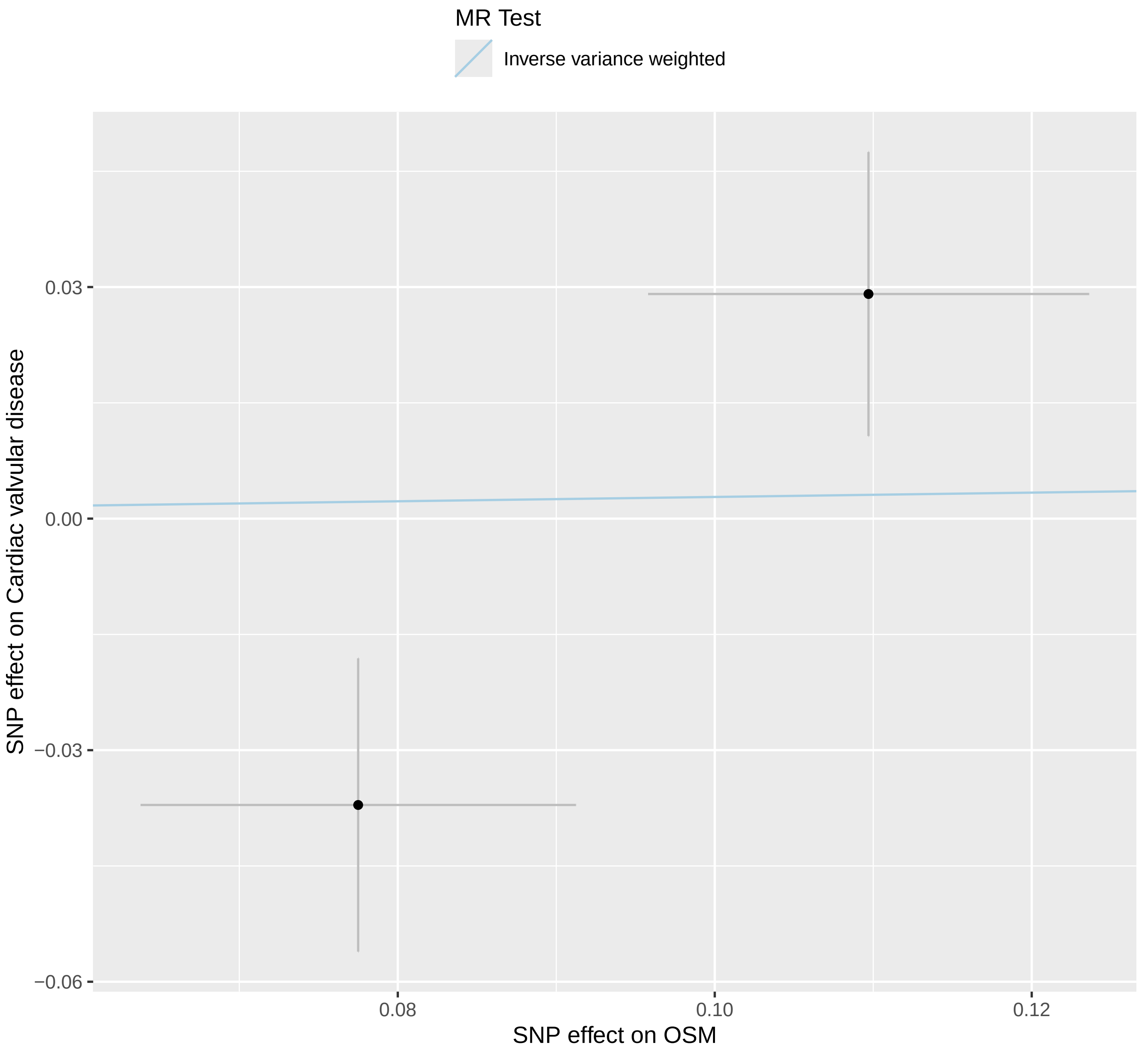

MR Test
Inverse variance weighted
SNP effect on Cardiac valvular disease
0.03
0.00
−0.03
−0.06
0.08
0.10
0.12
SNP effect on OSM

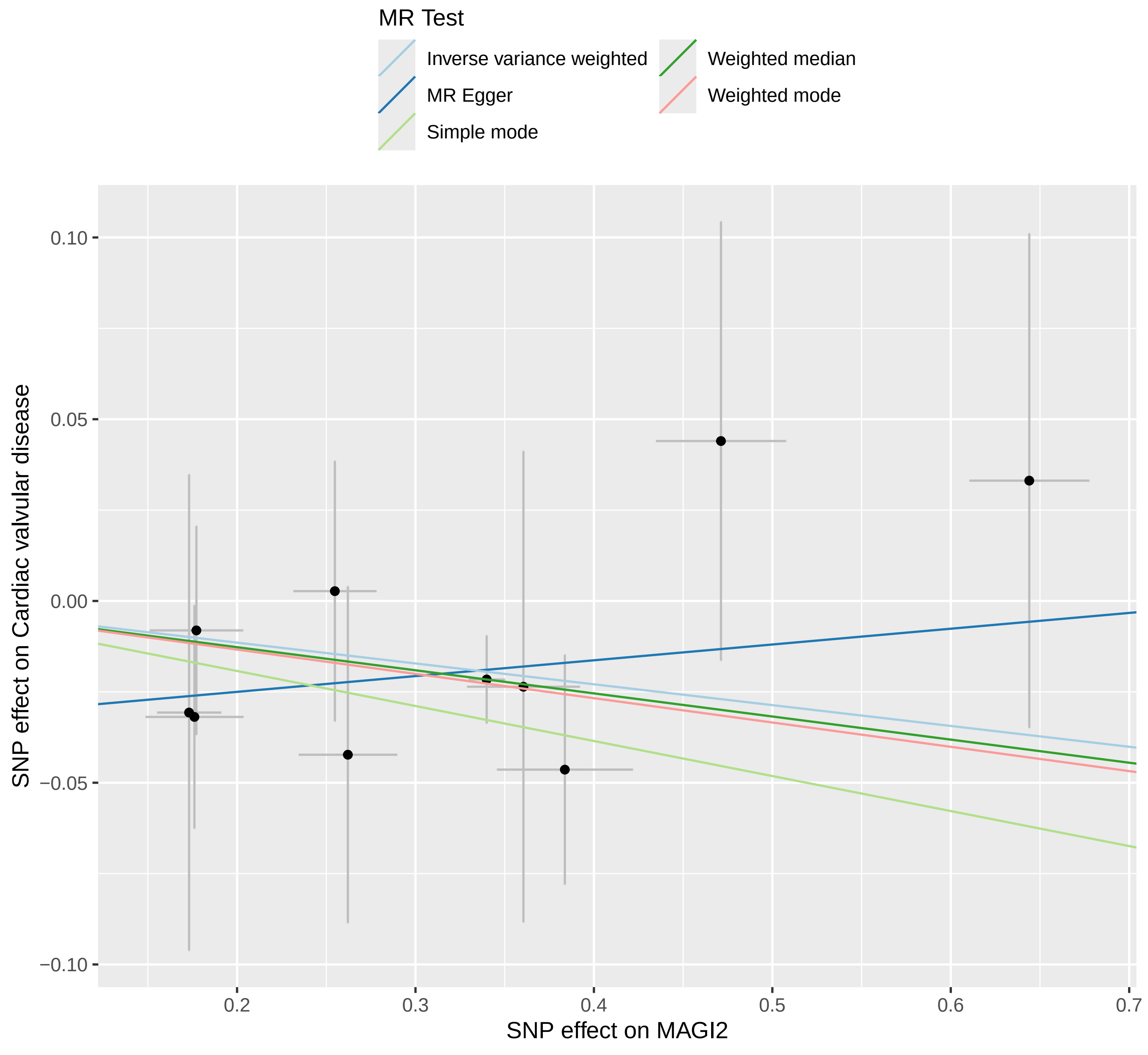

MR Test
Inverse variance weighted
MR Egger
Simple mode
Weighted median
Weighted mode
0.10
0.05
0.00
−0.05
−0.10
SNP effect on Cardiac valvular disease
0.2
0.3
0.4
0.5
0.6
0.7
SNP effect on MAGI2

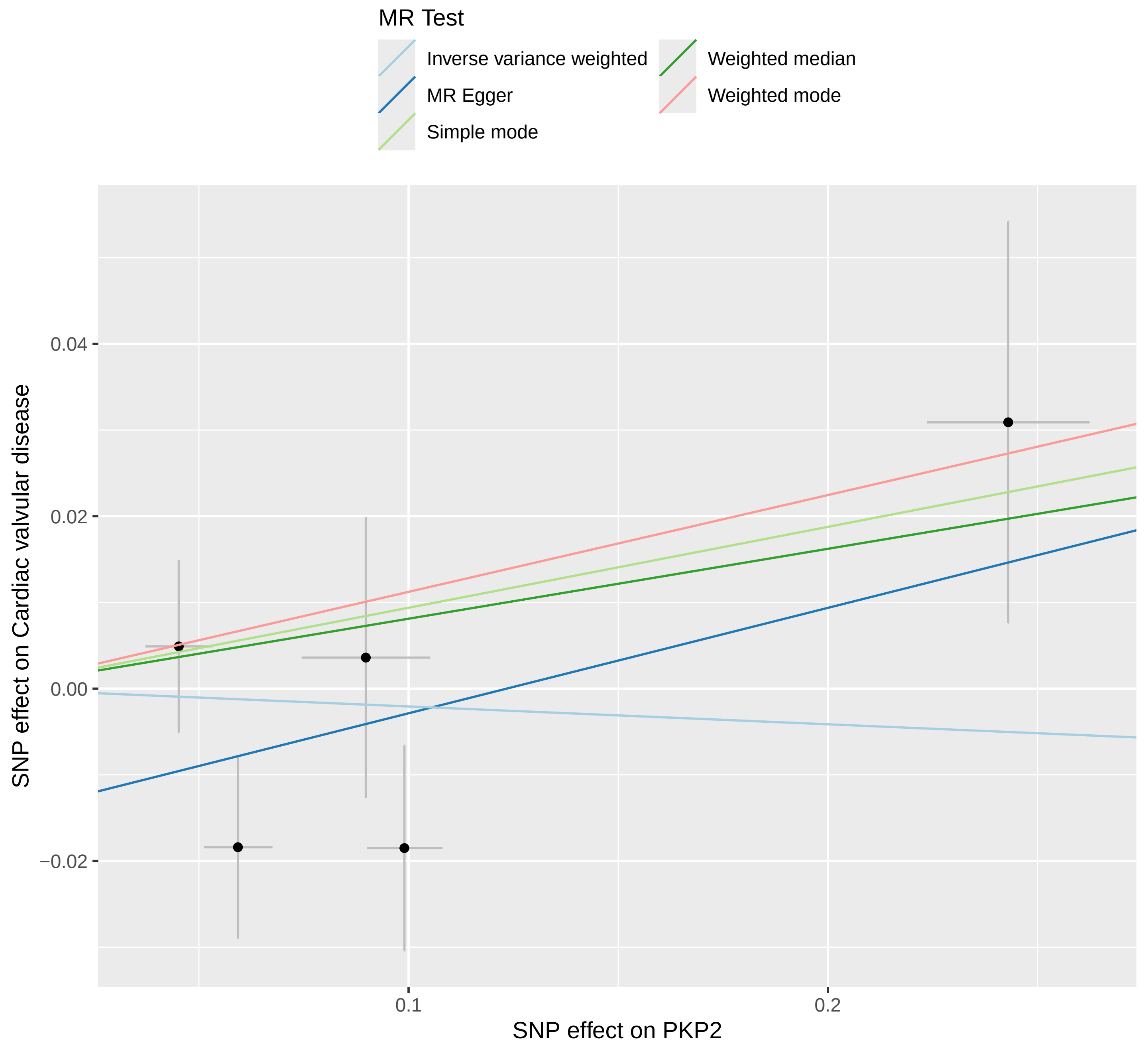
MR Test
Inverse variance weighted
MR Egger
Simple mode
Weighted median
Weighted mode
0.04
0.02
0.00
−0.02
0.1
0.2
SNP effect on Cardiac valvular disease
SNP effect on PKP2

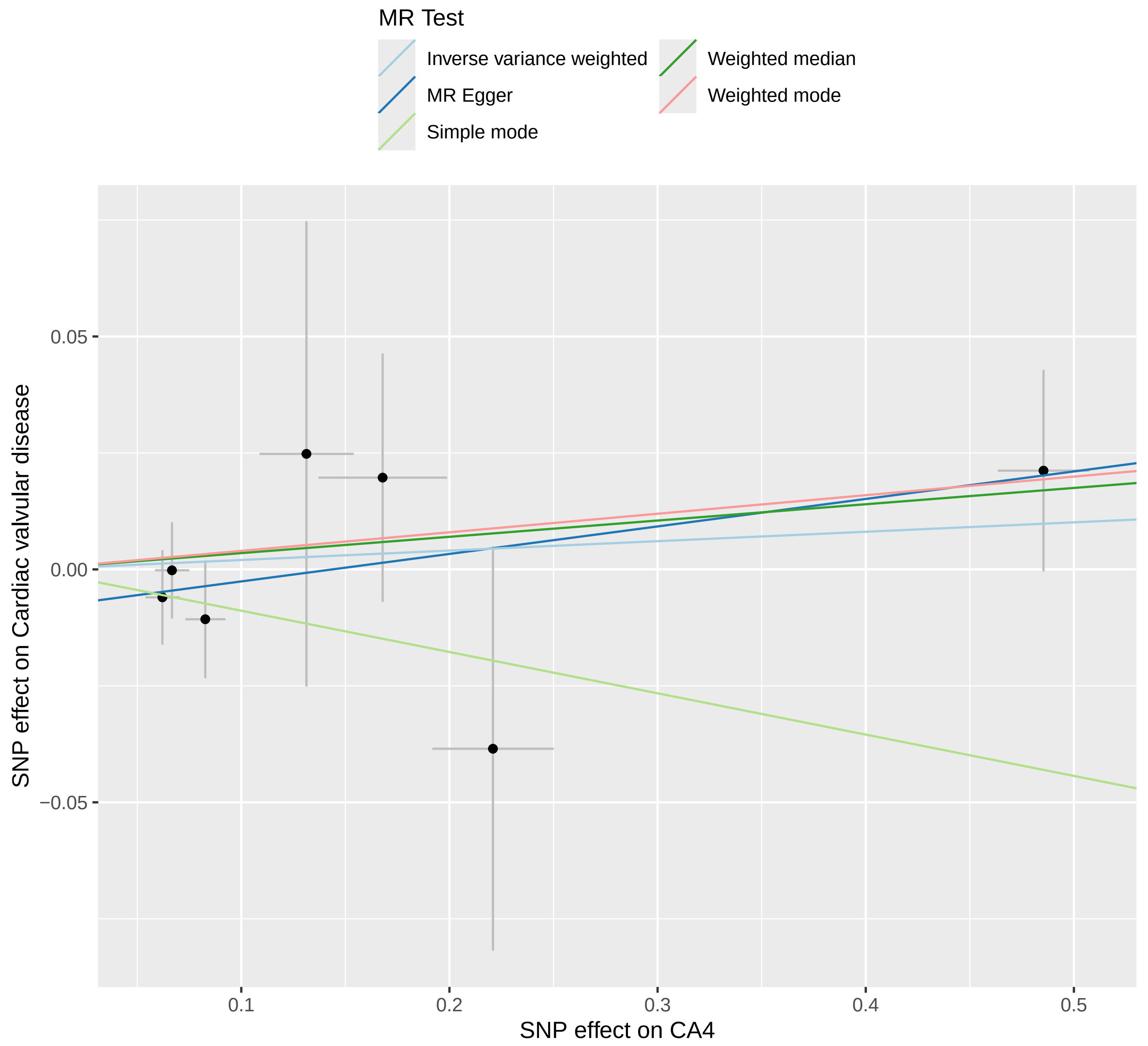
MR Test
Inverse variance weighted
MR Egger
Simple mode
Weighted median
Weighted mode
0.05
0.00
−0.05
0.1
0.2
0.3
0.4
0.5
SNP effect on Cardiac valvular disease
SNP effect on CA4

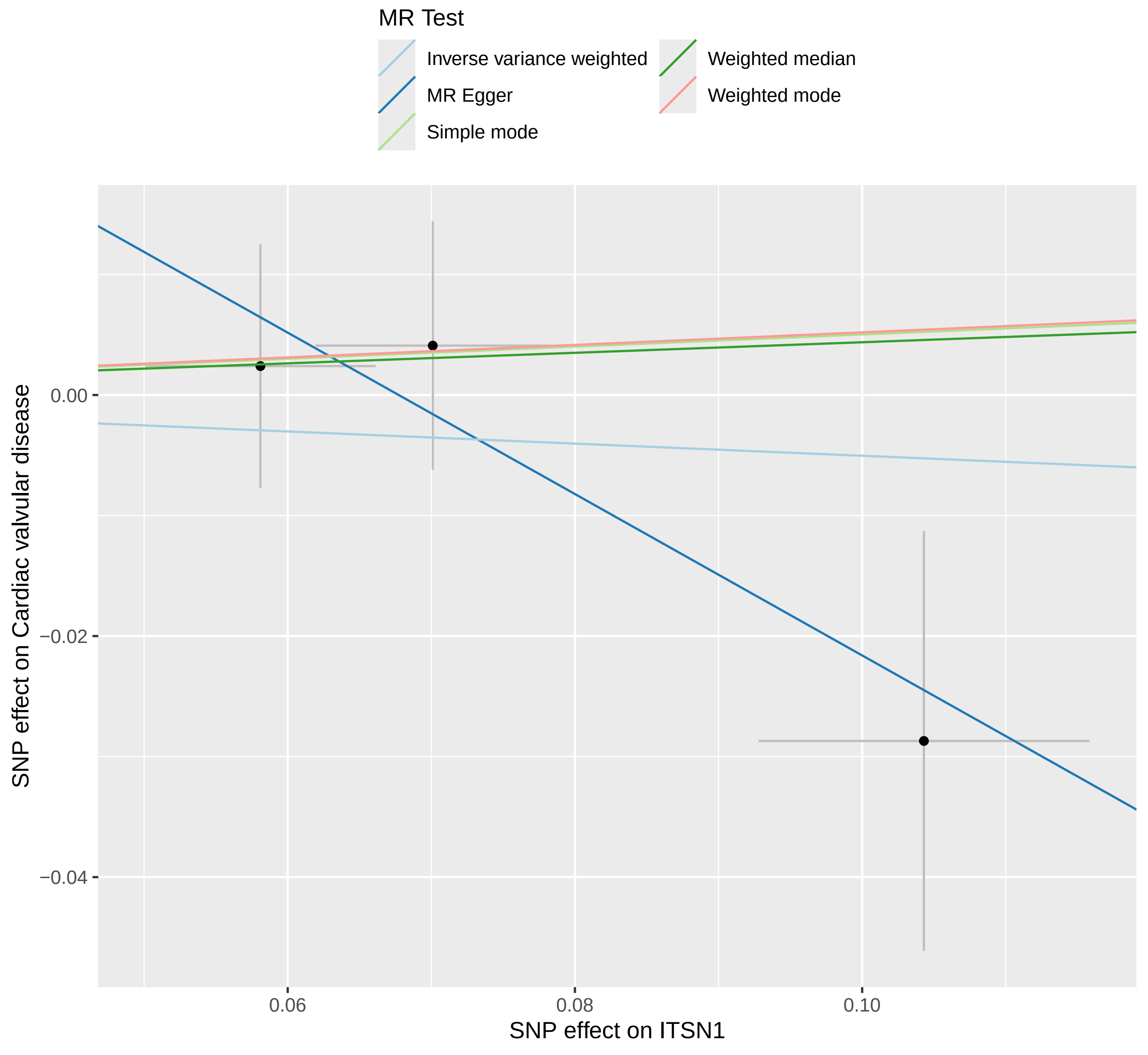

MR Test
Inverse variance weighted
MR Egger
Simple mode
Weighted median
Weighted mode
SNP effect on Cardiac valvular disease
0.00
−0.02
−0.04
0.06
0.08
0.10
SNP effect on ITSN1

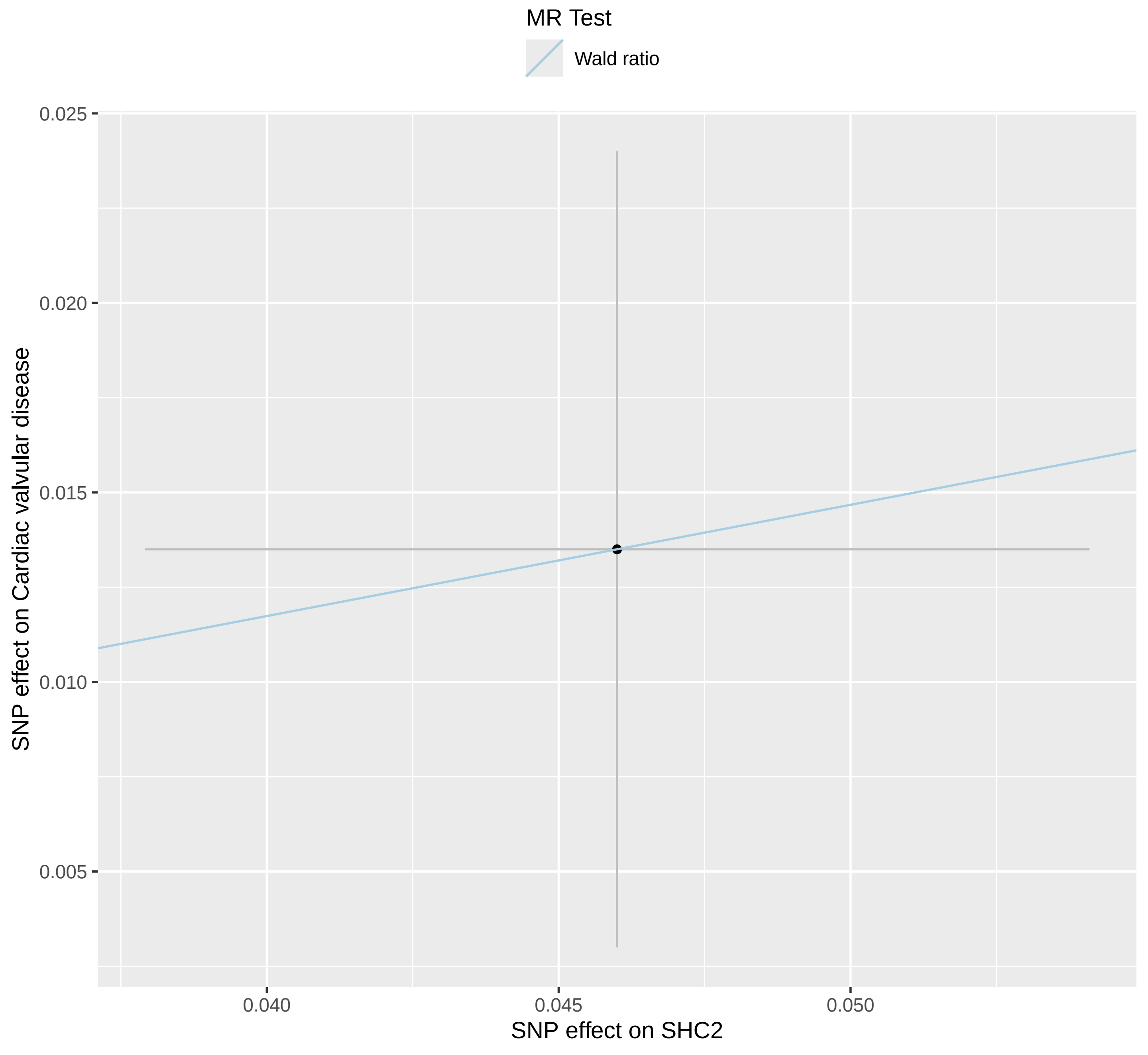
MR Test
Wald ratio
0.025
0.020
0.015
0.010
0.005
SNP effect on Cardiac valvular disease
0.040
0.045
0.050
SNP effect on SHC2

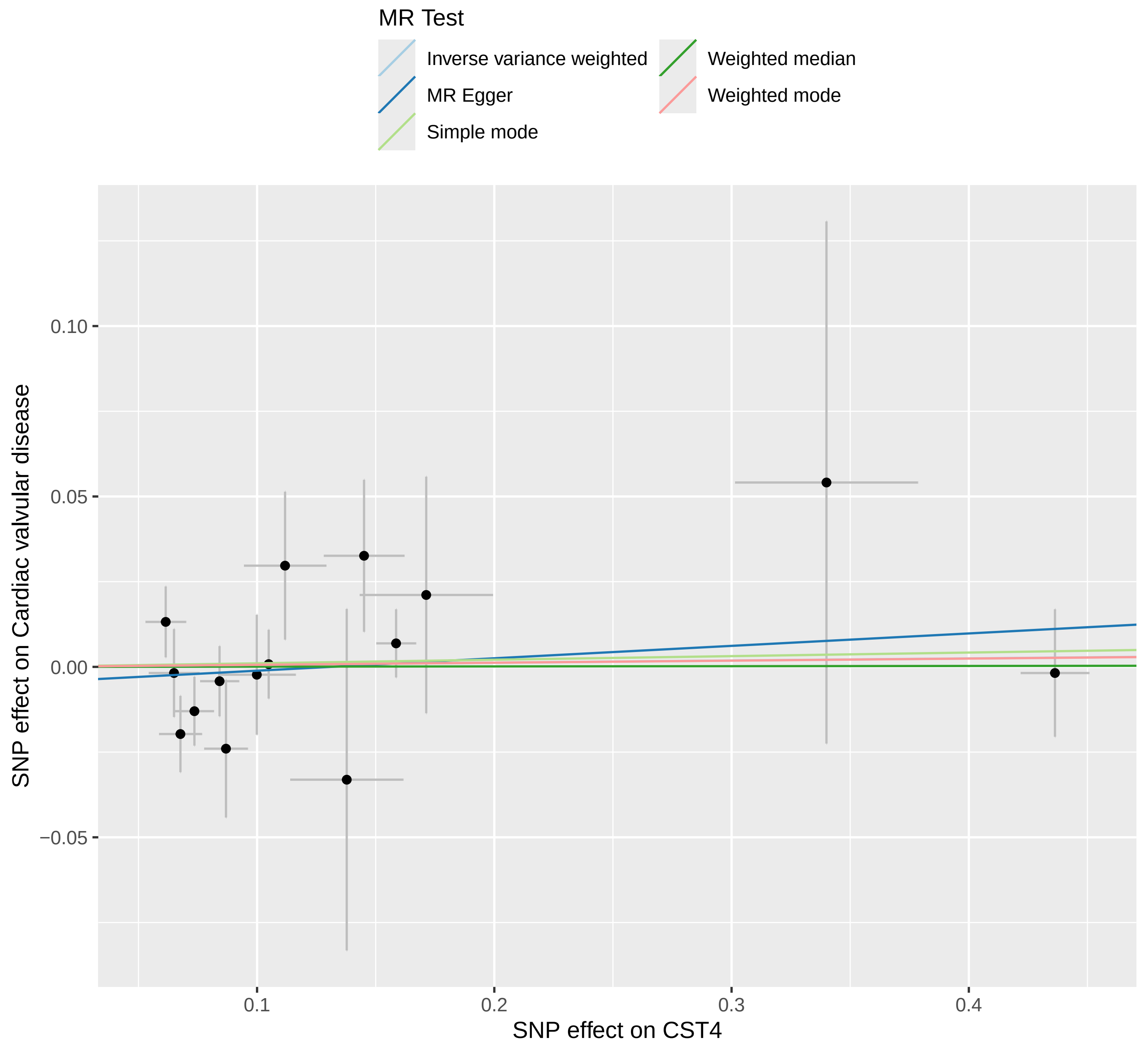
MR Test
Inverse variance weighted
MR Egger
Simple mode
Weighted median
Weighted mode
0.10
0.05
0.00
−0.05
0.1
0.2
0.3
0.4
SNP effect on Cardiac valvular disease
SNP effect on CST4

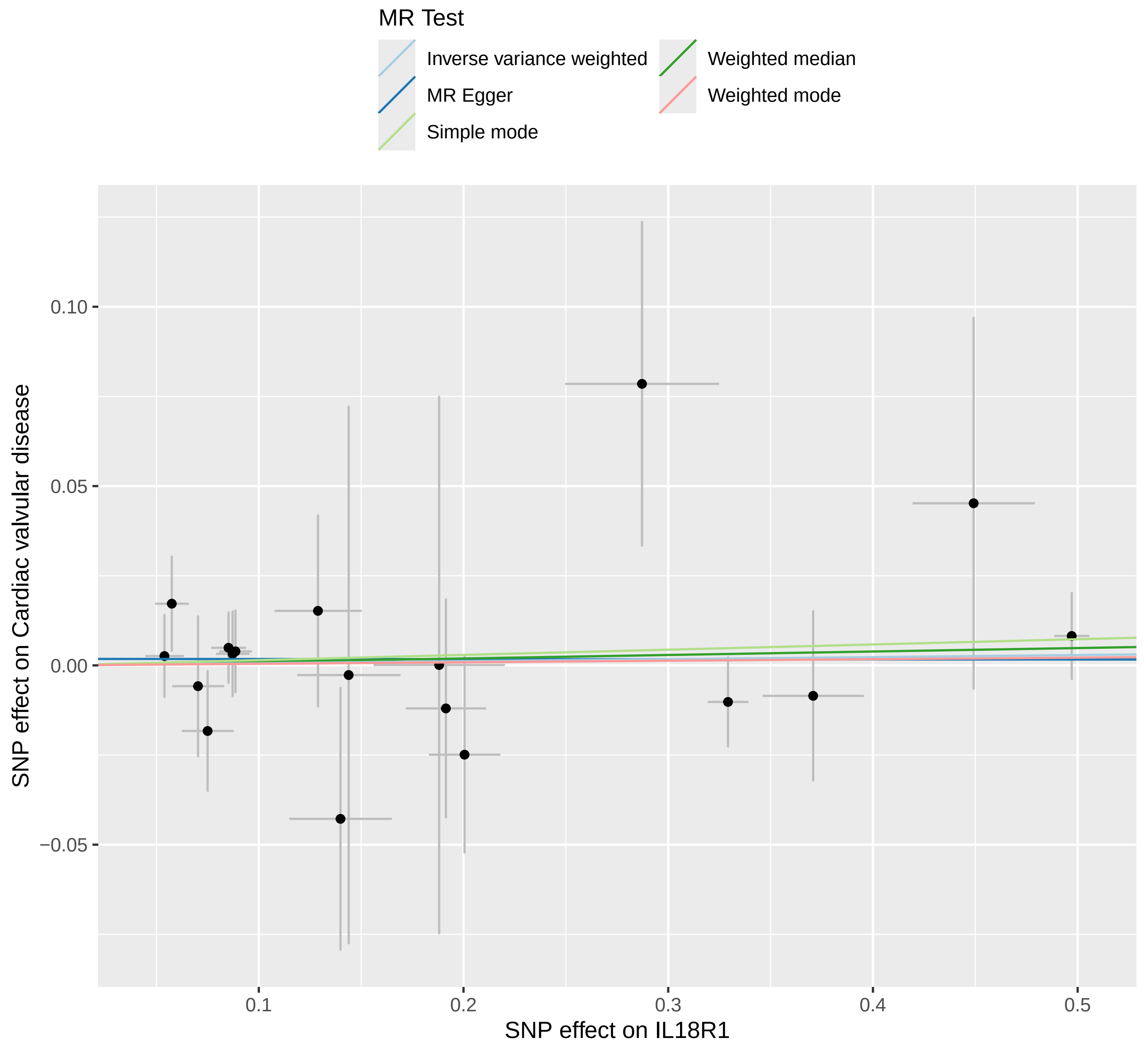
MR Test
Inverse variance weighted
MR Egger
Simple mode
Weighted median
Weighted mode
SNP effect on Cardiac valvular disease
0.10
0.05
0.00
−0.05
0.1
0.2
0.3
0.4
0.5
SNP effect on IL18R1

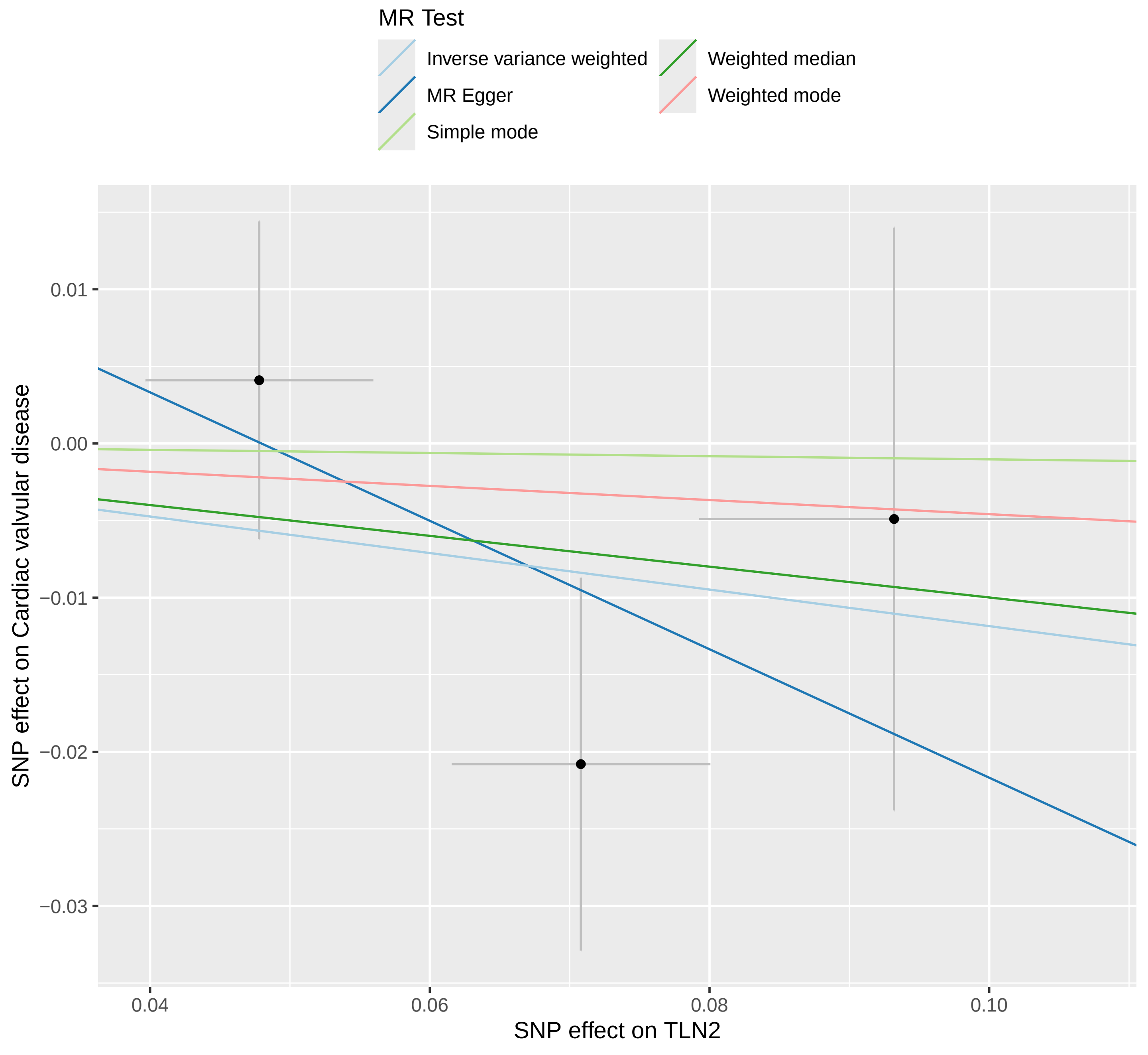

MR Test
Inverse variance weighted
MR Egger
Simple mode
Weighted median
Weighted mode
0.01
0.00
−0.01
−0.02
−0.03
SNP effect on Cardiac valvular disease
0.04
0.06
0.08
0.10
SNP effect on TLN2

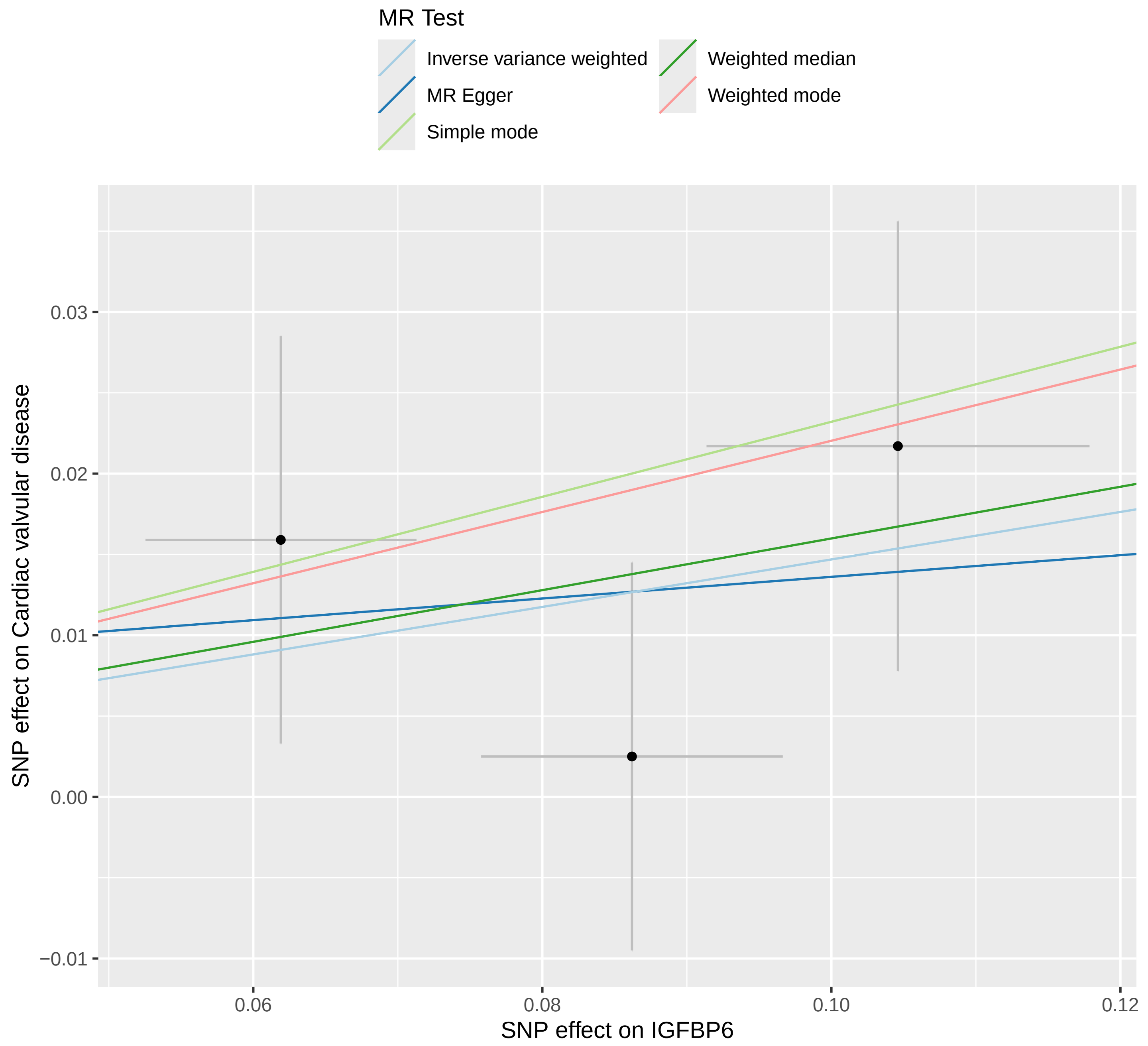
MR Test
Inverse variance weighted
MR Egger
Simple mode
Weighted median
Weighted mode
0.03
0.02
0.01
0.00
−0.01
0.06
0.08
0.10
0.12
SNP effect on Cardiac valvular disease
SNP effect on IGFBP6

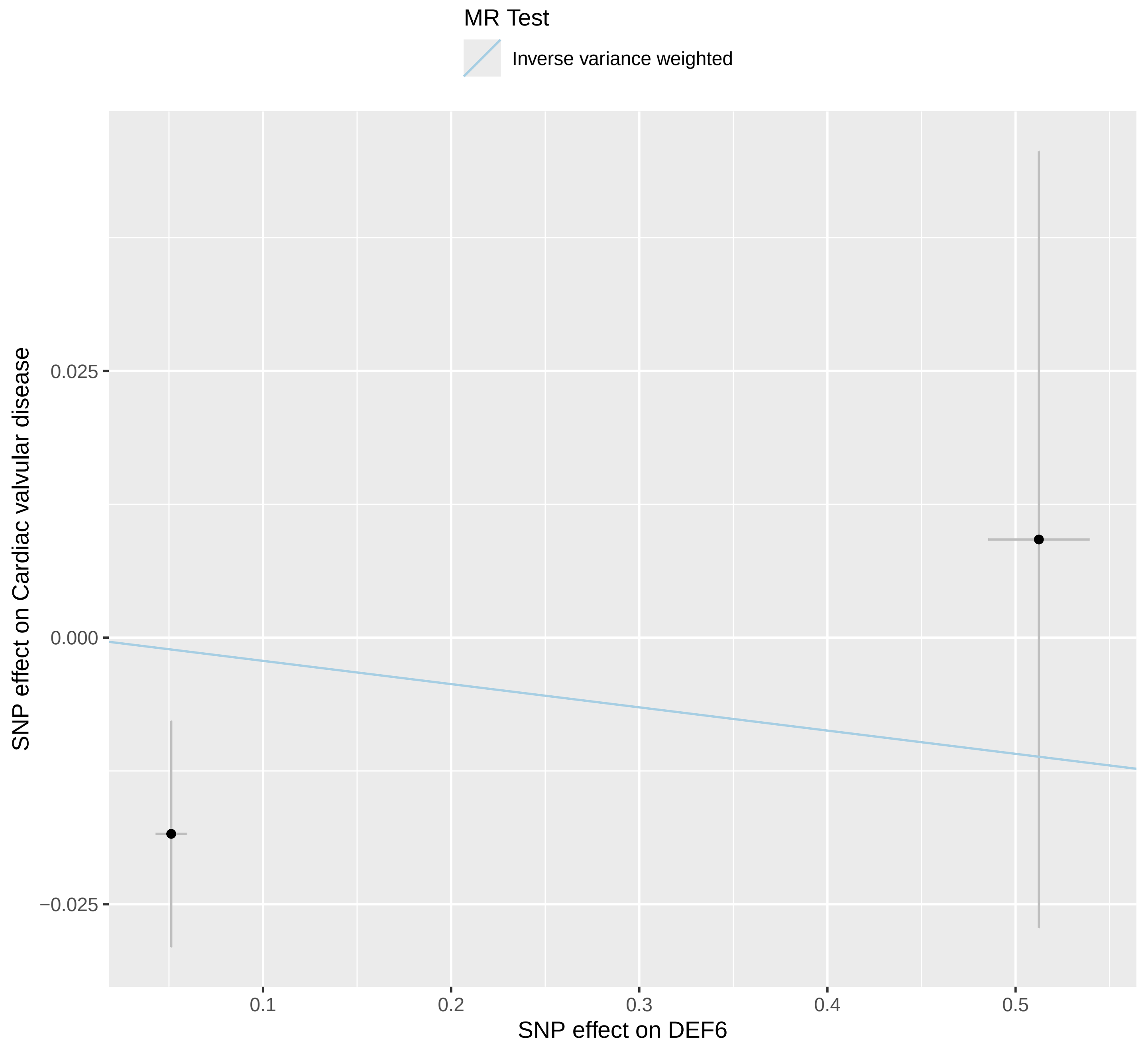

MR Test
Inverse variance weighted
0.025
0.000
−0.025
0.1
0.2
0.3
0.4
0.5
SNP effect on DEF6
SNP effect on Cardiac valvular disease

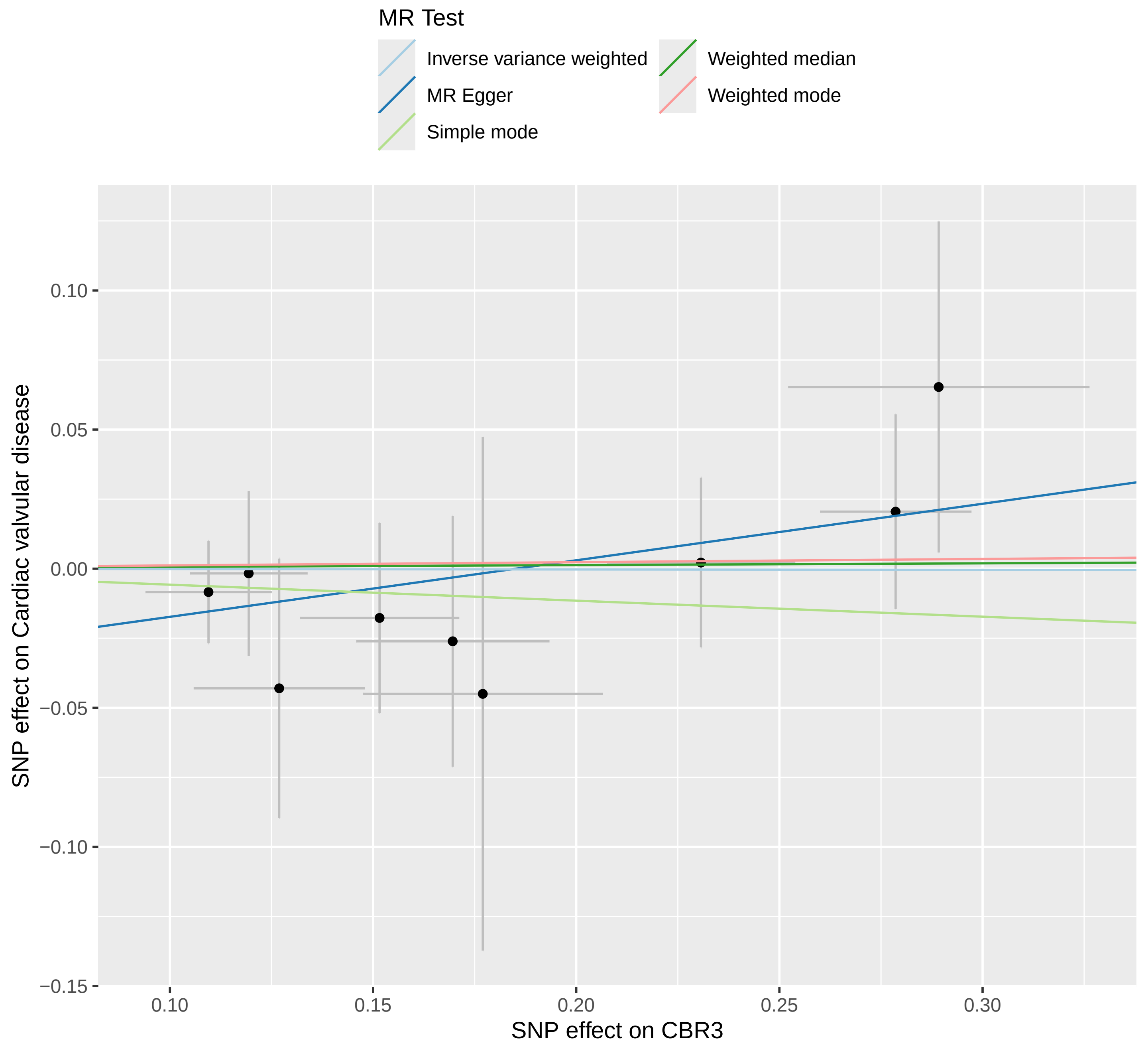

MR Test
Inverse variance weighted
MR Egger
Simple mode
Weighted median
Weighted mode
0.10
0.05
0.00
−0.05
−0.10
−0.15
SNP effect on Cardiac valvular disease
0.10
0.15
0.20
0.25
0.30
SNP effect on CBR3

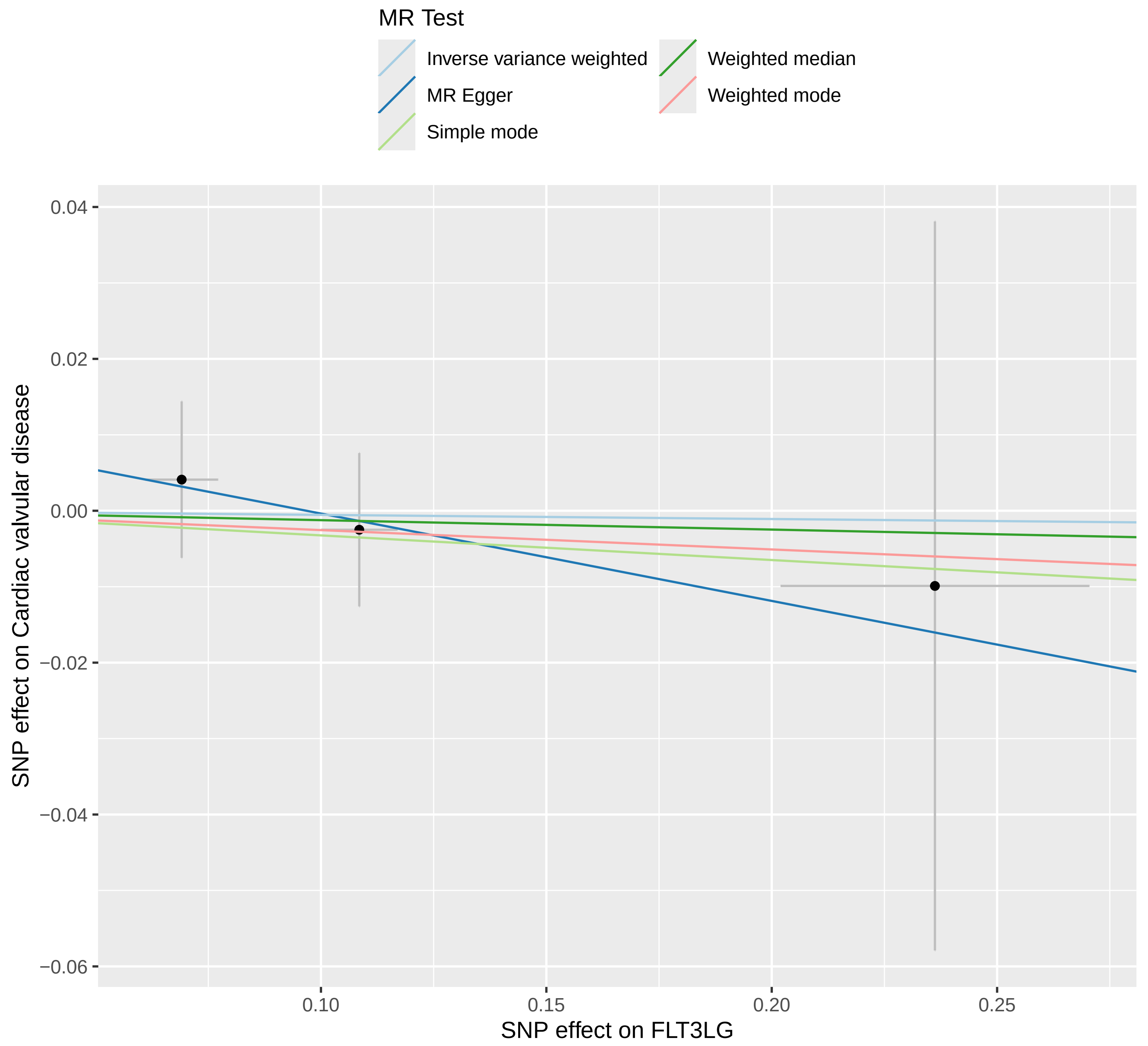
MR Test
Inverse variance weighted
MR Egger
Simple mode
Weighted median
Weighted mode
0.04
0.02
0.00
−0.02
−0.04
−0.06
SNP effect on Cardiac valvular disease
0.10
0.15
0.20
0.25
SNP effect on FLT3LG

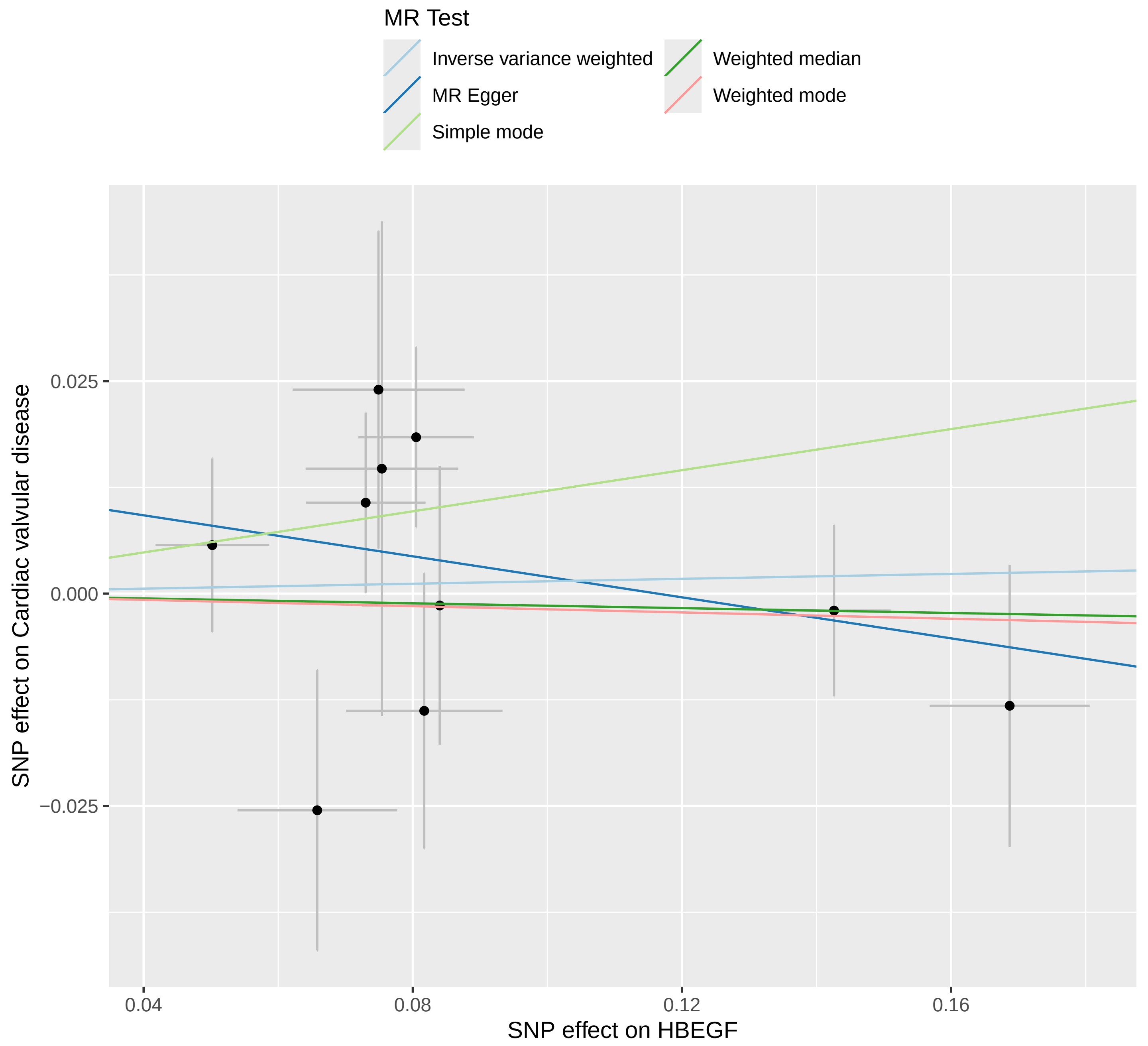
MR Test
Inverse variance weighted
MR Egger
Simple mode
Weighted median
Weighted mode
0.025
0.000
−0.025
SNP effect on Cardiac valvular disease
0.04
0.08
0.12
0.16
SNP effect on HBEGF

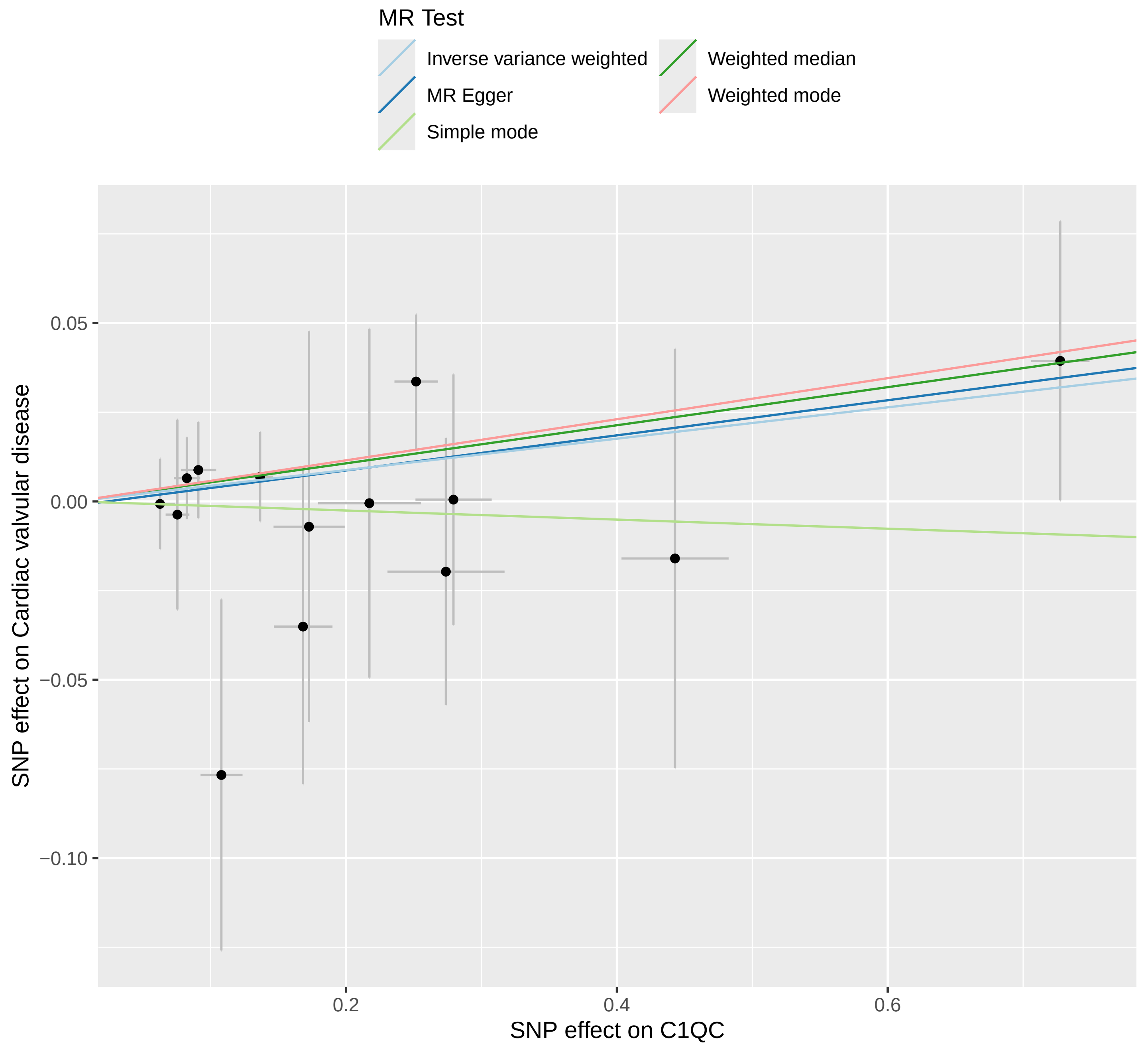
MR Test
Inverse variance weighted
MR Egger
Simple mode
Weighted median
Weighted mode
SNP effect on Cardiac valvular disease
0.05
0.00
−0.05
−0.10
0.2
0.4
0.6
SNP effect on C1QC

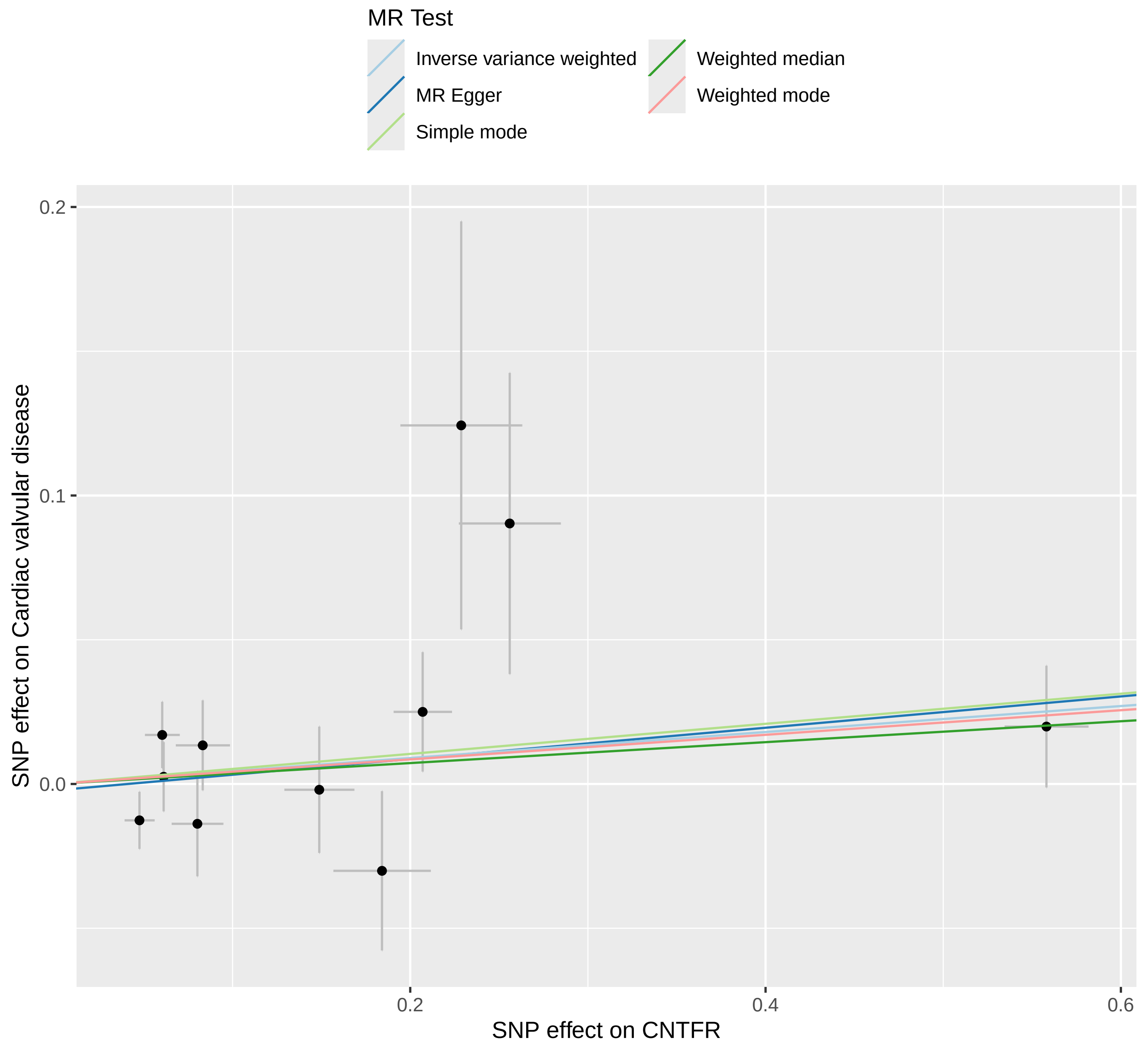
MR Test
Inverse variance weighted
MR Egger
Simple mode
Weighted median
Weighted mode
0.2
0.1
0.0
0.2
0.4
0.6
SNP effect on Cardiac valvular disease
SNP effect on CNTFR

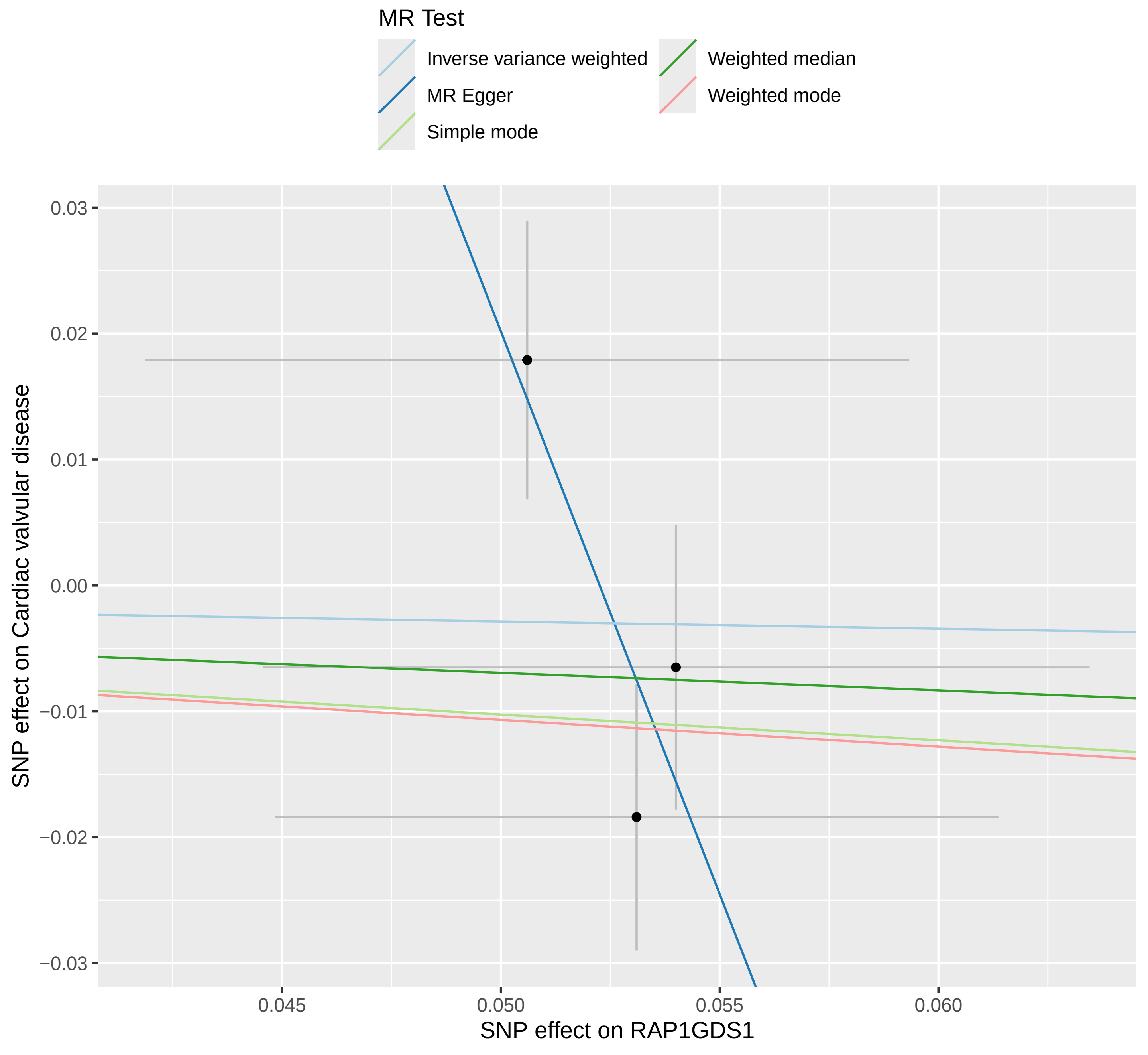
MR Test
Inverse variance weighted
MR Egger
Simple mode
Weighted median
Weighted mode
0.03
0.02
0.01
0.00
−0.01
−0.02
−0.03
SNP effect on Cardiac valvular disease
0.045
0.050
0.055
0.060
SNP effect on RAP1GDS1

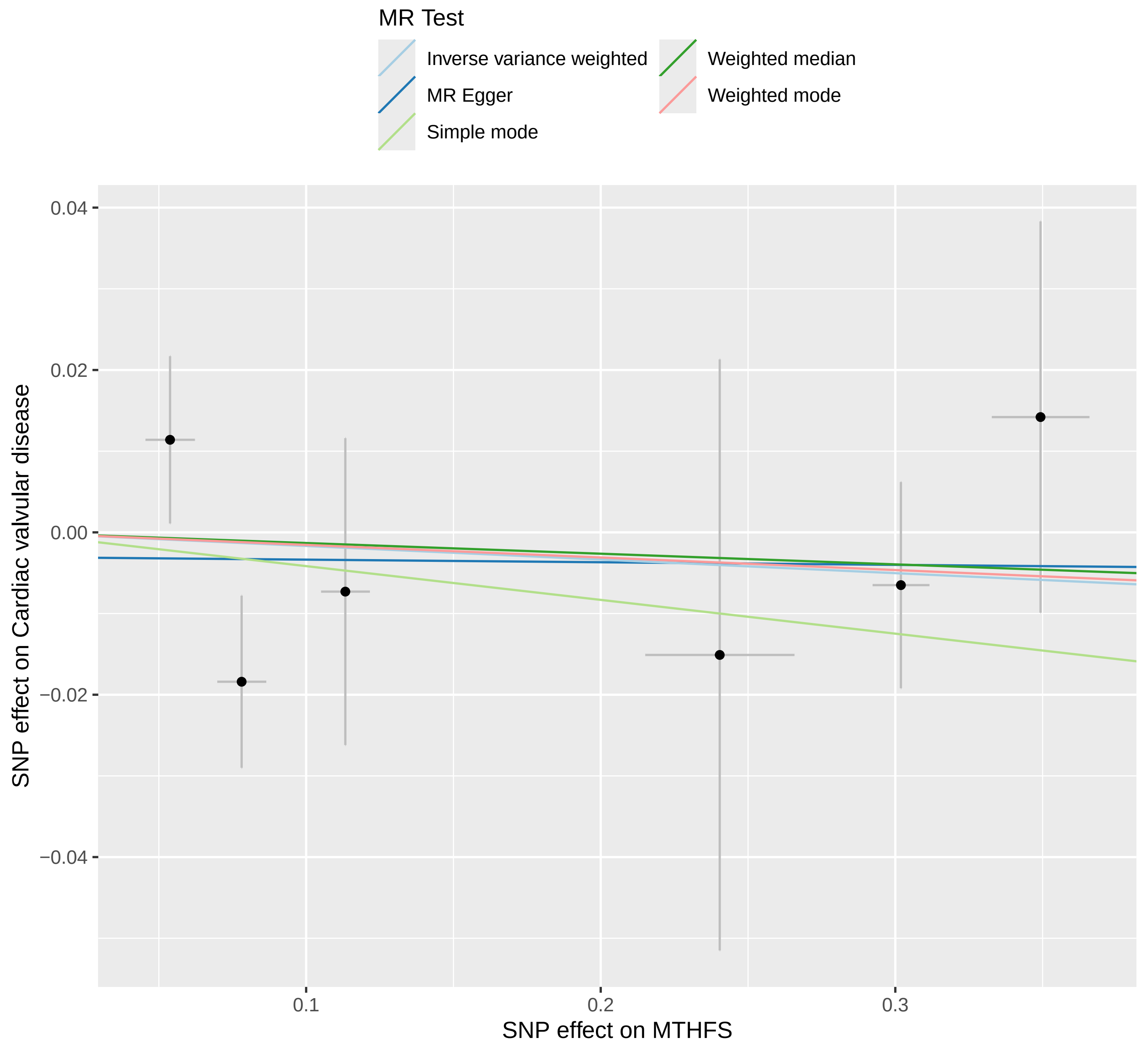

MR Test
Inverse variance weighted
MR Egger
Simple mode
Weighted median
Weighted mode
0.04
0.02
0.00
−0.02
−0.04
SNP effect on Cardiac valvular disease
0.1
0.2
0.3
SNP effect on MTHFS

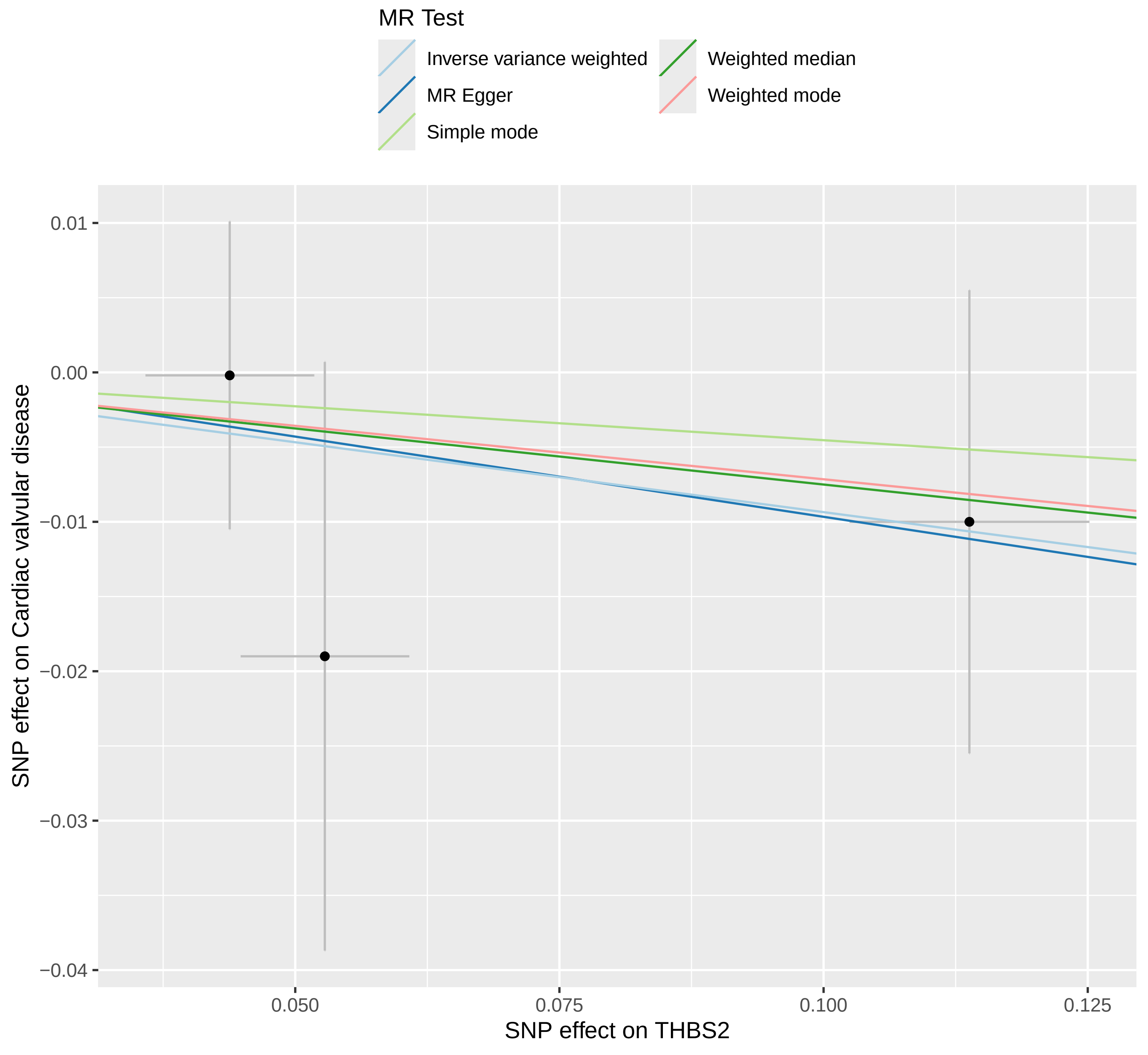
MR Test
Inverse variance weighted
MR Egger
Simple mode
Weighted median
Weighted mode
SNP effect on Cardiac valvular disease
0.01
0.00
−0.01
−0.02
−0.03
−0.04
0.050
0.075
0.100
0.125
SNP effect on THBS2

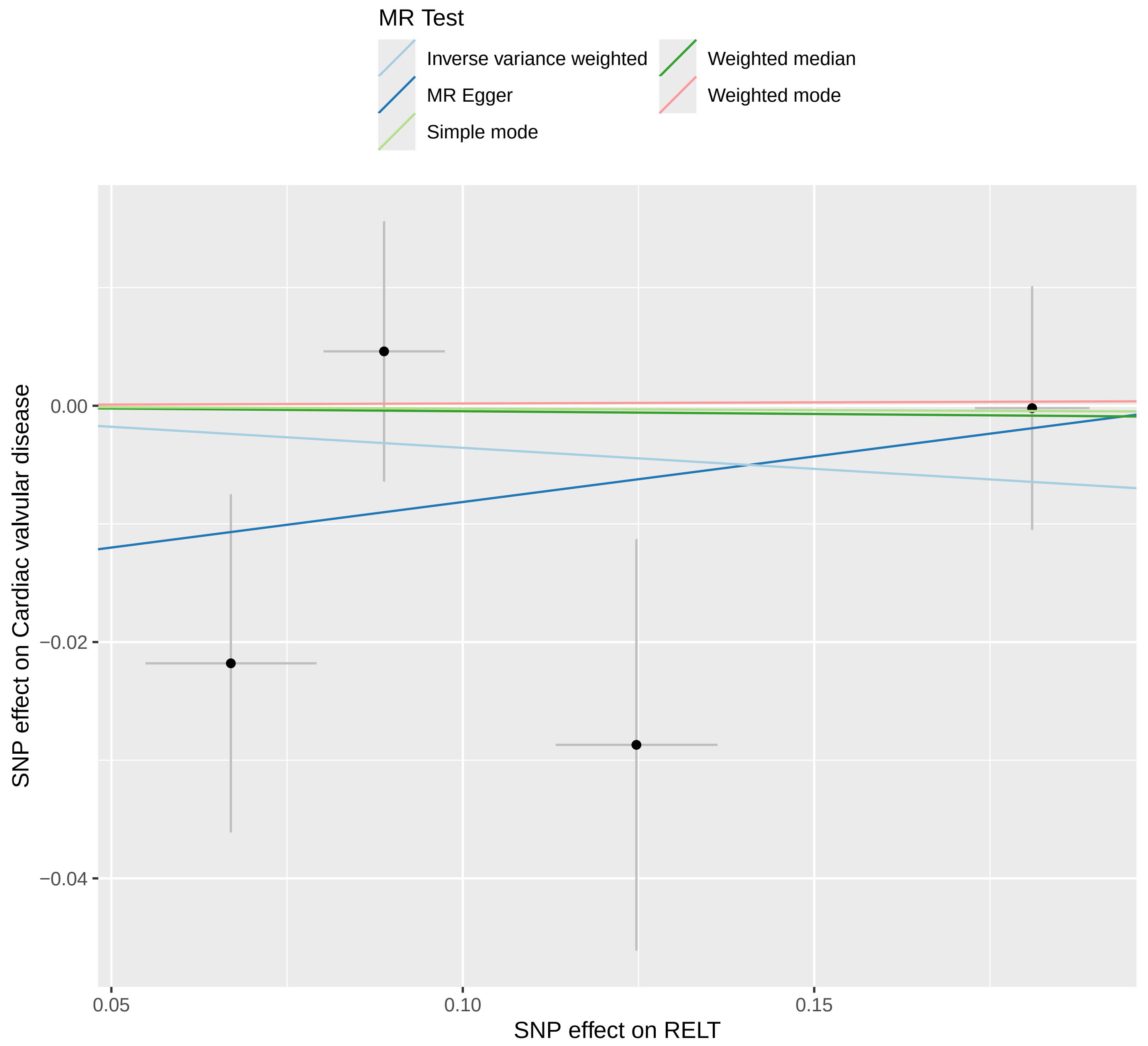

MR Test
Inverse variance weighted
MR Egger
Simple mode
Weighted median
Weighted mode
SNP effect on Cardiac valvular disease
0.00
−0.02
−0.04
0.05
0.10
0.15
SNP effect on RELT

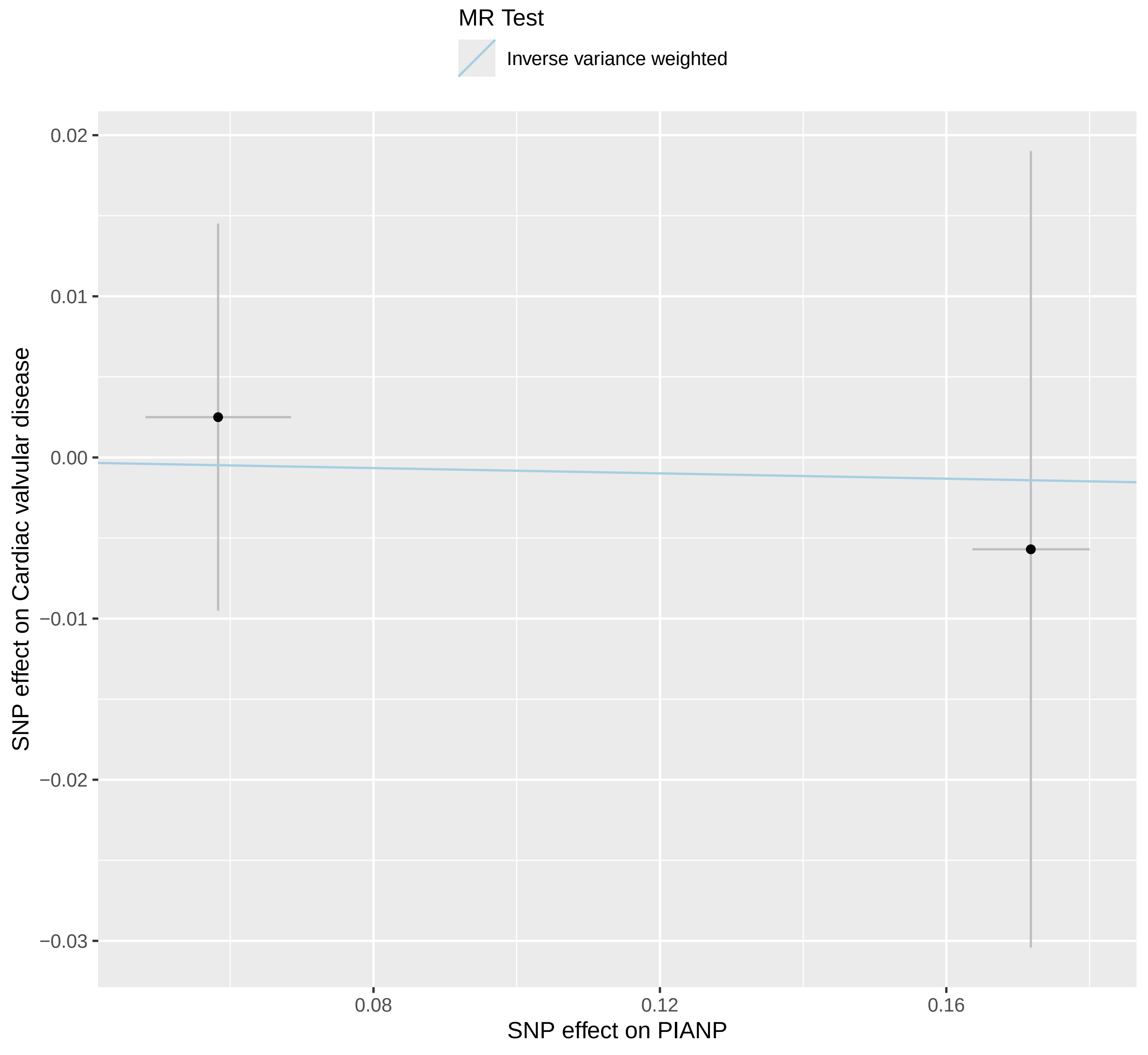
MR Test
Inverse variance weighted
0.02
0.01
0.00
−0.01
−0.02
−0.03
SNP effect on Cardiac valvular disease
0.08
0.12
0.16
SNP effect on PIANP

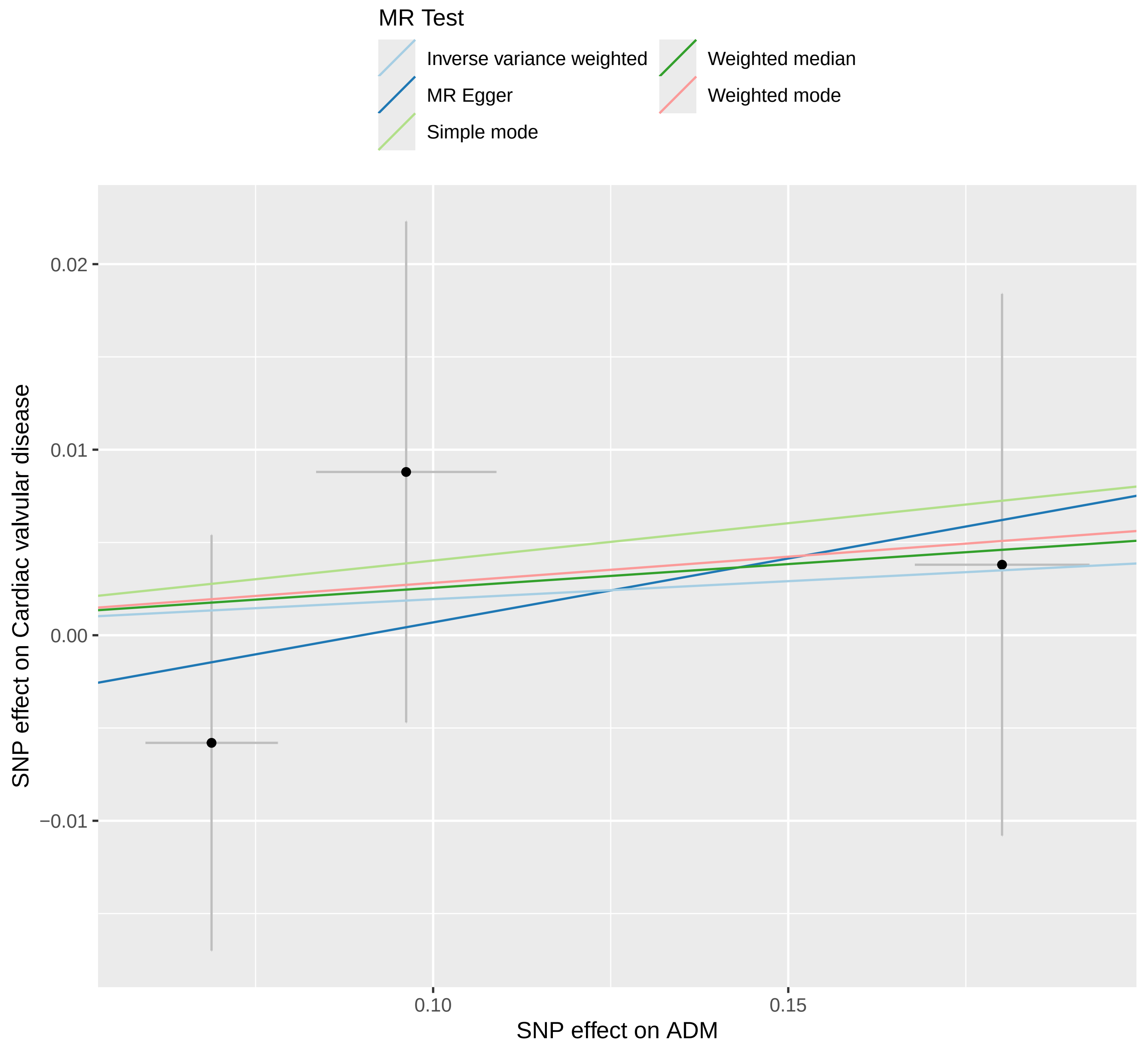
MR Test
Inverse variance weighted
MR Egger
Simple mode
Weighted median
Weighted mode
0.02
0.01
0.00
−0.01
SNP effect on Cardiac valvular disease
0.10
0.15
SNP effect on ADM

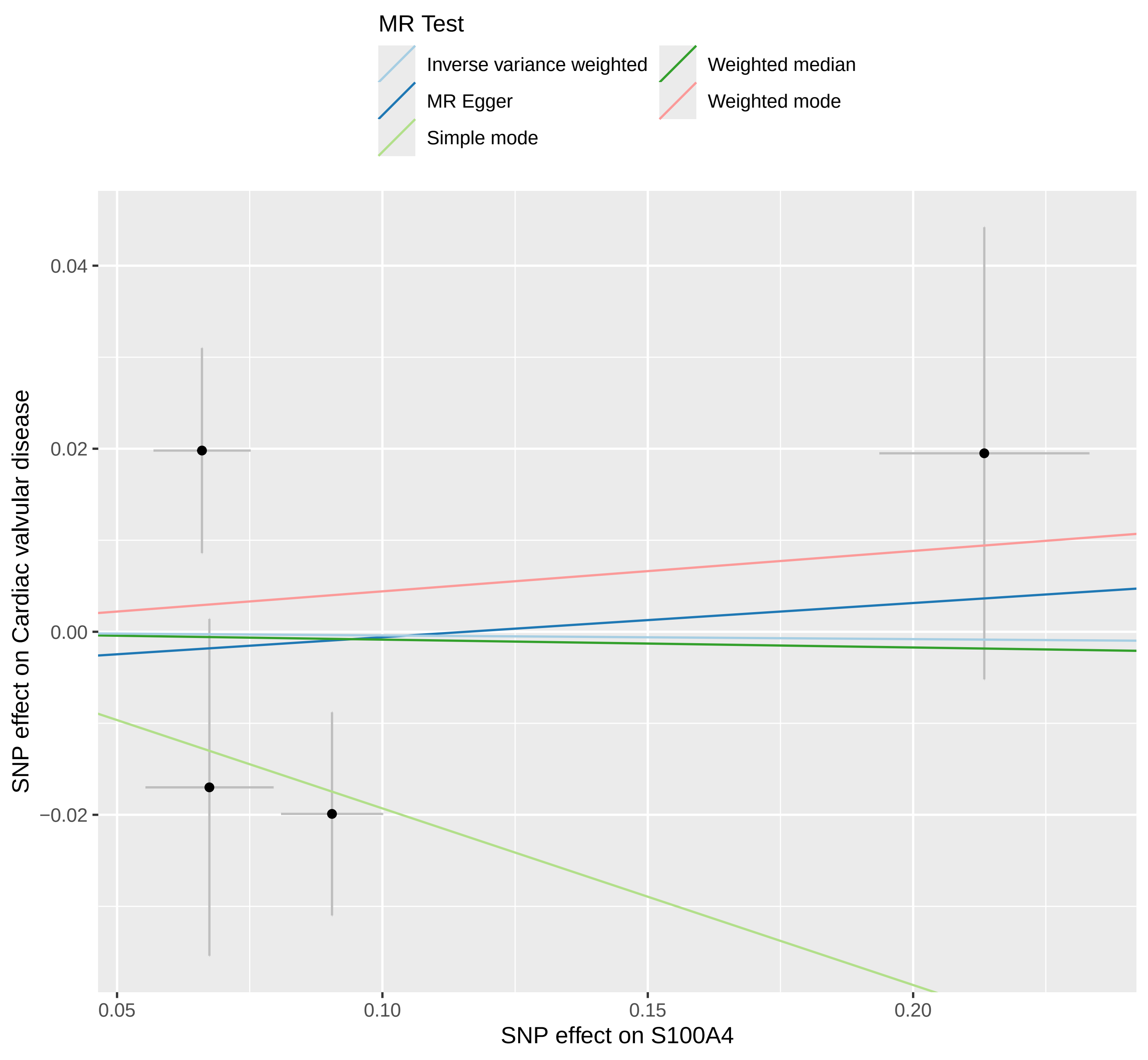
MR Test
Inverse variance weighted
MR Egger
Simple mode
Weighted median
Weighted mode
0.04
0.02
0.00
−0.02
SNP effect on Cardiac valvular disease
0.05
0.10
0.15
0.20
SNP effect on S100A4

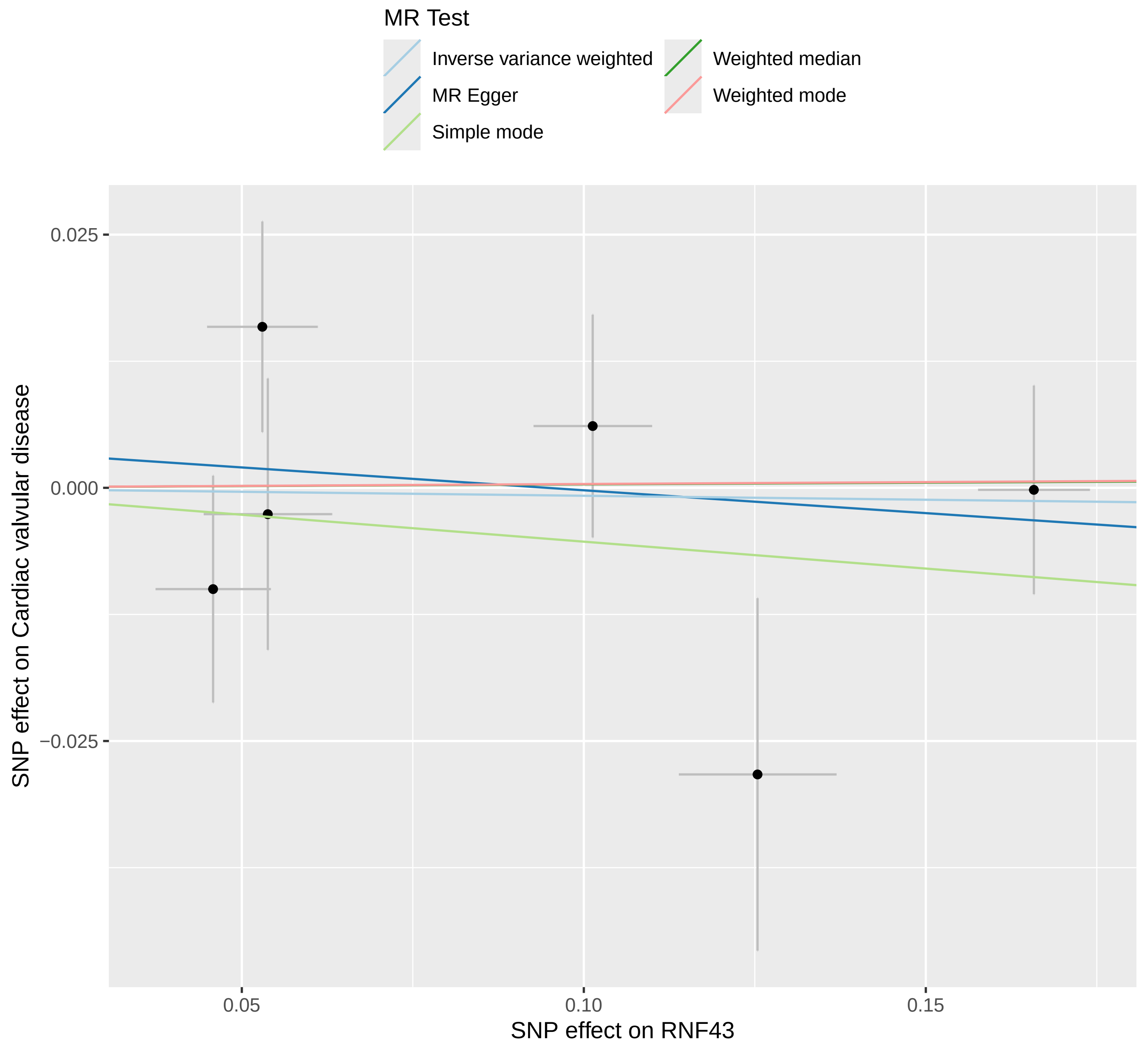

MR Test
Inverse variance weighted
MR Egger
Simple mode
Weighted median
Weighted mode
0.025
0.000
−0.025
SNP effect on Cardiac valvular disease
0.05
0.10
0.15
SNP effect on RNF43

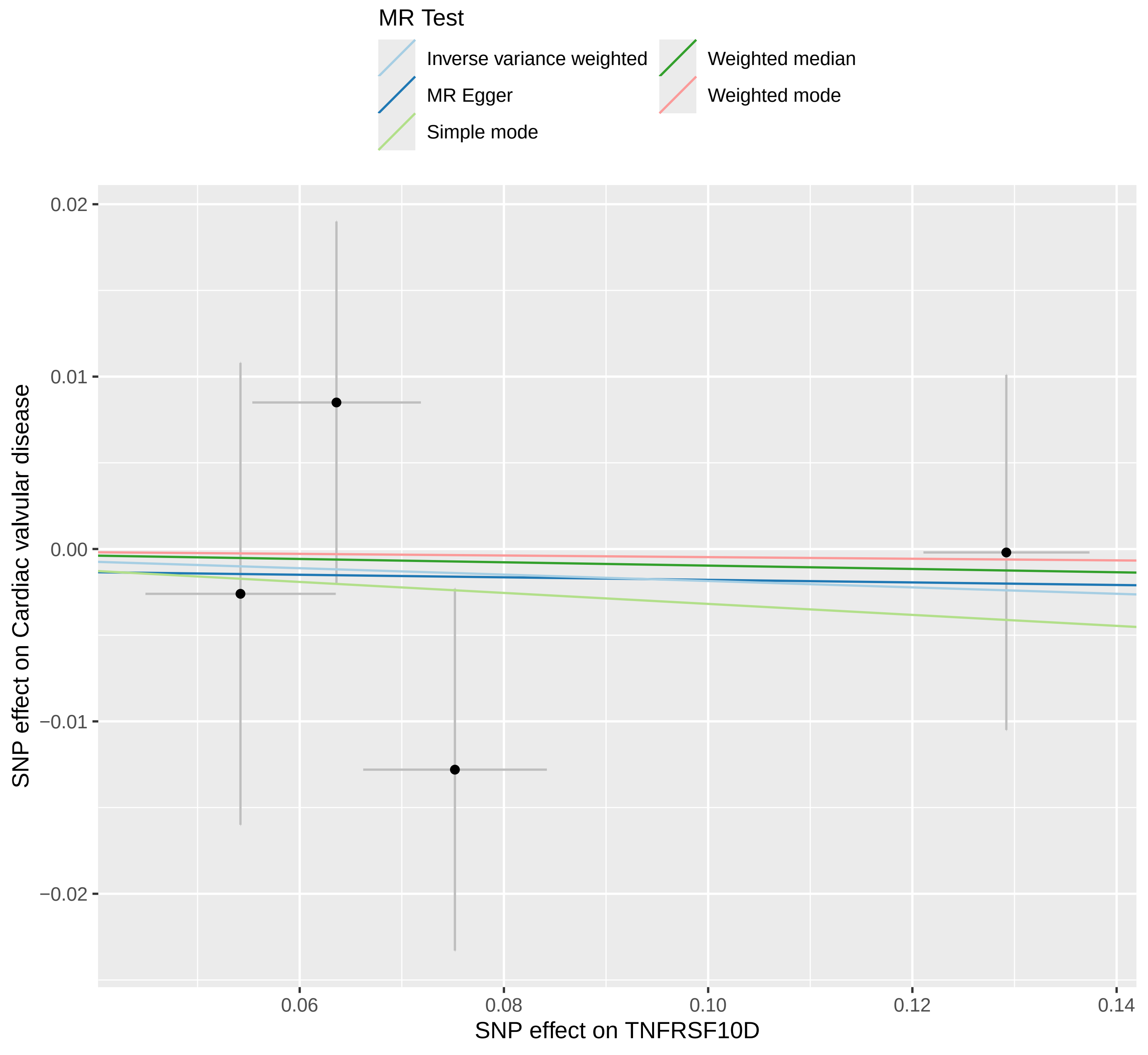
MR Test
Inverse variance weighted
MR Egger
Simple mode
Weighted median
Weighted mode
SNP effect on Cardiac valvular disease
0.02
0.01
0.00
−0.01
−0.02
0.06
0.08
0.10
0.12
0.14
SNP effect on TNFRSF10D

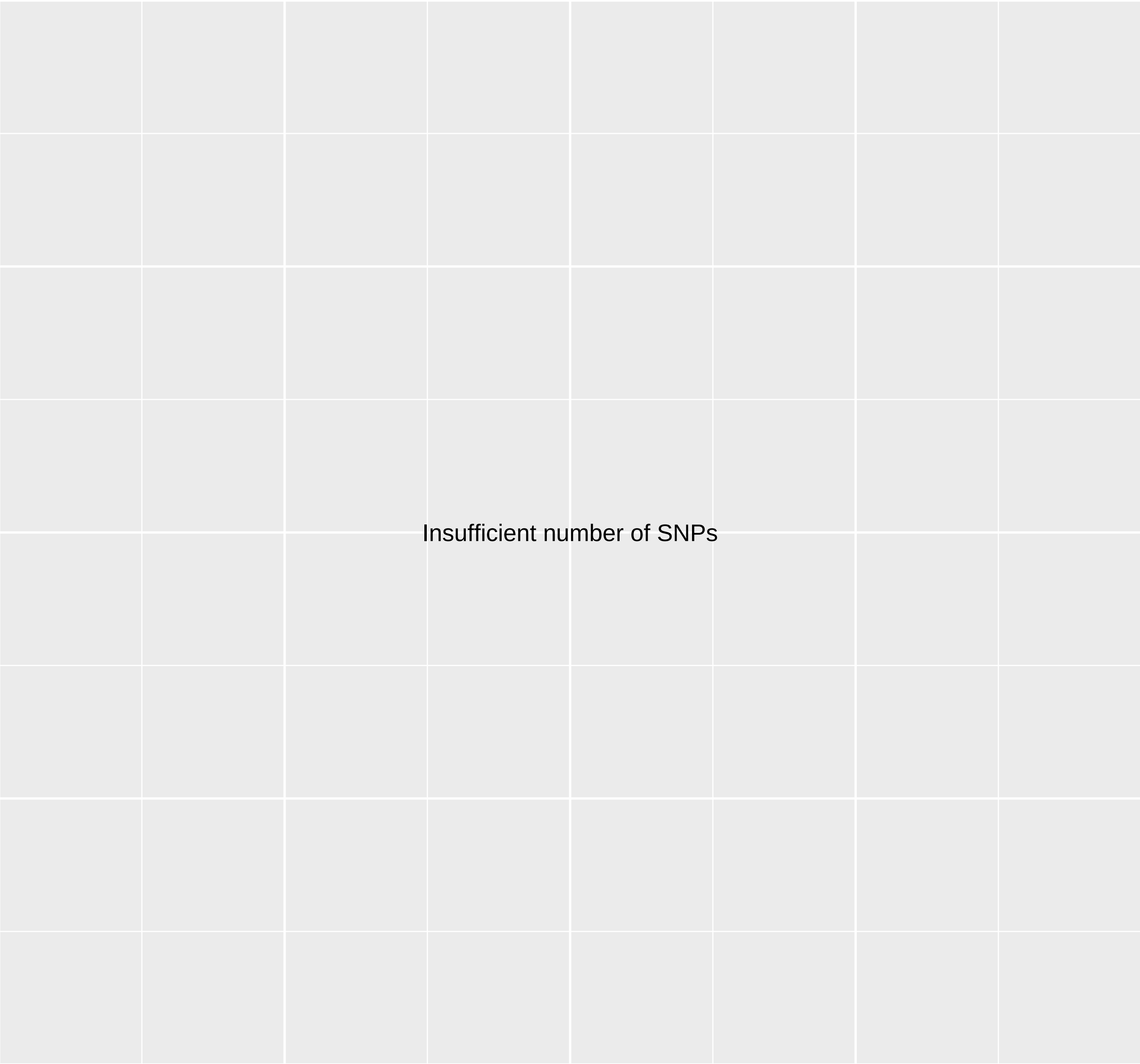
Insufficient number of SNPs

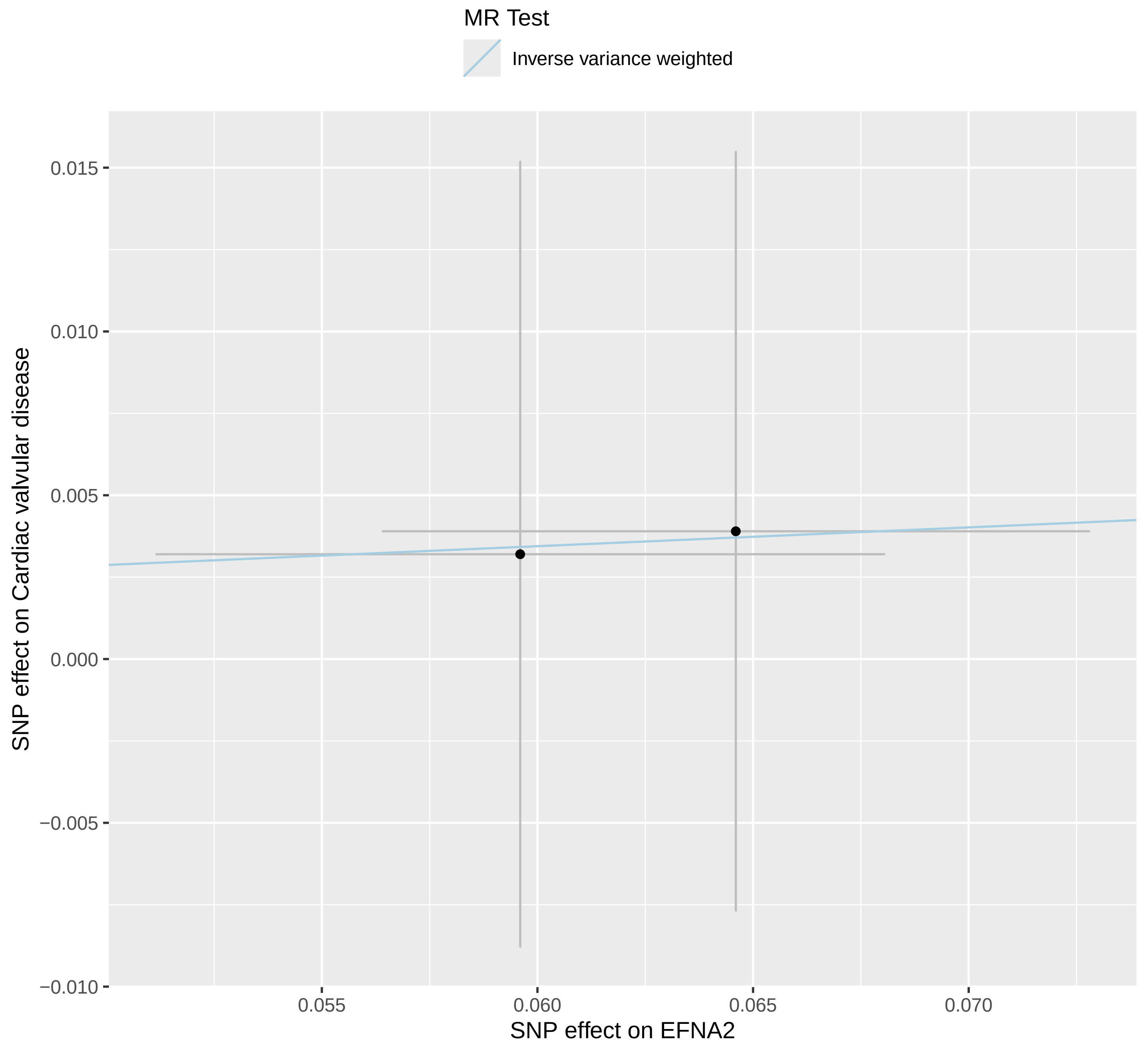
MR Test
Inverse variance weighted
0.015
0.010
0.005
0.000
−0.005
−0.010
0.055
0.060
0.065
0.070
SNP effect on EFNA2
SNP effect on Cardiac valvular disease

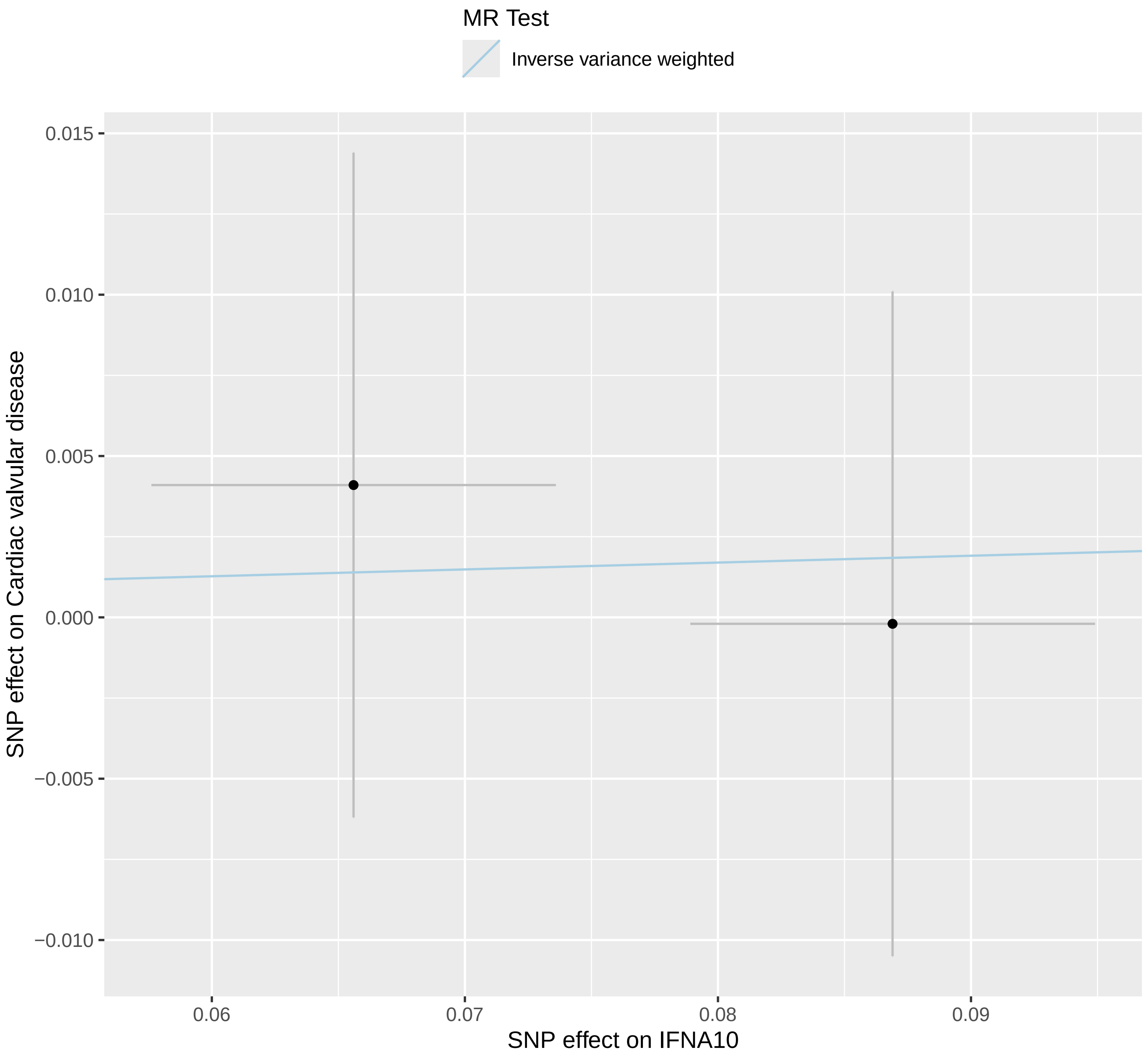
MR Test
Inverse variance weighted
0.015
0.010
0.005
0.000
−0.005
−0.010
SNP effect on Cardiac valvular disease
0.06
0.07
0.08
0.09
SNP effect on IFNA10

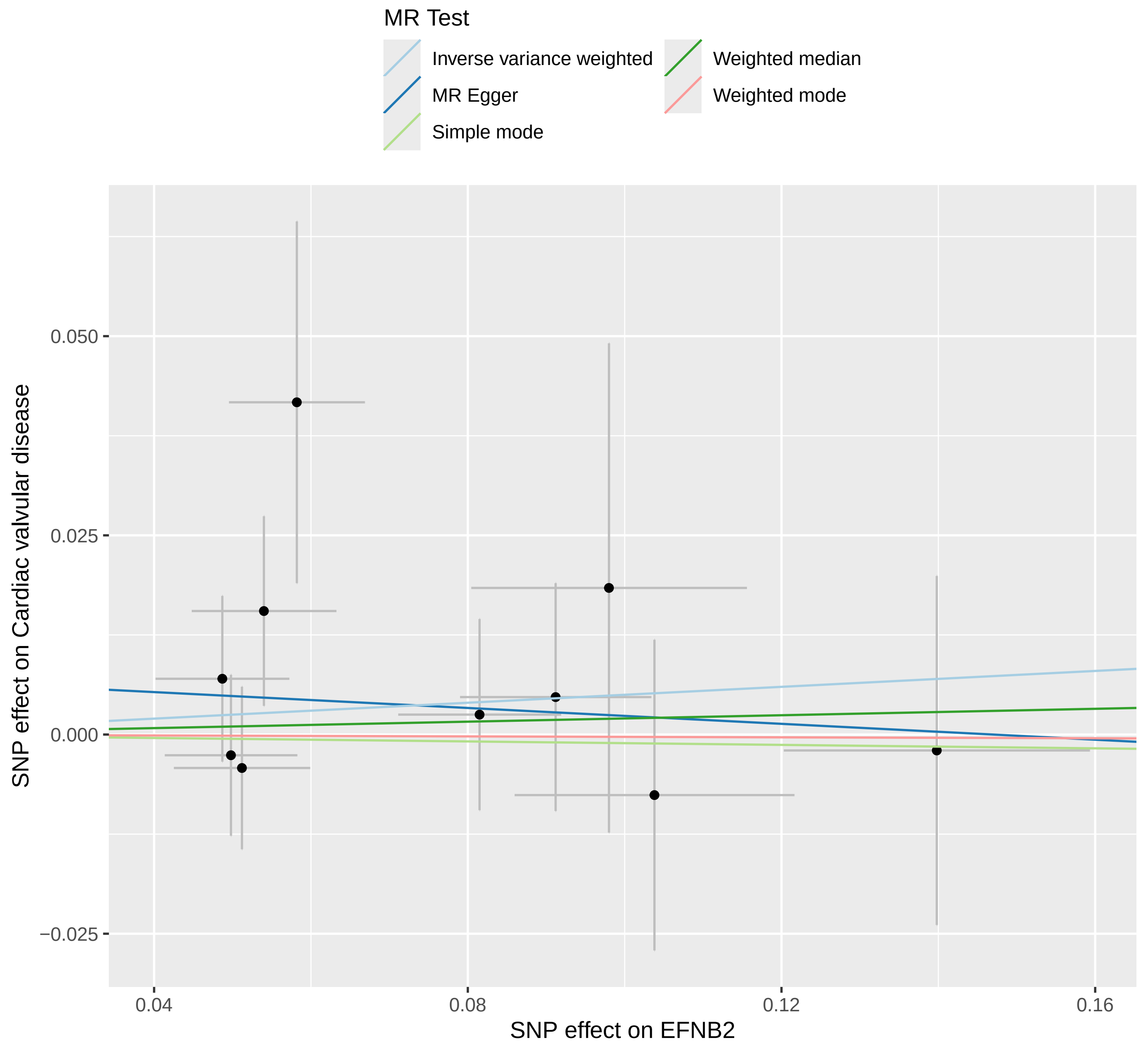

MR Test
Inverse variance weighted
MR Egger
Simple mode
Weighted median
Weighted mode
0.050
0.025
0.000
−0.025
0.04
0.08
0.12
0.16
SNP effect on Cardiac valvular disease
SNP effect on EFNB2

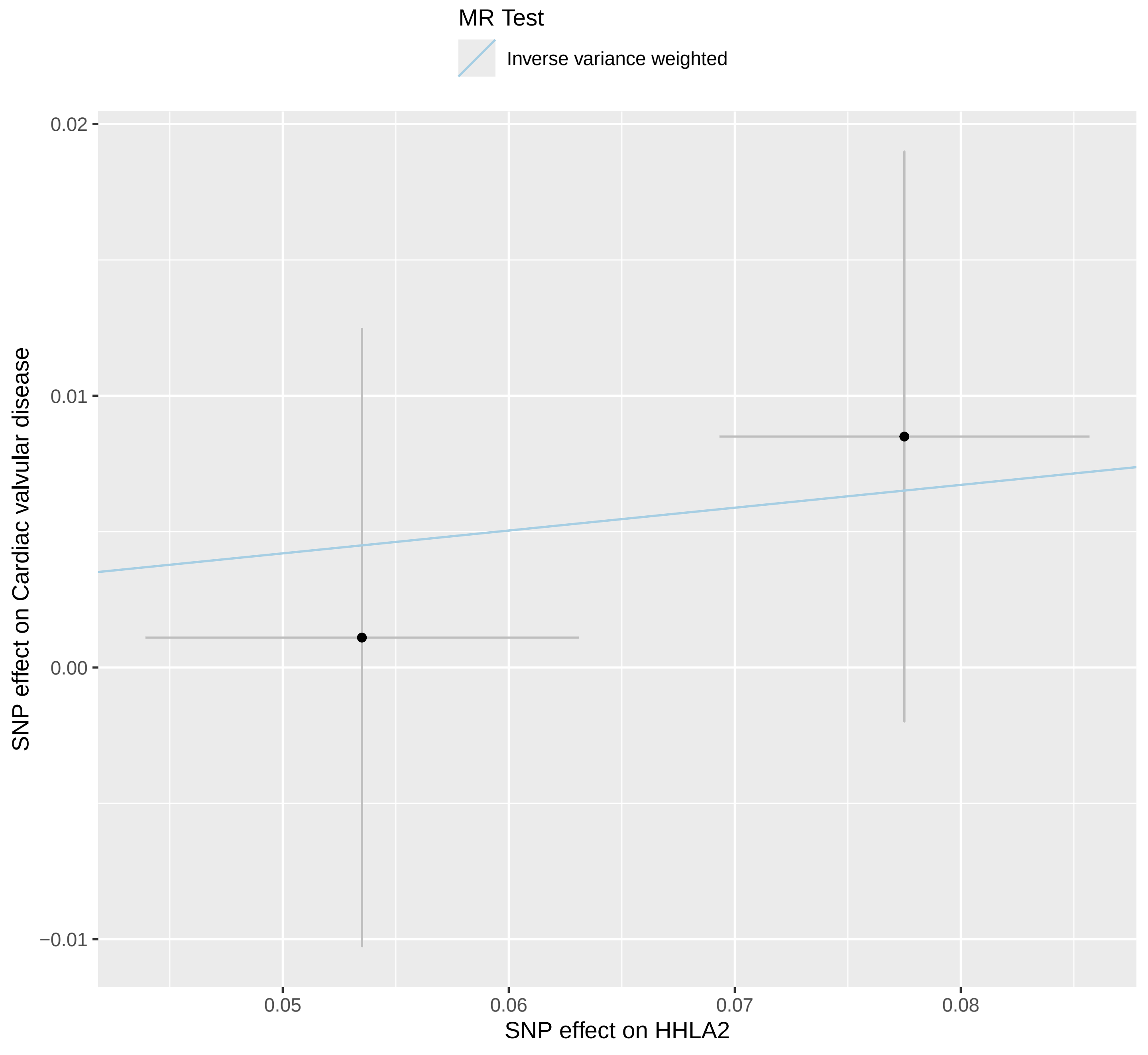

MR Test
Inverse variance weighted
0.02
0.01
0.00
−0.01
0.05
0.06
0.07
0.08
SNP effect on HHLA2
SNP effect on Cardiac valvular disease

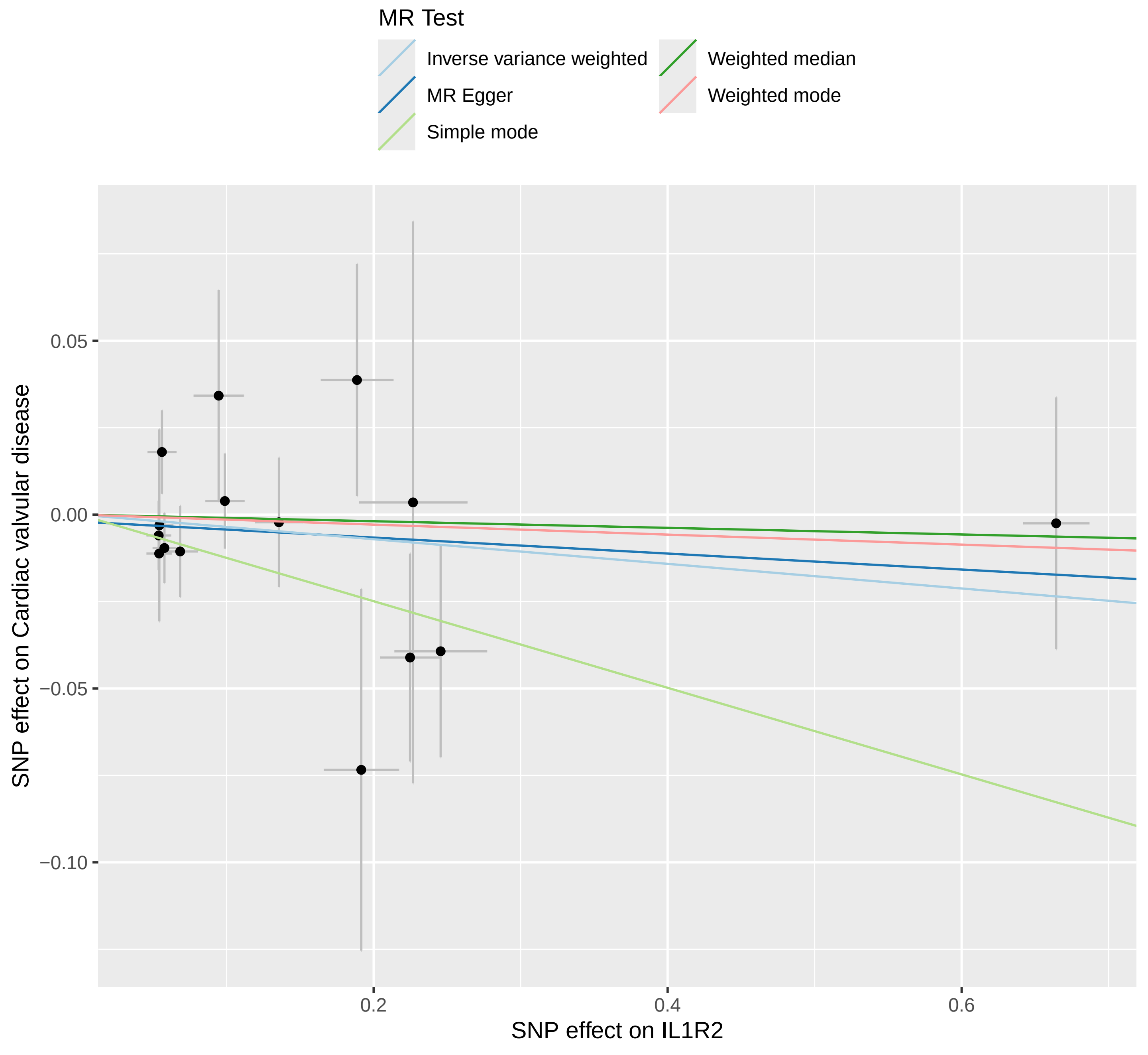

MR Test
Inverse variance weighted
MR Egger
Simple mode
Weighted median
Weighted mode
0.05
0.00
−0.05
−0.10
0.2
0.4
0.6
SNP effect on Cardiac valvular disease
SNP effect on IL1R2

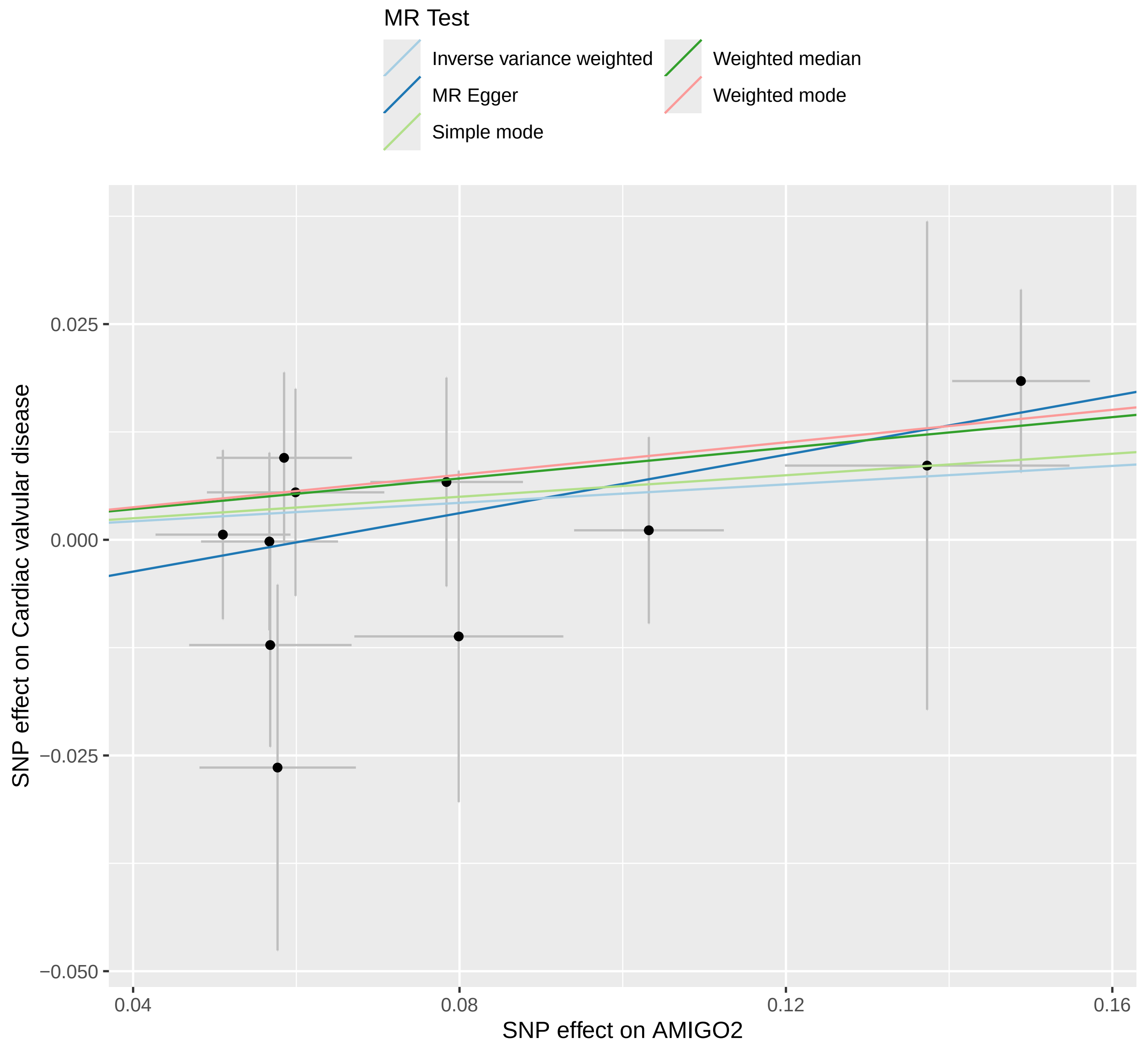
MR Test
Inverse variance weighted
MR Egger
Simple mode
Weighted median
Weighted mode
SNP effect on Cardiac valvular disease
0.025
0.000
−0.025
−0.050
0.04
0.08
0.12
0.16
SNP effect on AMIGO2

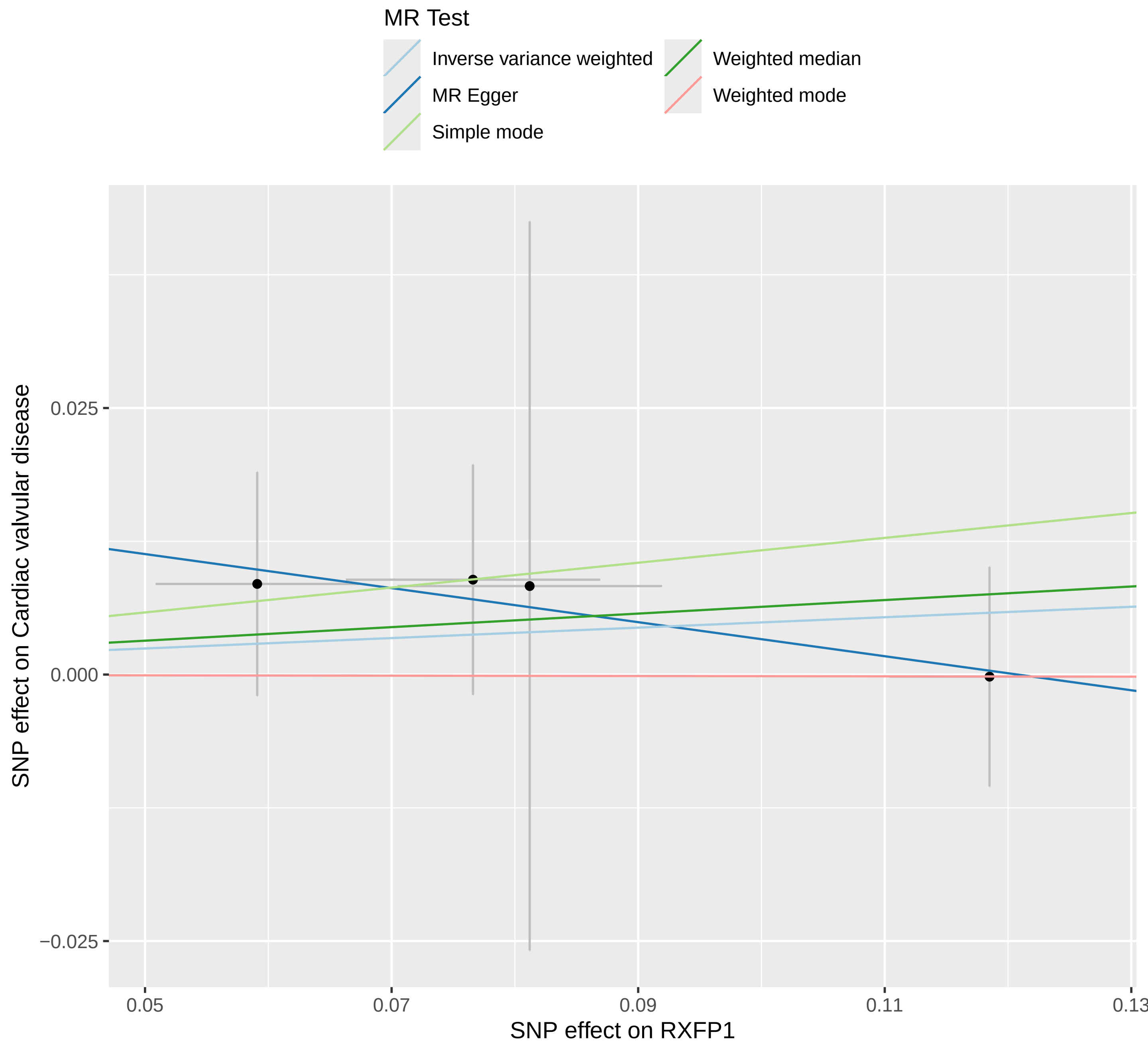
MR Test
Inverse variance weighted
MR Egger
Simple mode
Weighted median
Weighted mode
0.025
0.000
−0.025
SNP effect on Cardiac valvular disease
0.05
0.07
0.09
0.11
0.13
SNP effect on RXFP1

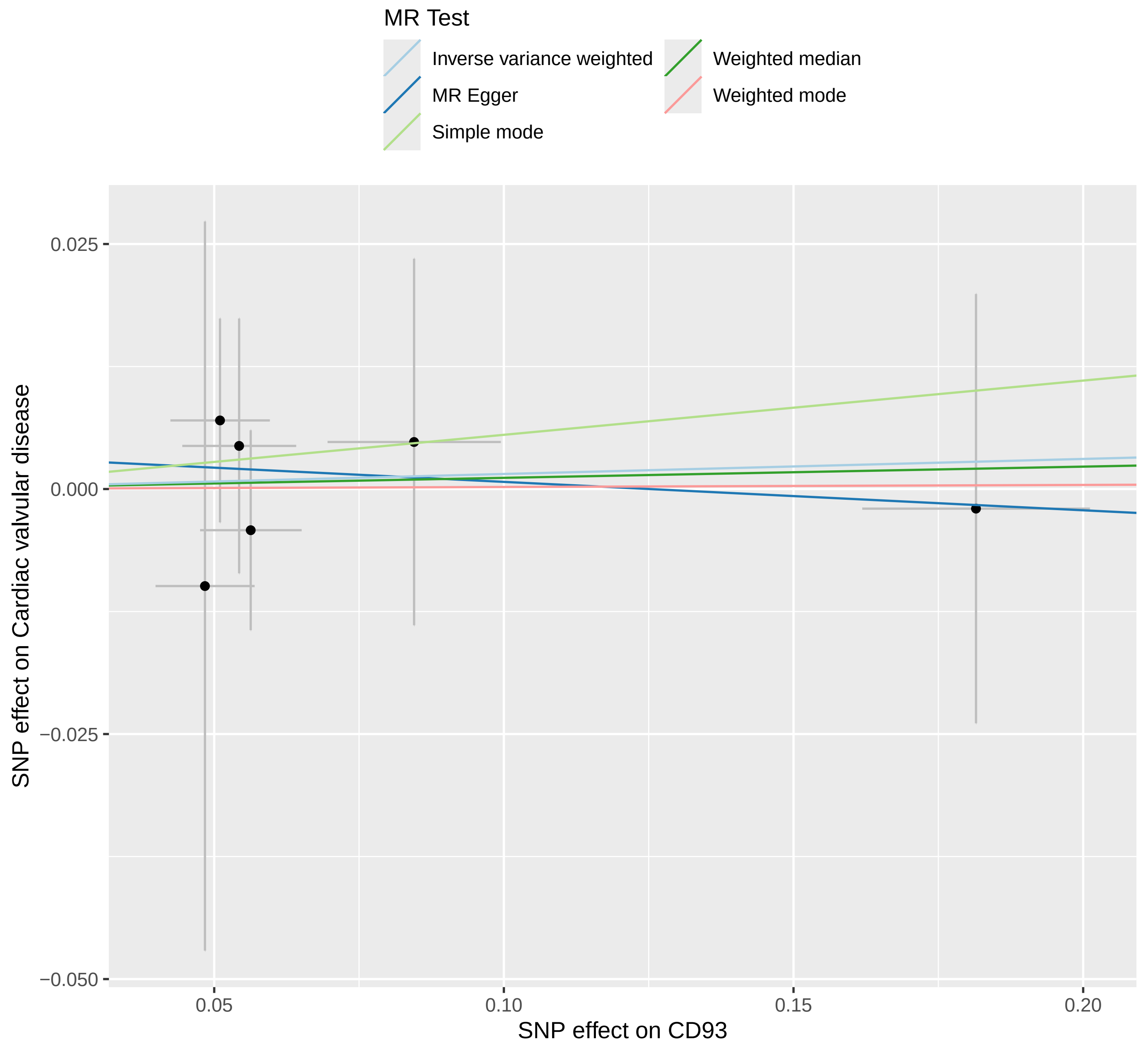
MR Test
Inverse variance weighted
MR Egger
Simple mode
Weighted median
Weighted mode
0.025
0.000
−0.025
−0.050
SNP effect on Cardiac valvular disease
0.05
0.10
0.15
0.20
SNP effect on CD93

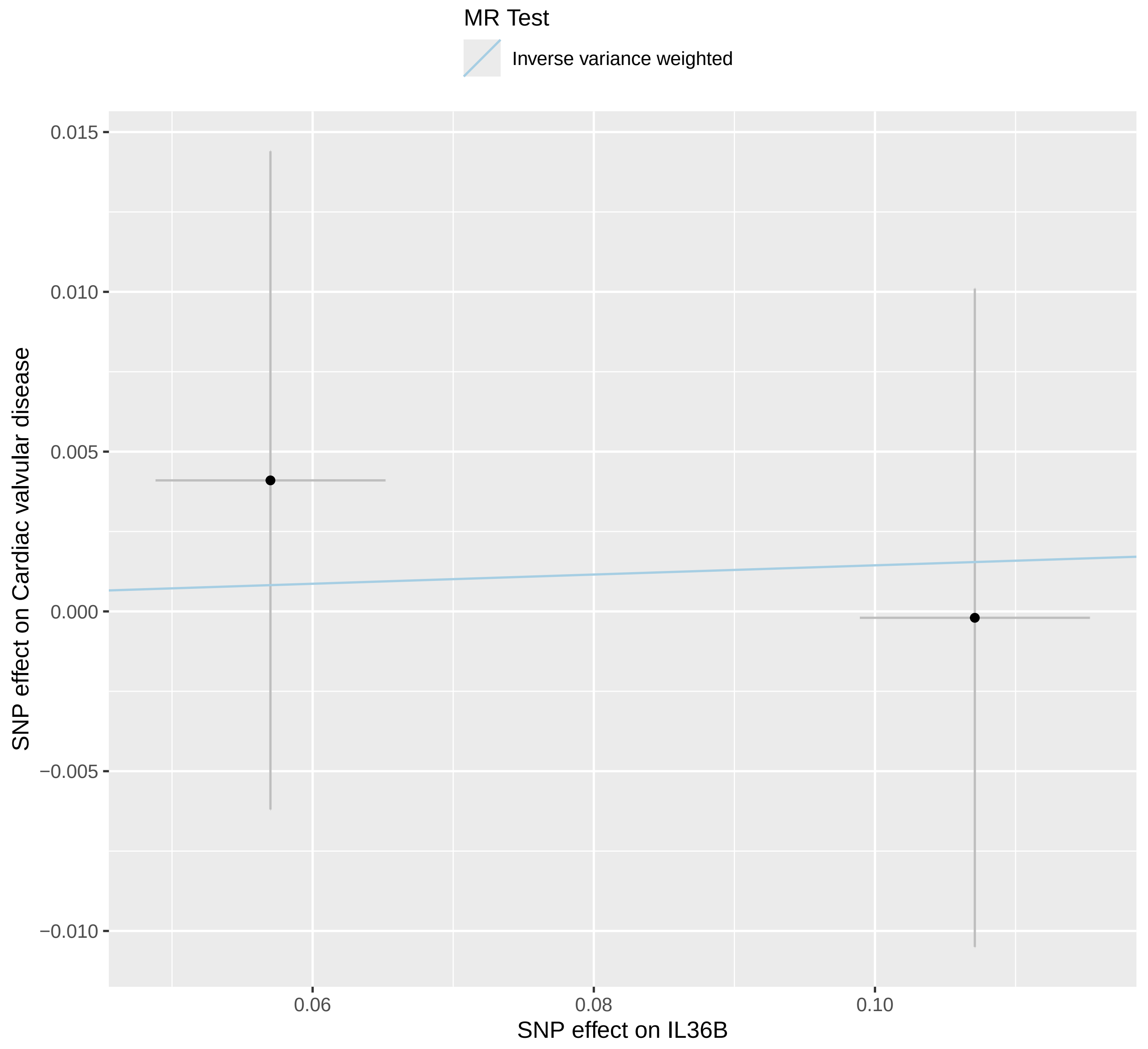
MR Test
Inverse variance weighted
0.015
0.010
0.005
0.000
−0.005
−0.010
SNP effect on Cardiac valvular disease
0.06
0.08
0.10
SNP effect on IL36B

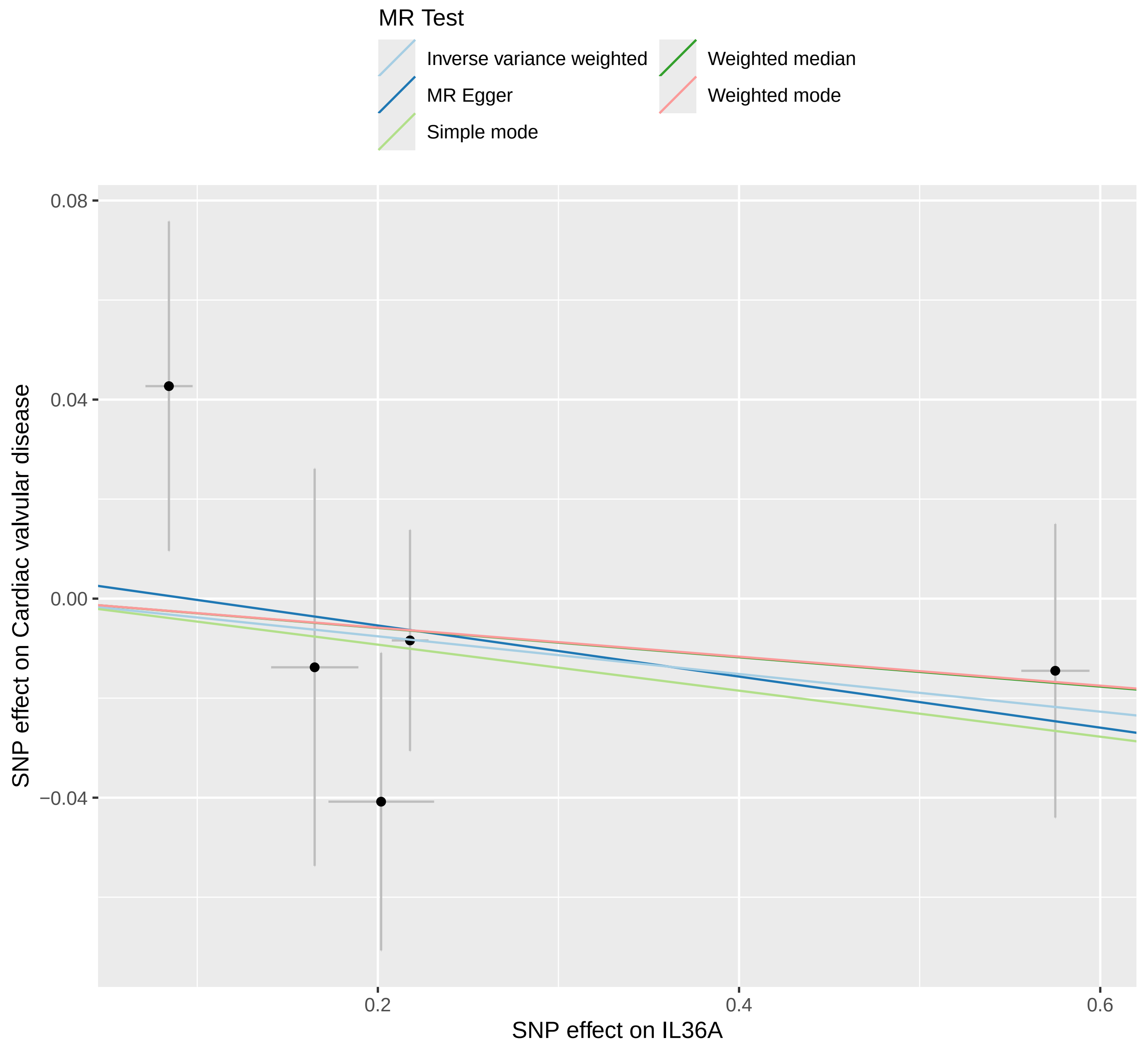

MR Test
Inverse variance weighted
MR Egger
Simple mode
Weighted median
Weighted mode
0.08
0.04
0.00
−0.04
0.2
0.4
0.6
SNP effect on Cardiac valvular disease
SNP effect on IL36A

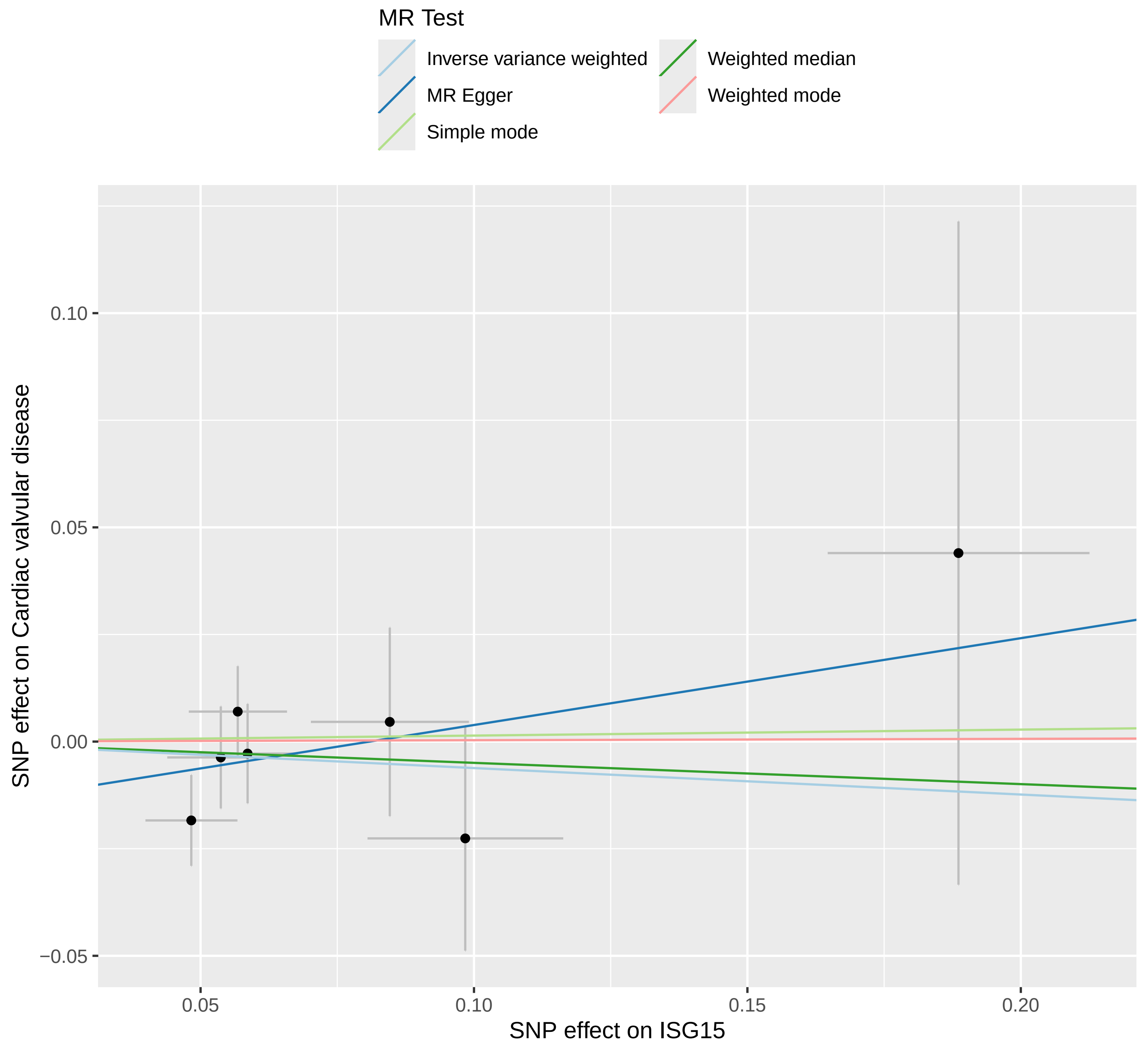

MR Test
Inverse variance weighted
MR Egger
Simple mode
Weighted median
Weighted mode
0.10
0.05
0.00
−0.05
SNP effect on Cardiac valvular disease
0.05
0.10
0.15
0.20
SNP effect on ISG15

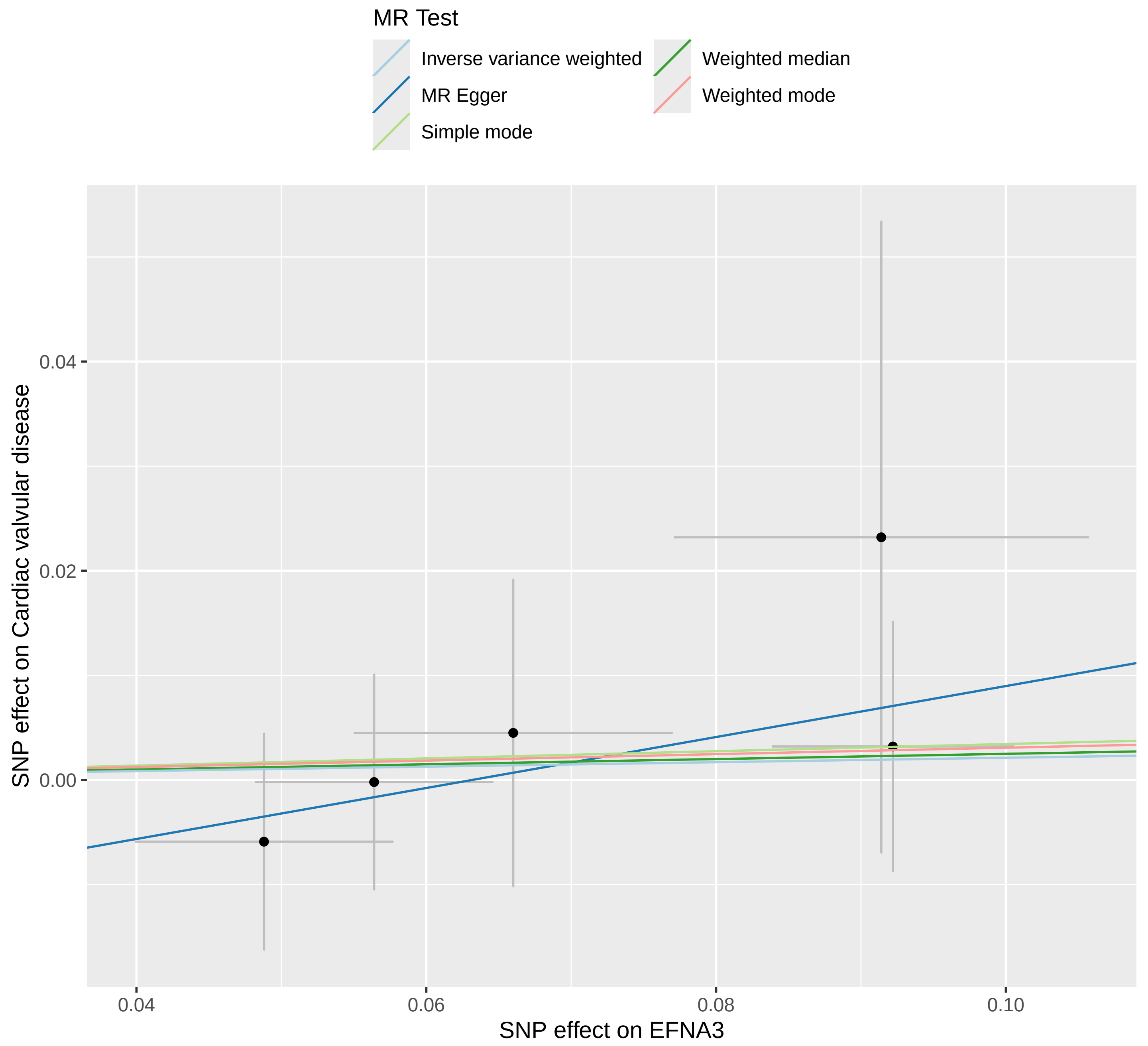
MR Test
Inverse variance weighted
MR Egger
Simple mode
Weighted median
Weighted mode
SNP effect on Cardiac valvular disease
0.04
0.02
0.00
0.04
0.06
0.08
0.10
SNP effect on EFNA3

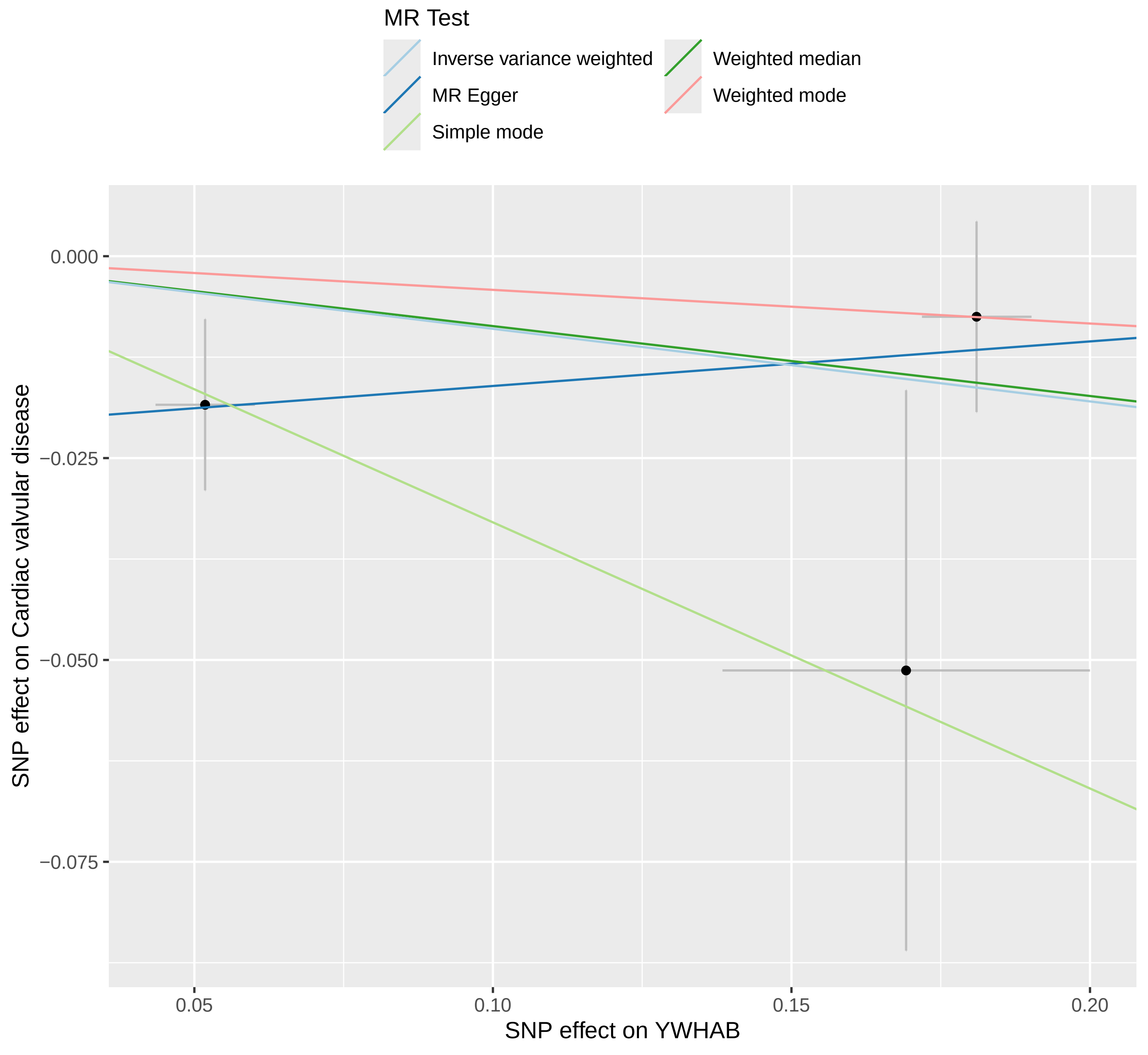
MR Test
Inverse variance weighted
MR Egger
Simple mode
Weighted median
Weighted mode
0.000
−0.025
−0.050
−0.075
0.05
0.10
0.15
0.20
SNP effect on Cardiac valvular disease
SNP effect on YWHAB

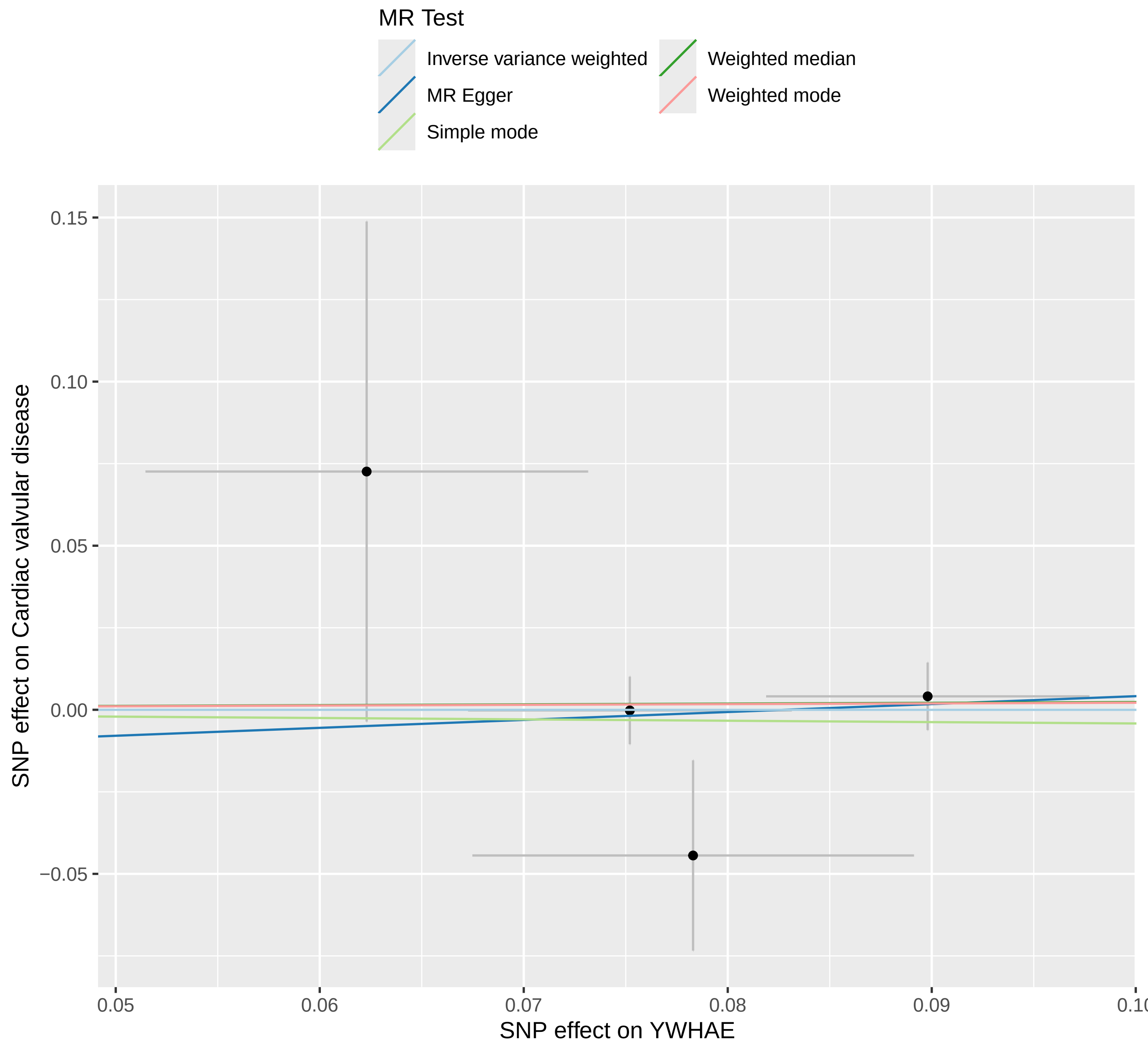
MR Test
Inverse variance weighted
MR Egger
Simple mode
Weighted median
Weighted mode
0.15
0.10
0.05
0.00
−0.05
SNP effect on Cardiac valvular disease
0.05
0.06
0.07
0.08
0.09
0.10
SNP effect on YWHAE

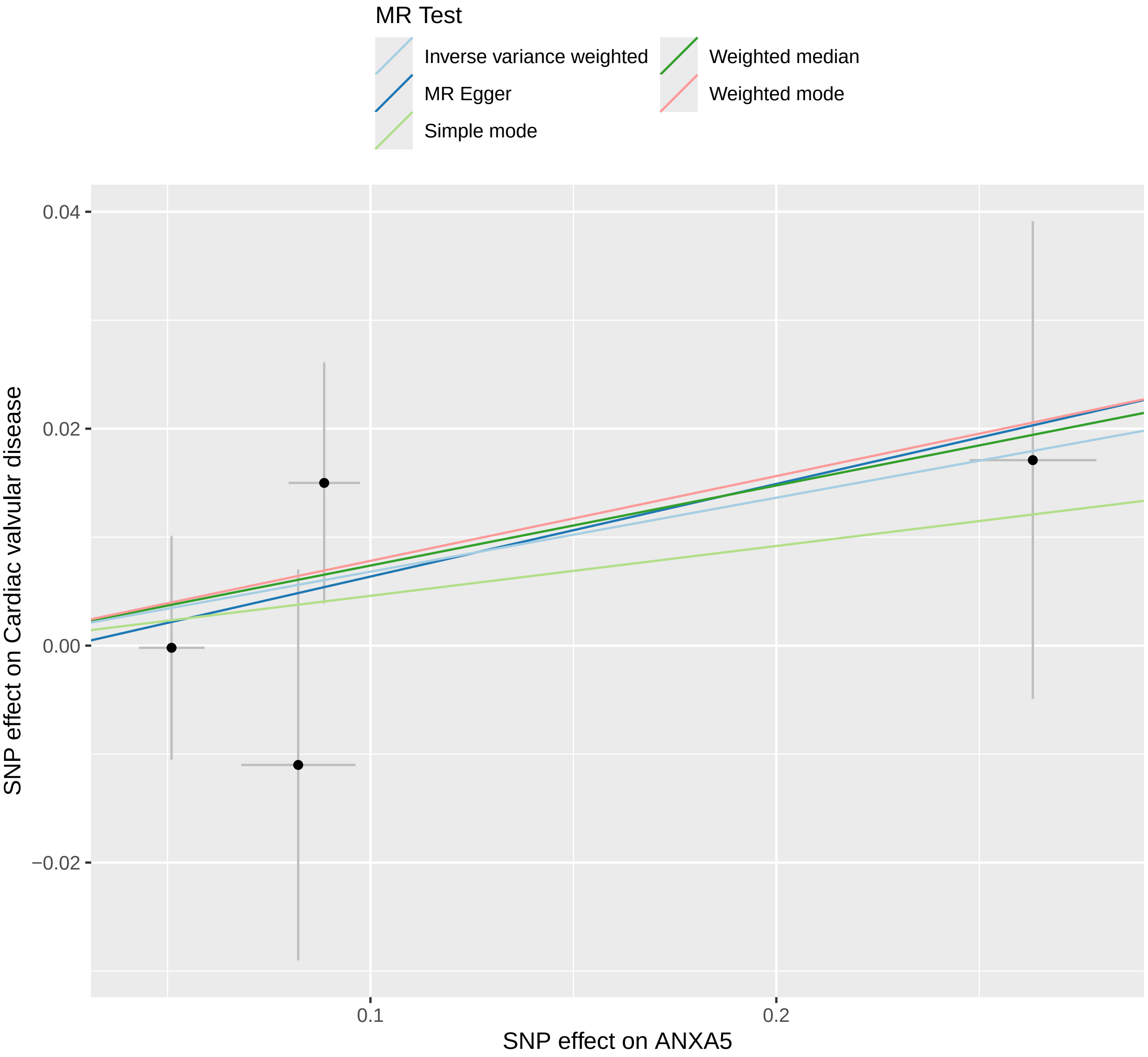

MR Test
Inverse variance weighted
MR Egger
Simple mode
Weighted median
Weighted mode
0.04
0.02
0.00
−0.02
SNP effect on Cardiac valvular disease
0.1
0.2
SNP effect on ANXA5

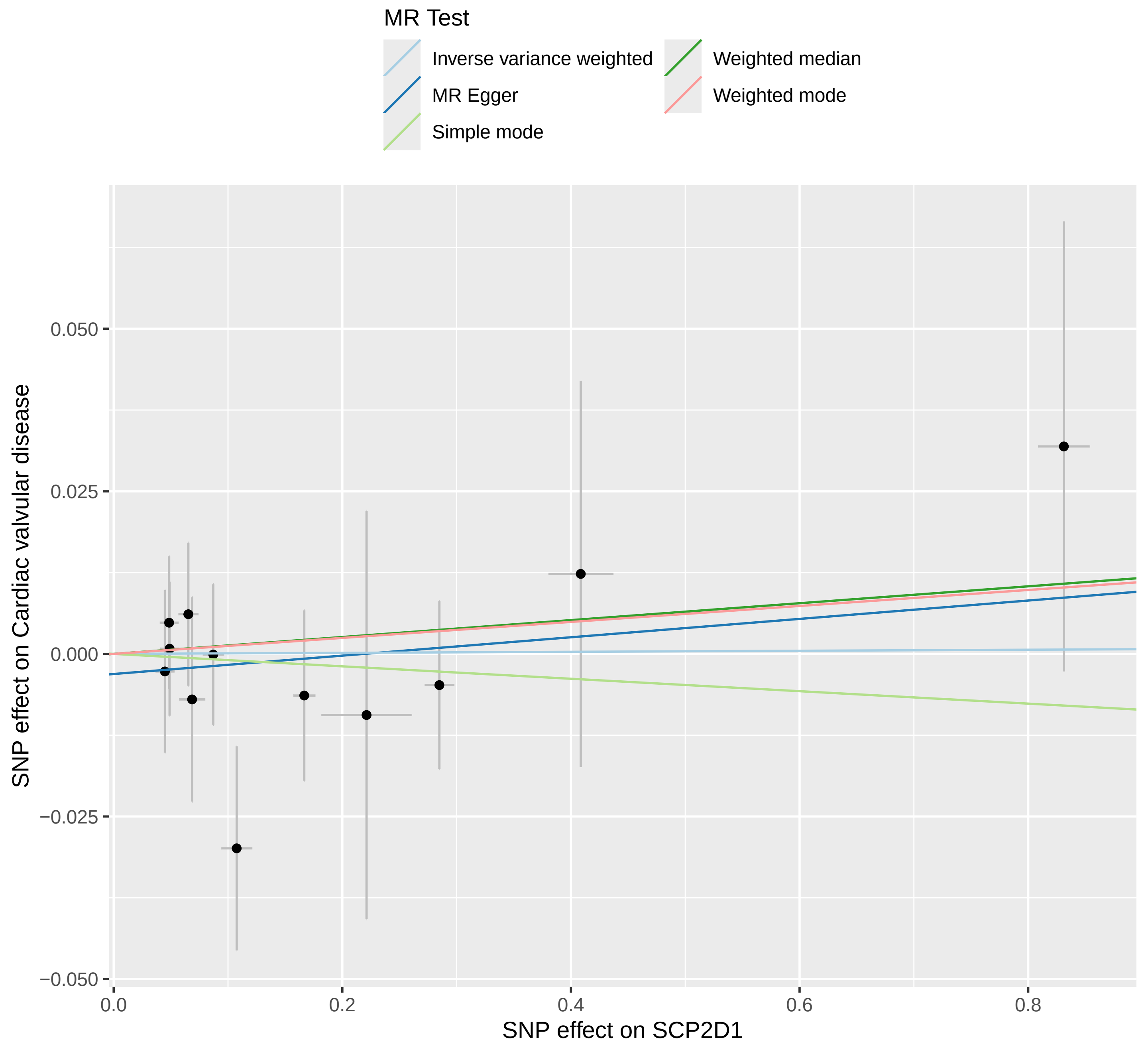

MR Test
Inverse variance weighted
MR Egger
Simple mode
Weighted median
Weighted mode
0.050
0.025
0.000
−0.025
−0.050
0.0
0.2
0.4
0.6
0.8
SNP effect on Cardiac valvular disease
SNP effect on SCP2D1

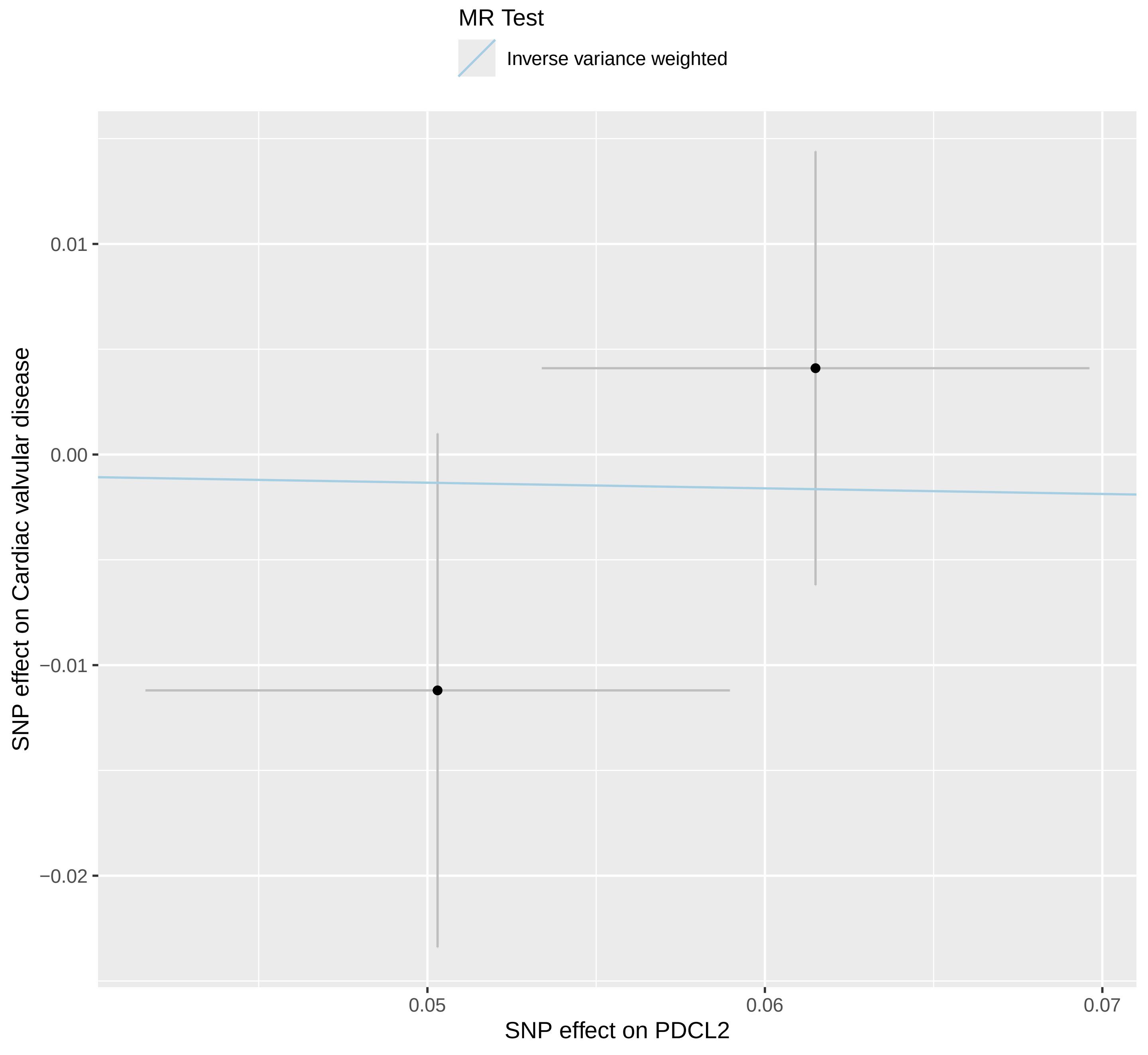

MR Test
Inverse variance weighted
SNP effect on Cardiac valvular disease
0.01
0.00
−0.01
−0.02
0.05
0.06
0.07
SNP effect on PDCL2

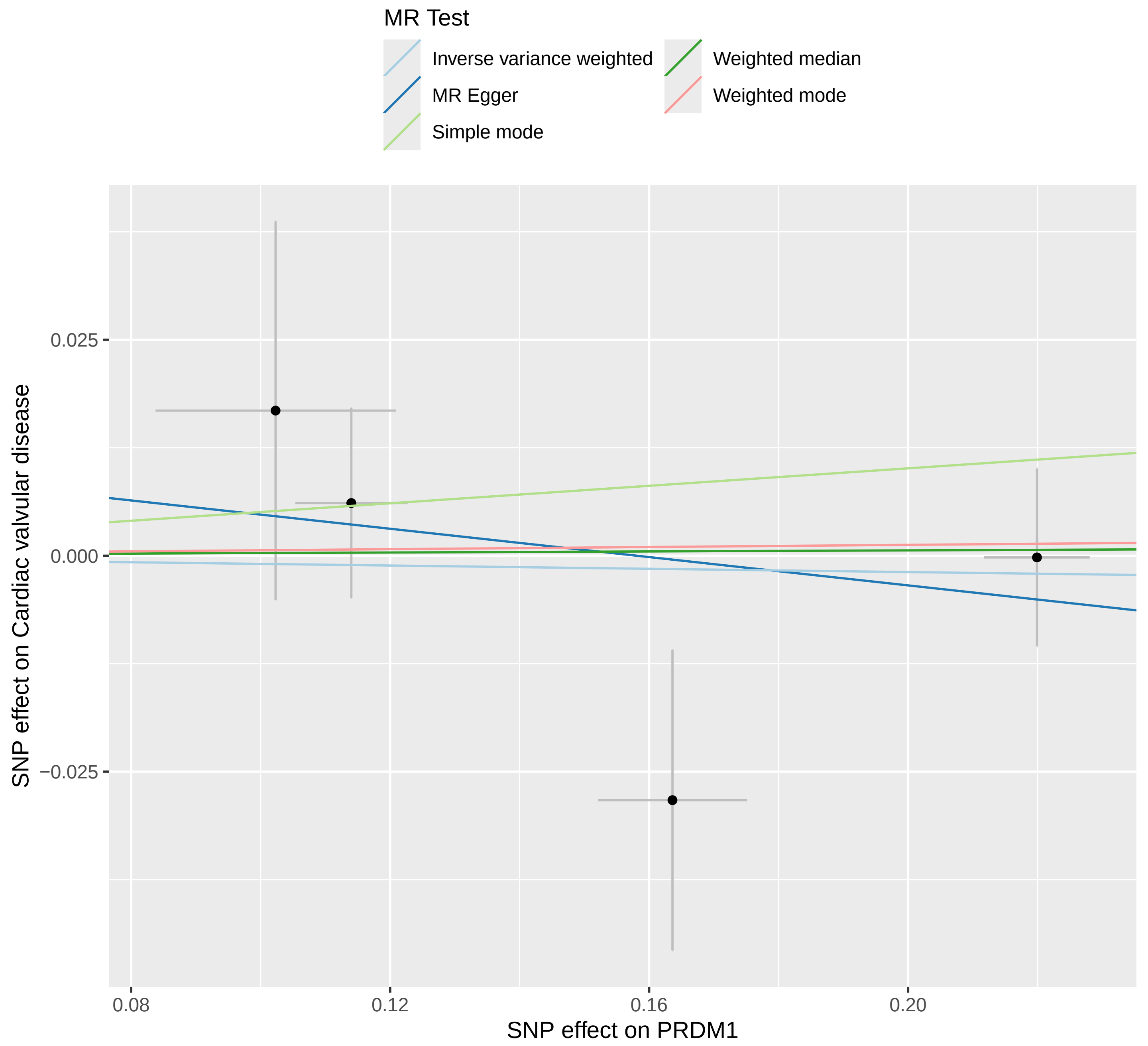

MR Test
Inverse variance weighted
MR Egger
Simple mode
Weighted median
Weighted mode
SNP effect on Cardiac valvular disease
0.025
0.000
−0.025
0.08
0.12
0.16
0.20
SNP effect on PRDM1

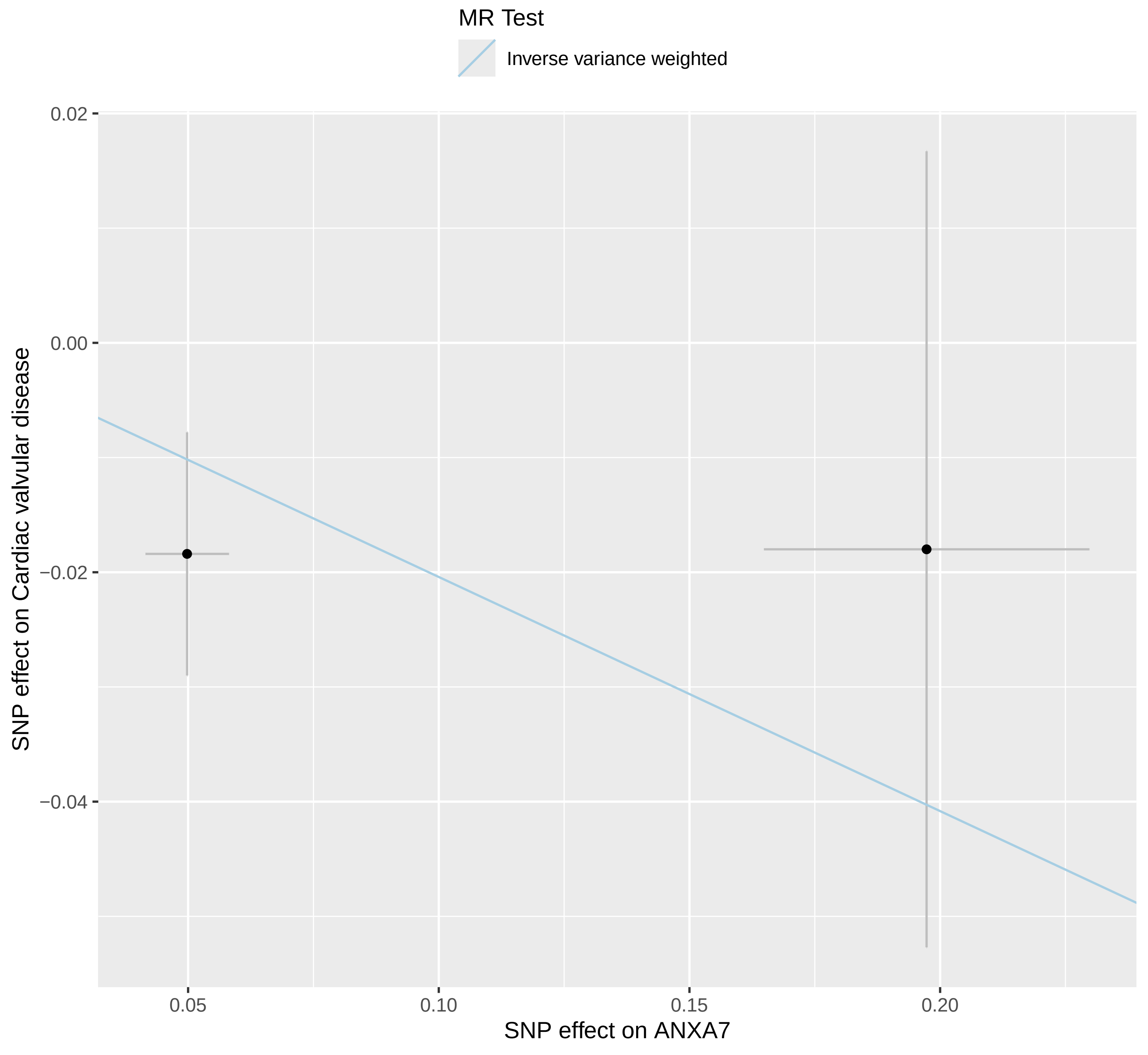

MR Test
Inverse variance weighted
SNP effect on Cardiac valvular disease
0.02
0.00
−0.02
−0.04
0.05
0.10
0.15
0.20
SNP effect on ANXA7

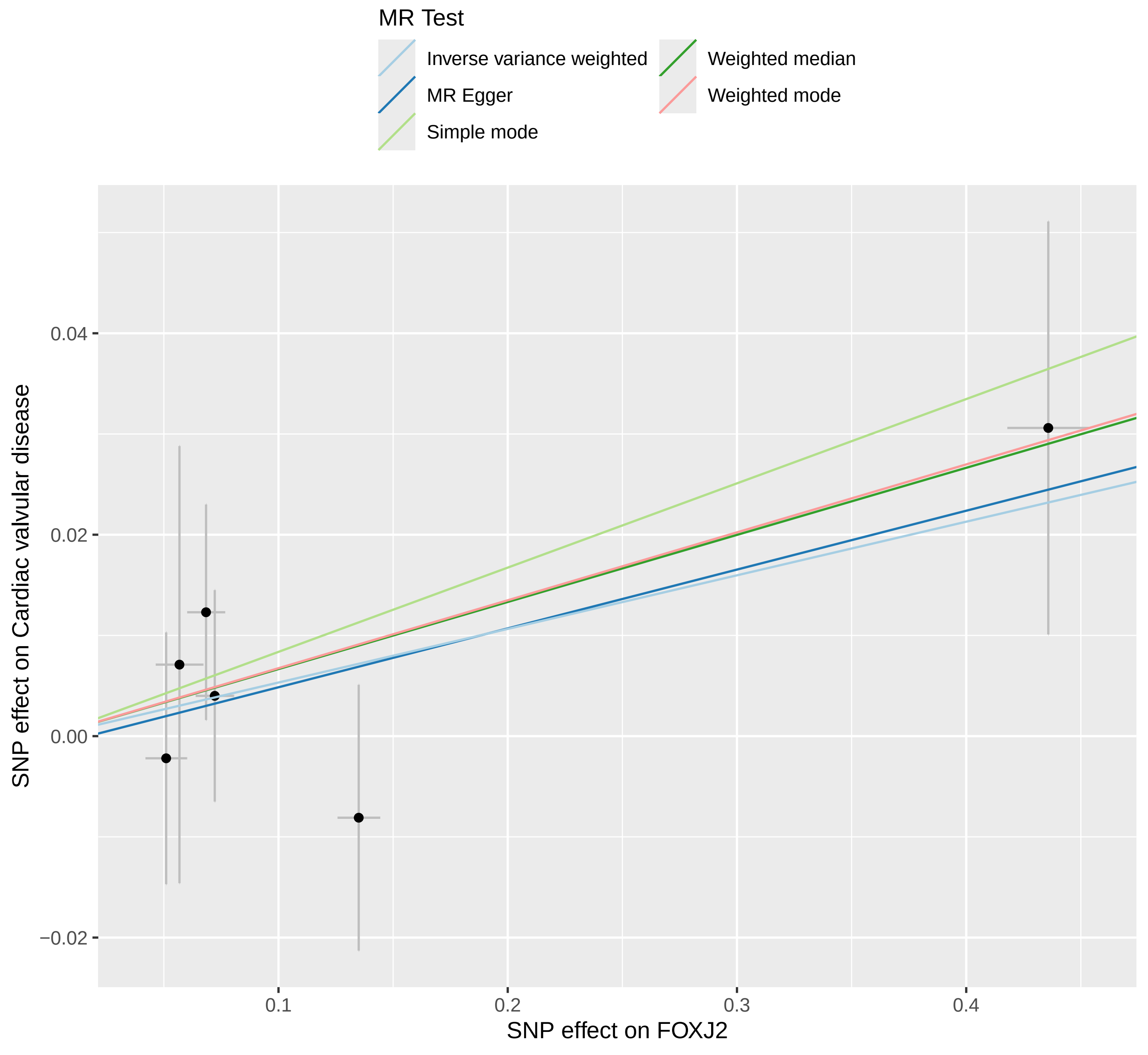
MR Test
Inverse variance weighted
MR Egger
Simple mode
Weighted median
Weighted mode
0.04
0.02
0.00
−0.02
0.1
0.2
0.3
0.4
SNP effect on FOXJ2
SNP effect on Cardiac valvular disease

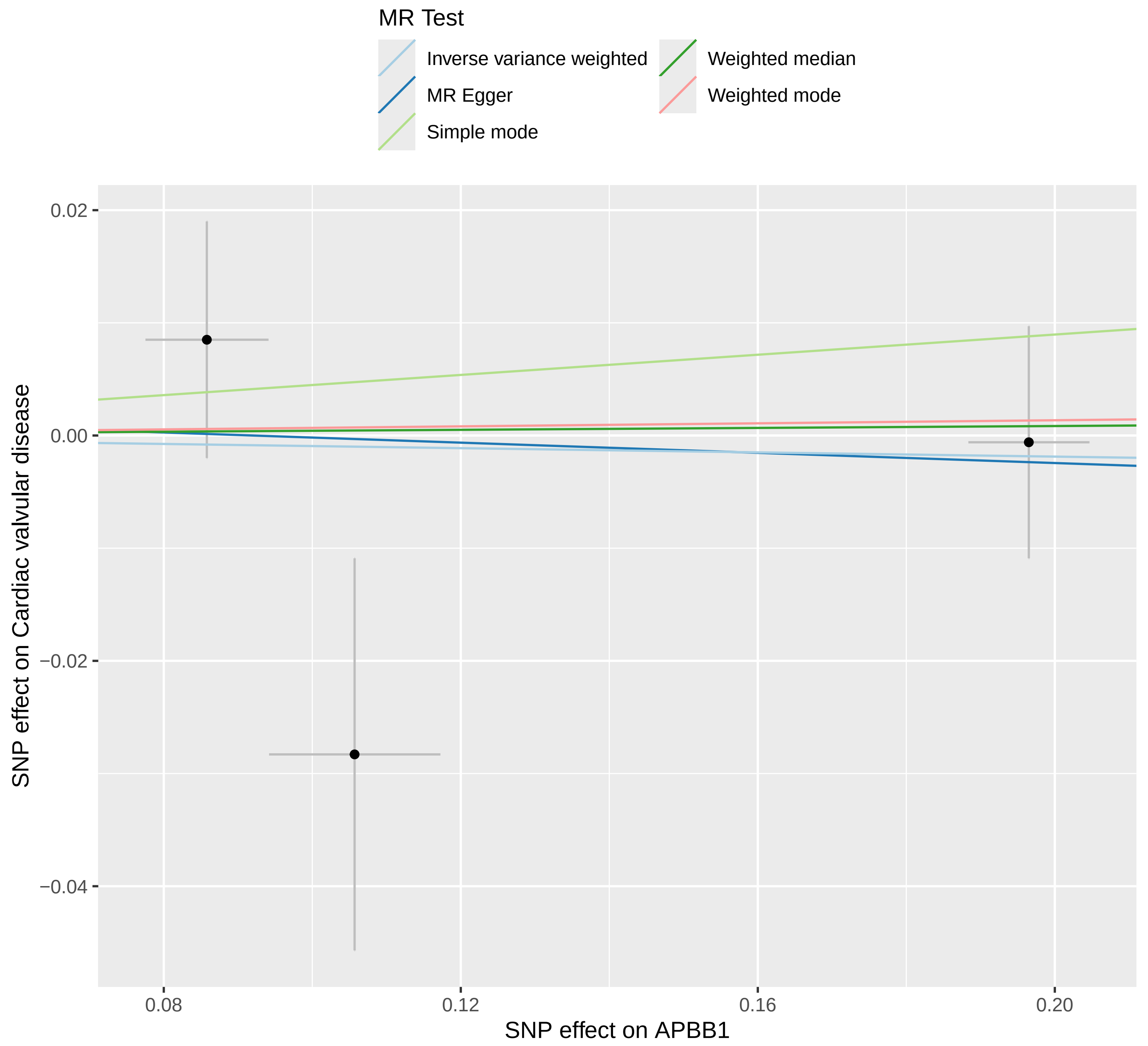
MR Test
Inverse variance weighted
MR Egger
Simple mode
Weighted median
Weighted mode
0.02
0.00
−0.02
−0.04
0.08
0.12
0.16
0.20
SNP effect on APBB1
SNP effect on Cardiac valvular disease

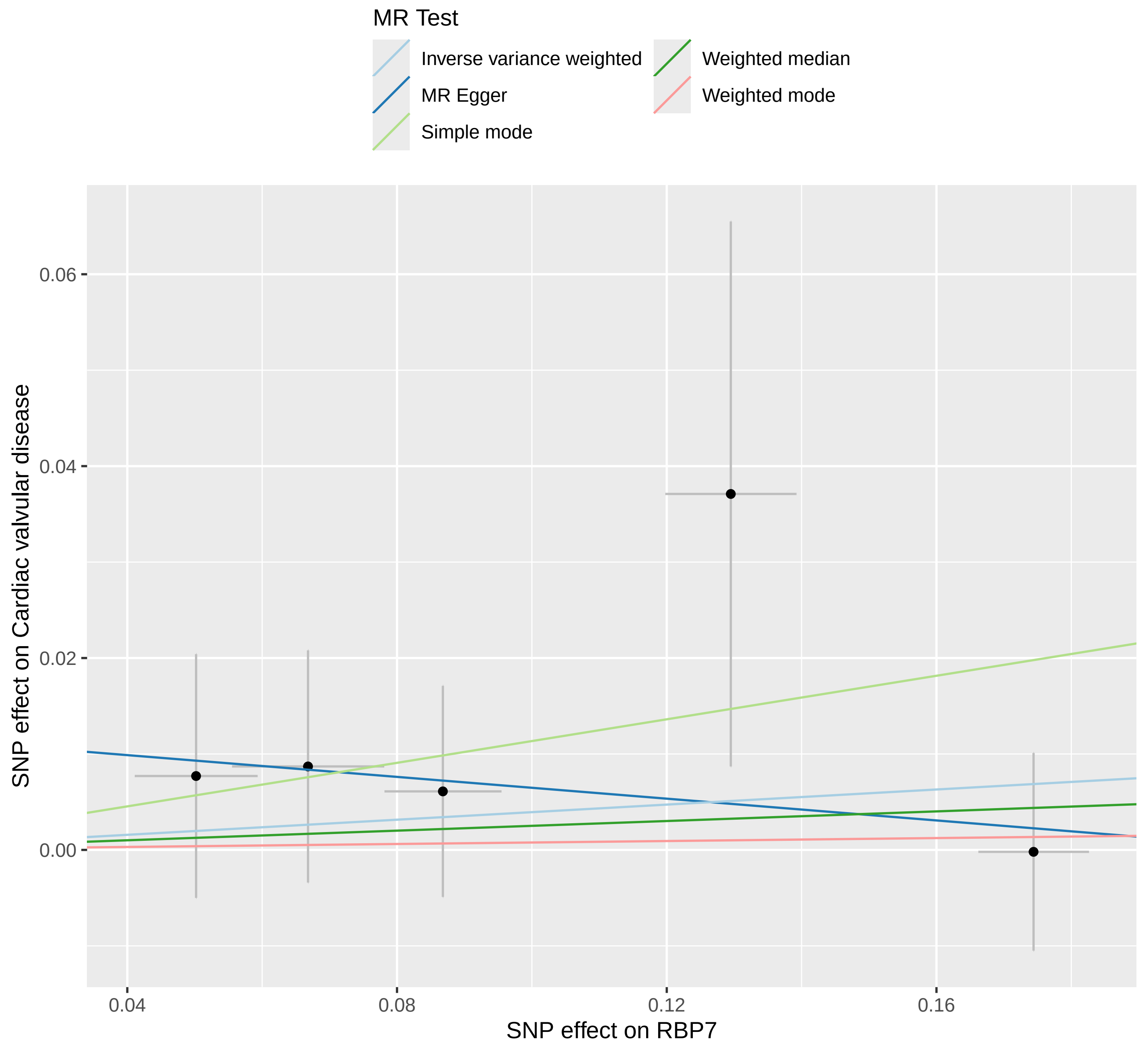
MR Test
Inverse variance weighted
MR Egger
Simple mode
Weighted median
Weighted mode
0.06
0.04
0.02
0.00
SNP effect on Cardiac valvular disease
0.04
0.08
0.12
0.16
SNP effect on RBP7

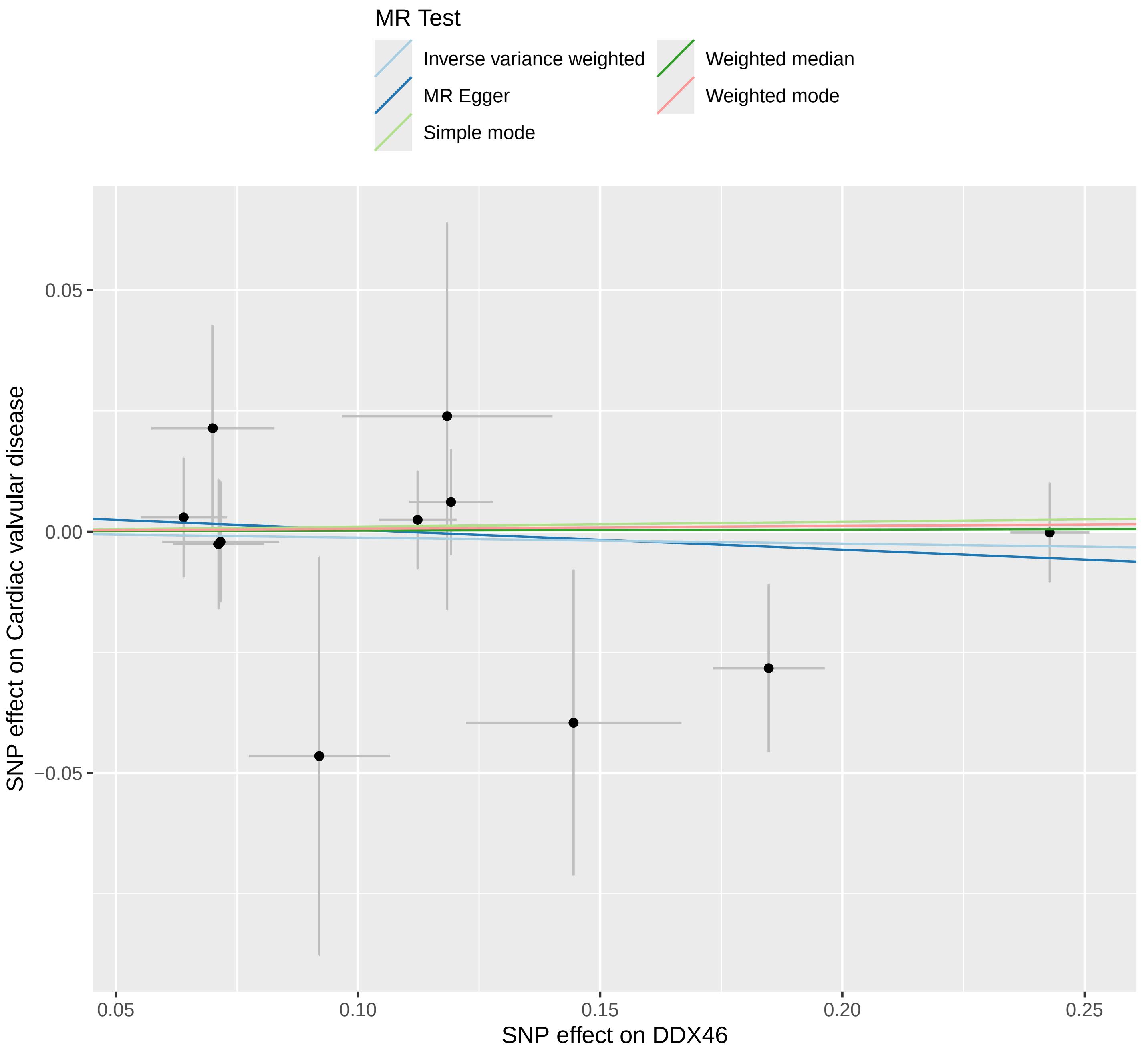

MR Test
Inverse variance weighted
MR Egger
Simple mode
Weighted median
Weighted mode
0.05
0.00
−0.05
SNP effect on Cardiac valvular disease
0.05
0.10
0.15
0.20
0.25
SNP effect on DDX46

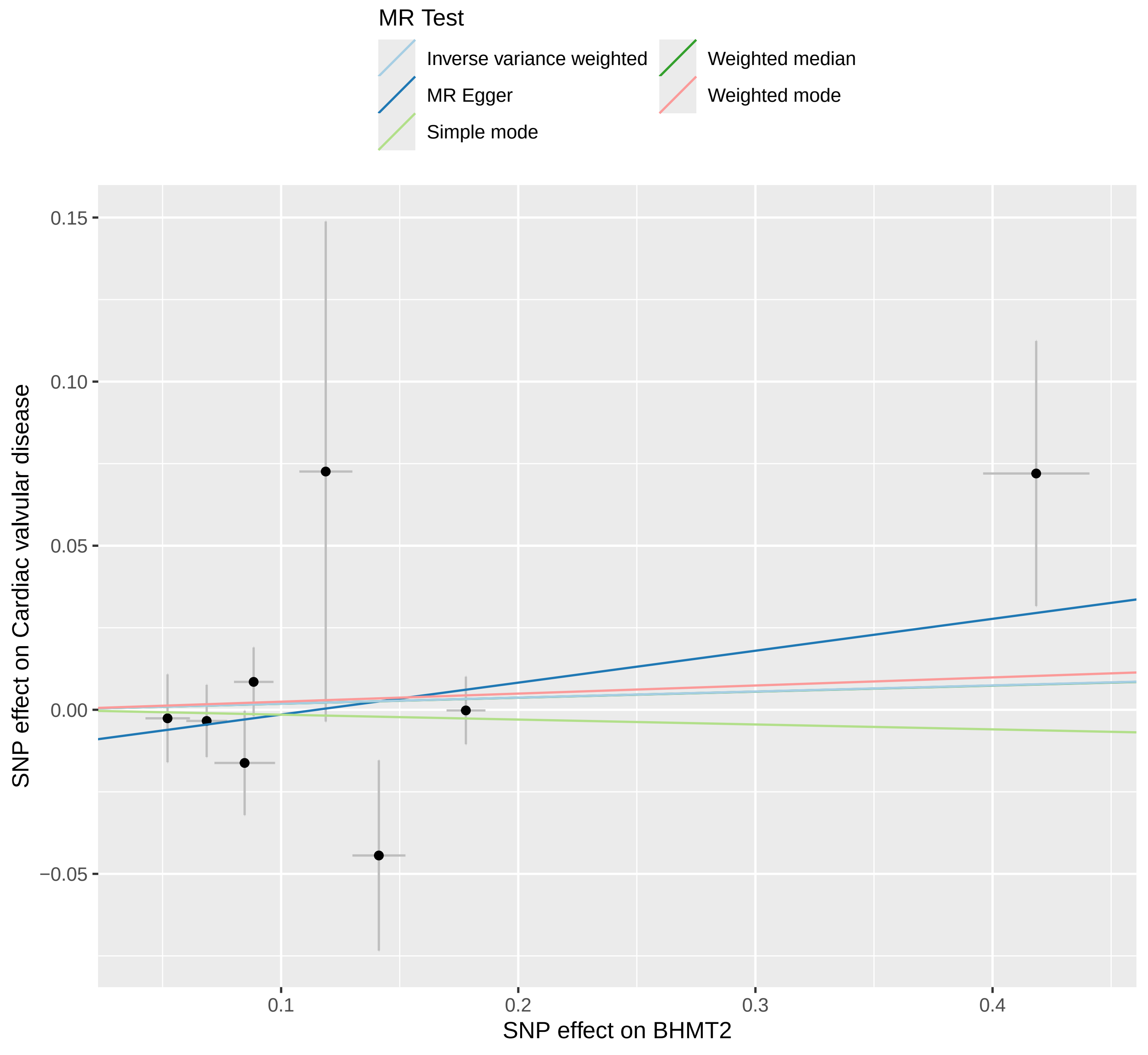
MR Test
Inverse variance weighted
MR Egger
Simple mode
Weighted median
Weighted mode
0.15
0.10
0.05
0.00
−0.05
0.1
0.2
0.3
0.4
SNP effect on Cardiac valvular disease
SNP effect on BHMT2

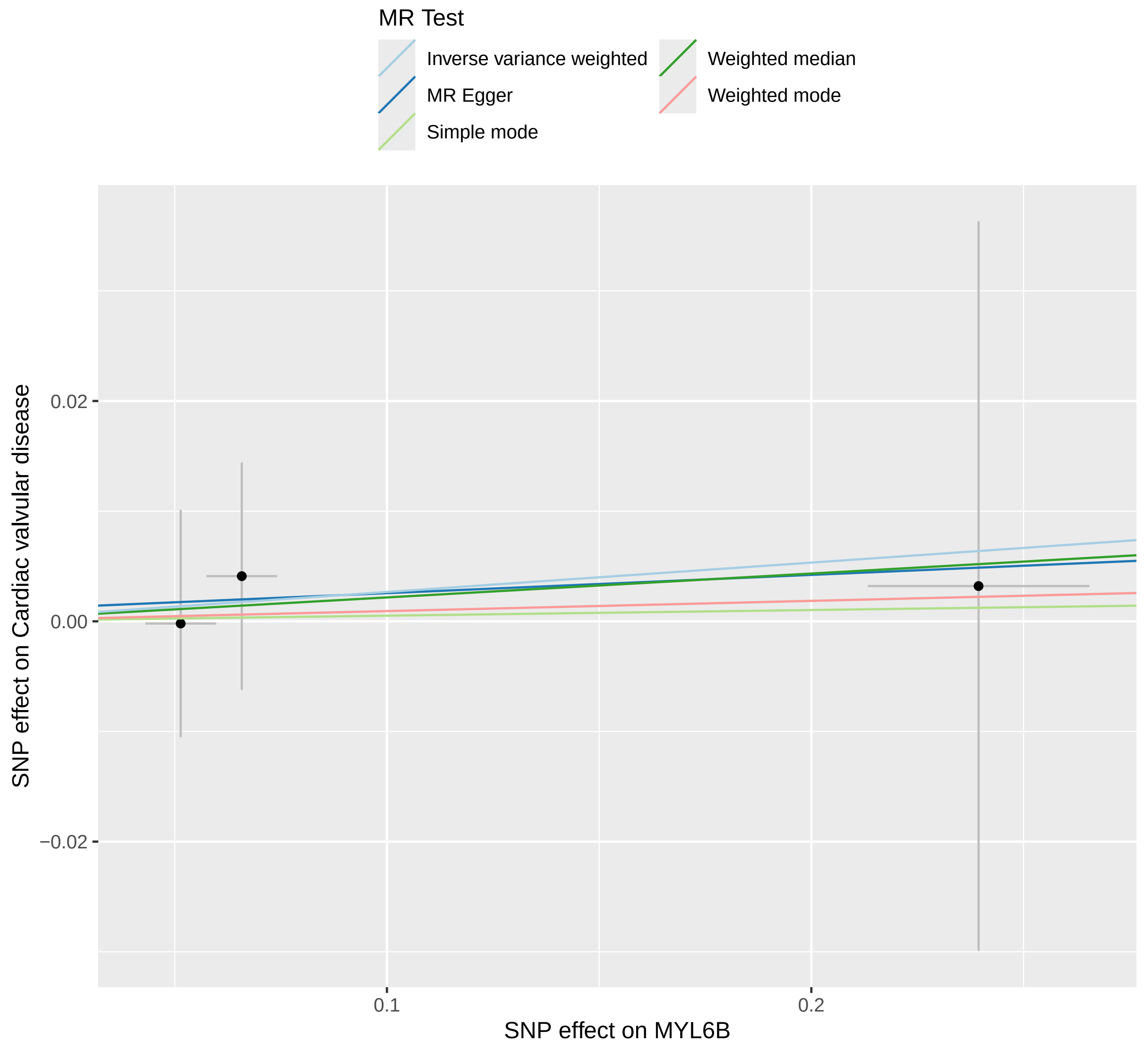
MR Test
Inverse variance weighted
MR Egger
Simple mode
Weighted median
Weighted mode
0.02
0.00
−0.02
0.1
0.2
SNP effect on Cardiac valvular disease
SNP effect on MYL6B

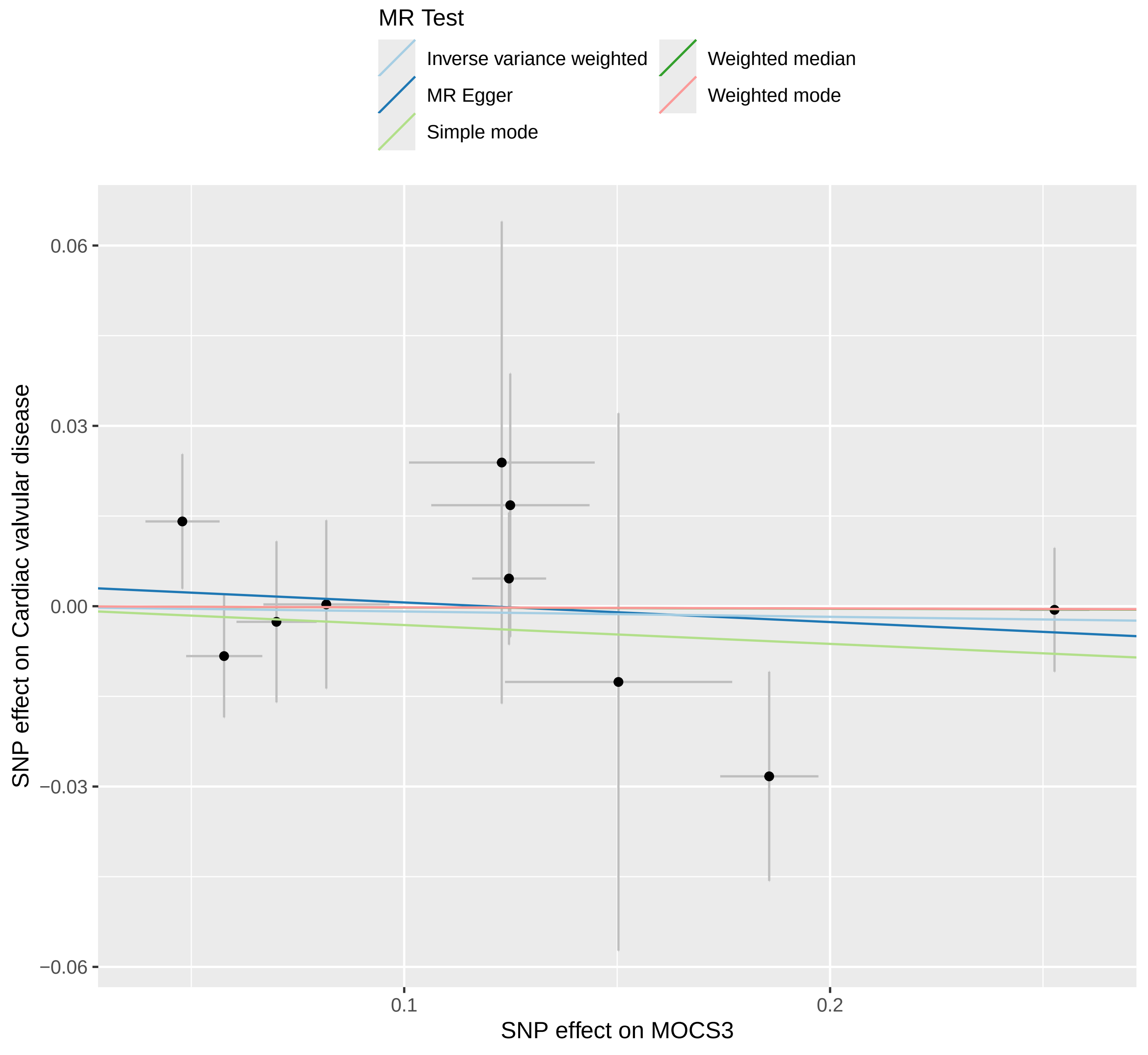
MR Test
Inverse variance weighted
MR Egger
Simple mode
Weighted median
Weighted mode
0.06
0.03
0.00
−0.03
−0.06
SNP effect on Cardiac valvular disease
0.1
0.2
SNP effect on MOCS3

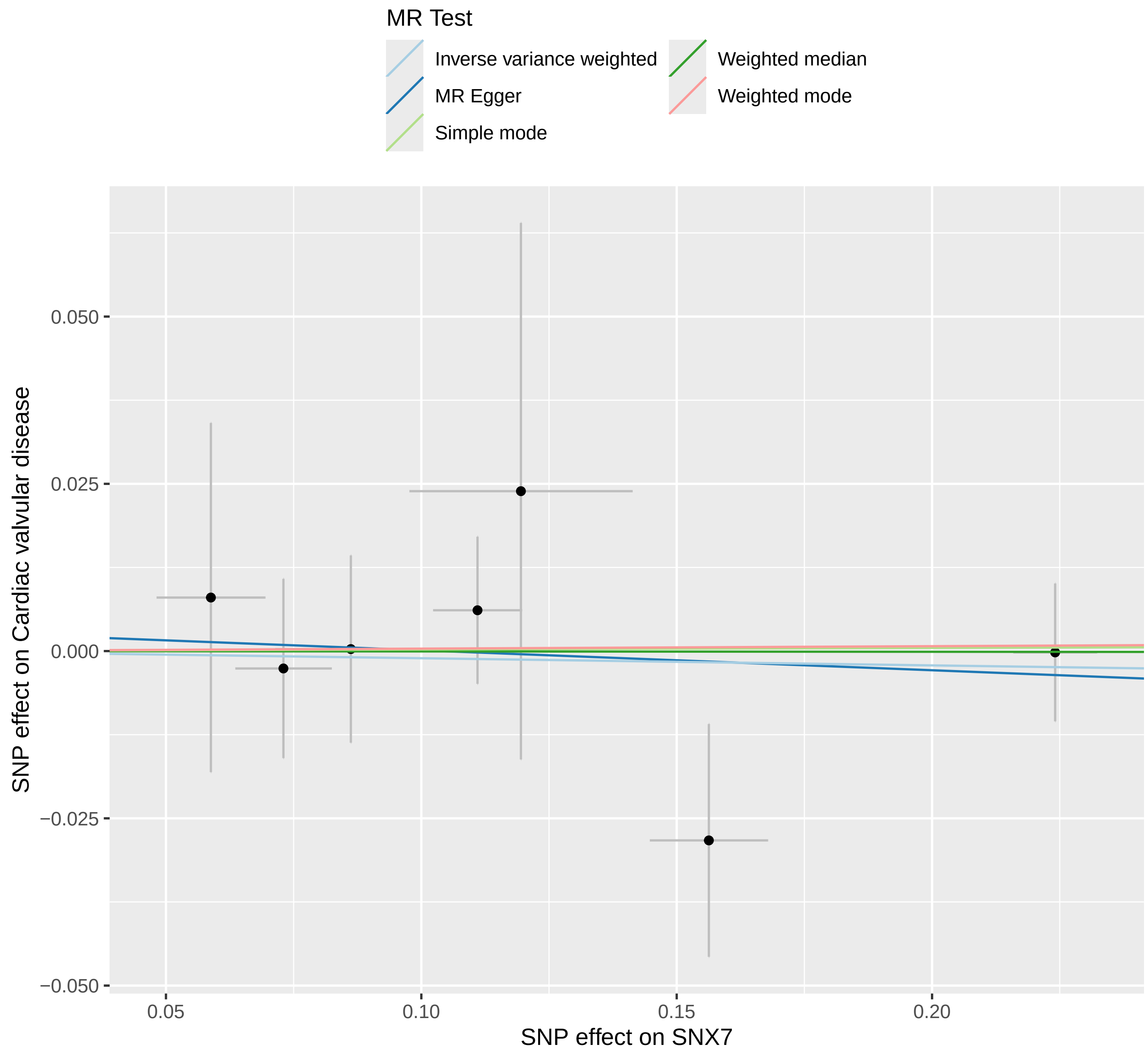
MR Test
Inverse variance weighted
MR Egger
Simple mode
Weighted median
Weighted mode
SNP effect on Cardiac valvular disease
0.050
0.025
0.000
−0.025
−0.050
0.05
0.10
0.15
0.20
SNP effect on SNX7

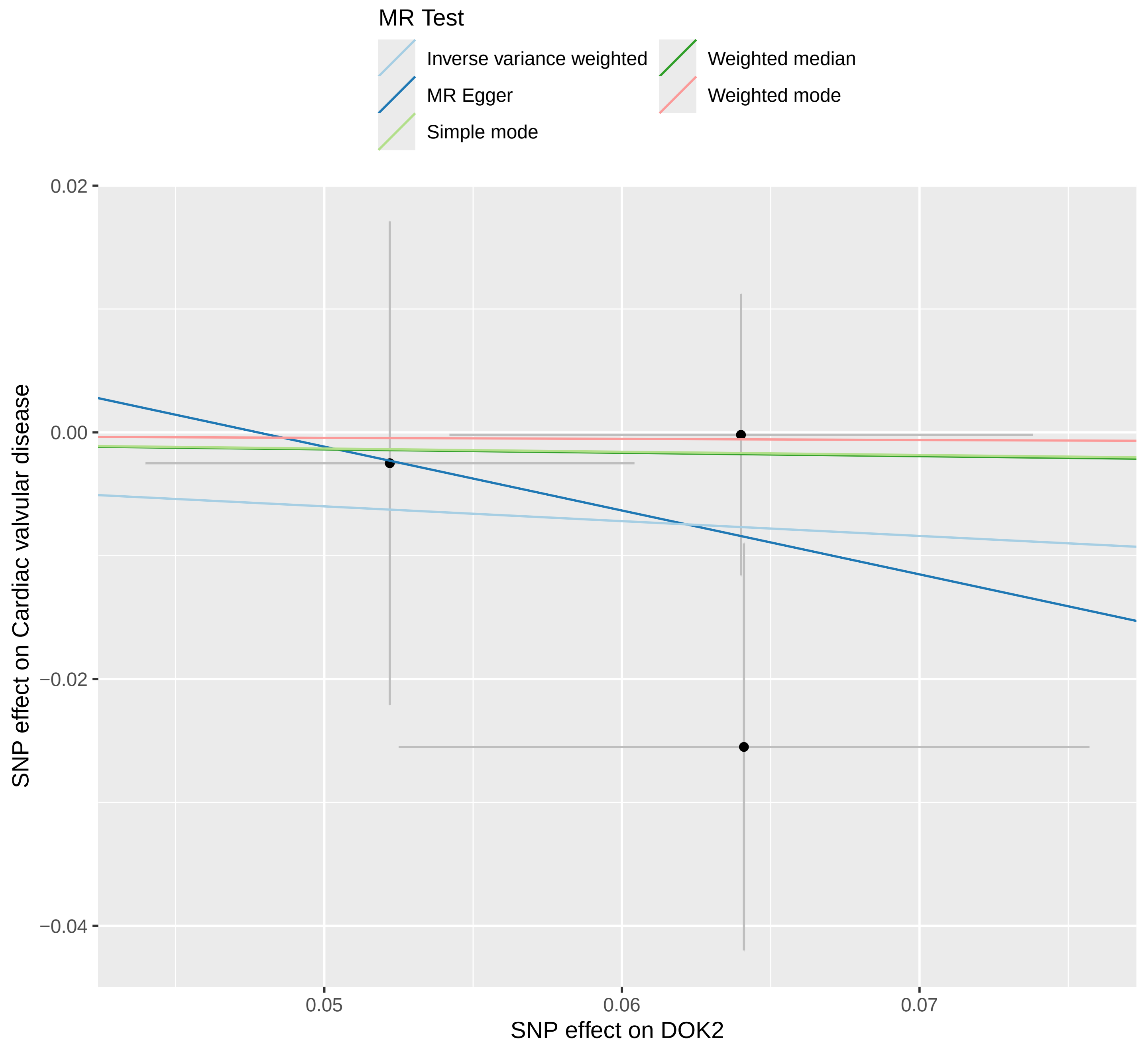

MR Test
Inverse variance weighted
MR Egger
Simple mode
Weighted median
Weighted mode
0.02
0.00
−0.02
−0.04
0.05
0.06
0.07
SNP effect on Cardiac valvular disease
SNP effect on DOK2

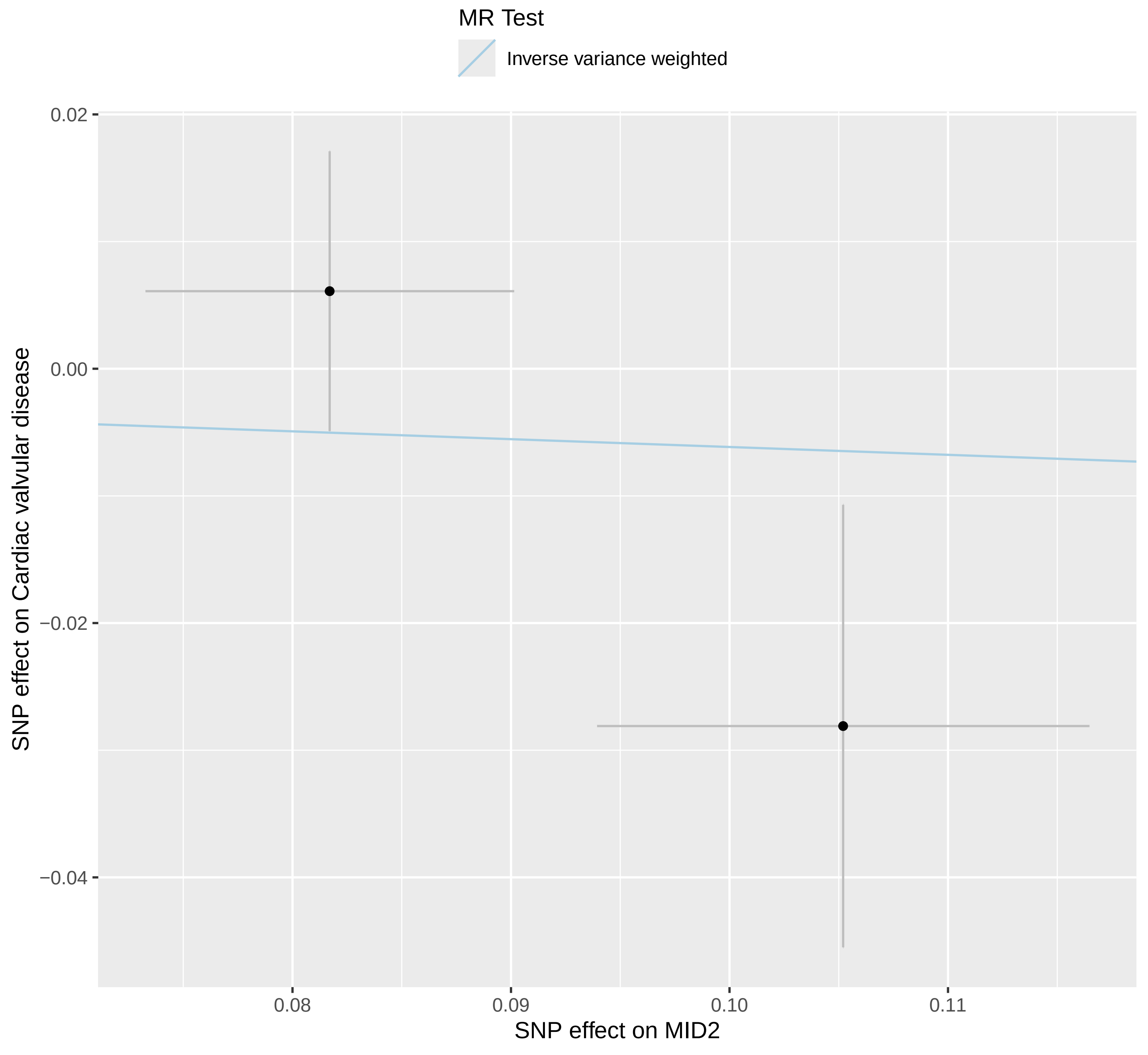
MR Test
Inverse variance weighted
0.02
0.00
−0.02
−0.04
SNP effect on Cardiac valvular disease
0.08
0.09
0.10
0.11
SNP effect on MID2

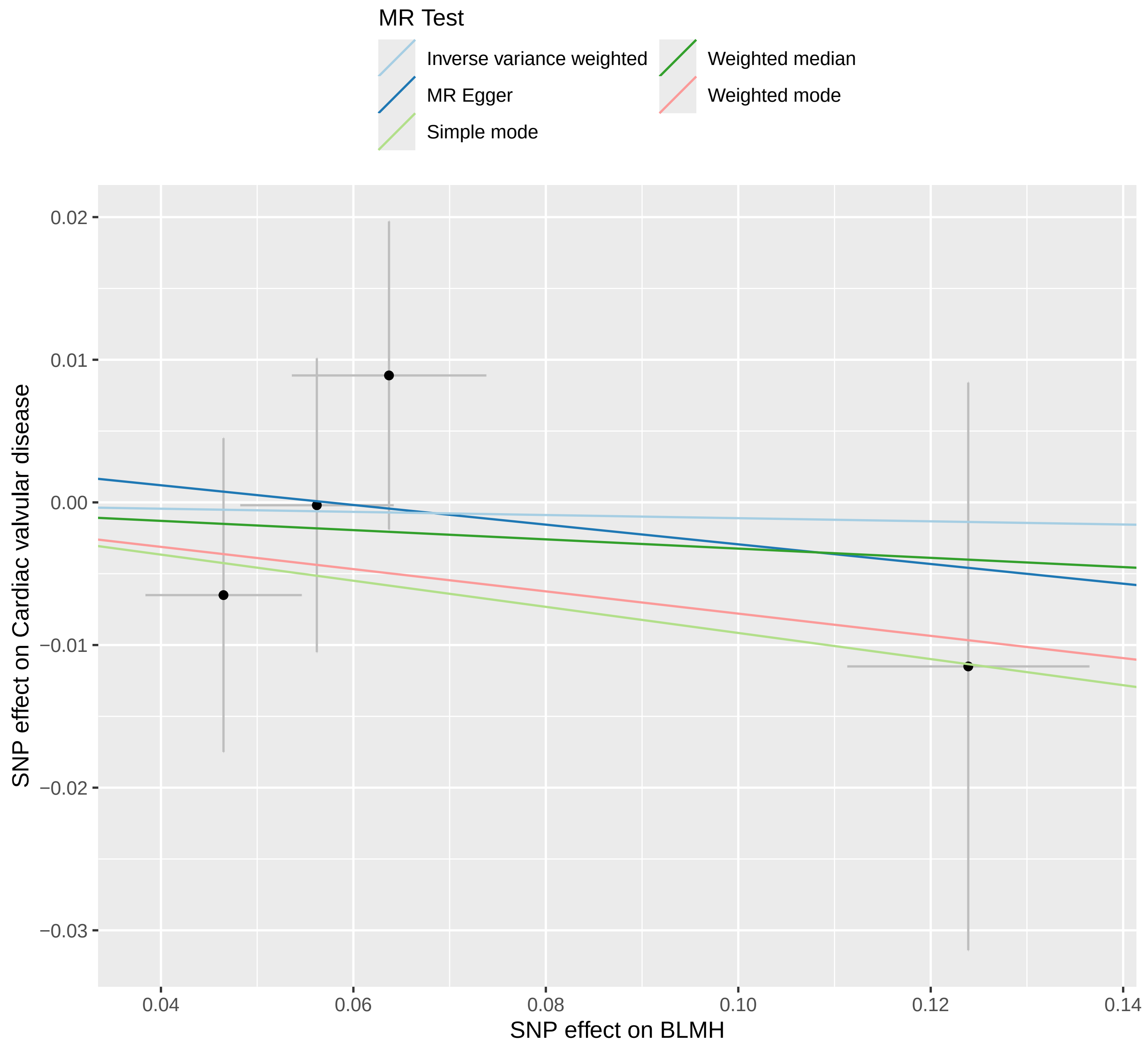
MR Test
Inverse variance weighted
MR Egger
Simple mode
Weighted median
Weighted mode
0.02
0.01
0.00
−0.01
−0.02
−0.03
SNP effect on Cardiac valvular disease
0.04
0.06
0.08
0.10
0.12
0.14
SNP effect on BLMH

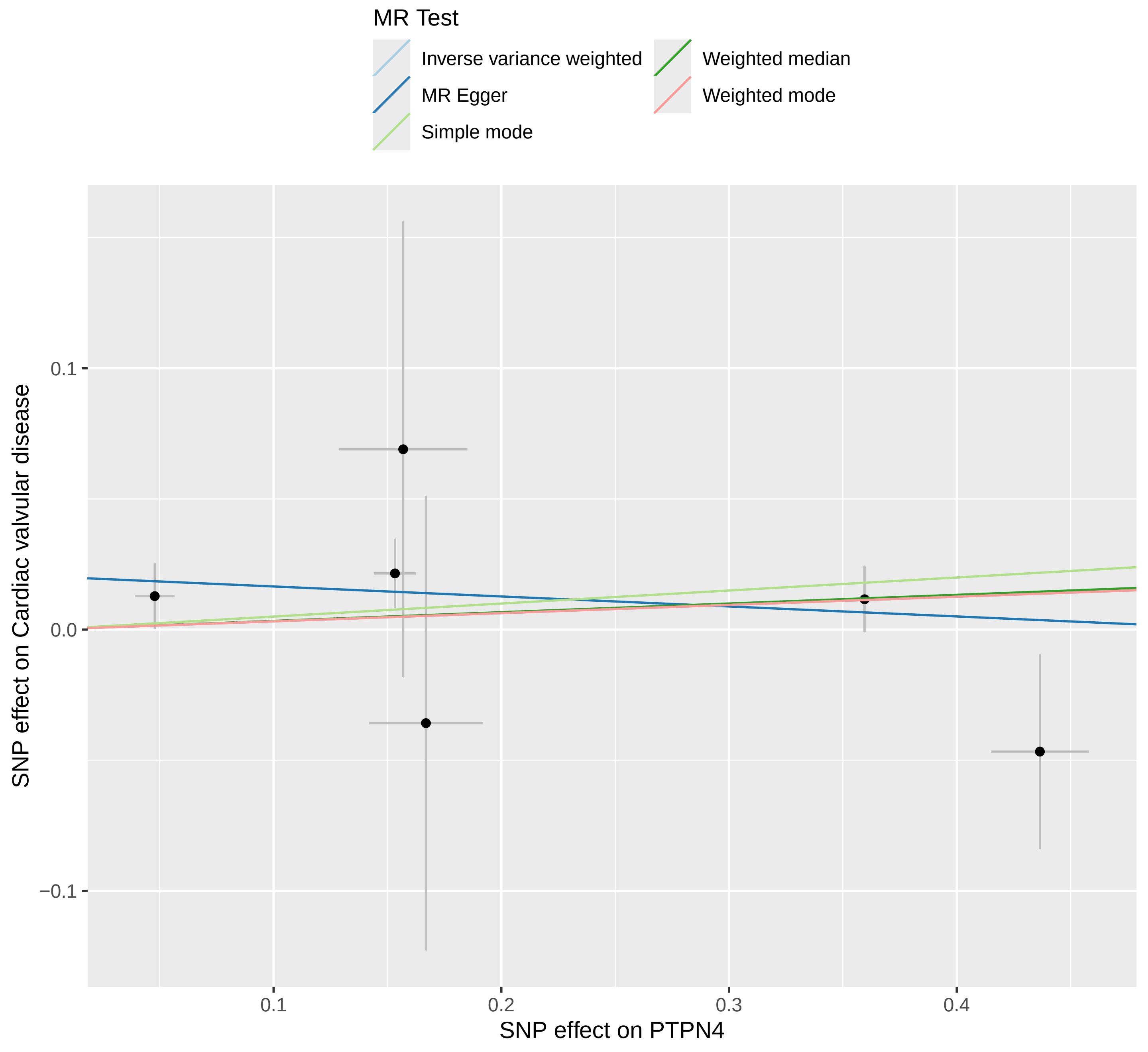
MR Test
Inverse variance weighted
MR Egger
Simple mode
Weighted median
Weighted mode
SNP effect on Cardiac valvular disease
0.1
0.0
−0.1
0.1
0.2
0.3
0.4
SNP effect on PTPN4

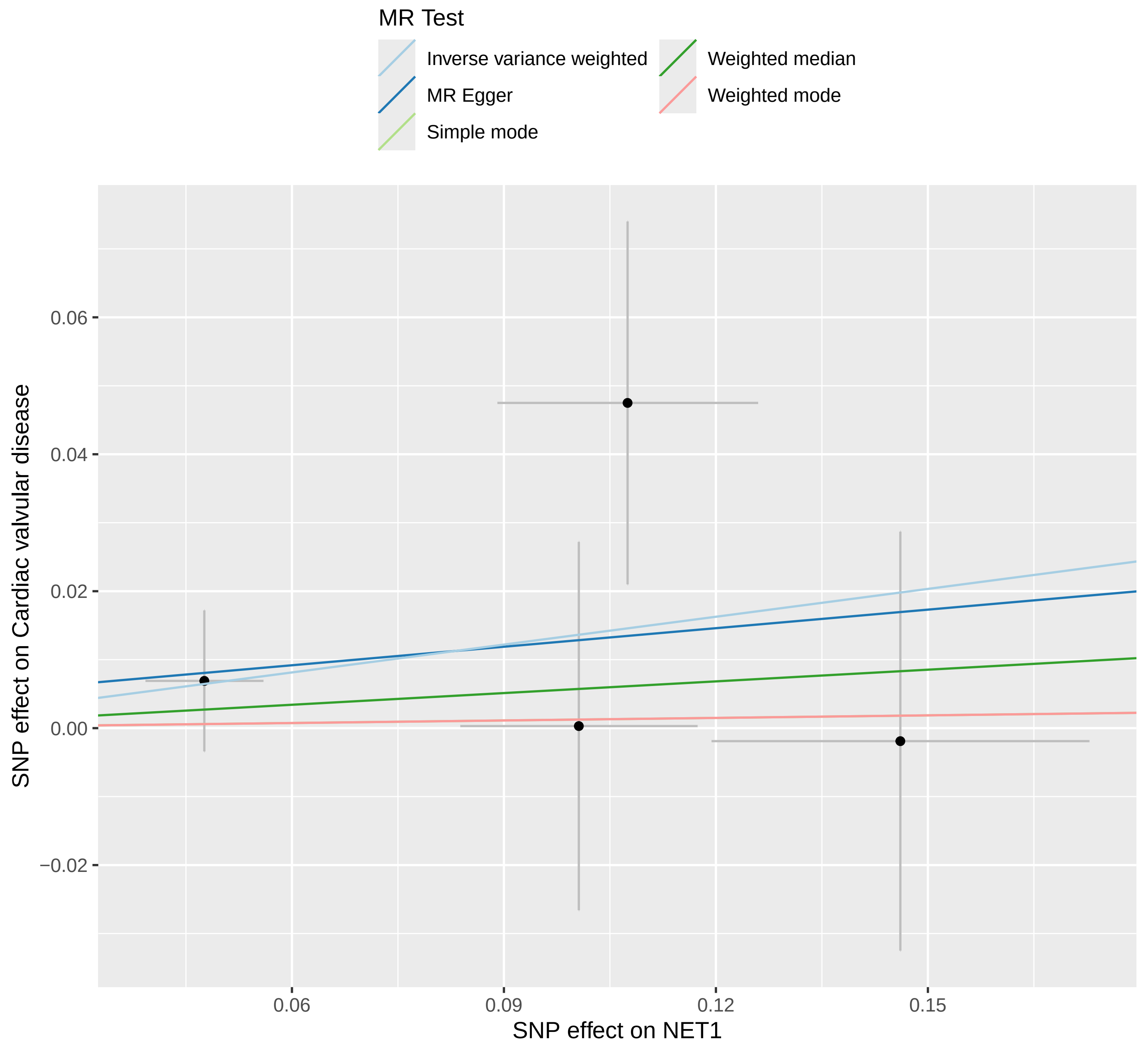
MR Test
Inverse variance weighted
MR Egger
Simple mode
Weighted median
Weighted mode
0.06
0.04
0.02
0.00
−0.02
0.06
0.09
0.12
0.15
SNP effect on Cardiac valvular disease
SNP effect on NET1

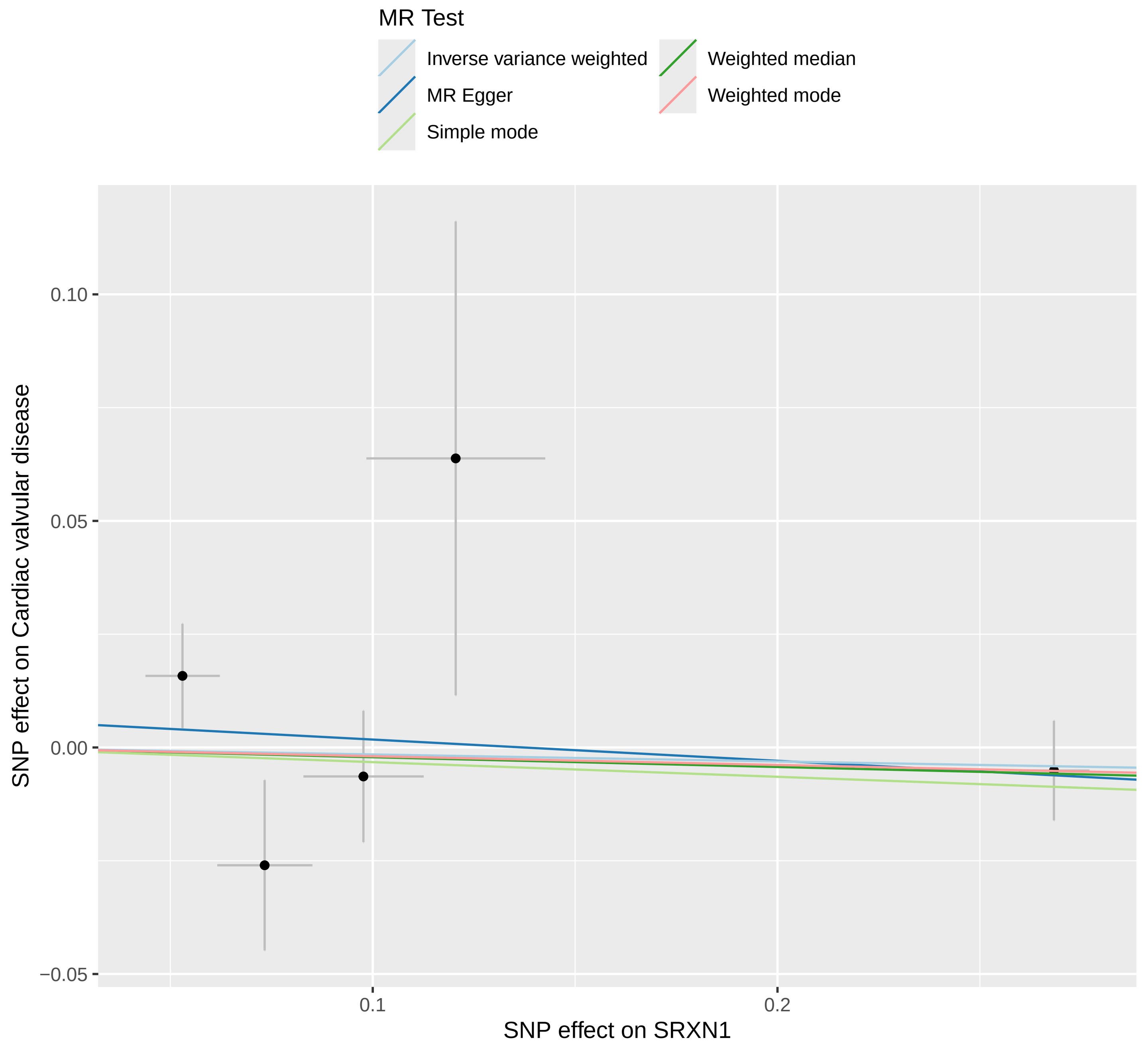

MR Test
Inverse variance weighted
MR Egger
Simple mode
Weighted median
Weighted mode
0.10
0.05
0.00
−0.05
0.1
0.2
SNP effect on Cardiac valvular disease
SNP effect on SRXN1

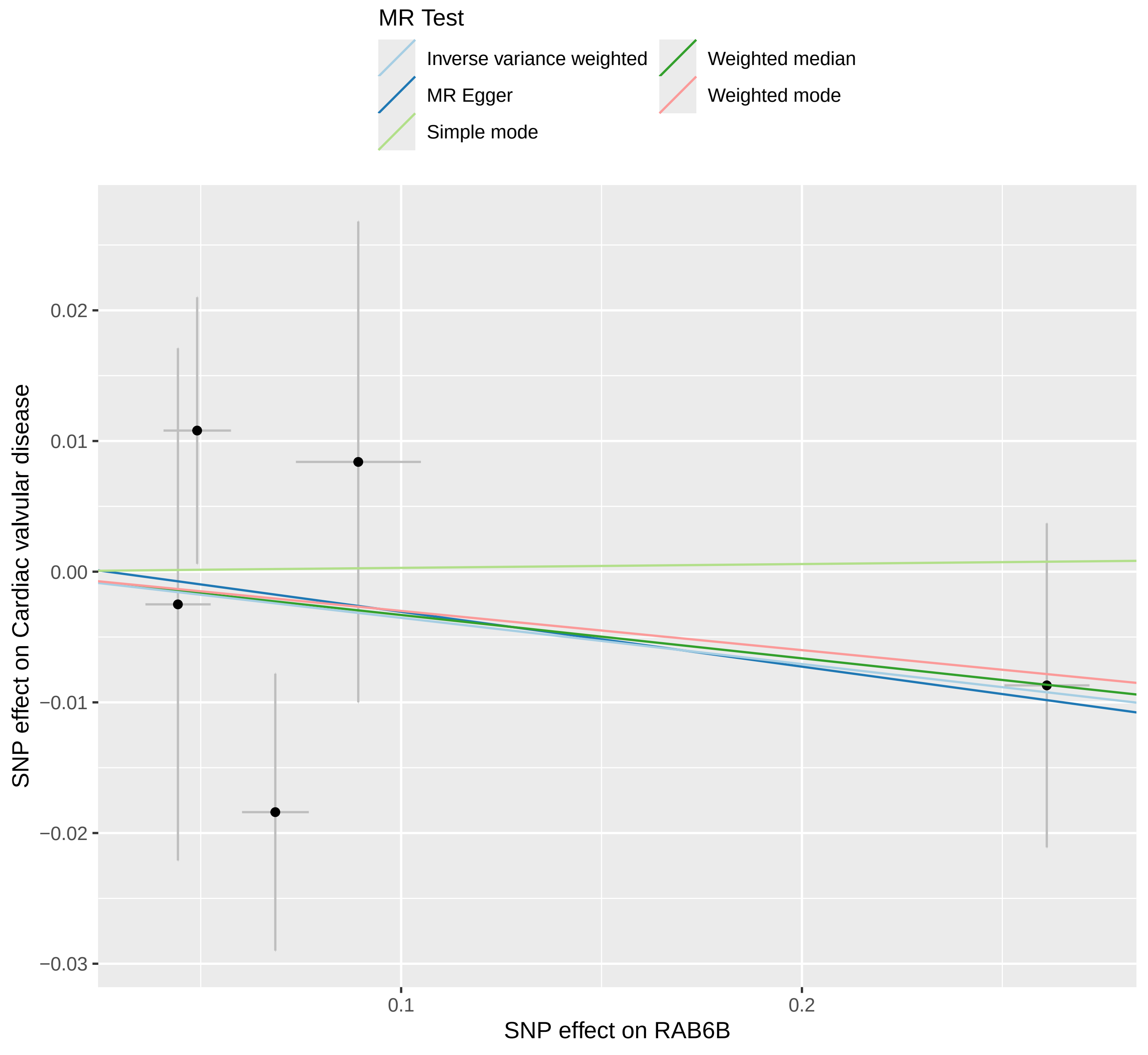
MR Test
Inverse variance weighted
MR Egger
Simple mode
Weighted median
Weighted mode
0.02
0.01
0.00
−0.01
−0.02
−0.03
SNP effect on Cardiac valvular disease
0.1
0.2
SNP effect on RAB6B

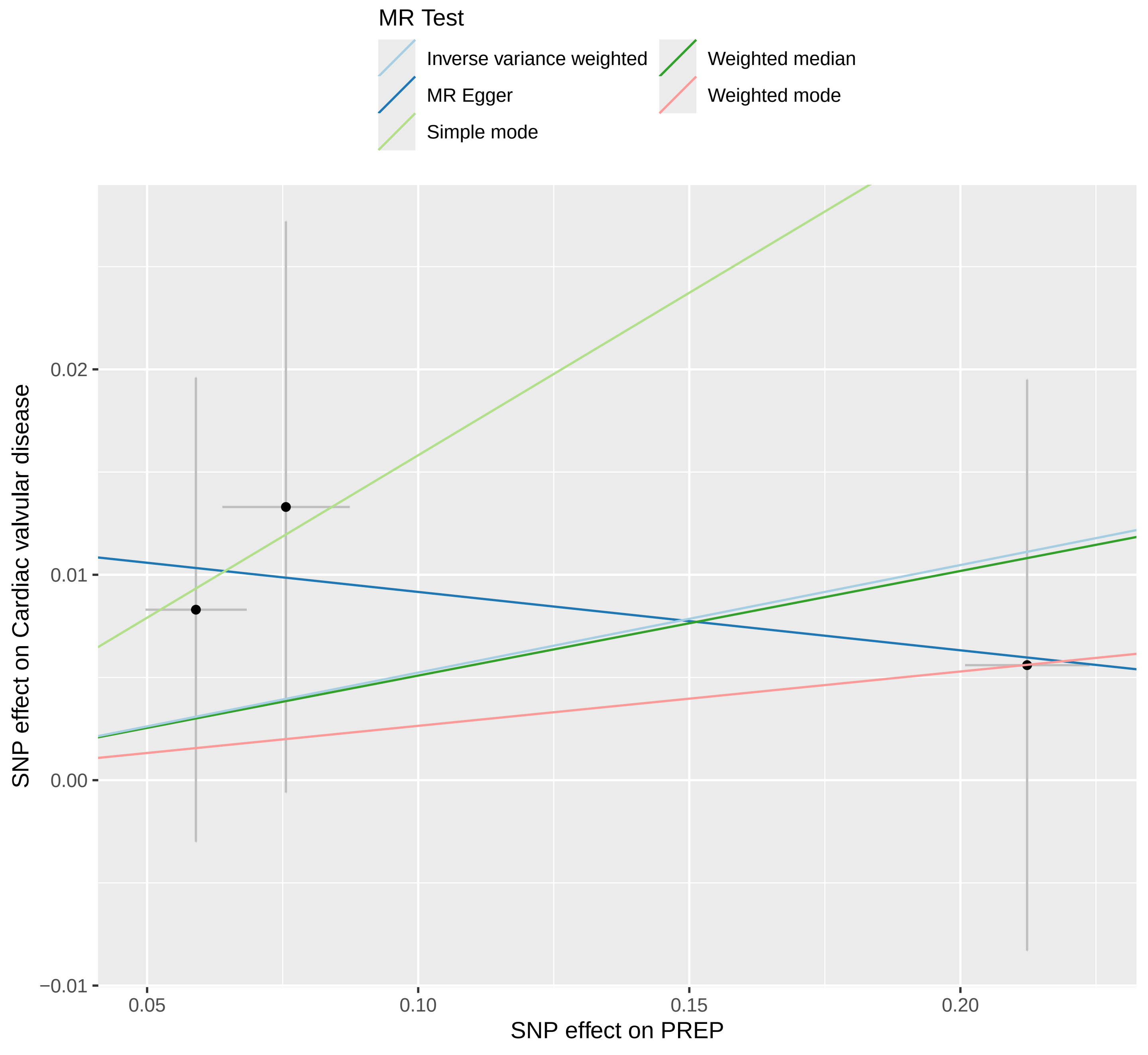
MR Test
Inverse variance weighted
MR Egger
Simple mode
Weighted median
Weighted mode
0.02
0.01
0.00
−0.01
0.05
0.10
0.15
0.20
SNP effect on Cardiac valvular disease
SNP effect on PREP

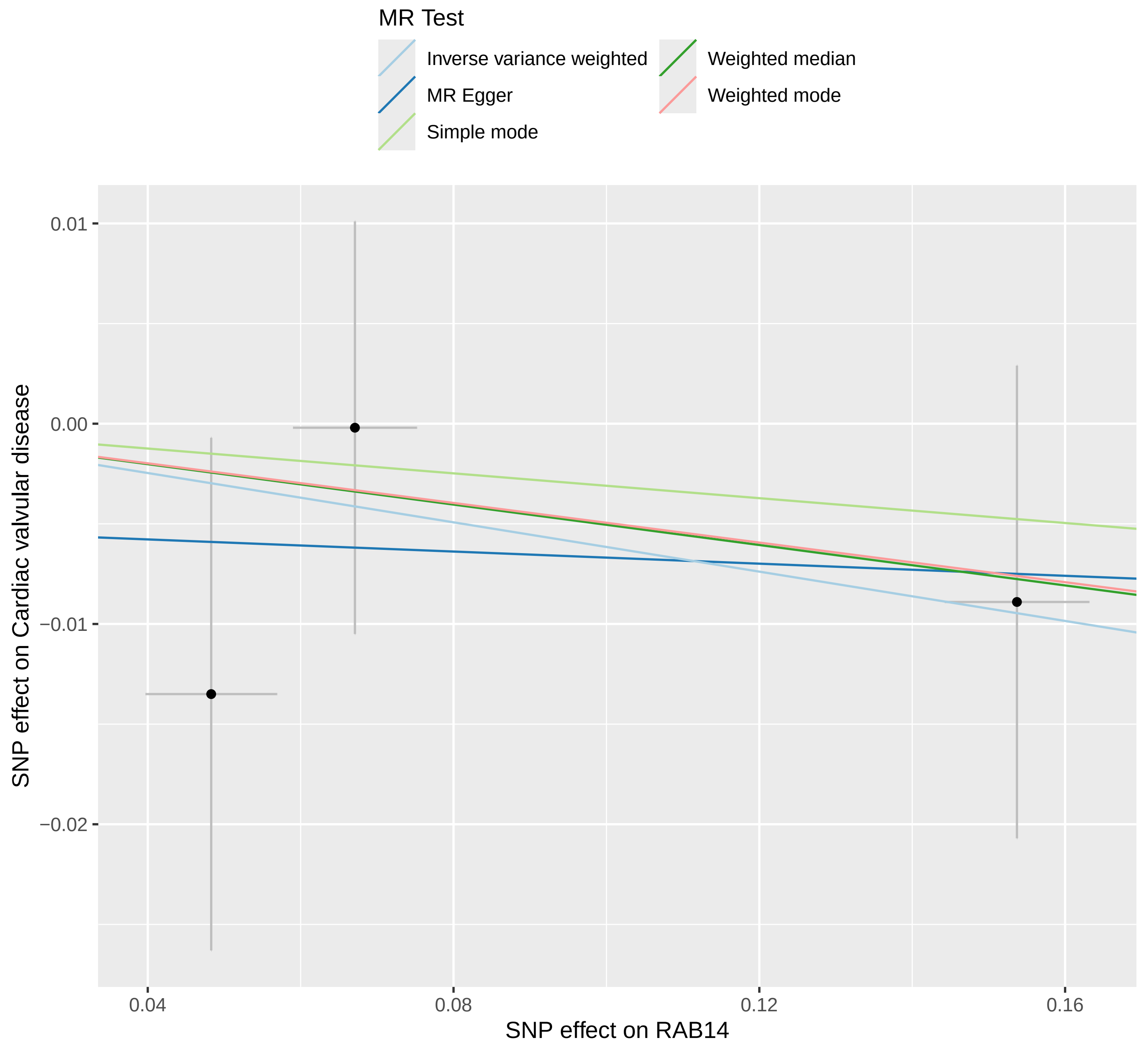
MR Test
Inverse variance weighted
MR Egger
Simple mode
Weighted median
Weighted mode
0.01
0.00
−0.01
−0.02
SNP effect on Cardiac valvular disease
0.04
0.08
0.12
0.16
SNP effect on RAB14

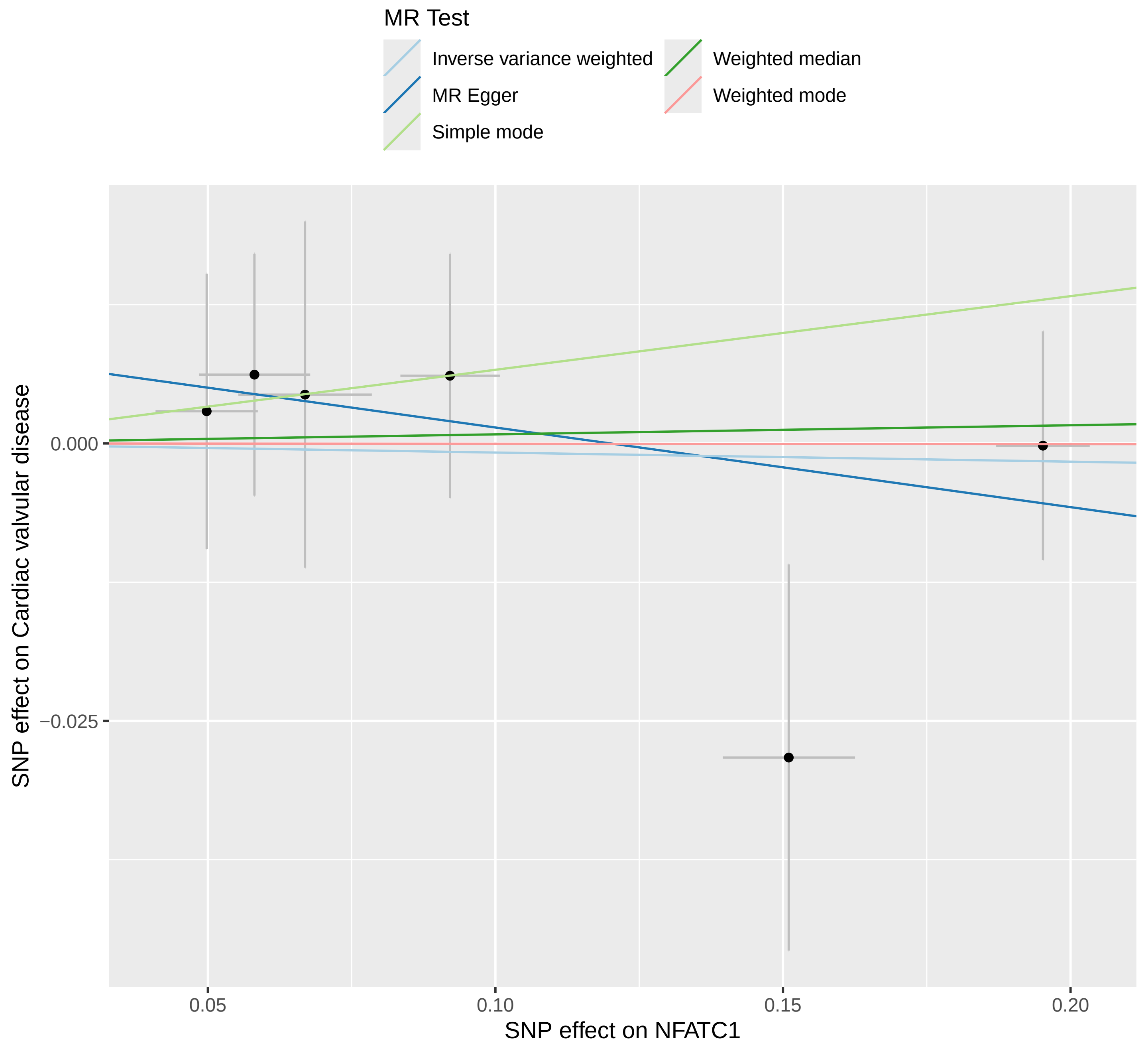
MR Test
Inverse variance weighted
MR Egger
Simple mode
Weighted median
Weighted mode
0.000
−0.025
0.05
0.10
0.15
0.20
SNP effect on NFATC1
SNP effect on Cardiac valvular disease

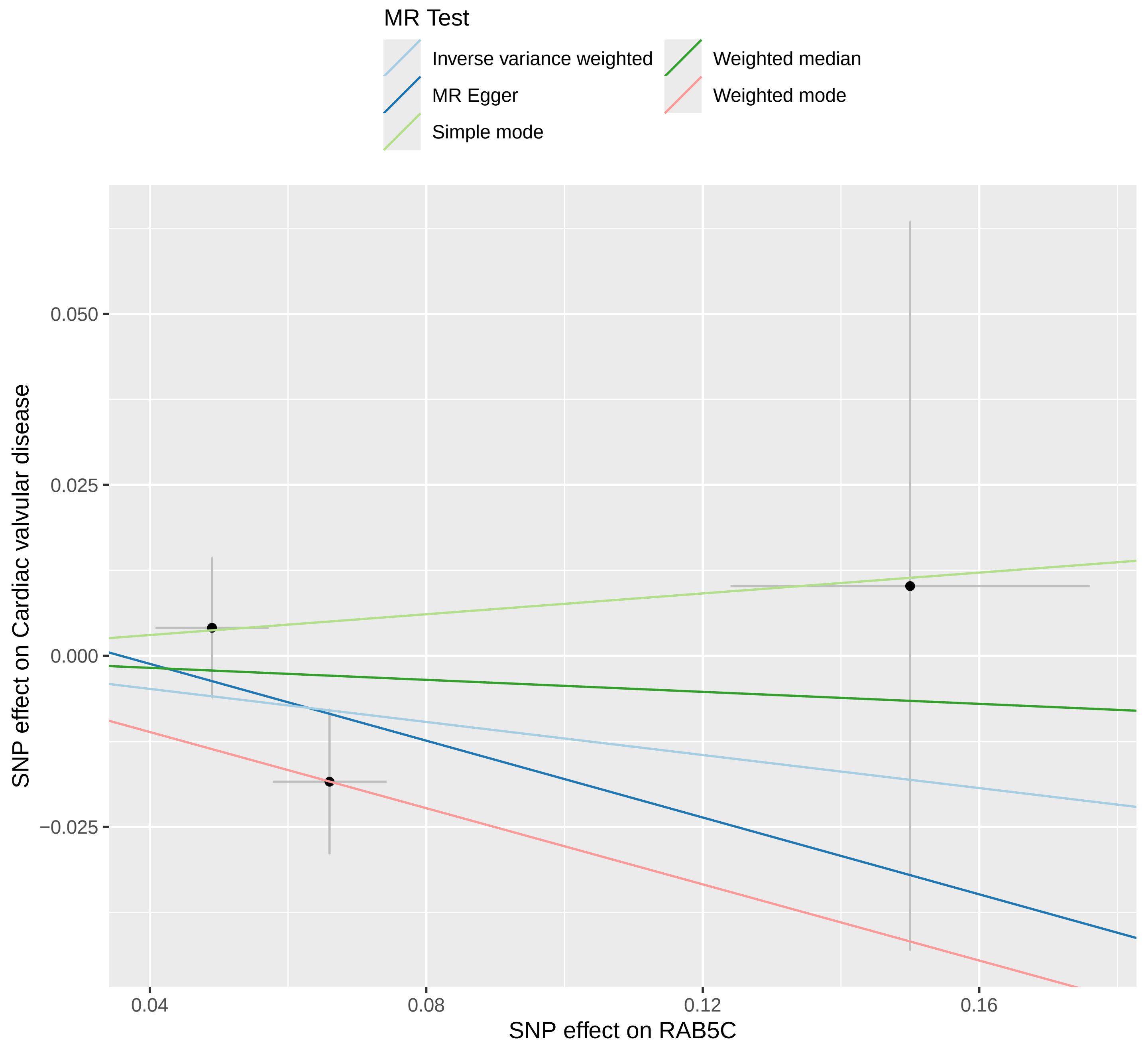

MR Test
Inverse variance weighted
MR Egger
Simple mode
Weighted median
Weighted mode
0.050
0.025
0.000
−0.025
SNP effect on Cardiac valvular disease
0.04
0.08
0.12
0.16
SNP effect on RAB5C

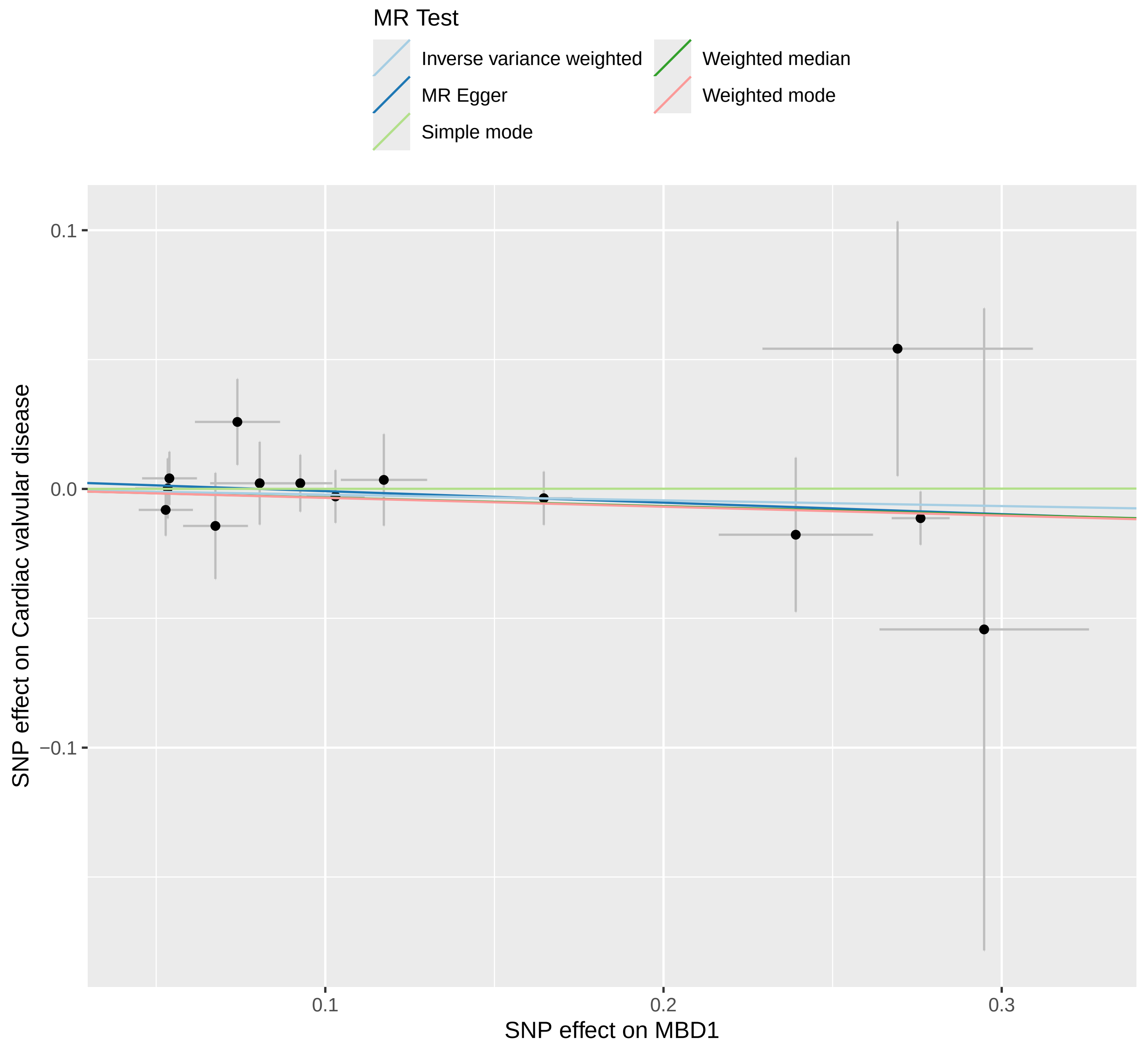
MR Test
Inverse variance weighted
MR Egger
Simple mode
Weighted median
Weighted mode
0.1
0.0
−0.1
SNP effect on Cardiac valvular disease
0.1
0.2
0.3
SNP effect on MBD1

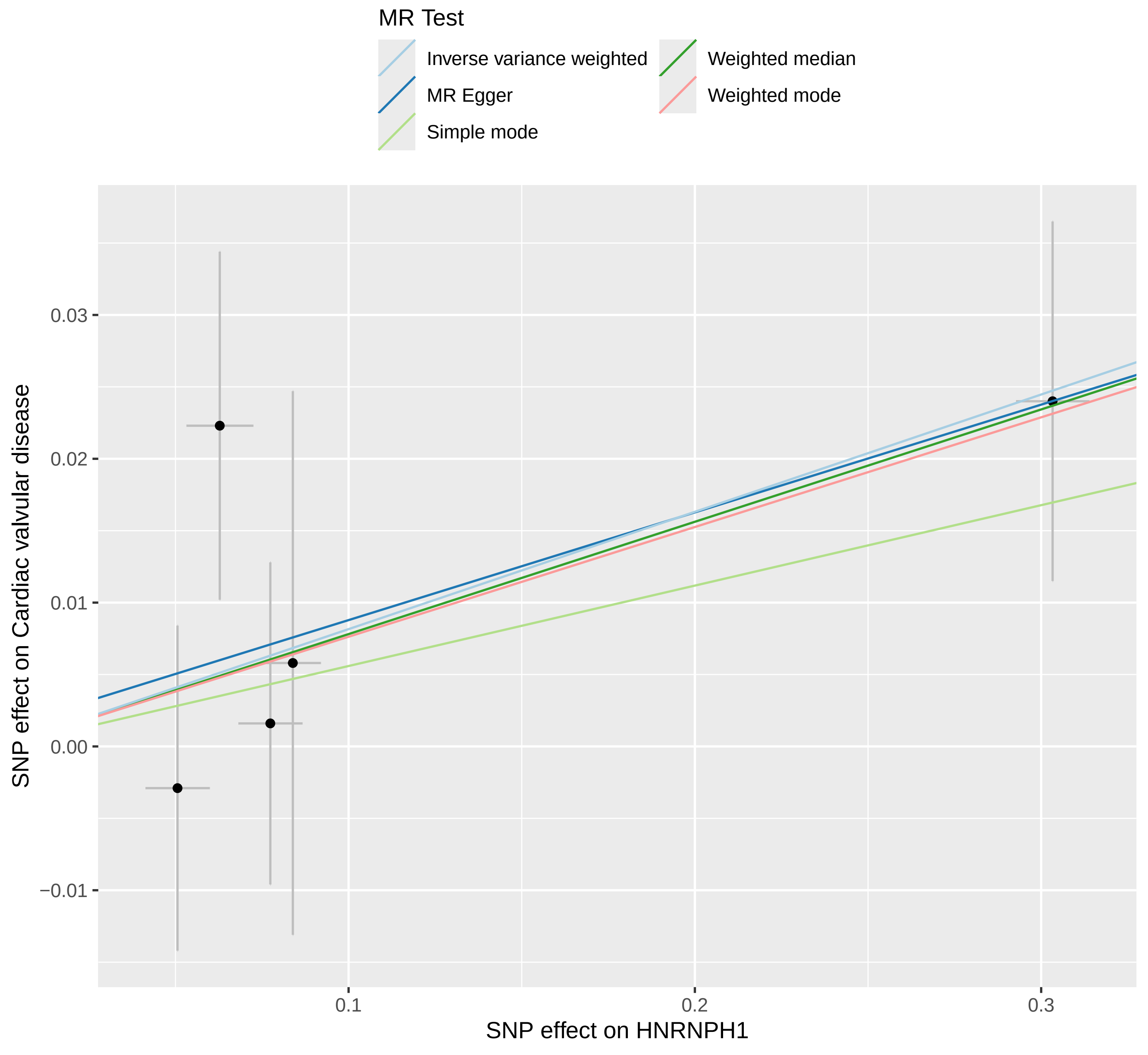
MR Test
Inverse variance weighted
MR Egger
Simple mode
Weighted median
Weighted mode
0.03
0.02
0.01
0.00
−0.01
SNP effect on Cardiac valvular disease
0.1
0.2
0.3
SNP effect on HNRNPH1

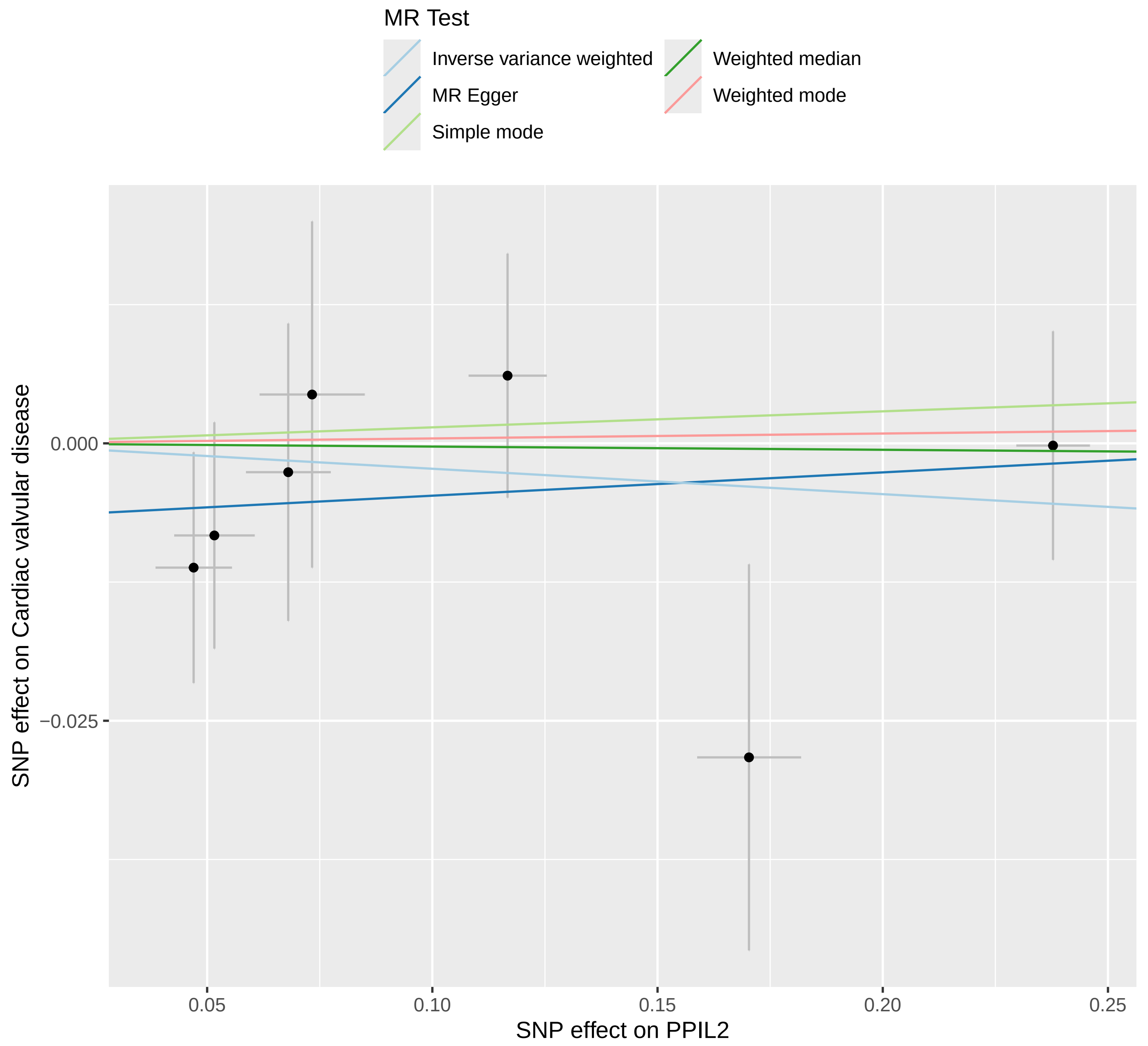

MR Test
Inverse variance weighted
MR Egger
Simple mode
Weighted median
Weighted mode
0.000
−0.025
0.05
0.10
0.15
0.20
0.25
SNP effect on PPIL2
SNP effect on Cardiac valvular disease

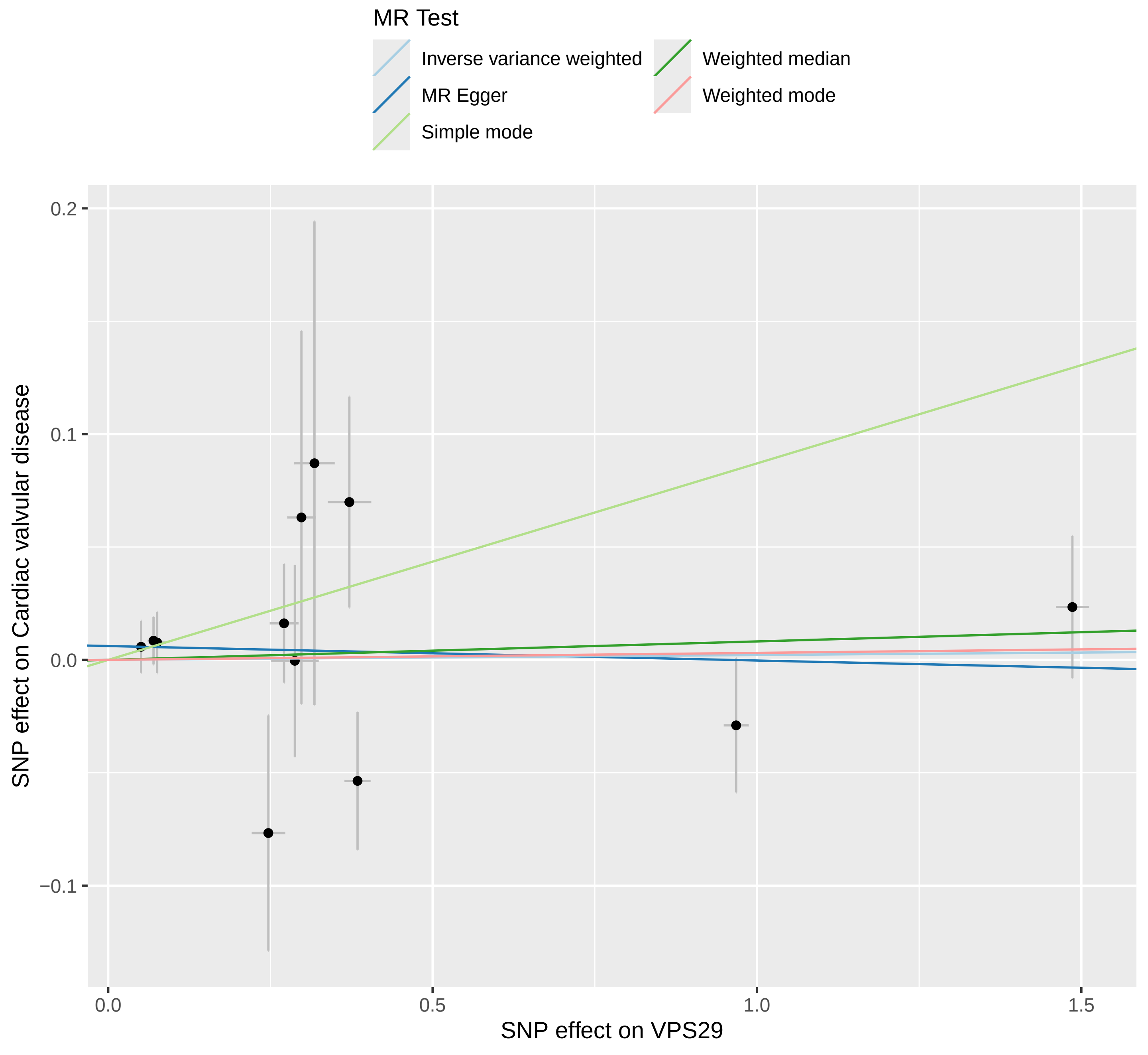
MR Test
Inverse variance weighted
MR Egger
Simple mode
Weighted median
Weighted mode
0.2
0.1
0.0
−0.1
SNP effect on Cardiac valvular disease
0.0
0.5
1.0
1.5
SNP effect on VPS29

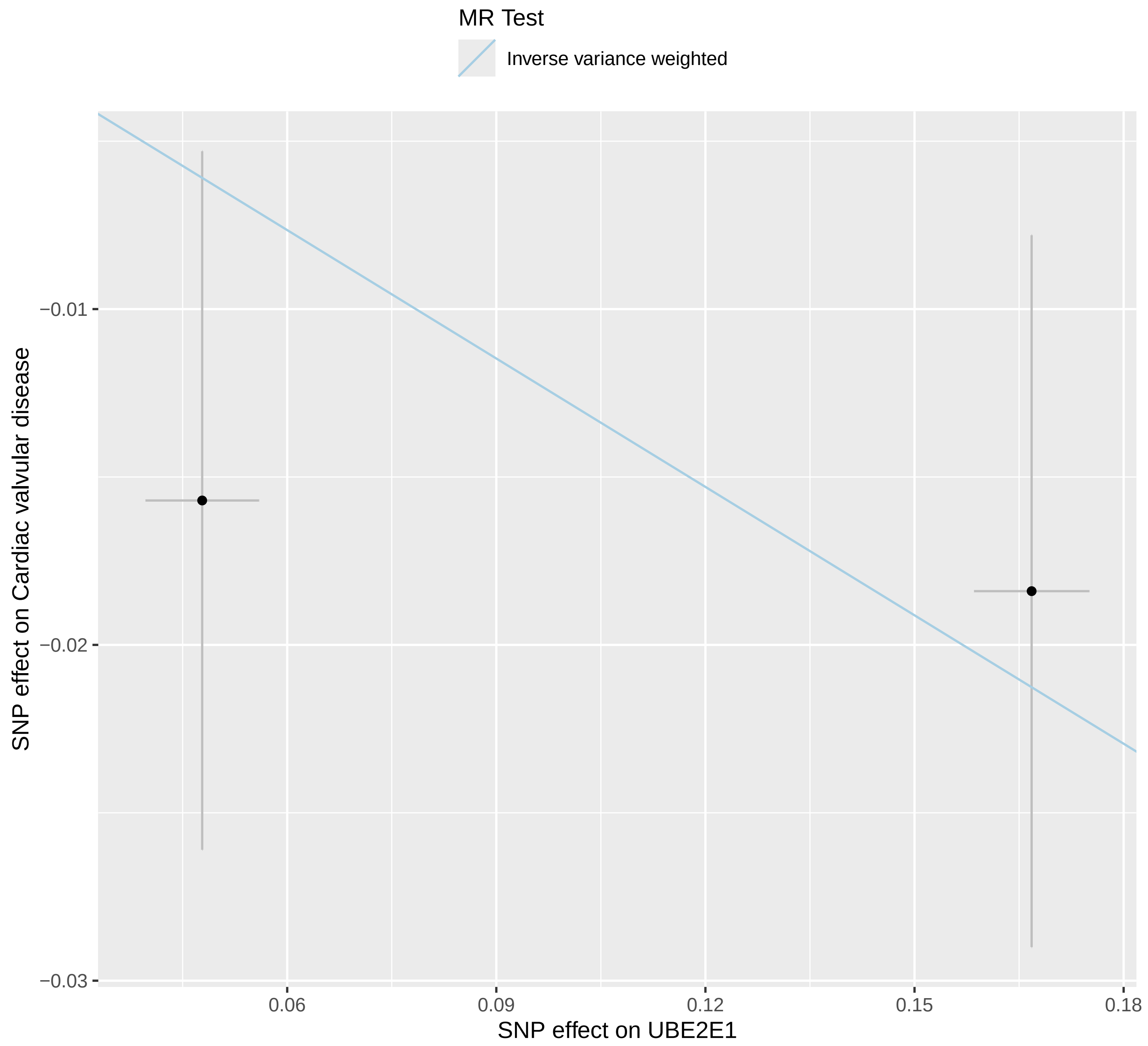

MR Test
Inverse variance weighted
SNP effect on Cardiac valvular disease
−0.01
−0.02
−0.03
0.06
0.09
0.12
0.15
0.18
SNP effect on UBE2E1

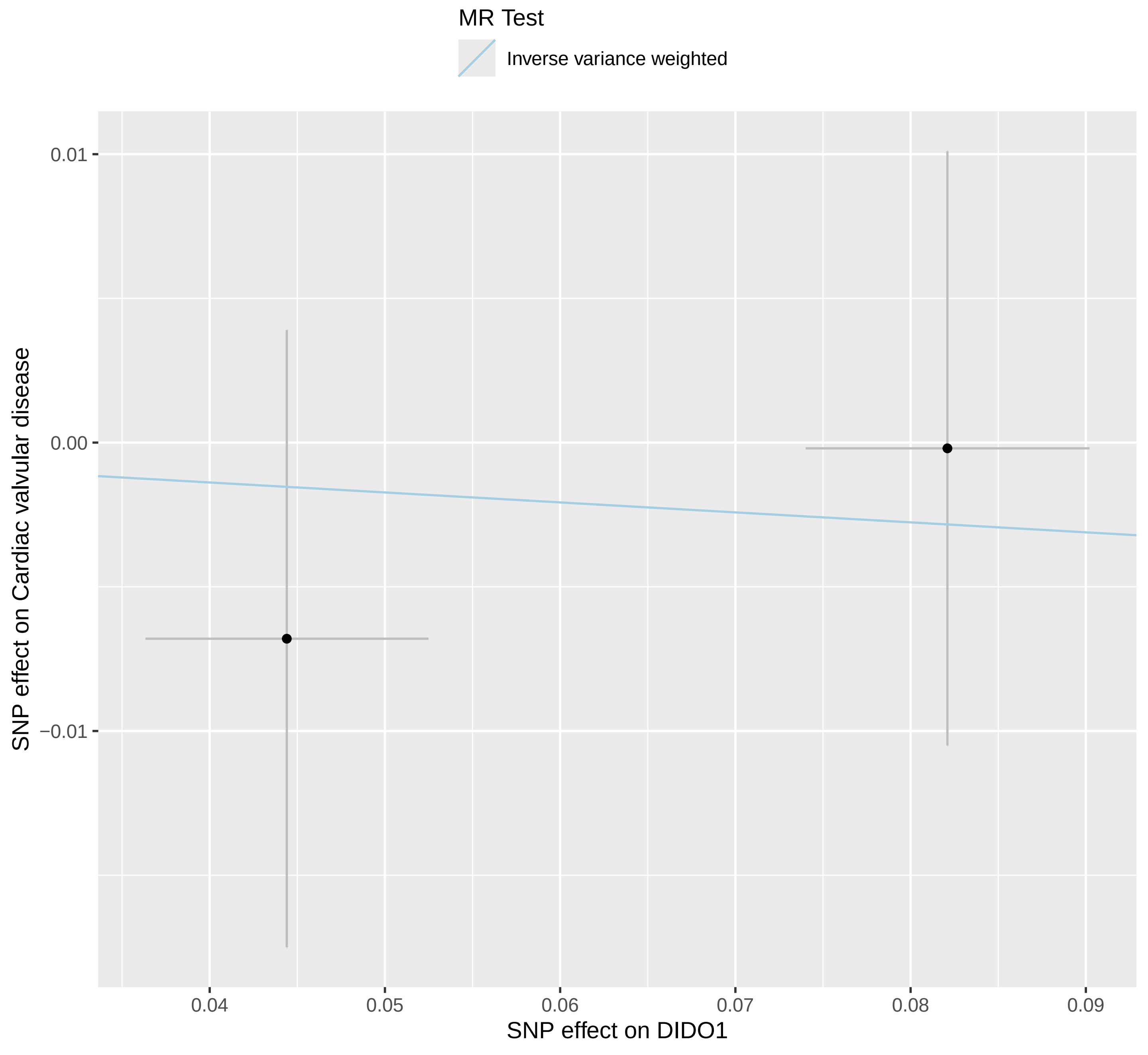
MR Test
Inverse variance weighted
0.01
0.00
−0.01
SNP effect on Cardiac valvular disease
0.04
0.05
0.06
0.07
0.08
0.09
SNP effect on DIDO1

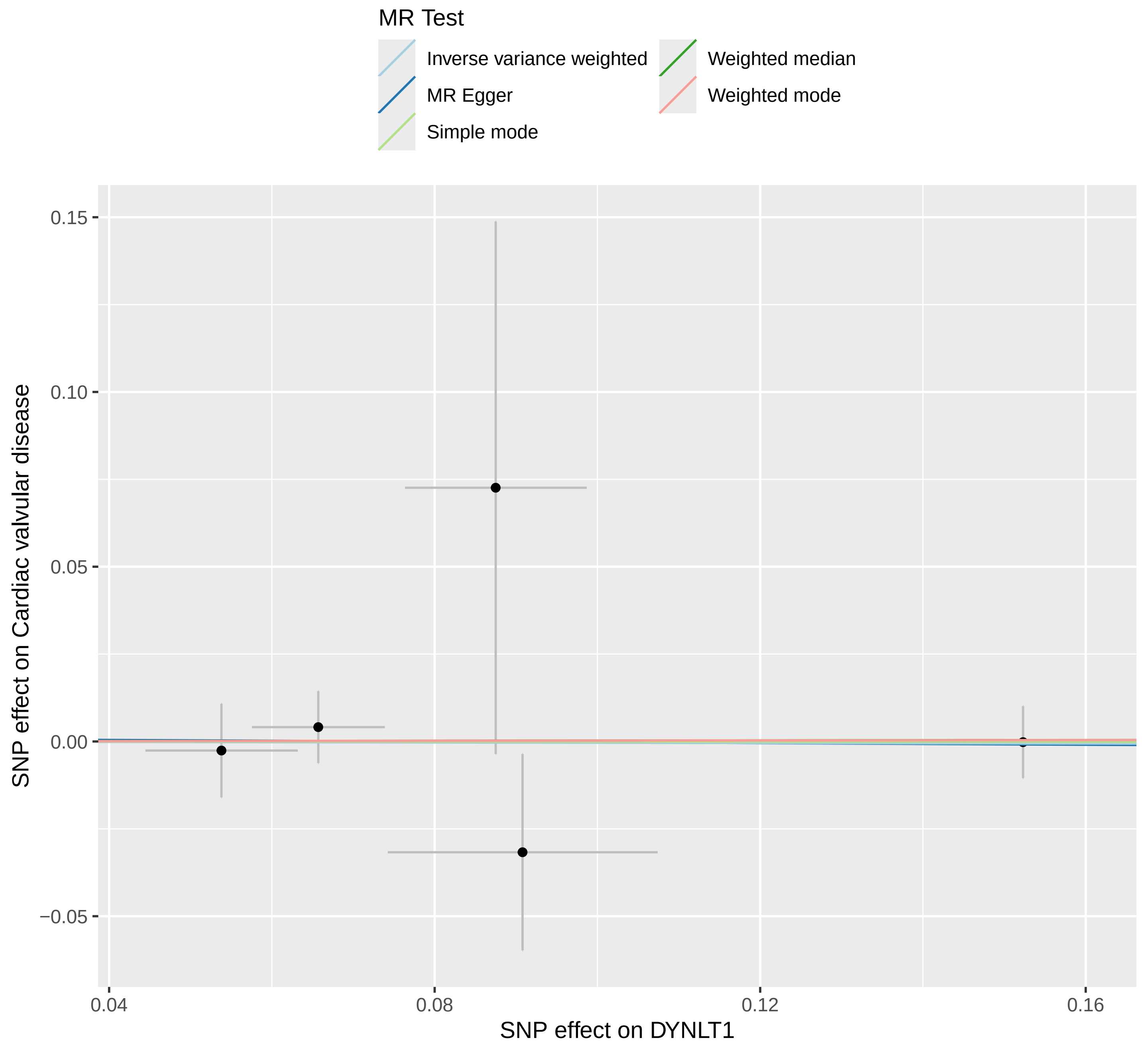

MR Test
Inverse variance weighted
MR Egger
Simple mode
Weighted median
Weighted mode
SNP effect on Cardiac valvular disease
0.15
0.10
0.05
0.00
−0.05
0.04
0.08
0.12
0.16
SNP effect on DYNLT1

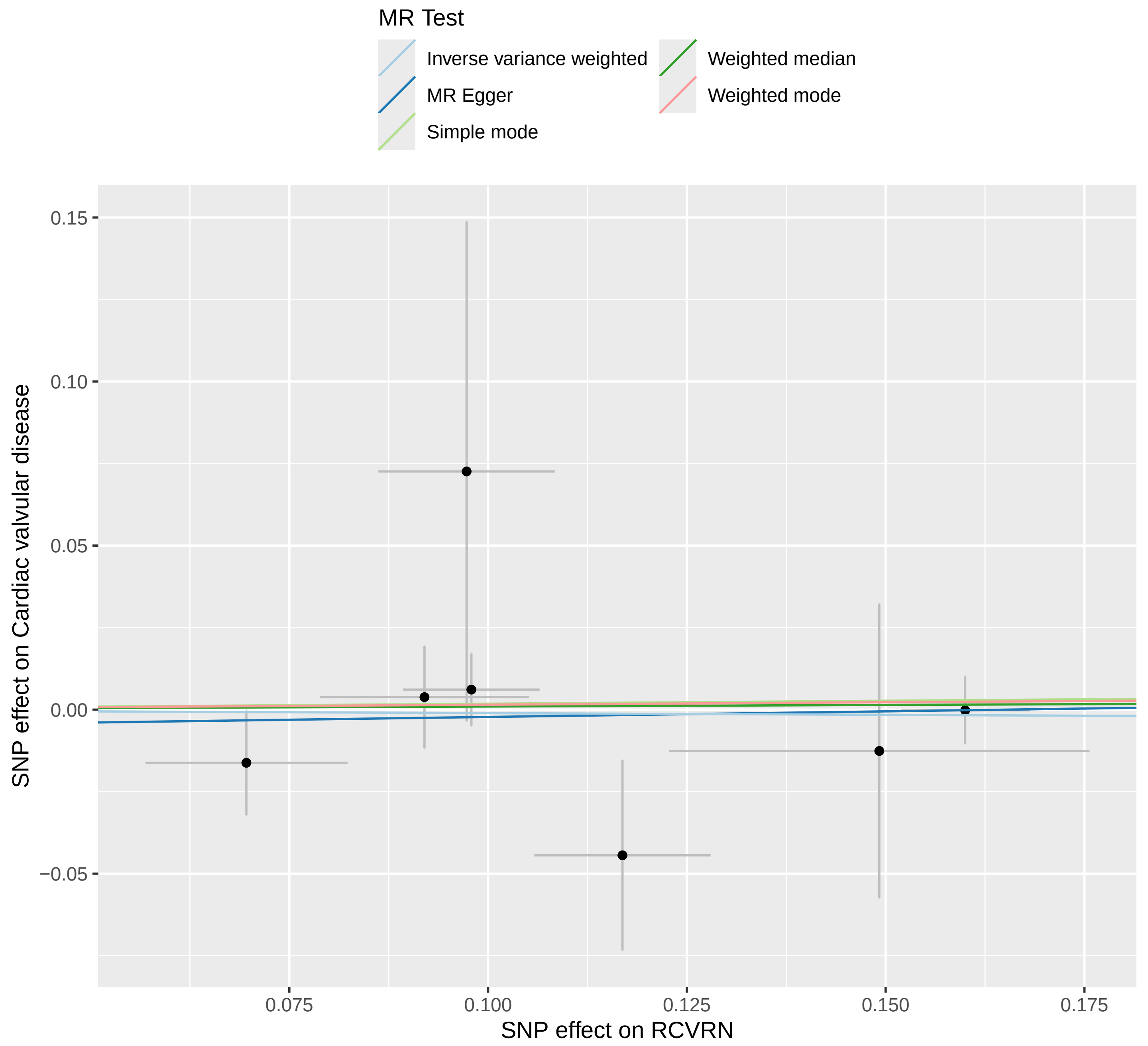

MR Test
Inverse variance weighted
MR Egger
Simple mode
Weighted median
Weighted mode
0.15
0.10
0.05
0.00
−0.05
0.075
0.100
0.125
0.150
0.175
SNP effect on Cardiac valvular disease
SNP effect on RCVRN

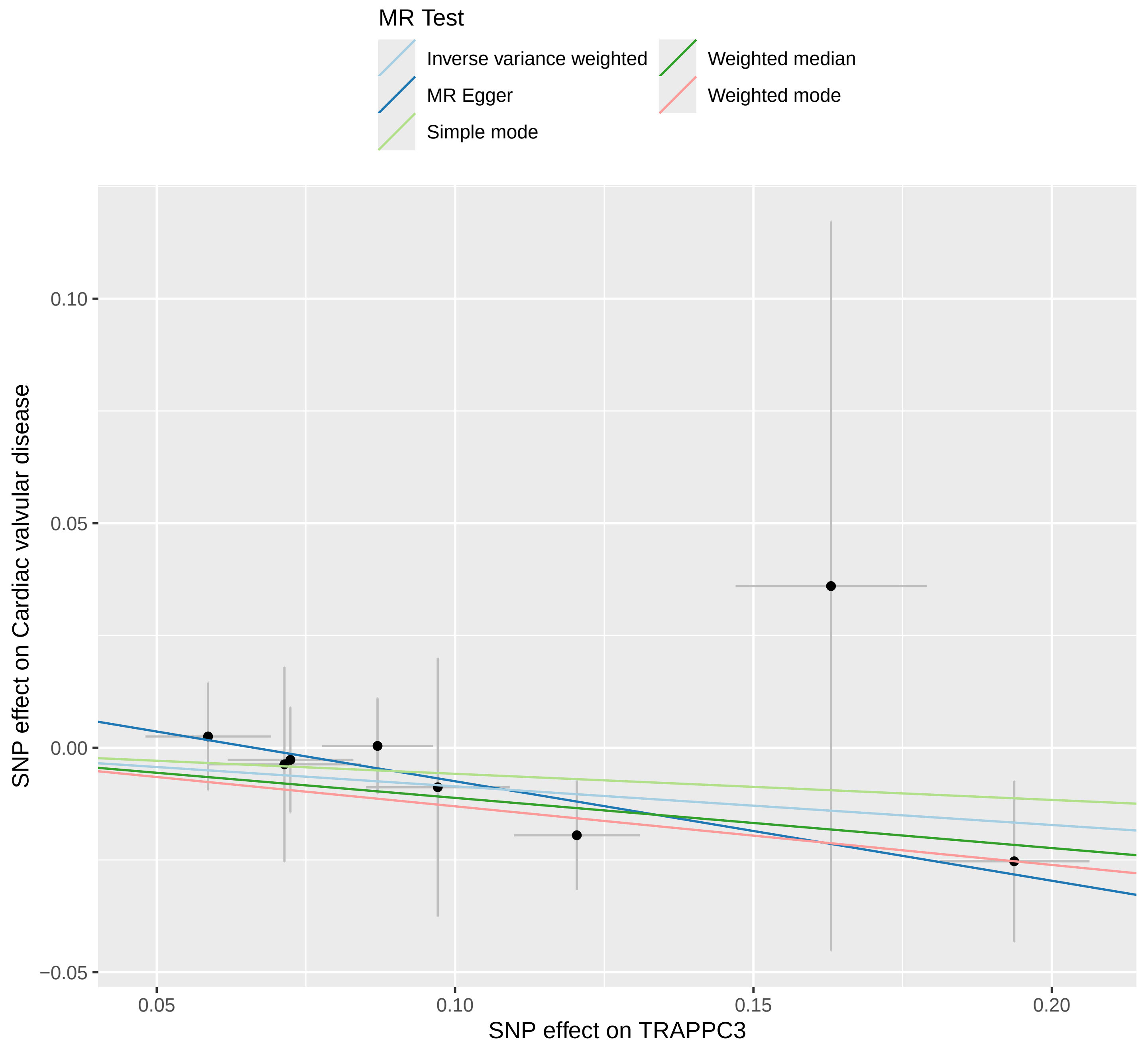
MR Test
Inverse variance weighted
MR Egger
Simple mode
Weighted median
Weighted mode
SNP effect on Cardiac valvular disease
SNP effect on TRAPPC3
0.10
0.05
0.00
−0.05
0.05
0.10
0.15
0.20

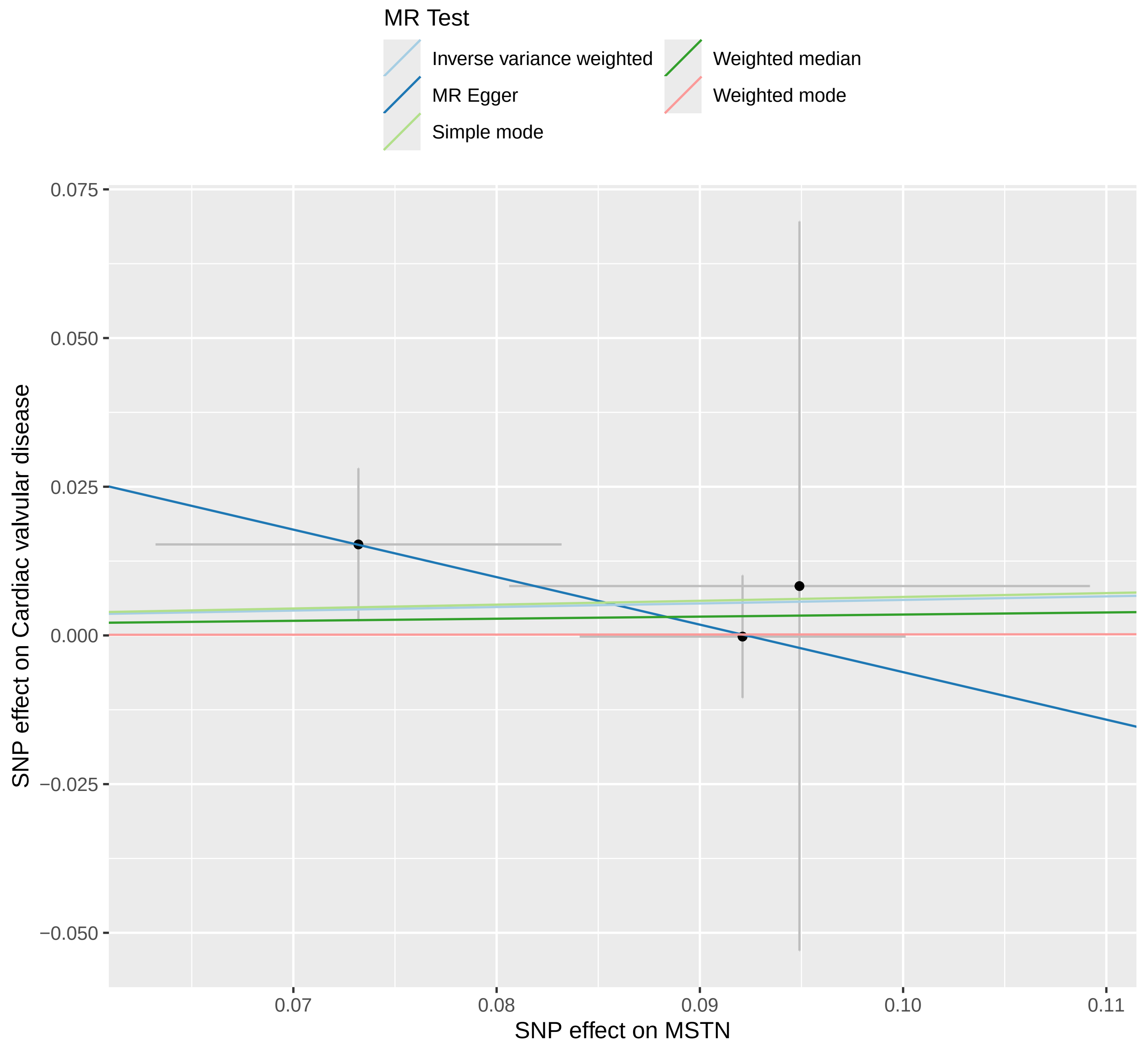
MR Test
Inverse variance weighted
MR Egger
Simple mode
Weighted median
Weighted mode
0.075
0.050
0.025
0.000
−0.025
−0.050
0.07
0.08
0.09
0.10
0.11
SNP effect on MSTN
SNP effect on Cardiac valvular disease

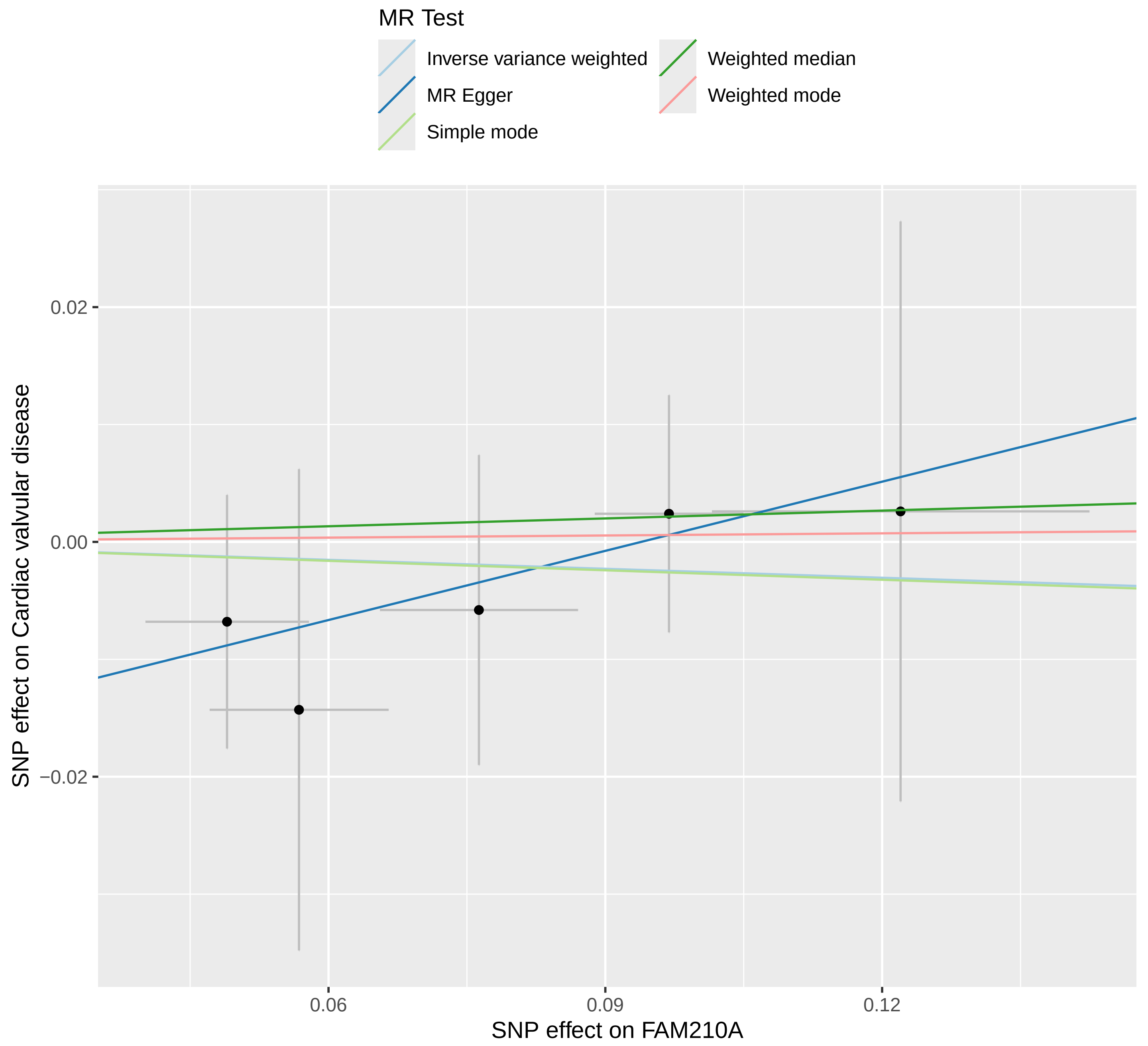

MR Test
Inverse variance weighted
MR Egger
Simple mode
Weighted median
Weighted mode
0.02
0.00
−0.02
0.06
0.09
0.12
SNP effect on Cardiac valvular disease
SNP effect on FAM210A

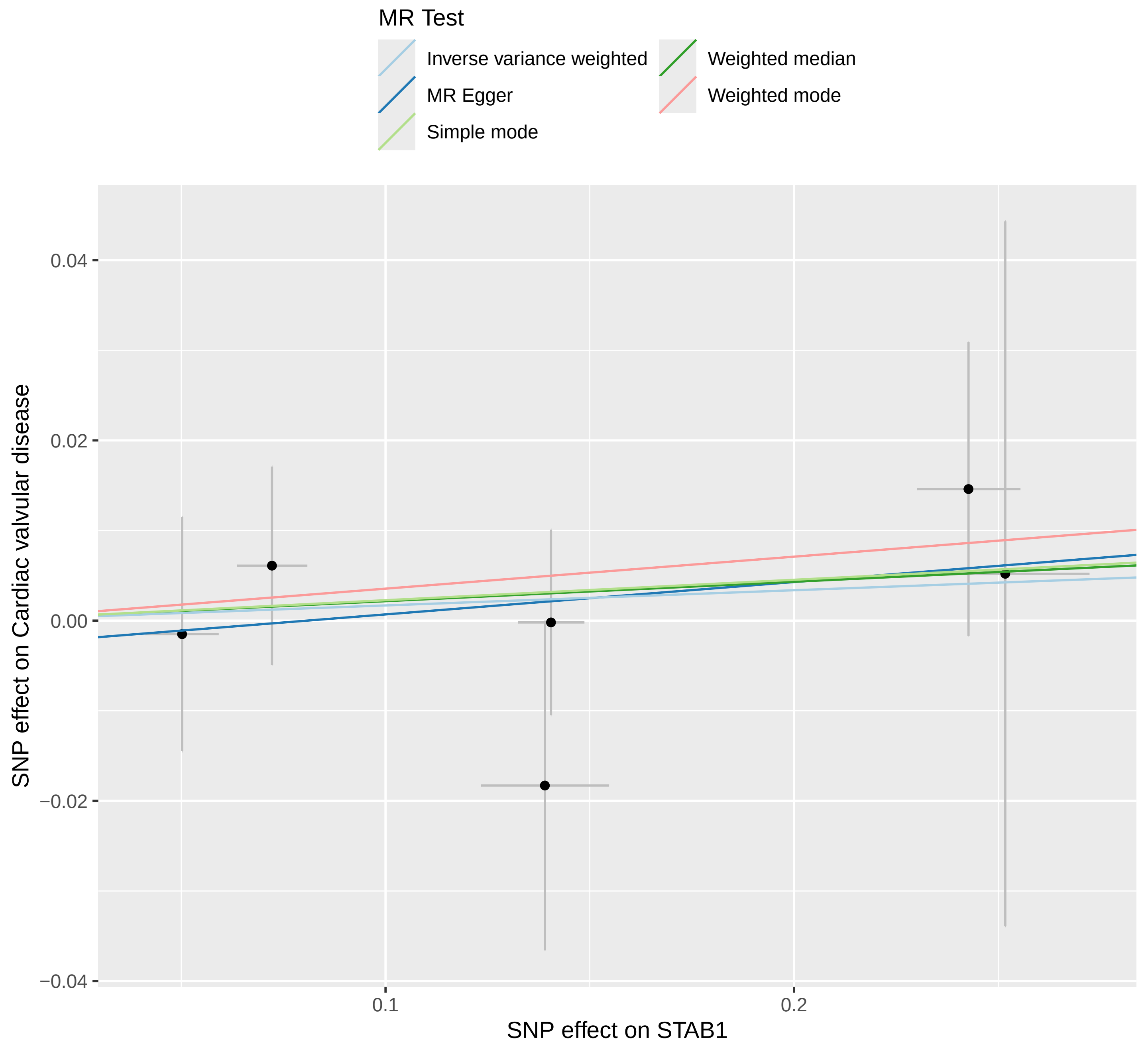
MR Test
Inverse variance weighted
MR Egger
Simple mode
Weighted median
Weighted mode
0.04
0.02
0.00
−0.02
−0.04
0.1
0.2
SNP effect on Cardiac valvular disease
SNP effect on STAB1

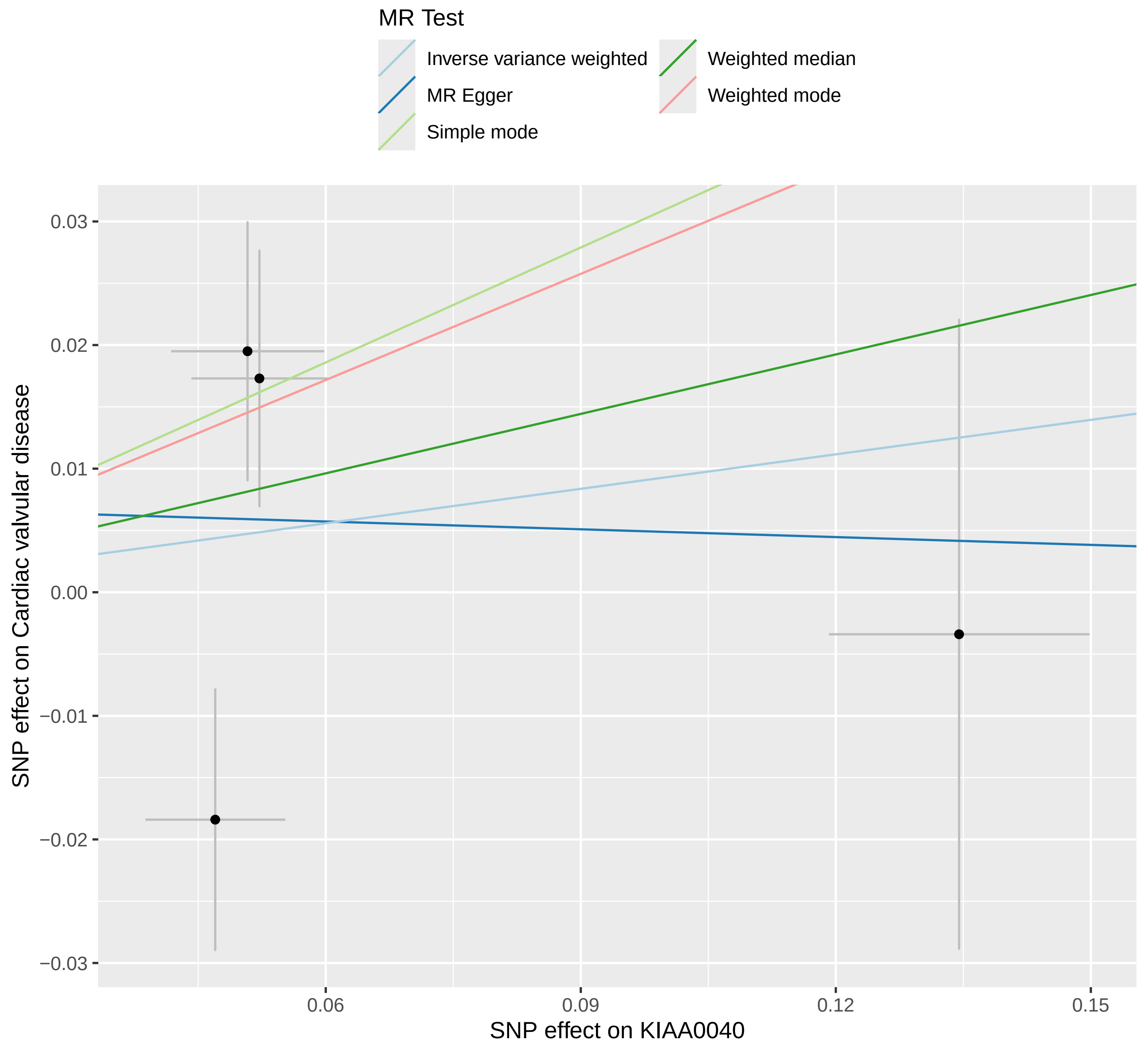

MR Test
Inverse variance weighted
MR Egger
Simple mode
Weighted median
Weighted mode
0.03
0.02
0.01
0.00
−0.01
−0.02
−0.03
0.06
0.09
0.12
0.15
SNP effect on Cardiac valvular disease
SNP effect on KIAA0040

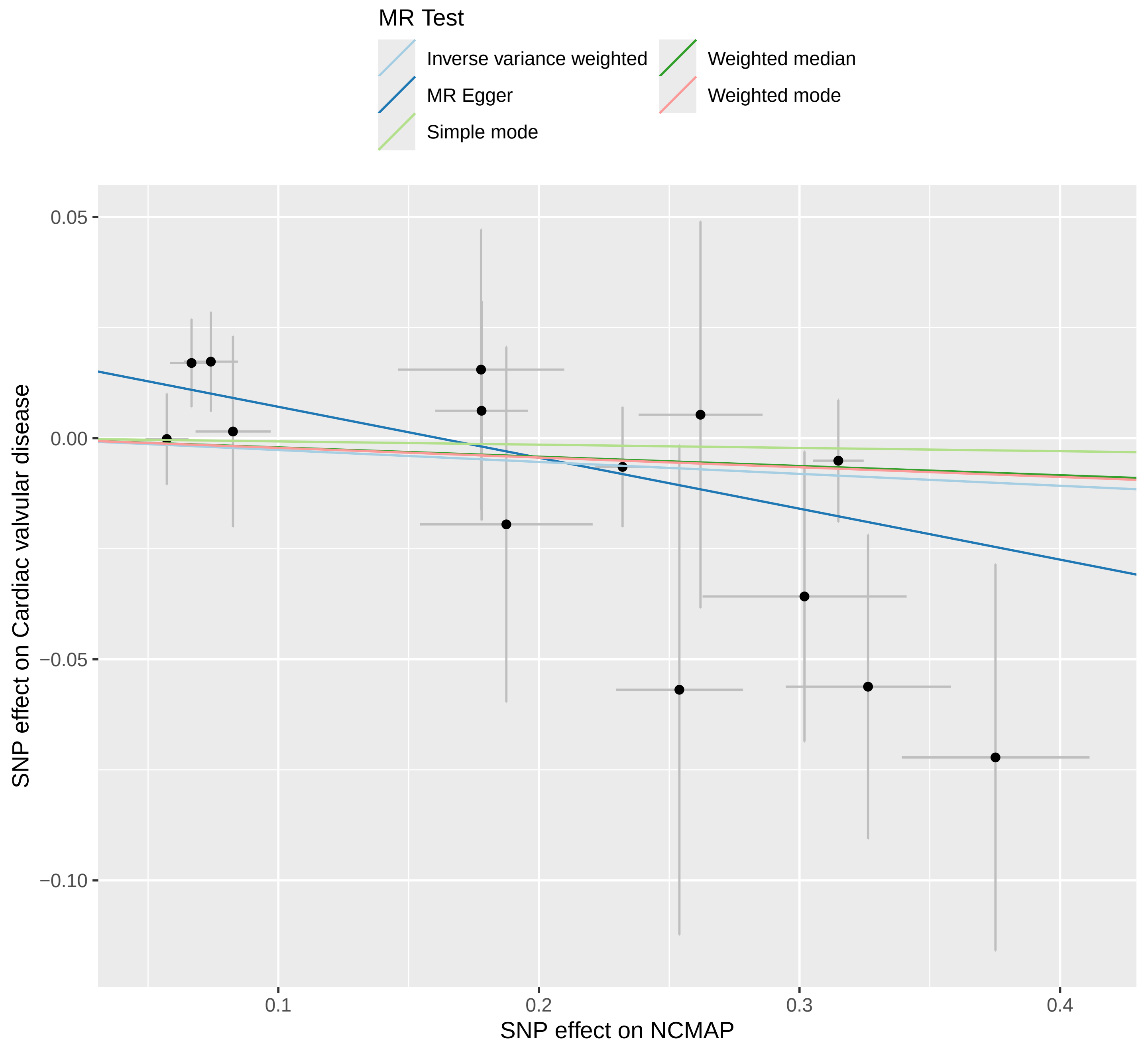
MR Test
Inverse variance weighted
MR Egger
Simple mode
Weighted median
Weighted mode
0.05
0.00
−0.05
−0.10
0.1
0.2
0.3
0.4
SNP effect on NCMAP
SNP effect on Cardiac valvular disease

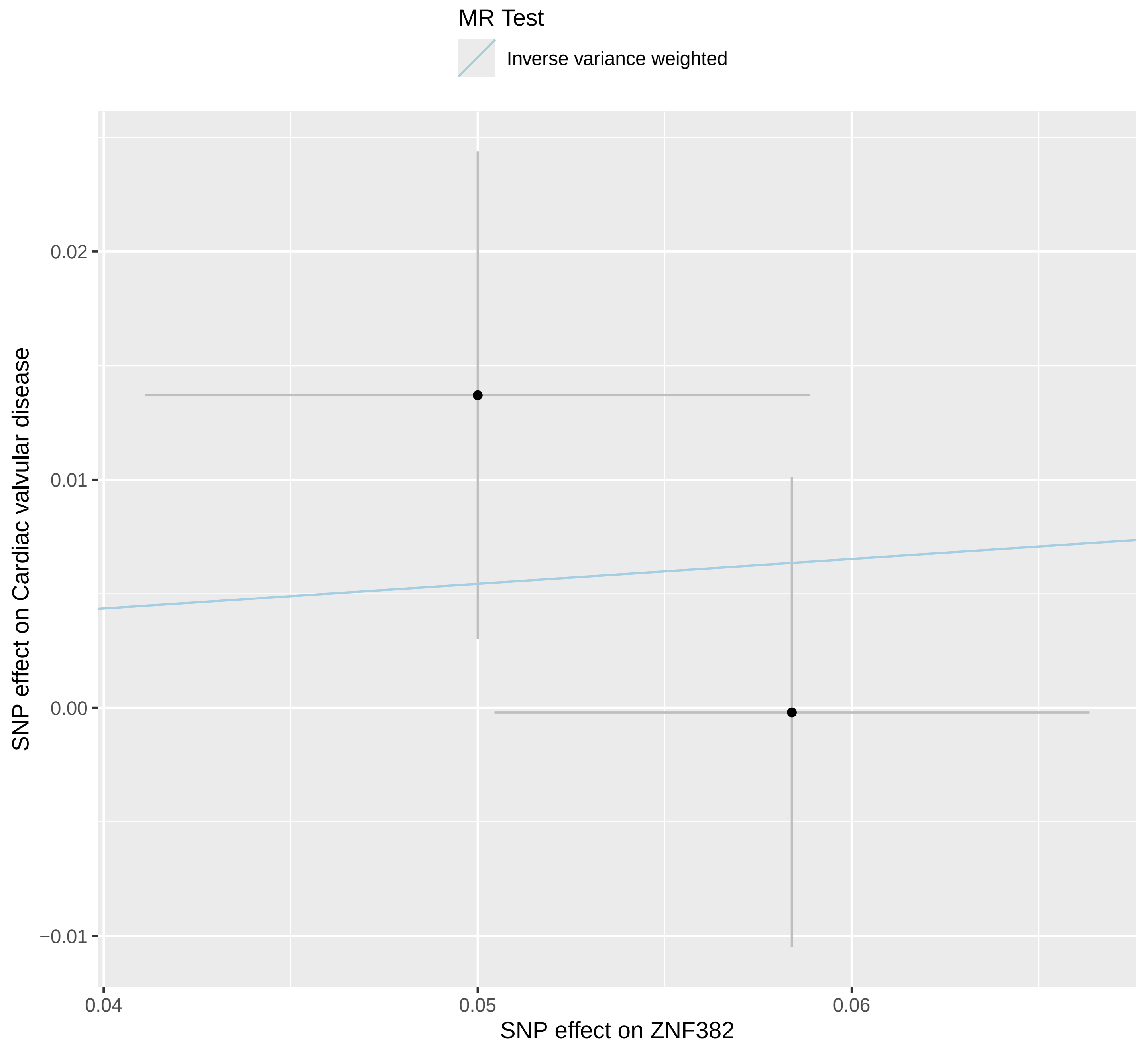

MR Test
Inverse variance weighted
0.02
0.01
0.00
−0.01
0.04
0.05
0.06
SNP effect on Cardiac valvular disease
SNP effect on ZNF382

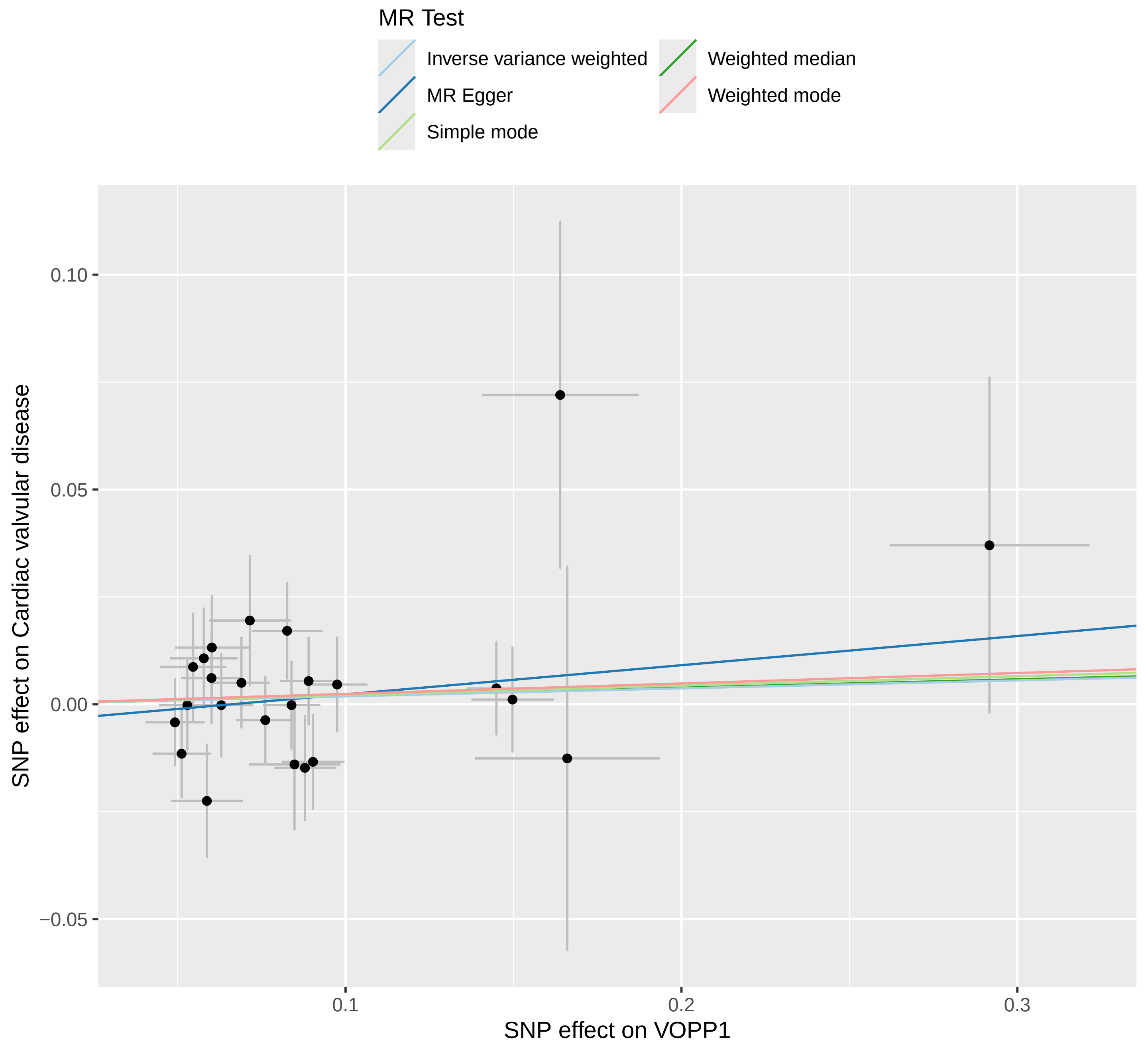

MR Test
Inverse variance weighted
MR Egger
Simple mode
Weighted median
Weighted mode
0.10
0.05
0.00
−0.05
0.1
0.2
0.3
SNP effect on Cardiac valvular disease
SNP effect on VOPP1

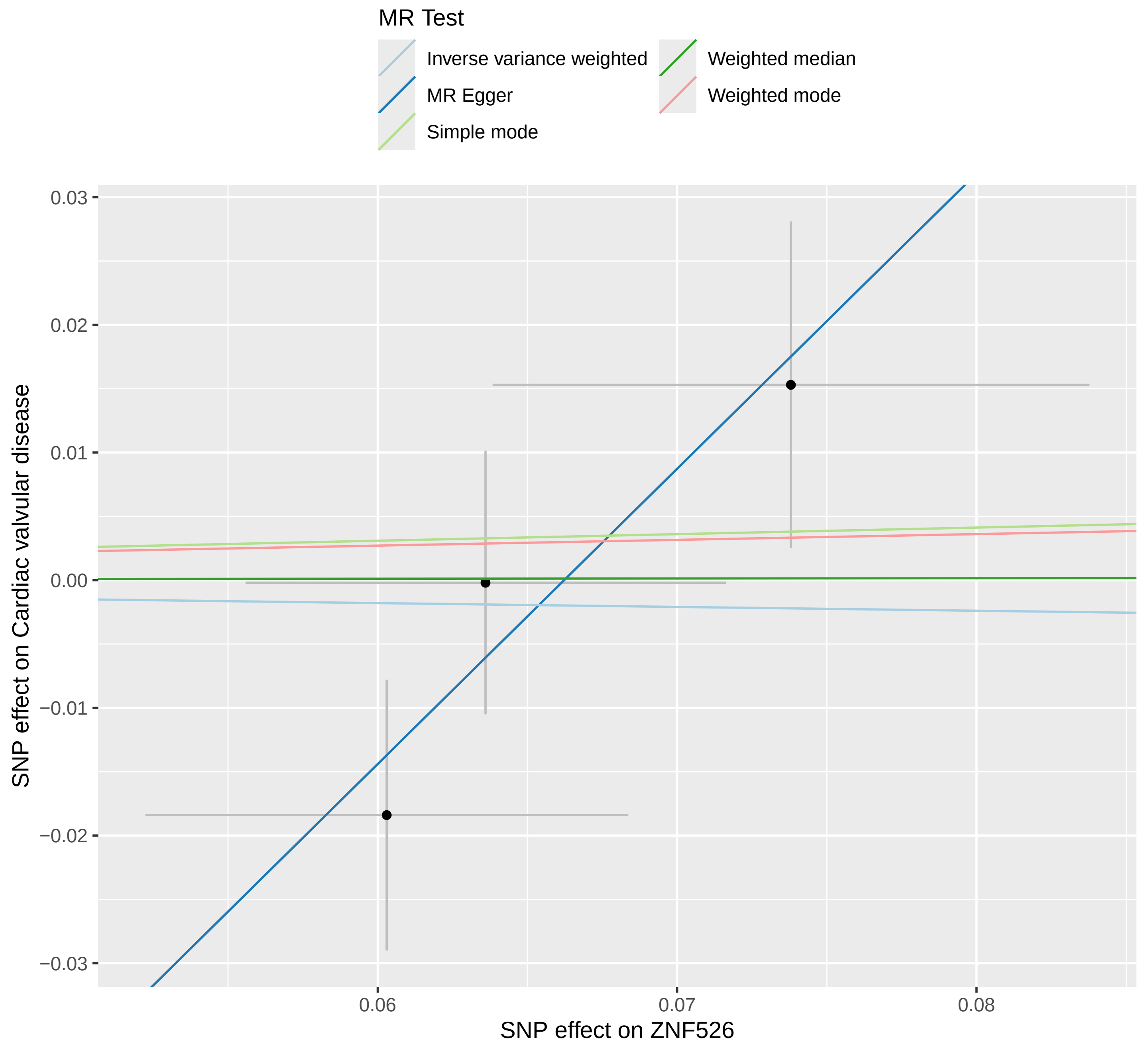
MR Test
Inverse variance weighted
MR Egger
Simple mode
Weighted median
Weighted mode
0.03
0.02
0.01
0.00
−0.01
−0.02
−0.03
SNP effect on Cardiac valvular disease
0.06
0.07
0.08
SNP effect on ZNF526

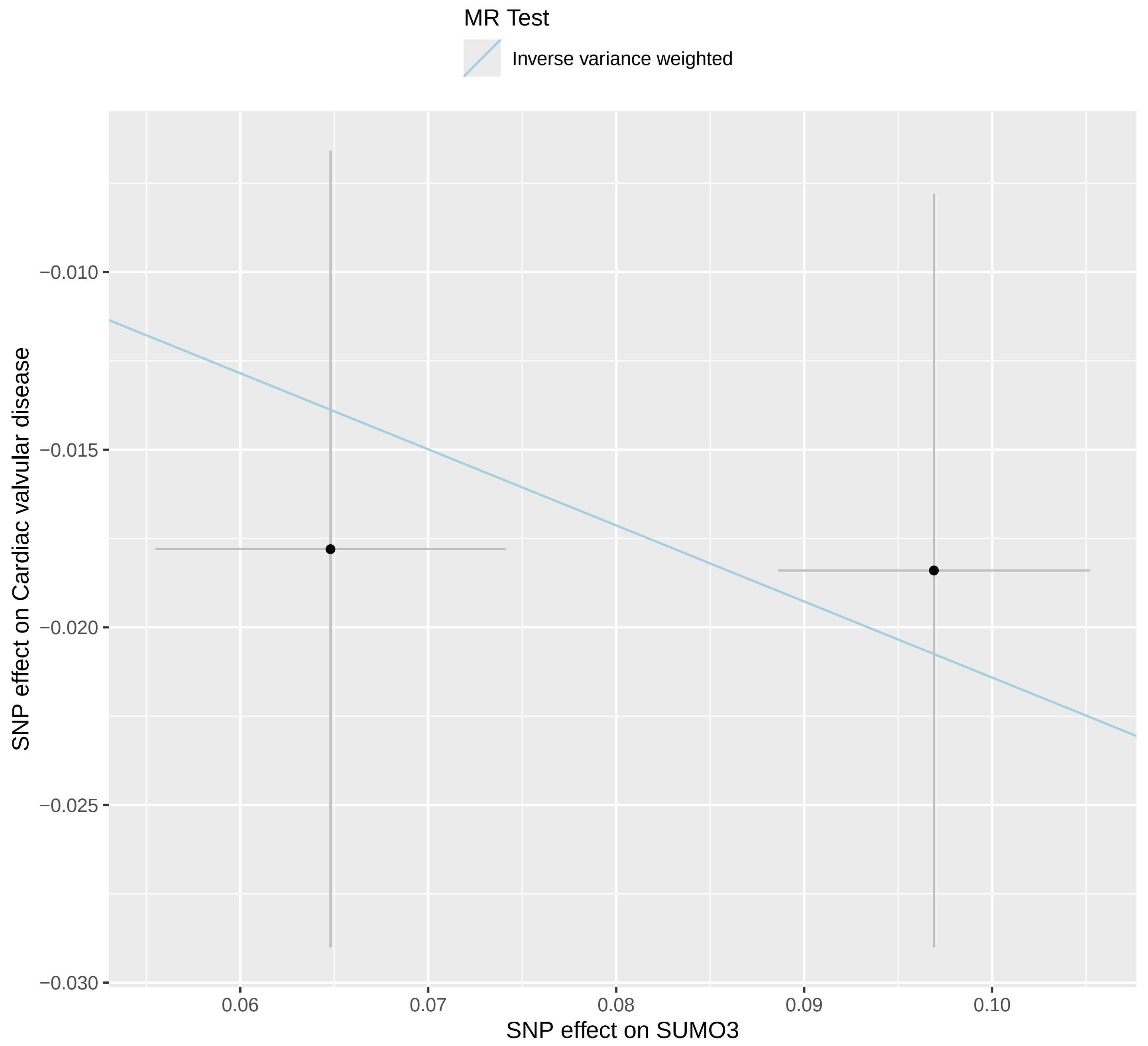
MR Test
Inverse variance weighted
−0.010
−0.015
−0.020
−0.025
−0.030
SNP effect on Cardiac valvular disease
0.06
0.07
0.08
0.09
0.10
SNP effect on SUMO3

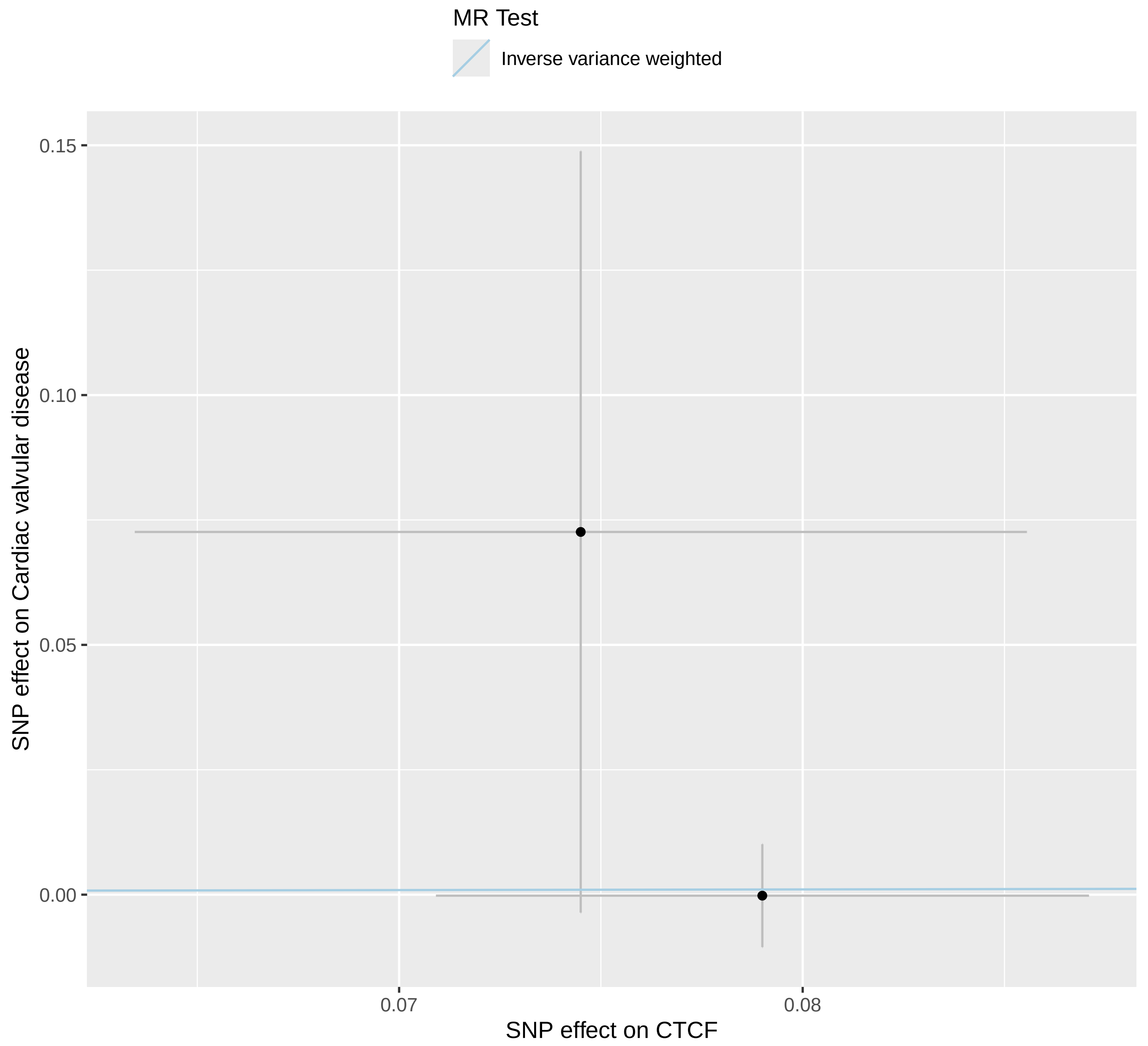

MR Test
Inverse variance weighted
0.15
0.10
0.05
0.00
0.07
0.08
SNP effect on Cardiac valvular disease
SNP effect on CTCF

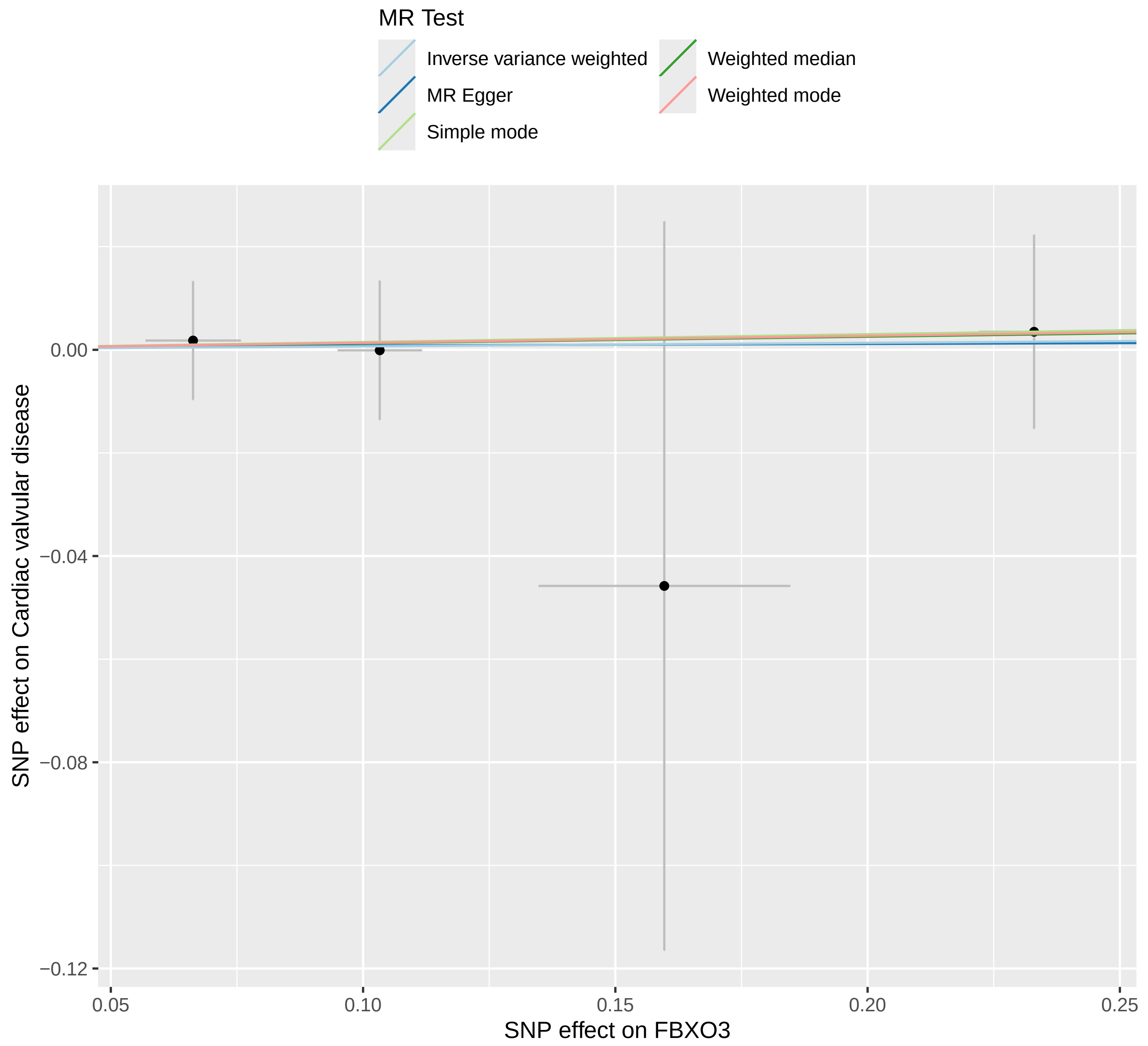
MR Test
Inverse variance weighted
MR Egger
Simple mode
Weighted median
Weighted mode
SNP effect on Cardiac valvular disease
0.00
−0.04
−0.08
−0.12
0.05
0.10
0.15
0.20
0.25
SNP effect on FBXO3

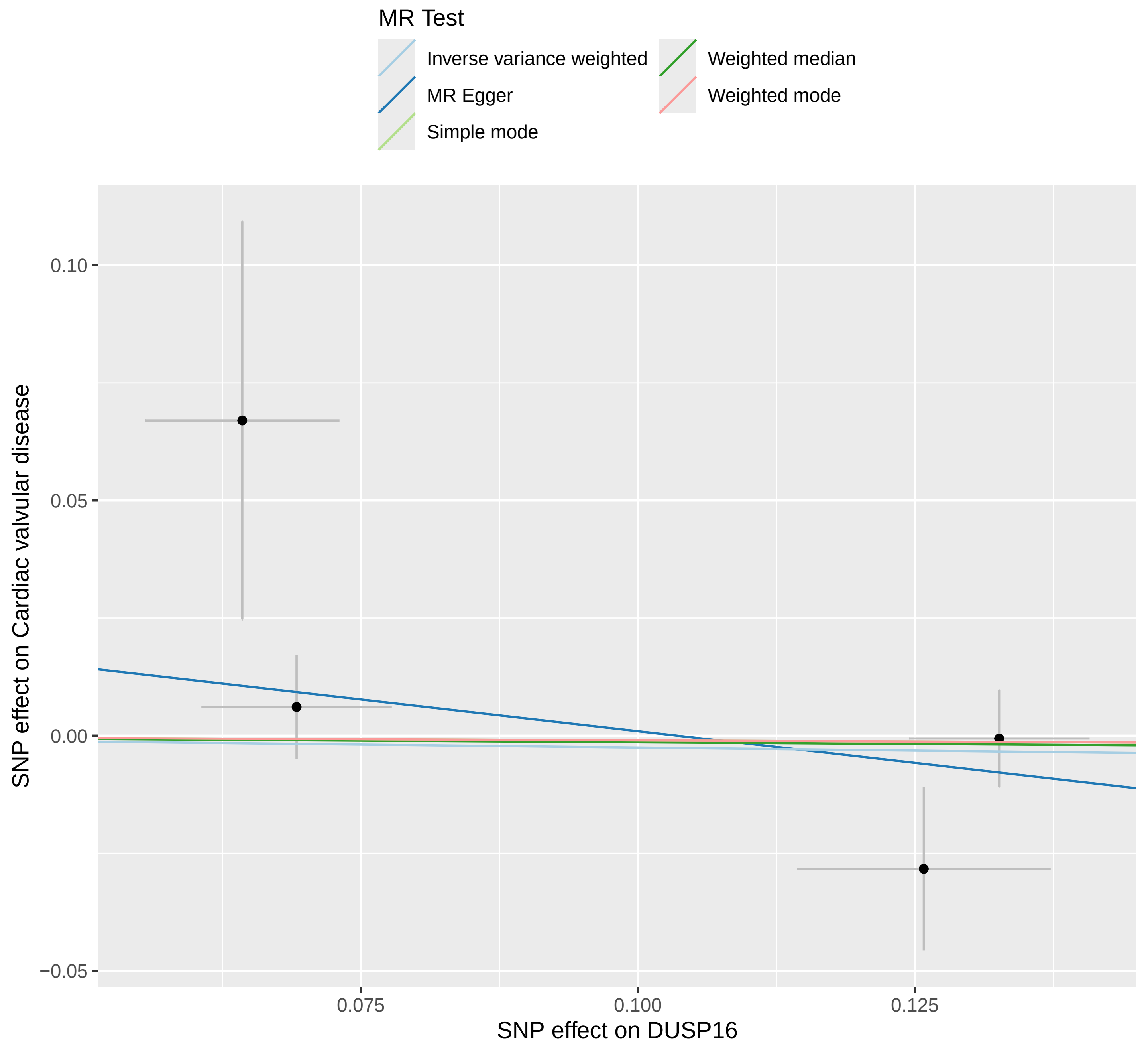

MR Test
Inverse variance weighted
MR Egger
Simple mode
Weighted median
Weighted mode
0.10
0.05
0.00
−0.05
0.075
0.100
0.125
SNP effect on DUSP16
SNP effect on Cardiac valvular disease

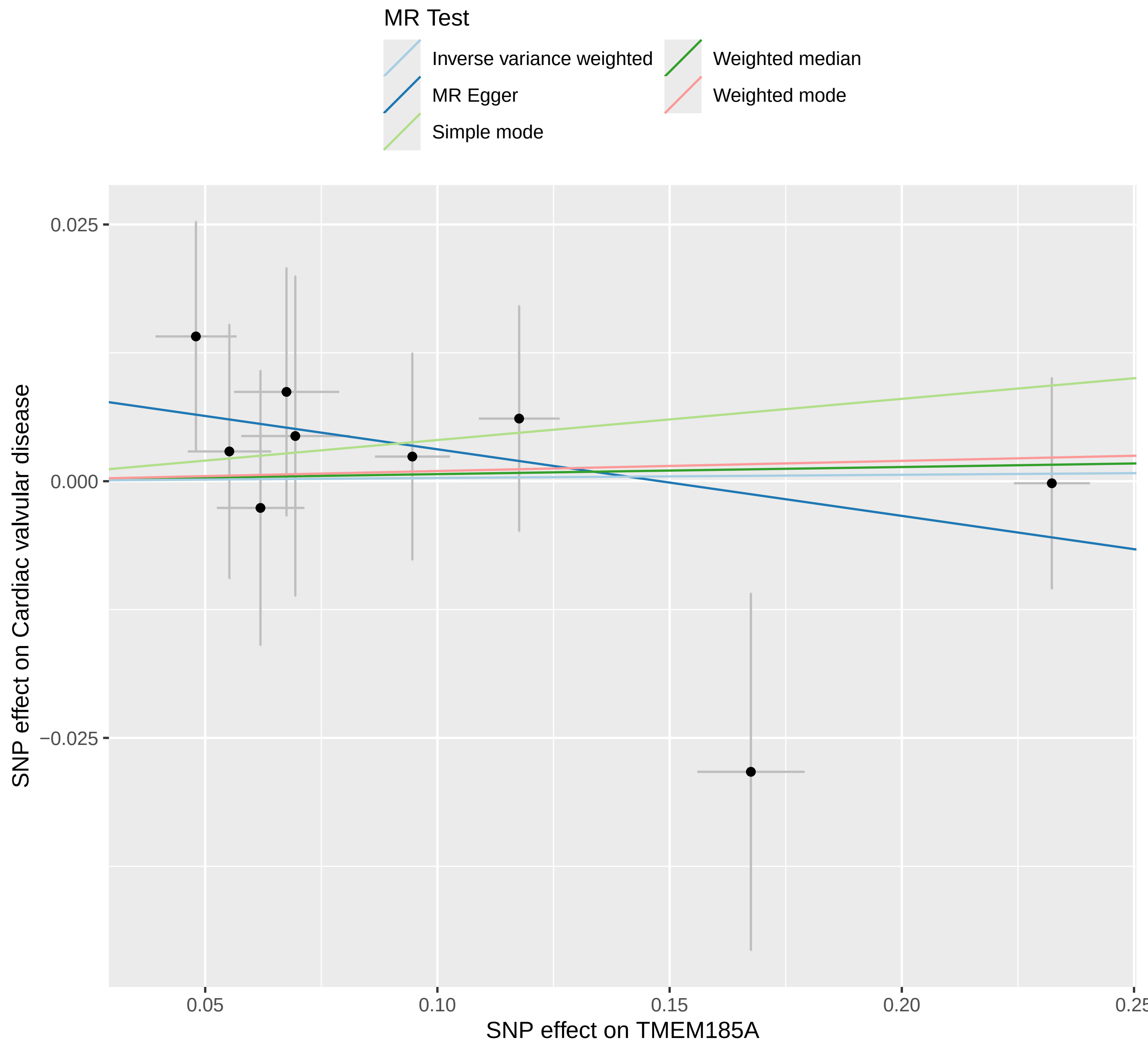

MR Test
Inverse variance weighted
MR Egger
Simple mode
Weighted median
Weighted mode
0.025
0.000
−0.025
0.05
0.10
0.15
0.20
0.25
SNP effect on TMEM185A
SNP effect on Cardiac valvular disease

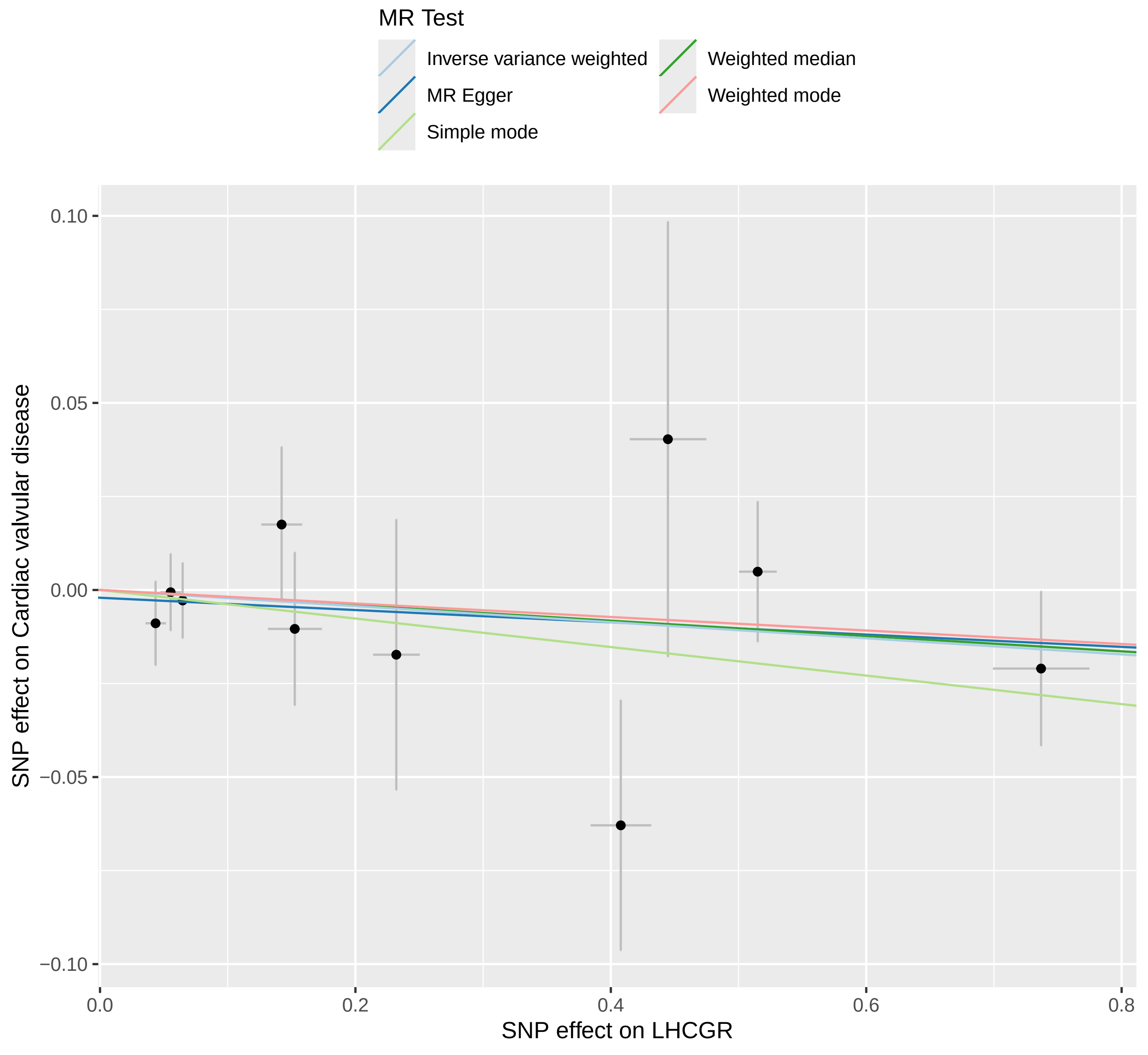
MR Test
Inverse variance weighted
MR Egger
Simple mode
Weighted median
Weighted mode
SNP effect on Cardiac valvular disease
0.10
0.05
0.00
−0.05
−0.10
0.0
0.2
0.4
0.6
0.8
SNP effect on LHCGR

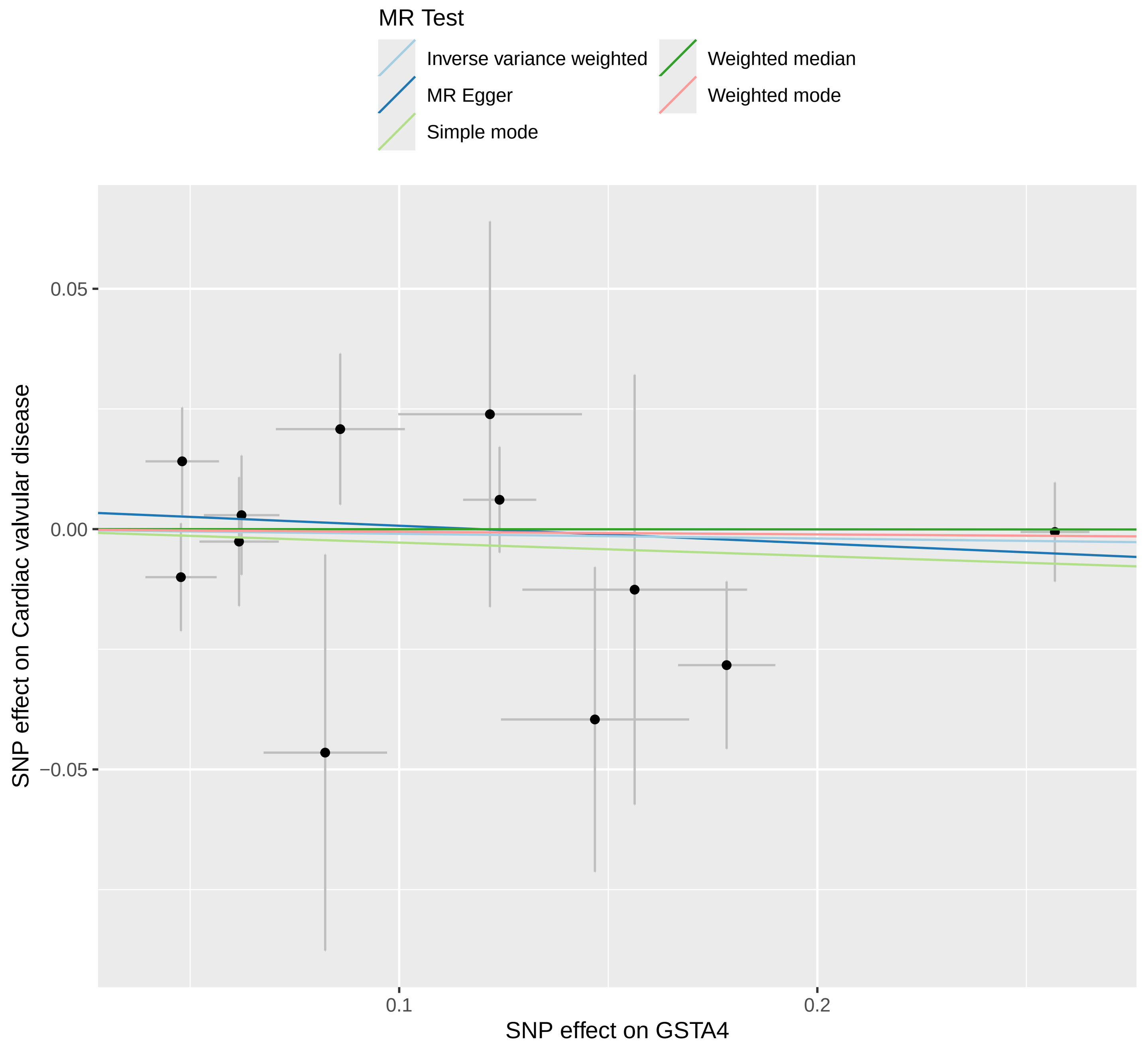

MR Test
Inverse variance weighted
MR Egger
Simple mode
Weighted median
Weighted mode
0.05
0.00
−0.05
0.1
0.2
SNP effect on GSTA4
SNP effect on Cardiac valvular disease

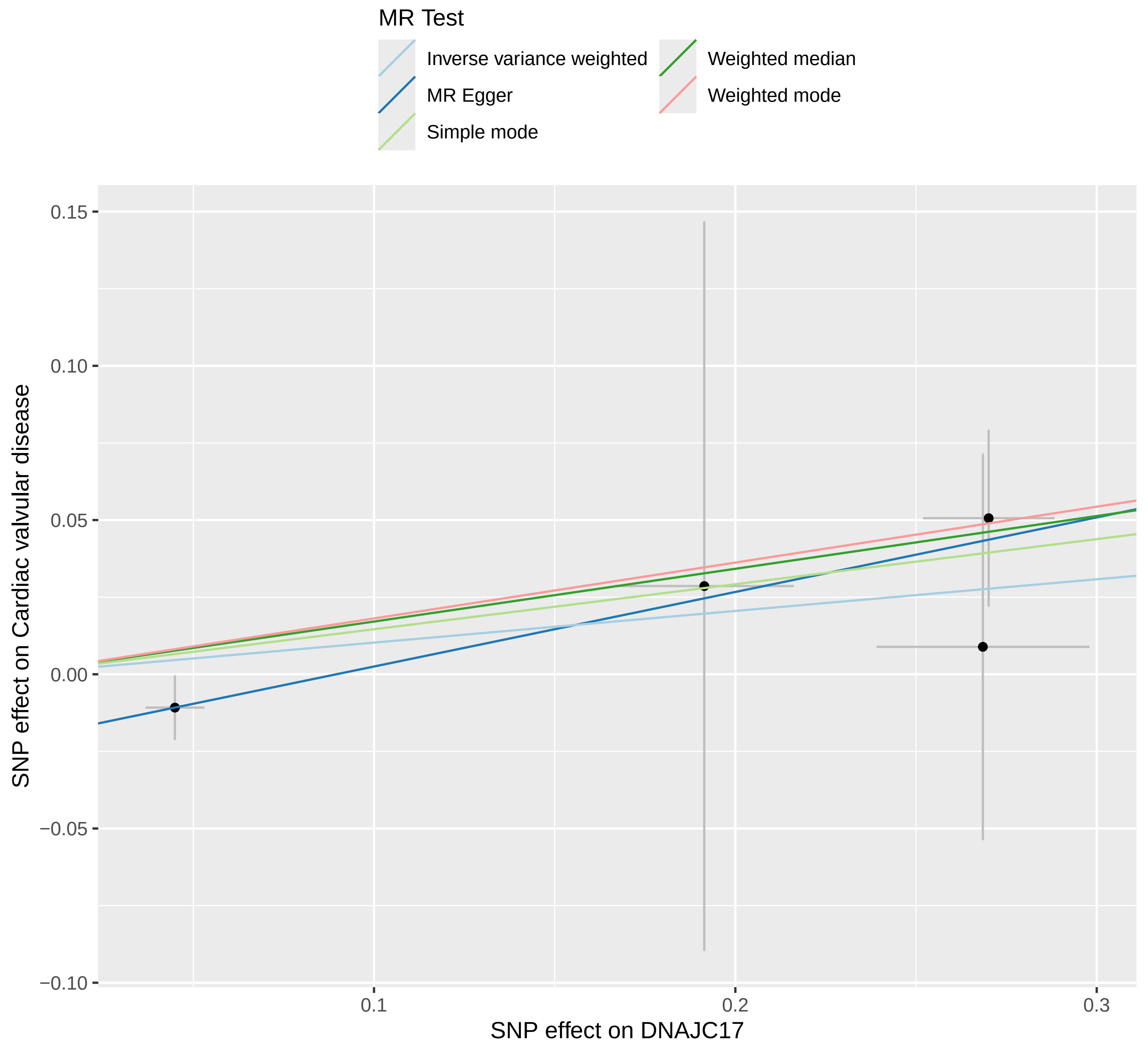
MR Test
Inverse variance weighted
MR Egger
Simple mode
Weighted median
Weighted mode
0.15
0.10
0.05
0.00
−0.05
−0.10
0.1
0.2
0.3
SNP effect on Cardiac valvular disease
SNP effect on DNAJC17

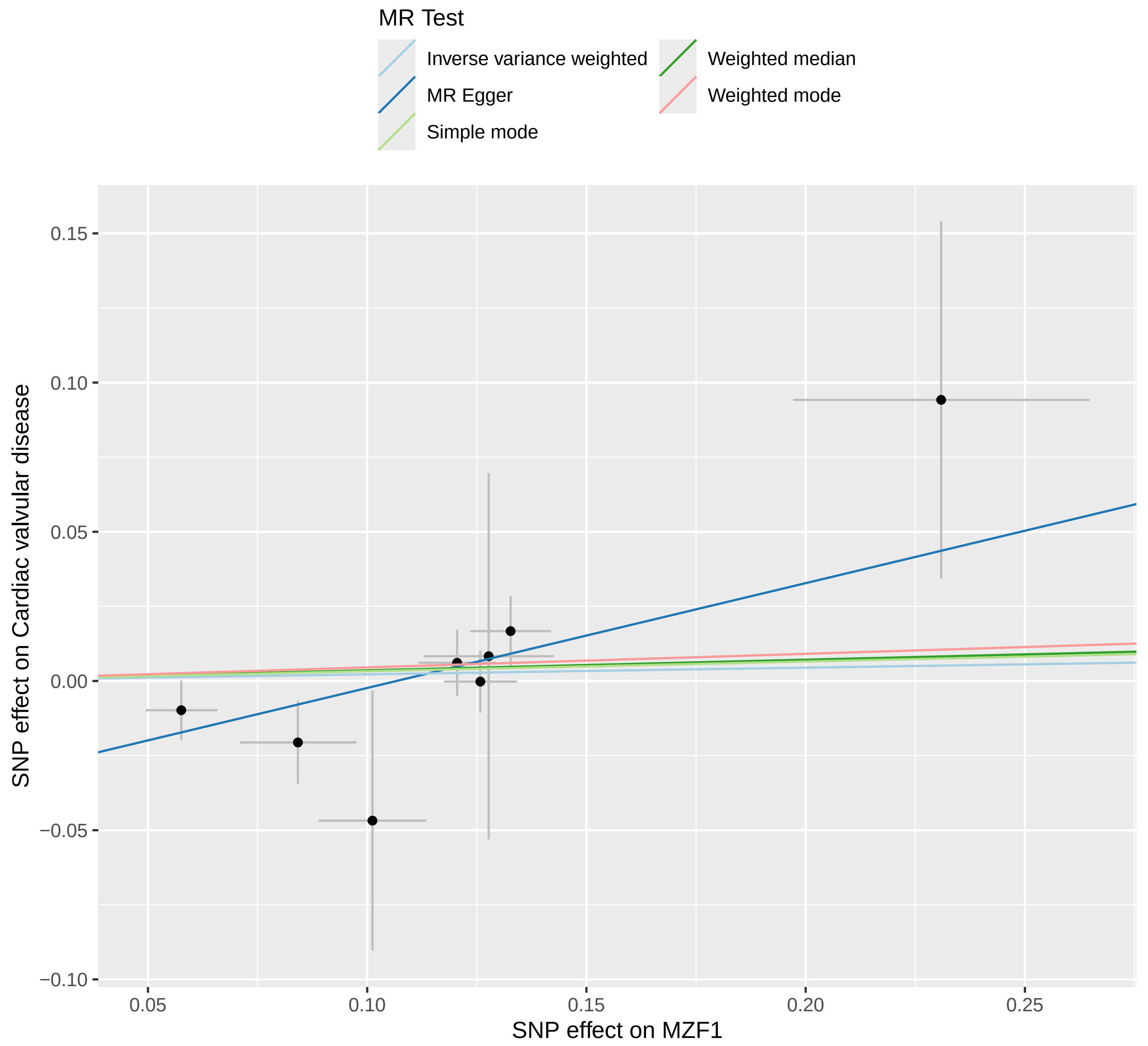
MR Test
Inverse variance weighted
MR Egger
Simple mode
Weighted median
Weighted mode
0.15
0.10
0.05
0.00
−0.05
−0.10
SNP effect on Cardiac valvular disease
0.05
0.10
0.15
0.20
0.25
SNP effect on MZF1

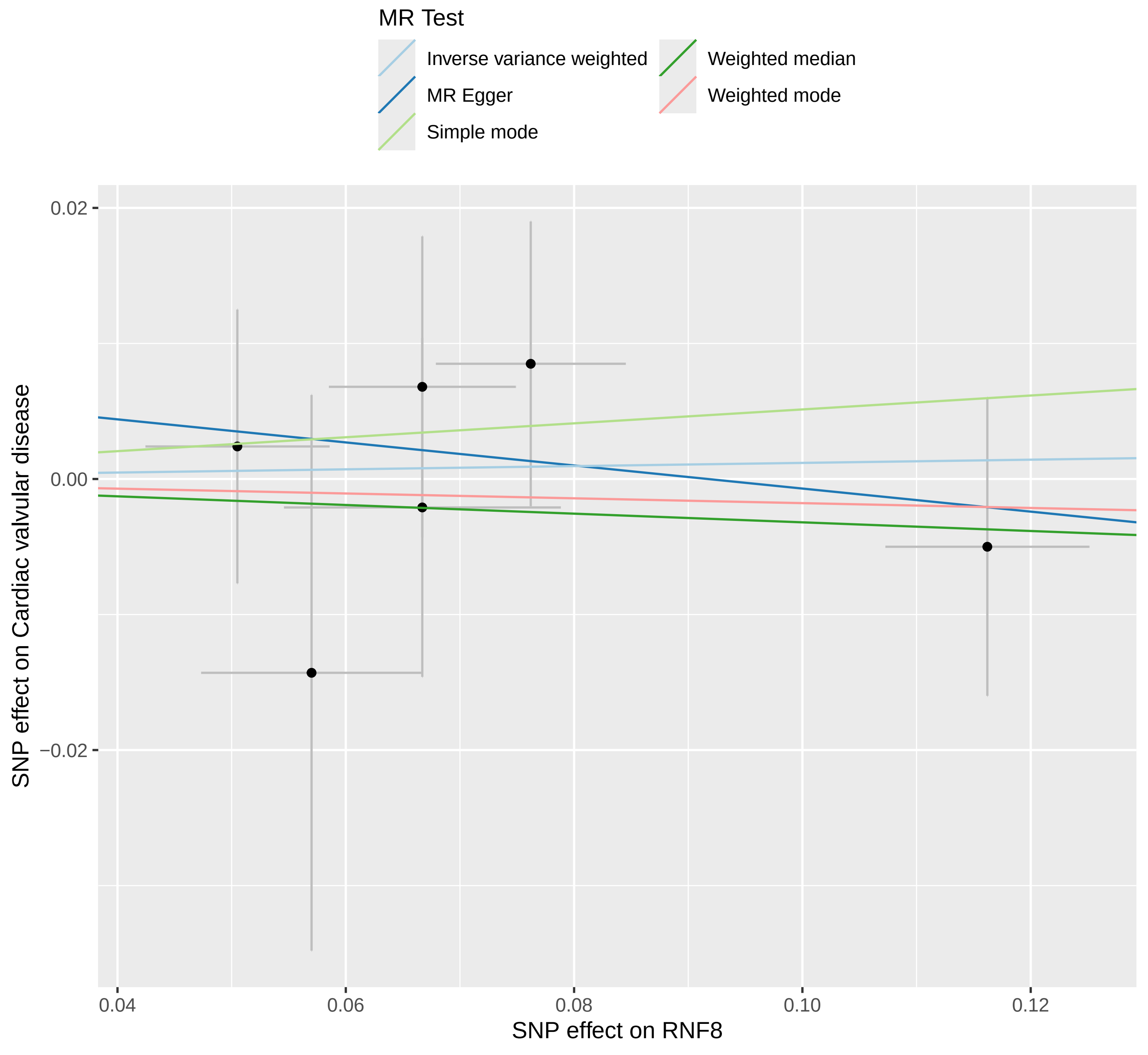

MR Test
Inverse variance weighted
MR Egger
Simple mode
Weighted median
Weighted mode
0.02
0.00
−0.02
0.04
0.06
0.08
0.10
0.12
SNP effect on RNF8
SNP effect on Cardiac valvular disease

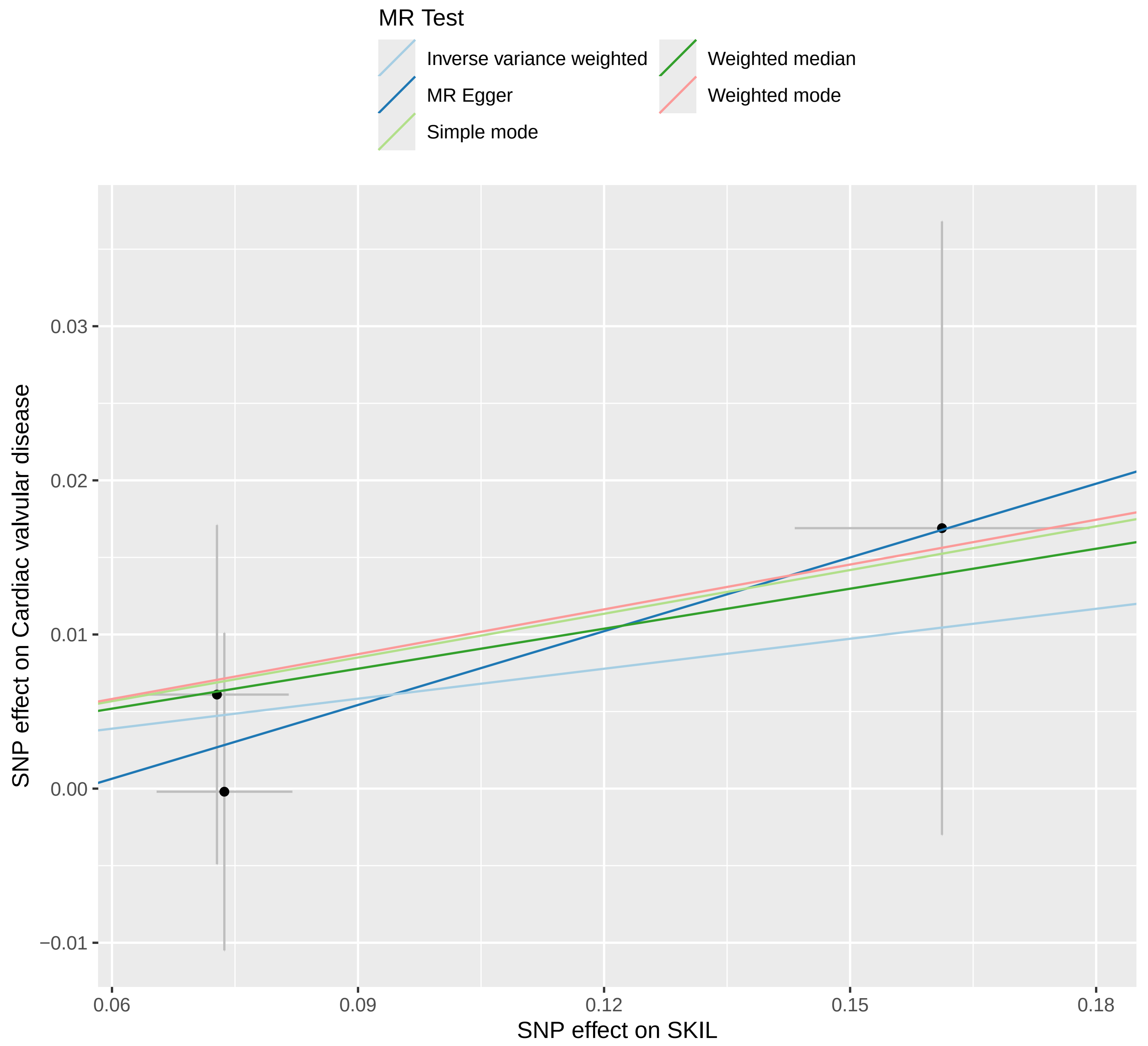
MR Test
Inverse variance weighted
MR Egger
Simple mode
Weighted median
Weighted mode
SNP effect on Cardiac valvular disease
0.03
0.02
0.01
0.00
−0.01
0.06
0.09
0.12
0.15
0.18
SNP effect on SKIL

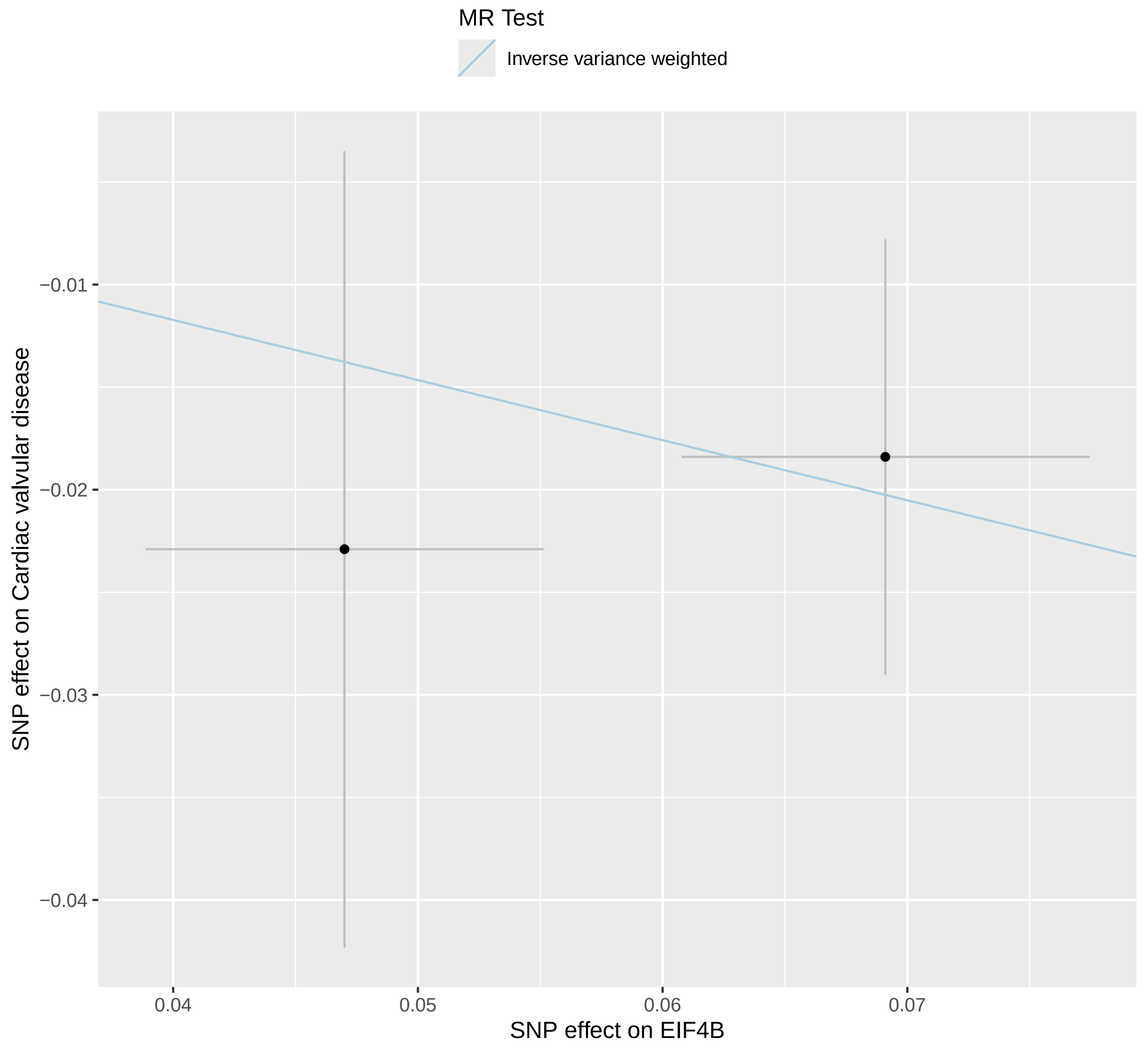
MR Test
Inverse variance weighted
−0.01
−0.02
−0.03
−0.04
0.04
0.05
0.06
0.07
SNP effect on EIF4B
SNP effect on Cardiac valvular disease

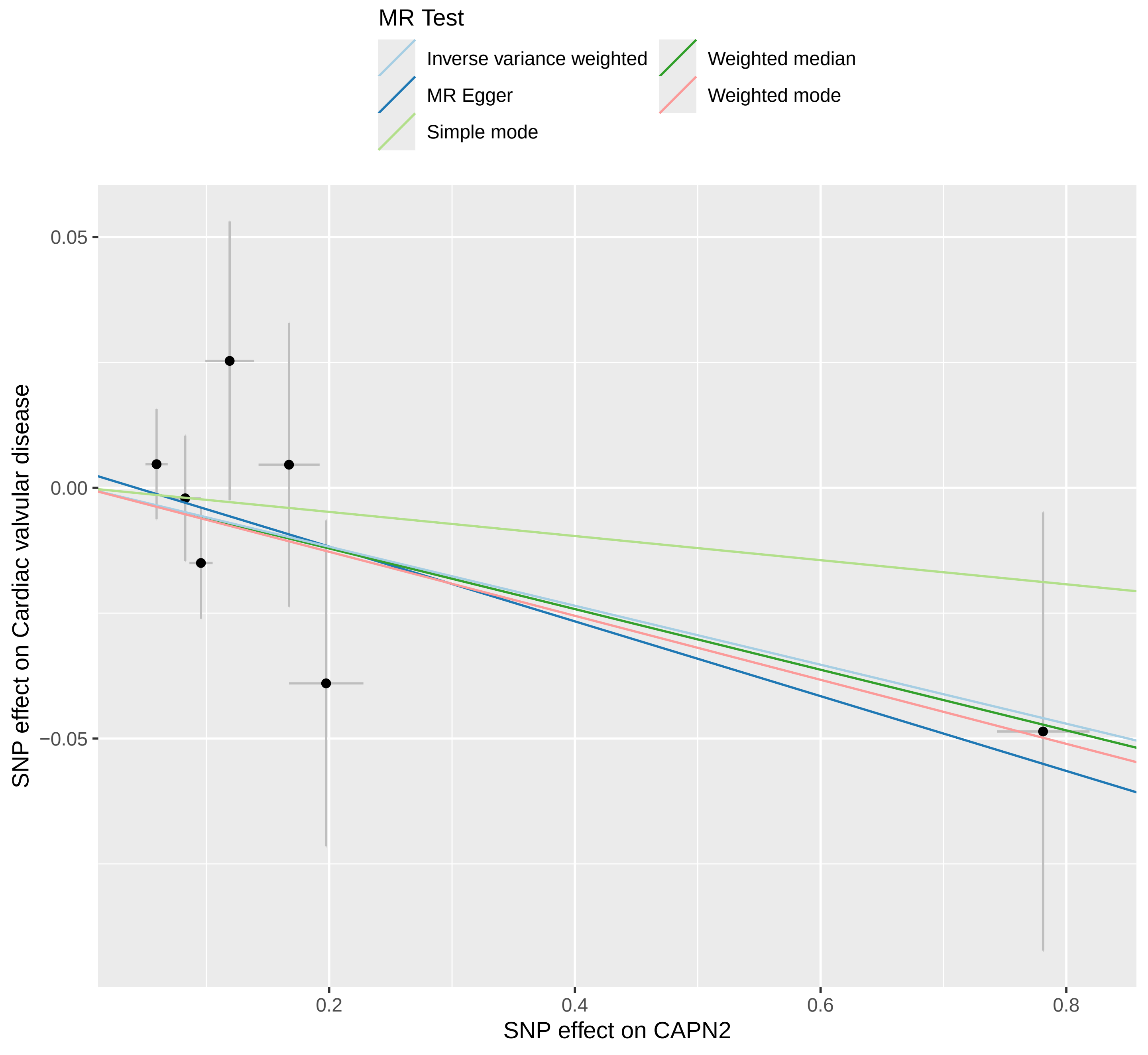
MR Test
Inverse variance weighted
MR Egger
Simple mode
Weighted median
Weighted mode
0.05
0.00
−0.05
0.2
0.4
0.6
0.8
SNP effect on Cardiac valvular disease
SNP effect on CAPN2

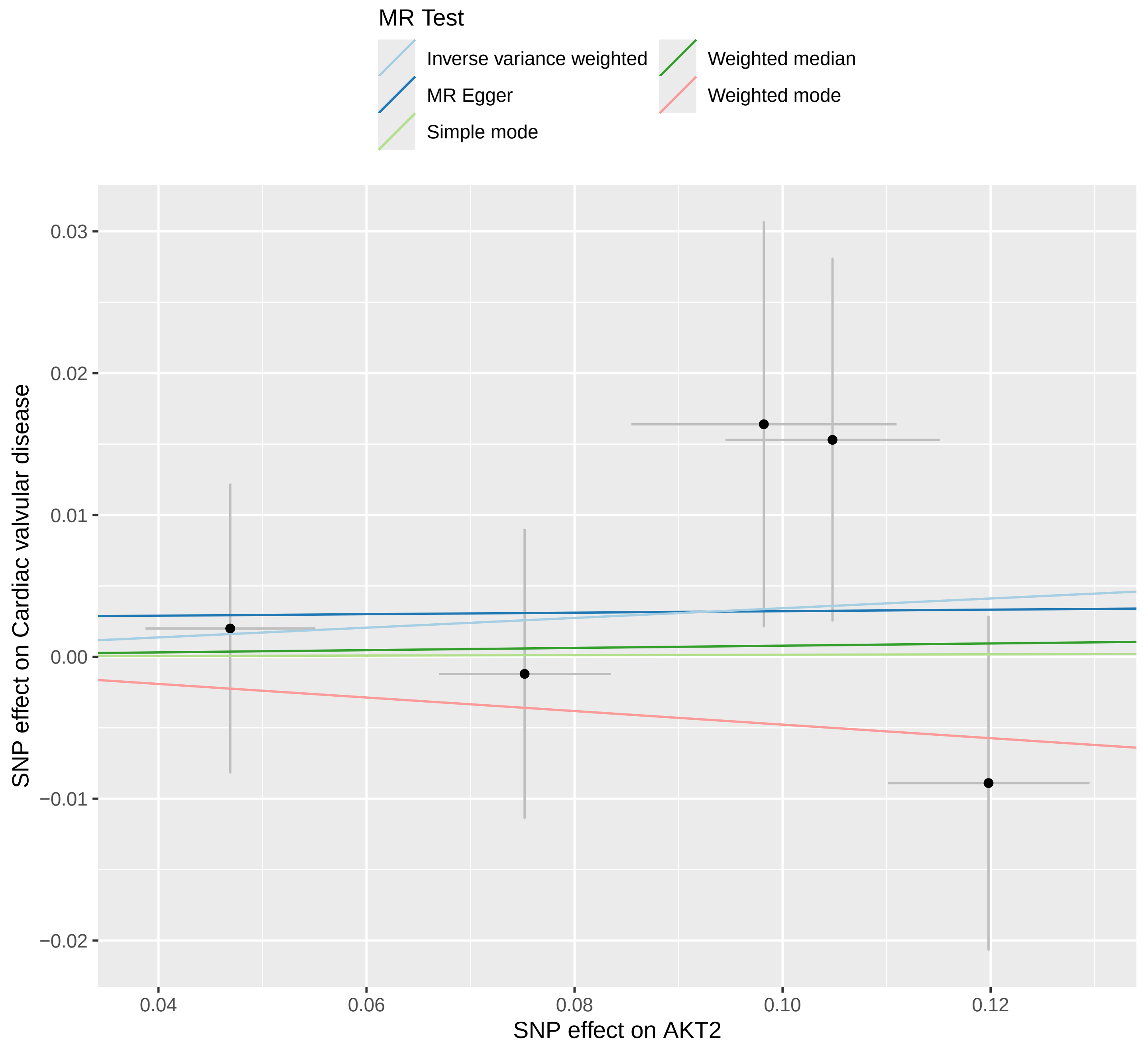
MR Test
Inverse variance weighted
MR Egger
Simple mode
Weighted median
Weighted mode
0.03
0.02
0.01
0.00
−0.01
−0.02
0.04
0.06
0.08
0.10
0.12
SNP effect on AKT2
SNP effect on Cardiac valvular disease

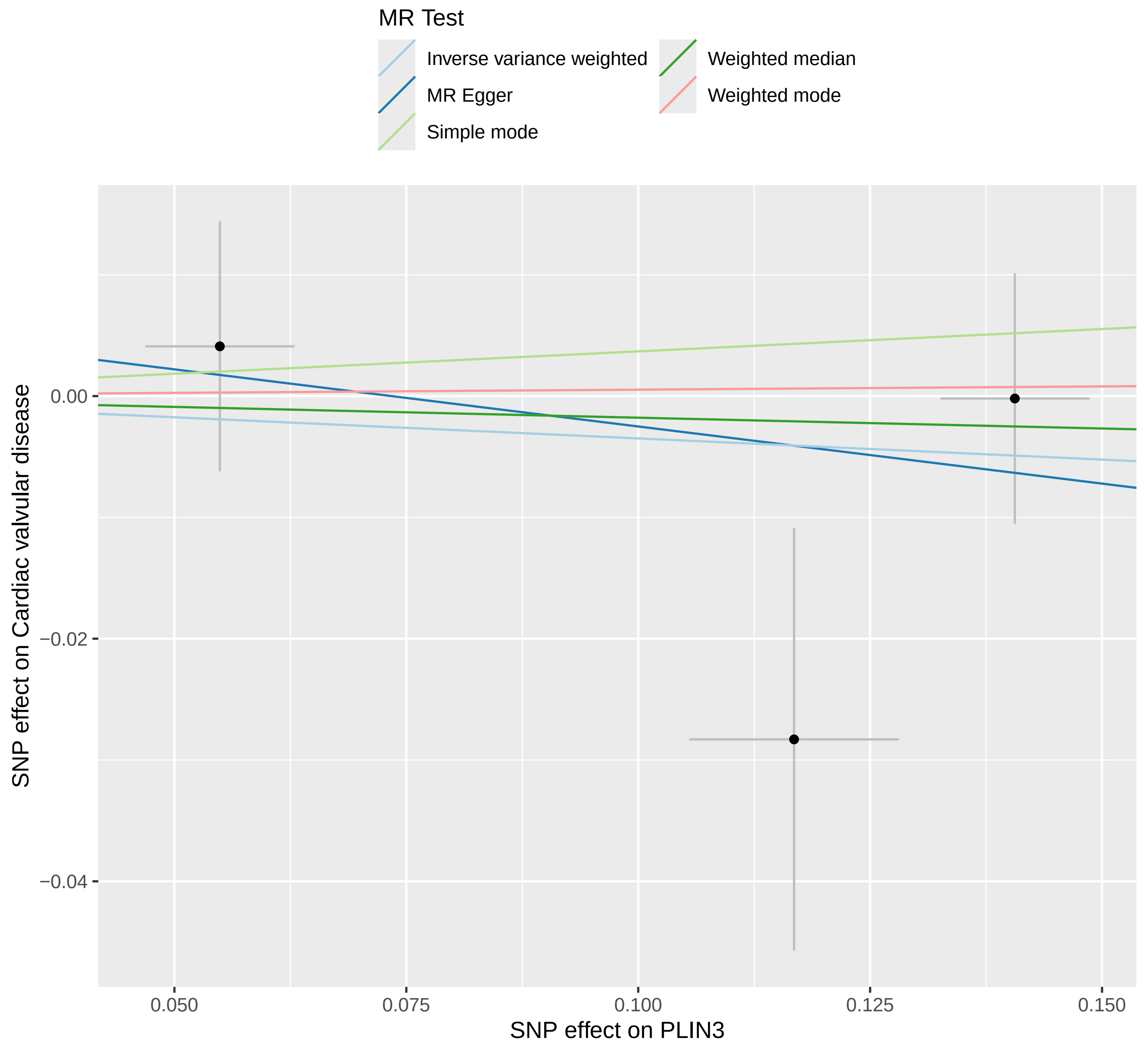
MR Test
Inverse variance weighted
MR Egger
Simple mode
Weighted median
Weighted mode
SNP effect on Cardiac valvular disease
0.00
−0.02
−0.04
0.050
0.075
0.100
0.125
0.150
SNP effect on PLIN3

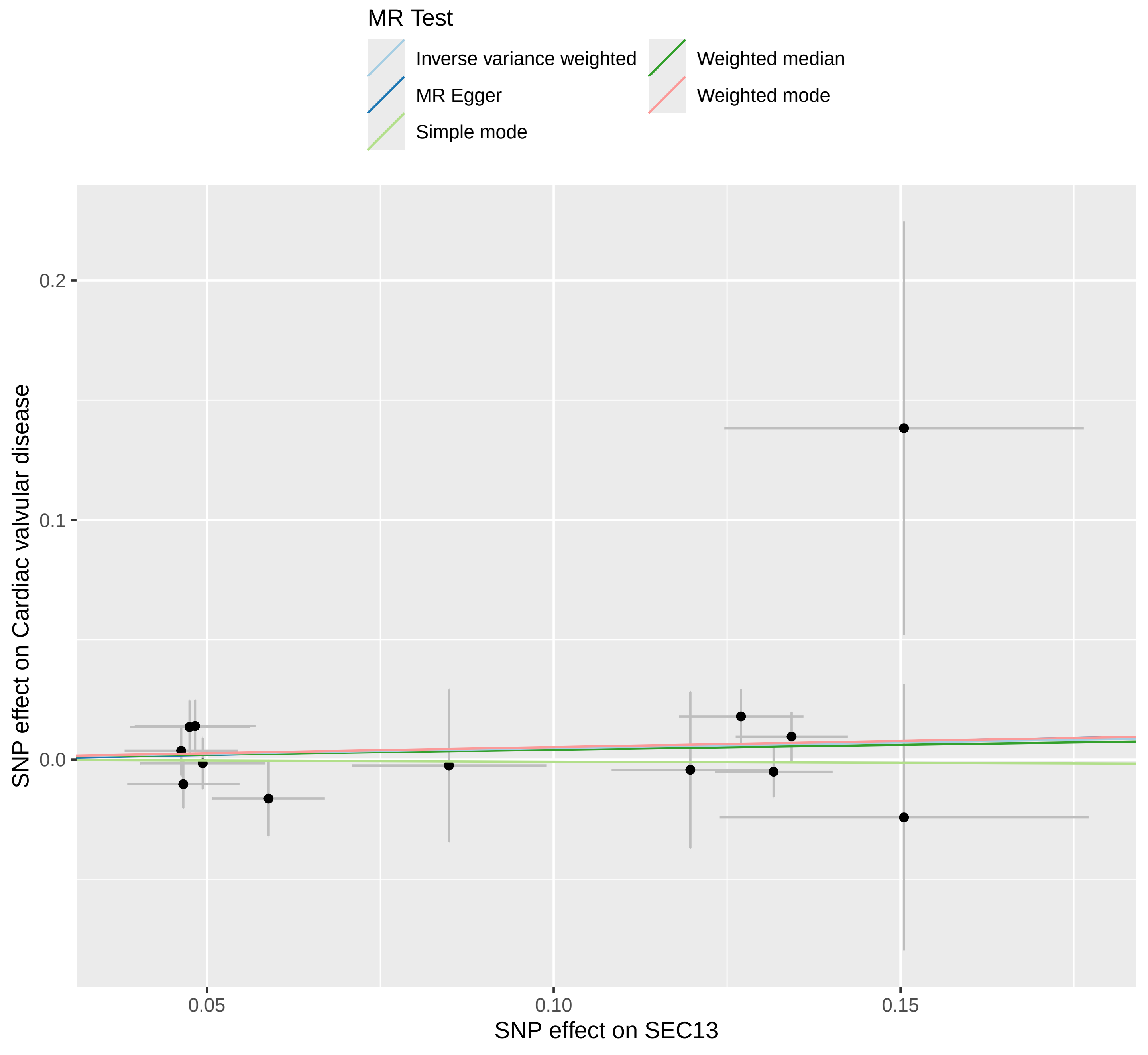
MR Test
Inverse variance weighted
MR Egger
Simple mode
Weighted median
Weighted mode
0.2
0.1
0.0
0.05
0.10
0.15
SNP effect on Cardiac valvular disease
SNP effect on SEC13

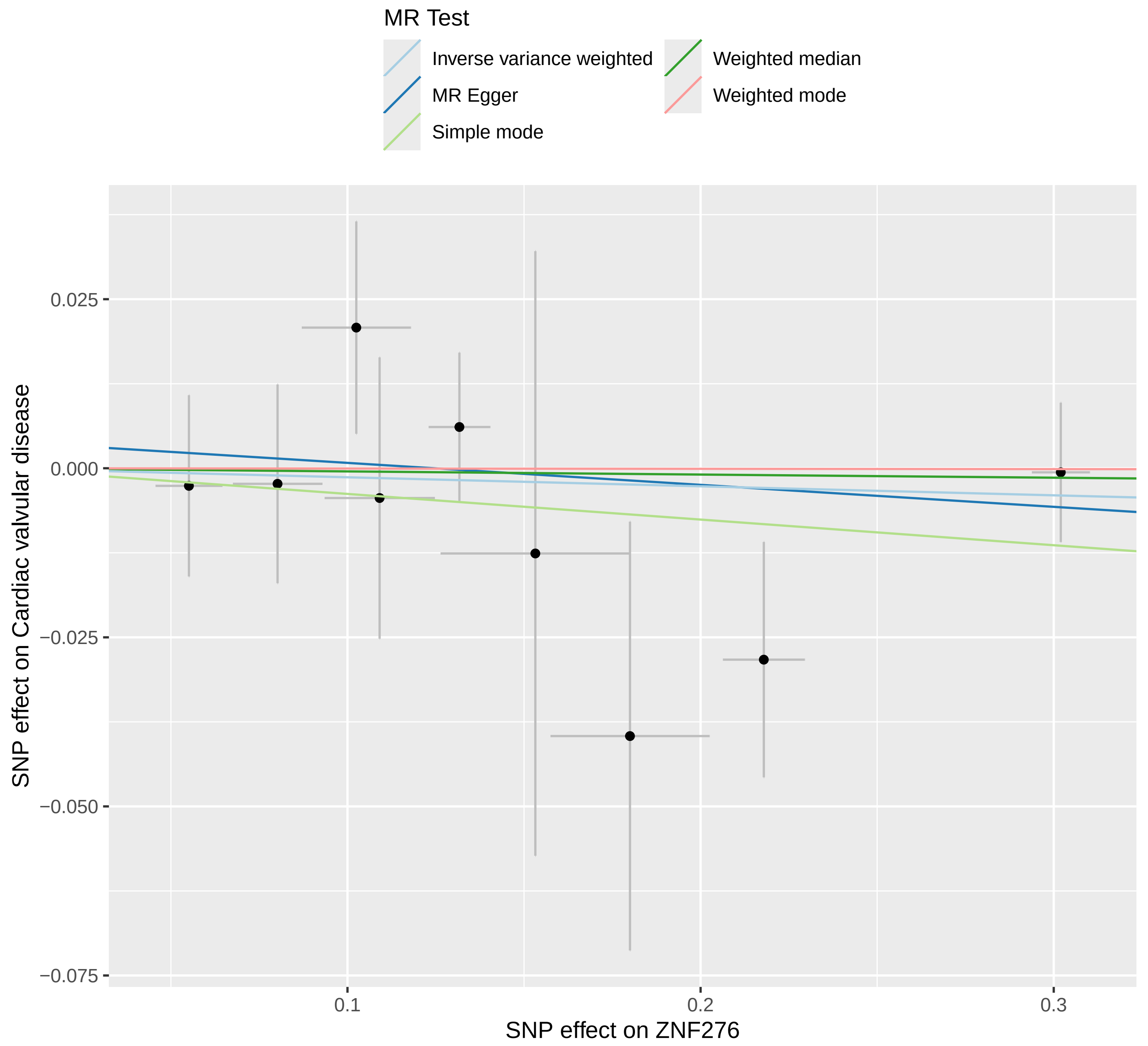
MR Test
Inverse variance weighted
MR Egger
Simple mode
Weighted median
Weighted mode
0.025
0.000
−0.025
−0.050
−0.075
0.1
0.2
0.3
SNP effect on Cardiac valvular disease
SNP effect on ZNF276

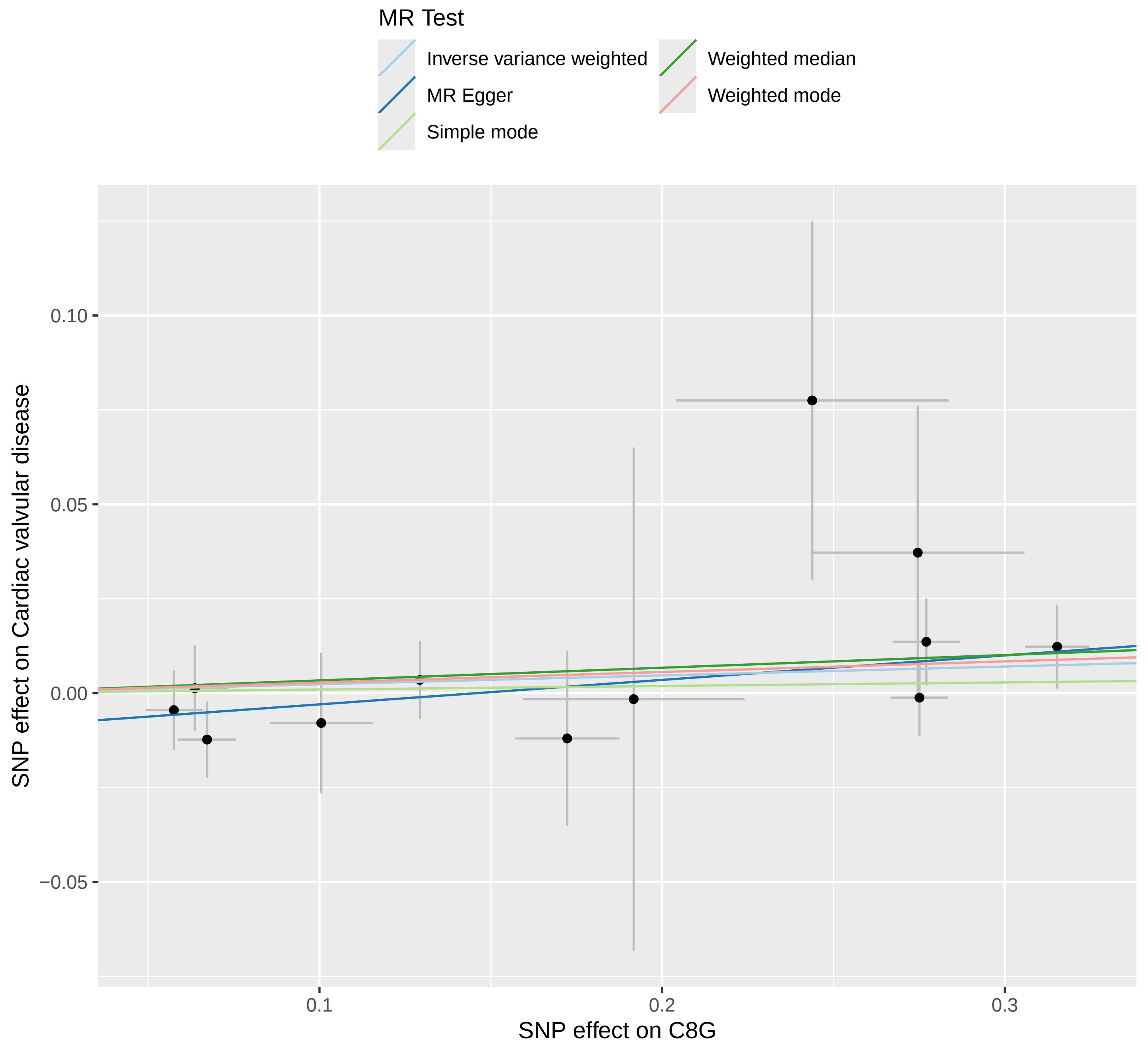
MR Test
Inverse variance weighted
MR Egger
Simple mode
Weighted median
Weighted mode
0.10
0.05
0.00
−0.05
0.1
0.2
0.3
SNP effect on Cardiac valvular disease
SNP effect on C8G

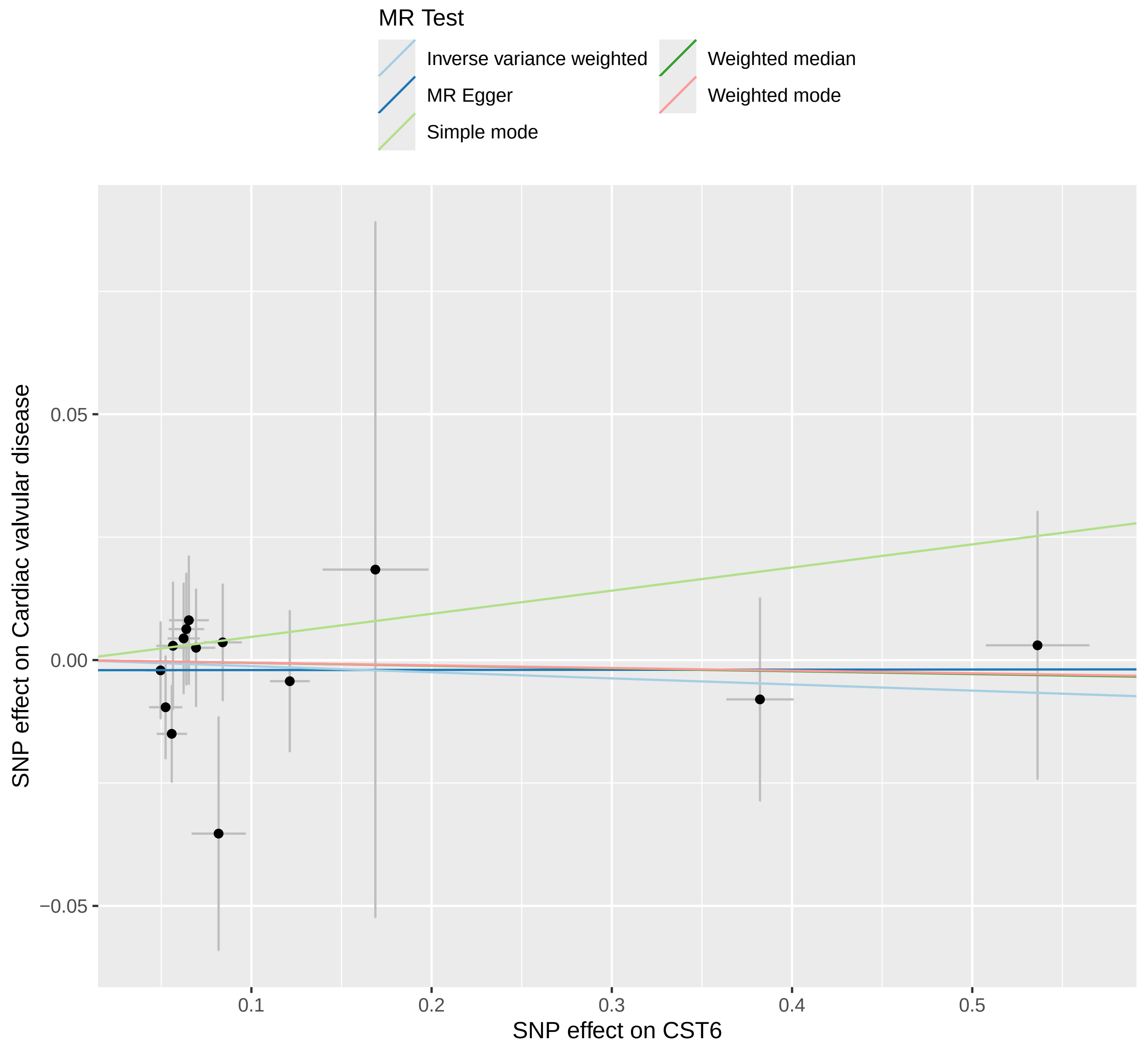

MR Test
Inverse variance weighted
MR Egger
Simple mode
Weighted median
Weighted mode
SNP effect on Cardiac valvular disease
0.05
0.00
−0.05
0.1
0.2
0.3
0.4
0.5
SNP effect on CST6

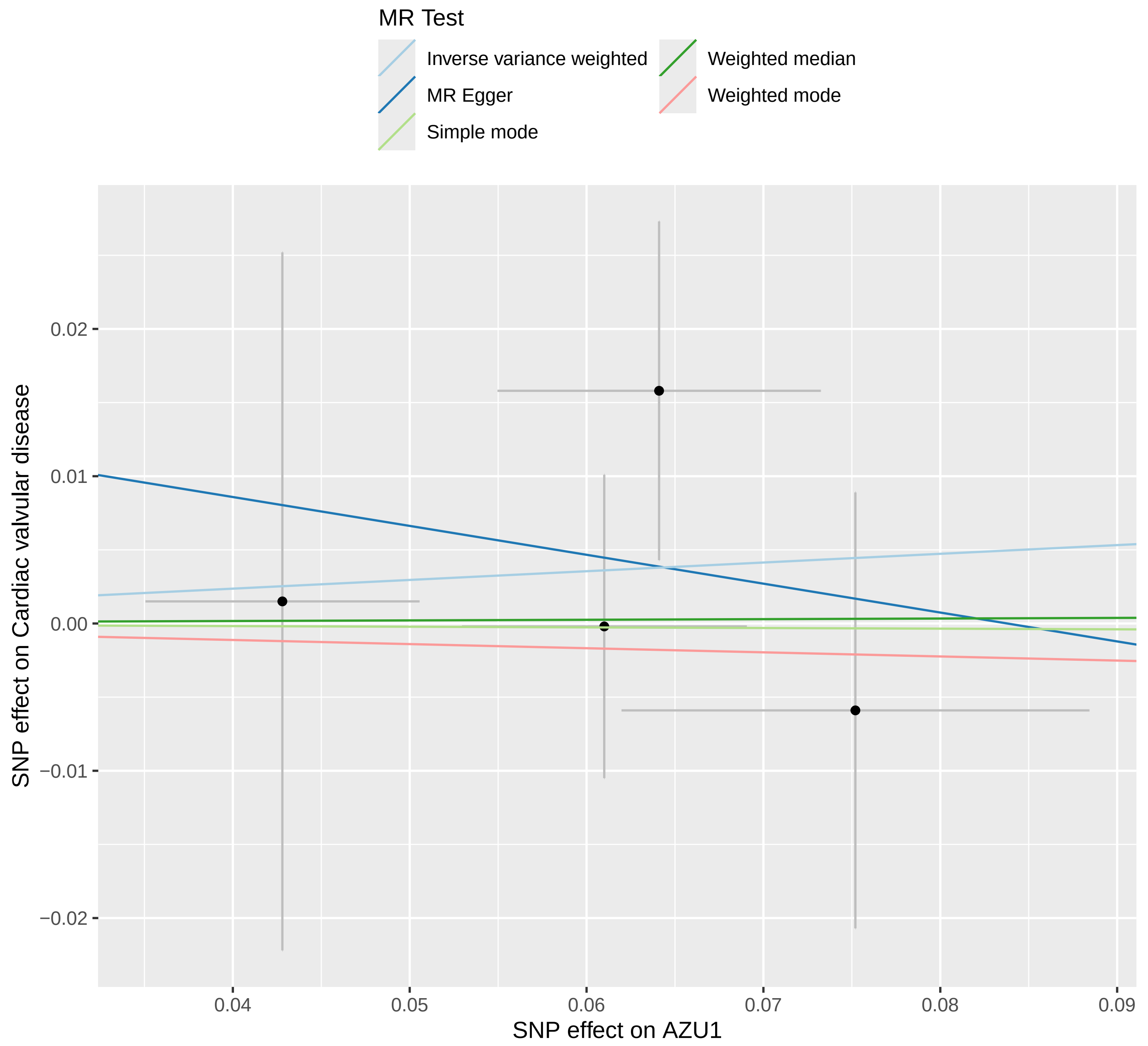
MR Test
Inverse variance weighted
MR Egger
Simple mode
Weighted median
Weighted mode
0.02
0.01
0.00
−0.01
−0.02
SNP effect on Cardiac valvular disease
0.04
0.05
0.06
0.07
0.08
0.09
SNP effect on AZU1

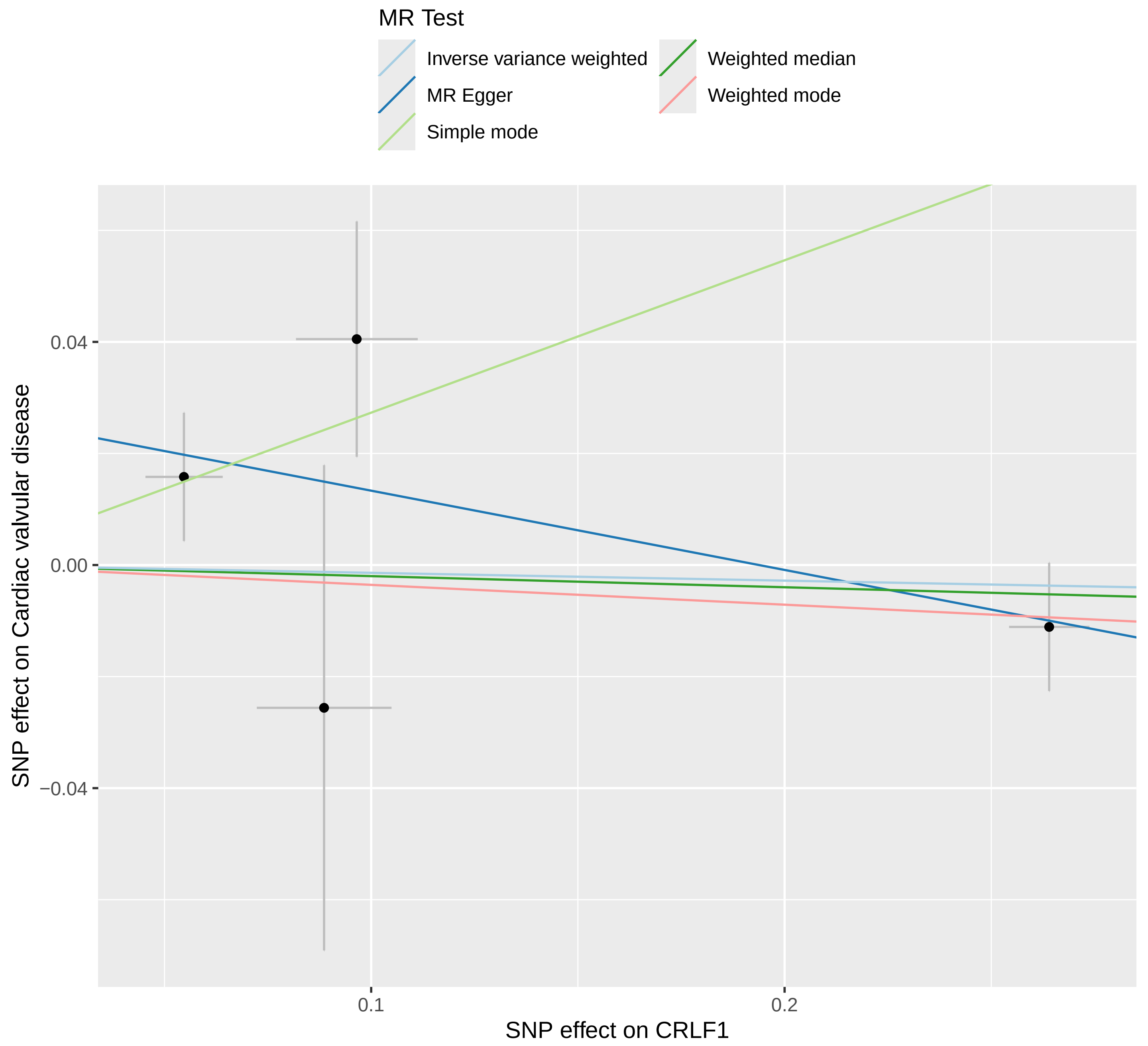
MR Test
Inverse variance weighted
MR Egger
Simple mode
Weighted median
Weighted mode
0.04
0.00
−0.04
SNP effect on Cardiac valvular disease
0.1
0.2
SNP effect on CRLF1

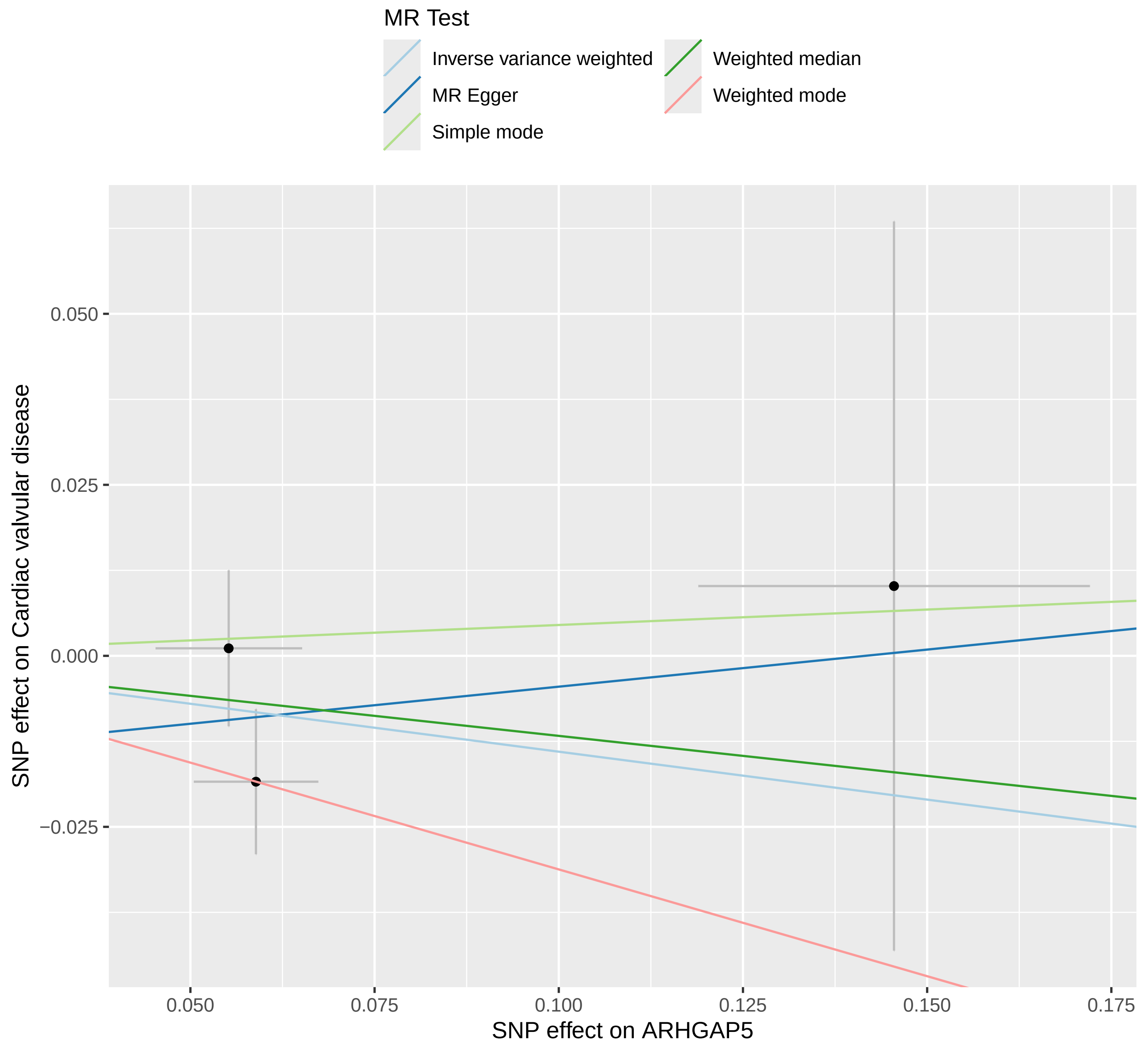
MR Test
Inverse variance weighted
MR Egger
Simple mode
Weighted median
Weighted mode
0.050
0.025
0.000
−0.025
SNP effect on Cardiac valvular disease
0.050
0.075
0.100
0.125
0.150
0.175
SNP effect on ARHGAP5

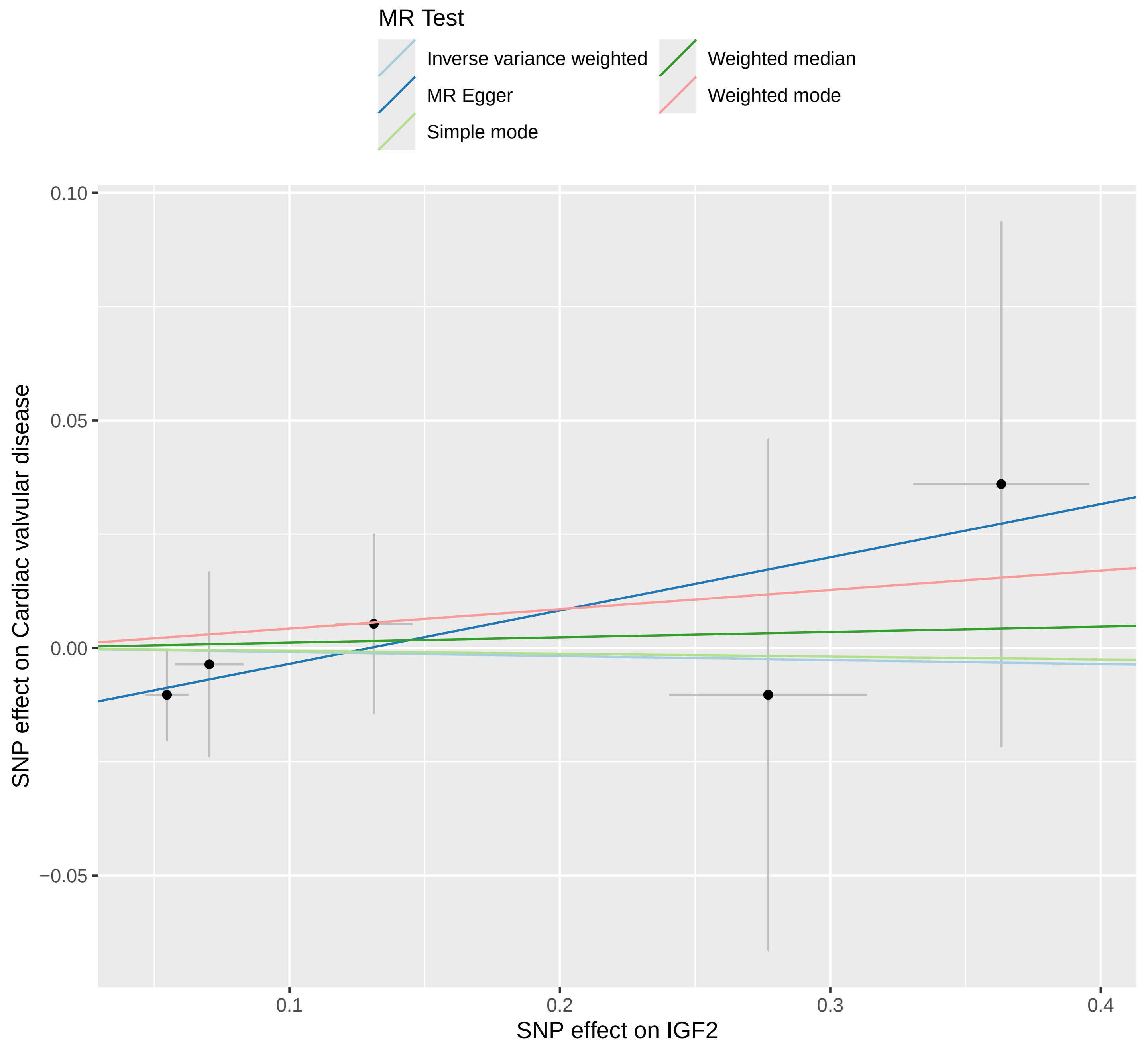
MR Test
Inverse variance weighted
MR Egger
Simple mode
Weighted median
Weighted mode
0.10
0.05
0.00
−0.05
0.1
0.2
0.3
0.4
SNP effect on Cardiac valvular disease
SNP effect on IGF2

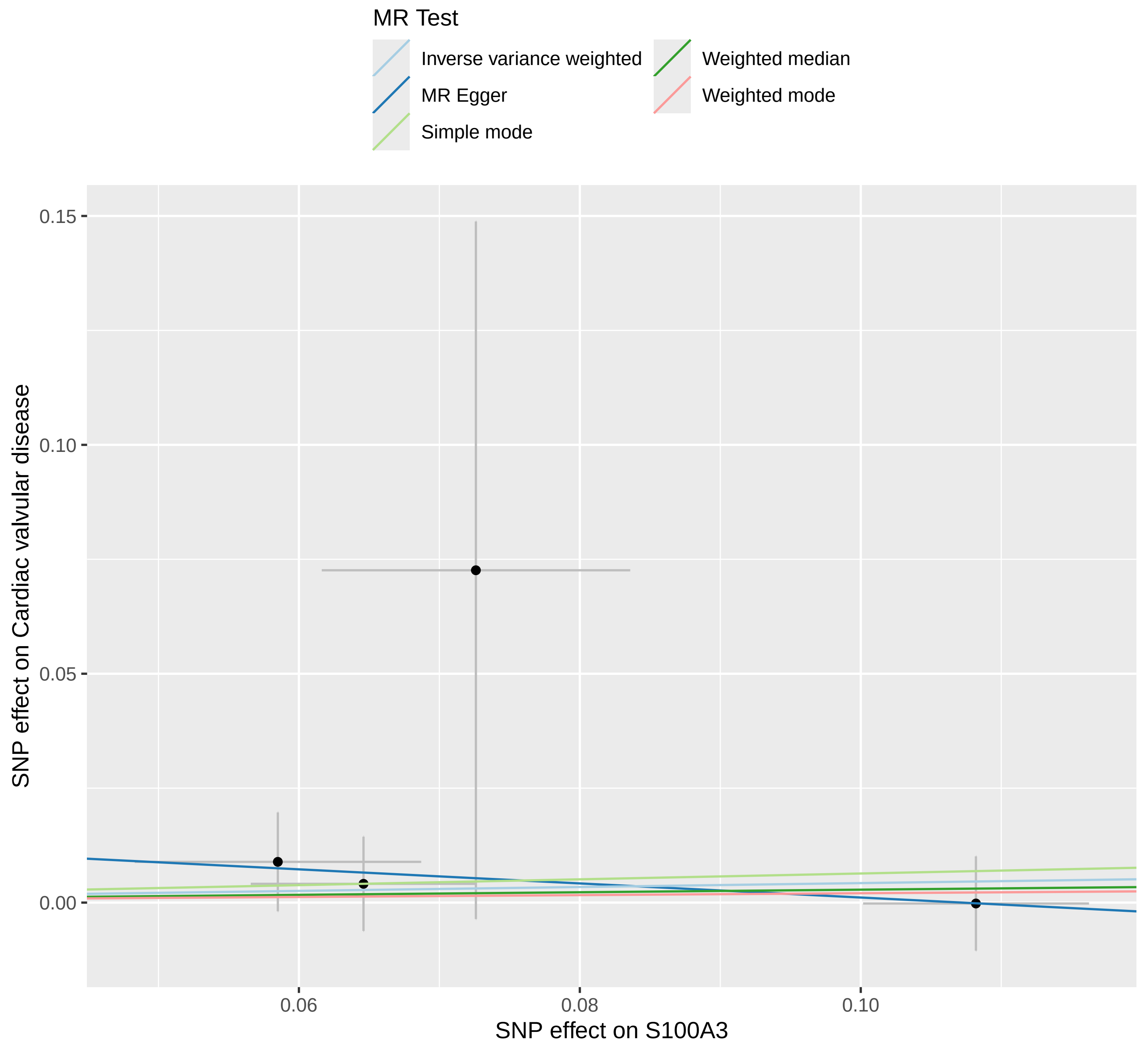
MR Test
Inverse variance weighted
MR Egger
Simple mode
Weighted median
Weighted mode
0.15
0.10
0.05
0.00
SNP effect on Cardiac valvular disease
0.06
0.08
0.10
SNP effect on S100A3

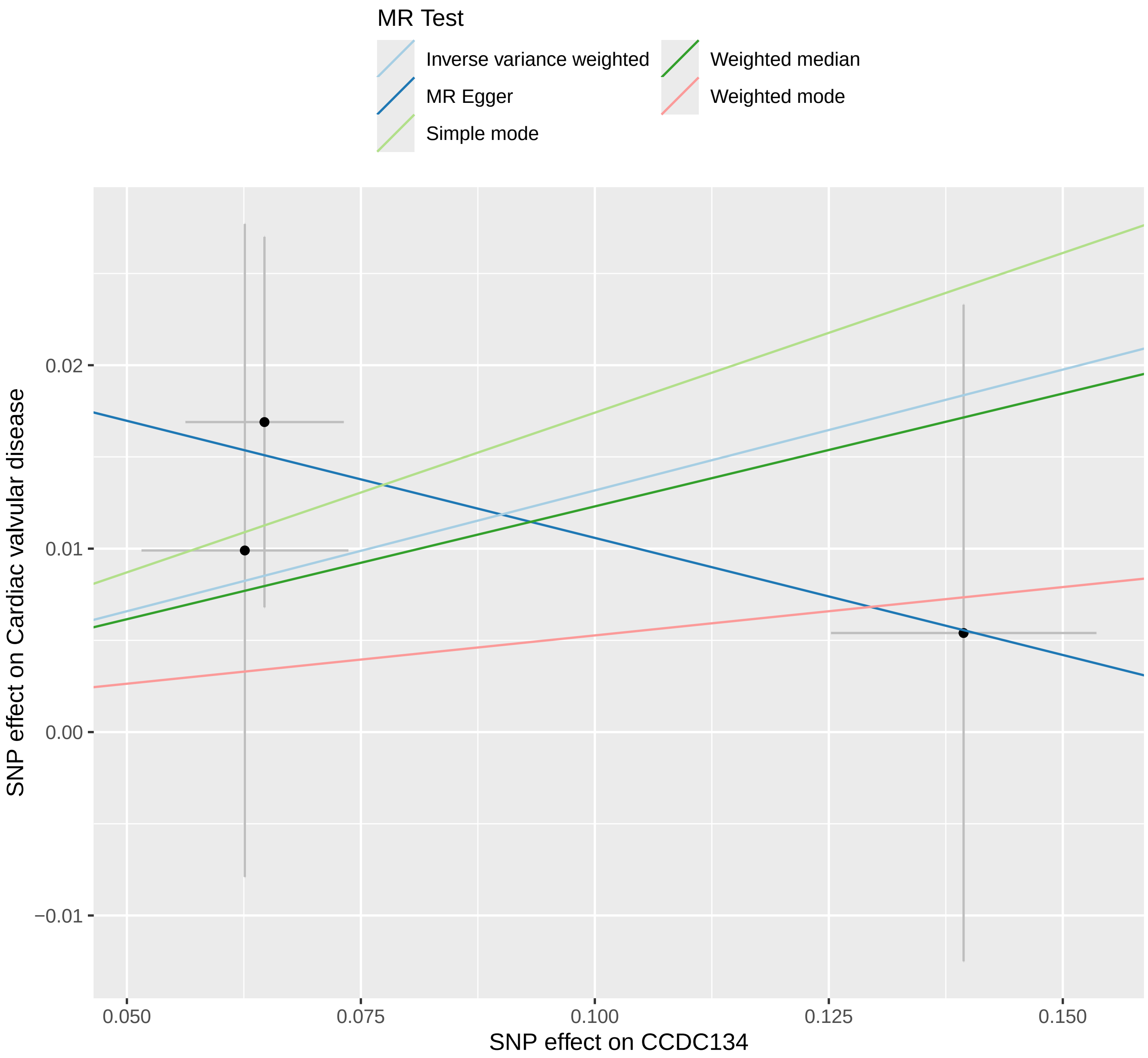

MR Test
Inverse variance weighted
MR Egger
Simple mode
Weighted median
Weighted mode
SNP effect on Cardiac valvular disease
0.02
0.01
0.00
−0.01
0.050
0.075
0.100
0.125
0.150
SNP effect on CCDC134

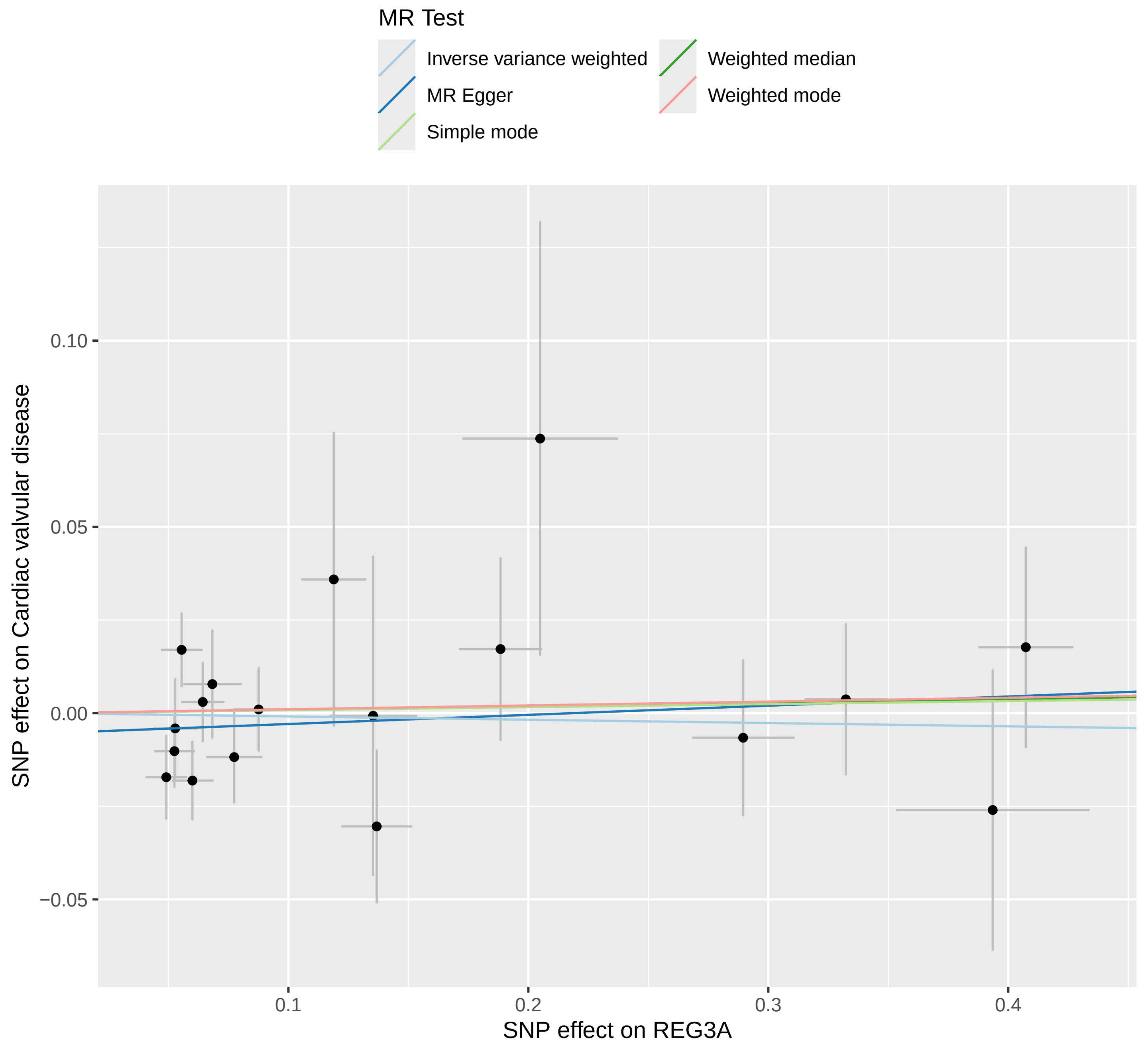
MR Test
Inverse variance weighted
MR Egger
Simple mode
Weighted median
Weighted mode
0.10
0.05
0.00
−0.05
0.1
0.2
0.3
0.4
SNP effect on Cardiac valvular disease
SNP effect on REG3A

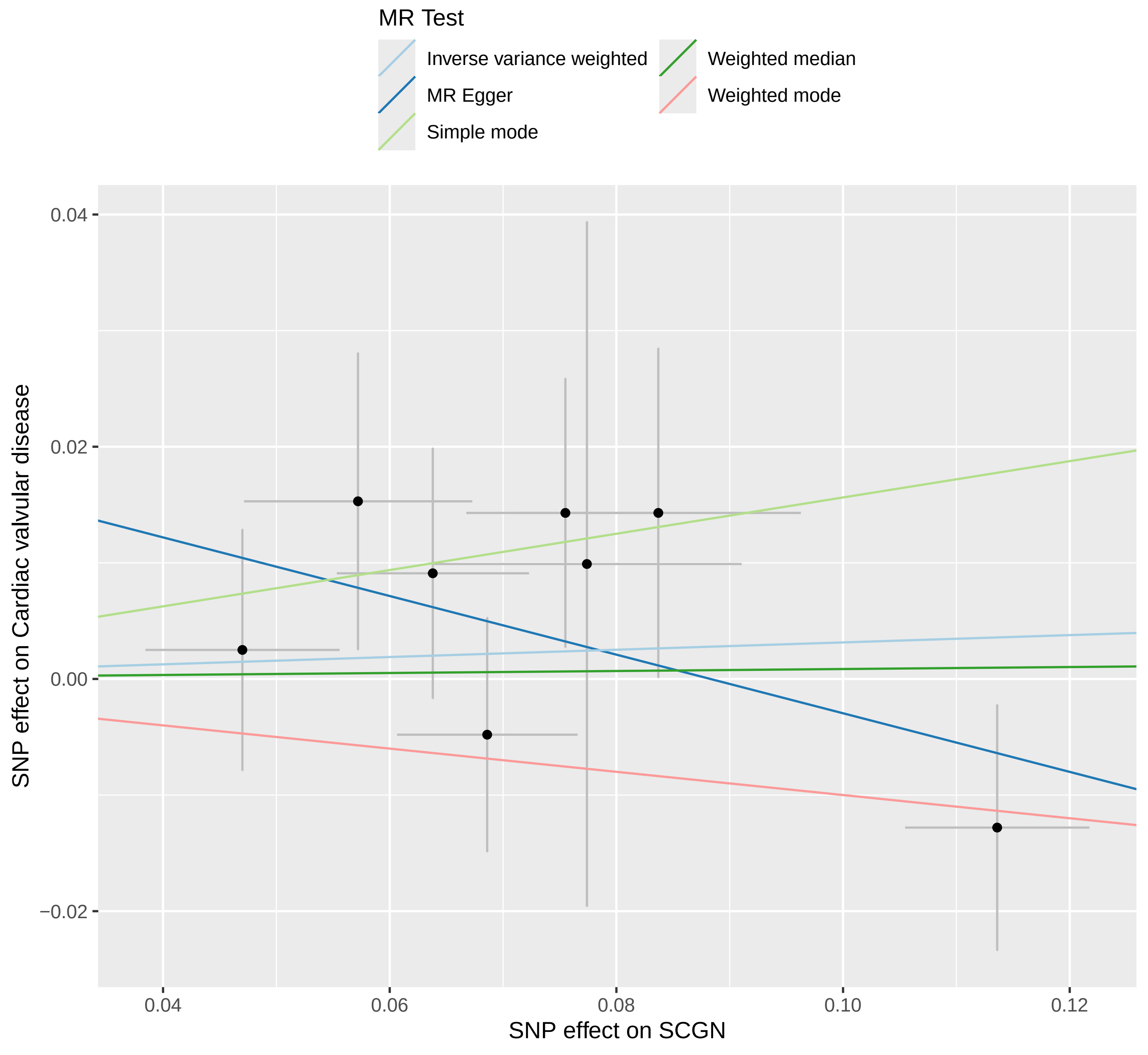
MR Test
Inverse variance weighted
MR Egger
Simple mode
Weighted median
Weighted mode
0.04
0.02
0.00
−0.02
SNP effect on Cardiac valvular disease
0.04
0.06
0.08
0.10
0.12
SNP effect on SCGN

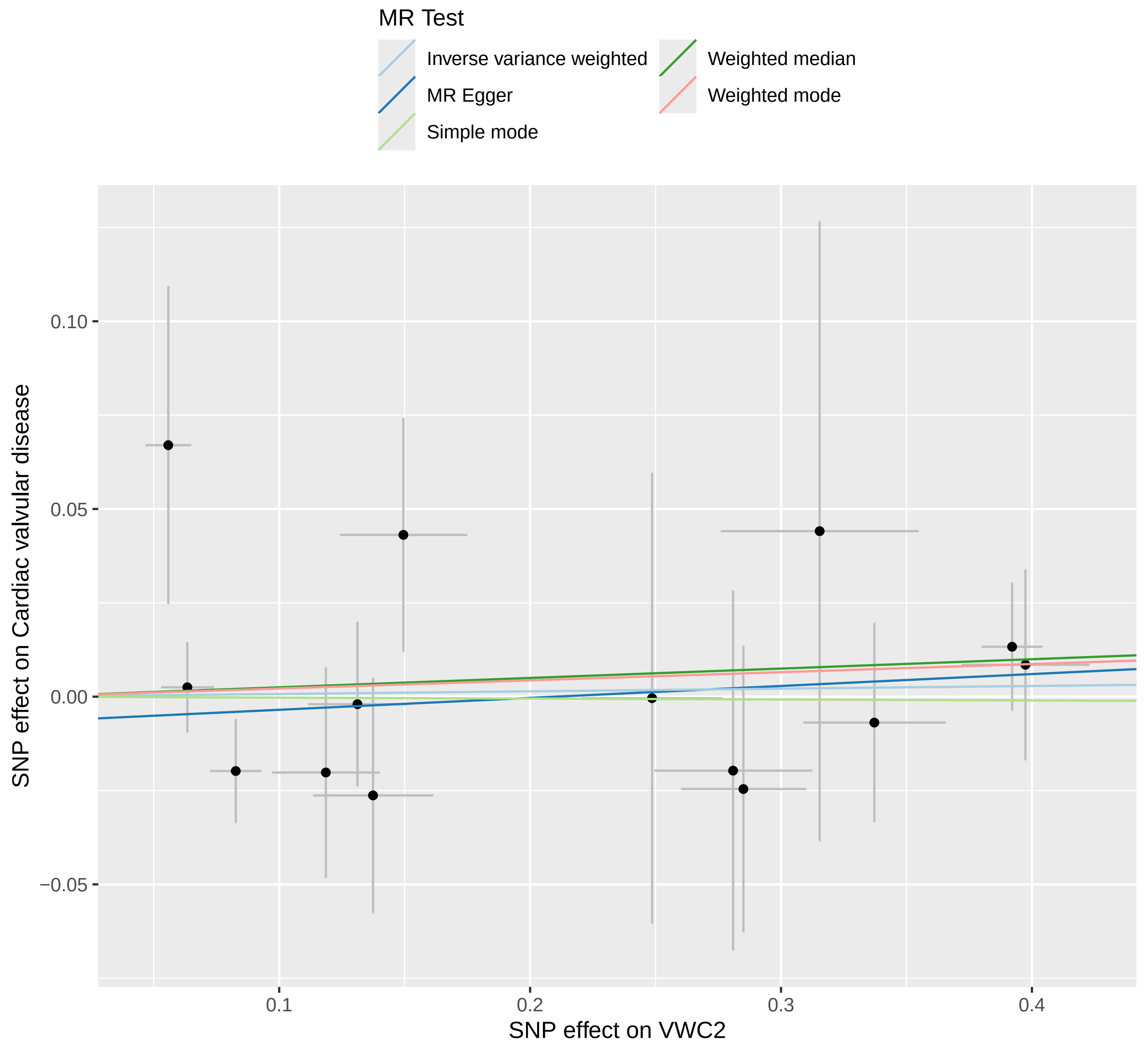
MR Test
Inverse variance weighted
MR Egger
Simple mode
Weighted median
Weighted mode
0.10
0.05
0.00
−0.05
0.1
0.2
0.3
0.4
SNP effect on VWC2
SNP effect on Cardiac valvular disease

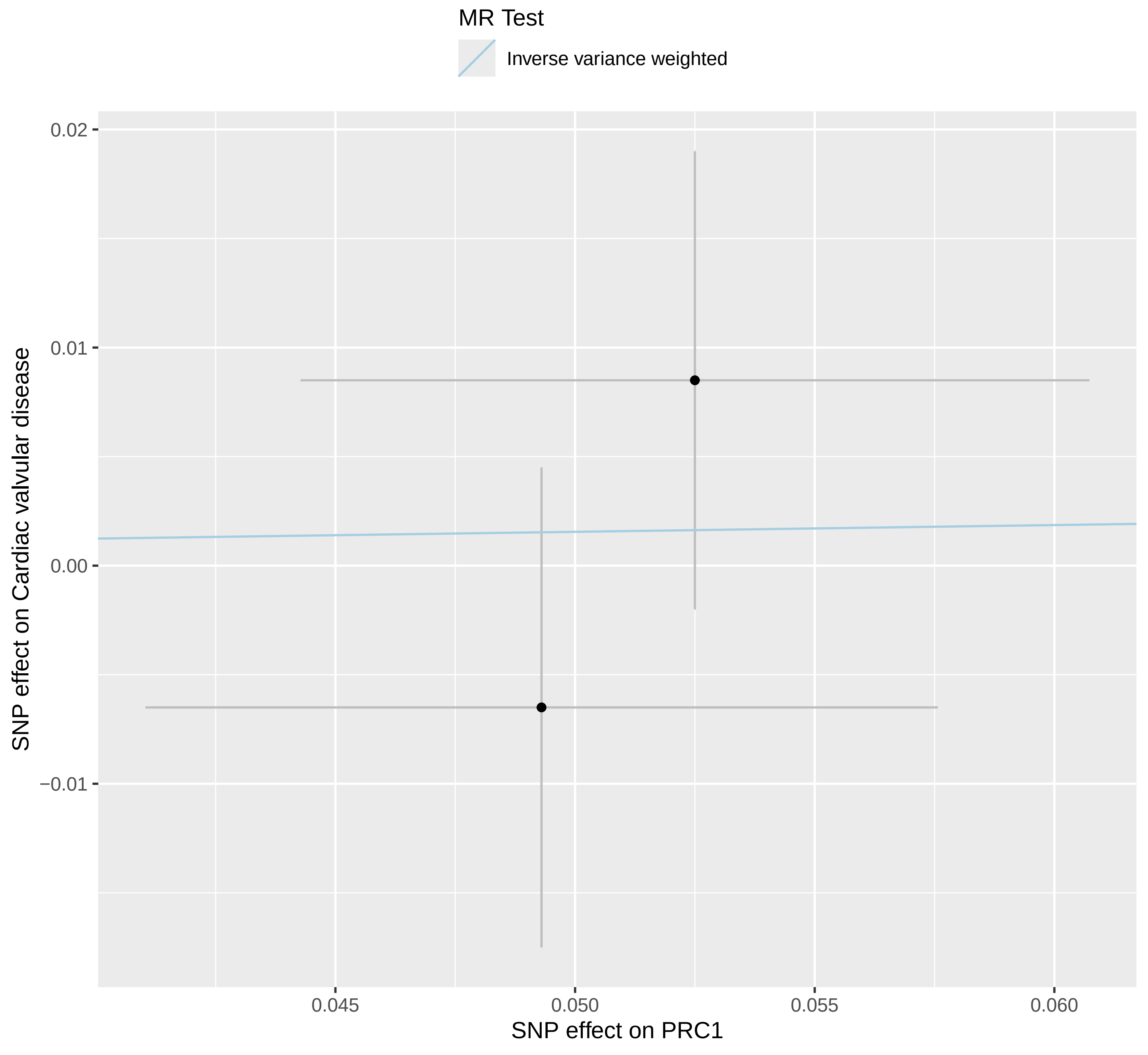
MR Test
Inverse variance weighted
0.02
0.01
0.00
−0.01
SNP effect on Cardiac valvular disease
0.045
0.050
0.055
0.060
SNP effect on PRC1

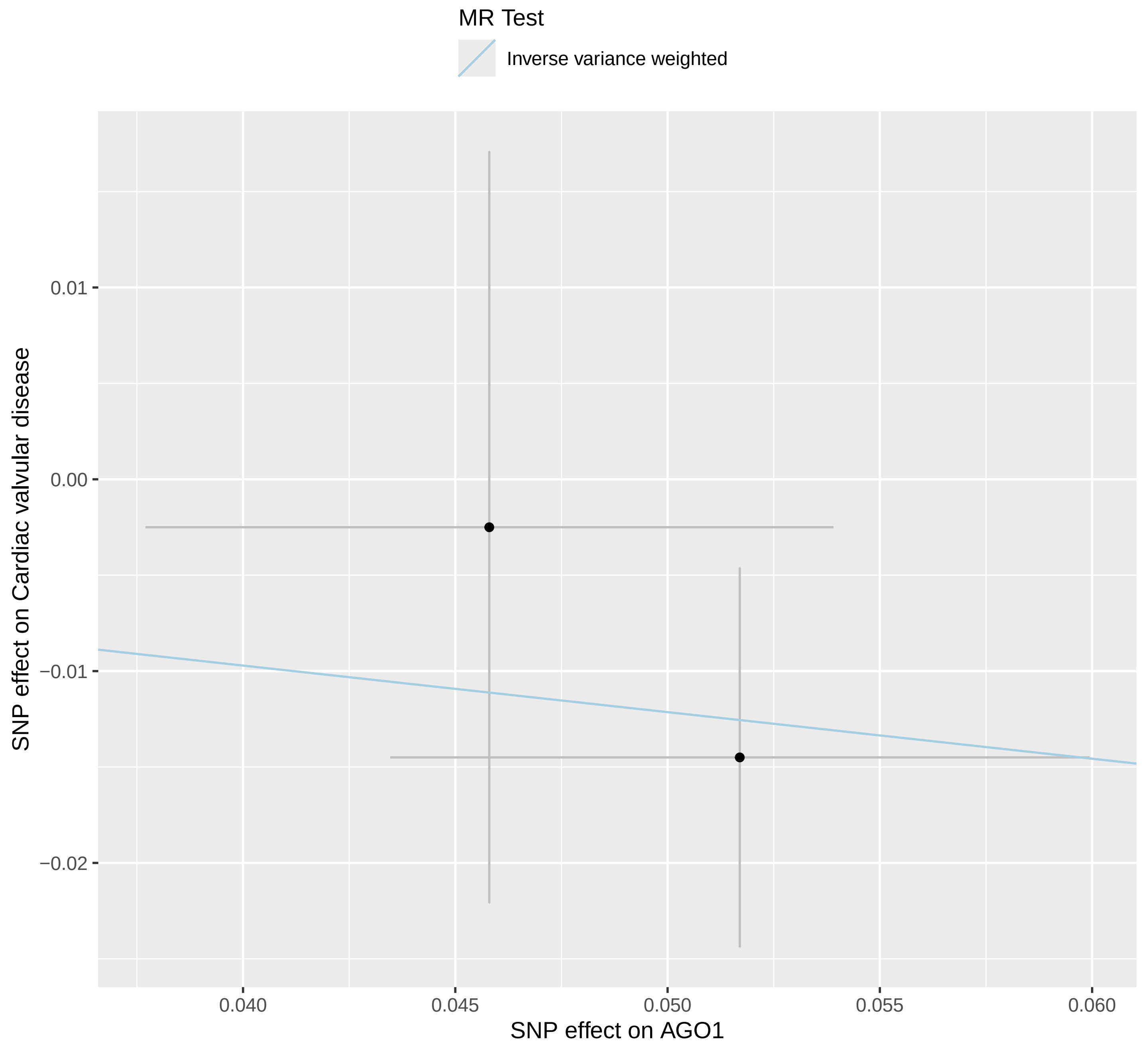

MR Test
Inverse variance weighted
0.01
0.00
−0.01
−0.02
SNP effect on Cardiac valvular disease
0.040
0.045
0.050
0.055
0.060
SNP effect on AGO1

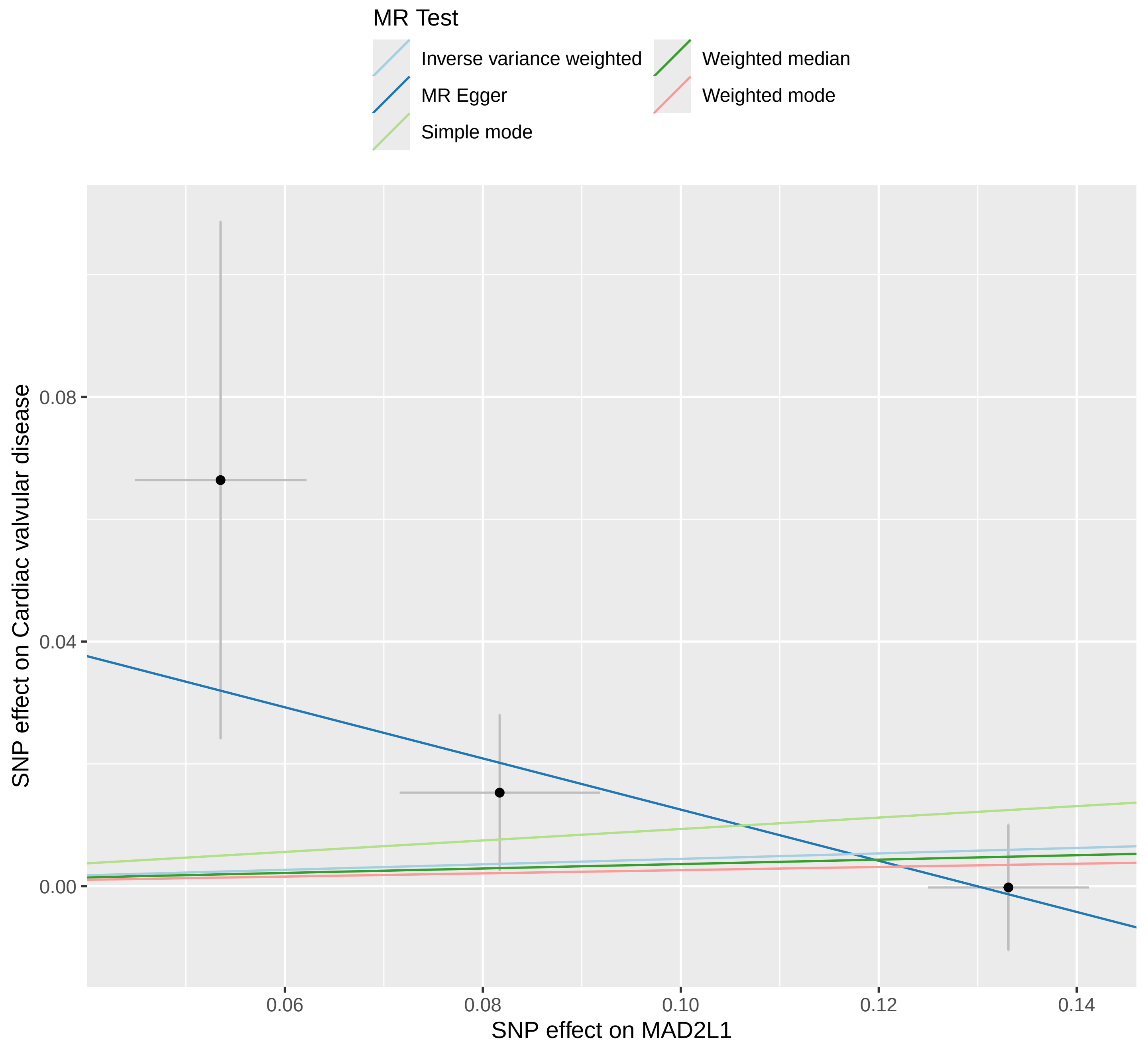

MR Test
Inverse variance weighted
MR Egger
Simple mode
Weighted median
Weighted mode
0.08
0.04
0.00
SNP effect on Cardiac valvular disease
0.06
0.08
0.10
0.12
0.14
SNP effect on MAD2L1

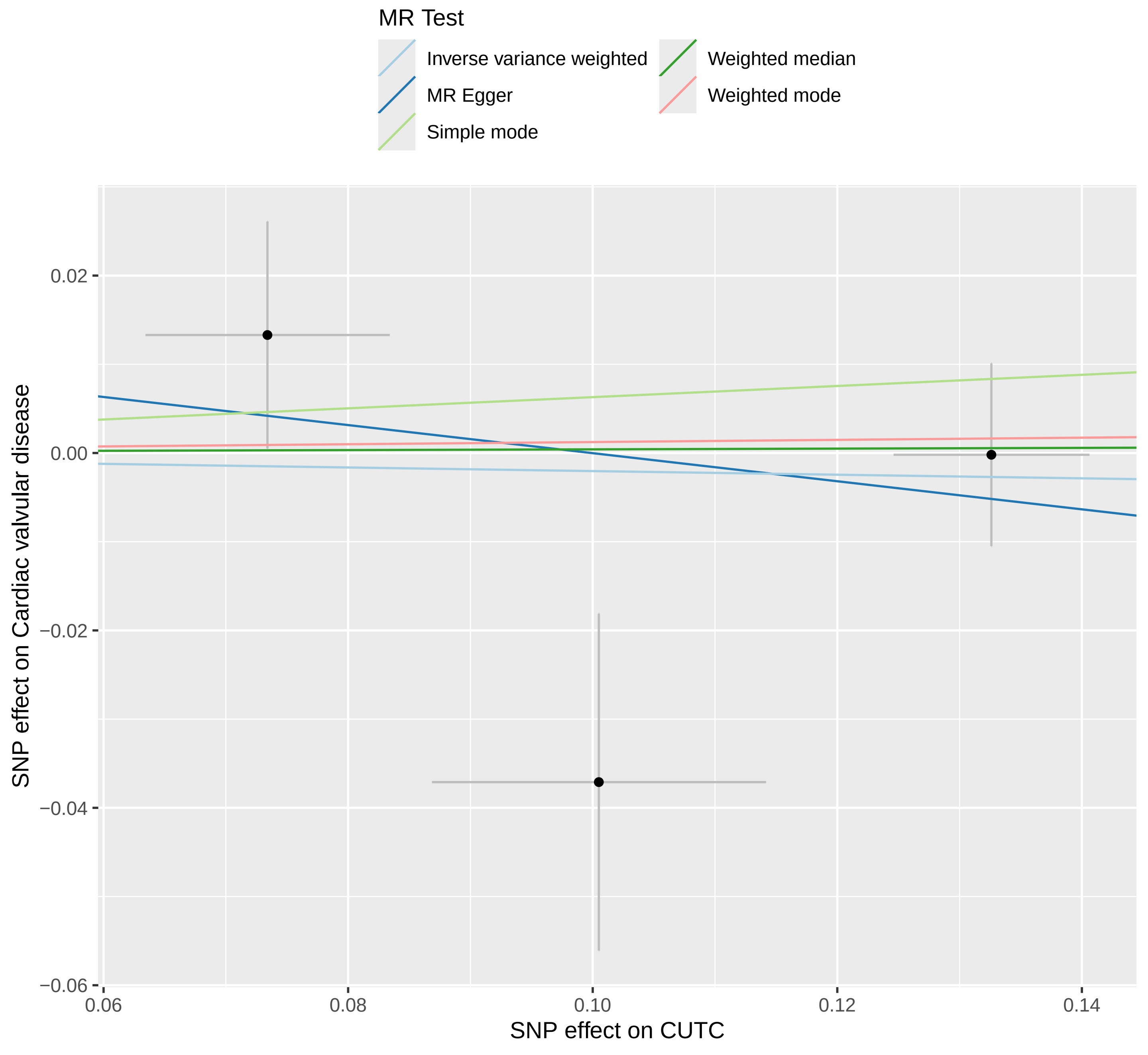

MR Test
Inverse variance weighted
MR Egger
Simple mode
Weighted median
Weighted mode
0.02
0.00
−0.02
−0.04
−0.06
0.06
0.08
0.10
0.12
0.14
SNP effect on Cardiac valvular disease
SNP effect on CUTC

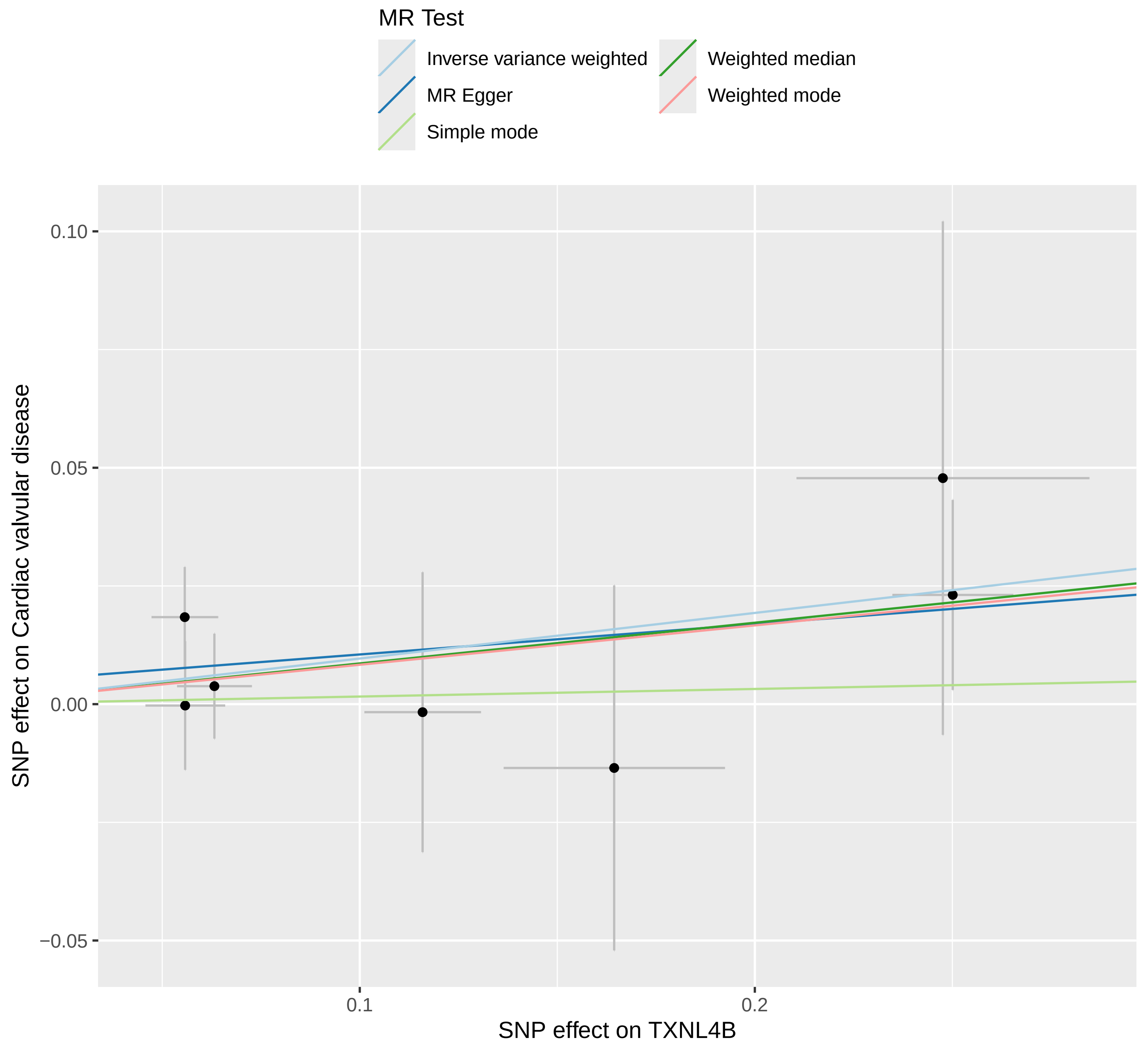
MR Test
Inverse variance weighted
MR Egger
Simple mode
Weighted median
Weighted mode
0.10
0.05
0.00
−0.05
0.1
0.2
SNP effect on TXNL4B
SNP effect on Cardiac valvular disease

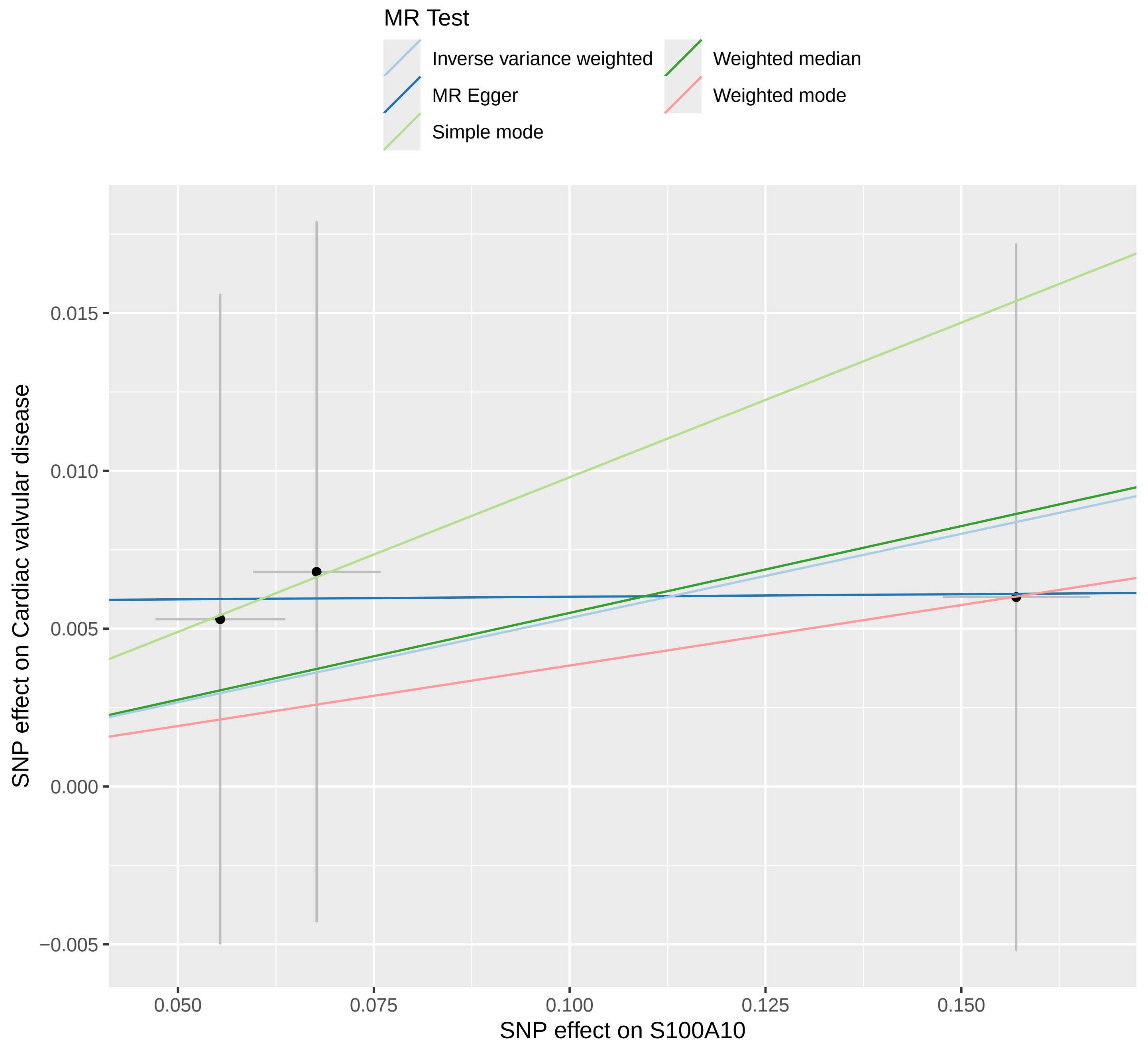

MR Test
Inverse variance weighted
MR Egger
Simple mode
Weighted median
Weighted mode
SNP effect on Cardiac valvular disease
0.015
0.010
0.005
0.000
−0.005
0.050
0.075
0.100
0.125
0.150
SNP effect on S100A10

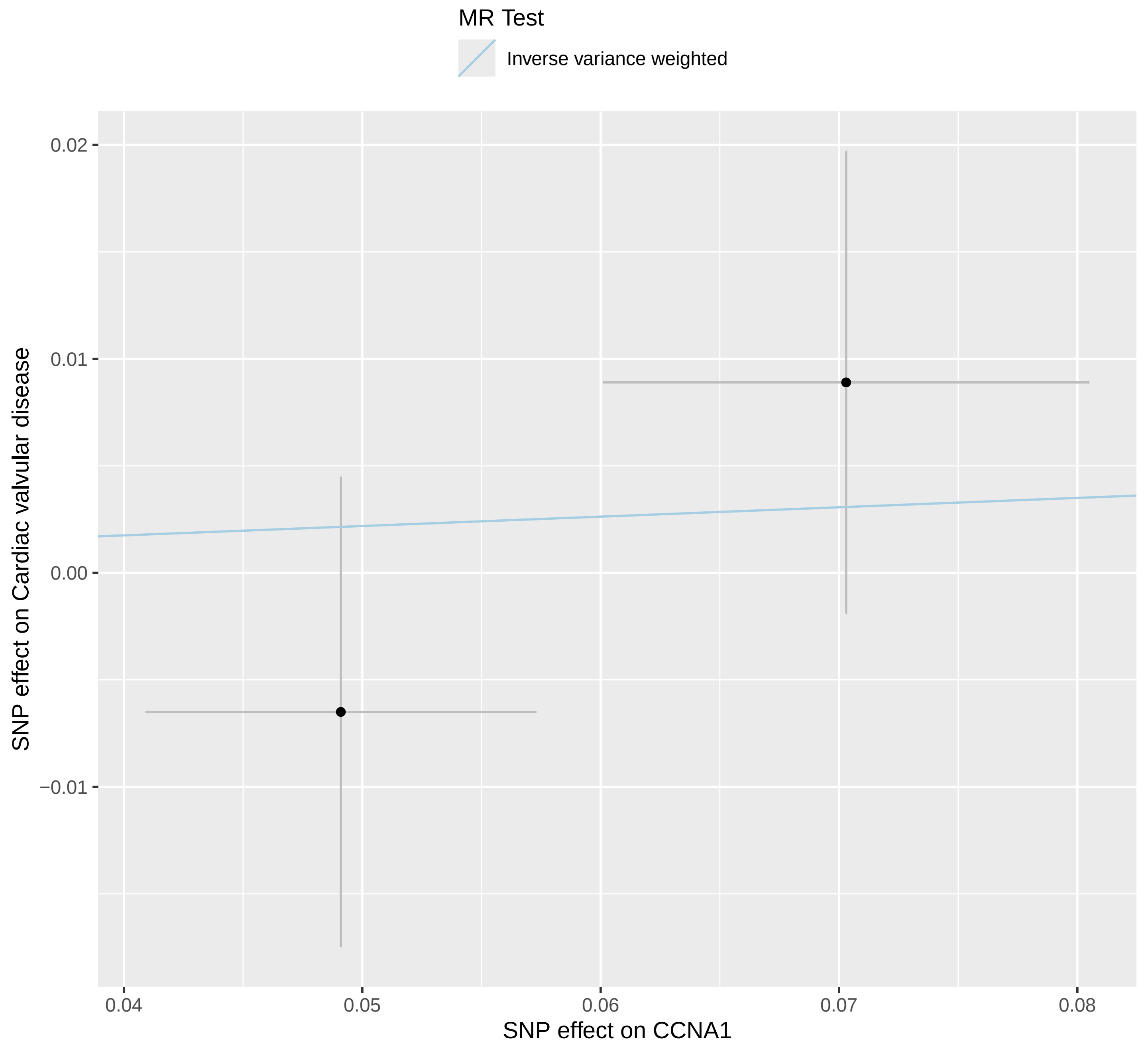

MR Test
Inverse variance weighted
0.02
0.01
0.00
−0.01
0.04
0.05
0.06
0.07
0.08
SNP effect on Cardiac valvular disease
SNP effect on CCNA1

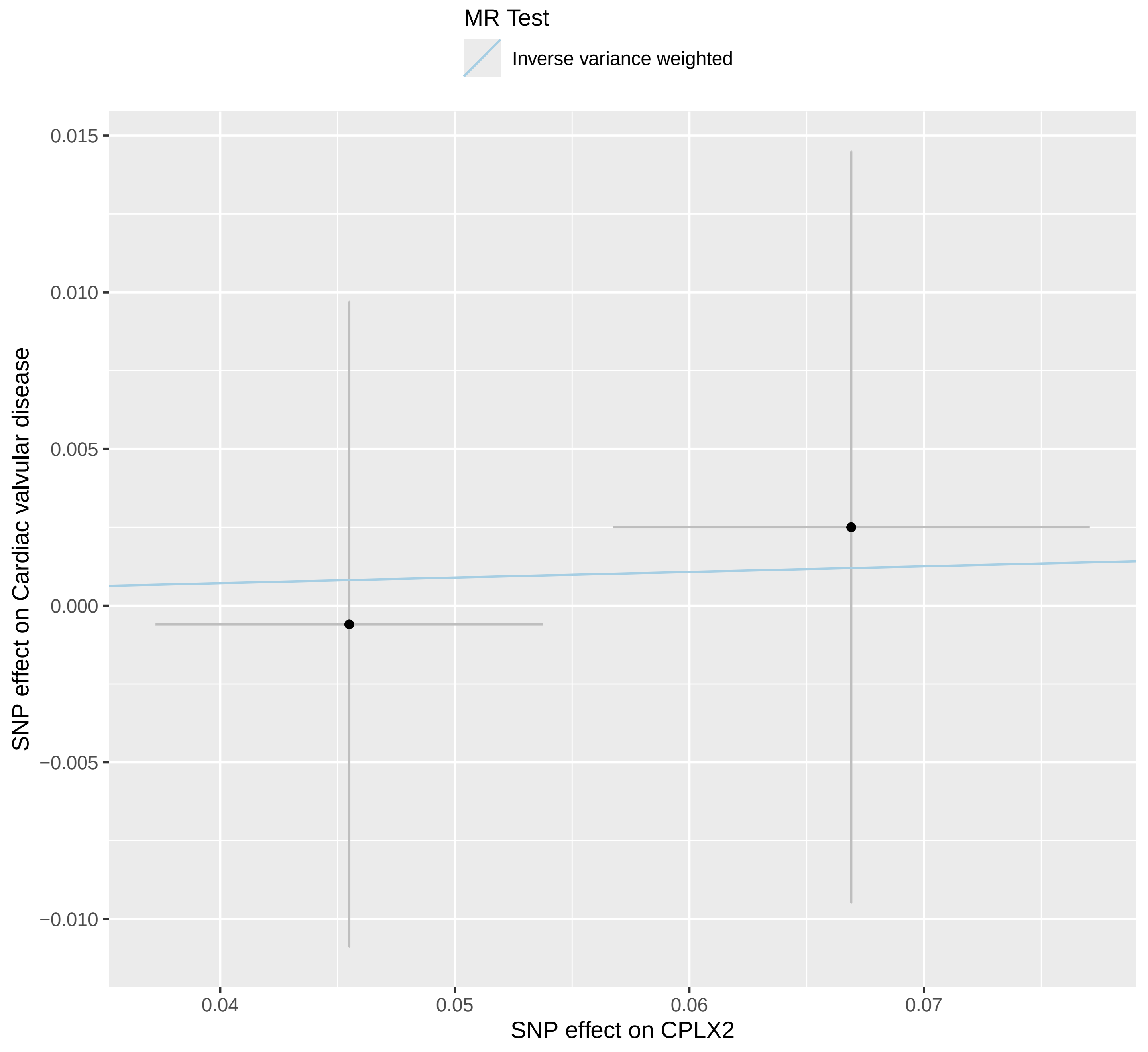
MR Test
Inverse variance weighted
0.015
0.010
0.005
0.000
−0.005
−0.010
SNP effect on Cardiac valvular disease
0.04
0.05
0.06
0.07
SNP effect on CPLX2

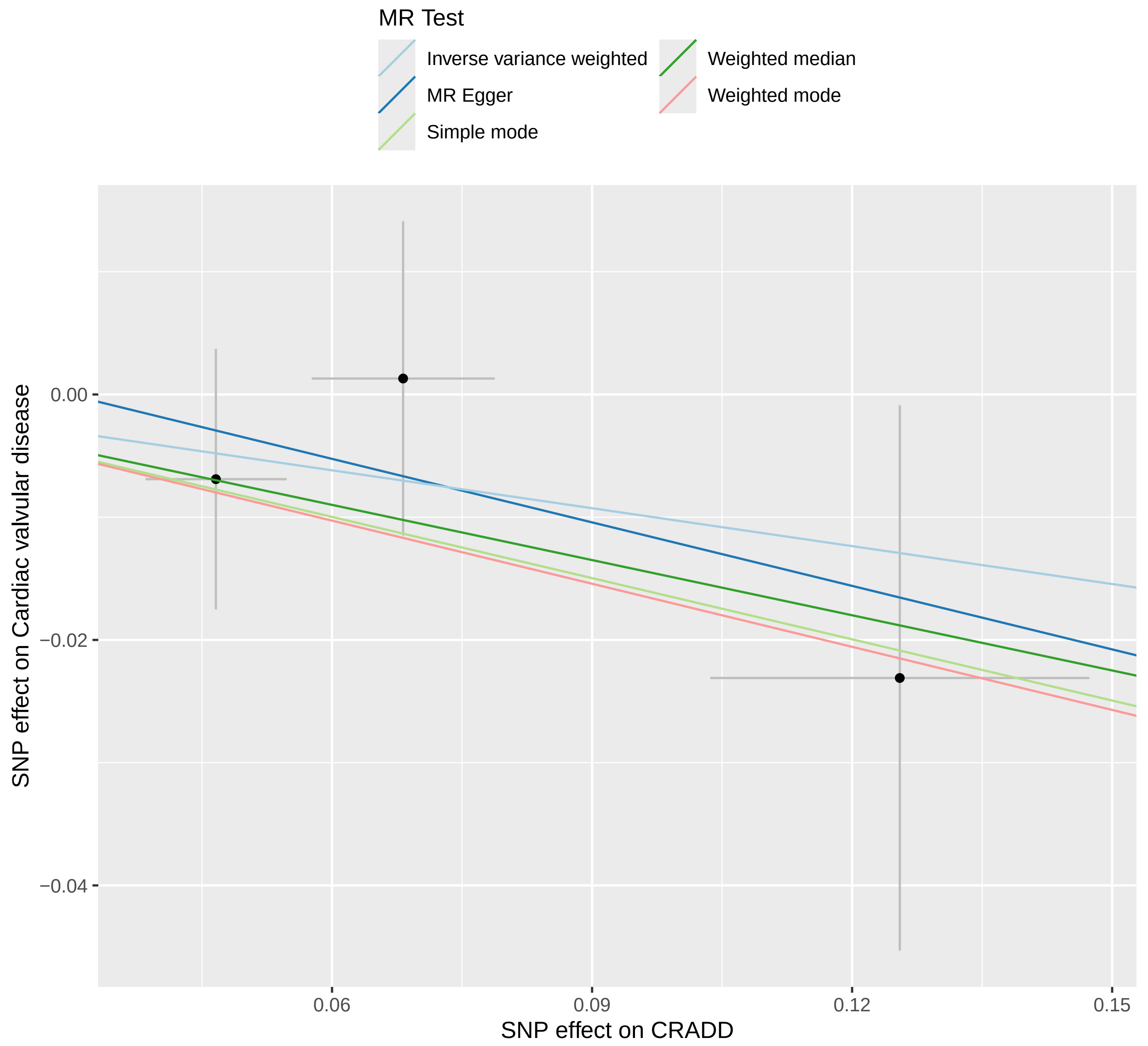
MR Test
Inverse variance weighted
MR Egger
Simple mode
Weighted median
Weighted mode
0.00
−0.02
−0.04
0.06
0.09
0.12
0.15
SNP effect on Cardiac valvular disease
SNP effect on CRADD

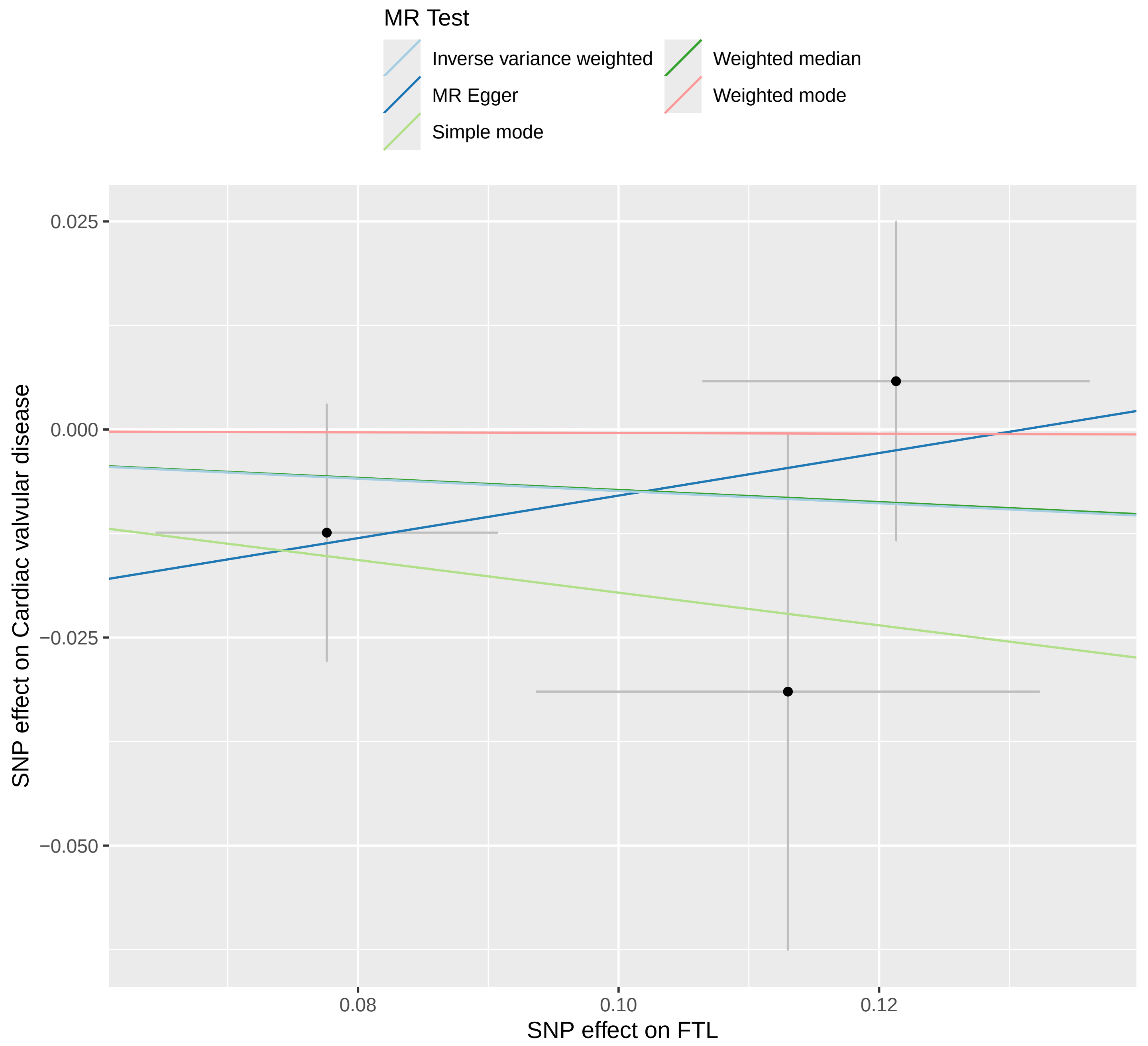
MR Test
Inverse variance weighted
MR Egger
Simple mode
Weighted median
Weighted mode
0.025
0.000
−0.025
−0.050
0.08
0.10
0.12
SNP effect on Cardiac valvular disease
SNP effect on FTL

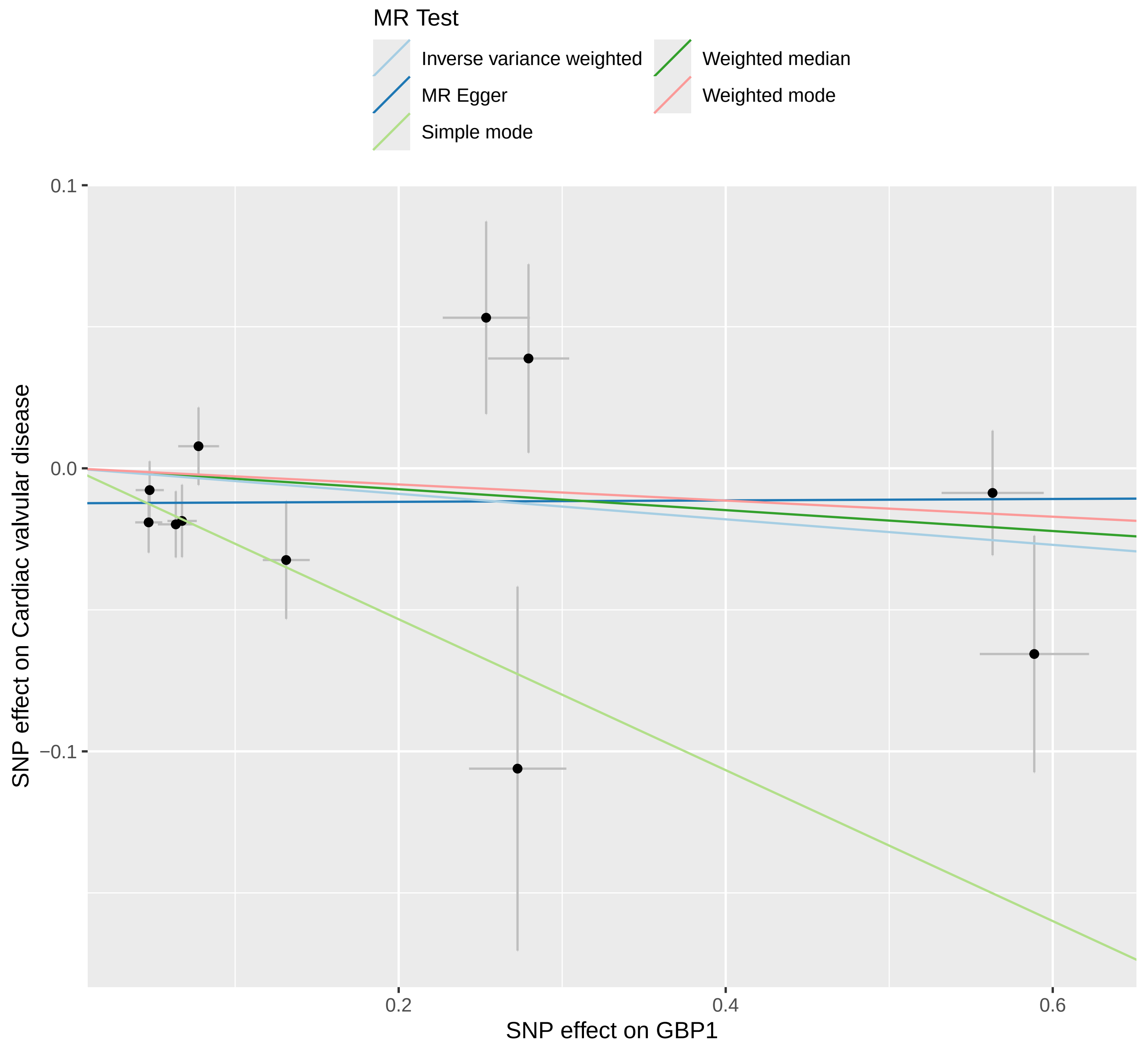
MR Test
Inverse variance weighted
MR Egger
Simple mode
Weighted median
Weighted mode
0.1
0.0
−0.1
0.2
0.4
0.6
SNP effect on Cardiac valvular disease
SNP effect on GBP1

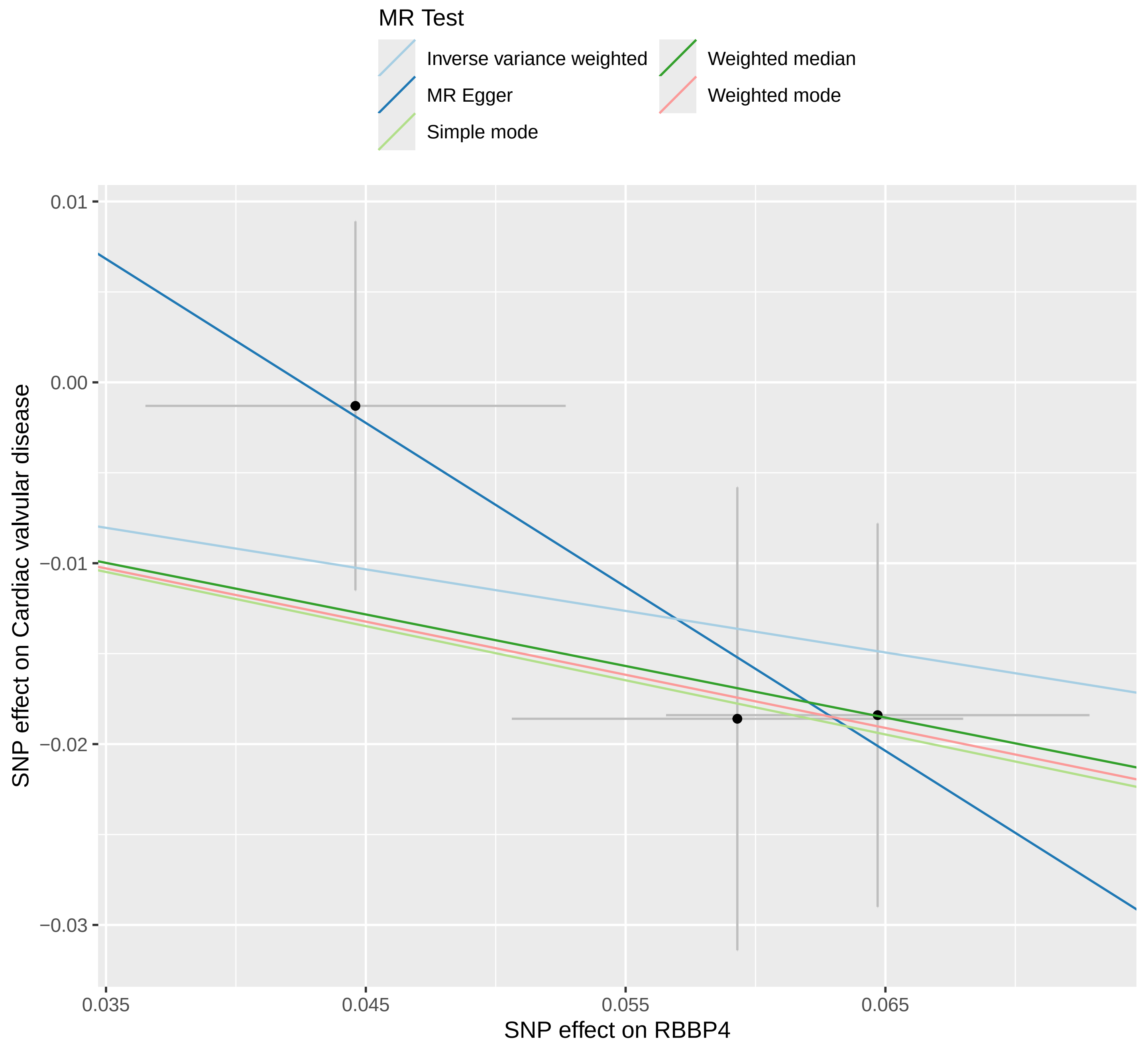
MR Test
Inverse variance weighted
MR Egger
Simple mode
Weighted median
Weighted mode
0.01
0.00
−0.01
−0.02
−0.03
SNP effect on Cardiac valvular disease
0.035
0.045
0.055
0.065
SNP effect on RBBP4

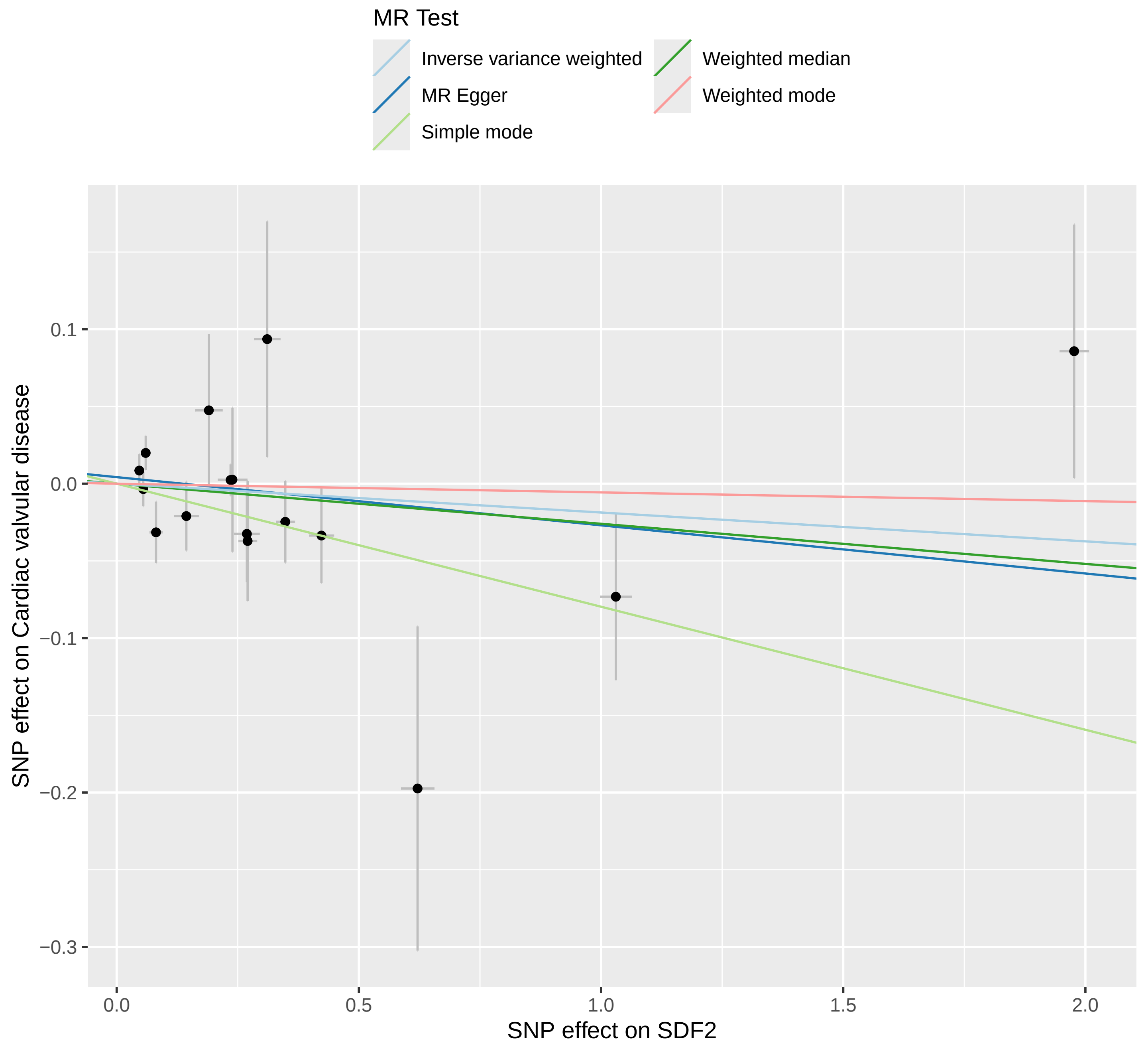
MR Test
Inverse variance weighted
MR Egger
Simple mode
Weighted median
Weighted mode
SNP effect on Cardiac valvular disease
0.1
0.0
−0.1
−0.2
−0.3
0.0
0.5
1.0
1.5
2.0
SNP effect on SDF2

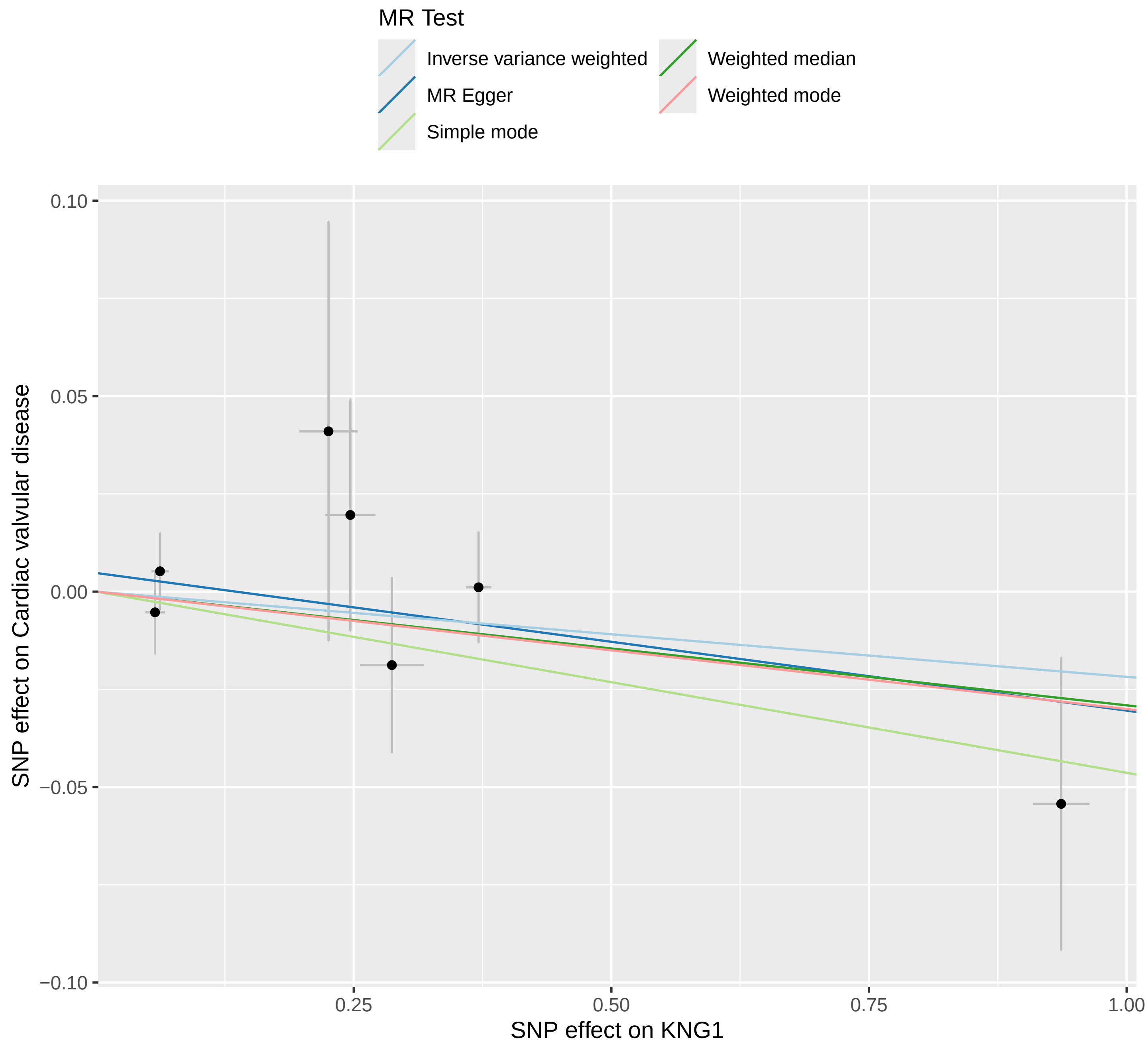

MR Test
Inverse variance weighted
MR Egger
Simple mode
Weighted median
Weighted mode
0.10
0.05
0.00
−0.05
−0.10
SNP effect on Cardiac valvular disease
0.25
0.50
0.75
1.00
SNP effect on KNG1

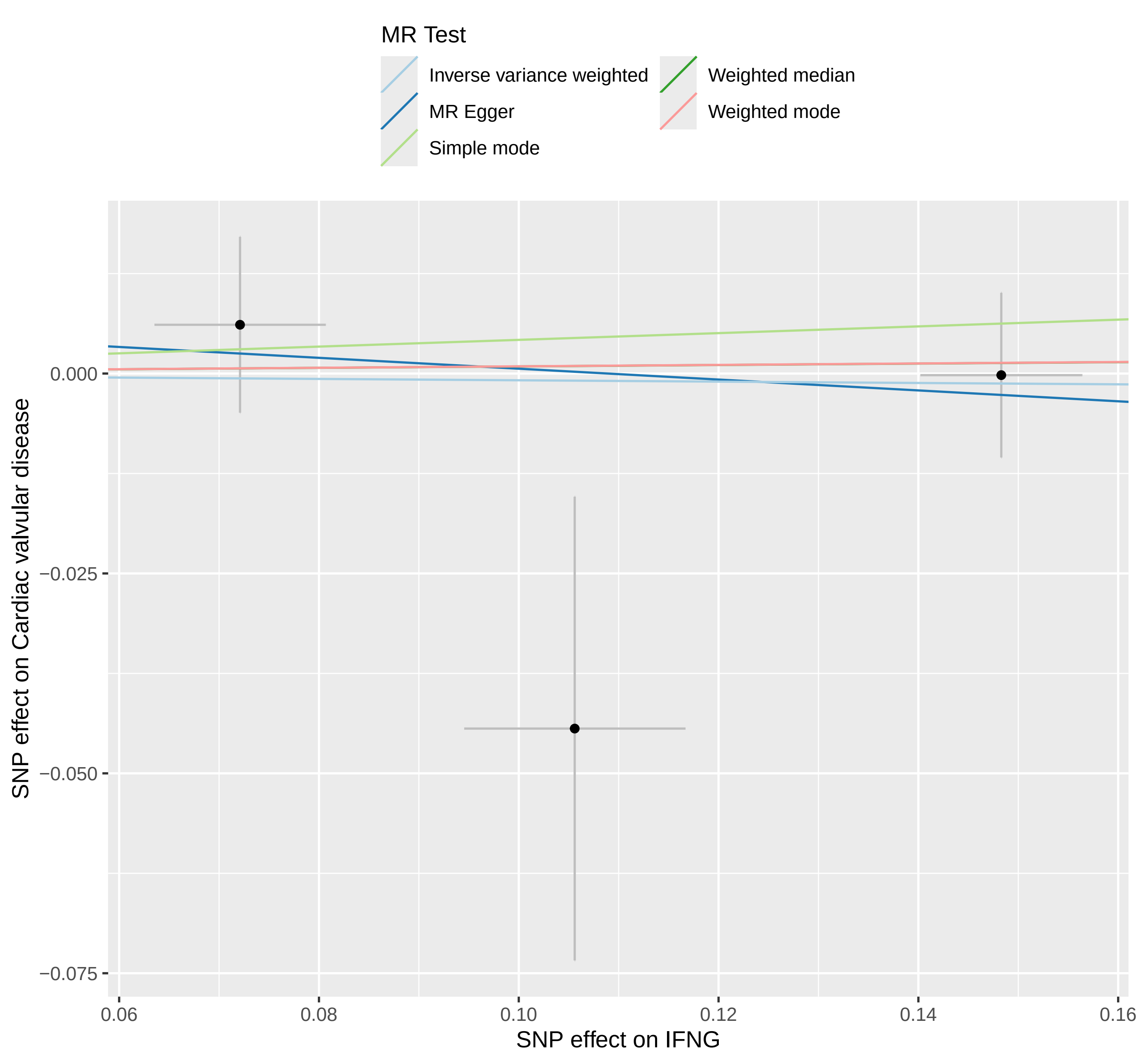
MR Test
Inverse variance weighted
MR Egger
Simple mode
Weighted median
Weighted mode
0.000
−0.025
−0.050
−0.075
SNP effect on Cardiac valvular disease
0.06
0.08
0.10
0.12
0.14
0.16
SNP effect on IFNG

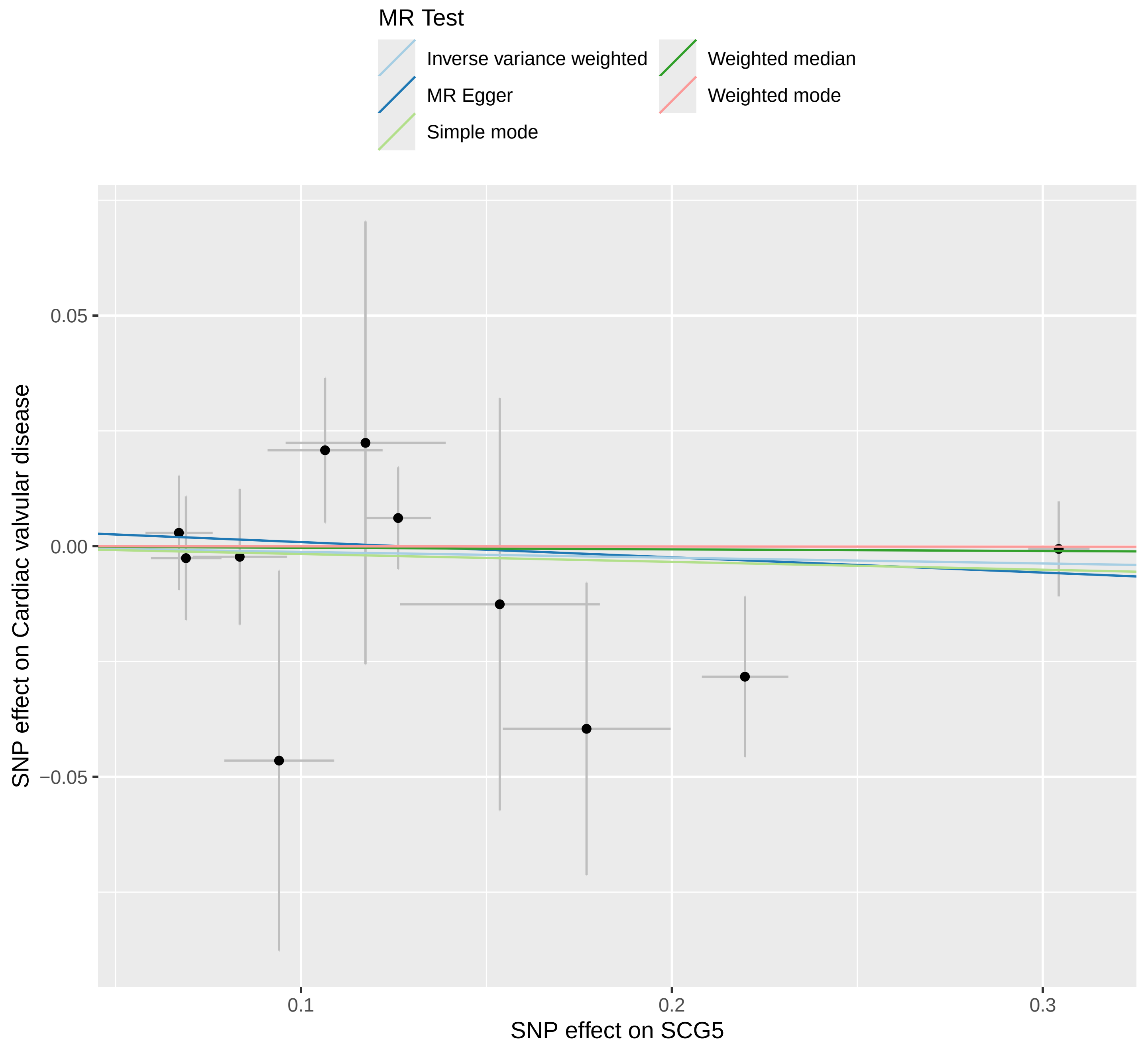

MR Test
Inverse variance weighted
MR Egger
Simple mode
Weighted median
Weighted mode
0.05
0.00
−0.05
0.1
0.2
0.3
SNP effect on Cardiac valvular disease
SNP effect on SCG5

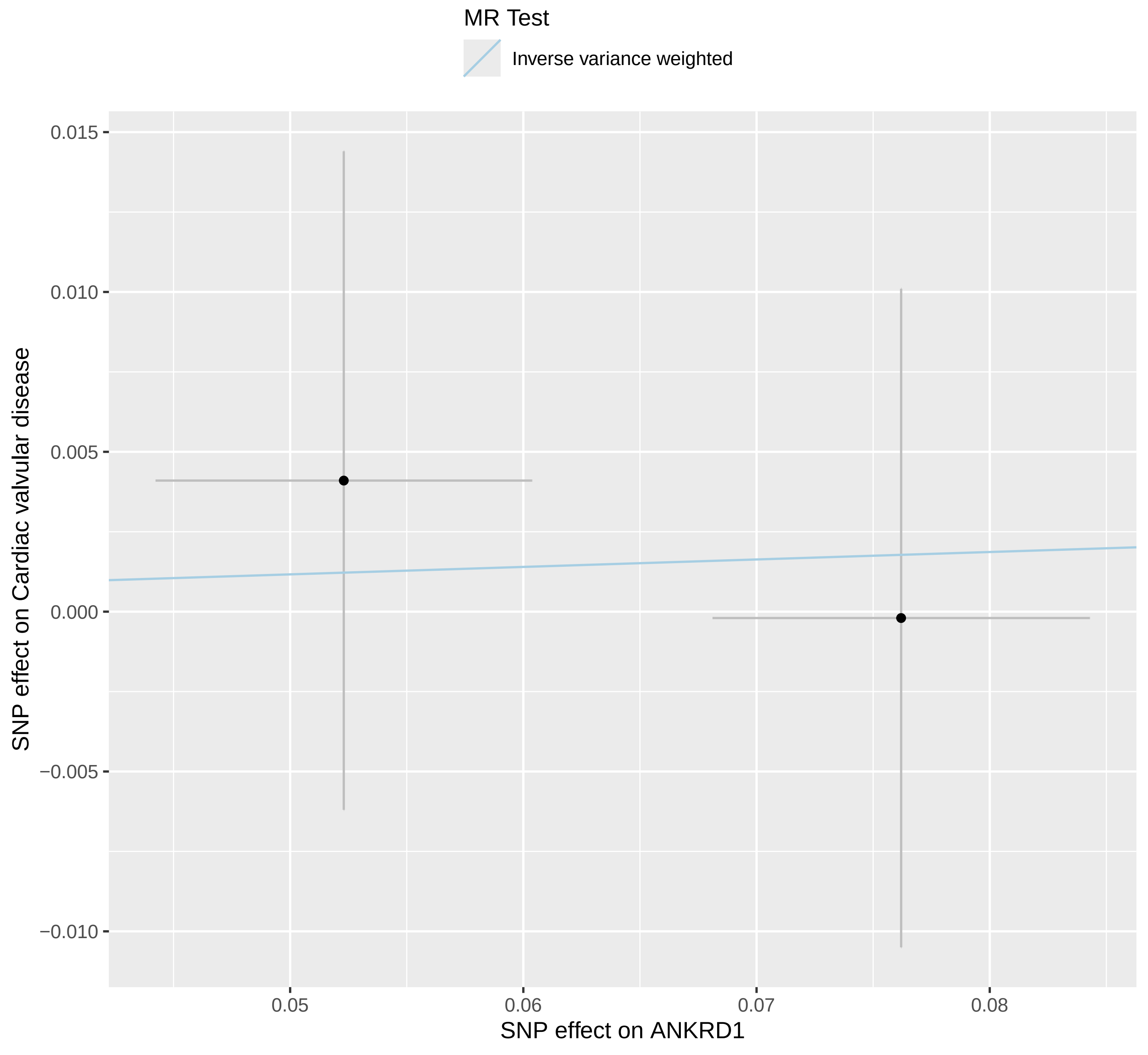
MR Test
Inverse variance weighted
0.015
0.010
0.005
0.000
−0.005
−0.010
SNP effect on Cardiac valvular disease
0.05
0.06
0.07
0.08
SNP effect on ANKRD1

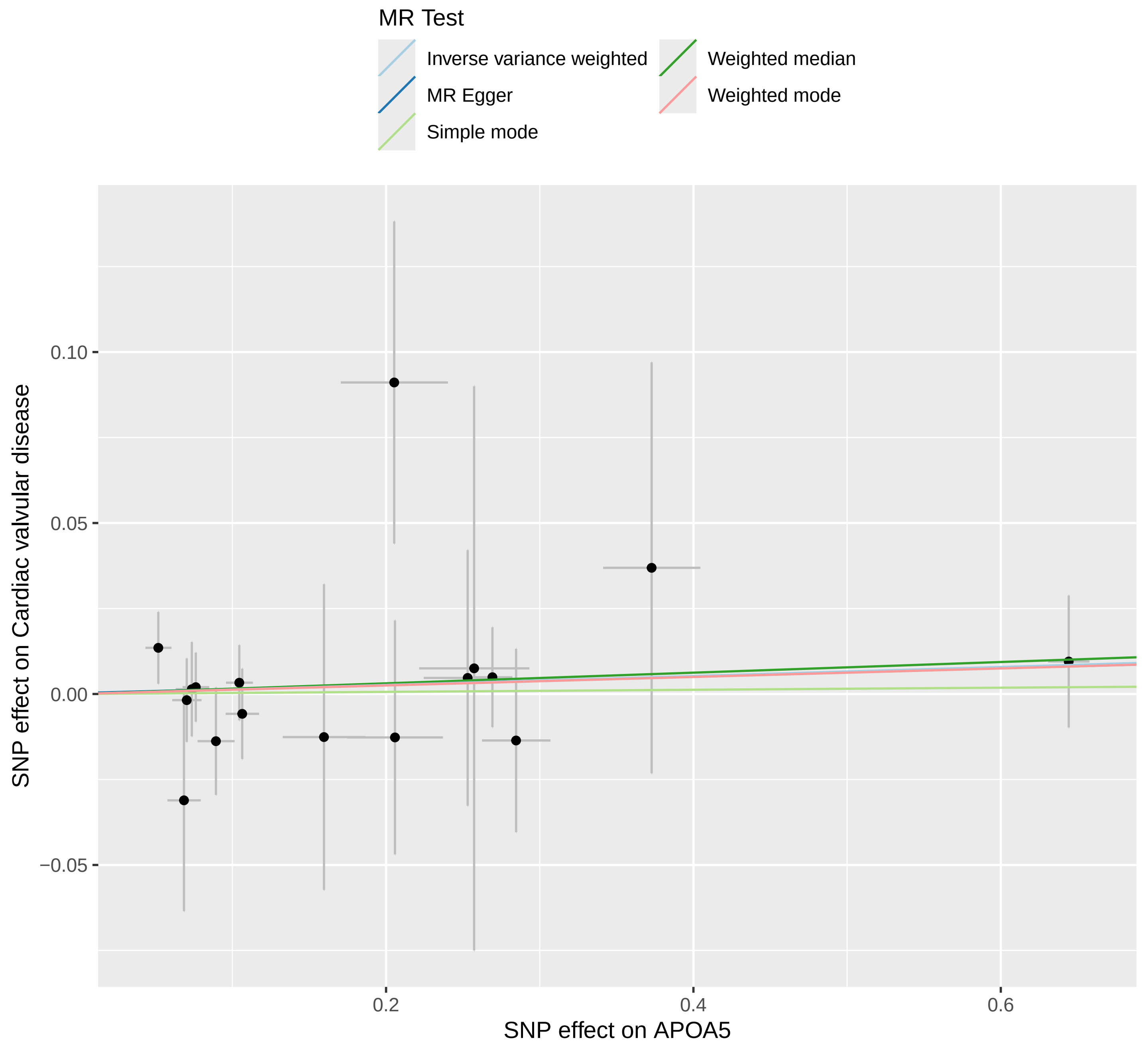

MR Test
Inverse variance weighted
MR Egger
Simple mode
Weighted median
Weighted mode
0.10
0.05
0.00
−0.05
SNP effect on Cardiac valvular disease
0.2
0.4
0.6
SNP effect on APOA5

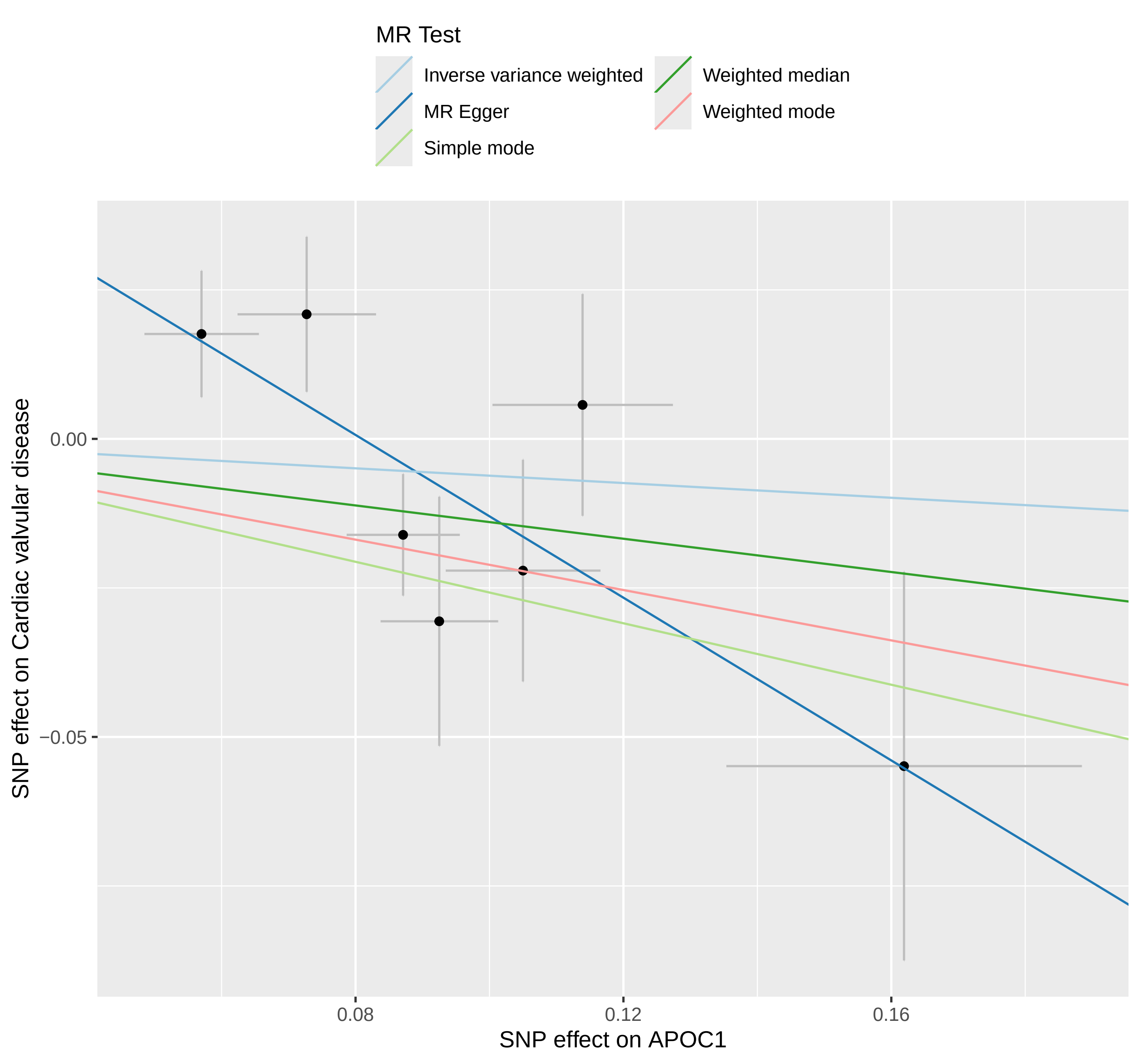
MR Test
Inverse variance weighted
MR Egger
Simple mode
Weighted median
Weighted mode
0.00
−0.05
SNP effect on Cardiac valvular disease
0.08
0.12
0.16
SNP effect on APOC1

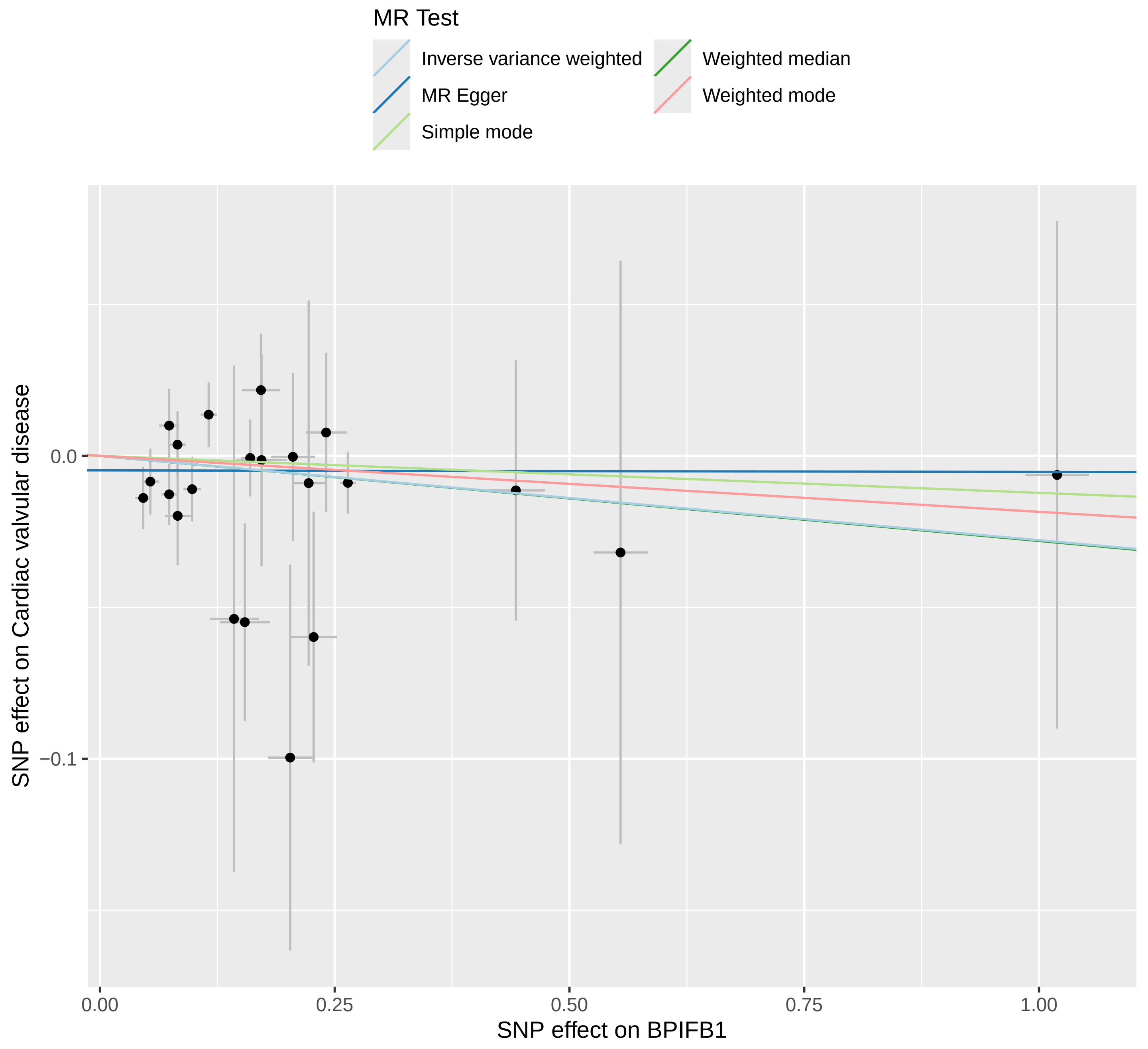

MR Test
Inverse variance weighted
MR Egger
Simple mode
Weighted median
Weighted mode
SNP effect on Cardiac valvular disease
0.0
−0.1
0.00
0.25
0.50
0.75
1.00
SNP effect on BPIFB1

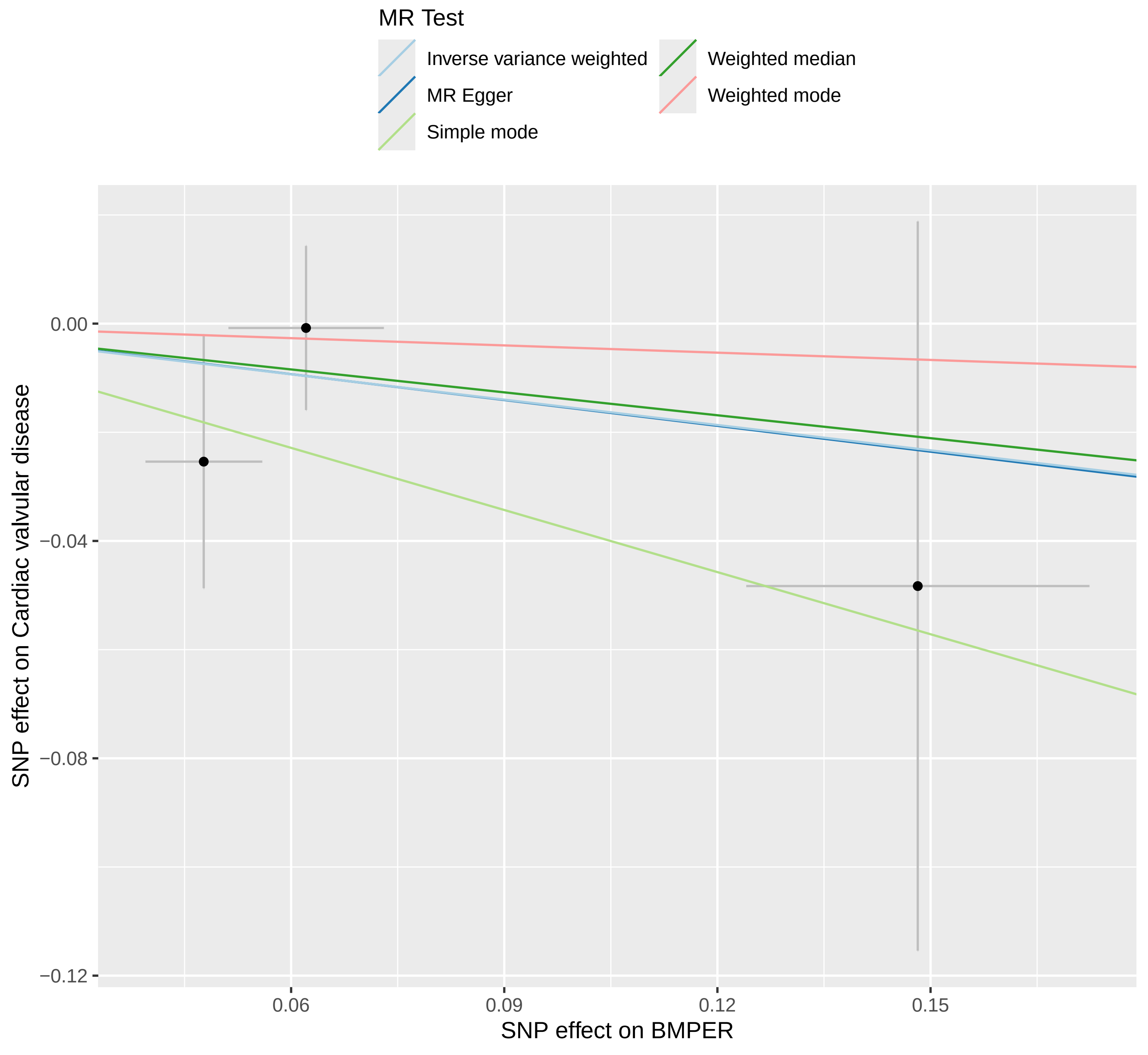
MR Test
Inverse variance weighted
MR Egger
Simple mode
Weighted median
Weighted mode
0.00
−0.04
−0.08
−0.12
0.06
0.09
0.12
0.15
SNP effect on Cardiac valvular disease
SNP effect on BMPER

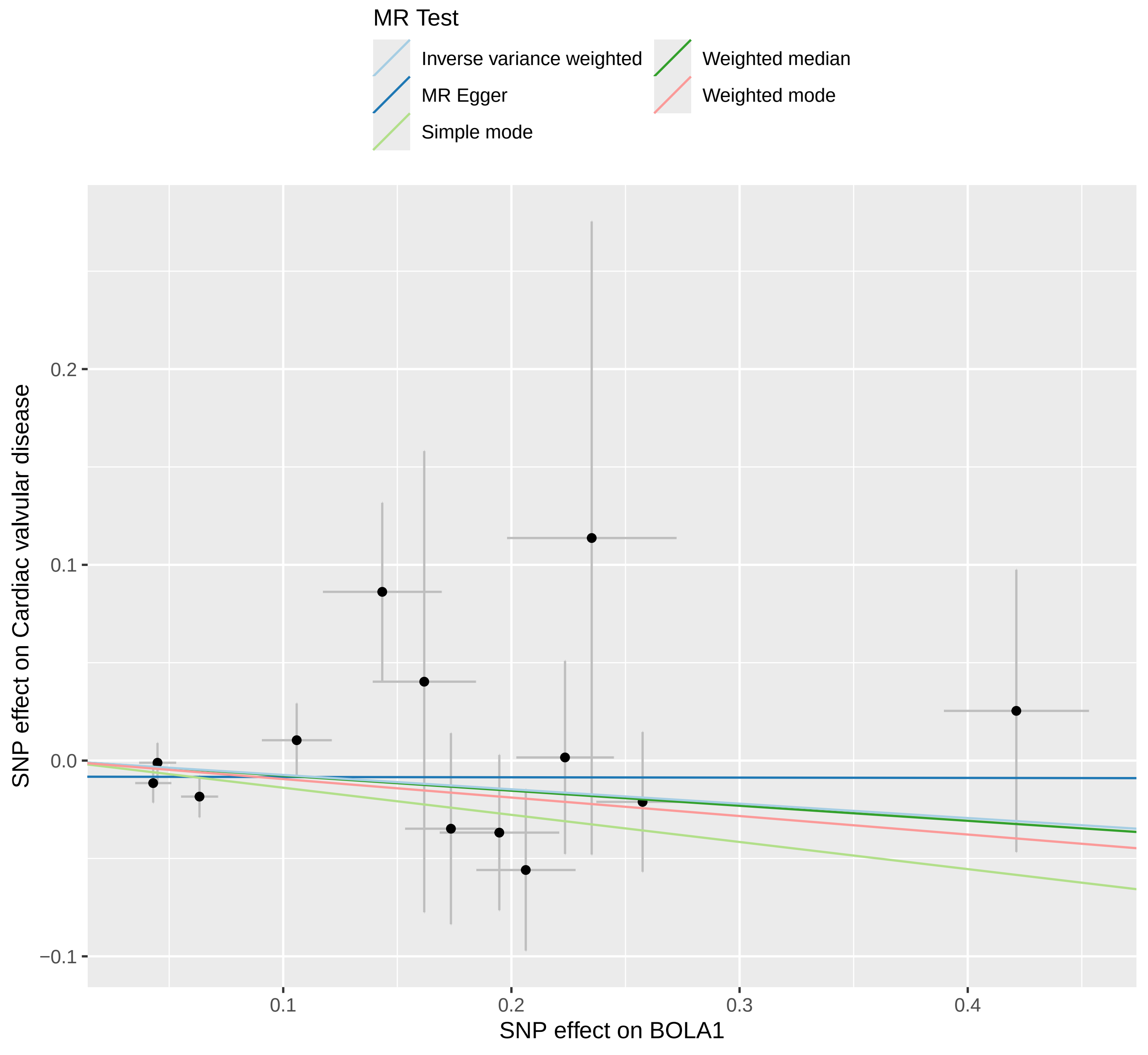

MR Test
Inverse variance weighted
MR Egger
Simple mode
Weighted median
Weighted mode
SNP effect on Cardiac valvular disease
0.2
0.1
0.0
−0.1
0.1
0.2
0.3
0.4
SNP effect on BOLA1

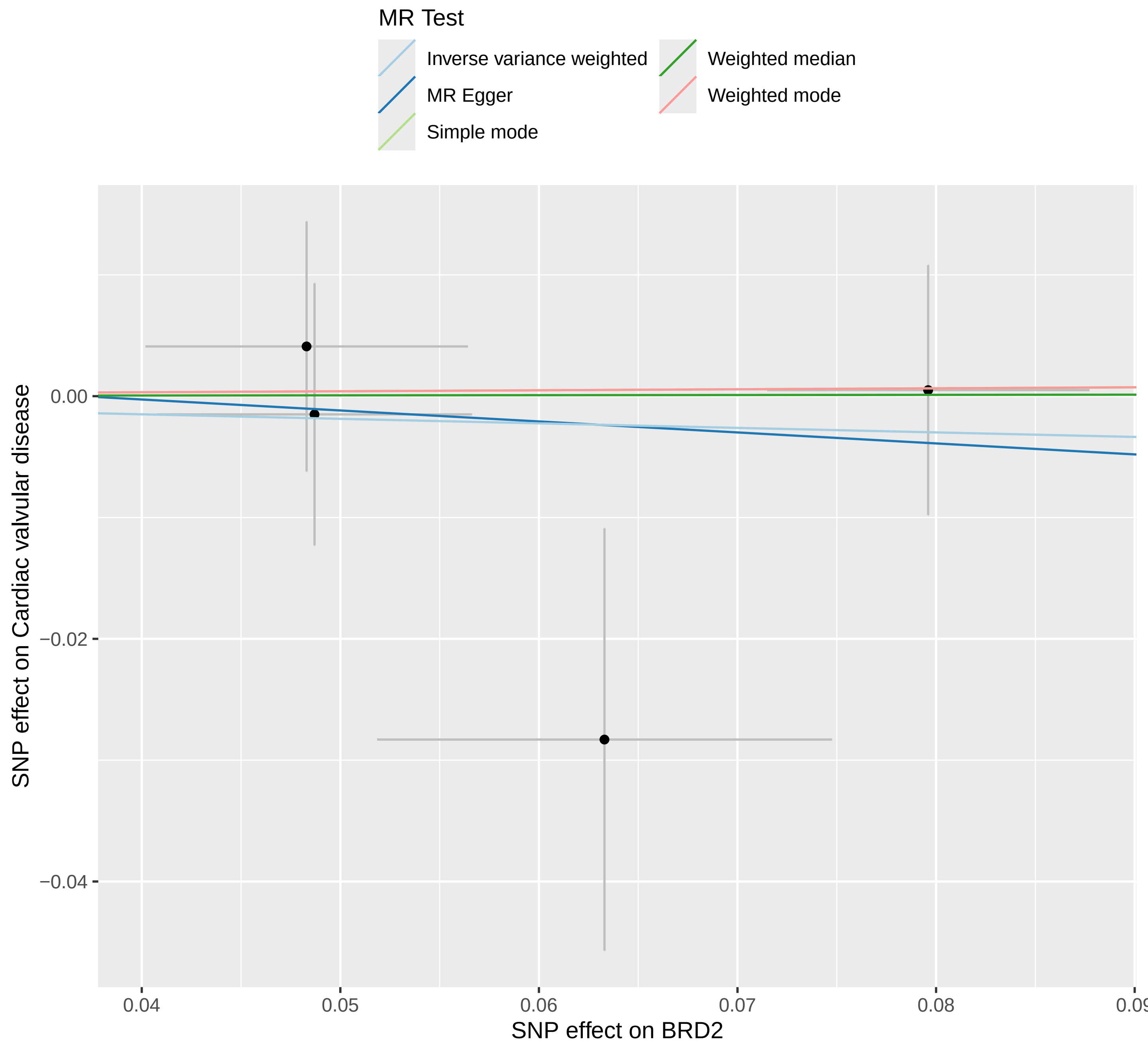
MR Test
Inverse variance weighted
MR Egger
Simple mode
Weighted median
Weighted mode
SNP effect on Cardiac valvular disease
0.00
−0.02
−0.04
0.04
0.05
0.06
0.07
0.08
SNP effect on BRD2

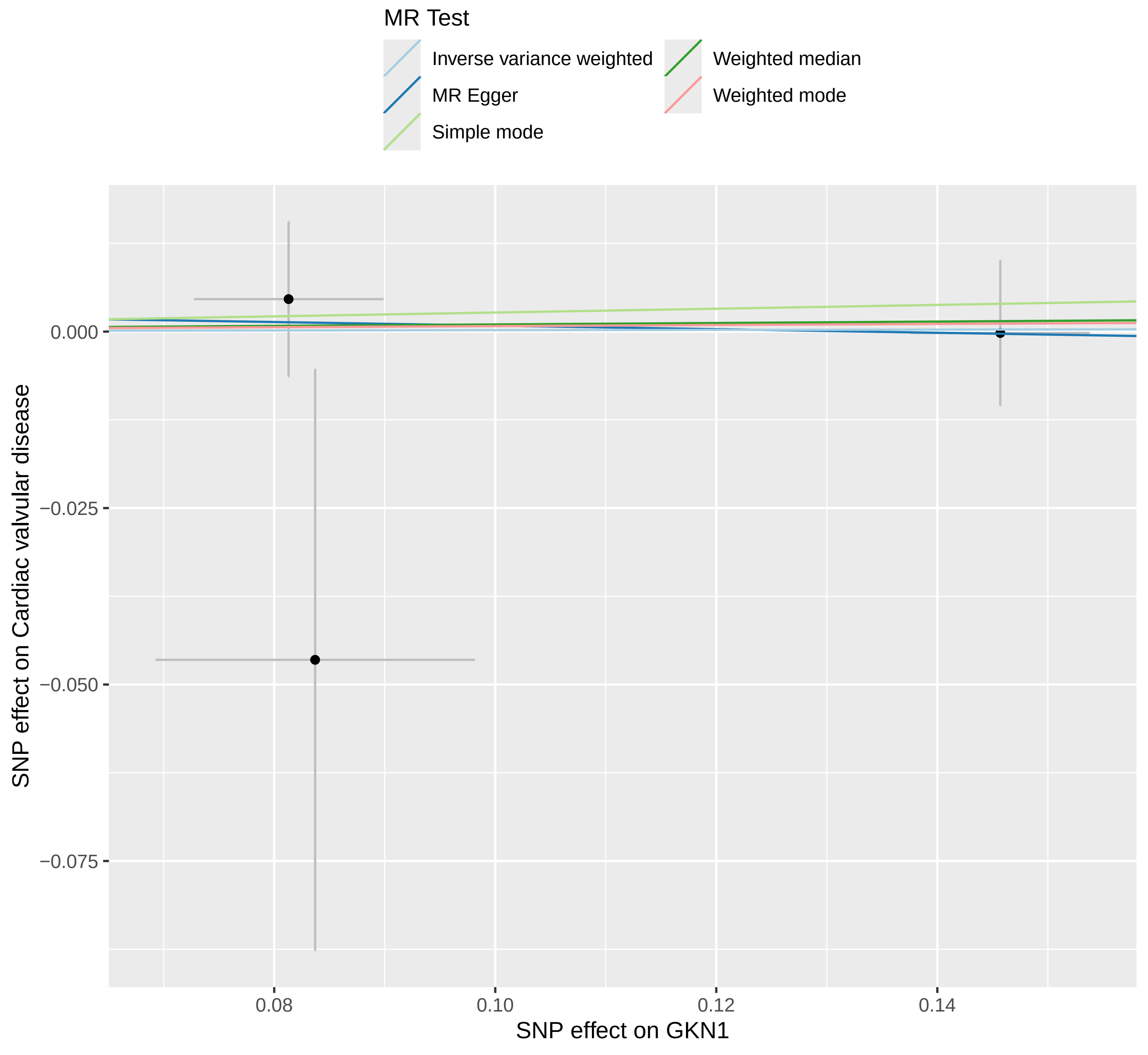
MR Test
Inverse variance weighted
MR Egger
Simple mode
Weighted median
Weighted mode
0.000
−0.025
−0.050
−0.075
0.08
0.10
0.12
0.14
SNP effect on Cardiac valvular disease
SNP effect on GKN1

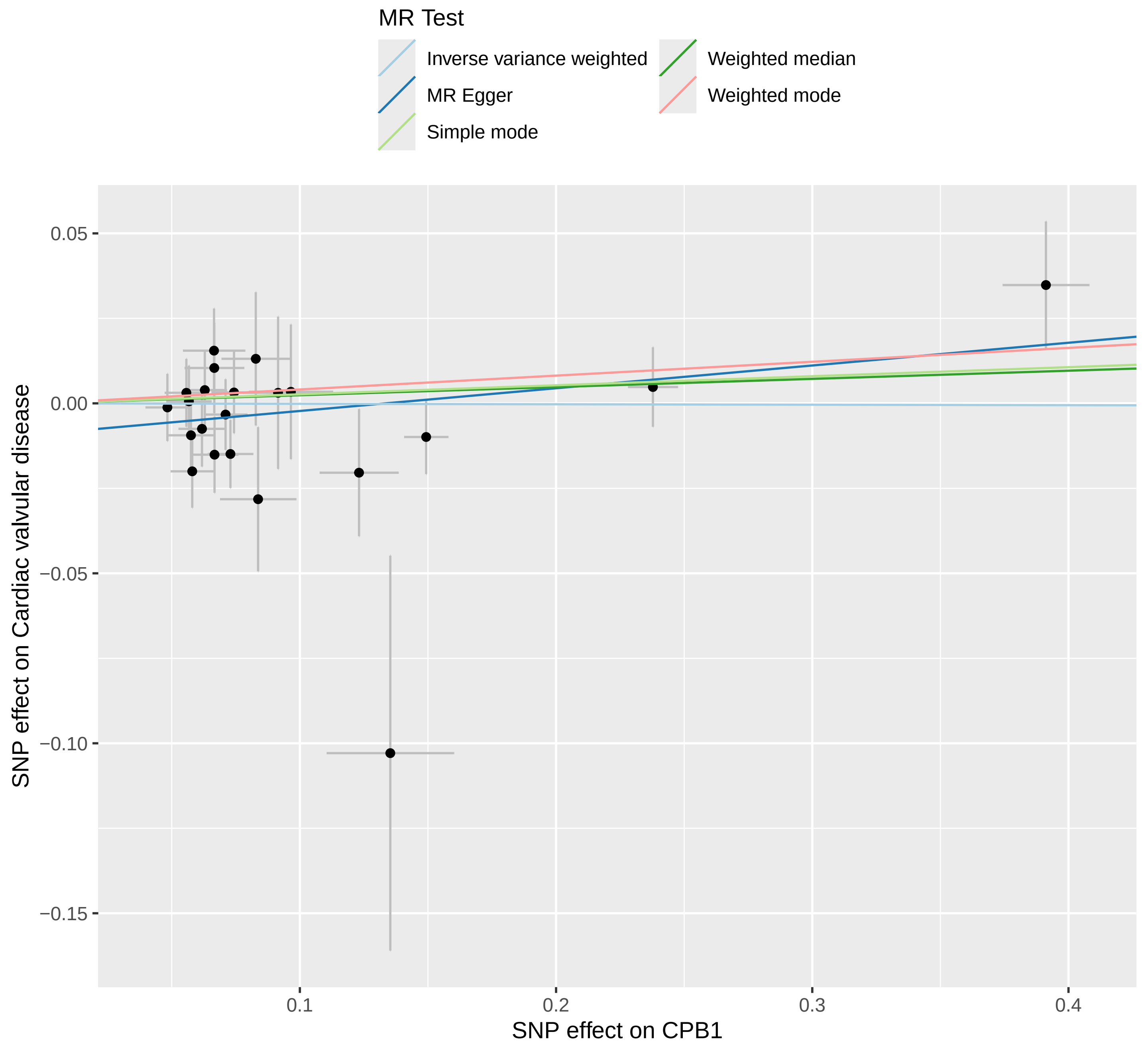
MR Test
Inverse variance weighted
MR Egger
Simple mode
Weighted median
Weighted mode
0.05
0.00
−0.05
−0.10
−0.15
0.1
0.2
0.3
0.4
SNP effect on CPB1
SNP effect on Cardiac valvular disease

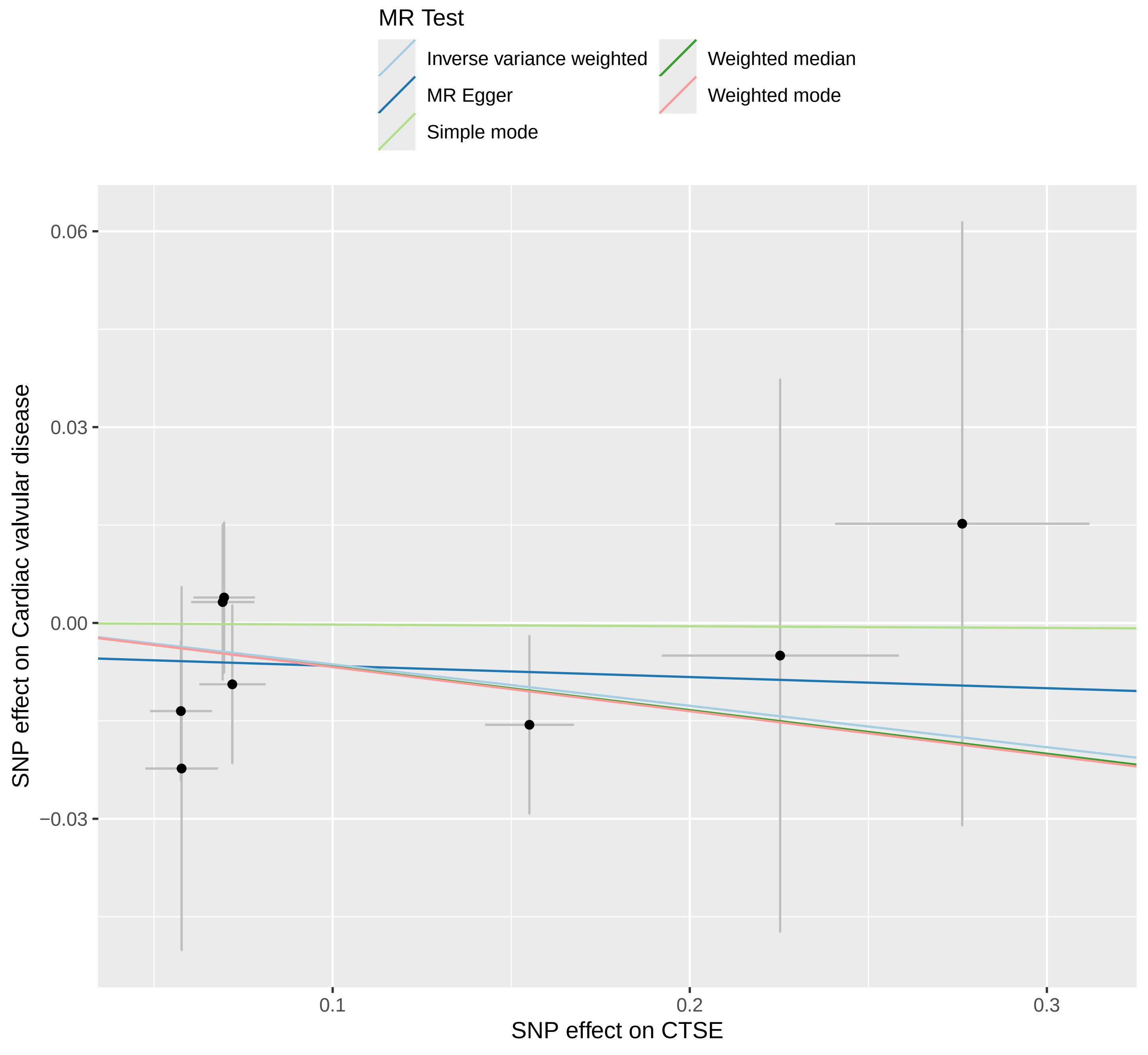
MR Test
Inverse variance weighted
MR Egger
Simple mode
Weighted median
Weighted mode
SNP effect on Cardiac valvular disease
0.06
0.03
0.00
−0.03
0.1
0.2
0.3
SNP effect on CTSE

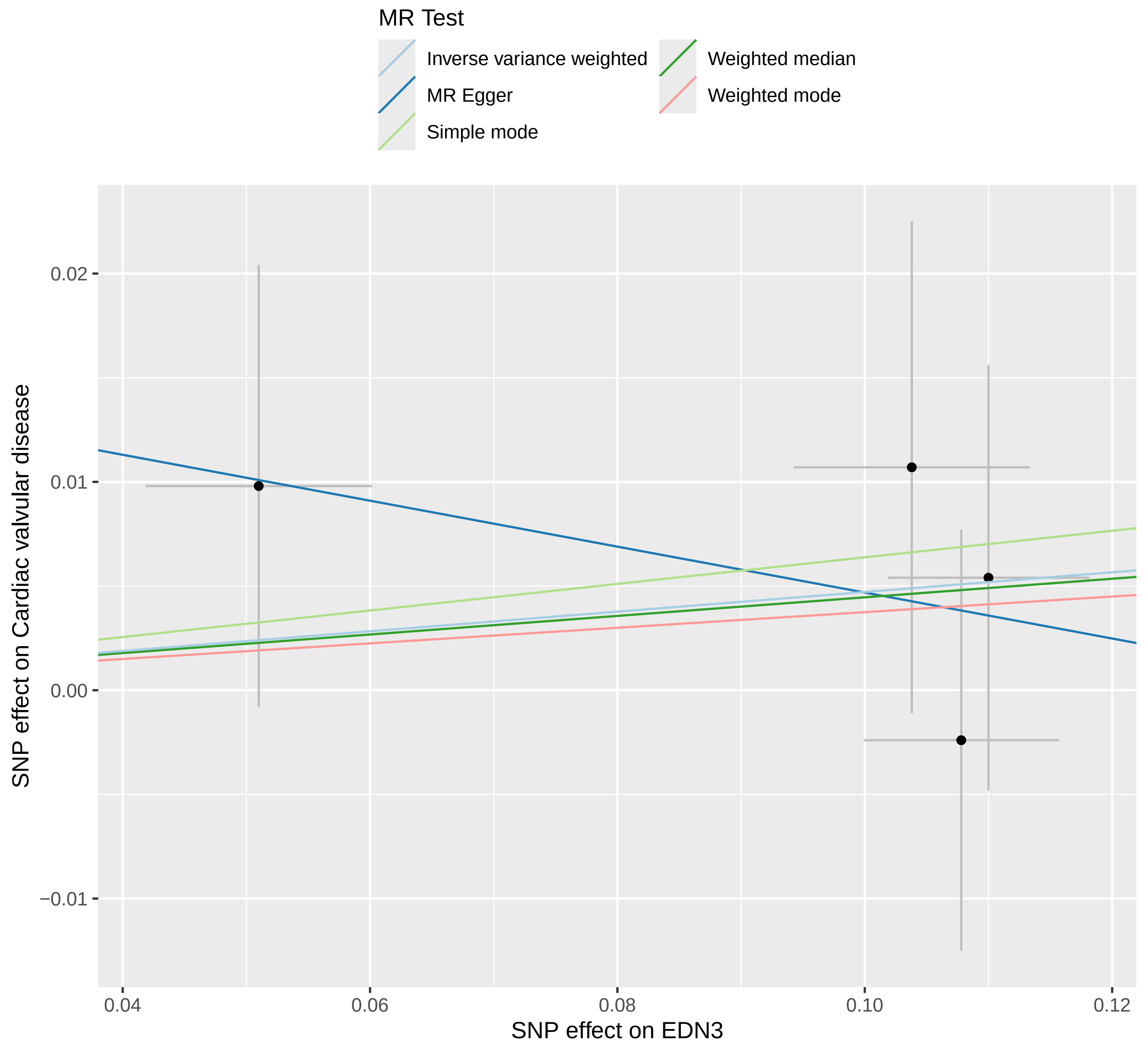

MR Test
Inverse variance weighted
MR Egger
Simple mode
Weighted median
Weighted mode
SNP effect on Cardiac valvular disease
0.02
0.01
0.00
−0.01
0.04
0.06
0.08
0.10
0.12
SNP effect on EDN3

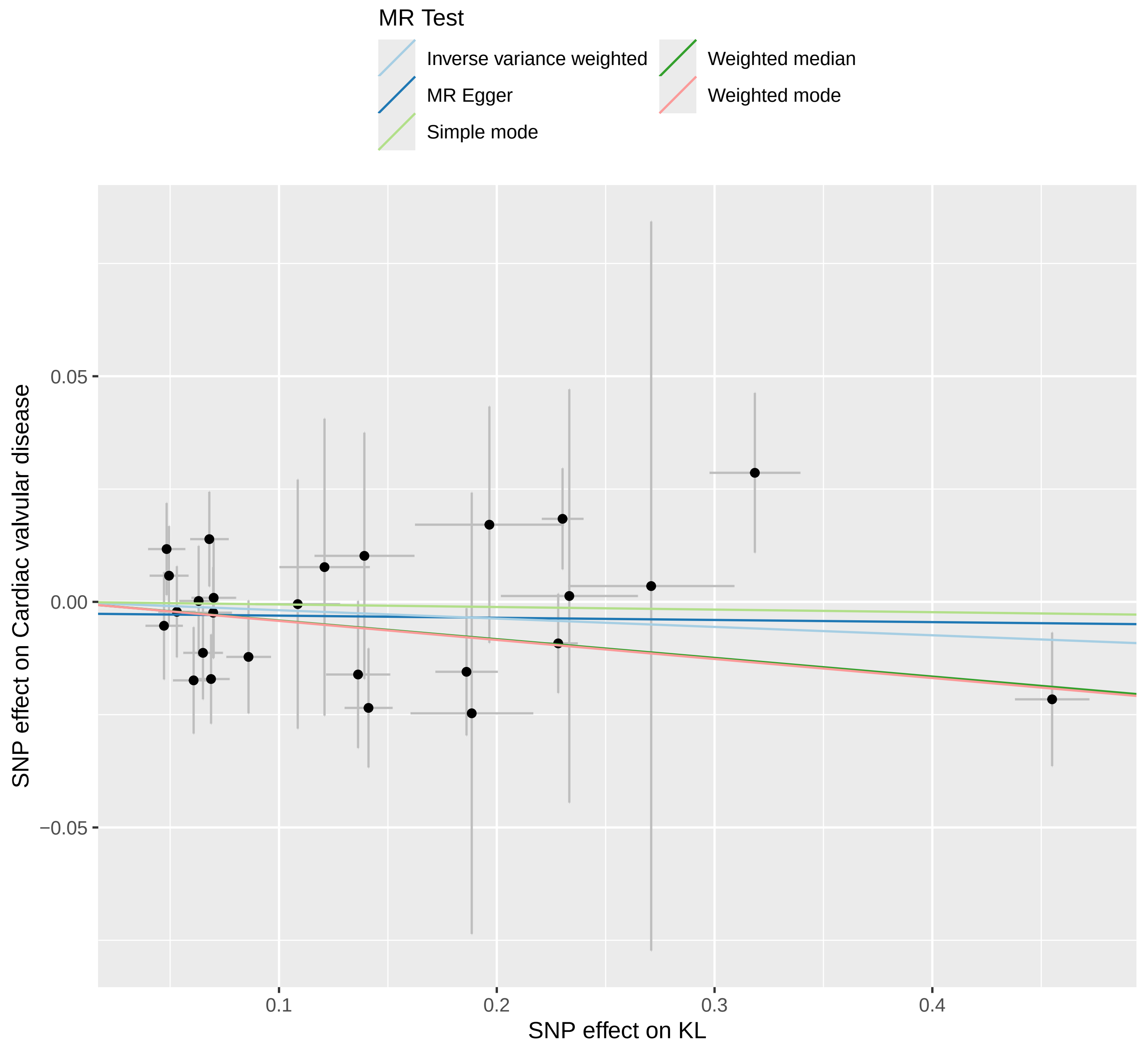

MR Test
Inverse variance weighted
MR Egger
Simple mode
Weighted median
Weighted mode
SNP effect on Cardiac valvular disease
0.05
0.00
−0.05
0.1
0.2
0.3
0.4
SNP effect on KL

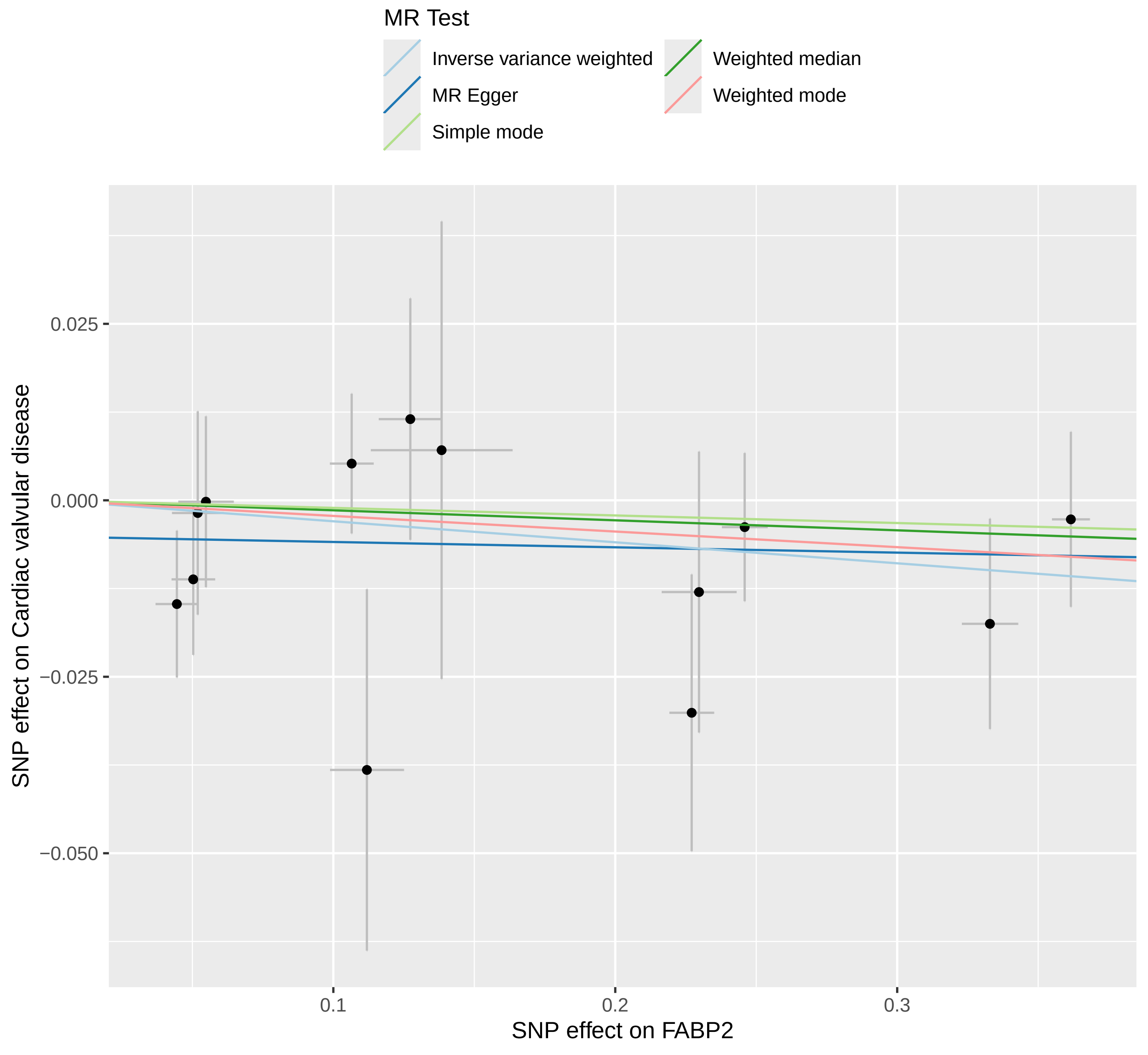

MR Test
Inverse variance weighted
MR Egger
Simple mode
Weighted median
Weighted mode
0.025
0.000
−0.025
−0.050
0.1
0.2
0.3
SNP effect on Cardiac valvular disease
SNP effect on FABP2

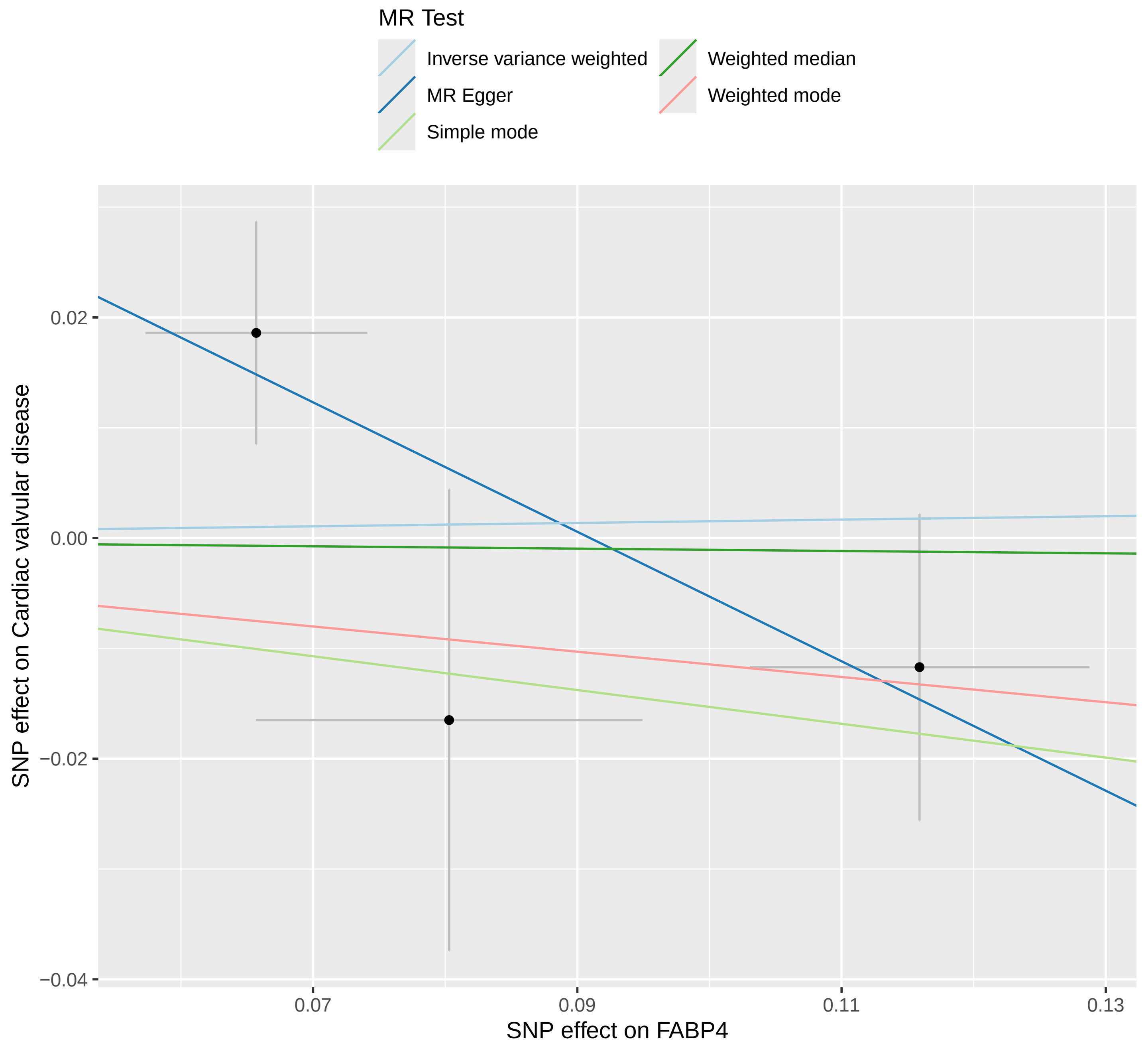
MR Test
Inverse variance weighted
MR Egger
Simple mode
Weighted median
Weighted mode
SNP effect on Cardiac valvular disease
0.02
0.00
−0.02
−0.04
0.07
0.09
0.11
0.13
SNP effect on FABP4

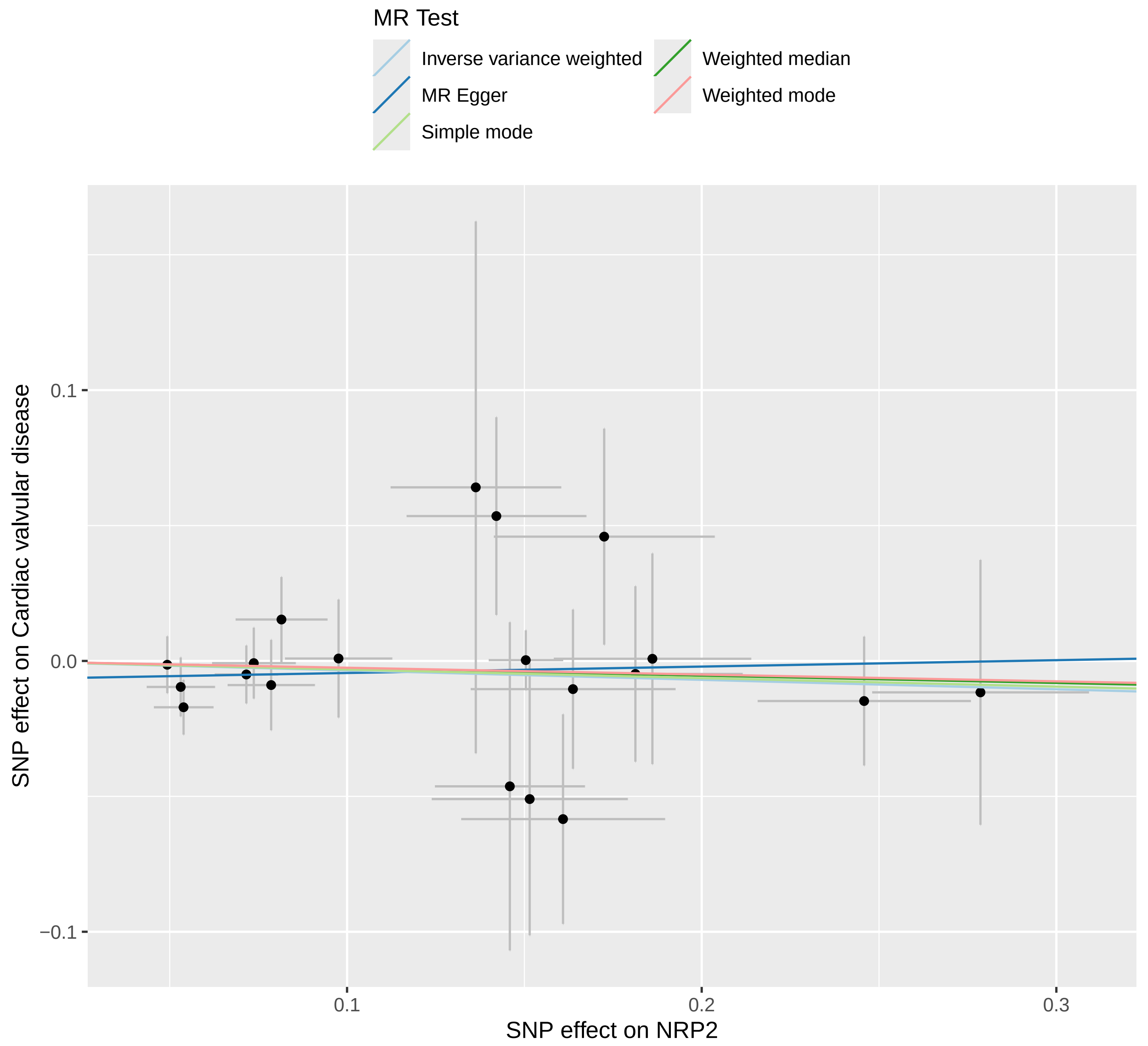
MR Test
Inverse variance weighted
MR Egger
Simple mode
Weighted median
Weighted mode
0.1
0.0
−0.1
SNP effect on Cardiac valvular disease
0.1
0.2
0.3
SNP effect on NRP2

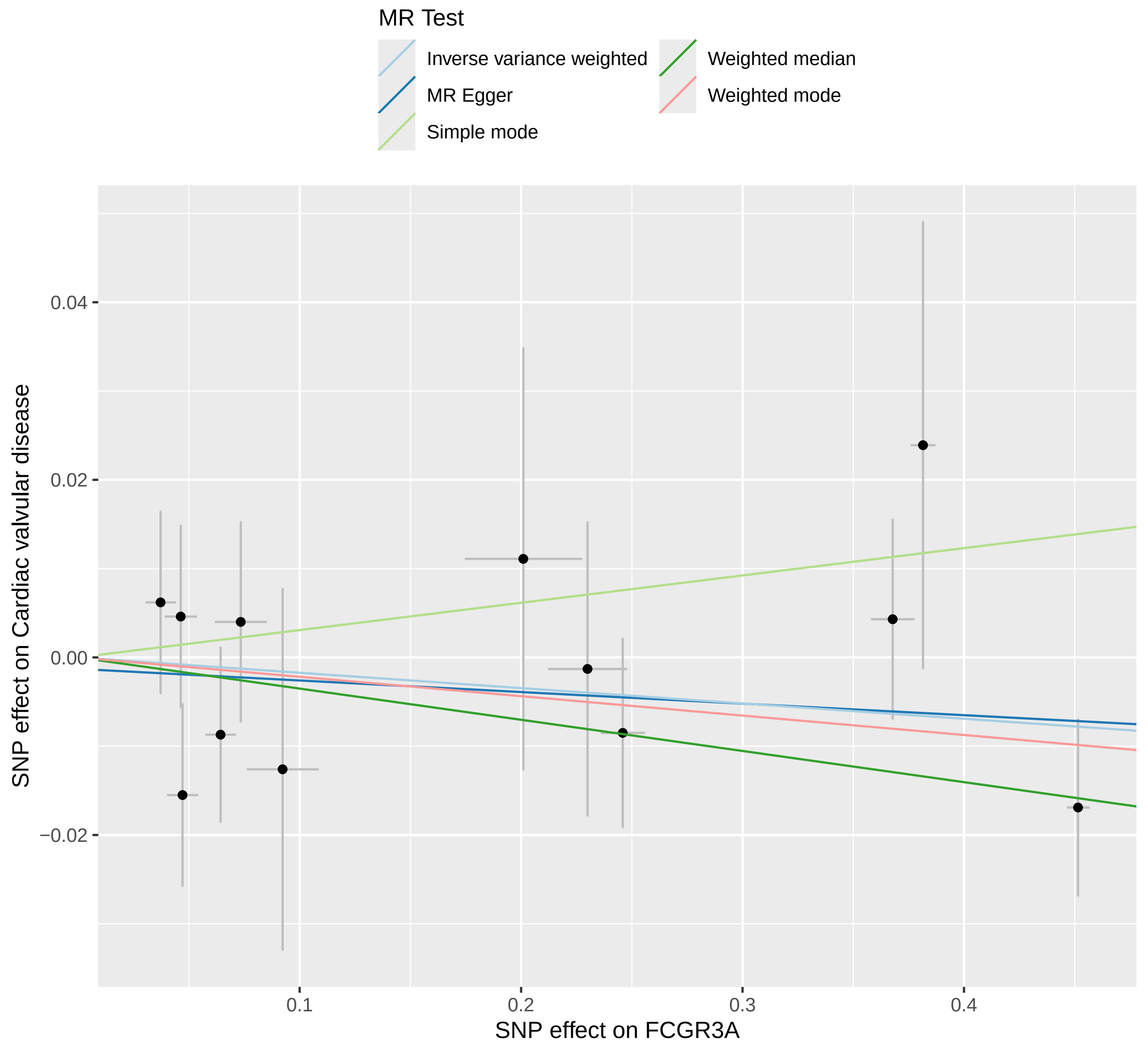
MR Test
Inverse variance weighted
MR Egger
Simple mode
Weighted median
Weighted mode
0.04
0.02
0.00
−0.02
SNP effect on Cardiac valvular disease
0.1
0.2
0.3
0.4
SNP effect on FCGR3A

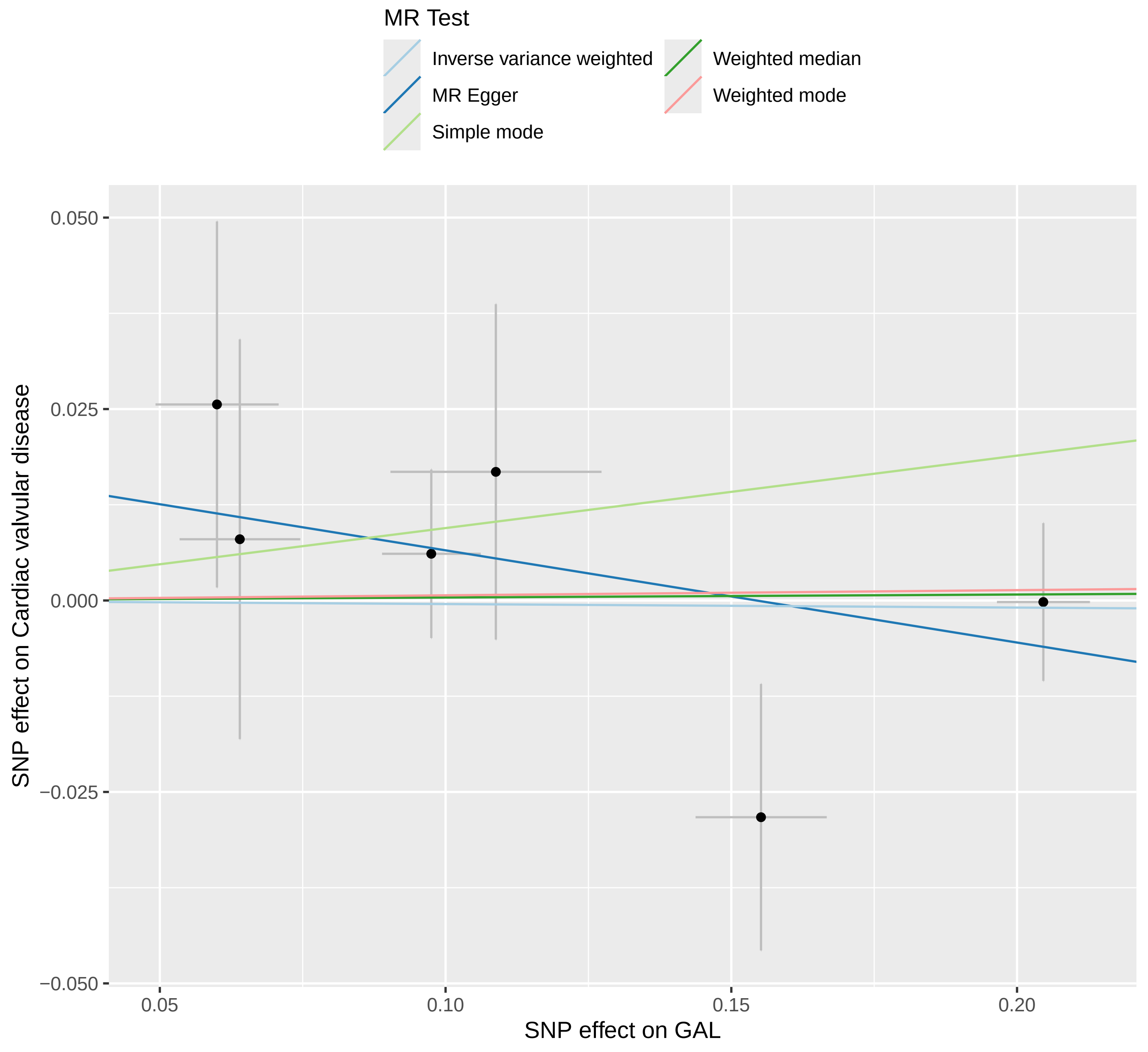
MR Test
Inverse variance weighted
MR Egger
Simple mode
Weighted median
Weighted mode
0.050
0.025
0.000
−0.025
−0.050
SNP effect on Cardiac valvular disease
0.05
0.10
0.15
0.20
SNP effect on GAL

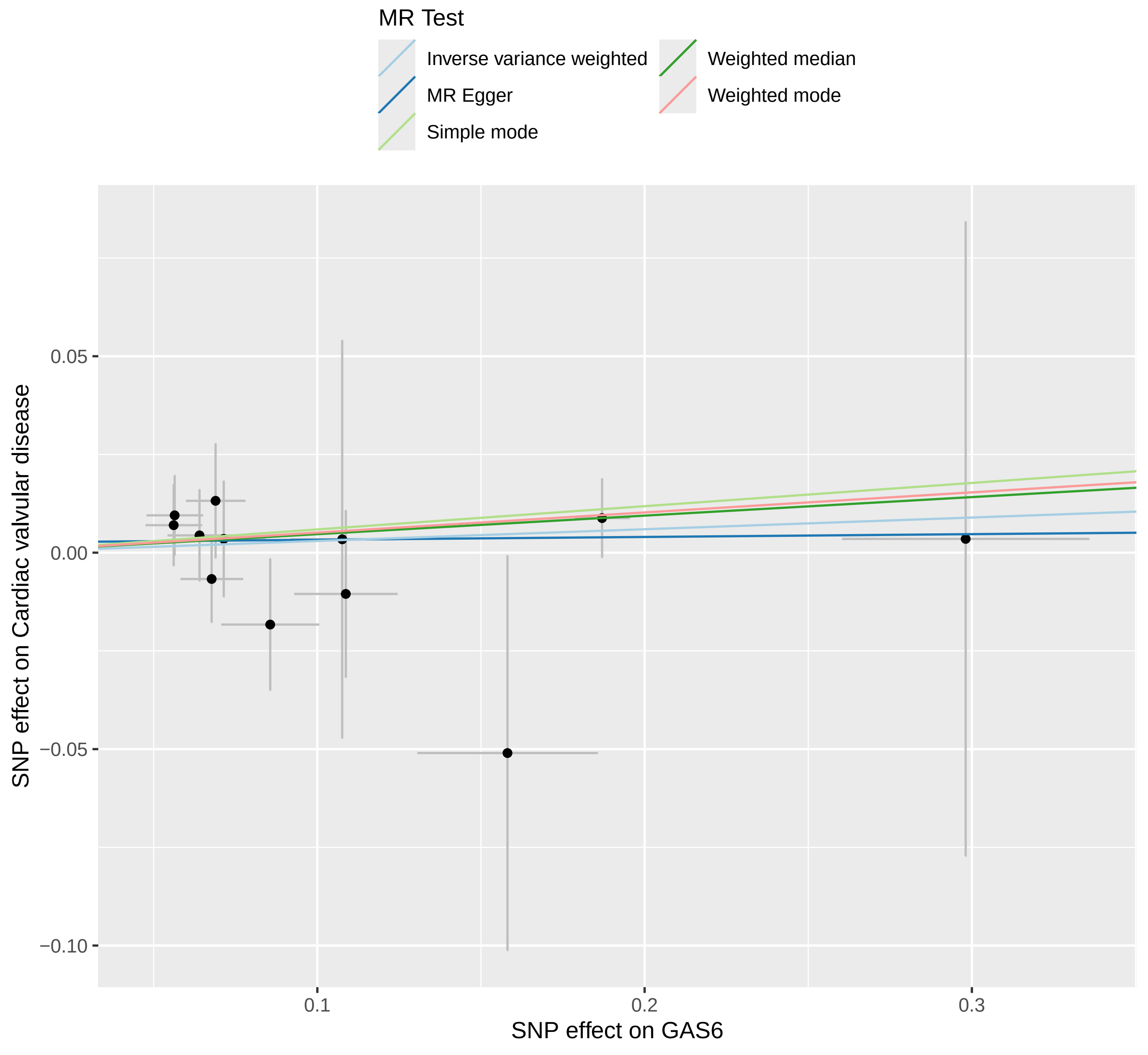

MR Test
Inverse variance weighted
MR Egger
Simple mode
Weighted median
Weighted mode
0.05
0.00
−0.05
−0.10
0.1
0.2
0.3
SNP effect on Cardiac valvular disease
SNP effect on GAS6

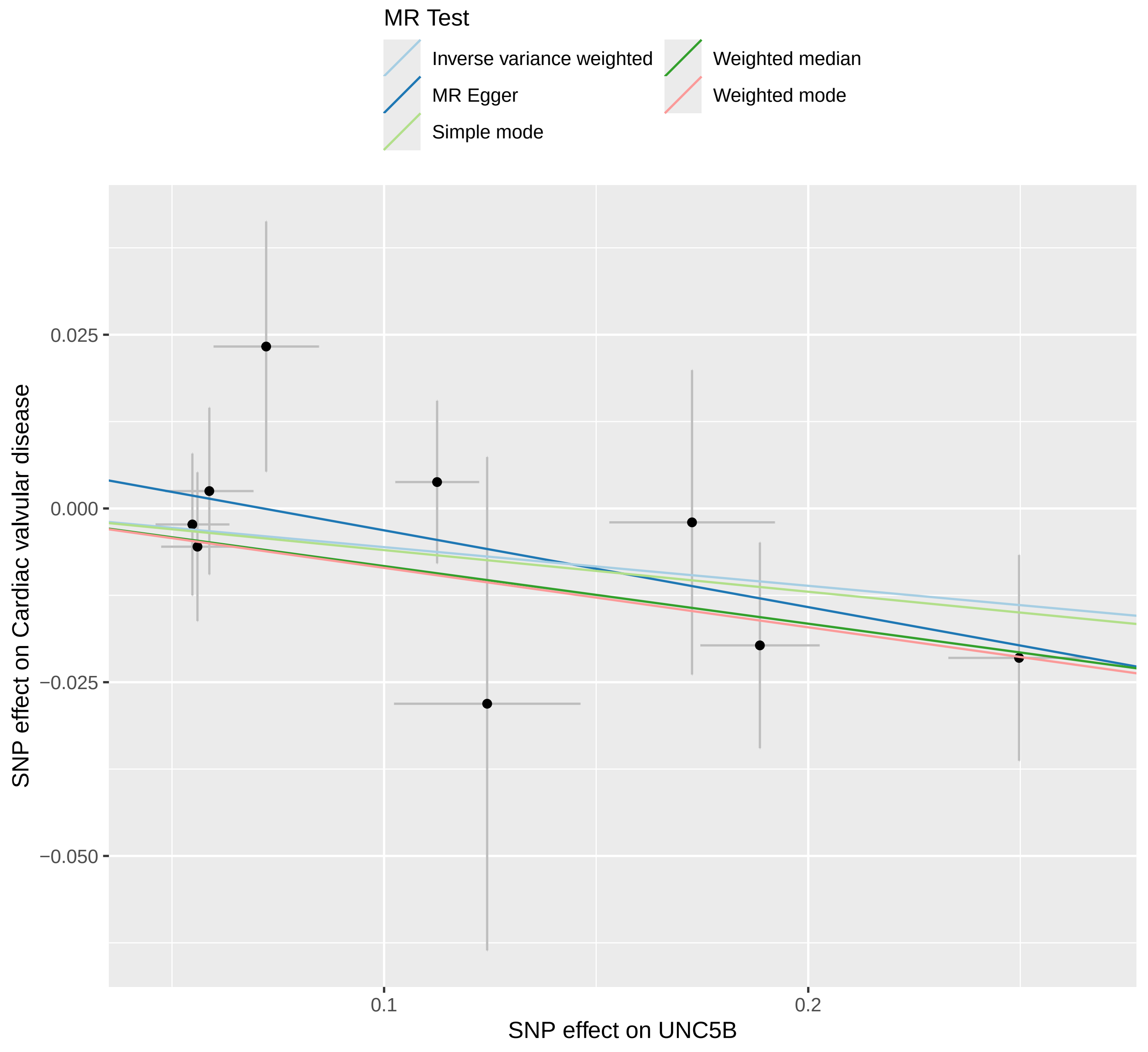

MR Test
Inverse variance weighted
MR Egger
Simple mode
Weighted median
Weighted mode
0.025
0.000
−0.025
−0.050
0.1
0.2
SNP effect on Cardiac valvular disease
SNP effect on UNC5B

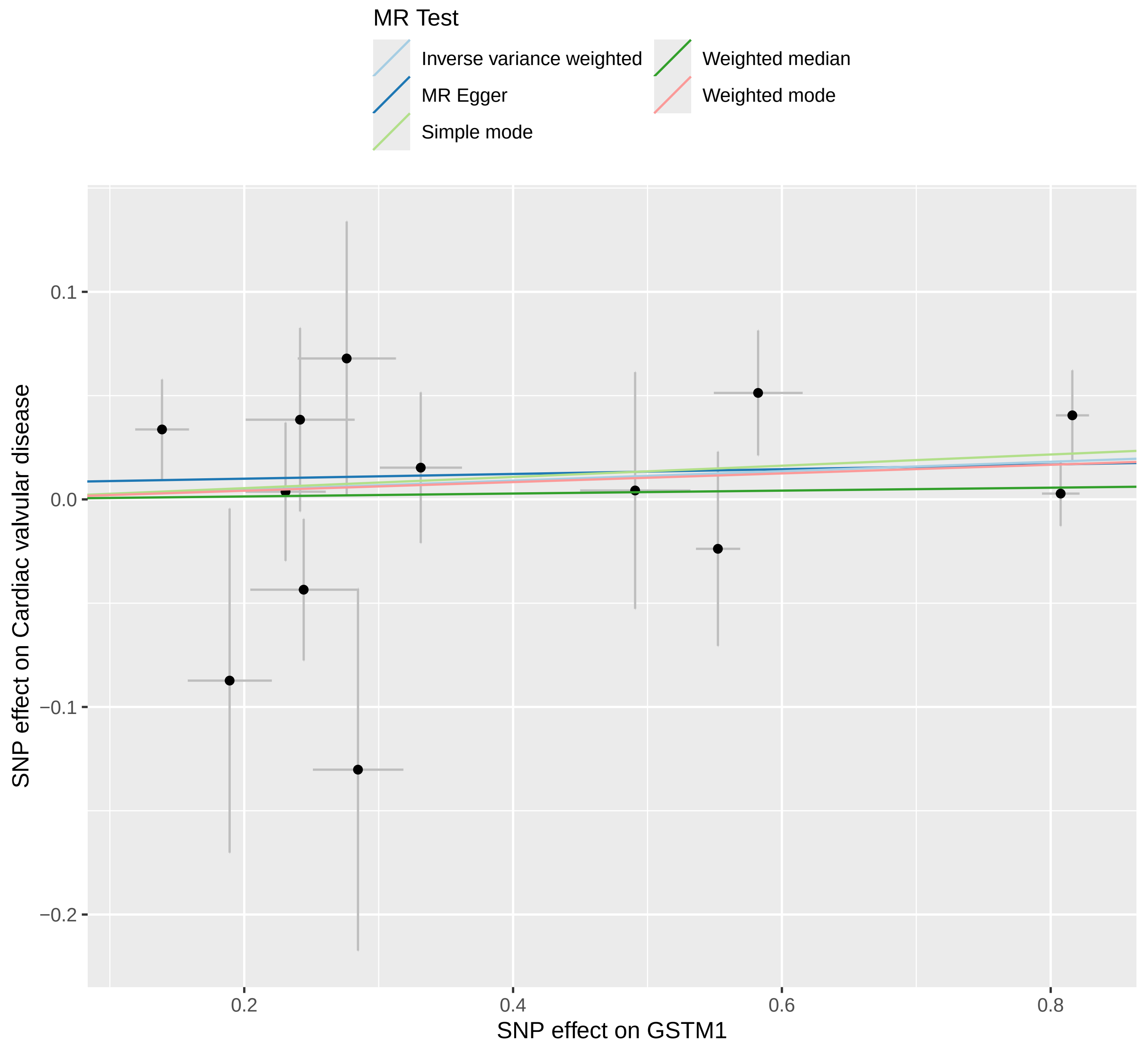

MR Test
Inverse variance weighted
MR Egger
Simple mode
Weighted median
Weighted mode
0.1
0.0
−0.1
−0.2
0.2
0.4
0.6
0.8
SNP effect on GSTM1
SNP effect on Cardiac valvular disease

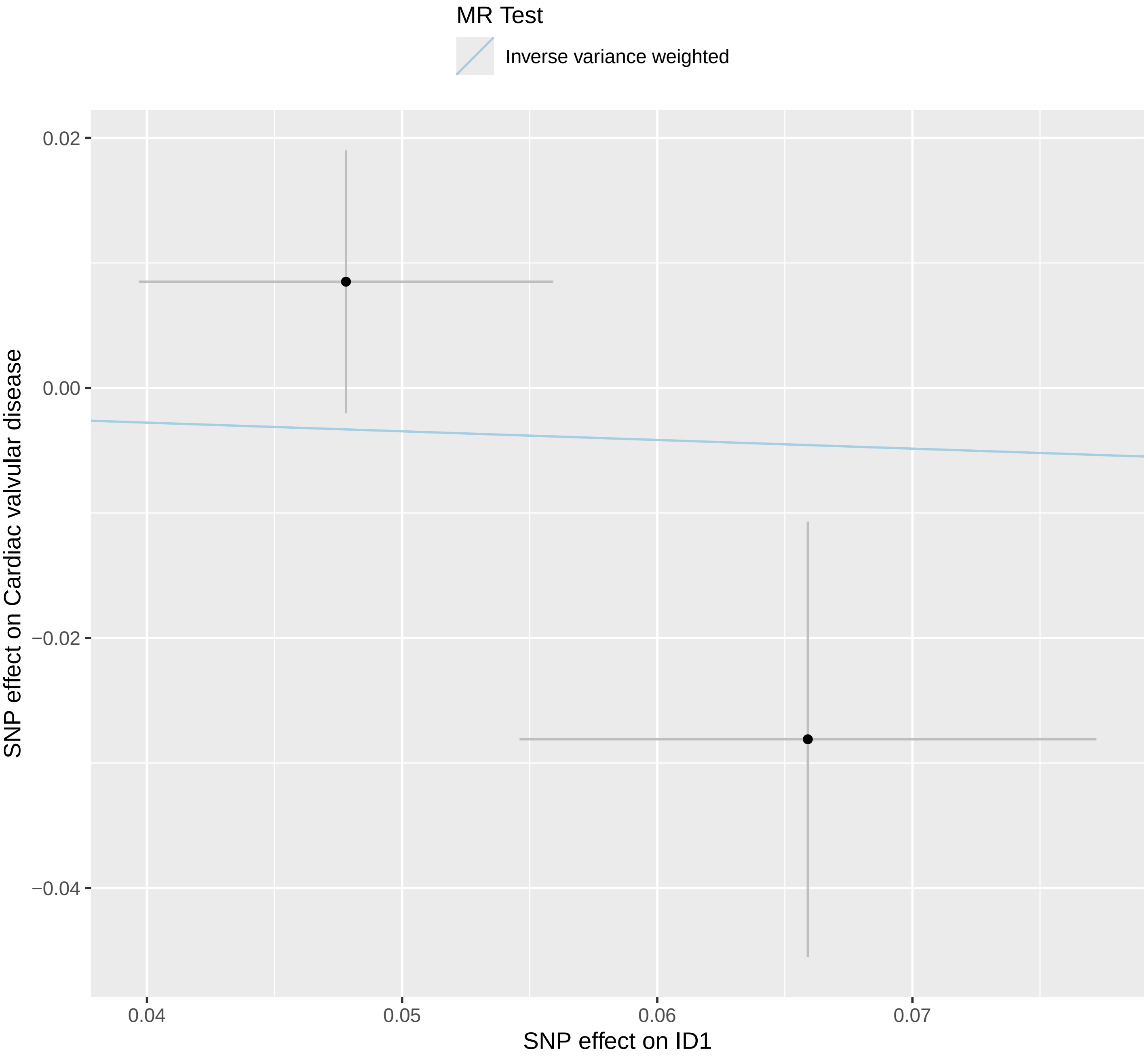

MR Test
Inverse variance weighted
0.02
0.00
−0.02
−0.04
SNP effect on Cardiac valvular disease
0.04
0.05
0.06
0.07
SNP effect on ID1

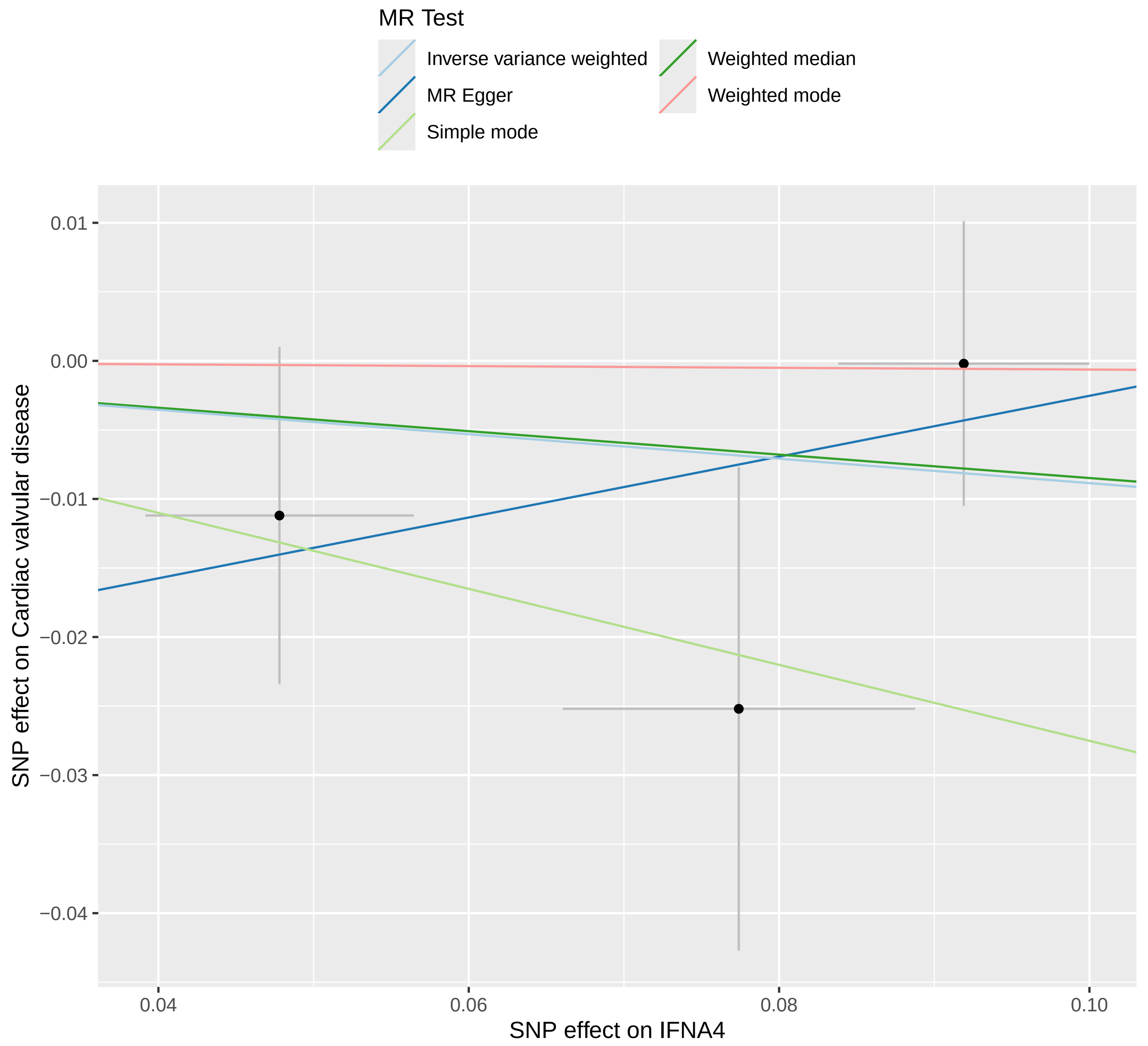
MR Test
Inverse variance weighted
MR Egger
Simple mode
Weighted median
Weighted mode
SNP effect on Cardiac valvular disease
0.01
0.00
−0.01
−0.02
−0.03
−0.04
0.04
0.06
0.08
0.10
SNP effect on IFNA4

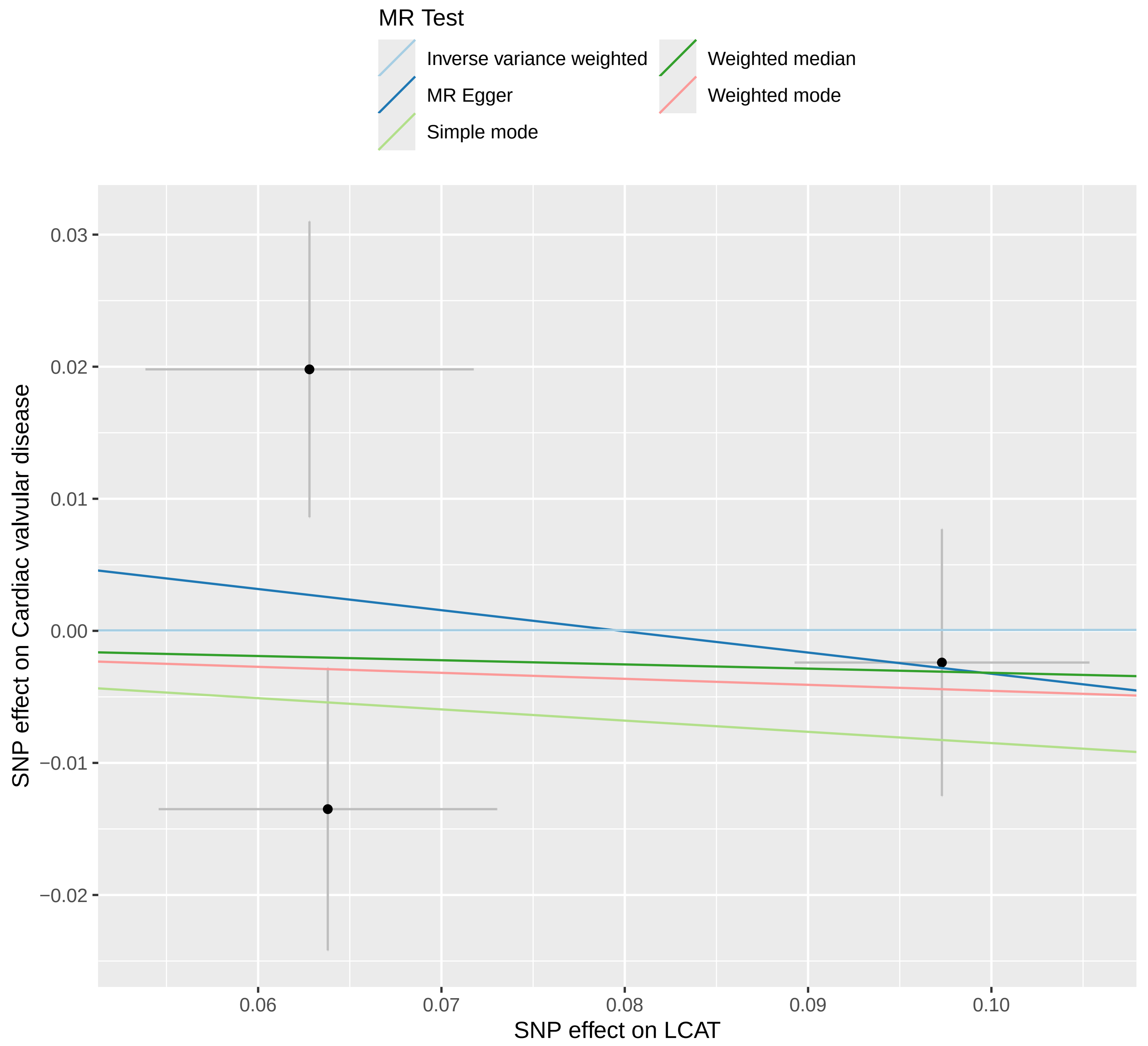
MR Test
Inverse variance weighted
MR Egger
Simple mode
Weighted median
Weighted mode
0.03
0.02
0.01
0.00
−0.01
−0.02
SNP effect on Cardiac valvular disease
0.06
0.07
0.08
0.09
0.10
SNP effect on LCAT

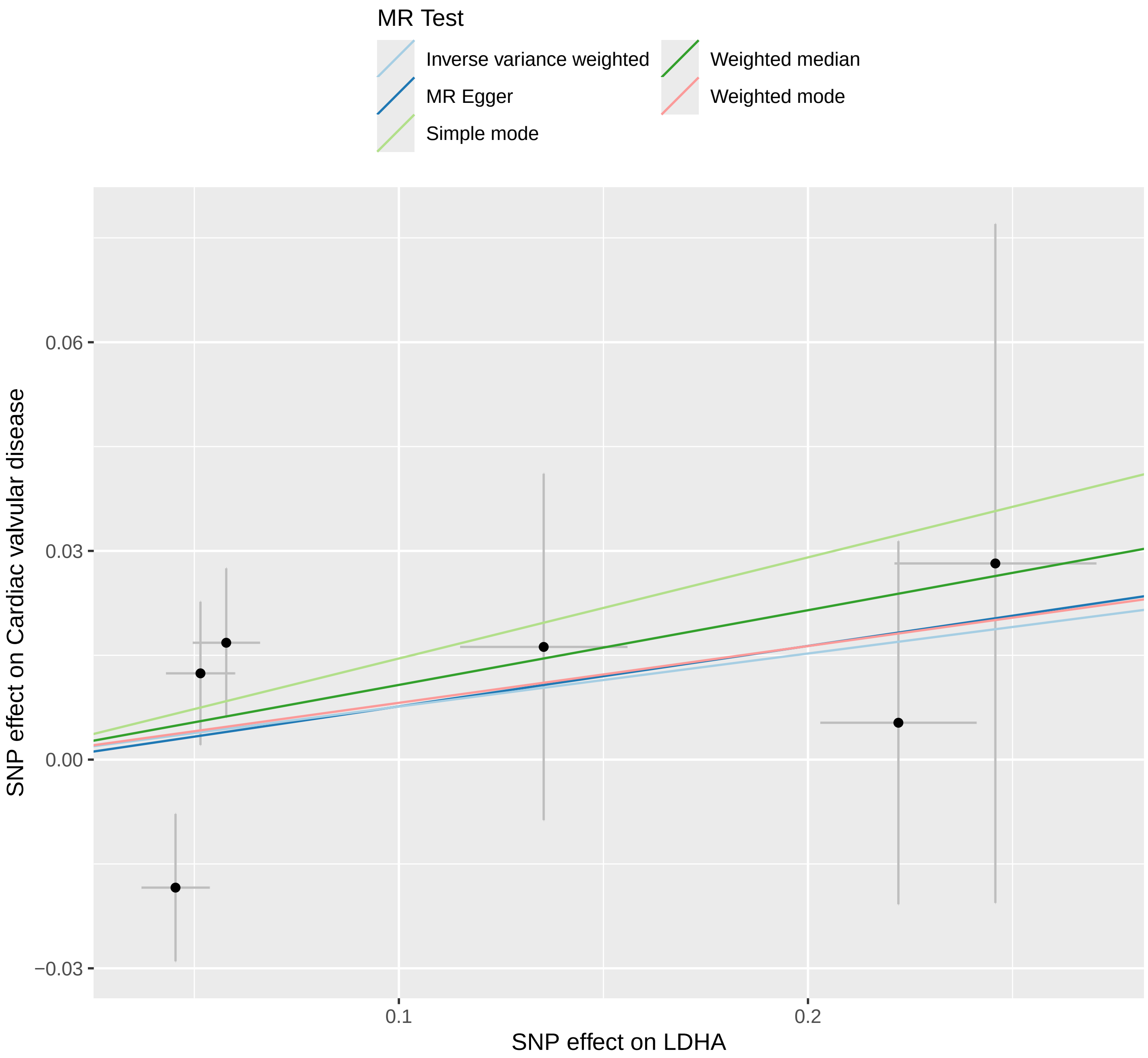

MR Test
Inverse variance weighted
MR Egger
Simple mode
Weighted median
Weighted mode
0.06
0.03
0.00
−0.03
0.1
0.2
SNP effect on Cardiac valvular disease
SNP effect on LDHA

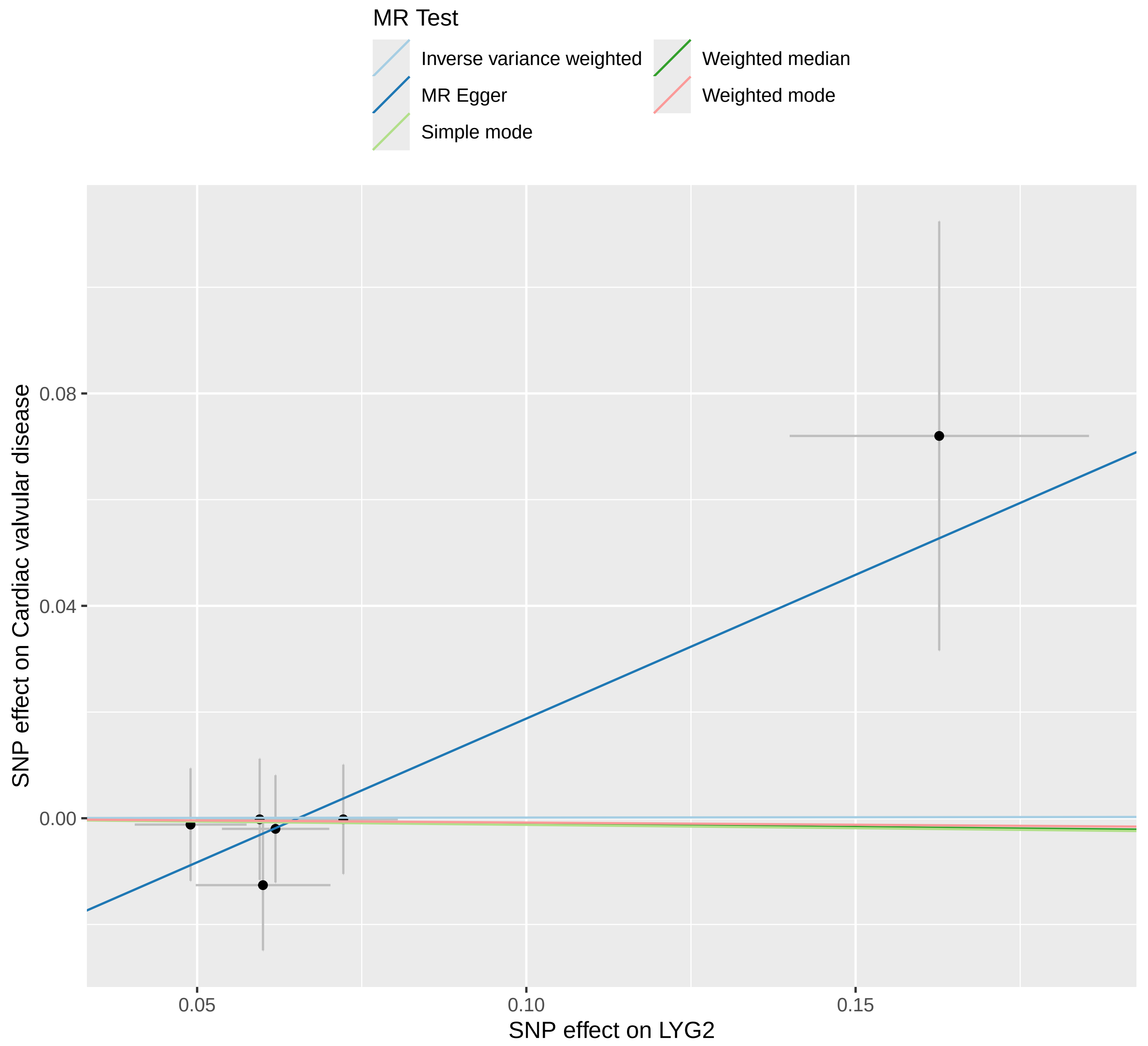

MR Test
Inverse variance weighted
MR Egger
Simple mode
Weighted median
Weighted mode
0.08
0.04
0.00
0.05
0.10
0.15
SNP effect on Cardiac valvular disease
SNP effect on LYG2

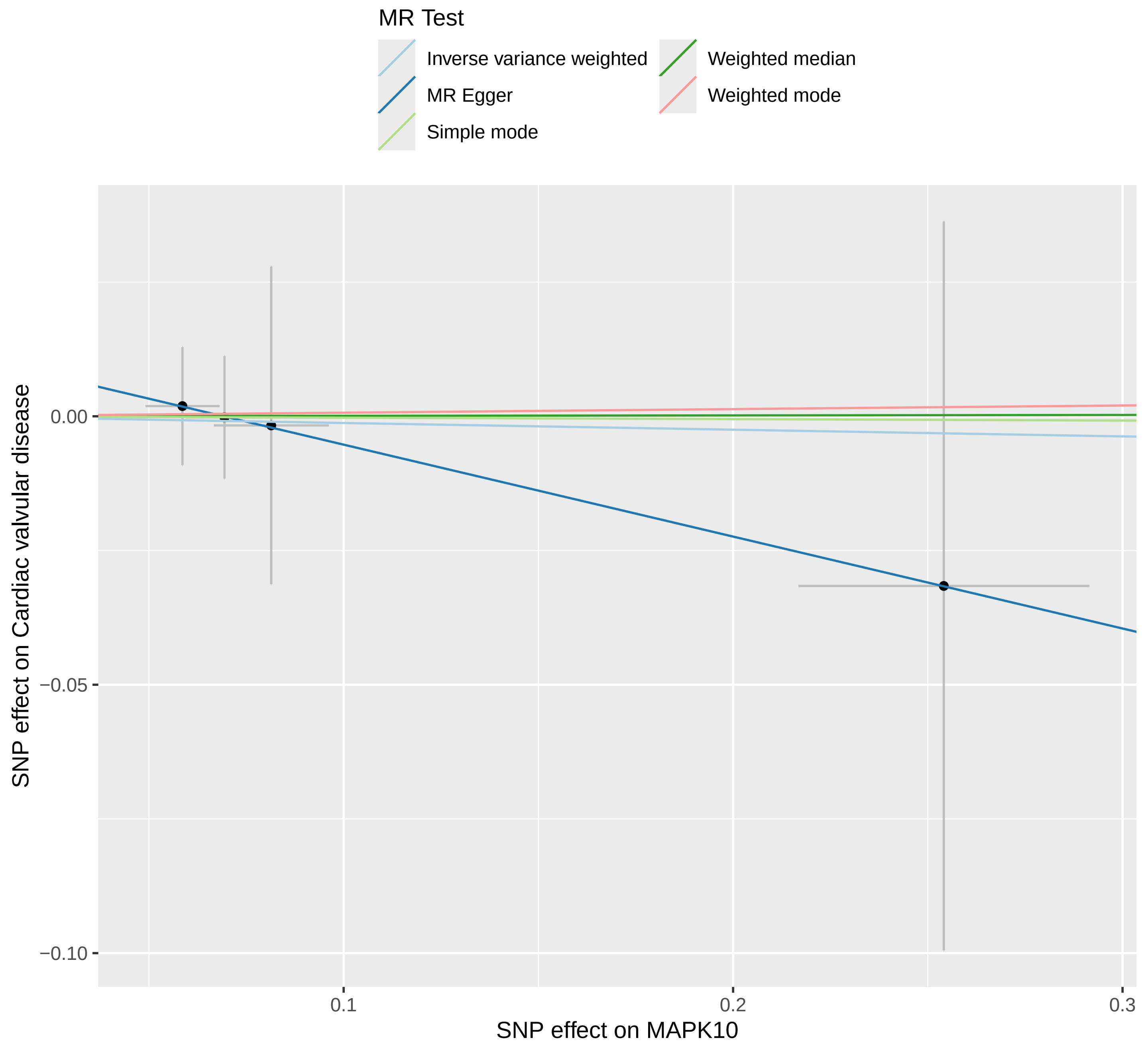

MR Test
Inverse variance weighted
MR Egger
Simple mode
Weighted median
Weighted mode
SNP effect on Cardiac valvular disease
0.00
−0.05
−0.10
0.1
0.2
0.3
SNP effect on MAPK10

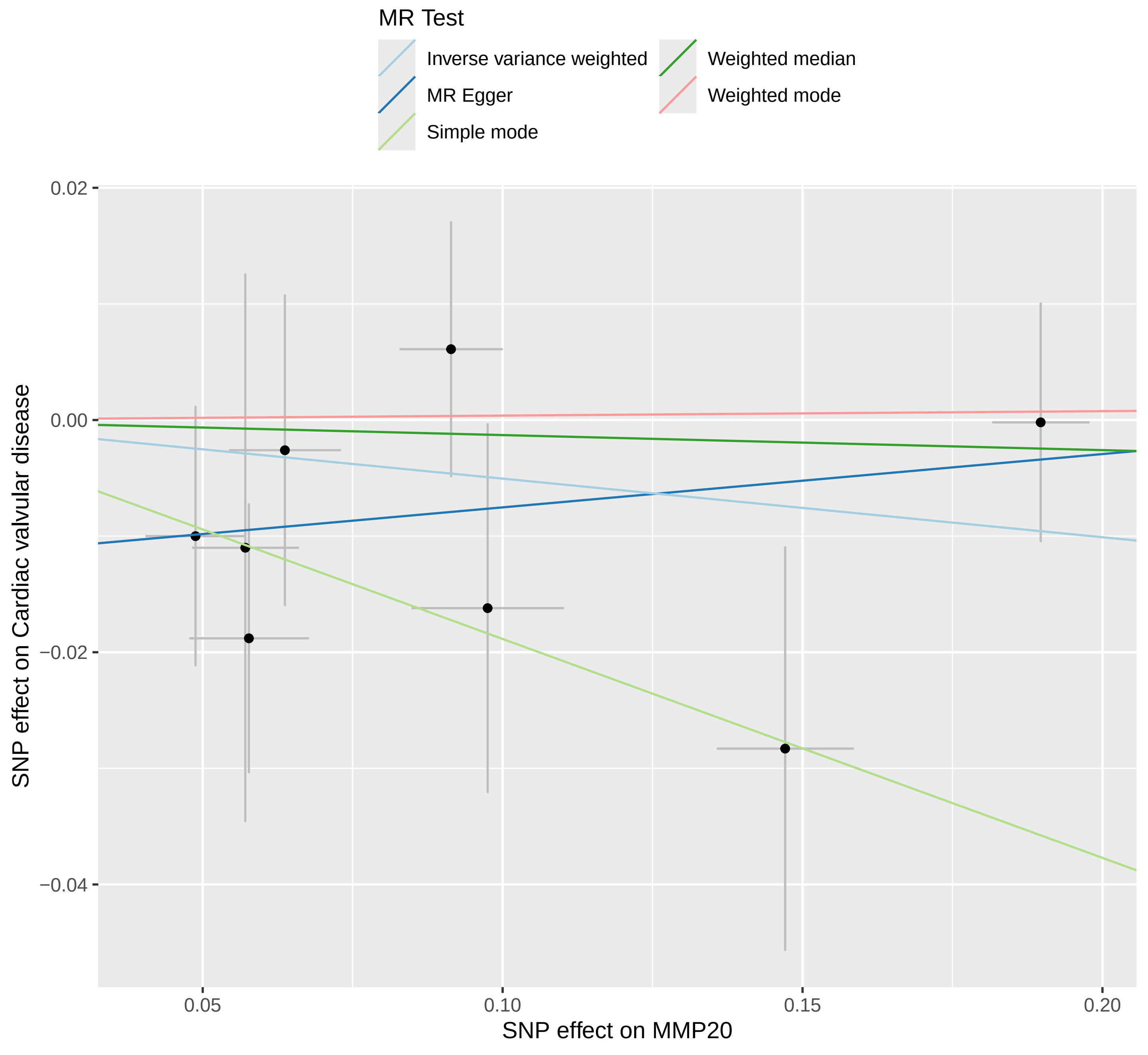

MR Test
Inverse variance weighted
MR Egger
Simple mode
Weighted median
Weighted mode
0.02
0.00
−0.02
−0.04
SNP effect on Cardiac valvular disease
0.05
0.10
0.15
0.20
SNP effect on MMP20

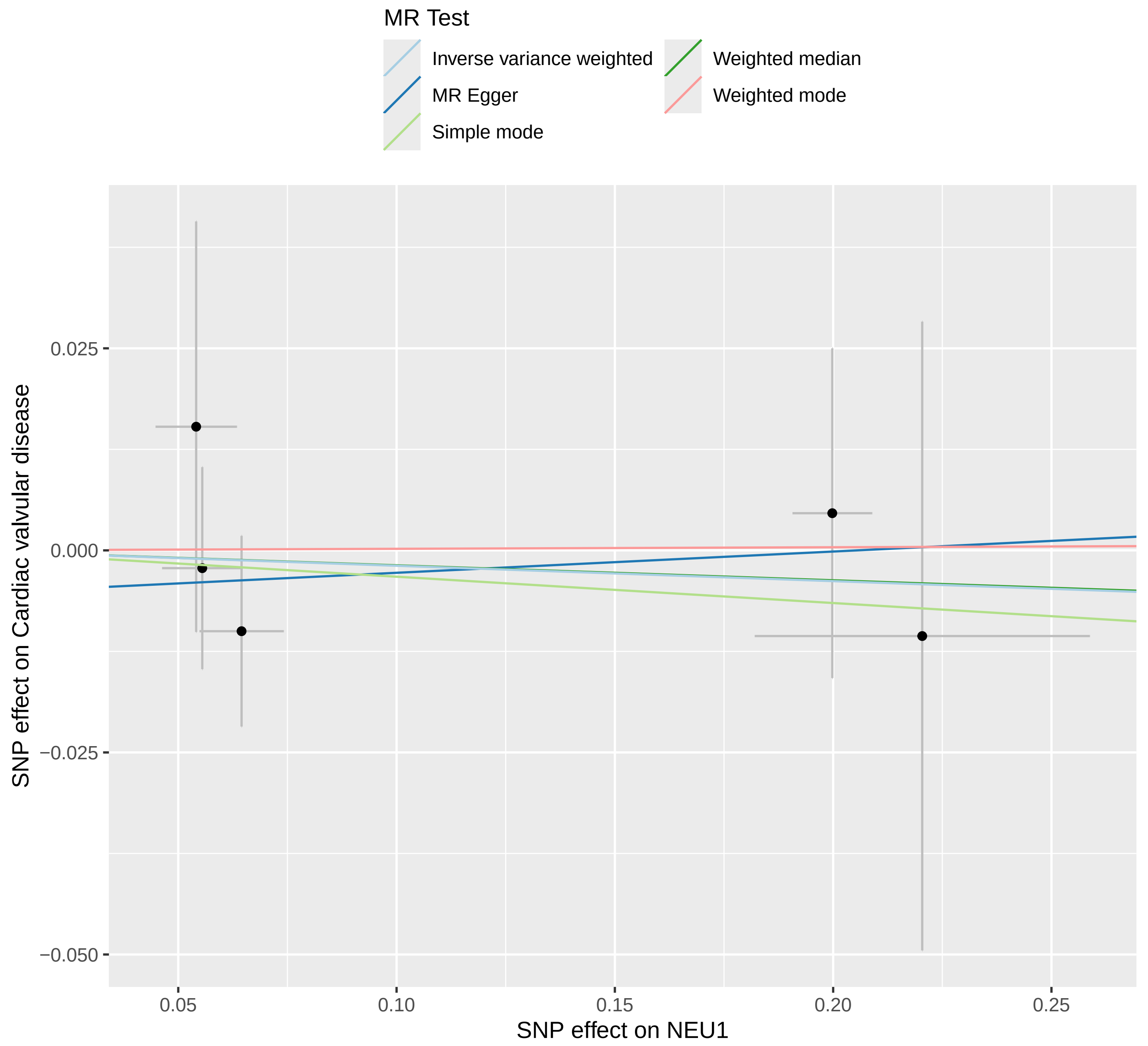

MR Test
Inverse variance weighted
MR Egger
Simple mode
Weighted median
Weighted mode
0.025
0.000
−0.025
−0.050
SNP effect on Cardiac valvular disease
0.05
0.10
0.15
0.20
0.25
SNP effect on NEU1

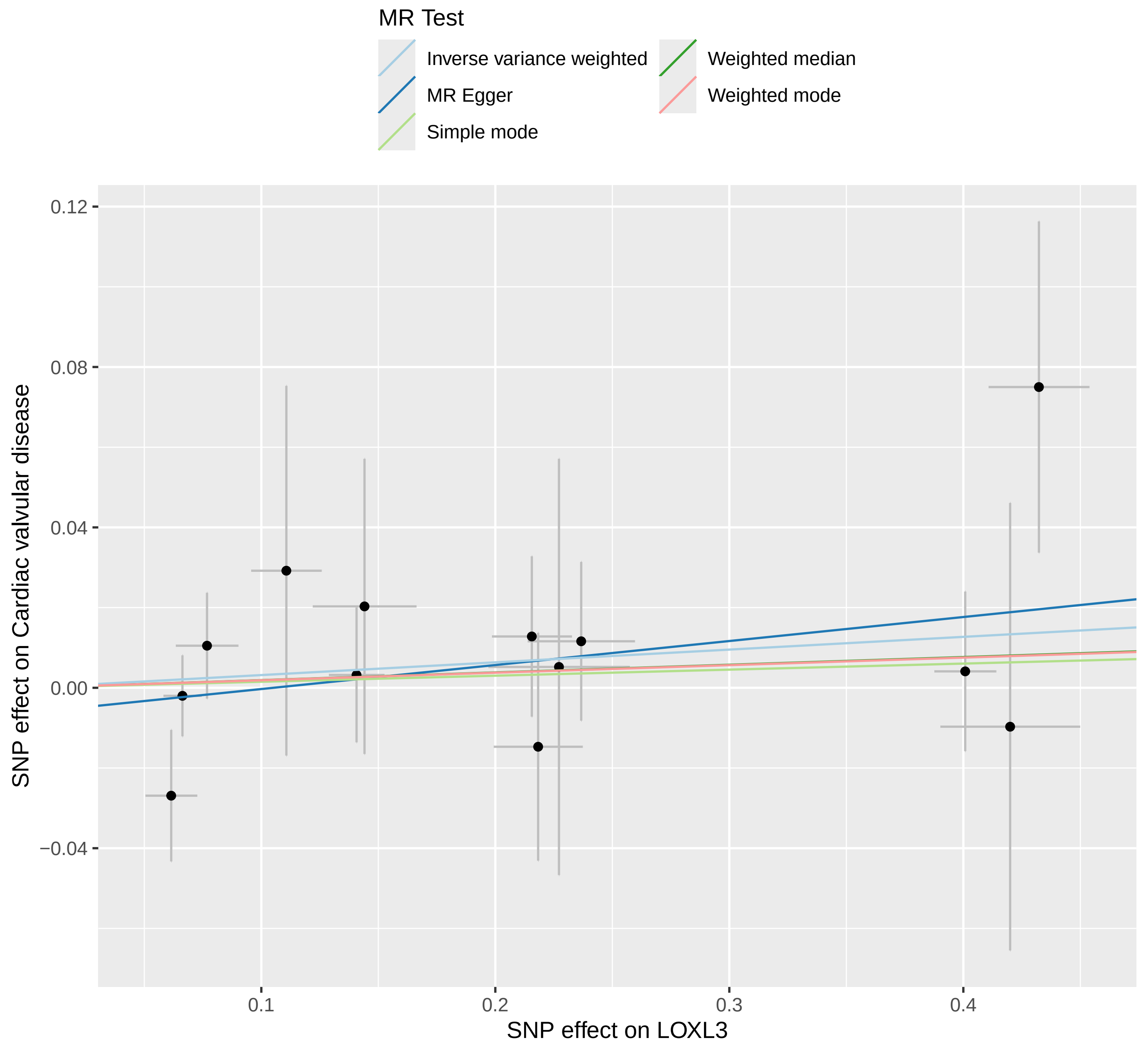
MR Test
Inverse variance weighted
MR Egger
Simple mode
Weighted median
Weighted mode
SNP effect on Cardiac valvular disease
0.12
0.08
0.04
0.00
−0.04
0.1
0.2
0.3
0.4
SNP effect on LOXL3

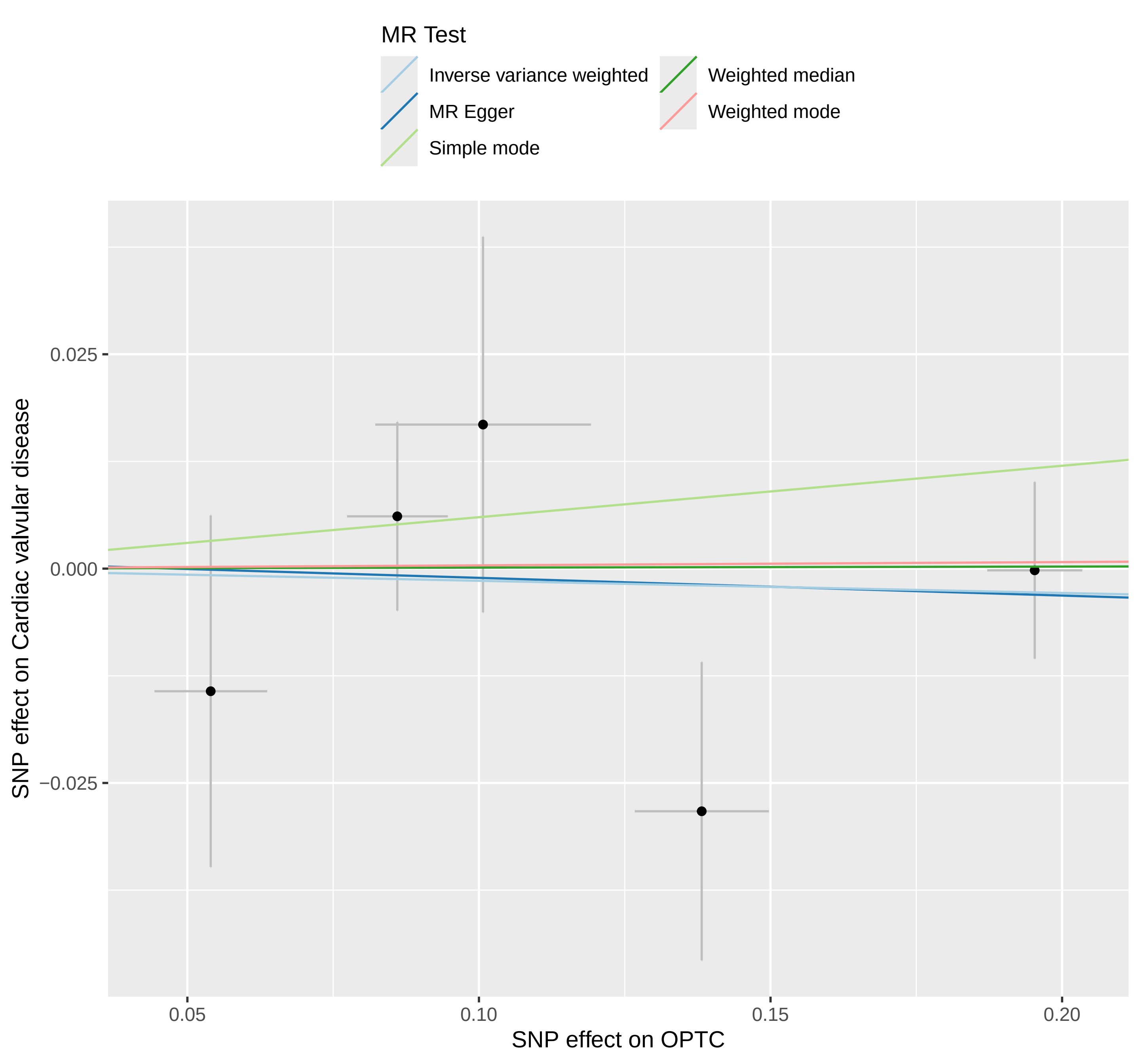
MR Test
Inverse variance weighted
MR Egger
Simple mode
Weighted median
Weighted mode
0.025
0.000
−0.025
0.05
0.10
0.15
0.20
SNP effect on Cardiac valvular disease
SNP effect on OPTC

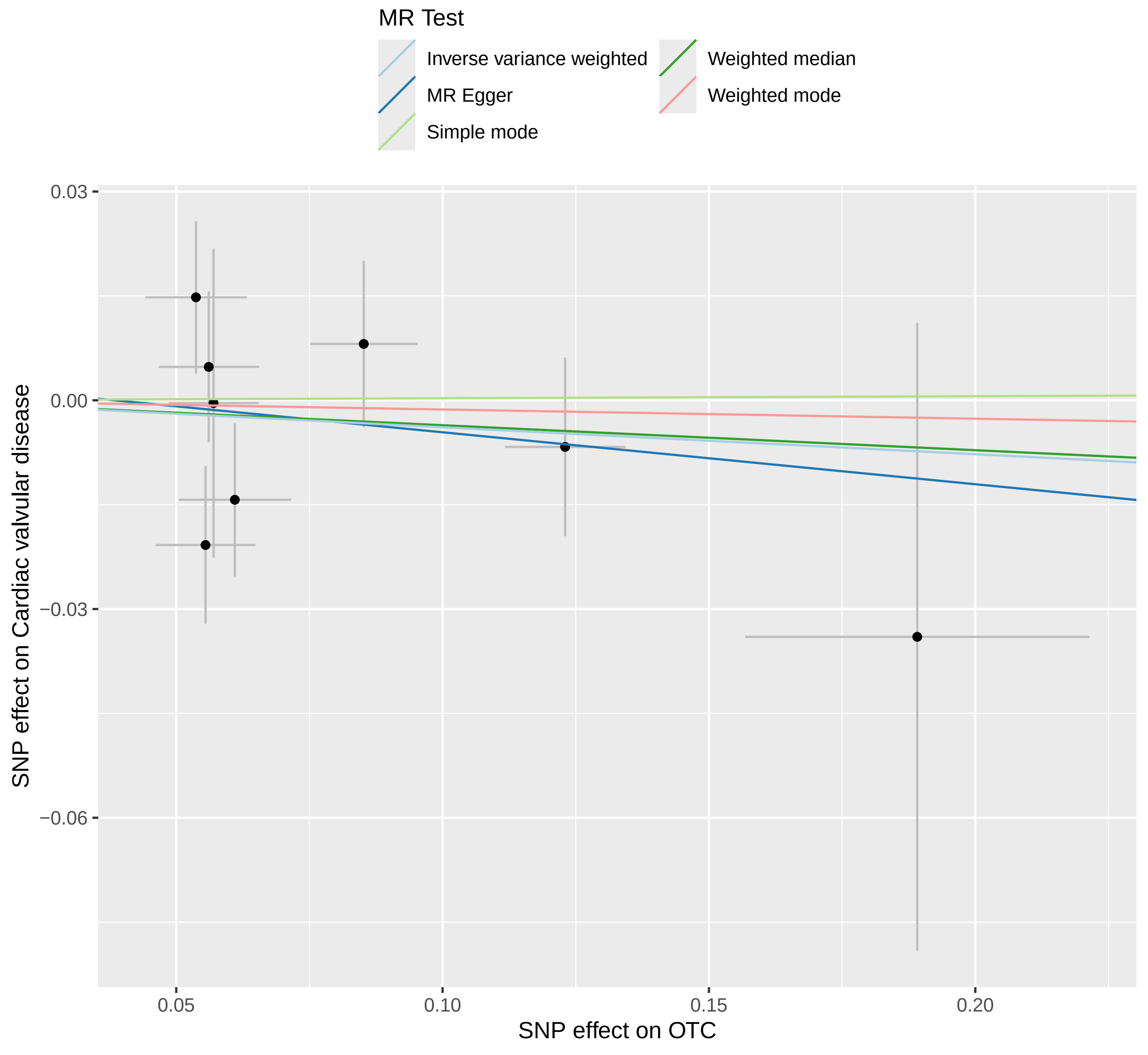
MR Test
Inverse variance weighted
MR Egger
Simple mode
Weighted median
Weighted mode
0.03
0.00
−0.03
−0.06
0.05
0.10
0.15
0.20
SNP effect on OTC
SNP effect on Cardiac valvular disease

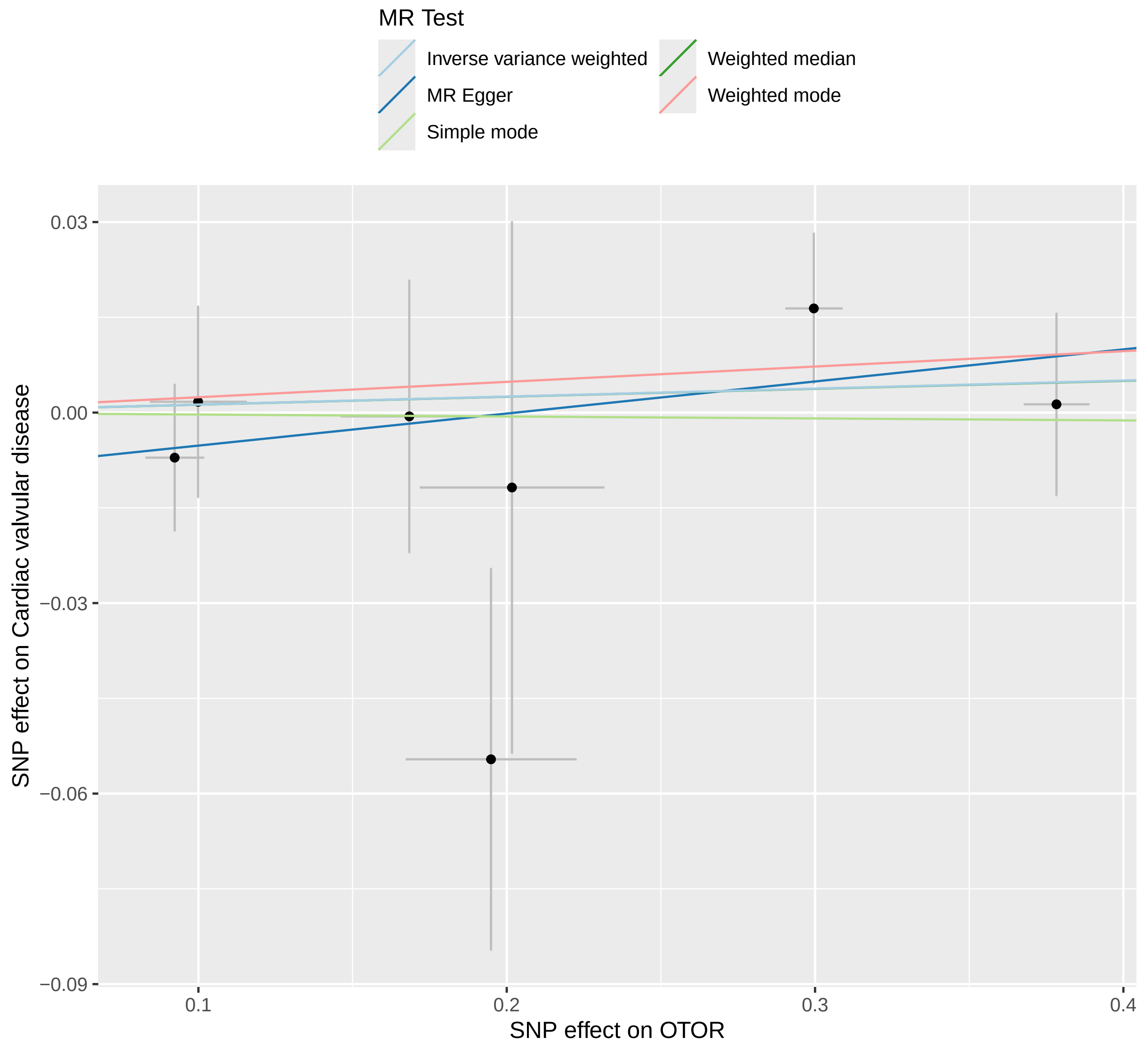
MR Test
Inverse variance weighted
MR Egger
Simple mode
Weighted median
Weighted mode
0.03
0.00
−0.03
−0.06
−0.09
SNP effect on Cardiac valvular disease
0.1
0.2
0.3
0.4
SNP effect on OTOR

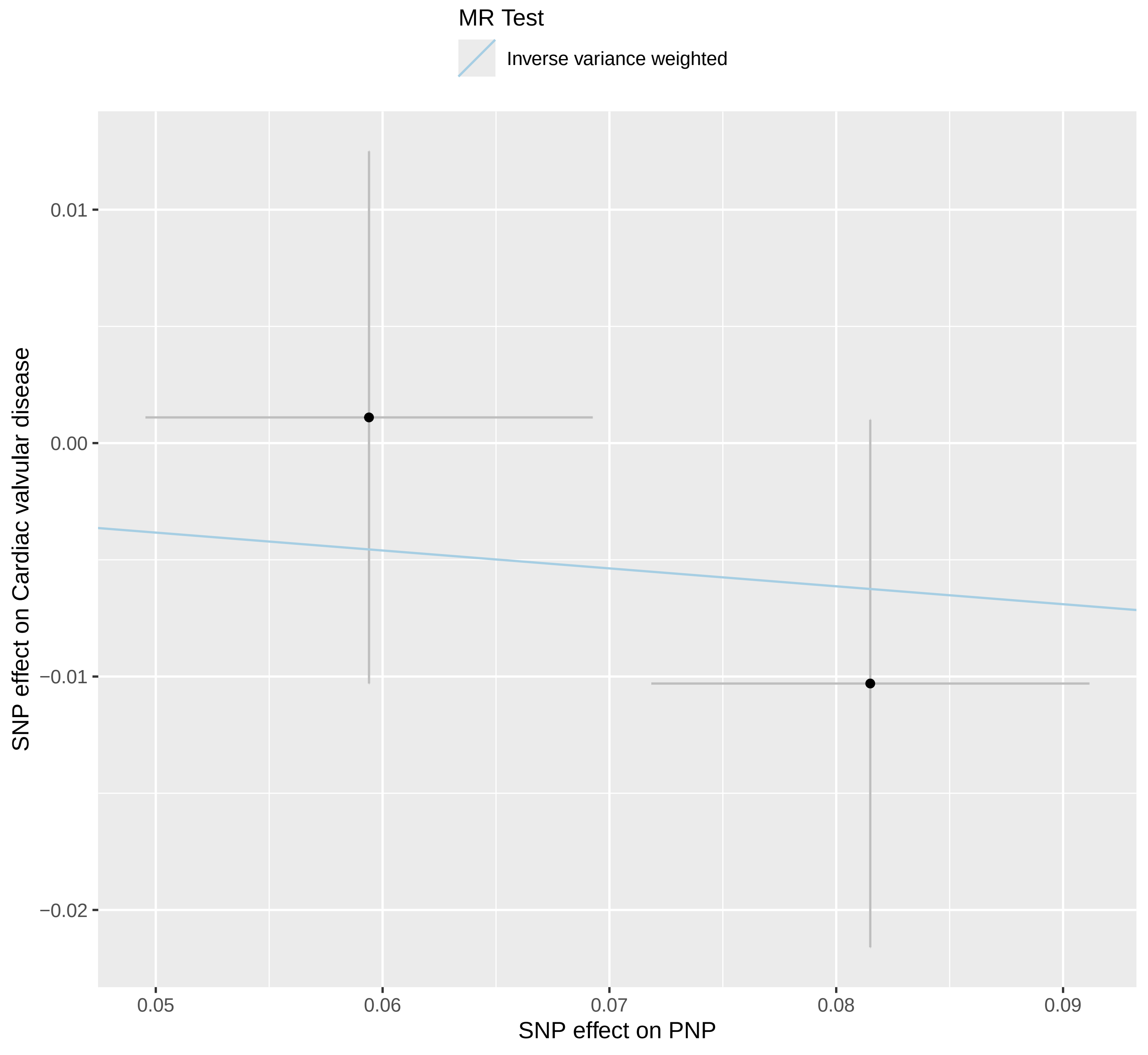

MR Test
Inverse variance weighted
0.01
0.00
−0.01
−0.02
SNP effect on Cardiac valvular disease
0.05
0.06
0.07
0.08
0.09
SNP effect on PNP

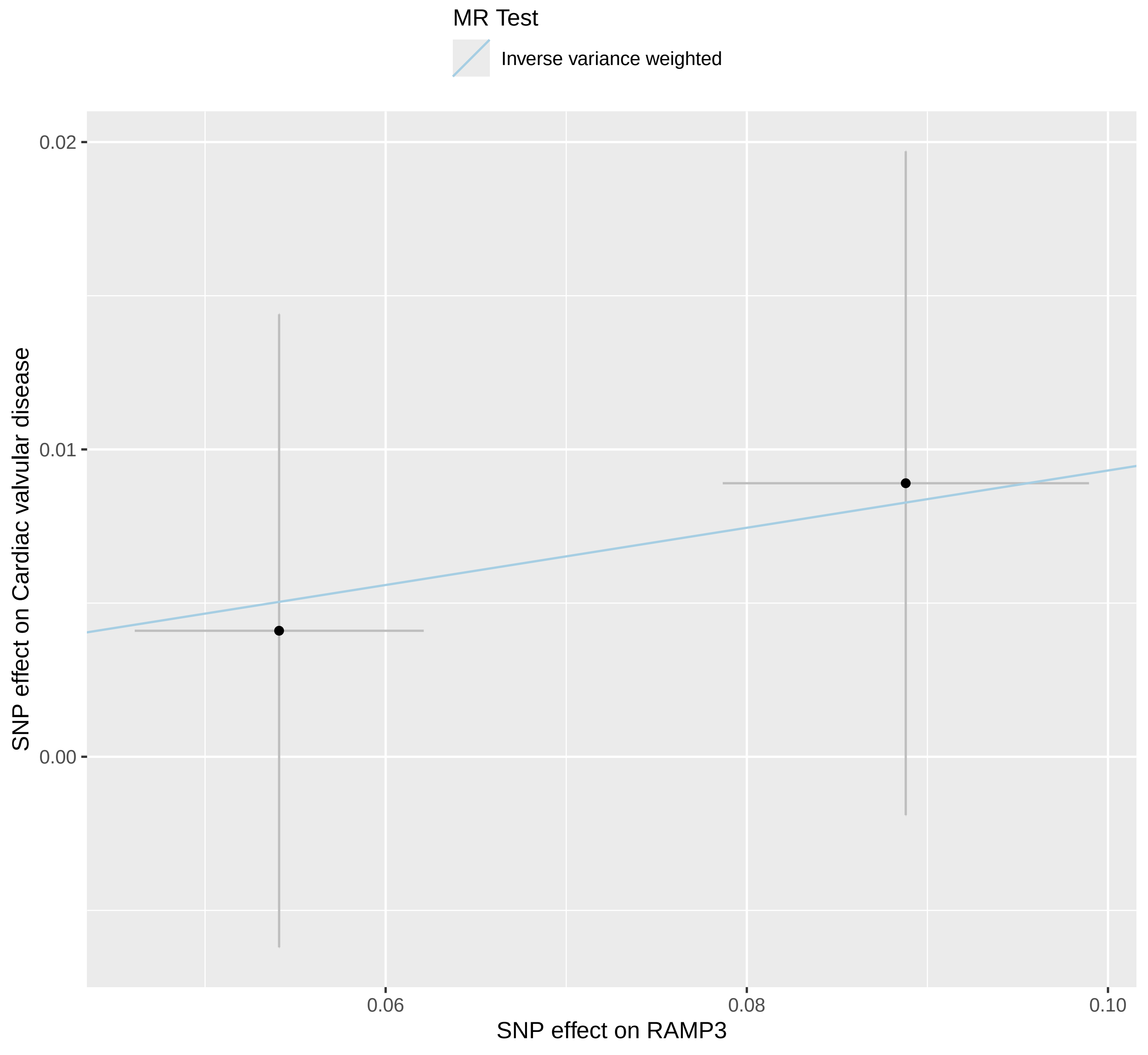
MR Test
Inverse variance weighted
0.02
0.01
0.00
0.06
0.08
0.10
SNP effect on Cardiac valvular disease
SNP effect on RAMP3

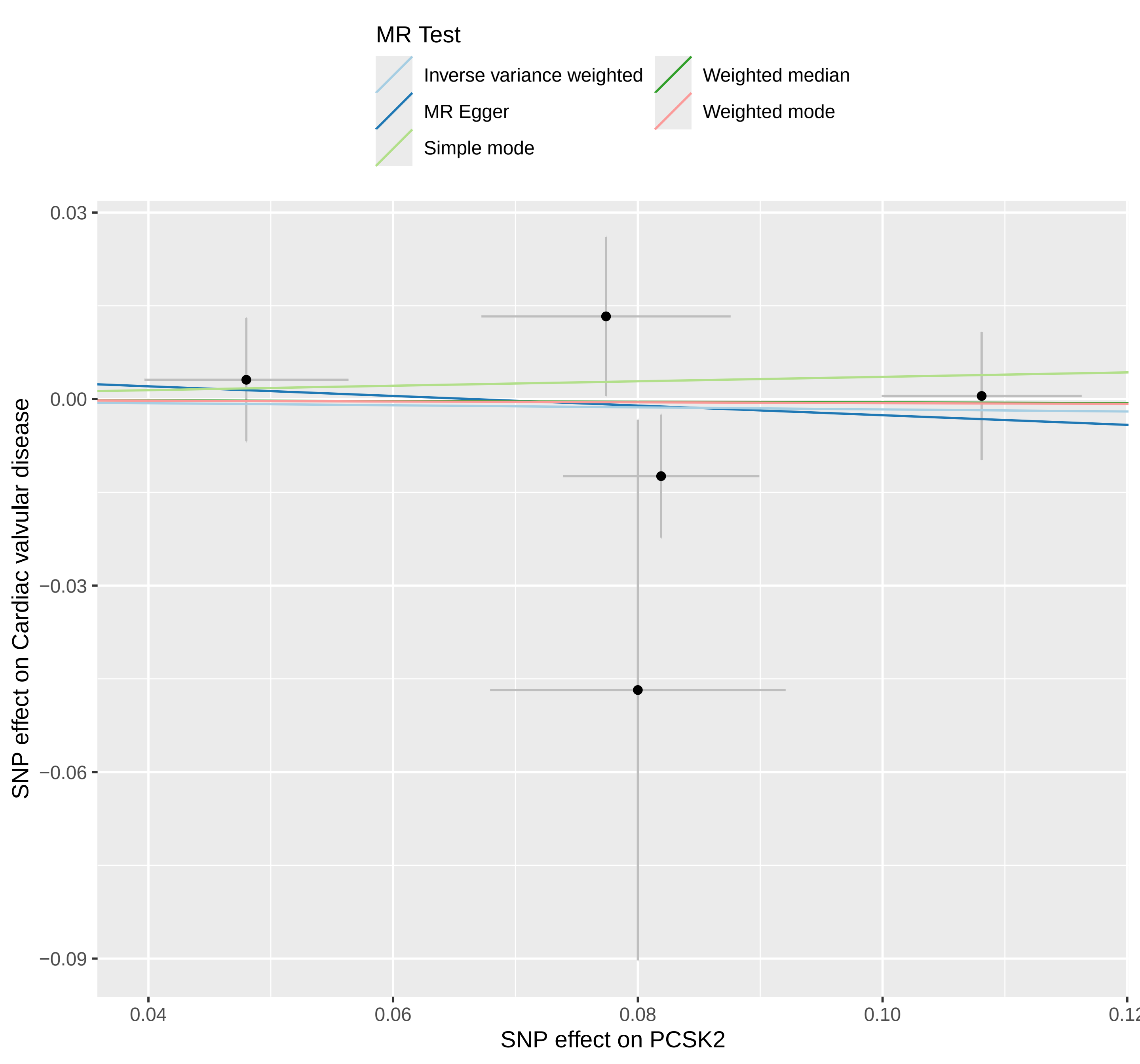
MR Test
Inverse variance weighted
MR Egger
Simple mode
Weighted median
Weighted mode
0.03
0.00
−0.03
−0.06
−0.09
SNP effect on Cardiac valvular disease
0.04
0.06
0.08
0.10
0.12
SNP effect on PCSK2

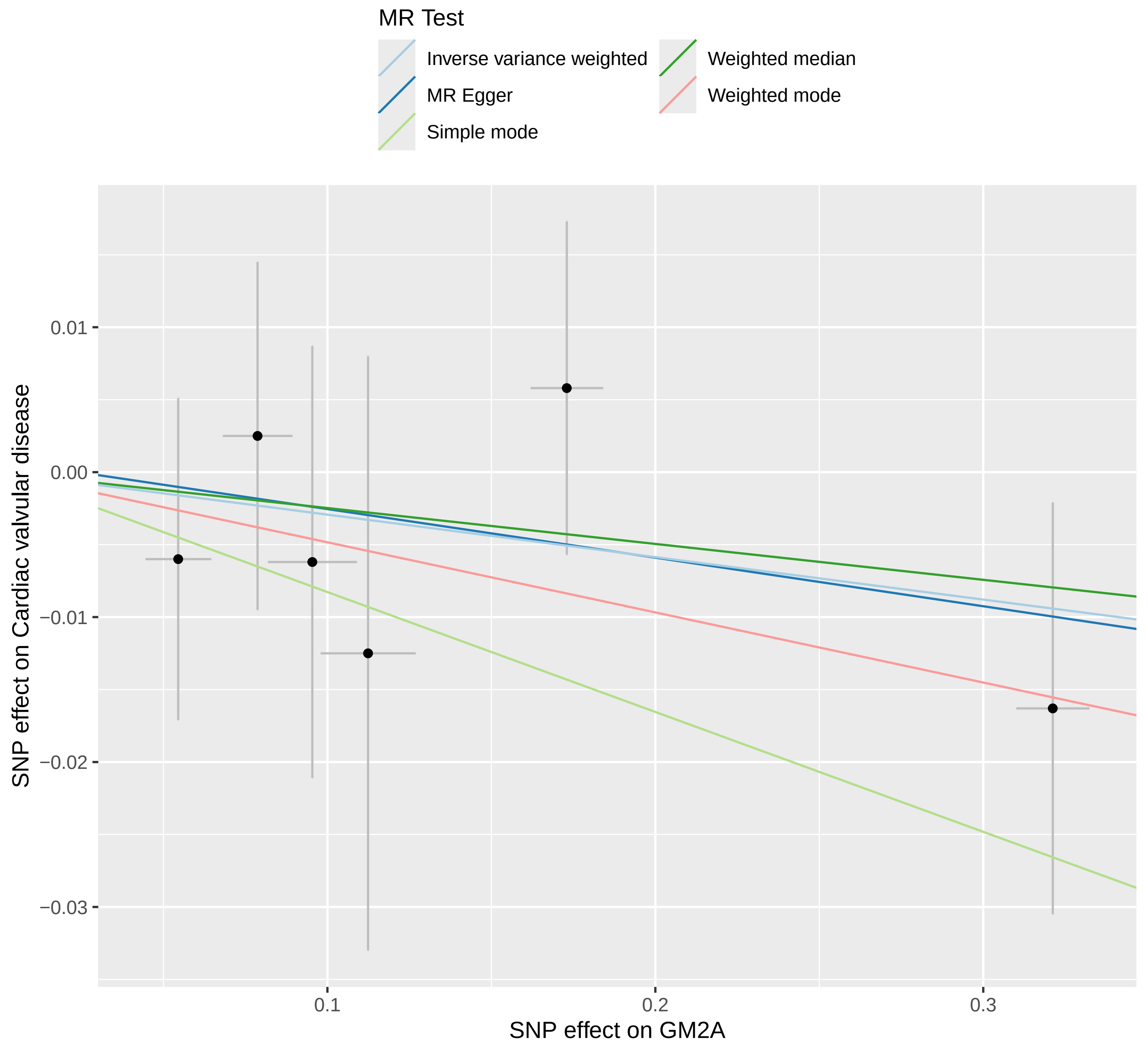
MR Test
Inverse variance weighted
MR Egger
Simple mode
Weighted median
Weighted mode
0.01
0.00
−0.01
−0.02
−0.03
0.1
0.2
0.3
SNP effect on Cardiac valvular disease
SNP effect on GM2A

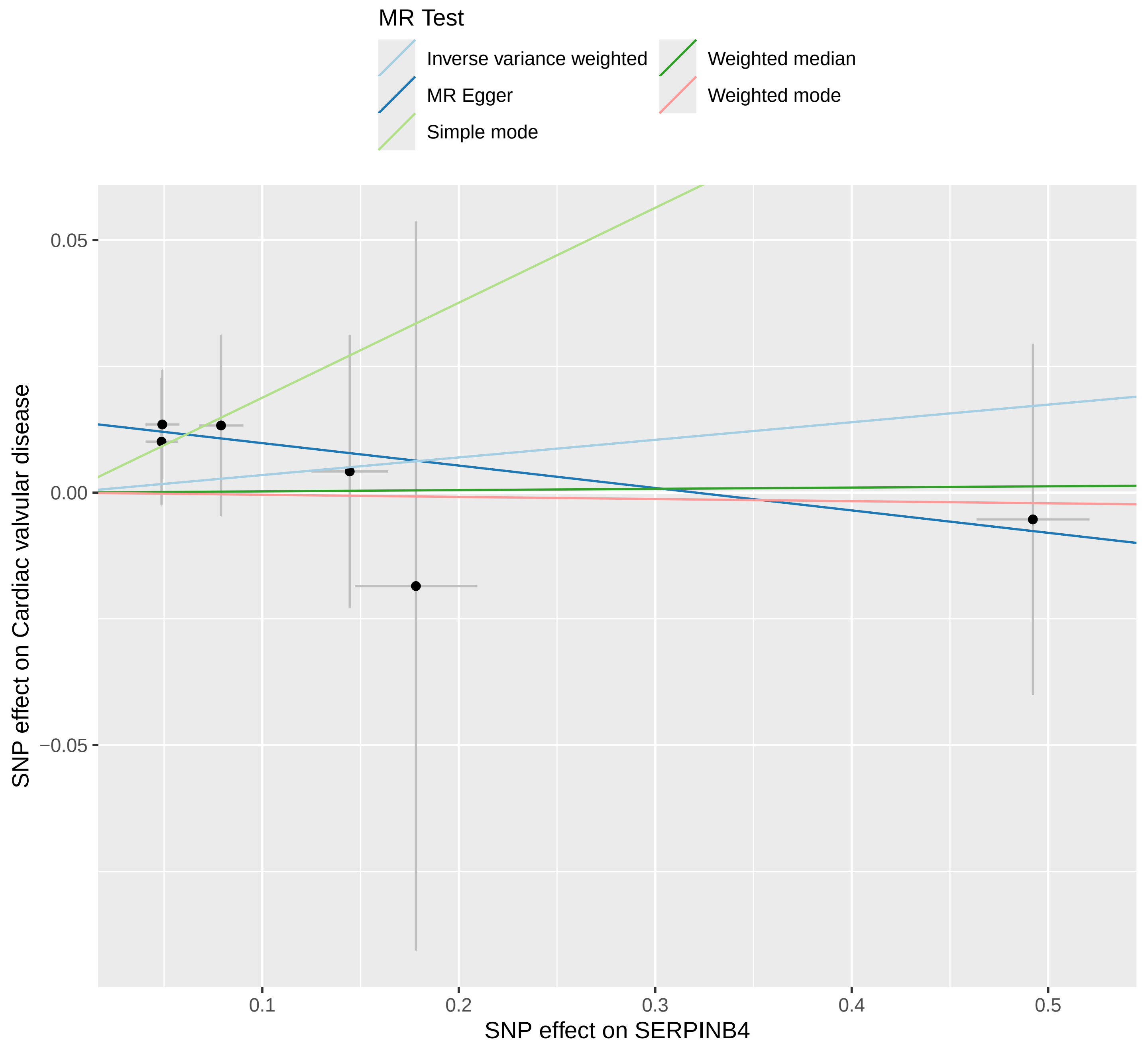
MR Test
Inverse variance weighted
MR Egger
Simple mode
Weighted median
Weighted mode
0.05
0.00
−0.05
SNP effect on Cardiac valvular disease
0.1
0.2
0.3
0.4
0.5
SNP effect on SERPINB4

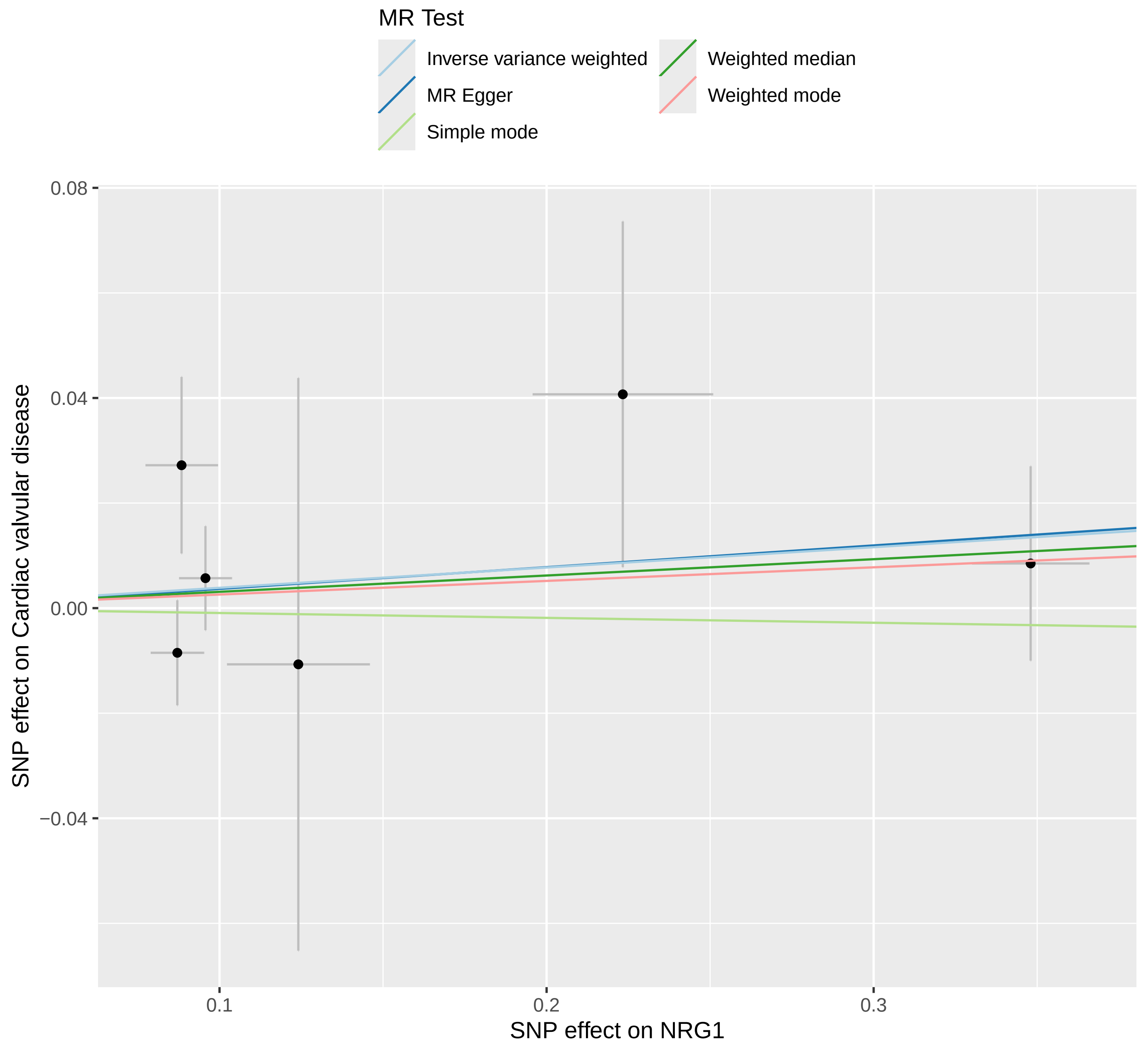
MR Test
Inverse variance weighted
MR Egger
Simple mode
Weighted median
Weighted mode
0.08
0.04
0.00
−0.04
0.1
0.2
0.3
SNP effect on Cardiac valvular disease
SNP effect on NRG1

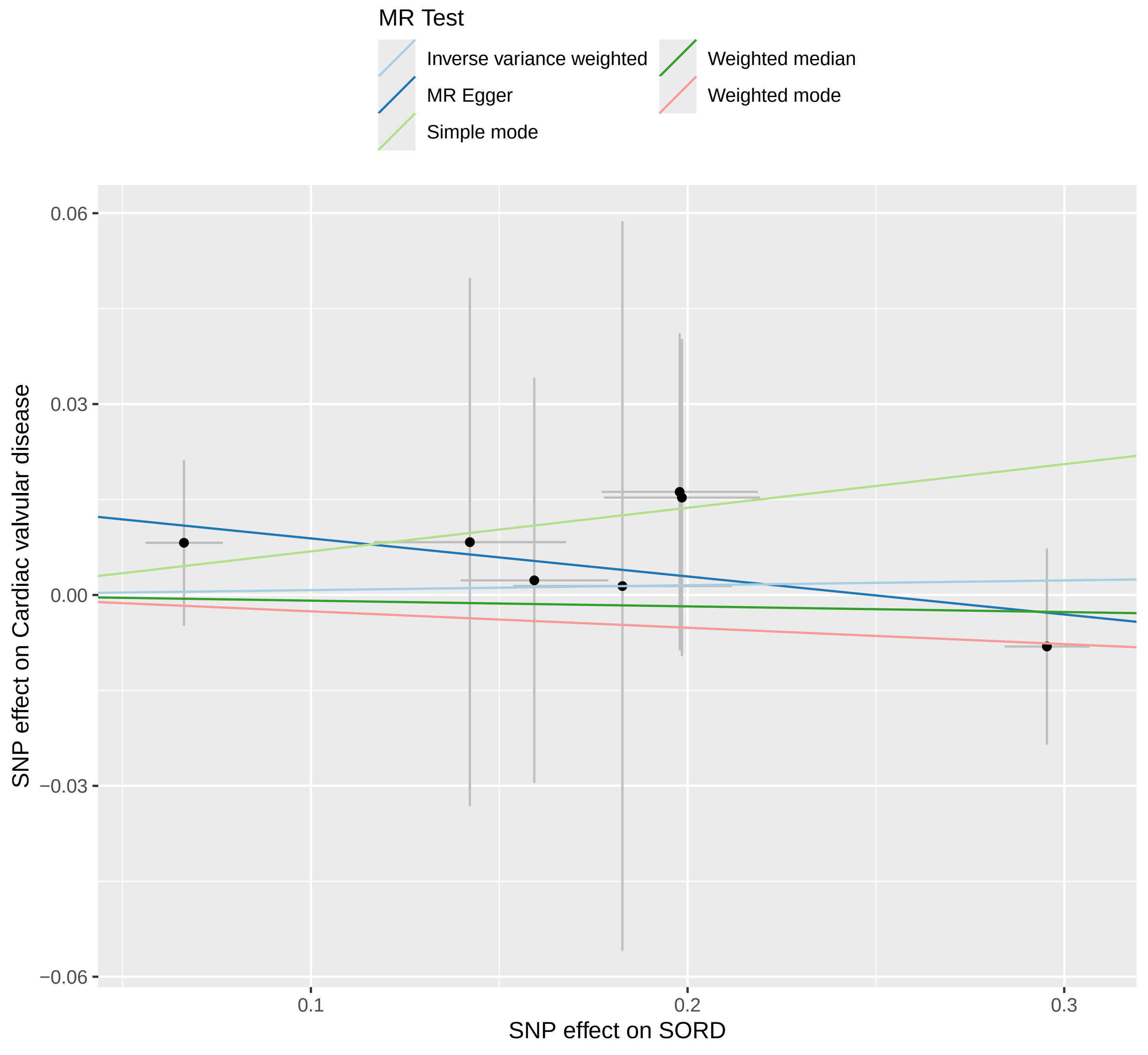

MR Test
Inverse variance weighted
MR Egger
Simple mode
Weighted median
Weighted mode
0.06
0.03
0.00
−0.03
−0.06
SNP effect on Cardiac valvular disease
0.1
0.2
0.3
SNP effect on SORD

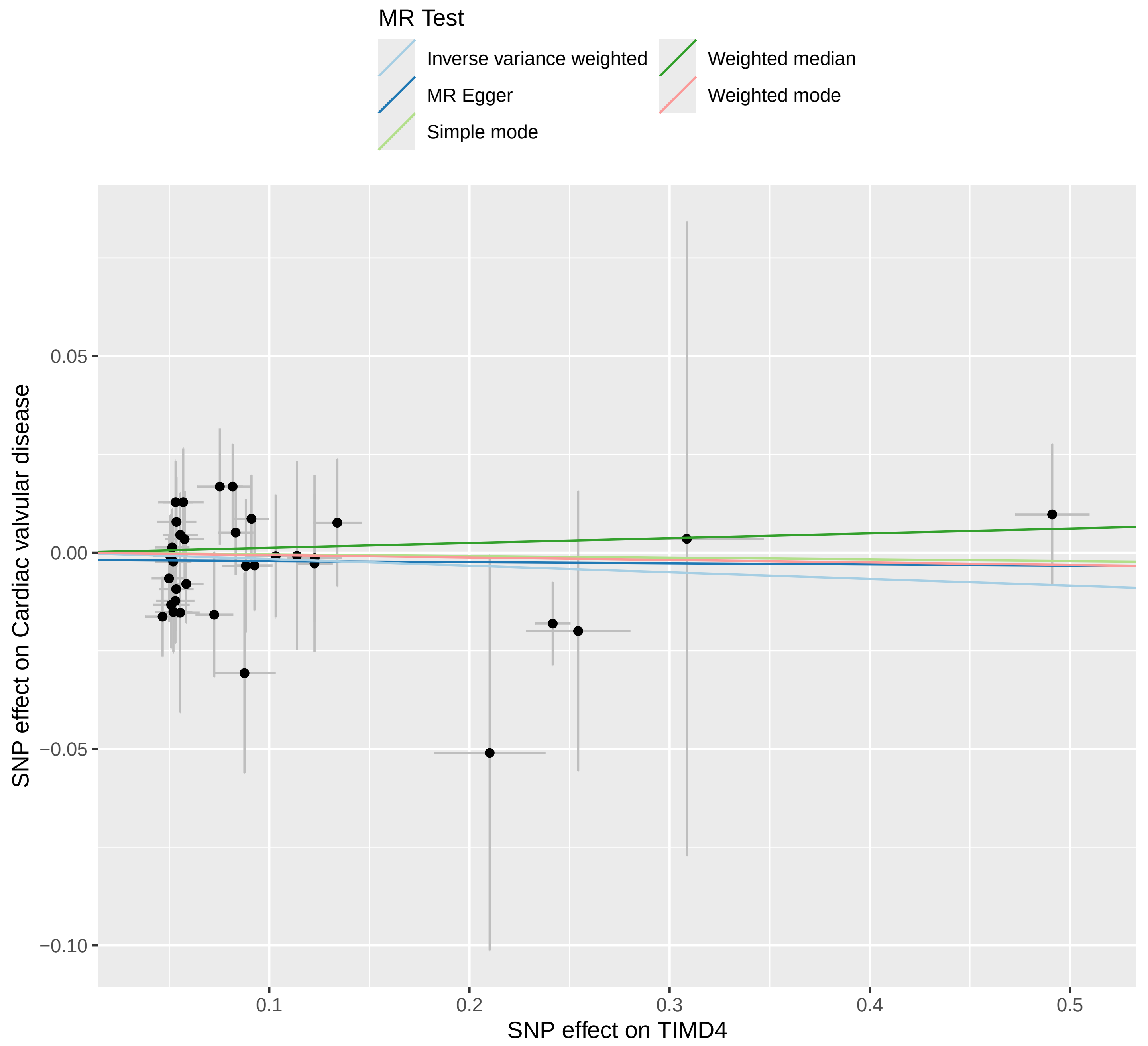

MR Test
Inverse variance weighted
MR Egger
Simple mode
Weighted median
Weighted mode
0.05
0.00
−0.05
−0.10
0.1
0.2
0.3
0.4
0.5
SNP effect on Cardiac valvular disease
SNP effect on TIMD4

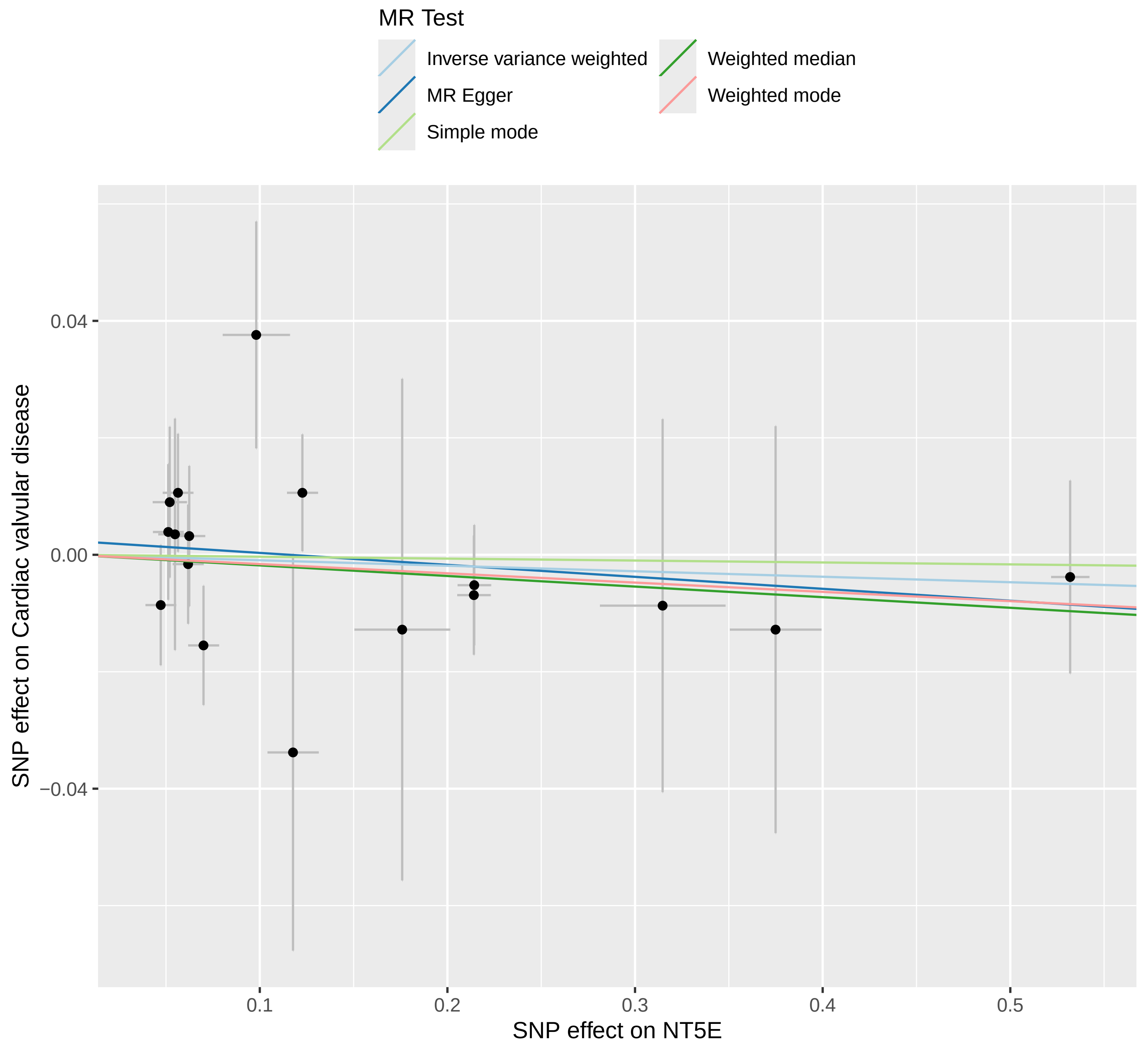

MR Test
Inverse variance weighted
MR Egger
Simple mode
Weighted median
Weighted mode
0.04
0.00
−0.04
0.1
0.2
0.3
0.4
0.5
SNP effect on Cardiac valvular disease
SNP effect on NT5E

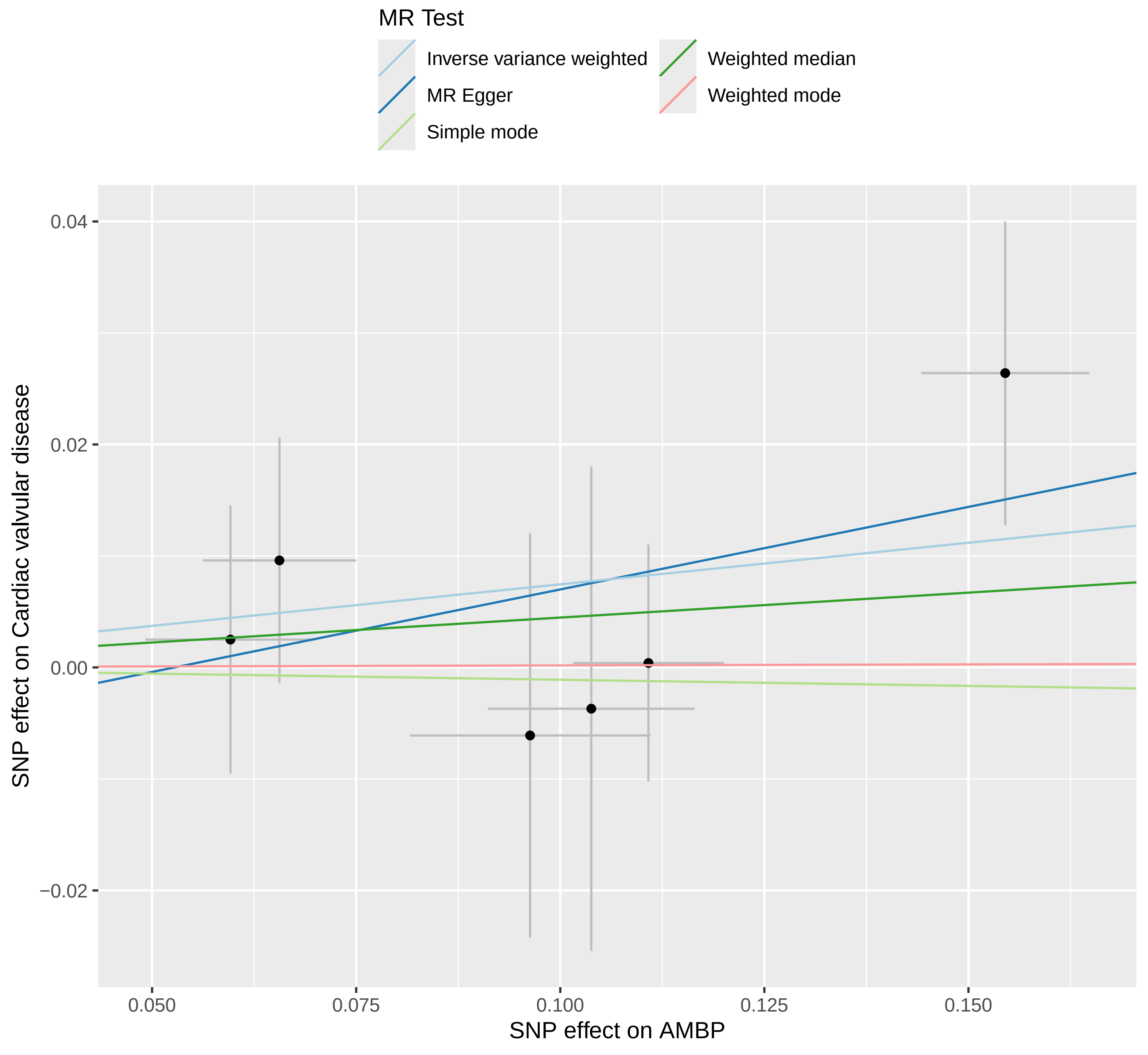
MR Test
Inverse variance weighted
MR Egger
Simple mode
Weighted median
Weighted mode
0.04
0.02
0.00
−0.02
0.050
0.075
0.100
0.125
0.150
SNP effect on Cardiac valvular disease
SNP effect on AMBP

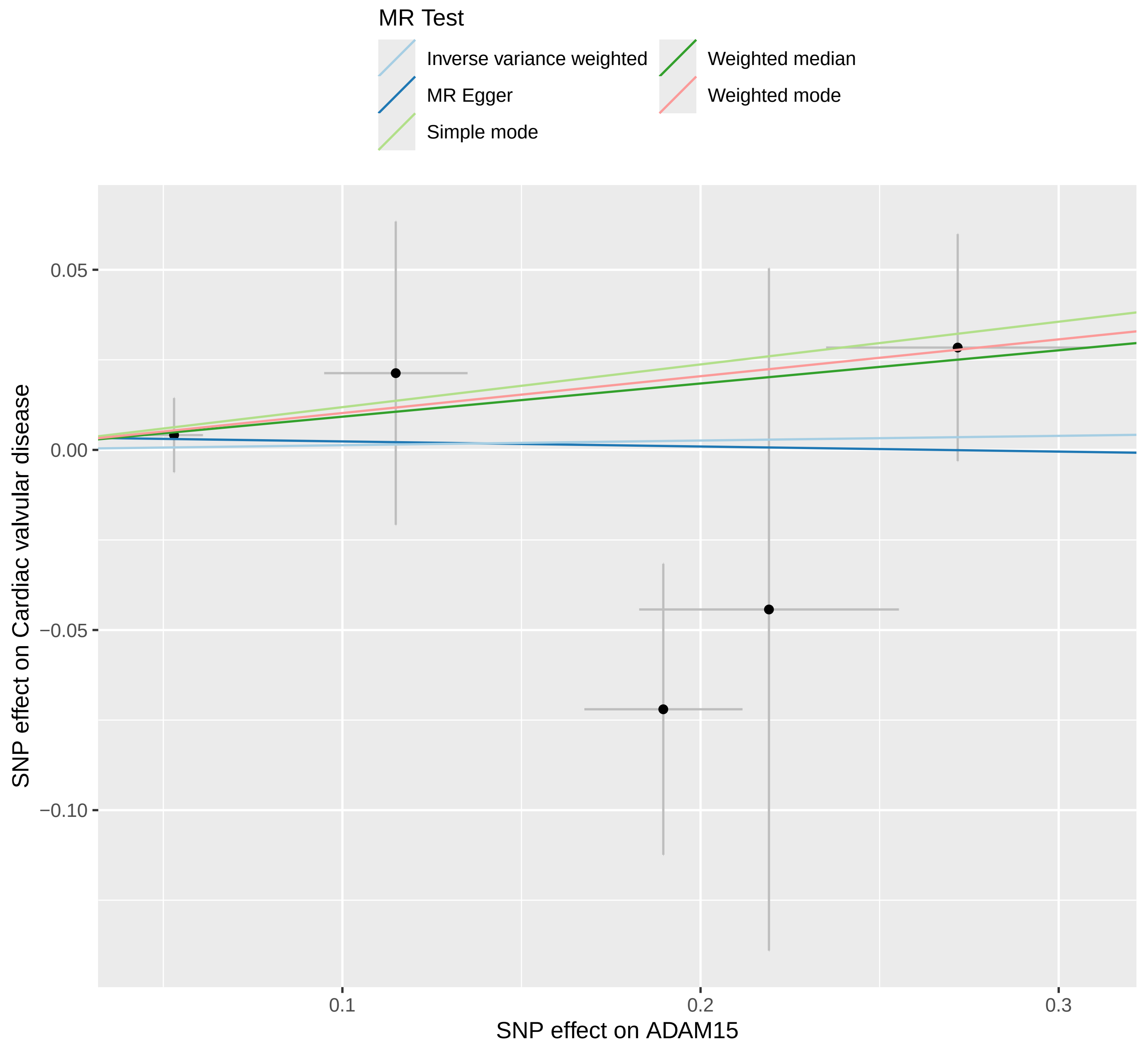

MR Test
Inverse variance weighted
MR Egger
Simple mode
Weighted median
Weighted mode
0.05
0.00
−0.05
−0.10
0.1
0.2
0.3
SNP effect on Cardiac valvular disease
SNP effect on ADAM15

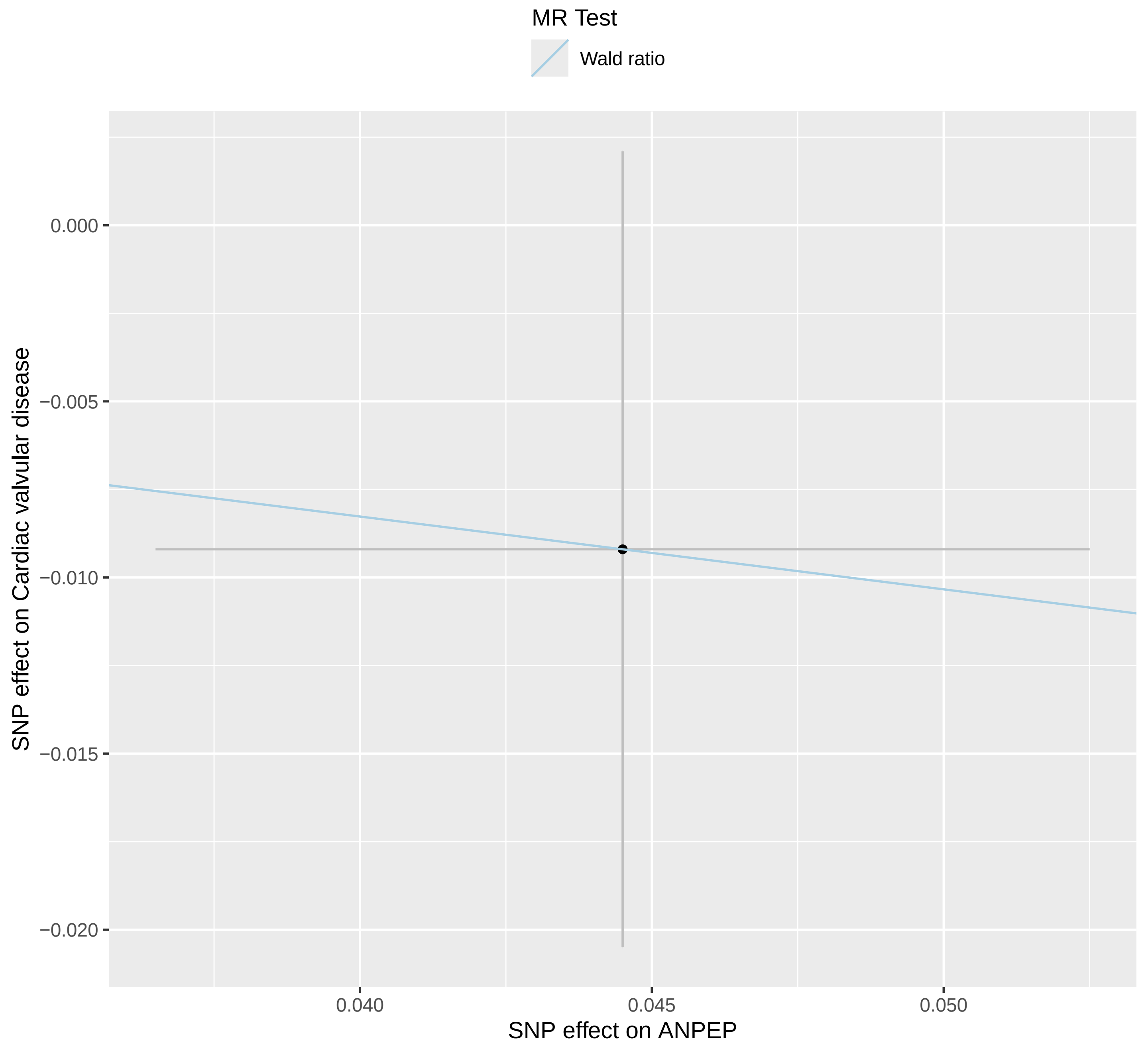
MR Test
Wald ratio
0.000
−0.005
−0.010
−0.015
−0.020
SNP effect on Cardiac valvular disease
0.040
0.045
0.050
SNP effect on ANPEP

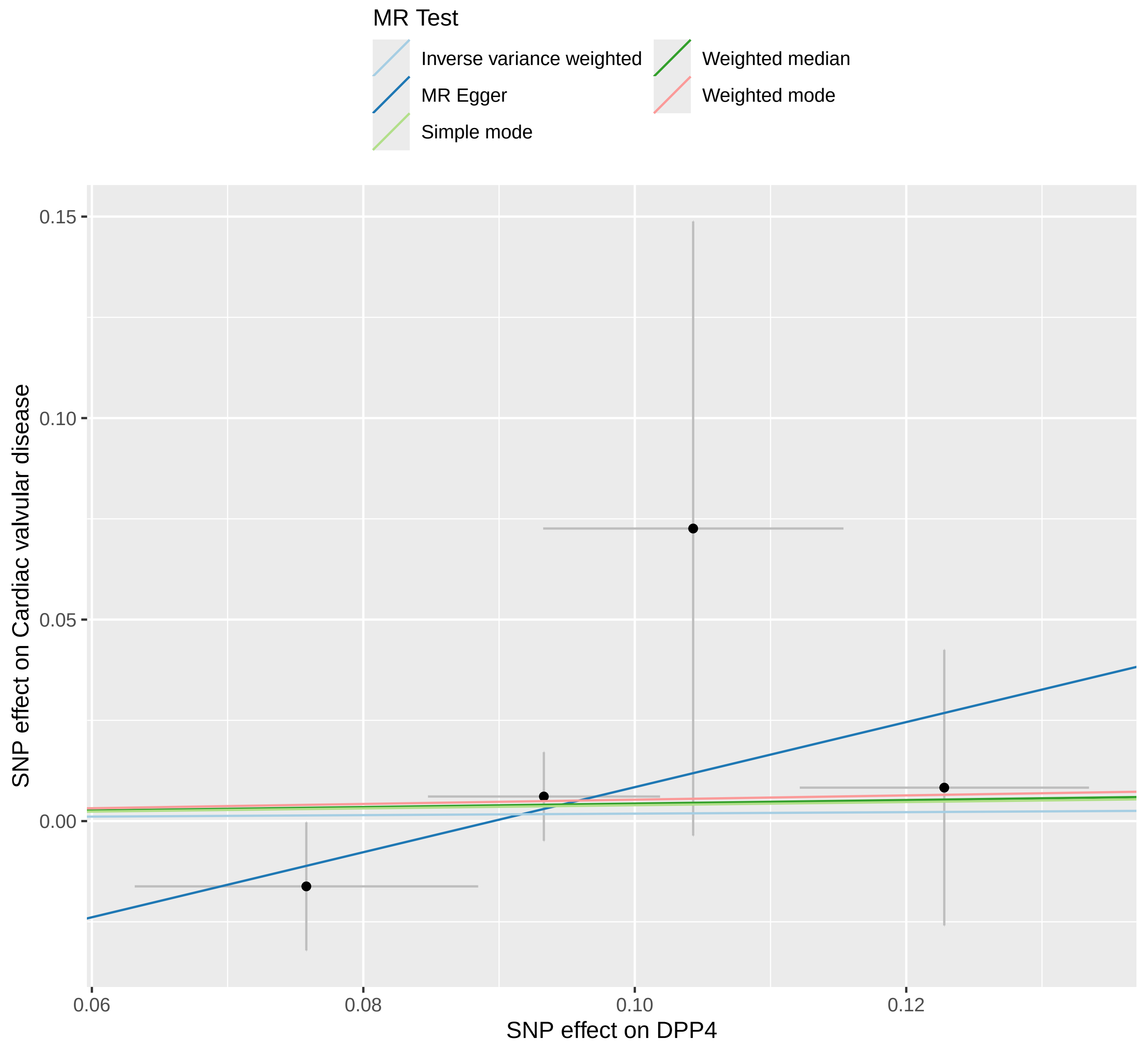
MR Test
Inverse variance weighted
MR Egger
Simple mode
Weighted median
Weighted mode
0.15
0.10
0.05
0.00
0.06
0.08
0.10
0.12
SNP effect on Cardiac valvular disease
SNP effect on DPP4

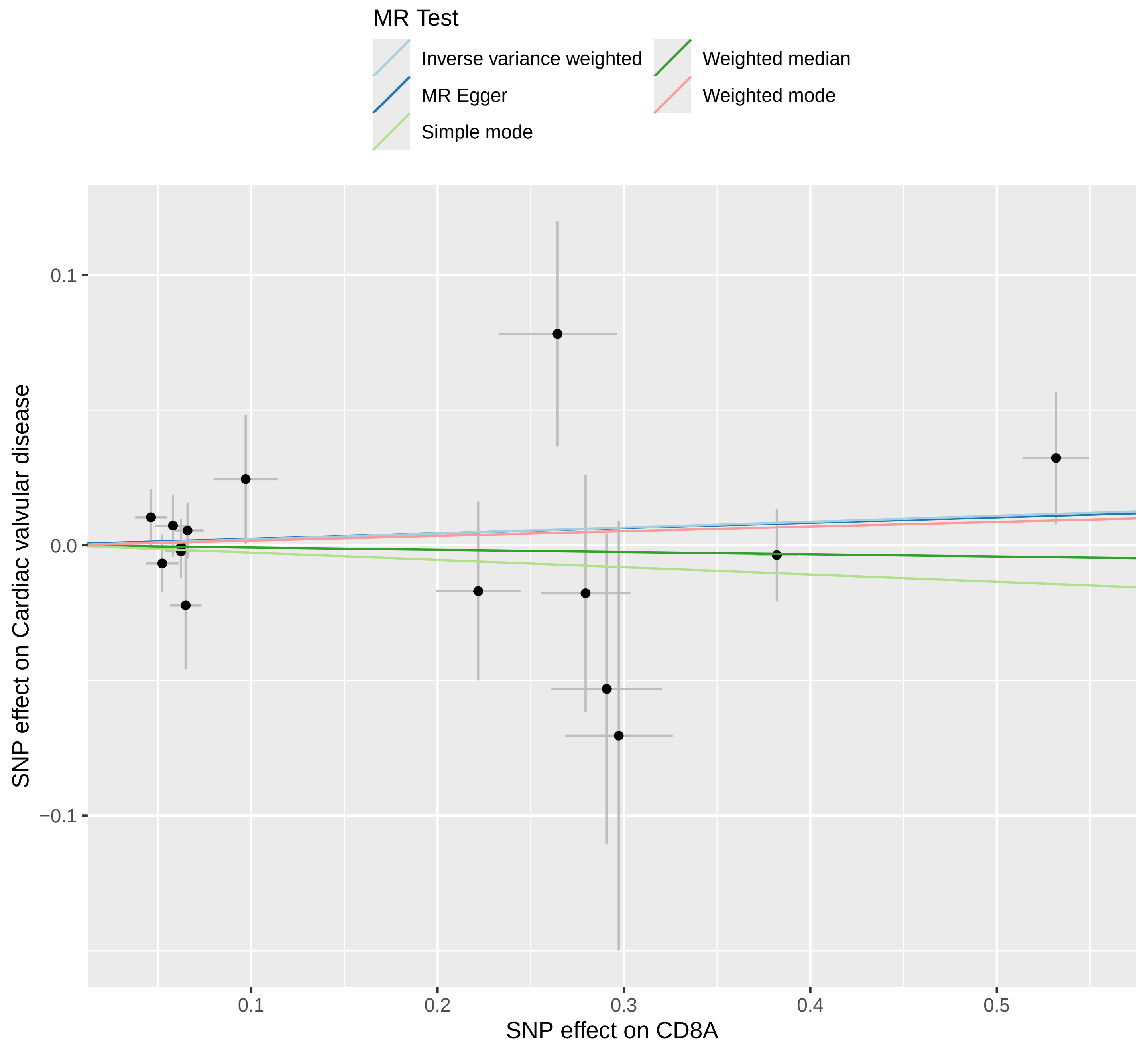
MR Test
Inverse variance weighted
MR Egger
Simple mode
Weighted median
Weighted mode
0.1
0.0
−0.1
SNP effect on Cardiac valvular disease
0.1
0.2
0.3
0.4
0.5
SNP effect on CD8A

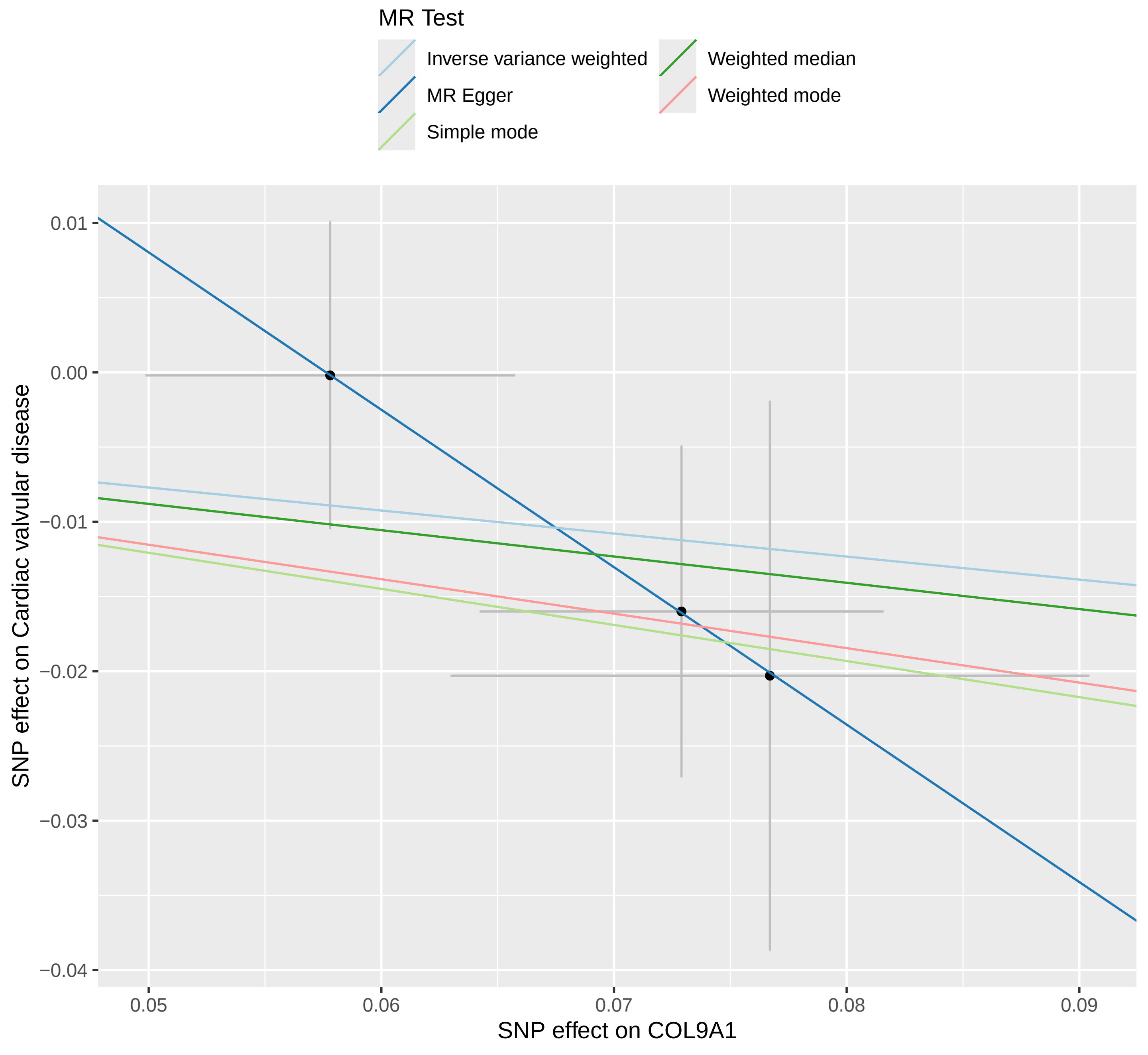
MR Test
Inverse variance weighted
MR Egger
Simple mode
Weighted median
Weighted mode
0.01
0.00
−0.01
−0.02
−0.03
−0.04
SNP effect on Cardiac valvular disease
0.05
0.06
0.07
0.08
0.09
SNP effect on COL9A1

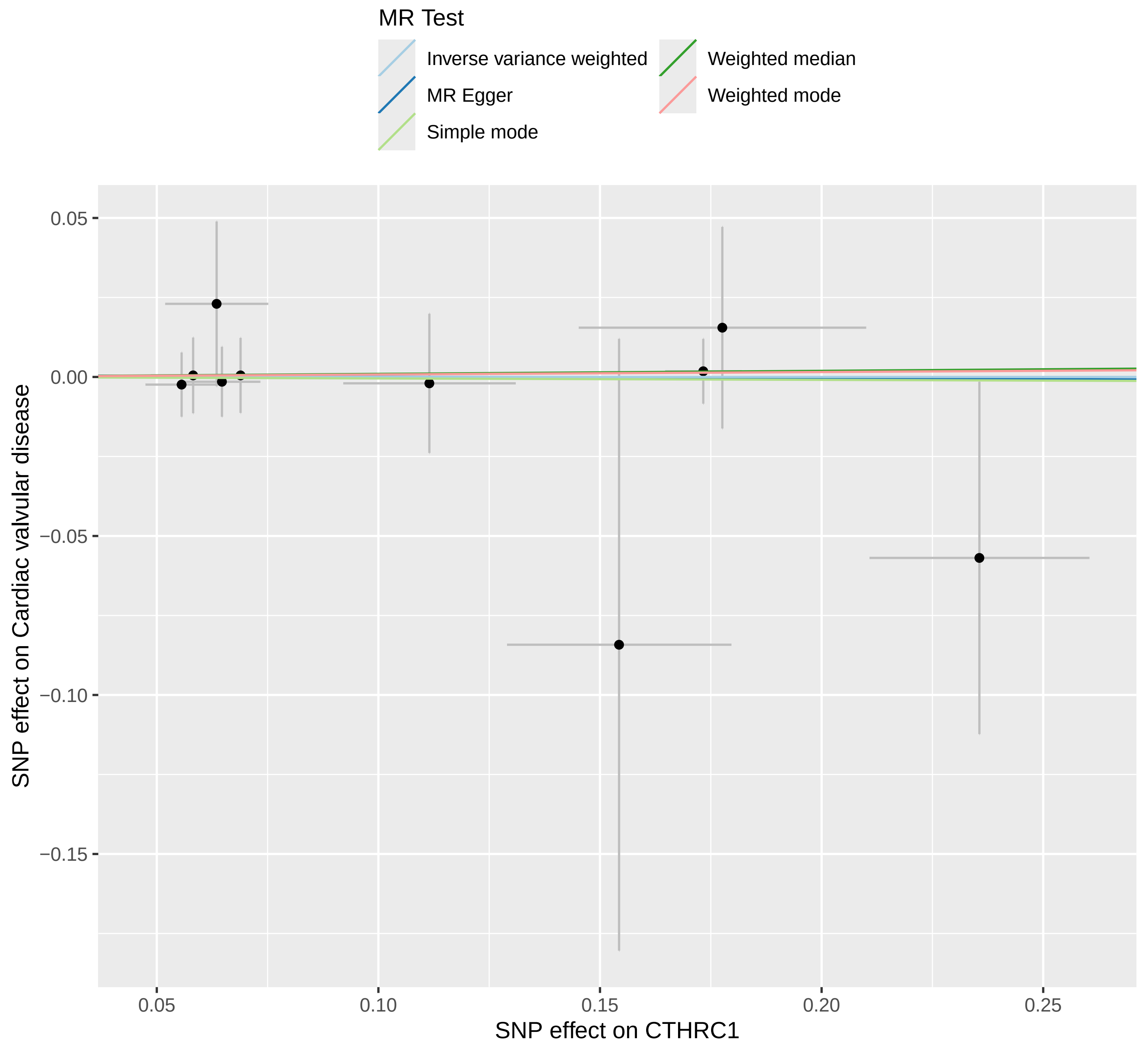
MR Test
Inverse variance weighted
MR Egger
Simple mode
Weighted median
Weighted mode
0.05
0.00
−0.05
−0.10
−0.15
0.05
0.10
0.15
0.20
0.25
SNP effect on Cardiac valvular disease
SNP effect on CTHRC1

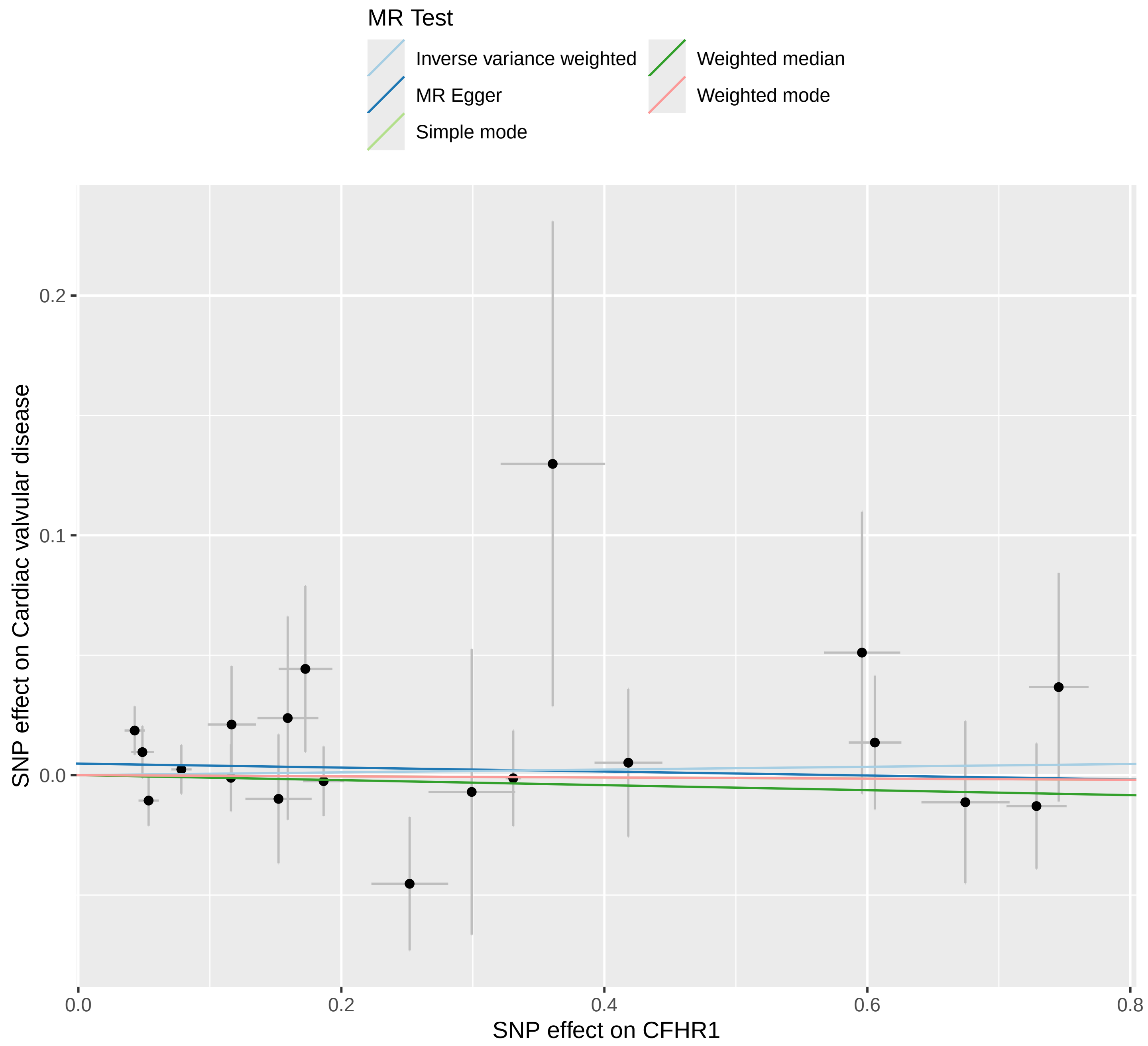
MR Test
Inverse variance weighted
MR Egger
Simple mode
Weighted median
Weighted mode
0.2
0.1
0.0
0.0
0.2
0.4
0.6
0.8
SNP effect on Cardiac valvular disease
SNP effect on CFHR1

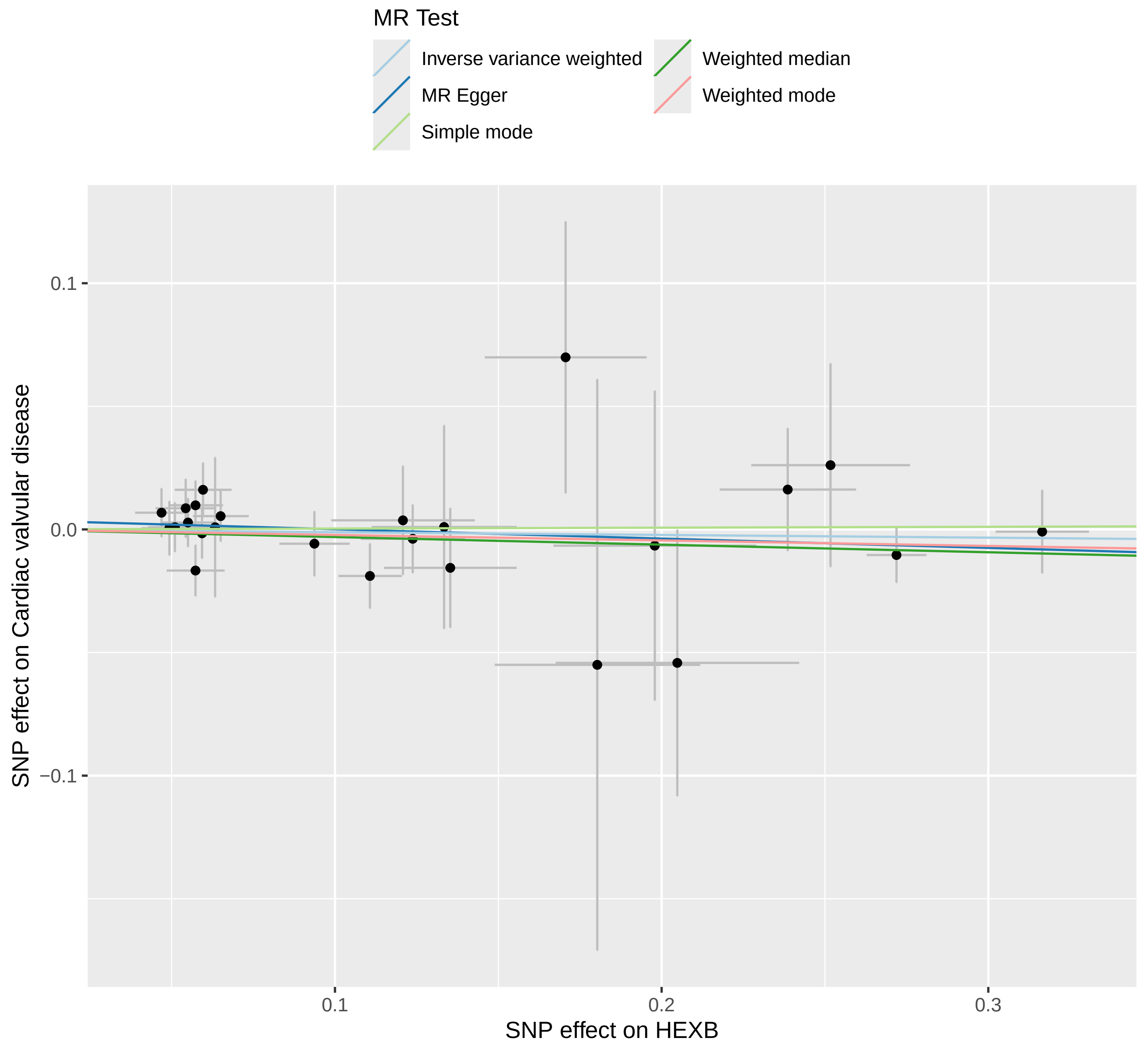

MR Test
Inverse variance weighted
MR Egger
Simple mode
Weighted median
Weighted mode
0.1
0.0
−0.1
0.1
0.2
0.3
SNP effect on Cardiac valvular disease
SNP effect on HEXB

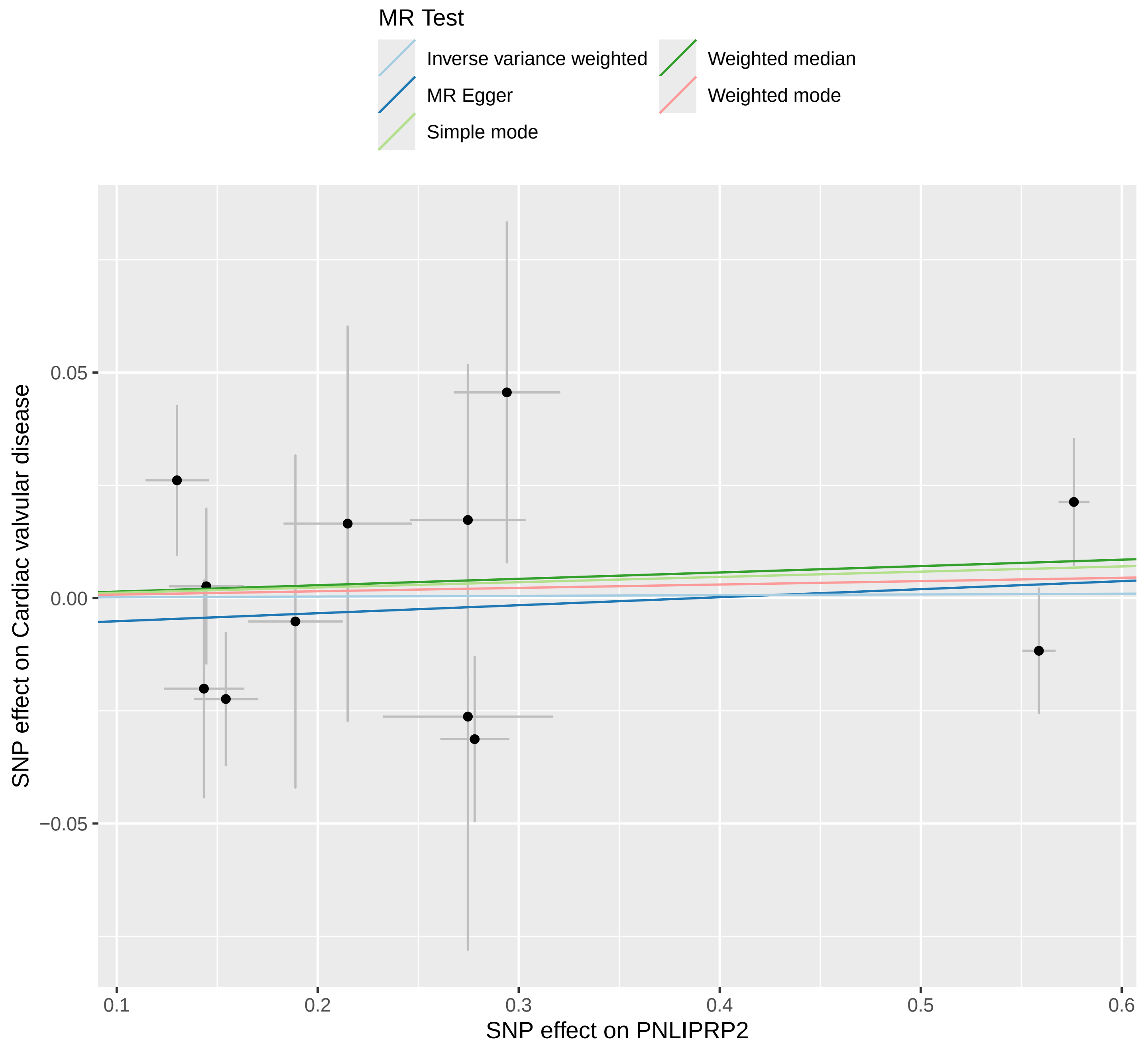

MR Test
Inverse variance weighted
MR Egger
Simple mode
Weighted median
Weighted mode
0.05
0.00
−0.05
0.1
0.2
0.3
0.4
0.5
0.6
SNP effect on Cardiac valvular disease
SNP effect on PNLIPRP2

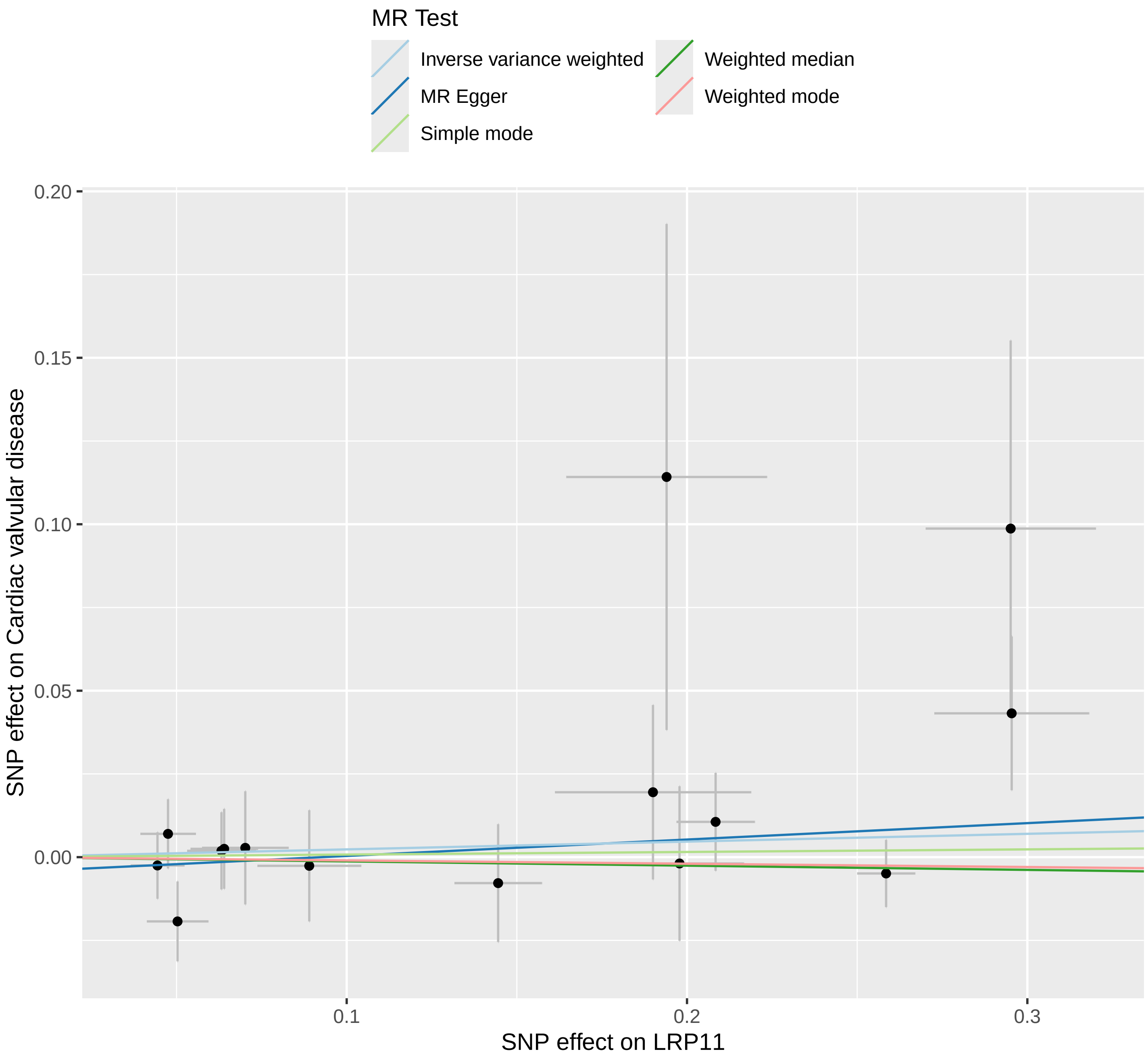
MR Test
Inverse variance weighted
MR Egger
Simple mode
Weighted median
Weighted mode
0.20
0.15
0.10
0.05
0.00
SNP effect on Cardiac valvular disease
0.1
0.2
0.3
SNP effect on LRP11

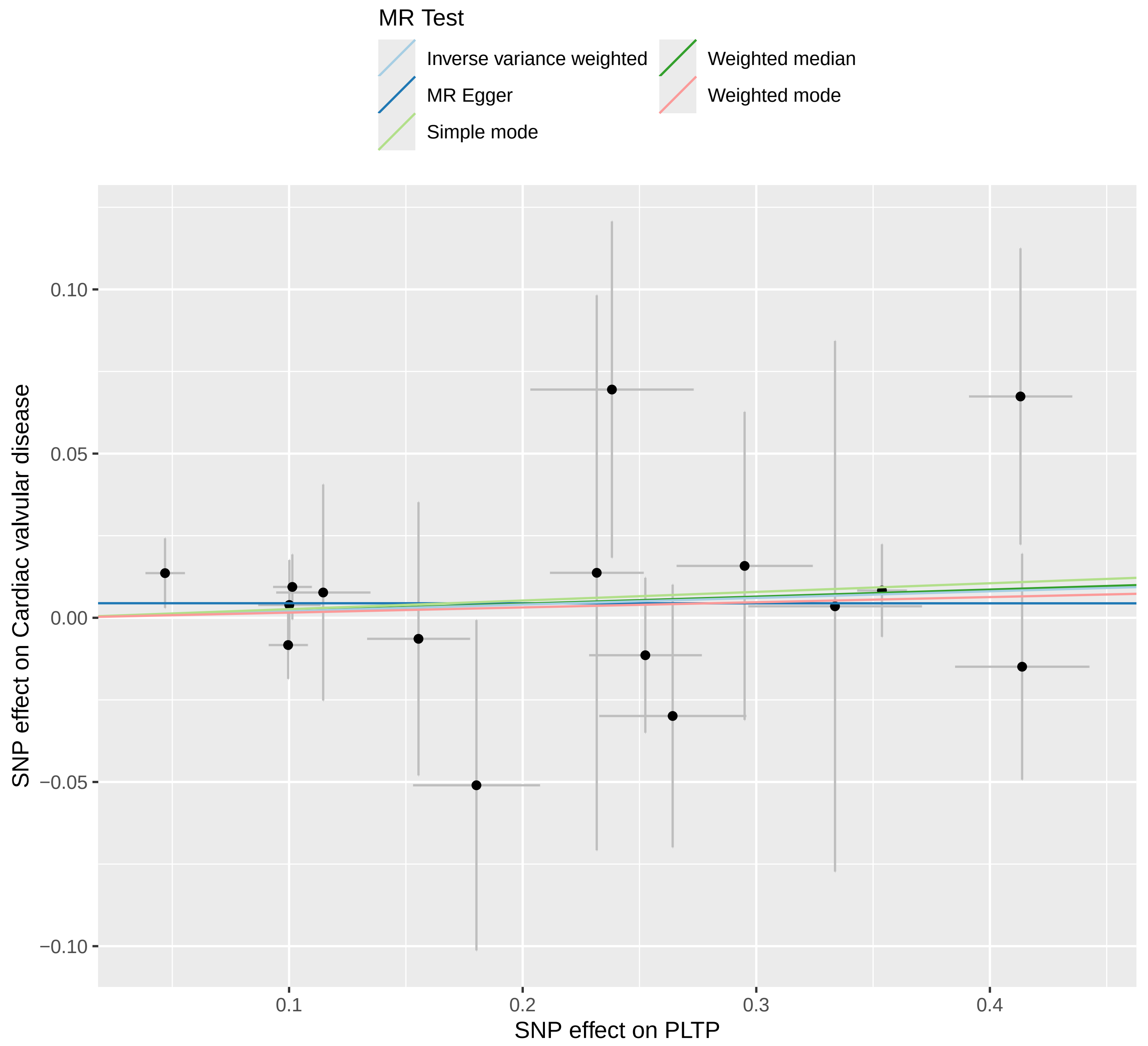
MR Test
Inverse variance weighted
MR Egger
Simple mode
Weighted median
Weighted mode
0.10
0.05
0.00
−0.05
−0.10
SNP effect on Cardiac valvular disease
0.1
0.2
0.3
0.4
SNP effect on PLTP

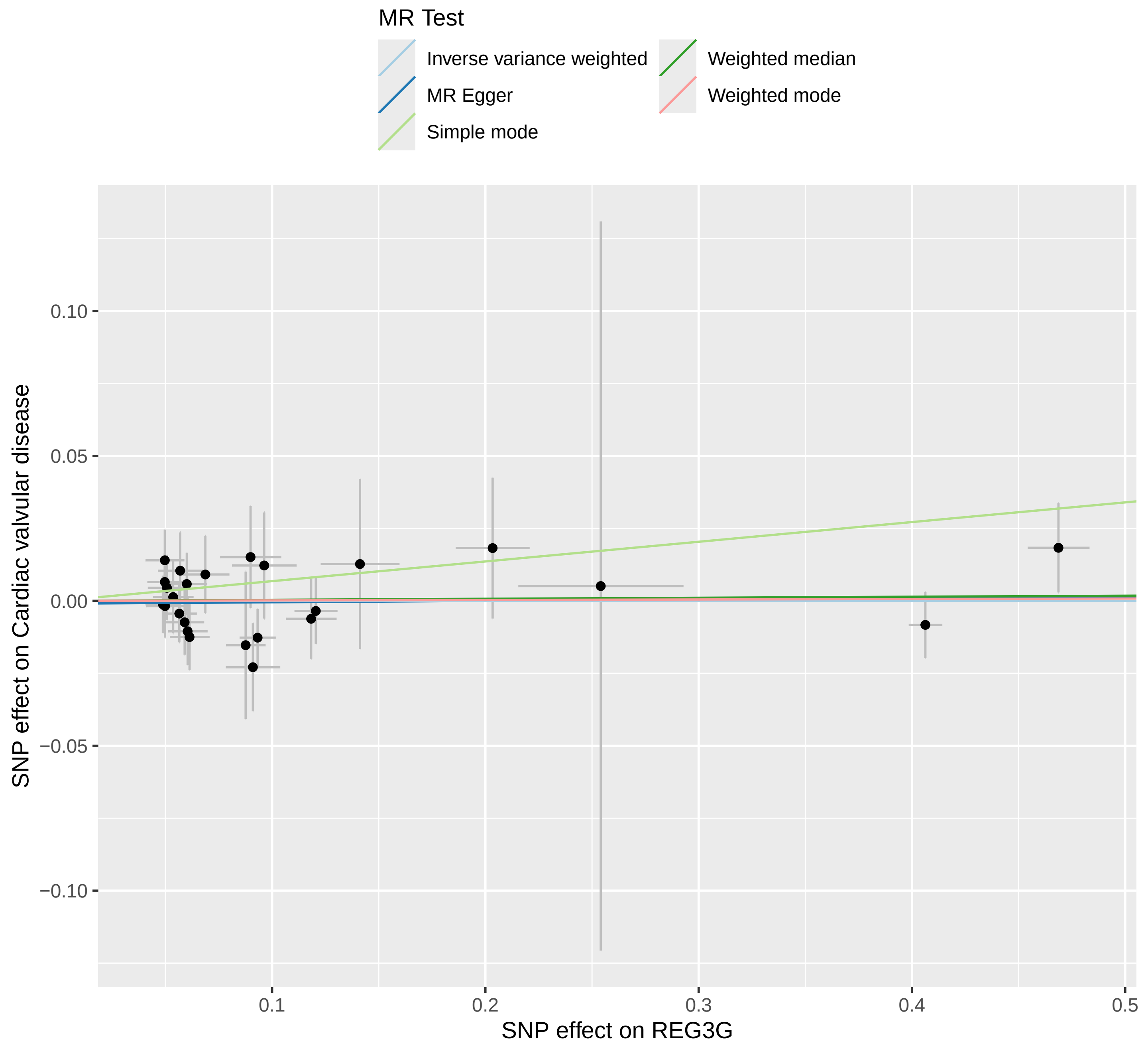

MR Test
Inverse variance weighted
MR Egger
Simple mode
Weighted median
Weighted mode
0.10
0.05
0.00
−0.05
−0.10
SNP effect on Cardiac valvular disease
0.1
0.2
0.3
0.4
0.5
SNP effect on REG3G

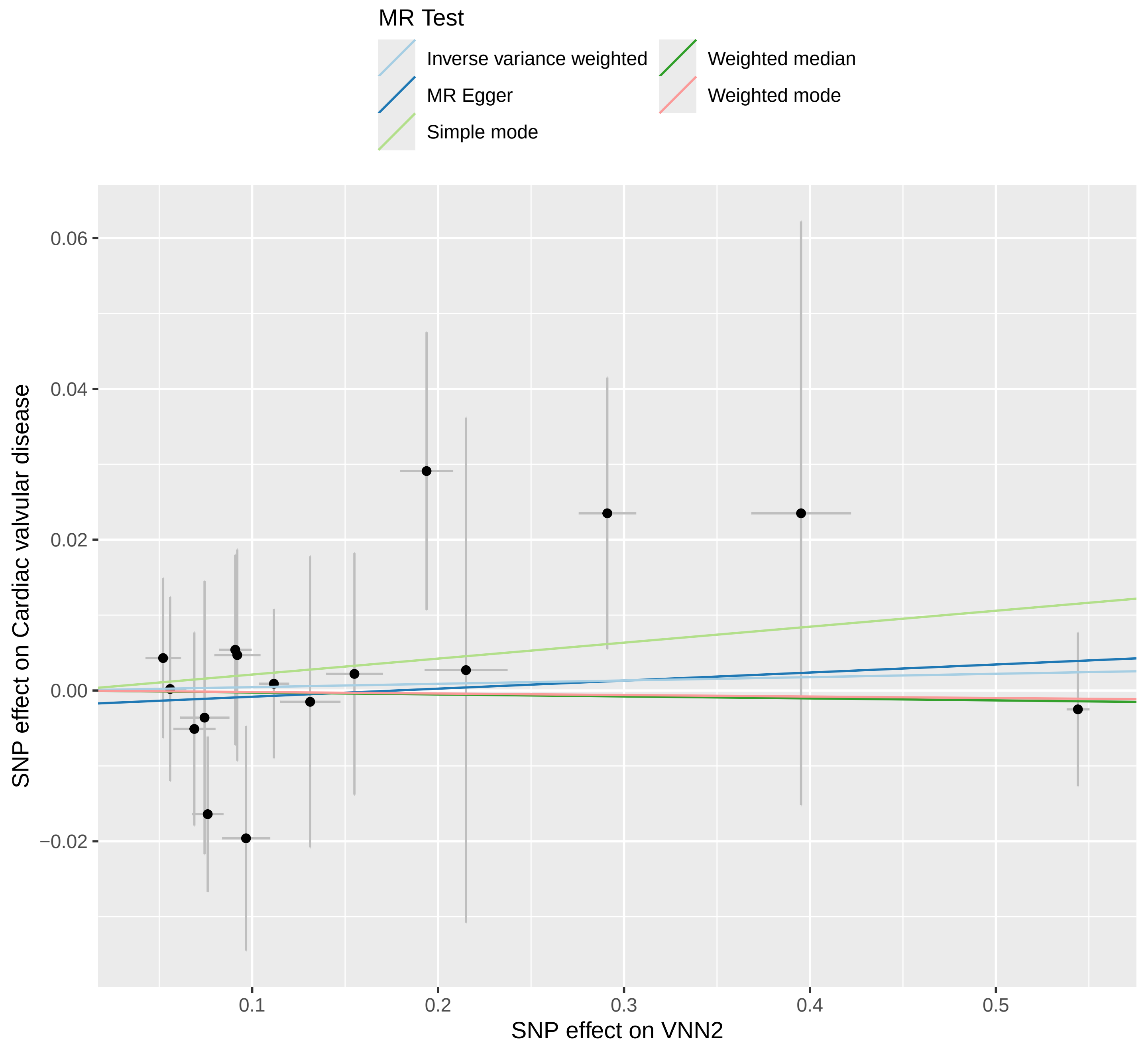

MR Test
Inverse variance weighted
MR Egger
Simple mode
Weighted median
Weighted mode
0.06
0.04
0.02
0.00
−0.02
SNP effect on Cardiac valvular disease
0.1
0.2
0.3
0.4
0.5
SNP effect on VNN2

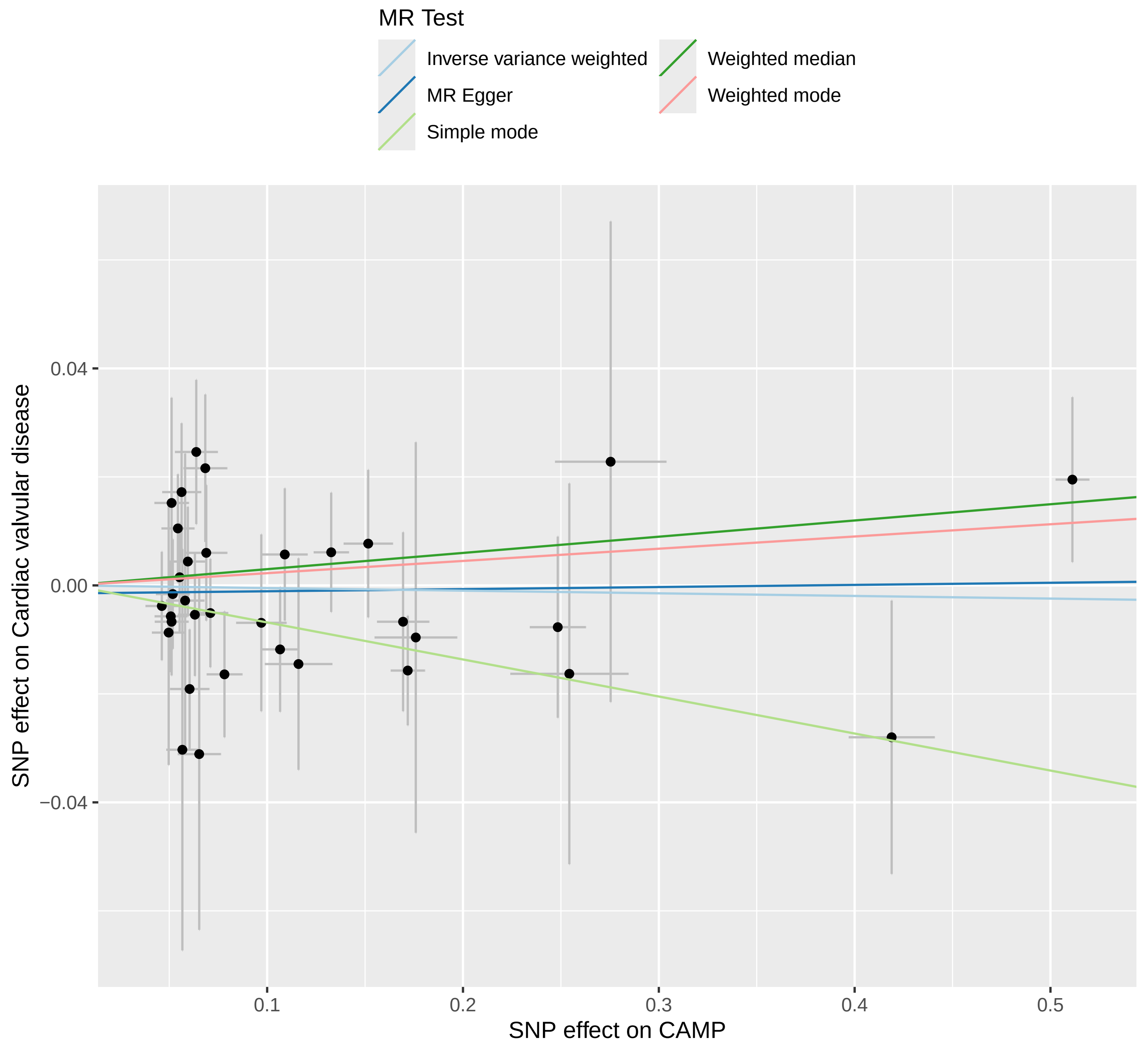
MR Test
Inverse variance weighted
MR Egger
Simple mode
Weighted median
Weighted mode
0.04
0.00
−0.04
0.1
0.2
0.3
0.4
0.5
SNP effect on Cardiac valvular disease
SNP effect on CAMP

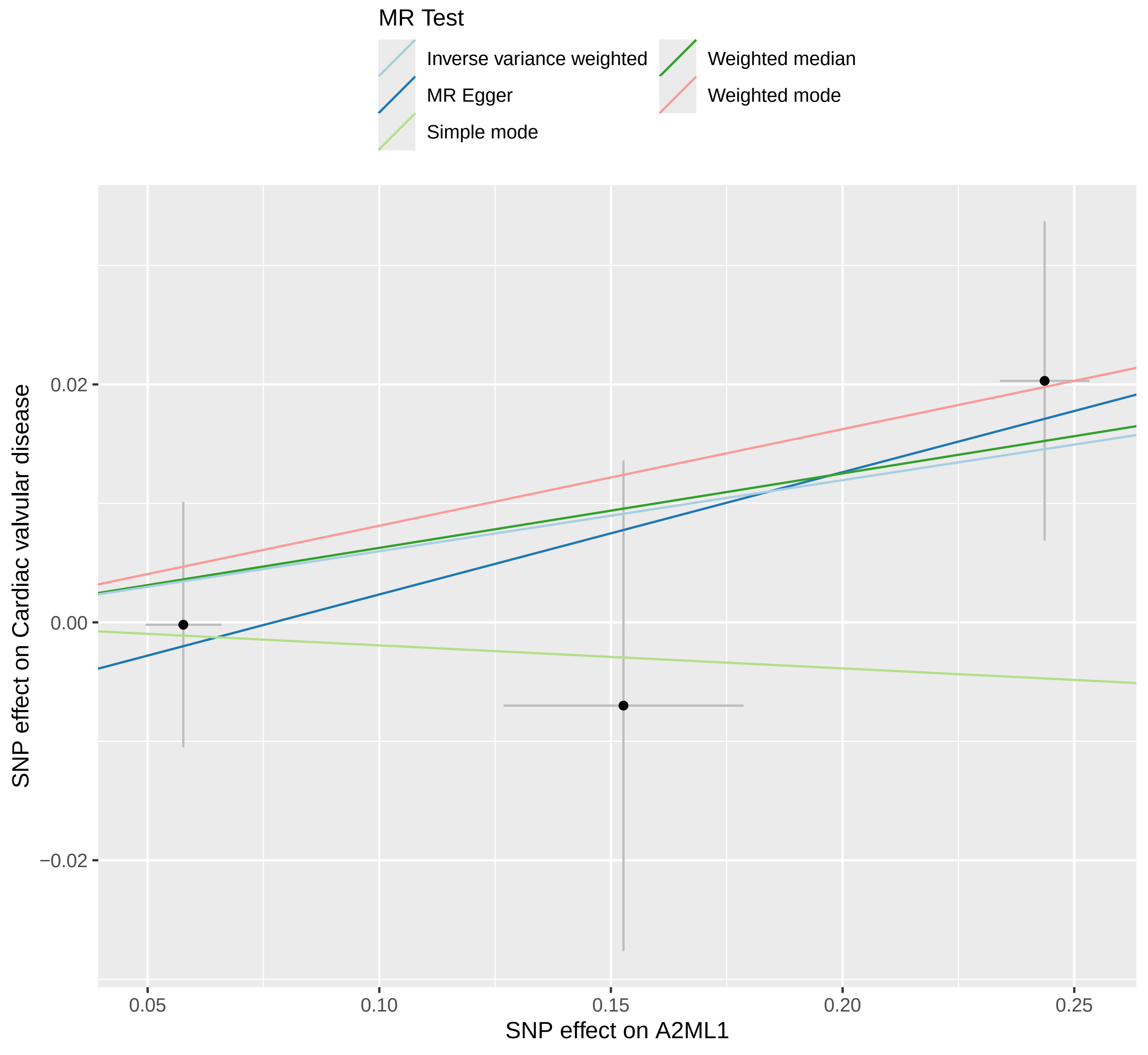

MR Test
Inverse variance weighted
MR Egger
Simple mode
Weighted median
Weighted mode
SNP effect on Cardiac valvular disease
0.02
0.00
−0.02
0.05
0.10
0.15
0.20
0.25
SNP effect on A2ML1

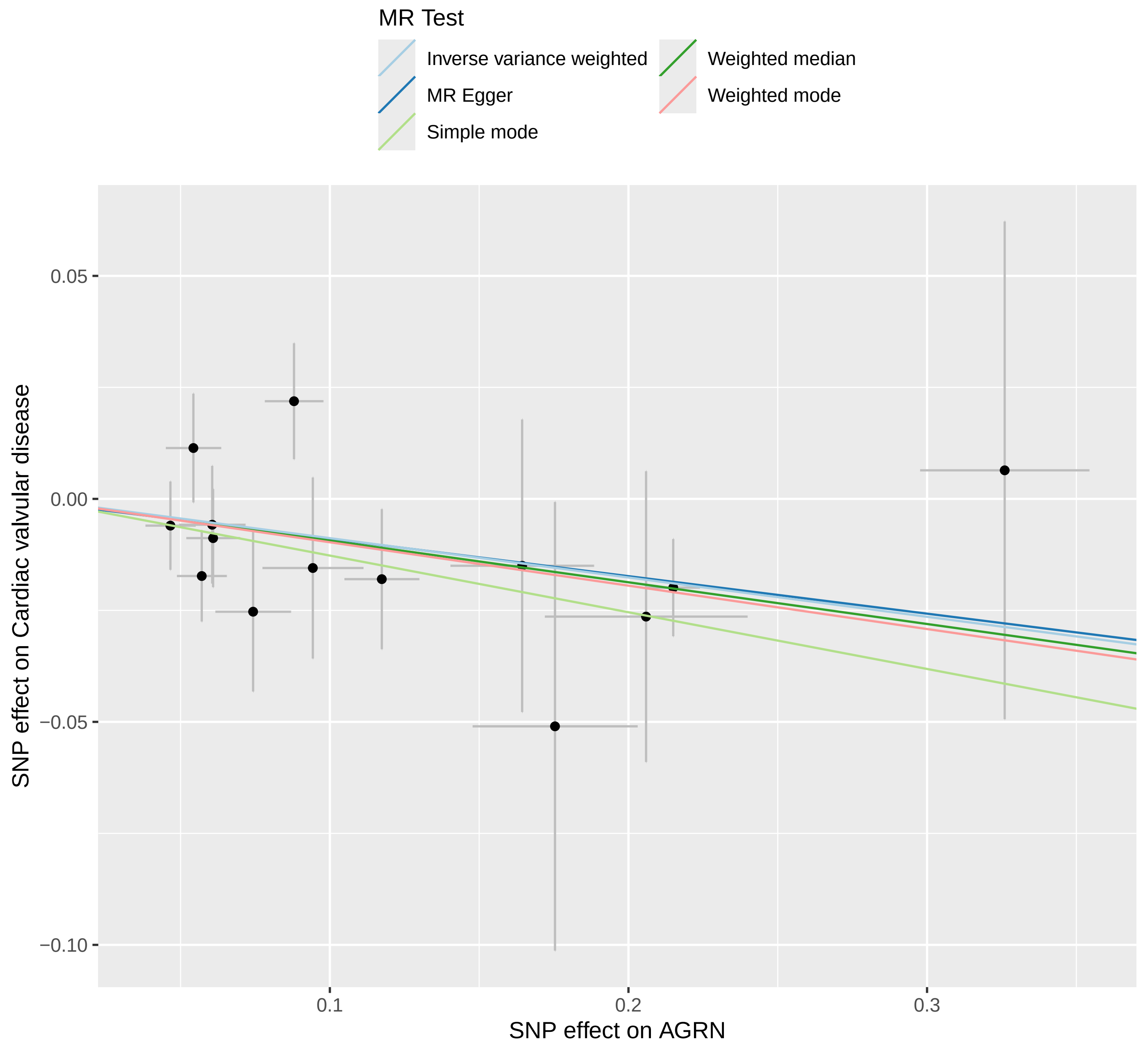
MR Test
Inverse variance weighted
MR Egger
Simple mode
Weighted median
Weighted mode
0.05
0.00
−0.05
−0.10
0.1
0.2
0.3
SNP effect on Cardiac valvular disease
SNP effect on AGRN

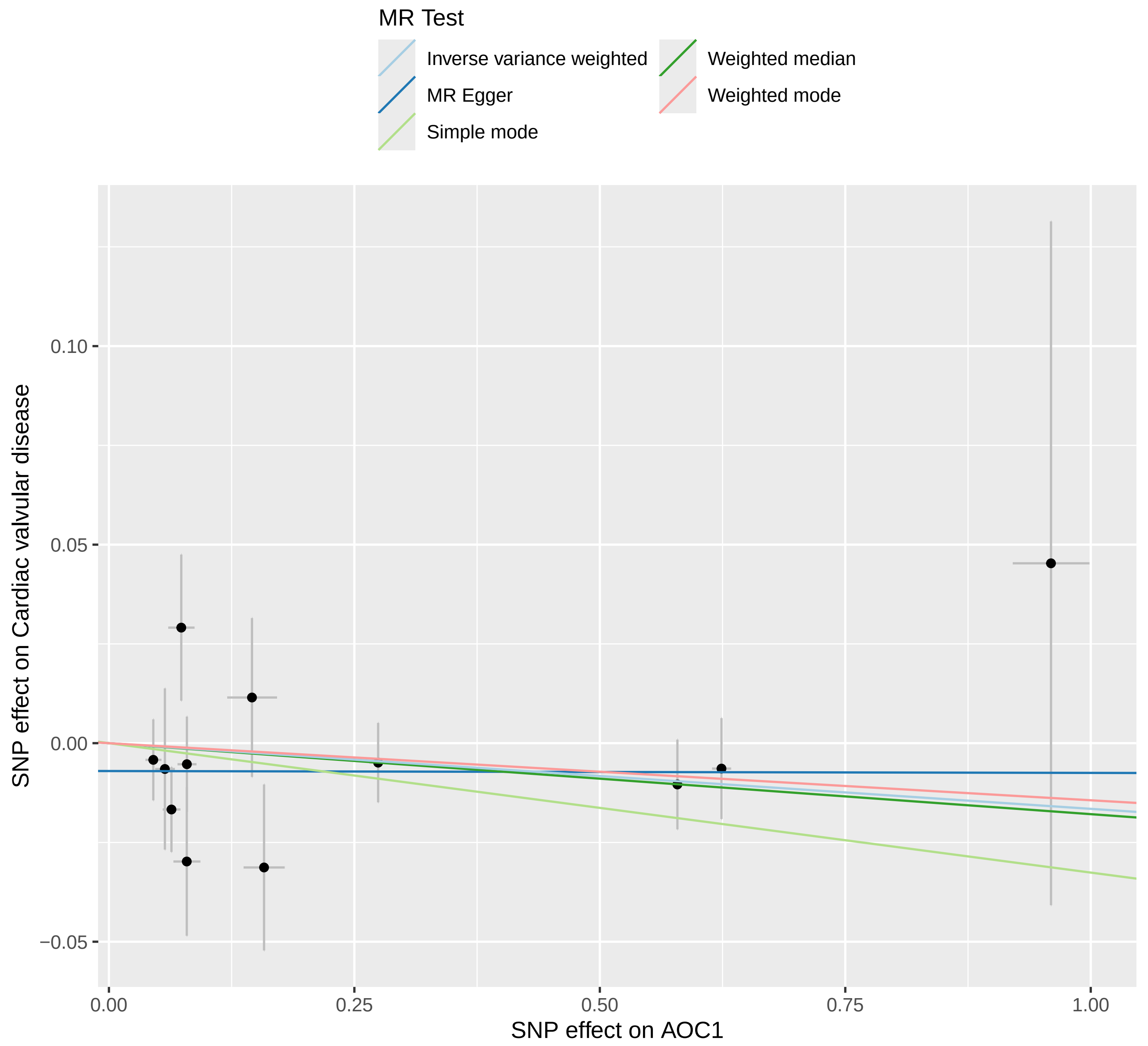
MR Test
Inverse variance weighted
MR Egger
Simple mode
Weighted median
Weighted mode
0.10
0.05
0.00
−0.05
SNP effect on Cardiac valvular disease
0.00
0.25
0.50
0.75
1.00
SNP effect on AOC1

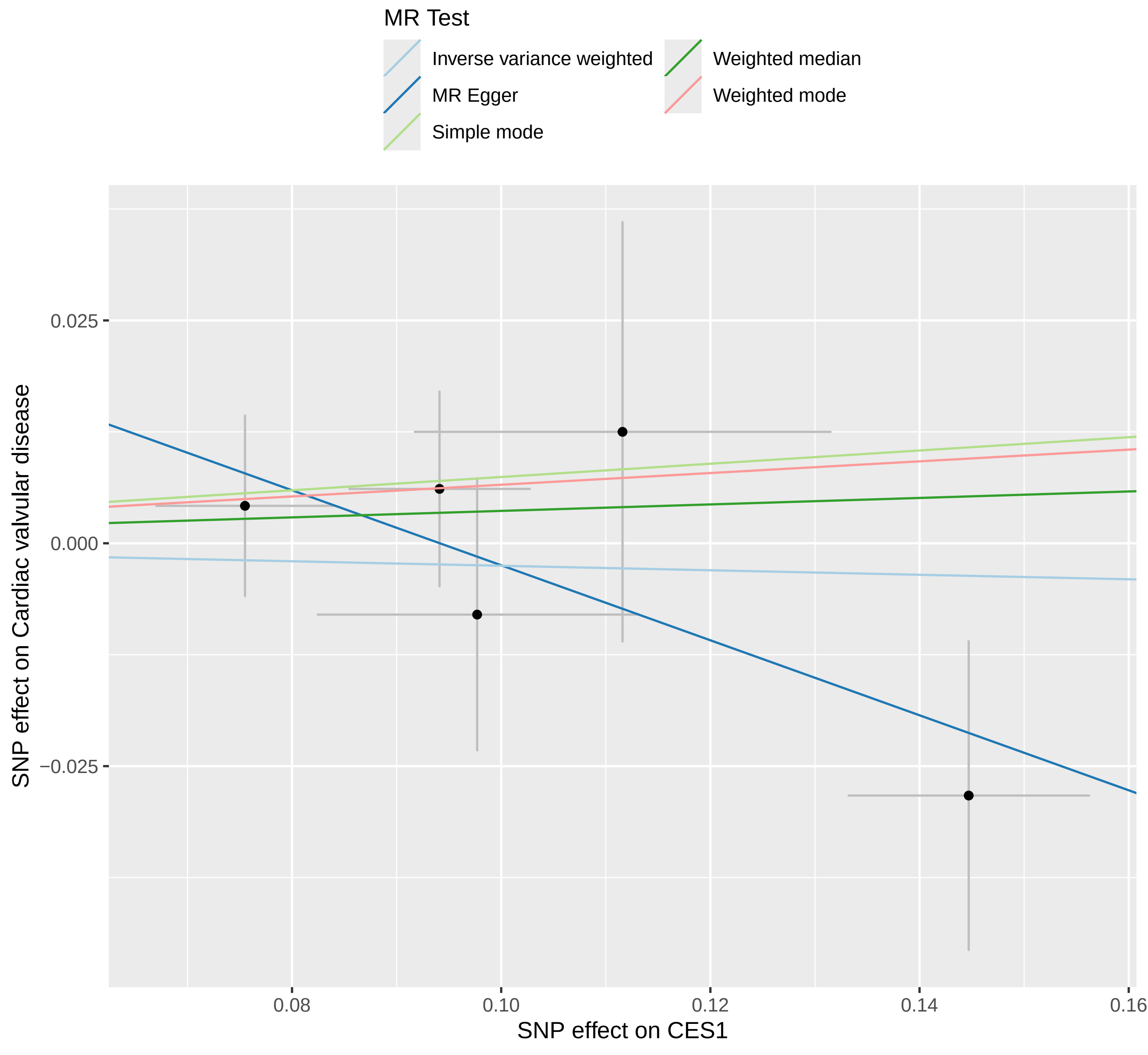

MR Test
Inverse variance weighted
MR Egger
Simple mode
Weighted median
Weighted mode
SNP effect on Cardiac valvular disease
0.025
0.000
−0.025
0.08
0.10
0.12
0.14
0.16
SNP effect on CES1

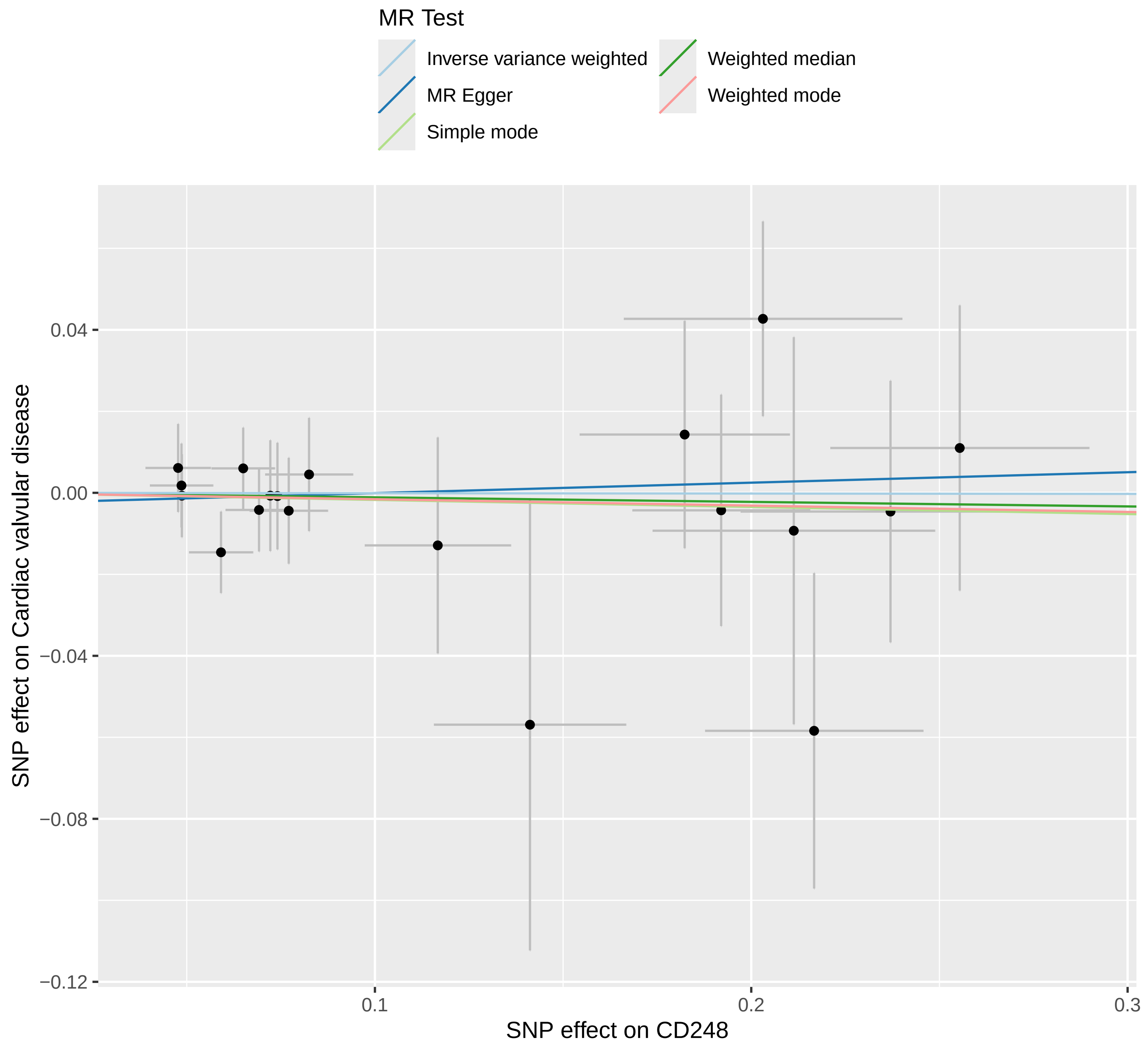

MR Test
Inverse variance weighted
MR Egger
Simple mode
Weighted median
Weighted mode
0.04
0.00
−0.04
−0.08
−0.12
0.1
0.2
0.3
SNP effect on Cardiac valvular disease
SNP effect on CD248

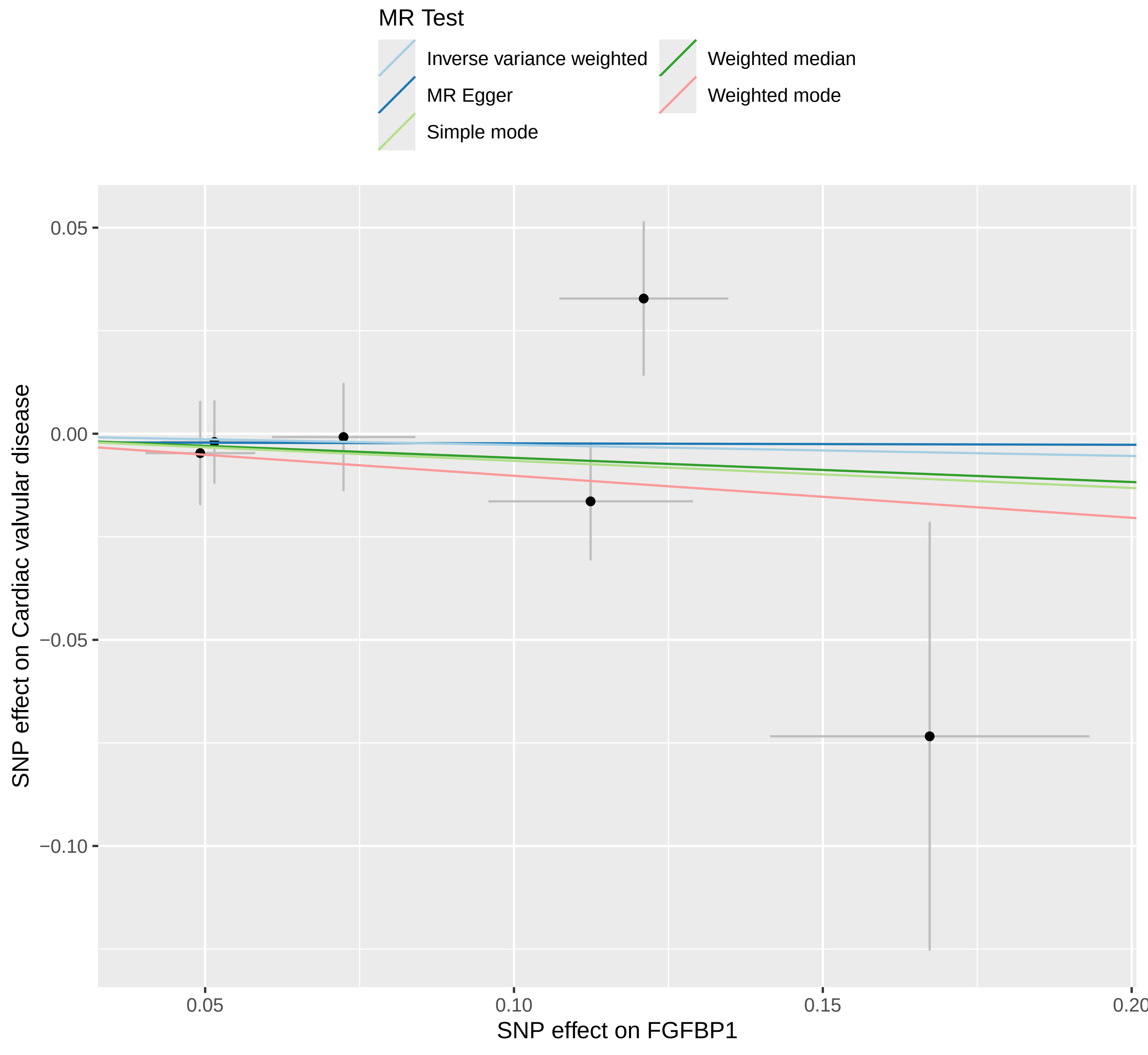
MR Test
Inverse variance weighted
MR Egger
Simple mode
Weighted median
Weighted mode
0.05
0.00
−0.05
−0.10
SNP effect on Cardiac valvular disease
0.05
0.10
0.15
0.20
SNP effect on FGFBP1

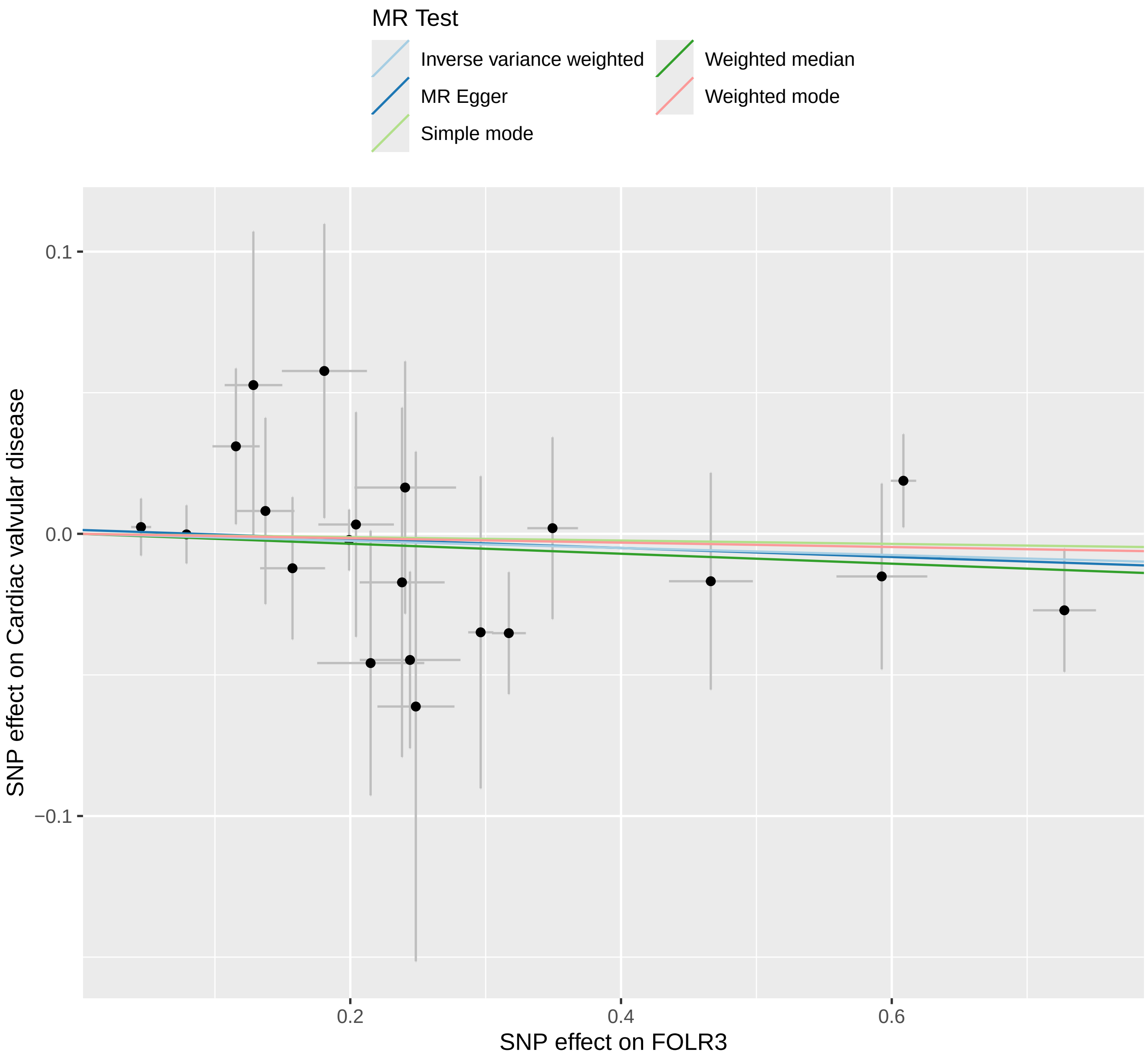

MR Test
Inverse variance weighted
MR Egger
Simple mode
Weighted median
Weighted mode
0.1
0.0
−0.1
SNP effect on Cardiac valvular disease
0.2
0.4
0.6
SNP effect on FOLR3

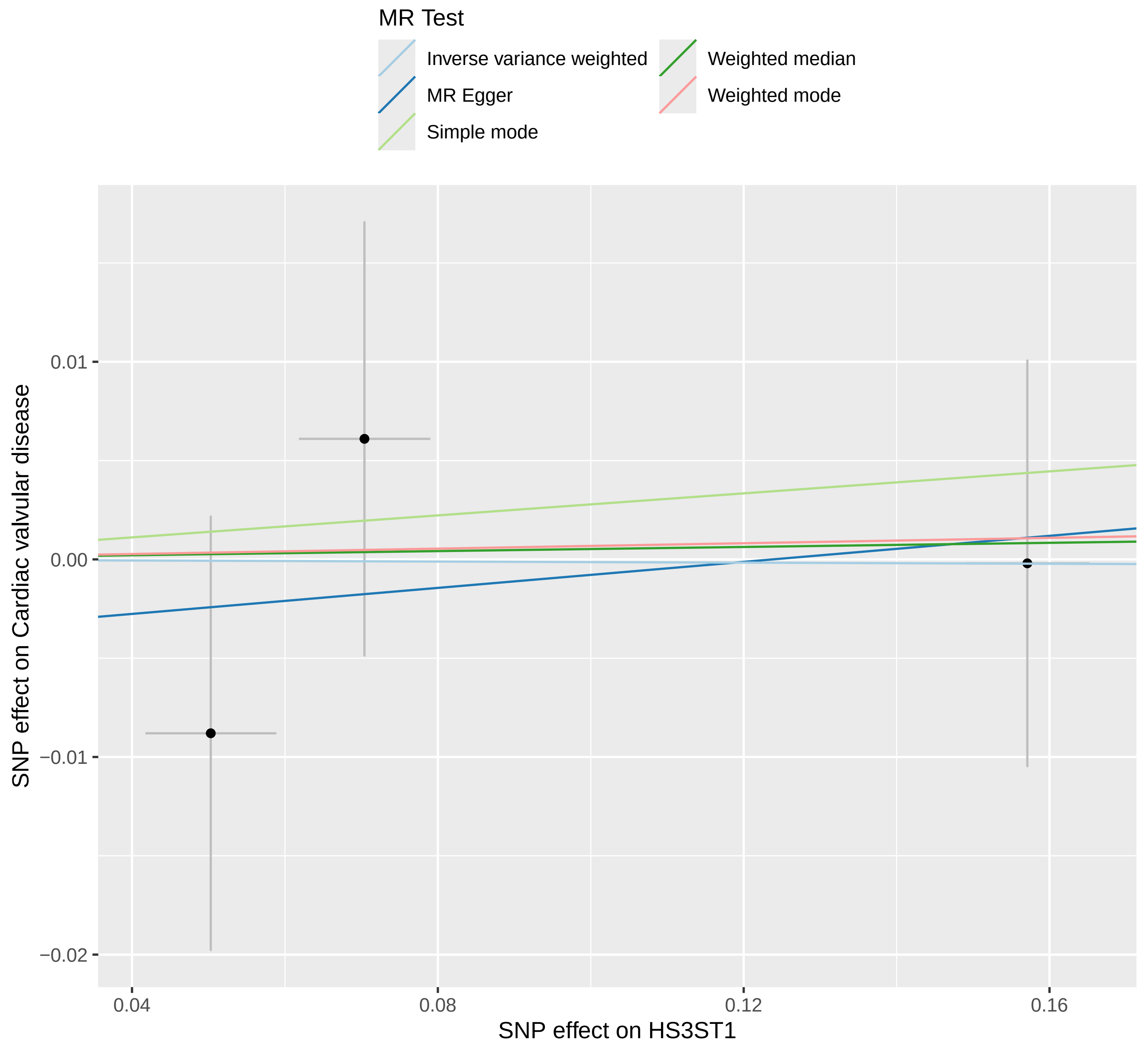
MR Test
Inverse variance weighted
MR Egger
Simple mode
Weighted median
Weighted mode
0.01
0.00
−0.01
−0.02
0.04
0.08
0.12
0.16
SNP effect on HS3ST1
SNP effect on Cardiac valvular disease

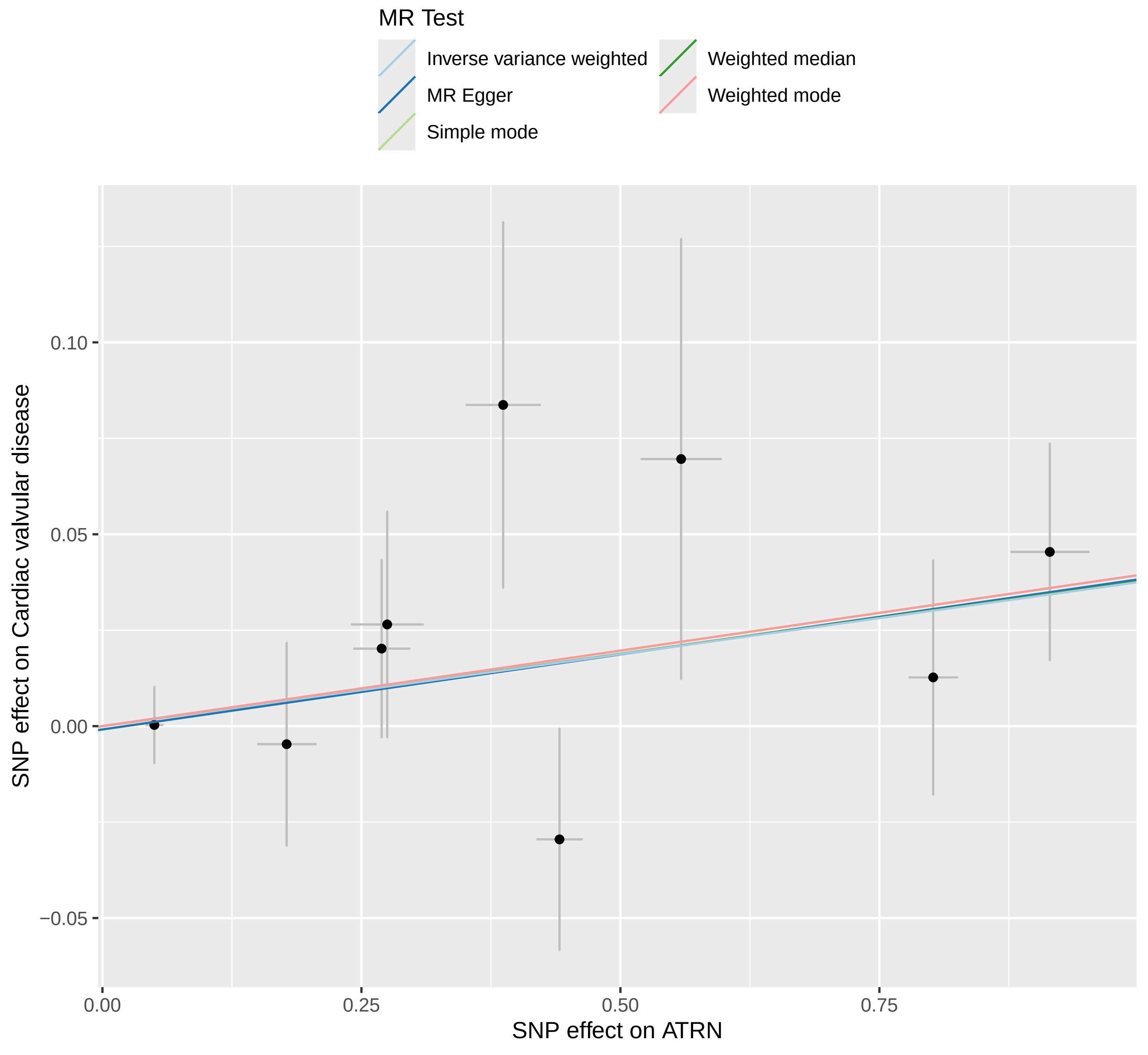

MR Test
Inverse variance weighted
MR Egger
Simple mode
Weighted median
Weighted mode
0.10
0.05
0.00
−0.05
0.00
0.25
0.50
0.75
SNP effect on Cardiac valvular disease
SNP effect on ATRN

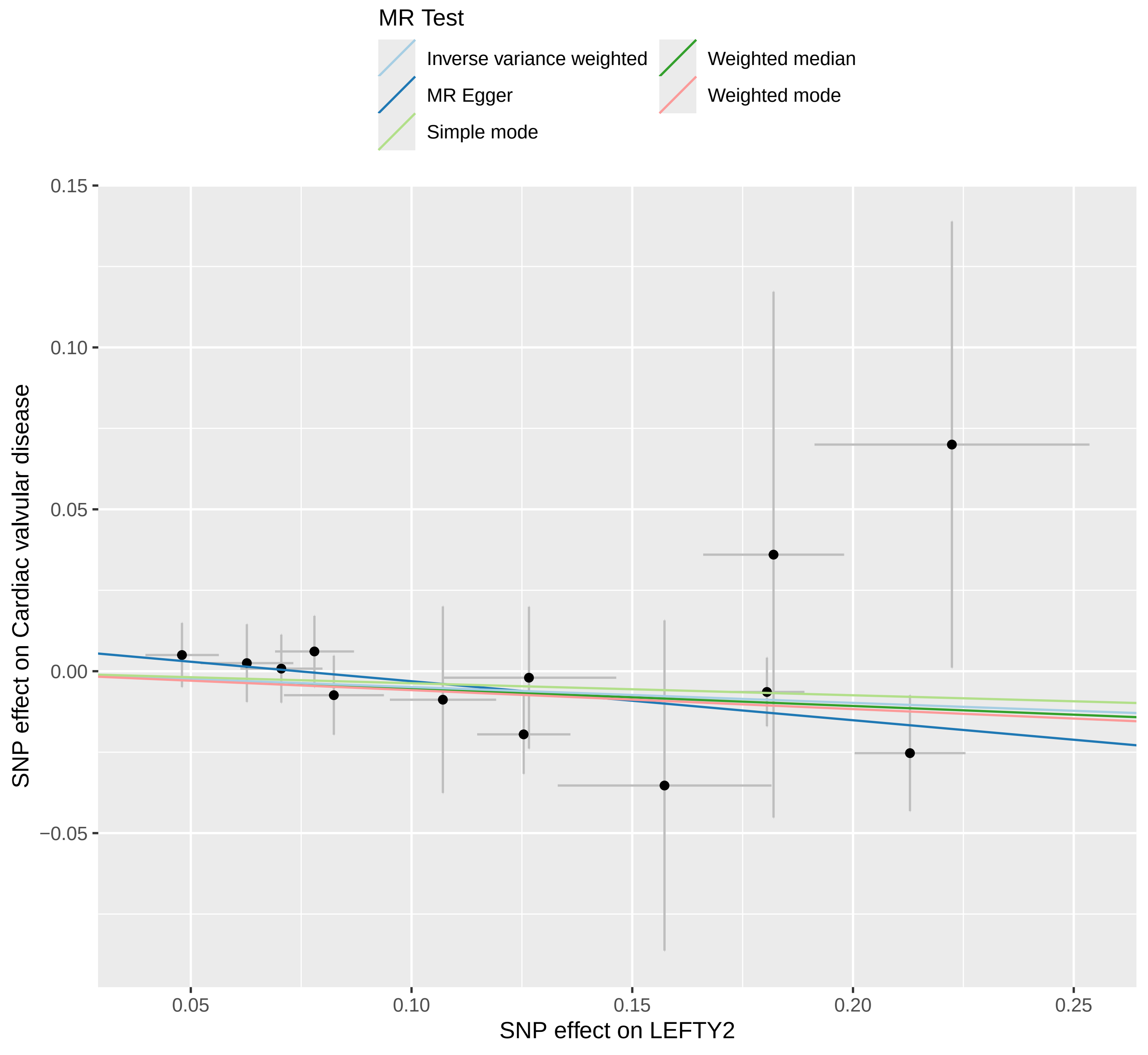

MR Test
Inverse variance weighted
MR Egger
Simple mode
Weighted median
Weighted mode
0.15
0.10
0.05
0.00
−0.05
SNP effect on Cardiac valvular disease
0.05
0.10
0.15
0.20
0.25
SNP effect on LEFTY2

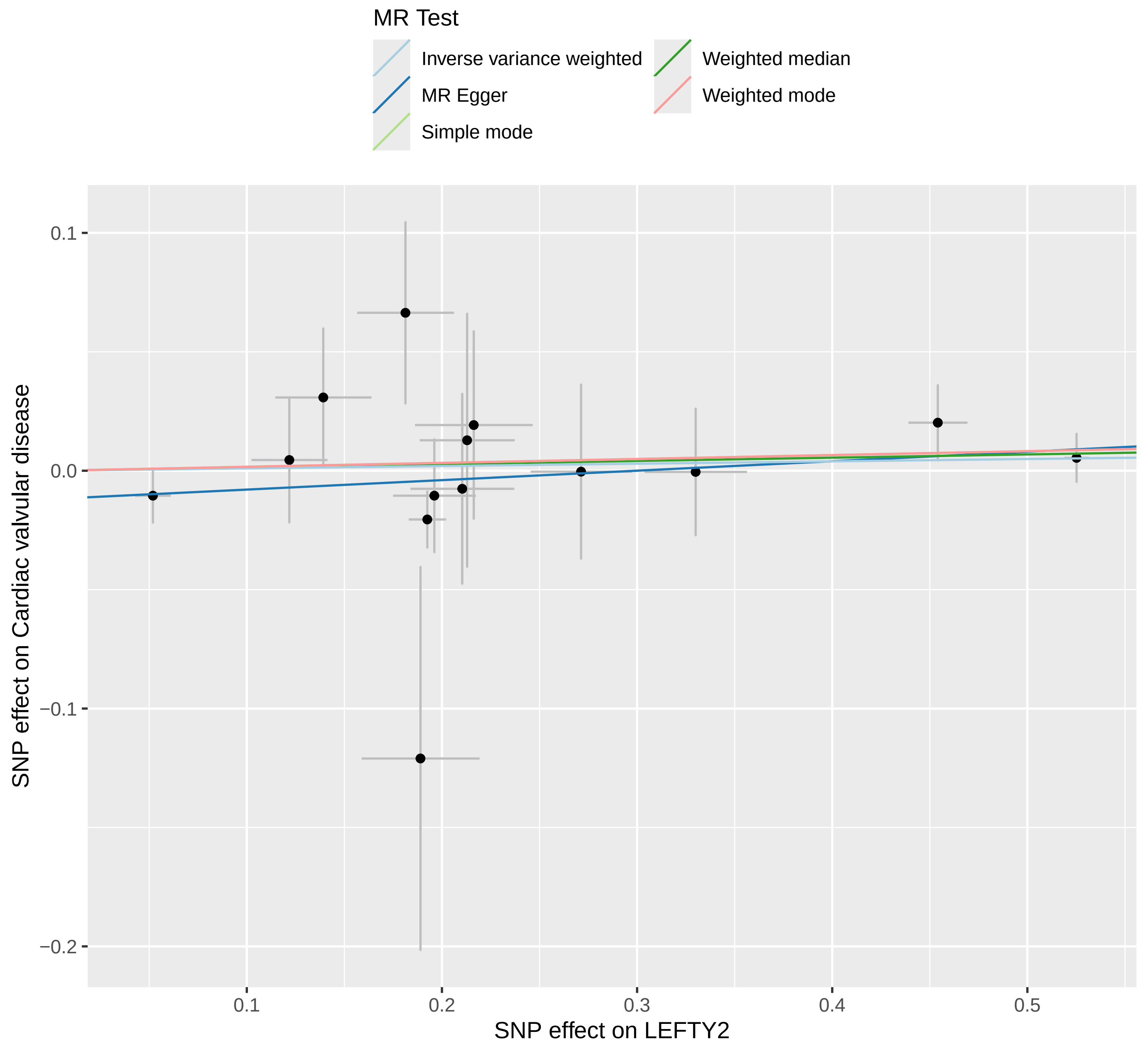

MR Test
Inverse variance weighted
MR Egger
Simple mode
Weighted median
Weighted mode
0.1
0.0
−0.1
−0.2
SNP effect on Cardiac valvular disease
0.1
0.2
0.3
0.4
0.5
SNP effect on LEFTY2

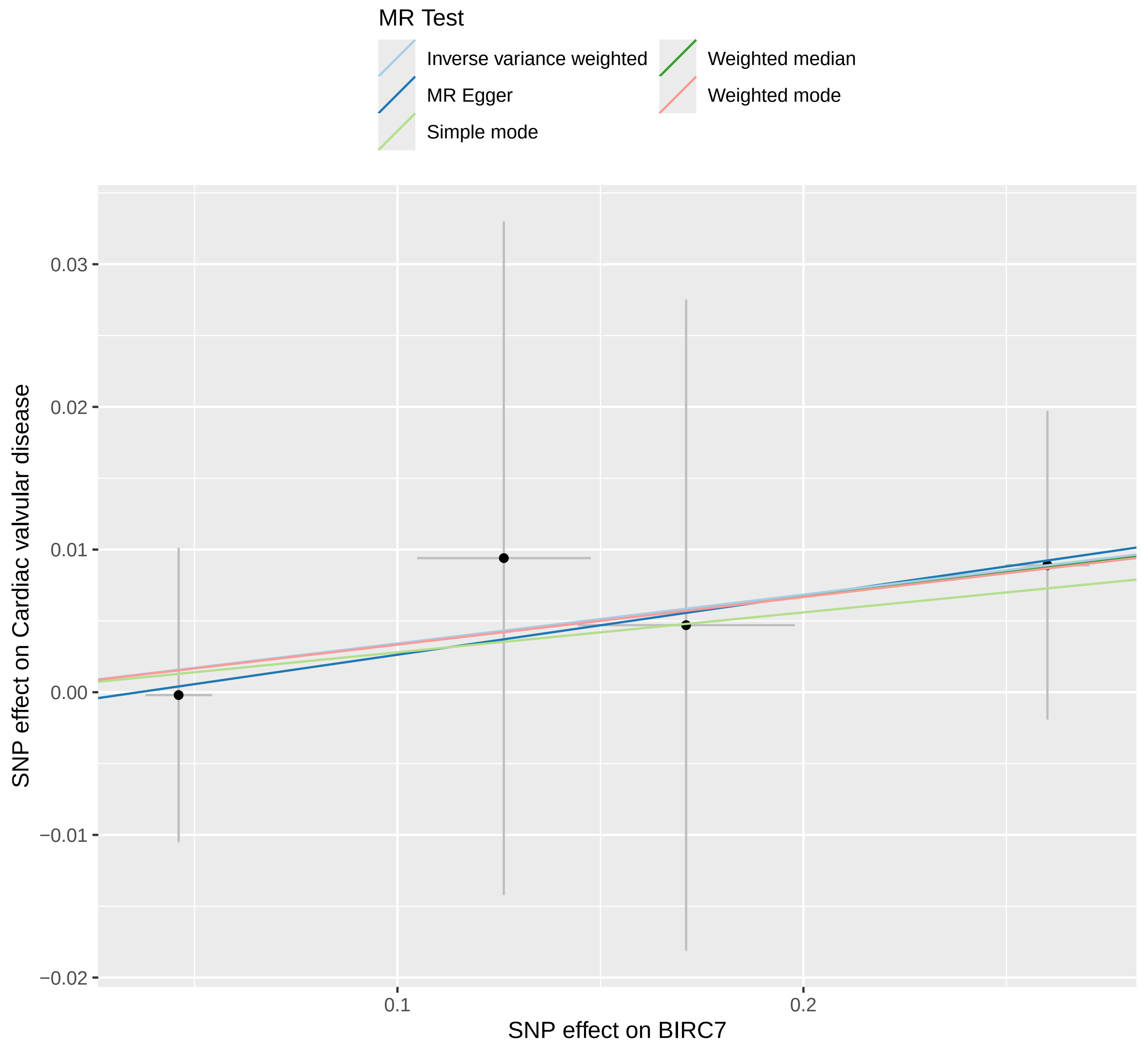
MR Test
Inverse variance weighted
MR Egger
Simple mode
Weighted median
Weighted mode
0.03
0.02
0.01
0.00
−0.01
−0.02
SNP effect on Cardiac valvular disease
0.1
0.2
SNP effect on BIRC7

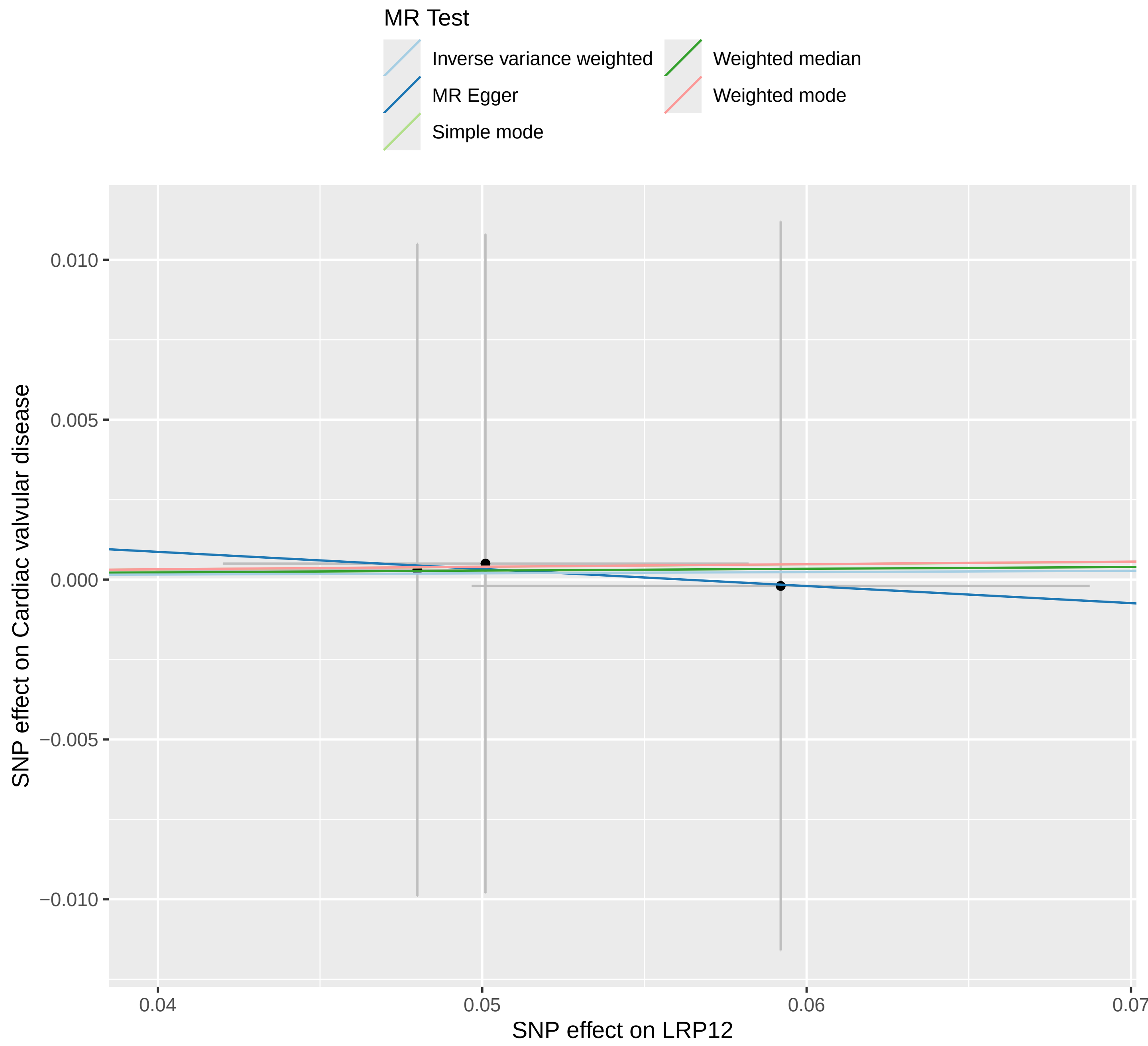
MR Test
Inverse variance weighted
MR Egger
Simple mode
Weighted median
Weighted mode
0.010
0.005
0.000
−0.005
−0.010
SNP effect on Cardiac valvular disease
0.04
0.05
0.06
0.07
SNP effect on LRP12

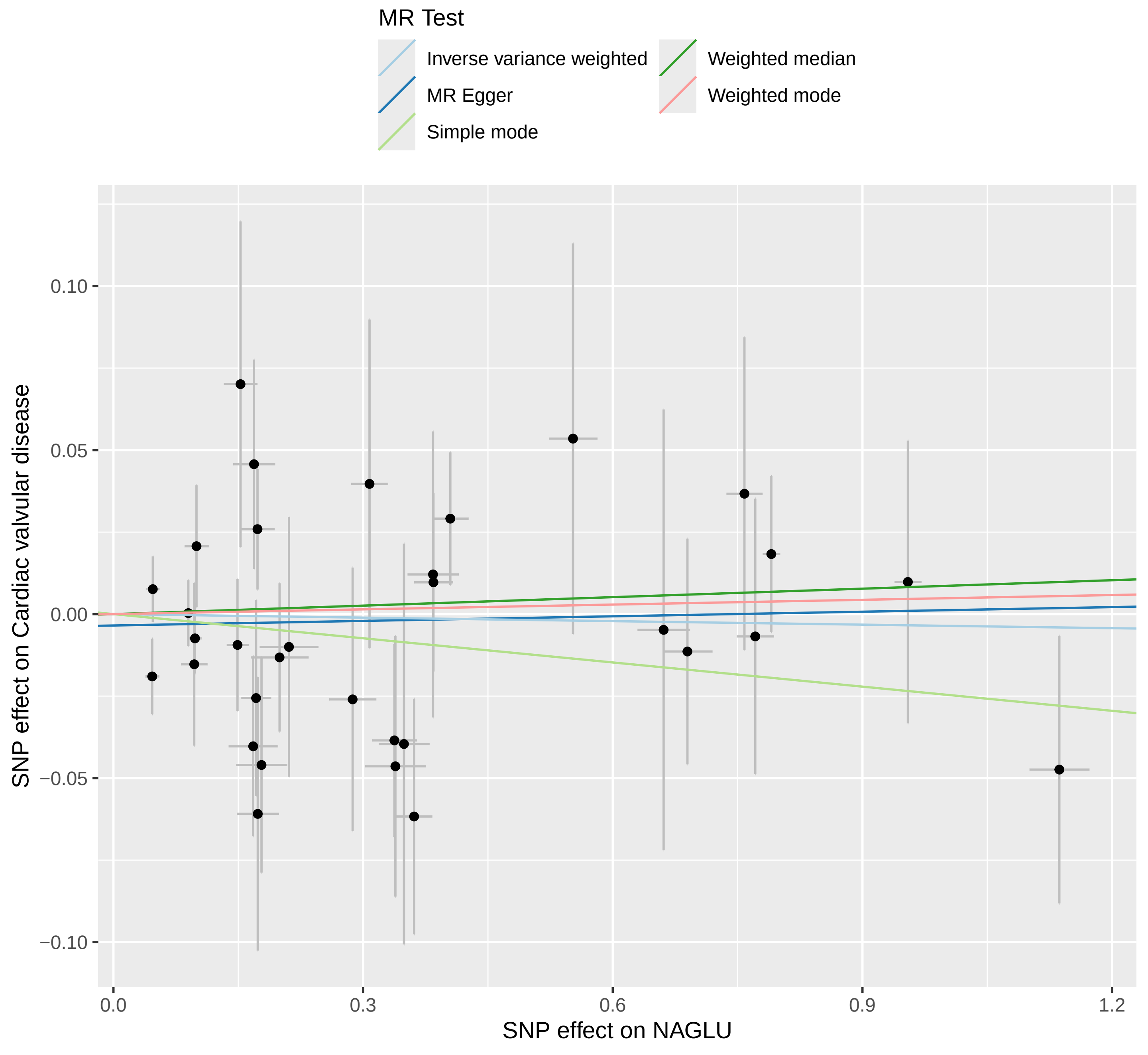
MR Test
Inverse variance weighted
MR Egger
Simple mode
Weighted median
Weighted mode
SNP effect on Cardiac valvular disease
0.10
0.05
0.00
−0.05
−0.10
0.0
0.3
0.6
0.9
1.2
SNP effect on NAGLU

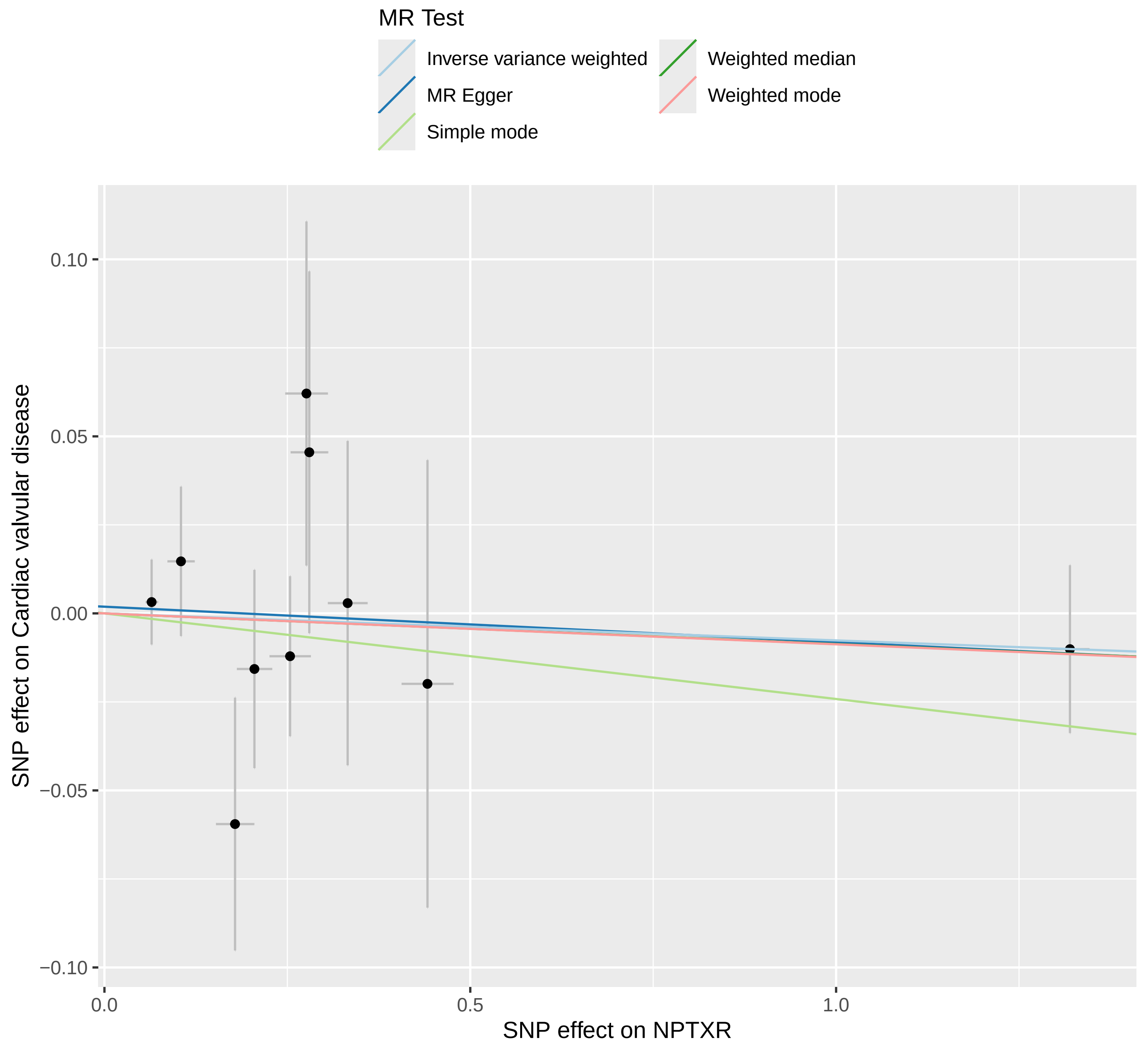
MR Test
Inverse variance weighted
MR Egger
Simple mode
Weighted median
Weighted mode
0.10
0.05
0.00
−0.05
−0.10
0.0
0.5
1.0
SNP effect on Cardiac valvular disease
SNP effect on NPTXR

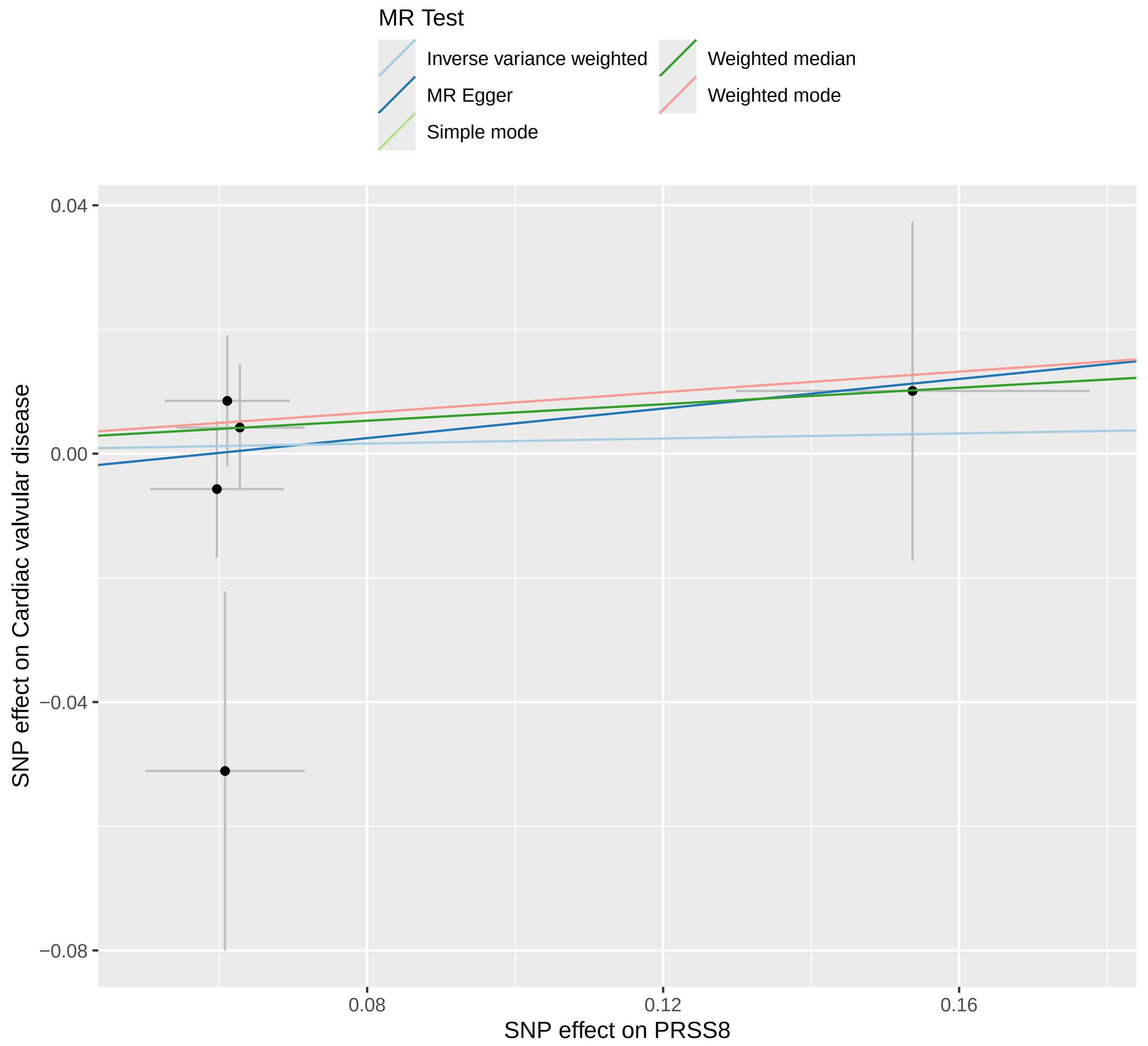
MR Test
Inverse variance weighted
MR Egger
Simple mode
Weighted median
Weighted mode
0.04
0.00
−0.04
−0.08
0.08
0.12
0.16
SNP effect on Cardiac valvular disease
SNP effect on PRSS8

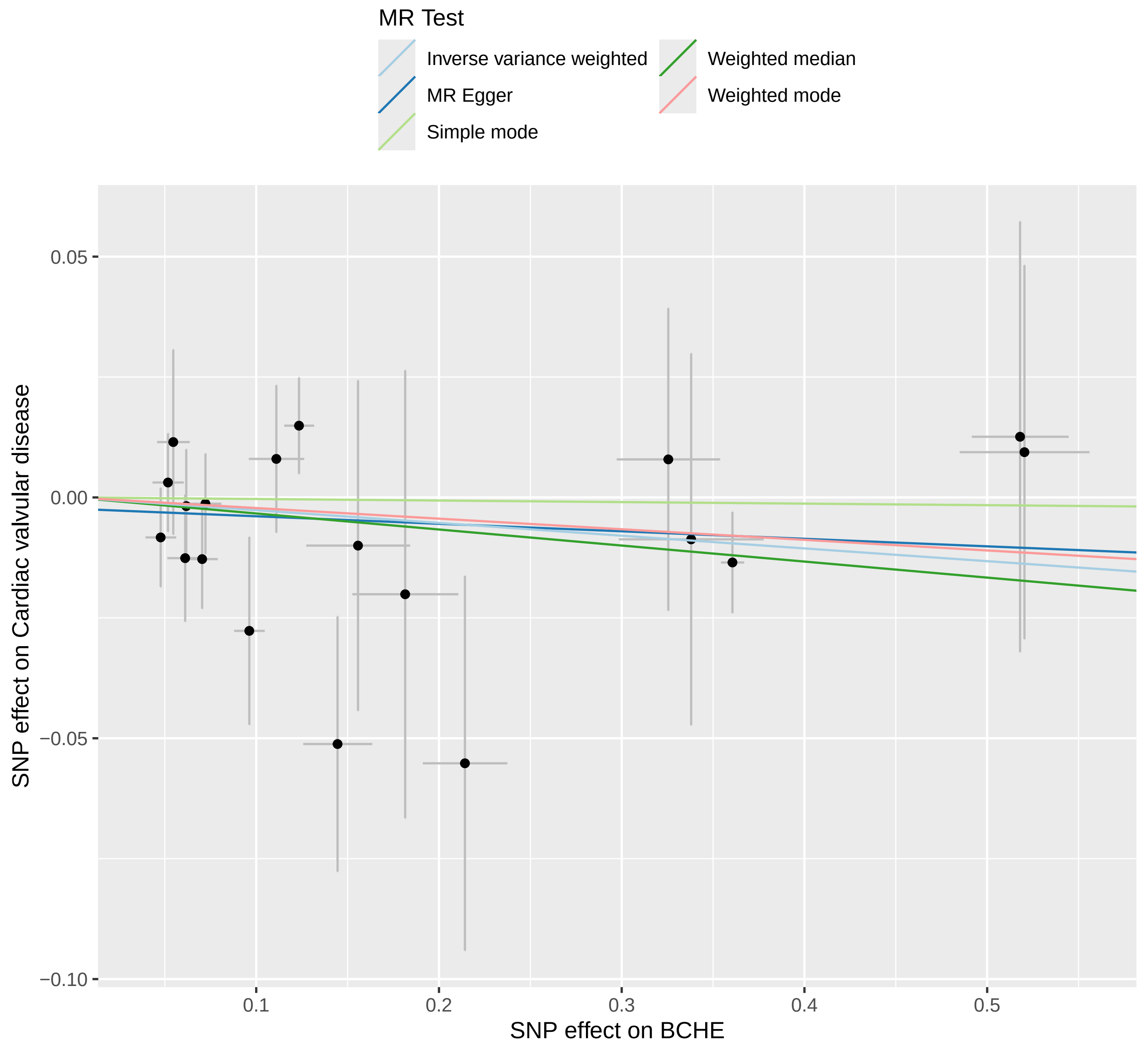
MR Test
Inverse variance weighted
MR Egger
Simple mode
Weighted median
Weighted mode
0.05
0.00
−0.05
−0.10
0.1
0.2
0.3
0.4
0.5
SNP effect on BCHE
SNP effect on Cardiac valvular disease

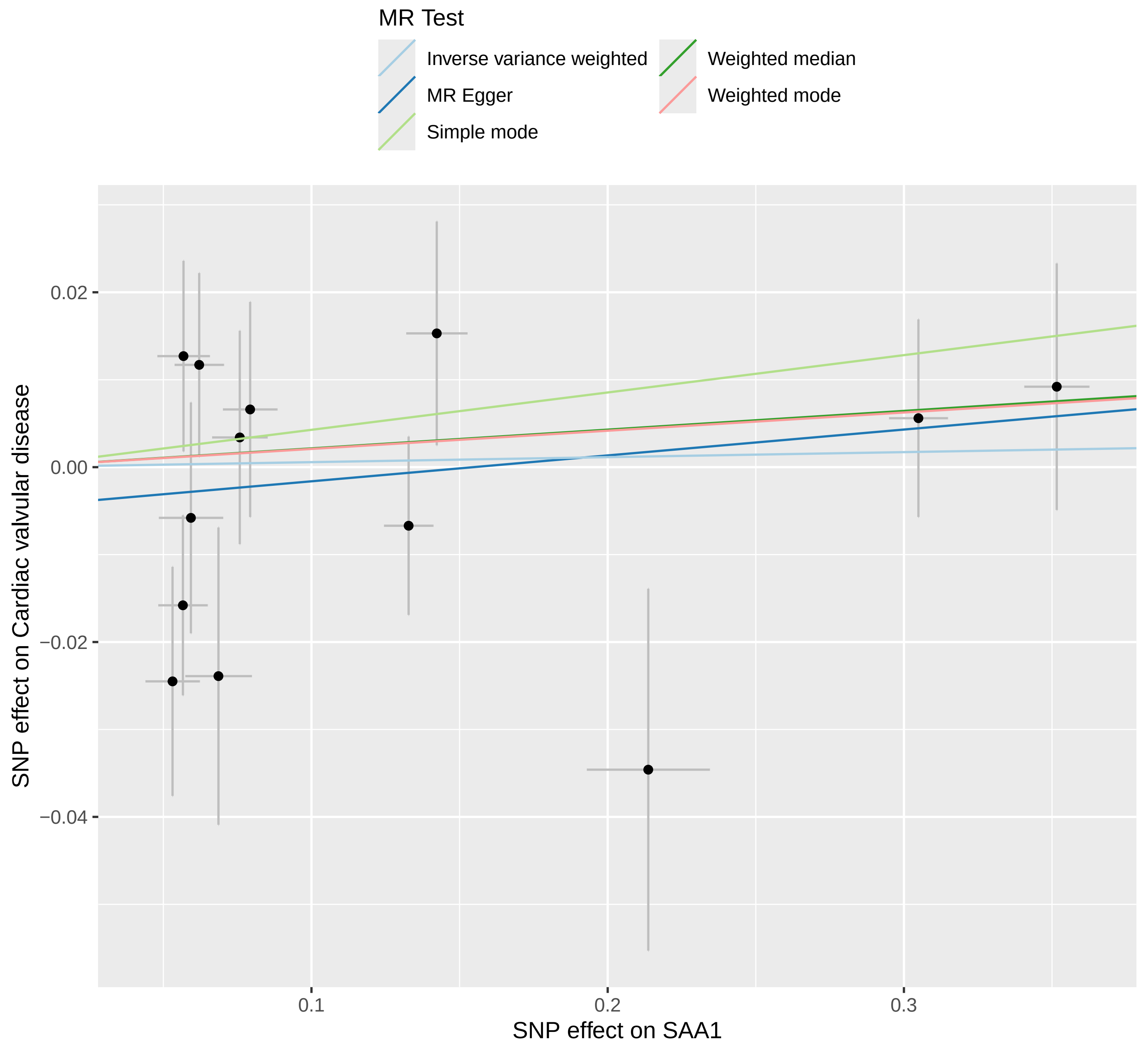
MR Test
Inverse variance weighted
MR Egger
Simple mode
Weighted median
Weighted mode
0.02
0.00
−0.02
−0.04
0.1
0.2
0.3
SNP effect on Cardiac valvular disease
SNP effect on SAA1

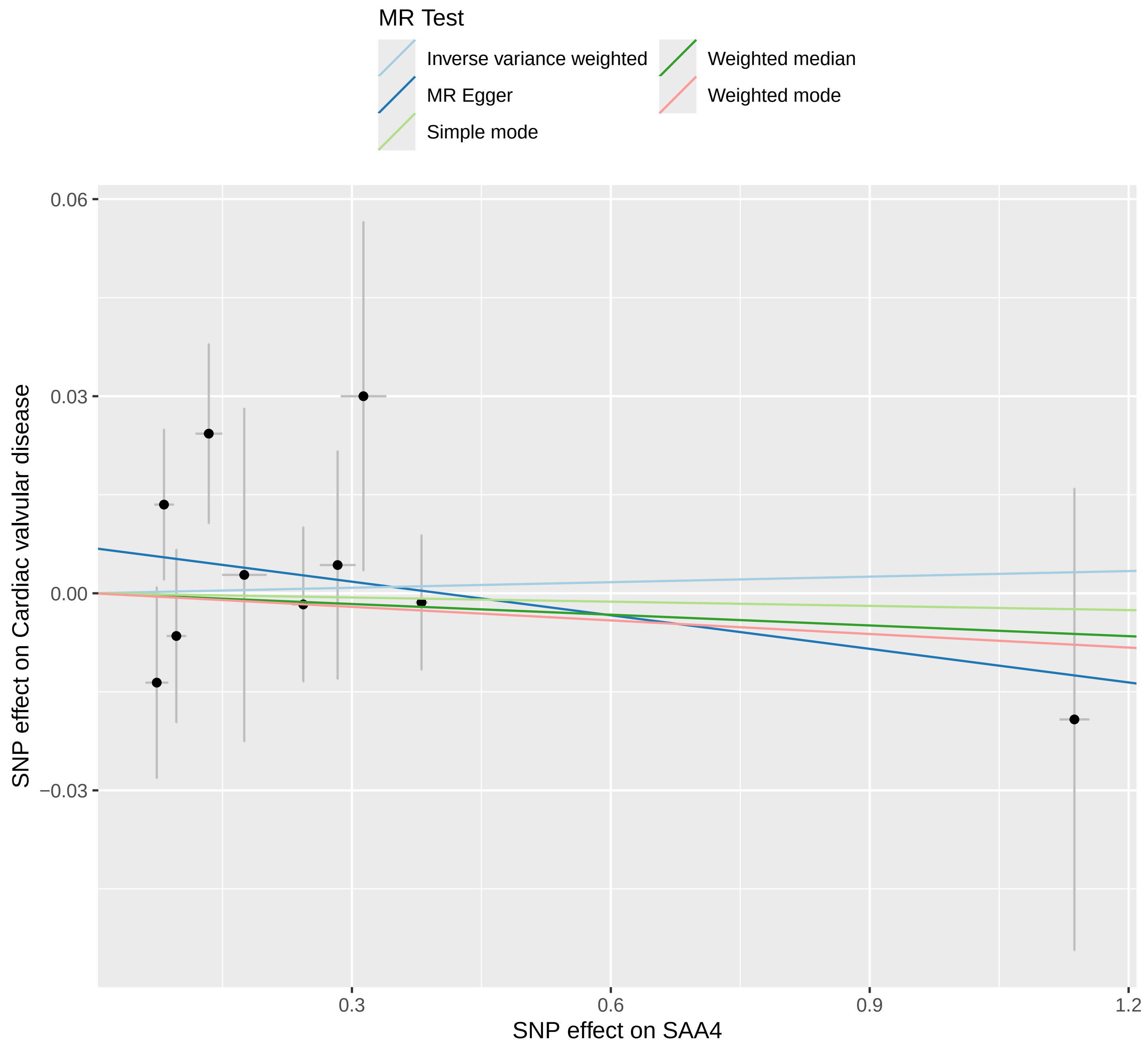
MR Test
Inverse variance weighted
MR Egger
Simple mode
Weighted median
Weighted mode
0.06
0.03
0.00
−0.03
0.3
0.6
0.9
1.2
SNP effect on Cardiac valvular disease
SNP effect on SAA4

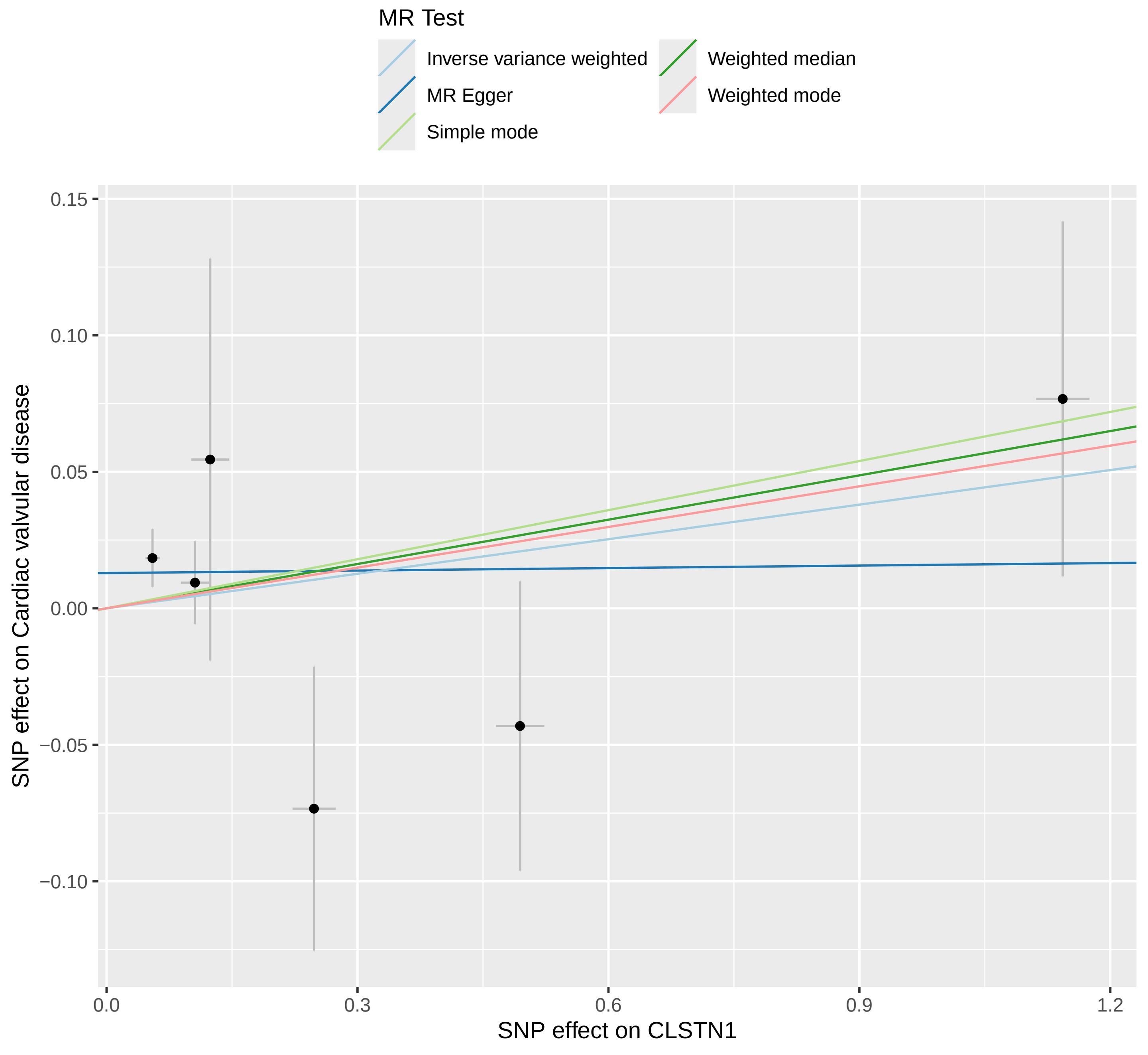
MR Test
Inverse variance weighted
MR Egger
Simple mode
Weighted median
Weighted mode
0.15
0.10
0.05
0.00
−0.05
−0.10
SNP effect on Cardiac valvular disease
0.0
0.3
0.6
0.9
1.2
SNP effect on CLSTN1

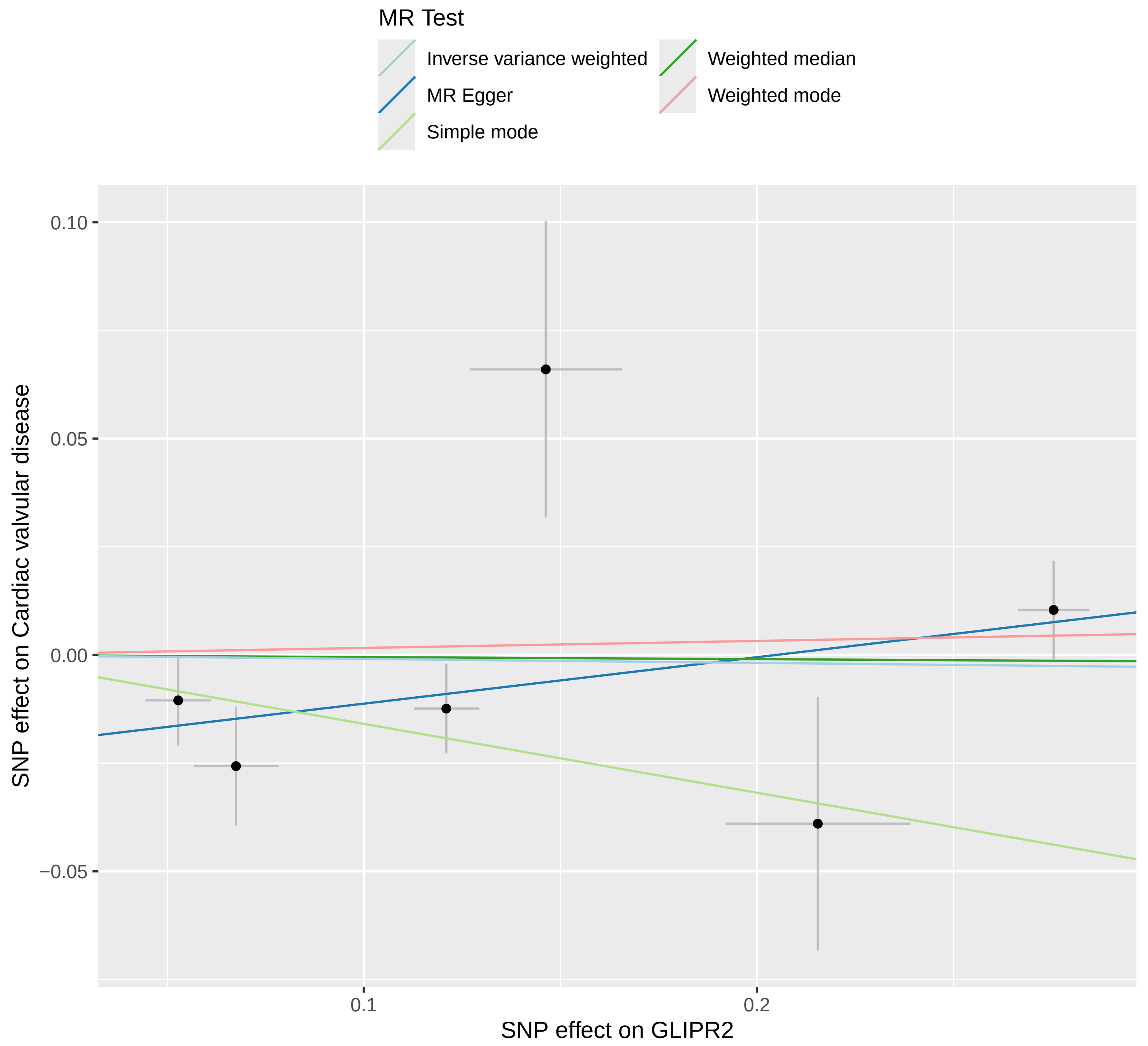
MR Test
Inverse variance weighted
MR Egger
Simple mode
Weighted median
Weighted mode
0.10
0.05
0.00
−0.05
0.1
0.2
SNP effect on Cardiac valvular disease
SNP effect on GLIPR2

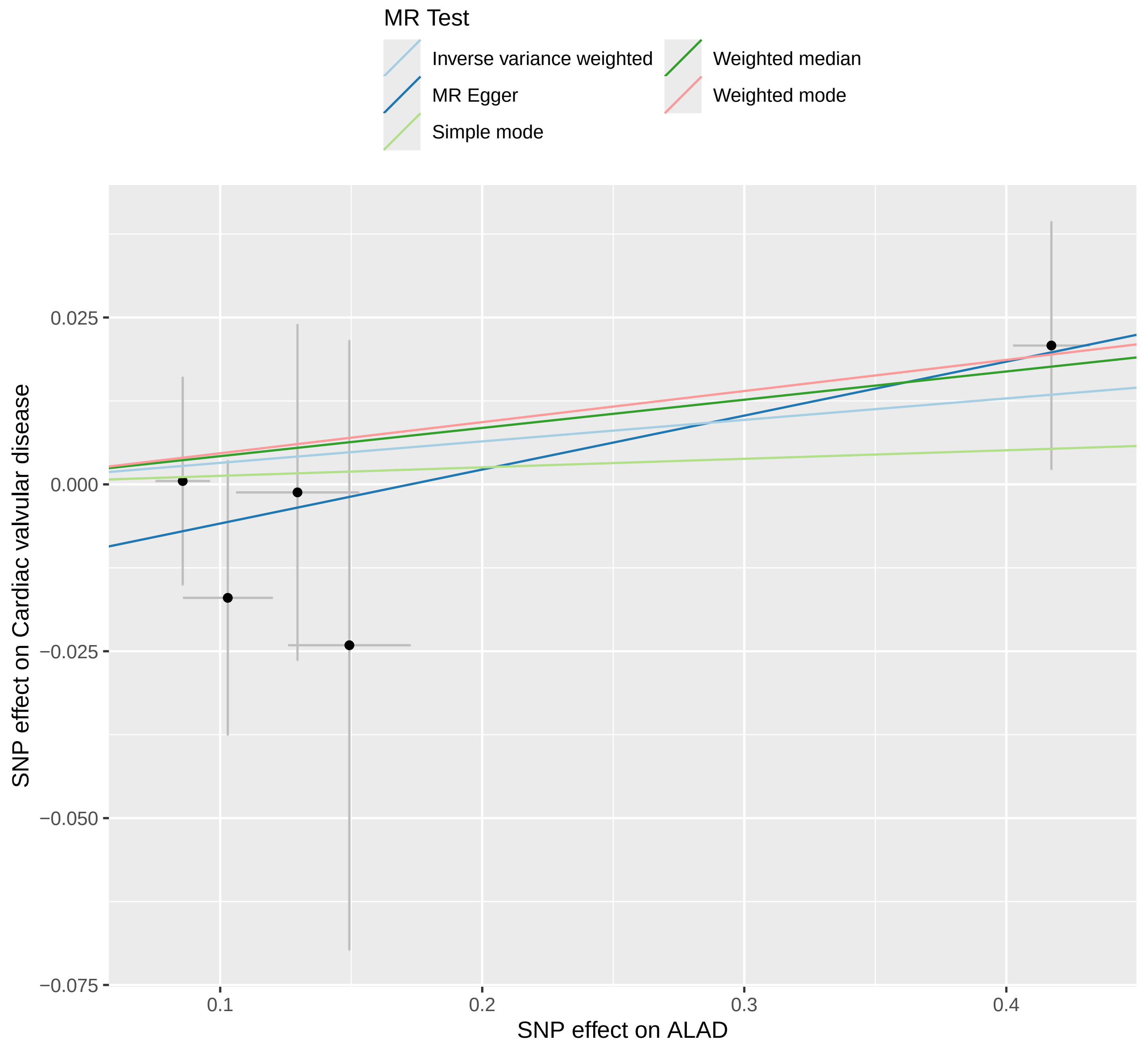
MR Test
Inverse variance weighted
MR Egger
Simple mode
Weighted median
Weighted mode
0.025
0.000
−0.025
−0.050
−0.075
0.1
0.2
0.3
0.4
SNP effect on Cardiac valvular disease
SNP effect on ALAD

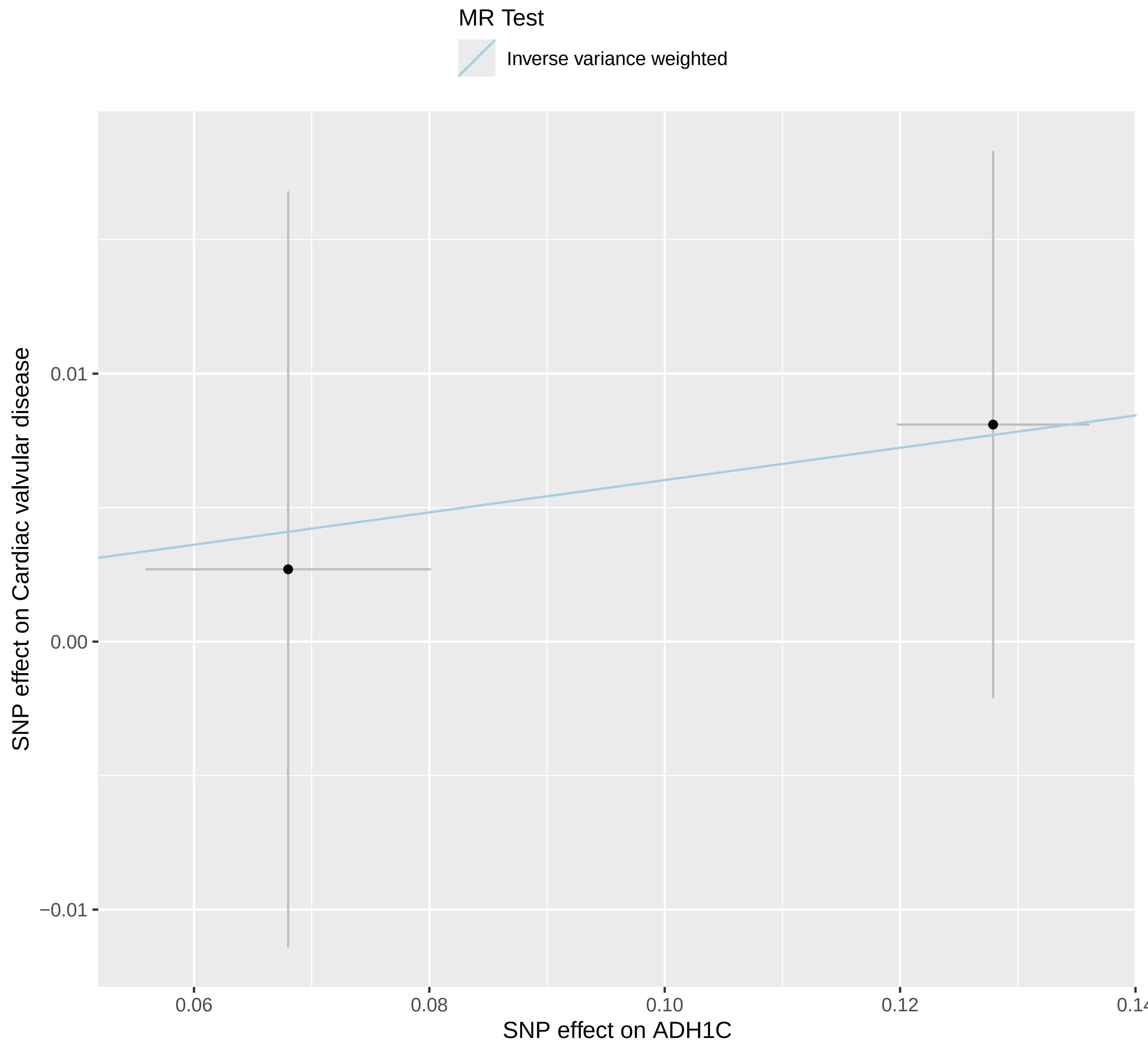

MR Test
Inverse variance weighted
SNP effect on Cardiac valvular disease
0.01
0.00
−0.01
0.06
0.08
0.10
0.12
0.14
SNP effect on ADH1C

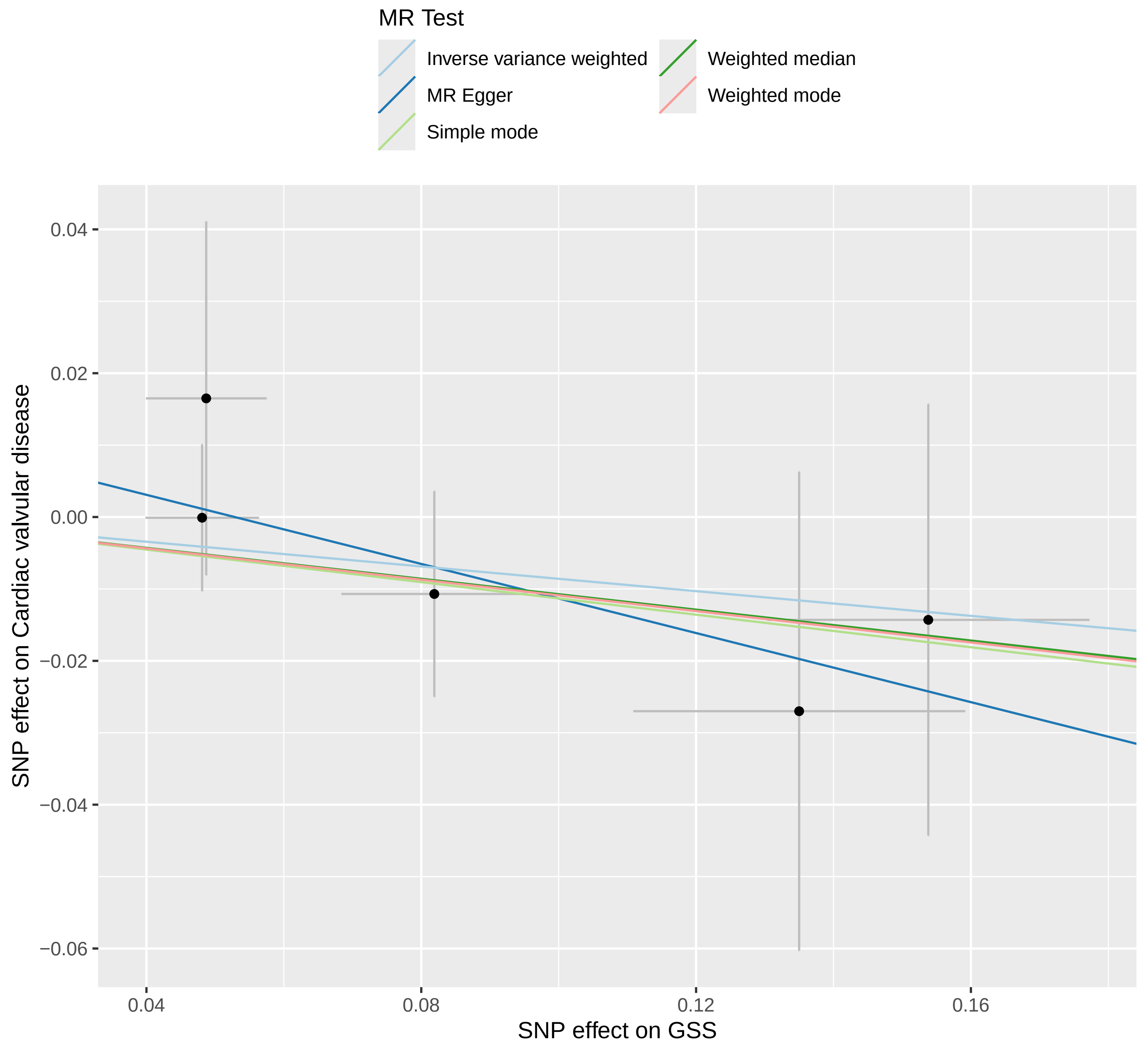

MR Test
Inverse variance weighted
MR Egger
Simple mode
Weighted median
Weighted mode
0.04
0.02
0.00
−0.02
−0.04
−0.06
SNP effect on Cardiac valvular disease
0.04
0.08
0.12
0.16
SNP effect on GSS

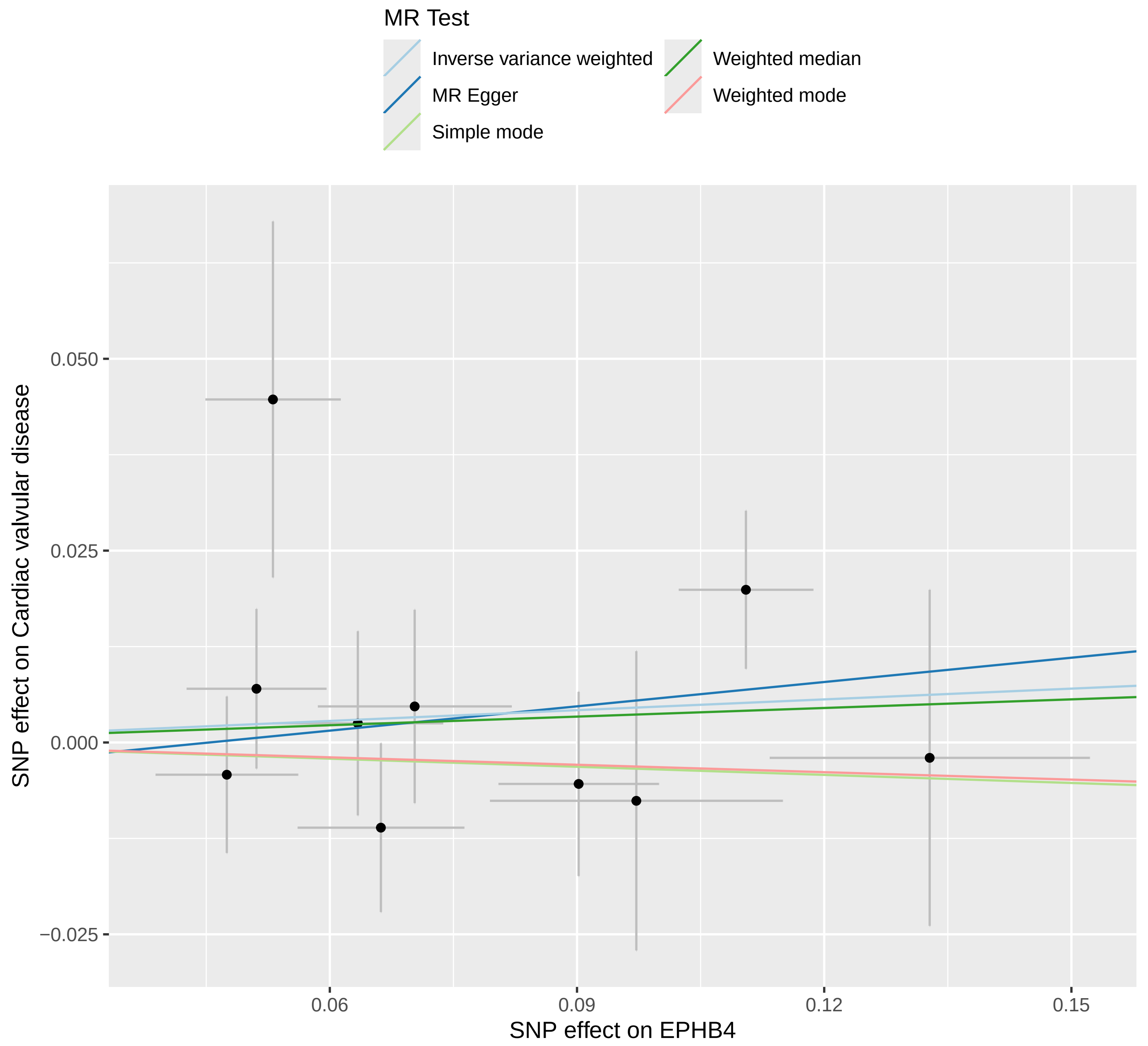
MR Test
Inverse variance weighted
MR Egger
Simple mode
Weighted median
Weighted mode
0.050
0.025
0.000
−0.025
SNP effect on Cardiac valvular disease
0.06
0.09
0.12
0.15
SNP effect on EPHB4

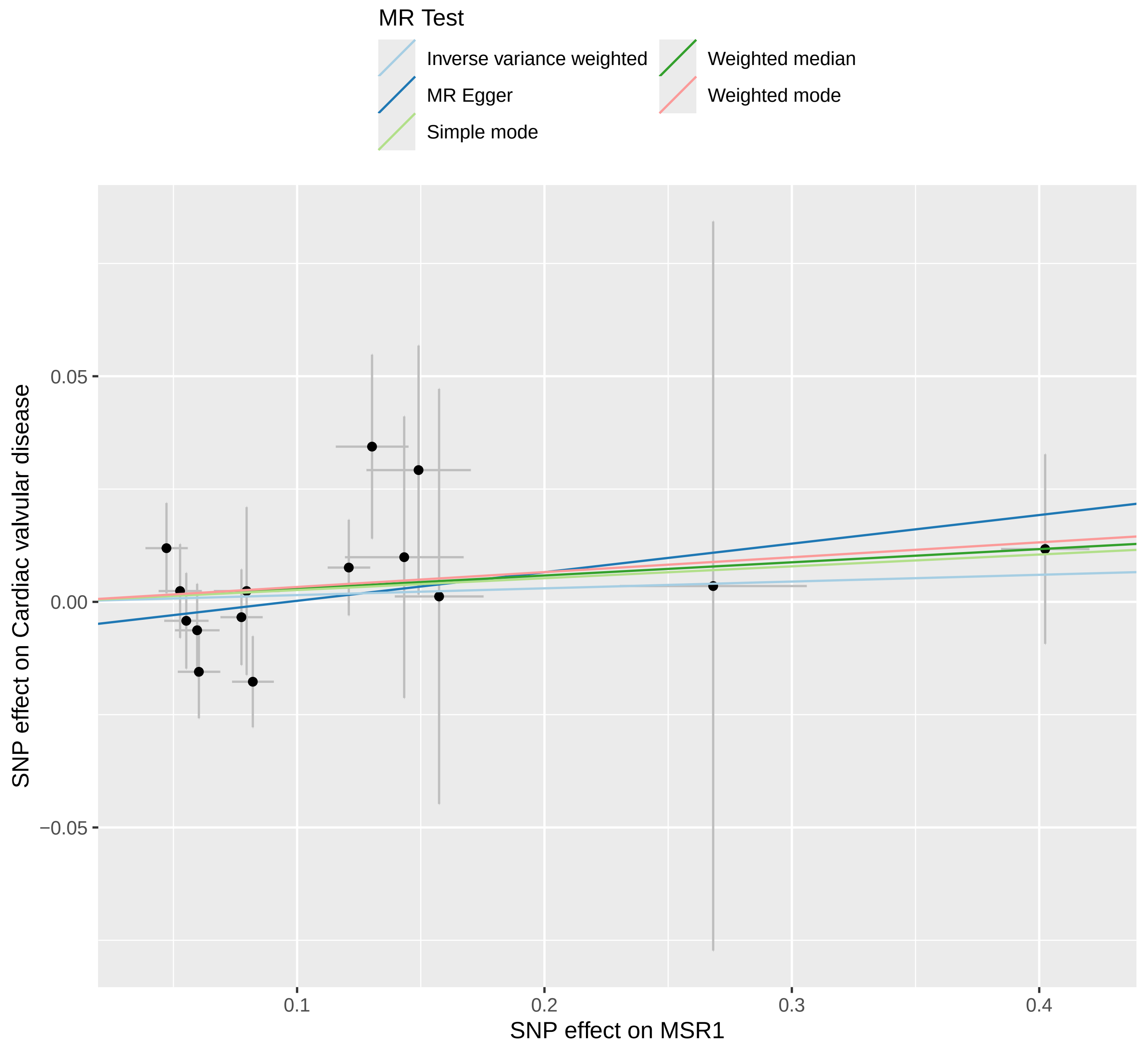

MR Test
Inverse variance weighted
MR Egger
Simple mode
Weighted median
Weighted mode
0.05
0.00
−0.05
0.1
0.2
0.3
0.4
SNP effect on MSR1
SNP effect on Cardiac valvular disease

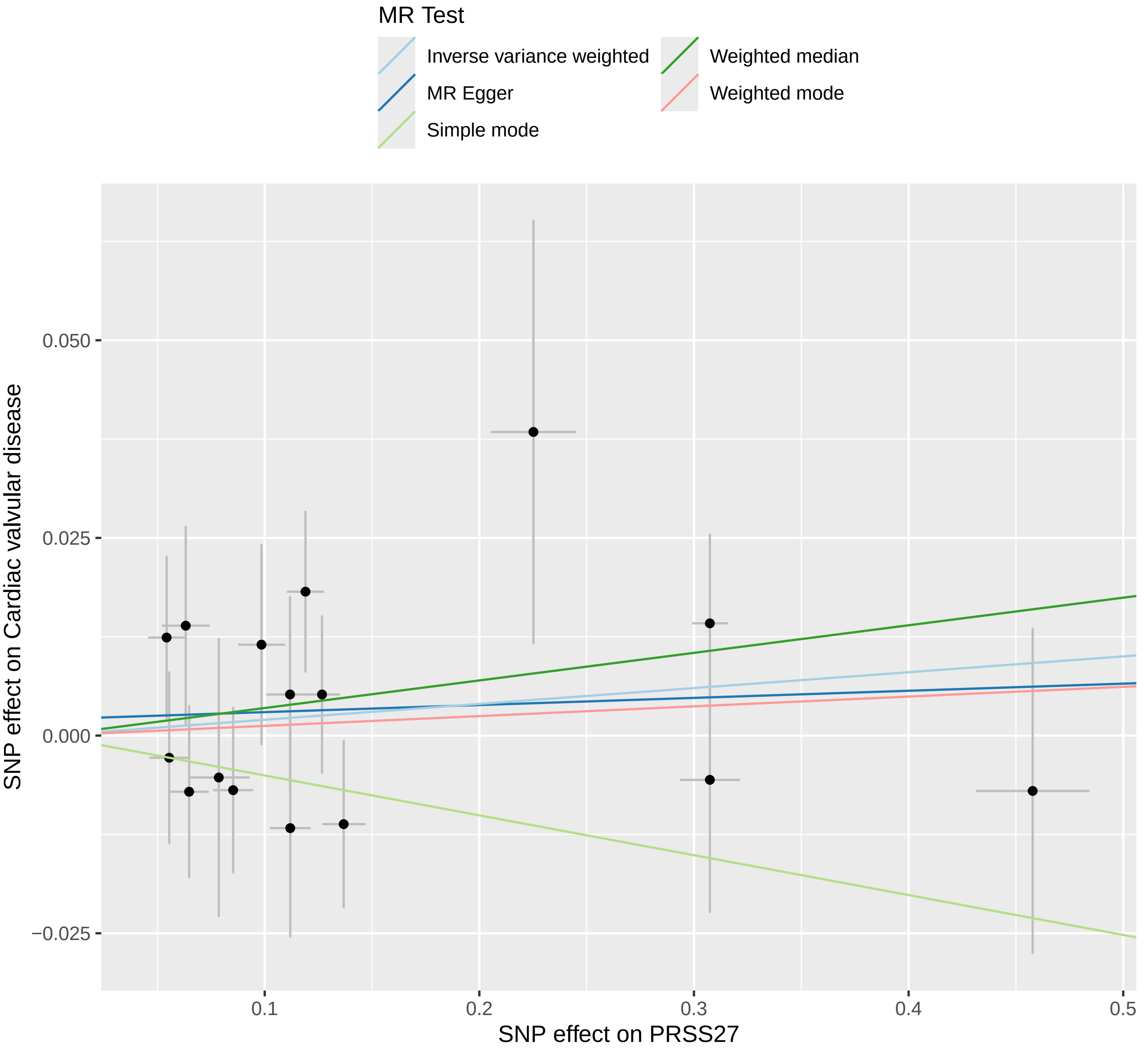
MR Test
Inverse variance weighted
MR Egger
Simple mode
Weighted median
Weighted mode
0.050
0.025
0.000
−0.025
0.1
0.2
0.3
0.4
0.5
SNP effect on Cardiac valvular disease
SNP effect on PRSS27

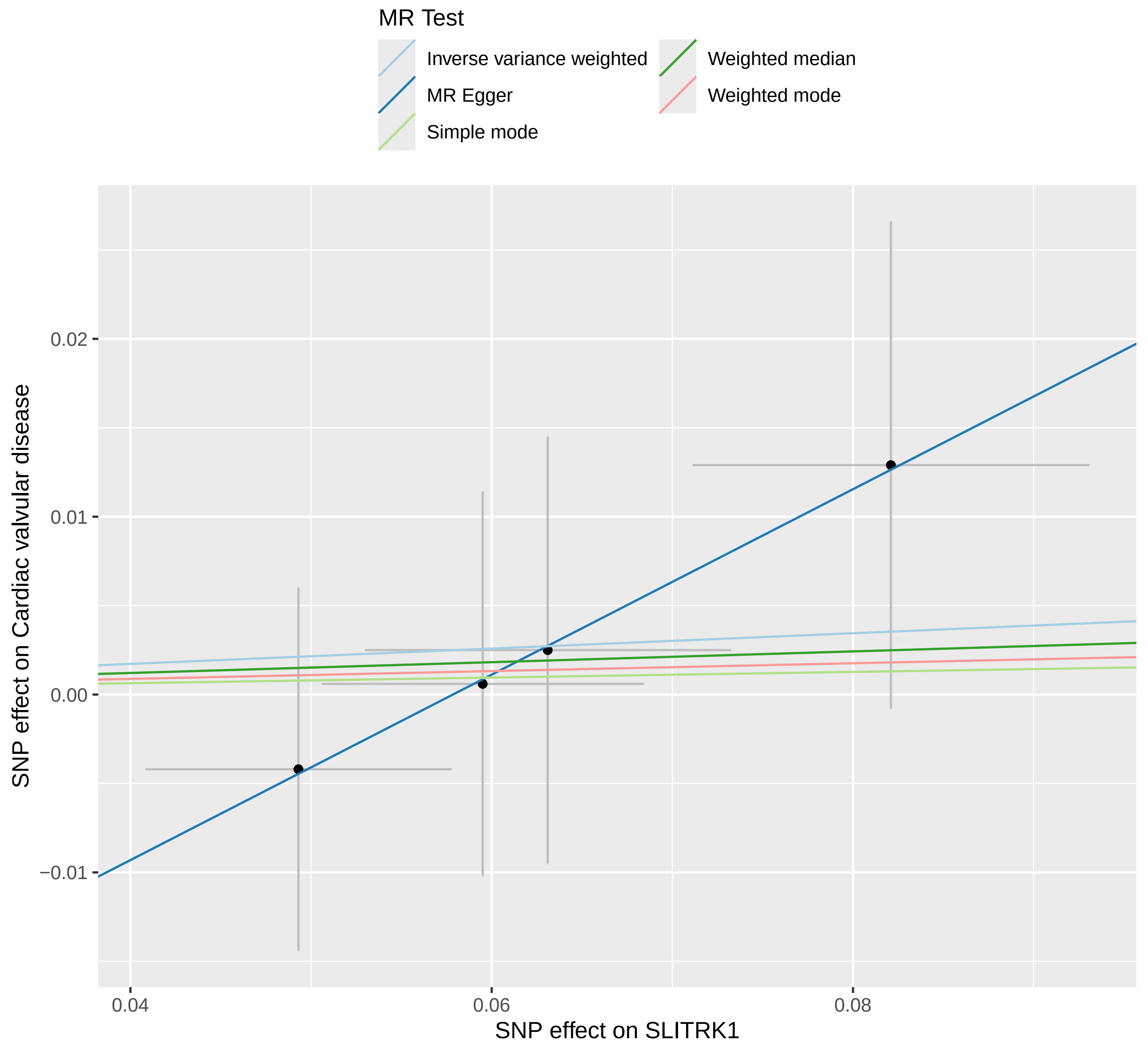

MR Test
Inverse variance weighted
MR Egger
Simple mode
Weighted median
Weighted mode
0.02
0.01
0.00
−0.01
0.04
0.06
0.08
SNP effect on Cardiac valvular disease
SNP effect on SLITRK1

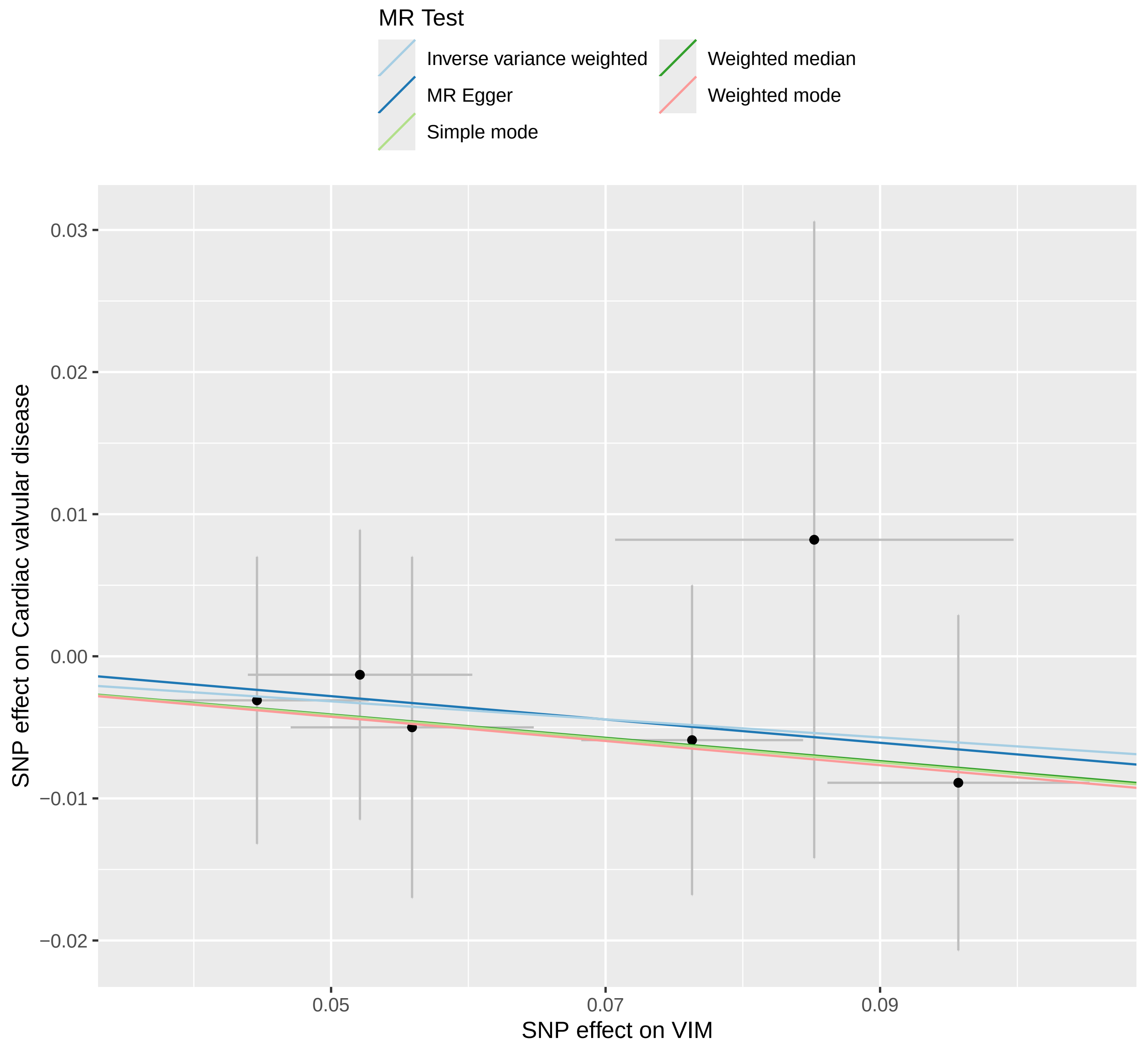
MR Test
Inverse variance weighted
MR Egger
Simple mode
Weighted median
Weighted mode
0.03
0.02
0.01
0.00
−0.01
−0.02
SNP effect on Cardiac valvular disease
0.05
0.07
0.09
SNP effect on VIM

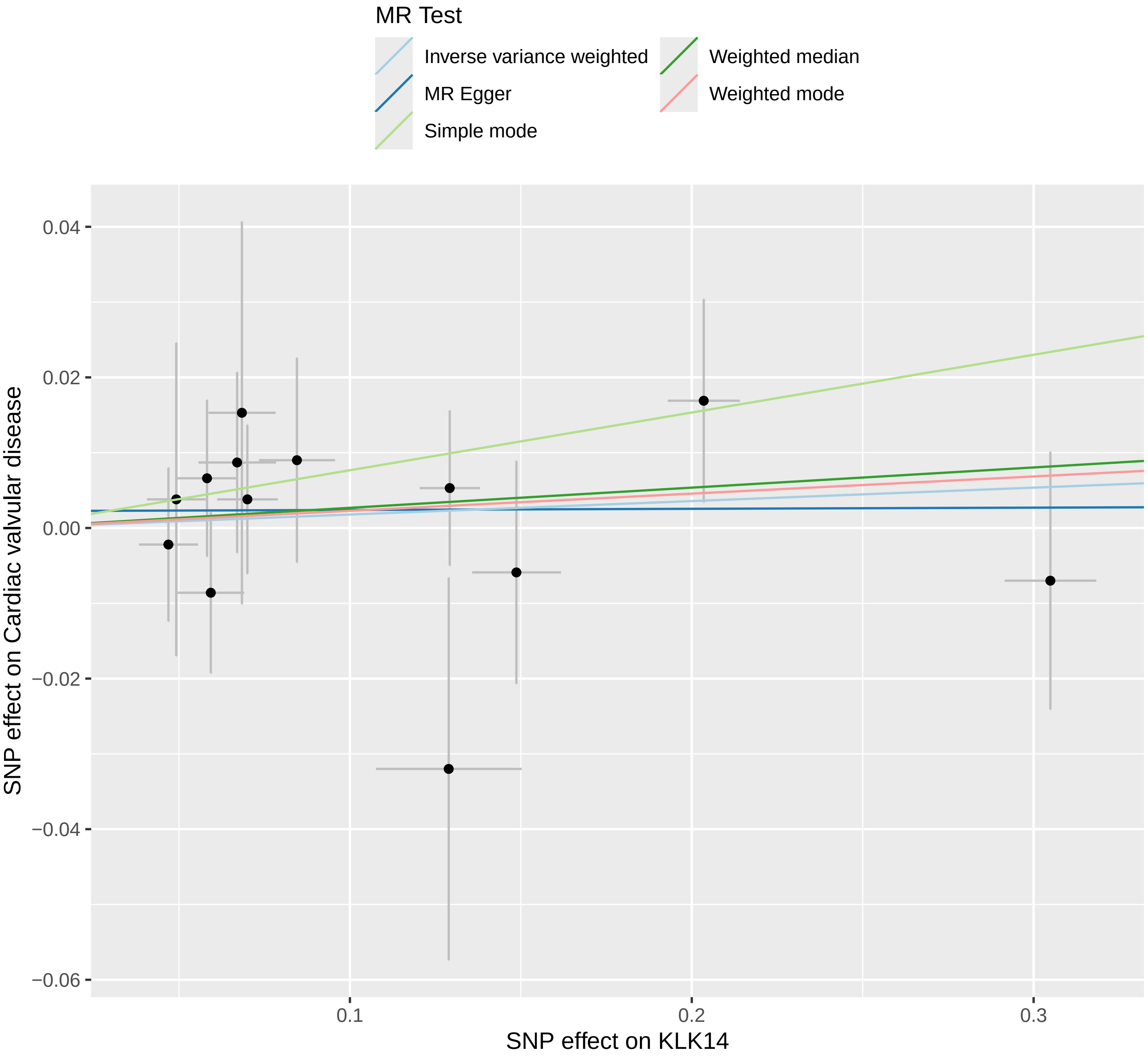
MR Test
Inverse variance weighted
MR Egger
Simple mode
Weighted median
Weighted mode
0.04
0.02
0.00
−0.02
−0.04
−0.06
SNP effect on Cardiac valvular disease
0.1
0.2
0.3
SNP effect on KLK14

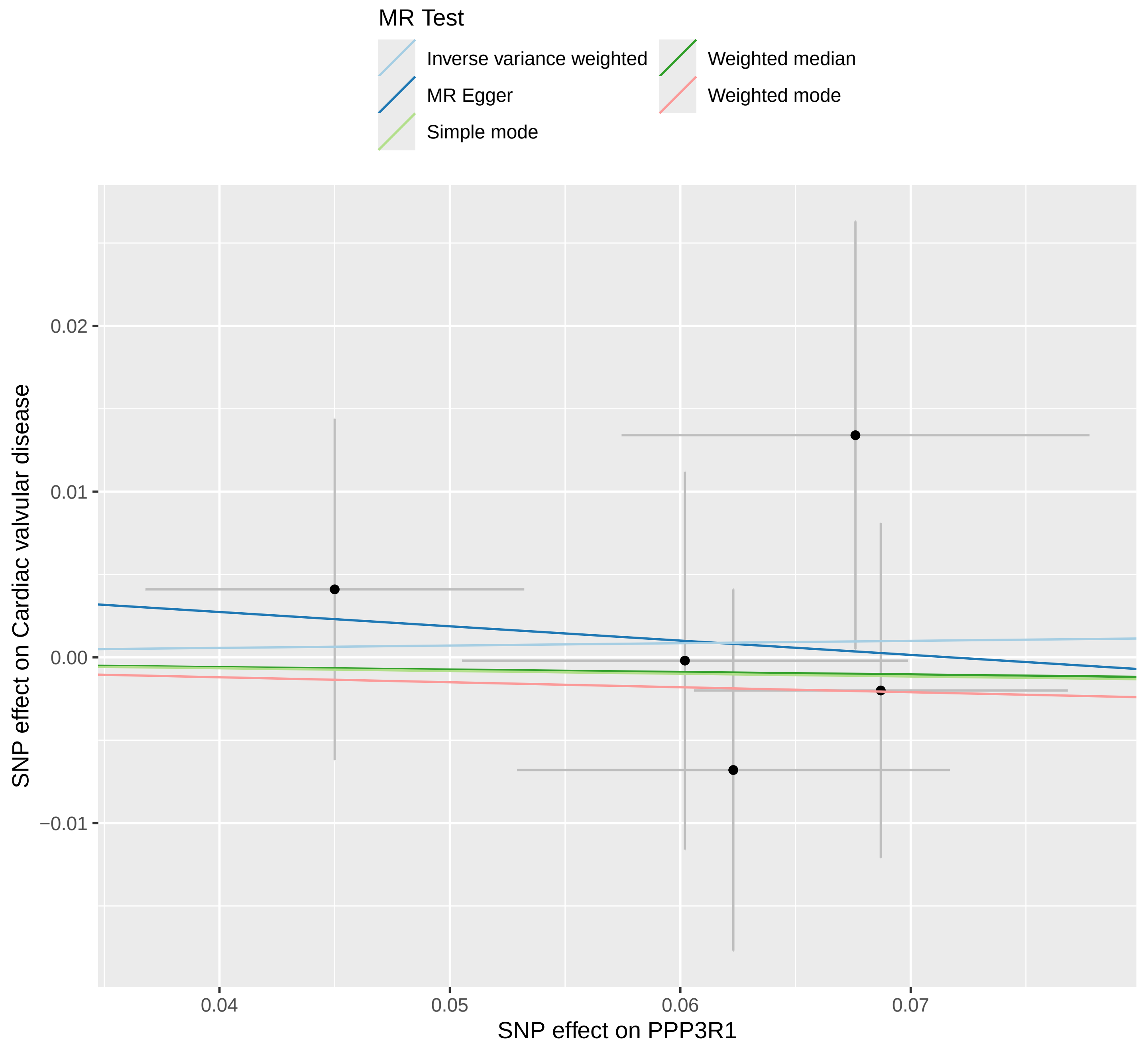
MR Test
Inverse variance weighted
MR Egger
Simple mode
Weighted median
Weighted mode
0.02
0.01
0.00
−0.01
0.04
0.05
0.06
0.07
SNP effect on Cardiac valvular disease
SNP effect on PPP3R1

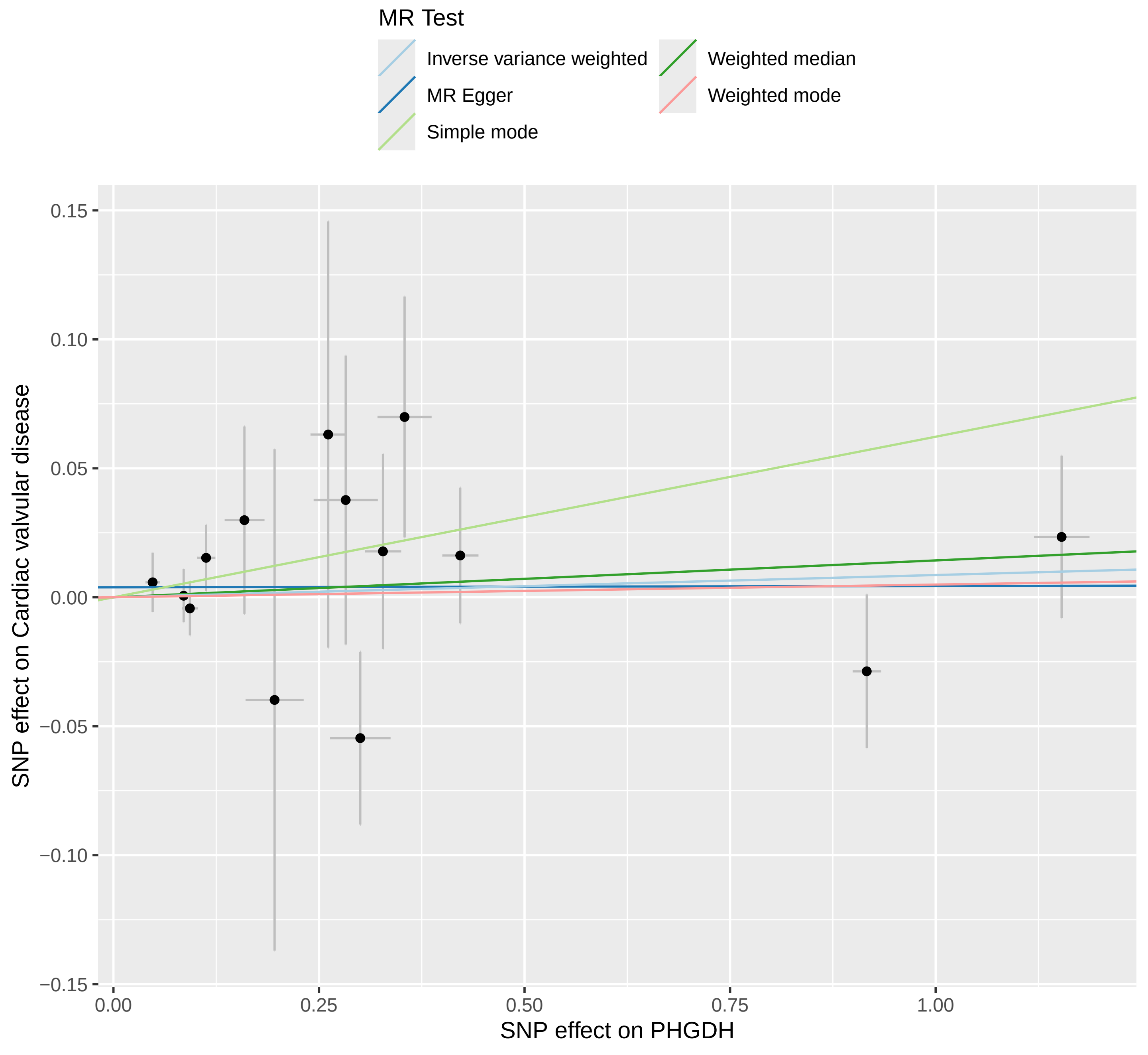

MR Test
Inverse variance weighted
MR Egger
Simple mode
Weighted median
Weighted mode
0.15
0.10
0.05
0.00
−0.05
−0.10
−0.15
SNP effect on Cardiac valvular disease
0.00
0.25
0.50
0.75
1.00
SNP effect on PHGDH

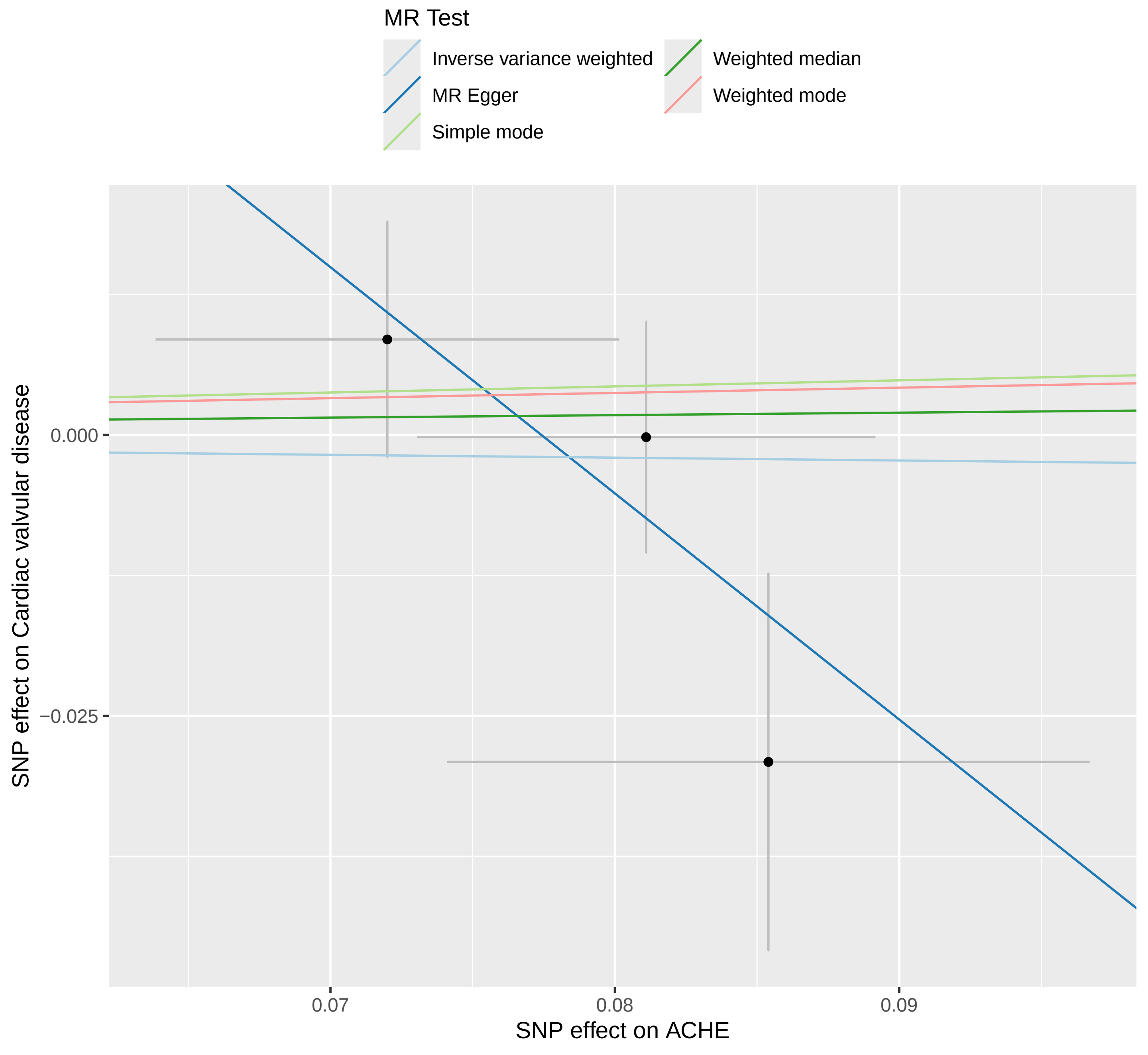
MR Test
Inverse variance weighted
MR Egger
Simple mode
Weighted median
Weighted mode
0.000
−0.025
0.07
0.08
0.09
SNP effect on ACHE
SNP effect on Cardiac valvular disease

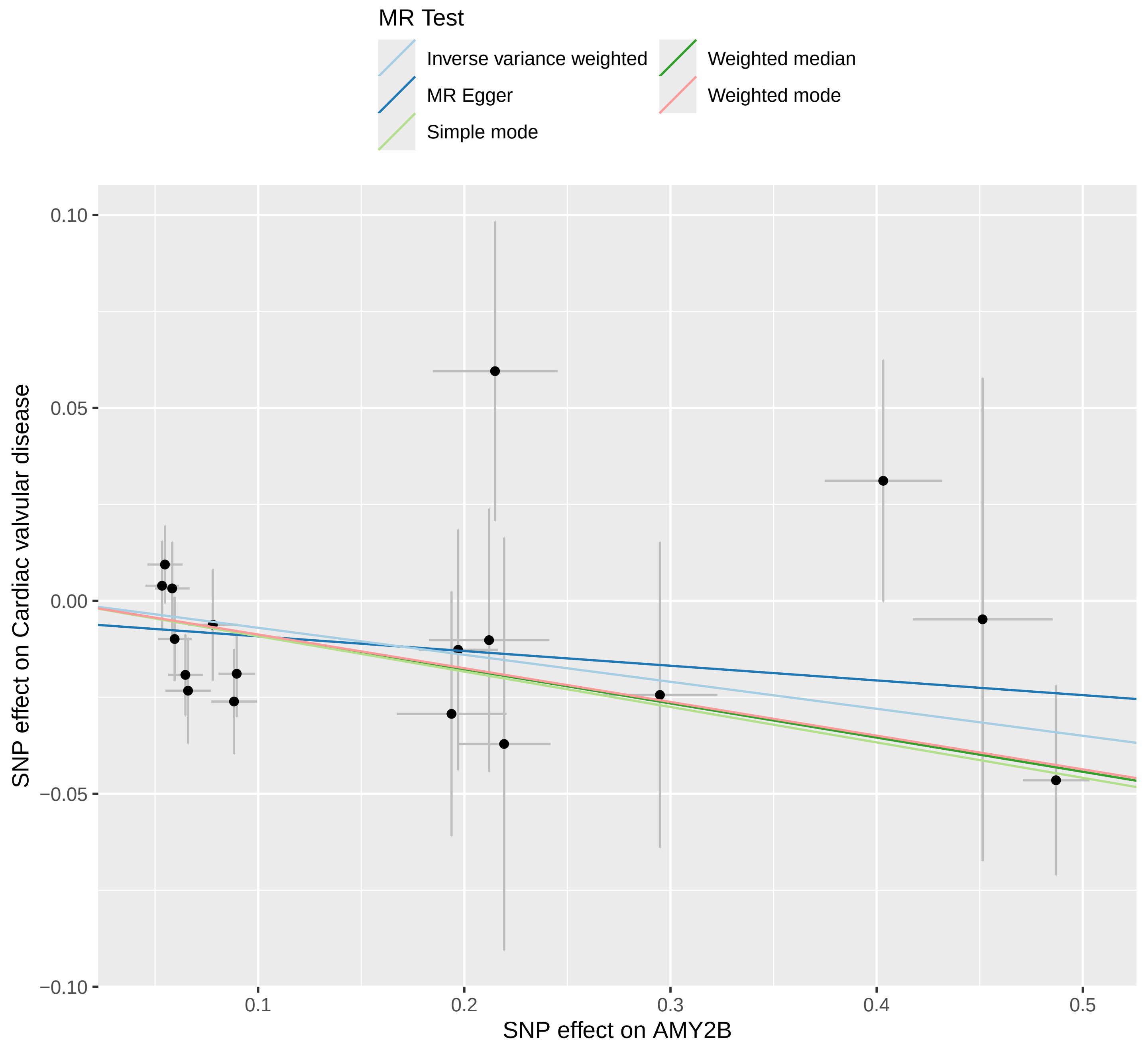
MR Test
Inverse variance weighted
MR Egger
Simple mode
Weighted median
Weighted mode
0.10
0.05
0.00
−0.05
−0.10
0.1
0.2
0.3
0.4
0.5
SNP effect on Cardiac valvular disease
SNP effect on AMY2B

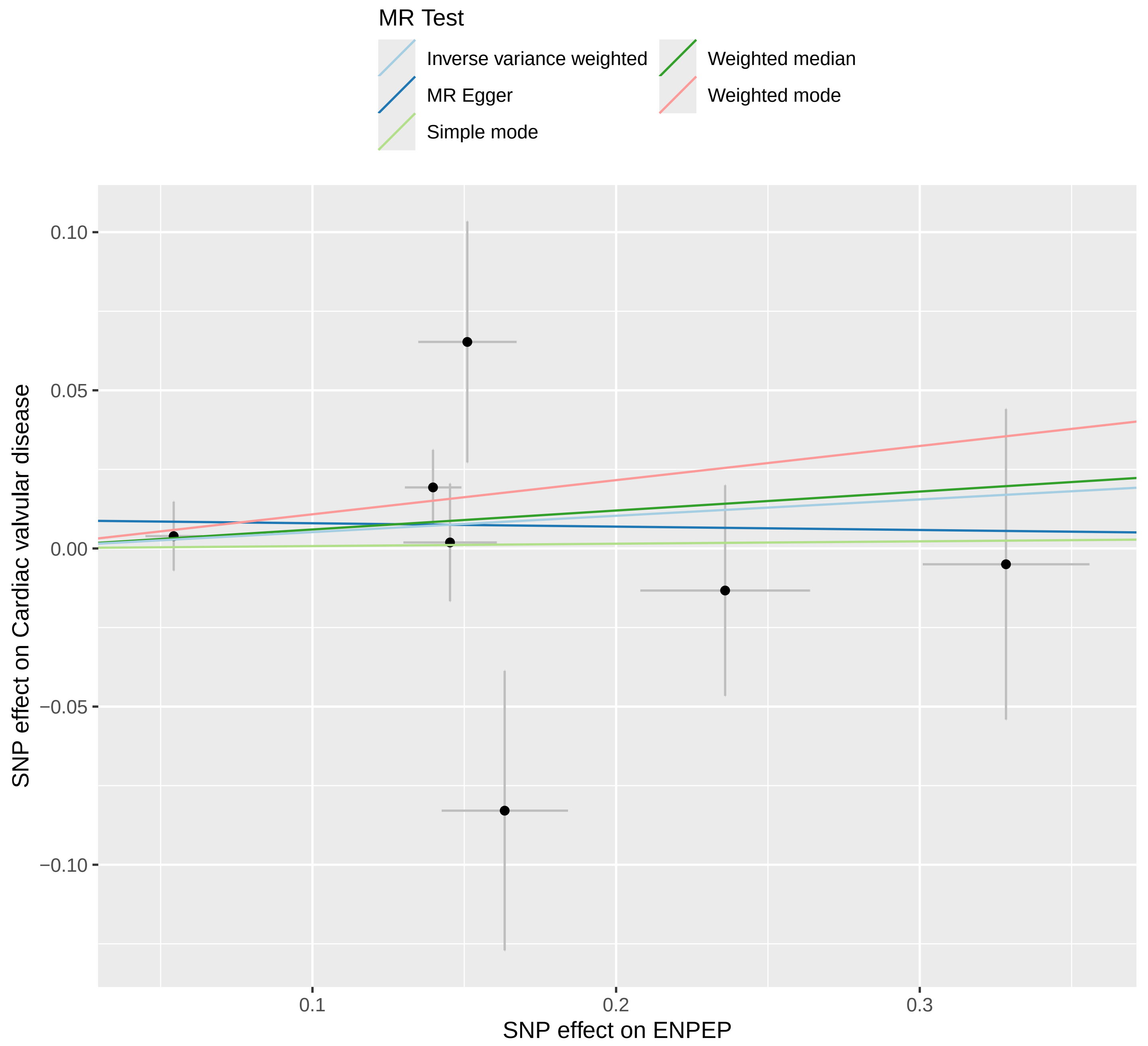
MR Test
Inverse variance weighted
MR Egger
Simple mode
Weighted median
Weighted mode
0.10
0.05
0.00
−0.05
−0.10
SNP effect on Cardiac valvular disease
0.1
0.2
0.3
SNP effect on ENPEP

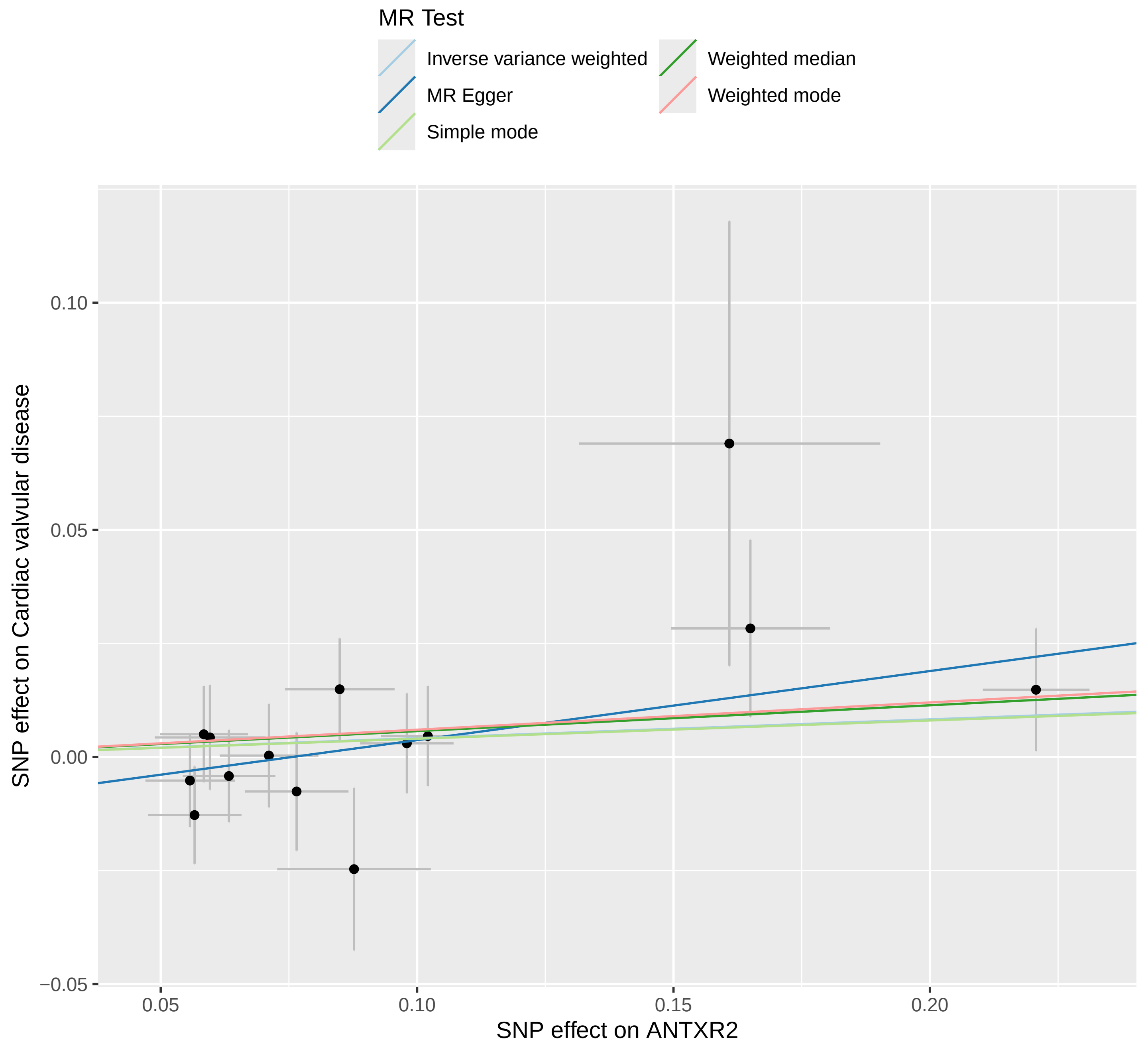

MR Test
Inverse variance weighted
MR Egger
Simple mode
Weighted median
Weighted mode
0.10
0.05
0.00
−0.05
SNP effect on Cardiac valvular disease
0.05
0.10
0.15
0.20
SNP effect on ANTXR2

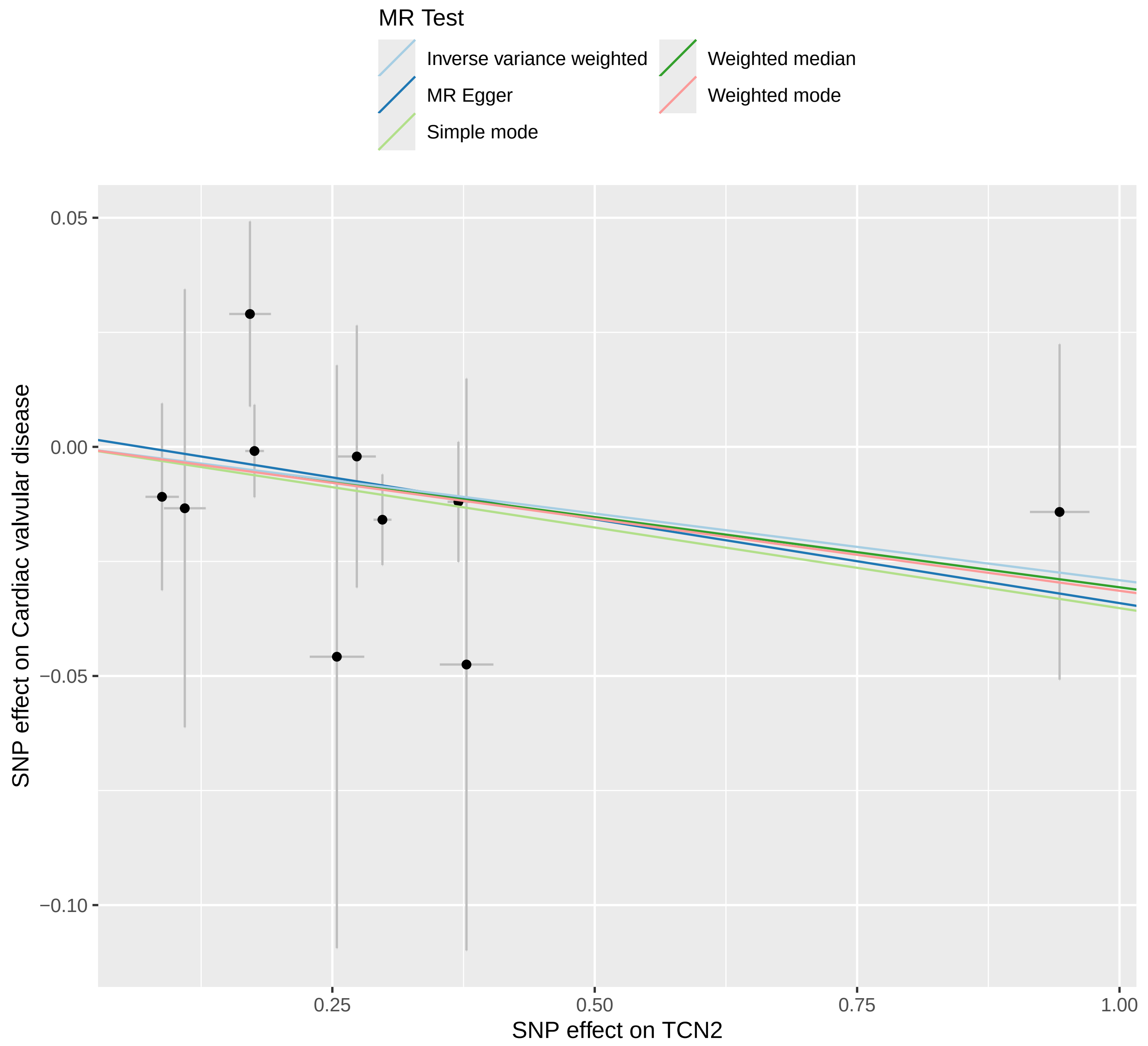
MR Test
Inverse variance weighted
MR Egger
Simple mode
Weighted median
Weighted mode
SNP effect on Cardiac valvular disease
0.05
0.00
−0.05
−0.10
0.25
0.50
0.75
1.00
SNP effect on TCN2

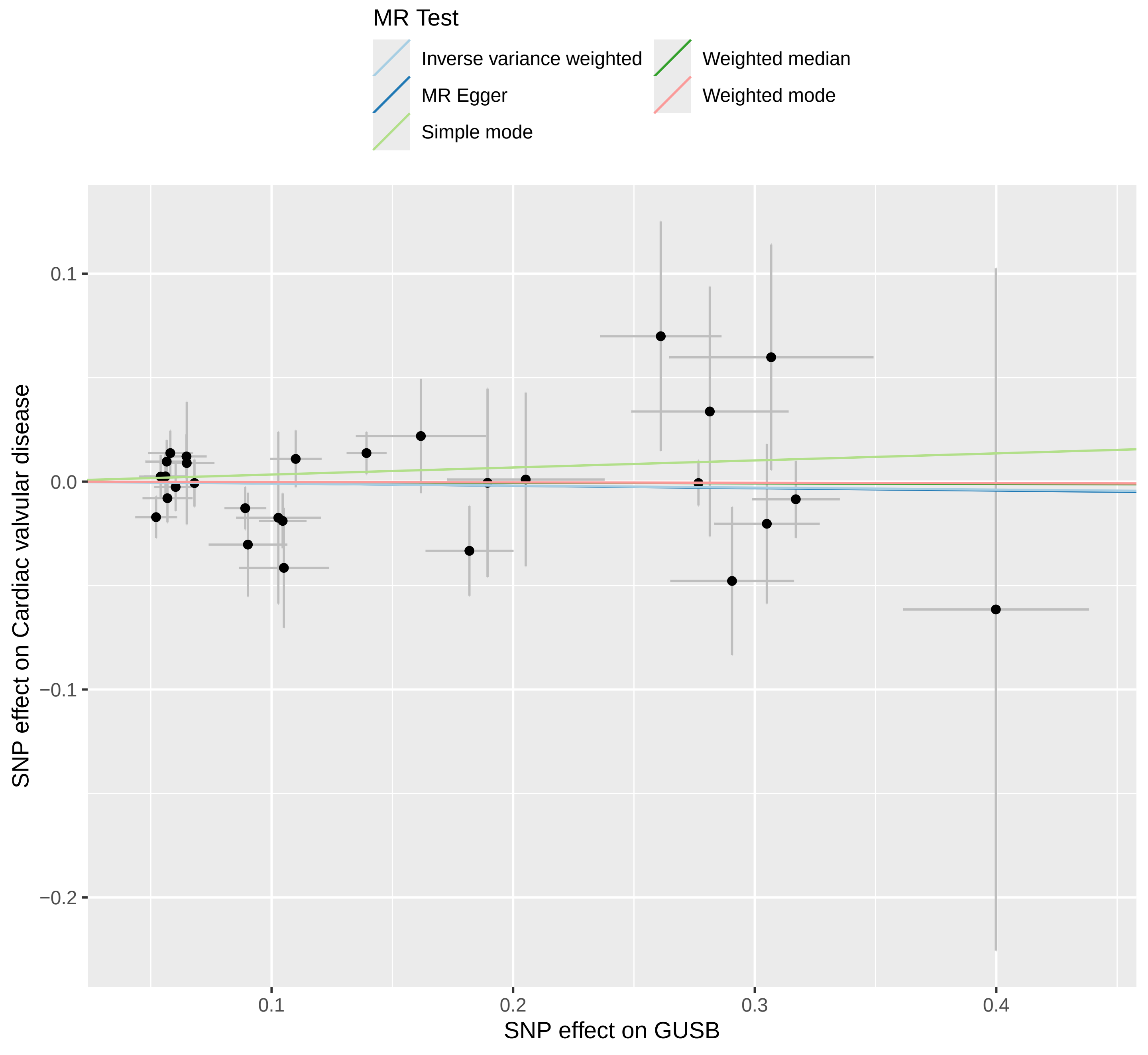
MR Test
Inverse variance weighted
MR Egger
Simple mode
Weighted median
Weighted mode
0.1
0.0
−0.1
−0.2
SNP effect on Cardiac valvular disease
0.1
0.2
0.3
0.4
SNP effect on GUSB

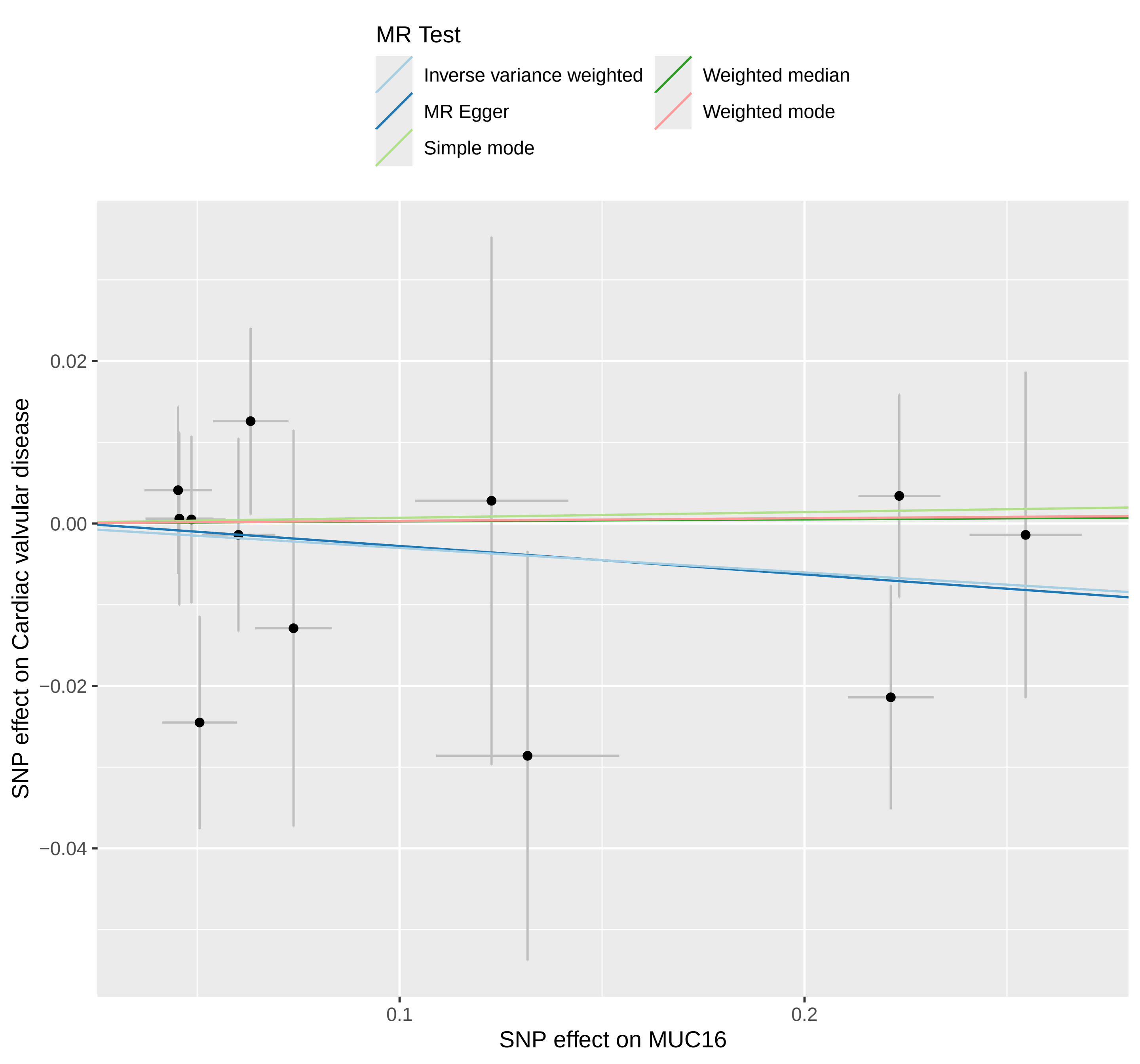
MR Test
Inverse variance weighted
MR Egger
Simple mode
Weighted median
Weighted mode
0.02
0.00
−0.02
−0.04
0.1
0.2
SNP effect on Cardiac valvular disease
SNP effect on MUC16

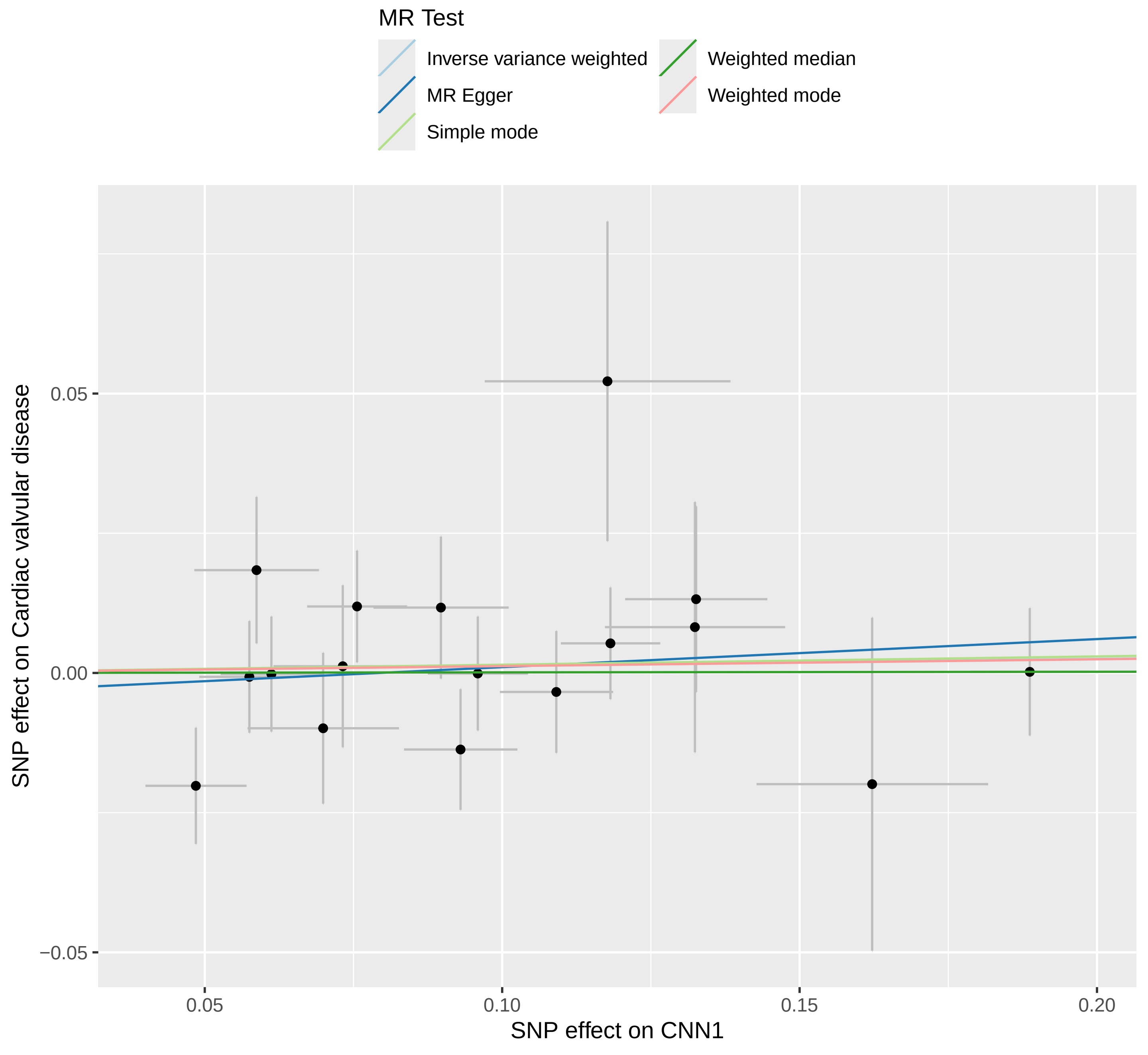
MR Test
Inverse variance weighted
MR Egger
Simple mode
Weighted median
Weighted mode
SNP effect on Cardiac valvular disease
0.05
0.00
−0.05
0.05
0.10
0.15
0.20
SNP effect on CNN1

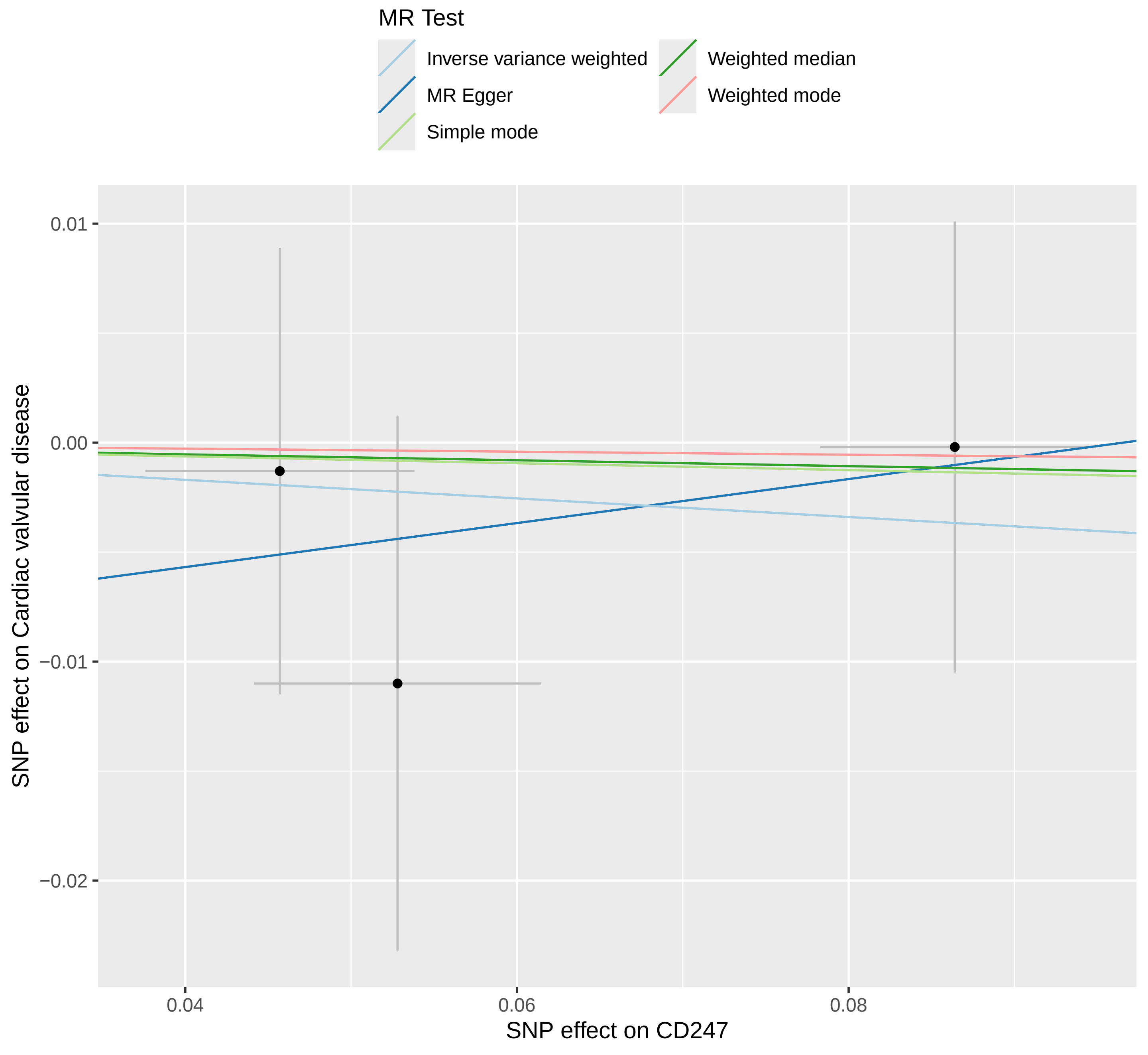
MR Test
Inverse variance weighted
MR Egger
Simple mode
Weighted median
Weighted mode
0.01
0.00
−0.01
−0.02
SNP effect on Cardiac valvular disease
0.04
0.06
0.08
SNP effect on CD247

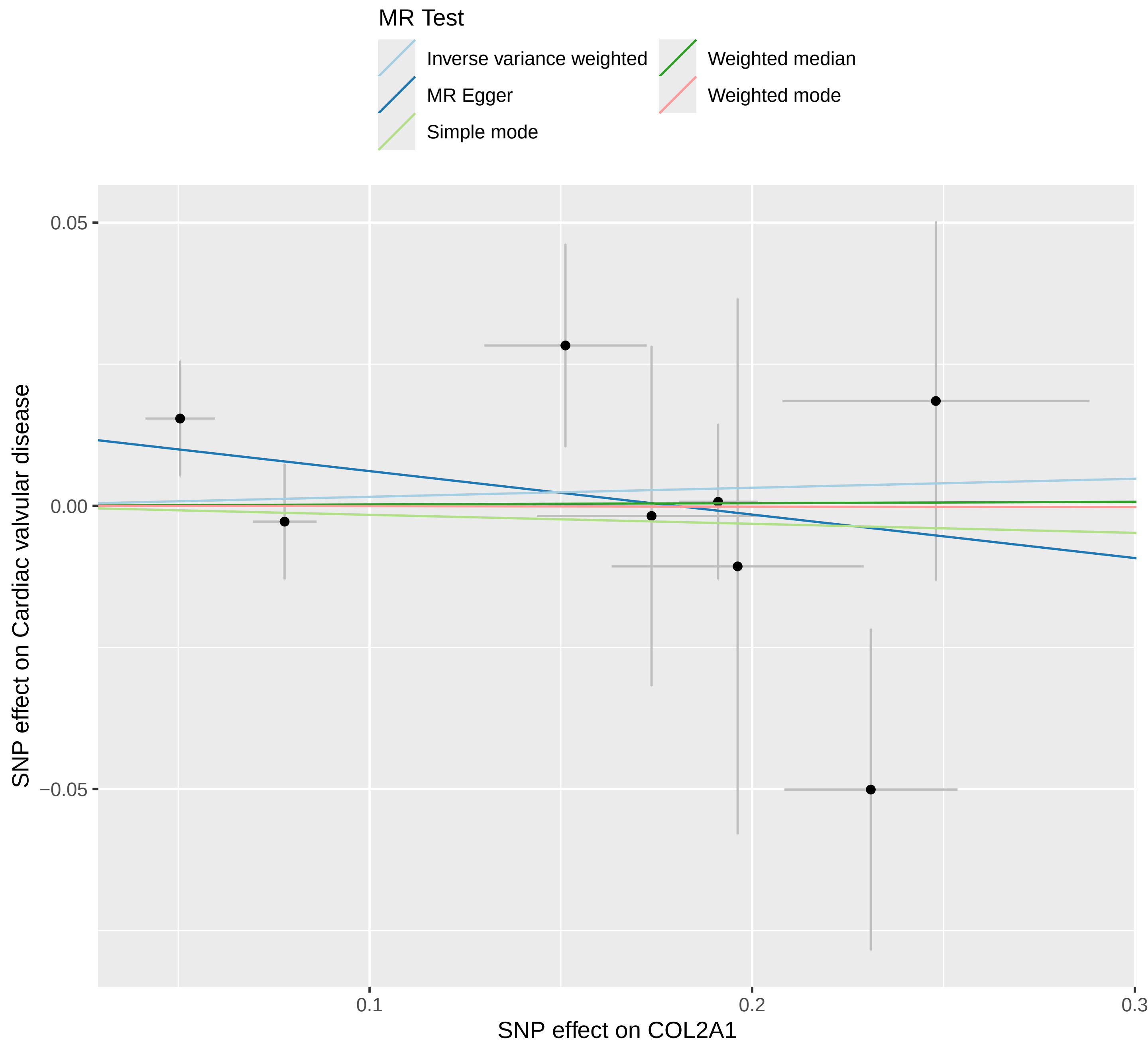
MR Test
Inverse variance weighted
MR Egger
Simple mode
Weighted median
Weighted mode
0.05
0.00
−0.05
0.1
0.2
0.3
SNP effect on COL2A1
SNP effect on Cardiac valvular disease

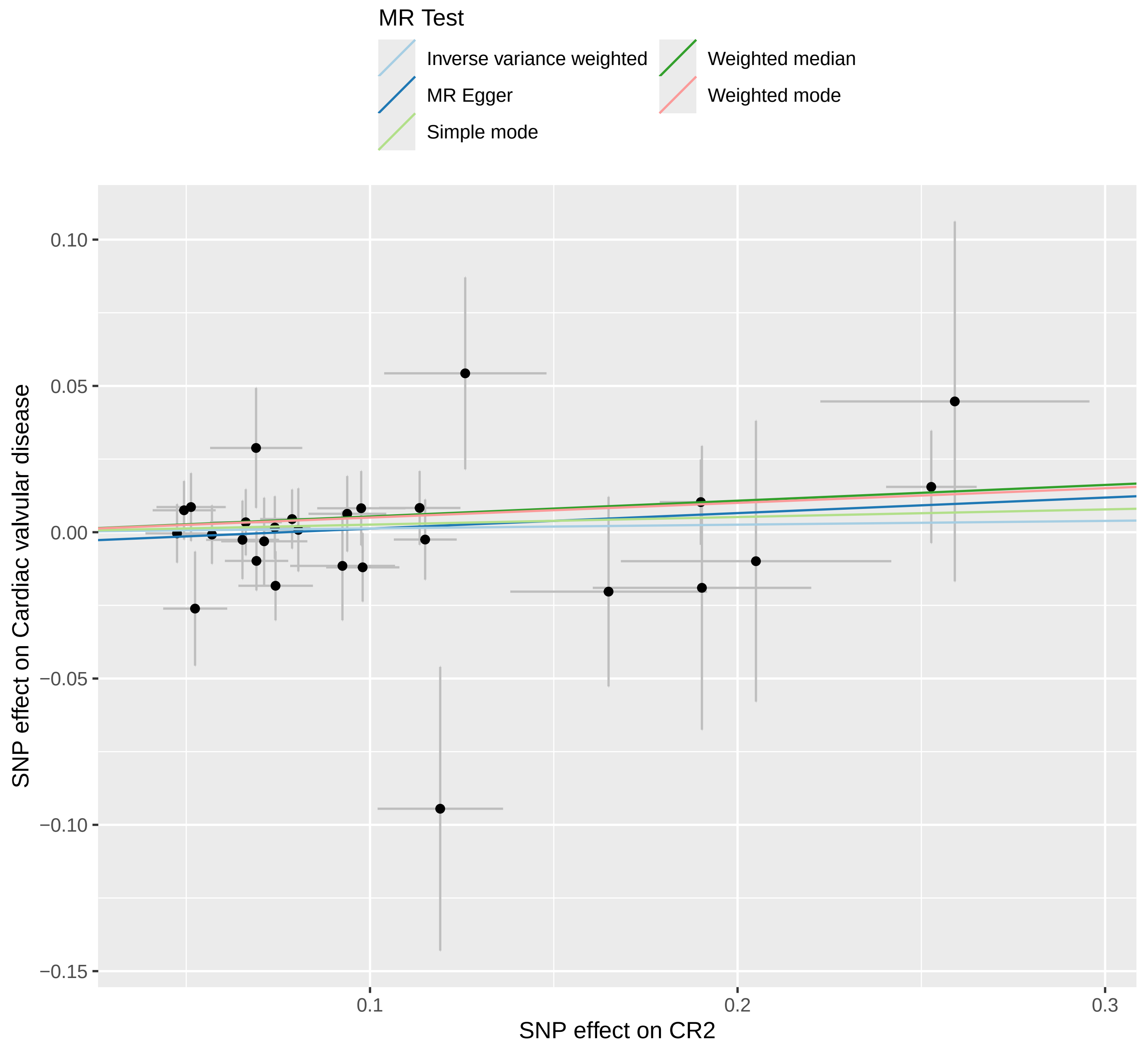
MR Test
Inverse variance weighted
MR Egger
Simple mode
Weighted median
Weighted mode
0.10
0.05
0.00
−0.05
−0.10
−0.15
SNP effect on Cardiac valvular disease
0.1
0.2
0.3
SNP effect on CR2

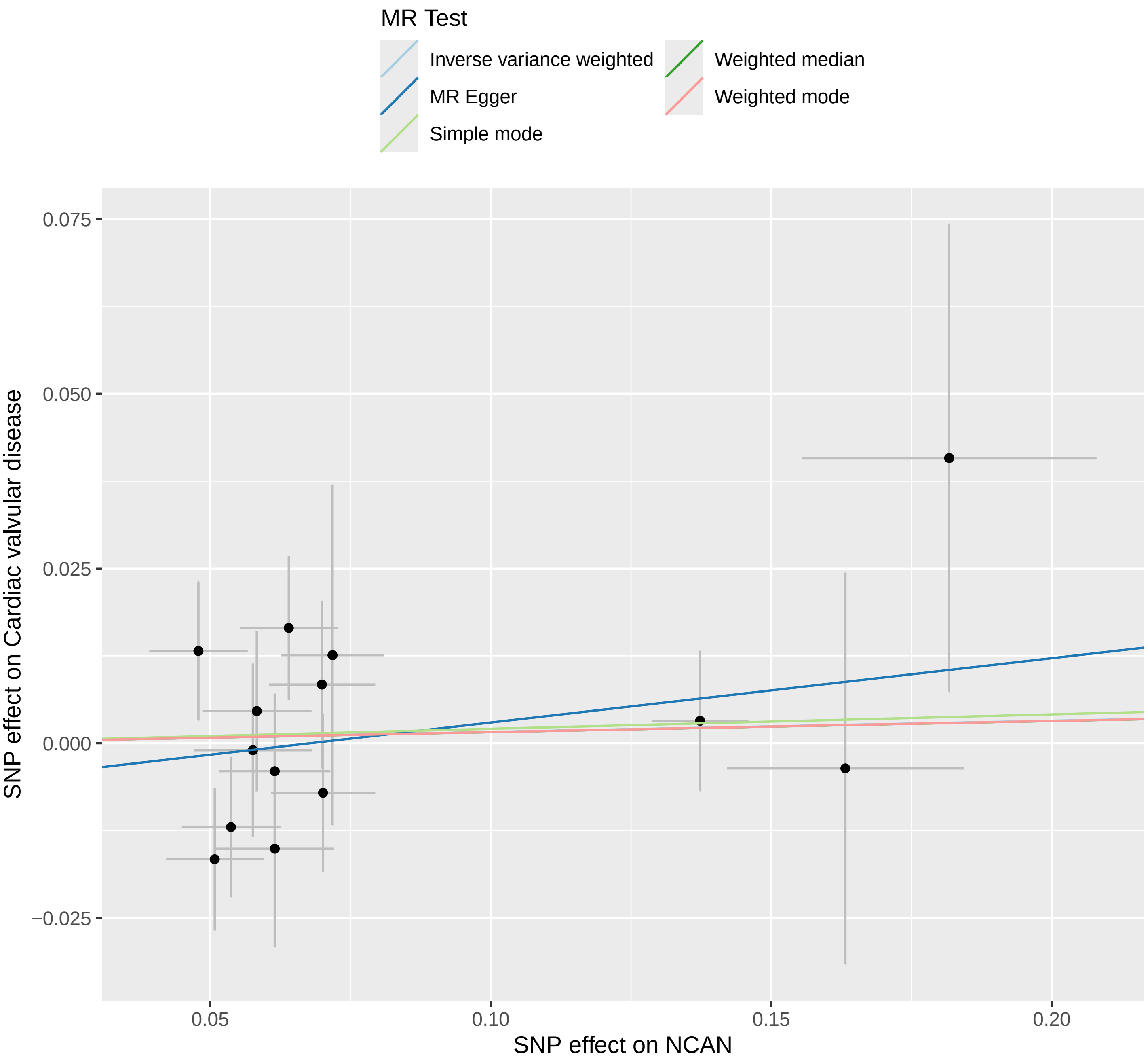

MR Test
Inverse variance weighted
MR Egger
Simple mode
Weighted median
Weighted mode
0.075
0.050
0.025
0.000
−0.025
SNP effect on Cardiac valvular disease
0.05
0.10
0.15
0.20
SNP effect on NCAN

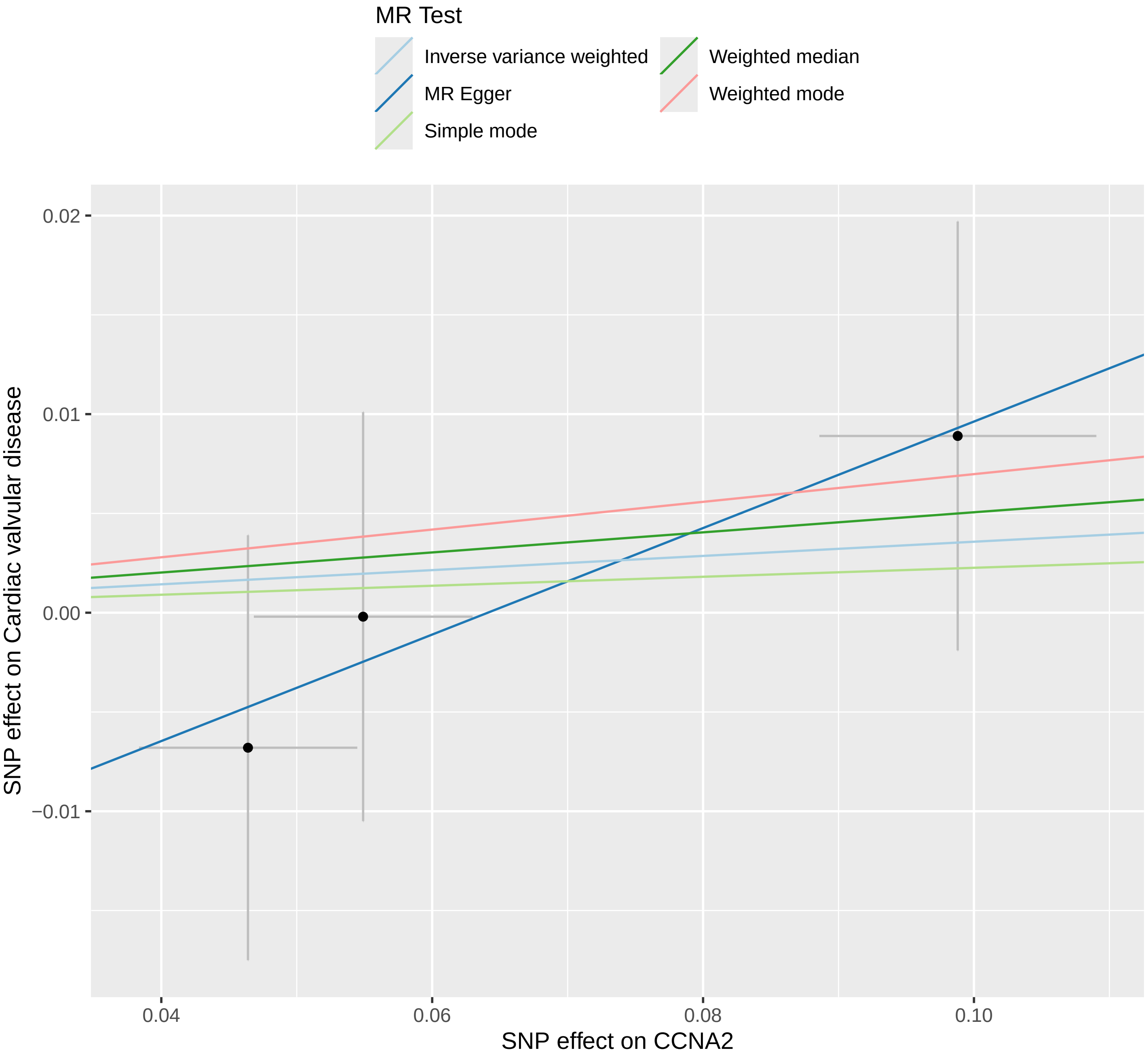

MR Test
Inverse variance weighted
MR Egger
Simple mode
Weighted median
Weighted mode
0.02
0.01
0.00
−0.01
SNP effect on Cardiac valvular disease
0.04
0.06
0.08
0.10
SNP effect on CCNA2

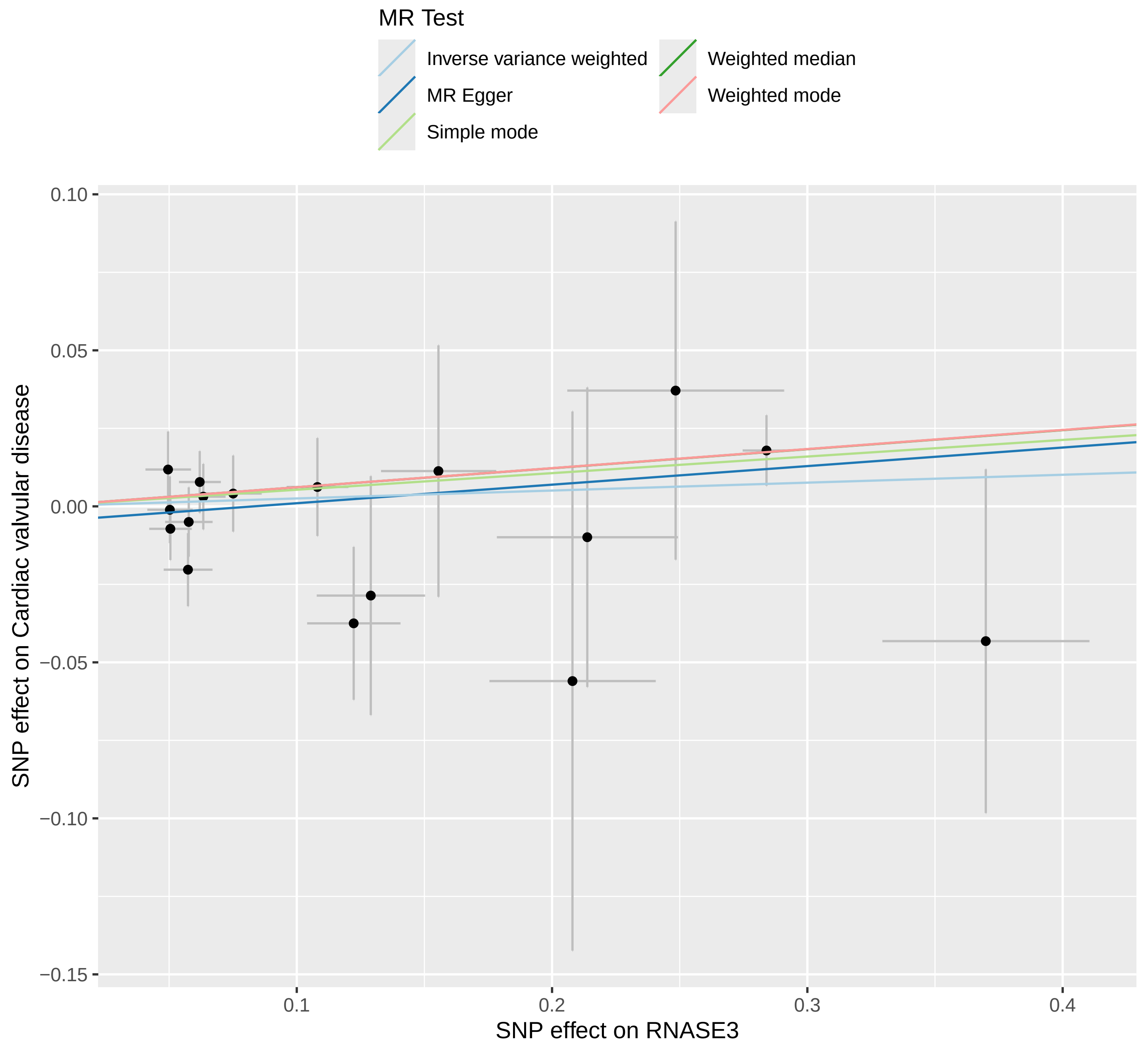
MR Test
Inverse variance weighted
MR Egger
Simple mode
Weighted median
Weighted mode
0.10
0.05
0.00
−0.05
−0.10
−0.15
SNP effect on Cardiac valvular disease
0.1
0.2
0.3
0.4
SNP effect on RNASE3

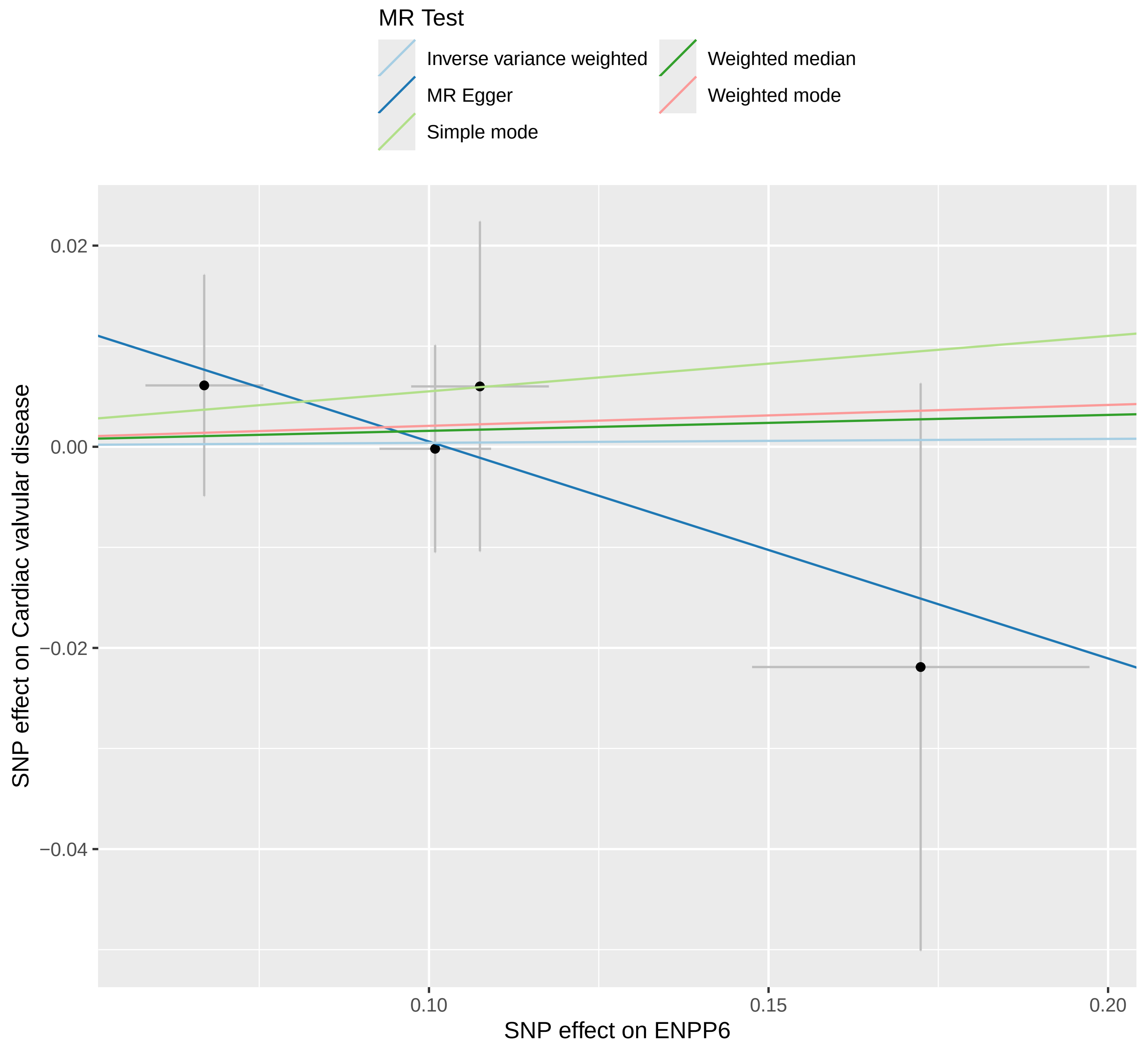

MR Test
Inverse variance weighted
MR Egger
Simple mode
Weighted median
Weighted mode
0.02
0.00
−0.02
−0.04
SNP effect on Cardiac valvular disease
0.10
0.15
0.20
SNP effect on ENPP6

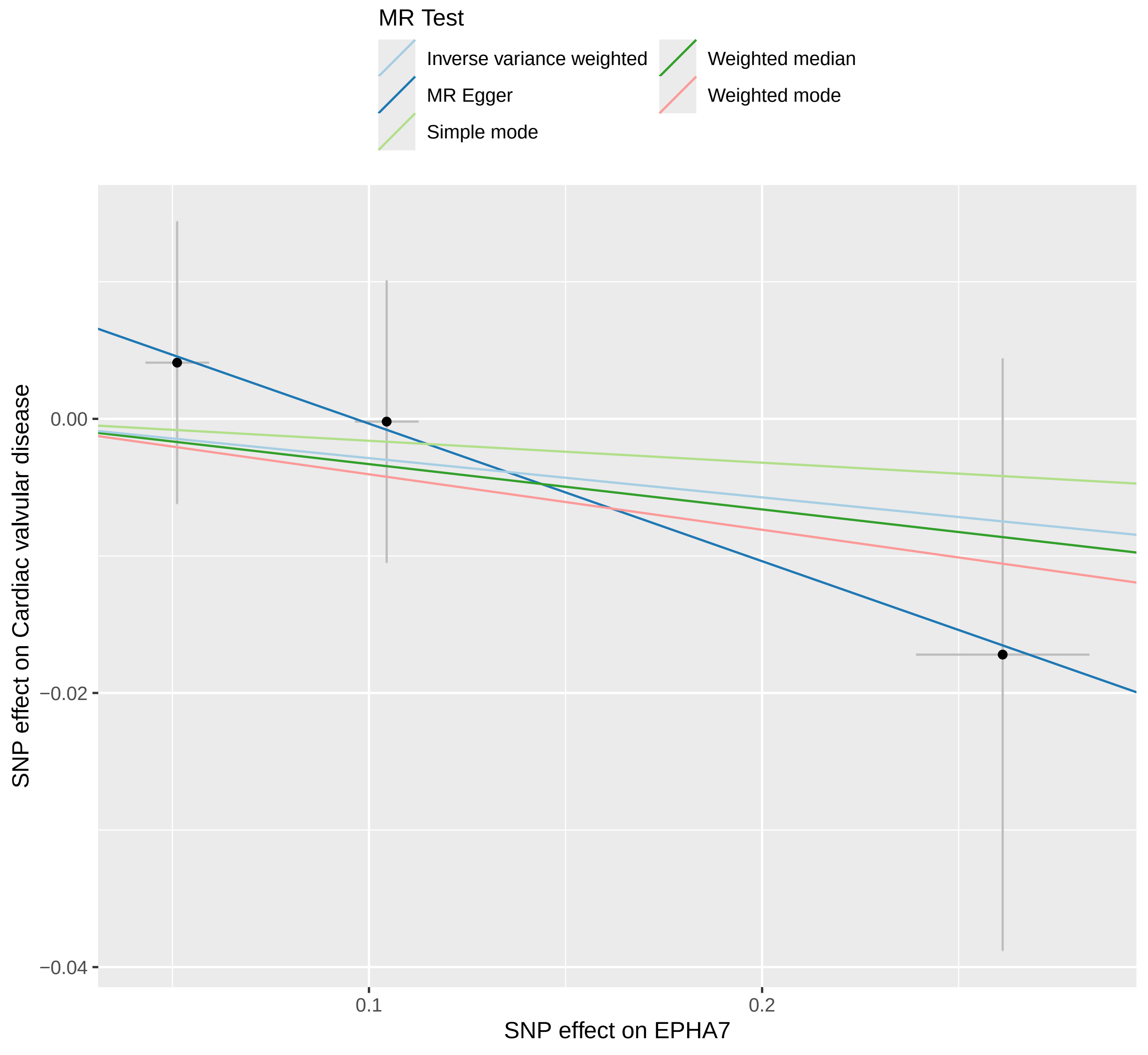
MR Test
Inverse variance weighted
MR Egger
Simple mode
Weighted median
Weighted mode
0.00
−0.02
−0.04
0.1
0.2
SNP effect on Cardiac valvular disease
SNP effect on EPHA7

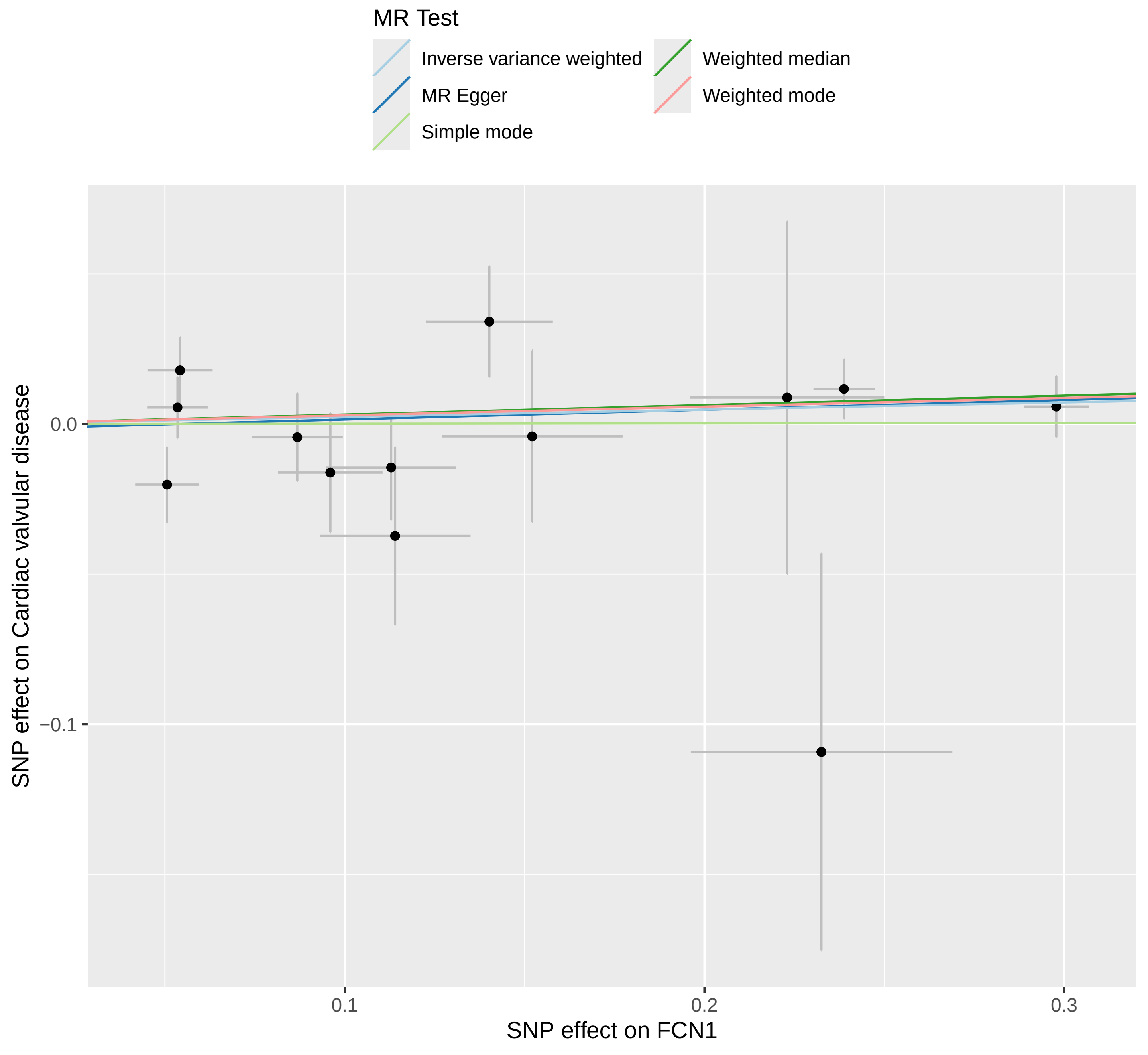
MR Test
Inverse variance weighted
MR Egger
Simple mode
Weighted median
Weighted mode
0.0
−0.1
0.1
0.2
0.3
SNP effect on FCN1
SNP effect on Cardiac valvular disease

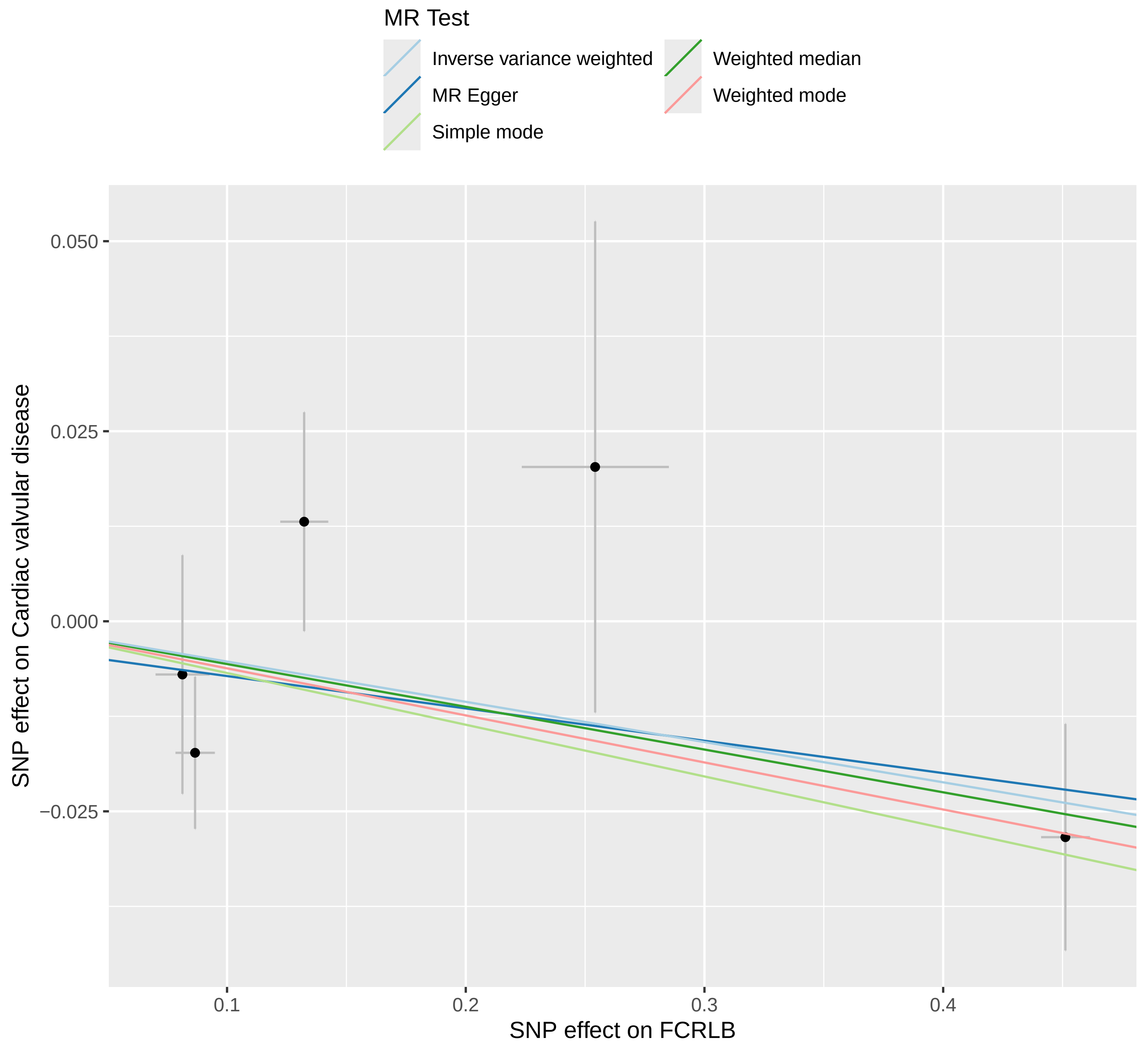
MR Test
Inverse variance weighted
MR Egger
Simple mode
Weighted median
Weighted mode
0.050
0.025
0.000
−0.025
0.1
0.2
0.3
0.4
SNP effect on Cardiac valvular disease
SNP effect on FCRLB

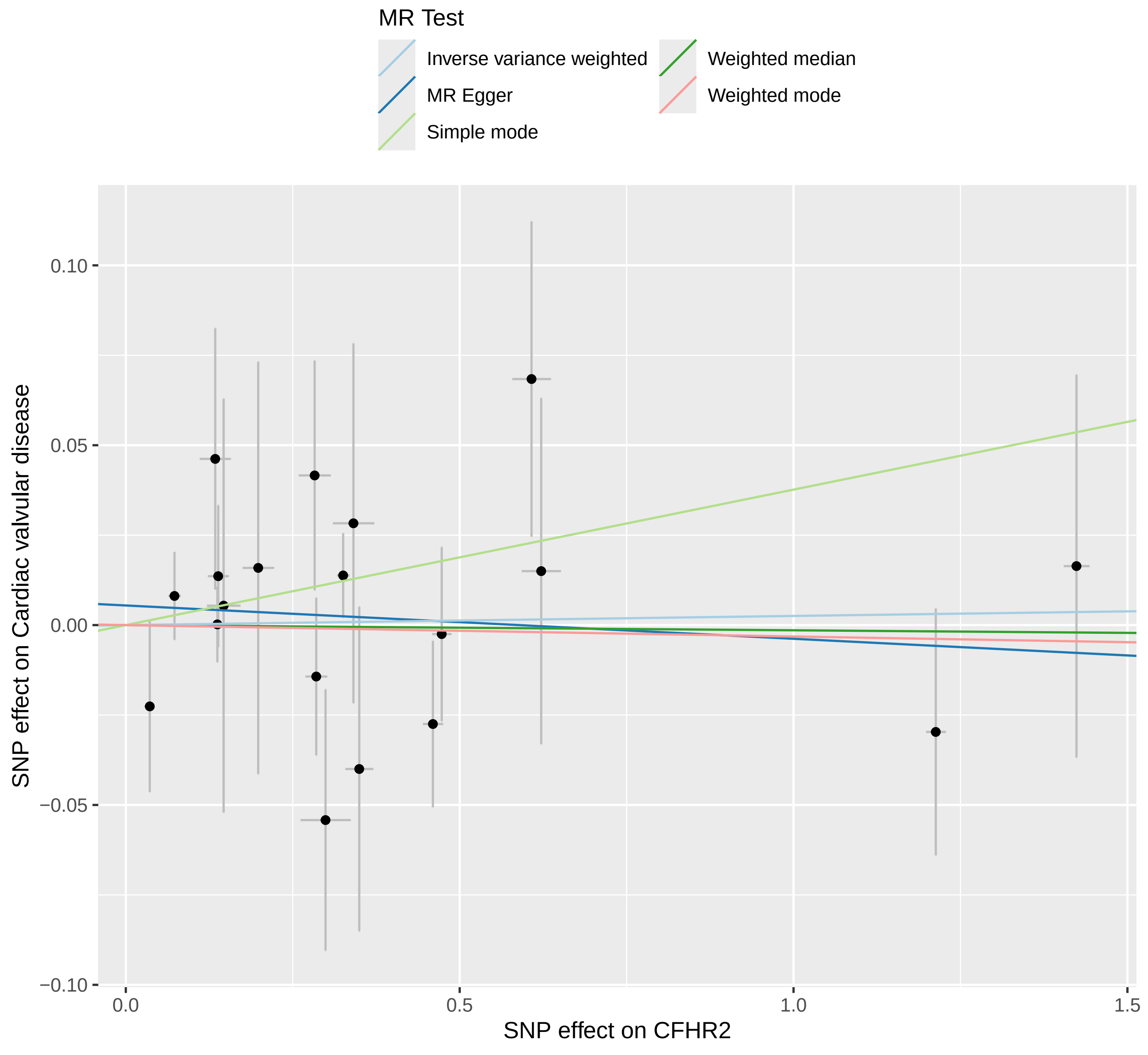

MR Test
Inverse variance weighted
MR Egger
Simple mode
Weighted median
Weighted mode
0.10
0.05
0.00
−0.05
−0.10
SNP effect on Cardiac valvular disease
0.0
0.5
1.0
1.5
SNP effect on CFHR2

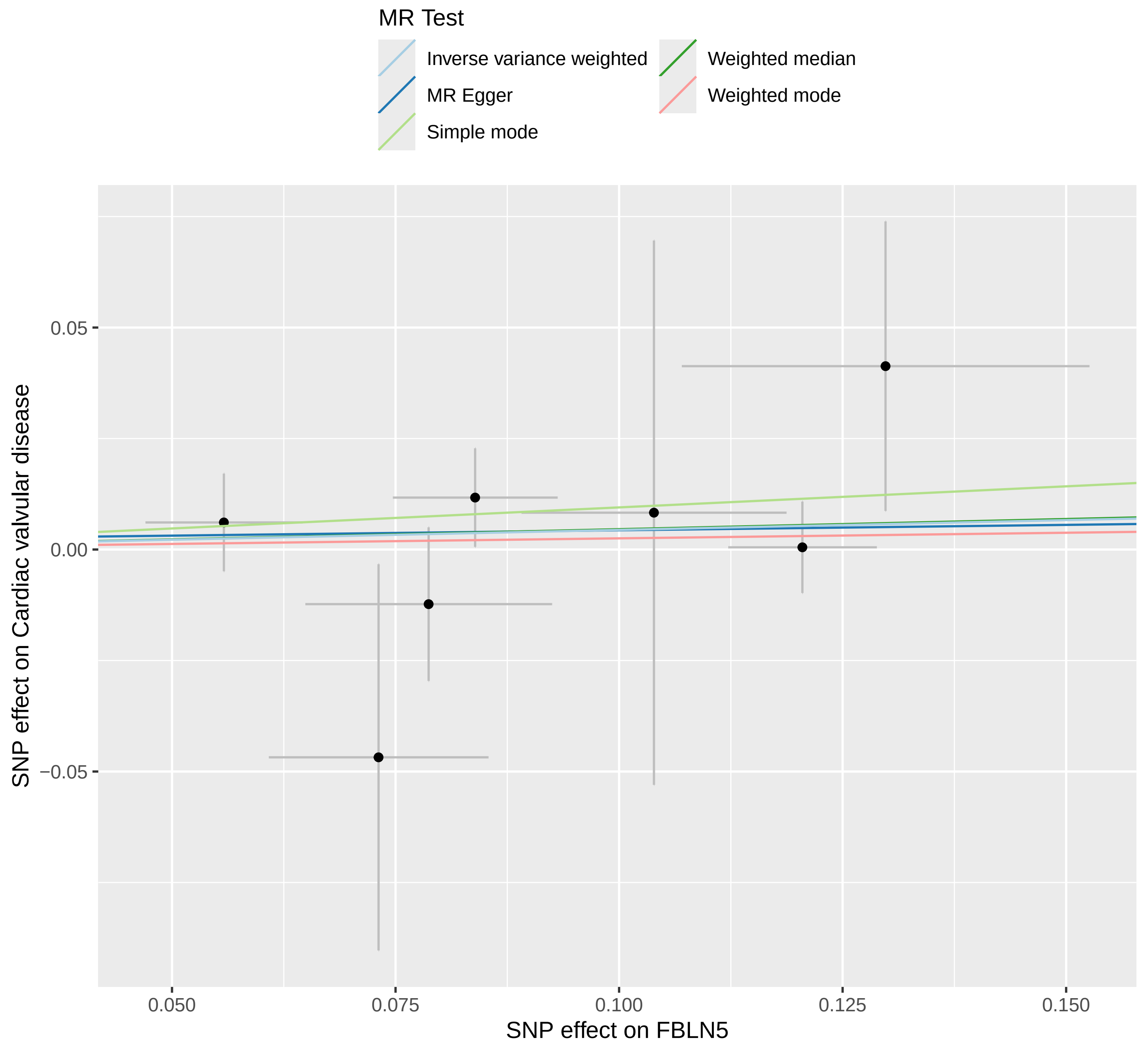
MR Test
Inverse variance weighted
MR Egger
Simple mode
Weighted median
Weighted mode
0.05
0.00
−0.05
0.050
0.075
0.100
0.125
0.150
SNP effect on FBLN5
SNP effect on Cardiac valvular disease

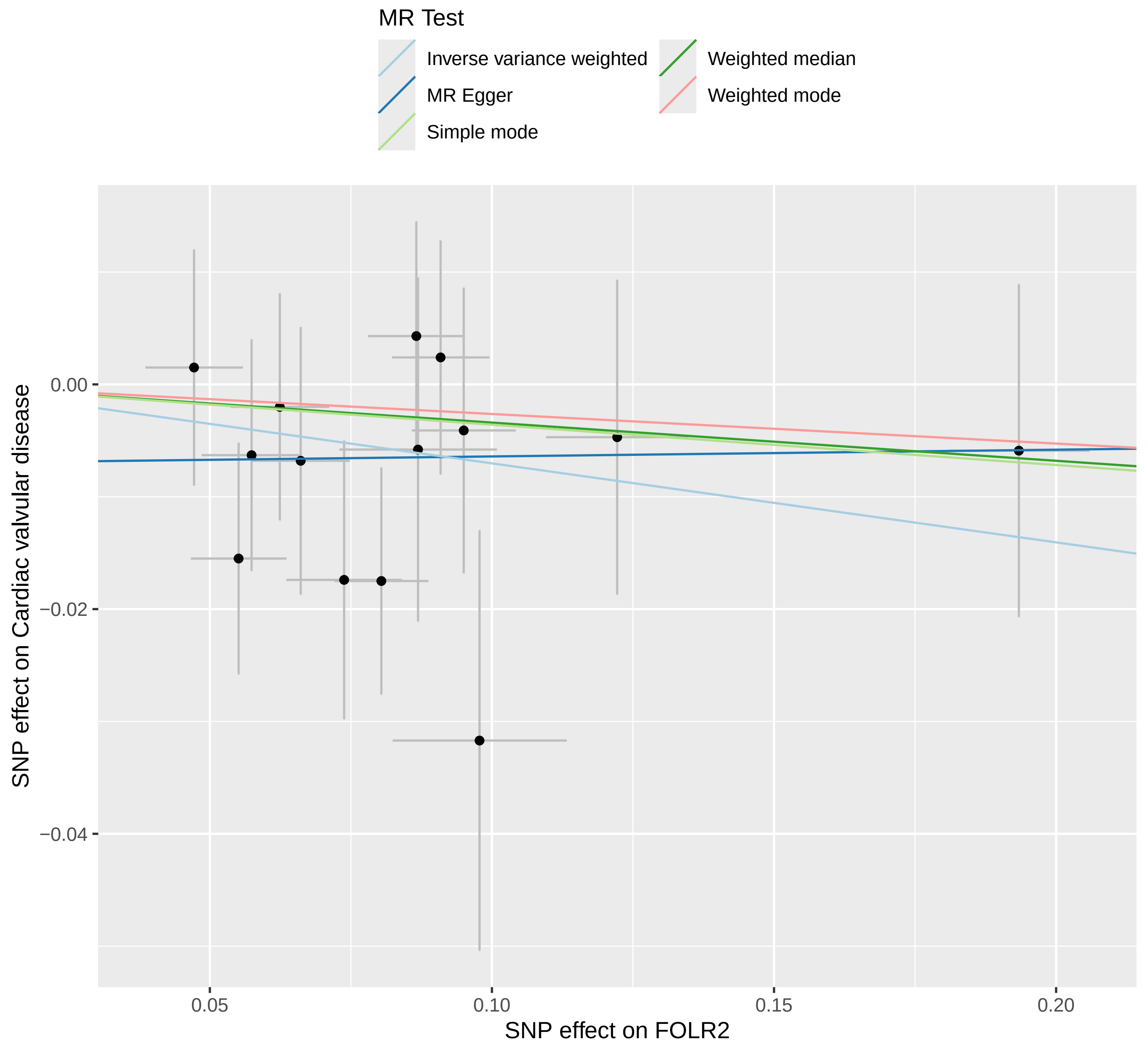
MR Test
Inverse variance weighted
MR Egger
Simple mode
Weighted median
Weighted mode
0.00
−0.02
−0.04
SNP effect on Cardiac valvular disease
0.05
0.10
0.15
0.20
SNP effect on FOLR2

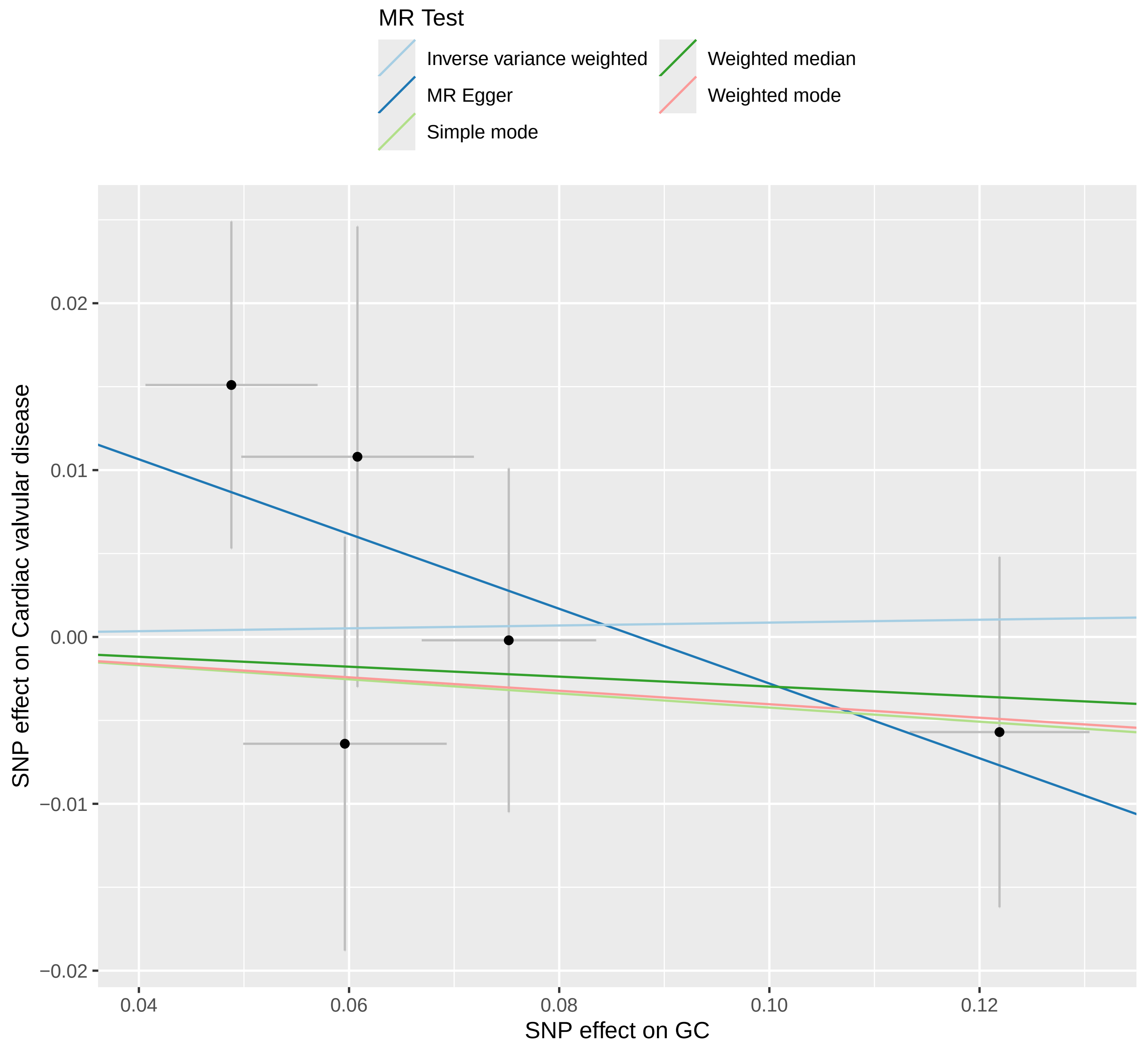
MR Test
Inverse variance weighted
MR Egger
Simple mode
Weighted median
Weighted mode
0.02
0.01
0.00
−0.01
−0.02
SNP effect on Cardiac valvular disease
0.04
0.06
0.08
0.10
0.12
SNP effect on GC

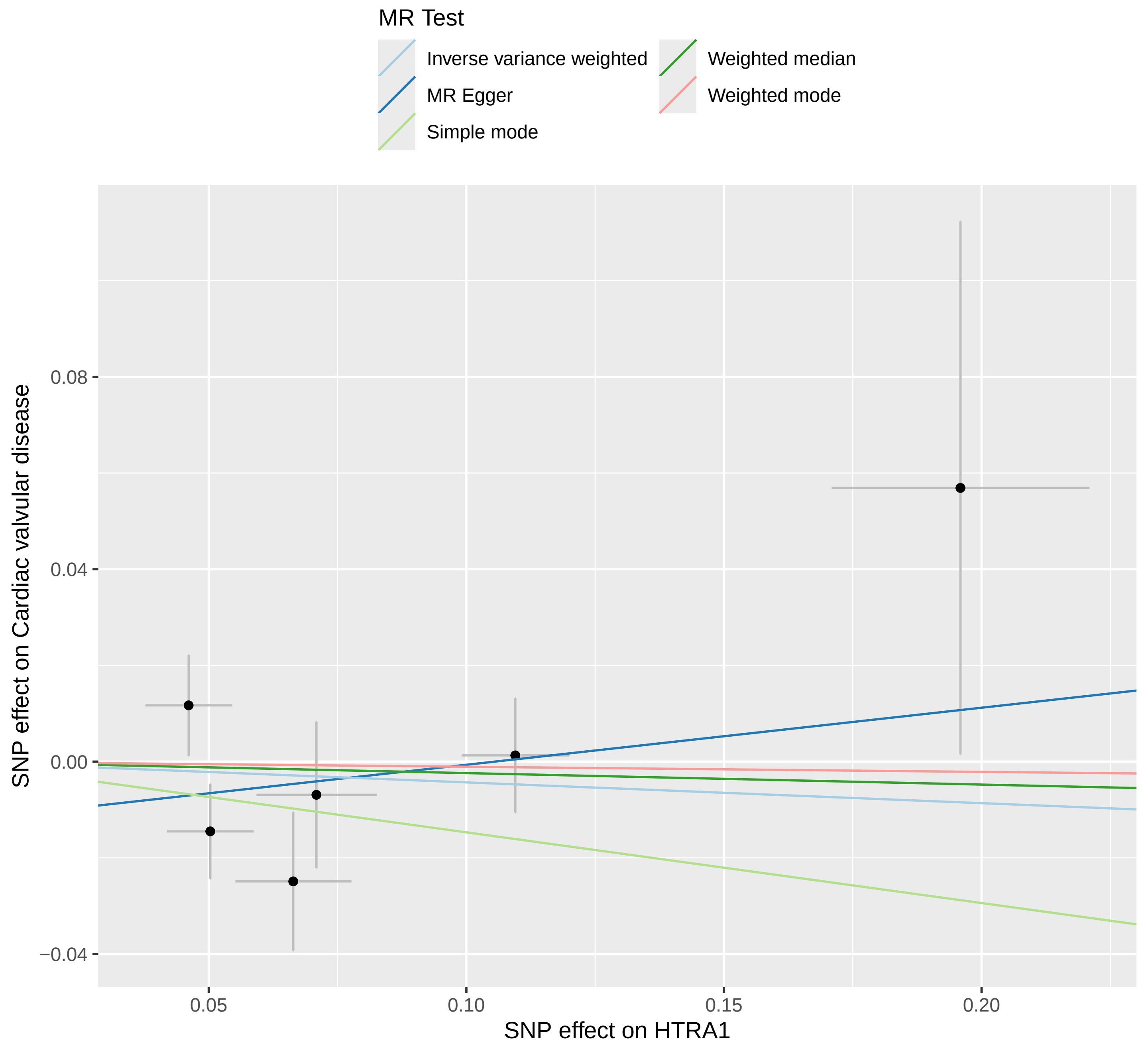
MR Test
Inverse variance weighted
MR Egger
Simple mode
Weighted median
Weighted mode
0.08
0.04
0.00
−0.04
SNP effect on Cardiac valvular disease
0.05
0.10
0.15
0.20
SNP effect on HTRA1

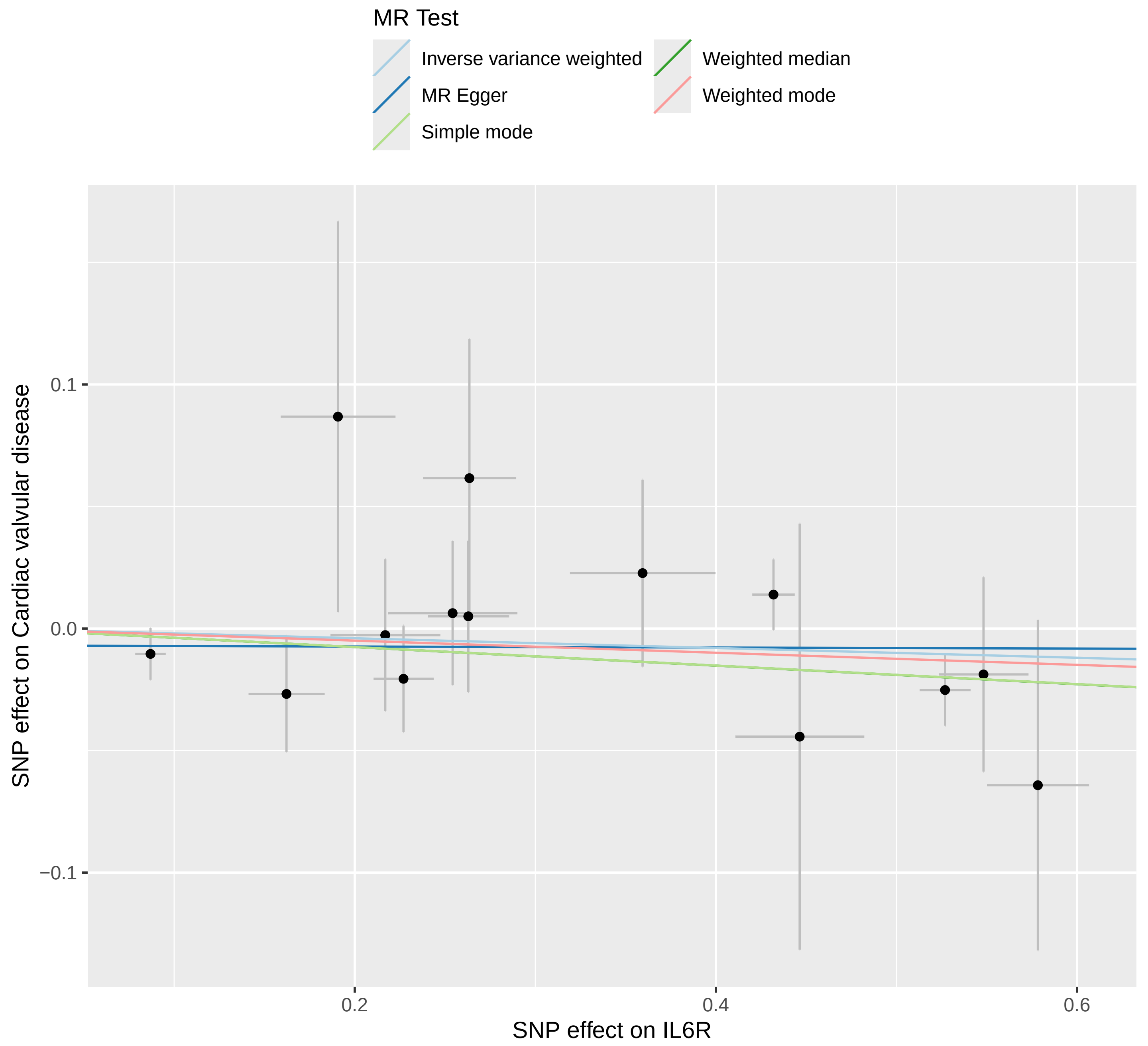
MR Test
Inverse variance weighted
MR Egger
Simple mode
Weighted median
Weighted mode
0.1
0.0
−0.1
0.2
0.4
0.6
SNP effect on Cardiac valvular disease
SNP effect on IL6R

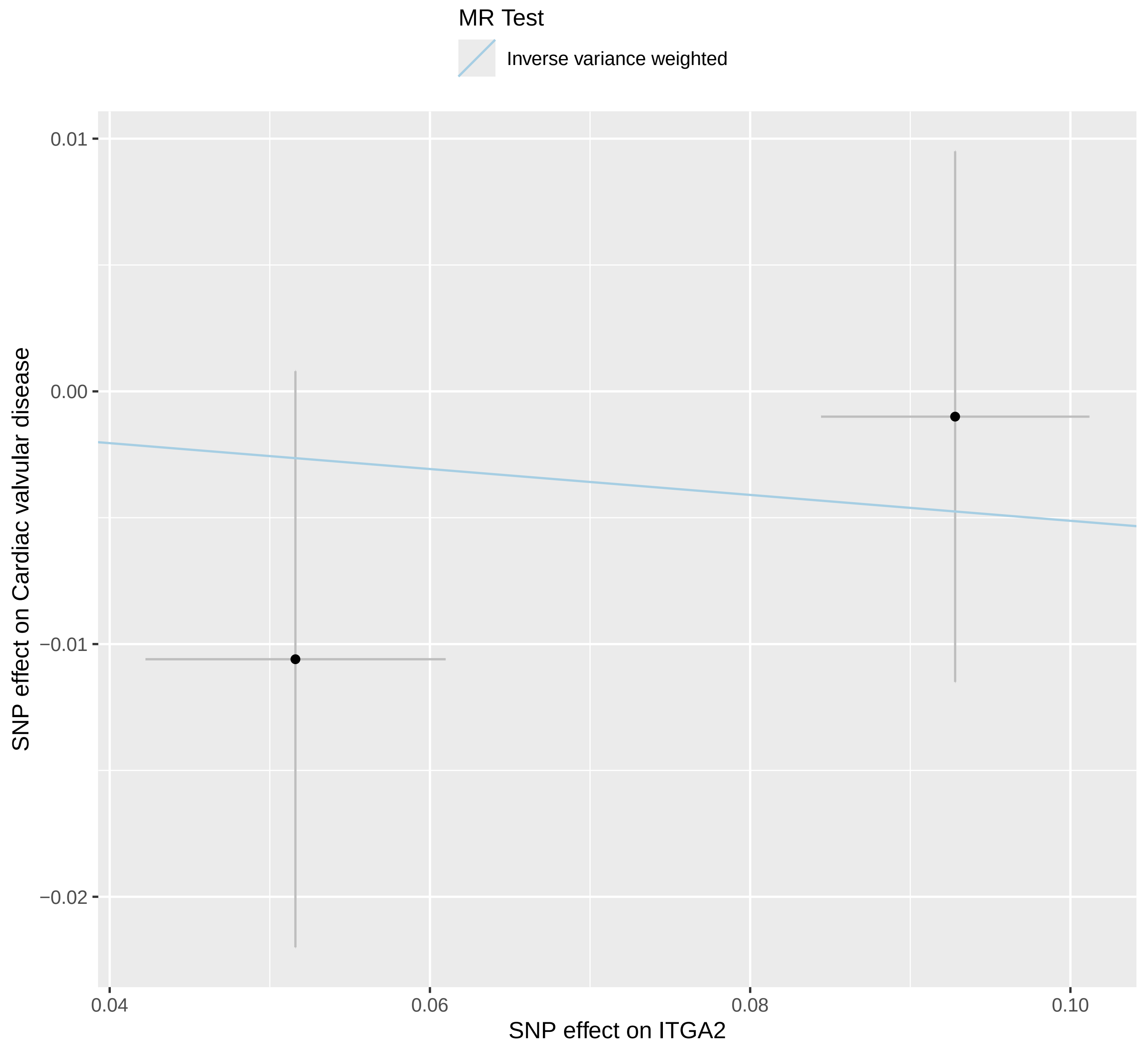
MR Test
Inverse variance weighted
0.01
0.00
−0.01
−0.02
0.04
0.06
0.08
0.10
SNP effect on ITGA2
SNP effect on Cardiac valvular disease

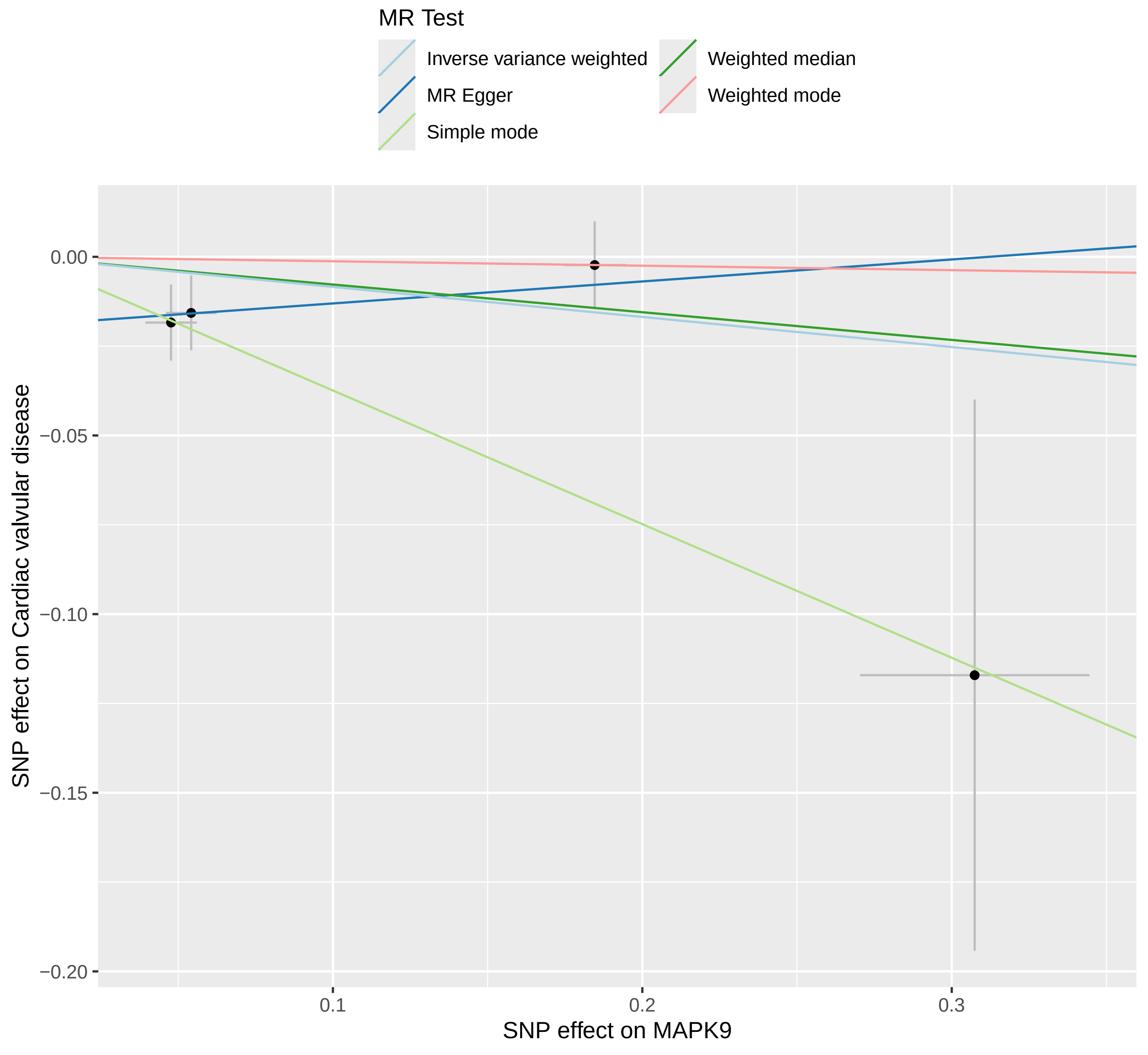
MR Test
Inverse variance weighted
MR Egger
Simple mode
Weighted median
Weighted mode
0.00
−0.05
−0.10
−0.15
−0.20
SNP effect on Cardiac valvular disease
0.1
0.2
0.3
SNP effect on MAPK9

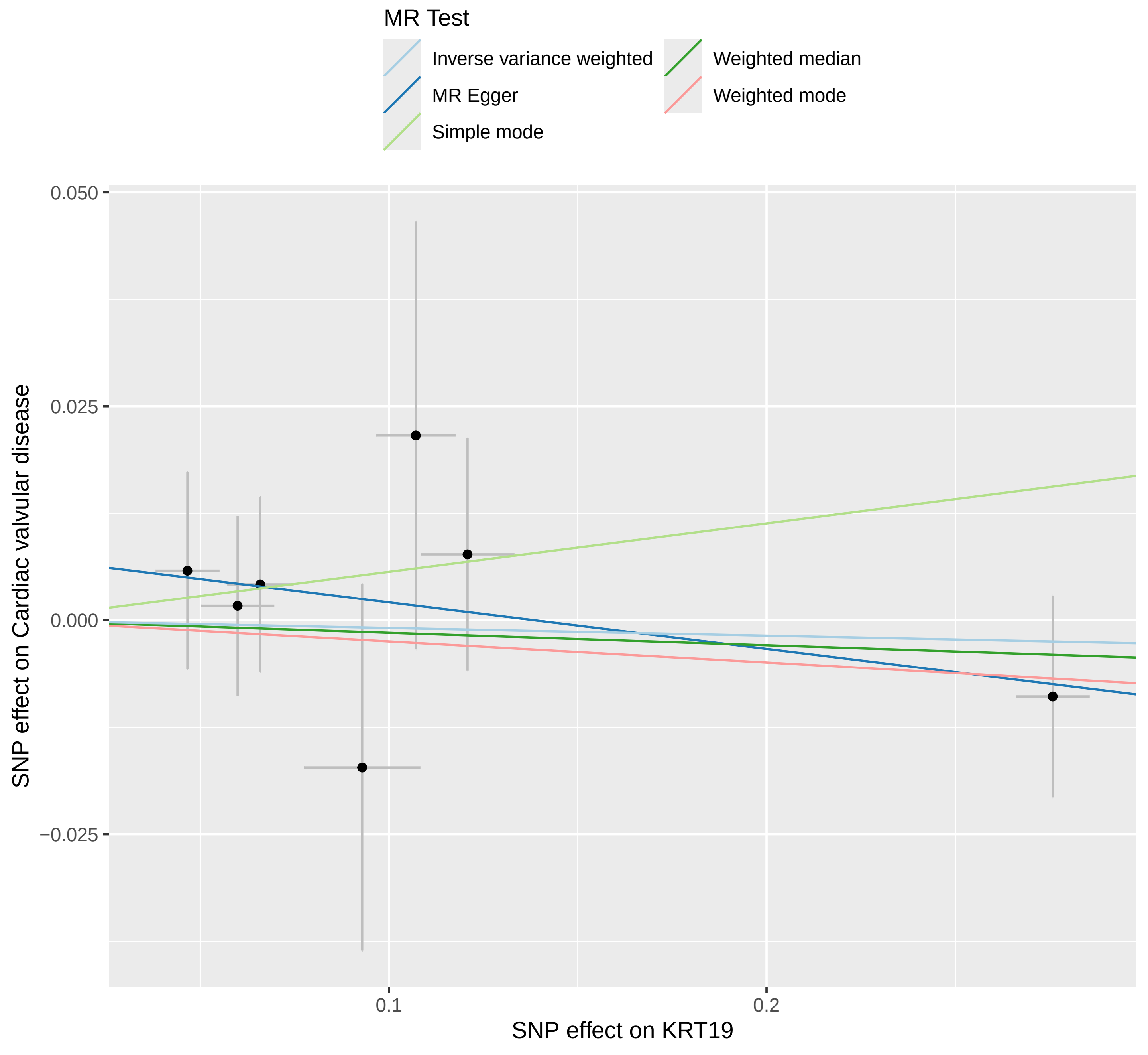
MR Test
Inverse variance weighted
MR Egger
Simple mode
Weighted median
Weighted mode
0.050
0.025
0.000
−0.025
0.1
0.2
SNP effect on Cardiac valvular disease
SNP effect on KRT19

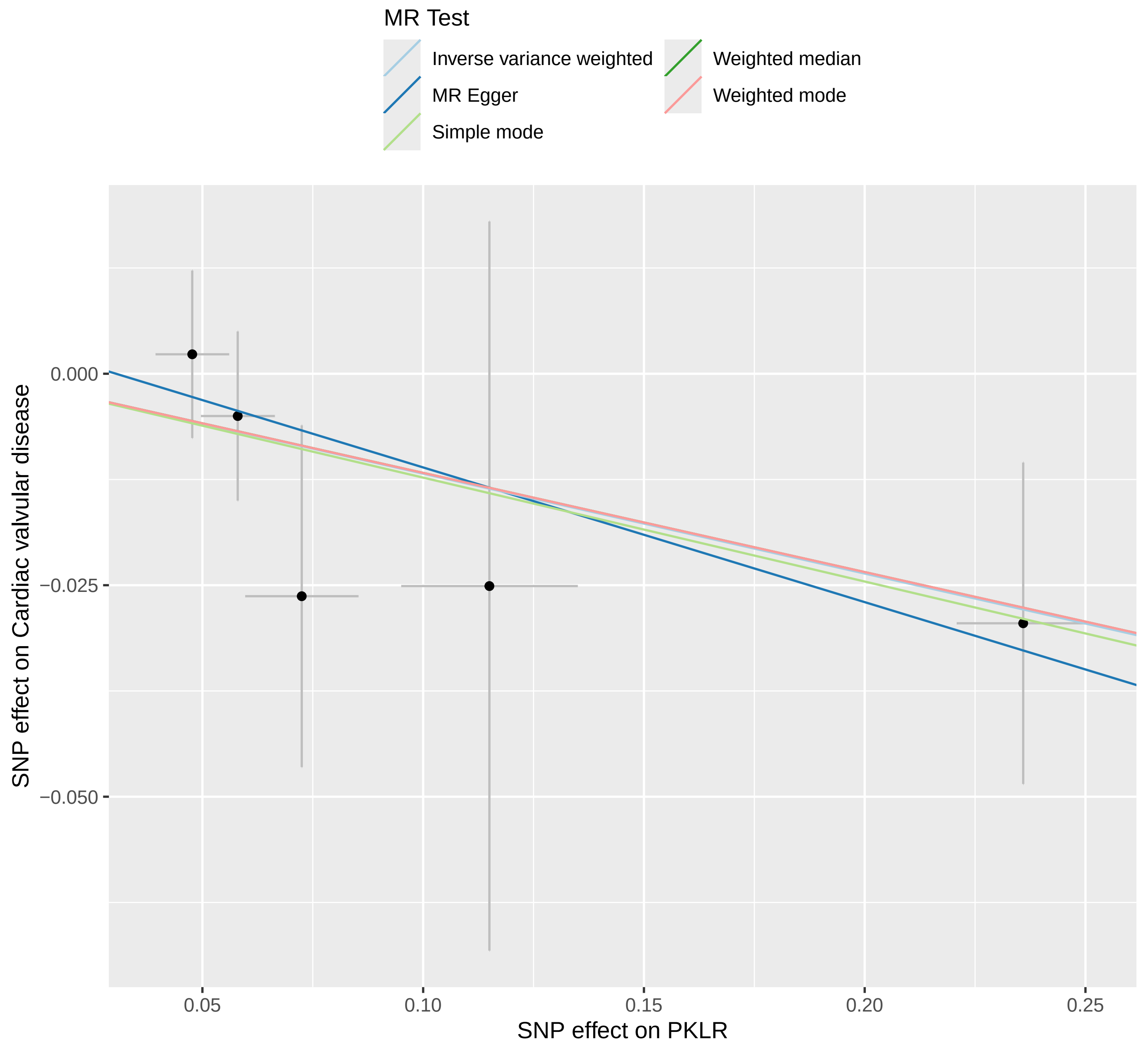
MR Test
Inverse variance weighted
MR Egger
Simple mode
Weighted median
Weighted mode
0.000
−0.025
−0.050
SNP effect on Cardiac valvular disease
0.05
0.10
0.15
0.20
0.25
SNP effect on PKLR

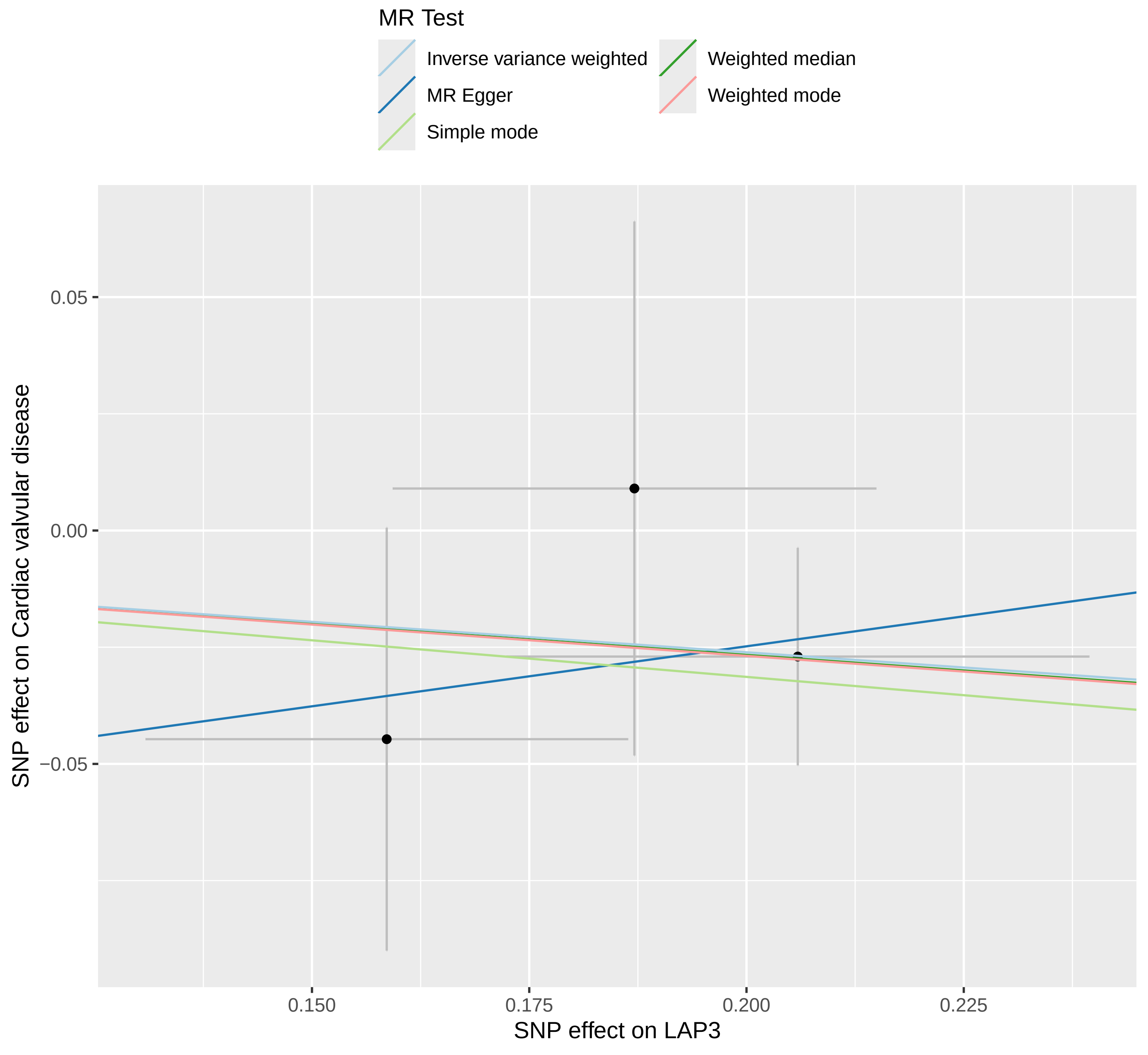

MR Test
Inverse variance weighted
MR Egger
Simple mode
Weighted median
Weighted mode
0.05
0.00
−0.05
0.150
0.175
0.200
0.225
SNP effect on Cardiac valvular disease
SNP effect on LAP3

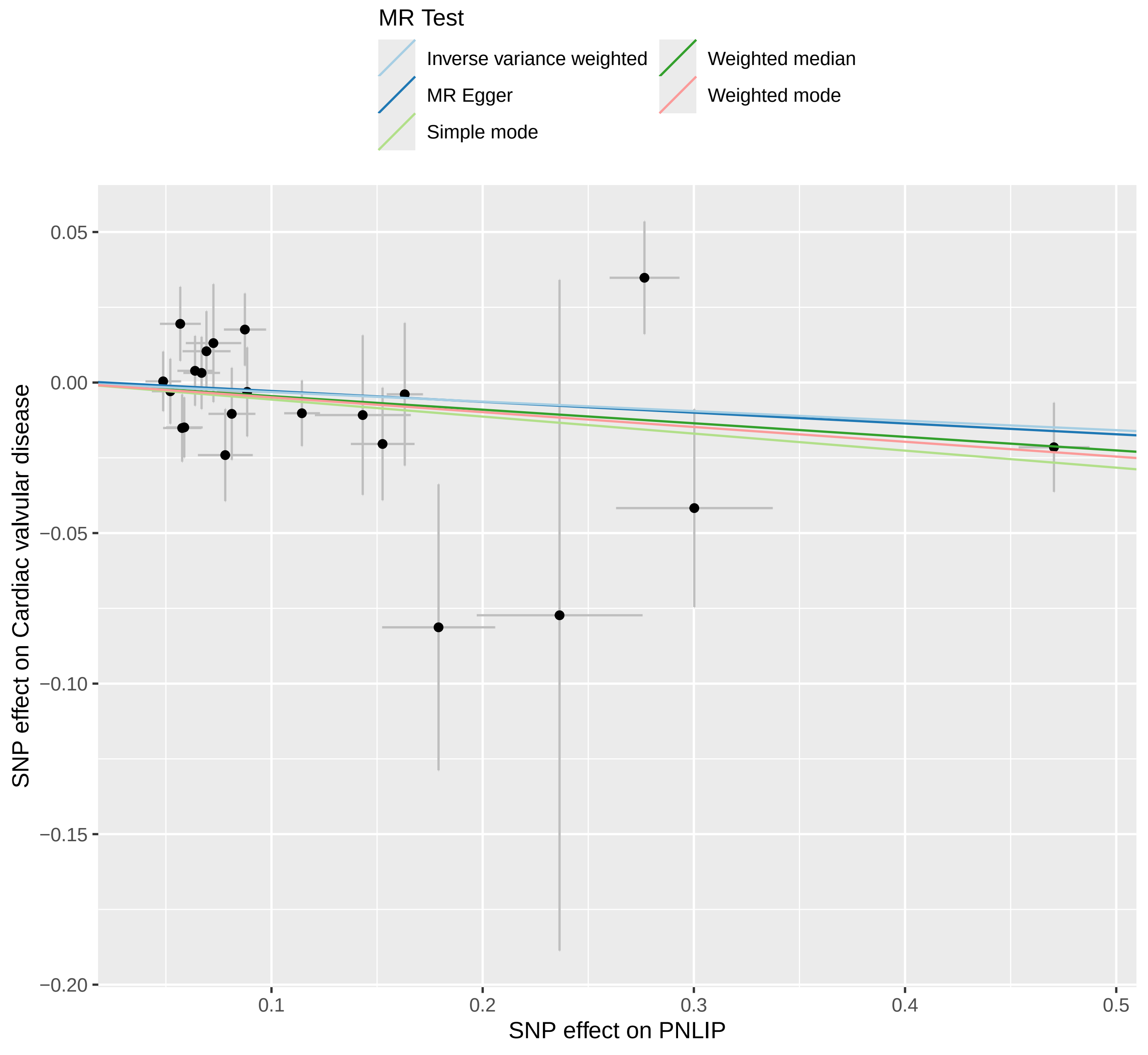

MR Test
Inverse variance weighted
MR Egger
Simple mode
Weighted median
Weighted mode
0.05
0.00
−0.05
−0.10
−0.15
−0.20
SNP effect on Cardiac valvular disease
0.1
0.2
0.3
0.4
0.5
SNP effect on PNLIP

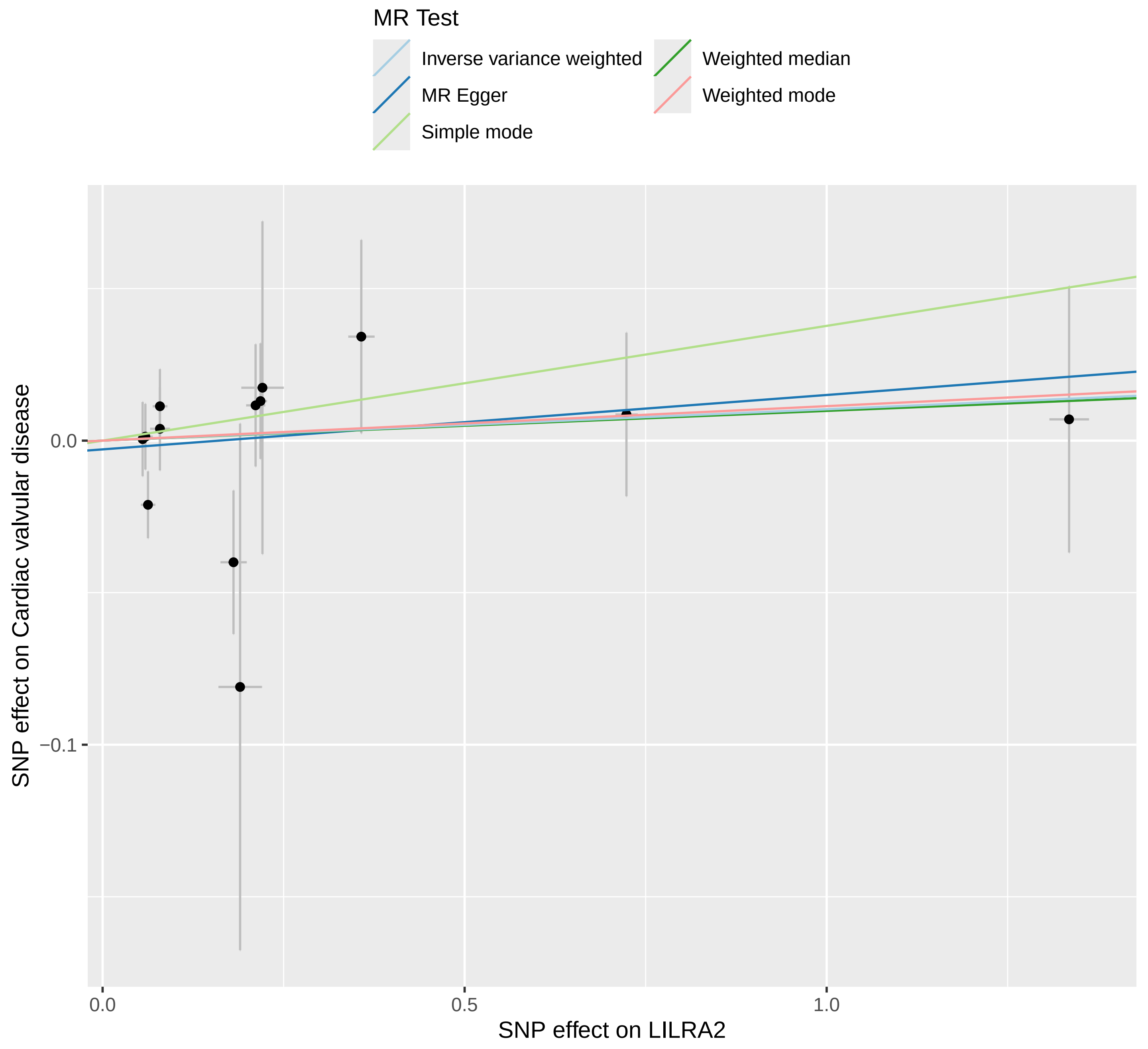
MR Test
Inverse variance weighted
MR Egger
Simple mode
Weighted median
Weighted mode
SNP effect on Cardiac valvular disease
0.0
−0.1
0.0
0.5
1.0
SNP effect on LILRA2

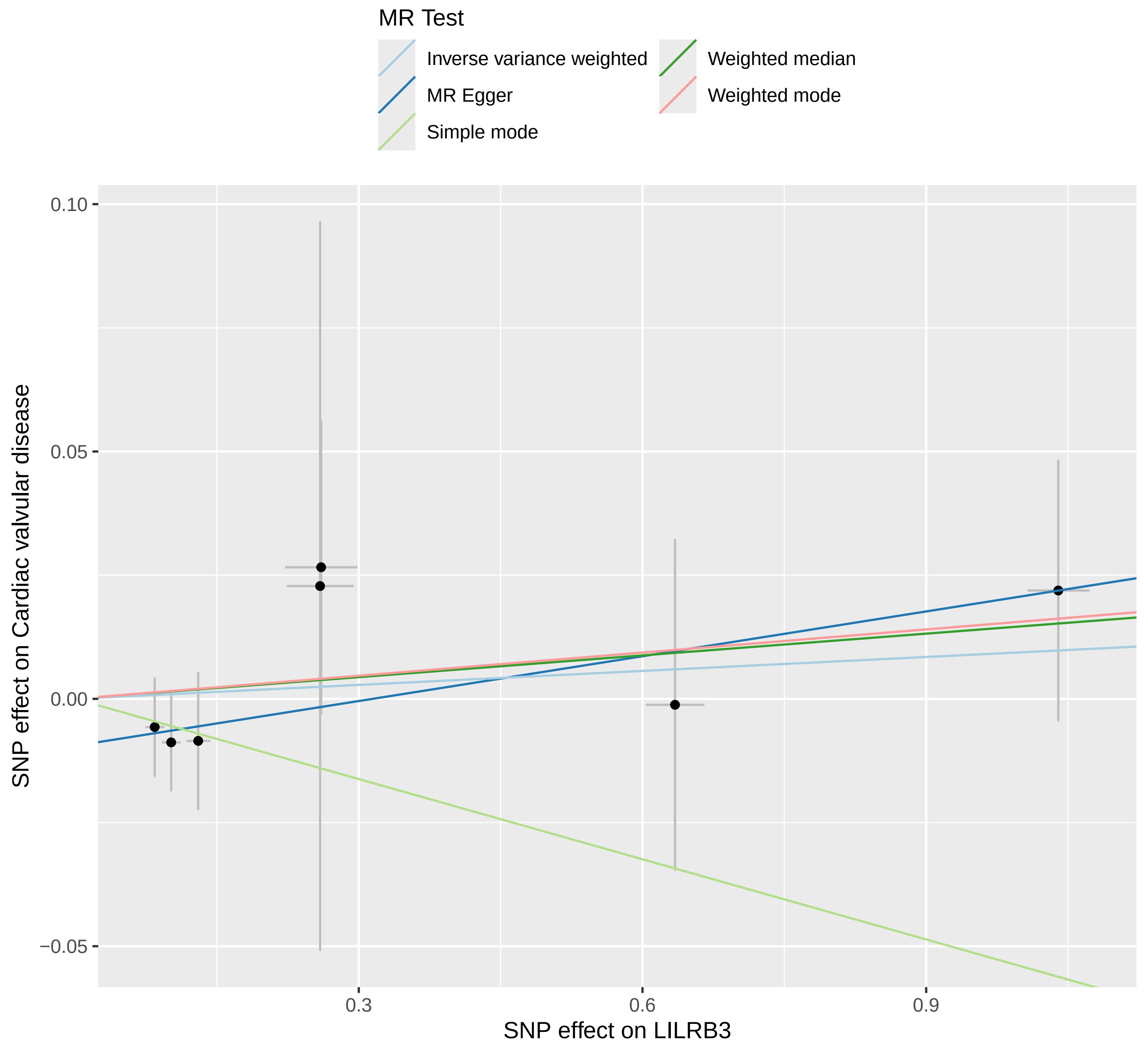
MR Test
Inverse variance weighted
MR Egger
Simple mode
Weighted median
Weighted mode
0.10
0.05
0.00
−0.05
0.3
0.6
0.9
SNP effect on Cardiac valvular disease
SNP effect on LILRB3

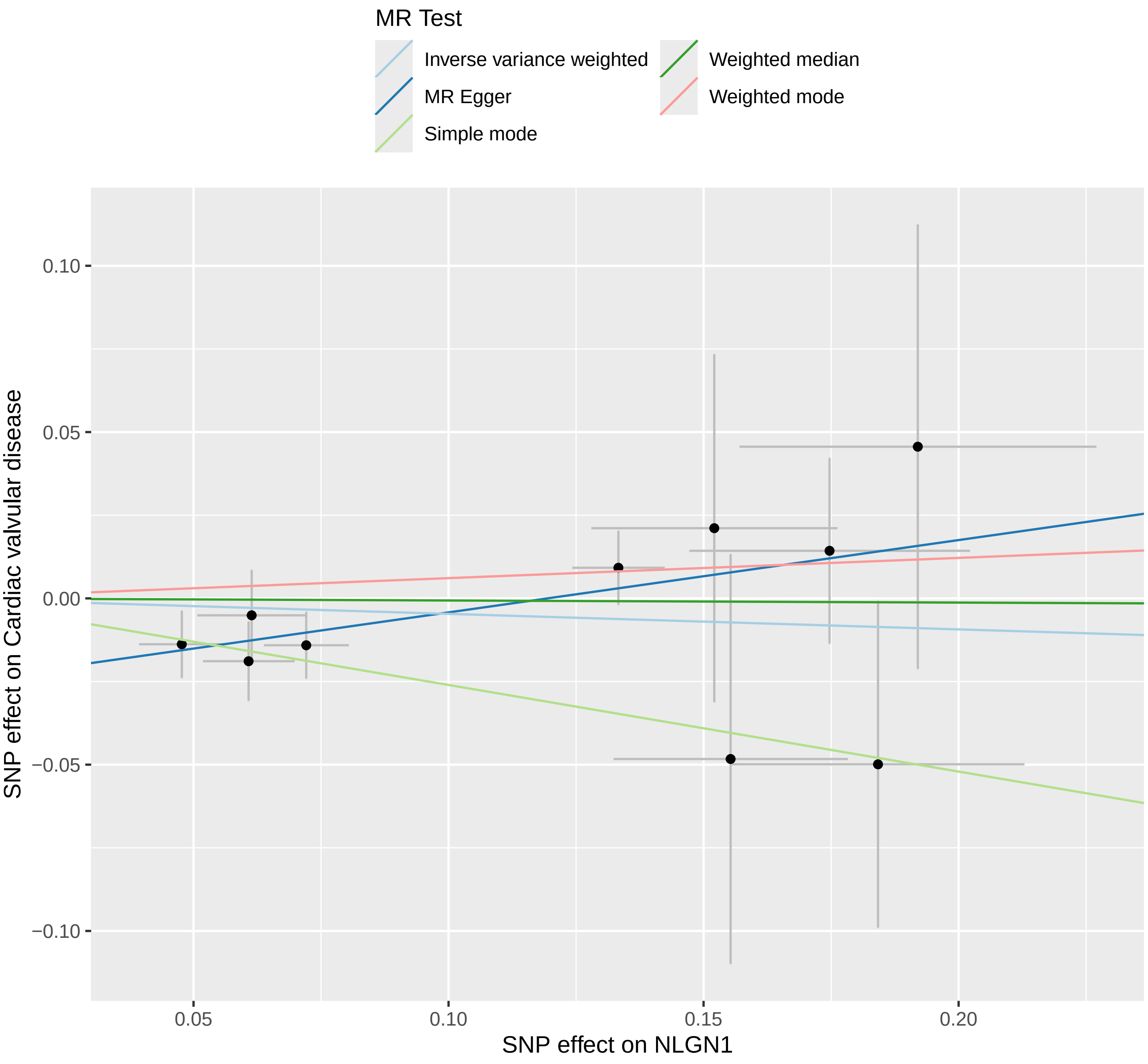

MR Test
Inverse variance weighted
MR Egger
Simple mode
Weighted median
Weighted mode
0.10
0.05
0.00
−0.05
−0.10
SNP effect on Cardiac valvular disease
0.05
0.10
0.15
0.20
SNP effect on NLGN1

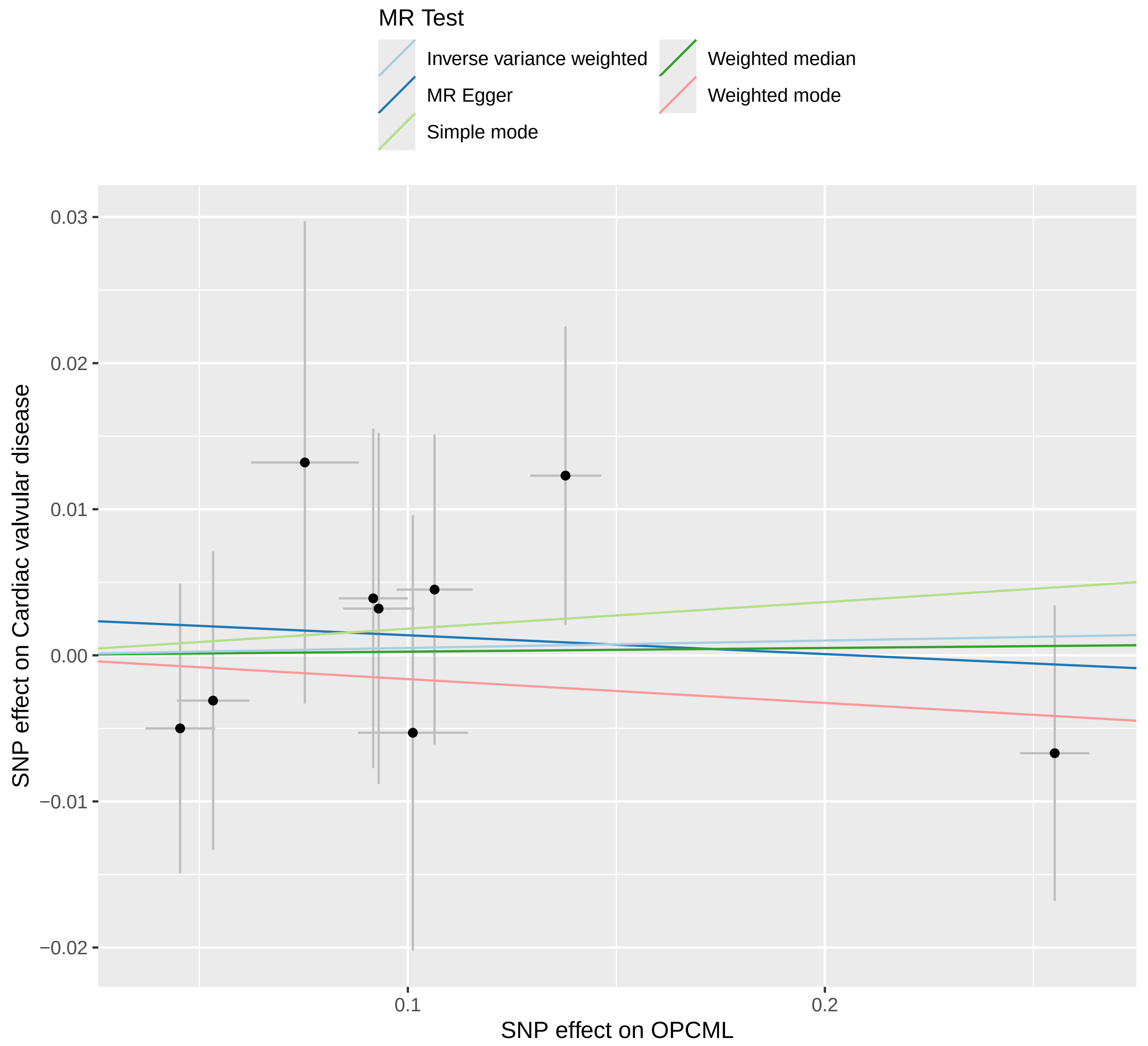
MR Test
Inverse variance weighted
MR Egger
Simple mode
Weighted median
Weighted mode
0.03
0.02
0.01
0.00
−0.01
−0.02
0.1
0.2
SNP effect on Cardiac valvular disease
SNP effect on OPCML

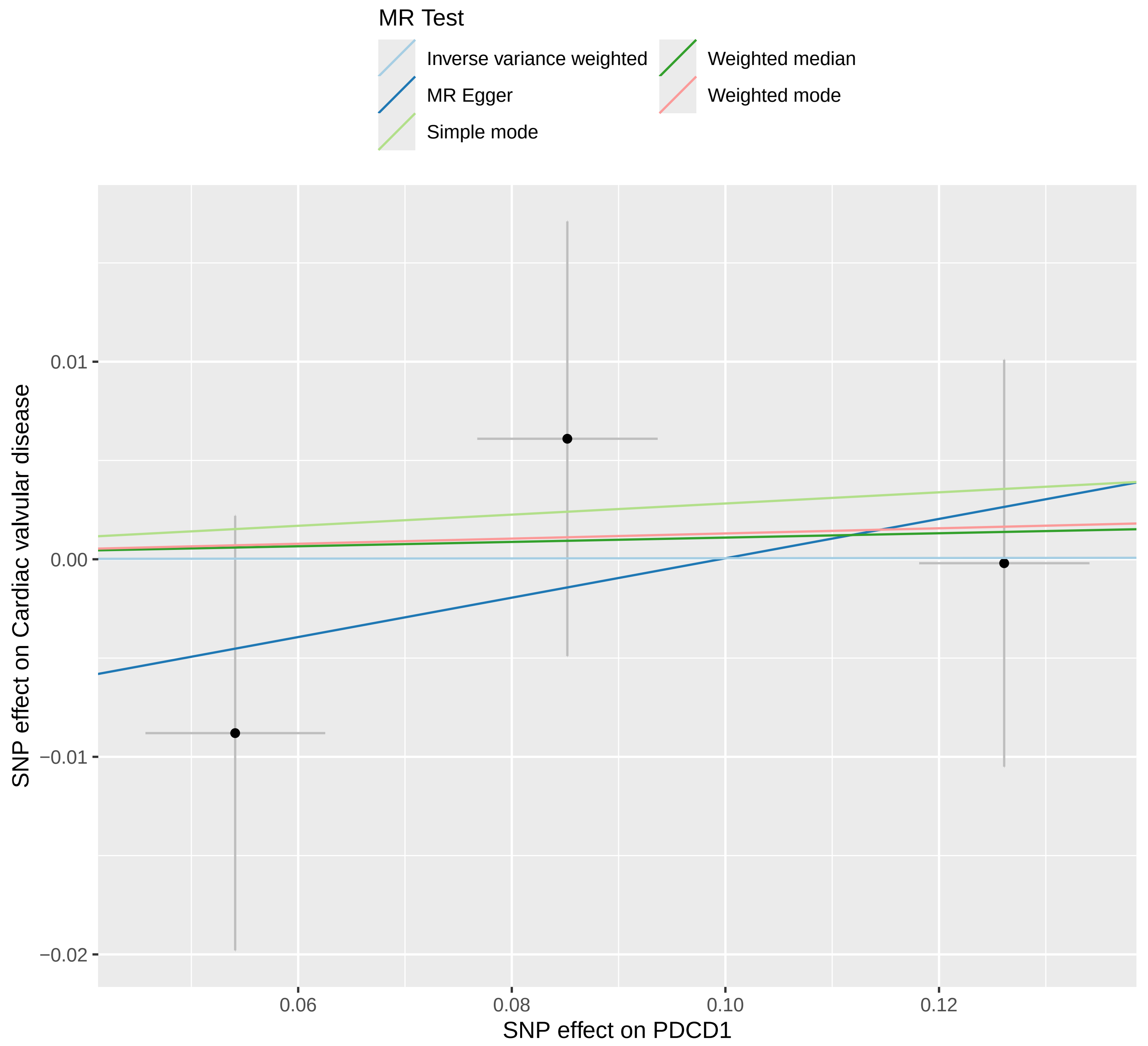

MR Test
Inverse variance weighted
MR Egger
Simple mode
Weighted median
Weighted mode
0.01
0.00
−0.01
−0.02
0.06
0.08
0.10
0.12
SNP effect on Cardiac valvular disease
SNP effect on PDCD1

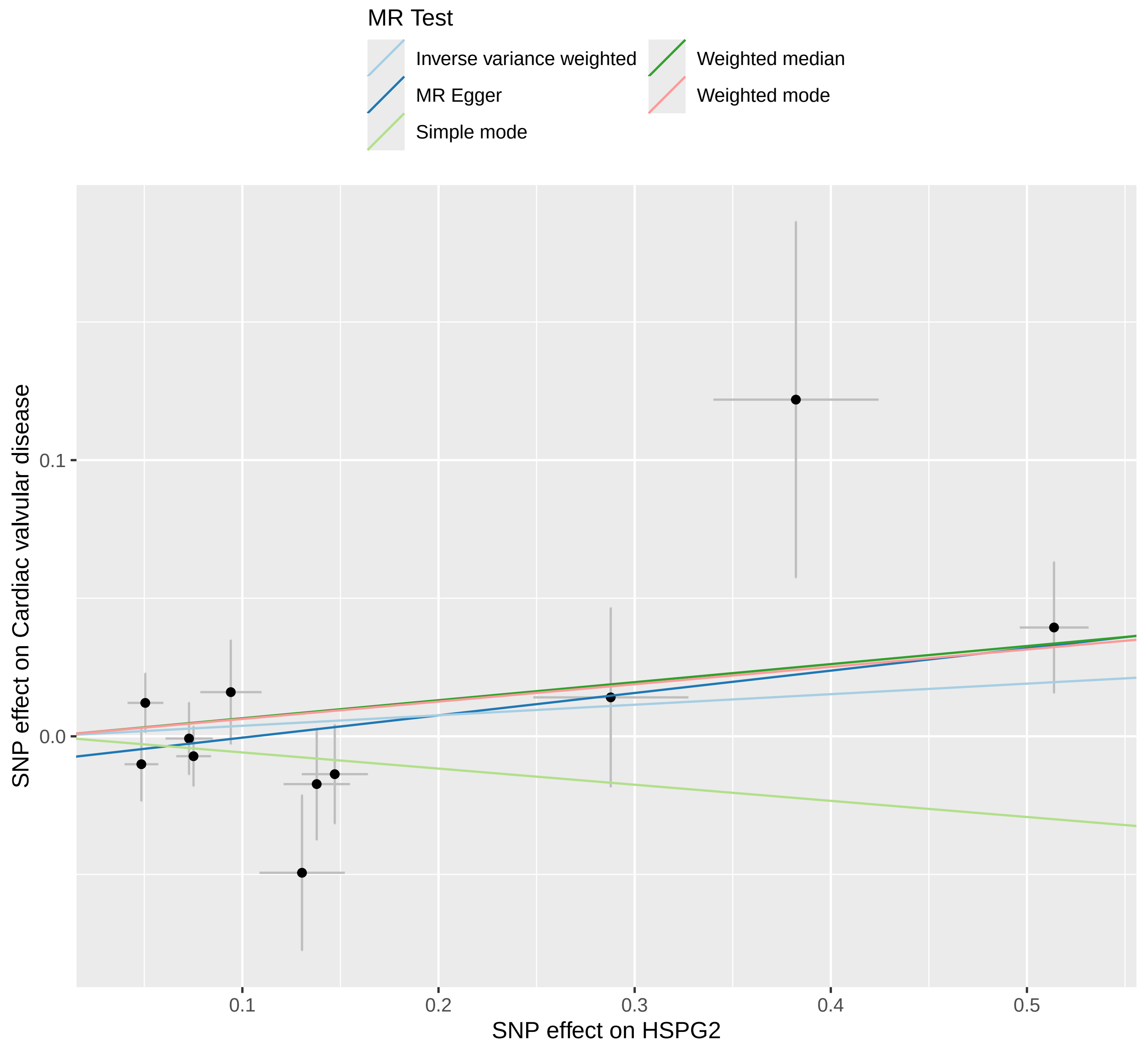

MR Test
Inverse variance weighted
MR Egger
Simple mode
Weighted median
Weighted mode
0.1
0.0
0.1
0.2
0.3
0.4
0.5
SNP effect on HSPG2
SNP effect on Cardiac valvular disease

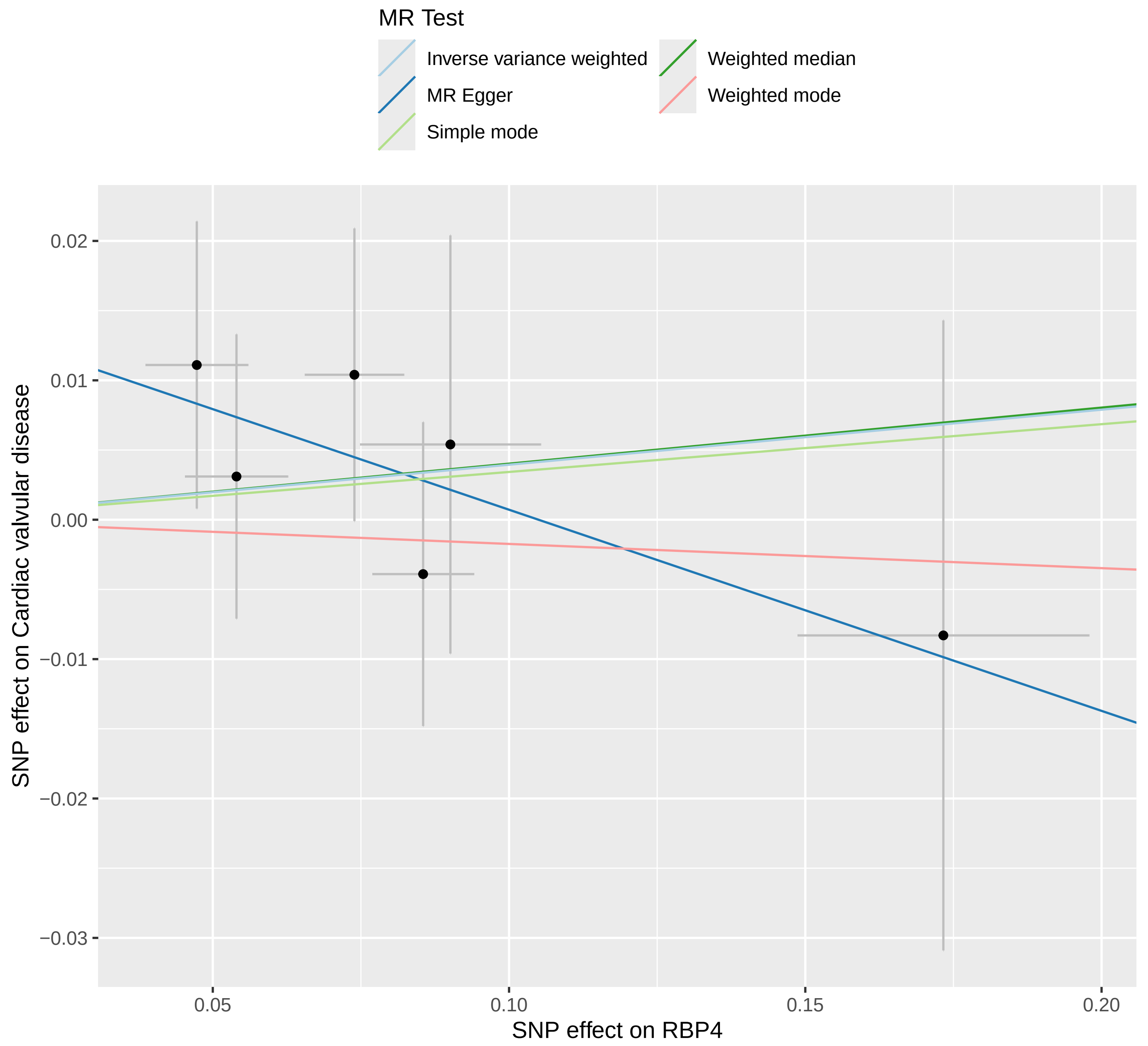
MR Test
Inverse variance weighted
MR Egger
Simple mode
Weighted median
Weighted mode
SNP effect on Cardiac valvular disease
0.02
0.01
0.00
−0.01
−0.02
−0.03
0.05
0.10
0.15
0.20
SNP effect on RBP4

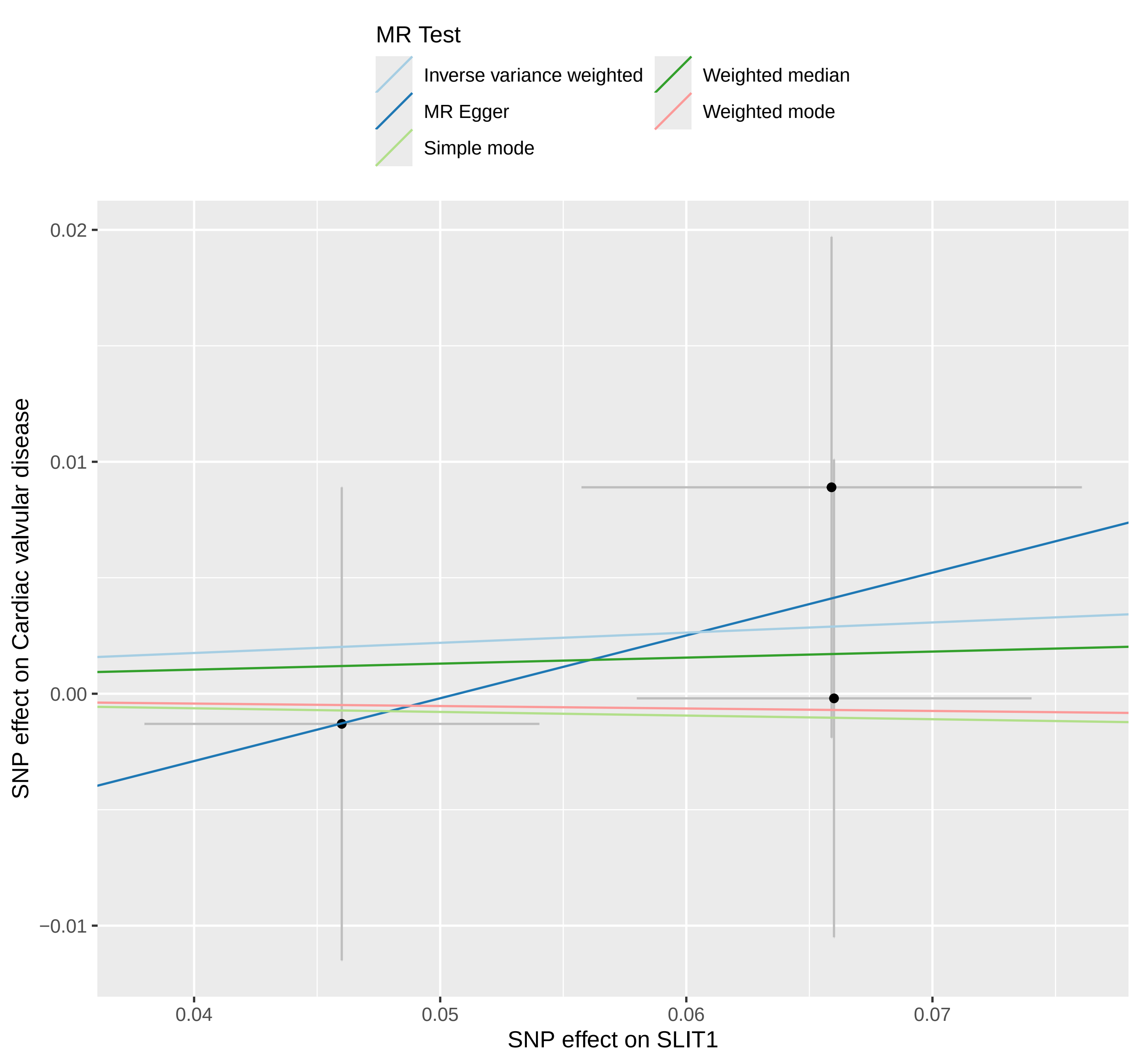

MR Test
Inverse variance weighted
MR Egger
Simple mode
Weighted median
Weighted mode
0.02
0.01
0.00
−0.01
0.04
0.05
0.06
0.07
SNP effect on Cardiac valvular disease
SNP effect on SLIT1

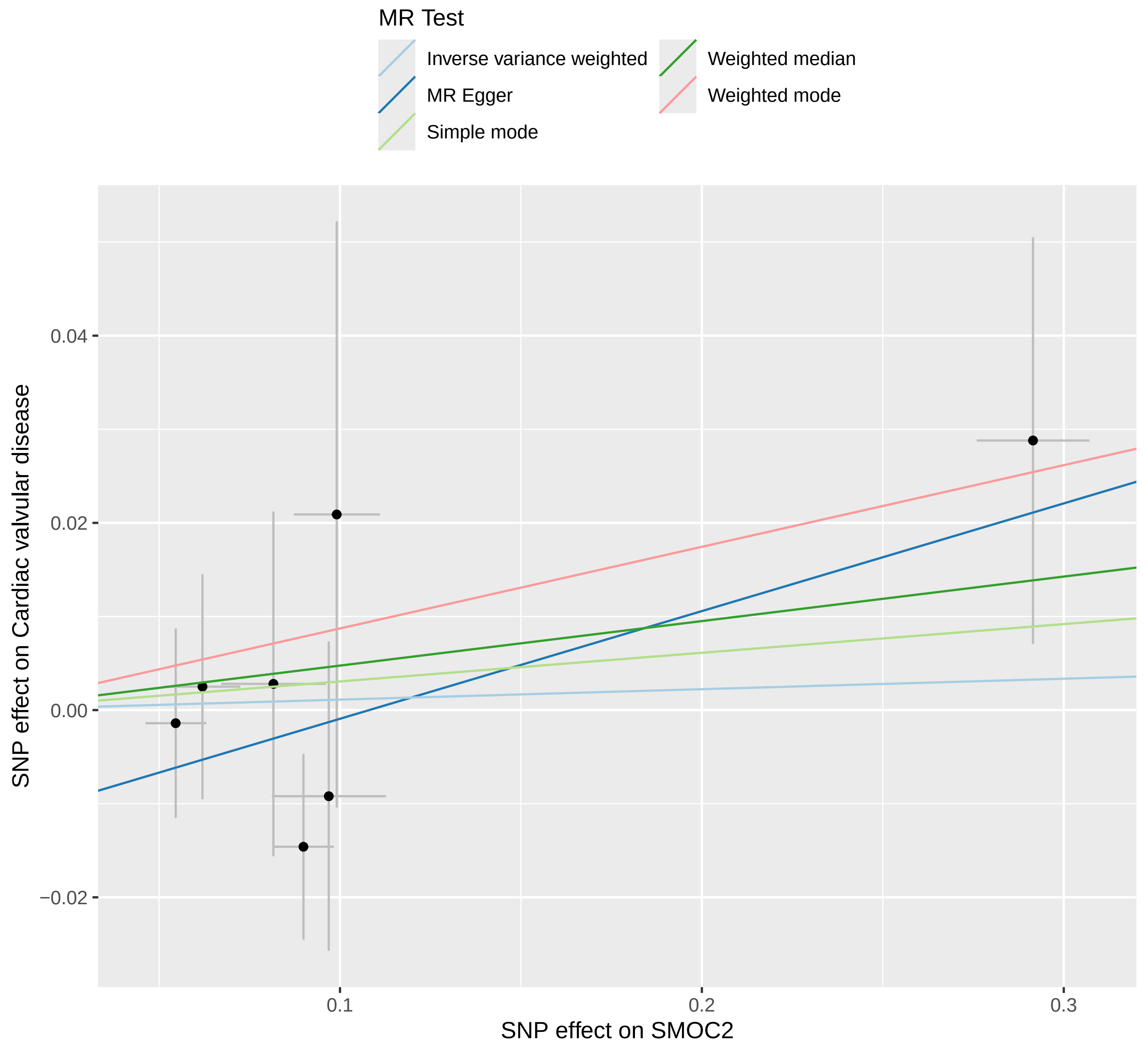
MR Test
Inverse variance weighted
MR Egger
Simple mode
Weighted median
Weighted mode
0.04
0.02
0.00
−0.02
SNP effect on Cardiac valvular disease
0.1
0.2
0.3
SNP effect on SMOC2

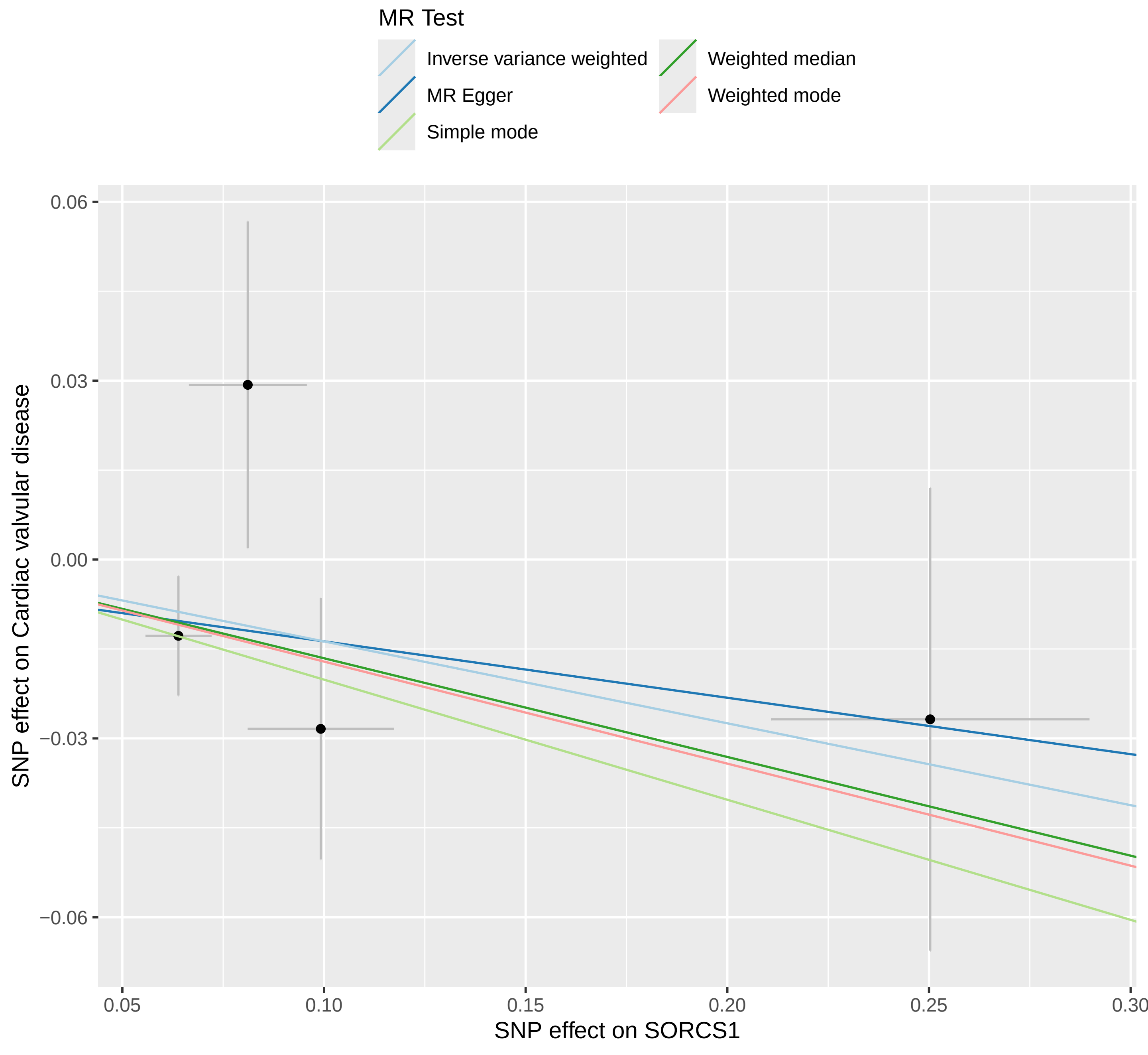
MR Test
Inverse variance weighted
MR Egger
Simple mode
Weighted median
Weighted mode
0.06
0.03
0.00
−0.03
−0.06
0.05
0.10
0.15
0.20
0.25
0.30
SNP effect on SORCS1
SNP effect on Cardiac valvular disease

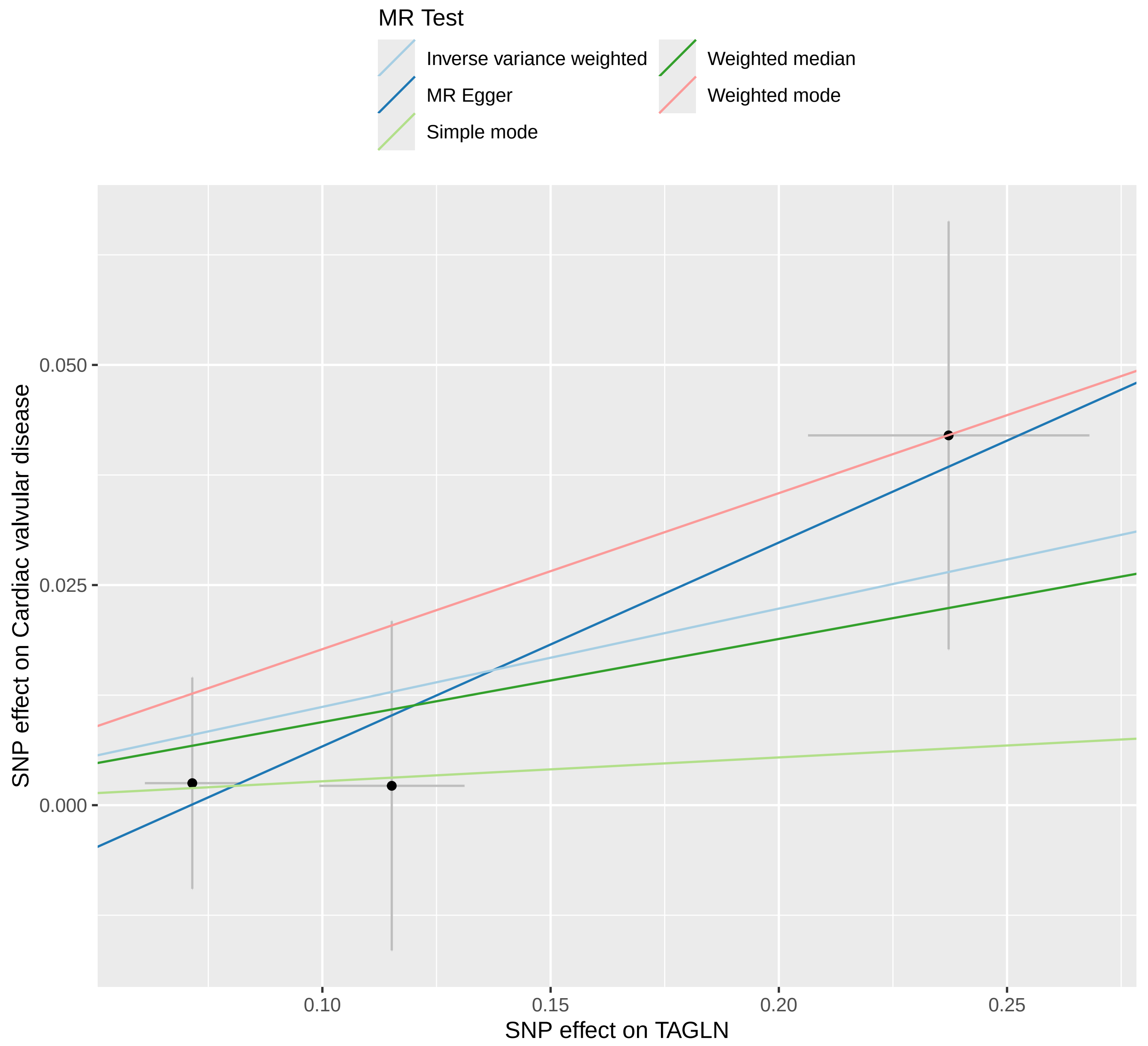

MR Test
Inverse variance weighted
MR Egger
Simple mode
Weighted median
Weighted mode
0.050
0.025
0.000
SNP effect on Cardiac valvular disease
0.10
0.15
0.20
0.25
SNP effect on TAGLN

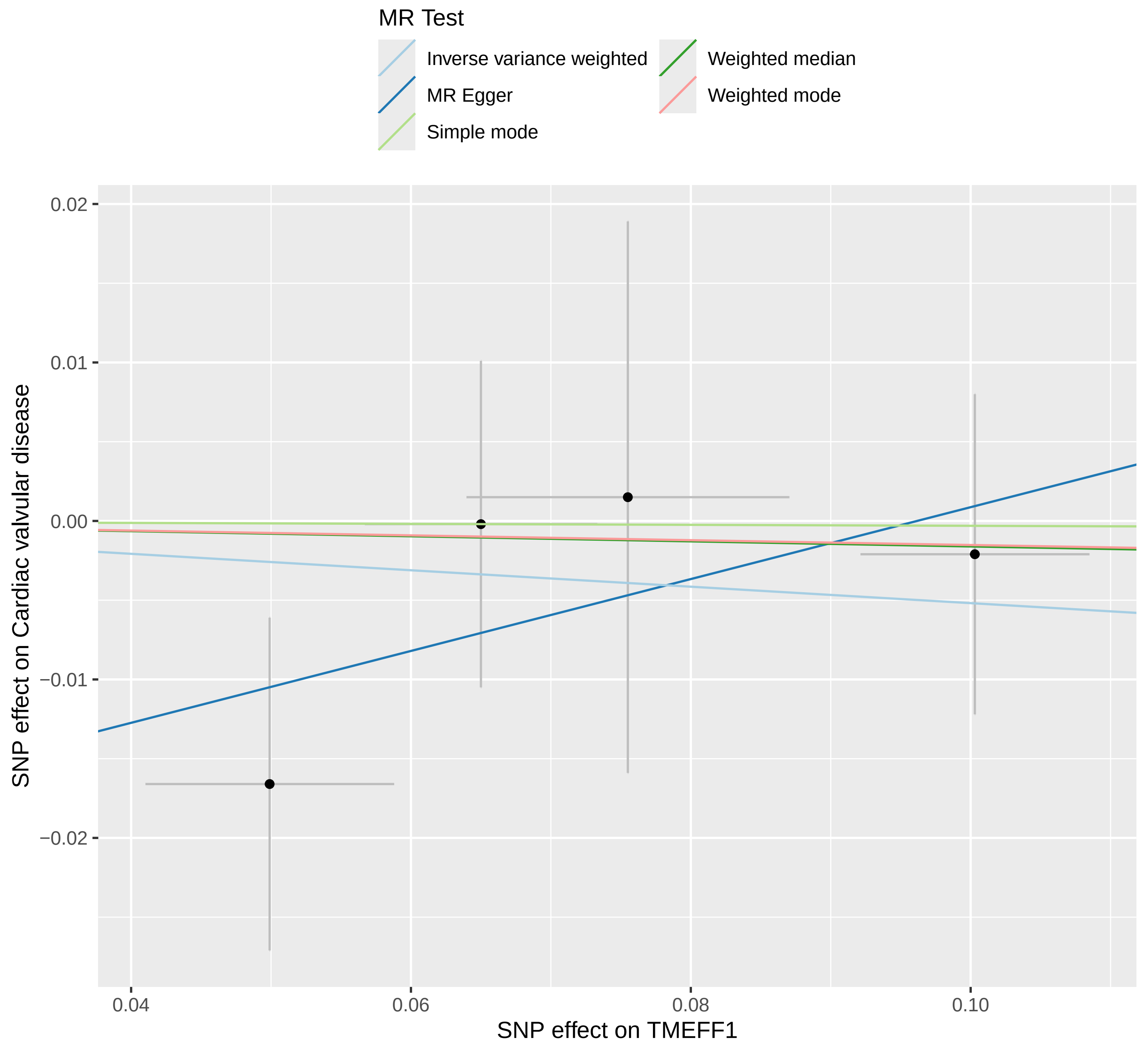
MR Test
Inverse variance weighted
MR Egger
Simple mode
Weighted median
Weighted mode
0.02
0.01
0.00
−0.01
−0.02
SNP effect on Cardiac valvular disease
0.04
0.06
0.08
0.10
SNP effect on TMEFF1

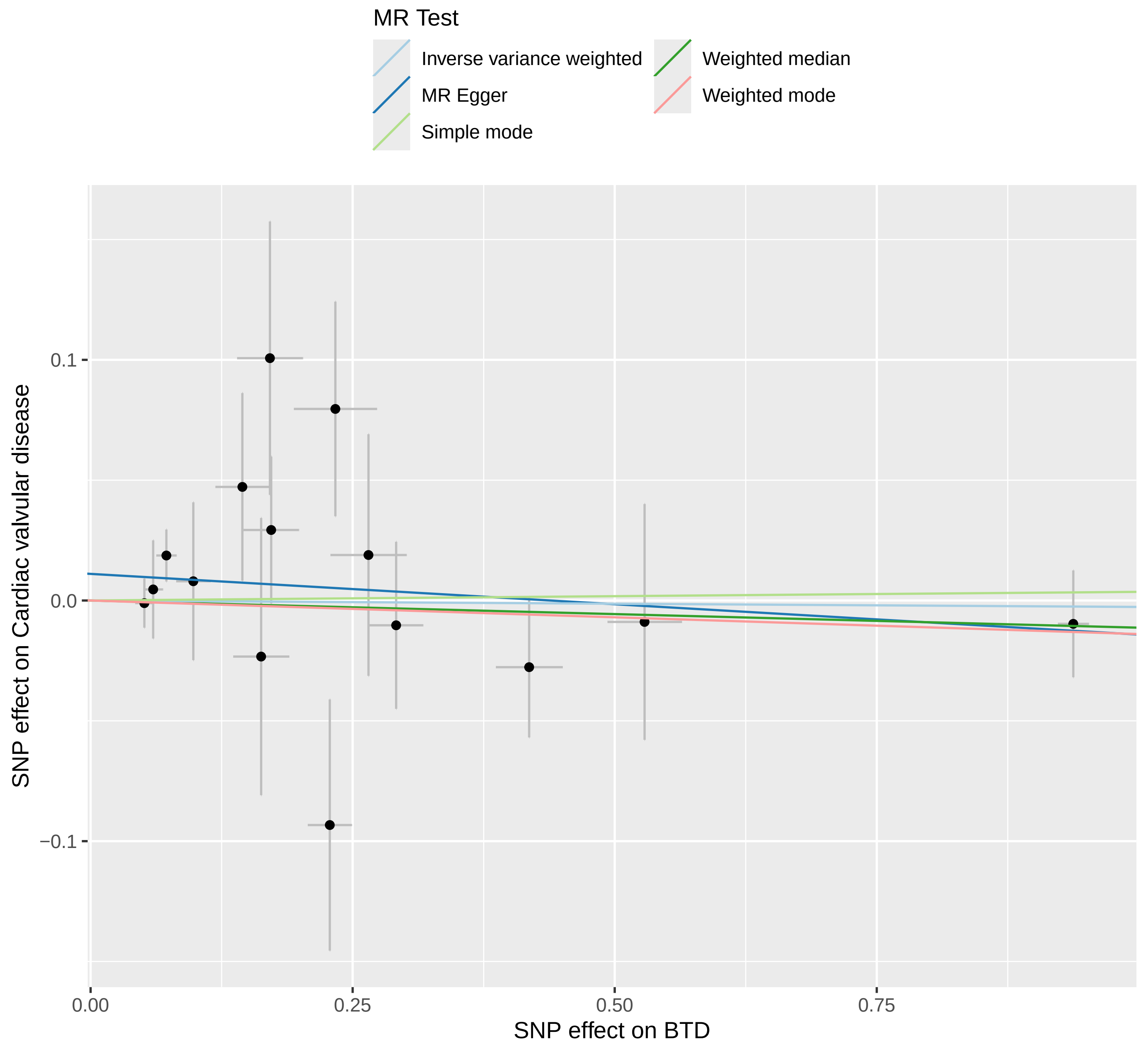

MR Test
Inverse variance weighted
MR Egger
Simple mode
Weighted median
Weighted mode
SNP effect on Cardiac valvular disease
0.1
0.0
−0.1
0.00
0.25
0.50
0.75
SNP effect on BTD

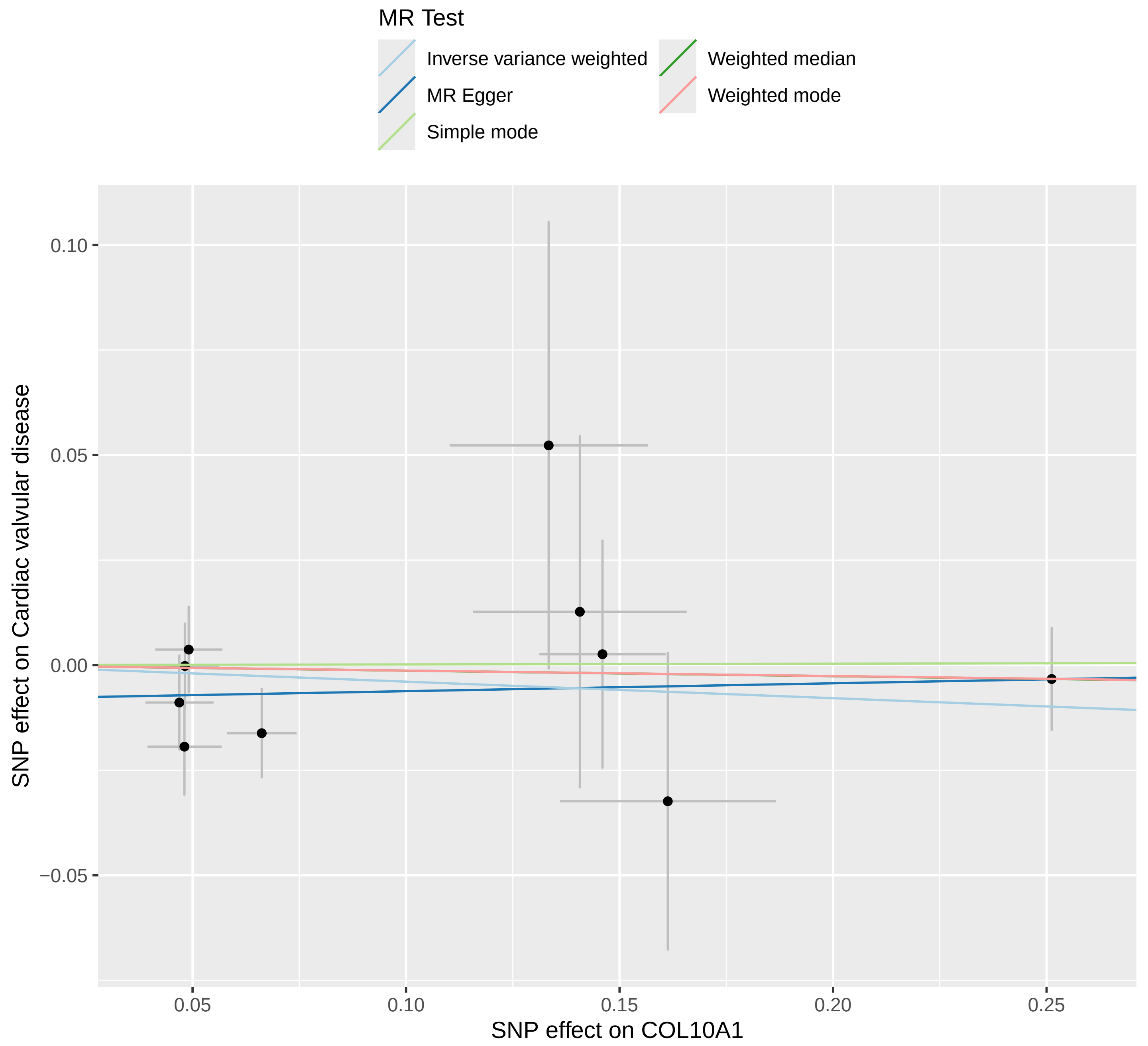
MR Test
Inverse variance weighted
MR Egger
Simple mode
Weighted median
Weighted mode
0.10
0.05
0.00
−0.05
SNP effect on Cardiac valvular disease
0.05
0.10
0.15
0.20
0.25
SNP effect on COL10A1

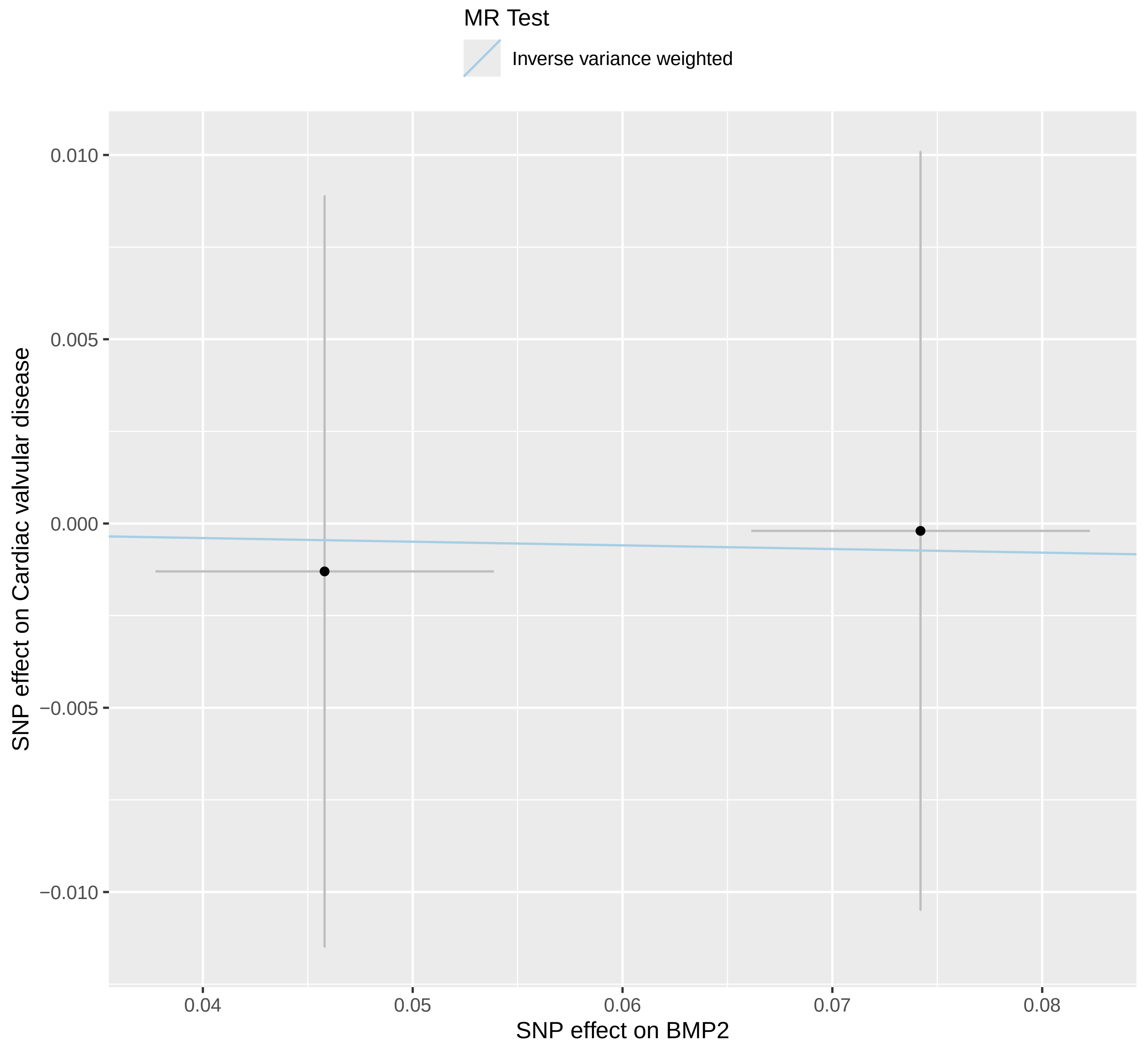

MR Test
Inverse variance weighted
0.010
0.005
0.000
−0.005
−0.010
SNP effect on Cardiac valvular disease
0.04
0.05
0.06
0.07
0.08
SNP effect on BMP2

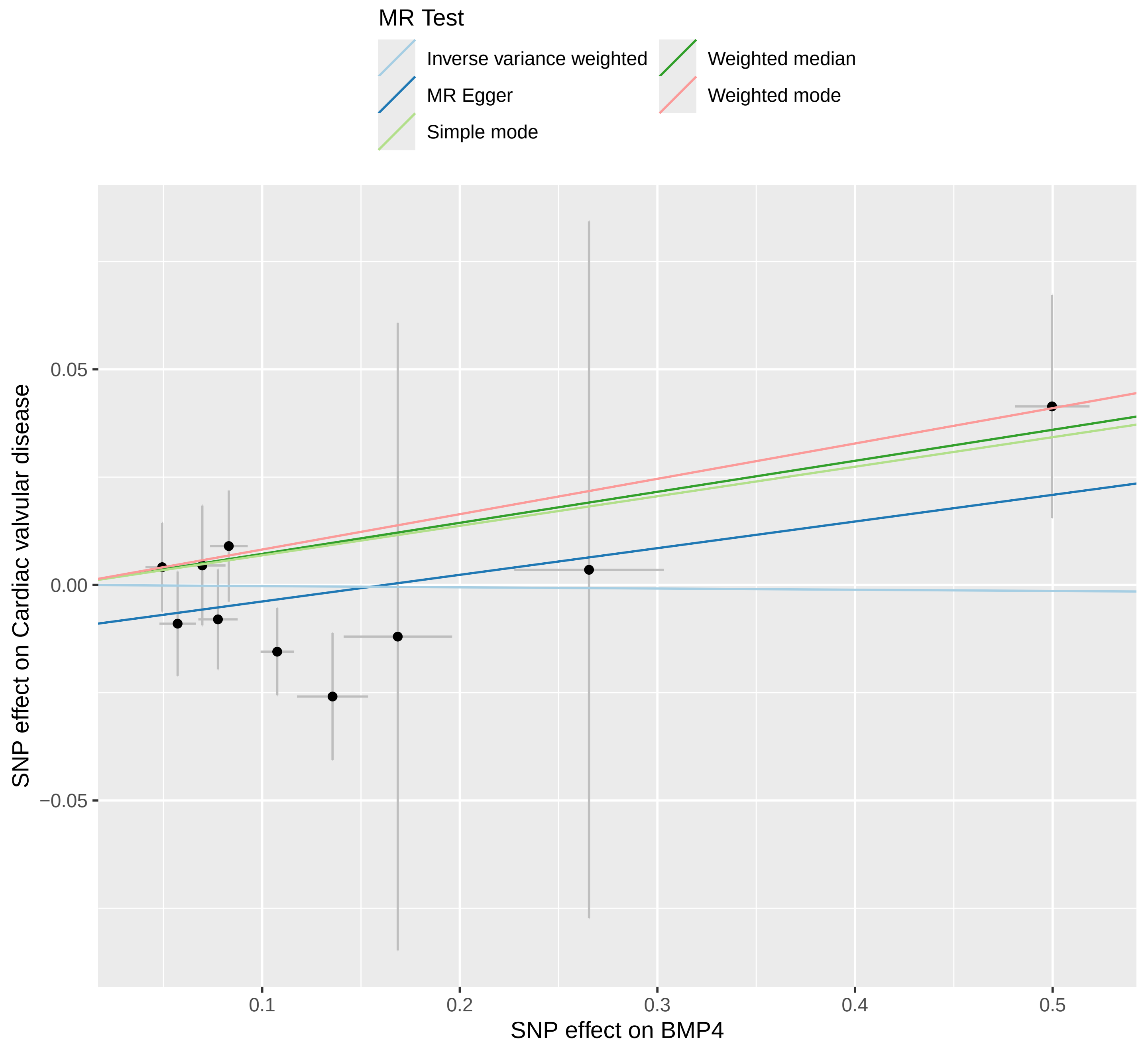

MR Test
Inverse variance weighted
MR Egger
Simple mode
Weighted median
Weighted mode
SNP effect on Cardiac valvular disease
0.05
0.00
−0.05
0.1
0.2
0.3
0.4
0.5
SNP effect on BMP4

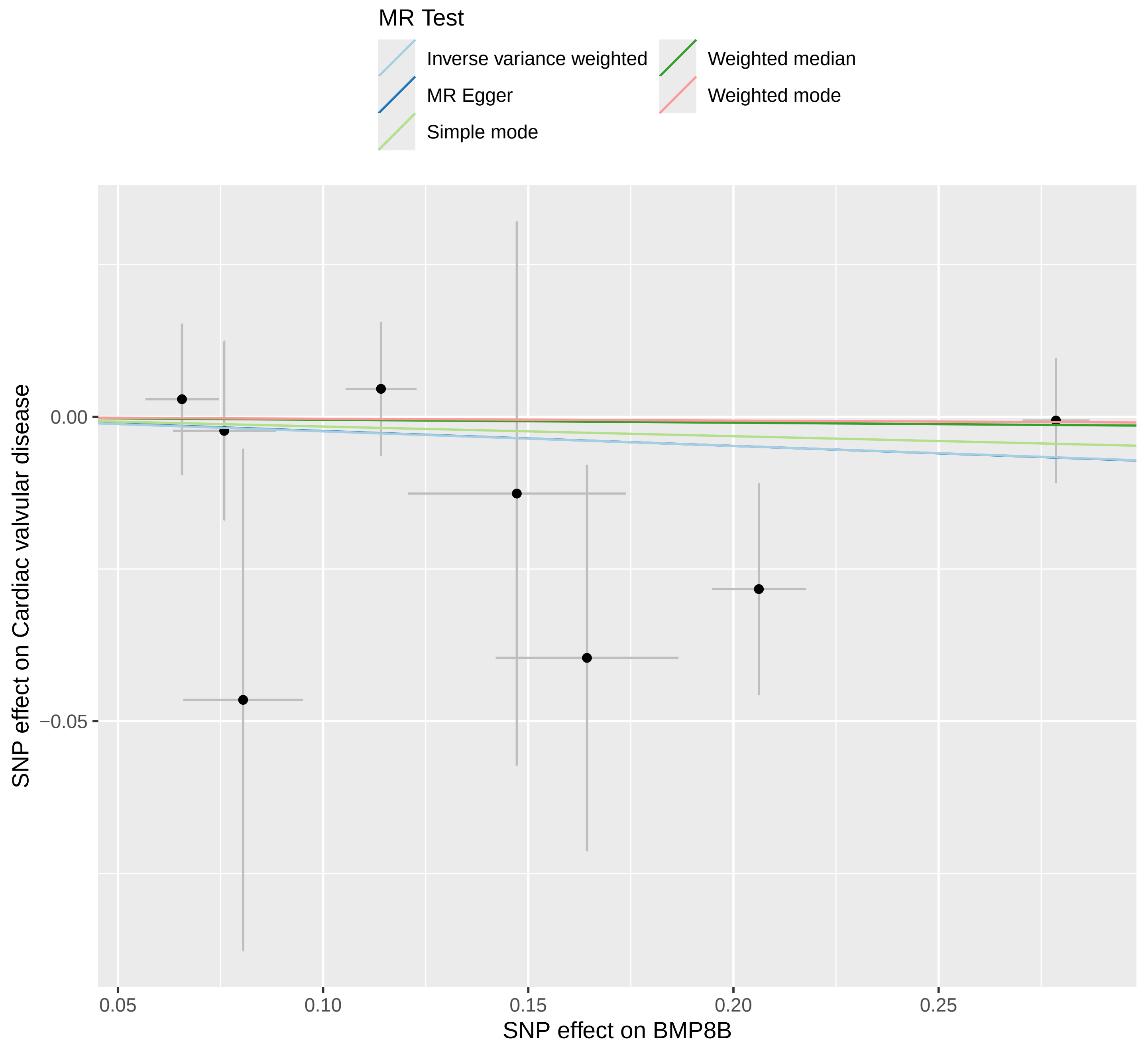
MR Test
Inverse variance weighted
MR Egger
Simple mode
Weighted median
Weighted mode
SNP effect on Cardiac valvular disease
0.00
−0.05
0.05
0.10
0.15
0.20
0.25
SNP effect on BMP8B

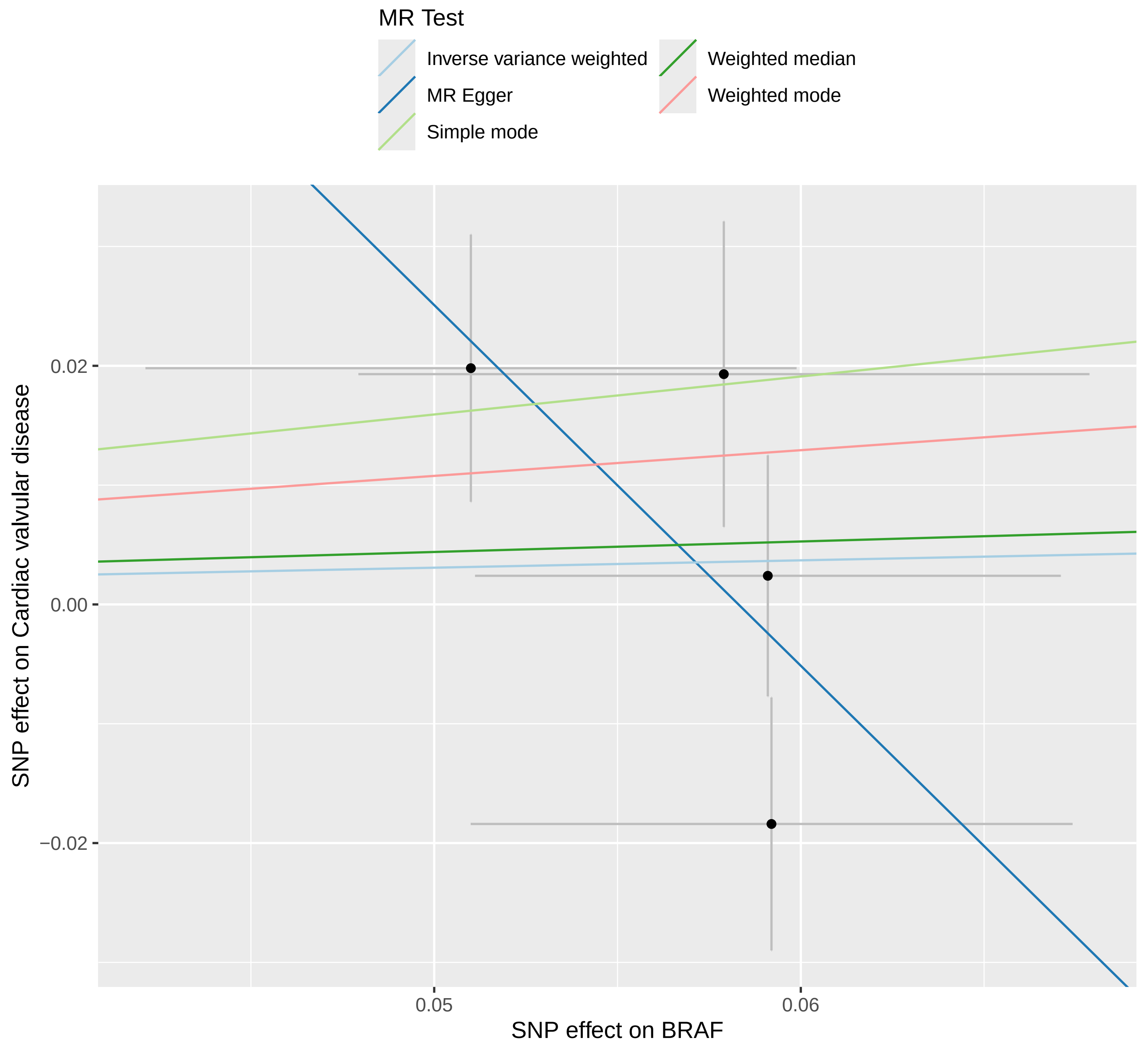

MR Test
Inverse variance weighted
MR Egger
Simple mode
Weighted median
Weighted mode
SNP effect on Cardiac valvular disease
0.02
0.00
−0.02
0.05
0.06
SNP effect on BRAF

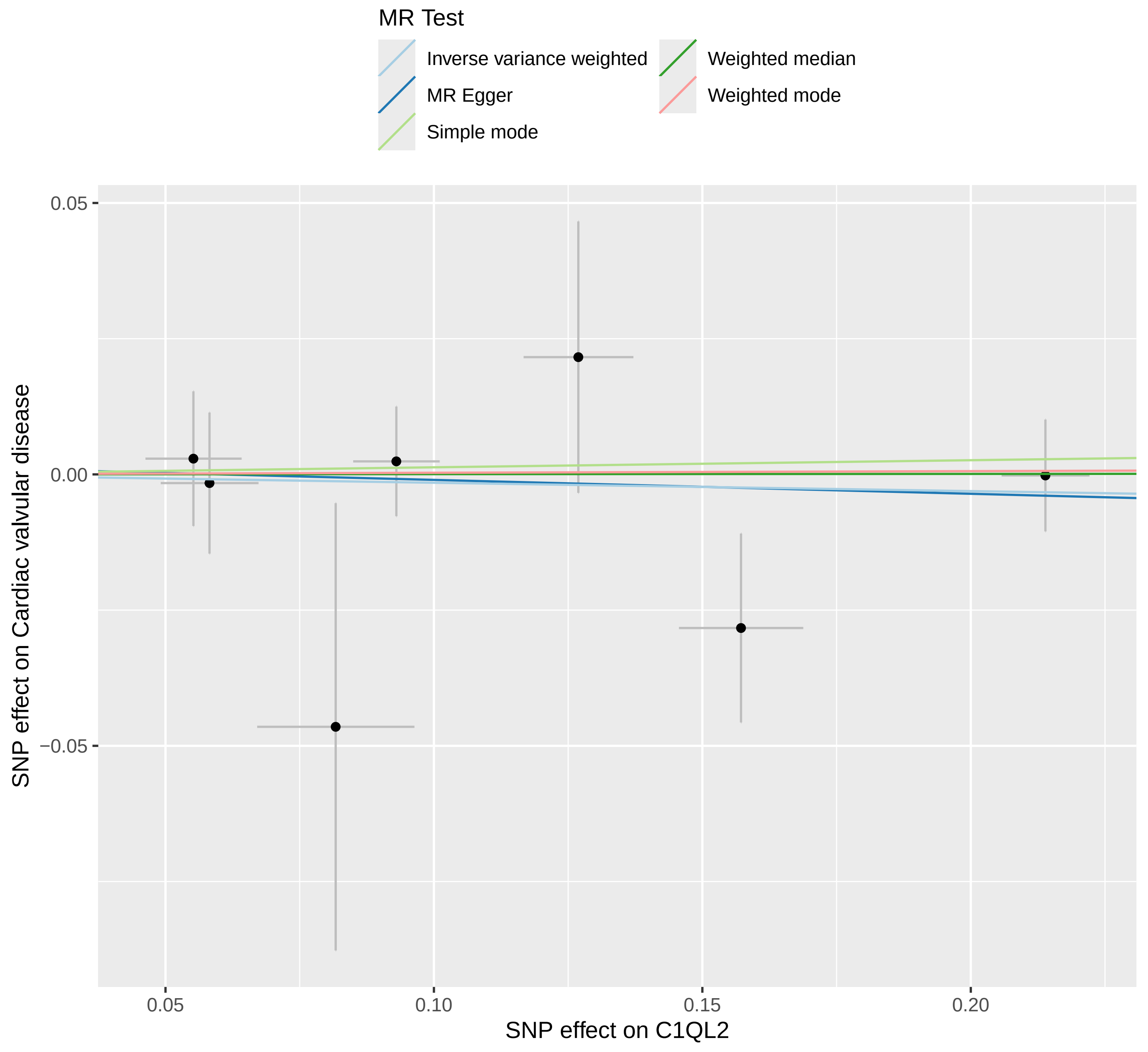
MR Test
Inverse variance weighted
MR Egger
Simple mode
Weighted median
Weighted mode
0.05
0.00
−0.05
SNP effect on Cardiac valvular disease
0.05
0.10
0.15
0.20
SNP effect on C1QL2

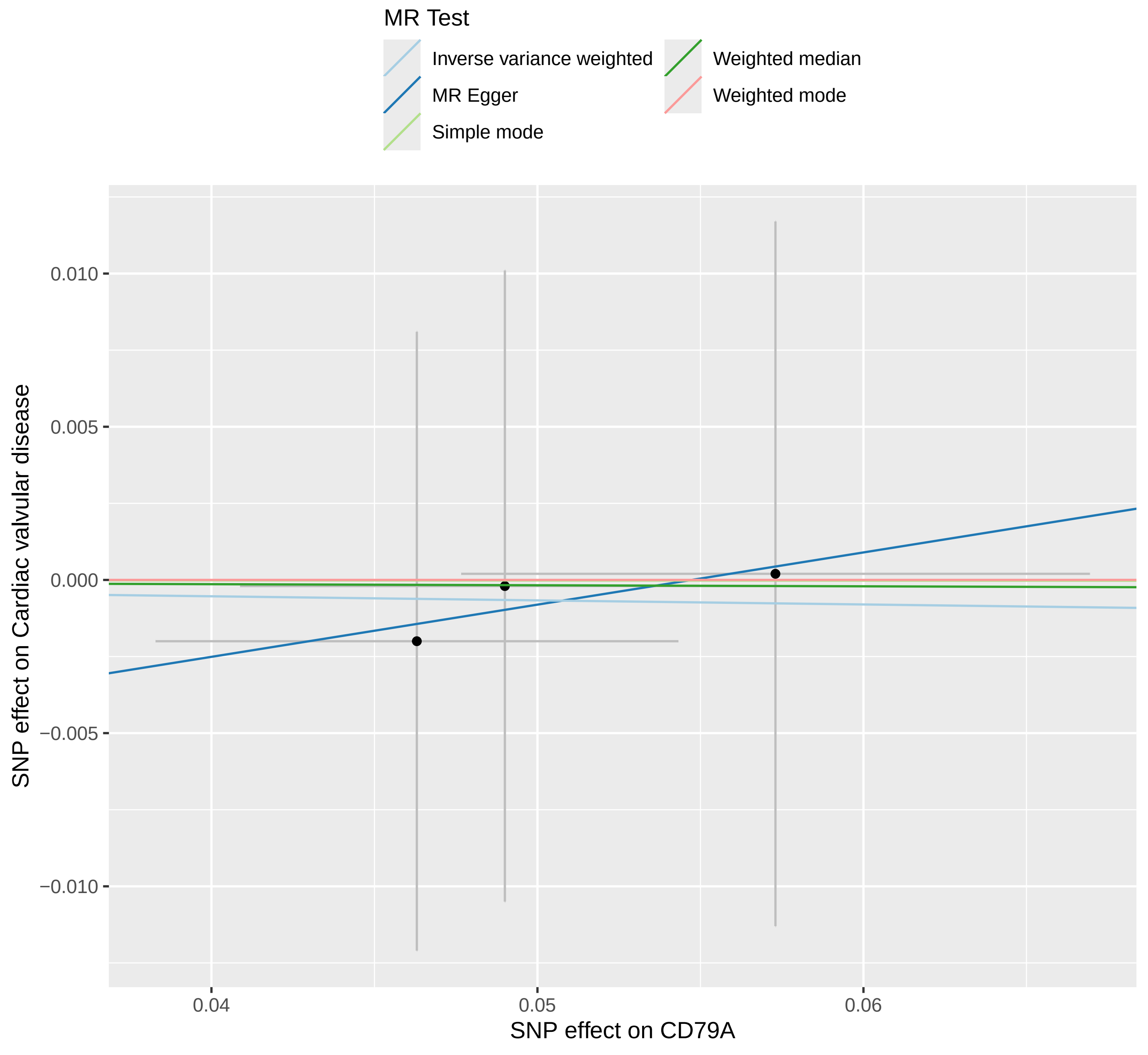

MR Test
Inverse variance weighted
MR Egger
Simple mode
Weighted median
Weighted mode
0.010
0.005
0.000
−0.005
−0.010
SNP effect on Cardiac valvular disease
0.04
0.05
0.06
SNP effect on CD79A

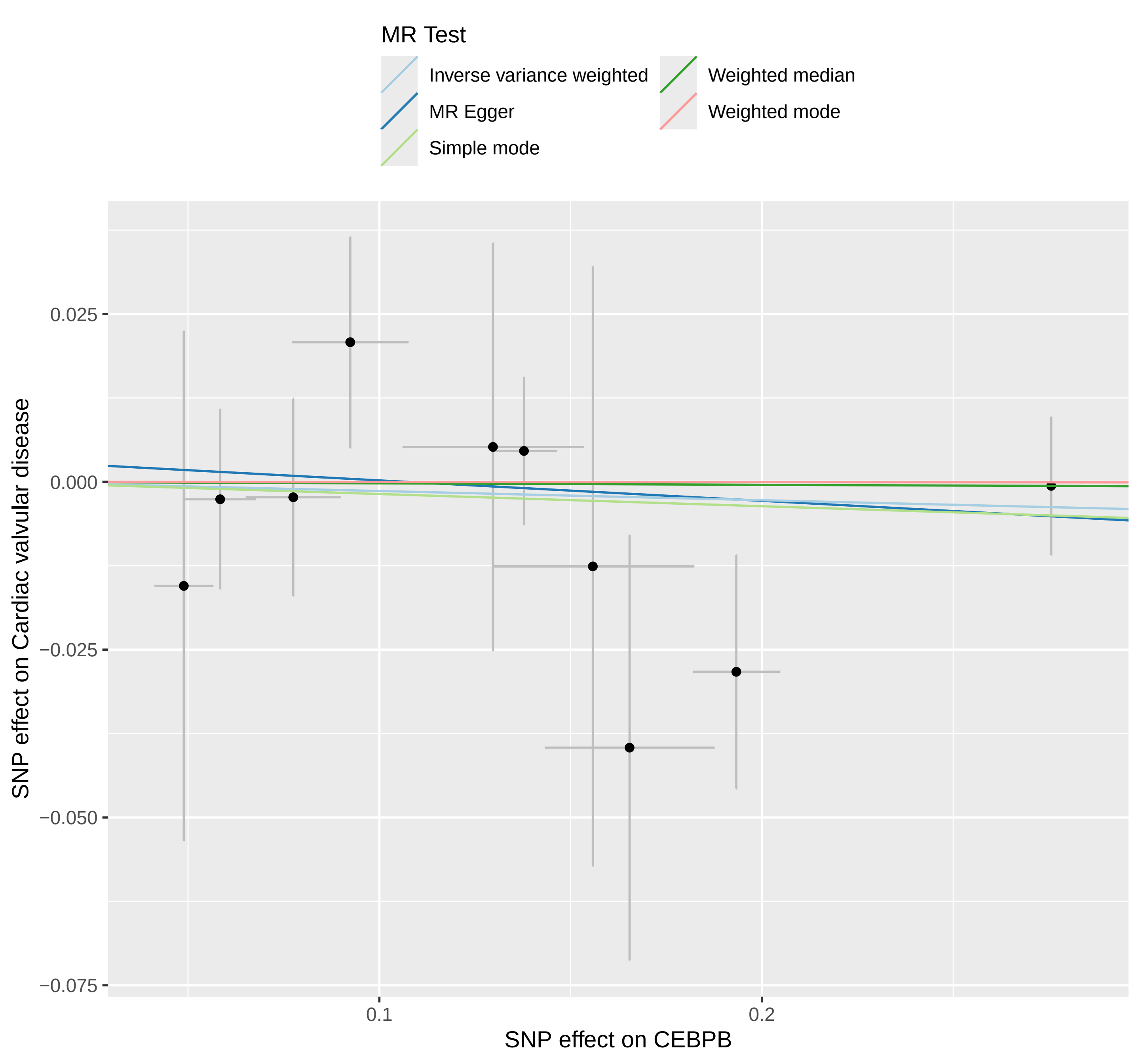
MR Test
Inverse variance weighted
MR Egger
Simple mode
Weighted median
Weighted mode
0.025
0.000
−0.025
−0.050
−0.075
0.1
0.2
SNP effect on Cardiac valvular disease
SNP effect on CEBPB

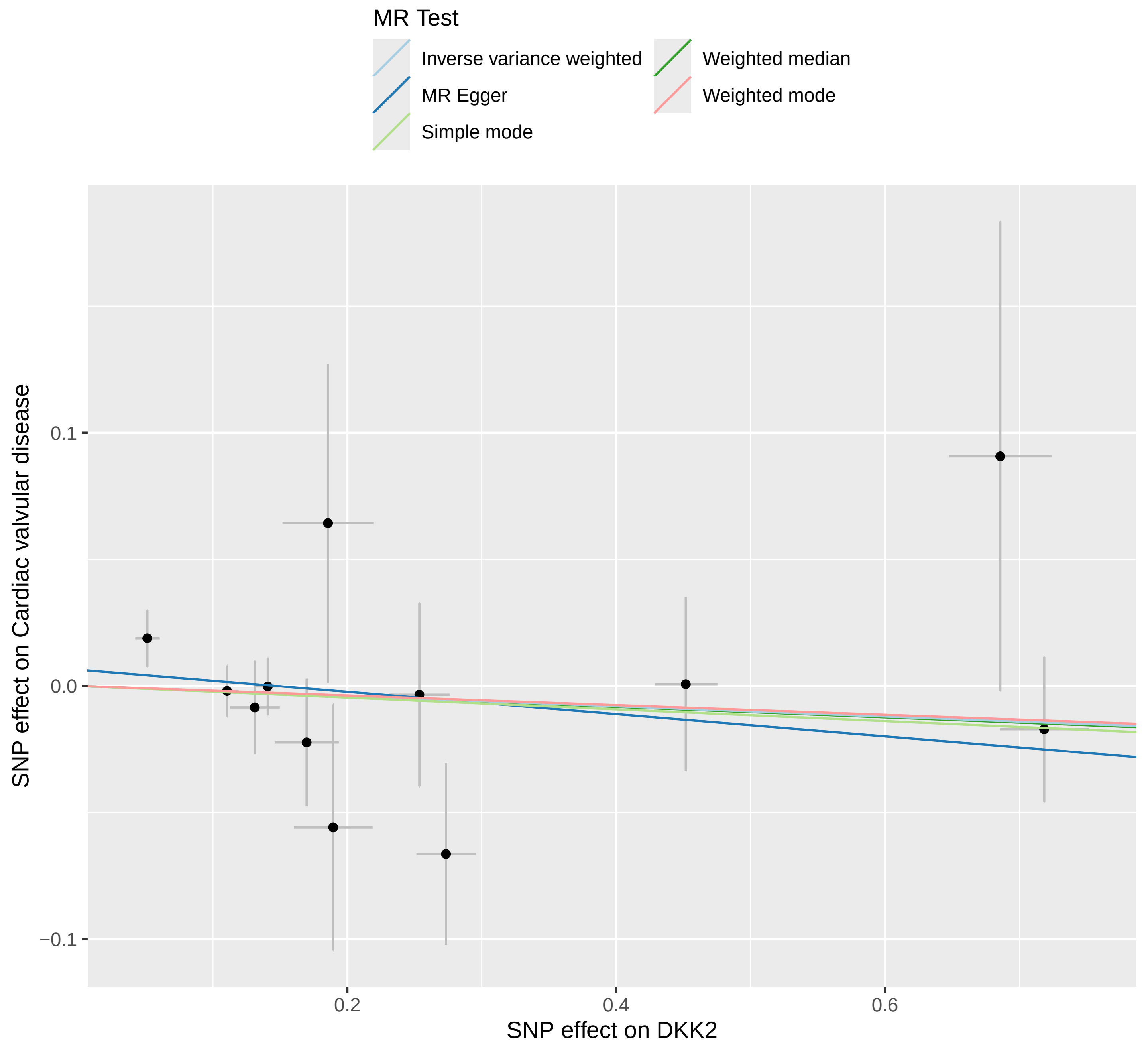
MR Test
Inverse variance weighted
MR Egger
Simple mode
Weighted median
Weighted mode
SNP effect on Cardiac valvular disease
0.1
0.0
−0.1
0.2
0.4
0.6
SNP effect on DKK2

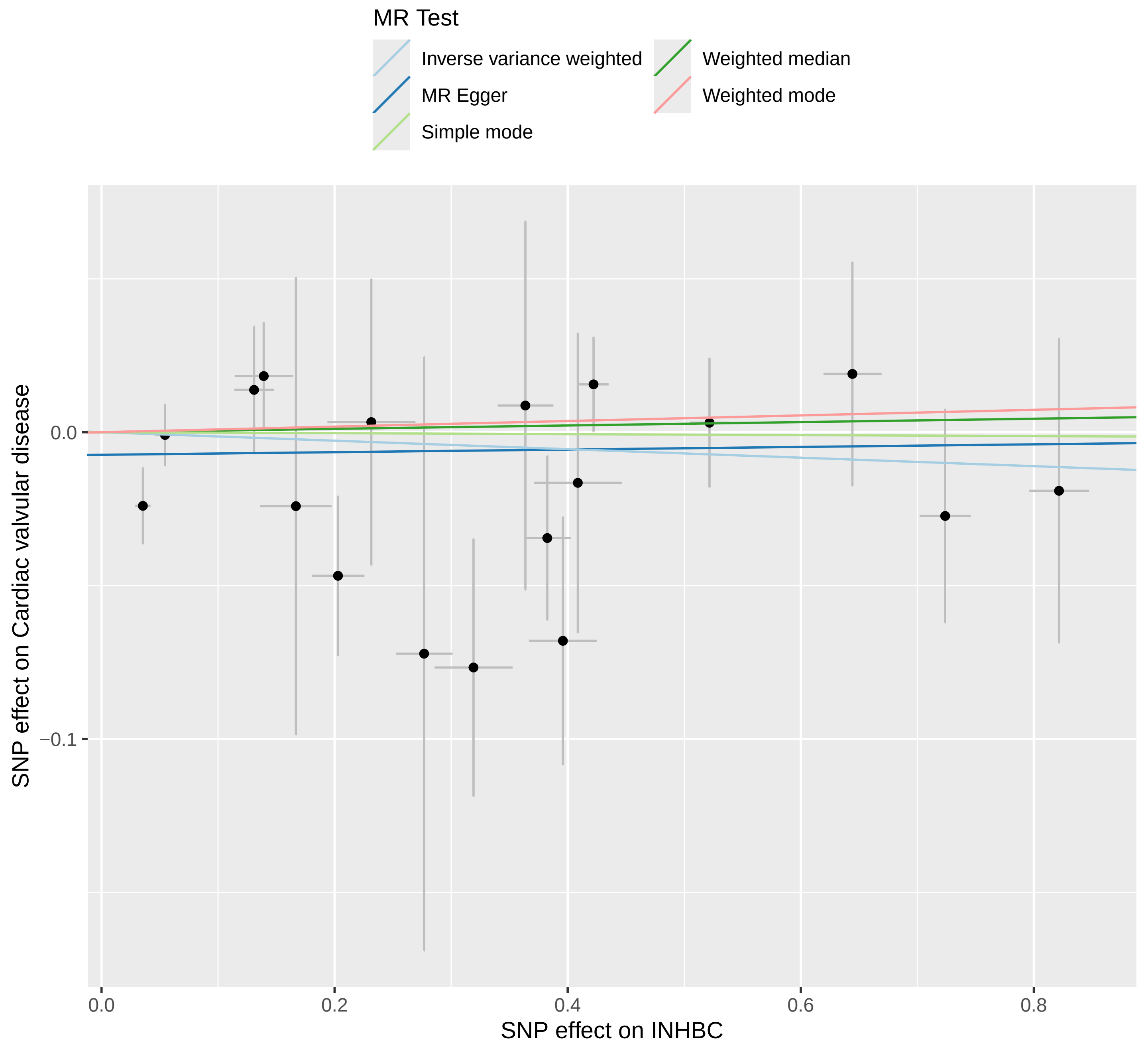
MR Test
Inverse variance weighted
MR Egger
Simple mode
Weighted median
Weighted mode
0.0
−0.1
SNP effect on Cardiac valvular disease
0.0
0.2
0.4
0.6
0.8
SNP effect on INHBC

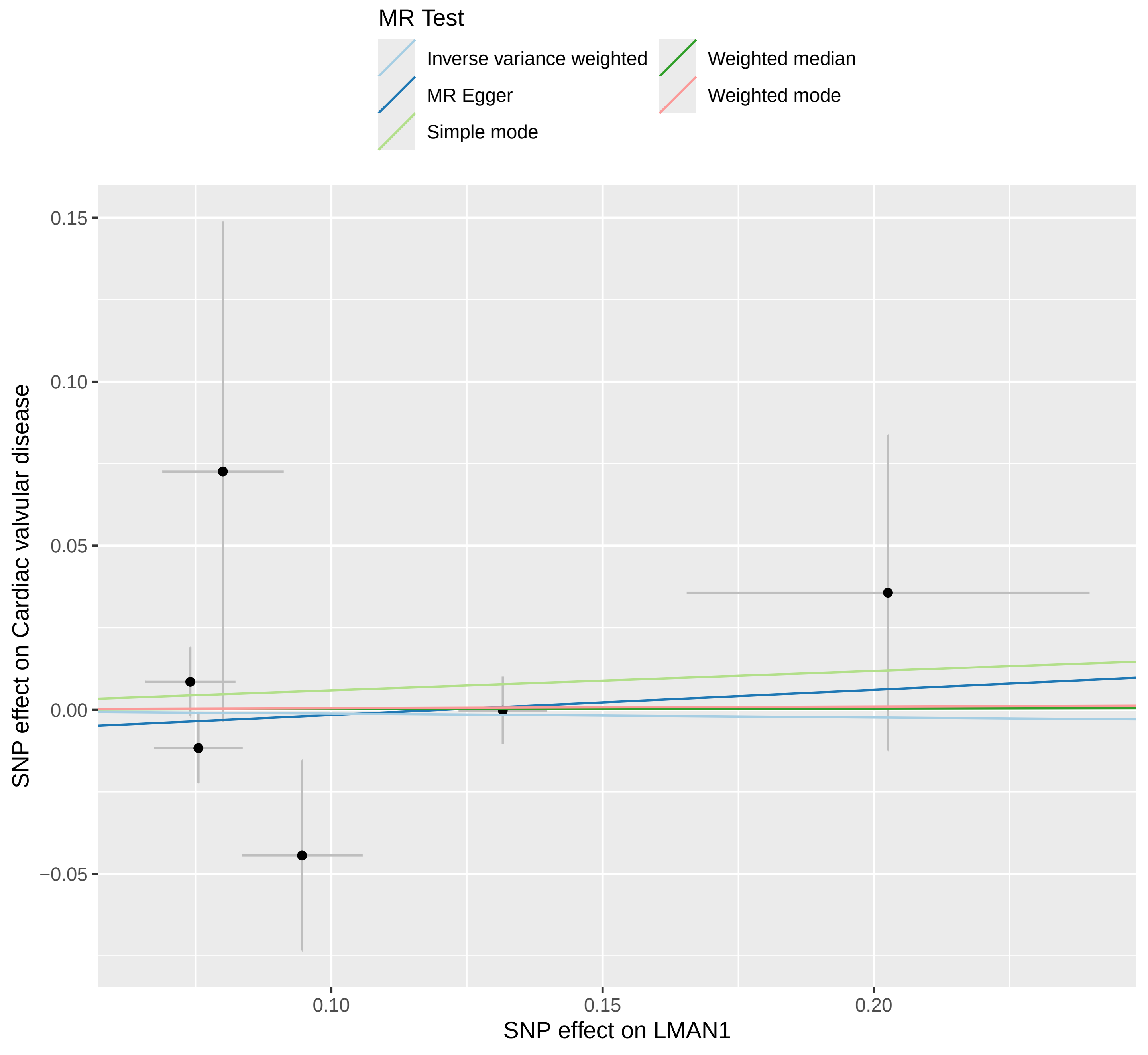
MR Test
Inverse variance weighted
MR Egger
Simple mode
Weighted median
Weighted mode
0.15
0.10
0.05
0.00
−0.05
0.10
0.15
0.20
SNP effect on Cardiac valvular disease
SNP effect on LMAN1

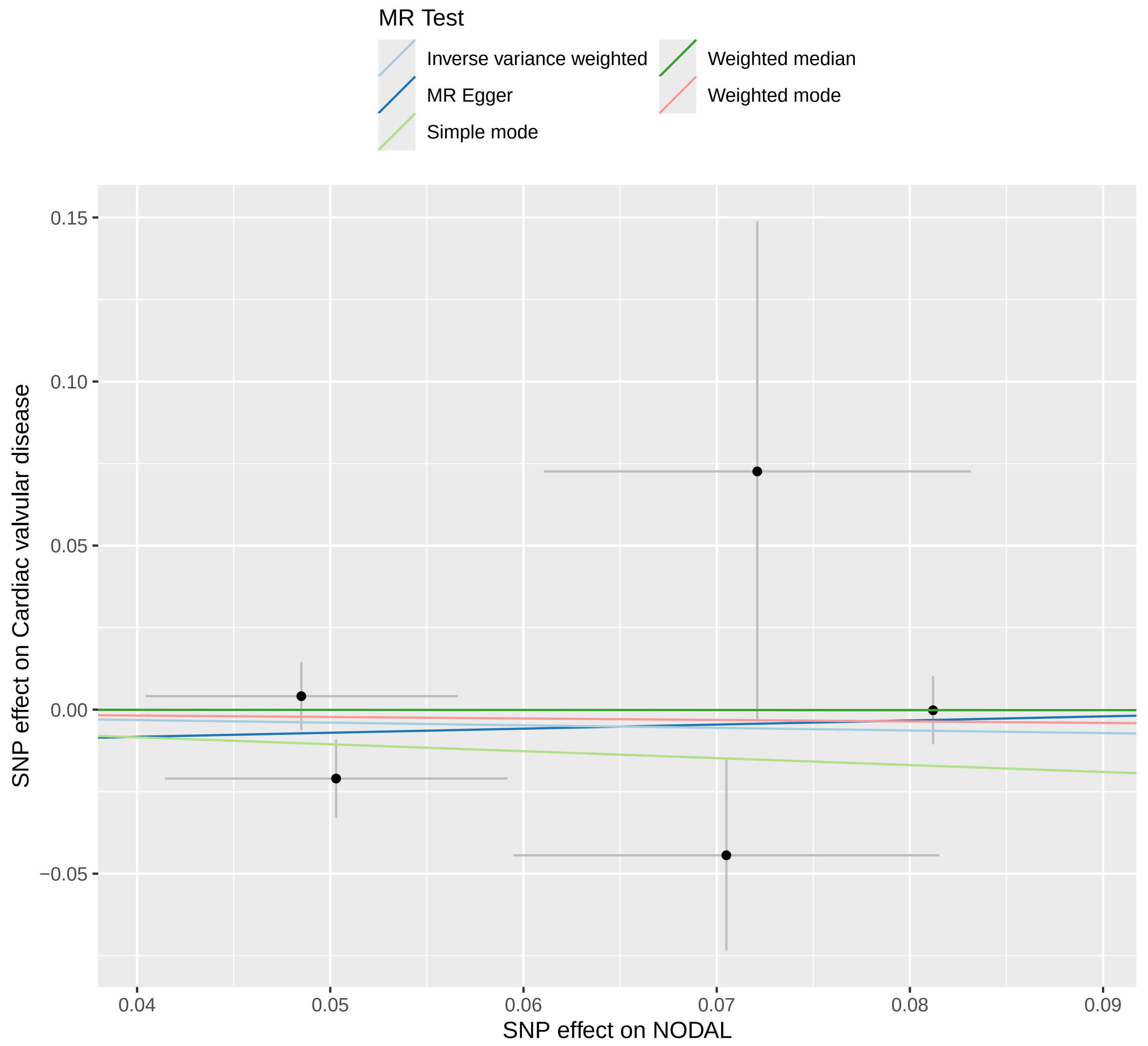

MR Test
Inverse variance weighted
MR Egger
Simple mode
Weighted median
Weighted mode
0.15
0.10
0.05
0.00
−0.05
0.04
0.05
0.06
0.07
0.08
0.09
SNP effect on NODAL
SNP effect on Cardiac valvular disease

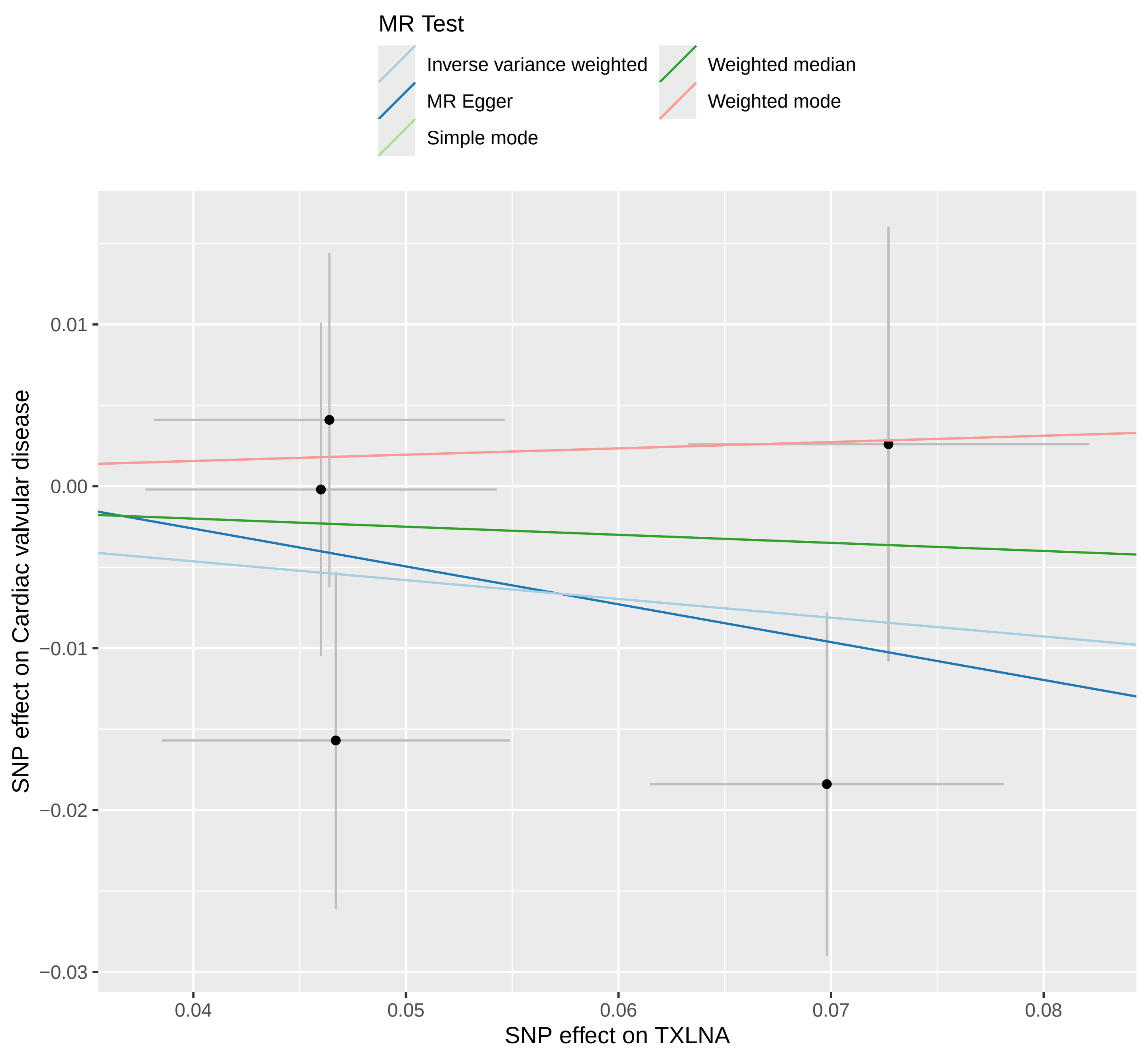

MR Test
Inverse variance weighted
MR Egger
Simple mode
Weighted median
Weighted mode
0.01
0.00
−0.01
−0.02
−0.03
SNP effect on Cardiac valvular disease
0.04
0.05
0.06
0.07
0.08
SNP effect on TXLNA

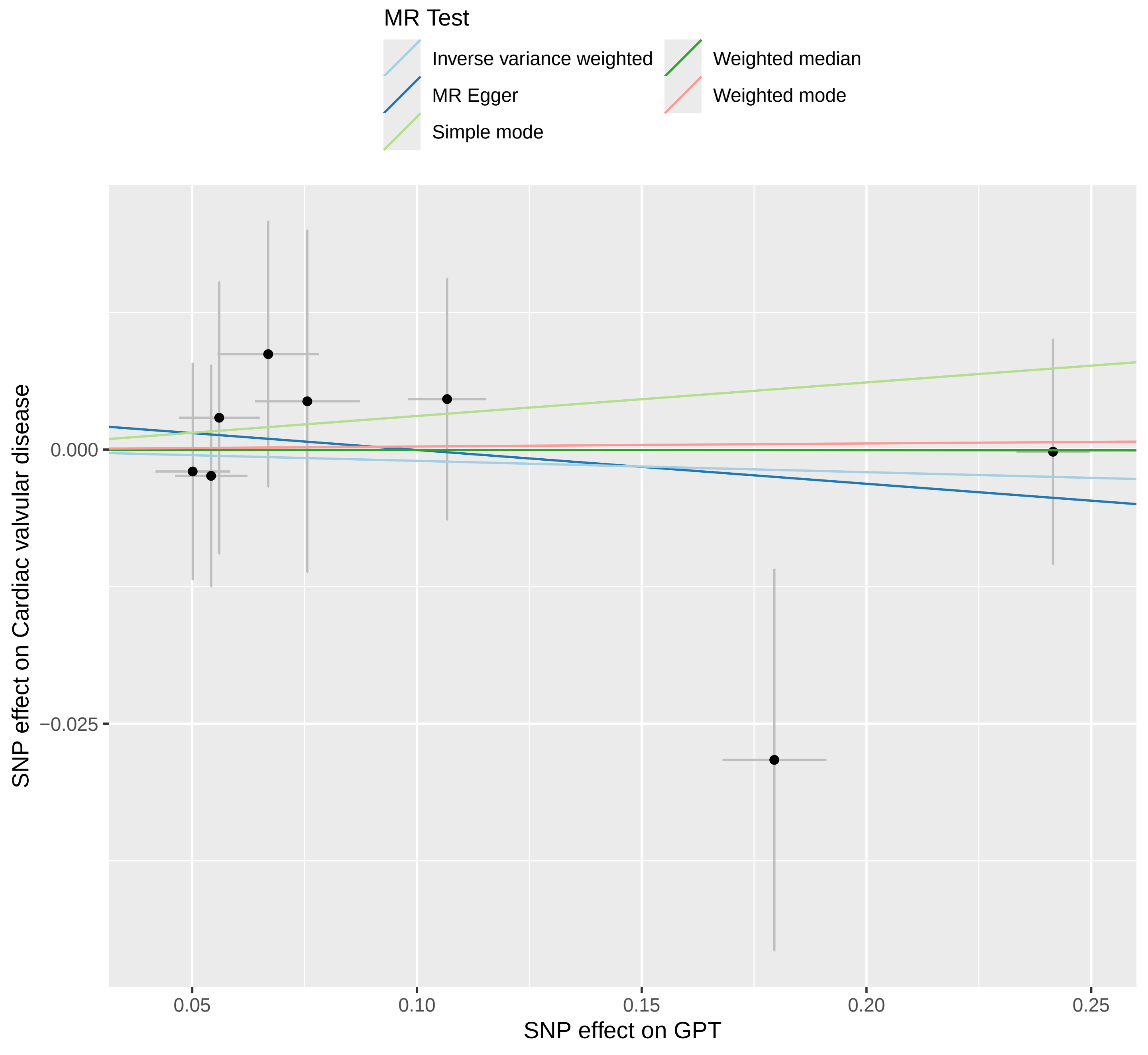
MR Test
Inverse variance weighted
MR Egger
Simple mode
Weighted median
Weighted mode
0.000
−0.025
0.05
0.10
0.15
0.20
0.25
SNP effect on GPT
SNP effect on Cardiac valvular disease

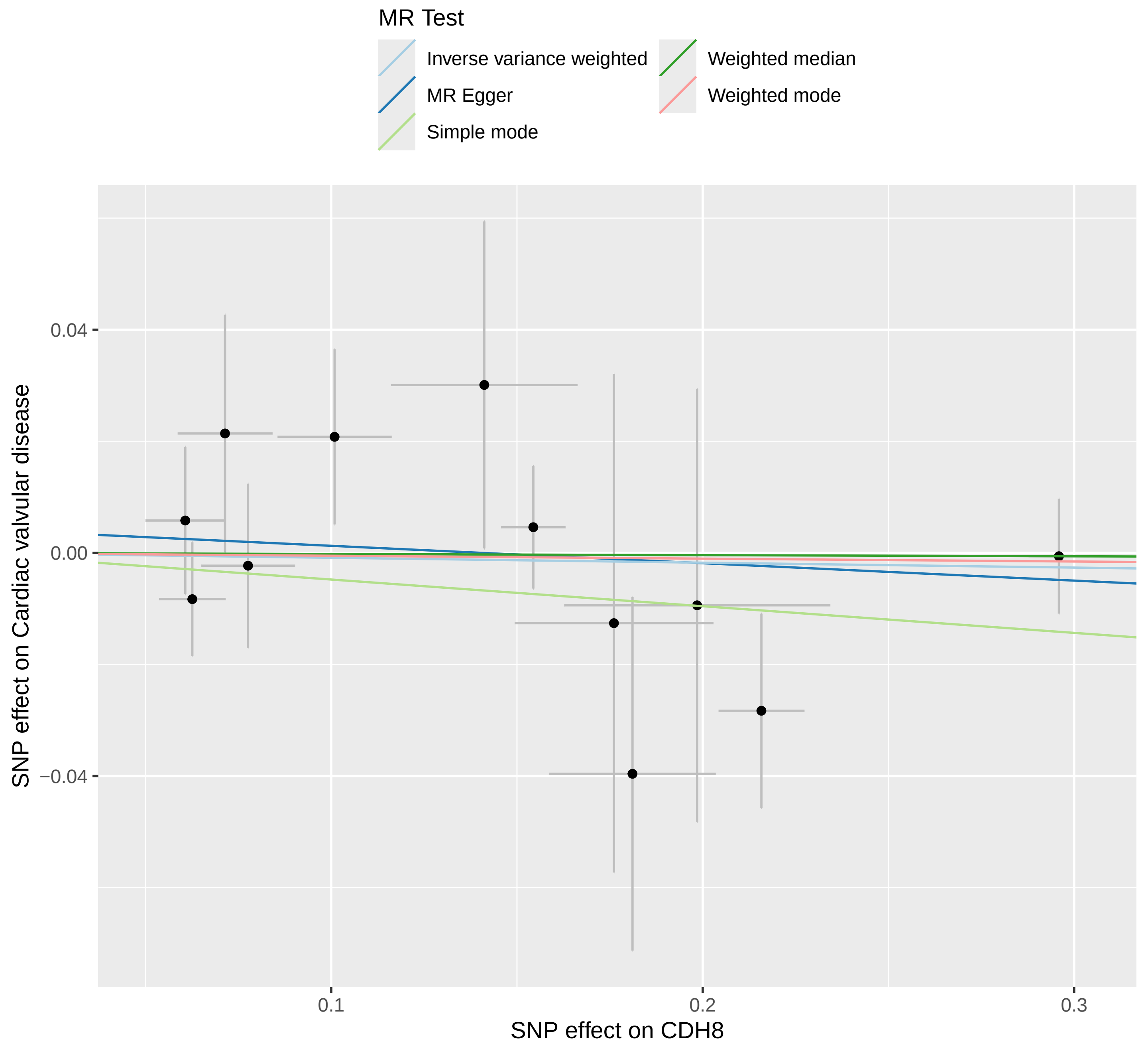
MR Test
Inverse variance weighted
MR Egger
Simple mode
Weighted median
Weighted mode
0.04
0.00
−0.04
0.1
0.2
0.3
SNP effect on Cardiac valvular disease
SNP effect on CDH8

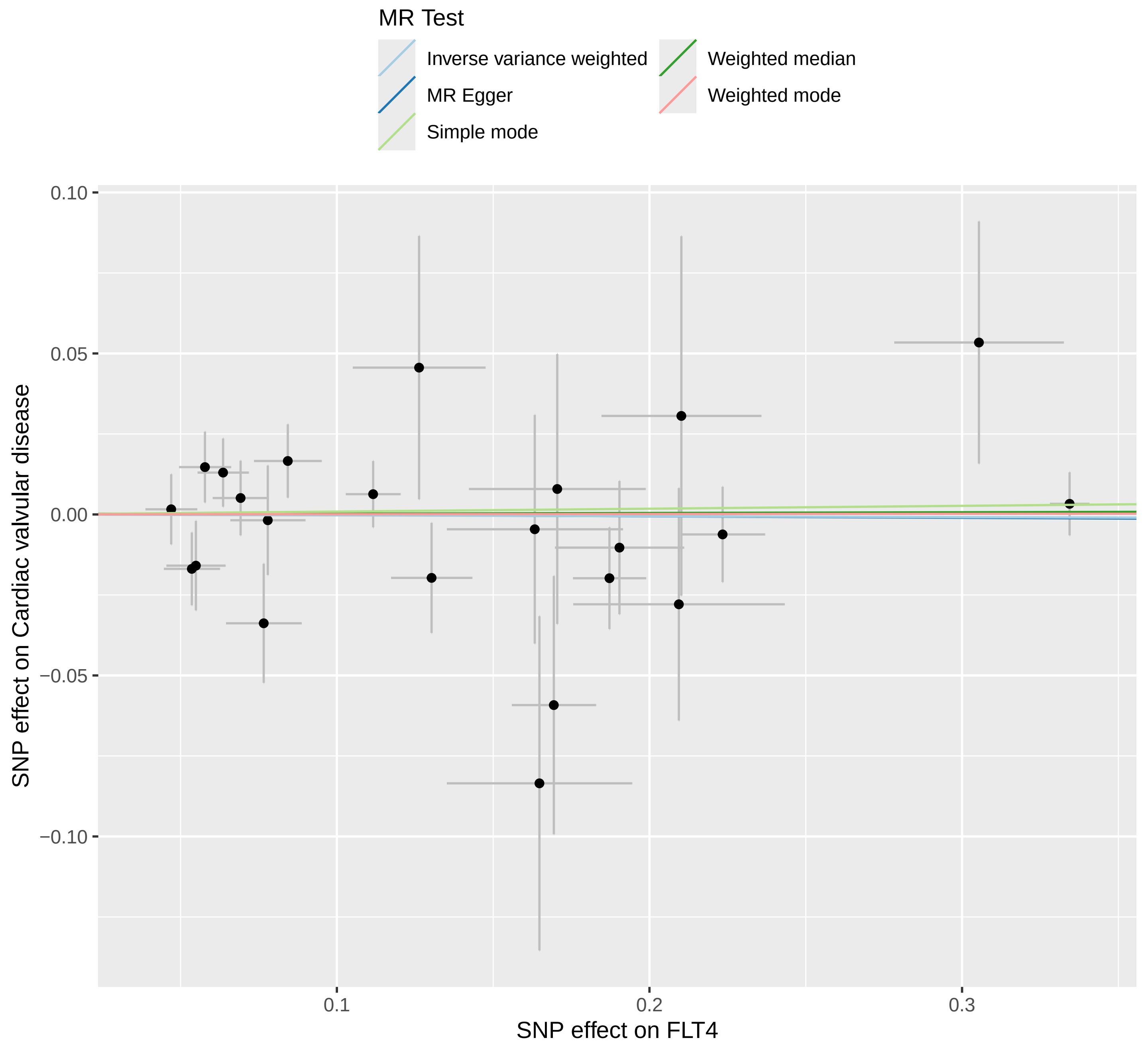
MR Test
Inverse variance weighted
MR Egger
Simple mode
Weighted median
Weighted mode
0.10
0.05
0.00
−0.05
−0.10
SNP effect on Cardiac valvular disease
0.1
0.2
0.3
SNP effect on FLT4

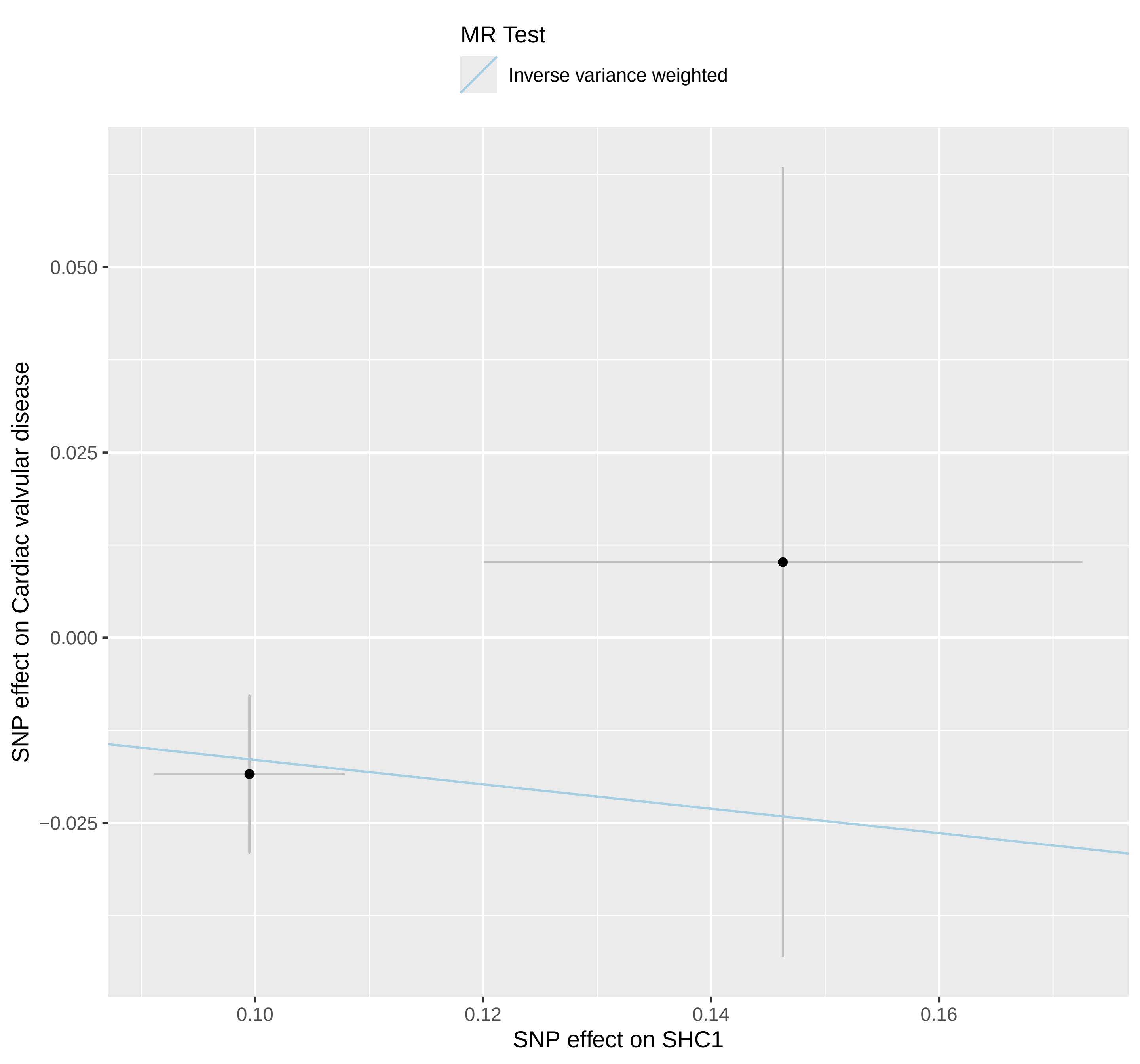
MR Test
Inverse variance weighted
0.050
0.025
0.000
−0.025
SNP effect on Cardiac valvular disease
0.10
0.12
0.14
0.16
SNP effect on SHC1

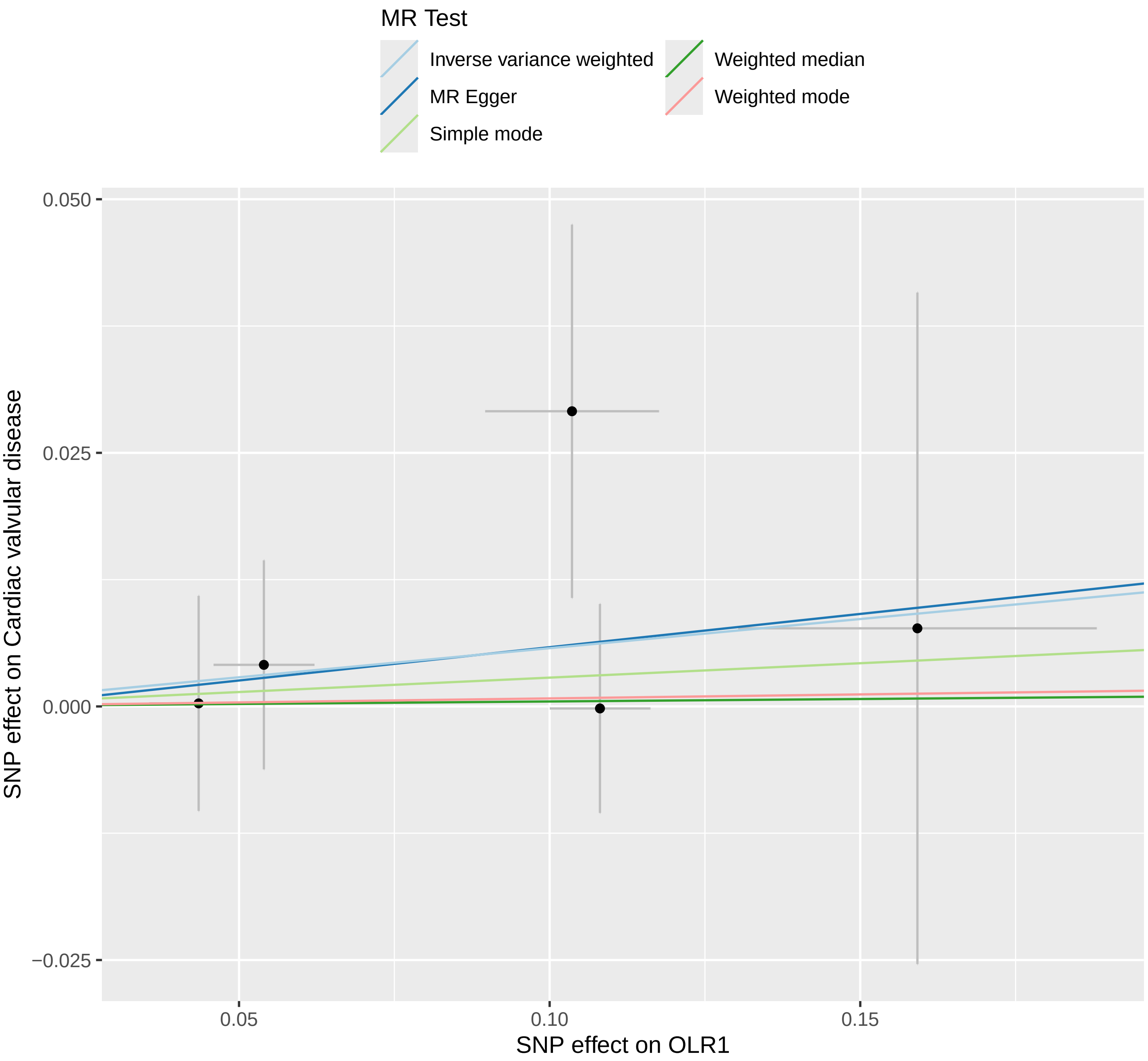

MR Test
Inverse variance weighted
MR Egger
Simple mode
Weighted median
Weighted mode
0.050
0.025
0.000
−0.025
0.05
0.10
0.15
SNP effect on OLR1
SNP effect on Cardiac valvular disease

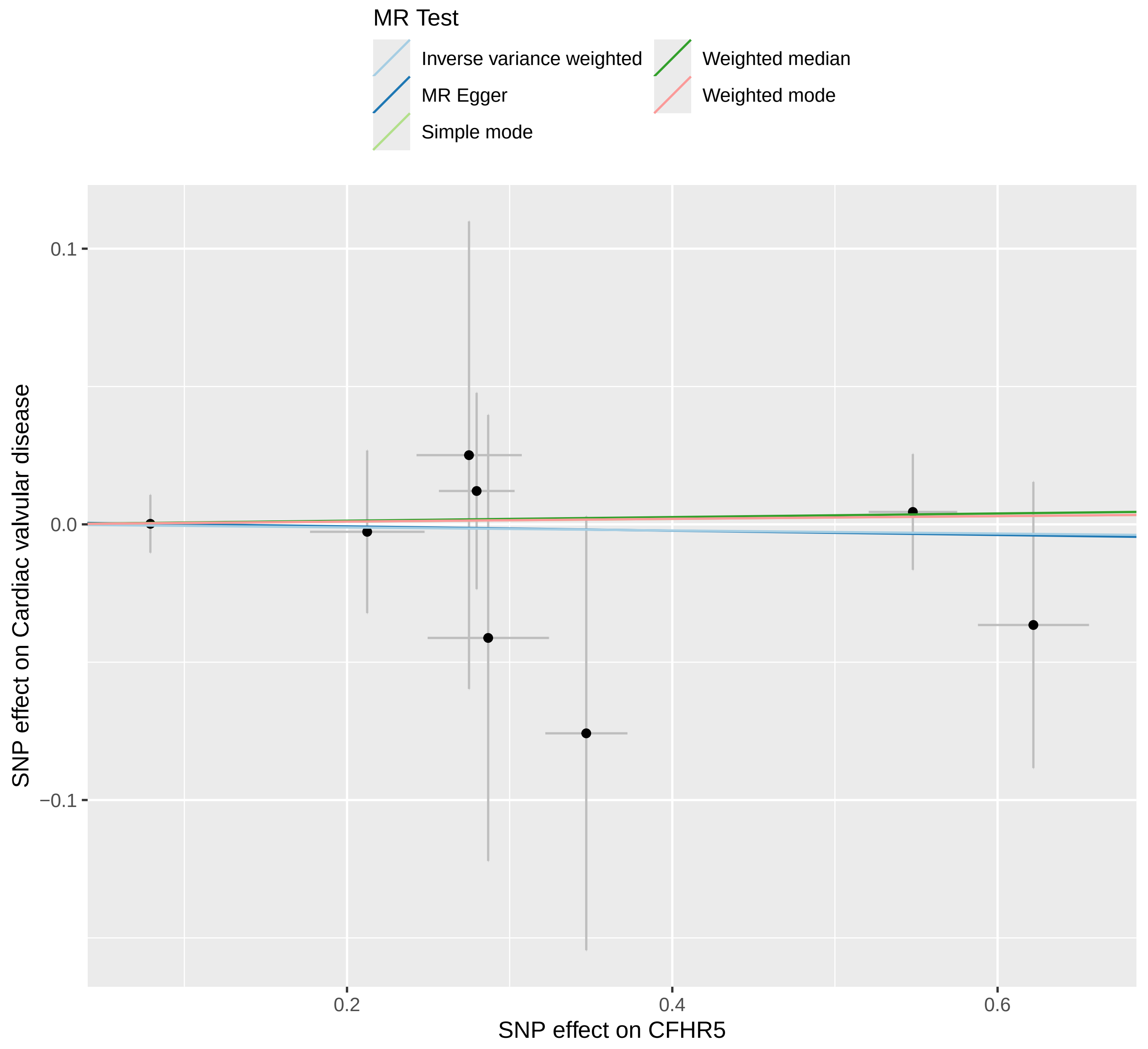
MR Test
Inverse variance weighted
MR Egger
Simple mode
Weighted median
Weighted mode
0.1
0.0
−0.1
0.2
0.4
0.6
SNP effect on Cardiac valvular disease
SNP effect on CFHR5

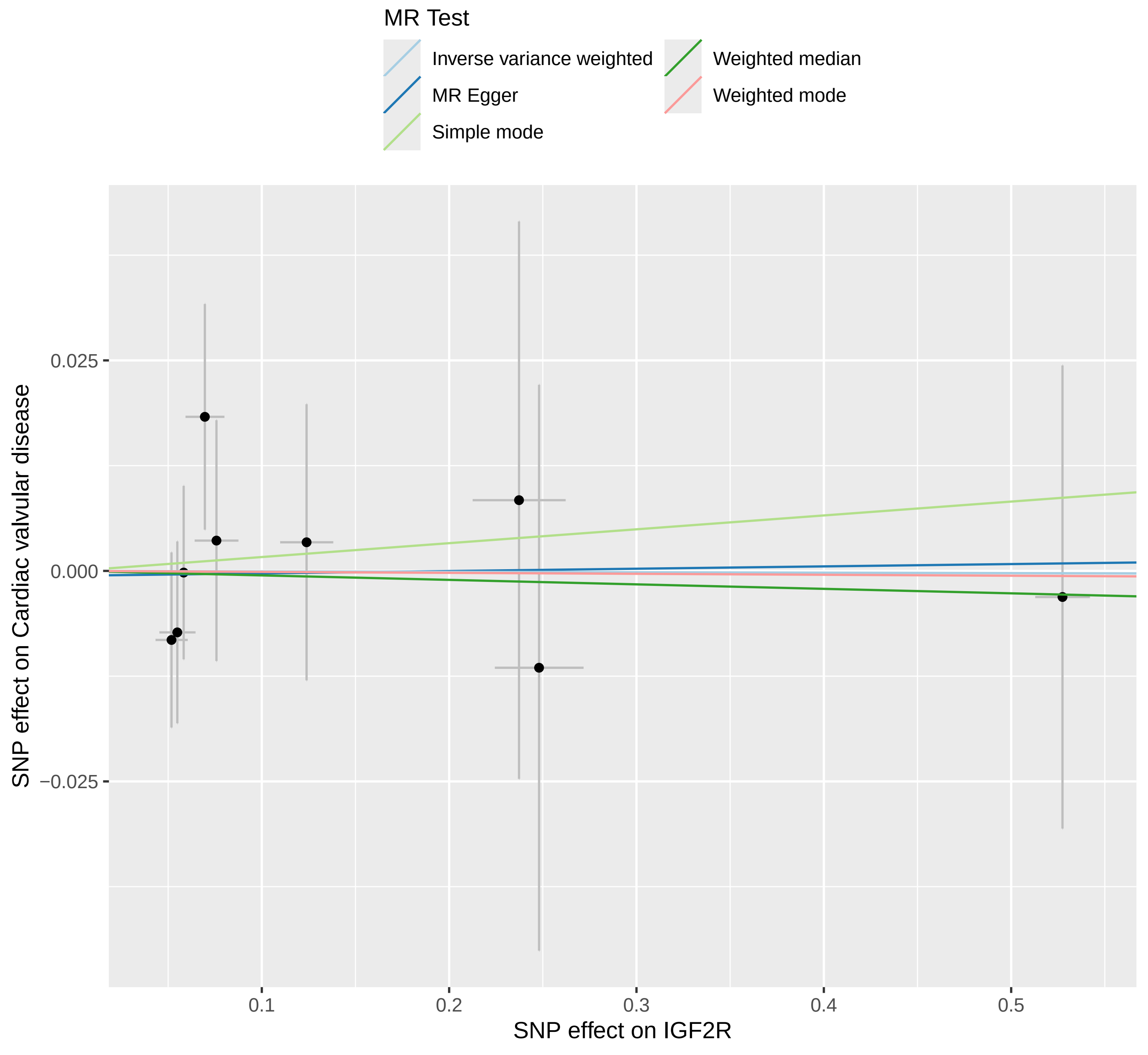
MR Test
Inverse variance weighted
MR Egger
Simple mode
Weighted median
Weighted mode
0.025
0.000
−0.025
0.1
0.2
0.3
0.4
0.5
SNP effect on Cardiac valvular disease
SNP effect on IGF2R

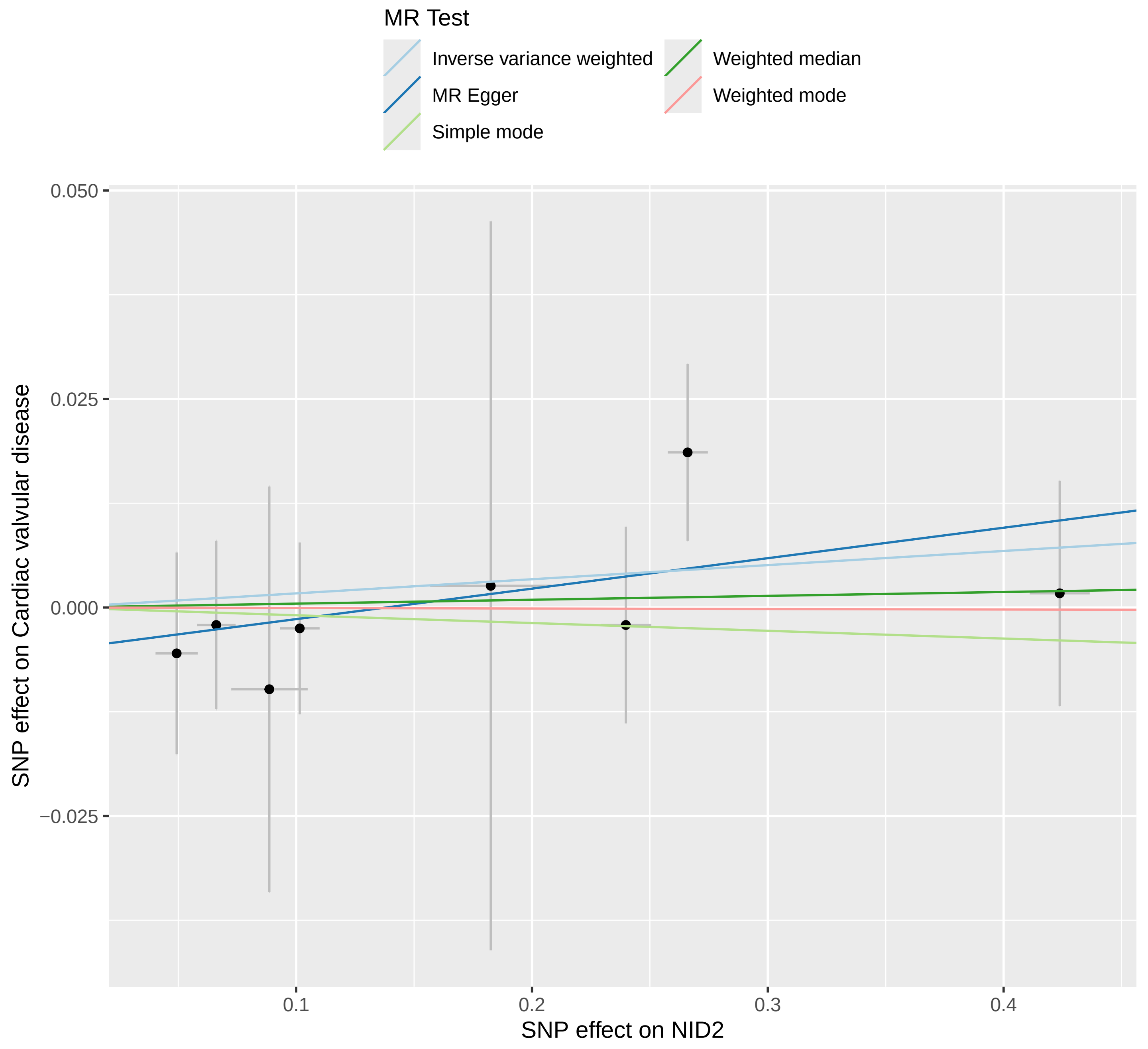
MR Test
Inverse variance weighted
MR Egger
Simple mode
Weighted median
Weighted mode
0.050
0.025
0.000
−0.025
SNP effect on Cardiac valvular disease
0.1
0.2
0.3
0.4
SNP effect on NID2

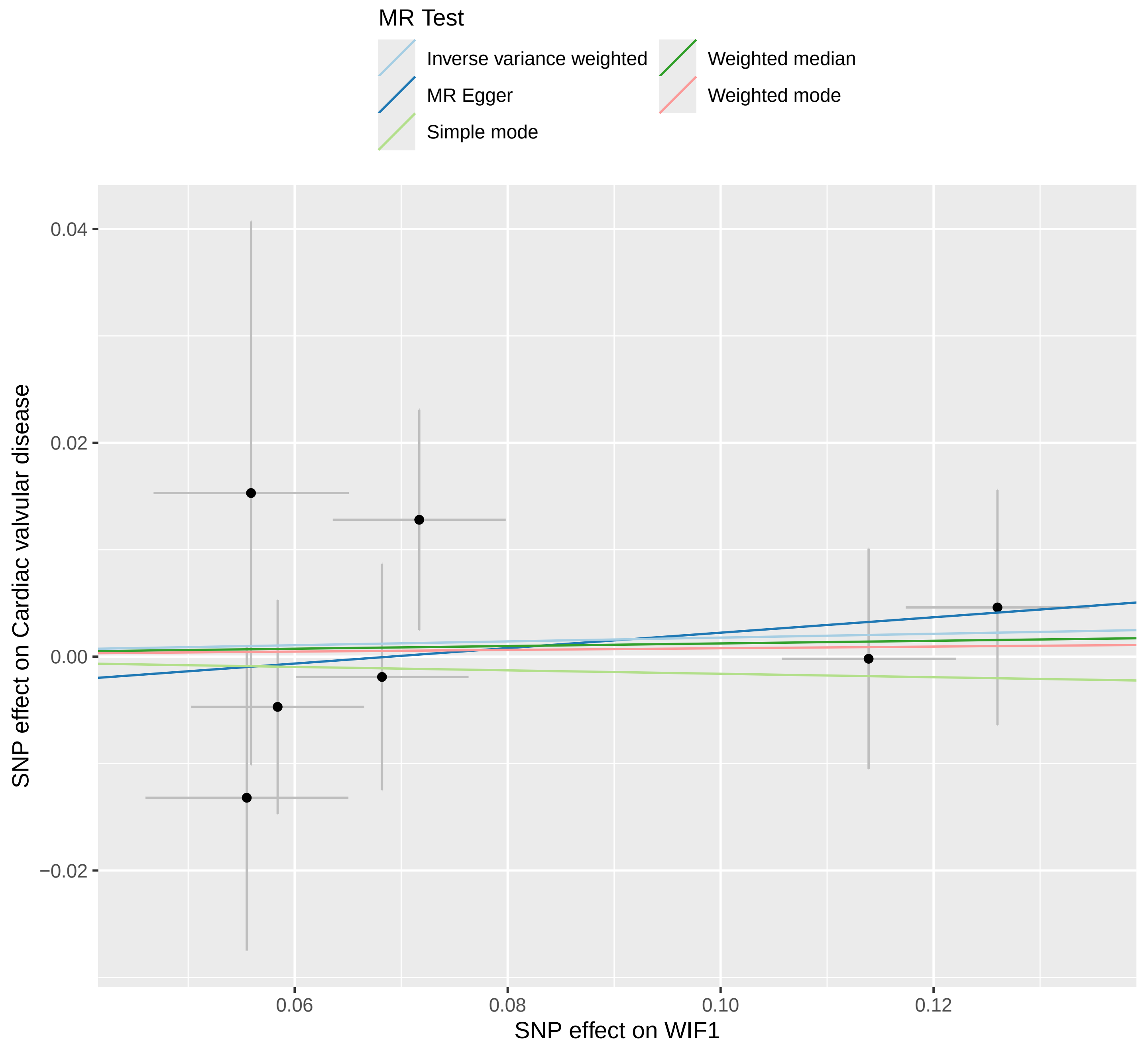

MR Test
Inverse variance weighted
MR Egger
Simple mode
Weighted median
Weighted mode
0.04
0.02
0.00
−0.02
0.06
0.08
0.10
0.12
SNP effect on Cardiac valvular disease
SNP effect on WIF1

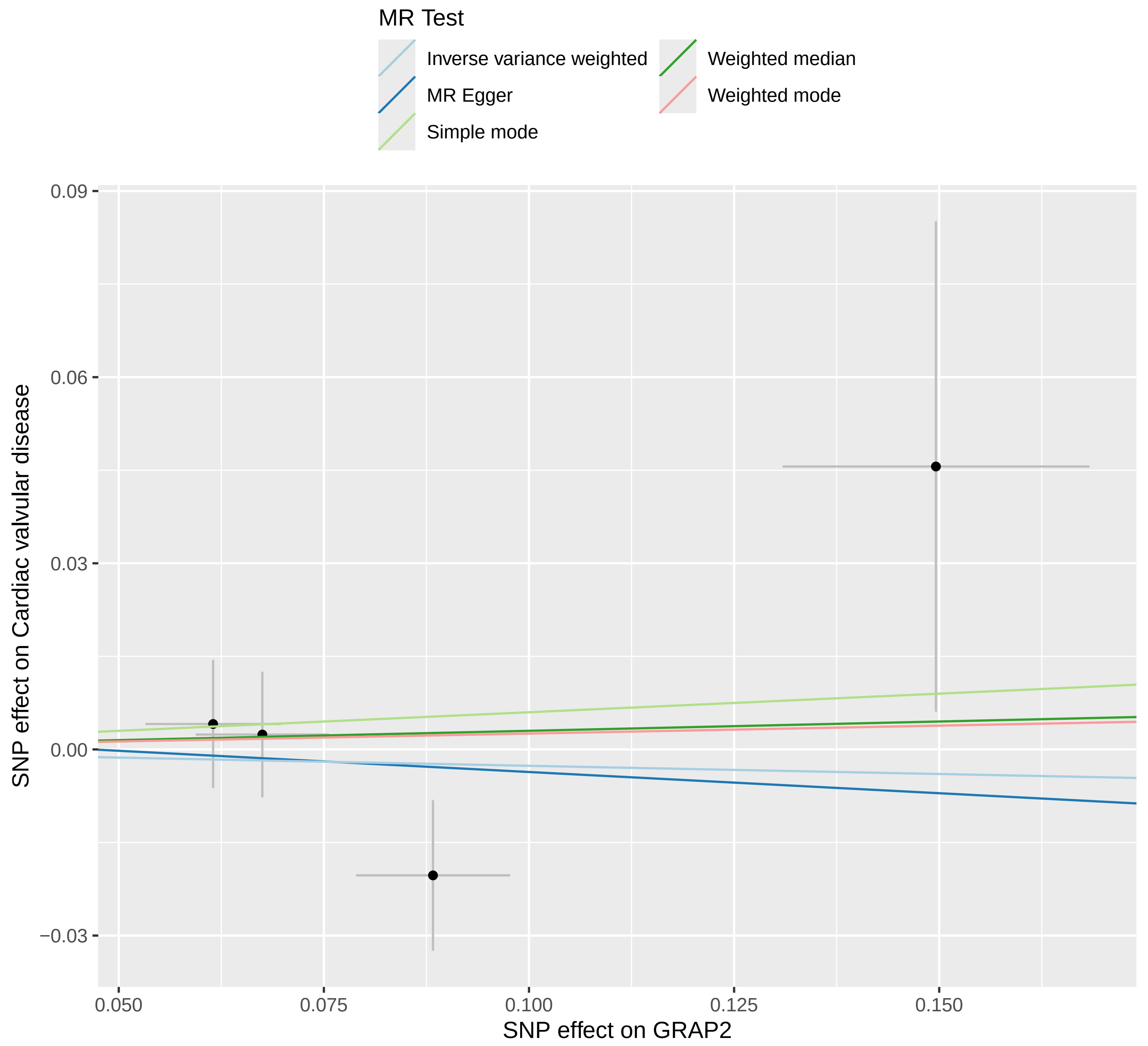

MR Test
Inverse variance weighted
MR Egger
Simple mode
Weighted median
Weighted mode
0.09
0.06
0.03
0.00
−0.03
0.050
0.075
0.100
0.125
0.150
SNP effect on GRAP2
SNP effect on Cardiac valvular disease

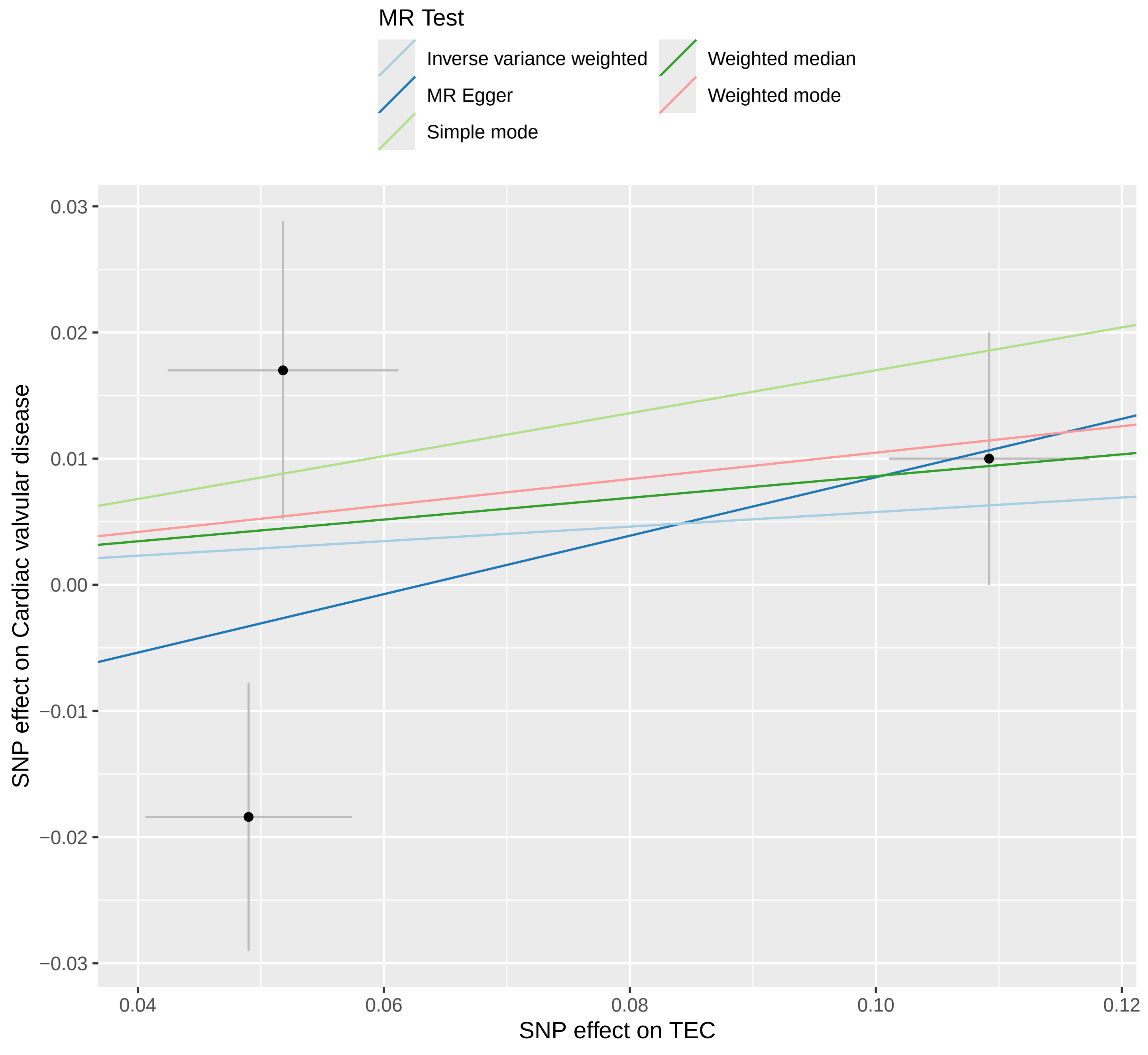

MR Test
Inverse variance weighted
MR Egger
Simple mode
Weighted median
Weighted mode
0.03
0.02
0.01
0.00
−0.01
−0.02
−0.03
SNP effect on Cardiac valvular disease
0.04
0.06
0.08
0.10
0.12
SNP effect on TEC

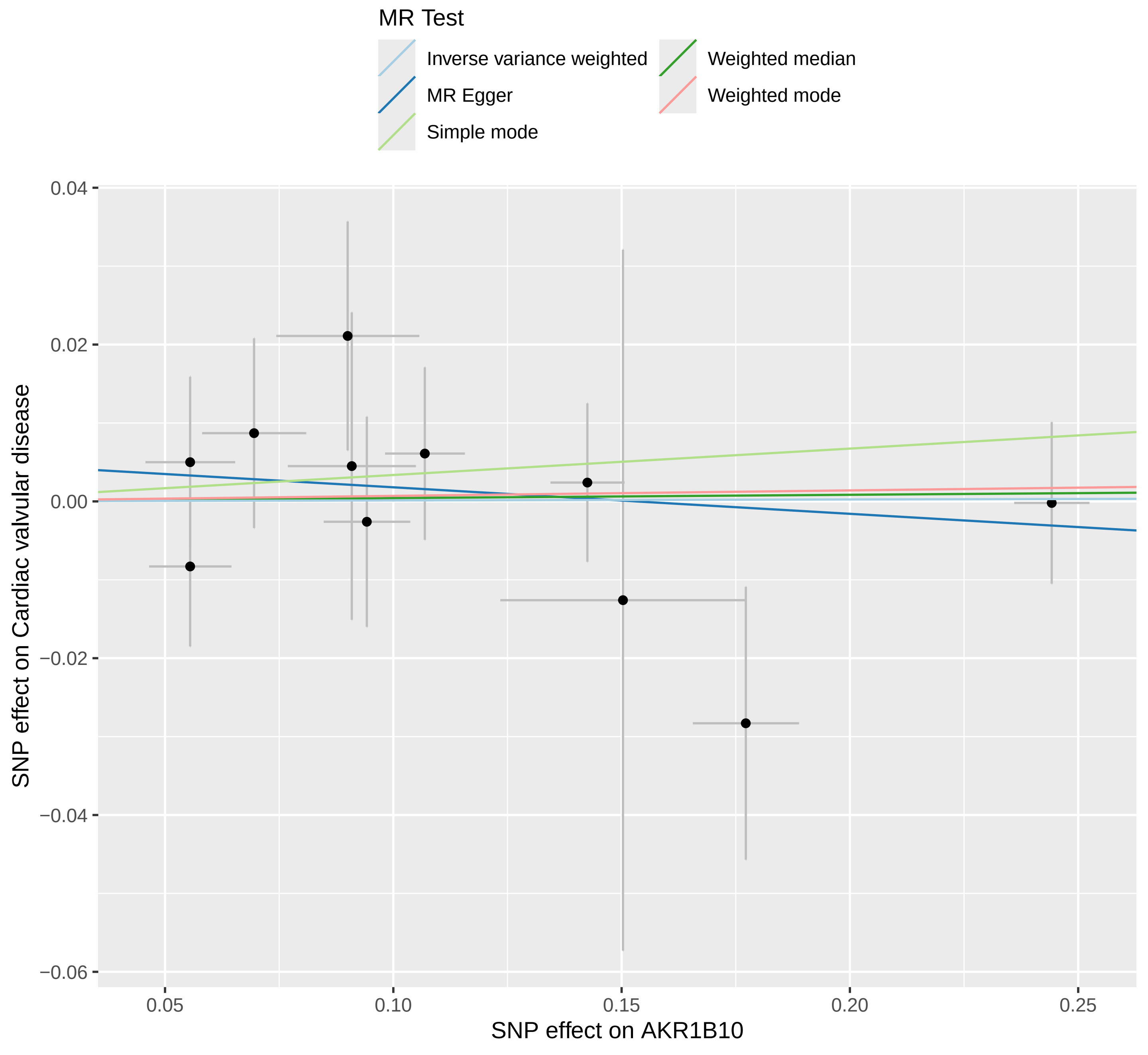

MR Test
Inverse variance weighted
MR Egger
Simple mode
Weighted median
Weighted mode
0.04
0.02
0.00
−0.02
−0.04
−0.06
SNP effect on Cardiac valvular disease
0.05
0.10
0.15
0.20
0.25
SNP effect on AKR1B10

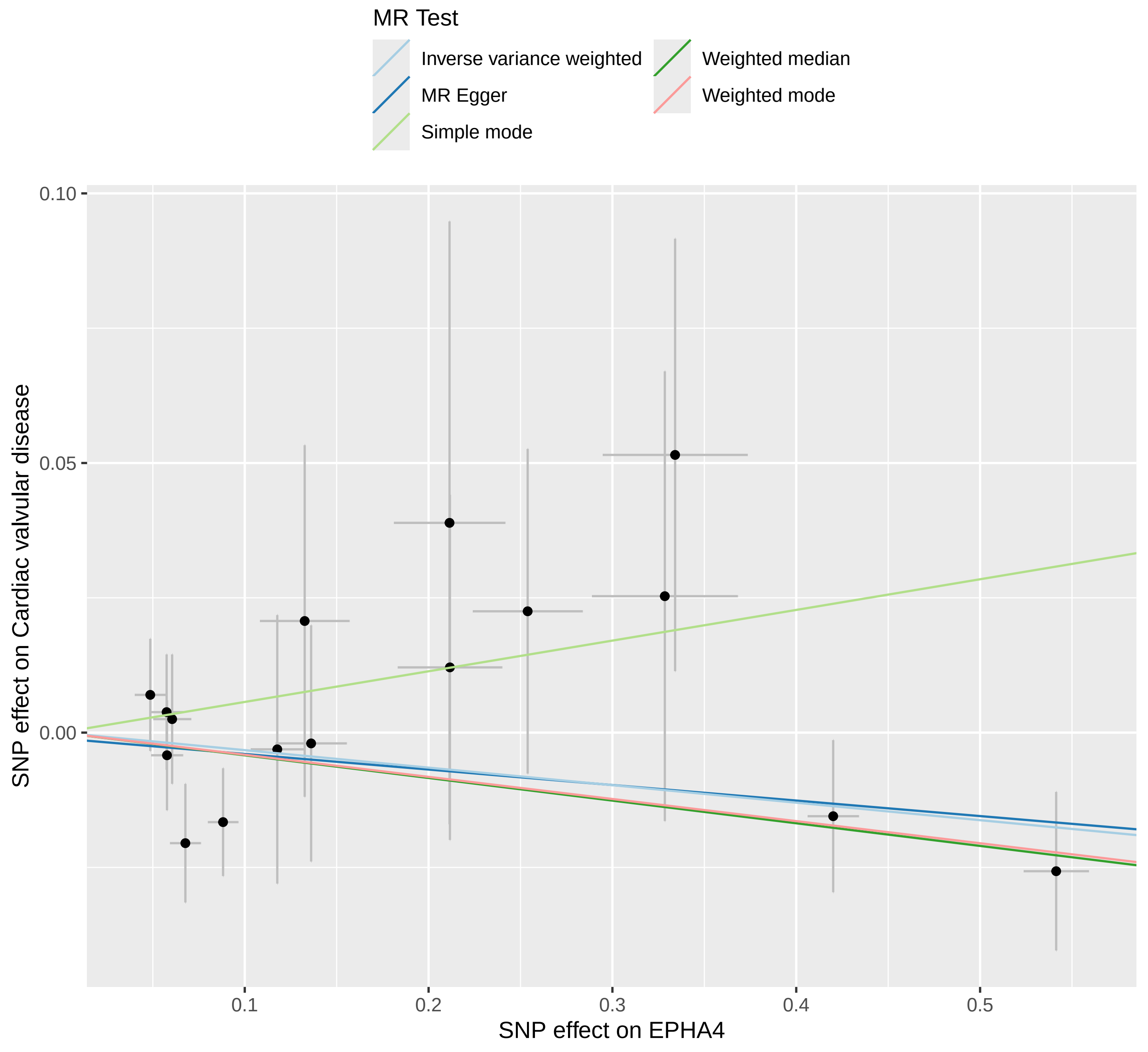
MR Test
Inverse variance weighted
MR Egger
Simple mode
Weighted median
Weighted mode
0.10
0.05
0.00
0.1
0.2
0.3
0.4
0.5
SNP effect on Cardiac valvular disease
SNP effect on EPHA4

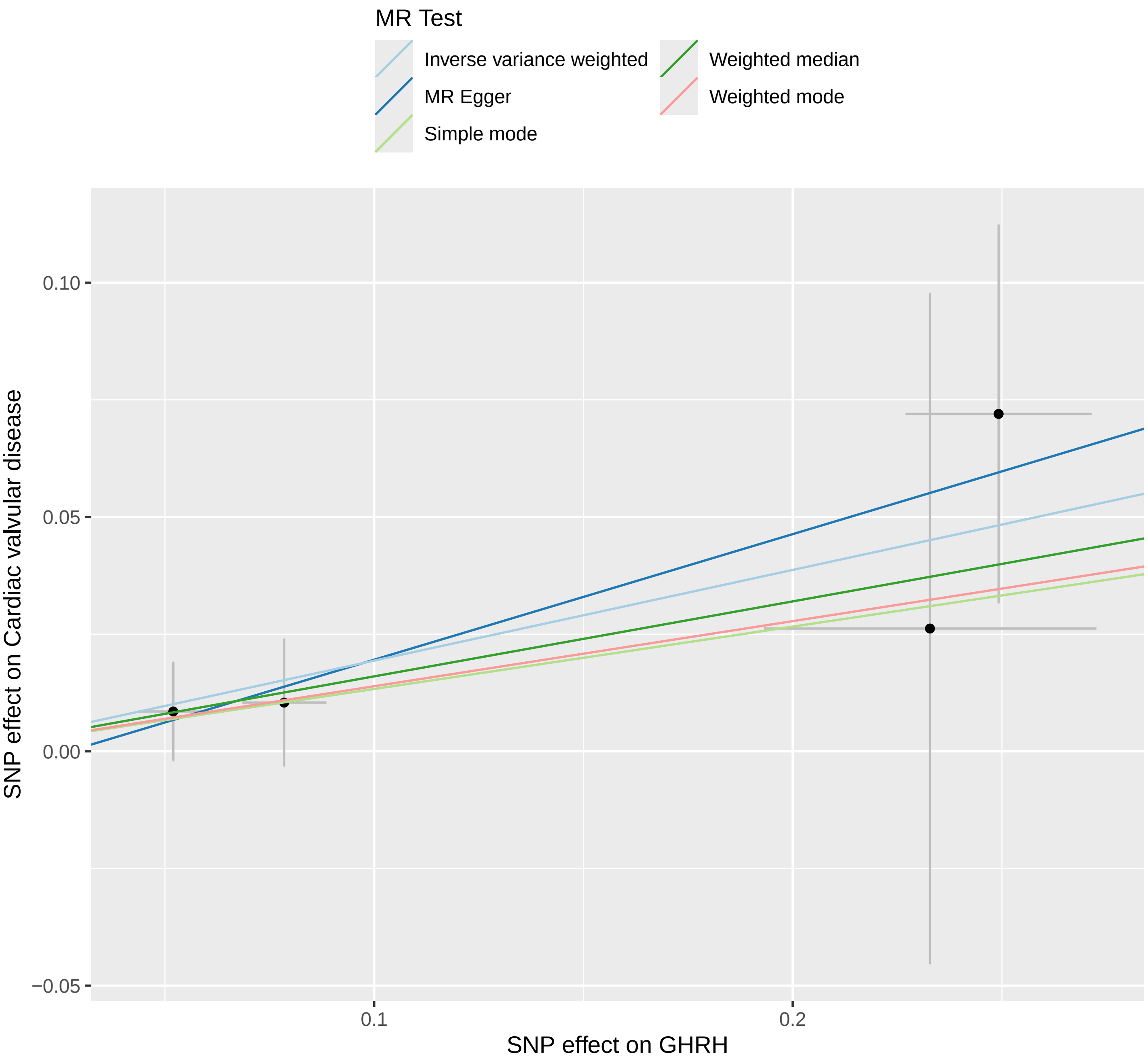
MR Test
Inverse variance weighted
MR Egger
Simple mode
Weighted median
Weighted mode
0.10
0.05
0.00
−0.05
0.1
0.2
SNP effect on Cardiac valvular disease
SNP effect on GHRH

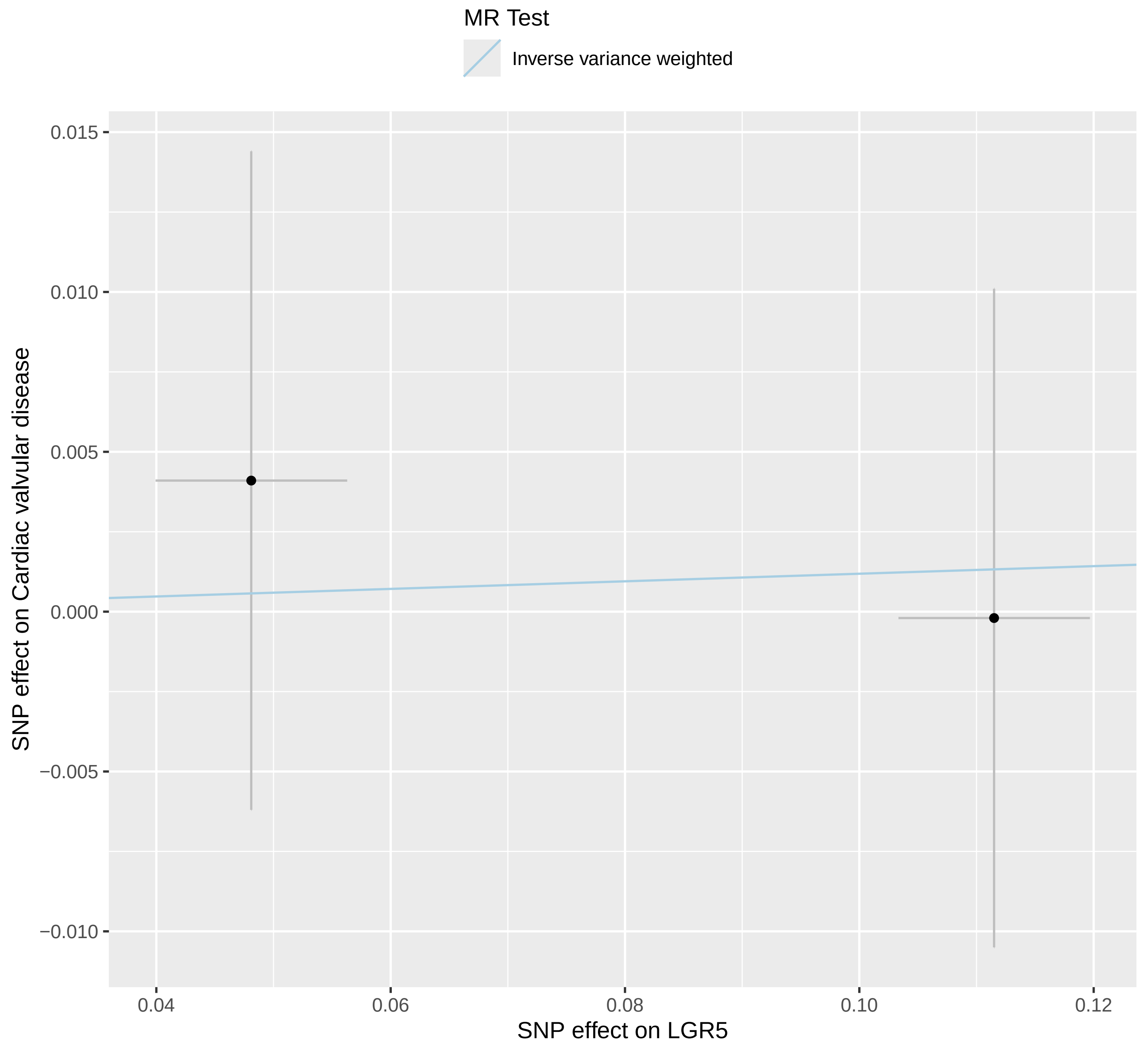
MR Test
Inverse variance weighted
0.015
0.010
0.005
0.000
−0.005
−0.010
SNP effect on Cardiac valvular disease
0.04
0.06
0.08
0.10
0.12
SNP effect on LGR5

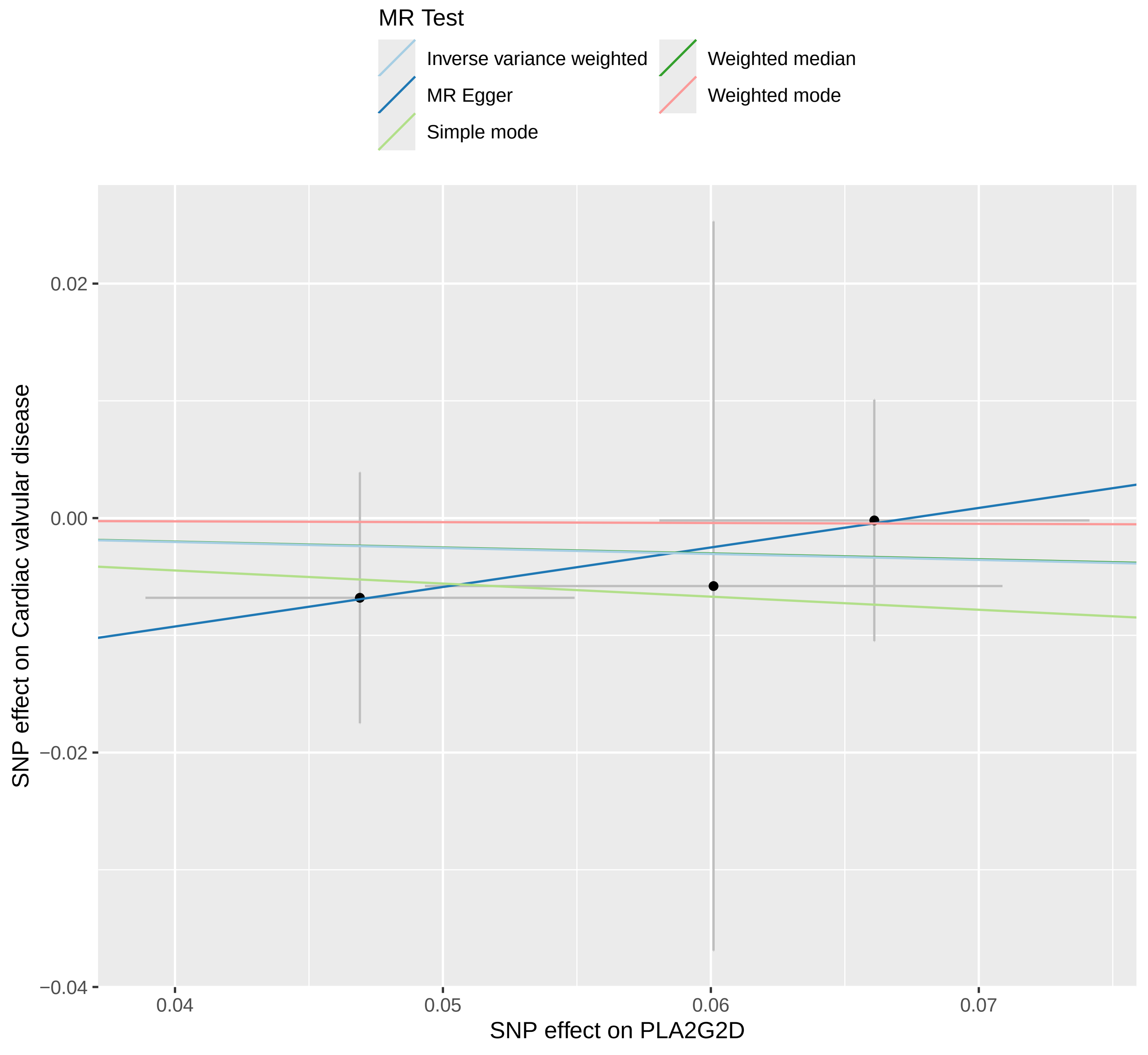

MR Test
Inverse variance weighted
MR Egger
Simple mode
Weighted median
Weighted mode
0.02
0.00
−0.02
−0.04
0.04
0.05
0.06
0.07
SNP effect on PLA2G2D
SNP effect on Cardiac valvular disease

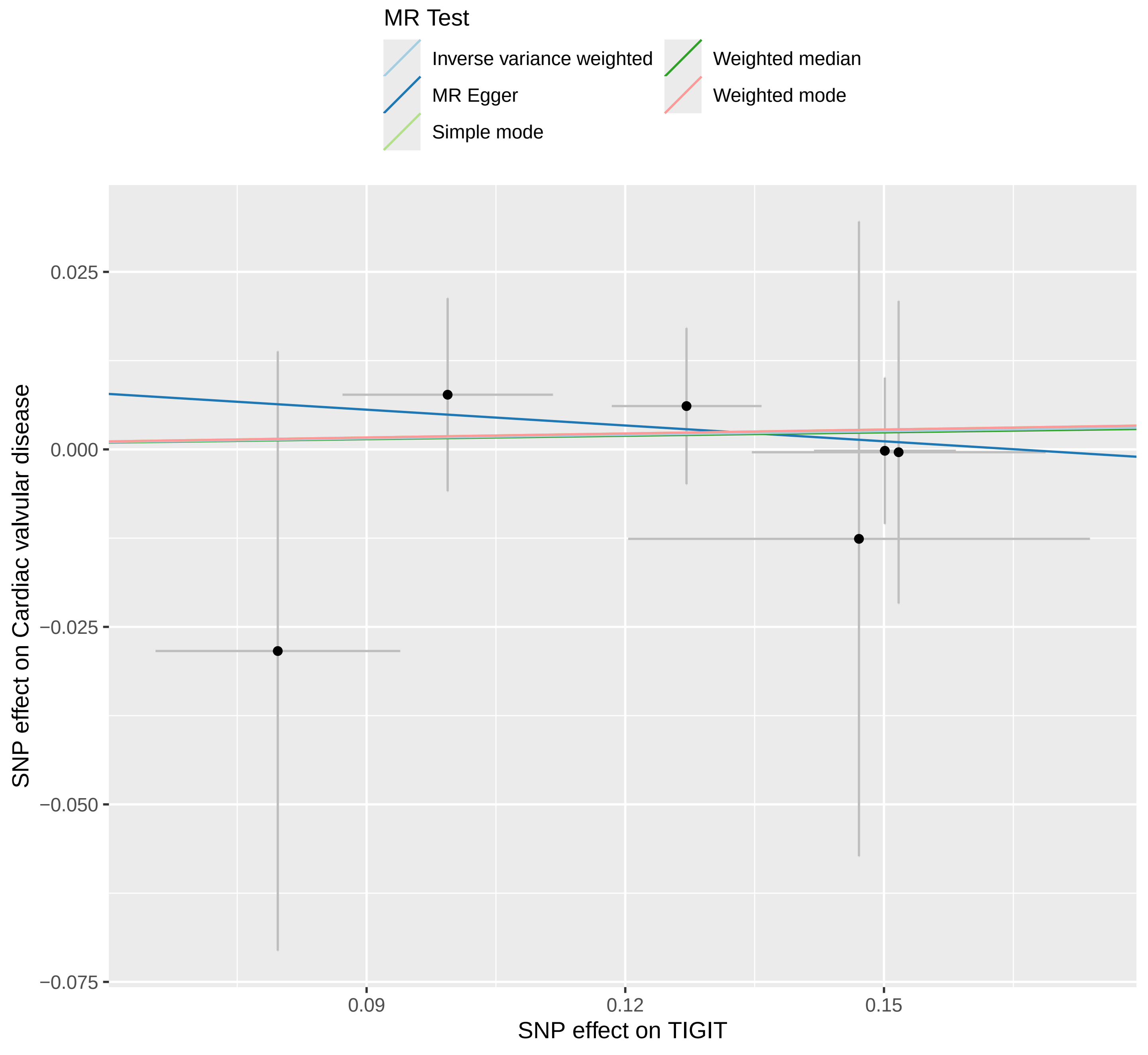
MR Test
Inverse variance weighted
MR Egger
Simple mode
Weighted median
Weighted mode
0.025
0.000
−0.025
−0.050
−0.075
0.09
0.12
0.15
SNP effect on Cardiac valvular disease
SNP effect on TIGIT

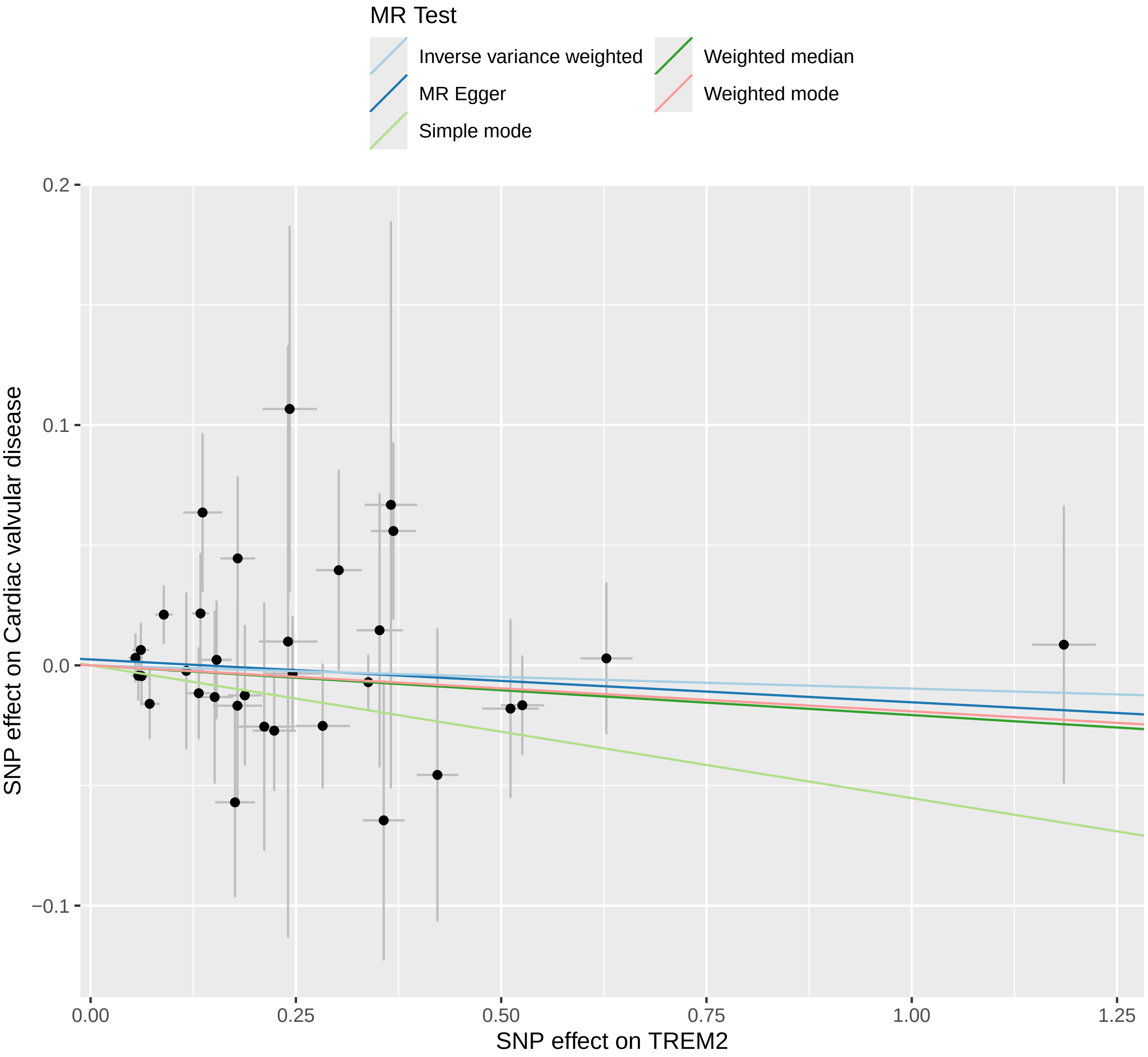

MR Test
Inverse variance weighted
MR Egger
Simple mode
Weighted median
Weighted mode
0.2
0.1
0.0
−0.1
SNP effect on Cardiac valvular disease
0.00
0.25
0.50
0.75
1.00
1.25
SNP effect on TREM2

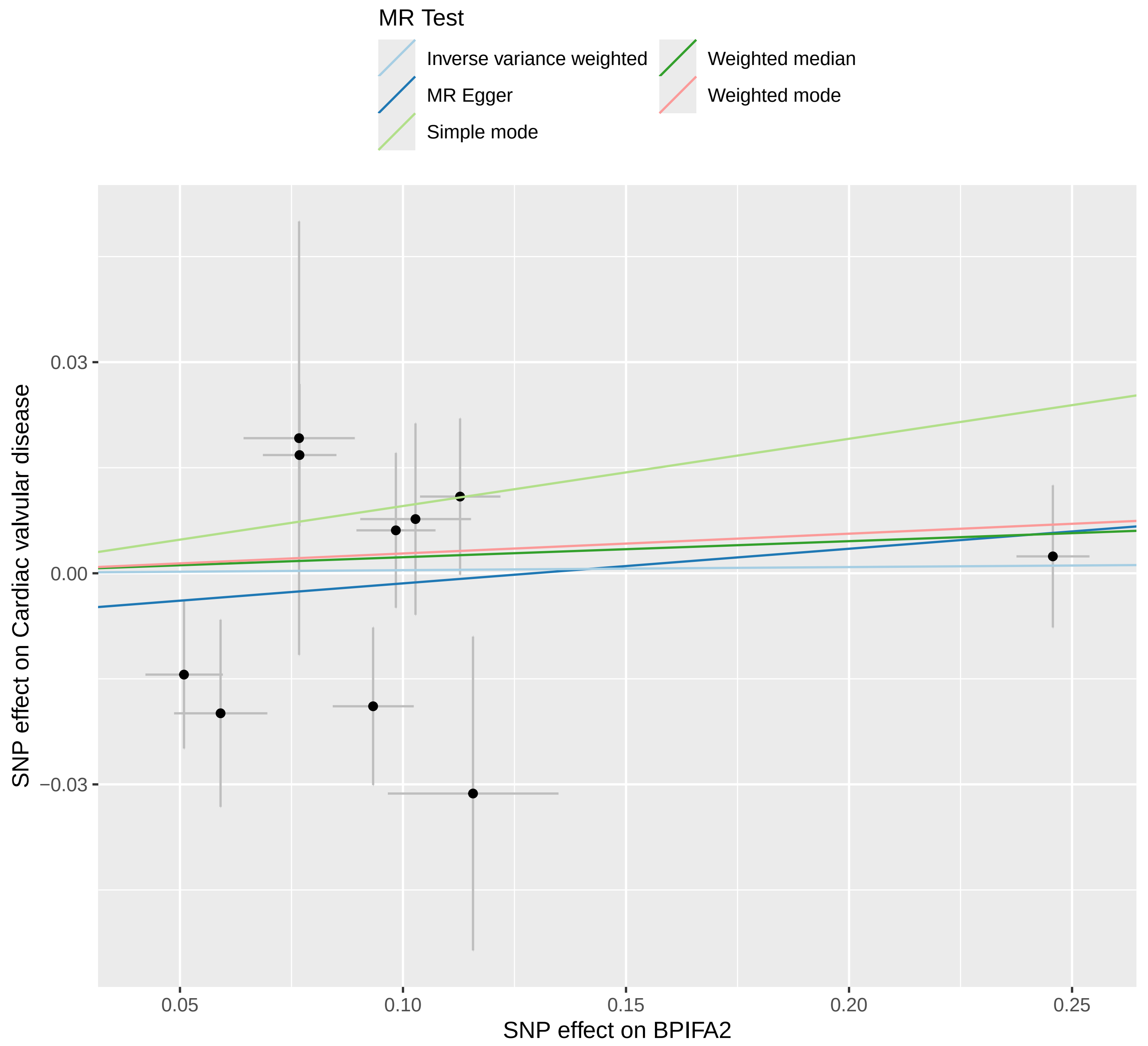

MR Test
Inverse variance weighted
MR Egger
Simple mode
Weighted median
Weighted mode
0.03
0.00
−0.03
SNP effect on Cardiac valvular disease
0.05
0.10
0.15
0.20
0.25
SNP effect on BPIFA2

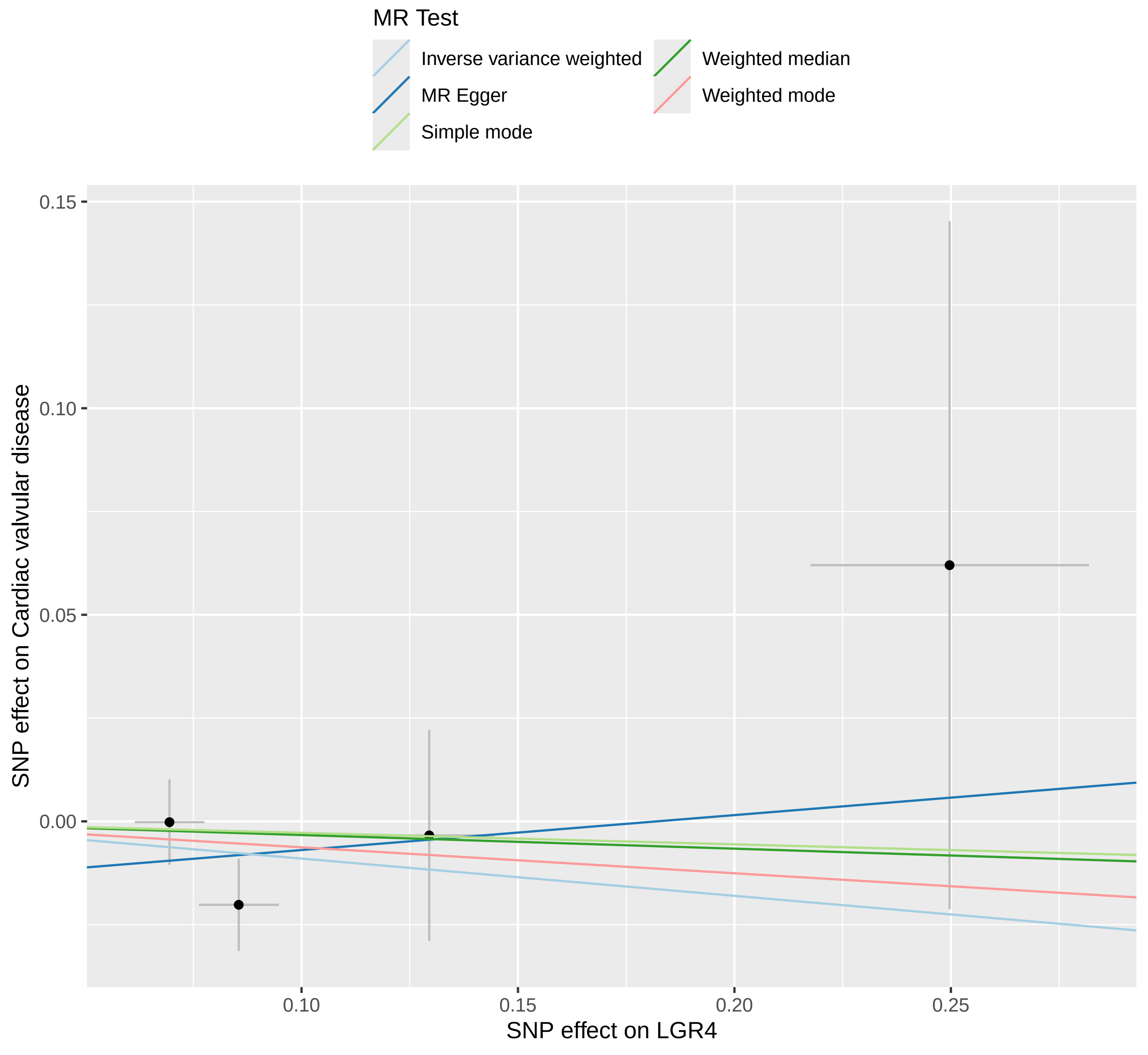
MR Test
Inverse variance weighted
MR Egger
Simple mode
Weighted median
Weighted mode
0.15
0.10
0.05
0.00
0.10
0.15
0.20
0.25
SNP effect on Cardiac valvular disease
SNP effect on LGR4

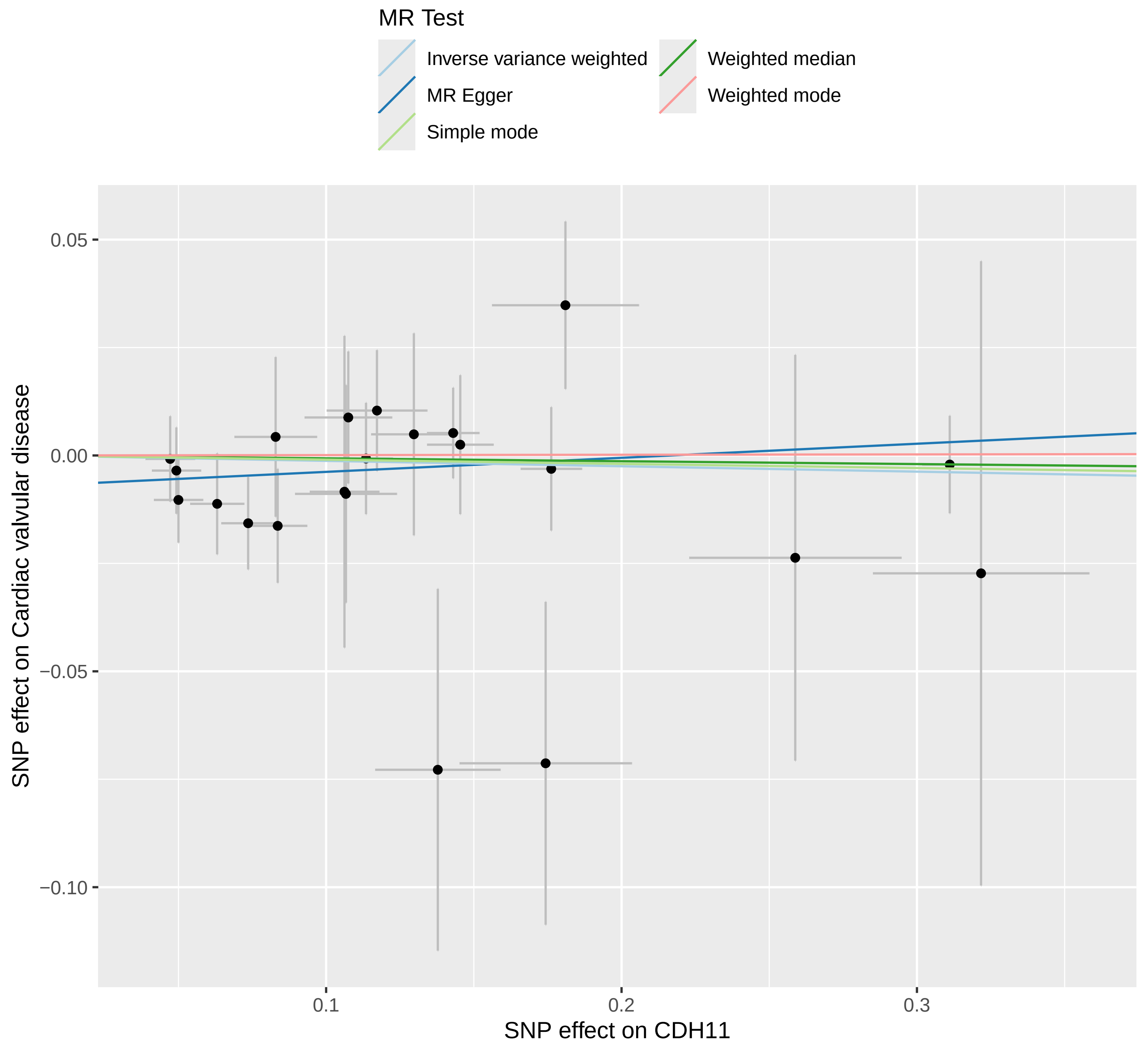
MR Test
Inverse variance weighted
MR Egger
Simple mode
Weighted median
Weighted mode
0.05
0.00
−0.05
−0.10
0.1
0.2
0.3
SNP effect on Cardiac valvular disease
SNP effect on CDH11

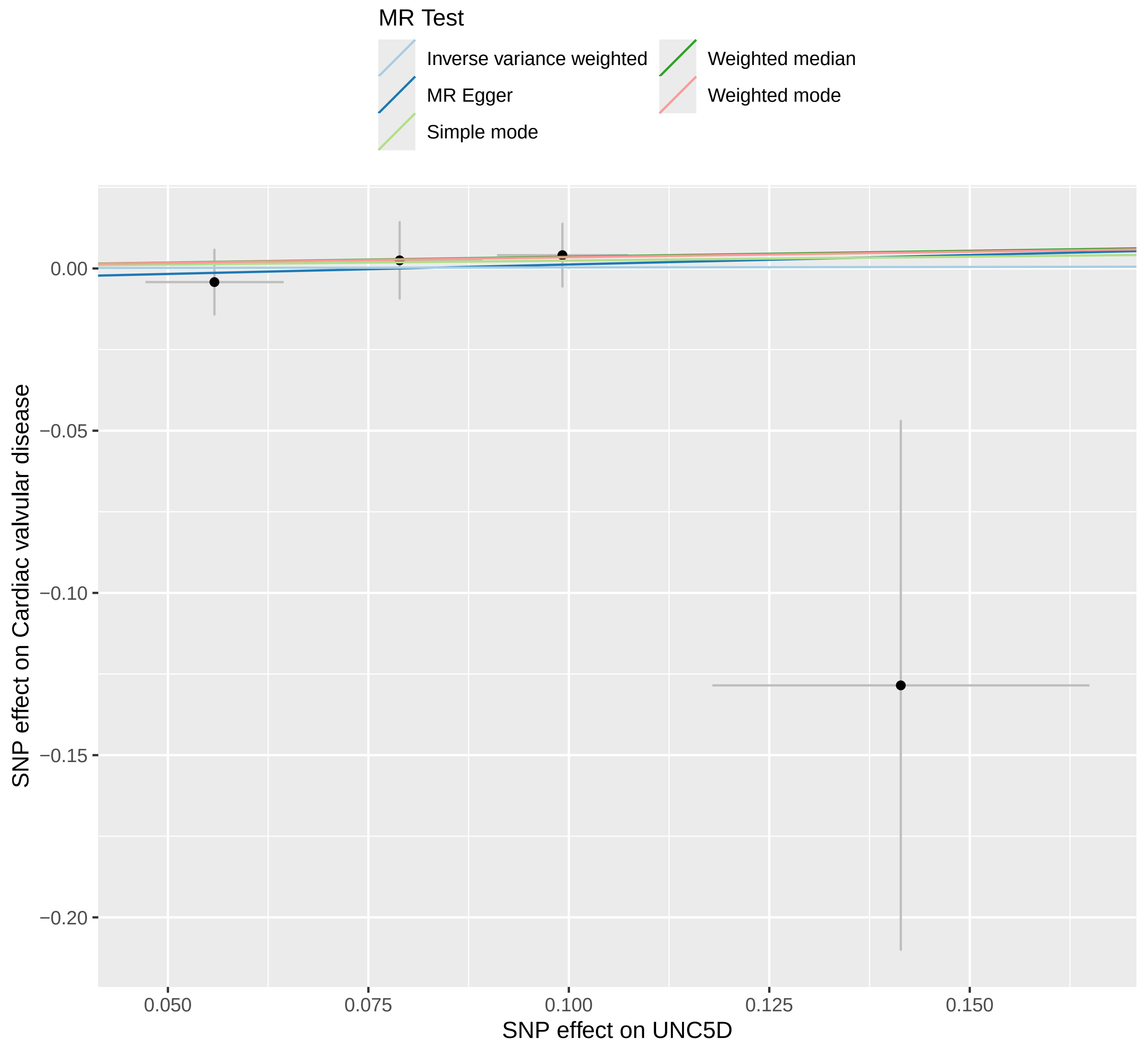
MR Test
Inverse variance weighted
MR Egger
Simple mode
Weighted median
Weighted mode
0.00
−0.05
−0.10
−0.15
−0.20
SNP effect on Cardiac valvular disease
0.050
0.075
0.100
0.125
0.150
SNP effect on UNC5D

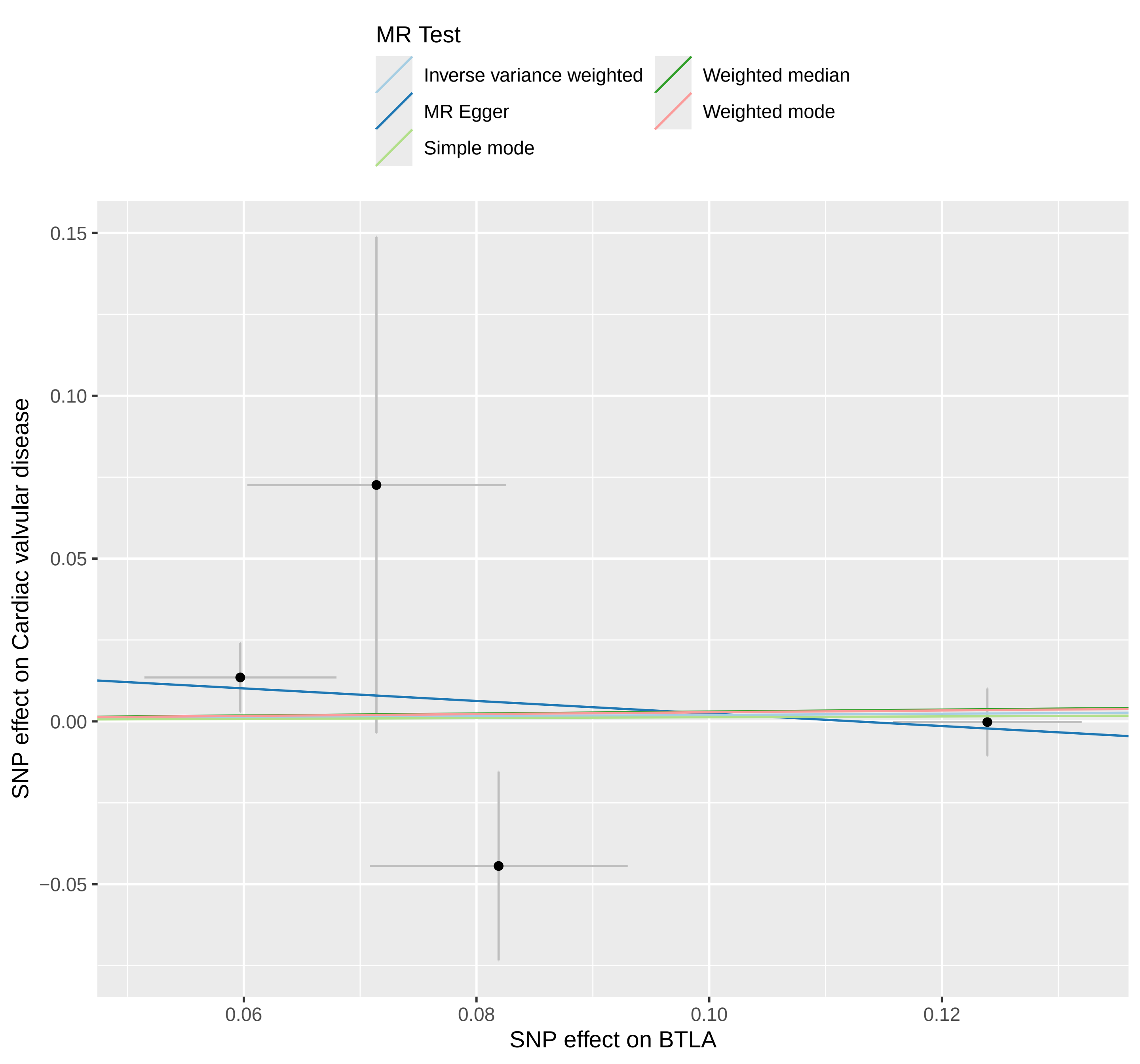
MR Test
Inverse variance weighted
MR Egger
Simple mode
Weighted median
Weighted mode
0.15
0.10
0.05
0.00
−0.05
SNP effect on Cardiac valvular disease
0.06
0.08
0.10
0.12
SNP effect on BTLA

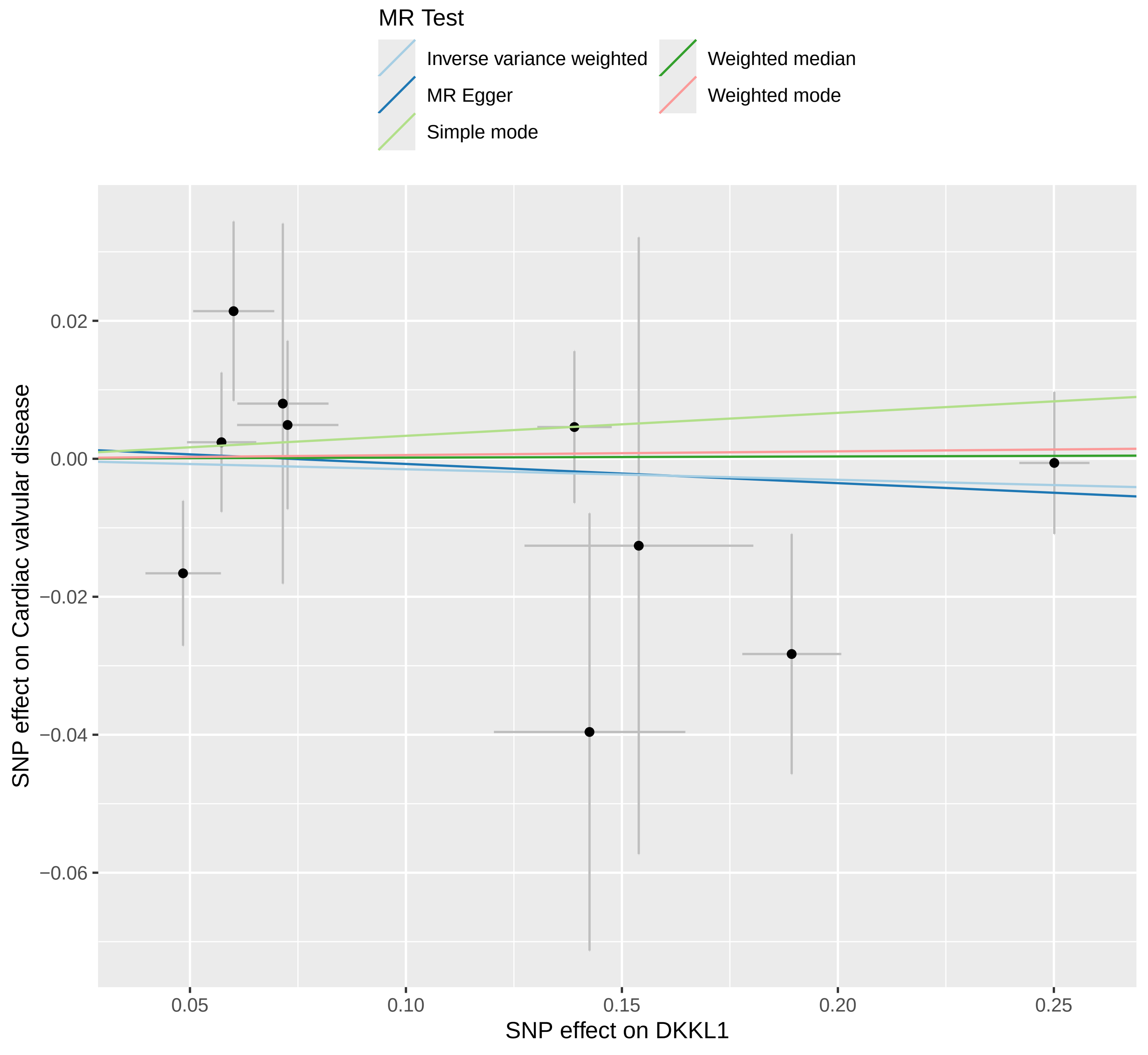

MR Test
Inverse variance weighted
MR Egger
Simple mode
Weighted median
Weighted mode
SNP effect on Cardiac valvular disease
0.02
0.00
−0.02
−0.04
−0.06
0.05
0.10
0.15
0.20
0.25
SNP effect on DKKL1

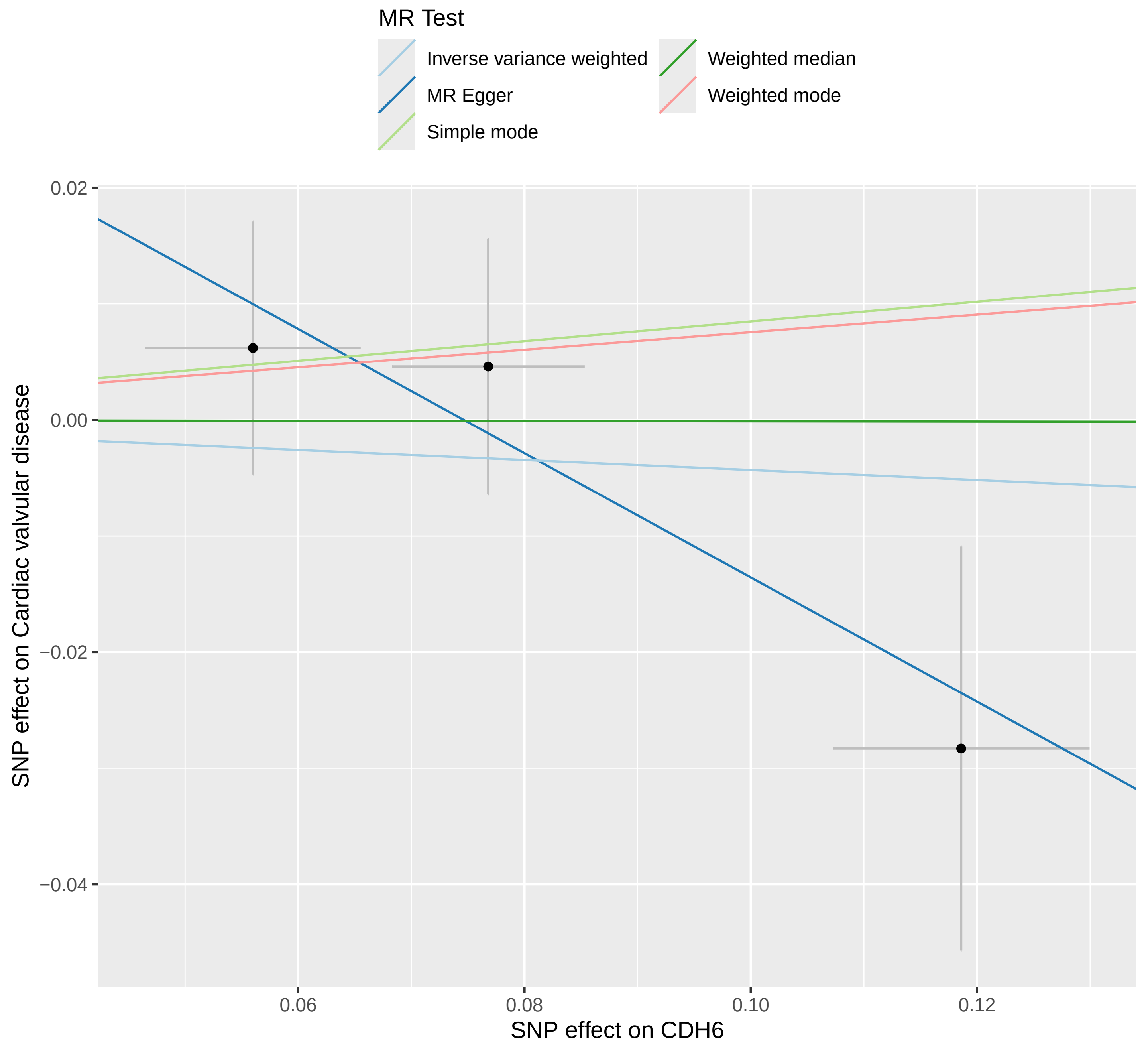

MR Test
Inverse variance weighted
MR Egger
Simple mode
Weighted median
Weighted mode
0.02
0.00
−0.02
−0.04
SNP effect on Cardiac valvular disease
0.06
0.08
0.10
0.12
SNP effect on CDH6

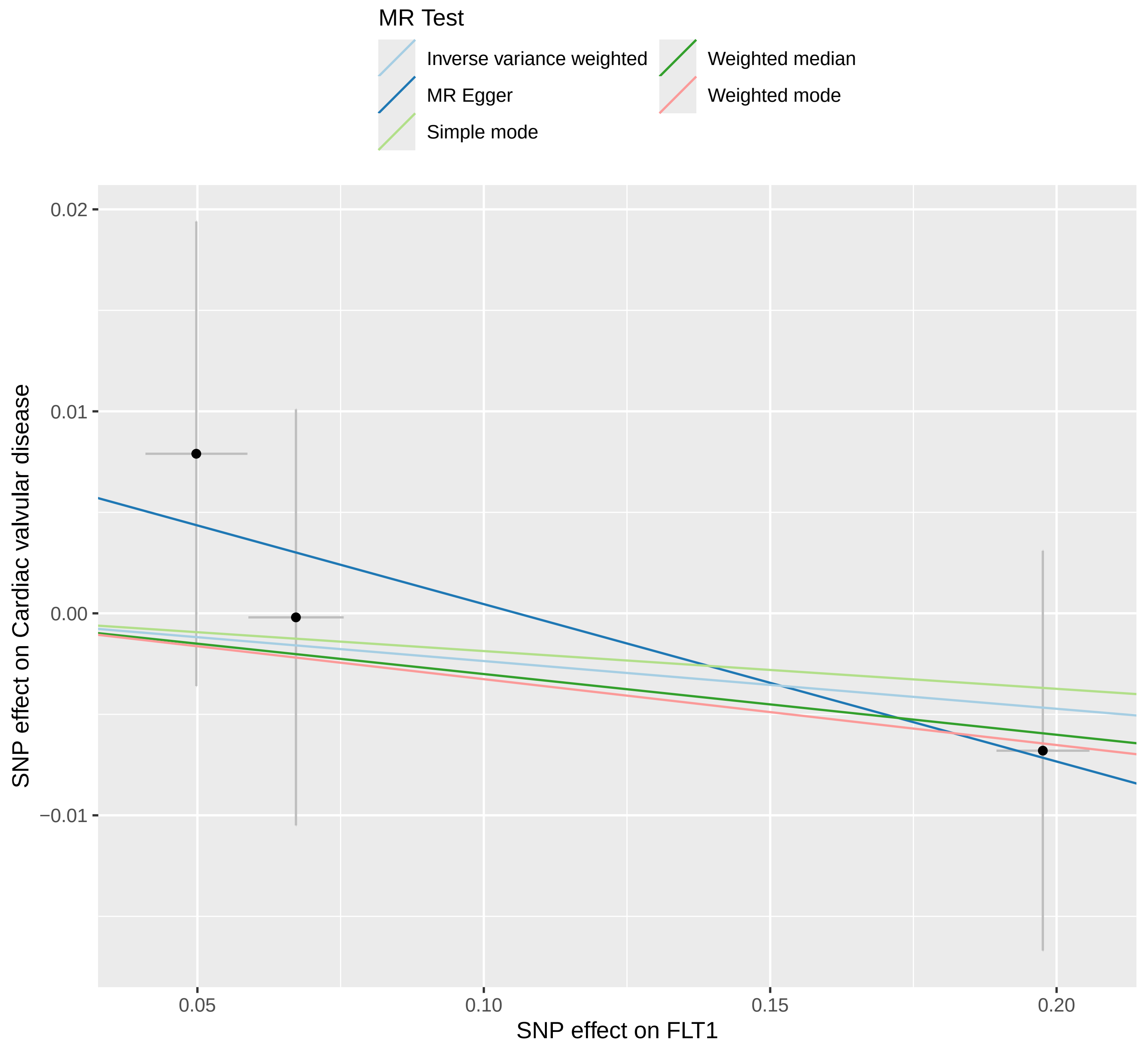
MR Test
Inverse variance weighted
MR Egger
Simple mode
Weighted median
Weighted mode
0.02
0.01
0.00
−0.01
0.05
0.10
0.15
0.20
SNP effect on Cardiac valvular disease
SNP effect on FLT1

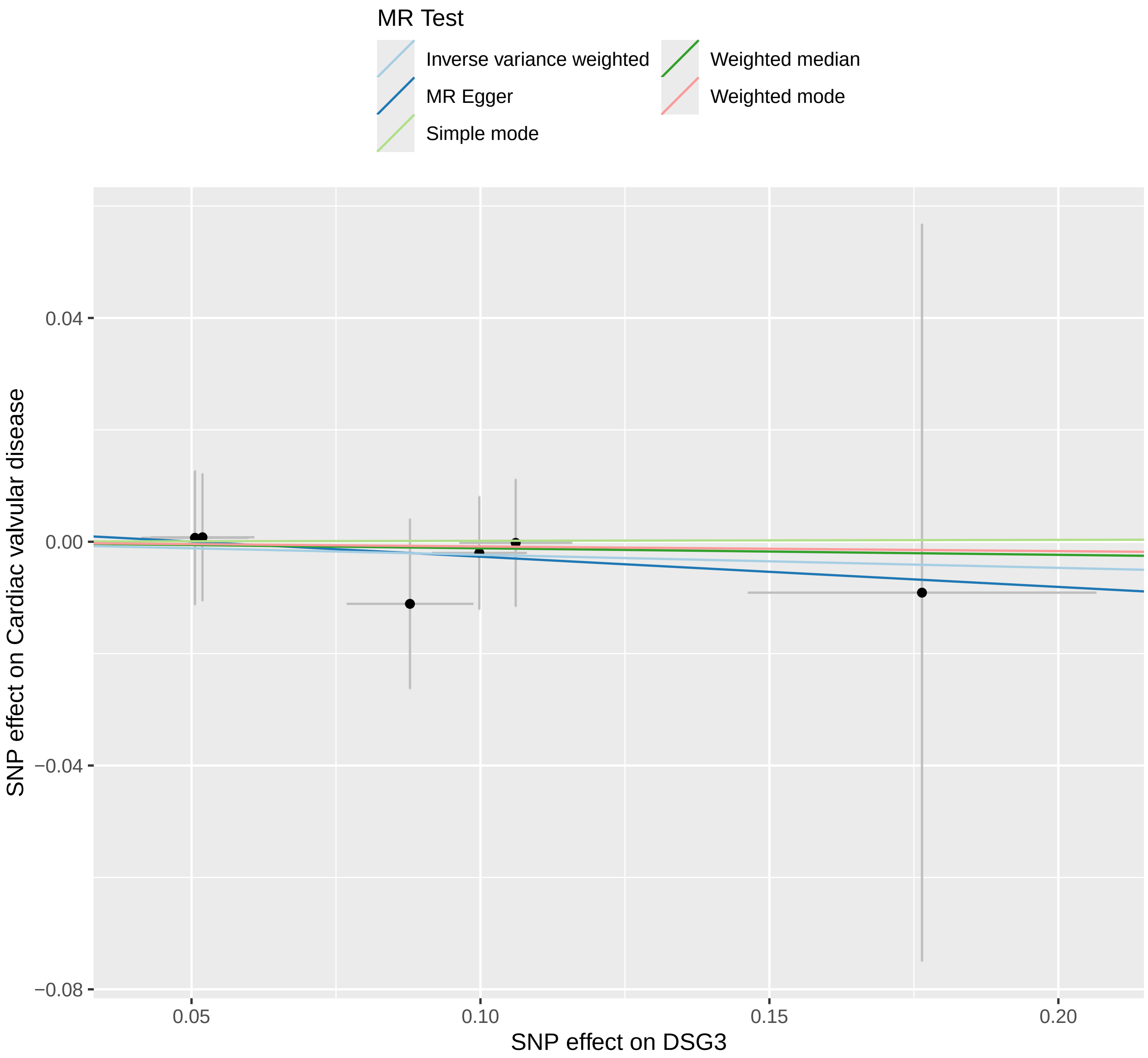
MR Test
Inverse variance weighted
MR Egger
Simple mode
Weighted median
Weighted mode
0.04
0.00
−0.04
−0.08
SNP effect on Cardiac valvular disease
0.05
0.10
0.15
0.20
SNP effect on DSG3

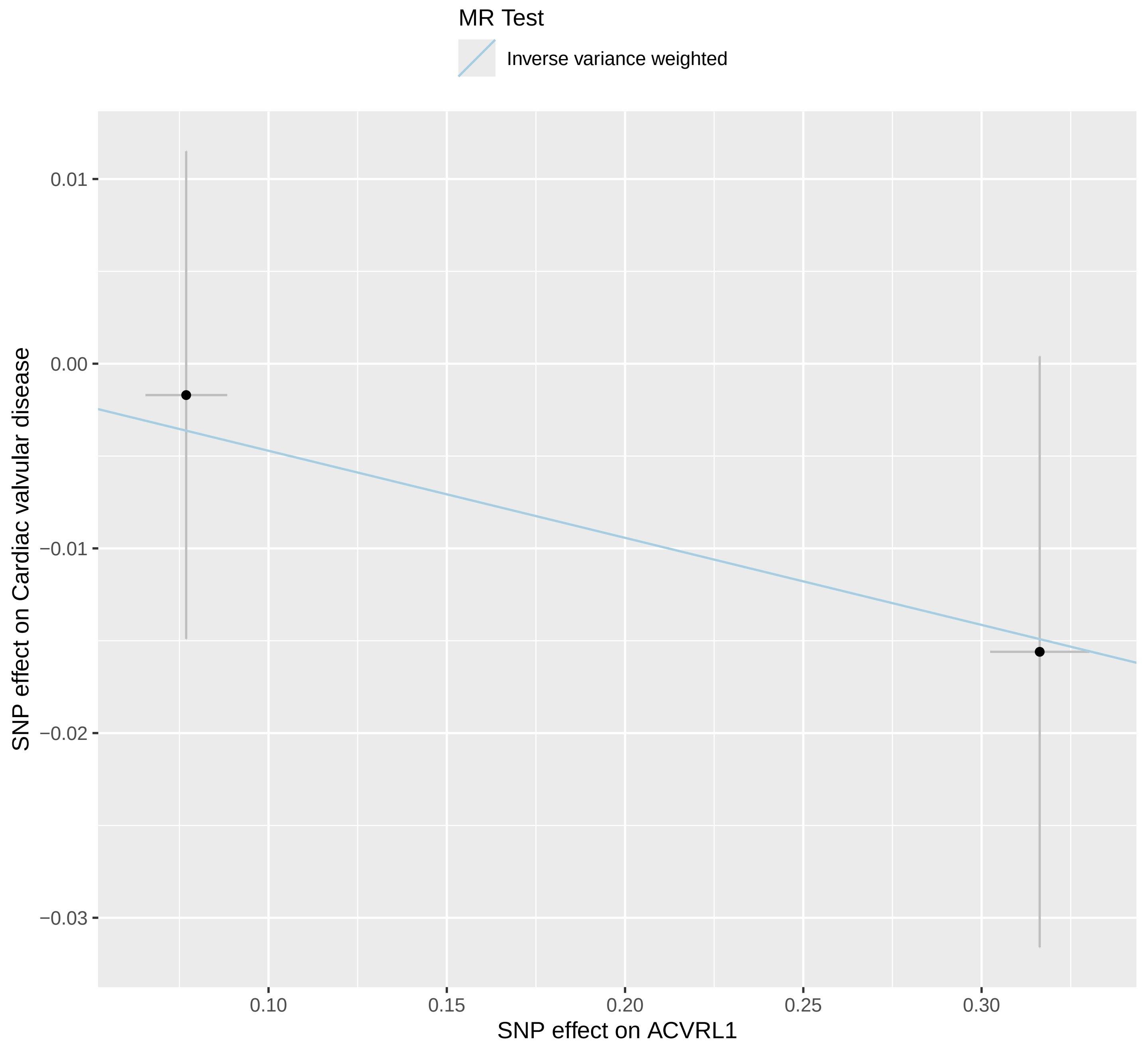

MR Test
Inverse variance weighted
0.01
0.00
−0.01
−0.02
−0.03
0.10
0.15
0.20
0.25
0.30
SNP effect on ACVRL1
SNP effect on Cardiac valvular disease

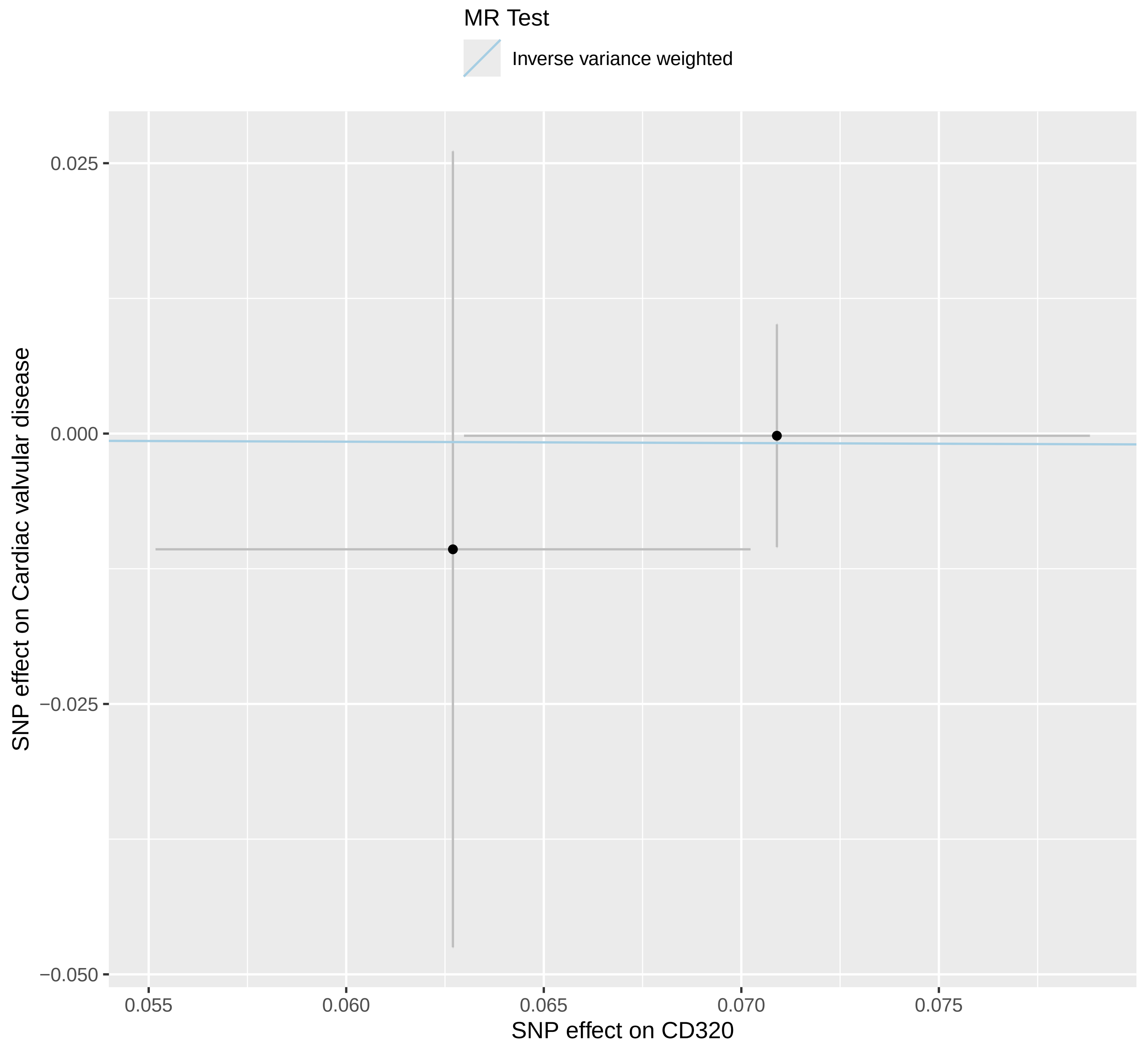

MR Test
Inverse variance weighted
0.025
0.000
−0.025
−0.050
0.055
0.060
0.065
0.070
0.075
SNP effect on CD320
SNP effect on Cardiac valvular disease

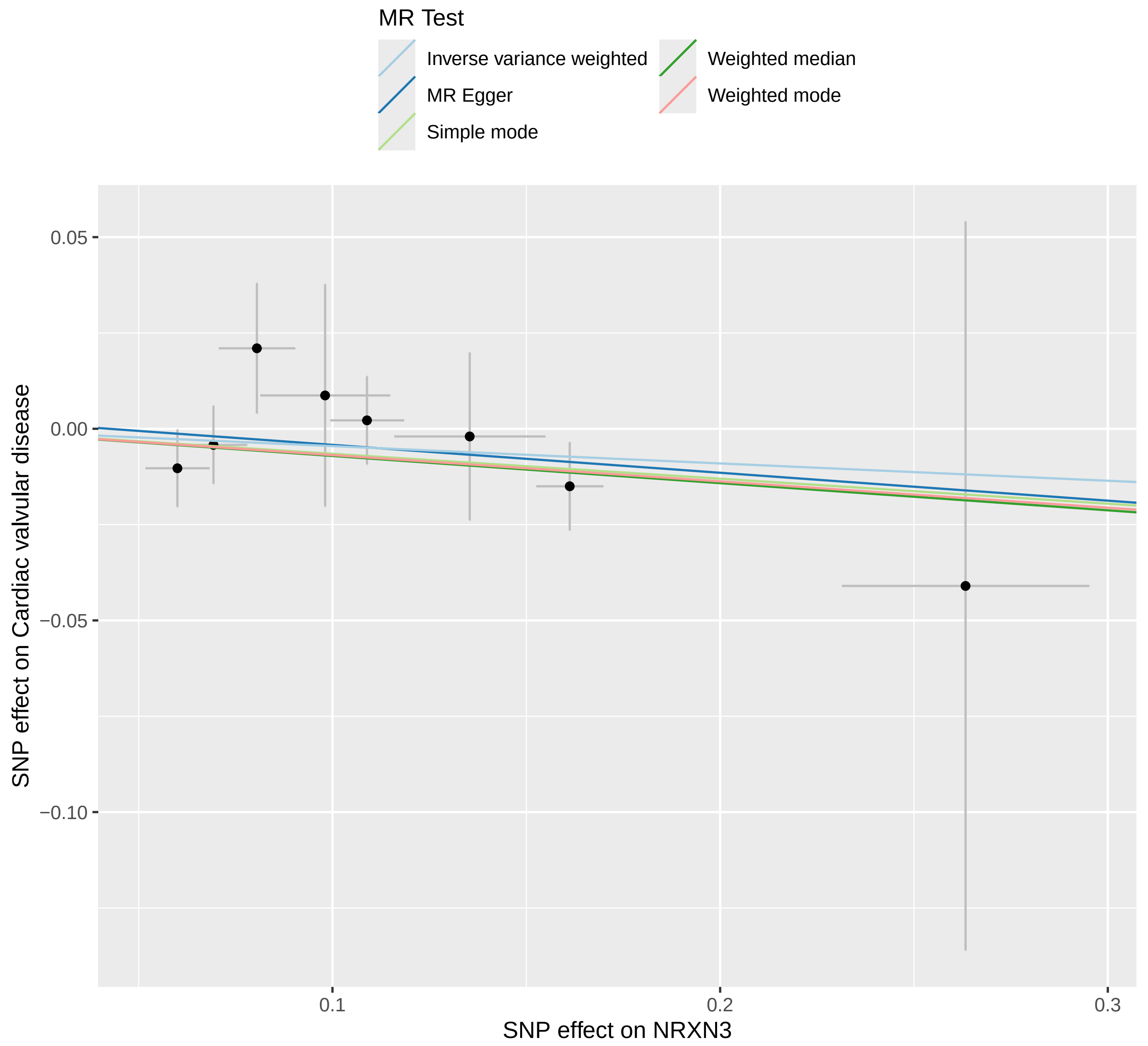
MR Test
Inverse variance weighted
MR Egger
Simple mode
Weighted median
Weighted mode
0.05
0.00
−0.05
−0.10
0.1
0.2
0.3
SNP effect on Cardiac valvular disease
SNP effect on NRXN3

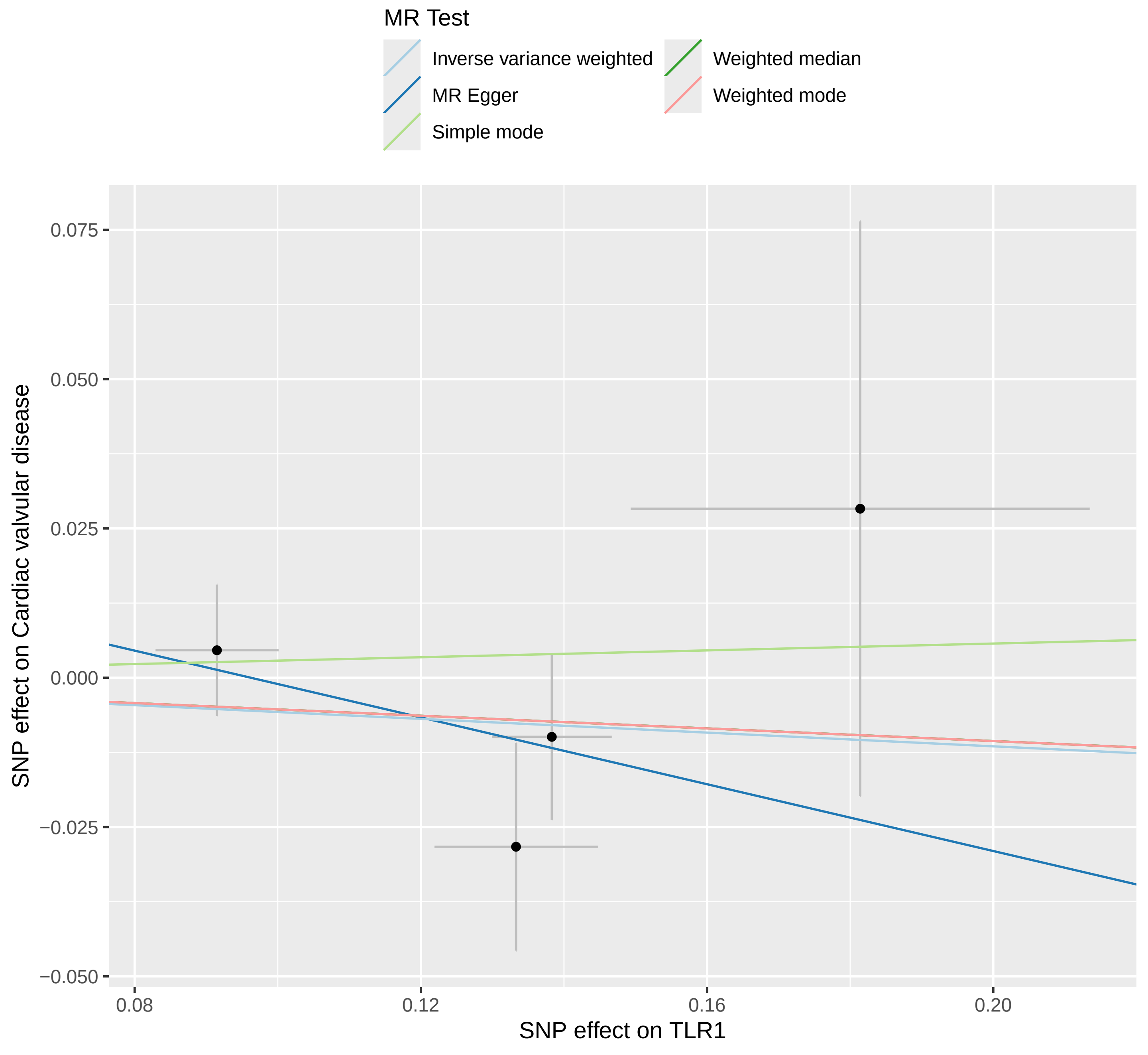
MR Test
Inverse variance weighted
MR Egger
Simple mode
Weighted median
Weighted mode
0.075
0.050
0.025
0.000
−0.025
−0.050
0.08
0.12
0.16
0.20
SNP effect on TLR1
SNP effect on Cardiac valvular disease

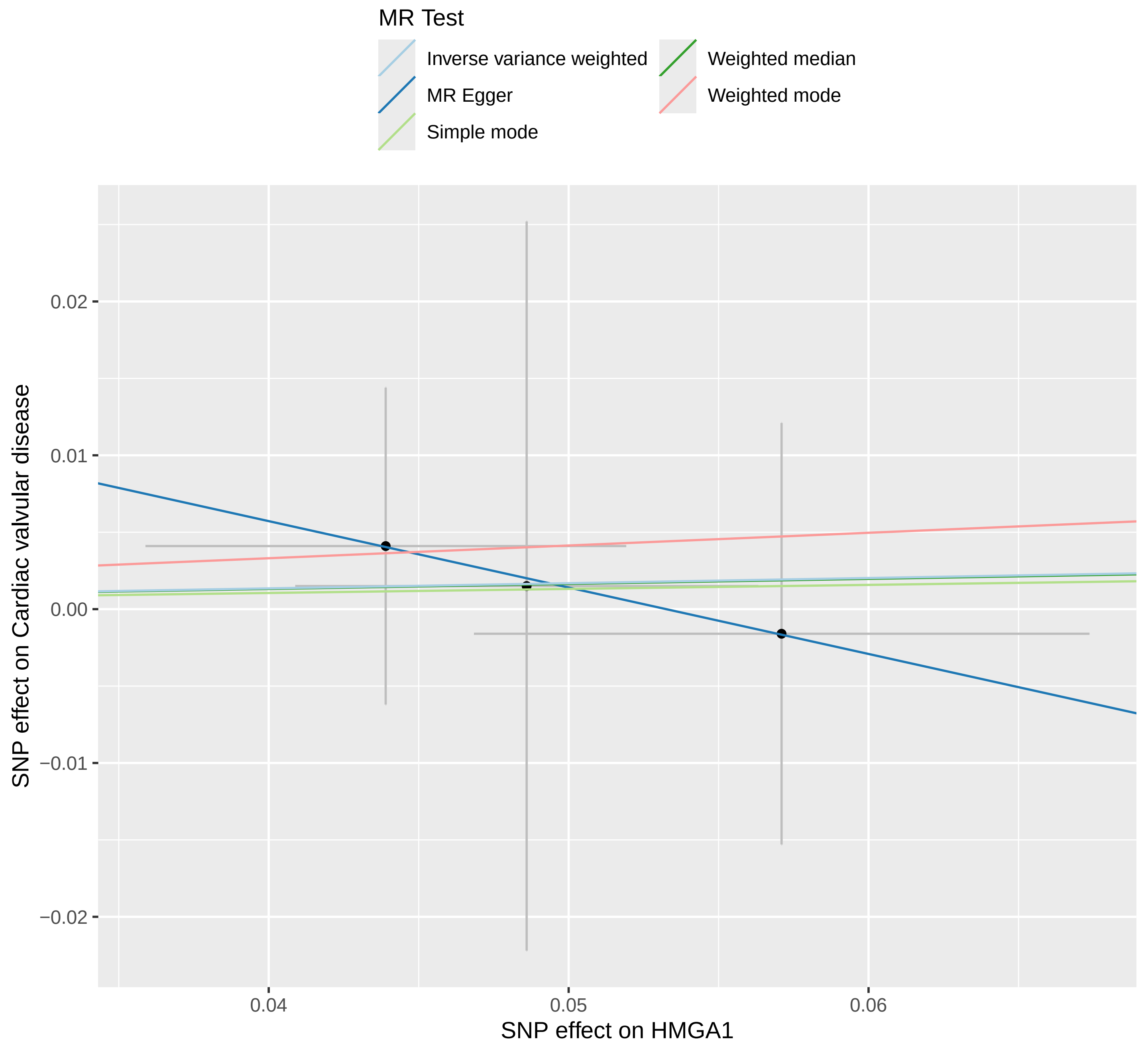
MR Test
Inverse variance weighted
MR Egger
Simple mode
Weighted median
Weighted mode
0.02
0.01
0.00
−0.01
−0.02
SNP effect on Cardiac valvular disease
0.04
0.05
0.06
SNP effect on HMGA1

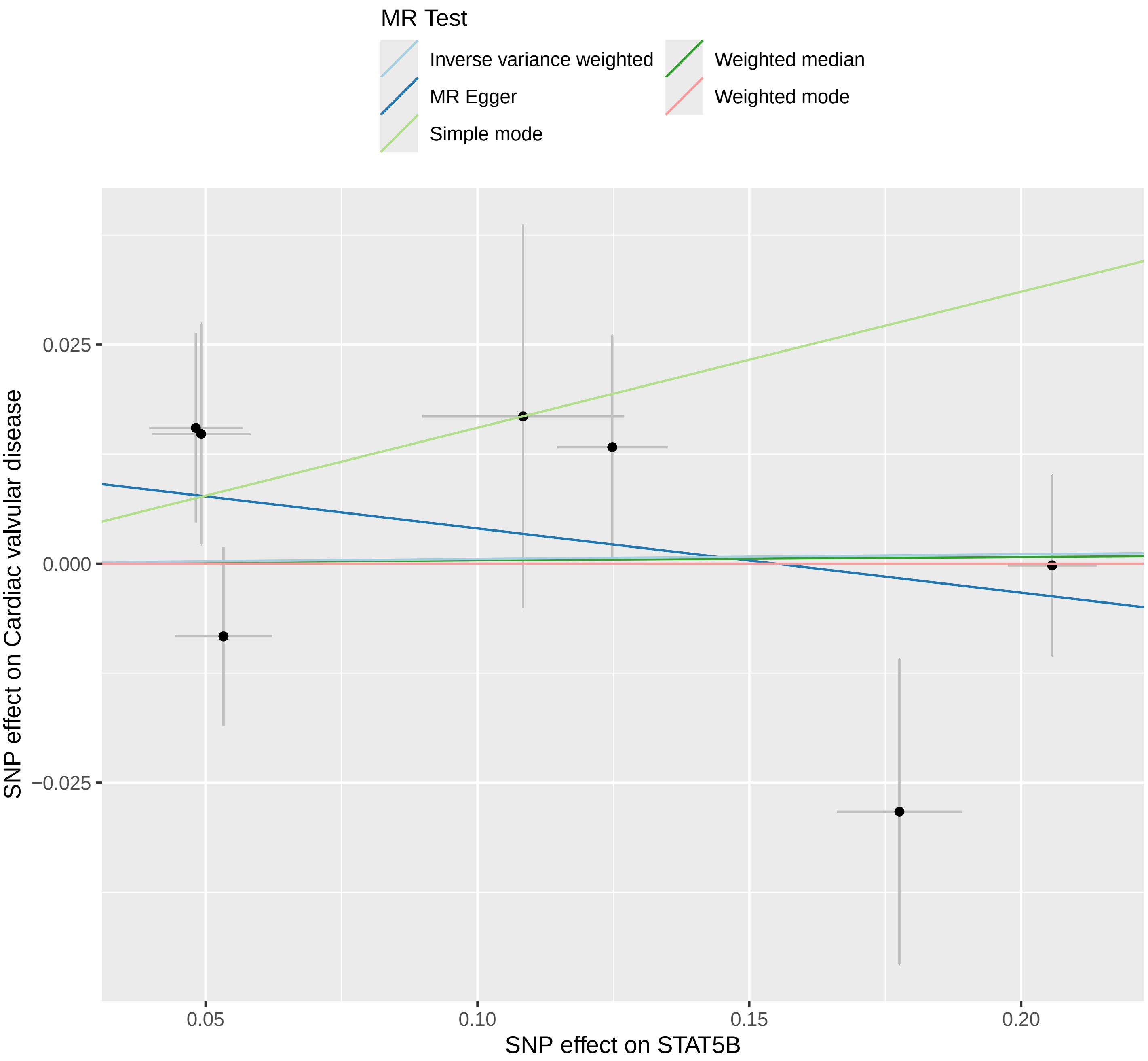
MR Test
Inverse variance weighted
MR Egger
Simple mode
Weighted median
Weighted mode
0.025
0.000
−0.025
0.05
0.10
0.15
0.20
SNP effect on Cardiac valvular disease
SNP effect on STAT5B

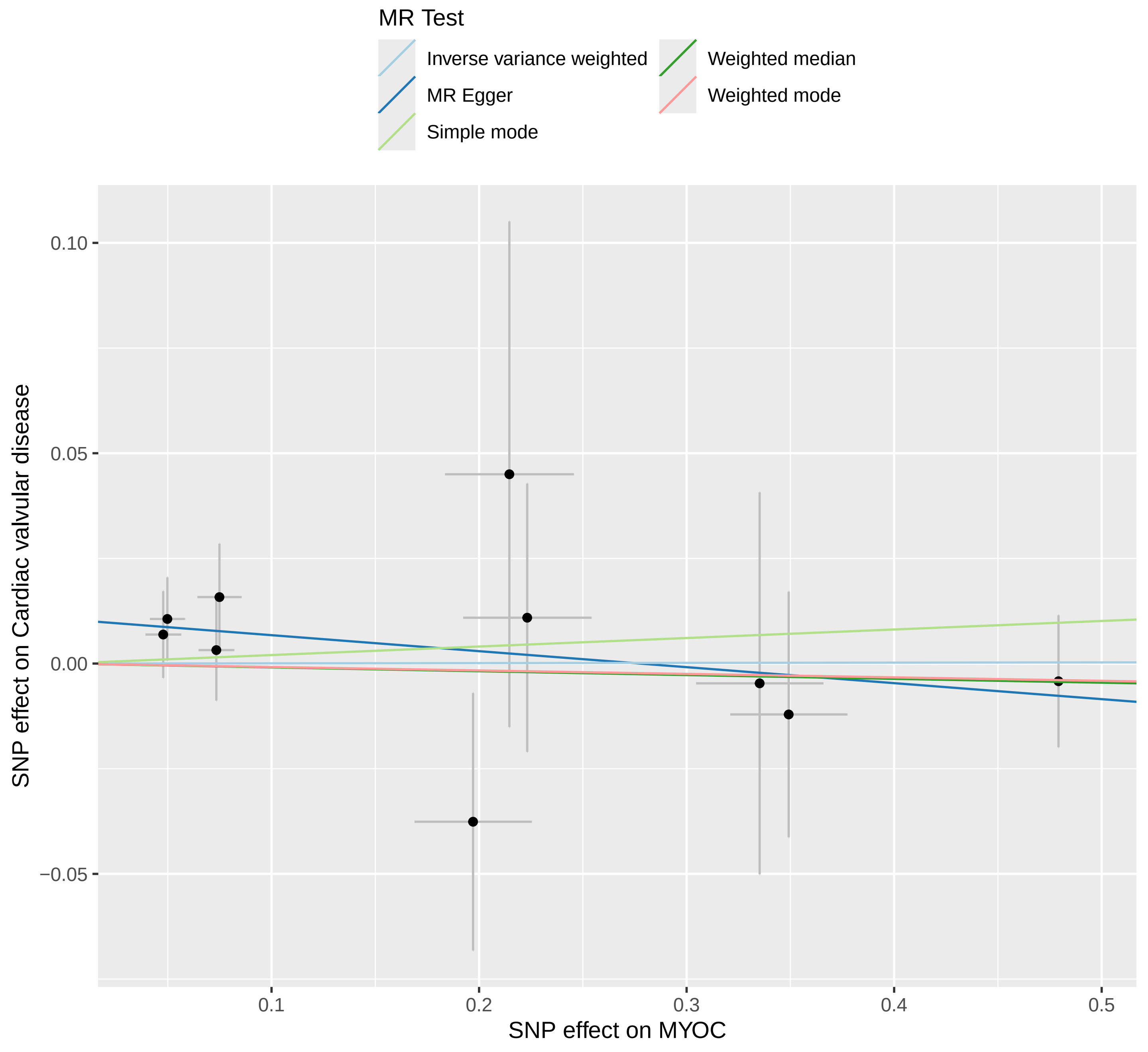
MR Test
Inverse variance weighted
MR Egger
Simple mode
Weighted median
Weighted mode
0.10
0.05
0.00
−0.05
SNP effect on Cardiac valvular disease
0.1
0.2
0.3
0.4
0.5
SNP effect on MYOC

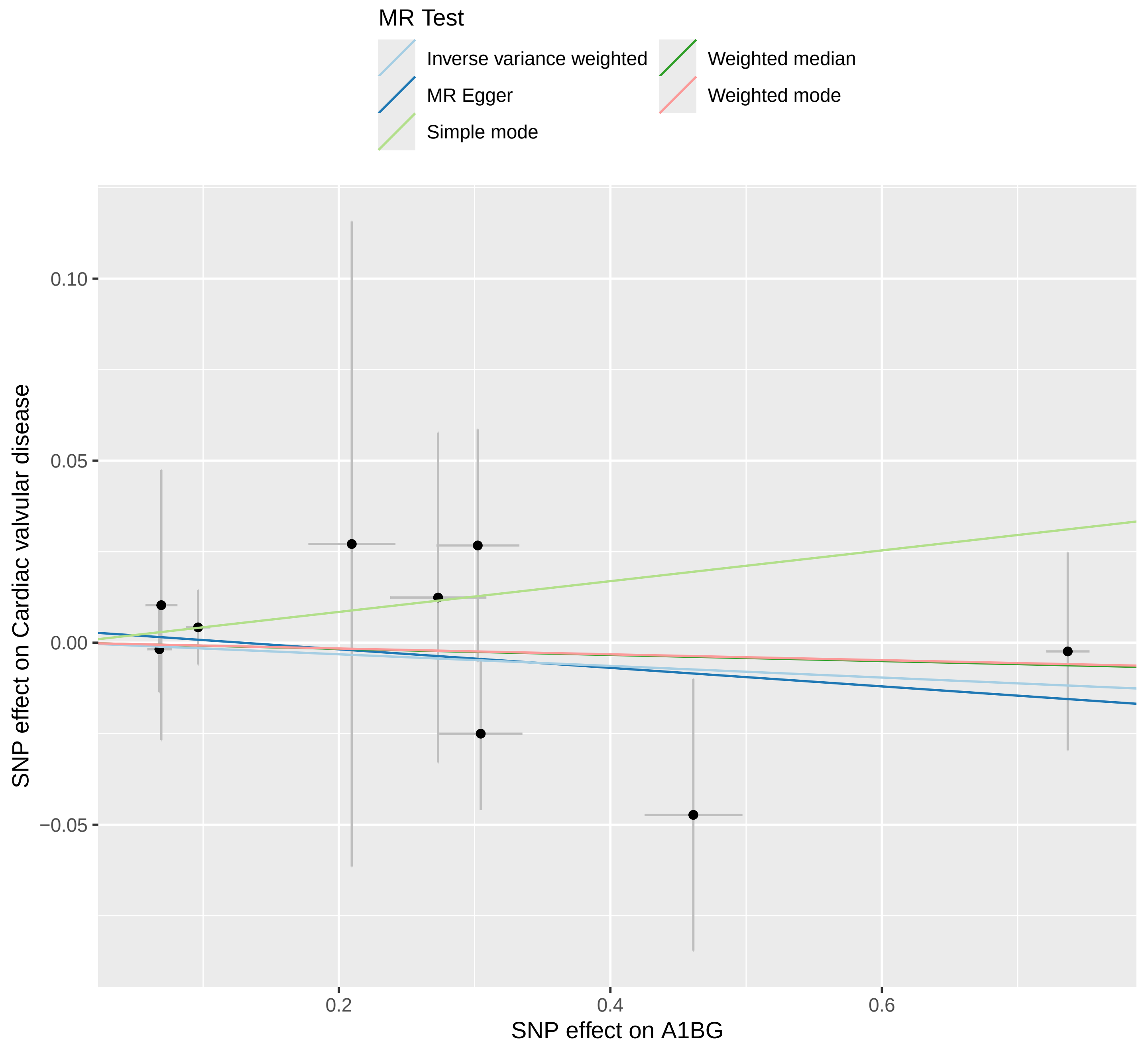
MR Test
Inverse variance weighted
MR Egger
Simple mode
Weighted median
Weighted mode
0.10
0.05
0.00
−0.05
SNP effect on Cardiac valvular disease
0.2
0.4
0.6
SNP effect on A1BG

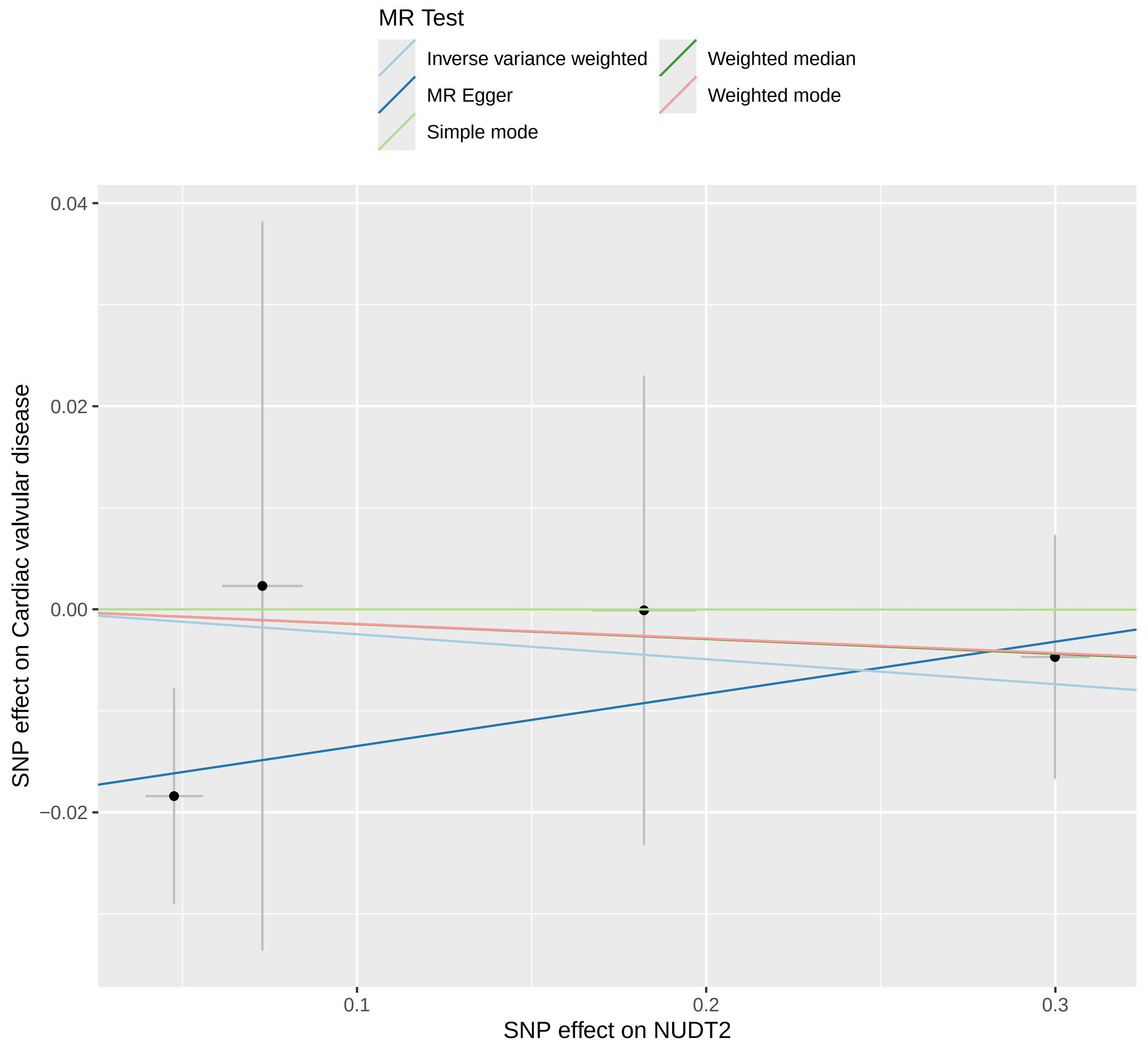
MR Test
Inverse variance weighted
MR Egger
Simple mode
Weighted median
Weighted mode
0.04
0.02
0.00
−0.02
0.1
0.2
0.3
SNP effect on Cardiac valvular disease
SNP effect on NUDT2

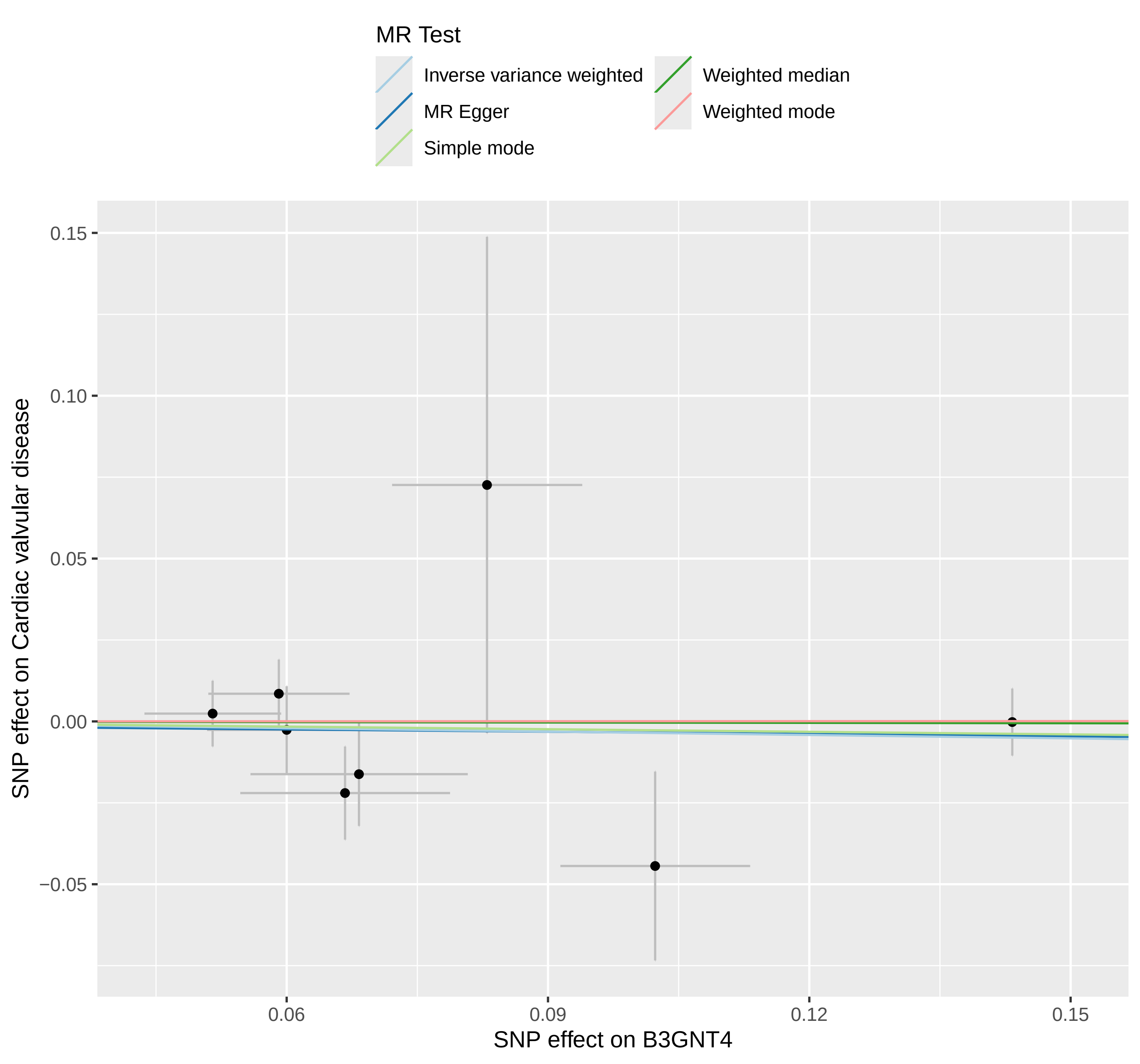

MR Test
Inverse variance weighted
MR Egger
Simple mode
Weighted median
Weighted mode
SNP effect on Cardiac valvular disease
0.15
0.10
0.05
0.00
−0.05
0.06
0.09
0.12
0.15
SNP effect on B3GNT4

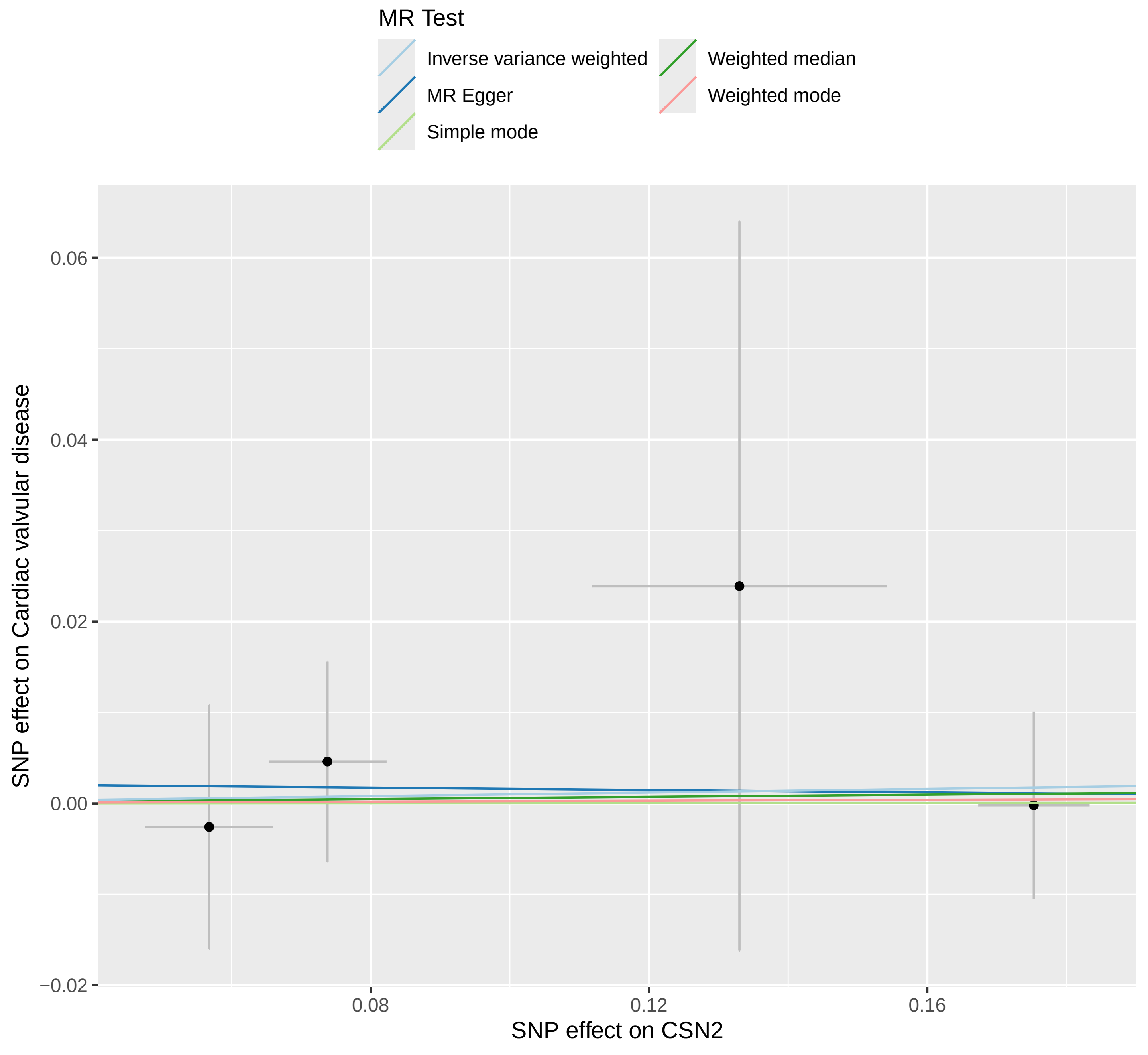
MR Test
Inverse variance weighted
MR Egger
Simple mode
Weighted median
Weighted mode
SNP effect on Cardiac valvular disease
0.06
0.04
0.02
0.00
−0.02
0.08
0.12
0.16
SNP effect on CSN2

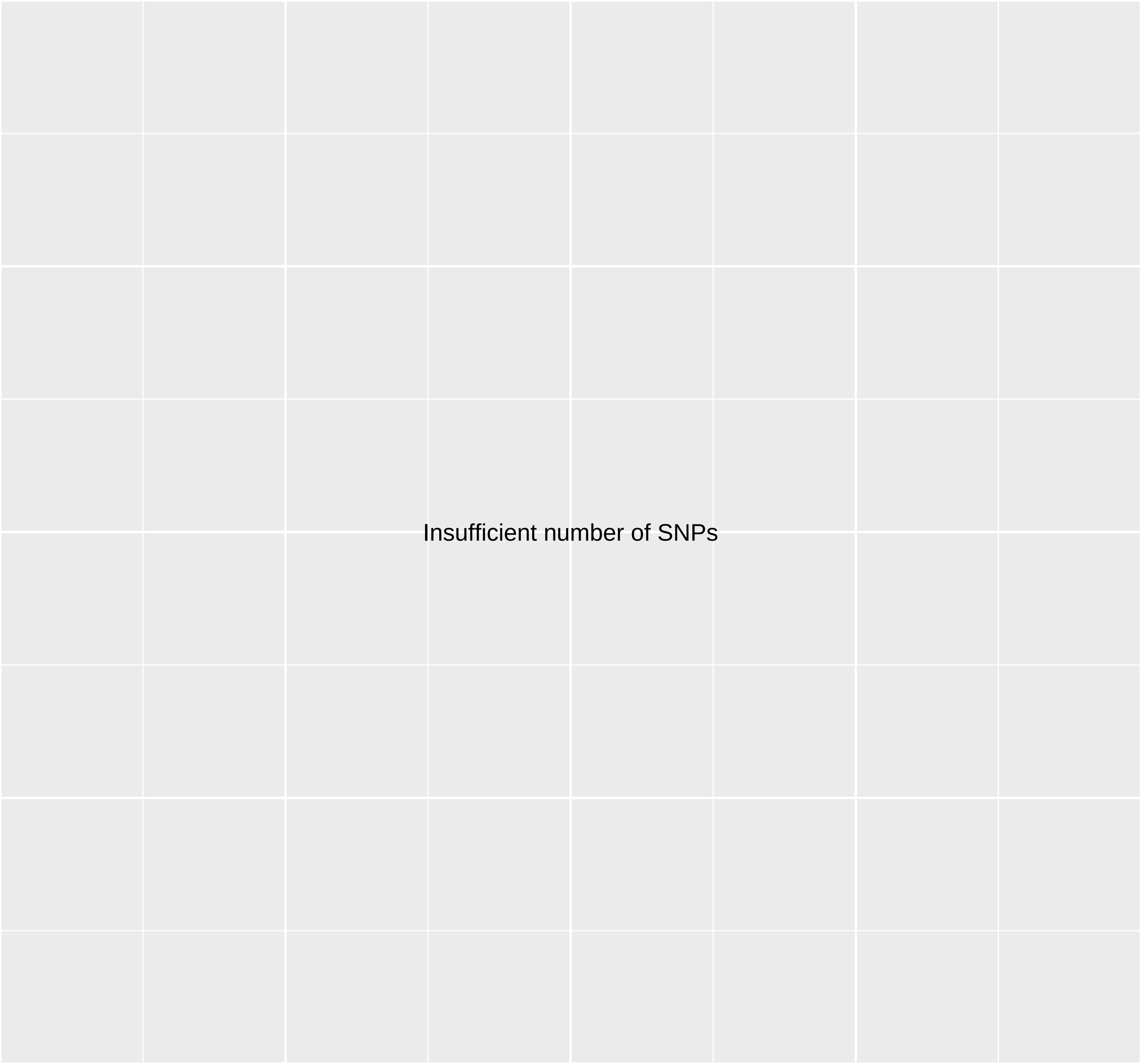
Insufficient number of SNPs

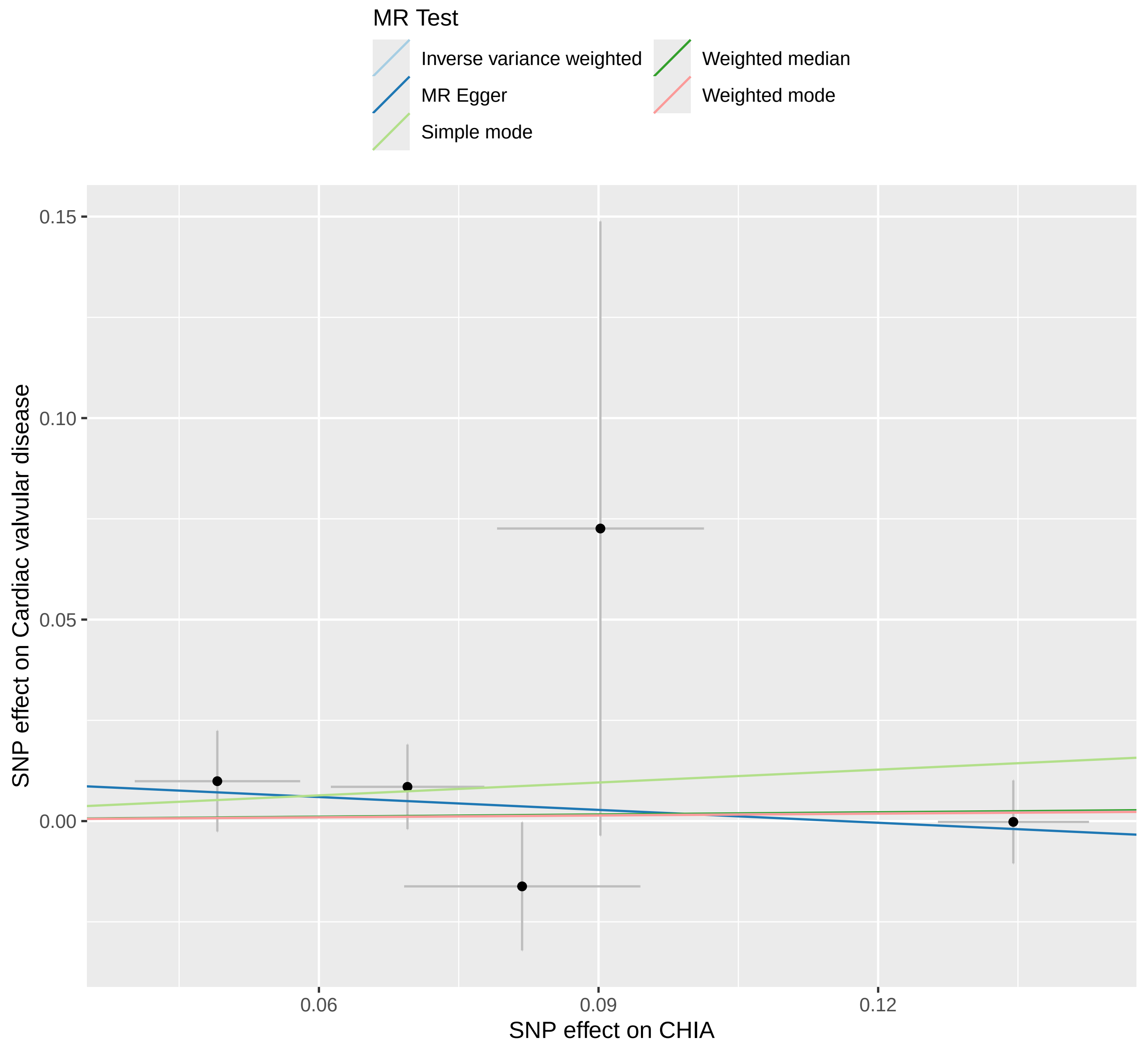
MR Test
Inverse variance weighted
MR Egger
Simple mode
Weighted median
Weighted mode
0.15
0.10
0.05
0.00
SNP effect on Cardiac valvular disease
0.06
0.09
0.12
SNP effect on CHIA

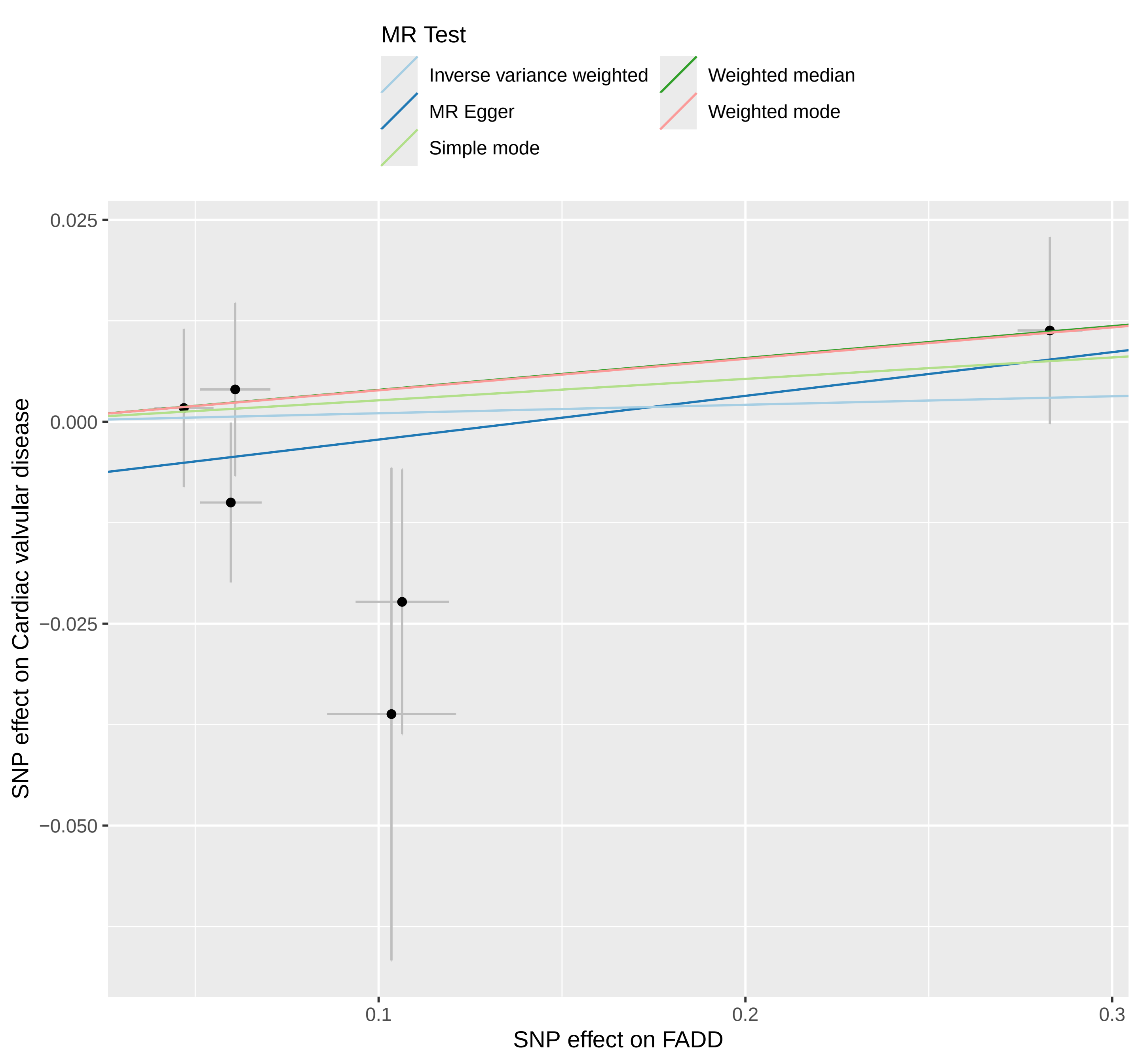
MR Test
Inverse variance weighted
MR Egger
Simple mode
Weighted median
Weighted mode
0.025
0.000
−0.025
−0.050
SNP effect on Cardiac valvular disease
0.1
0.2
0.3
SNP effect on FADD

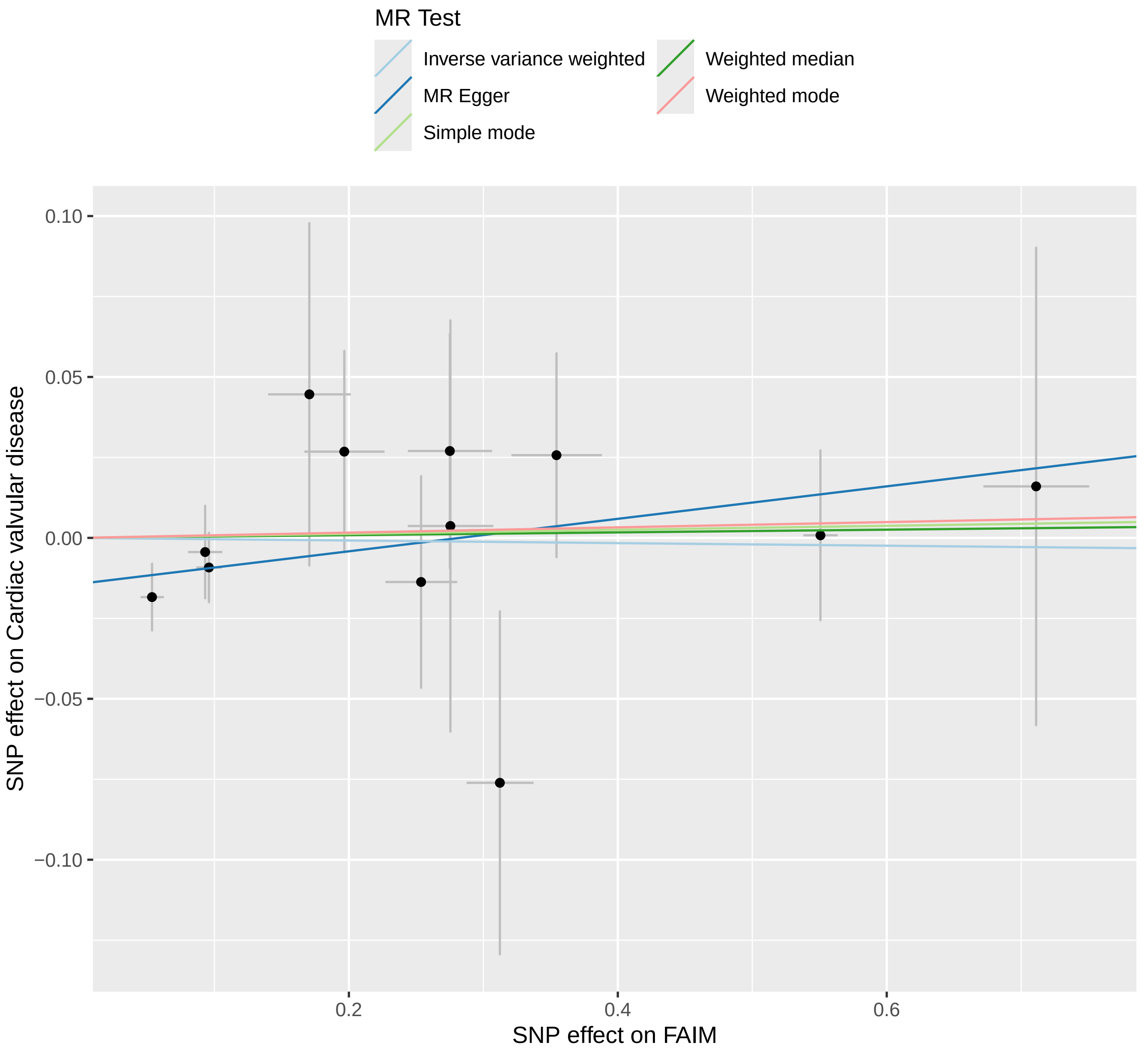
MR Test
Inverse variance weighted
MR Egger
Simple mode
Weighted median
Weighted mode
0.10
0.05
0.00
−0.05
−0.10
SNP effect on Cardiac valvular disease
0.2
0.4
0.6
SNP effect on FAIM

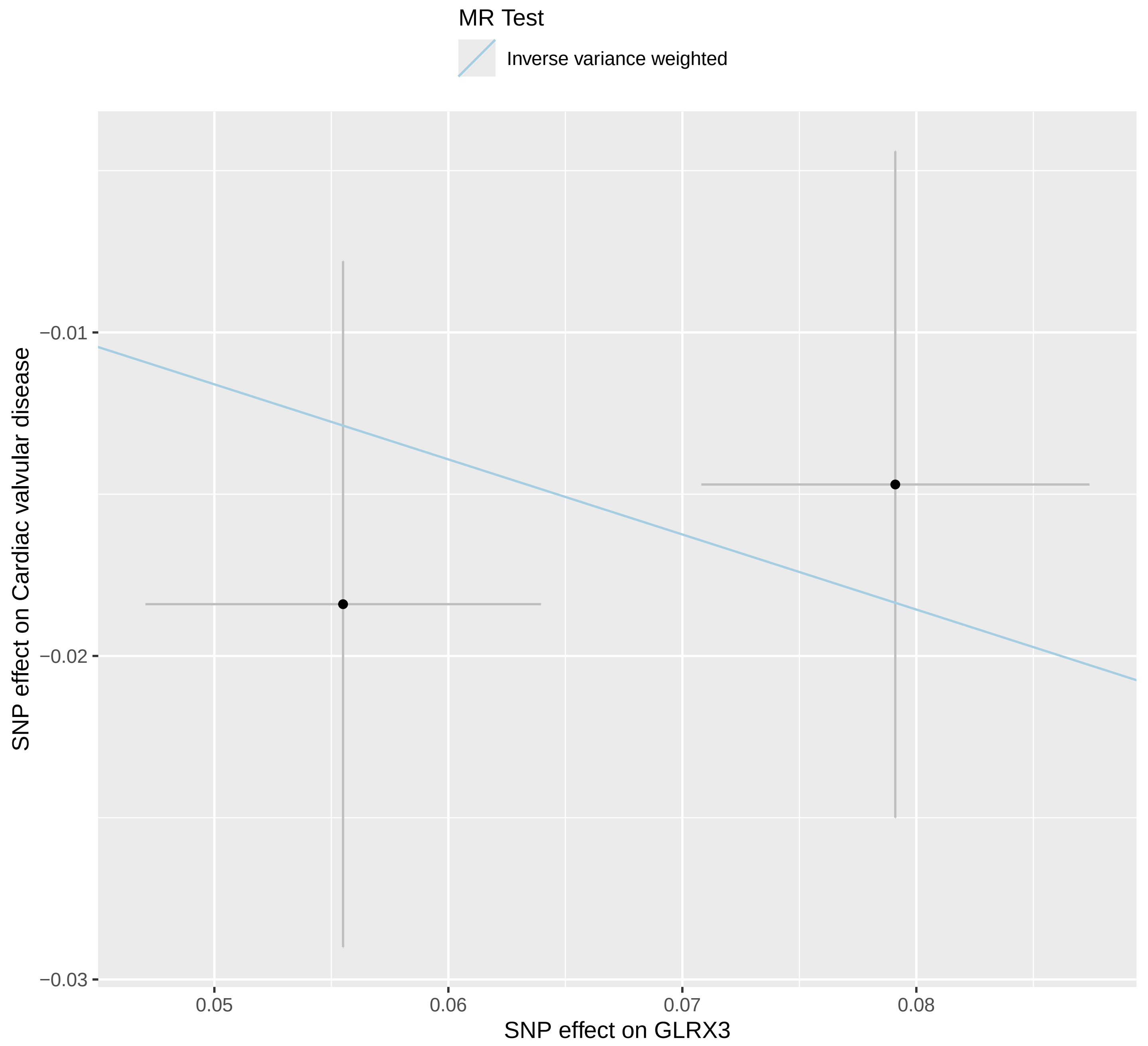
MR Test
Inverse variance weighted
−0.01
−0.02
−0.03
0.05
0.06
0.07
0.08
SNP effect on GLRX3
SNP effect on Cardiac valvular disease

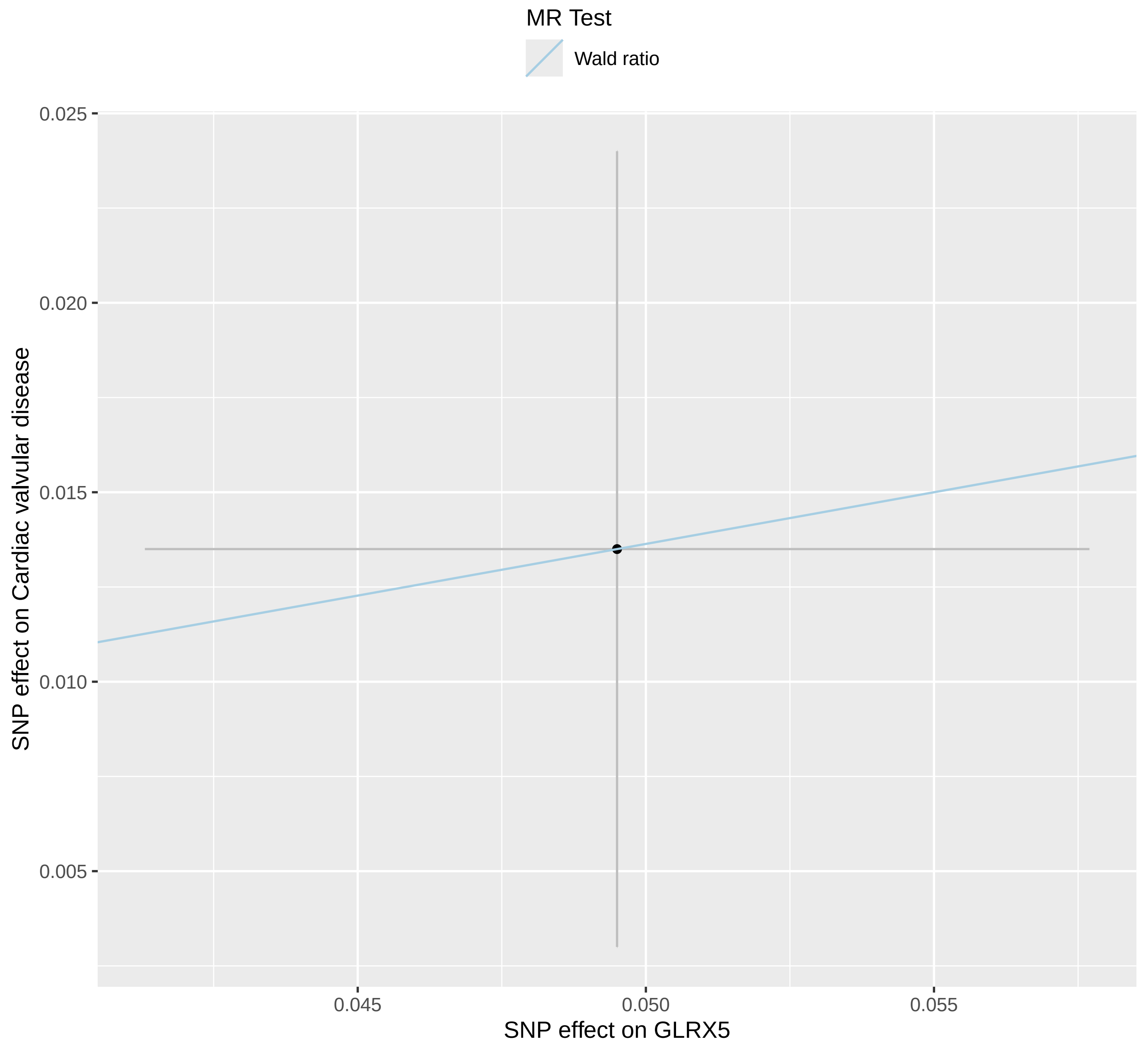
MR Test
Wald ratio
0.025
0.020
0.015
0.010
0.005
SNP effect on Cardiac valvular disease
0.045
0.050
0.055
SNP effect on GLRX5

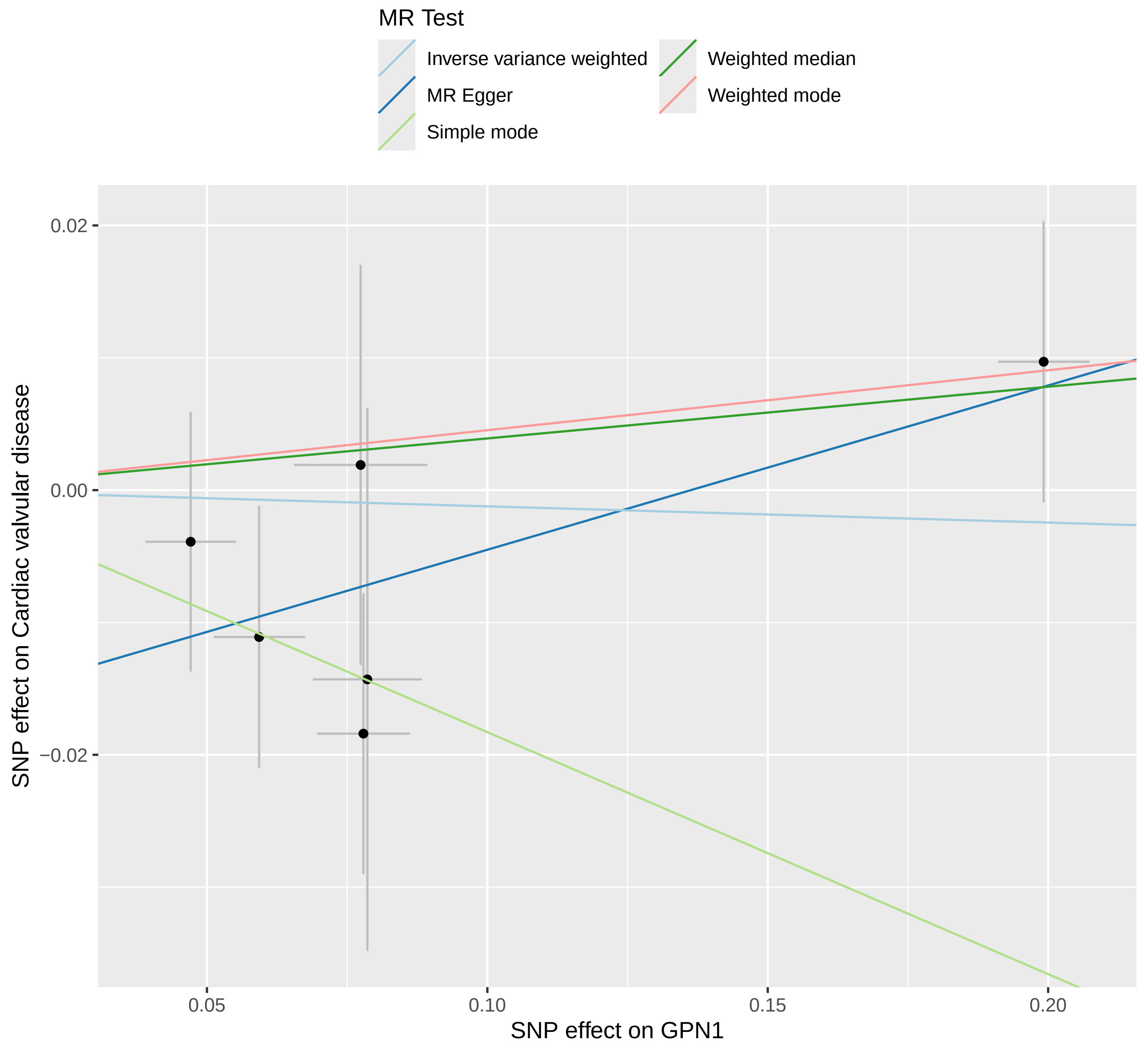
MR Test
Inverse variance weighted
MR Egger
Simple mode
Weighted median
Weighted mode
0.02
0.00
−0.02
0.05
0.10
0.15
0.20
SNP effect on GPN1
SNP effect on Cardiac valvular disease

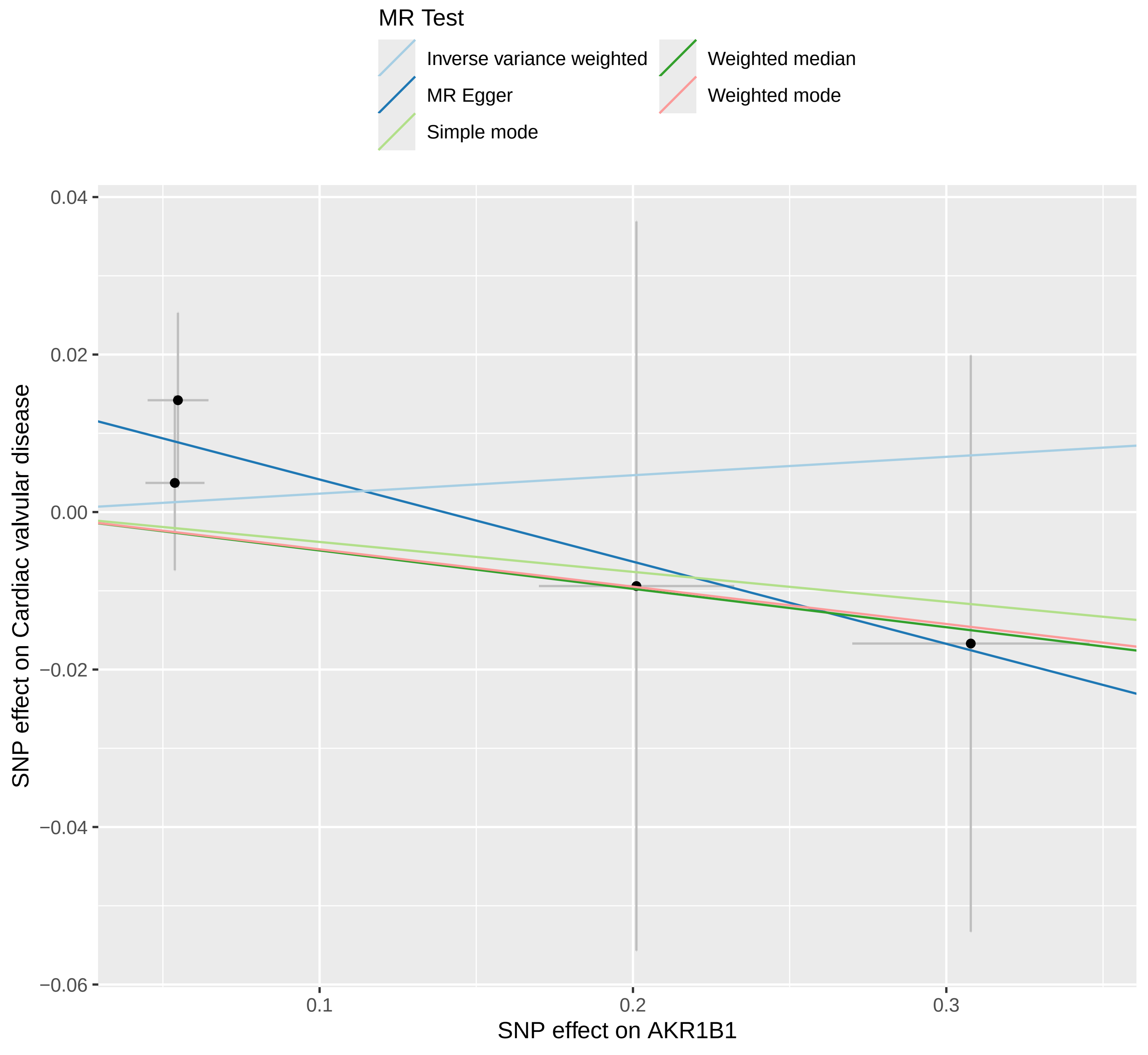
MR Test
Inverse variance weighted
MR Egger
Simple mode
Weighted median
Weighted mode
0.04
0.02
0.00
−0.02
−0.04
−0.06
0.1
0.2
0.3
SNP effect on Cardiac valvular disease
SNP effect on AKR1B1

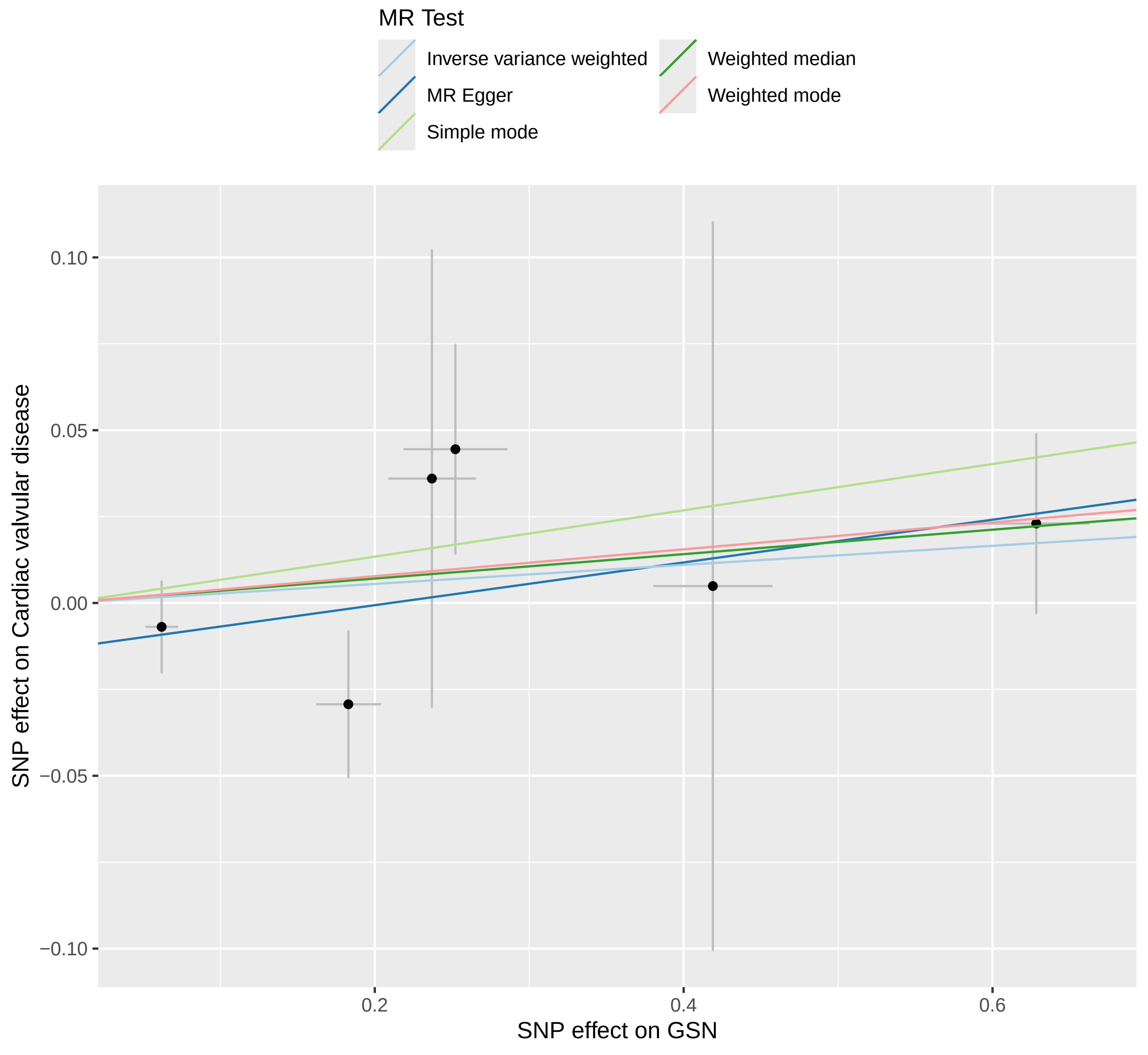
MR Test
Inverse variance weighted
MR Egger
Simple mode
Weighted median
Weighted mode
0.10
0.05
0.00
−0.05
−0.10
SNP effect on Cardiac valvular disease
0.2
0.4
0.6
SNP effect on GSN

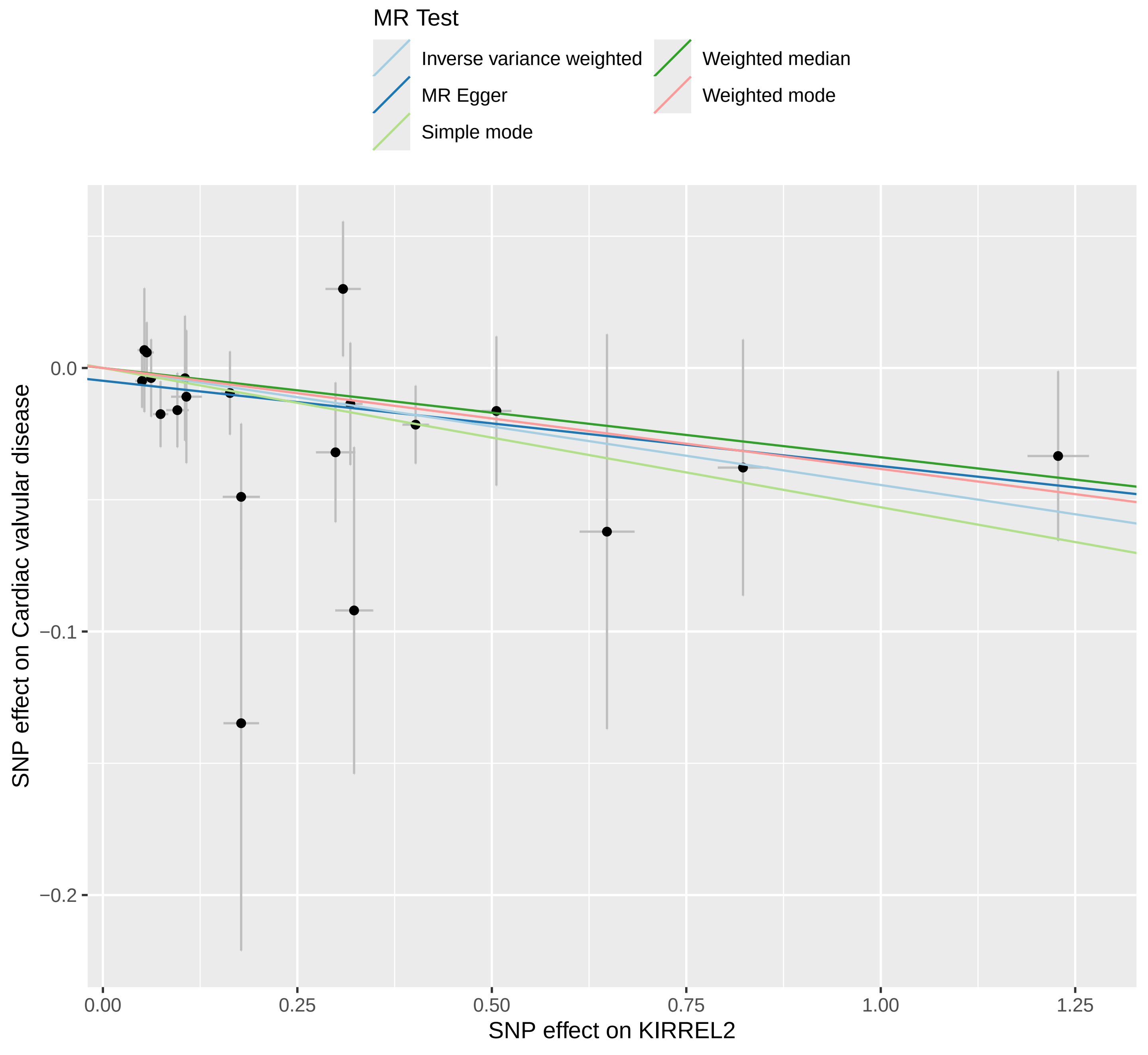
MR Test
Inverse variance weighted
MR Egger
Simple mode
Weighted median
Weighted mode
0.0
−0.1
−0.2
SNP effect on Cardiac valvular disease
0.00
0.25
0.50
0.75
1.00
1.25
SNP effect on KIRREL2

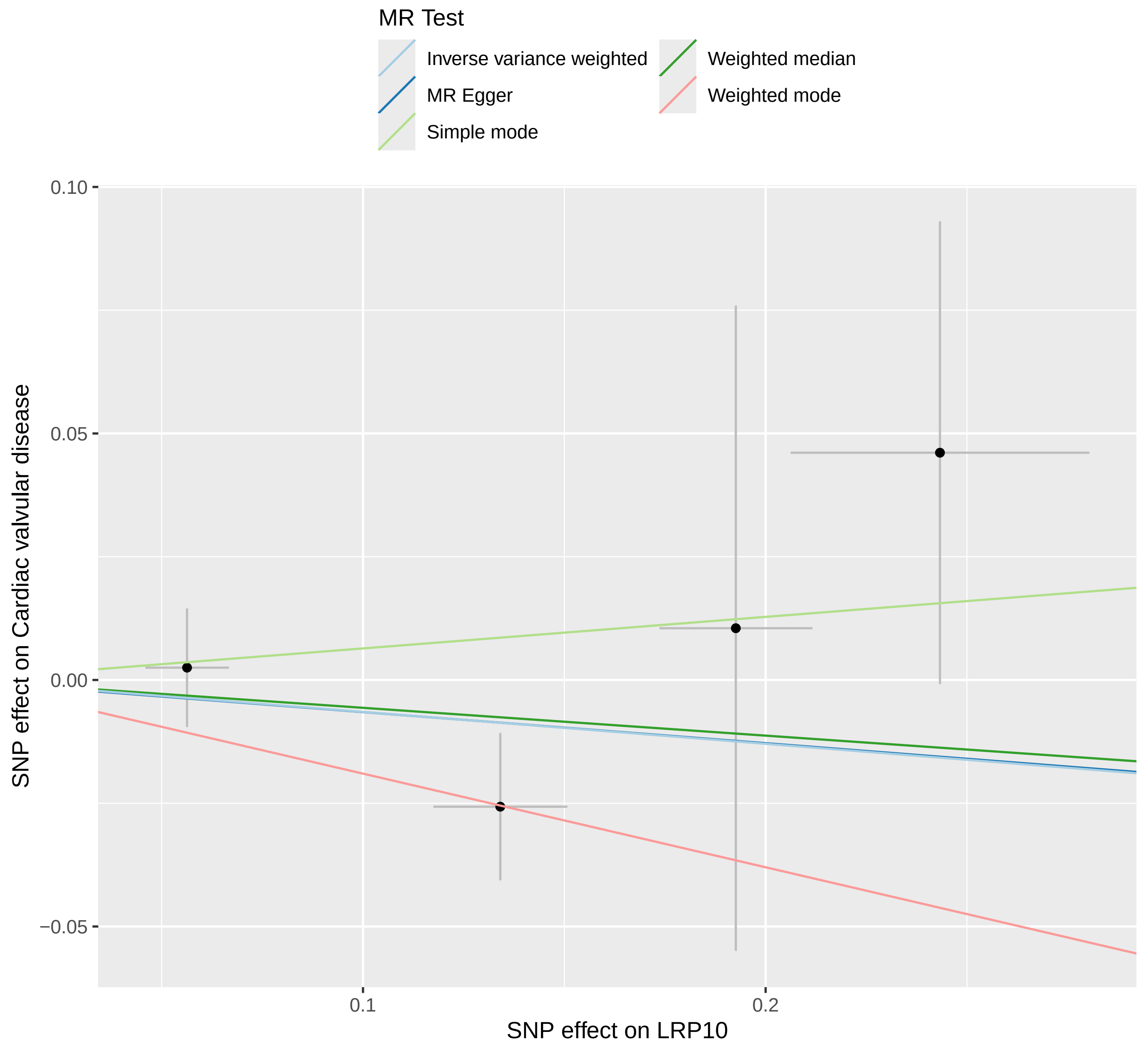
MR Test
Inverse variance weighted
MR Egger
Simple mode
Weighted median
Weighted mode
0.10
0.05
0.00
−0.05
0.1
0.2
SNP effect on Cardiac valvular disease
SNP effect on LRP10

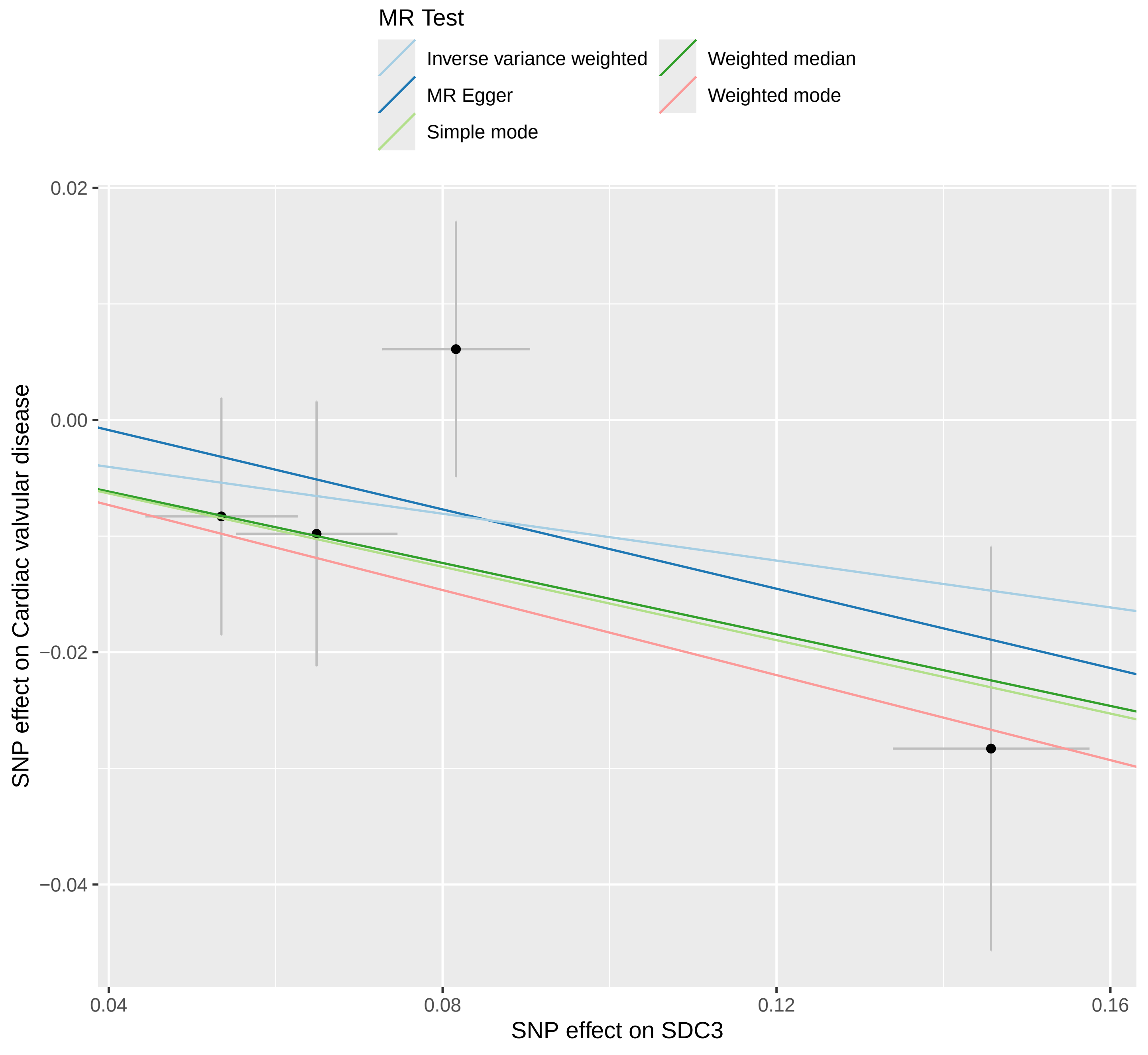
MR Test
Inverse variance weighted
MR Egger
Simple mode
Weighted median
Weighted mode
0.02
0.00
−0.02
−0.04
SNP effect on Cardiac valvular disease
0.04
0.08
0.12
0.16
SNP effect on SDC3

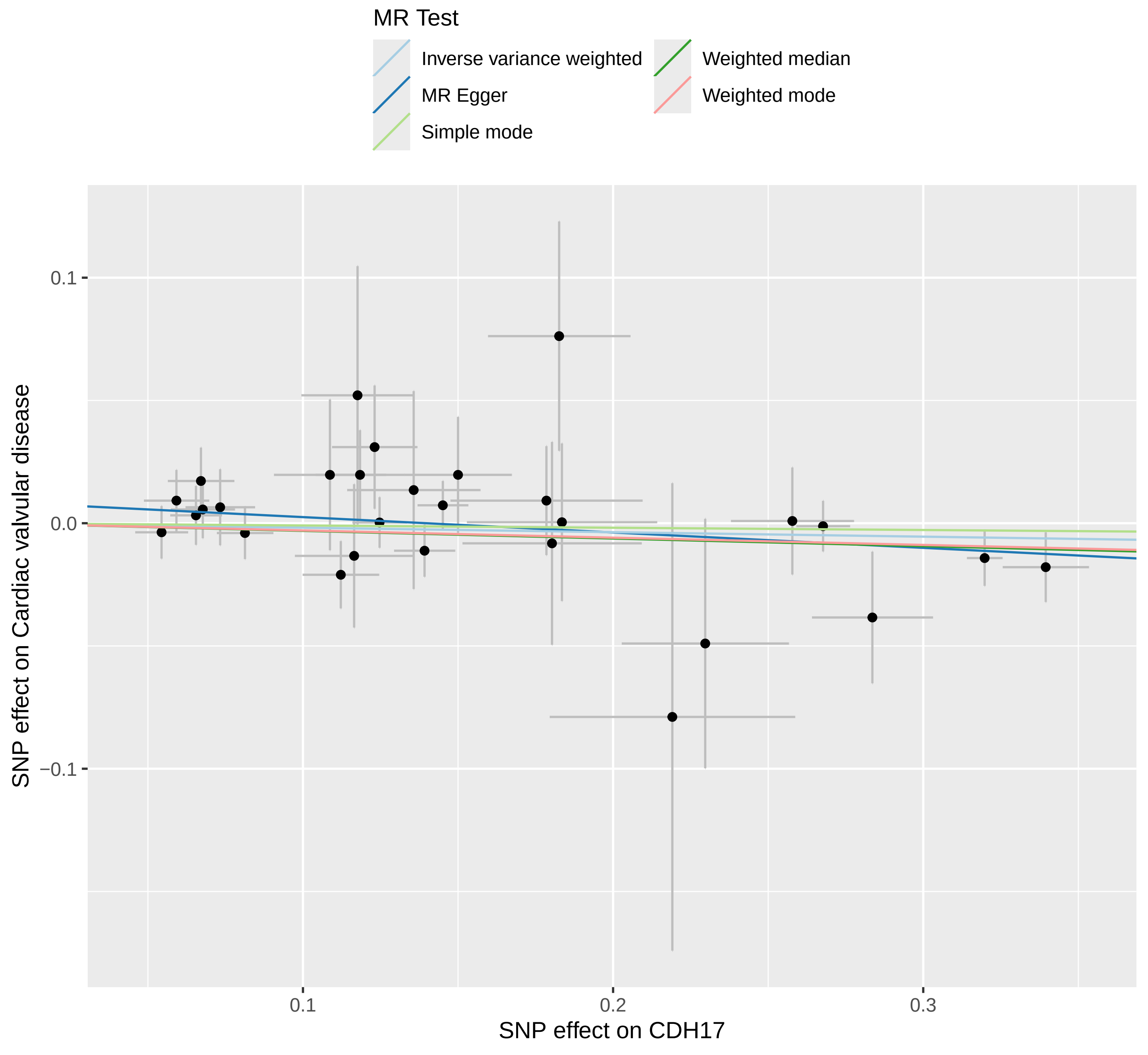

MR Test
Inverse variance weighted
MR Egger
Simple mode
Weighted median
Weighted mode
0.1
0.0
−0.1
SNP effect on Cardiac valvular disease
0.1
0.2
0.3
SNP effect on CDH17

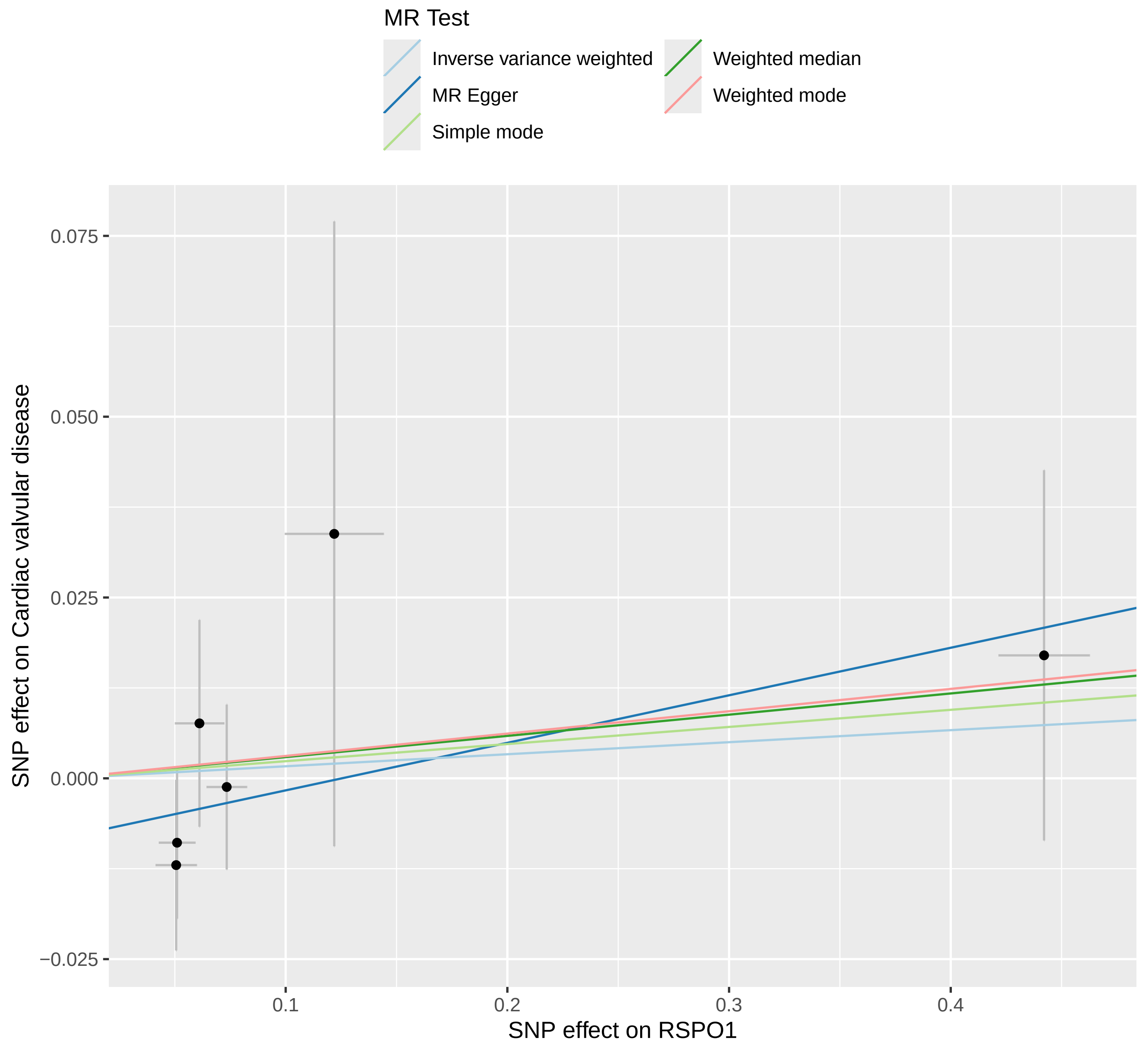
MR Test
Inverse variance weighted
MR Egger
Simple mode
Weighted median
Weighted mode
0.075
0.050
0.025
0.000
−0.025
SNP effect on Cardiac valvular disease
0.1
0.2
0.3
0.4
SNP effect on RSPO1

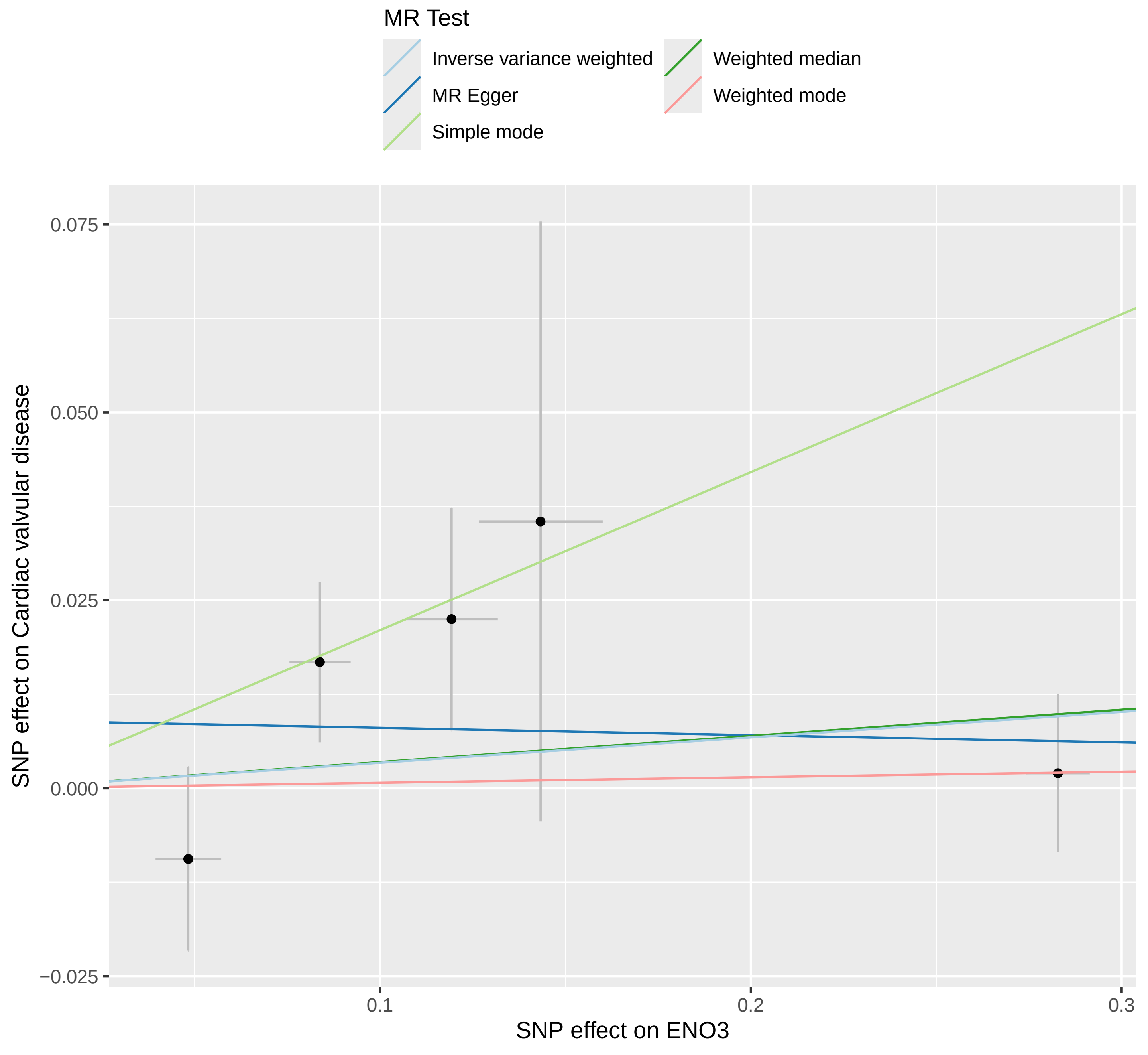
MR Test
Inverse variance weighted
MR Egger
Simple mode
Weighted median
Weighted mode
0.075
0.050
0.025
0.000
−0.025
SNP effect on Cardiac valvular disease
0.1
0.2
0.3
SNP effect on ENO3

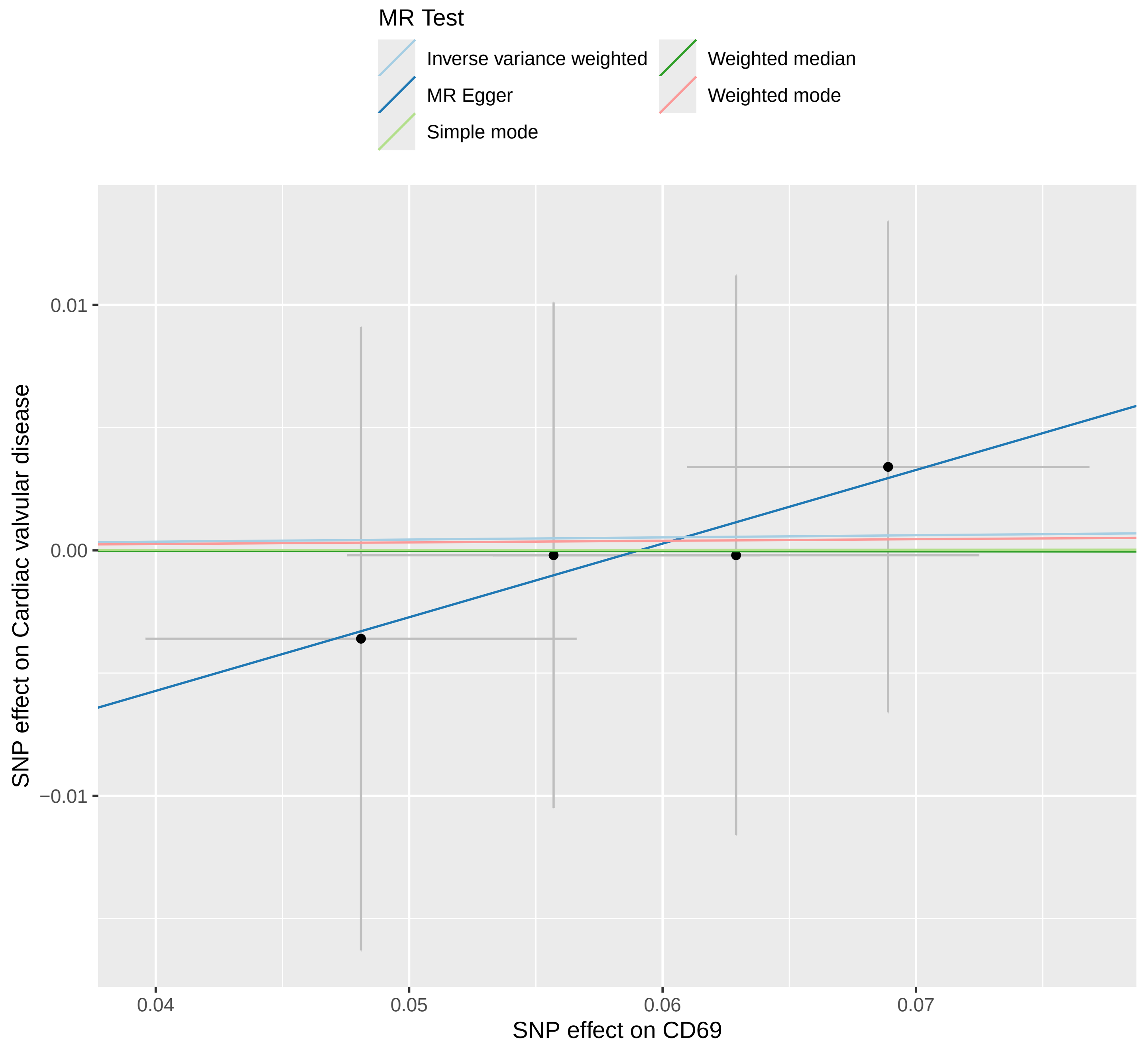
MR Test
Inverse variance weighted
MR Egger
Simple mode
Weighted median
Weighted mode
0.01
0.00
−0.01
0.04
0.05
0.06
0.07
SNP effect on Cardiac valvular disease
SNP effect on CD69

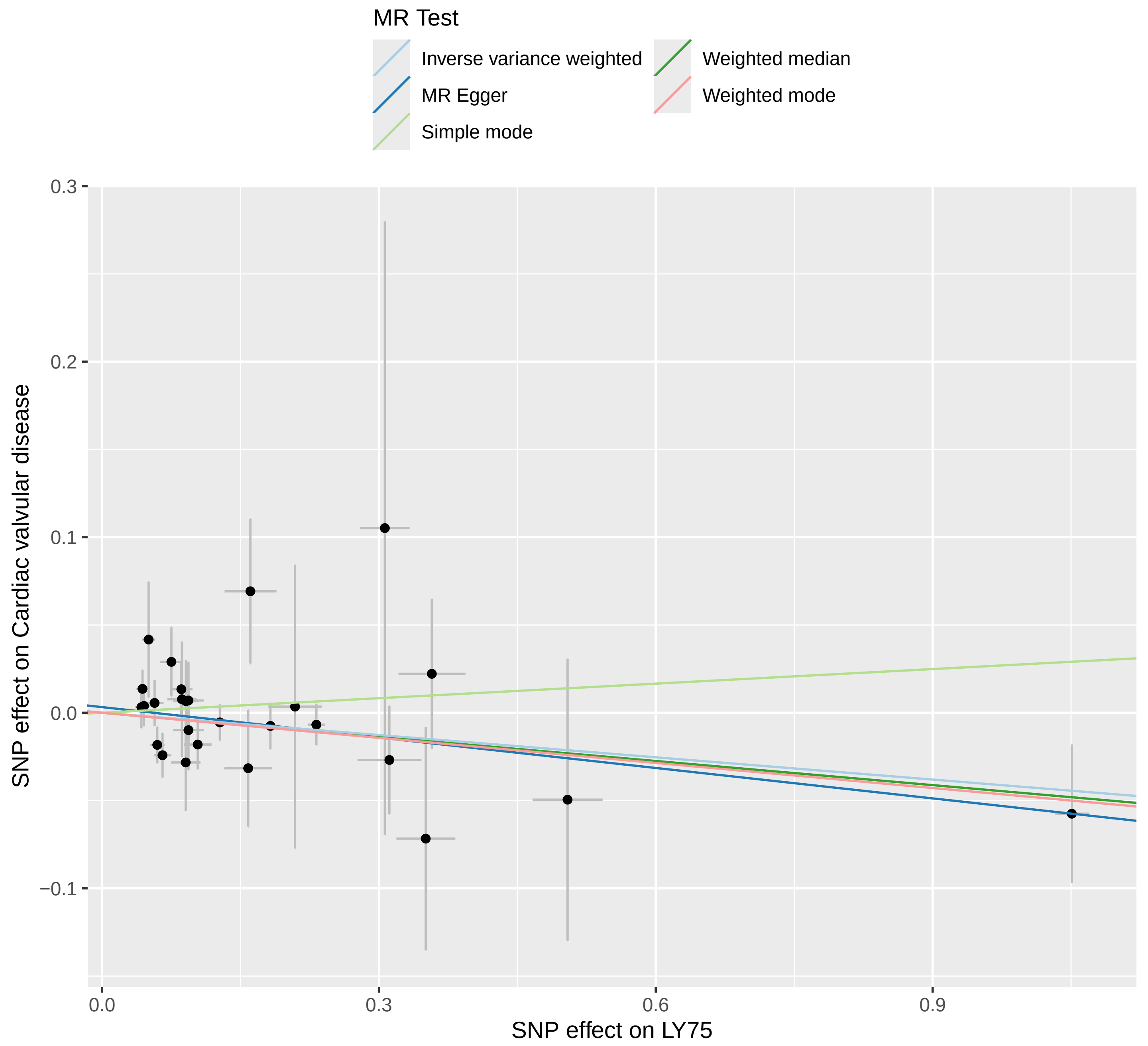

MR Test
Inverse variance weighted
MR Egger
Simple mode
Weighted median
Weighted mode
0.3
0.2
0.1
0.0
−0.1
SNP effect on Cardiac valvular disease
0.0
0.3
0.6
0.9
SNP effect on LY75

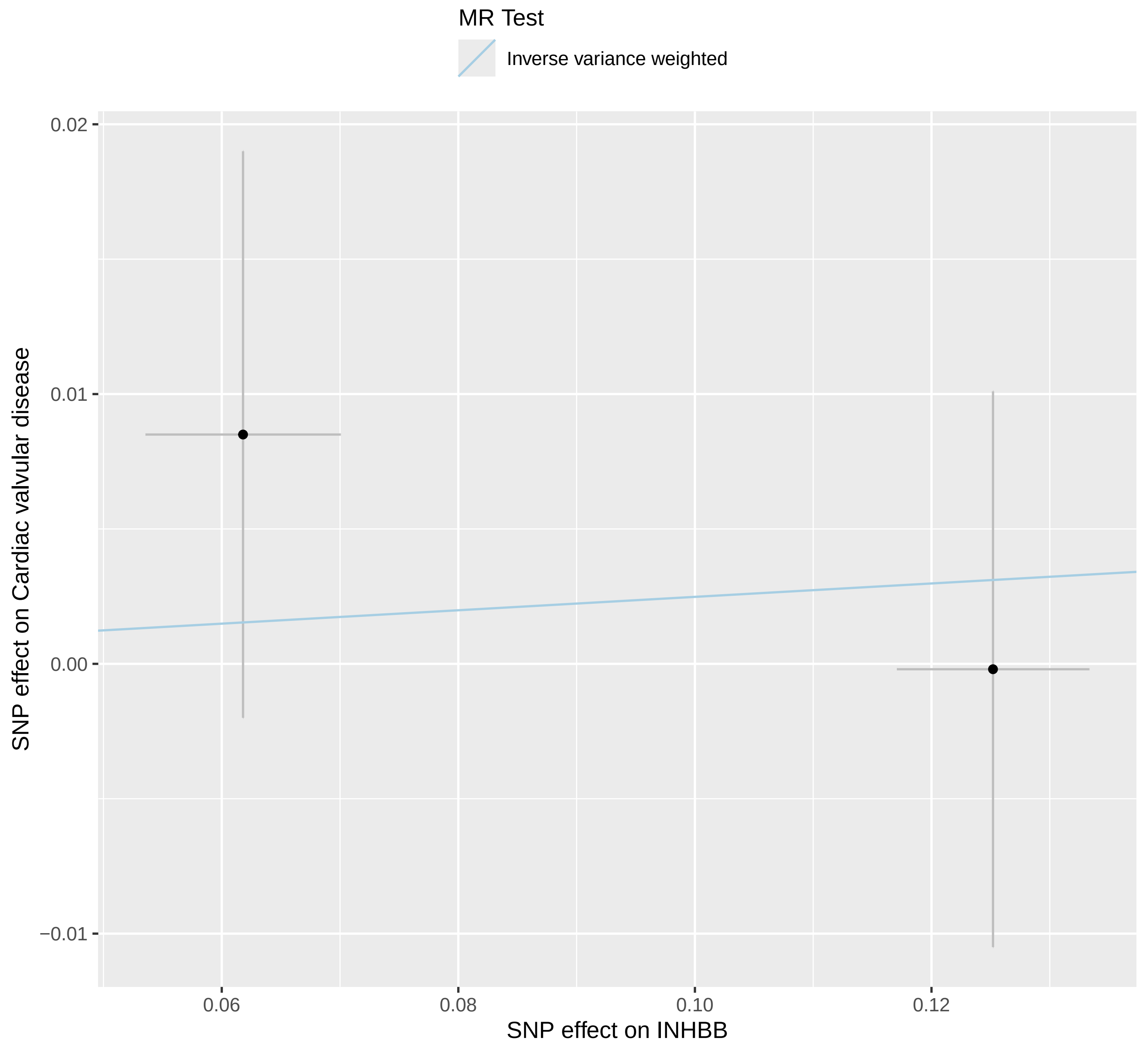

MR Test
Inverse variance weighted
0.02
0.01
0.00
−0.01
SNP effect on Cardiac valvular disease
0.06
0.08
0.10
0.12
SNP effect on INHBB

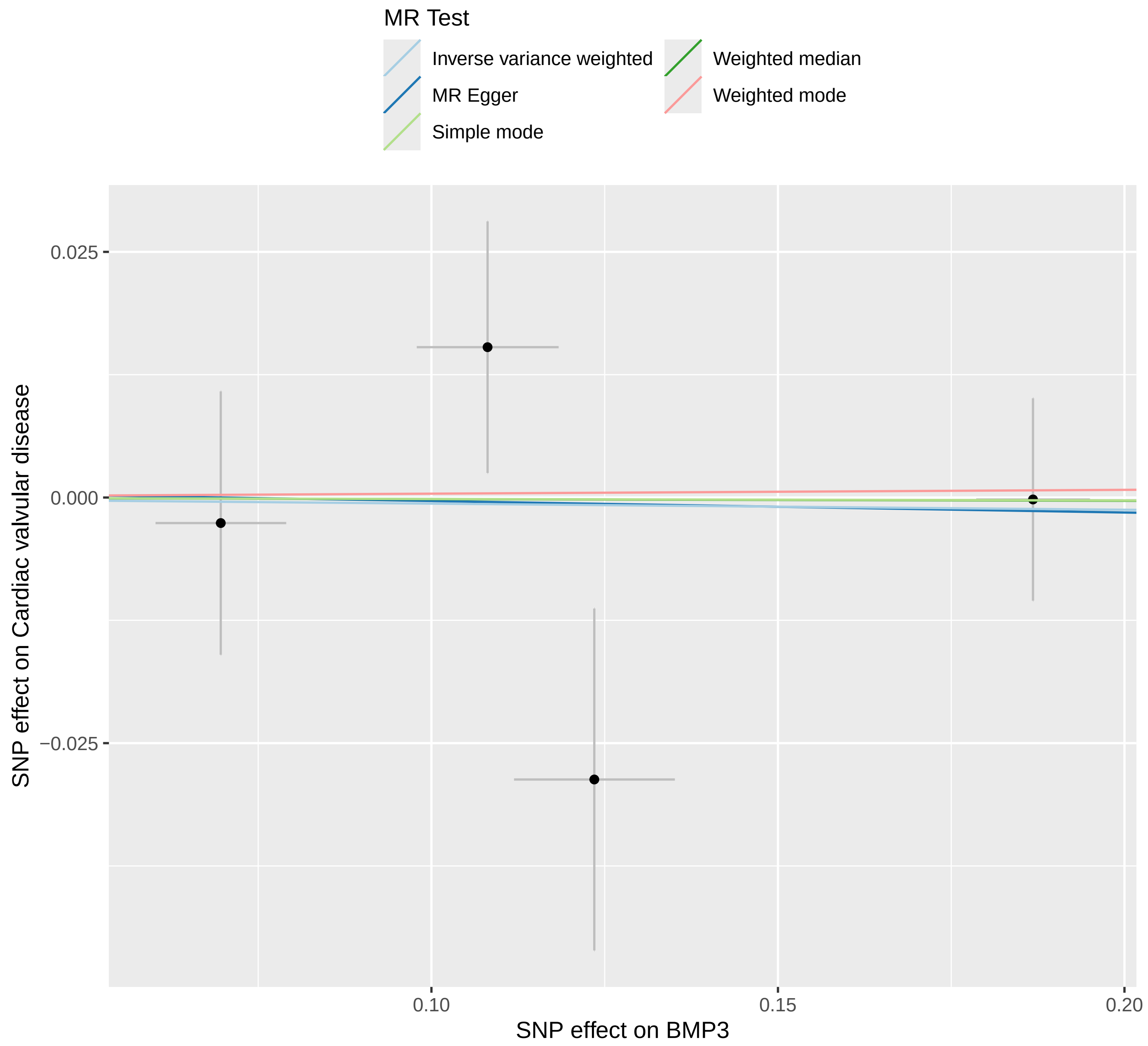
MR Test
Inverse variance weighted
MR Egger
Simple mode
Weighted median
Weighted mode
0.025
0.000
−0.025
0.10
0.15
0.20
SNP effect on Cardiac valvular disease
SNP effect on BMP3

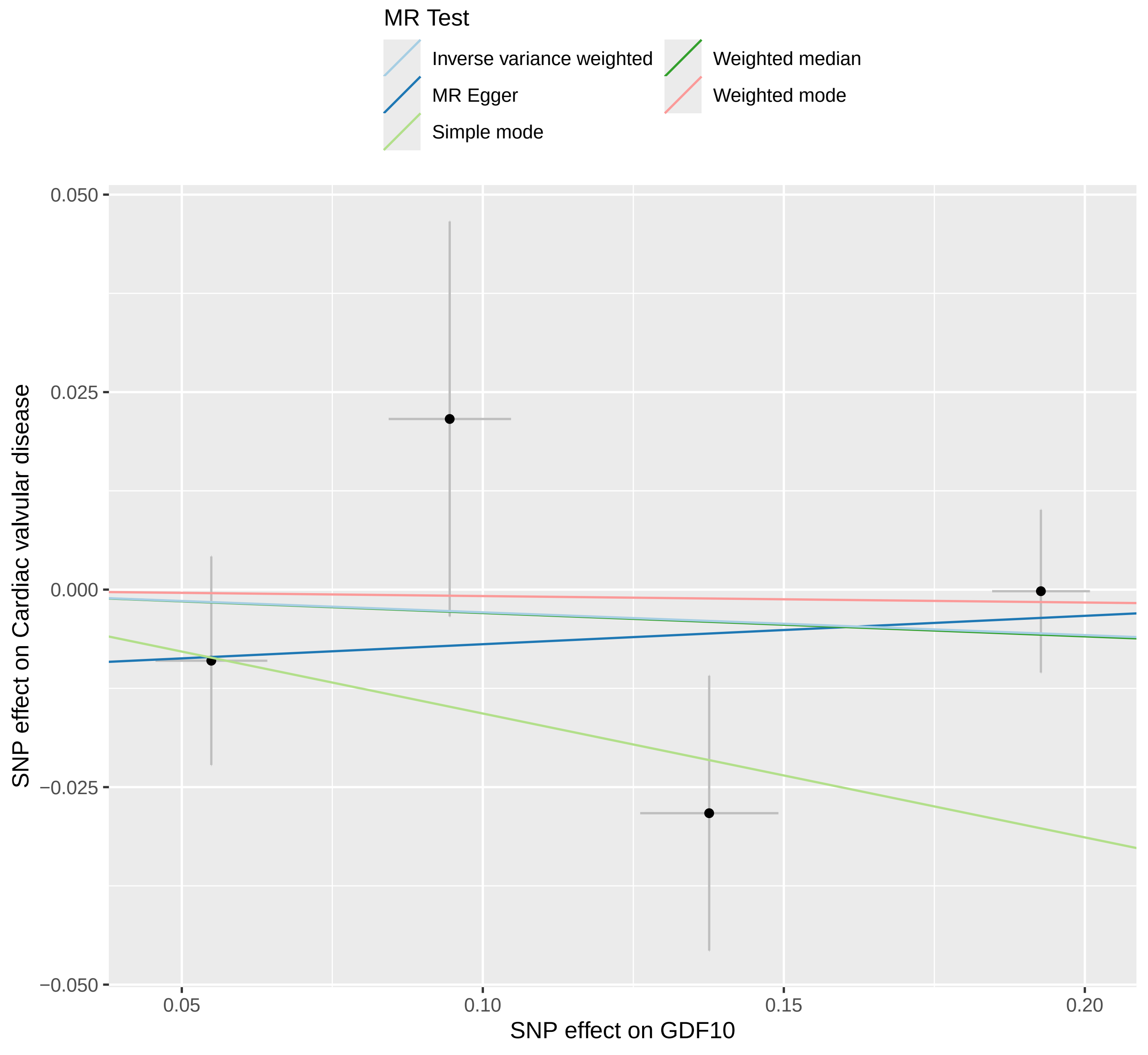
MR Test
Inverse variance weighted
MR Egger
Simple mode
Weighted median
Weighted mode
0.050
0.025
0.000
−0.025
−0.050
SNP effect on Cardiac valvular disease
0.05
0.10
0.15
0.20
SNP effect on GDF10

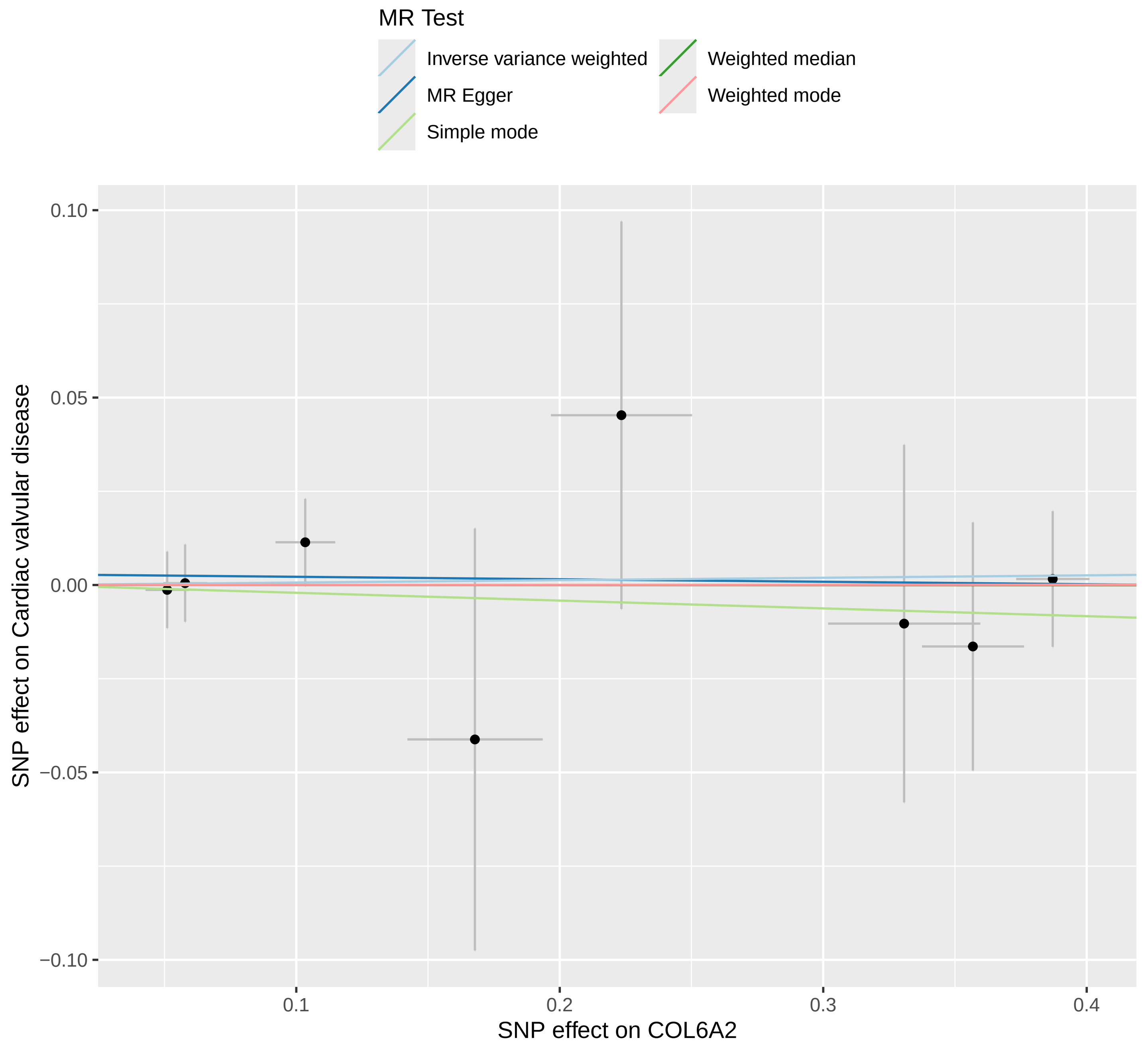
MR Test
Inverse variance weighted
MR Egger
Simple mode
Weighted median
Weighted mode
0.10
0.05
0.00
−0.05
−0.10
0.1
0.2
0.3
0.4
SNP effect on Cardiac valvular disease
SNP effect on COL6A2

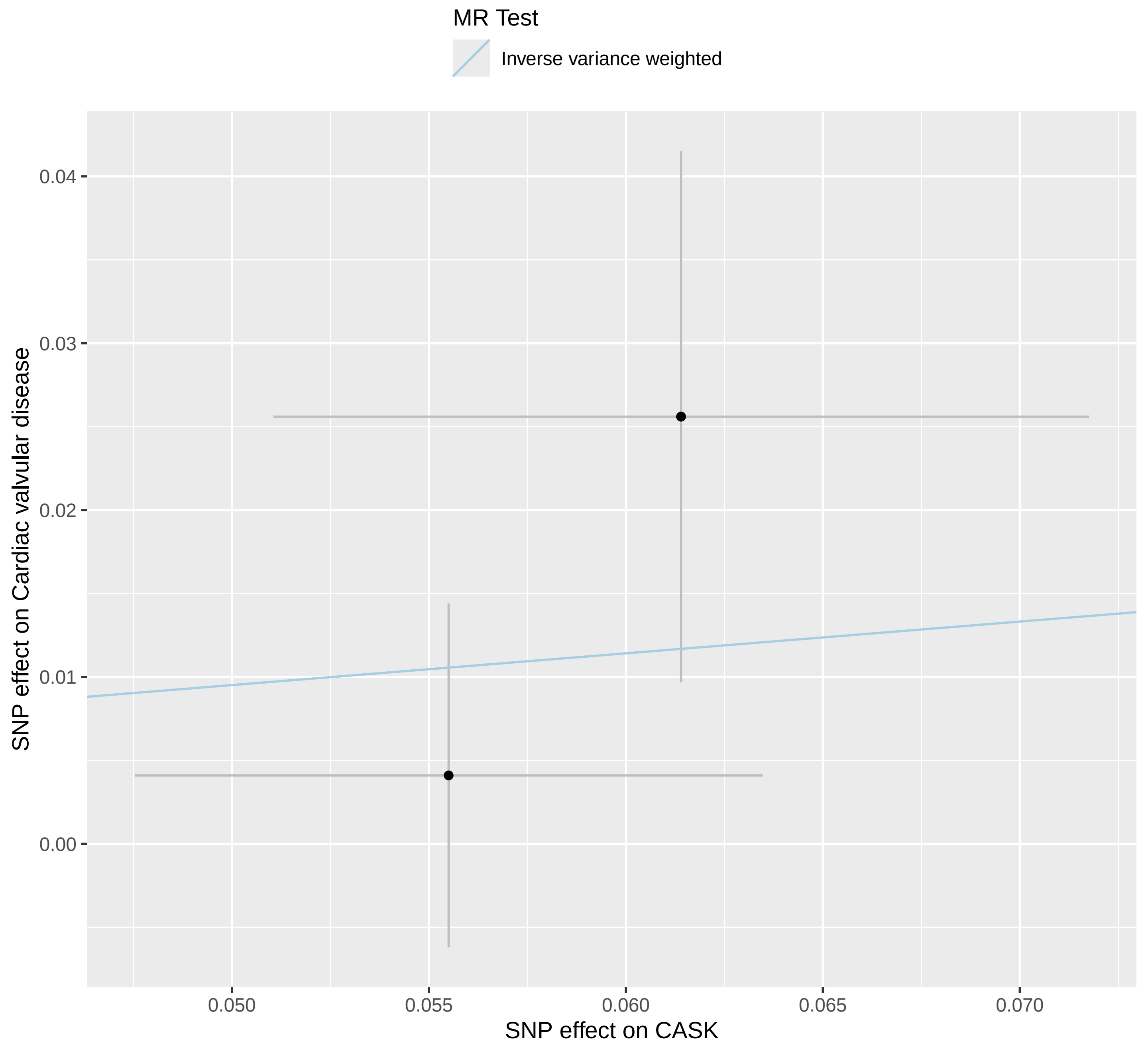
MR Test
Inverse variance weighted
0.04
0.03
0.02
0.01
0.00
SNP effect on Cardiac valvular disease
0.050
0.055
0.060
0.065
0.070
SNP effect on CASK

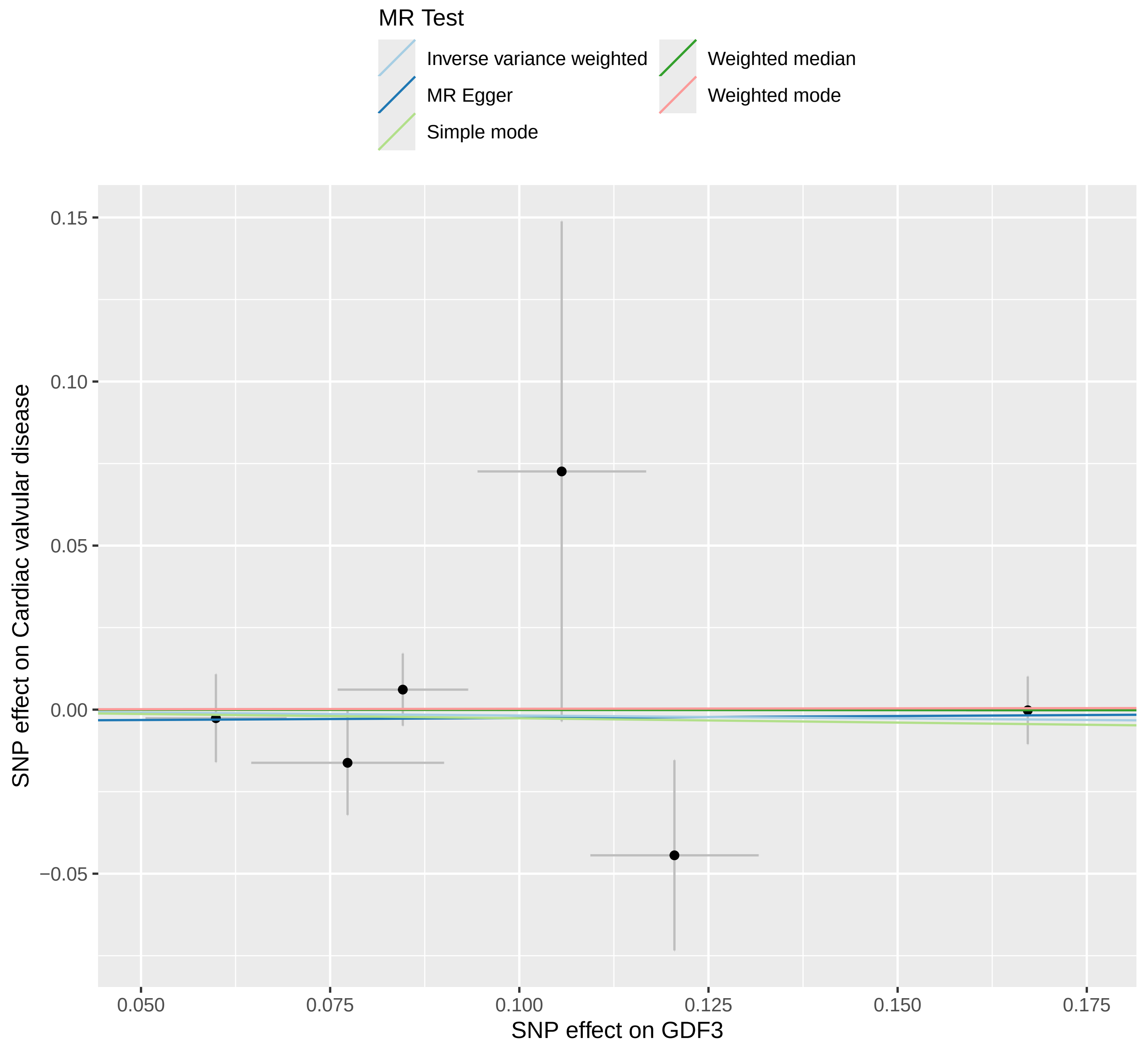
MR Test
Inverse variance weighted
MR Egger
Simple mode
Weighted median
Weighted mode
0.15
0.10
0.05
0.00
−0.05
0.050
0.075
0.100
0.125
0.150
0.175
SNP effect on Cardiac valvular disease
SNP effect on GDF3

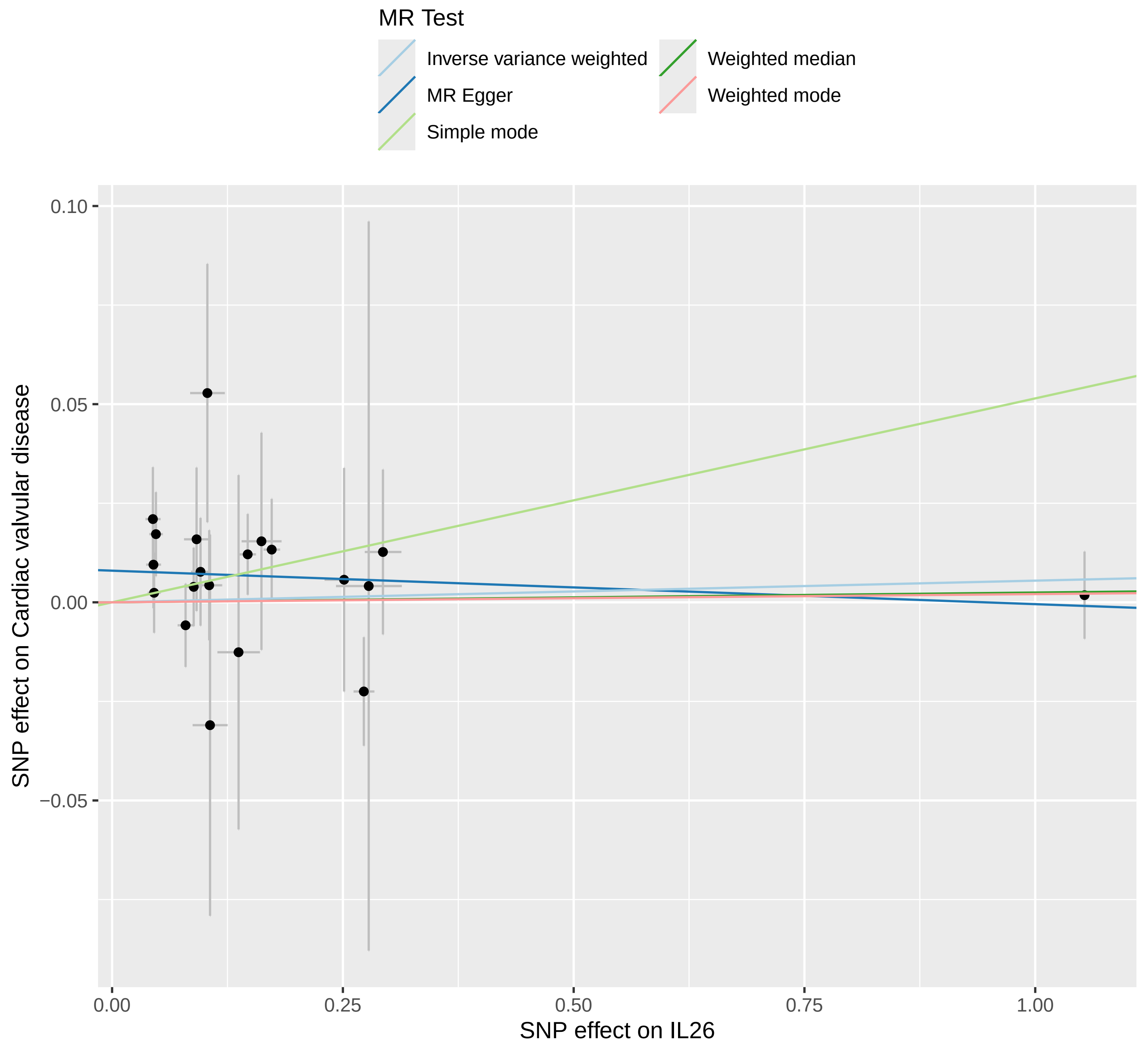

MR Test
Inverse variance weighted
MR Egger
Simple mode
Weighted median
Weighted mode
0.10
0.05
0.00
−0.05
SNP effect on Cardiac valvular disease
0.00
0.25
0.50
0.75
1.00
SNP effect on IL26

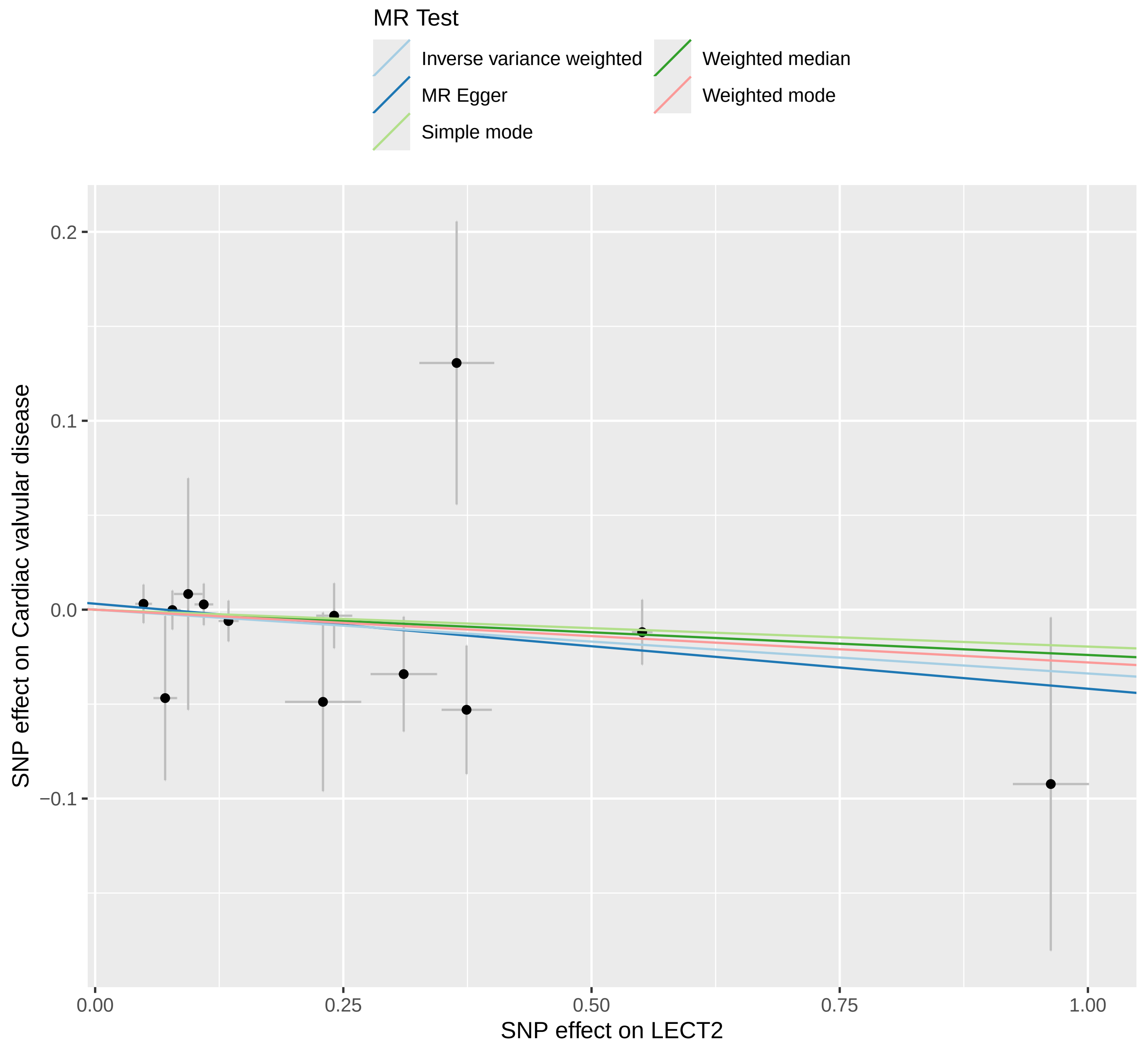
MR Test
Inverse variance weighted
MR Egger
Simple mode
Weighted median
Weighted mode
0.2
0.1
0.0
−0.1
0.00
0.25
0.50
0.75
1.00
SNP effect on Cardiac valvular disease
SNP effect on LECT2

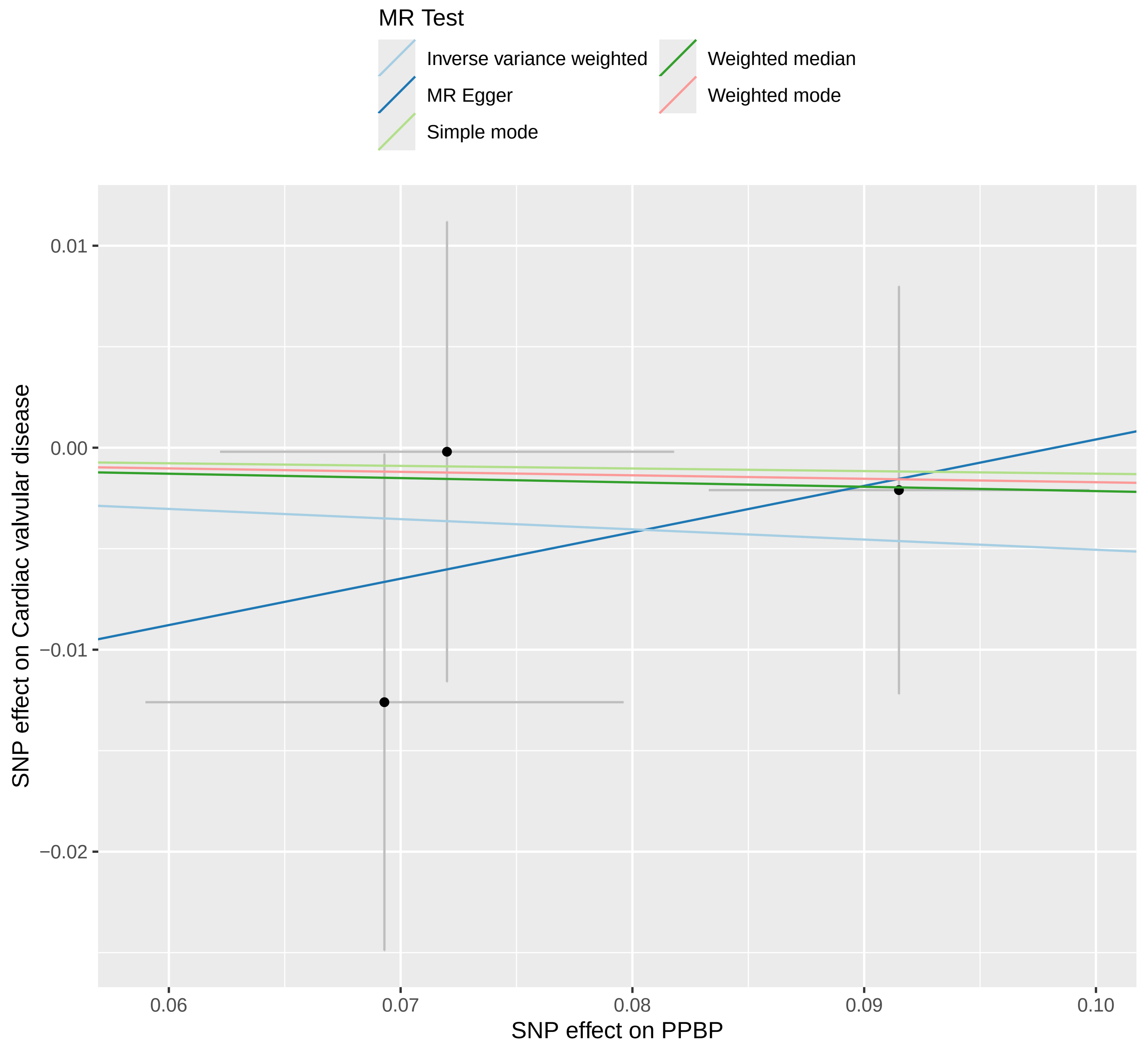
MR Test
Inverse variance weighted
MR Egger
Simple mode
Weighted median
Weighted mode
0.01
0.00
−0.01
−0.02
0.06
0.07
0.08
0.09
0.10
SNP effect on Cardiac valvular disease
SNP effect on PPBP

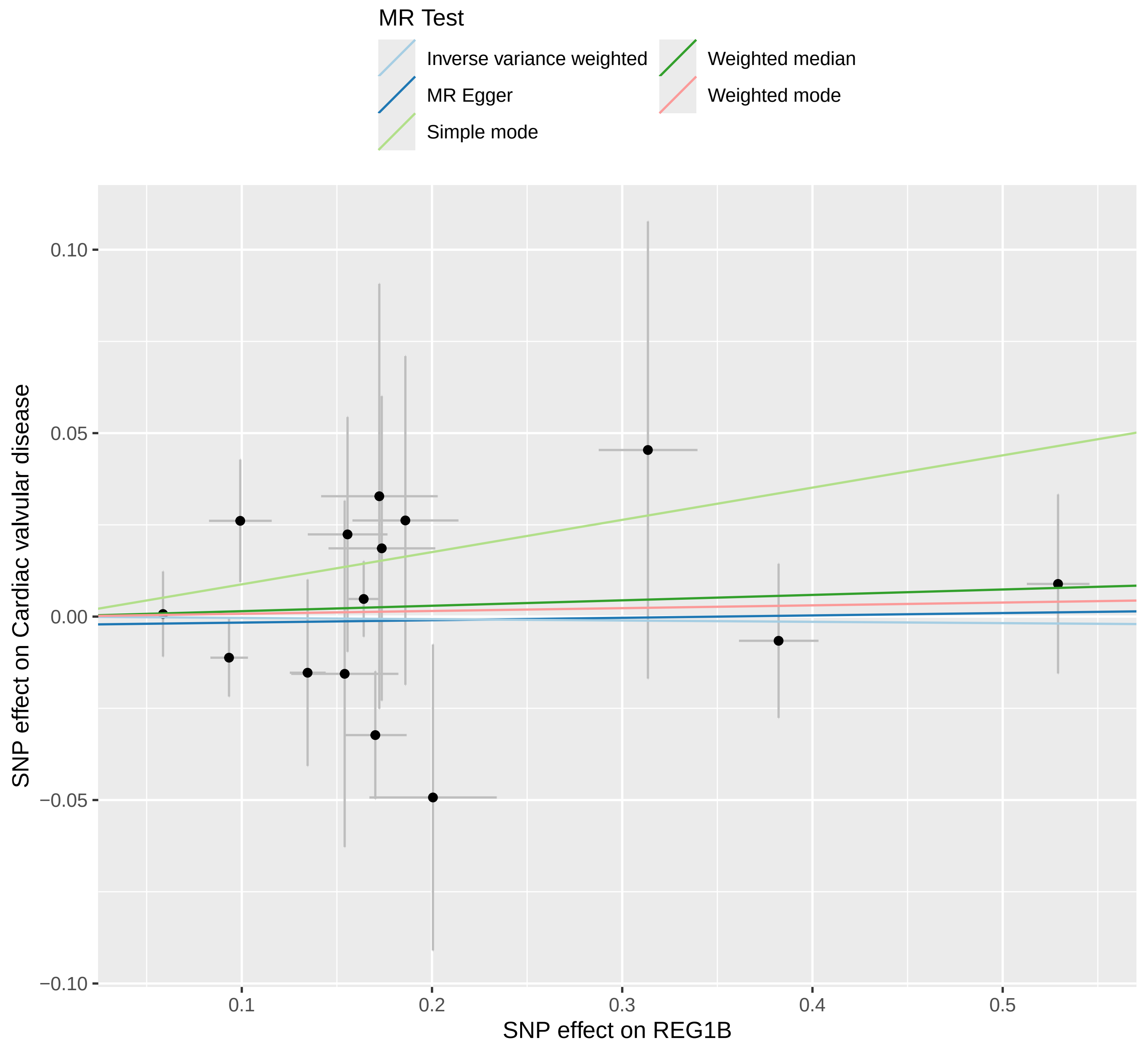
MR Test
Inverse variance weighted
MR Egger
Simple mode
Weighted median
Weighted mode
0.10
0.05
0.00
−0.05
−0.10
0.1
0.2
0.3
0.4
0.5
SNP effect on REG1B
SNP effect on Cardiac valvular disease

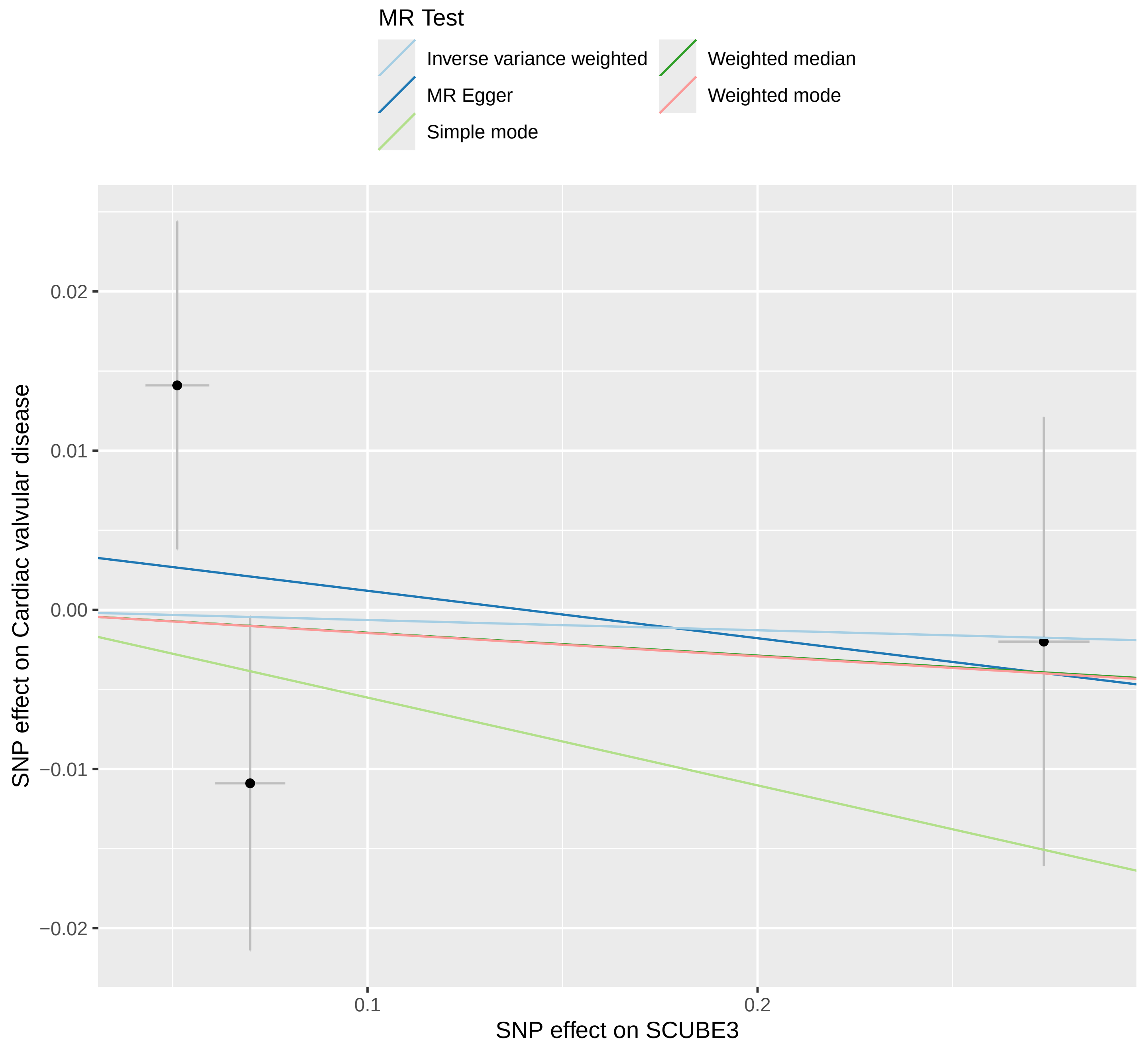

MR Test
Inverse variance weighted
MR Egger
Simple mode
Weighted median
Weighted mode
0.02
0.01
0.00
−0.01
−0.02
SNP effect on Cardiac valvular disease
0.1
0.2
SNP effect on SCUBE3

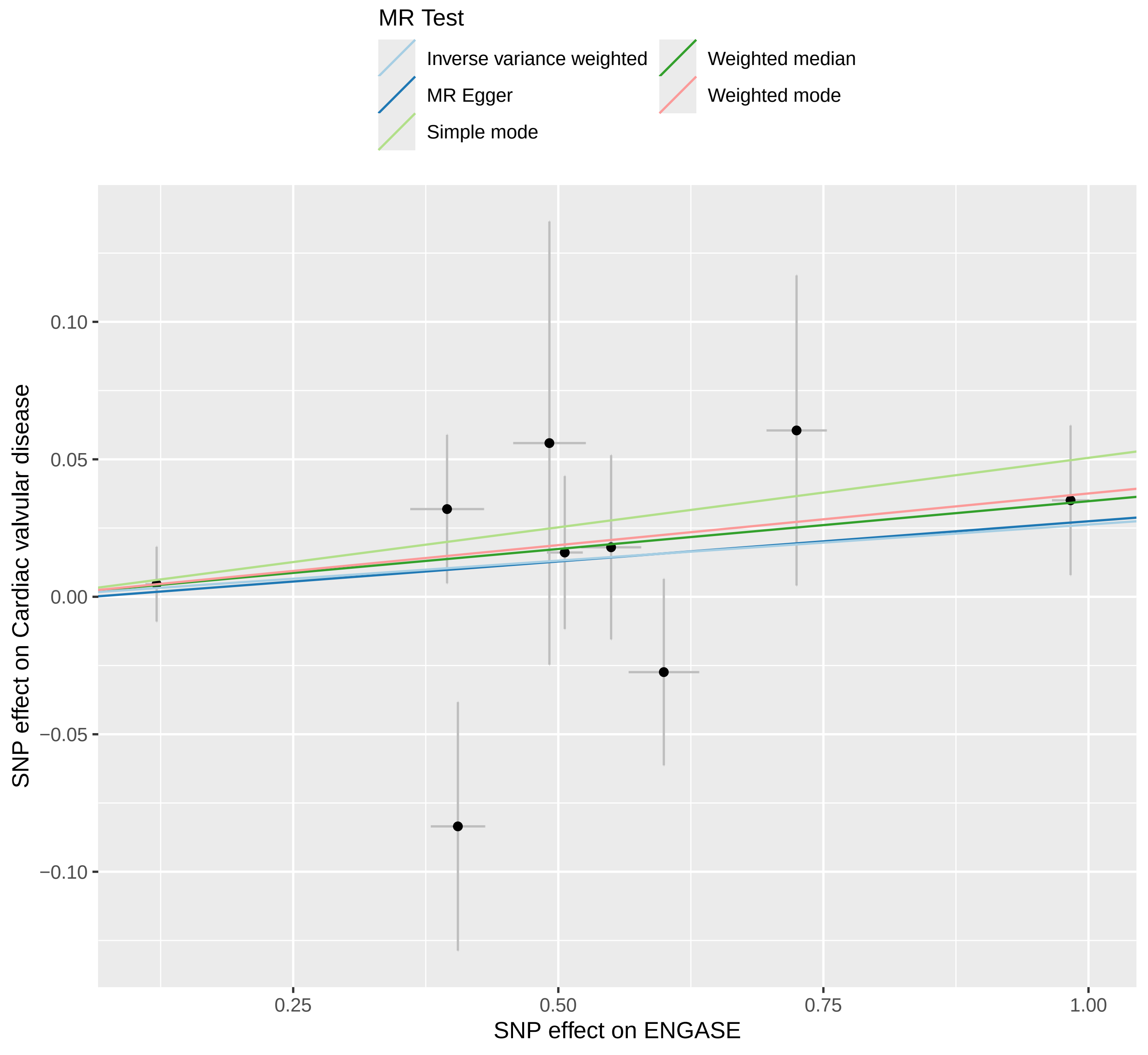
MR Test
Inverse variance weighted
MR Egger
Simple mode
Weighted median
Weighted mode
0.10
0.05
0.00
−0.05
−0.10
SNP effect on Cardiac valvular disease
0.25
0.50
0.75
1.00
SNP effect on ENGASE

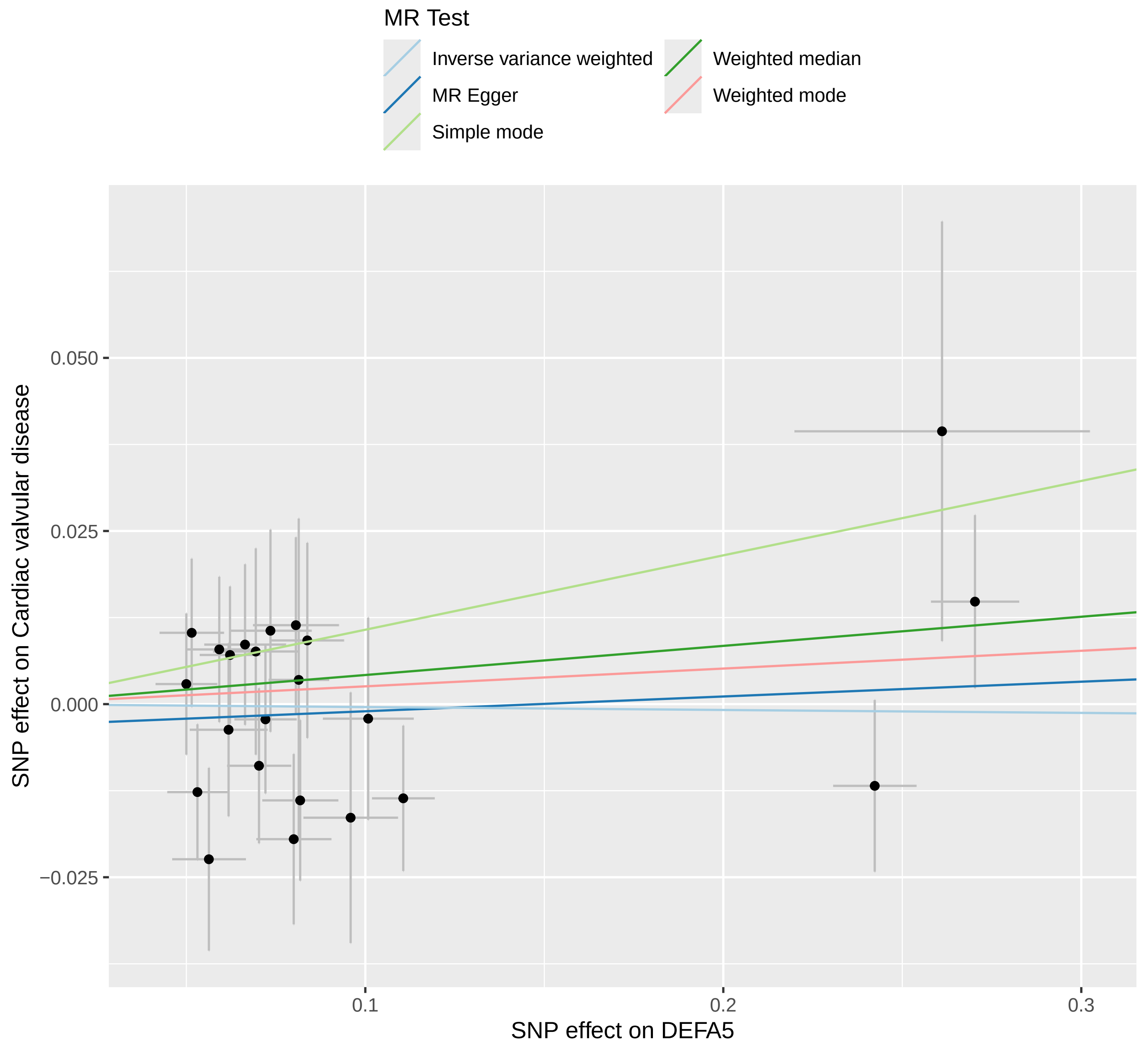
MR Test
Inverse variance weighted
MR Egger
Simple mode
Weighted median
Weighted mode
0.050
0.025
0.000
−0.025
SNP effect on Cardiac valvular disease
0.1
0.2
0.3
SNP effect on DEFA5

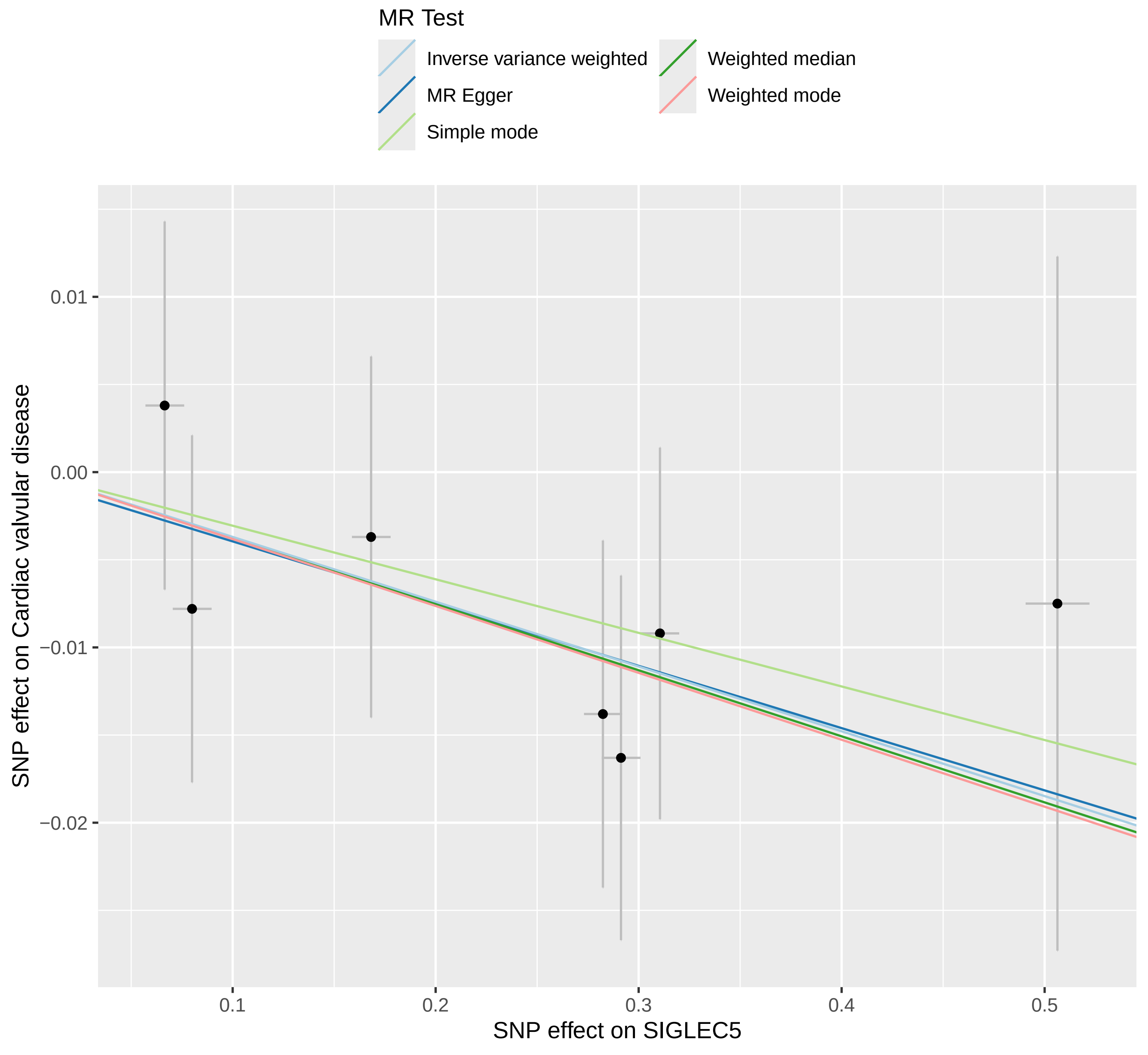
MR Test
Inverse variance weighted
MR Egger
Simple mode
Weighted median
Weighted mode
0.01
0.00
−0.01
−0.02
0.1
0.2
0.3
0.4
0.5
SNP effect on Cardiac valvular disease
SNP effect on SIGLEC5

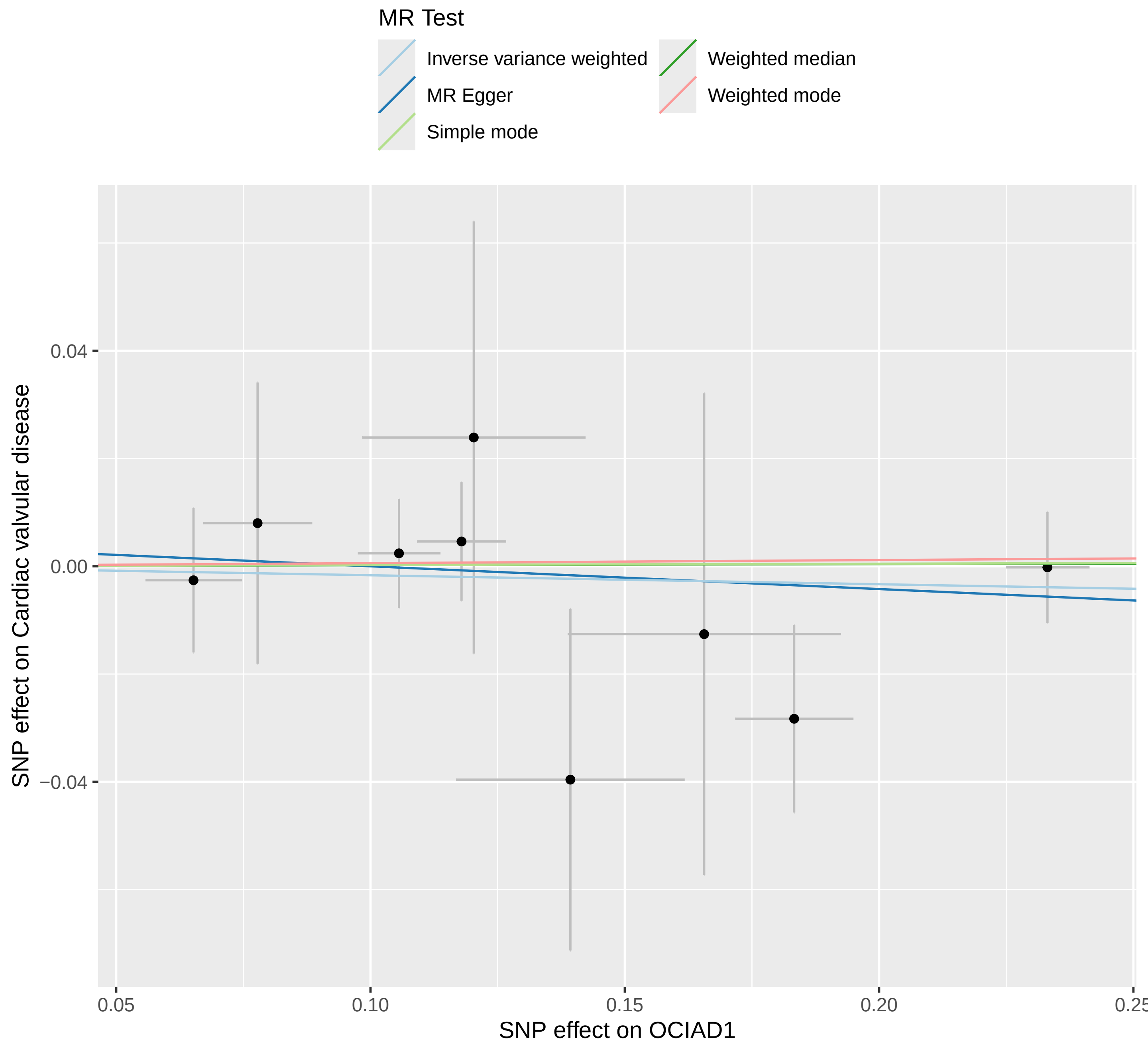
MR Test
Inverse variance weighted
MR Egger
Simple mode
Weighted median
Weighted mode
0.04
0.00
−0.04
0.05
0.10
0.15
0.20
0.25
SNP effect on OCIAD1
SNP effect on Cardiac valvular disease

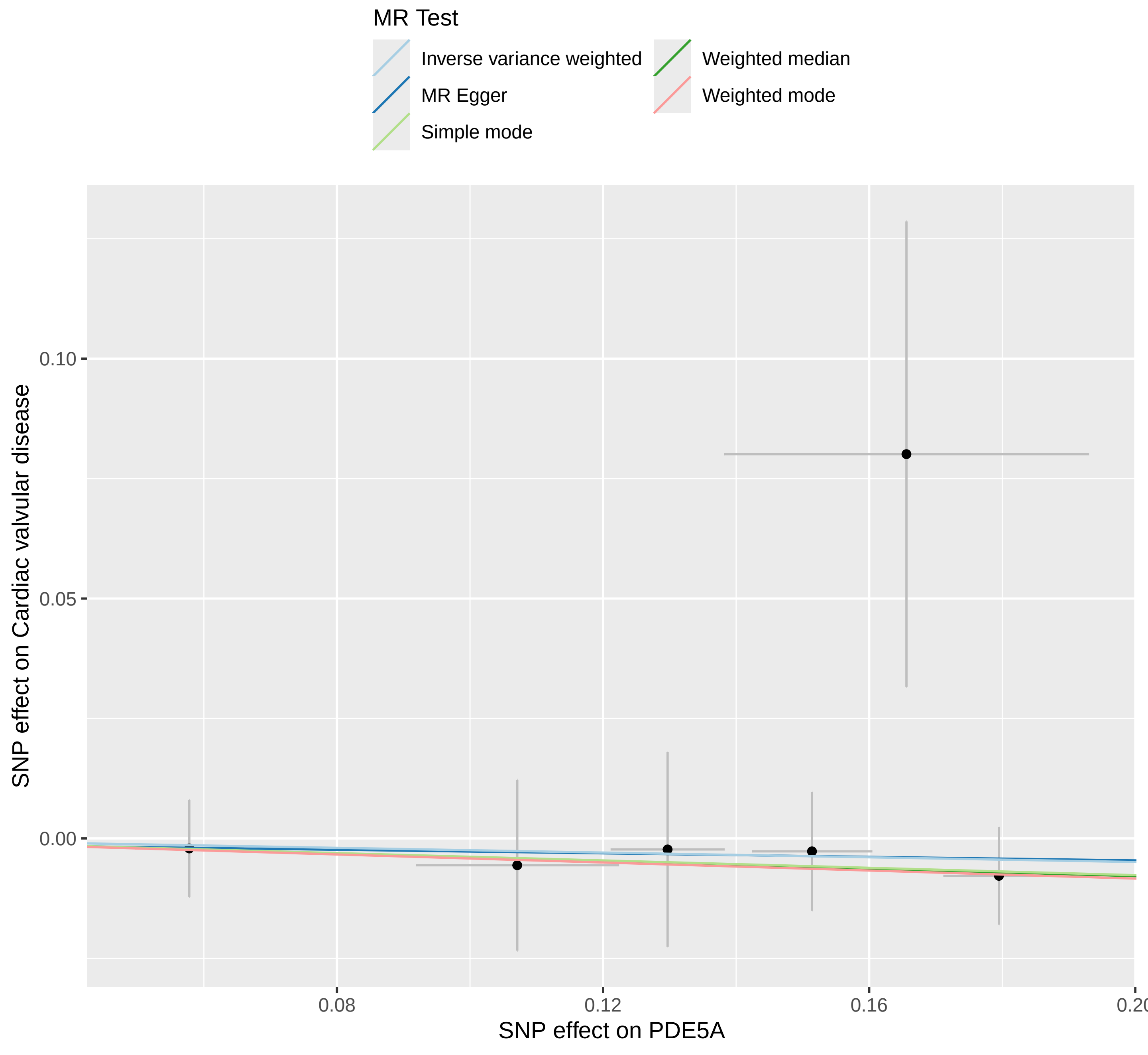

MR Test
Inverse variance weighted
MR Egger
Simple mode
Weighted median
Weighted mode
0.10
0.05
0.00
0.08
0.12
0.16
0.20
SNP effect on PDE5A
SNP effect on Cardiac valvular disease

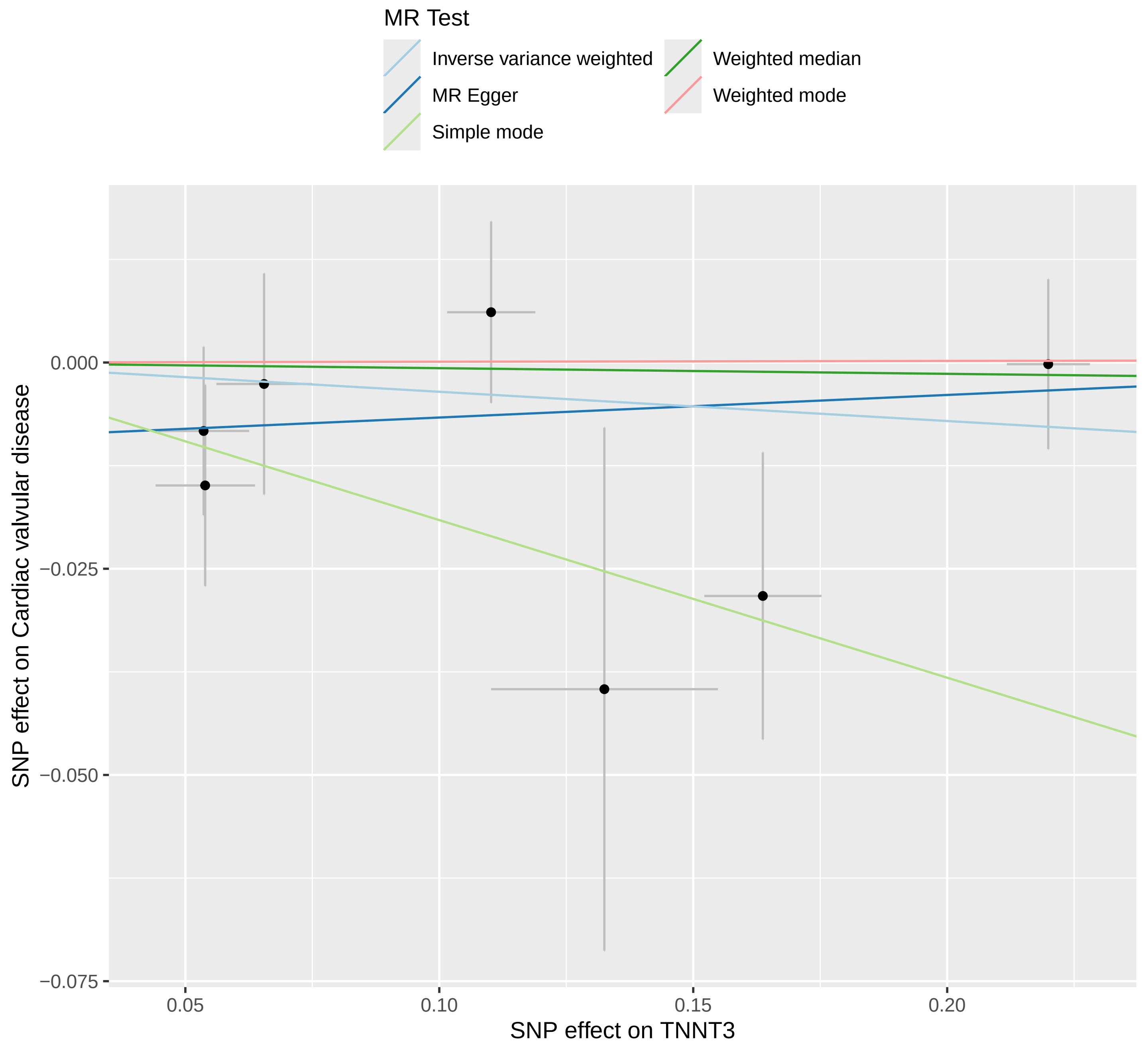

MR Test
Inverse variance weighted
MR Egger
Simple mode
Weighted median
Weighted mode
0.000
−0.025
−0.050
−0.075
SNP effect on Cardiac valvular disease
0.05
0.10
0.15
0.20
SNP effect on TNNT3

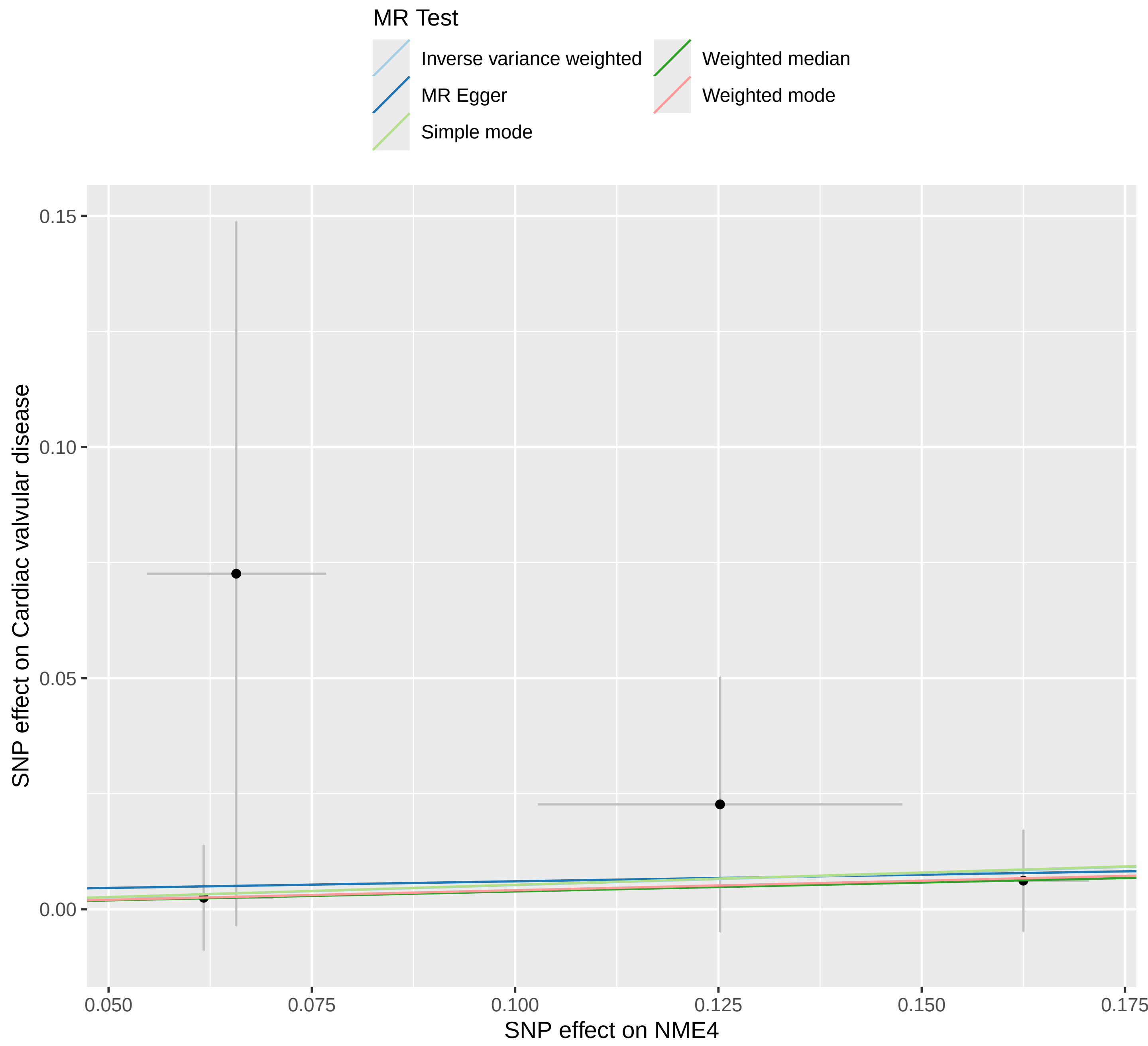
MR Test
Inverse variance weighted
MR Egger
Simple mode
Weighted median
Weighted mode
0.15
0.10
0.05
0.00
SNP effect on Cardiac valvular disease
0.050
0.075
0.100
0.125
0.150
0.175
SNP effect on NME4

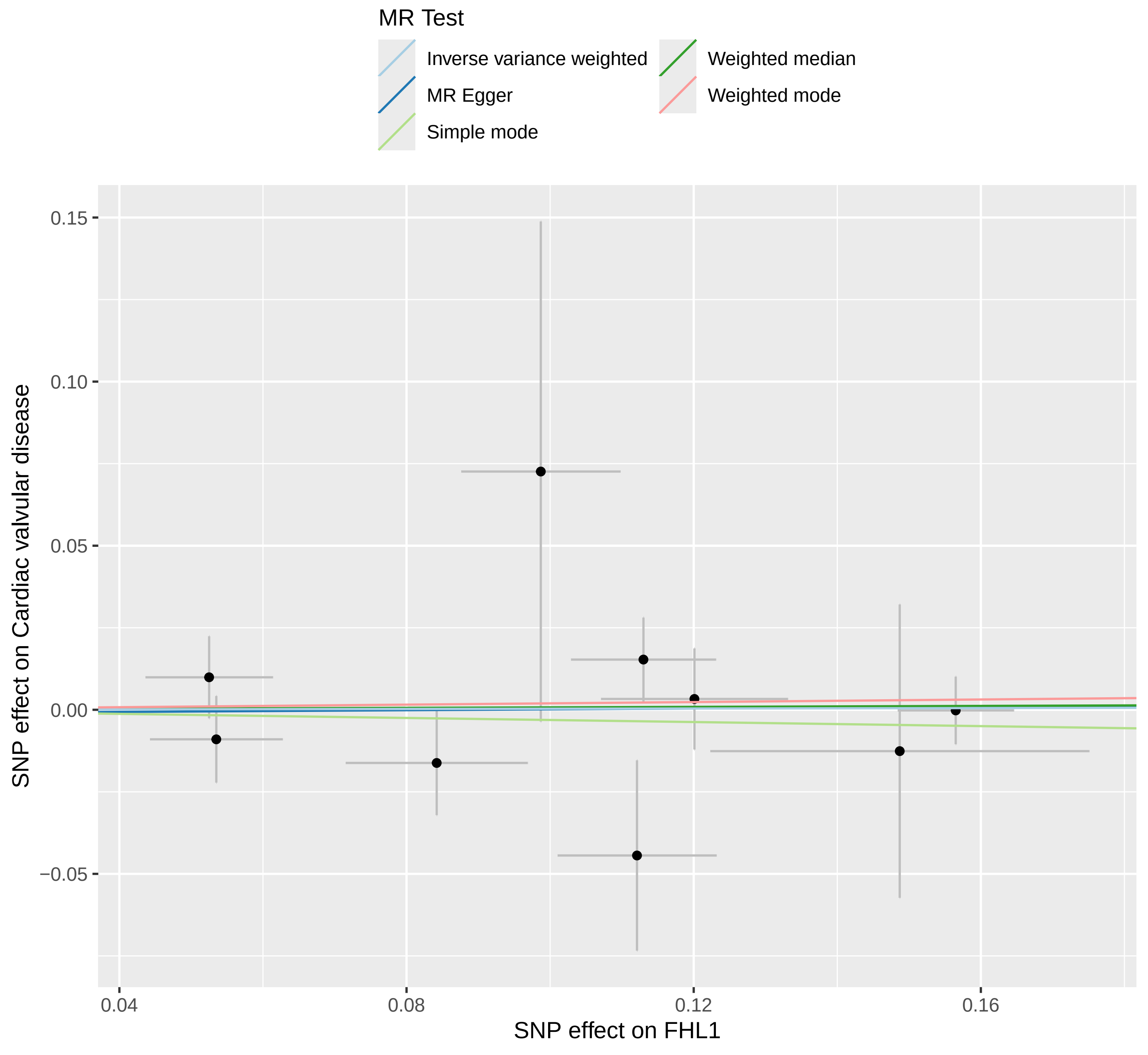
MR Test
Inverse variance weighted
MR Egger
Simple mode
Weighted median
Weighted mode
0.15
0.10
0.05
0.00
−0.05
SNP effect on Cardiac valvular disease
0.04
0.08
0.12
0.16
SNP effect on FHL1

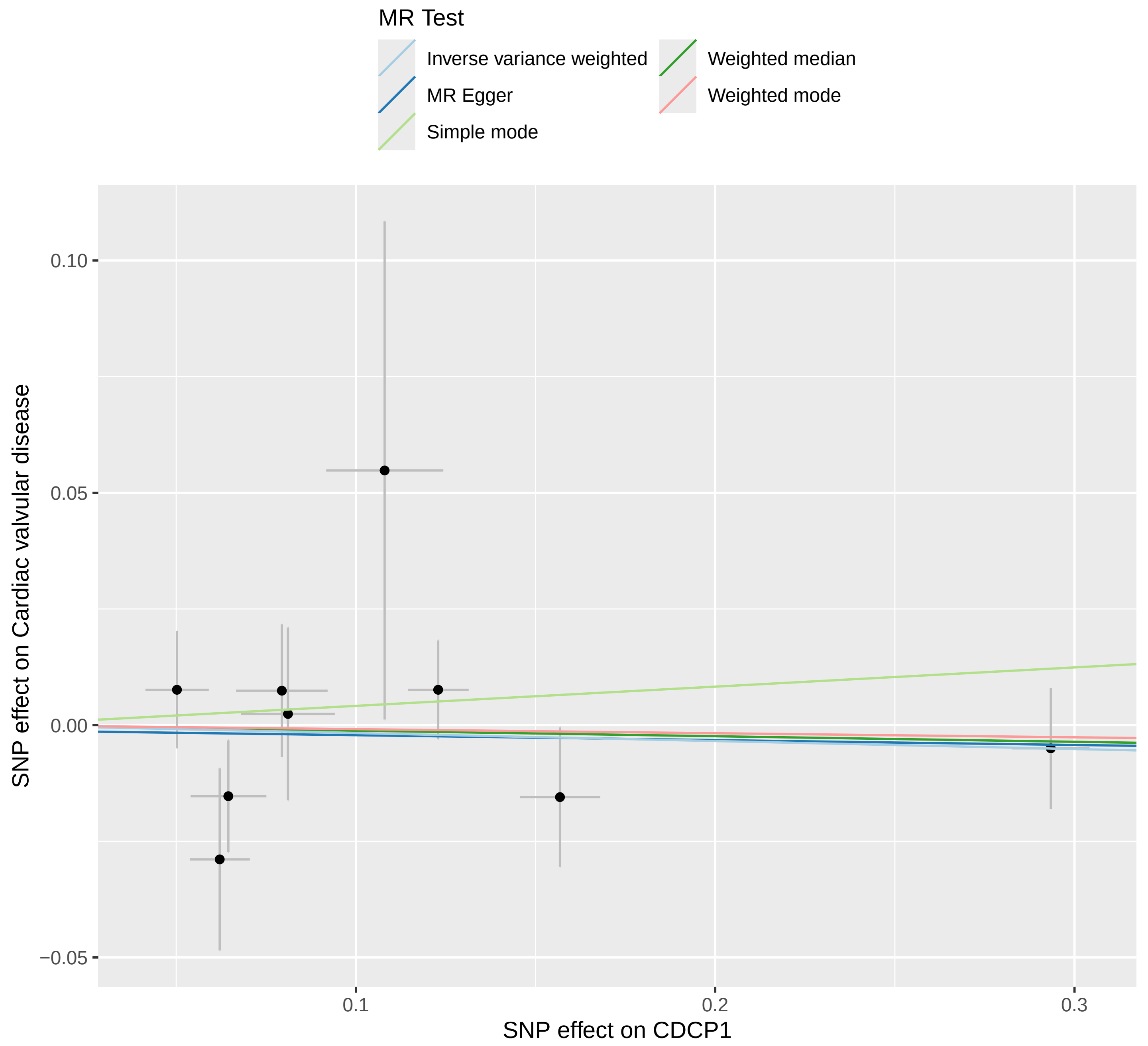
MR Test
Inverse variance weighted
MR Egger
Simple mode
Weighted median
Weighted mode
SNP effect on Cardiac valvular disease
0.10
0.05
0.00
−0.05
0.1
0.2
0.3
SNP effect on CDCP1

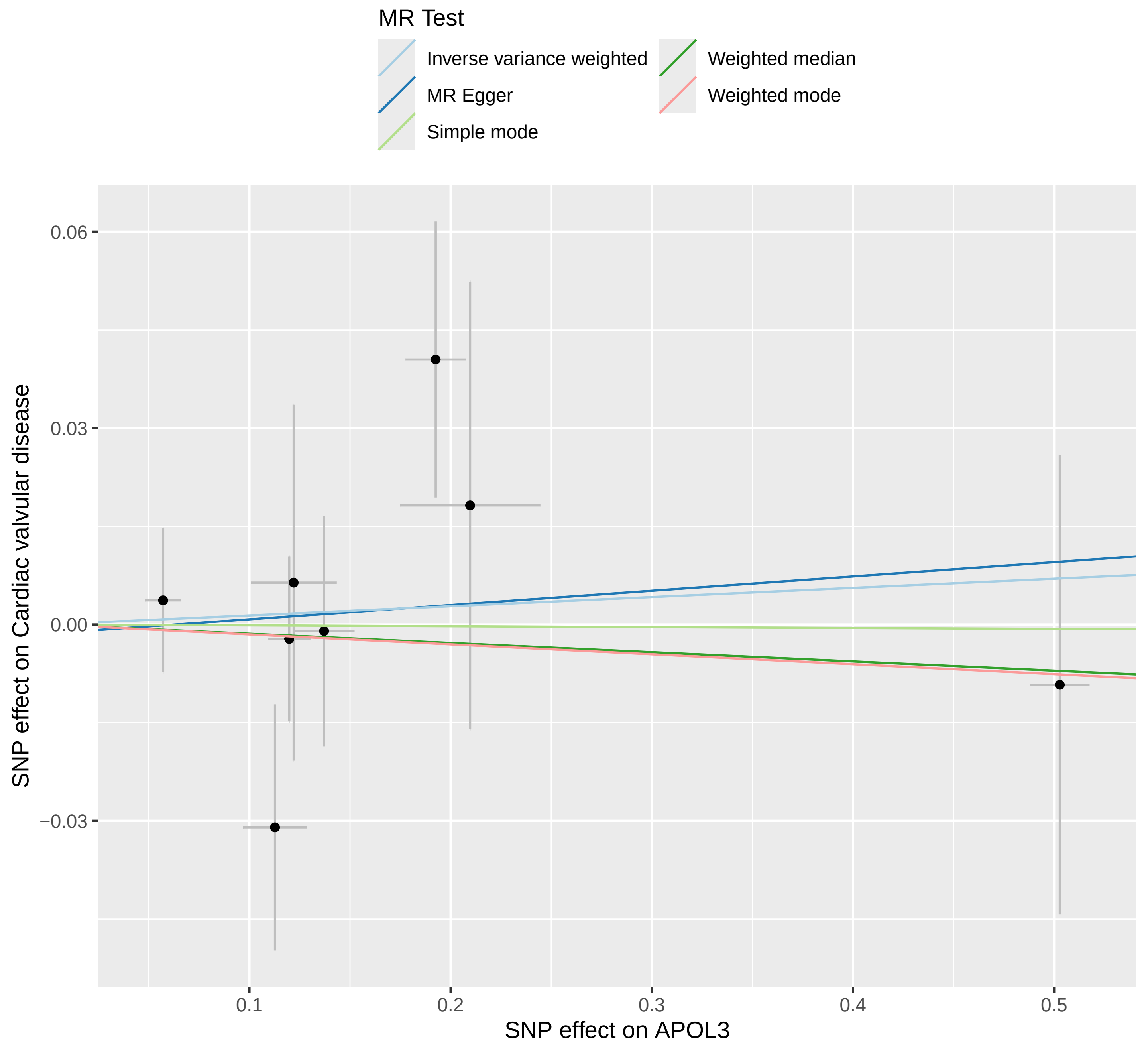

MR Test
Inverse variance weighted
MR Egger
Simple mode
Weighted median
Weighted mode
SNP effect on Cardiac valvular disease
0.06
0.03
0.00
−0.03
0.1
0.2
0.3
0.4
0.5
SNP effect on APOL3

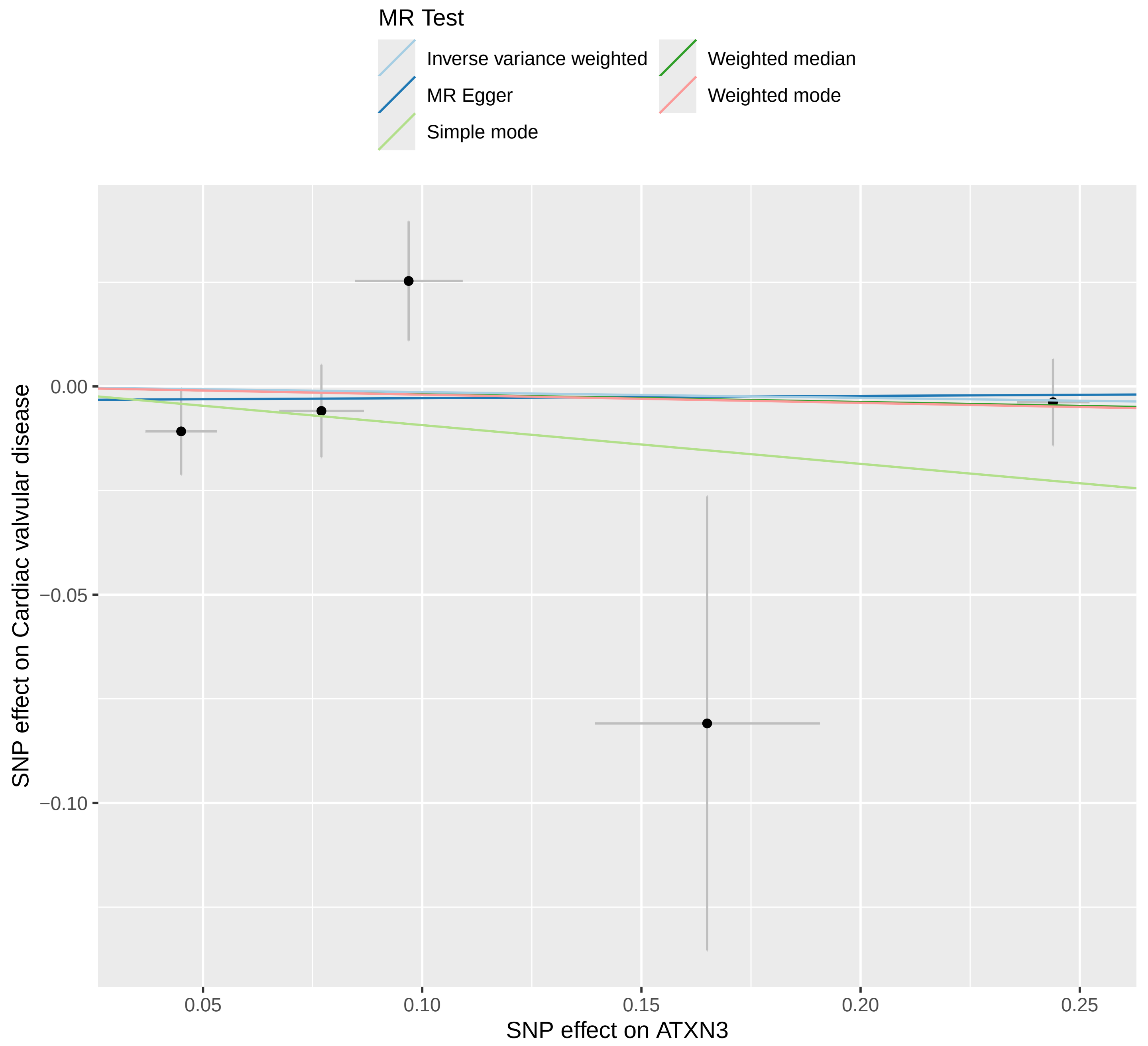
MR Test
Inverse variance weighted
MR Egger
Simple mode
Weighted median
Weighted mode
SNP effect on Cardiac valvular disease
0.00
−0.05
−0.10
0.05
0.10
0.15
0.20
0.25
SNP effect on ATXN3

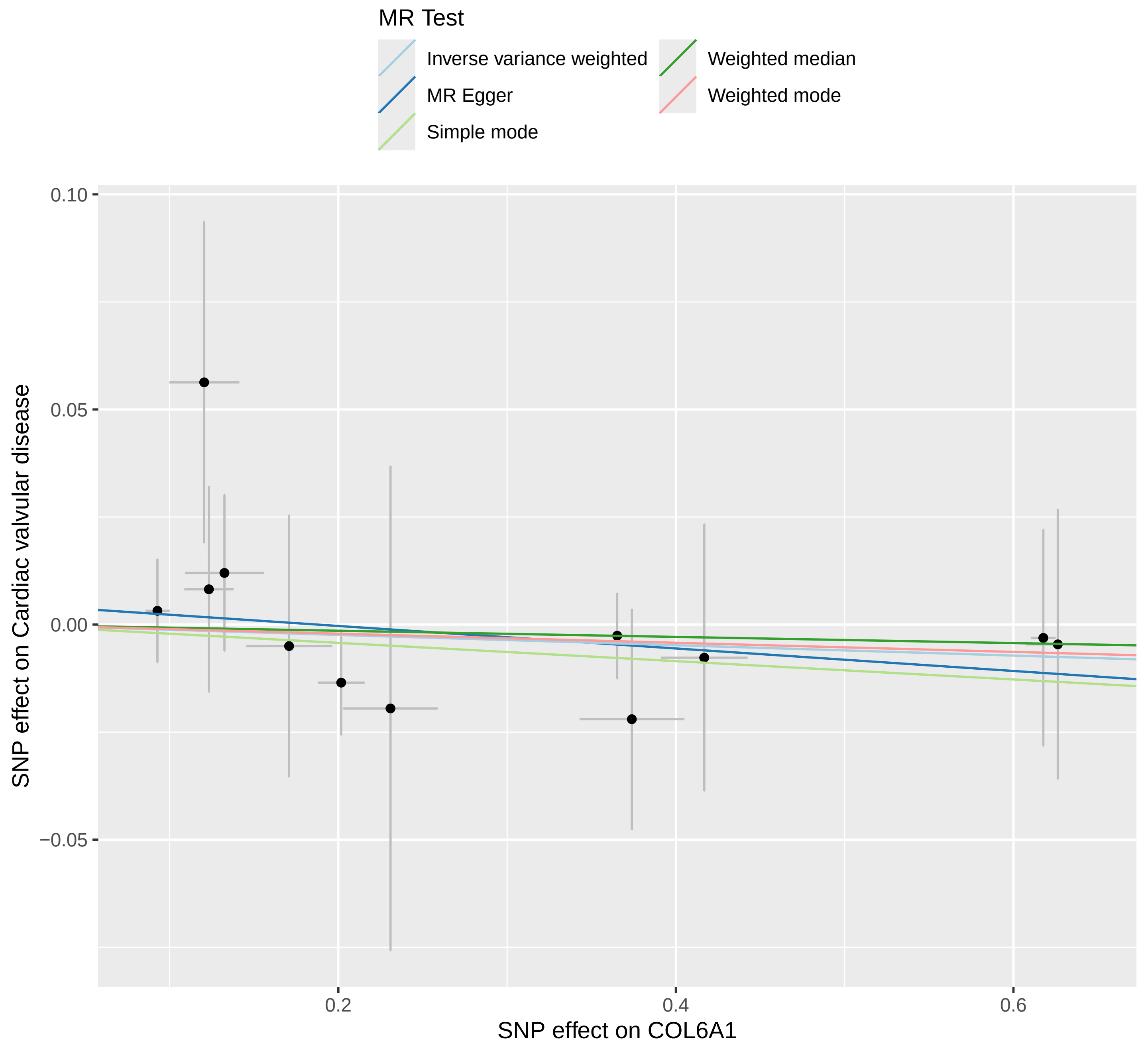
MR Test
Inverse variance weighted
MR Egger
Simple mode
Weighted median
Weighted mode
0.10
0.05
0.00
−0.05
0.2
0.4
0.6
SNP effect on Cardiac valvular disease
SNP effect on COL6A1

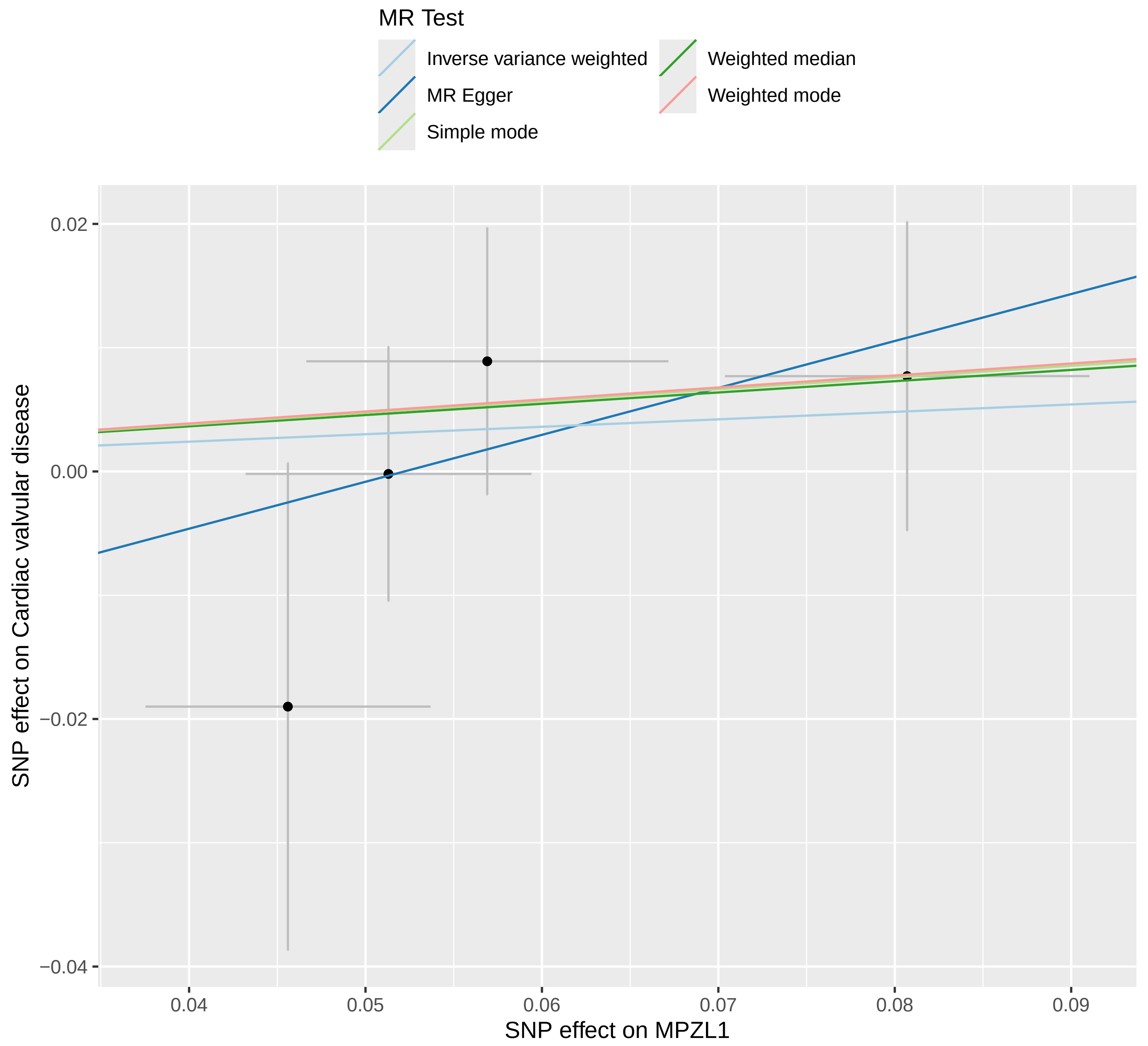

MR Test
Inverse variance weighted
MR Egger
Simple mode
Weighted median
Weighted mode
0.02
0.00
−0.02
−0.04
0.04
0.05
0.06
0.07
0.08
0.09
SNP effect on Cardiac valvular disease
SNP effect on MPZL1

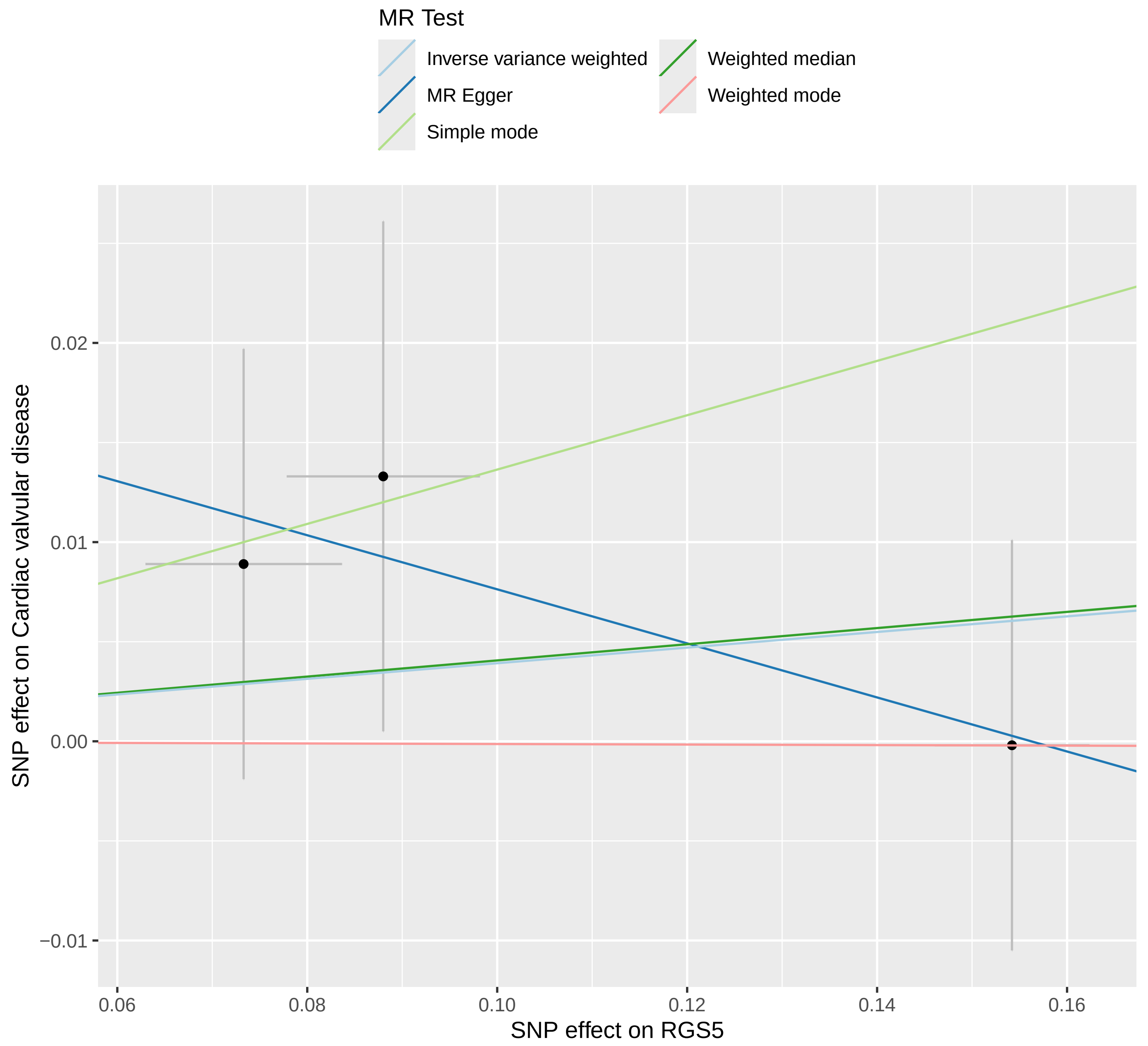

MR Test
Inverse variance weighted
MR Egger
Simple mode
Weighted median
Weighted mode
SNP effect on Cardiac valvular disease
0.02
0.01
0.00
−0.01
0.06
0.08
0.10
0.12
0.14
0.16
SNP effect on RGS5

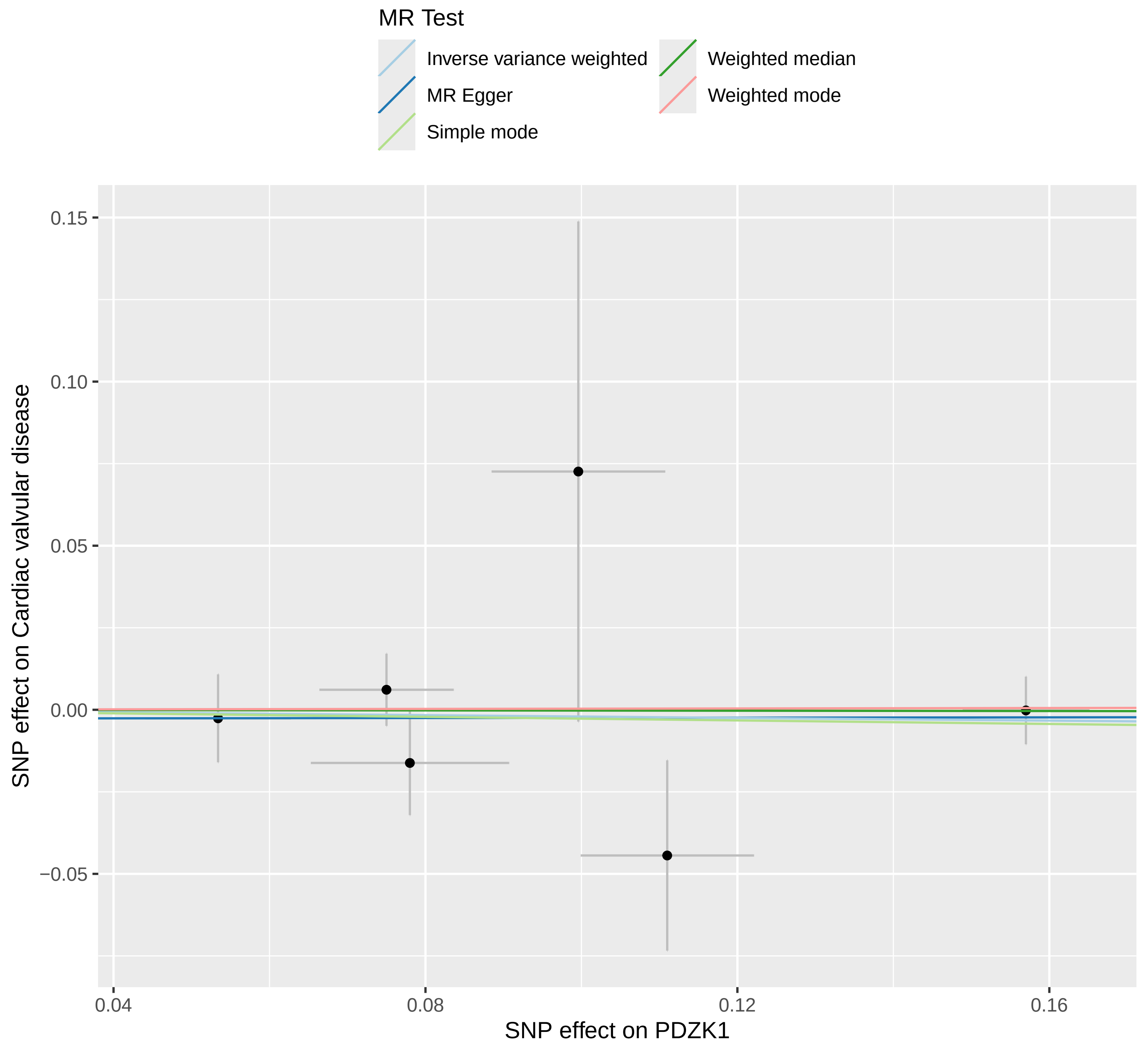

MR Test
Inverse variance weighted
MR Egger
Simple mode
Weighted median
Weighted mode
SNP effect on Cardiac valvular disease
0.15
0.10
0.05
0.00
−0.05
0.04
0.08
0.12
0.16
SNP effect on PDZK1

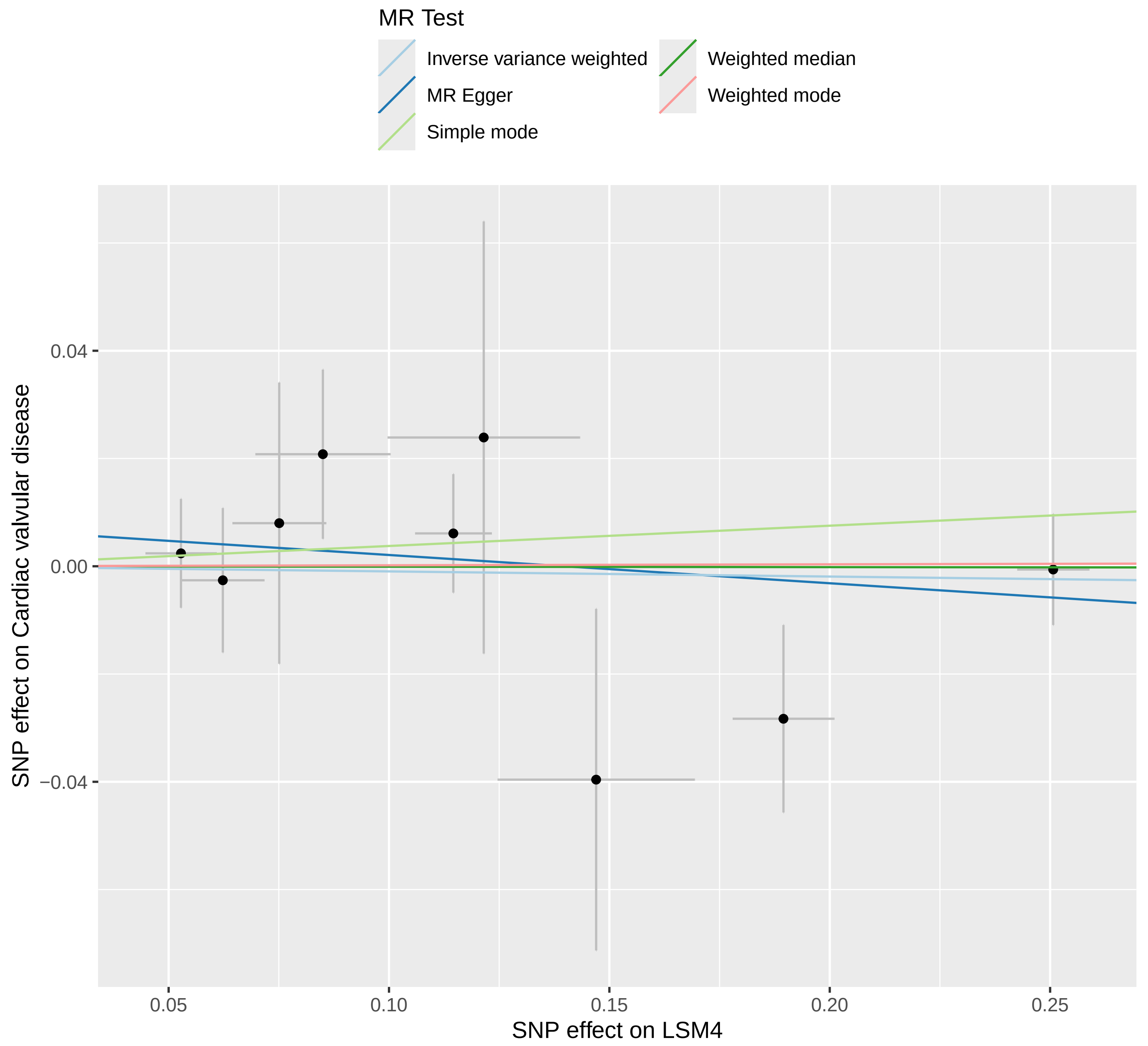

MR Test
Inverse variance weighted
MR Egger
Simple mode
Weighted median
Weighted mode
0.04
0.00
−0.04
0.05
0.10
0.15
0.20
0.25
SNP effect on Cardiac valvular disease
SNP effect on LSM4

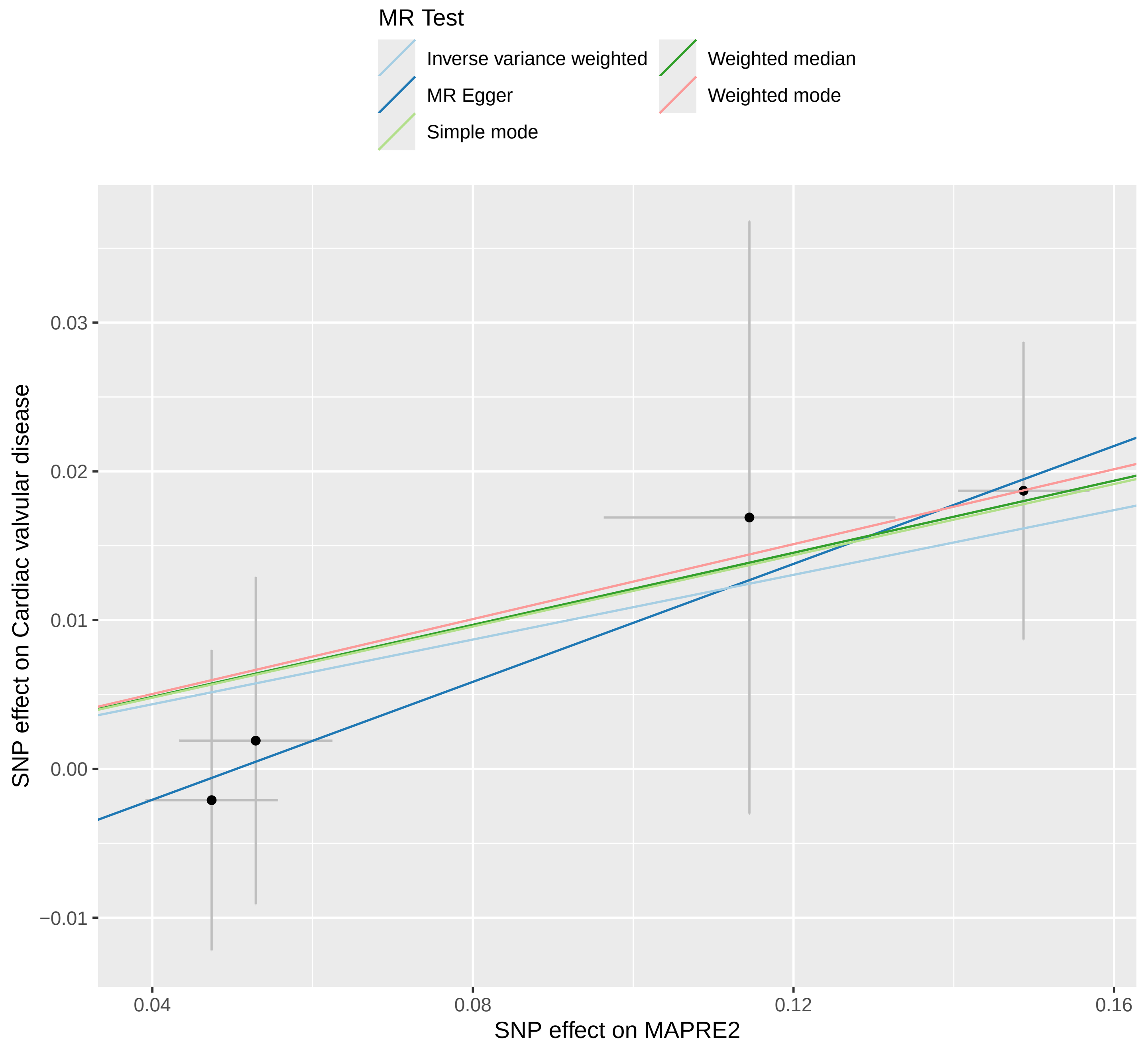

MR Test
Inverse variance weighted
MR Egger
Simple mode
Weighted median
Weighted mode
SNP effect on Cardiac valvular disease
0.03
0.02
0.01
0.00
−0.01
0.04
0.08
0.12
0.16
SNP effect on MAPRE2

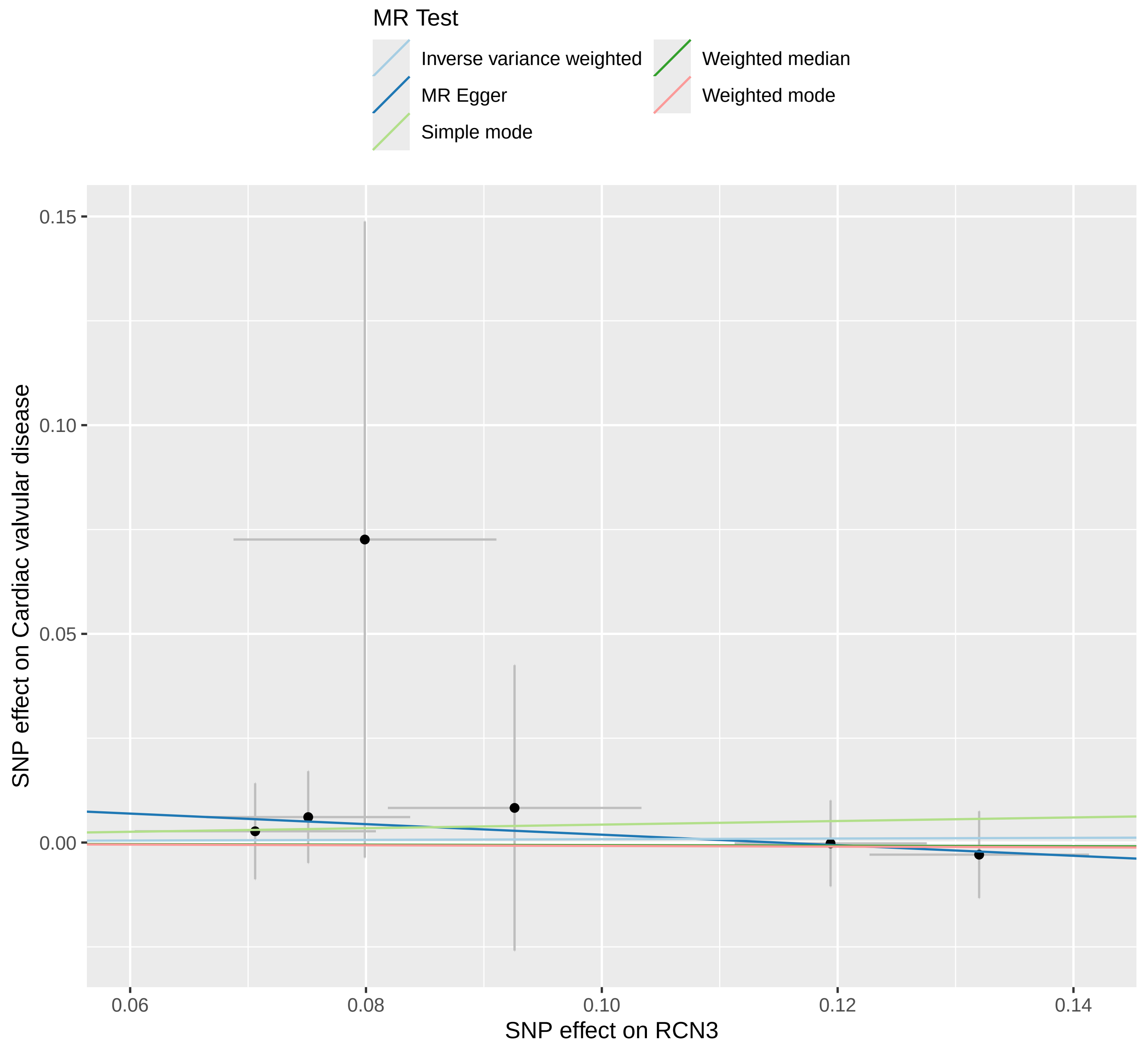
MR Test
Inverse variance weighted
MR Egger
Simple mode
Weighted median
Weighted mode
SNP effect on Cardiac valvular disease
0.15
0.10
0.05
0.00
0.06
0.08
0.10
0.12
0.14
SNP effect on RCN3

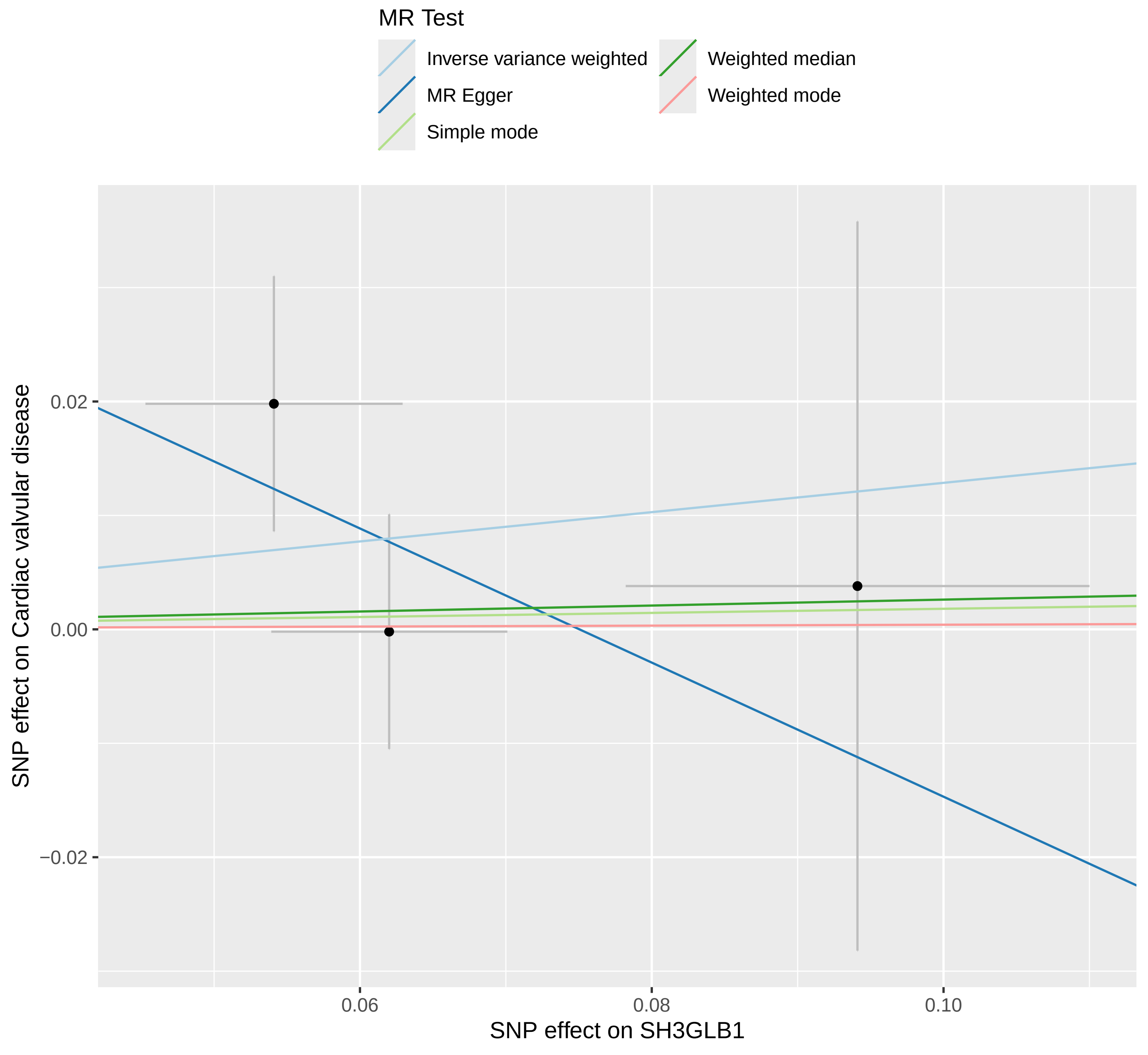

MR Test
Inverse variance weighted
MR Egger
Simple mode
Weighted median
Weighted mode
SNP effect on Cardiac valvular disease
0.02
0.00
−0.02
0.06
0.08
0.10
SNP effect on SH3GLB1

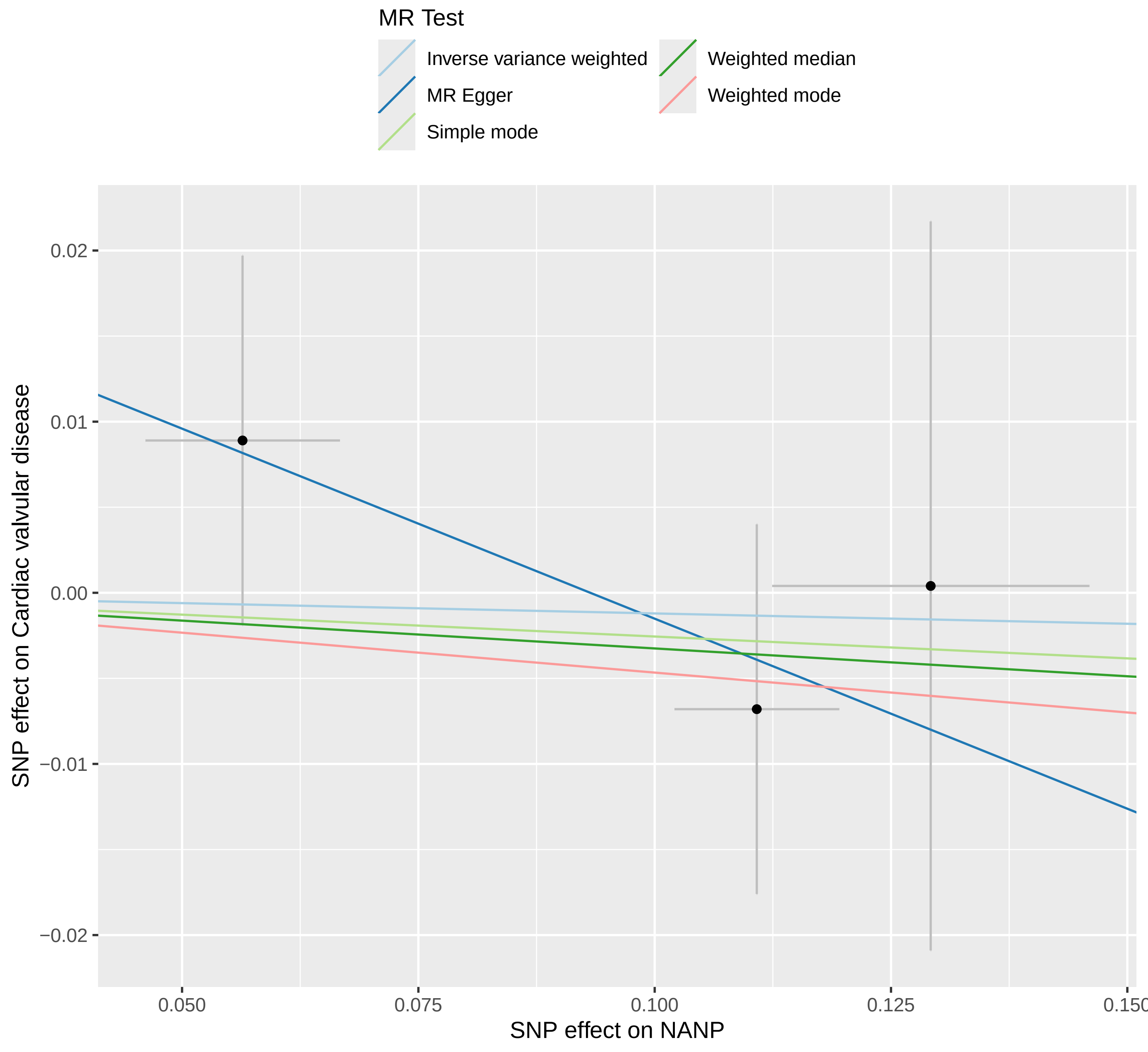
MR Test
Inverse variance weighted
MR Egger
Simple mode
Weighted median
Weighted mode
0.02
0.01
0.00
−0.01
−0.02
SNP effect on Cardiac valvular disease
0.050
0.075
0.100
0.125
0.150
SNP effect on NANP

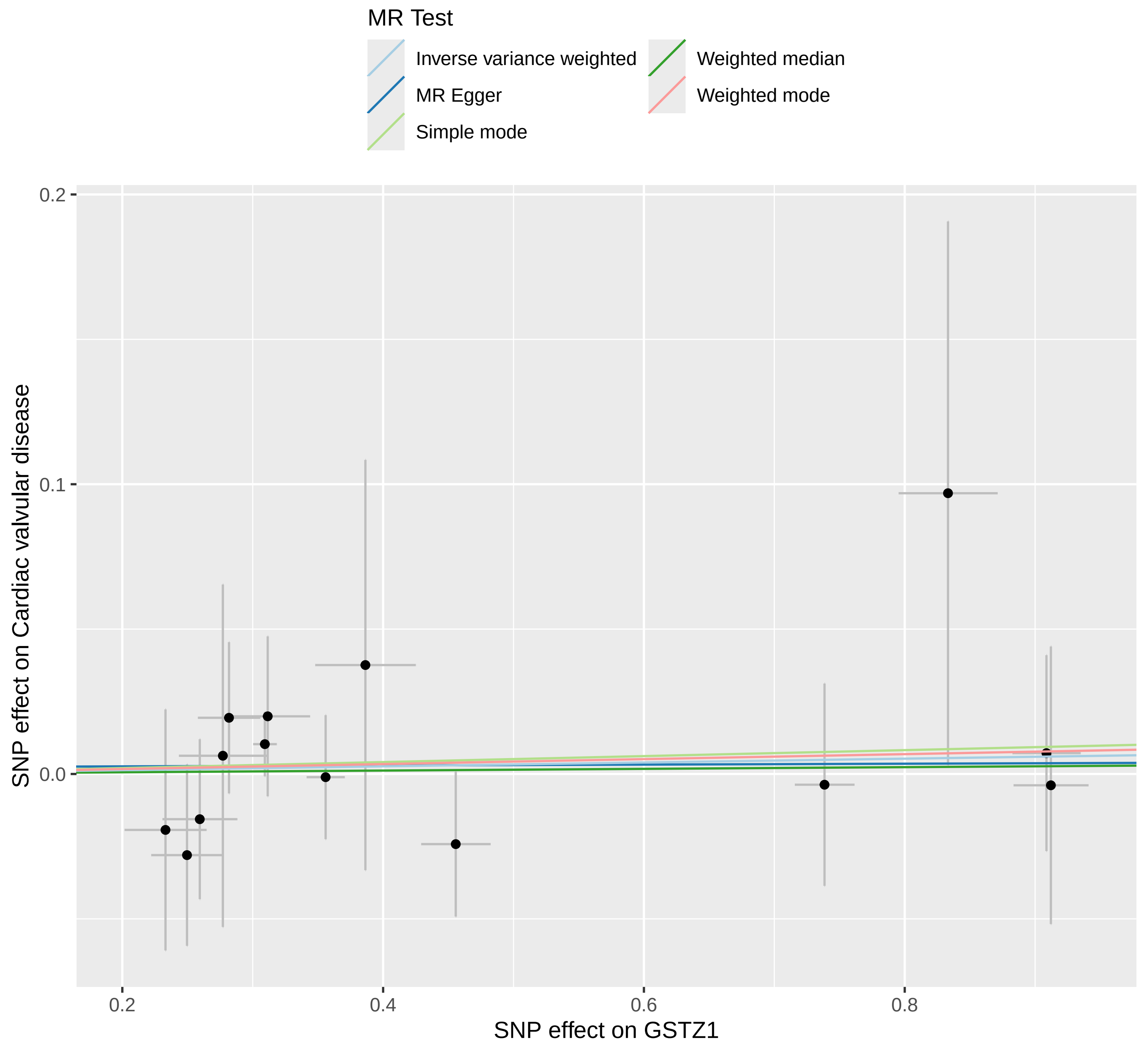
MR Test
Inverse variance weighted
MR Egger
Simple mode
Weighted median
Weighted mode
0.2
0.1
0.0
SNP effect on Cardiac valvular disease
0.2
0.4
0.6
0.8
SNP effect on GSTZ1

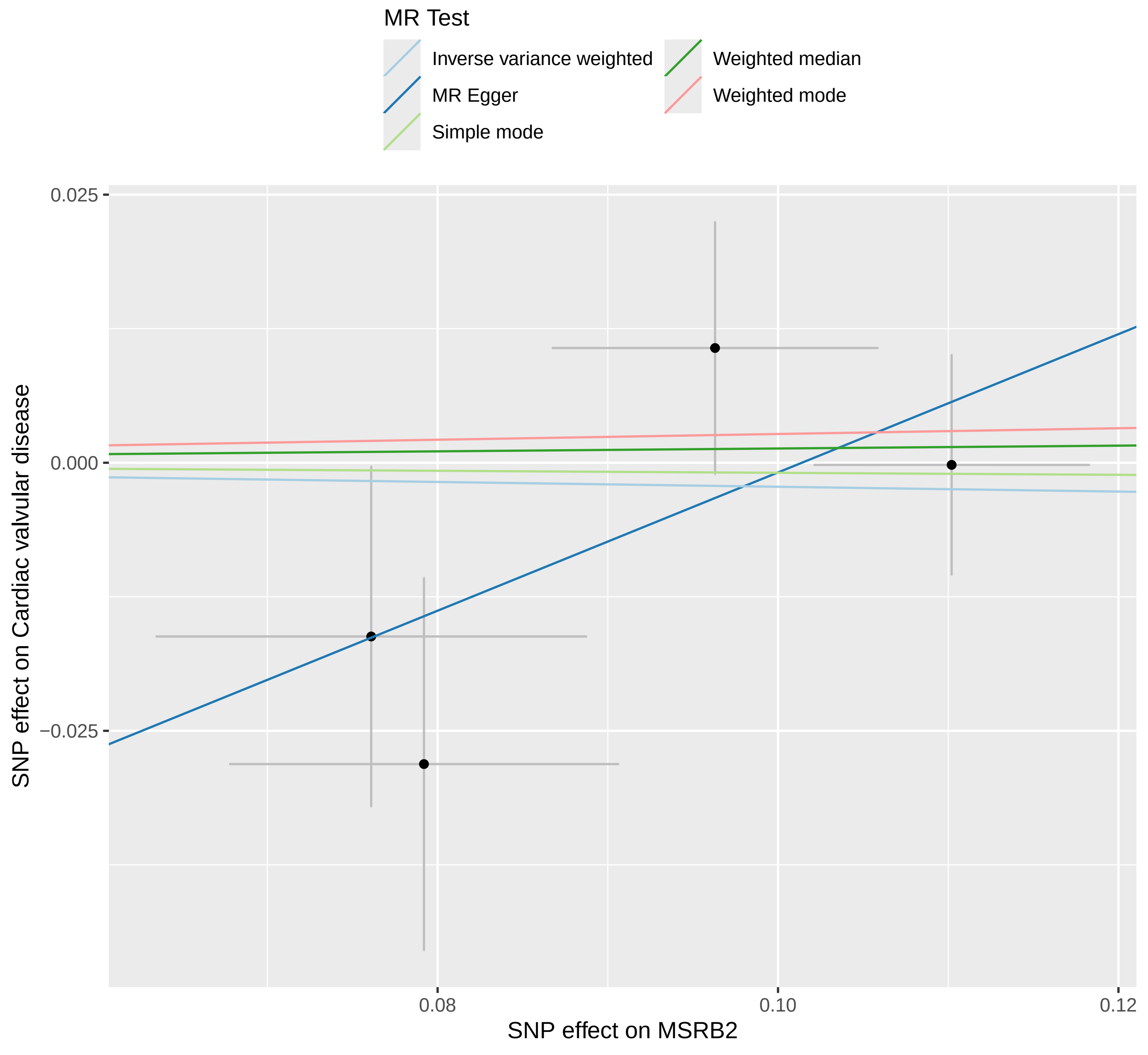

MR Test
Inverse variance weighted
MR Egger
Simple mode
Weighted median
Weighted mode
0.025
0.000
−0.025
0.08
0.10
0.12
SNP effect on MSRB2
SNP effect on Cardiac valvular disease

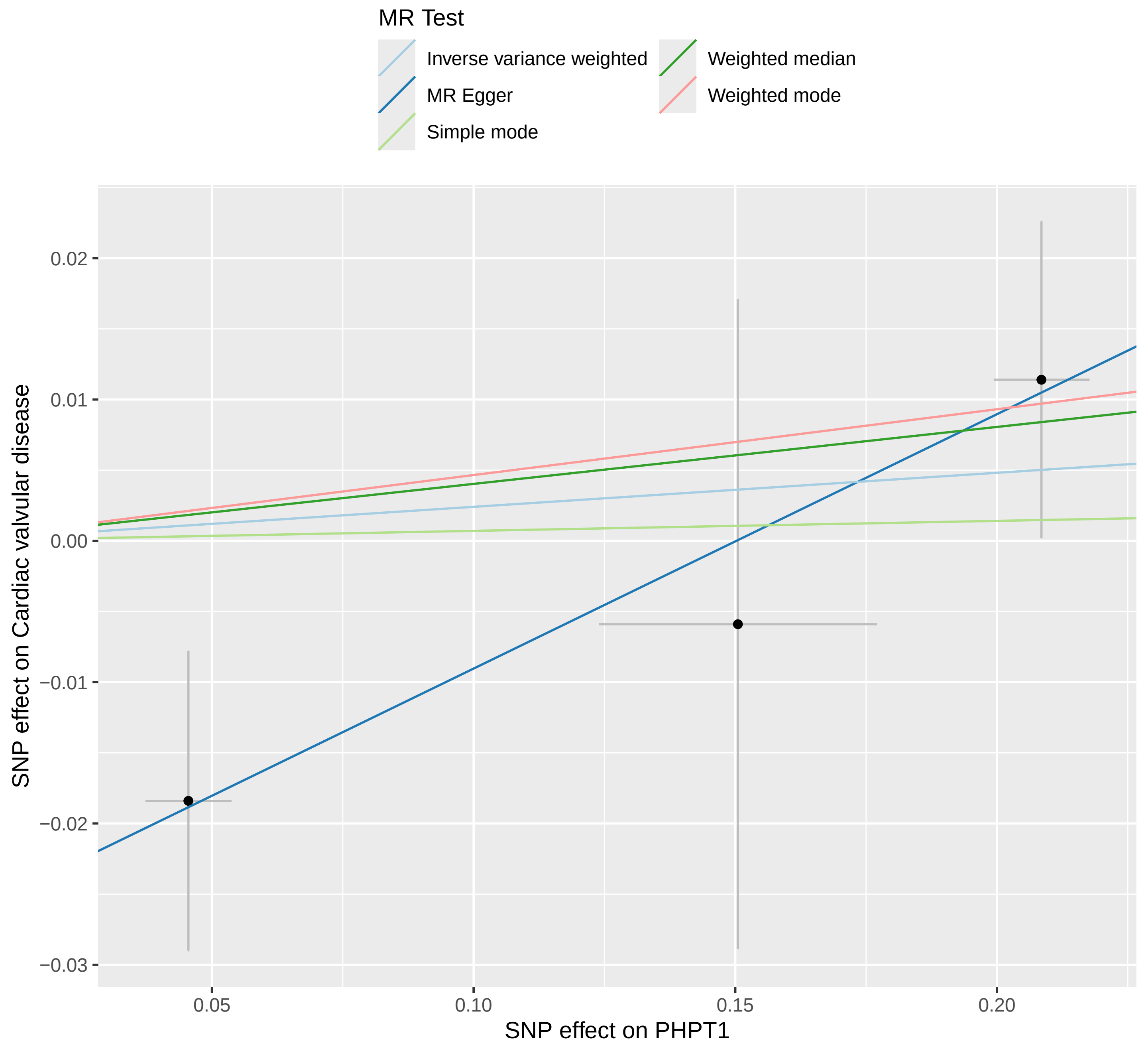

MR Test
Inverse variance weighted
MR Egger
Simple mode
Weighted median
Weighted mode
0.02
0.01
0.00
−0.01
−0.02
−0.03
0.05
0.10
0.15
0.20
SNP effect on Cardiac valvular disease
SNP effect on PHPT1

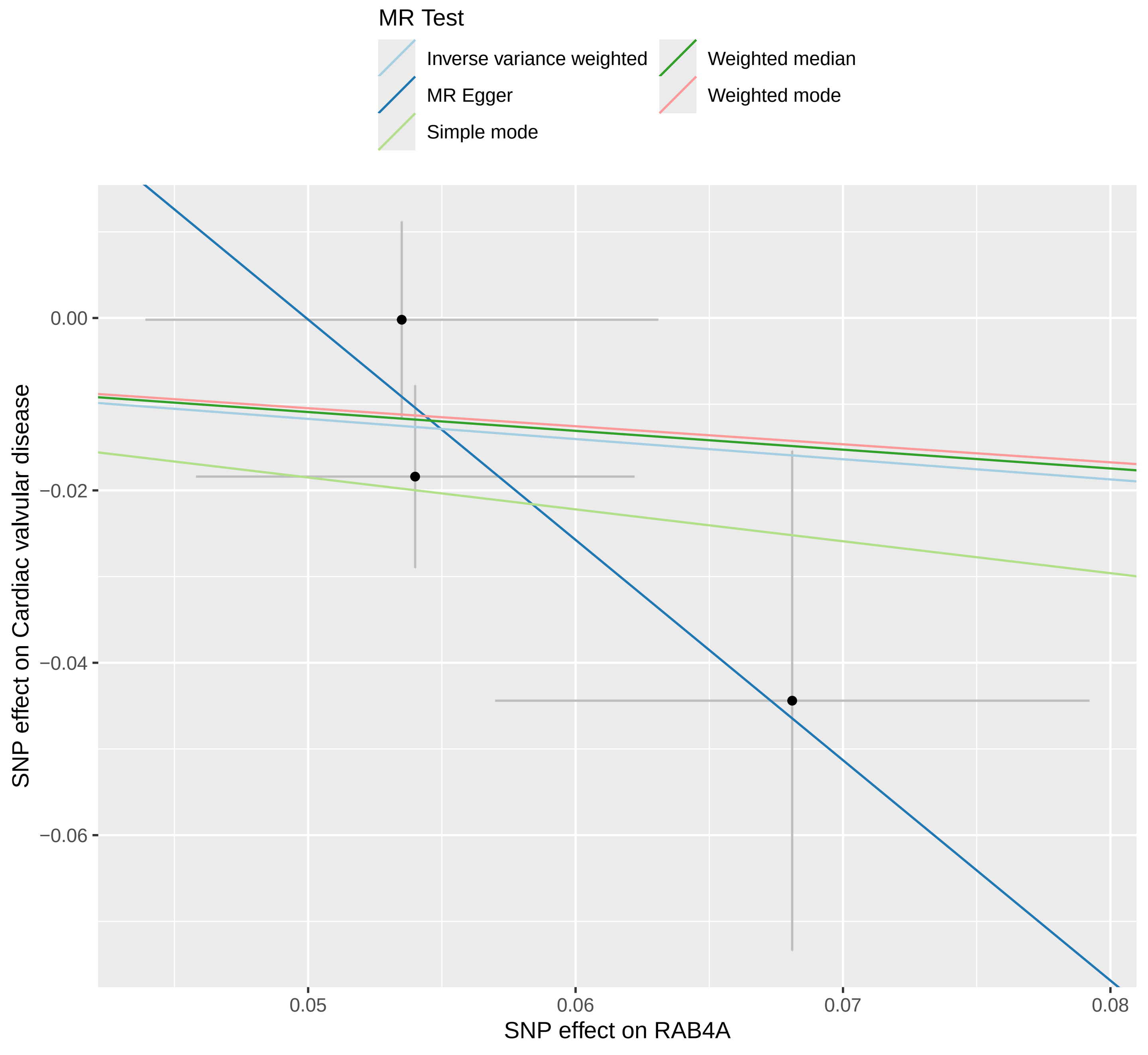
MR Test
Inverse variance weighted
MR Egger
Simple mode
Weighted median
Weighted mode
0.00
−0.02
−0.04
−0.06
0.05
0.06
0.07
0.08
SNP effect on Cardiac valvular disease
SNP effect on RAB4A

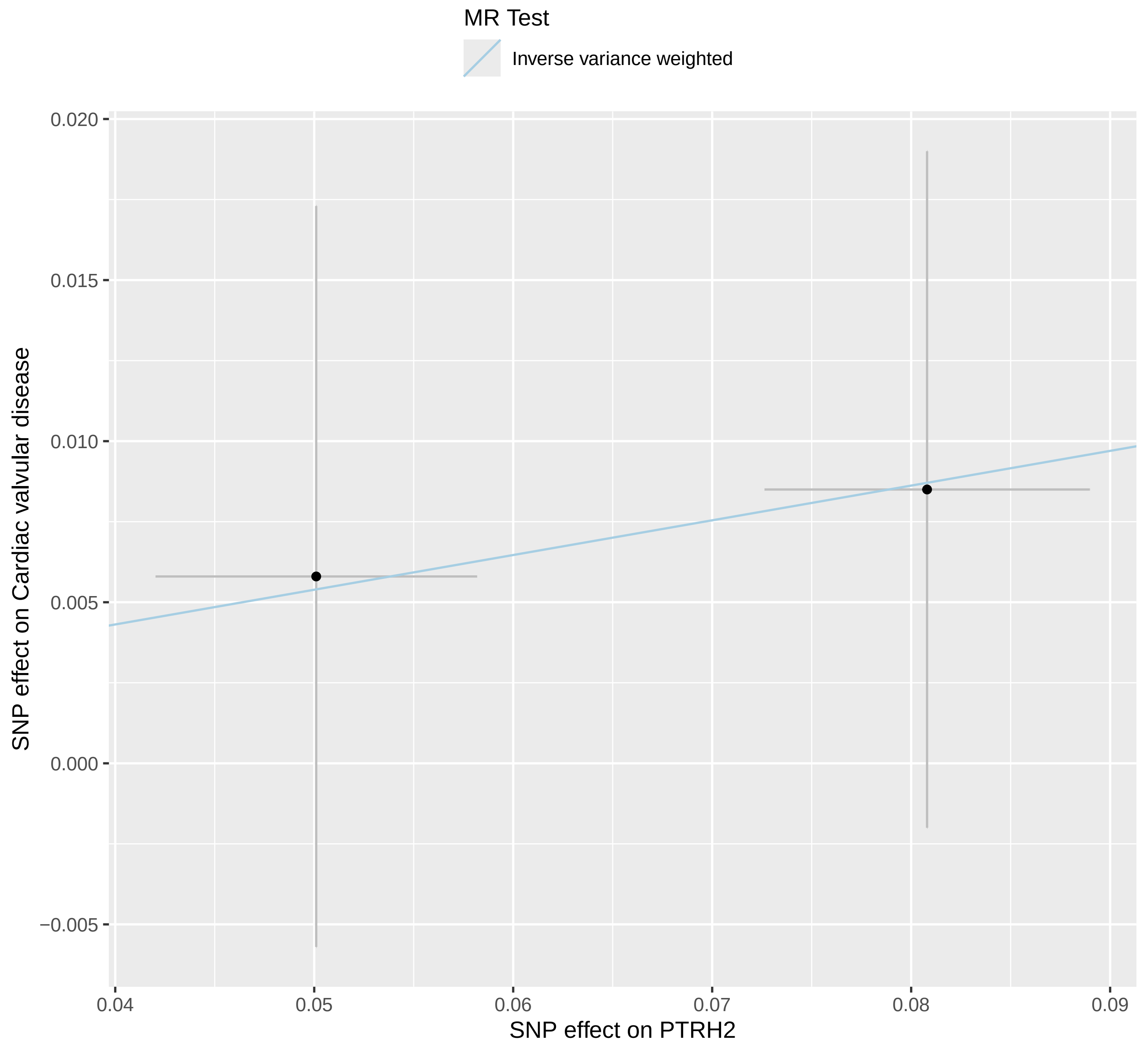

MR Test
Inverse variance weighted
0.020
0.015
0.010
0.005
0.000
−0.005
SNP effect on Cardiac valvular disease
0.04
0.05
0.06
0.07
0.08
0.09
SNP effect on PTRH2

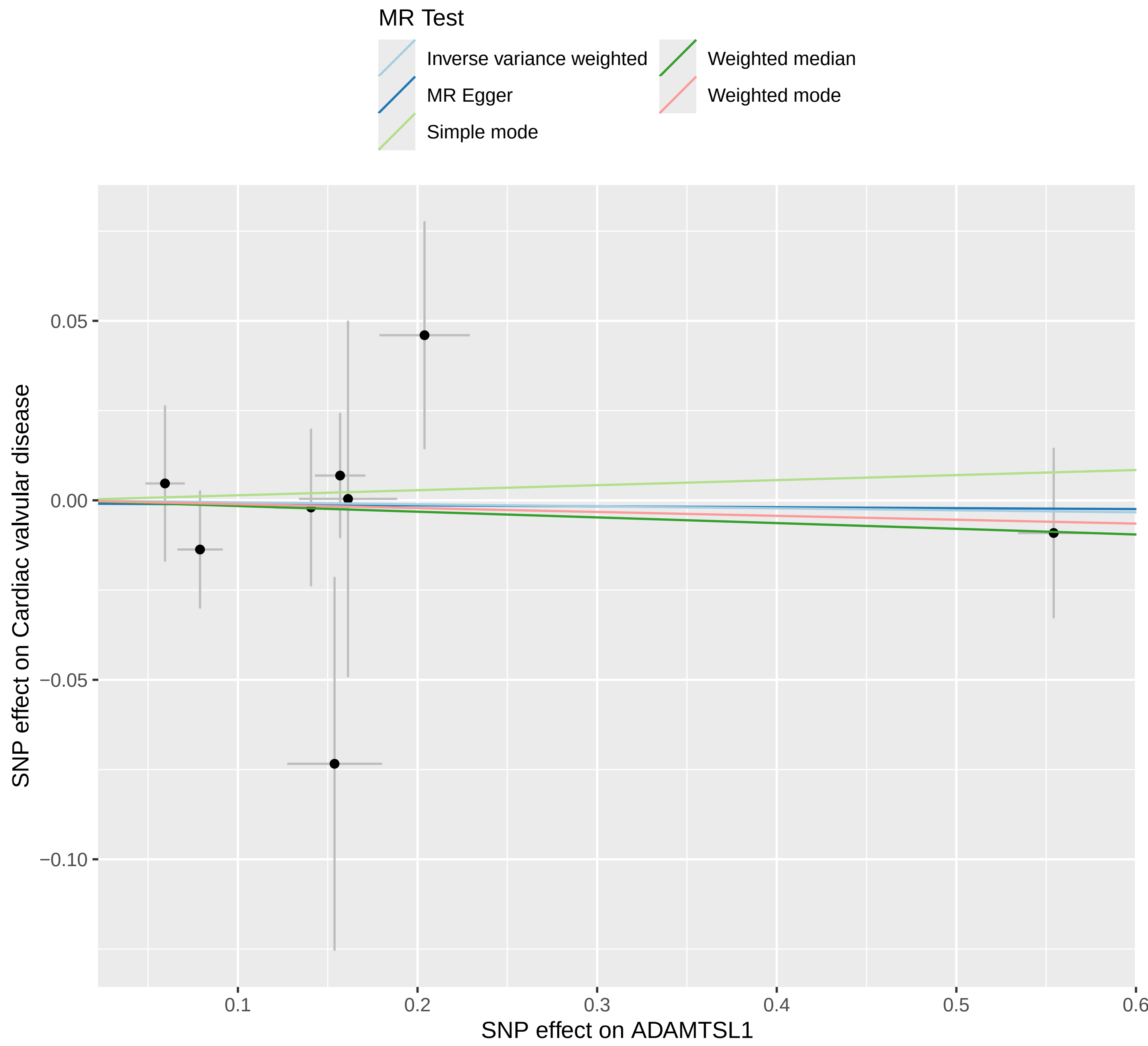

MR Test
Inverse variance weighted
MR Egger
Simple mode
Weighted median
Weighted mode
0.05
0.00
−0.05
−0.10
0.1
0.2
0.3
0.4
0.5
0.6
SNP effect on Cardiac valvular disease
SNP effect on ADAMTSL1

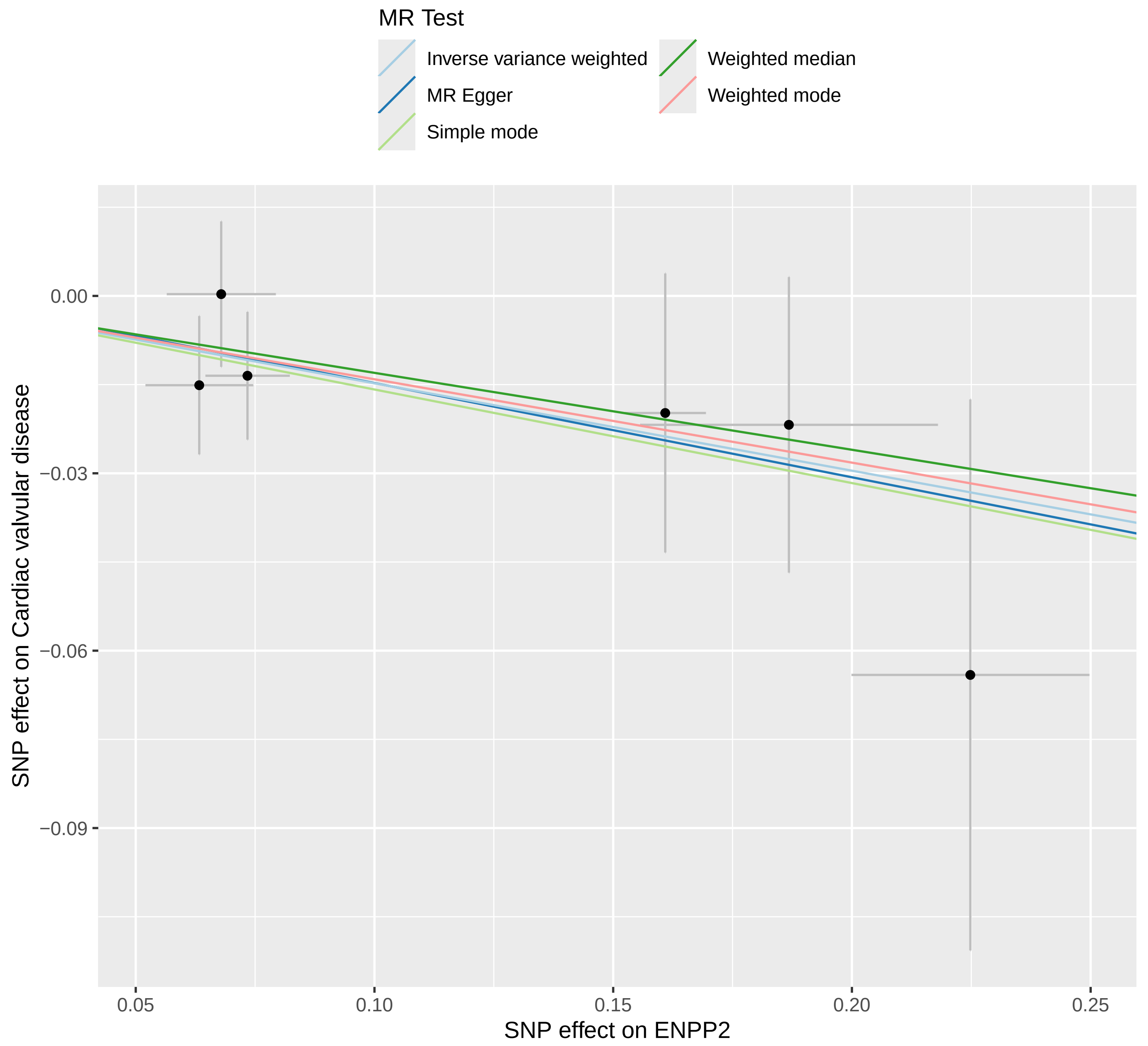
MR Test
Inverse variance weighted
MR Egger
Simple mode
Weighted median
Weighted mode
0.00
−0.03
−0.06
−0.09
0.05
0.10
0.15
0.20
0.25
SNP effect on ENPP2
SNP effect on Cardiac valvular disease

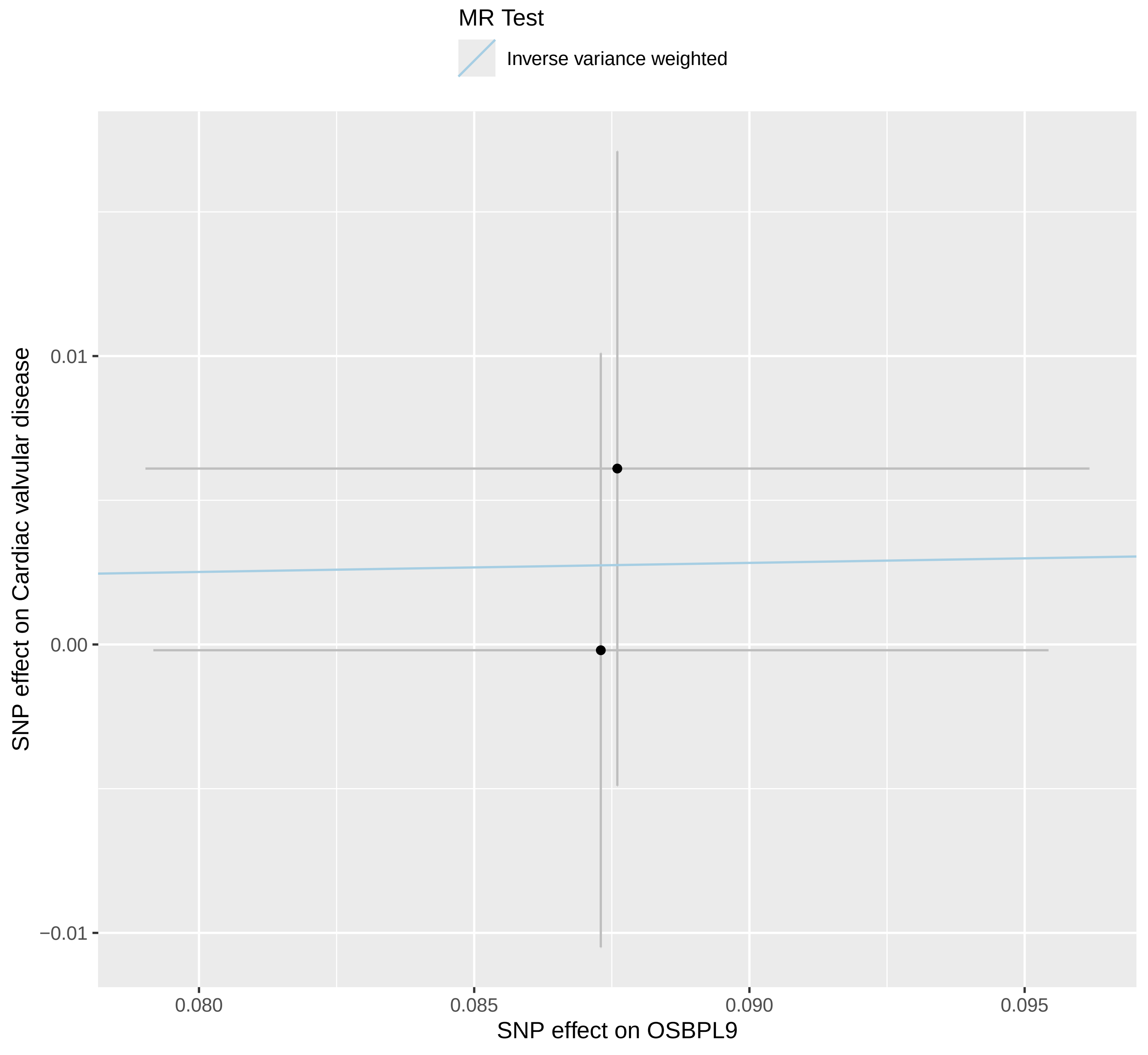

MR Test
Inverse variance weighted
0.01
0.00
−0.01
0.080
0.085
0.090
0.095
SNP effect on OSBPL9
SNP effect on Cardiac valvular disease

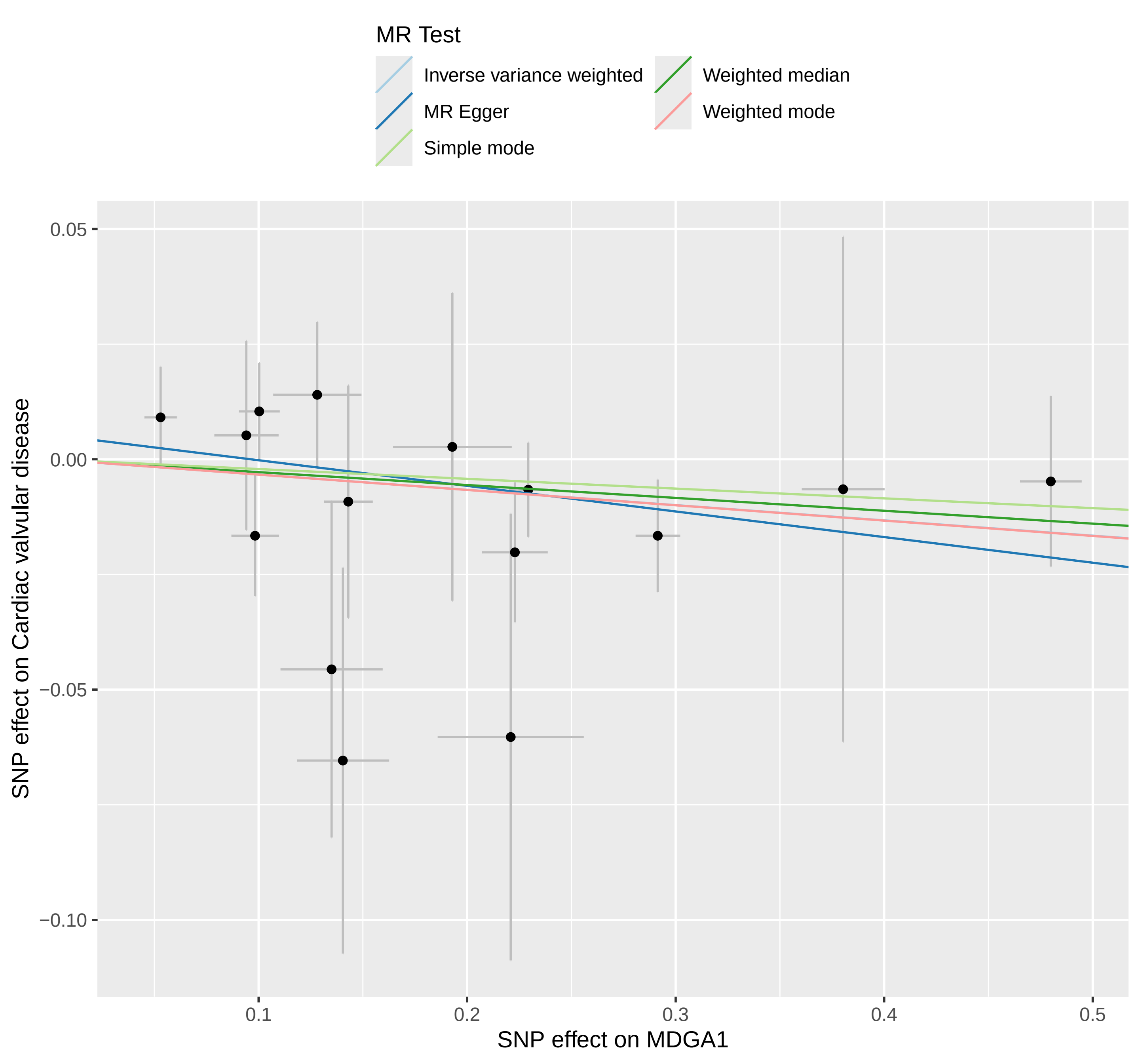
MR Test
Inverse variance weighted
MR Egger
Simple mode
Weighted median
Weighted mode
0.05
0.00
−0.05
−0.10
0.1
0.2
0.3
0.4
0.5
SNP effect on MDGA1
SNP effect on Cardiac valvular disease

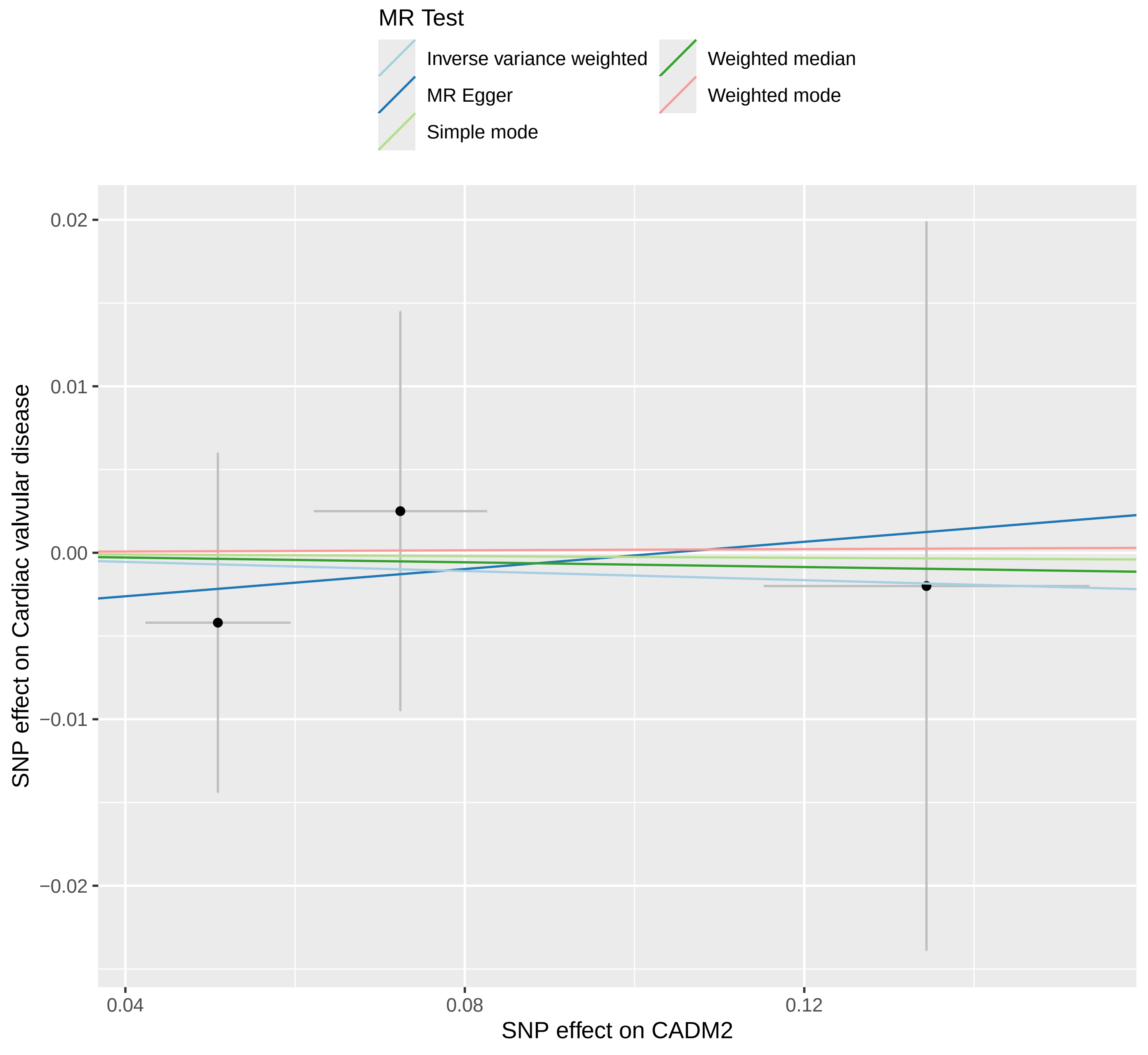
MR Test
Inverse variance weighted
MR Egger
Simple mode
Weighted median
Weighted mode
0.02
0.01
0.00
−0.01
−0.02
SNP effect on Cardiac valvular disease
0.04
0.08
0.12
SNP effect on CADM2

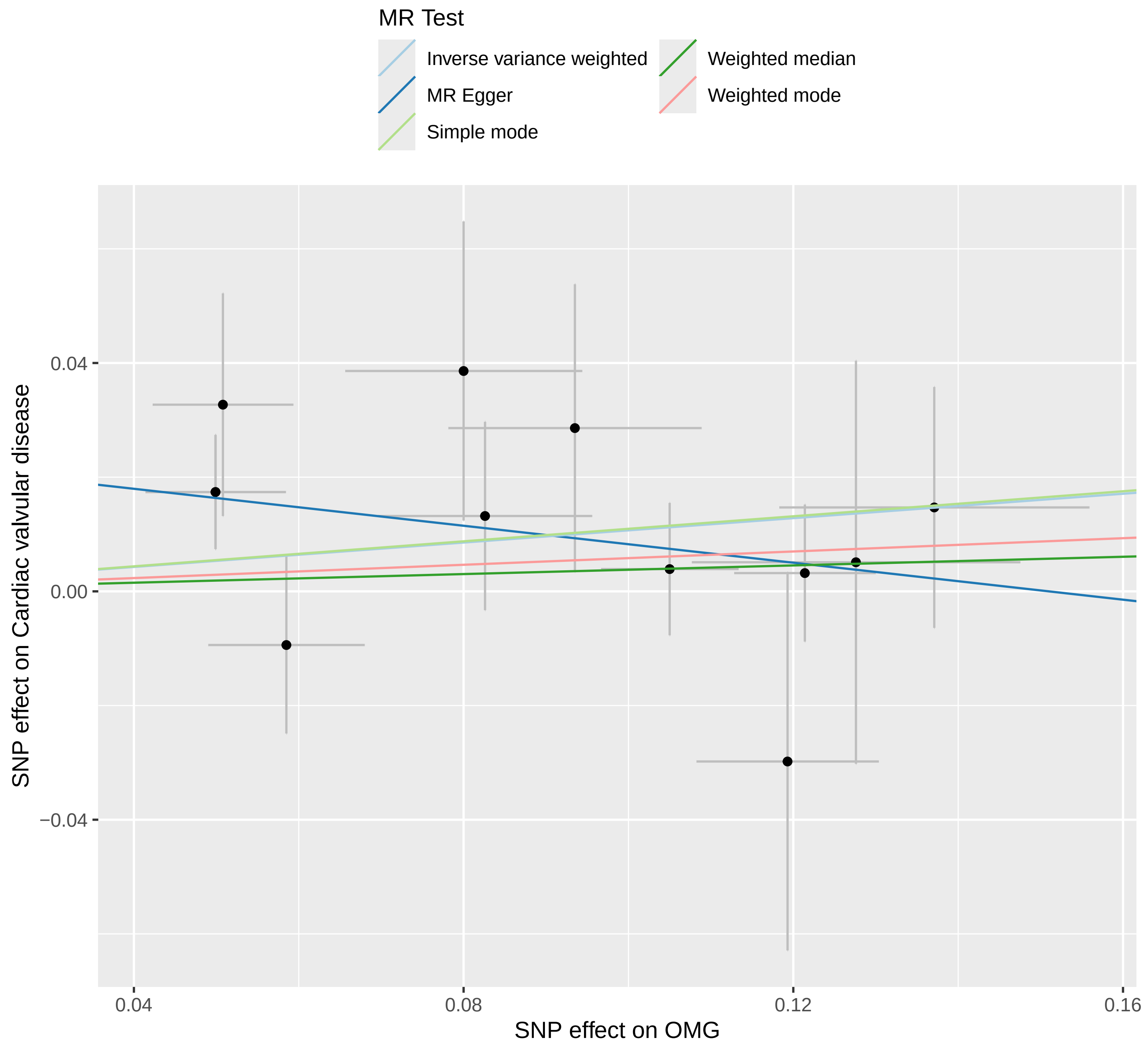

MR Test
Inverse variance weighted
MR Egger
Simple mode
Weighted median
Weighted mode
0.04
0.00
−0.04
SNP effect on Cardiac valvular disease
0.04
0.08
0.12
0.16
SNP effect on OMG

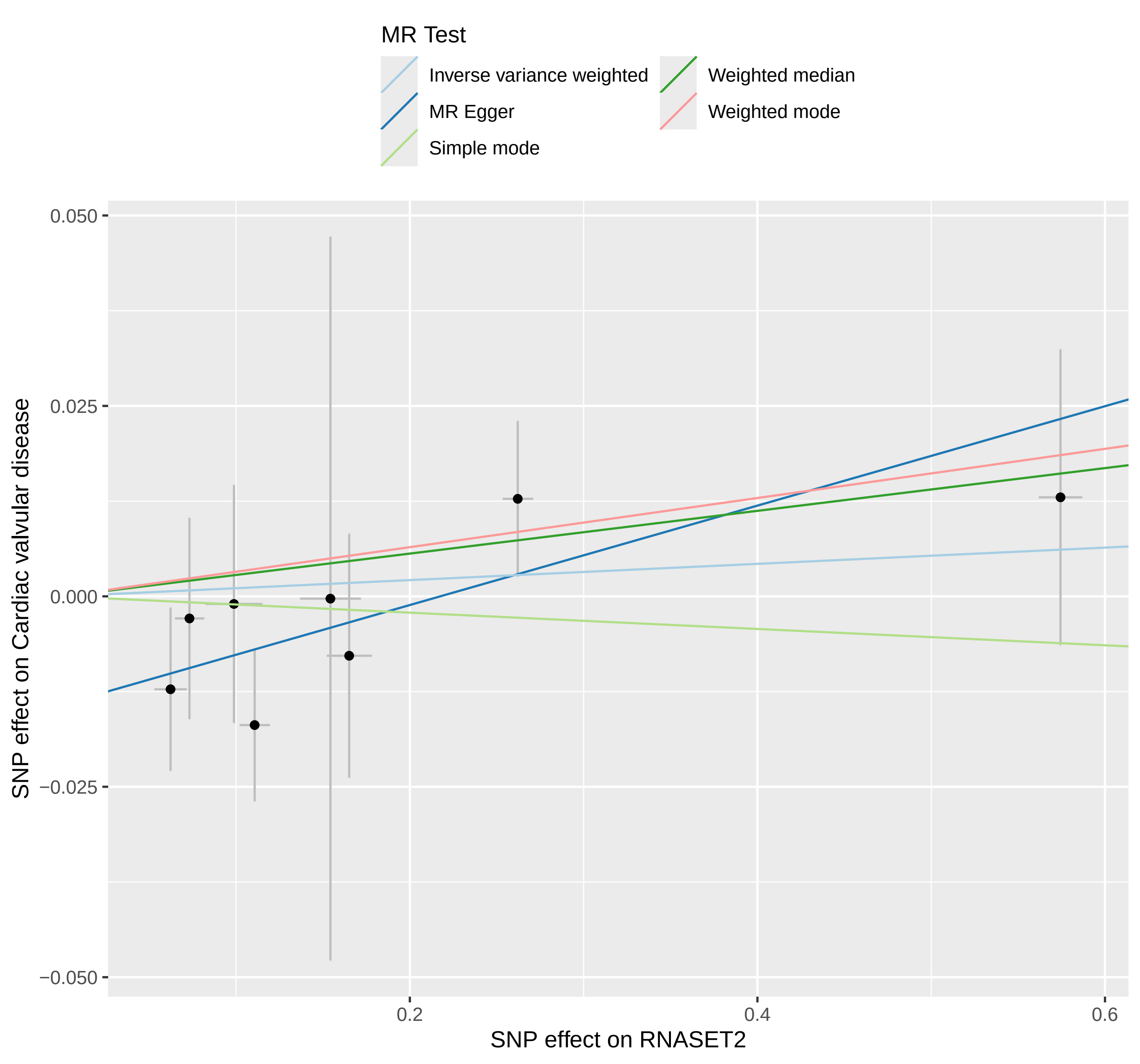
MR Test
Inverse variance weighted
MR Egger
Simple mode
Weighted median
Weighted mode
0.050
0.025
0.000
−0.025
−0.050
SNP effect on Cardiac valvular disease
0.2
0.4
0.6
SNP effect on RNASET2

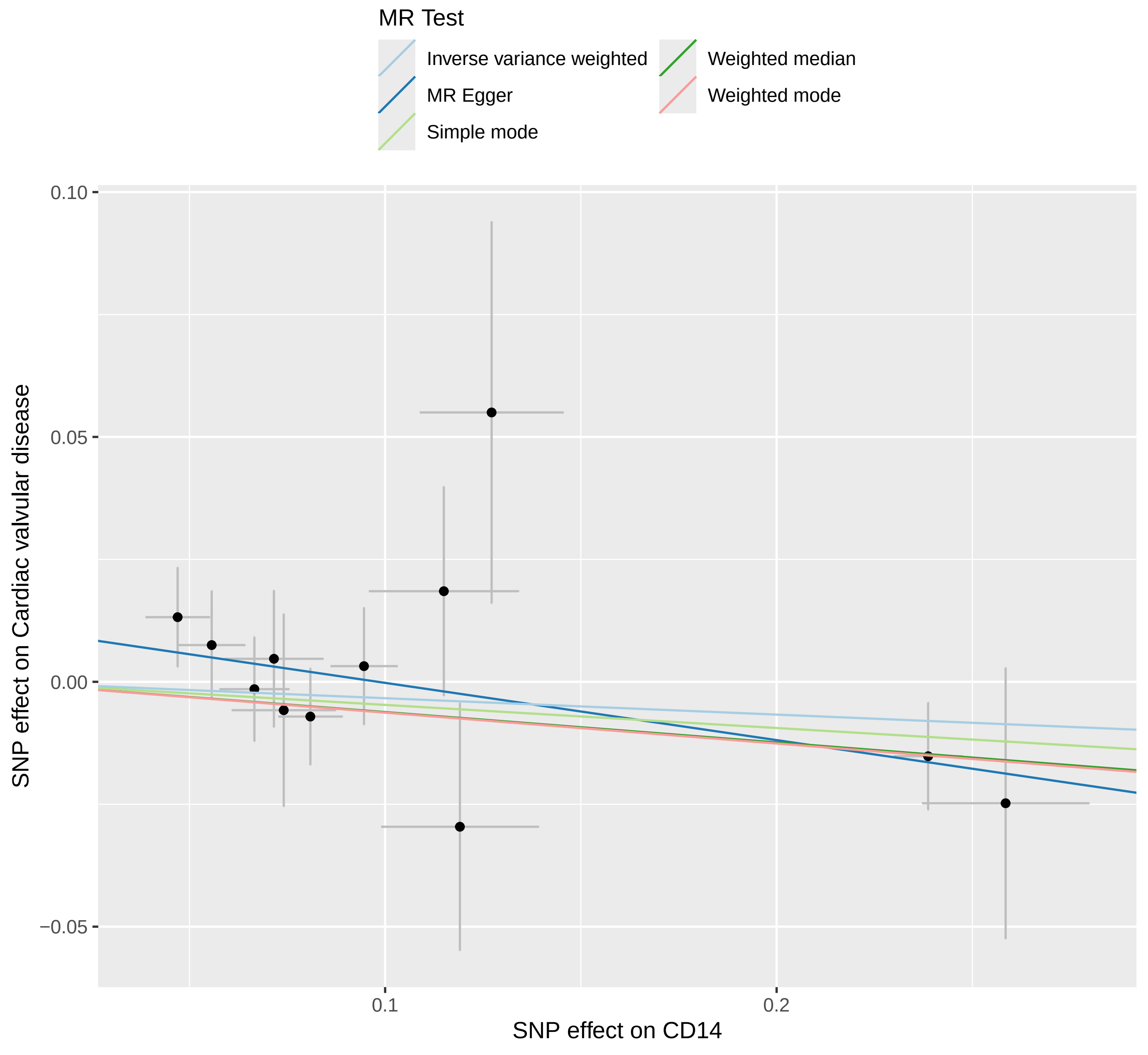

MR Test
Inverse variance weighted
MR Egger
Simple mode
Weighted median
Weighted mode
0.10
0.05
0.00
−0.05
0.1
0.2
SNP effect on CD14
SNP effect on Cardiac valvular disease

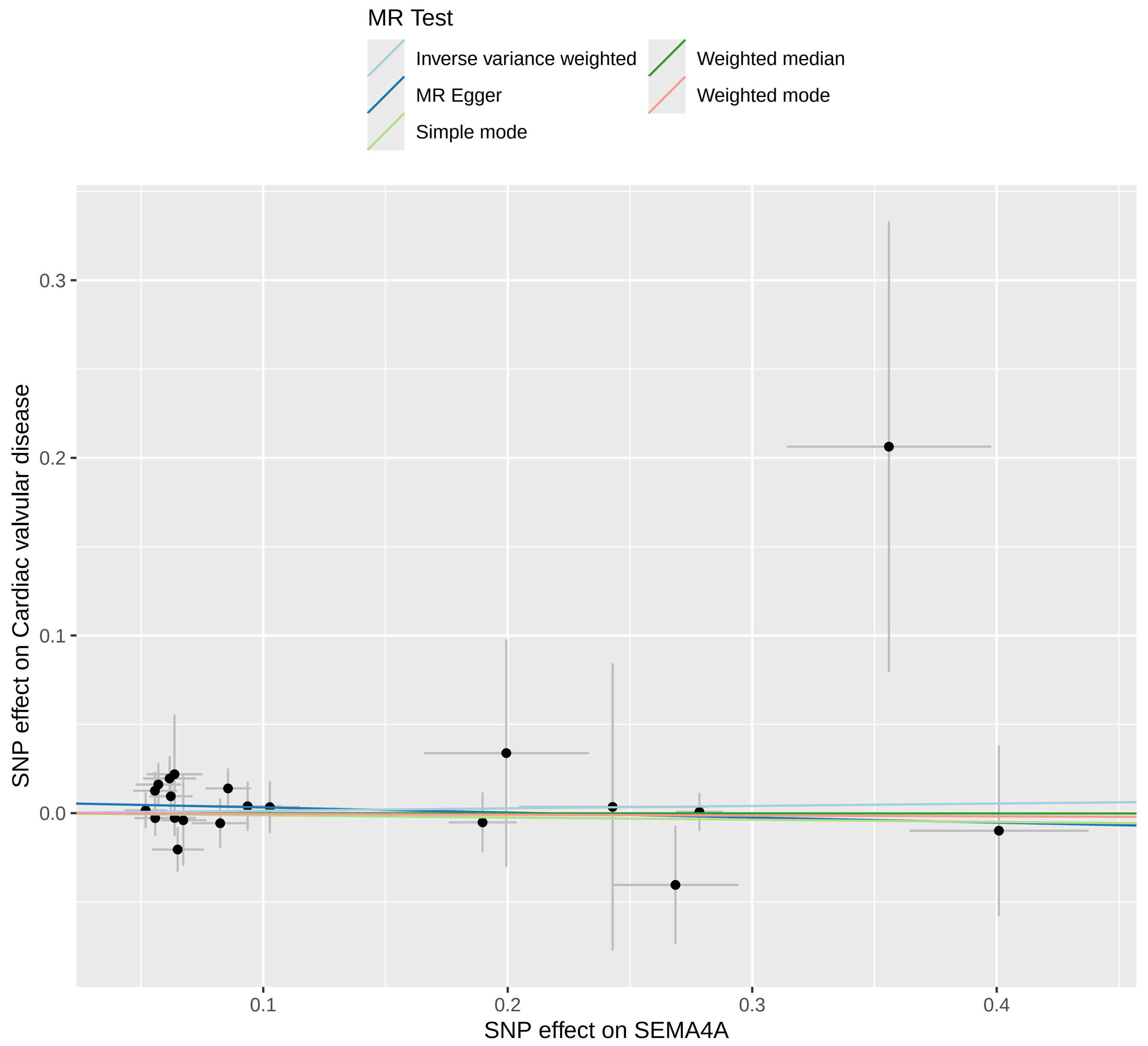
MR Test
Inverse variance weighted
MR Egger
Simple mode
Weighted median
Weighted mode
0.3
0.2
0.1
0.0
0.1
0.2
0.3
0.4
SNP effect on Cardiac valvular disease
SNP effect on SEMA4A

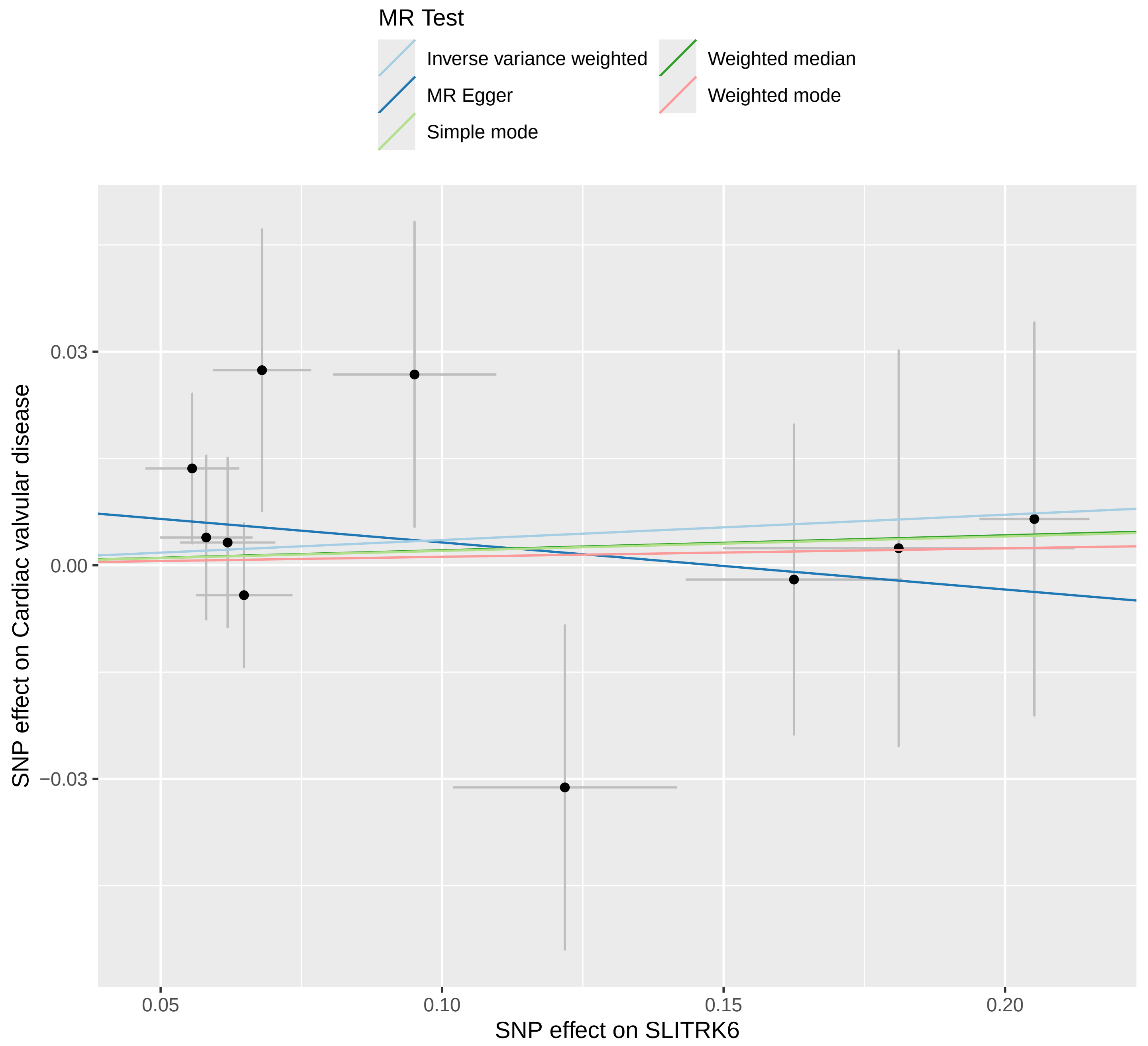

MR Test
Inverse variance weighted
MR Egger
Simple mode
Weighted median
Weighted mode
SNP effect on Cardiac valvular disease
0.03
0.00
−0.03
0.05
0.10
0.15
0.20
SNP effect on SLITRK6

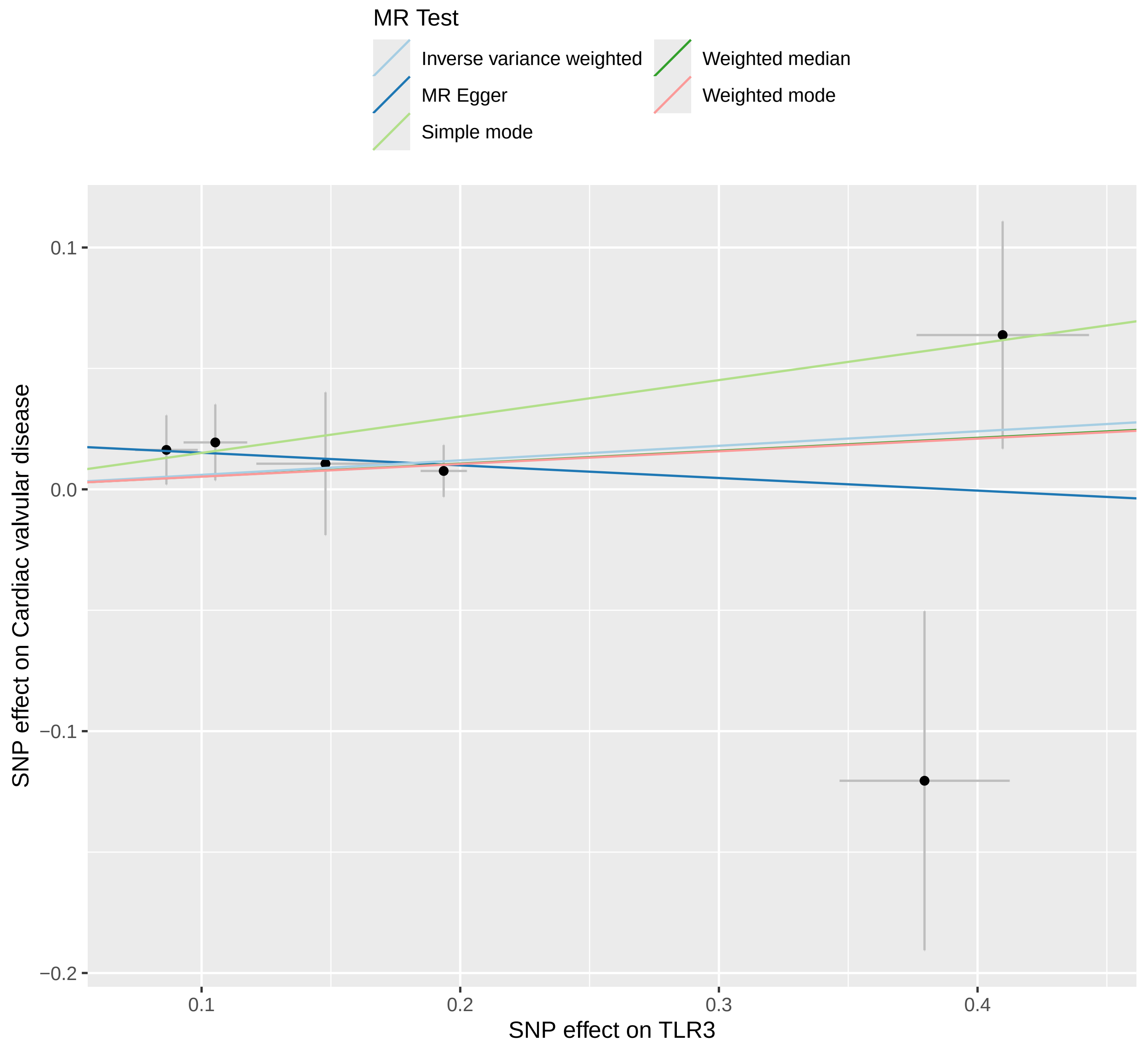
MR Test
Inverse variance weighted
MR Egger
Simple mode
Weighted median
Weighted mode
0.1
0.0
−0.1
−0.2
SNP effect on Cardiac valvular disease
0.1
0.2
0.3
0.4
SNP effect on TLR3

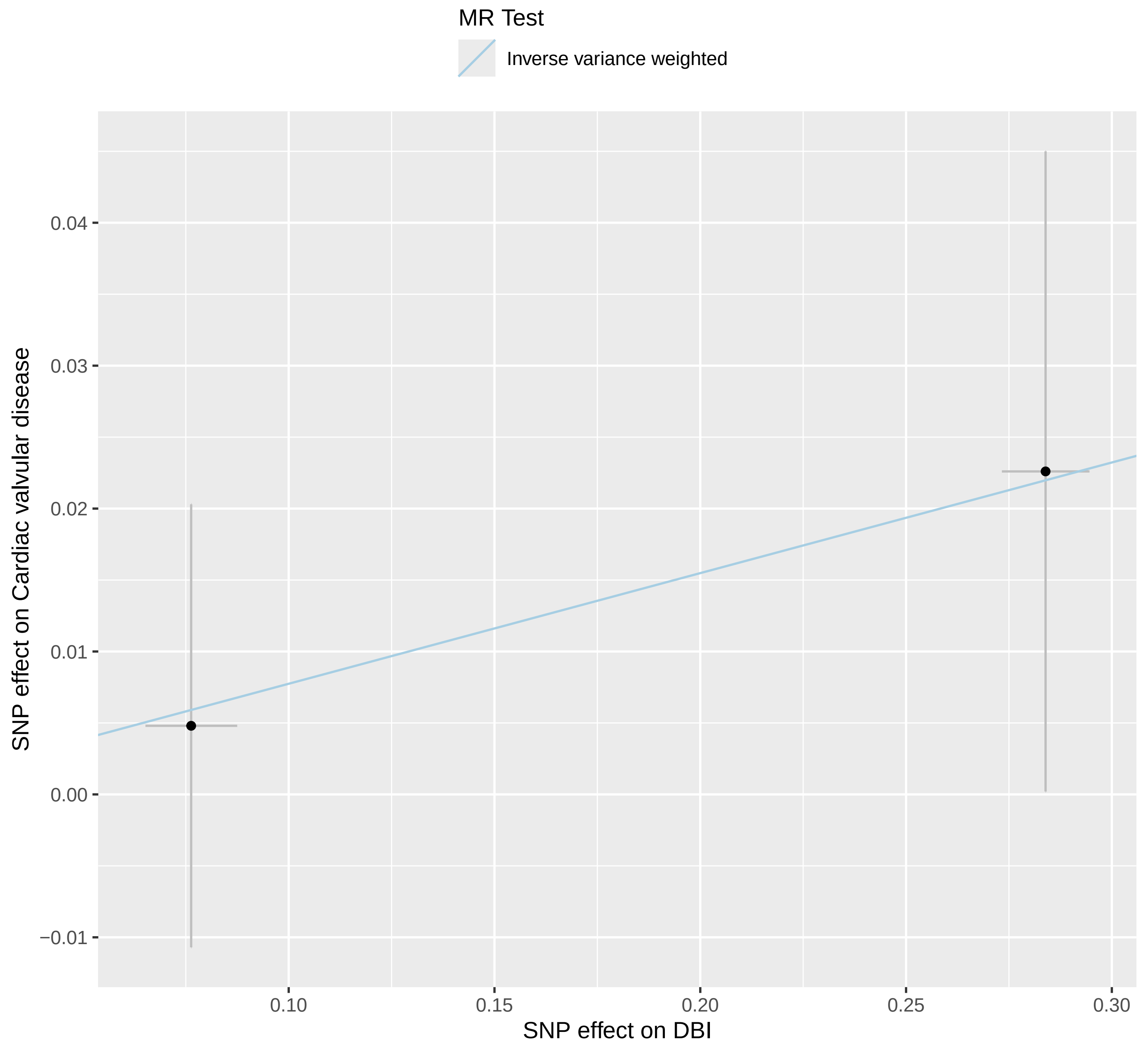
MR Test
Inverse variance weighted
SNP effect on Cardiac valvular disease
0.04
0.03
0.02
0.01
0.00
−0.01
0.10
0.15
0.20
0.25
0.30
SNP effect on DBI

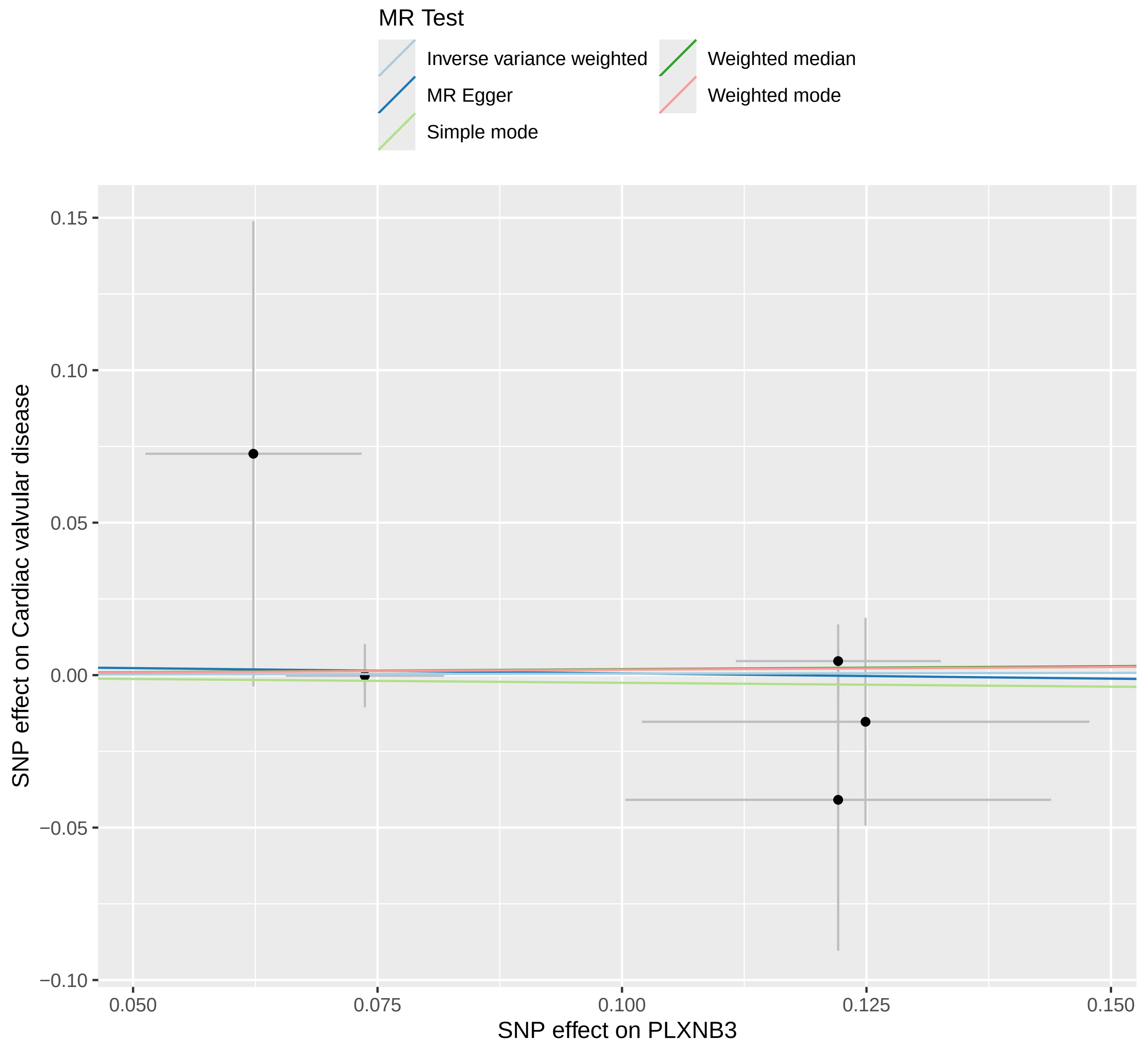
MR Test
Inverse variance weighted
MR Egger
Simple mode
Weighted median
Weighted mode
0.15
0.10
0.05
0.00
−0.05
−0.10
SNP effect on Cardiac valvular disease
0.050
0.075
0.100
0.125
0.150
SNP effect on PLXNB3

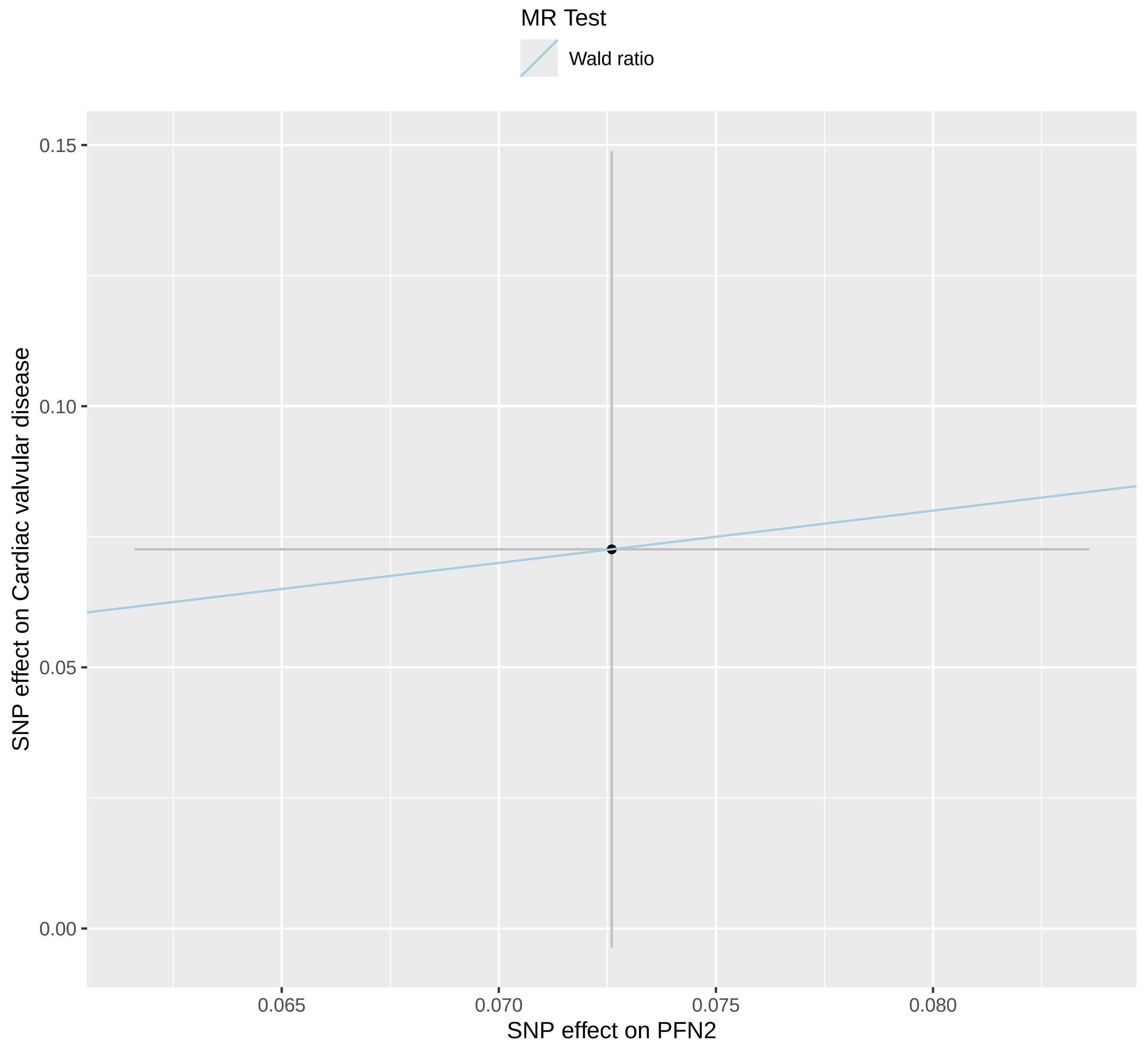

MR Test
Wald ratio
0.15
0.10
0.05
0.00
0.065
0.070
0.075
0.080
SNP effect on Cardiac valvular disease
SNP effect on PFN2

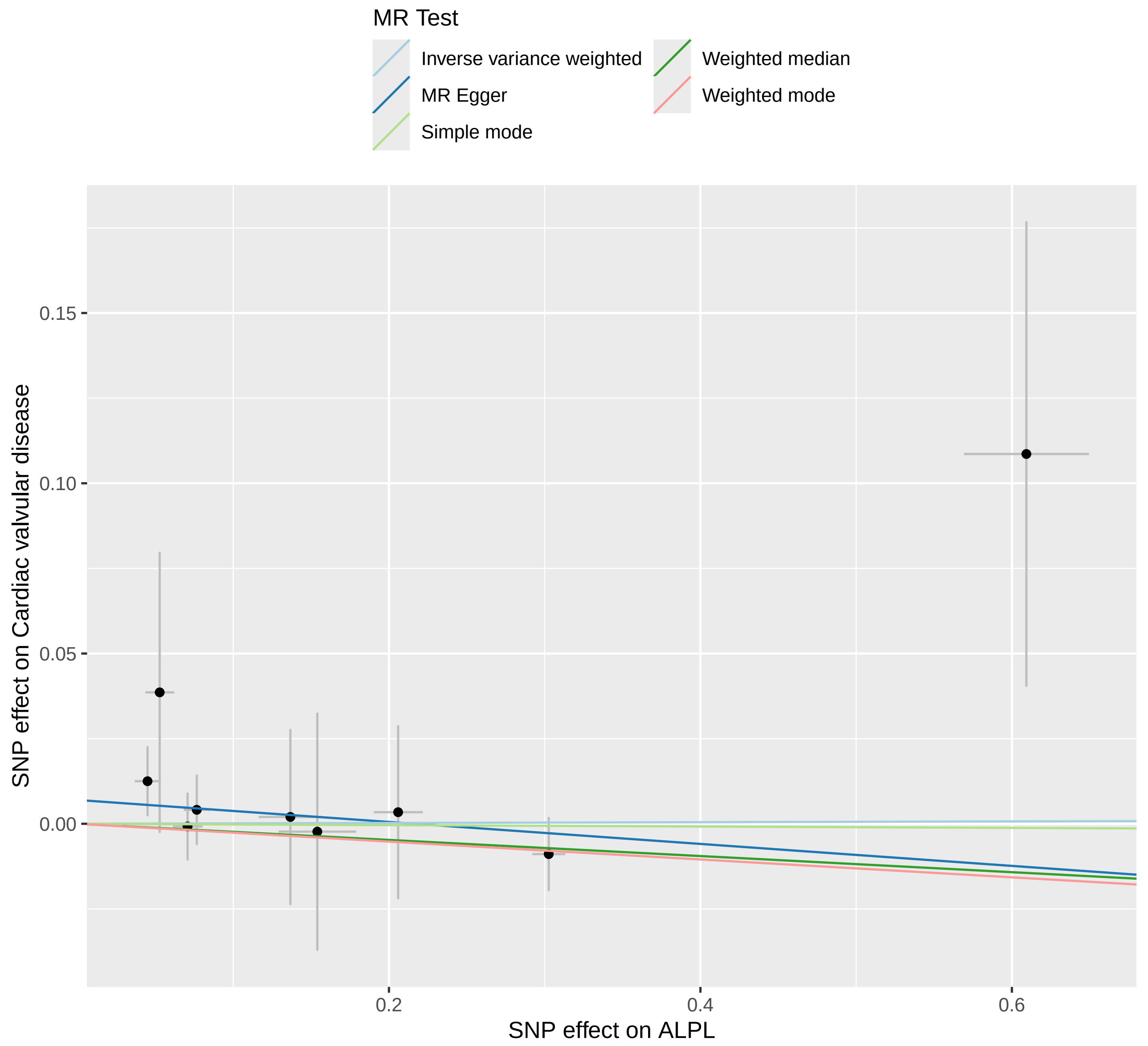
MR Test
Inverse variance weighted
MR Egger
Simple mode
Weighted median
Weighted mode
SNP effect on Cardiac valvular disease
0.15
0.10
0.05
0.00
0.2
0.4
0.6
SNP effect on ALPL

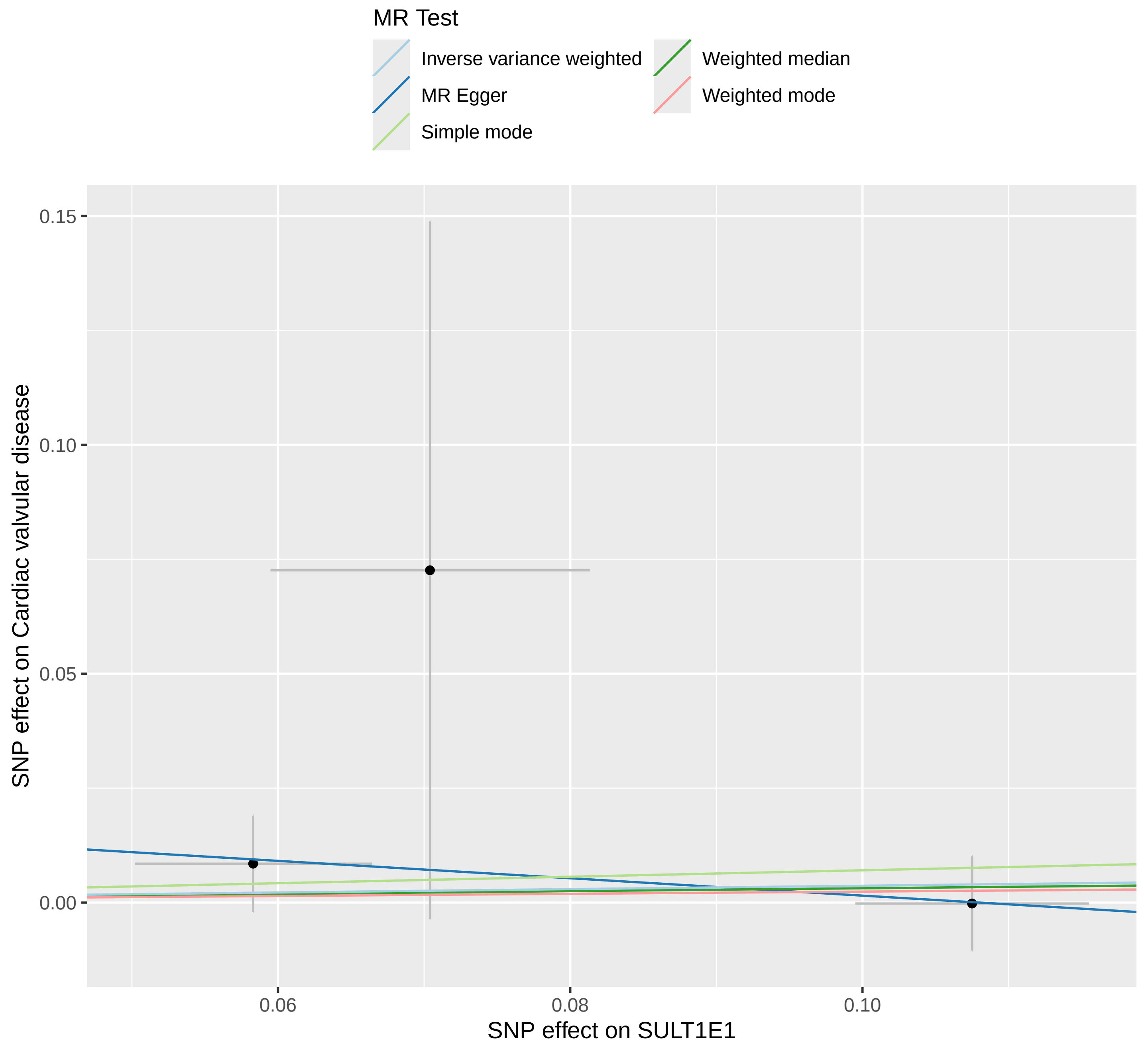
MR Test
Inverse variance weighted
MR Egger
Simple mode
Weighted median
Weighted mode
0.15
0.10
0.05
0.00
0.06
0.08
0.10
SNP effect on Cardiac valvular disease
SNP effect on SULT1E1

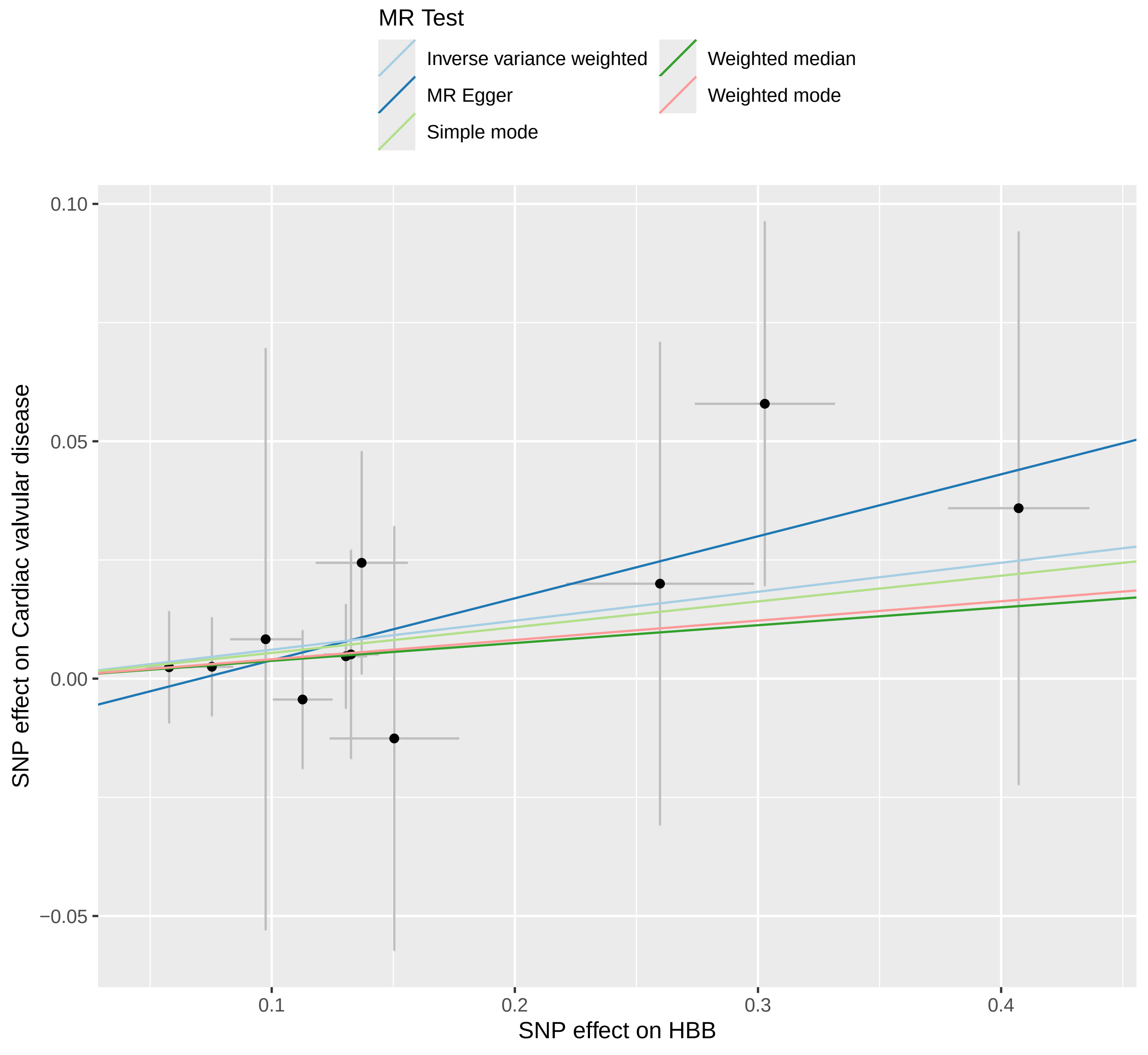

MR Test
Inverse variance weighted
MR Egger
Simple mode
Weighted median
Weighted mode
0.10
0.05
0.00
−0.05
0.1
0.2
0.3
0.4
SNP effect on Cardiac valvular disease
SNP effect on HBB

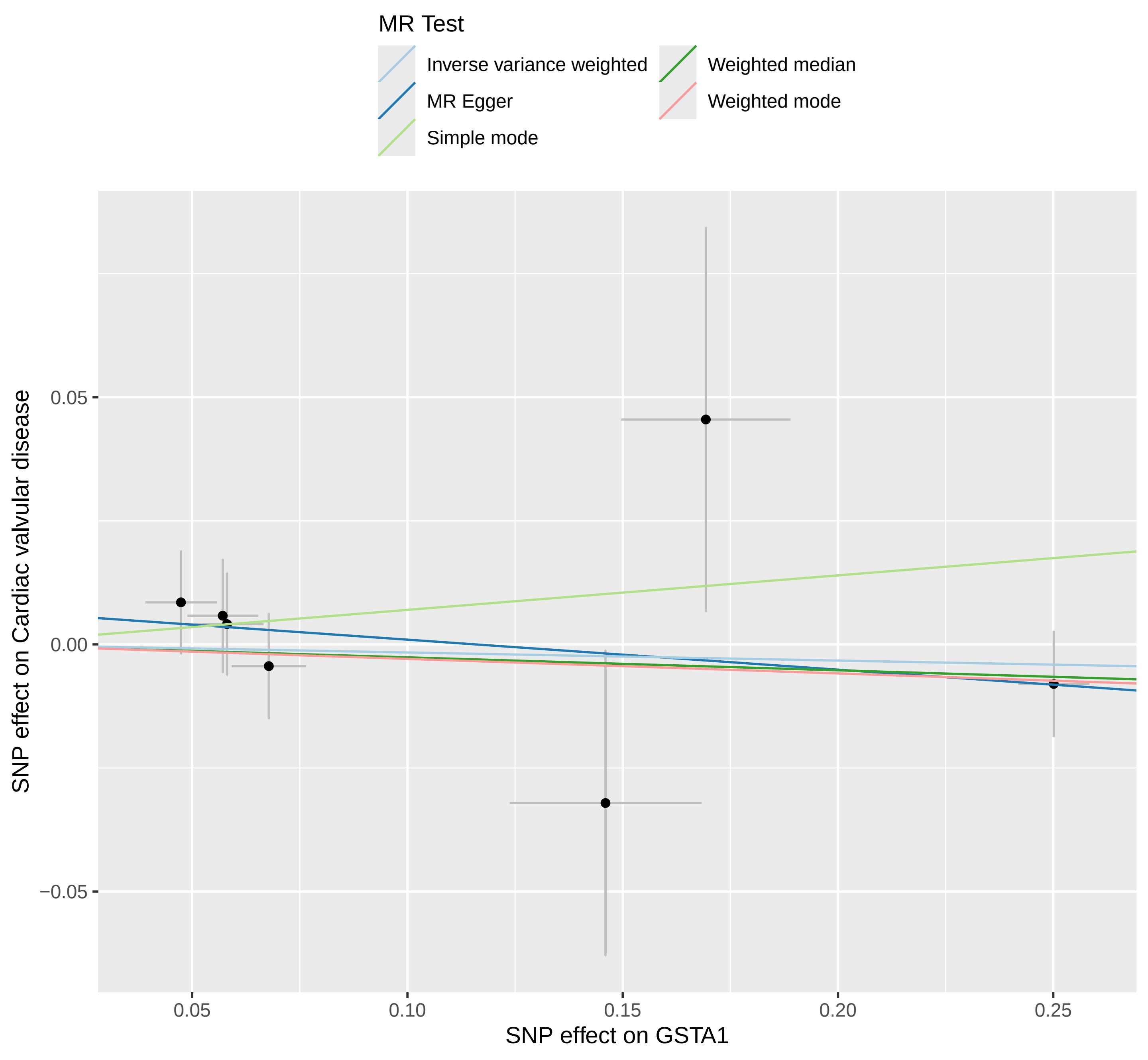
MR Test
Inverse variance weighted
MR Egger
Simple mode
Weighted median
Weighted mode
0.05
0.00
−0.05
0.05
0.10
0.15
0.20
0.25
SNP effect on GSTA1
SNP effect on Cardiac valvular disease

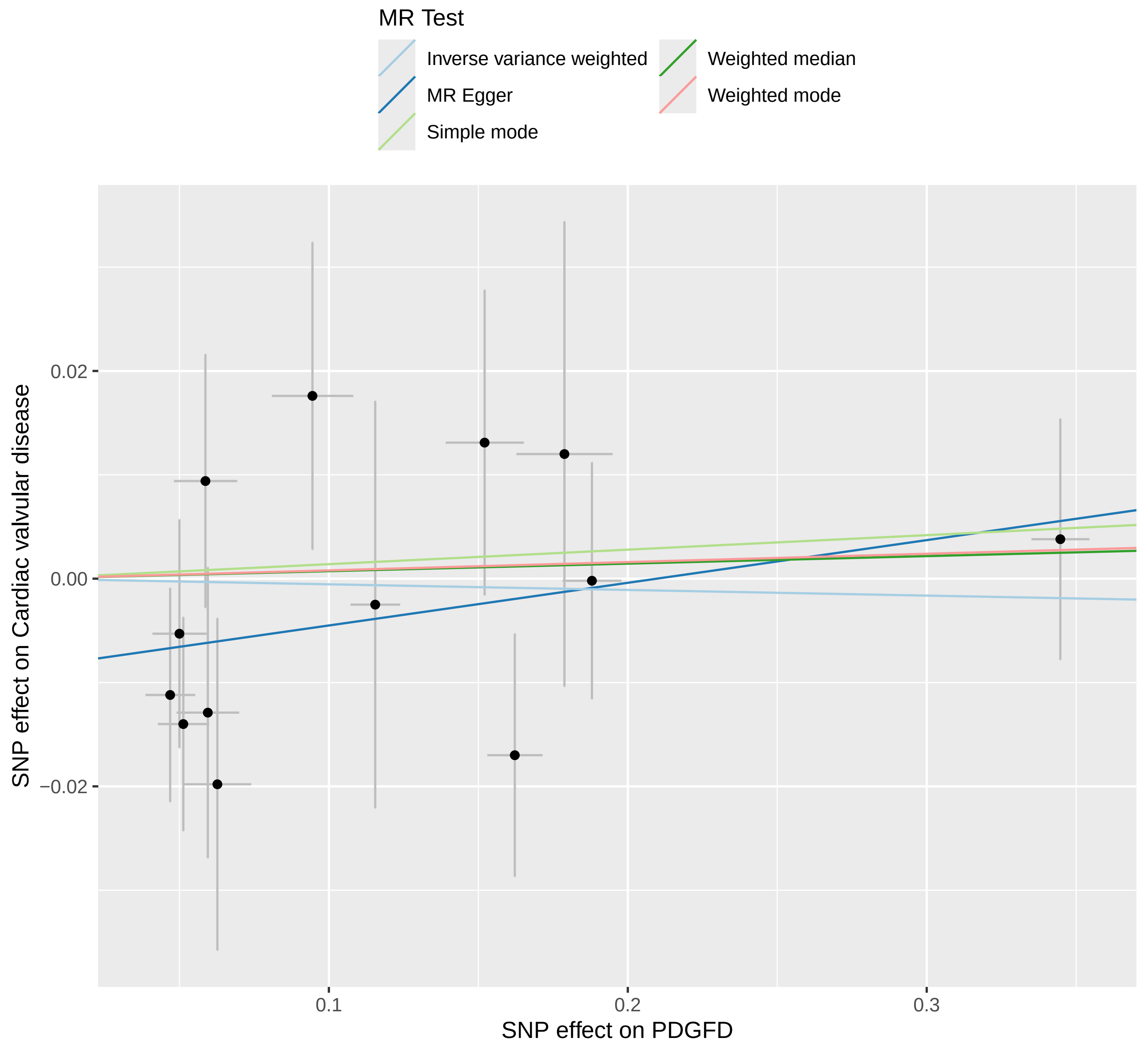

MR Test
Inverse variance weighted
MR Egger
Simple mode
Weighted median
Weighted mode
0.02
0.00
−0.02
0.1
0.2
0.3
SNP effect on Cardiac valvular disease
SNP effect on PDGFD

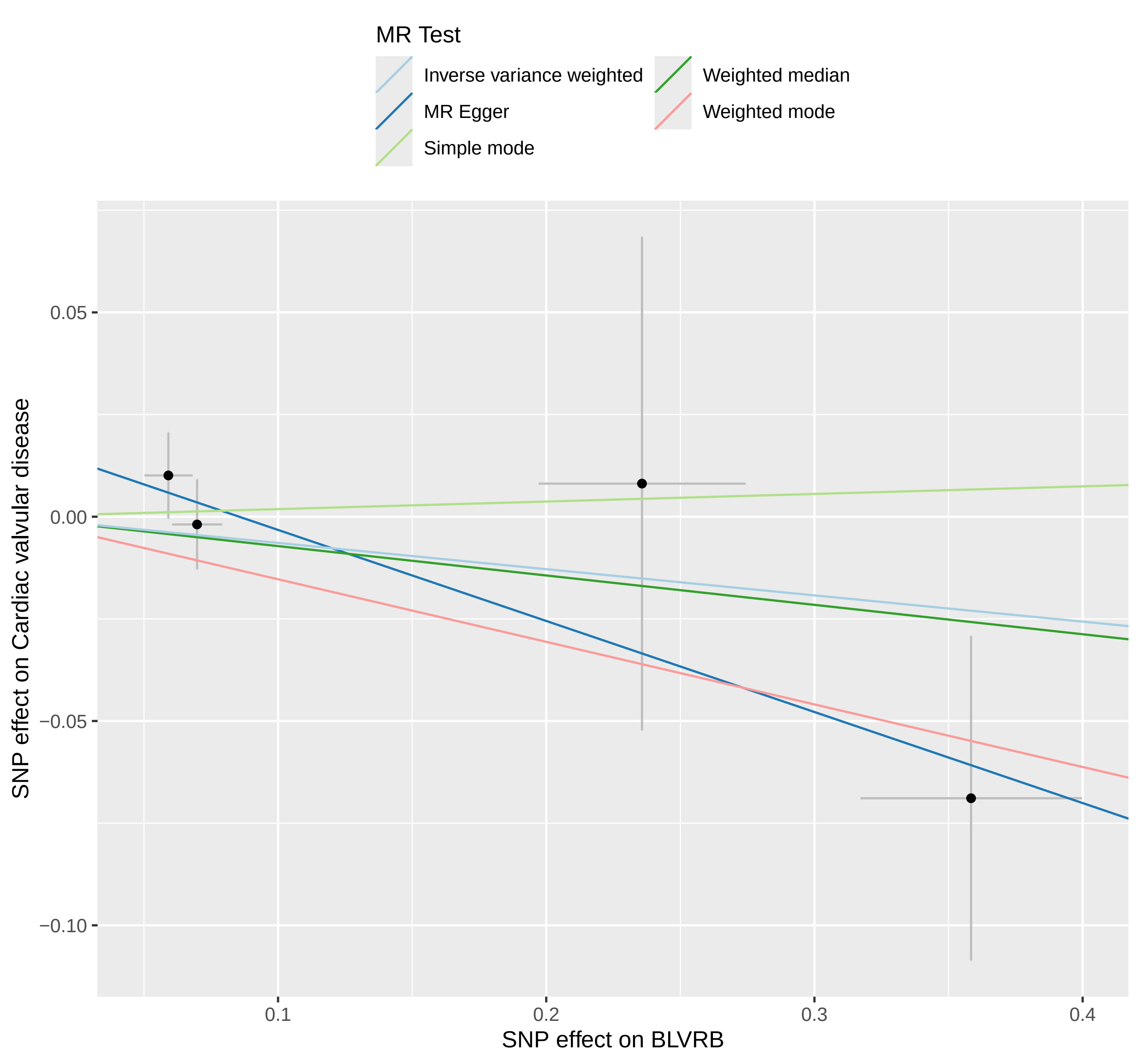

MR Test
Inverse variance weighted
MR Egger
Simple mode
Weighted median
Weighted mode
0.05
0.00
−0.05
−0.10
0.1
0.2
0.3
0.4
SNP effect on BLVRB
SNP effect on Cardiac valvular disease

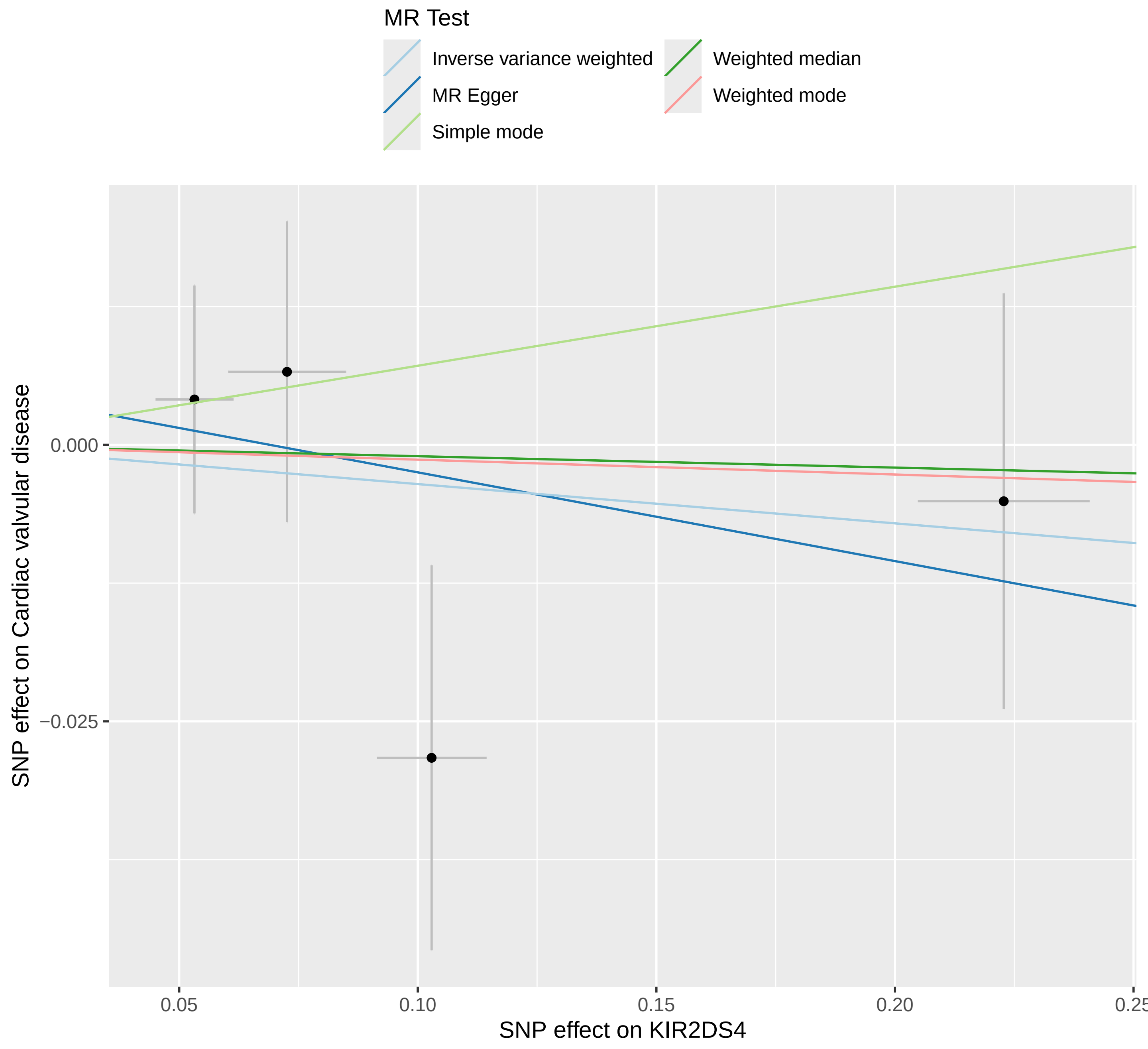
MR Test
Inverse variance weighted
MR Egger
Simple mode
Weighted median
Weighted mode
0.000
−0.025
0.05
0.10
0.15
0.20
0.25
SNP effect on KIR2DS4
SNP effect on Cardiac valvular disease

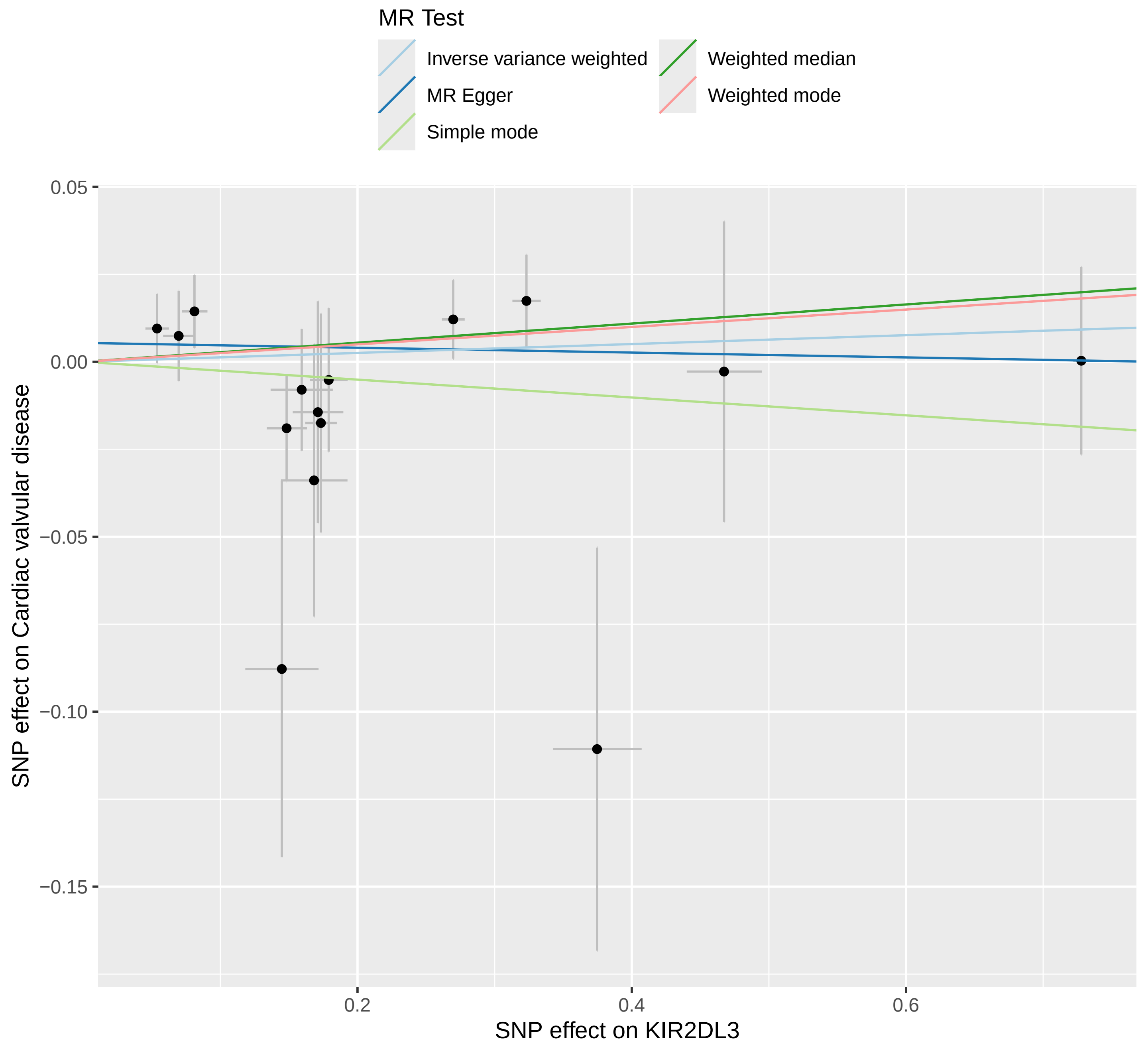
MR Test
Inverse variance weighted
MR Egger
Simple mode
Weighted median
Weighted mode
0.05
0.00
−0.05
−0.10
−0.15
0.2
0.4
0.6
SNP effect on Cardiac valvular disease
SNP effect on KIR2DL3

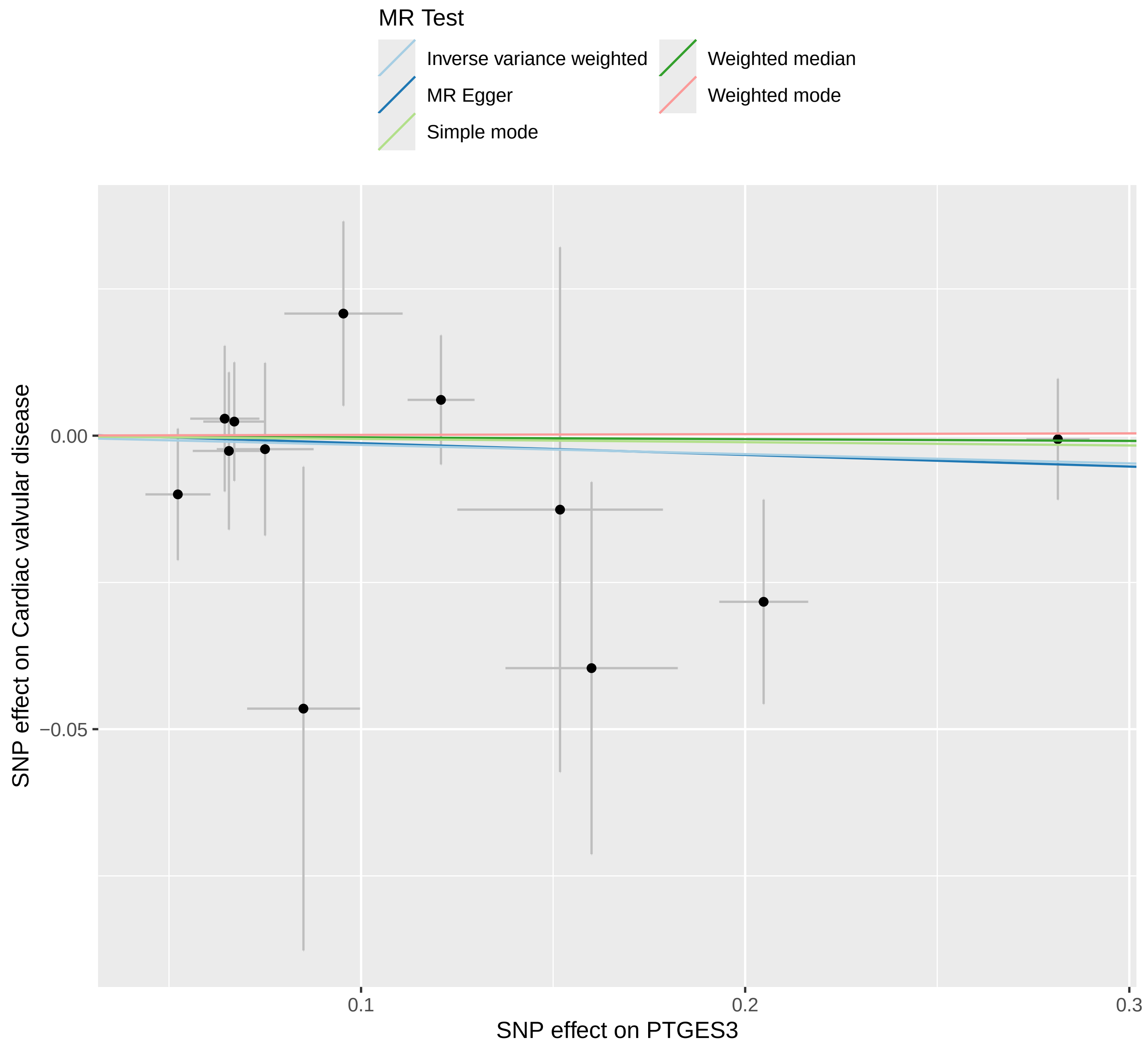
MR Test
Inverse variance weighted
MR Egger
Simple mode
Weighted median
Weighted mode
SNP effect on Cardiac valvular disease
0.00
−0.05
0.1
0.2
0.3
SNP effect on PTGES3

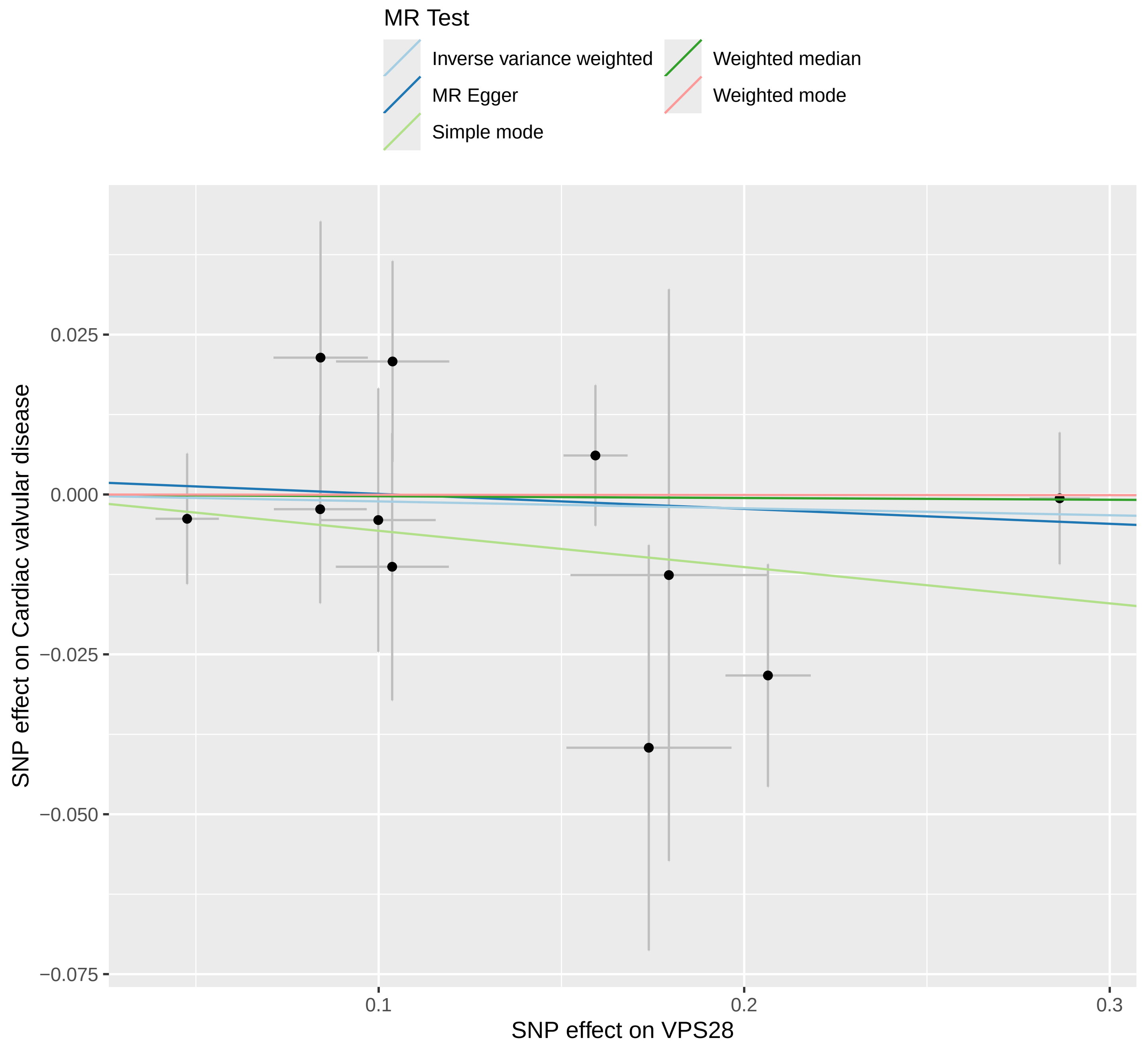
MR Test
Inverse variance weighted
MR Egger
Simple mode
Weighted median
Weighted mode
SNP effect on Cardiac valvular disease
0.025
0.000
−0.025
−0.050
−0.075
0.1
0.2
0.3
SNP effect on VPS28

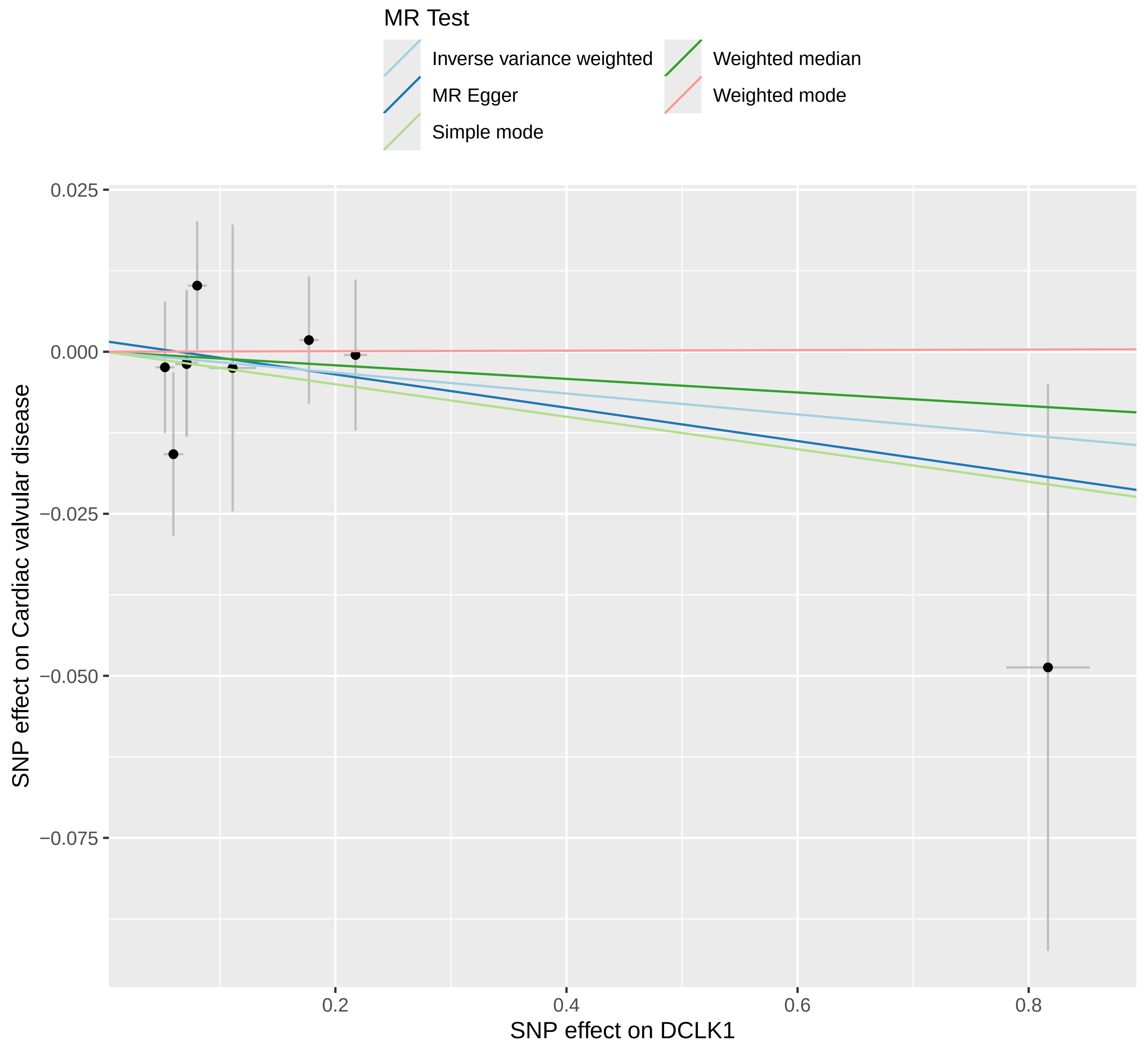

MR Test
Inverse variance weighted
MR Egger
Simple mode
Weighted median
Weighted mode
0.025
0.000
−0.025
−0.050
−0.075
0.2
0.4
0.6
0.8
SNP effect on DCLK1
SNP effect on Cardiac valvular disease

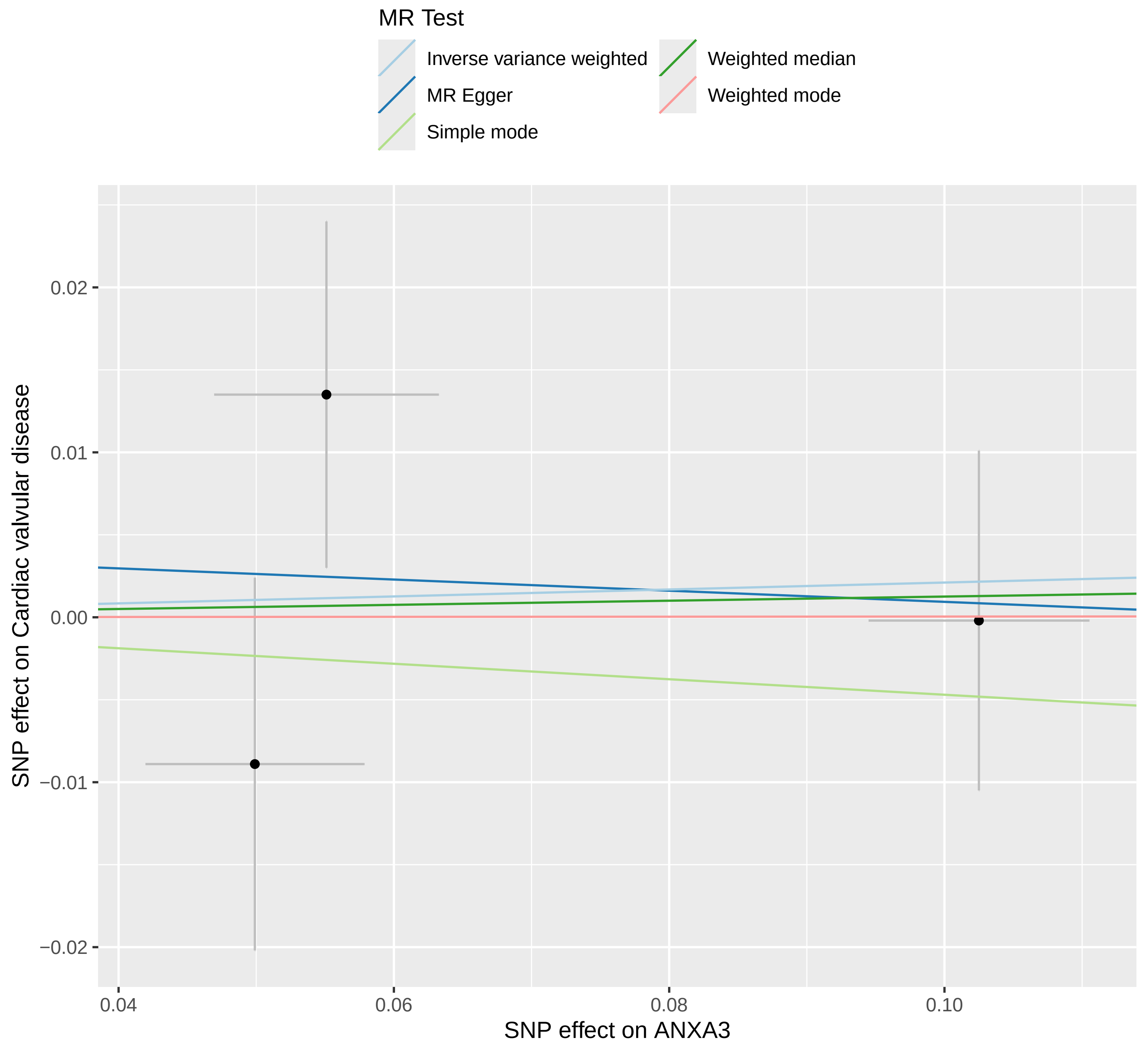

MR Test
Inverse variance weighted
MR Egger
Simple mode
Weighted median
Weighted mode
0.02
0.01
0.00
−0.01
−0.02
SNP effect on Cardiac valvular disease
0.04
0.06
0.08
0.10
SNP effect on ANXA3

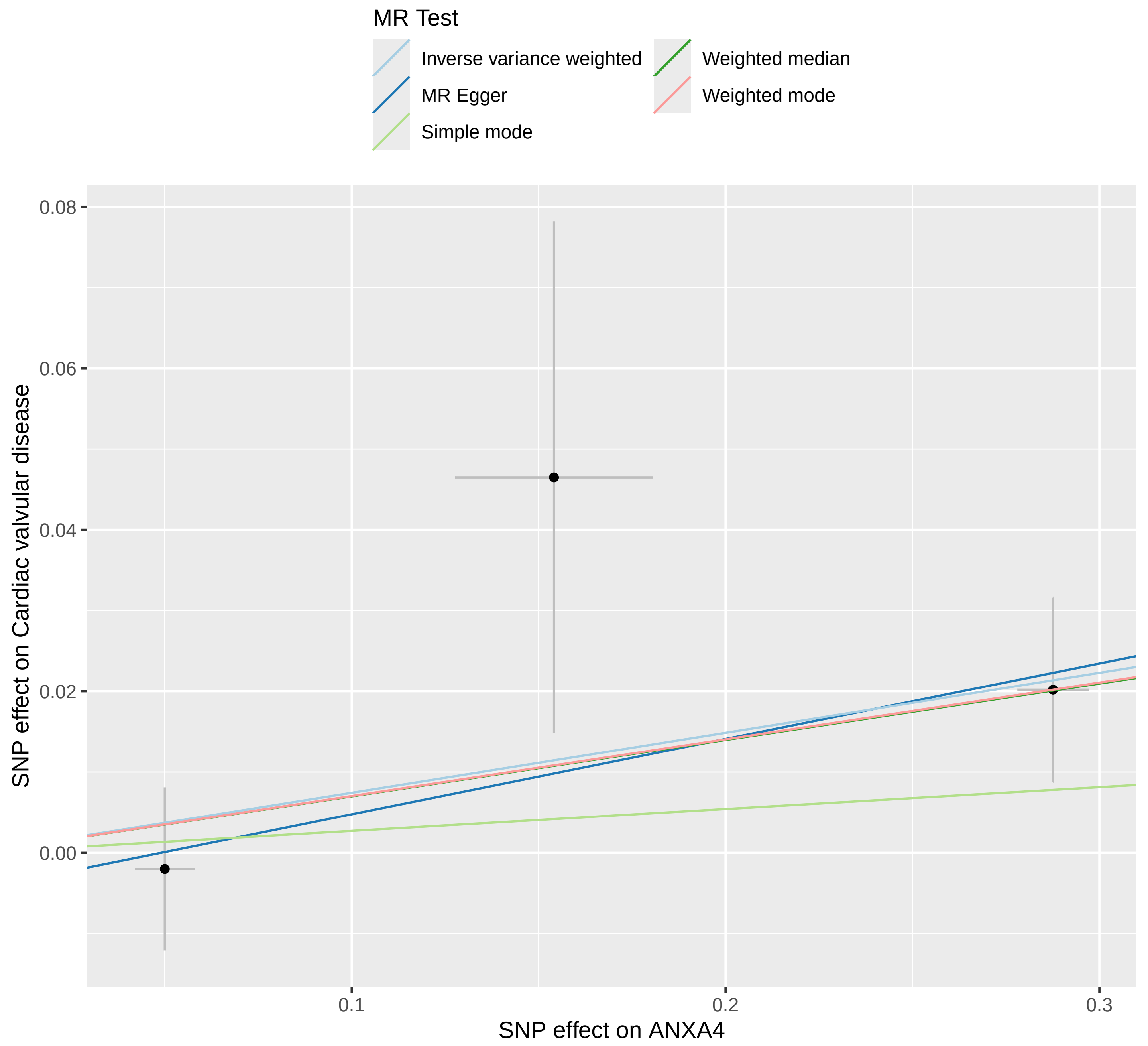
MR Test
Inverse variance weighted
MR Egger
Simple mode
Weighted median
Weighted mode
0.08
0.06
0.04
0.02
0.00
SNP effect on Cardiac valvular disease
0.1
0.2
0.3
SNP effect on ANXA4

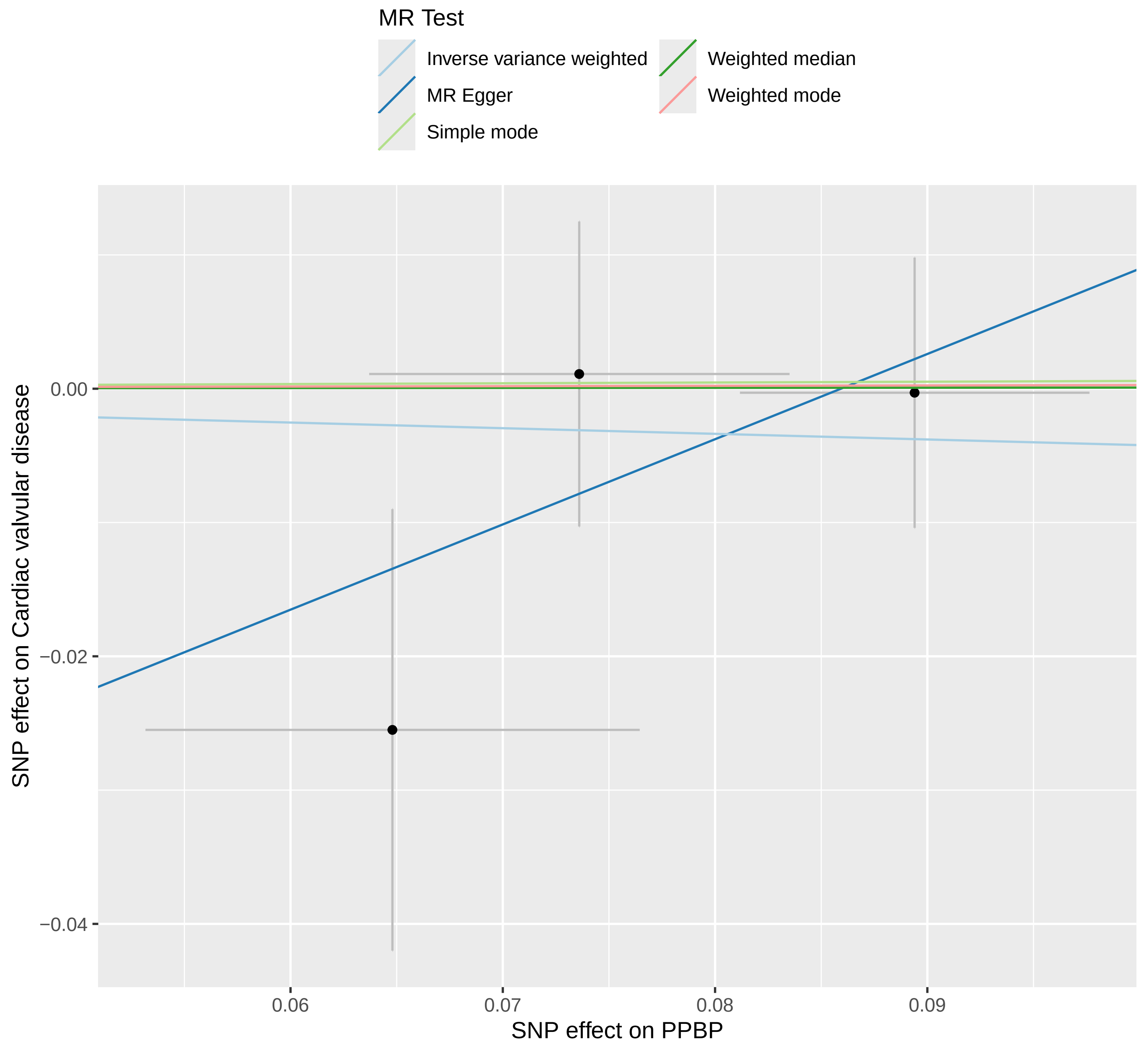
MR Test
Inverse variance weighted
MR Egger
Simple mode
Weighted median
Weighted mode
0.00
−0.02
−0.04
0.06
0.07
0.08
0.09
SNP effect on PPBP
SNP effect on Cardiac valvular disease

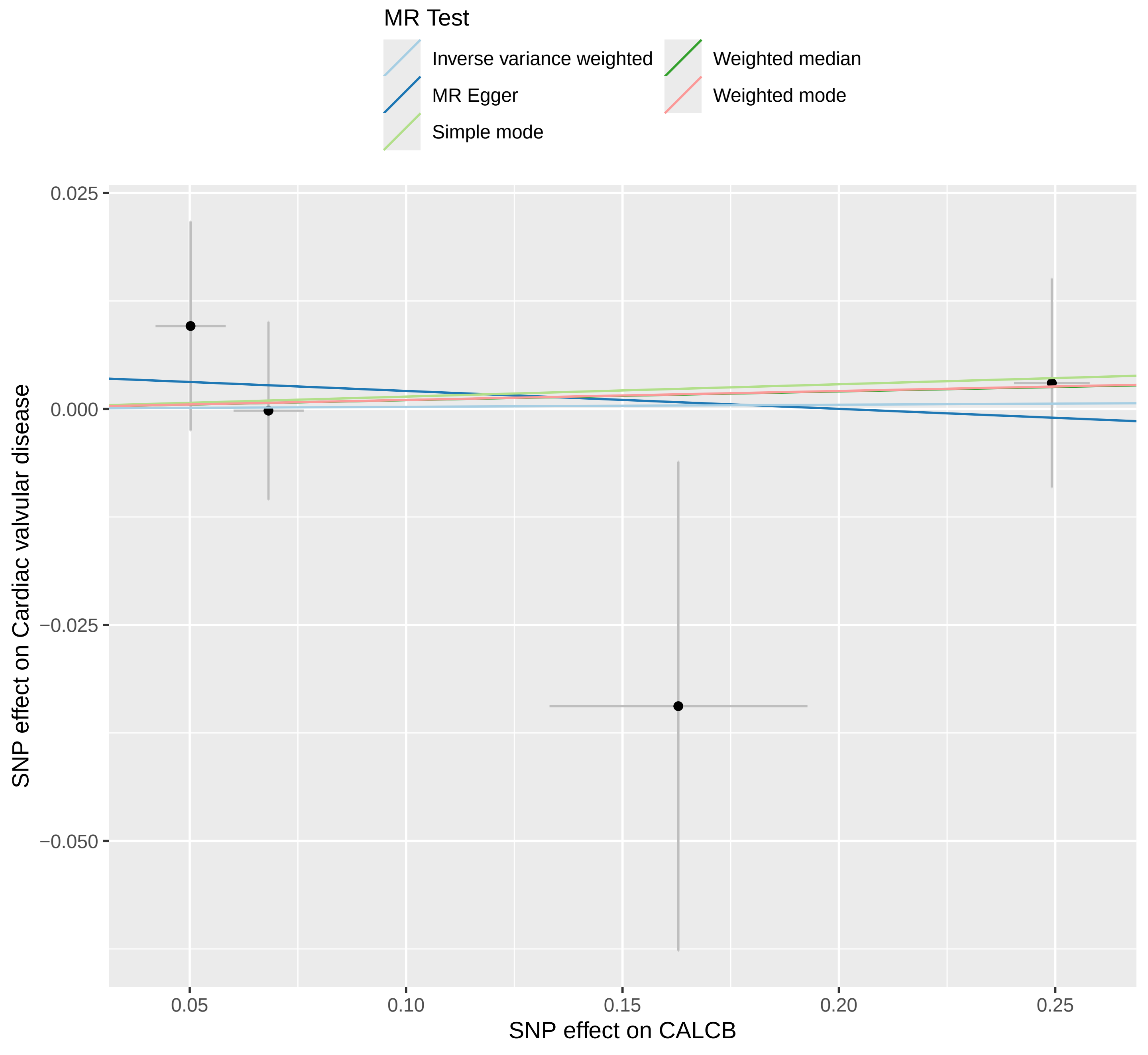
MR Test
Inverse variance weighted
MR Egger
Simple mode
Weighted median
Weighted mode
0.025
0.000
−0.025
−0.050
SNP effect on Cardiac valvular disease
0.05
0.10
0.15
0.20
0.25
SNP effect on CALCB

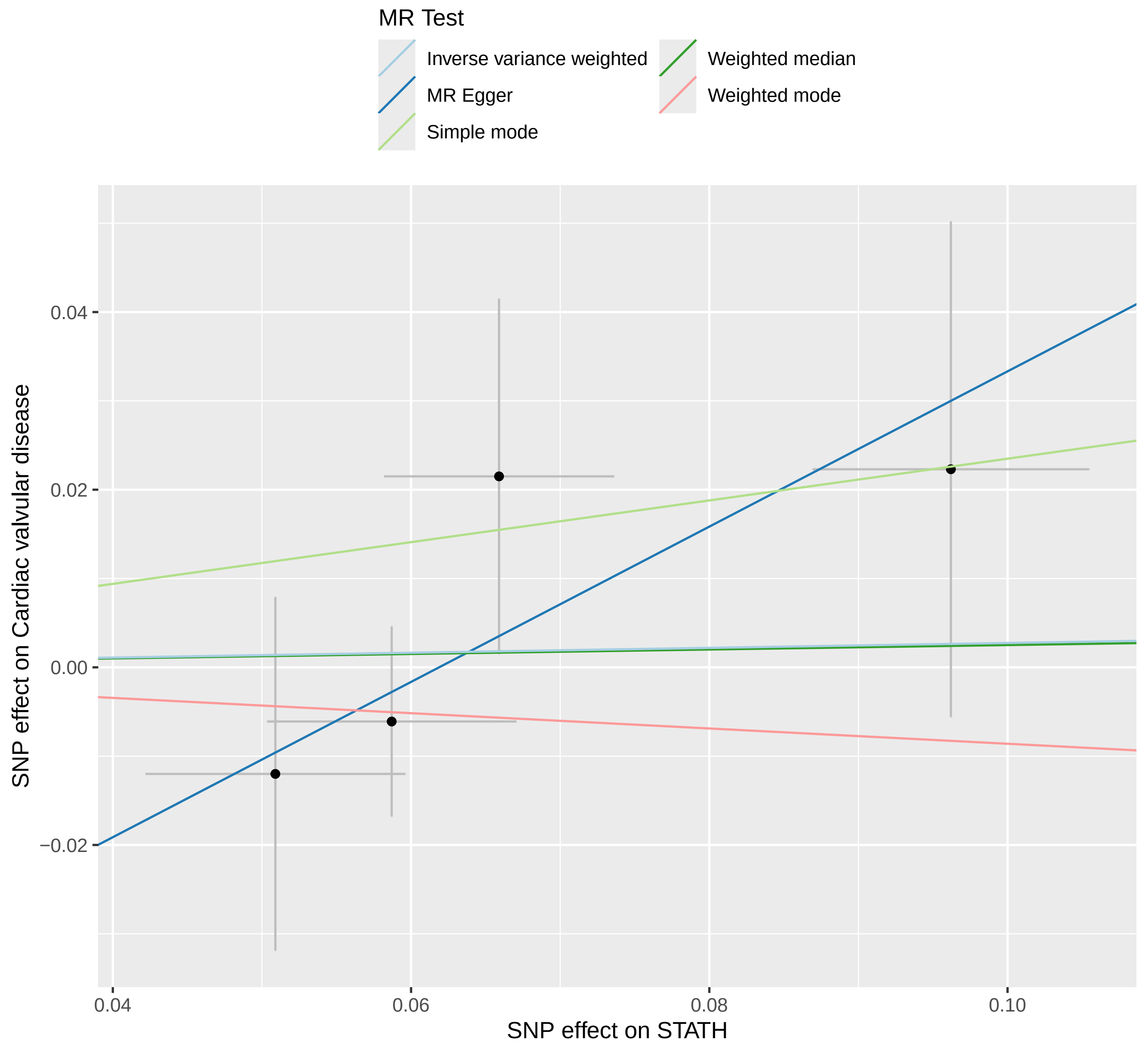
MR Test
Inverse variance weighted
MR Egger
Simple mode
Weighted median
Weighted mode
0.04
0.02
0.00
−0.02
0.04
0.06
0.08
0.10
SNP effect on Cardiac valvular disease
SNP effect on STATH

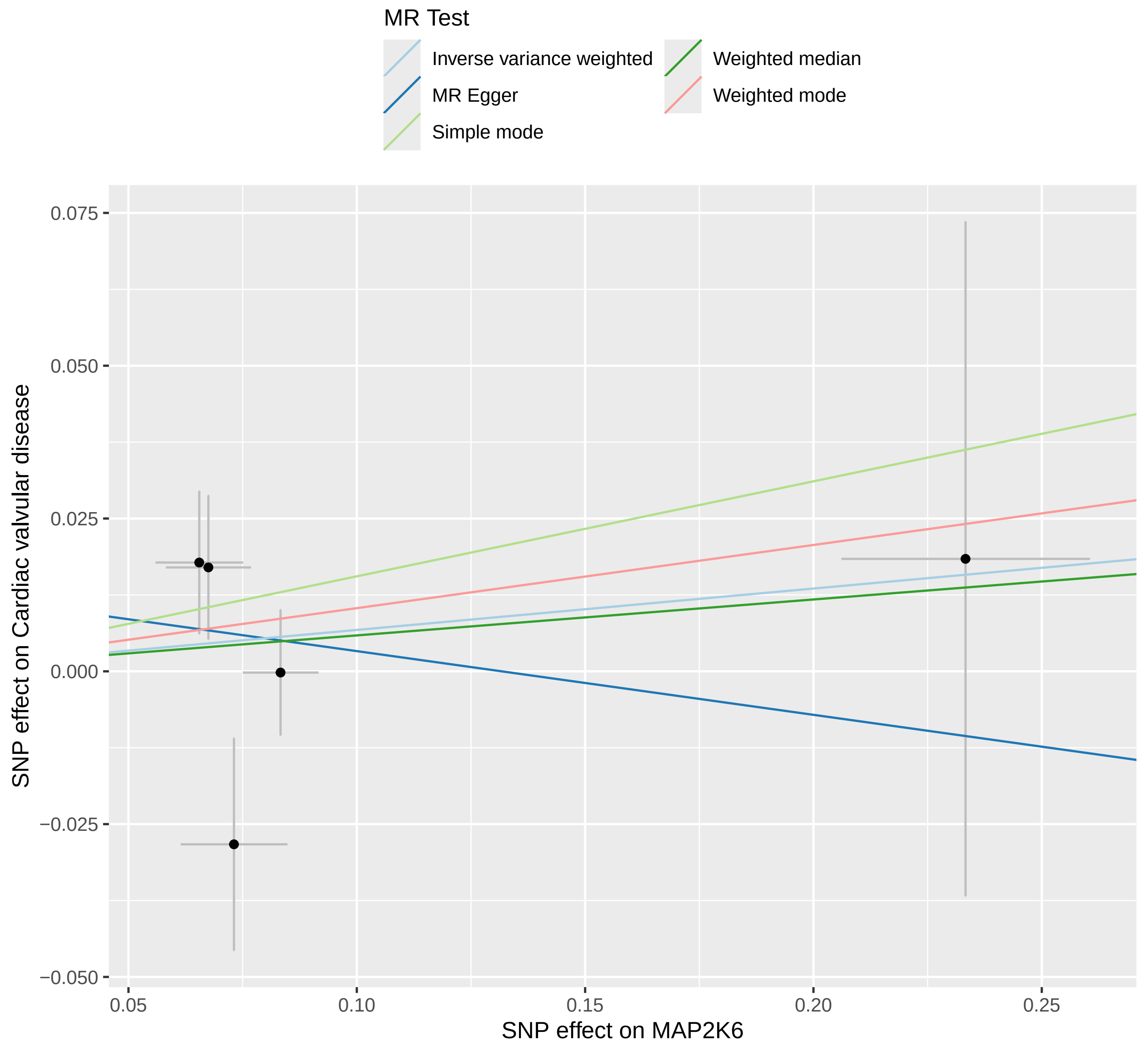
MR Test
Inverse variance weighted
MR Egger
Simple mode
Weighted median
Weighted mode
0.075
0.050
0.025
0.000
−0.025
−0.050
SNP effect on Cardiac valvular disease
0.05
0.10
0.15
0.20
0.25
SNP effect on MAP2K6

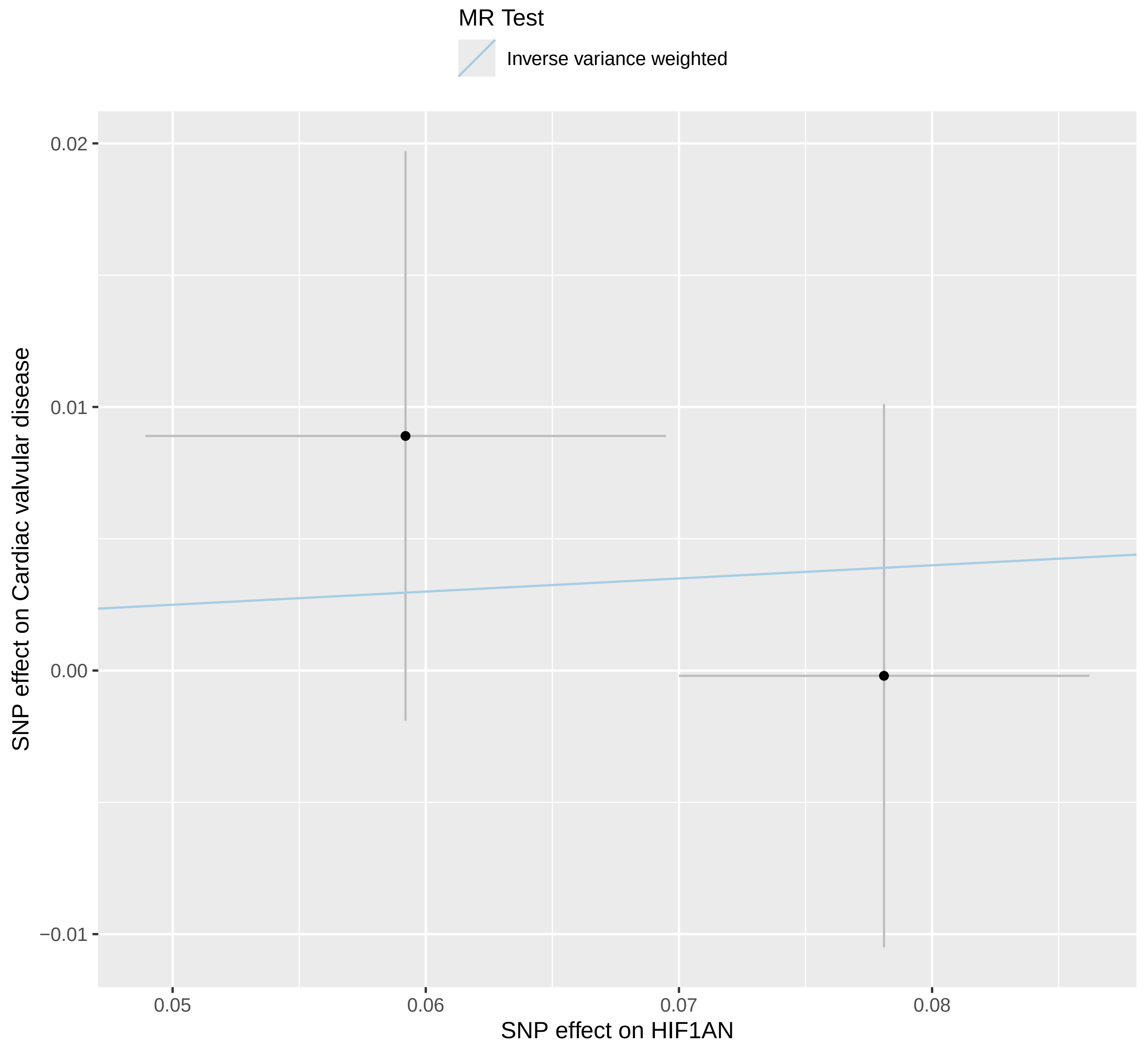

MR Test
Inverse variance weighted
0.02
0.01
0.00
−0.01
0.05
0.06
0.07
0.08
SNP effect on HIF1AN
SNP effect on Cardiac valvular disease

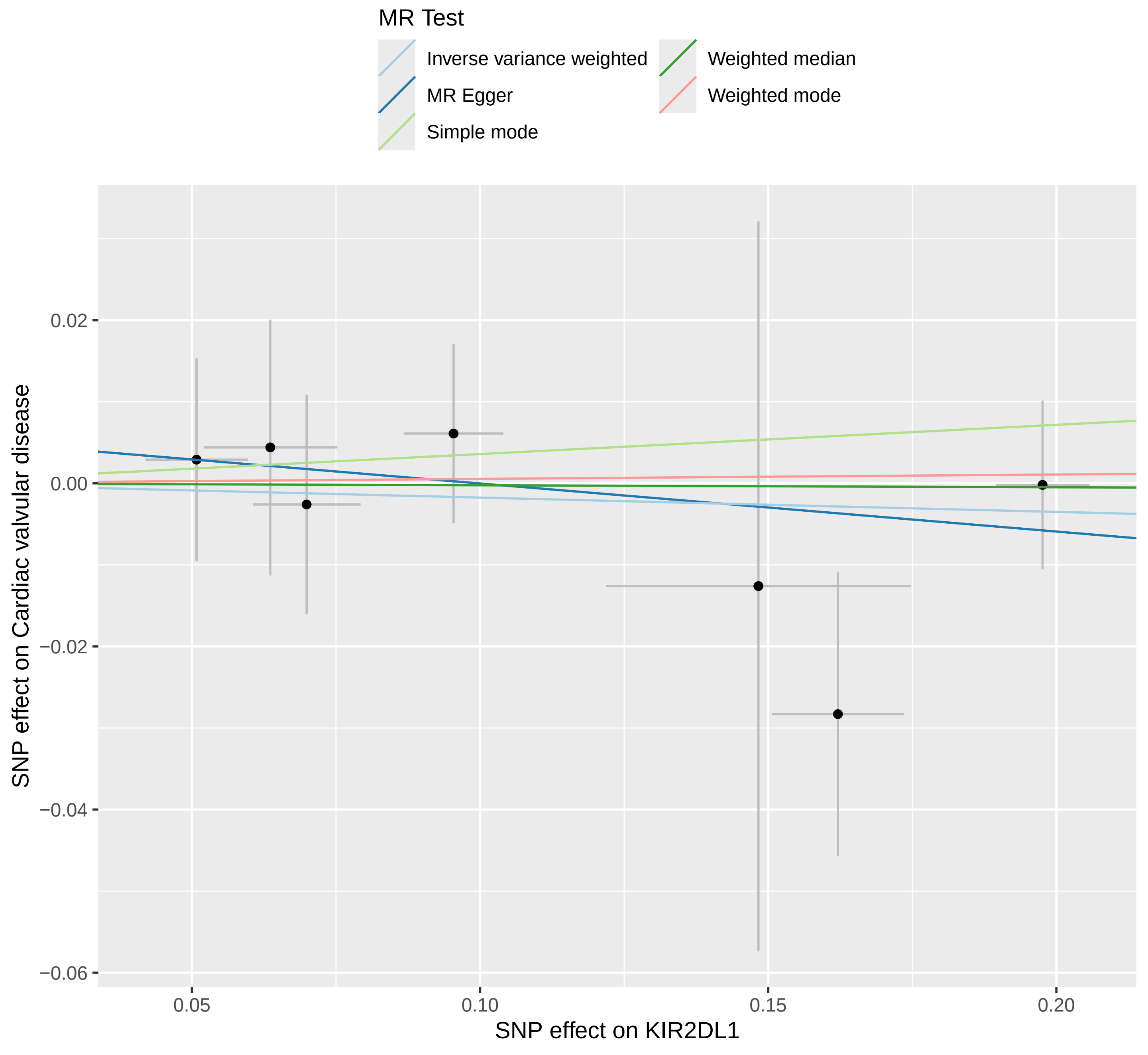
MR Test
Inverse variance weighted
MR Egger
Simple mode
Weighted median
Weighted mode
0.02
0.00
−0.02
−0.04
−0.06
SNP effect on Cardiac valvular disease
0.05
0.10
0.15
0.20
SNP effect on KIR2DL1

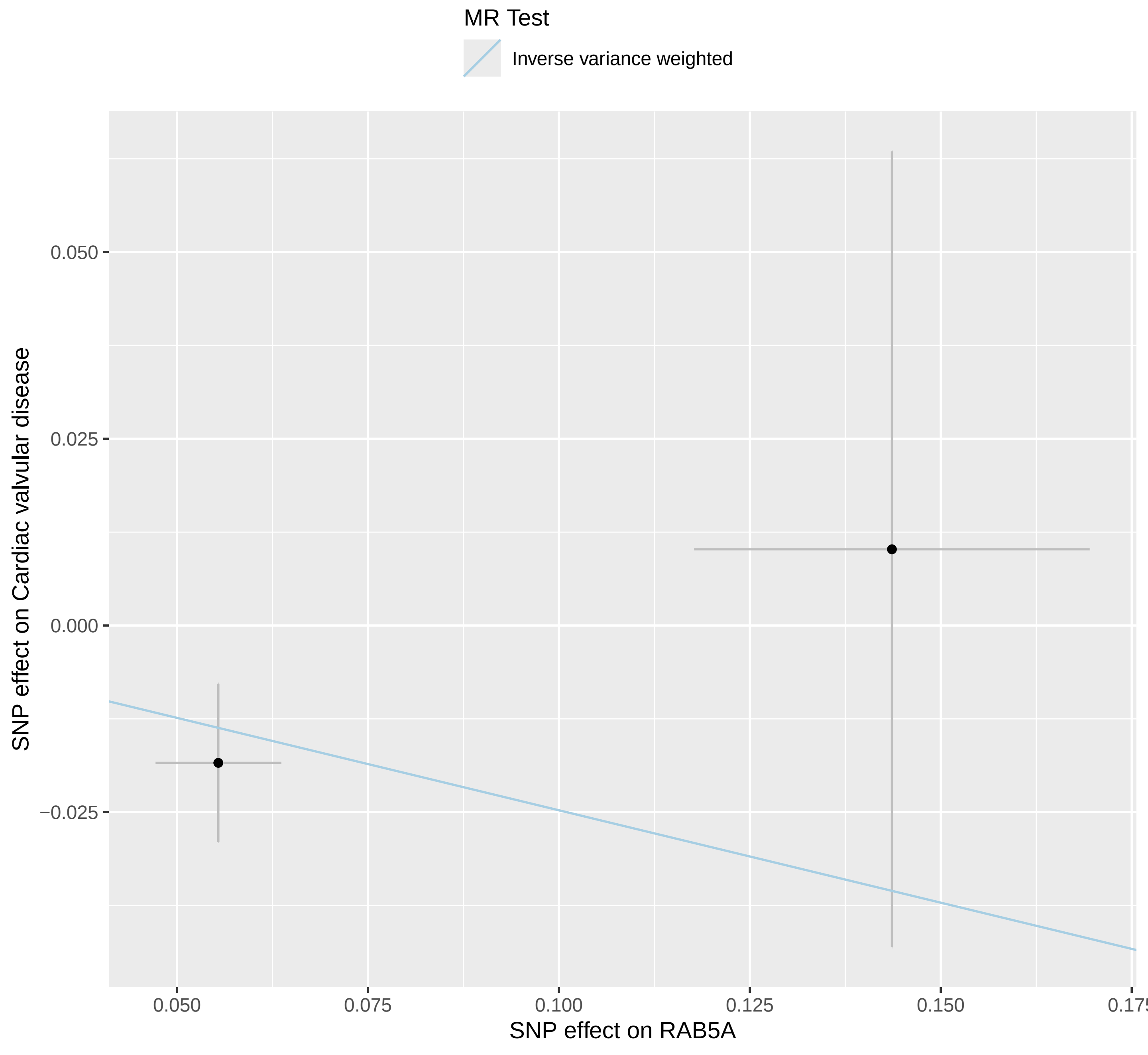
MR Test
Inverse variance weighted
0.050
0.025
0.000
−0.025
SNP effect on Cardiac valvular disease
0.050
0.075
0.100
0.125
0.150
0.175
SNP effect on RAB5A

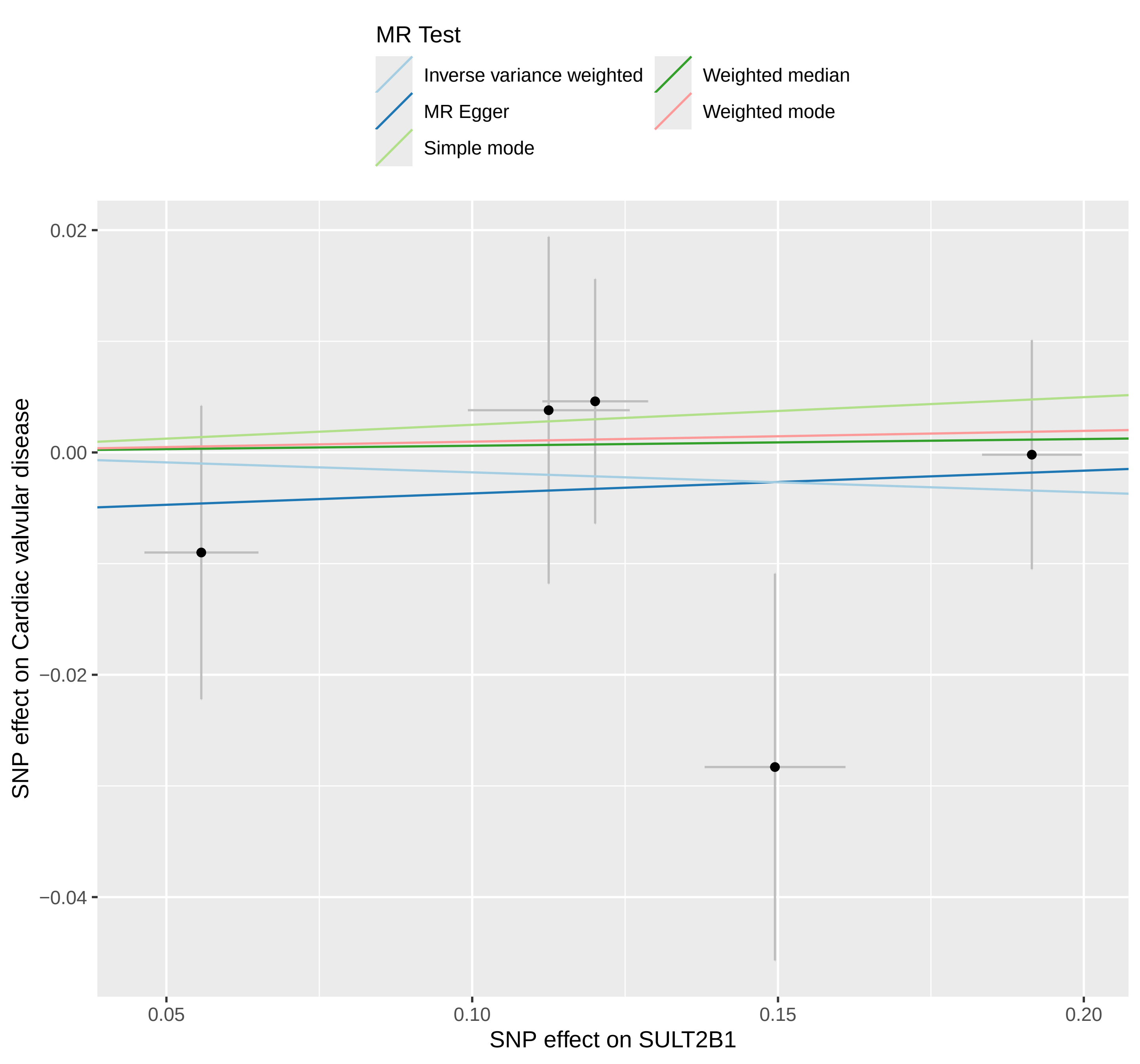
MR Test
Inverse variance weighted
MR Egger
Simple mode
Weighted median
Weighted mode
0.02
0.00
−0.02
−0.04
0.05
0.10
0.15
0.20
SNP effect on Cardiac valvular disease
SNP effect on SULT2B1

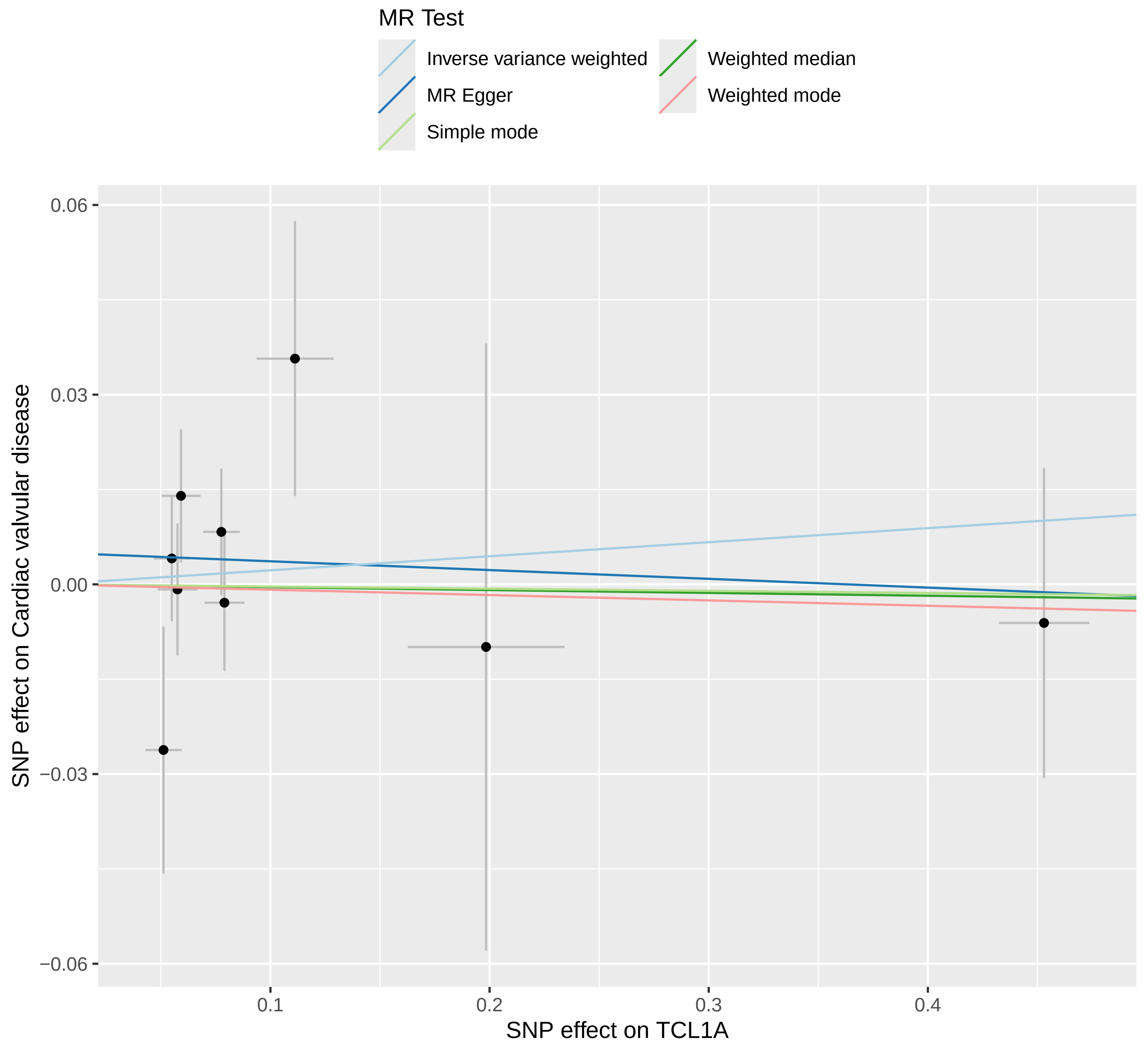
MR Test
Inverse variance weighted
MR Egger
Simple mode
Weighted median
Weighted mode
0.06
0.03
0.00
−0.03
−0.06
0.1
0.2
0.3
0.4
SNP effect on Cardiac valvular disease
SNP effect on TCL1A

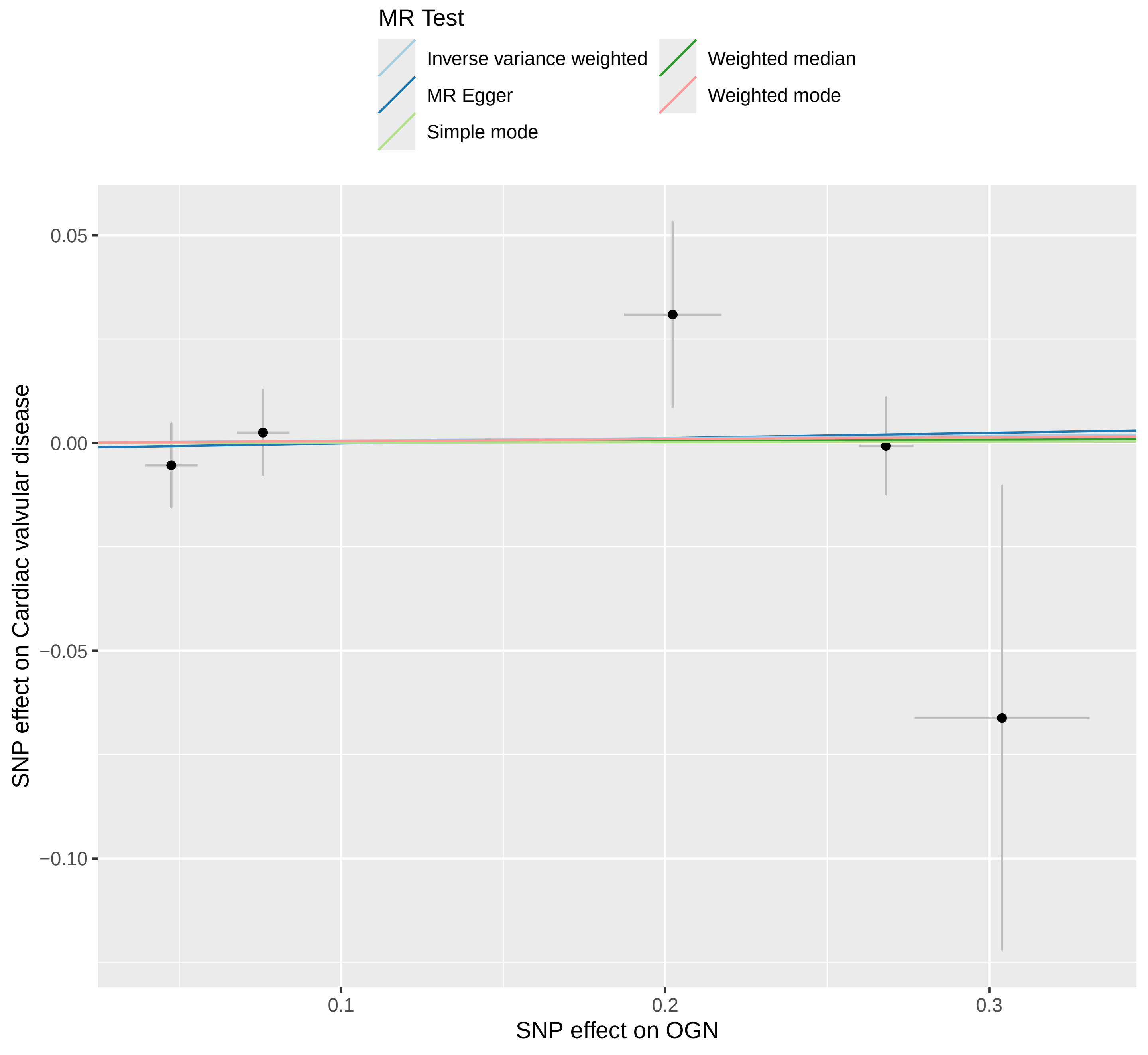

MR Test
Inverse variance weighted
MR Egger
Simple mode
Weighted median
Weighted mode
0.05
0.00
−0.05
−0.10
0.1
0.2
0.3
SNP effect on Cardiac valvular disease
SNP effect on OGN

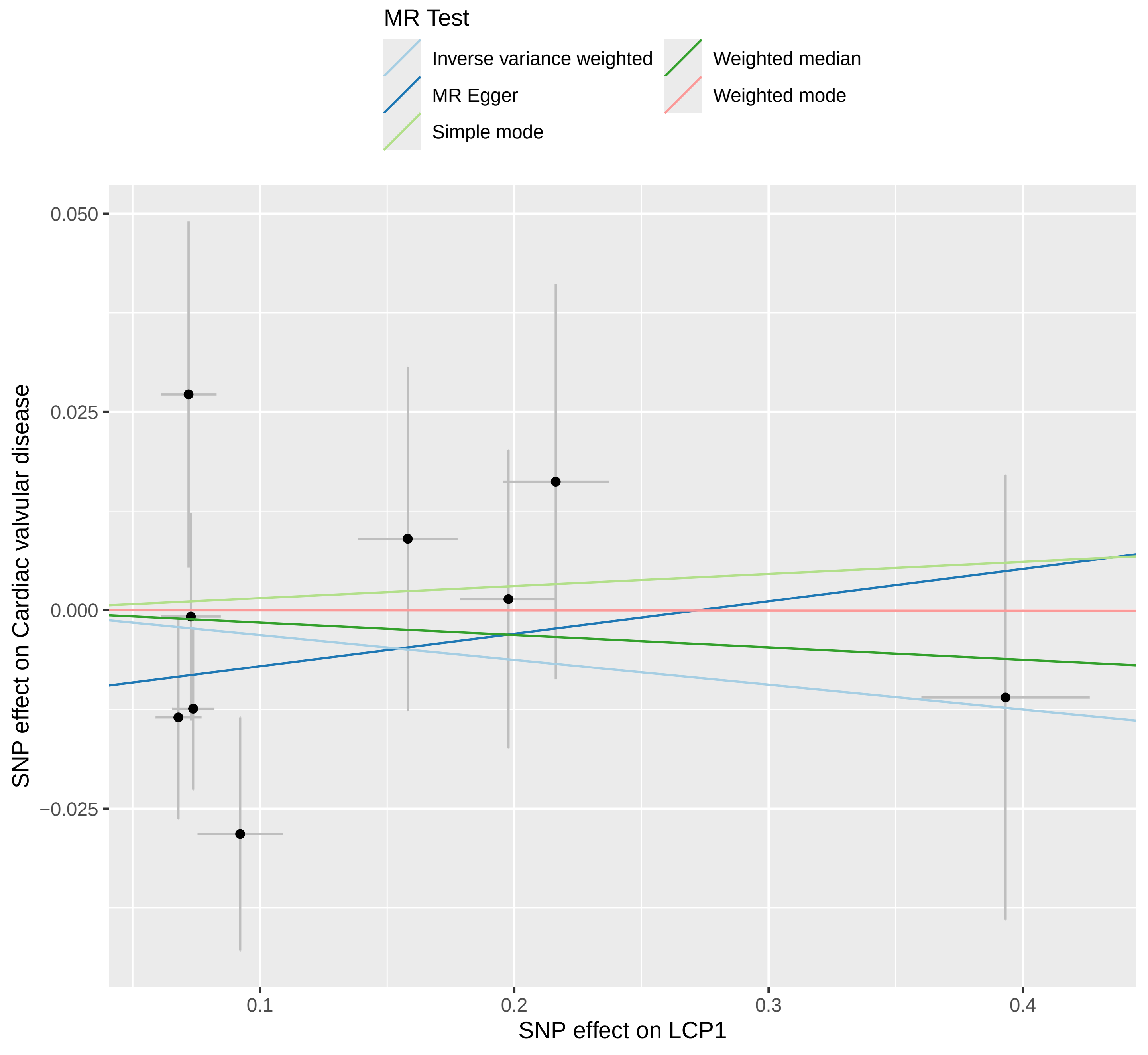

MR Test
Inverse variance weighted
MR Egger
Simple mode
Weighted median
Weighted mode
0.050
0.025
0.000
−0.025
0.1
0.2
0.3
0.4
SNP effect on Cardiac valvular disease
SNP effect on LCP1

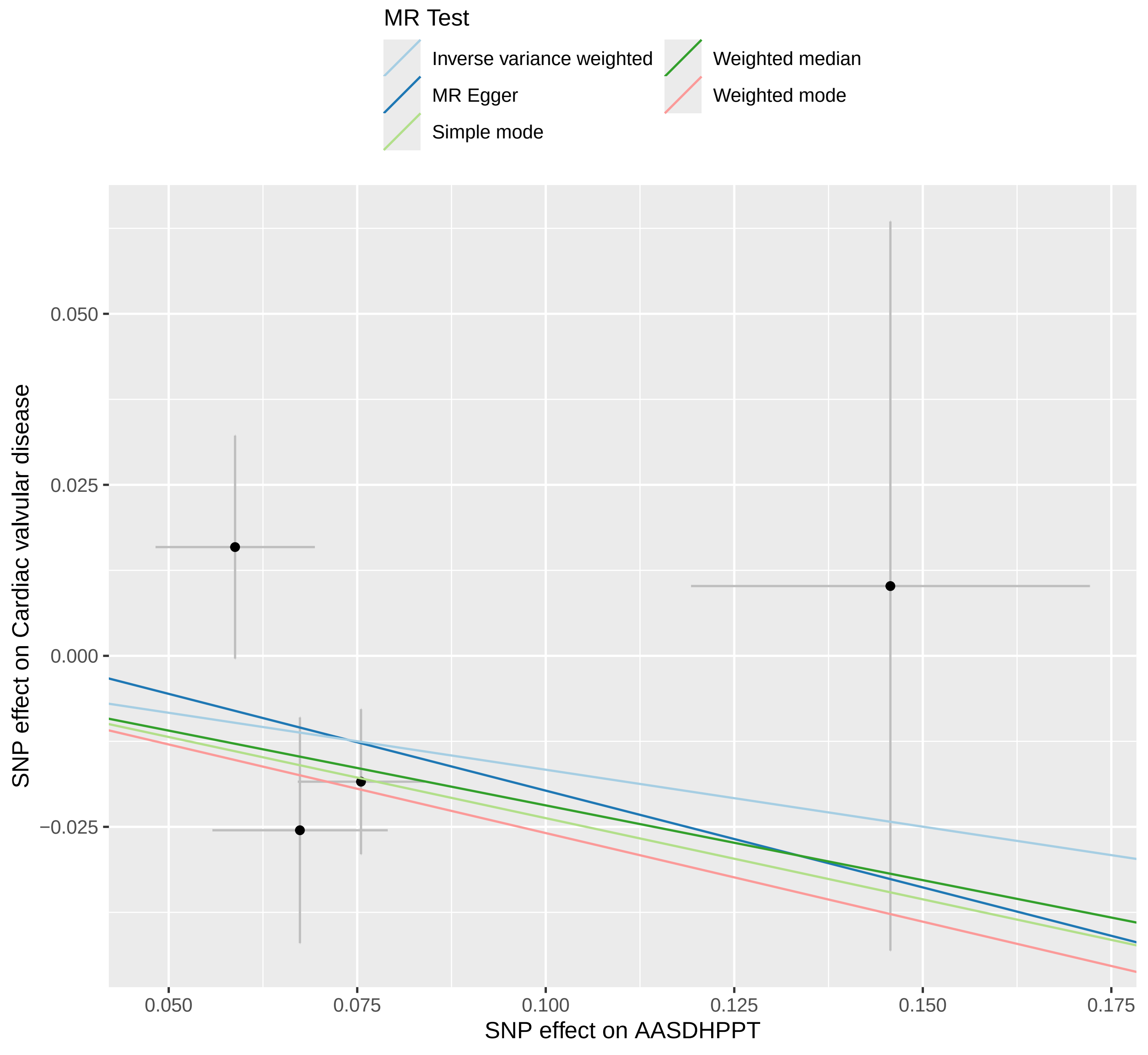

MR Test
Inverse variance weighted
MR Egger
Simple mode
Weighted median
Weighted mode
SNP effect on Cardiac valvular disease
0.050
0.025
0.000
−0.025
0.050
0.075
0.100
0.125
0.150
0.175
SNP effect on AASDHPPT

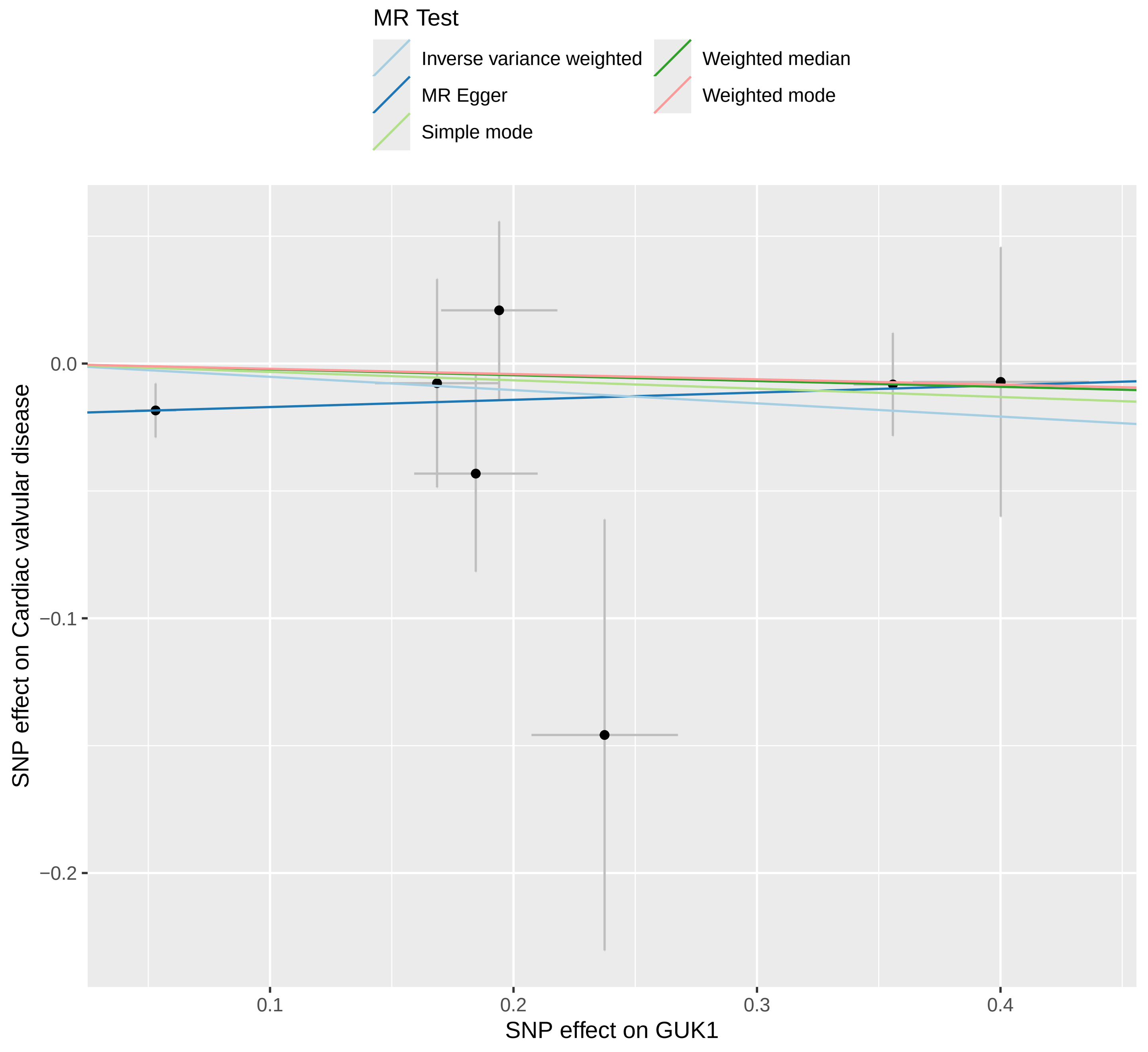
MR Test
Inverse variance weighted
MR Egger
Simple mode
Weighted median
Weighted mode
0.0
−0.1
−0.2
0.1
0.2
0.3
0.4
SNP effect on Cardiac valvular disease
SNP effect on GUK1

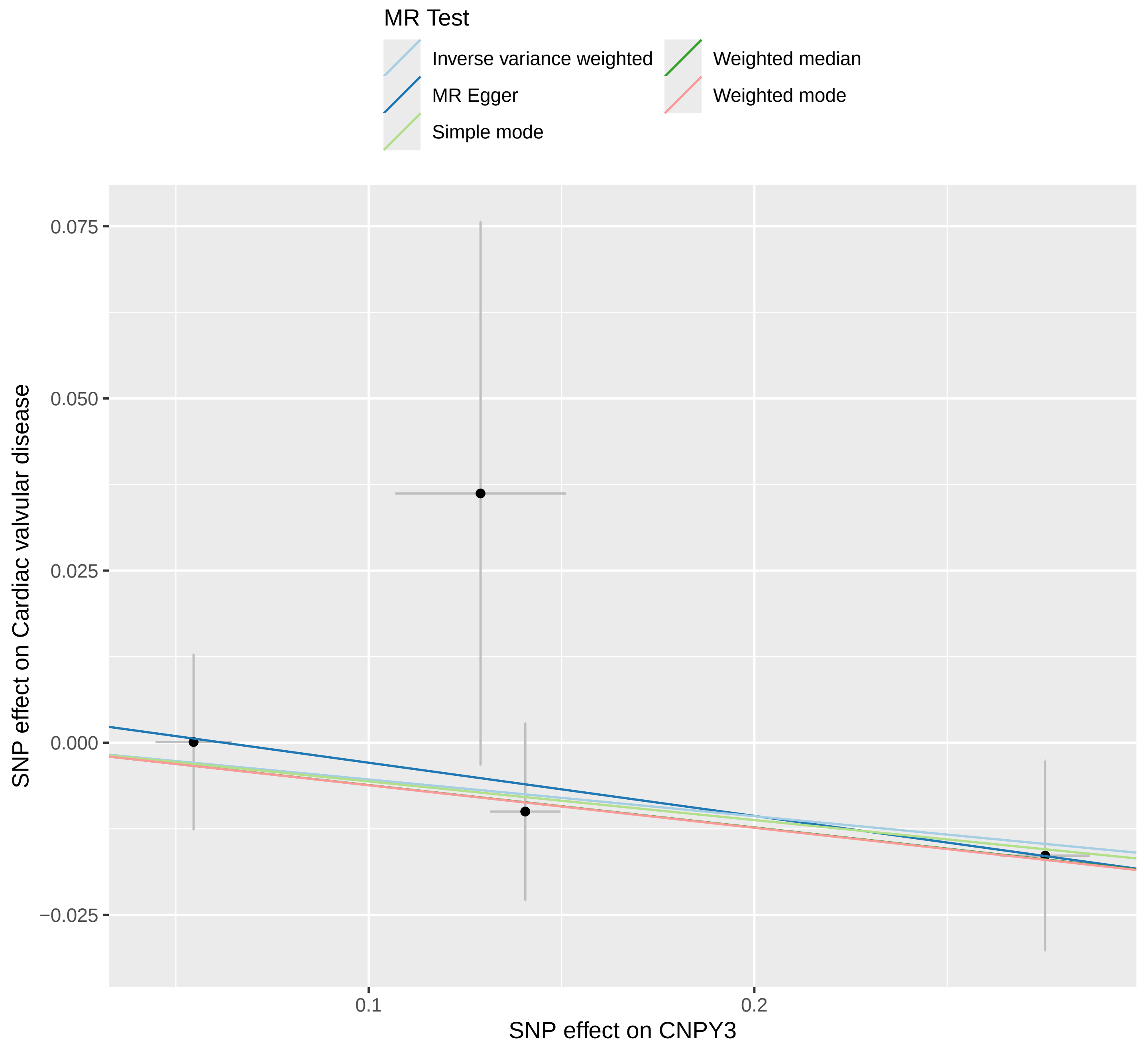

MR Test
Inverse variance weighted
MR Egger
Simple mode
Weighted median
Weighted mode
0.075
0.050
0.025
0.000
−0.025
0.1
0.2
SNP effect on Cardiac valvular disease
SNP effect on CNPY3

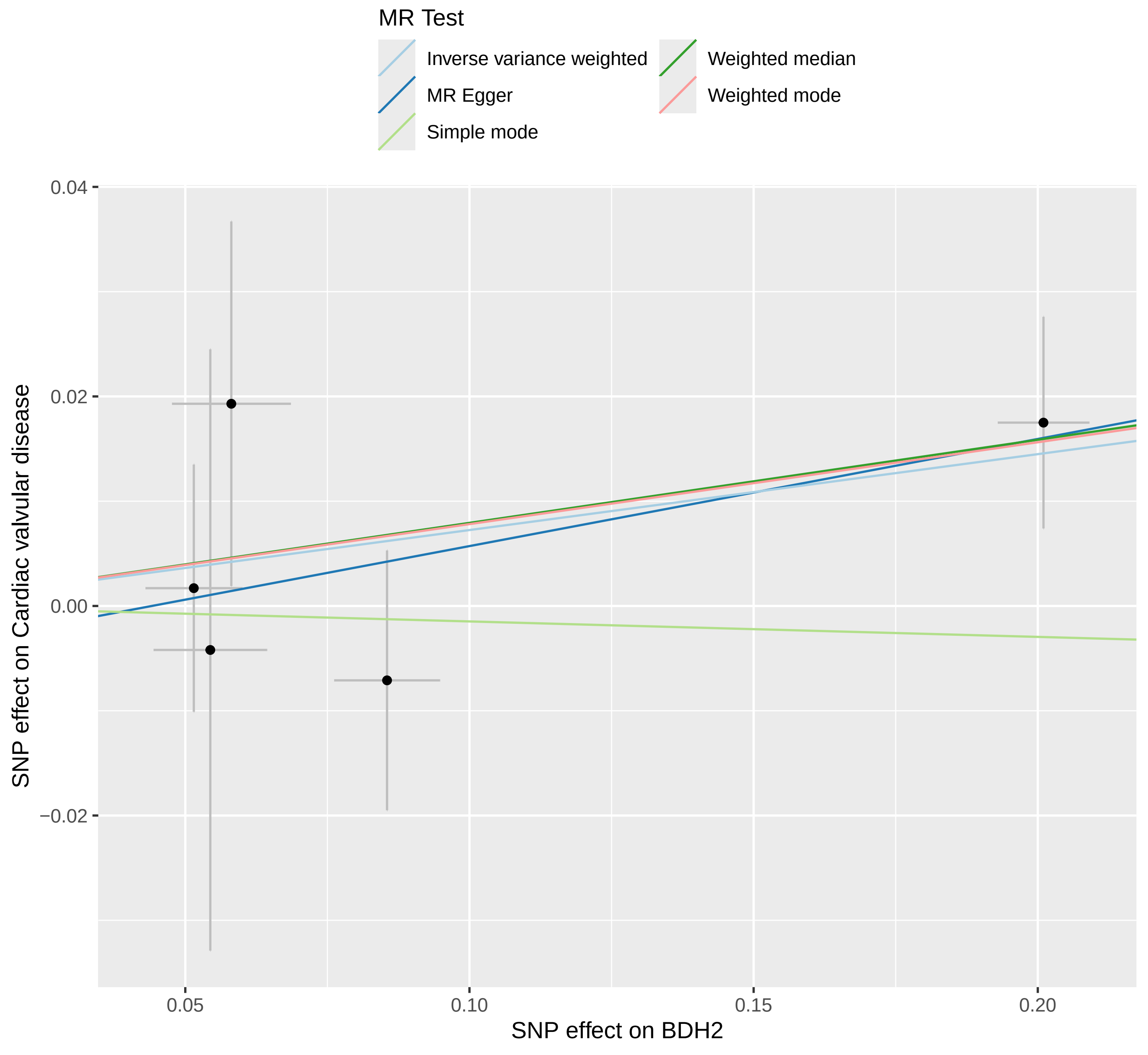
MR Test
Inverse variance weighted
MR Egger
Simple mode
Weighted median
Weighted mode
0.04
0.02
0.00
−0.02
0.05
0.10
0.15
0.20
SNP effect on Cardiac valvular disease
SNP effect on BDH2

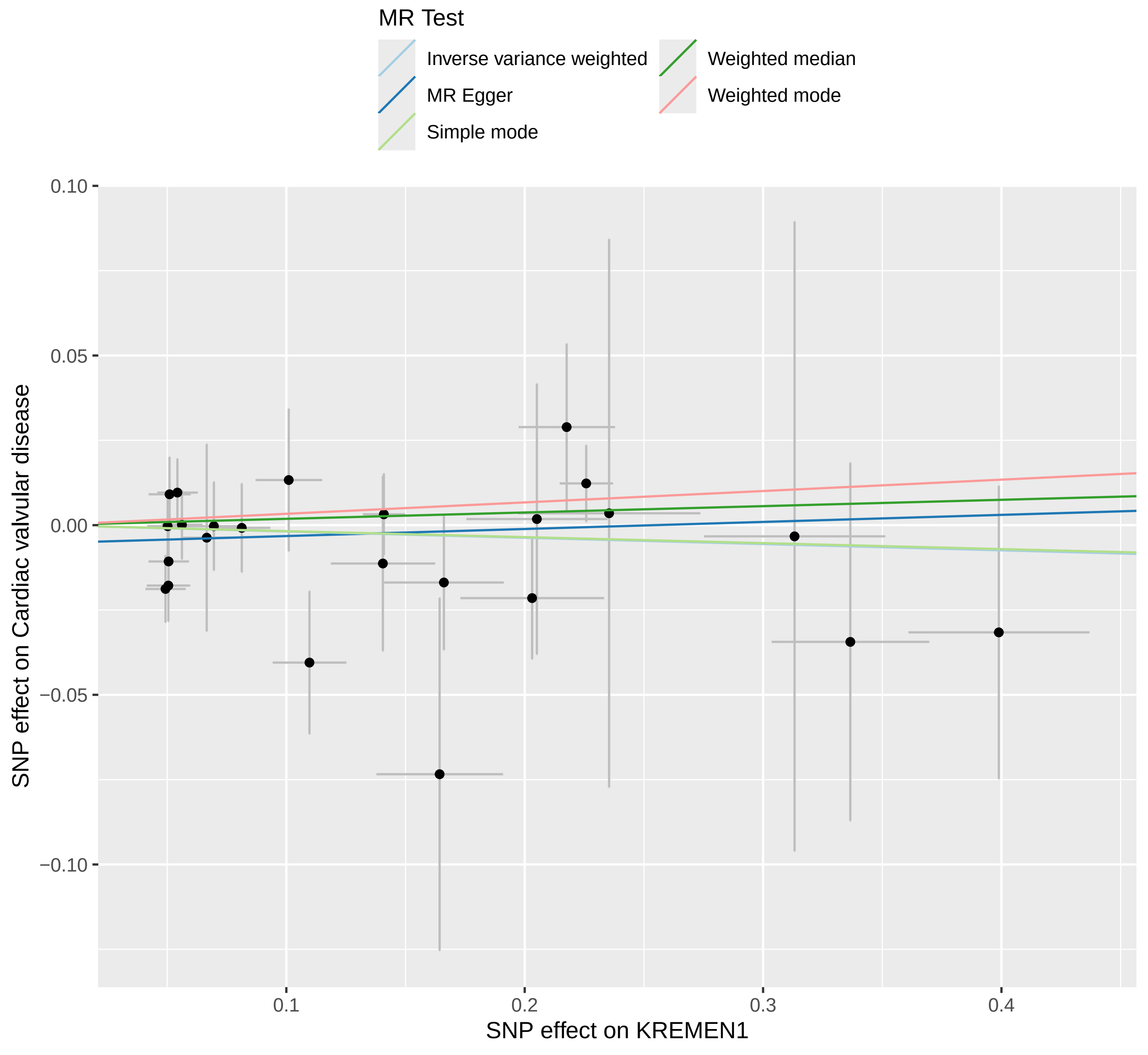

MR Test
Inverse variance weighted
MR Egger
Simple mode
Weighted median
Weighted mode
0.10
0.05
0.00
−0.05
−0.10
0.1
0.2
0.3
0.4
SNP effect on Cardiac valvular disease
SNP effect on KREMEN1

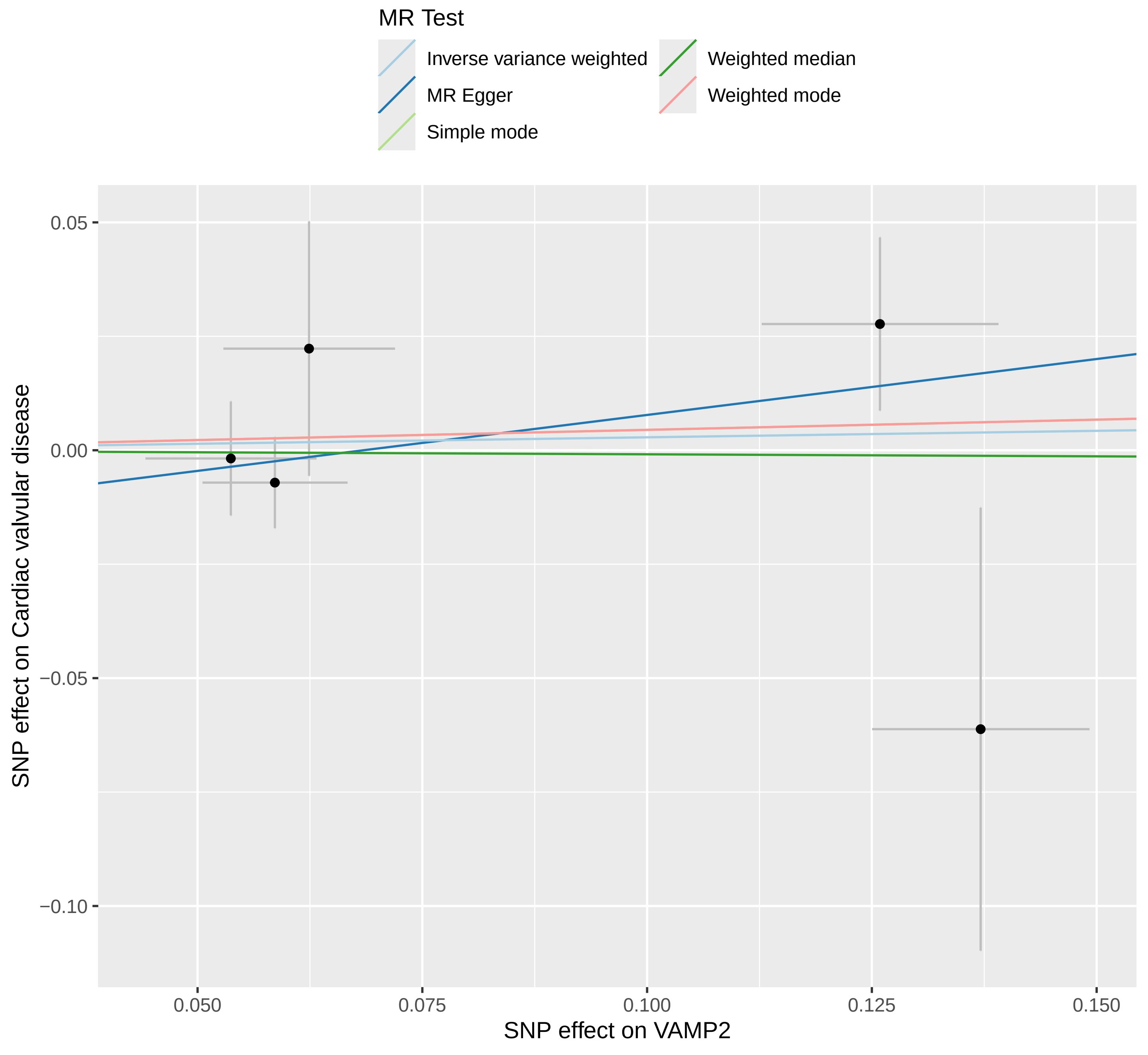

MR Test
Inverse variance weighted
MR Egger
Simple mode
Weighted median
Weighted mode
0.05
0.00
−0.05
−0.10
SNP effect on Cardiac valvular disease
0.050
0.075
0.100
0.125
0.150
SNP effect on VAMP2

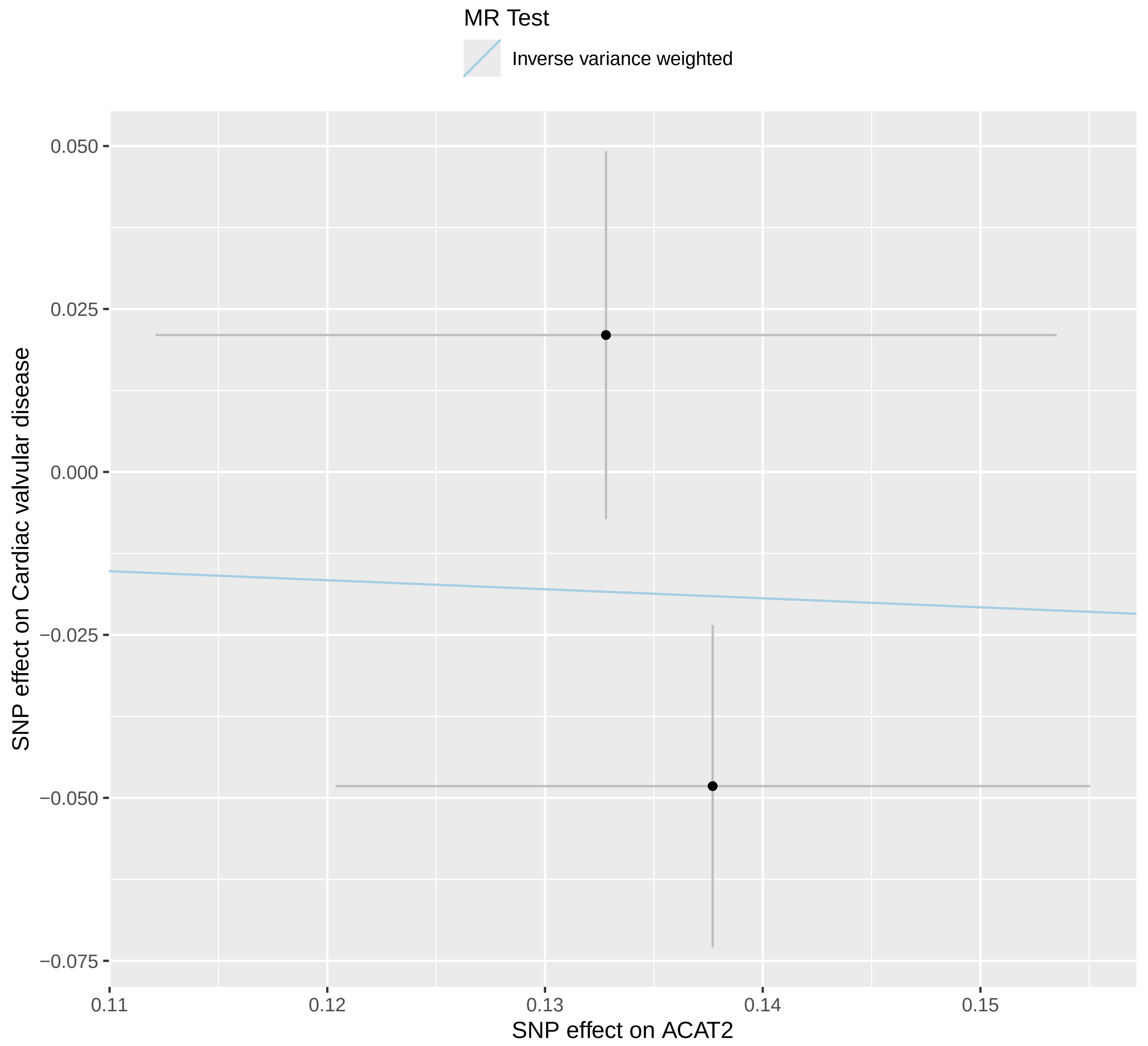
MR Test
Inverse variance weighted
0.050
0.025
0.000
−0.025
−0.050
−0.075
0.11
0.12
0.13
0.14
0.15
SNP effect on Cardiac valvular disease
SNP effect on ACAT2

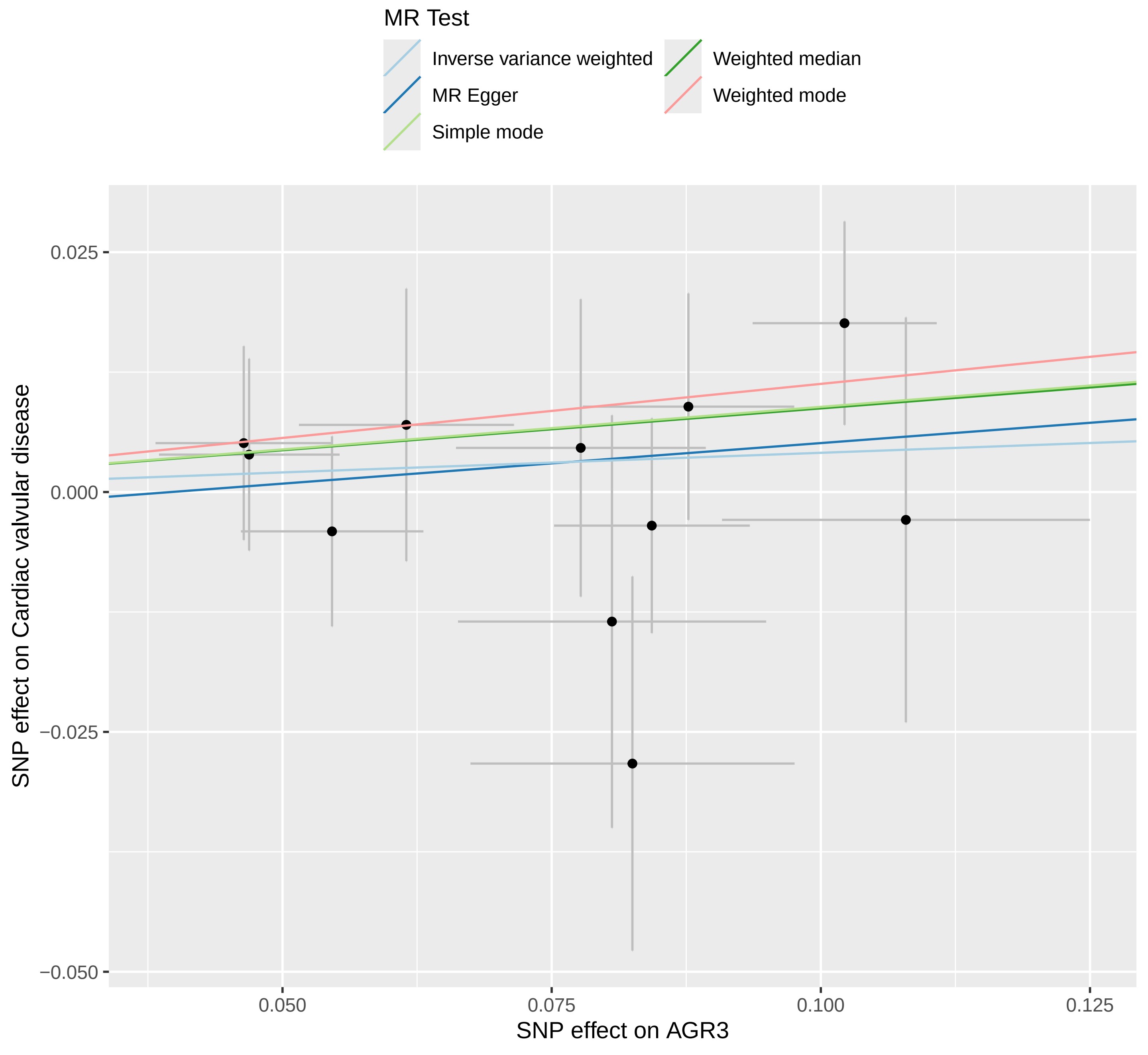

MR Test
Inverse variance weighted
MR Egger
Simple mode
Weighted median
Weighted mode
0.025
0.000
−0.025
−0.050
0.050
0.075
0.100
0.125
SNP effect on Cardiac valvular disease
SNP effect on AGR3

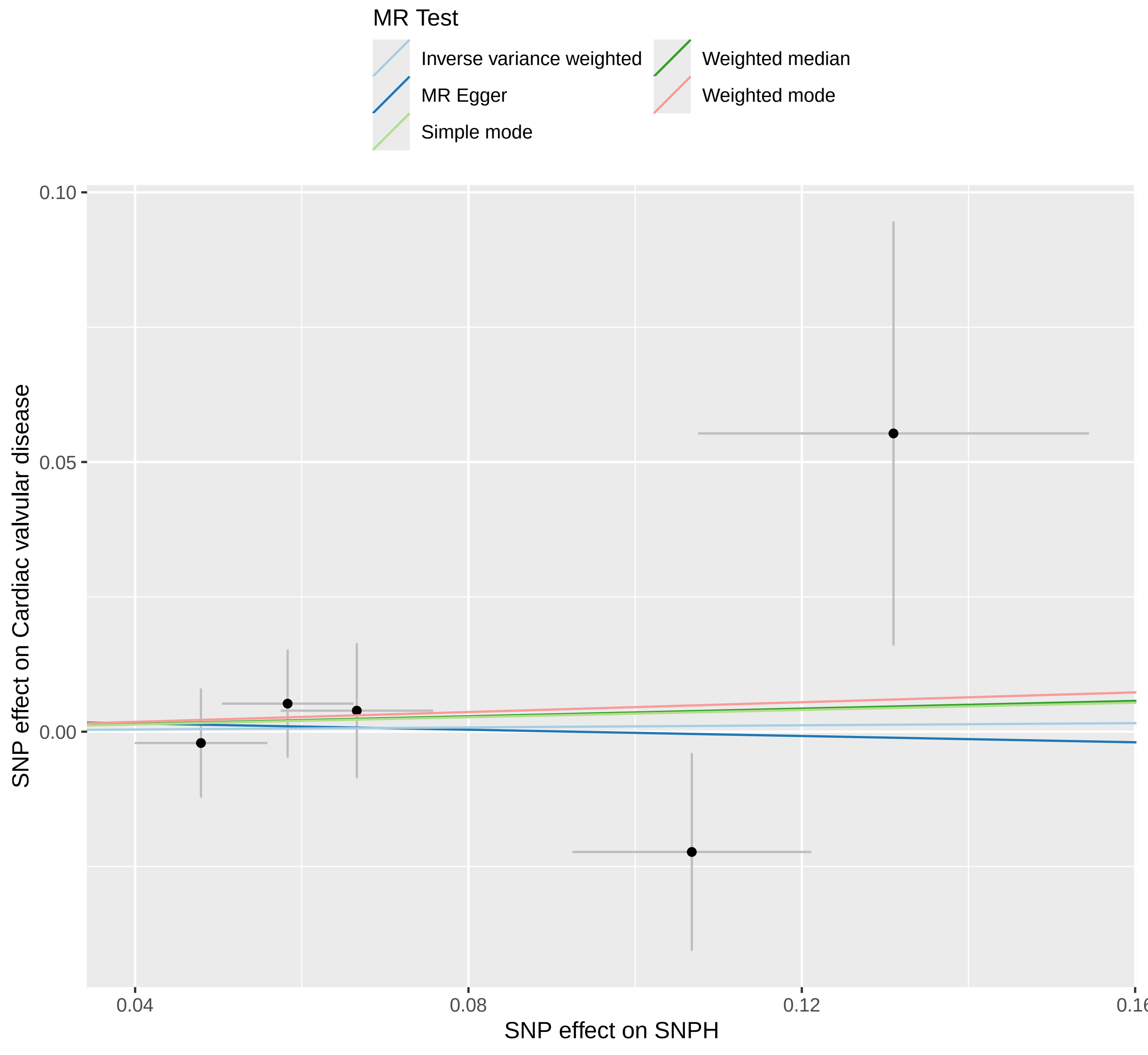

MR Test
Inverse variance weighted
MR Egger
Simple mode
Weighted median
Weighted mode
0.10
0.05
0.00
0.04
0.08
0.12
0.16
SNP effect on Cardiac valvular disease
SNP effect on SNPH

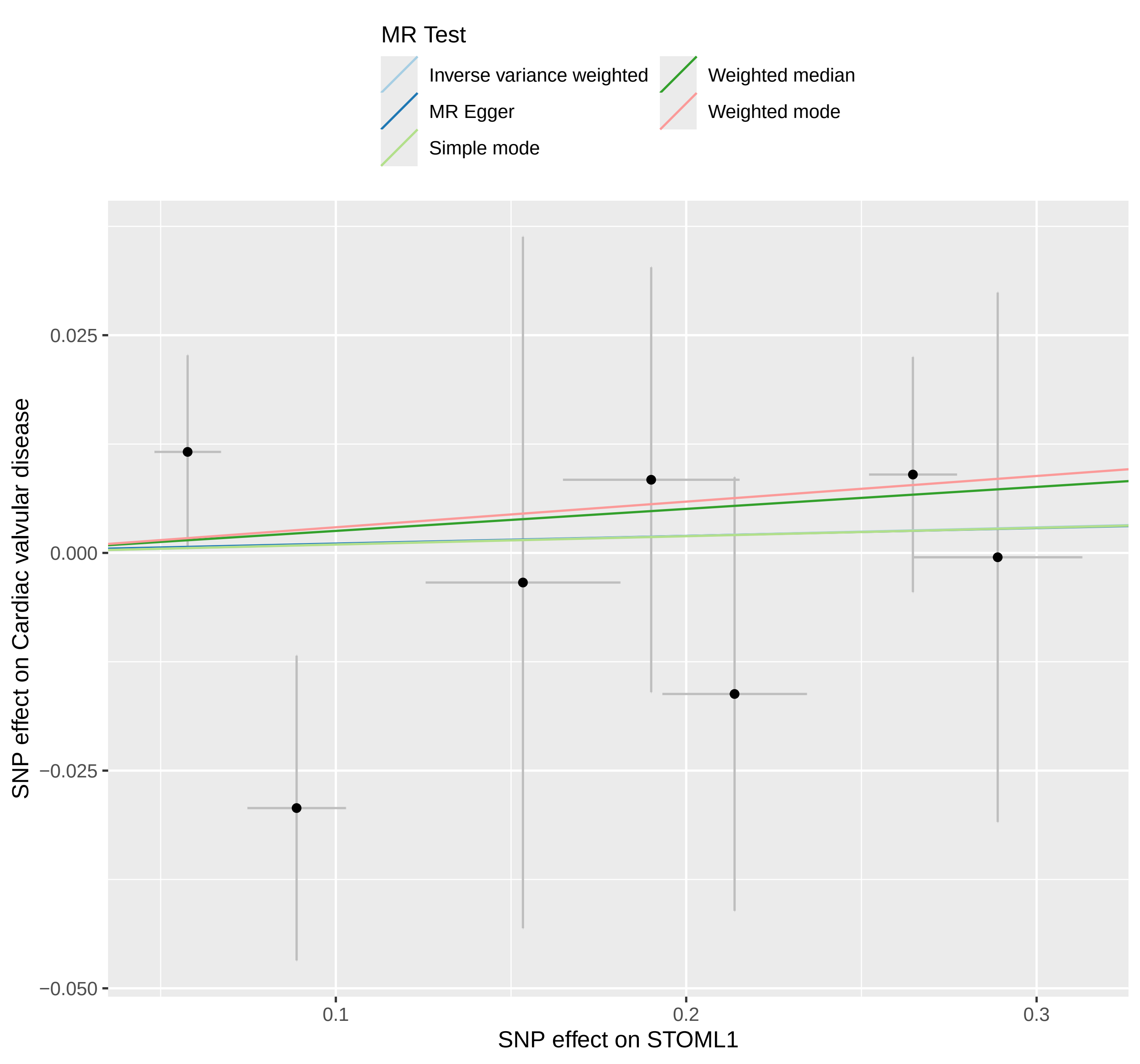

MR Test
Inverse variance weighted
MR Egger
Simple mode
Weighted median
Weighted mode
SNP effect on Cardiac valvular disease
0.025
0.000
−0.025
−0.050
0.1
0.2
0.3
SNP effect on STOML1

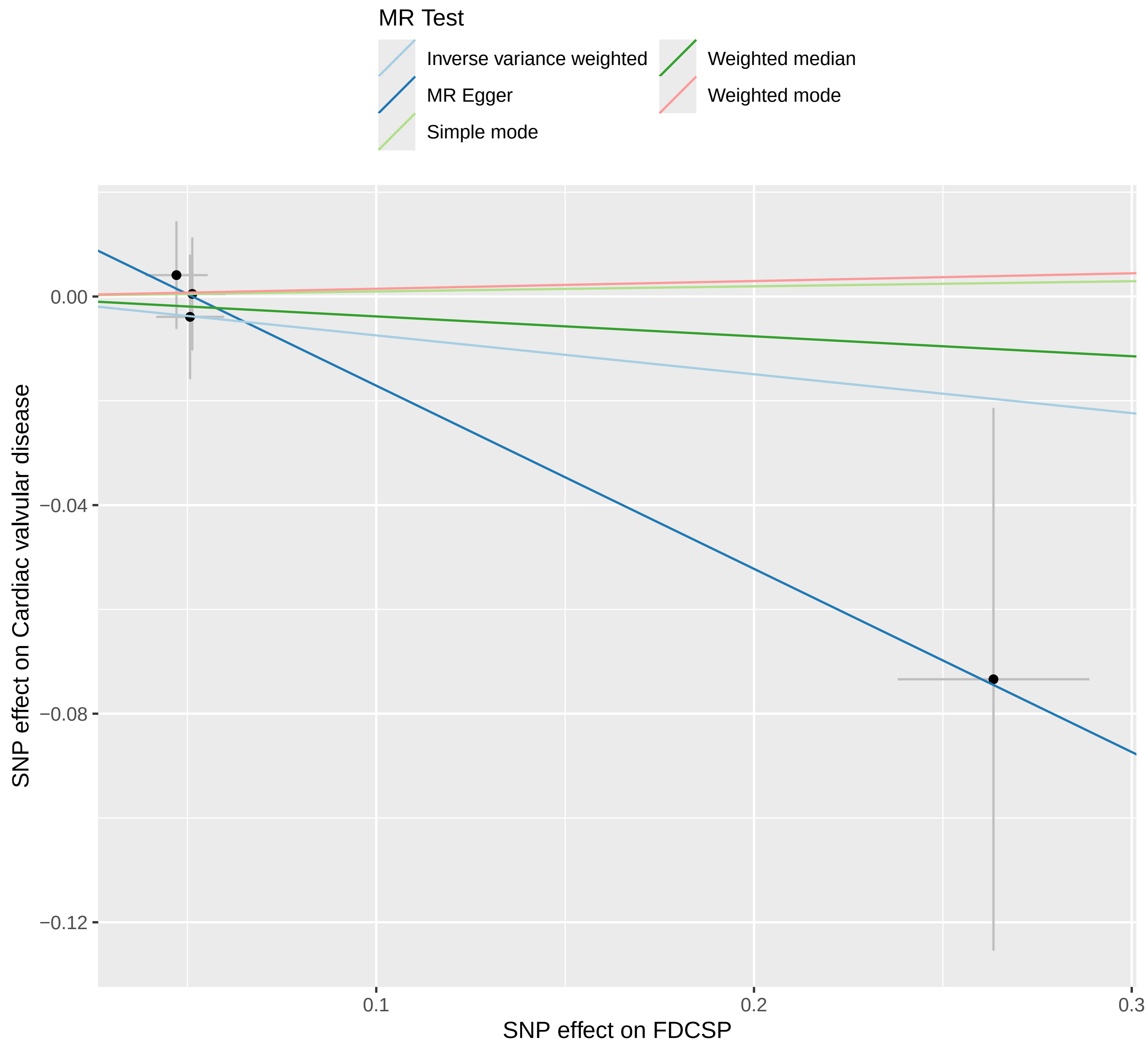
MR Test
Inverse variance weighted
MR Egger
Simple mode
Weighted median
Weighted mode
0.00
−0.04
−0.08
−0.12
SNP effect on Cardiac valvular disease
0.1
0.2
0.3
SNP effect on FDCSP

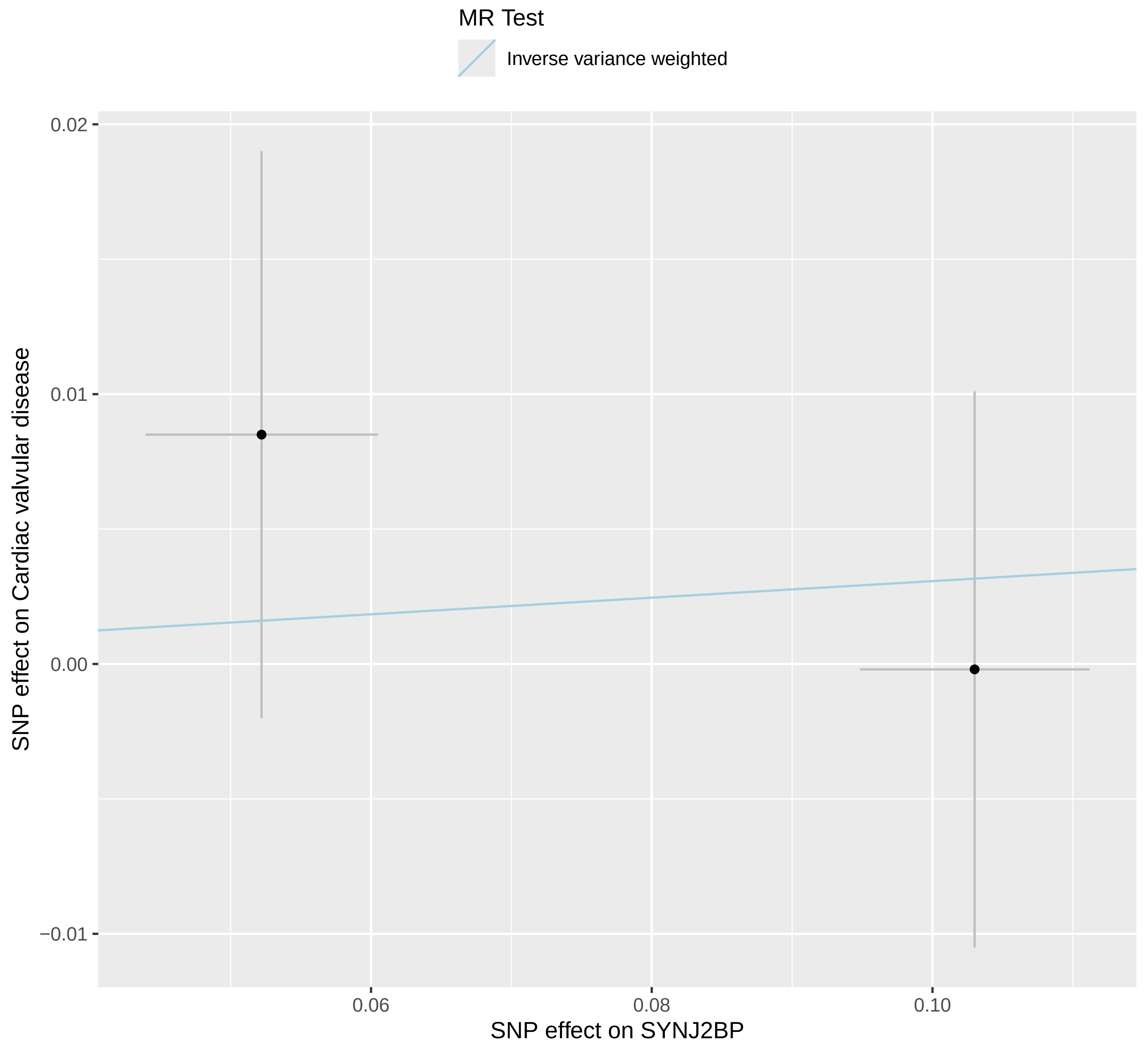
MR Test
Inverse variance weighted
0.02
0.01
0.00
−0.01
0.06
0.08
0.10
SNP effect on Cardiac valvular disease
SNP effect on SYNJ2BP

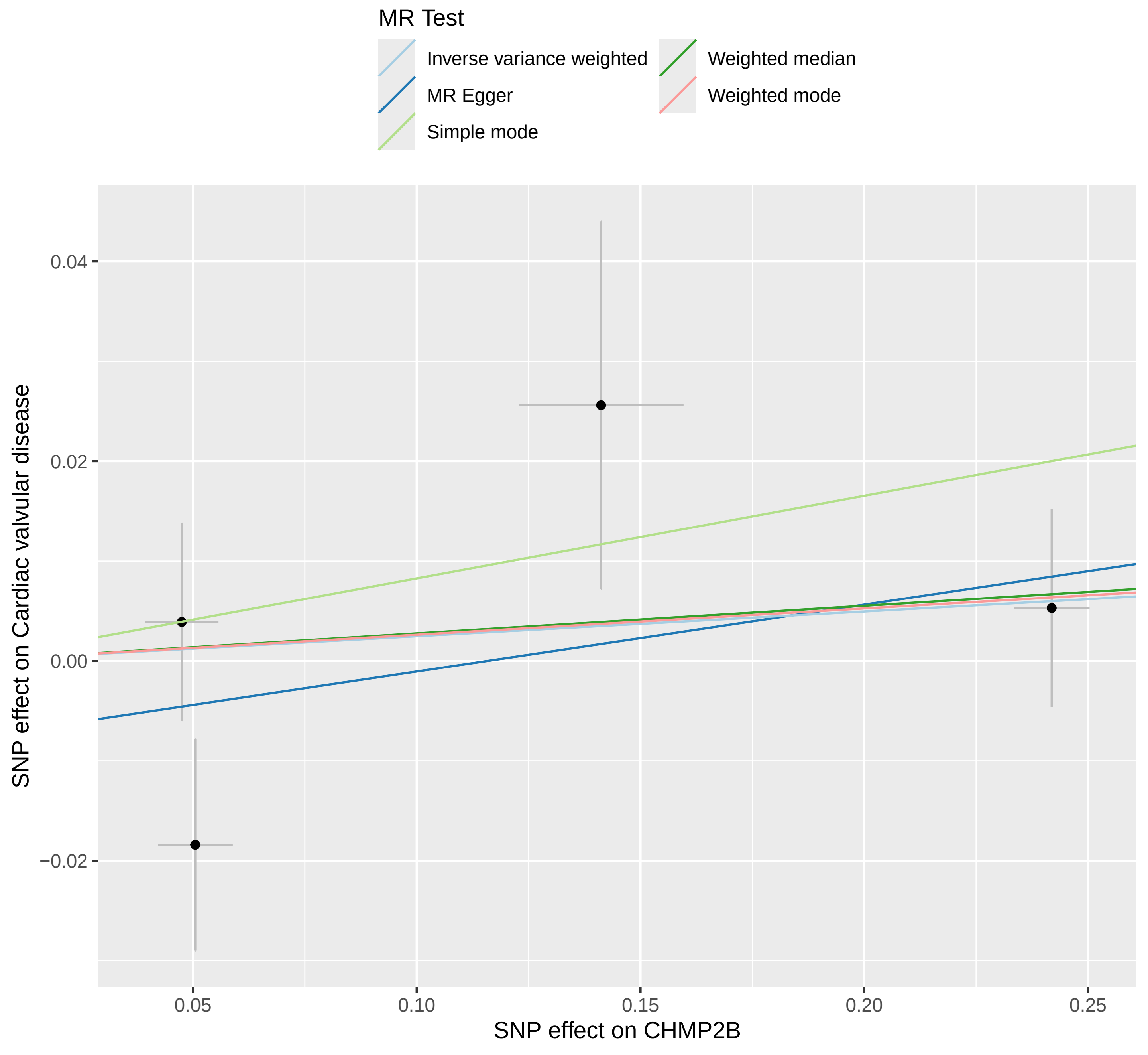
MR Test
Inverse variance weighted
MR Egger
Simple mode
Weighted median
Weighted mode
SNP effect on Cardiac valvular disease
0.04
0.02
0.00
−0.02
0.05
0.10
0.15
0.20
0.25
SNP effect on CHMP2B

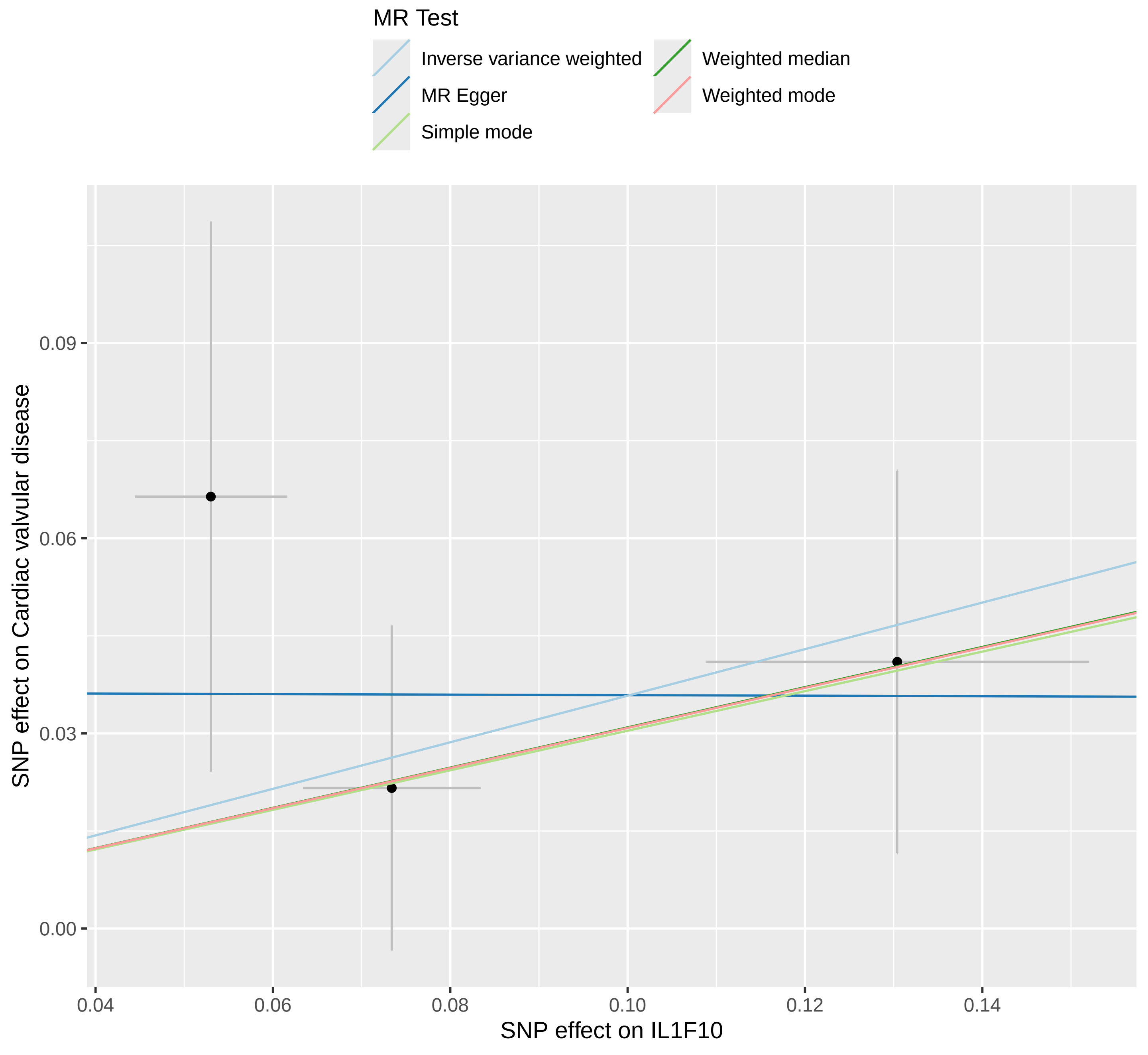
MR Test
Inverse variance weighted
MR Egger
Simple mode
Weighted median
Weighted mode
0.09
0.06
0.03
0.00
SNP effect on Cardiac valvular disease
0.04
0.06
0.08
0.10
0.12
0.14
SNP effect on IL1F10

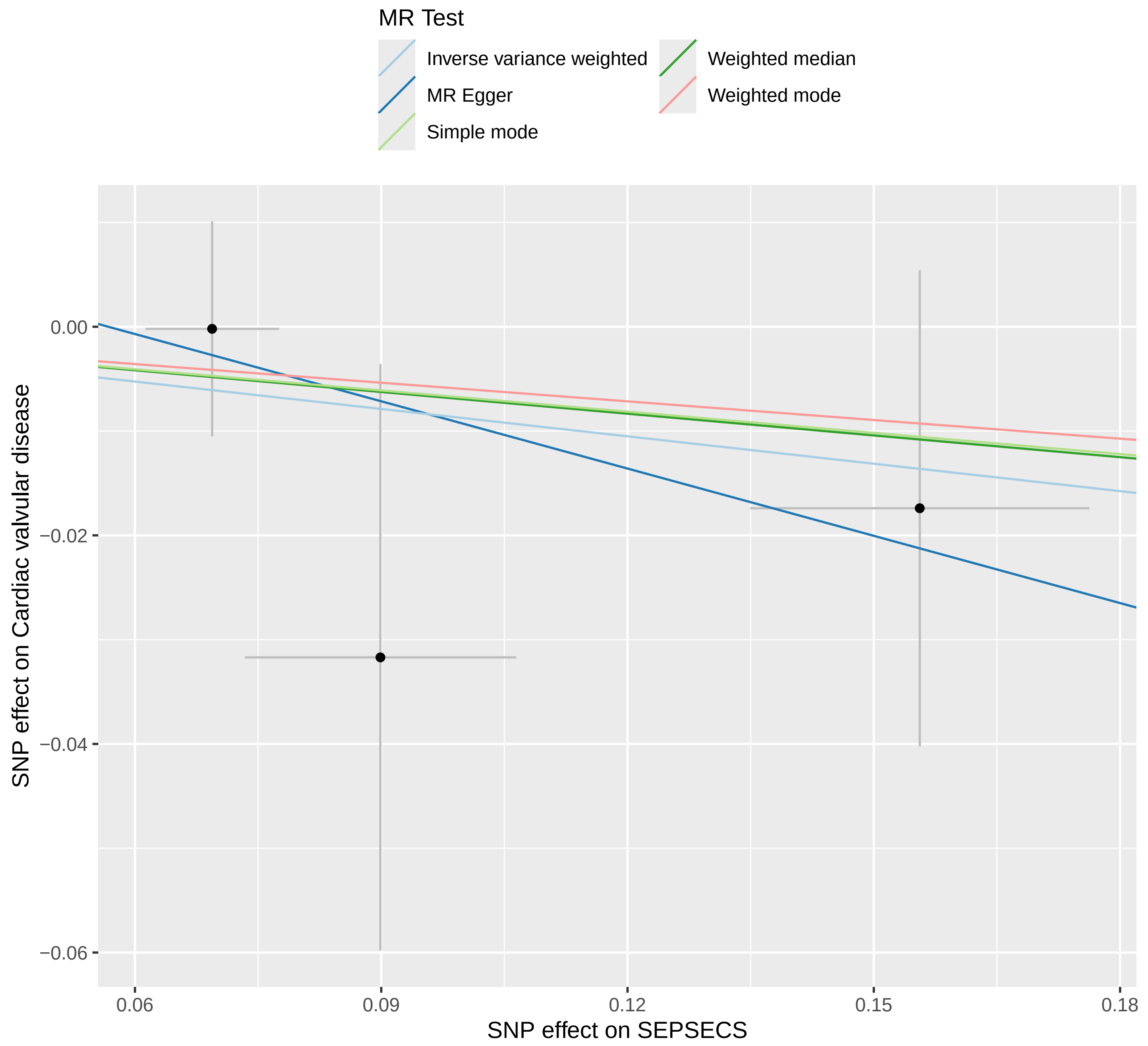
MR Test
Inverse variance weighted
MR Egger
Simple mode
Weighted median
Weighted mode
0.00
−0.02
−0.04
−0.06
0.06
0.09
0.12
0.15
0.18
SNP effect on SEPSECS
SNP effect on Cardiac valvular disease

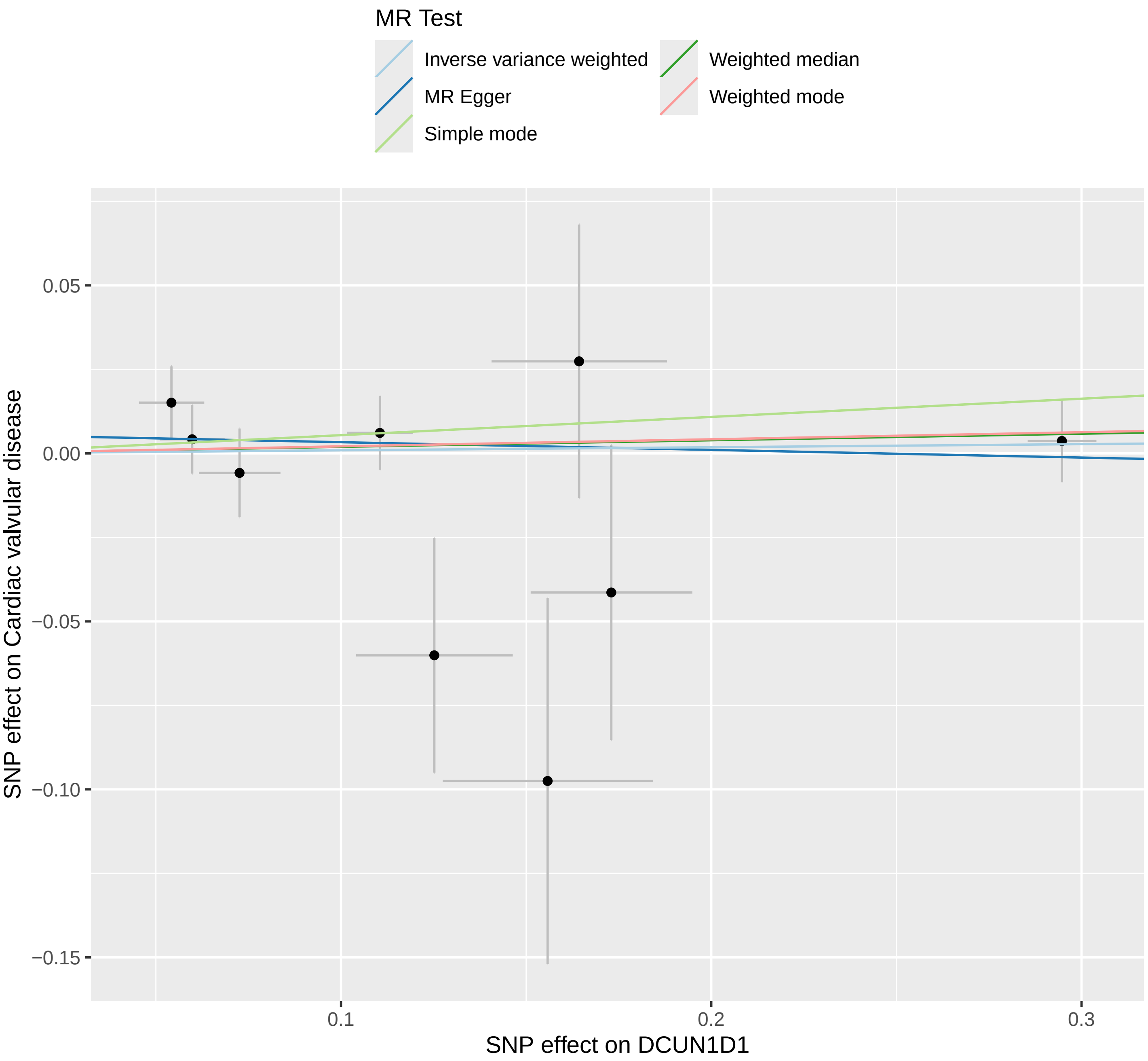
MR Test
Inverse variance weighted
MR Egger
Simple mode
Weighted median
Weighted mode
0.05
0.00
−0.05
−0.10
−0.15
SNP effect on Cardiac valvular disease
0.1
0.2
0.3
SNP effect on DCUN1D1

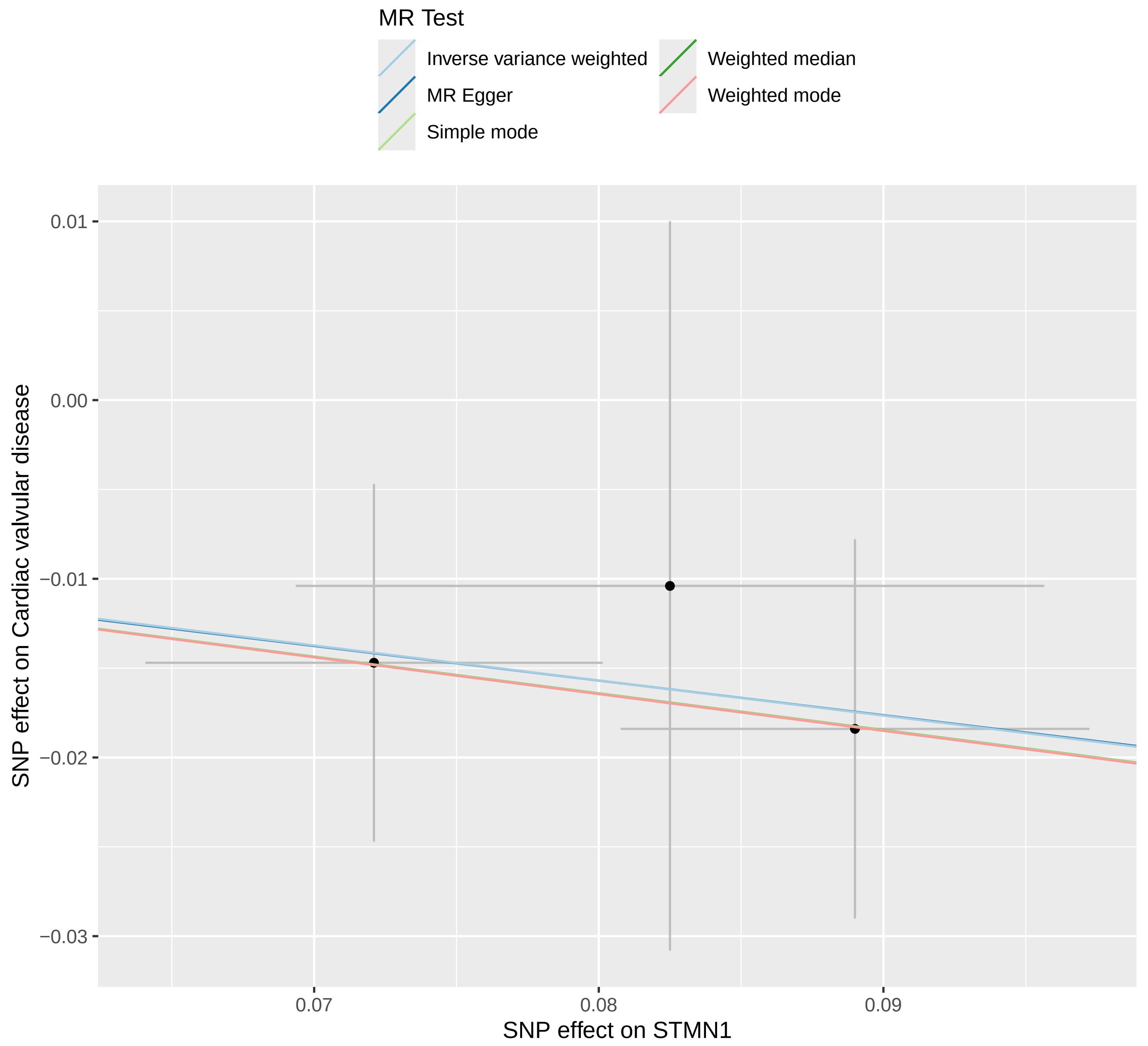
MR Test
Inverse variance weighted
MR Egger
Simple mode
Weighted median
Weighted mode
0.01
0.00
−0.01
−0.02
−0.03
0.07
0.08
0.09
SNP effect on Cardiac valvular disease
SNP effect on STMN1

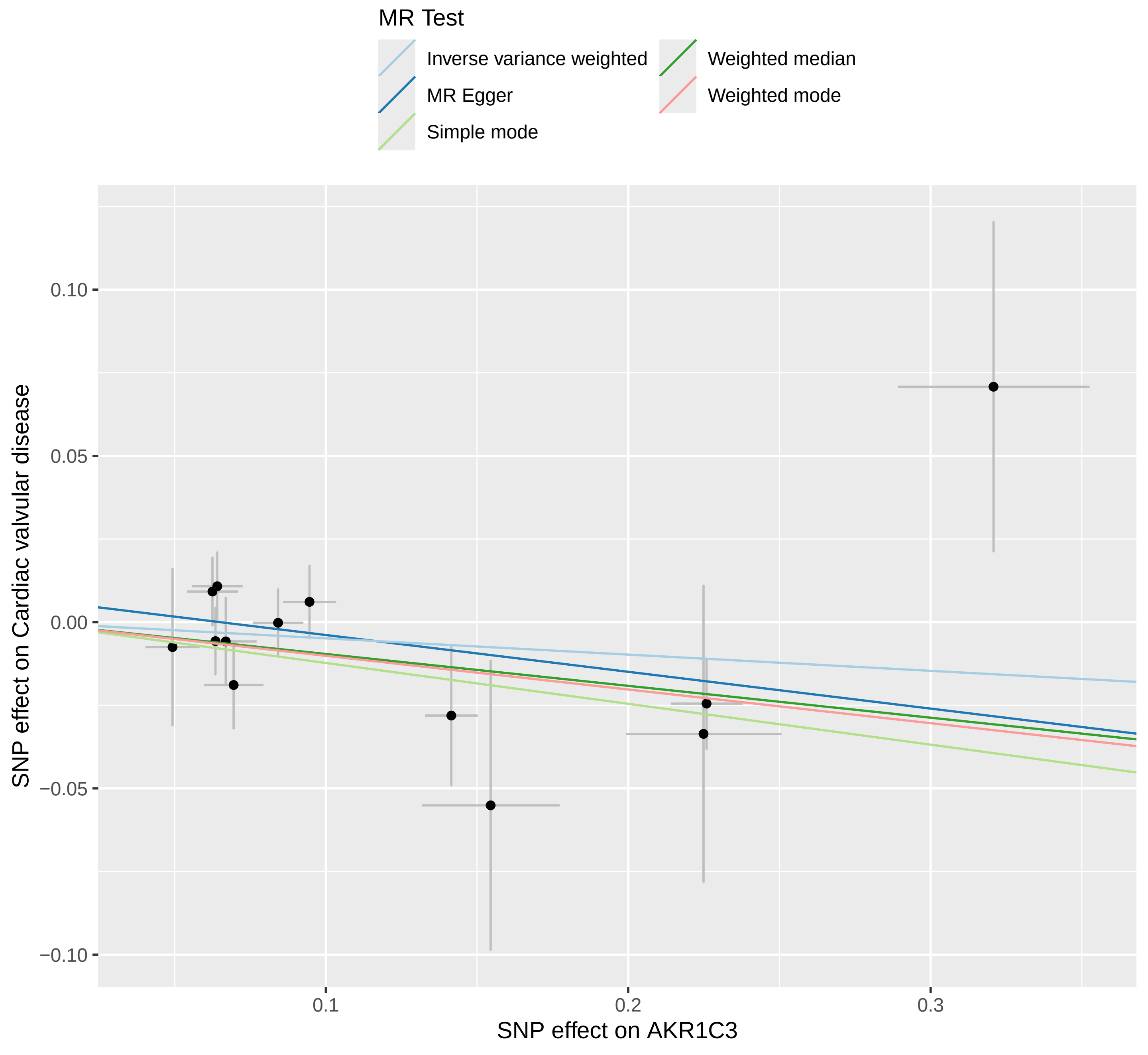
MR Test
Inverse variance weighted
MR Egger
Simple mode
Weighted median
Weighted mode
0.10
0.05
0.00
−0.05
−0.10
0.1
0.2
0.3
SNP effect on Cardiac valvular disease
SNP effect on AKR1C3

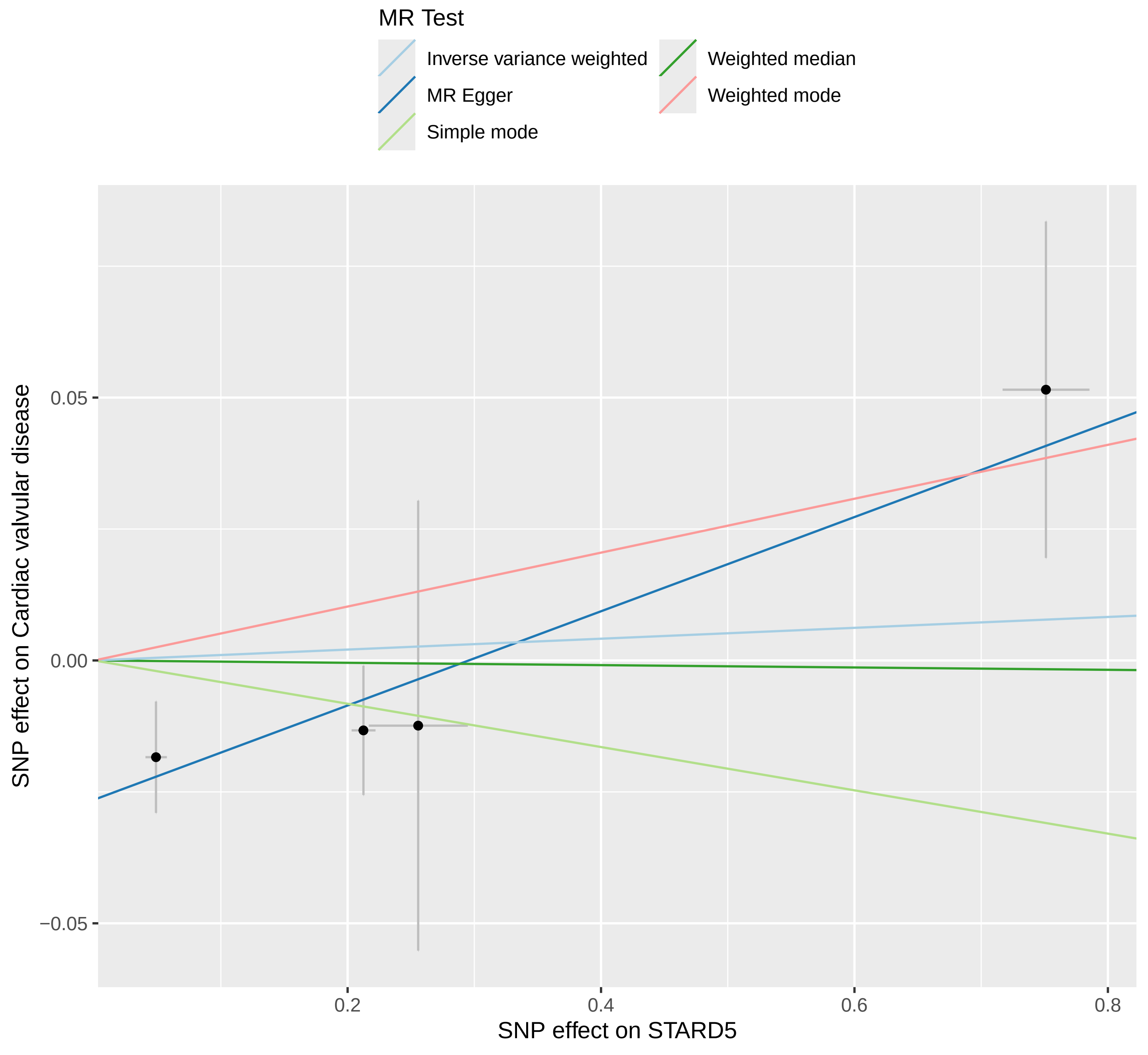
MR Test
Inverse variance weighted
MR Egger
Simple mode
Weighted median
Weighted mode
0.05
0.00
−0.05
SNP effect on Cardiac valvular disease
0.2
0.4
0.6
0.8
SNP effect on STARD5

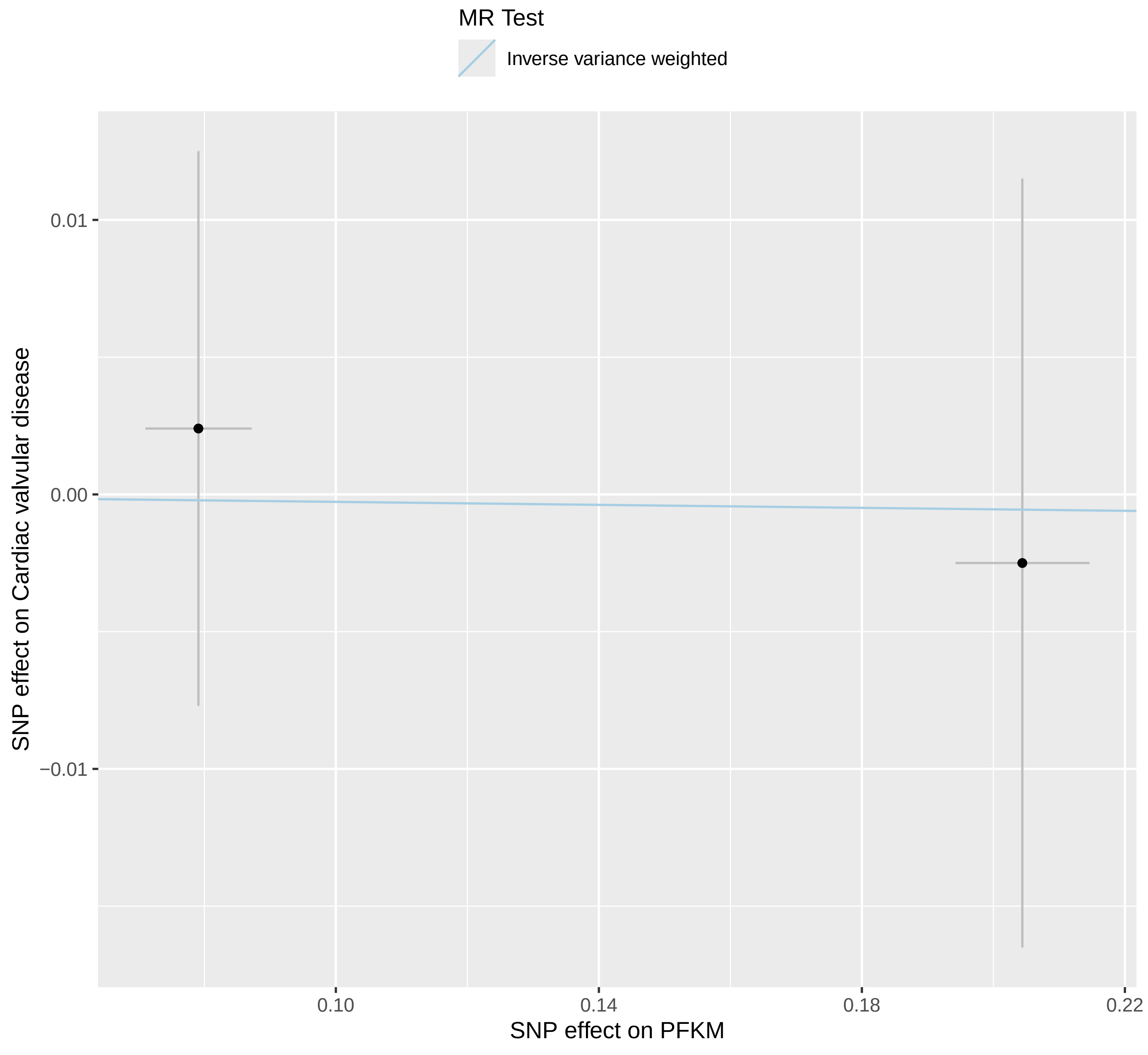
MR Test
Inverse variance weighted
0.01
0.00
−0.01
0.10
0.14
0.18
0.22
SNP effect on PFKM
SNP effect on Cardiac valvular disease

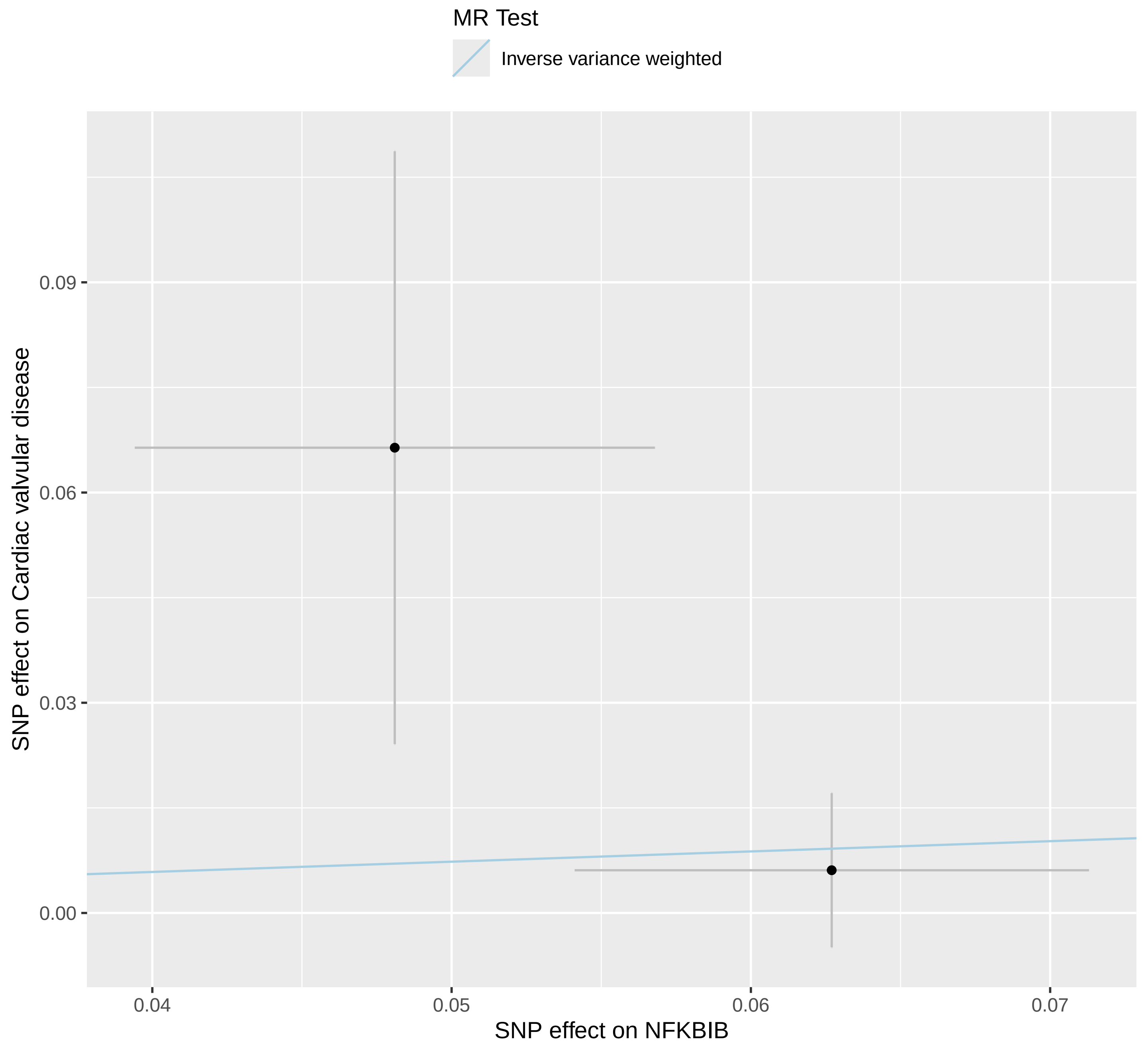
MR Test
Inverse variance weighted
0.09
0.06
0.03
0.00
SNP effect on Cardiac valvular disease
0.04
0.05
0.06
0.07
SNP effect on NFKBIB

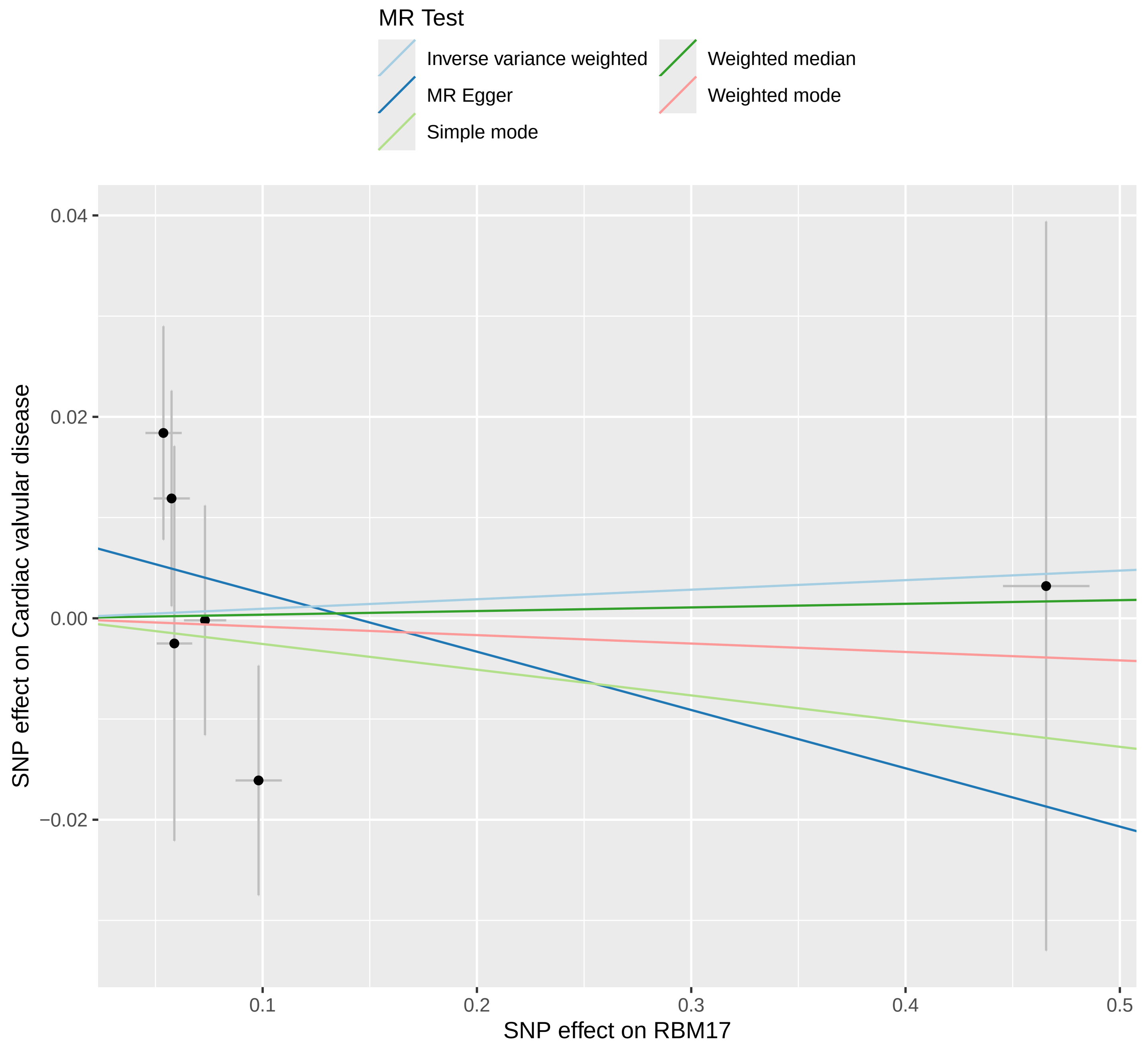

MR Test
Inverse variance weighted
MR Egger
Simple mode
Weighted median
Weighted mode
0.04
0.02
0.00
−0.02
0.1
0.2
0.3
0.4
0.5
SNP effect on Cardiac valvular disease
SNP effect on RBM17

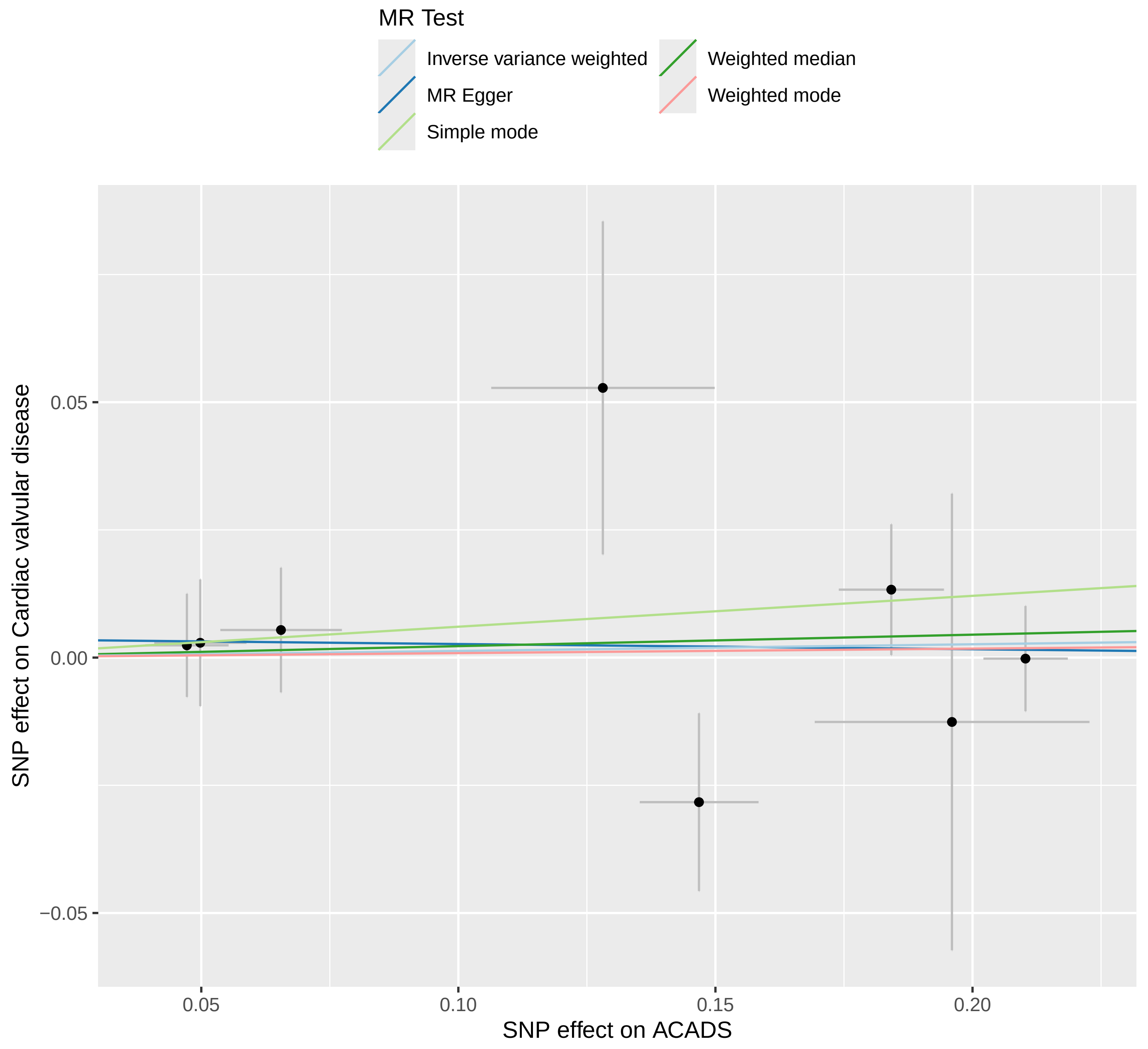
MR Test
Inverse variance weighted
MR Egger
Simple mode
Weighted median
Weighted mode
SNP effect on Cardiac valvular disease
0.05
0.00
−0.05
0.05
0.10
0.15
0.20
SNP effect on ACADS

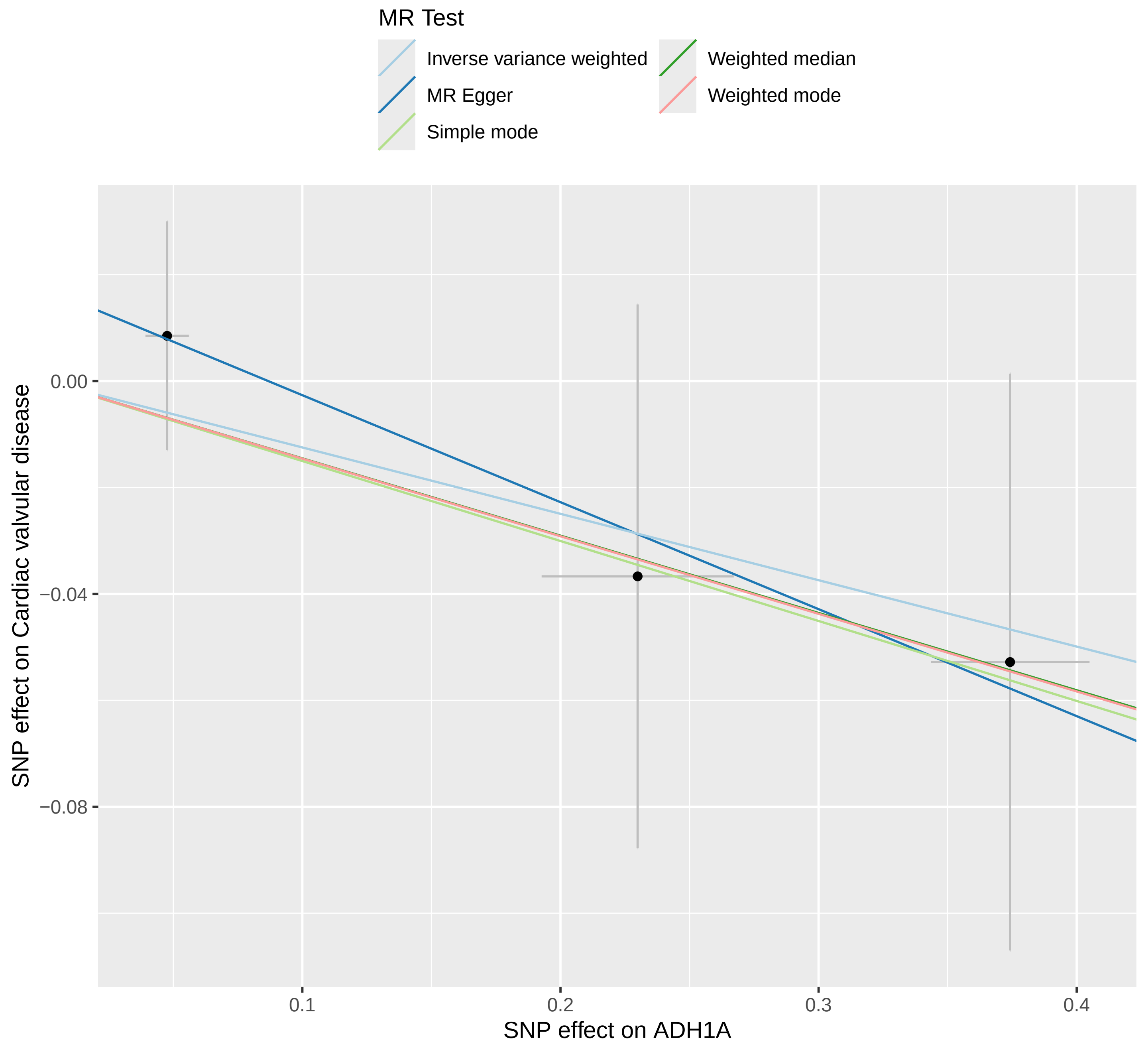
MR Test
Inverse variance weighted
MR Egger
Simple mode
Weighted median
Weighted mode
0.00
−0.04
−0.08
0.1
0.2
0.3
0.4
SNP effect on Cardiac valvular disease
SNP effect on ADH1A

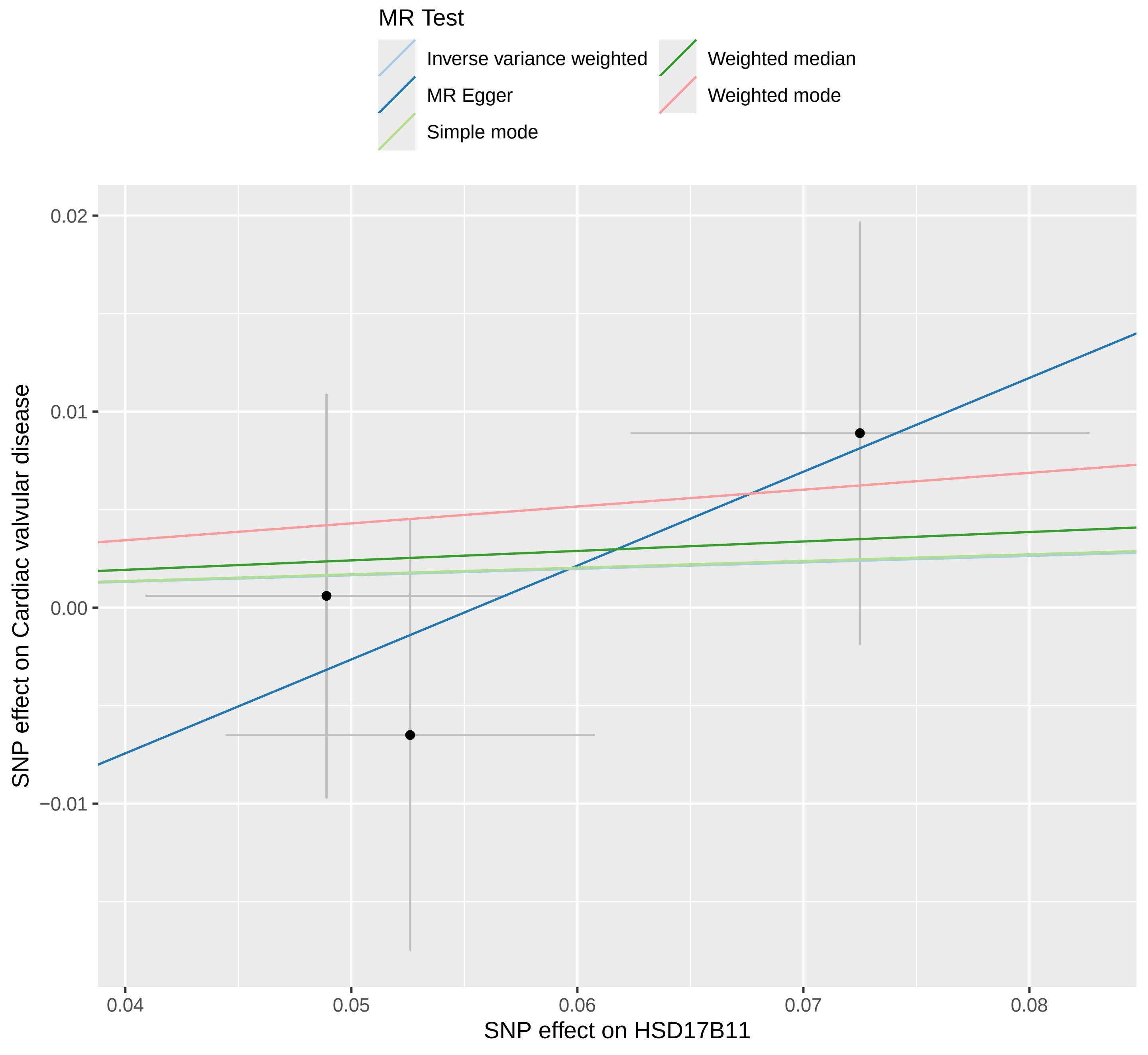

MR Test
Inverse variance weighted
MR Egger
Simple mode
Weighted median
Weighted mode
SNP effect on Cardiac valvular disease
0.02
0.01
0.00
−0.01
0.04
0.05
0.06
0.07
0.08
SNP effect on HSD17B11

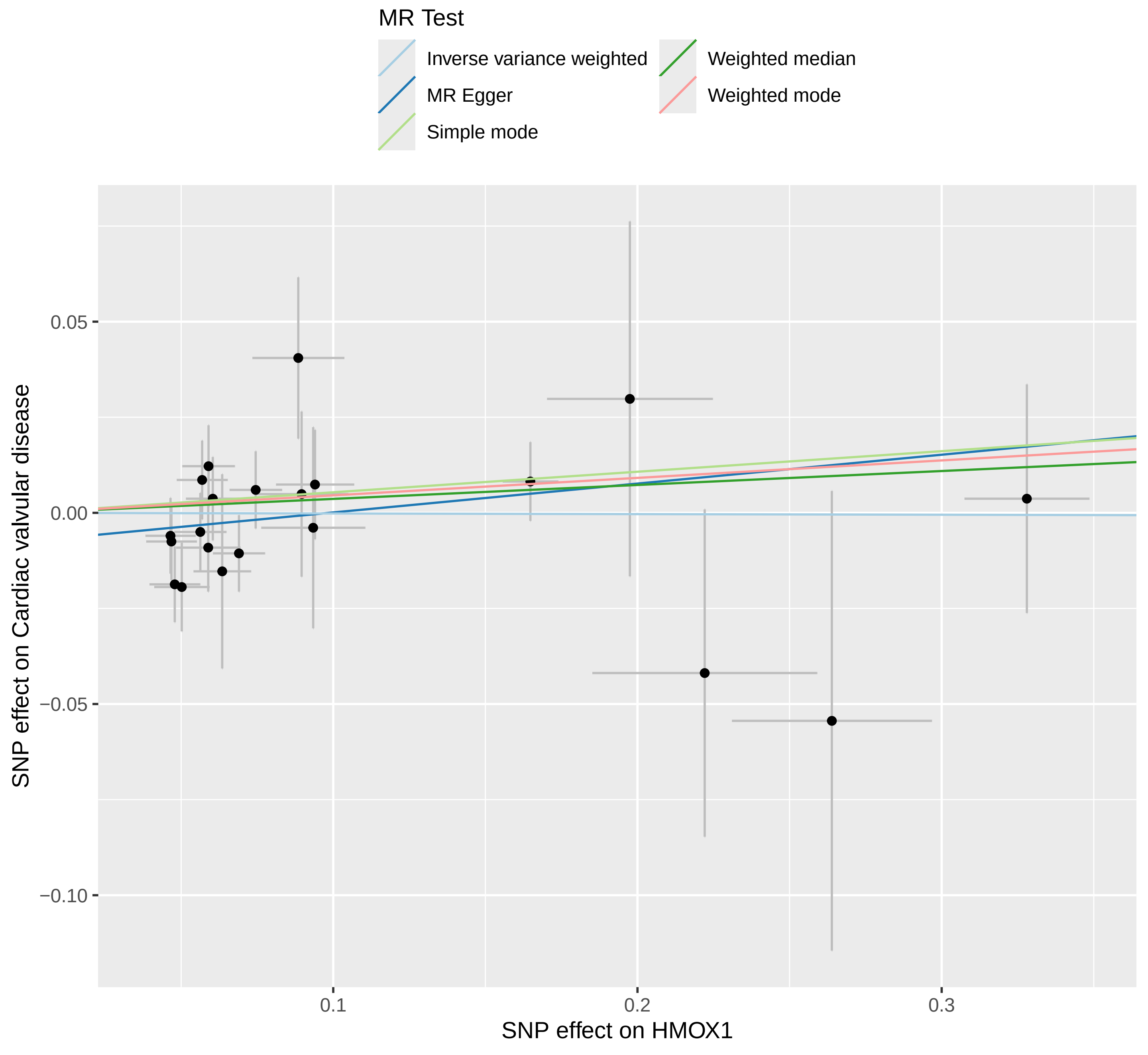

MR Test
Inverse variance weighted
MR Egger
Simple mode
Weighted median
Weighted mode
0.05
0.00
−0.05
−0.10
0.1
0.2
0.3
SNP effect on Cardiac valvular disease
SNP effect on HMOX1

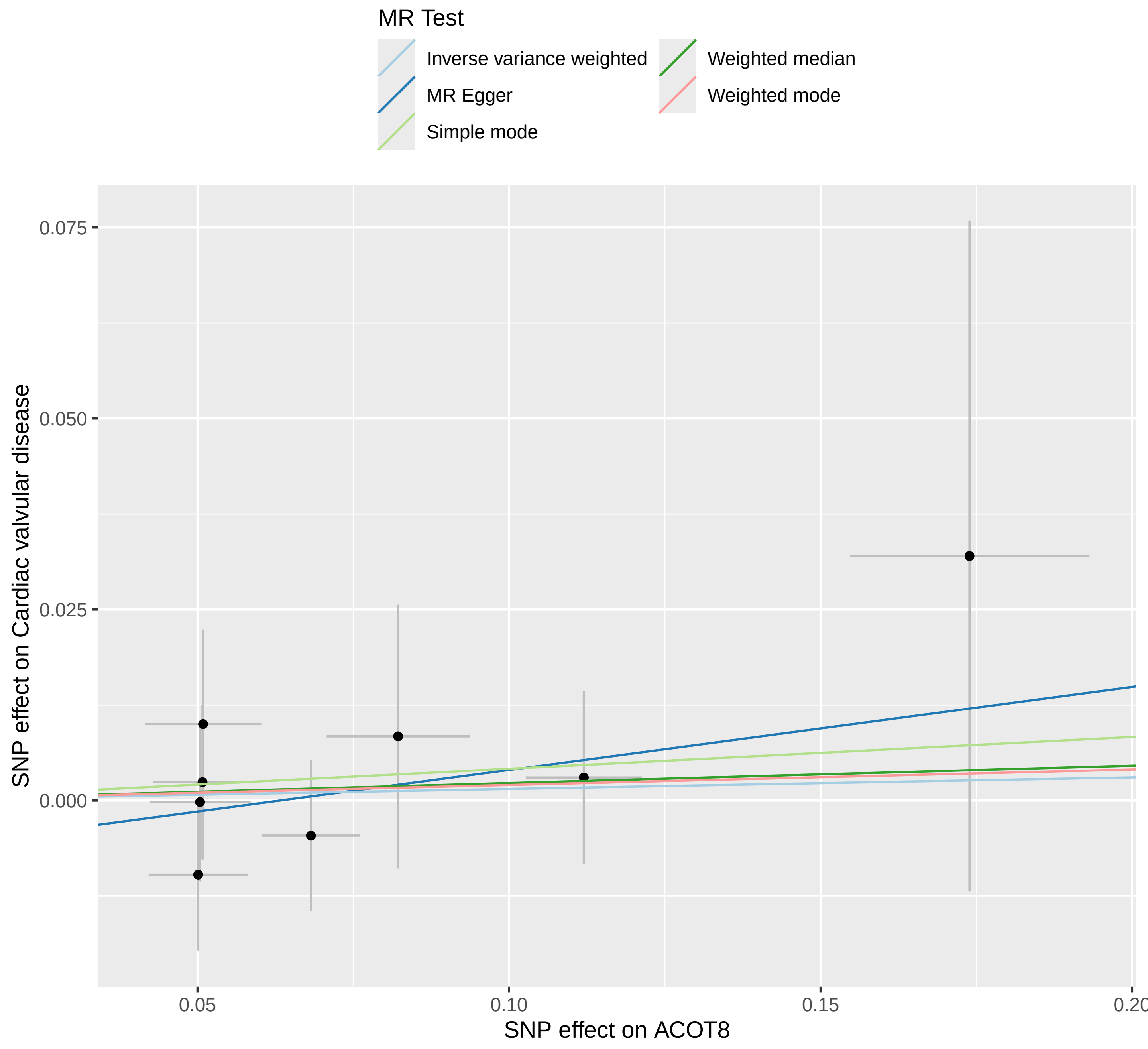
MR Test
Inverse variance weighted
MR Egger
Simple mode
Weighted median
Weighted mode
0.075
0.050
0.025
0.000
0.05
0.10
0.15
0.20
SNP effect on Cardiac valvular disease
SNP effect on ACOT8

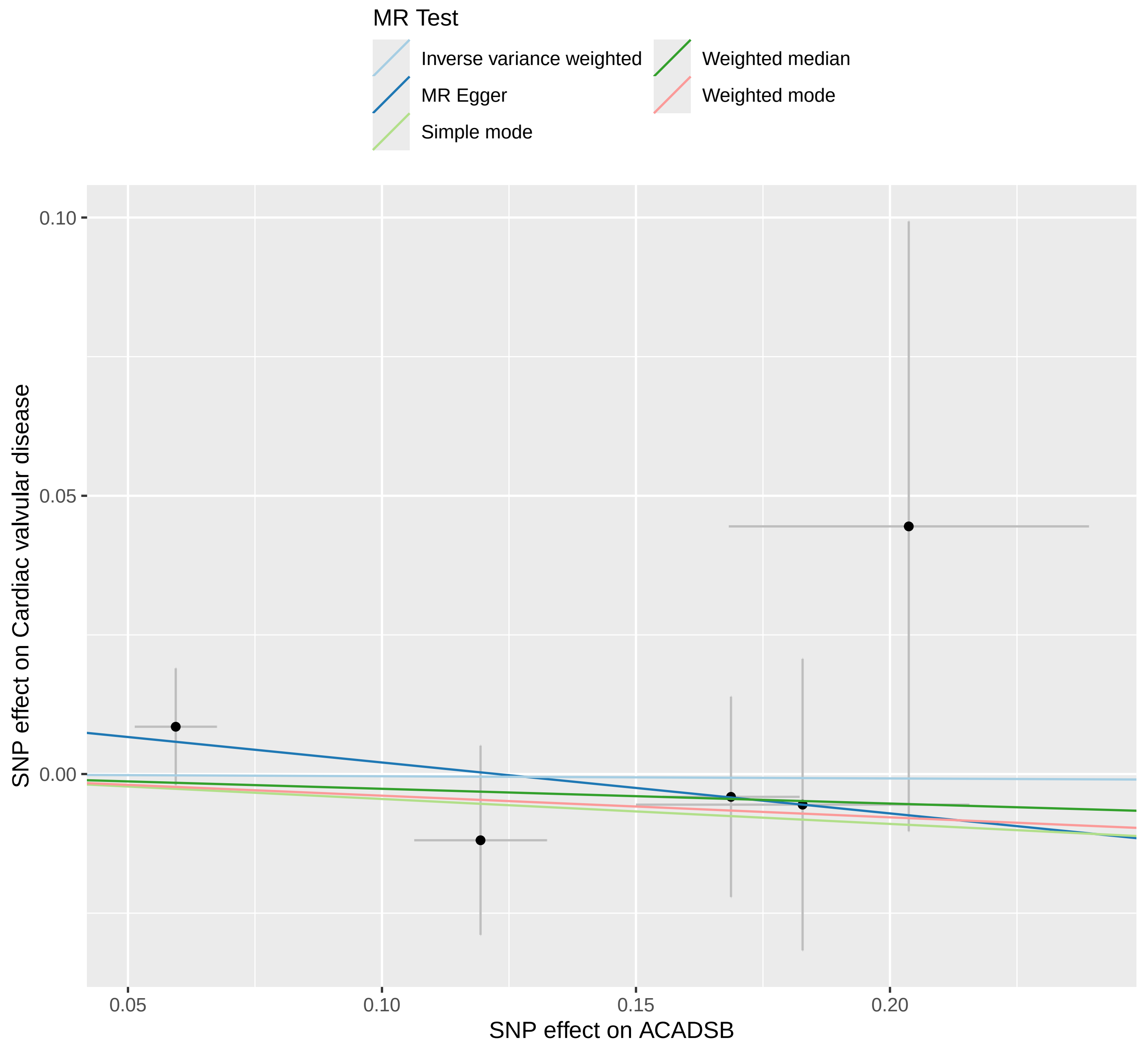

MR Test
Inverse variance weighted
MR Egger
Simple mode
Weighted median
Weighted mode
0.10
0.05
0.00
SNP effect on Cardiac valvular disease
0.05
0.10
0.15
0.20
SNP effect on ACADSB

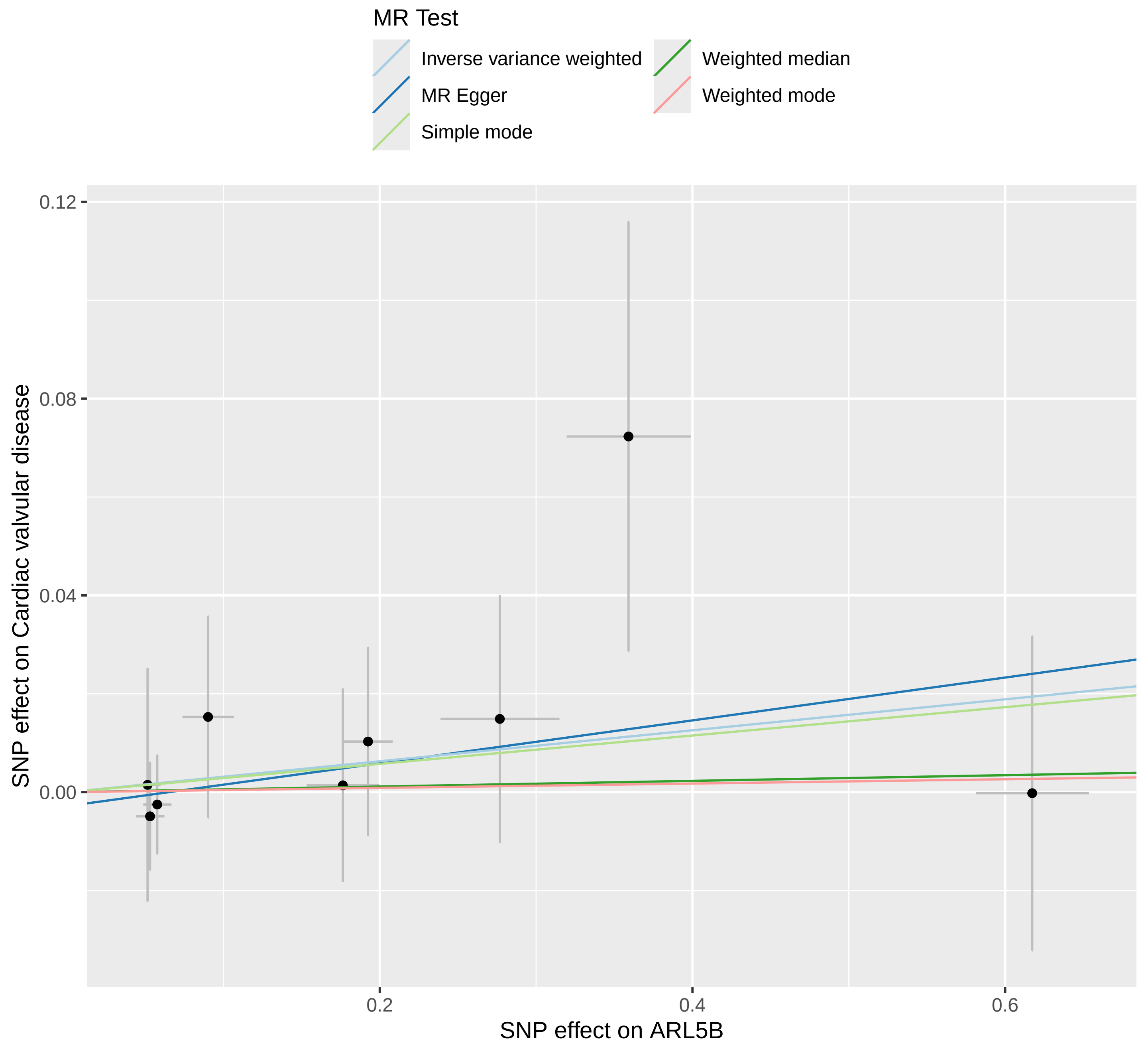

MR Test
Inverse variance weighted
MR Egger
Simple mode
Weighted median
Weighted mode
0.12
0.08
0.04
0.00
0.2
0.4
0.6
SNP effect on Cardiac valvular disease
SNP effect on ARL5B

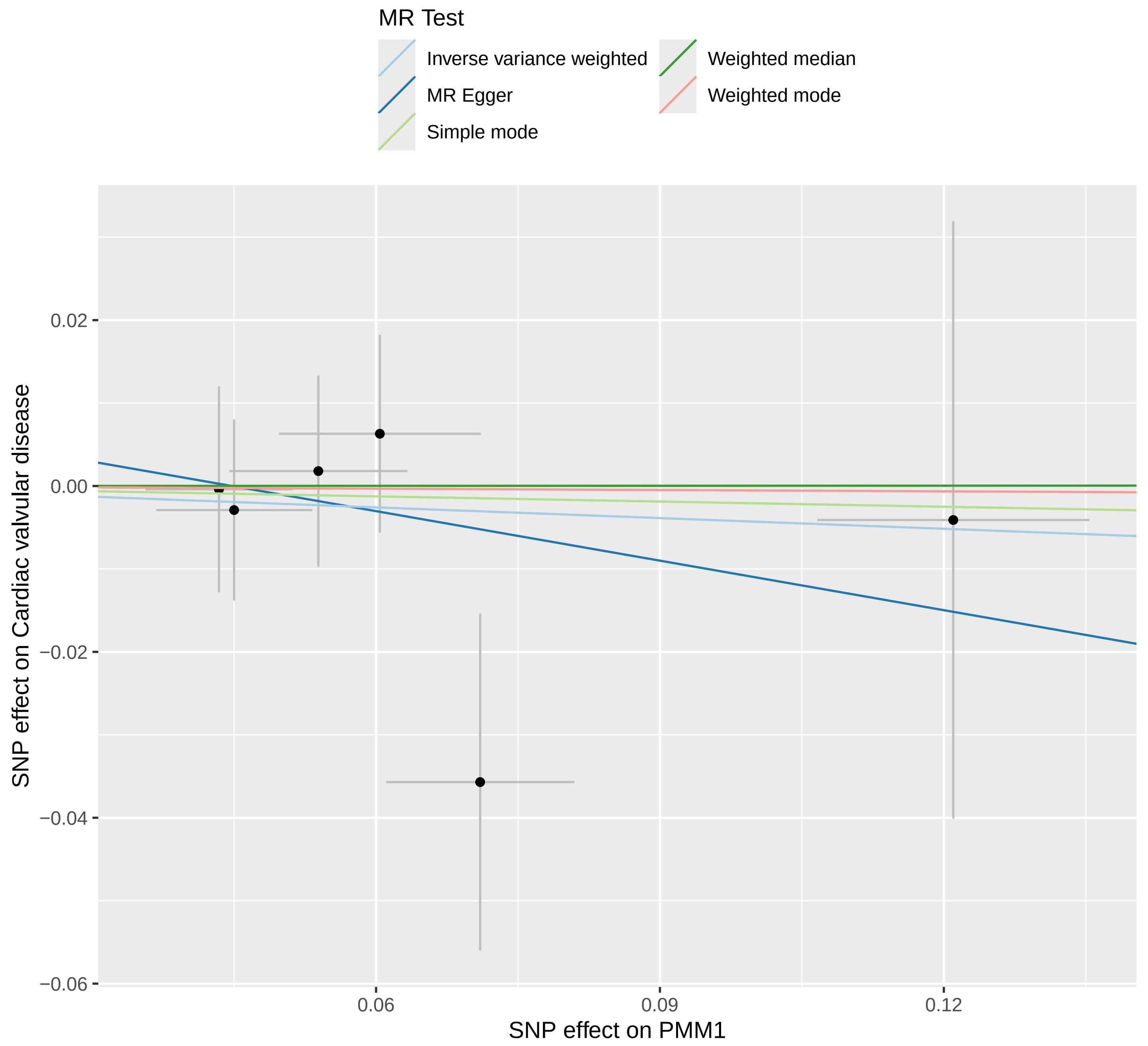

MR Test
Inverse variance weighted
MR Egger
Simple mode
Weighted median
Weighted mode
0.02
0.00
−0.02
−0.04
−0.06
SNP effect on Cardiac valvular disease
0.06
0.09
0.12
SNP effect on PMM1

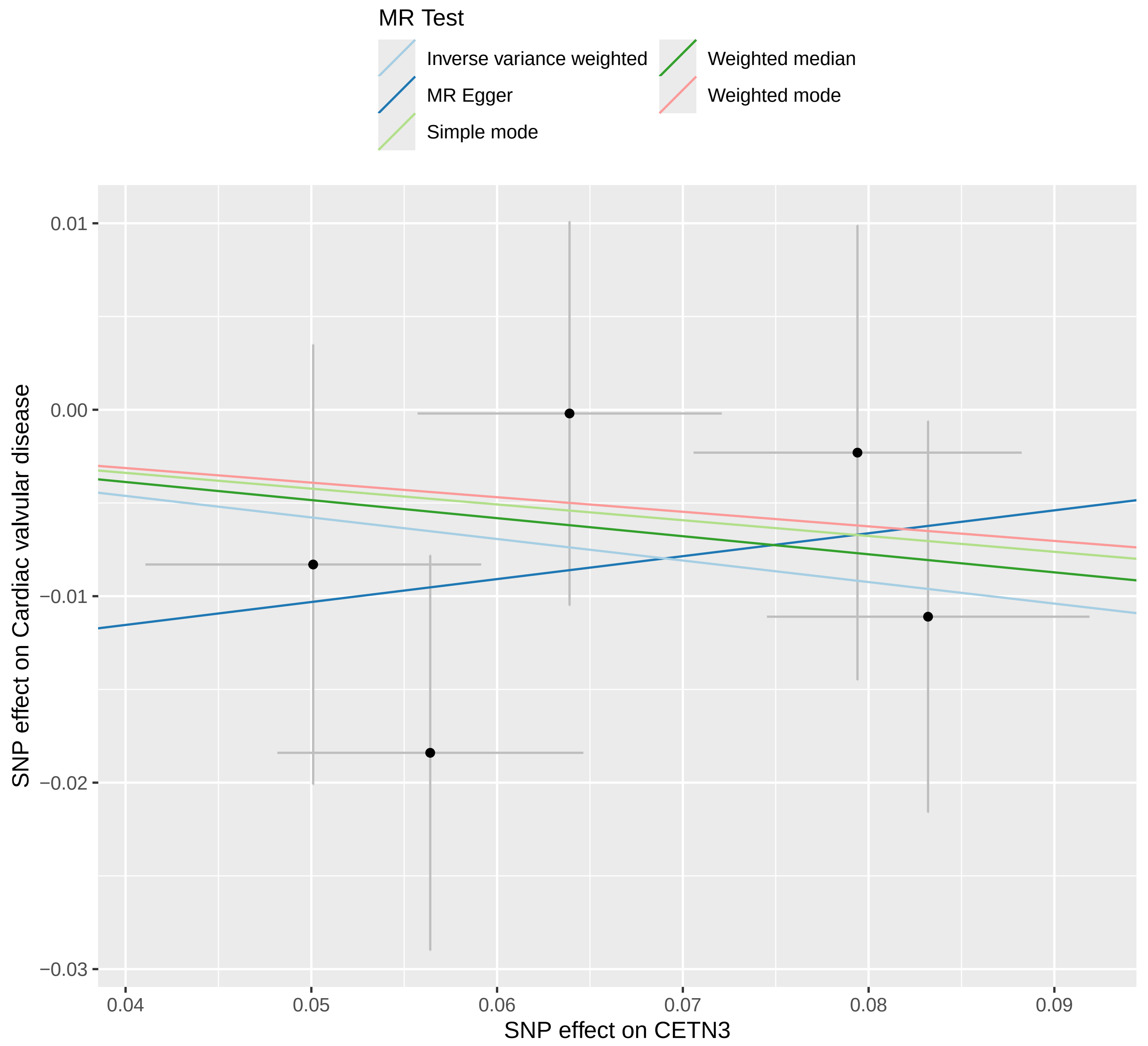
MR Test
Inverse variance weighted
MR Egger
Simple mode
Weighted median
Weighted mode
0.01
0.00
−0.01
−0.02
−0.03
SNP effect on Cardiac valvular disease
0.04
0.05
0.06
0.07
0.08
0.09
SNP effect on CETN3

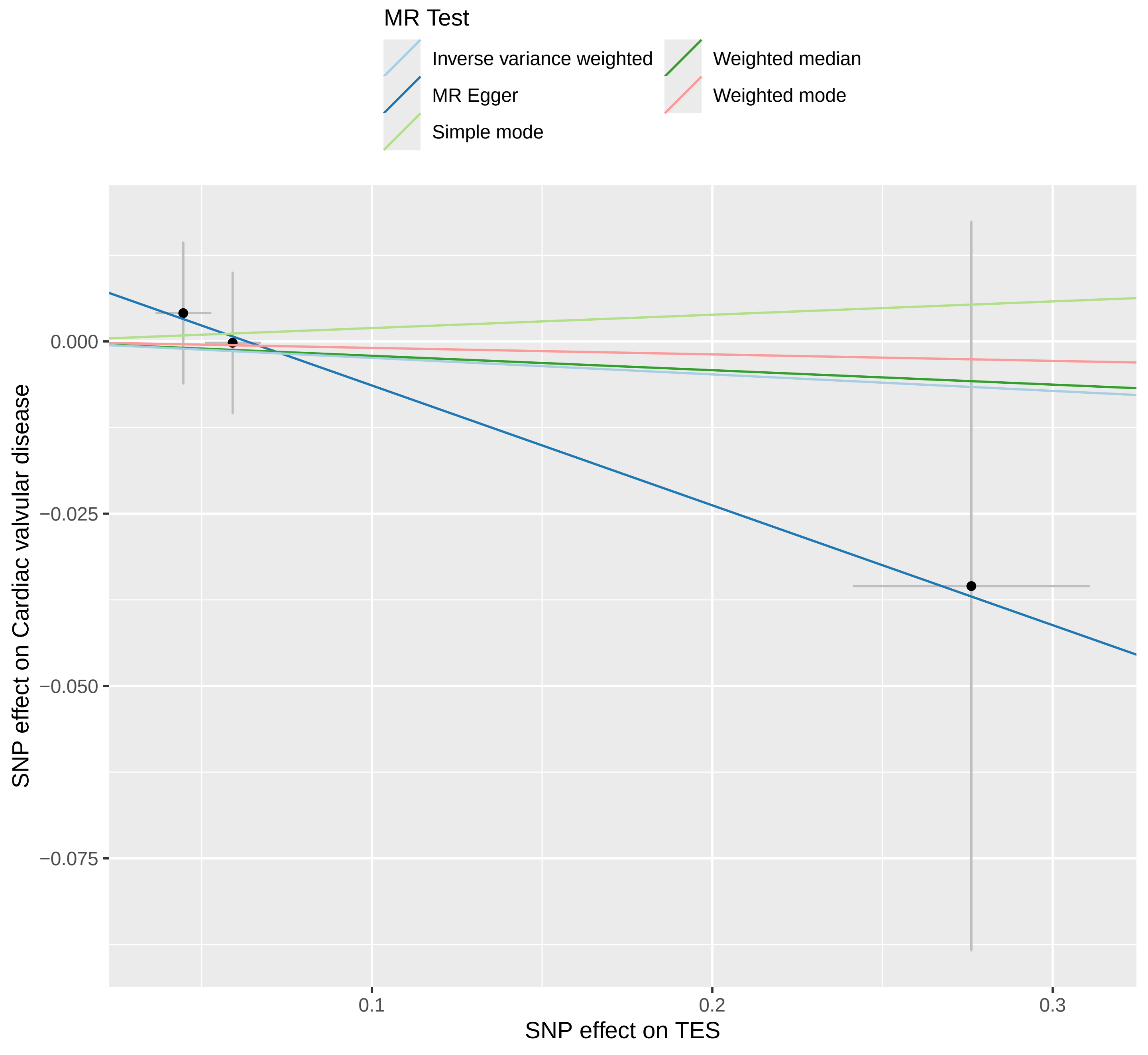

MR Test
Inverse variance weighted
MR Egger
Simple mode
Weighted median
Weighted mode
0.000
−0.025
−0.050
−0.075
0.1
0.2
0.3
SNP effect on Cardiac valvular disease
SNP effect on TES

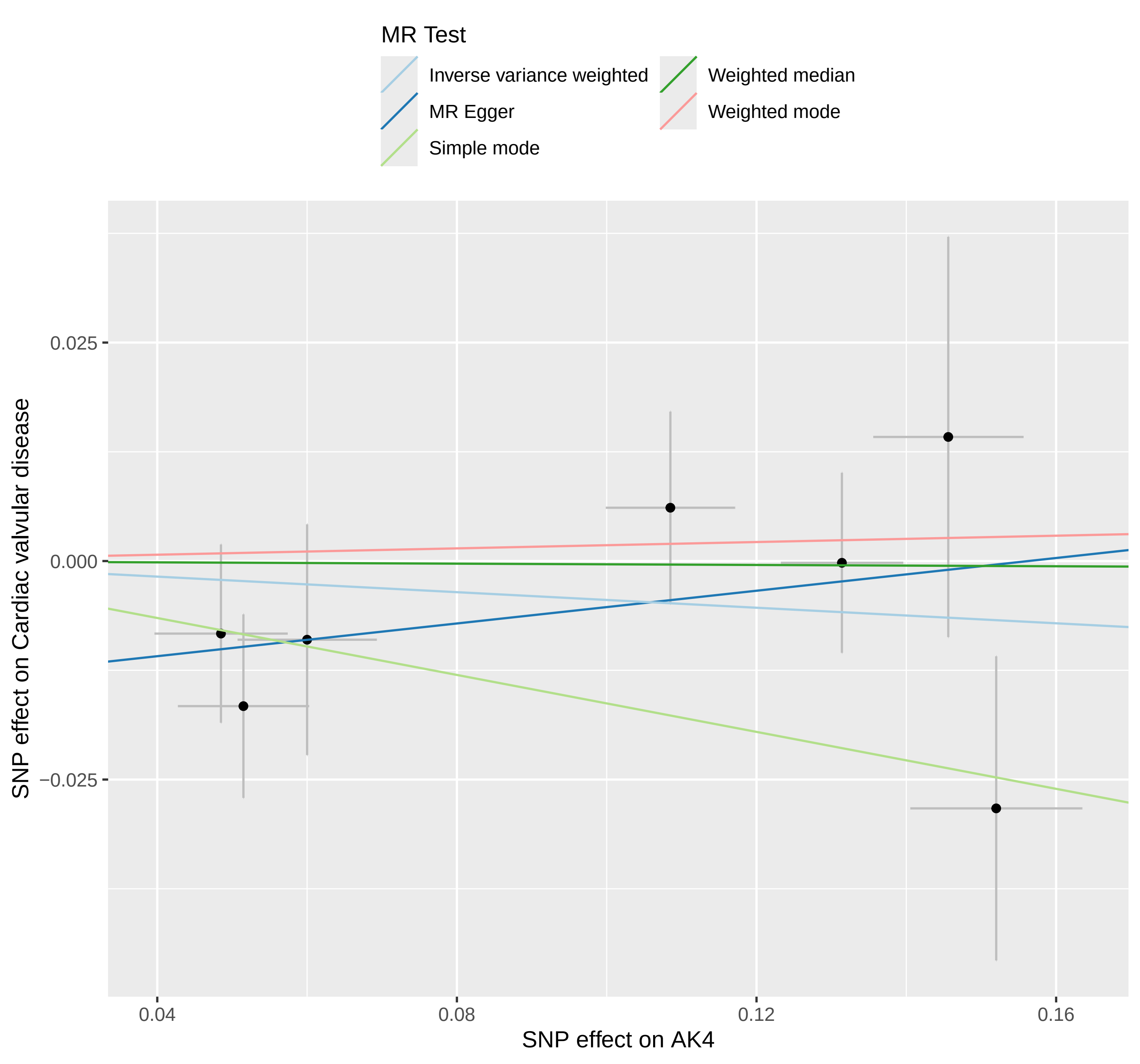

MR Test
Inverse variance weighted
MR Egger
Simple mode
Weighted median
Weighted mode
0.025
0.000
−0.025
SNP effect on Cardiac valvular disease
0.04
0.08
0.12
0.16
SNP effect on AK4

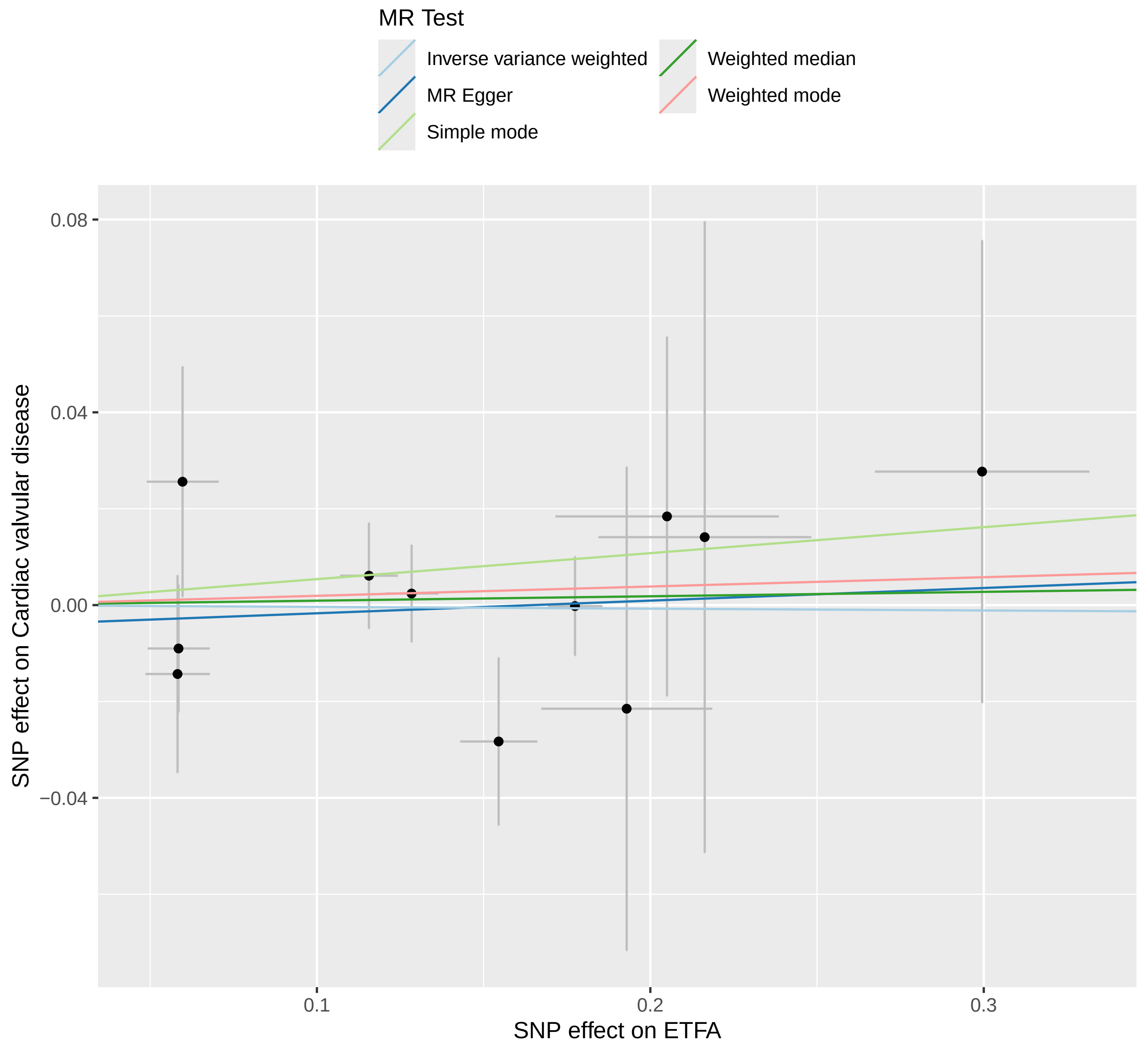
MR Test
Inverse variance weighted
MR Egger
Simple mode
Weighted median
Weighted mode
0.08
0.04
0.00
−0.04
0.1
0.2
0.3
SNP effect on Cardiac valvular disease
SNP effect on ETFA

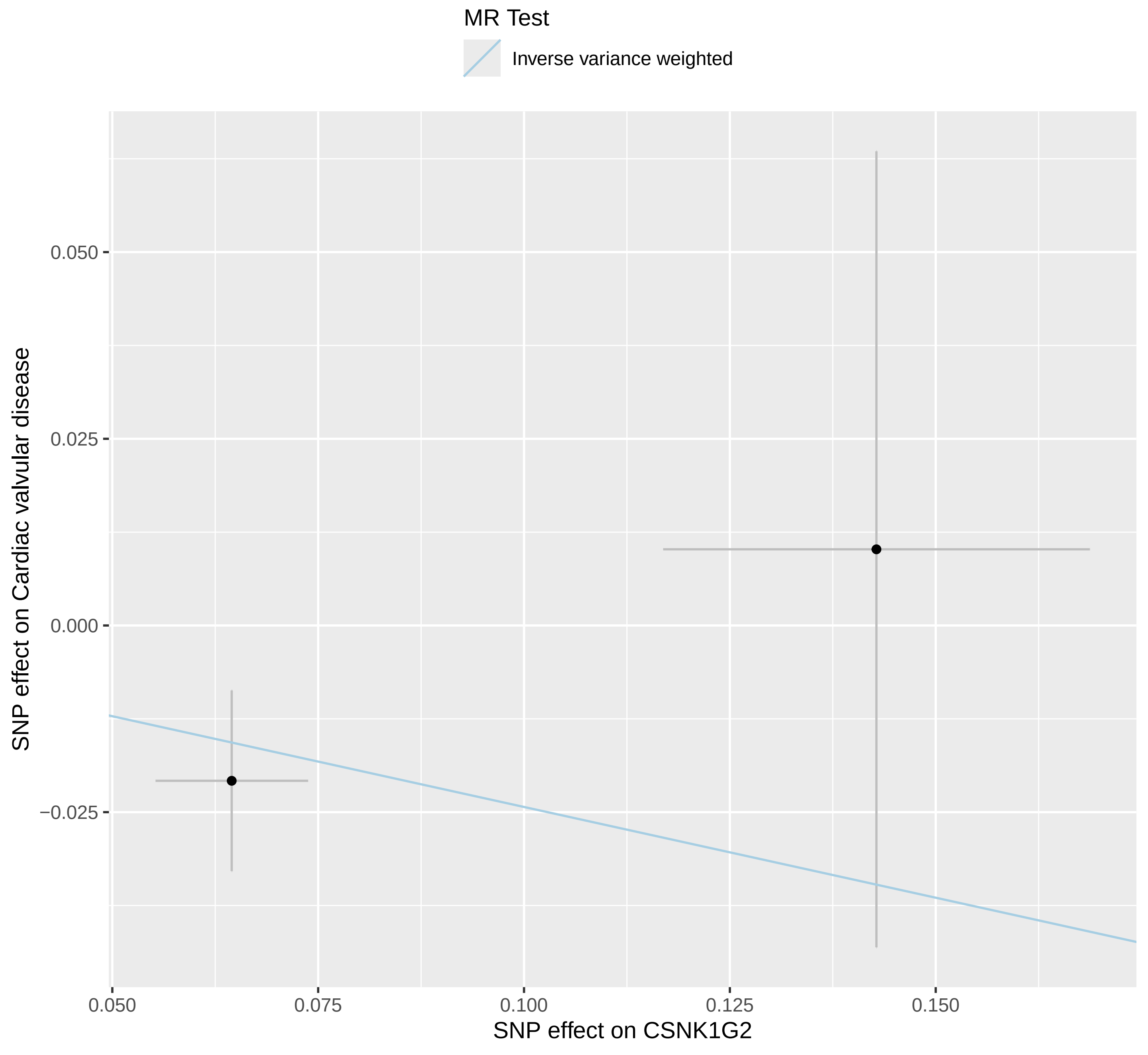

MR Test
Inverse variance weighted
SNP effect on Cardiac valvular disease
0.050
0.025
0.000
−0.025
0.050
0.075
0.100
0.125
0.150
SNP effect on CSNK1G2

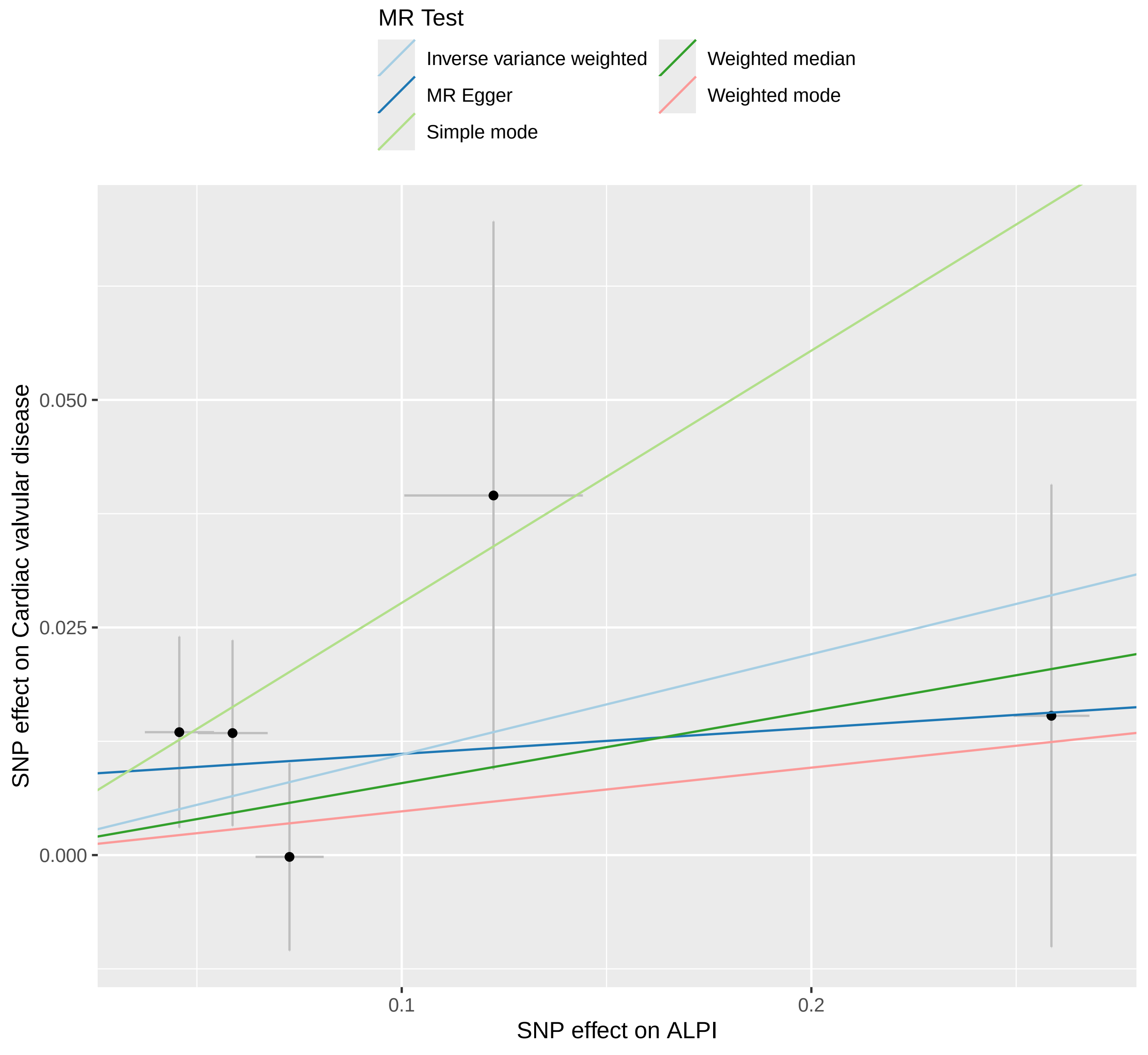
MR Test
Inverse variance weighted
MR Egger
Simple mode
Weighted median
Weighted mode
0.050
0.025
0.000
0.1
0.2
SNP effect on Cardiac valvular disease
SNP effect on ALPI

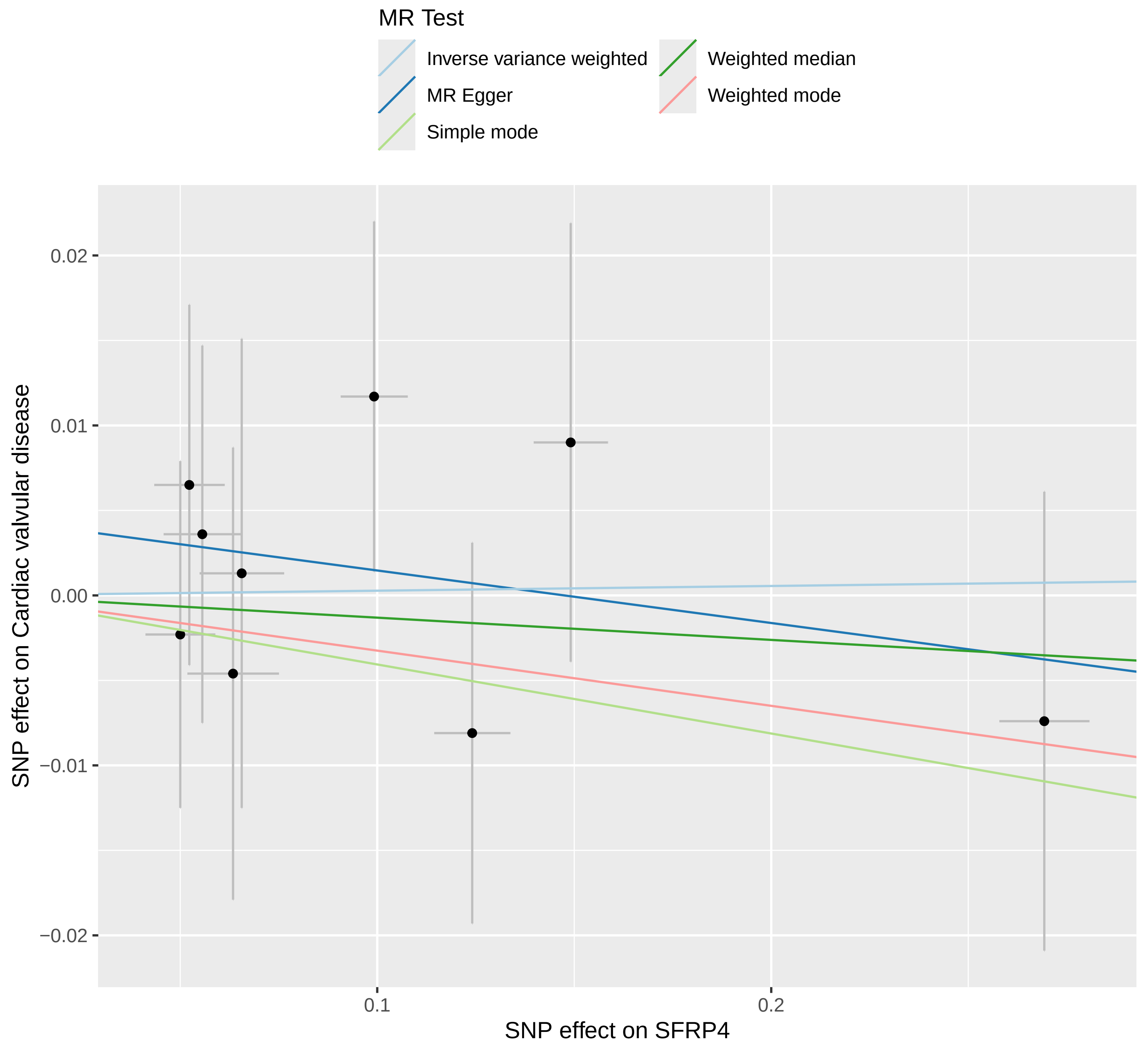
MR Test
Inverse variance weighted
MR Egger
Simple mode
Weighted median
Weighted mode
0.02
0.01
0.00
−0.01
−0.02
SNP effect on Cardiac valvular disease
0.1
0.2
SNP effect on SFRP4

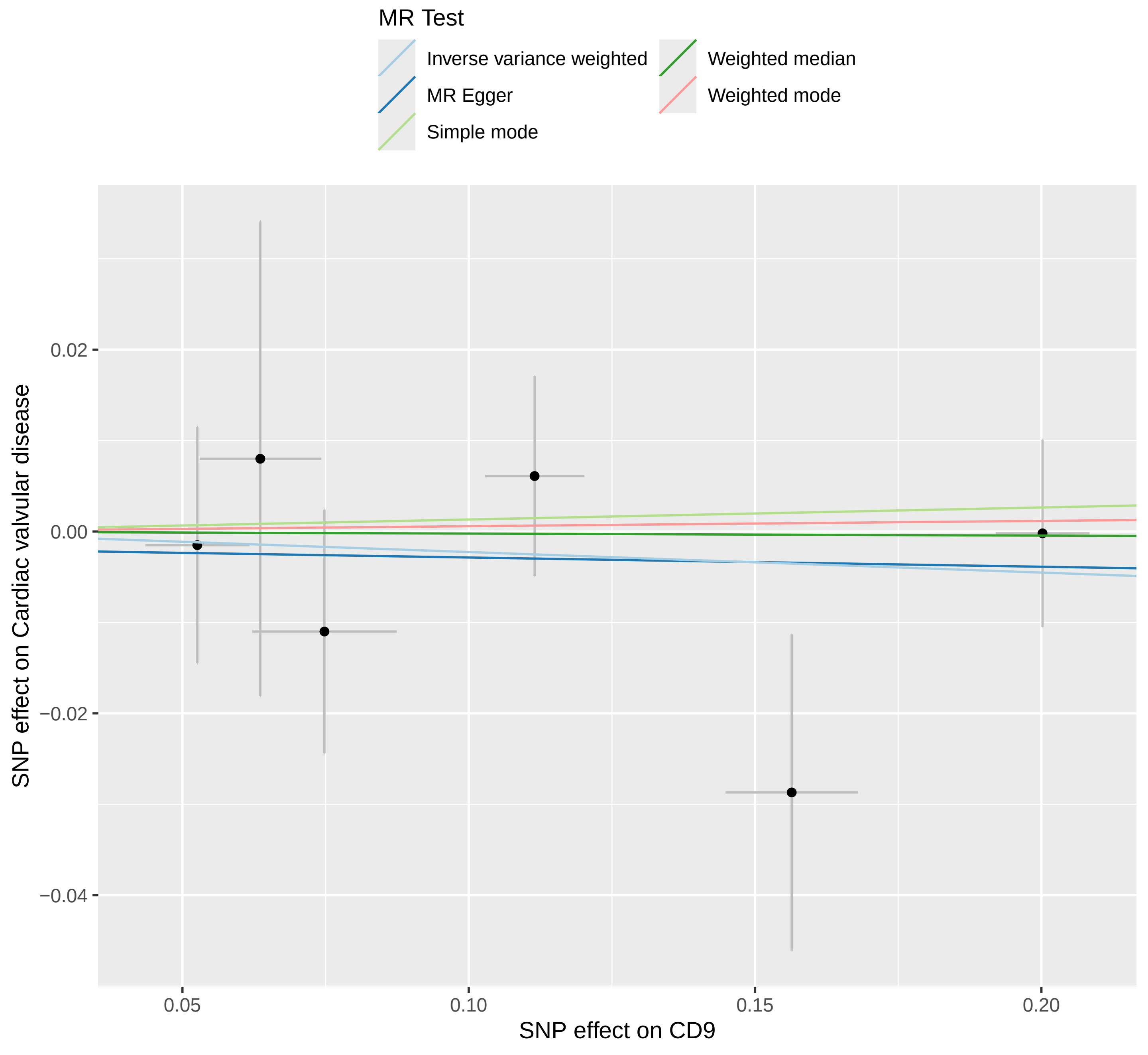

MR Test
Inverse variance weighted
MR Egger
Simple mode
Weighted median
Weighted mode
0.02
0.00
−0.02
−0.04
SNP effect on Cardiac valvular disease
0.05
0.10
0.15
0.20
SNP effect on CD9

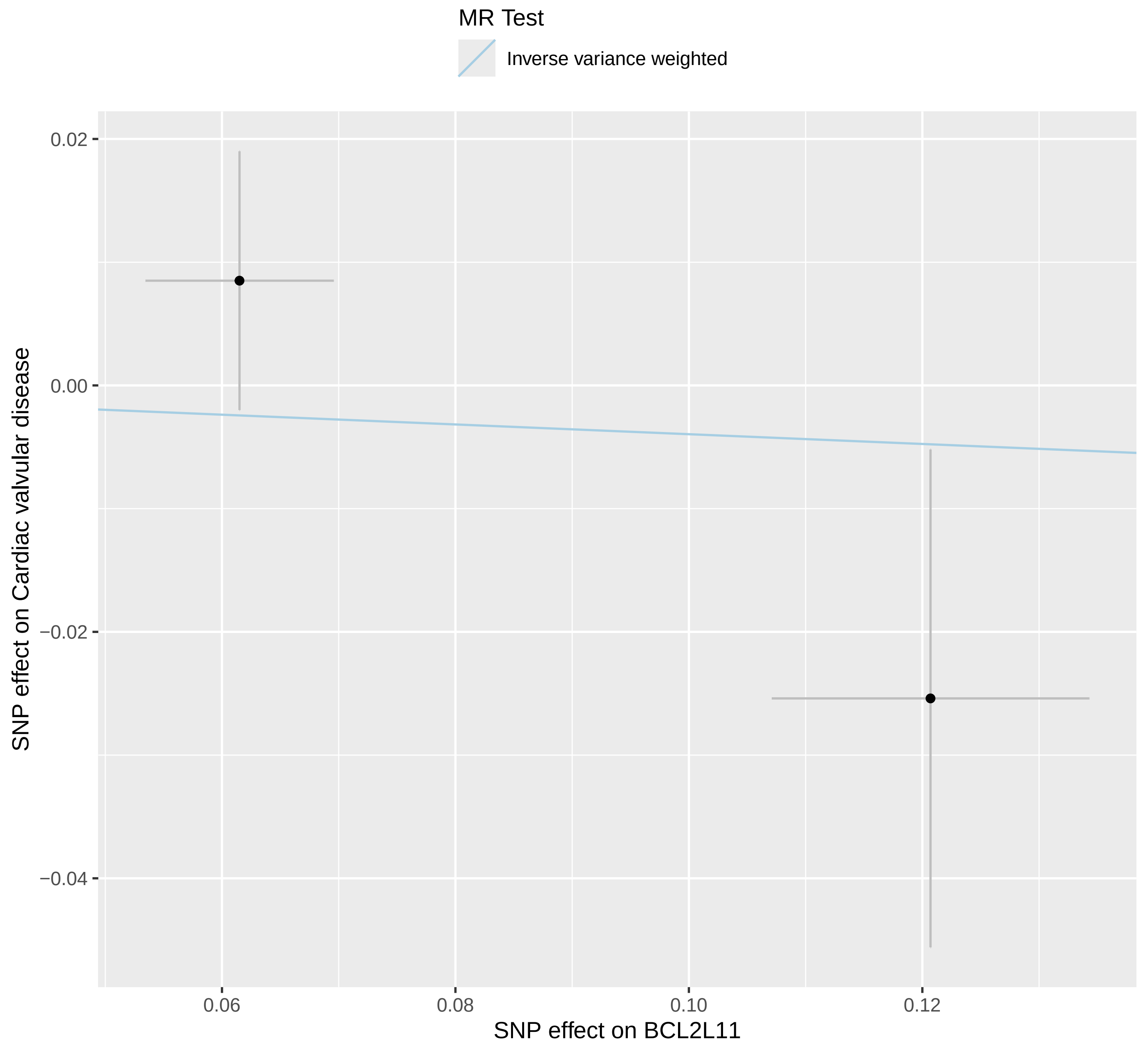
MR Test
Inverse variance weighted
0.02
0.00
−0.02
−0.04
SNP effect on Cardiac valvular disease
0.06
0.08
0.10
0.12
SNP effect on BCL2L11

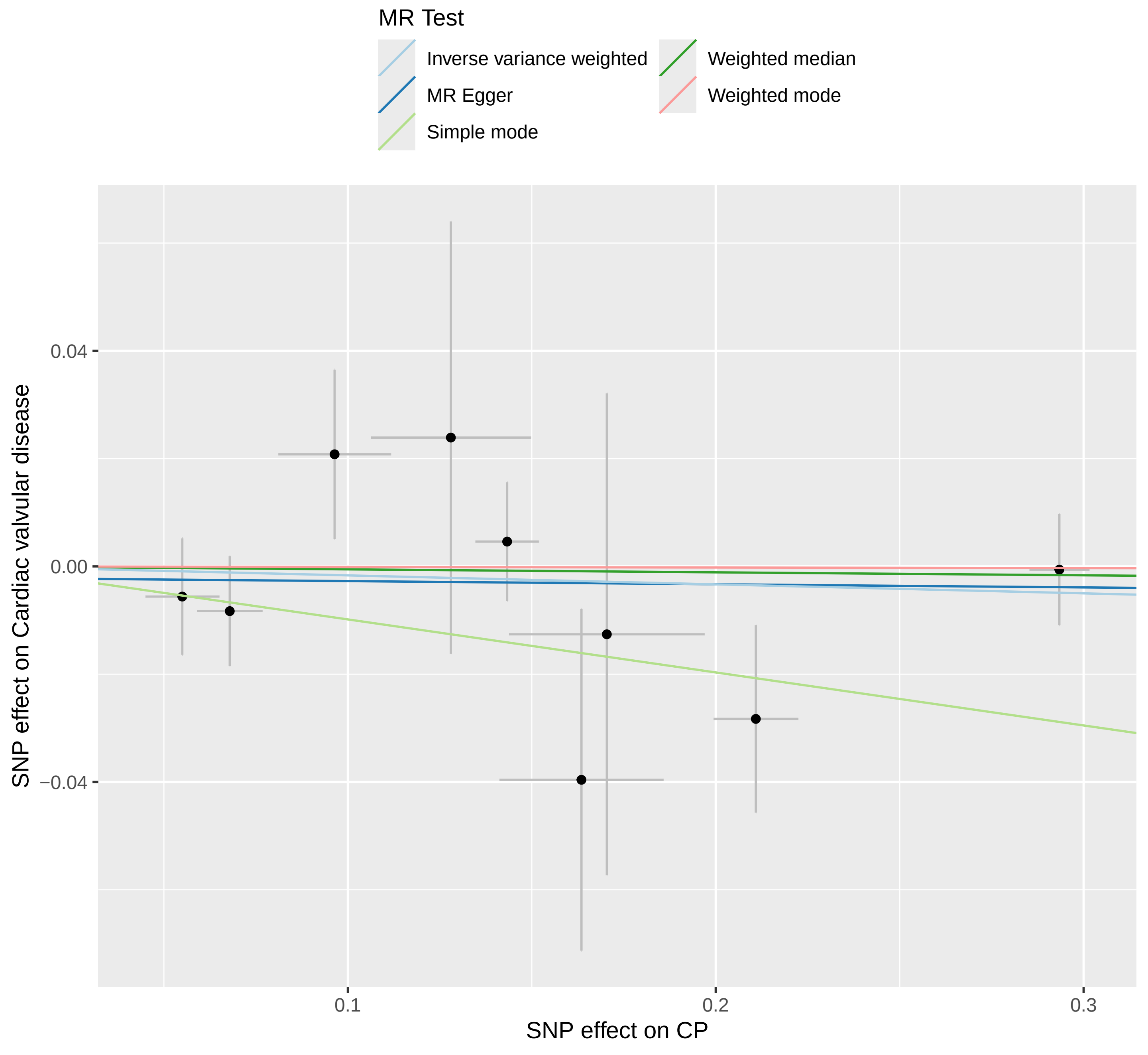

MR Test
Inverse variance weighted
MR Egger
Simple mode
Weighted median
Weighted mode
0.04
0.00
−0.04
0.1
0.2
0.3
SNP effect on Cardiac valvular disease
SNP effect on CP

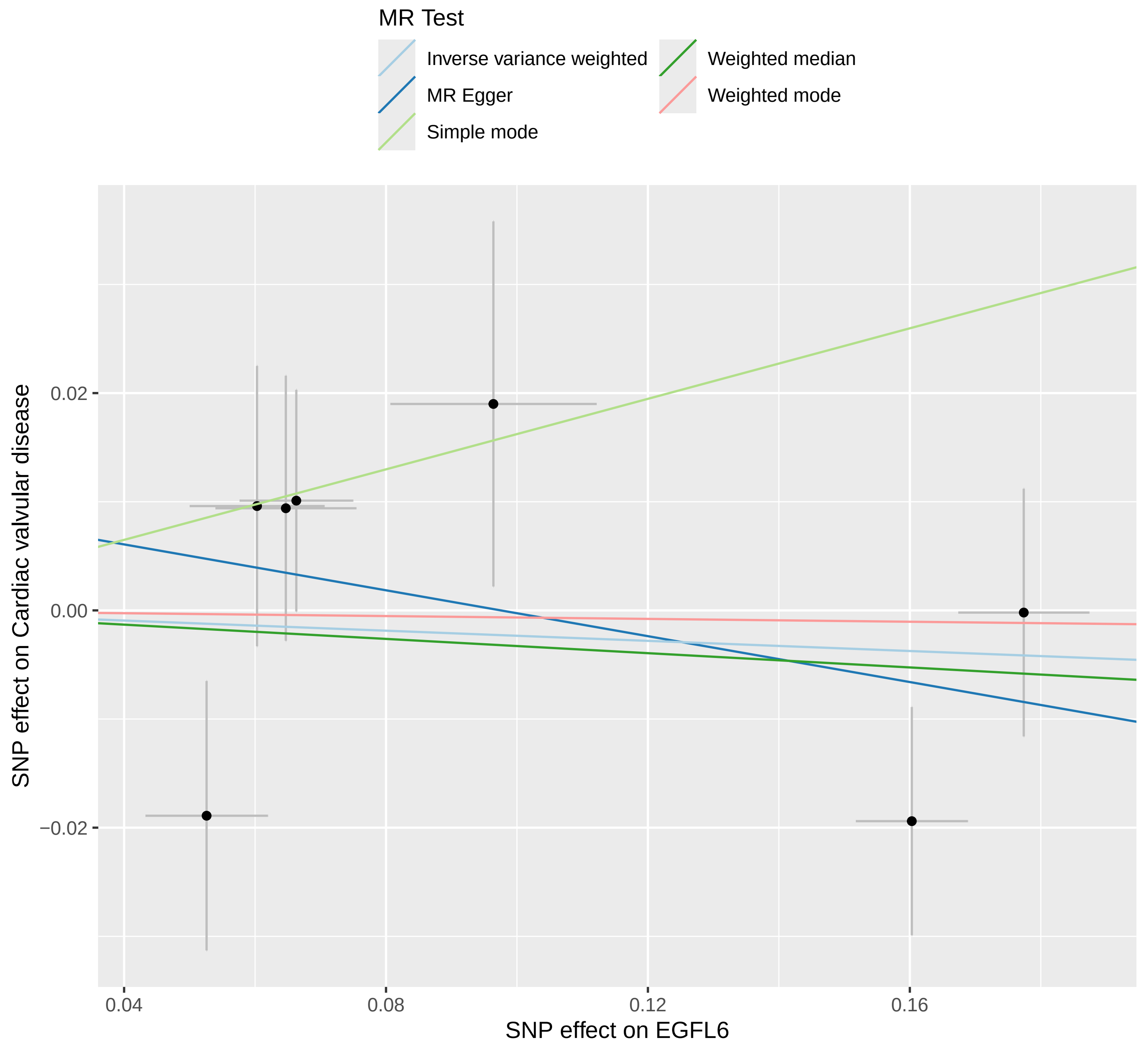
MR Test
Inverse variance weighted
MR Egger
Simple mode
Weighted median
Weighted mode
0.02
0.00
−0.02
0.04
0.08
0.12
0.16
SNP effect on Cardiac valvular disease
SNP effect on EGFL6

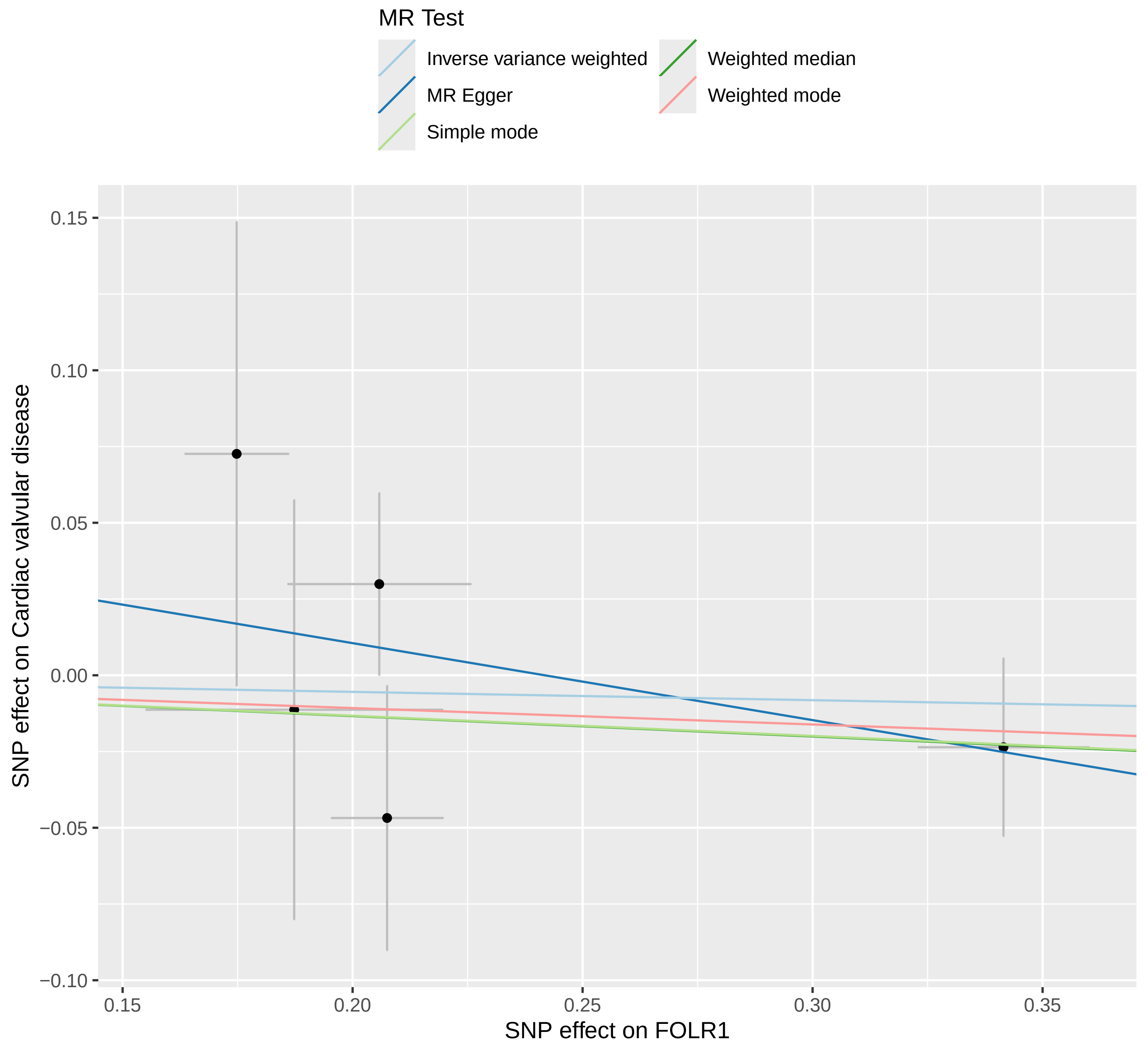
MR Test
Inverse variance weighted
MR Egger
Simple mode
Weighted median
Weighted mode
0.15
0.10
0.05
0.00
−0.05
−0.10
SNP effect on Cardiac valvular disease
0.15
0.20
0.25
0.30
0.35
SNP effect on FOLR1

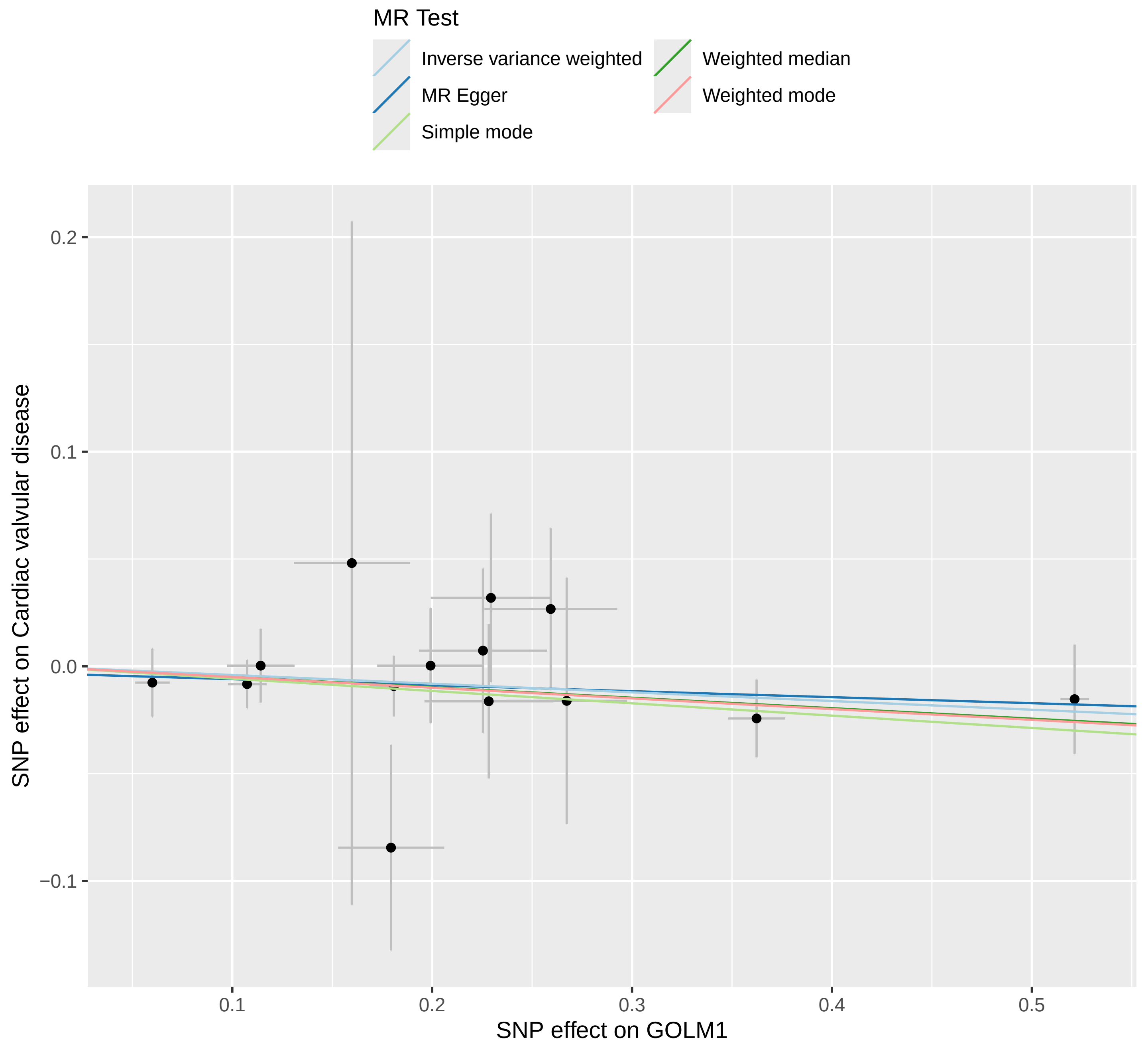

MR Test
Inverse variance weighted
MR Egger
Simple mode
Weighted median
Weighted mode
0.2
0.1
0.0
−0.1
SNP effect on Cardiac valvular disease
0.1
0.2
0.3
0.4
0.5
SNP effect on GOLM1

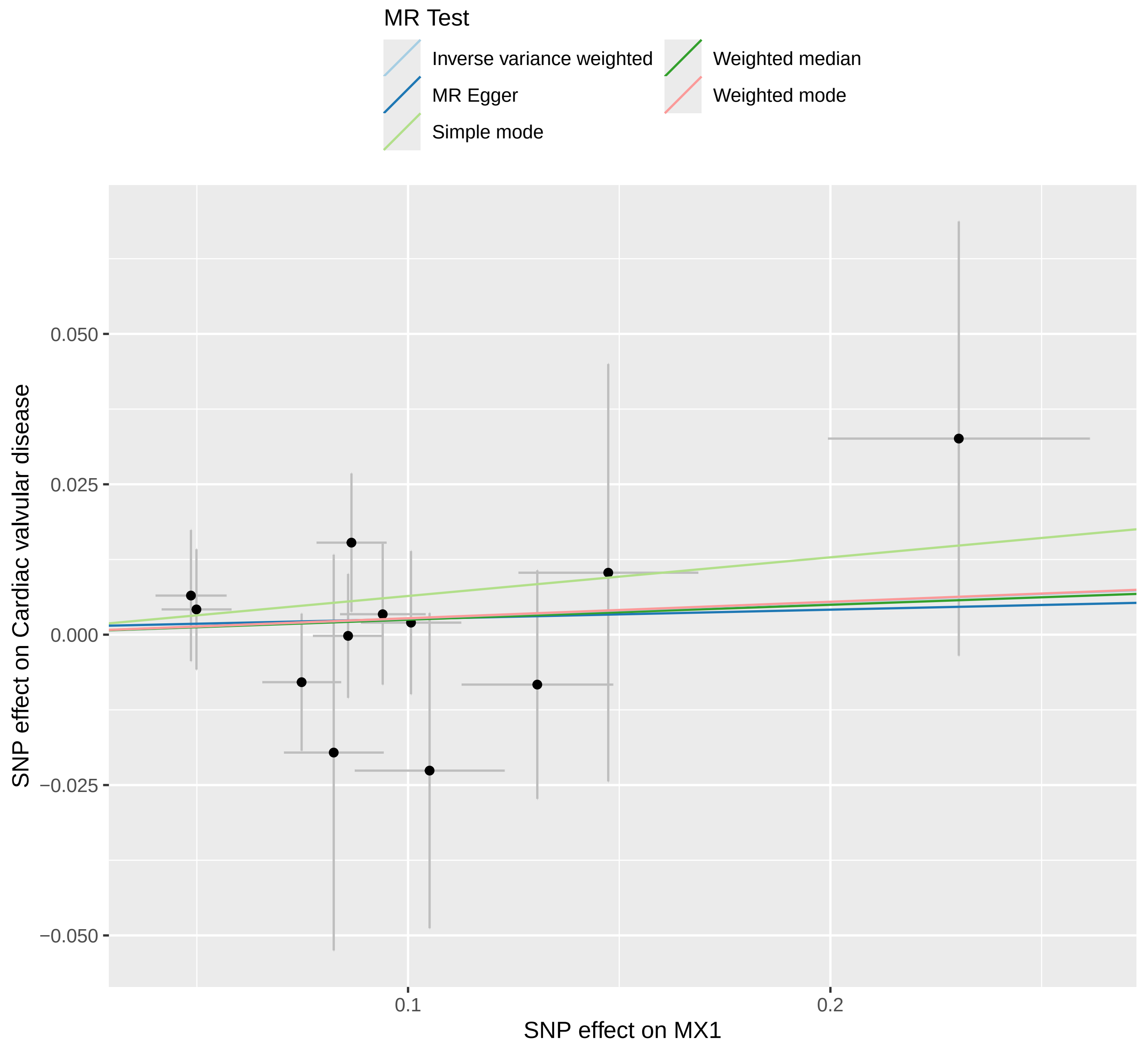
MR Test
Inverse variance weighted
MR Egger
Simple mode
Weighted median
Weighted mode
0.050
0.025
0.000
−0.025
−0.050
SNP effect on Cardiac valvular disease
0.1
0.2
SNP effect on MX1

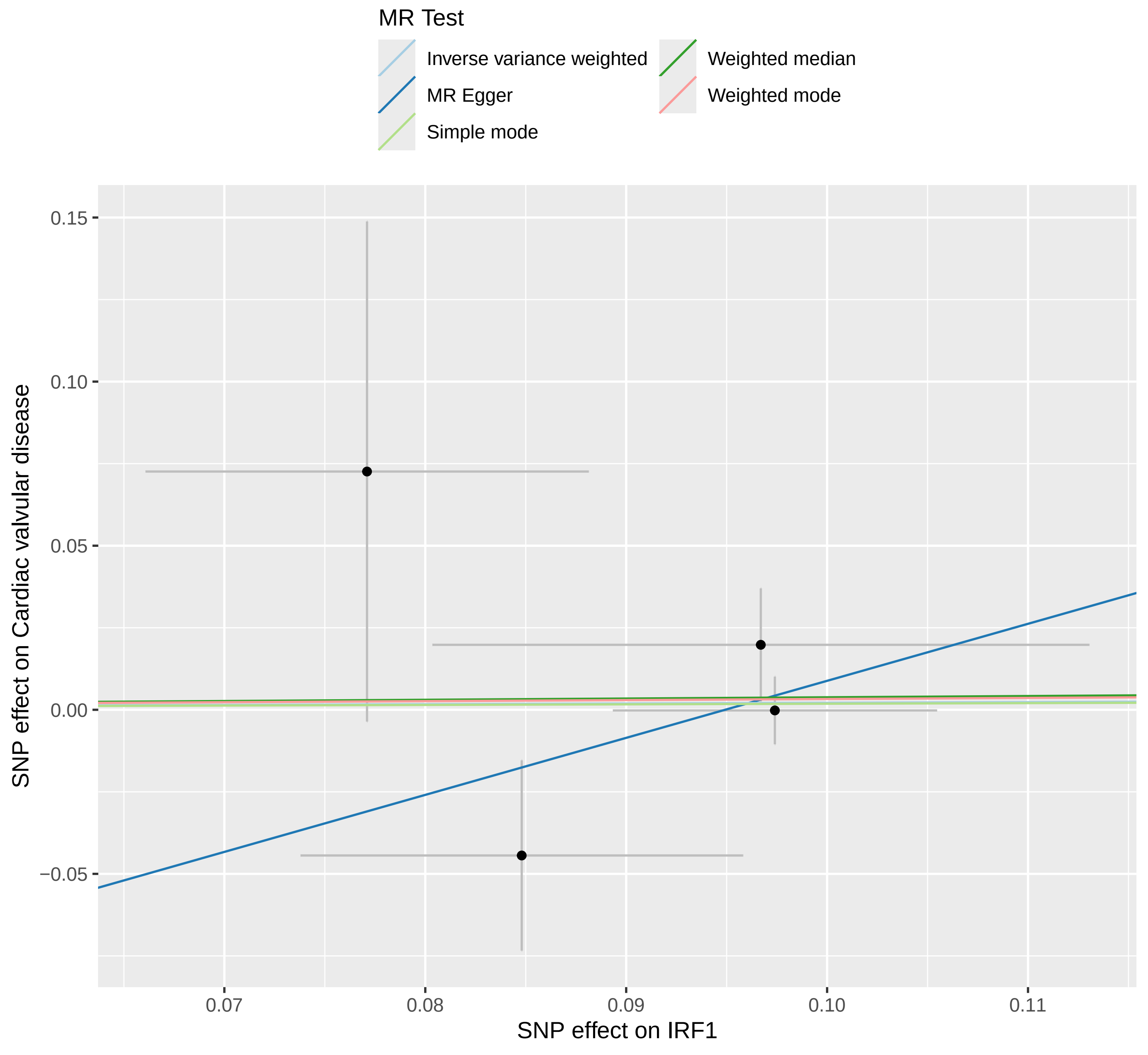
MR Test
Inverse variance weighted
MR Egger
Simple mode
Weighted median
Weighted mode
0.15
0.10
0.05
0.00
−0.05
SNP effect on Cardiac valvular disease
0.07
0.08
0.09
0.10
0.11
SNP effect on IRF1

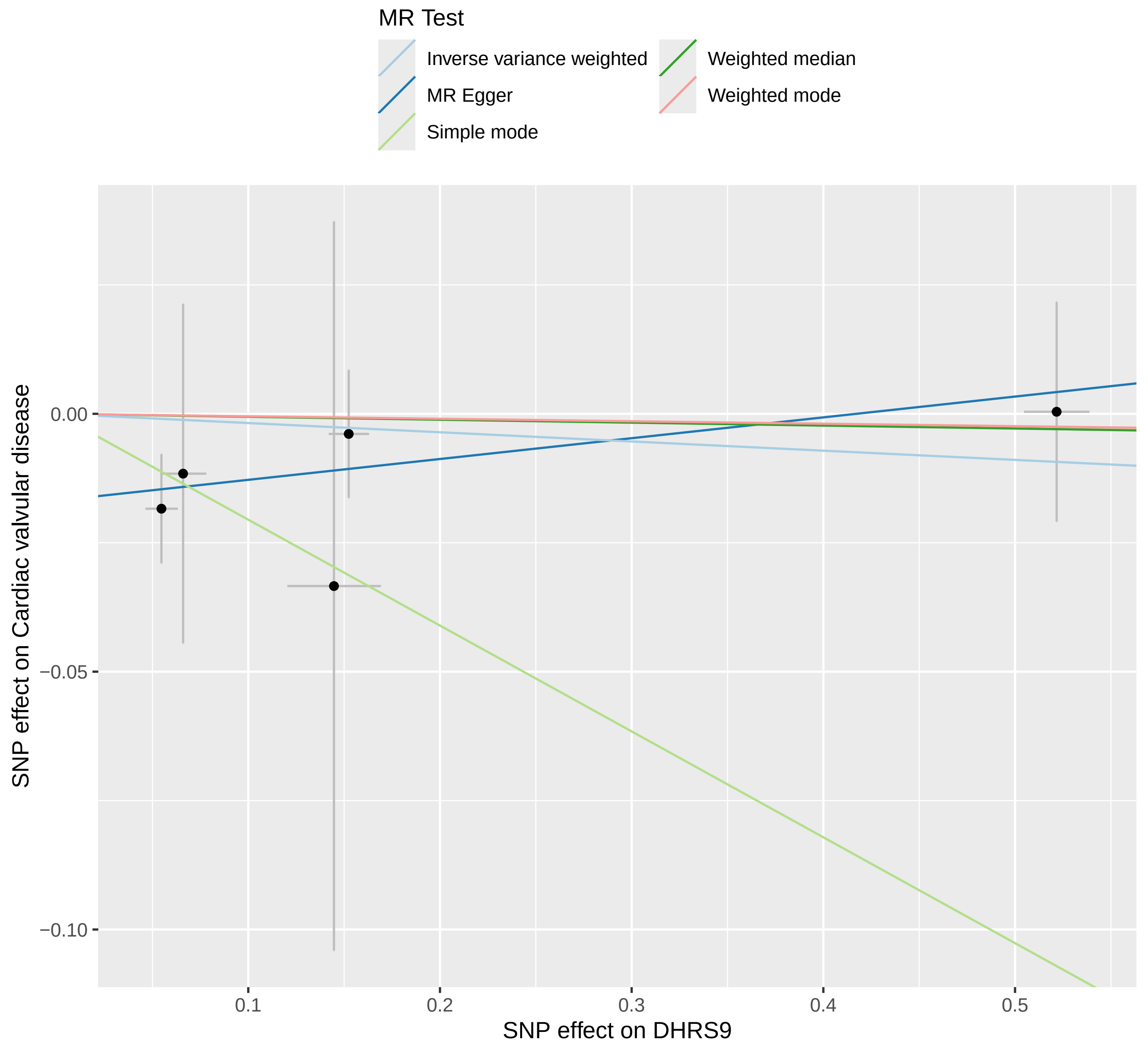
MR Test
Inverse variance weighted
MR Egger
Simple mode
Weighted median
Weighted mode
SNP effect on Cardiac valvular disease
0.00
−0.05
−0.10
0.1
0.2
0.3
0.4
0.5
SNP effect on DHRS9

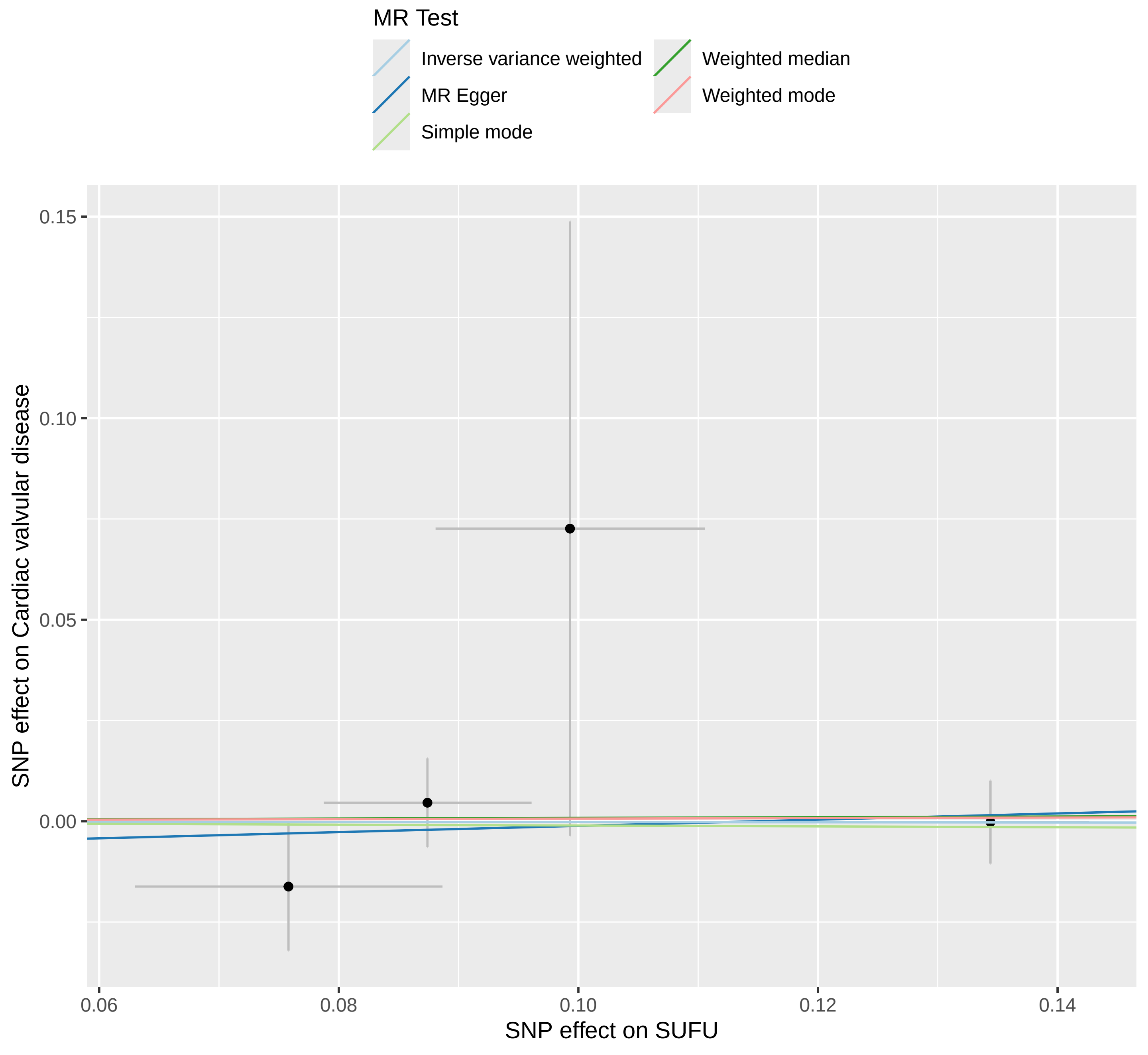
MR Test
Inverse variance weighted
MR Egger
Simple mode
Weighted median
Weighted mode
SNP effect on Cardiac valvular disease
0.15
0.10
0.05
0.00
0.06
0.08
0.10
0.12
0.14
SNP effect on SUFU

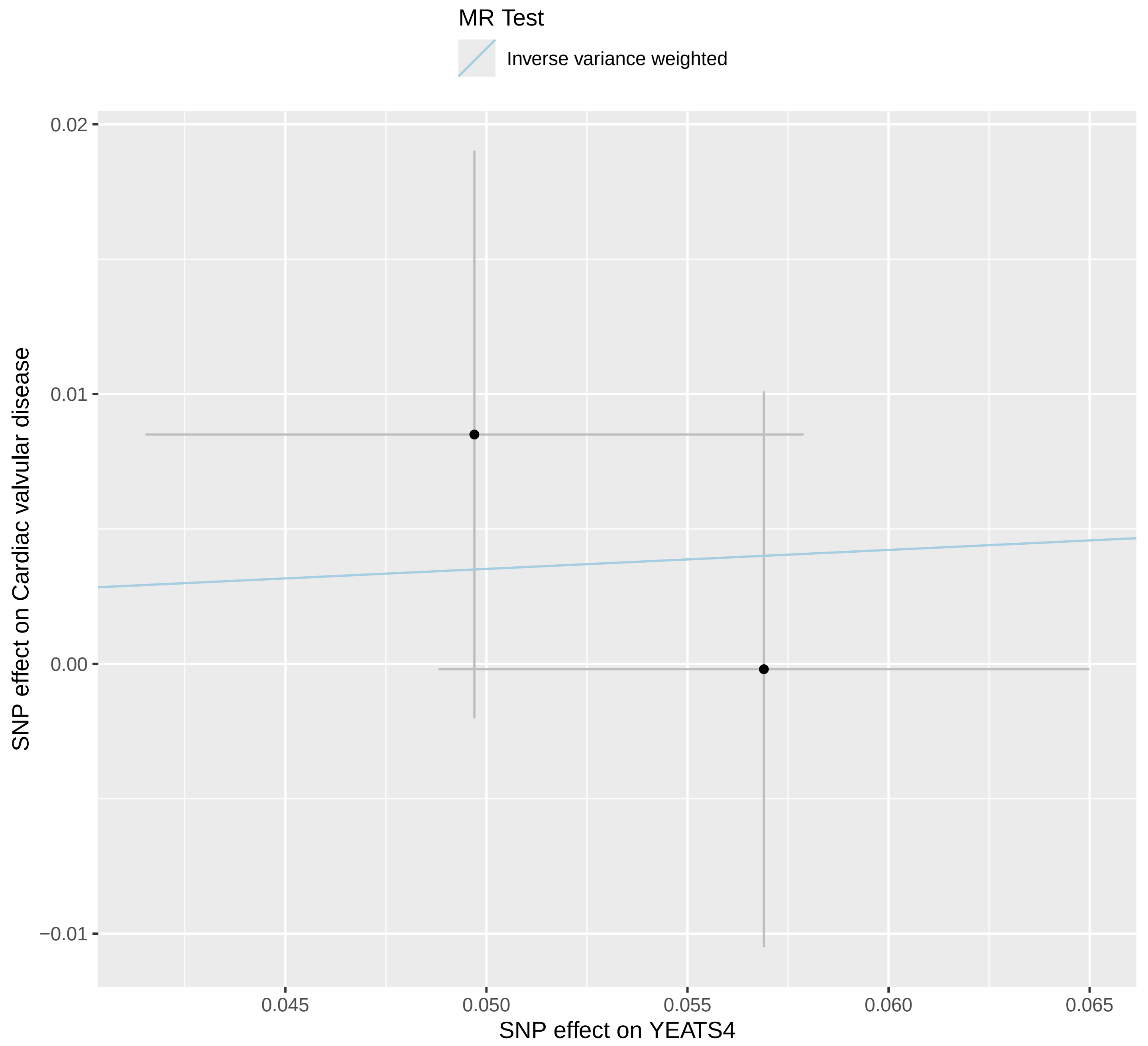
MR Test
Inverse variance weighted
0.02
0.01
0.00
−0.01
SNP effect on Cardiac valvular disease
0.045
0.050
0.055
0.060
0.065
SNP effect on YEATS4

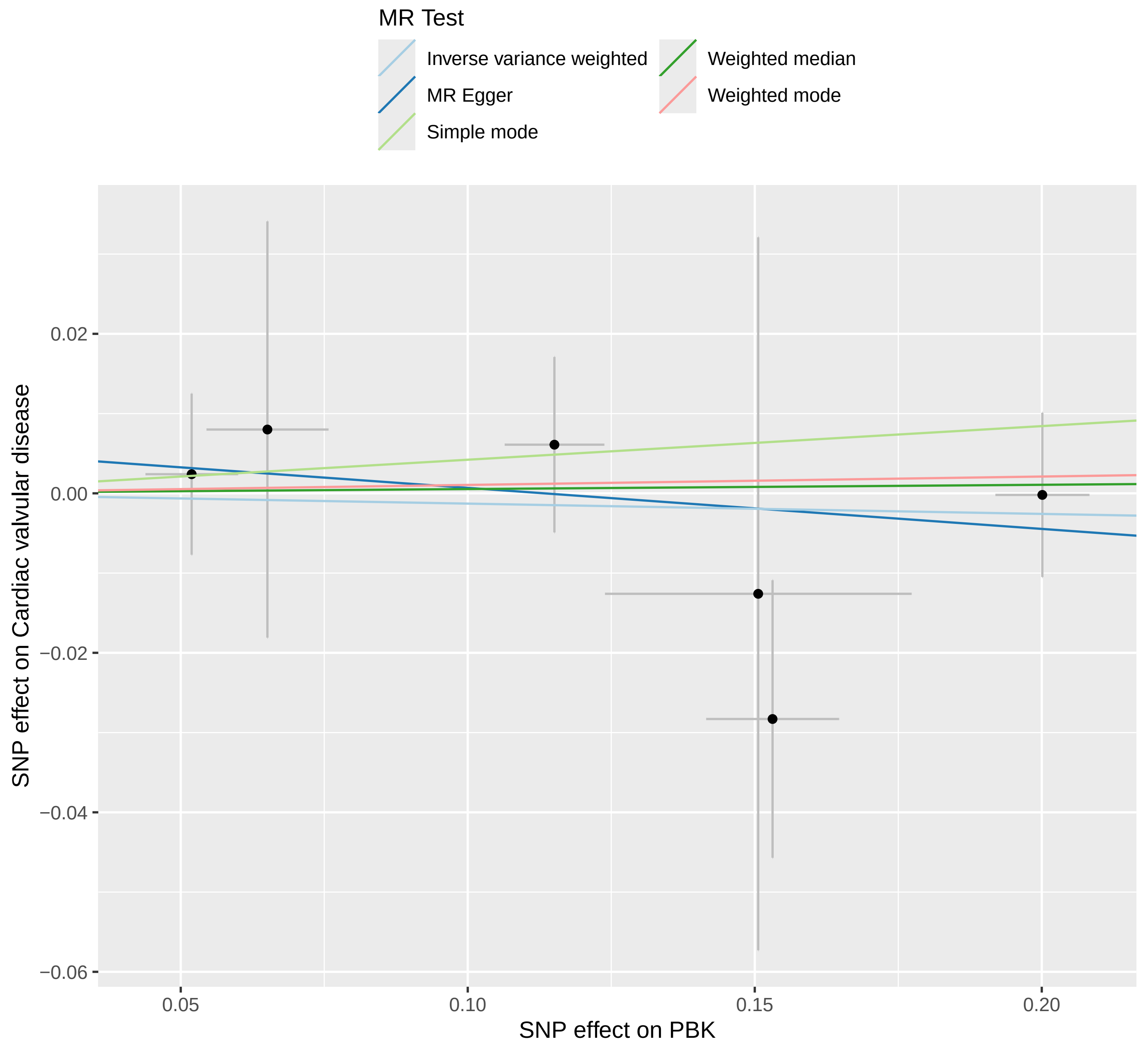
MR Test
Inverse variance weighted
MR Egger
Simple mode
Weighted median
Weighted mode
0.02
0.00
−0.02
−0.04
−0.06
SNP effect on Cardiac valvular disease
0.05
0.10
0.15
0.20
SNP effect on PBK

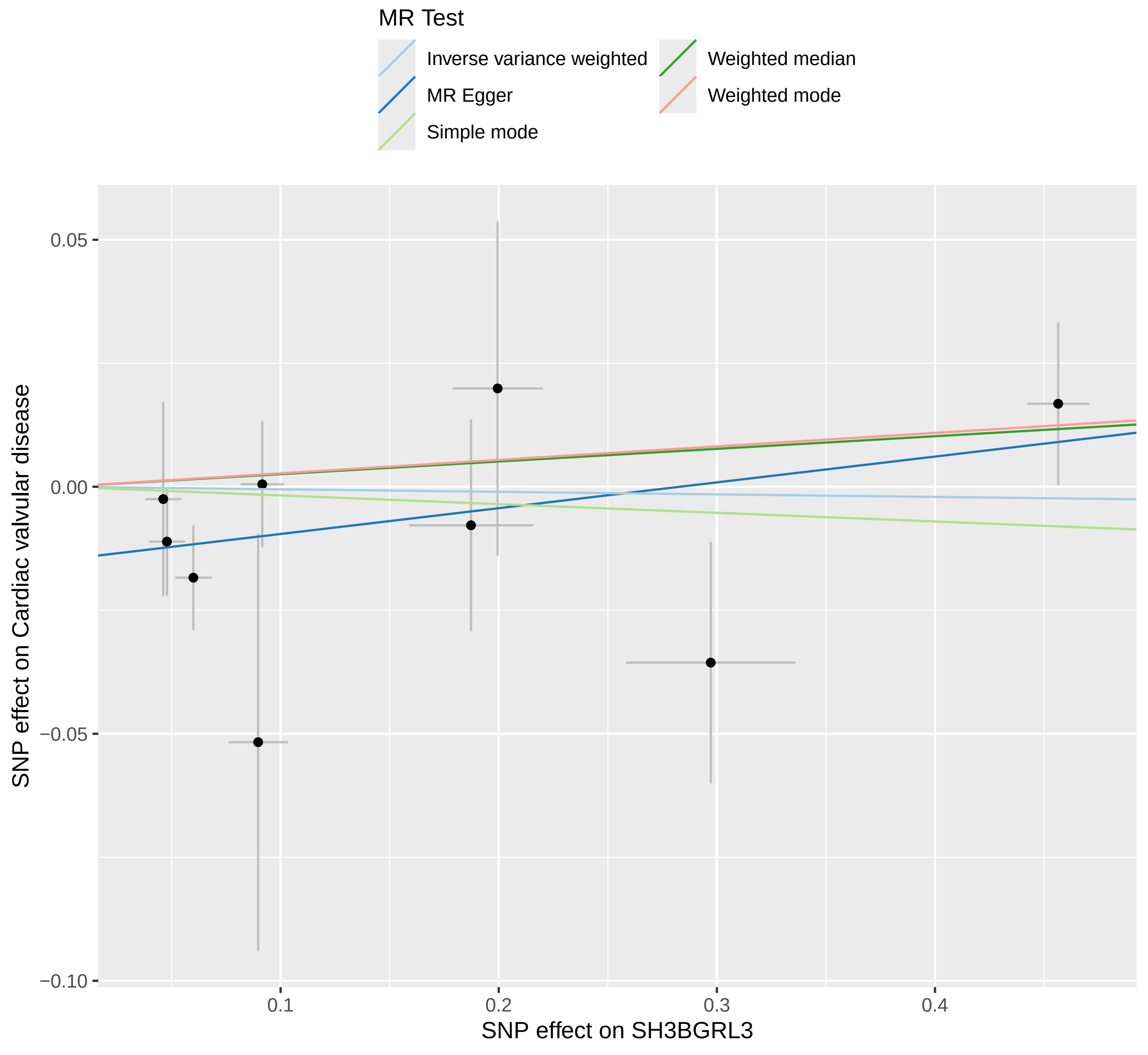
MR Test
Inverse variance weighted
MR Egger
Simple mode
Weighted median
Weighted mode
0.05
0.00
−0.05
−0.10
0.1
0.2
0.3
0.4
SNP effect on SH3BGRL3
SNP effect on Cardiac valvular disease

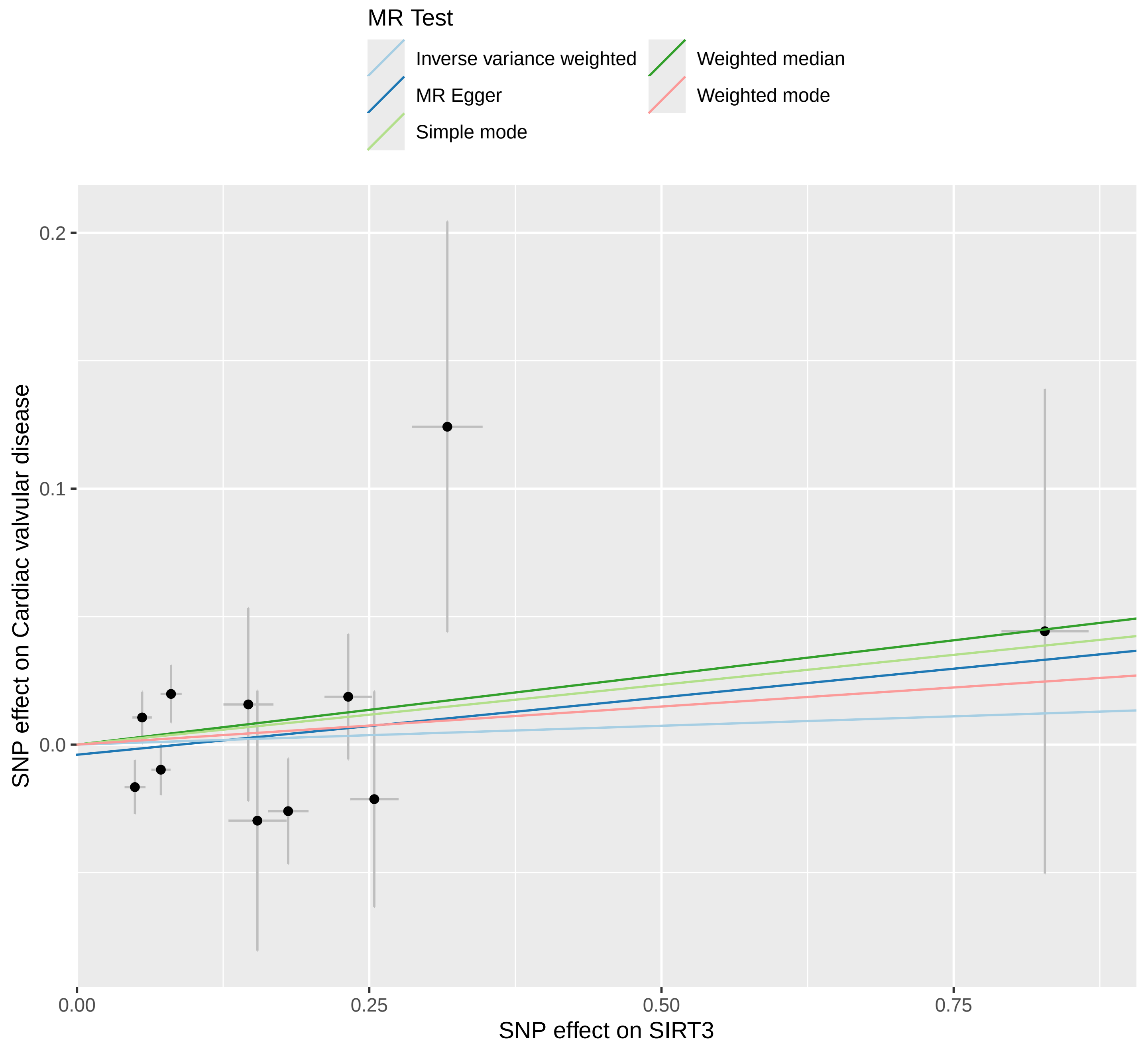

MR Test
Inverse variance weighted
MR Egger
Simple mode
Weighted median
Weighted mode
0.2
0.1
0.0
0.00
0.25
0.50
0.75
SNP effect on Cardiac valvular disease
SNP effect on SIRT3

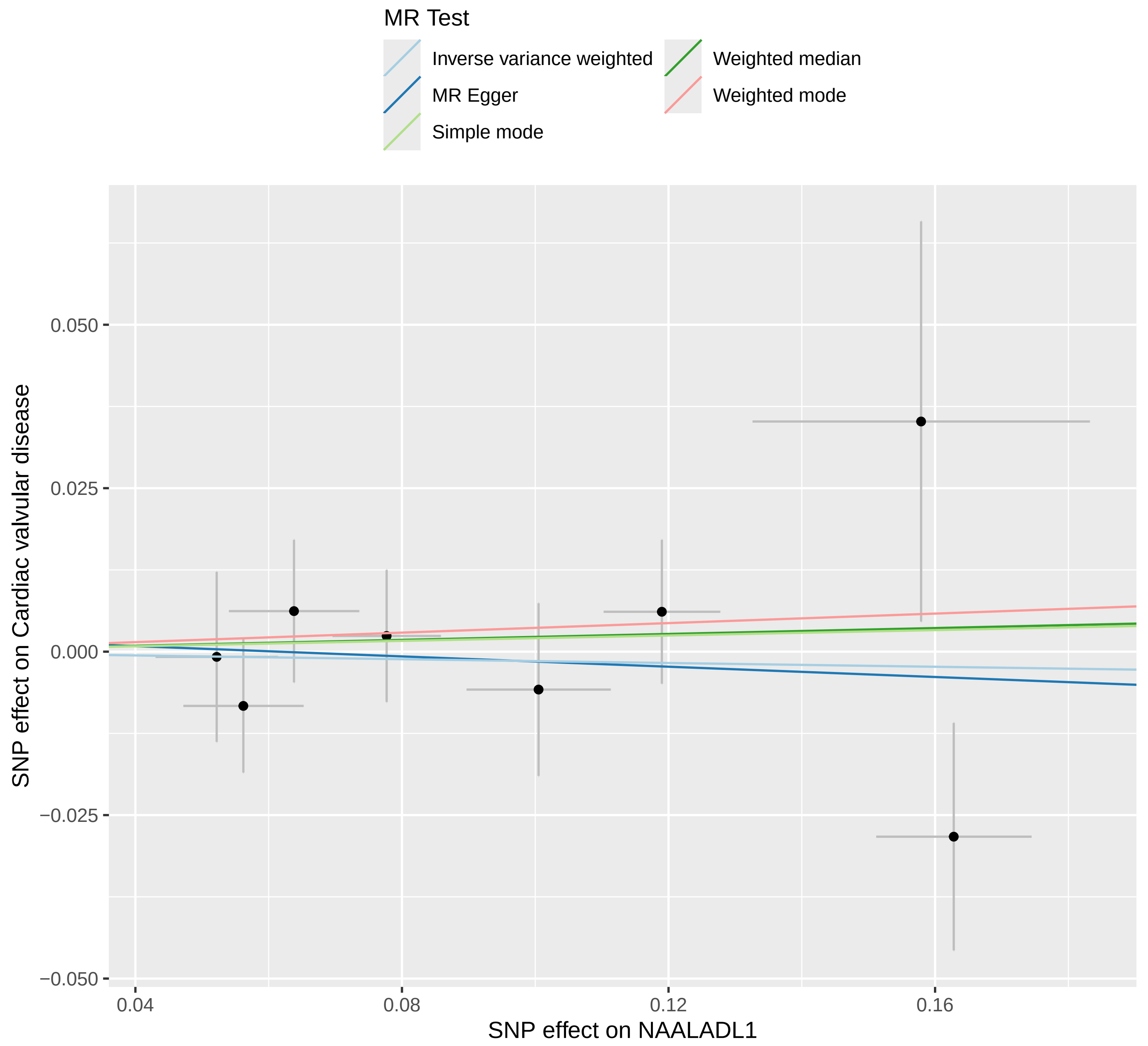
MR Test
Inverse variance weighted
MR Egger
Simple mode
Weighted median
Weighted mode
SNP effect on Cardiac valvular disease
0.050
0.025
0.000
−0.025
−0.050
0.04
0.08
0.12
0.16
SNP effect on NAALADL1

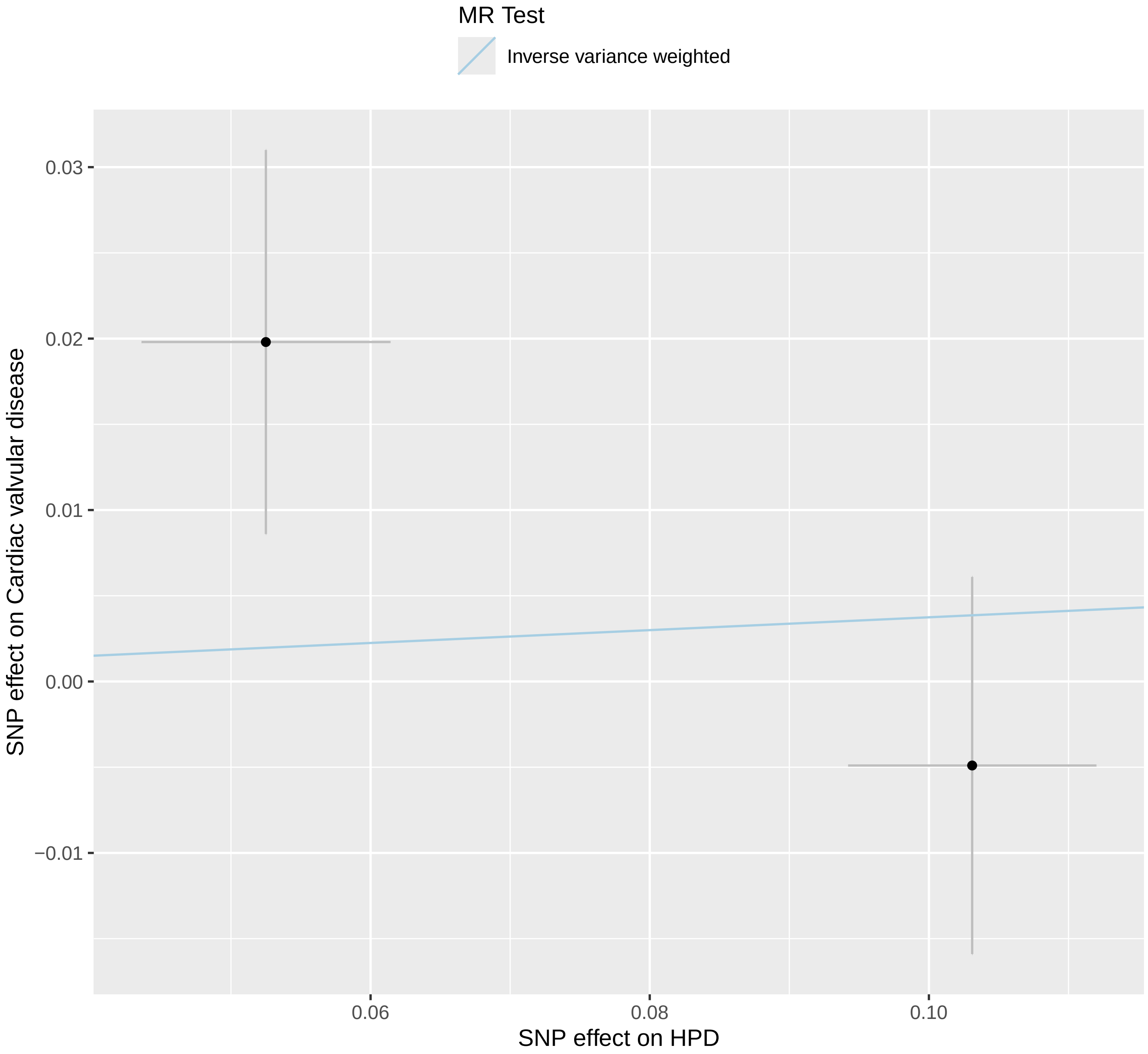

MR Test
Inverse variance weighted
0.03
0.02
0.01
0.00
−0.01
0.06
0.08
0.10
SNP effect on HPD
SNP effect on Cardiac valvular disease

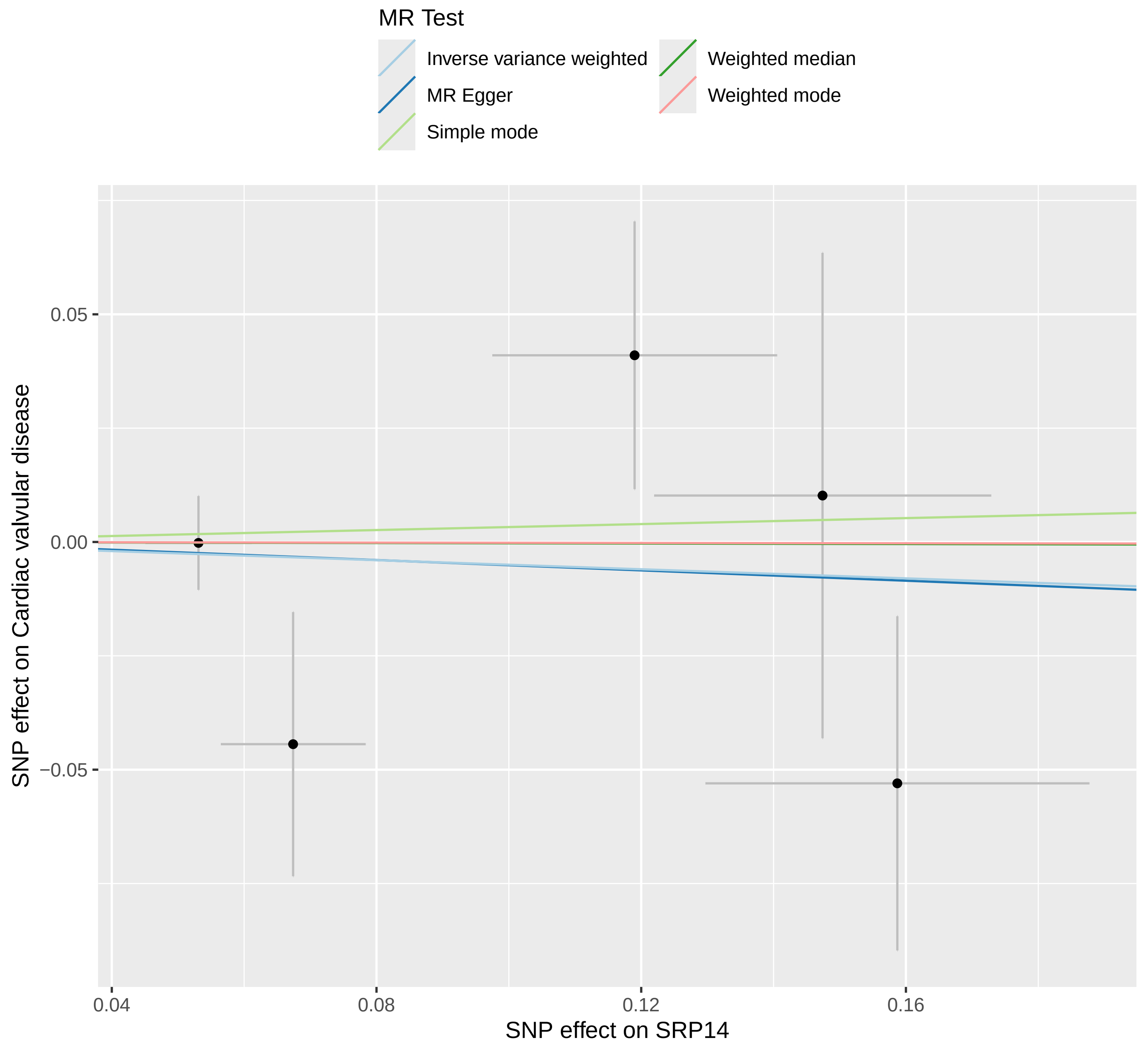
MR Test
Inverse variance weighted
MR Egger
Simple mode
Weighted median
Weighted mode
0.05
0.00
−0.05
SNP effect on Cardiac valvular disease
0.04
0.08
0.12
0.16
SNP effect on SRP14

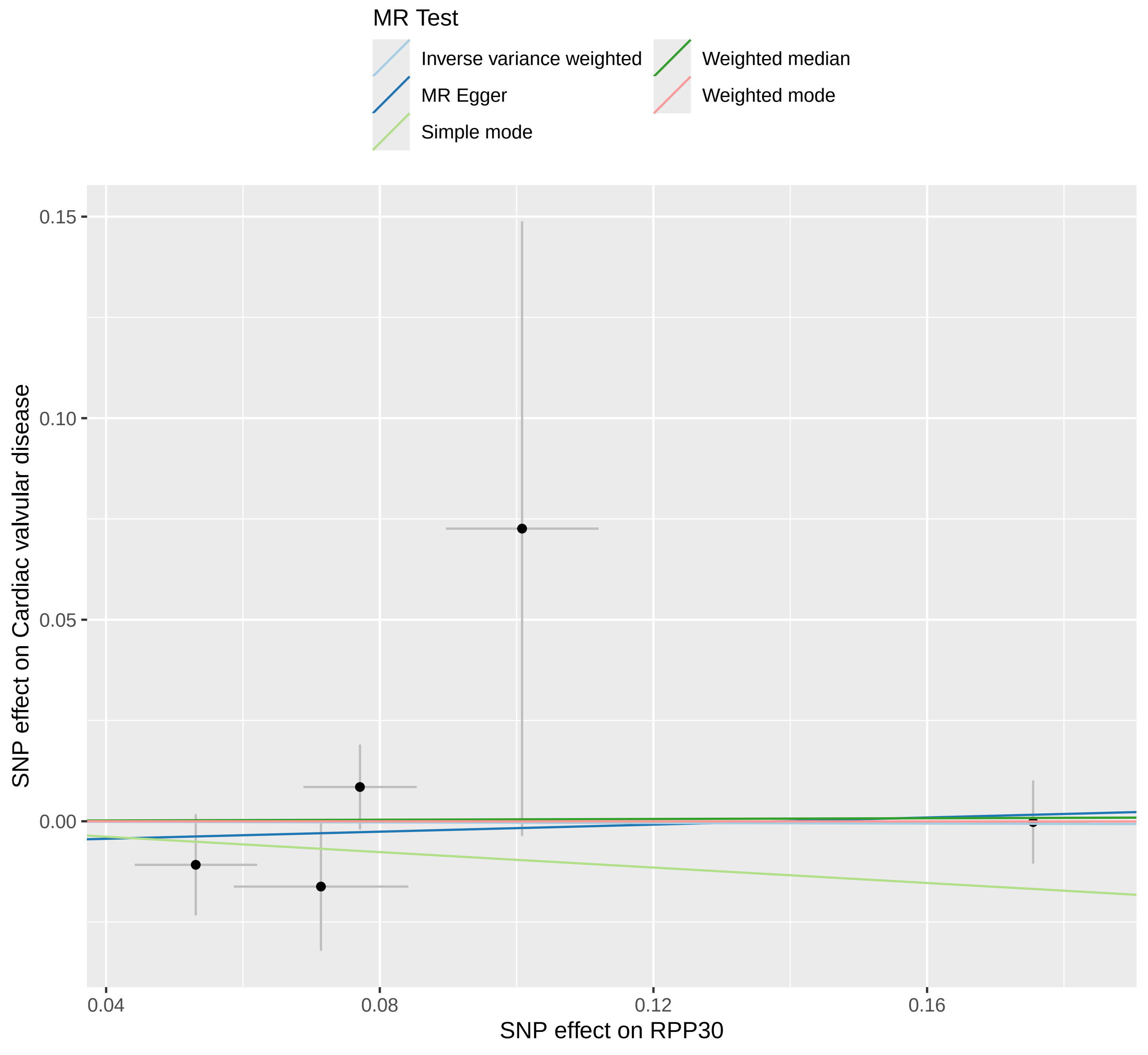
MR Test
Inverse variance weighted
MR Egger
Simple mode
Weighted median
Weighted mode
SNP effect on Cardiac valvular disease
0.15
0.10
0.05
0.00
0.04
0.08
0.12
0.16
SNP effect on RPP30

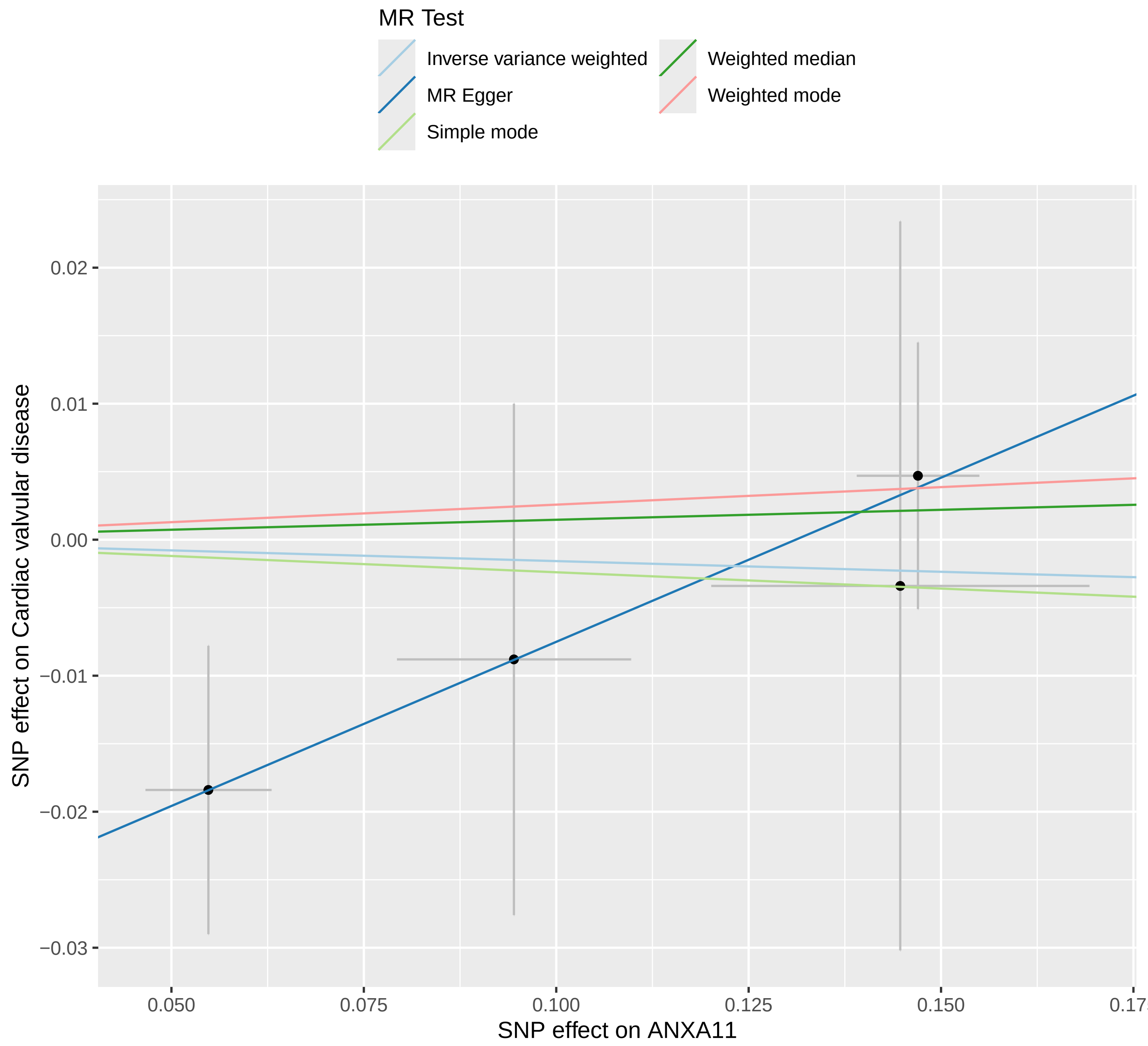
MR Test
Inverse variance weighted
MR Egger
Simple mode
Weighted median
Weighted mode
SNP effect on Cardiac valvular disease
0.02
0.01
0.00
−0.01
−0.02
−0.03
0.050
0.075
0.100
0.125
0.150
SNP effect on ANXA11

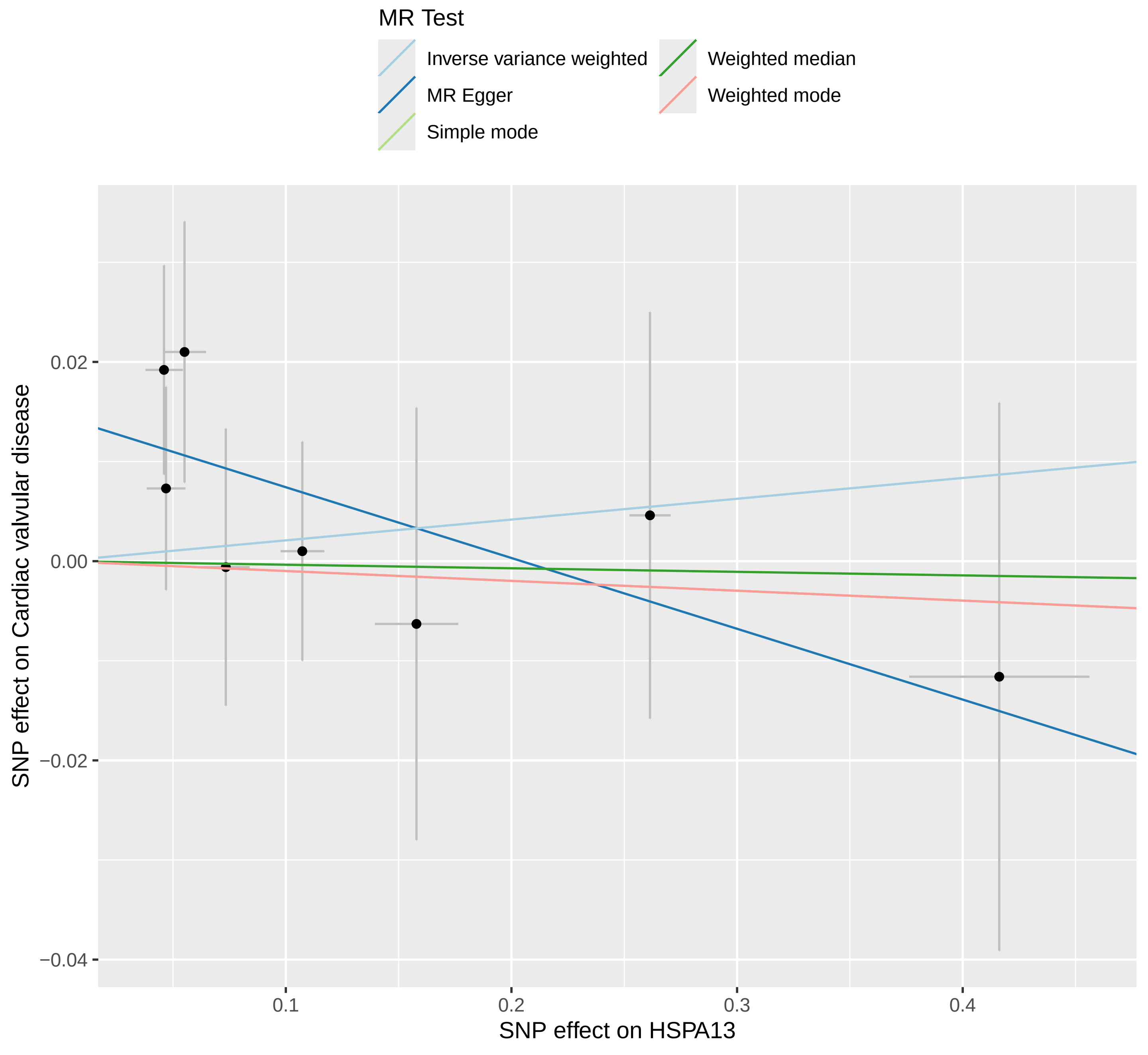
MR Test
Inverse variance weighted
MR Egger
Simple mode
Weighted median
Weighted mode
0.02
0.00
−0.02
−0.04
0.1
0.2
0.3
0.4
SNP effect on Cardiac valvular disease
SNP effect on HSPA13

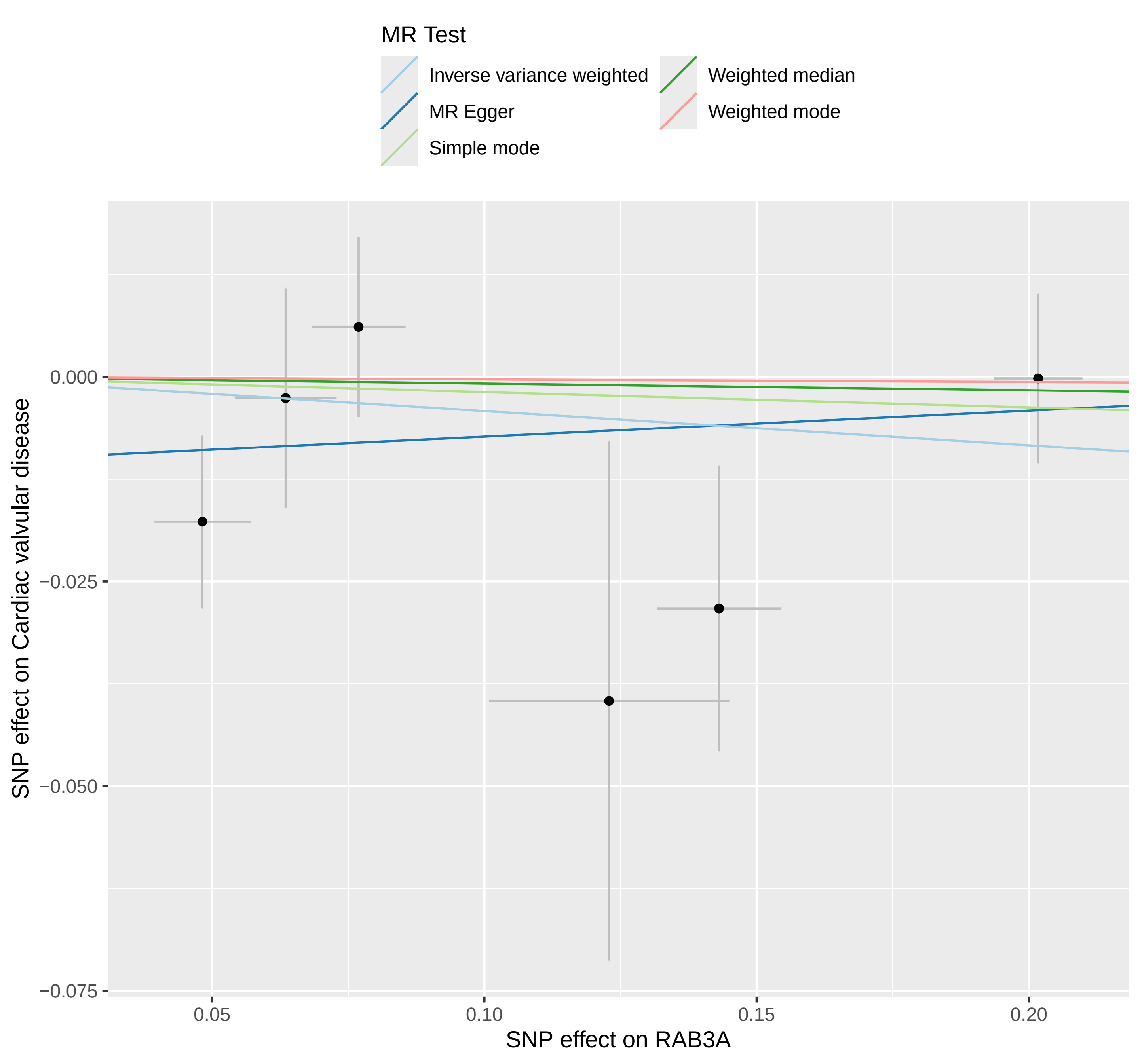
MR Test
Inverse variance weighted
MR Egger
Simple mode
Weighted median
Weighted mode
0.000
−0.025
−0.050
−0.075
SNP effect on Cardiac valvular disease
0.05
0.10
0.15
0.20
SNP effect on RAB3A

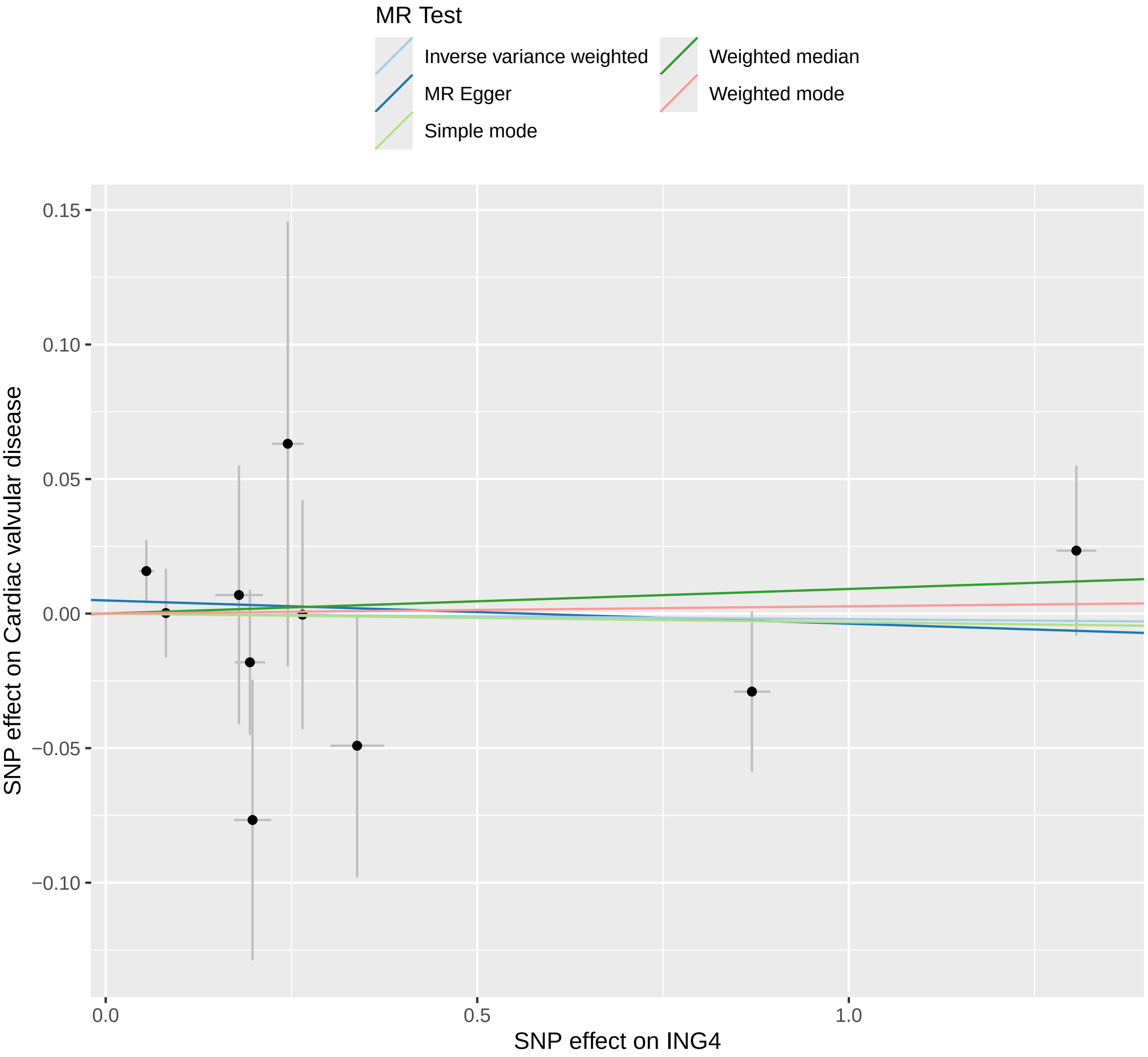
MR Test
Inverse variance weighted
MR Egger
Simple mode
Weighted median
Weighted mode
0.15
0.10
0.05
0.00
−0.05
−0.10
SNP effect on Cardiac valvular disease
0.0
0.5
1.0
SNP effect on ING4

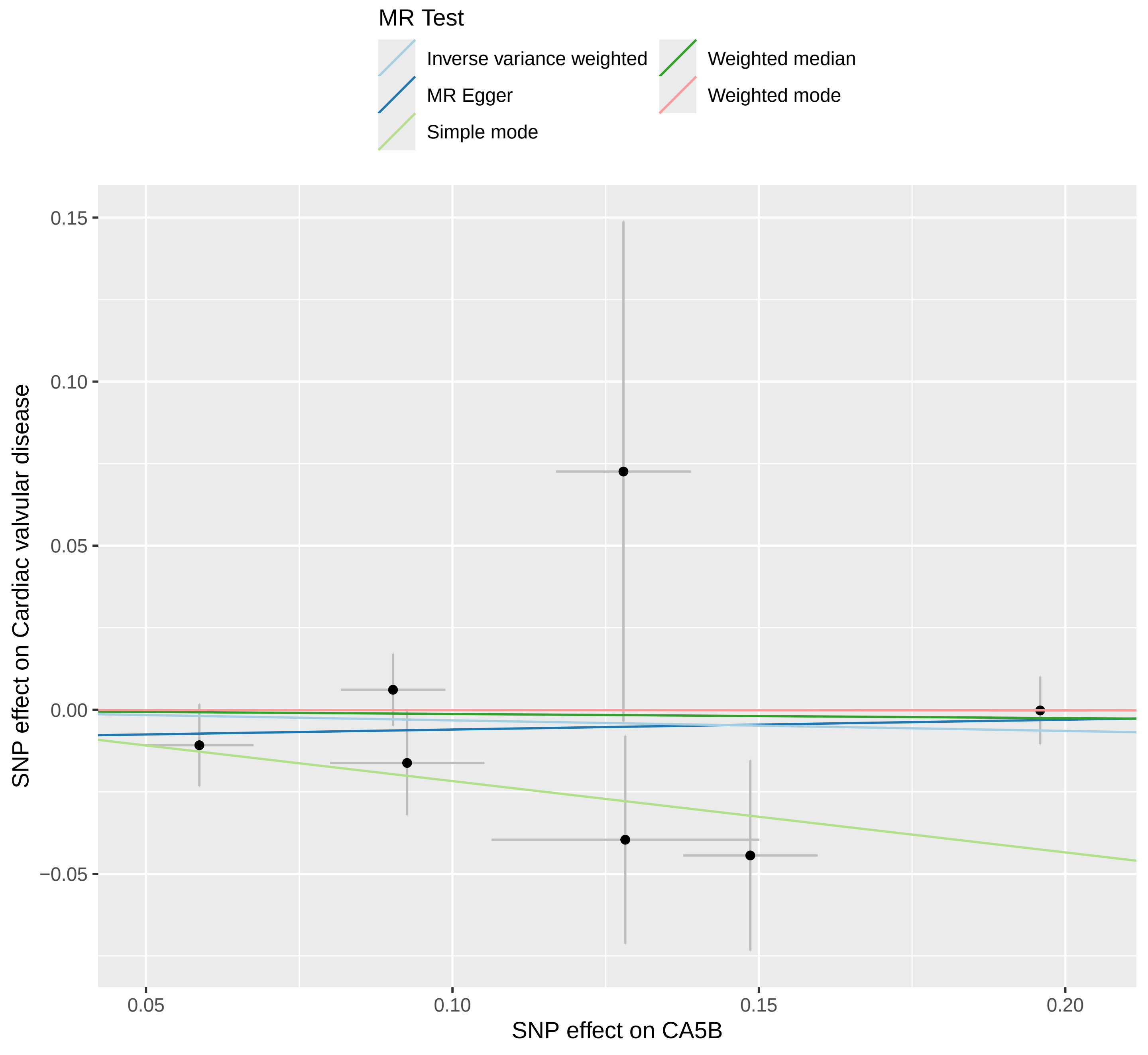
MR Test
Inverse variance weighted
MR Egger
Simple mode
Weighted median
Weighted mode
0.15
0.10
0.05
0.00
−0.05
0.05
0.10
0.15
0.20
SNP effect on Cardiac valvular disease
SNP effect on CA5B

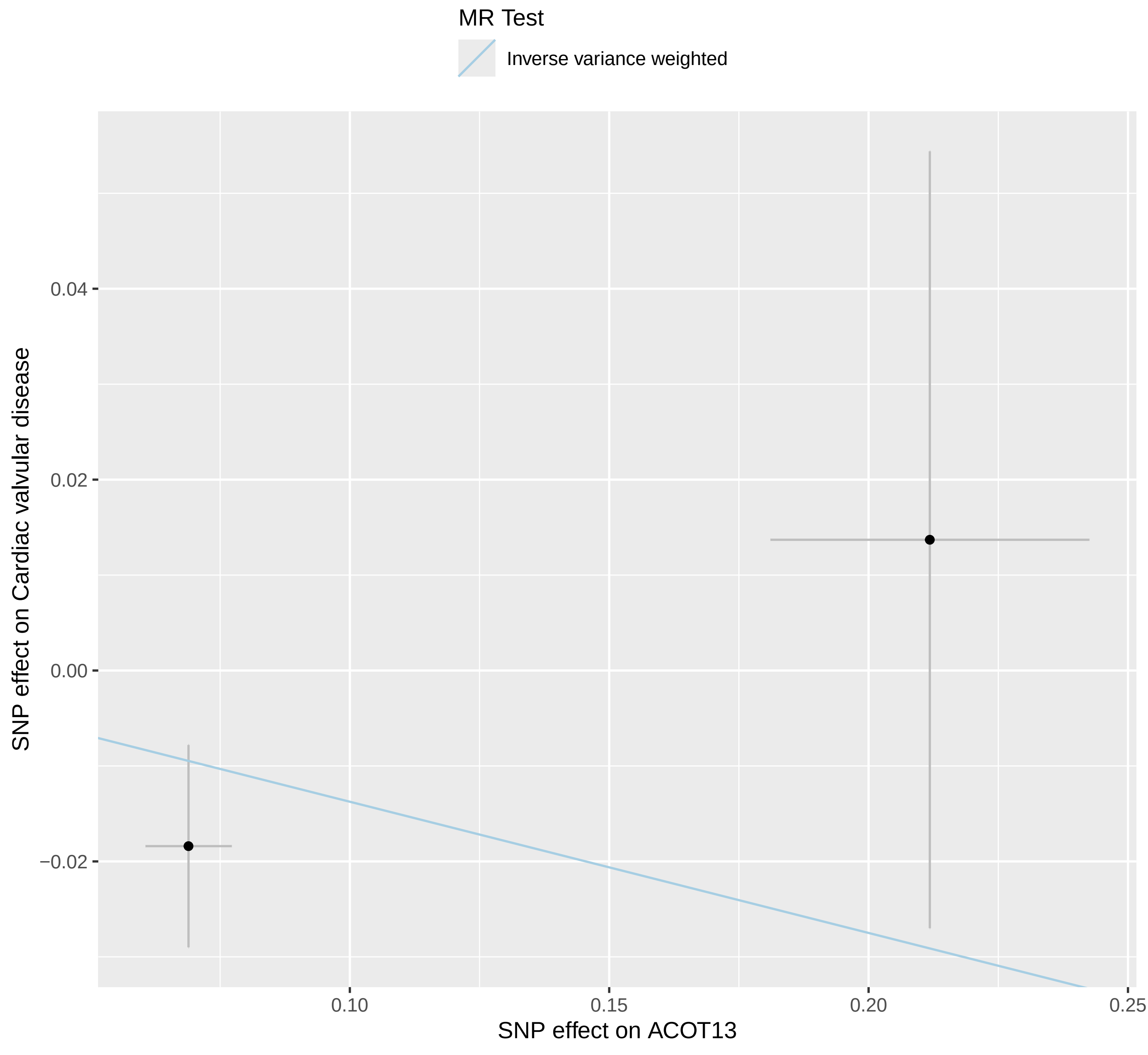
MR Test
Inverse variance weighted
0.04
0.02
0.00
−0.02
SNP effect on Cardiac valvular disease
0.10
0.15
0.20
0.25
SNP effect on ACOT13

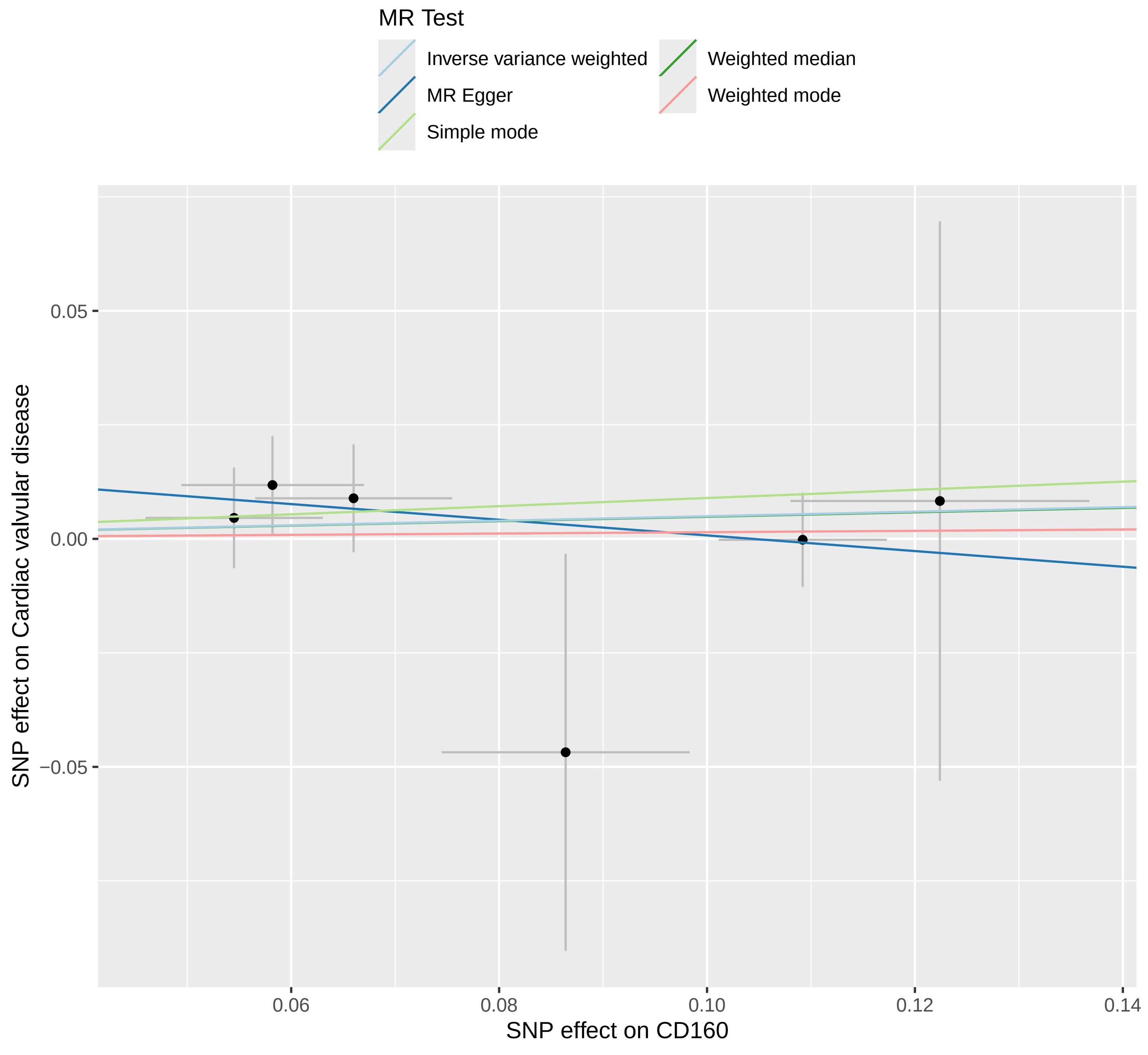

MR Test
Inverse variance weighted
MR Egger
Simple mode
Weighted median
Weighted mode
0.05
0.00
−0.05
0.06
0.08
0.10
0.12
0.14
SNP effect on CD160
SNP effect on Cardiac valvular disease

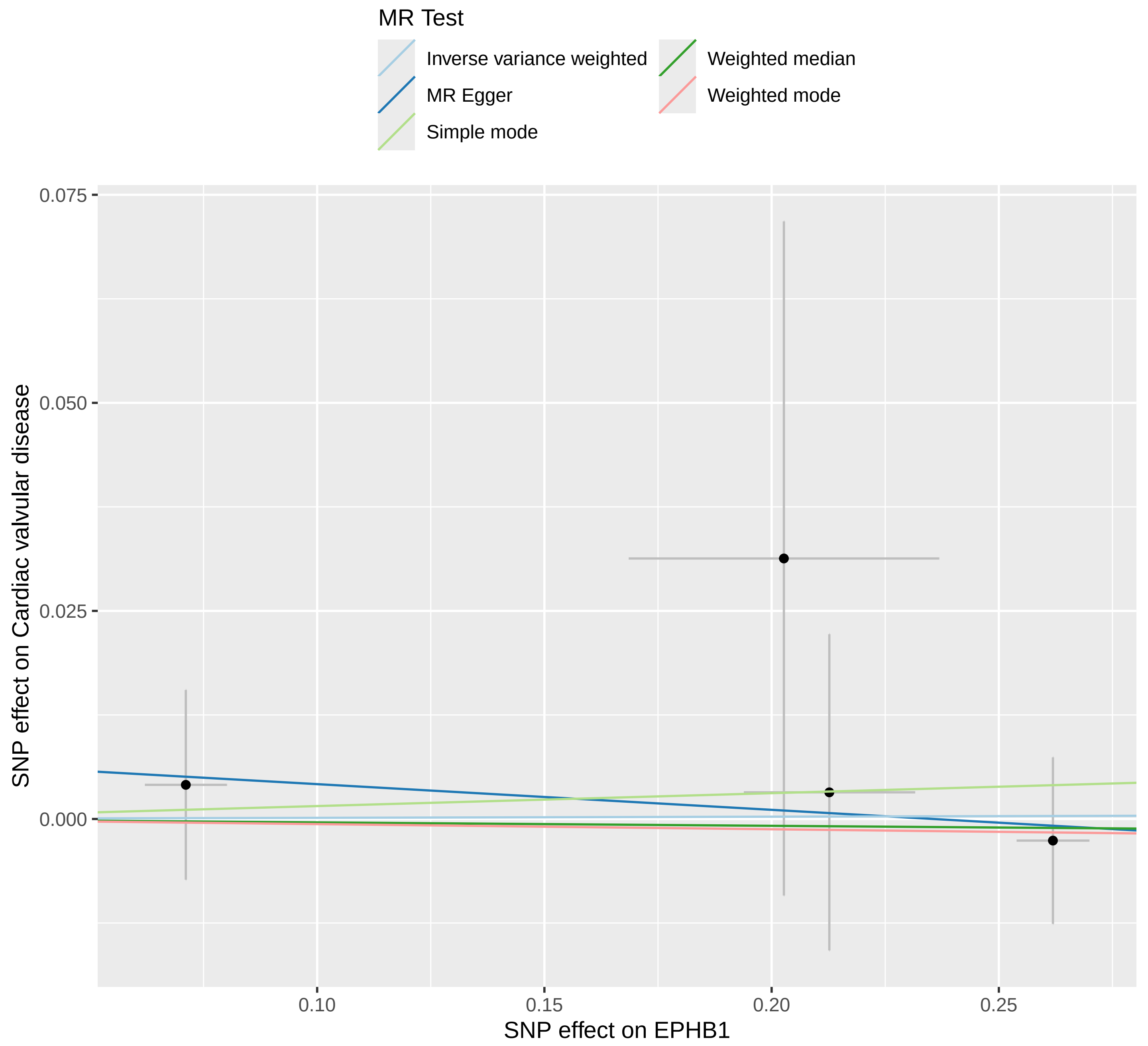

MR Test
Inverse variance weighted
MR Egger
Simple mode
Weighted median
Weighted mode
0.075
0.050
0.025
0.000
0.10
0.15
0.20
0.25
SNP effect on Cardiac valvular disease
SNP effect on EPHB1

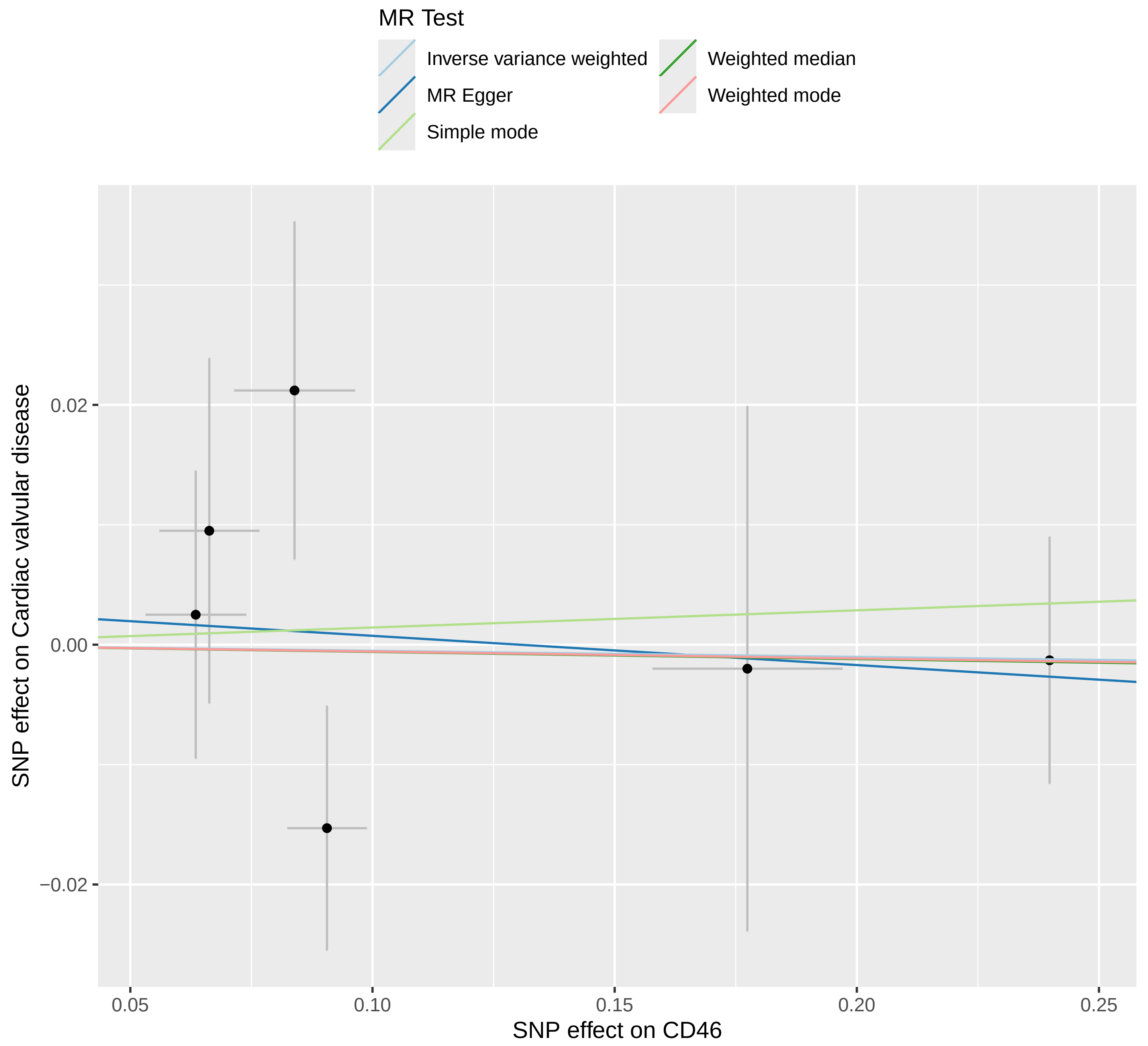
MR Test
Inverse variance weighted
MR Egger
Simple mode
Weighted median
Weighted mode
0.02
0.00
−0.02
0.05
0.10
0.15
0.20
0.25
SNP effect on CD46
SNP effect on Cardiac valvular disease

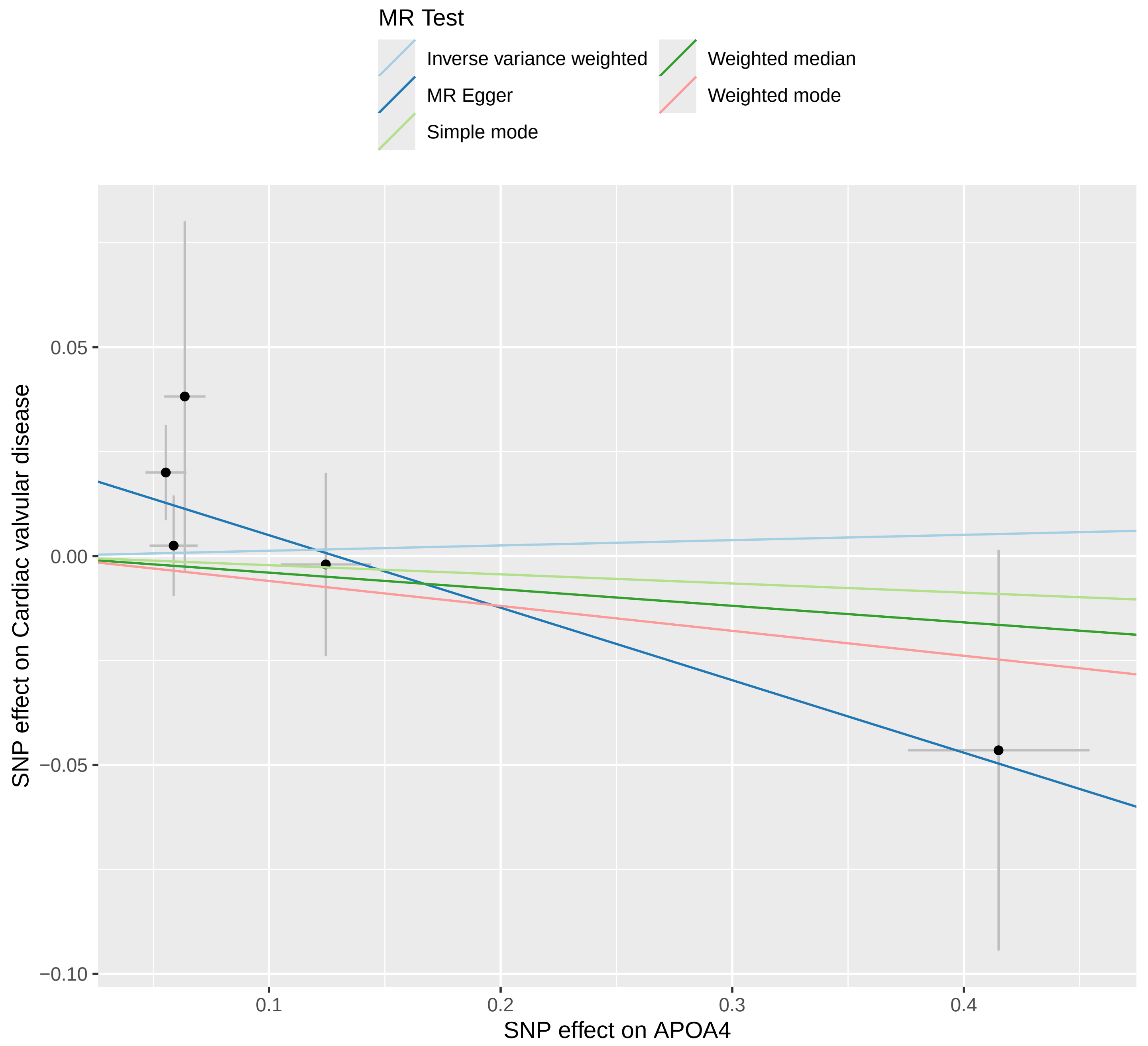

MR Test
Inverse variance weighted
MR Egger
Simple mode
Weighted median
Weighted mode
0.05
0.00
−0.05
−0.10
0.1
0.2
0.3
0.4
SNP effect on Cardiac valvular disease
SNP effect on APOA4

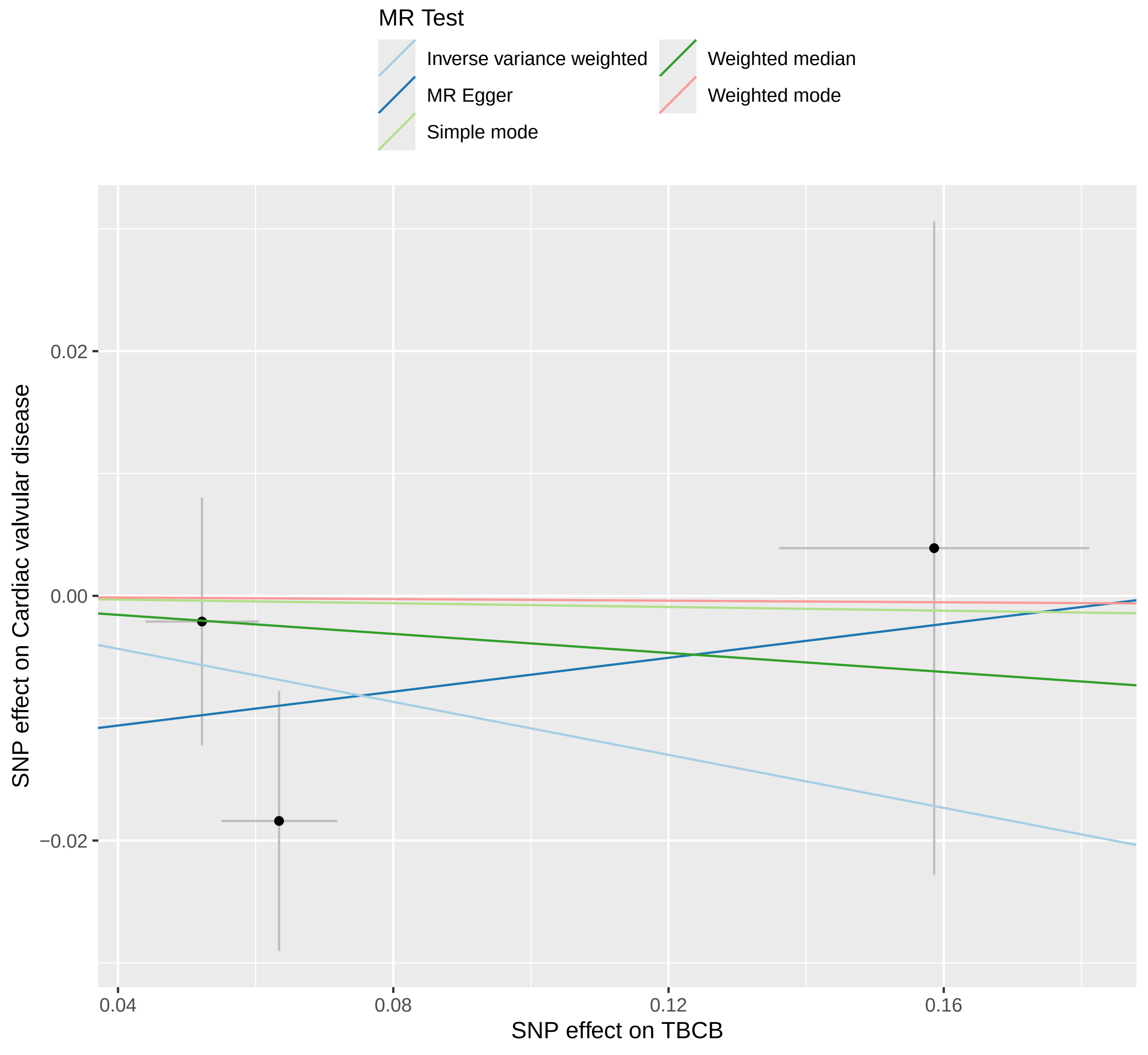

MR Test
Inverse variance weighted
MR Egger
Simple mode
Weighted median
Weighted mode
0.02
0.00
−0.02
SNP effect on Cardiac valvular disease
0.04
0.08
0.12
0.16
SNP effect on TBCB

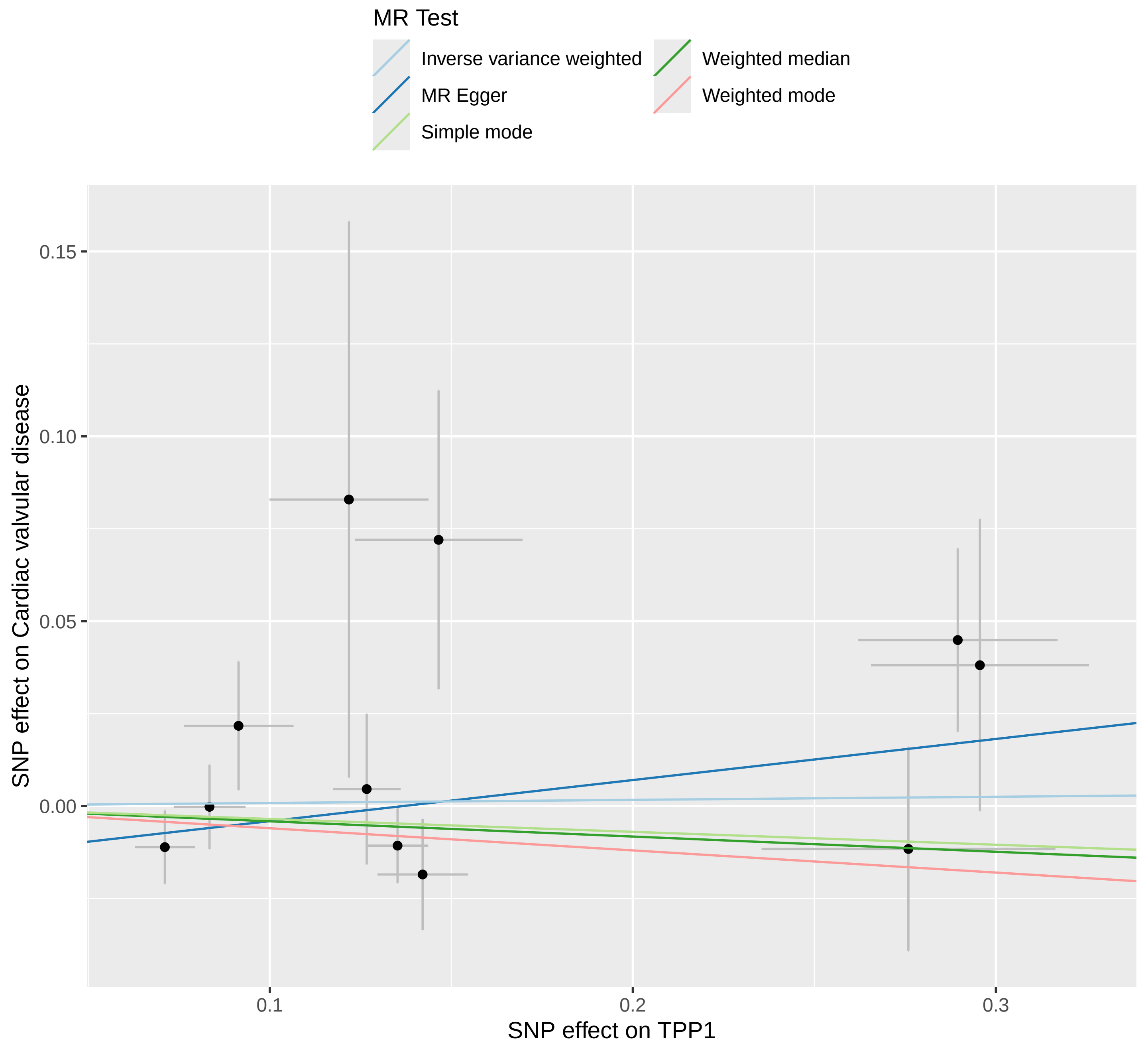
MR Test
Inverse variance weighted
MR Egger
Simple mode
Weighted median
Weighted mode
0.15
0.10
0.05
0.00
SNP effect on Cardiac valvular disease
0.1
0.2
0.3
SNP effect on TPP1

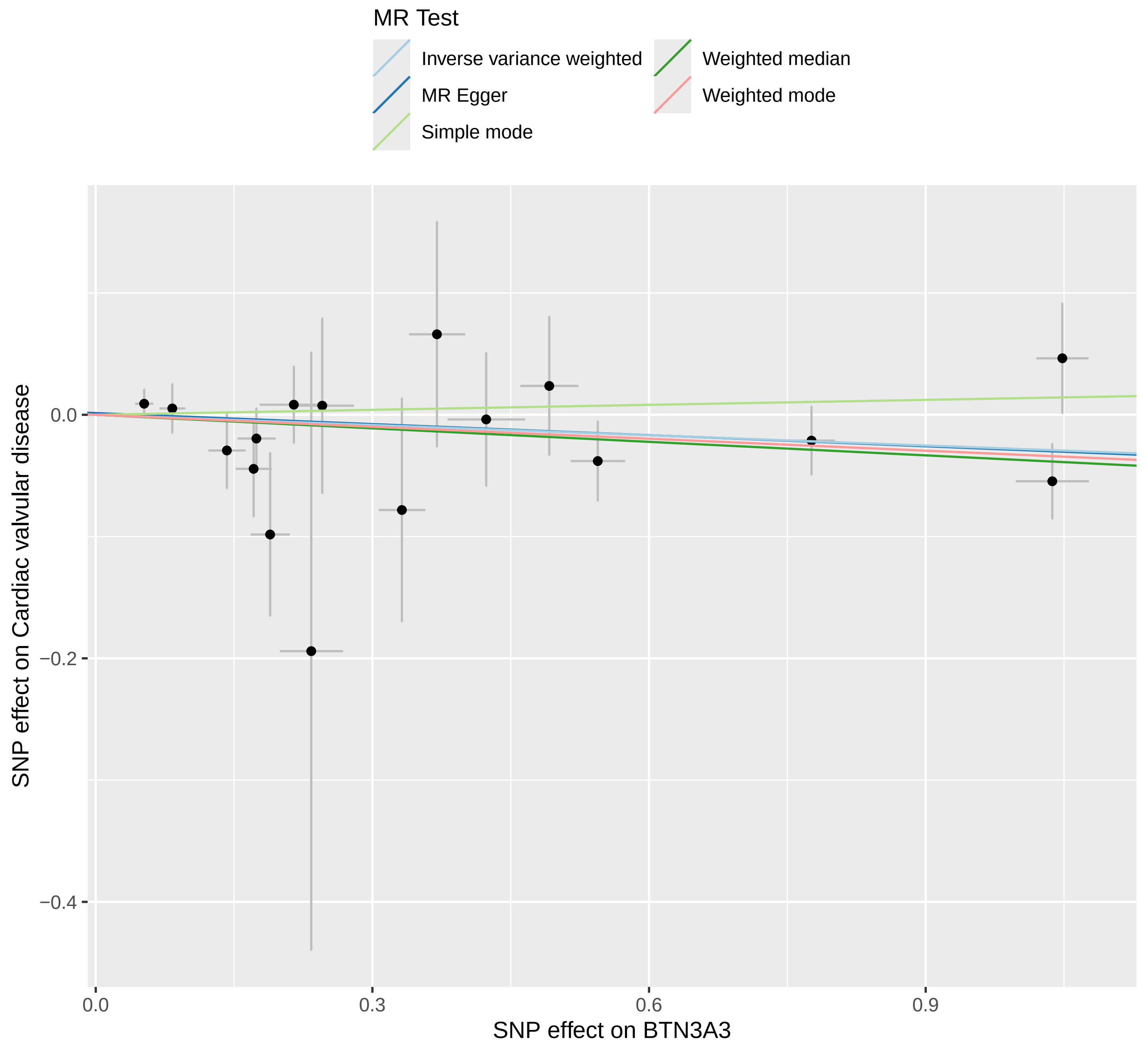
MR Test
Inverse variance weighted
MR Egger
Simple mode
Weighted median
Weighted mode
SNP effect on Cardiac valvular disease
0.0
−0.2
−0.4
0.0
0.3
0.6
0.9
SNP effect on BTN3A3

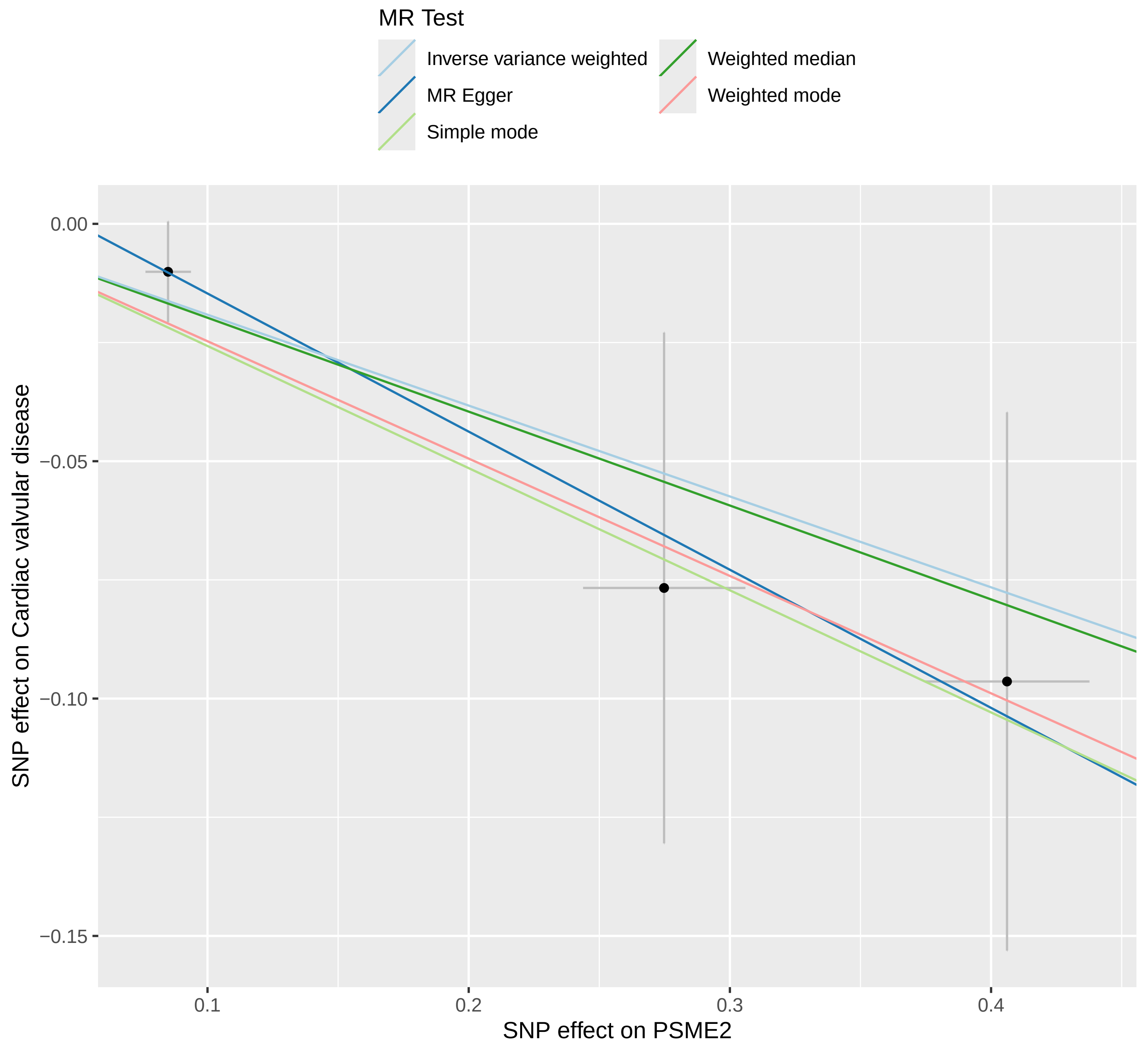
MR Test
Inverse variance weighted
MR Egger
Simple mode
Weighted median
Weighted mode
0.00
−0.05
−0.10
−0.15
0.1
0.2
0.3
0.4
SNP effect on Cardiac valvular disease
SNP effect on PSME2

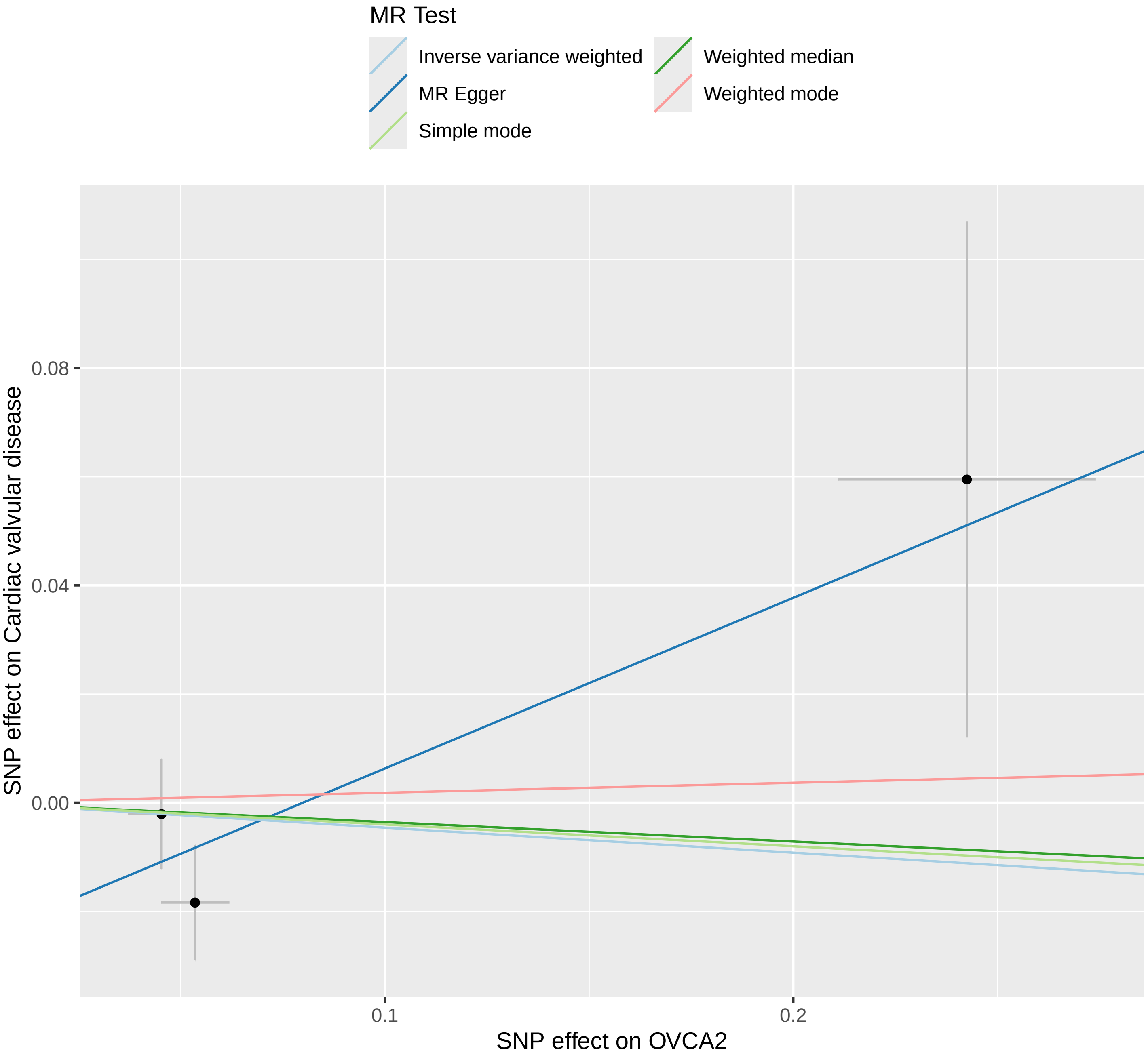
MR Test
Inverse variance weighted
MR Egger
Simple mode
Weighted median
Weighted mode
SNP effect on Cardiac valvular disease
0.08
0.04
0.00
0.1
0.2
SNP effect on OVCA2

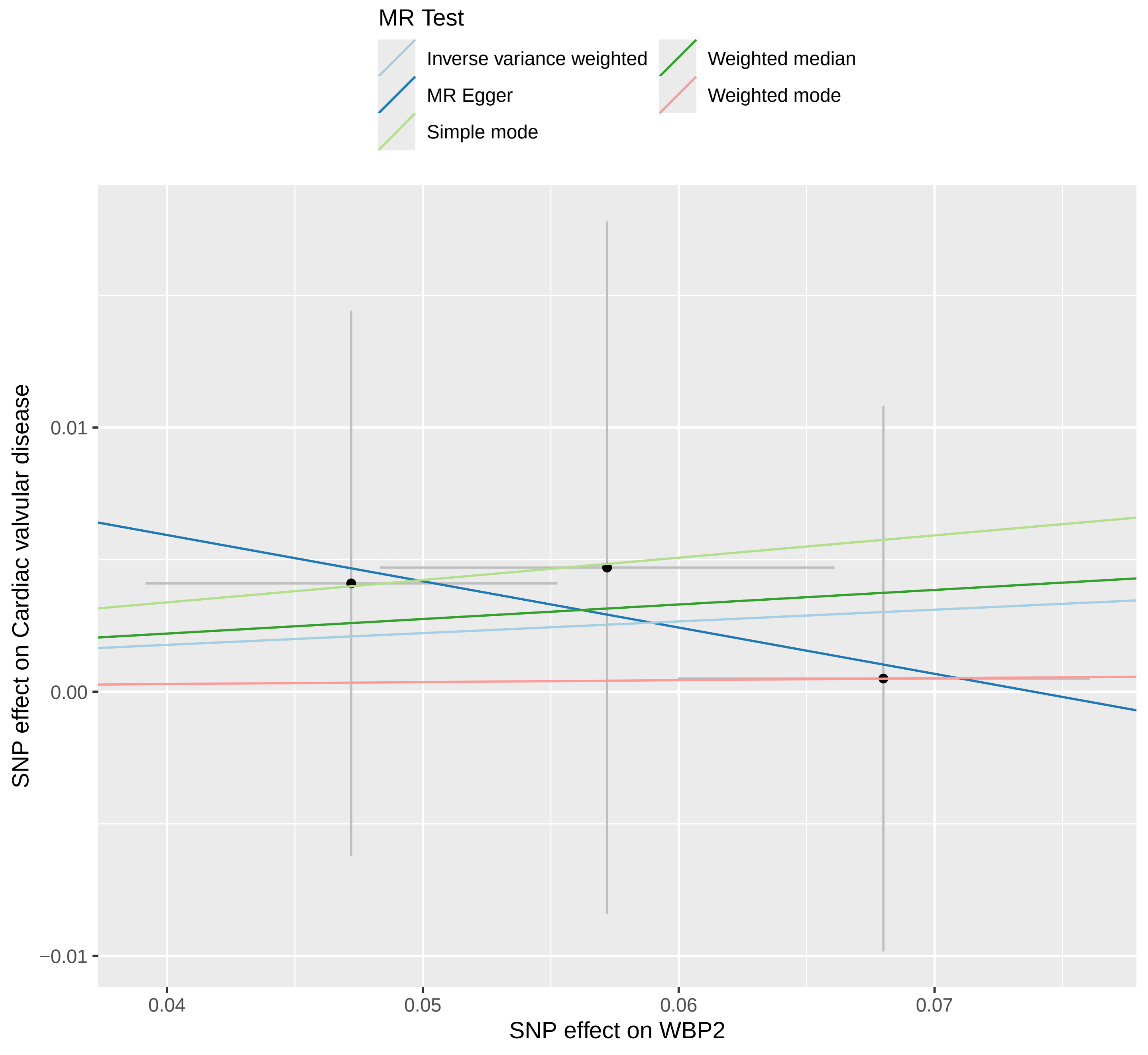

MR Test
Inverse variance weighted
MR Egger
Simple mode
Weighted median
Weighted mode
0.01
0.00
−0.01
0.04
0.05
0.06
0.07
SNP effect on WBP2
SNP effect on Cardiac valvular disease

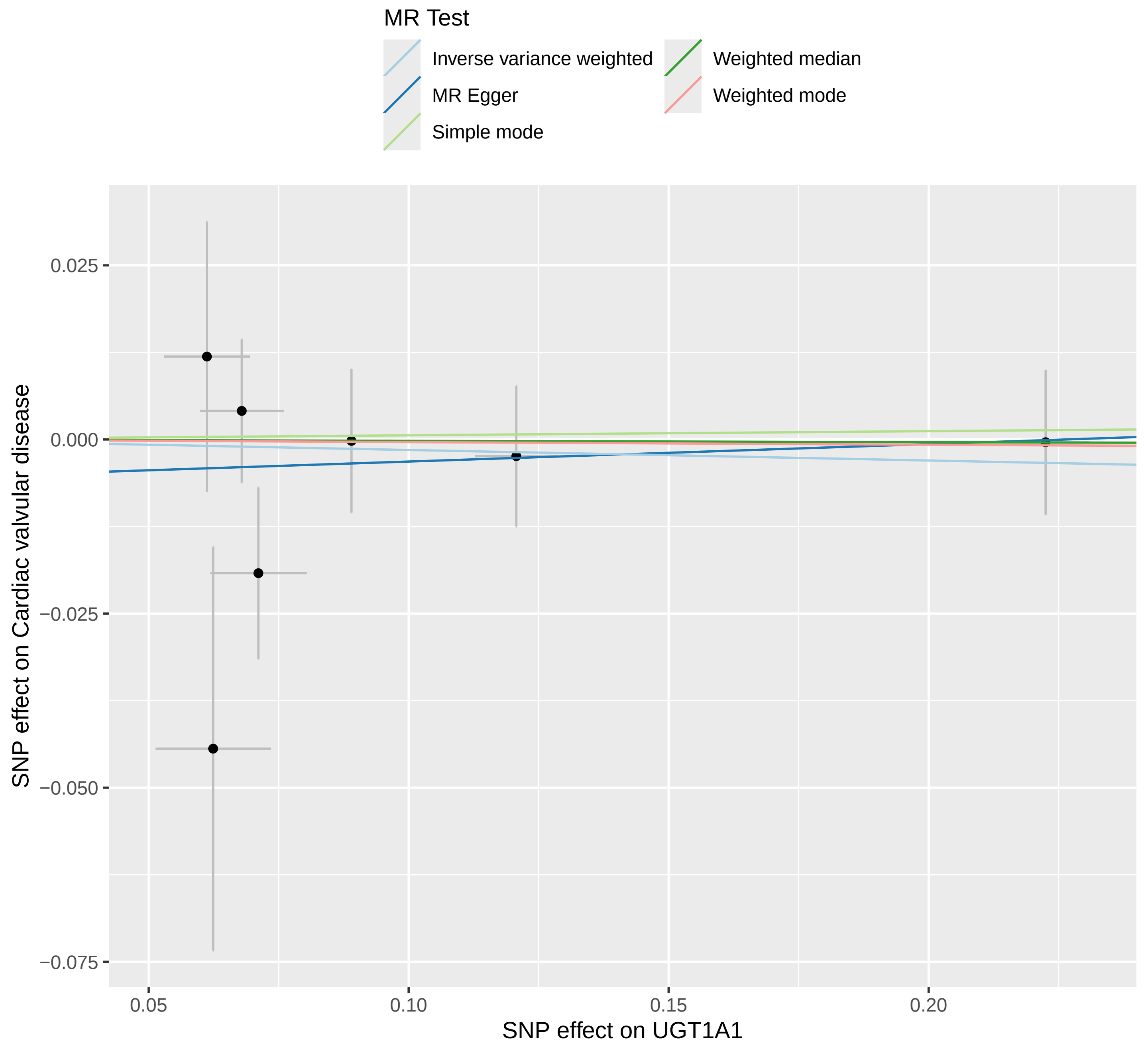
MR Test
Inverse variance weighted
MR Egger
Simple mode
Weighted median
Weighted mode
0.025
0.000
−0.025
−0.050
−0.075
SNP effect on Cardiac valvular disease
0.05
0.10
0.15
0.20
SNP effect on UGT1A1

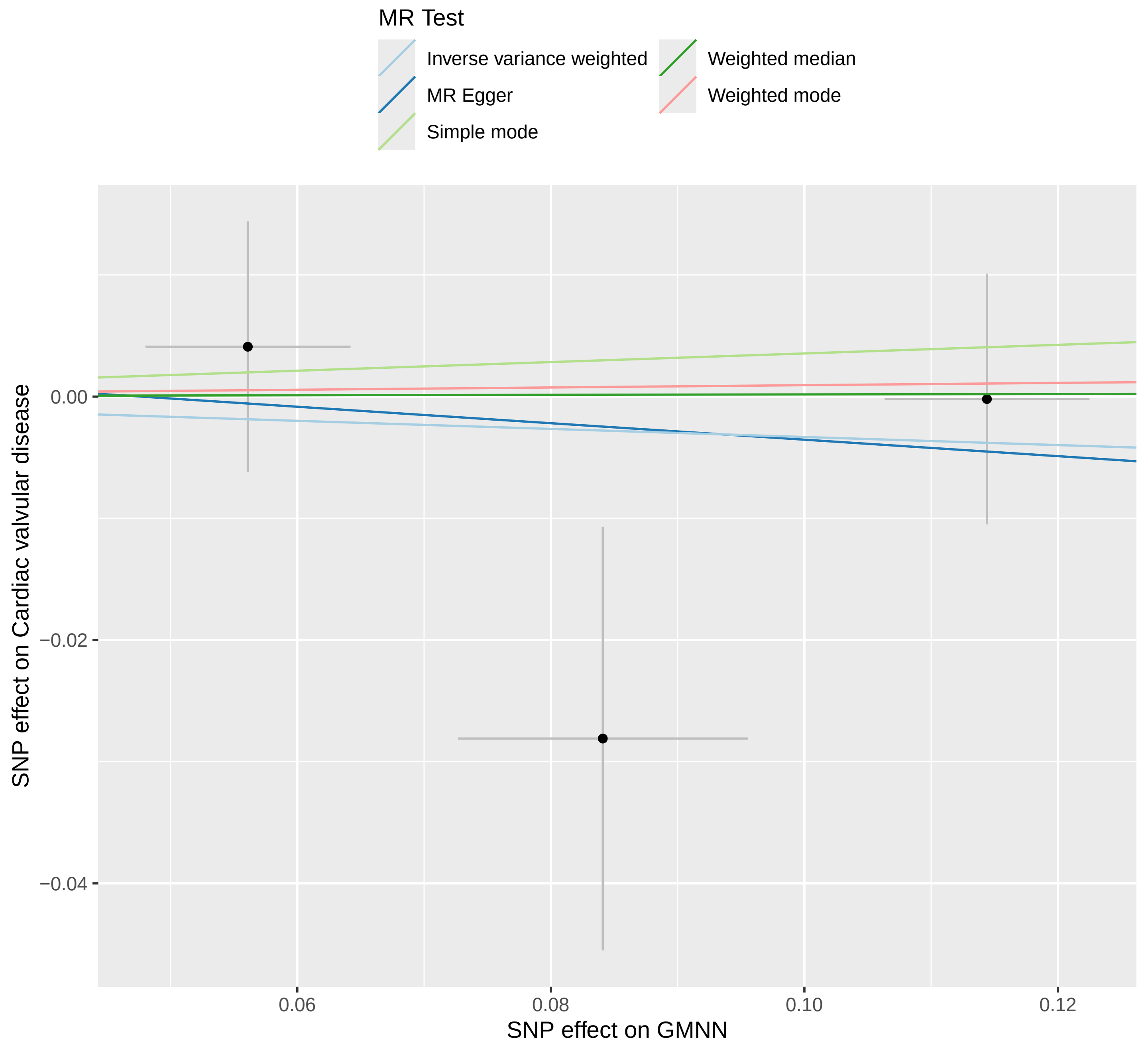
MR Test
Inverse variance weighted
MR Egger
Simple mode
Weighted median
Weighted mode
0.00
−0.02
−0.04
SNP effect on Cardiac valvular disease
0.06
0.08
0.10
0.12
SNP effect on GMNN

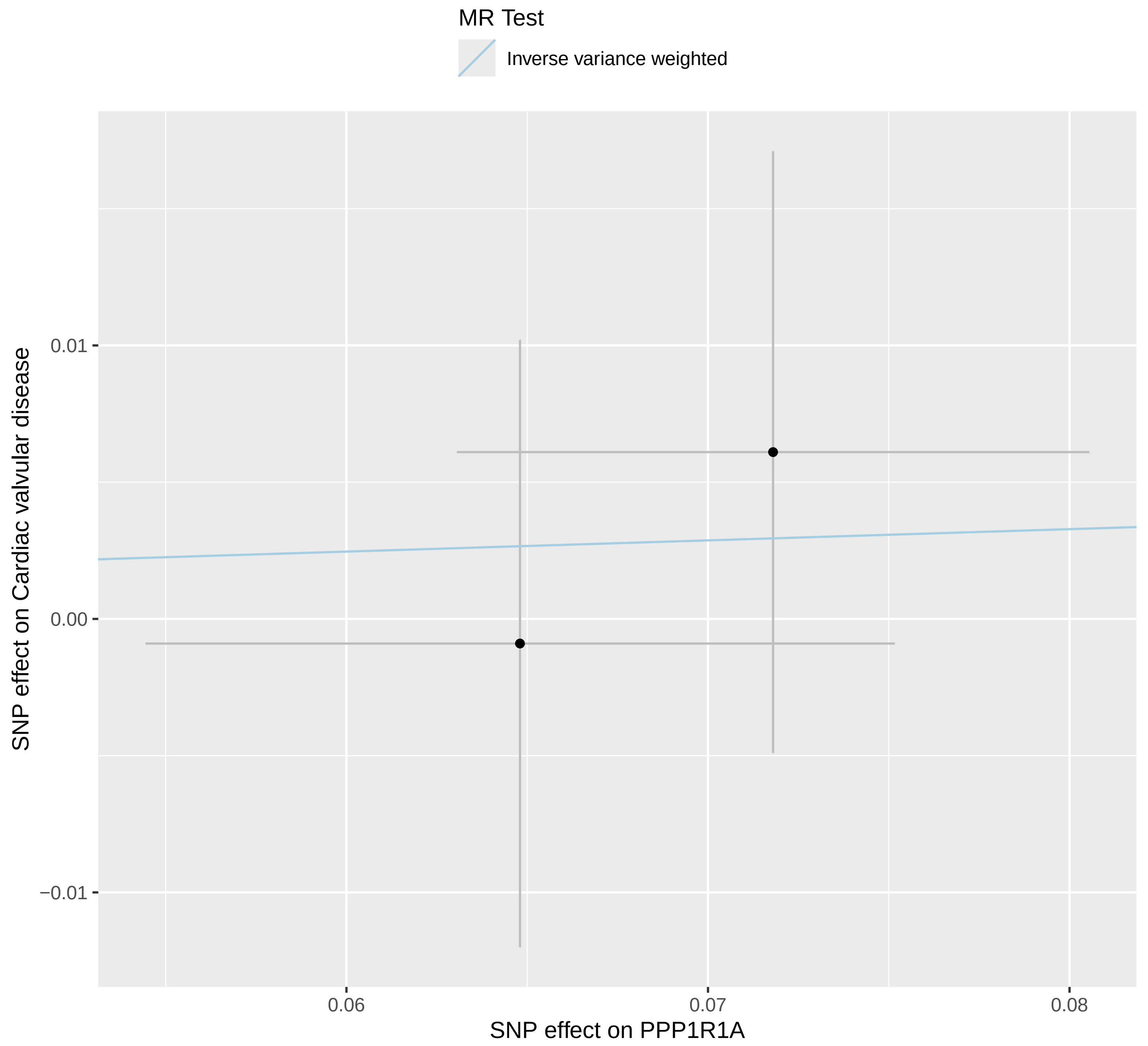

MR Test
Inverse variance weighted
SNP effect on Cardiac valvular disease
0.01
0.00
−0.01
0.06
0.07
0.08
SNP effect on PPP1R1A

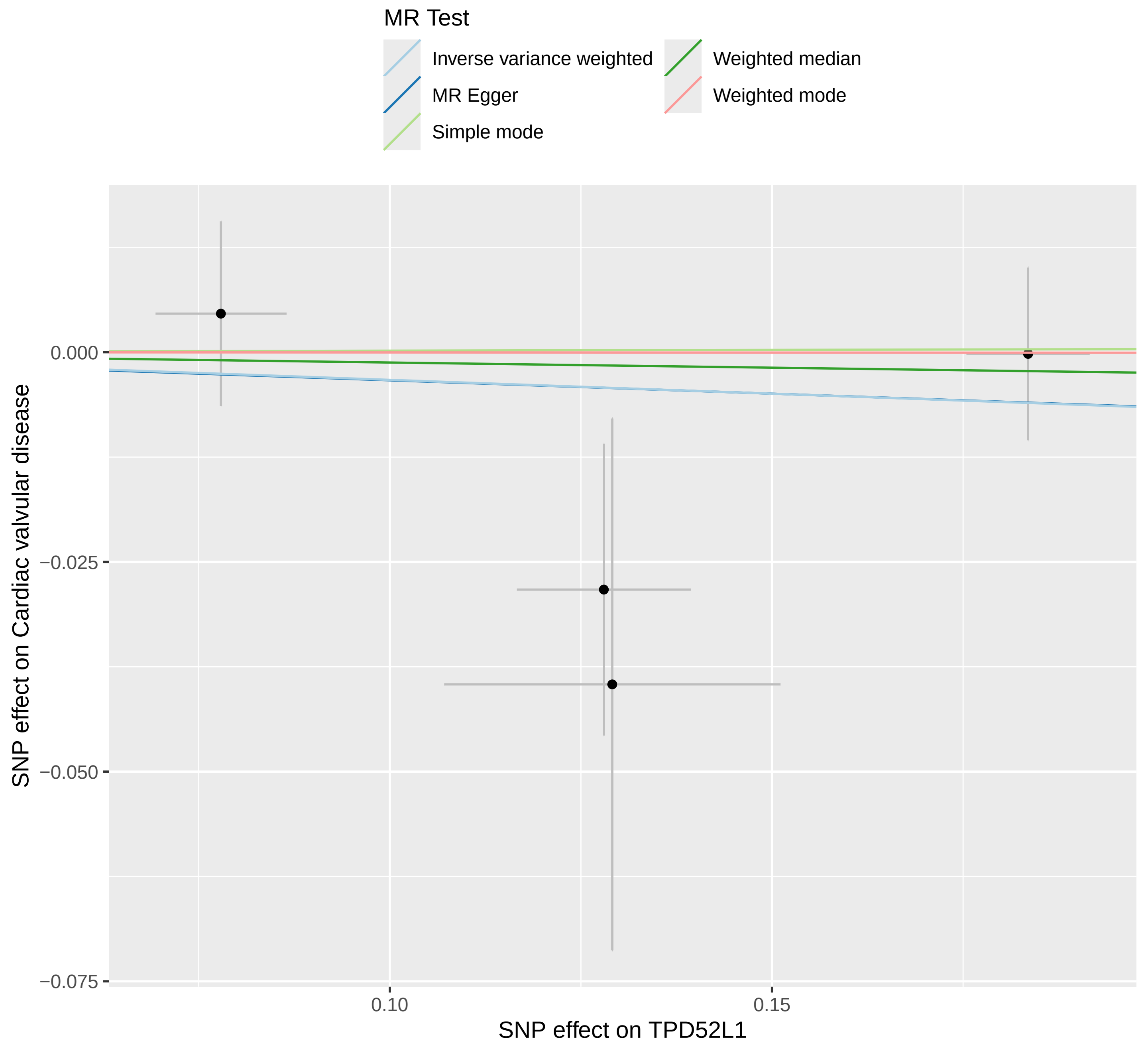
MR Test
Inverse variance weighted
MR Egger
Simple mode
Weighted median
Weighted mode
0.000
−0.025
−0.050
−0.075
0.10
0.15
SNP effect on Cardiac valvular disease
SNP effect on TPD52L1

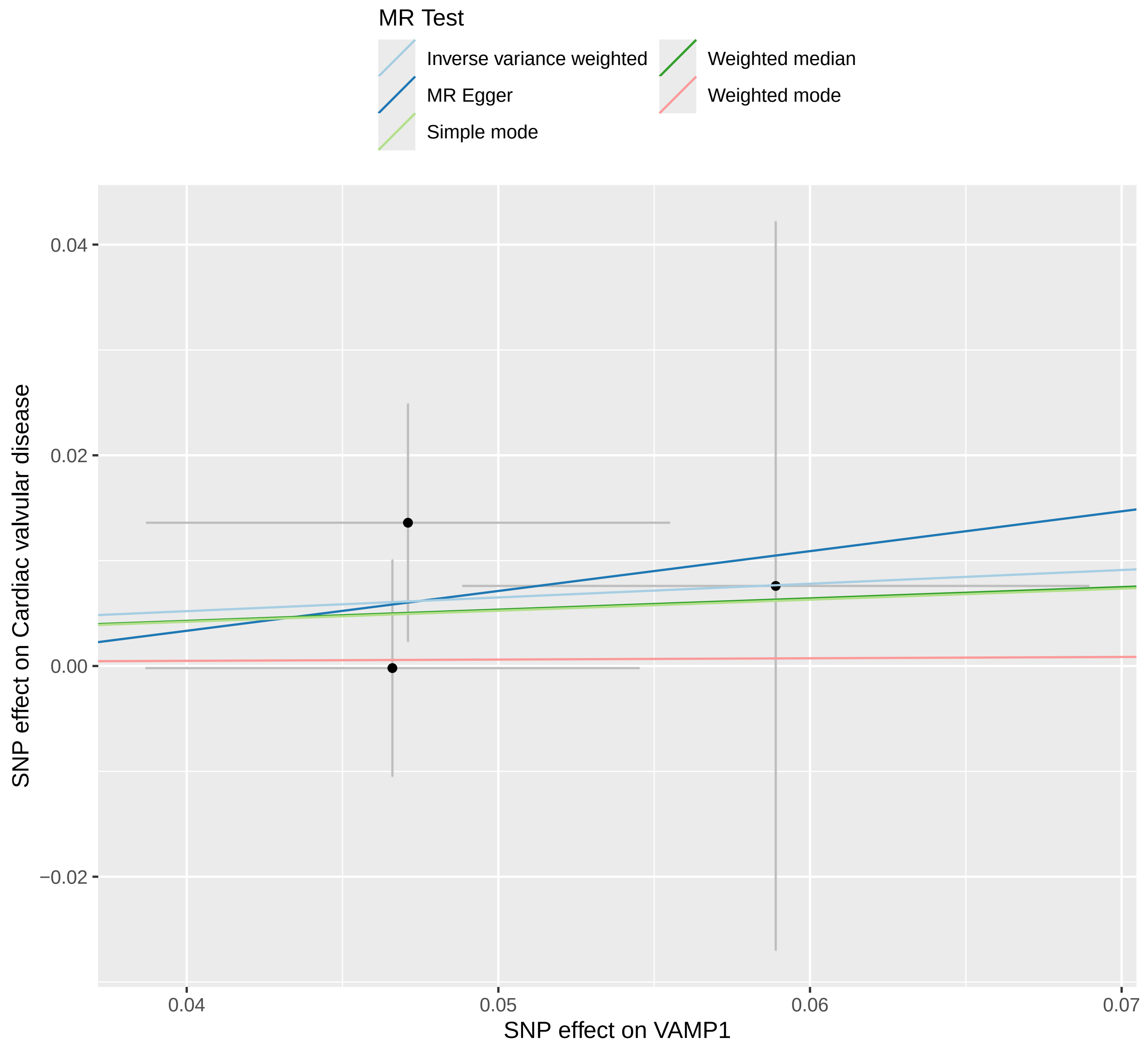

MR Test
Inverse variance weighted
MR Egger
Simple mode
Weighted median
Weighted mode
0.04
0.02
0.00
−0.02
SNP effect on Cardiac valvular disease
0.04
0.05
0.06
0.07
SNP effect on VAMP1

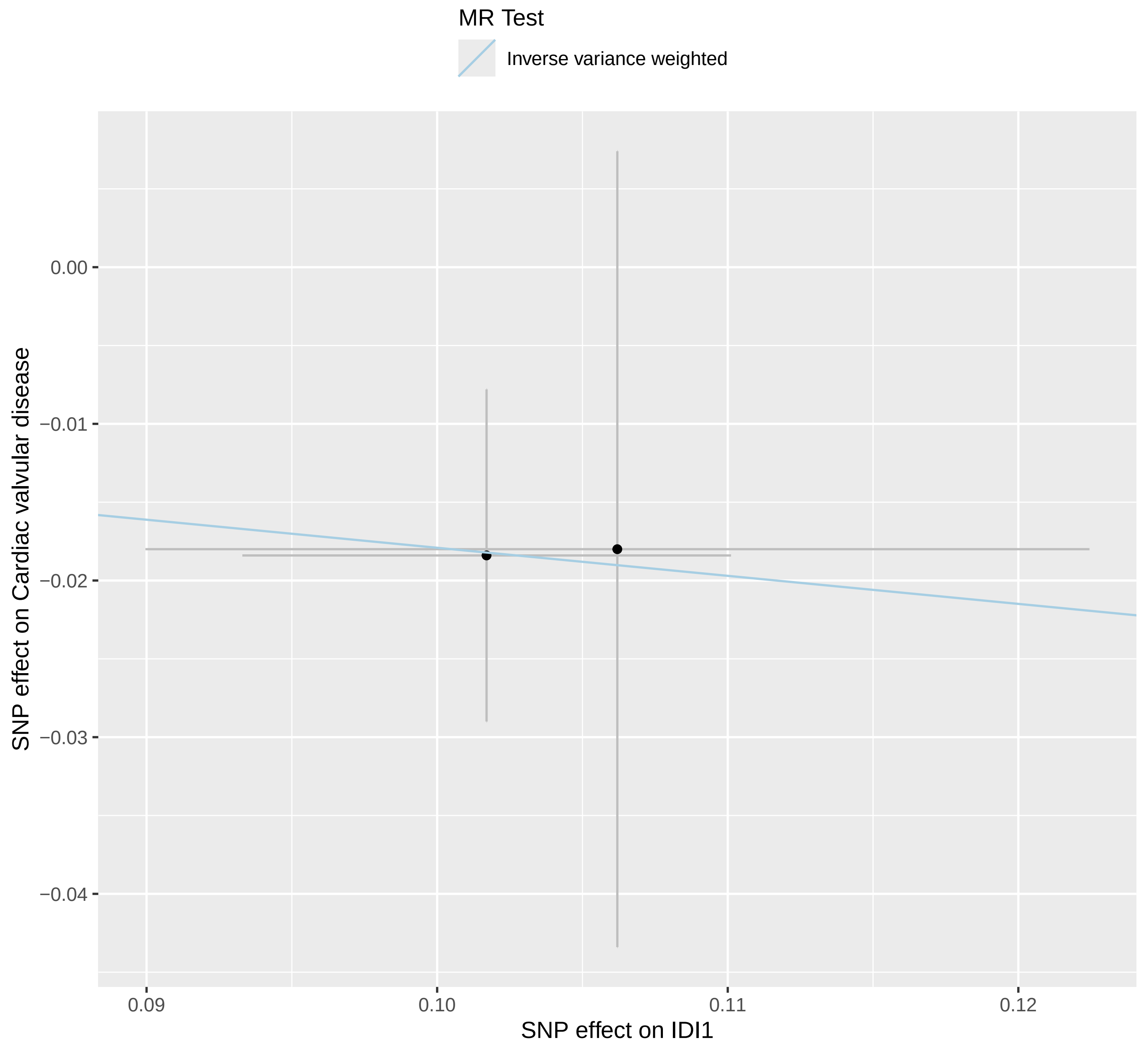
MR Test
Inverse variance weighted
0.00
−0.01
−0.02
−0.03
−0.04
SNP effect on Cardiac valvular disease
0.09
0.10
0.11
0.12
SNP effect on IDI1

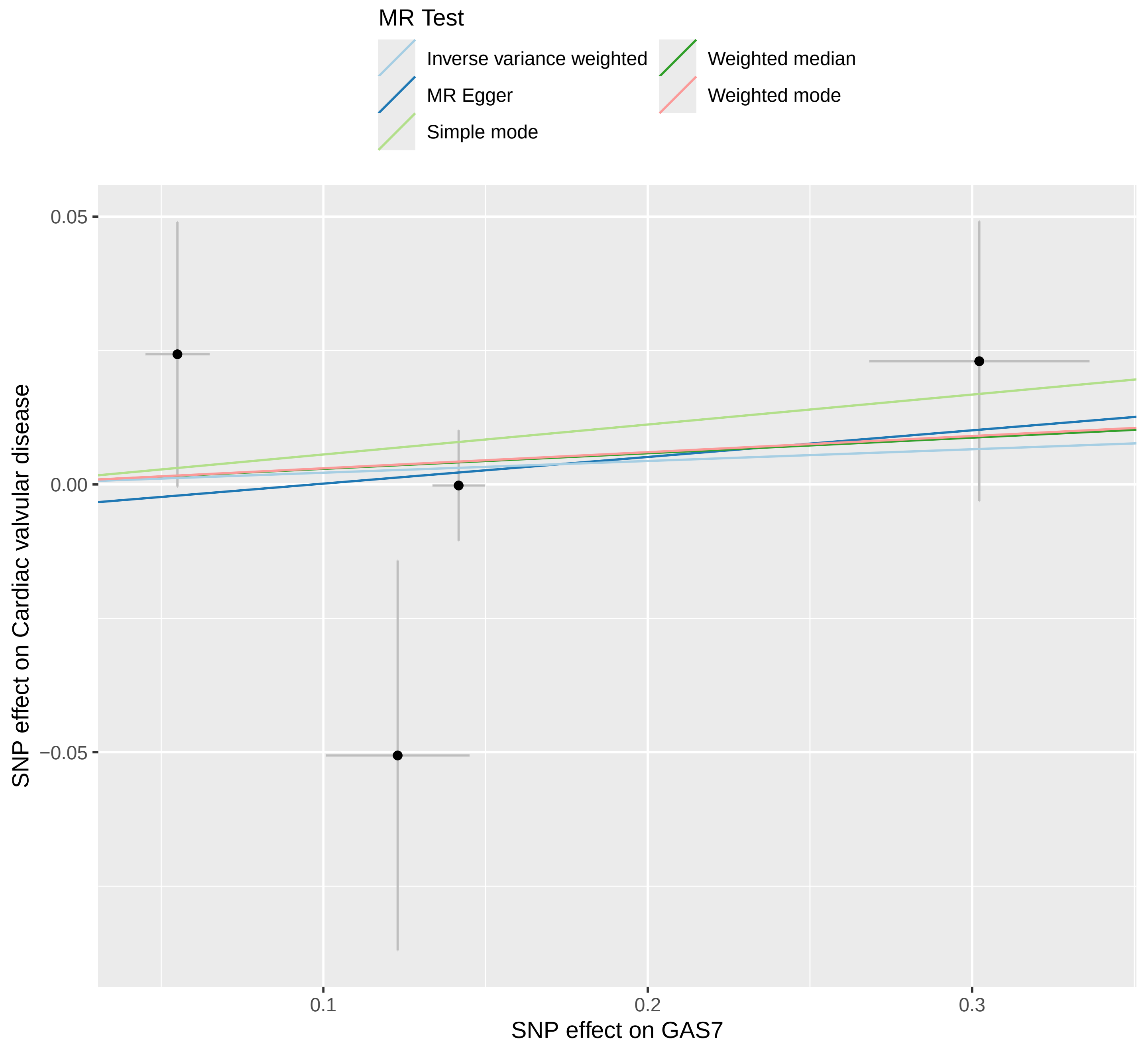
MR Test
Inverse variance weighted
MR Egger
Simple mode
Weighted median
Weighted mode
0.05
0.00
−0.05
0.1
0.2
0.3
SNP effect on Cardiac valvular disease
SNP effect on GAS7

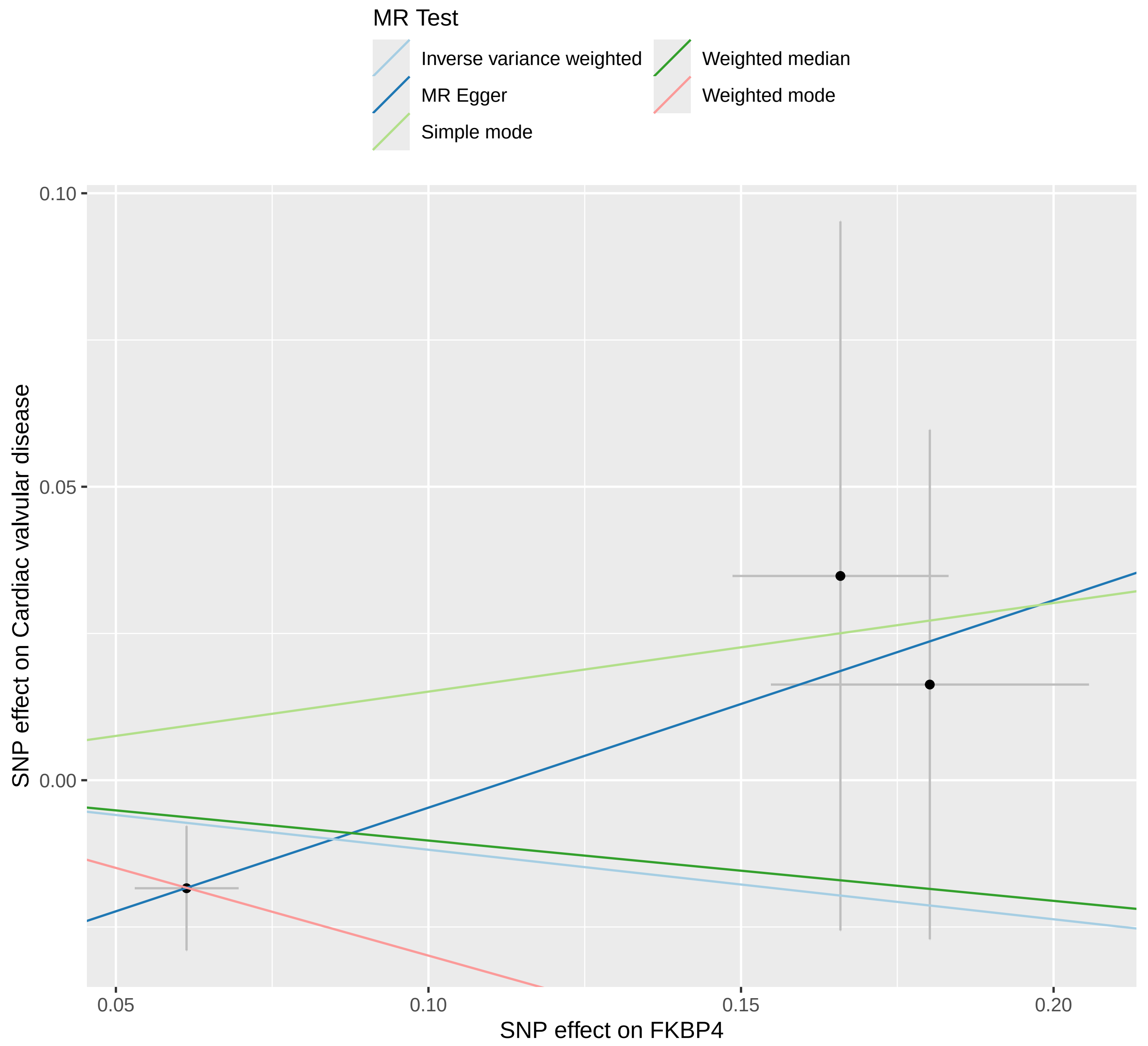

MR Test
Inverse variance weighted
MR Egger
Simple mode
Weighted median
Weighted mode
0.10
0.05
0.00
0.05
0.10
0.15
0.20
SNP effect on Cardiac valvular disease
SNP effect on FKBP4

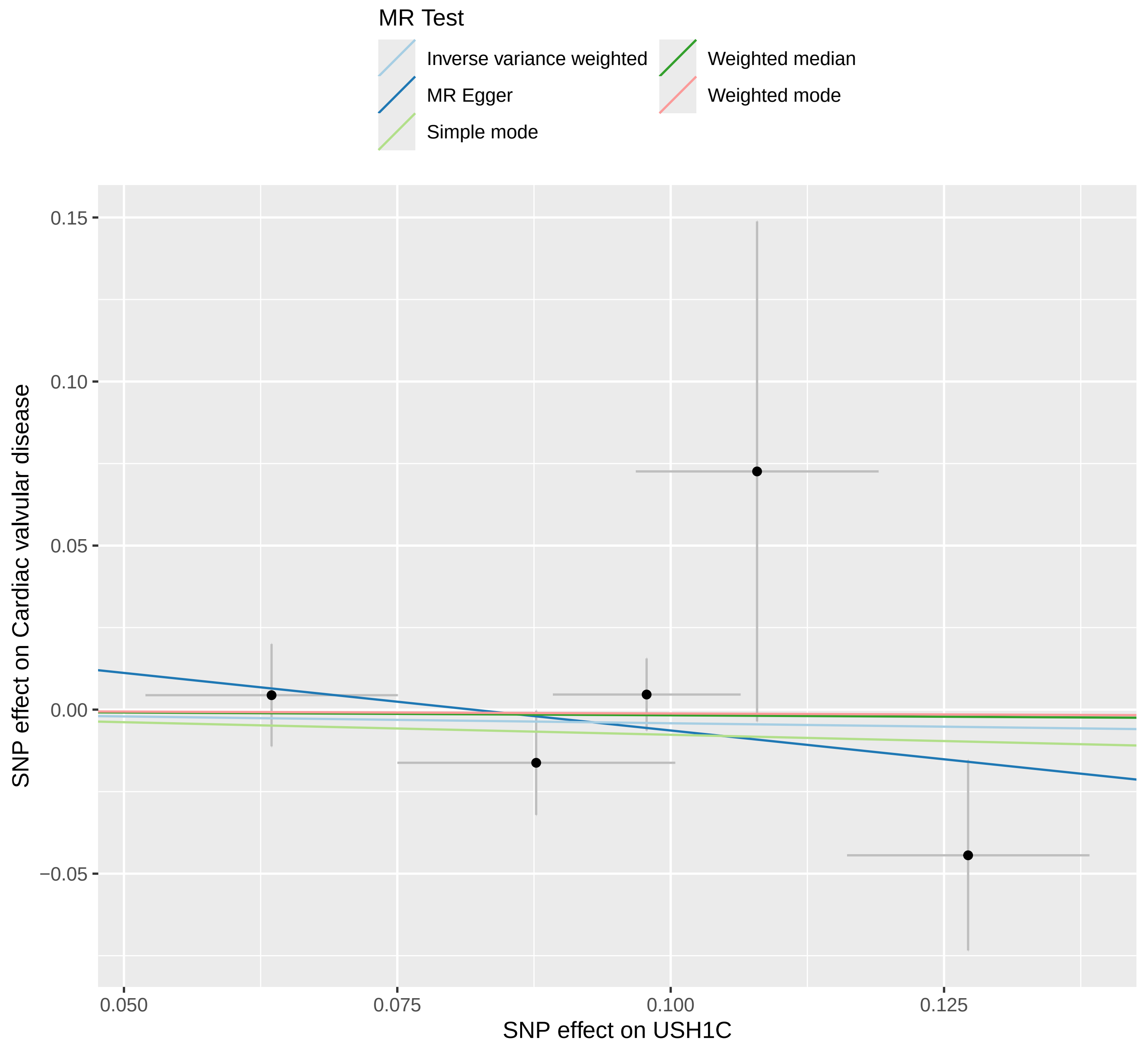

MR Test
Inverse variance weighted
MR Egger
Simple mode
Weighted median
Weighted mode
0.15
0.10
0.05
0.00
−0.05
0.050
0.075
0.100
0.125
SNP effect on Cardiac valvular disease
SNP effect on USH1C

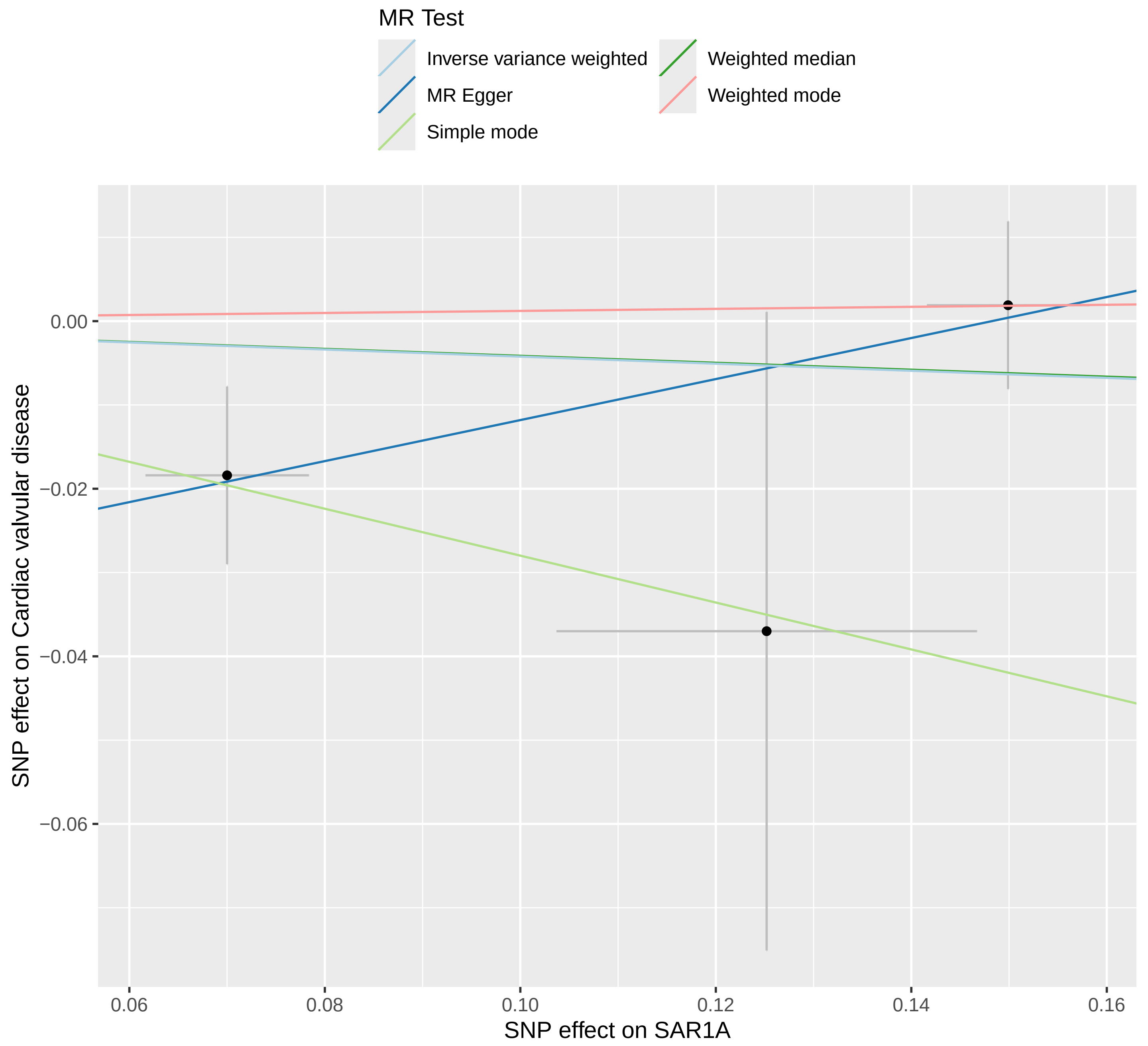
MR Test
Inverse variance weighted
MR Egger
Simple mode
Weighted median
Weighted mode
0.00
−0.02
−0.04
−0.06
SNP effect on Cardiac valvular disease
0.06
0.08
0.10
0.12
0.14
0.16
SNP effect on SAR1A

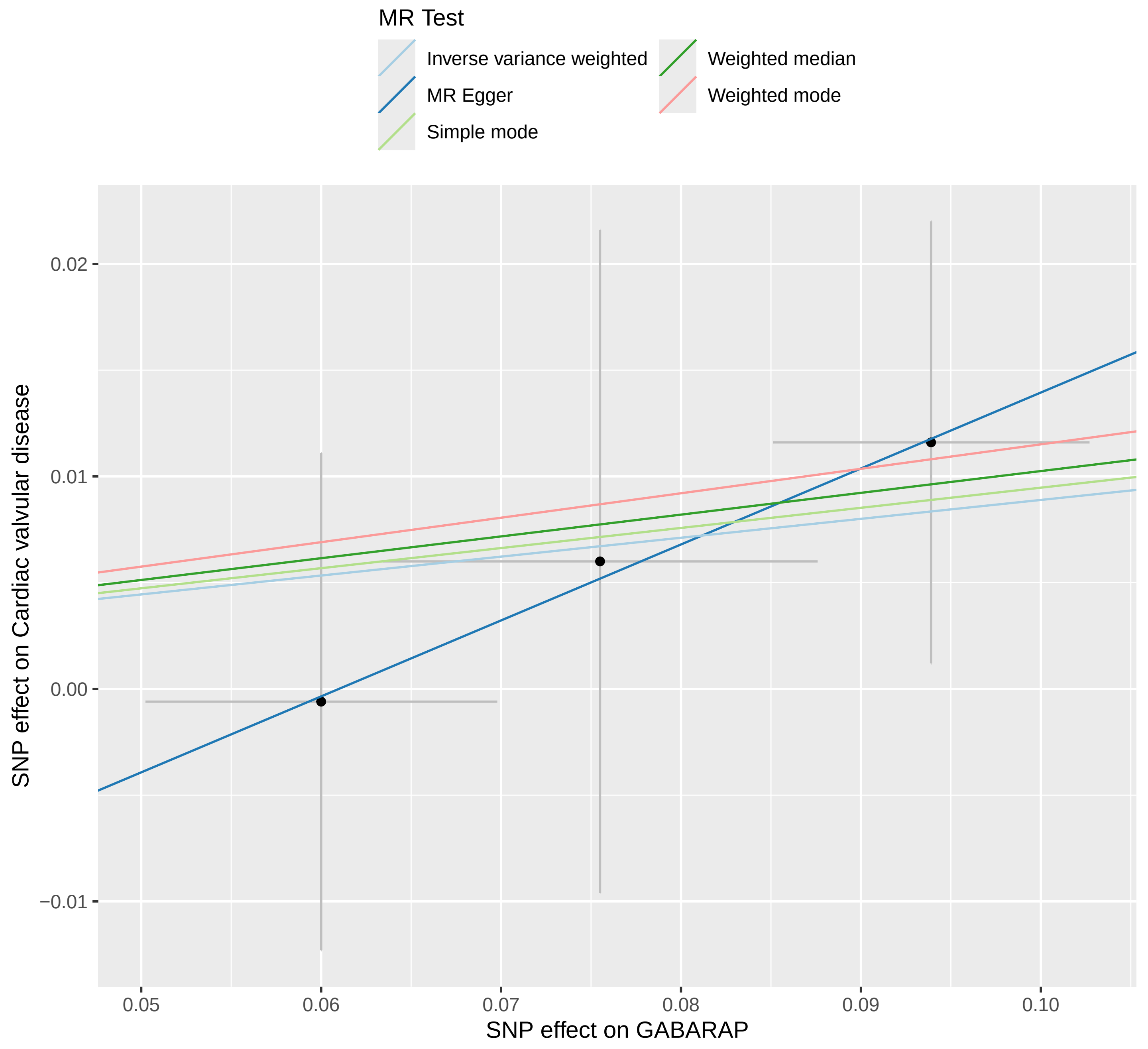

MR Test
Inverse variance weighted
MR Egger
Simple mode
Weighted median
Weighted mode
SNP effect on Cardiac valvular disease
0.02
0.01
0.00
−0.01
0.05
0.06
0.07
0.08
0.09
0.10
SNP effect on GABARAP

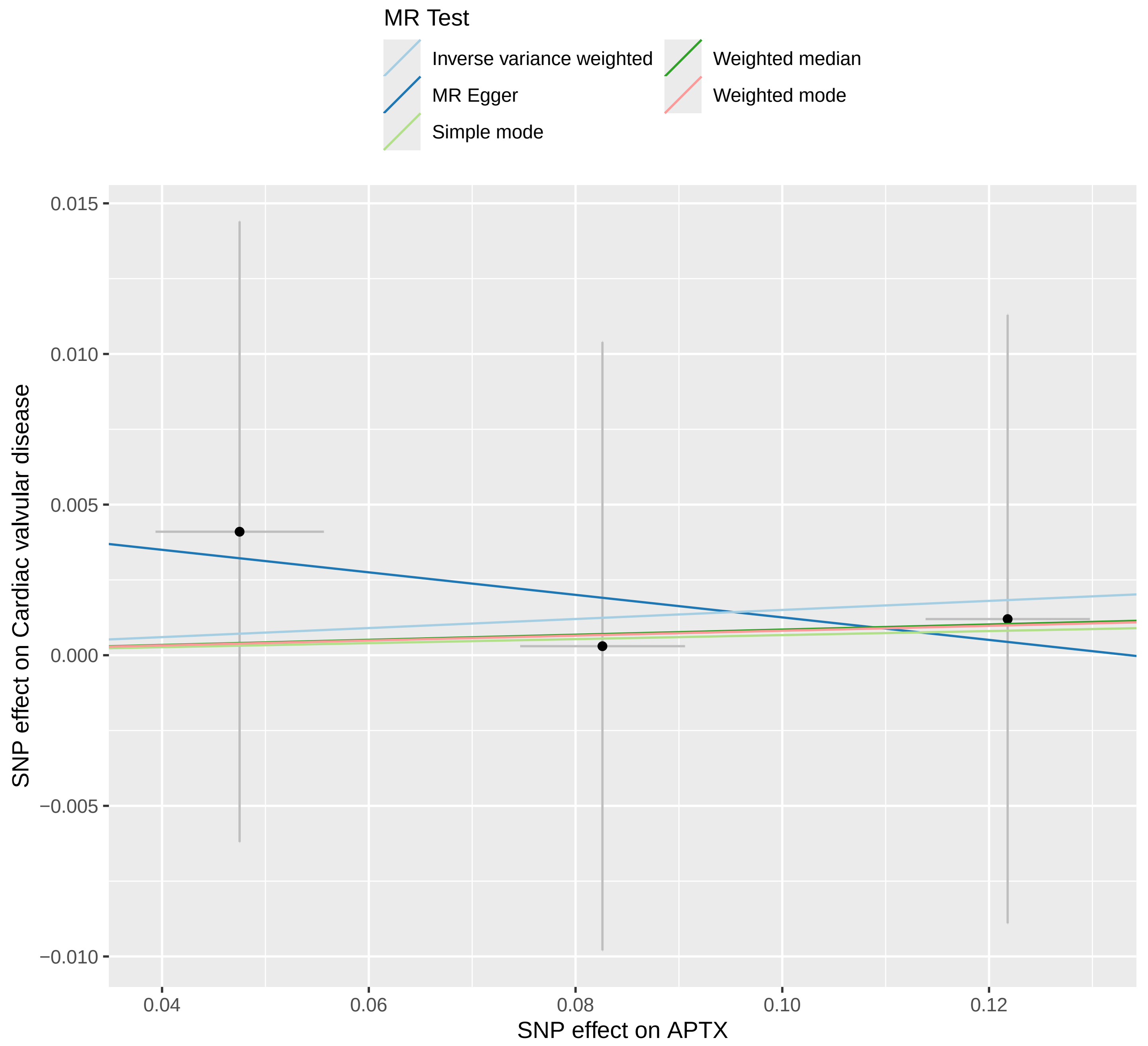
MR Test
Inverse variance weighted
MR Egger
Simple mode
Weighted median
Weighted mode
0.015
0.010
0.005
0.000
−0.005
−0.010
SNP effect on Cardiac valvular disease
0.04
0.06
0.08
0.10
0.12
SNP effect on APTX

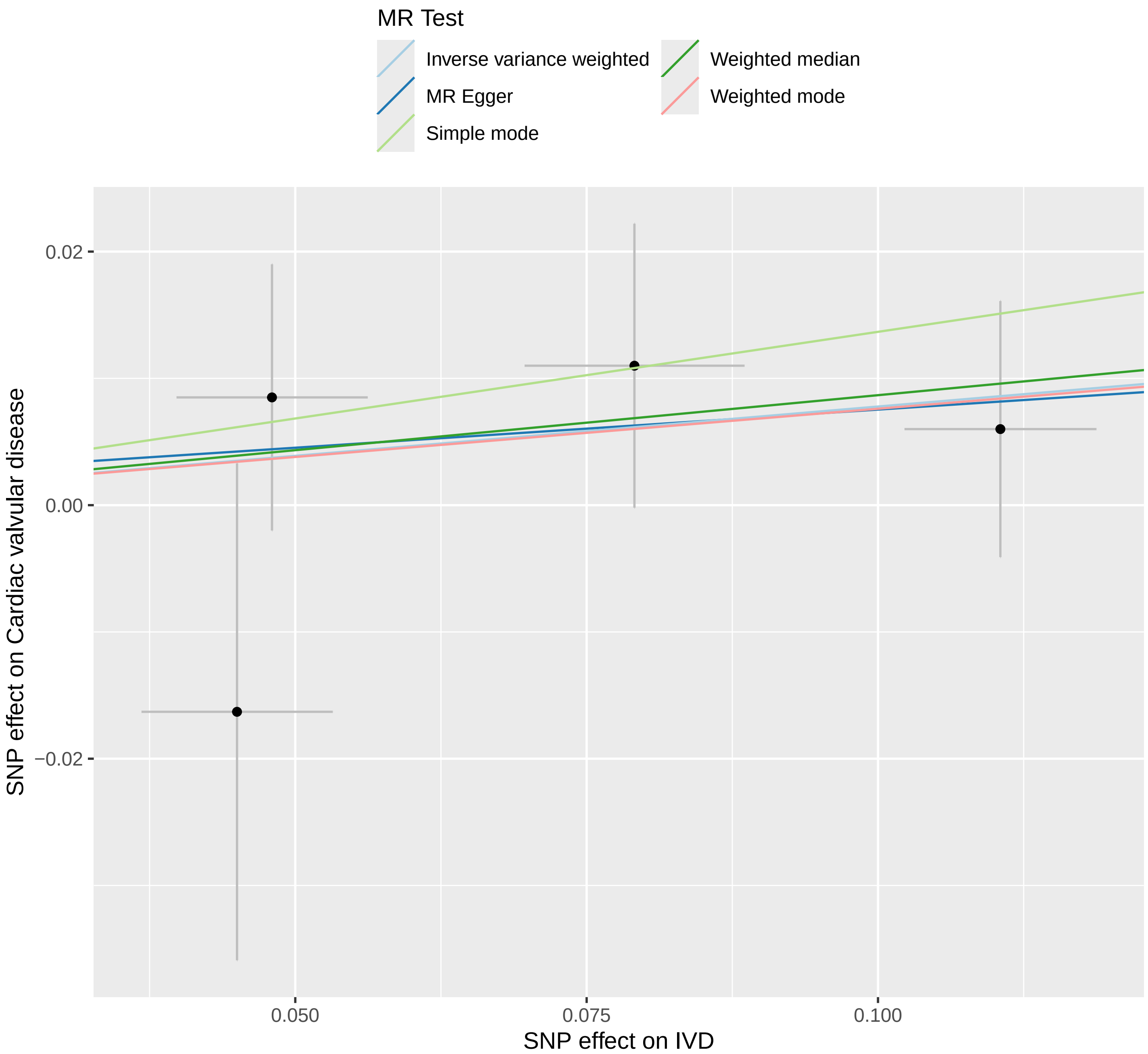
MR Test
Inverse variance weighted
MR Egger
Simple mode
Weighted median
Weighted mode
0.02
0.00
−0.02
0.050
0.075
0.100
SNP effect on Cardiac valvular disease
SNP effect on IVD

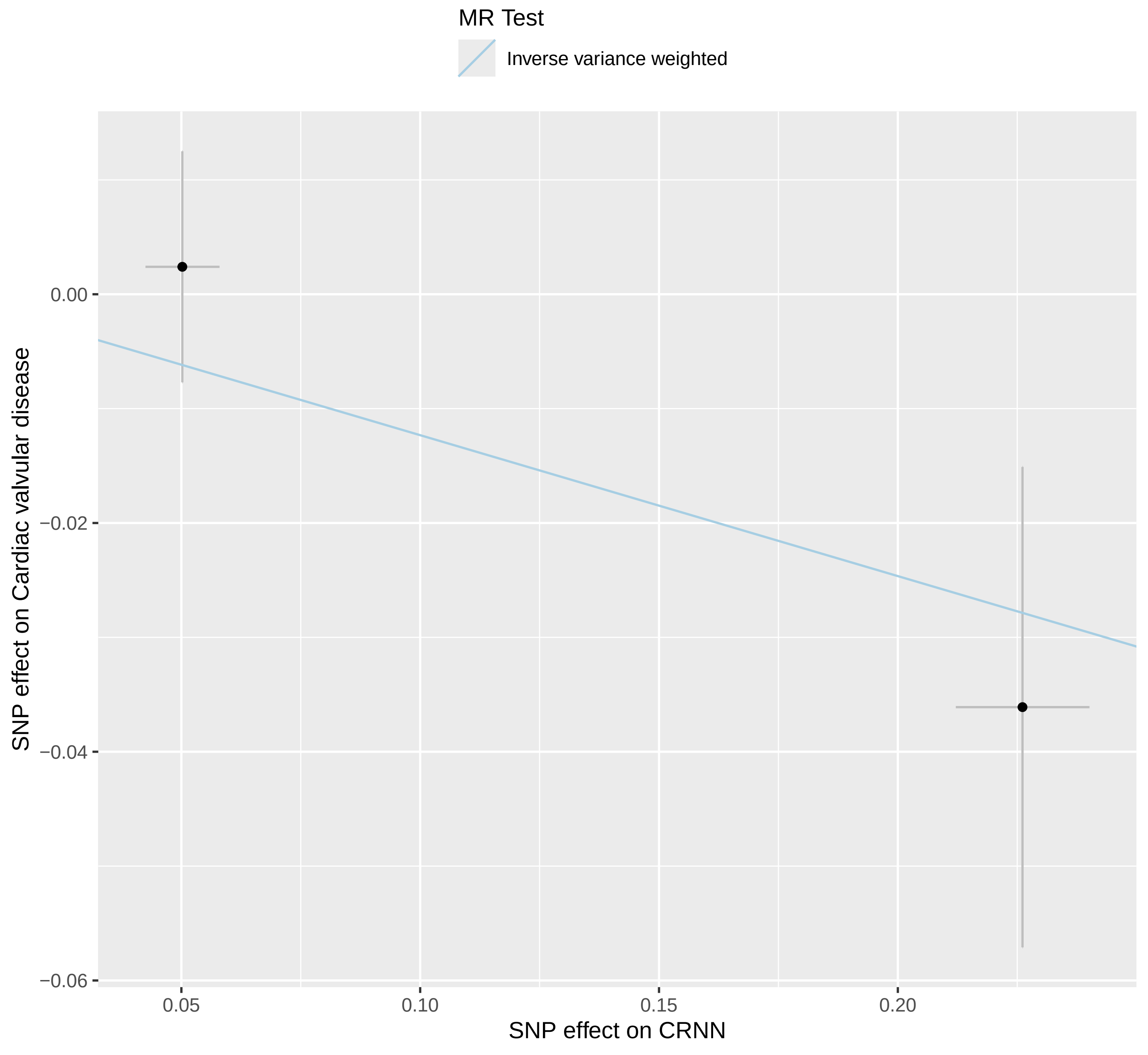
MR Test
Inverse variance weighted
0.00
−0.02
−0.04
−0.06
SNP effect on Cardiac valvular disease
0.05
0.10
0.15
0.20
SNP effect on CRNN

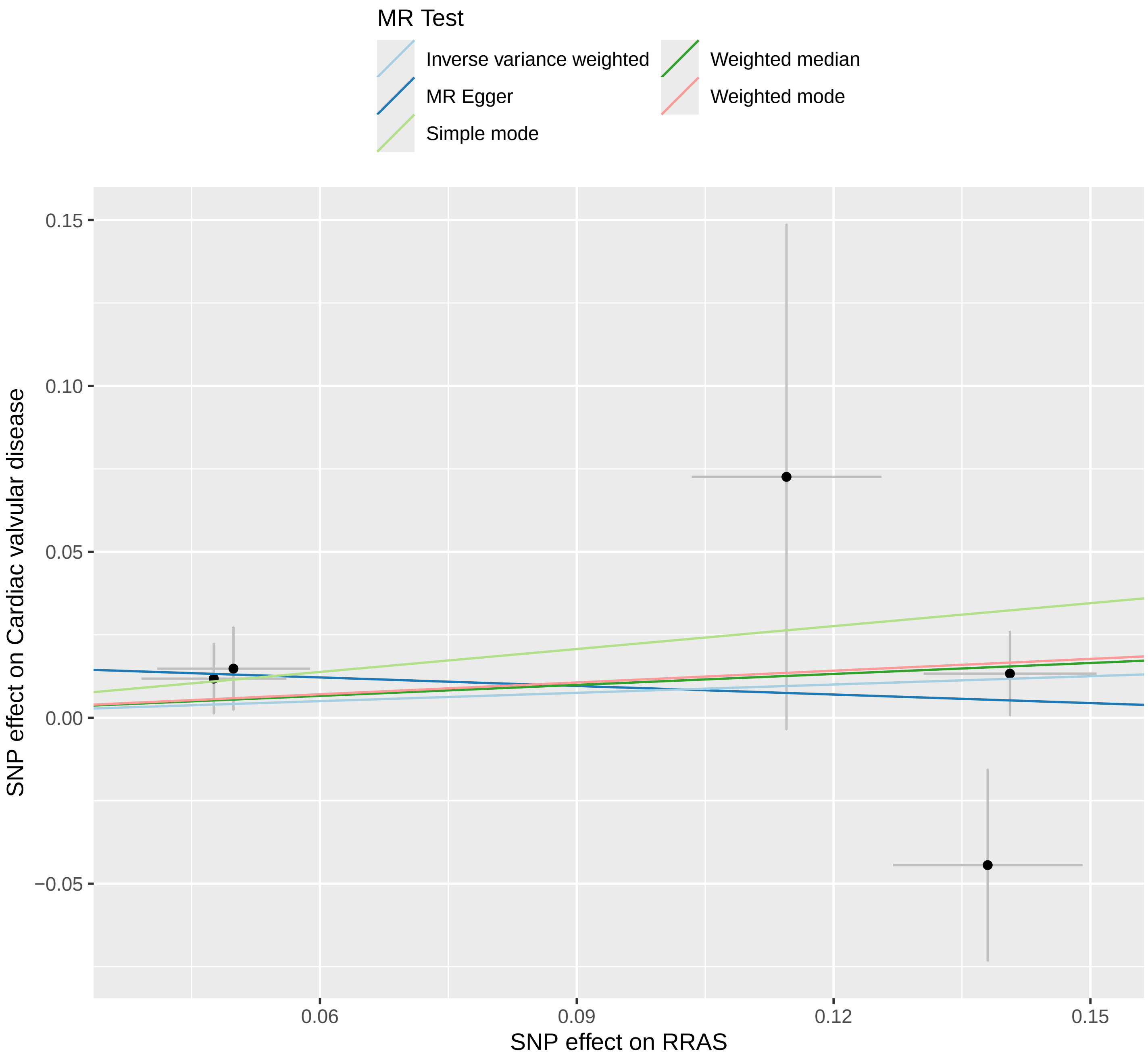
MR Test
Inverse variance weighted
MR Egger
Simple mode
Weighted median
Weighted mode
0.15
0.10
0.05
0.00
−0.05
SNP effect on Cardiac valvular disease
0.06
0.09
0.12
0.15
SNP effect on RRAS

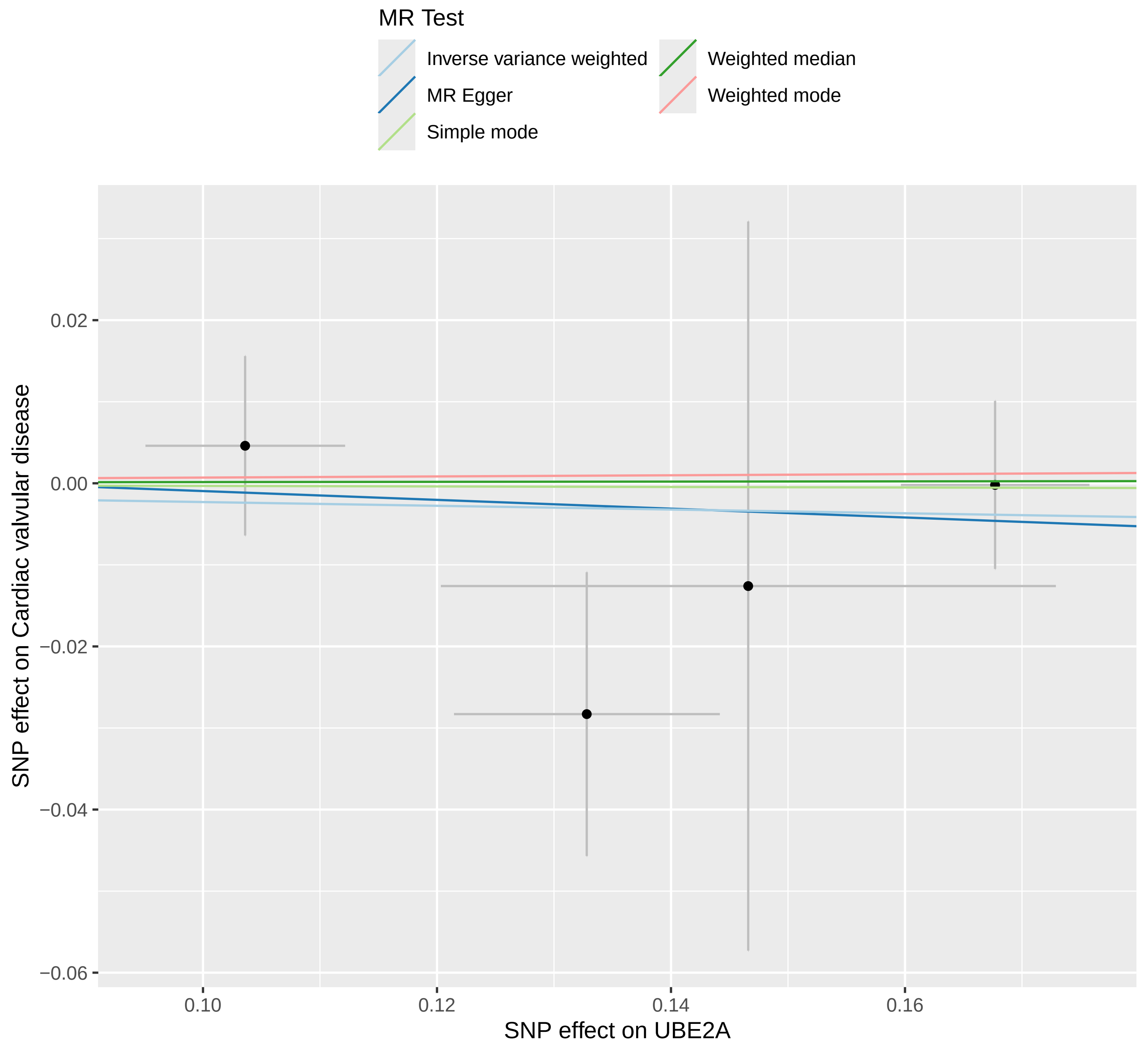

MR Test
Inverse variance weighted
MR Egger
Simple mode
Weighted median
Weighted mode
0.02
0.00
−0.02
−0.04
−0.06
0.10
0.12
0.14
0.16
SNP effect on Cardiac valvular disease
SNP effect on UBE2A

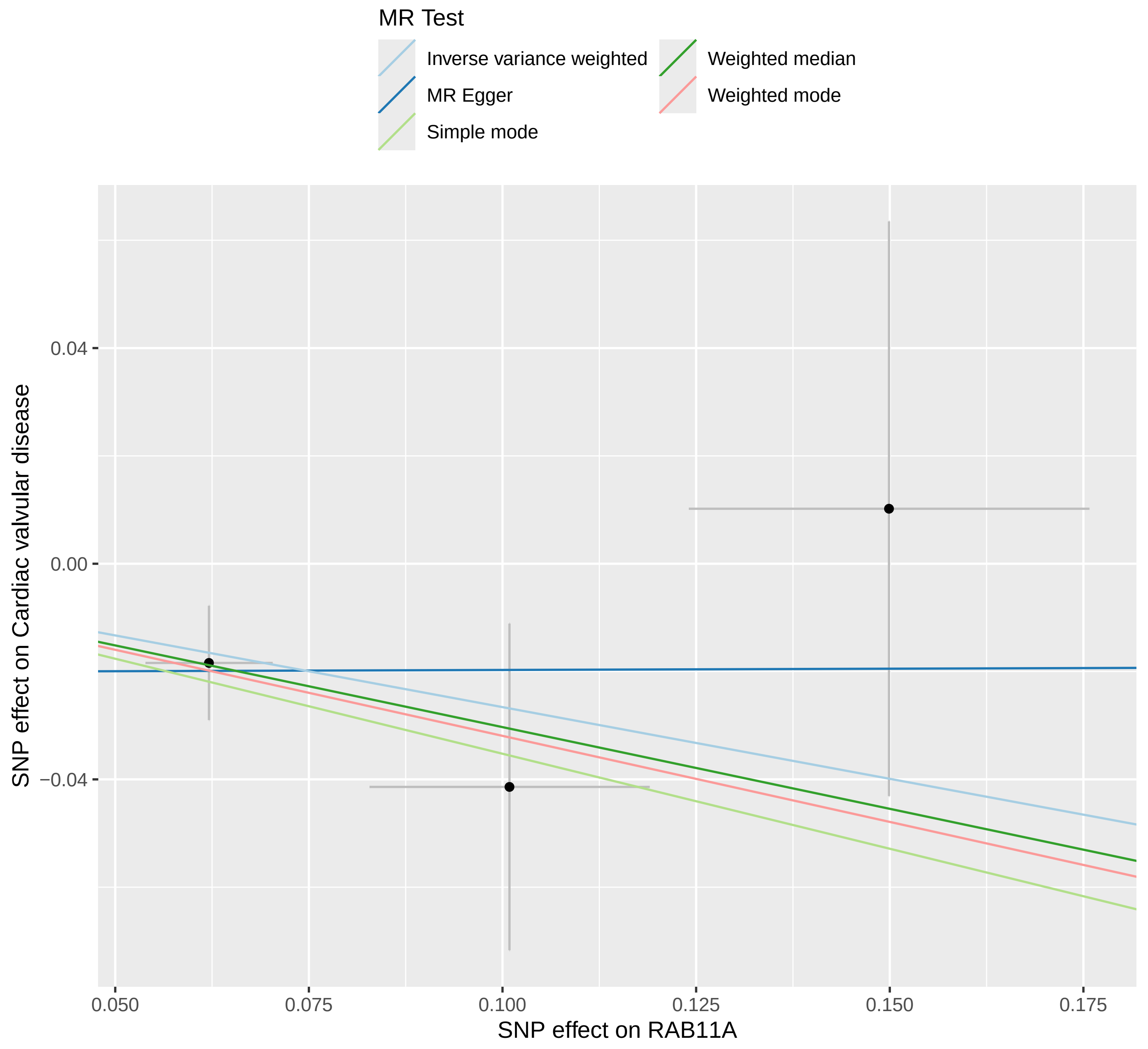
MR Test
Inverse variance weighted
MR Egger
Simple mode
Weighted median
Weighted mode
0.04
0.00
−0.04
SNP effect on Cardiac valvular disease
0.050
0.075
0.100
0.125
0.150
0.175
SNP effect on RAB11A

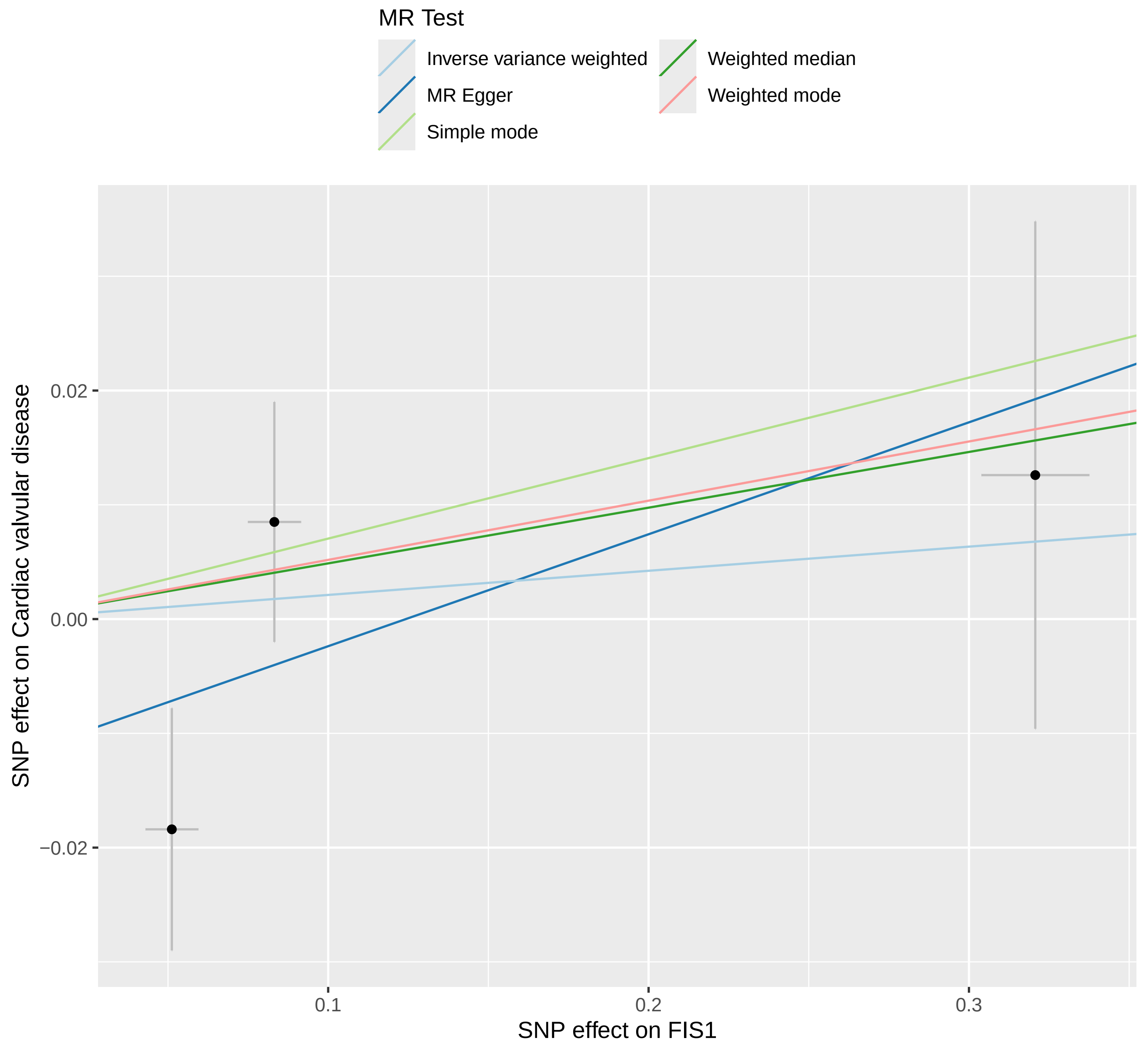

MR Test
Inverse variance weighted
MR Egger
Simple mode
Weighted median
Weighted mode
0.02
0.00
−0.02
SNP effect on Cardiac valvular disease
0.1
0.2
0.3
SNP effect on FIS1

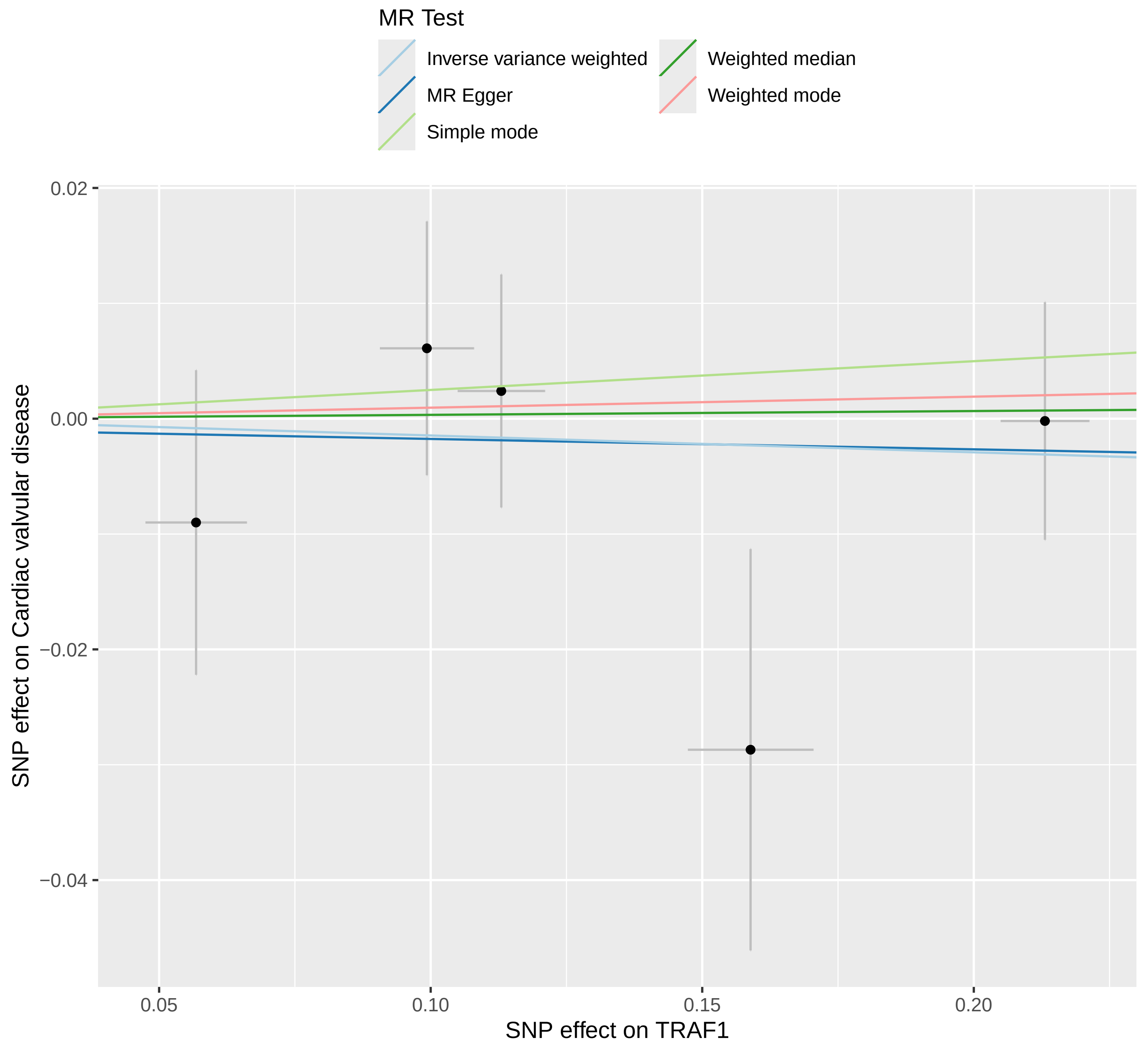
MR Test
Inverse variance weighted
MR Egger
Simple mode
Weighted median
Weighted mode
0.02
0.00
−0.02
−0.04
SNP effect on Cardiac valvular disease
0.05
0.10
0.15
0.20
SNP effect on TRAF1

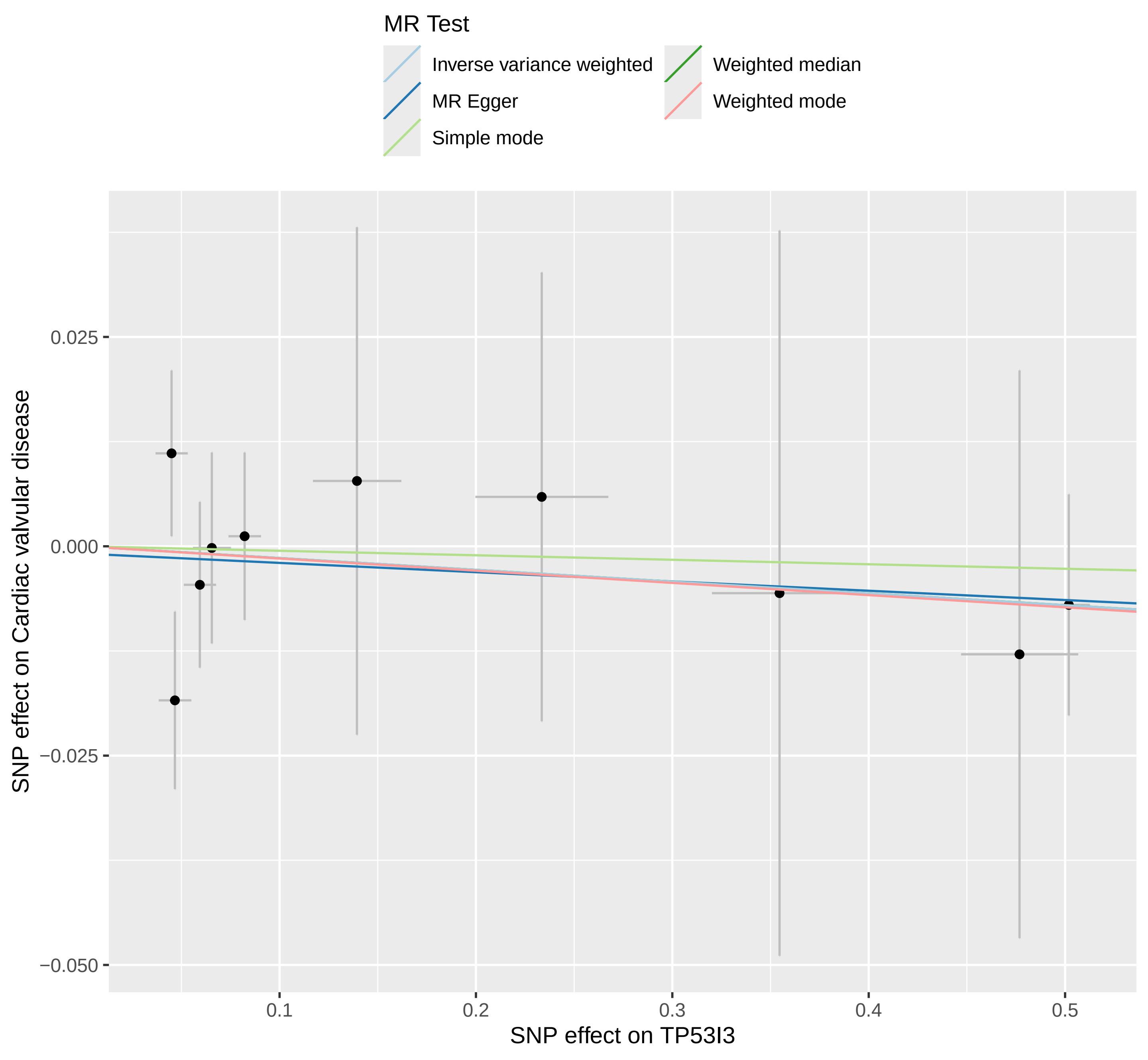
MR Test
Inverse variance weighted
MR Egger
Simple mode
Weighted median
Weighted mode
0.025
0.000
−0.025
−0.050
0.1
0.2
0.3
0.4
0.5
SNP effect on Cardiac valvular disease
SNP effect on TP53I3

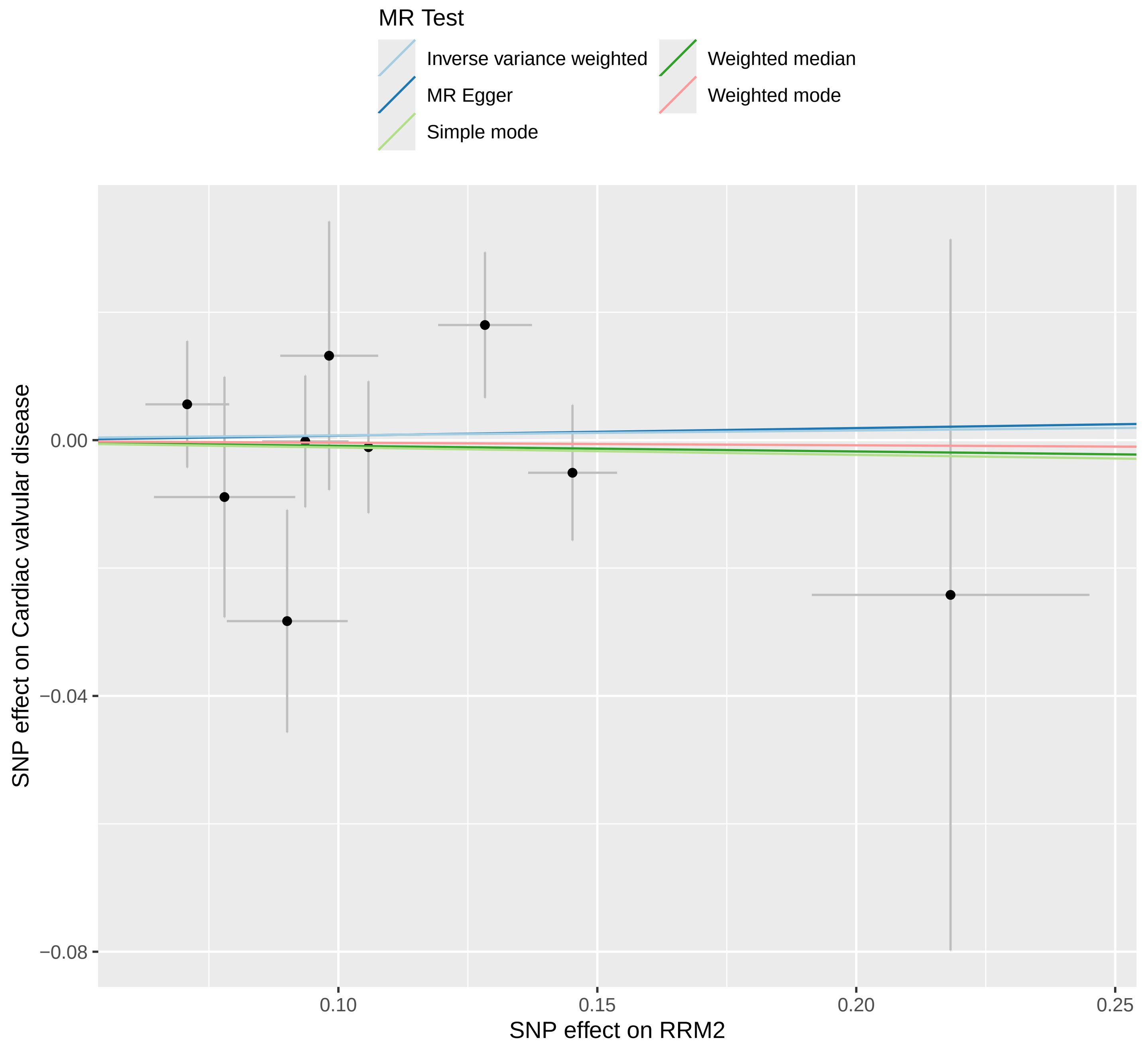
MR Test
Inverse variance weighted
MR Egger
Simple mode
Weighted median
Weighted mode
SNP effect on Cardiac valvular disease
0.00
−0.04
−0.08
0.10
0.15
0.20
0.25
SNP effect on RRM2

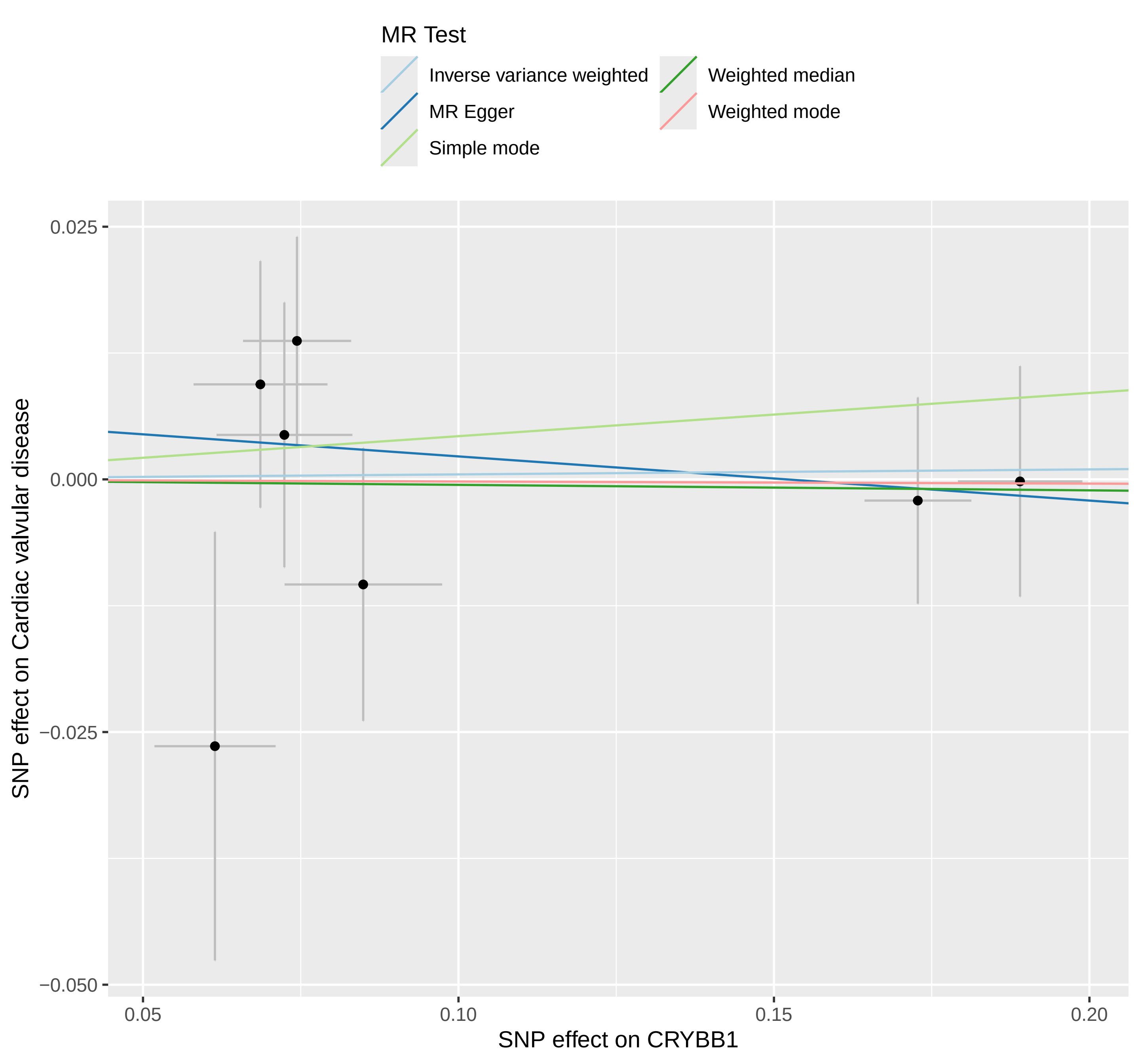
MR Test
Inverse variance weighted
MR Egger
Simple mode
Weighted median
Weighted mode
0.025
0.000
−0.025
−0.050
0.05
0.10
0.15
0.20
SNP effect on CRYBB1
SNP effect on Cardiac valvular disease

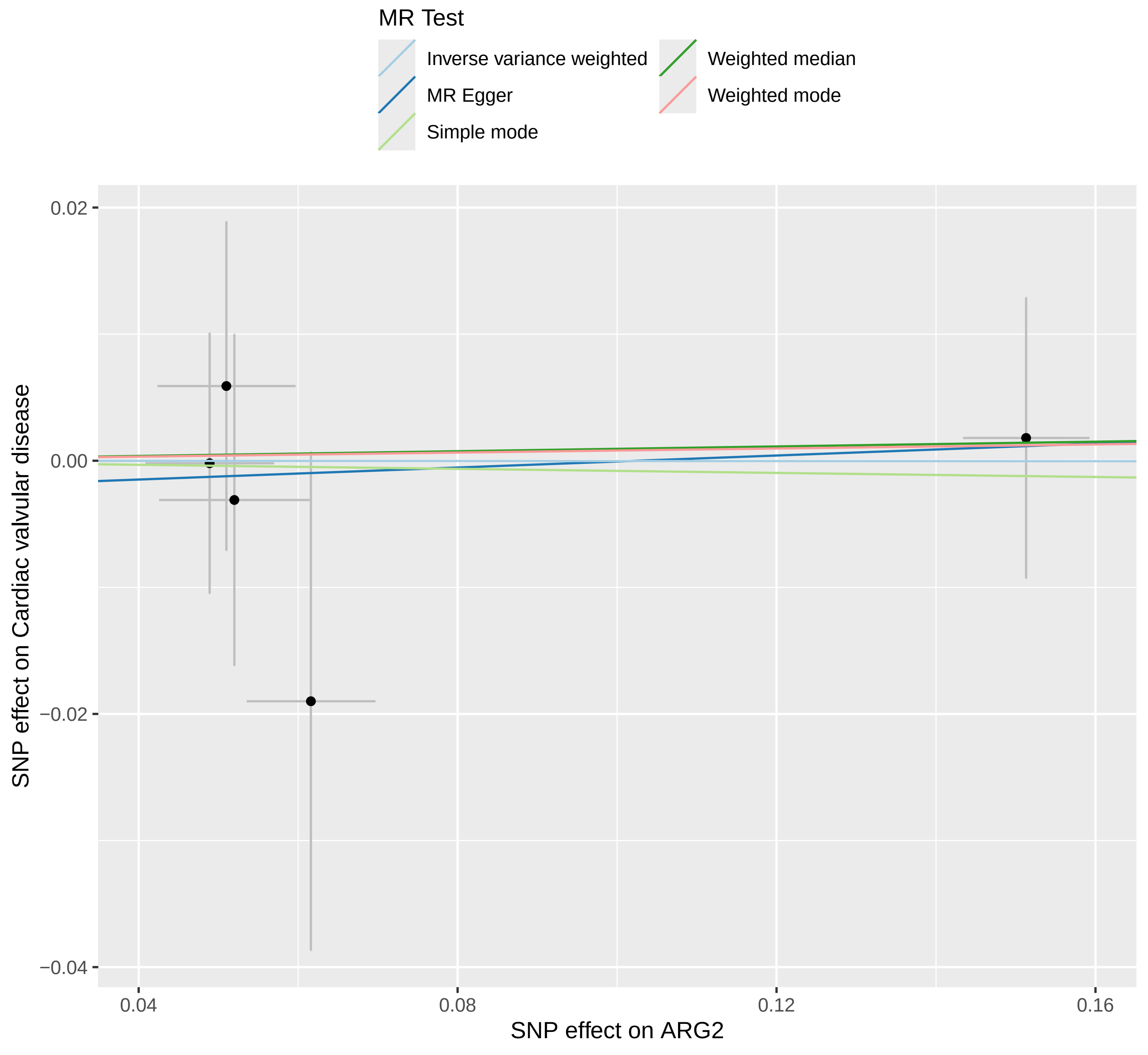
MR Test
Inverse variance weighted
MR Egger
Simple mode
Weighted median
Weighted mode
0.02
0.00
−0.02
−0.04
SNP effect on Cardiac valvular disease
0.04
0.08
0.12
0.16
SNP effect on ARG2

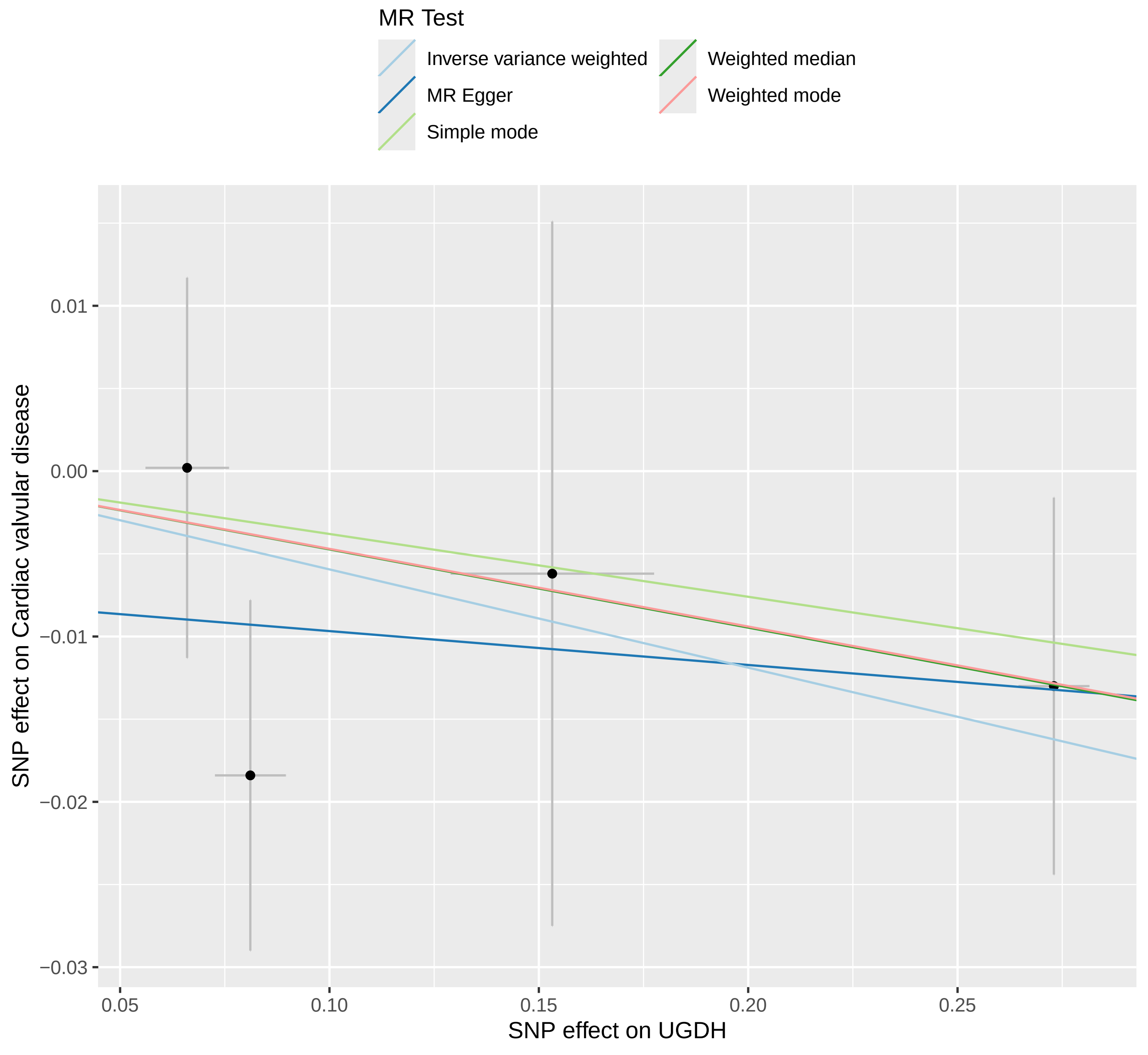
MR Test
Inverse variance weighted
MR Egger
Simple mode
Weighted median
Weighted mode
0.01
0.00
−0.01
−0.02
−0.03
0.05
0.10
0.15
0.20
0.25
SNP effect on Cardiac valvular disease
SNP effect on UGDH

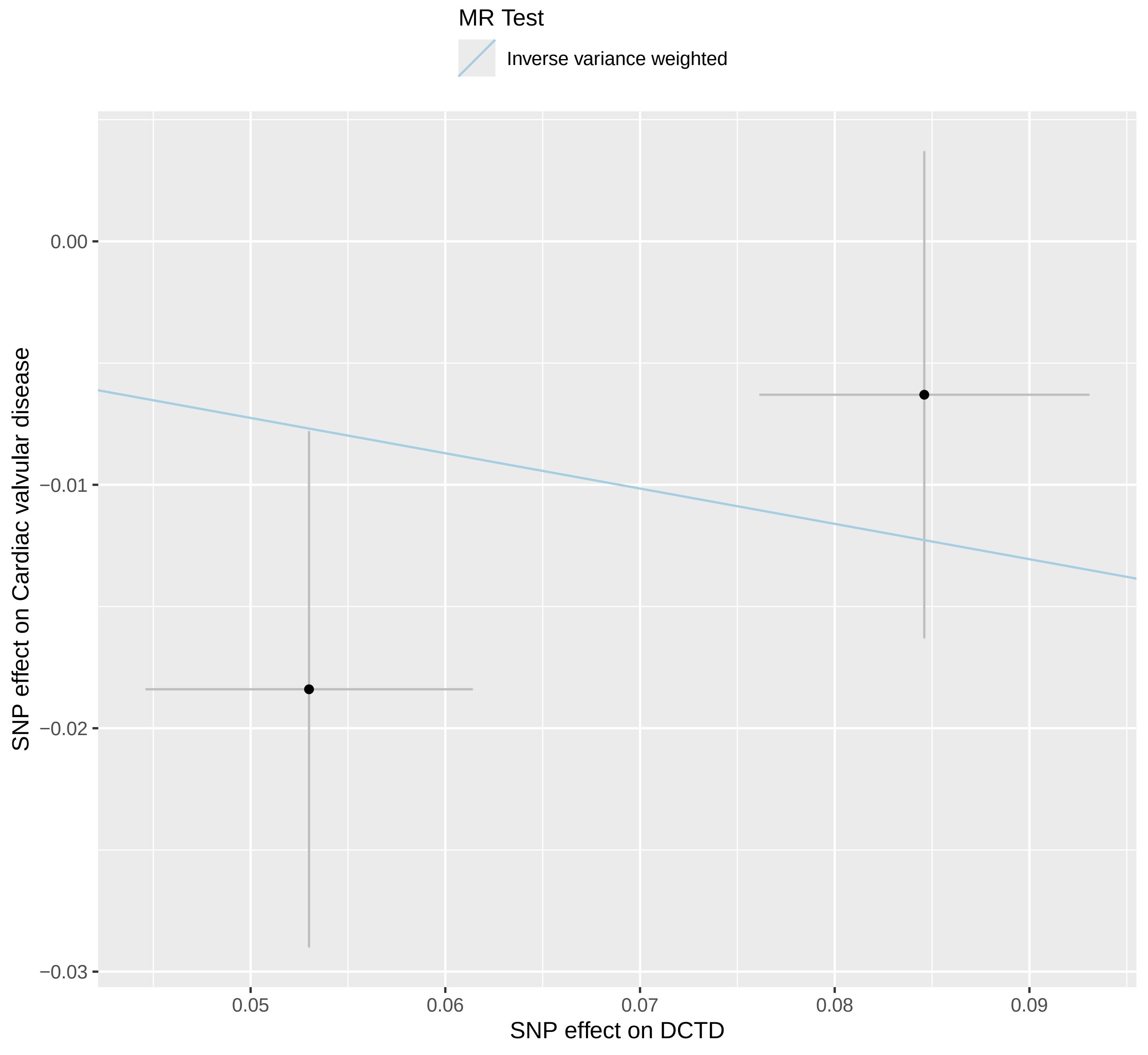
MR Test
Inverse variance weighted
0.00
−0.01
−0.02
−0.03
0.05
0.06
0.07
0.08
0.09
SNP effect on Cardiac valvular disease
SNP effect on DCTD

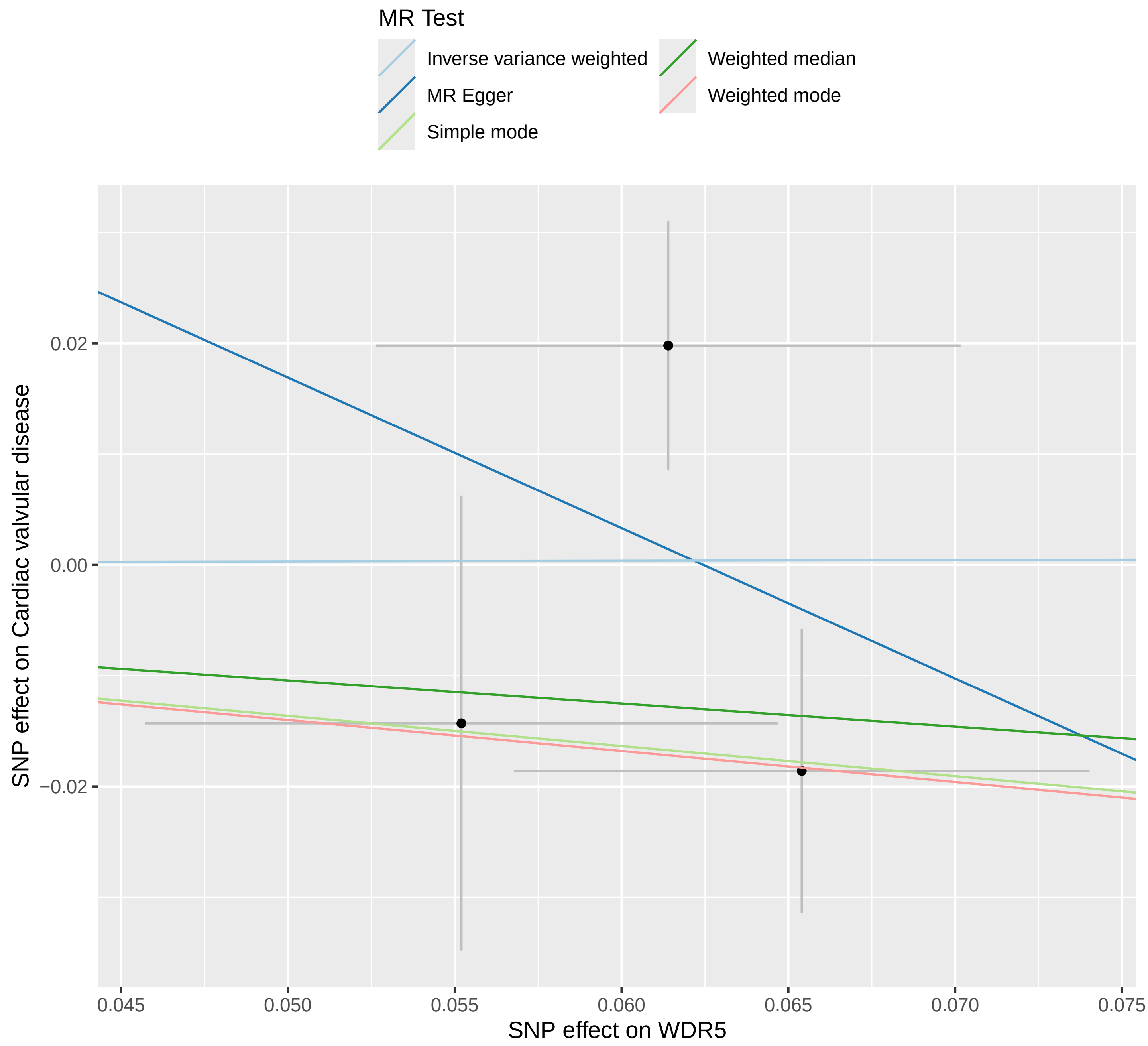
MR Test
Inverse variance weighted
MR Egger
Simple mode
Weighted median
Weighted mode
0.02
0.00
−0.02
SNP effect on Cardiac valvular disease
0.045
0.050
0.055
0.060
0.065
0.070
0.075
SNP effect on WDR5

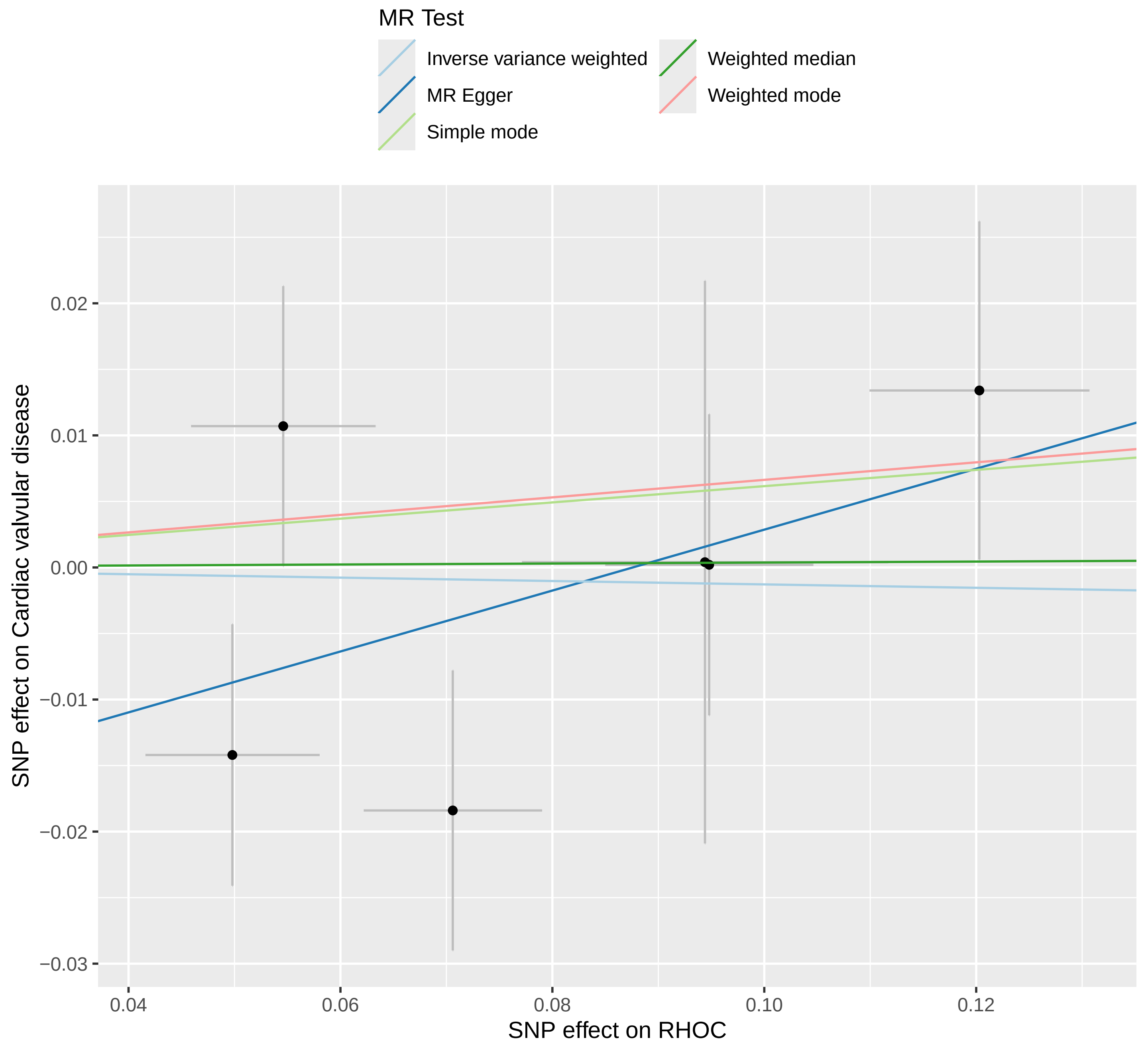

MR Test
Inverse variance weighted
MR Egger
Simple mode
Weighted median
Weighted mode
0.02
0.01
0.00
−0.01
−0.02
−0.03
SNP effect on Cardiac valvular disease
0.04
0.06
0.08
0.10
0.12
SNP effect on RHOC

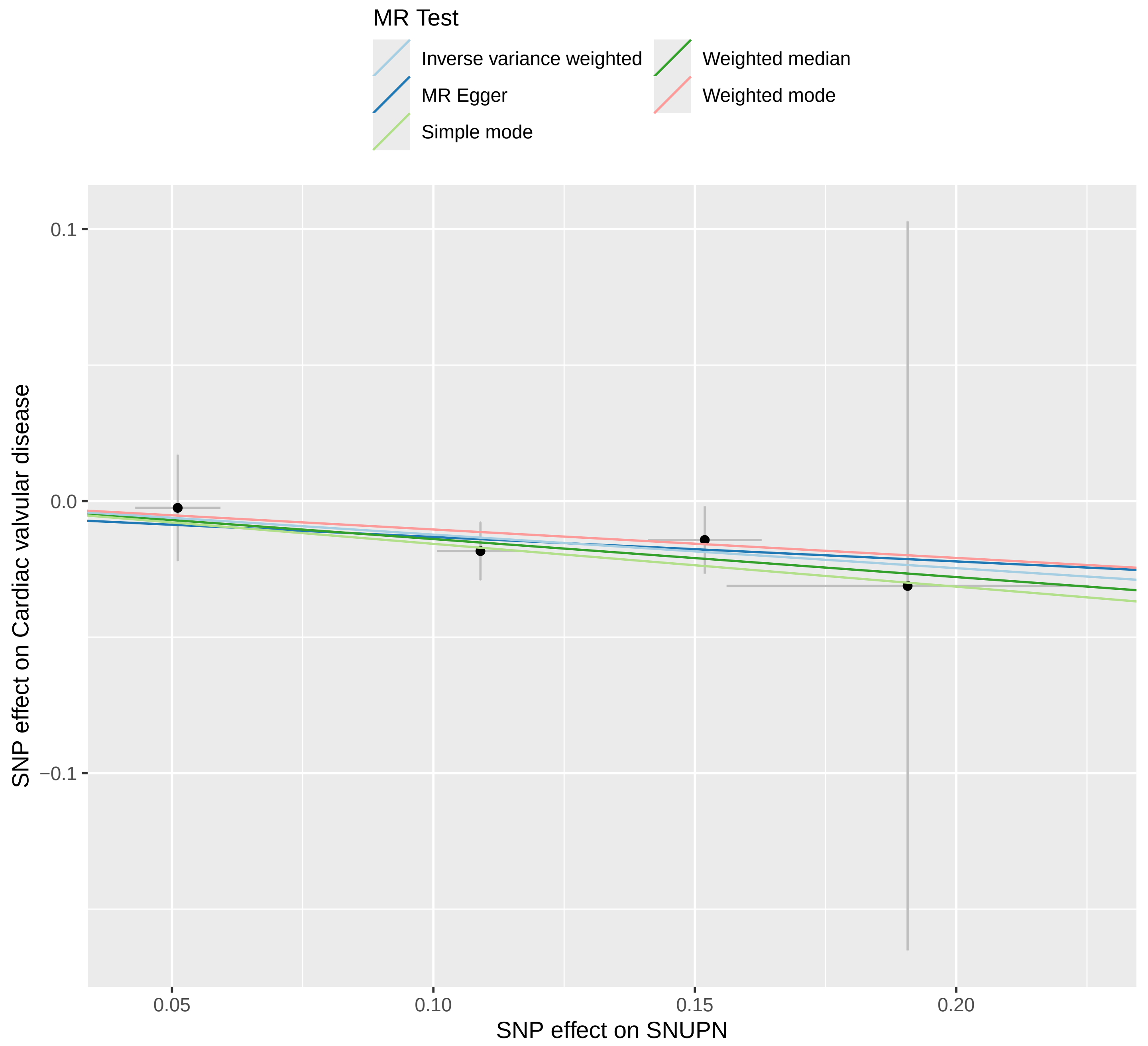
MR Test
Inverse variance weighted
MR Egger
Simple mode
Weighted median
Weighted mode
0.1
0.0
−0.1
0.05
0.10
0.15
0.20
SNP effect on Cardiac valvular disease
SNP effect on SNUPN

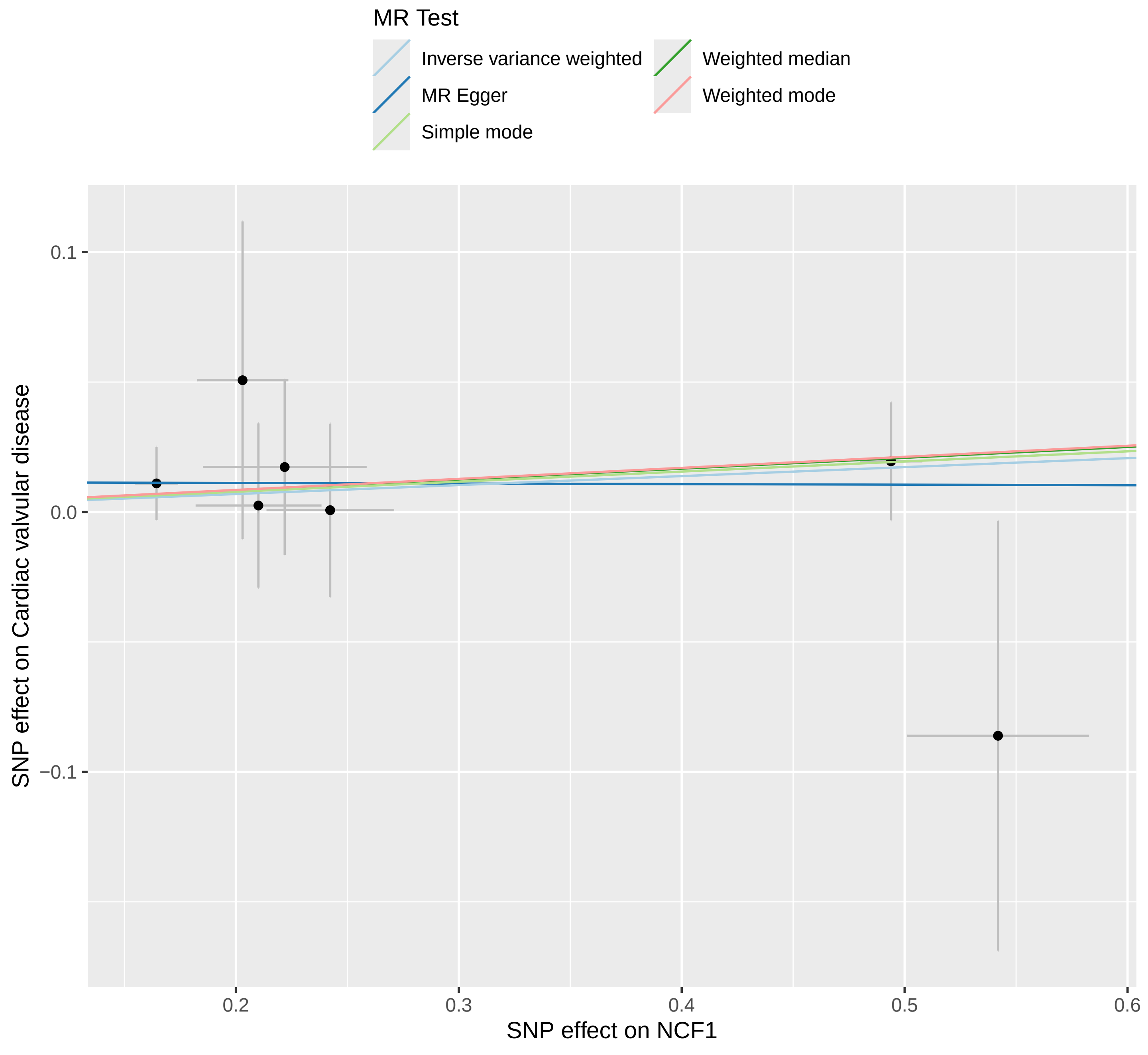
MR Test
Inverse variance weighted
MR Egger
Simple mode
Weighted median
Weighted mode
SNP effect on Cardiac valvular disease
0.1
0.0
−0.1
0.2
0.3
0.4
0.5
0.6
SNP effect on NCF1

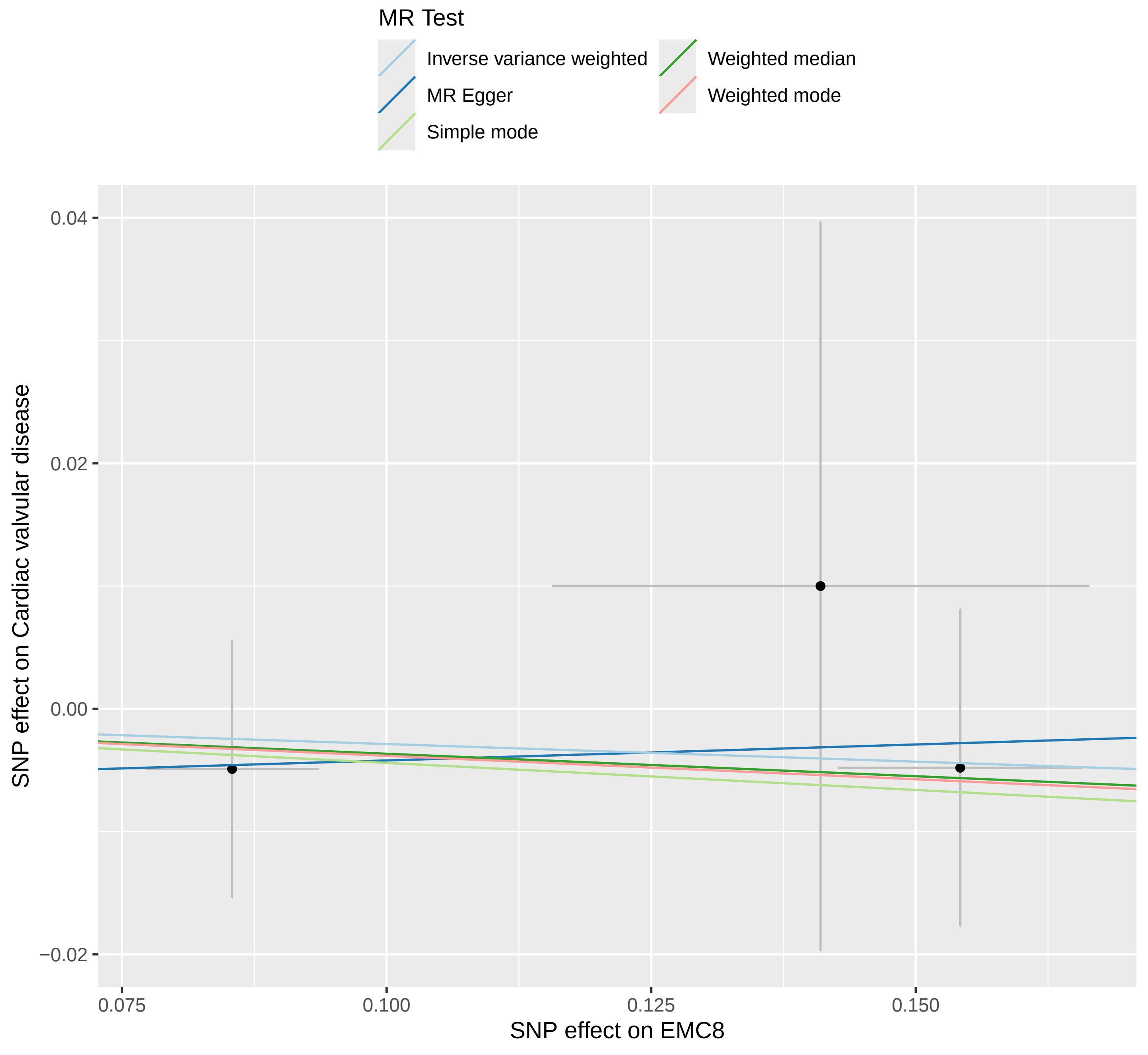
MR Test
Inverse variance weighted
MR Egger
Simple mode
Weighted median
Weighted mode
SNP effect on Cardiac valvular disease
0.04
0.02
0.00
−0.02
0.075
0.100
0.125
0.150
SNP effect on EMC8

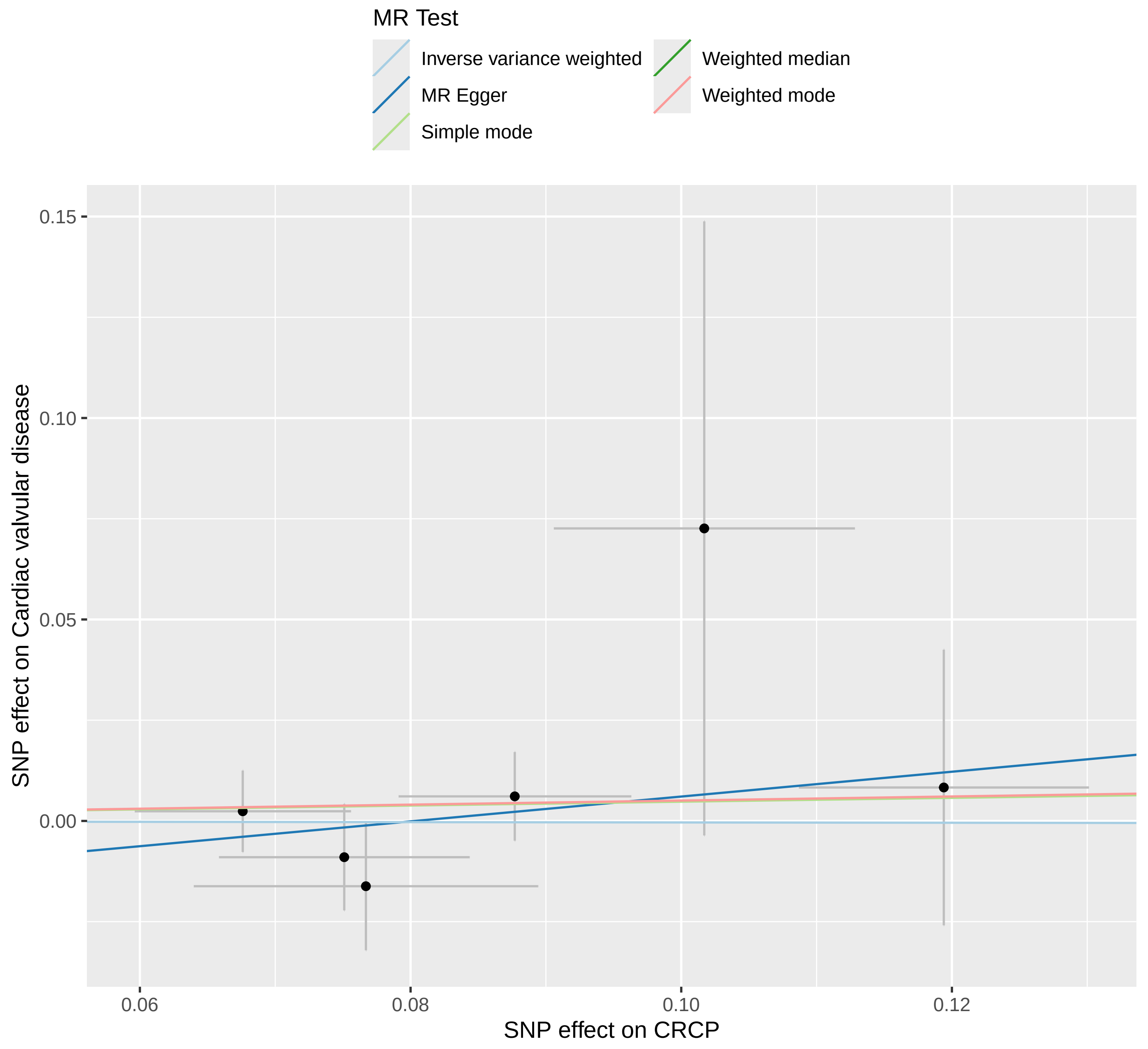
MR Test
Inverse variance weighted
MR Egger
Simple mode
Weighted median
Weighted mode
0.15
0.10
0.05
0.00
SNP effect on Cardiac valvular disease
0.06
0.08
0.10
0.12
SNP effect on CRCP

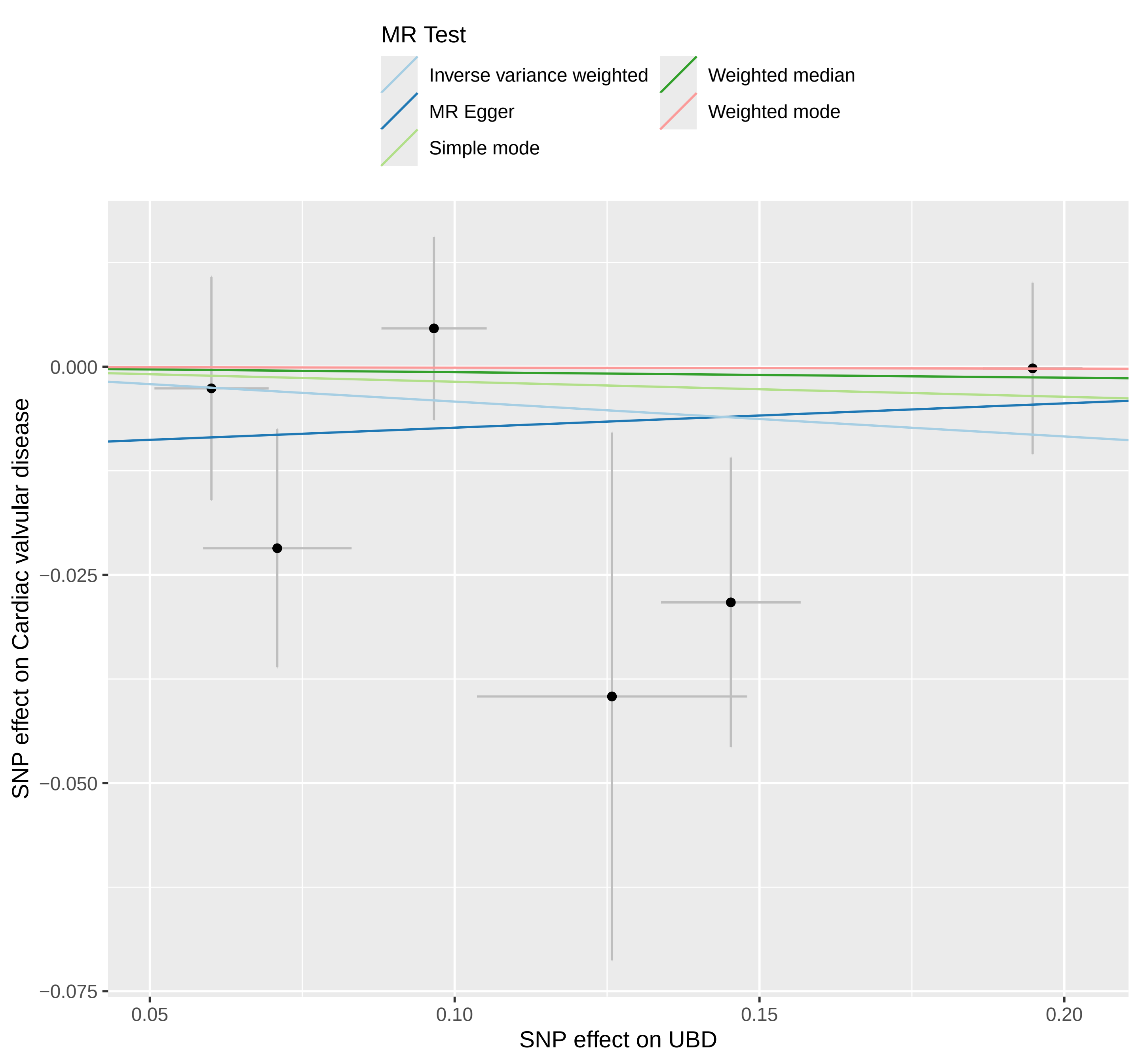
MR Test
Inverse variance weighted
MR Egger
Simple mode
Weighted median
Weighted mode
SNP effect on Cardiac valvular disease
0.000
−0.025
−0.050
−0.075
0.05
0.10
0.15
0.20
SNP effect on UBD

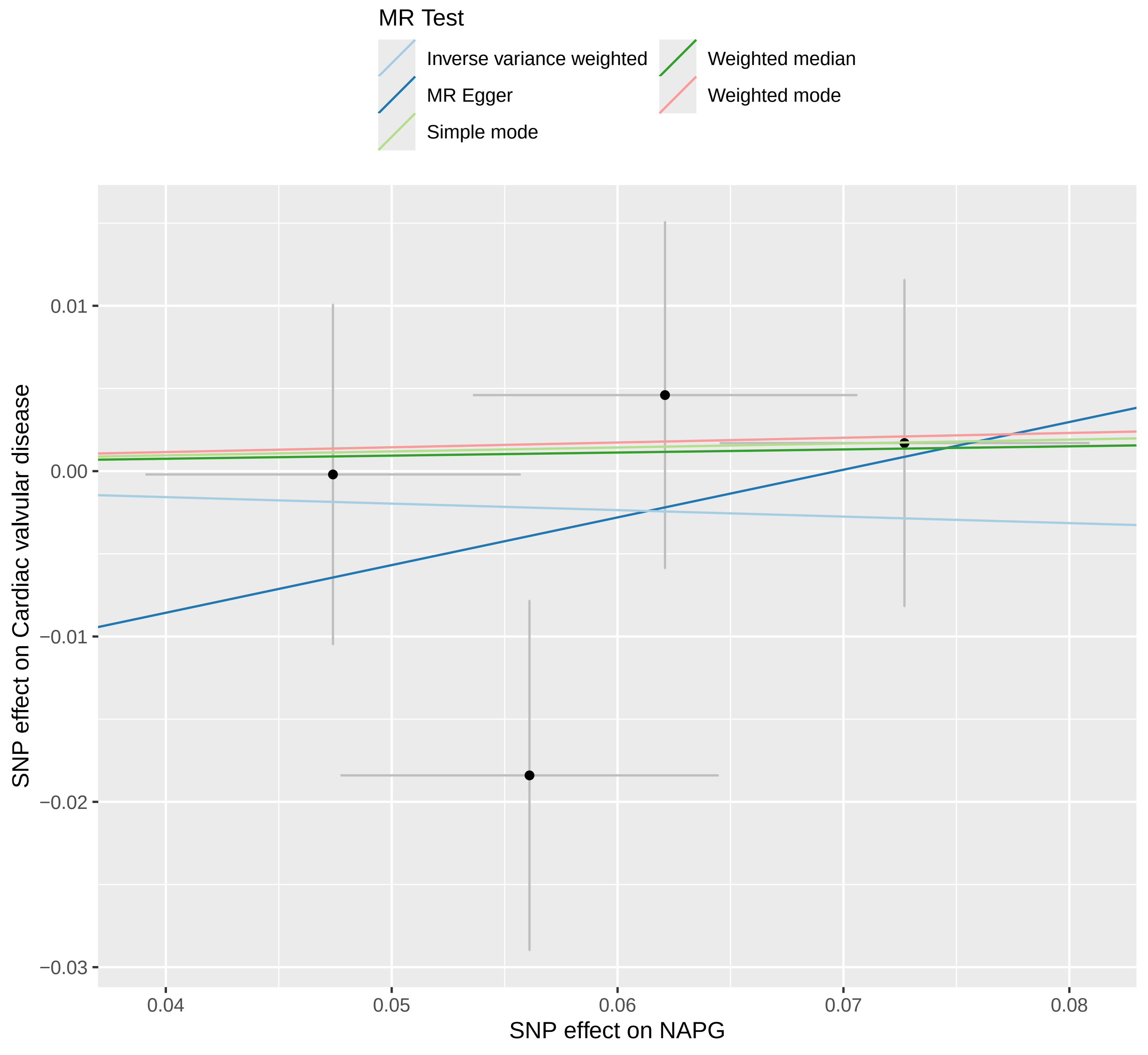
MR Test
Inverse variance weighted
MR Egger
Simple mode
Weighted median
Weighted mode
0.01
0.00
−0.01
−0.02
−0.03
0.04
0.05
0.06
0.07
0.08
SNP effect on Cardiac valvular disease
SNP effect on NAPG

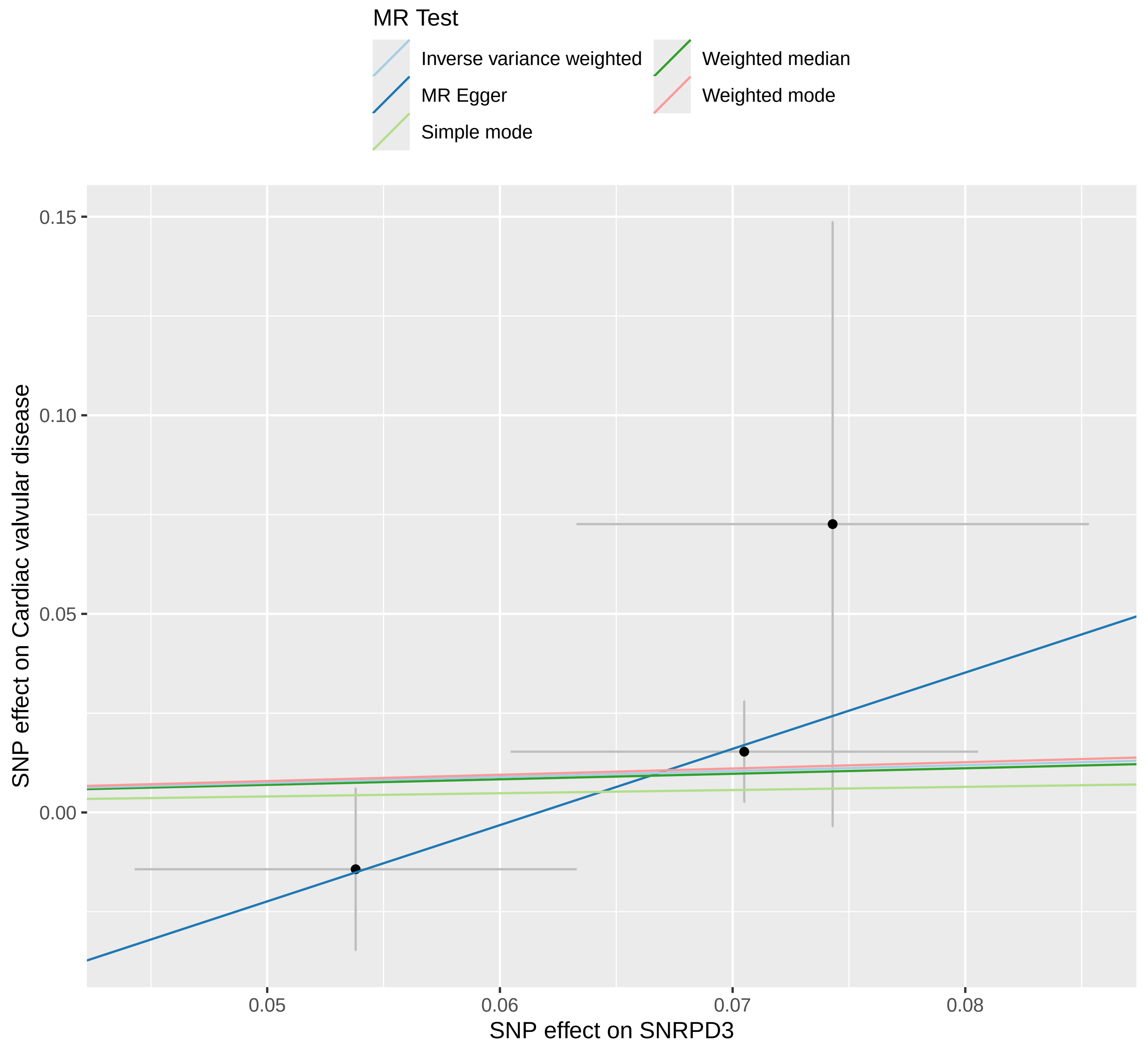

MR Test
Inverse variance weighted
MR Egger
Simple mode
Weighted median
Weighted mode
0.15
0.10
0.05
0.00
0.05
0.06
0.07
0.08
SNP effect on Cardiac valvular disease
SNP effect on SNRPD3

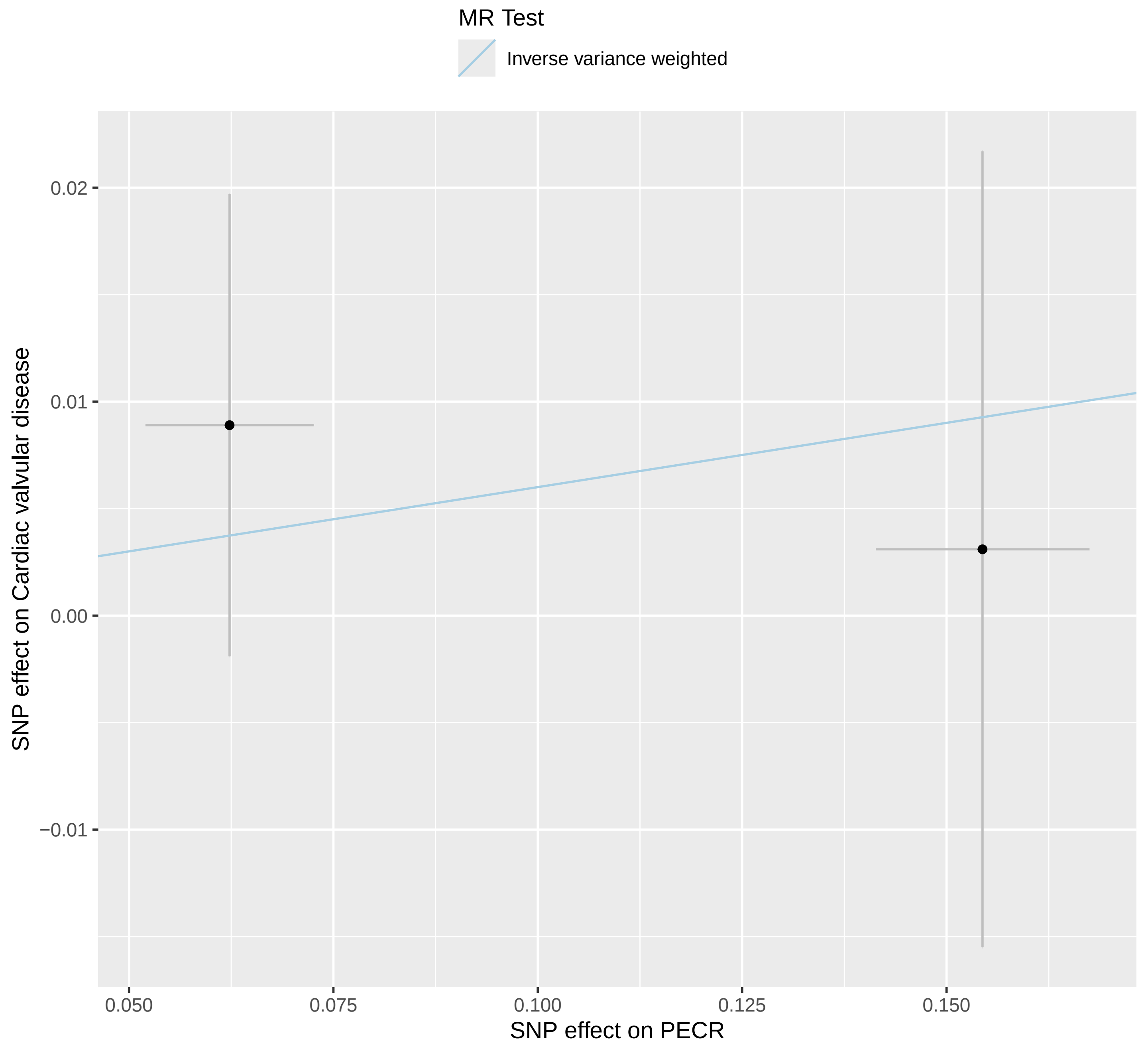
MR Test
Inverse variance weighted
0.02
0.01
0.00
−0.01
SNP effect on Cardiac valvular disease
0.050
0.075
0.100
0.125
0.150
SNP effect on PECR

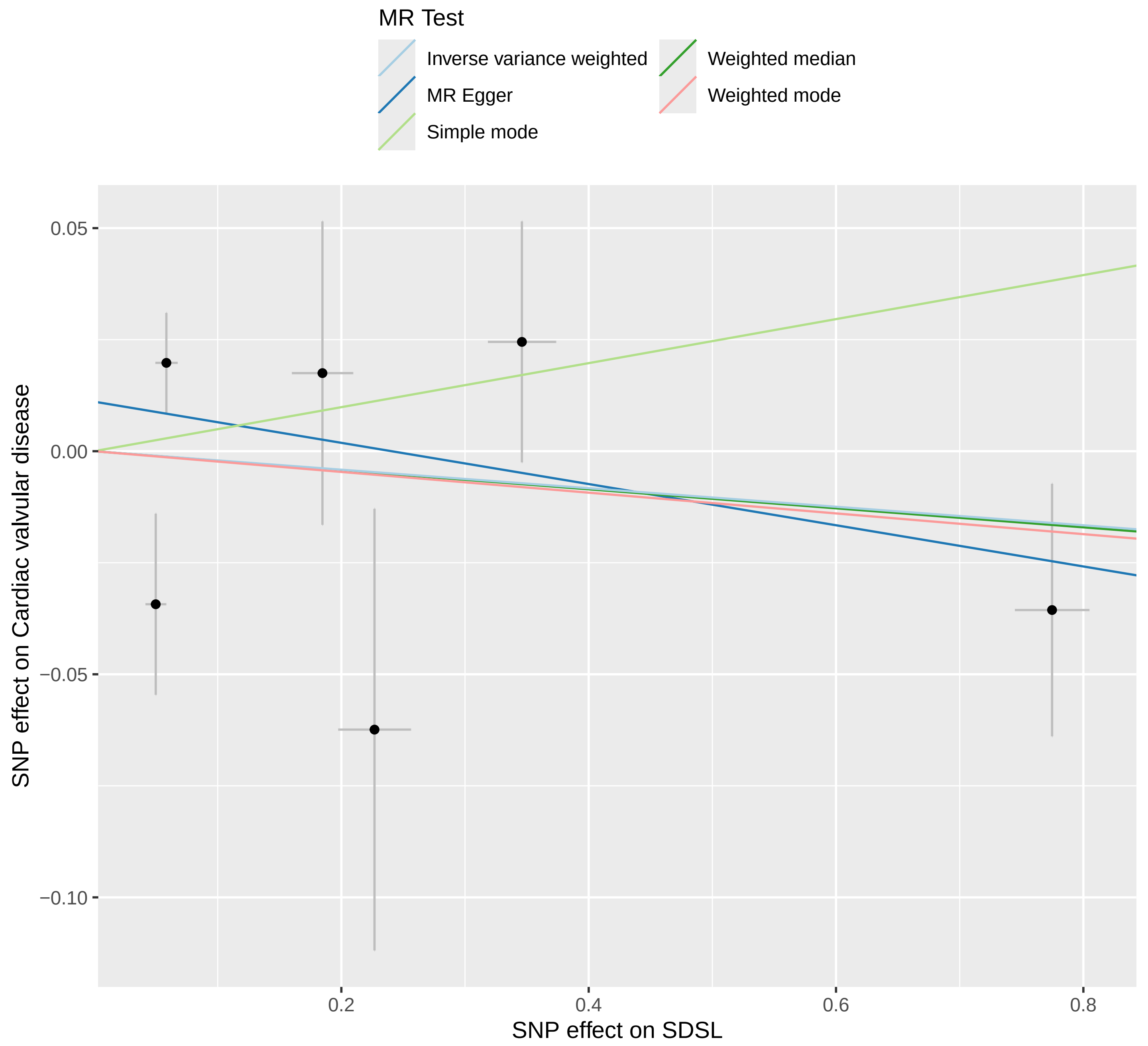
MR Test
Inverse variance weighted
MR Egger
Simple mode
Weighted median
Weighted mode
0.05
0.00
−0.05
−0.10
SNP effect on Cardiac valvular disease
0.2
0.4
0.6
0.8
SNP effect on SDSL

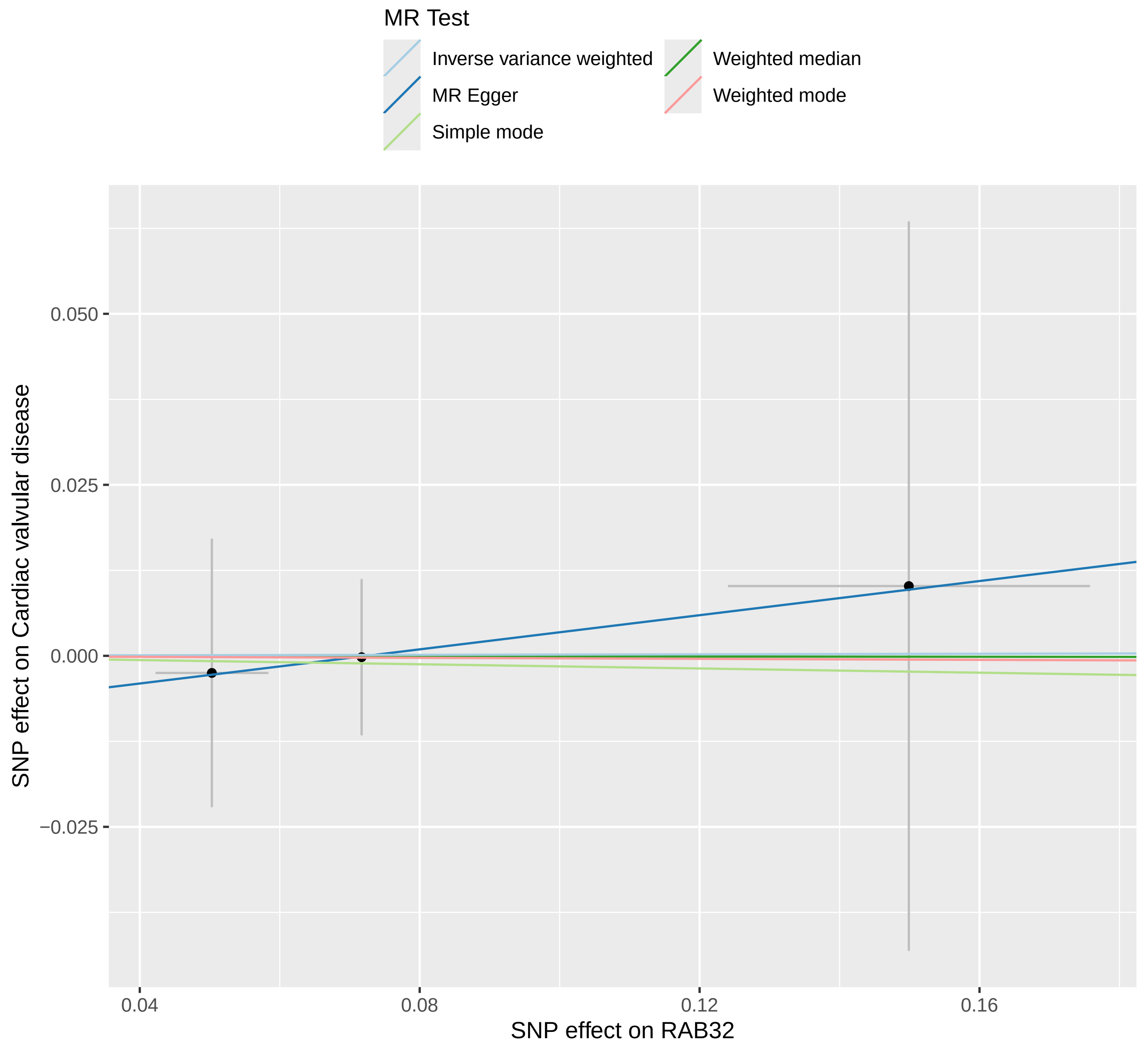

MR Test
Inverse variance weighted
MR Egger
Simple mode
Weighted median
Weighted mode
0.050
0.025
0.000
−0.025
0.04
0.08
0.12
0.16
SNP effect on Cardiac valvular disease
SNP effect on RAB32

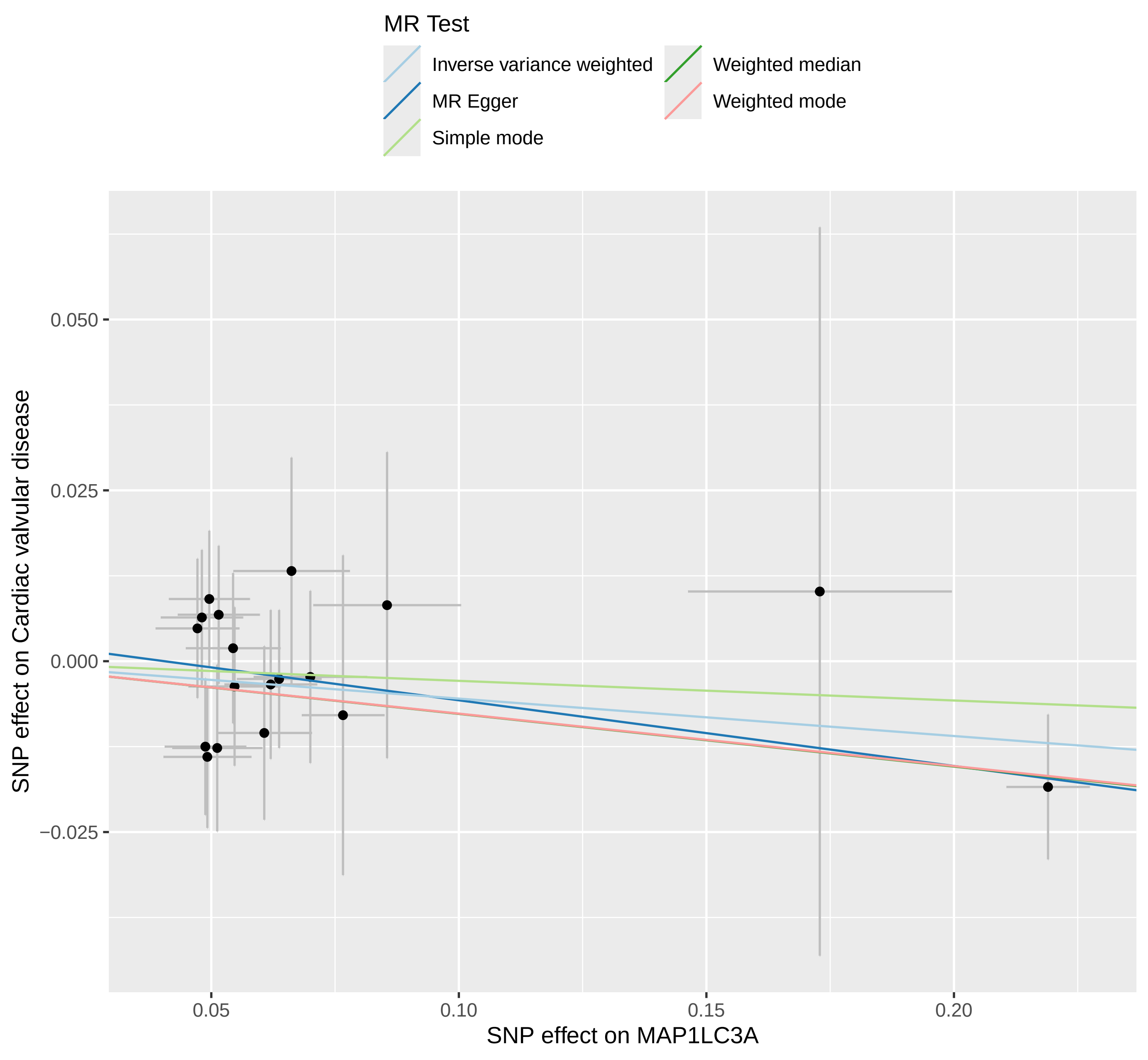
MR Test
Inverse variance weighted
MR Egger
Simple mode
Weighted median
Weighted mode
0.050
0.025
0.000
−0.025
0.05
0.10
0.15
0.20
SNP effect on MAP1LC3A
SNP effect on Cardiac valvular disease

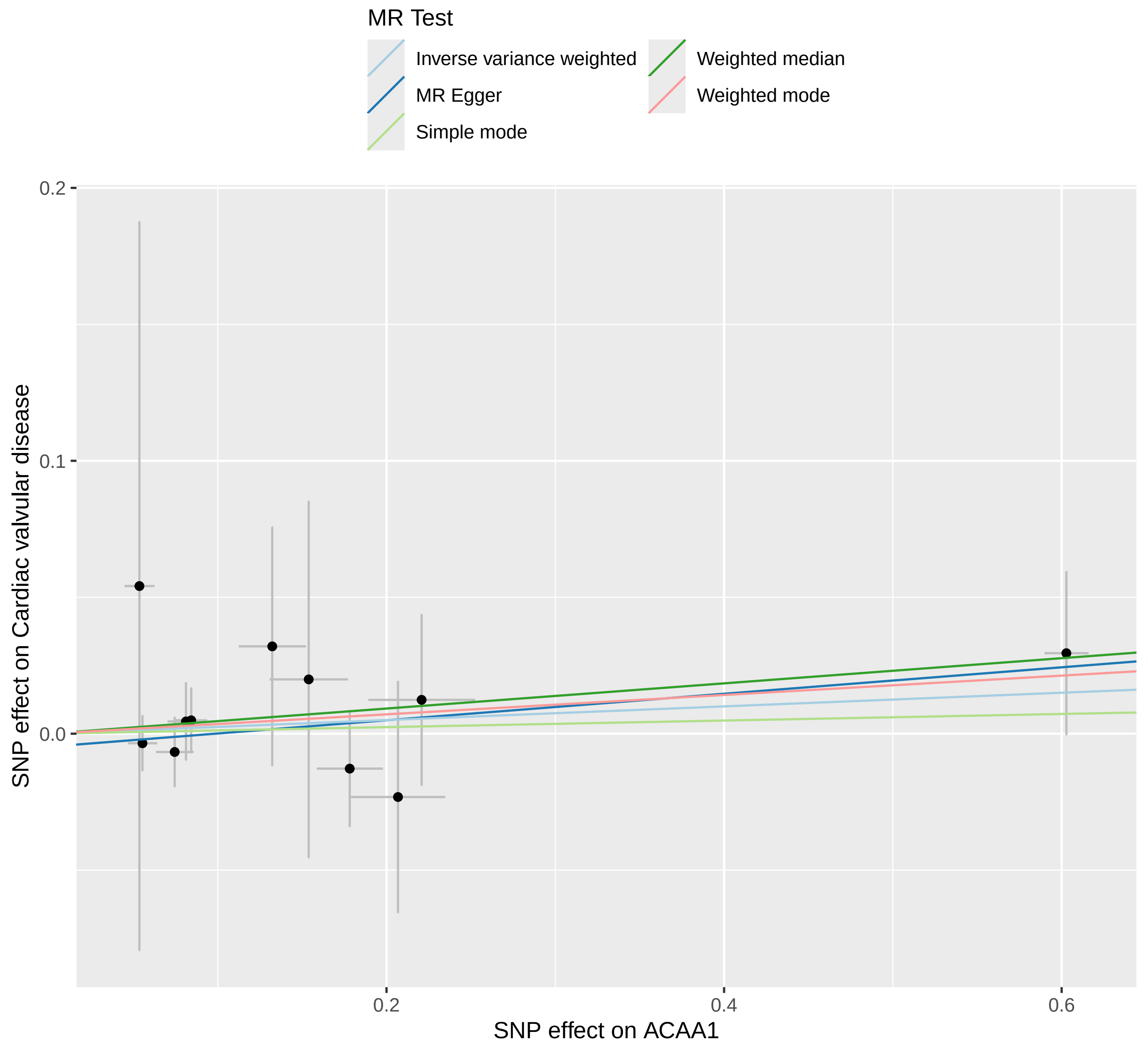

MR Test
Inverse variance weighted
MR Egger
Simple mode
Weighted median
Weighted mode
0.2
0.1
0.0
SNP effect on Cardiac valvular disease
0.2
0.4
0.6
SNP effect on ACAA1

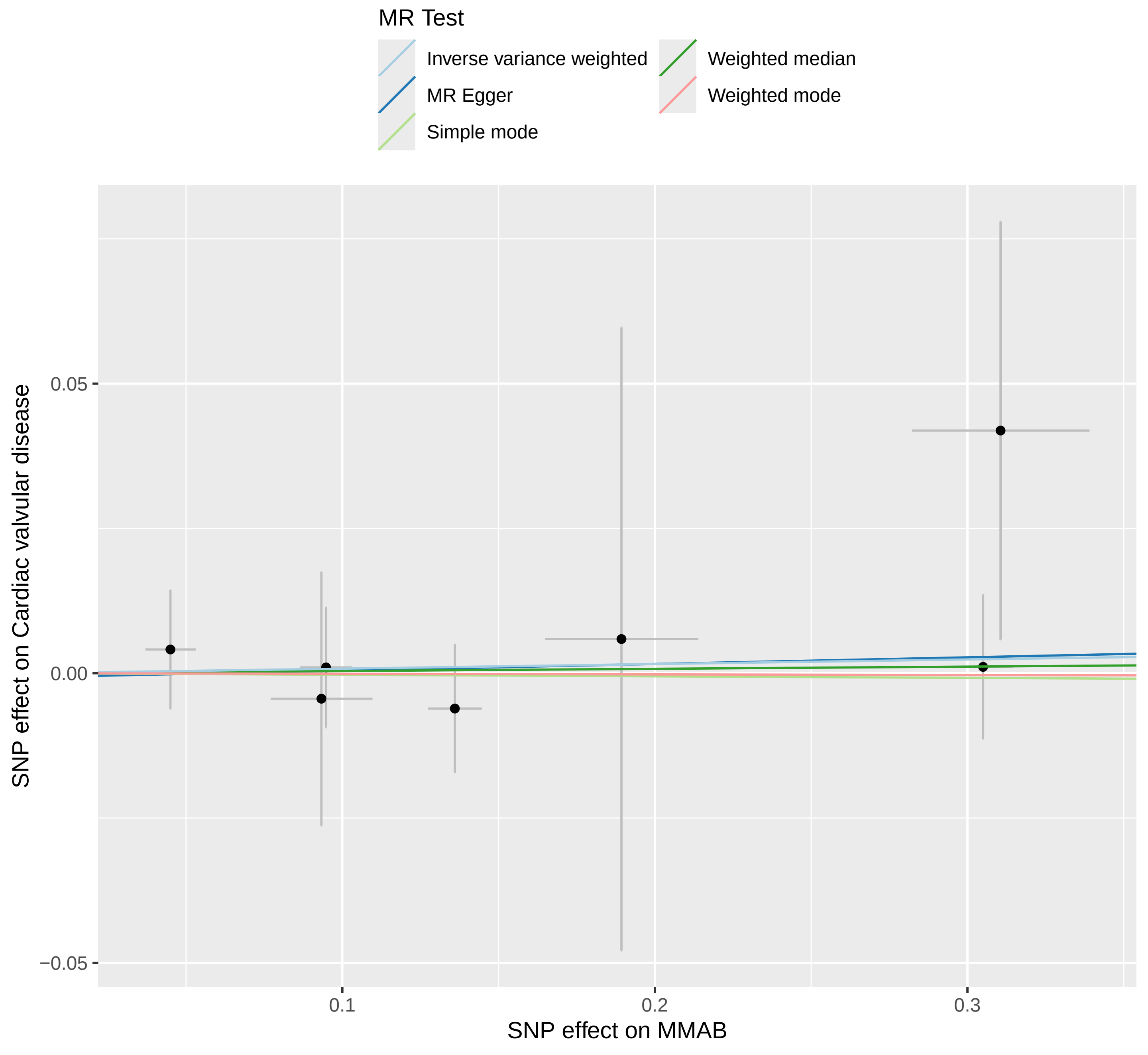
MR Test
Inverse variance weighted
MR Egger
Simple mode
Weighted median
Weighted mode
0.05
0.00
−0.05
0.1
0.2
0.3
SNP effect on MMAB
SNP effect on Cardiac valvular disease

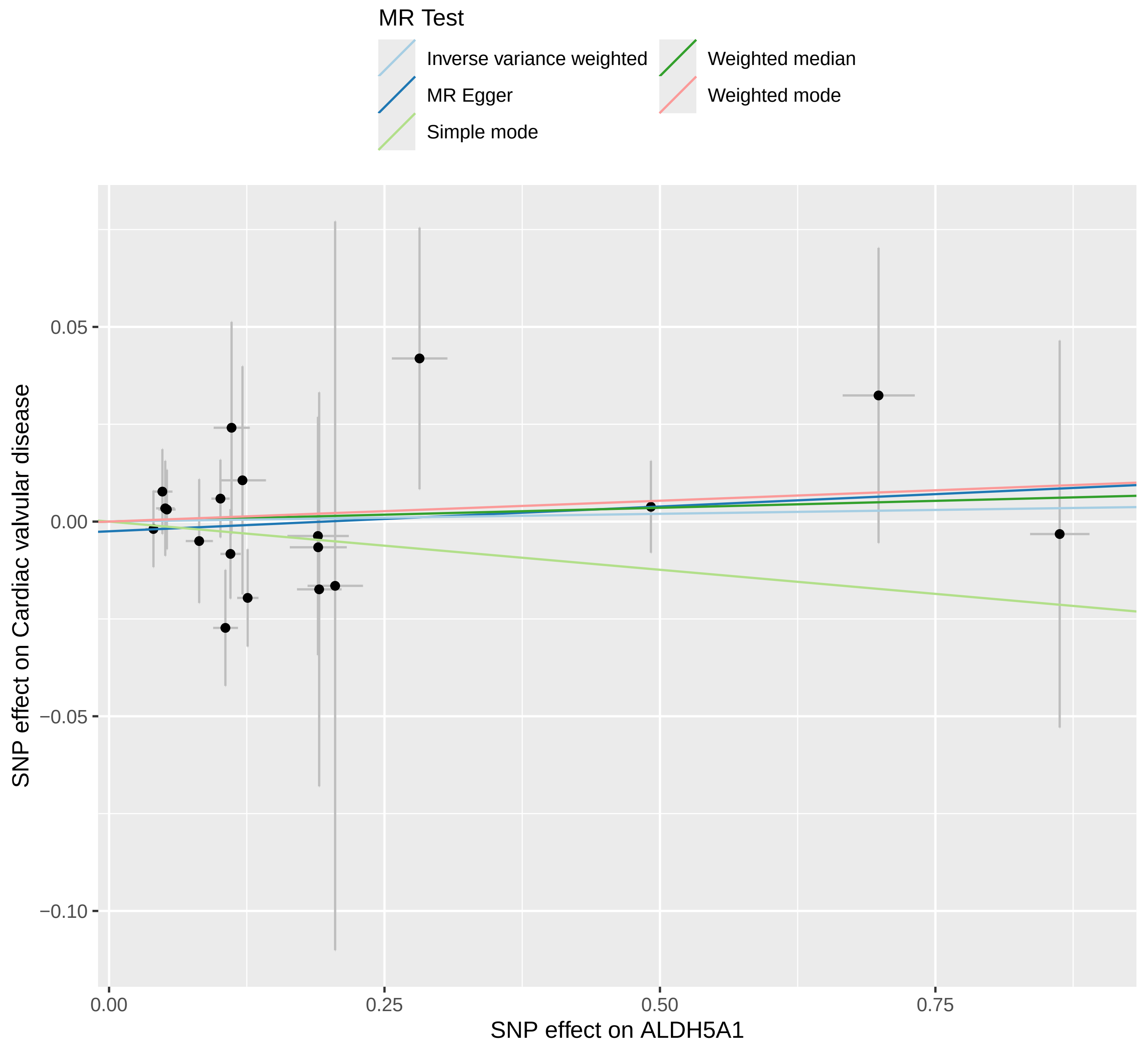

MR Test
Inverse variance weighted
MR Egger
Simple mode
Weighted median
Weighted mode
0.05
0.00
−0.05
−0.10
SNP effect on Cardiac valvular disease
0.00
0.25
0.50
0.75
SNP effect on ALDH5A1

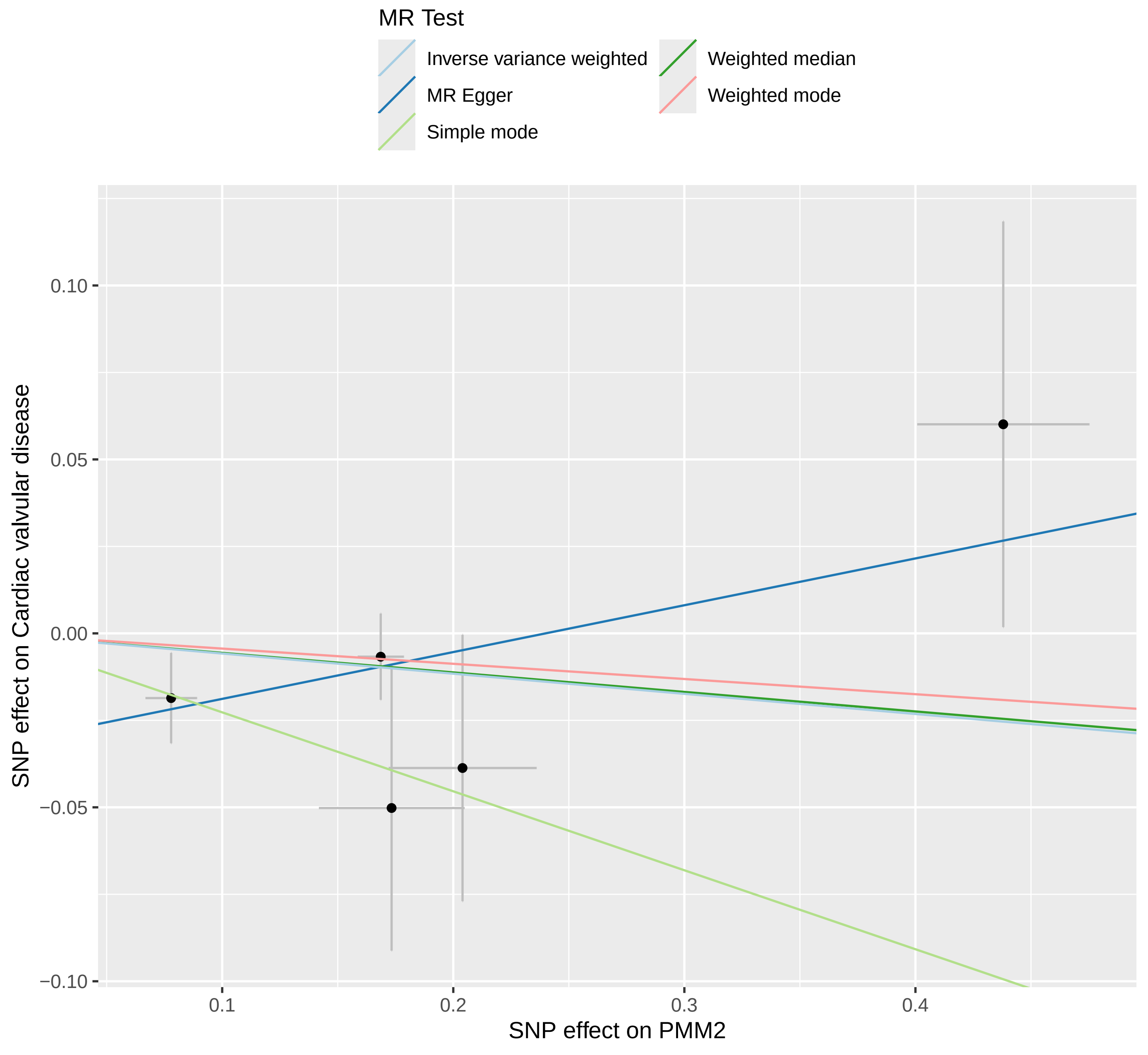
MR Test
Inverse variance weighted
MR Egger
Simple mode
Weighted median
Weighted mode
SNP effect on Cardiac valvular disease
0.10
0.05
0.00
−0.05
−0.10
0.1
0.2
0.3
0.4
SNP effect on PMM2

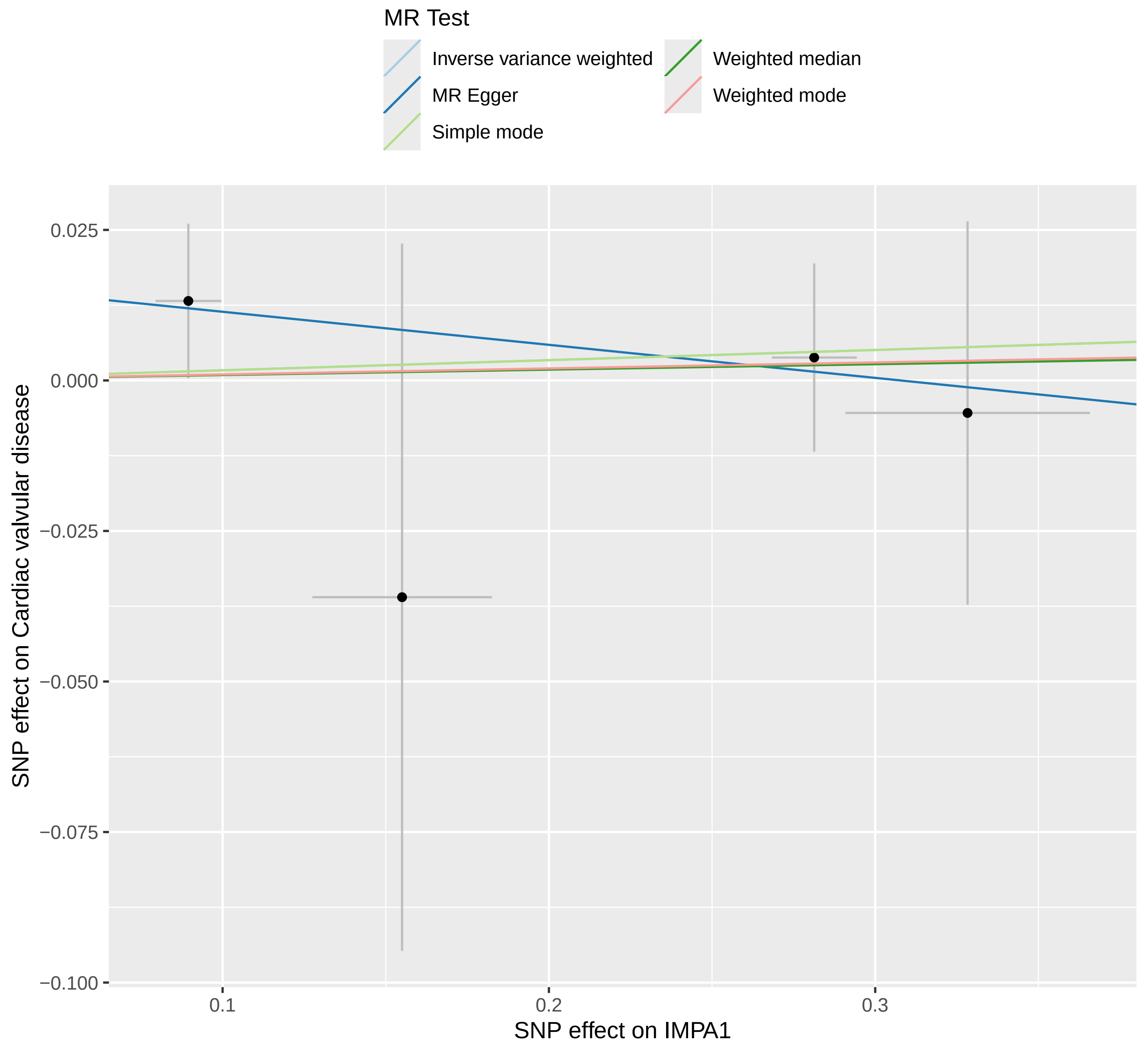

MR Test
Inverse variance weighted
MR Egger
Simple mode
Weighted median
Weighted mode
0.025
0.000
−0.025
−0.050
−0.075
−0.100
0.1
0.2
0.3
SNP effect on IMPA1
SNP effect on Cardiac valvular disease

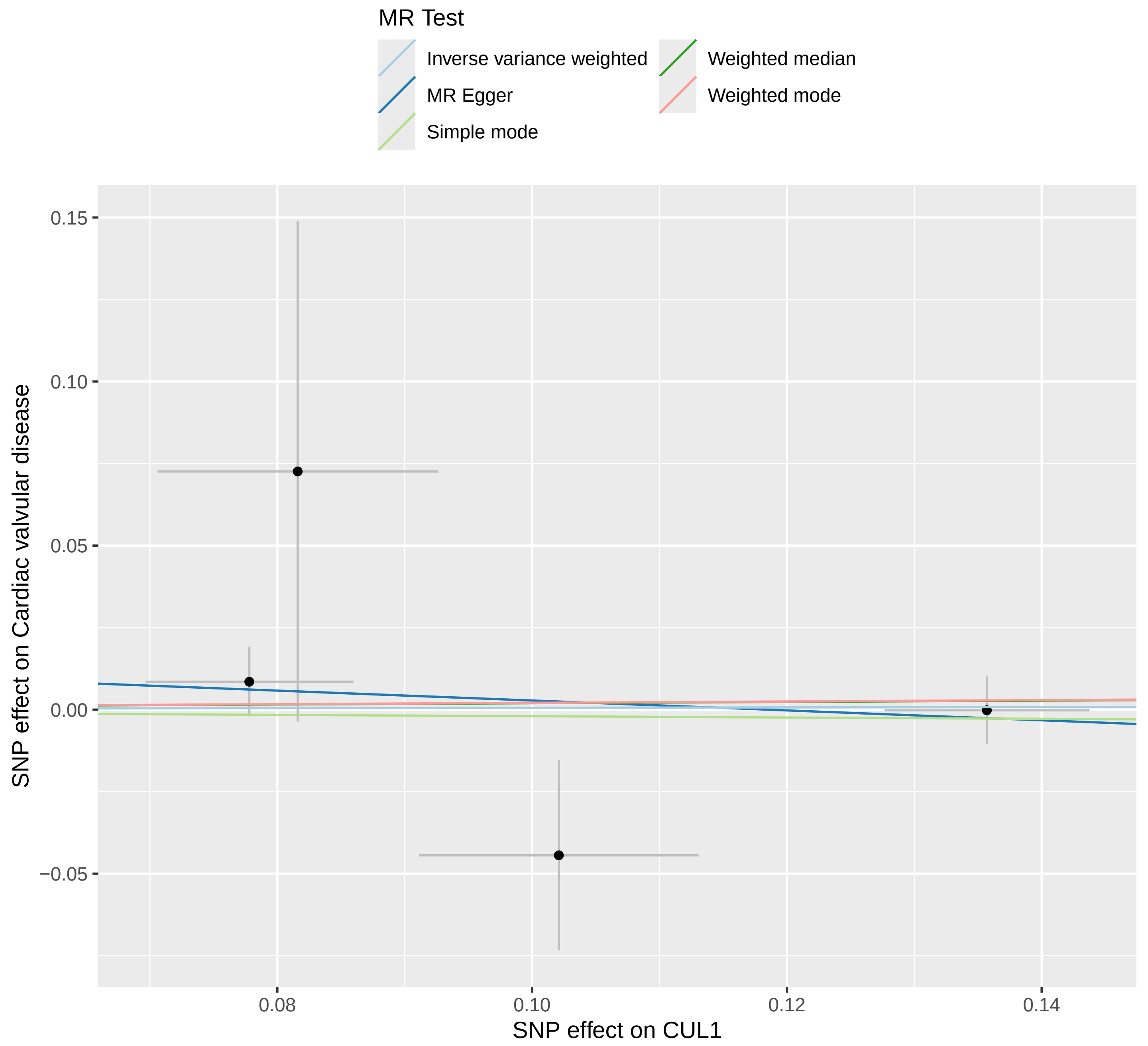
MR Test
Inverse variance weighted
MR Egger
Simple mode
Weighted median
Weighted mode
0.15
0.10
0.05
0.00
−0.05
0.08
0.10
0.12
0.14
SNP effect on Cardiac valvular disease
SNP effect on CUL1

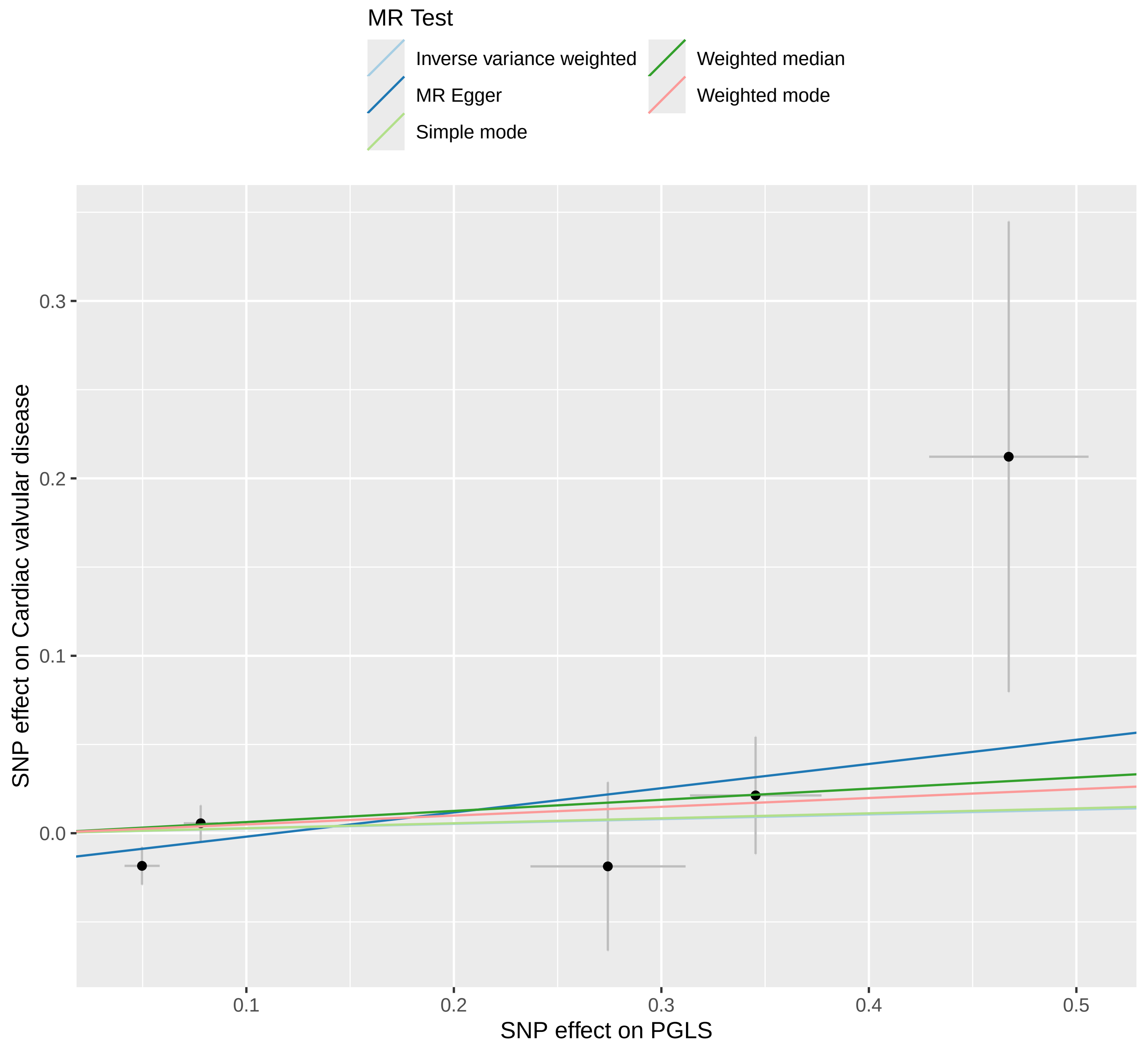
MR Test
Inverse variance weighted
MR Egger
Simple mode
Weighted median
Weighted mode
0.3
0.2
0.1
0.0
0.1
0.2
0.3
0.4
0.5
SNP effect on Cardiac valvular disease
SNP effect on PGLS

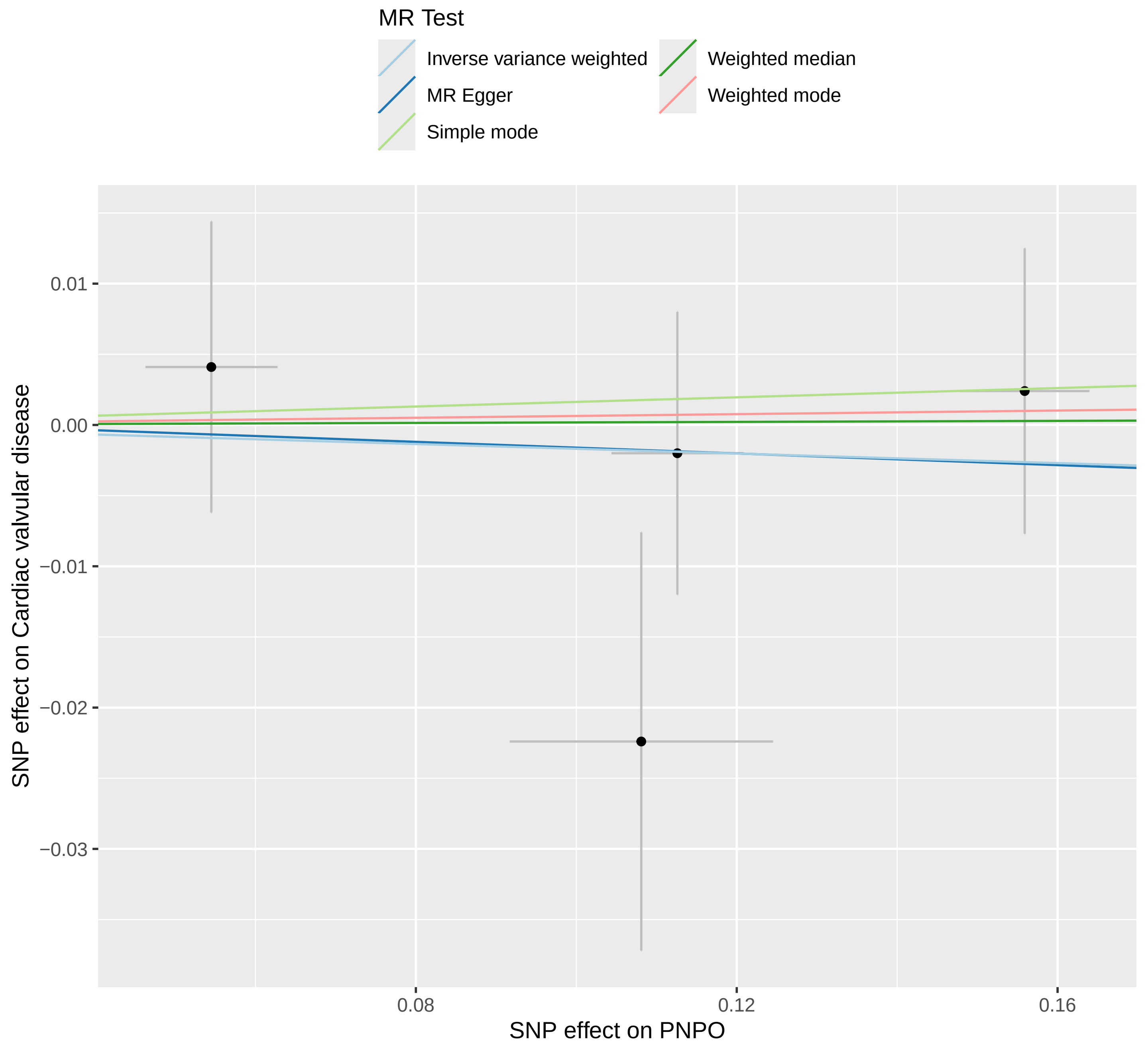

MR Test
Inverse variance weighted
MR Egger
Simple mode
Weighted median
Weighted mode
0.01
0.00
−0.01
−0.02
−0.03
SNP effect on Cardiac valvular disease
0.08
0.12
0.16
SNP effect on PNPO

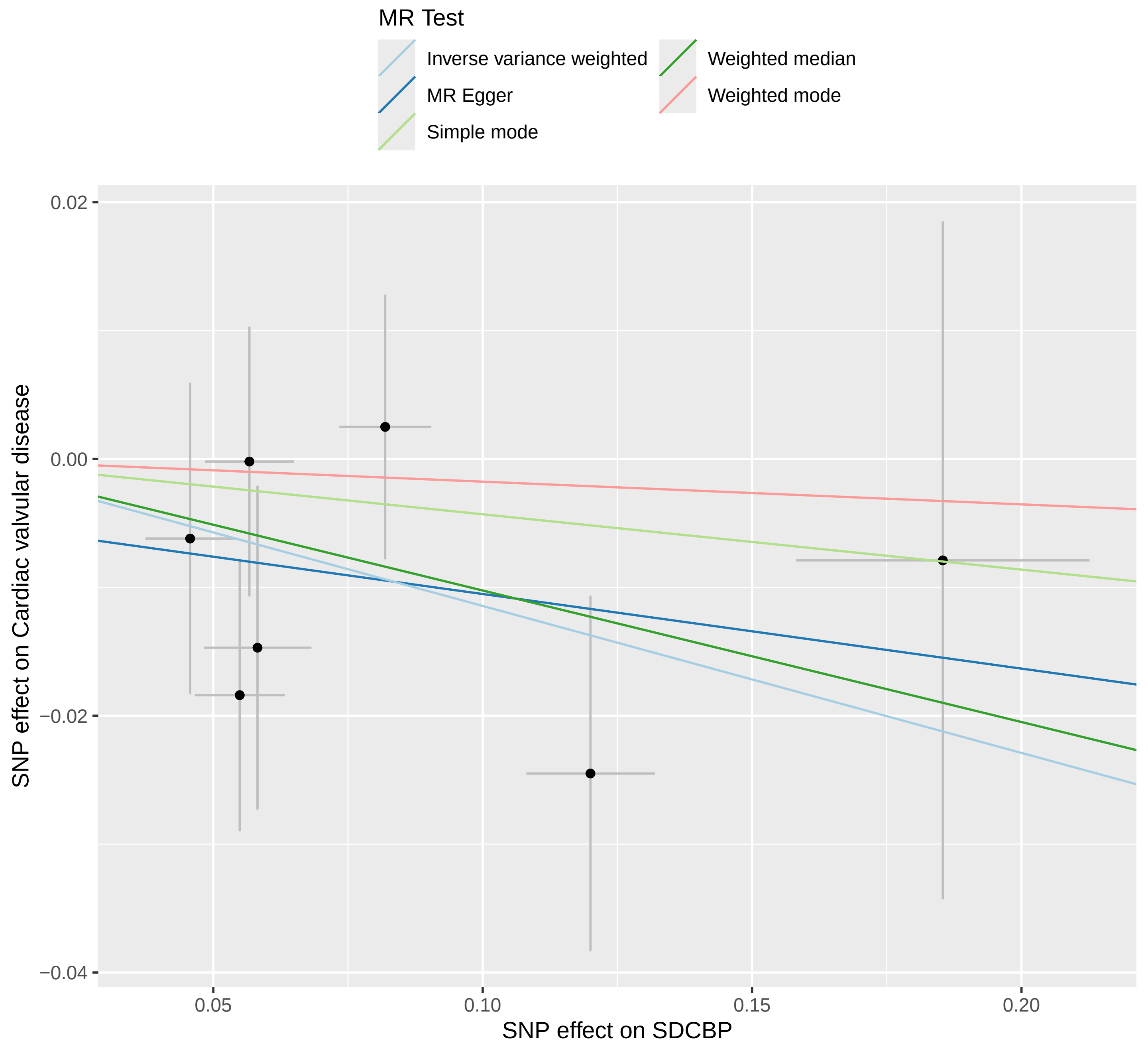
MR Test
Inverse variance weighted
MR Egger
Simple mode
Weighted median
Weighted mode
0.02
0.00
−0.02
−0.04
0.05
0.10
0.15
0.20
SNP effect on SDCBP
SNP effect on Cardiac valvular disease

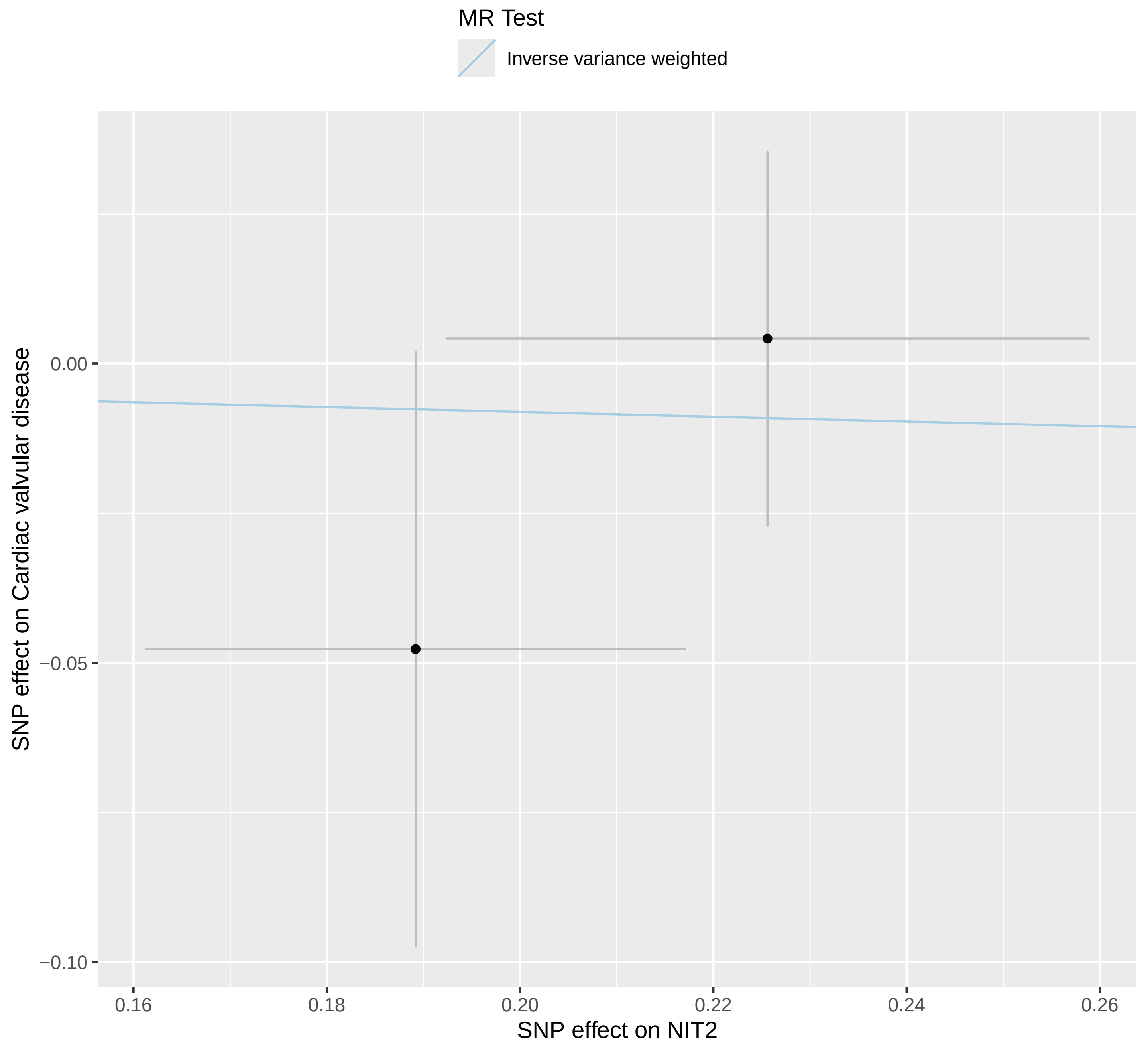
MR Test
Inverse variance weighted
SNP effect on Cardiac valvular disease
0.00
−0.05
−0.10
0.16
0.18
0.20
0.22
0.24
0.26
SNP effect on NIT2

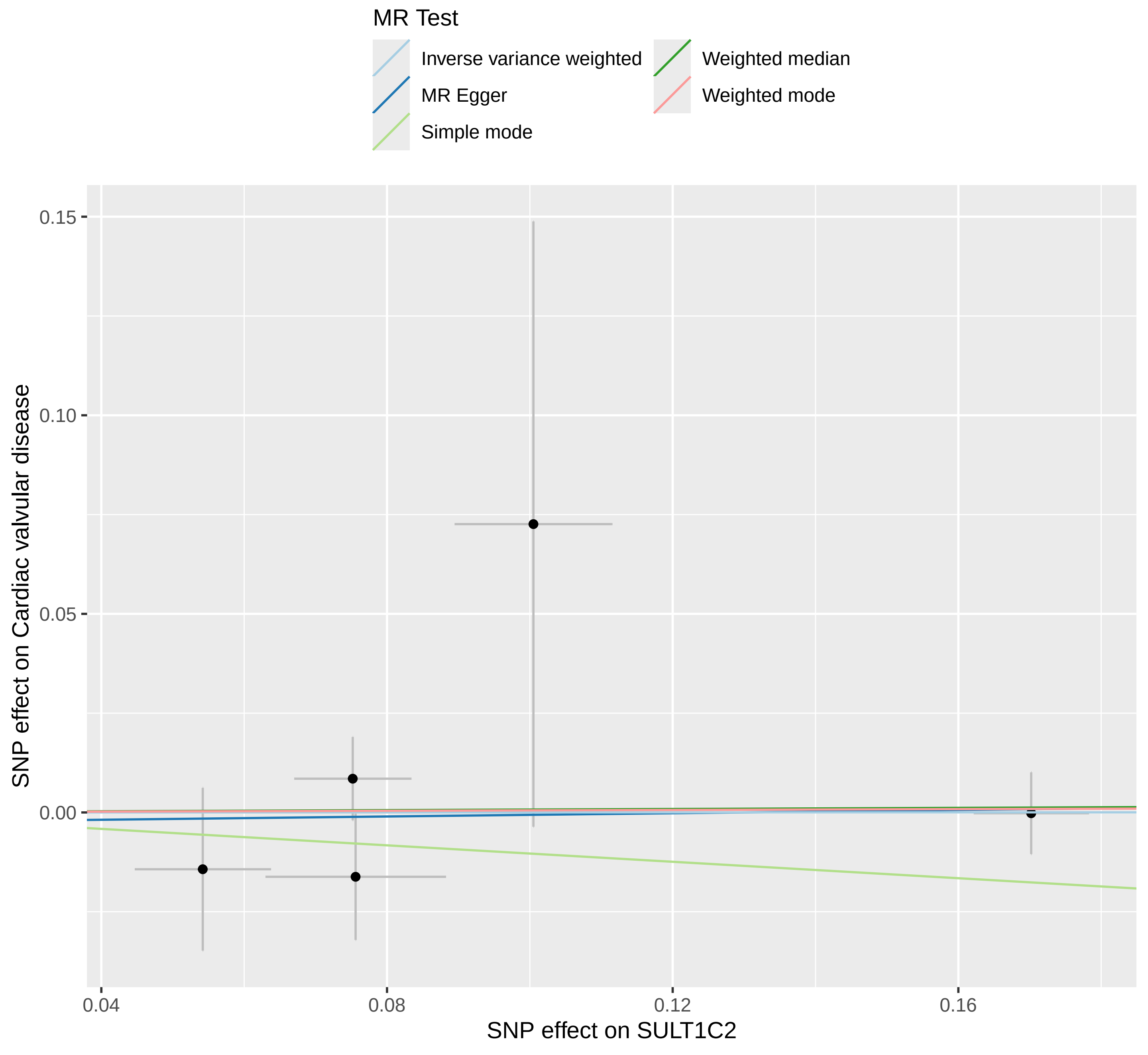
MR Test
Inverse variance weighted
MR Egger
Simple mode
Weighted median
Weighted mode
0.15
0.10
0.05
0.00
SNP effect on Cardiac valvular disease
0.04
0.08
0.12
0.16
SNP effect on SULT1C2

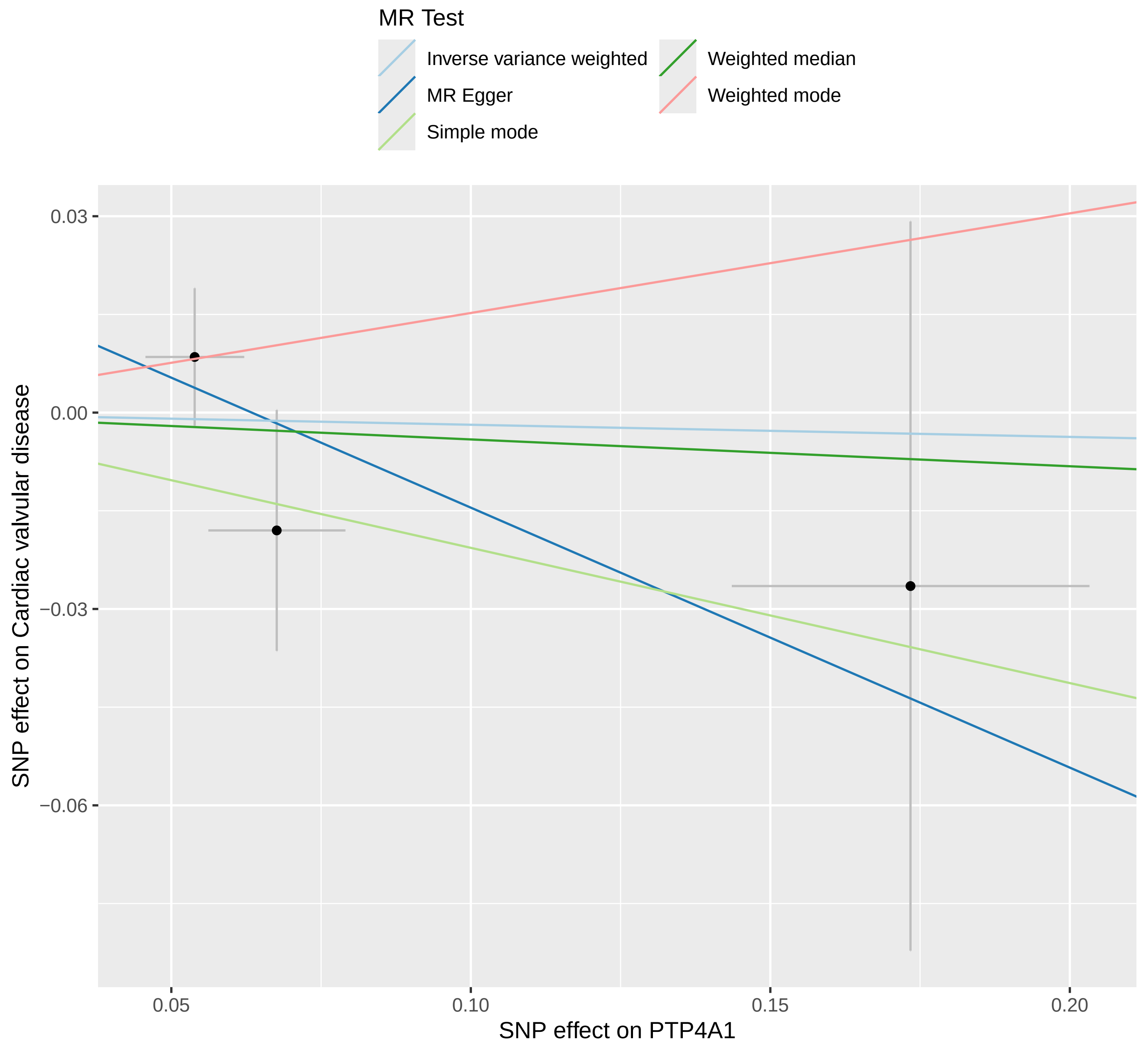
MR Test
Inverse variance weighted
MR Egger
Simple mode
Weighted median
Weighted mode
0.03
0.00
−0.03
−0.06
0.05
0.10
0.15
0.20
SNP effect on Cardiac valvular disease
SNP effect on PTP4A1

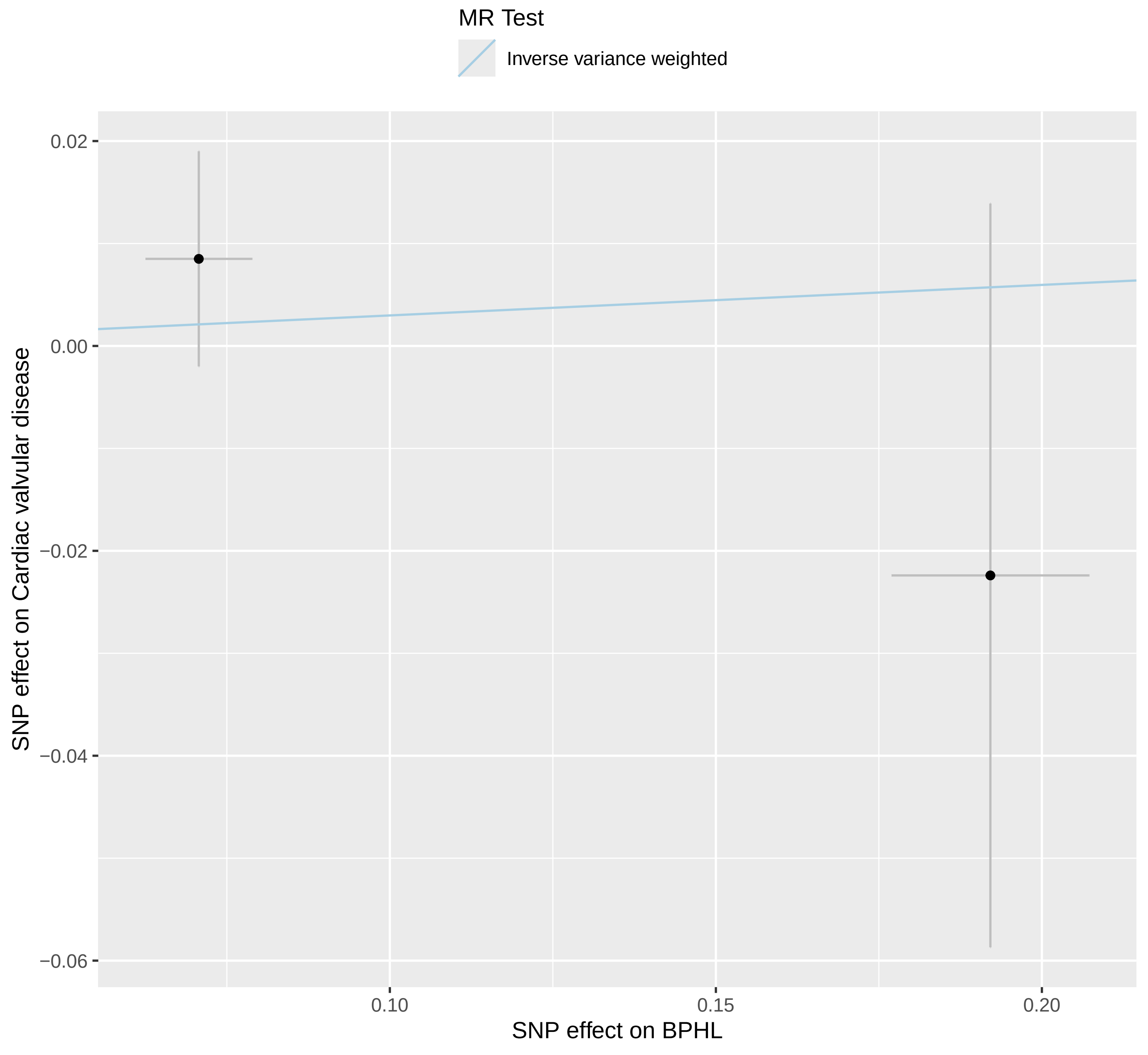
MR Test
Inverse variance weighted
0.02
0.00
−0.02
−0.04
−0.06
0.10
0.15
0.20
SNP effect on BPHL
SNP effect on Cardiac valvular disease

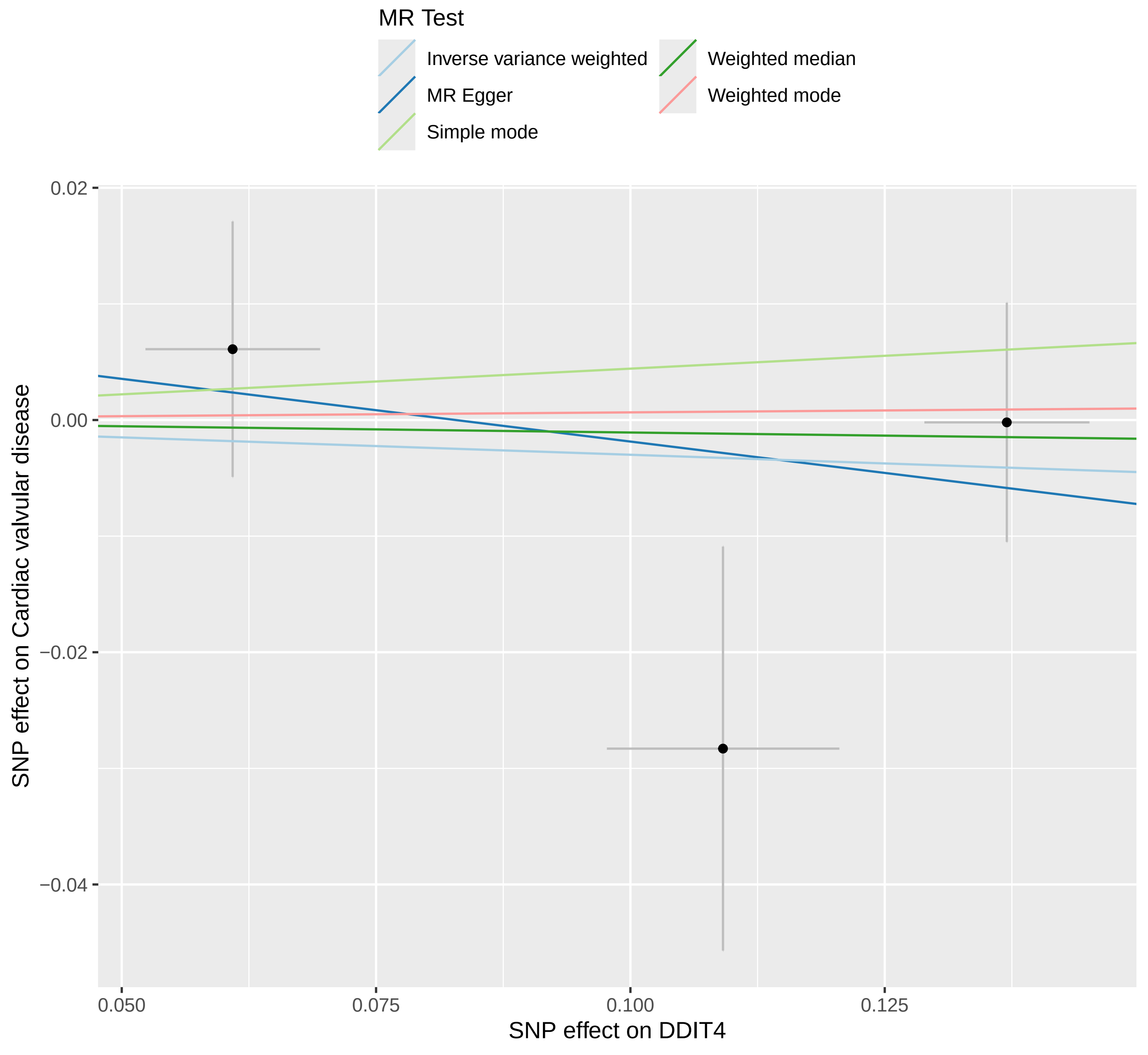

MR Test
Inverse variance weighted
MR Egger
Simple mode
Weighted median
Weighted mode
0.02
0.00
−0.02
−0.04
0.050
0.075
0.100
0.125
SNP effect on DDIT4
SNP effect on Cardiac valvular disease

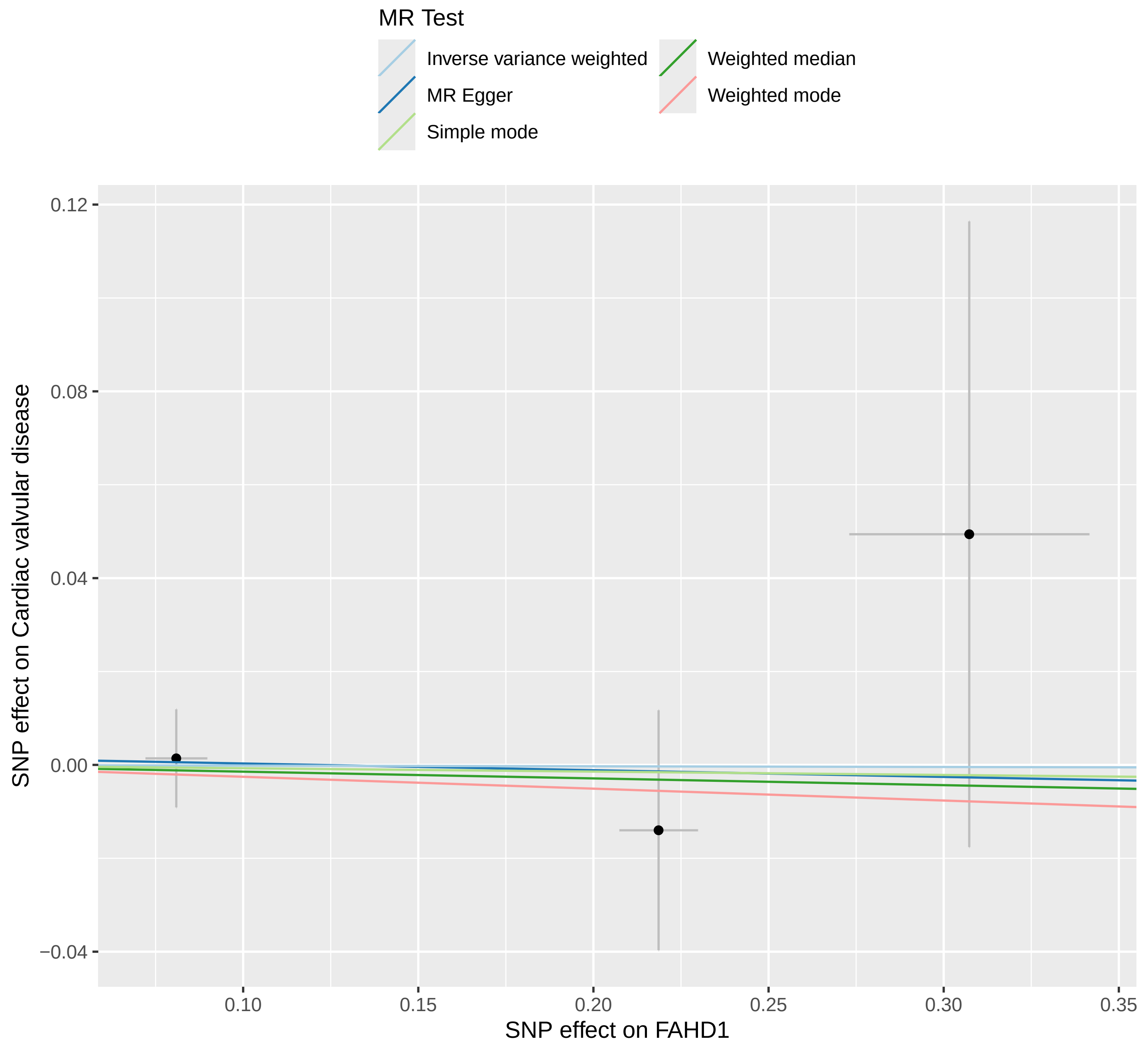
MR Test
Inverse variance weighted
MR Egger
Simple mode
Weighted median
Weighted mode
0.12
0.08
0.04
0.00
−0.04
0.10
0.15
0.20
0.25
0.30
0.35
SNP effect on Cardiac valvular disease
SNP effect on FAHD1

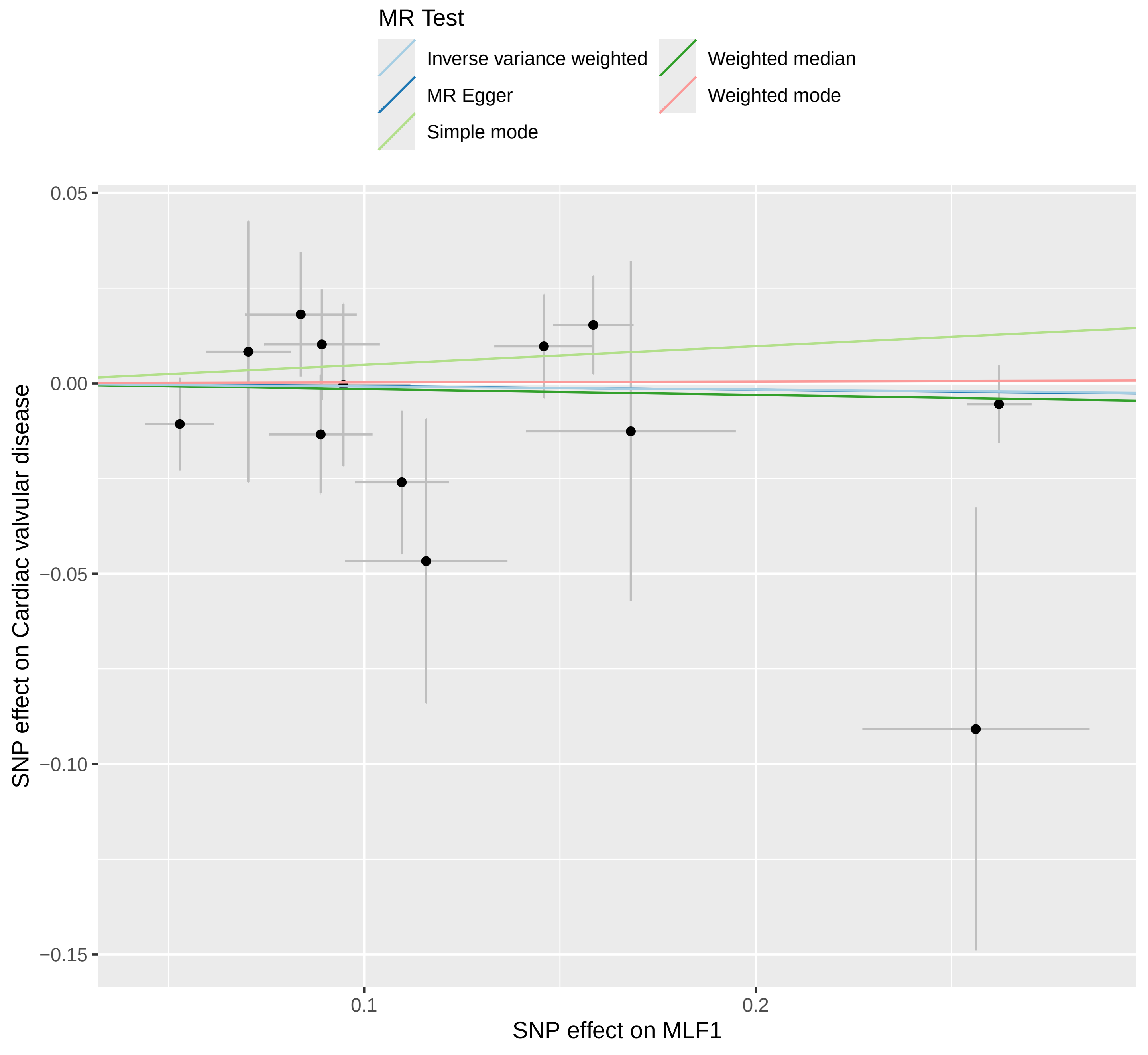
MR Test
Inverse variance weighted
MR Egger
Simple mode
Weighted median
Weighted mode
0.05
0.00
−0.05
−0.10
−0.15
0.1
0.2
SNP effect on Cardiac valvular disease
SNP effect on MLF1

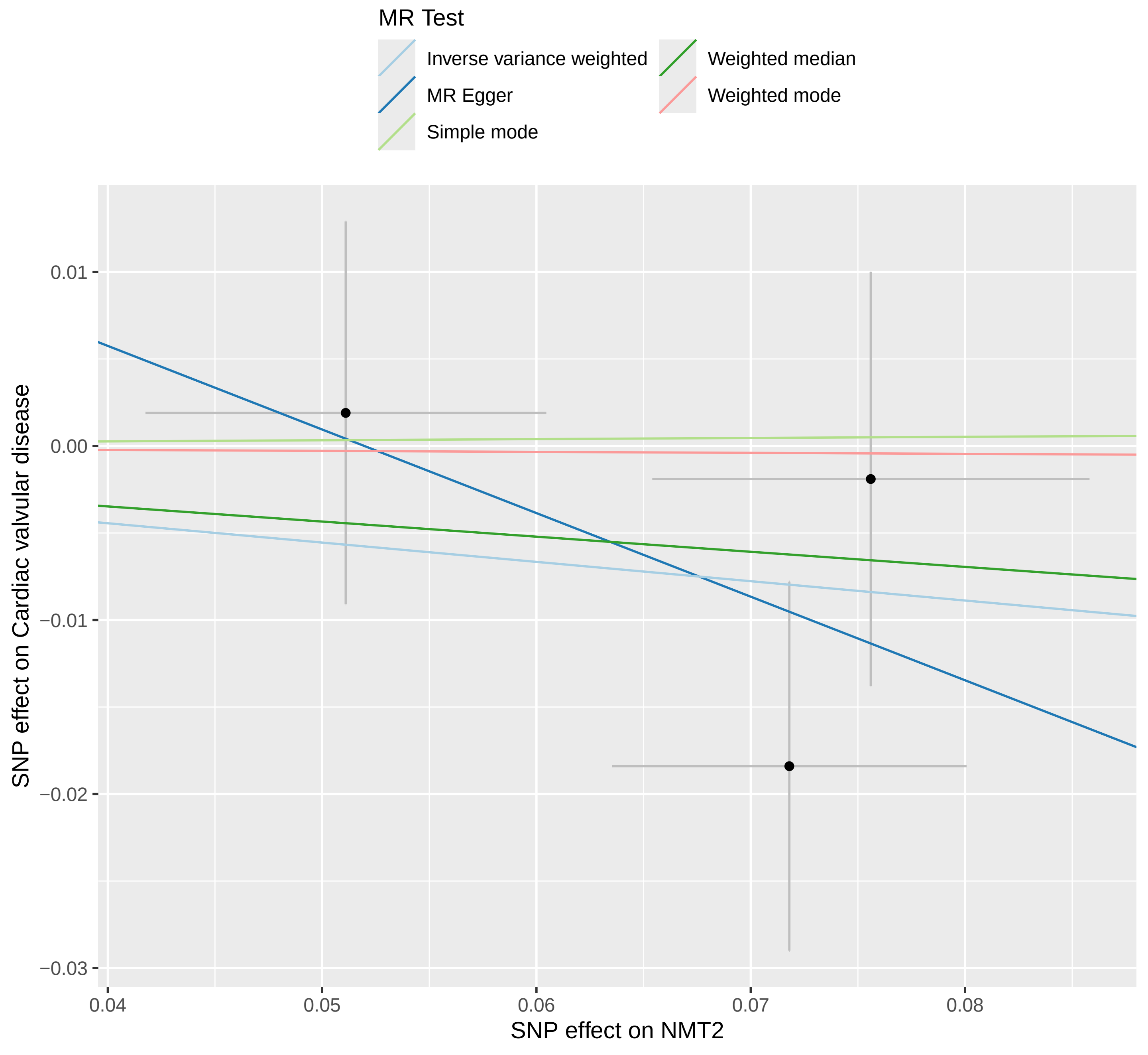
MR Test
Inverse variance weighted
MR Egger
Simple mode
Weighted median
Weighted mode
0.01
0.00
−0.01
−0.02
−0.03
SNP effect on Cardiac valvular disease
0.04
0.05
0.06
0.07
0.08
SNP effect on NMT2

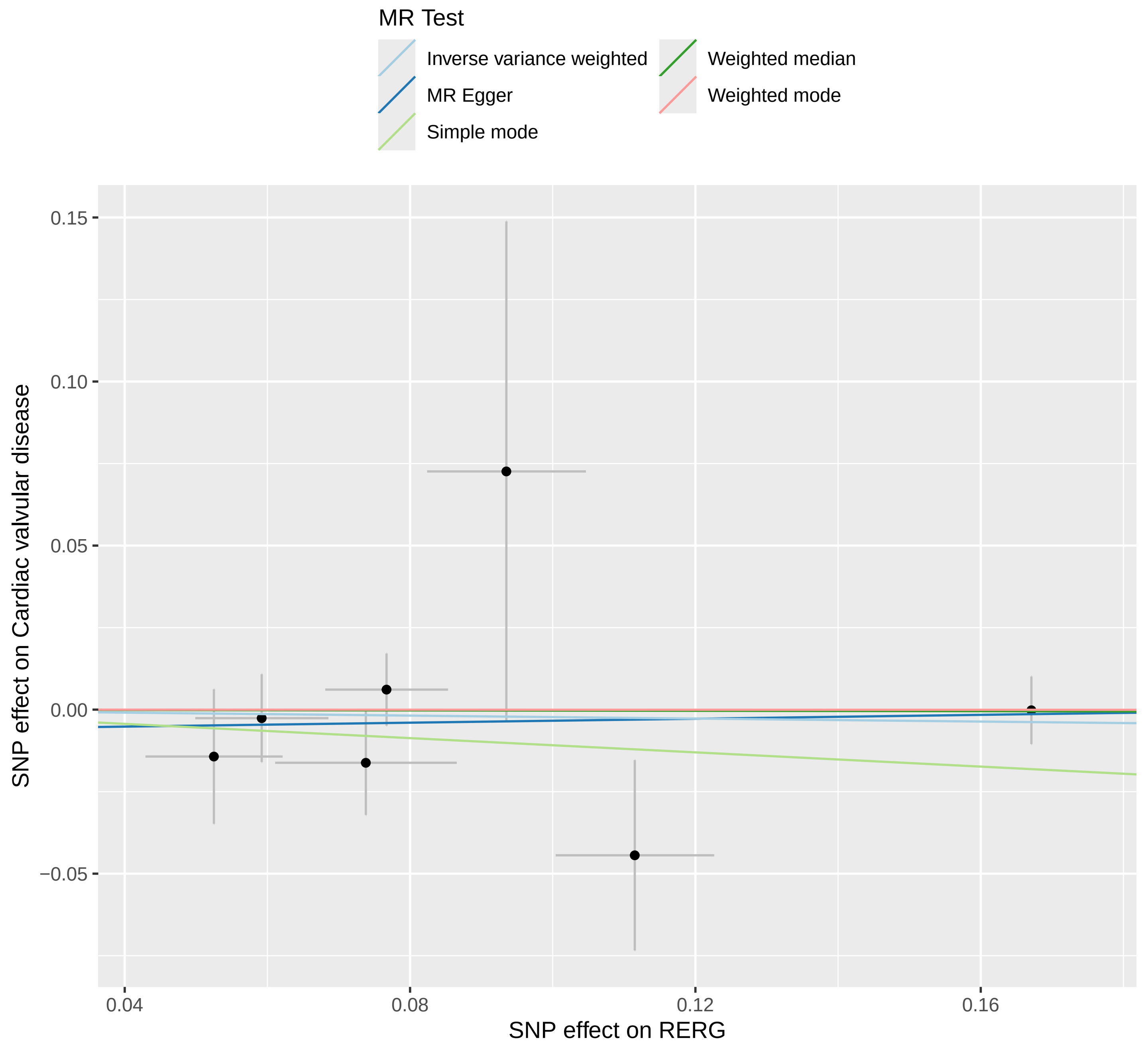
MR Test
Inverse variance weighted
MR Egger
Simple mode
Weighted median
Weighted mode
0.15
0.10
0.05
0.00
−0.05
0.04
0.08
0.12
0.16
SNP effect on Cardiac valvular disease
SNP effect on RERG

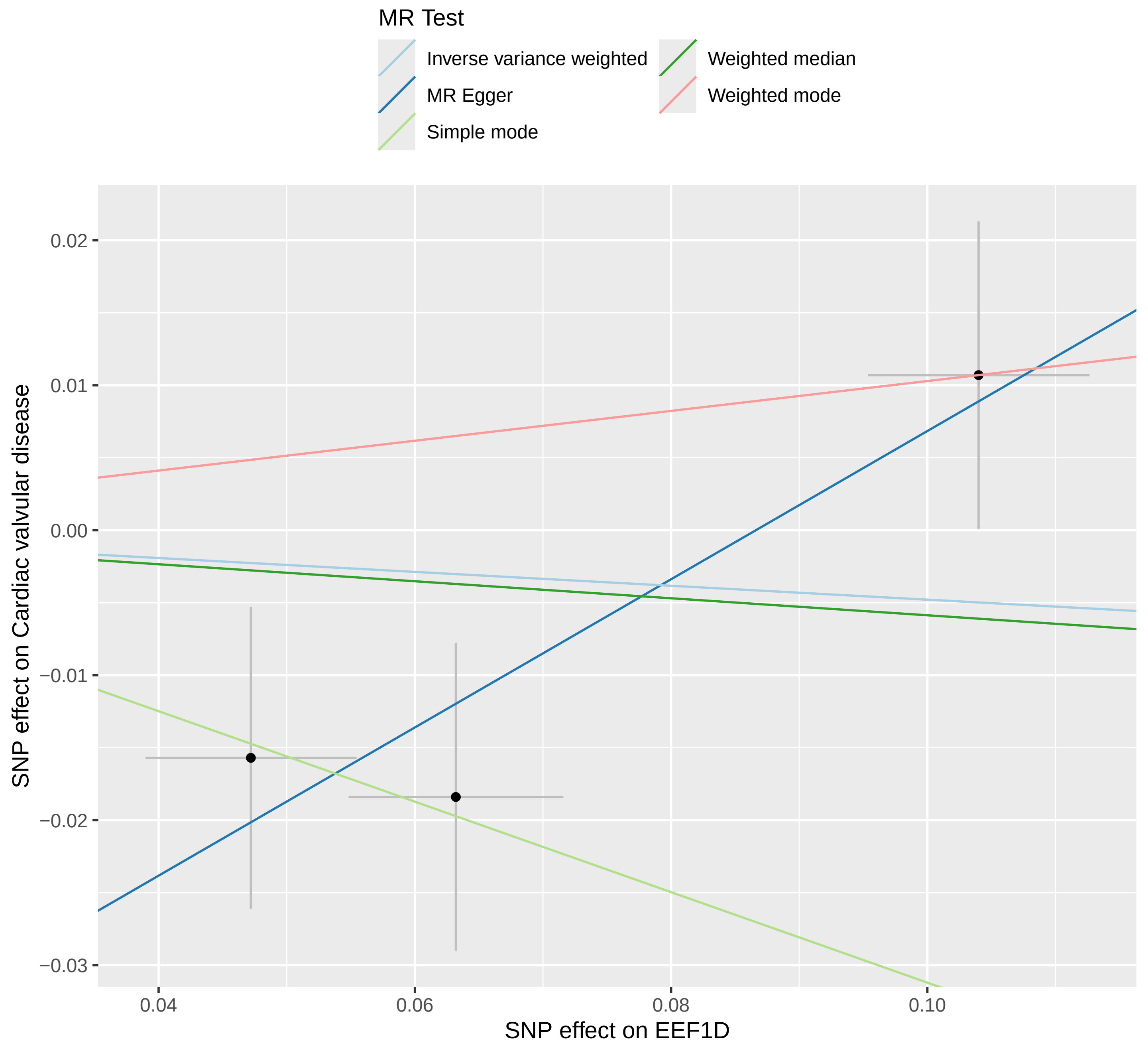

MR Test
Inverse variance weighted
MR Egger
Simple mode
Weighted median
Weighted mode
0.02
0.01
0.00
−0.01
−0.02
−0.03
SNP effect on Cardiac valvular disease
0.04
0.06
0.08
0.10
SNP effect on EEF1D

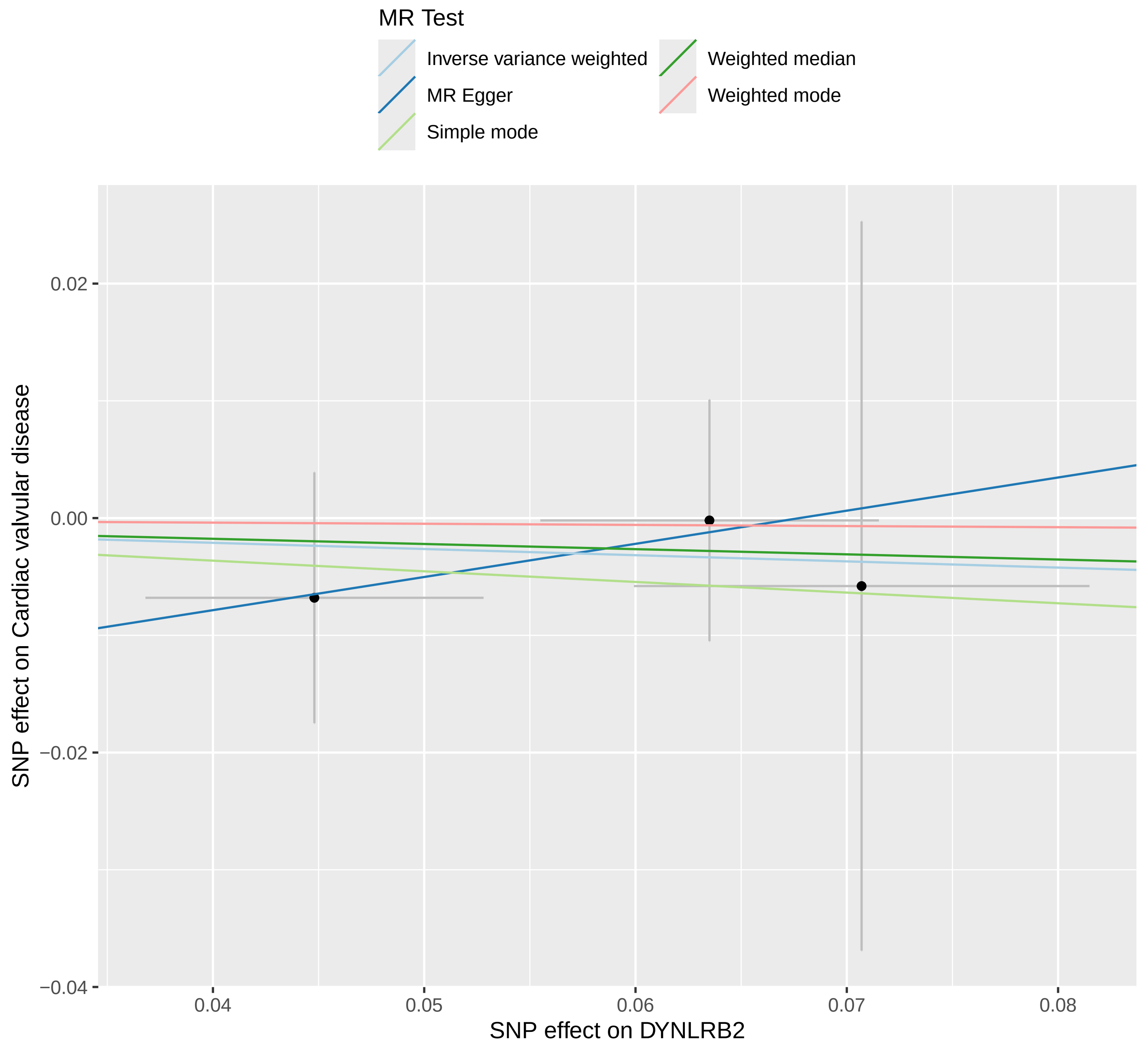
MR Test
Inverse variance weighted
MR Egger
Simple mode
Weighted median
Weighted mode
0.02
0.00
−0.02
−0.04
0.04
0.05
0.06
0.07
0.08
SNP effect on Cardiac valvular disease
SNP effect on DYNLRB2

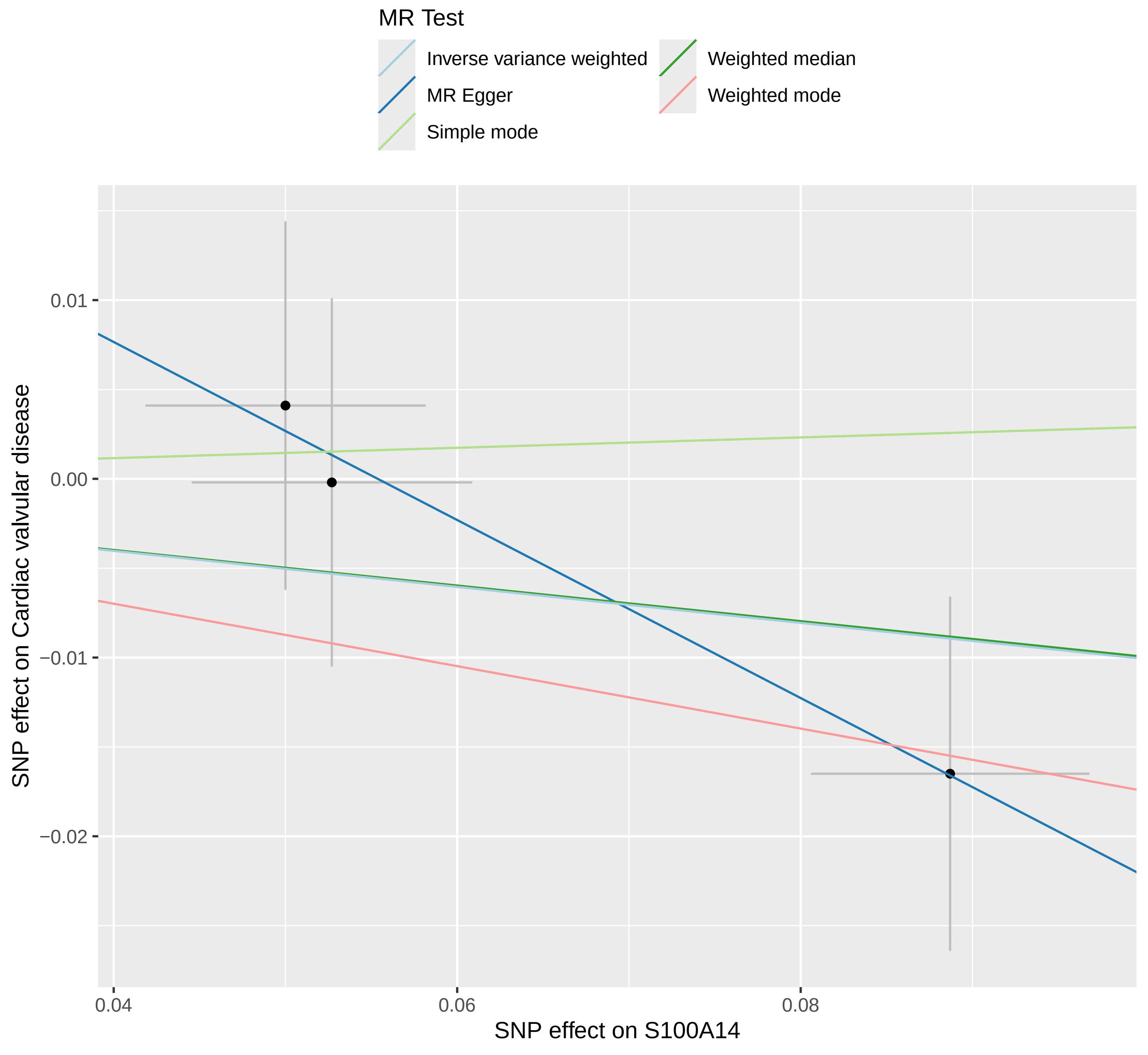
MR Test
Inverse variance weighted
MR Egger
Simple mode
Weighted median
Weighted mode
0.01
0.00
−0.01
−0.02
SNP effect on Cardiac valvular disease
0.04
0.06
0.08
SNP effect on S100A14

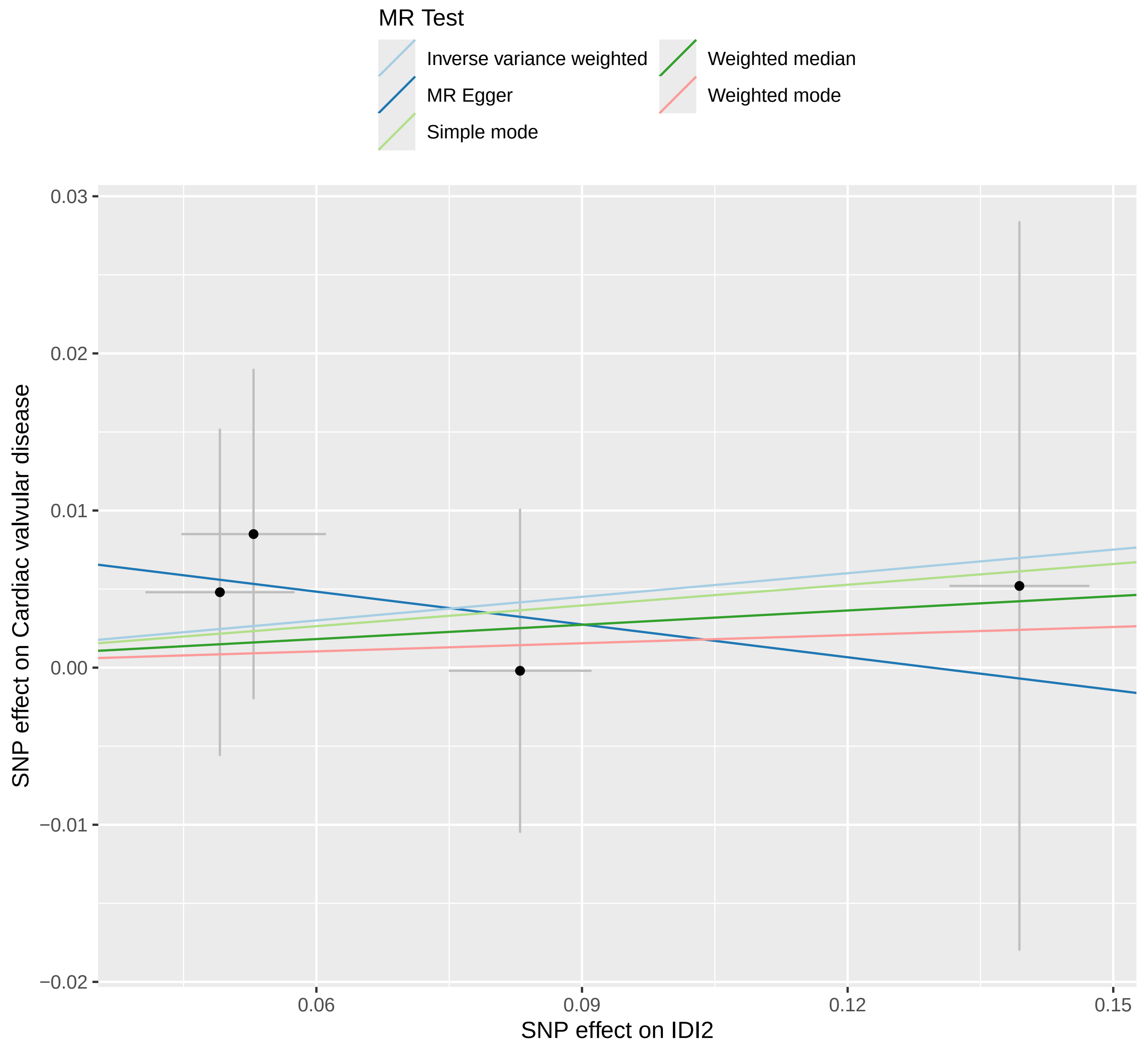
MR Test
Inverse variance weighted
MR Egger
Simple mode
Weighted median
Weighted mode
SNP effect on Cardiac valvular disease
0.03
0.02
0.01
0.00
−0.01
−0.02
0.06
0.09
0.12
0.15
SNP effect on IDI2

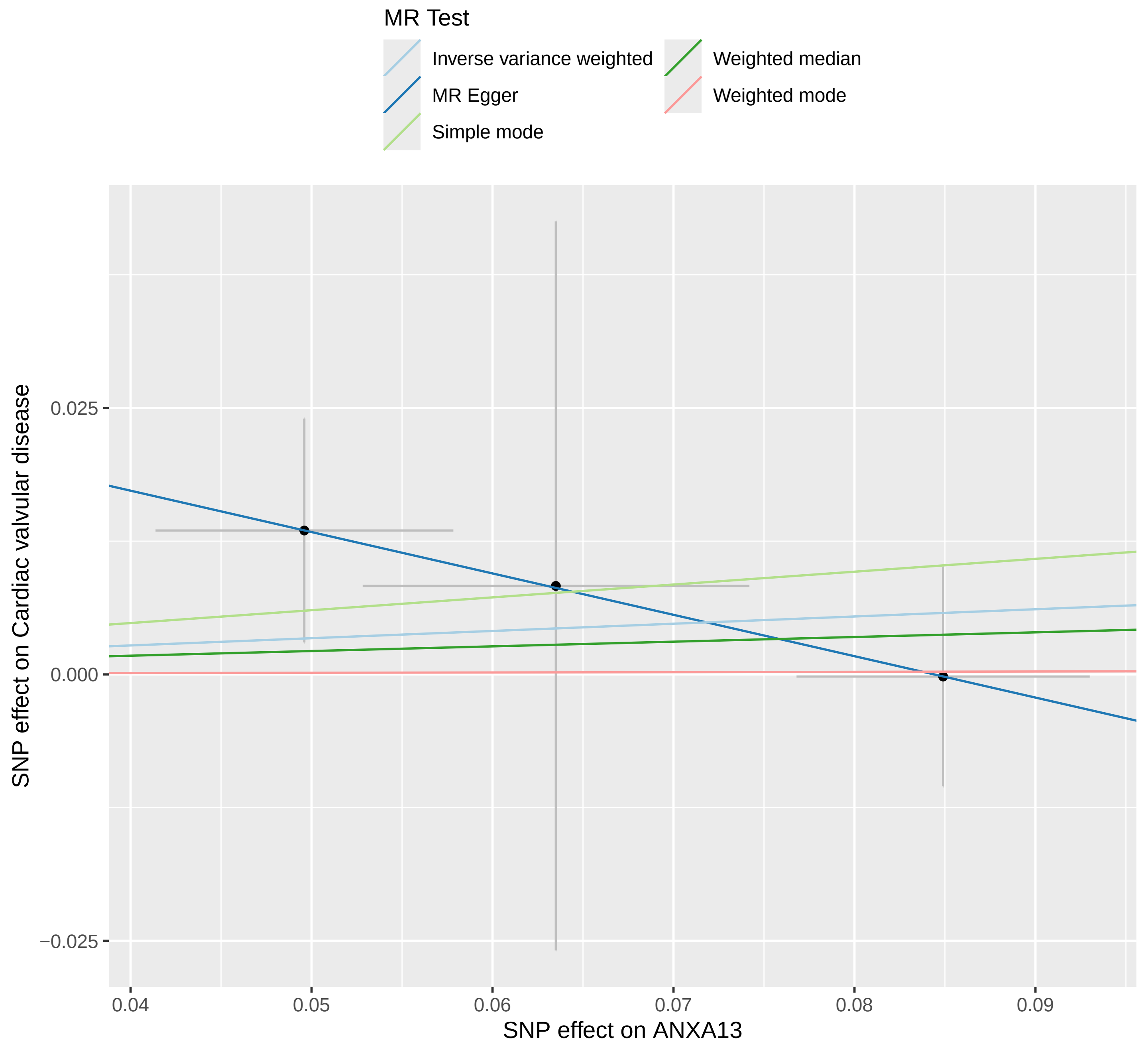
MR Test
Inverse variance weighted
MR Egger
Simple mode
Weighted median
Weighted mode
0.025
0.000
−0.025
0.04
0.05
0.06
0.07
0.08
0.09
SNP effect on Cardiac valvular disease
SNP effect on ANXA13

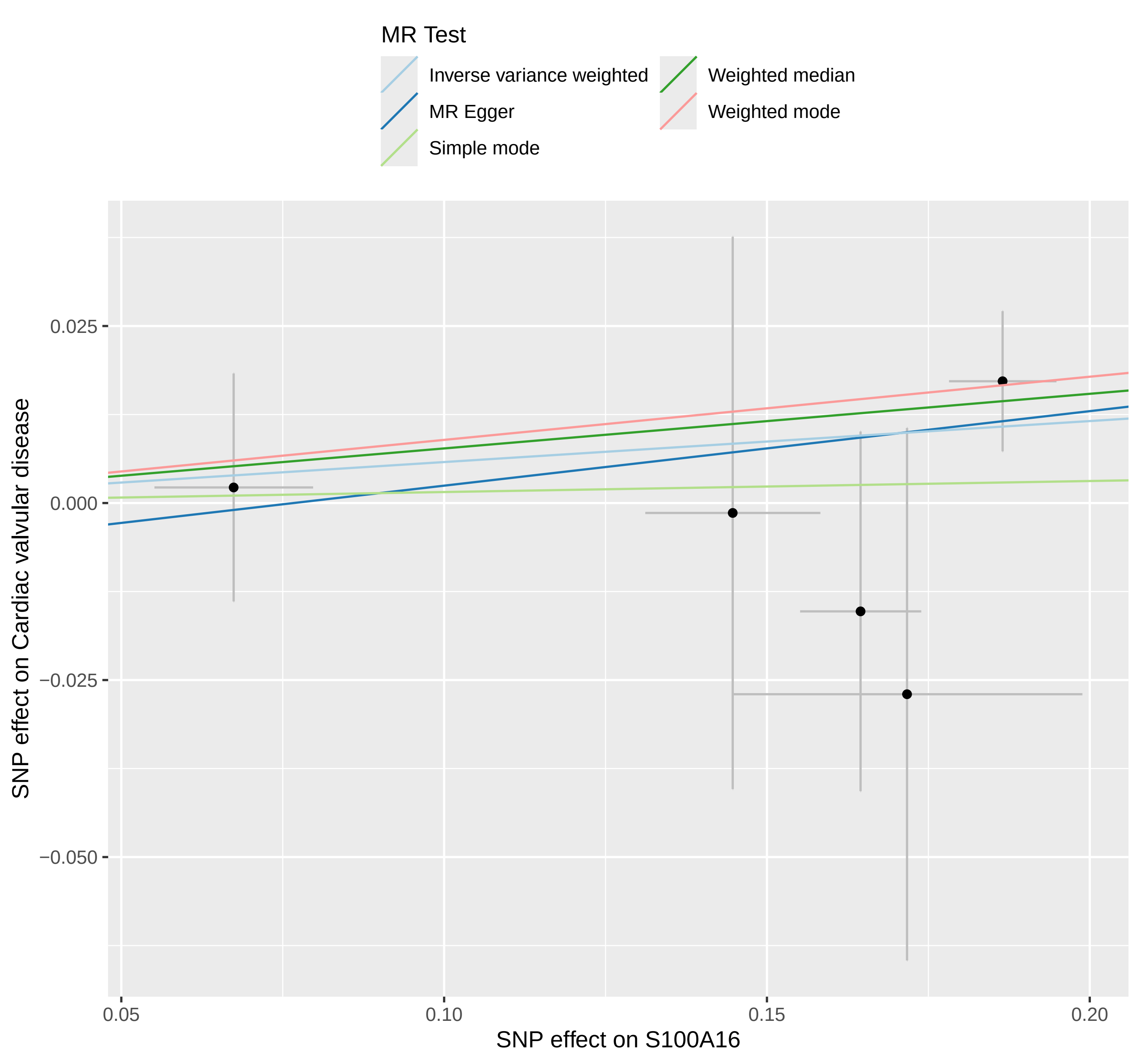
MR Test
Inverse variance weighted
MR Egger
Simple mode
Weighted median
Weighted mode
0.025
0.000
−0.025
−0.050
SNP effect on Cardiac valvular disease
0.05
0.10
0.15
0.20
SNP effect on S100A16

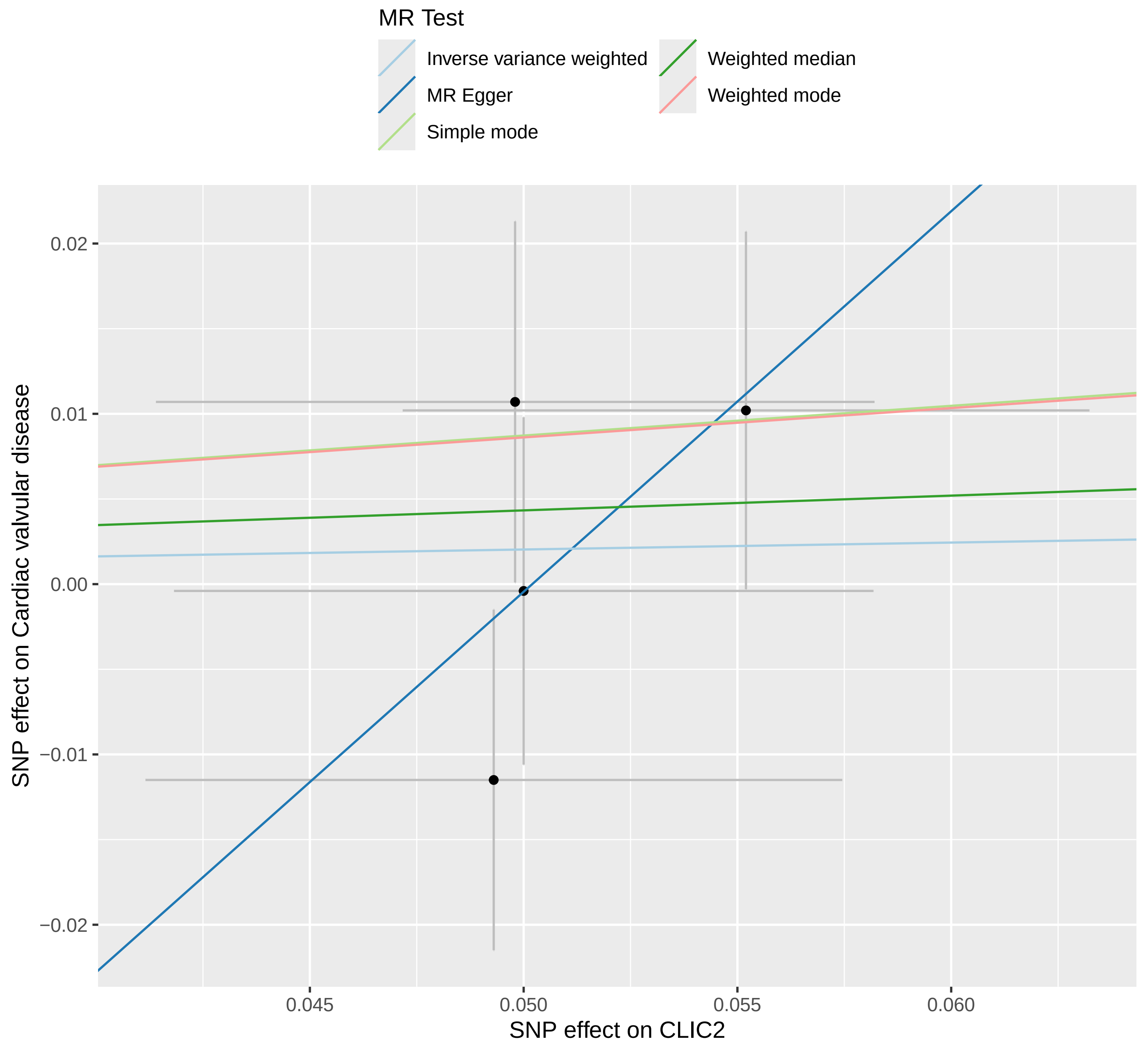
MR Test
Inverse variance weighted
MR Egger
Simple mode
Weighted median
Weighted mode
0.02
0.01
0.00
−0.01
−0.02
SNP effect on Cardiac valvular disease
0.045
0.050
0.055
0.060
SNP effect on CLIC2

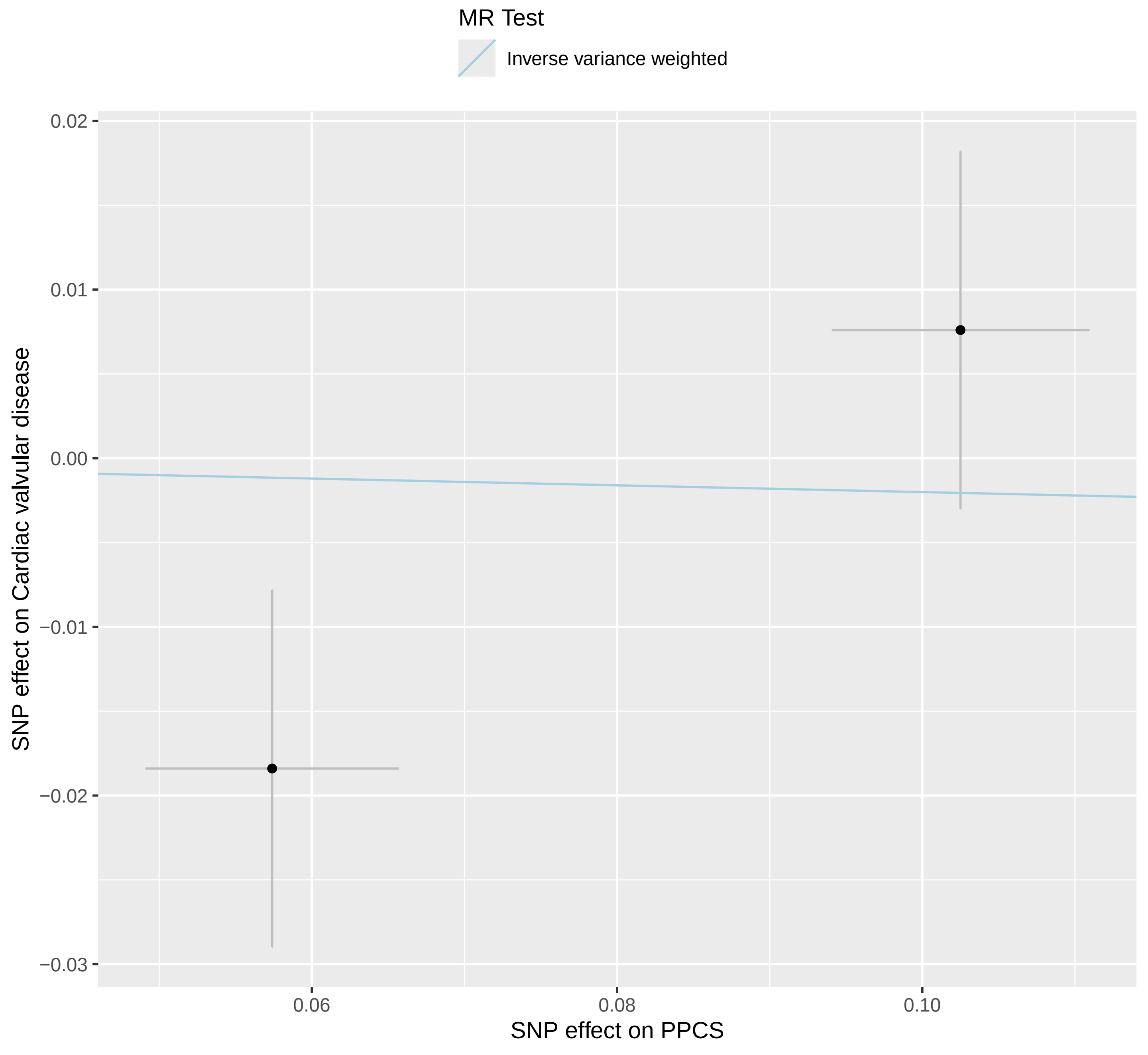
MR Test
Inverse variance weighted
0.02
0.01
0.00
−0.01
−0.02
−0.03
SNP effect on Cardiac valvular disease
0.06
0.08
0.10
SNP effect on PPCS

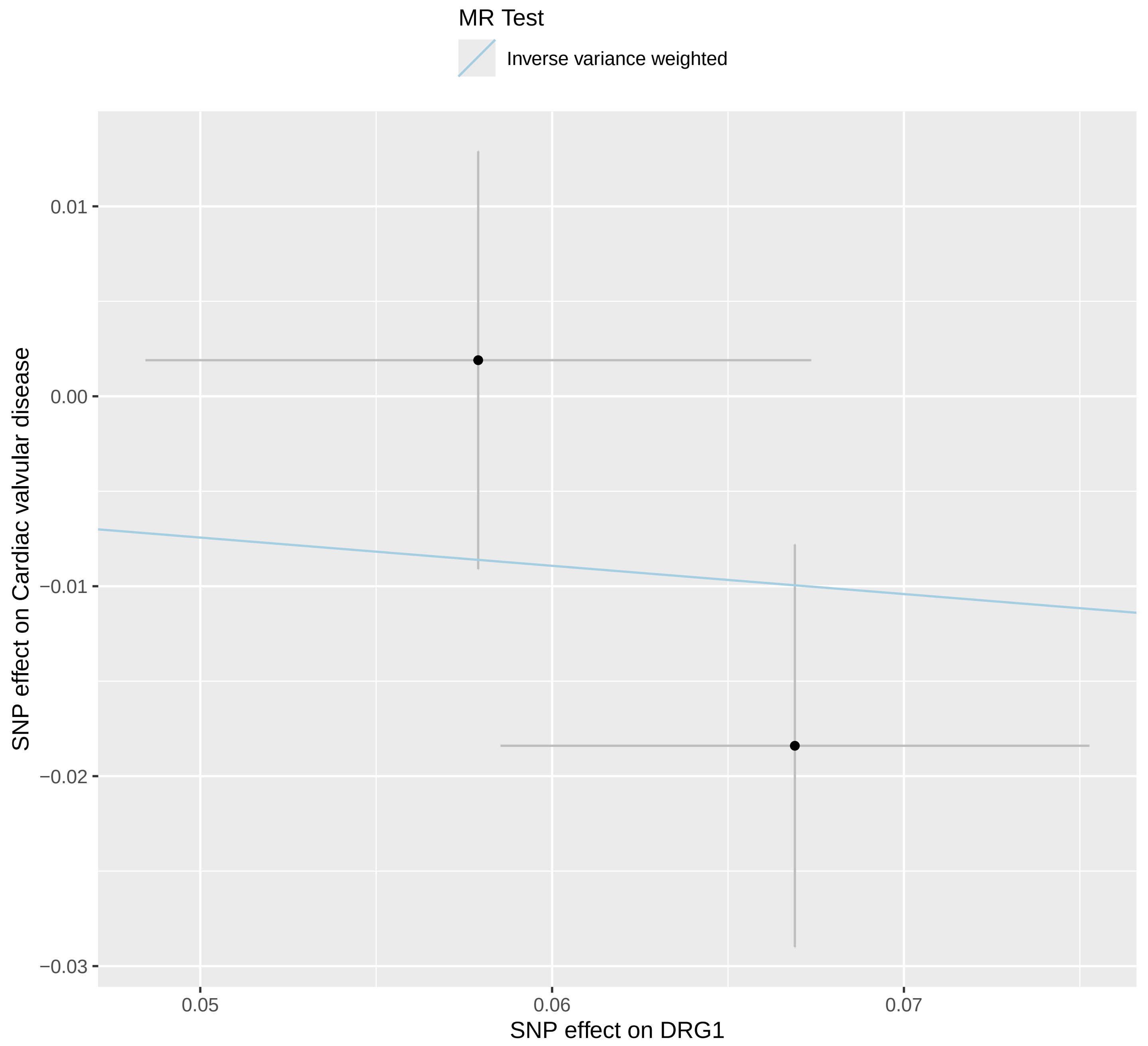
MR Test
Inverse variance weighted
SNP effect on Cardiac valvular disease
0.01
0.00
−0.01
−0.02
−0.03
0.05
0.06
0.07
SNP effect on DRG1

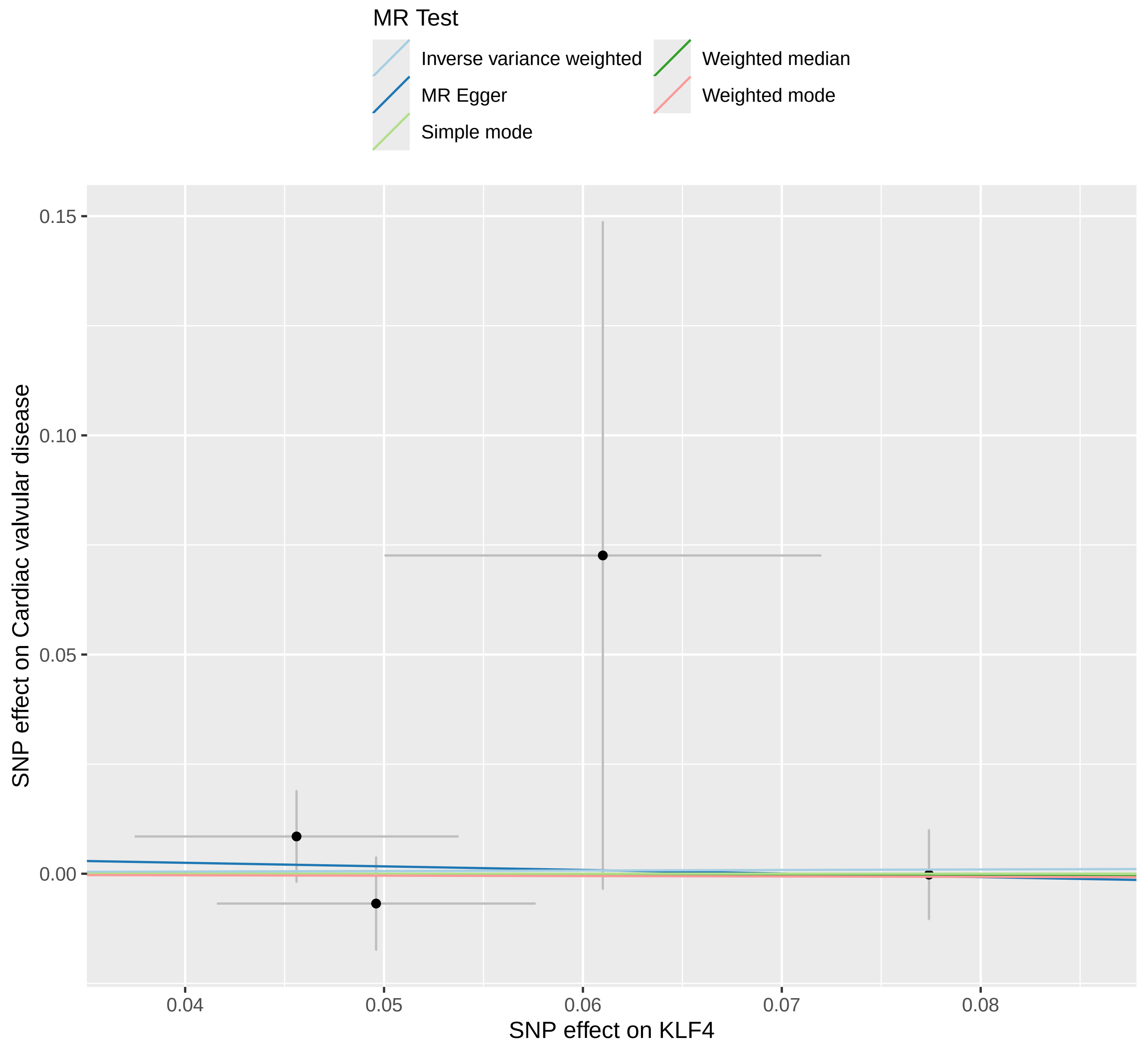
MR Test
Inverse variance weighted
MR Egger
Simple mode
Weighted median
Weighted mode
SNP effect on Cardiac valvular disease
0.15
0.10
0.05
0.00
0.04
0.05
0.06
0.07
0.08
SNP effect on KLF4

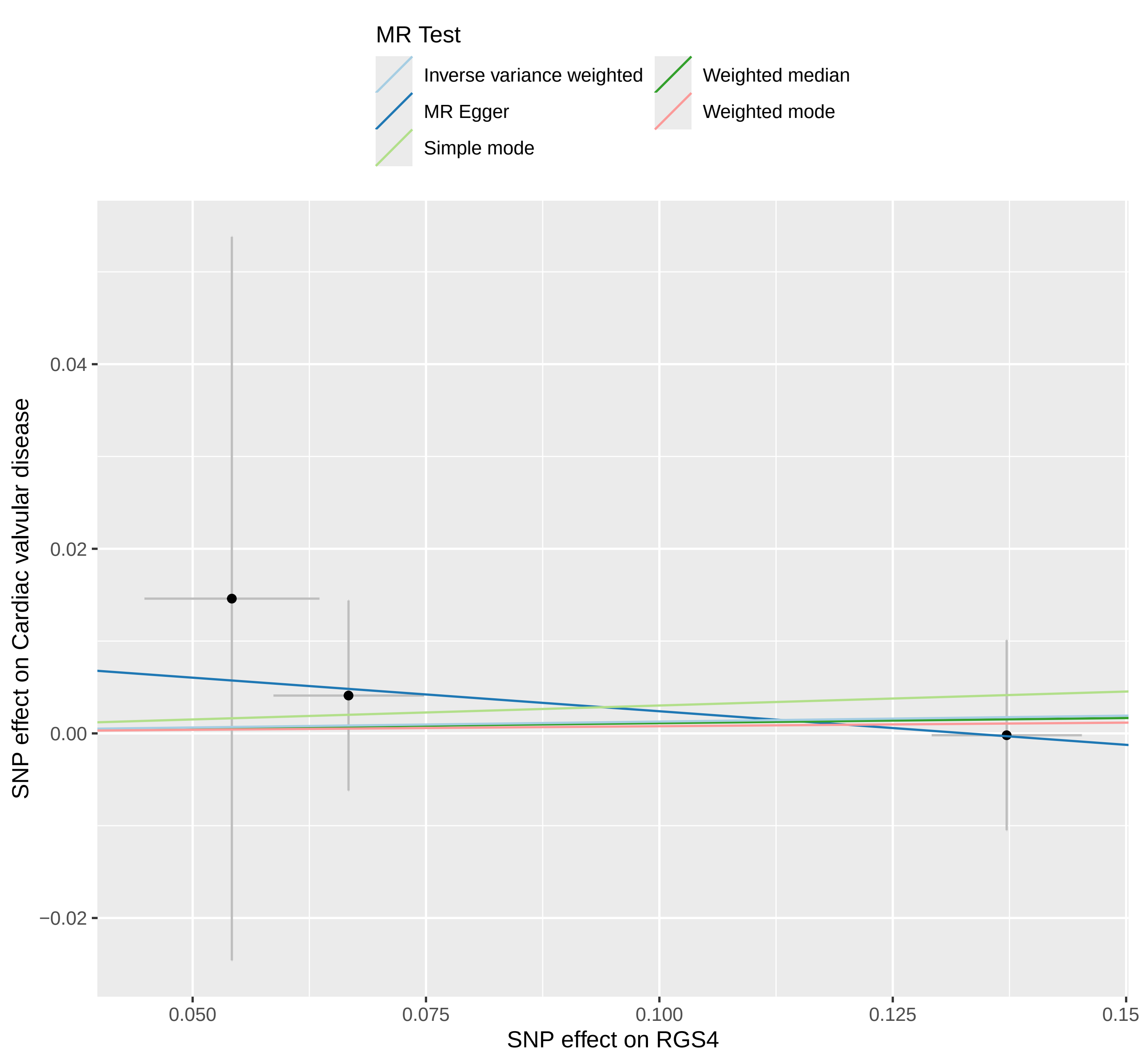

MR Test
Inverse variance weighted
MR Egger
Simple mode
Weighted median
Weighted mode
SNP effect on Cardiac valvular disease
0.04
0.02
0.00
−0.02
0.050
0.075
0.100
0.125
0.15
SNP effect on RGS4

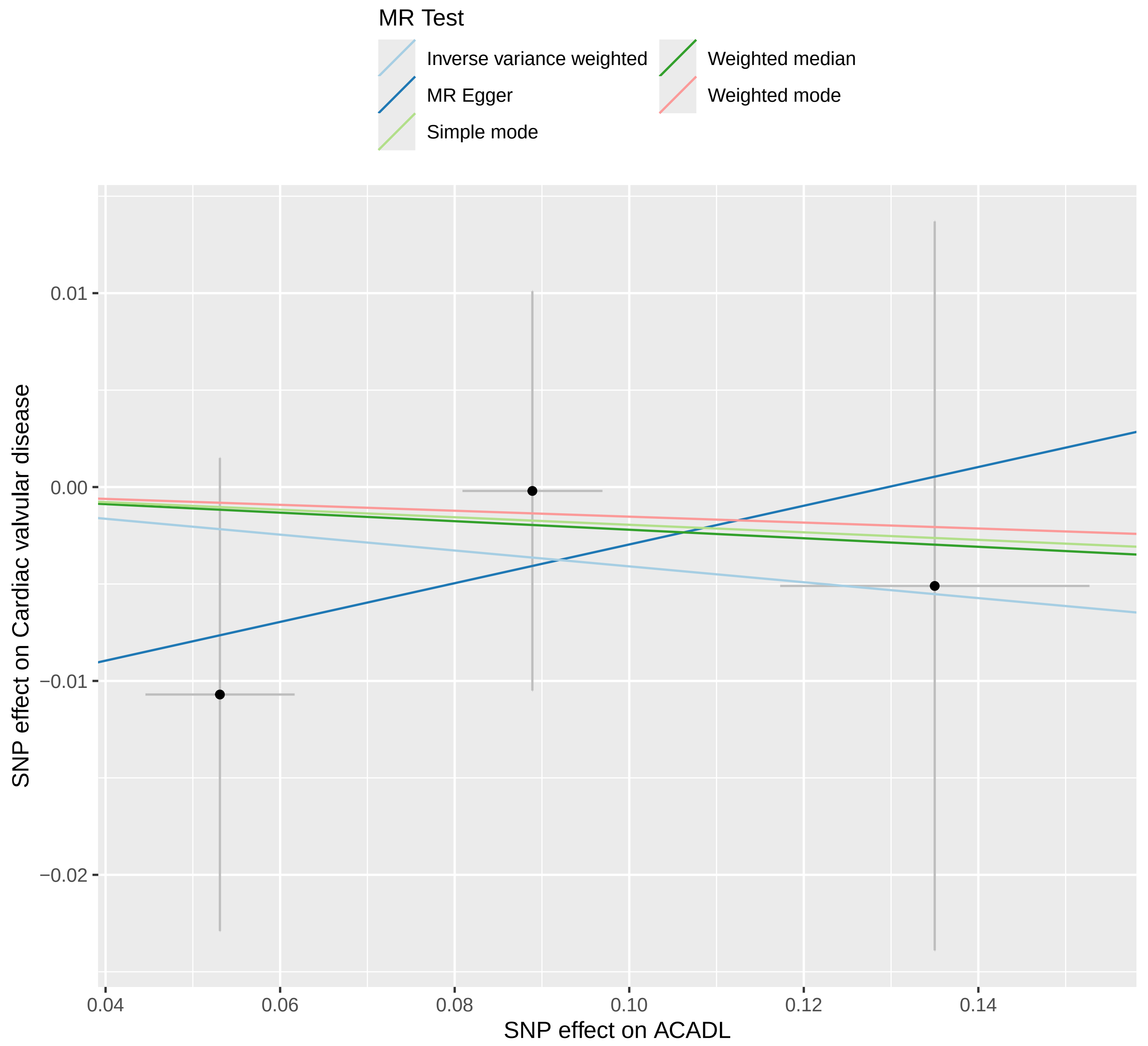
MR Test
Inverse variance weighted
MR Egger
Simple mode
Weighted median
Weighted mode
0.01
0.00
−0.01
−0.02
SNP effect on Cardiac valvular disease
0.04
0.06
0.08
0.10
0.12
0.14
SNP effect on ACADL

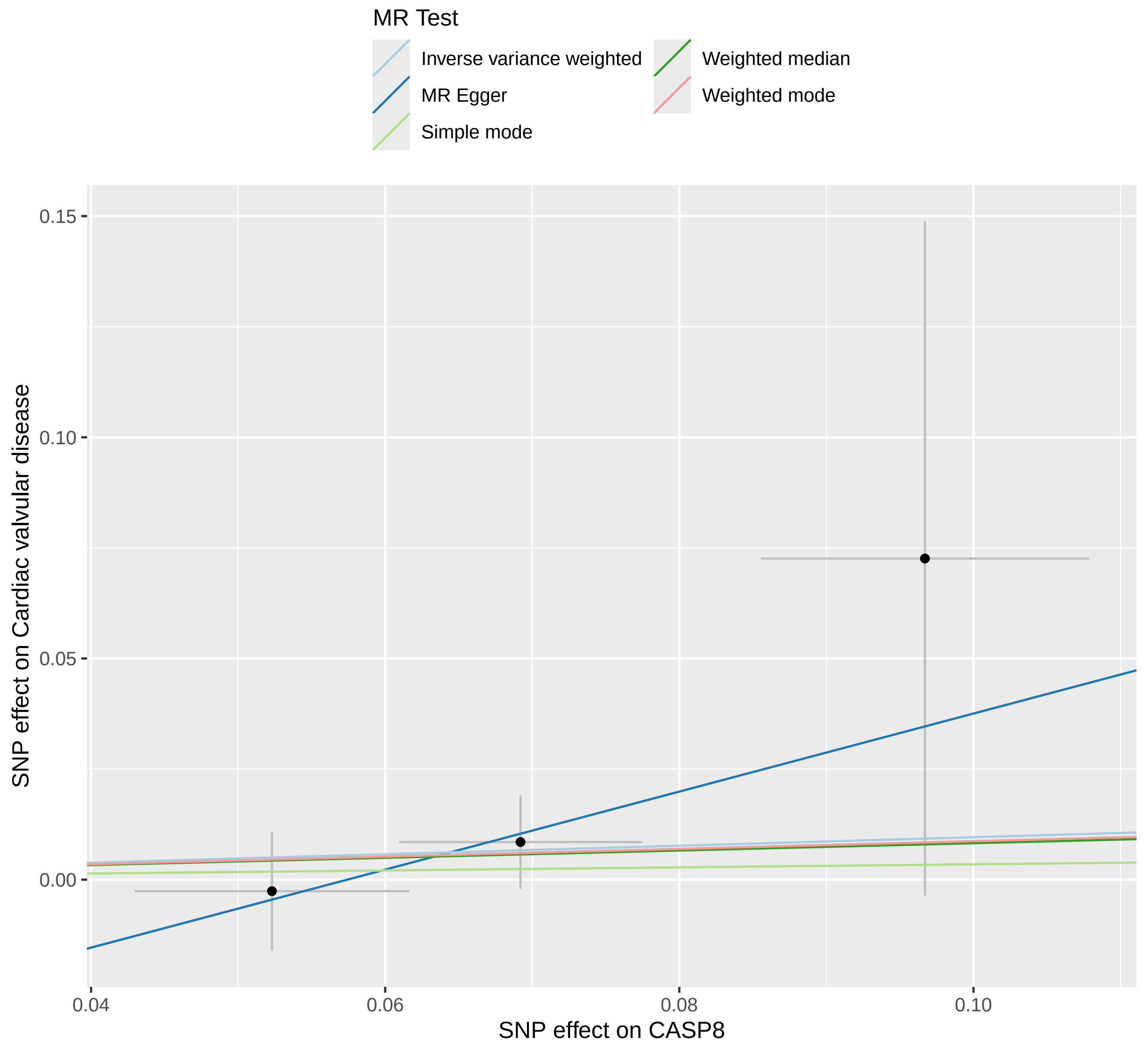

MR Test
Inverse variance weighted
MR Egger
Simple mode
Weighted median
Weighted mode
0.15
0.10
0.05
0.00
SNP effect on Cardiac valvular disease
0.04
0.06
0.08
0.10
SNP effect on CASP8

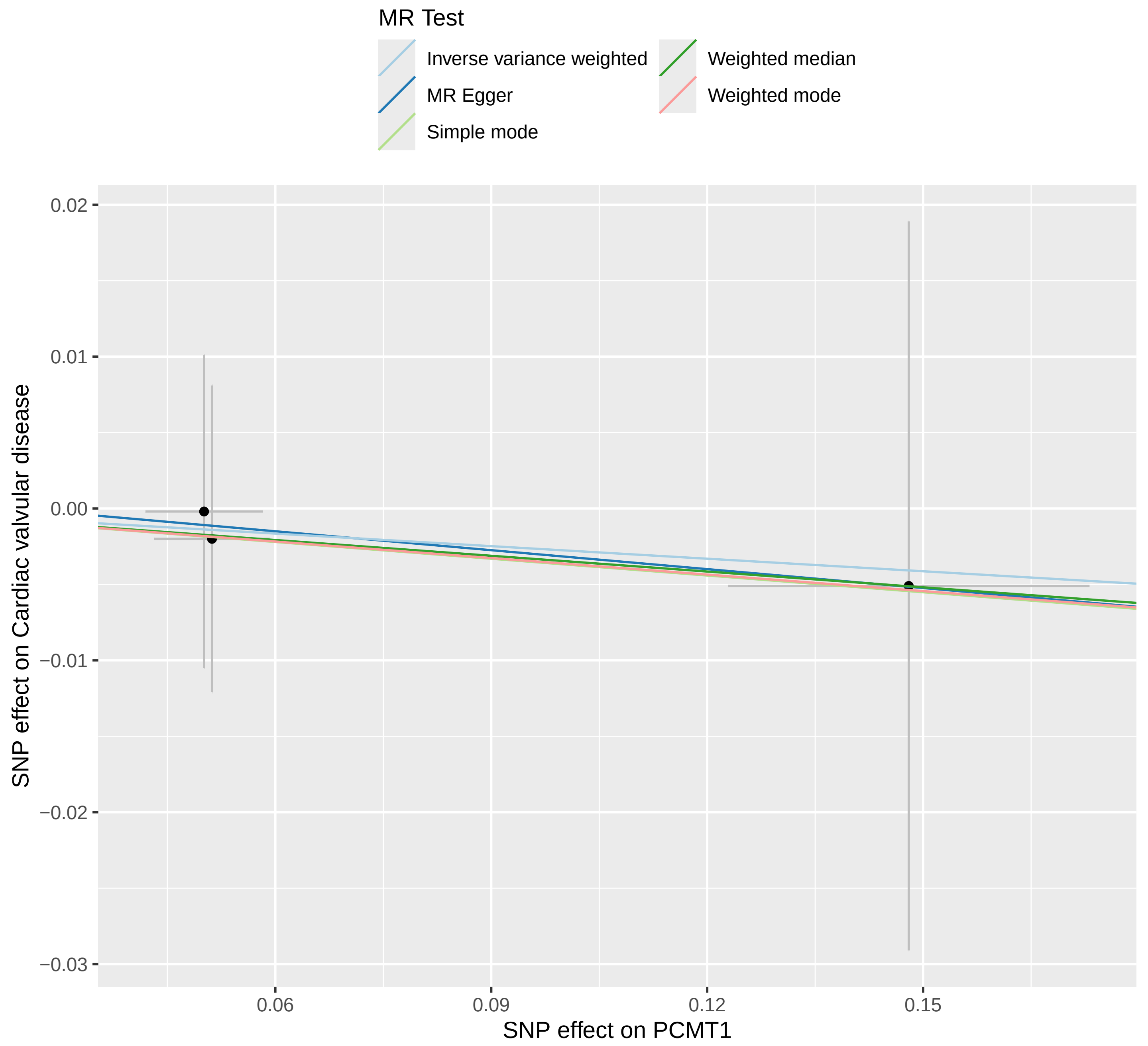

MR Test
Inverse variance weighted
MR Egger
Simple mode
Weighted median
Weighted mode
0.02
0.01
0.00
−0.01
−0.02
−0.03
SNP effect on Cardiac valvular disease
0.06
0.09
0.12
0.15
SNP effect on PCMT1

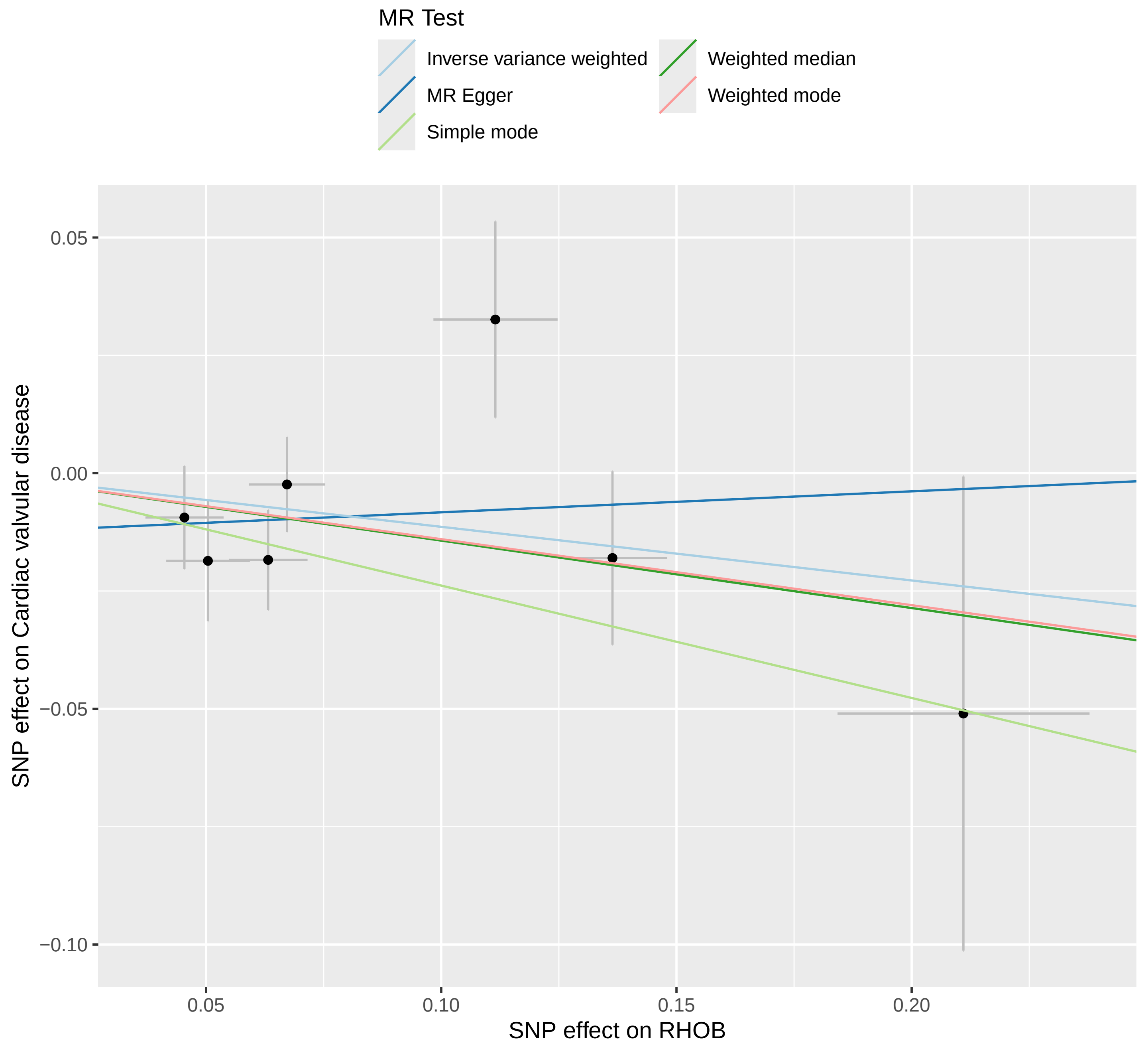
MR Test
Inverse variance weighted
MR Egger
Simple mode
Weighted median
Weighted mode
0.05
0.00
−0.05
−0.10
0.05
0.10
0.15
0.20
SNP effect on Cardiac valvular disease
SNP effect on RHOB

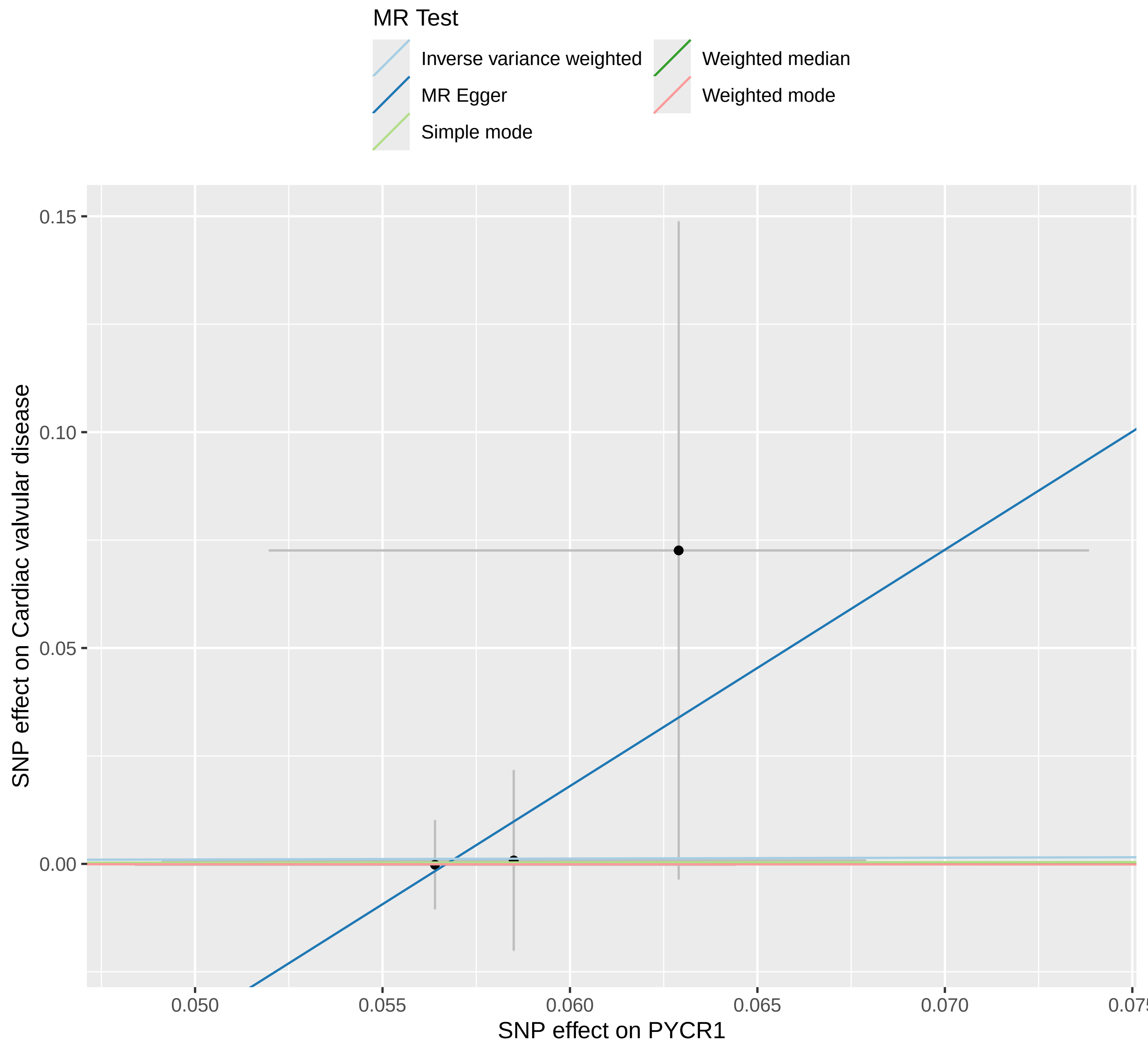

MR Test
Inverse variance weighted
MR Egger
Simple mode
Weighted median
Weighted mode
0.15
0.10
0.05
0.00
SNP effect on Cardiac valvular disease
0.050
0.055
0.060
0.065
0.070
SNP effect on PYCR1

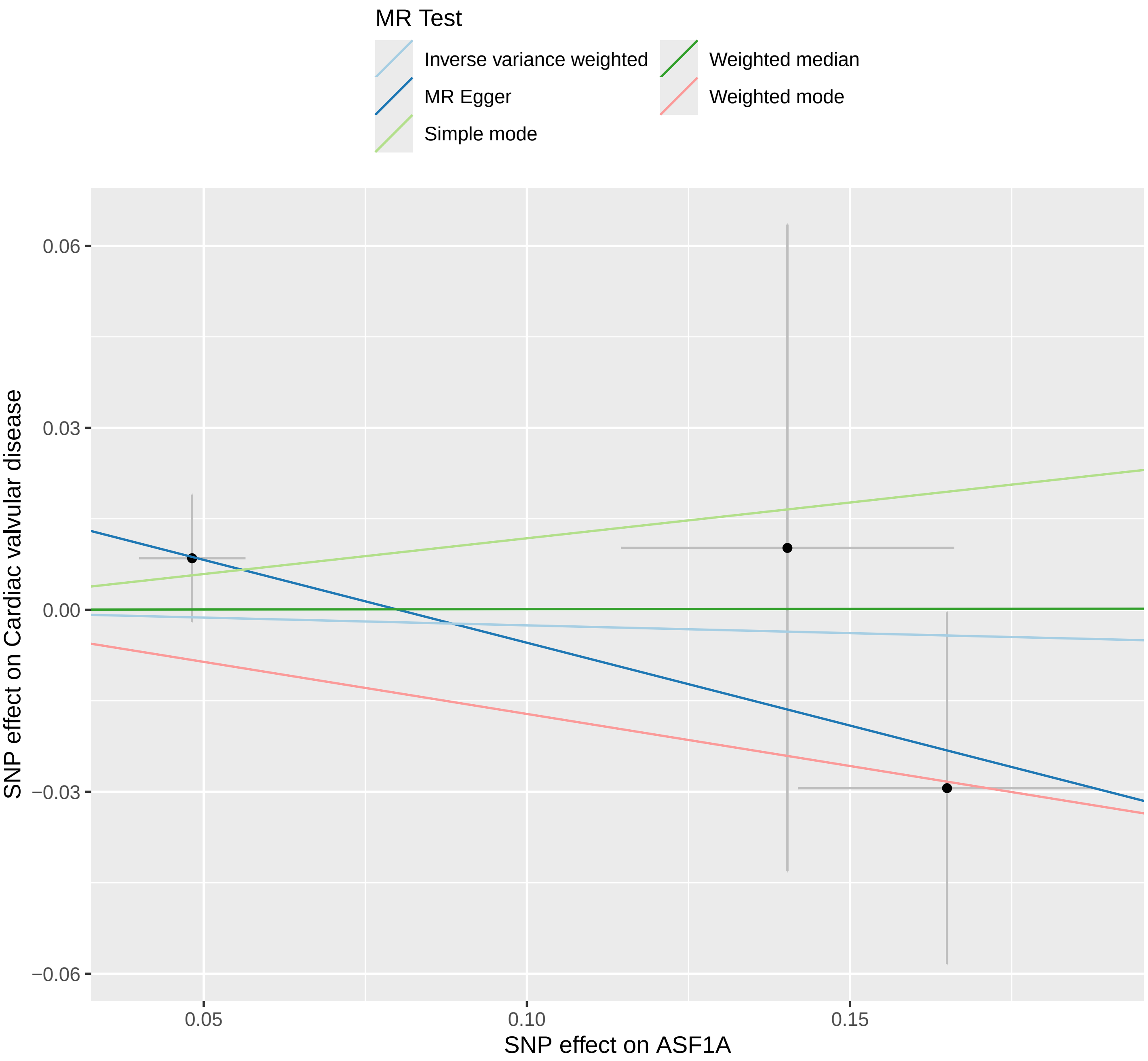
MR Test
Inverse variance weighted
MR Egger
Simple mode
Weighted median
Weighted mode
0.06
0.03
0.00
−0.03
−0.06
SNP effect on Cardiac valvular disease
0.05
0.10
0.15
SNP effect on ASF1A

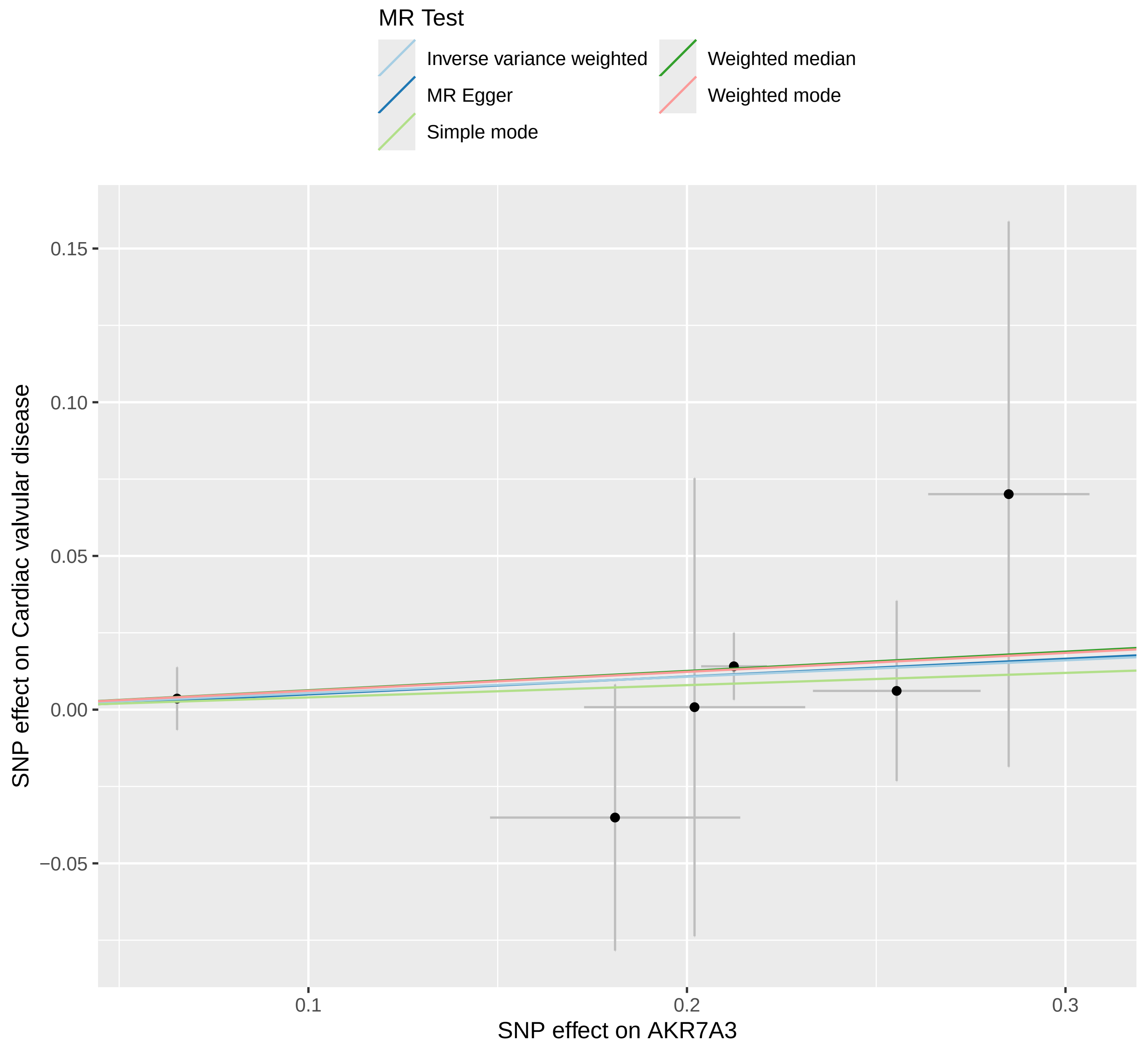
MR Test
Inverse variance weighted
MR Egger
Simple mode
Weighted median
Weighted mode
0.15
0.10
0.05
0.00
−0.05
SNP effect on Cardiac valvular disease
0.1
0.2
0.3
SNP effect on AKR7A3

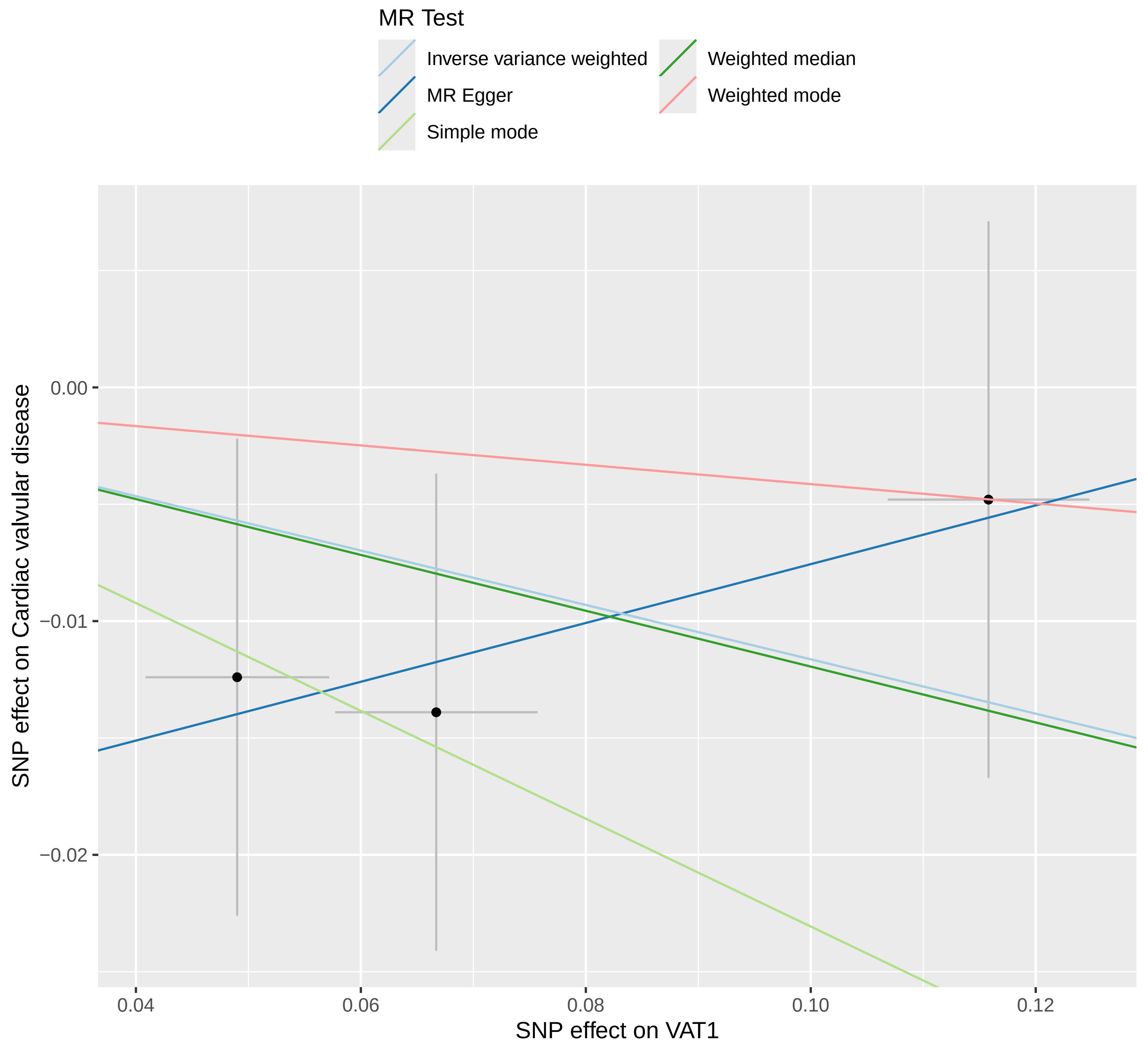
MR Test
Inverse variance weighted
MR Egger
Simple mode
Weighted median
Weighted mode
0.00
−0.01
−0.02
0.04
0.06
0.08
0.10
0.12
SNP effect on Cardiac valvular disease
SNP effect on VAT1

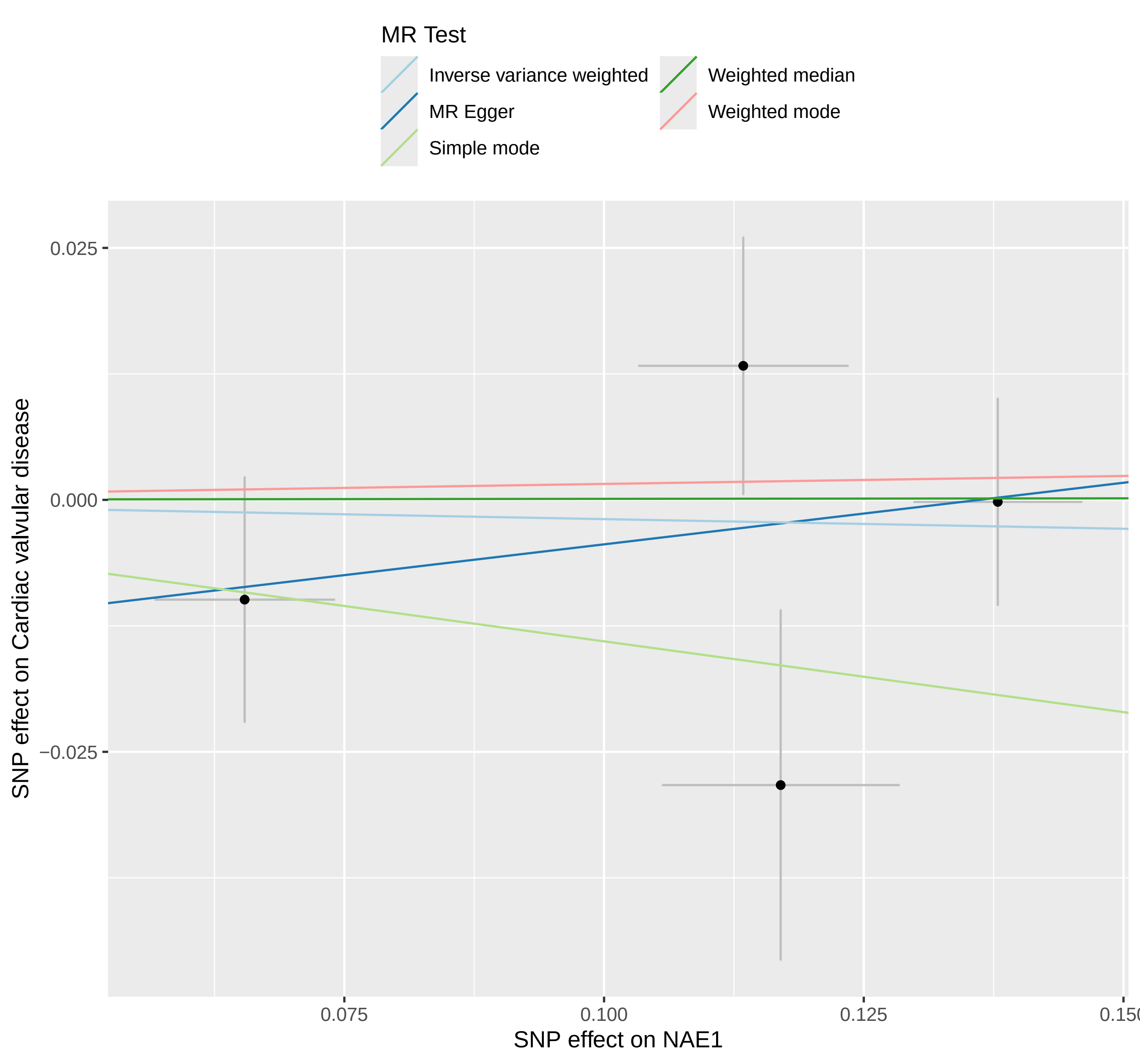
MR Test
Inverse variance weighted
MR Egger
Simple mode
Weighted median
Weighted mode
0.025
0.000
−0.025
SNP effect on Cardiac valvular disease
0.075
0.100
0.125
0.150
SNP effect on NAE1

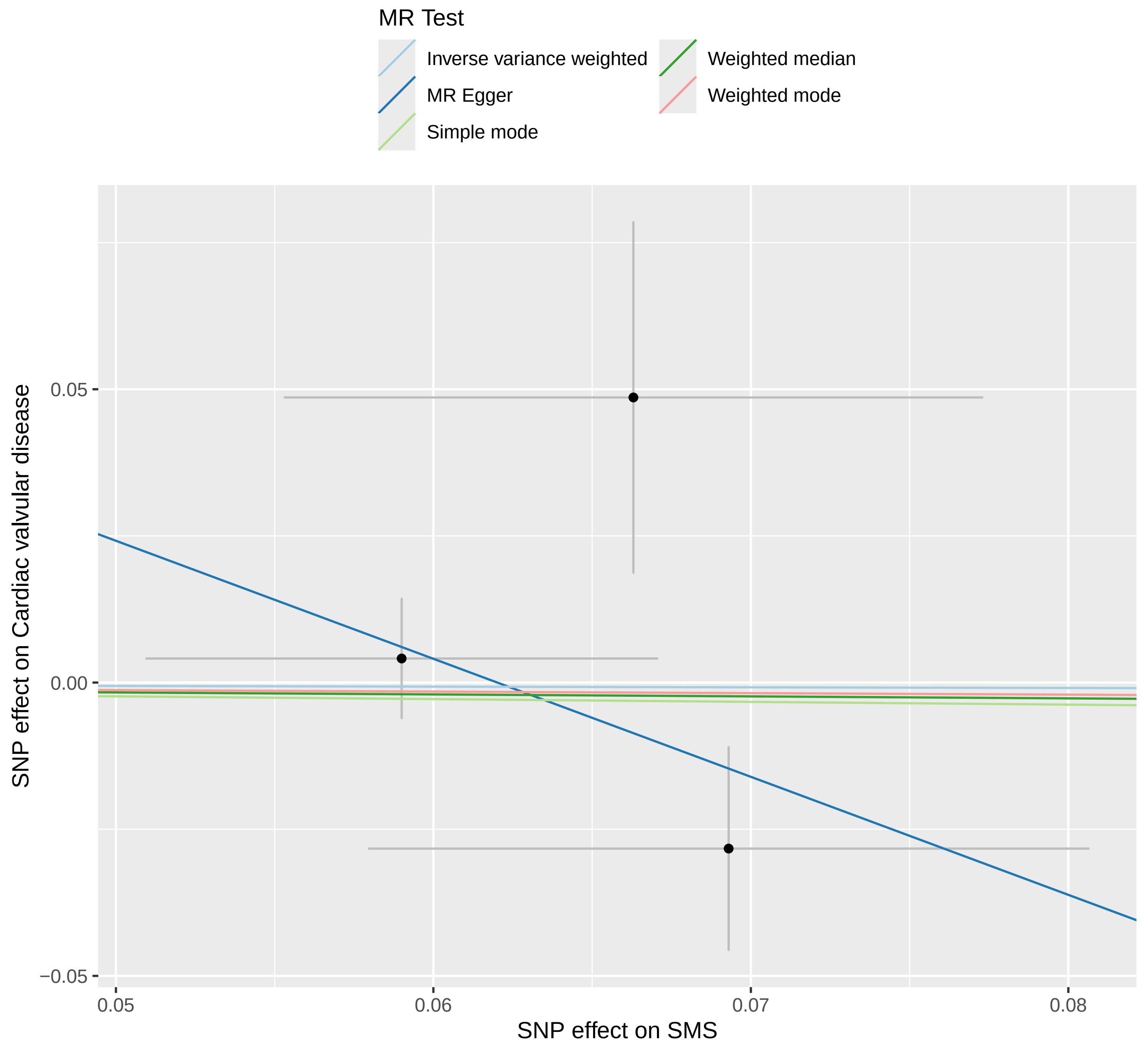
MR Test
Inverse variance weighted
MR Egger
Simple mode
Weighted median
Weighted mode
0.05
0.00
−0.05
0.05
0.06
0.07
0.08
SNP effect on SMS
SNP effect on Cardiac valvular disease

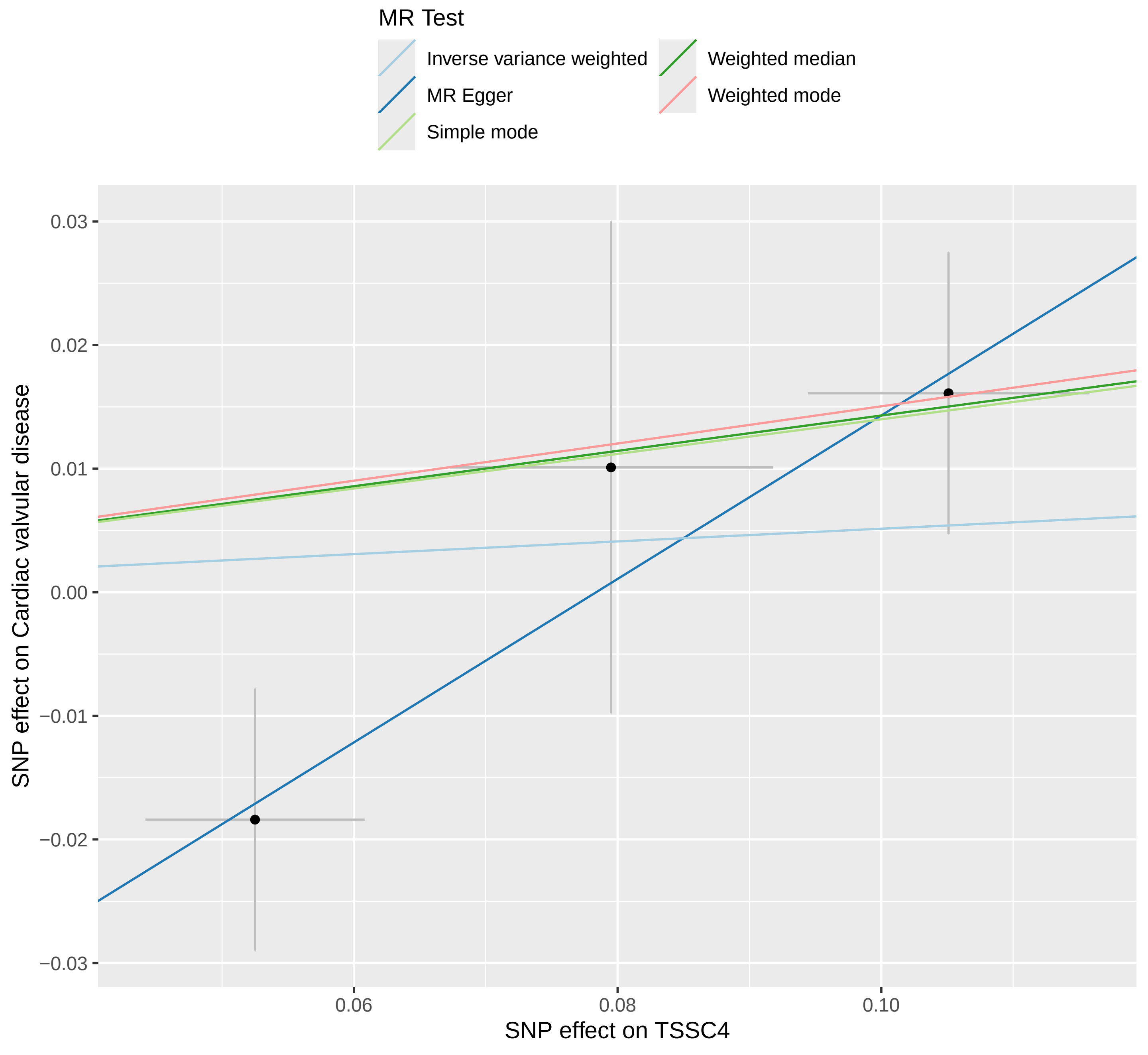

MR Test
Inverse variance weighted
MR Egger
Simple mode
Weighted median
Weighted mode
SNP effect on Cardiac valvular disease
0.03
0.02
0.01
0.00
−0.01
−0.02
−0.03
0.06
0.08
0.10
SNP effect on TSSC4

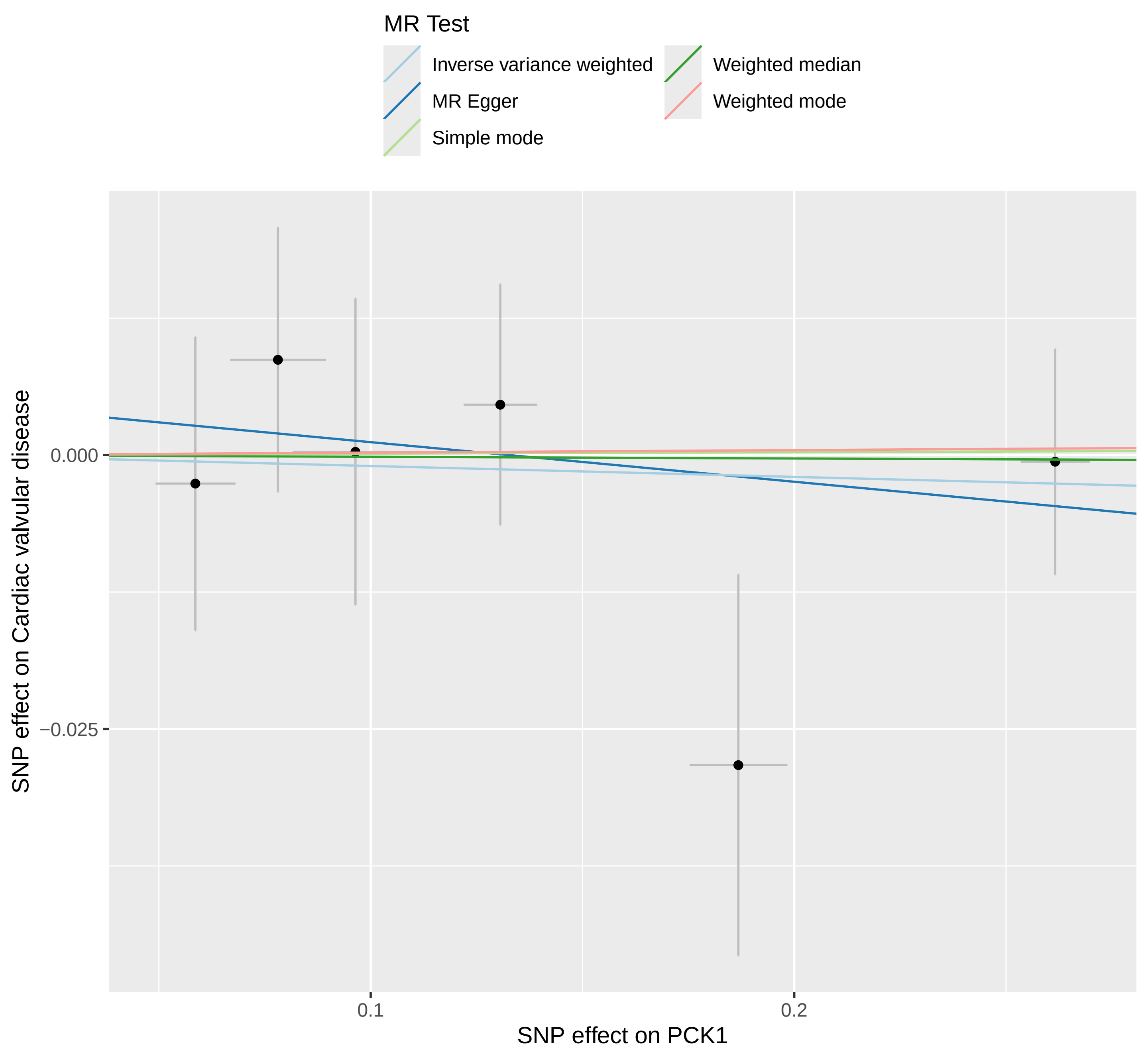

MR Test
Inverse variance weighted
MR Egger
Simple mode
Weighted median
Weighted mode
0.000
−0.025
0.1
0.2
SNP effect on PCK1
SNP effect on Cardiac valvular disease

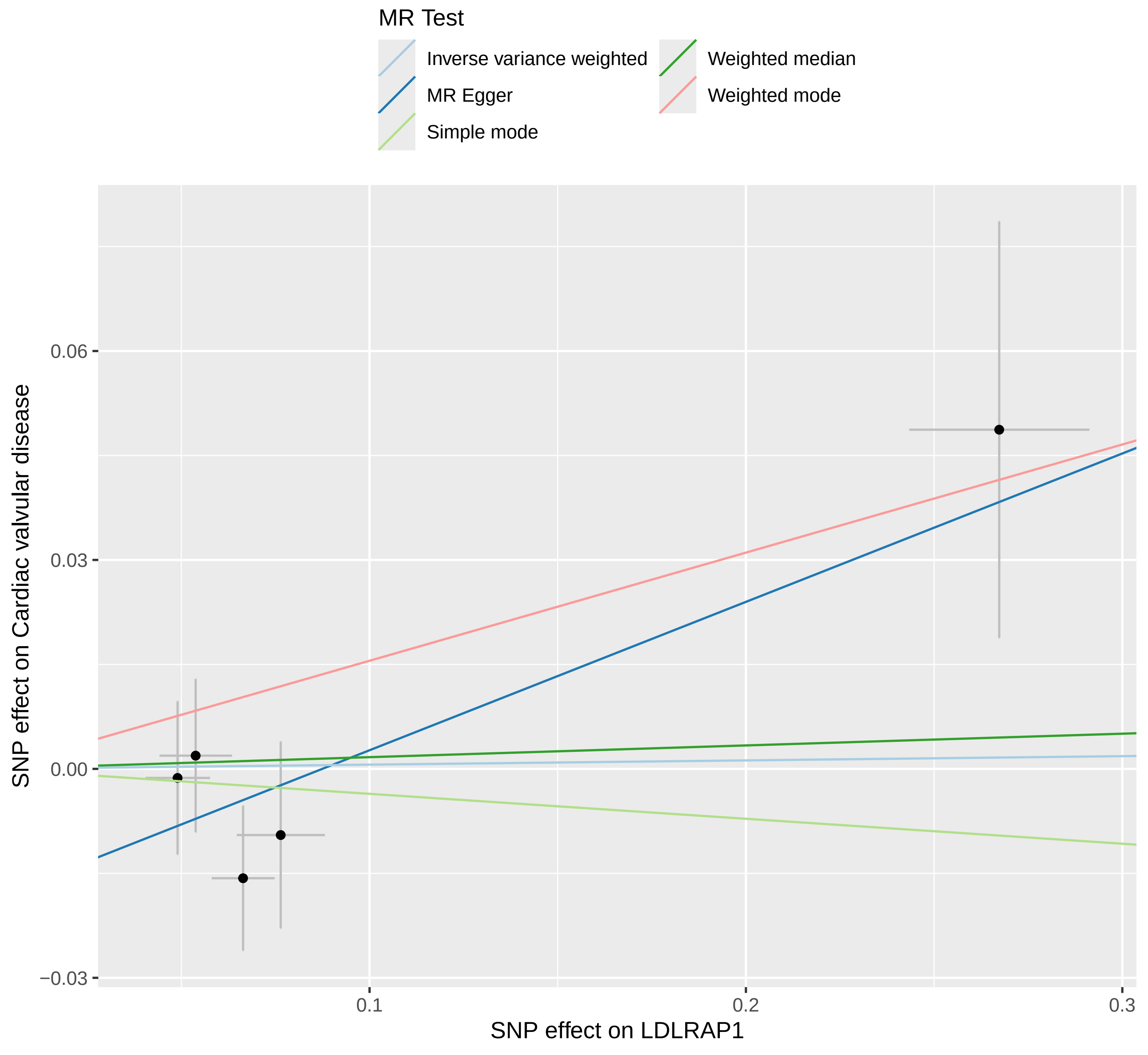
MR Test
Inverse variance weighted
MR Egger
Simple mode
Weighted median
Weighted mode
SNP effect on Cardiac valvular disease
0.06
0.03
0.00
−0.03
0.1
0.2
0.3
SNP effect on LDLRAP1

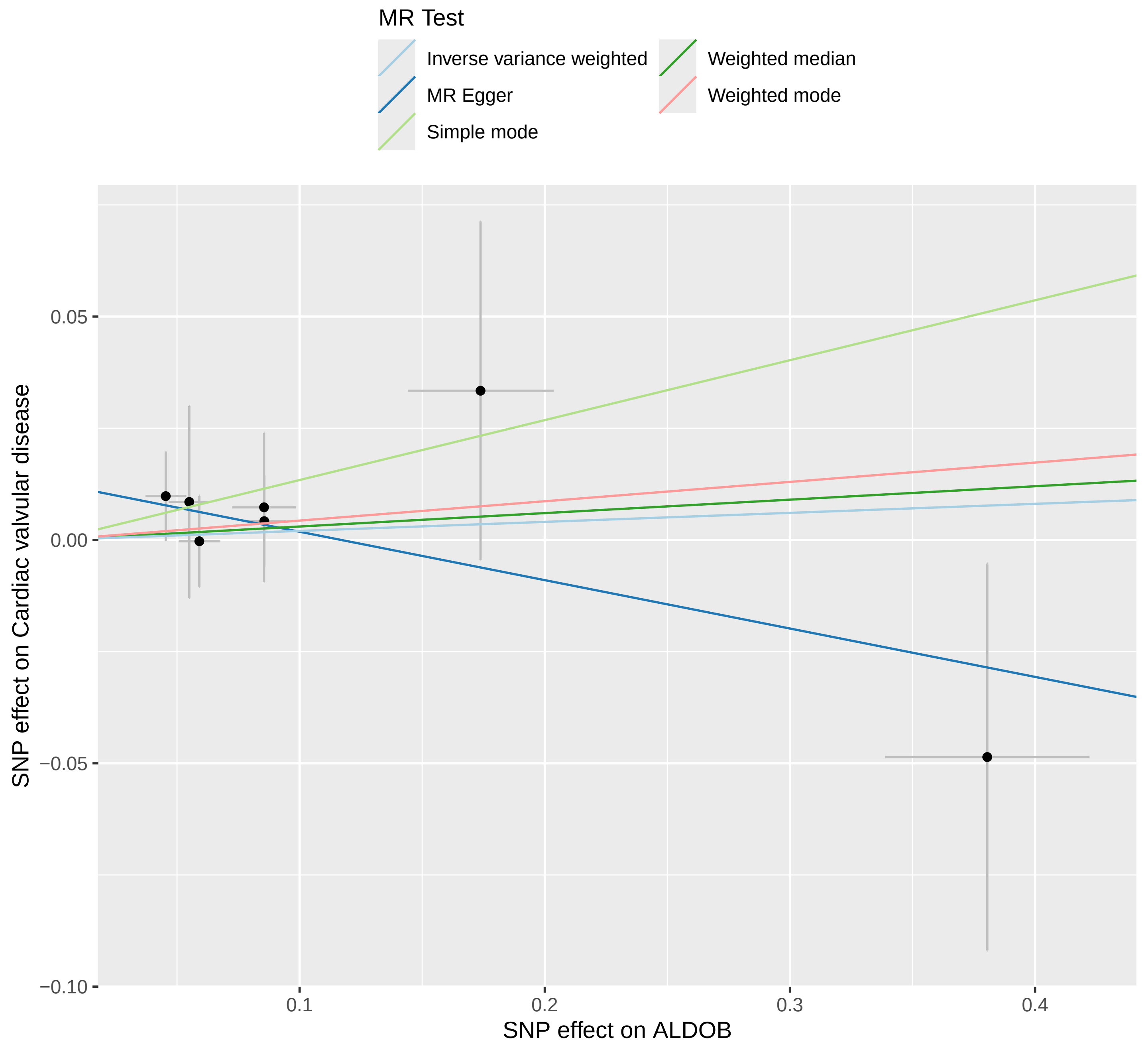
MR Test
Inverse variance weighted
MR Egger
Simple mode
Weighted median
Weighted mode
0.05
0.00
−0.05
−0.10
0.1
0.2
0.3
0.4
SNP effect on Cardiac valvular disease
SNP effect on ALDOB

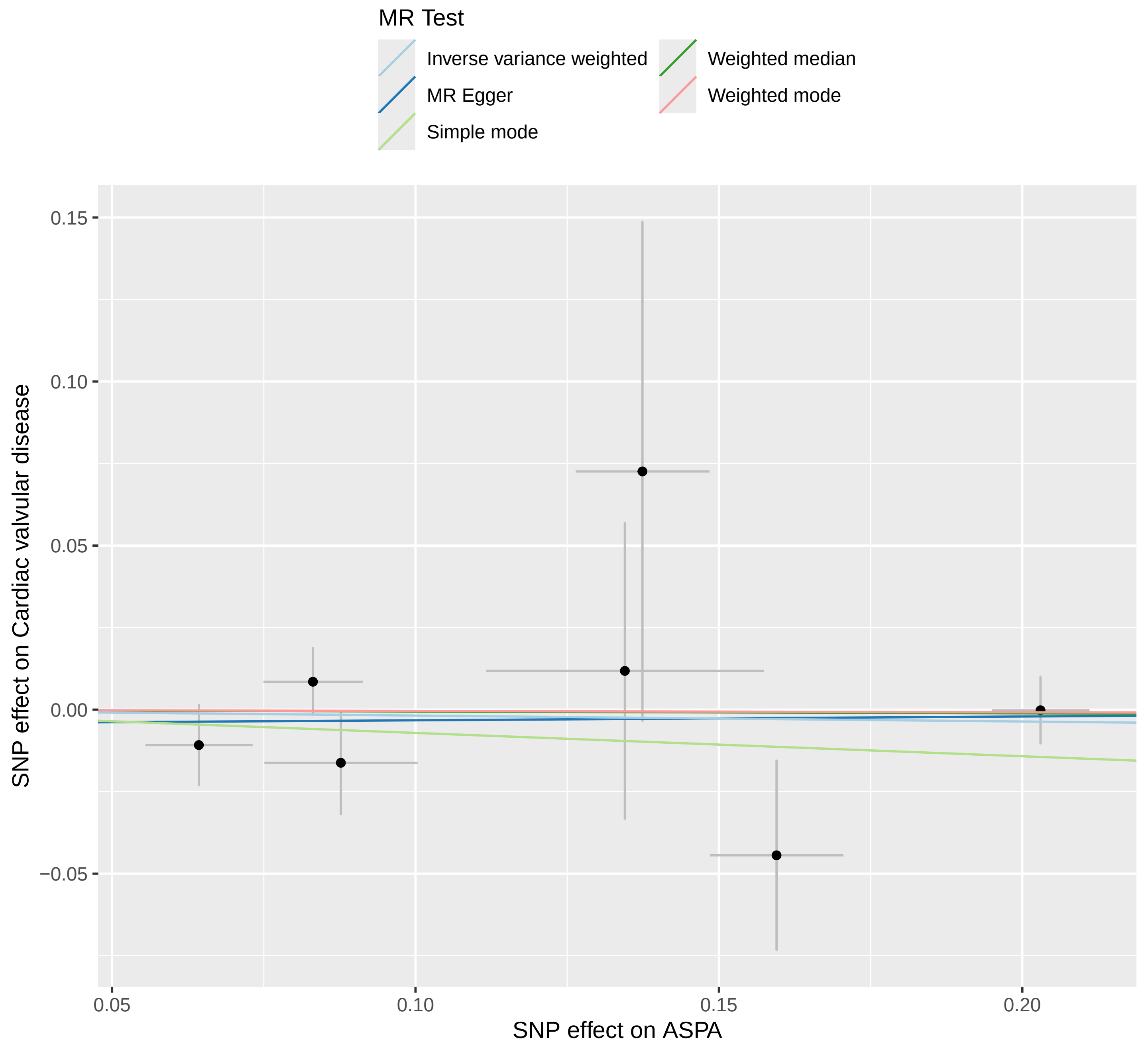

MR Test
Inverse variance weighted
MR Egger
Simple mode
Weighted median
Weighted mode
0.15
0.10
0.05
0.00
−0.05
0.05
0.10
0.15
0.20
SNP effect on Cardiac valvular disease
SNP effect on ASPA

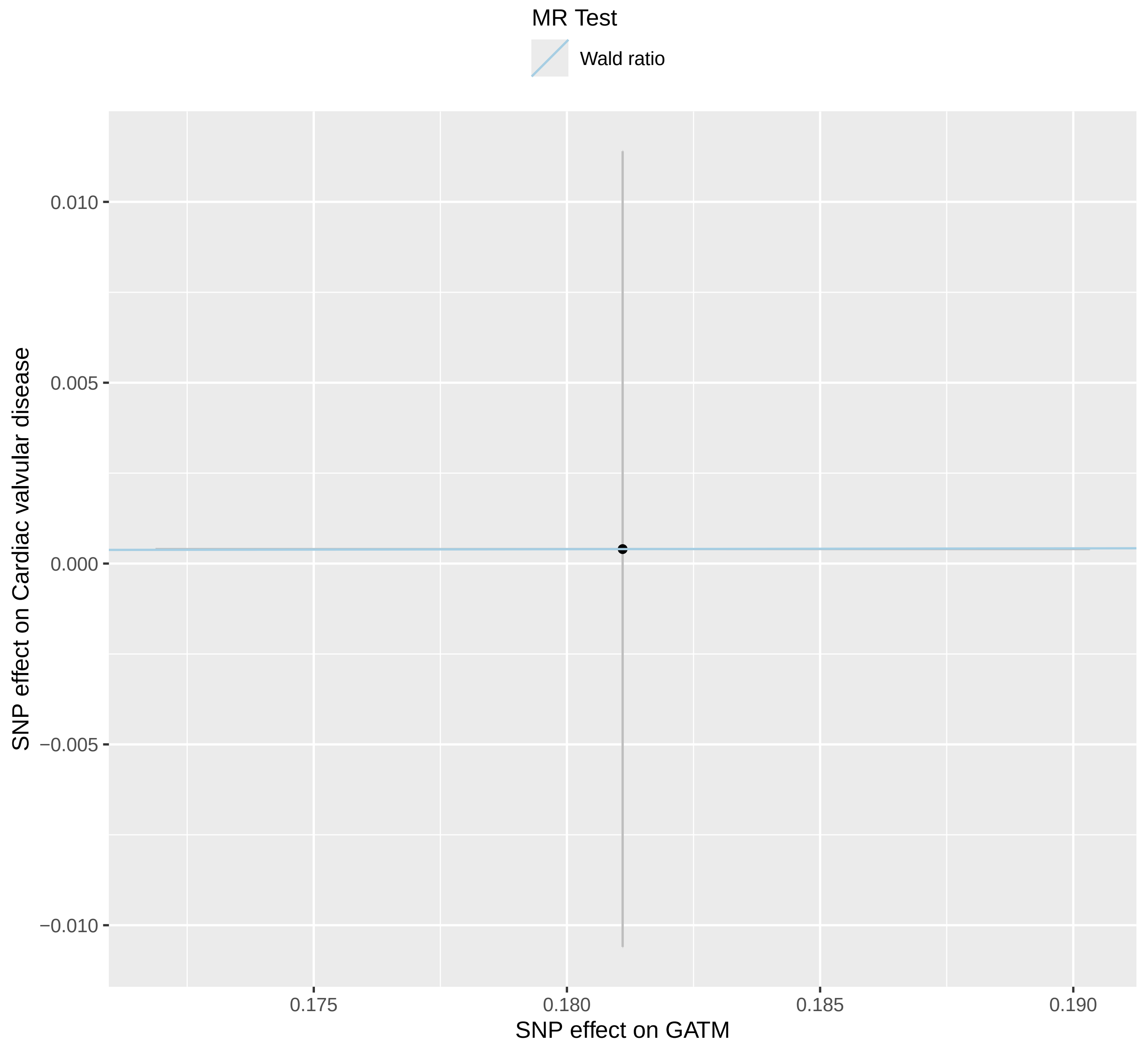
MR Test
Wald ratio
0.010
0.005
0.000
−0.005
−0.010
SNP effect on Cardiac valvular disease
0.175
0.180
0.185
0.190
SNP effect on GATM

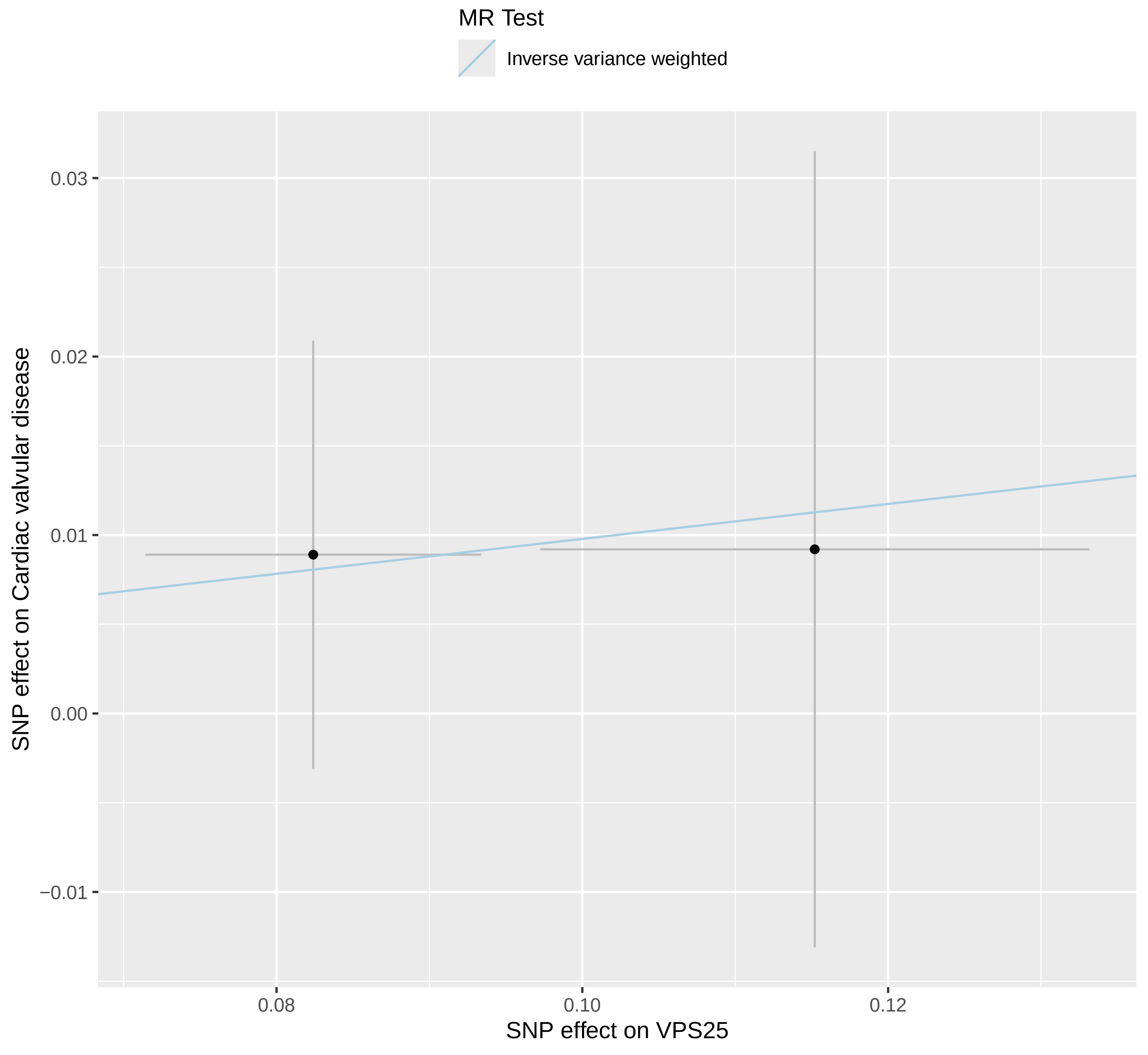

MR Test
Inverse variance weighted
0.03
0.02
0.01
0.00
−0.01
SNP effect on Cardiac valvular disease
0.08
0.10
0.12
SNP effect on VPS25

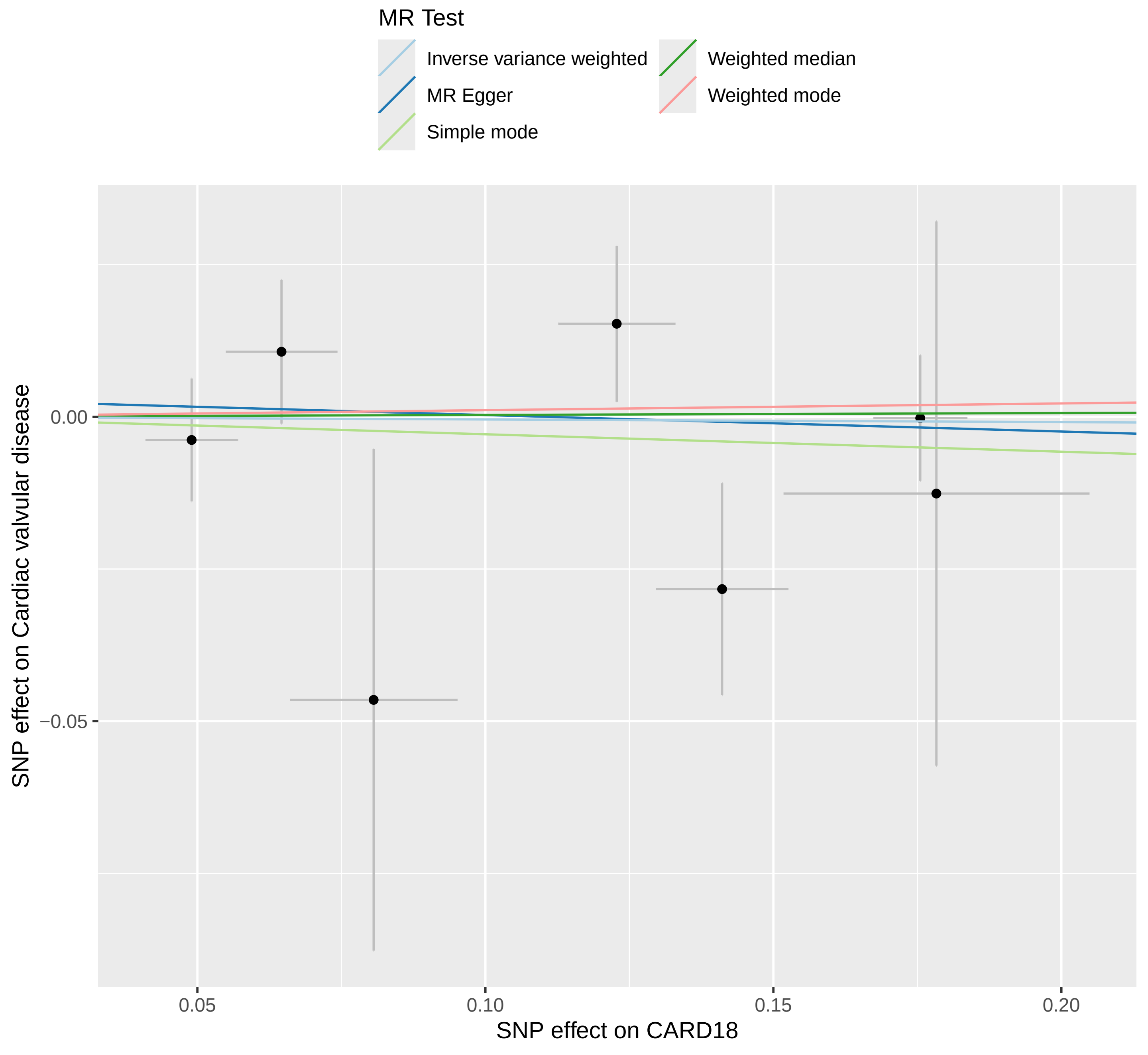
MR Test
Inverse variance weighted
MR Egger
Simple mode
Weighted median
Weighted mode
SNP effect on Cardiac valvular disease
0.00
−0.05
0.05
0.10
0.15
0.20
SNP effect on CARD18

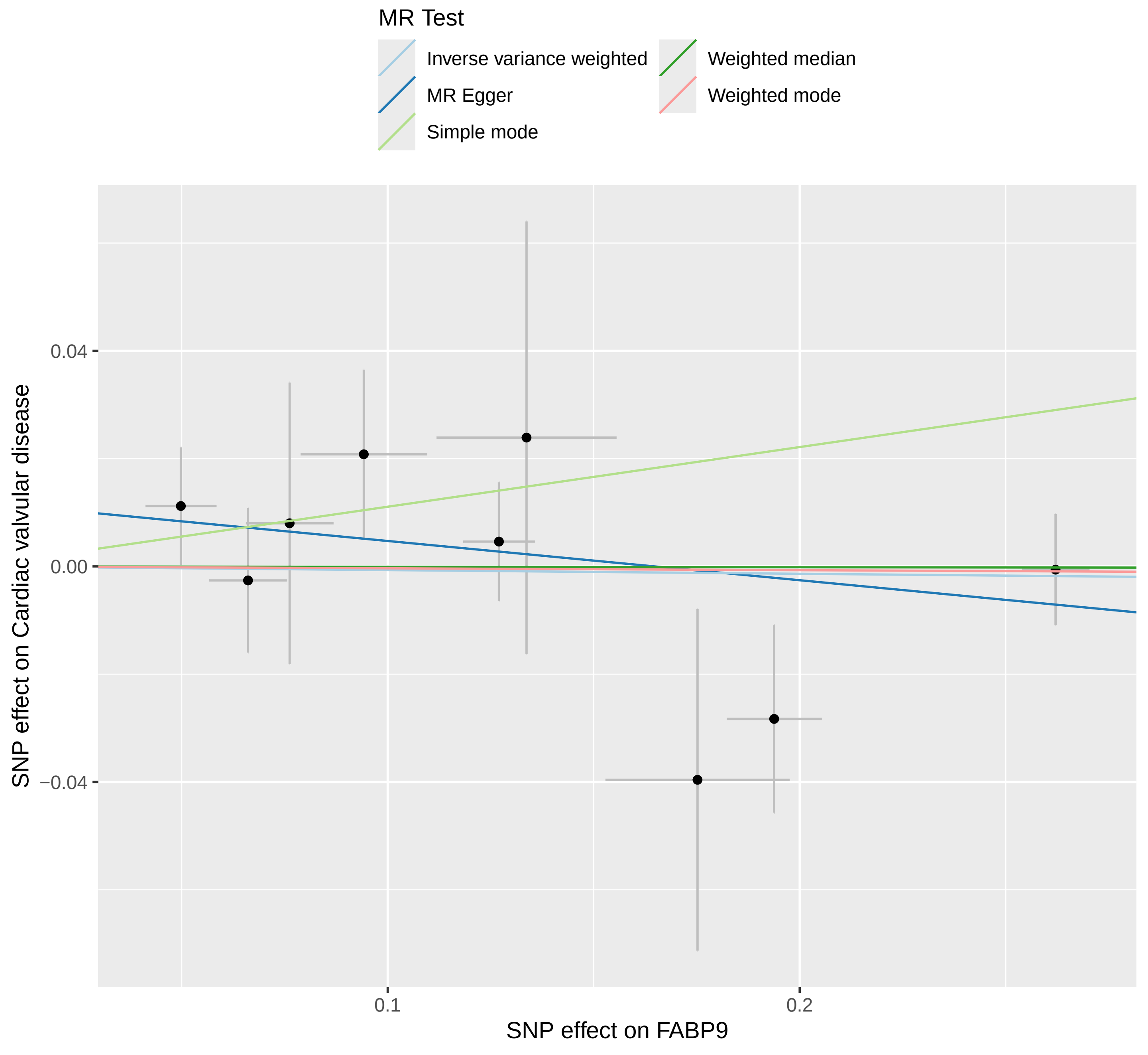
MR Test
Inverse variance weighted
MR Egger
Simple mode
Weighted median
Weighted mode
0.04
0.00
−0.04
SNP effect on Cardiac valvular disease
0.1
0.2
SNP effect on FABP9

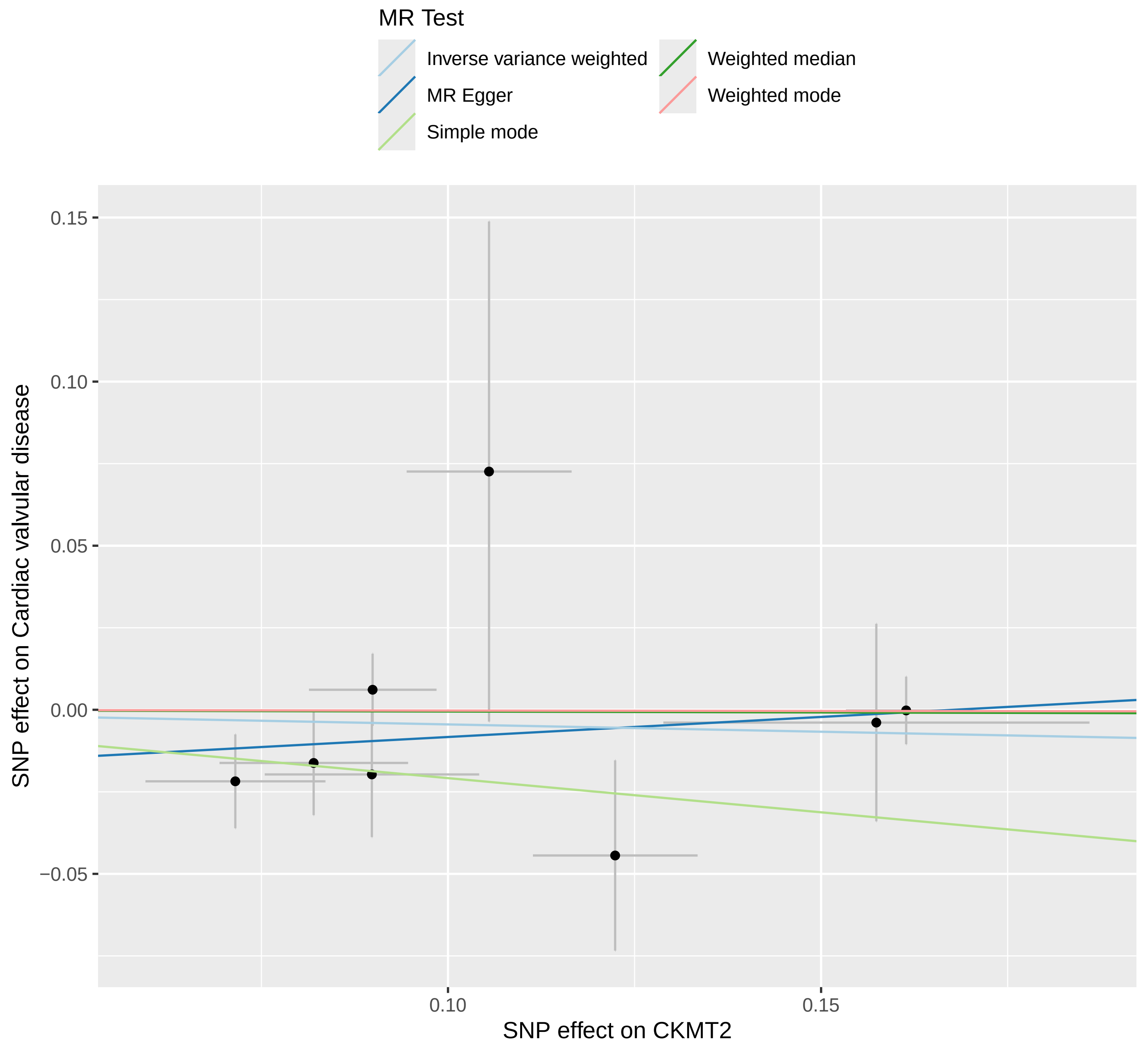

MR Test
Inverse variance weighted
MR Egger
Simple mode
Weighted median
Weighted mode
0.15
0.10
0.05
0.00
−0.05
0.10
0.15
SNP effect on Cardiac valvular disease
SNP effect on CKMT2

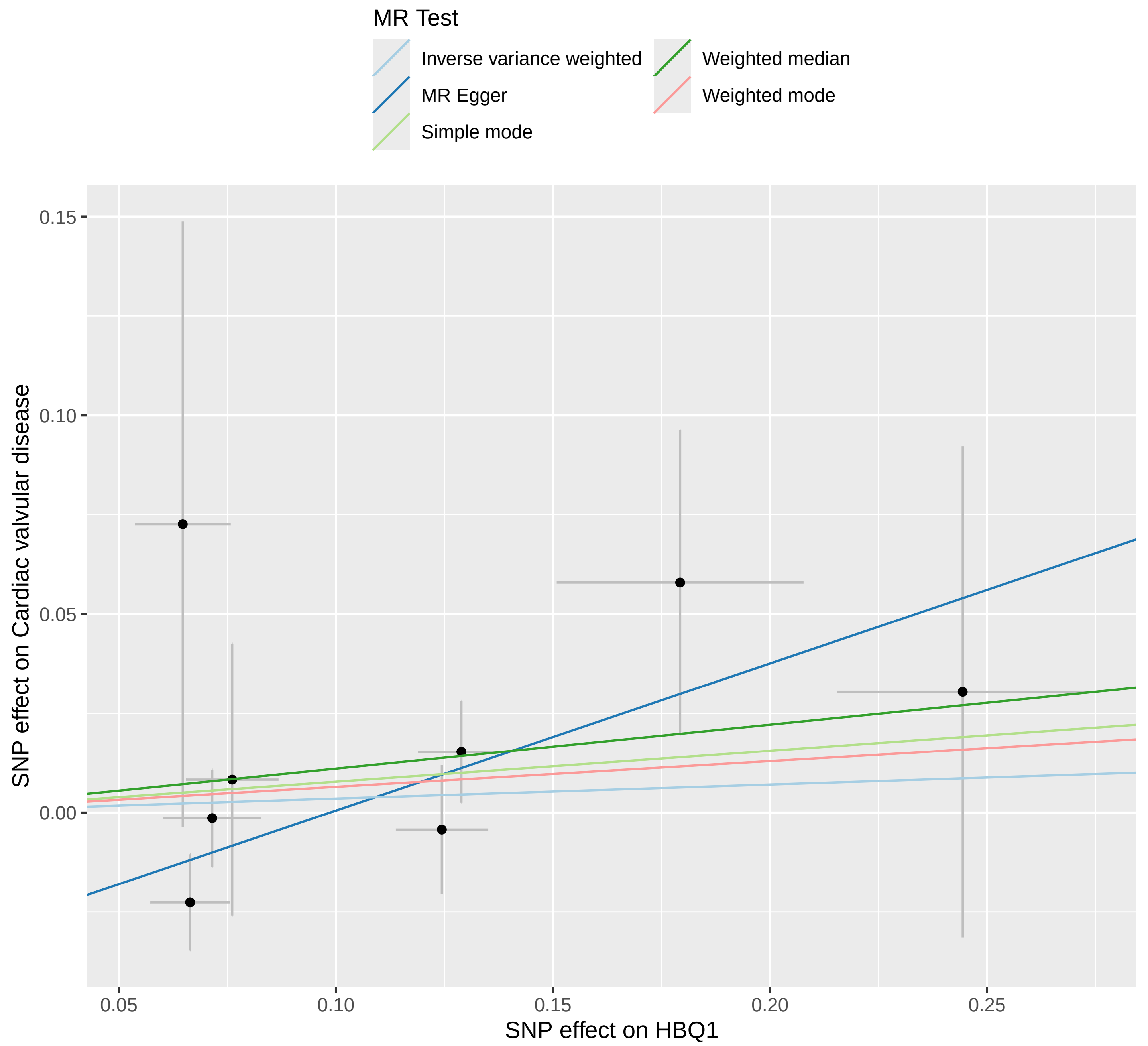

MR Test
Inverse variance weighted
MR Egger
Simple mode
Weighted median
Weighted mode
0.15
0.10
0.05
0.00
SNP effect on Cardiac valvular disease
0.05
0.10
0.15
0.20
0.25
SNP effect on HBQ1

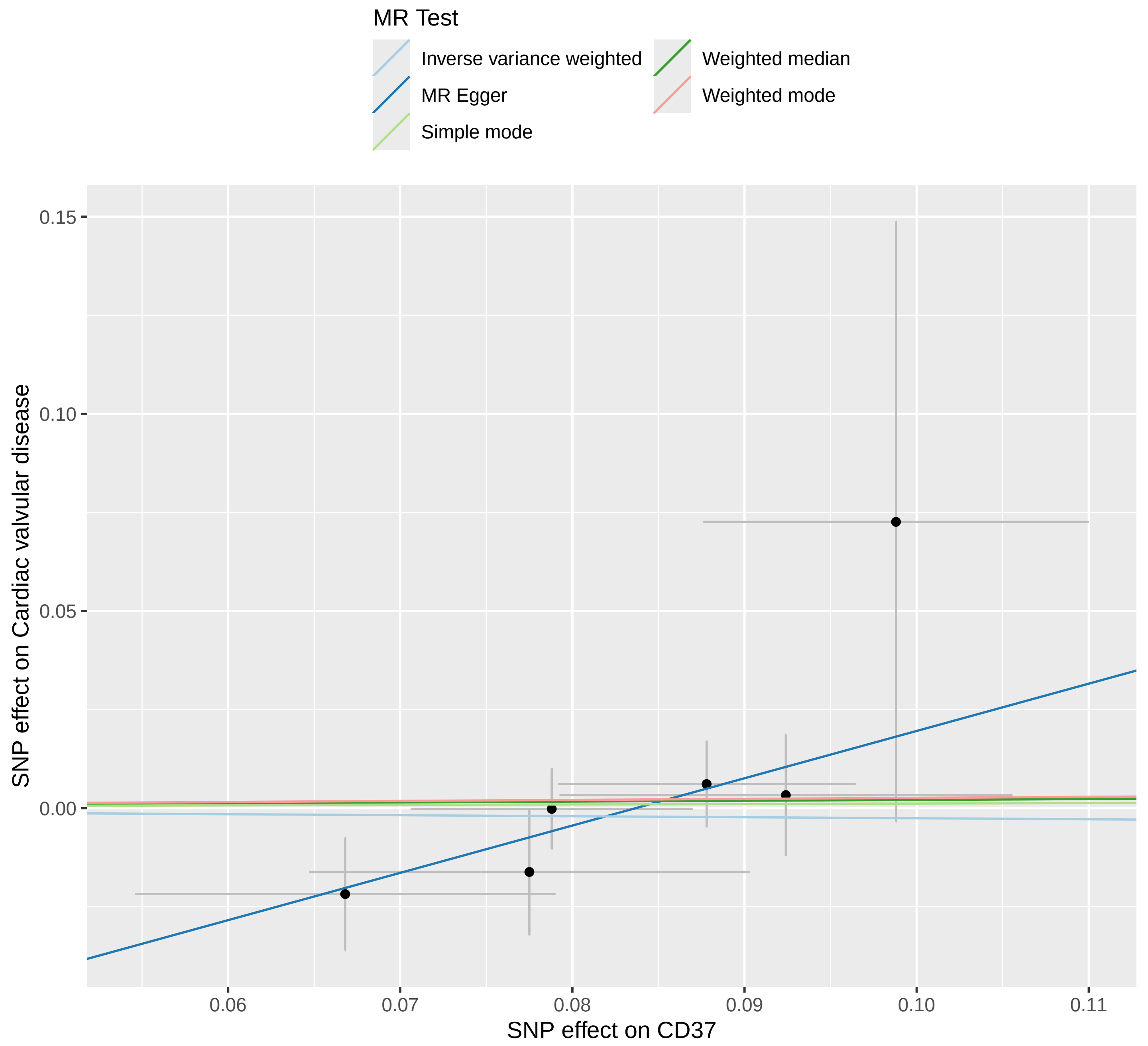
MR Test
Inverse variance weighted
MR Egger
Simple mode
Weighted median
Weighted mode
0.15
0.10
0.05
0.00
0.06
0.07
0.08
0.09
0.10
0.11
SNP effect on Cardiac valvular disease
SNP effect on CD37

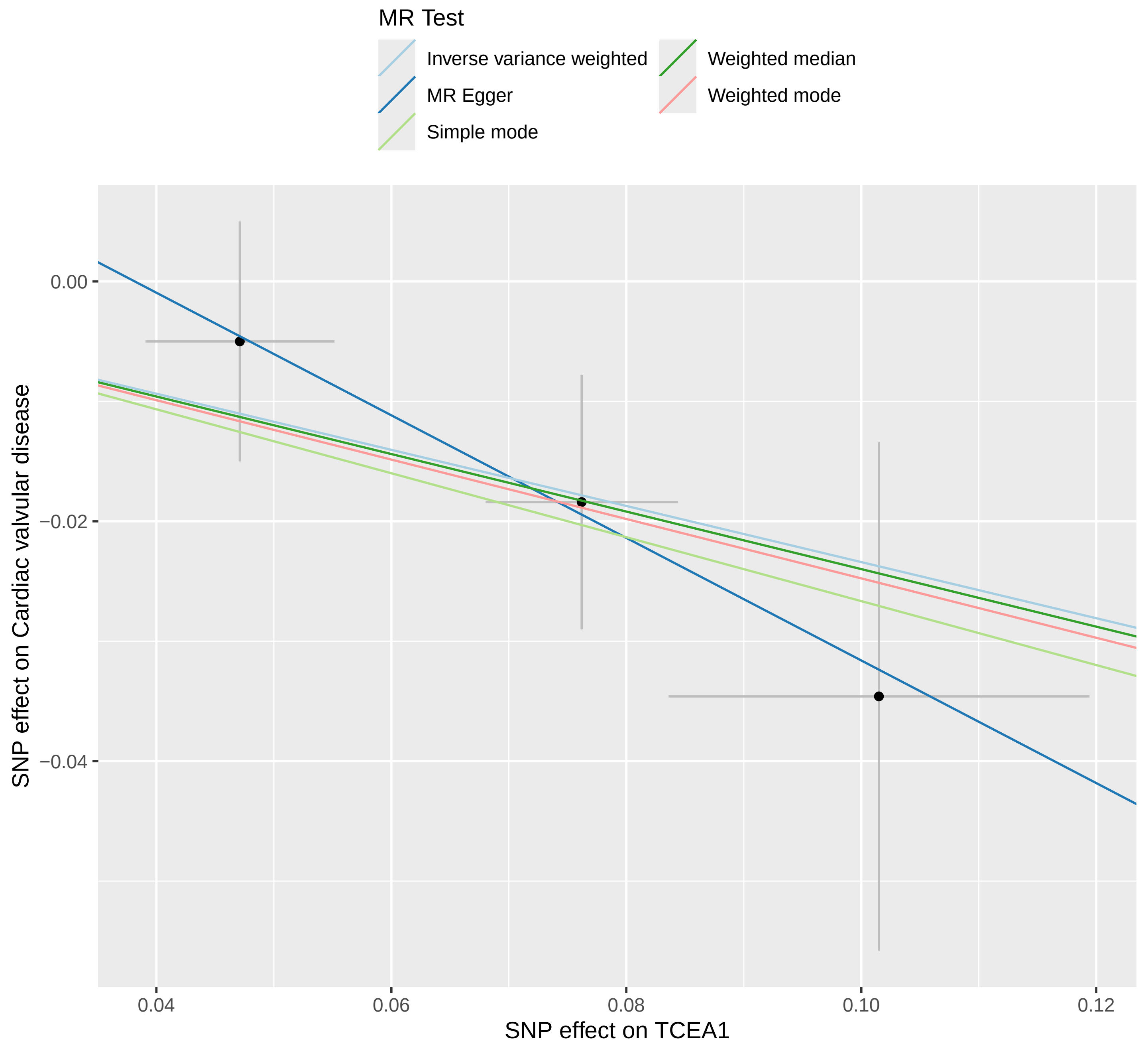
MR Test
Inverse variance weighted
MR Egger
Simple mode
Weighted median
Weighted mode
0.00
−0.02
−0.04
0.04
0.06
0.08
0.10
0.12
SNP effect on Cardiac valvular disease
SNP effect on TCEA1

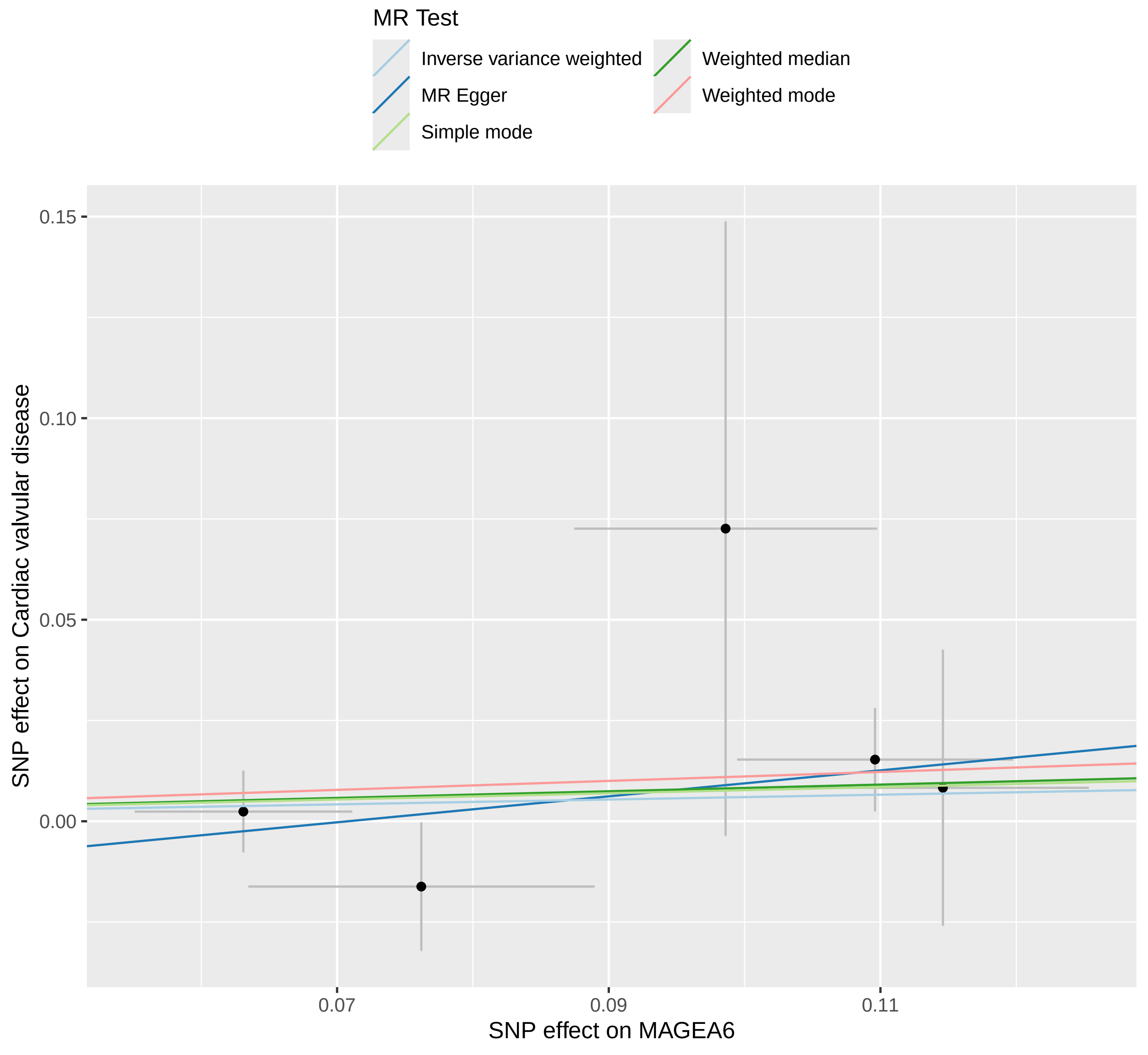
MR Test
Inverse variance weighted
MR Egger
Simple mode
Weighted median
Weighted mode
SNP effect on Cardiac valvular disease
0.15
0.10
0.05
0.00
0.07
0.09
0.11
SNP effect on MAGEA6

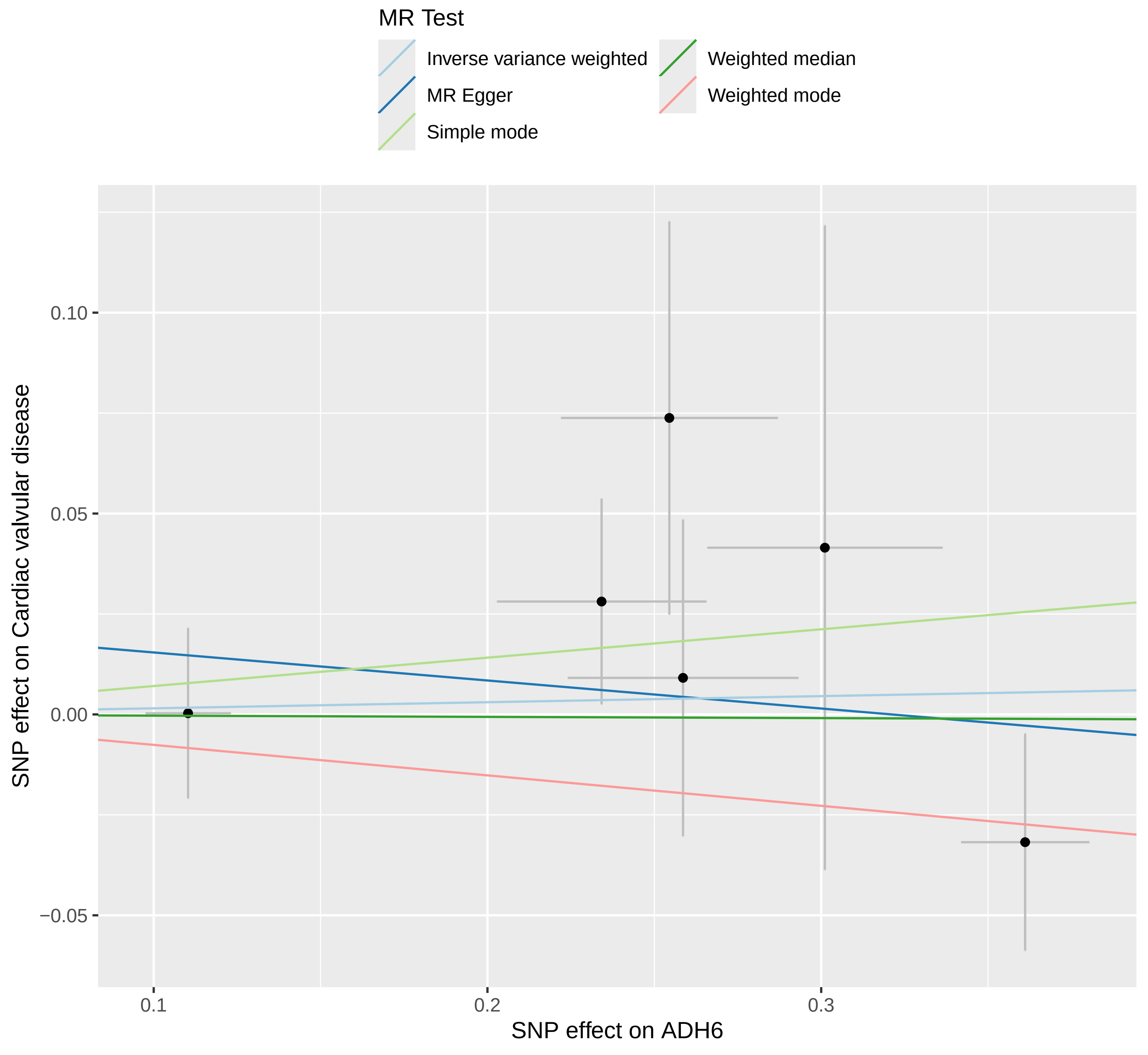
MR Test
Inverse variance weighted
MR Egger
Simple mode
Weighted median
Weighted mode
0.10
0.05
0.00
−0.05
SNP effect on Cardiac valvular disease
0.1
0.2
0.3
SNP effect on ADH6

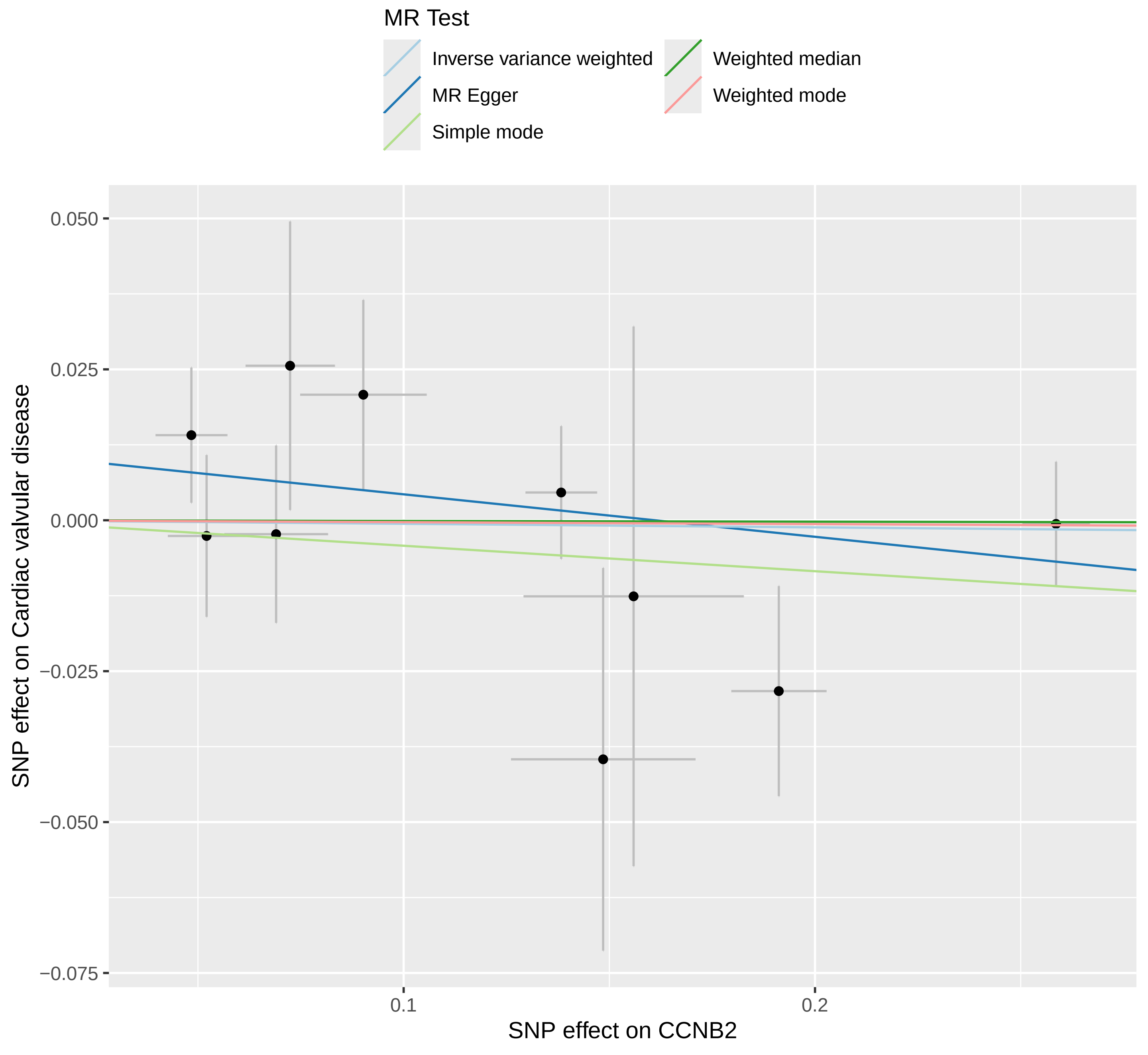
MR Test
Inverse variance weighted
MR Egger
Simple mode
Weighted median
Weighted mode
0.050
0.025
0.000
−0.025
−0.050
−0.075
0.1
0.2
SNP effect on Cardiac valvular disease
SNP effect on CCNB2

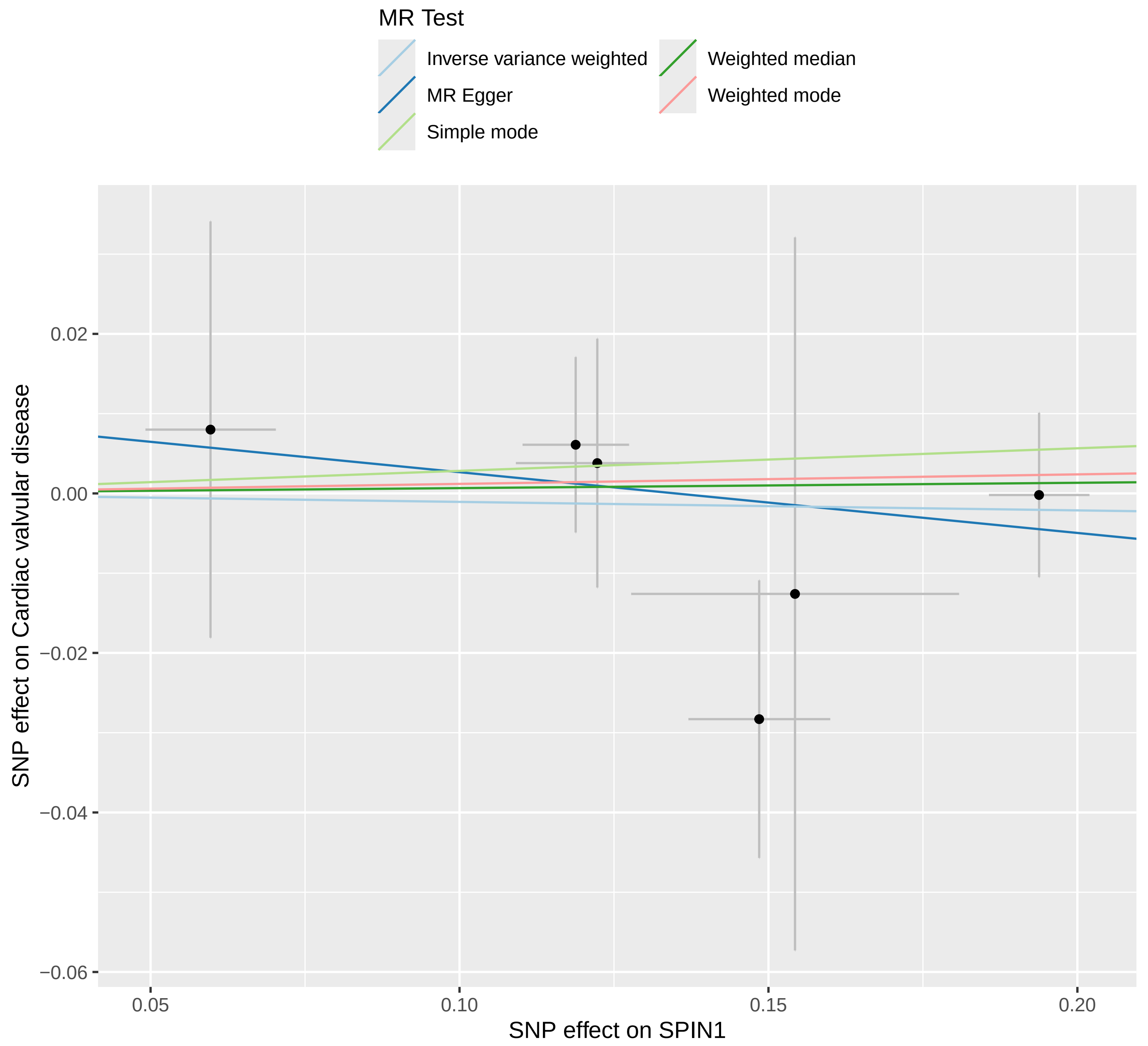

MR Test
Inverse variance weighted
MR Egger
Simple mode
Weighted median
Weighted mode
0.02
0.00
−0.02
−0.04
−0.06
SNP effect on Cardiac valvular disease
0.05
0.10
0.15
0.20
SNP effect on SPIN1

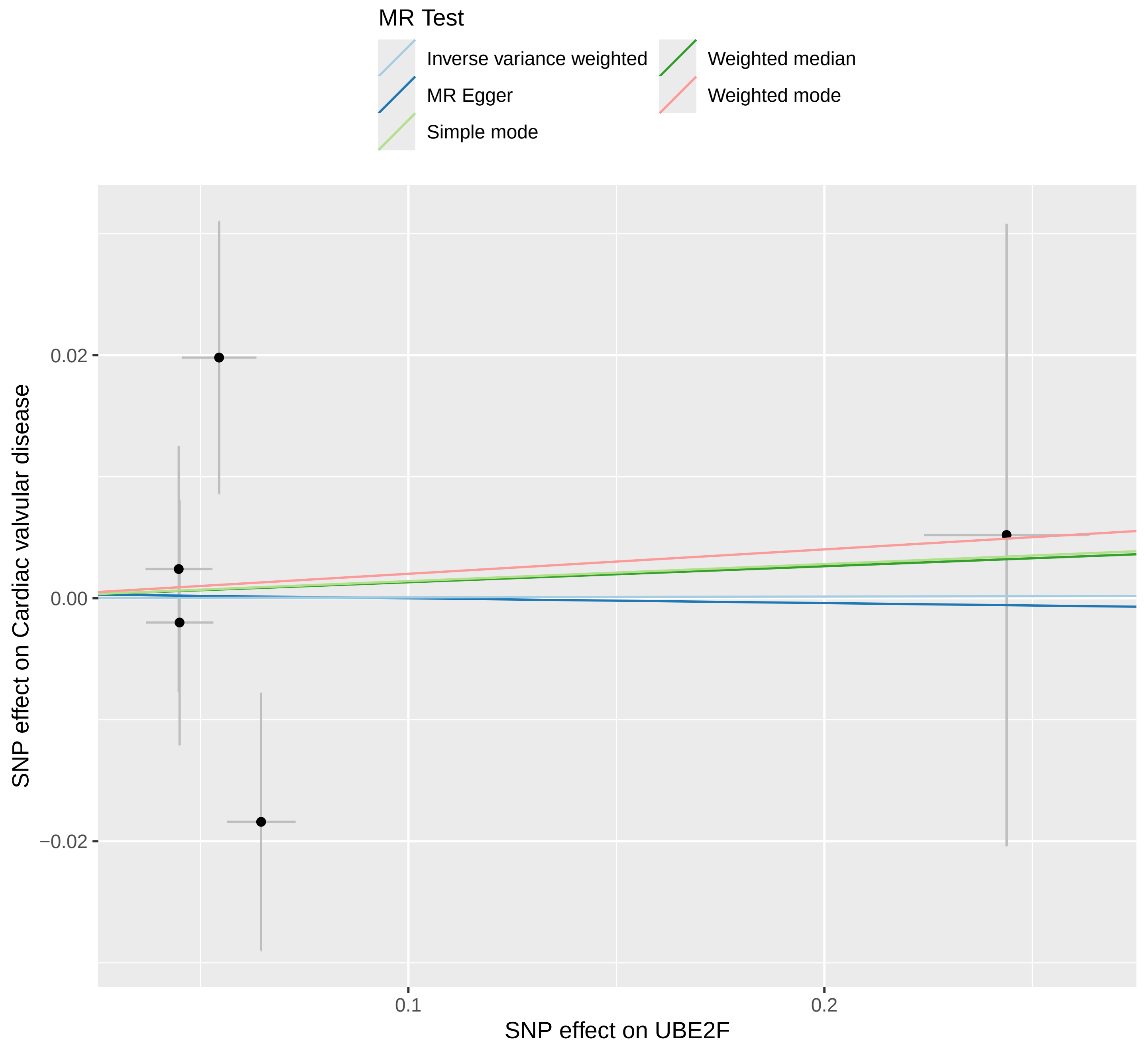
MR Test
Inverse variance weighted
MR Egger
Simple mode
Weighted median
Weighted mode
0.02
0.00
−0.02
0.1
0.2
SNP effect on UBE2F
SNP effect on Cardiac valvular disease

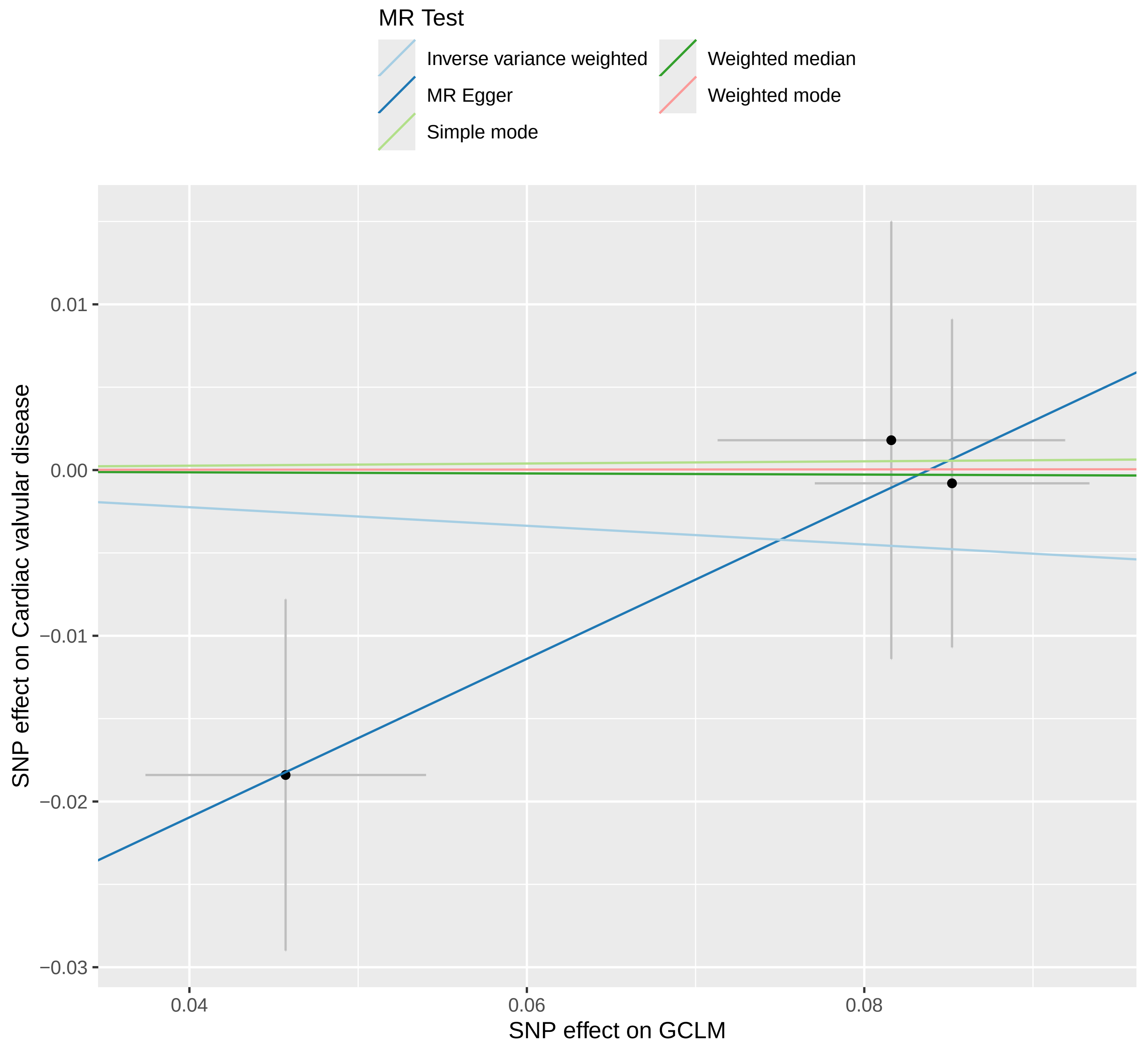
MR Test
Inverse variance weighted
MR Egger
Simple mode
Weighted median
Weighted mode
0.01
0.00
−0.01
−0.02
−0.03
SNP effect on Cardiac valvular disease
0.04
0.06
0.08
SNP effect on GCLM

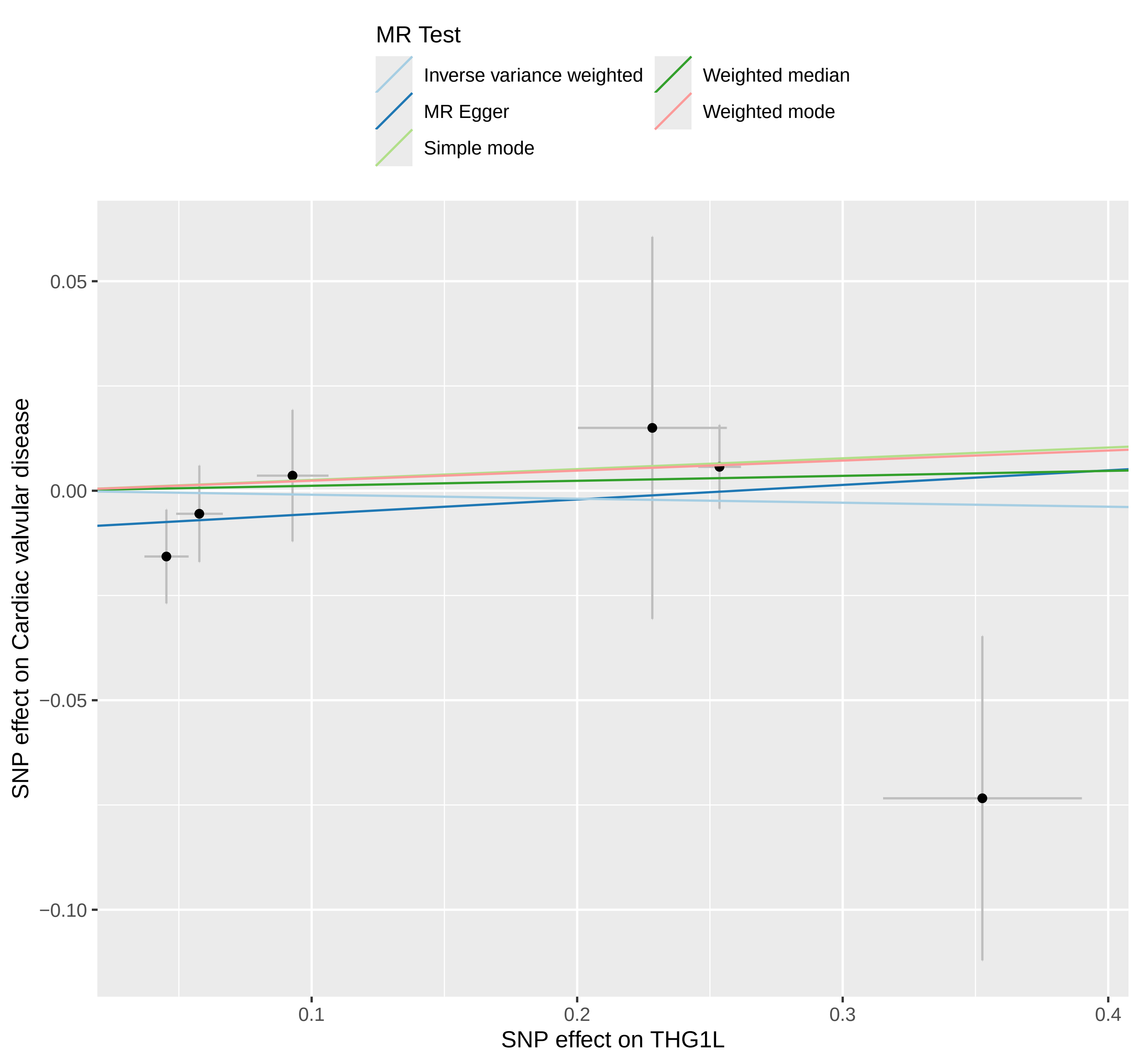

MR Test
Inverse variance weighted
MR Egger
Simple mode
Weighted median
Weighted mode
0.05
0.00
−0.05
−0.10
0.1
0.2
0.3
0.4
SNP effect on Cardiac valvular disease
SNP effect on THG1L

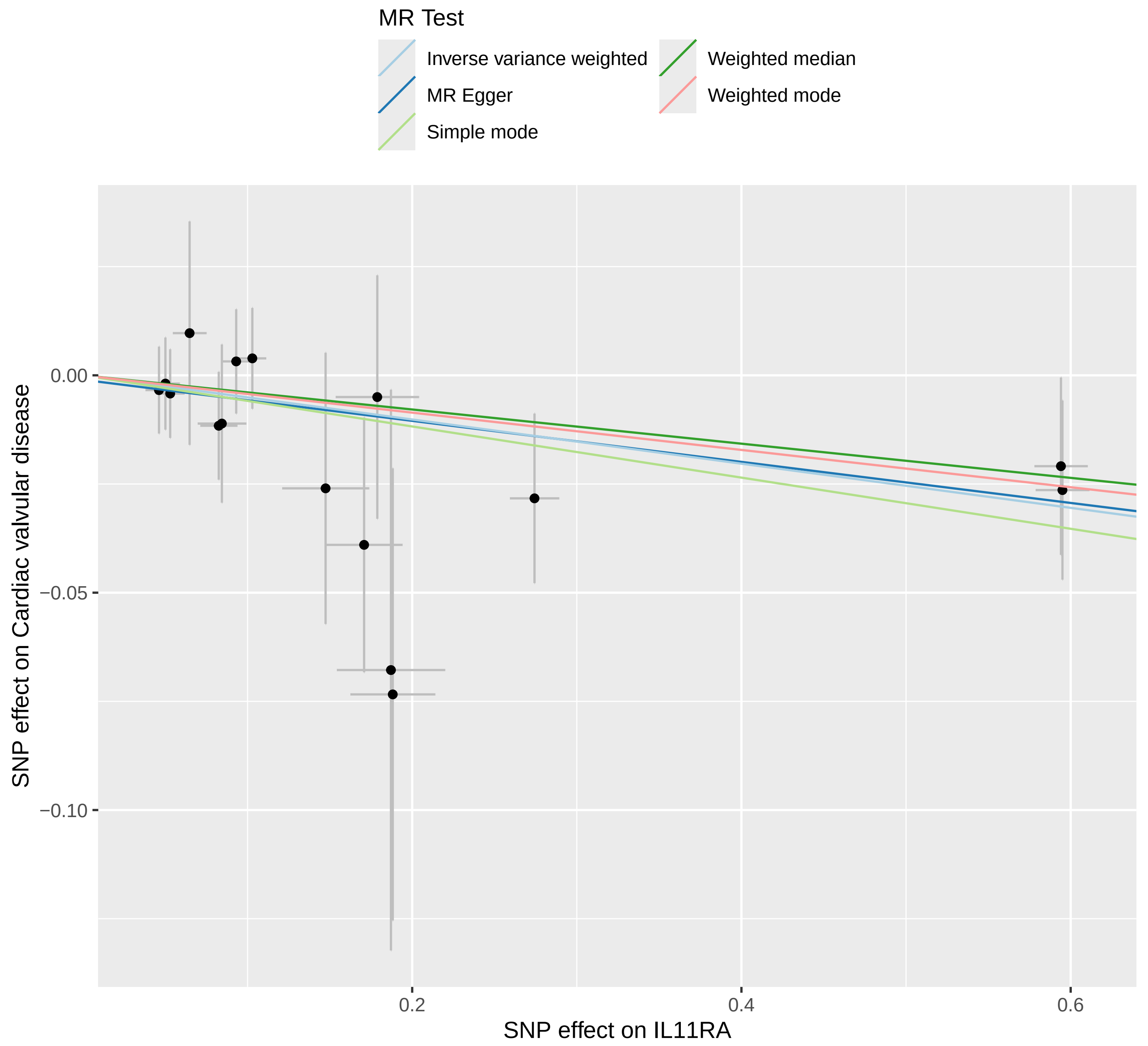
MR Test
Inverse variance weighted
MR Egger
Simple mode
Weighted median
Weighted mode
0.00
−0.05
−0.10
0.2
0.4
0.6
SNP effect on IL11RA
SNP effect on Cardiac valvular disease

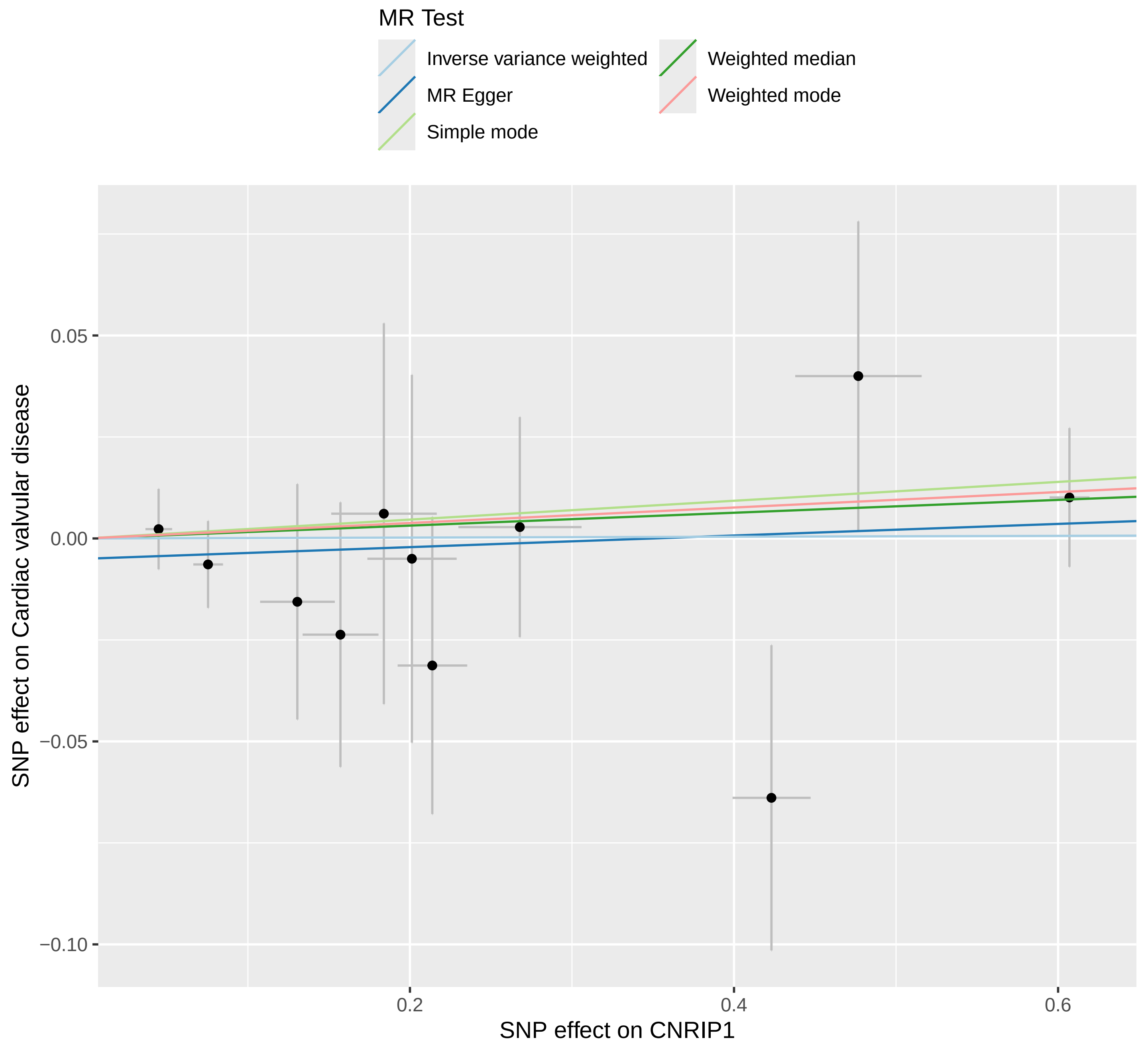
MR Test
Inverse variance weighted
MR Egger
Simple mode
Weighted median
Weighted mode
0.05
0.00
−0.05
−0.10
SNP effect on Cardiac valvular disease
0.2
0.4
0.6
SNP effect on CNRIP1

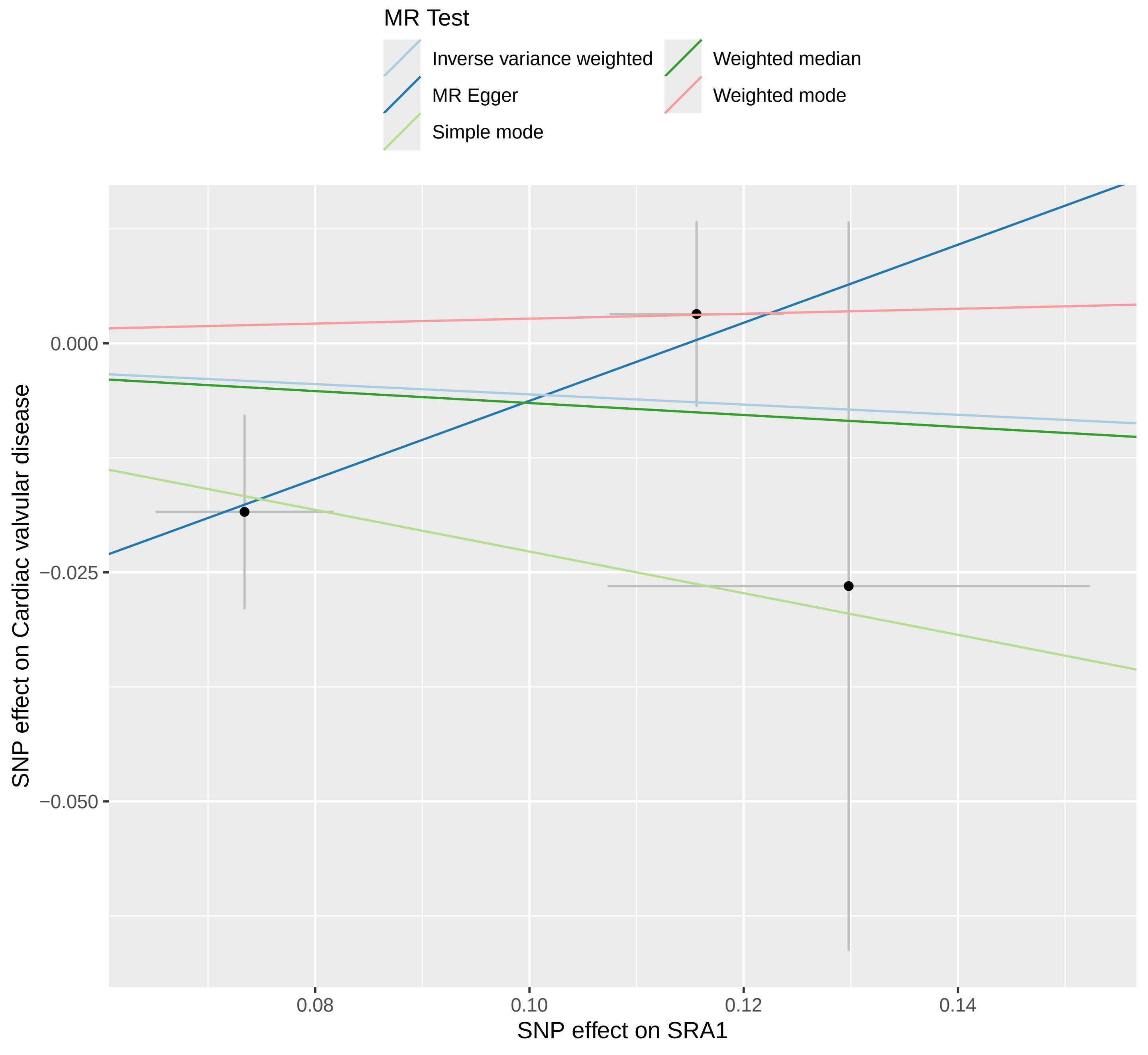

MR Test
Inverse variance weighted
MR Egger
Simple mode
Weighted median
Weighted mode
0.000
−0.025
−0.050
0.08
0.10
0.12
0.14
SNP effect on Cardiac valvular disease
SNP effect on SRA1

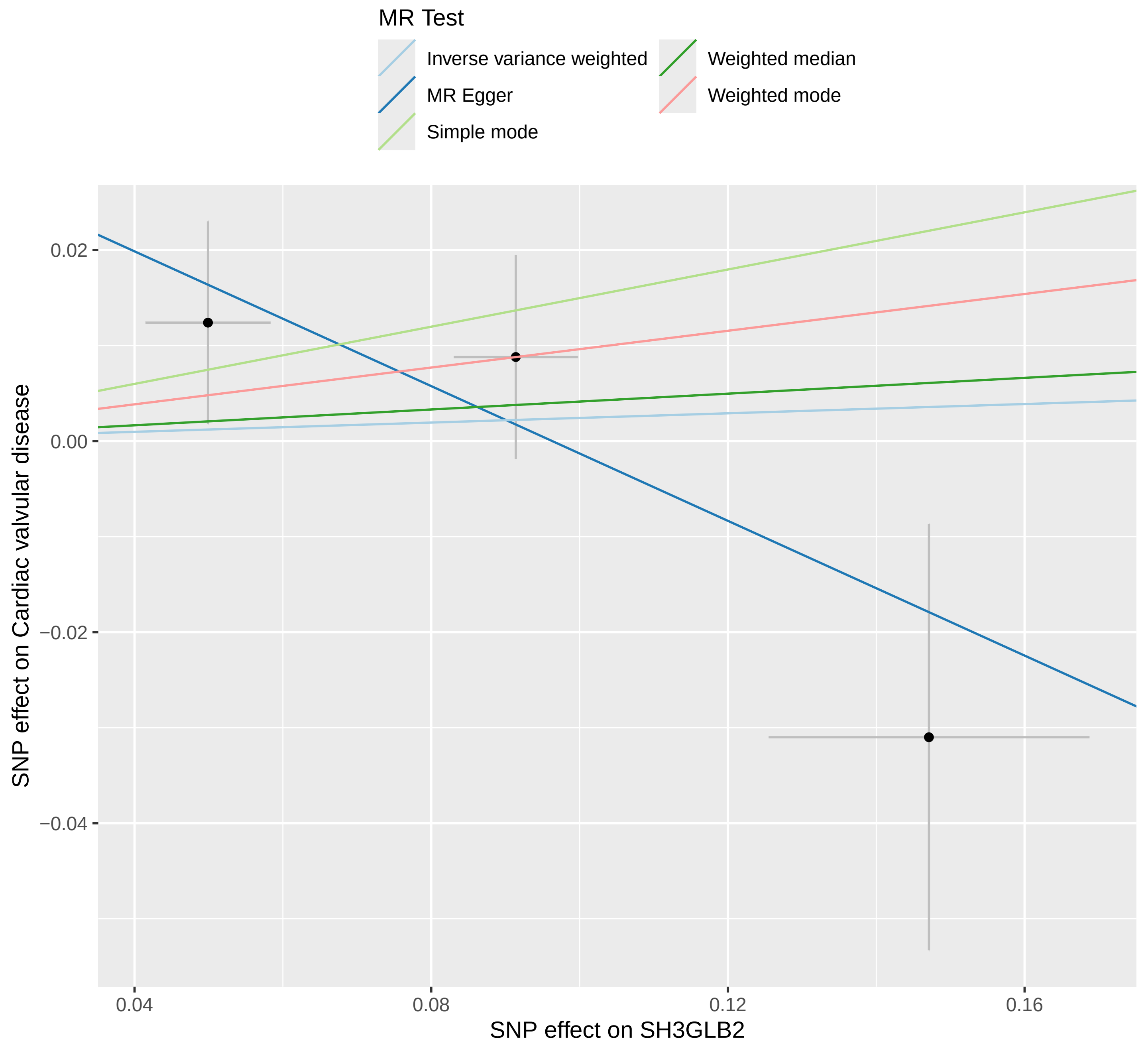

MR Test
Inverse variance weighted
MR Egger
Simple mode
Weighted median
Weighted mode
0.02
0.00
−0.02
−0.04
0.04
0.08
0.12
0.16
SNP effect on SH3GLB2
SNP effect on Cardiac valvular disease

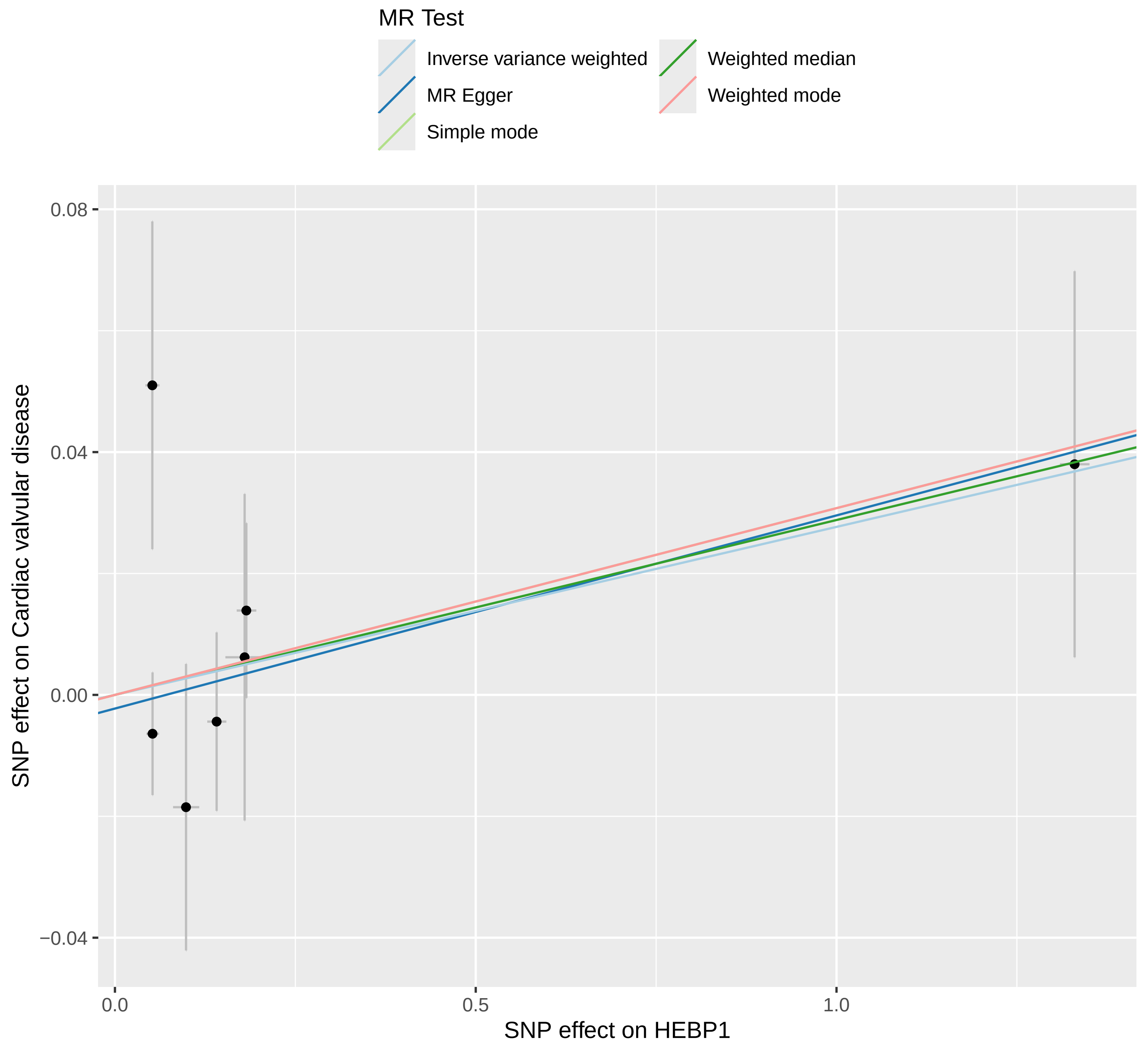

MR Test
Inverse variance weighted
MR Egger
Simple mode
Weighted median
Weighted mode
0.08
0.04
0.00
−0.04
0.0
0.5
1.0
SNP effect on Cardiac valvular disease
SNP effect on HEBP1

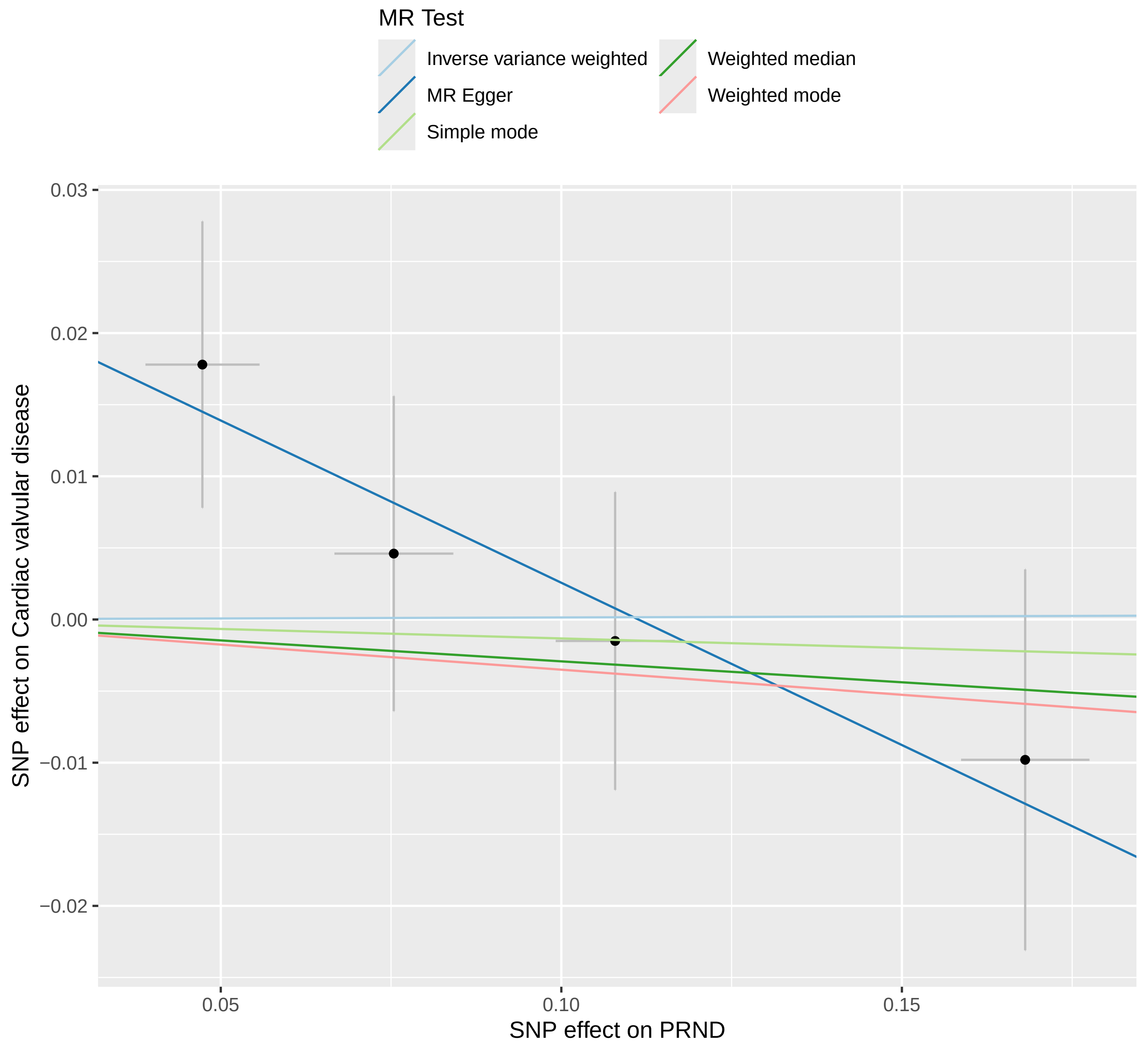

MR Test
Inverse variance weighted
MR Egger
Simple mode
Weighted median
Weighted mode
0.03
0.02
0.01
0.00
−0.01
−0.02
SNP effect on Cardiac valvular disease
0.05
0.10
0.15
SNP effect on PRND

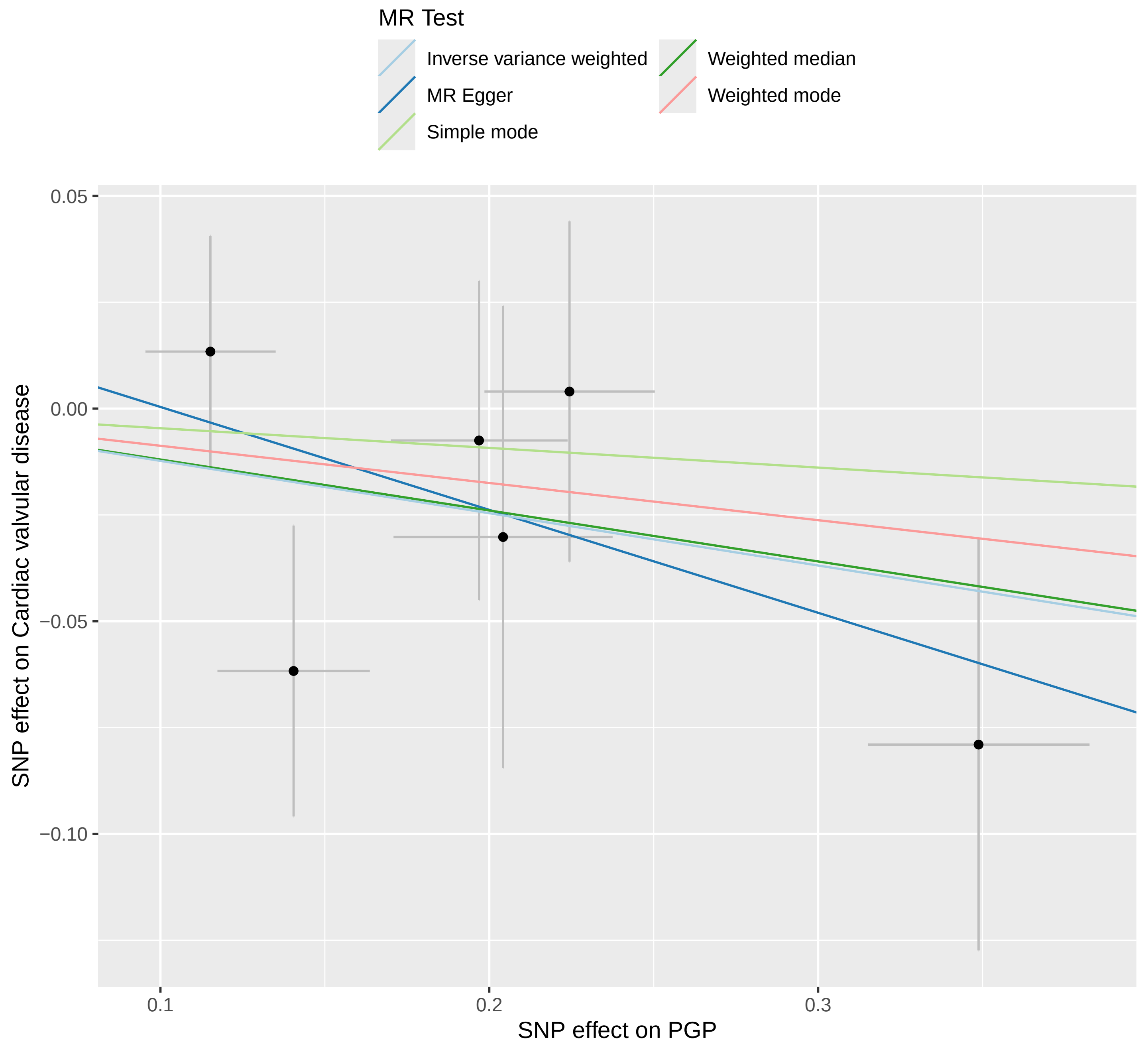

MR Test
Inverse variance weighted
MR Egger
Simple mode
Weighted median
Weighted mode
0.05
0.00
−0.05
−0.10
0.1
0.2
0.3
SNP effect on PGP
SNP effect on Cardiac valvular disease

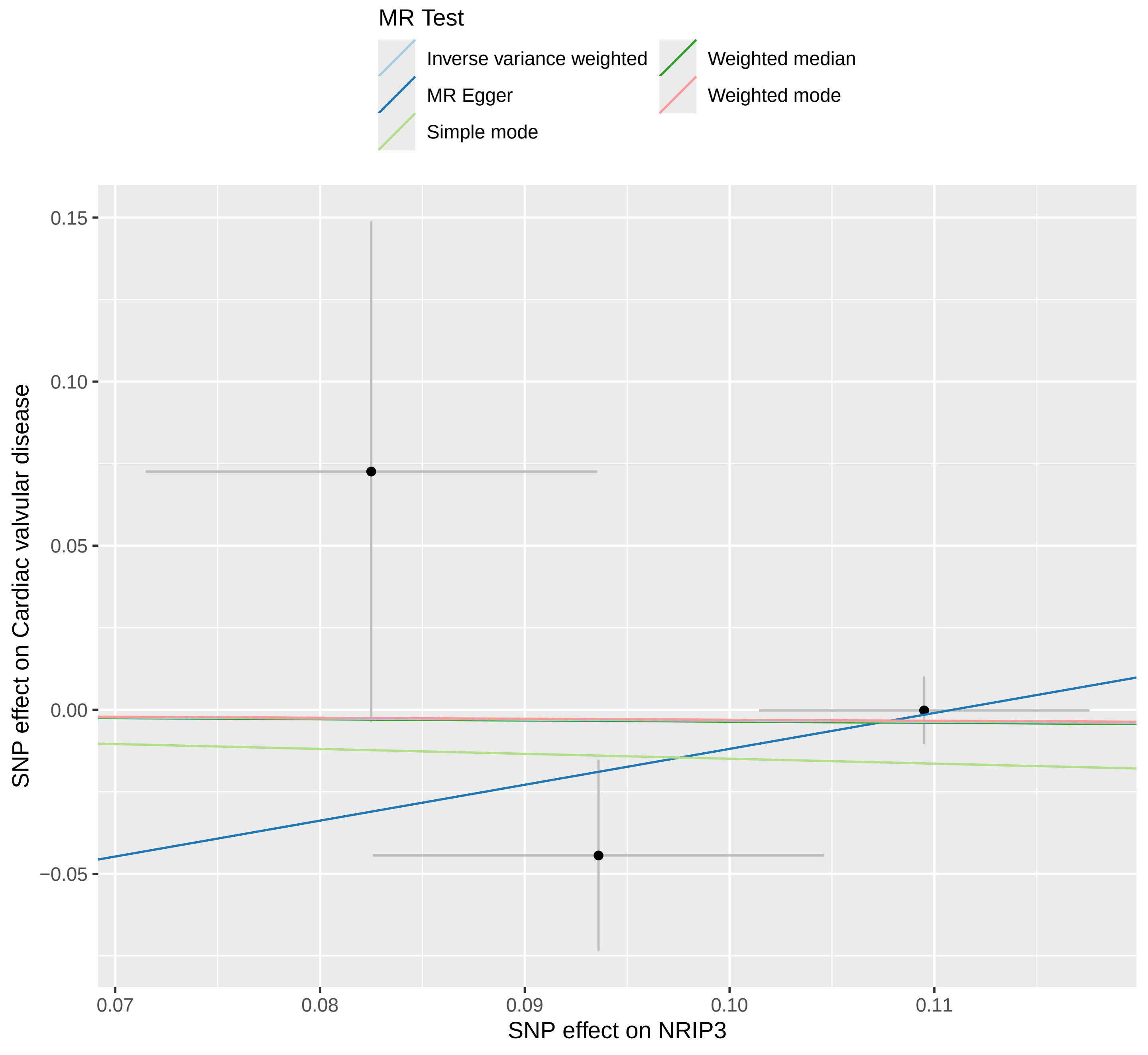
MR Test
Inverse variance weighted
MR Egger
Simple mode
Weighted median
Weighted mode
SNP effect on Cardiac valvular disease
0.15
0.10
0.05
0.00
−0.05
0.07
0.08
0.09
0.10
0.11
SNP effect on NRIP3

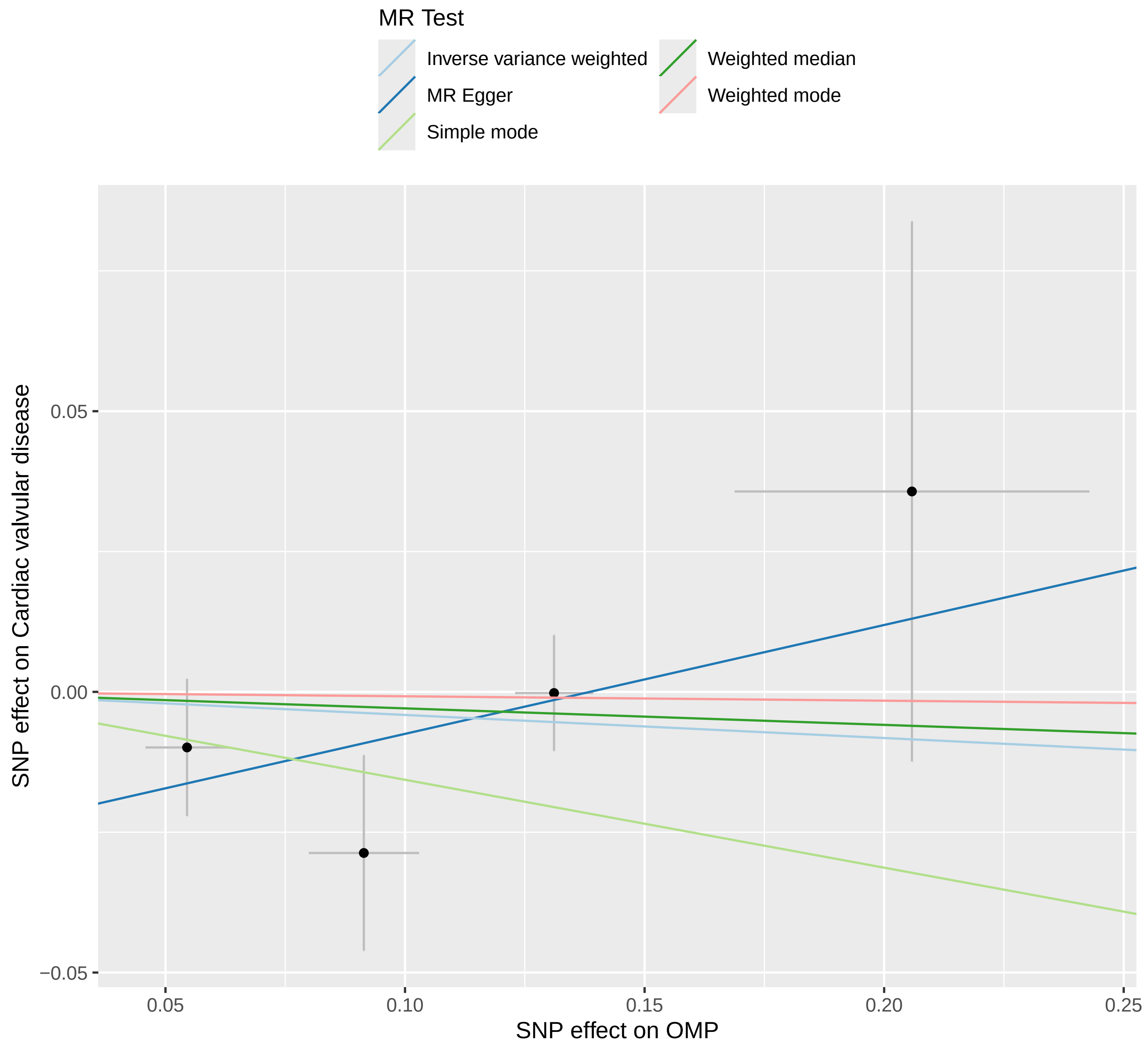

MR Test
Inverse variance weighted
MR Egger
Simple mode
Weighted median
Weighted mode
0.05
0.00
−0.05
SNP effect on Cardiac valvular disease
0.05
0.10
0.15
0.20
0.25
SNP effect on OMP

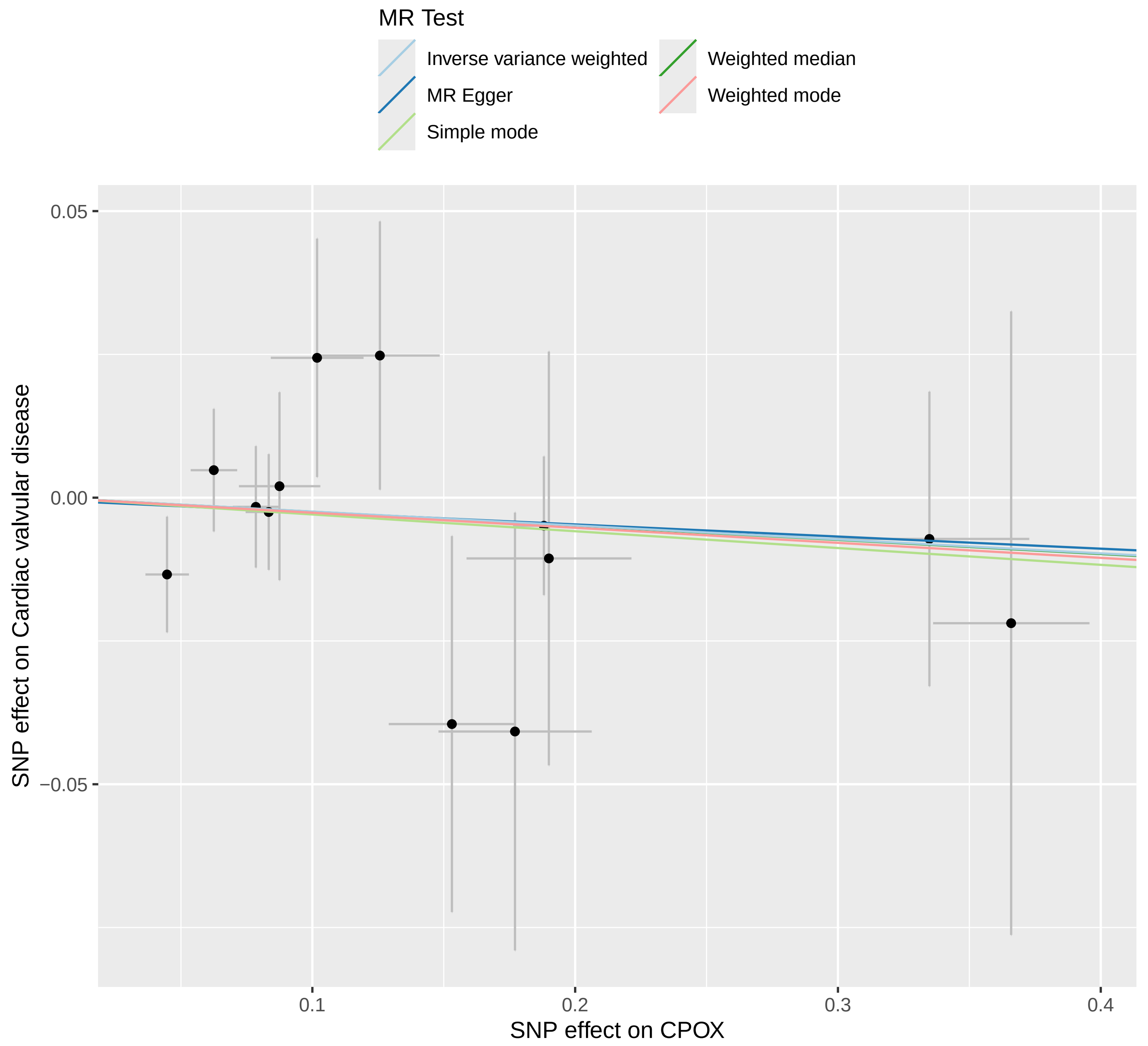

MR Test
Inverse variance weighted
MR Egger
Simple mode
Weighted median
Weighted mode
0.05
0.00
−0.05
SNP effect on Cardiac valvular disease
0.1
0.2
0.3
0.4
SNP effect on CPOX

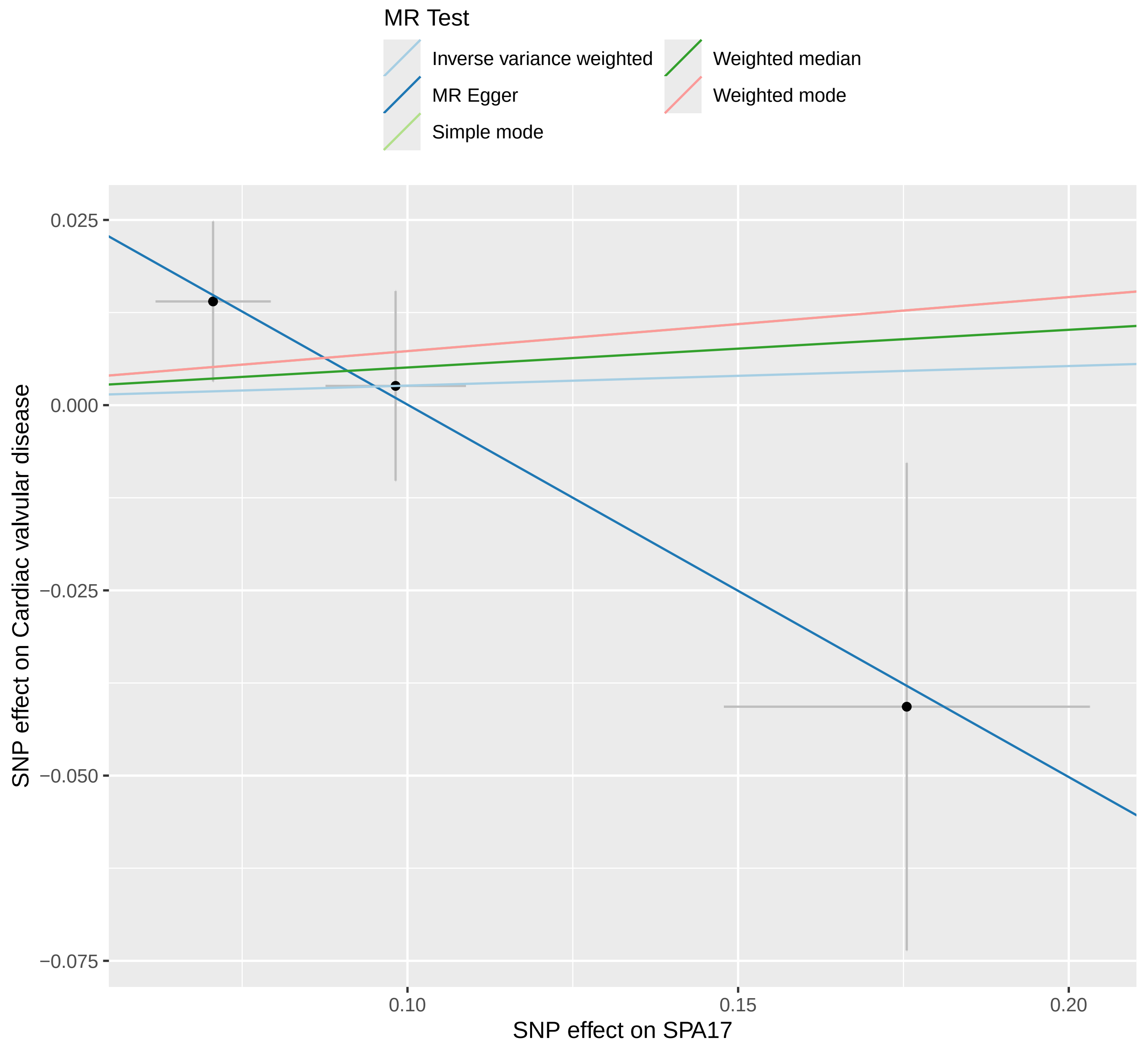

MR Test
Inverse variance weighted
MR Egger
Simple mode
Weighted median
Weighted mode
0.025
0.000
−0.025
−0.050
−0.075
0.10
0.15
0.20
SNP effect on Cardiac valvular disease
SNP effect on SPA17

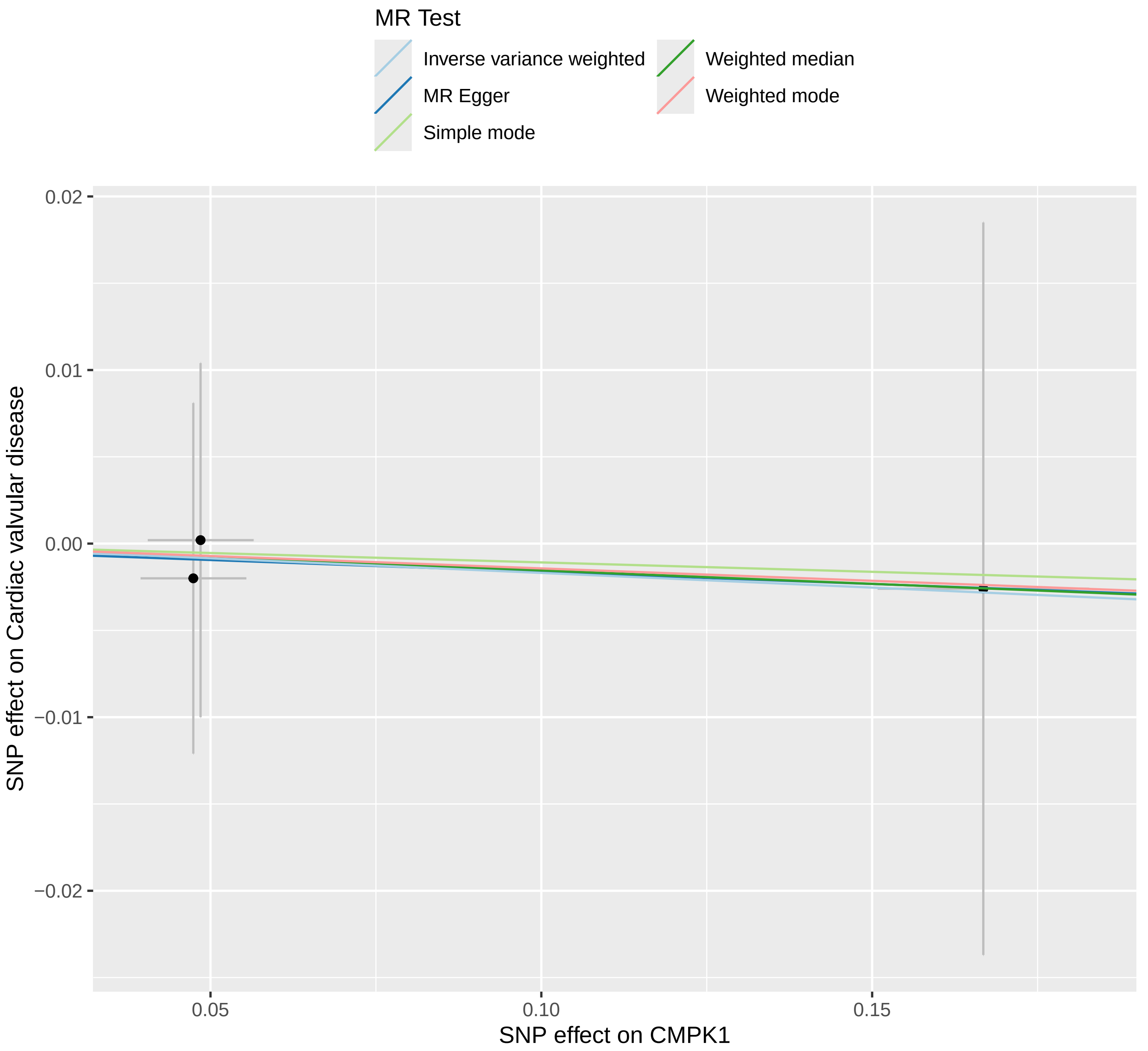
MR Test
Inverse variance weighted
MR Egger
Simple mode
Weighted median
Weighted mode
0.02
0.01
0.00
−0.01
−0.02
SNP effect on Cardiac valvular disease
0.05
0.10
0.15
SNP effect on CMPK1

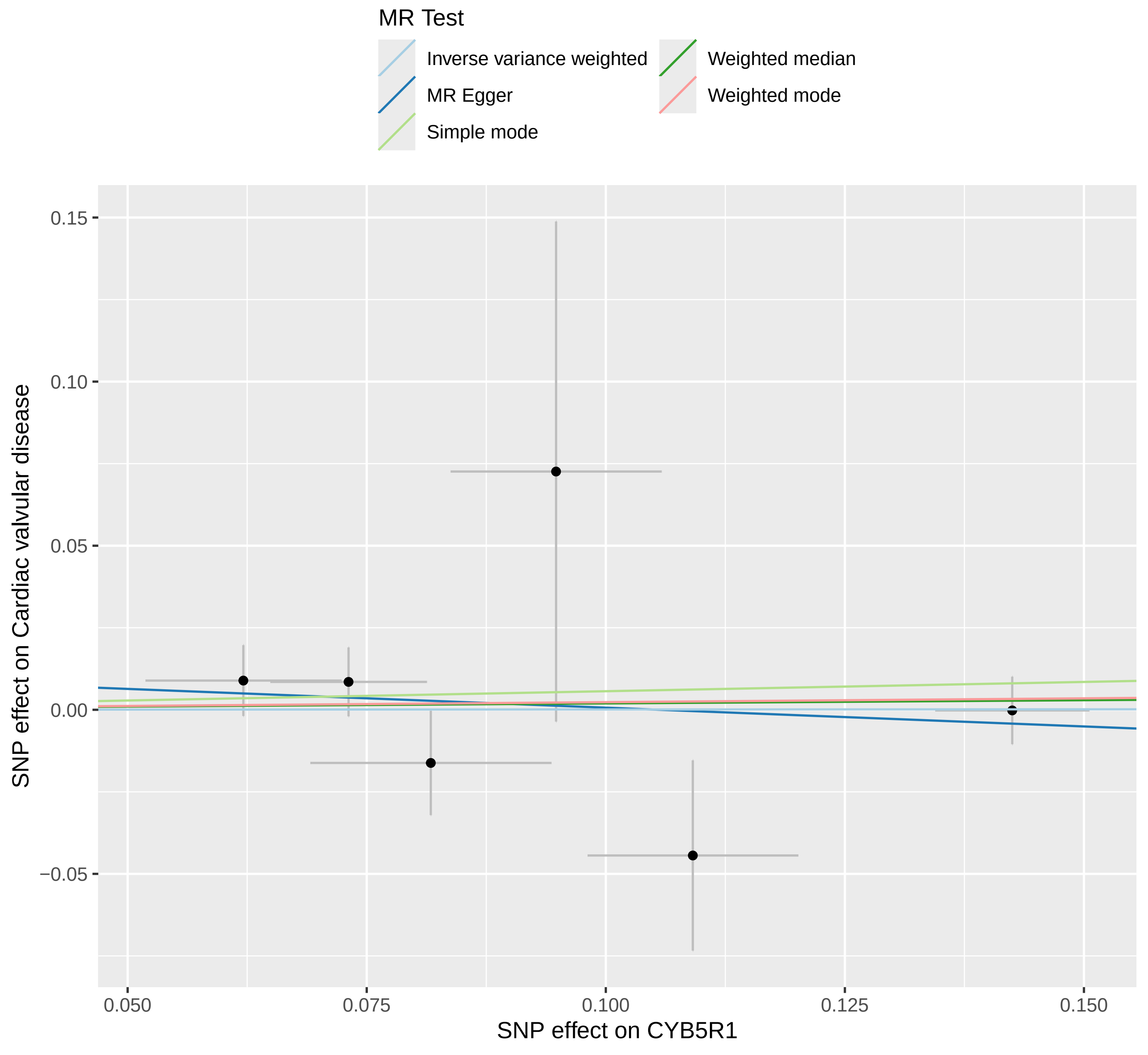
MR Test
Inverse variance weighted
MR Egger
Simple mode
Weighted median
Weighted mode
0.15
0.10
0.05
0.00
−0.05
SNP effect on Cardiac valvular disease
0.050
0.075
0.100
0.125
0.150
SNP effect on CYB5R1

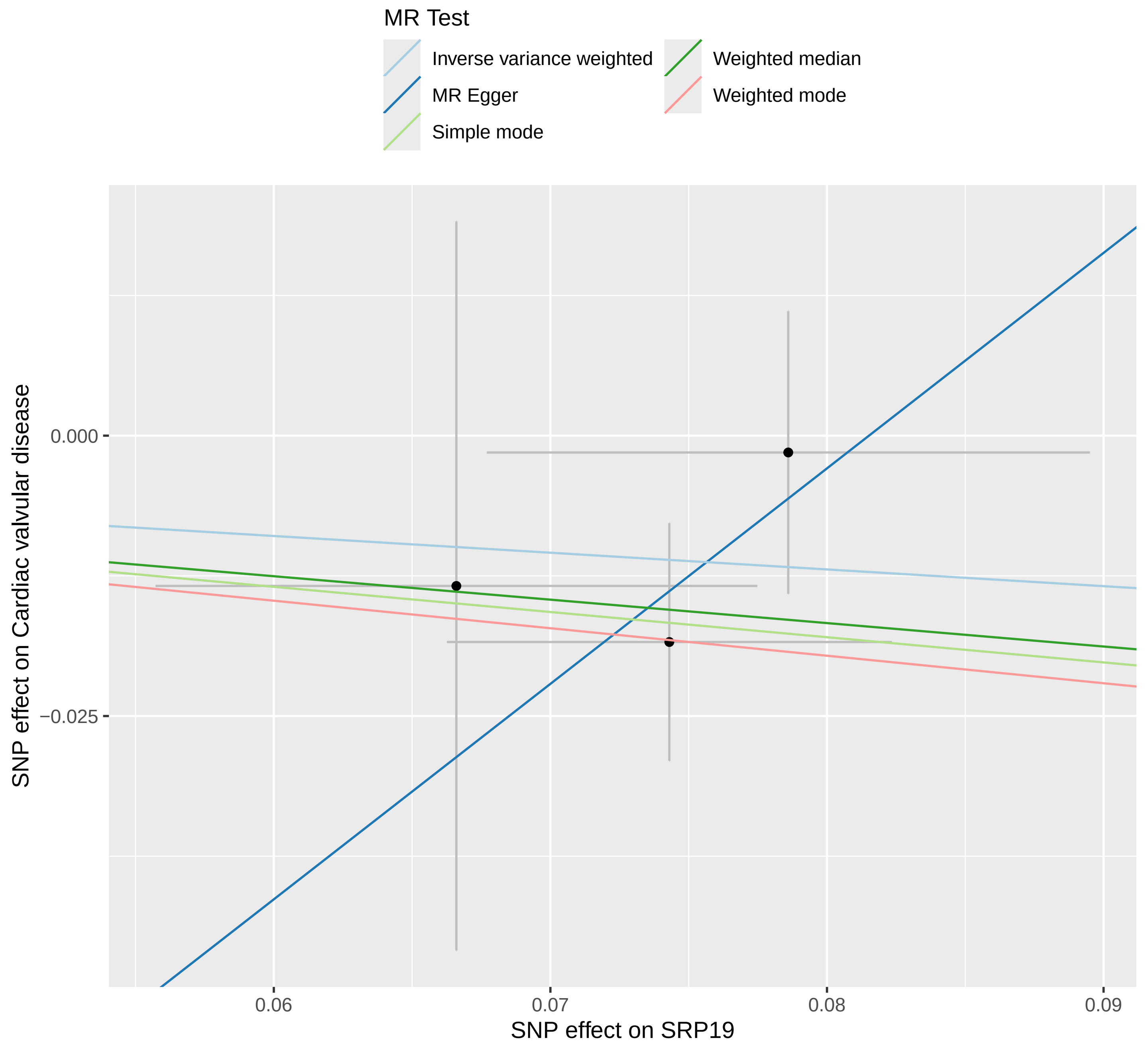
MR Test
Inverse variance weighted
MR Egger
Simple mode
Weighted median
Weighted mode
SNP effect on Cardiac valvular disease
0.000
−0.025
0.06
0.07
0.08
0.09
SNP effect on SRP19

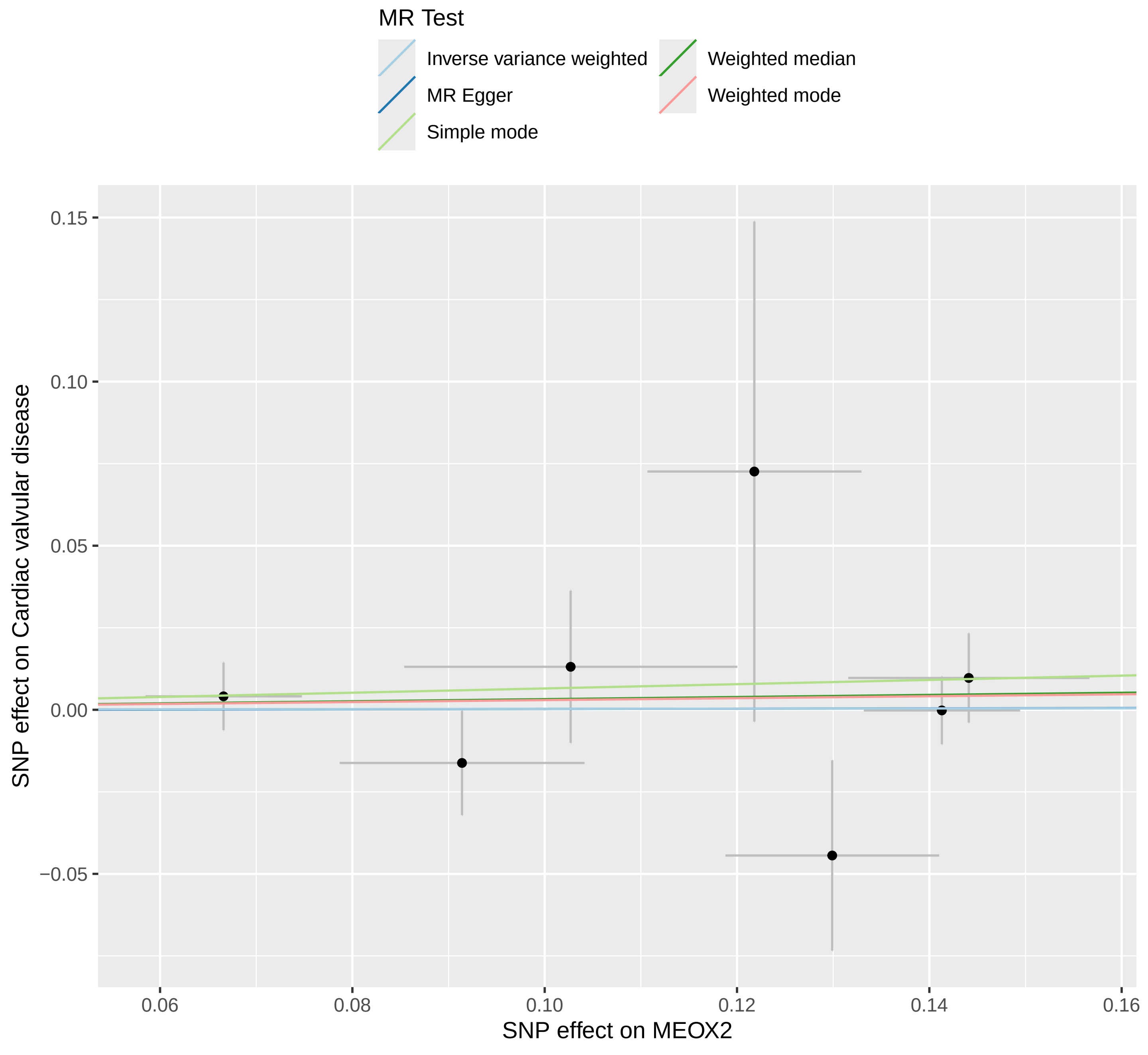

MR Test
Inverse variance weighted
MR Egger
Simple mode
Weighted median
Weighted mode
0.15
0.10
0.05
0.00
−0.05
SNP effect on Cardiac valvular disease
0.06
0.08
0.10
0.12
0.14
0.16
SNP effect on MEOX2

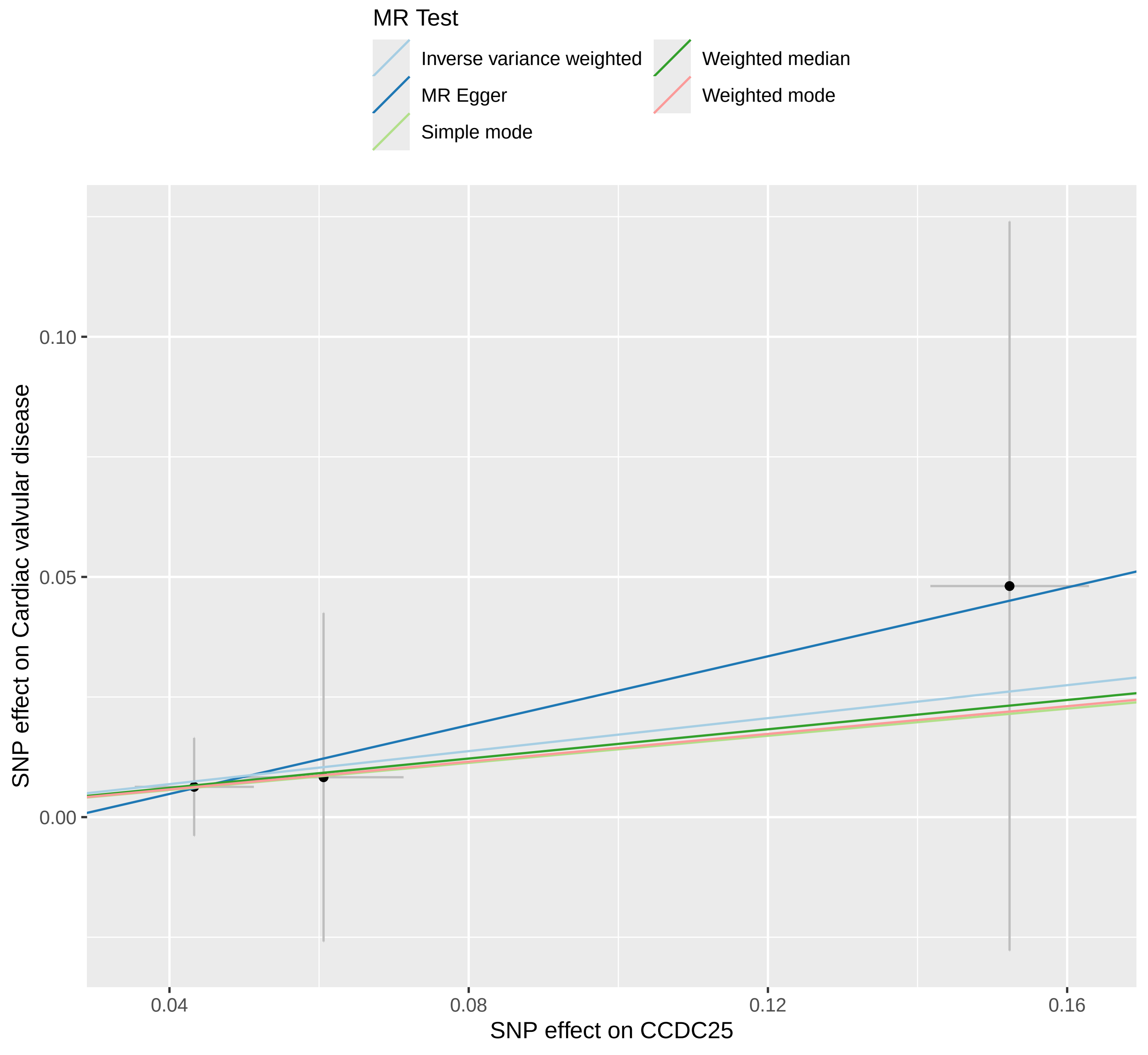

MR Test
Inverse variance weighted
MR Egger
Simple mode
Weighted median
Weighted mode
0.10
0.05
0.00
SNP effect on Cardiac valvular disease
0.04
0.08
0.12
0.16
SNP effect on CCDC25

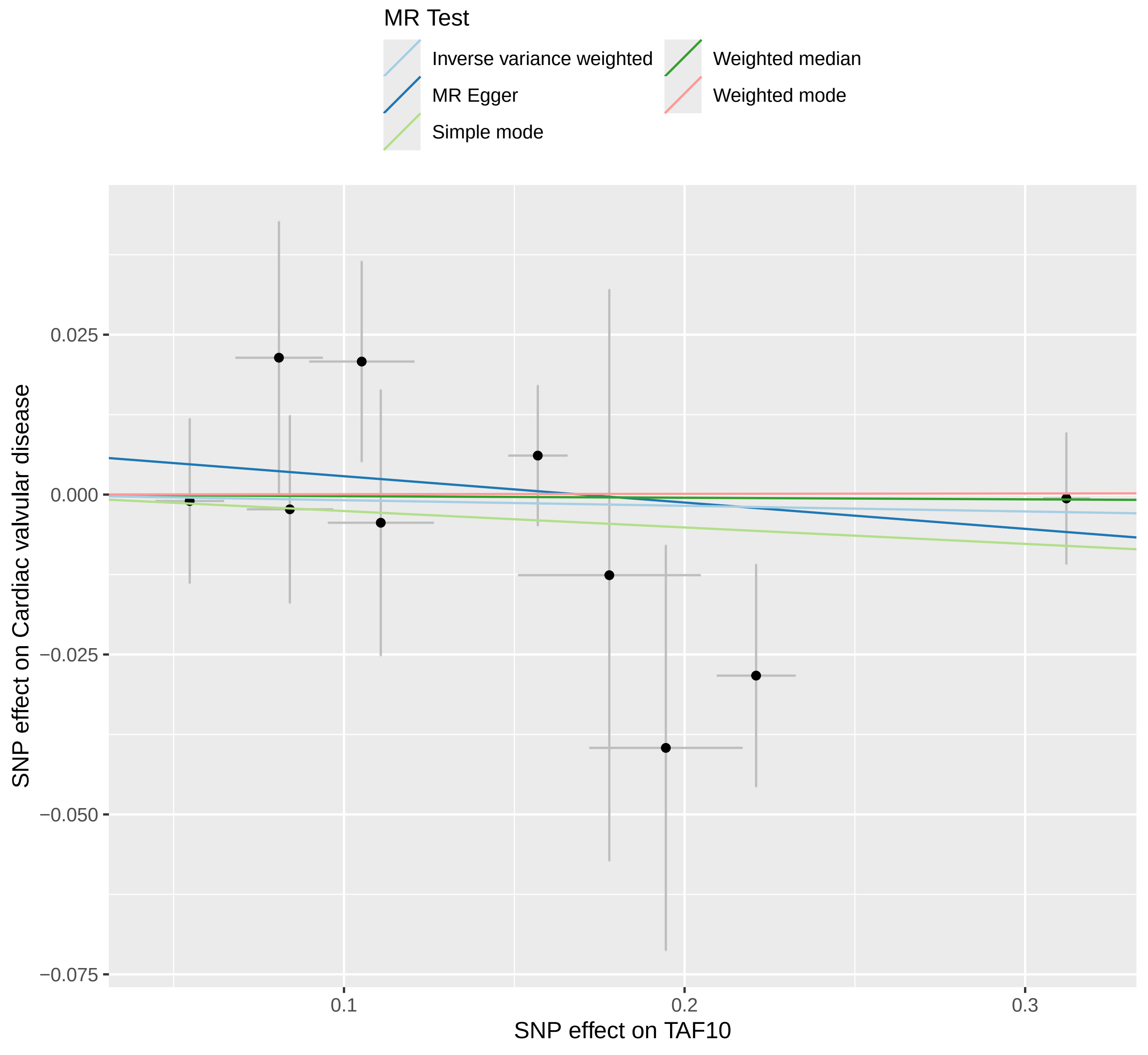

MR Test
Inverse variance weighted
MR Egger
Simple mode
Weighted median
Weighted mode
0.025
0.000
−0.025
−0.050
−0.075
0.1
0.2
0.3
SNP effect on Cardiac valvular disease
SNP effect on TAF10

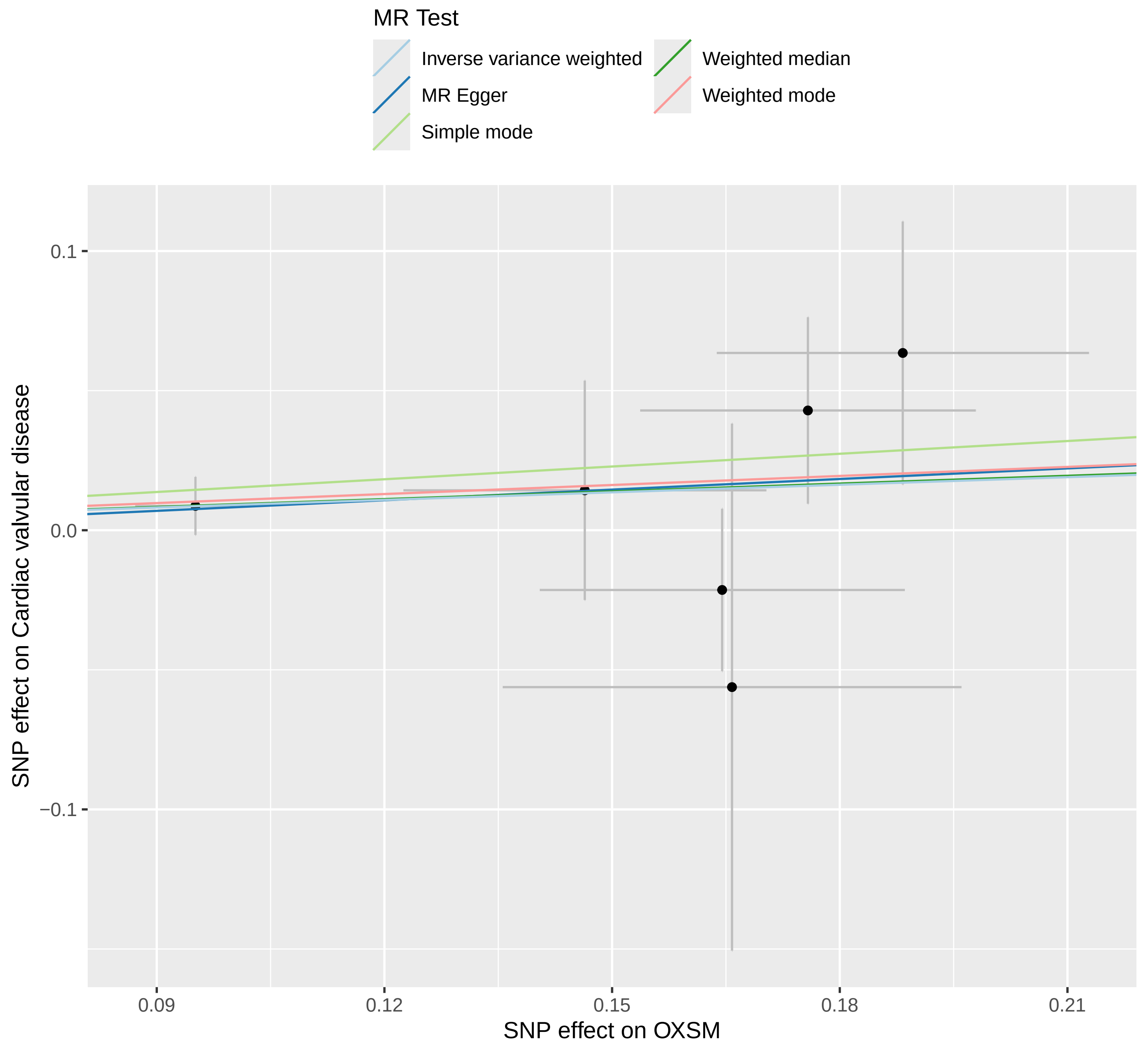

MR Test
Inverse variance weighted
MR Egger
Simple mode
Weighted median
Weighted mode
0.1
0.0
−0.1
SNP effect on Cardiac valvular disease
0.09
0.12
0.15
0.18
0.21
SNP effect on OXSM

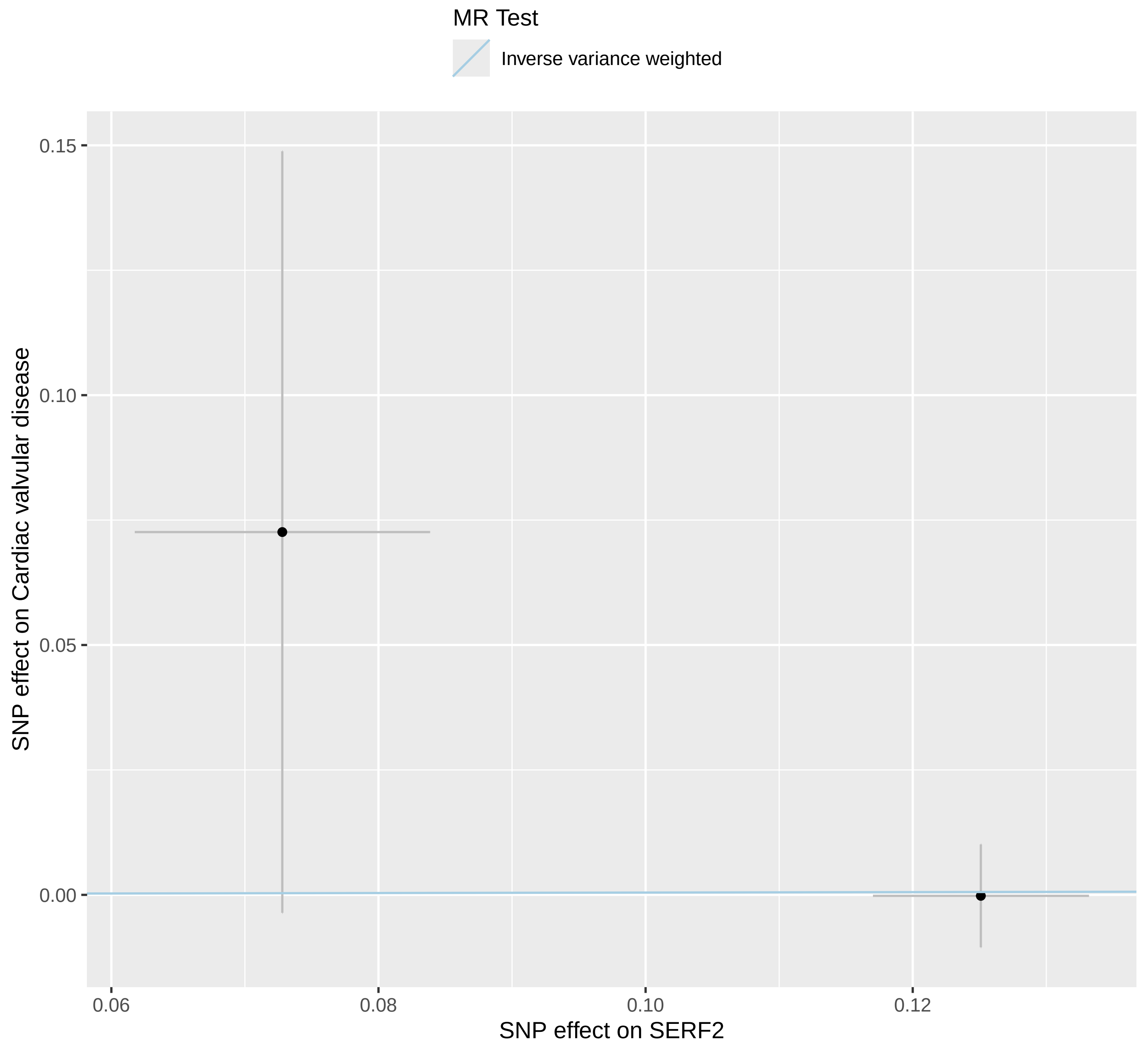

MR Test
Inverse variance weighted
SNP effect on Cardiac valvular disease
0.15
0.10
0.05
0.00
0.06
0.08
0.10
0.12
SNP effect on SERF2

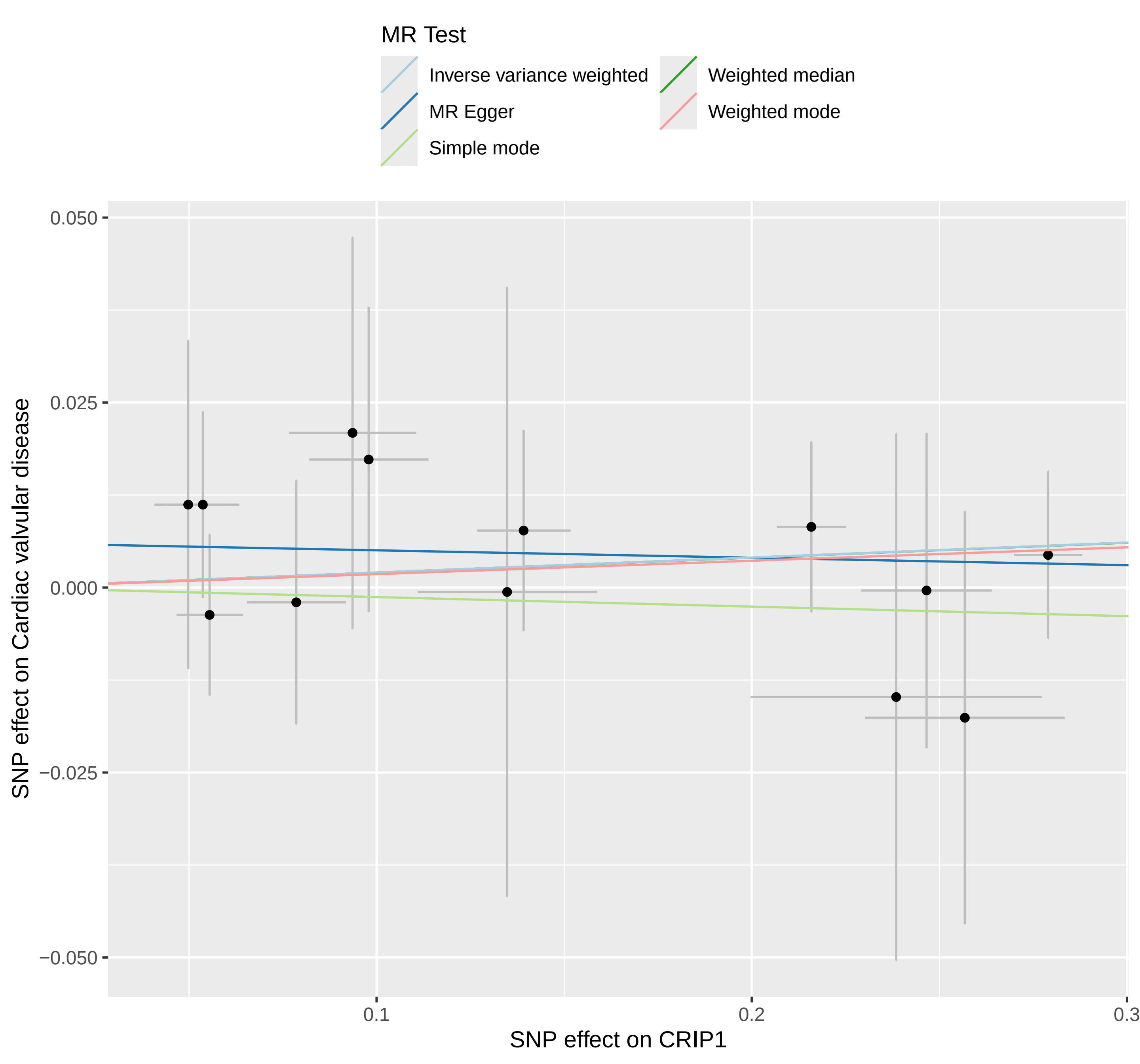
MR Test
Inverse variance weighted
MR Egger
Simple mode
Weighted median
Weighted mode
0.050
0.025
0.000
−0.025
−0.050
0.1
0.2
0.3
SNP effect on CRIP1
SNP effect on Cardiac valvular disease

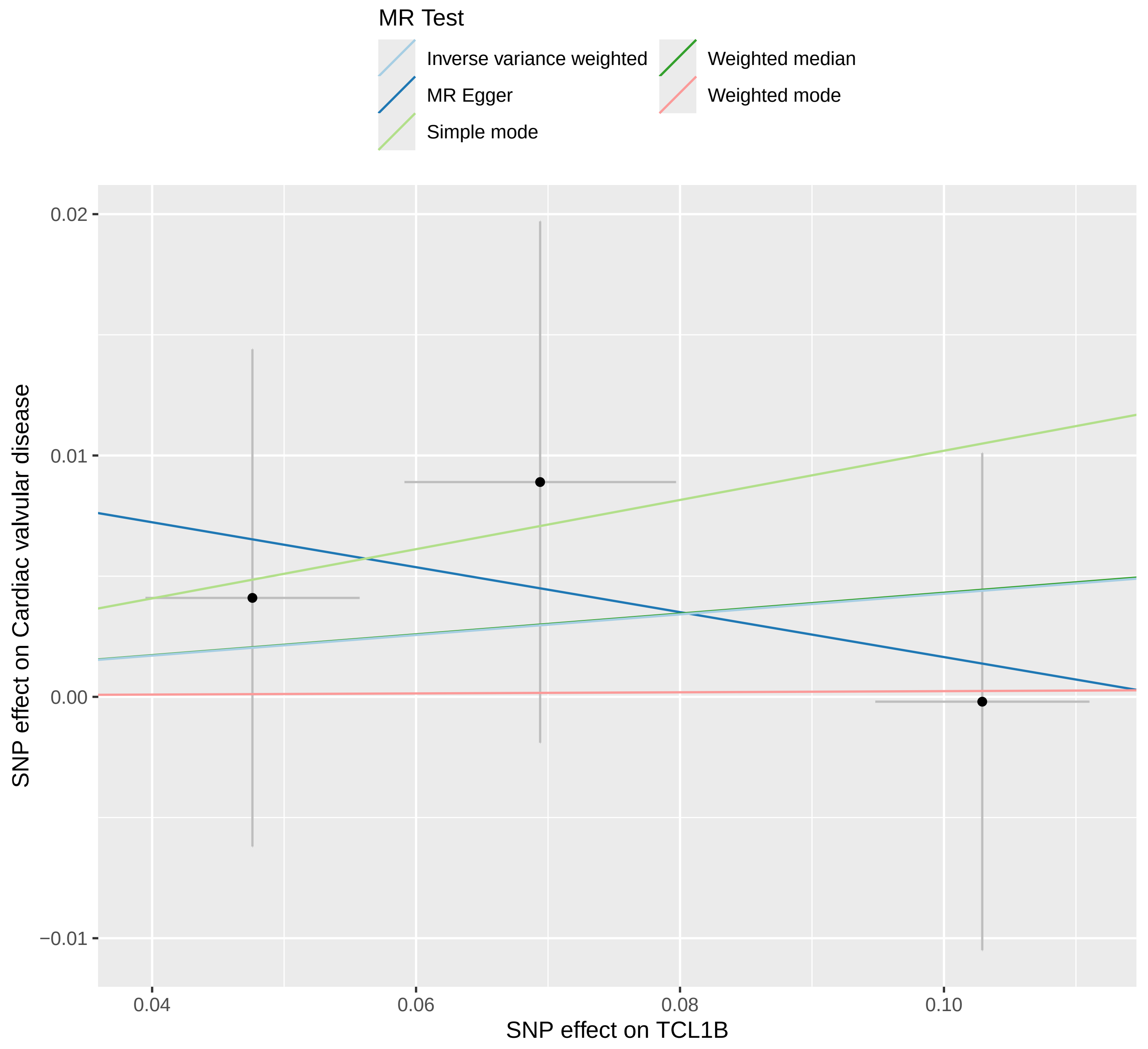
MR Test
Inverse variance weighted
MR Egger
Simple mode
Weighted median
Weighted mode
0.02
0.01
0.00
−0.01
0.04
0.06
0.08
0.10
SNP effect on Cardiac valvular disease
SNP effect on TCL1B

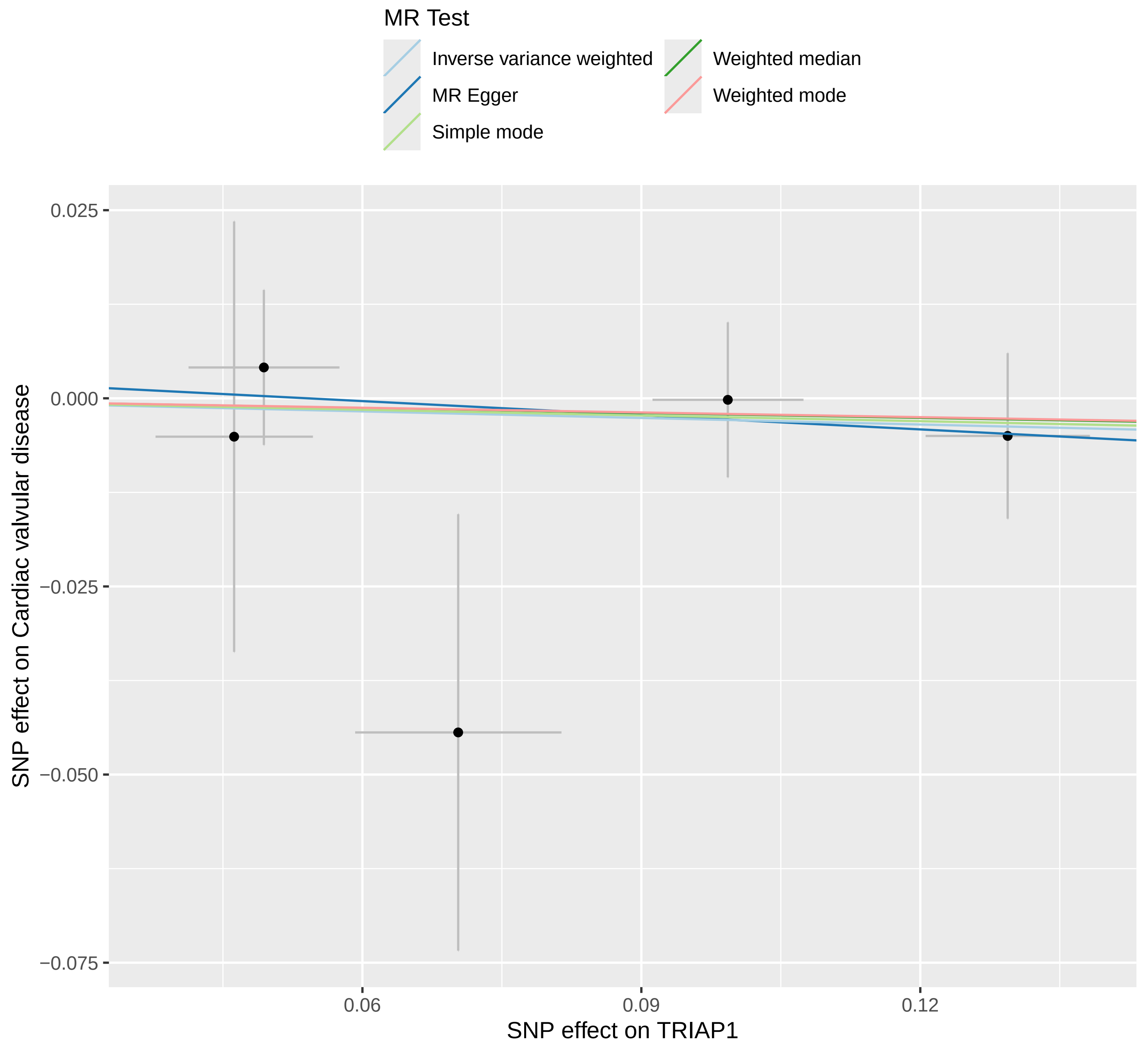
MR Test
Inverse variance weighted
MR Egger
Simple mode
Weighted median
Weighted mode
0.025
0.000
−0.025
−0.050
−0.075
0.06
0.09
0.12
SNP effect on Cardiac valvular disease
SNP effect on TRIAP1

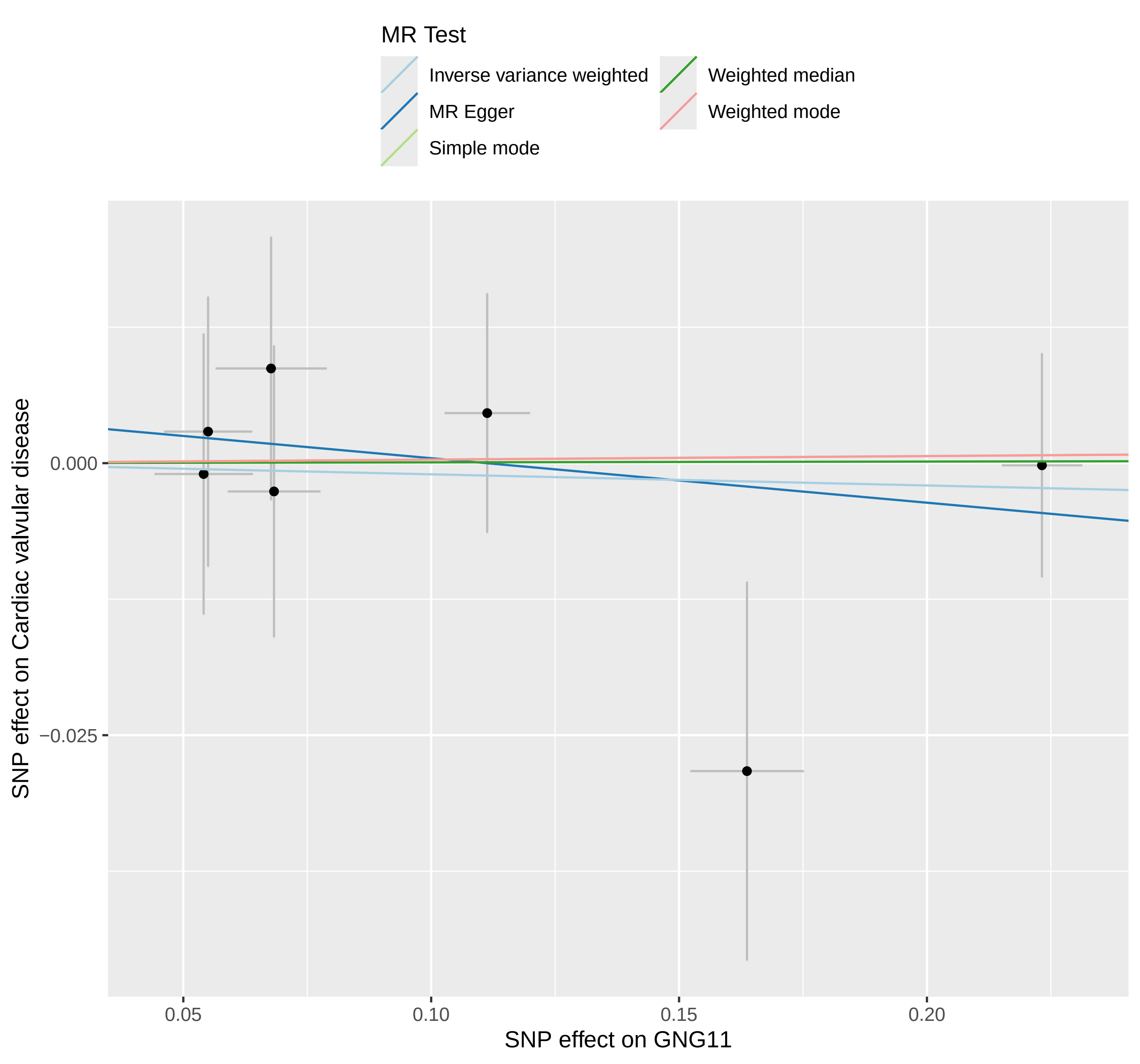
MR Test
Inverse variance weighted
MR Egger
Simple mode
Weighted median
Weighted mode
0.000
−0.025
0.05
0.10
0.15
0.20
SNP effect on GNG11
SNP effect on Cardiac valvular disease

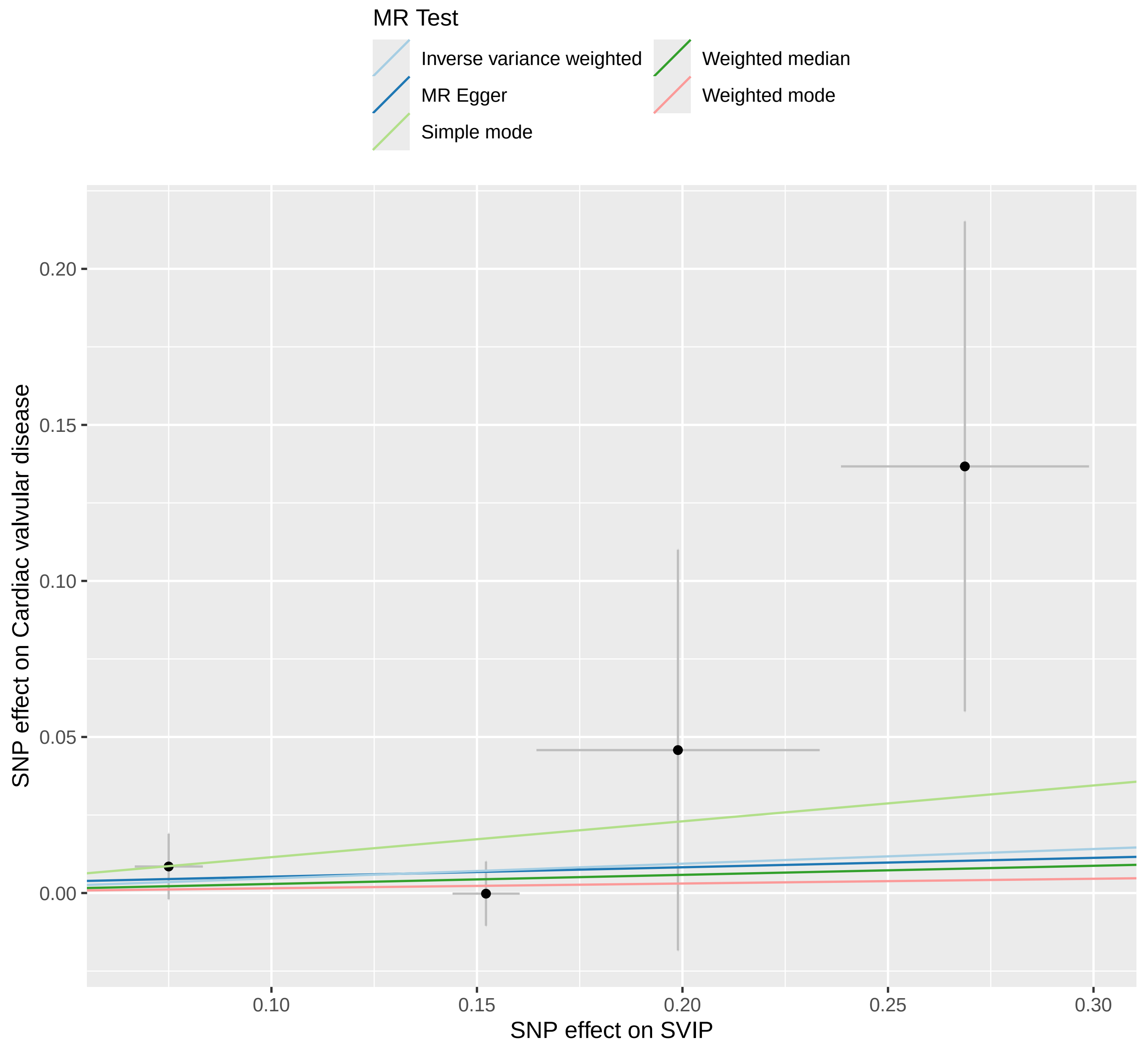

MR Test
Inverse variance weighted
MR Egger
Simple mode
Weighted median
Weighted mode
0.20
0.15
0.10
0.05
0.00
SNP effect on Cardiac valvular disease
0.10
0.15
0.20
0.25
0.30
SNP effect on SVIP

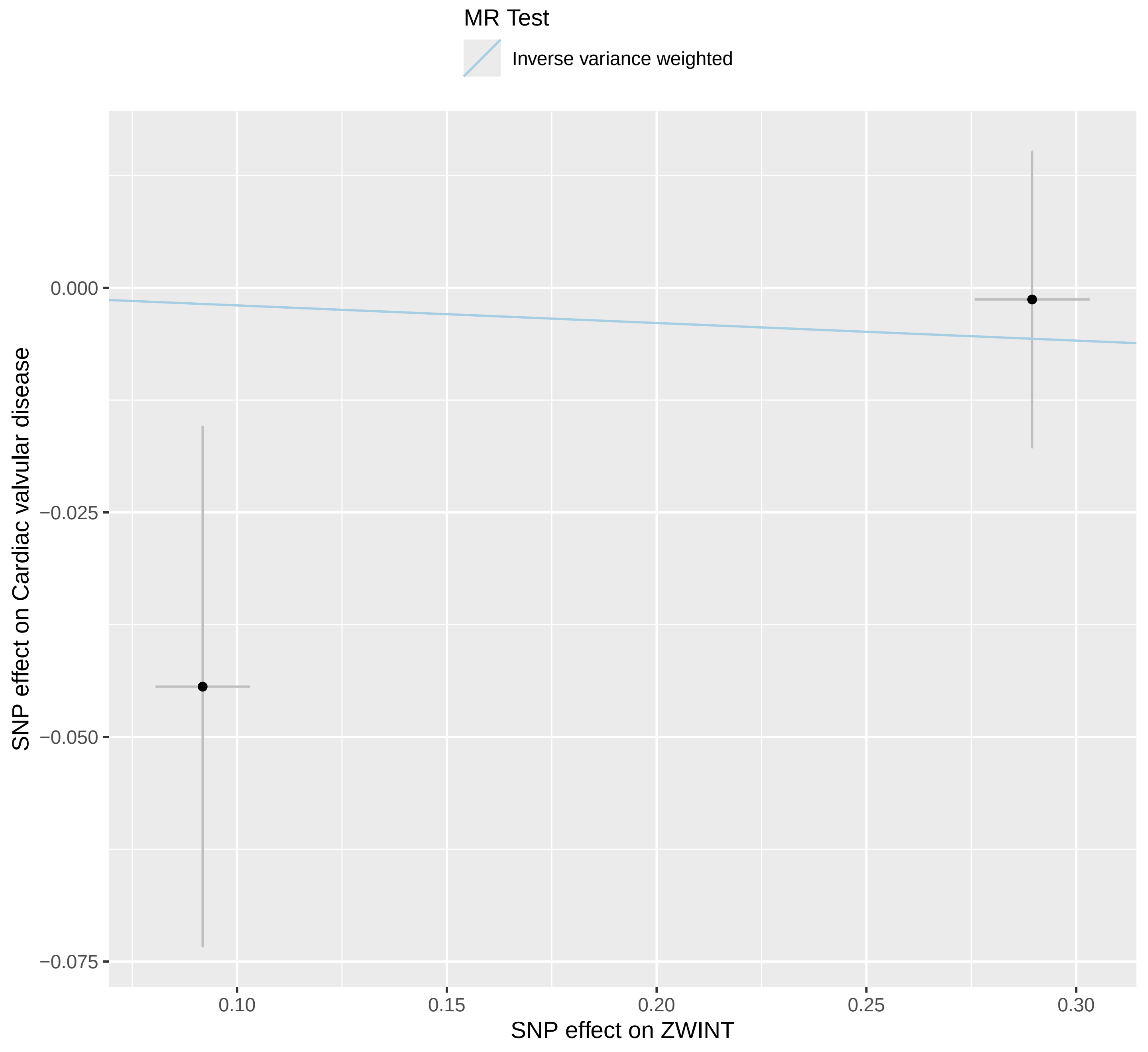

MR Test
Inverse variance weighted
0.000
−0.025
−0.050
−0.075
0.10
0.15
0.20
0.25
0.30
SNP effect on Cardiac valvular disease
SNP effect on ZWINT

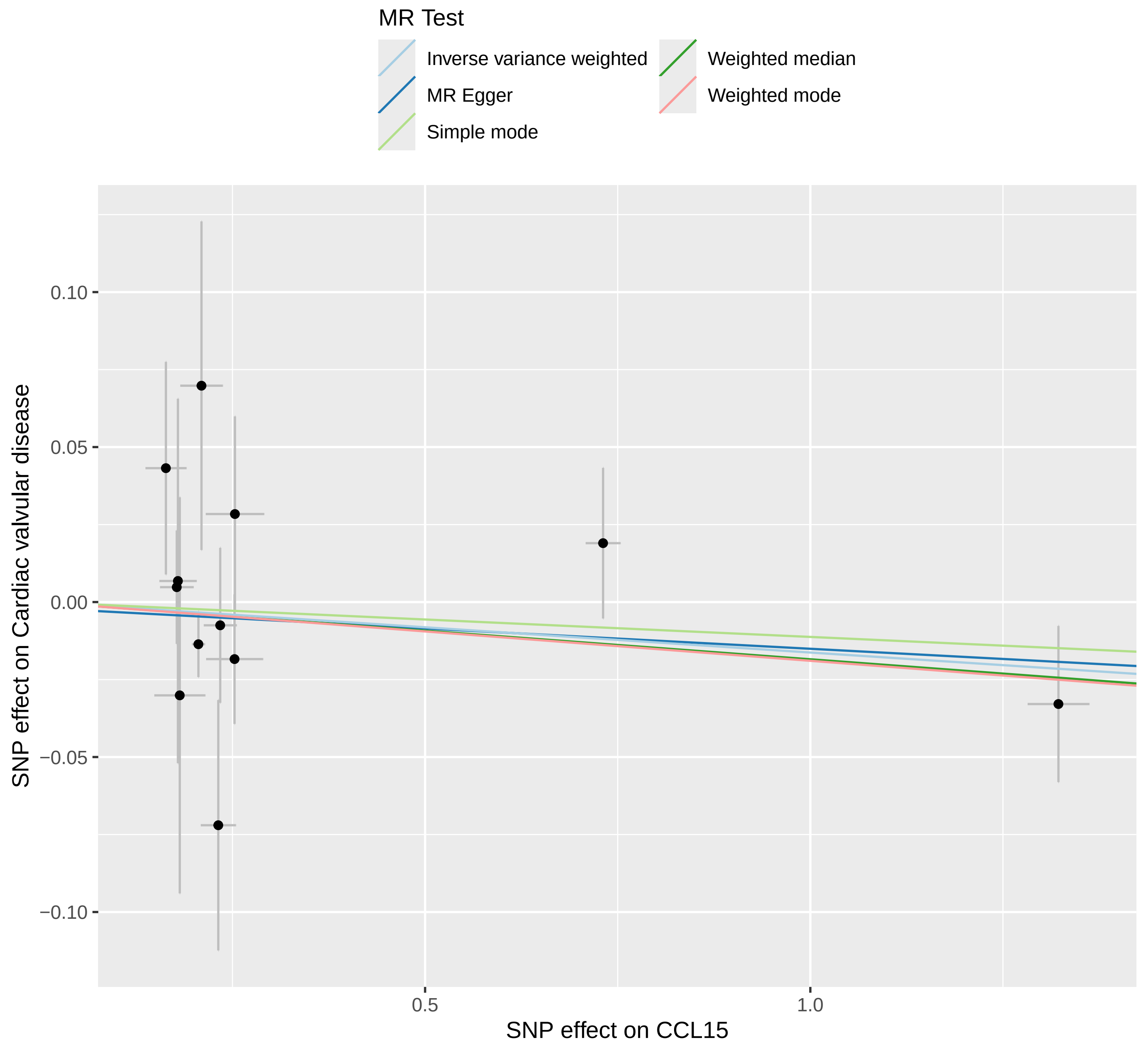

MR Test
Inverse variance weighted
MR Egger
Simple mode
Weighted median
Weighted mode
0.10
0.05
0.00
−0.05
−0.10
SNP effect on Cardiac valvular disease
0.5
1.0
SNP effect on CCL15

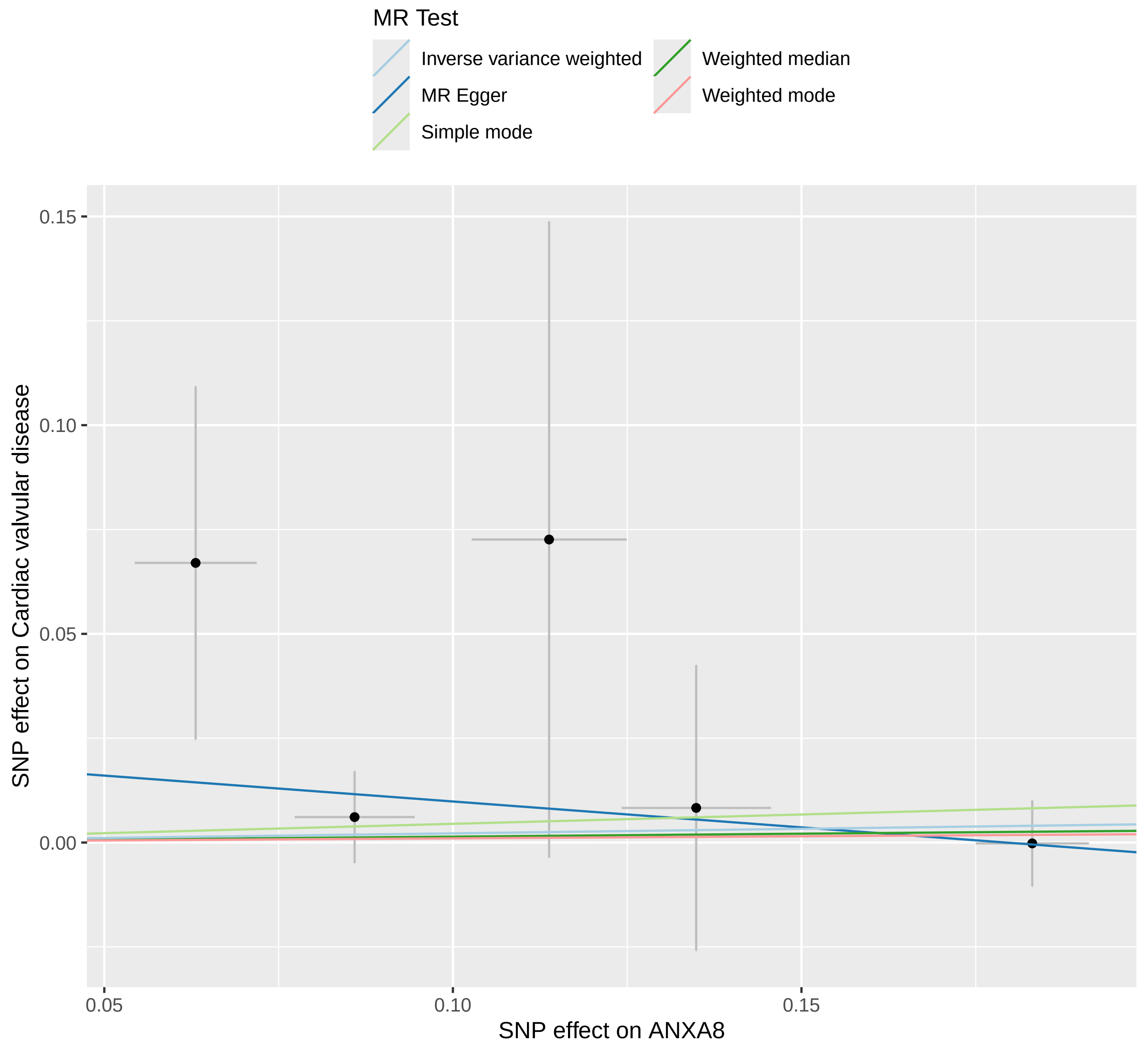
MR Test
Inverse variance weighted
MR Egger
Simple mode
Weighted median
Weighted mode
0.15
0.10
0.05
0.00
SNP effect on Cardiac valvular disease
0.05
0.10
0.15
SNP effect on ANXA8

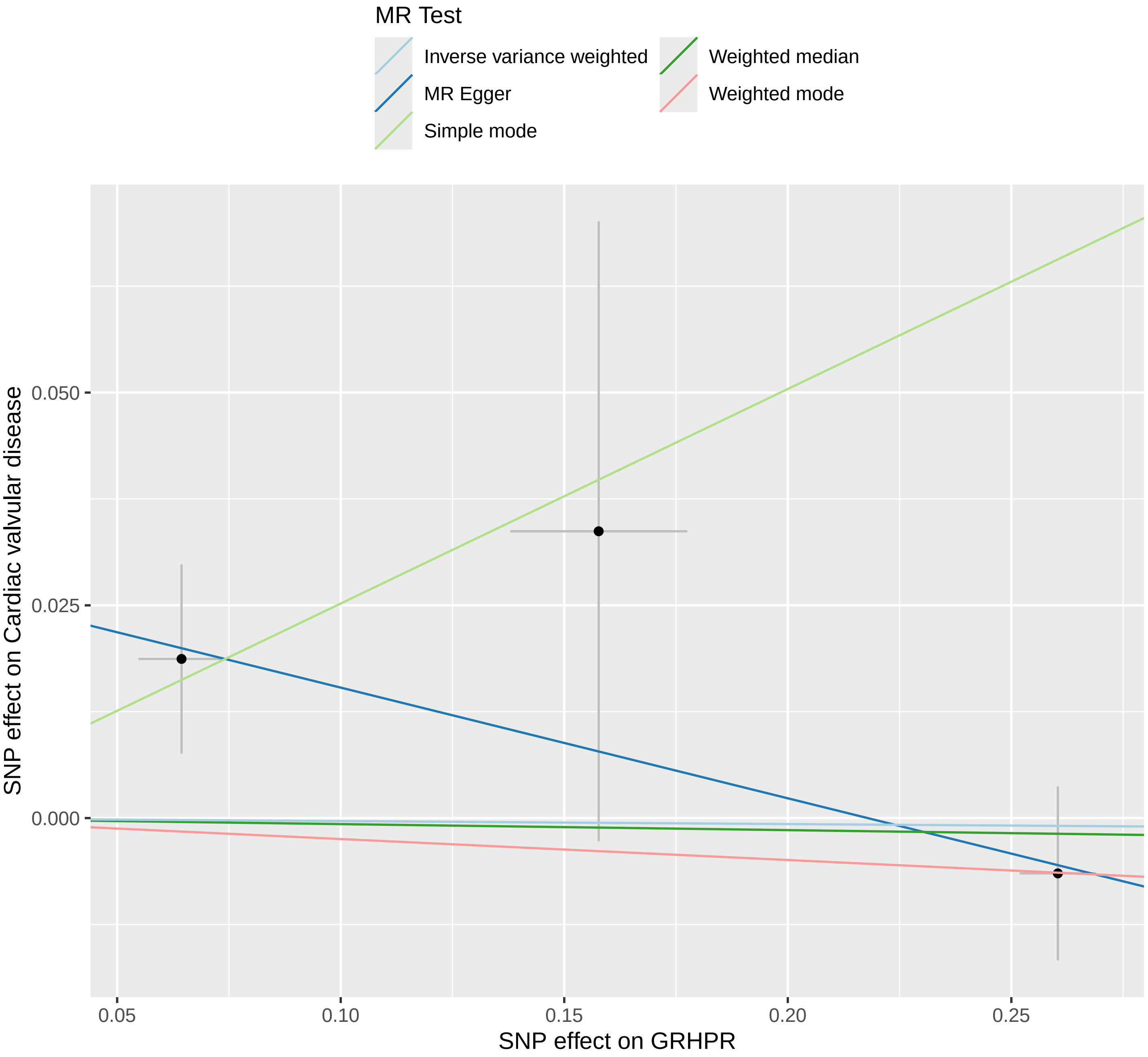
MR Test
Inverse variance weighted
MR Egger
Simple mode
Weighted median
Weighted mode
SNP effect on Cardiac valvular disease
0.050
0.025
0.000
0.05
0.10
0.15
0.20
0.25
SNP effect on GRHPR

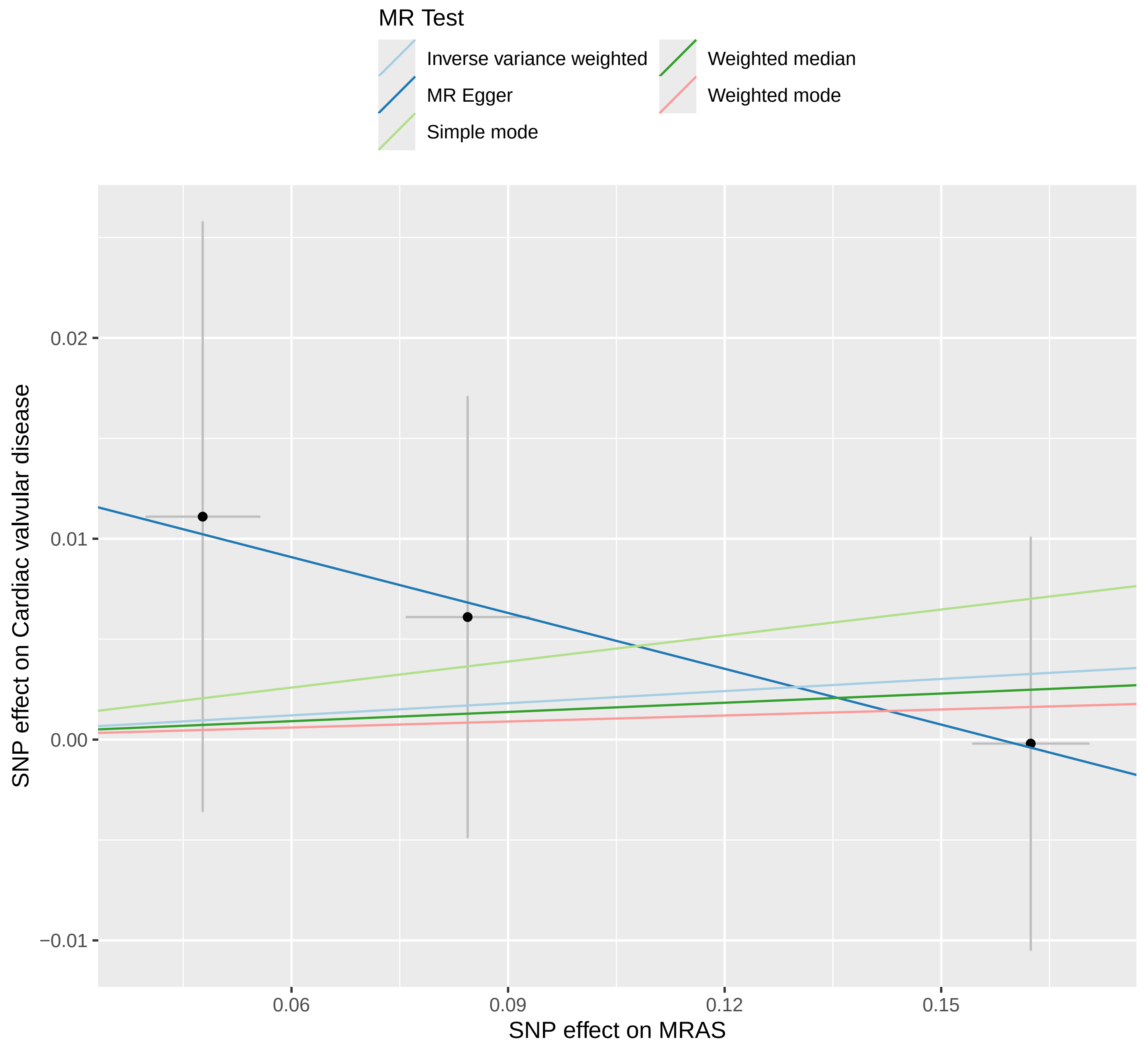
MR Test
Inverse variance weighted
MR Egger
Simple mode
Weighted median
Weighted mode
0.02
0.01
0.00
−0.01
0.06
0.09
0.12
0.15
SNP effect on MRAS
SNP effect on Cardiac valvular disease

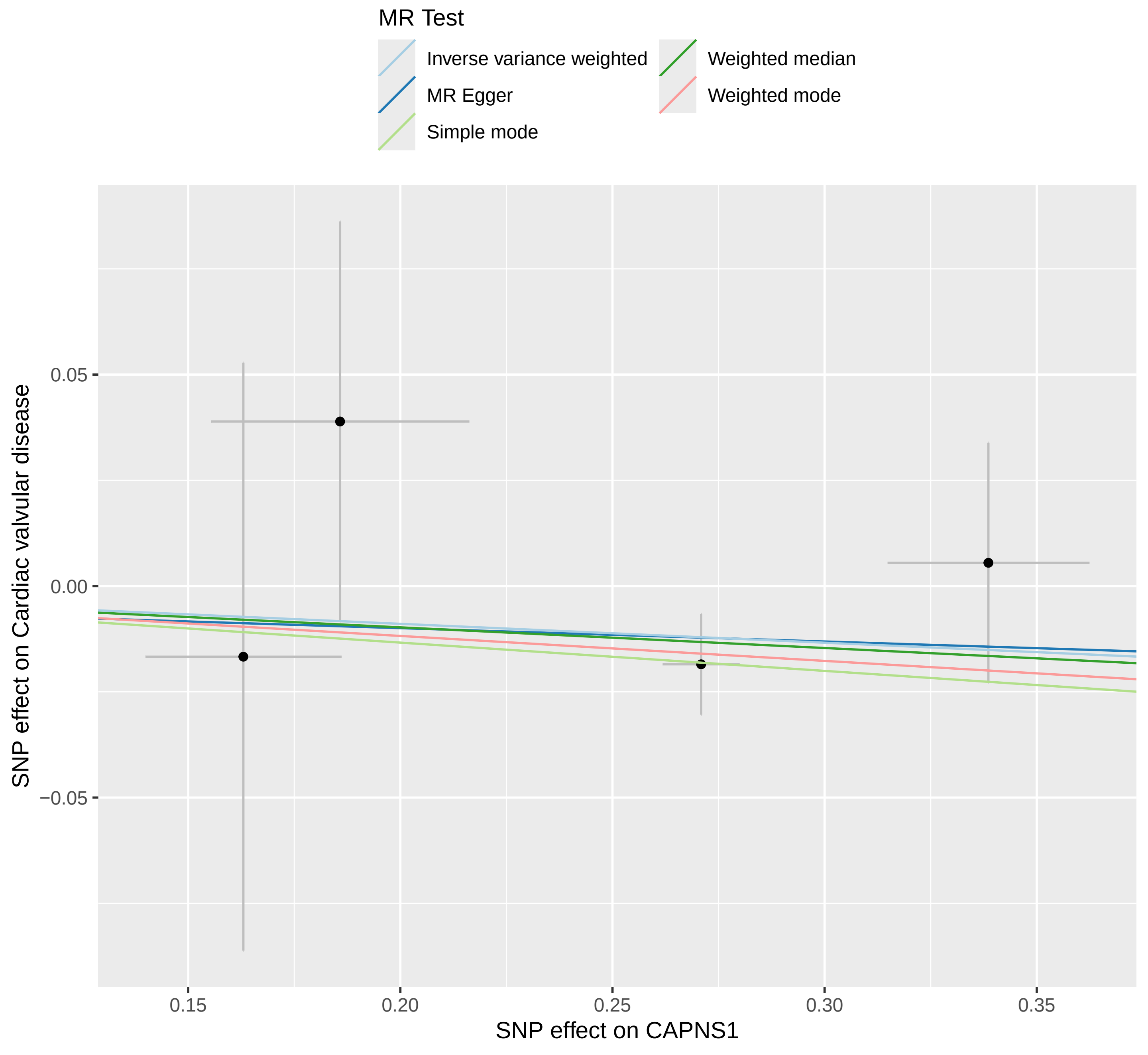
MR Test
Inverse variance weighted
MR Egger
Simple mode
Weighted median
Weighted mode
SNP effect on Cardiac valvular disease
0.05
0.00
−0.05
0.15
0.20
0.25
0.30
0.35
SNP effect on CAPNS1

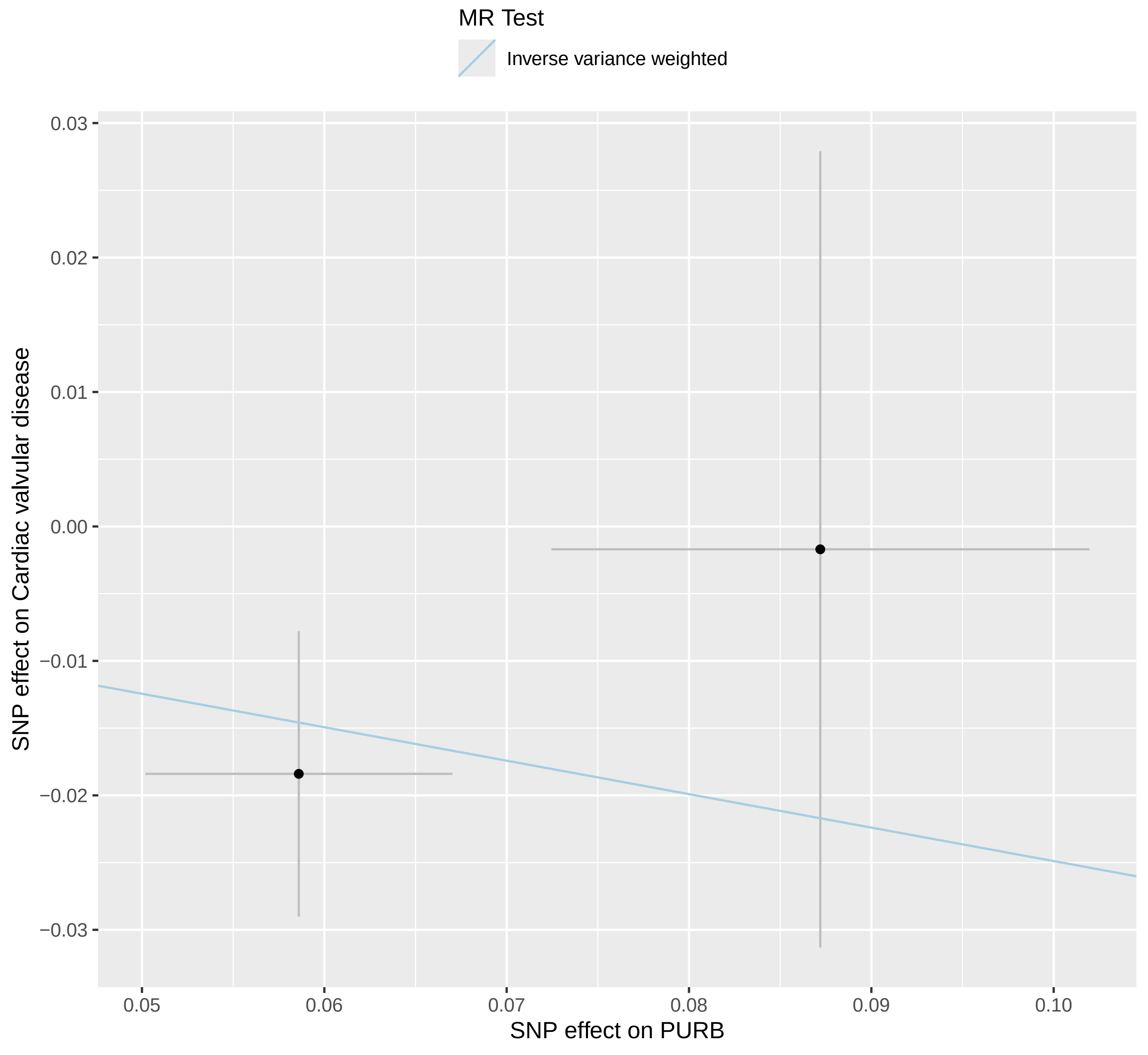

MR Test
Inverse variance weighted
0.03
0.02
0.01
0.00
−0.01
−0.02
−0.03
SNP effect on Cardiac valvular disease
0.05
0.06
0.07
0.08
0.09
0.10
SNP effect on PURB

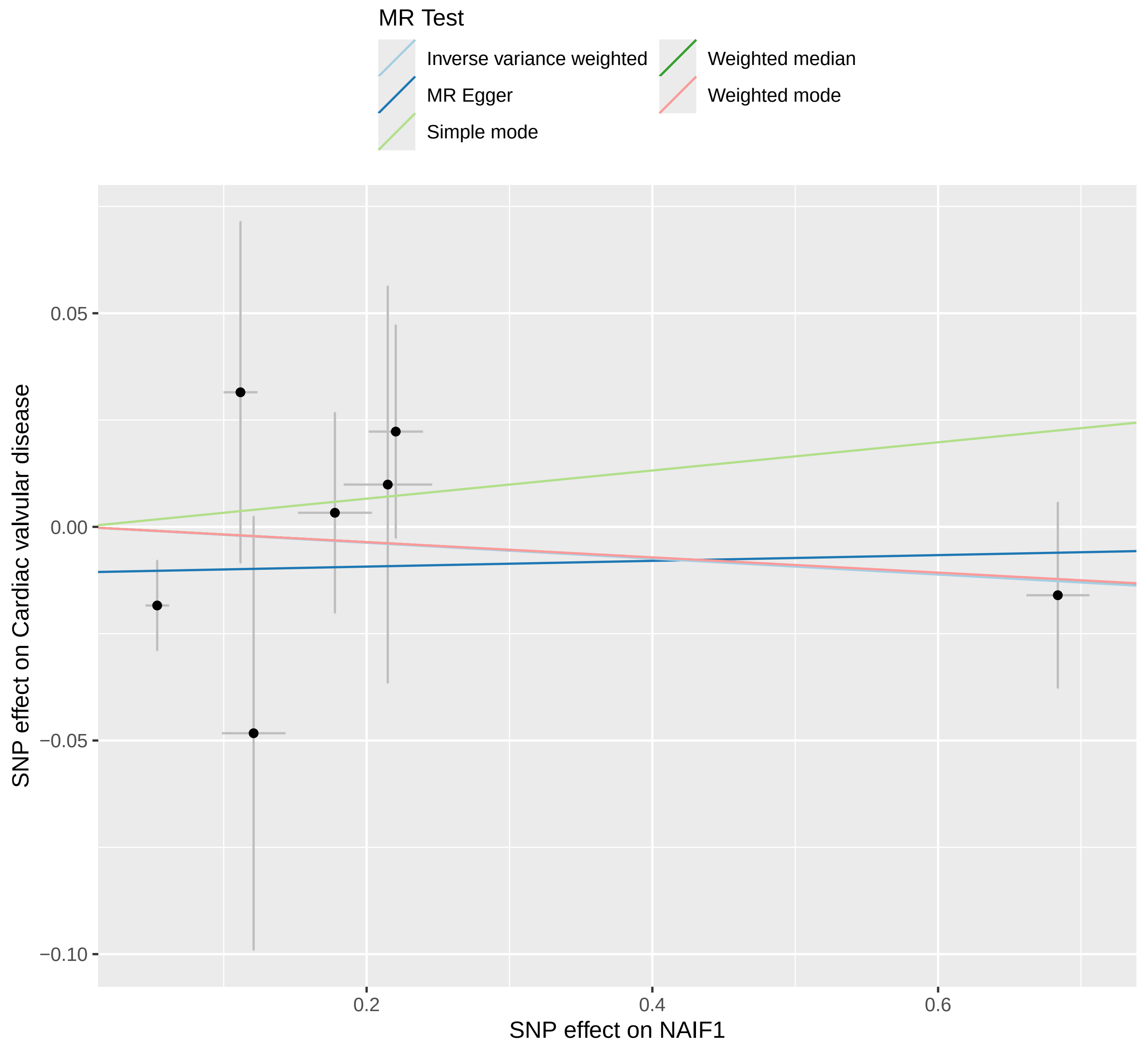
MR Test
Inverse variance weighted
MR Egger
Simple mode
Weighted median
Weighted mode
0.05
0.00
−0.05
−0.10
0.2
0.4
0.6
SNP effect on Cardiac valvular disease
SNP effect on NAIF1

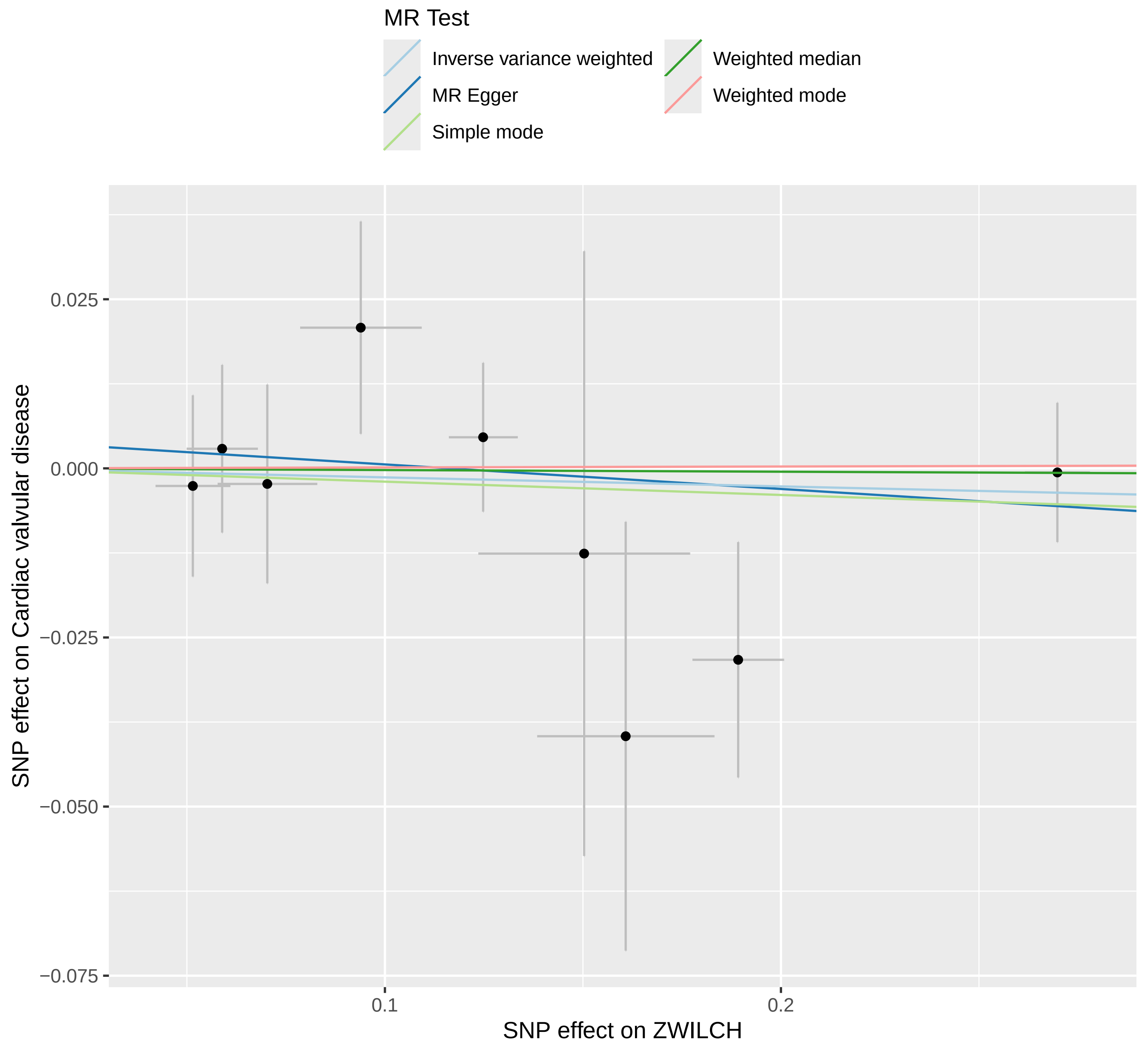

MR Test
Inverse variance weighted
MR Egger
Simple mode
Weighted median
Weighted mode
0.025
0.000
−0.025
−0.050
−0.075
0.1
0.2
SNP effect on Cardiac valvular disease
SNP effect on ZWILCH

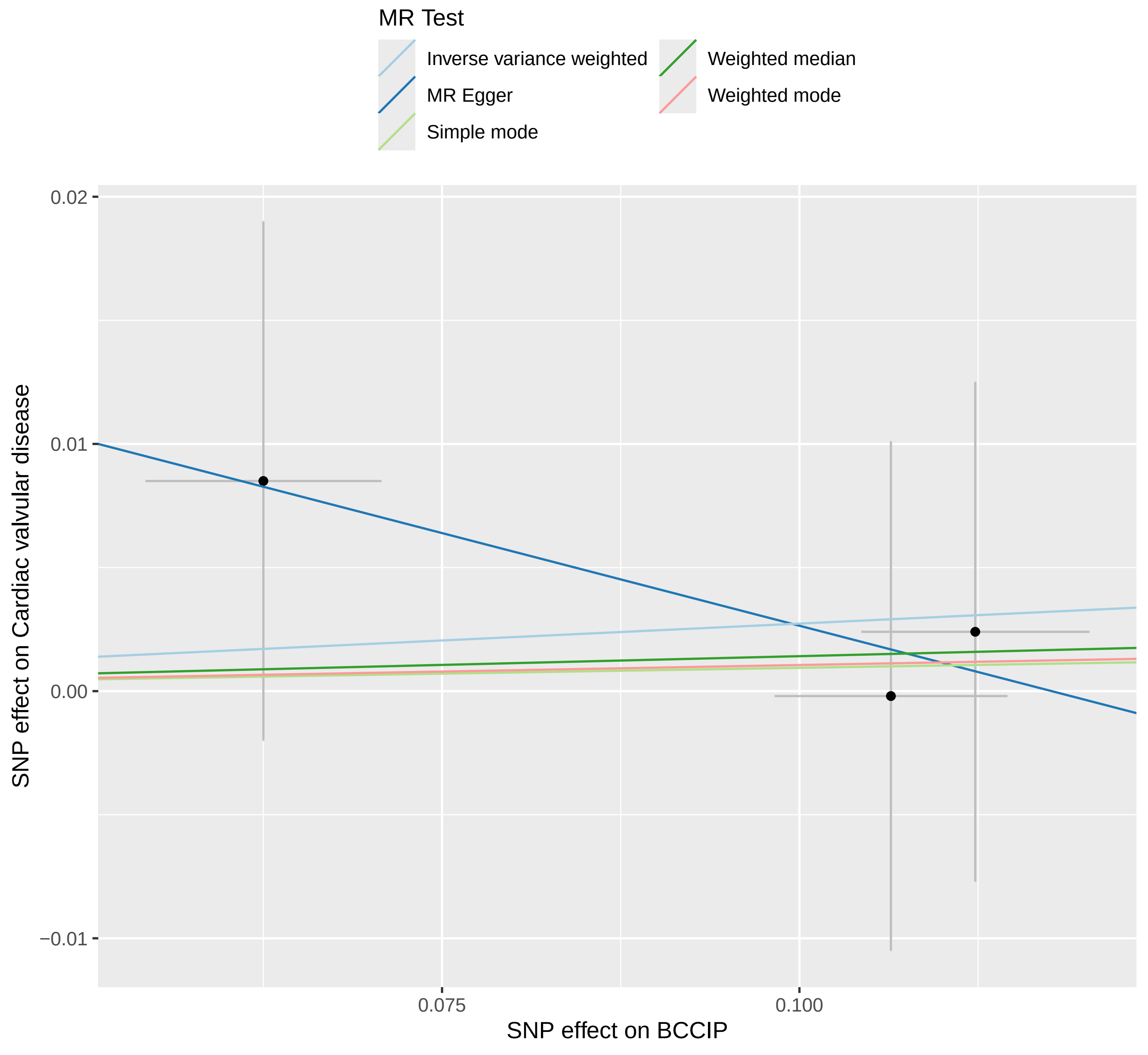

MR Test
Inverse variance weighted
MR Egger
Simple mode
Weighted median
Weighted mode
0.02
0.01
0.00
−0.01
SNP effect on Cardiac valvular disease
0.075
0.100
SNP effect on BCCIP

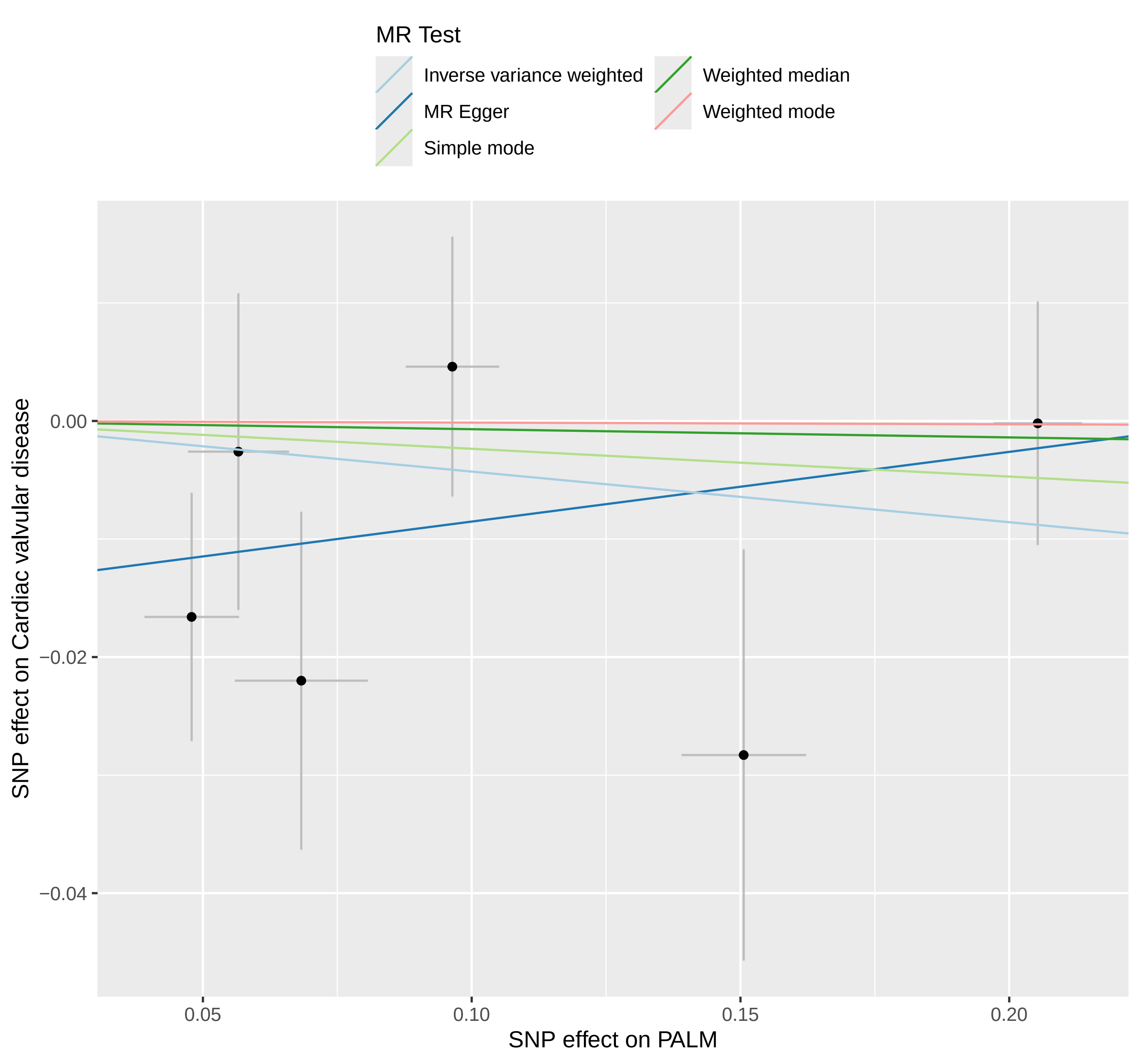
MR Test
Inverse variance weighted
MR Egger
Simple mode
Weighted median
Weighted mode
SNP effect on Cardiac valvular disease
0.00
−0.02
−0.04
0.05
0.10
0.15
0.20
SNP effect on PALM

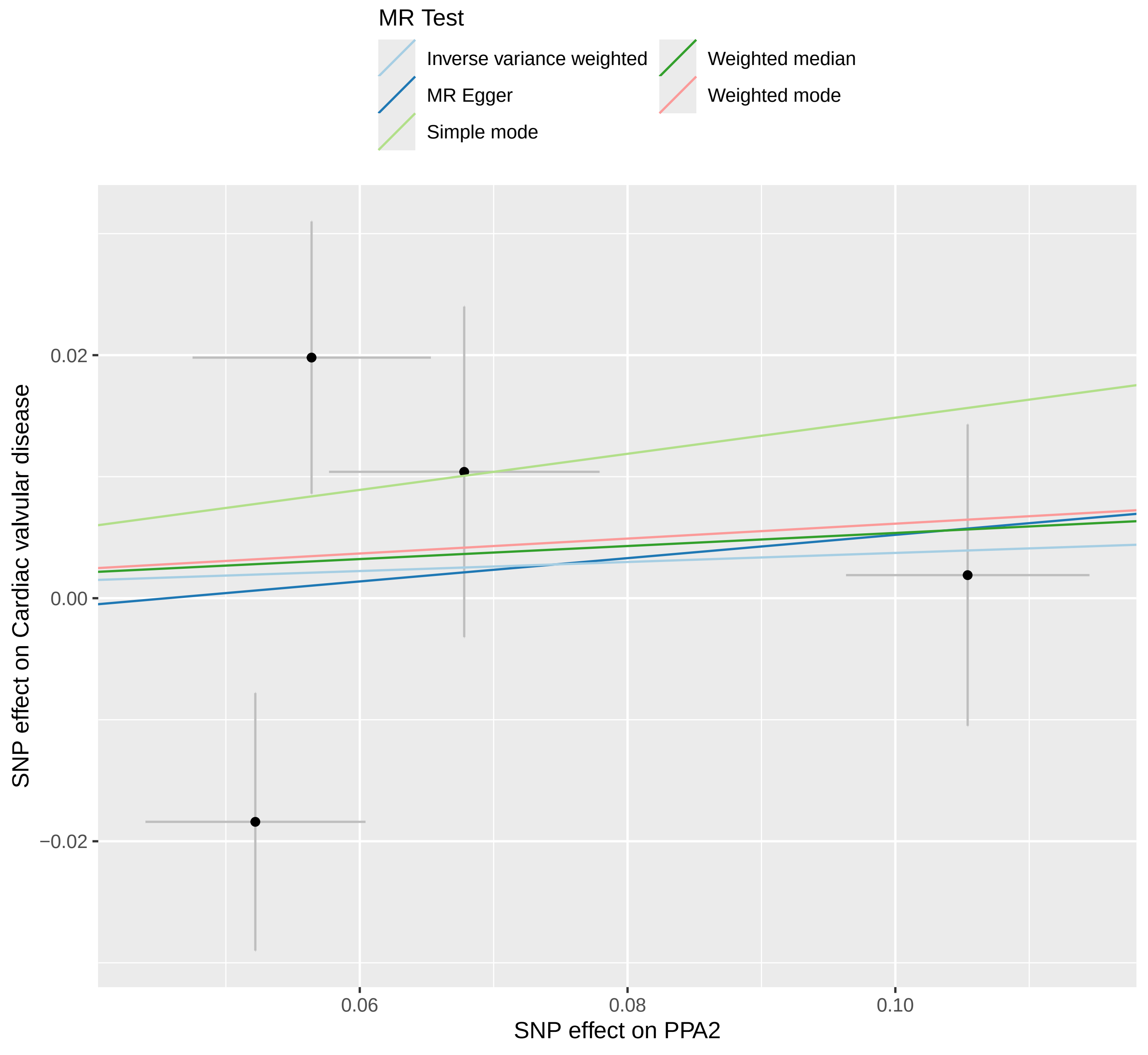

MR Test
Inverse variance weighted
MR Egger
Simple mode
Weighted median
Weighted mode
0.02
0.00
−0.02
0.06
0.08
0.10
SNP effect on Cardiac valvular disease
SNP effect on PPA2

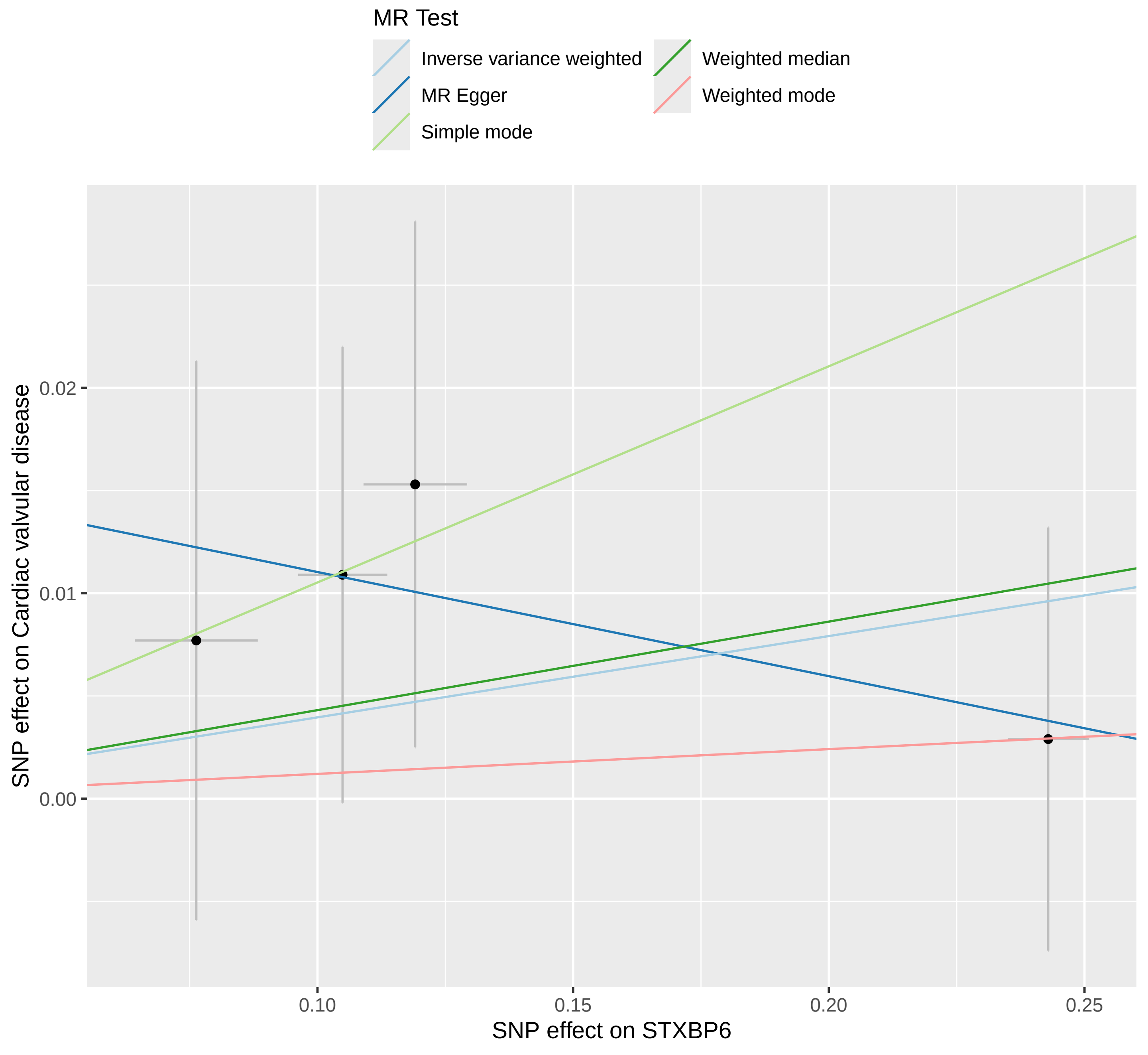

MR Test
Inverse variance weighted
MR Egger
Simple mode
Weighted median
Weighted mode
0.02
0.01
0.00
0.10
0.15
0.20
0.25
SNP effect on Cardiac valvular disease
SNP effect on STXBP6

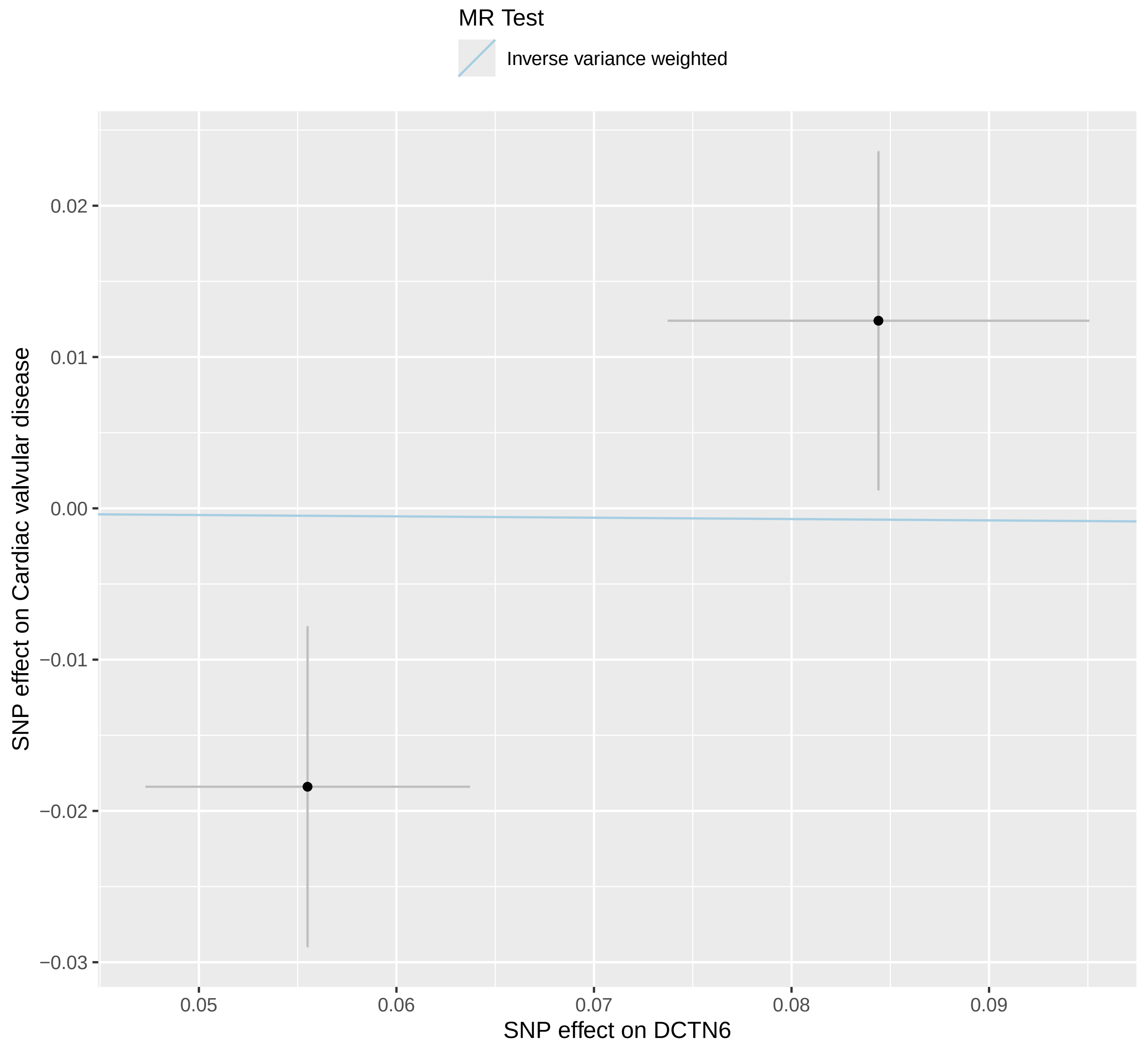

MR Test
Inverse variance weighted
0.02
0.01
0.00
−0.01
−0.02
−0.03
SNP effect on Cardiac valvular disease
0.05
0.06
0.07
0.08
0.09
SNP effect on DCTN6

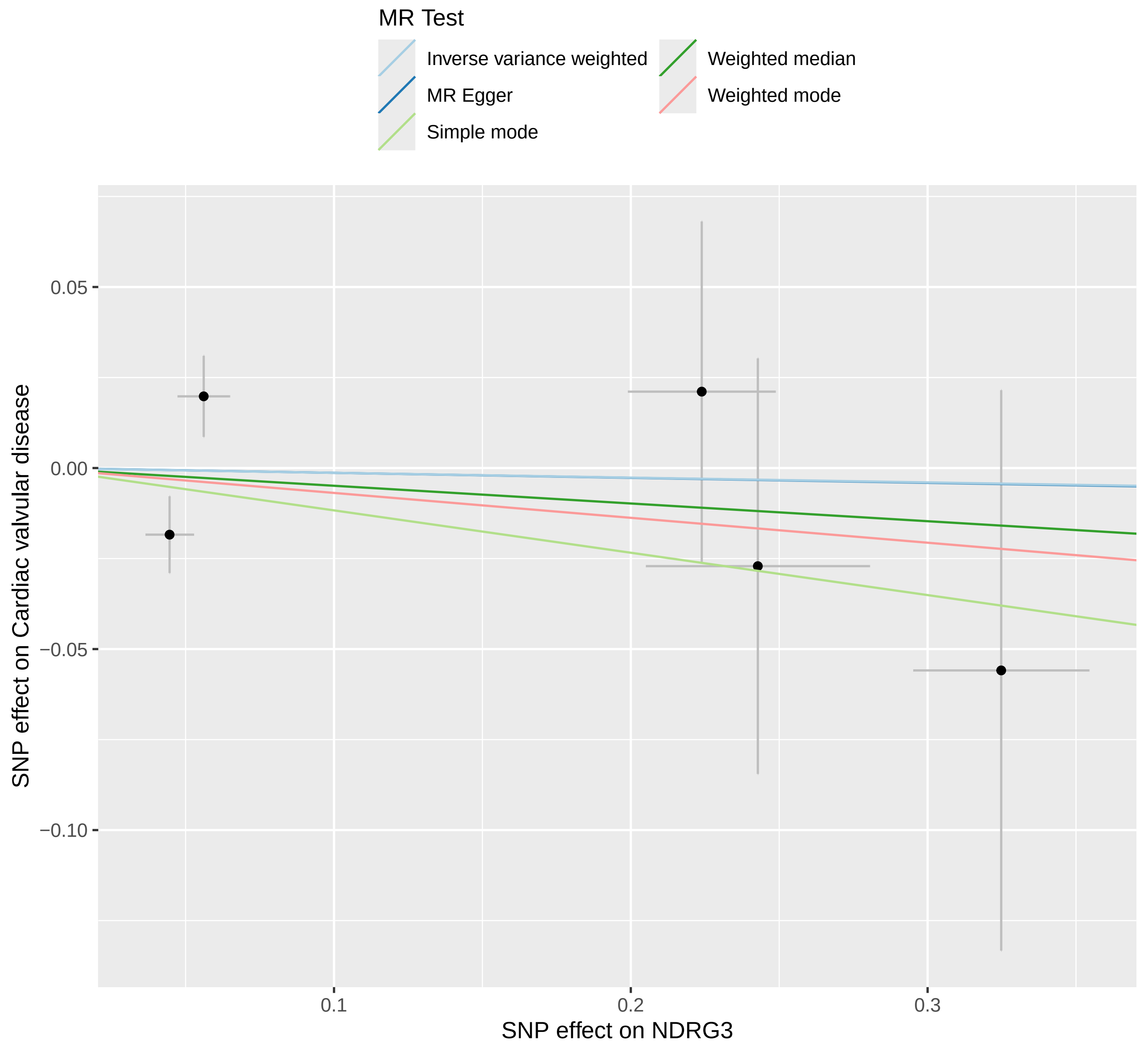

MR Test
Inverse variance weighted
MR Egger
Simple mode
Weighted median
Weighted mode
0.05
0.00
−0.05
−0.10
0.1
0.2
0.3
SNP effect on Cardiac valvular disease
SNP effect on NDRG3

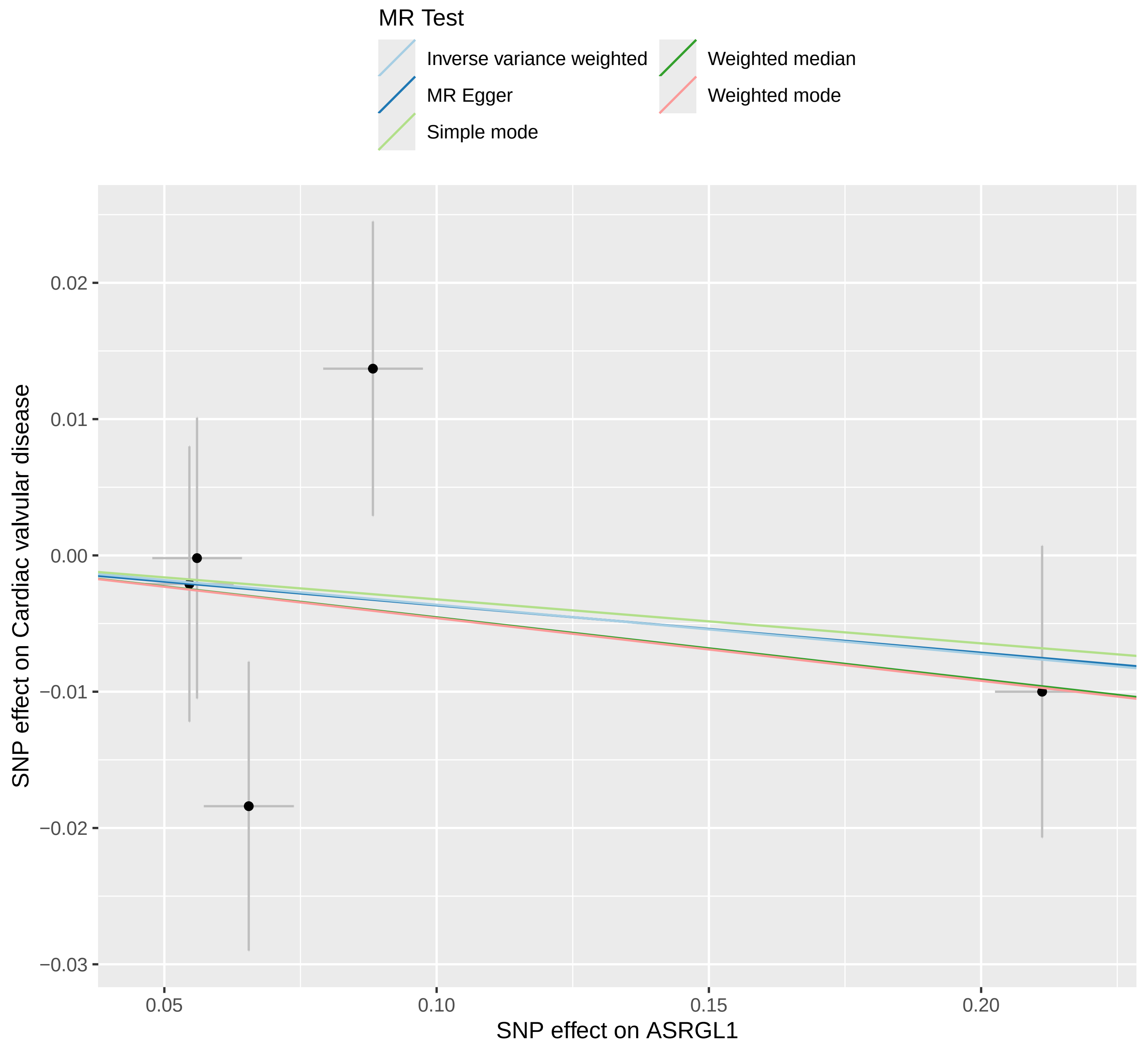
MR Test
Inverse variance weighted
MR Egger
Simple mode
Weighted median
Weighted mode
0.02
0.01
0.00
−0.01
−0.02
−0.03
0.05
0.10
0.15
0.20
SNP effect on Cardiac valvular disease
SNP effect on ASRGL1

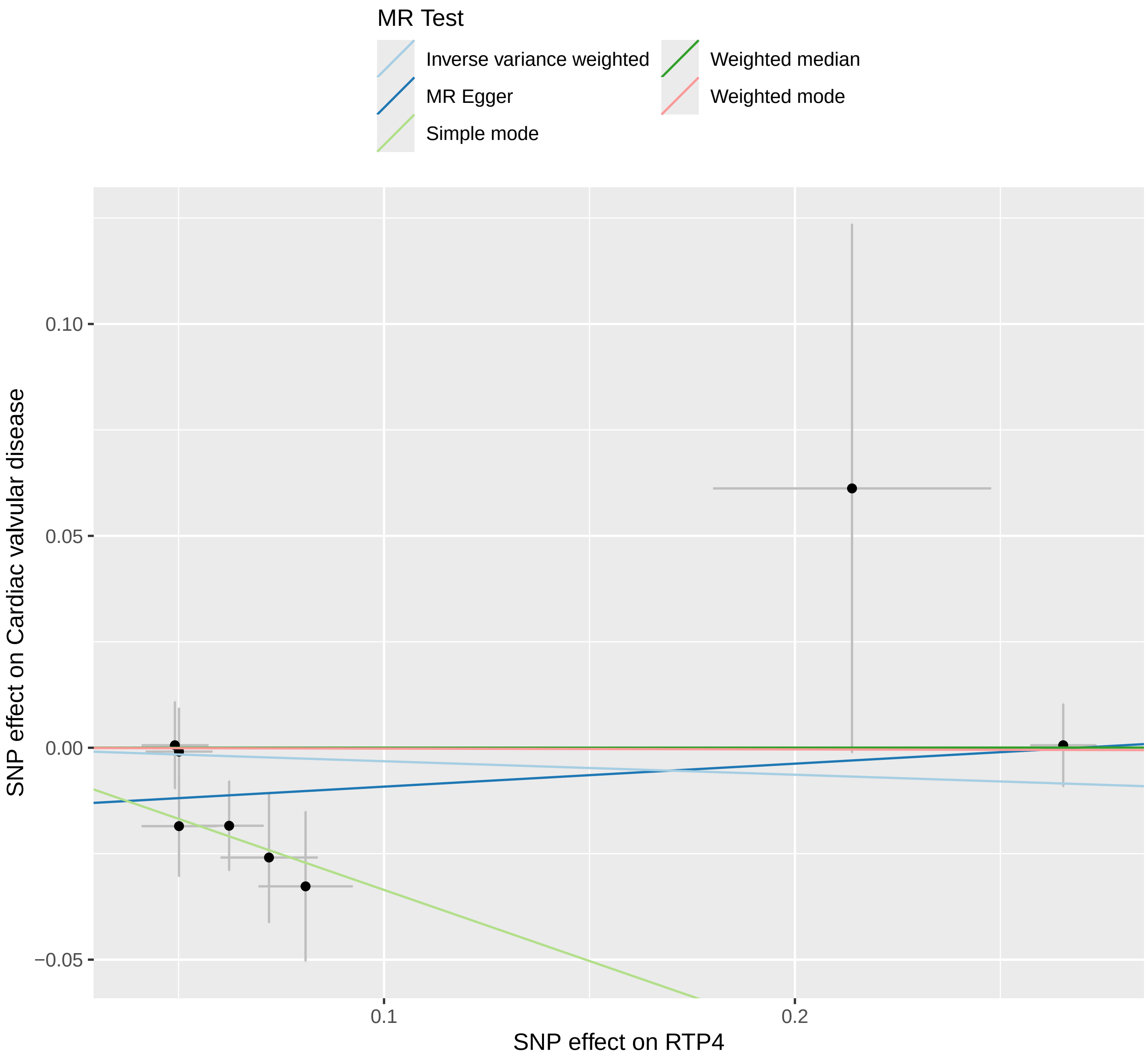

MR Test
Inverse variance weighted
MR Egger
Simple mode
Weighted median
Weighted mode
0.10
0.05
0.00
−0.05
0.1
0.2
SNP effect on Cardiac valvular disease
SNP effect on RTP4

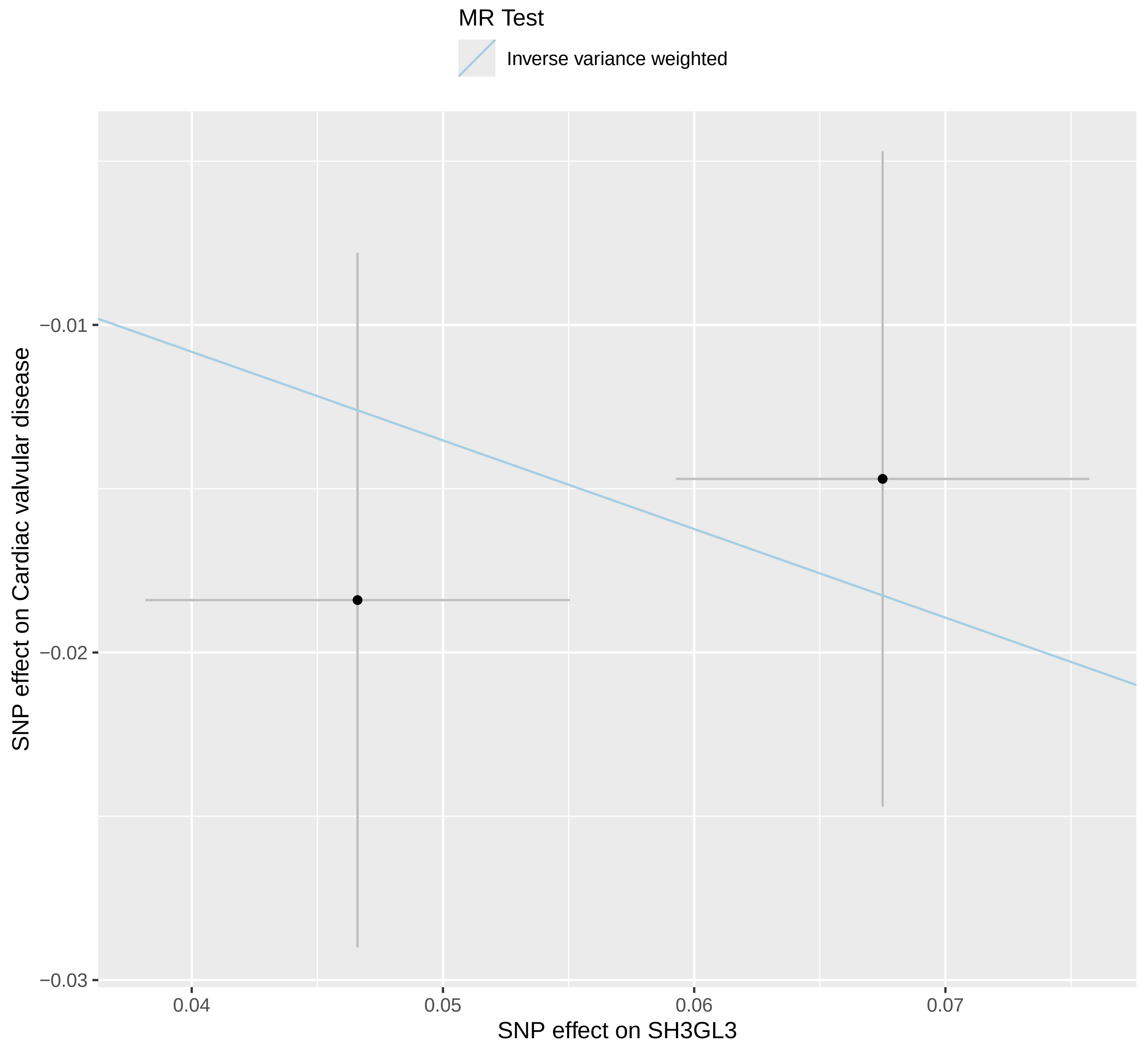
MR Test
Inverse variance weighted
−0.01
−0.02
−0.03
0.04
0.05
0.06
0.07
SNP effect on Cardiac valvular disease
SNP effect on SH3GL3

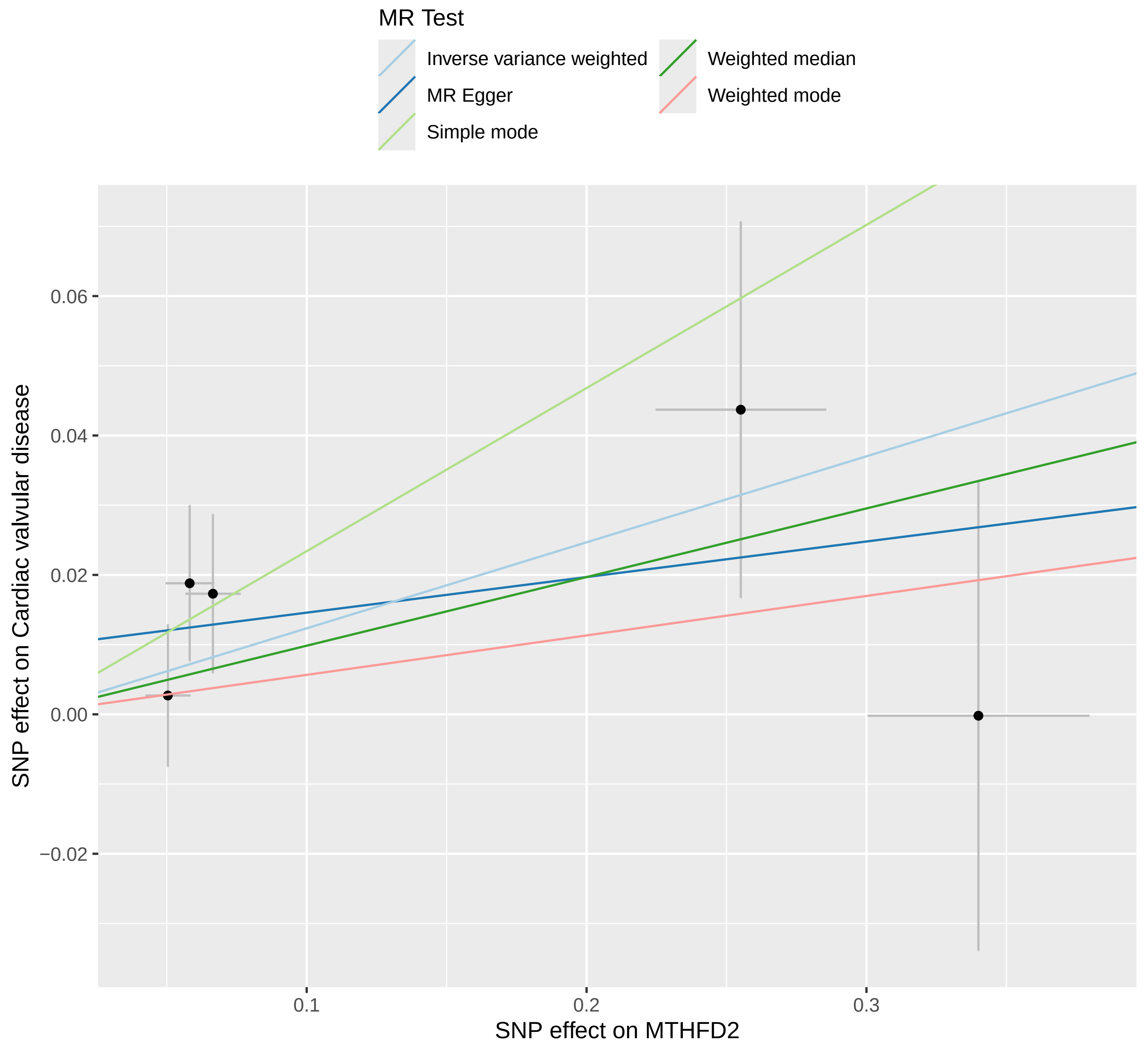

MR Test
Inverse variance weighted
MR Egger
Simple mode
Weighted median
Weighted mode
0.06
0.04
0.02
0.00
−0.02
0.1
0.2
0.3
SNP effect on Cardiac valvular disease
SNP effect on MTHFD2

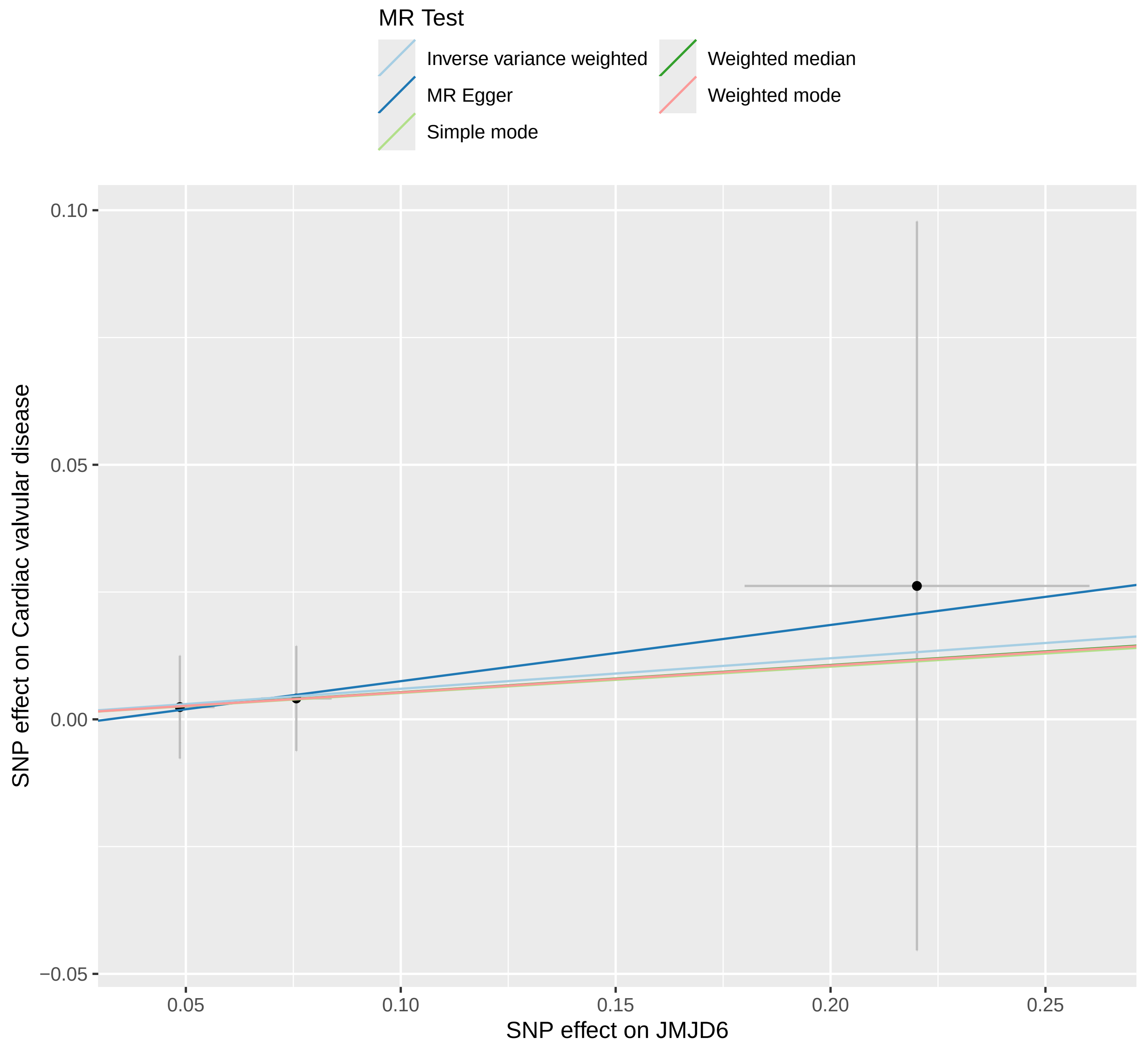
MR Test
Inverse variance weighted
MR Egger
Simple mode
Weighted median
Weighted mode
0.10
0.05
0.00
−0.05
SNP effect on Cardiac valvular disease
0.05
0.10
0.15
0.20
0.25
SNP effect on JMJD6

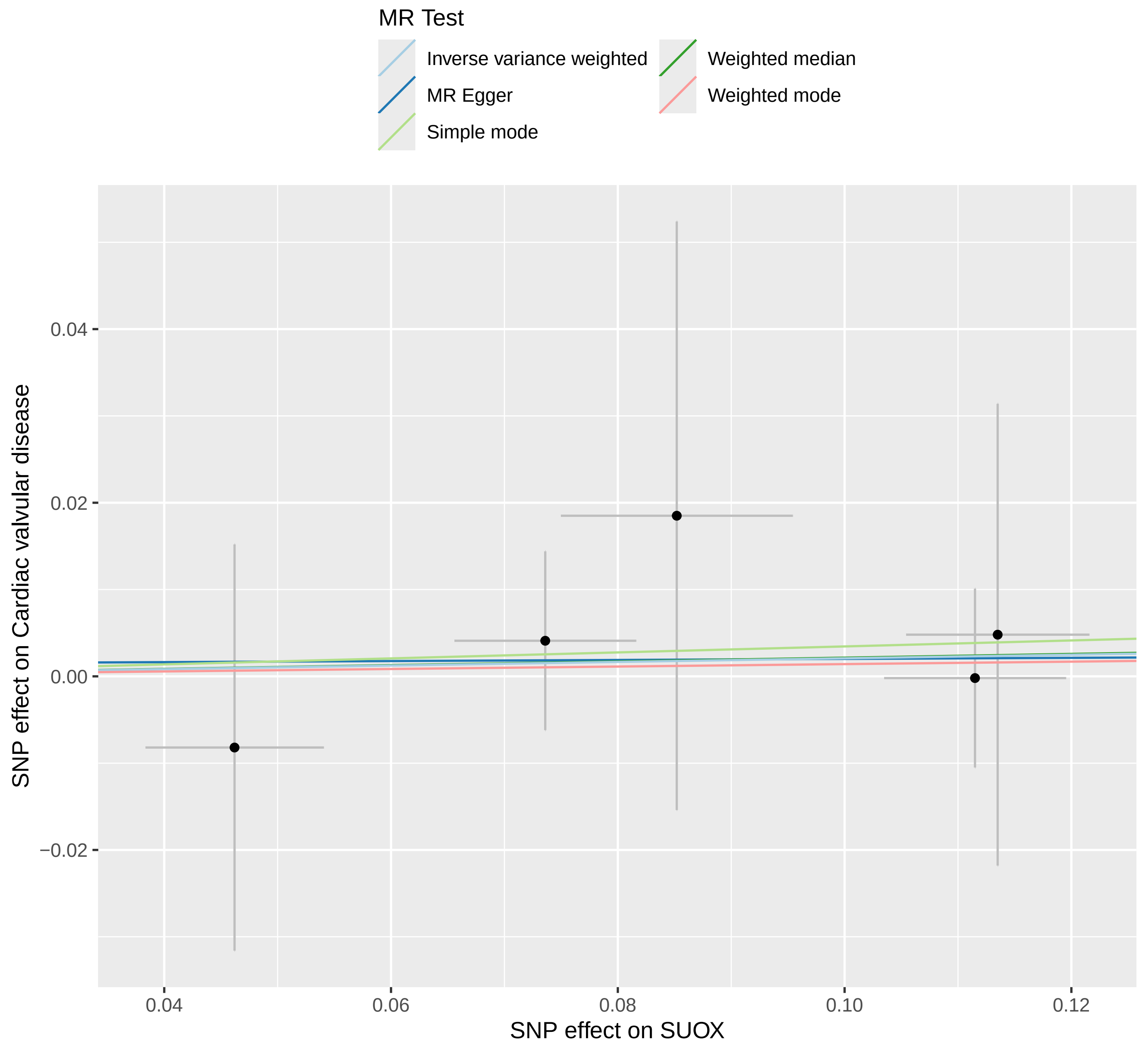

MR Test
Inverse variance weighted
MR Egger
Simple mode
Weighted median
Weighted mode
0.04
0.02
0.00
−0.02
SNP effect on Cardiac valvular disease
0.04
0.06
0.08
0.10
0.12
SNP effect on SUOX

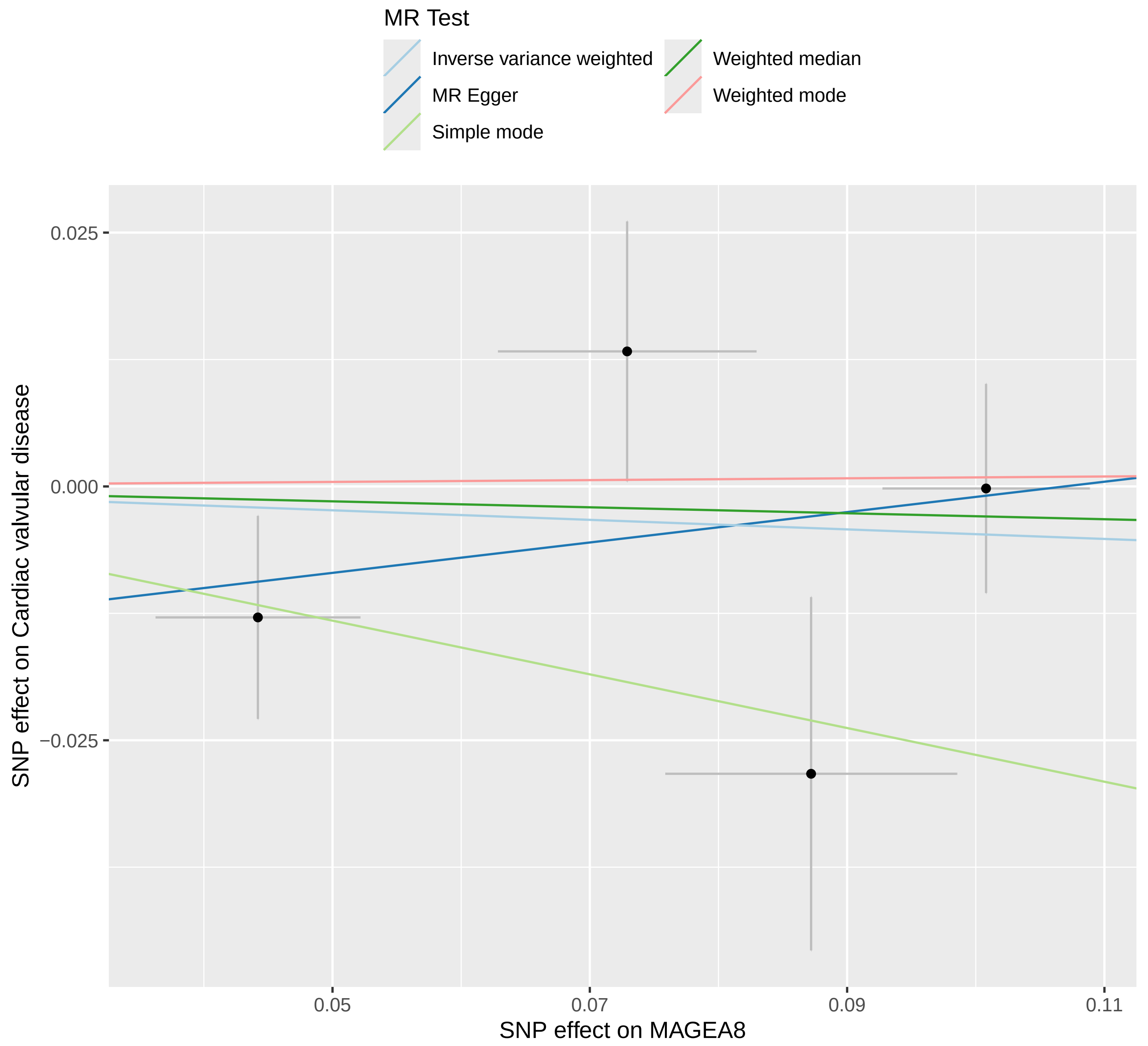
MR Test
Inverse variance weighted
MR Egger
Simple mode
Weighted median
Weighted mode
0.025
0.000
−0.025
0.05
0.07
0.09
0.11
SNP effect on Cardiac valvular disease
SNP effect on MAGEA8

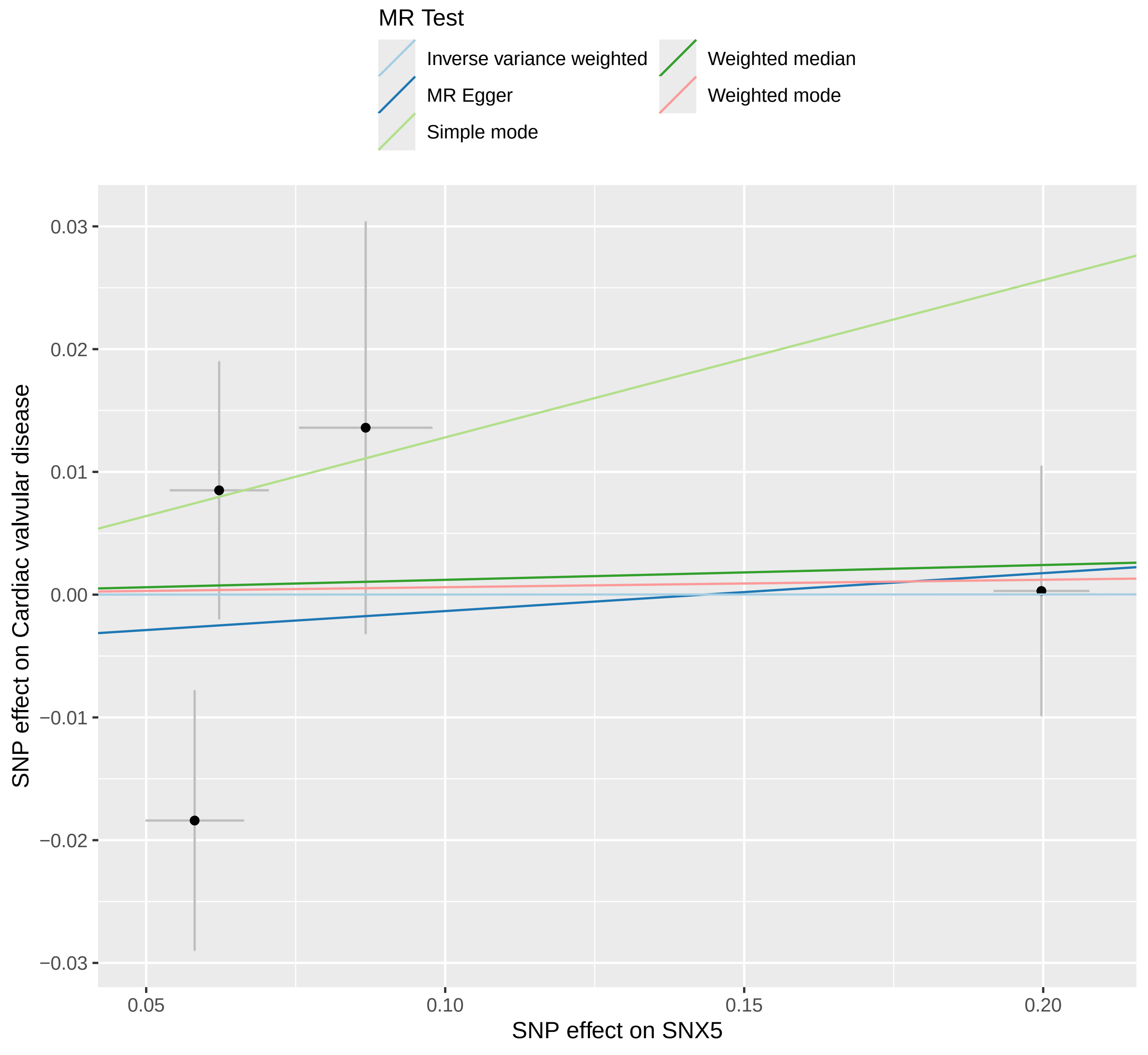
MR Test
Inverse variance weighted
MR Egger
Simple mode
Weighted median
Weighted mode
0.03
0.02
0.01
0.00
−0.01
−0.02
−0.03
SNP effect on Cardiac valvular disease
0.05
0.10
0.15
0.20
SNP effect on SNX5

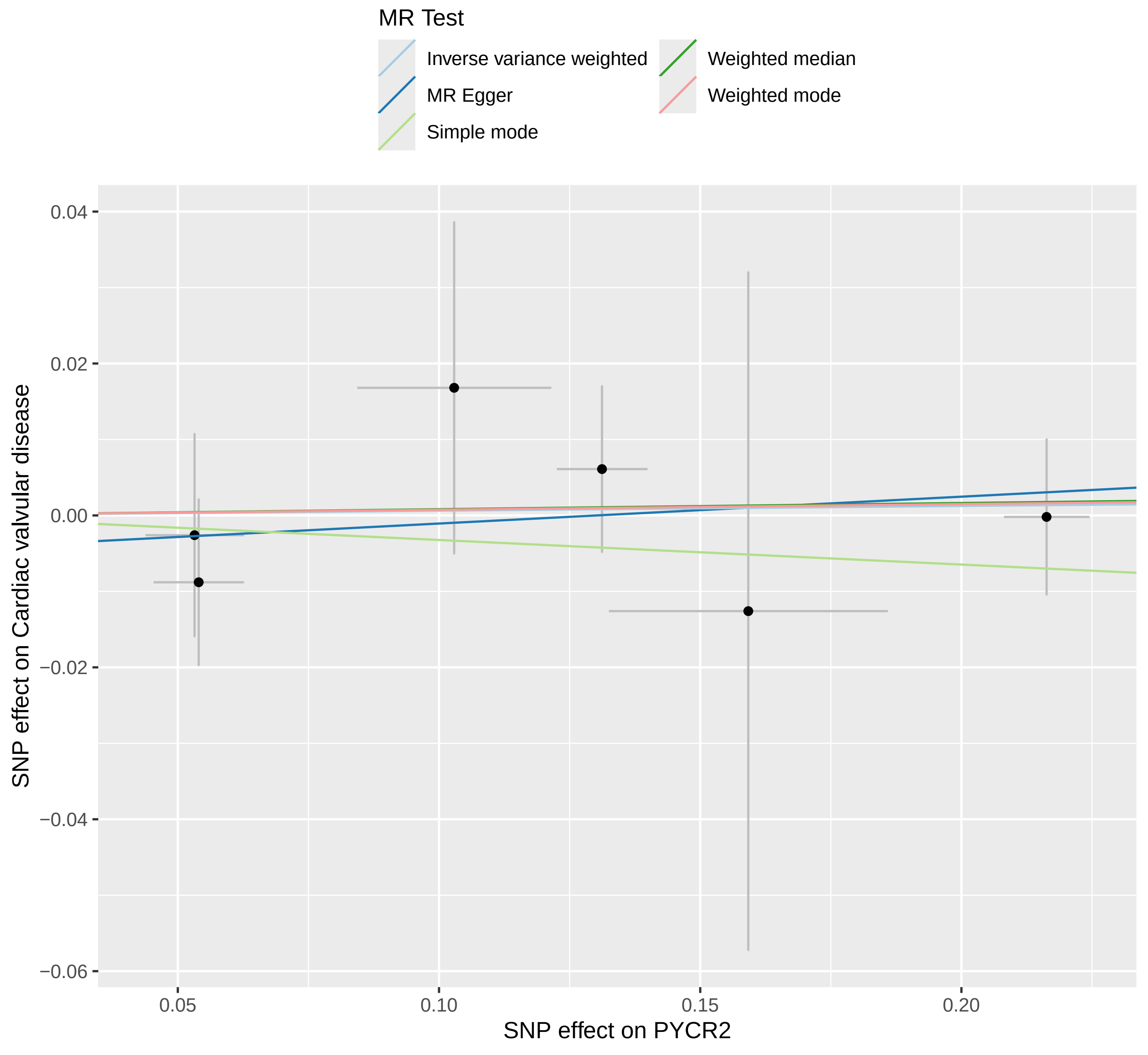
MR Test
Inverse variance weighted
MR Egger
Simple mode
Weighted median
Weighted mode
0.04
0.02
0.00
−0.02
−0.04
−0.06
SNP effect on Cardiac valvular disease
0.05
0.10
0.15
0.20
SNP effect on PYCR2

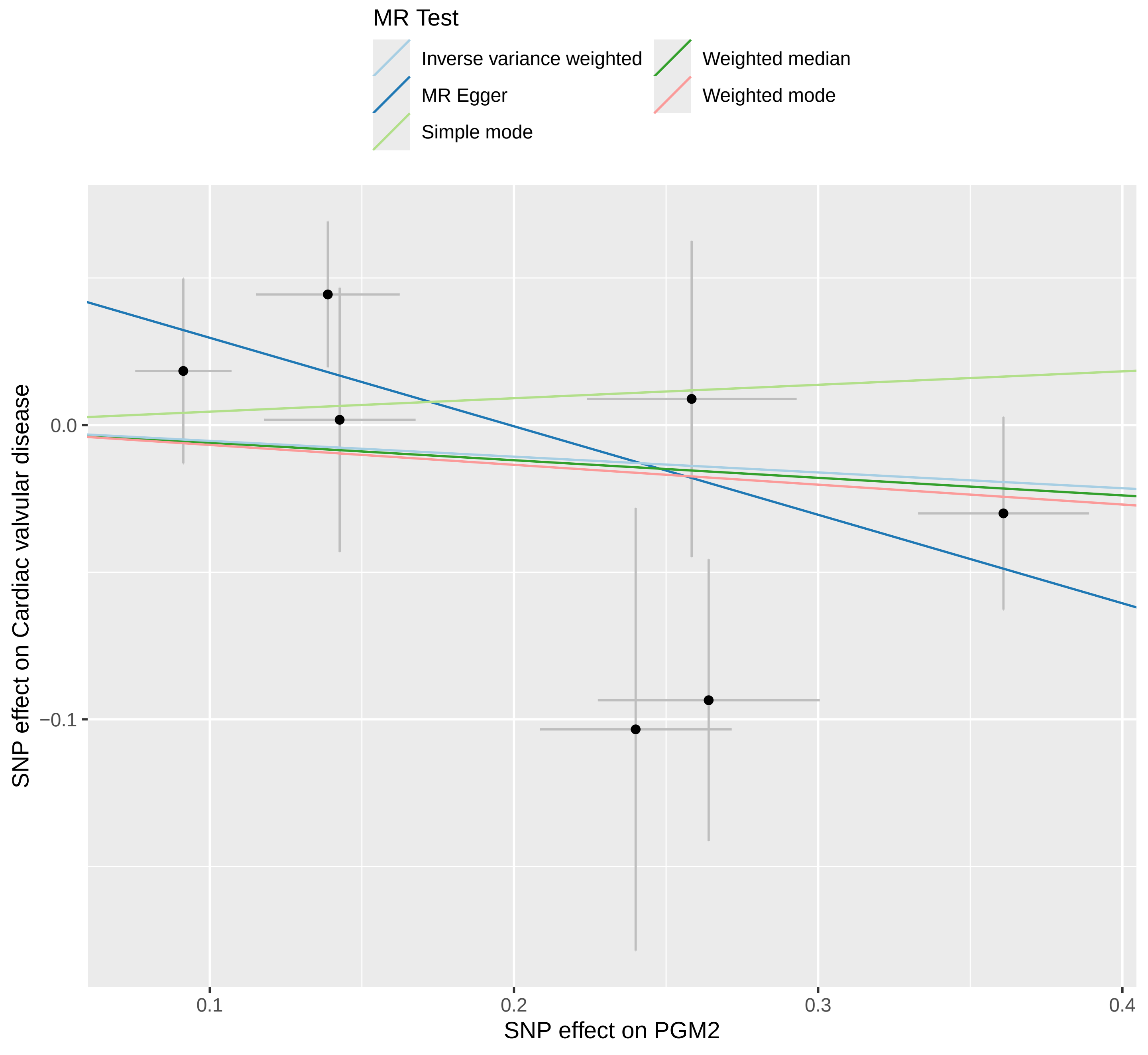

MR Test
Inverse variance weighted
MR Egger
Simple mode
Weighted median
Weighted mode
SNP effect on Cardiac valvular disease
0.0
−0.1
0.1
0.2
0.3
0.4
SNP effect on PGM2

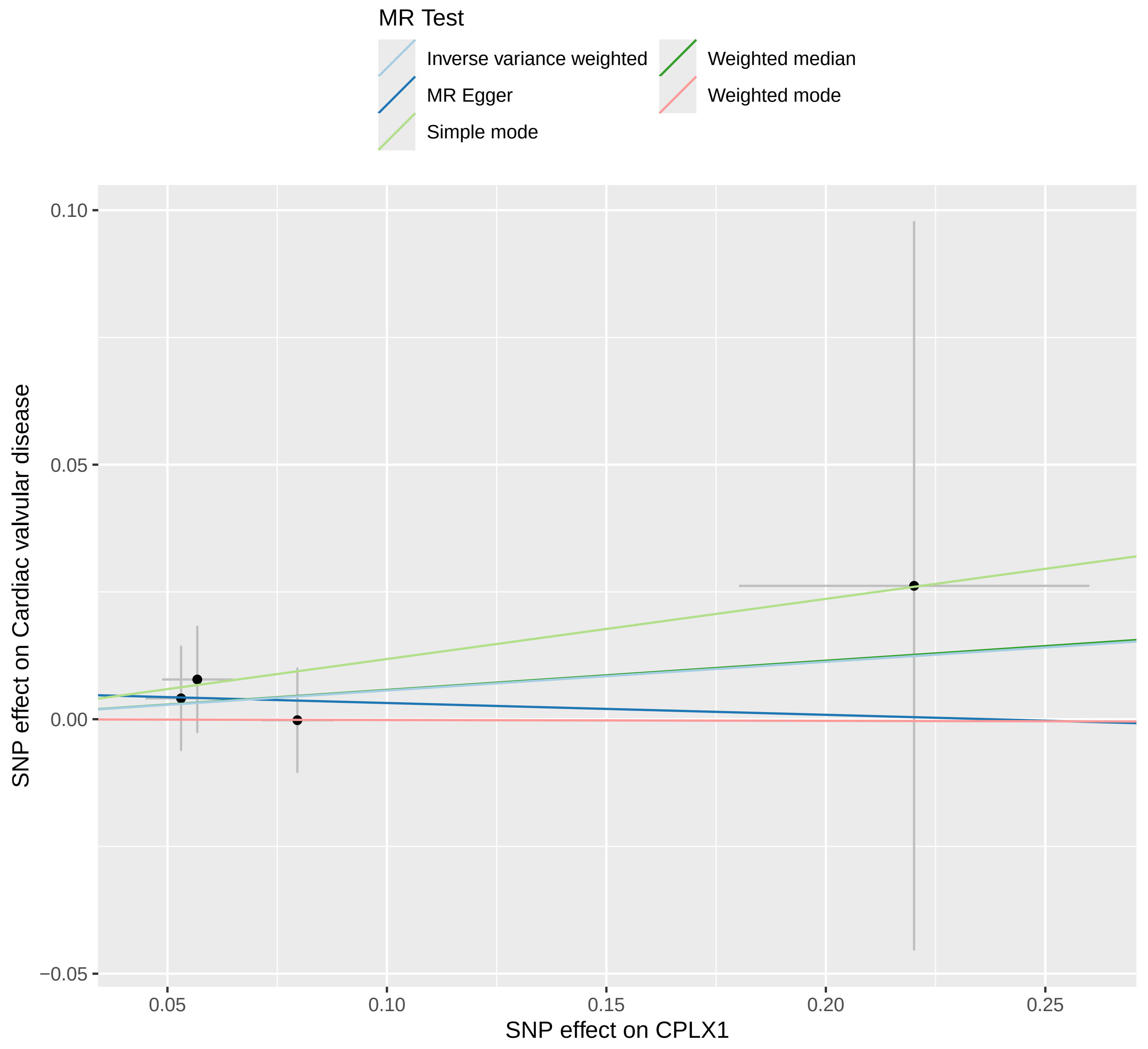
MR Test
Inverse variance weighted
MR Egger
Simple mode
Weighted median
Weighted mode
0.10
0.05
0.00
−0.05
0.05
0.10
0.15
0.20
0.25
SNP effect on Cardiac valvular disease
SNP effect on CPLX1

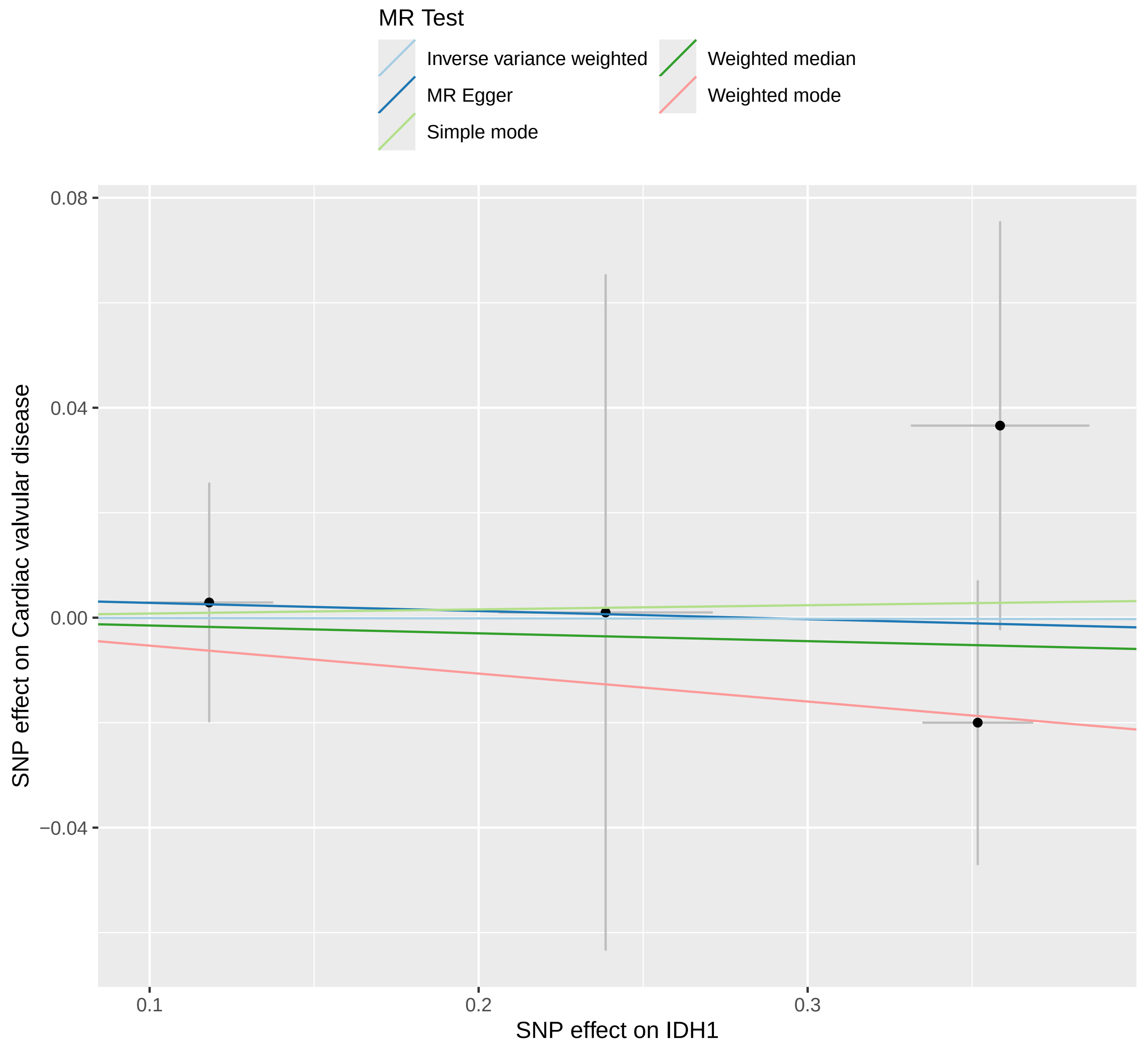

MR Test
Inverse variance weighted
MR Egger
Simple mode
Weighted median
Weighted mode
0.08
0.04
0.00
−0.04
SNP effect on Cardiac valvular disease
0.1
0.2
0.3
SNP effect on IDH1

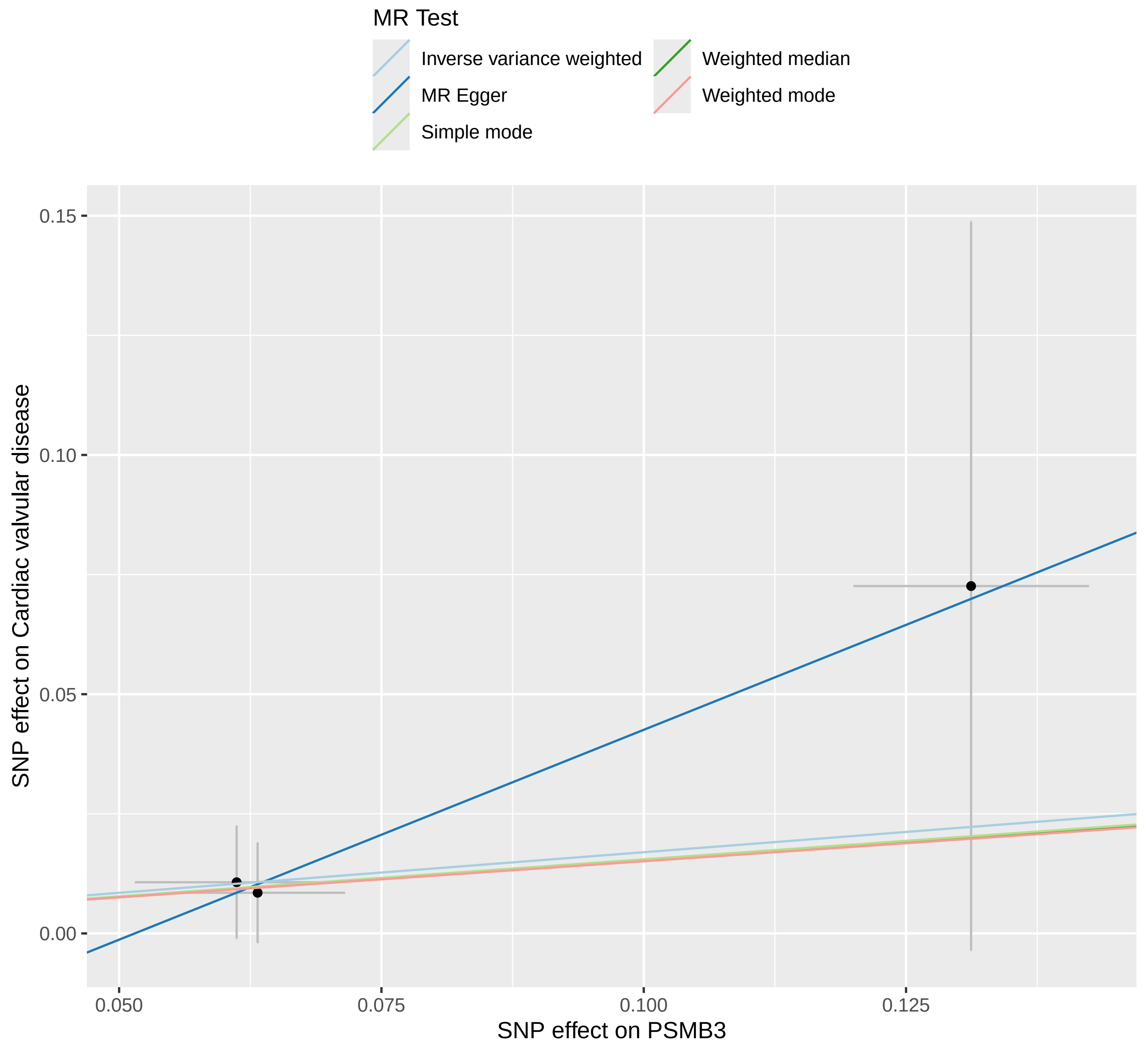
MR Test
Inverse variance weighted
MR Egger
Simple mode
Weighted median
Weighted mode
SNP effect on Cardiac valvular disease
0.15
0.10
0.05
0.00
0.050
0.075
0.100
0.125
SNP effect on PSMB3

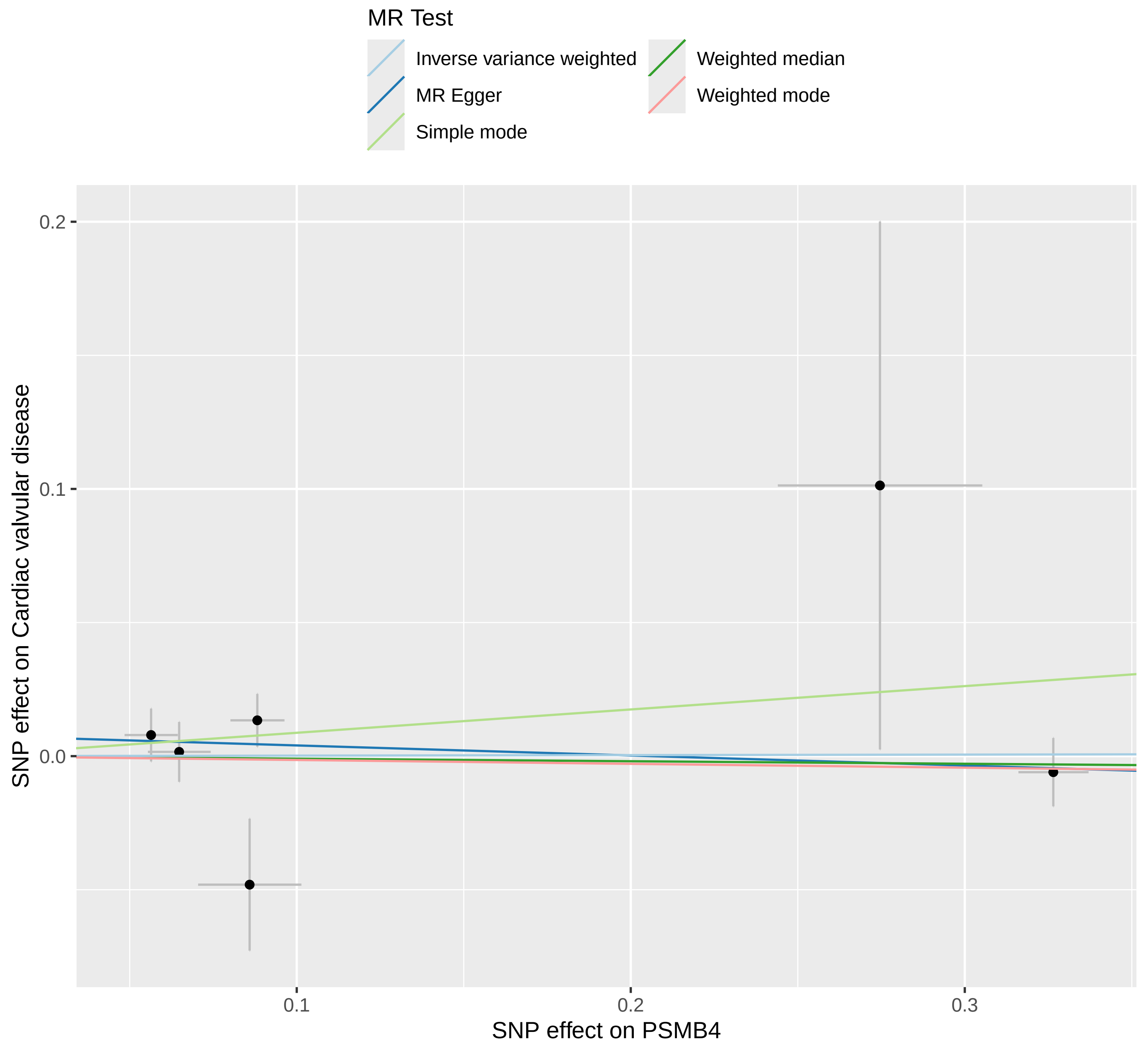
MR Test
Inverse variance weighted
MR Egger
Simple mode
Weighted median
Weighted mode
0.2
0.1
0.0
0.1
0.2
0.3
SNP effect on Cardiac valvular disease
SNP effect on PSMB4

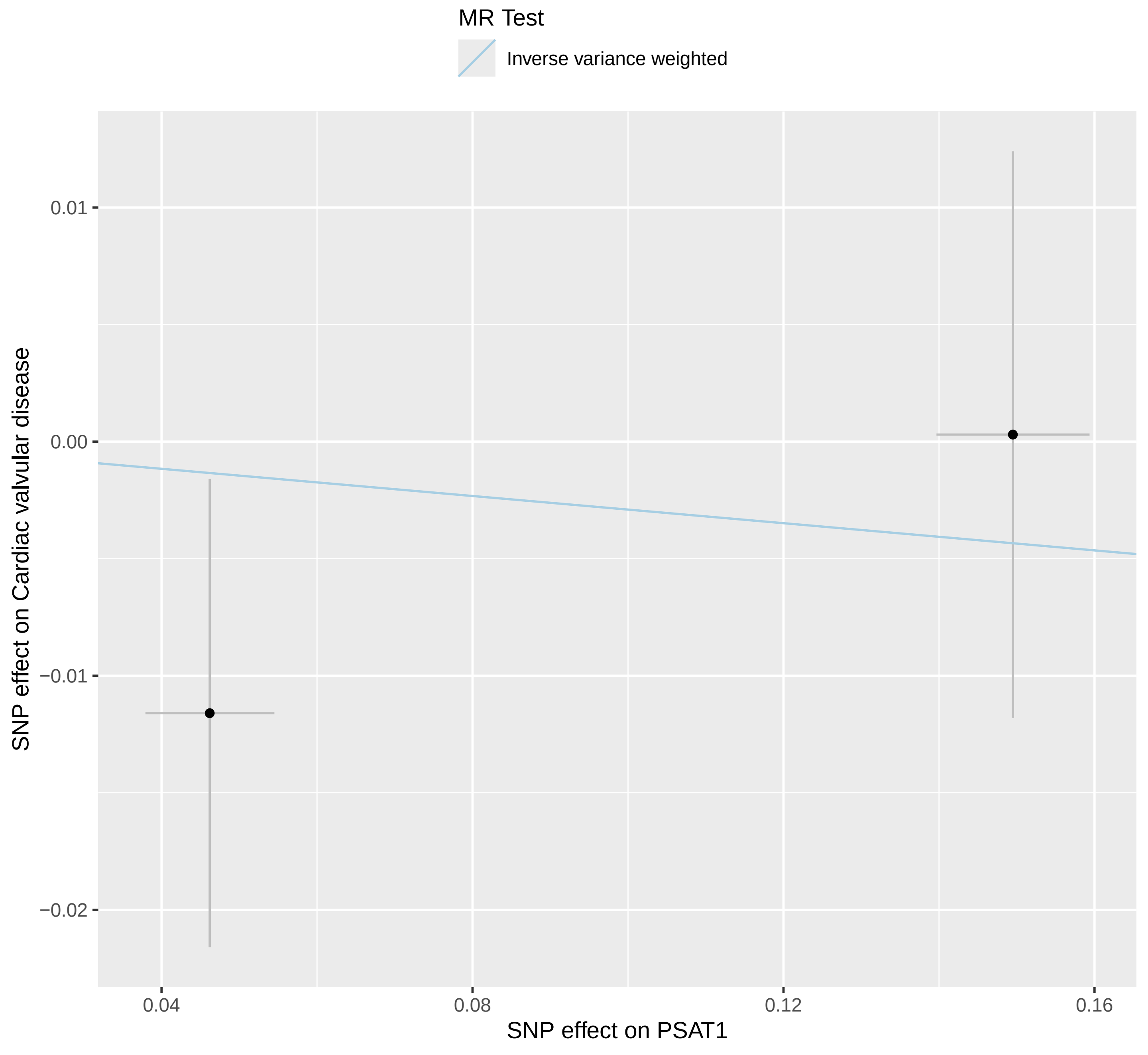
MR Test
Inverse variance weighted
SNP effect on Cardiac valvular disease
0.01
0.00
−0.01
−0.02
0.04
0.08
0.12
0.16
SNP effect on PSAT1

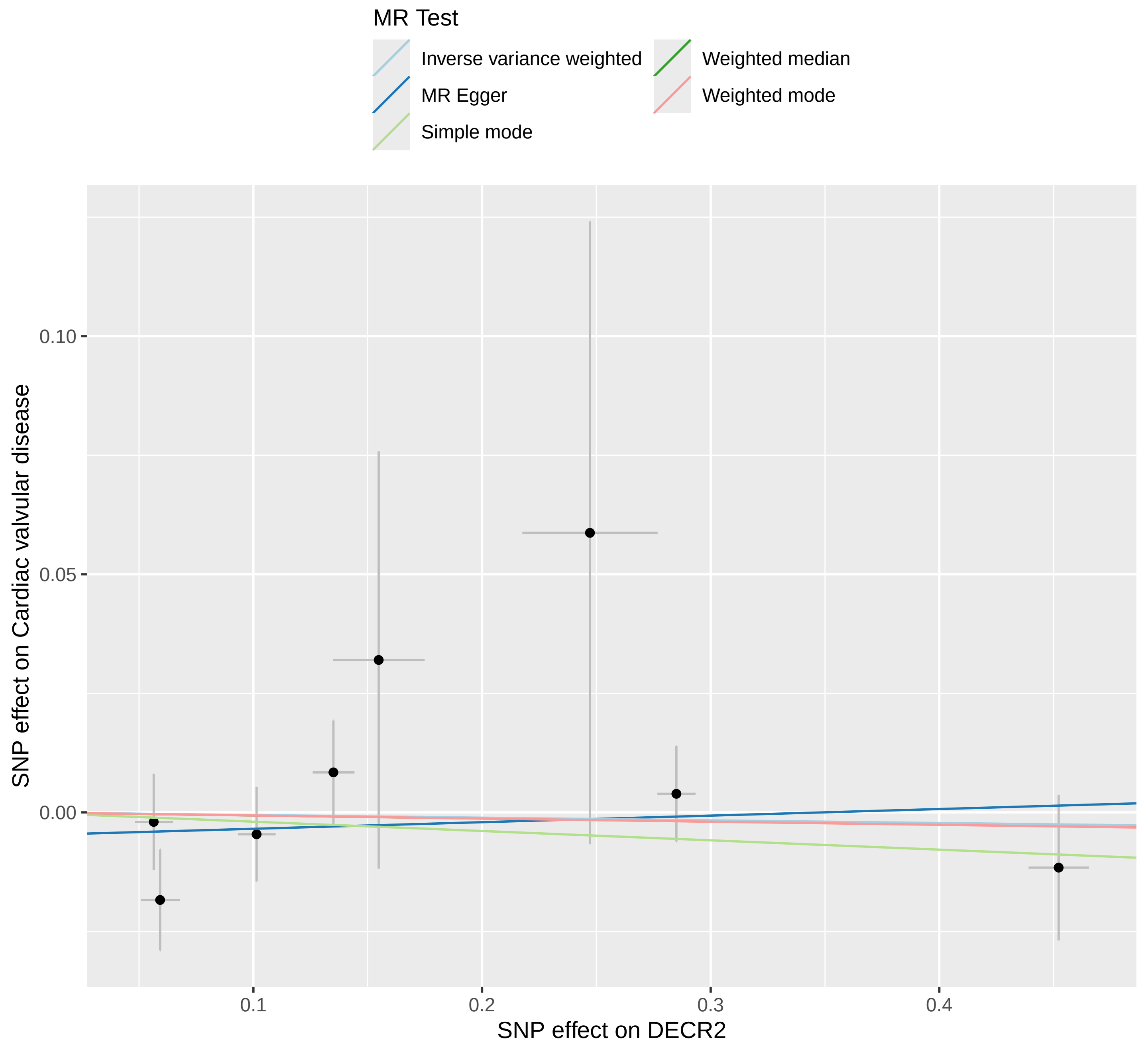
MR Test
Inverse variance weighted
MR Egger
Simple mode
Weighted median
Weighted mode
0.10
0.05
0.00
0.1
0.2
0.3
0.4
SNP effect on Cardiac valvular disease
SNP effect on DECR2

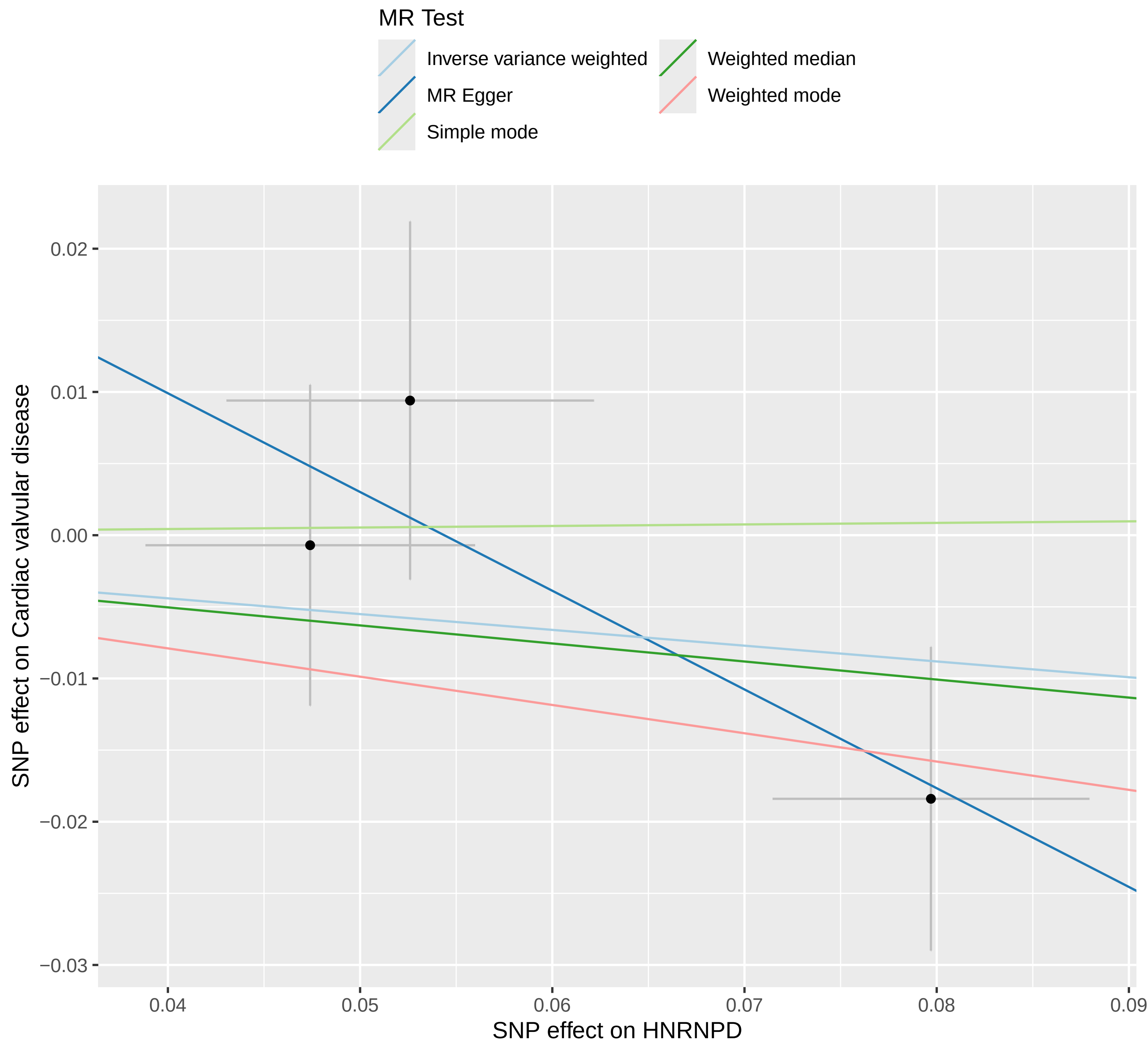
MR Test
Inverse variance weighted
MR Egger
Simple mode
Weighted median
Weighted mode
0.02
0.01
0.00
−0.01
−0.02
−0.03
SNP effect on Cardiac valvular disease
0.04
0.05
0.06
0.07
0.08
0.09
SNP effect on HNRNPD

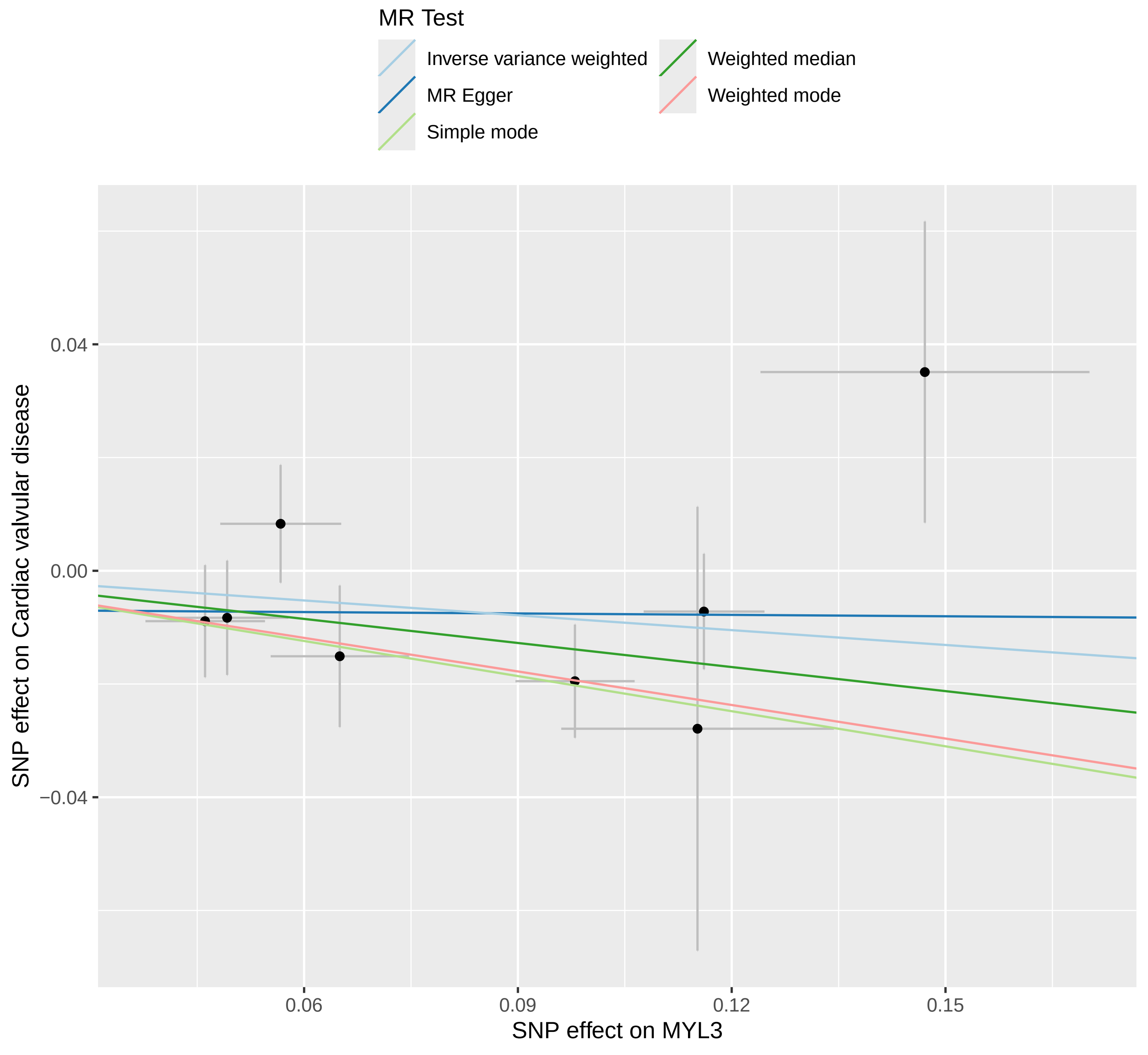

MR Test
Inverse variance weighted
MR Egger
Simple mode
Weighted median
Weighted mode
0.04
0.00
−0.04
SNP effect on Cardiac valvular disease
0.06
0.09
0.12
0.15
SNP effect on MYL3

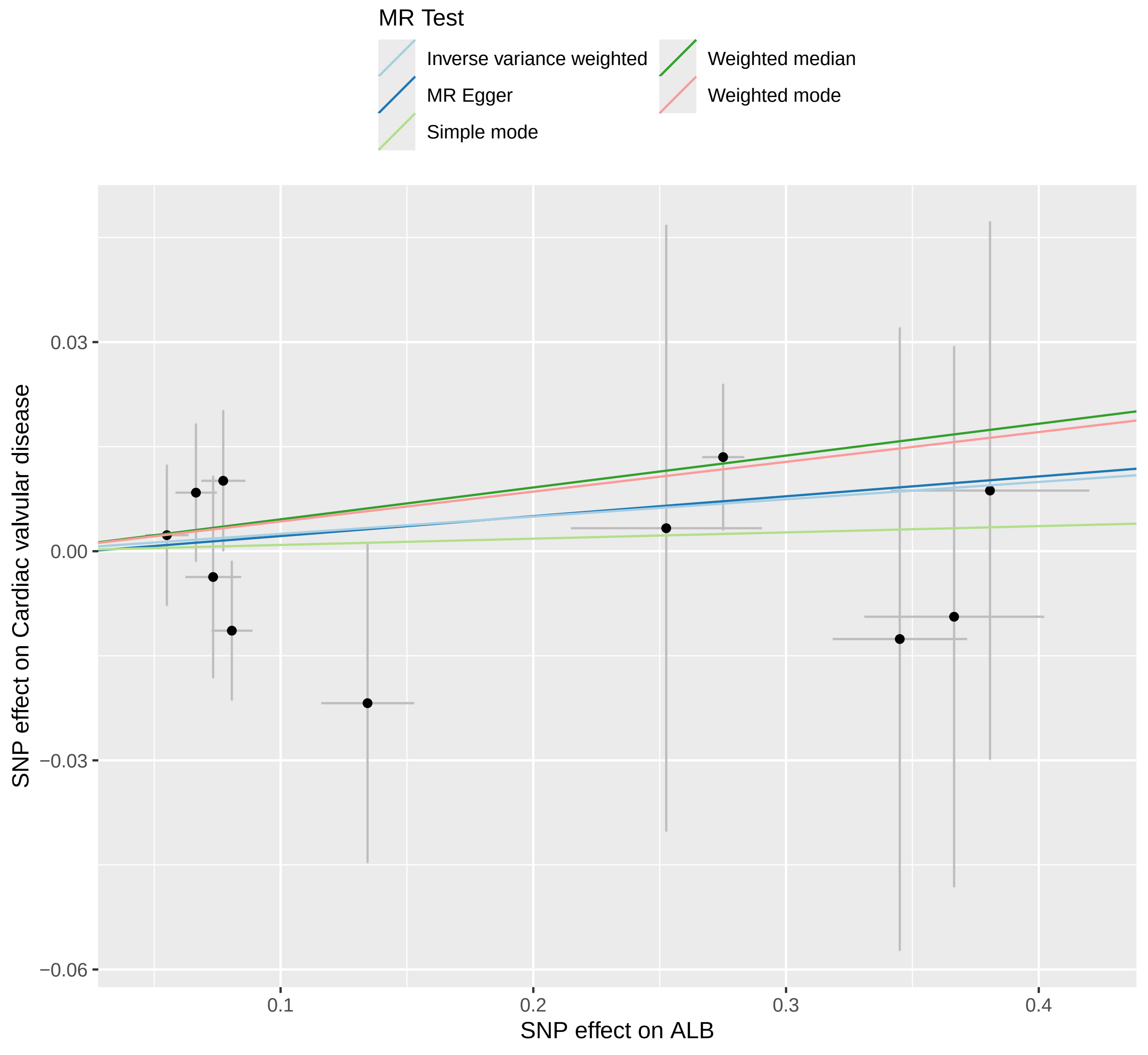
MR Test
Inverse variance weighted
MR Egger
Simple mode
Weighted median
Weighted mode
0.03
0.00
−0.03
−0.06
0.1
0.2
0.3
0.4
SNP effect on ALB
SNP effect on Cardiac valvular disease

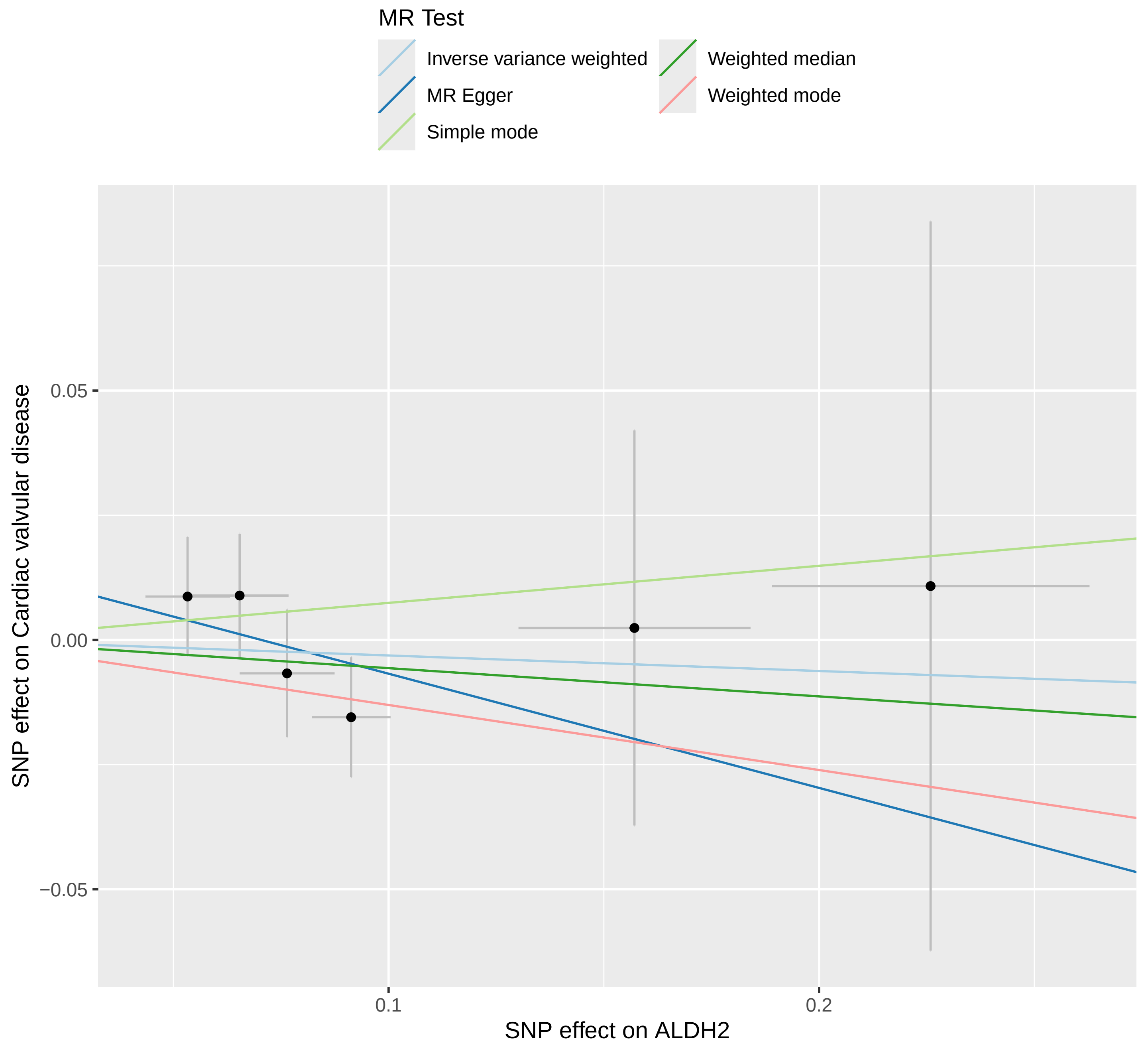

MR Test
Inverse variance weighted
MR Egger
Simple mode
Weighted median
Weighted mode
0.05
0.00
−0.05
0.1
0.2
SNP effect on Cardiac valvular disease
SNP effect on ALDH2

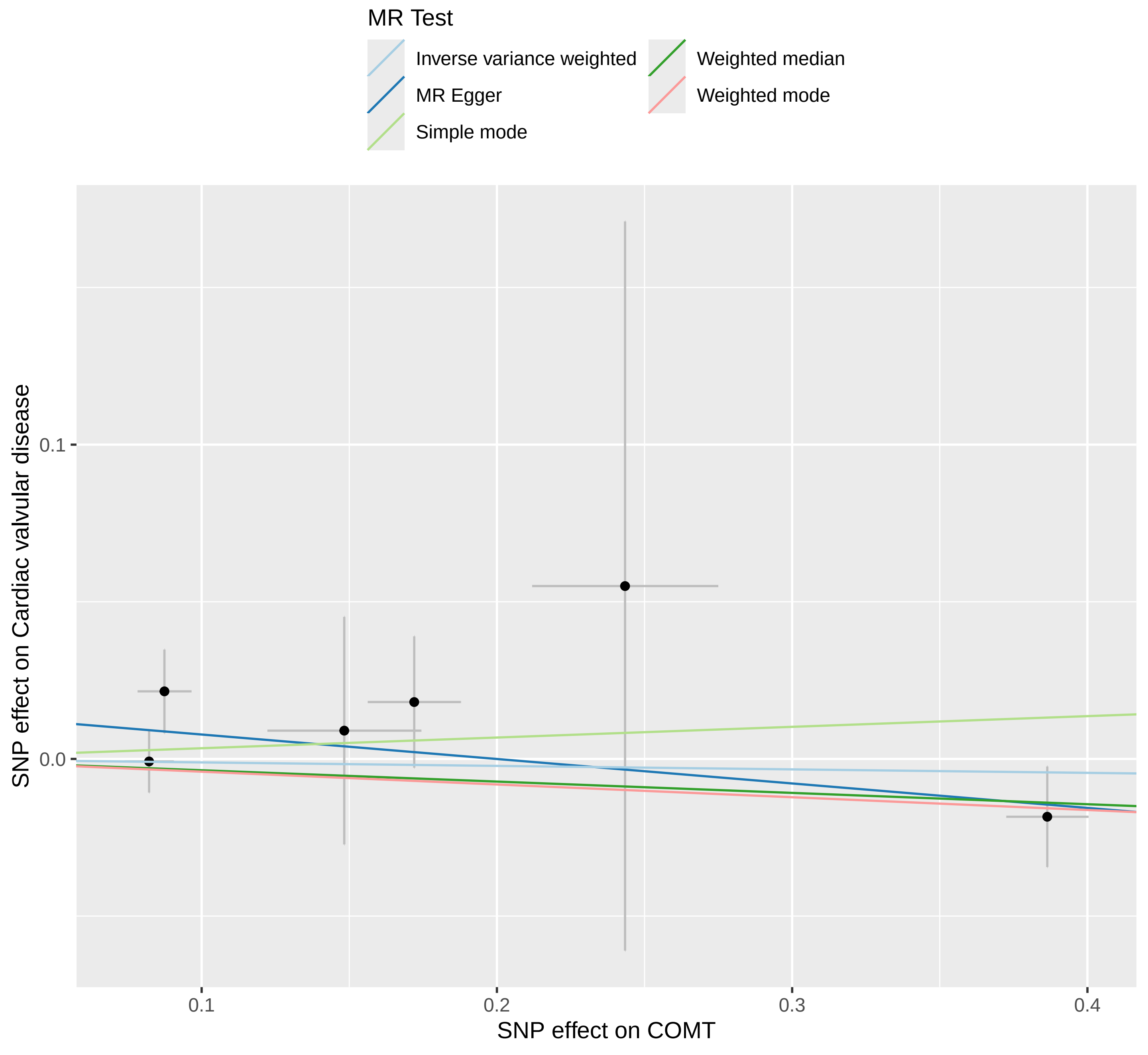
MR Test
Inverse variance weighted
MR Egger
Simple mode
Weighted median
Weighted mode
0.1
0.0
0.1
0.2
0.3
0.4
SNP effect on Cardiac valvular disease
SNP effect on COMT

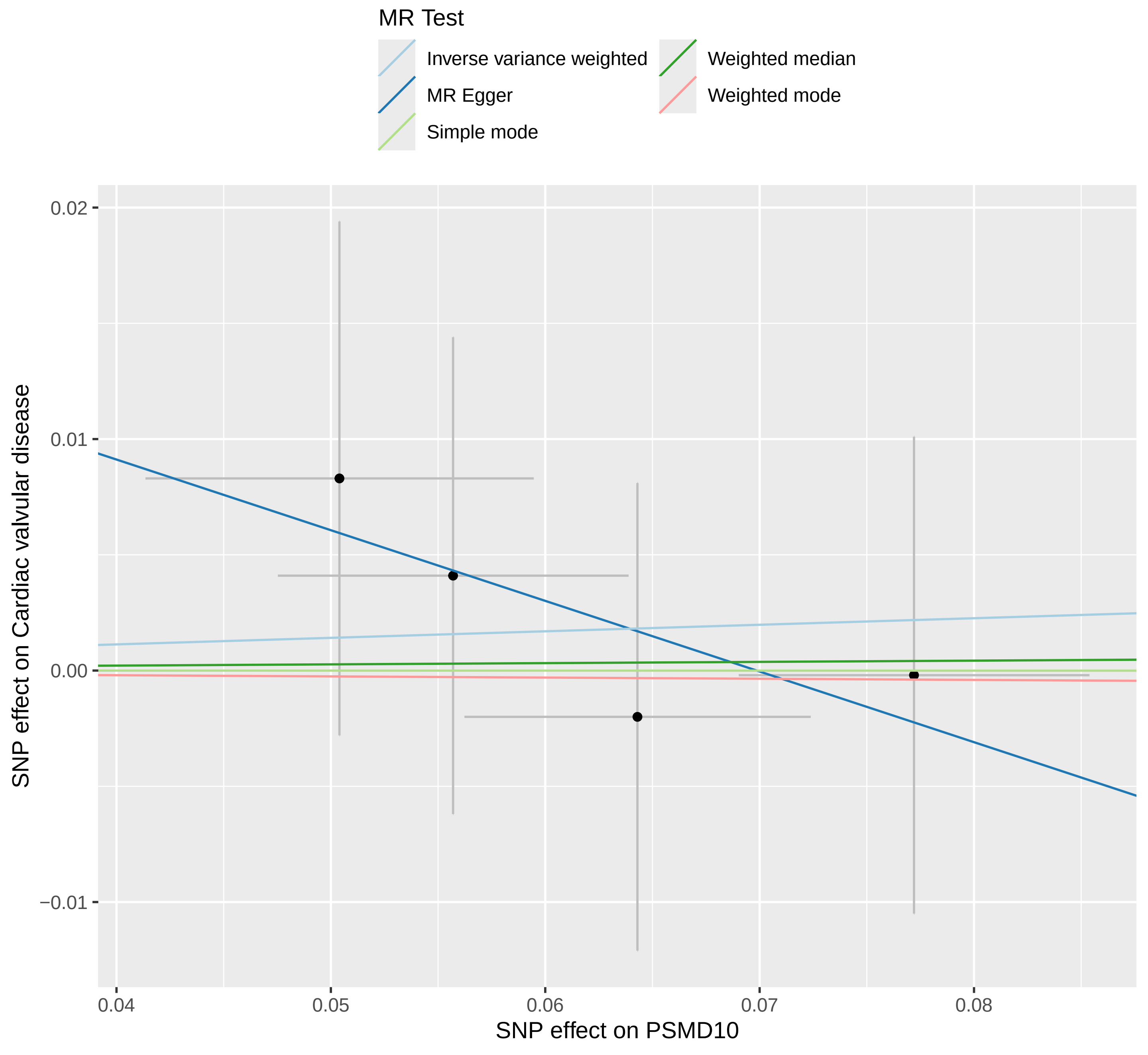
MR Test
Inverse variance weighted
MR Egger
Simple mode
Weighted median
Weighted mode
0.02
0.01
0.00
−0.01
0.04
0.05
0.06
0.07
0.08
SNP effect on Cardiac valvular disease
SNP effect on PSMD10

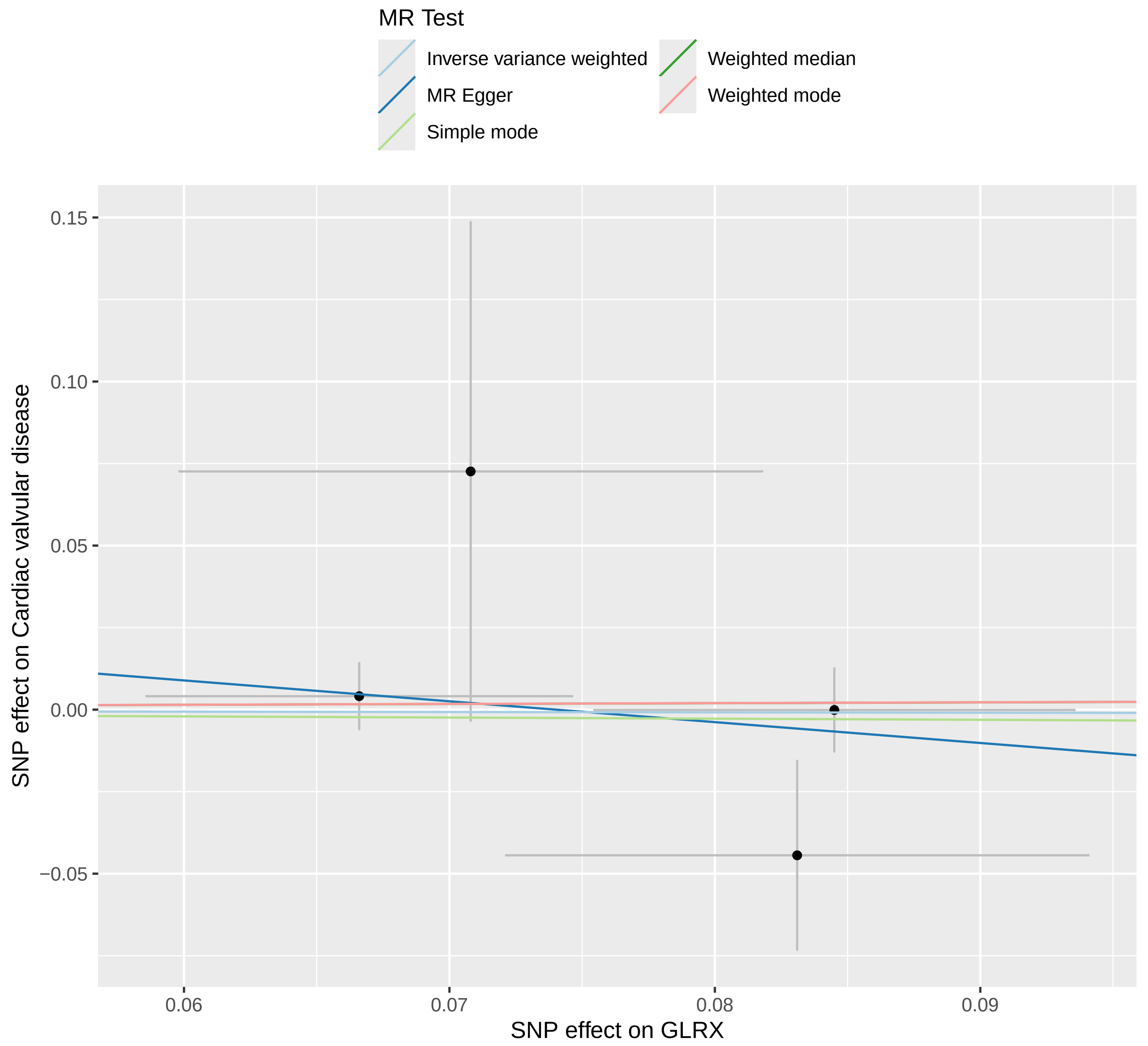
MR Test
Inverse variance weighted
MR Egger
Simple mode
Weighted median
Weighted mode
0.15
0.10
0.05
0.00
−0.05
SNP effect on Cardiac valvular disease
0.06
0.07
0.08
0.09
SNP effect on GLRX

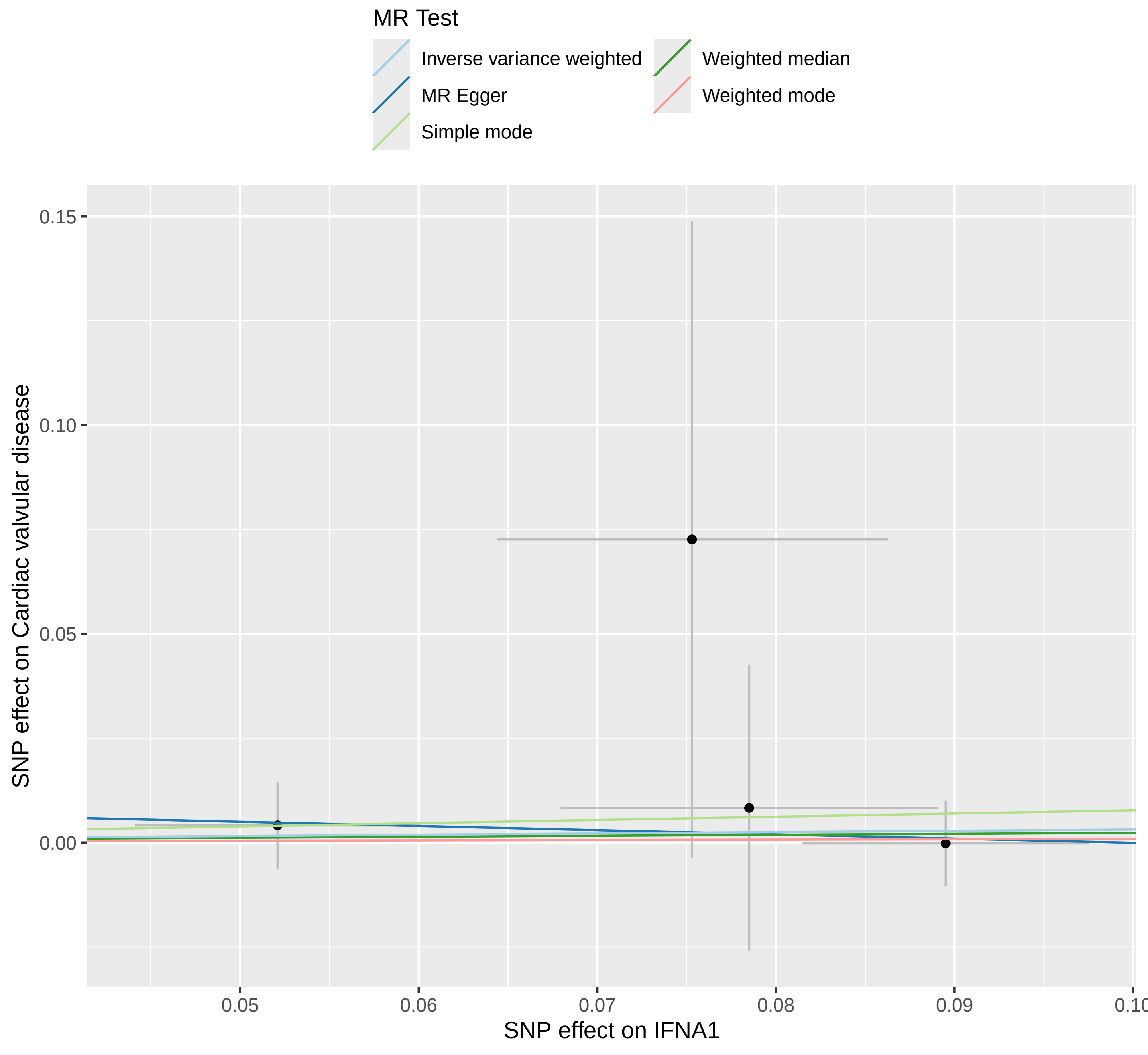

MR Test
Inverse variance weighted
MR Egger
Simple mode
Weighted median
Weighted mode
0.15
0.10
0.05
0.00
SNP effect on Cardiac valvular disease
0.05
0.06
0.07
0.08
0.09
SNP effect on IFNA1

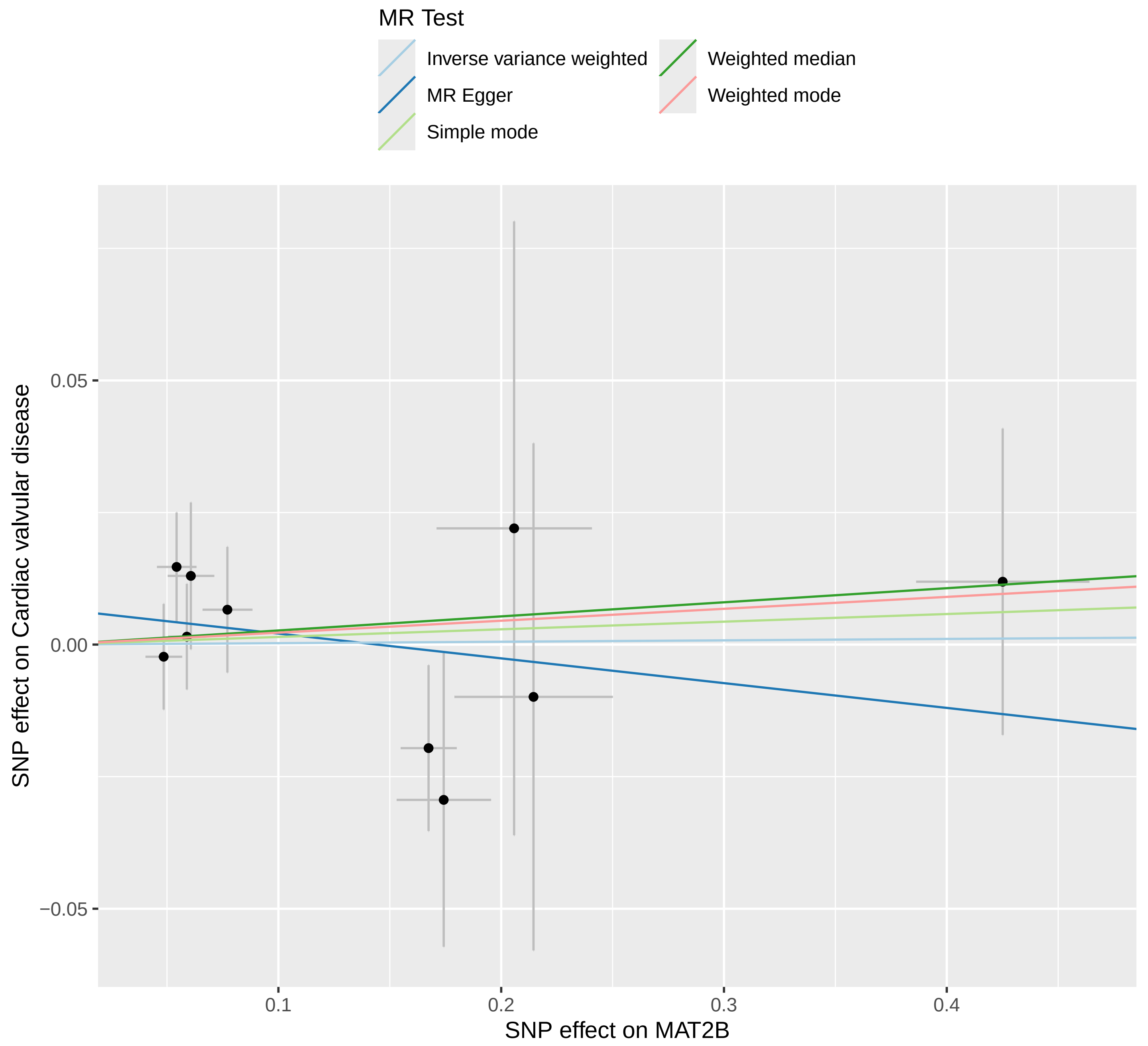
MR Test
Inverse variance weighted
MR Egger
Simple mode
Weighted median
Weighted mode
0.05
0.00
−0.05
0.1
0.2
0.3
0.4
SNP effect on Cardiac valvular disease
SNP effect on MAT2B

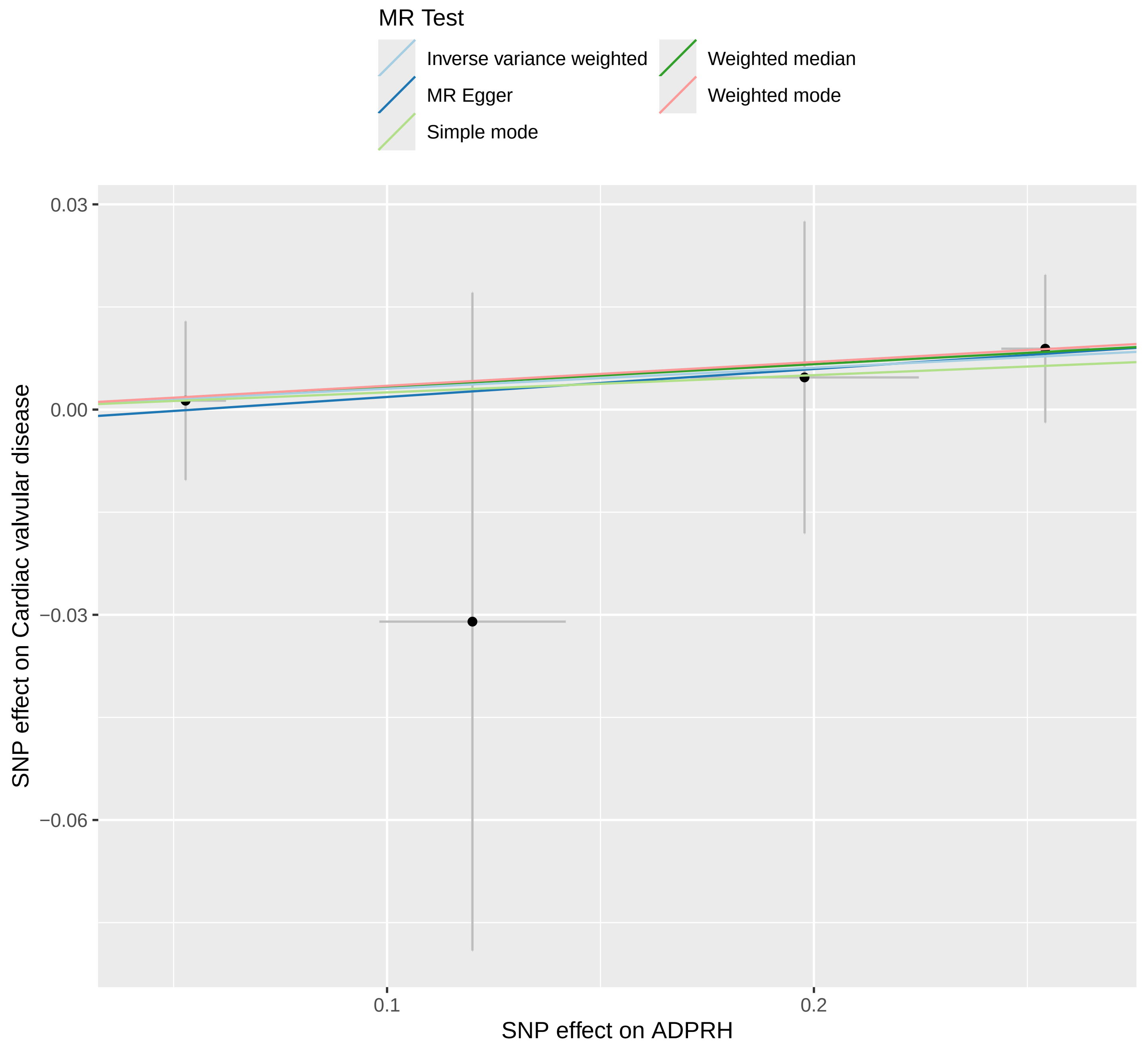

MR Test
Inverse variance weighted
MR Egger
Simple mode
Weighted median
Weighted mode
0.03
0.00
−0.03
−0.06
0.1
0.2
SNP effect on ADPRH
SNP effect on Cardiac valvular disease

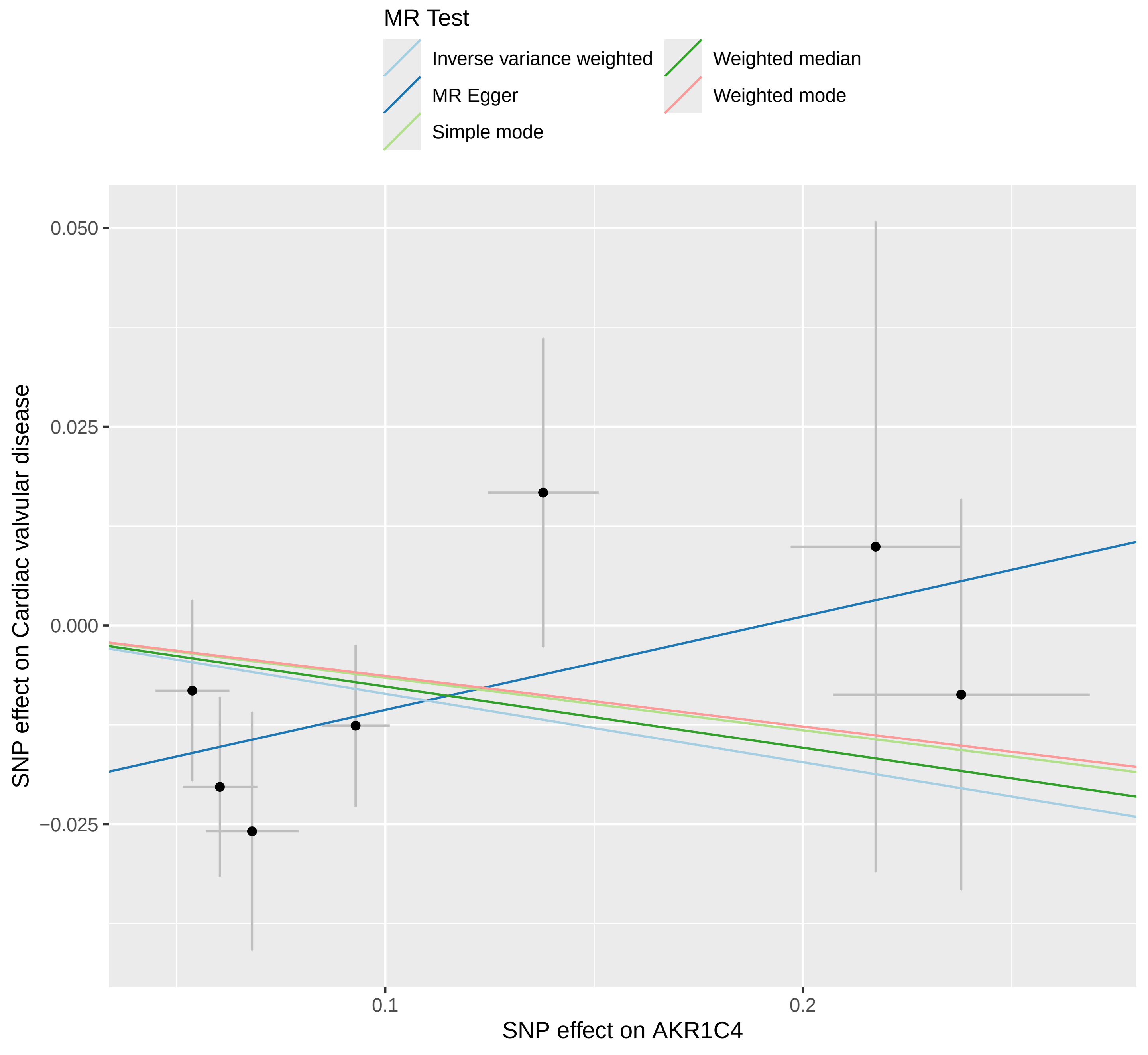

MR Test
Inverse variance weighted
MR Egger
Simple mode
Weighted median
Weighted mode
0.050
0.025
0.000
−0.025
0.1
0.2
SNP effect on AKR1C4
SNP effect on Cardiac valvular disease

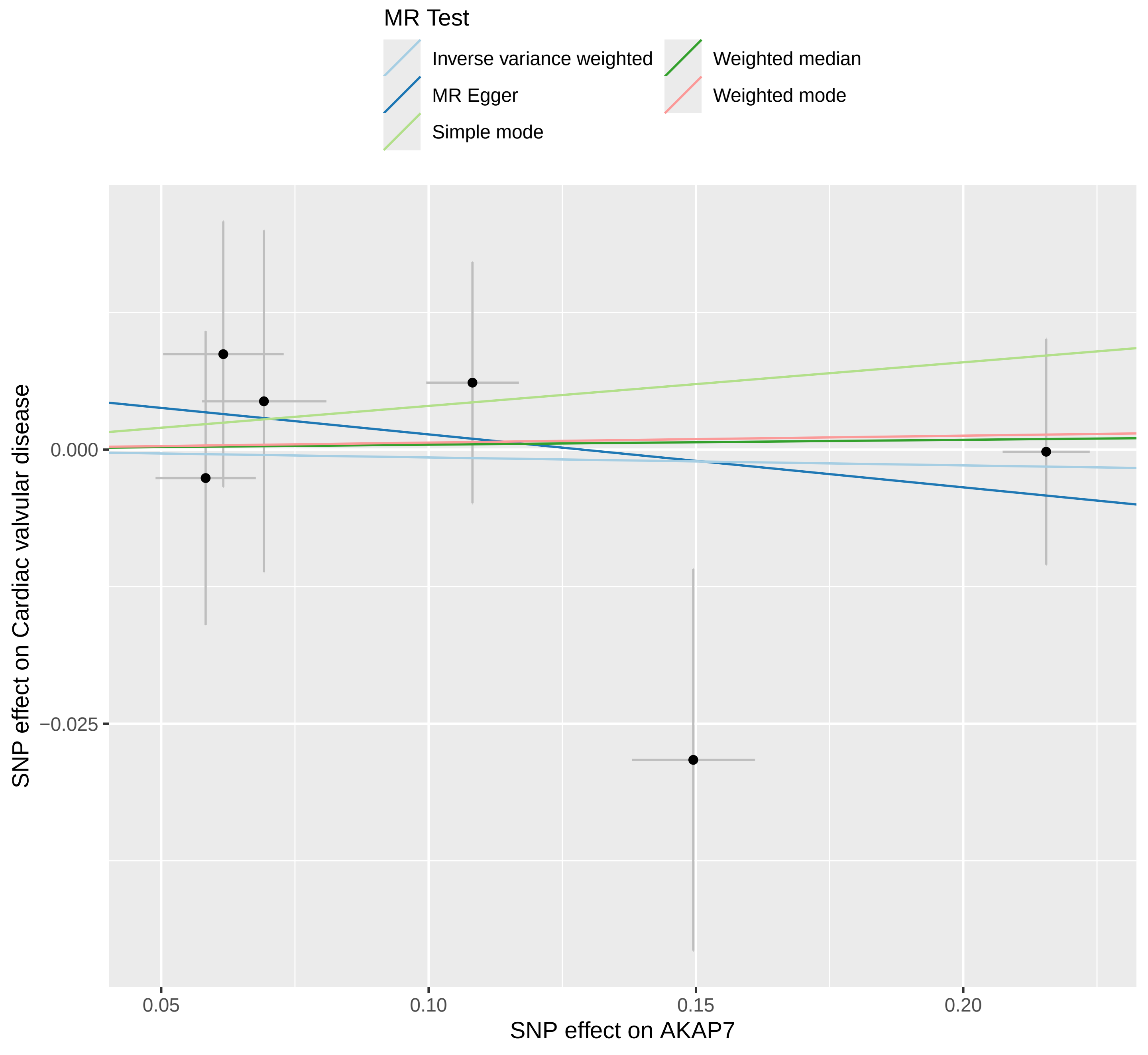

MR Test
Inverse variance weighted
MR Egger
Simple mode
Weighted median
Weighted mode
0.000
−0.025
0.05
0.10
0.15
0.20
SNP effect on Cardiac valvular disease
SNP effect on AKAP7

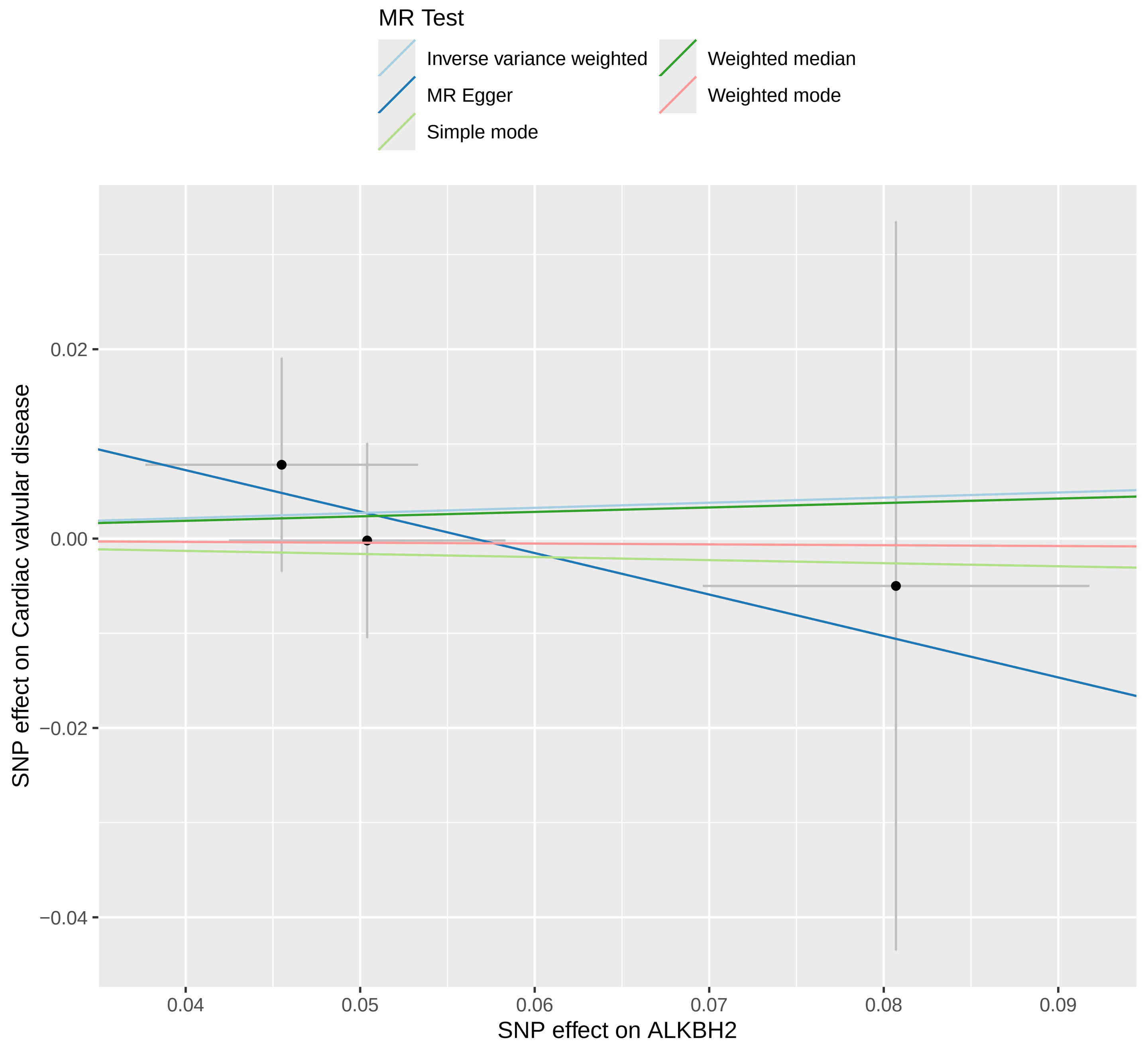

MR Test
Inverse variance weighted
MR Egger
Simple mode
Weighted median
Weighted mode
0.02
0.00
−0.02
−0.04
SNP effect on Cardiac valvular disease
0.04
0.05
0.06
0.07
0.08
0.09
SNP effect on ALKBH2

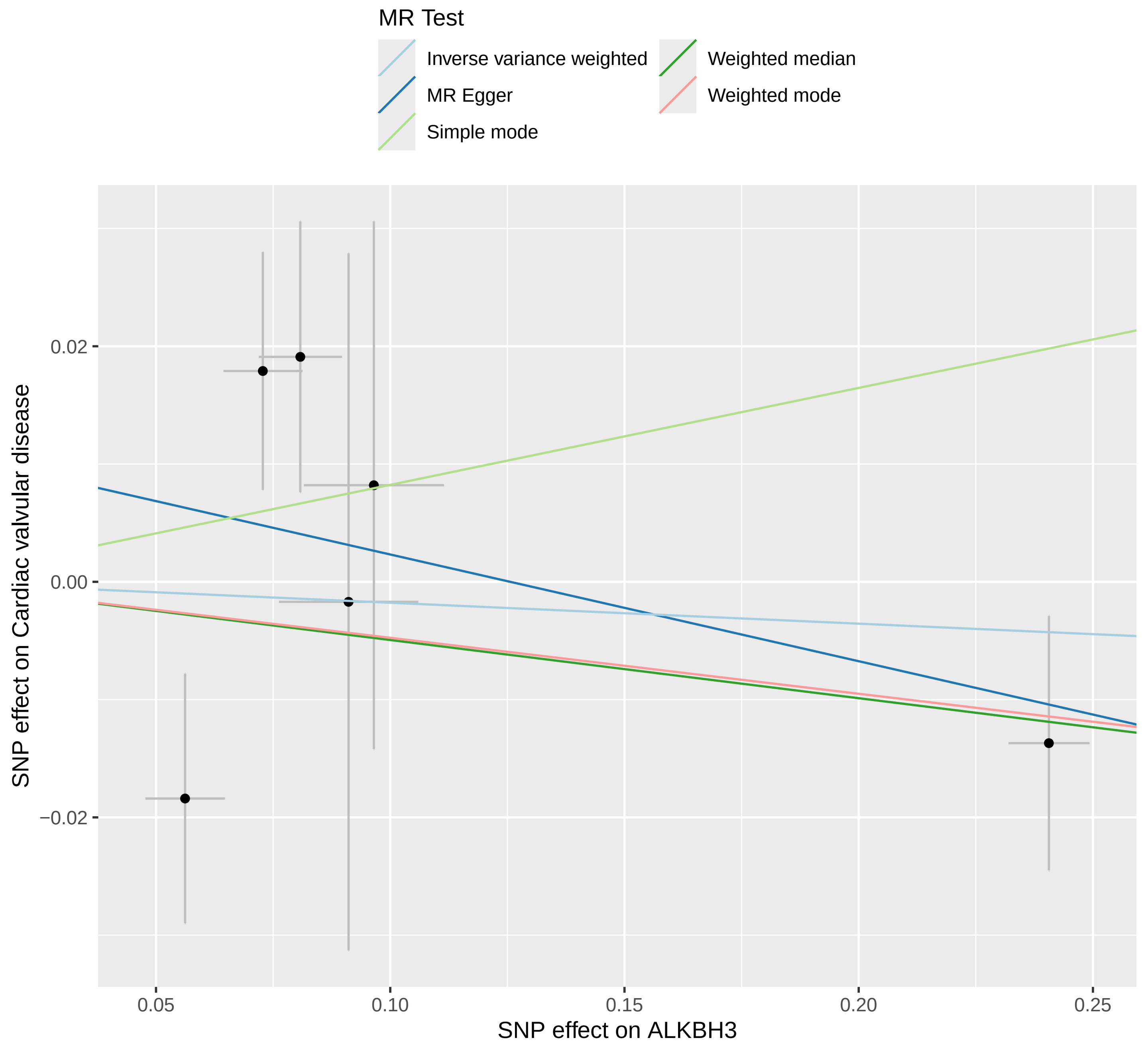
MR Test
Inverse variance weighted
MR Egger
Simple mode
Weighted median
Weighted mode
SNP effect on Cardiac valvular disease
0.02
0.00
−0.02
0.05
0.10
0.15
0.20
0.25
SNP effect on ALKBH3

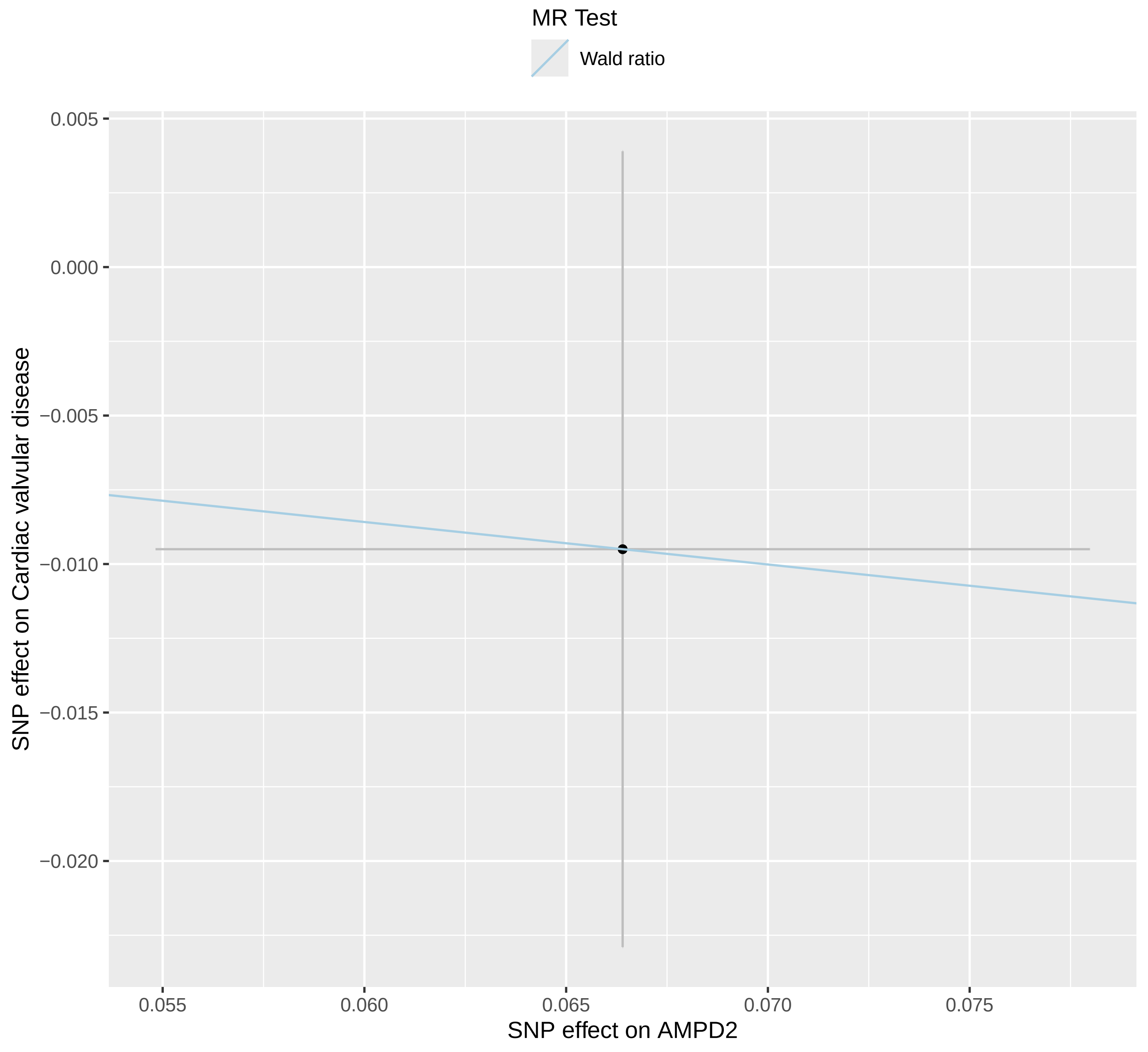
MR Test
Wald ratio
0.005
0.000
−0.005
−0.010
−0.015
−0.020
SNP effect on Cardiac valvular disease
0.055
0.060
0.065
0.070
0.075
SNP effect on AMPD2

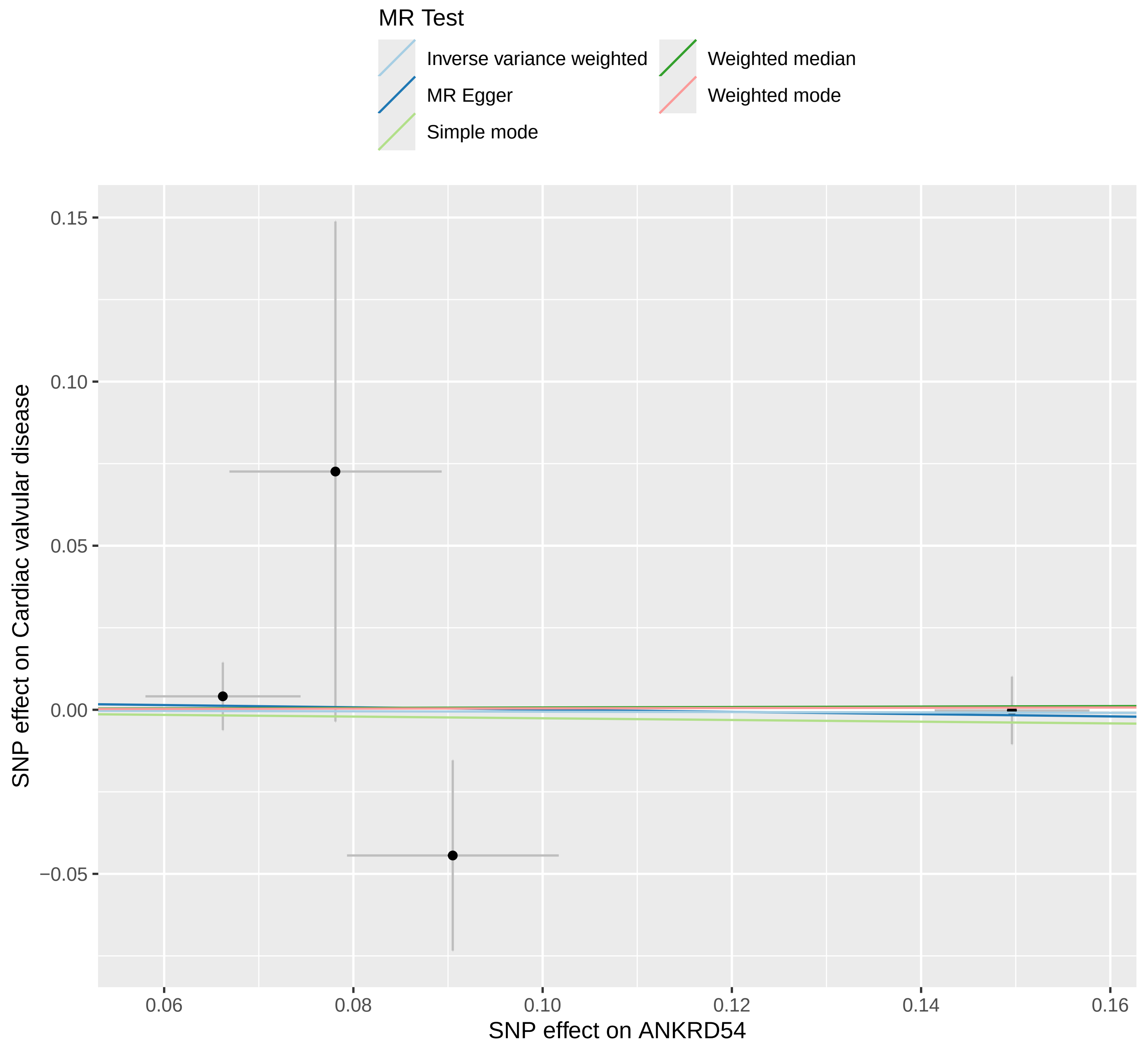
MR Test
Inverse variance weighted
MR Egger
Simple mode
Weighted median
Weighted mode
0.15
0.10
0.05
0.00
−0.05
SNP effect on Cardiac valvular disease
0.06
0.08
0.10
0.12
0.14
0.16
SNP effect on ANKRD54

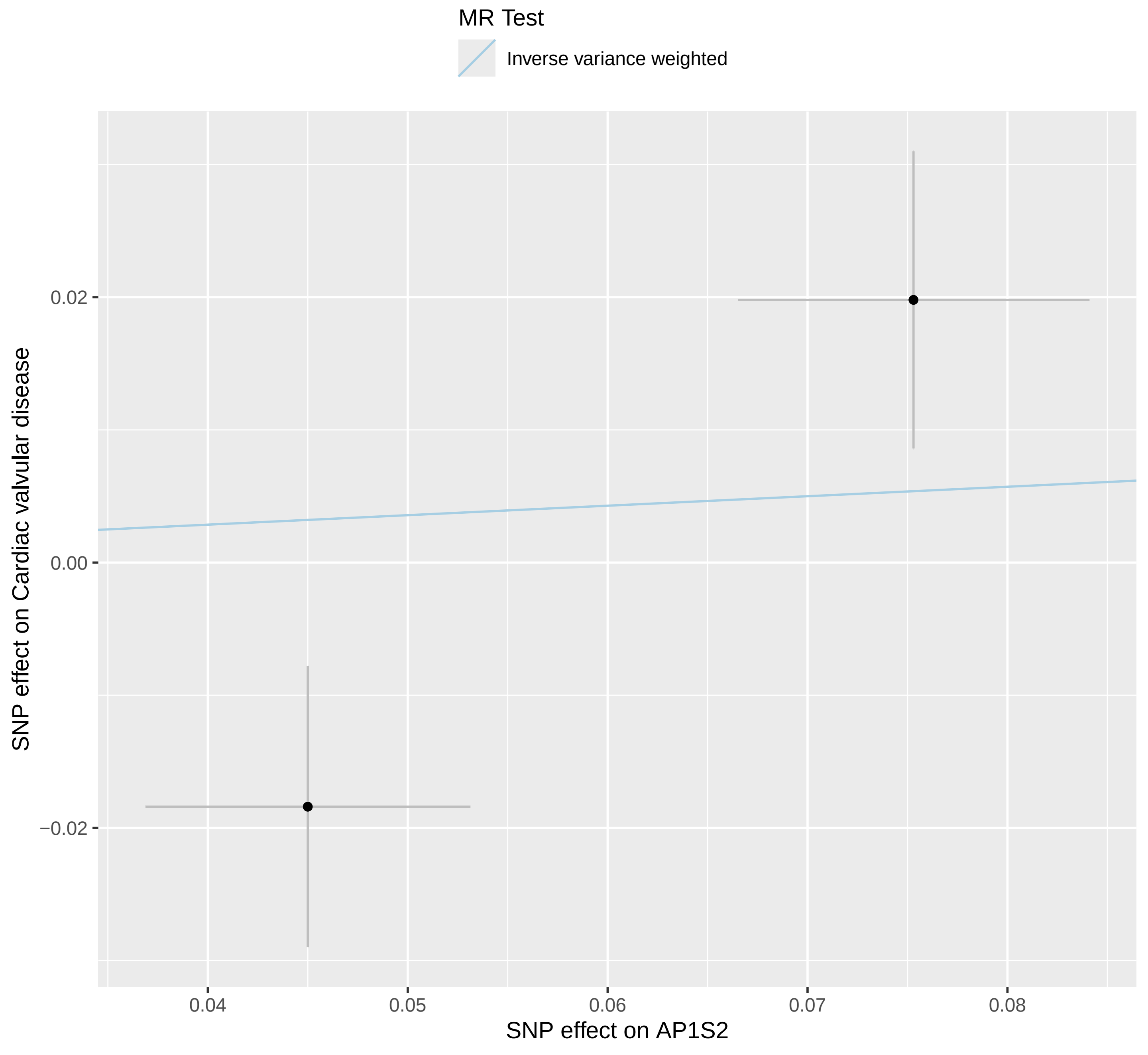

MR Test
Inverse variance weighted
0.02
0.00
−0.02
0.04
0.05
0.06
0.07
0.08
SNP effect on Cardiac valvular disease
SNP effect on AP1S2

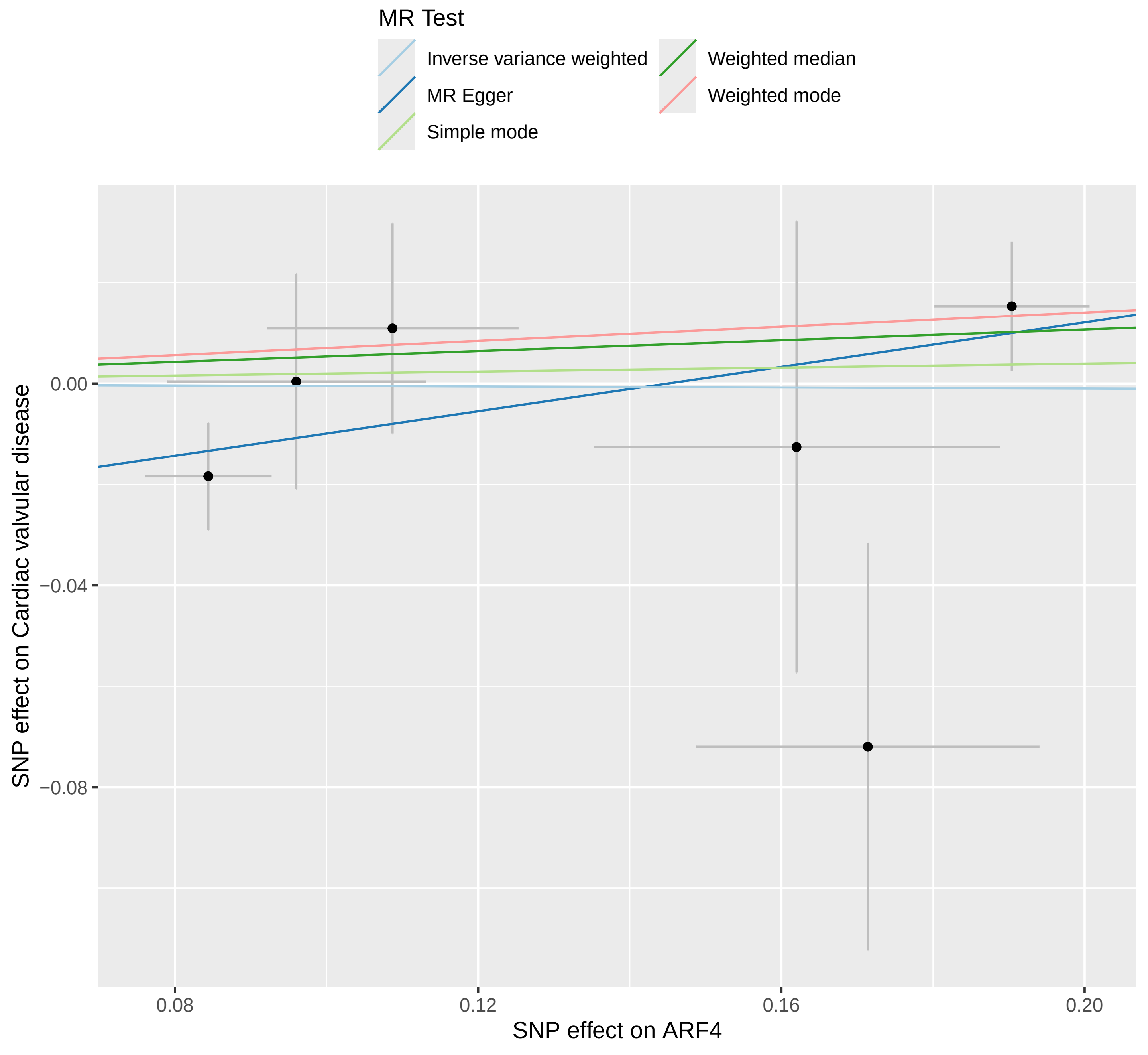
MR Test
Inverse variance weighted
MR Egger
Simple mode
Weighted median
Weighted mode
0.00
−0.04
−0.08
0.08
0.12
0.16
0.20
SNP effect on Cardiac valvular disease
SNP effect on ARF4

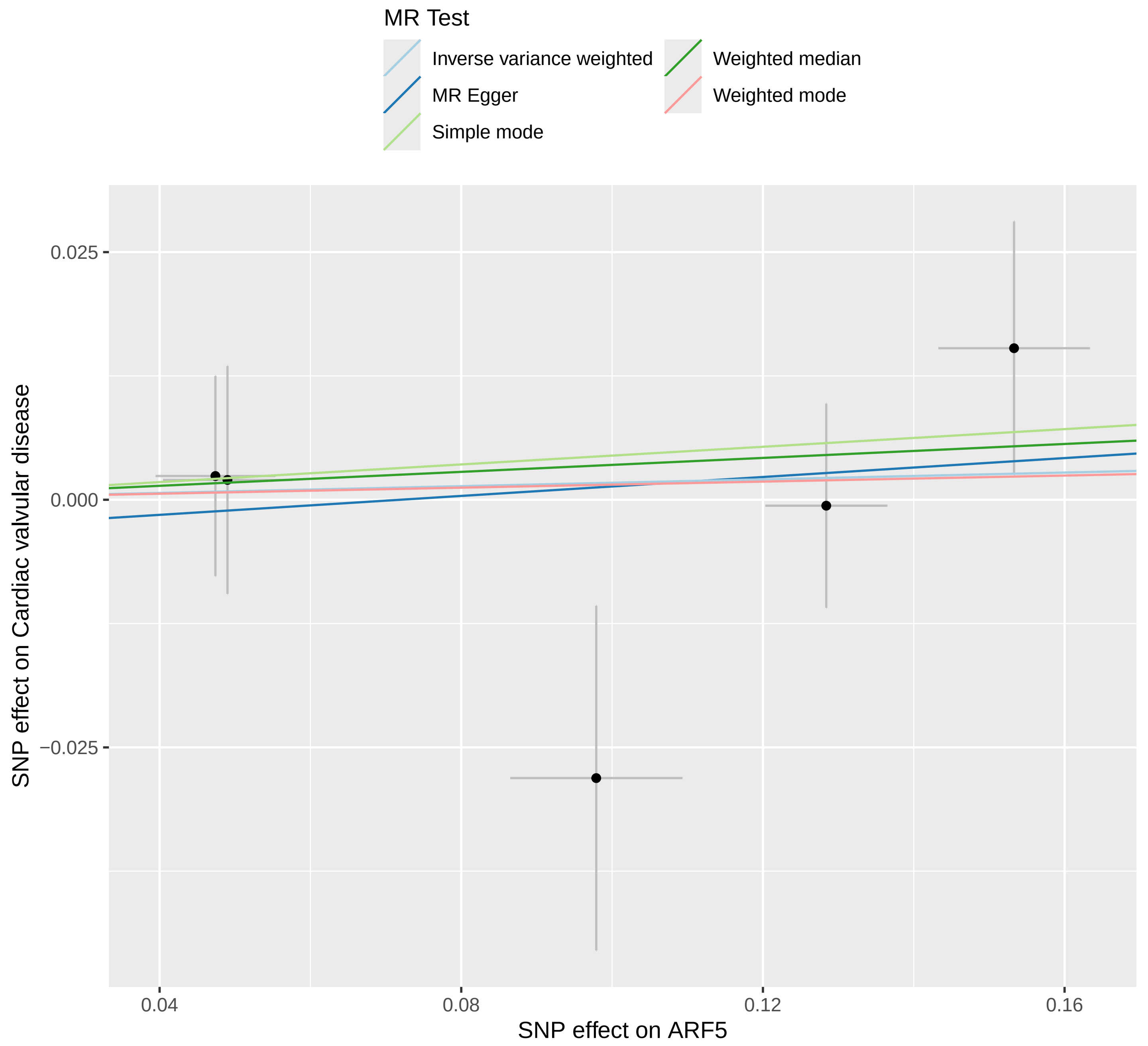
MR Test
Inverse variance weighted
MR Egger
Simple mode
Weighted median
Weighted mode
0.025
0.000
−0.025
0.04
0.08
0.12
0.16
SNP effect on Cardiac valvular disease
SNP effect on ARF5

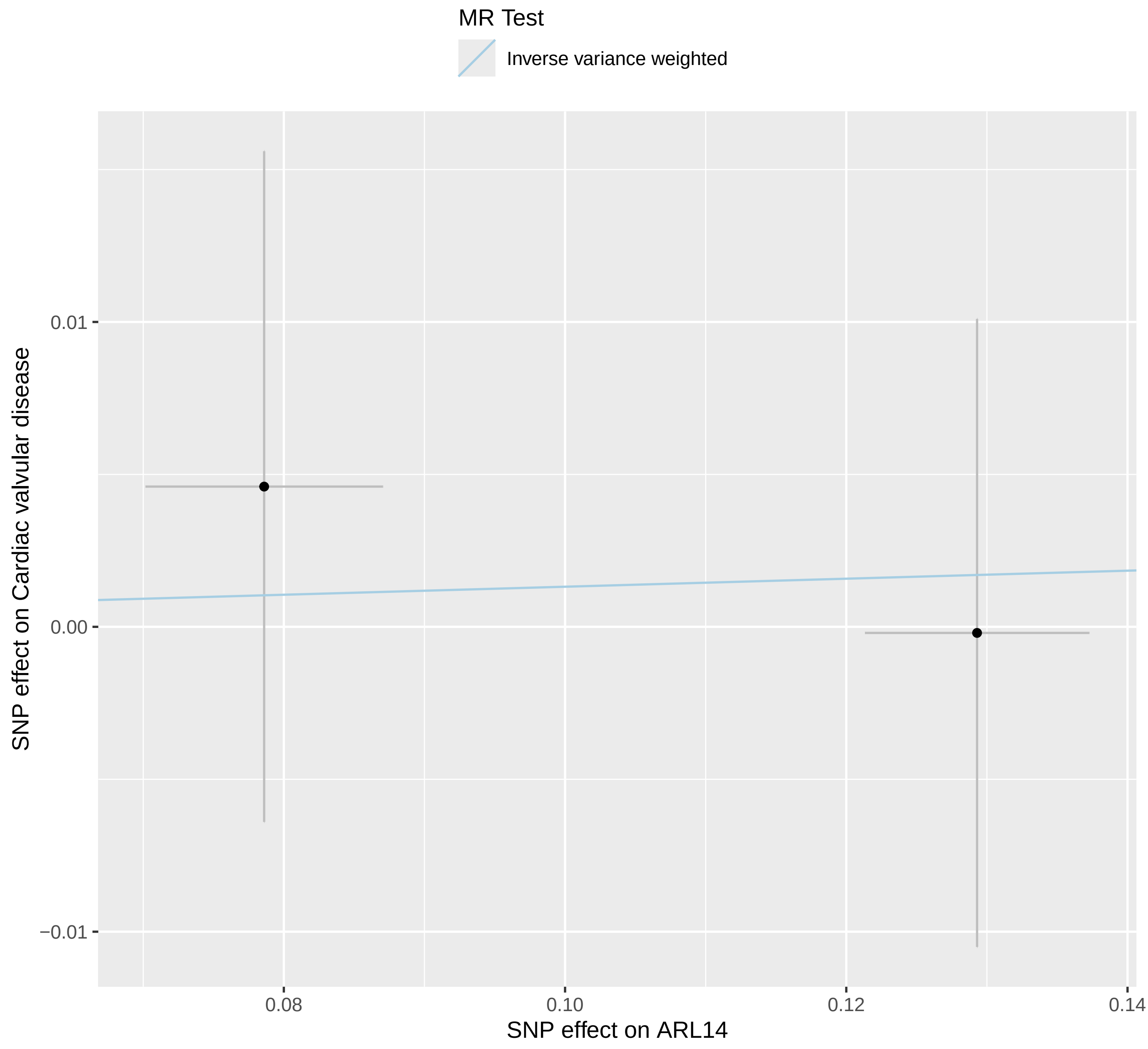
MR Test
Inverse variance weighted
0.01
0.00
−0.01
0.08
0.10
0.12
0.14
SNP effect on ARL14
SNP effect on Cardiac valvular disease

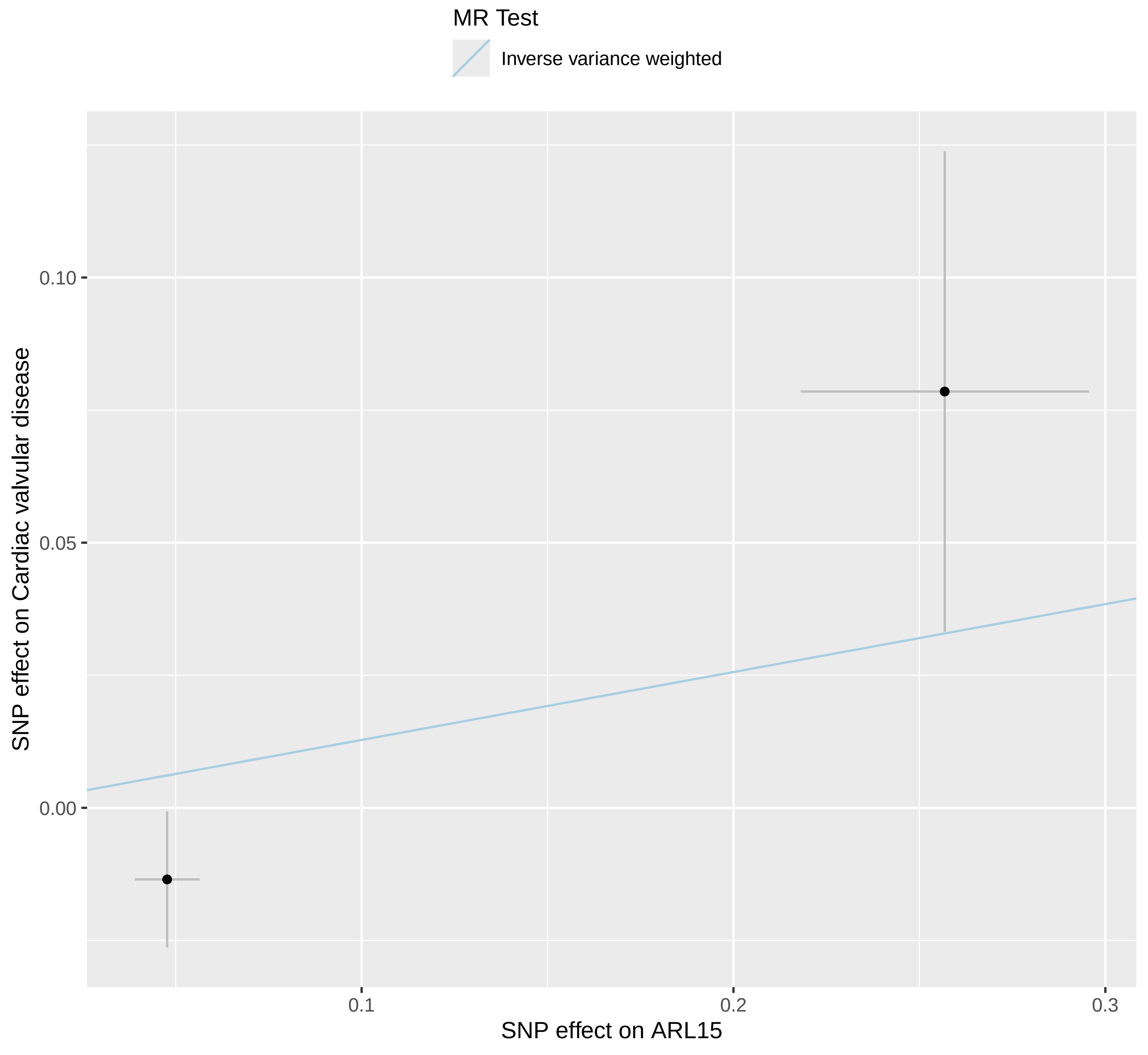
MR Test
Inverse variance weighted
0.10
0.05
0.00
0.1
0.2
0.3
SNP effect on ARL15
SNP effect on Cardiac valvular disease

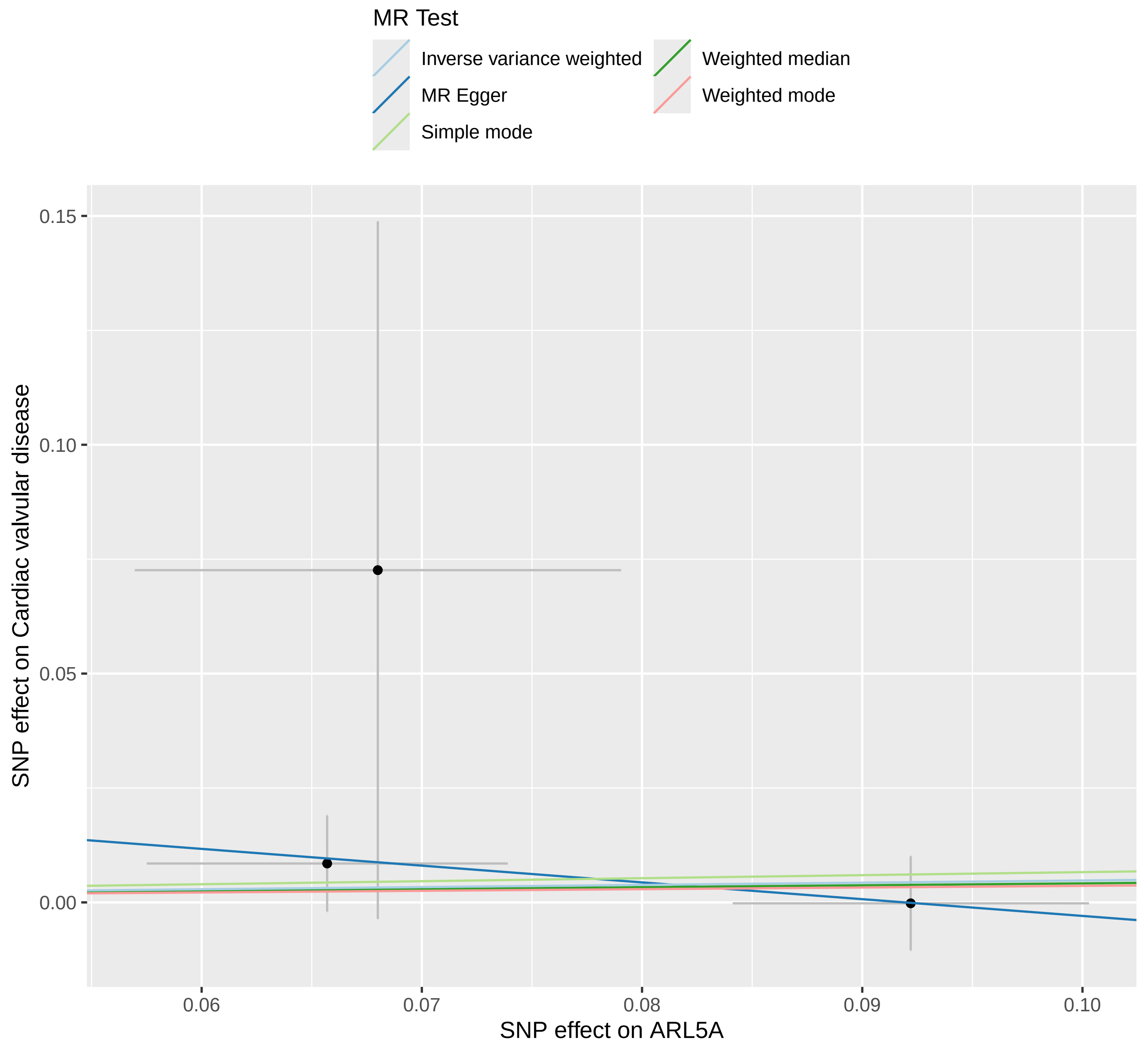
MR Test
Inverse variance weighted
MR Egger
Simple mode
Weighted median
Weighted mode
0.15
0.10
0.05
0.00
SNP effect on Cardiac valvular disease
0.06
0.07
0.08
0.09
0.10
SNP effect on ARL5A

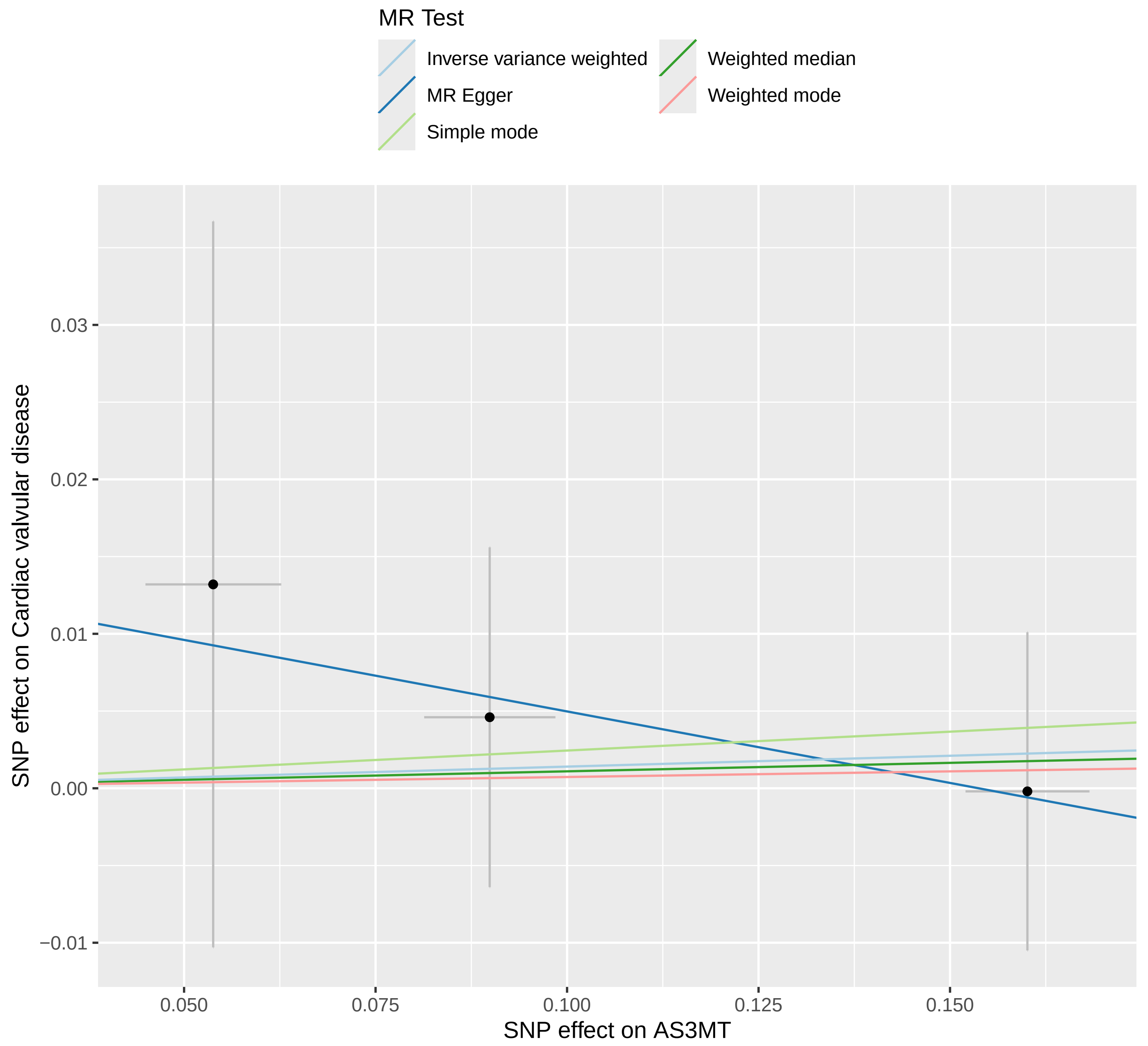
MR Test
Inverse variance weighted
MR Egger
Simple mode
Weighted median
Weighted mode
SNP effect on Cardiac valvular disease
0.03
0.02
0.01
0.00
−0.01
0.050
0.075
0.100
0.125
0.150
SNP effect on AS3MT

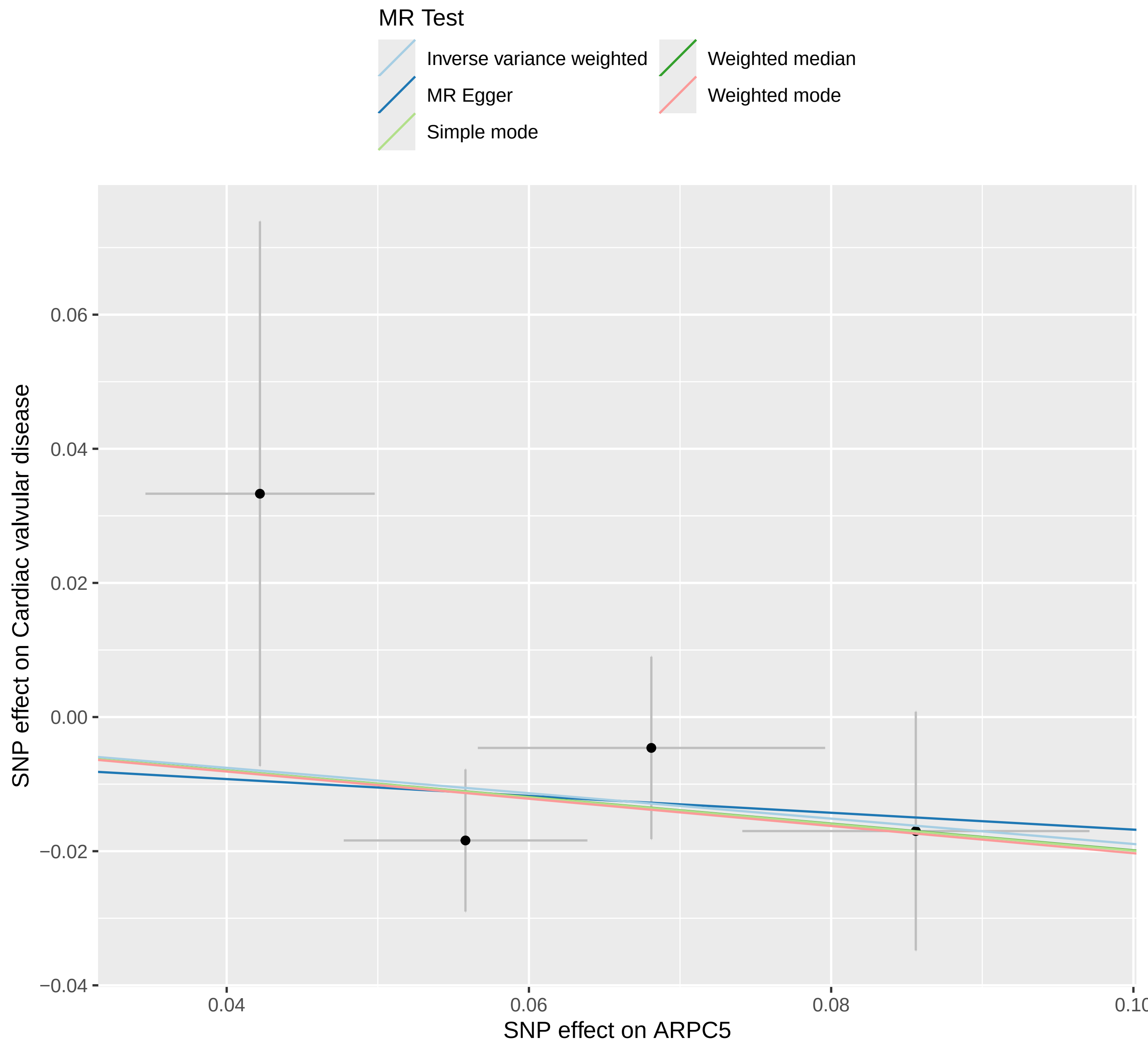
MR Test
Inverse variance weighted
MR Egger
Simple mode
Weighted median
Weighted mode
0.06
0.04
0.02
0.00
−0.02
−0.04
0.04
0.06
0.08
0.10
SNP effect on ARPC5
SNP effect on Cardiac valvular disease

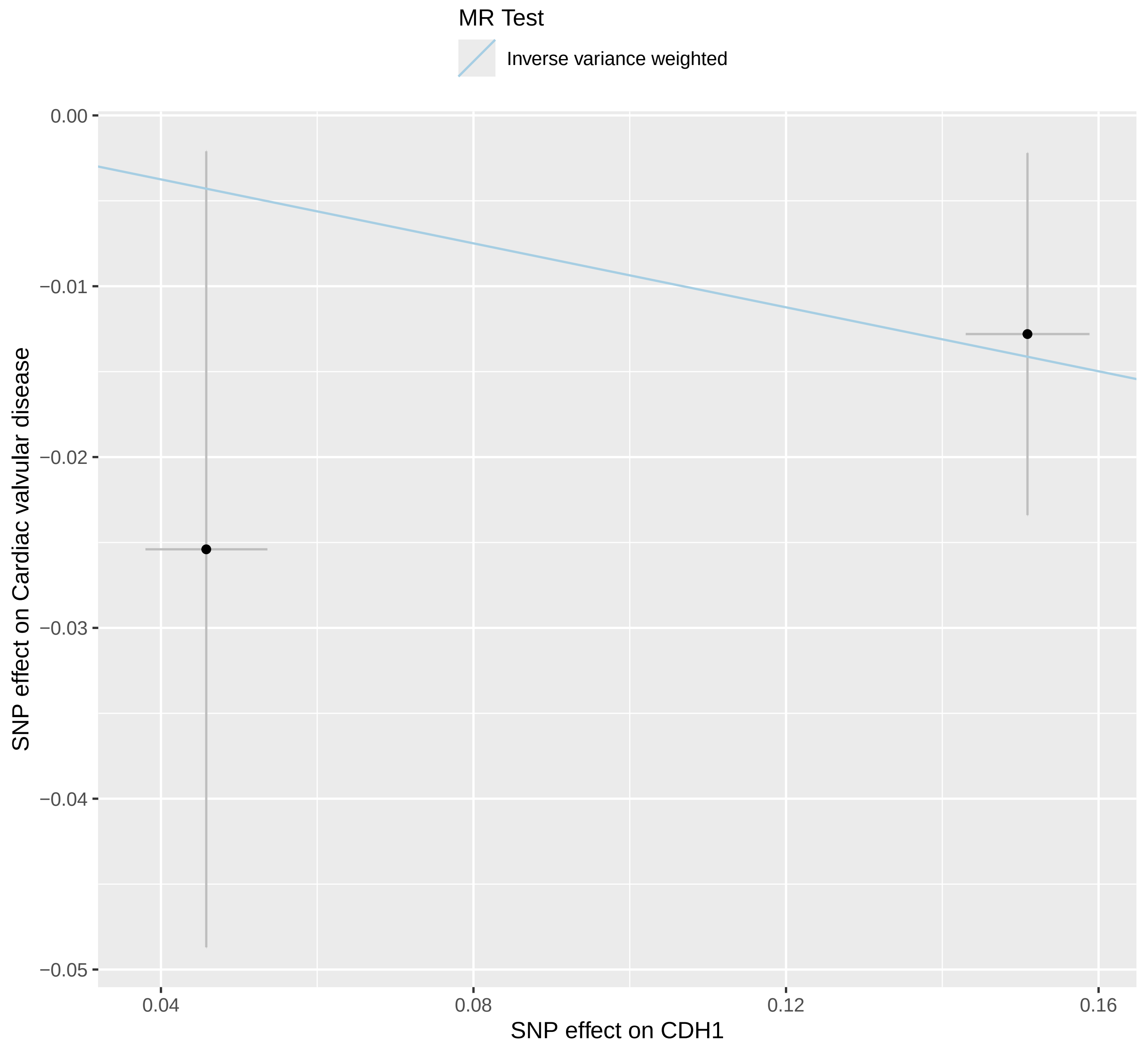
MR Test
Inverse variance weighted
0.00
−0.01
−0.02
−0.03
−0.04
−0.05
SNP effect on Cardiac valvular disease
0.04
0.08
0.12
0.16
SNP effect on CDH1

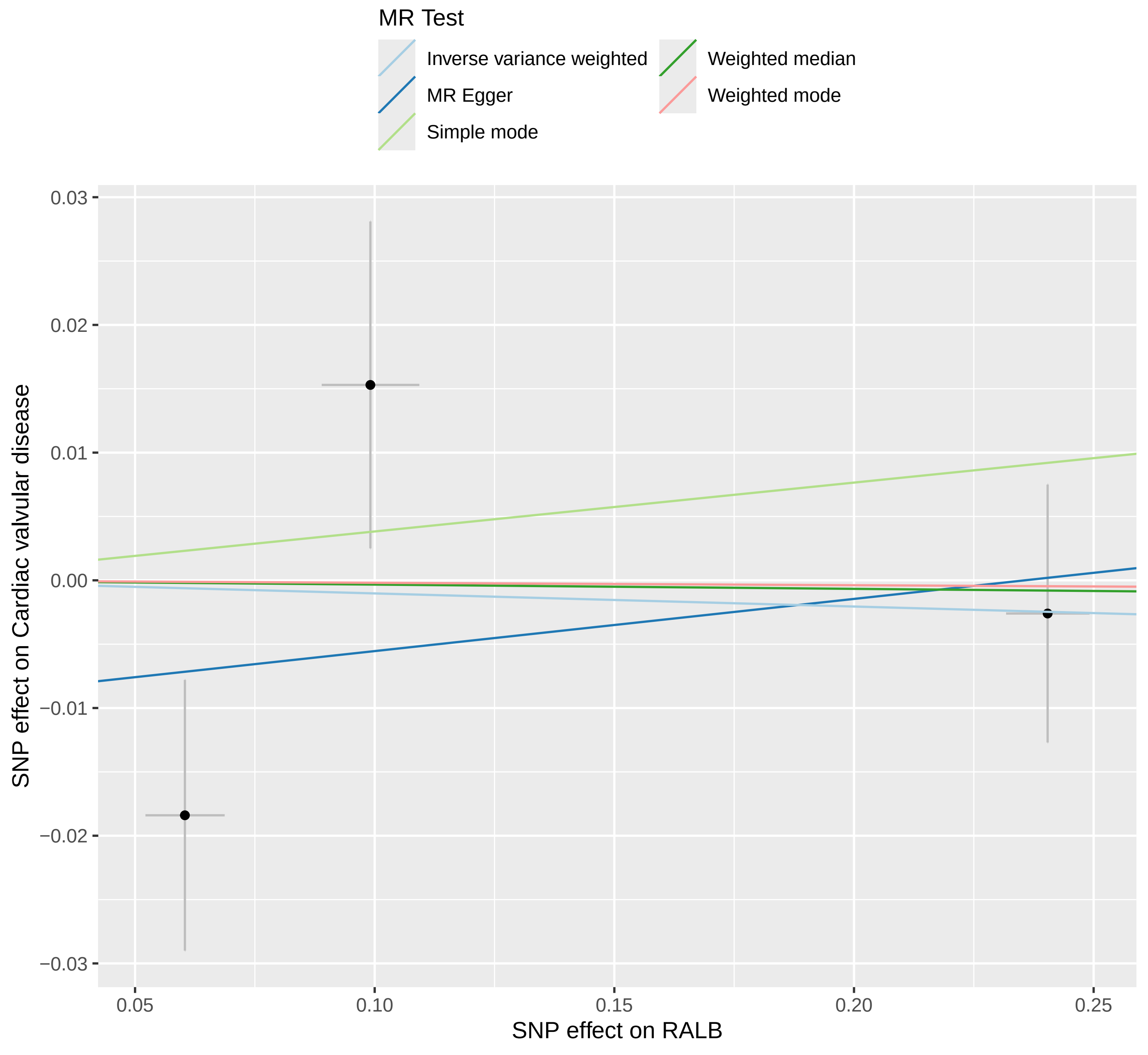

MR Test
Inverse variance weighted
MR Egger
Simple mode
Weighted median
Weighted mode
0.03
0.02
0.01
0.00
−0.01
−0.02
−0.03
SNP effect on Cardiac valvular disease
0.05
0.10
0.15
0.20
0.25
SNP effect on RALB

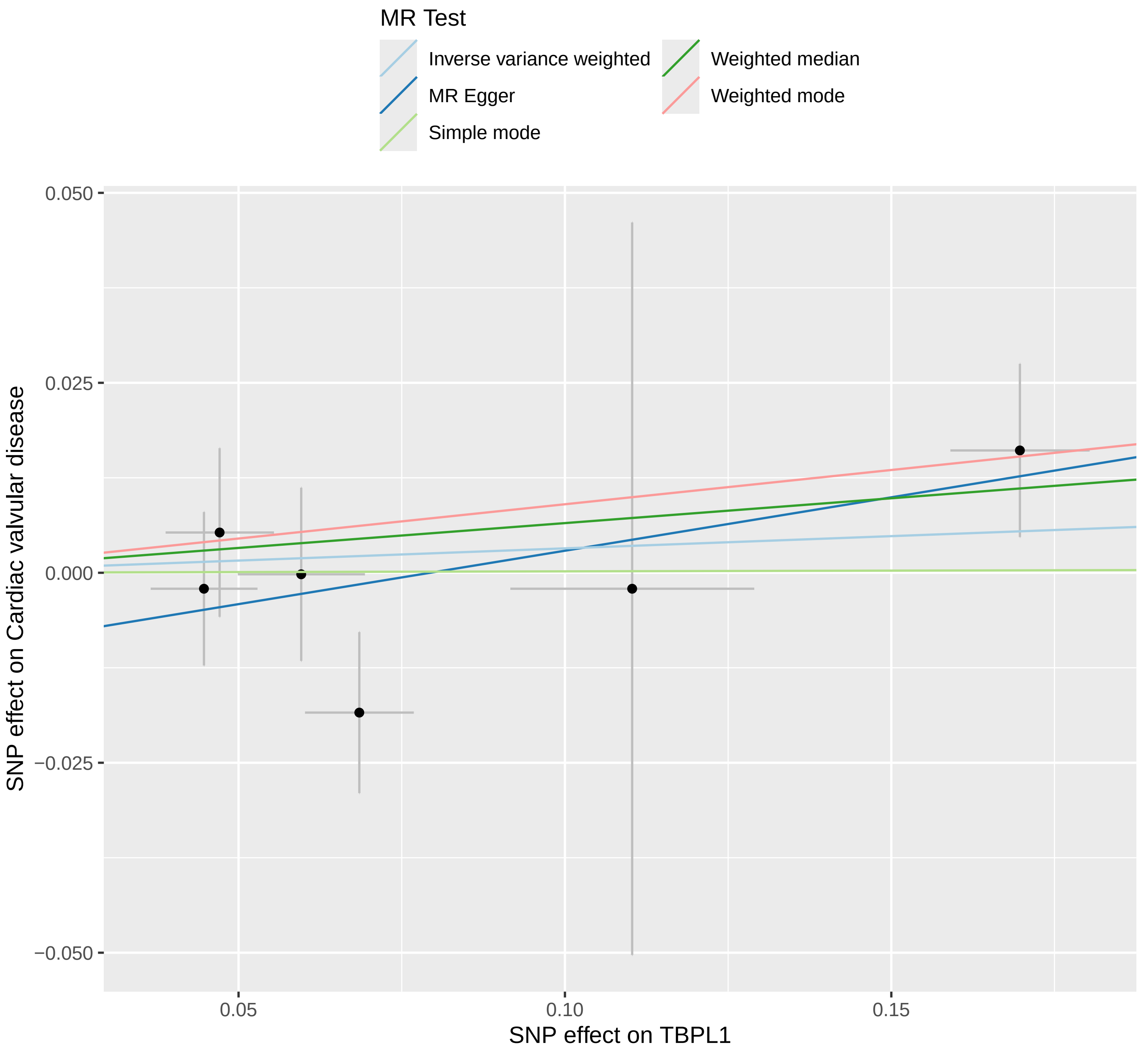
MR Test
Inverse variance weighted
MR Egger
Simple mode
Weighted median
Weighted mode
0.050
0.025
0.000
−0.025
−0.050
0.05
0.10
0.15
SNP effect on Cardiac valvular disease
SNP effect on TBPL1

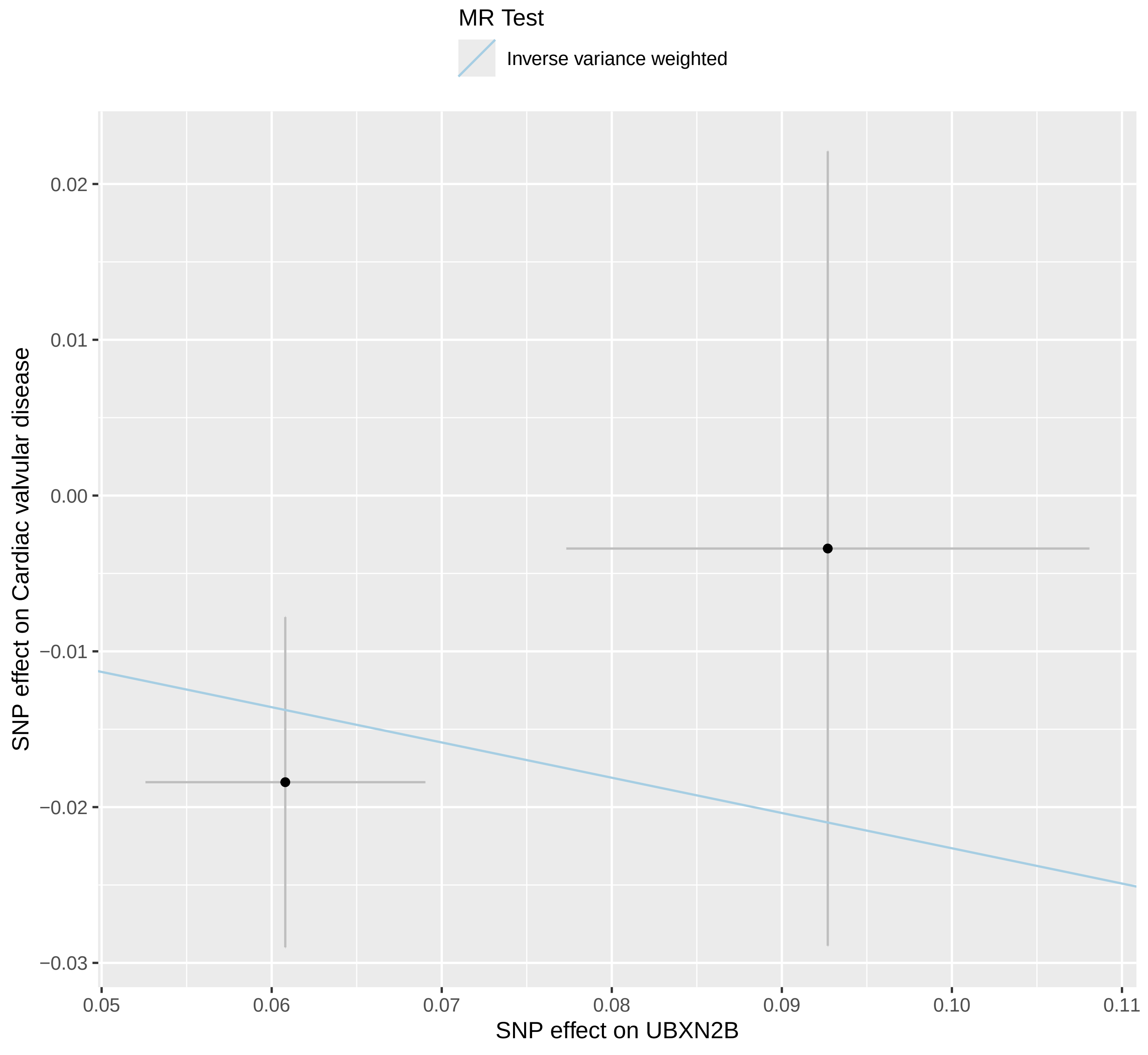
MR Test
Inverse variance weighted
SNP effect on Cardiac valvular disease
0.02
0.01
0.00
−0.01
−0.02
−0.03
0.05
0.06
0.07
0.08
0.09
0.10
0.11
SNP effect on UBXN2B

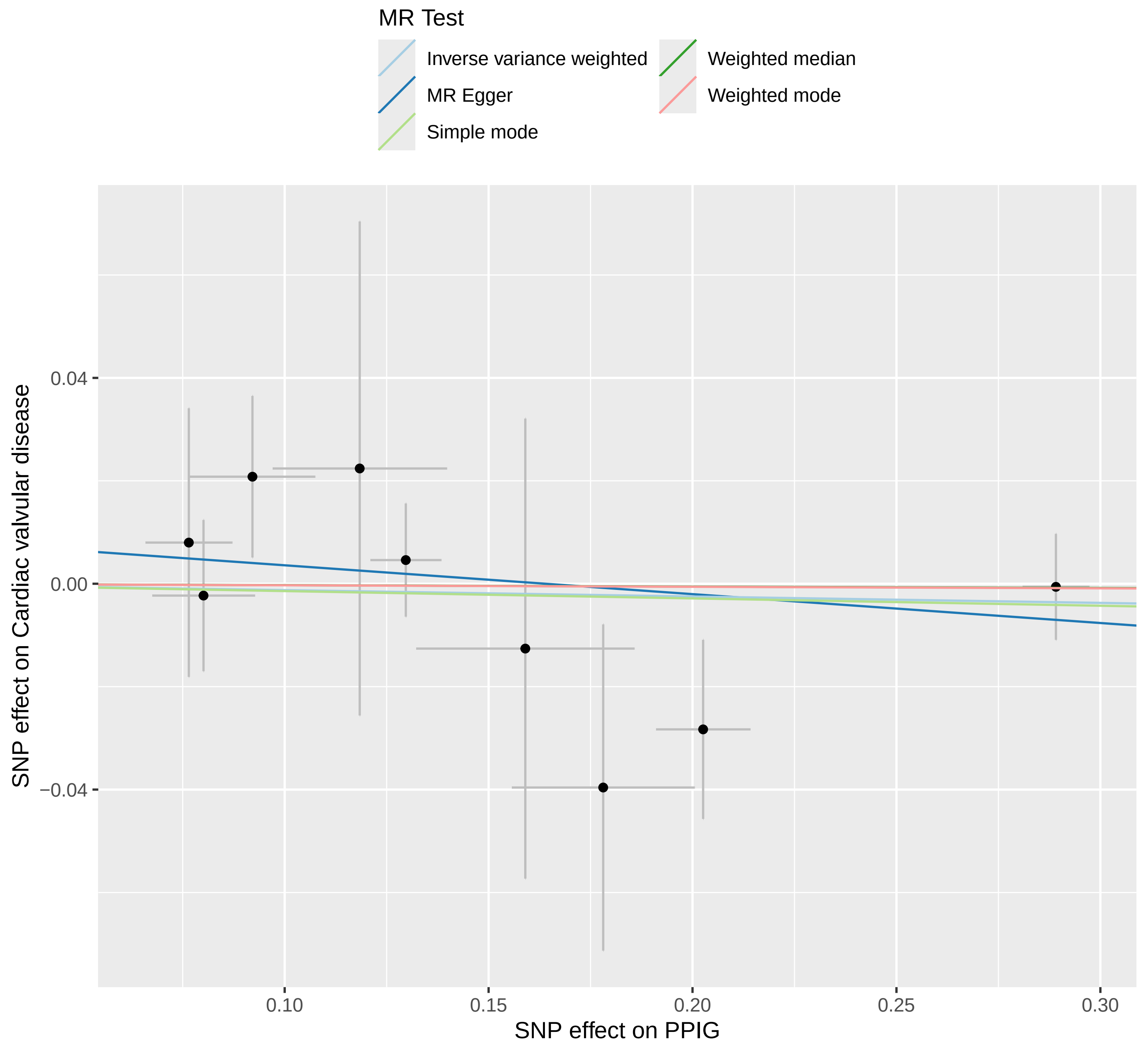

MR Test
Inverse variance weighted
MR Egger
Simple mode
Weighted median
Weighted mode
0.04
0.00
−0.04
0.10
0.15
0.20
0.25
0.30
SNP effect on PPIG
SNP effect on Cardiac valvular disease

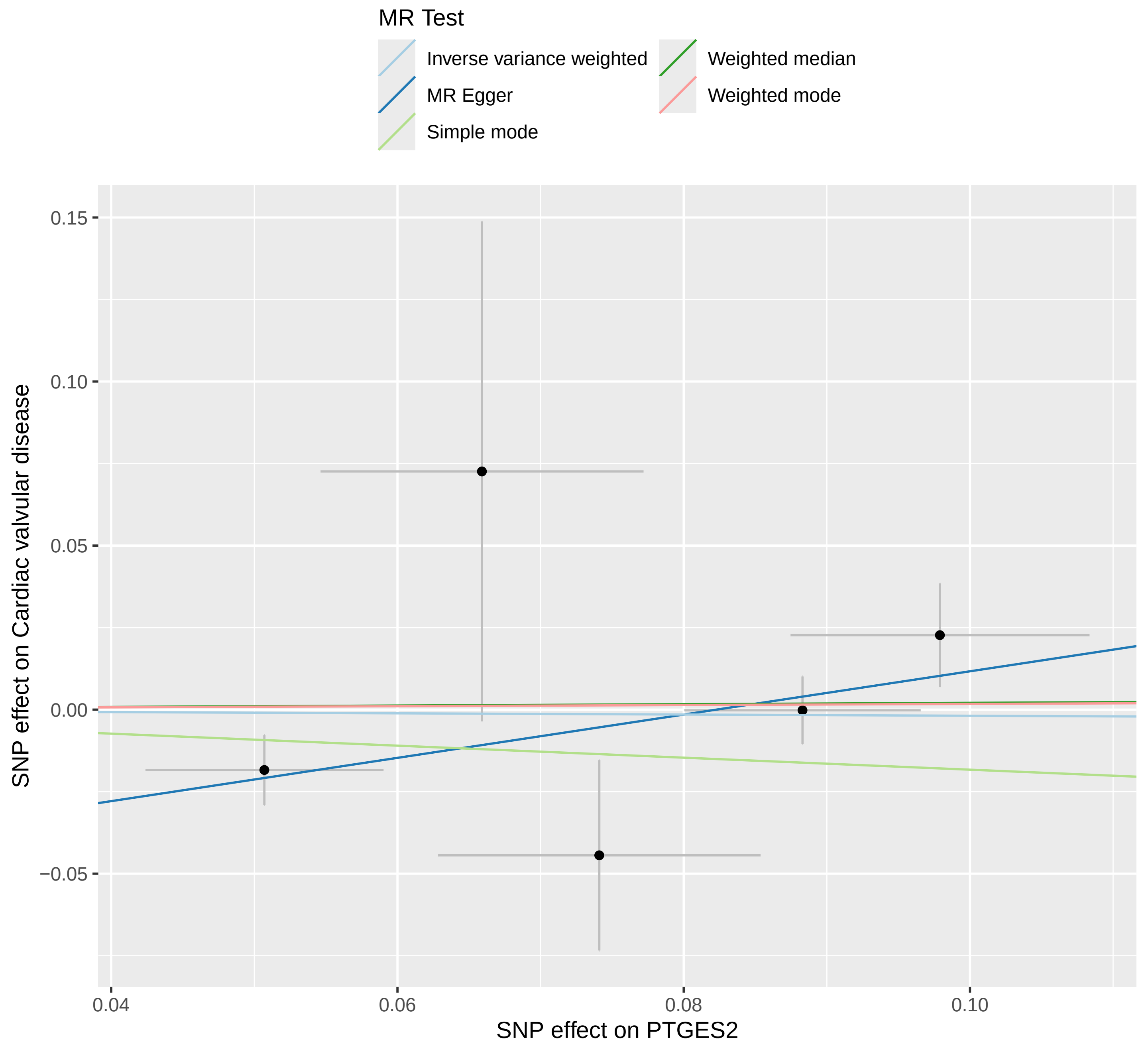

MR Test
Inverse variance weighted
MR Egger
Simple mode
Weighted median
Weighted mode
0.15
0.10
0.05
0.00
−0.05
SNP effect on Cardiac valvular disease
0.04
0.06
0.08
0.10
SNP effect on PTGES2

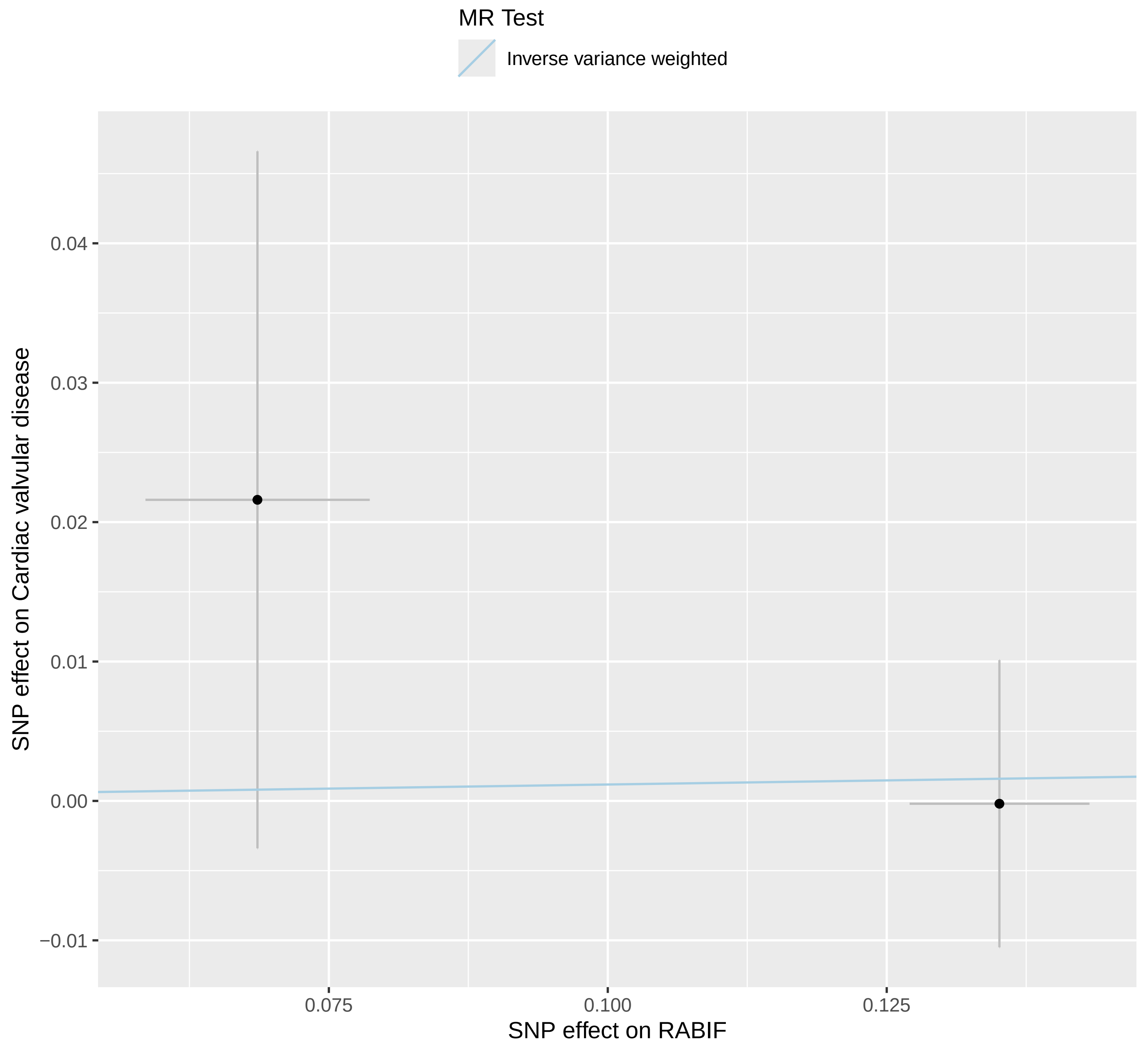
MR Test
Inverse variance weighted
0.04
0.03
0.02
0.01
0.00
−0.01
SNP effect on Cardiac valvular disease
0.075
0.100
0.125
SNP effect on RABIF

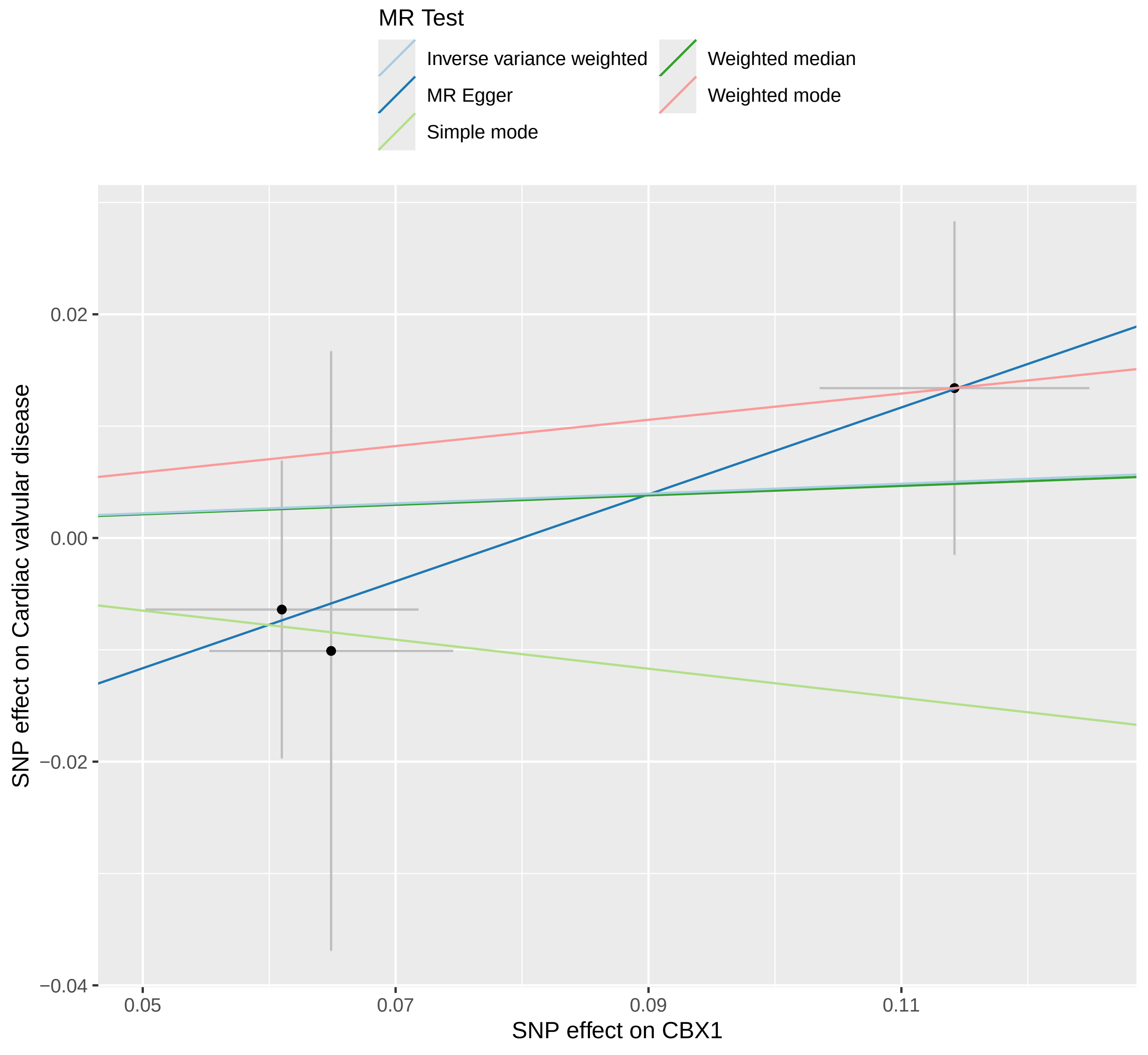
MR Test
Inverse variance weighted
MR Egger
Simple mode
Weighted median
Weighted mode
SNP effect on Cardiac valvular disease
0.02
0.00
−0.02
−0.04
0.05
0.07
0.09
0.11
SNP effect on CBX1

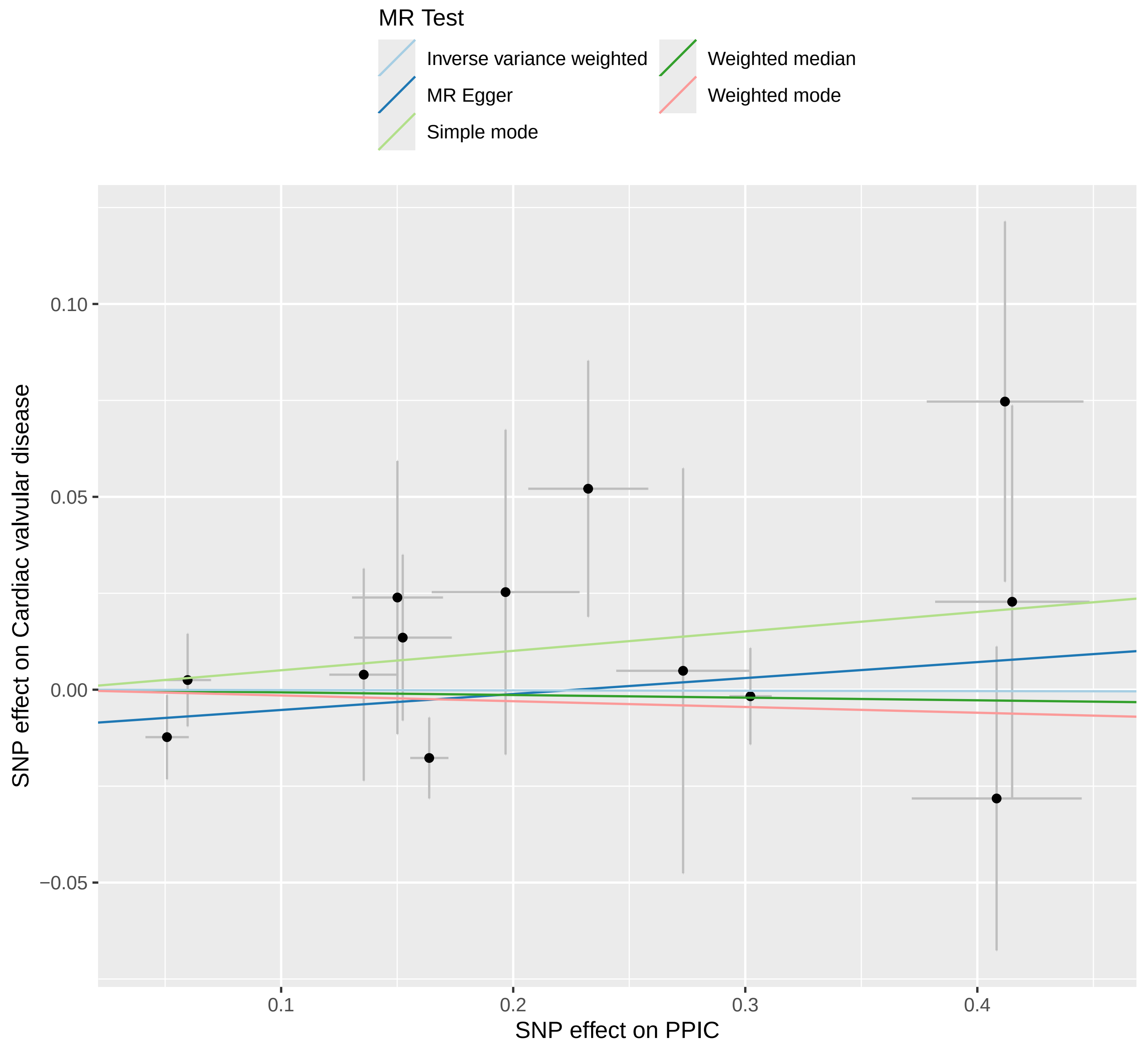
MR Test
Inverse variance weighted
MR Egger
Simple mode
Weighted median
Weighted mode
0.10
0.05
0.00
−0.05
0.1
0.2
0.3
0.4
SNP effect on Cardiac valvular disease
SNP effect on PPIC

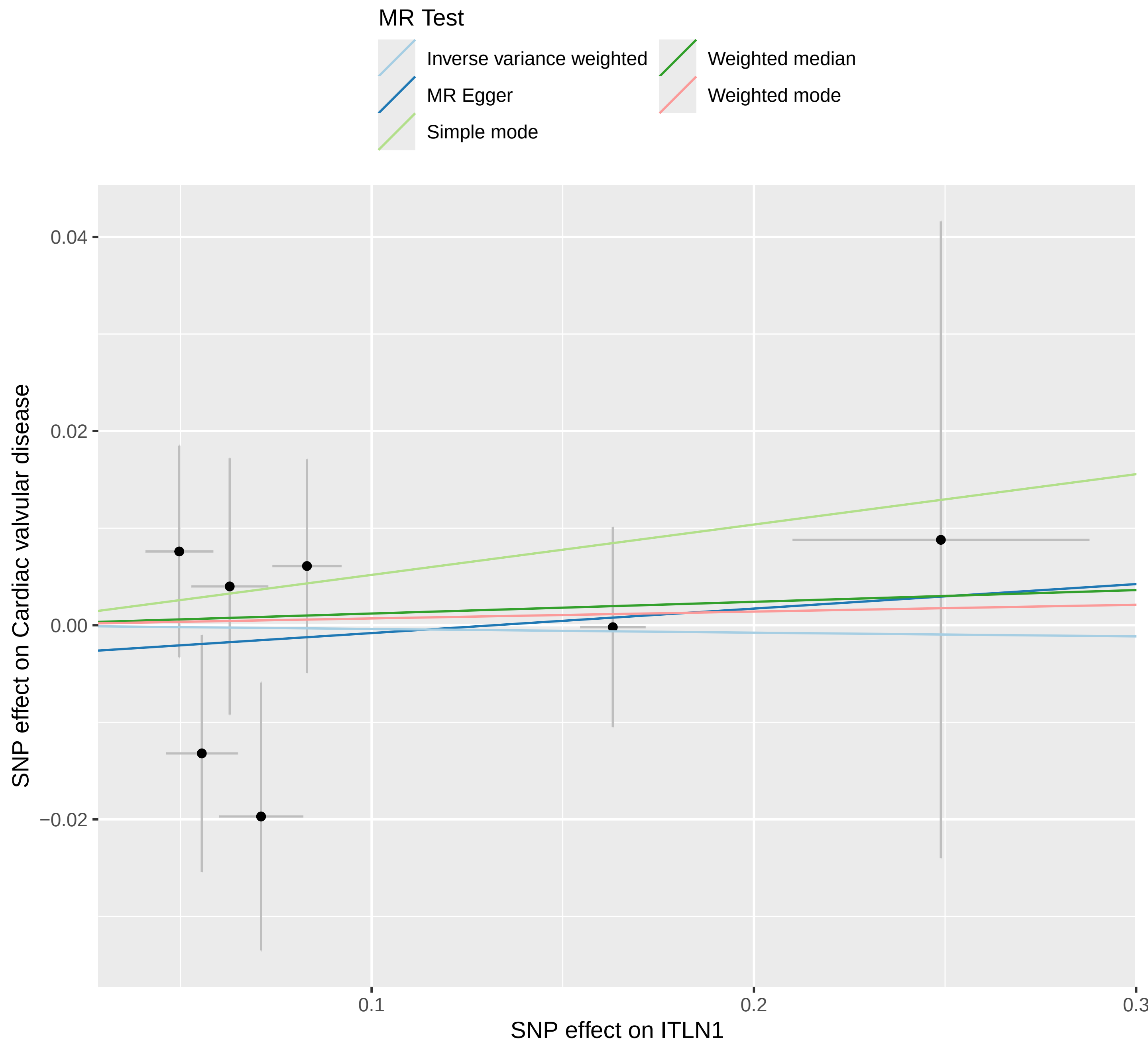
MR Test
Inverse variance weighted
MR Egger
Simple mode
Weighted median
Weighted mode
0.04
0.02
0.00
−0.02
0.1
0.2
0.3
SNP effect on ITLN1
SNP effect on Cardiac valvular disease

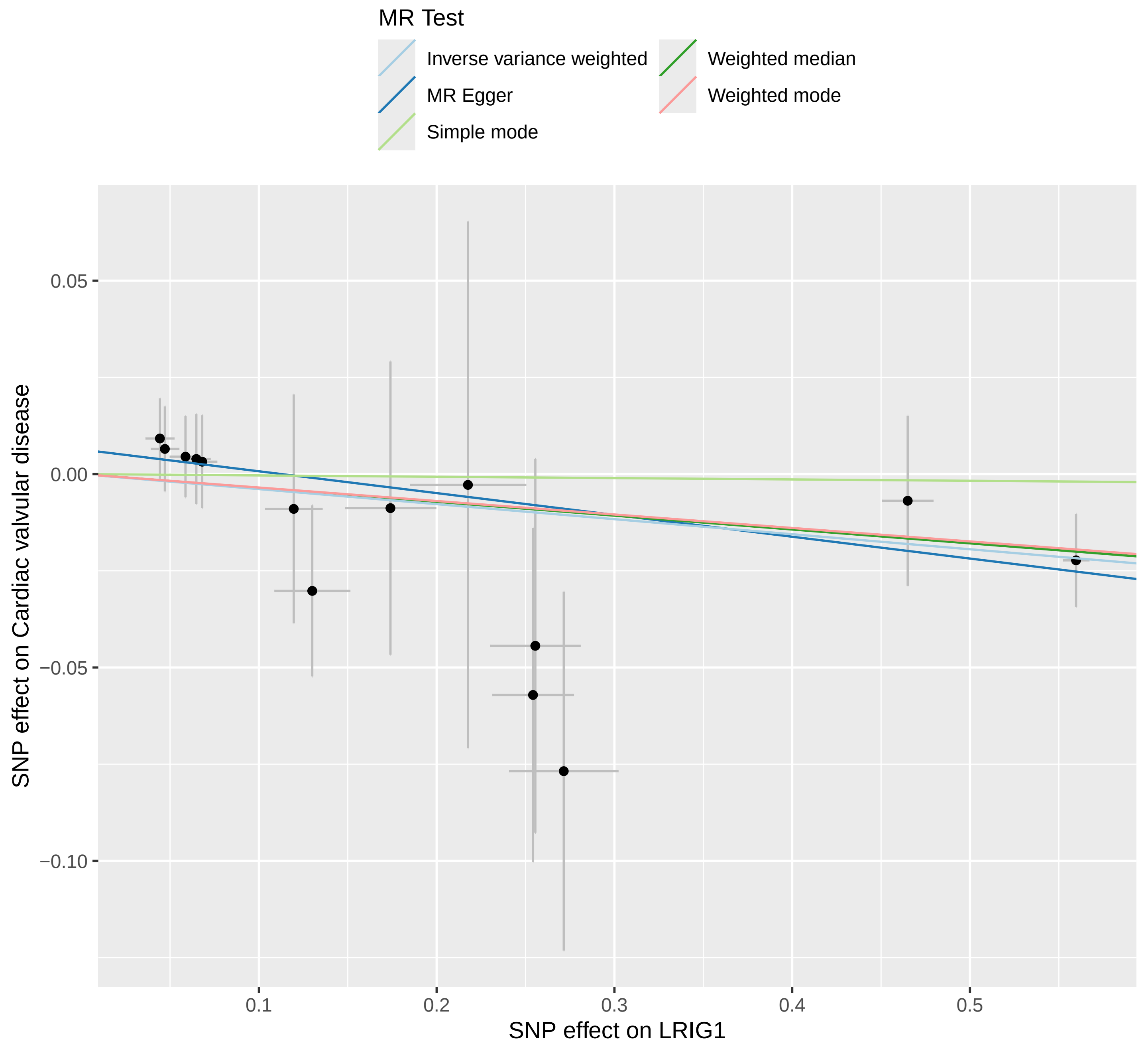
MR Test
Inverse variance weighted
MR Egger
Simple mode
Weighted median
Weighted mode
0.05
0.00
−0.05
−0.10
0.1
0.2
0.3
0.4
0.5
SNP effect on Cardiac valvular disease
SNP effect on LRIG1

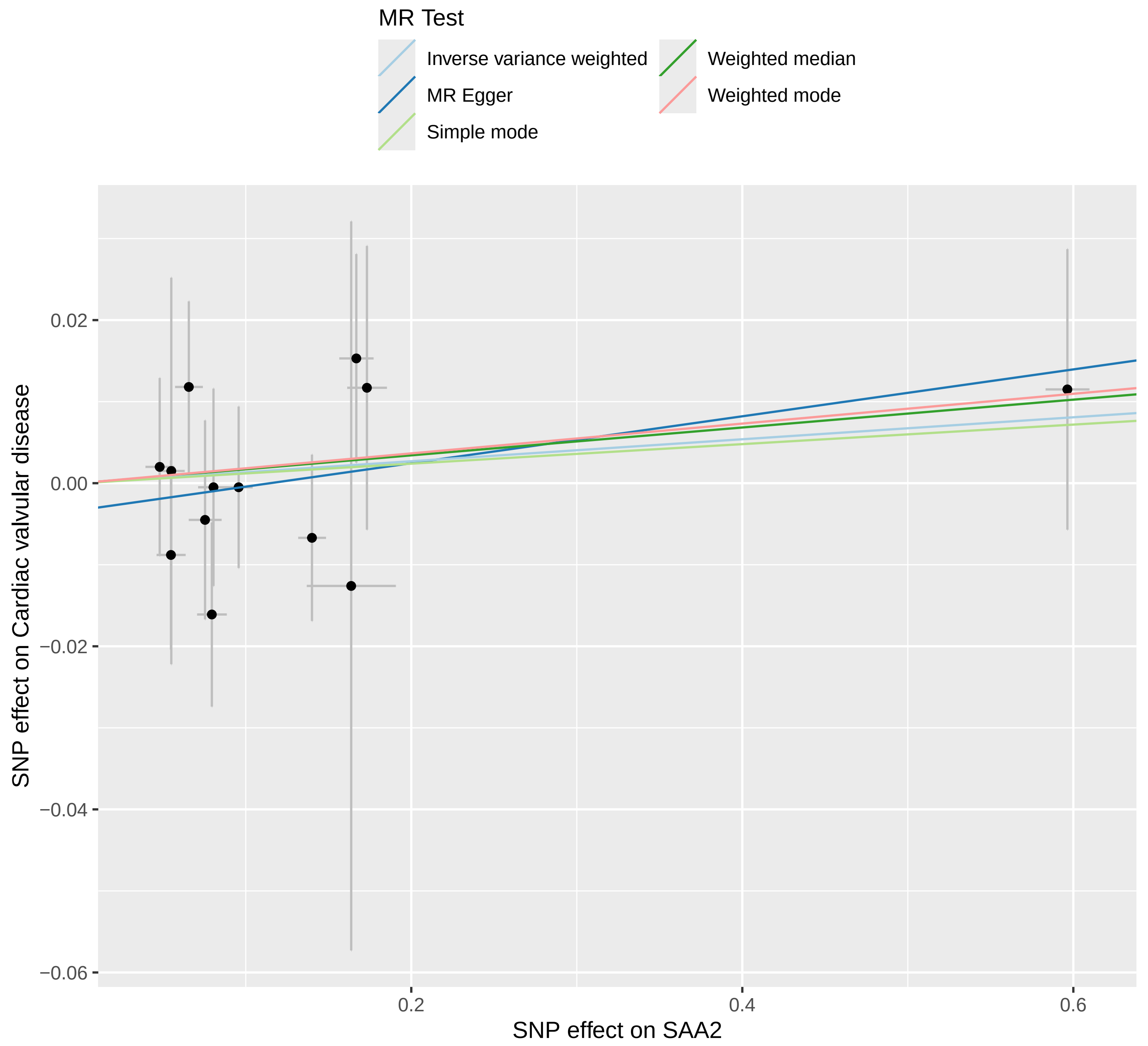

MR Test
Inverse variance weighted
MR Egger
Simple mode
Weighted median
Weighted mode
SNP effect on Cardiac valvular disease
0.02
0.00
−0.02
−0.04
−0.06
0.2
0.4
0.6
SNP effect on SAA2

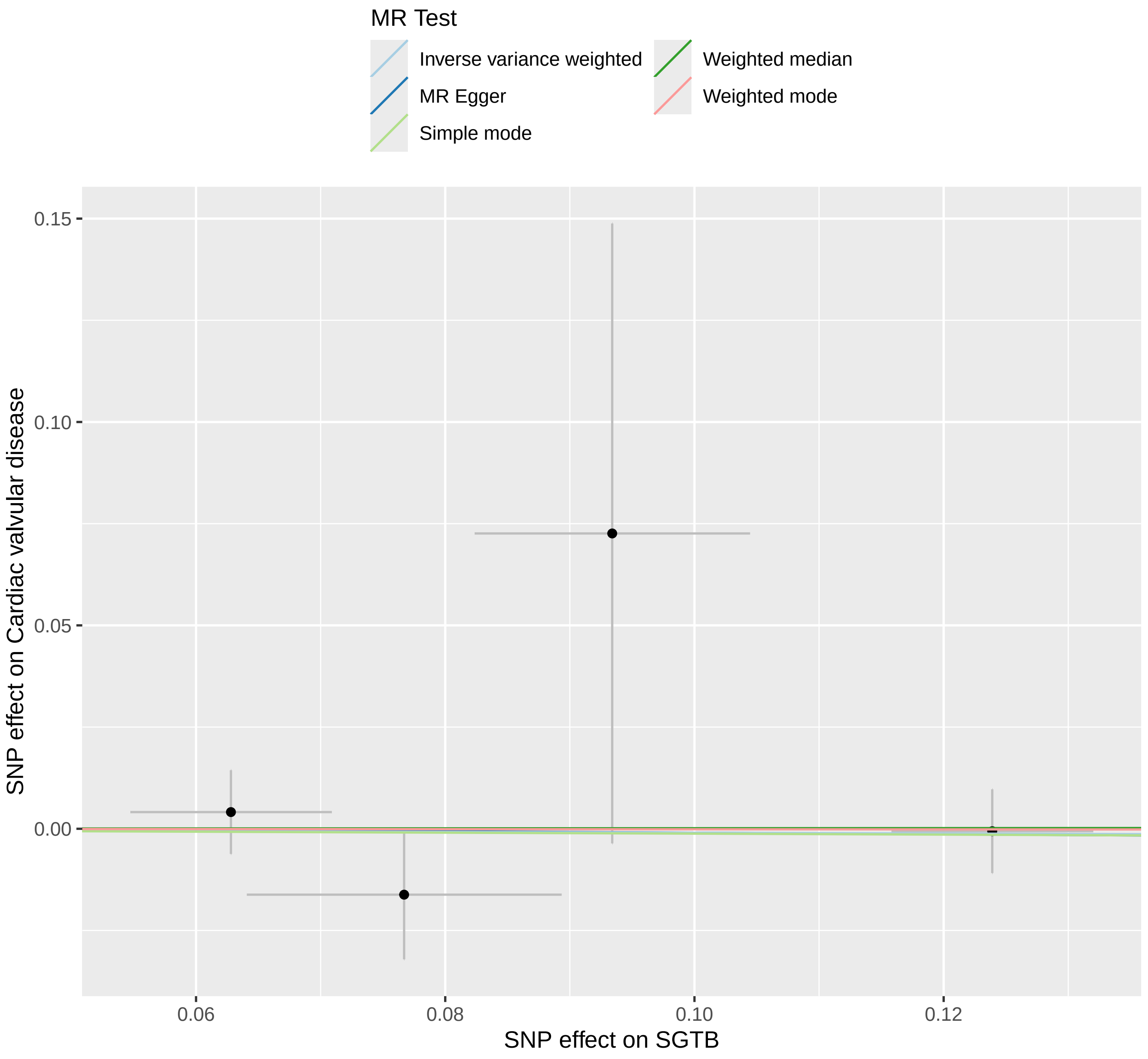
MR Test
Inverse variance weighted
MR Egger
Simple mode
Weighted median
Weighted mode
0.15
0.10
0.05
0.00
SNP effect on Cardiac valvular disease
0.06
0.08
0.10
0.12
SNP effect on SGTB

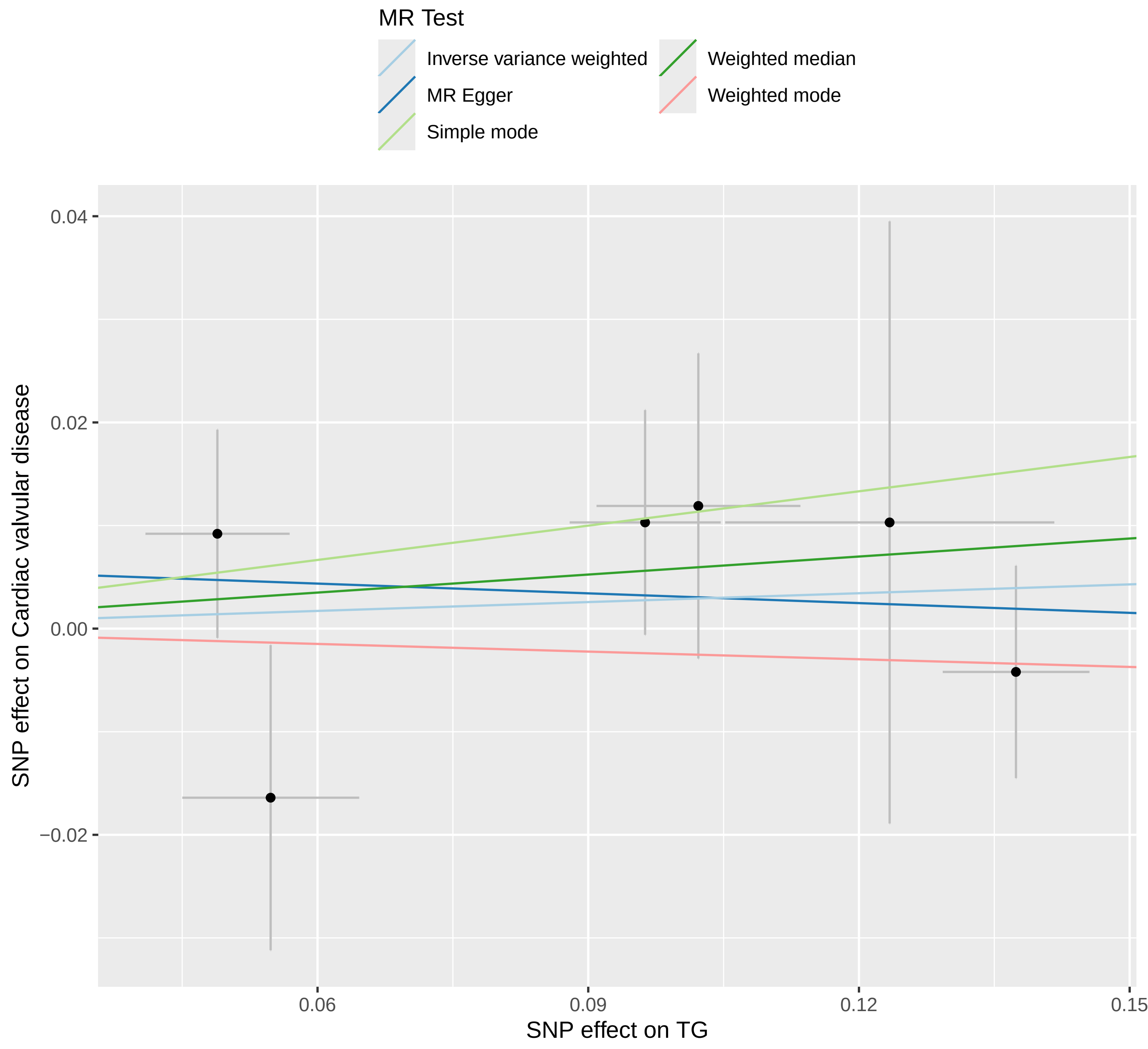

MR Test
Inverse variance weighted
MR Egger
Simple mode
Weighted median
Weighted mode
0.04
0.02
0.00
−0.02
0.06
0.09
0.12
0.15
SNP effect on TG
SNP effect on Cardiac valvular disease

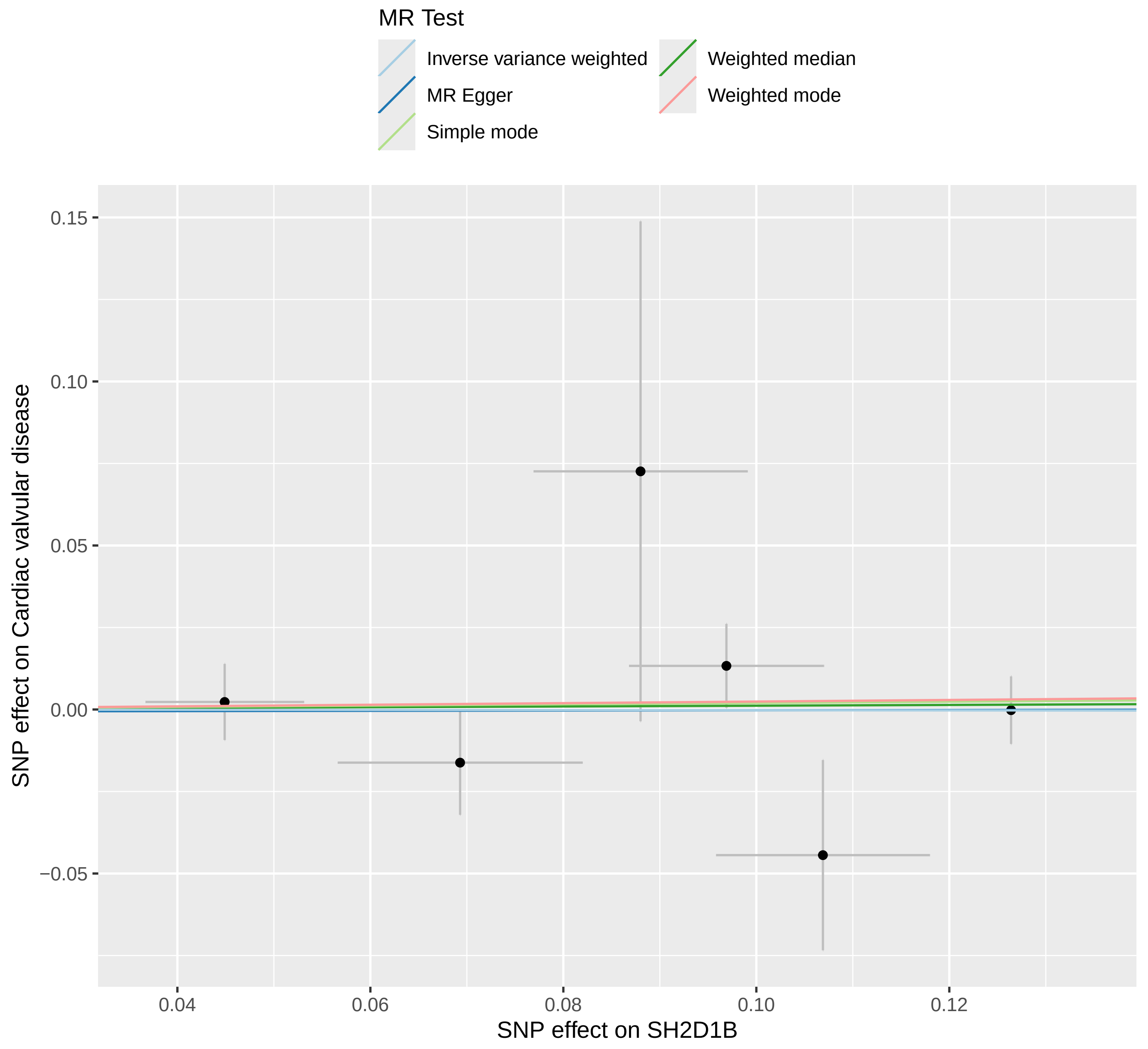

MR Test
Inverse variance weighted
MR Egger
Simple mode
Weighted median
Weighted mode
0.15
0.10
0.05
0.00
−0.05
SNP effect on Cardiac valvular disease
0.04
0.06
0.08
0.10
0.12
SNP effect on SH2D1B

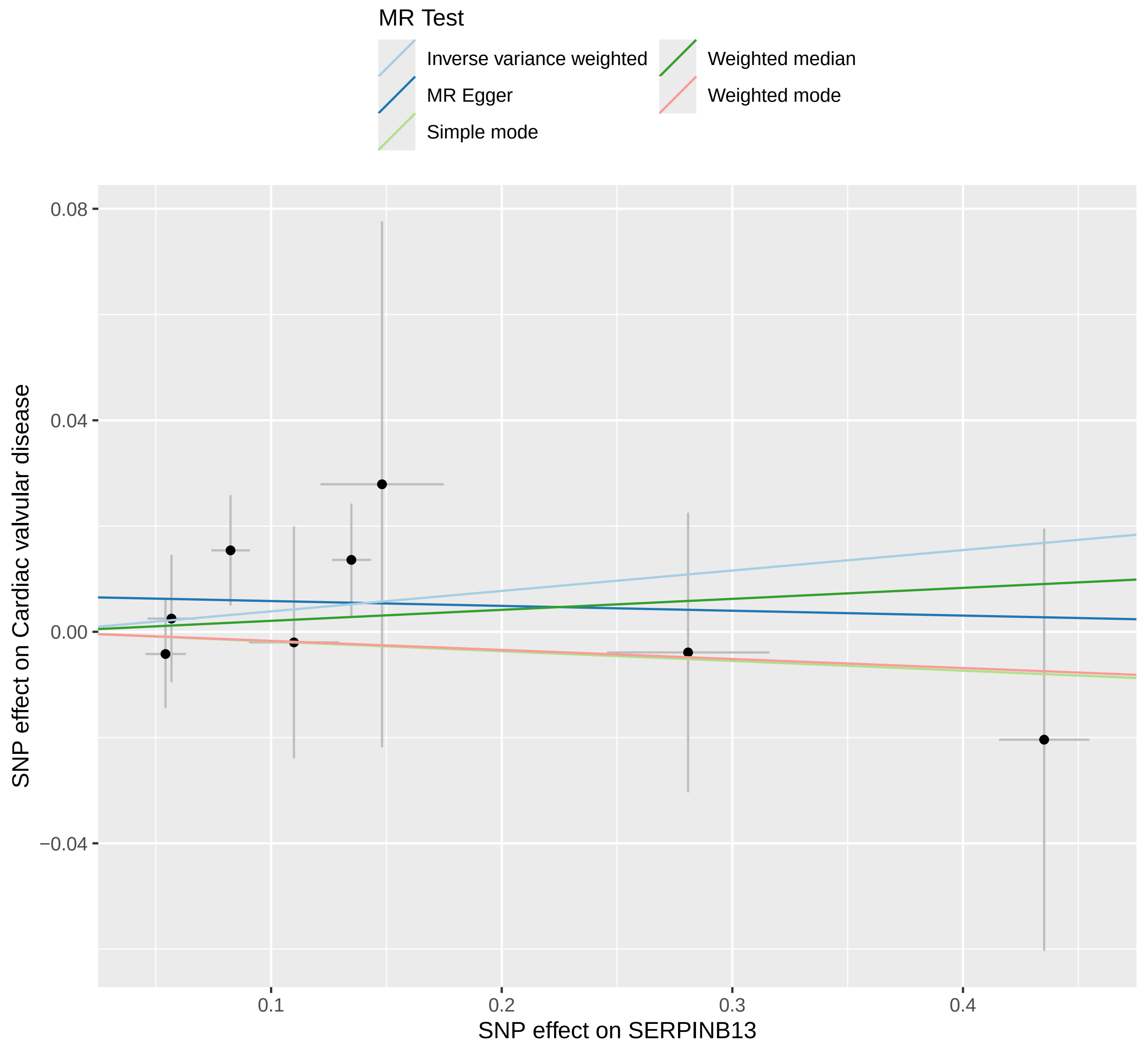
MR Test
Inverse variance weighted
MR Egger
Simple mode
Weighted median
Weighted mode
0.08
0.04
0.00
−0.04
0.1
0.2
0.3
0.4
SNP effect on SERPINB13
SNP effect on Cardiac valvular disease

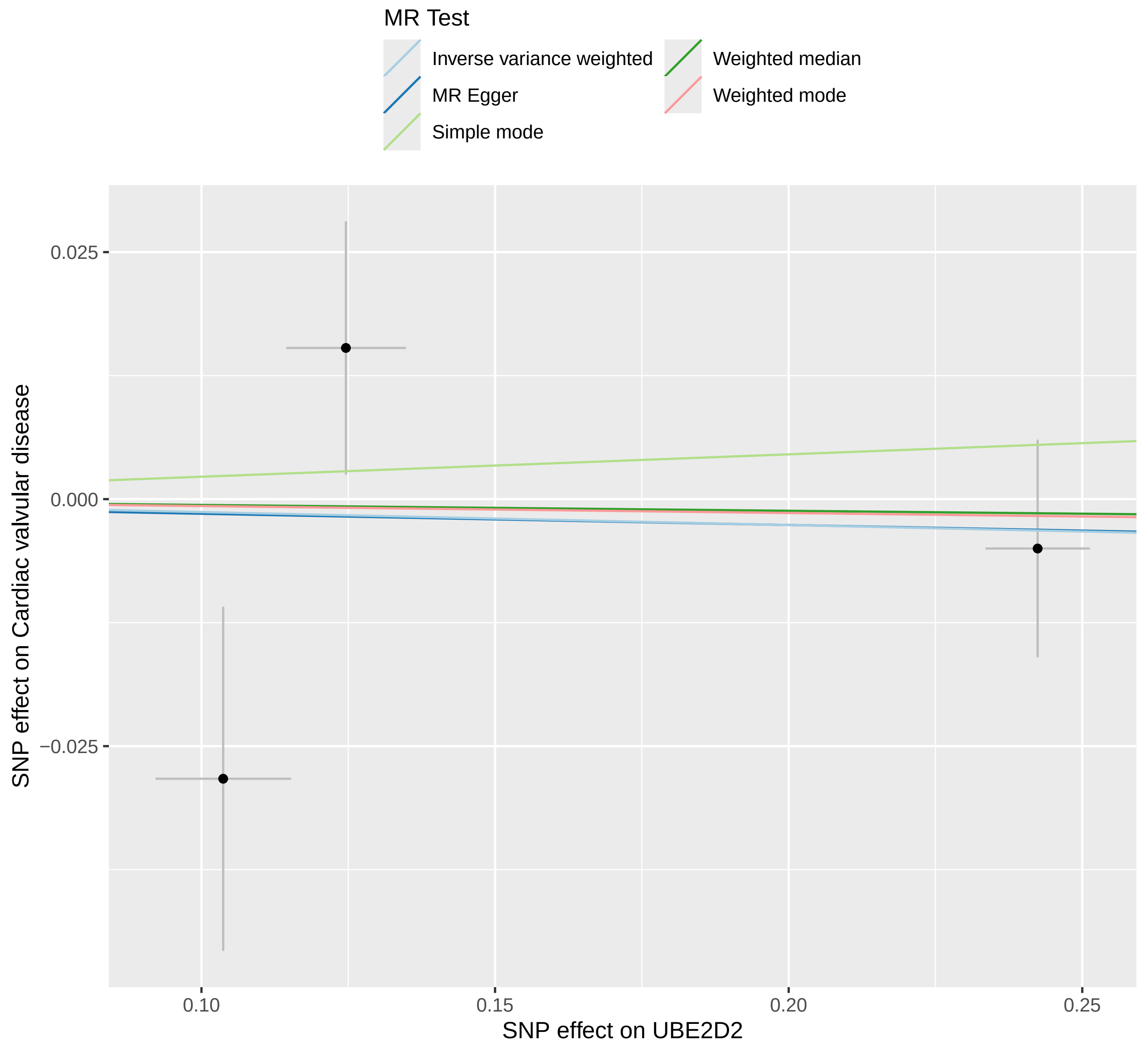
MR Test
Inverse variance weighted
MR Egger
Simple mode
Weighted median
Weighted mode
0.025
0.000
−0.025
0.10
0.15
0.20
0.25
SNP effect on Cardiac valvular disease
SNP effect on UBE2D2

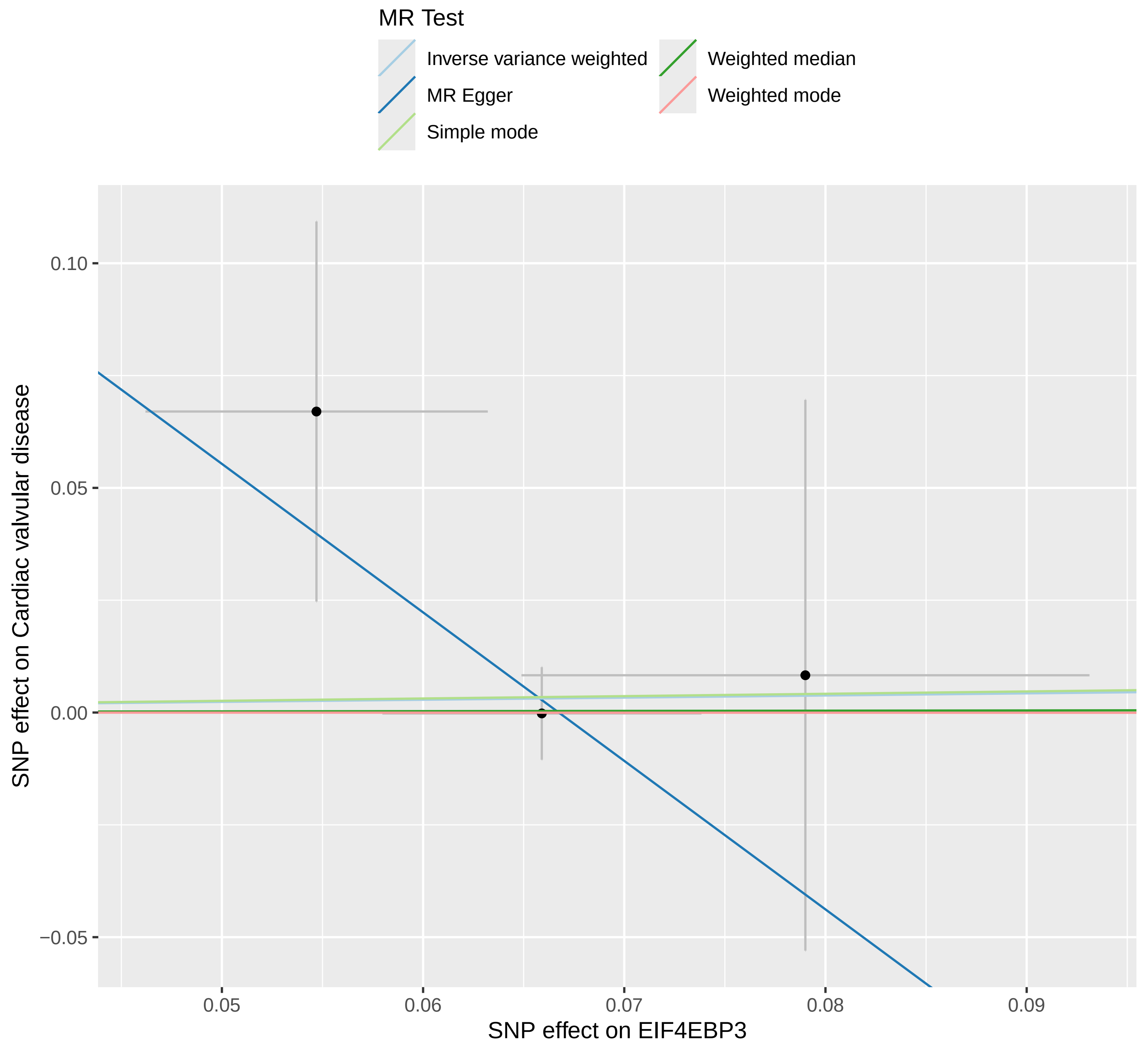

MR Test
Inverse variance weighted
MR Egger
Simple mode
Weighted median
Weighted mode
0.10
0.05
0.00
−0.05
SNP effect on Cardiac valvular disease
0.05
0.06
0.07
0.08
0.09
SNP effect on EIF4EBP3

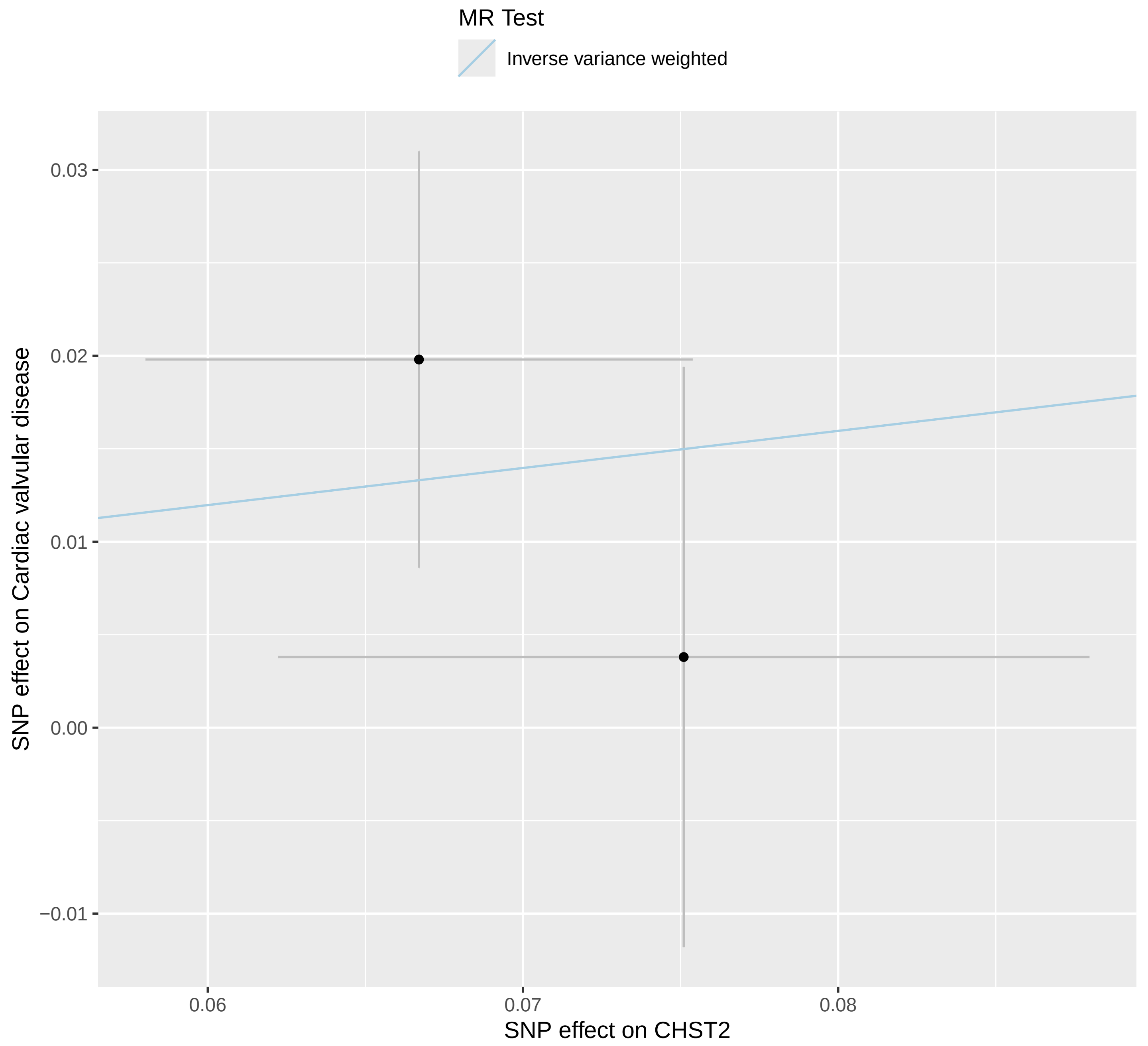
MR Test
Inverse variance weighted
0.03
0.02
0.01
0.00
−0.01
SNP effect on Cardiac valvular disease
0.06
0.07
0.08
SNP effect on CHST2

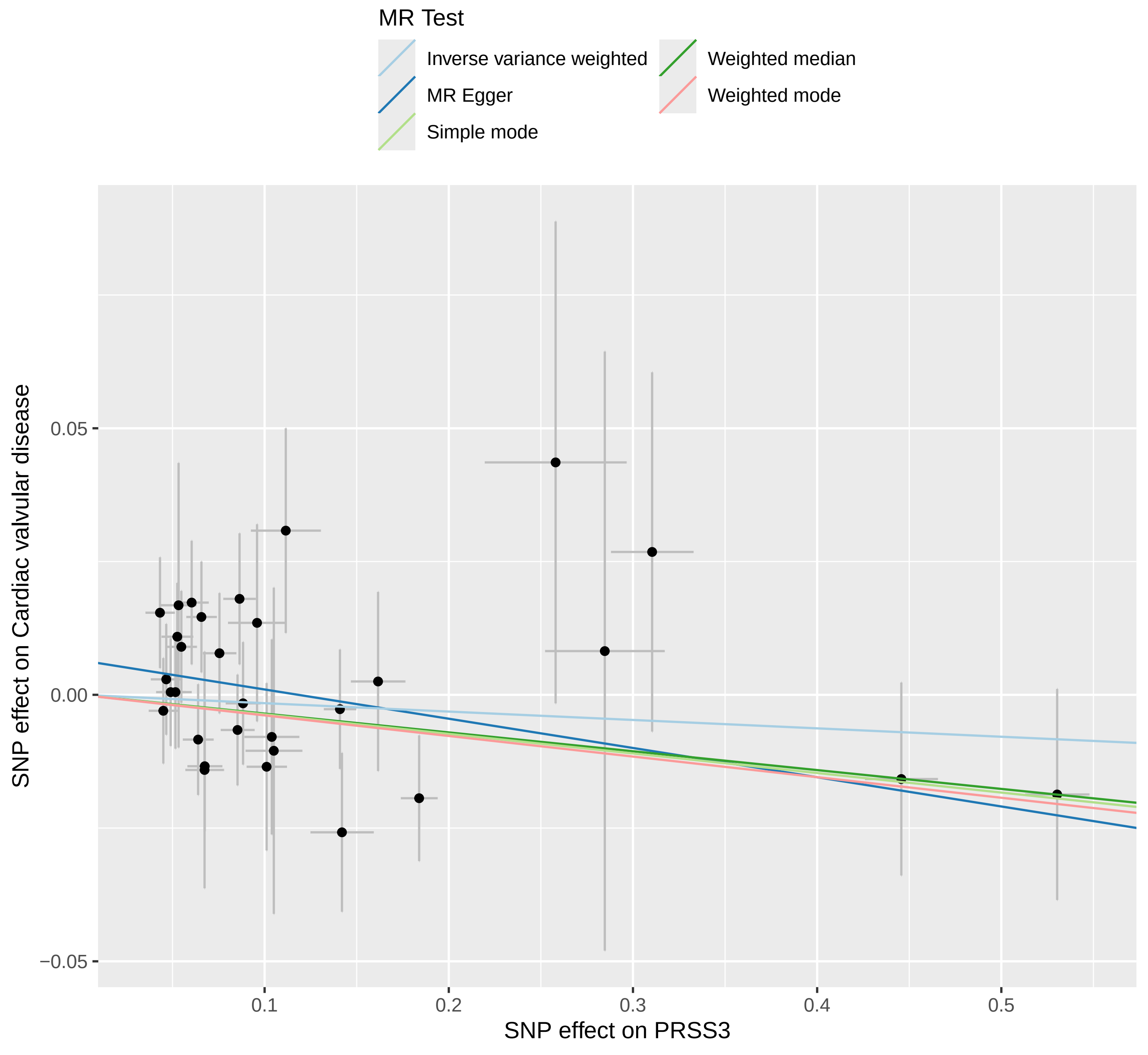

MR Test
Inverse variance weighted
MR Egger
Simple mode
Weighted median
Weighted mode
SNP effect on Cardiac valvular disease
0.05
0.00
−0.05
0.1
0.2
0.3
0.4
0.5
SNP effect on PRSS3

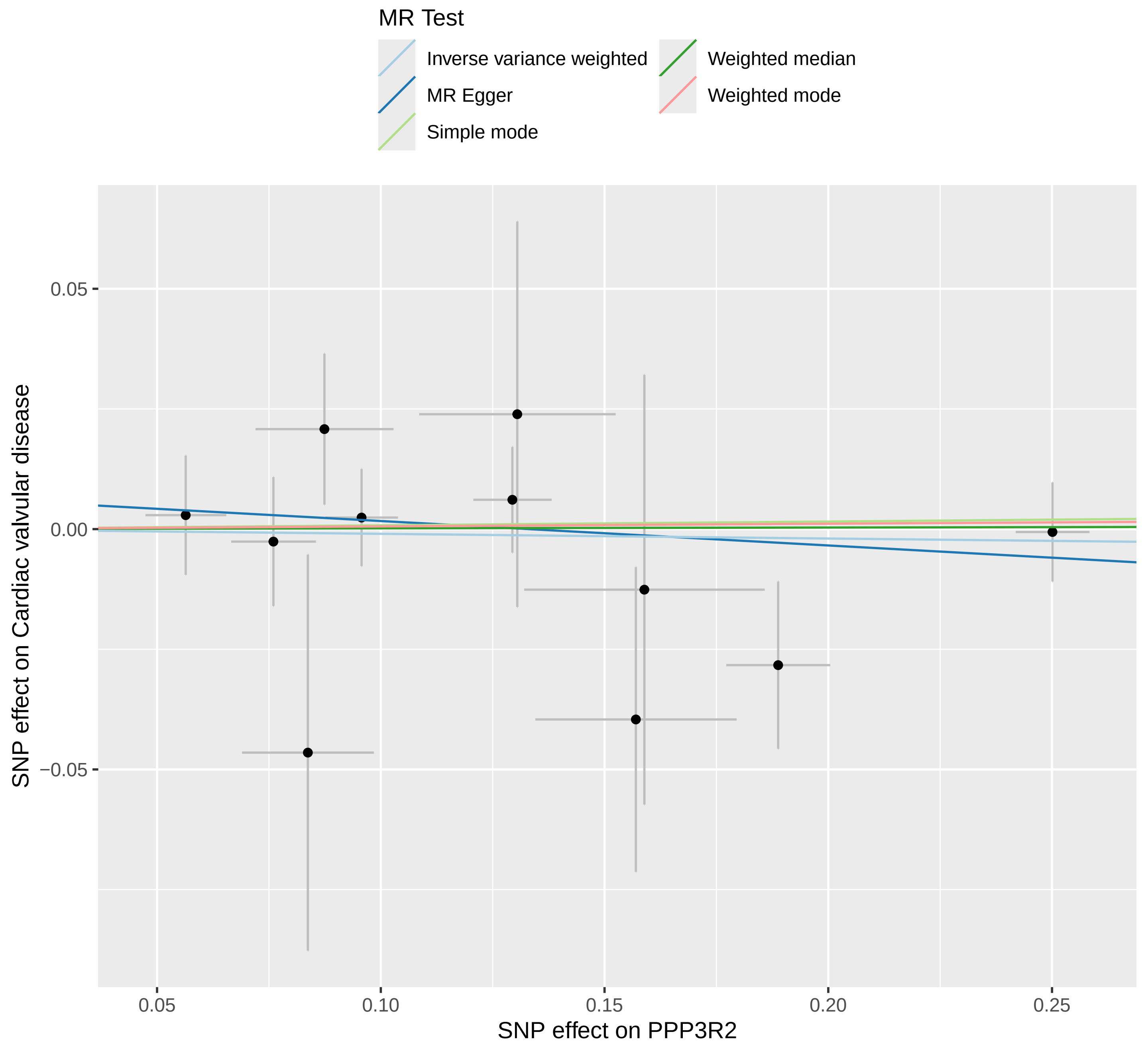

MR Test
Inverse variance weighted
MR Egger
Simple mode
Weighted median
Weighted mode
0.05
0.00
−0.05
0.05
0.10
0.15
0.20
0.25
SNP effect on PPP3R2
SNP effect on Cardiac valvular disease

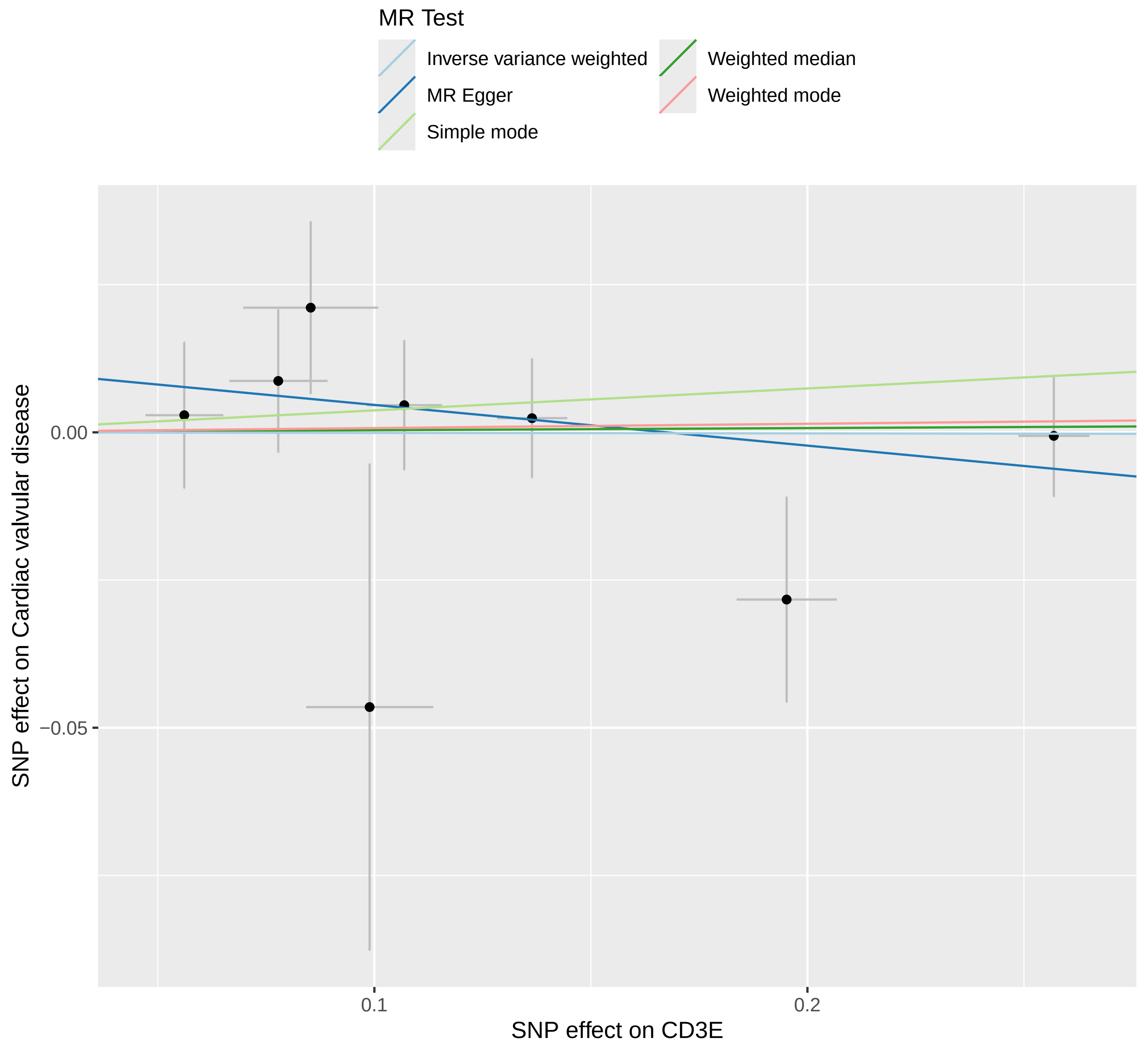
MR Test
Inverse variance weighted
MR Egger
Simple mode
Weighted median
Weighted mode
SNP effect on Cardiac valvular disease
0.00
−0.05
0.1
0.2
SNP effect on CD3E

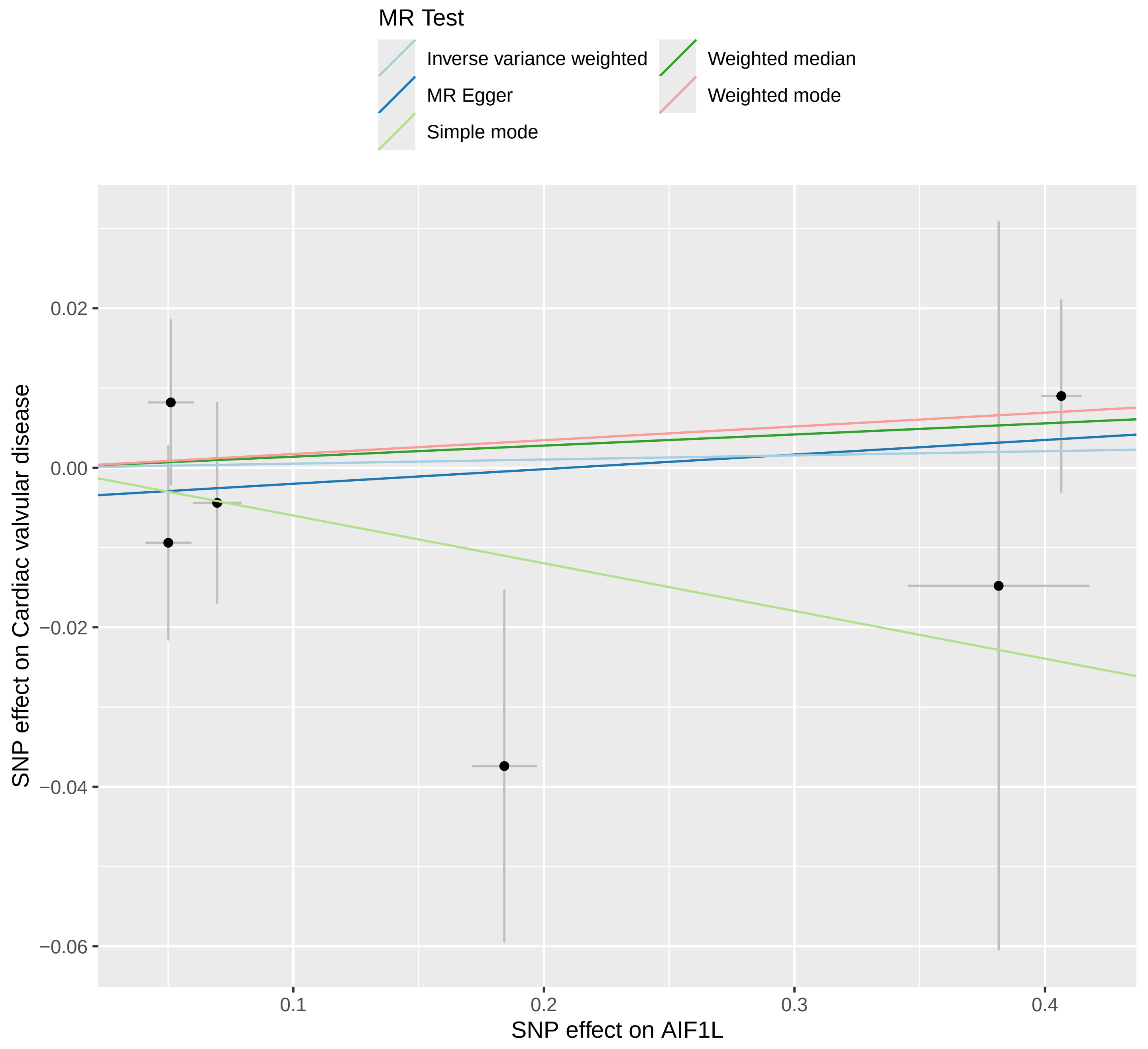
MR Test
Inverse variance weighted
MR Egger
Simple mode
Weighted median
Weighted mode
0.02
0.00
−0.02
−0.04
−0.06
0.1
0.2
0.3
0.4
SNP effect on Cardiac valvular disease
SNP effect on AIF1L

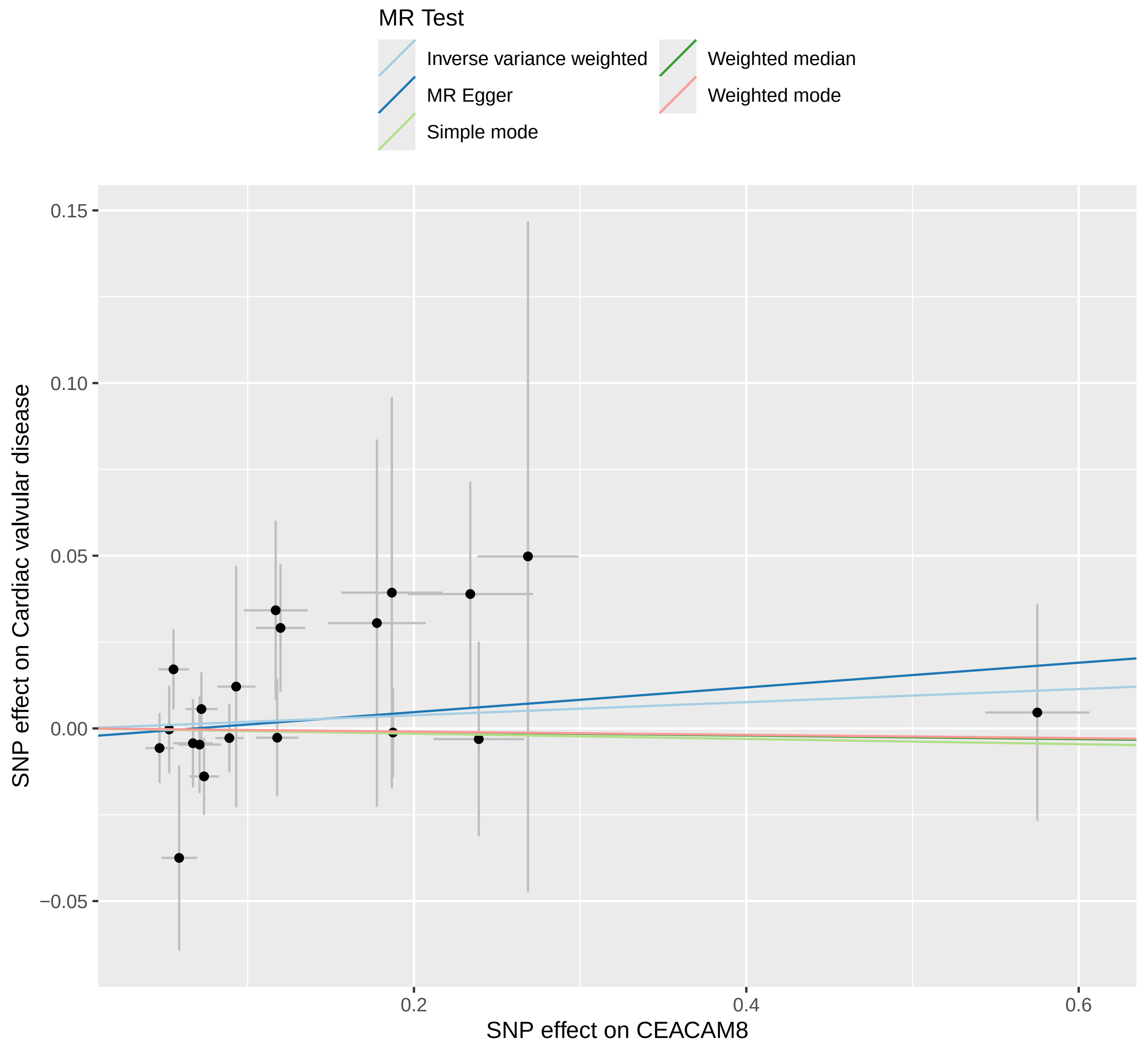
MR Test
Inverse variance weighted
MR Egger
Simple mode
Weighted median
Weighted mode
0.15
0.10
0.05
0.00
−0.05
0.2
0.4
0.6
SNP effect on Cardiac valvular disease
SNP effect on CEACAM8

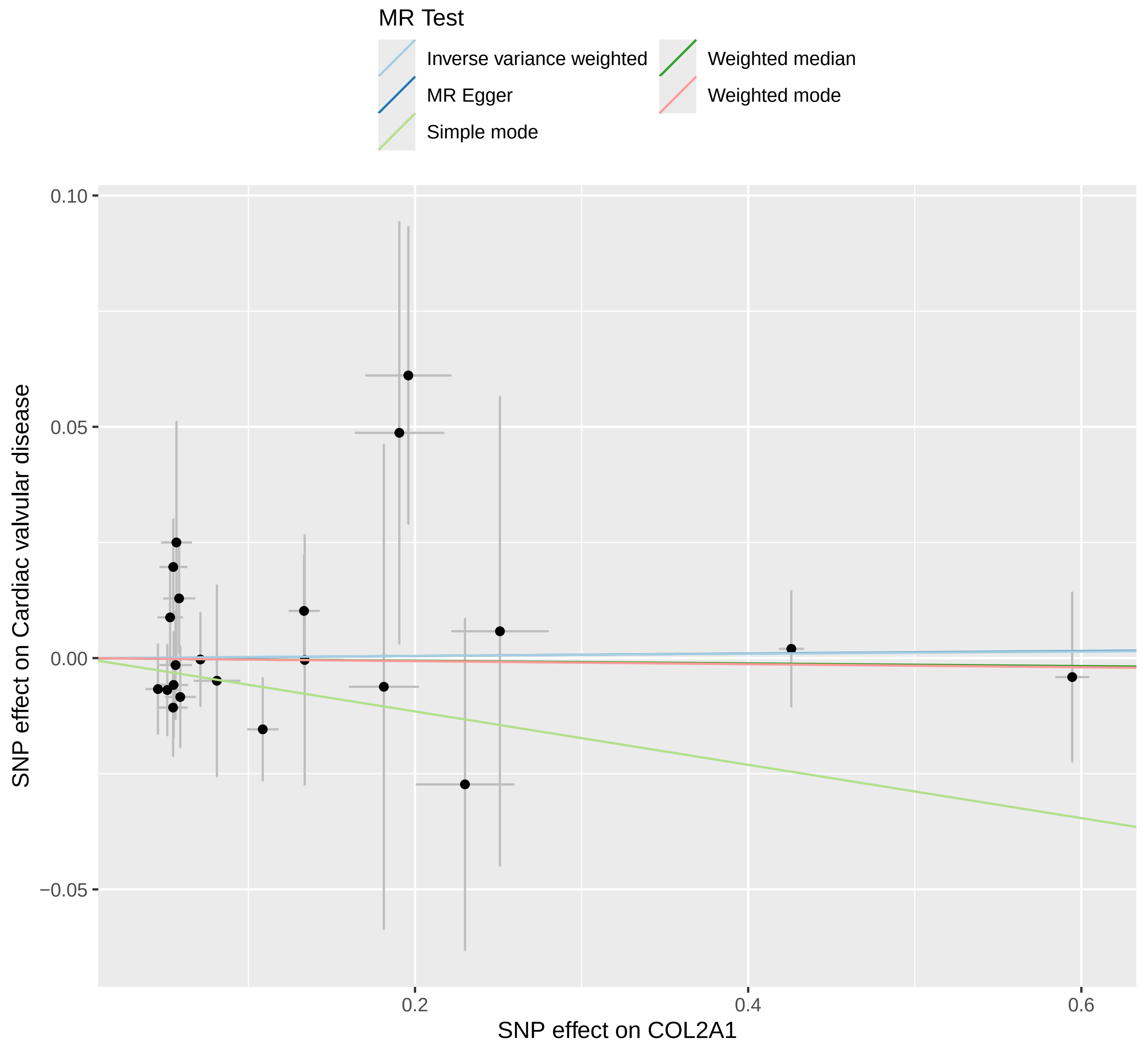
MR Test
Inverse variance weighted
MR Egger
Simple mode
Weighted median
Weighted mode
0.10
0.05
0.00
−0.05
0.2
0.4
0.6
SNP effect on Cardiac valvular disease
SNP effect on COL2A1

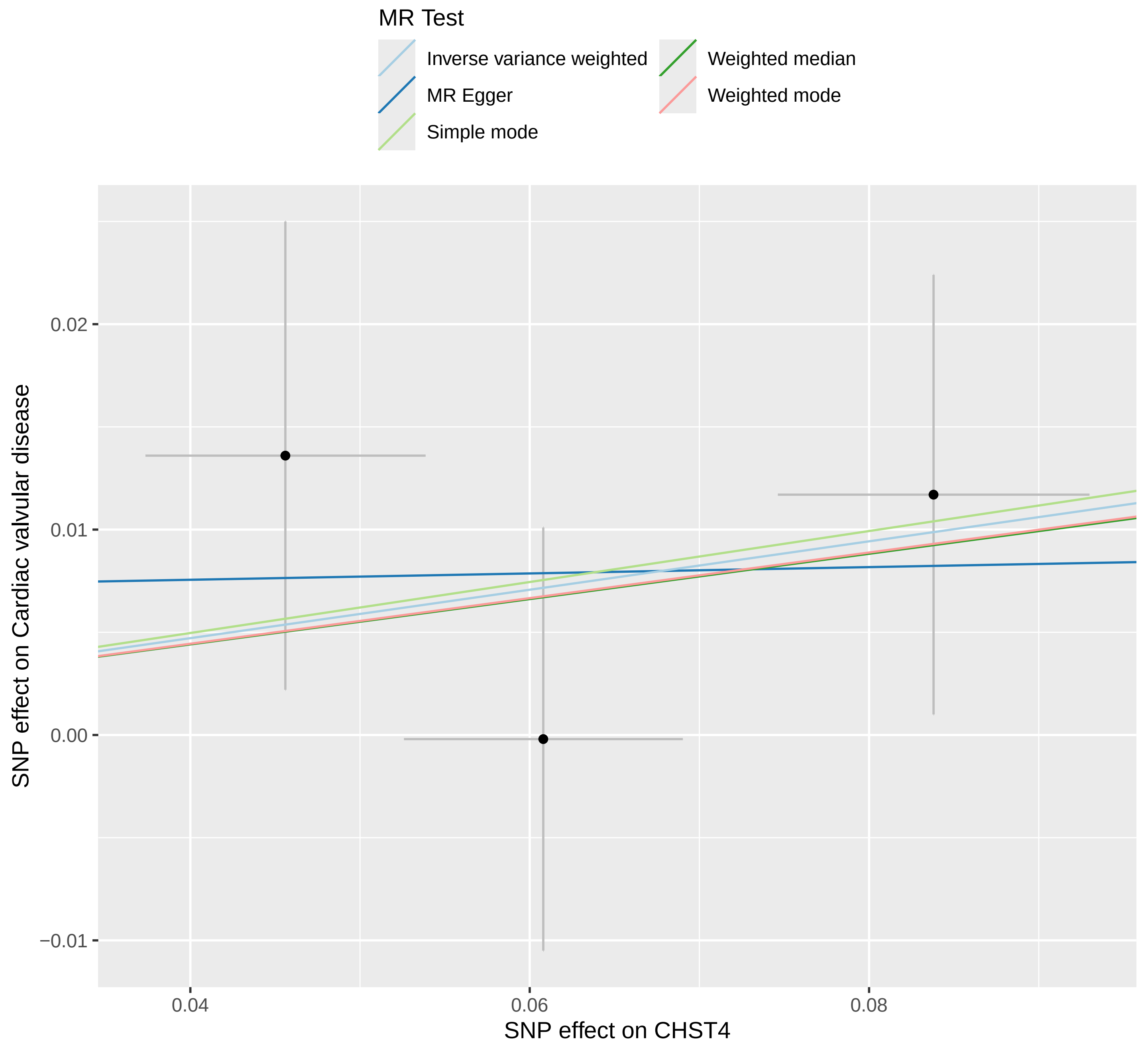
MR Test
Inverse variance weighted
MR Egger
Simple mode
Weighted median
Weighted mode
SNP effect on Cardiac valvular disease
0.02
0.01
0.00
−0.01
0.04
0.06
0.08
SNP effect on CHST4

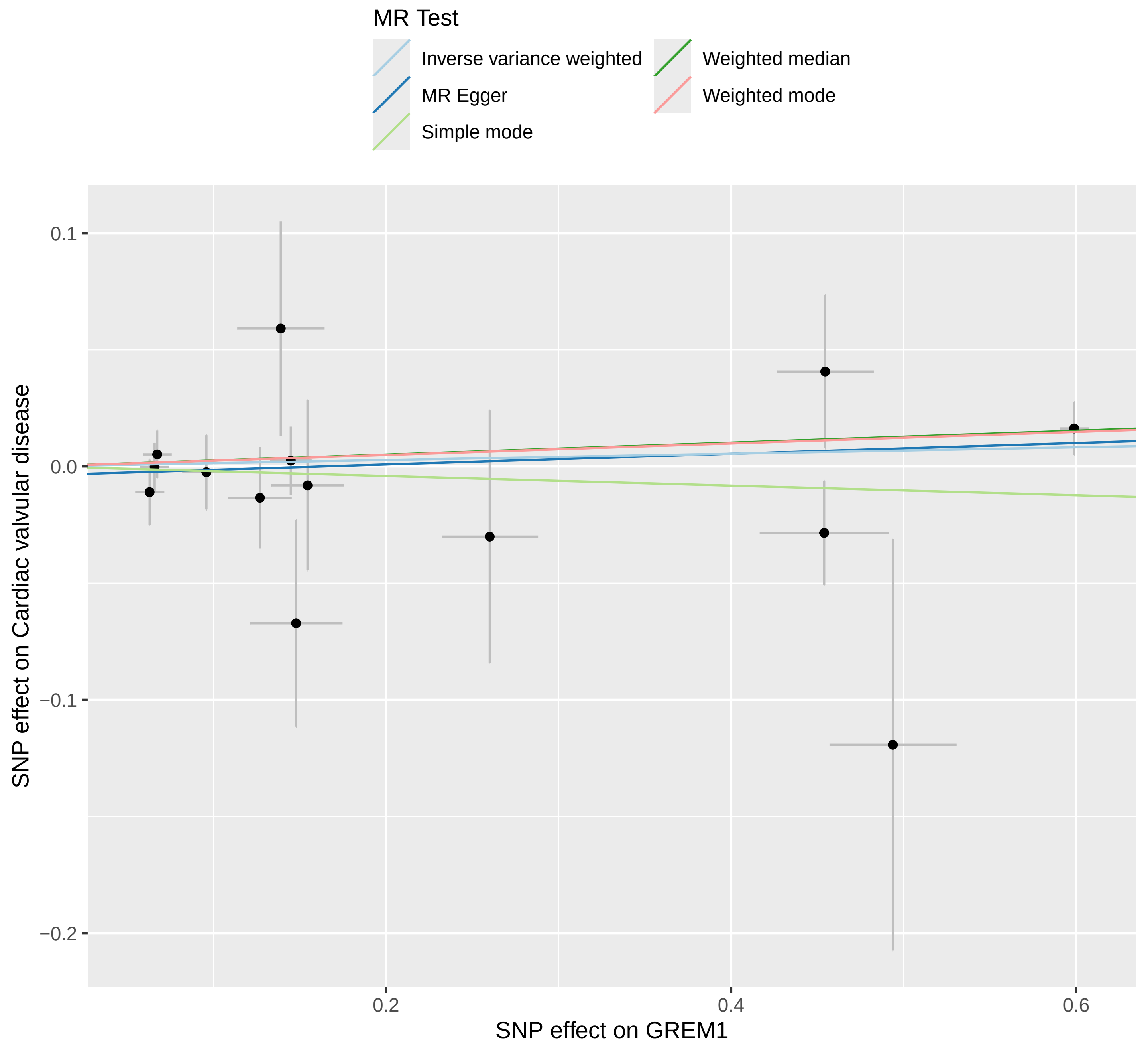

MR Test
Inverse variance weighted
MR Egger
Simple mode
Weighted median
Weighted mode
0.1
0.0
−0.1
−0.2
0.2
0.4
0.6
SNP effect on GREM1
SNP effect on Cardiac valvular disease

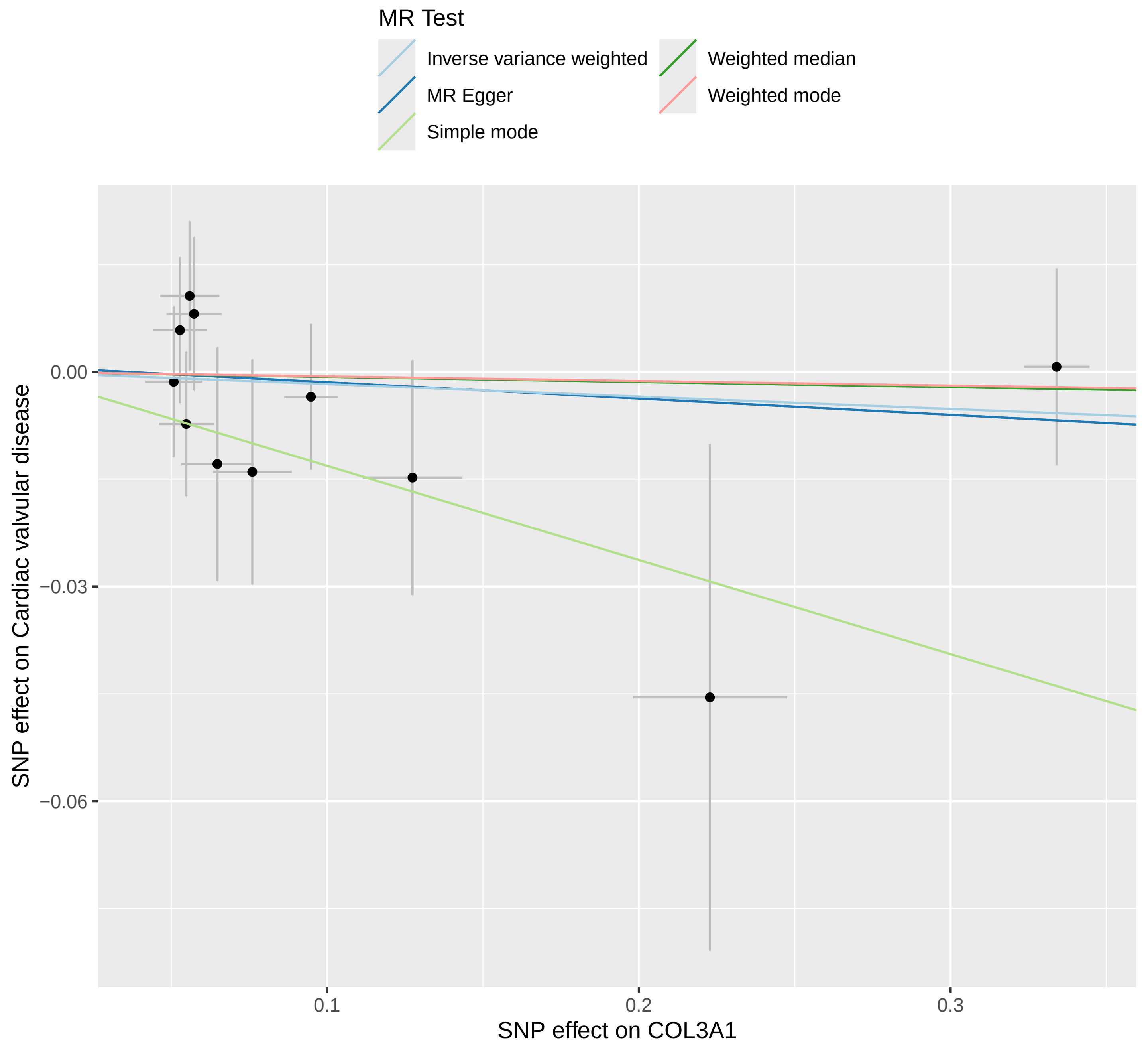
MR Test
Inverse variance weighted
MR Egger
Simple mode
Weighted median
Weighted mode
SNP effect on Cardiac valvular disease
0.00
−0.03
−0.06
0.1
0.2
0.3
SNP effect on COL3A1

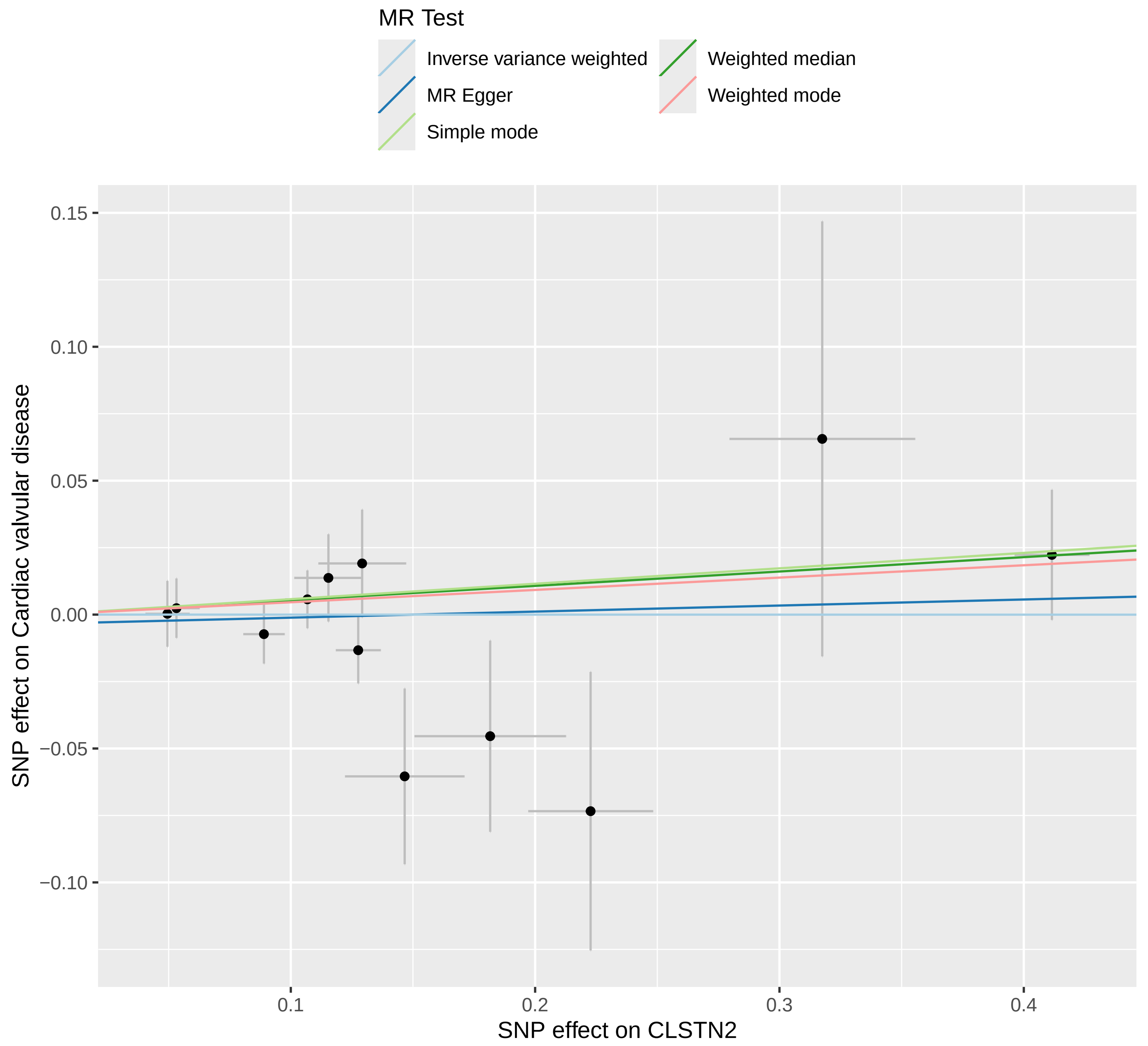
MR Test
Inverse variance weighted
MR Egger
Simple mode
Weighted median
Weighted mode
0.15
0.10
0.05
0.00
−0.05
−0.10
0.1
0.2
0.3
0.4
SNP effect on Cardiac valvular disease
SNP effect on CLSTN2

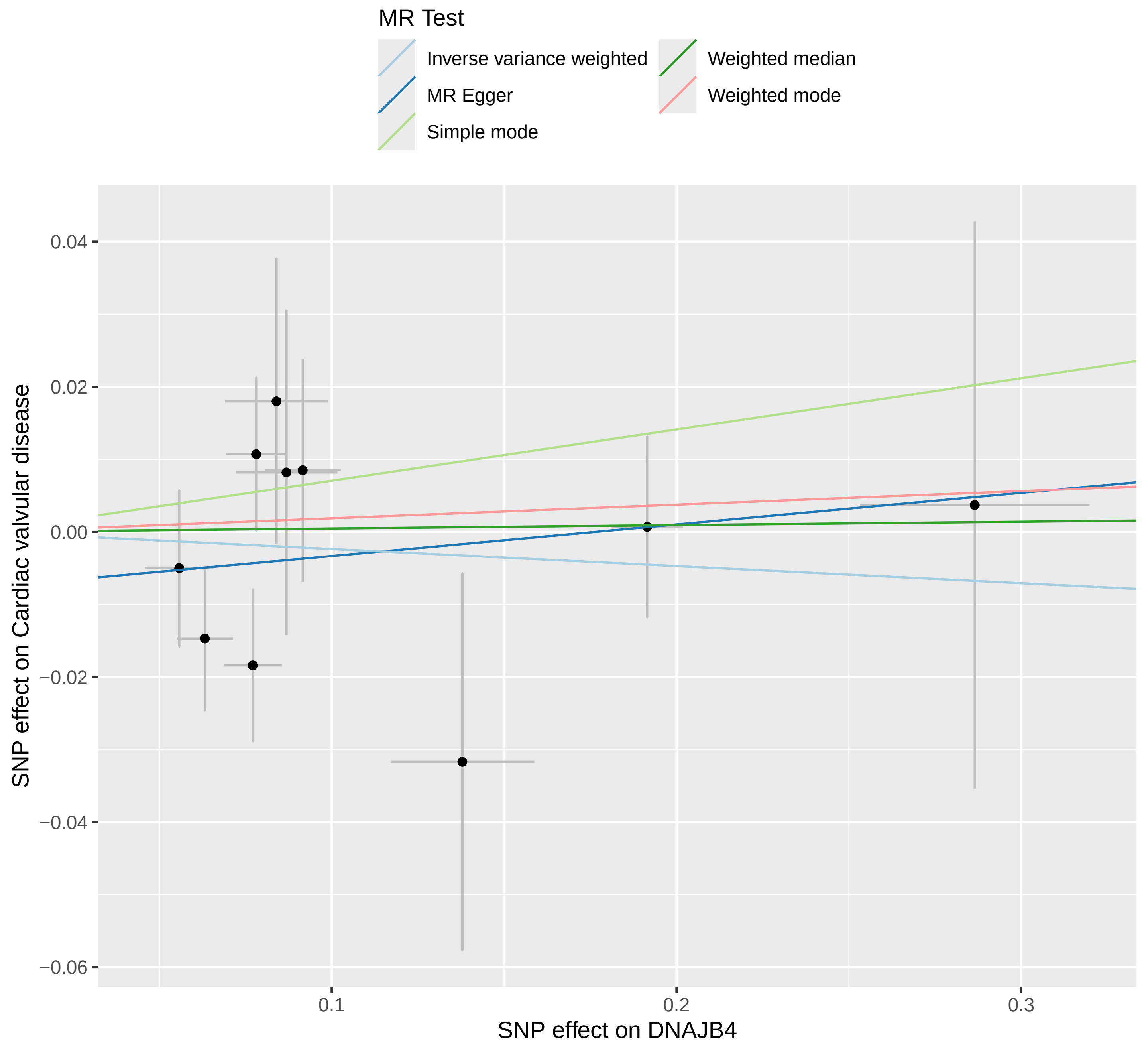

MR Test
Inverse variance weighted
MR Egger
Simple mode
Weighted median
Weighted mode
0.04
0.02
0.00
−0.02
−0.04
−0.06
SNP effect on Cardiac valvular disease
0.1
0.2
0.3
SNP effect on DNAJB4

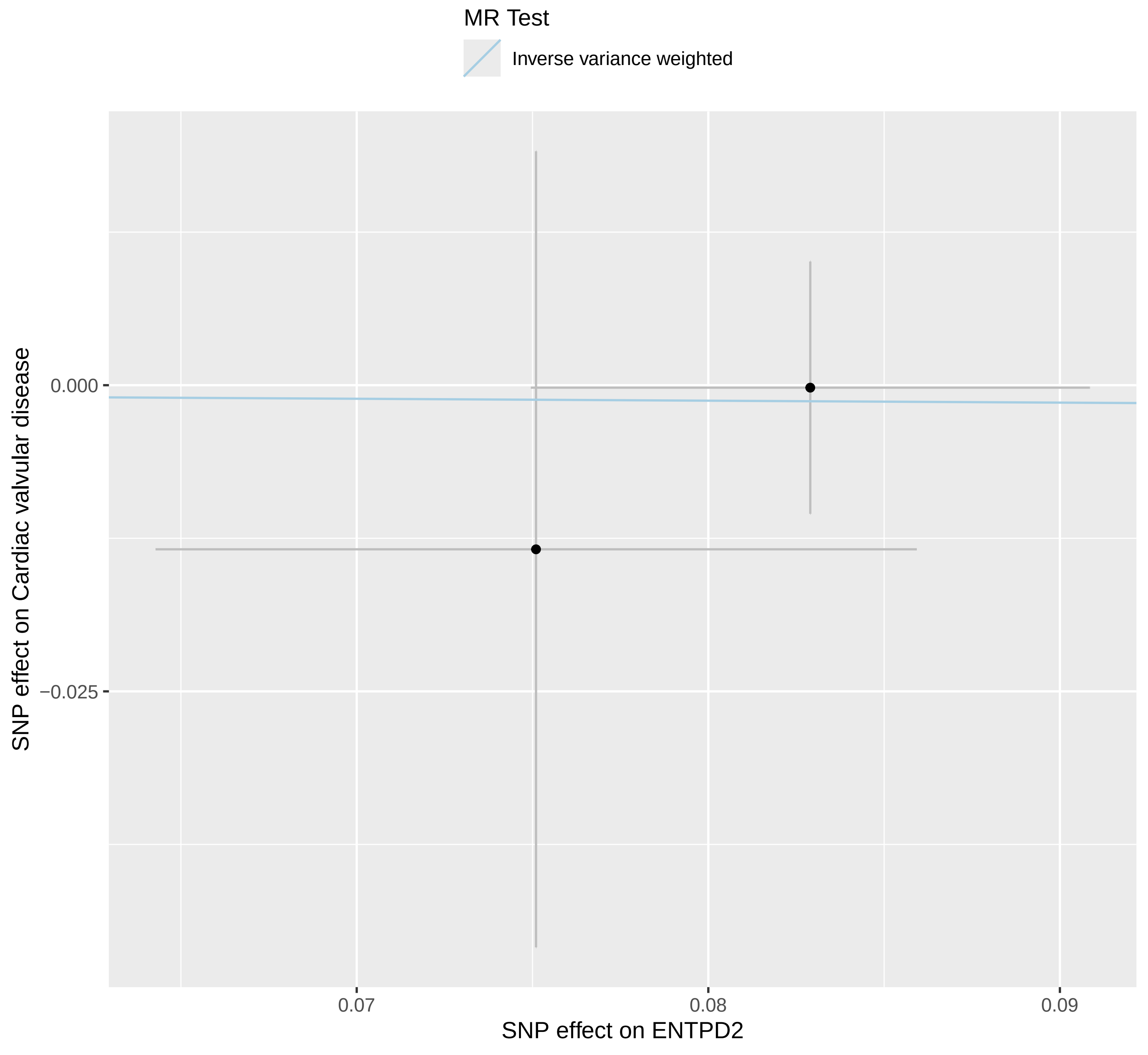

MR Test
Inverse variance weighted
SNP effect on Cardiac valvular disease
0.000
−0.025
0.07
0.08
0.09
SNP effect on ENTPD2

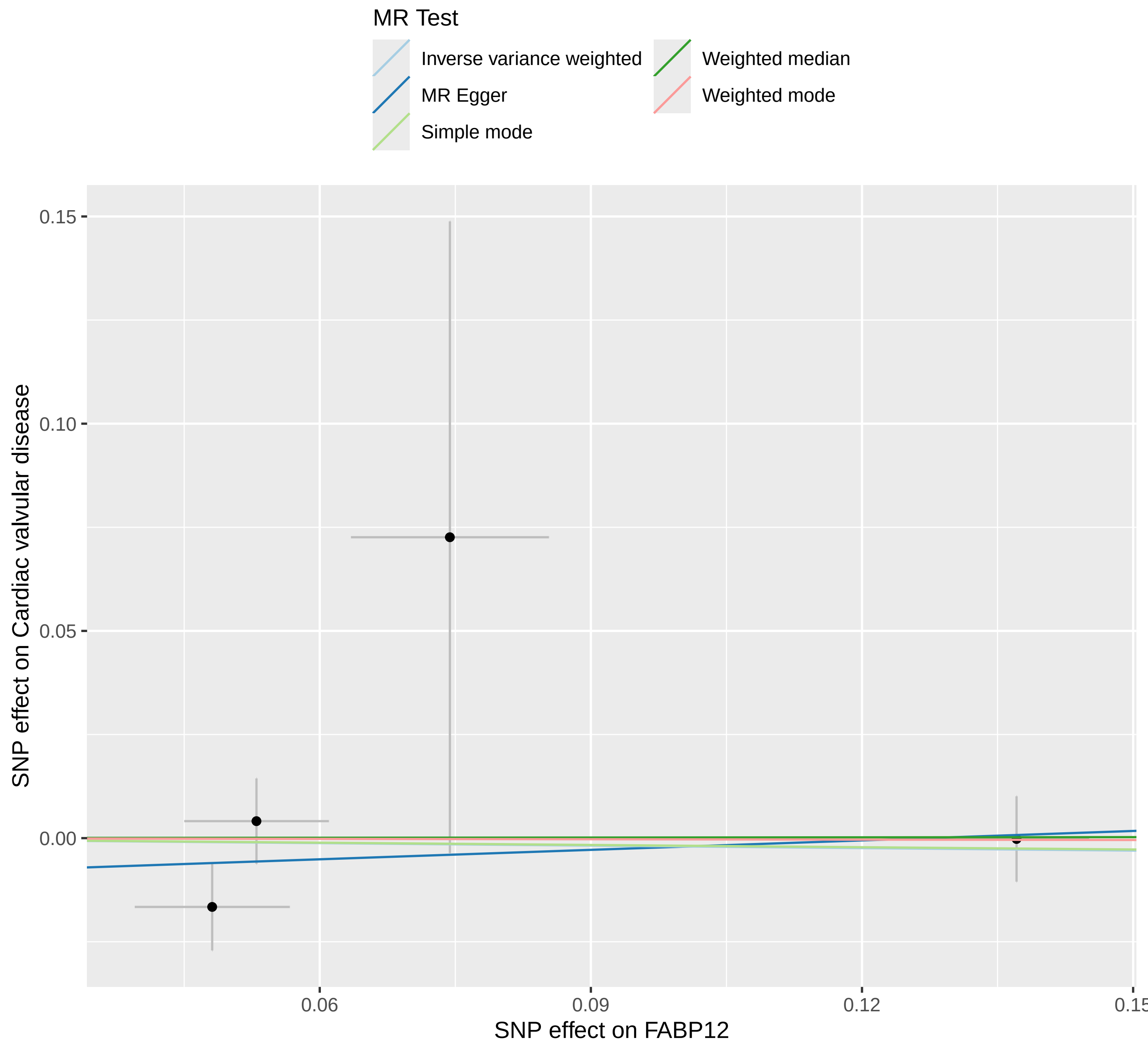
MR Test
Inverse variance weighted
MR Egger
Simple mode
Weighted median
Weighted mode
0.15
0.10
0.05
0.00
SNP effect on Cardiac valvular disease
0.06
0.09
0.12
0.15
SNP effect on FABP12

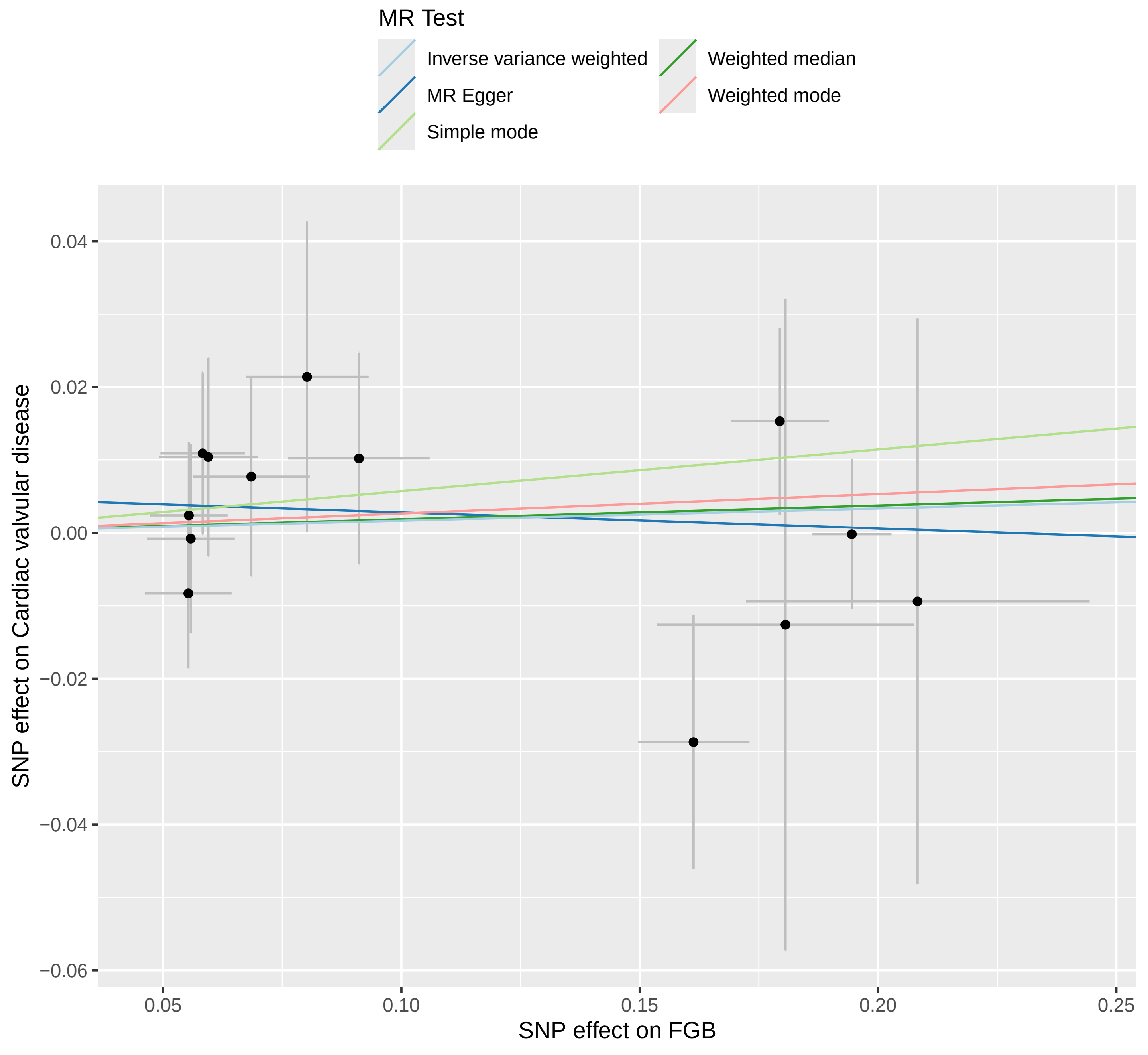

MR Test
Inverse variance weighted
MR Egger
Simple mode
Weighted median
Weighted mode
SNP effect on Cardiac valvular disease
0.04
0.02
0.00
−0.02
−0.04
−0.06
0.05
0.10
0.15
0.20
0.25
SNP effect on FGB

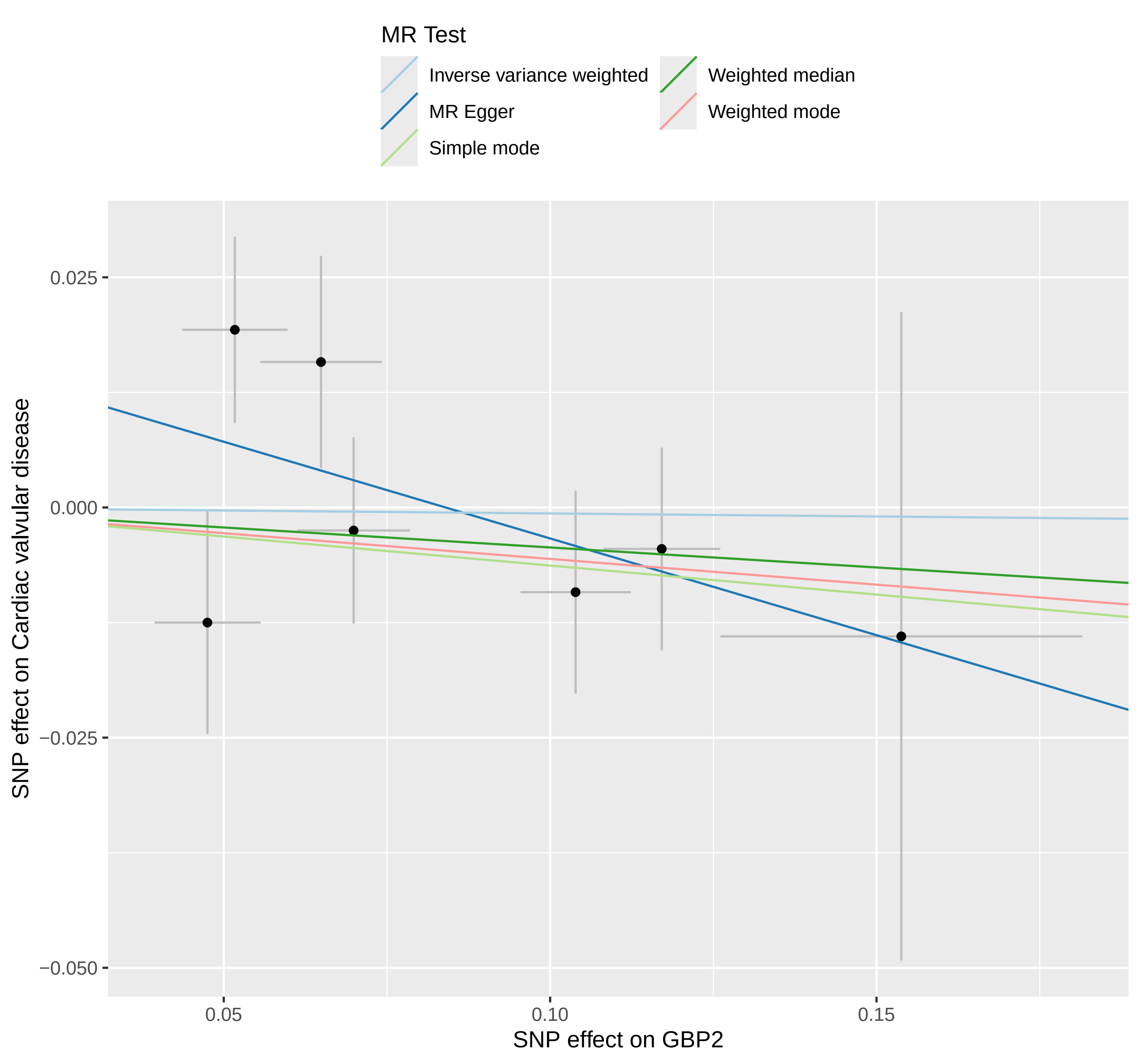
MR Test
Inverse variance weighted
MR Egger
Simple mode
Weighted median
Weighted mode
0.025
0.000
−0.025
−0.050
0.05
0.10
0.15
SNP effect on GBP2
SNP effect on Cardiac valvular disease

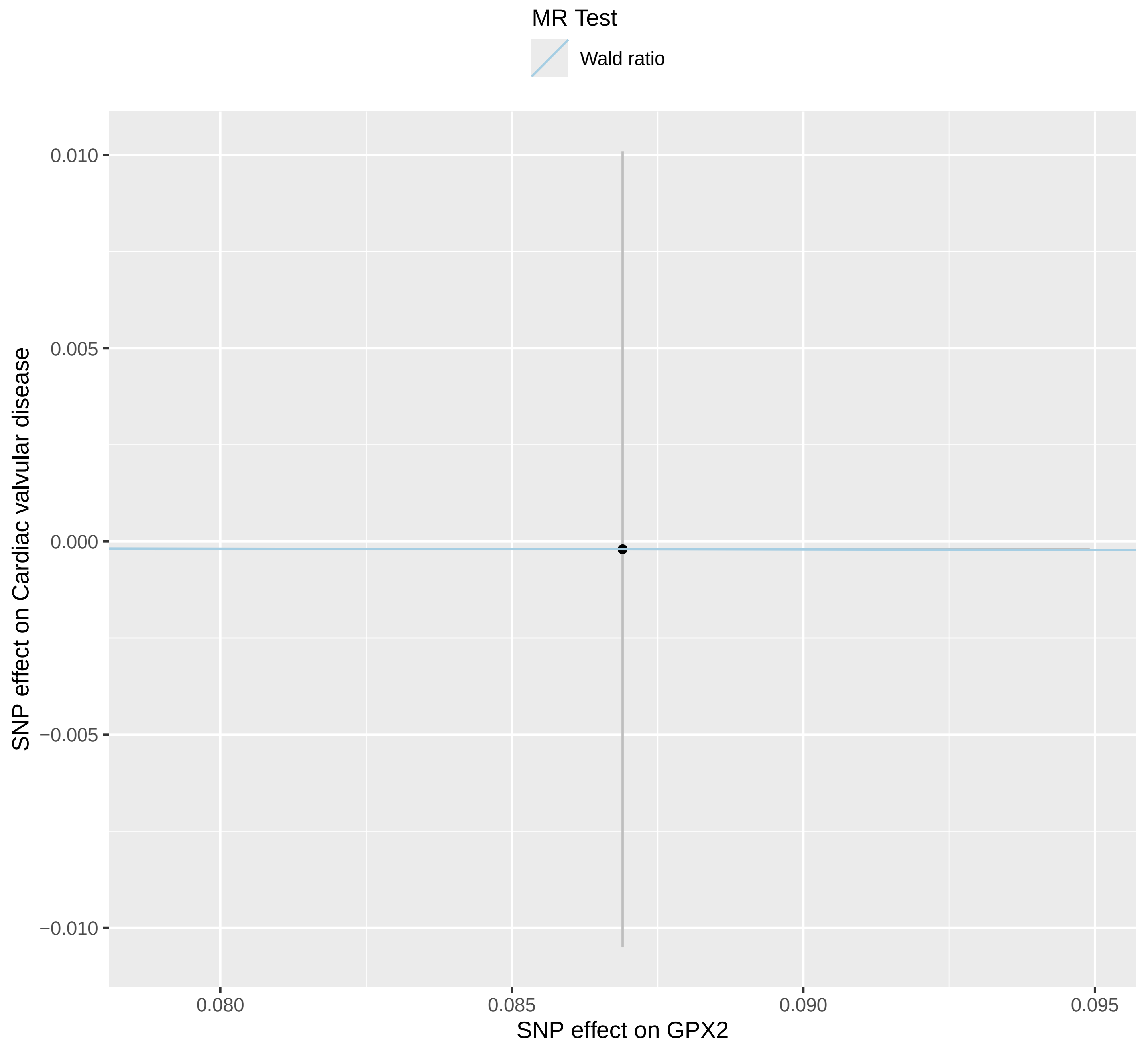

MR Test
Wald ratio
0.010
0.005
0.000
−0.005
−0.010
SNP effect on Cardiac valvular disease
0.080
0.085
0.090
0.095
SNP effect on GPX2

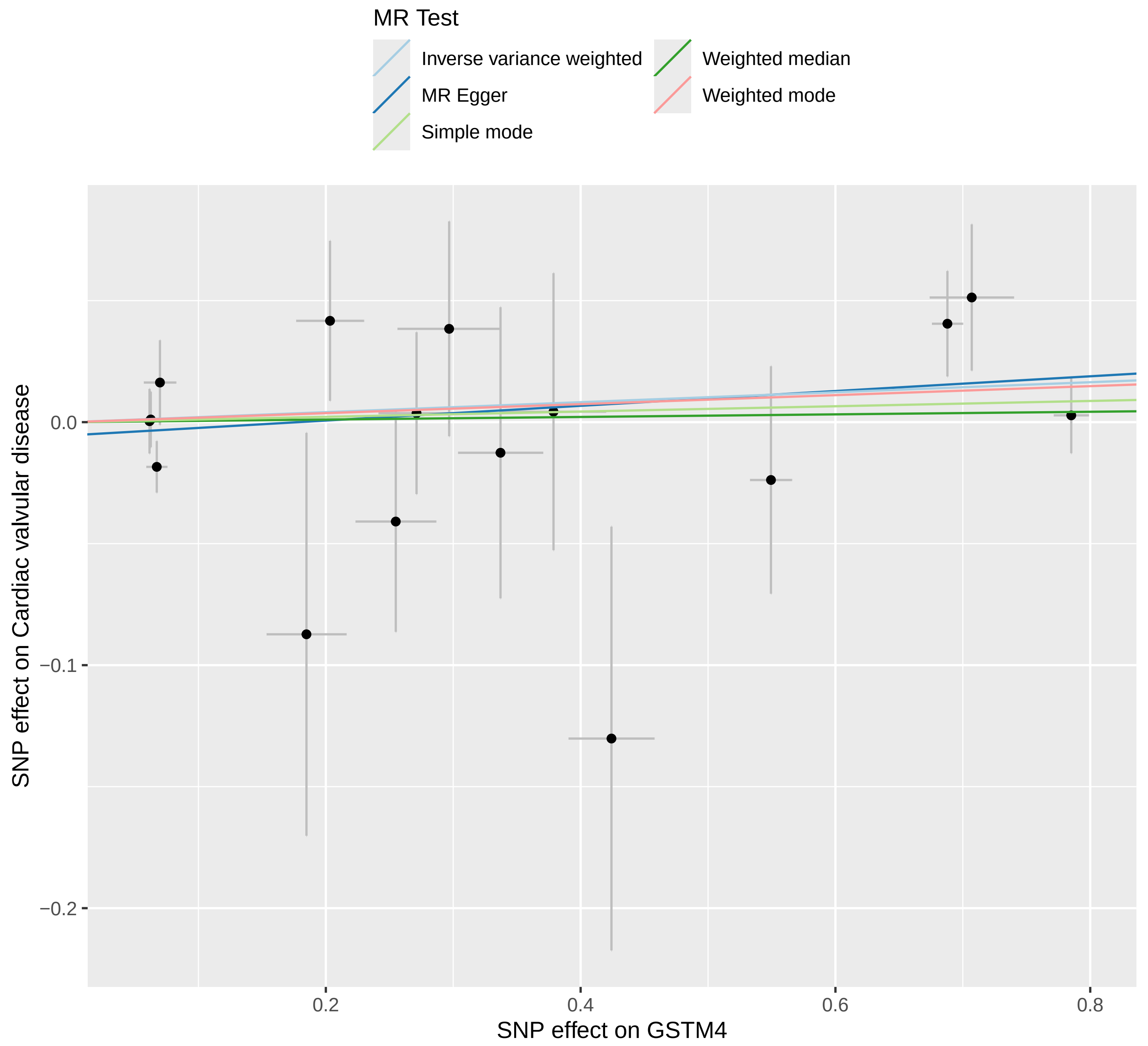
MR Test
Inverse variance weighted
MR Egger
Simple mode
Weighted median
Weighted mode
0.0
−0.1
−0.2
0.2
0.4
0.6
0.8
SNP effect on Cardiac valvular disease
SNP effect on GSTM4

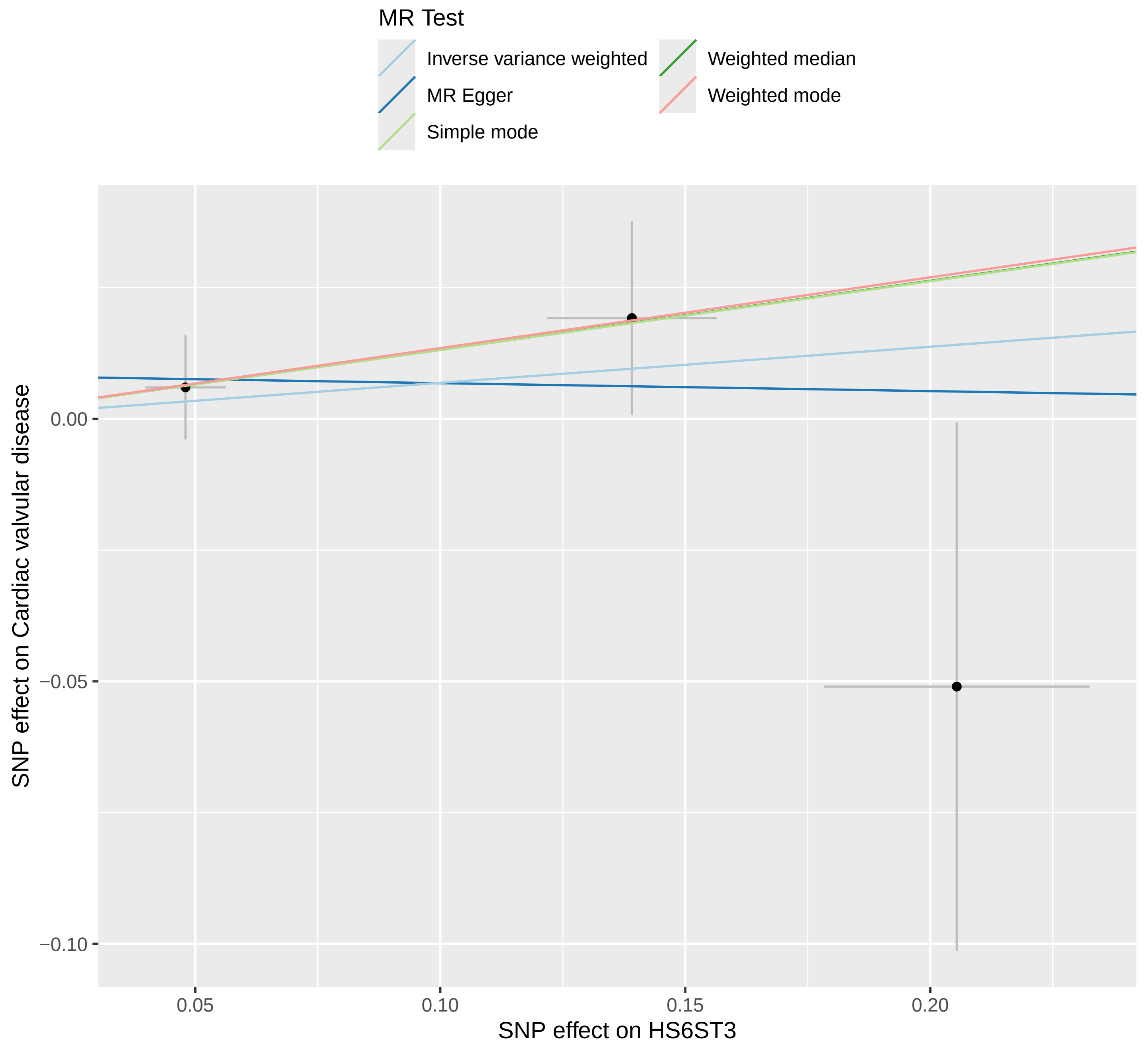
MR Test
Inverse variance weighted
MR Egger
Simple mode
Weighted median
Weighted mode
SNP effect on Cardiac valvular disease
0.00
−0.05
−0.10
0.05
0.10
0.15
0.20
SNP effect on HS6ST3

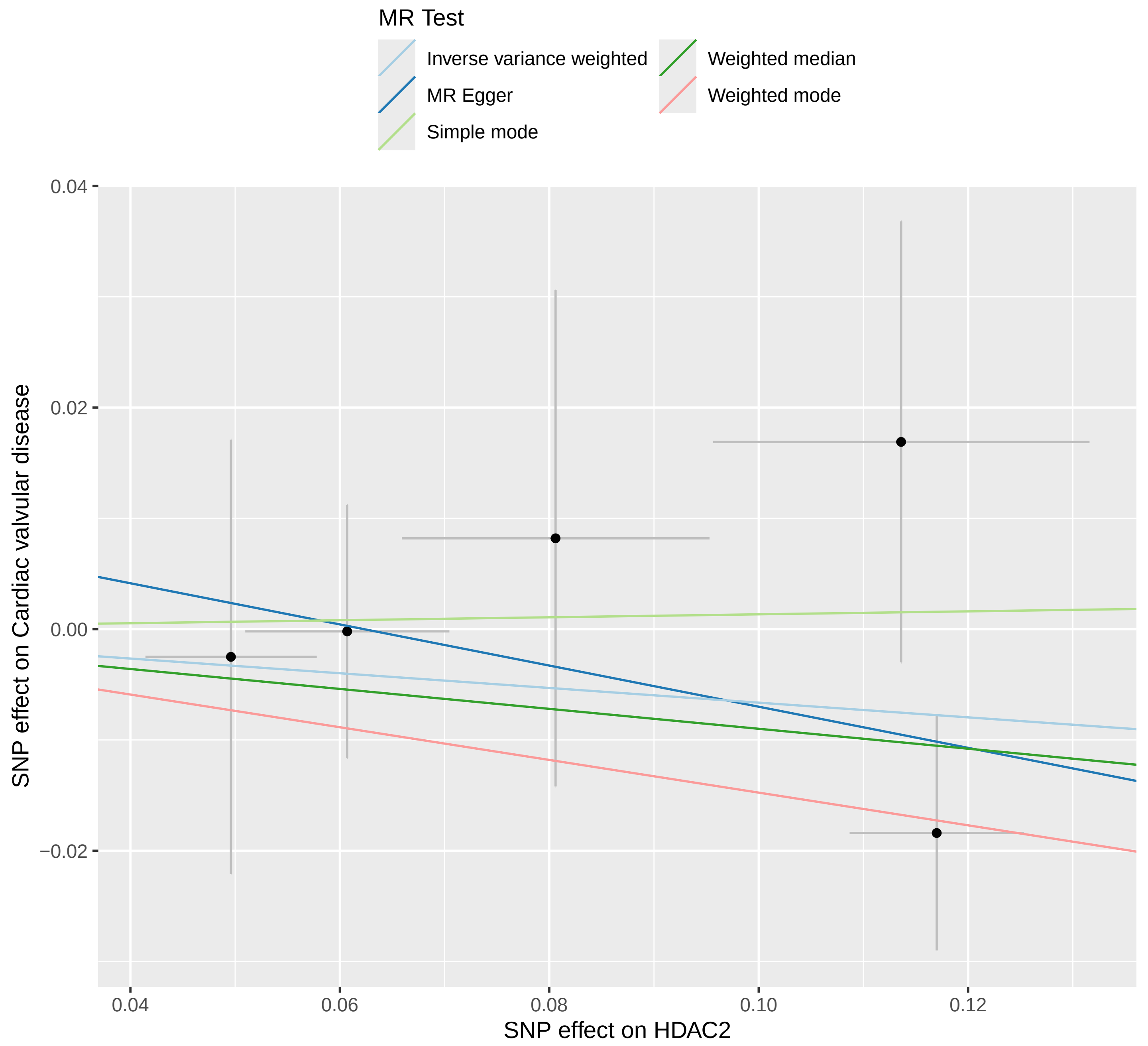

MR Test
Inverse variance weighted
MR Egger
Simple mode
Weighted median
Weighted mode
0.04
0.02
0.00
−0.02
SNP effect on Cardiac valvular disease
0.04
0.06
0.08
0.10
0.12
SNP effect on HDAC2

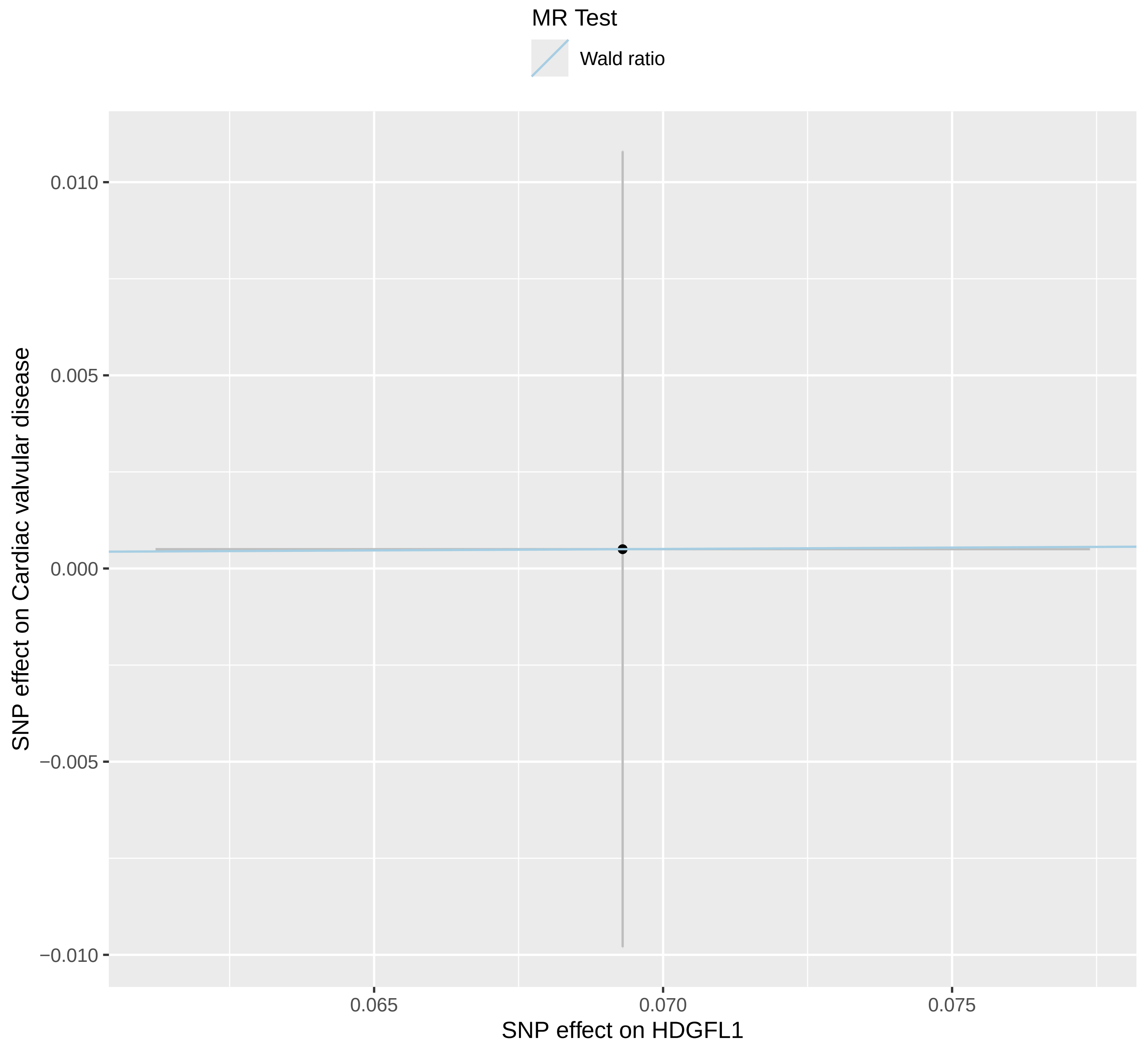
MR Test
Wald ratio
0.010
0.005
0.000
−0.005
−0.010
0.065
0.070
0.075
SNP effect on HDGFL1
SNP effect on Cardiac valvular disease

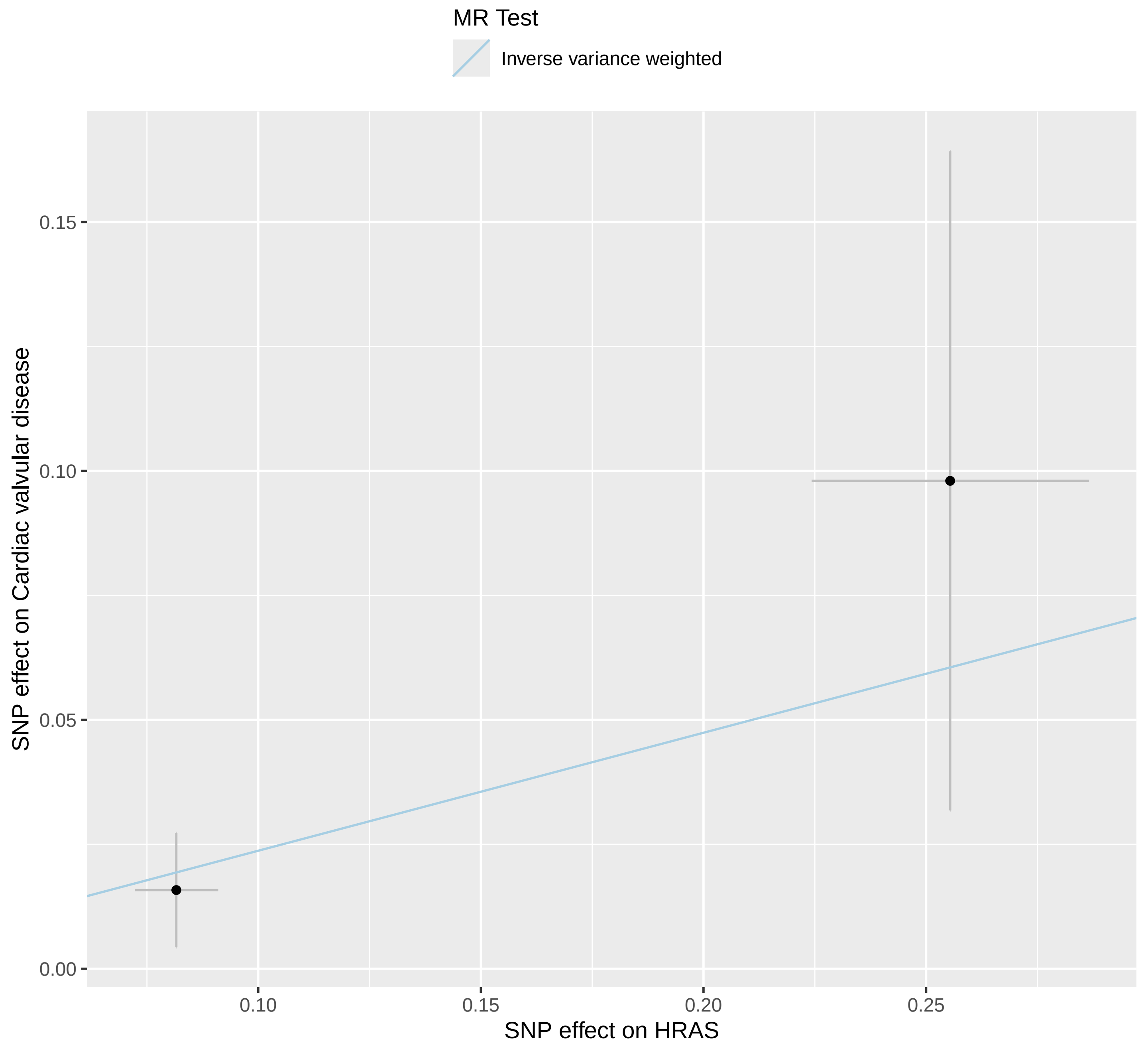
MR Test
Inverse variance weighted
0.15
0.10
0.05
0.00
0.10
0.15
0.20
0.25
SNP effect on Cardiac valvular disease
SNP effect on HRAS

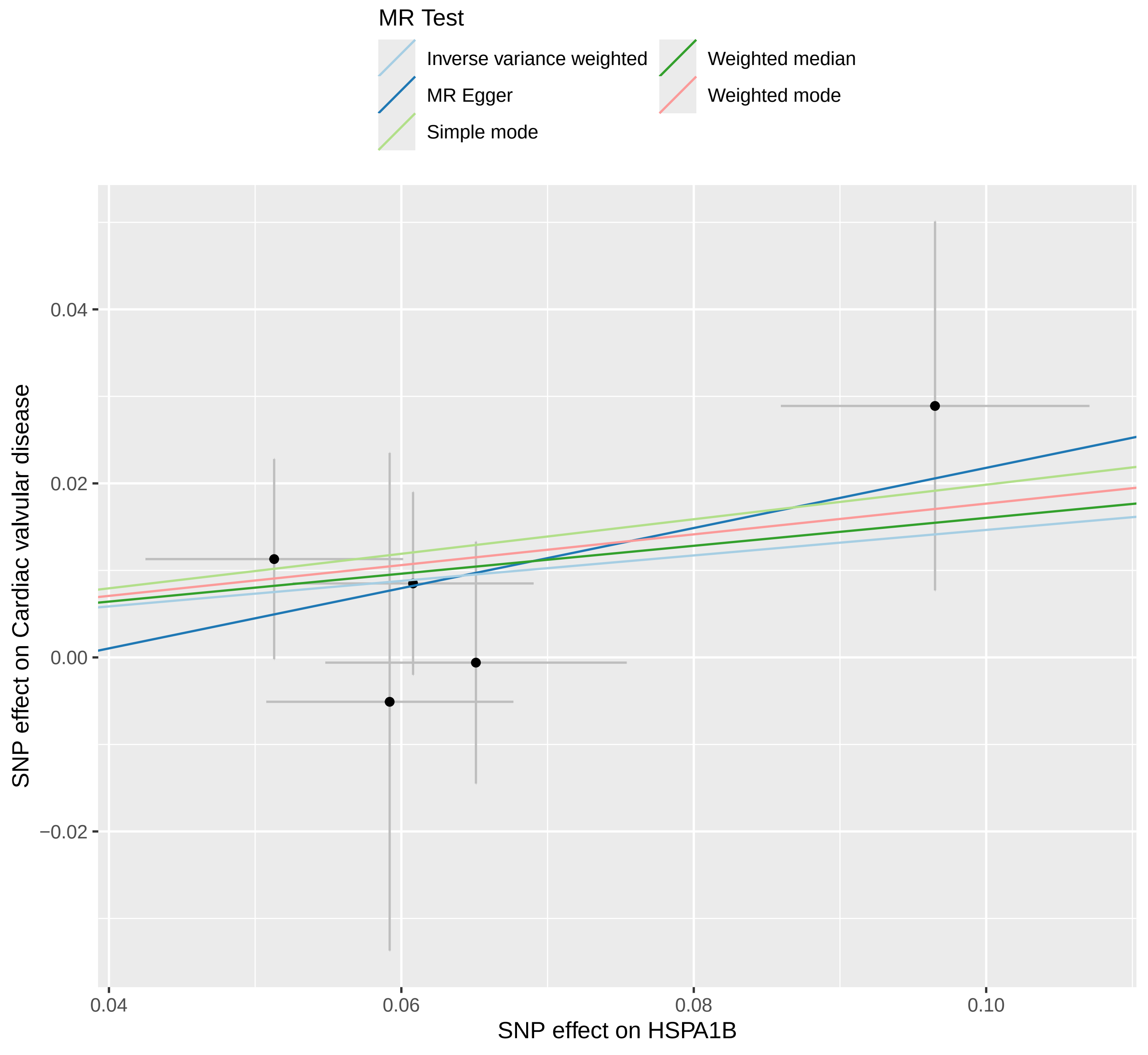
MR Test
Inverse variance weighted
MR Egger
Simple mode
Weighted median
Weighted mode
SNP effect on Cardiac valvular disease
0.04
0.02
0.00
−0.02
0.04
0.06
0.08
0.10
SNP effect on HSPA1B

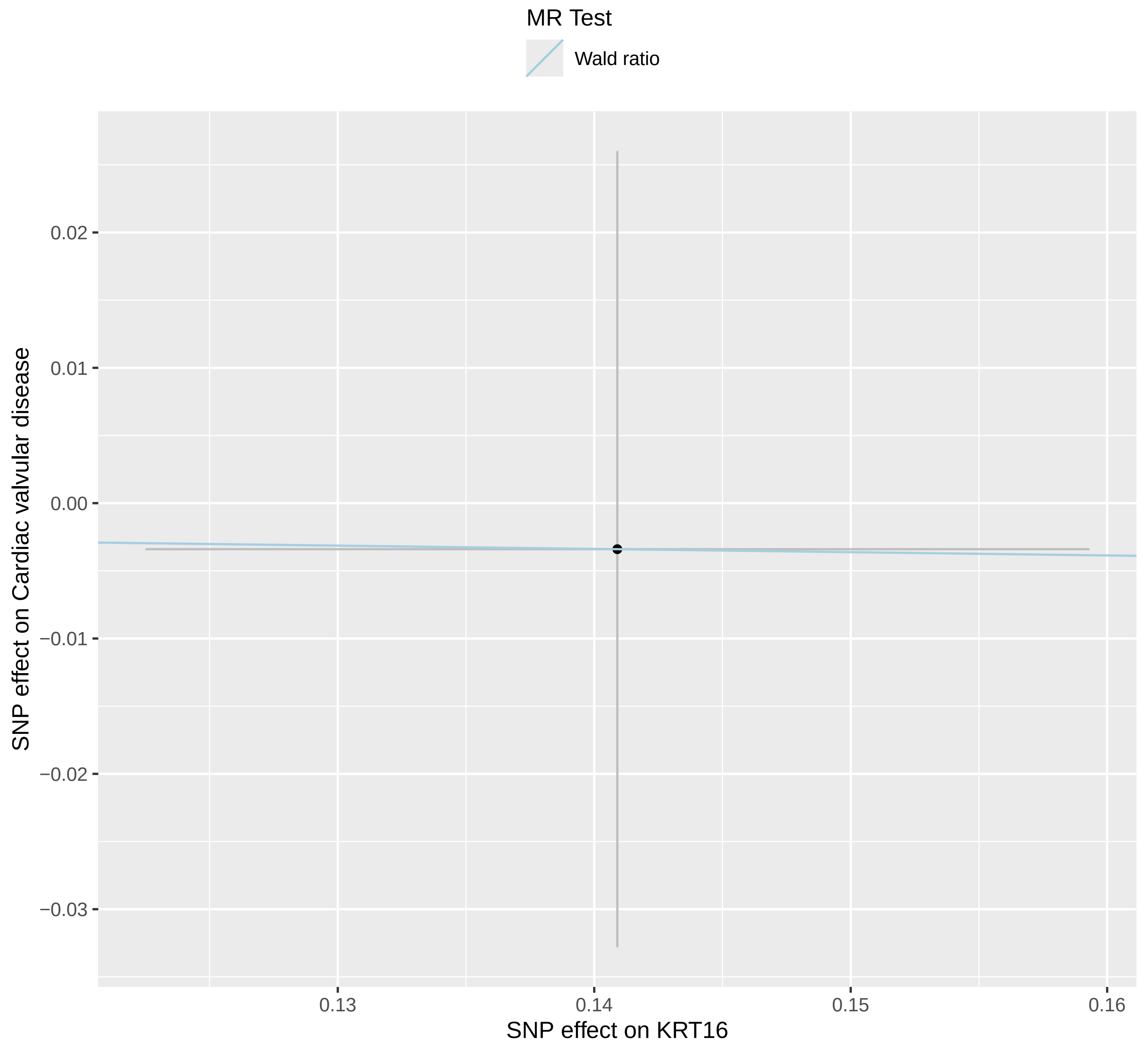

MR Test
Wald ratio
SNP effect on Cardiac valvular disease
0.02
0.01
0.00
−0.01
−0.02
−0.03
0.13
0.14
0.15
0.16
SNP effect on KRT16

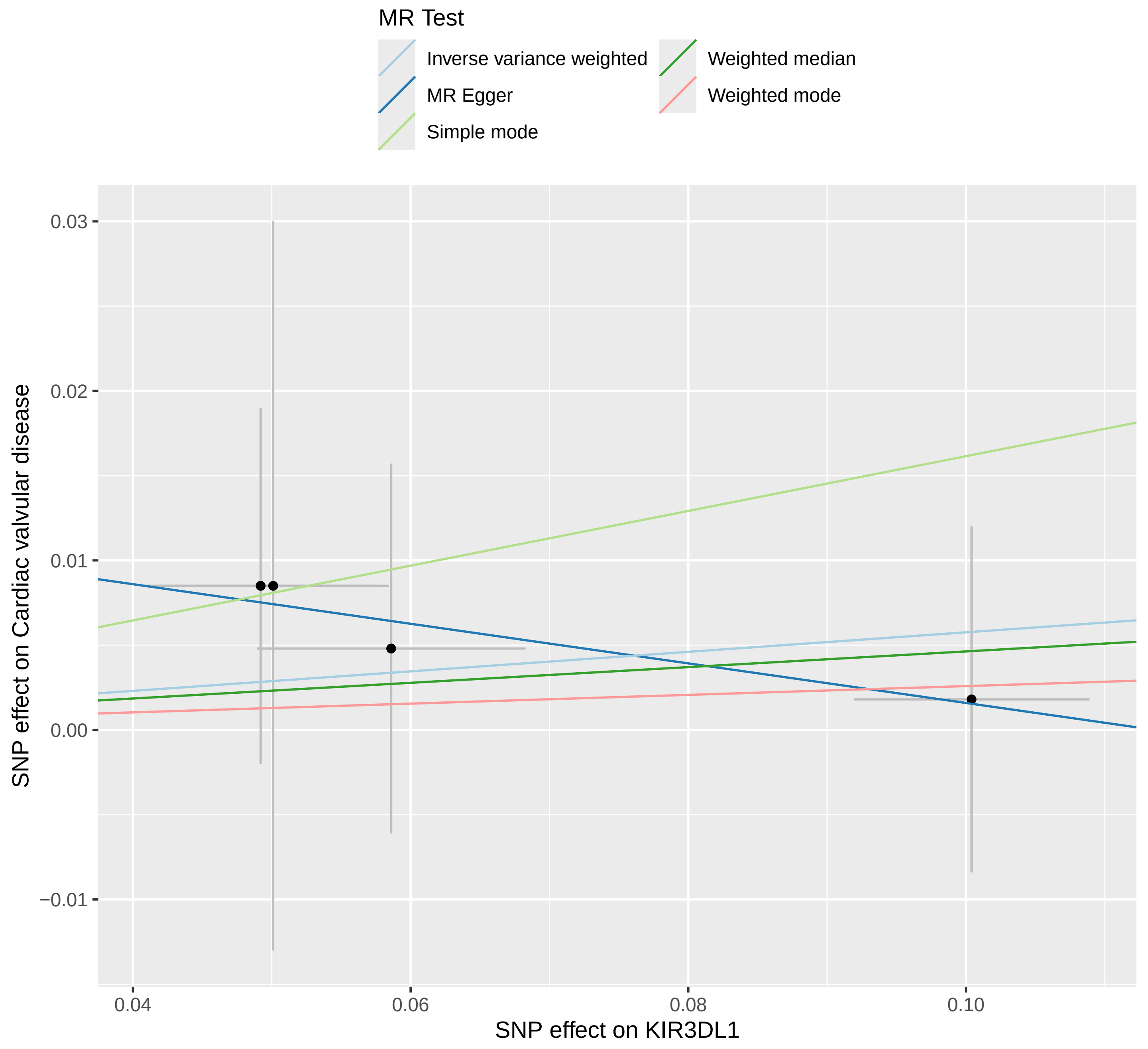
MR Test
Inverse variance weighted
MR Egger
Simple mode
Weighted median
Weighted mode
0.03
0.02
0.01
0.00
−0.01
0.04
0.06
0.08
0.10
SNP effect on Cardiac valvular disease
SNP effect on KIR3DL1

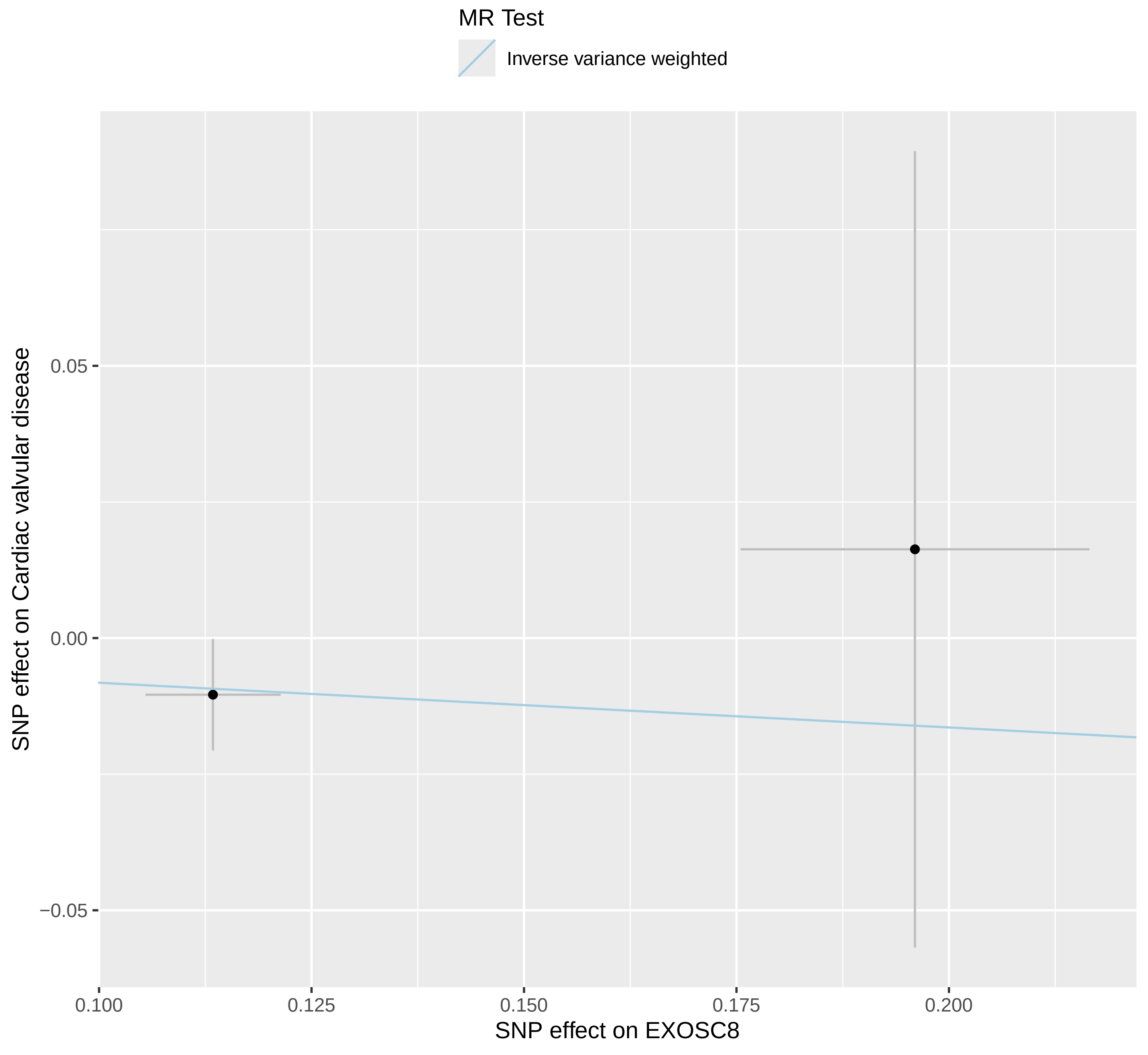
MR Test
Inverse variance weighted
SNP effect on Cardiac valvular disease
0.05
0.00
−0.05
0.100
0.125
0.150
0.175
0.200
SNP effect on EXOSC8

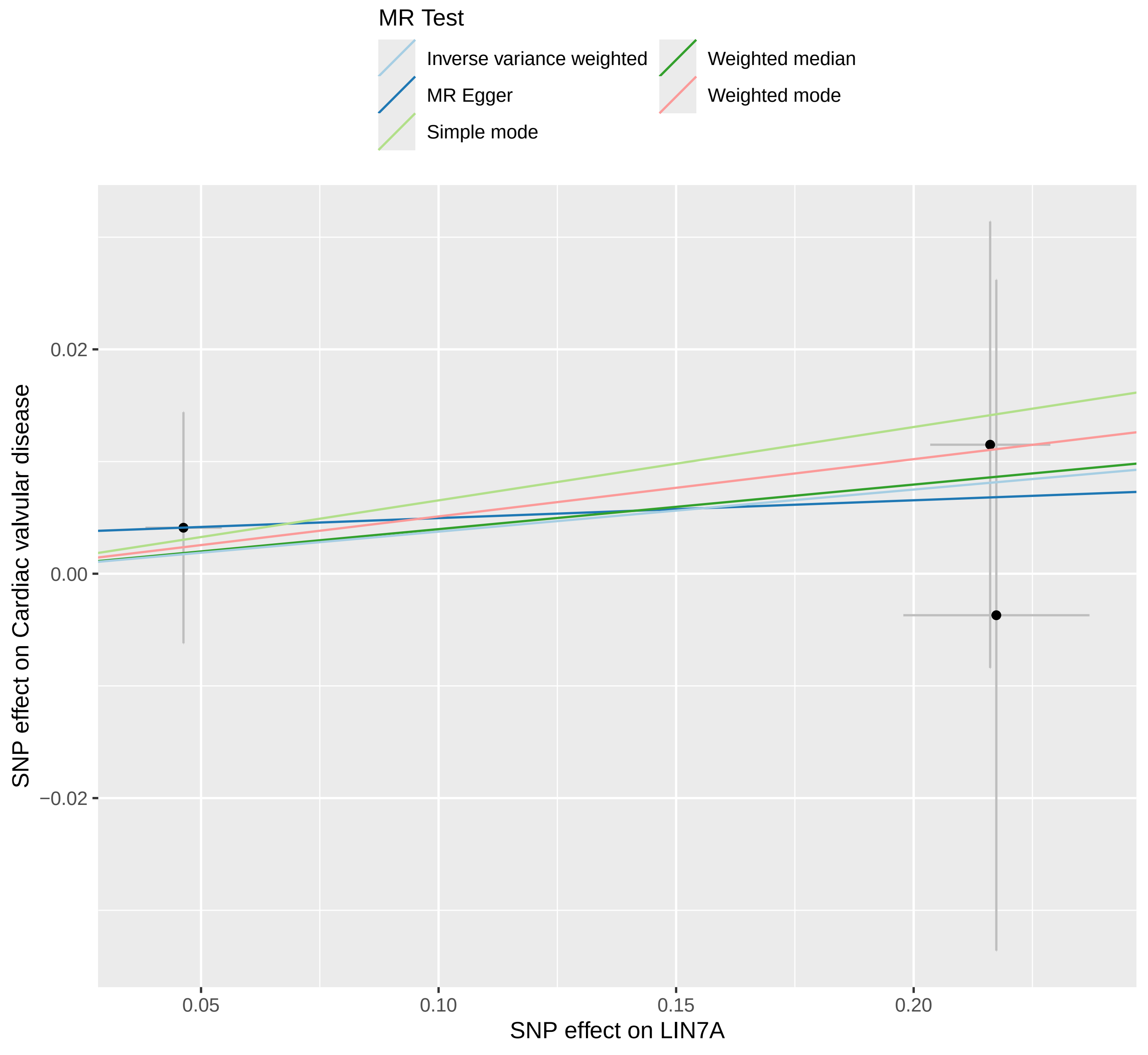

MR Test
Inverse variance weighted
MR Egger
Simple mode
Weighted median
Weighted mode
0.02
0.00
−0.02
SNP effect on Cardiac valvular disease
0.05
0.10
0.15
0.20
SNP effect on LIN7A

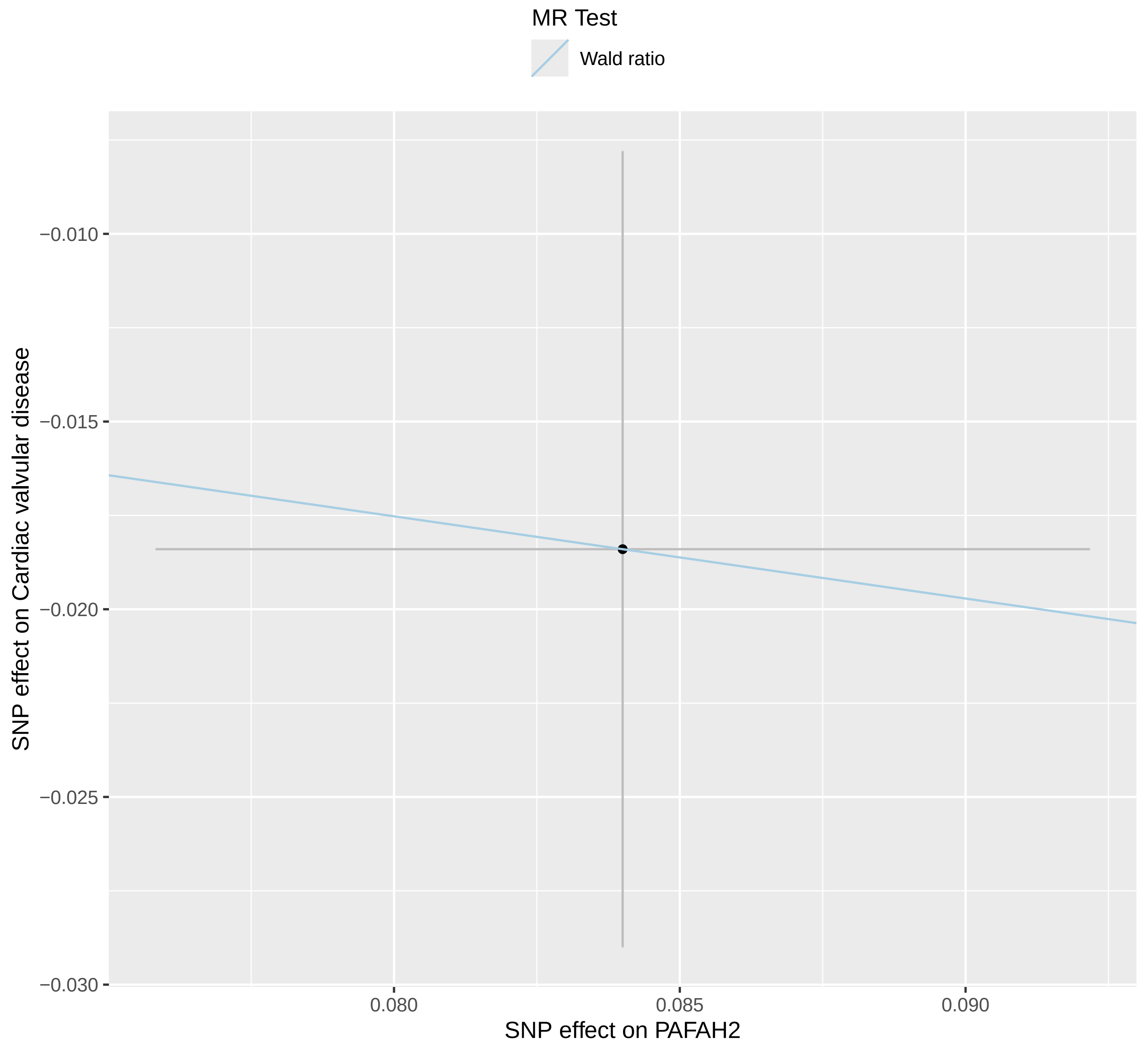
MR Test
Wald ratio
SNP effect on Cardiac valvular disease
SNP effect on PAFAH2
−0.010
−0.015
−0.020
−0.025
−0.030
0.080
0.085
0.090

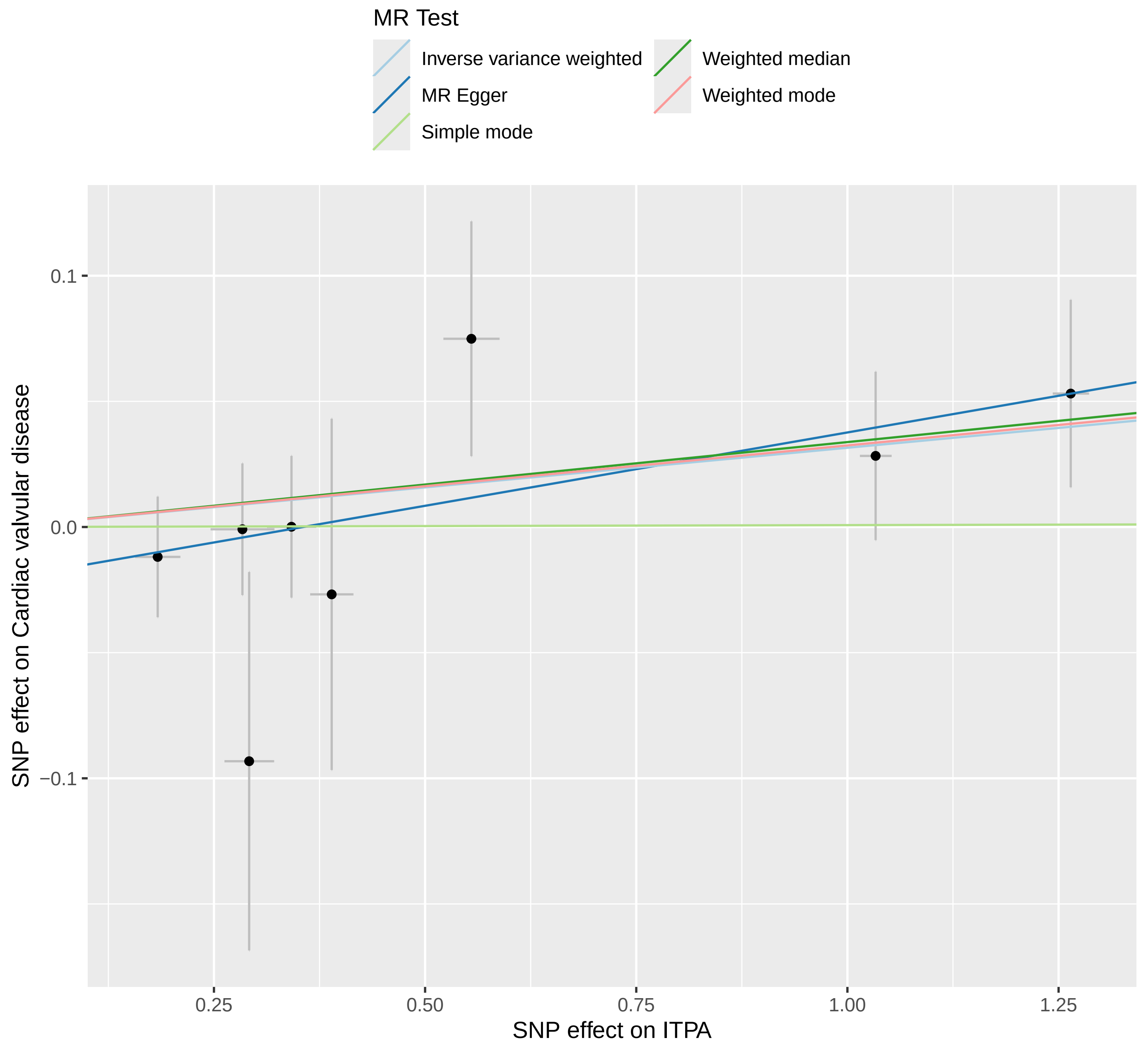

MR Test
Inverse variance weighted
MR Egger
Simple mode
Weighted median
Weighted mode
0.1
0.0
−0.1
0.25
0.50
0.75
1.00
1.25
SNP effect on Cardiac valvular disease
SNP effect on ITPA

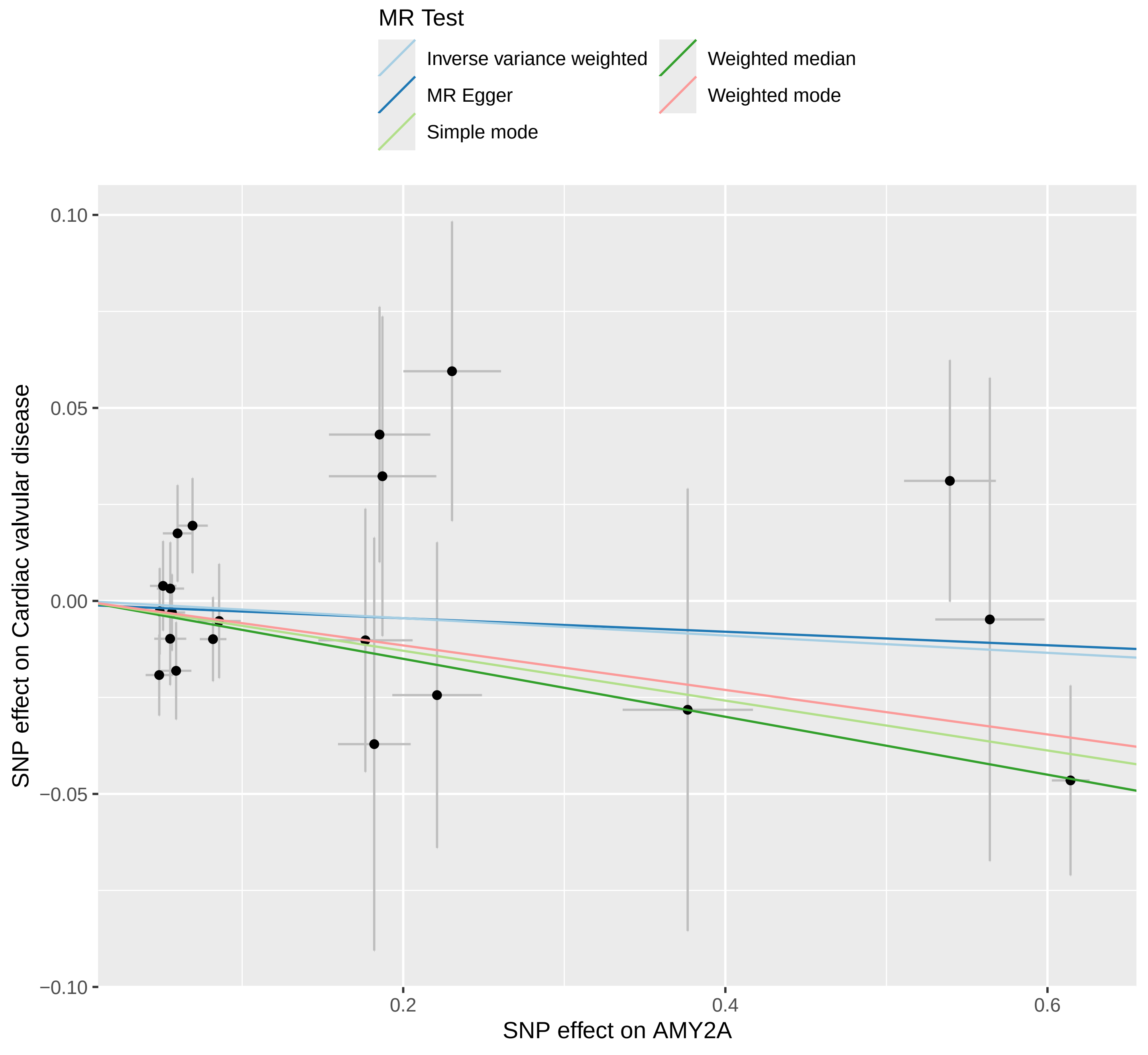
MR Test
Inverse variance weighted
MR Egger
Simple mode
Weighted median
Weighted mode
0.10
0.05
0.00
−0.05
−0.10
SNP effect on Cardiac valvular disease
0.2
0.4
0.6
SNP effect on AMY2A

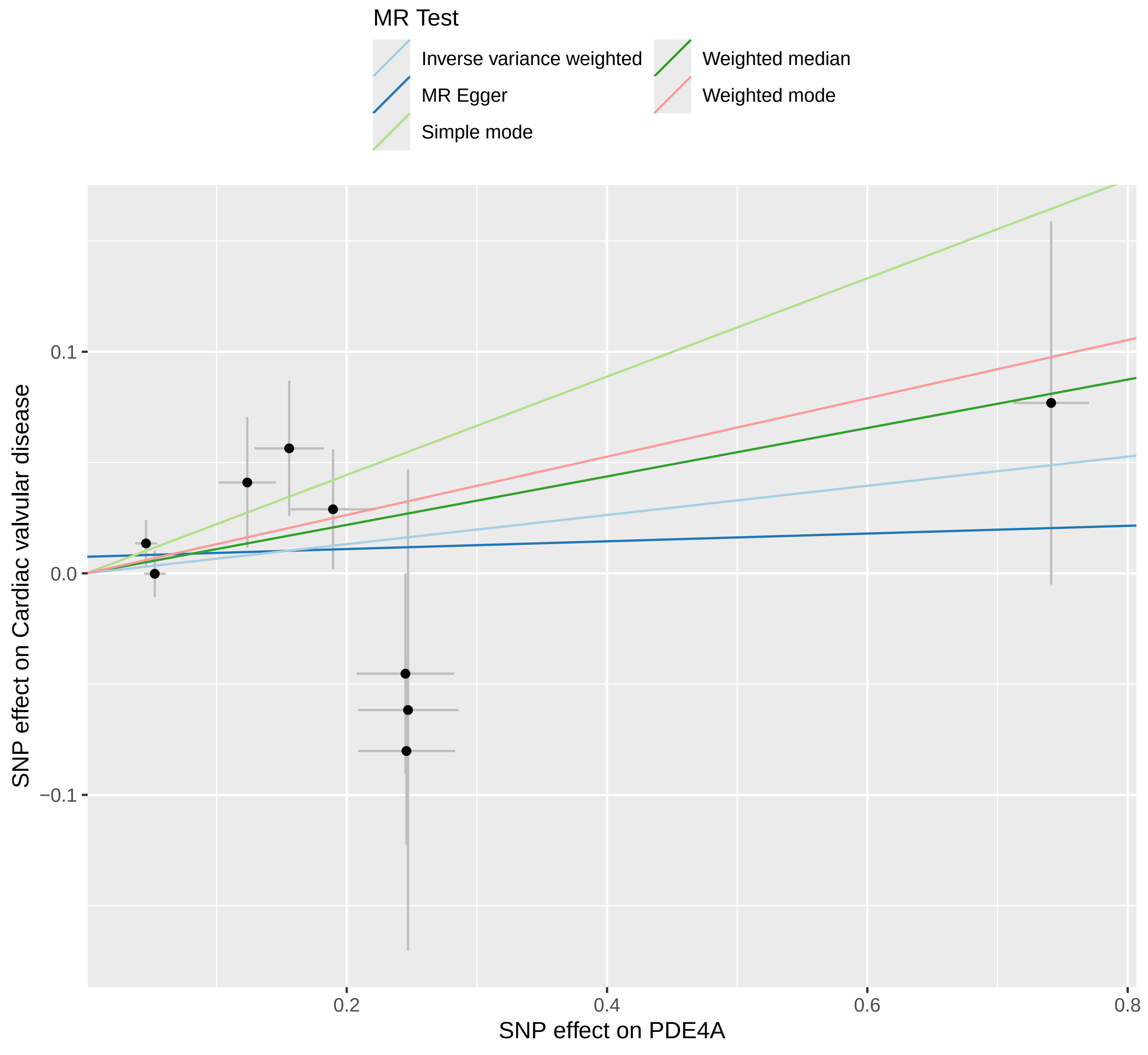
MR Test
Inverse variance weighted
MR Egger
Simple mode
Weighted median
Weighted mode
0.1
0.0
−0.1
0.2
0.4
0.6
0.8
SNP effect on Cardiac valvular disease
SNP effect on PDE4A

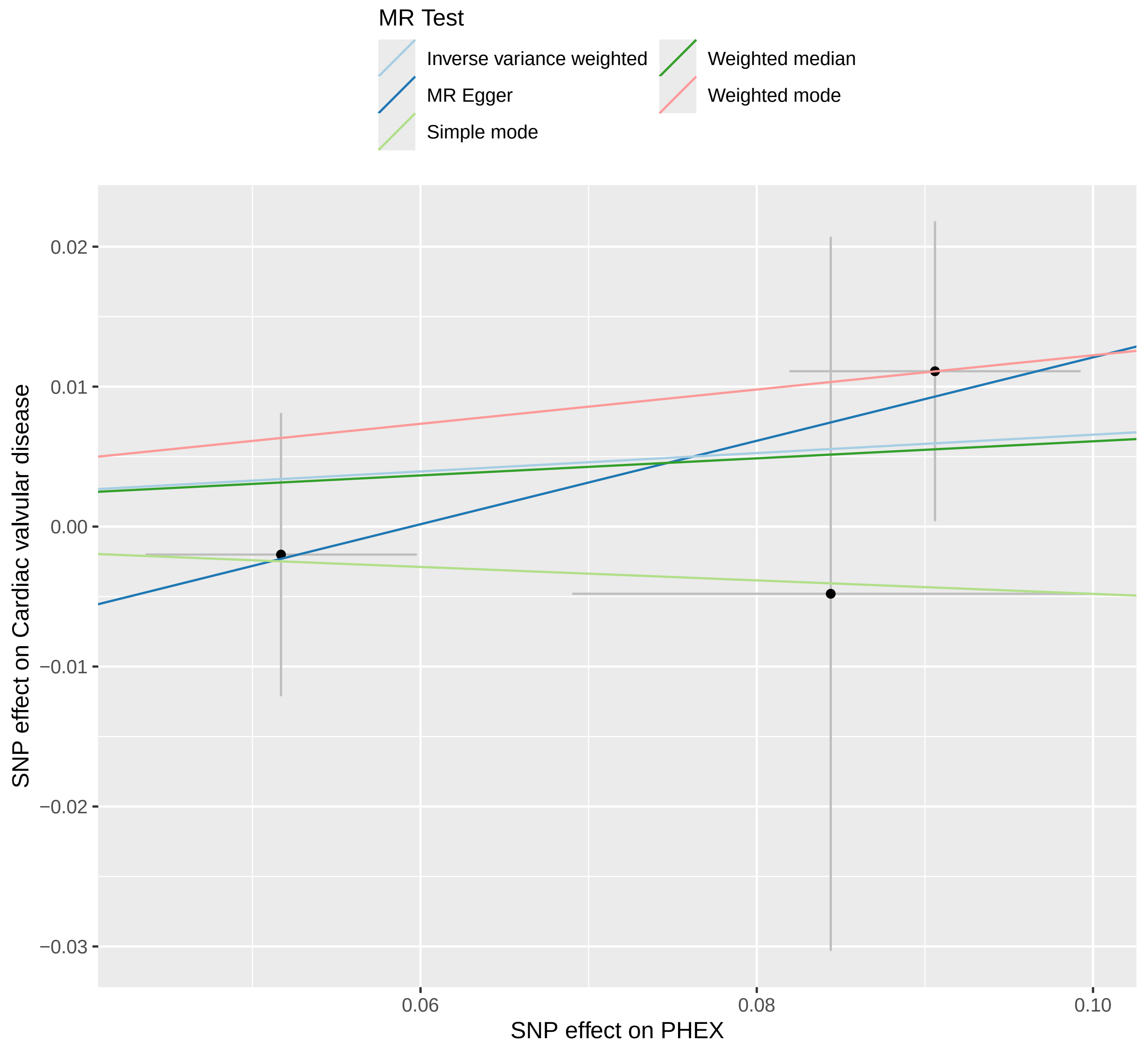

MR Test
Inverse variance weighted
MR Egger
Simple mode
Weighted median
Weighted mode
0.02
0.01
0.00
−0.01
−0.02
−0.03
0.06
0.08
0.10
SNP effect on Cardiac valvular disease
SNP effect on PHEX

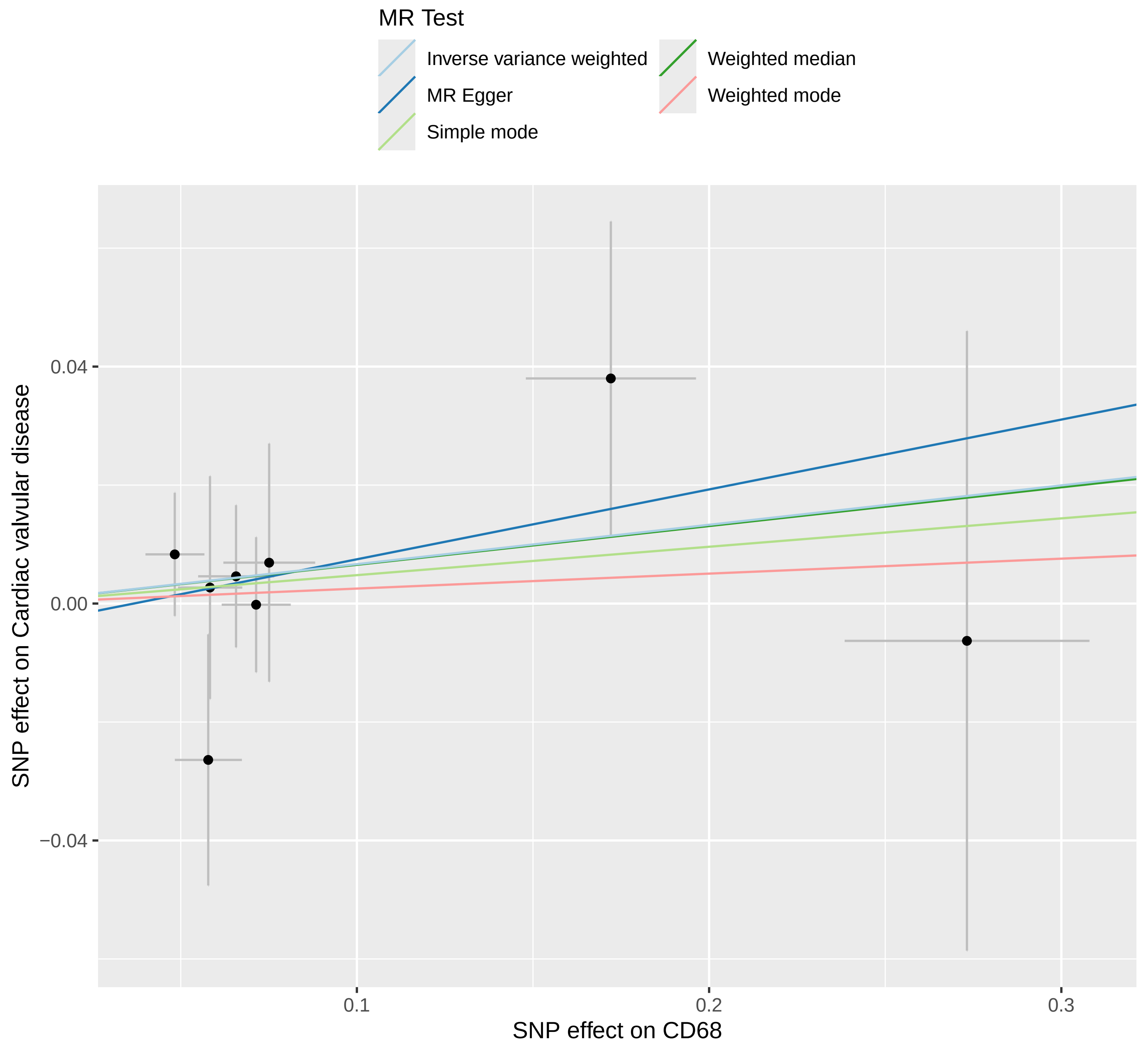

MR Test
Inverse variance weighted
MR Egger
Simple mode
Weighted median
Weighted mode
0.04
0.00
−0.04
0.1
0.2
0.3
SNP effect on Cardiac valvular disease
SNP effect on CD68

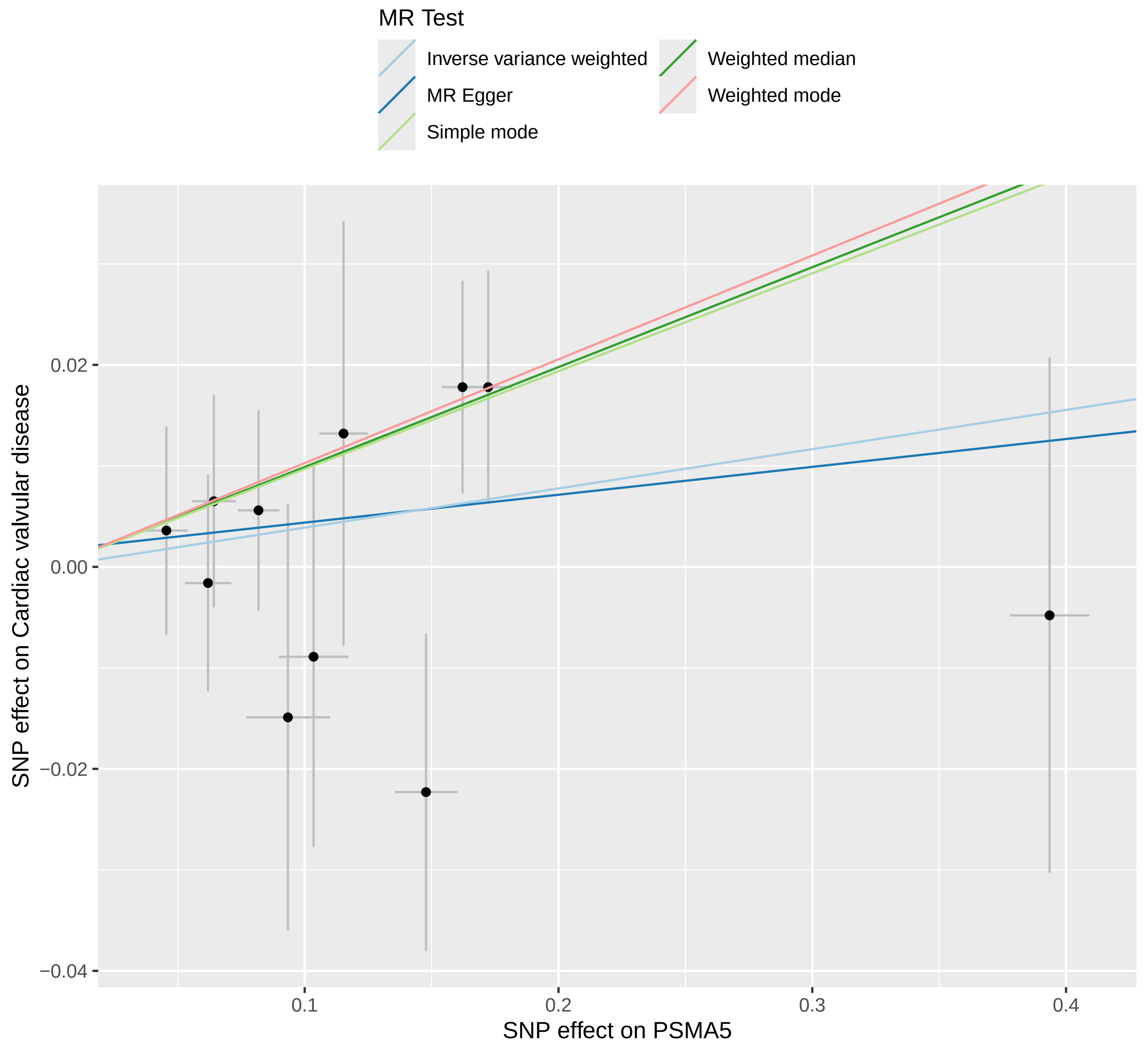
MR Test
Inverse variance weighted
MR Egger
Simple mode
Weighted median
Weighted mode
0.02
0.00
−0.02
−0.04
SNP effect on Cardiac valvular disease
0.1
0.2
0.3
0.4
SNP effect on PSMA5

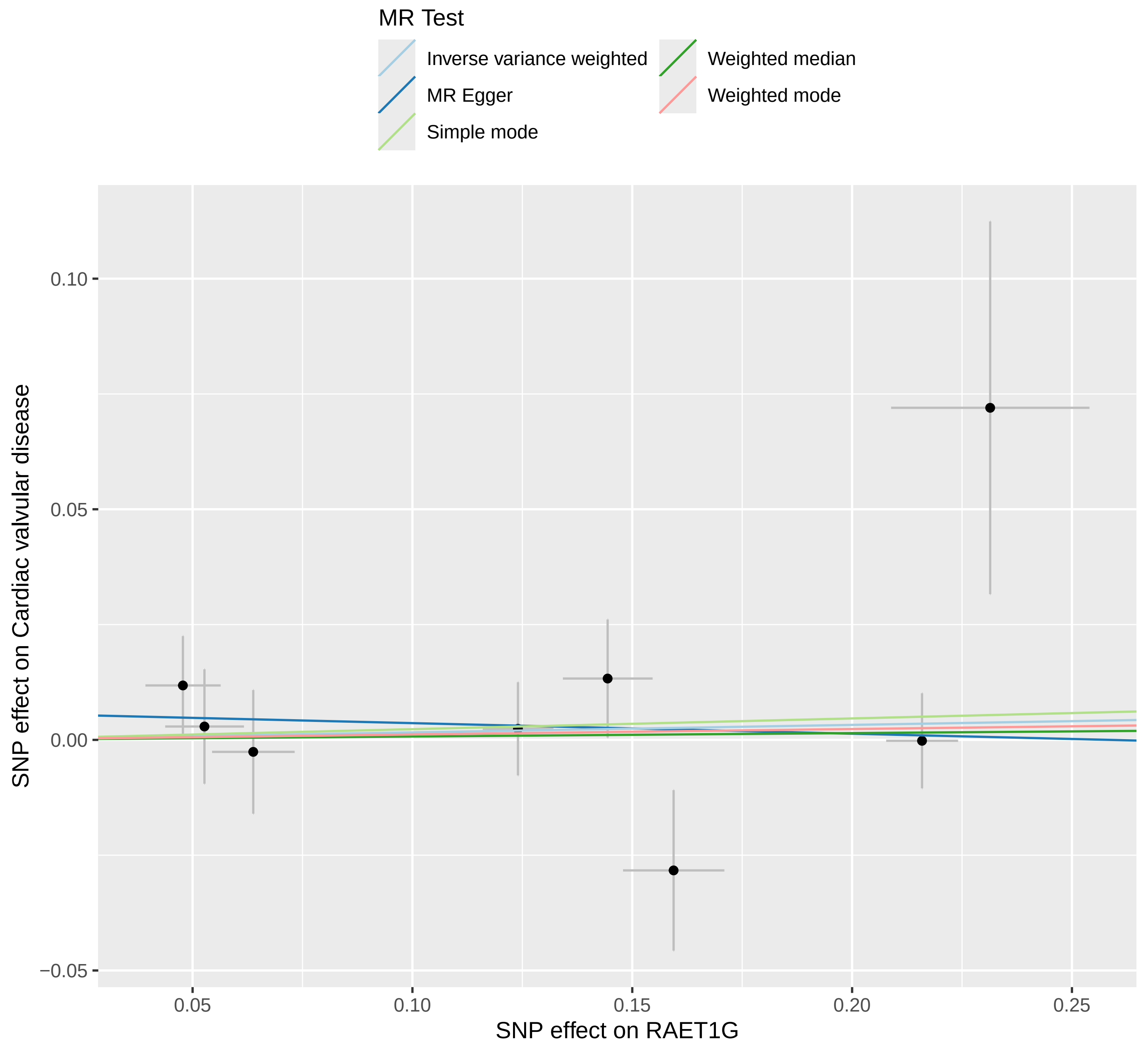
MR Test
Inverse variance weighted
MR Egger
Simple mode
Weighted median
Weighted mode
SNP effect on Cardiac valvular disease
0.10
0.05
0.00
−0.05
0.05
0.10
0.15
0.20
0.25
SNP effect on RAET1G

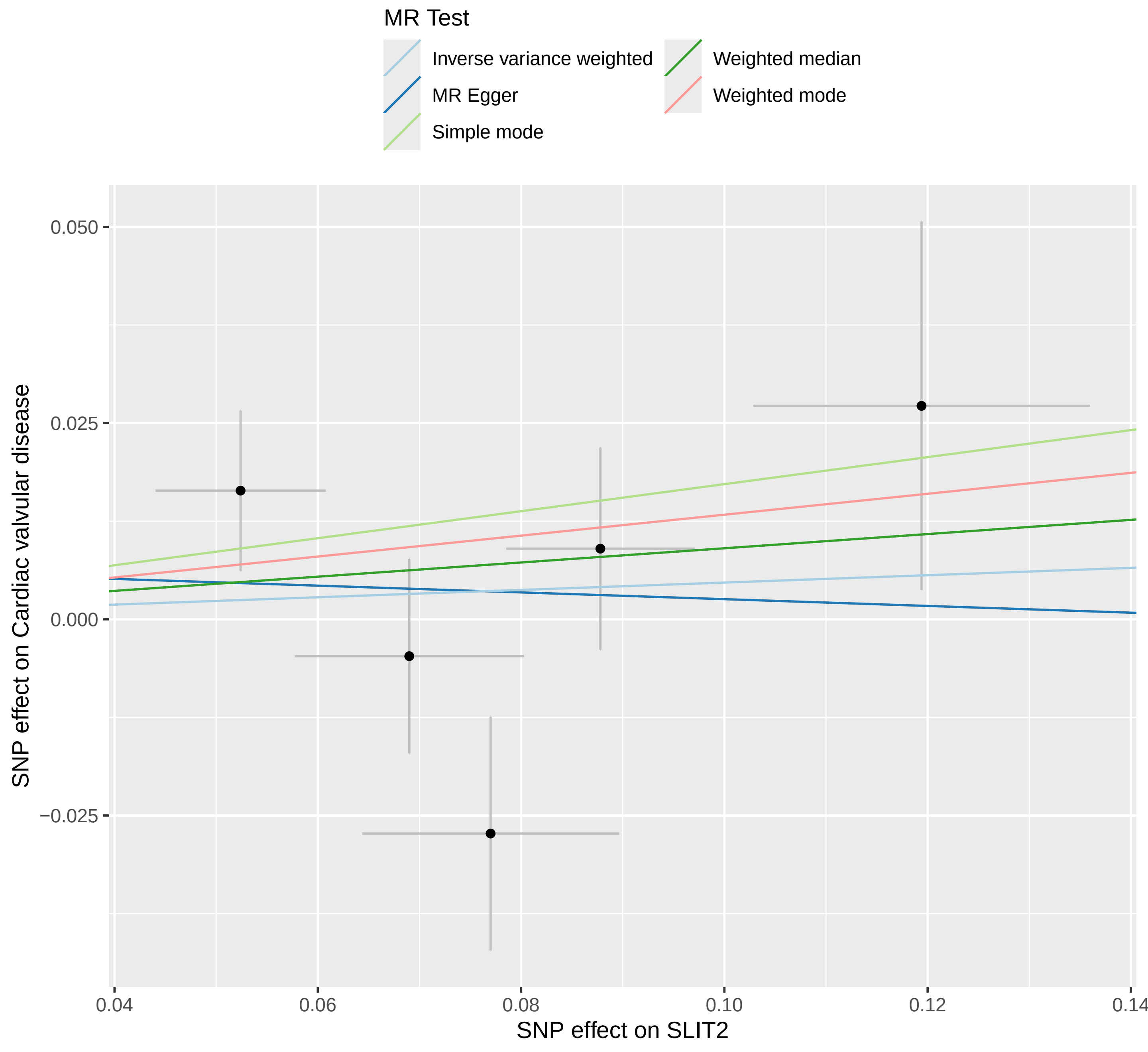
MR Test
Inverse variance weighted
MR Egger
Simple mode
Weighted median
Weighted mode
0.050
0.025
0.000
−0.025
SNP effect on Cardiac valvular disease
0.04
0.06
0.08
0.10
0.12
0.14
SNP effect on SLIT2

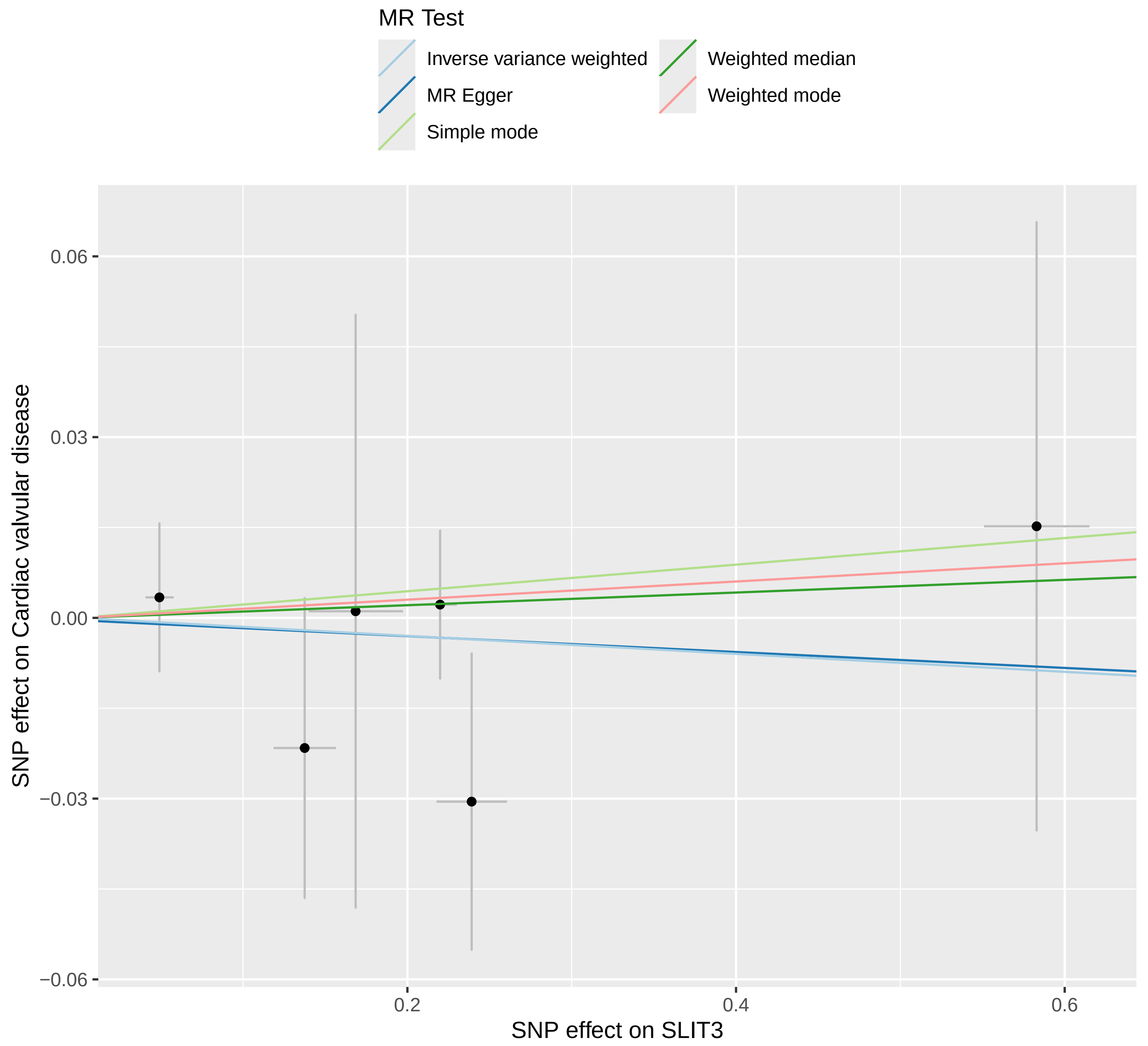
MR Test
Inverse variance weighted
MR Egger
Simple mode
Weighted median
Weighted mode
0.06
0.03
0.00
−0.03
−0.06
SNP effect on Cardiac valvular disease
0.2
0.4
0.6
SNP effect on SLIT3

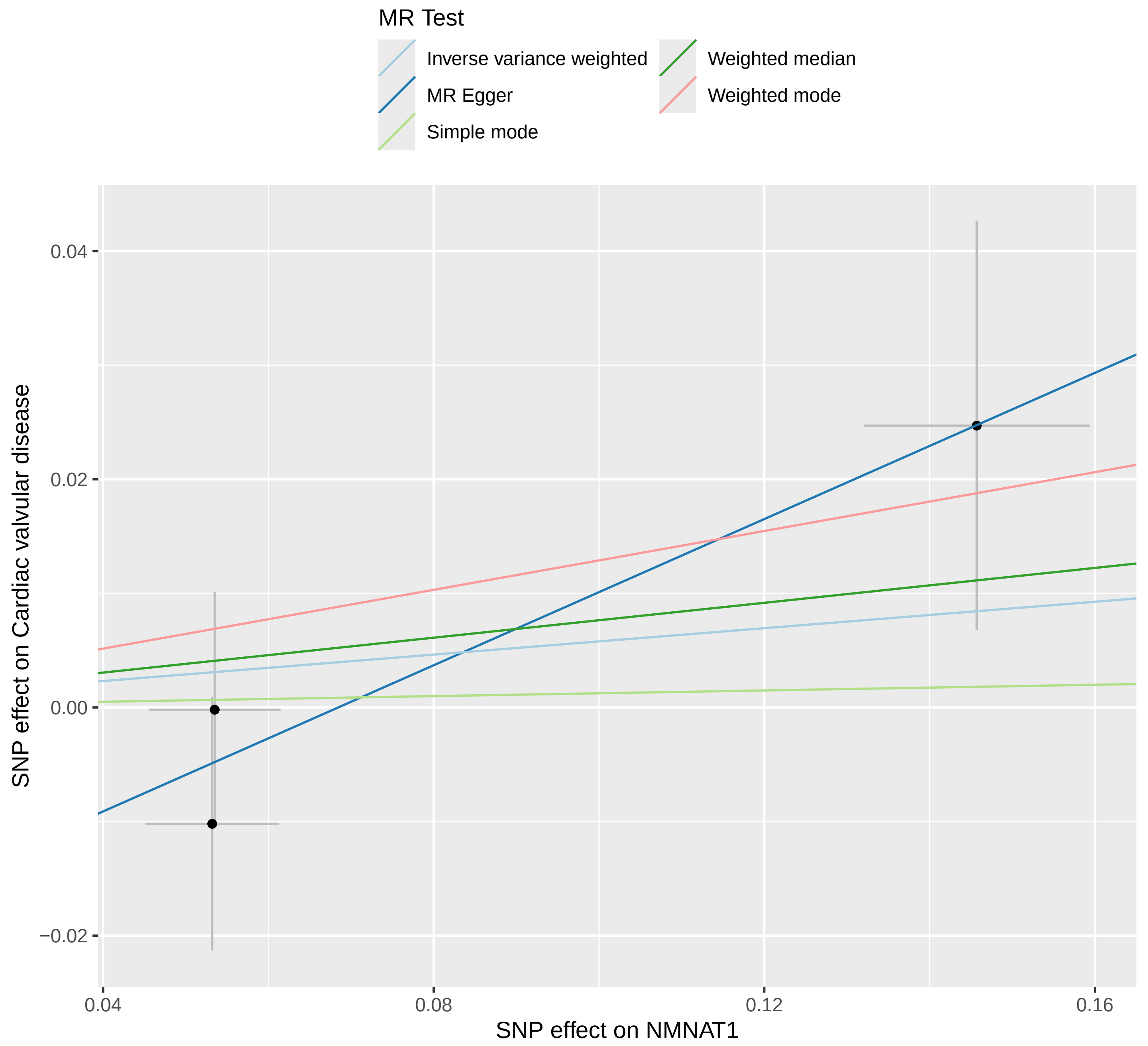
MR Test
Inverse variance weighted
MR Egger
Simple mode
Weighted median
Weighted mode
0.04
0.02
0.00
−0.02
SNP effect on Cardiac valvular disease
0.04
0.08
0.12
0.16
SNP effect on NMNAT1

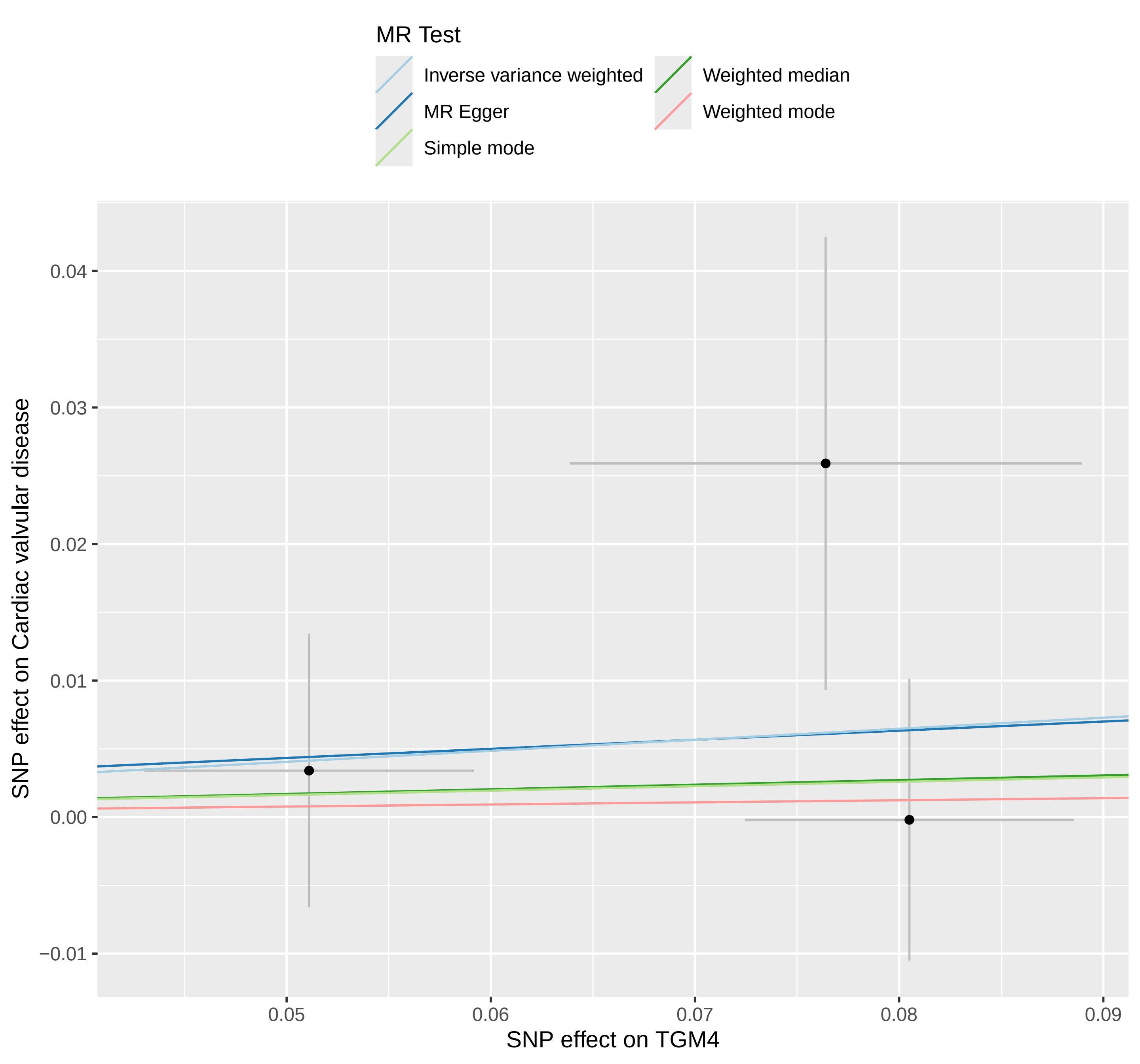
MR Test
Inverse variance weighted
MR Egger
Simple mode
Weighted median
Weighted mode
SNP effect on Cardiac valvular disease
0.04
0.03
0.02
0.01
0.00
−0.01
0.05
0.06
0.07
0.08
0.09
SNP effect on TGM4

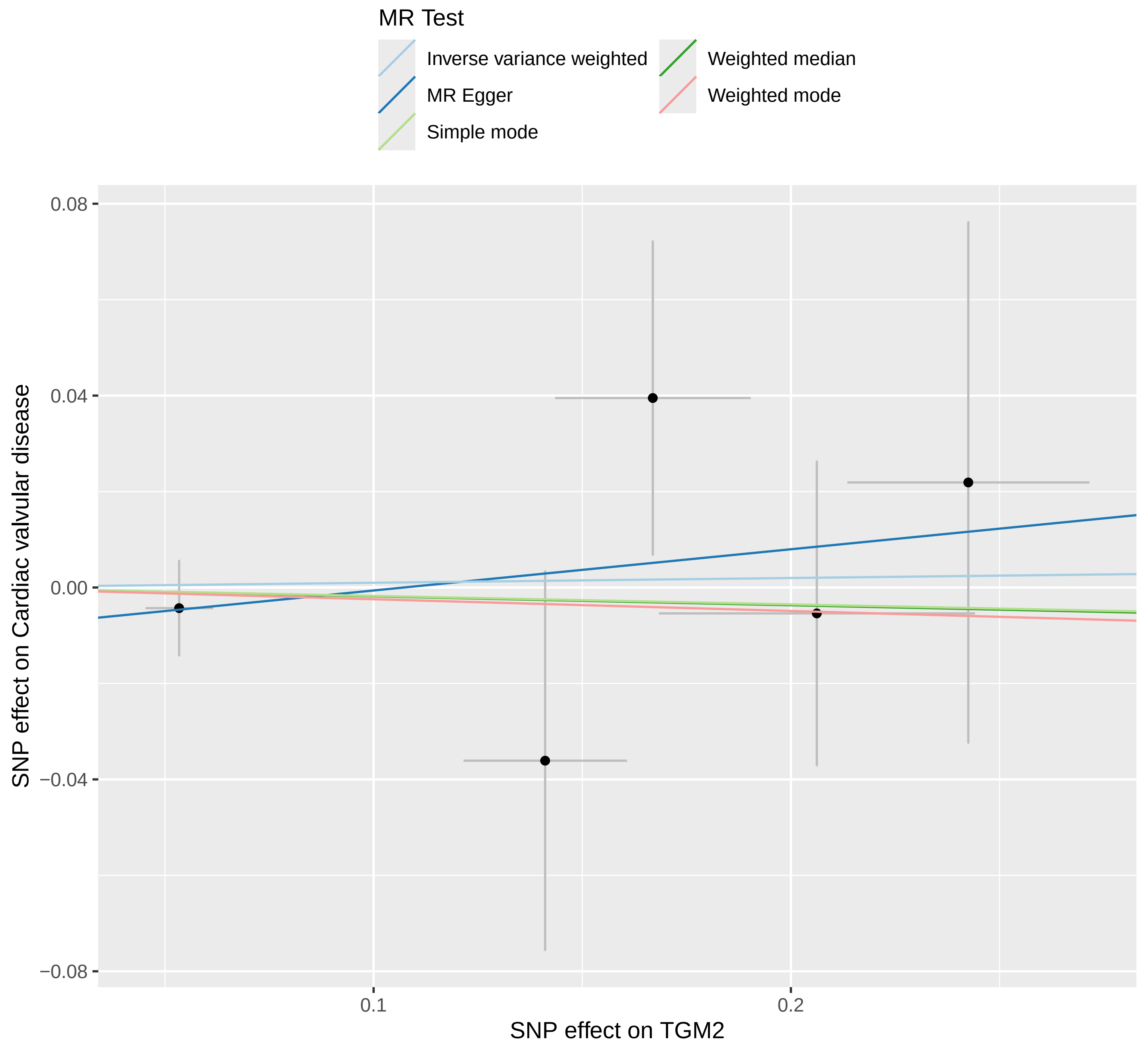

MR Test
Inverse variance weighted
MR Egger
Simple mode
Weighted median
Weighted mode
0.08
0.04
0.00
−0.04
−0.08
SNP effect on Cardiac valvular disease
0.1
0.2
SNP effect on TGM2

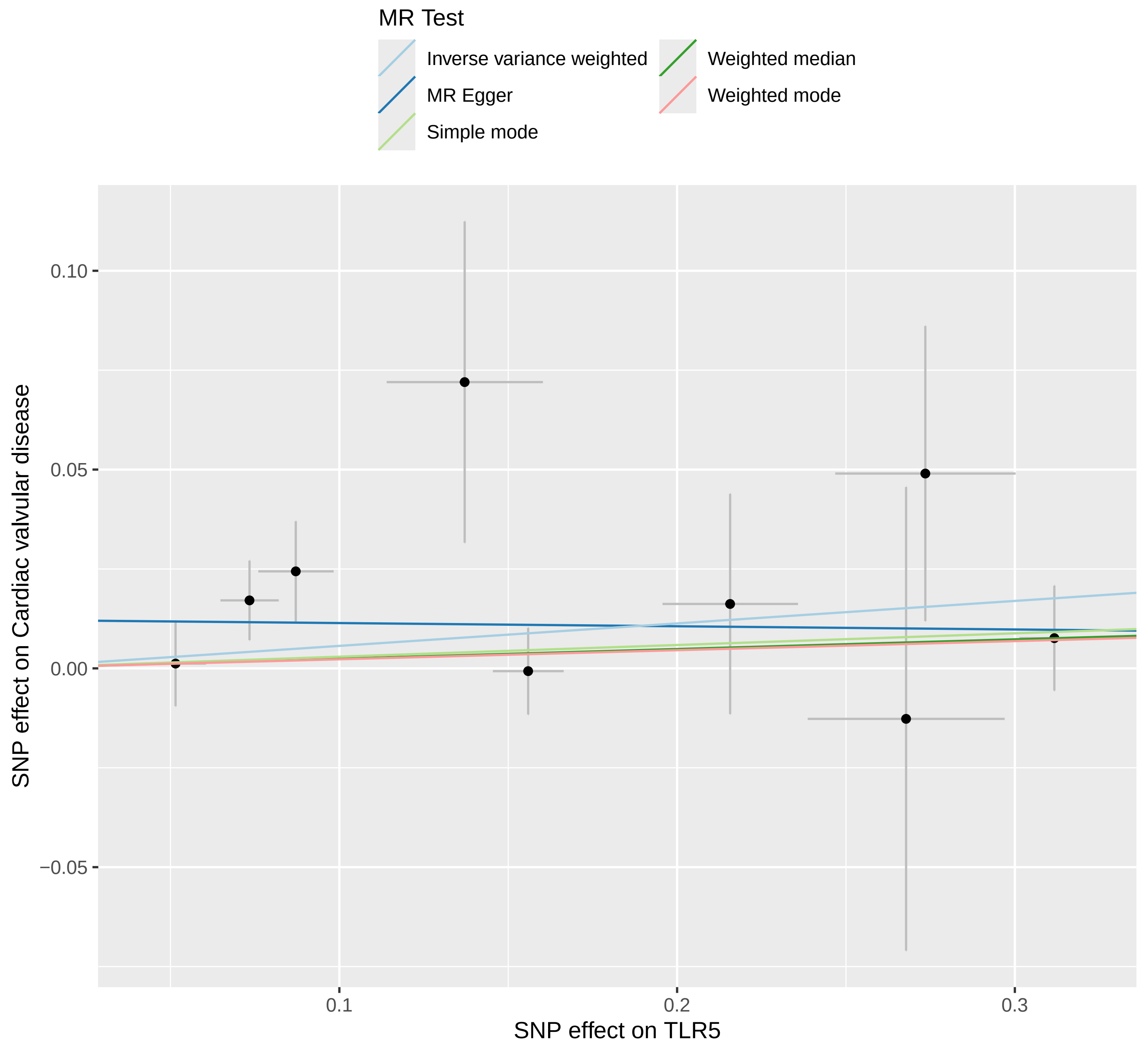

MR Test
Inverse variance weighted
MR Egger
Simple mode
Weighted median
Weighted mode
0.10
0.05
0.00
−0.05
0.1
0.2
0.3
SNP effect on Cardiac valvular disease
SNP effect on TLR5

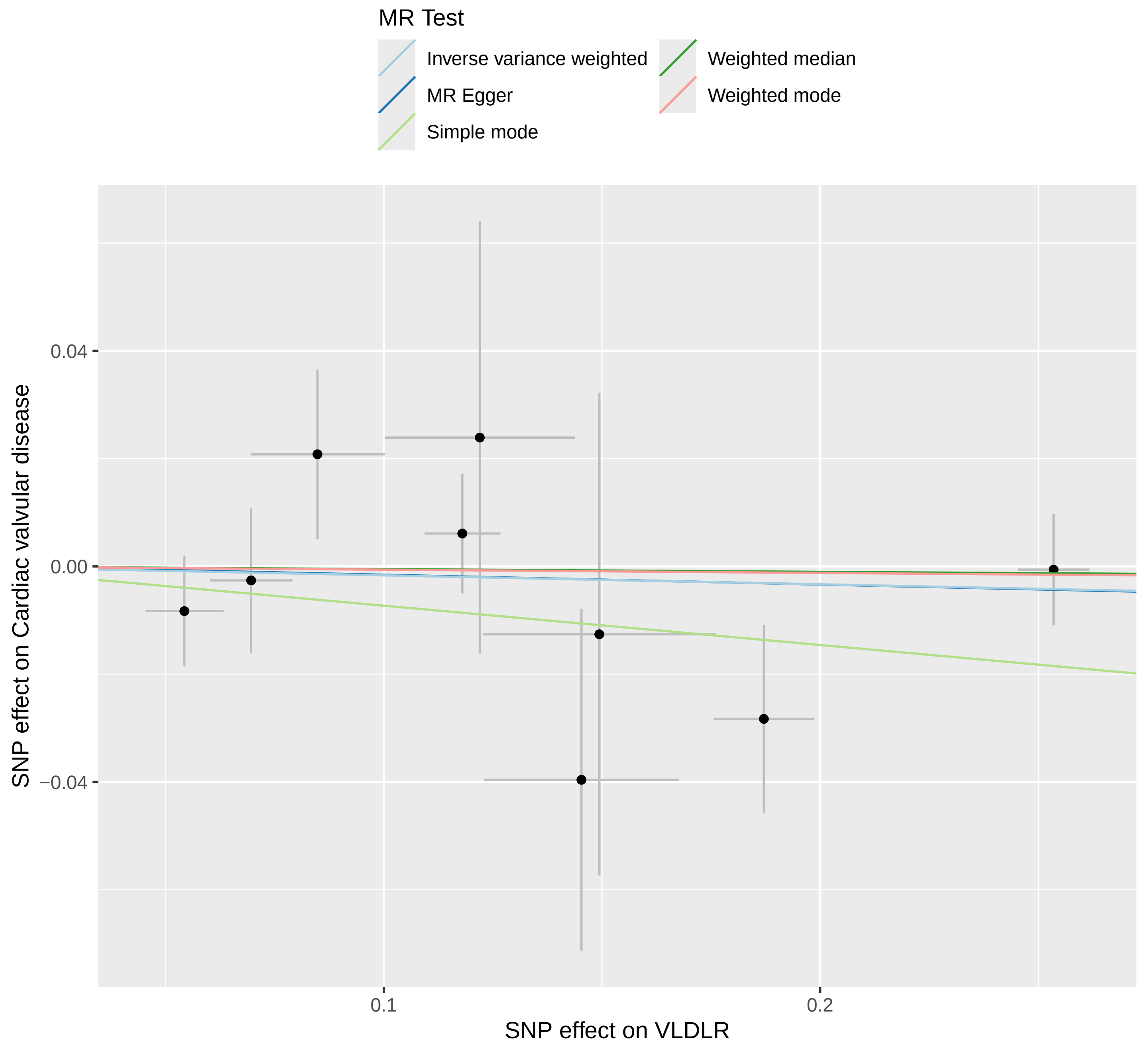
MR Test
Inverse variance weighted
MR Egger
Simple mode
Weighted median
Weighted mode
0.04
0.00
−0.04
0.1
0.2
SNP effect on VLDLR
SNP effect on Cardiac valvular disease

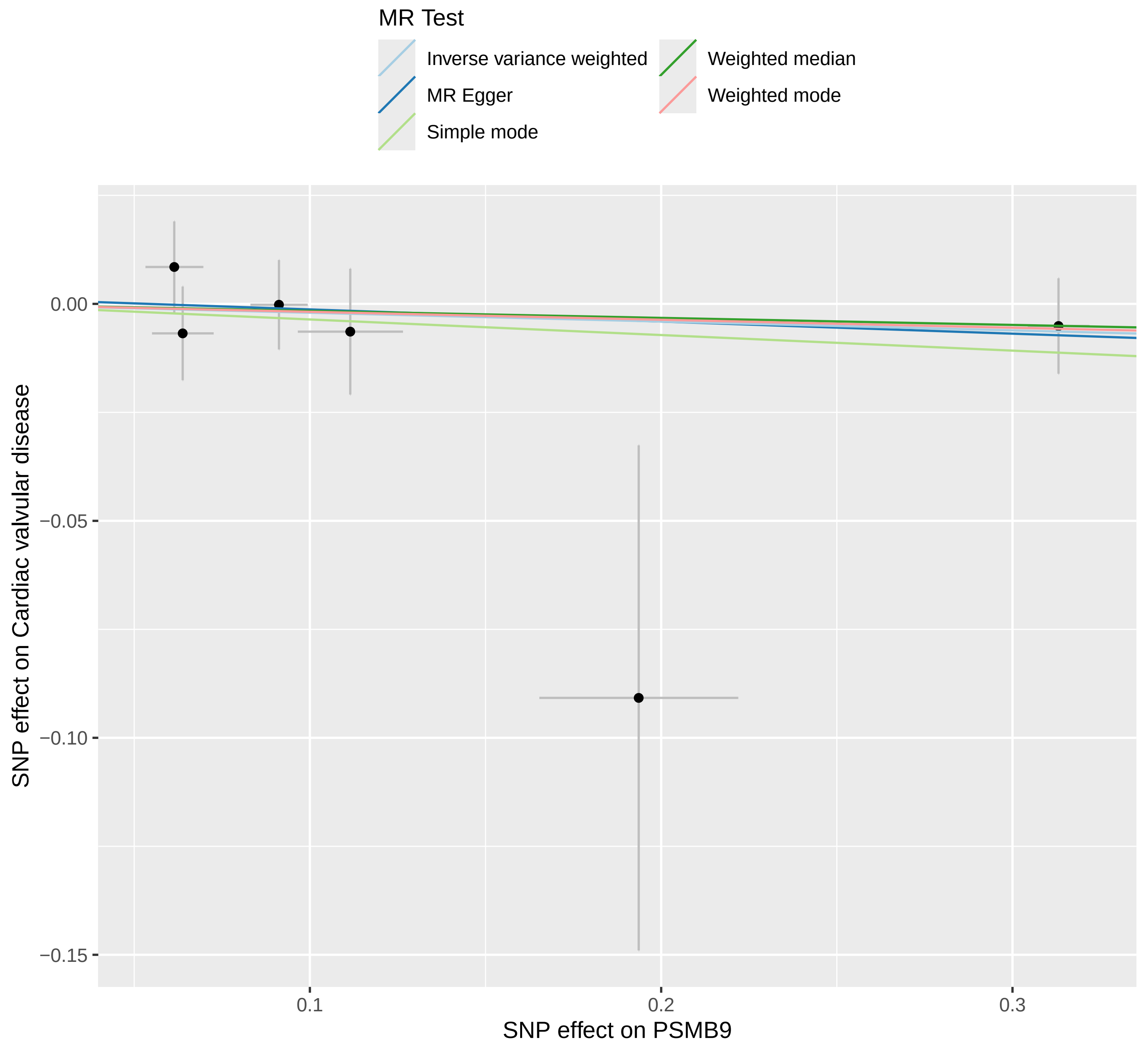
MR Test
Inverse variance weighted
MR Egger
Simple mode
Weighted median
Weighted mode
0.00
−0.05
−0.10
−0.15
0.1
0.2
0.3
SNP effect on Cardiac valvular disease
SNP effect on PSMB9

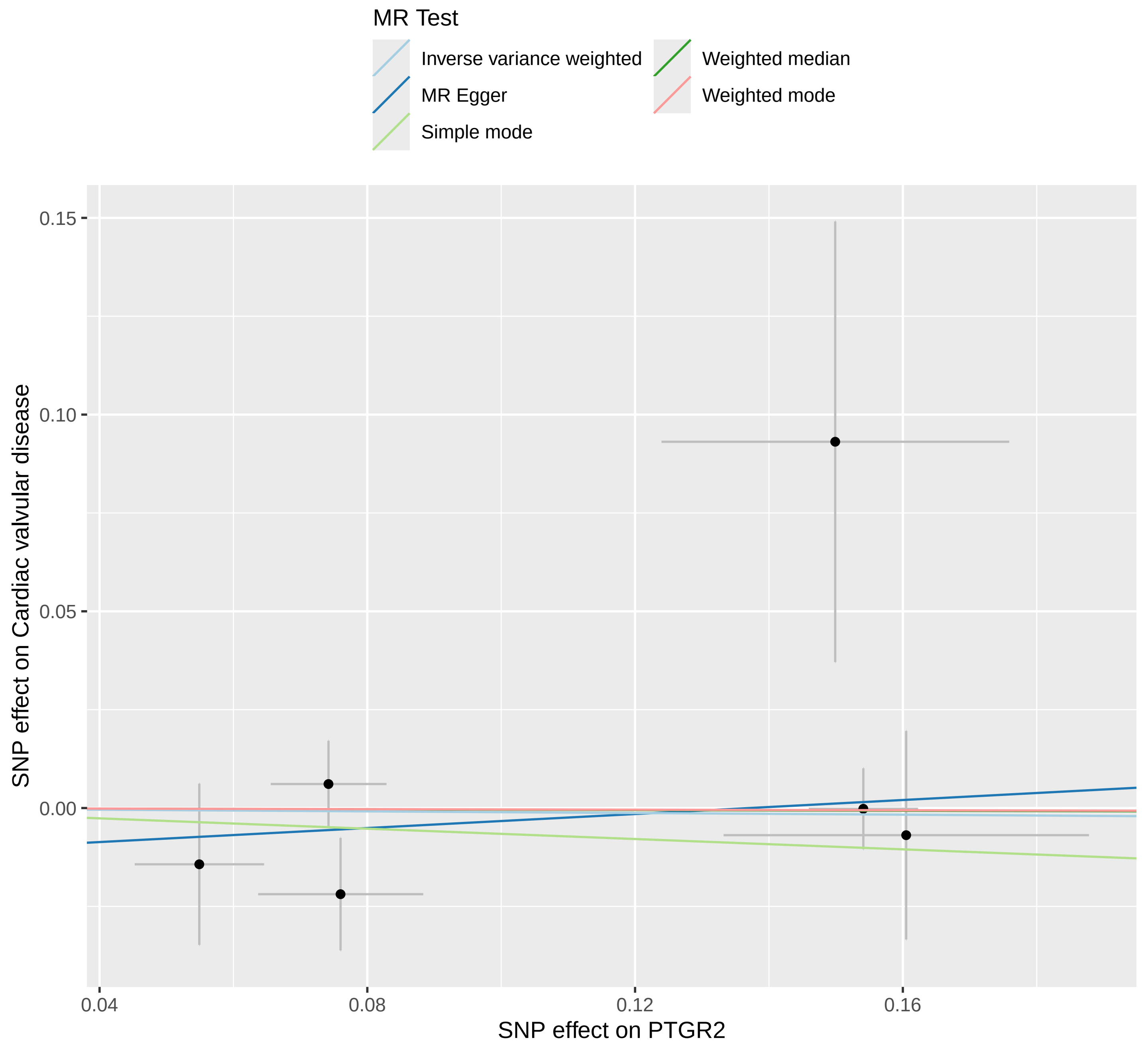
MR Test
Inverse variance weighted
MR Egger
Simple mode
Weighted median
Weighted mode
SNP effect on Cardiac valvular disease
0.15
0.10
0.05
0.00
0.04
0.08
0.12
0.16
SNP effect on PTGR2

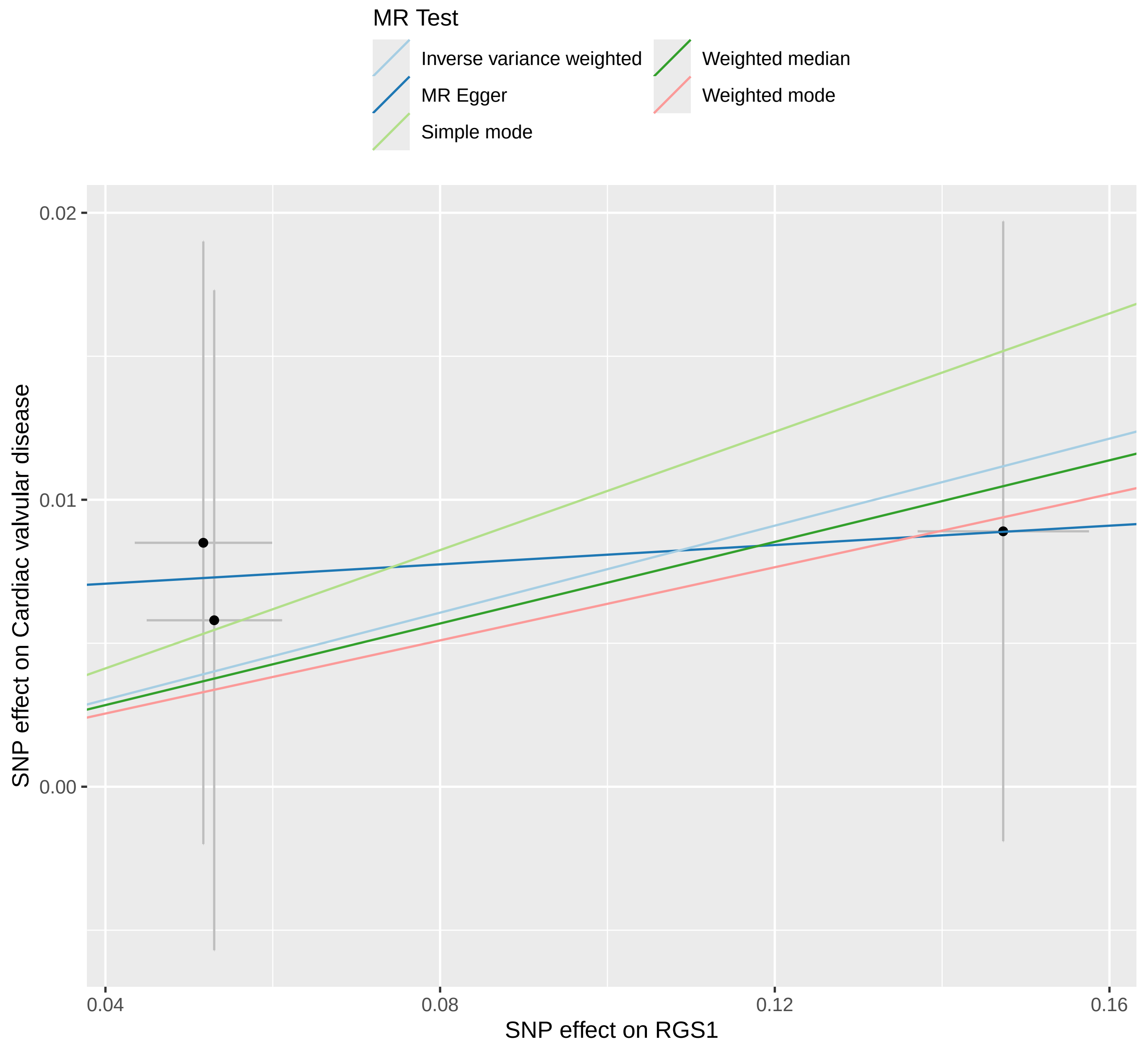
MR Test
Inverse variance weighted
MR Egger
Simple mode
Weighted median
Weighted mode
0.02
0.01
0.00
SNP effect on Cardiac valvular disease
0.04
0.08
0.12
0.16
SNP effect on RGS1

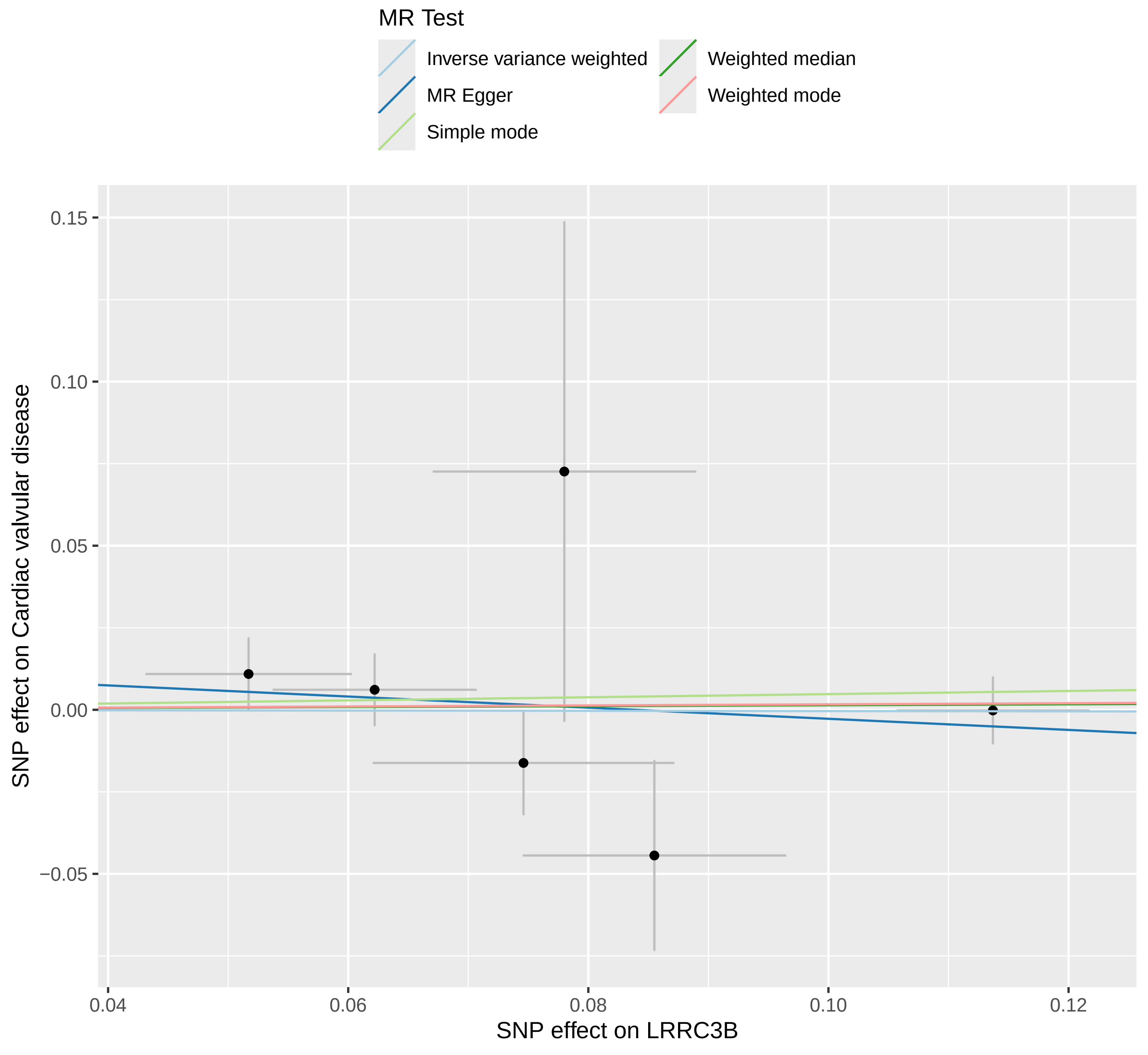

MR Test
Inverse variance weighted
MR Egger
Simple mode
Weighted median
Weighted mode
0.15
0.10
0.05
0.00
−0.05
SNP effect on Cardiac valvular disease
0.04
0.06
0.08
0.10
0.12
SNP effect on LRRC3B

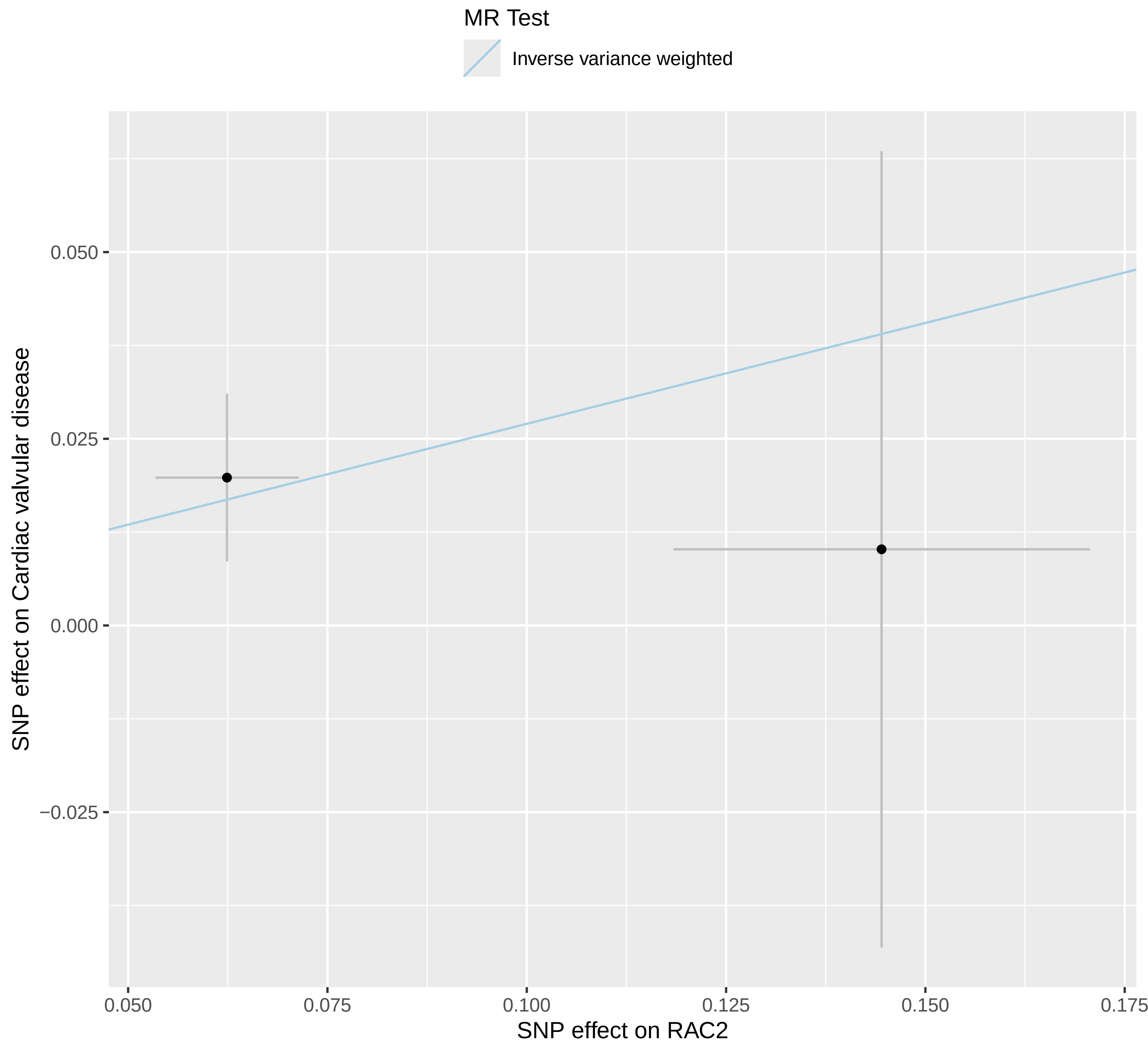

MR Test
Inverse variance weighted
SNP effect on Cardiac valvular disease
0.050
0.025
0.000
−0.025
0.050
0.075
0.100
0.125
0.150
0.175
SNP effect on RAC2

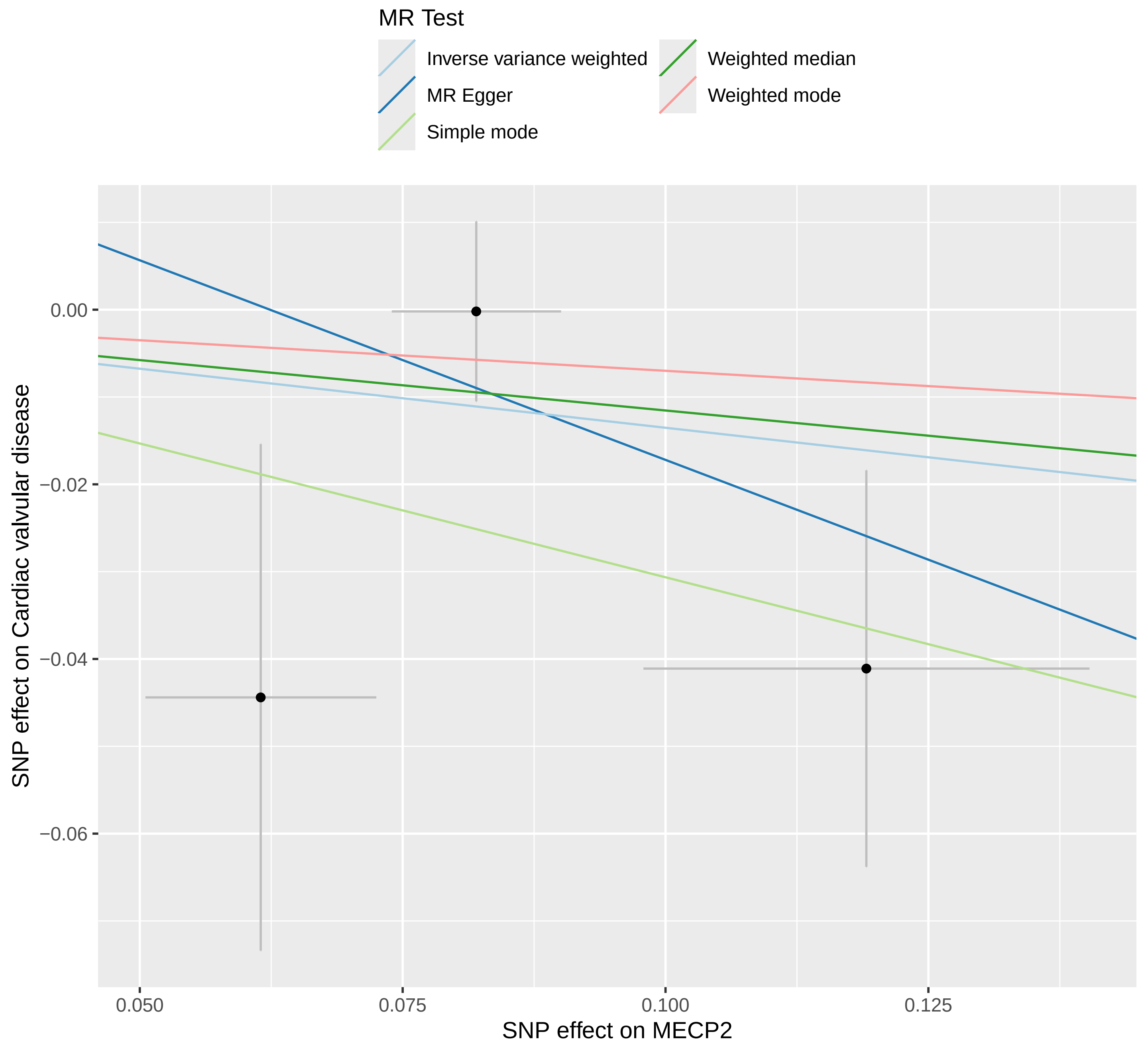
MR Test
Inverse variance weighted
MR Egger
Simple mode
Weighted median
Weighted mode
SNP effect on Cardiac valvular disease
0.00
−0.02
−0.04
−0.06
0.050
0.075
0.100
0.125
SNP effect on MECP2

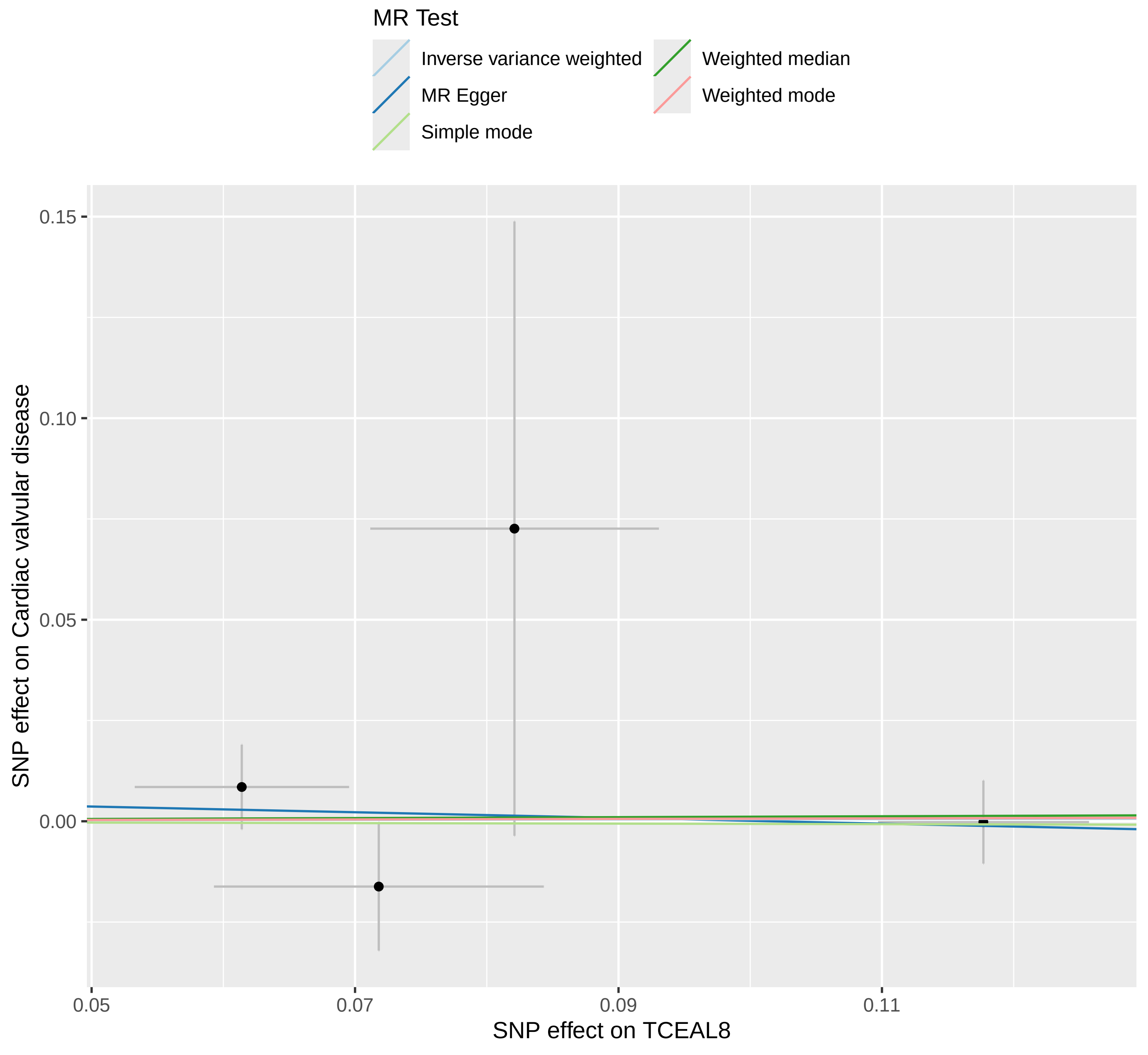

MR Test
Inverse variance weighted
MR Egger
Simple mode
Weighted median
Weighted mode
0.15
0.10
0.05
0.00
0.05
0.07
0.09
0.11
SNP effect on Cardiac valvular disease
SNP effect on TCEAL8

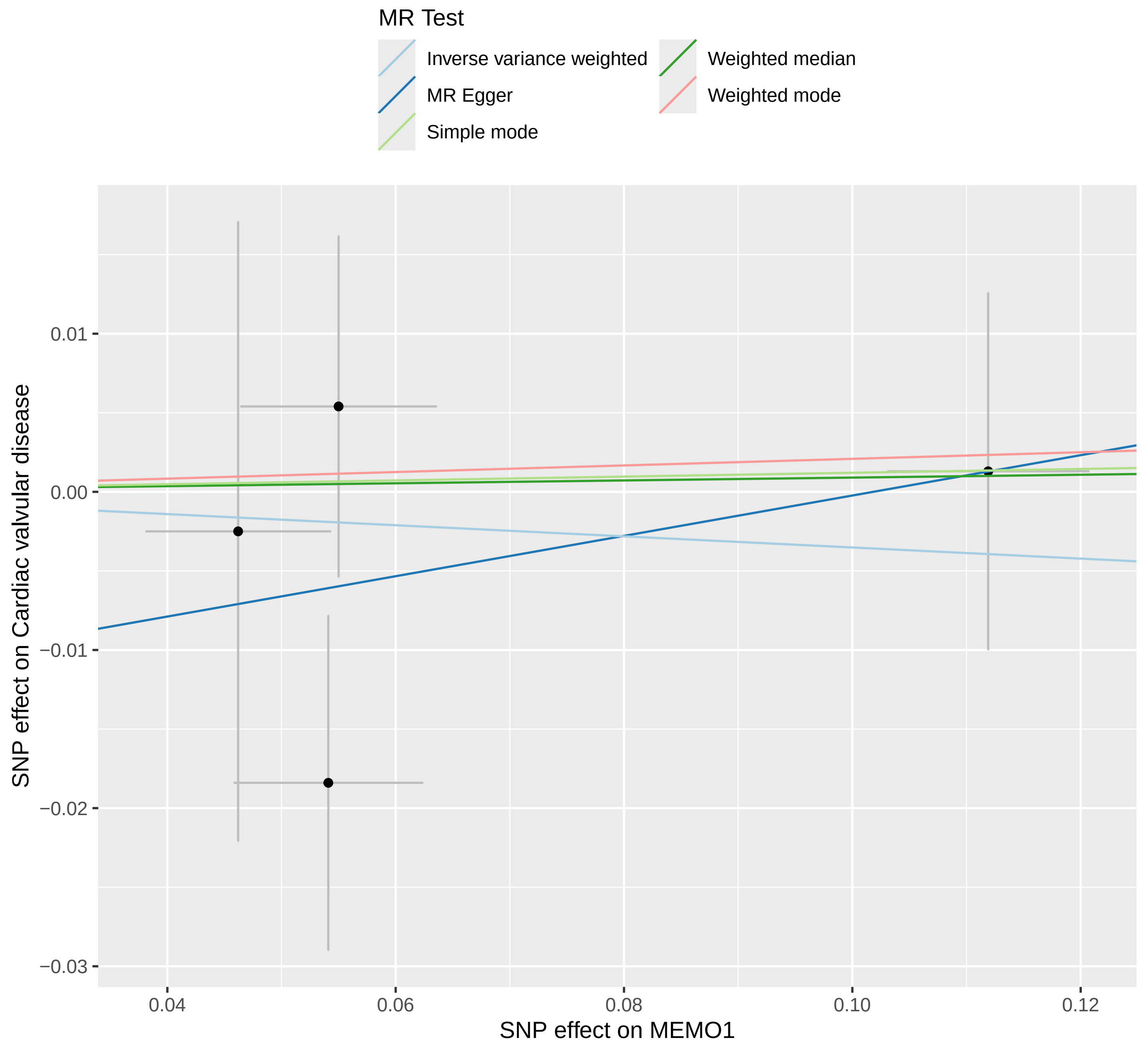
MR Test
Inverse variance weighted
MR Egger
Simple mode
Weighted median
Weighted mode
SNP effect on Cardiac valvular disease
0.01
0.00
−0.01
−0.02
−0.03
0.04
0.06
0.08
0.10
0.12
SNP effect on MEMO1

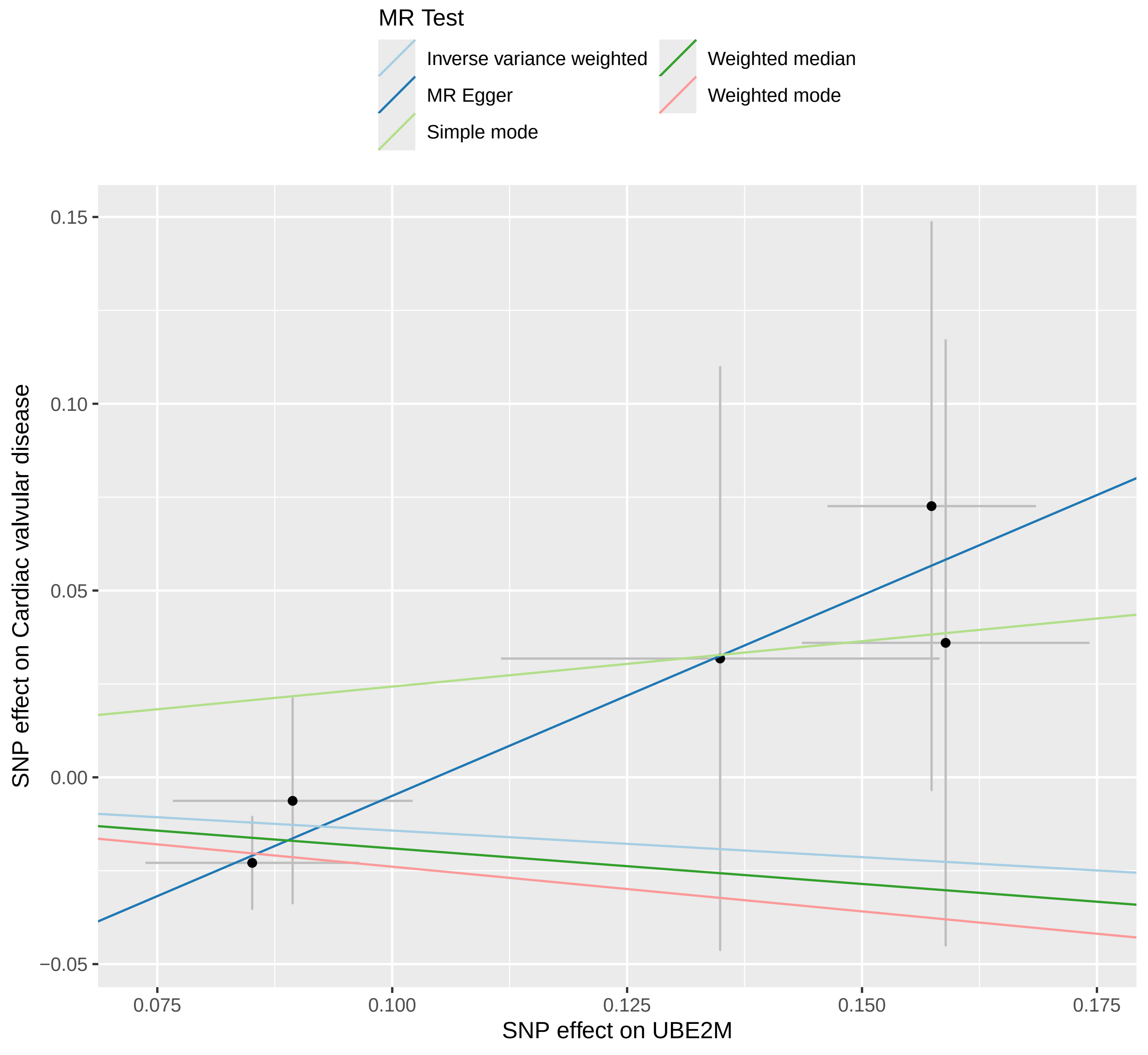

MR Test
Inverse variance weighted
MR Egger
Simple mode
Weighted median
Weighted mode
0.15
0.10
0.05
0.00
−0.05
SNP effect on Cardiac valvular disease
0.075
0.100
0.125
0.150
0.175
SNP effect on UBE2M

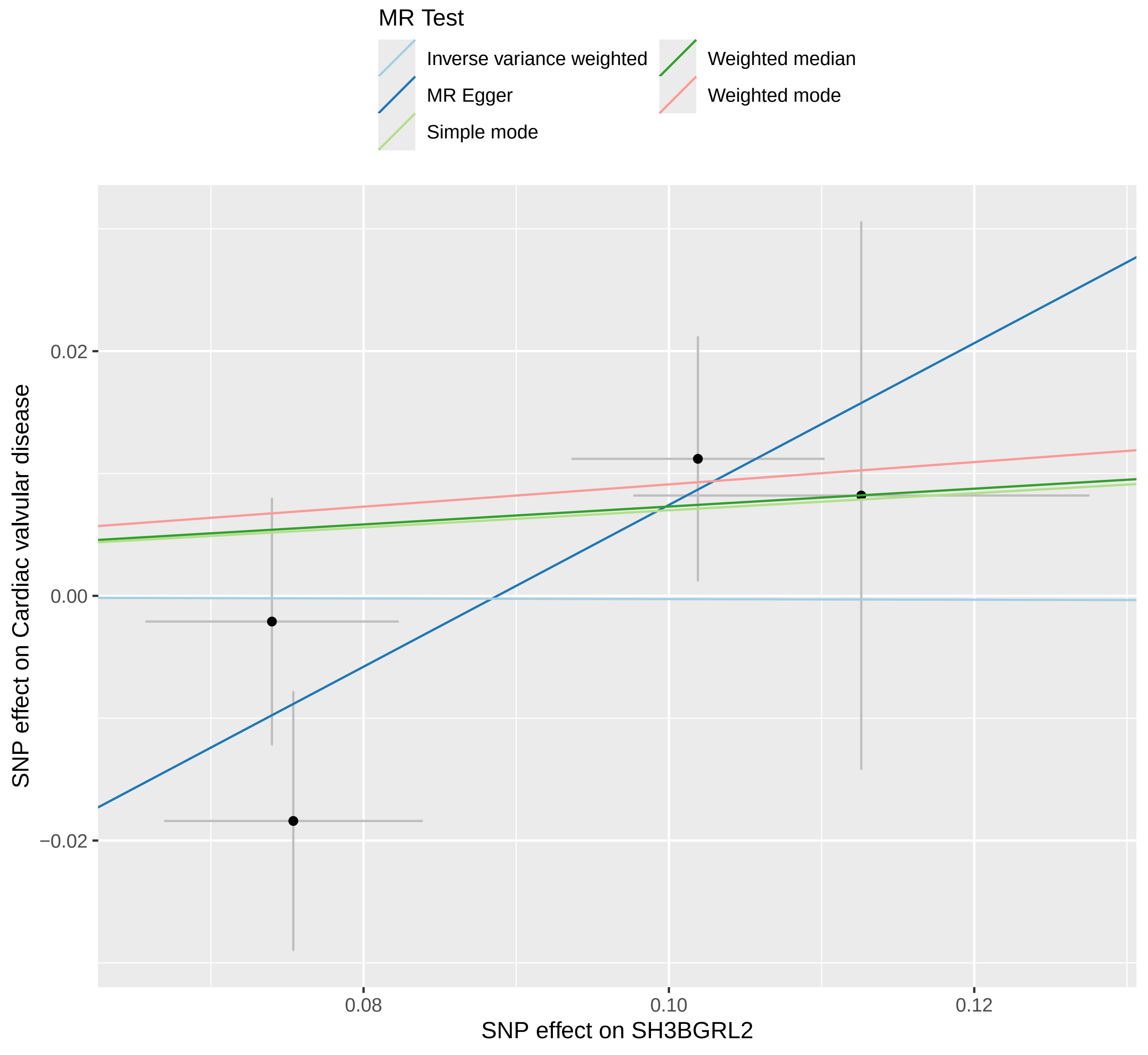
MR Test
Inverse variance weighted
MR Egger
Simple mode
Weighted median
Weighted mode
SNP effect on Cardiac valvular disease
0.02
0.00
−0.02
0.08
0.10
0.12
SNP effect on SH3BGRL2

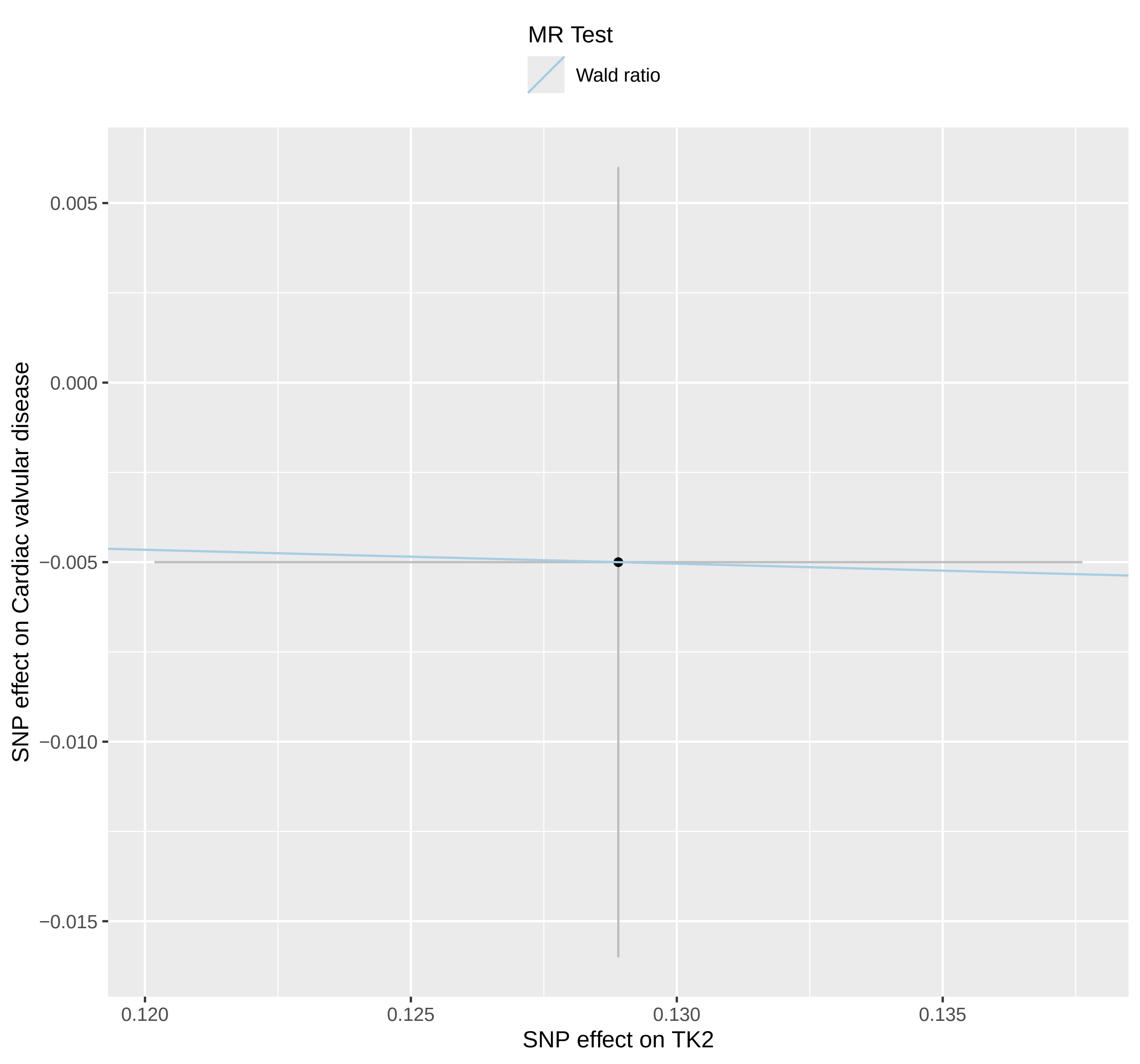
MR Test
Wald ratio
0.005
0.000
−0.005
−0.010
−0.015
0.120
0.125
0.130
0.135
SNP effect on TK2
SNP effect on Cardiac valvular disease

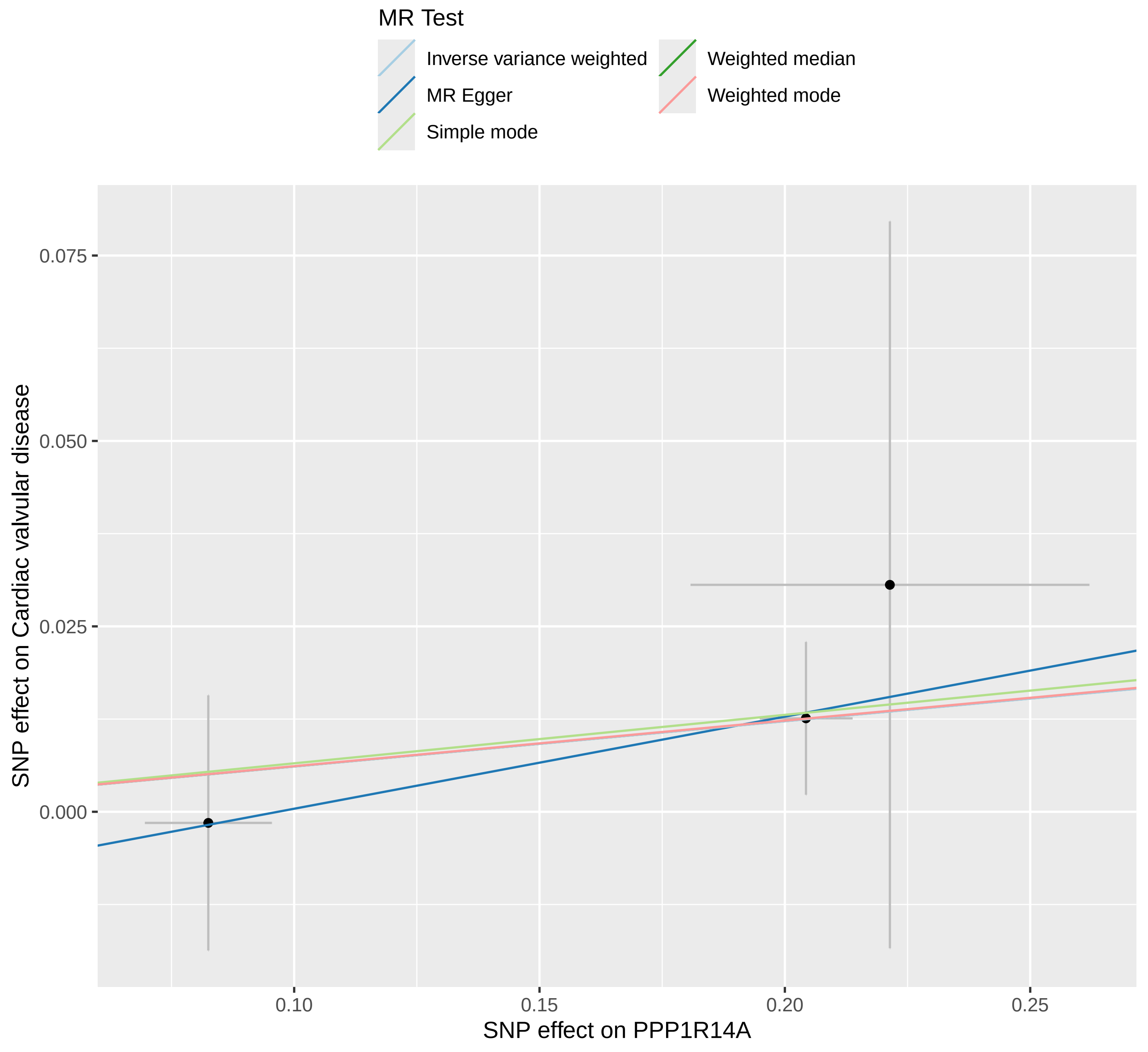
MR Test
Inverse variance weighted
MR Egger
Simple mode
Weighted median
Weighted mode
SNP effect on Cardiac valvular disease
0.075
0.050
0.025
0.000
0.10
0.15
0.20
0.25
SNP effect on PPP1R14A

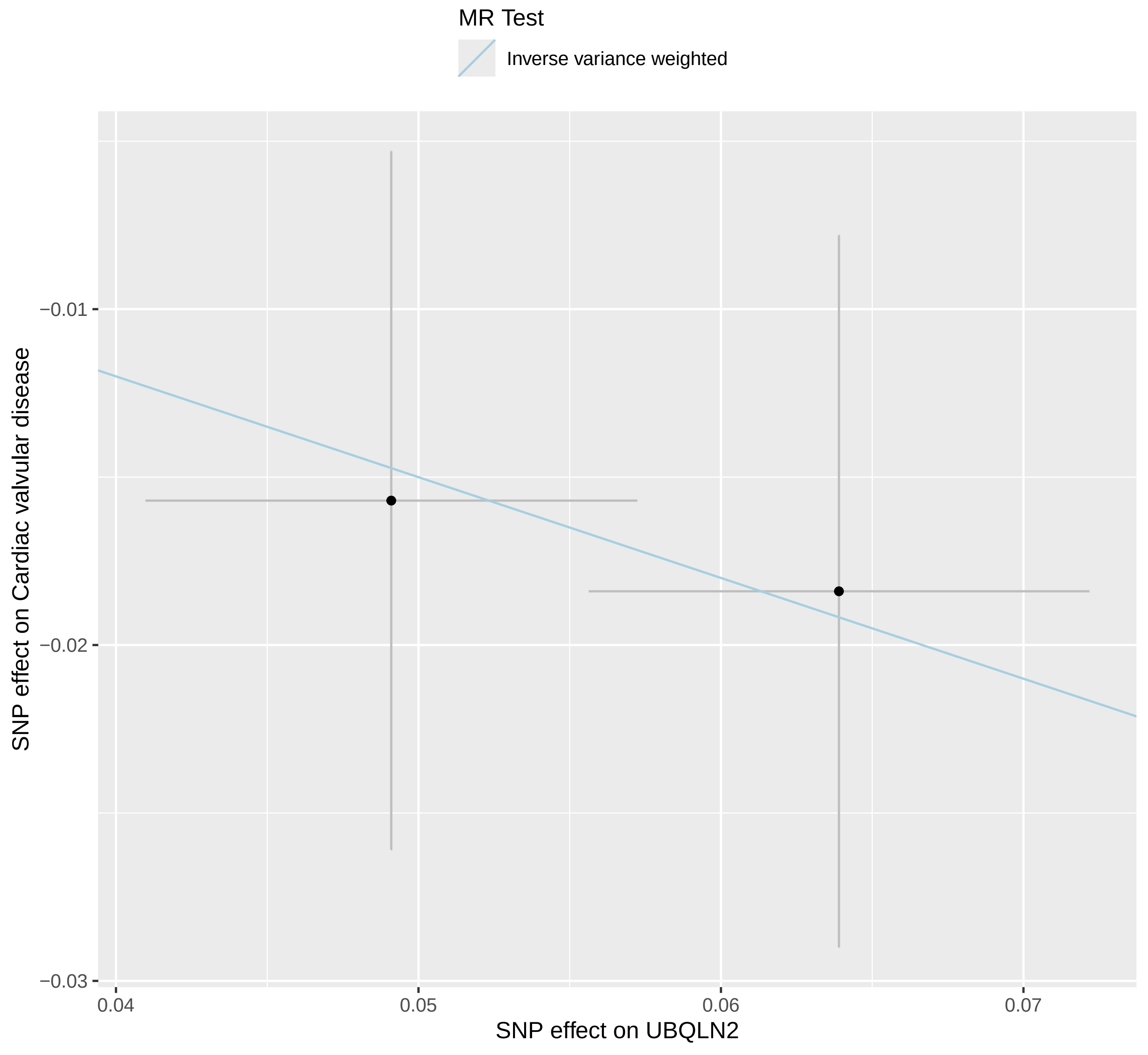
MR Test
Inverse variance weighted
−0.01
−0.02
−0.03
0.04
0.05
0.06
0.07
SNP effect on Cardiac valvular disease
SNP effect on UBQLN2

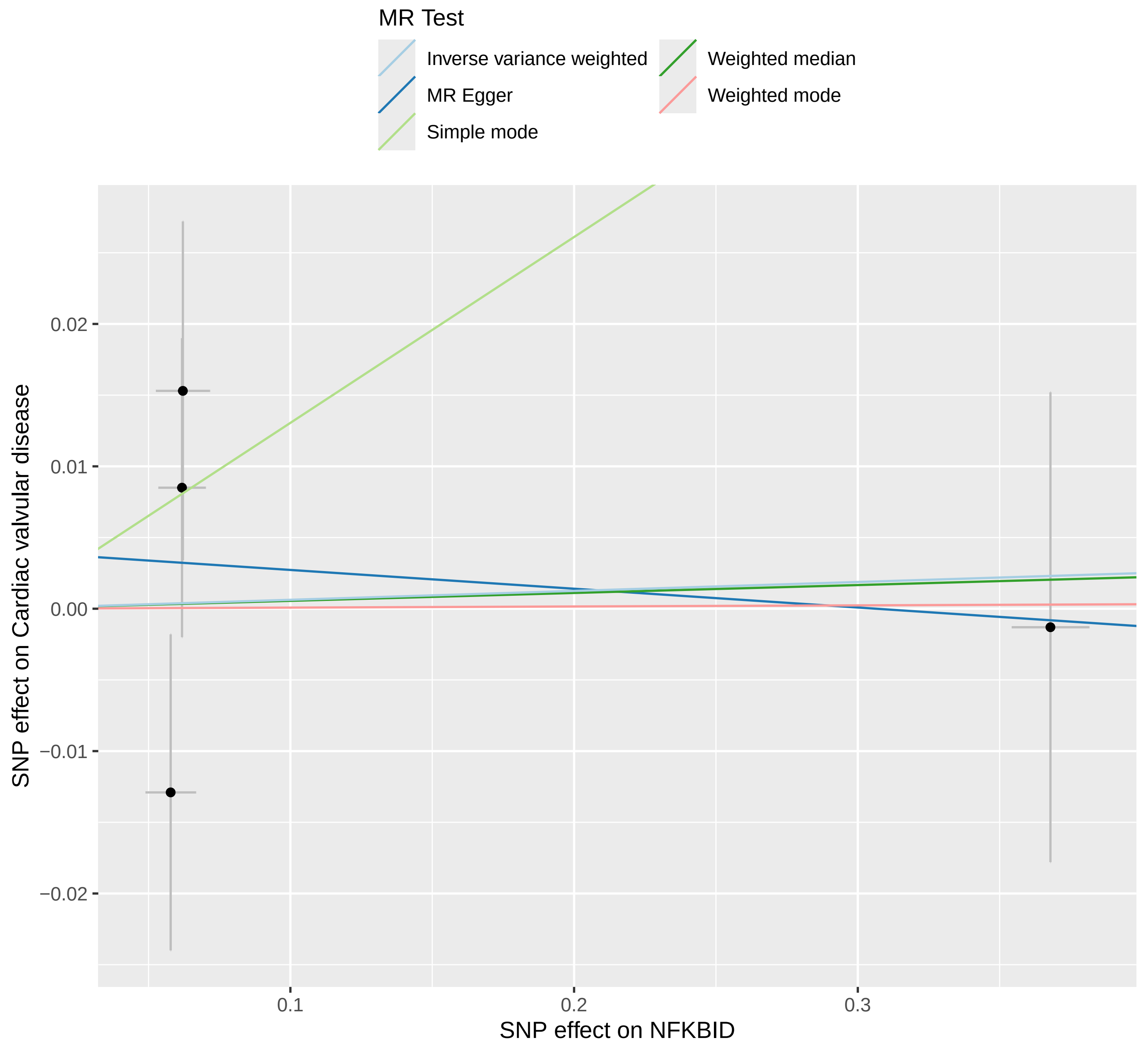

MR Test
Inverse variance weighted
MR Egger
Simple mode
Weighted median
Weighted mode
0.02
0.01
0.00
−0.01
−0.02
SNP effect on Cardiac valvular disease
0.1
0.2
0.3
SNP effect on NFKBID

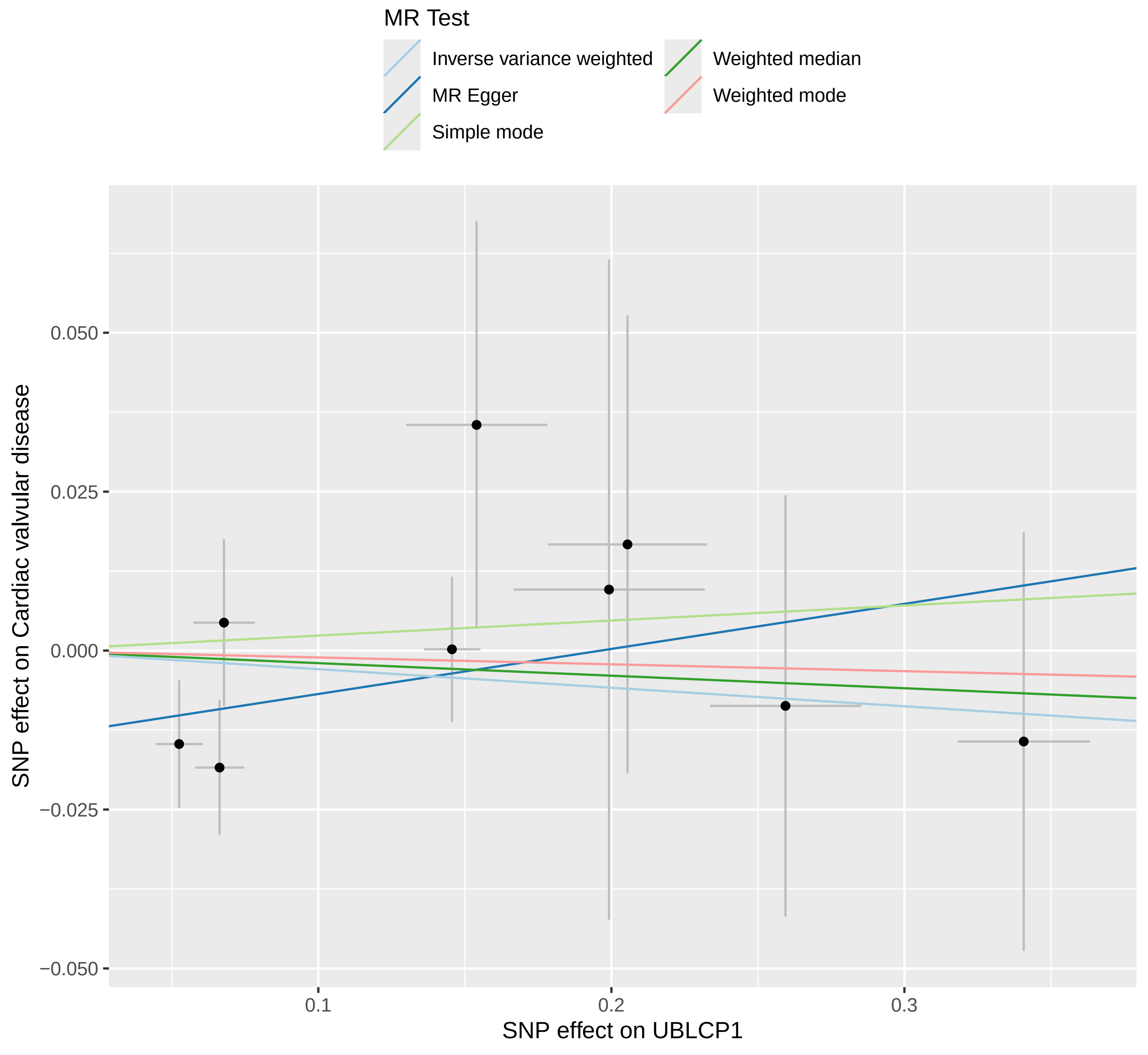

MR Test
Inverse variance weighted
MR Egger
Simple mode
Weighted median
Weighted mode
0.050
0.025
0.000
−0.025
−0.050
SNP effect on Cardiac valvular disease
0.1
0.2
0.3
SNP effect on UBLCP1

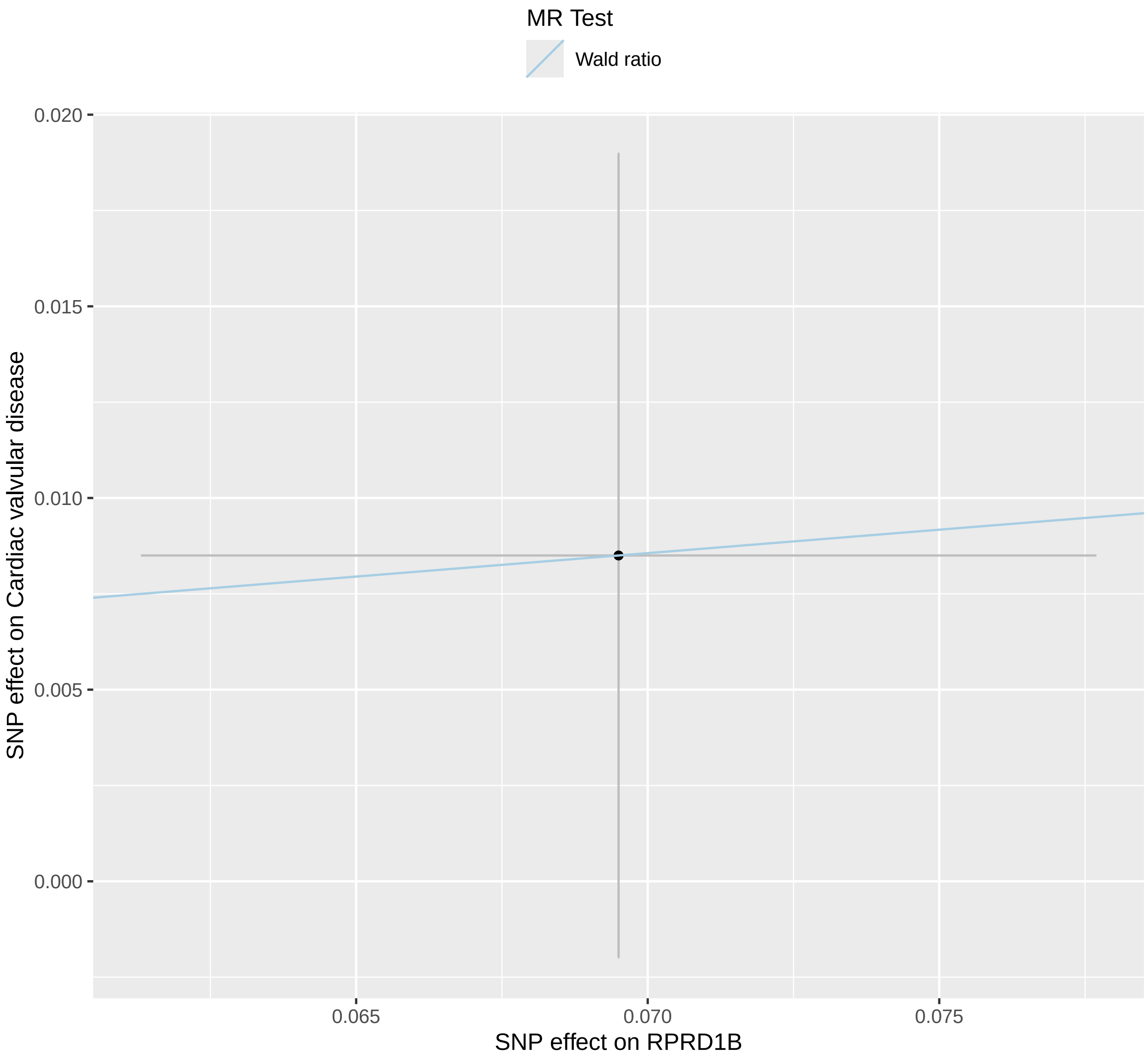
MR Test
Wald ratio
0.020
0.015
0.010
0.005
0.000
SNP effect on Cardiac valvular disease
0.065
0.070
0.075
SNP effect on RPRD1B

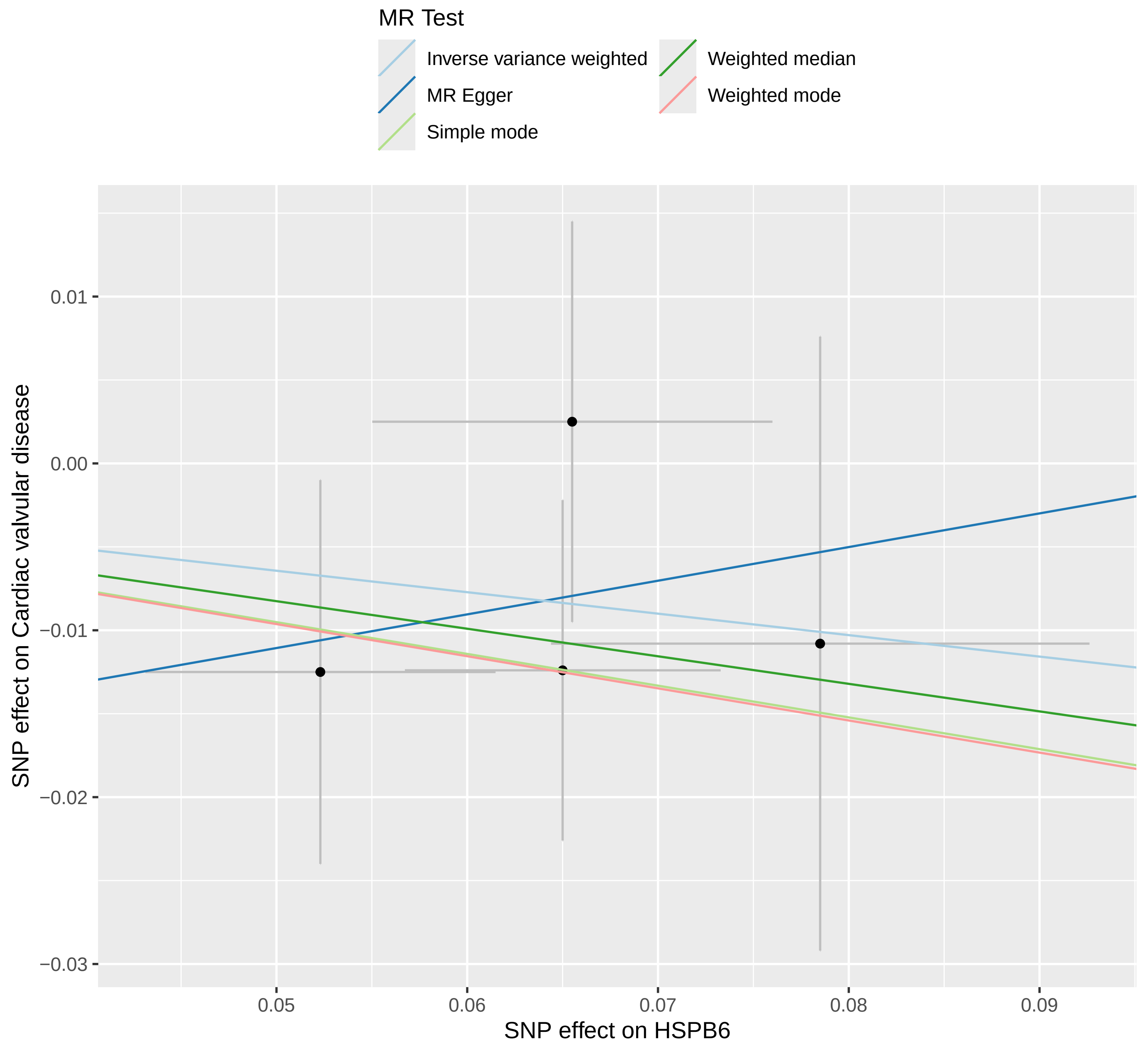
MR Test
Inverse variance weighted
MR Egger
Simple mode
Weighted median
Weighted mode
0.01
0.00
−0.01
−0.02
−0.03
SNP effect on Cardiac valvular disease
0.05
0.06
0.07
0.08
0.09
SNP effect on HSPB6

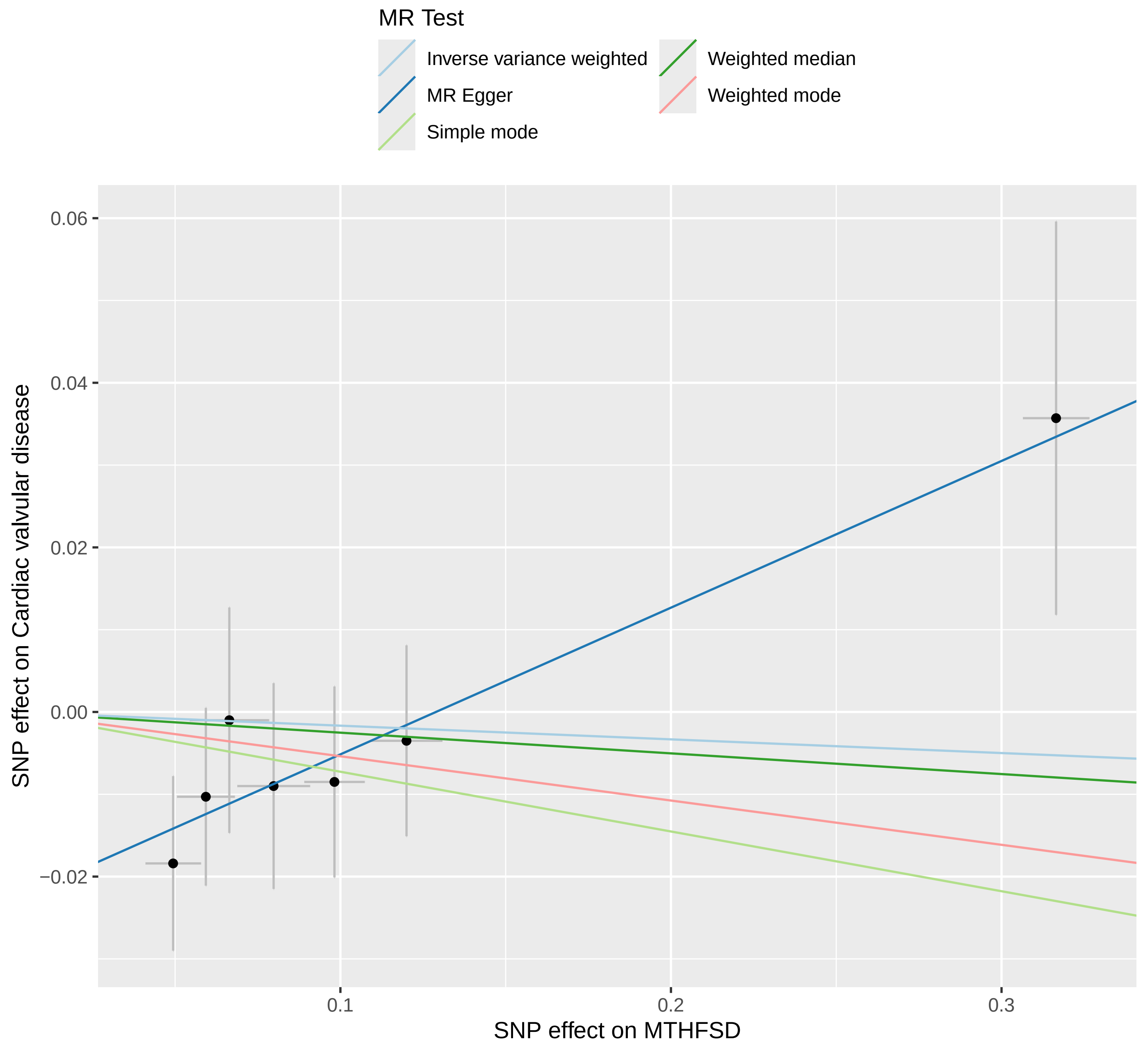

MR Test
Inverse variance weighted
MR Egger
Simple mode
Weighted median
Weighted mode
0.06
0.04
0.02
0.00
−0.02
SNP effect on Cardiac valvular disease
0.1
0.2
0.3
SNP effect on MTHFSD

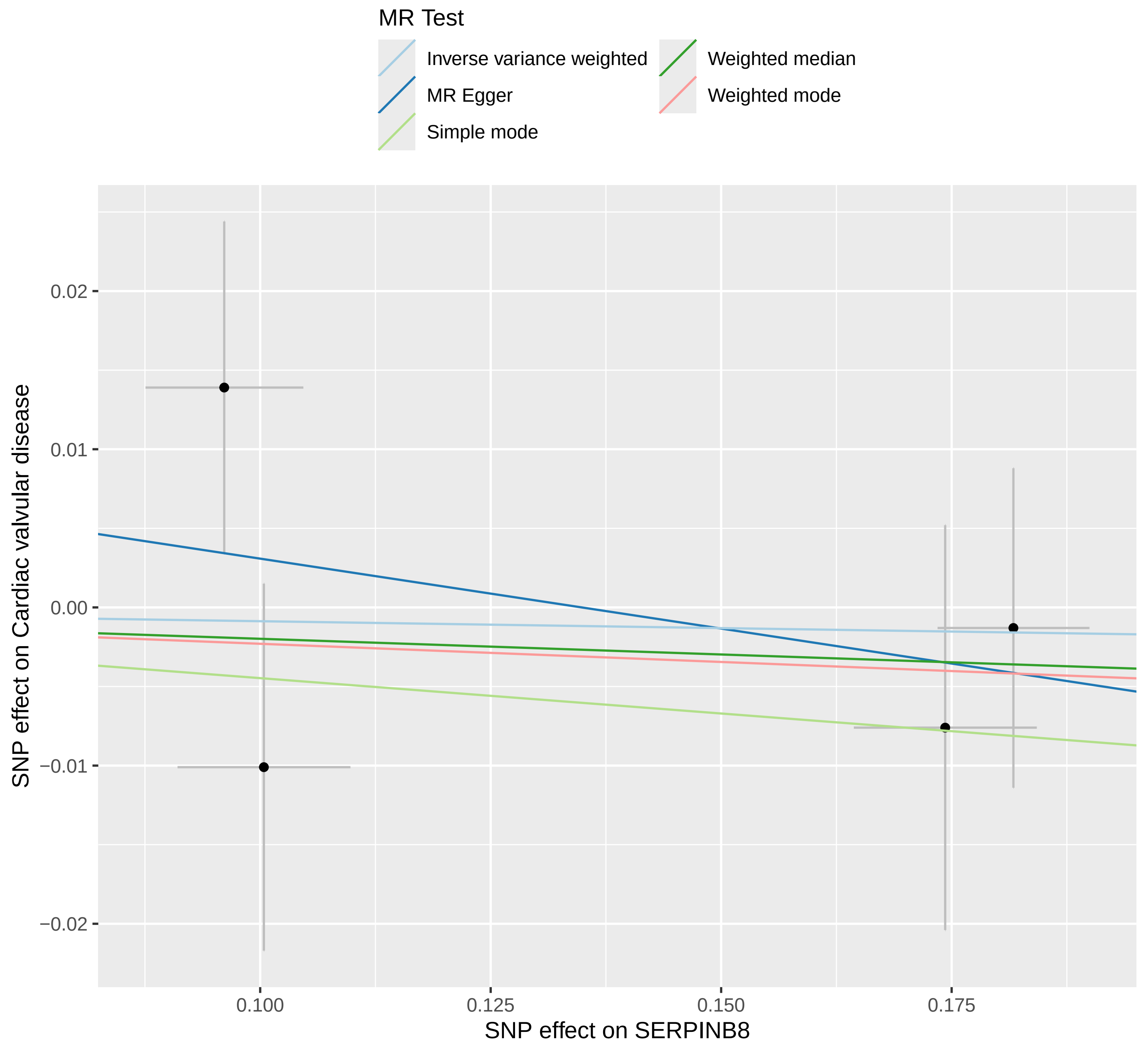
MR Test
Inverse variance weighted
MR Egger
Simple mode
Weighted median
Weighted mode
0.02
0.01
0.00
−0.01
−0.02
SNP effect on Cardiac valvular disease
0.100
0.125
0.150
0.175
SNP effect on SERPINB8

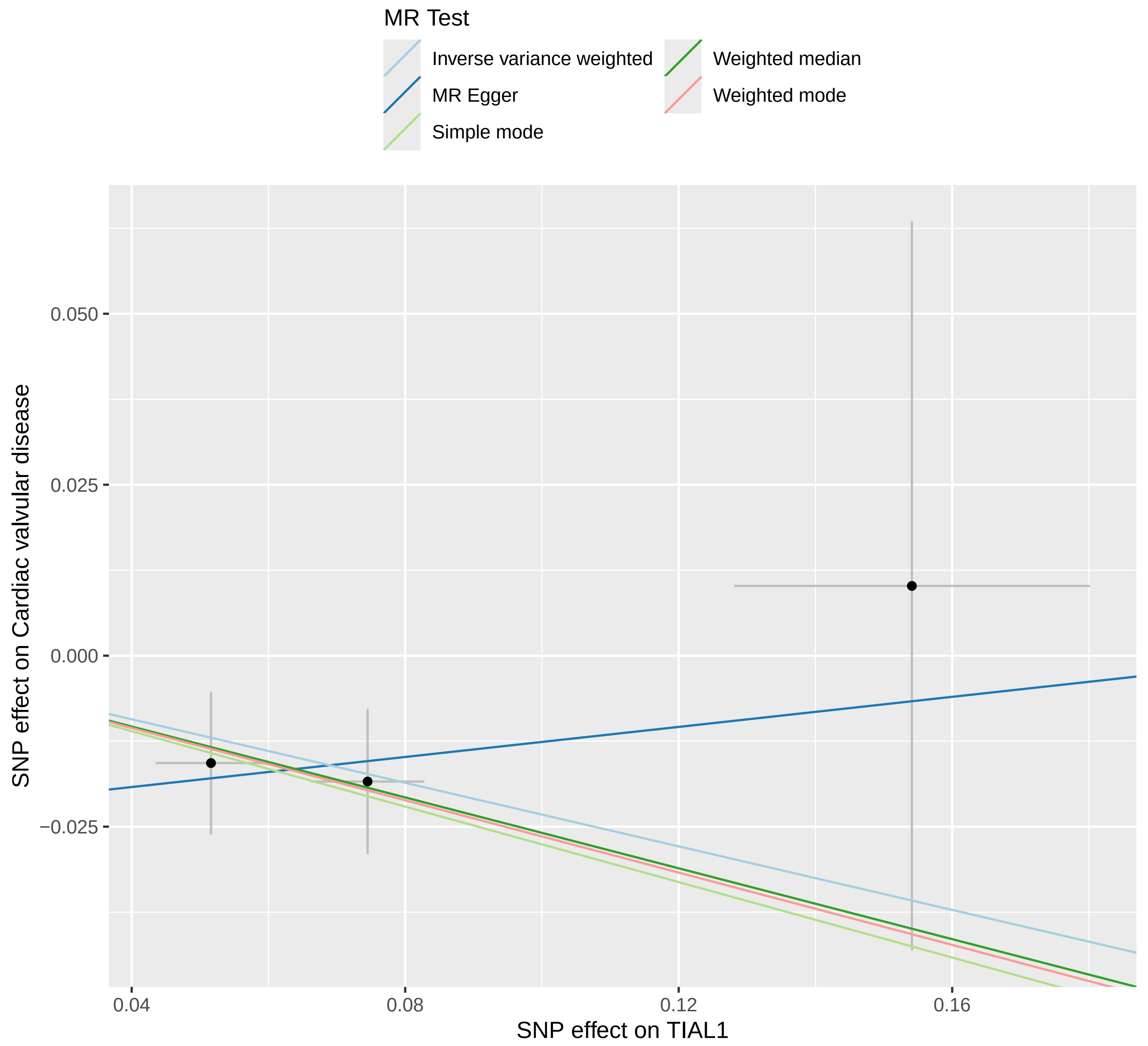
MR Test
Inverse variance weighted
MR Egger
Simple mode
Weighted median
Weighted mode
0.050
0.025
0.000
−0.025
SNP effect on Cardiac valvular disease
0.04
0.08
0.12
0.16
SNP effect on TIAL1

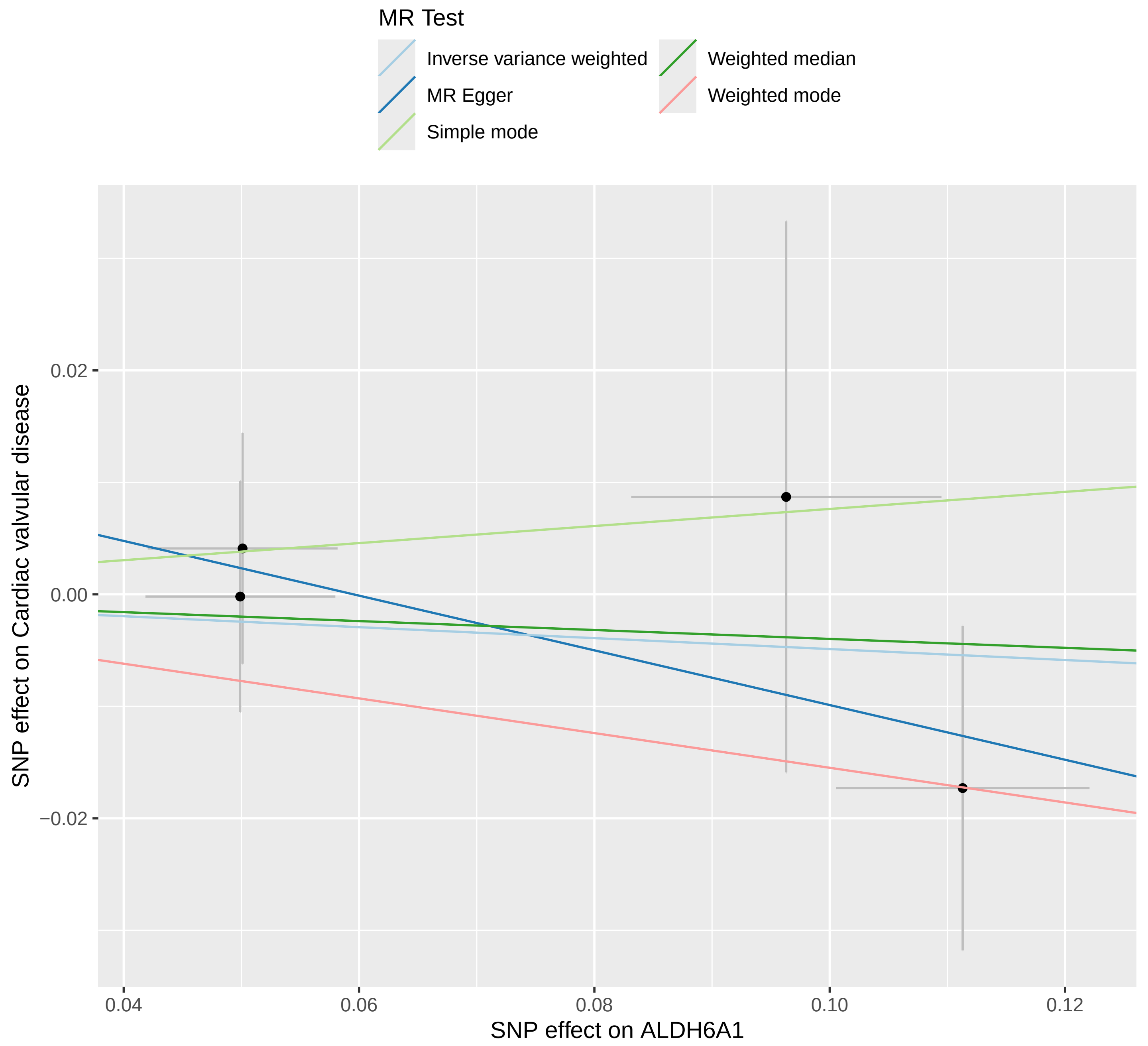
MR Test
Inverse variance weighted
MR Egger
Simple mode
Weighted median
Weighted mode
0.02
0.00
−0.02
0.04
0.06
0.08
0.10
0.12
SNP effect on Cardiac valvular disease
SNP effect on ALDH6A1

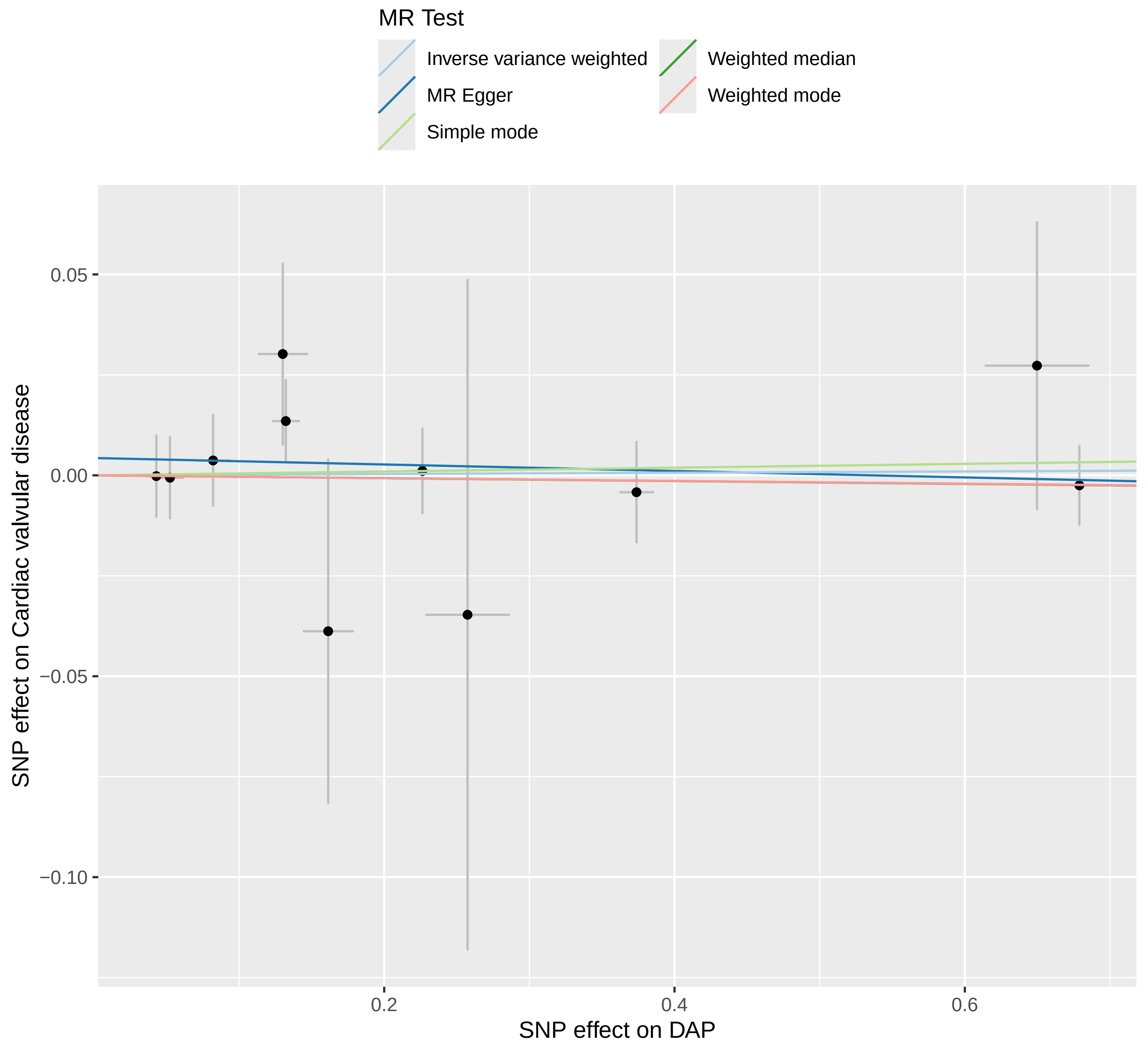

MR Test
Inverse variance weighted
MR Egger
Simple mode
Weighted median
Weighted mode
0.05
0.00
−0.05
−0.10
0.2
0.4
0.6
SNP effect on DAP
SNP effect on Cardiac valvular disease

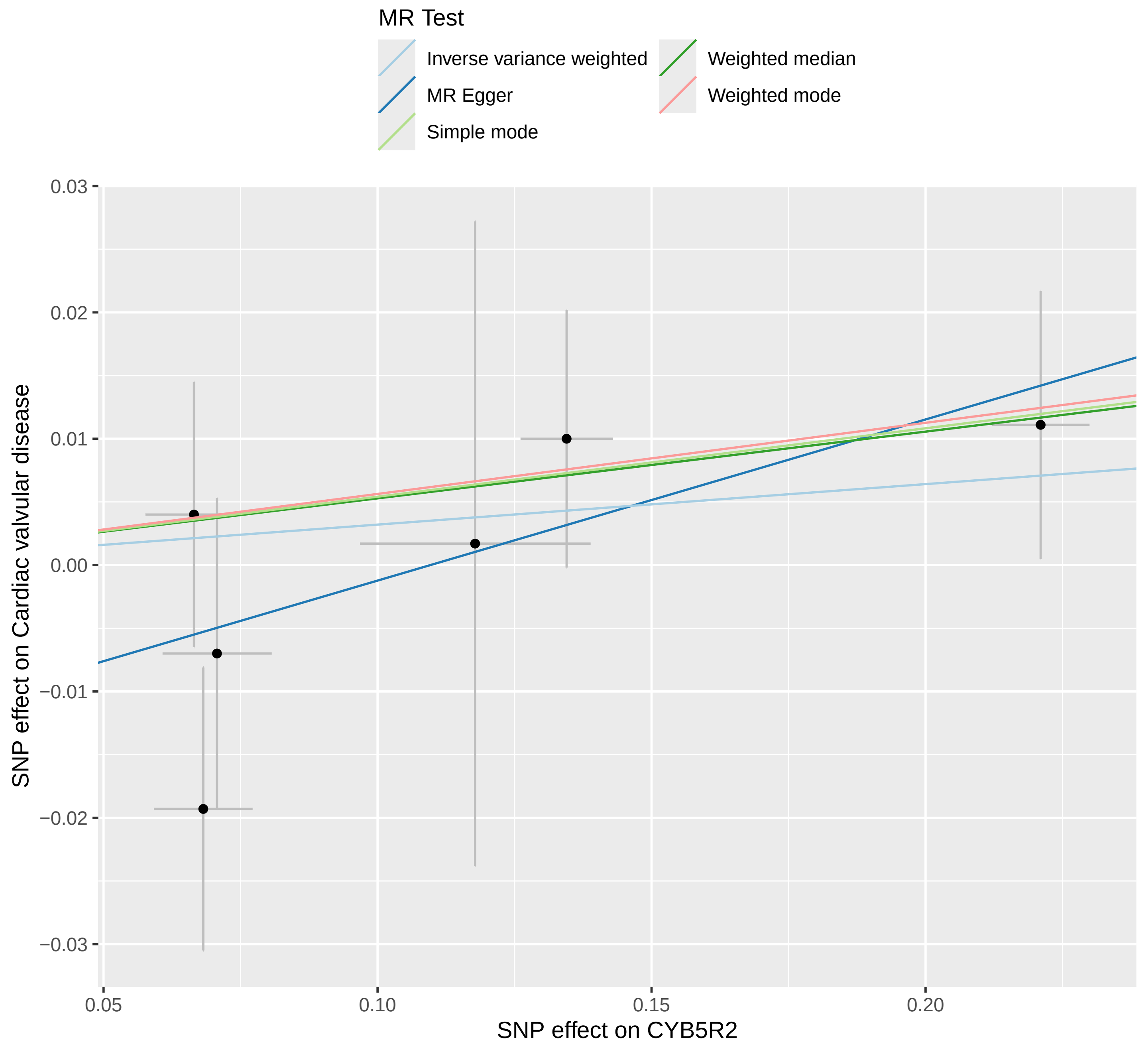
MR Test
Inverse variance weighted
MR Egger
Simple mode
Weighted median
Weighted mode
0.03
0.02
0.01
0.00
−0.01
−0.02
−0.03
0.05
0.10
0.15
0.20
SNP effect on Cardiac valvular disease
SNP effect on CYB5R2

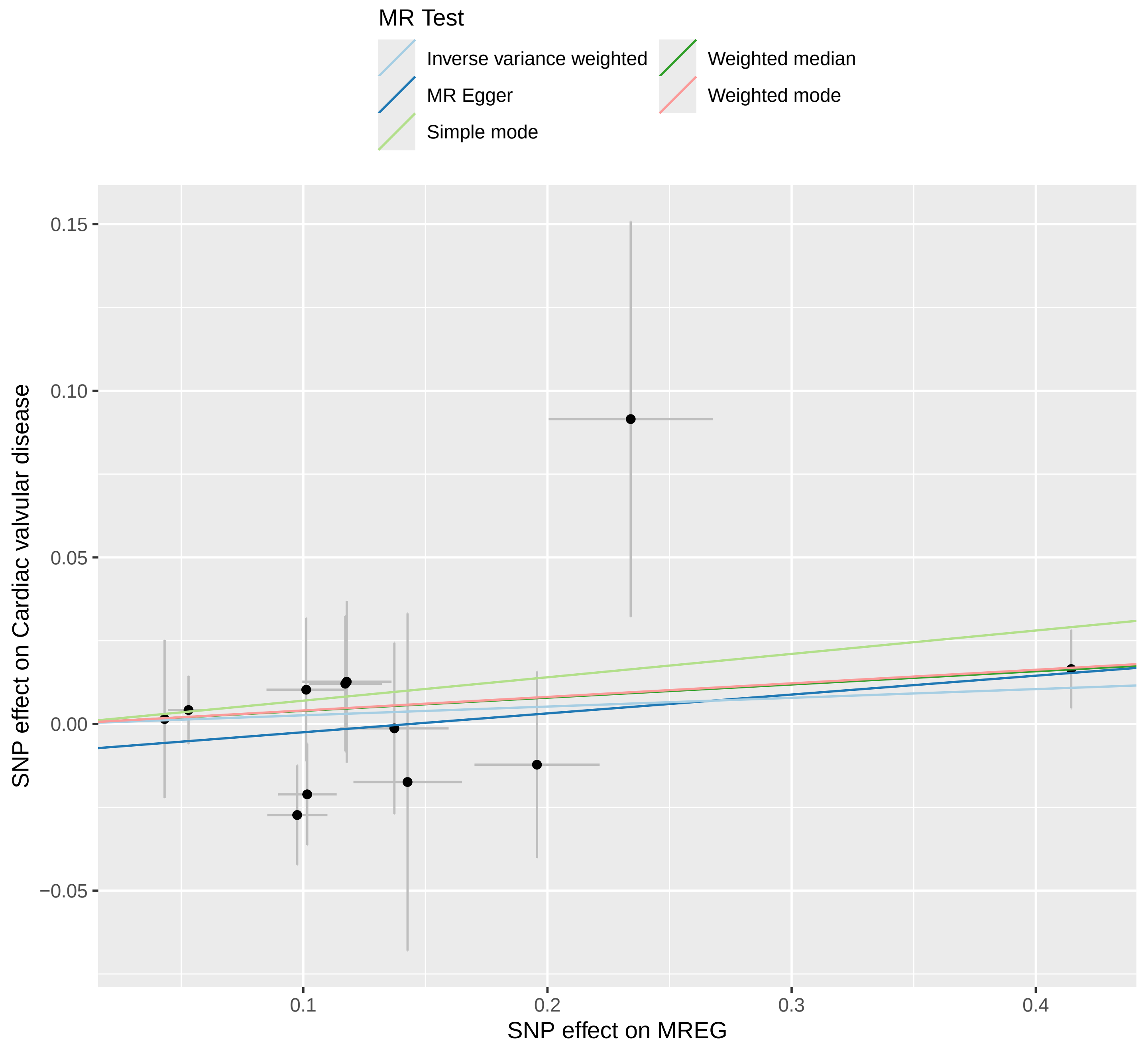
MR Test
Inverse variance weighted
MR Egger
Simple mode
Weighted median
Weighted mode
0.15
0.10
0.05
0.00
−0.05
0.1
0.2
0.3
0.4
SNP effect on Cardiac valvular disease
SNP effect on MREG

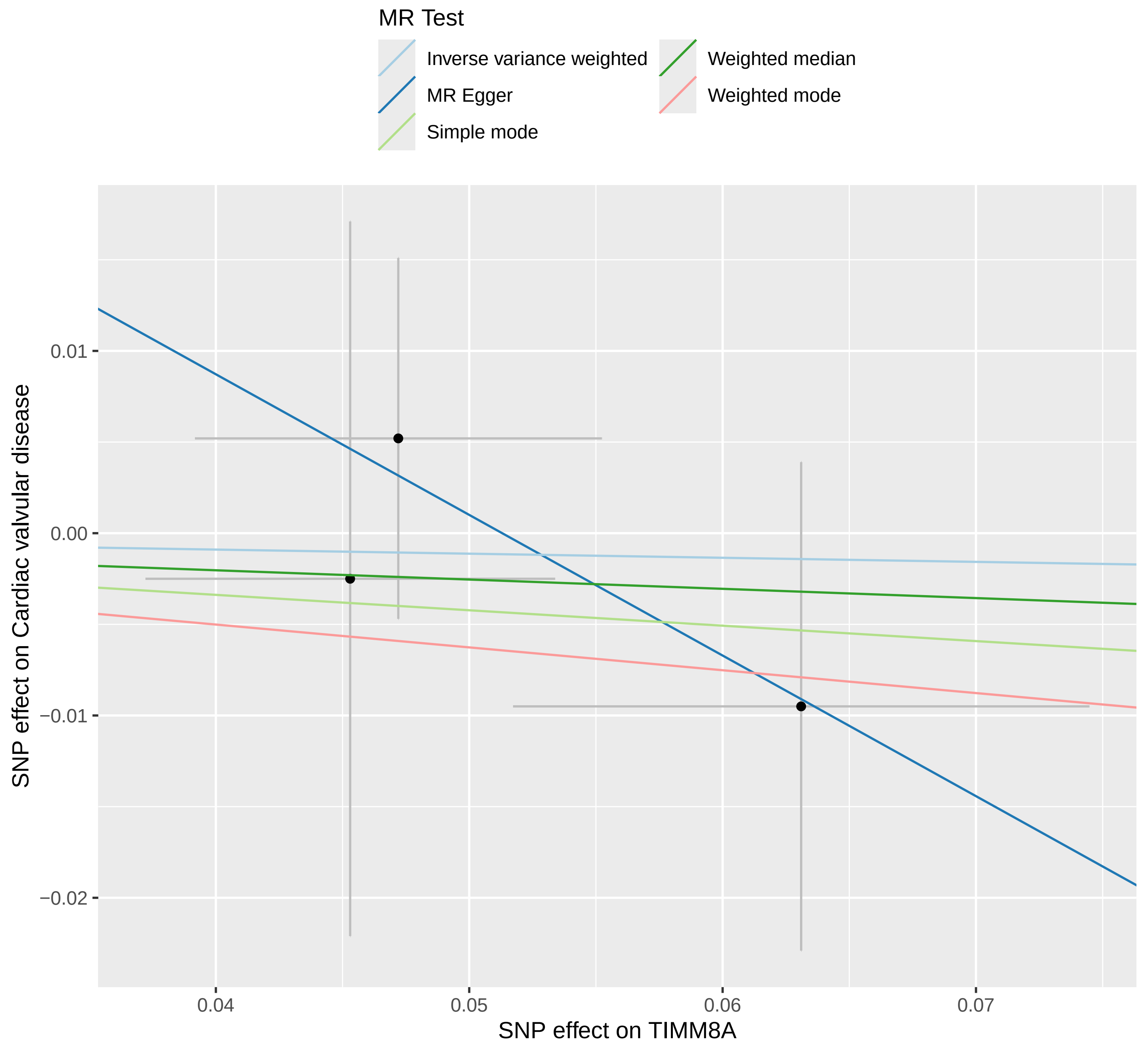
MR Test
Inverse variance weighted
MR Egger
Simple mode
Weighted median
Weighted mode
0.01
0.00
−0.01
−0.02
0.04
0.05
0.06
0.07
SNP effect on TIMM8A
SNP effect on Cardiac valvular disease

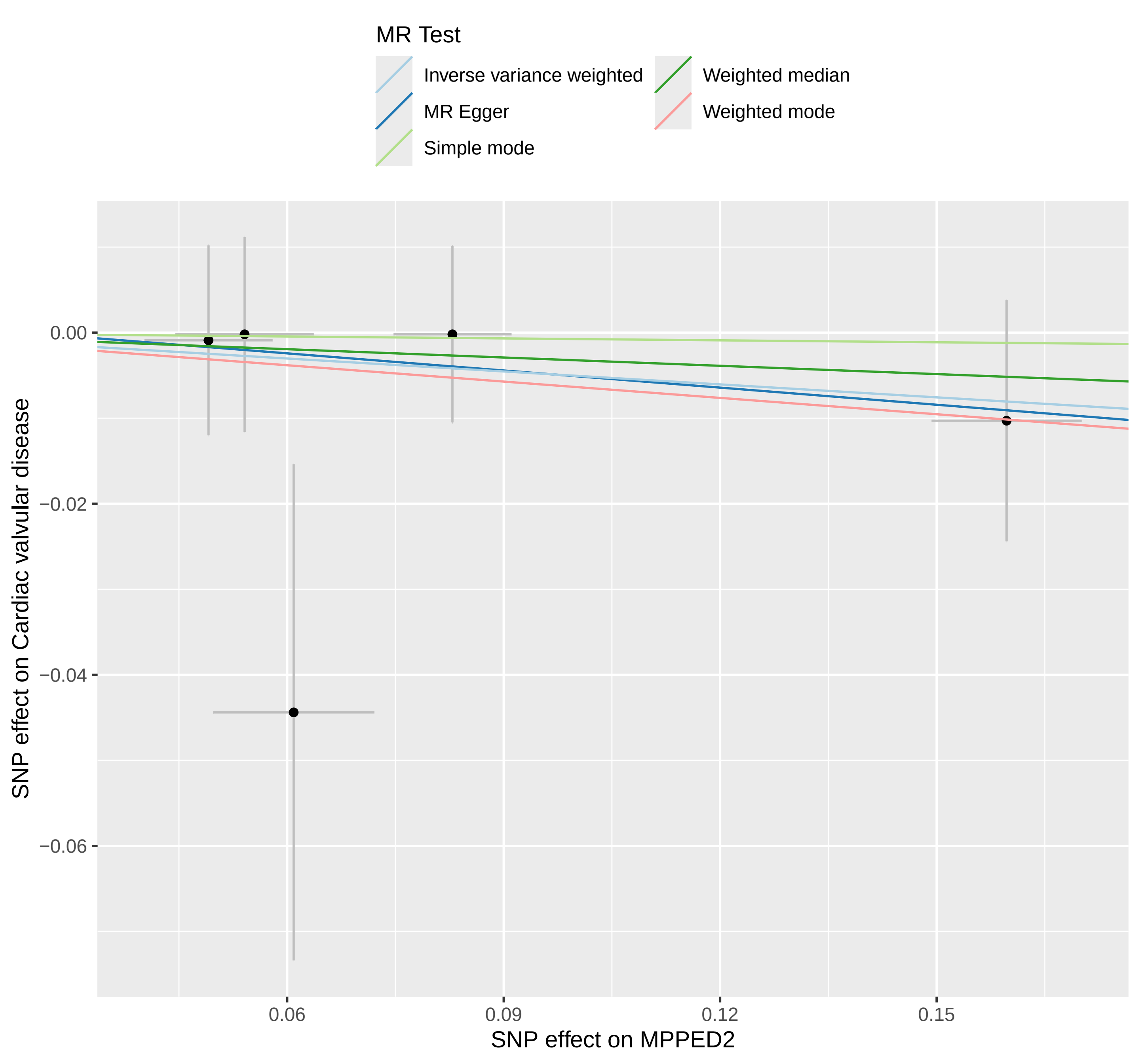

MR Test
Inverse variance weighted
MR Egger
Simple mode
Weighted median
Weighted mode
0.00
−0.02
−0.04
−0.06
0.06
0.09
0.12
0.15
SNP effect on MPPED2
SNP effect on Cardiac valvular disease

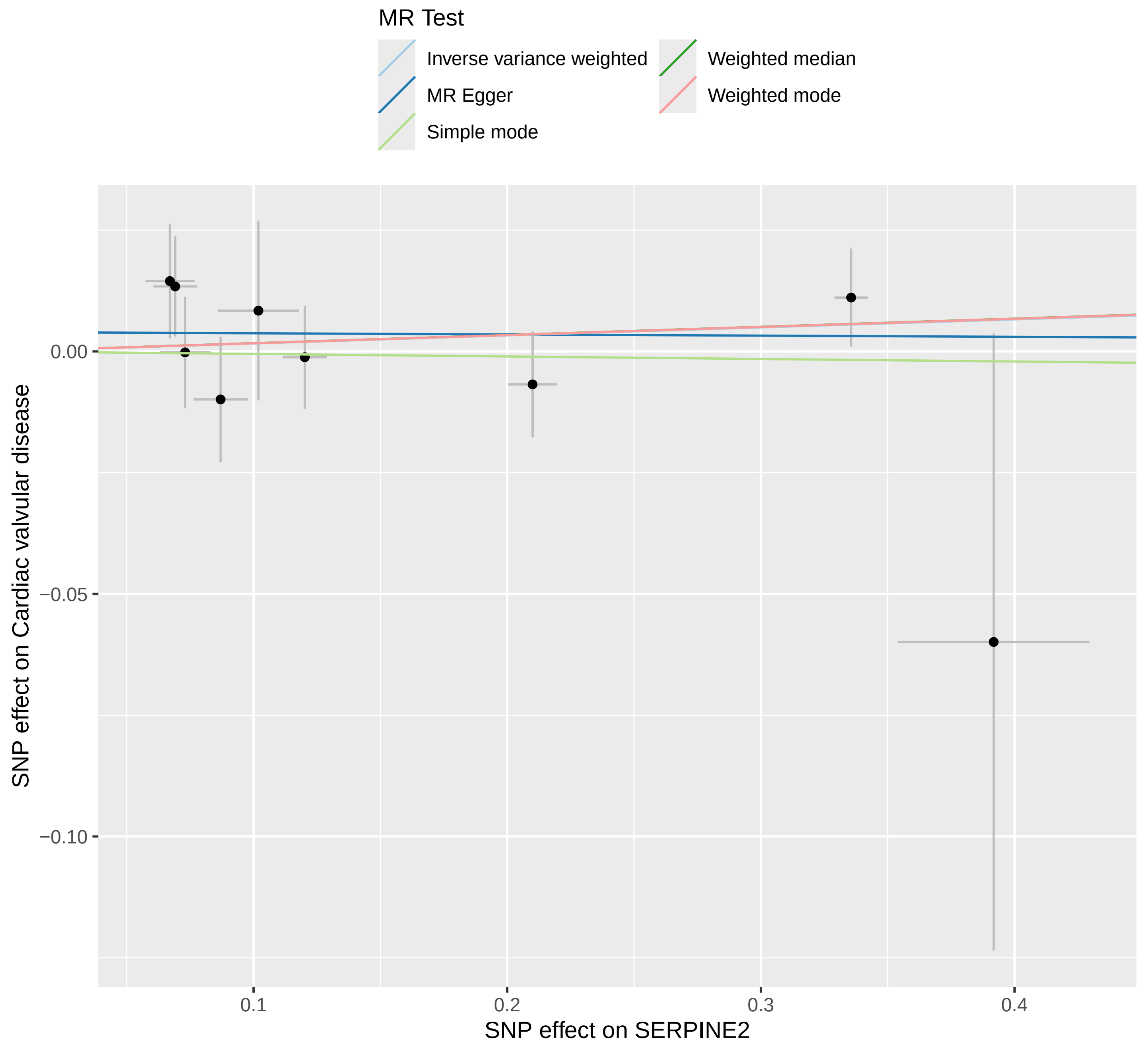
MR Test
Inverse variance weighted
MR Egger
Simple mode
Weighted median
Weighted mode
0.00
−0.05
−0.10
SNP effect on Cardiac valvular disease
0.1
0.2
0.3
0.4
SNP effect on SERPINE2

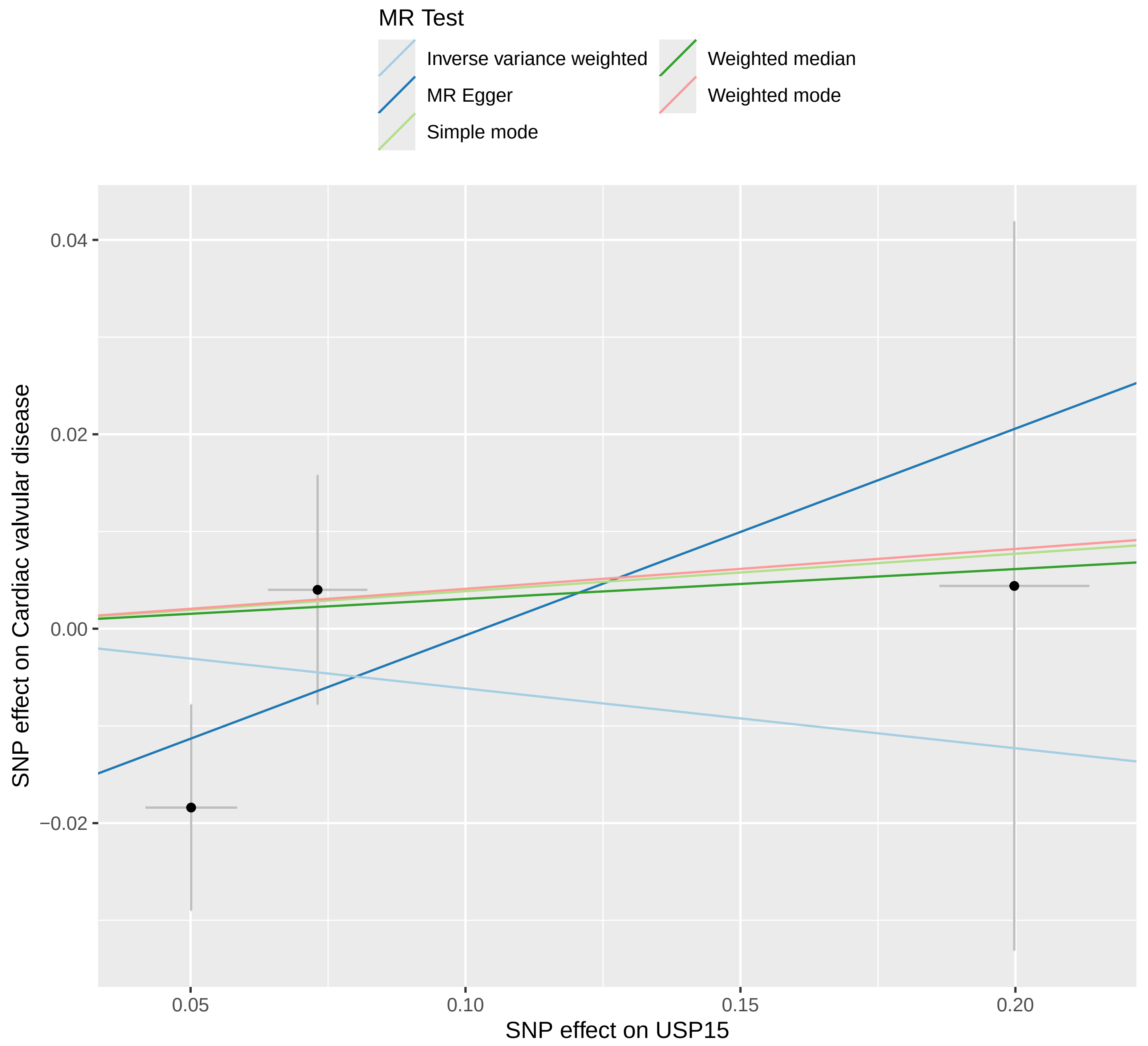

MR Test
Inverse variance weighted
MR Egger
Simple mode
Weighted median
Weighted mode
0.04
0.02
0.00
−0.02
0.05
0.10
0.15
0.20
SNP effect on USP15
SNP effect on Cardiac valvular disease

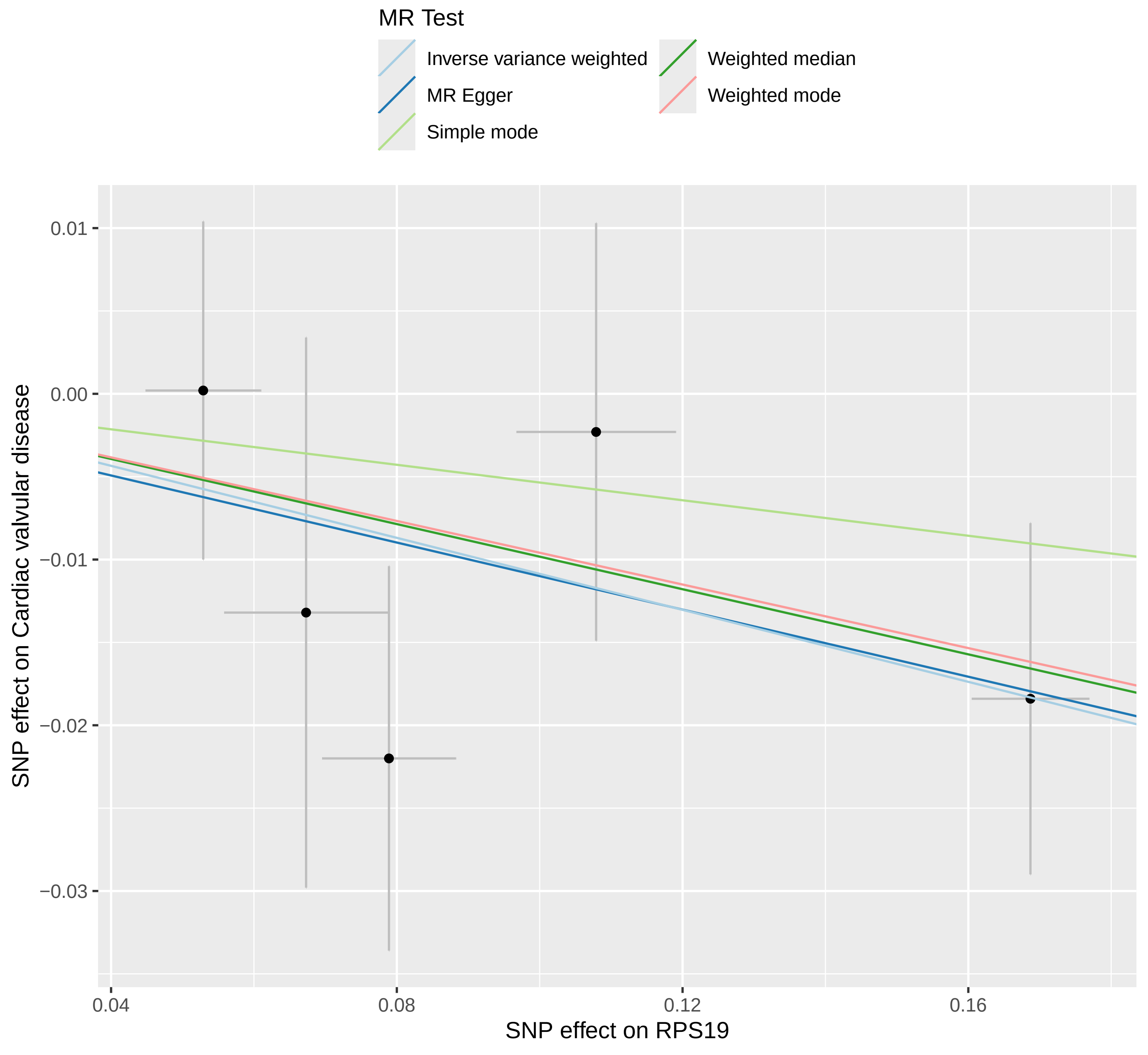

MR Test
Inverse variance weighted
MR Egger
Simple mode
Weighted median
Weighted mode
0.01
0.00
−0.01
−0.02
−0.03
SNP effect on Cardiac valvular disease
0.04
0.08
0.12
0.16
SNP effect on RPS19

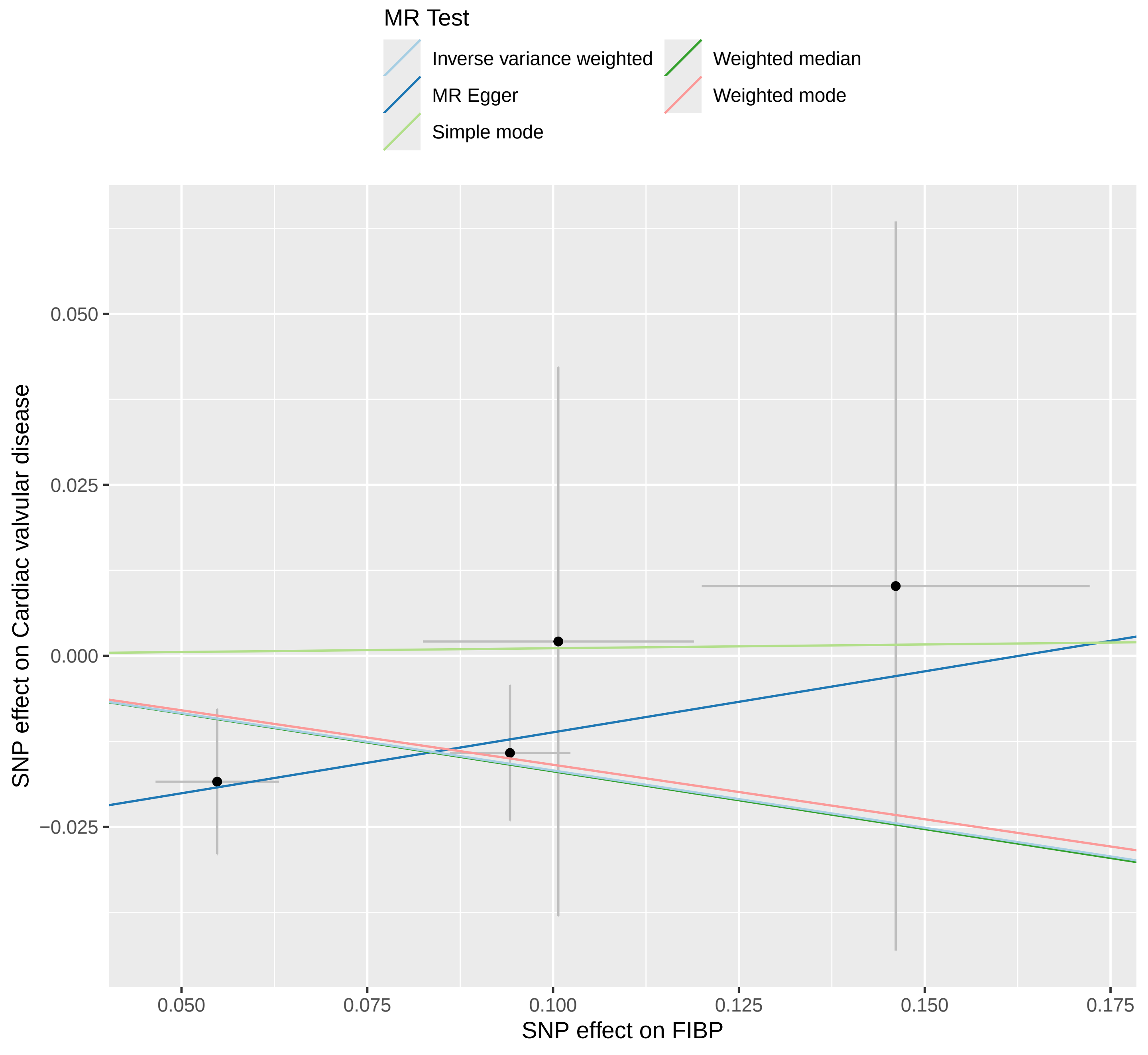
MR Test
Inverse variance weighted
MR Egger
Simple mode
Weighted median
Weighted mode
SNP effect on Cardiac valvular disease
0.050
0.025
0.000
−0.025
0.050
0.075
0.100
0.125
0.150
0.175
SNP effect on FIBP

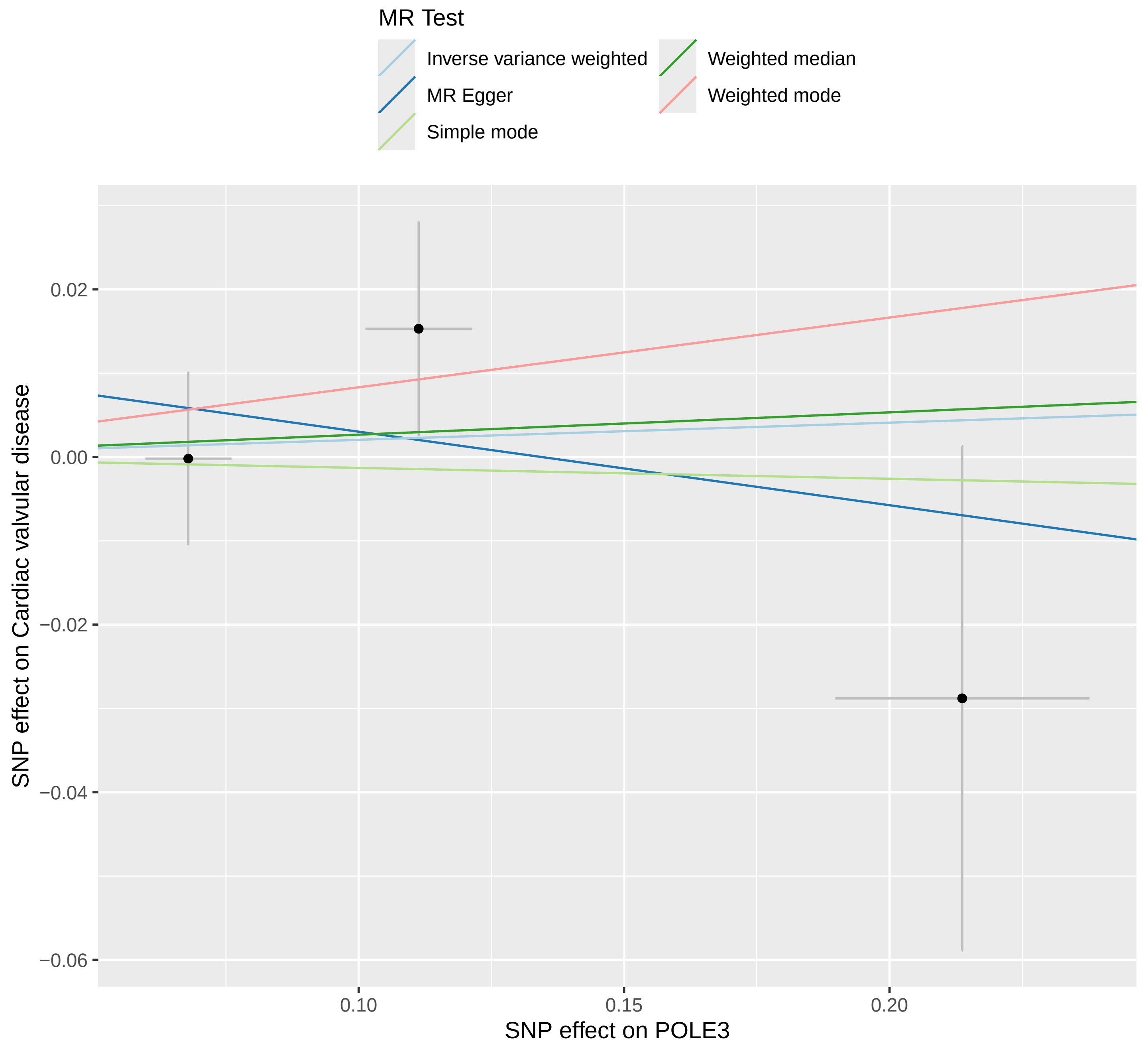

MR Test
Inverse variance weighted
MR Egger
Simple mode
Weighted median
Weighted mode
0.02
0.00
−0.02
−0.04
−0.06
0.10
0.15
0.20
SNP effect on Cardiac valvular disease
SNP effect on POLE3

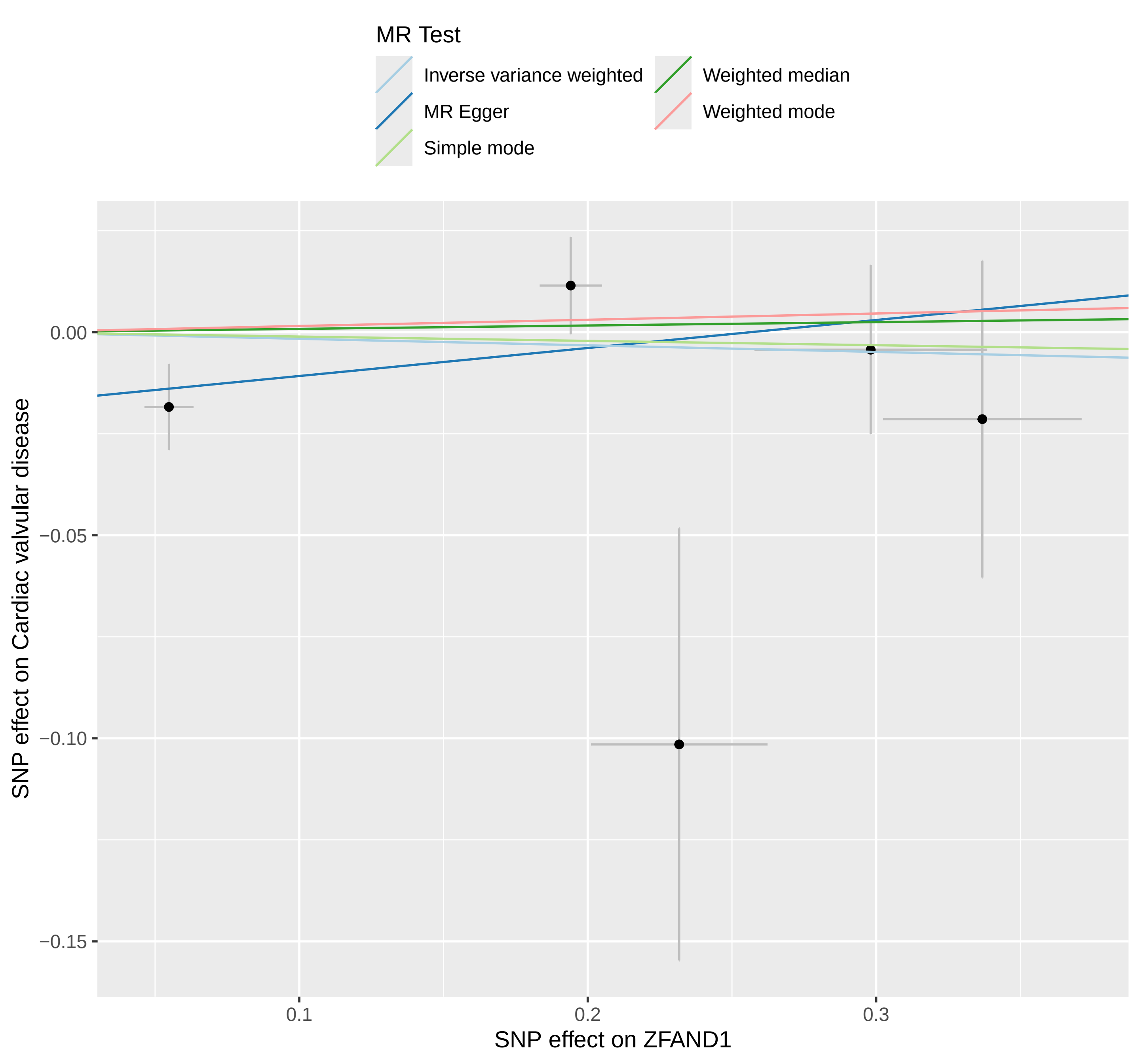
MR Test
Inverse variance weighted
MR Egger
Simple mode
Weighted median
Weighted mode
SNP effect on Cardiac valvular disease
0.00
−0.05
−0.10
−0.15
0.1
0.2
0.3
SNP effect on ZFAND1

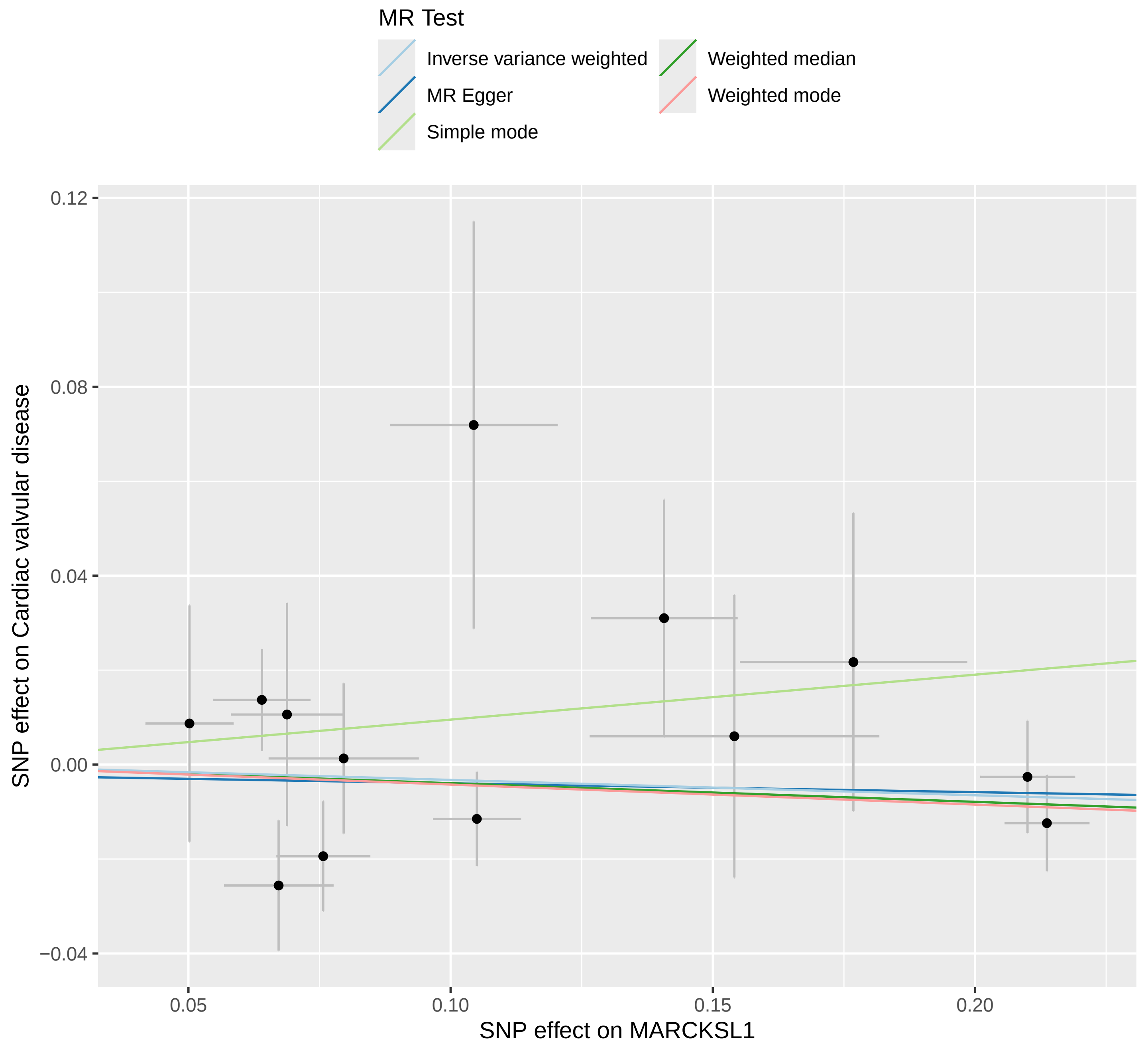
MR Test
Inverse variance weighted
MR Egger
Simple mode
Weighted median
Weighted mode
0.12
0.08
0.04
0.00
−0.04
0.05
0.10
0.15
0.20
SNP effect on Cardiac valvular disease
SNP effect on MARCKSL1

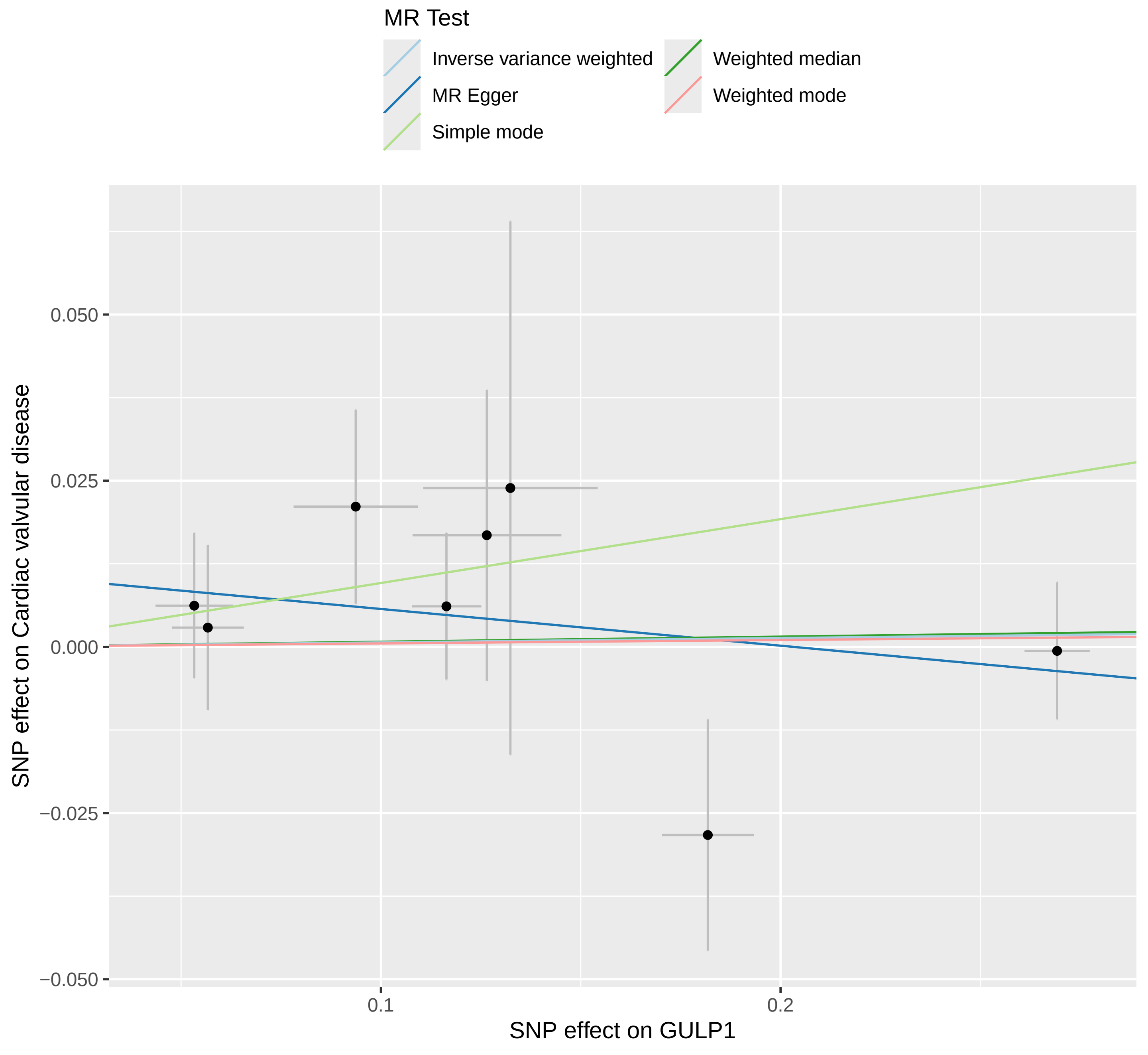

MR Test
Inverse variance weighted
MR Egger
Simple mode
Weighted median
Weighted mode
0.050
0.025
0.000
−0.025
−0.050
0.1
0.2
SNP effect on Cardiac valvular disease
SNP effect on GULP1

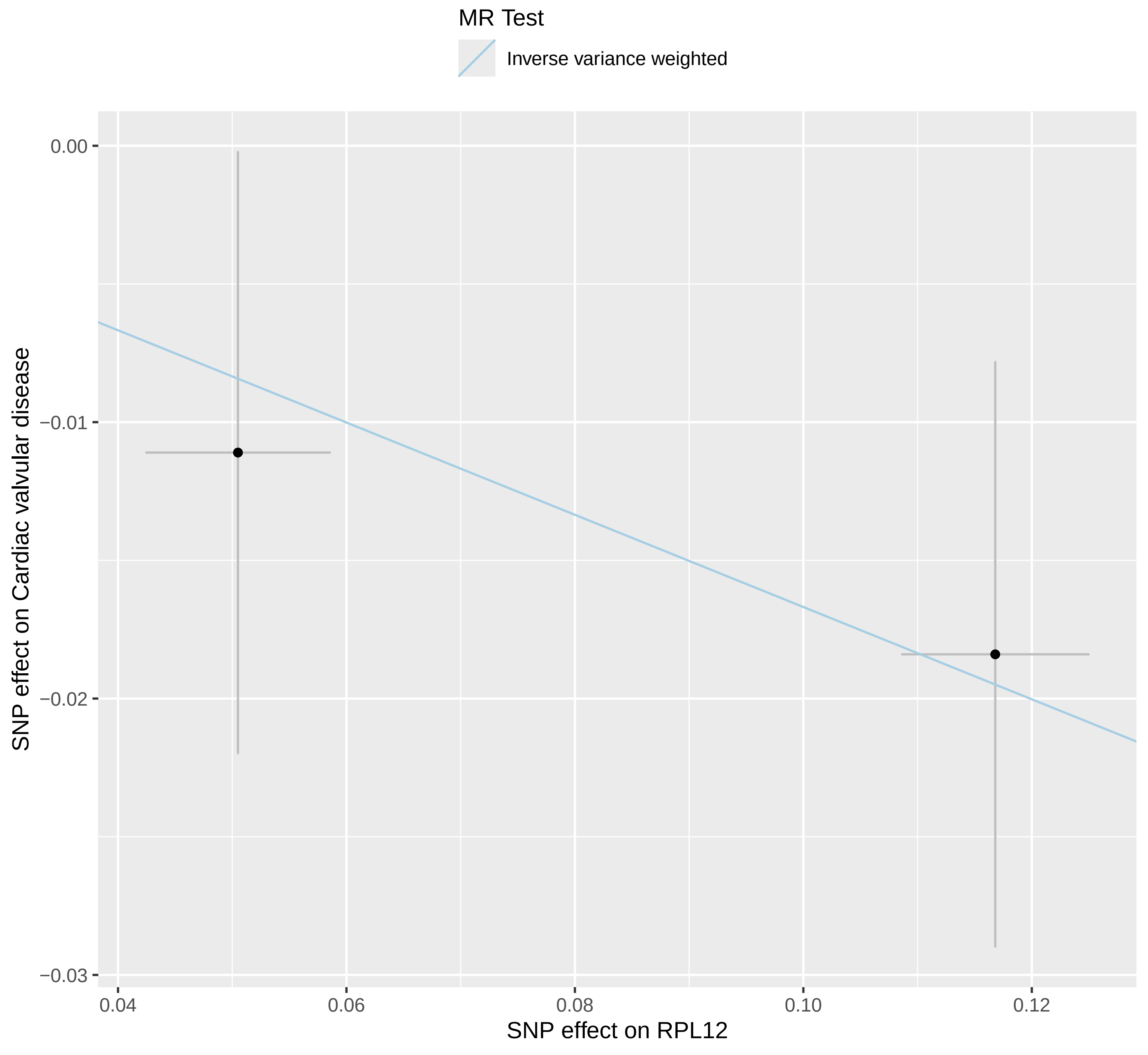

MR Test
Inverse variance weighted
0.00
−0.01
−0.02
−0.03
0.04
0.06
0.08
0.10
0.12
SNP effect on Cardiac valvular disease
SNP effect on RPL12

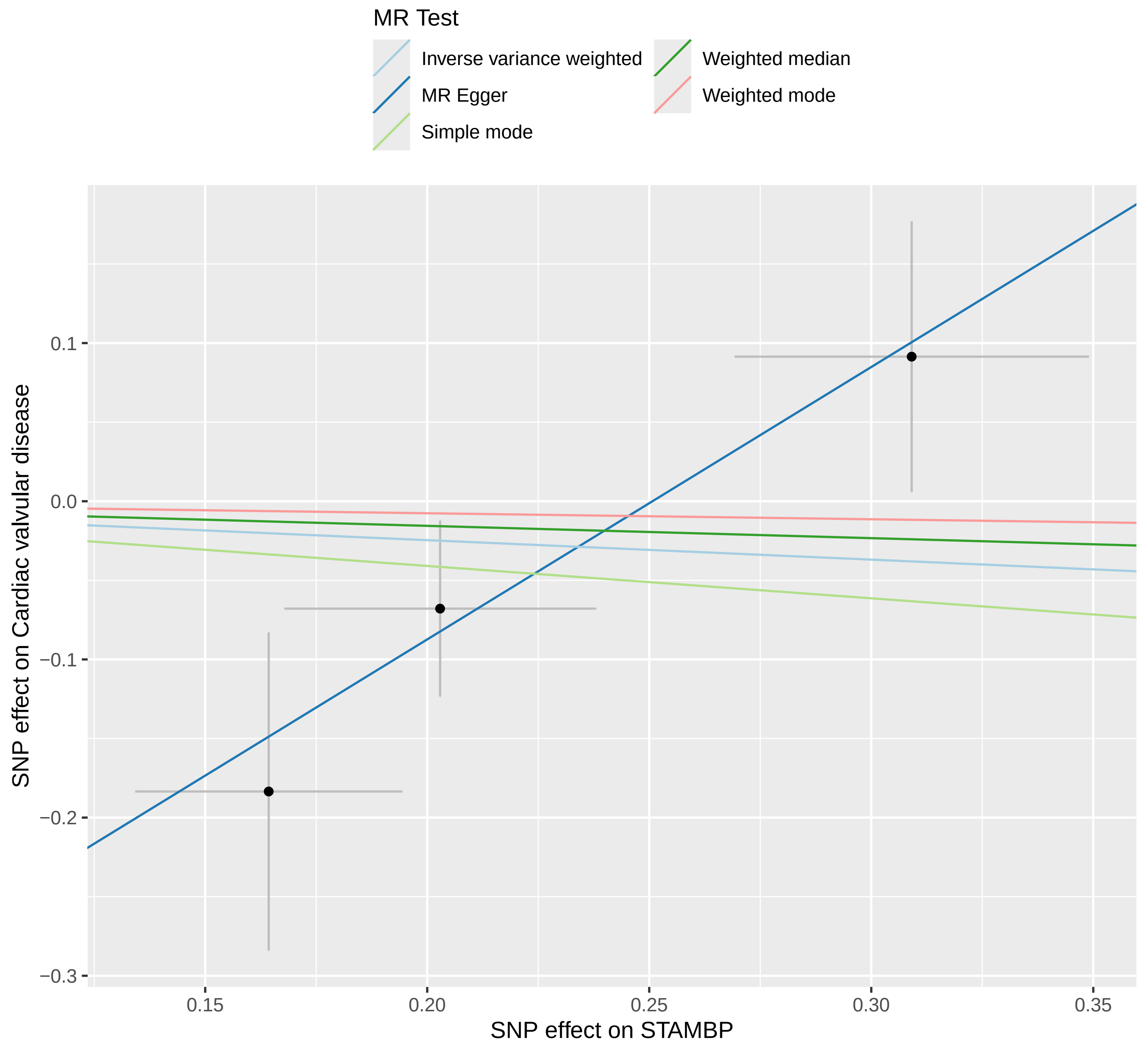
MR Test
Inverse variance weighted
MR Egger
Simple mode
Weighted median
Weighted mode
SNP effect on Cardiac valvular disease
0.1
0.0
−0.1
−0.2
−0.3
0.15
0.20
0.25
0.30
0.35
SNP effect on STAMBP

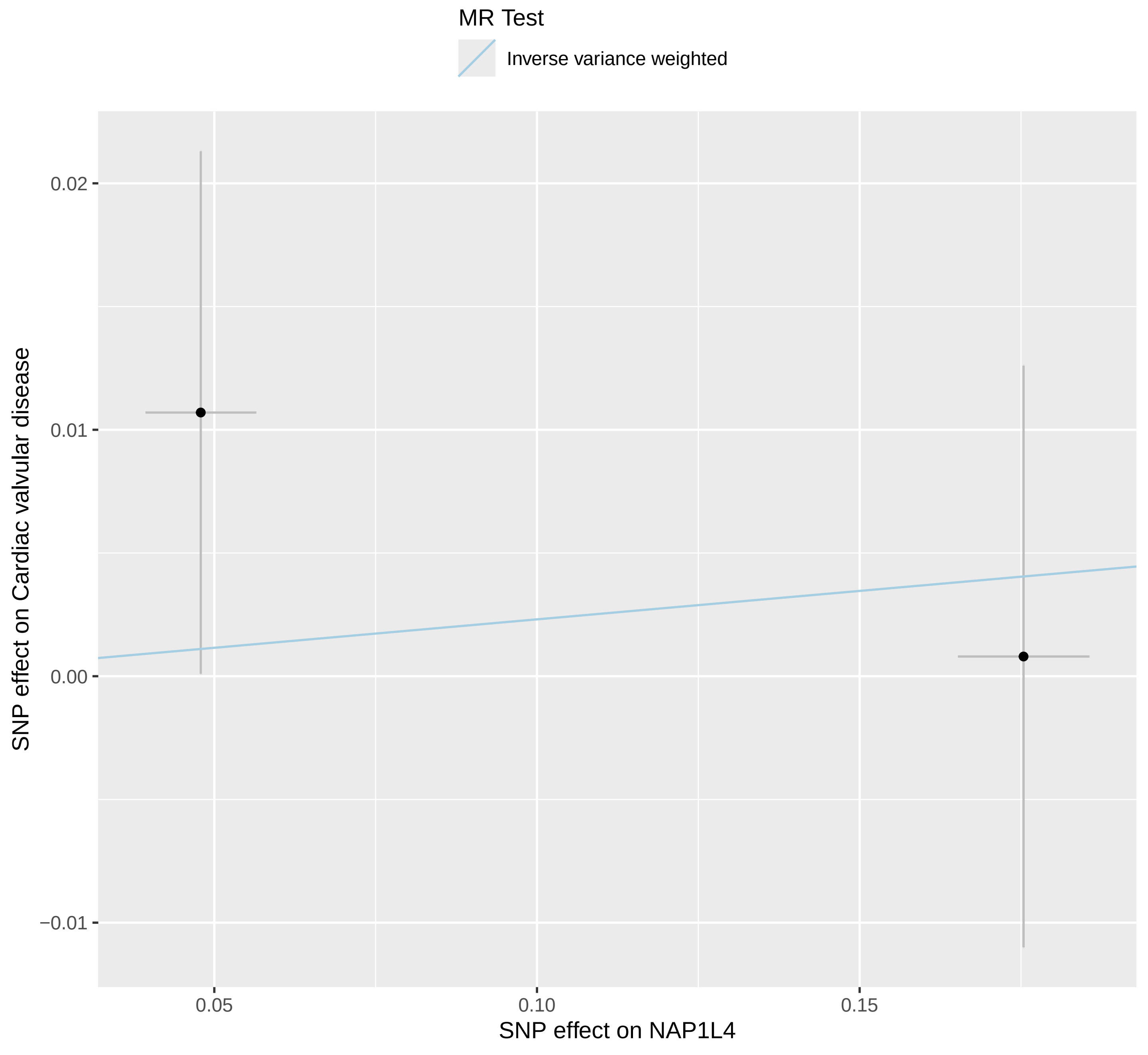
MR Test
Inverse variance weighted
0.02
0.01
0.00
−0.01
0.05
0.10
0.15
SNP effect on NAP1L4
SNP effect on Cardiac valvular disease

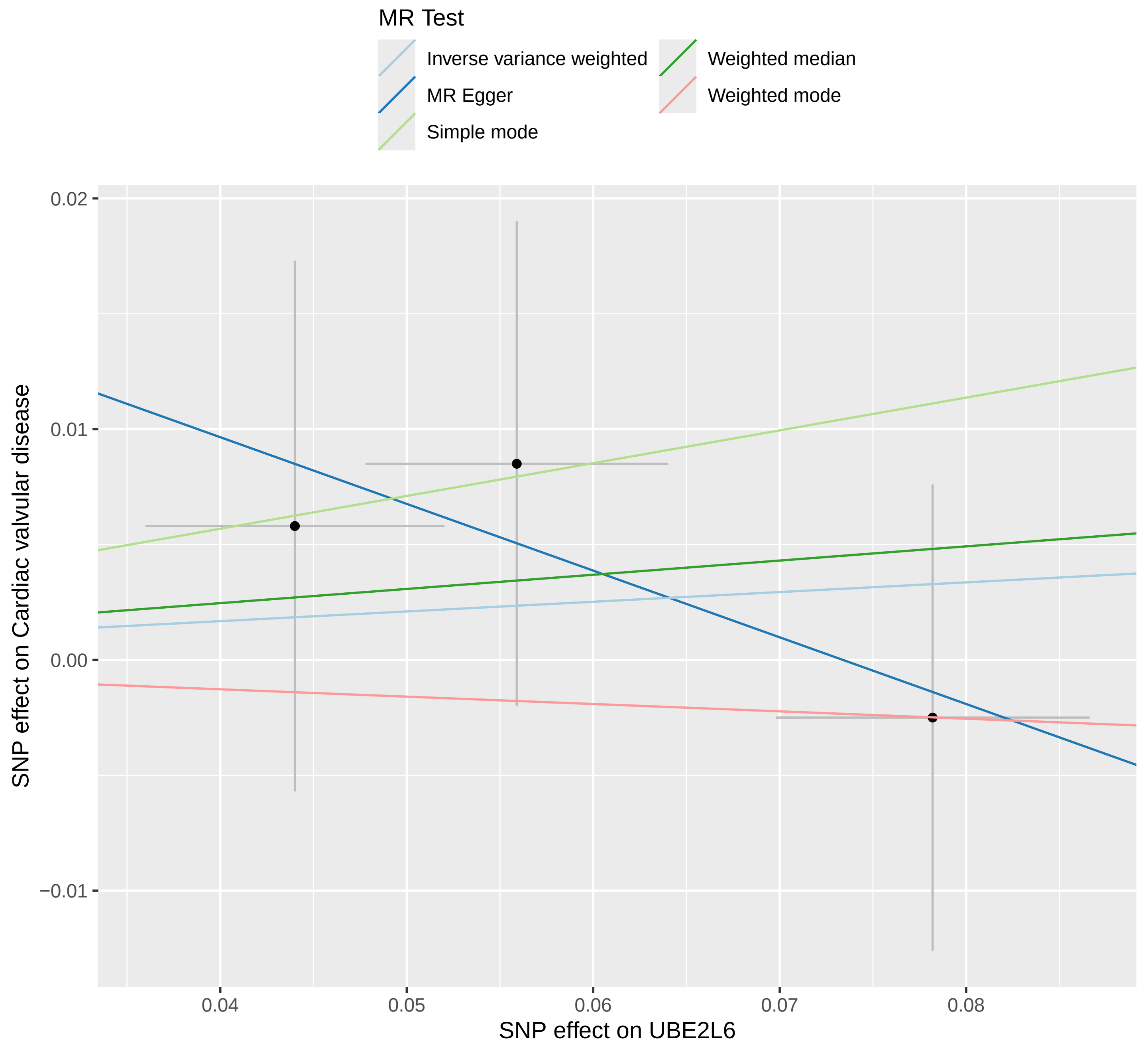
MR Test
Inverse variance weighted
MR Egger
Simple mode
Weighted median
Weighted mode
0.02
0.01
0.00
−0.01
0.04
0.05
0.06
0.07
0.08
SNP effect on UBE2L6
SNP effect on Cardiac valvular disease

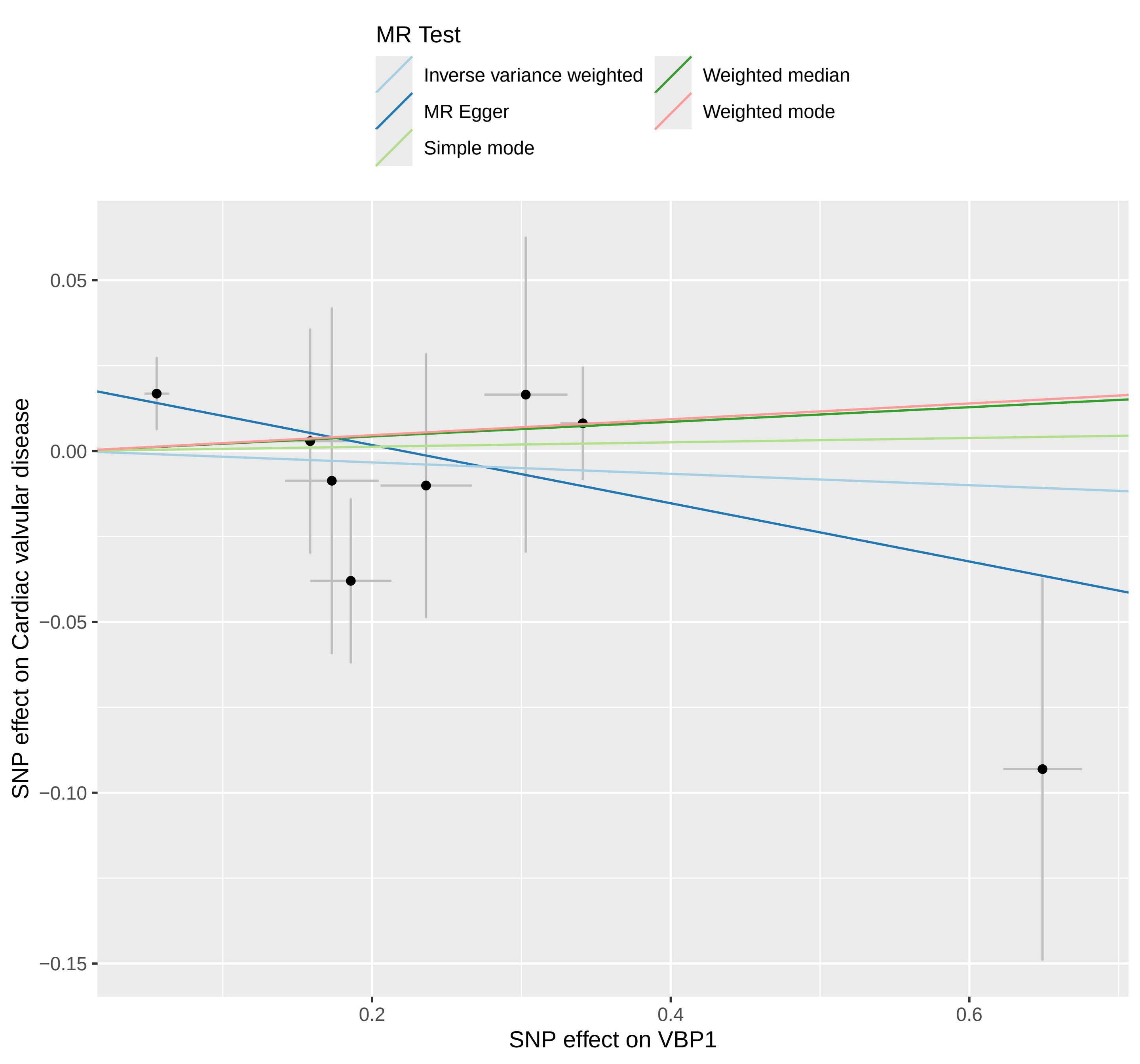

MR Test
Inverse variance weighted
MR Egger
Simple mode
Weighted median
Weighted mode
SNP effect on Cardiac valvular disease
0.05
0.00
−0.05
−0.10
−0.15
0.2
0.4
0.6
SNP effect on VBP1

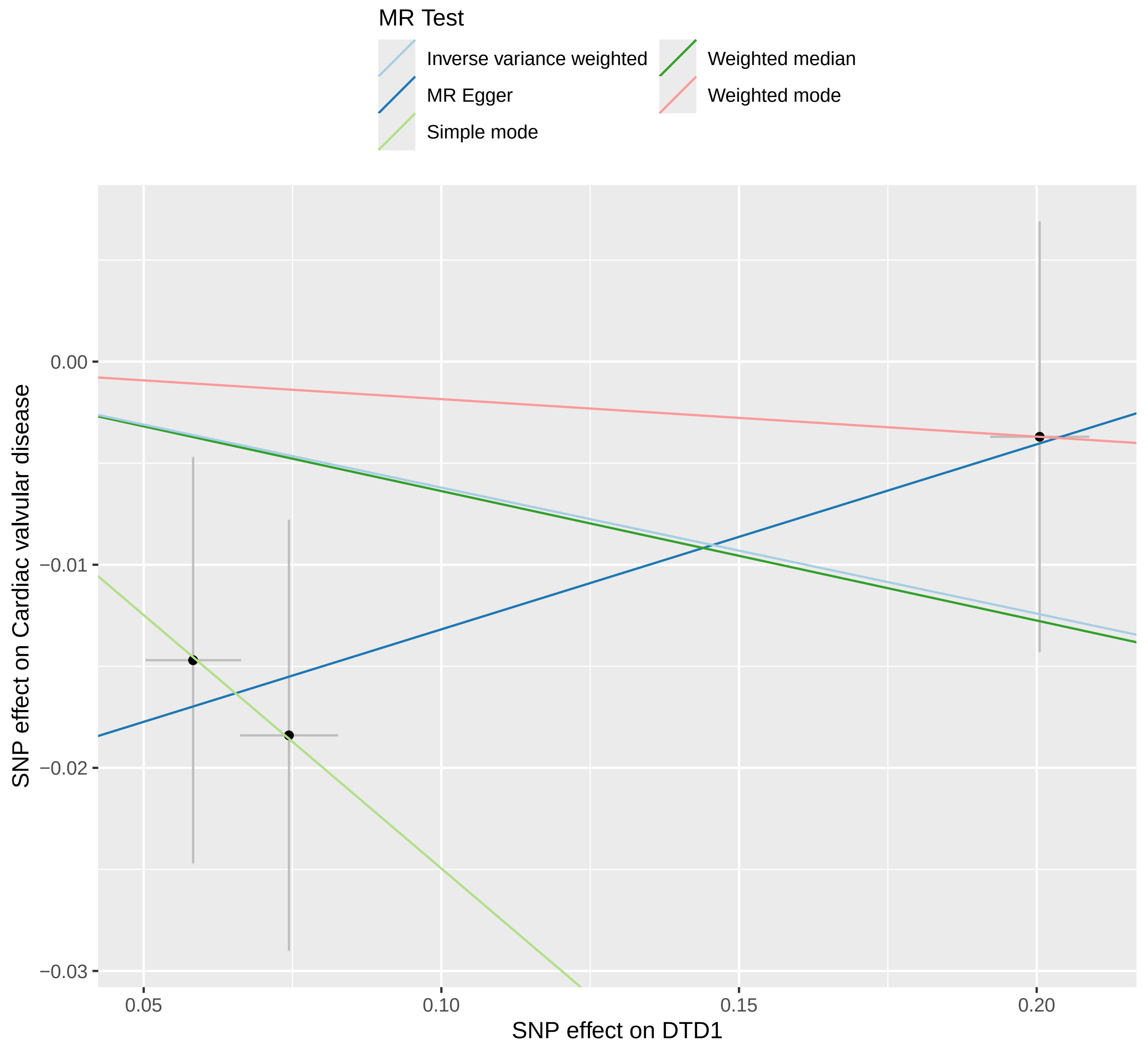
MR Test
Inverse variance weighted
MR Egger
Simple mode
Weighted median
Weighted mode
0.00
−0.01
−0.02
−0.03
SNP effect on Cardiac valvular disease
0.05
0.10
0.15
0.20
SNP effect on DTD1

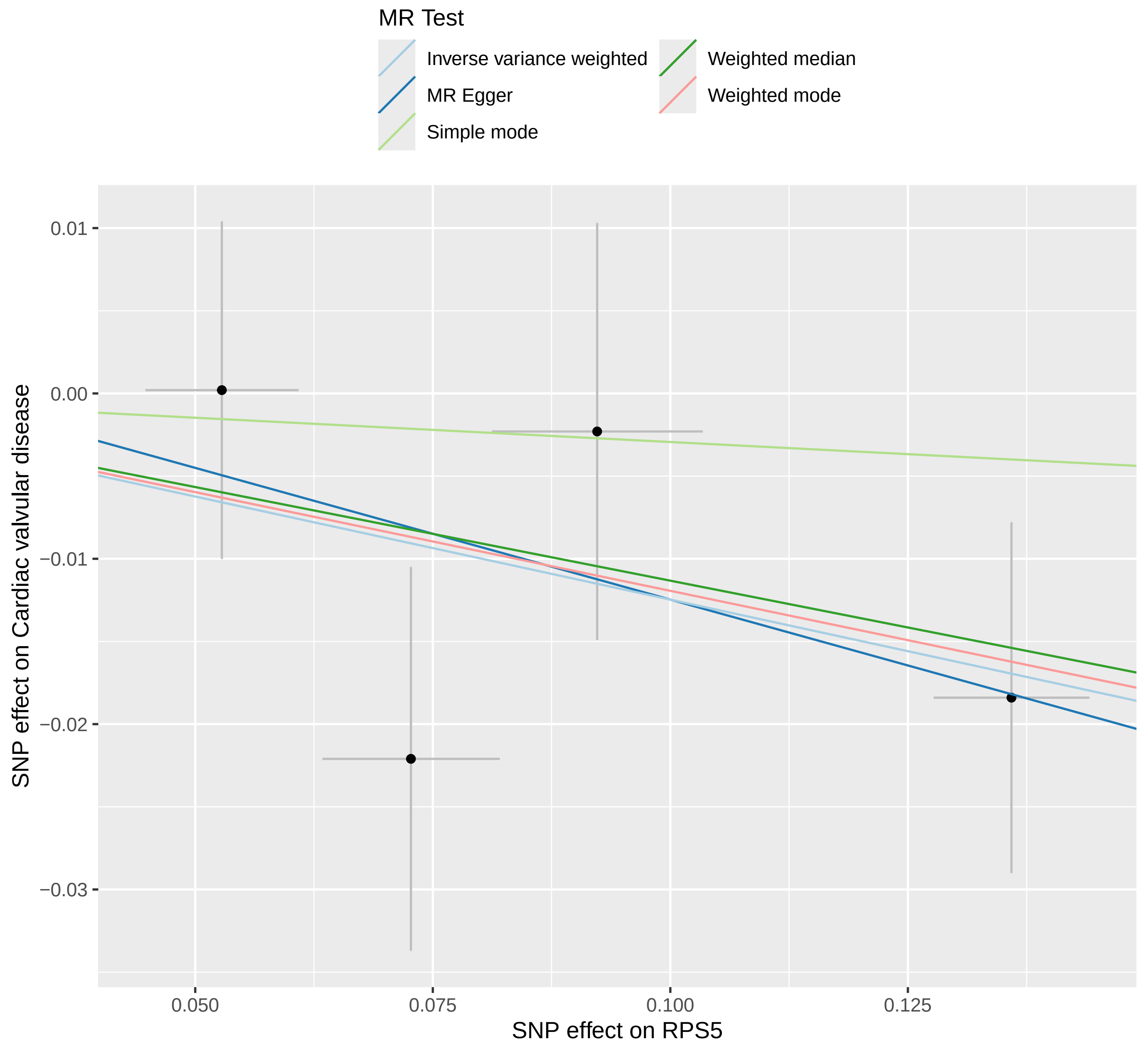

MR Test
Inverse variance weighted
MR Egger
Simple mode
Weighted median
Weighted mode
0.01
0.00
−0.01
−0.02
−0.03
SNP effect on Cardiac valvular disease
0.050
0.075
0.100
0.125
SNP effect on RPS5

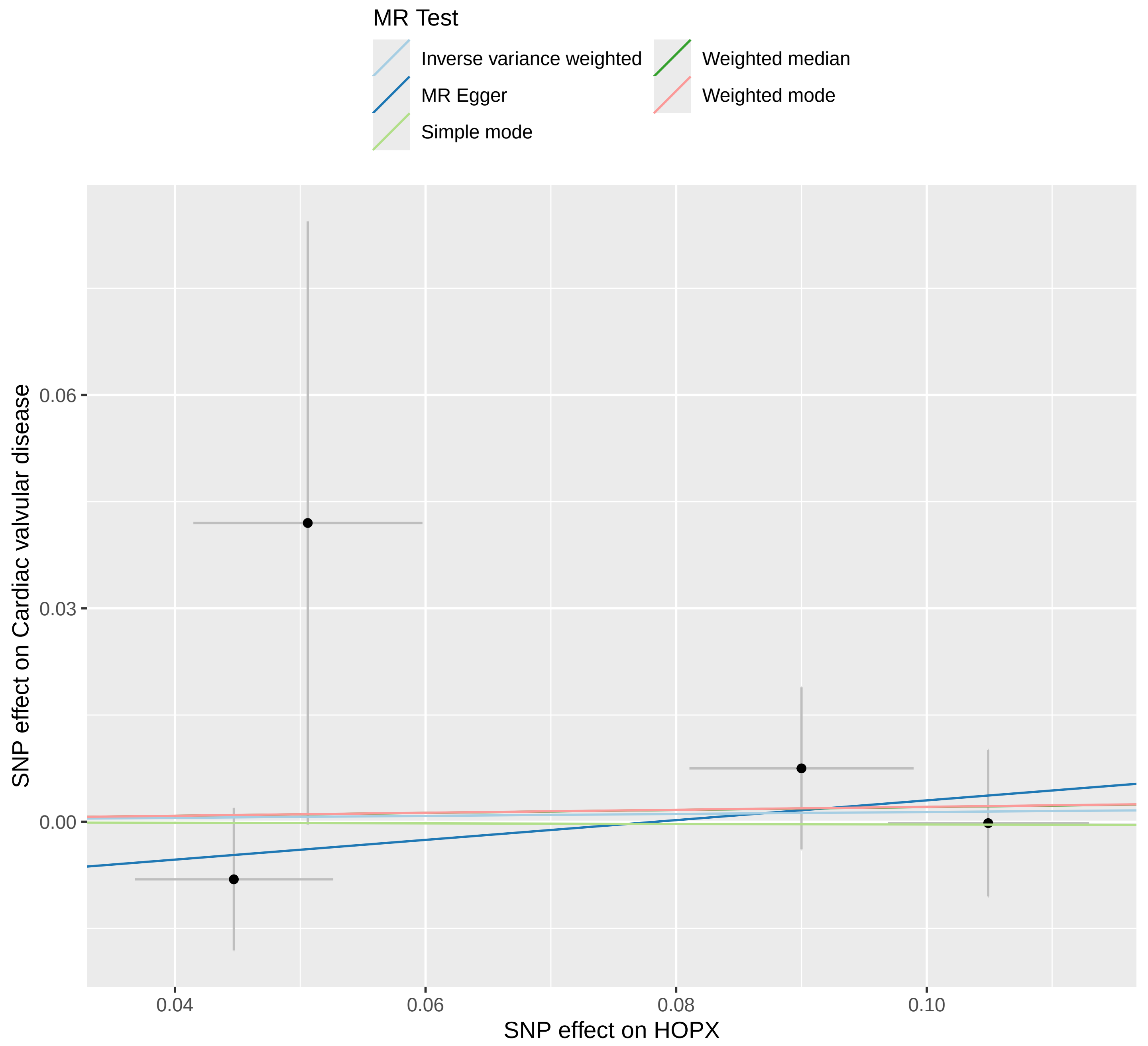
MR Test
Inverse variance weighted
MR Egger
Simple mode
Weighted median
Weighted mode
SNP effect on Cardiac valvular disease
0.06
0.03
0.00
0.04
0.06
0.08
0.10
SNP effect on HOPX

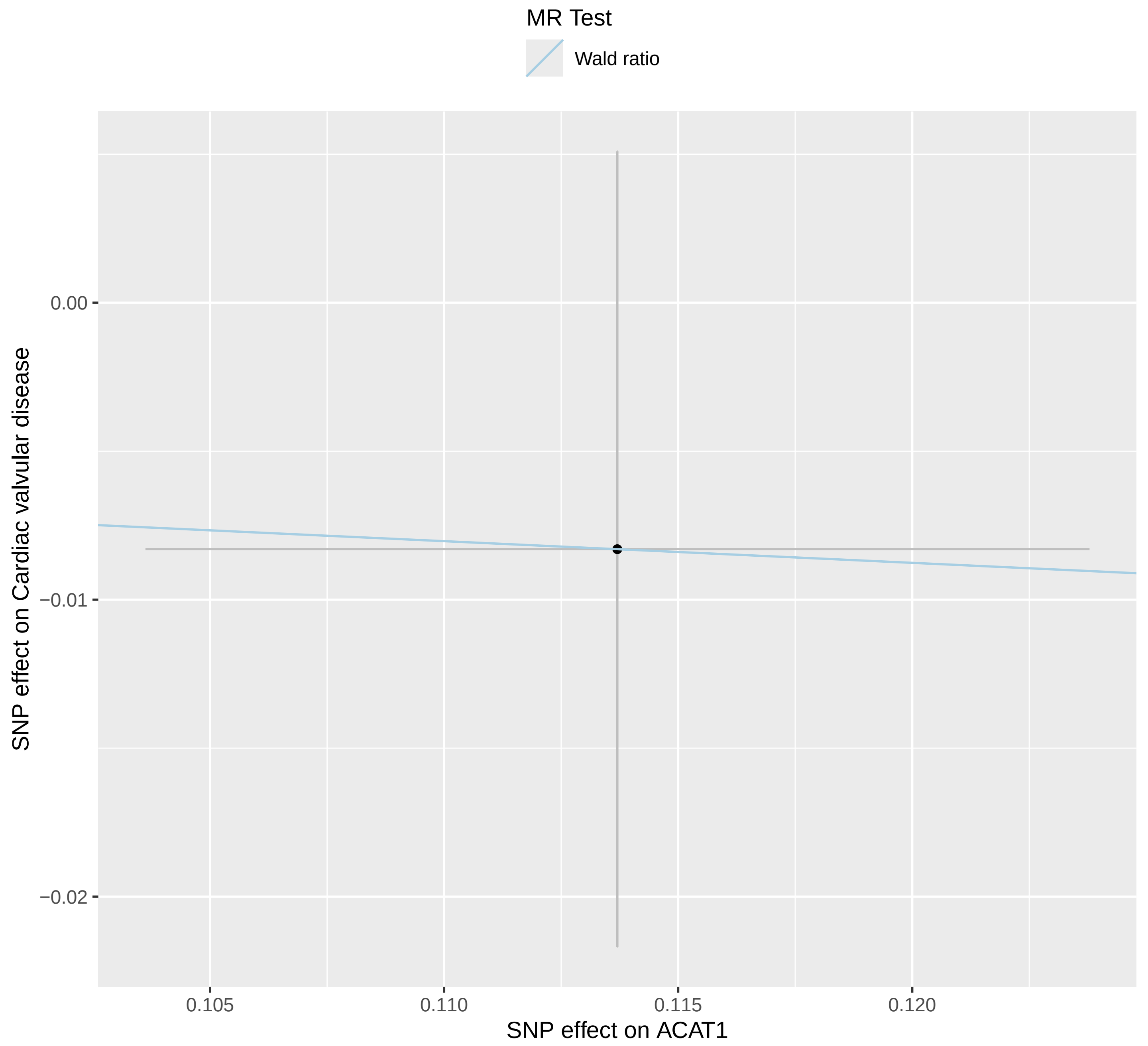
MR Test
Wald ratio
0.00
−0.01
−0.02
SNP effect on Cardiac valvular disease
0.105
0.110
0.115
0.120
SNP effect on ACAT1

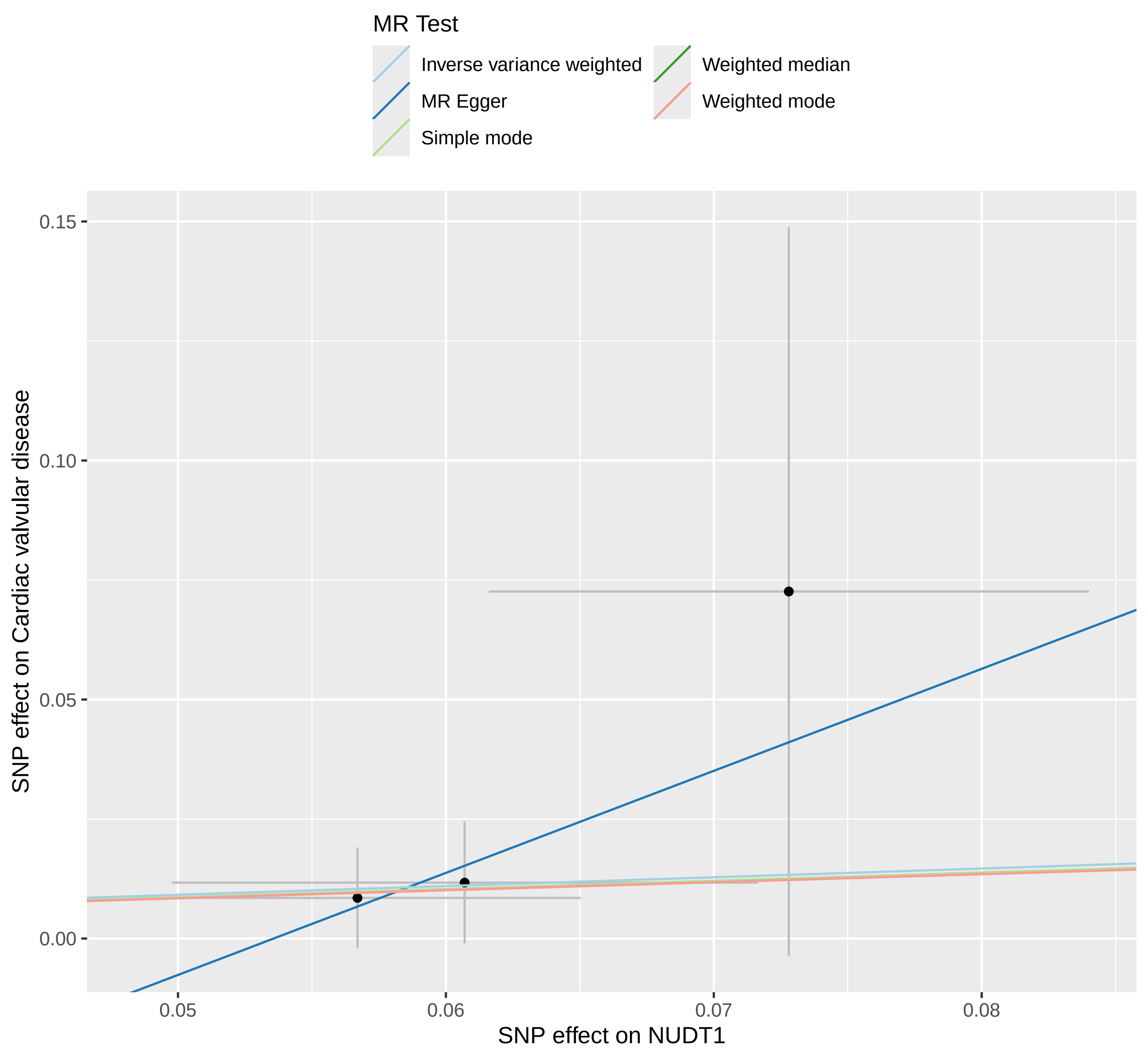
MR Test
Inverse variance weighted
MR Egger
Simple mode
Weighted median
Weighted mode
0.15
0.10
0.05
0.00
SNP effect on Cardiac valvular disease
0.05
0.06
0.07
0.08
SNP effect on NUDT1

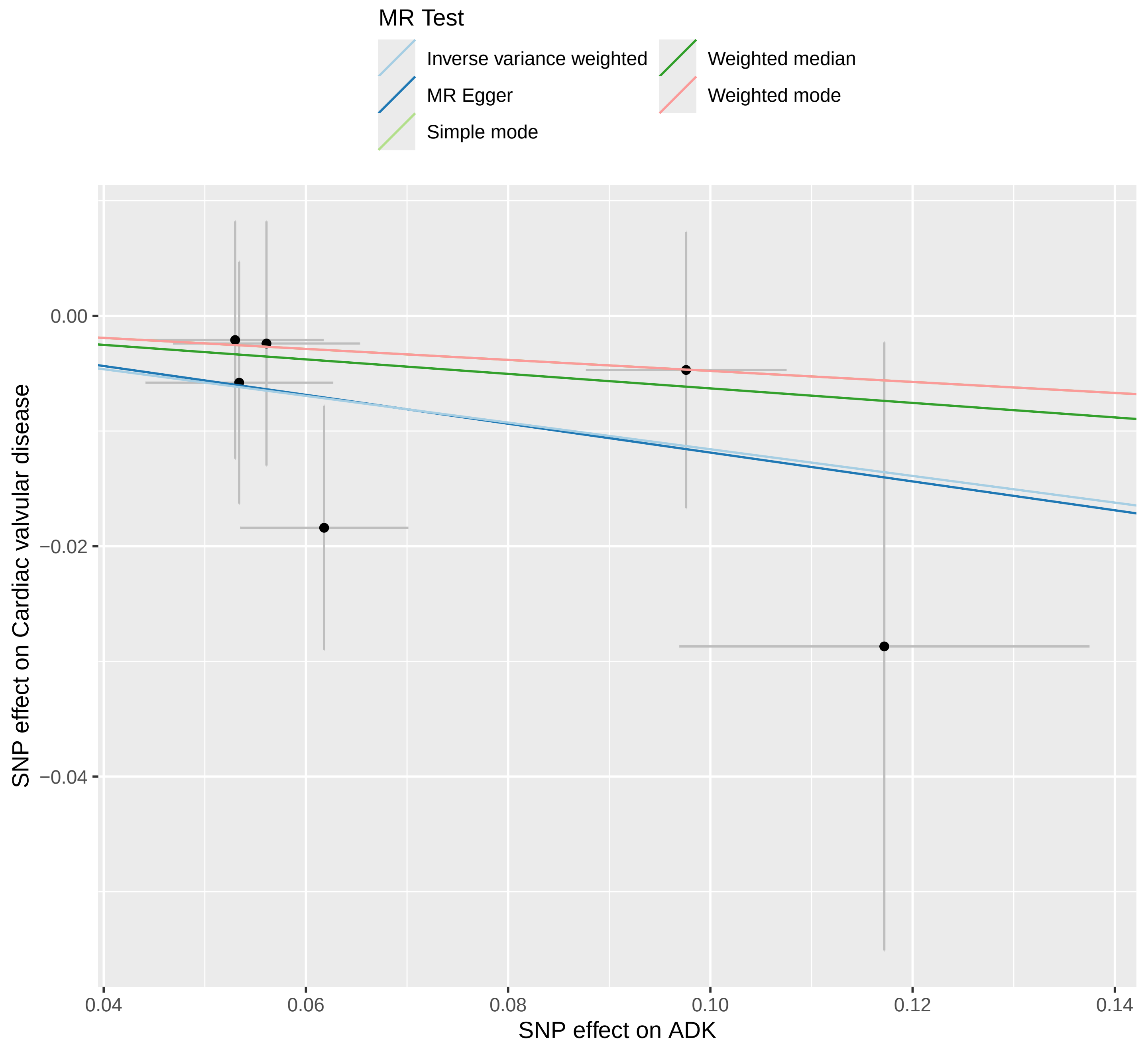
MR Test
Inverse variance weighted
MR Egger
Simple mode
Weighted median
Weighted mode
SNP effect on Cardiac valvular disease
0.00
−0.02
−0.04
0.04
0.06
0.08
0.10
0.12
0.14
SNP effect on ADK

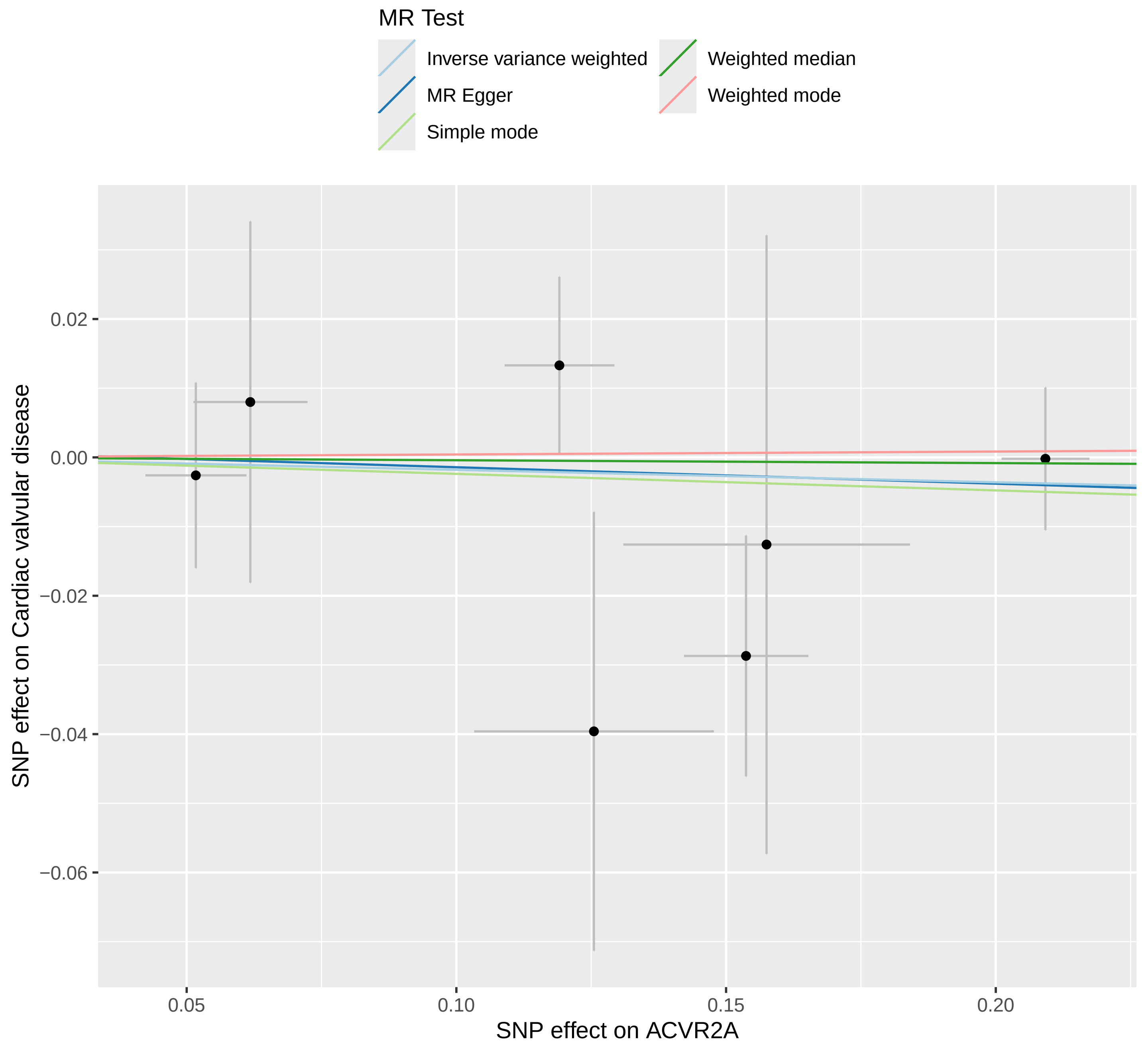

MR Test
Inverse variance weighted
MR Egger
Simple mode
Weighted median
Weighted mode
0.02
0.00
−0.02
−0.04
−0.06
0.05
0.10
0.15
0.20
SNP effect on ACVR2A
SNP effect on Cardiac valvular disease

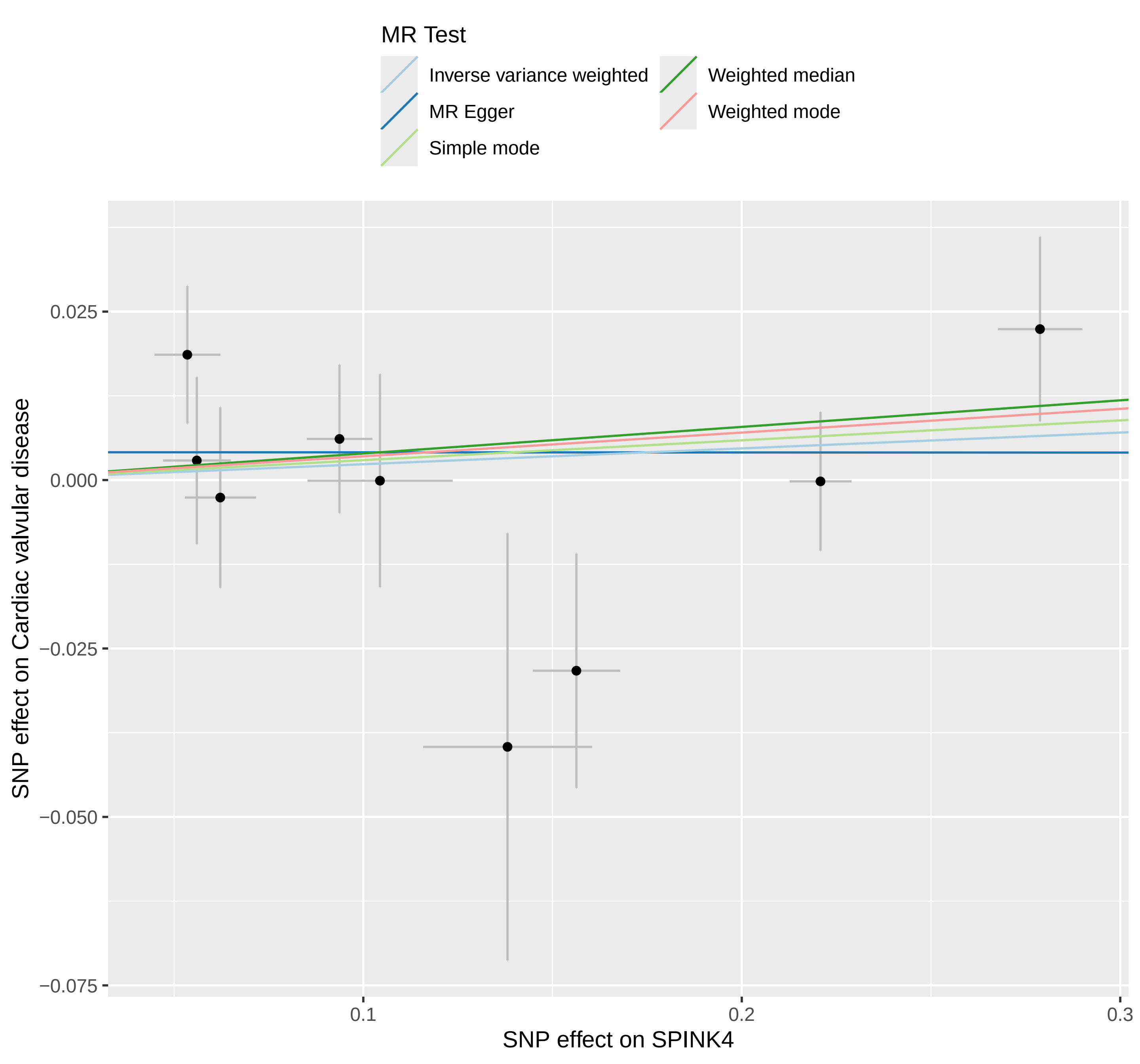

MR Test
Inverse variance weighted
MR Egger
Simple mode
Weighted median
Weighted mode
0.025
0.000
−0.025
−0.050
−0.075
SNP effect on Cardiac valvular disease
0.1
0.2
0.3
SNP effect on SPINK4

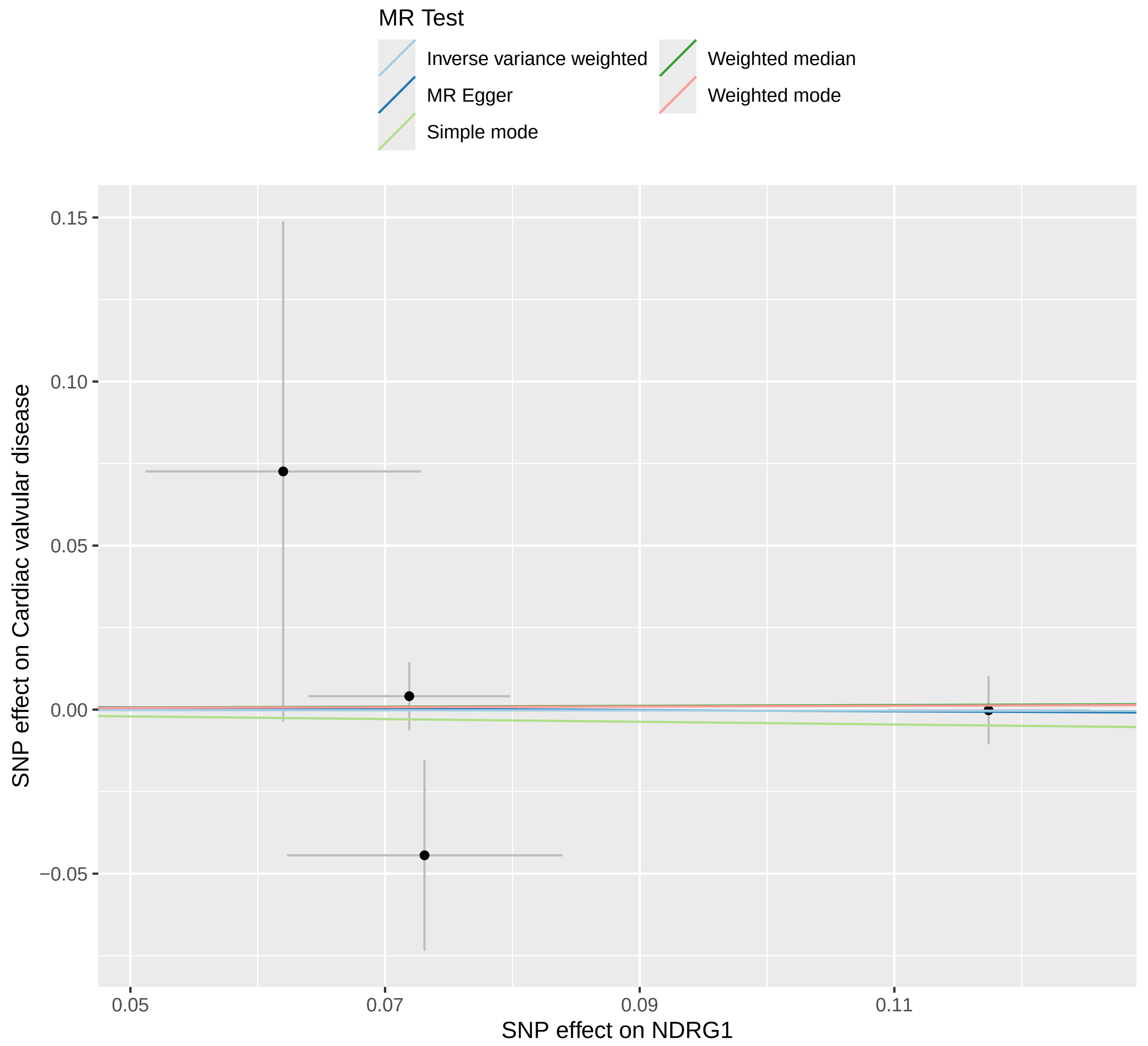

MR Test
Inverse variance weighted
MR Egger
Simple mode
Weighted median
Weighted mode
0.15
0.10
0.05
0.00
−0.05
0.05
0.07
0.09
0.11
SNP effect on Cardiac valvular disease
SNP effect on NDRG1

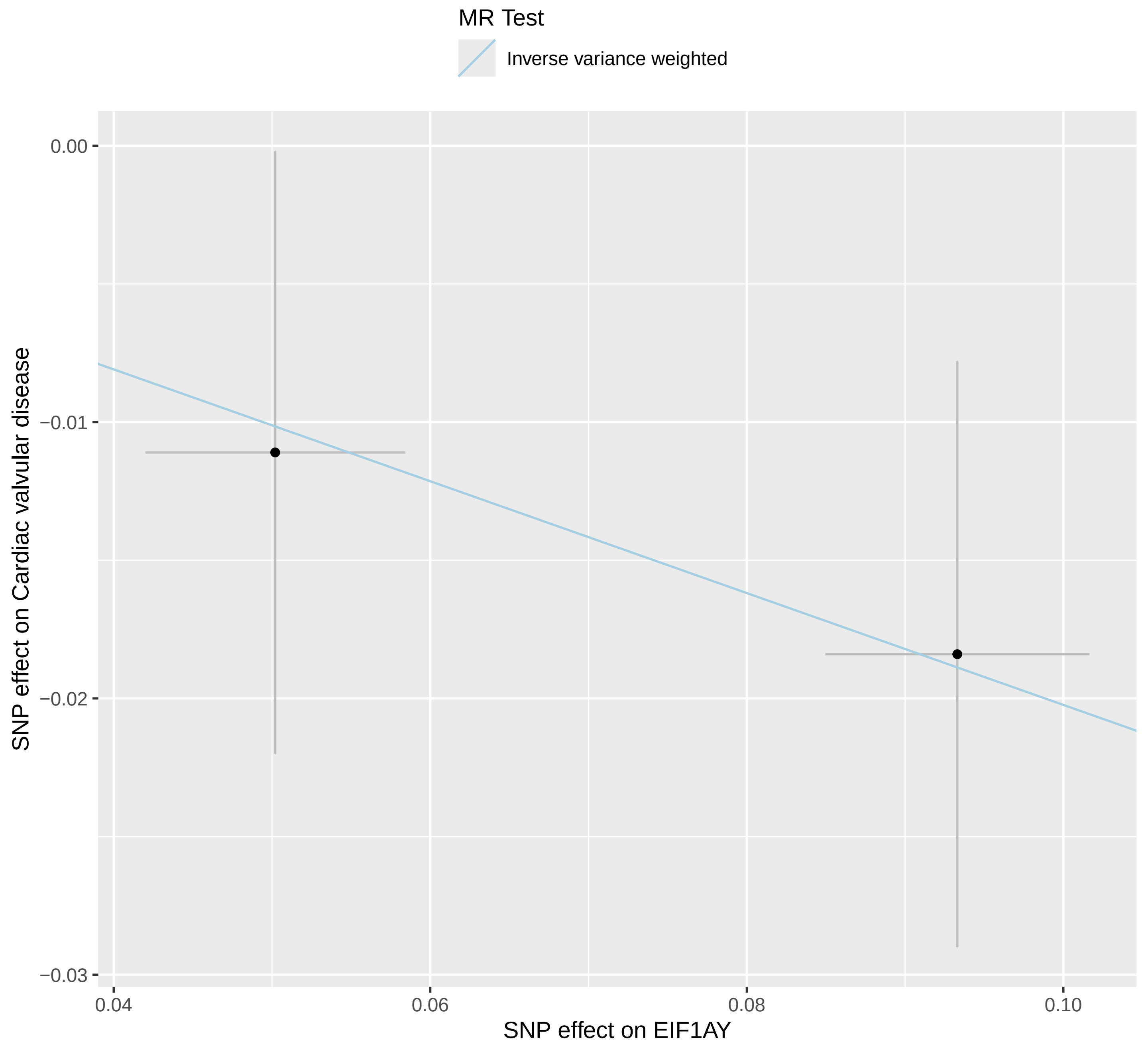
MR Test
Inverse variance weighted
0.00
−0.01
−0.02
−0.03
0.04
0.06
0.08
0.10
SNP effect on Cardiac valvular disease
SNP effect on EIF1AY

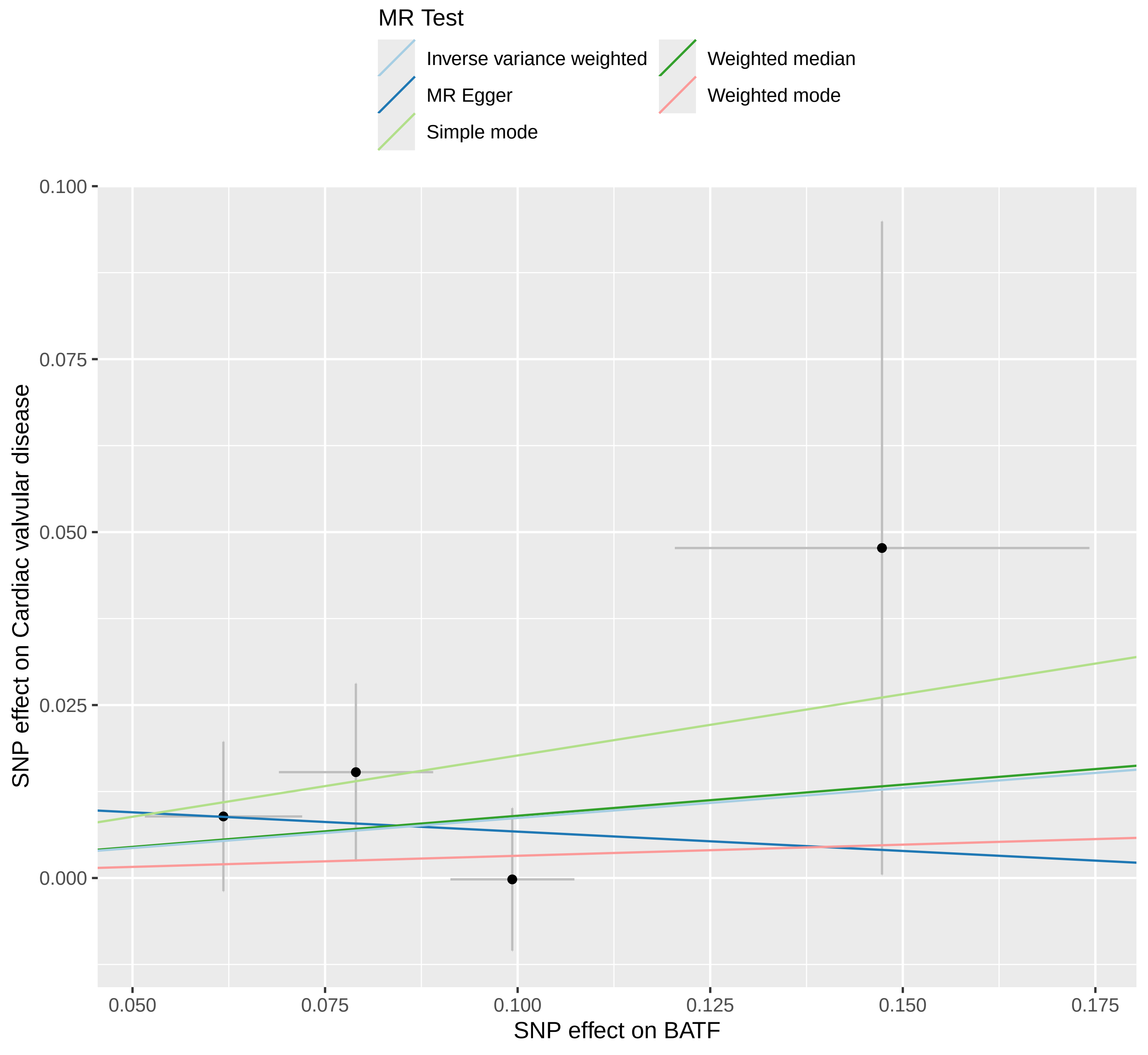
MR Test
Inverse variance weighted
MR Egger
Simple mode
Weighted median
Weighted mode
0.100
0.075
0.050
0.025
0.000
SNP effect on Cardiac valvular disease
0.050
0.075
0.100
0.125
0.150
0.175
SNP effect on BATF

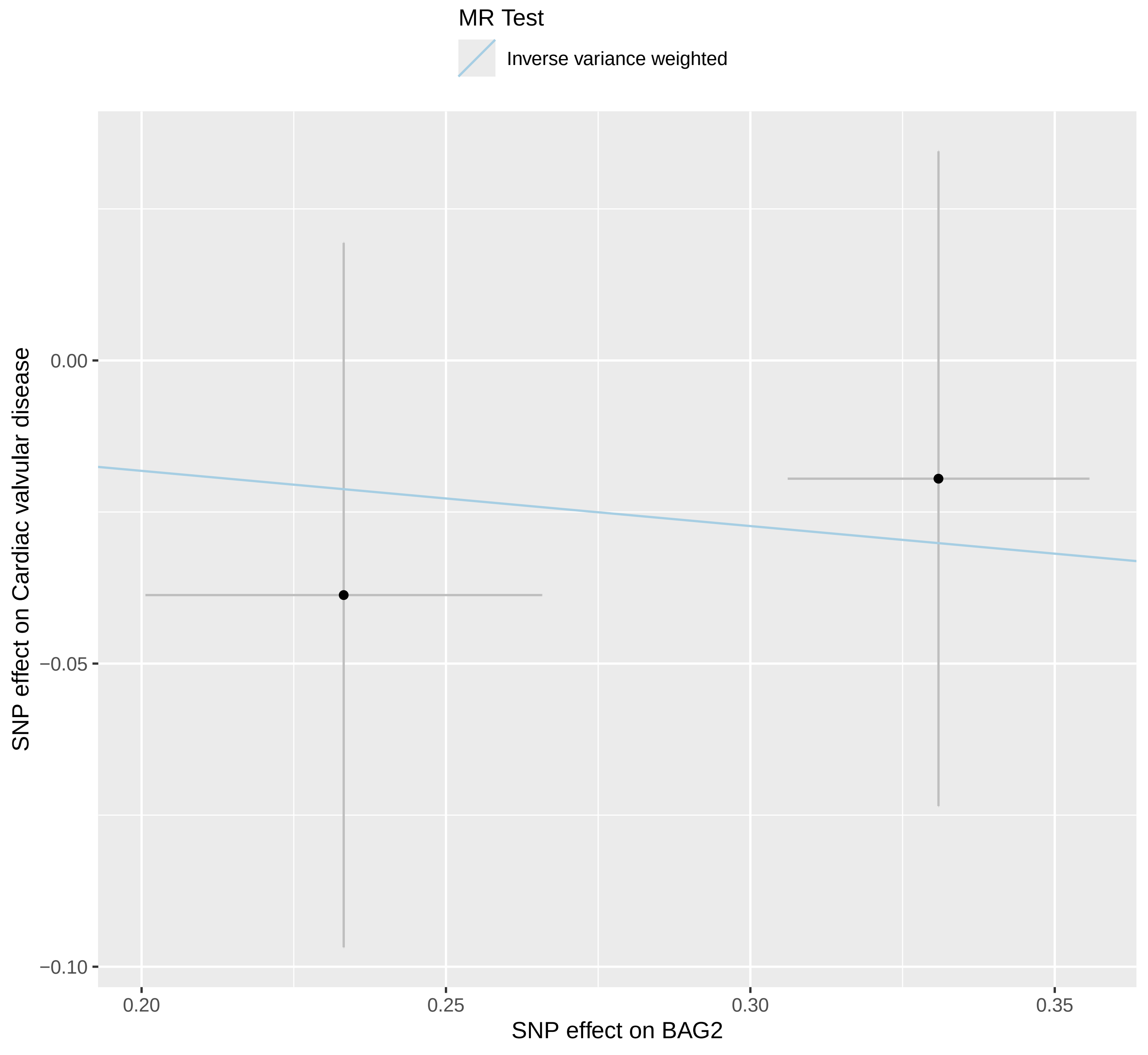

MR Test
Inverse variance weighted
0.00
−0.05
−0.10
0.20
0.25
0.30
0.35
SNP effect on BAG2
SNP effect on Cardiac valvular disease

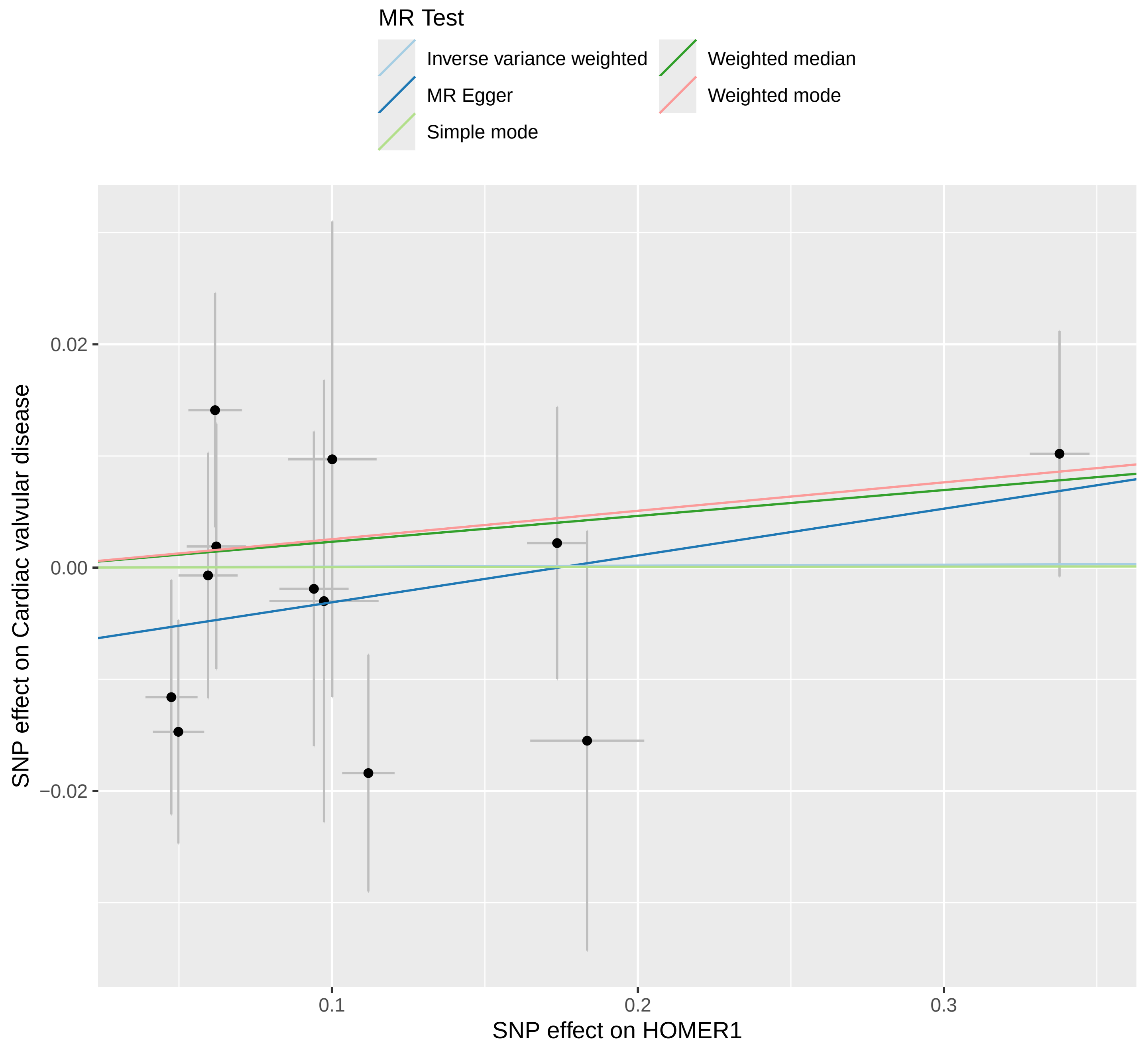

MR Test
Inverse variance weighted
MR Egger
Simple mode
Weighted median
Weighted mode
0.02
0.00
−0.02
0.1
0.2
0.3
SNP effect on Cardiac valvular disease
SNP effect on HOMER1

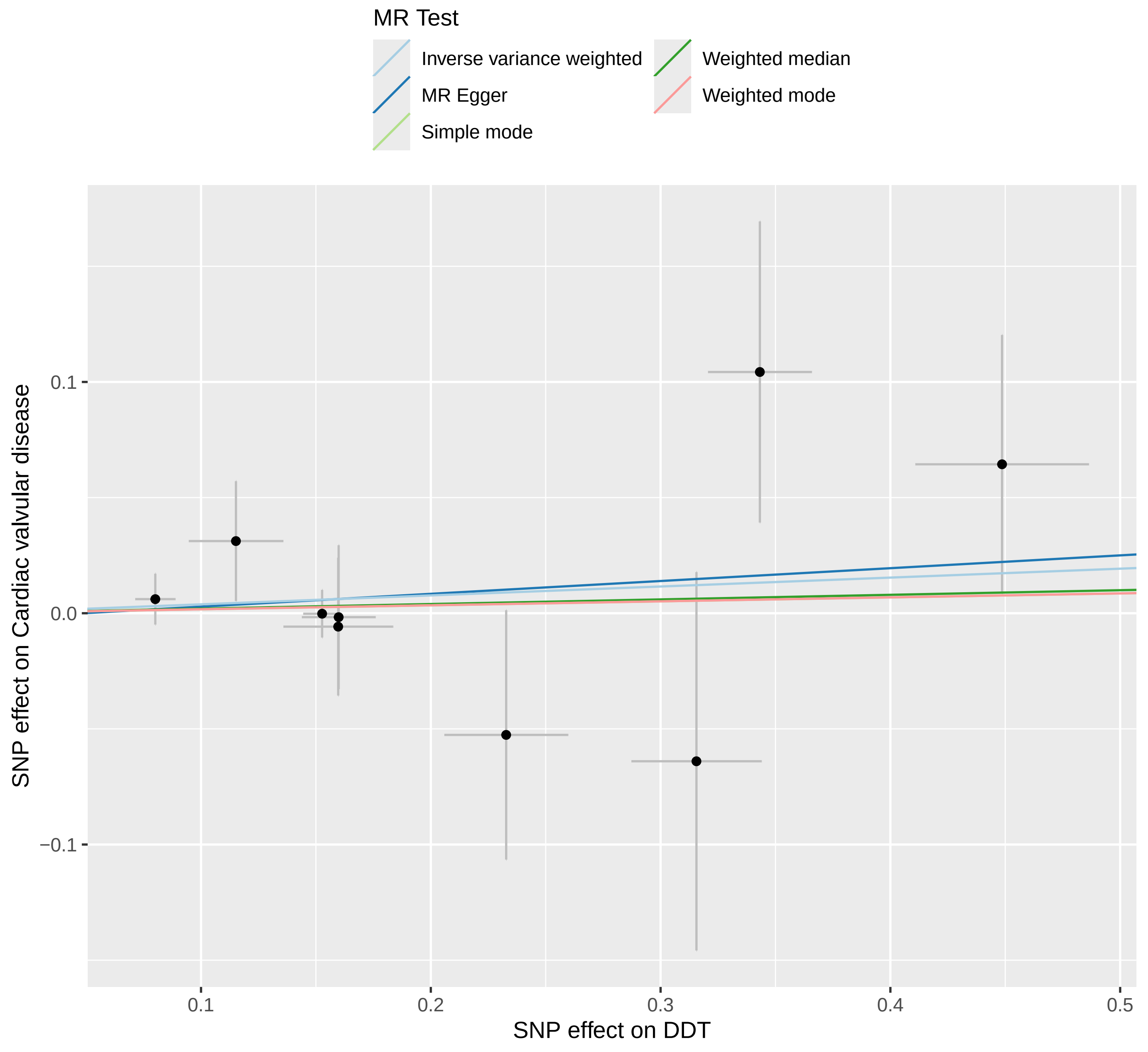

MR Test
Inverse variance weighted
MR Egger
Simple mode
Weighted median
Weighted mode
0.1
0.0
−0.1
SNP effect on Cardiac valvular disease
0.1
0.2
0.3
0.4
0.5
SNP effect on DDT

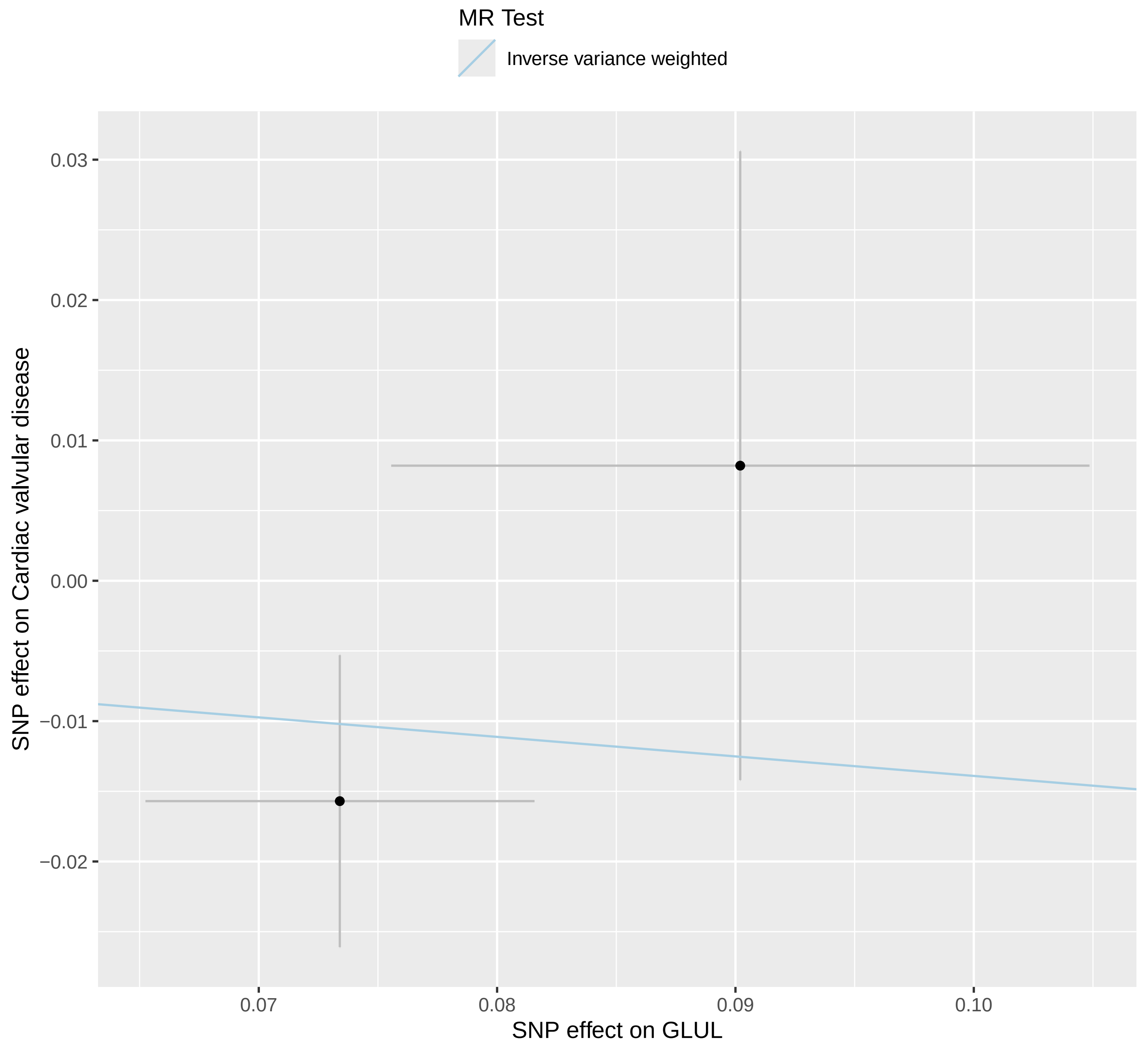
MR Test
Inverse variance weighted
SNP effect on Cardiac valvular disease
0.03
0.02
0.01
0.00
−0.01
−0.02
0.07
0.08
0.09
0.10
SNP effect on GLUL

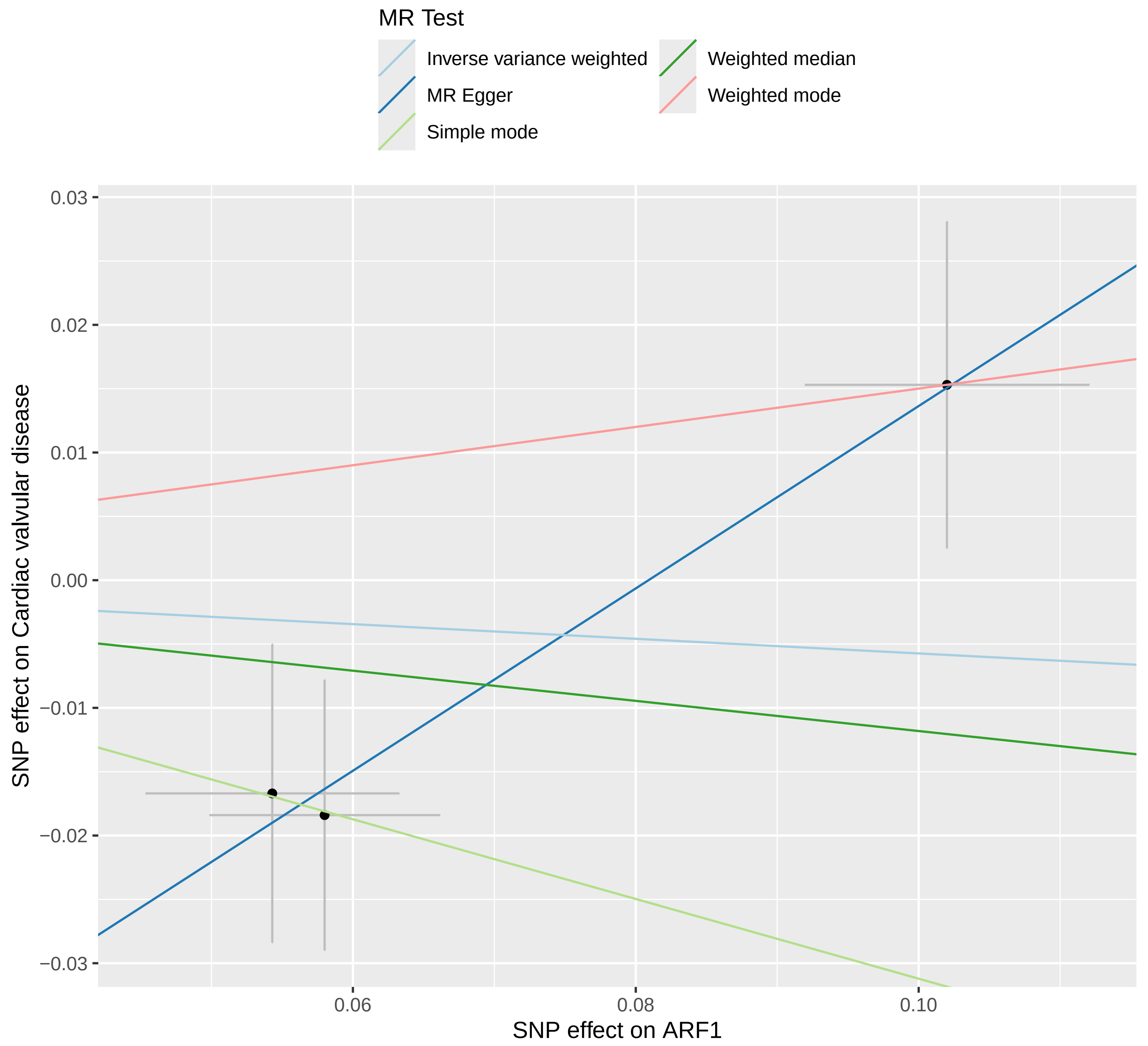
MR Test
Inverse variance weighted
MR Egger
Simple mode
Weighted median
Weighted mode
0.03
0.02
0.01
0.00
−0.01
−0.02
−0.03
0.06
0.08
0.10
SNP effect on Cardiac valvular disease
SNP effect on ARF1

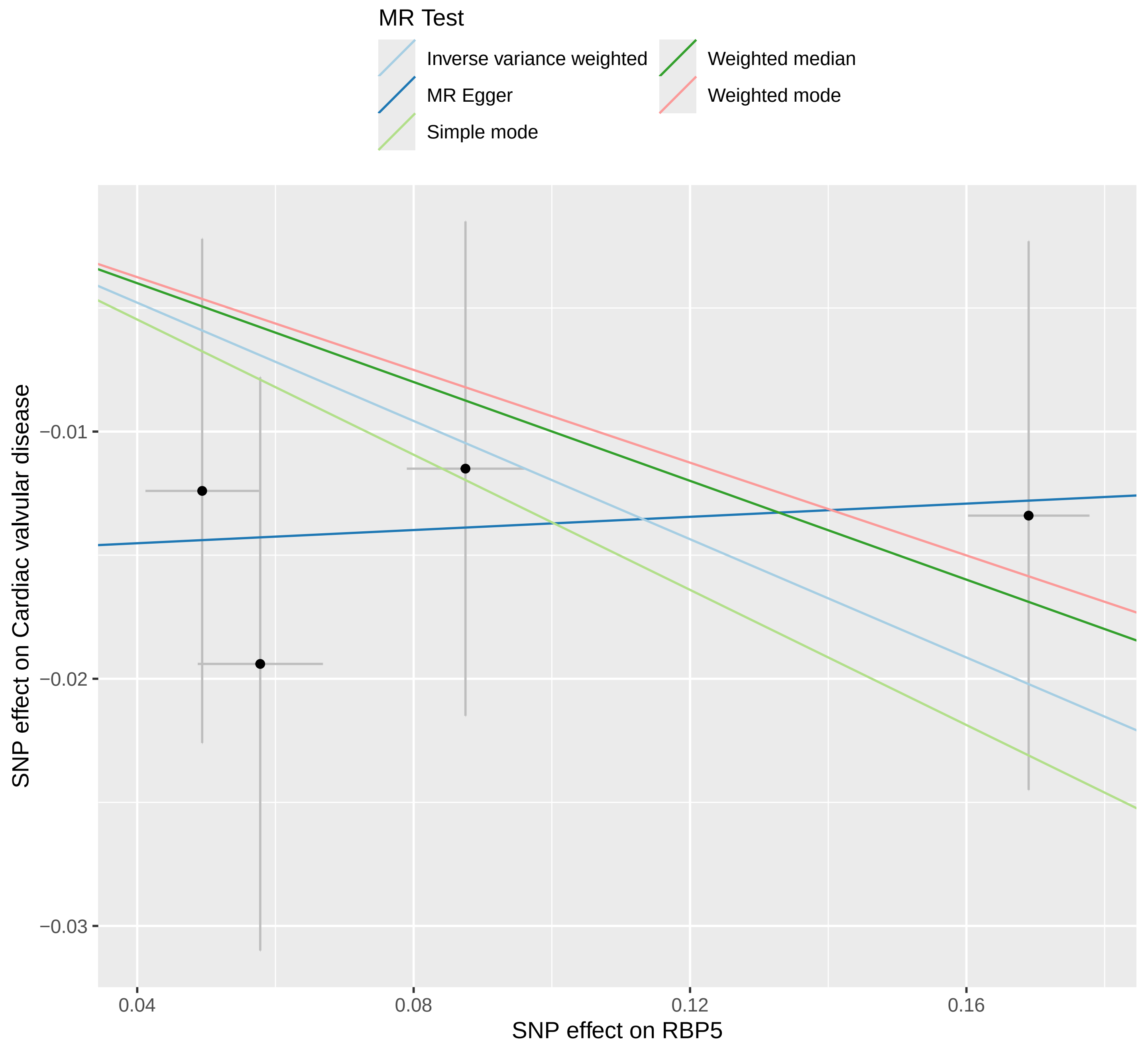
MR Test
Inverse variance weighted
MR Egger
Simple mode
Weighted median
Weighted mode
−0.01
−0.02
−0.03
0.04
0.08
0.12
0.16
SNP effect on RBP5
SNP effect on Cardiac valvular disease

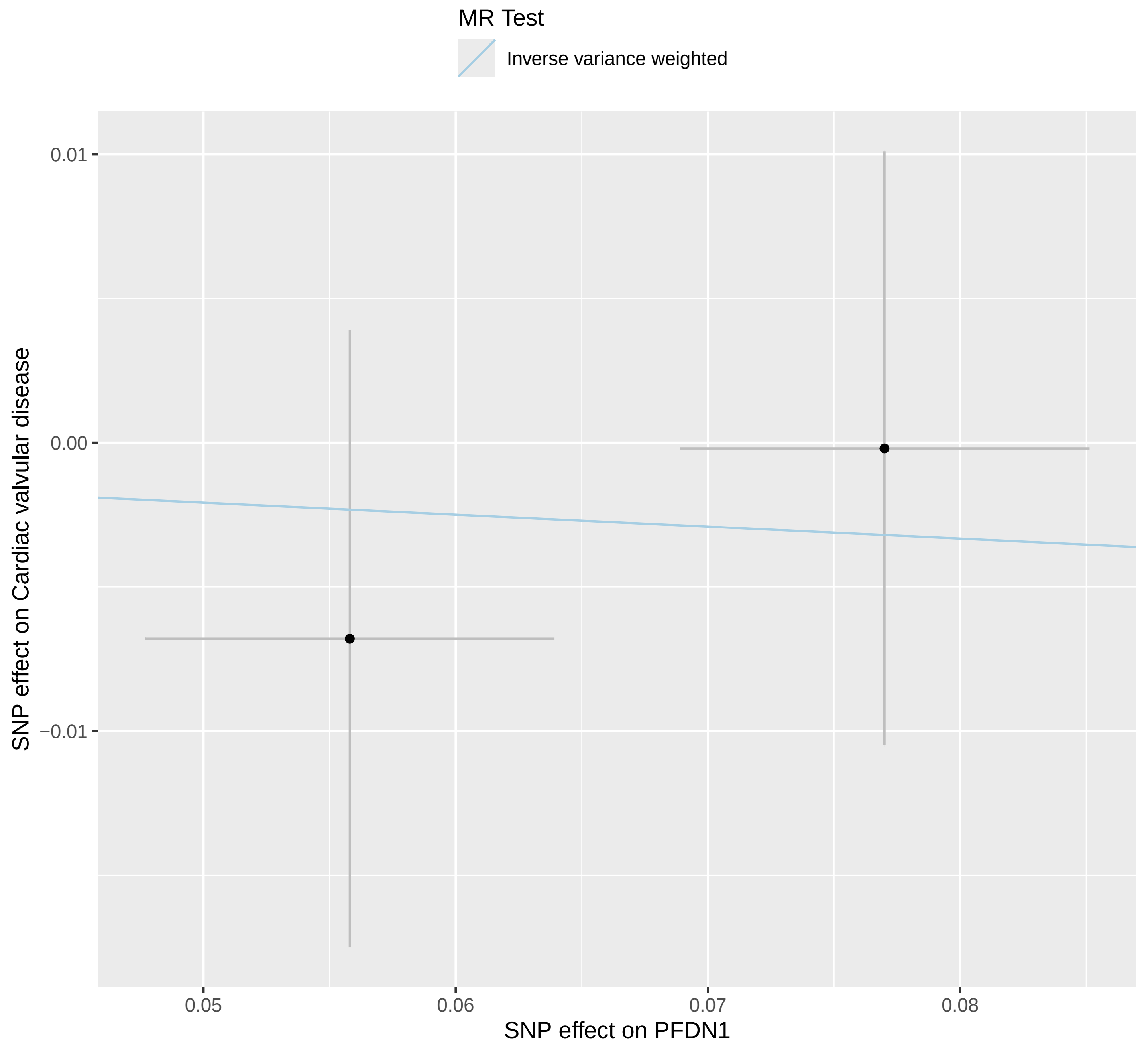

MR Test
Inverse variance weighted
0.01
0.00
−0.01
0.05
0.06
0.07
0.08
SNP effect on Cardiac valvular disease
SNP effect on PFDN1

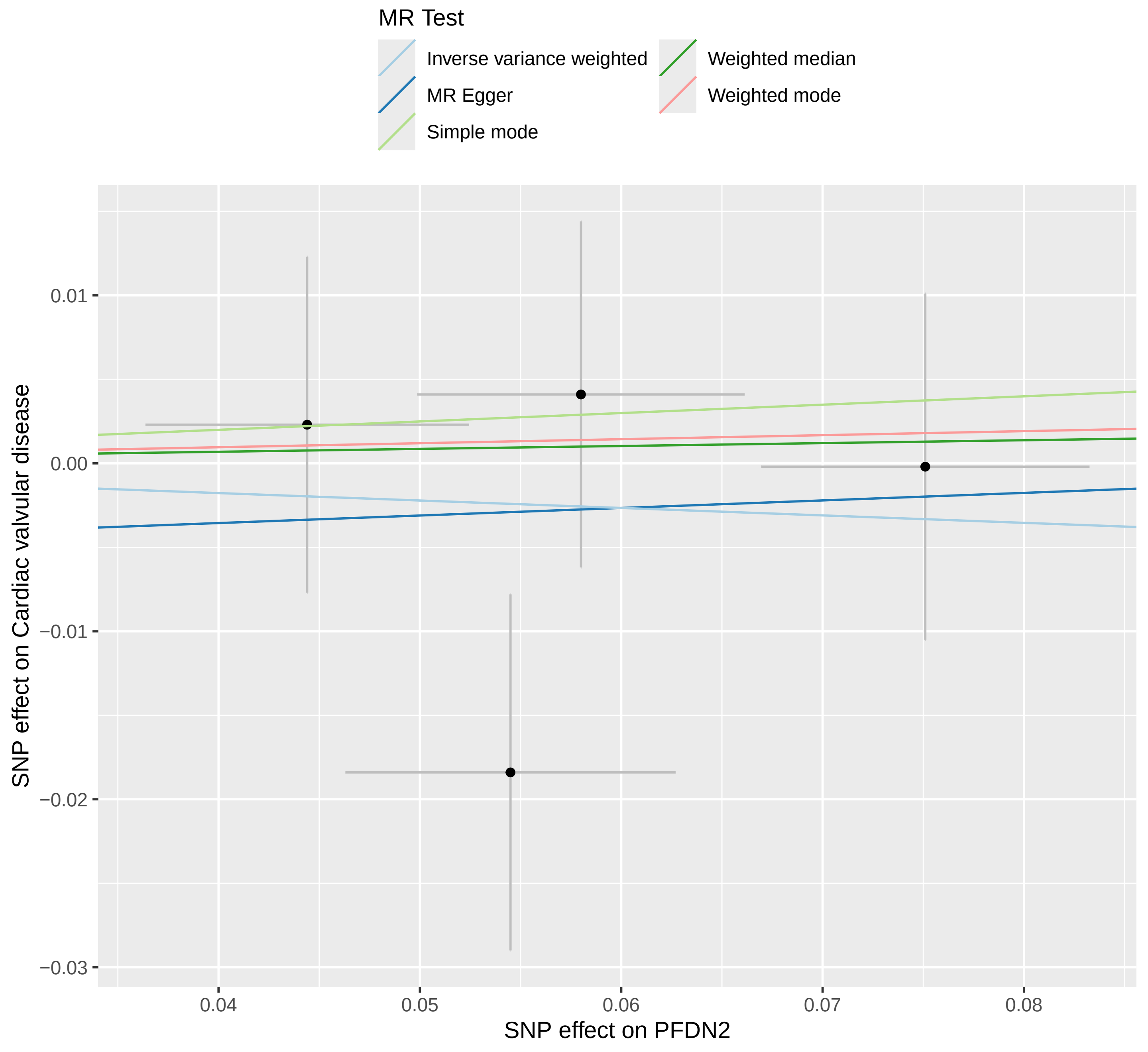

MR Test
Inverse variance weighted
MR Egger
Simple mode
Weighted median
Weighted mode
0.01
0.00
−0.01
−0.02
−0.03
0.04
0.05
0.06
0.07
0.08
SNP effect on Cardiac valvular disease
SNP effect on PFDN2

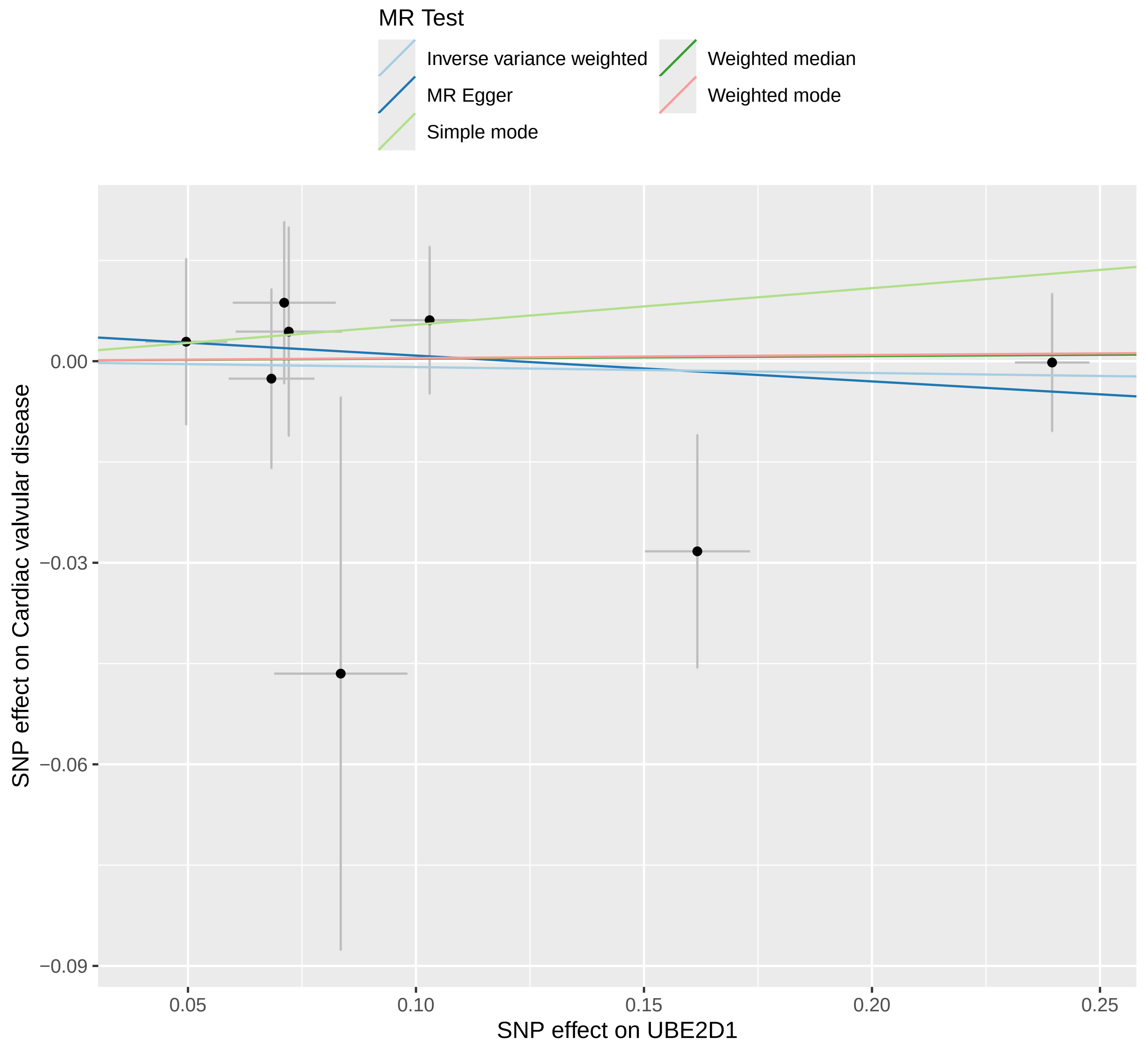
MR Test
Inverse variance weighted
MR Egger
Simple mode
Weighted median
Weighted mode
0.00
−0.03
−0.06
−0.09
0.05
0.10
0.15
0.20
0.25
SNP effect on Cardiac valvular disease
SNP effect on UBE2D1

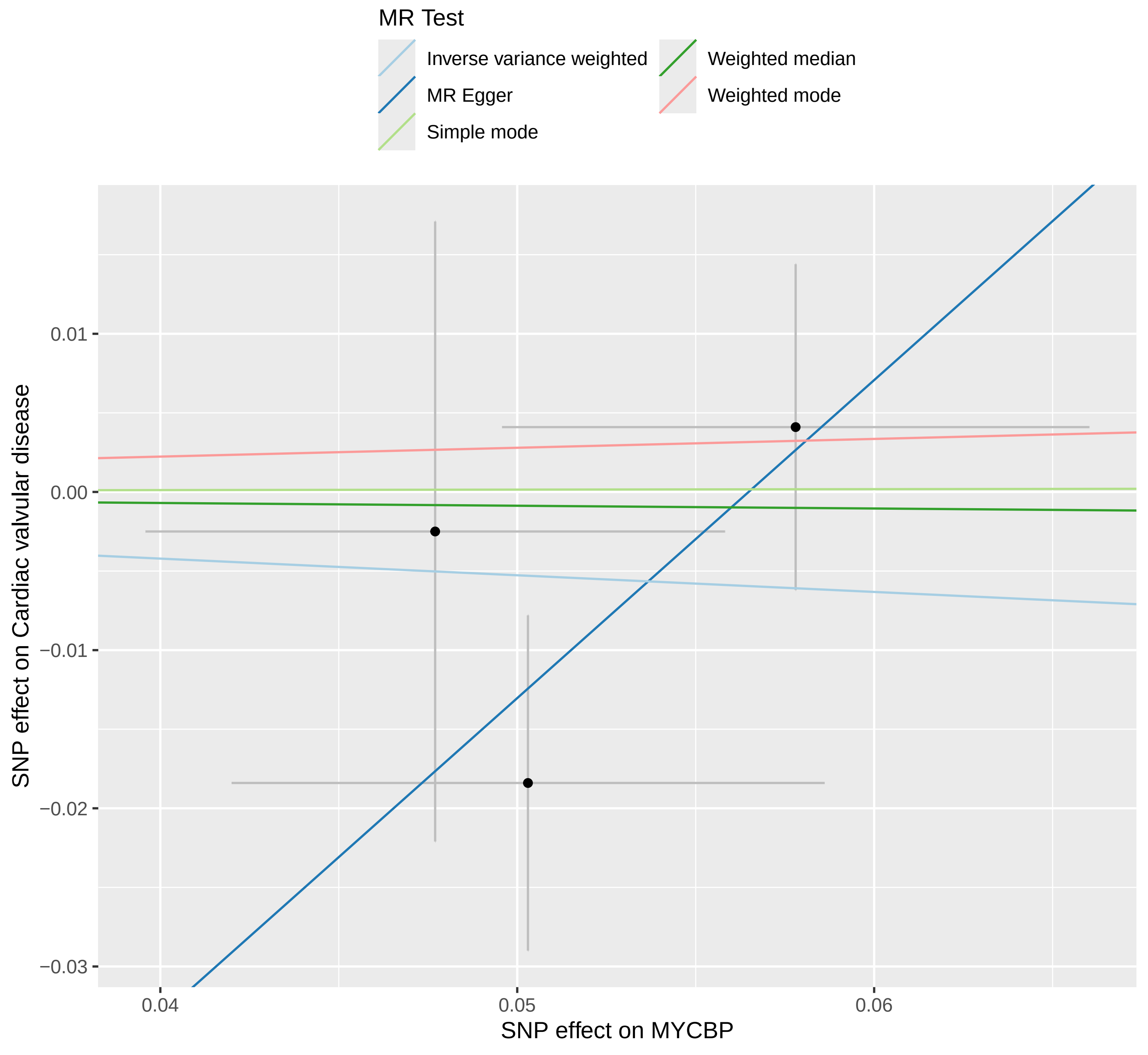
MR Test
Inverse variance weighted
MR Egger
Simple mode
Weighted median
Weighted mode
0.01
0.00
−0.01
−0.02
−0.03
0.04
0.05
0.06
SNP effect on Cardiac valvular disease
SNP effect on MYCBP

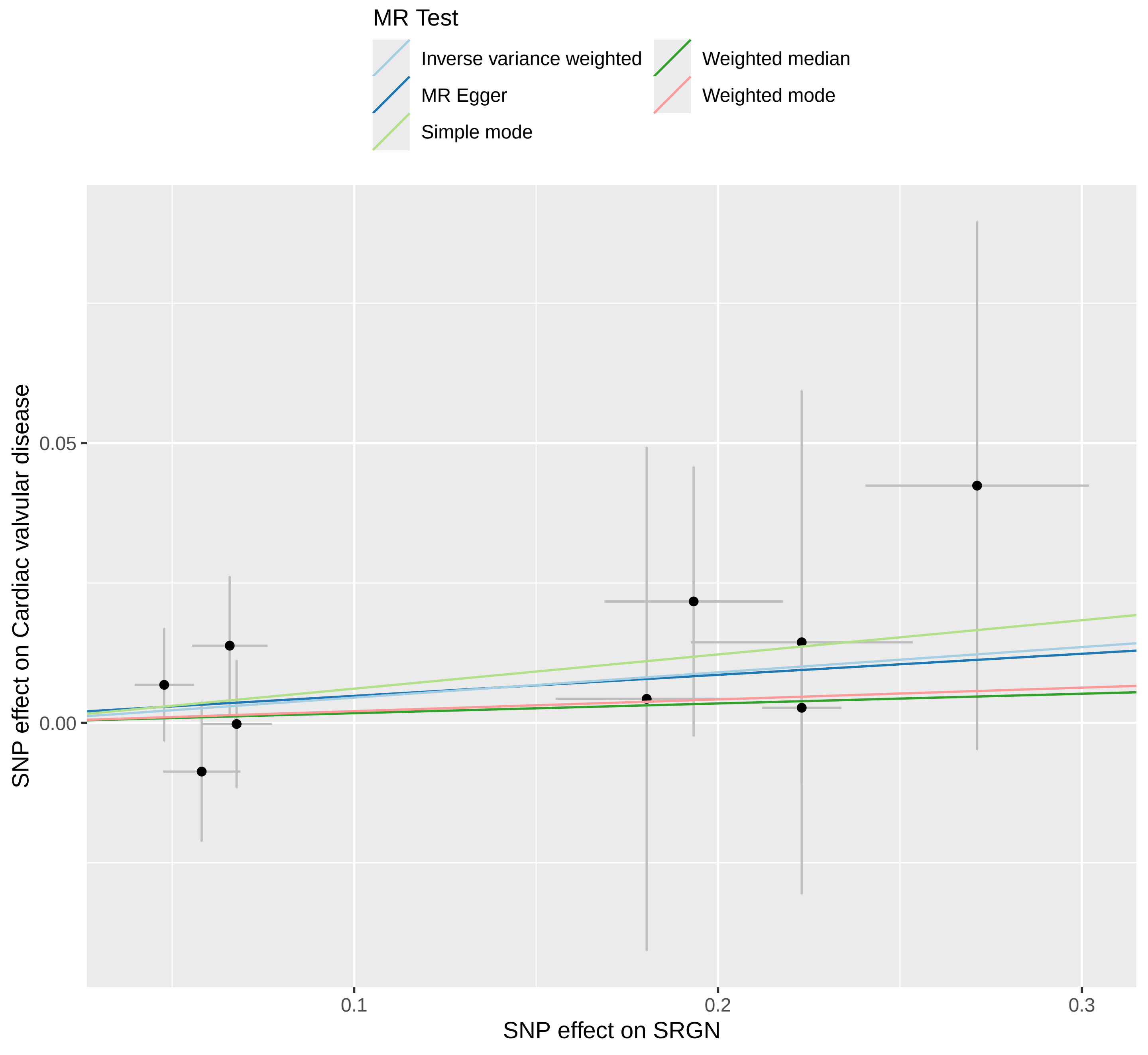

MR Test
Inverse variance weighted
MR Egger
Simple mode
Weighted median
Weighted mode
SNP effect on Cardiac valvular disease
0.05
0.00
0.1
0.2
0.3
SNP effect on SRGN

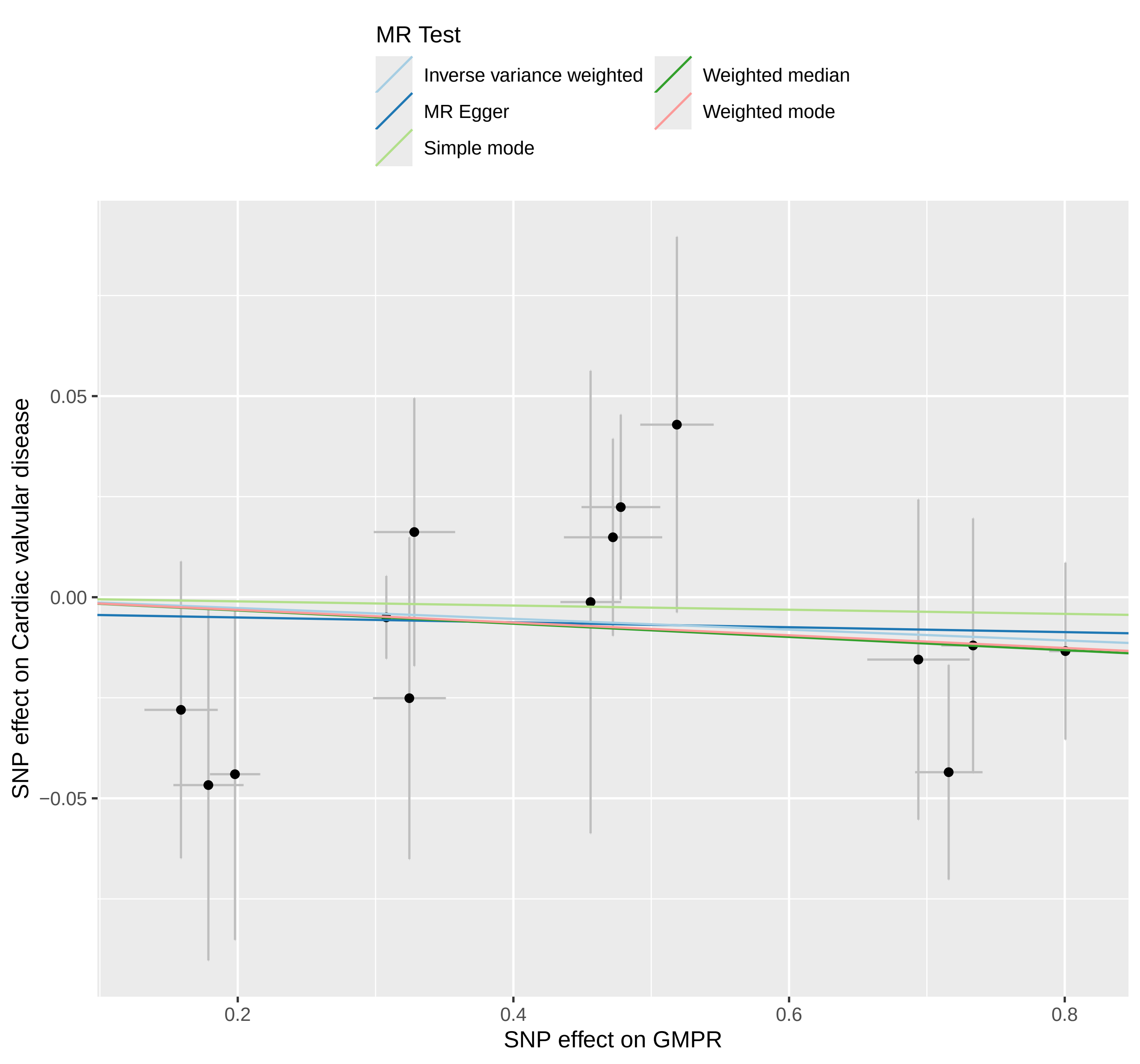
MR Test
Inverse variance weighted
MR Egger
Simple mode
Weighted median
Weighted mode
0.05
0.00
−0.05
0.2
0.4
0.6
0.8
SNP effect on Cardiac valvular disease
SNP effect on GMPR

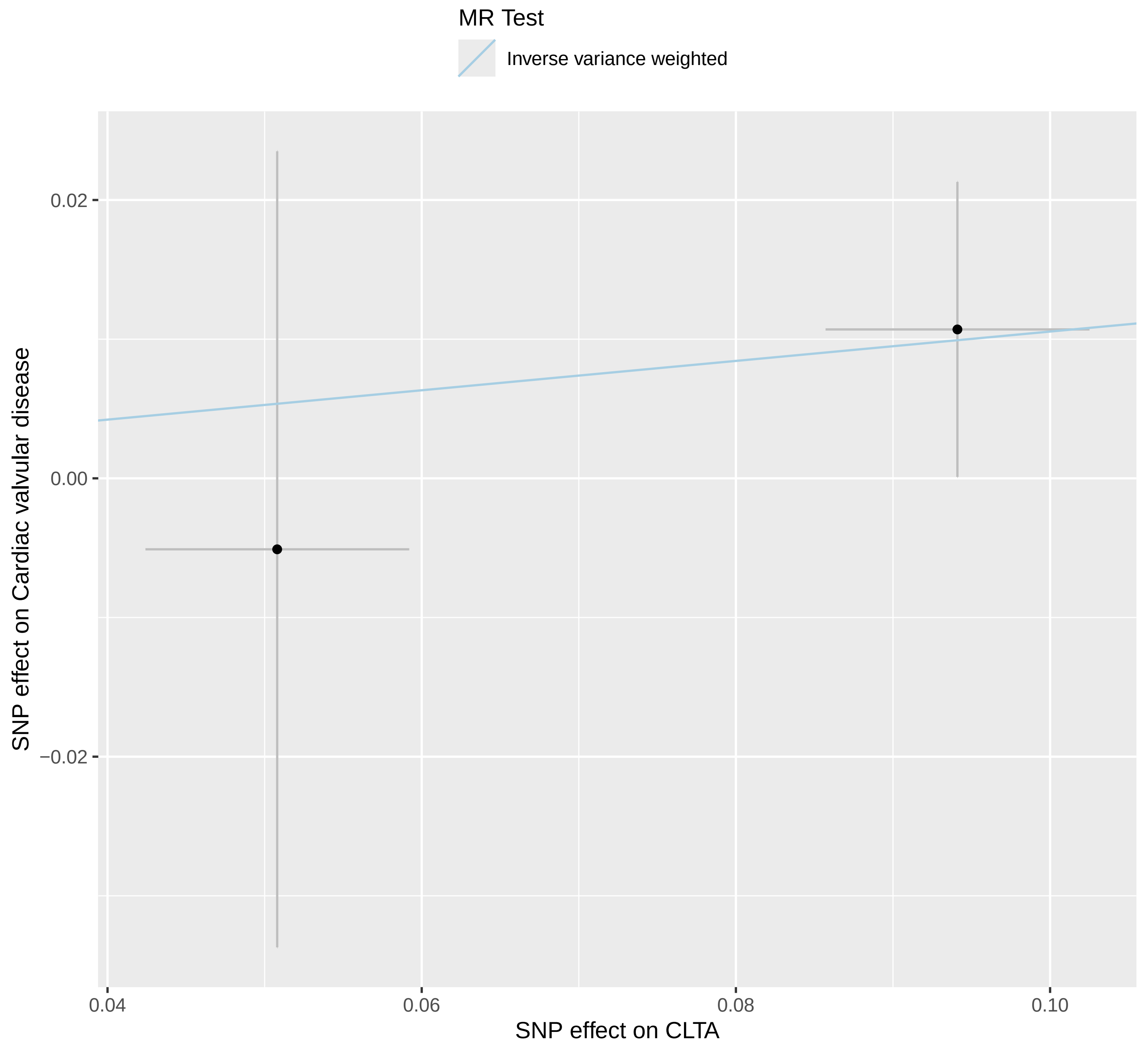
MR Test
Inverse variance weighted
0.02
0.00
−0.02
0.04
0.06
0.08
0.10
SNP effect on Cardiac valvular disease
SNP effect on CLTA

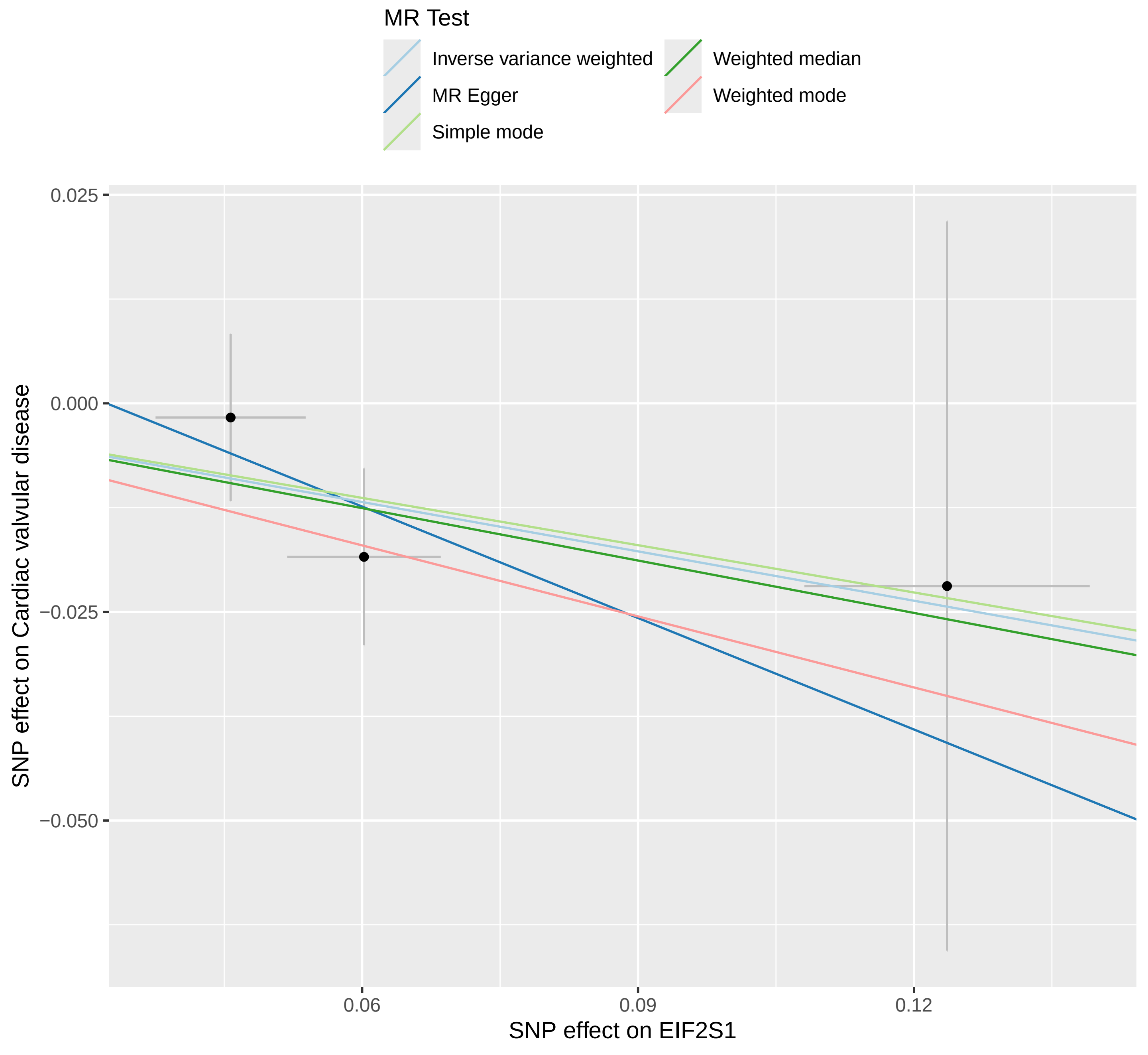
MR Test
Inverse variance weighted
MR Egger
Simple mode
Weighted median
Weighted mode
0.025
0.000
−0.025
−0.050
SNP effect on Cardiac valvular disease
0.06
0.09
0.12
SNP effect on EIF2S1

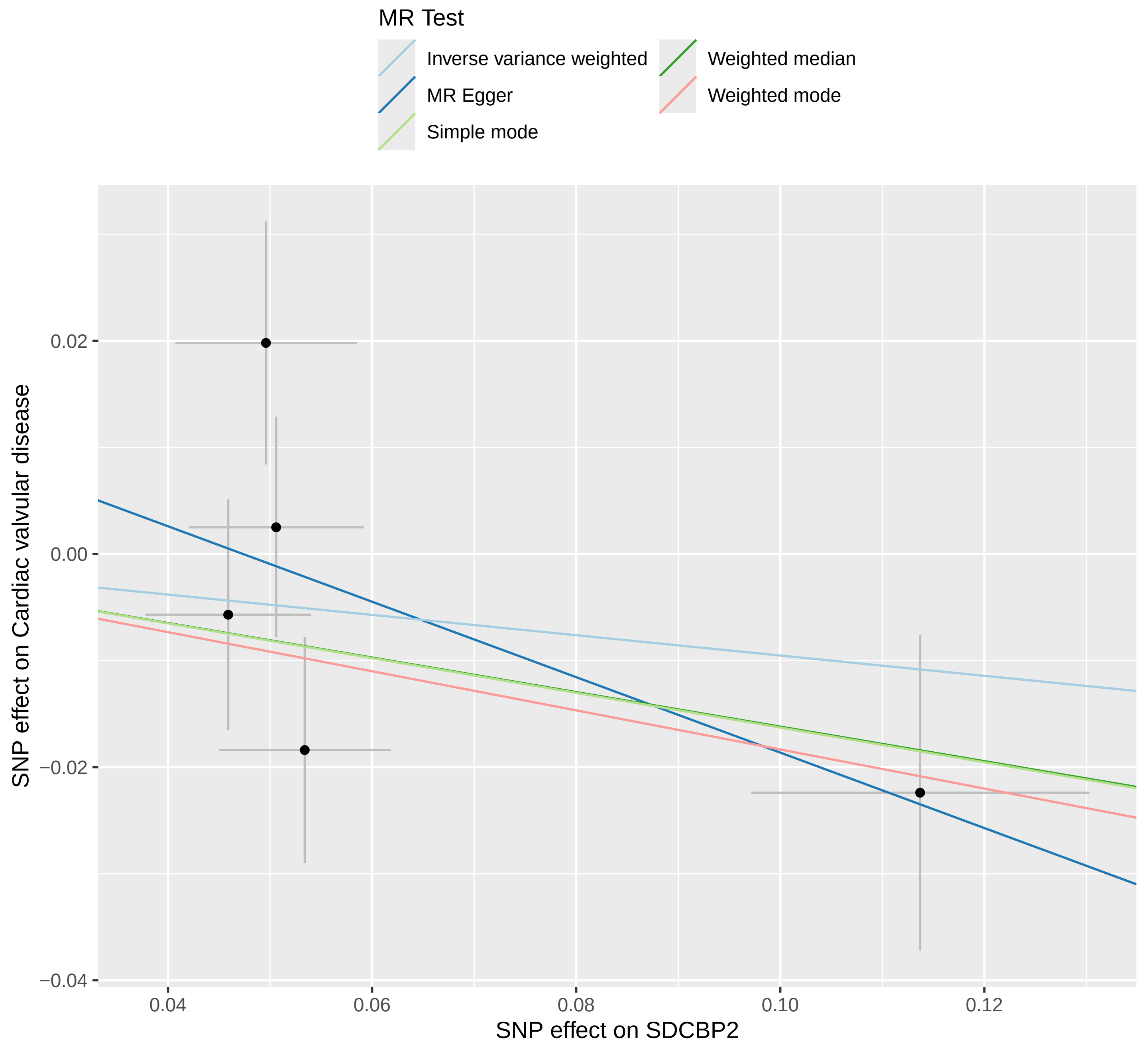

MR Test
Inverse variance weighted
MR Egger
Simple mode
Weighted median
Weighted mode
0.02
0.00
−0.02
−0.04
SNP effect on Cardiac valvular disease
0.04
0.06
0.08
0.10
0.12
SNP effect on SDCBP2

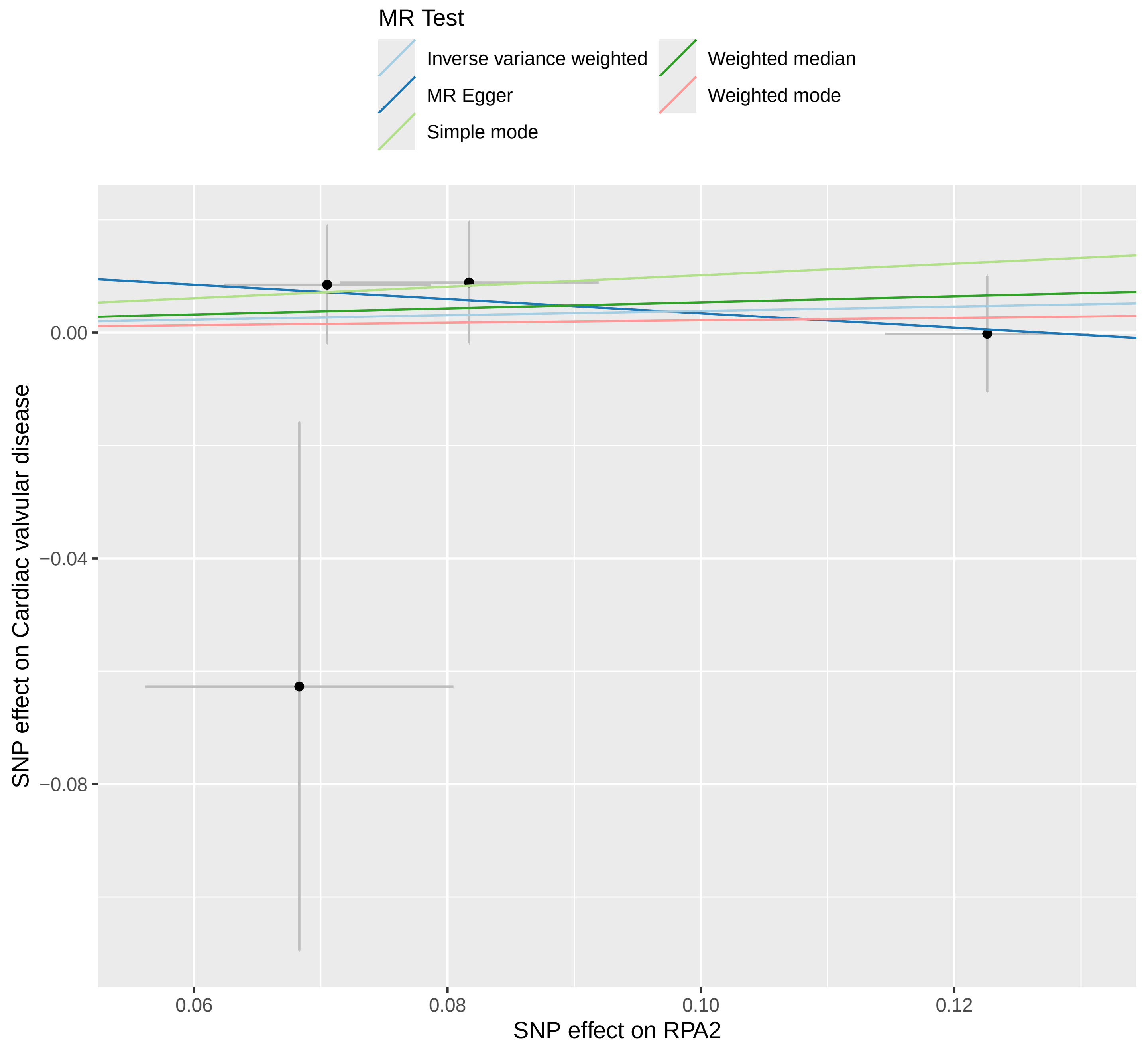
MR Test
Inverse variance weighted
MR Egger
Simple mode
Weighted median
Weighted mode
0.00
−0.04
−0.08
SNP effect on Cardiac valvular disease
0.06
0.08
0.10
0.12
SNP effect on RPA2

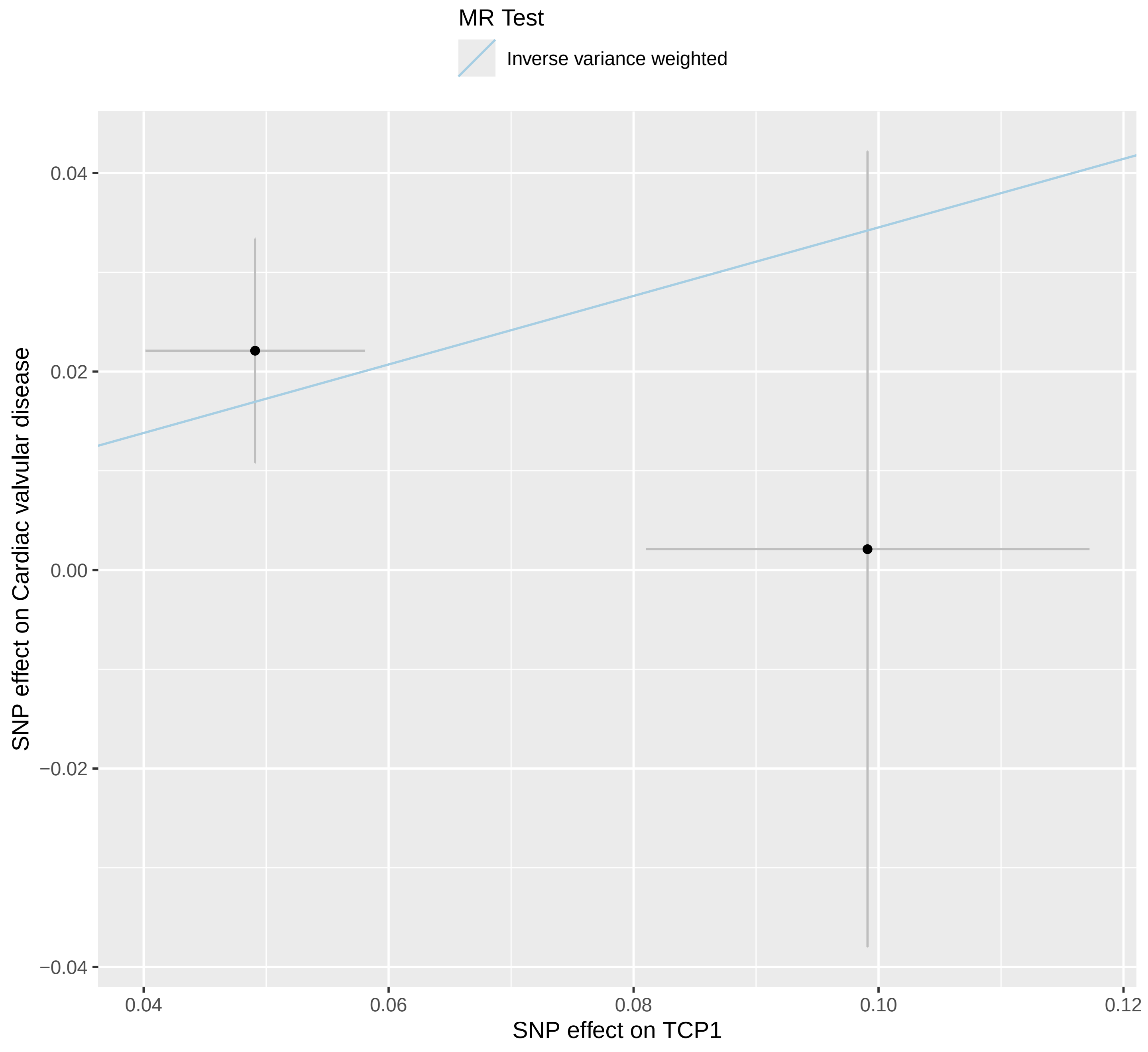
MR Test
Inverse variance weighted
SNP effect on Cardiac valvular disease
0.04
0.02
0.00
−0.02
−0.04
0.04
0.06
0.08
0.10
0.12
SNP effect on TCP1

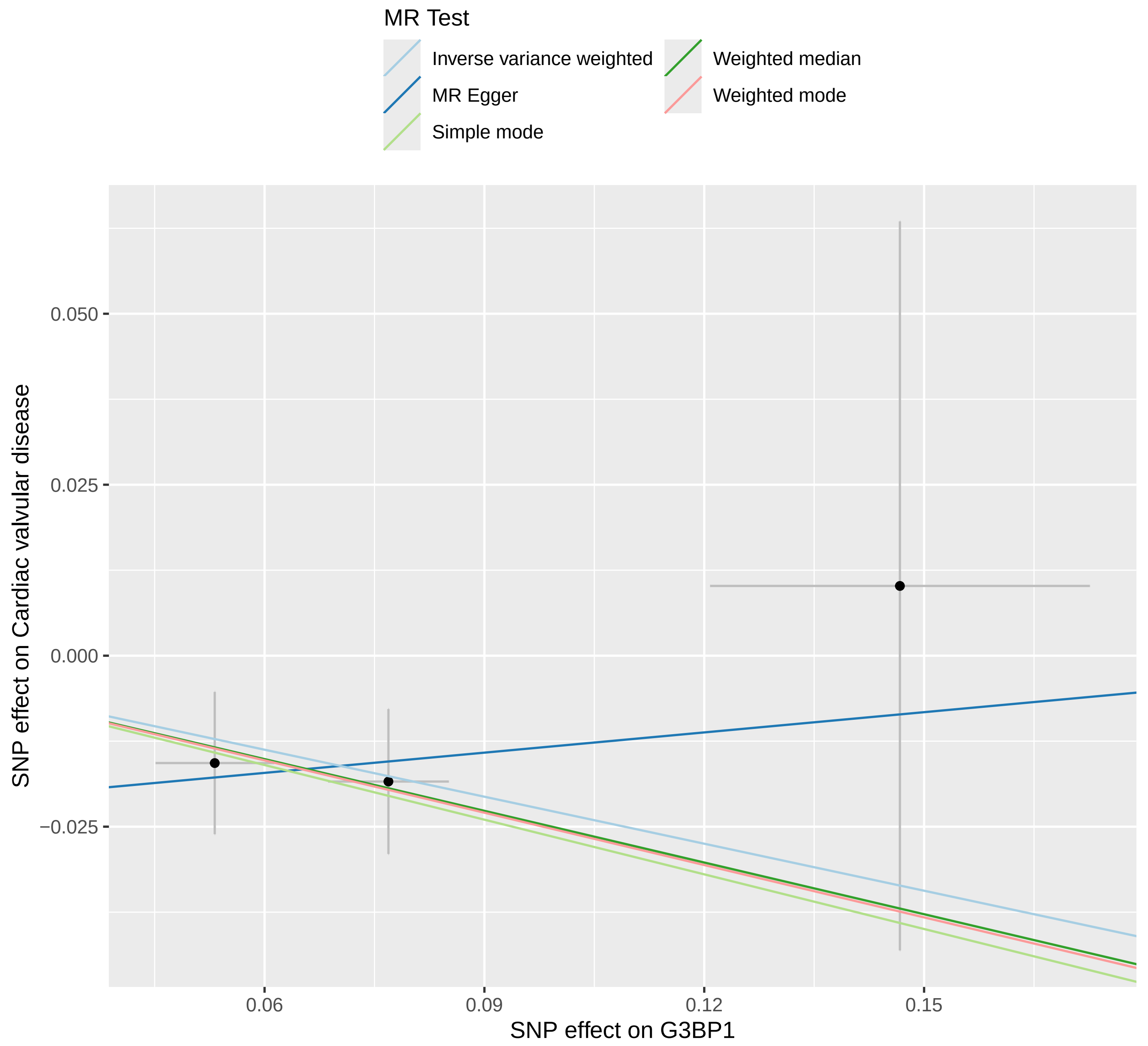
MR Test
Inverse variance weighted
MR Egger
Simple mode
Weighted median
Weighted mode
0.050
0.025
0.000
−0.025
SNP effect on Cardiac valvular disease
0.06
0.09
0.12
0.15
SNP effect on G3BP1

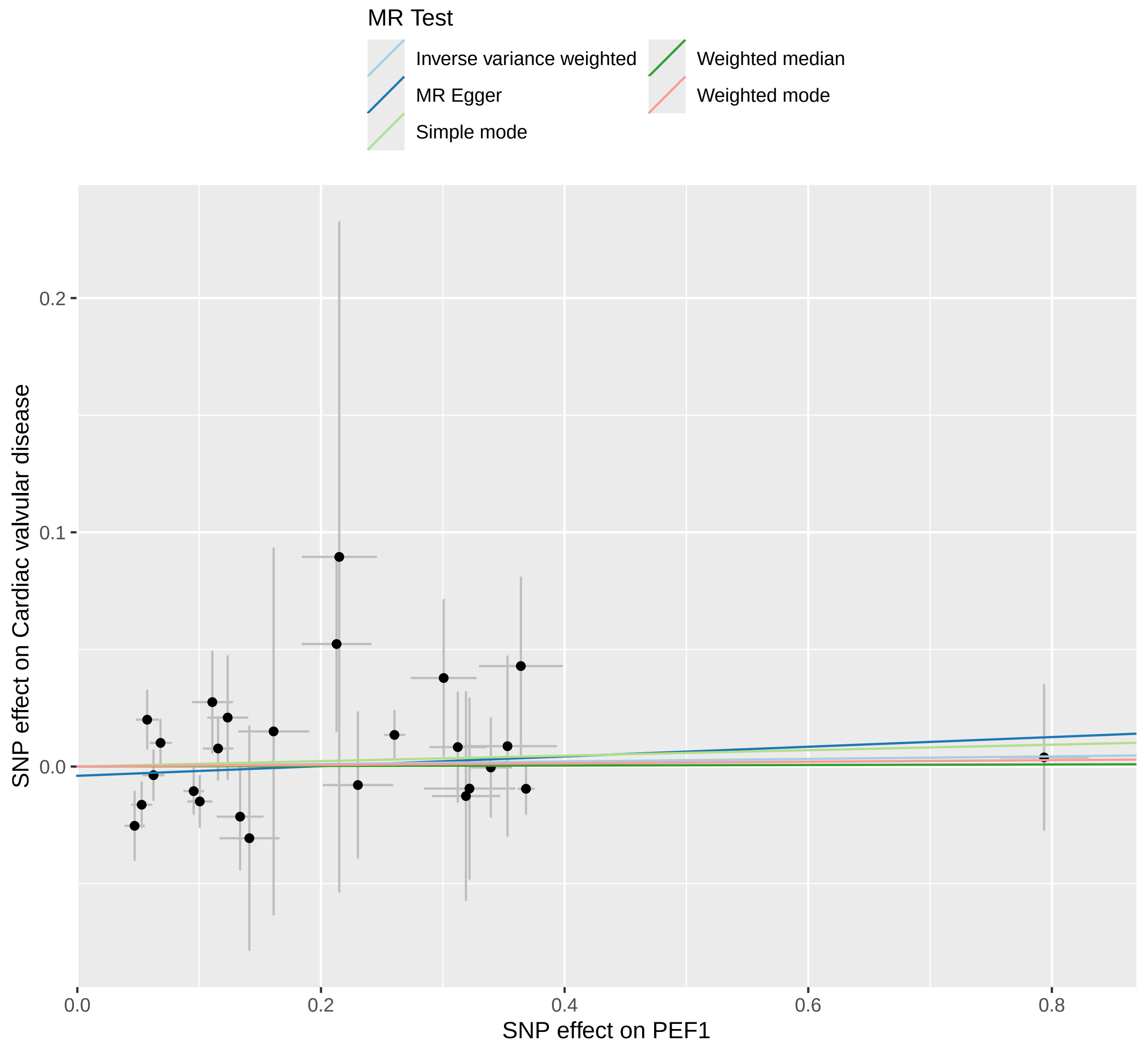

MR Test
Inverse variance weighted
MR Egger
Simple mode
Weighted median
Weighted mode
SNP effect on Cardiac valvular disease
0.2
0.1
0.0
0.0
0.2
0.4
0.6
0.8
SNP effect on PEF1

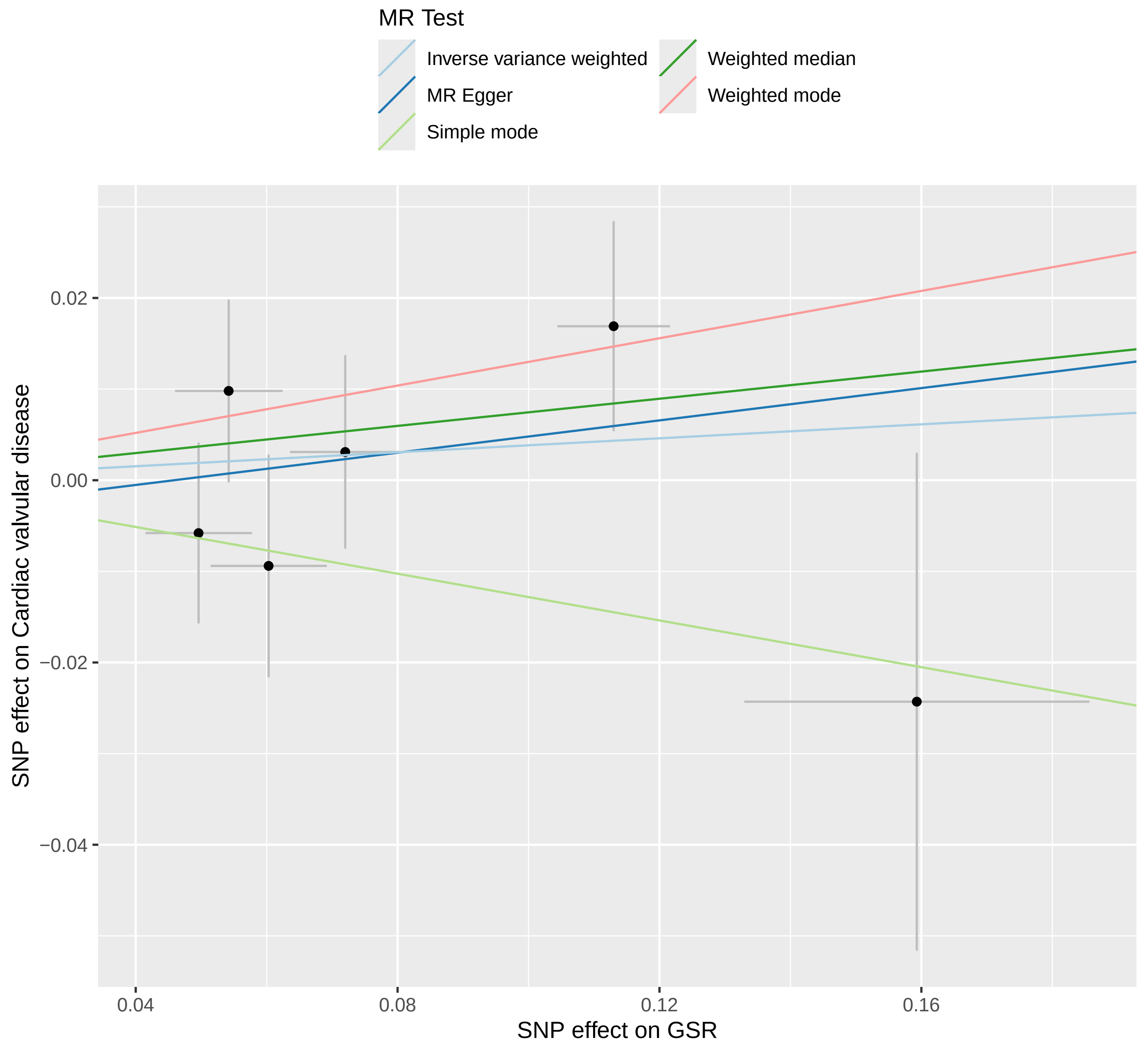

MR Test
Inverse variance weighted
MR Egger
Simple mode
Weighted median
Weighted mode
0.02
0.00
−0.02
−0.04
SNP effect on Cardiac valvular disease
0.04
0.08
0.12
0.16
SNP effect on GSR

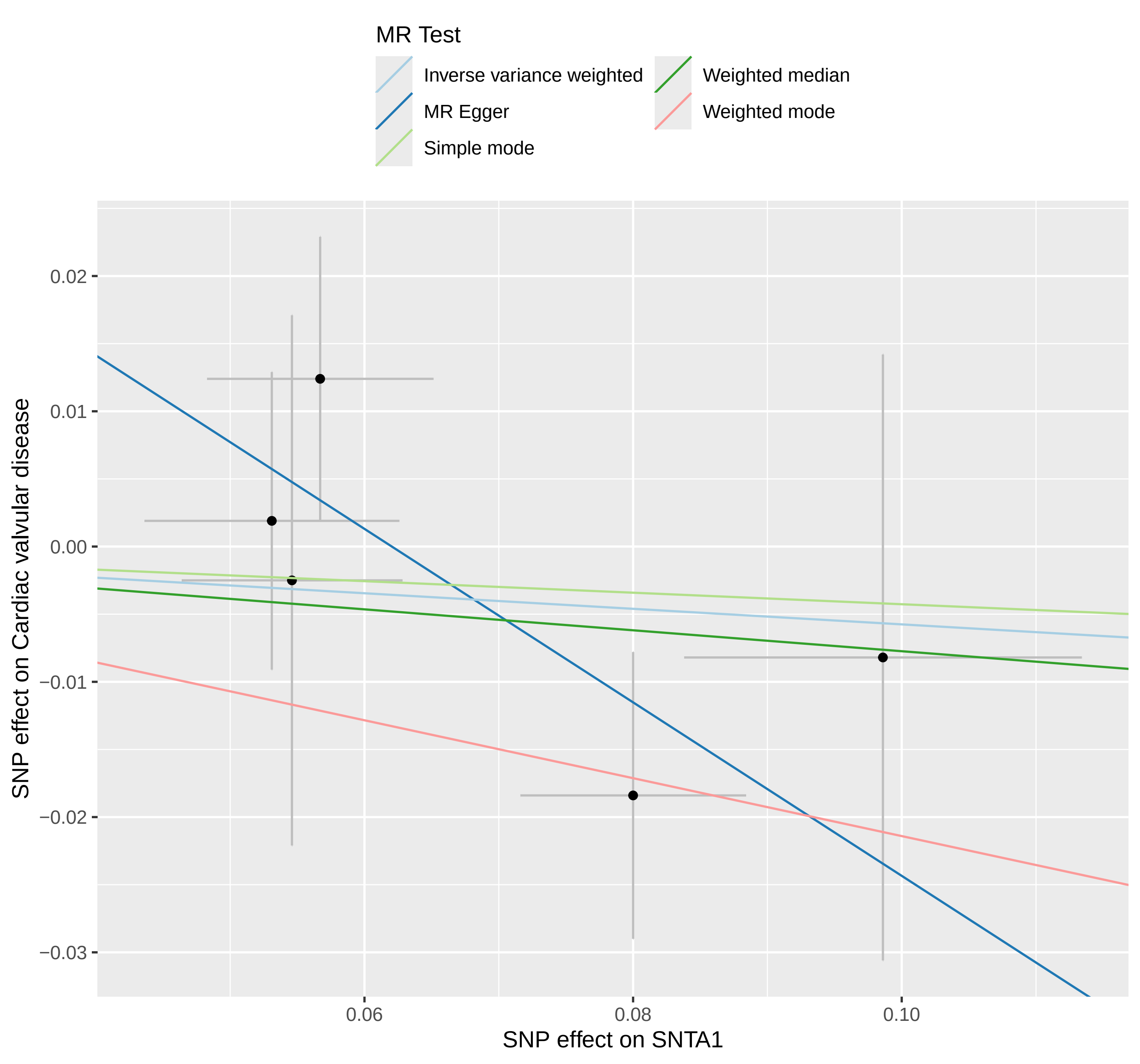
MR Test
Inverse variance weighted
MR Egger
Simple mode
Weighted median
Weighted mode
0.02
0.01
0.00
−0.01
−0.02
−0.03
SNP effect on Cardiac valvular disease
0.06
0.08
0.10
SNP effect on SNTA1

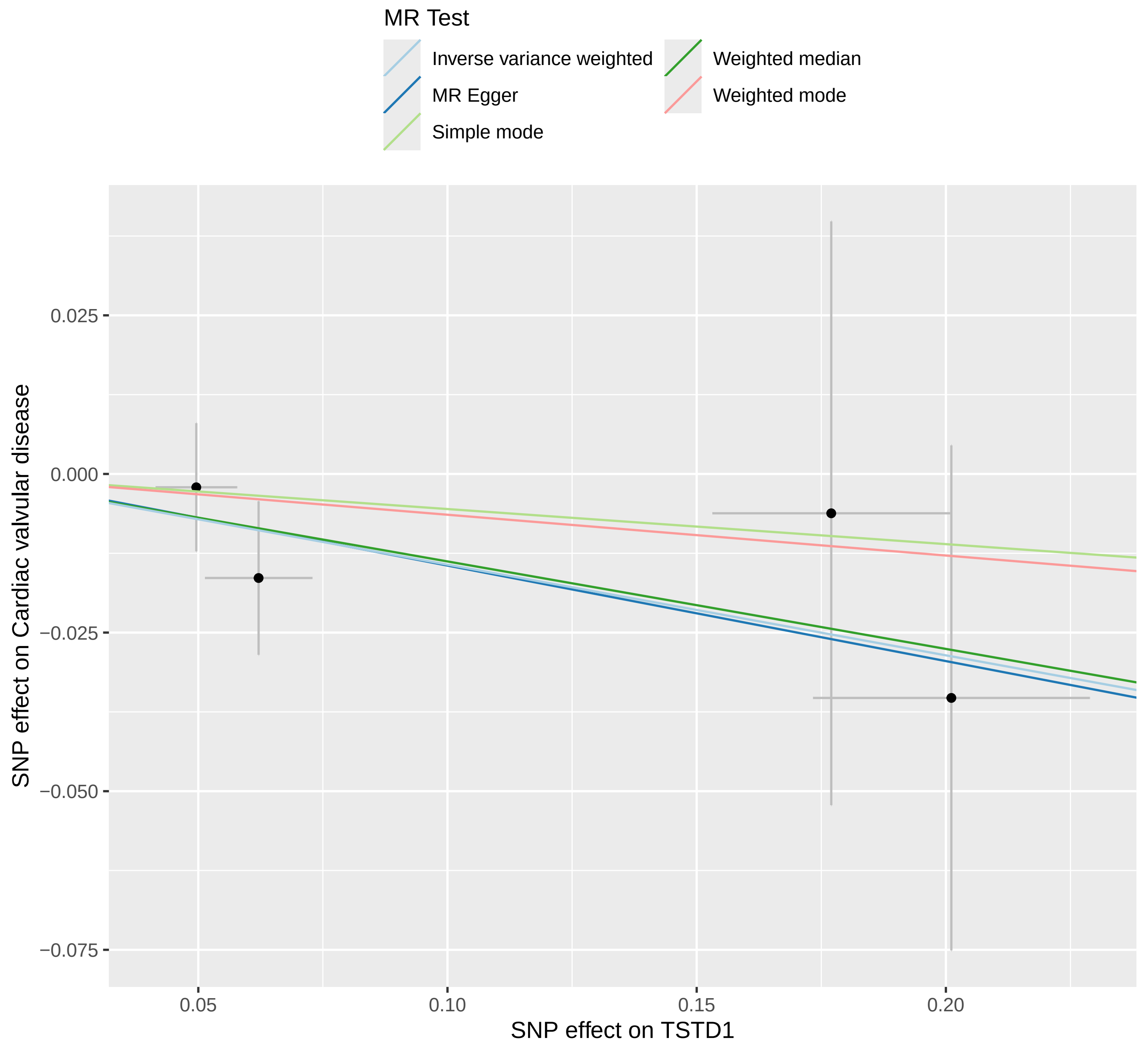

MR Test
Inverse variance weighted
MR Egger
Simple mode
Weighted median
Weighted mode
0.025
0.000
−0.025
−0.050
−0.075
SNP effect on Cardiac valvular disease
0.05
0.10
0.15
0.20
SNP effect on TSTD1

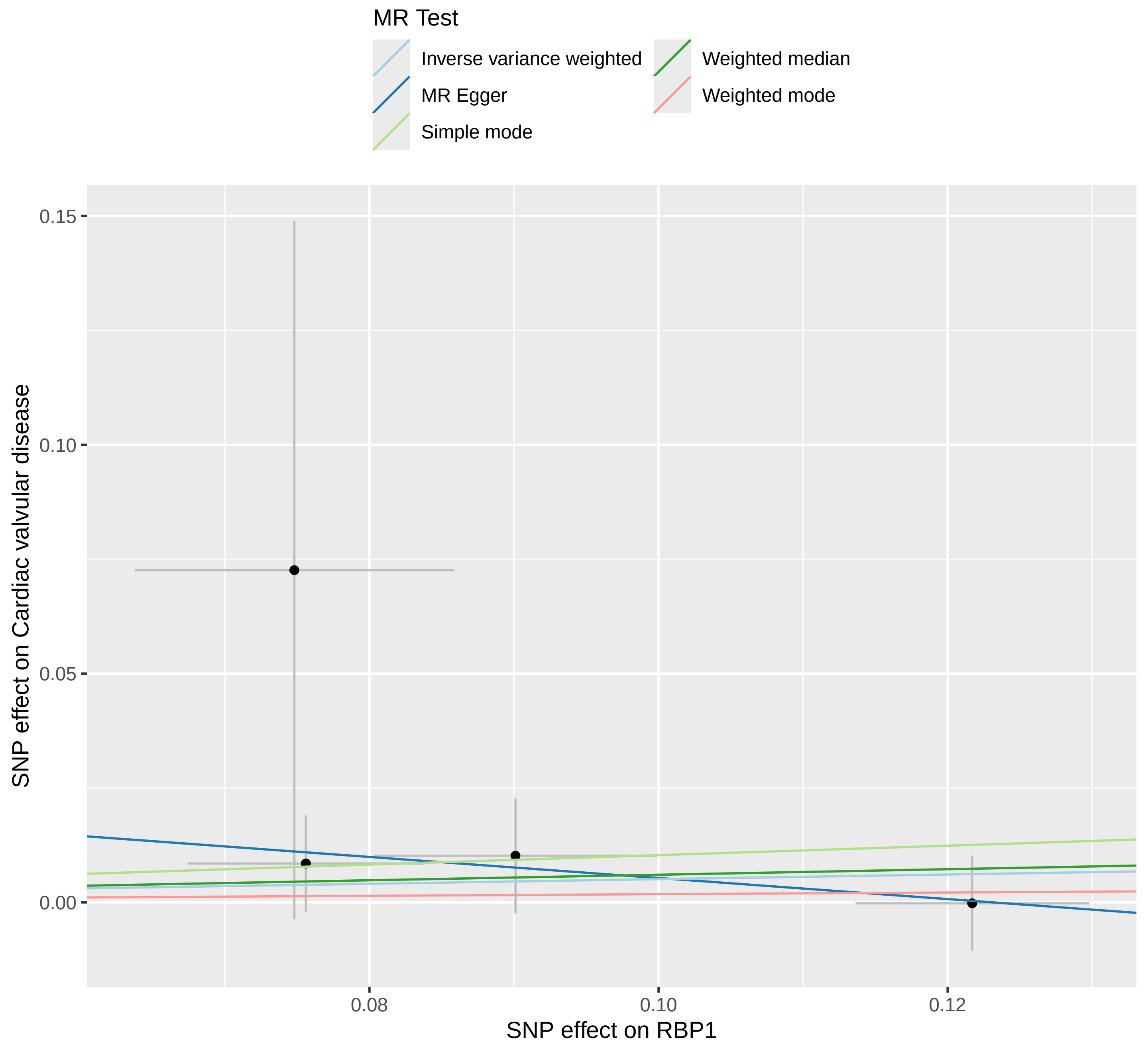
MR Test
Inverse variance weighted
MR Egger
Simple mode
Weighted median
Weighted mode
0.15
0.10
0.05
0.00
0.08
0.10
0.12
SNP effect on Cardiac valvular disease
SNP effect on RBP1

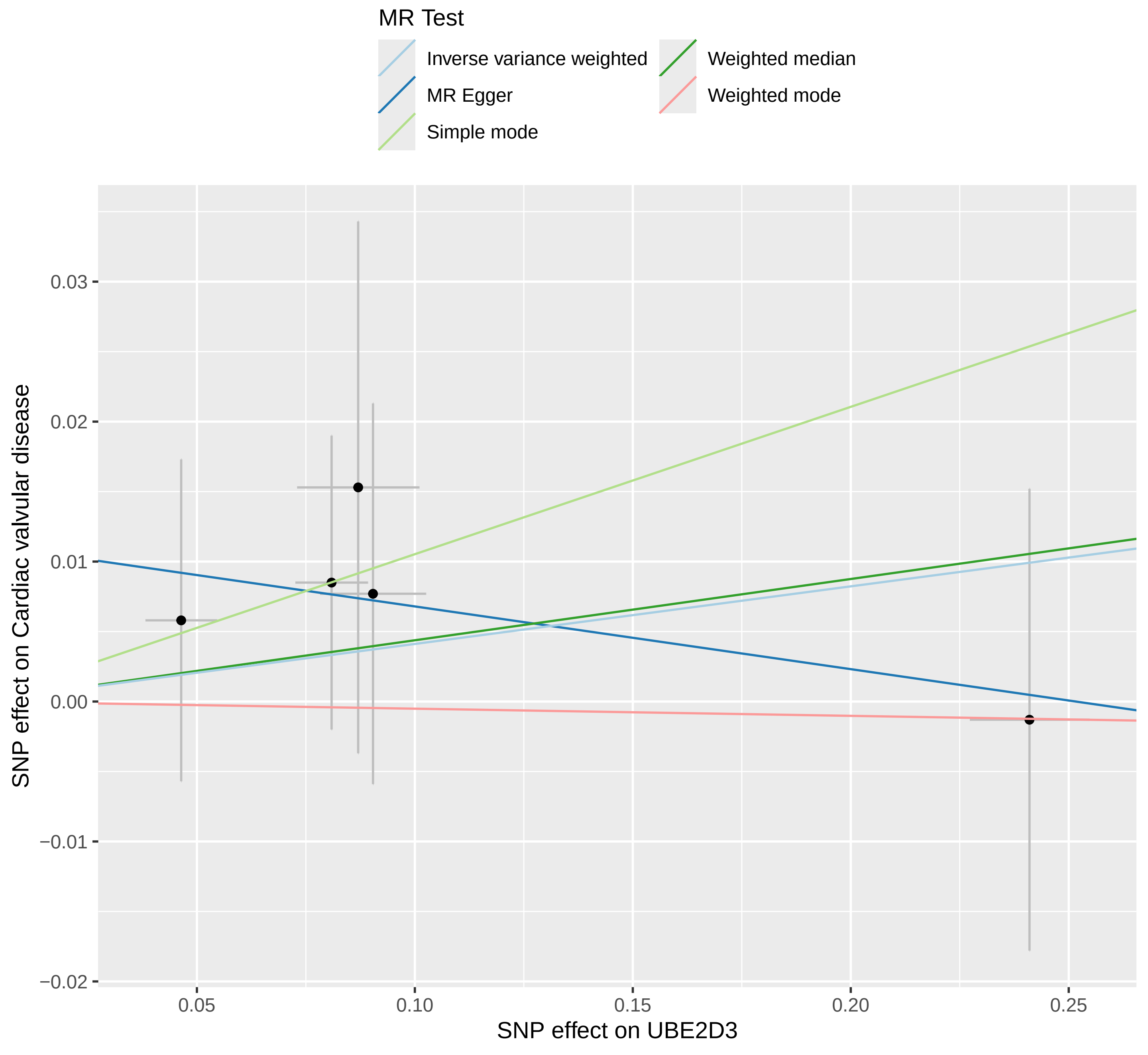
MR Test
Inverse variance weighted
MR Egger
Simple mode
Weighted median
Weighted mode
0.03
0.02
0.01
0.00
−0.01
−0.02
SNP effect on Cardiac valvular disease
0.05
0.10
0.15
0.20
0.25
SNP effect on UBE2D3

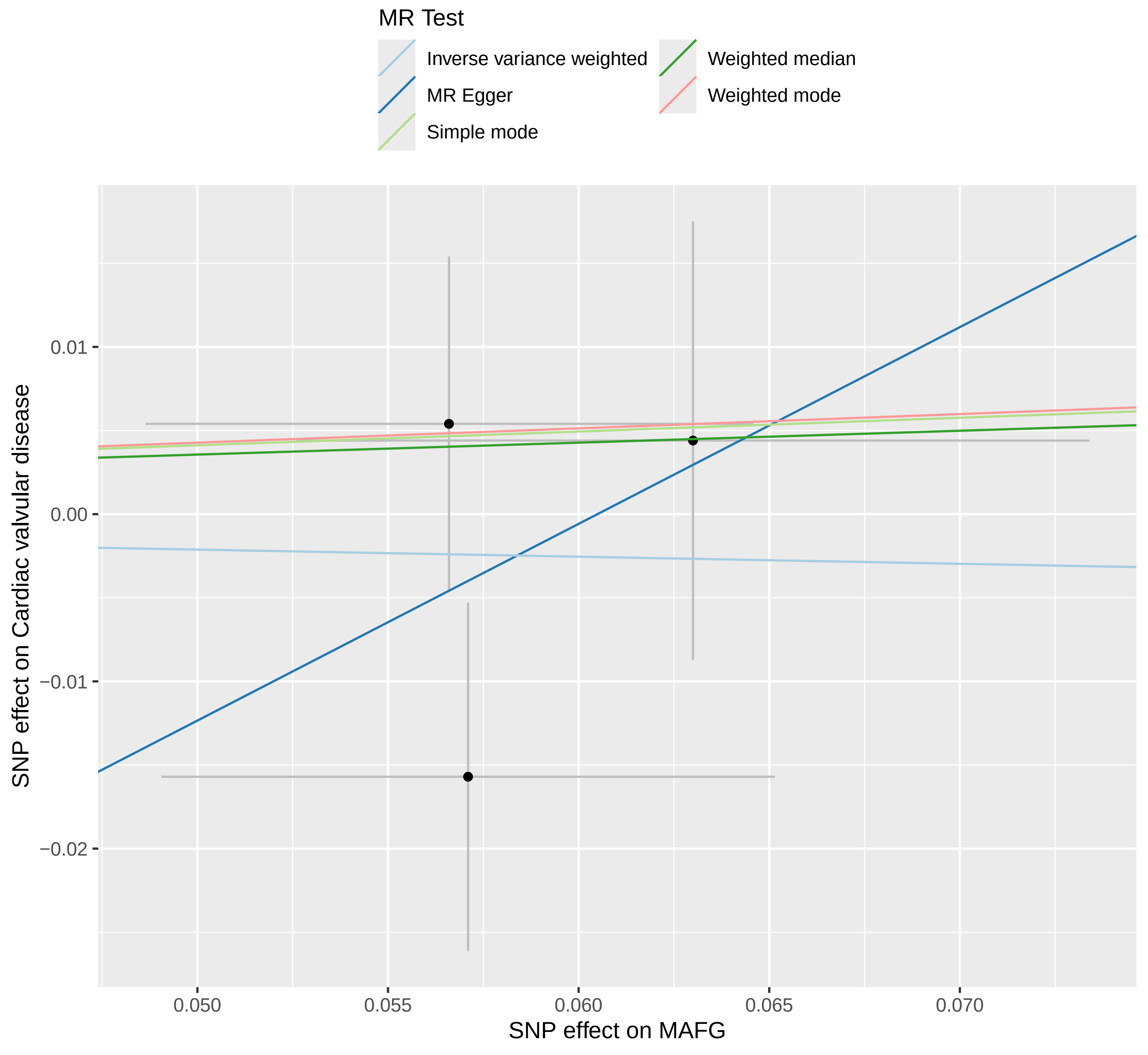
MR Test
Inverse variance weighted
MR Egger
Simple mode
Weighted median
Weighted mode
0.01
0.00
−0.01
−0.02
0.050
0.055
0.060
0.065
0.070
SNP effect on MAFG
SNP effect on Cardiac valvular disease

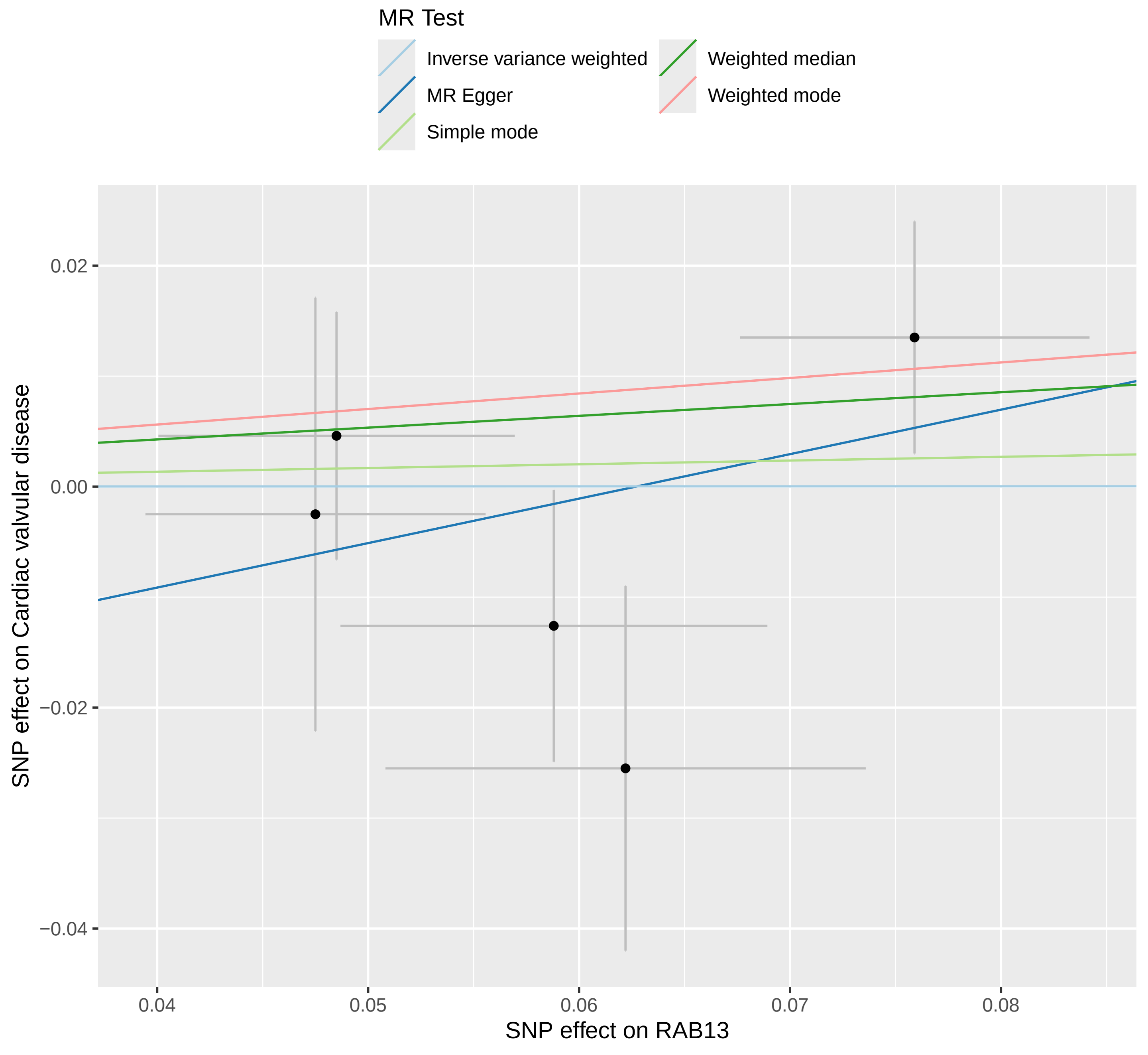

MR Test
Inverse variance weighted
MR Egger
Simple mode
Weighted median
Weighted mode
0.02
0.00
−0.02
−0.04
0.04
0.05
0.06
0.07
0.08
SNP effect on Cardiac valvular disease
SNP effect on RAB13

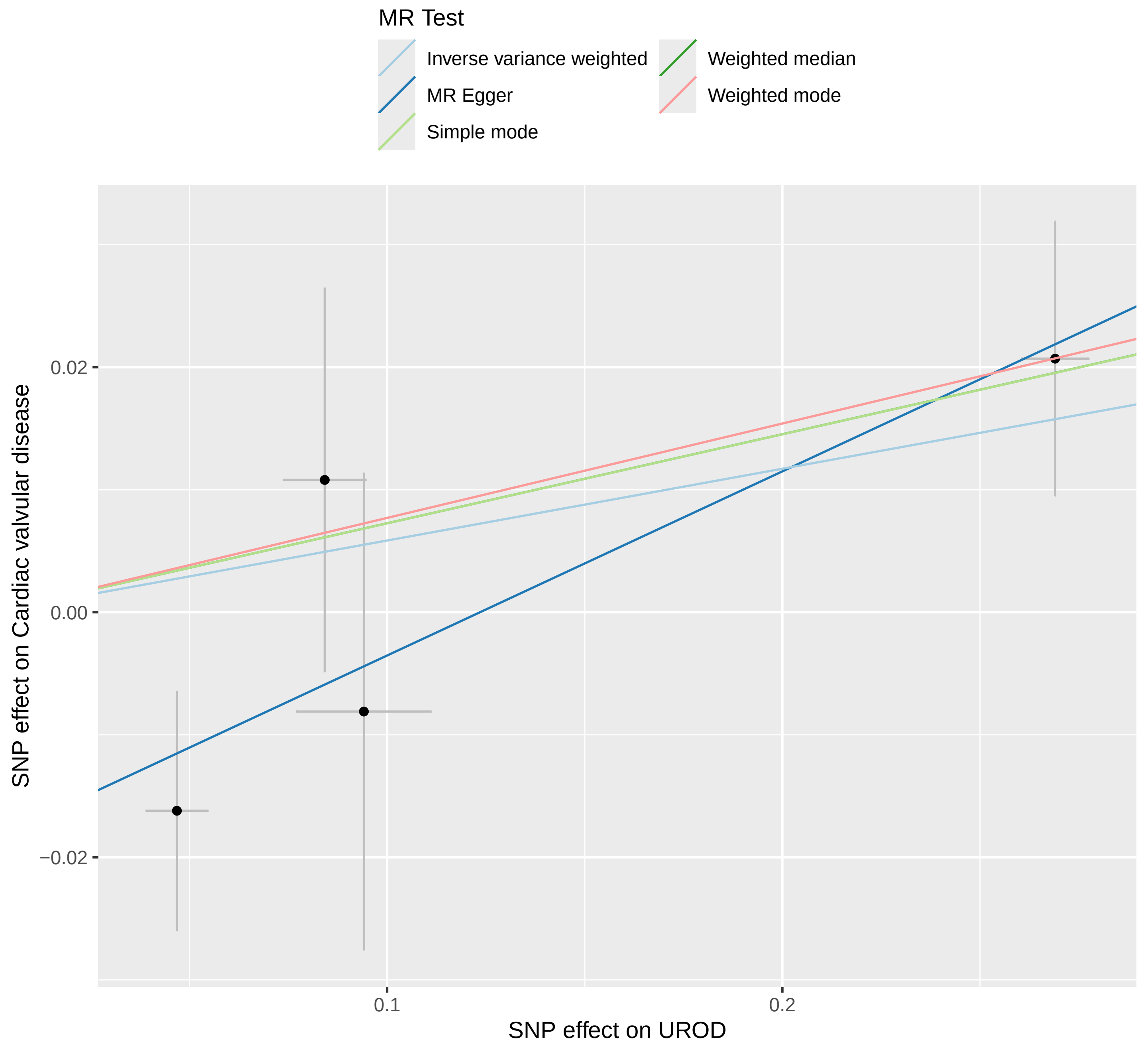
MR Test
Inverse variance weighted
MR Egger
Simple mode
Weighted median
Weighted mode
0.02
0.00
−0.02
0.1
0.2
SNP effect on Cardiac valvular disease
SNP effect on UROD

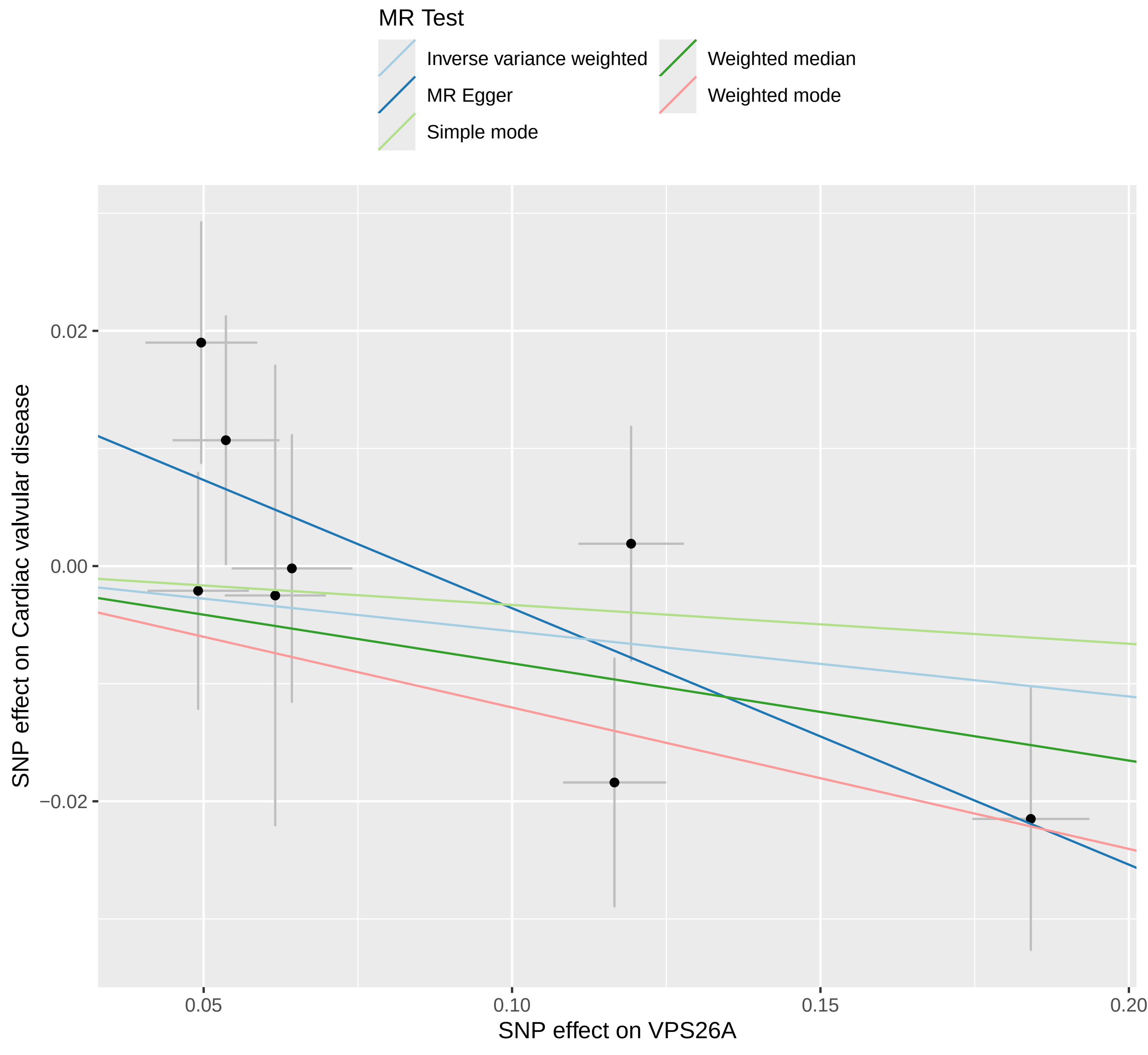

MR Test
Inverse variance weighted
MR Egger
Simple mode
Weighted median
Weighted mode
0.02
0.00
−0.02
0.05
0.10
0.15
0.20
SNP effect on Cardiac valvular disease
SNP effect on VPS26A

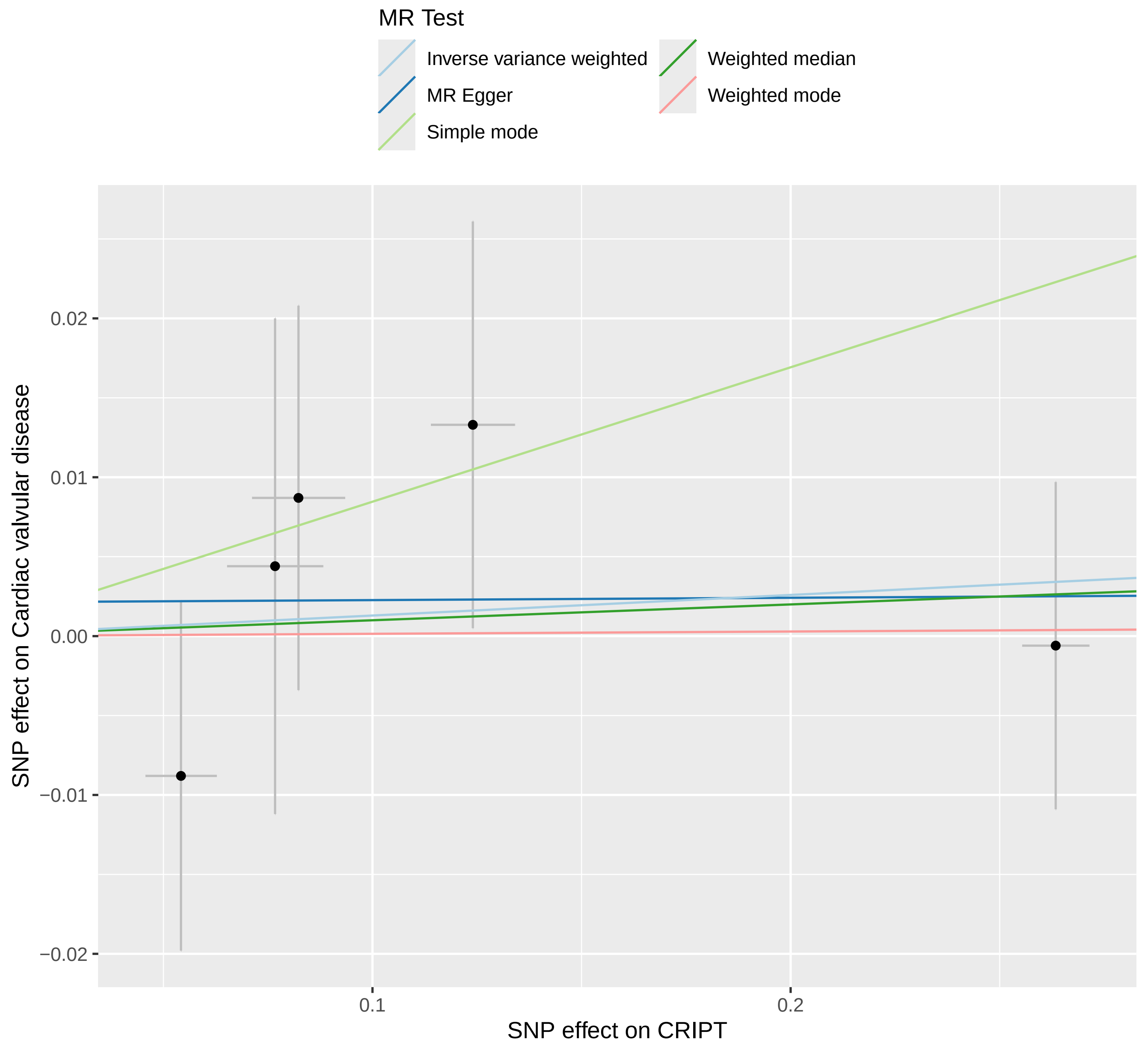

MR Test
Inverse variance weighted
MR Egger
Simple mode
Weighted median
Weighted mode
0.02
0.01
0.00
−0.01
−0.02
SNP effect on Cardiac valvular disease
0.1
0.2
SNP effect on CRIPT

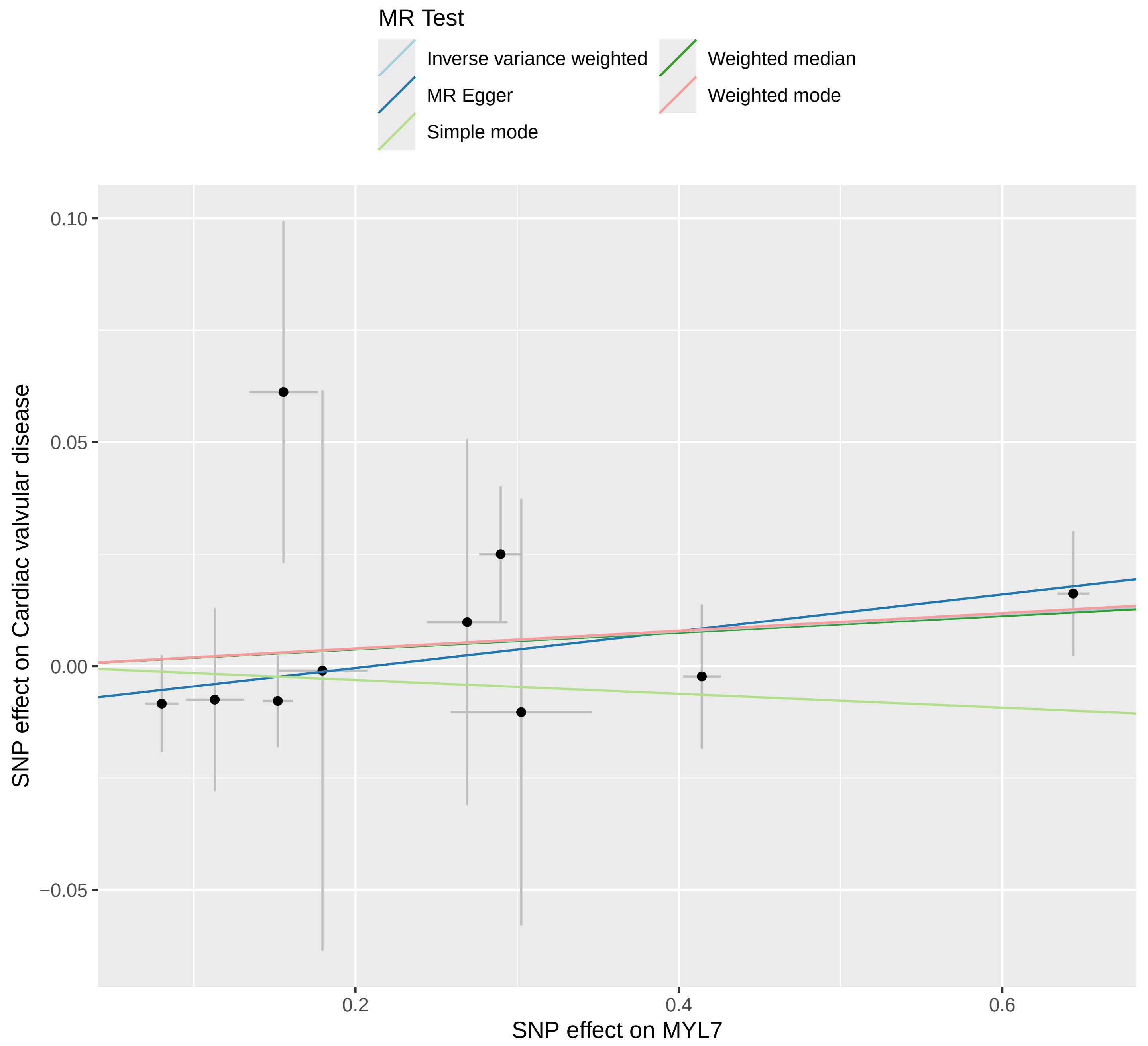
MR Test
Inverse variance weighted
MR Egger
Simple mode
Weighted median
Weighted mode
0.10
0.05
0.00
−0.05
0.2
0.4
0.6
SNP effect on Cardiac valvular disease
SNP effect on MYL7

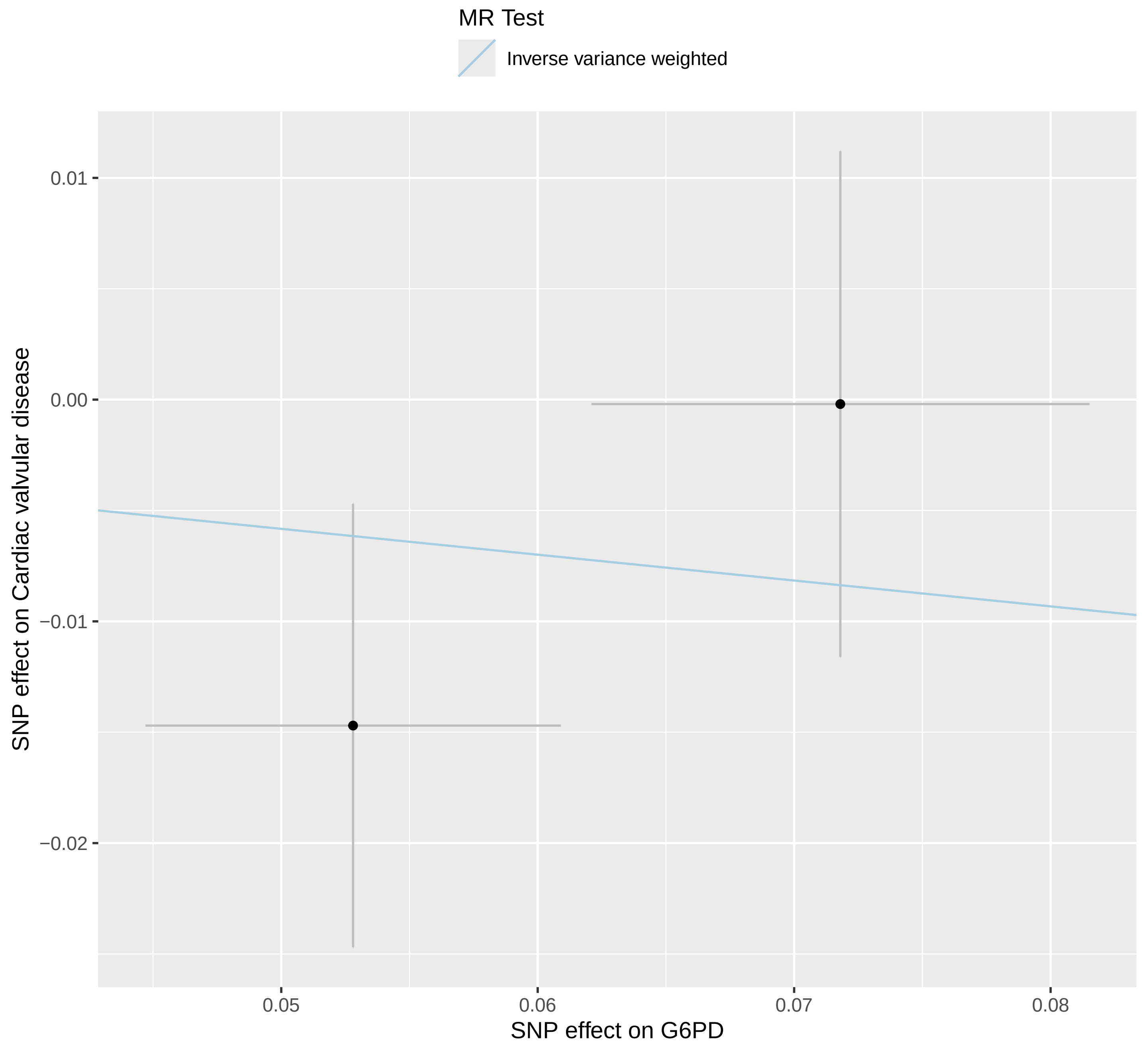
MR Test
Inverse variance weighted
0.01
0.00
−0.01
−0.02
0.05
0.06
0.07
0.08
SNP effect on G6PD
SNP effect on Cardiac valvular disease

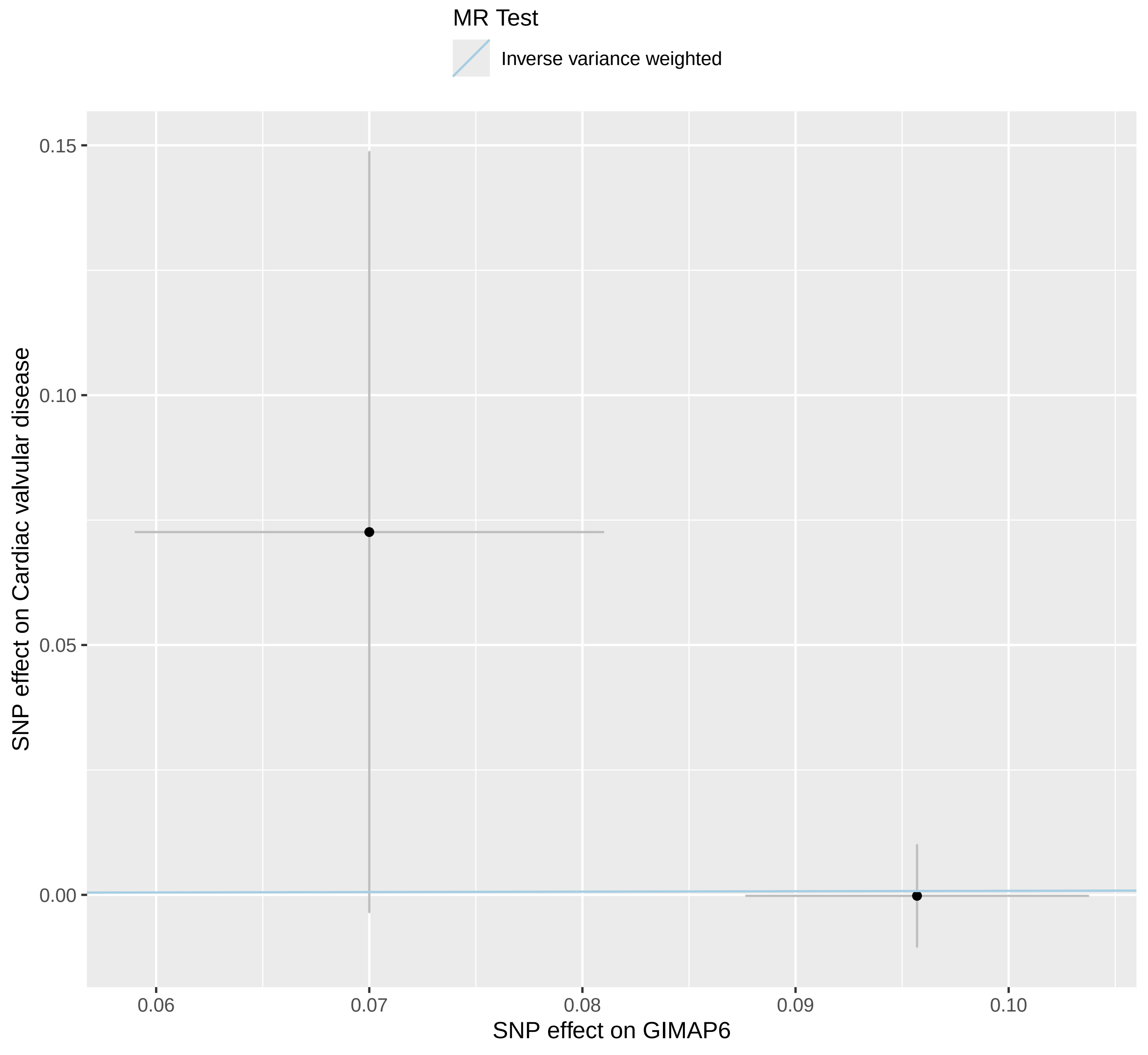

MR Test
Inverse variance weighted
0.15
0.10
0.05
0.00
0.06
0.07
0.08
0.09
0.10
SNP effect on Cardiac valvular disease
SNP effect on GIMAP6

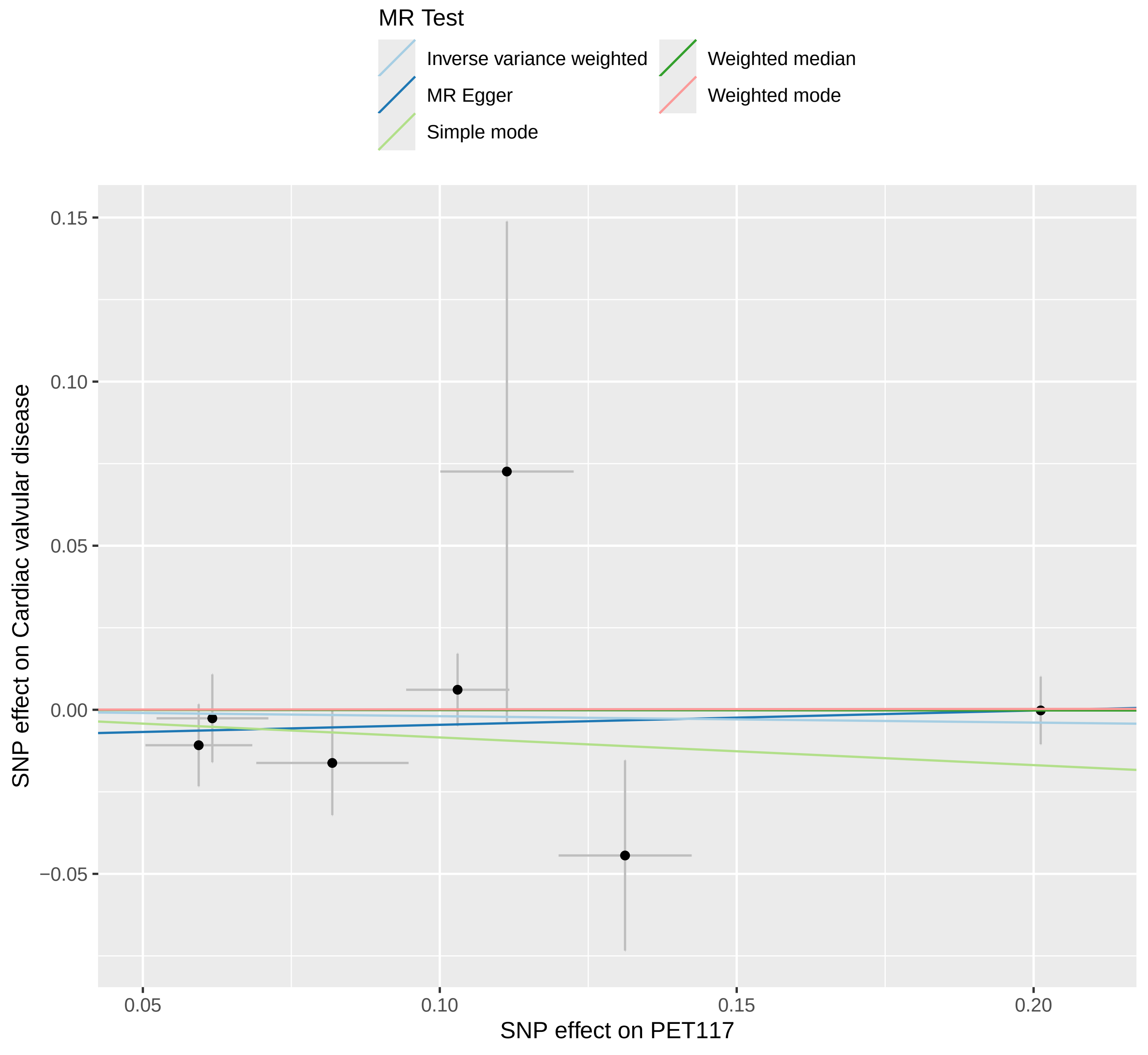
MR Test
Inverse variance weighted
MR Egger
Simple mode
Weighted median
Weighted mode
0.15
0.10
0.05
0.00
−0.05
SNP effect on Cardiac valvular disease
0.05
0.10
0.15
0.20
SNP effect on PET117

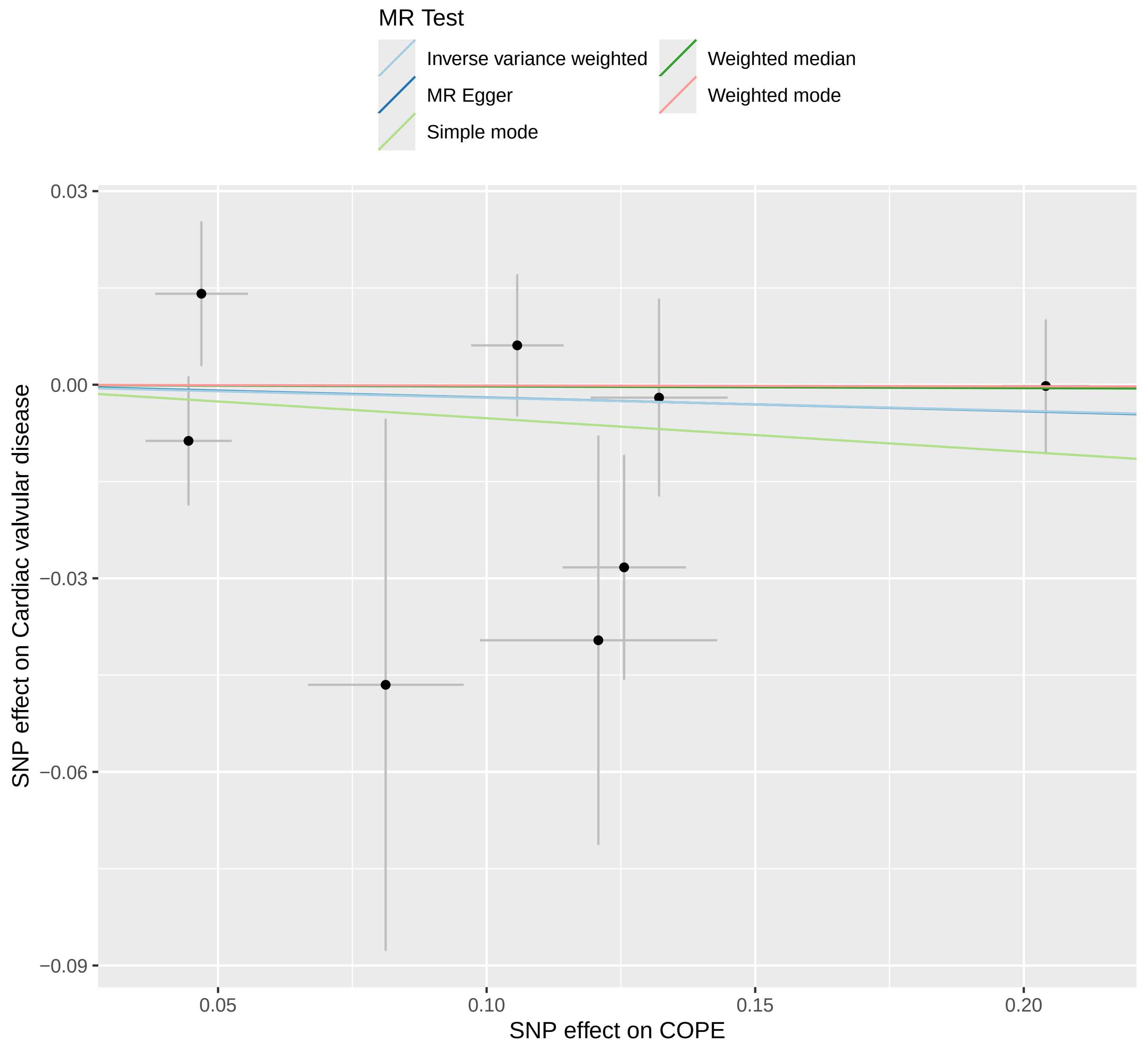

MR Test
Inverse variance weighted
MR Egger
Simple mode
Weighted median
Weighted mode
0.03
0.00
−0.03
−0.06
−0.09
SNP effect on Cardiac valvular disease
0.05
0.10
0.15
0.20
SNP effect on COPE

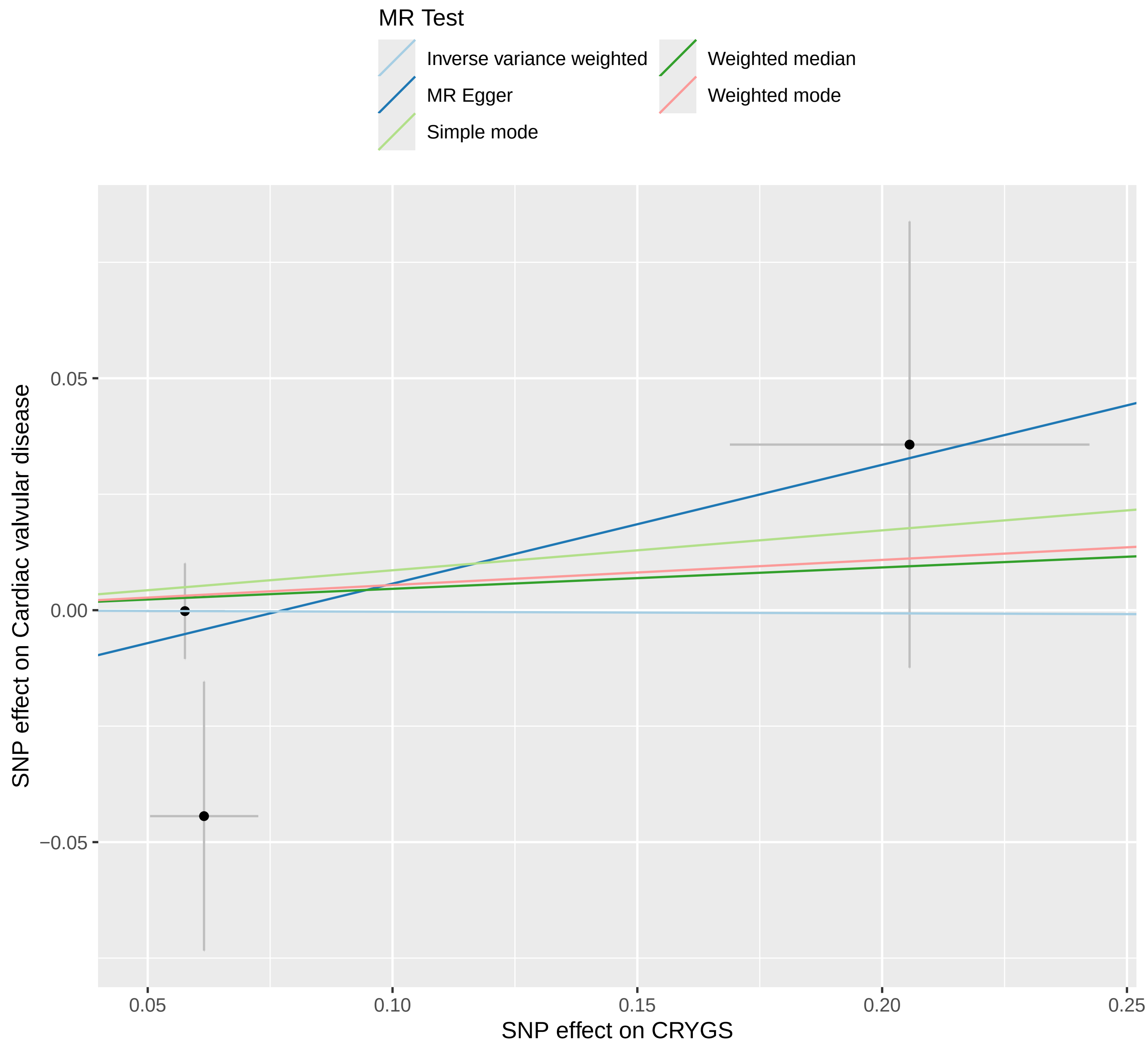
MR Test
Inverse variance weighted
MR Egger
Simple mode
Weighted median
Weighted mode
0.05
0.00
−0.05
SNP effect on Cardiac valvular disease
0.05
0.10
0.15
0.20
0.25
SNP effect on CRYGS

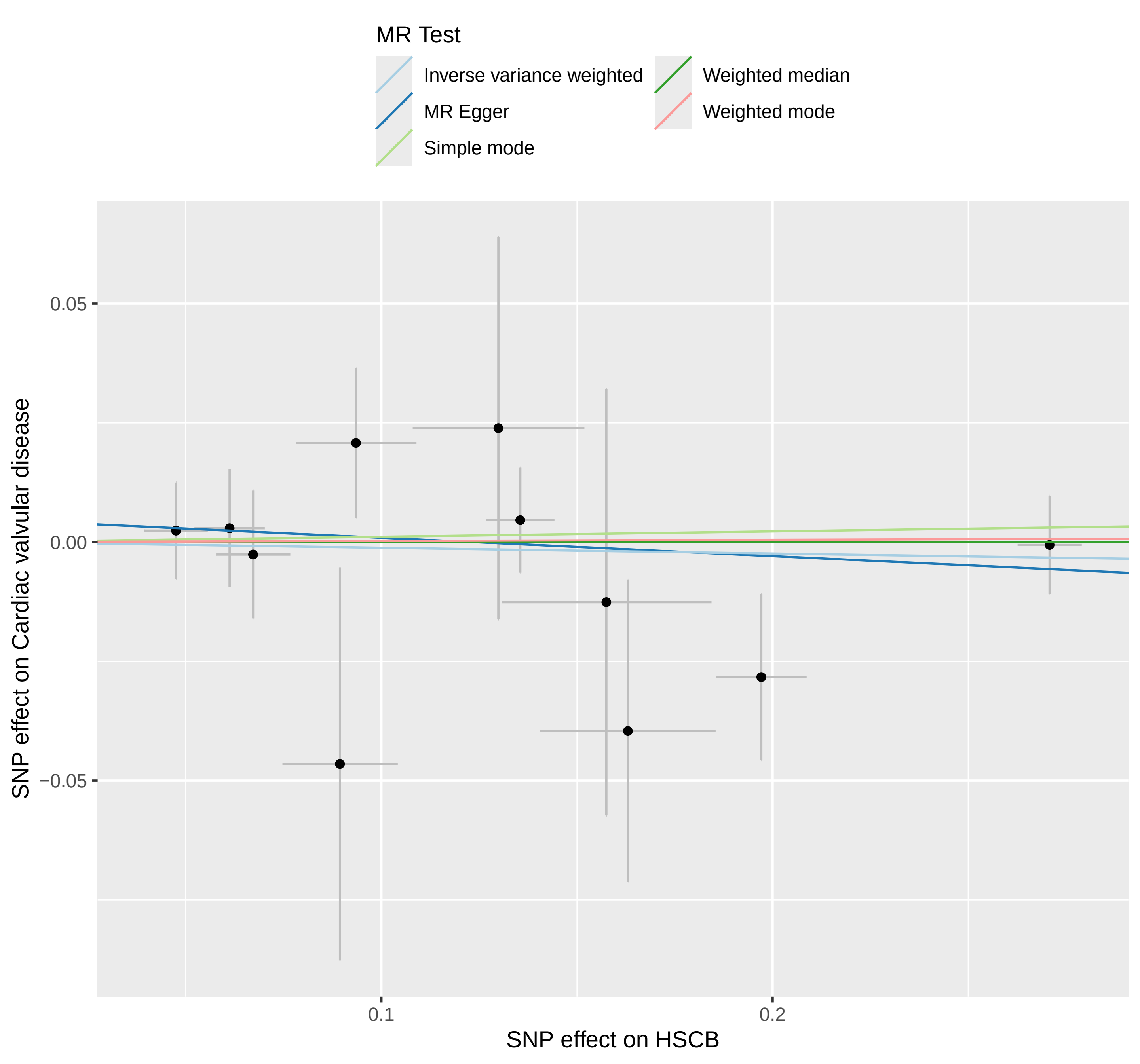
MR Test
Inverse variance weighted
MR Egger
Simple mode
Weighted median
Weighted mode
0.05
0.00
−0.05
0.1
0.2
SNP effect on Cardiac valvular disease
SNP effect on HSCB

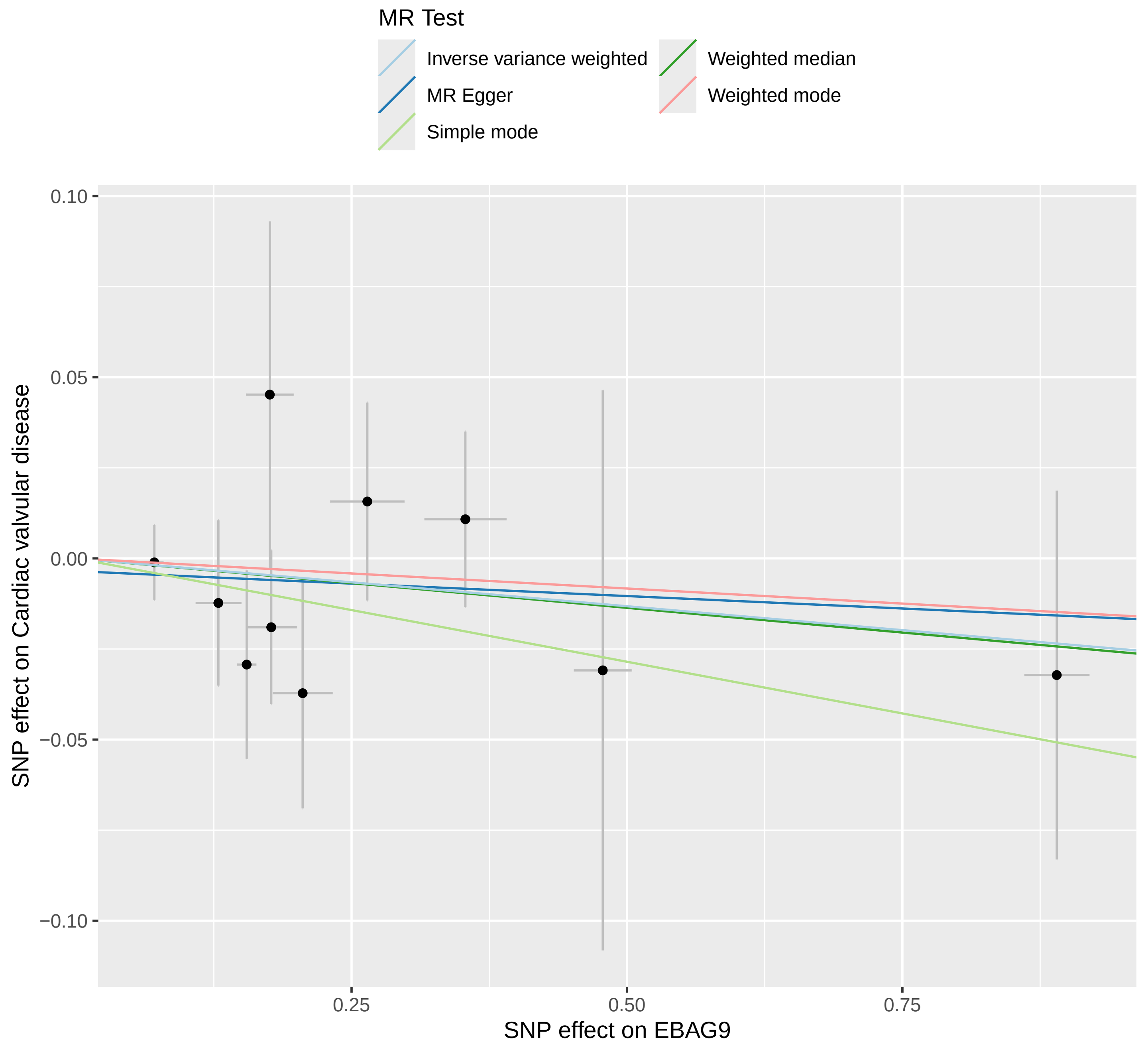
MR Test
Inverse variance weighted
MR Egger
Simple mode
Weighted median
Weighted mode
0.10
0.05
0.00
−0.05
−0.10
SNP effect on Cardiac valvular disease
0.25
0.50
0.75
SNP effect on EBAG9

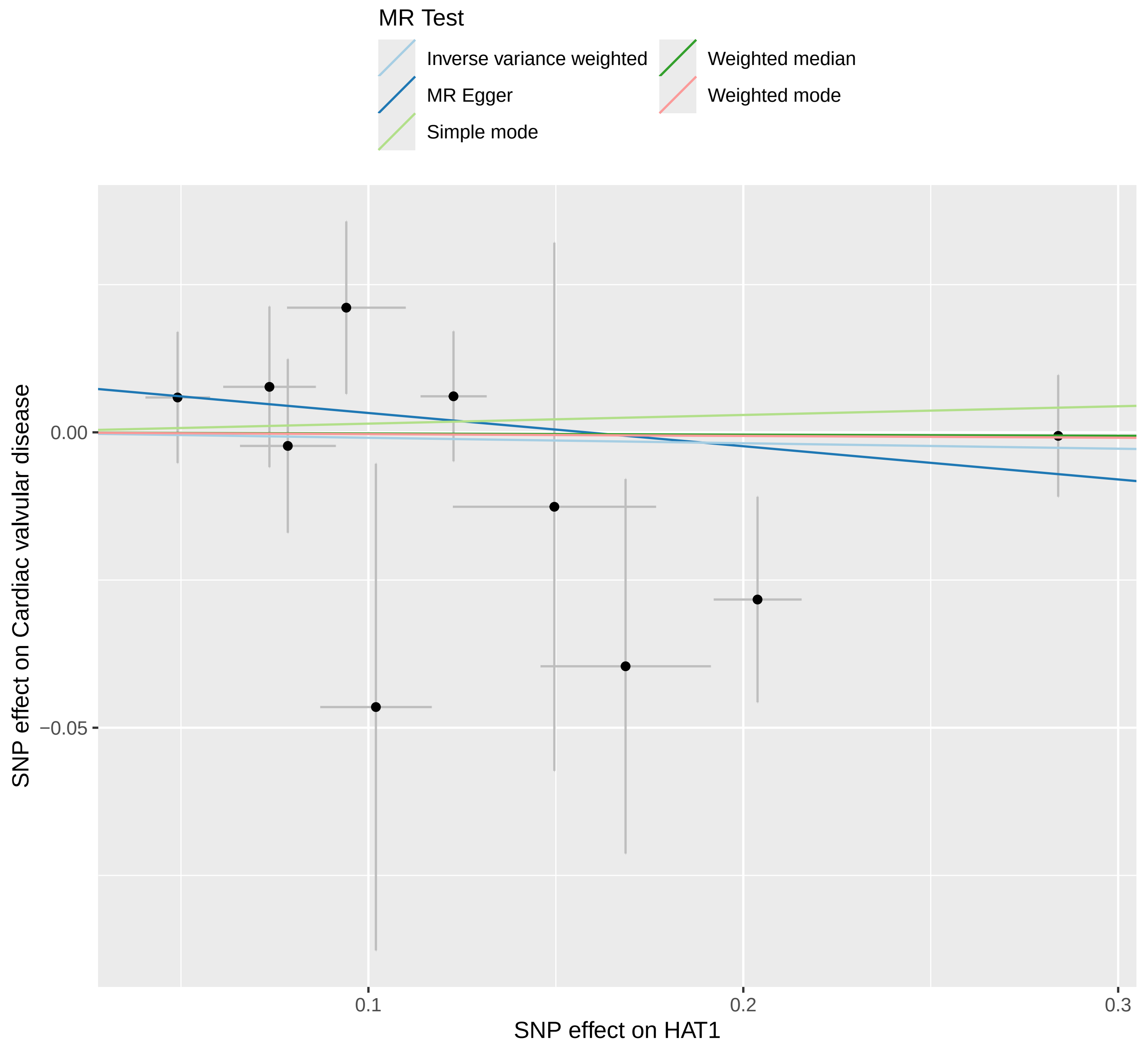
MR Test
Inverse variance weighted
MR Egger
Simple mode
Weighted median
Weighted mode
0.00
−0.05
0.1
0.2
0.3
SNP effect on HAT1
SNP effect on Cardiac valvular disease

Insufficient number of SNPs

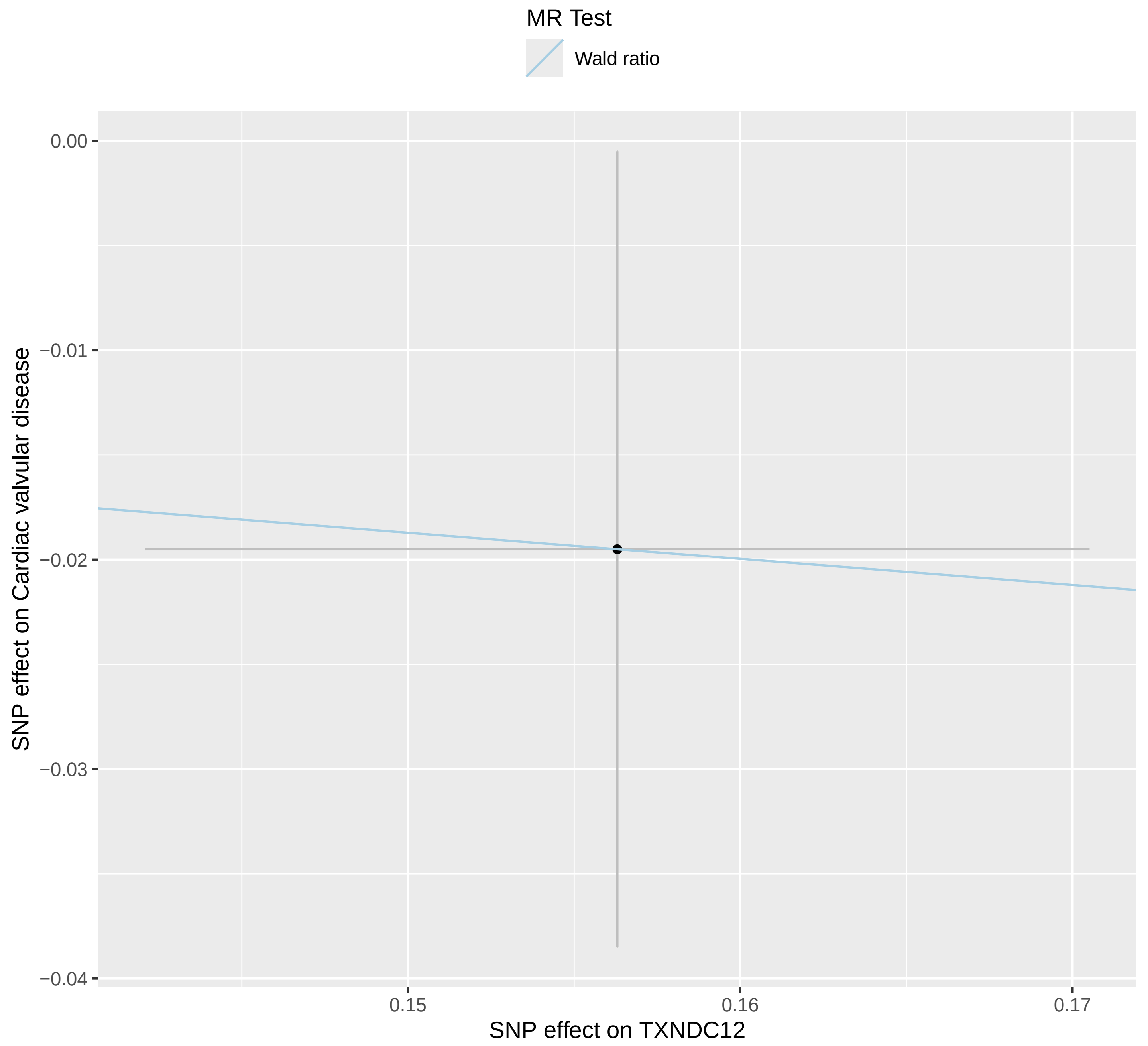
MR Test
Wald ratio
0.00
−0.01
−0.02
−0.03
−0.04
SNP effect on Cardiac valvular disease
0.15
0.16
0.17
SNP effect on TXNDC12

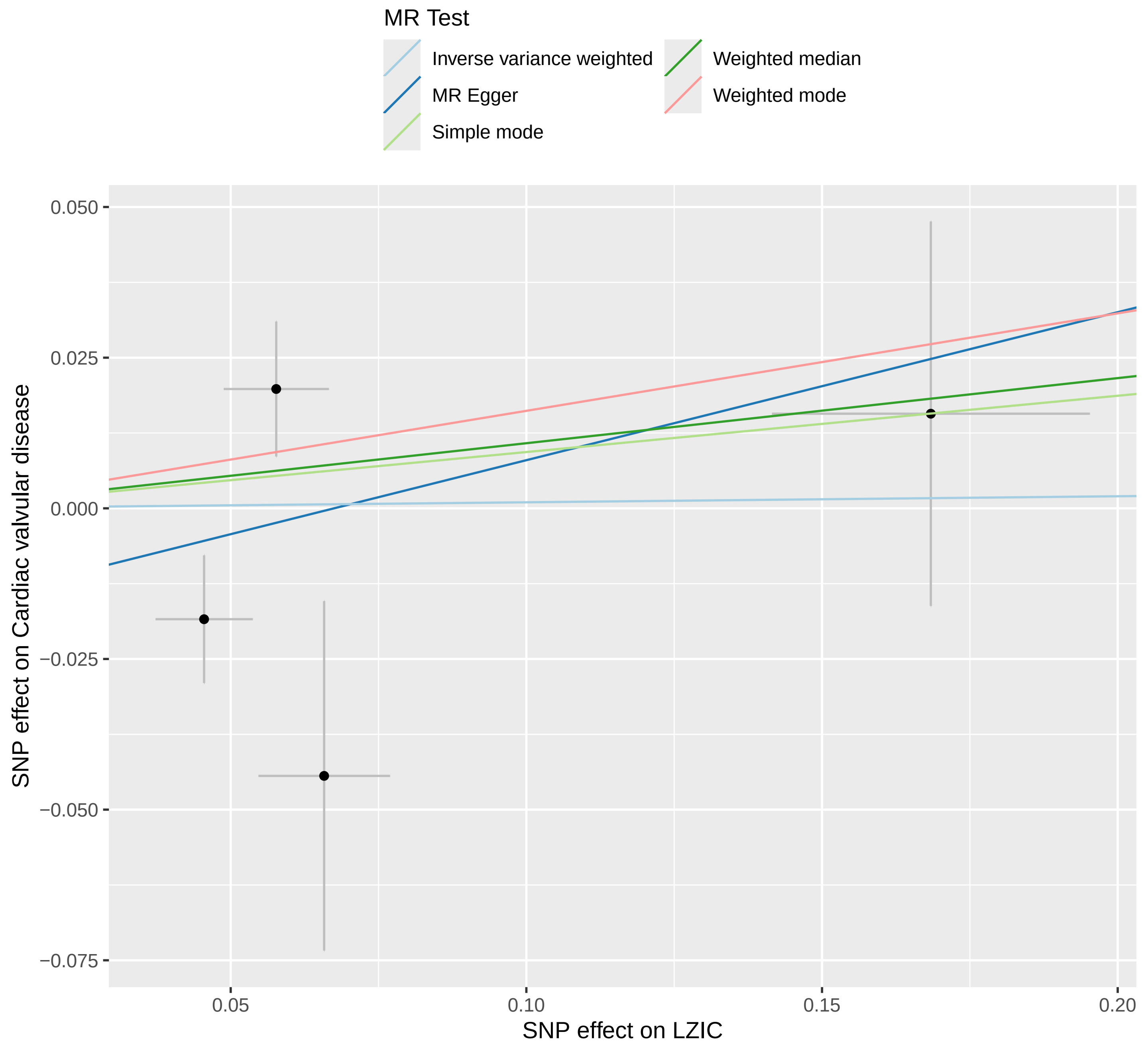

MR Test
Inverse variance weighted
MR Egger
Simple mode
Weighted median
Weighted mode
0.050
0.025
0.000
−0.025
−0.050
−0.075
SNP effect on Cardiac valvular disease
0.05
0.10
0.15
0.20
SNP effect on LZIC

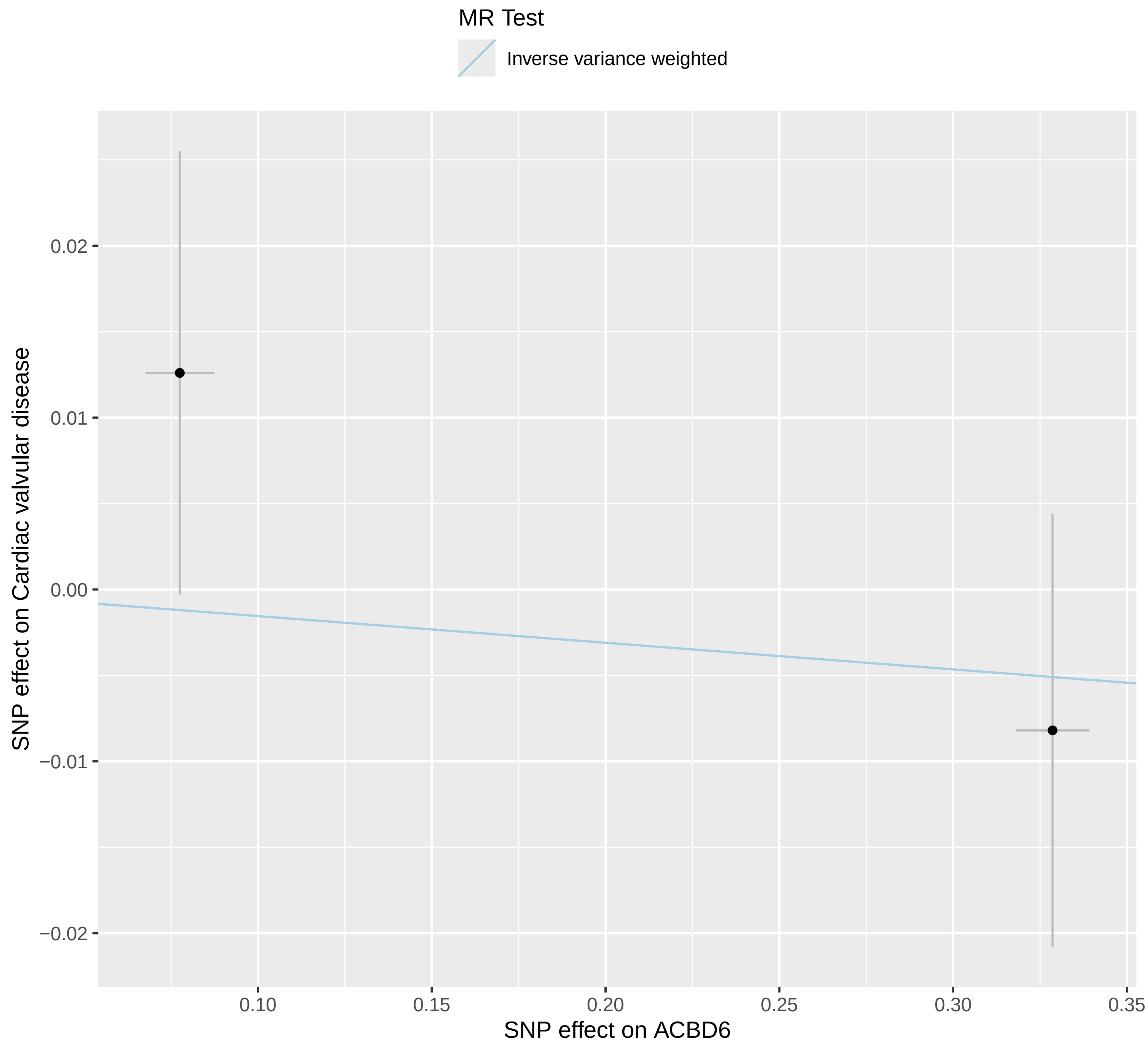

MR Test
Inverse variance weighted
SNP effect on Cardiac valvular disease
0.02
0.01
0.00
−0.01
−0.02
0.10
0.15
0.20
0.25
0.30
0.35
SNP effect on ACBD6

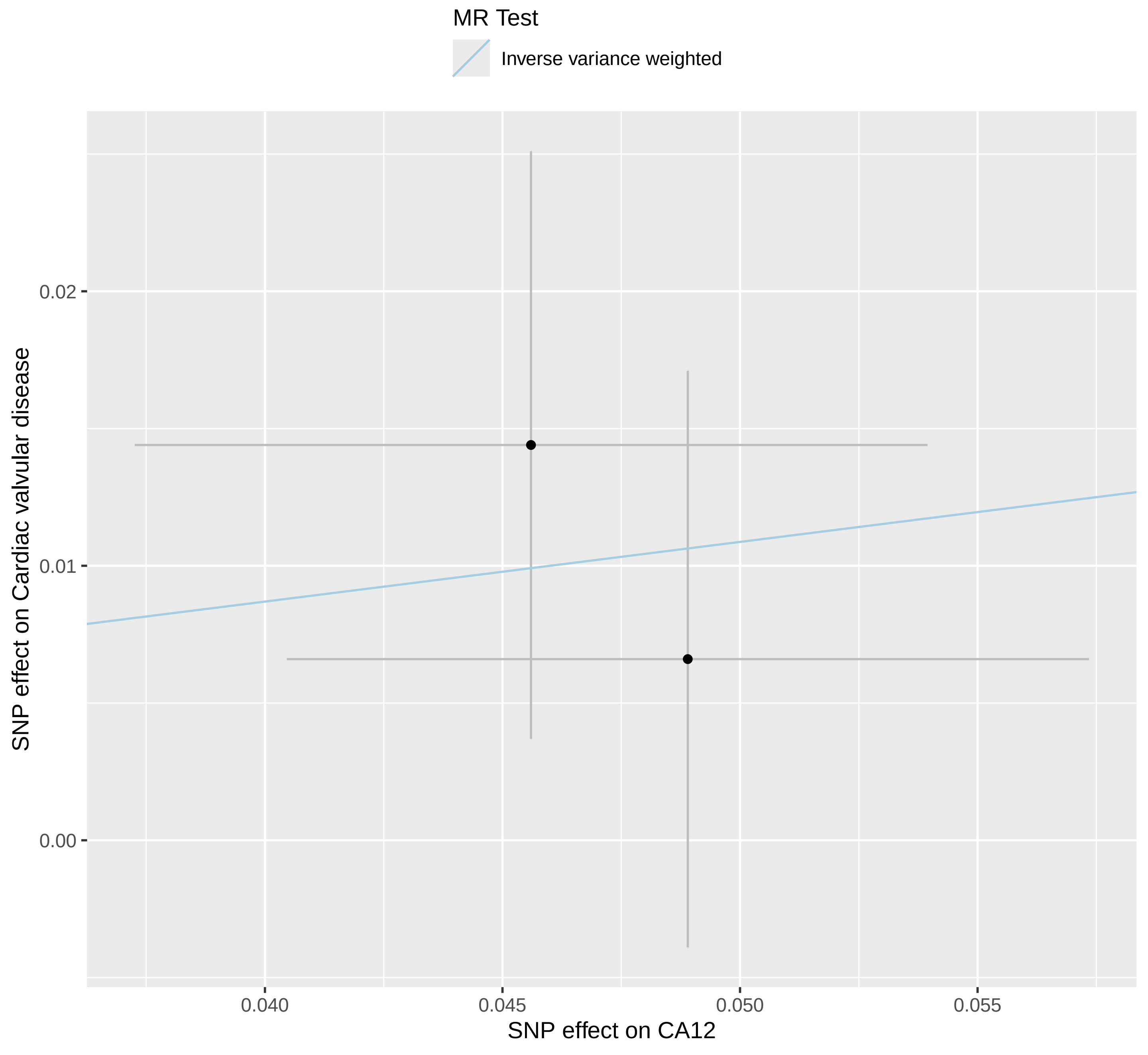
MR Test
Inverse variance weighted
SNP effect on Cardiac valvular disease
0.02
0.01
0.00
0.040
0.045
0.050
0.055
SNP effect on CA12

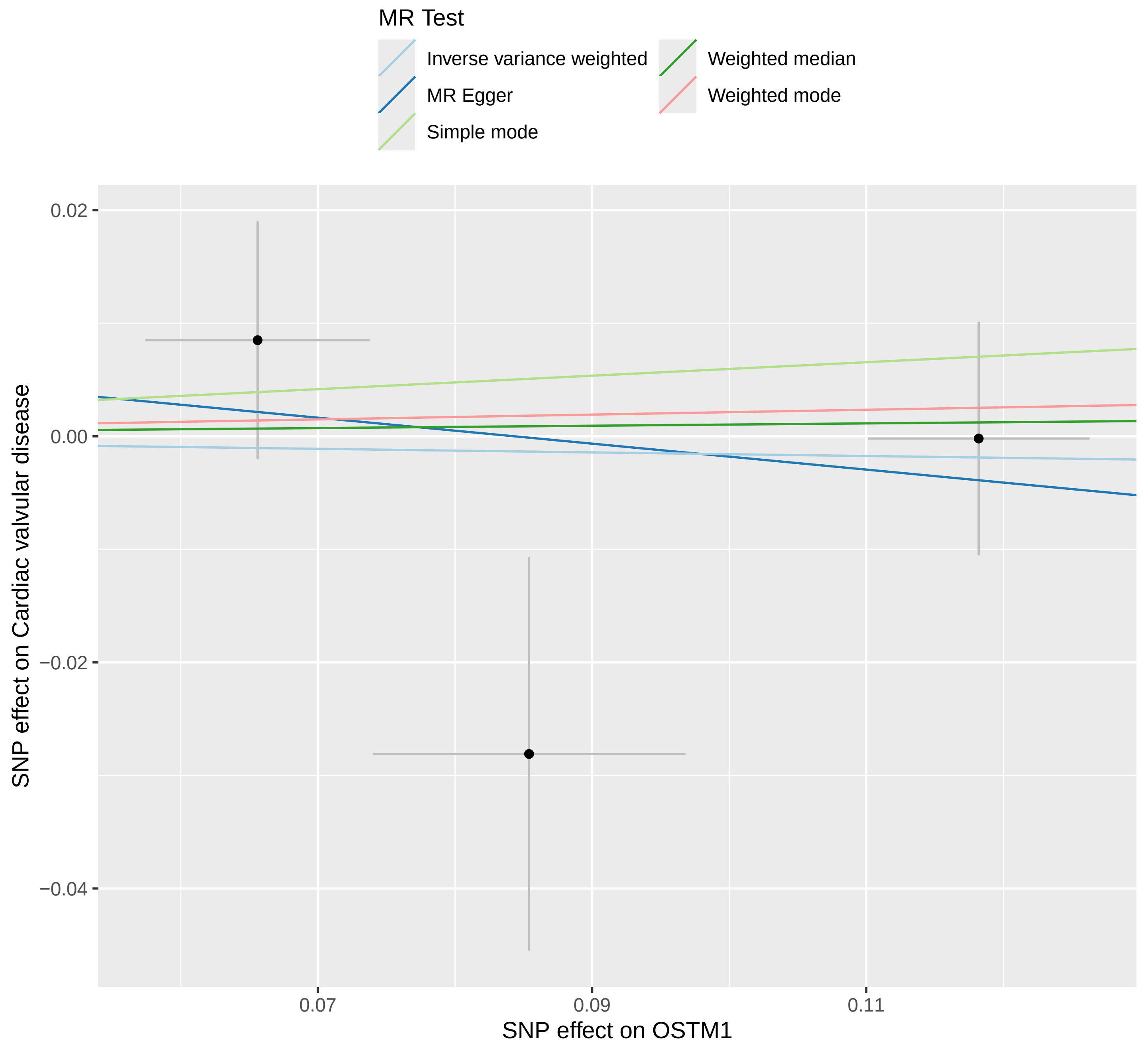
MR Test
Inverse variance weighted
MR Egger
Simple mode
Weighted median
Weighted mode
0.02
0.00
−0.02
−0.04
0.07
0.09
0.11
SNP effect on Cardiac valvular disease
SNP effect on OSTM1

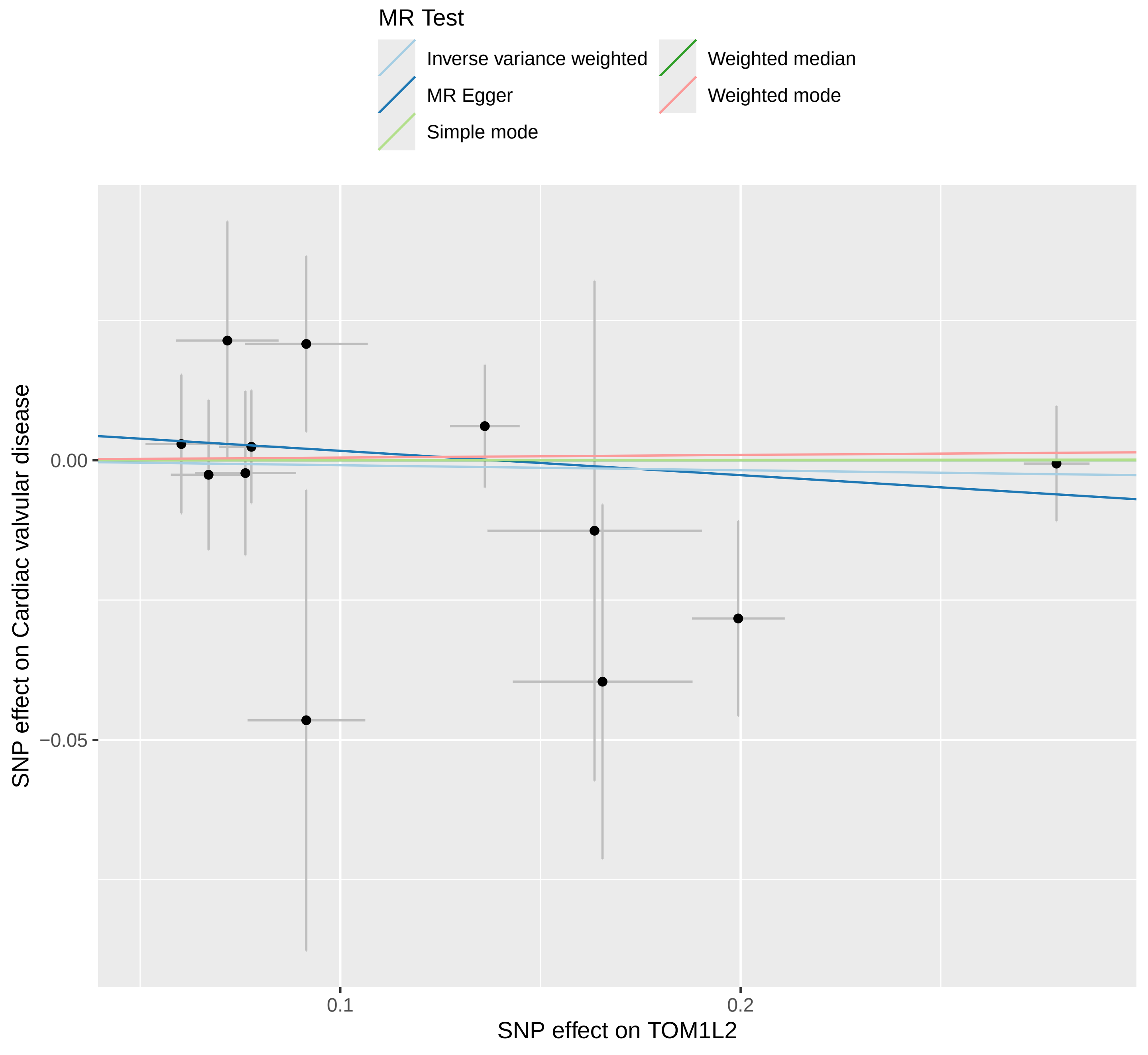
MR Test
Inverse variance weighted
MR Egger
Simple mode
Weighted median
Weighted mode
SNP effect on Cardiac valvular disease
0.00
−0.05
0.1
0.2
SNP effect on TOM1L2

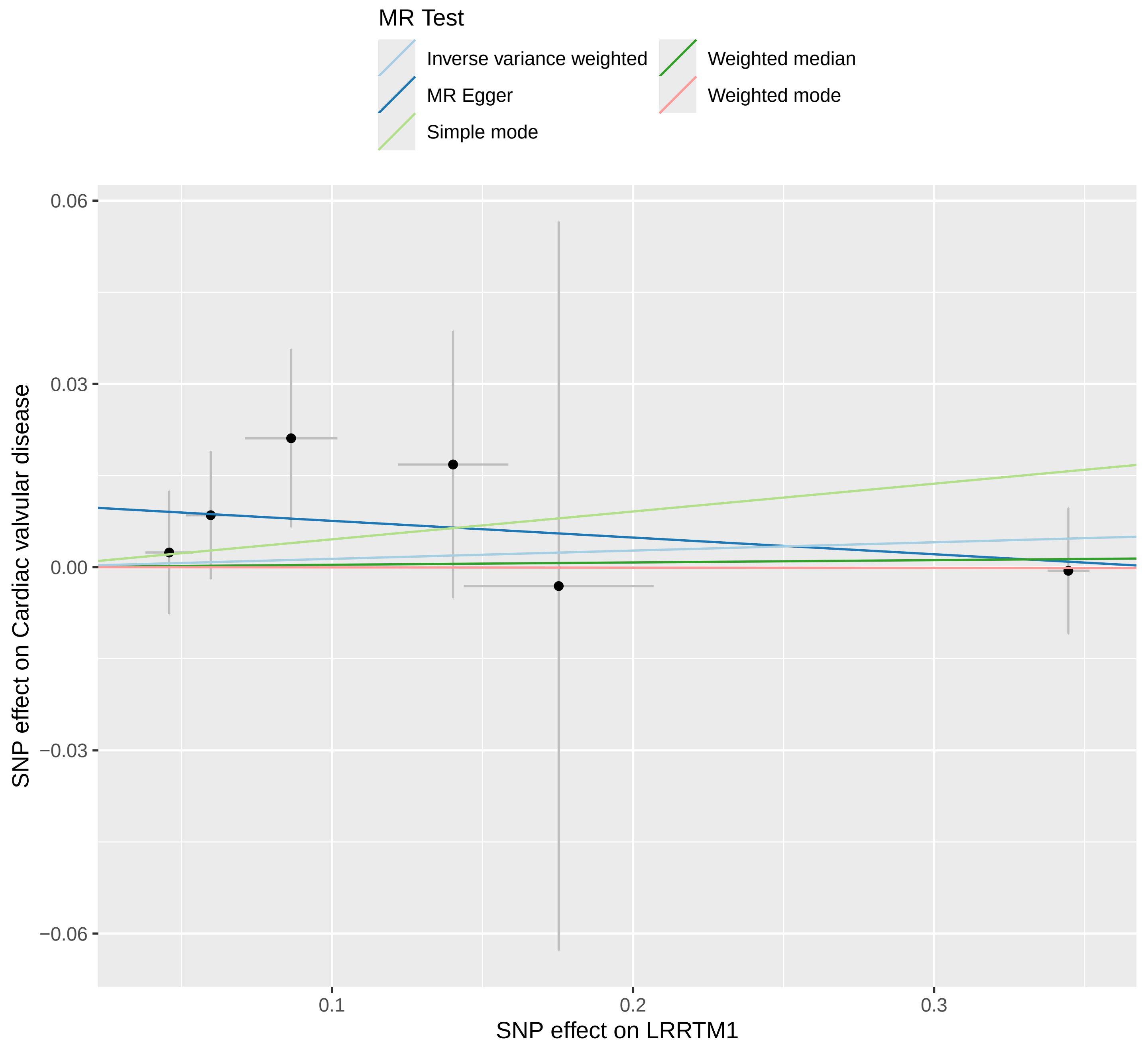
MR Test
Inverse variance weighted
MR Egger
Simple mode
Weighted median
Weighted mode
0.06
0.03
0.00
−0.03
−0.06
0.1
0.2
0.3
SNP effect on Cardiac valvular disease
SNP effect on LRRTM1

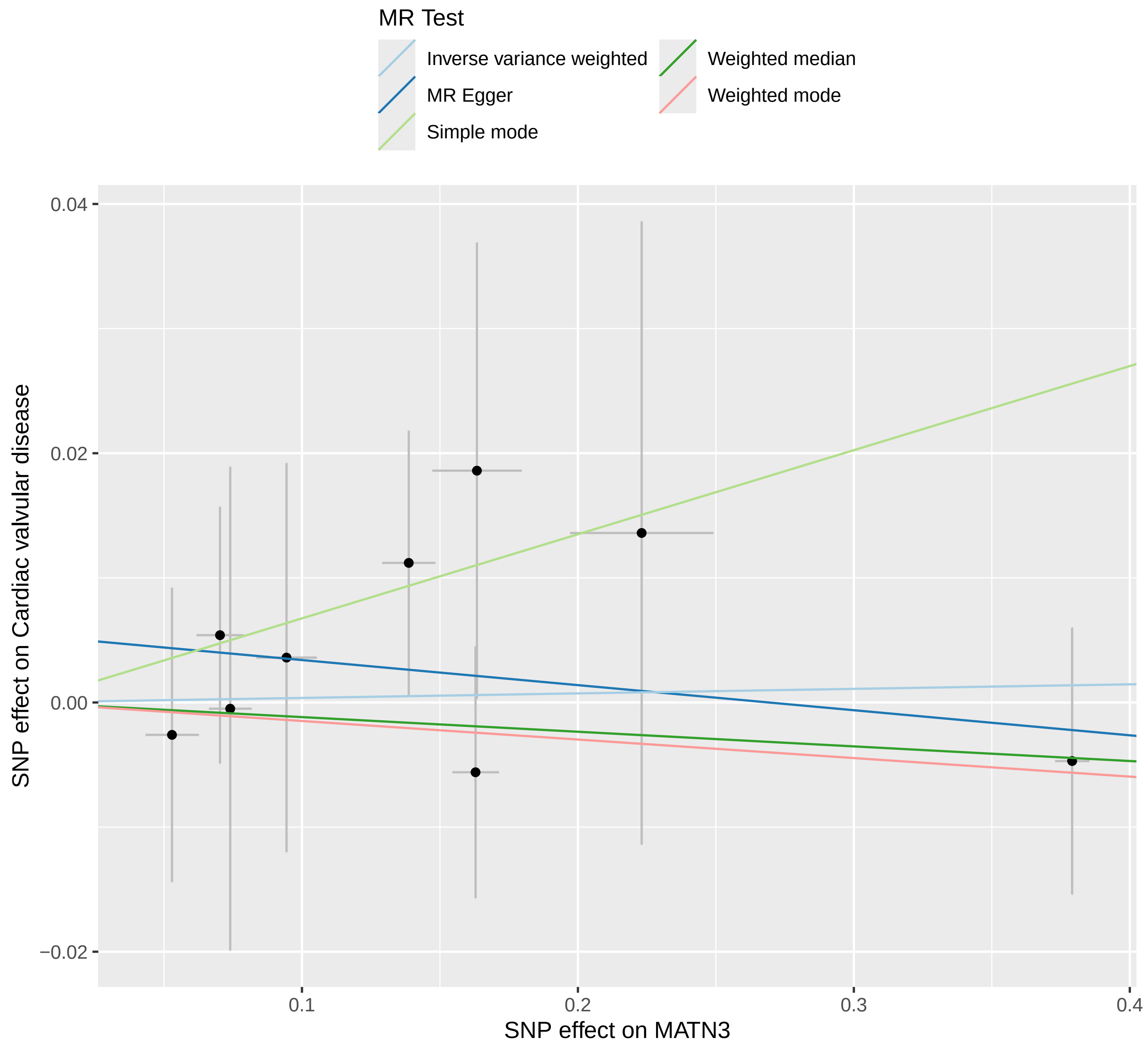
MR Test
Inverse variance weighted
MR Egger
Simple mode
Weighted median
Weighted mode
0.04
0.02
0.00
−0.02
0.1
0.2
0.3
0.4
SNP effect on Cardiac valvular disease
SNP effect on MATN3

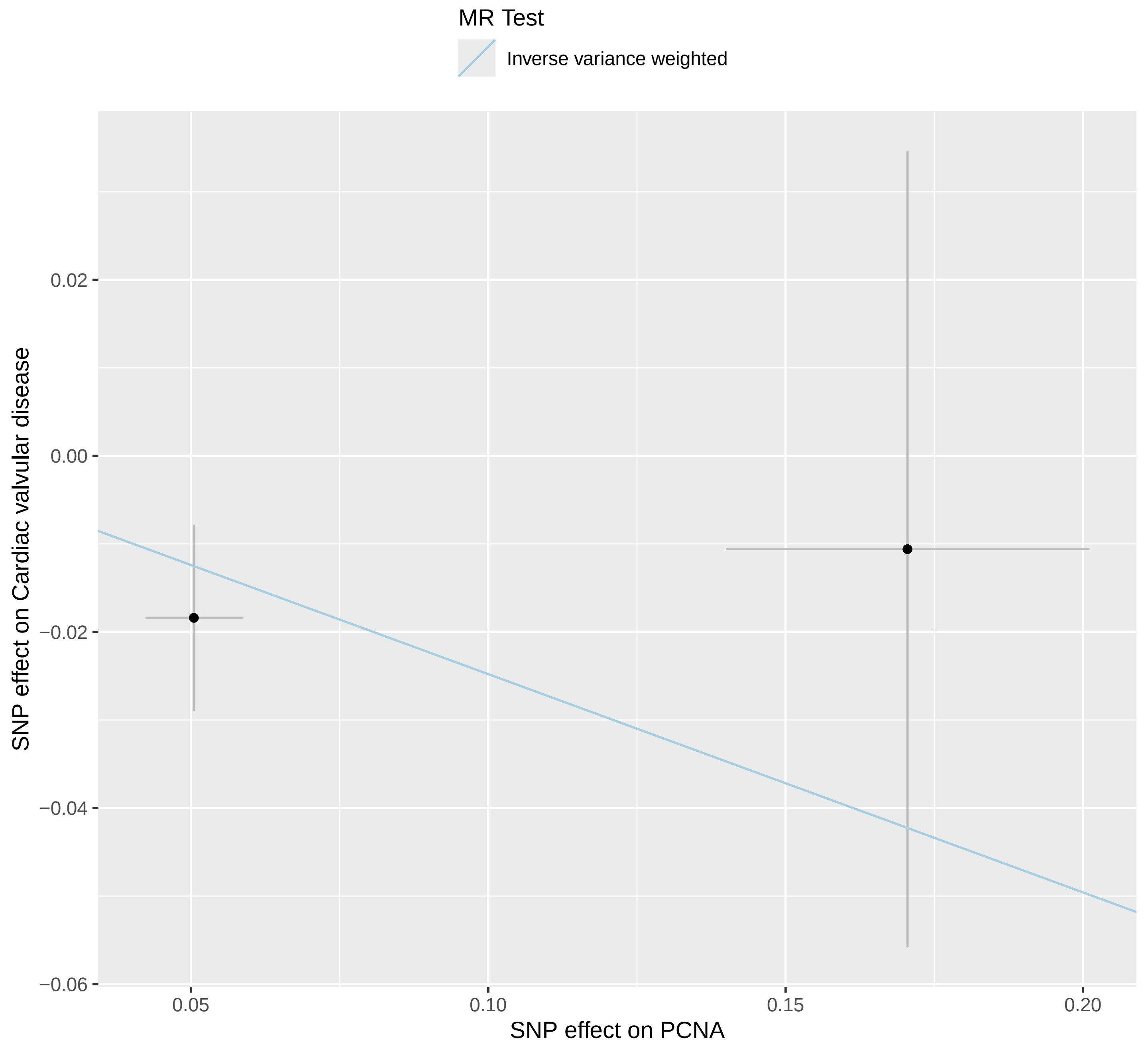
MR Test
Inverse variance weighted
SNP effect on Cardiac valvular disease
0.02
0.00
−0.02
−0.04
−0.06
0.05
0.10
0.15
0.20
SNP effect on PCNA

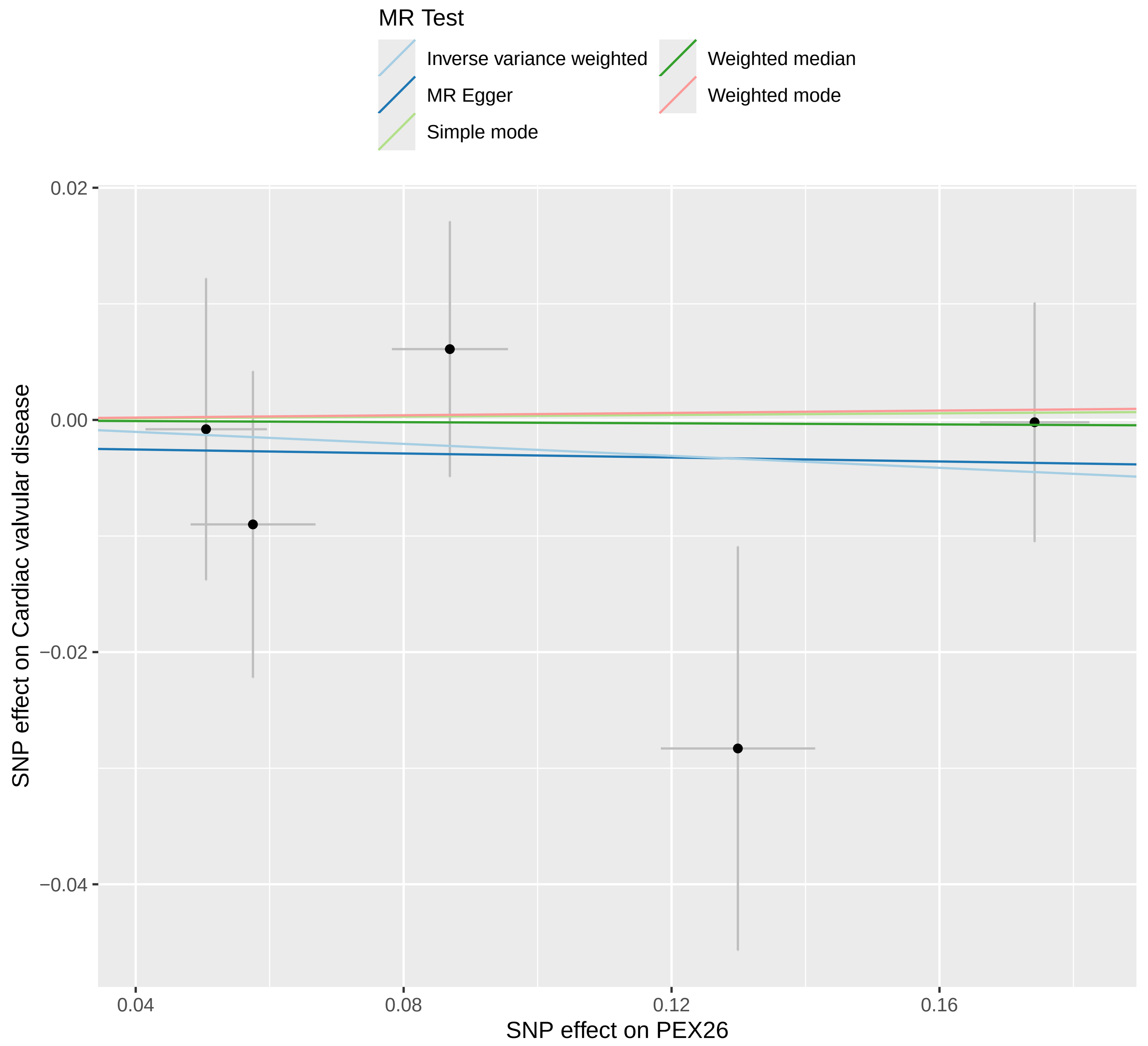
MR Test
Inverse variance weighted
MR Egger
Simple mode
Weighted median
Weighted mode
0.02
0.00
−0.02
−0.04
SNP effect on Cardiac valvular disease
0.04
0.08
0.12
0.16
SNP effect on PEX26

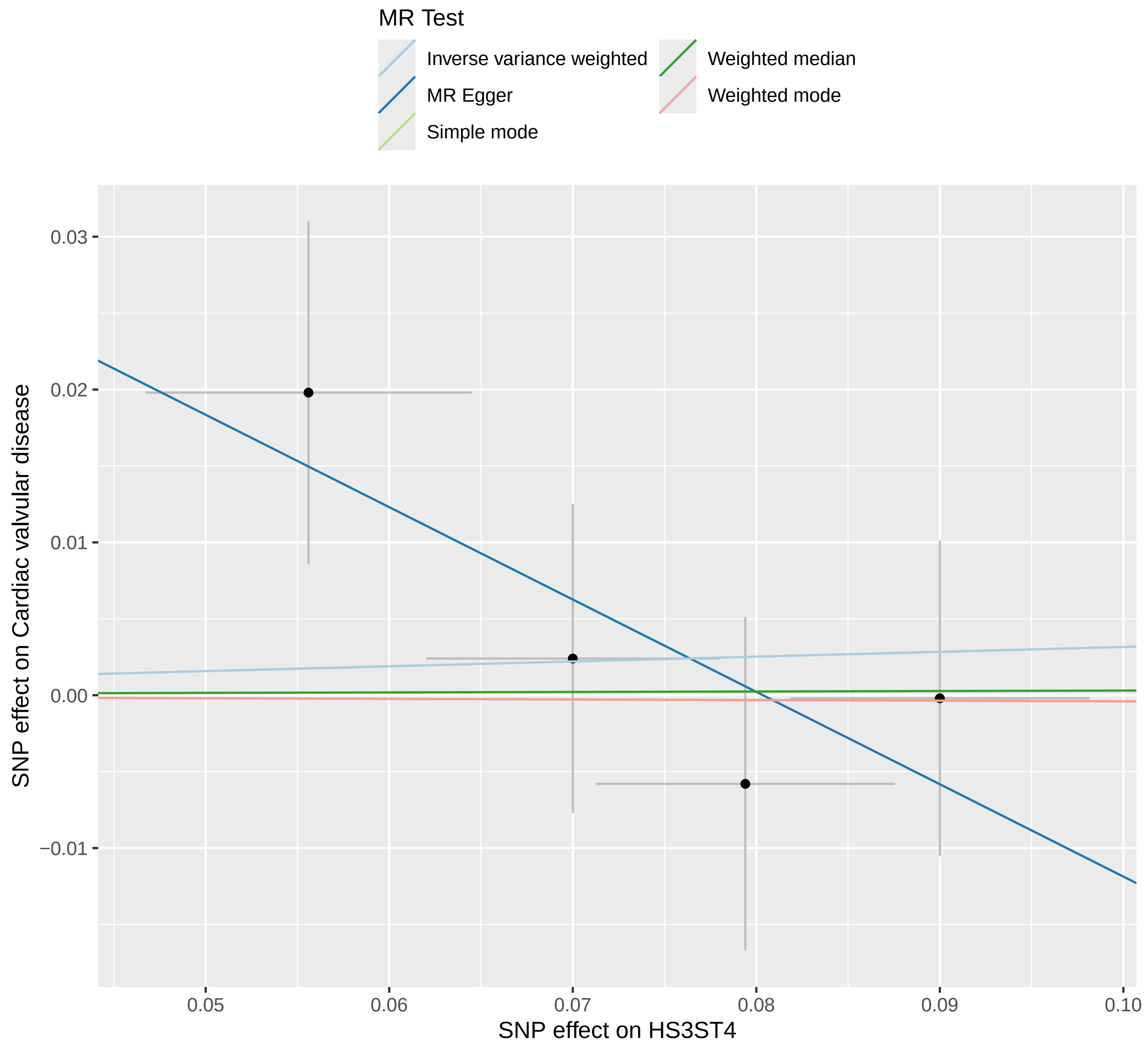

MR Test
Inverse variance weighted
MR Egger
Simple mode
Weighted median
Weighted mode
0.03
0.02
0.01
0.00
−0.01
0.05
0.06
0.07
0.08
0.09
0.10
SNP effect on Cardiac valvular disease
SNP effect on HS3ST4

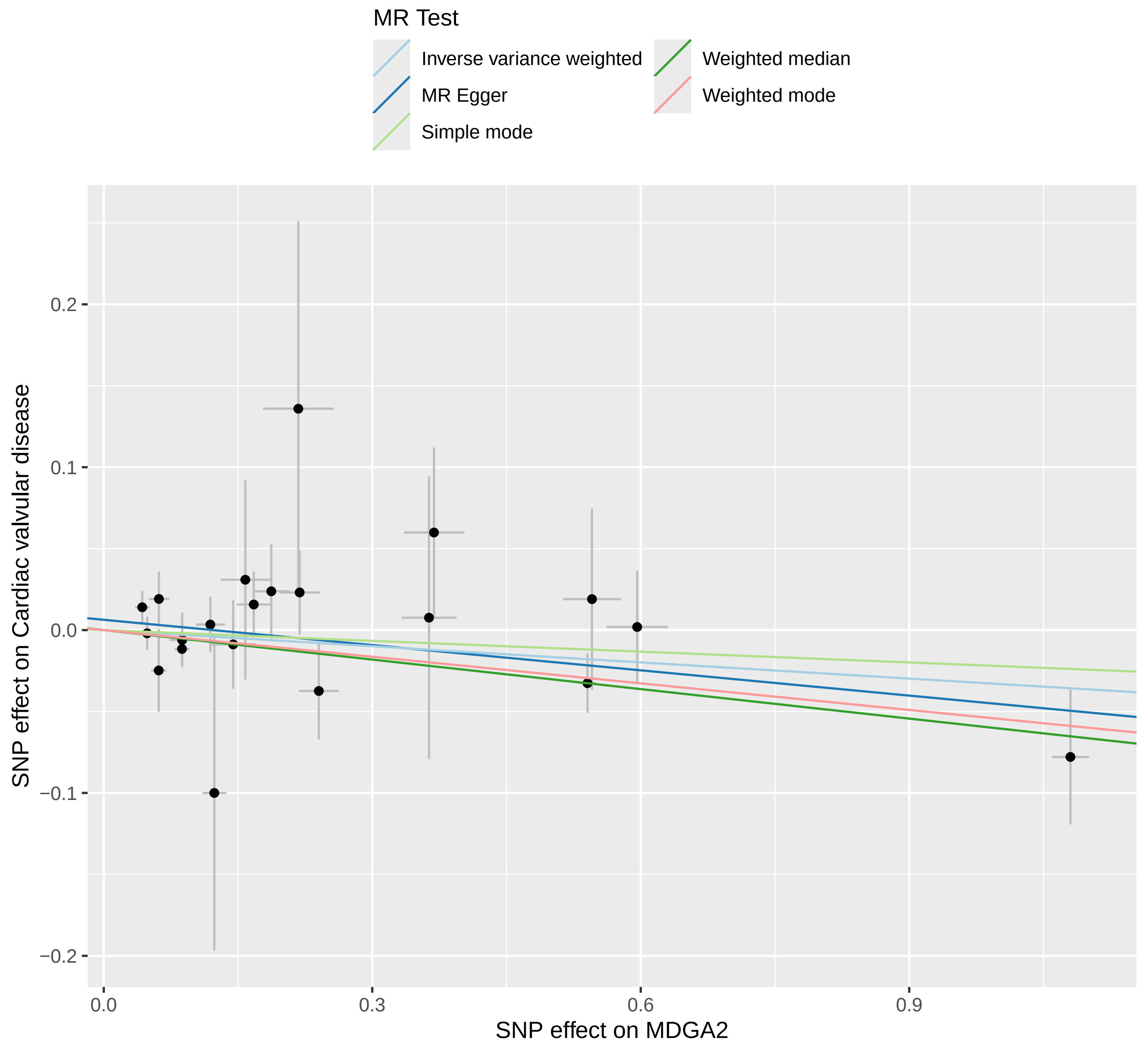

MR Test
Inverse variance weighted
MR Egger
Simple mode
Weighted median
Weighted mode
0.2
0.1
0.0
−0.1
−0.2
SNP effect on Cardiac valvular disease
0.0
0.3
0.6
0.9
SNP effect on MDGA2

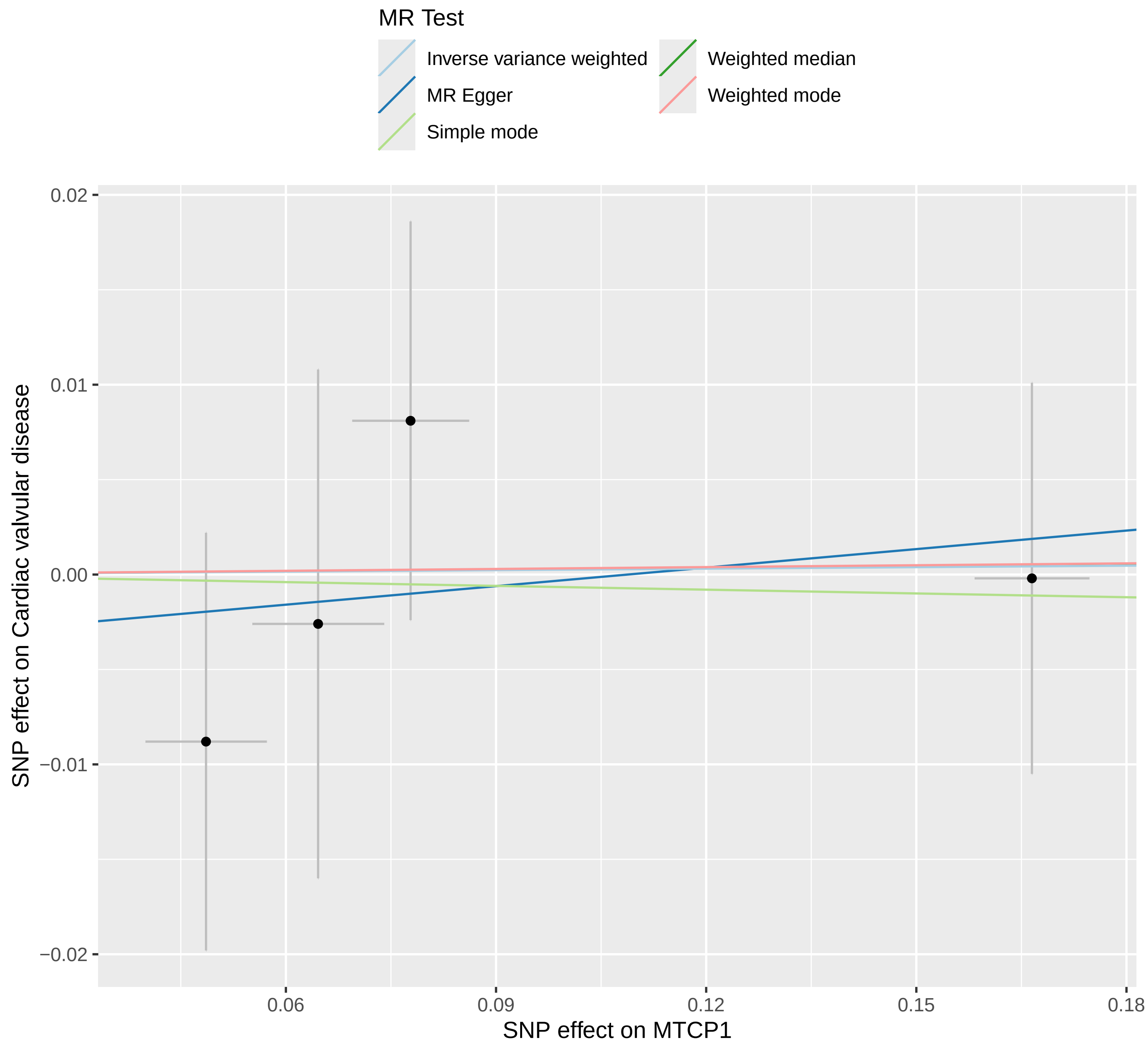

MR Test
Inverse variance weighted
MR Egger
Simple mode
Weighted median
Weighted mode
0.02
0.01
0.00
−0.01
−0.02
0.06
0.09
0.12
0.15
0.18
SNP effect on Cardiac valvular disease
SNP effect on MTCP1

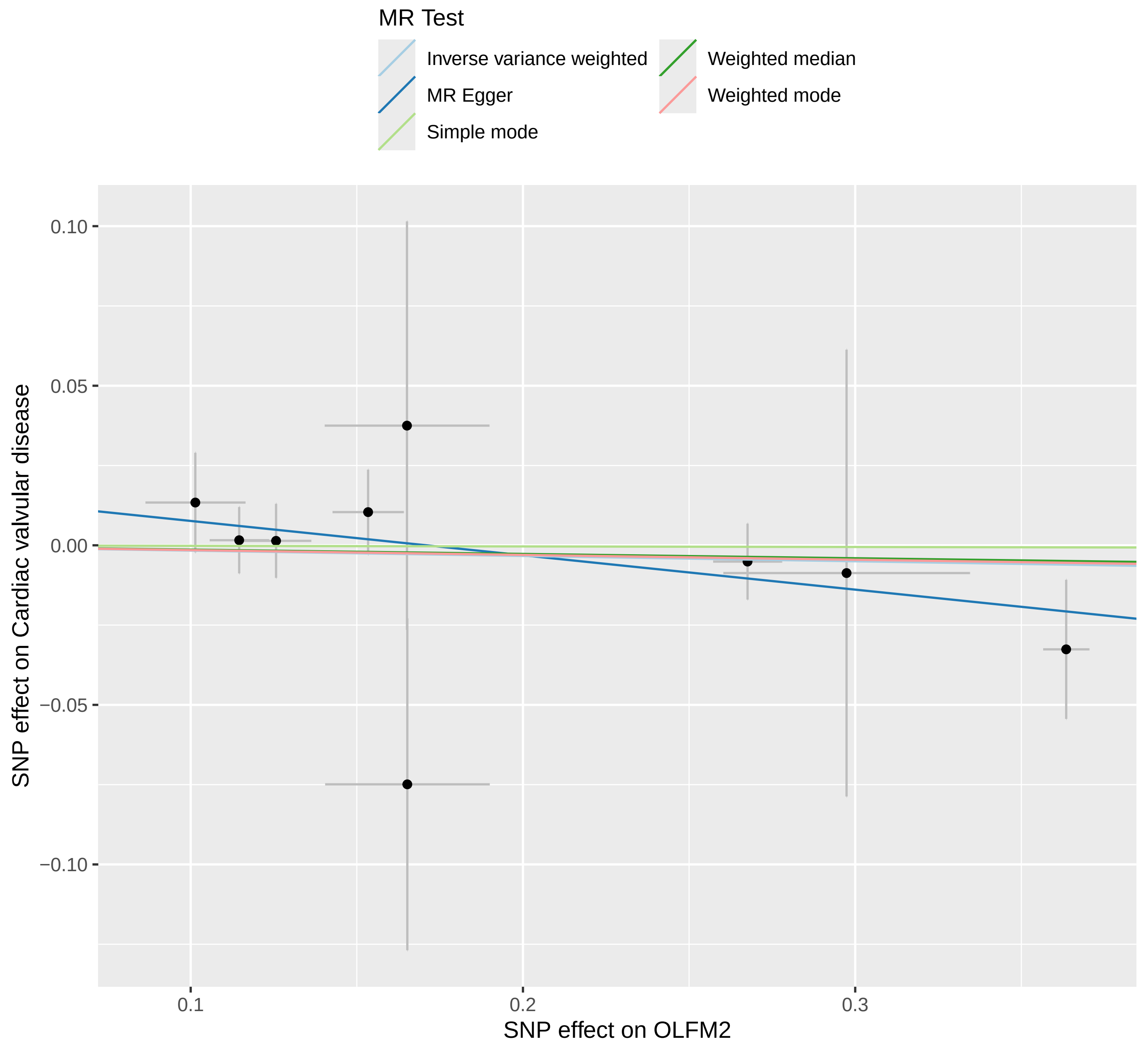

MR Test
Inverse variance weighted
MR Egger
Simple mode
Weighted median
Weighted mode
0.10
0.05
0.00
−0.05
−0.10
0.1
0.2
0.3
SNP effect on Cardiac valvular disease
SNP effect on OLFM2

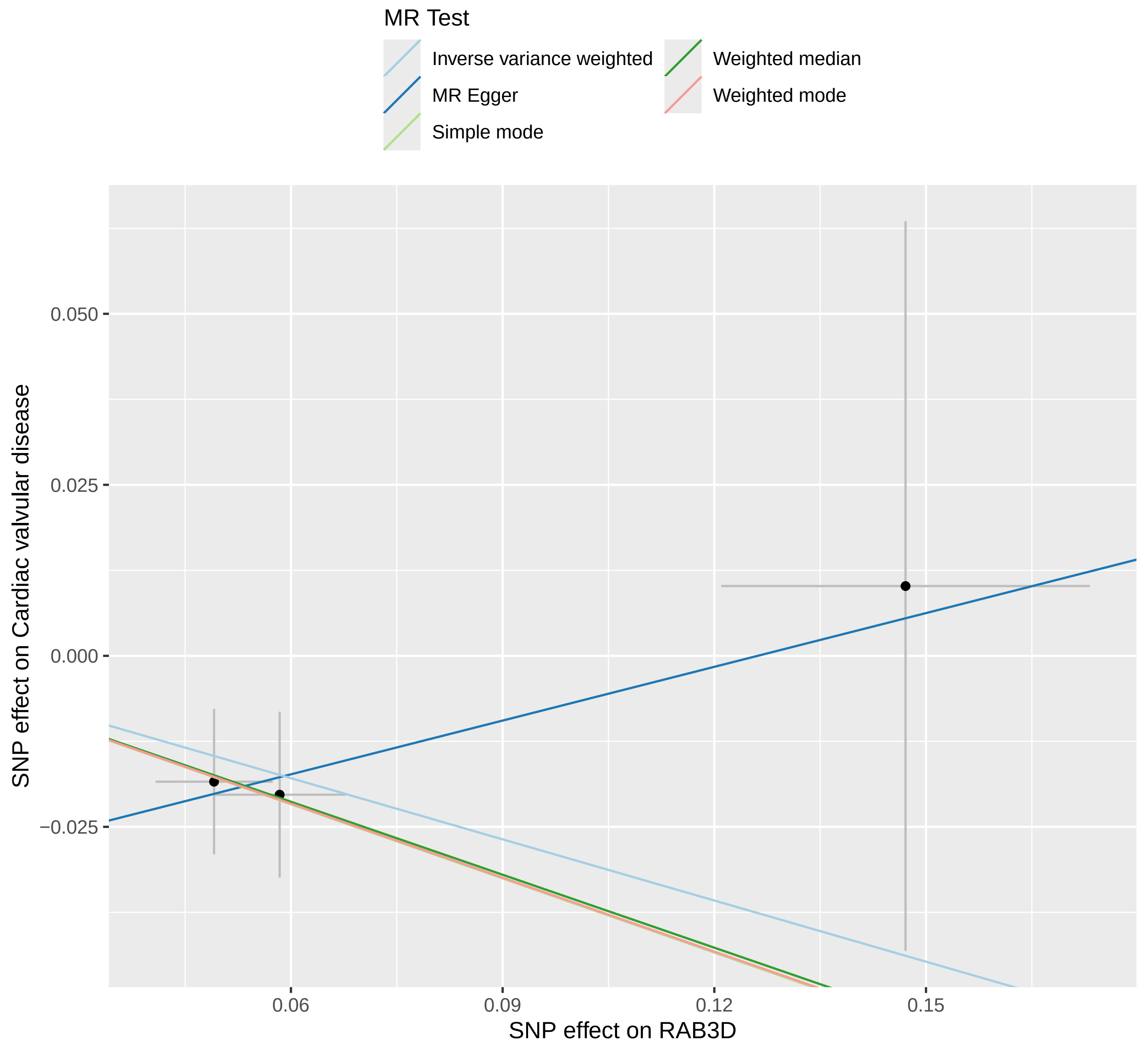
MR Test
Inverse variance weighted
MR Egger
Simple mode
Weighted median
Weighted mode
0.050
0.025
0.000
−0.025
SNP effect on Cardiac valvular disease
0.06
0.09
0.12
0.15
SNP effect on RAB3D

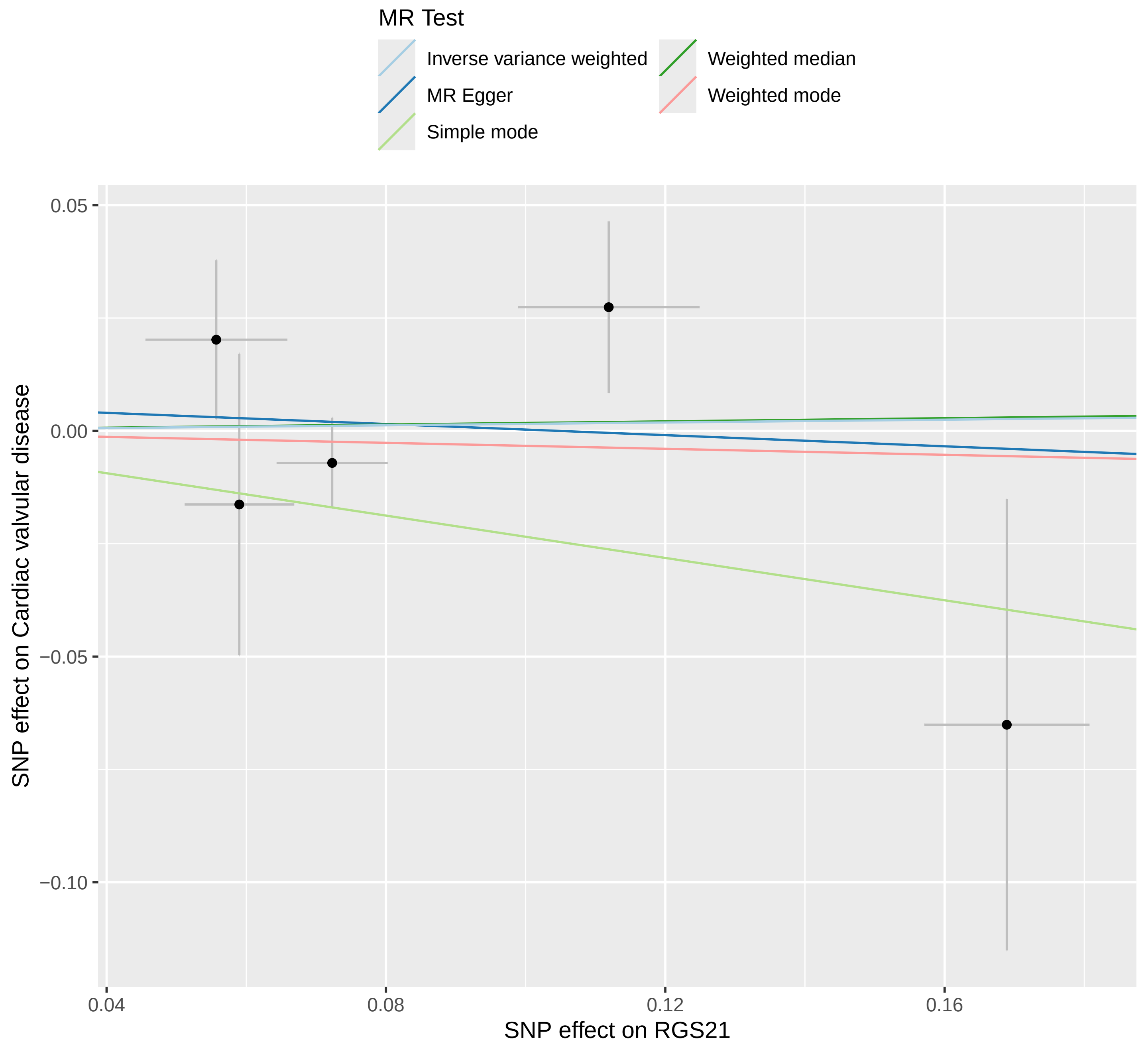
MR Test
Inverse variance weighted
MR Egger
Simple mode
Weighted median
Weighted mode
0.05
0.00
−0.05
−0.10
0.04
0.08
0.12
0.16
SNP effect on RGS21
SNP effect on Cardiac valvular disease

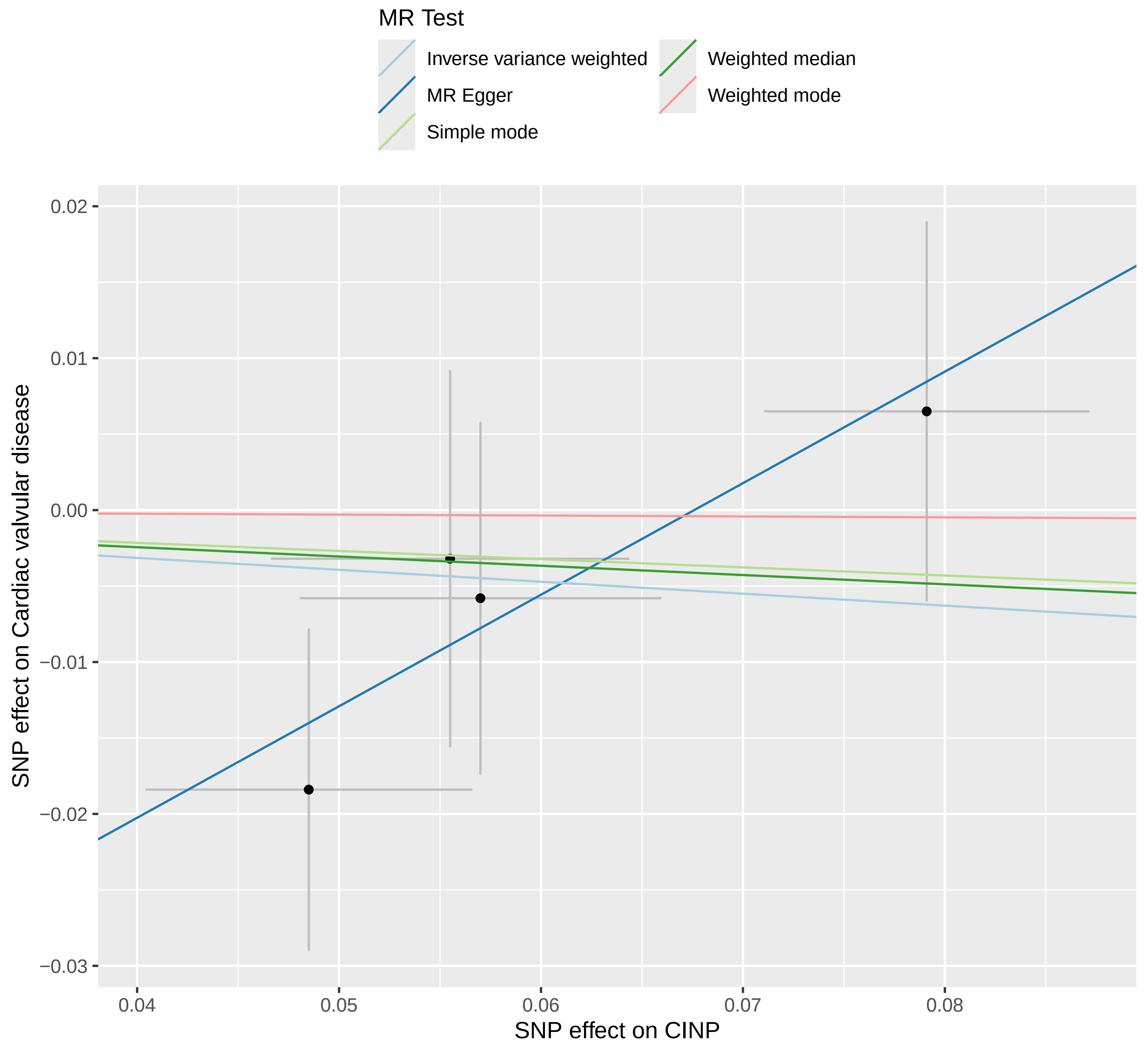
MR Test
Inverse variance weighted
MR Egger
Simple mode
Weighted median
Weighted mode
0.02
0.01
0.00
−0.01
−0.02
−0.03
SNP effect on Cardiac valvular disease
0.04
0.05
0.06
0.07
0.08
SNP effect on CINP

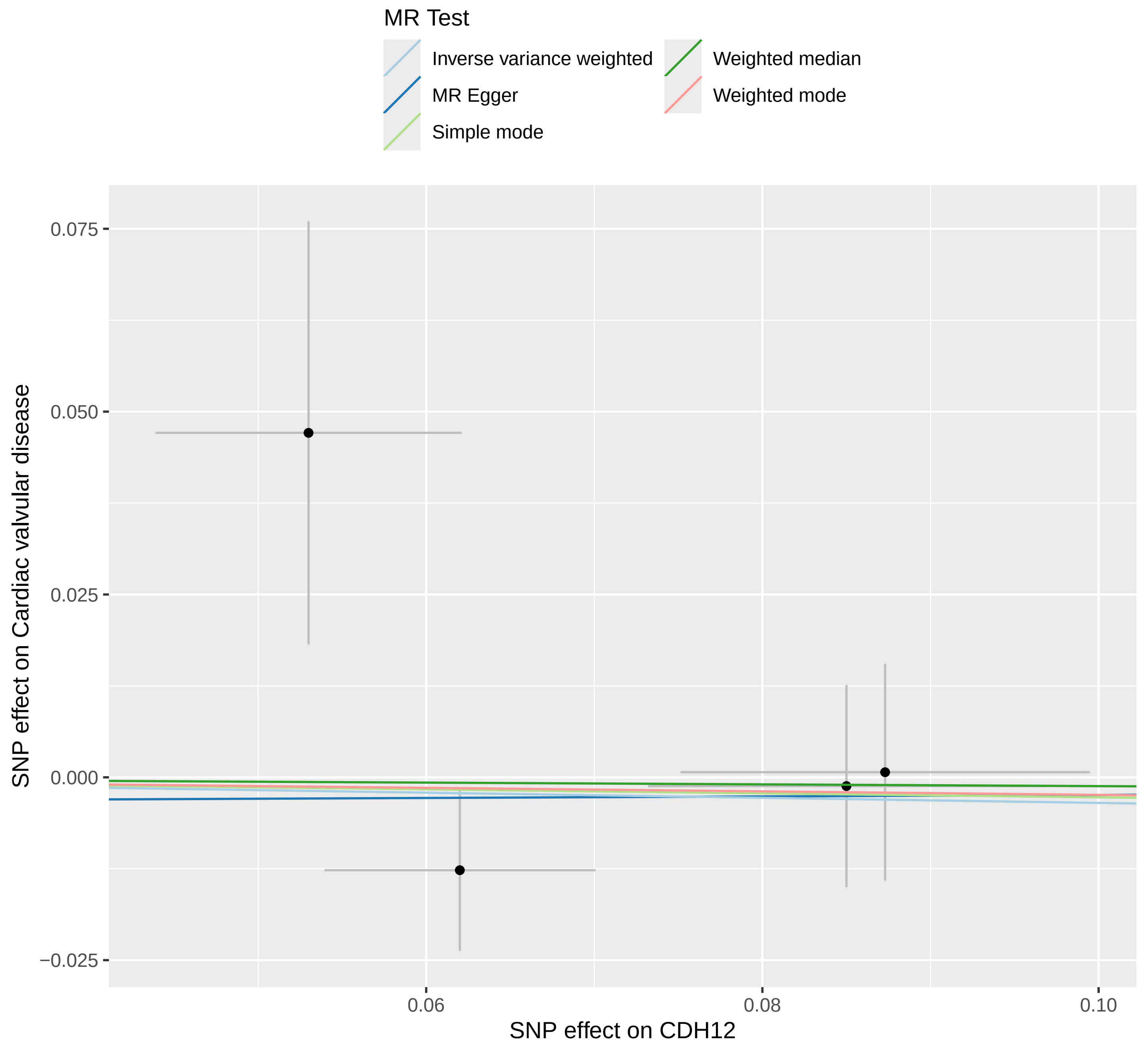

MR Test
Inverse variance weighted
MR Egger
Simple mode
Weighted median
Weighted mode
0.075
0.050
0.025
0.000
−0.025
0.06
0.08
0.10
SNP effect on CDH12
SNP effect on Cardiac valvular disease

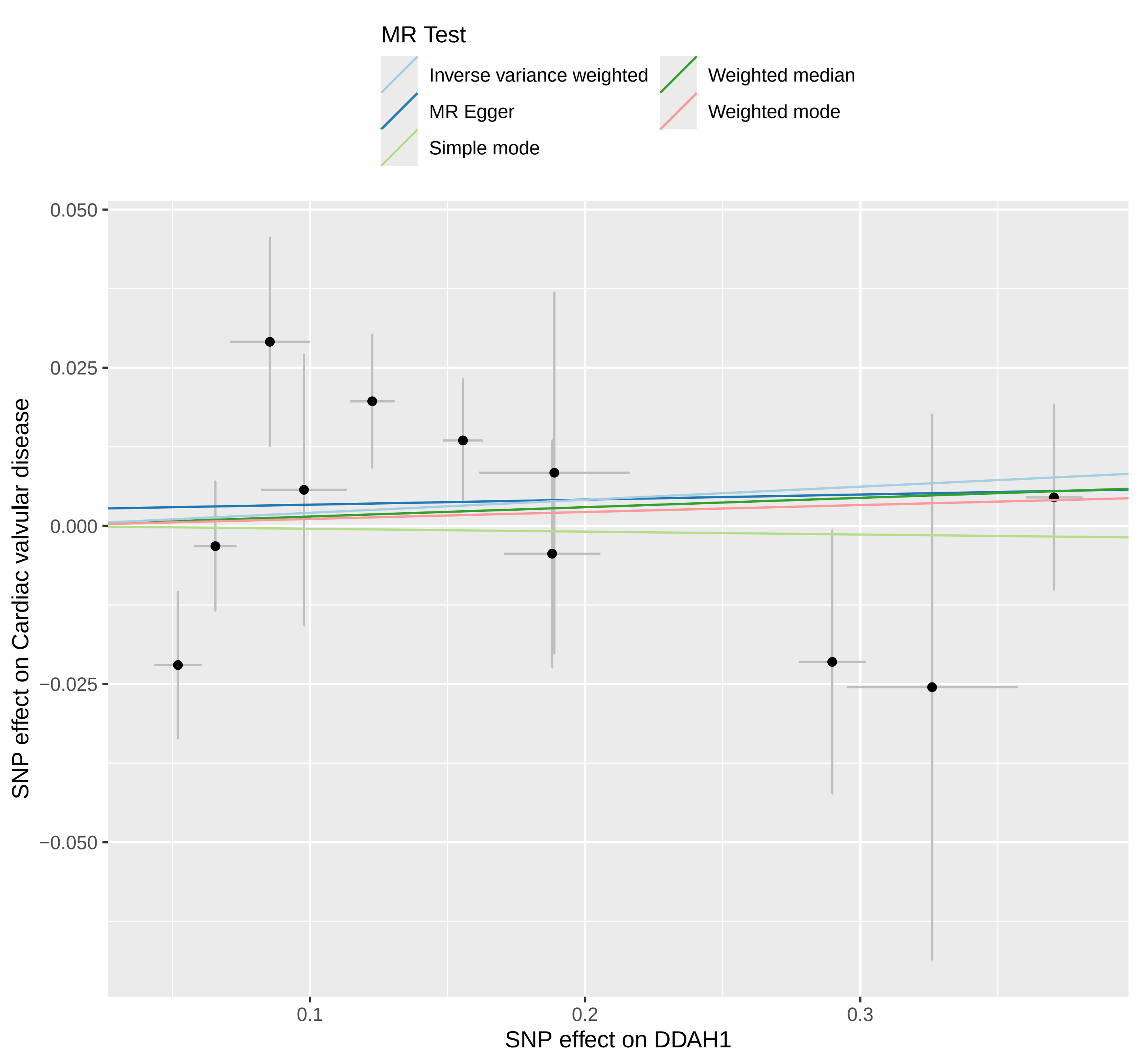

MR Test
Inverse variance weighted
MR Egger
Simple mode
Weighted median
Weighted mode
0.050
0.025
0.000
−0.025
−0.050
SNP effect on Cardiac valvular disease
0.1
0.2
0.3
SNP effect on DDAH1

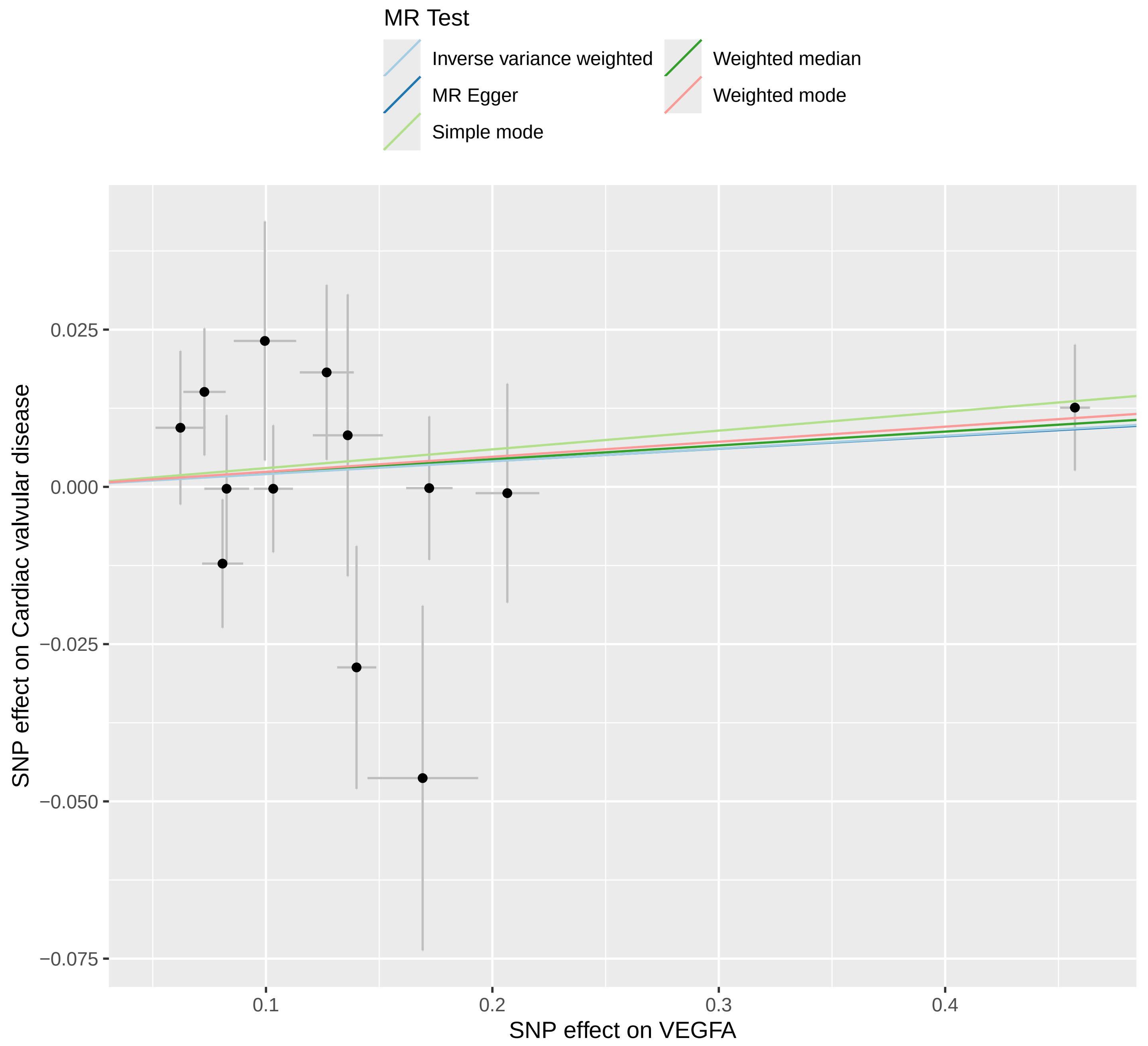

MR Test
Inverse variance weighted
MR Egger
Simple mode
Weighted median
Weighted mode
0.025
0.000
−0.025
−0.050
−0.075
0.1
0.2
0.3
0.4
SNP effect on Cardiac valvular disease
SNP effect on VEGFA

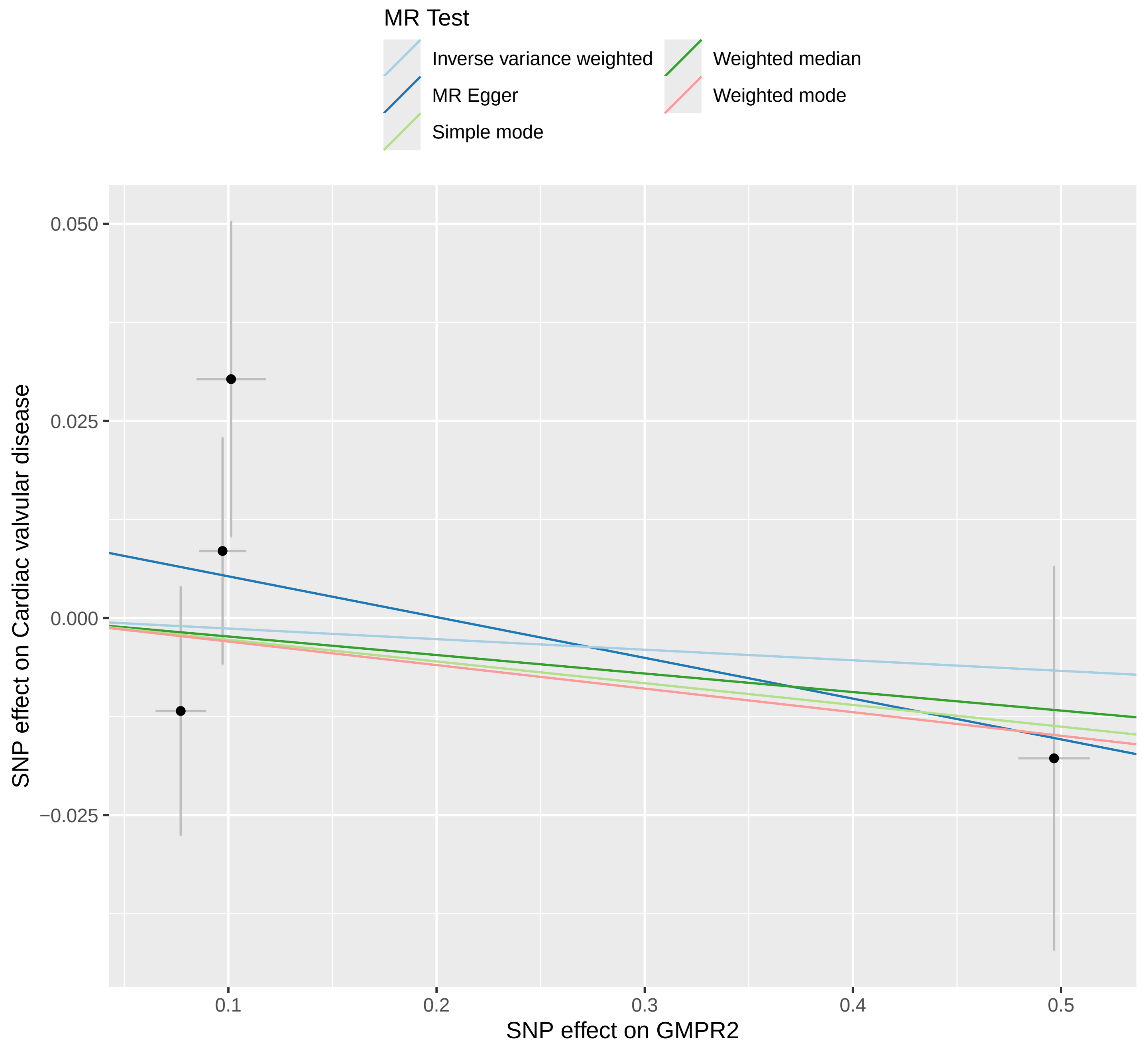
MR Test
Inverse variance weighted
MR Egger
Simple mode
Weighted median
Weighted mode
0.050
0.025
0.000
−0.025
0.1
0.2
0.3
0.4
0.5
SNP effect on Cardiac valvular disease
SNP effect on GMPR2

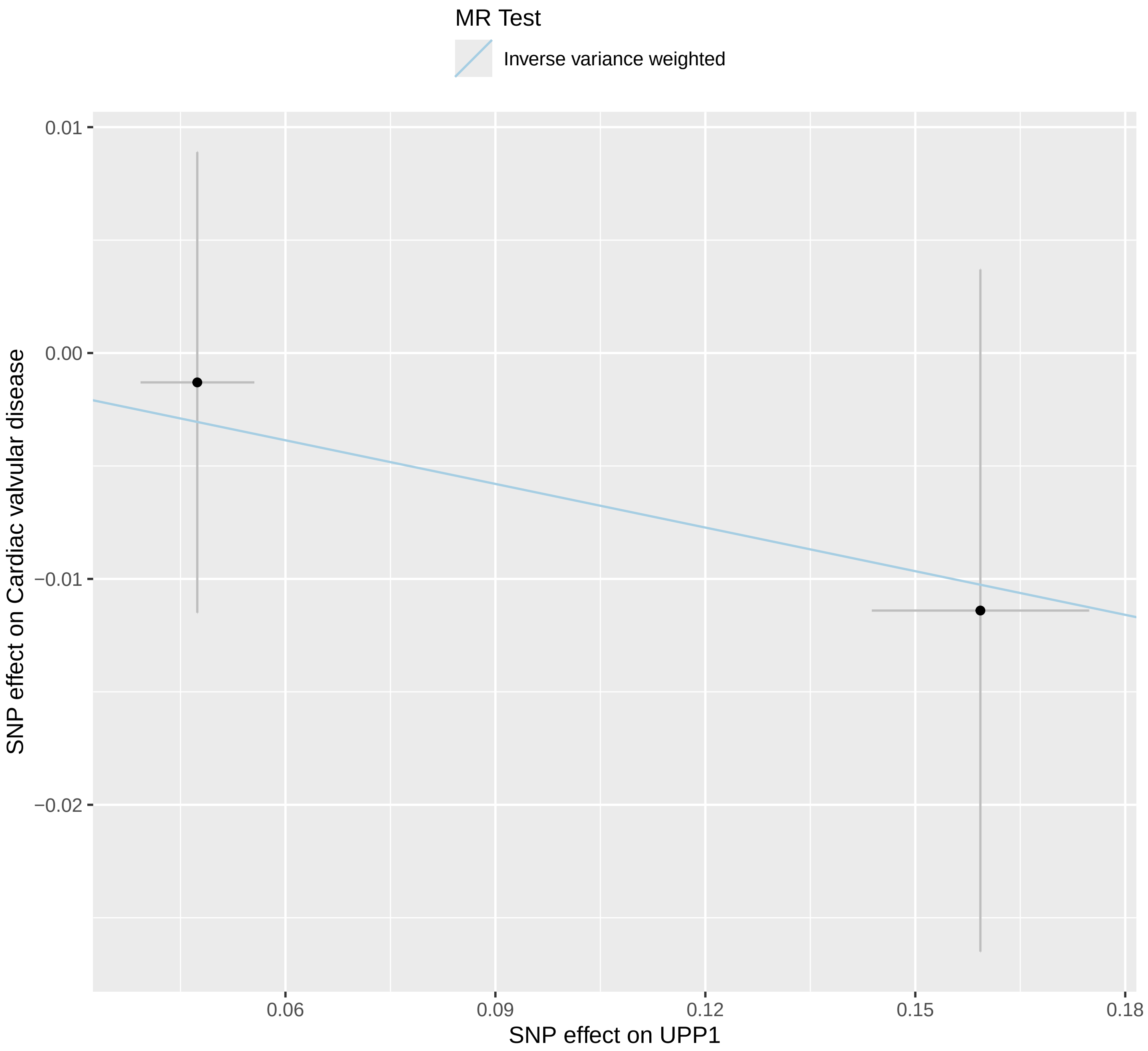

MR Test
Inverse variance weighted
0.01
0.00
−0.01
−0.02
SNP effect on Cardiac valvular disease
0.06
0.09
0.12
0.15
0.18
SNP effect on UPP1

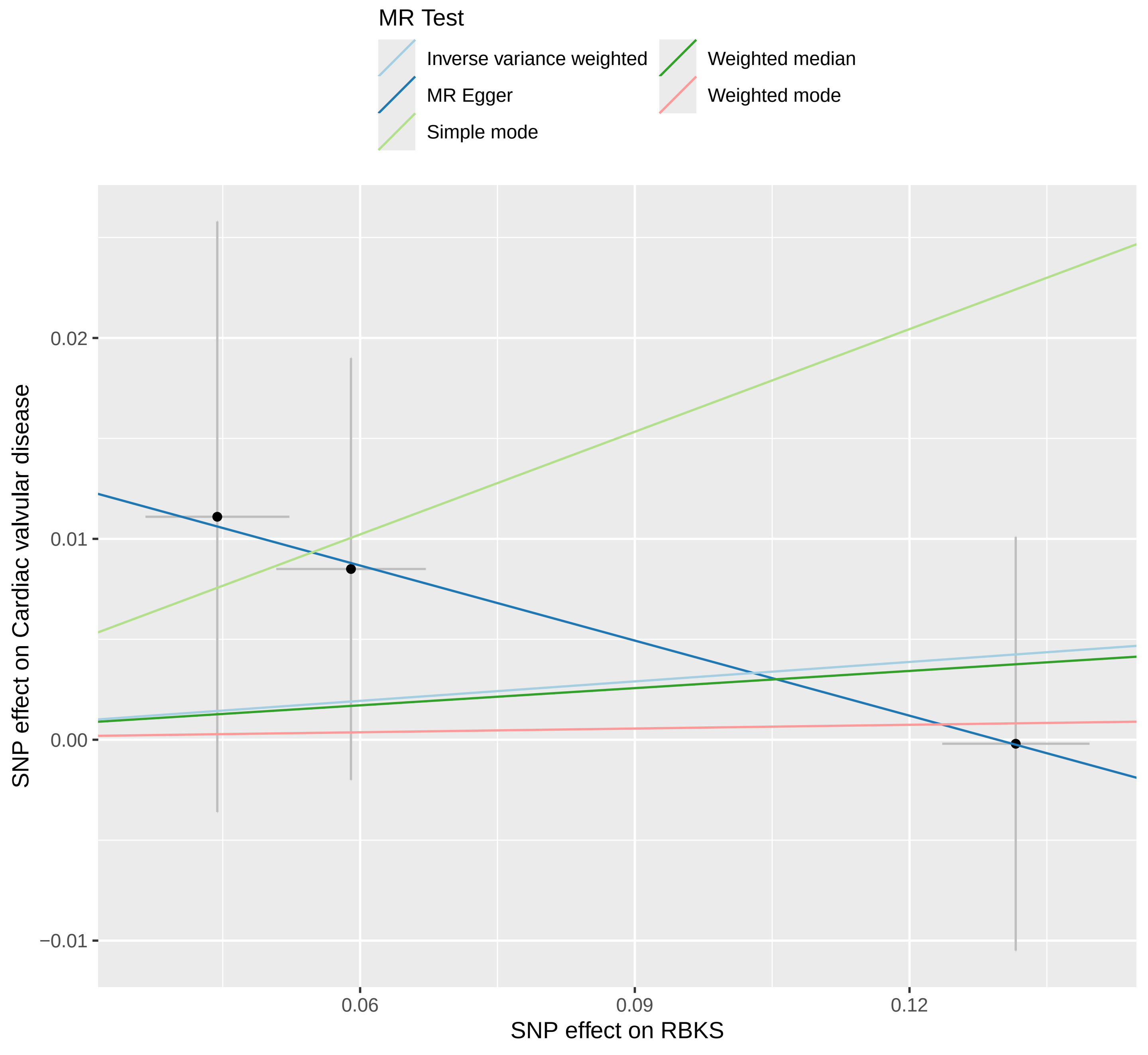

MR Test
Inverse variance weighted
MR Egger
Simple mode
Weighted median
Weighted mode
0.02
0.01
0.00
−0.01
0.06
0.09
0.12
SNP effect on Cardiac valvular disease
SNP effect on RBKS

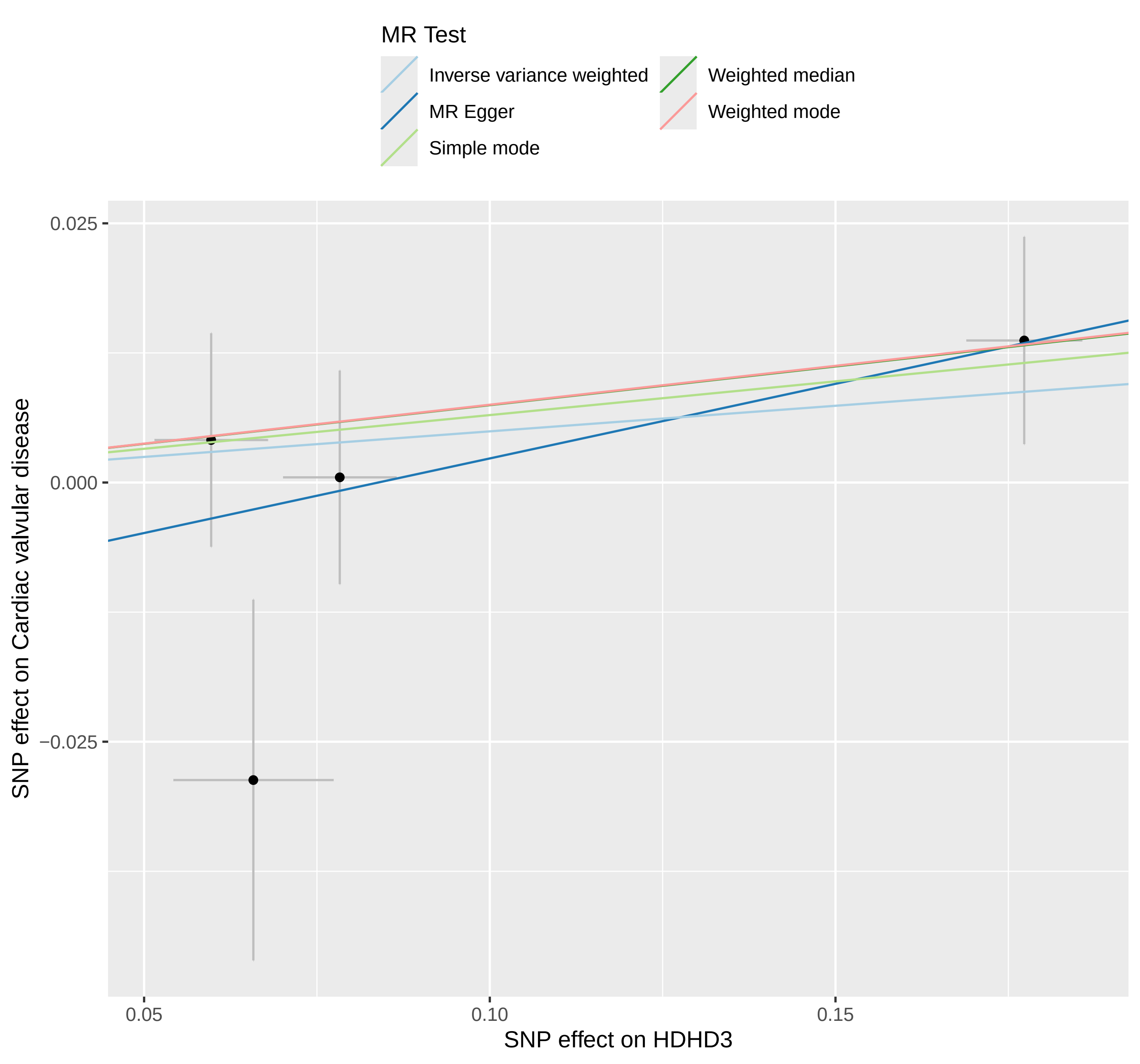
MR Test
Inverse variance weighted
MR Egger
Simple mode
Weighted median
Weighted mode
0.025
0.000
−0.025
0.05
0.10
0.15
SNP effect on Cardiac valvular disease
SNP effect on HDHD3

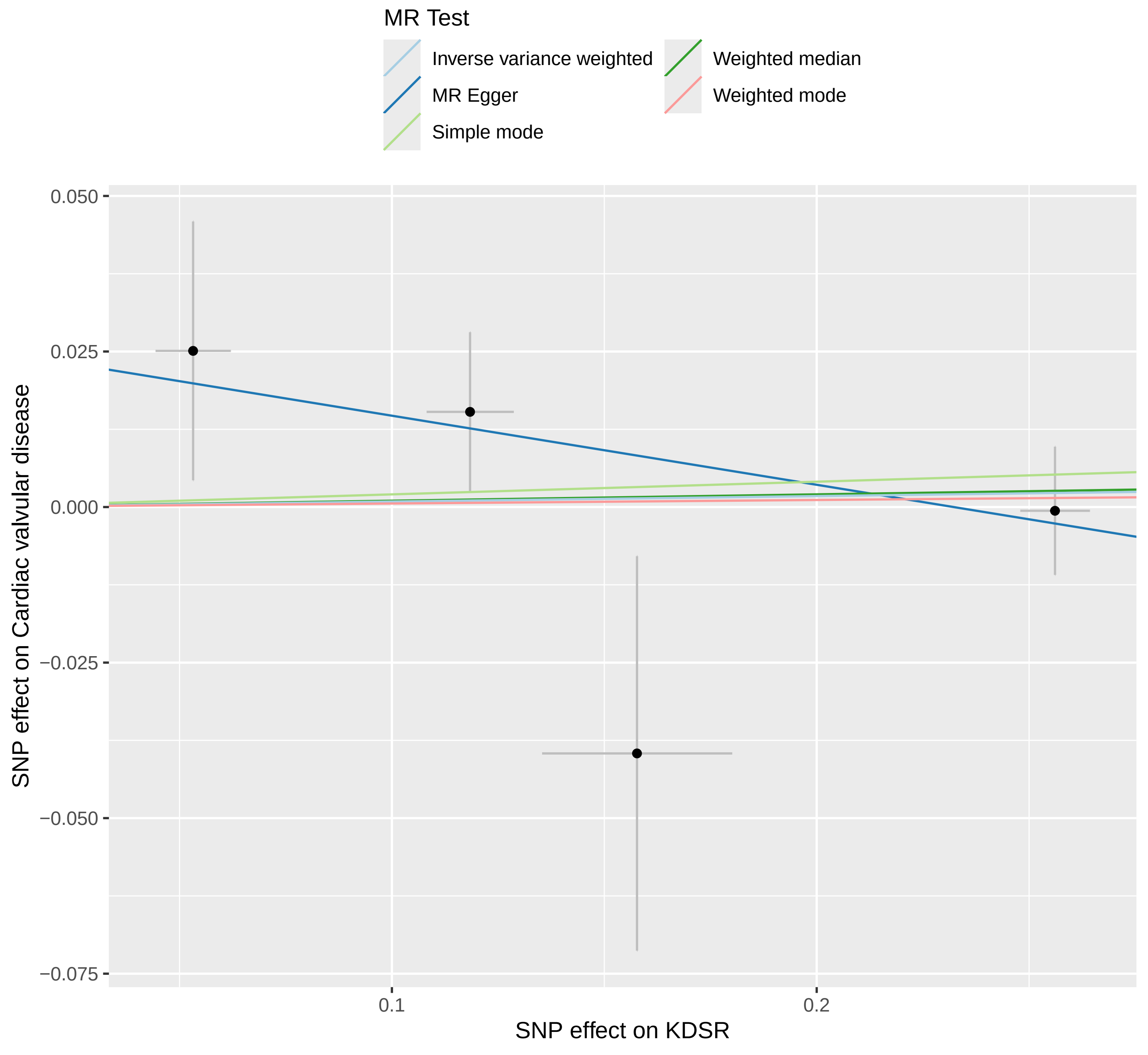

MR Test
Inverse variance weighted
MR Egger
Simple mode
Weighted median
Weighted mode
0.050
0.025
0.000
−0.025
−0.050
−0.075
SNP effect on Cardiac valvular disease
0.1
0.2
SNP effect on KDSR

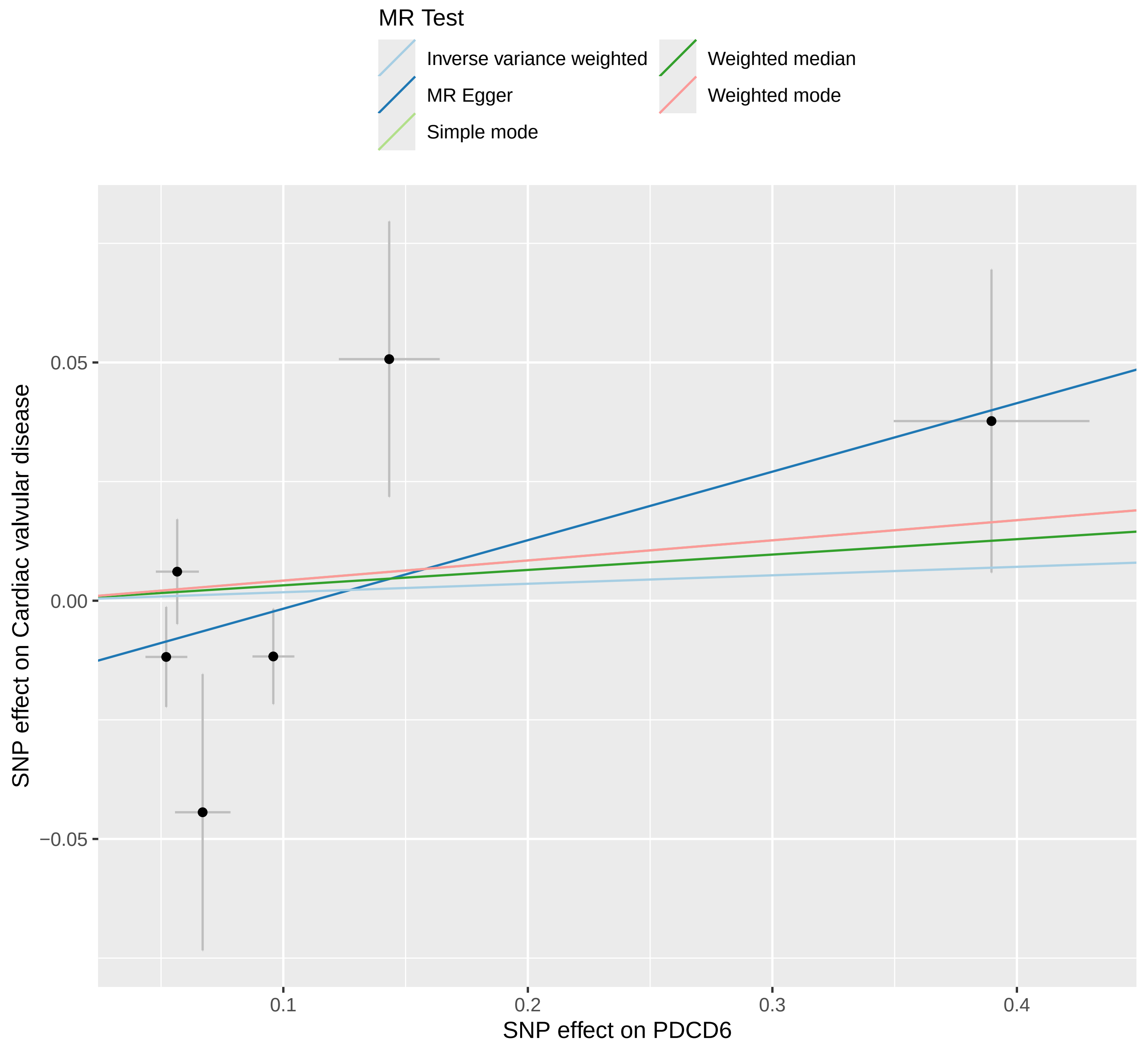
MR Test
Inverse variance weighted
MR Egger
Simple mode
Weighted median
Weighted mode
0.05
0.00
−0.05
0.1
0.2
0.3
0.4
SNP effect on PDCD6
SNP effect on Cardiac valvular disease

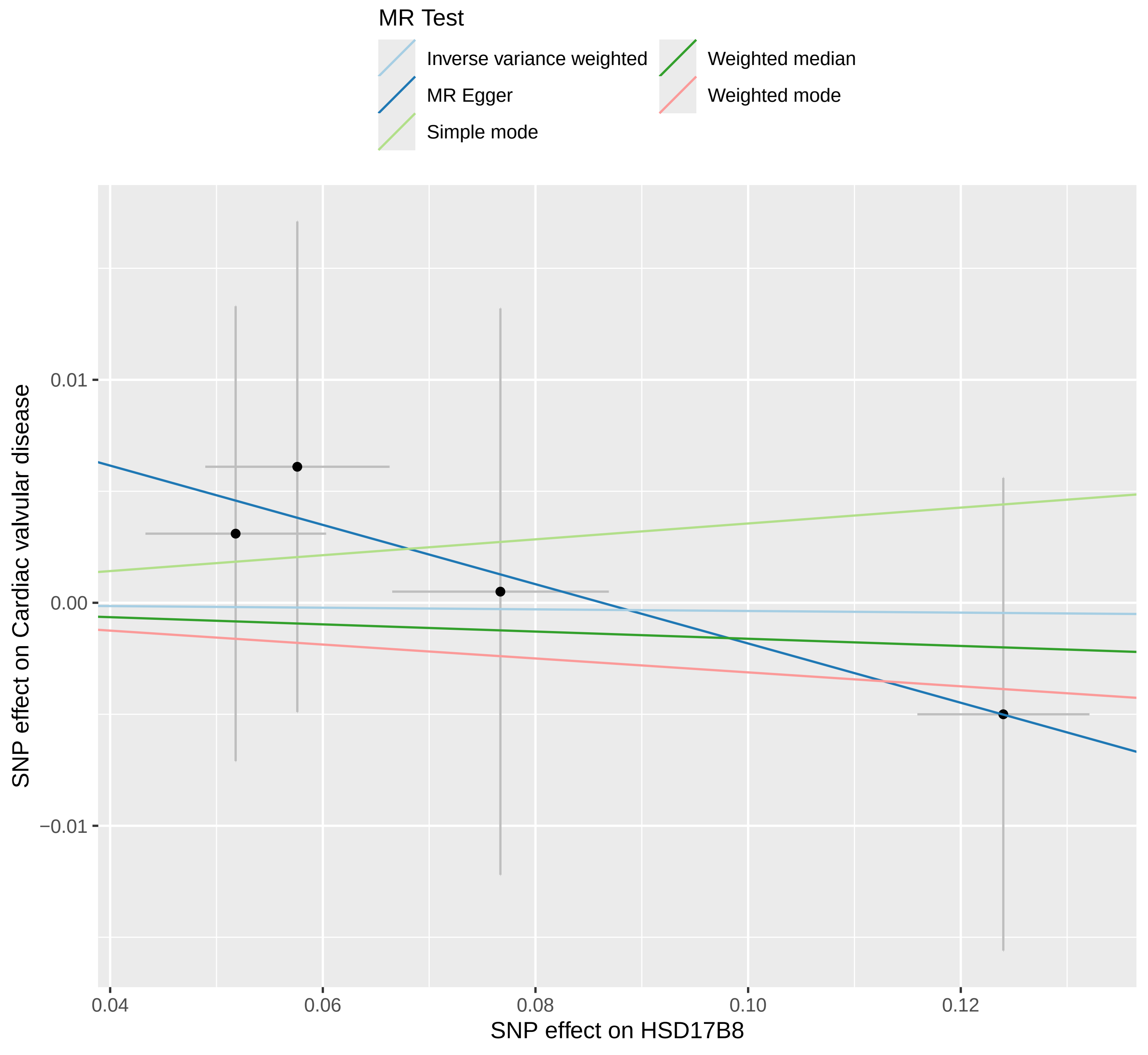

MR Test
Inverse variance weighted
MR Egger
Simple mode
Weighted median
Weighted mode
0.01
0.00
−0.01
0.04
0.06
0.08
0.10
0.12
SNP effect on Cardiac valvular disease
SNP effect on HSD17B8

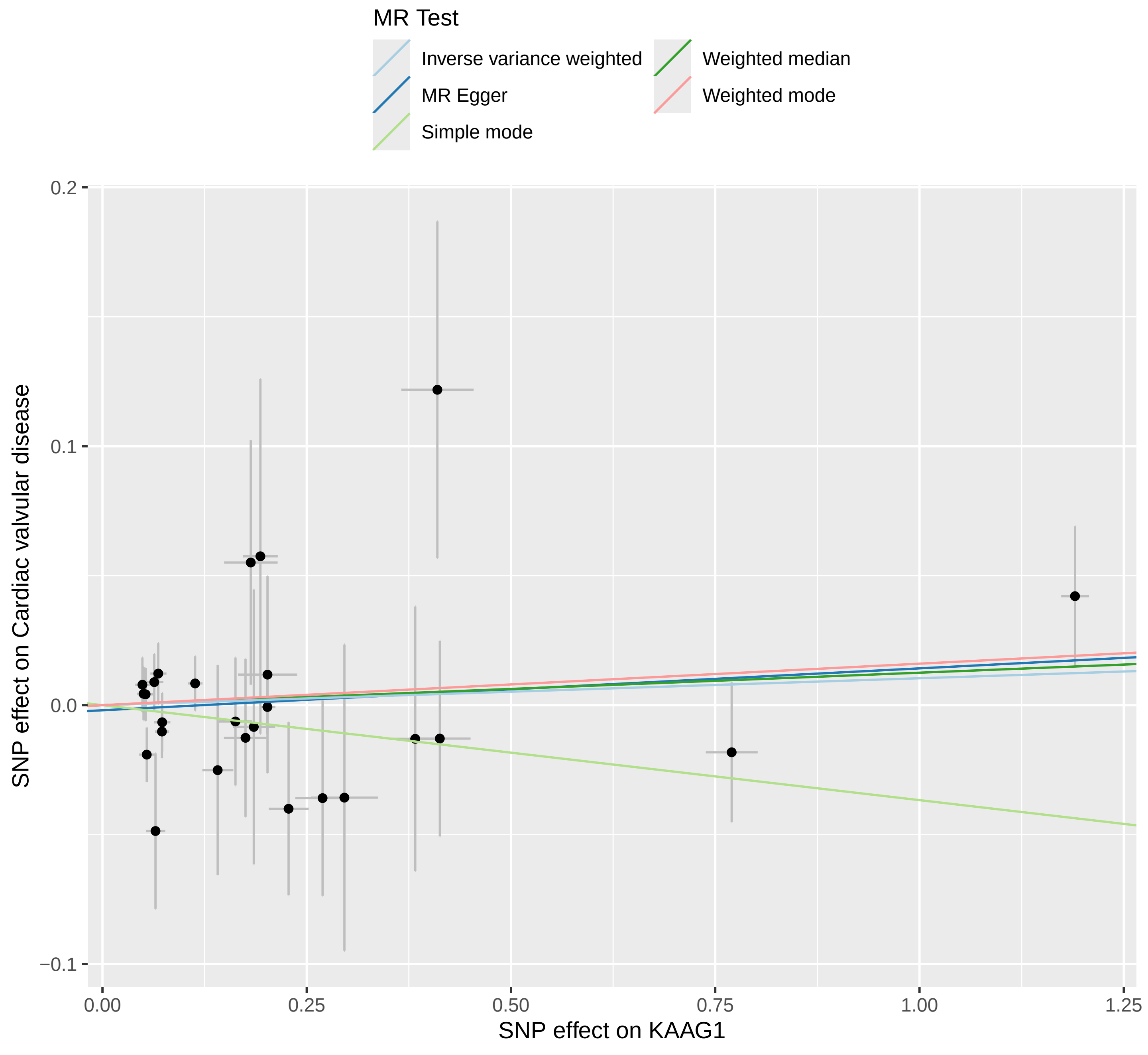

MR Test
Inverse variance weighted
MR Egger
Simple mode
Weighted median
Weighted mode
0.2
0.1
0.0
−0.1
0.00
0.25
0.50
0.75
1.00
1.25
SNP effect on Cardiac valvular disease
SNP effect on KAAG1

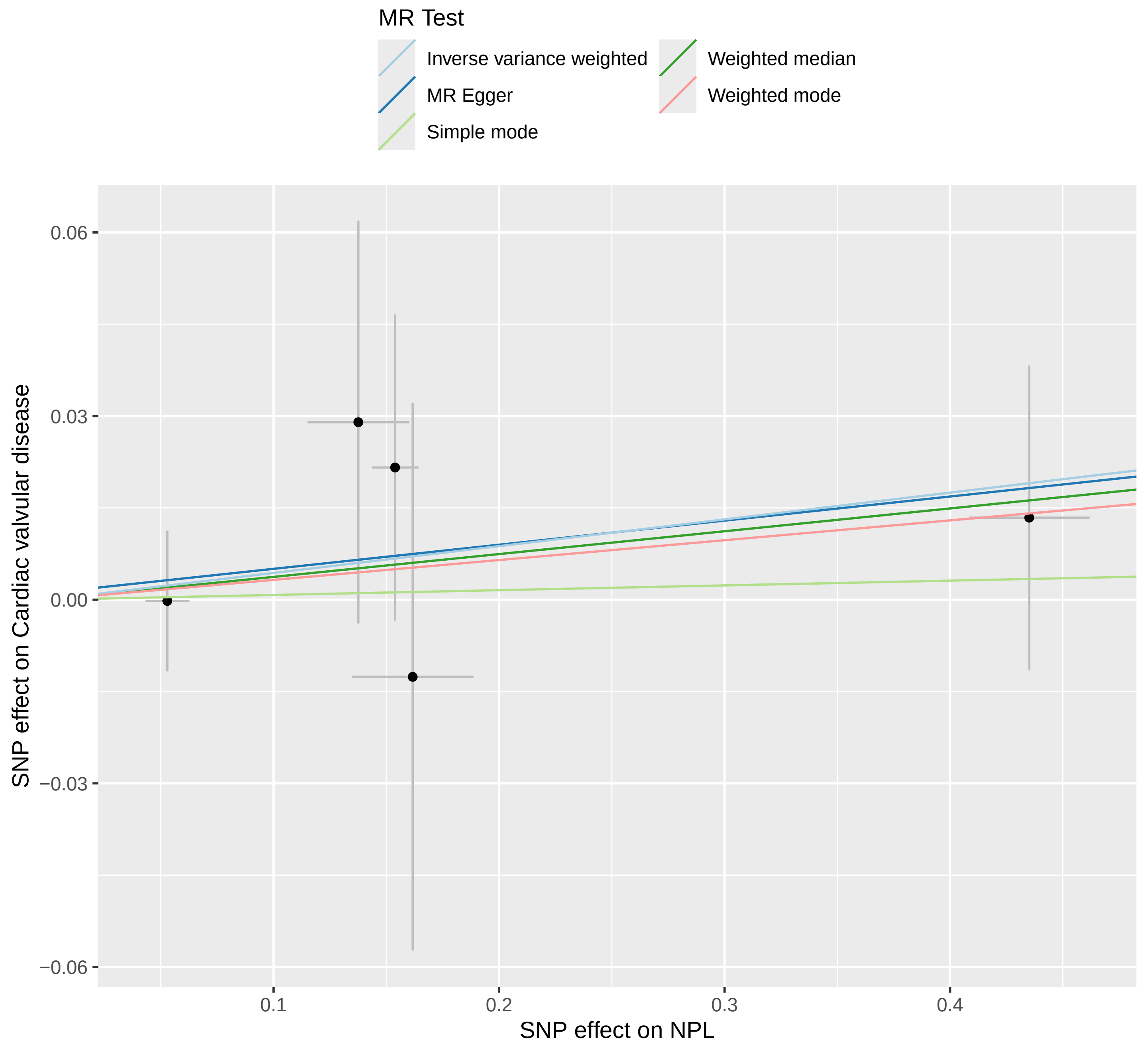
MR Test
Inverse variance weighted
MR Egger
Simple mode
Weighted median
Weighted mode
0.06
0.03
0.00
−0.03
−0.06
SNP effect on Cardiac valvular disease
0.1
0.2
0.3
0.4
SNP effect on NPL

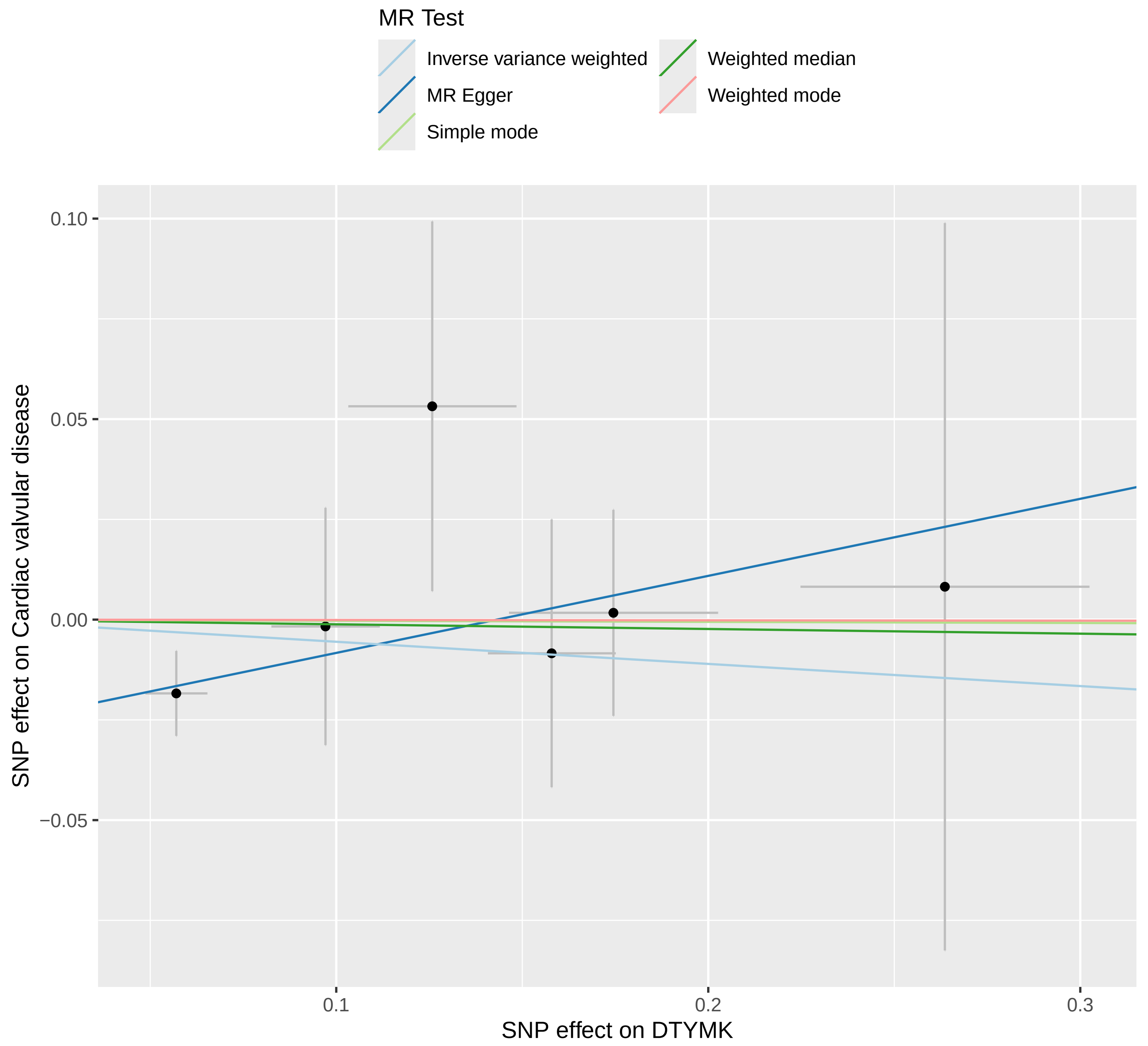
MR Test
Inverse variance weighted
MR Egger
Simple mode
Weighted median
Weighted mode
0.10
0.05
0.00
−0.05
SNP effect on Cardiac valvular disease
0.1
0.2
0.3
SNP effect on DTYMK

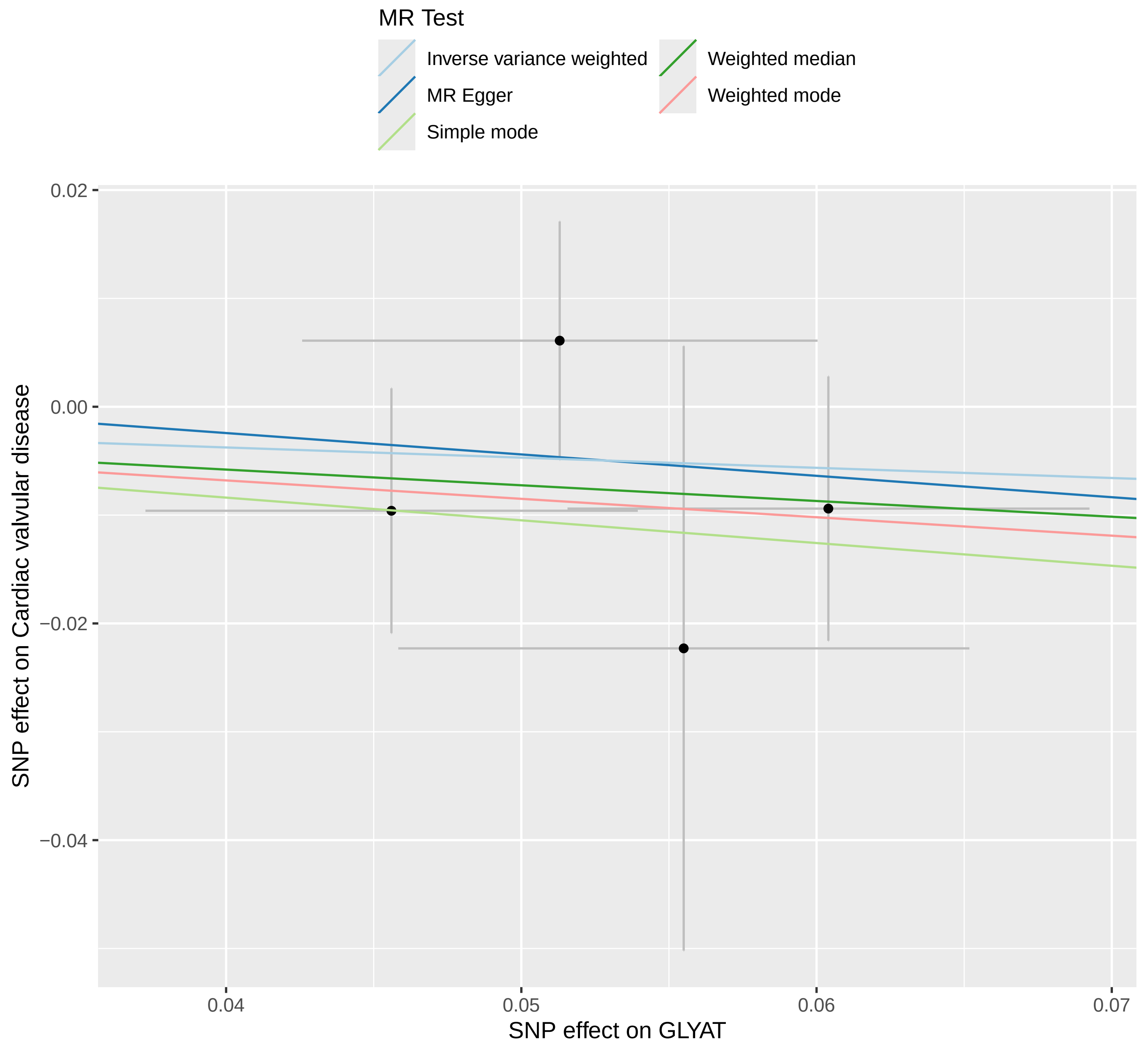

MR Test
Inverse variance weighted
MR Egger
Simple mode
Weighted median
Weighted mode
0.02
0.00
−0.02
−0.04
0.04
0.05
0.06
0.07
SNP effect on GLYAT
SNP effect on Cardiac valvular disease

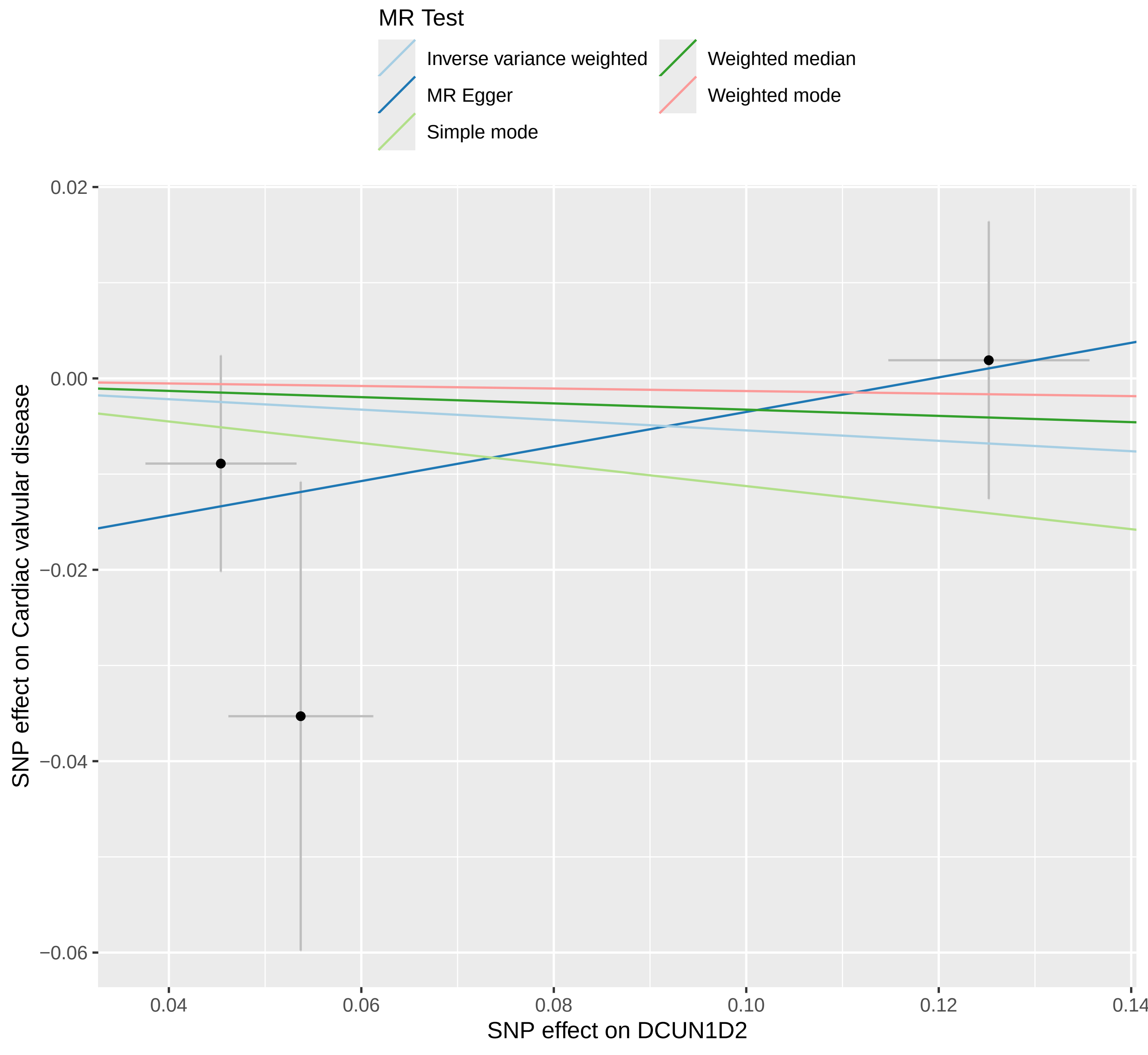
MR Test
Inverse variance weighted
MR Egger
Simple mode
Weighted median
Weighted mode
0.02
0.00
−0.02
−0.04
−0.06
SNP effect on Cardiac valvular disease
0.04
0.06
0.08
0.10
0.12
0.14
SNP effect on DCUN1D2

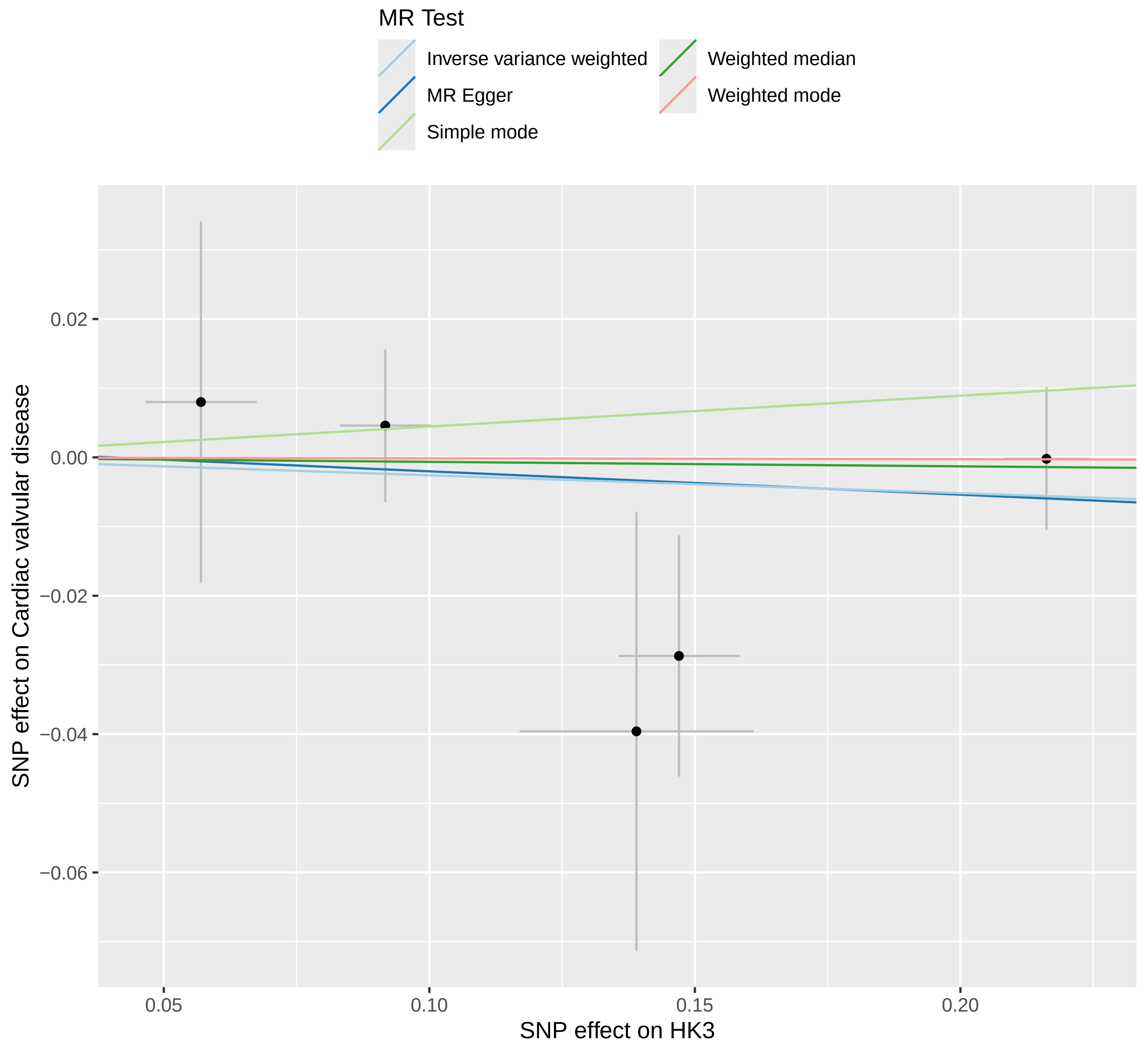
MR Test
Inverse variance weighted
MR Egger
Simple mode
Weighted median
Weighted mode
0.02
0.00
−0.02
−0.04
−0.06
SNP effect on Cardiac valvular disease
0.05
0.10
0.15
0.20
SNP effect on HK3

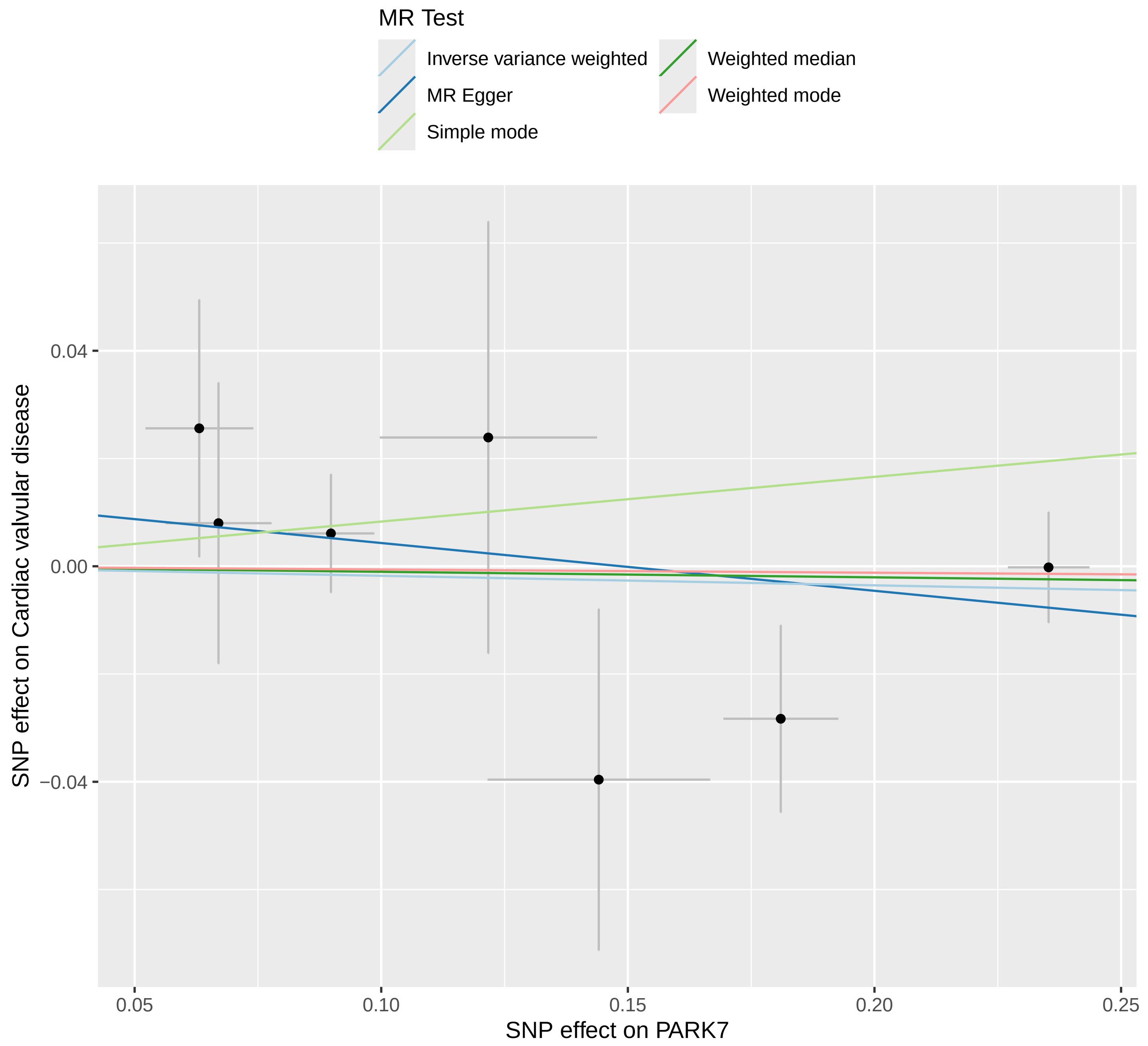
MR Test
Inverse variance weighted
MR Egger
Simple mode
Weighted median
Weighted mode
0.04
0.00
−0.04
SNP effect on Cardiac valvular disease
0.05
0.10
0.15
0.20
0.25
SNP effect on PARK7

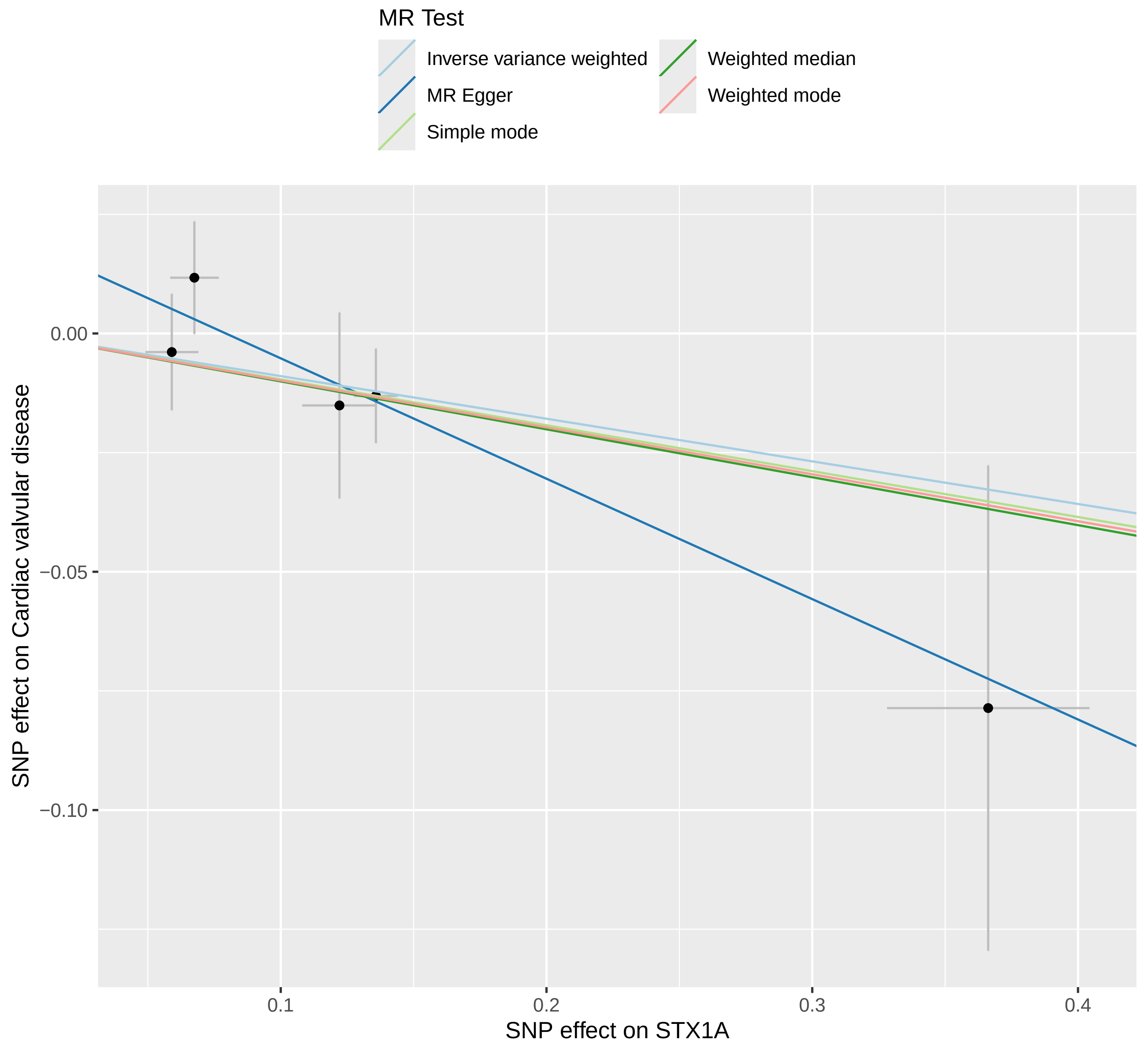
MR Test
Inverse variance weighted
MR Egger
Simple mode
Weighted median
Weighted mode
0.00
−0.05
−0.10
0.1
0.2
0.3
0.4
SNP effect on Cardiac valvular disease
SNP effect on STX1A

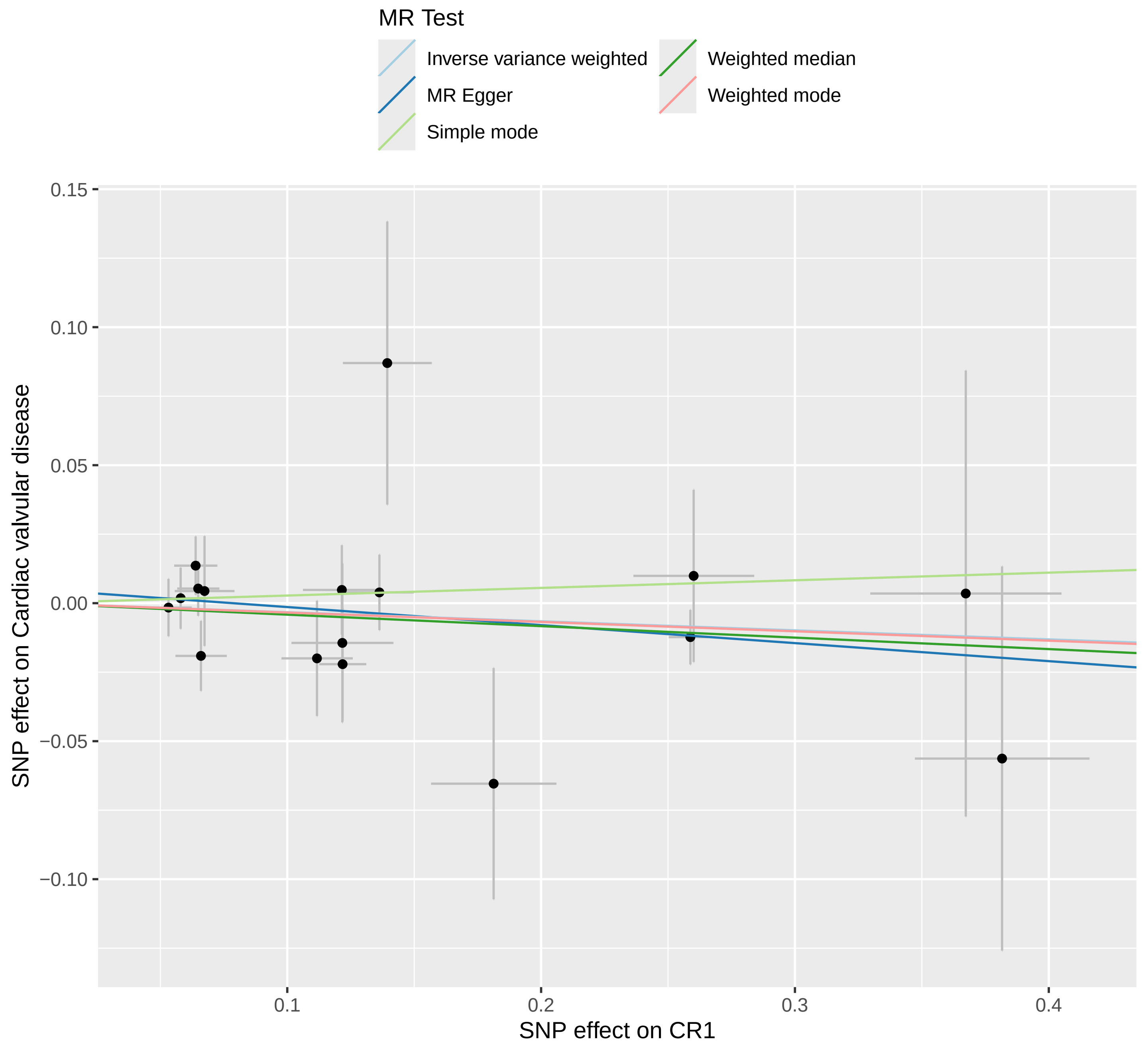
MR Test
Inverse variance weighted
MR Egger
Simple mode
Weighted median
Weighted mode
0.15
0.10
0.05
0.00
−0.05
−0.10
SNP effect on Cardiac valvular disease
0.1
0.2
0.3
0.4
SNP effect on CR1

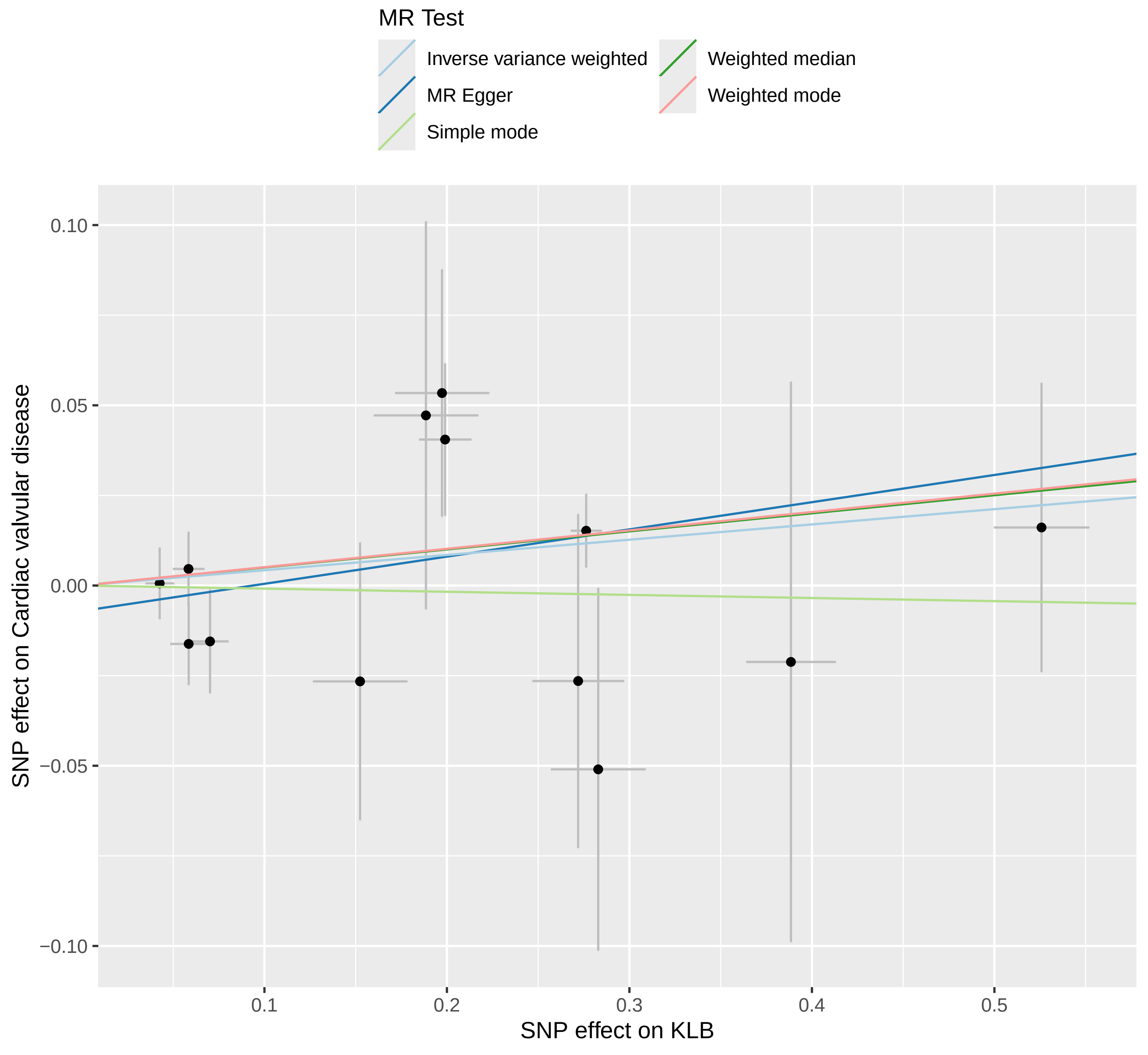
MR Test
Inverse variance weighted
MR Egger
Simple mode
Weighted median
Weighted mode
0.10
0.05
0.00
−0.05
−0.10
SNP effect on Cardiac valvular disease
0.1
0.2
0.3
0.4
0.5
SNP effect on KLB

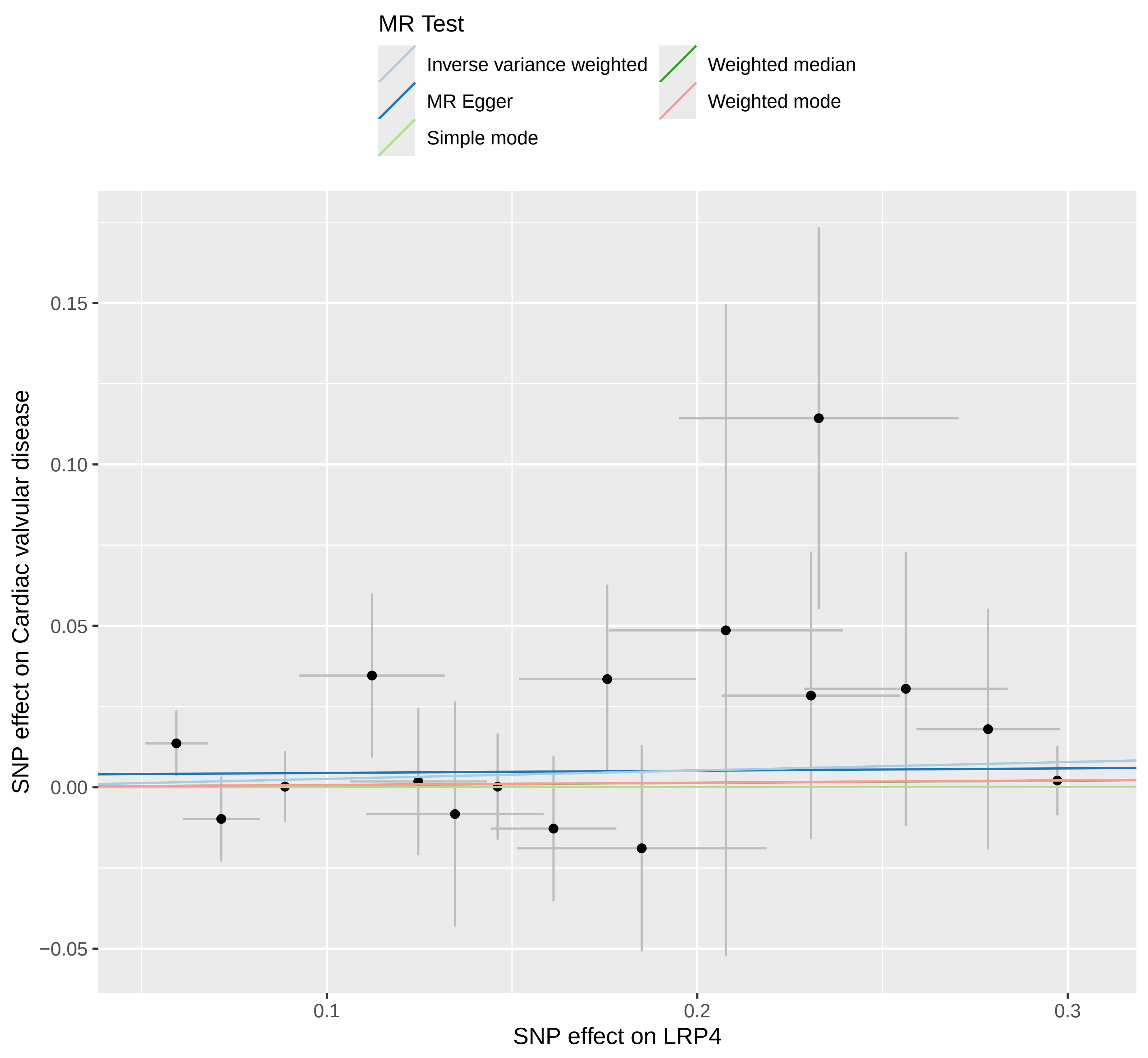
MR Test
Inverse variance weighted
MR Egger
Simple mode
Weighted median
Weighted mode
0.15
0.10
0.05
0.00
−0.05
SNP effect on Cardiac valvular disease
0.1
0.2
0.3
SNP effect on LRP4

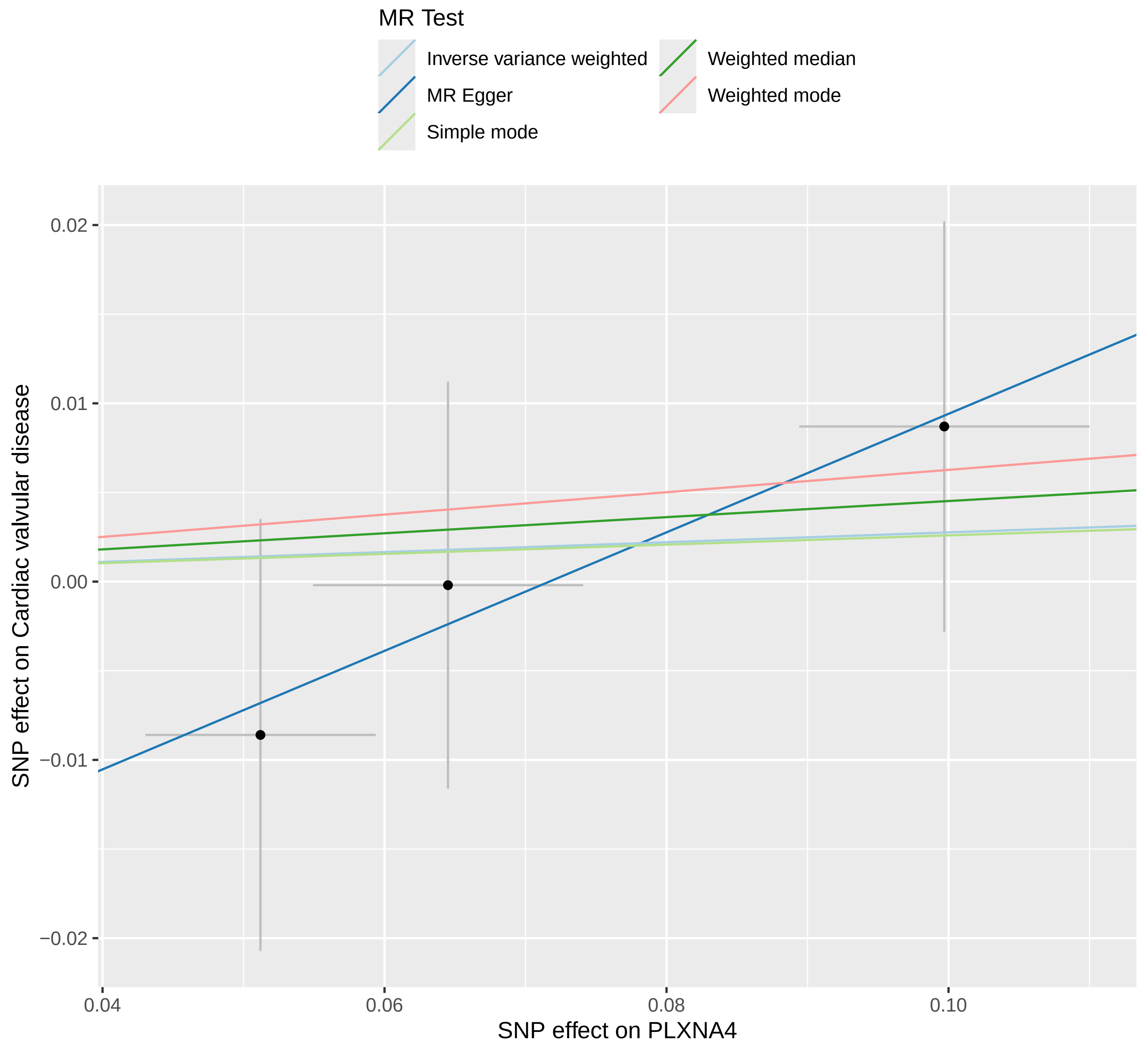
MR Test
Inverse variance weighted
MR Egger
Simple mode
Weighted median
Weighted mode
0.02
0.01
0.00
−0.01
−0.02
SNP effect on Cardiac valvular disease
0.04
0.06
0.08
0.10
SNP effect on PLXNA4

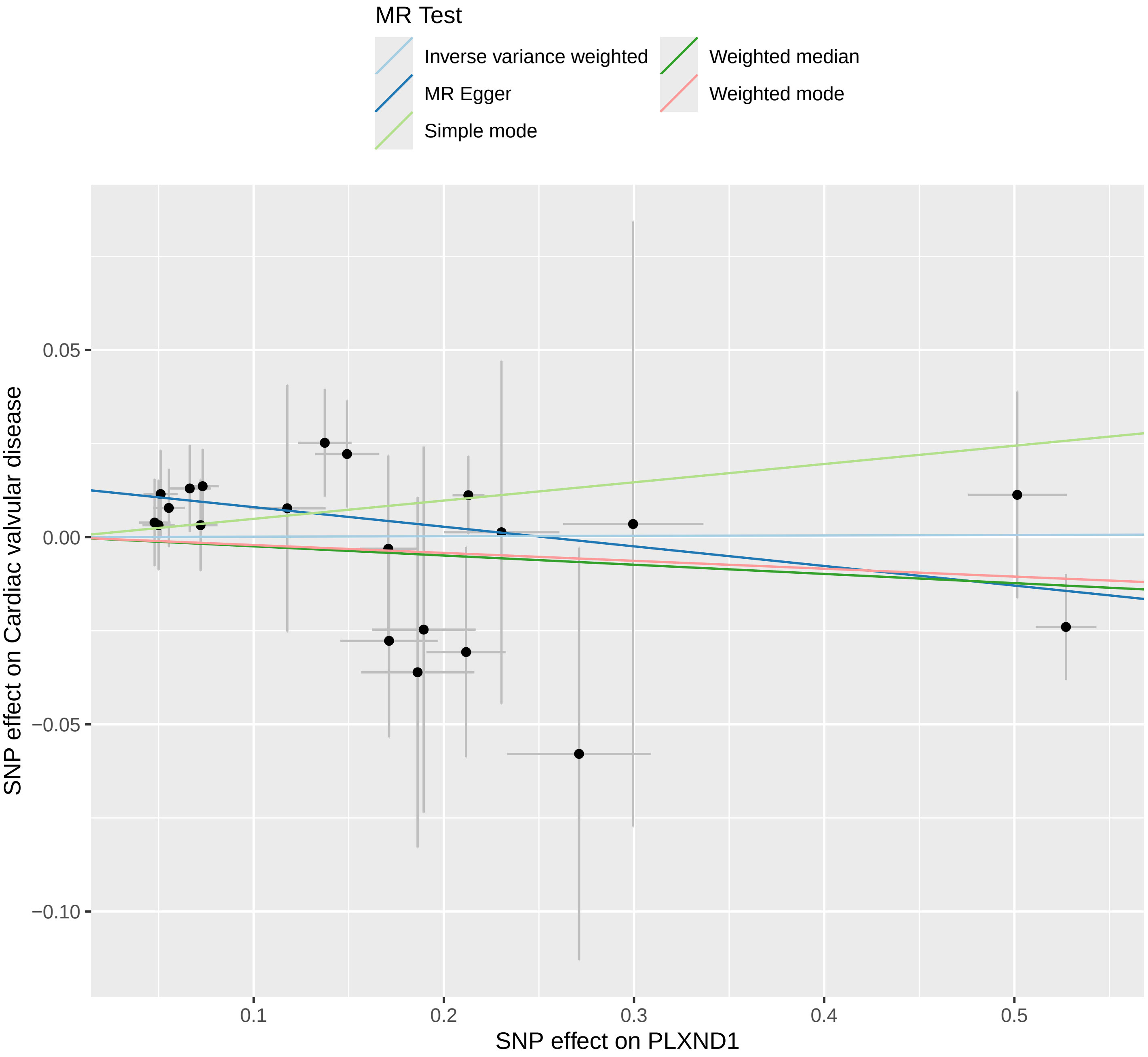
MR Test
Inverse variance weighted
MR Egger
Simple mode
Weighted median
Weighted mode
0.05
0.00
−0.05
−0.10
SNP effect on Cardiac valvular disease
0.1
0.2
0.3
0.4
0.5
SNP effect on PLXND1

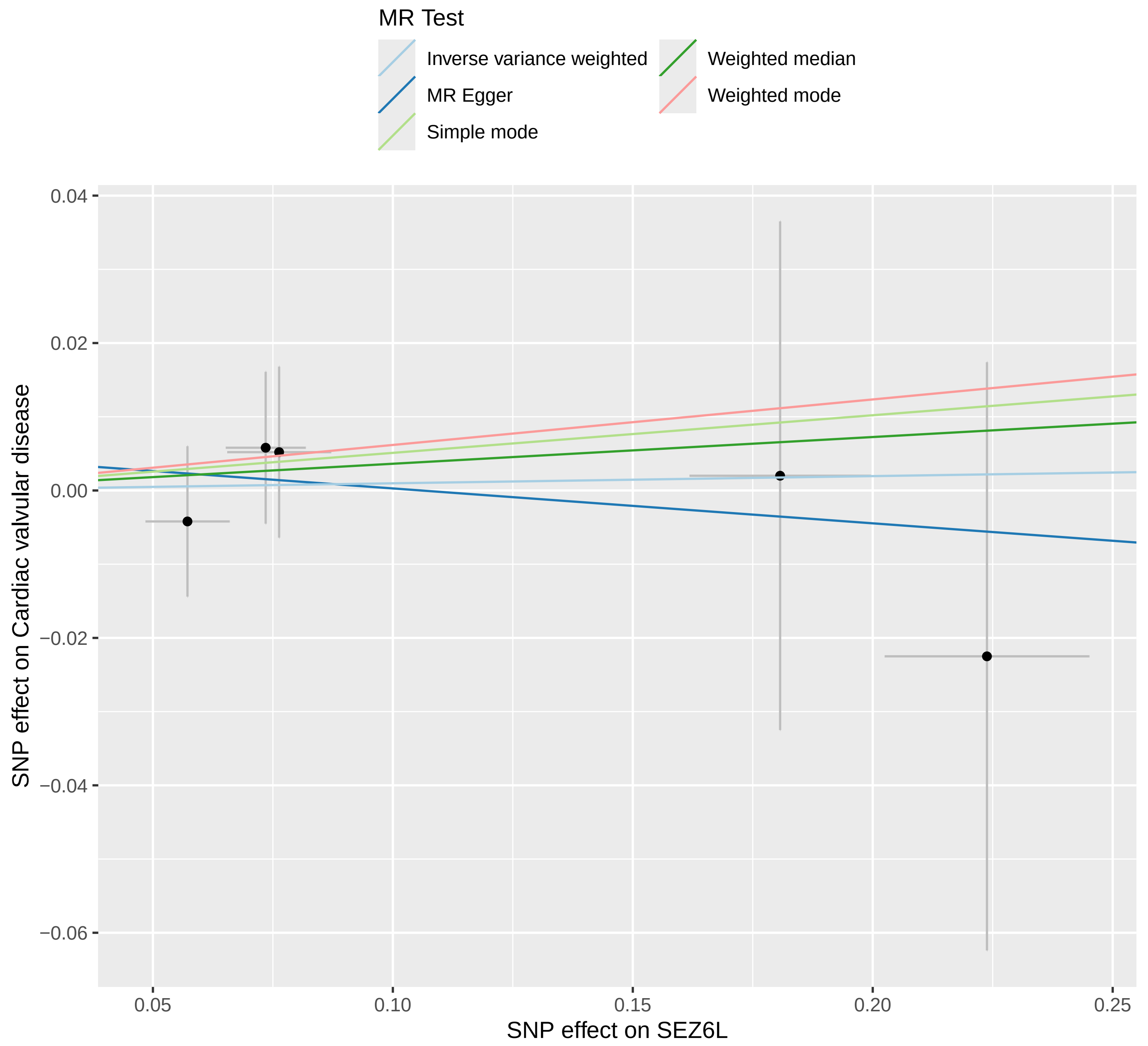
MR Test
Inverse variance weighted
MR Egger
Simple mode
Weighted median
Weighted mode
0.04
0.02
0.00
−0.02
−0.04
−0.06
SNP effect on Cardiac valvular disease
0.05
0.10
0.15
0.20
0.25
SNP effect on SEZ6L

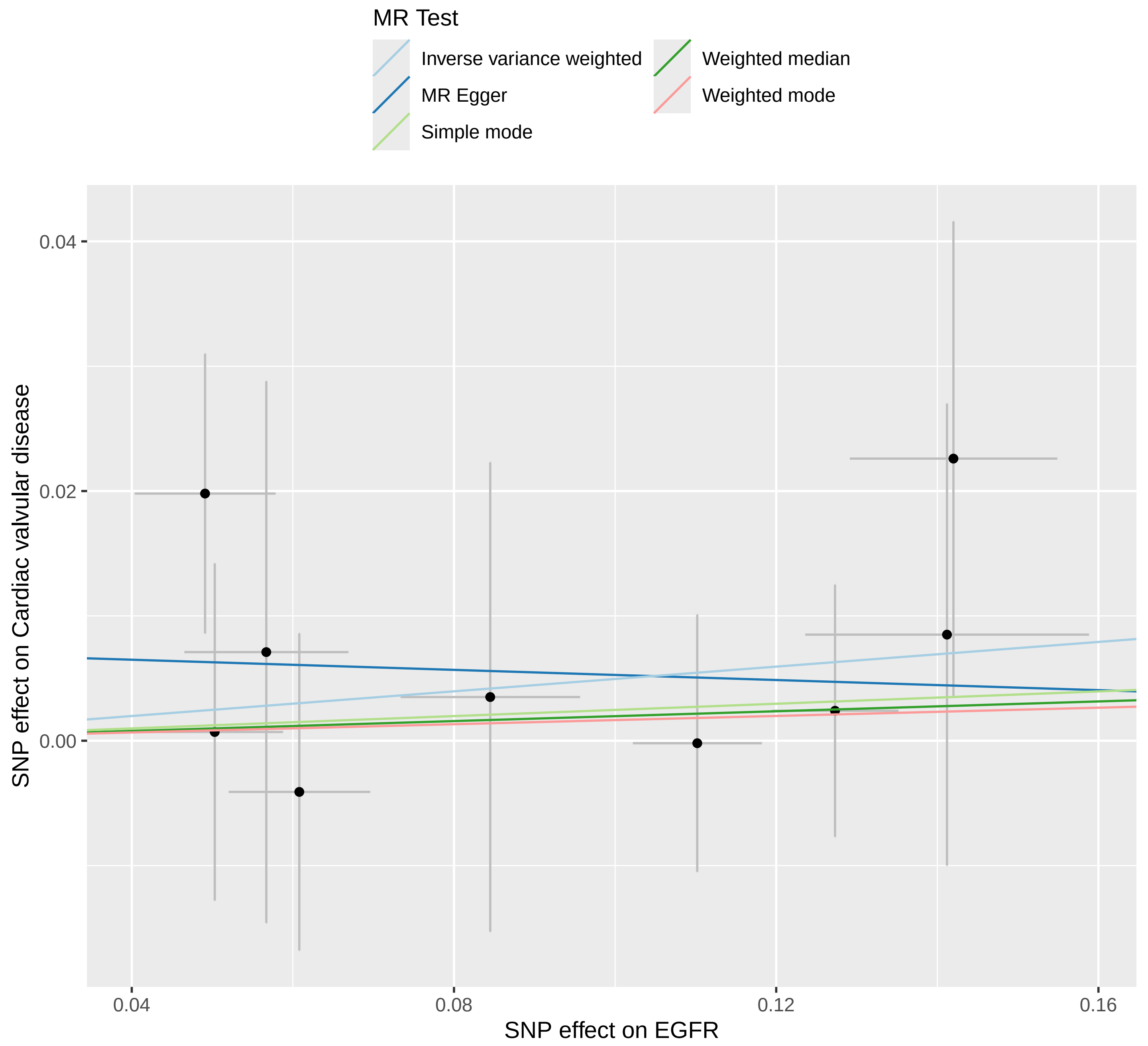

MR Test
Inverse variance weighted
MR Egger
Simple mode
Weighted median
Weighted mode
SNP effect on Cardiac valvular disease
0.04
0.02
0.00
0.04
0.08
0.12
0.16
SNP effect on EGFR

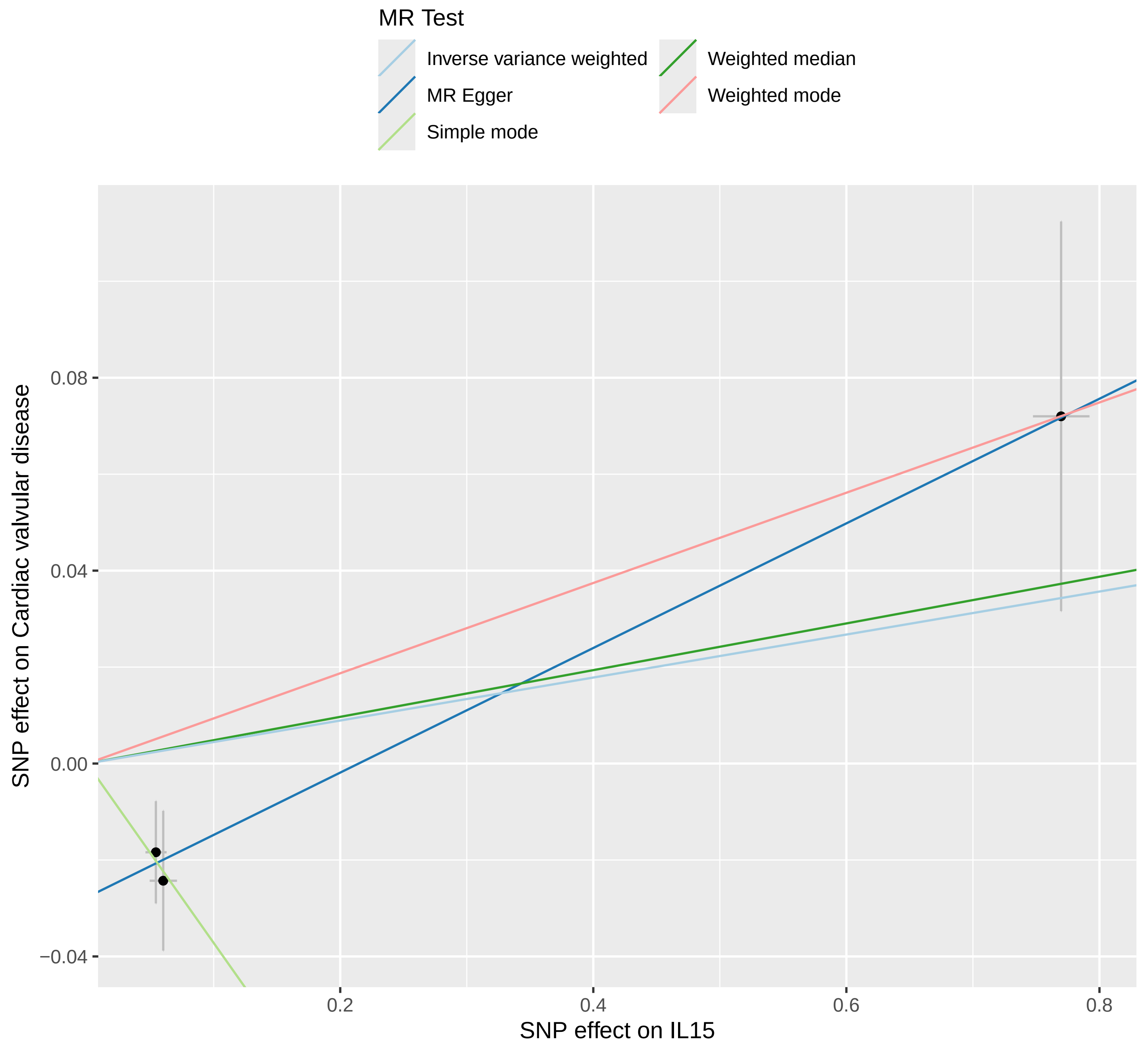

MR Test
Inverse variance weighted
MR Egger
Simple mode
Weighted median
Weighted mode
SNP effect on Cardiac valvular disease
0.08
0.04
0.00
−0.04
0.2
0.4
0.6
0.8
SNP effect on IL15

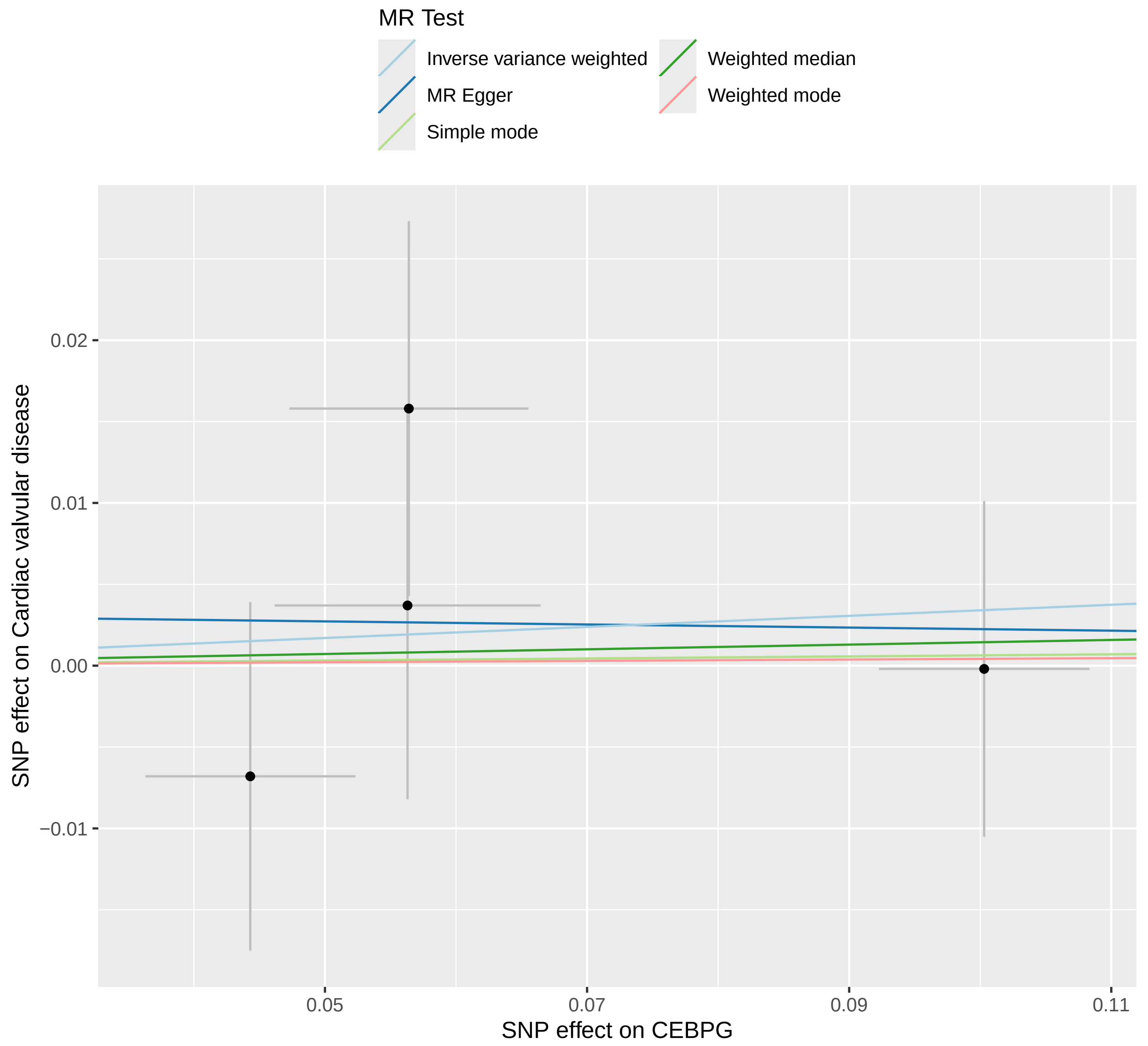
MR Test
Inverse variance weighted
MR Egger
Simple mode
Weighted median
Weighted mode
SNP effect on Cardiac valvular disease
0.02
0.01
0.00
−0.01
0.05
0.07
0.09
0.11
SNP effect on CEBPG

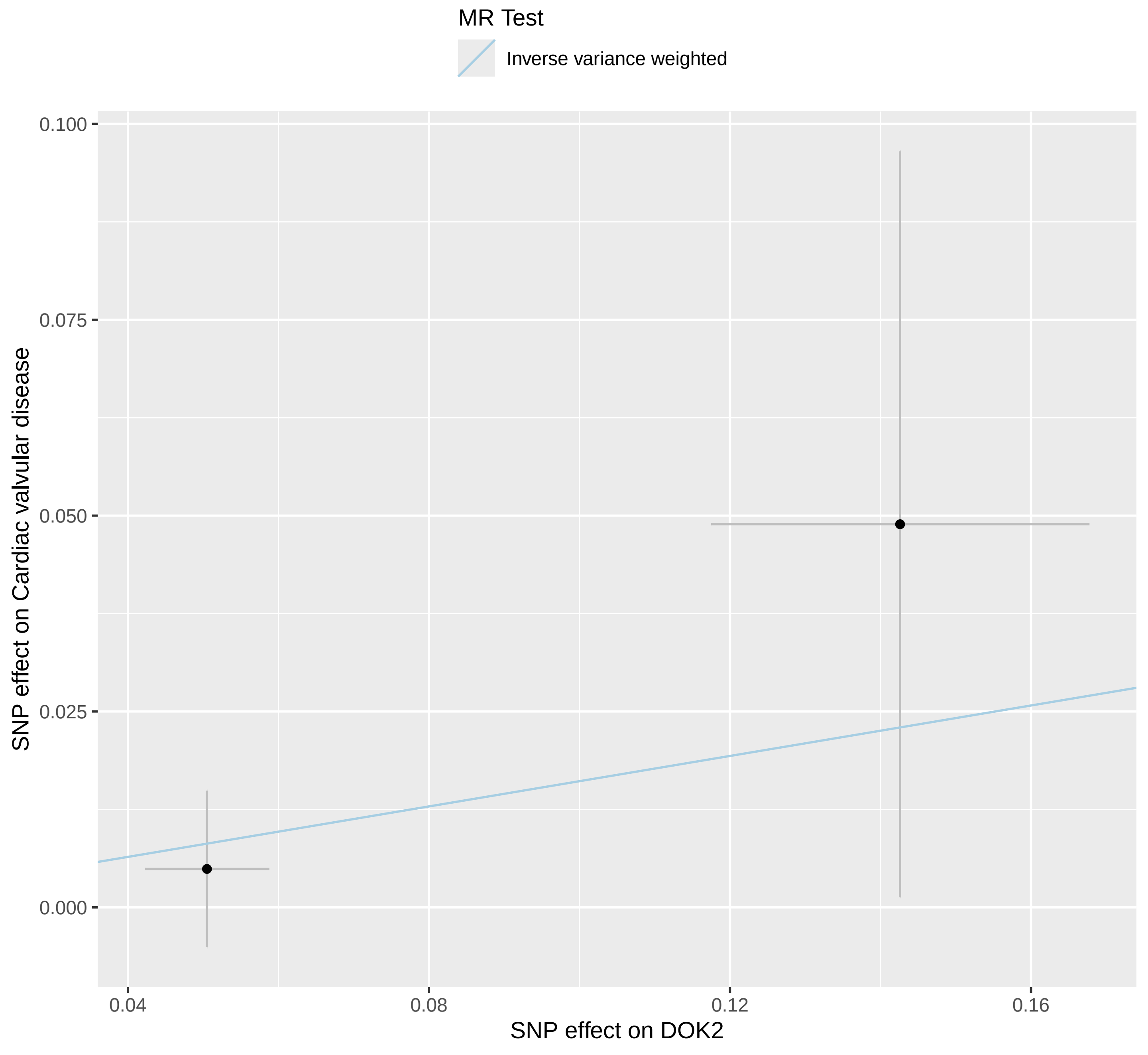
MR Test
Inverse variance weighted
0.100
0.075
0.050
0.025
0.000
SNP effect on Cardiac valvular disease
0.04
0.08
0.12
0.16
SNP effect on DOK2

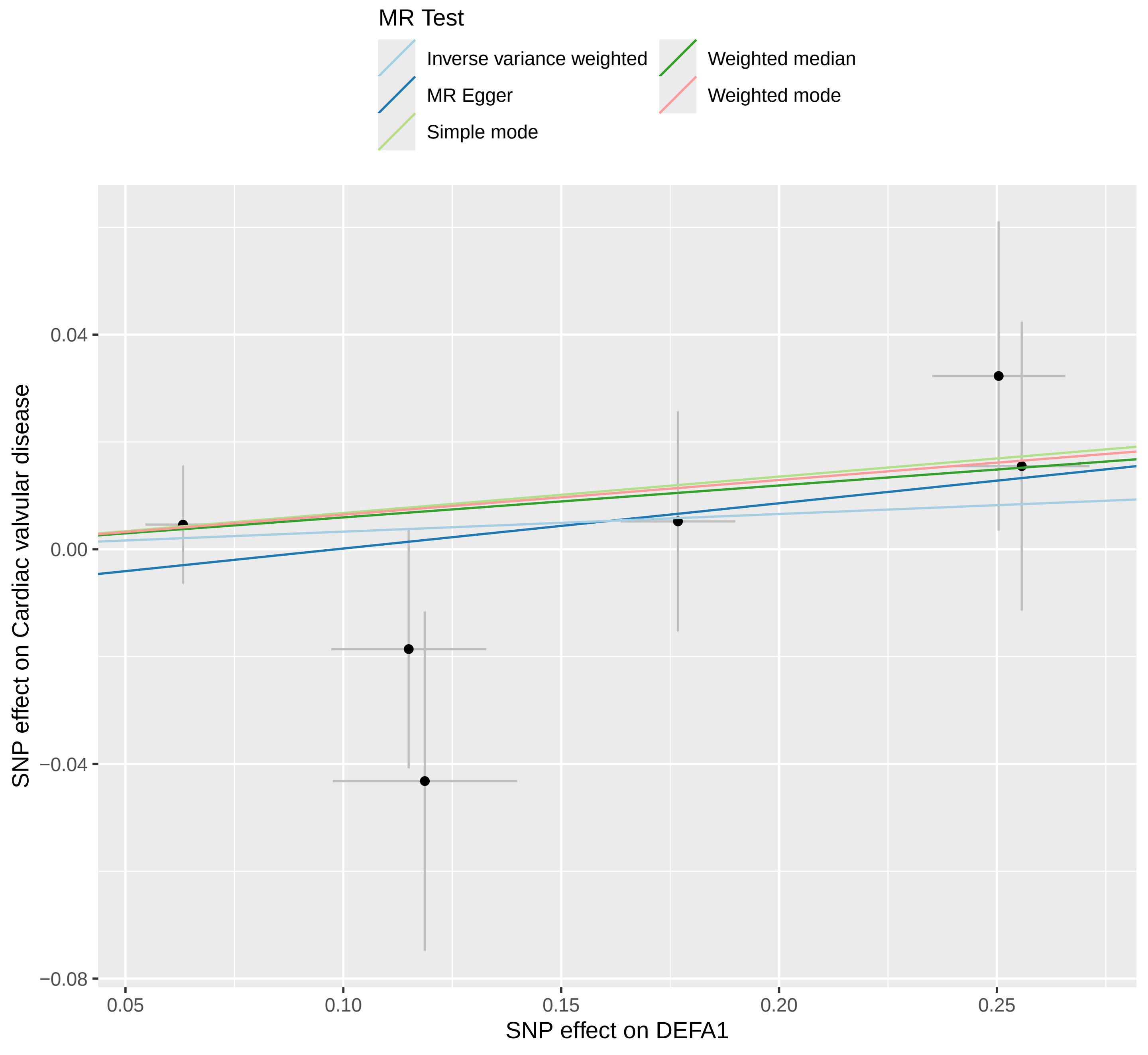
MR Test
Inverse variance weighted
MR Egger
Simple mode
Weighted median
Weighted mode
SNP effect on Cardiac valvular disease
0.04
0.00
−0.04
−0.08
0.05
0.10
0.15
0.20
0.25
SNP effect on DEFA1

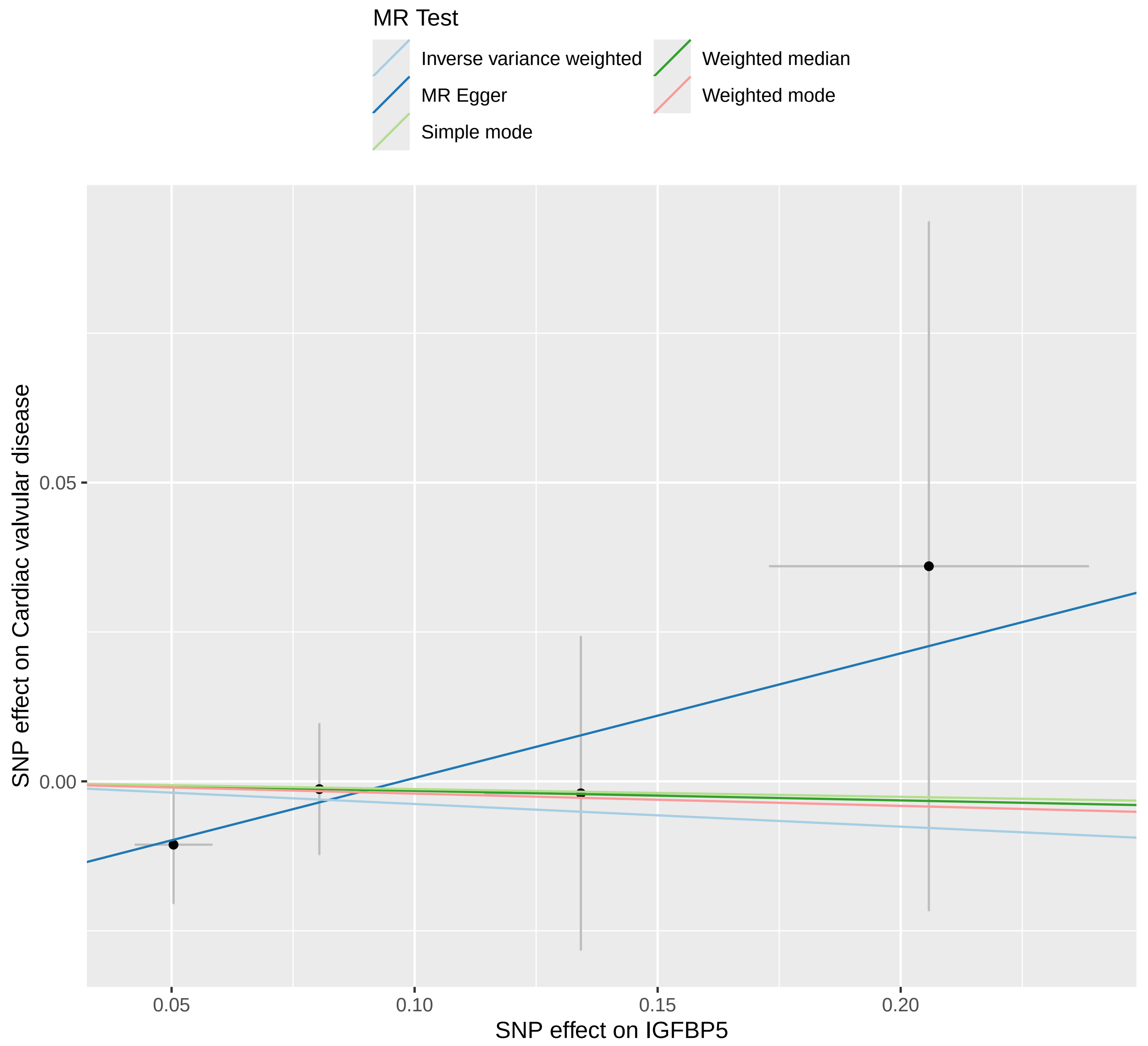

MR Test
Inverse variance weighted
MR Egger
Simple mode
Weighted median
Weighted mode
SNP effect on Cardiac valvular disease
0.05
0.00
0.05
0.10
0.15
0.20
SNP effect on IGFBP5

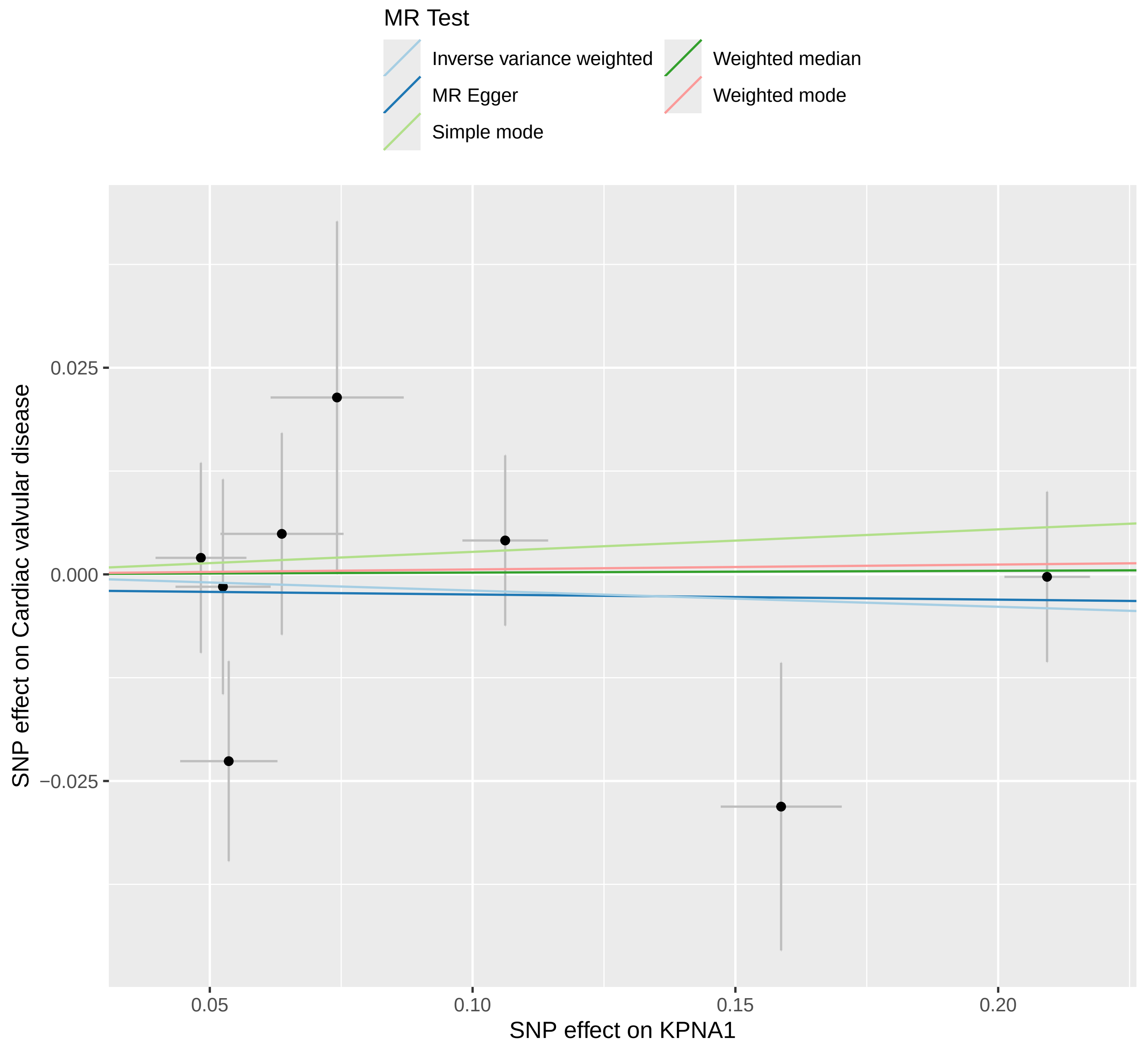
MR Test
Inverse variance weighted
MR Egger
Simple mode
Weighted median
Weighted mode
0.025
0.000
−0.025
SNP effect on Cardiac valvular disease
0.05
0.10
0.15
0.20
SNP effect on KPNA1

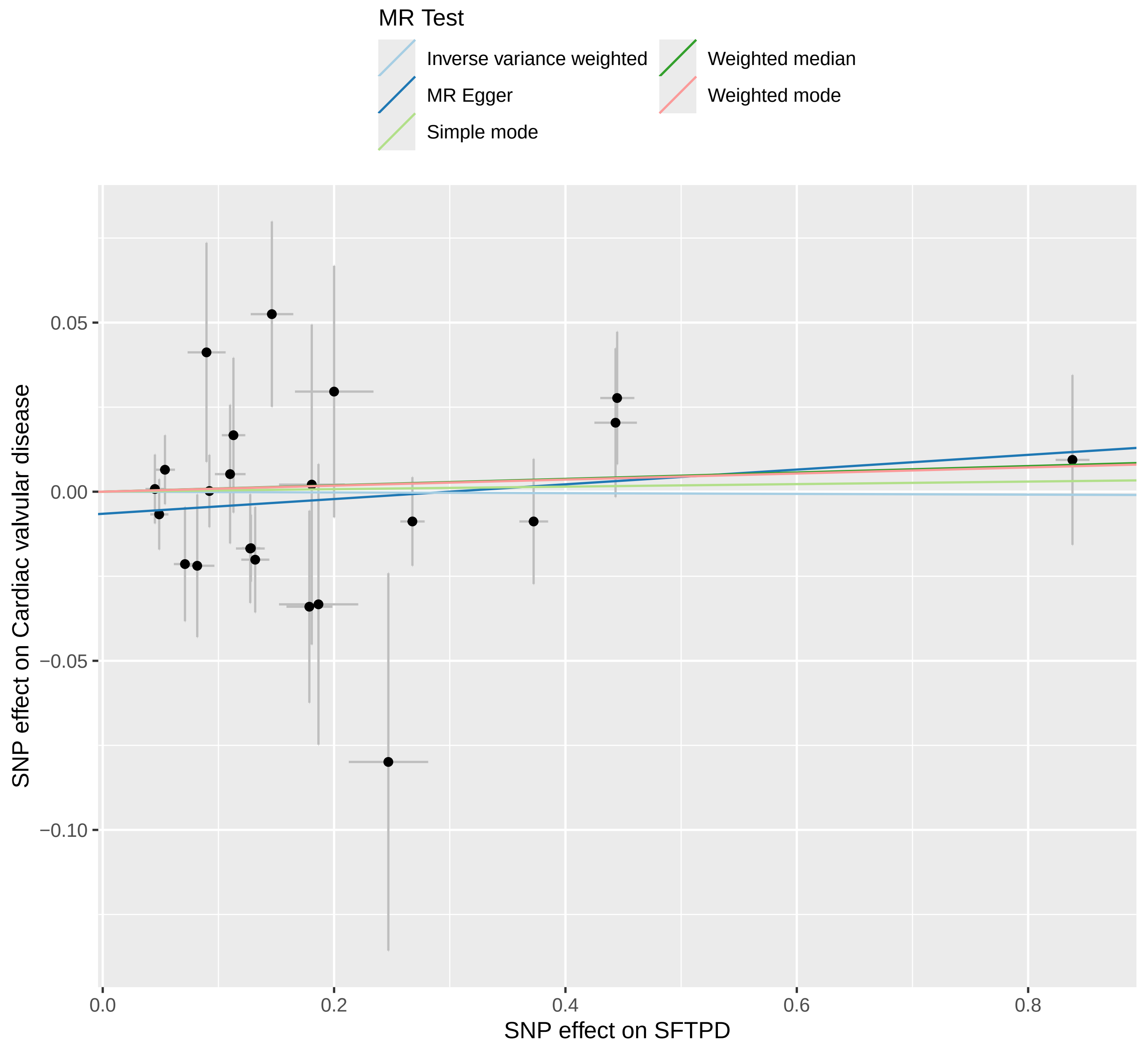
MR Test
Inverse variance weighted
MR Egger
Simple mode
Weighted median
Weighted mode
0.05
0.00
−0.05
−0.10
SNP effect on Cardiac valvular disease
0.0
0.2
0.4
0.6
0.8
SNP effect on SFTPD

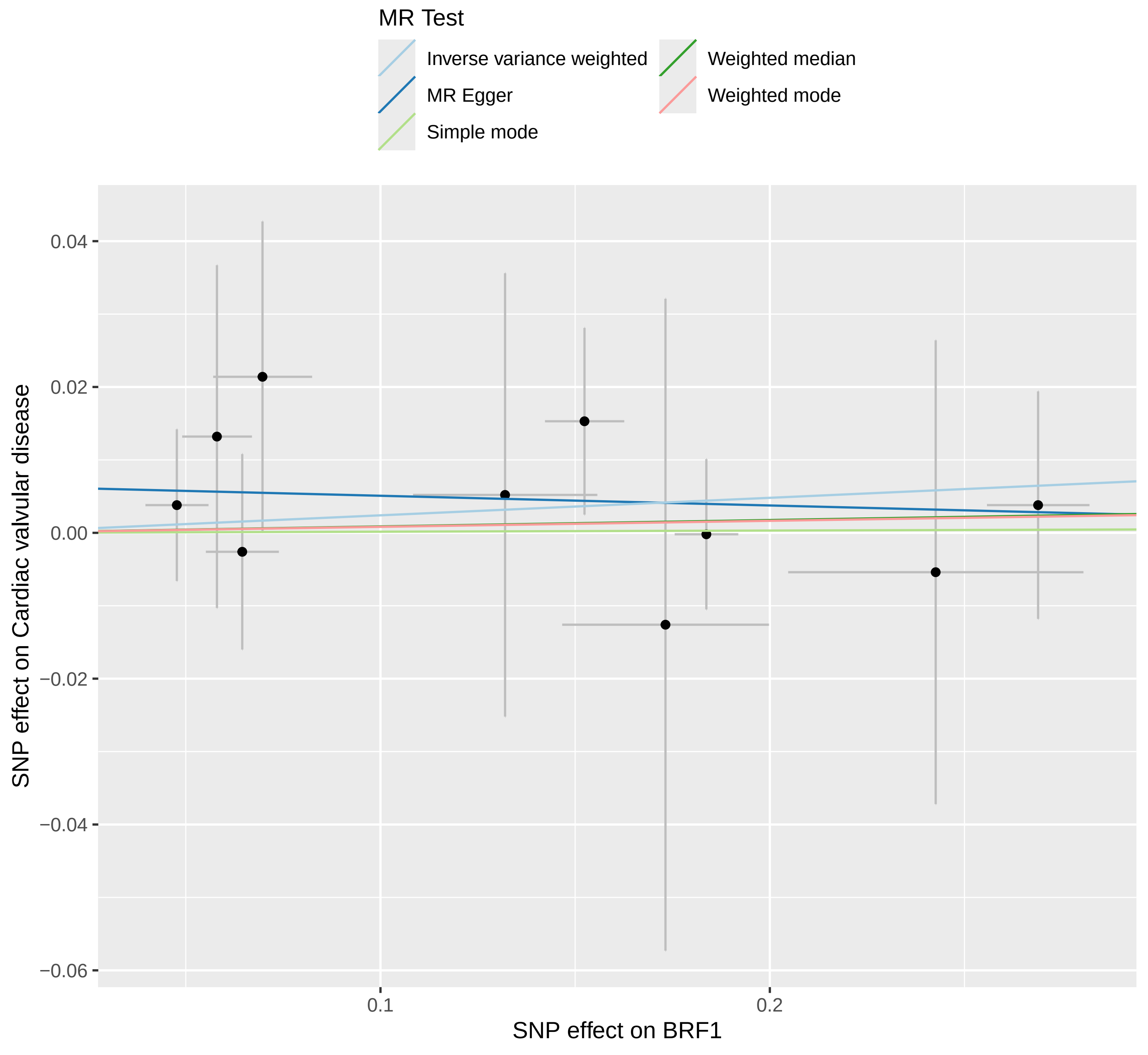
MR Test
Inverse variance weighted
MR Egger
Simple mode
Weighted median
Weighted mode
SNP effect on Cardiac valvular disease
0.04
0.02
0.00
−0.02
−0.04
−0.06
0.1
0.2
SNP effect on BRF1

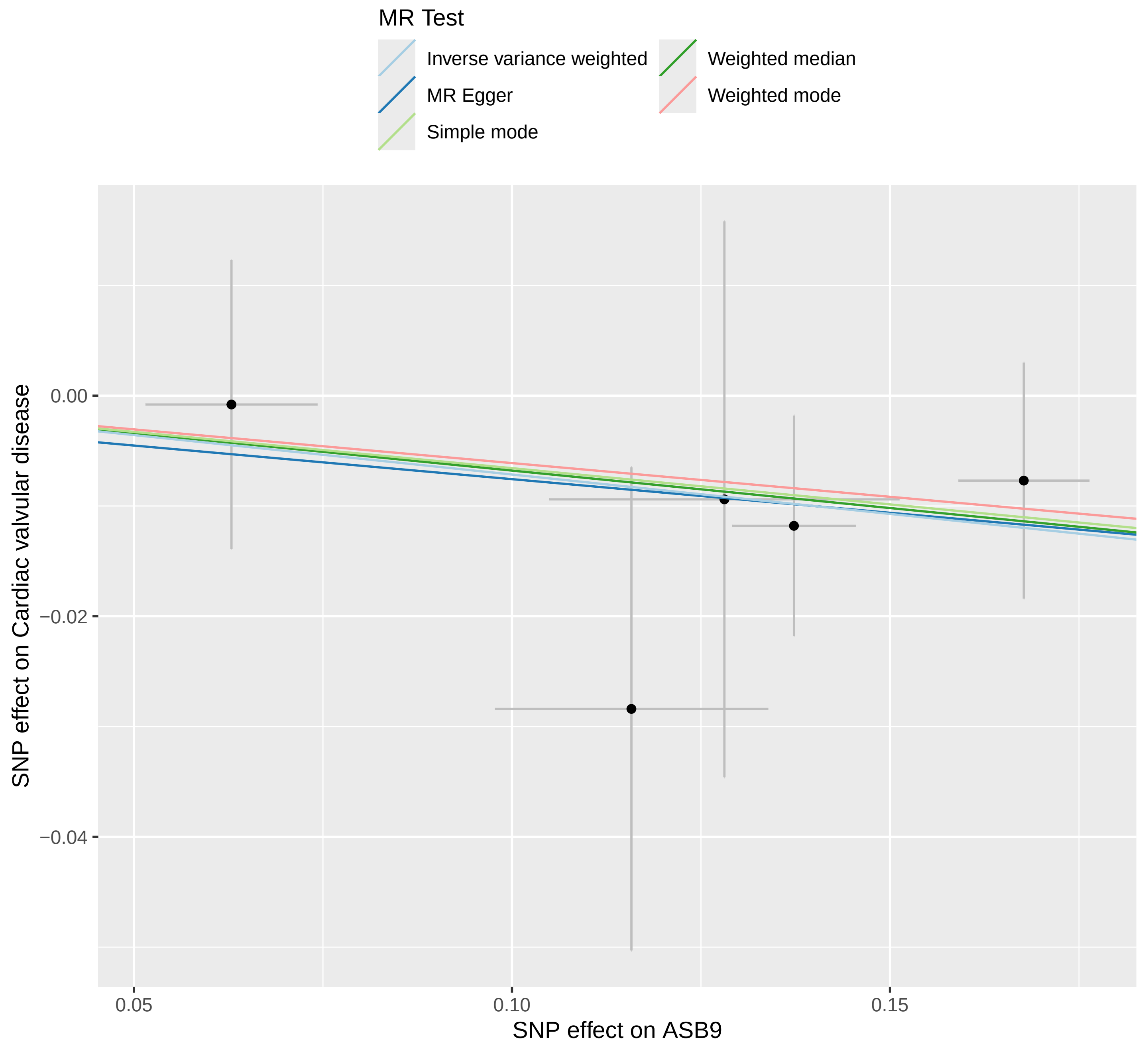
MR Test
Inverse variance weighted
MR Egger
Simple mode
Weighted median
Weighted mode
SNP effect on Cardiac valvular disease
0.00
−0.02
−0.04
0.05
0.10
0.15
SNP effect on ASB9

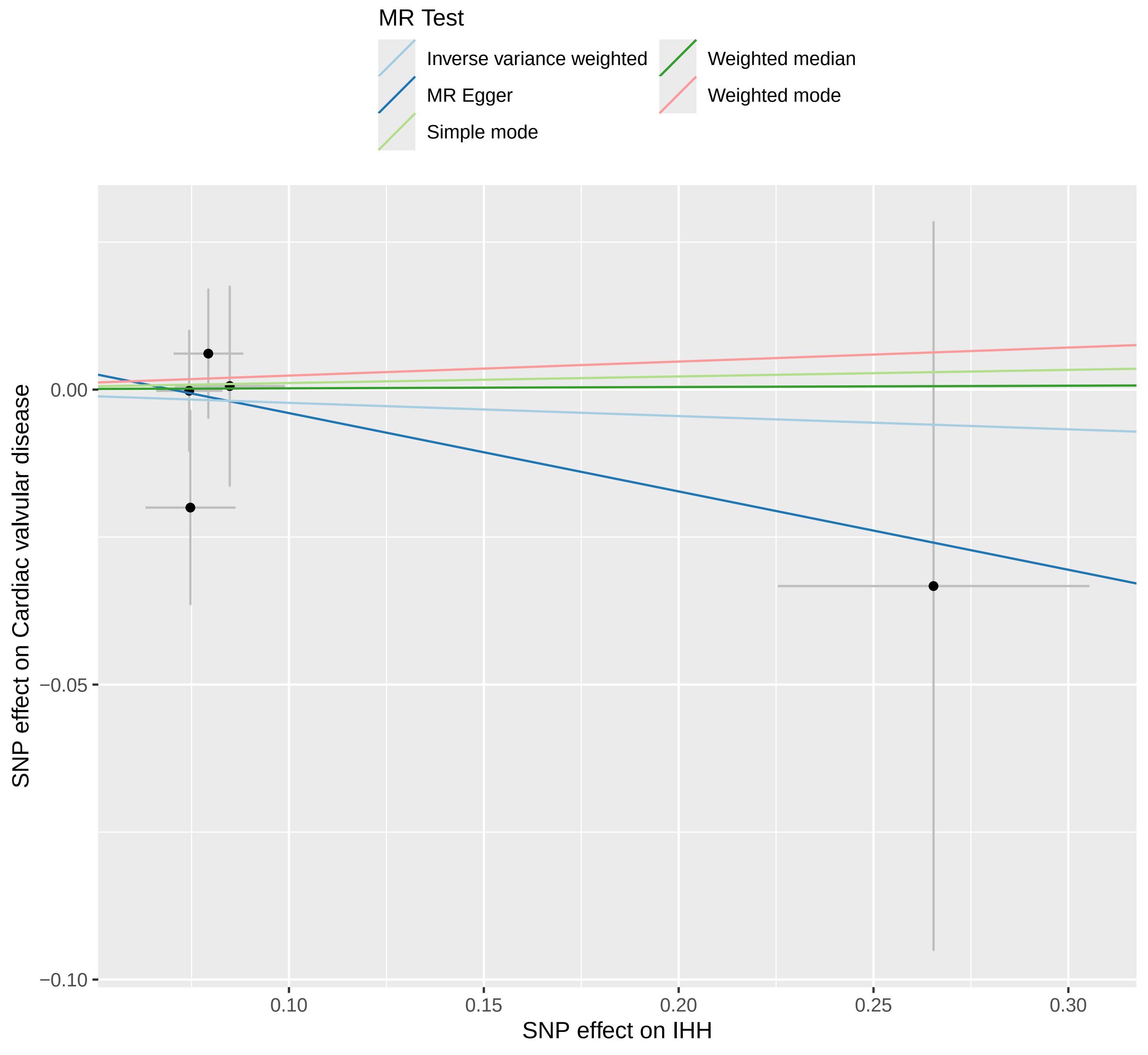
MR Test
Inverse variance weighted
MR Egger
Simple mode
Weighted median
Weighted mode
0.00
−0.05
−0.10
0.10
0.15
0.20
0.25
0.30
SNP effect on IHH
SNP effect on Cardiac valvular disease

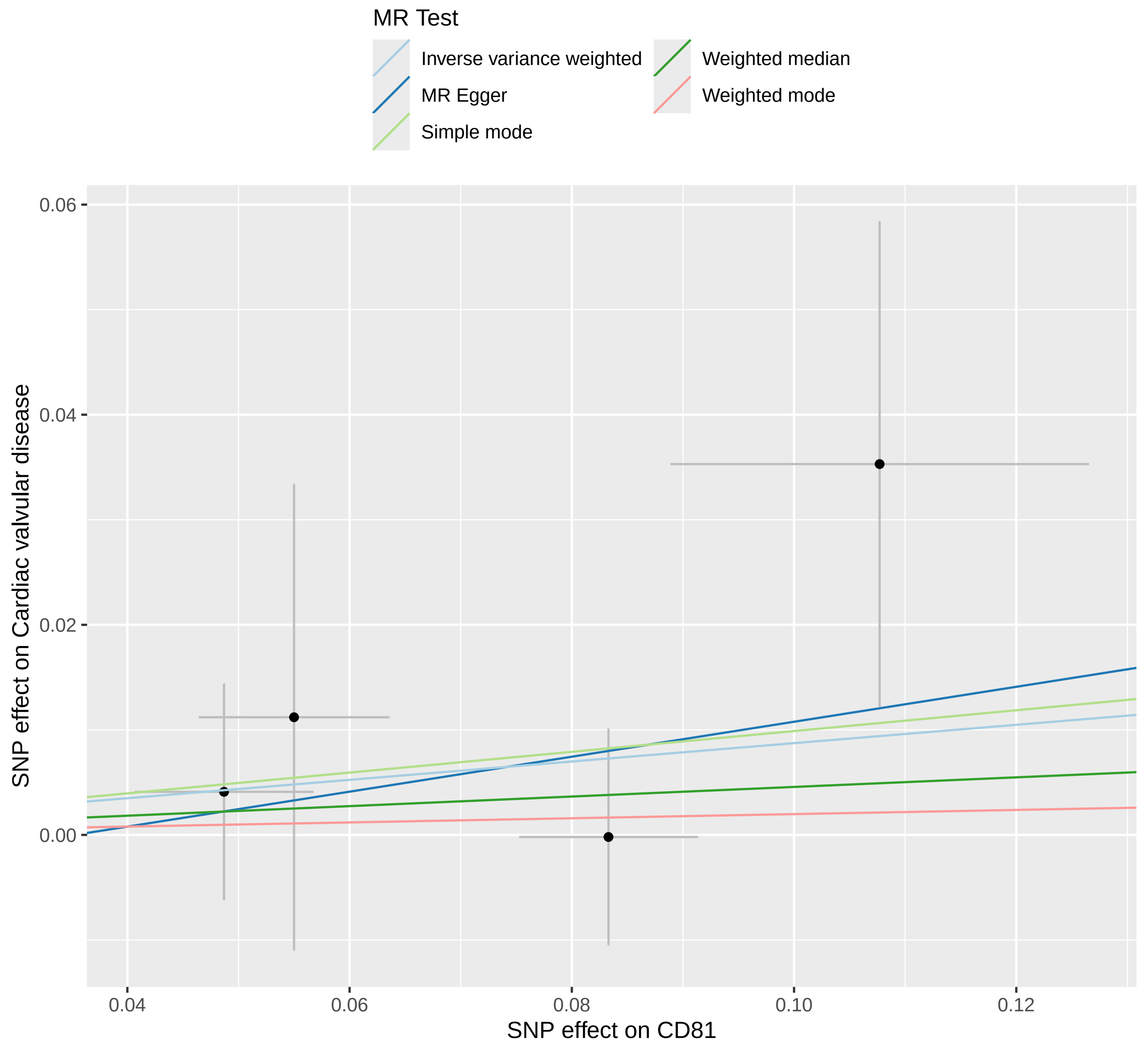
MR Test
Inverse variance weighted
MR Egger
Simple mode
Weighted median
Weighted mode
0.06
0.04
0.02
0.00
SNP effect on Cardiac valvular disease
0.04
0.06
0.08
0.10
0.12
SNP effect on CD81

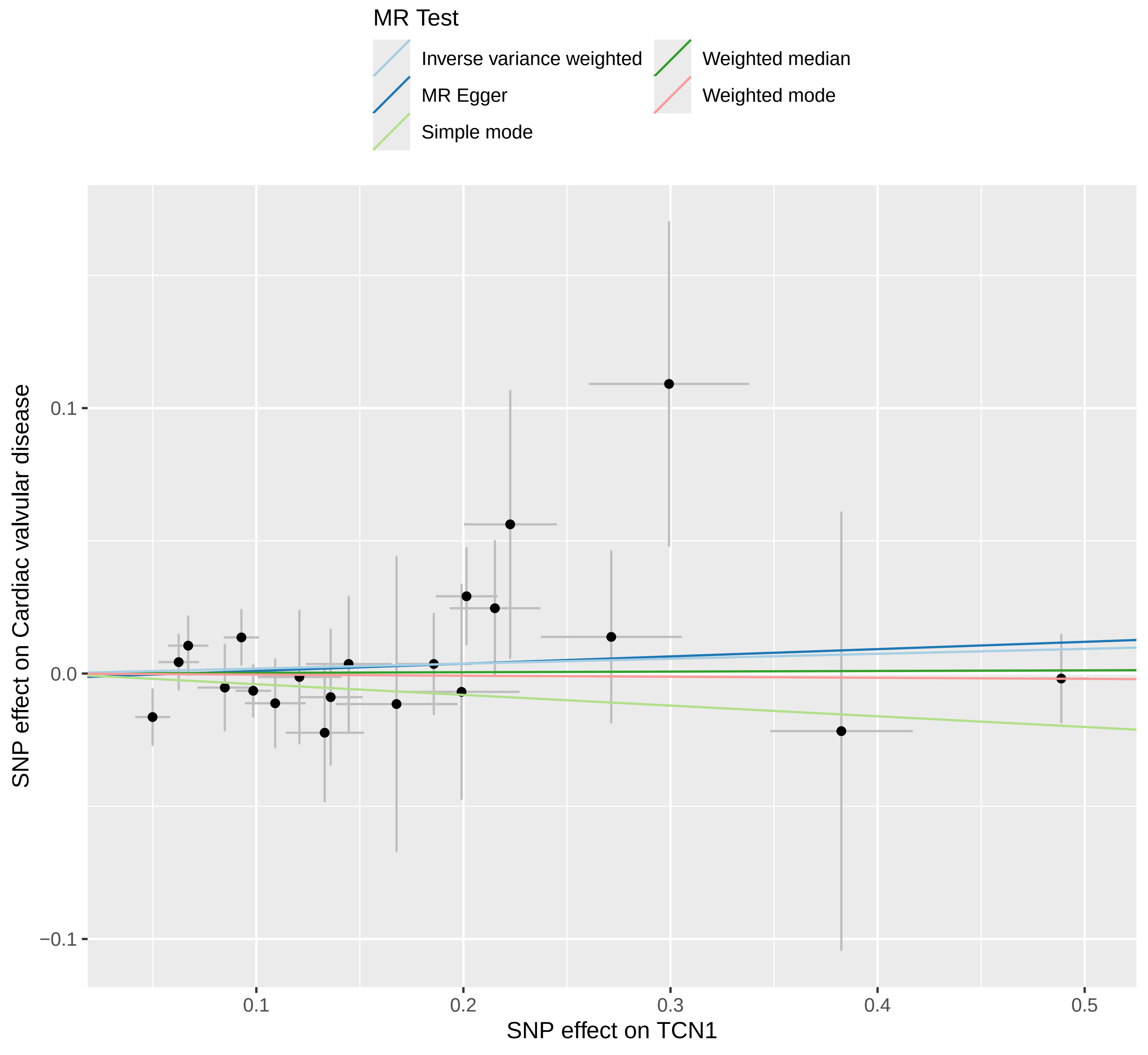

MR Test
Inverse variance weighted
MR Egger
Simple mode
Weighted median
Weighted mode
0.1
0.0
−0.1
0.1
0.2
0.3
0.4
0.5
SNP effect on TCN1
SNP effect on Cardiac valvular disease

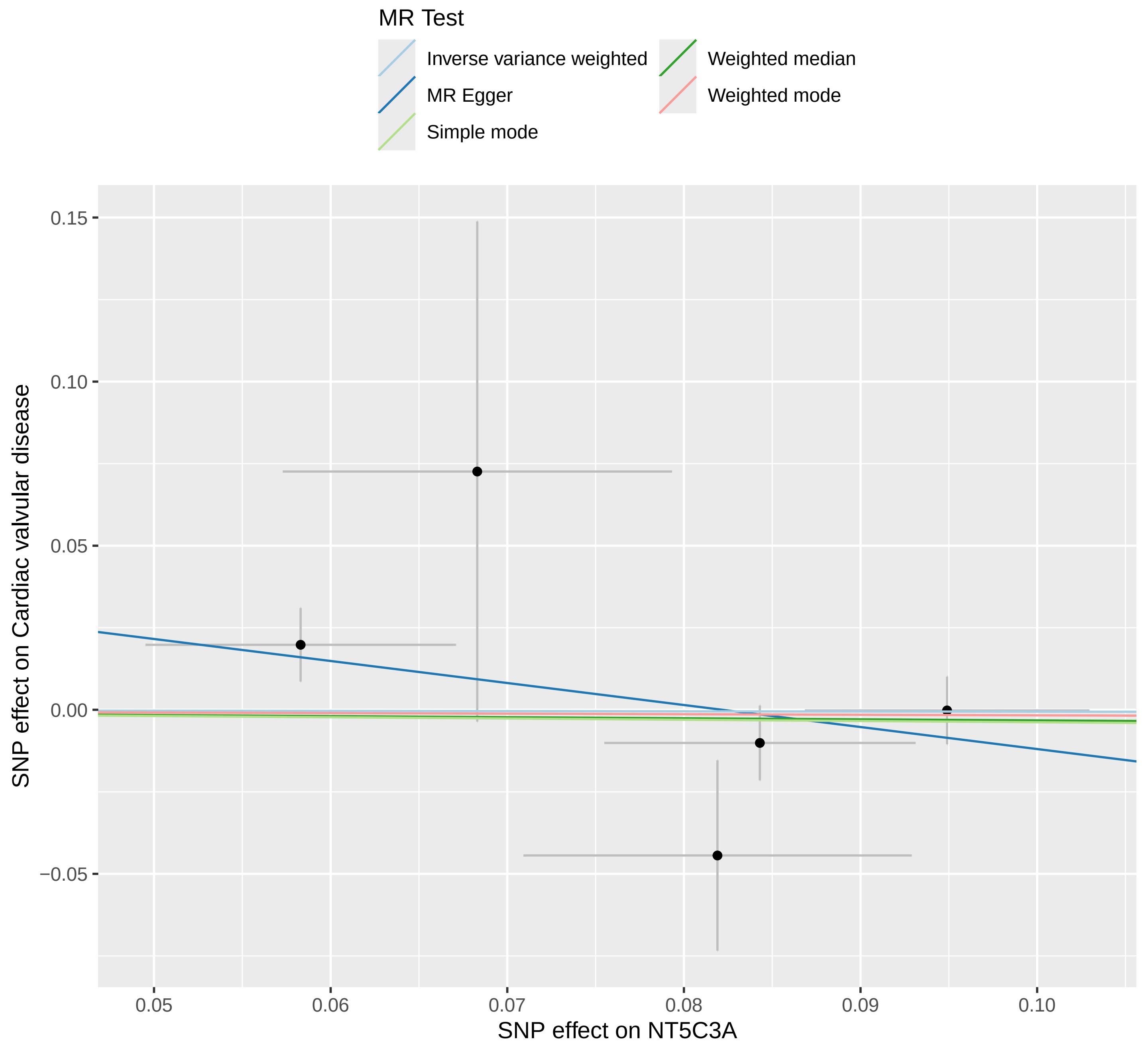
MR Test
Inverse variance weighted
MR Egger
Simple mode
Weighted median
Weighted mode
0.15
0.10
0.05
0.00
−0.05
0.05
0.06
0.07
0.08
0.09
0.10
SNP effect on Cardiac valvular disease
SNP effect on NT5C3A

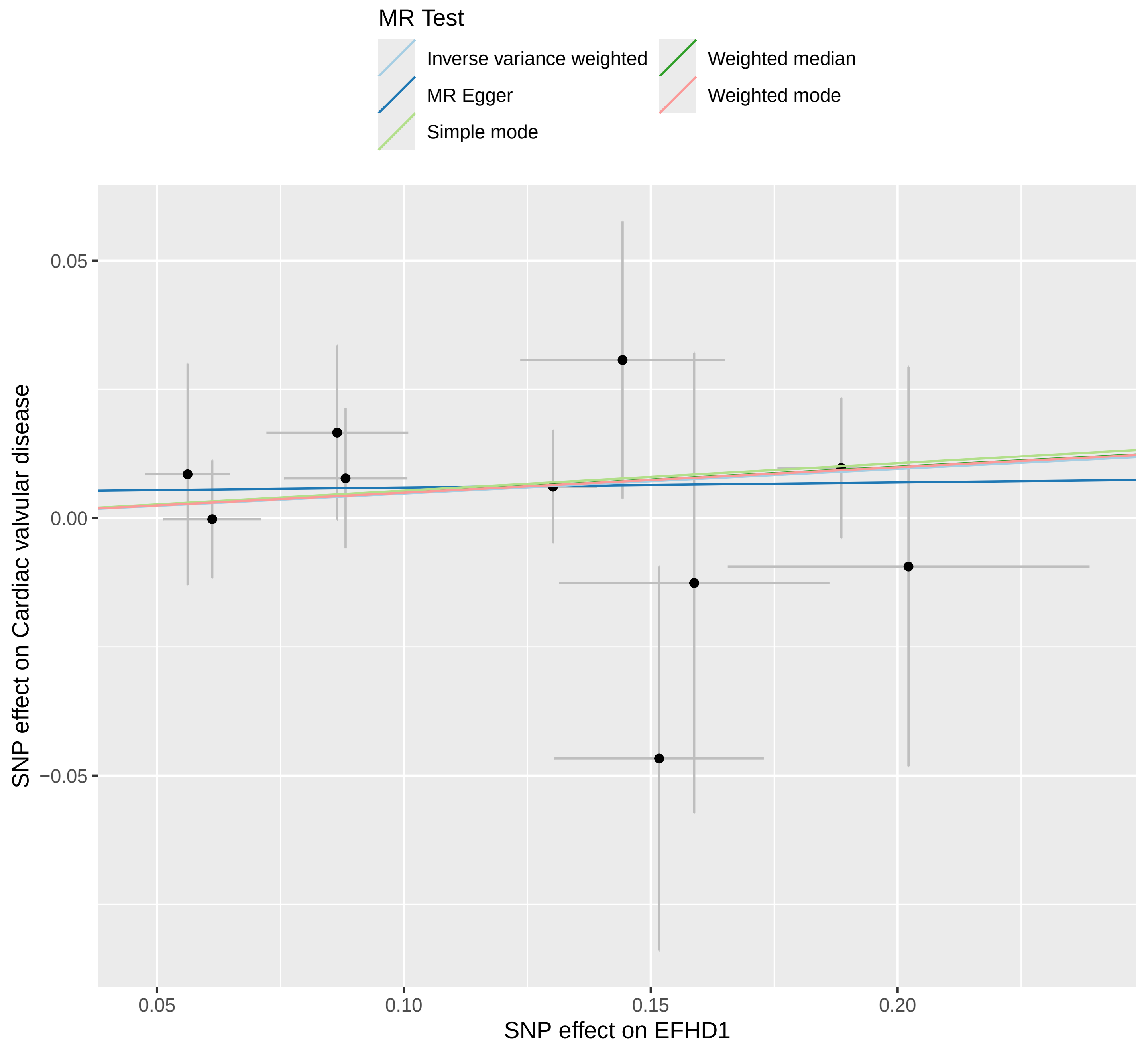
MR Test
Inverse variance weighted
MR Egger
Simple mode
Weighted median
Weighted mode
0.05
0.00
−0.05
0.05
0.10
0.15
0.20
SNP effect on Cardiac valvular disease
SNP effect on EFHD1

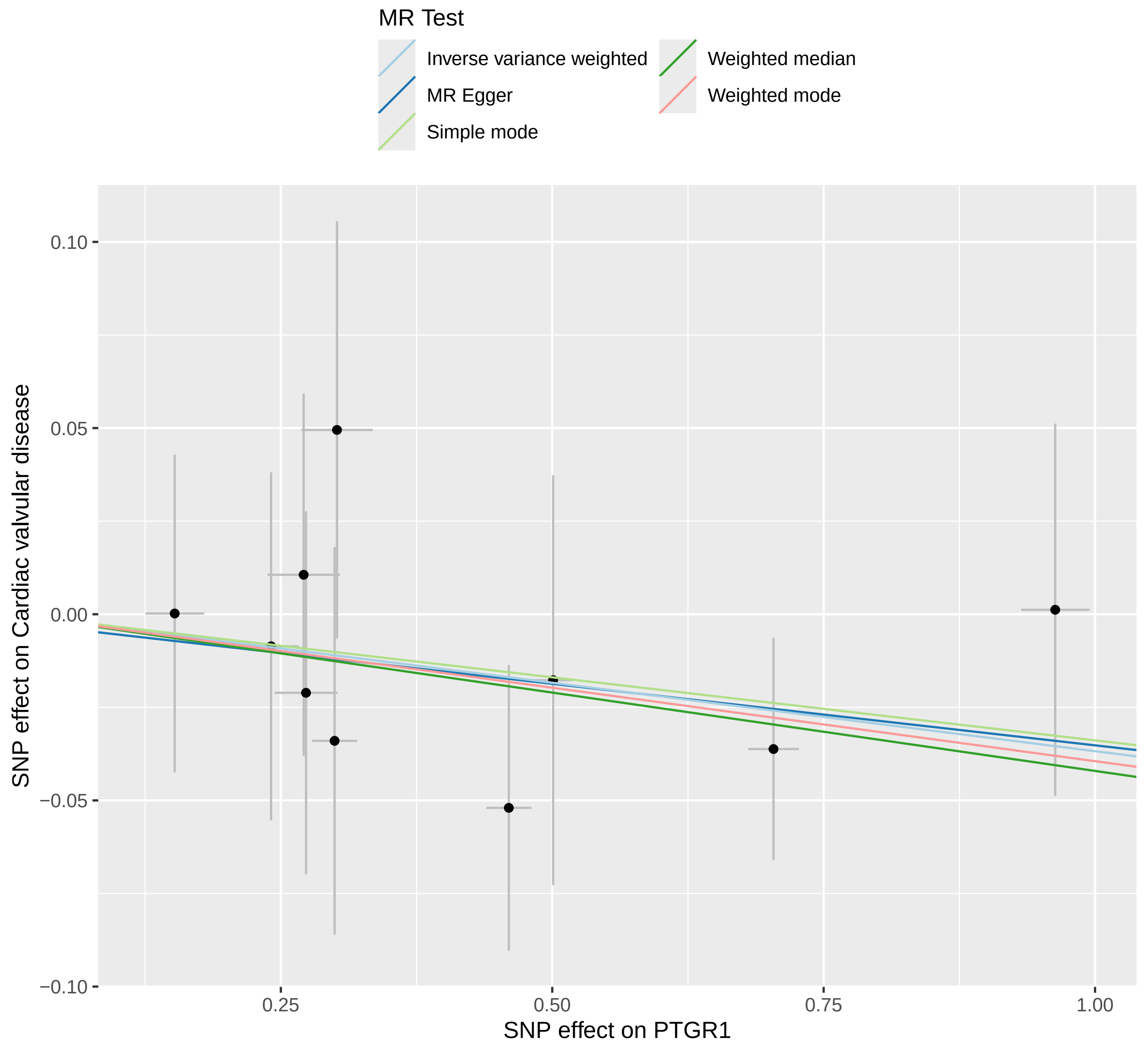

MR Test
Inverse variance weighted
MR Egger
Simple mode
Weighted median
Weighted mode
0.10
0.05
0.00
−0.05
−0.10
0.25
0.50
0.75
1.00
SNP effect on Cardiac valvular disease
SNP effect on PTGR1

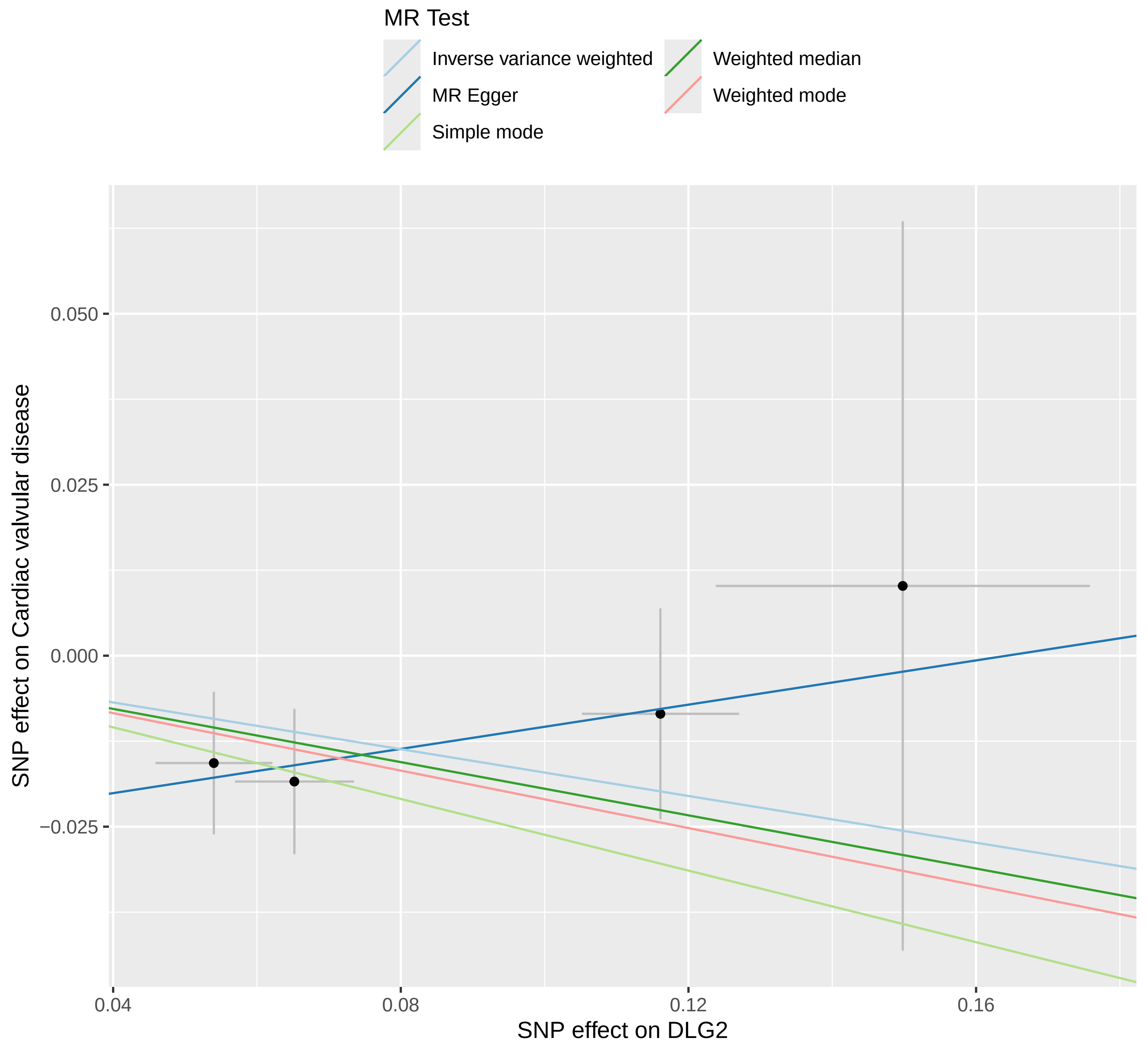
MR Test
Inverse variance weighted
MR Egger
Simple mode
Weighted median
Weighted mode
0.050
0.025
0.000
−0.025
SNP effect on Cardiac valvular disease
0.04
0.08
0.12
0.16
SNP effect on DLG2

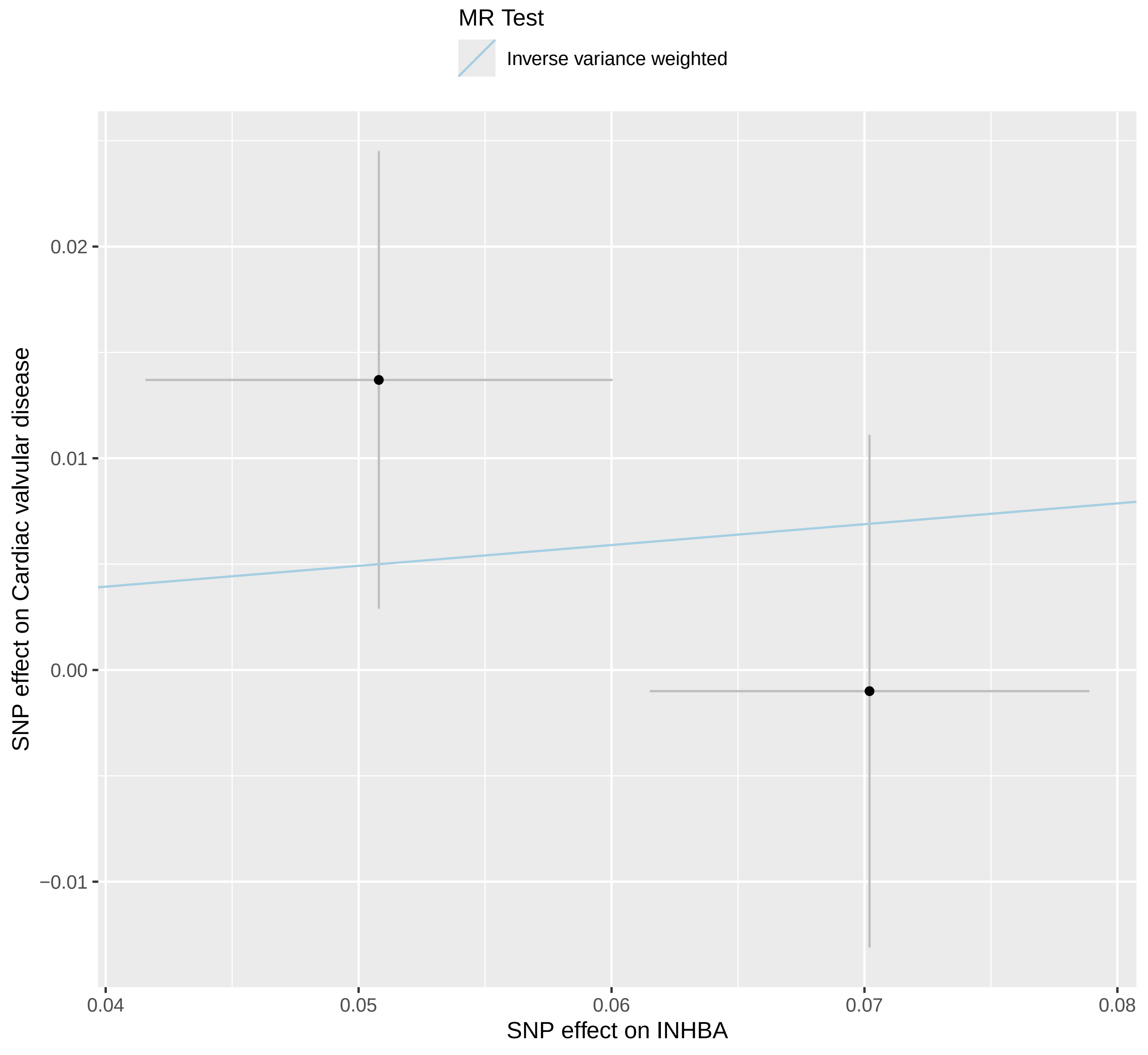
MR Test
Inverse variance weighted
0.02
0.01
0.00
−0.01
SNP effect on Cardiac valvular disease
0.04
0.05
0.06
0.07
0.08
SNP effect on INHBA

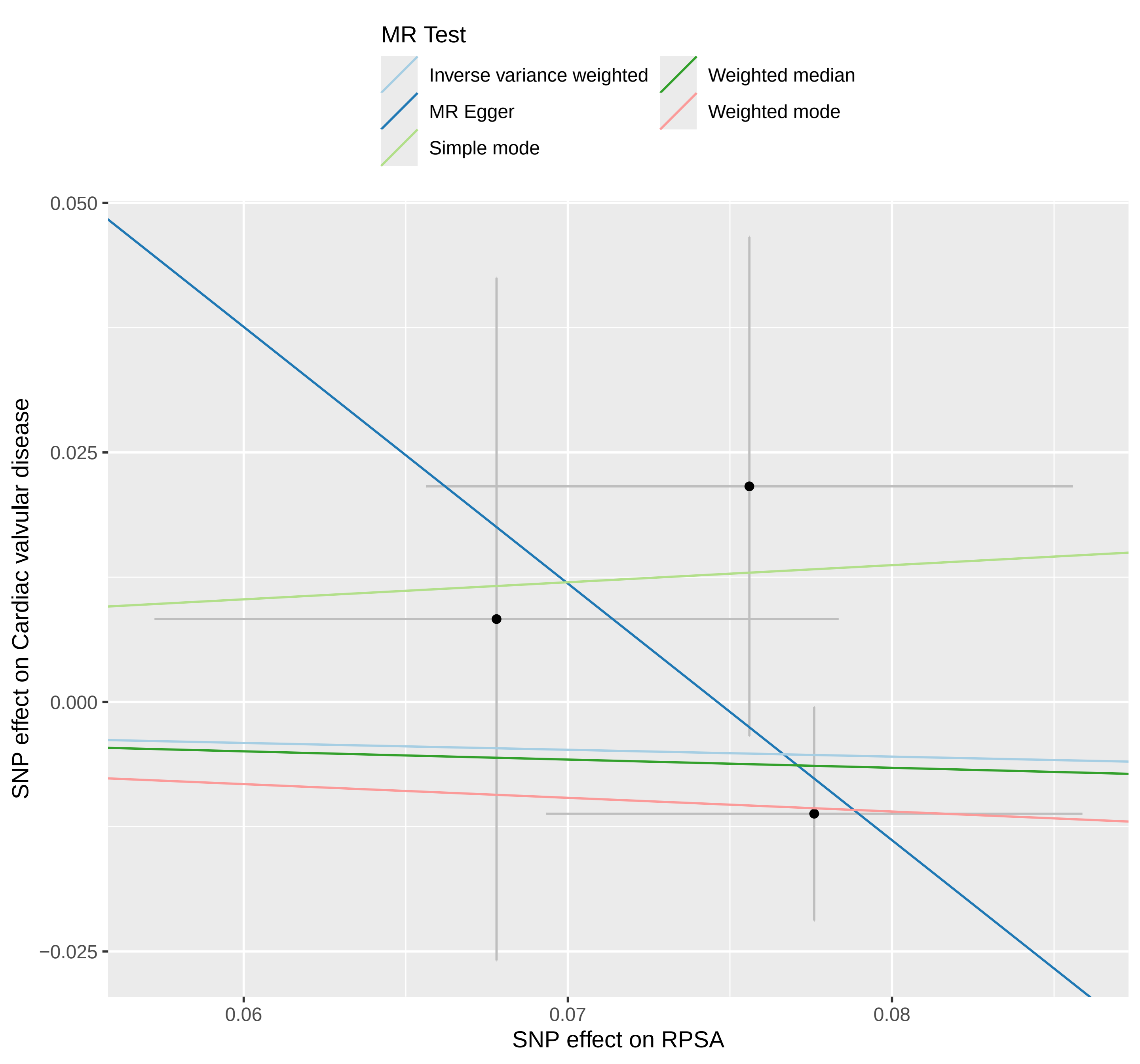
MR Test
Inverse variance weighted
MR Egger
Simple mode
Weighted median
Weighted mode
0.050
0.025
0.000
−0.025
0.06
0.07
0.08
SNP effect on RPSA
SNP effect on Cardiac valvular disease

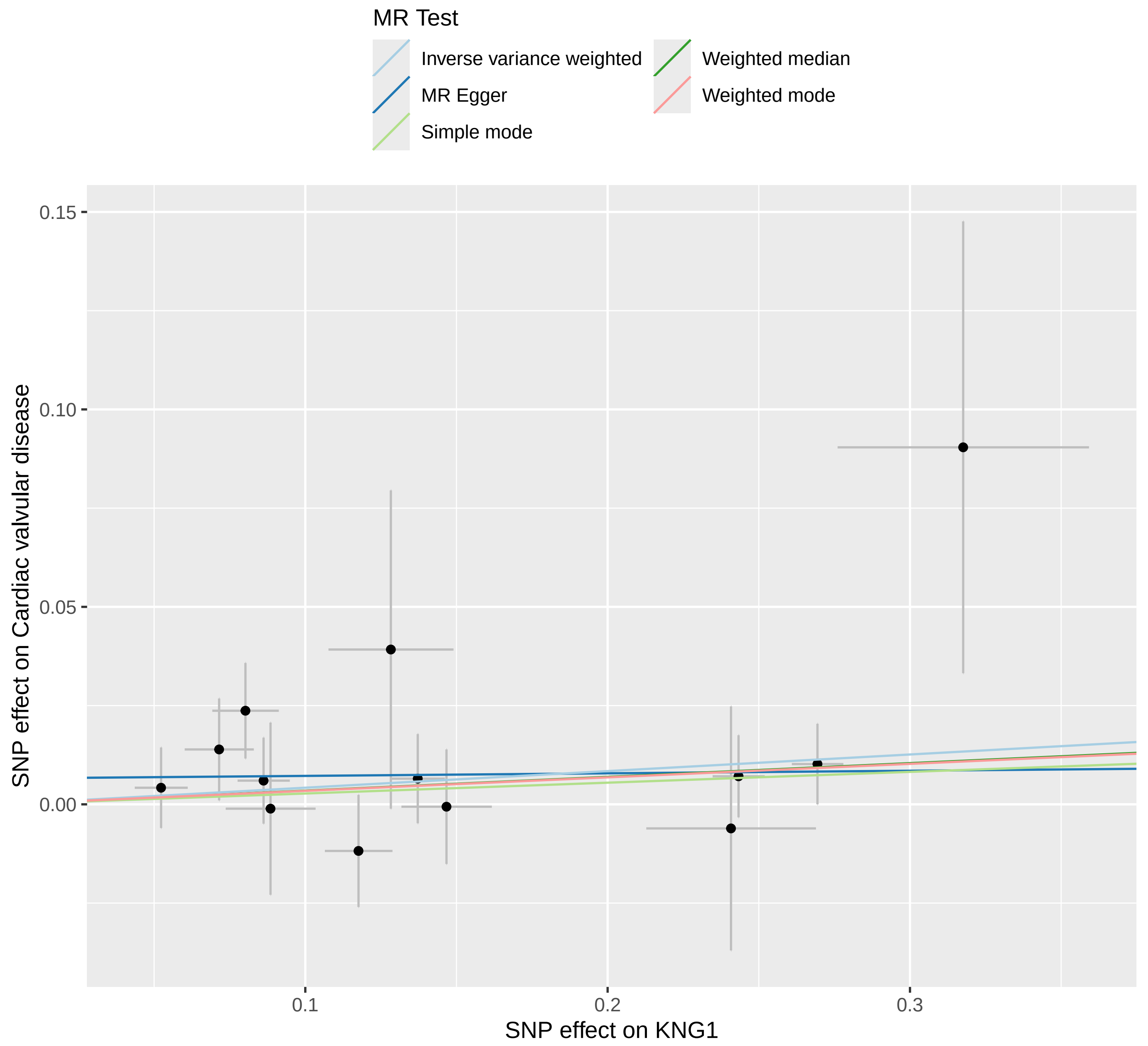
MR Test
Inverse variance weighted
MR Egger
Simple mode
Weighted median
Weighted mode
0.15
0.10
0.05
0.00
0.1
0.2
0.3
SNP effect on Cardiac valvular disease
SNP effect on KNG1

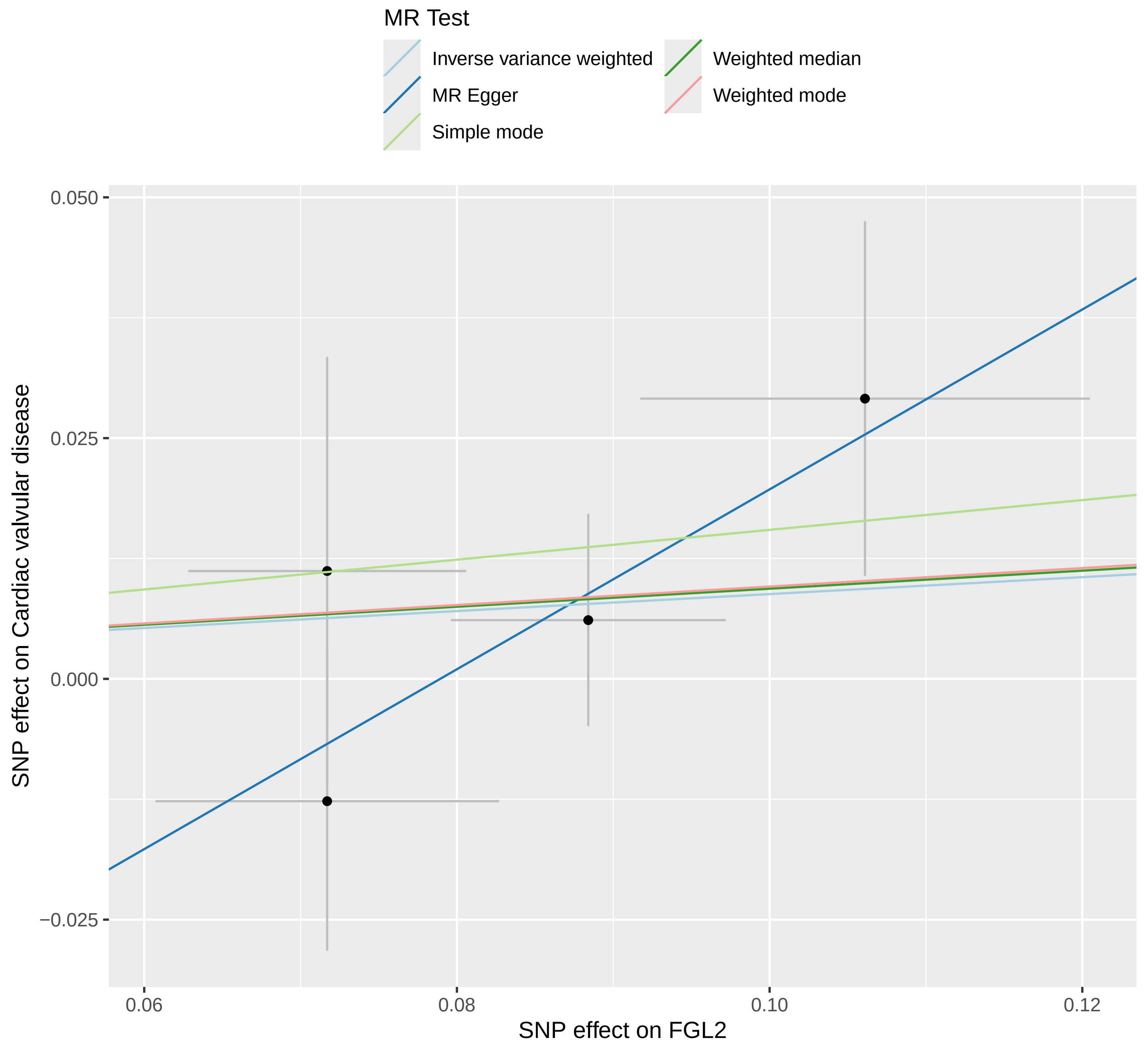
MR Test
Inverse variance weighted
MR Egger
Simple mode
Weighted median
Weighted mode
0.050
0.025
0.000
−0.025
SNP effect on Cardiac valvular disease
0.06
0.08
0.10
0.12
SNP effect on FGL2

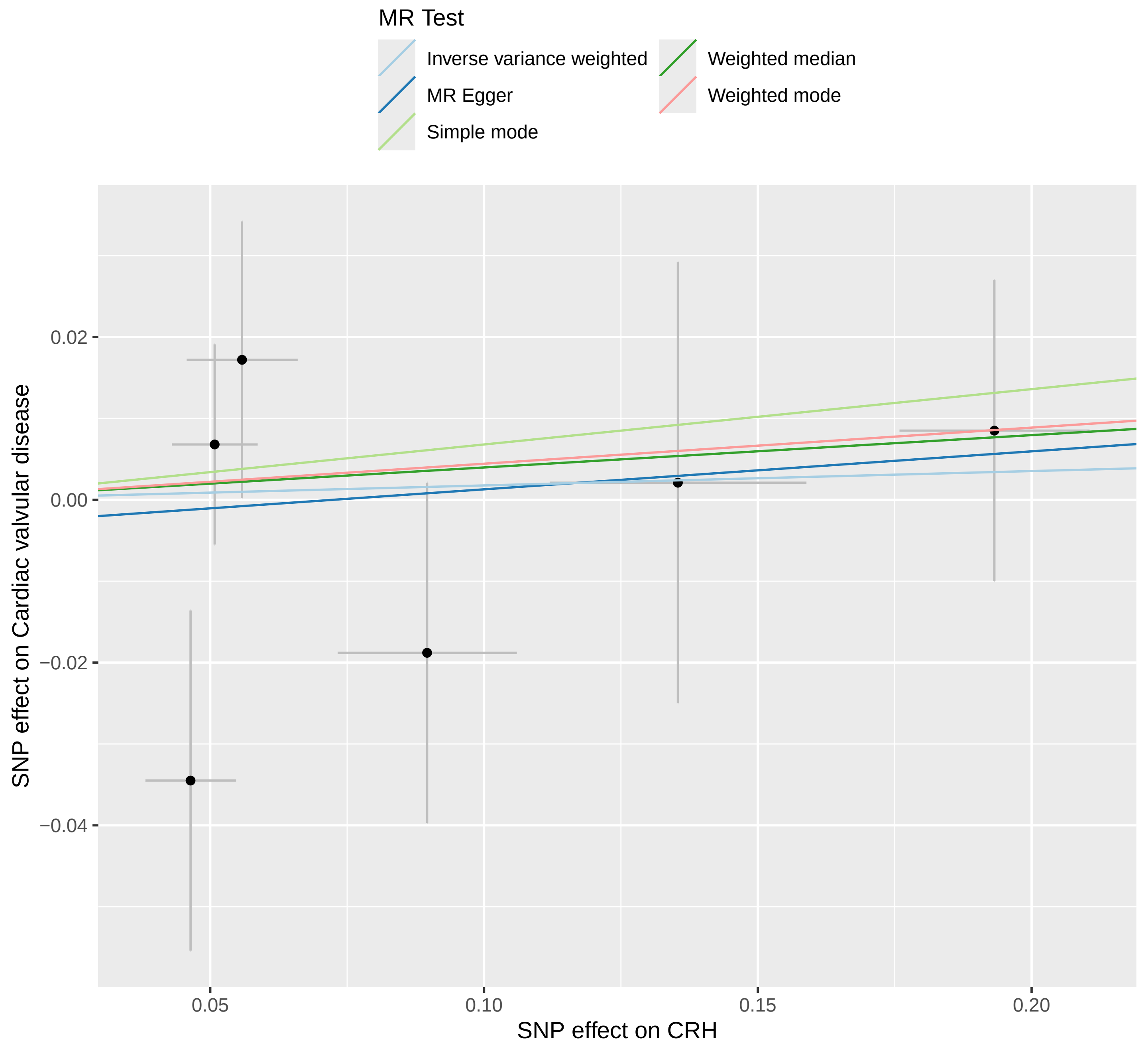
MR Test
Inverse variance weighted
MR Egger
Simple mode
Weighted median
Weighted mode
0.02
0.00
−0.02
−0.04
SNP effect on Cardiac valvular disease
0.05
0.10
0.15
0.20
SNP effect on CRH

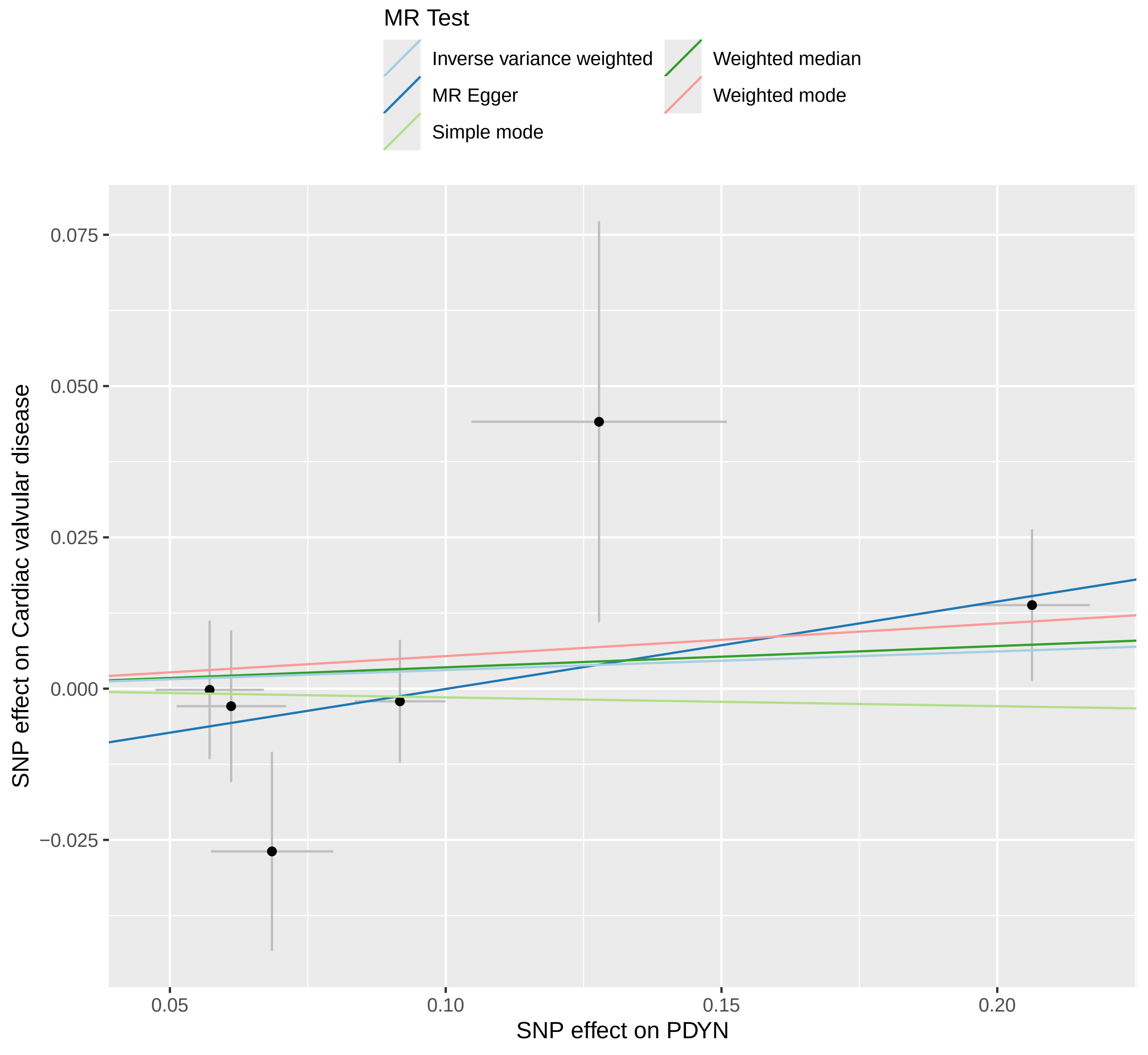
MR Test
Inverse variance weighted
MR Egger
Simple mode
Weighted median
Weighted mode
0.075
0.050
0.025
0.000
−0.025
SNP effect on Cardiac valvular disease
0.05
0.10
0.15
0.20
SNP effect on PDYN

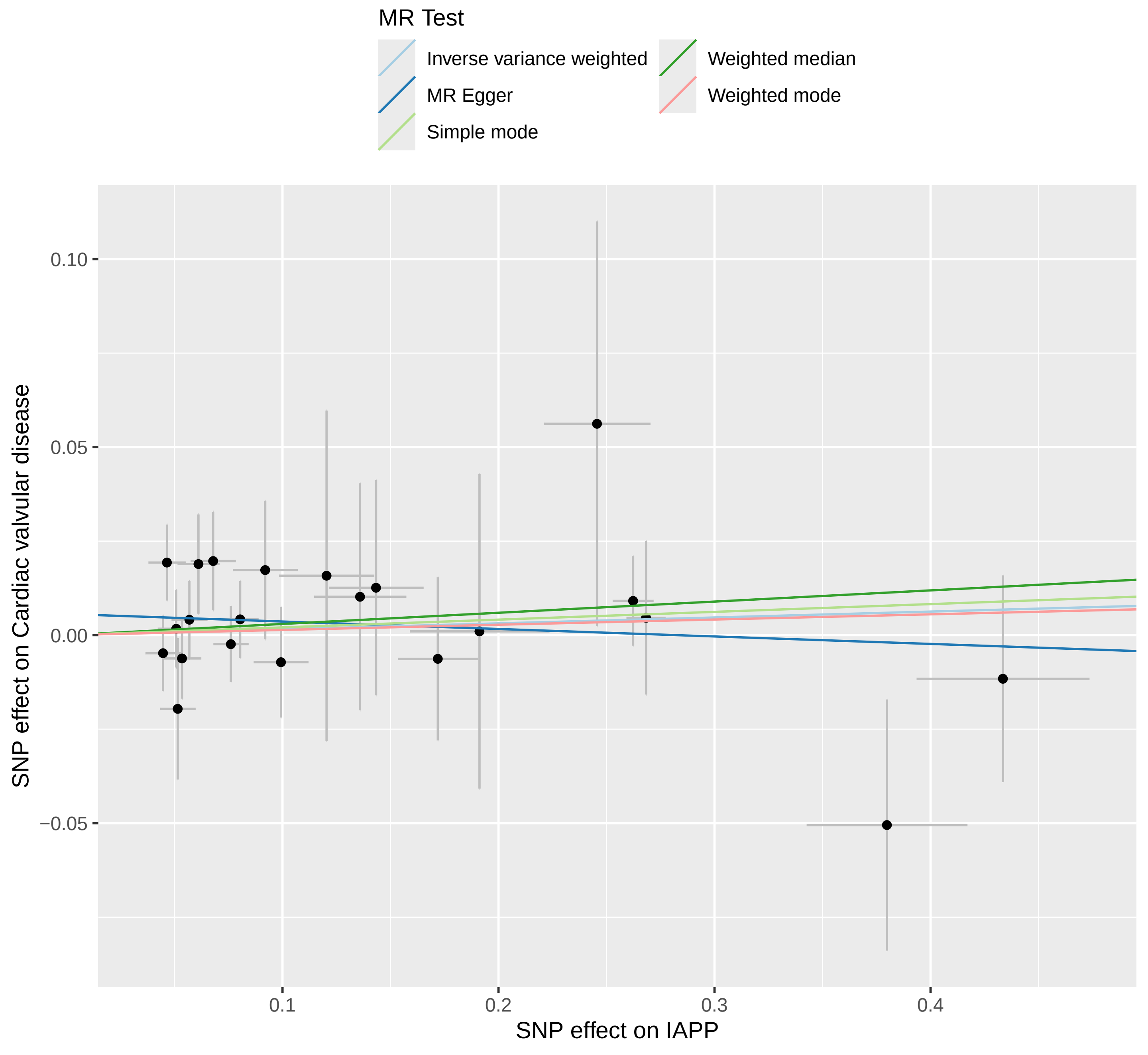
MR Test
Inverse variance weighted
MR Egger
Simple mode
Weighted median
Weighted mode
0.10
0.05
0.00
−0.05
SNP effect on Cardiac valvular disease
0.1
0.2
0.3
0.4
SNP effect on IAPP

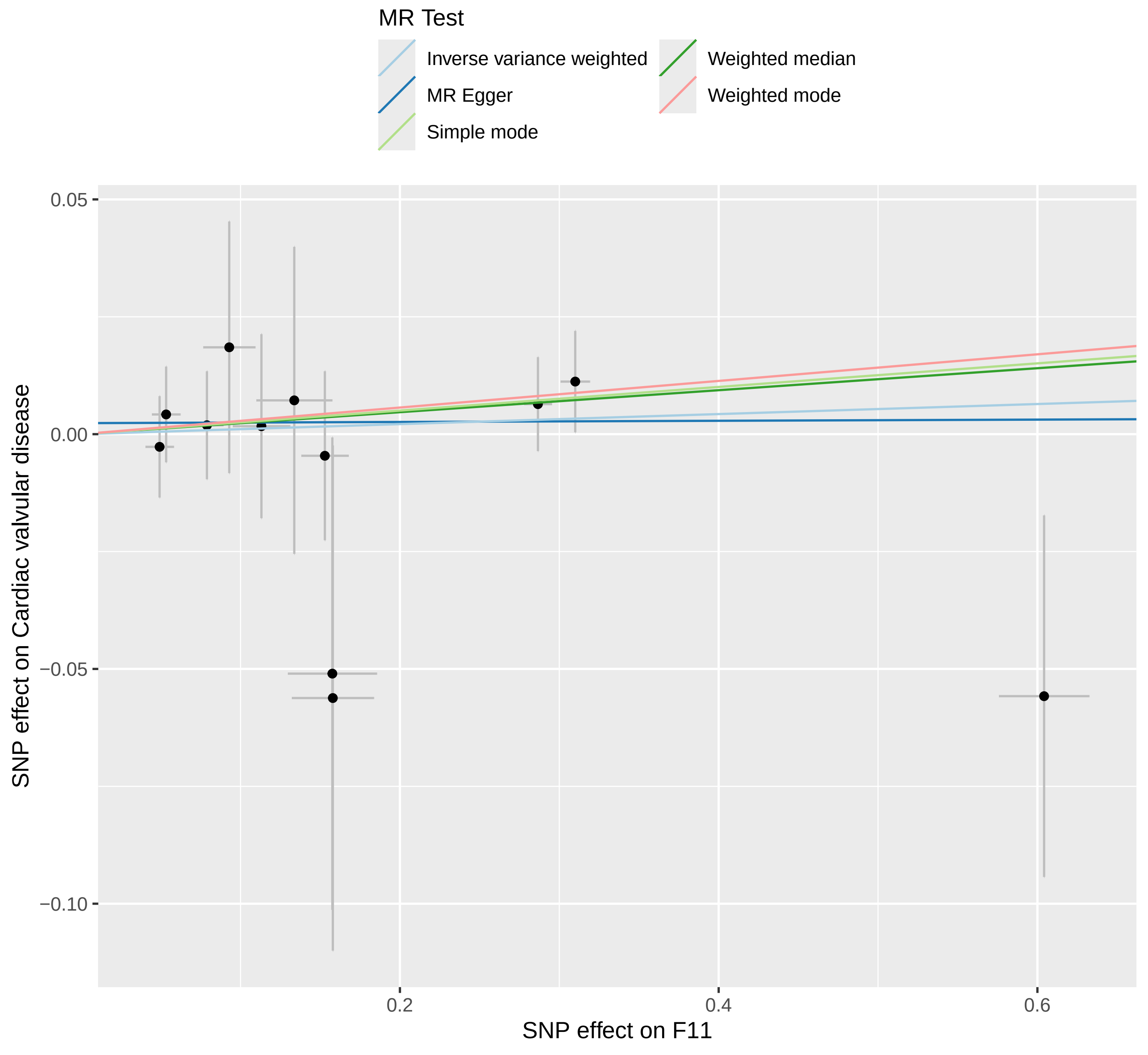
MR Test
Inverse variance weighted
MR Egger
Simple mode
Weighted median
Weighted mode
0.05
0.00
−0.05
−0.10
0.2
0.4
0.6
SNP effect on Cardiac valvular disease
SNP effect on F11

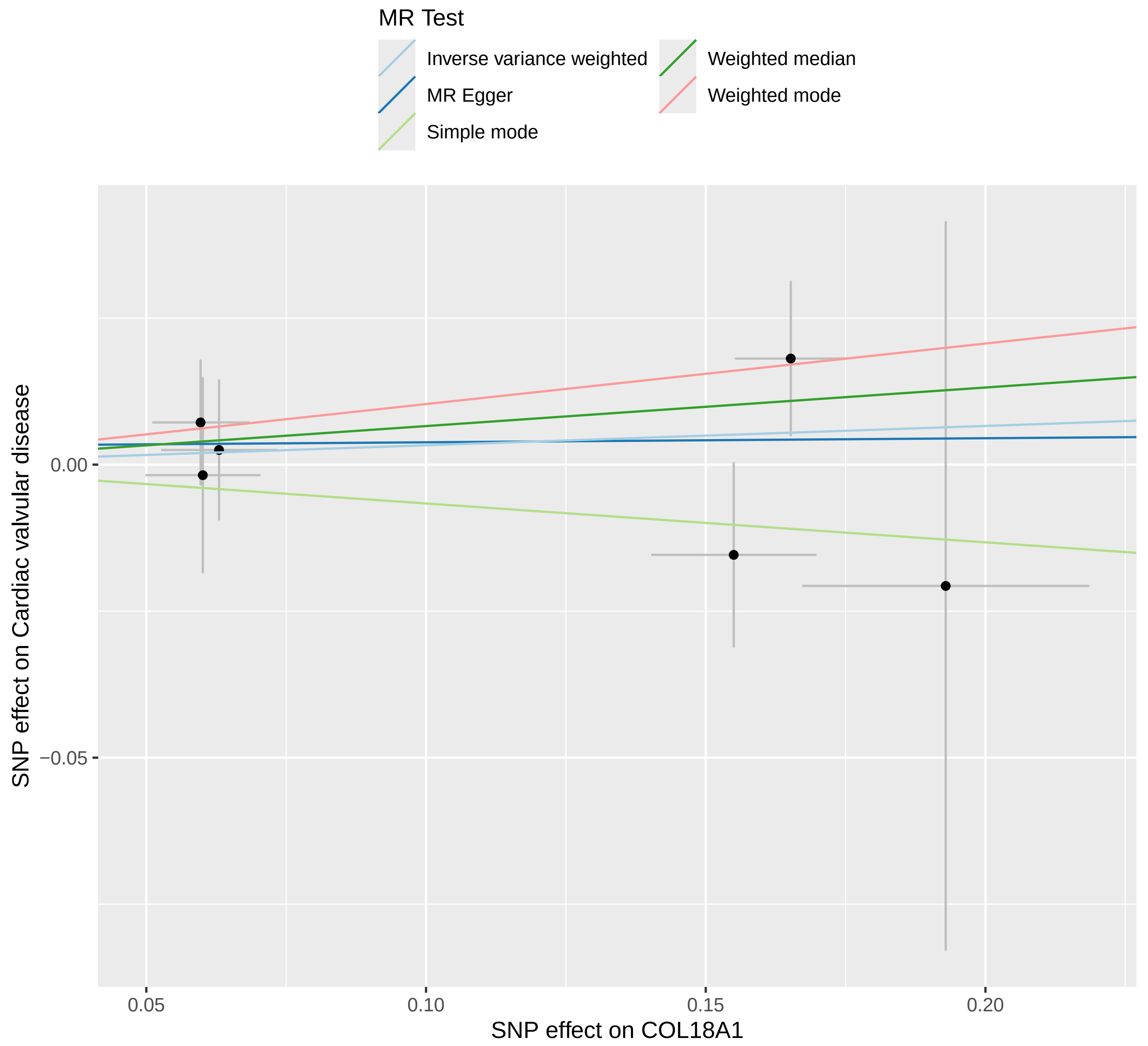

MR Test
Inverse variance weighted
MR Egger
Simple mode
Weighted median
Weighted mode
SNP effect on Cardiac valvular disease
0.00
−0.05
0.05
0.10
0.15
0.20
SNP effect on COL18A1

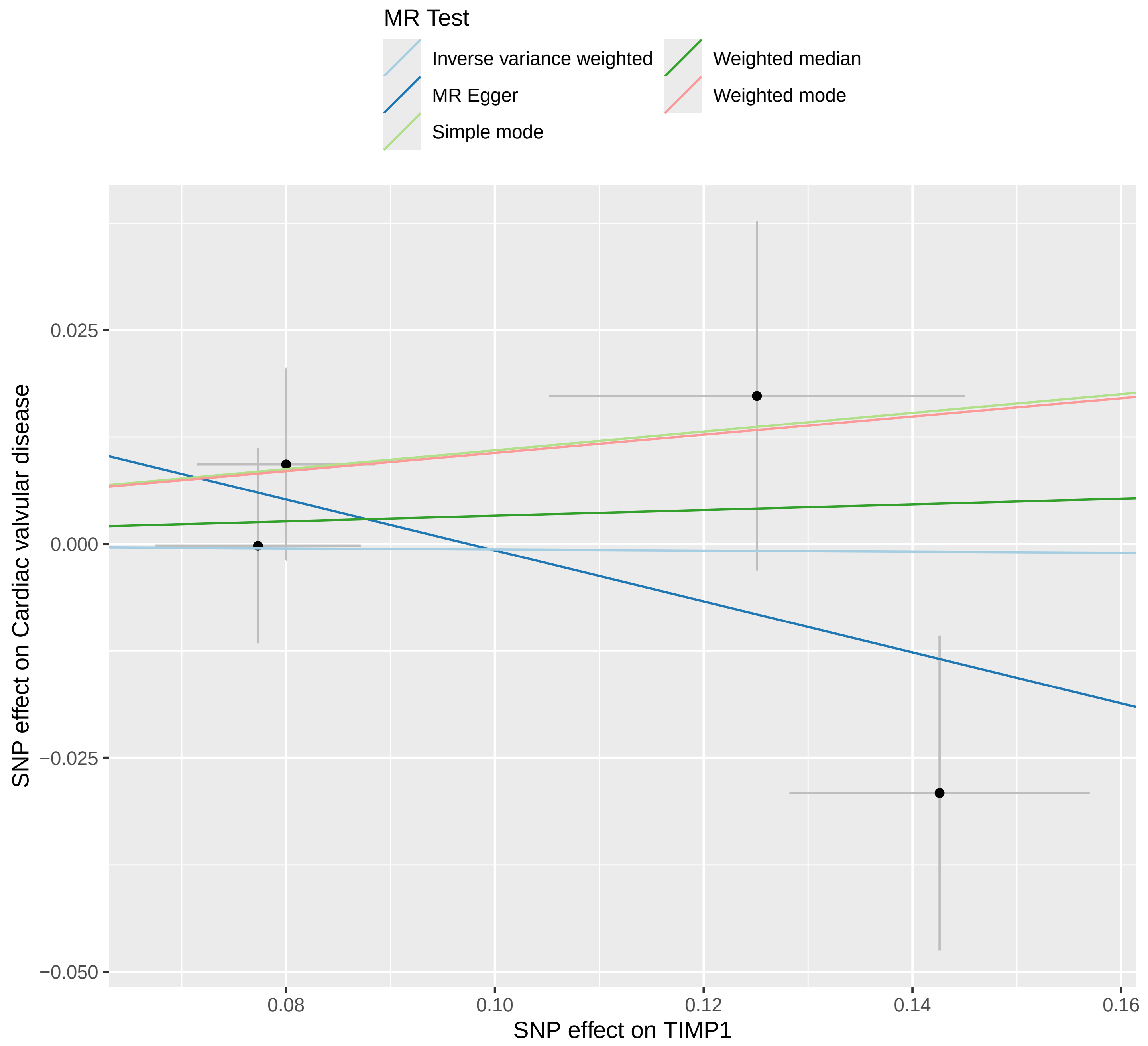
MR Test
Inverse variance weighted
MR Egger
Simple mode
Weighted median
Weighted mode
SNP effect on Cardiac valvular disease
0.025
0.000
−0.025
−0.050
0.08
0.10
0.12
0.14
0.16
SNP effect on TIMP1

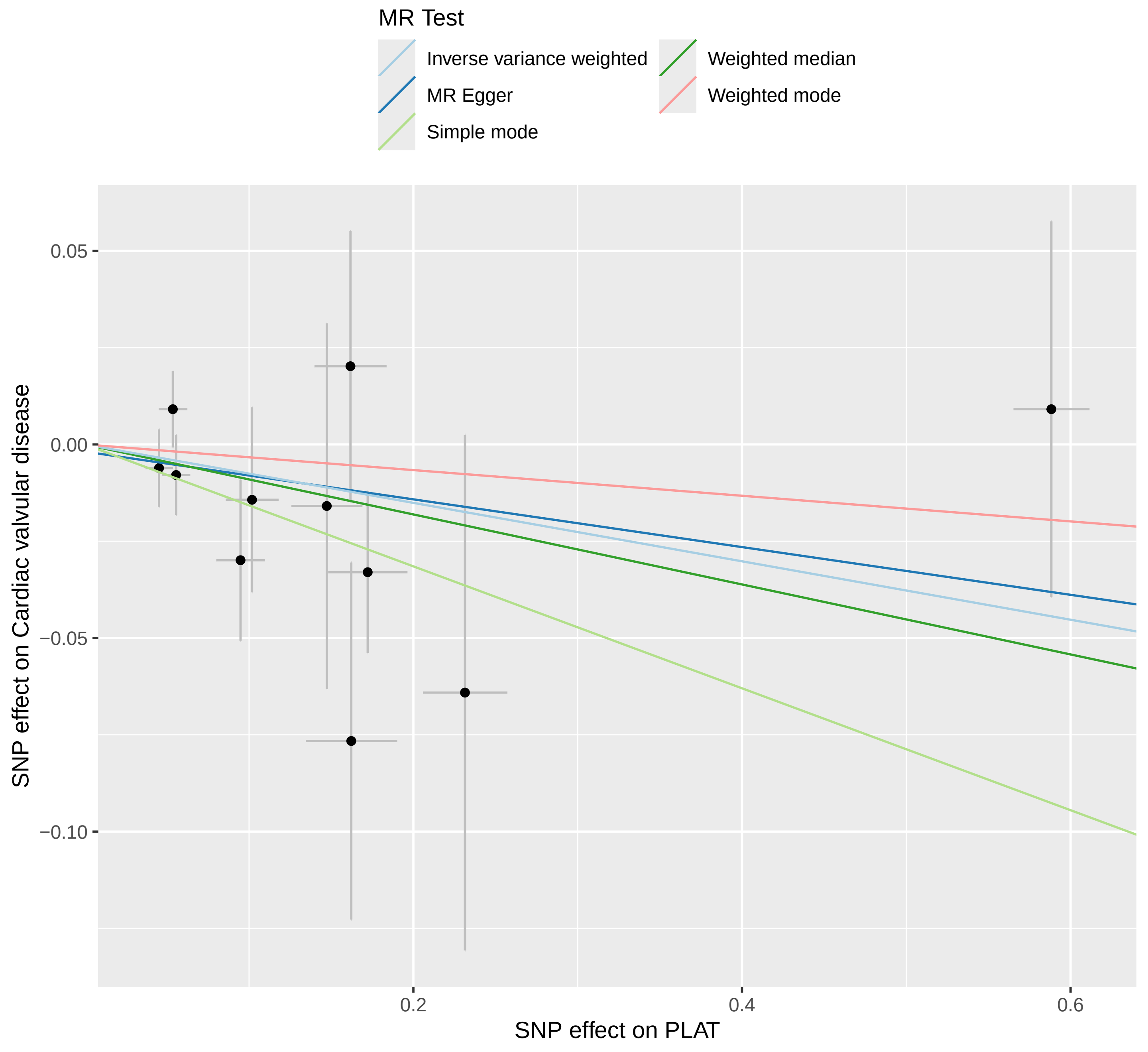

MR Test
Inverse variance weighted
MR Egger
Simple mode
Weighted median
Weighted mode
0.05
0.00
−0.05
−0.10
SNP effect on Cardiac valvular disease
0.2
0.4
0.6
SNP effect on PLAT

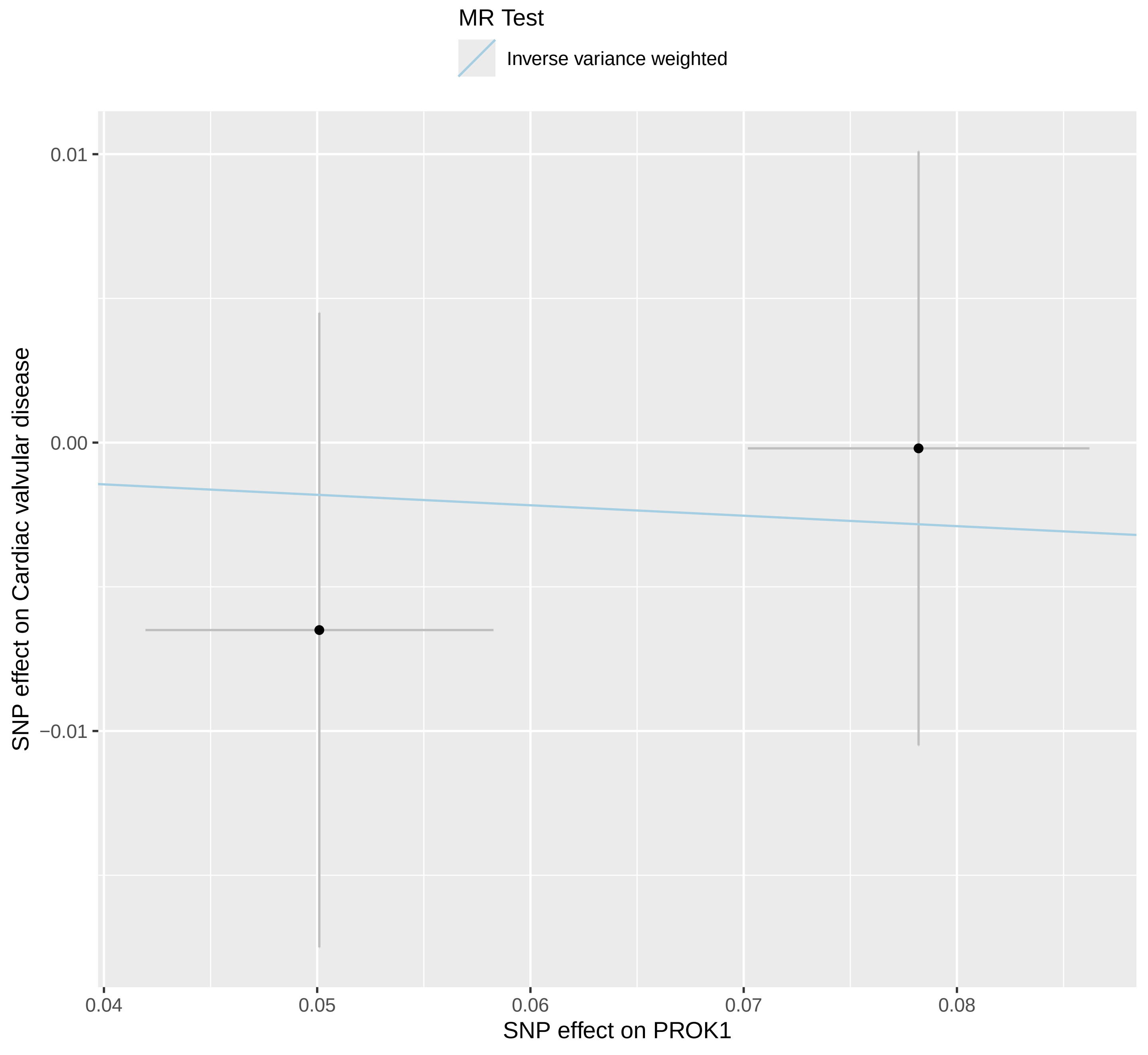

MR Test
Inverse variance weighted
0.01
0.00
−0.01
0.04
0.05
0.06
0.07
0.08
SNP effect on PROK1
SNP effect on Cardiac valvular disease

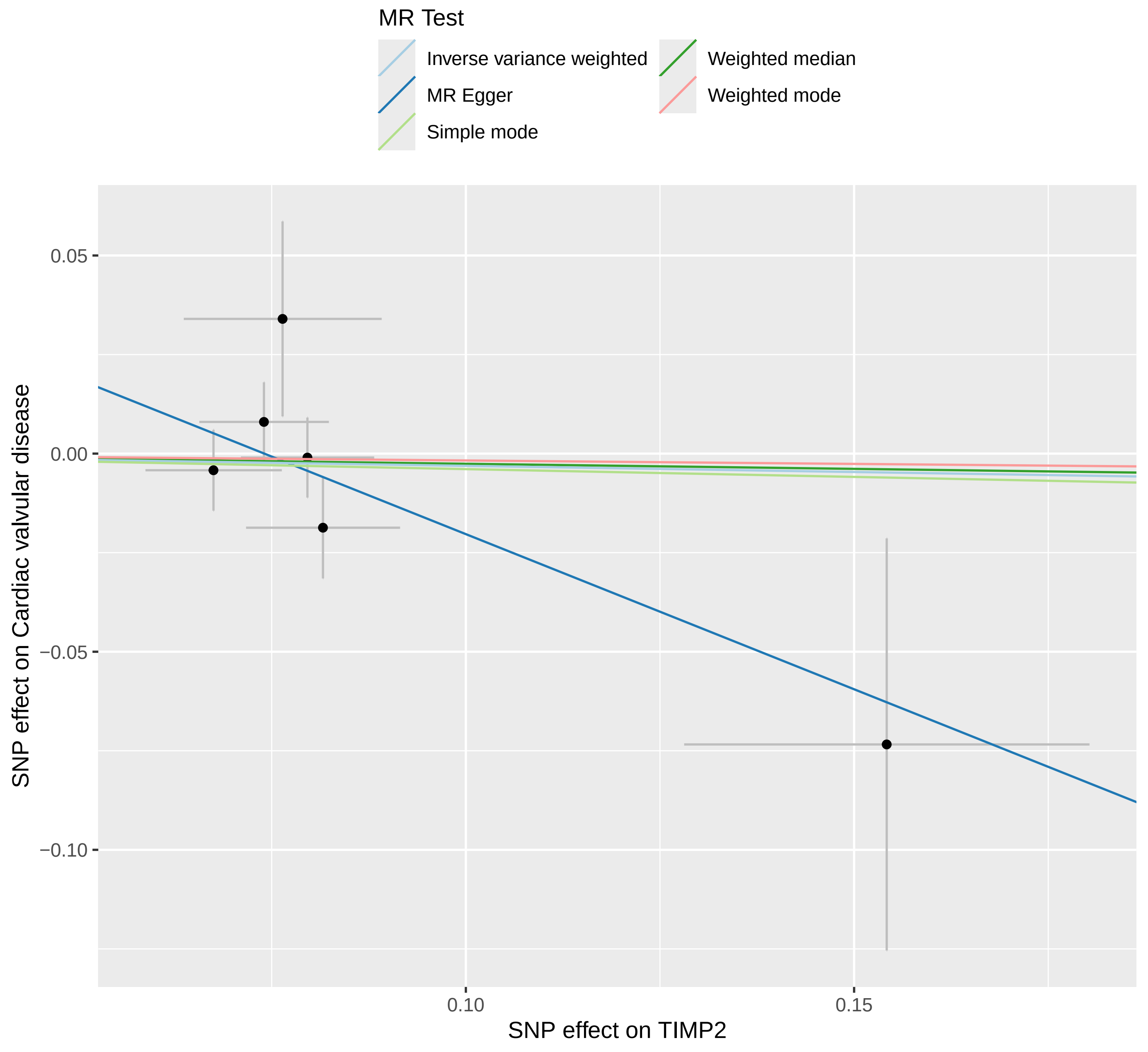
MR Test
Inverse variance weighted
MR Egger
Simple mode
Weighted median
Weighted mode
0.05
0.00
−0.05
−0.10
0.10
0.15
SNP effect on Cardiac valvular disease
SNP effect on TIMP2

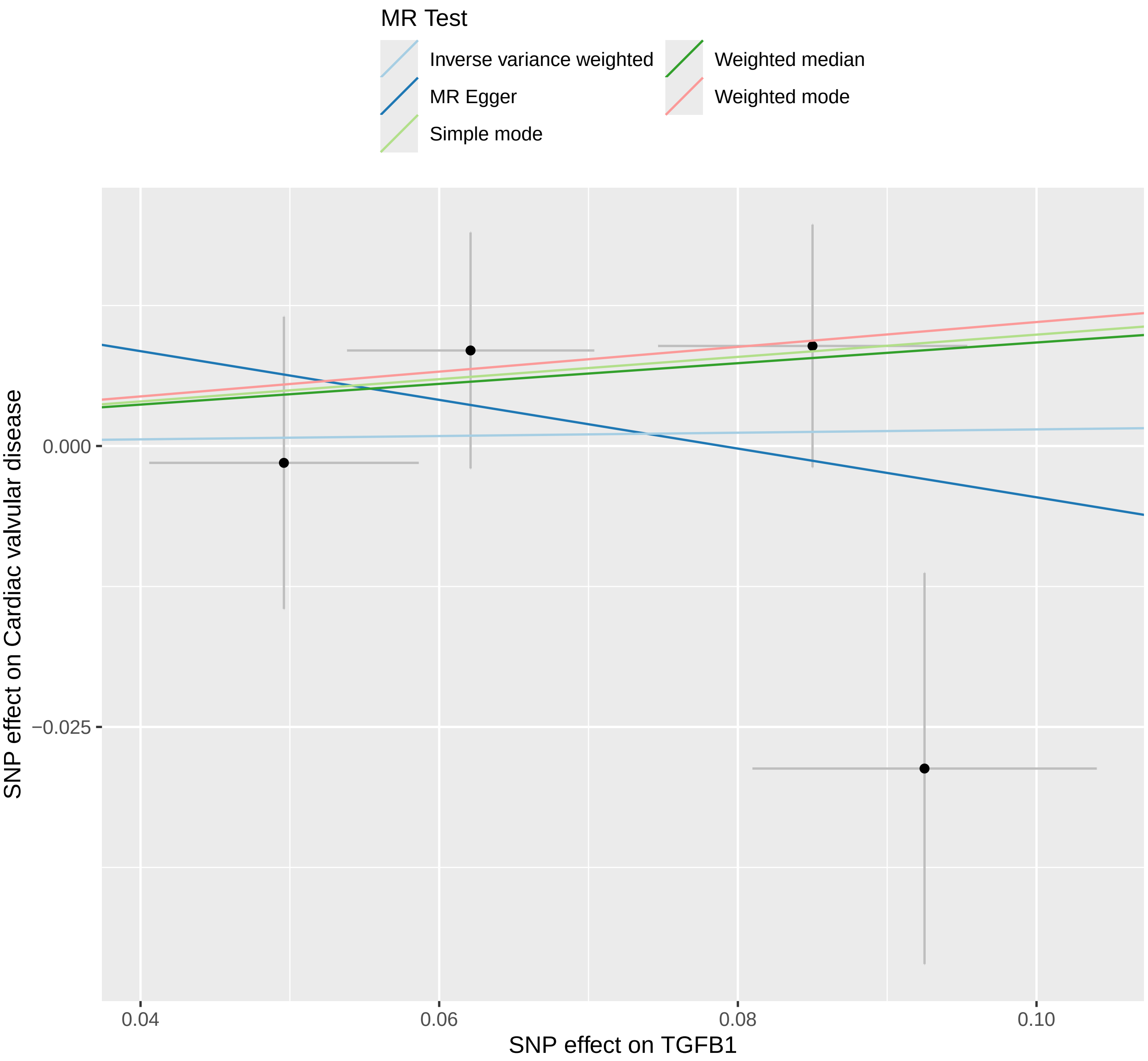
MR Test
Inverse variance weighted
MR Egger
Simple mode
Weighted median
Weighted mode
SNP effect on Cardiac valvular disease
0.000
−0.025
0.04
0.06
0.08
0.10
SNP effect on TGFB1

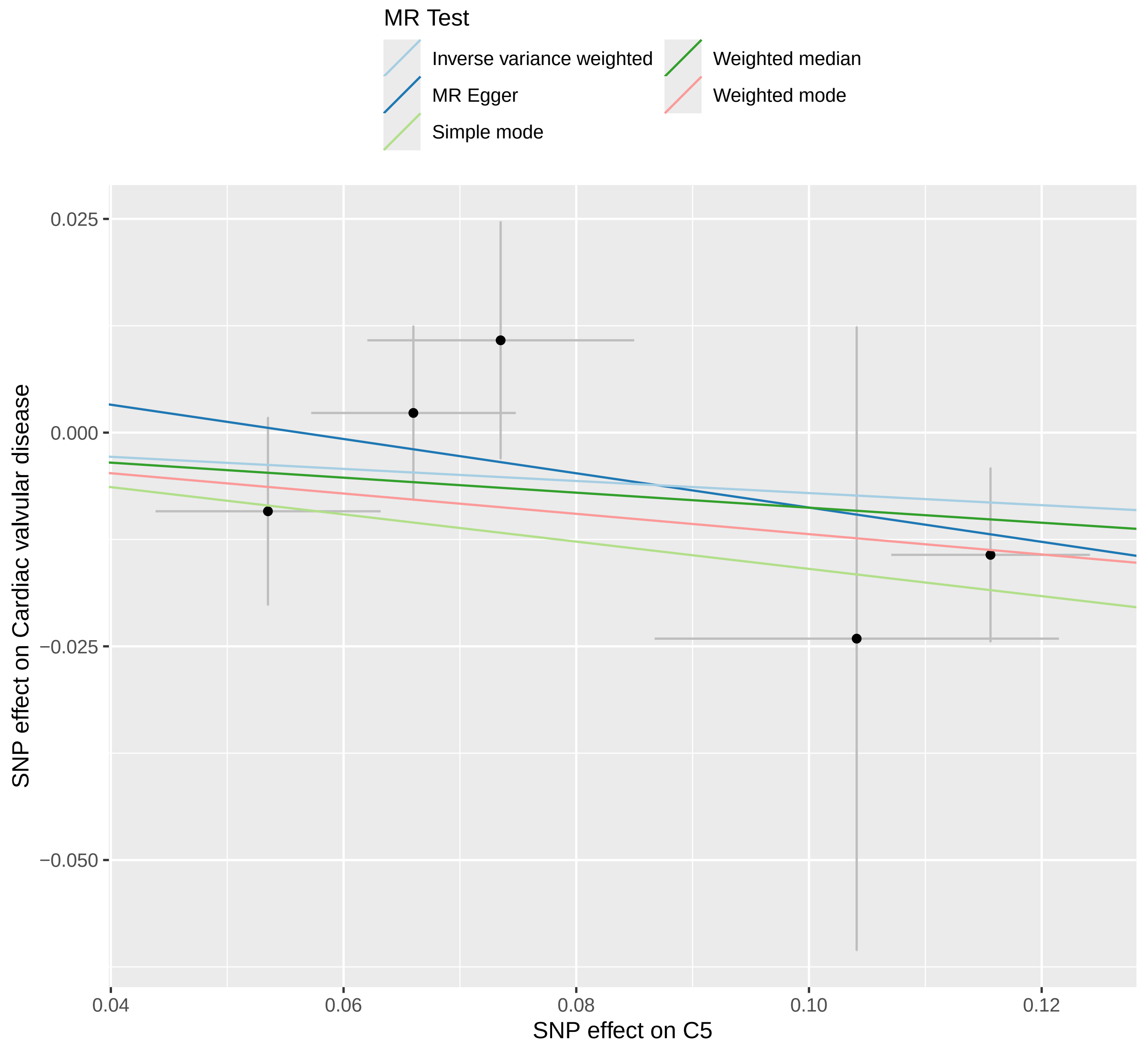
MR Test
Inverse variance weighted
MR Egger
Simple mode
Weighted median
Weighted mode
0.025
0.000
−0.025
−0.050
SNP effect on Cardiac valvular disease
0.04
0.06
0.08
0.10
0.12
SNP effect on C5

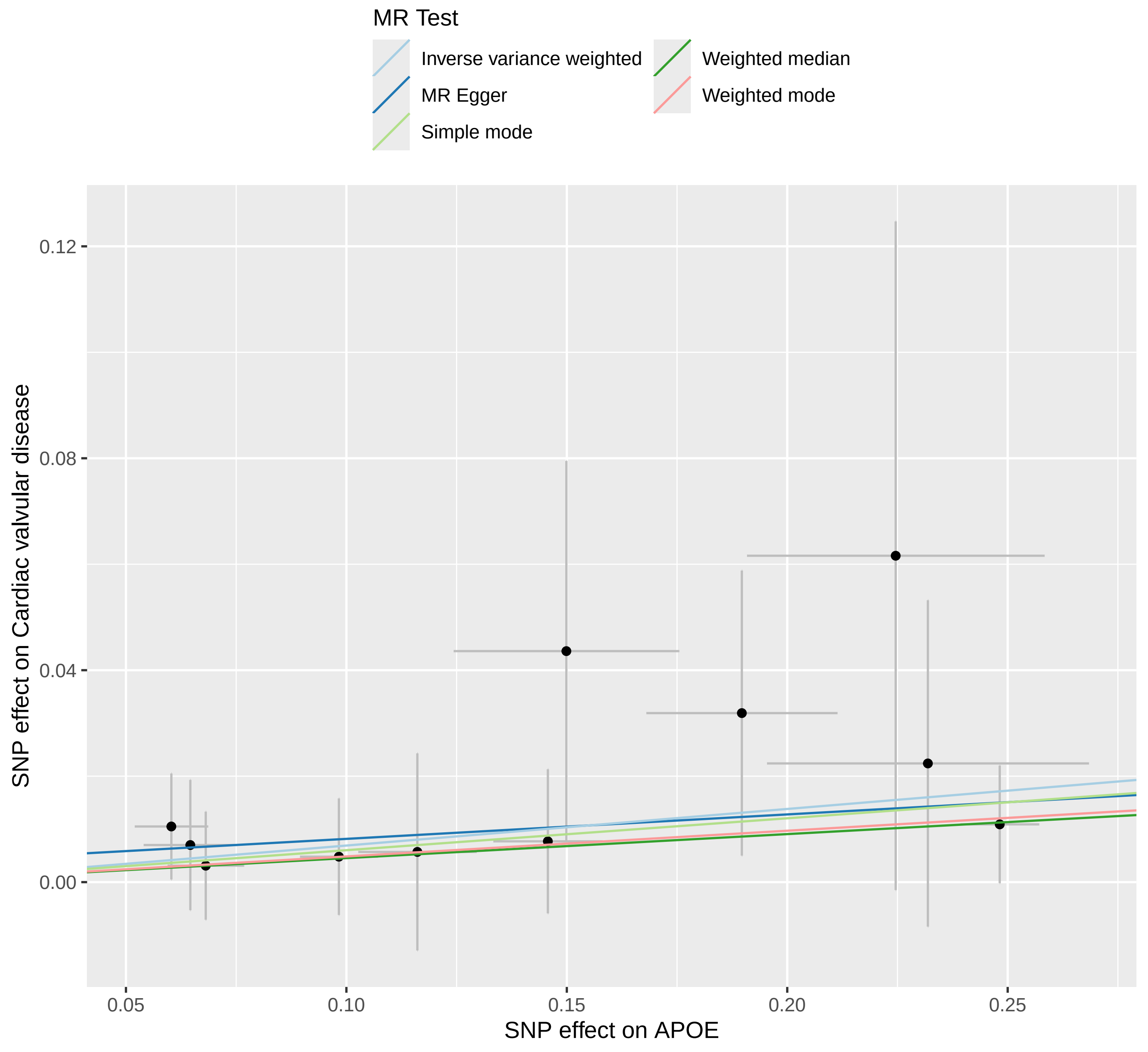
MR Test
Inverse variance weighted
MR Egger
Simple mode
Weighted median
Weighted mode
SNP effect on Cardiac valvular disease
0.12
0.08
0.04
0.00
0.05
0.10
0.15
0.20
0.25
SNP effect on APOE

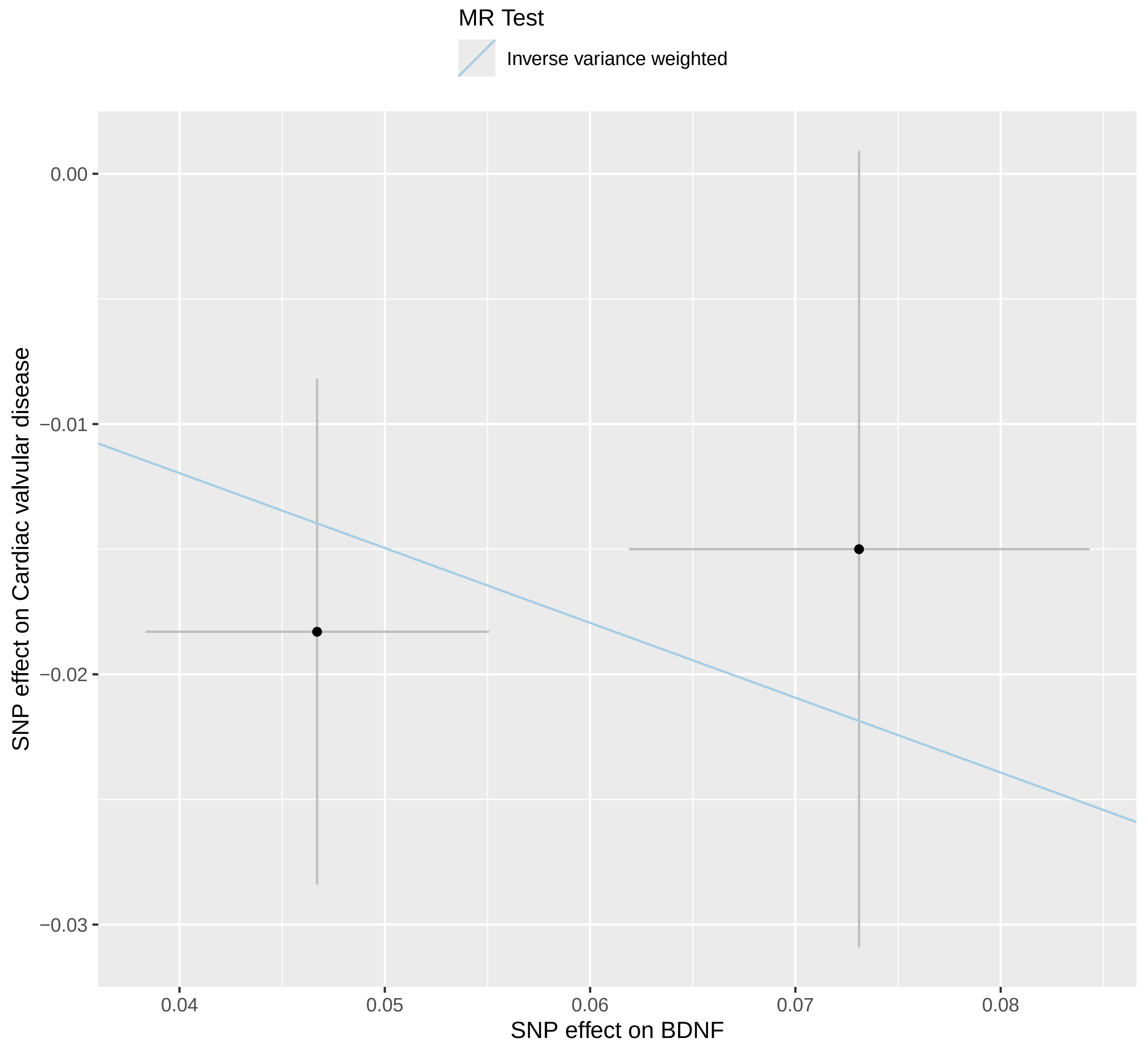

MR Test
Inverse variance weighted
0.00
−0.01
−0.02
−0.03
SNP effect on Cardiac valvular disease
0.04
0.05
0.06
0.07
0.08
SNP effect on BDNF

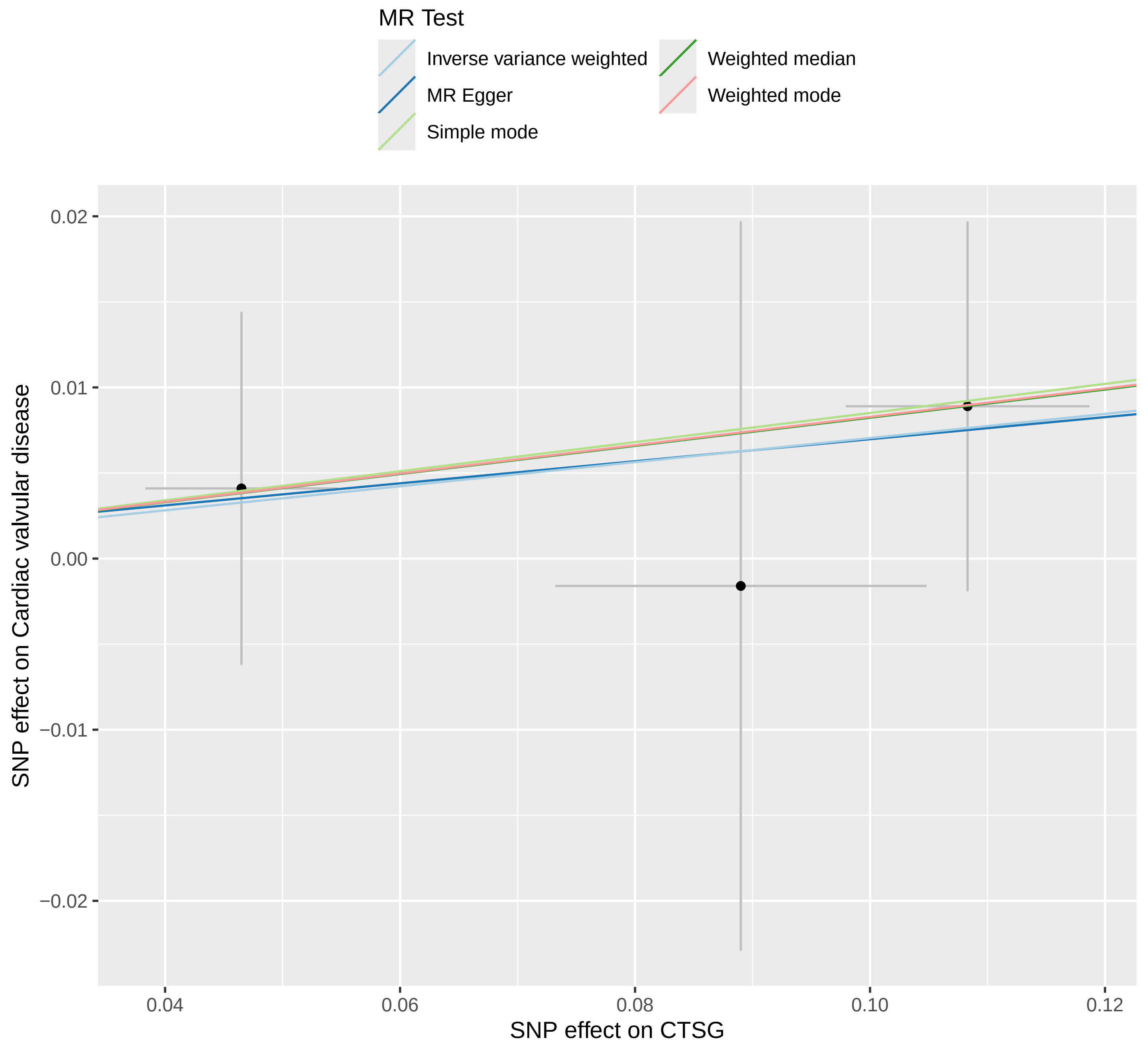

MR Test
Inverse variance weighted
MR Egger
Simple mode
Weighted median
Weighted mode
0.02
0.01
0.00
−0.01
−0.02
SNP effect on Cardiac valvular disease
0.04
0.06
0.08
0.10
0.12
SNP effect on CTSG

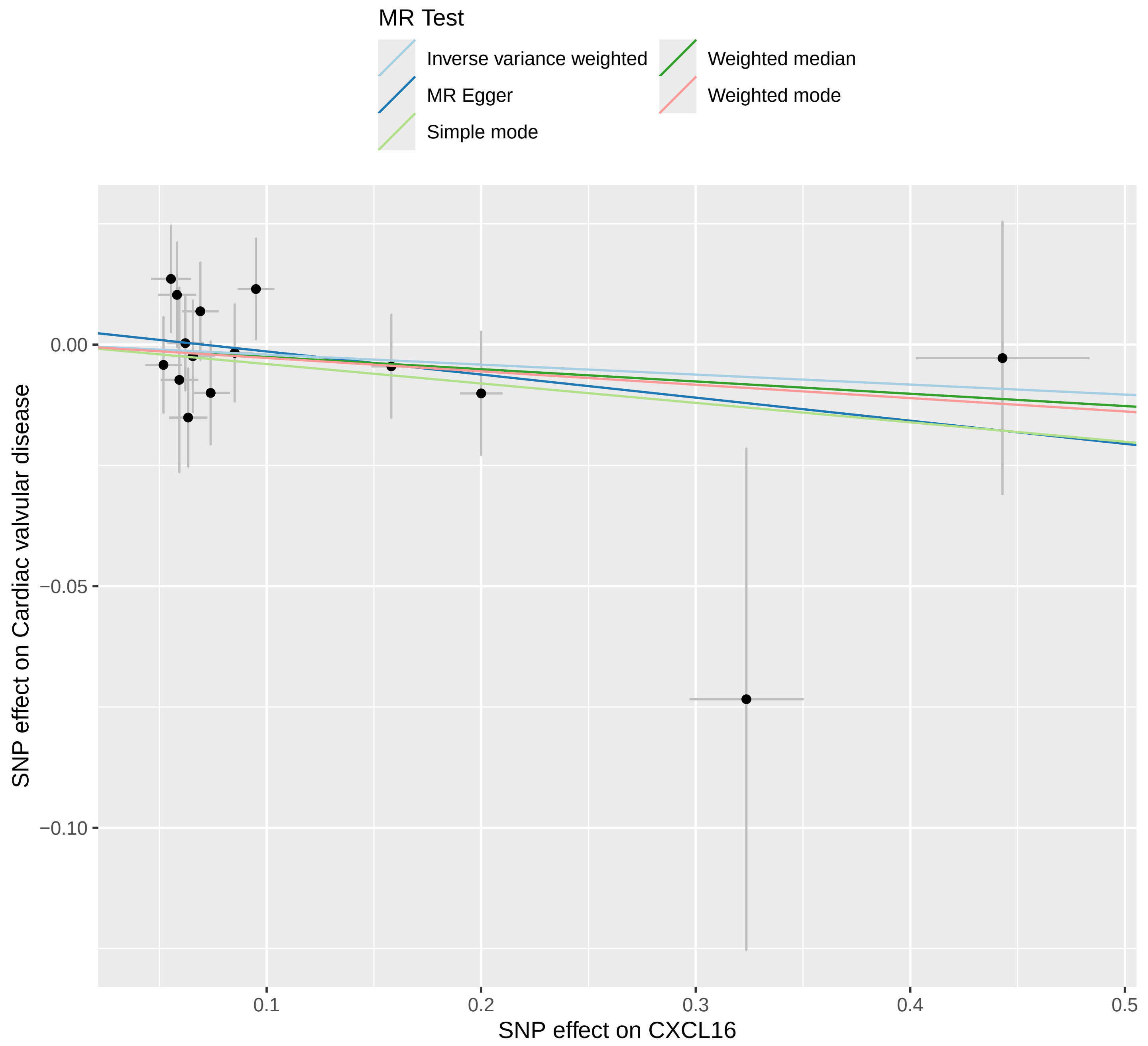
MR Test
Inverse variance weighted
MR Egger
Simple mode
Weighted median
Weighted mode
0.00
−0.05
−0.10
0.1
0.2
0.3
0.4
0.5
SNP effect on CXCL16
SNP effect on Cardiac valvular disease

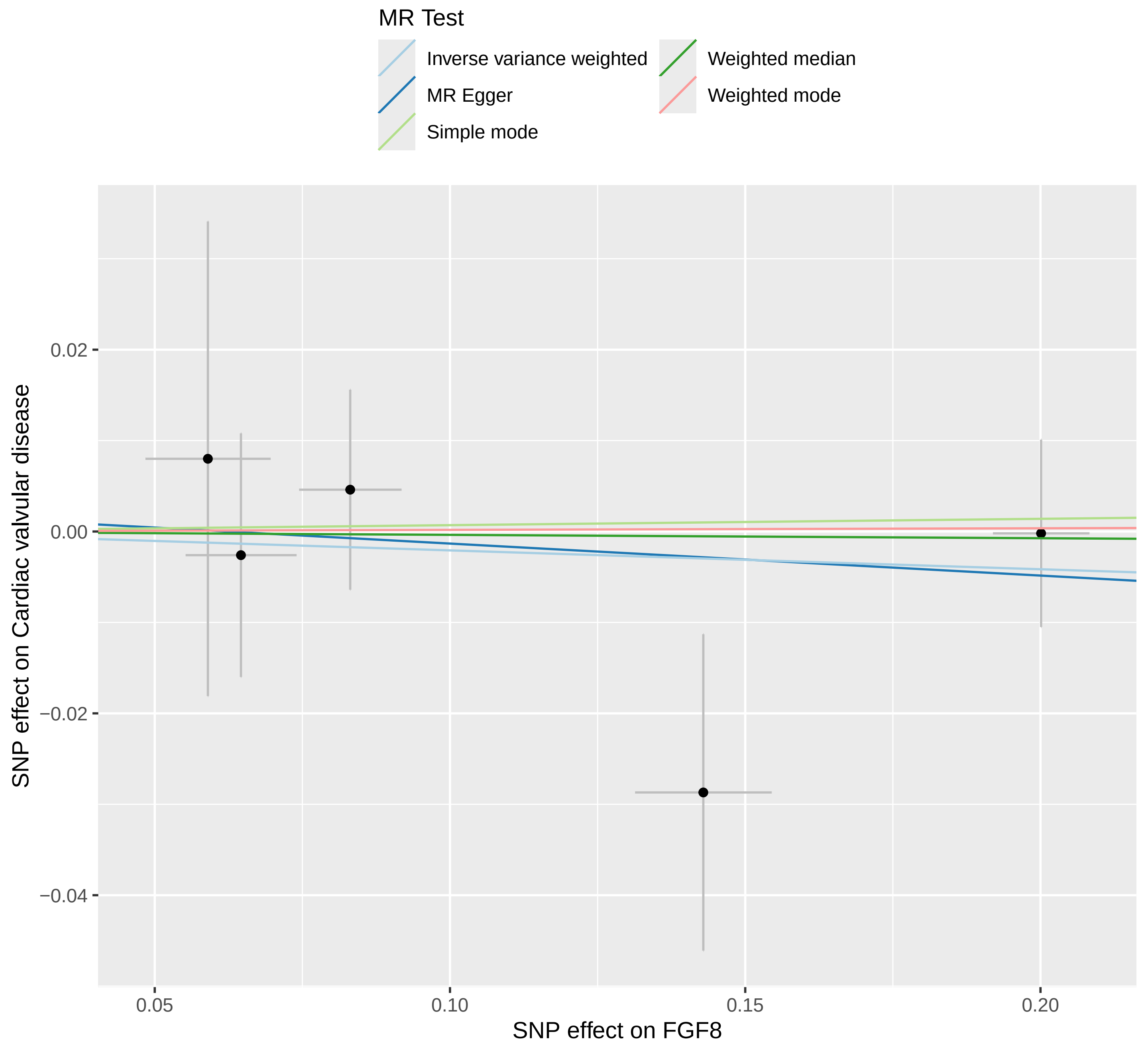

MR Test
Inverse variance weighted
MR Egger
Simple mode
Weighted median
Weighted mode
0.02
0.00
−0.02
−0.04
SNP effect on Cardiac valvular disease
0.05
0.10
0.15
0.20
SNP effect on FGF8

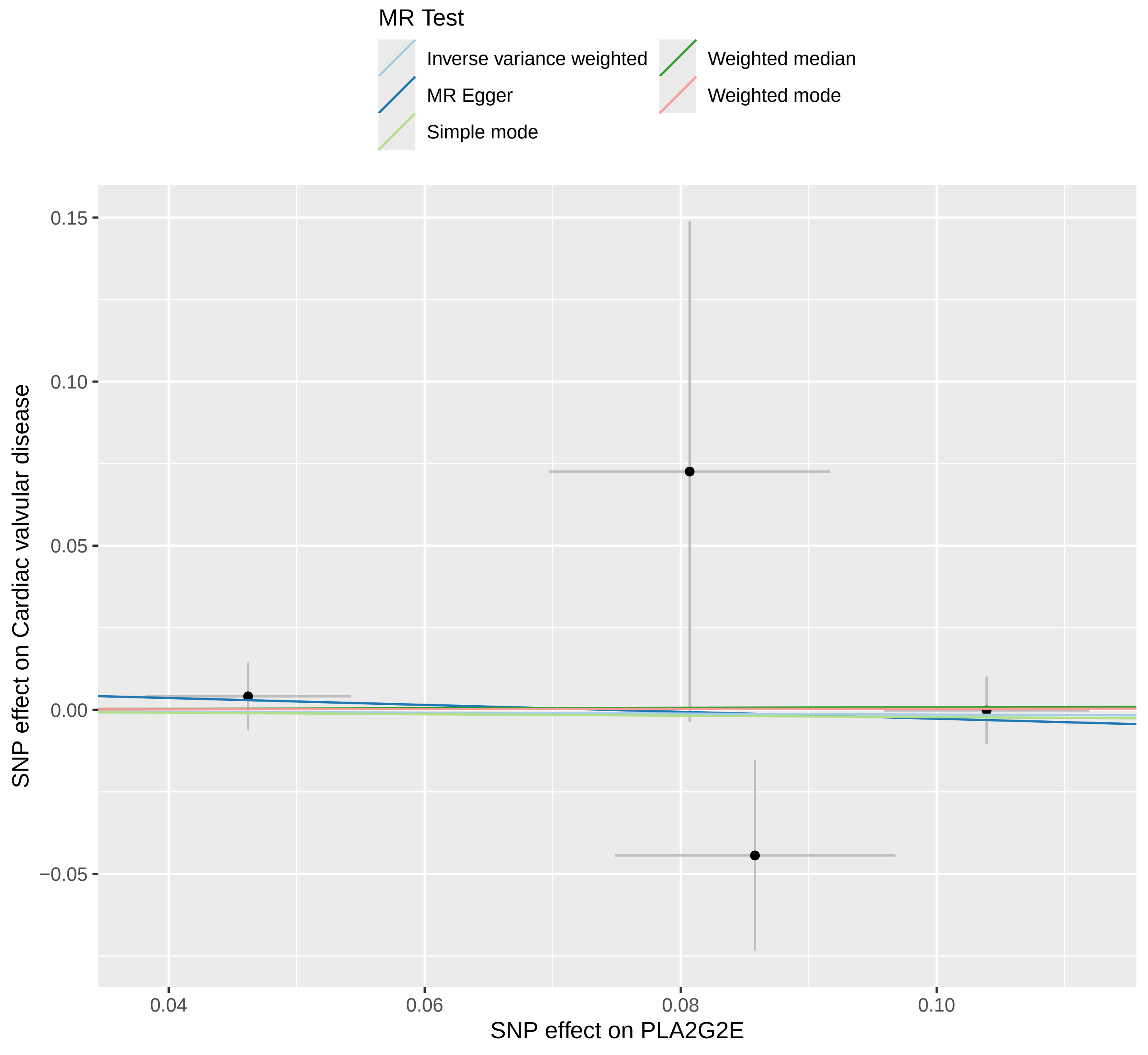
MR Test
Inverse variance weighted
MR Egger
Simple mode
Weighted median
Weighted mode
0.15
0.10
0.05
0.00
−0.05
SNP effect on Cardiac valvular disease
0.04
0.06
0.08
0.10
SNP effect on PLA2G2E

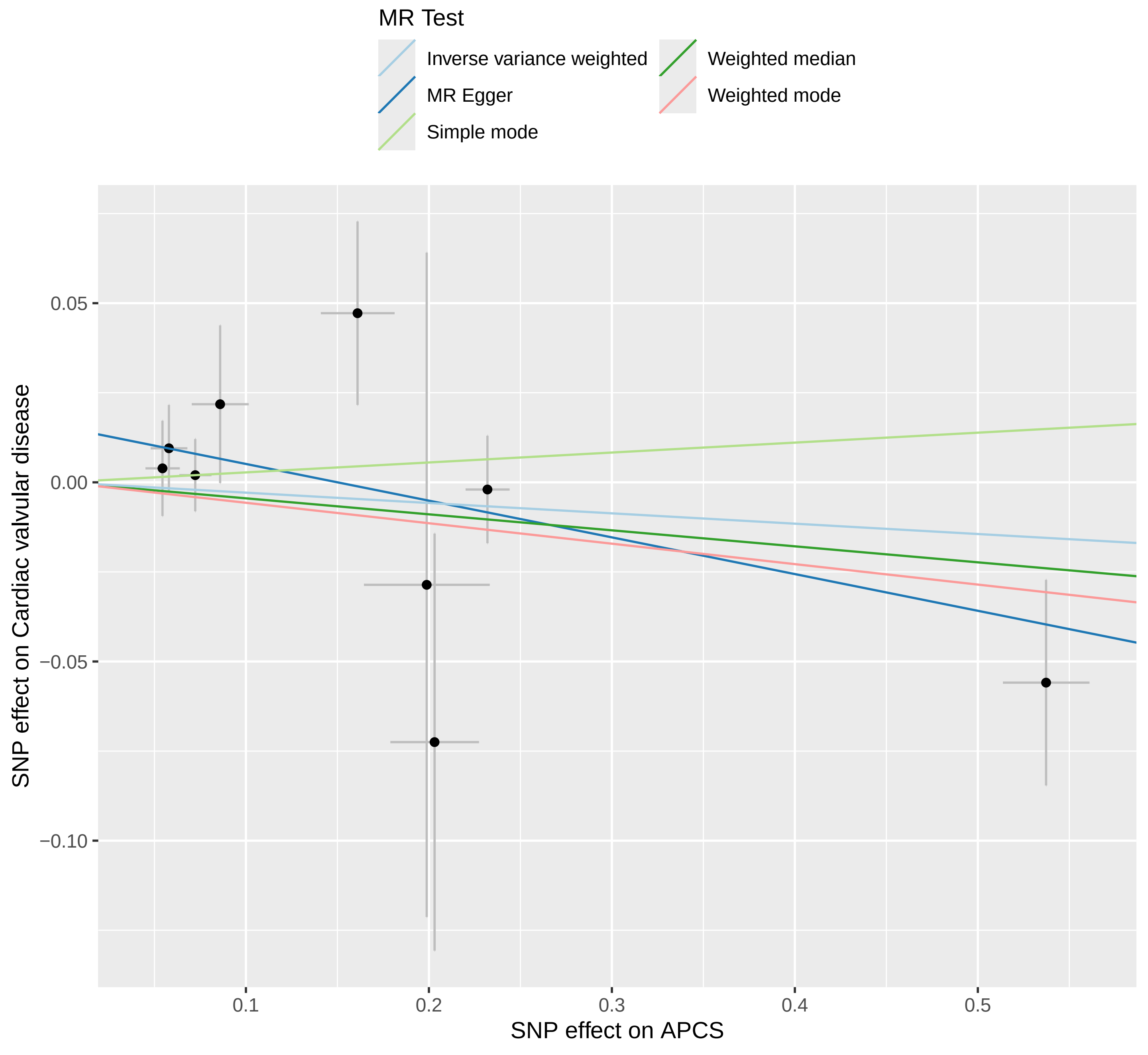
MR Test
Inverse variance weighted
MR Egger
Simple mode
Weighted median
Weighted mode
0.05
0.00
−0.05
−0.10
0.1
0.2
0.3
0.4
0.5
SNP effect on APCS
SNP effect on Cardiac valvular disease

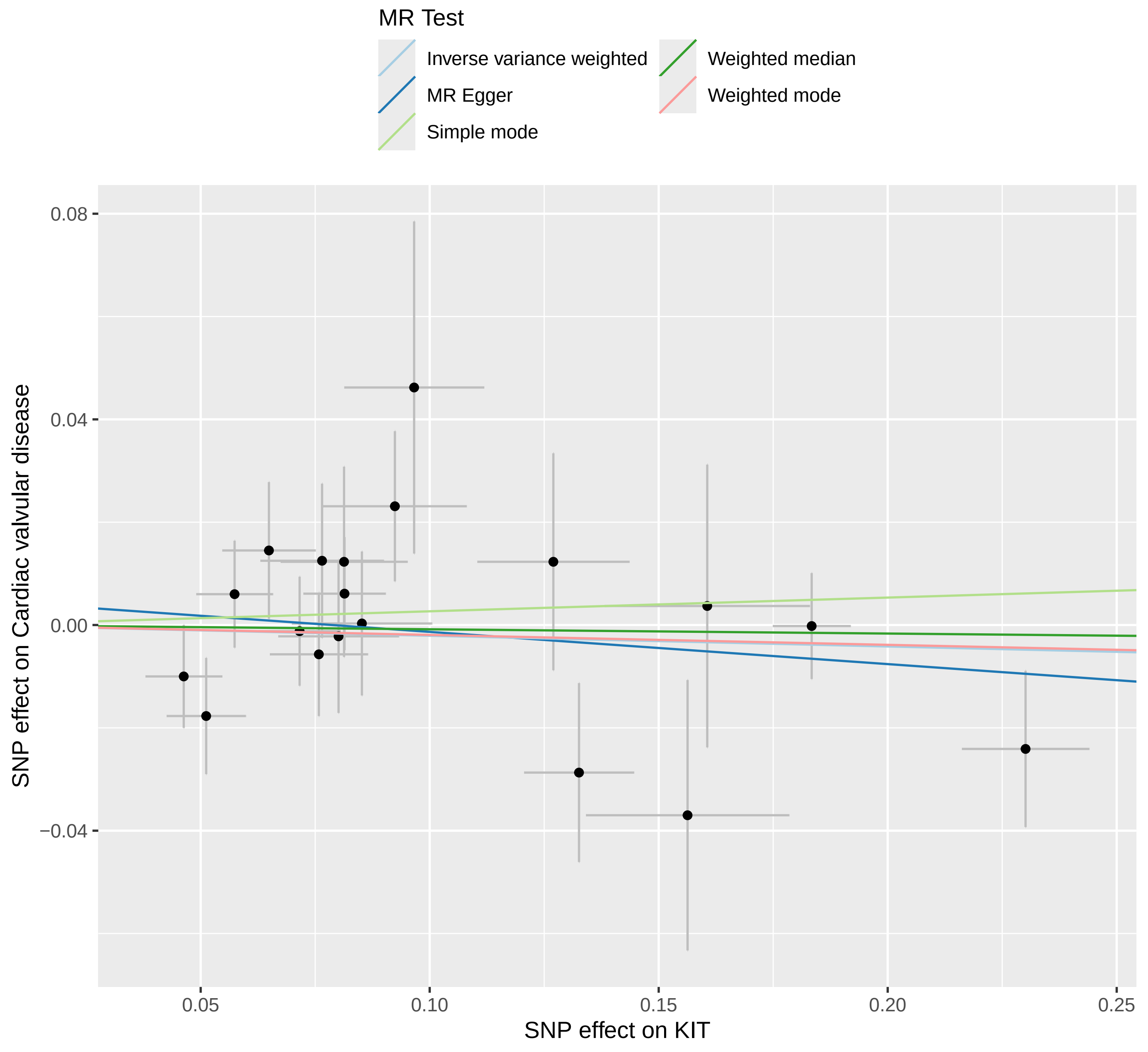

MR Test
Inverse variance weighted
MR Egger
Simple mode
Weighted median
Weighted mode
0.08
0.04
0.00
−0.04
SNP effect on Cardiac valvular disease
0.05
0.10
0.15
0.20
0.25
SNP effect on KIT

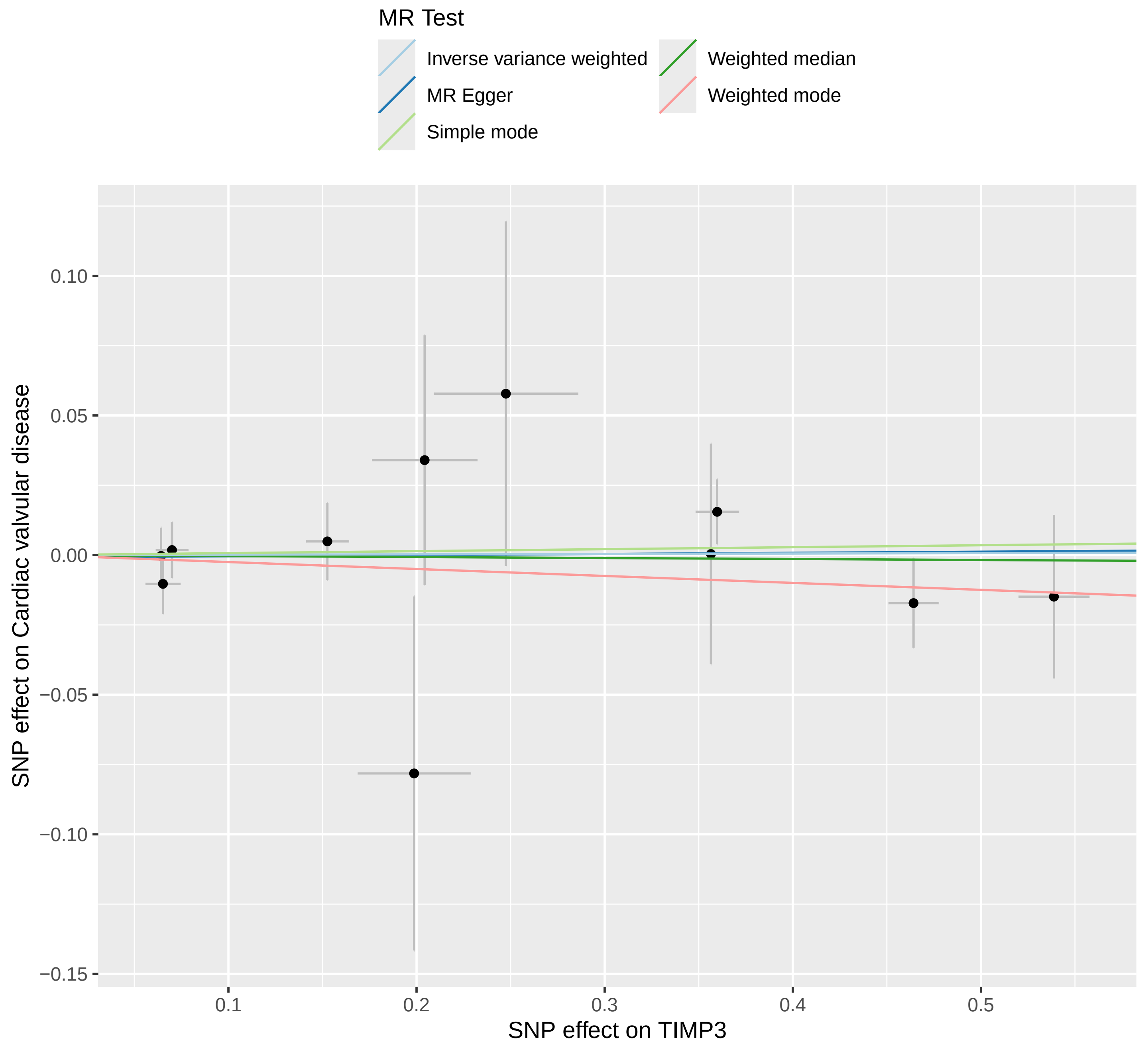
MR Test
Inverse variance weighted
MR Egger
Simple mode
Weighted median
Weighted mode
0.10
0.05
0.00
−0.05
−0.10
−0.15
SNP effect on Cardiac valvular disease
0.1
0.2
0.3
0.4
0.5
SNP effect on TIMP3

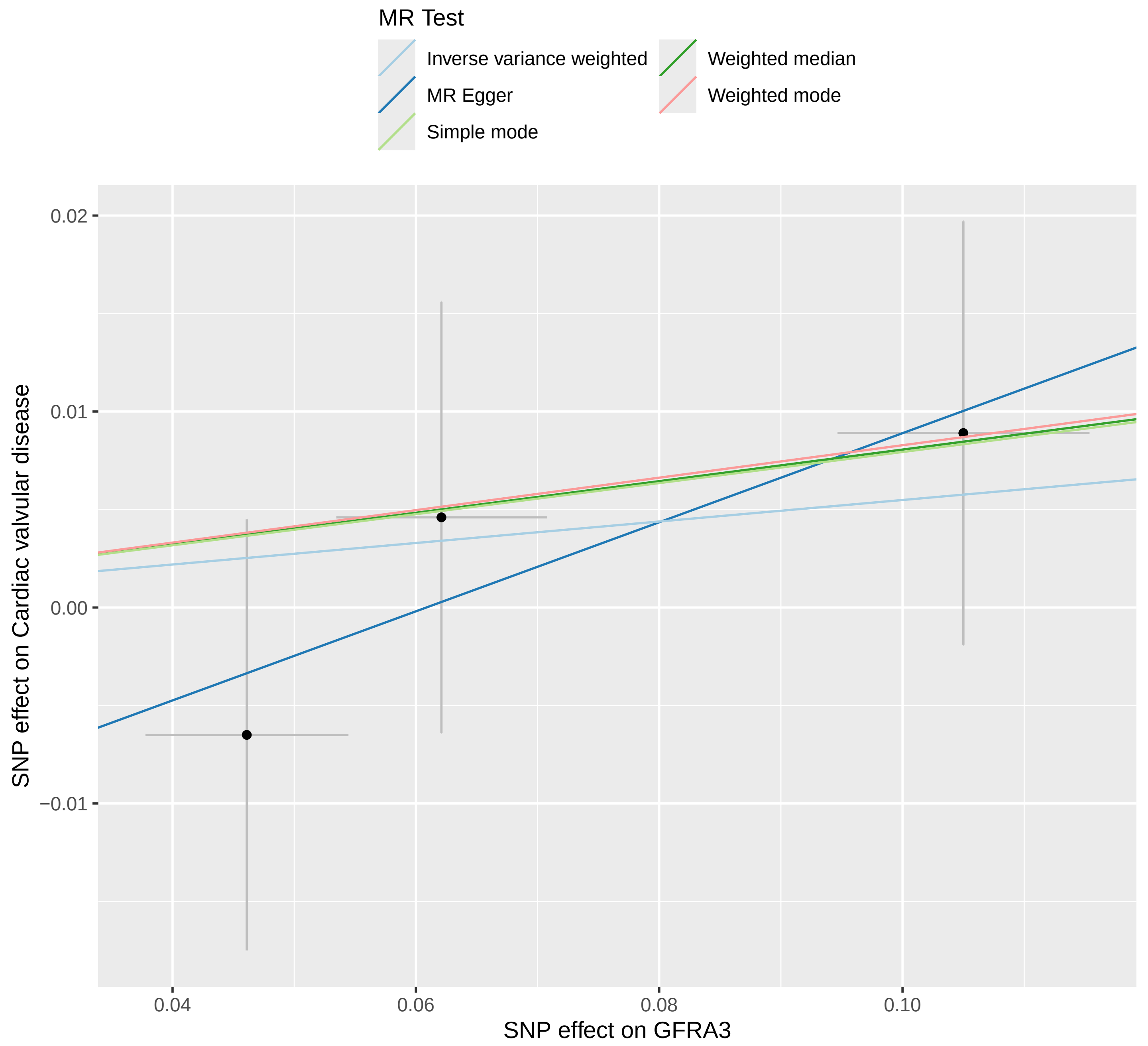
MR Test
Inverse variance weighted
MR Egger
Simple mode
Weighted median
Weighted mode
0.02
0.01
0.00
−0.01
SNP effect on Cardiac valvular disease
0.04
0.06
0.08
0.10
SNP effect on GFRA3

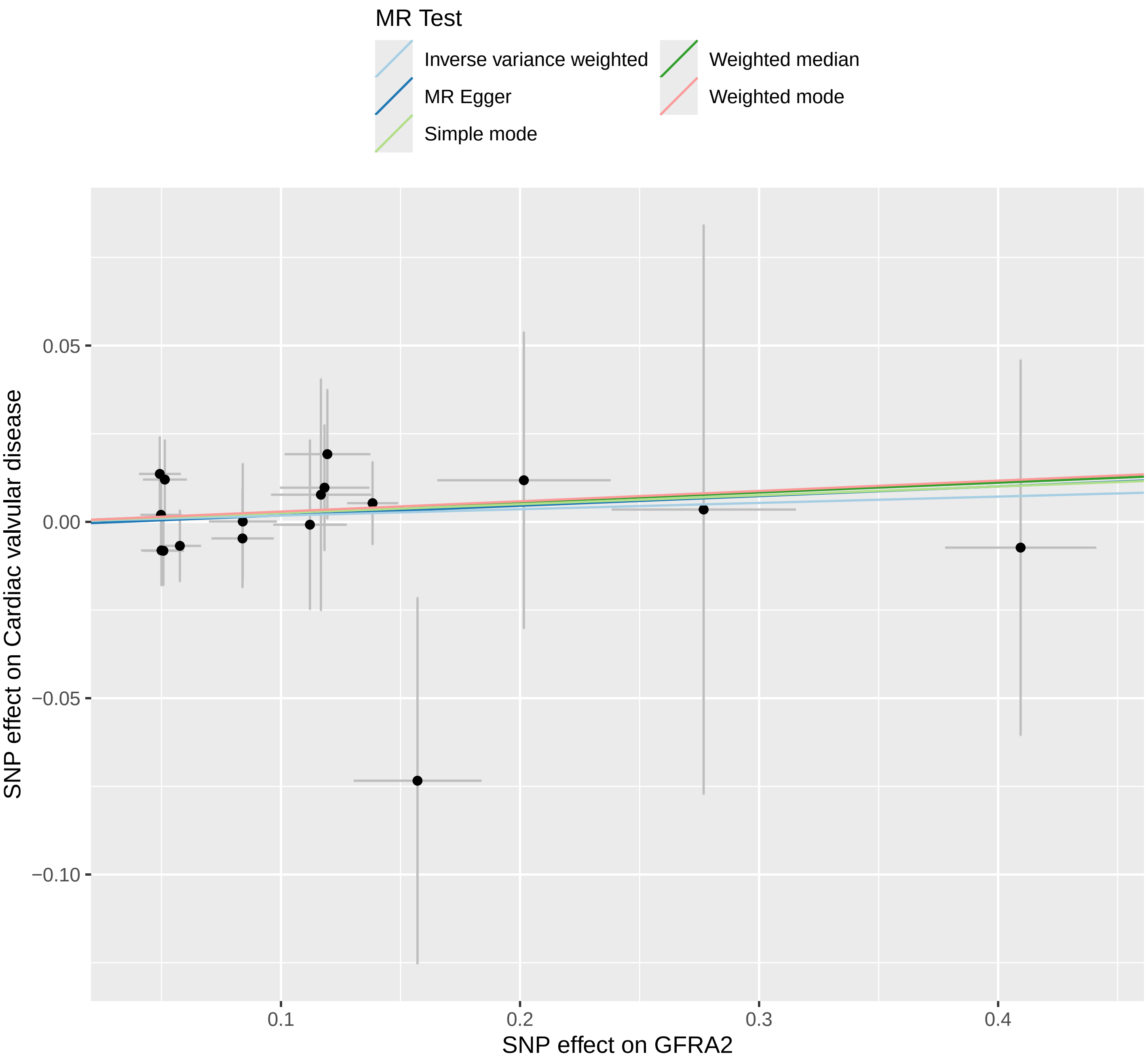

MR Test
Inverse variance weighted
MR Egger
Simple mode
Weighted median
Weighted mode
0.05
0.00
−0.05
−0.10
0.1
0.2
0.3
0.4
SNP effect on Cardiac valvular disease
SNP effect on GFRA2

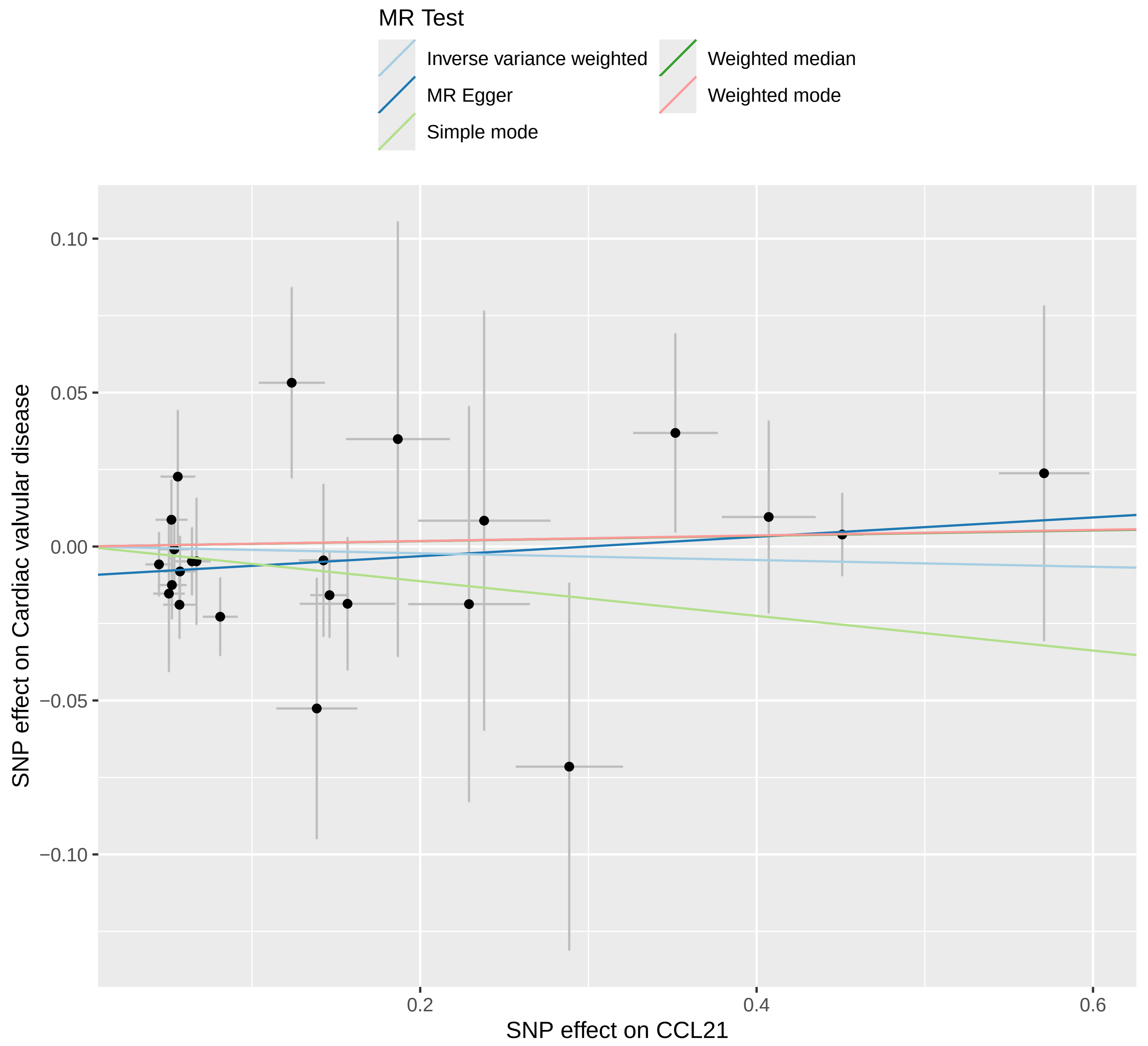
MR Test
Inverse variance weighted
MR Egger
Simple mode
Weighted median
Weighted mode
0.10
0.05
0.00
−0.05
−0.10
0.2
0.4
0.6
SNP effect on Cardiac valvular disease
SNP effect on CCL21

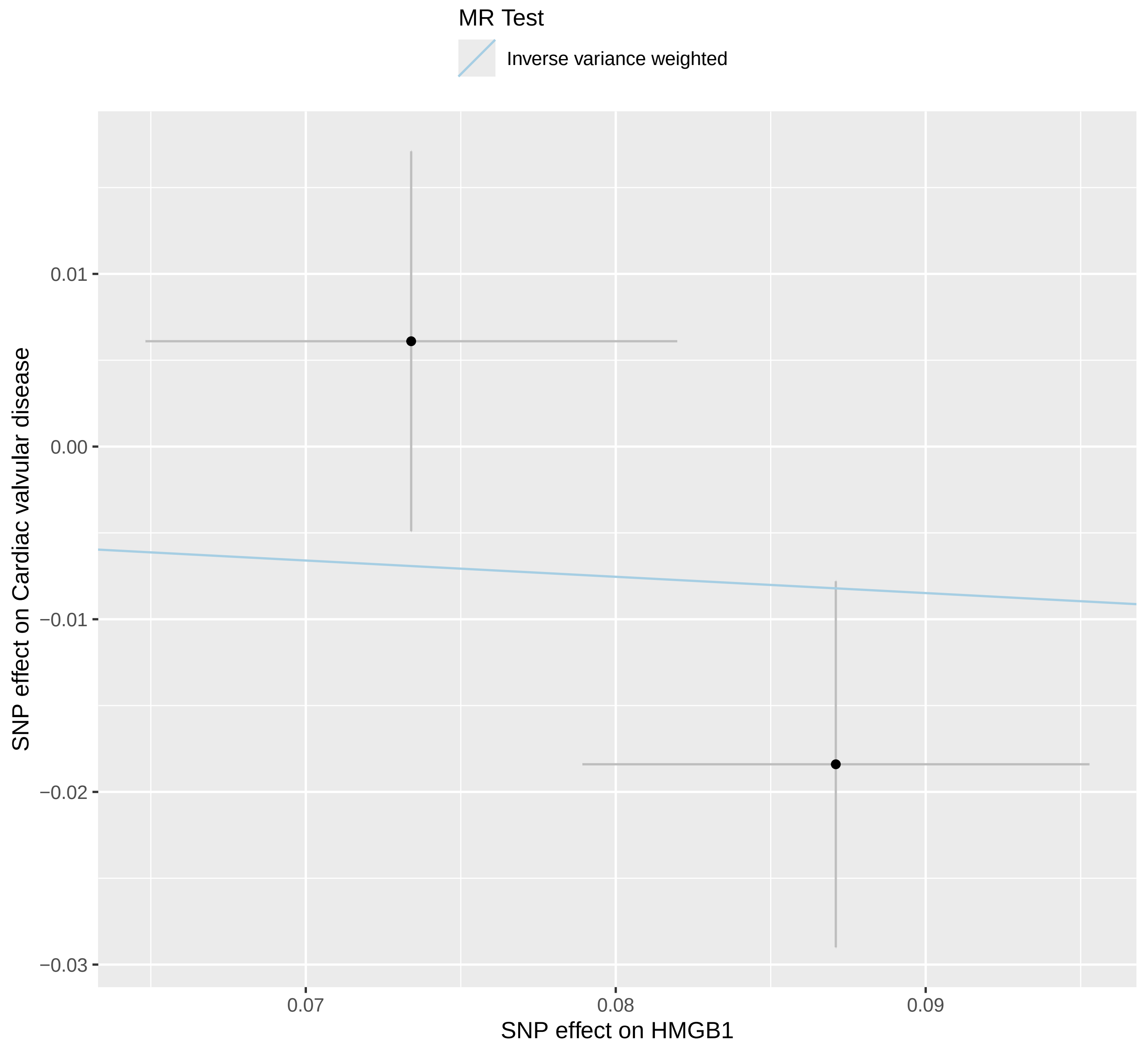
MR Test
Inverse variance weighted
0.01
0.00
−0.01
−0.02
−0.03
0.07
0.08
0.09
SNP effect on Cardiac valvular disease
SNP effect on HMGB1

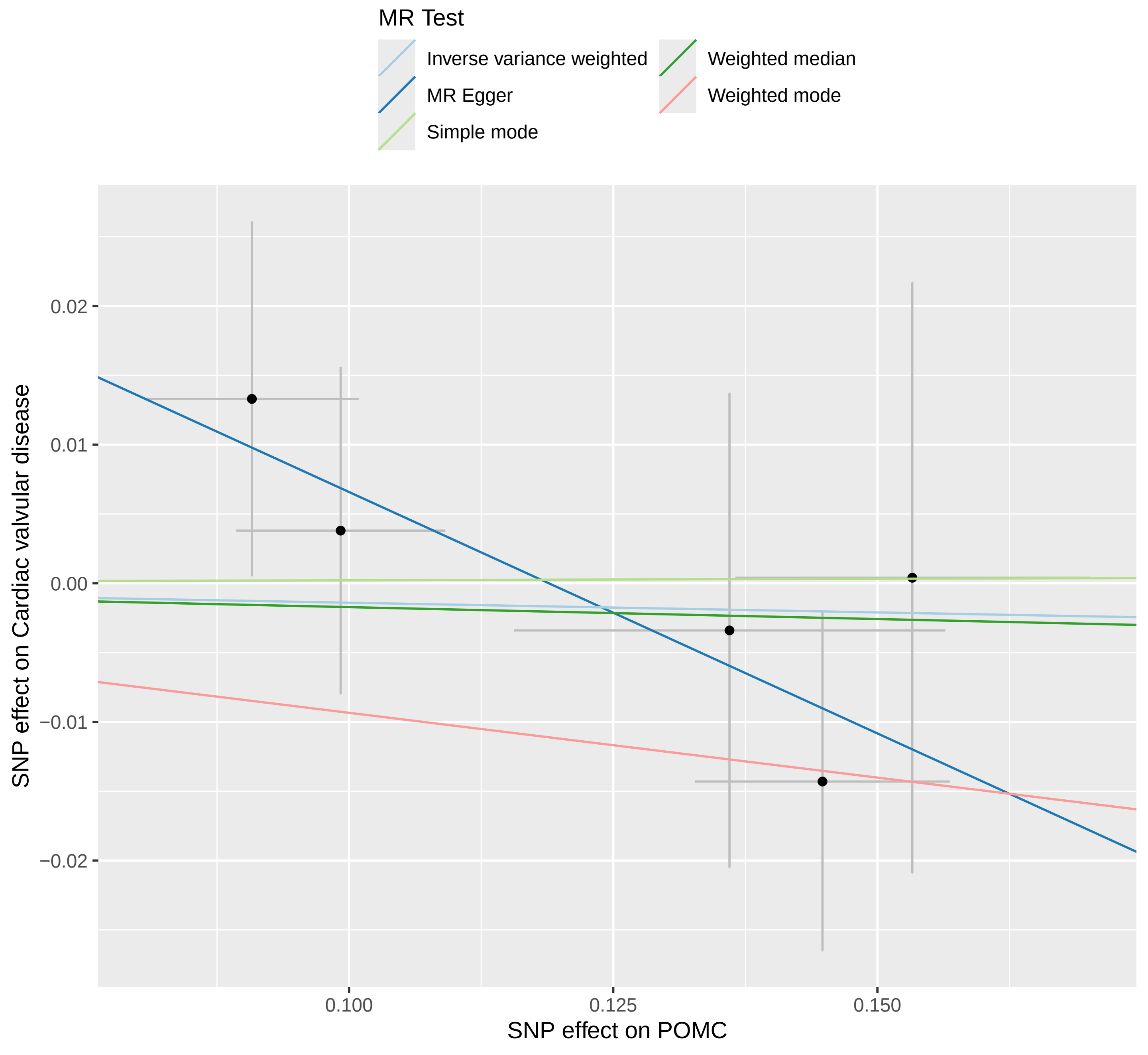
MR Test
Inverse variance weighted
MR Egger
Simple mode
Weighted median
Weighted mode
0.02
0.01
0.00
−0.01
−0.02
0.100
0.125
0.150
SNP effect on Cardiac valvular disease
SNP effect on POMC

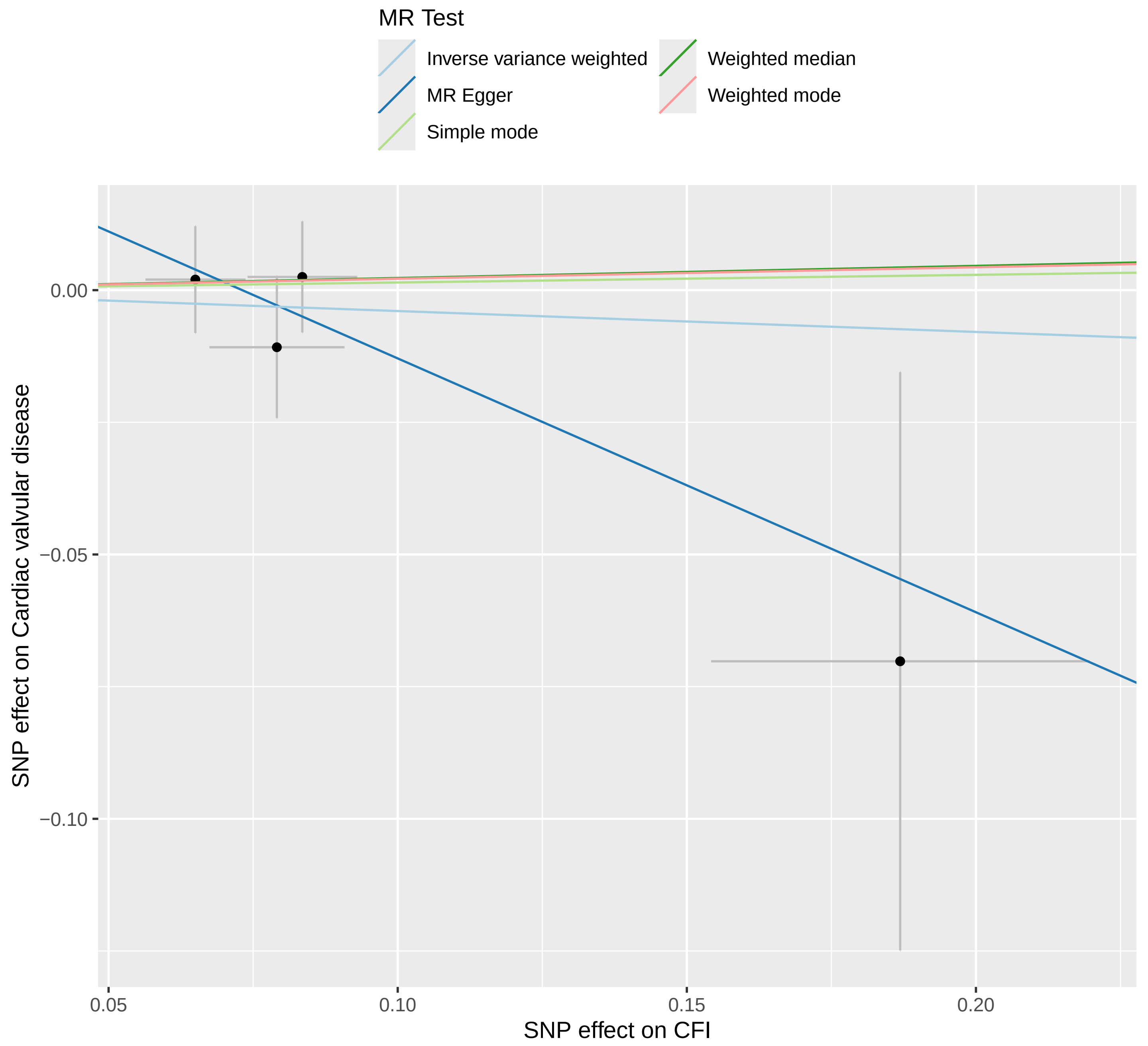
MR Test
Inverse variance weighted
MR Egger
Simple mode
Weighted median
Weighted mode
0.00
−0.05
−0.10
SNP effect on Cardiac valvular disease
0.05
0.10
0.15
0.20
SNP effect on CFI

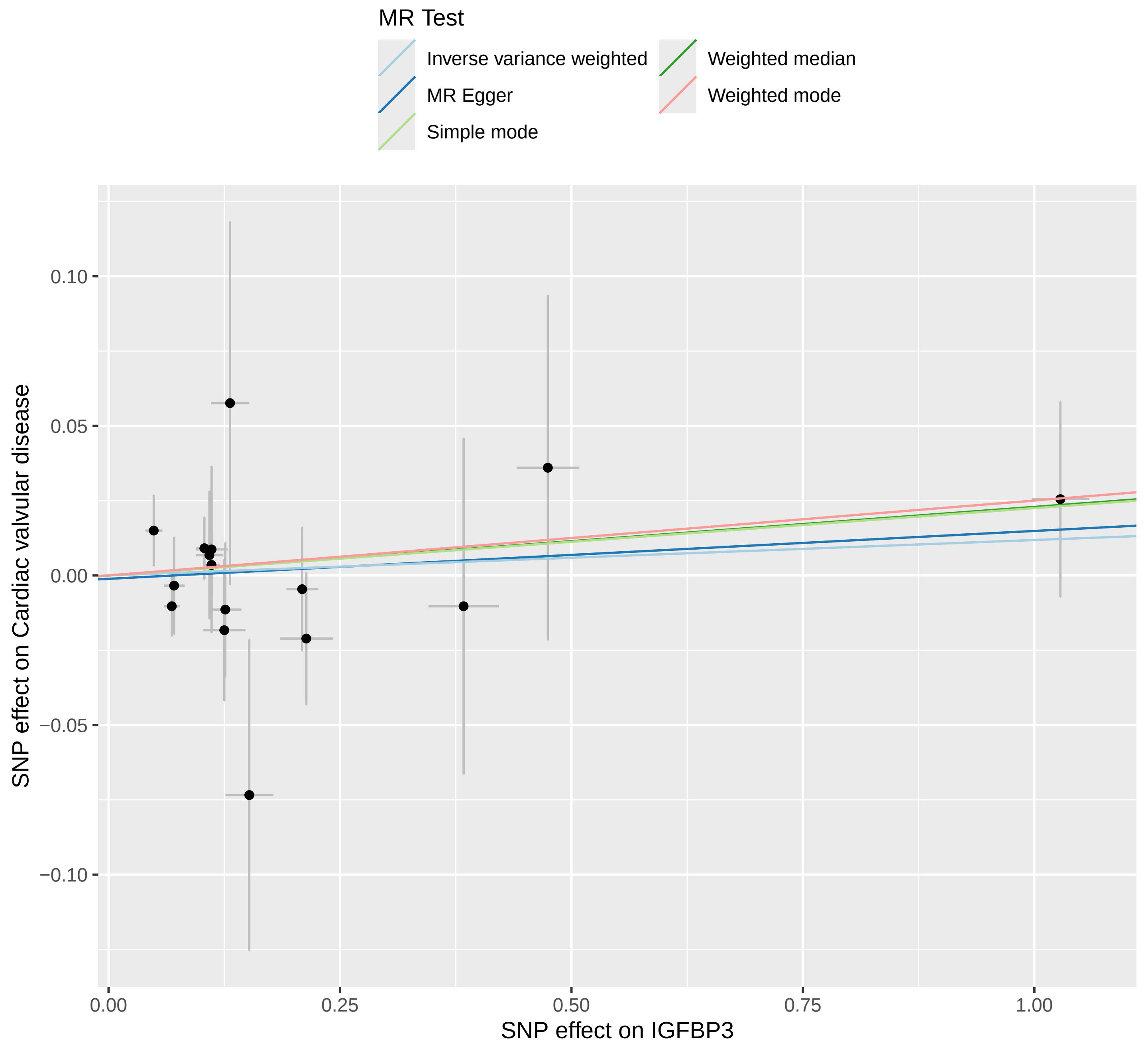
MR Test
Inverse variance weighted
MR Egger
Simple mode
Weighted median
Weighted mode
0.10
0.05
0.00
−0.05
−0.10
SNP effect on Cardiac valvular disease
0.00
0.25
0.50
0.75
1.00
SNP effect on IGFBP3

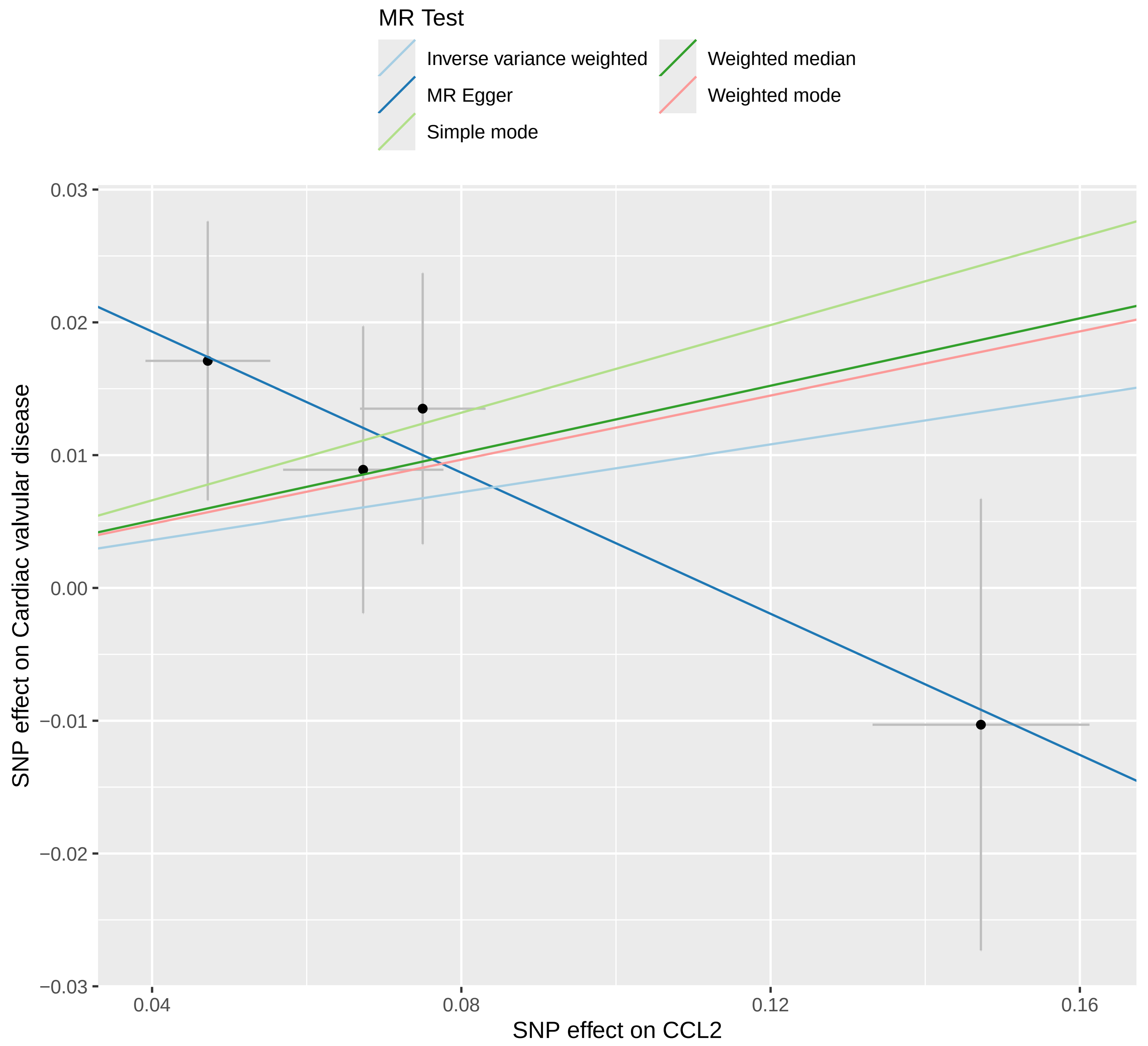

MR Test
Inverse variance weighted
MR Egger
Simple mode
Weighted median
Weighted mode
0.03
0.02
0.01
0.00
−0.01
−0.02
−0.03
SNP effect on Cardiac valvular disease
0.04
0.08
0.12
0.16
SNP effect on CCL2

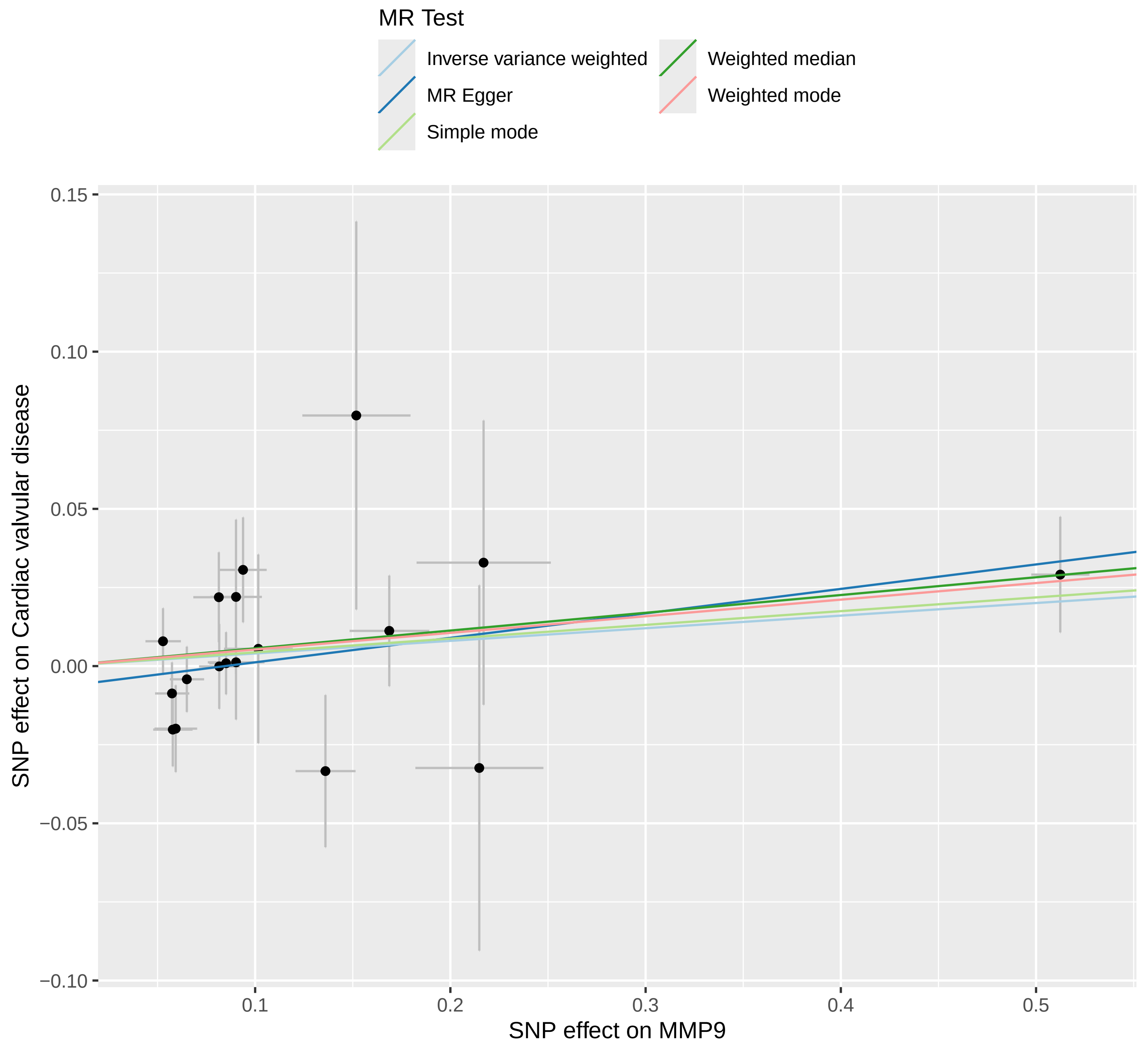

MR Test
Inverse variance weighted
MR Egger
Simple mode
Weighted median
Weighted mode
SNP effect on Cardiac valvular disease
0.15
0.10
0.05
0.00
−0.05
−0.10
0.1
0.2
0.3
0.4
0.5
SNP effect on MMP9

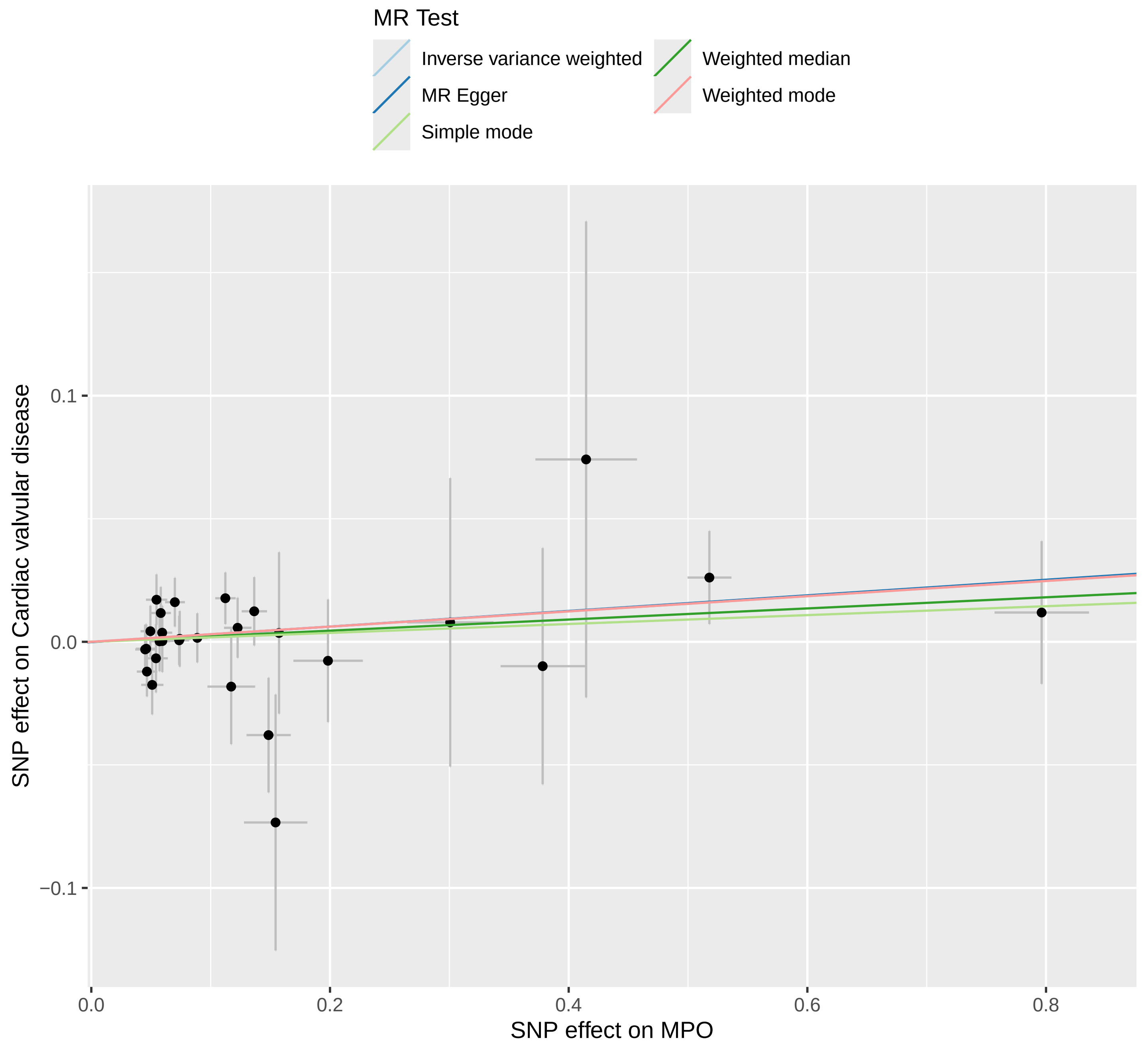

MR Test
Inverse variance weighted
MR Egger
Simple mode
Weighted median
Weighted mode
0.1
0.0
−0.1
0.0
0.2
0.4
0.6
0.8
SNP effect on Cardiac valvular disease
SNP effect on MPO

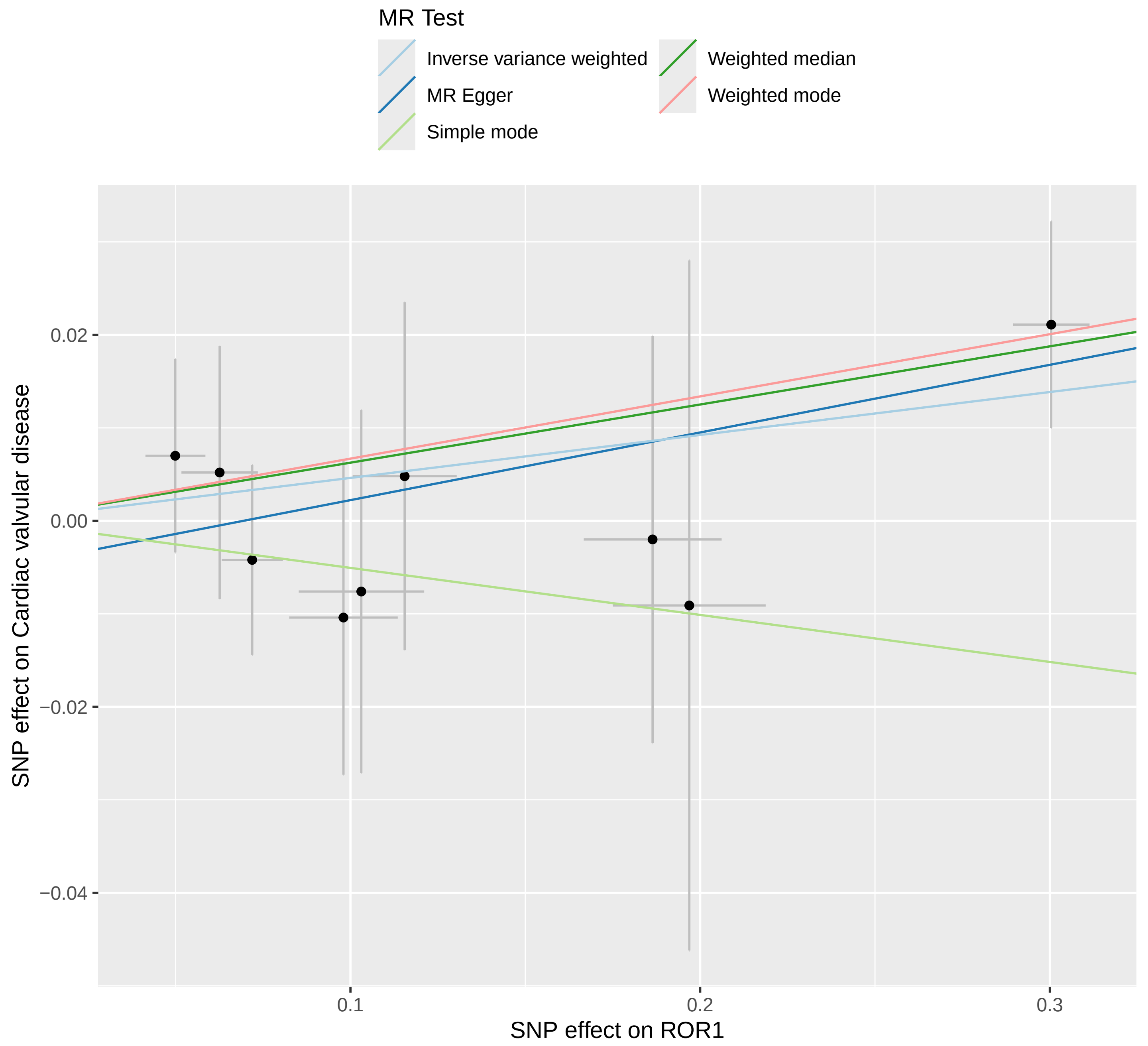
MR Test
Inverse variance weighted
MR Egger
Simple mode
Weighted median
Weighted mode
SNP effect on Cardiac valvular disease
0.02
0.00
−0.02
−0.04
0.1
0.2
0.3
SNP effect on ROR1

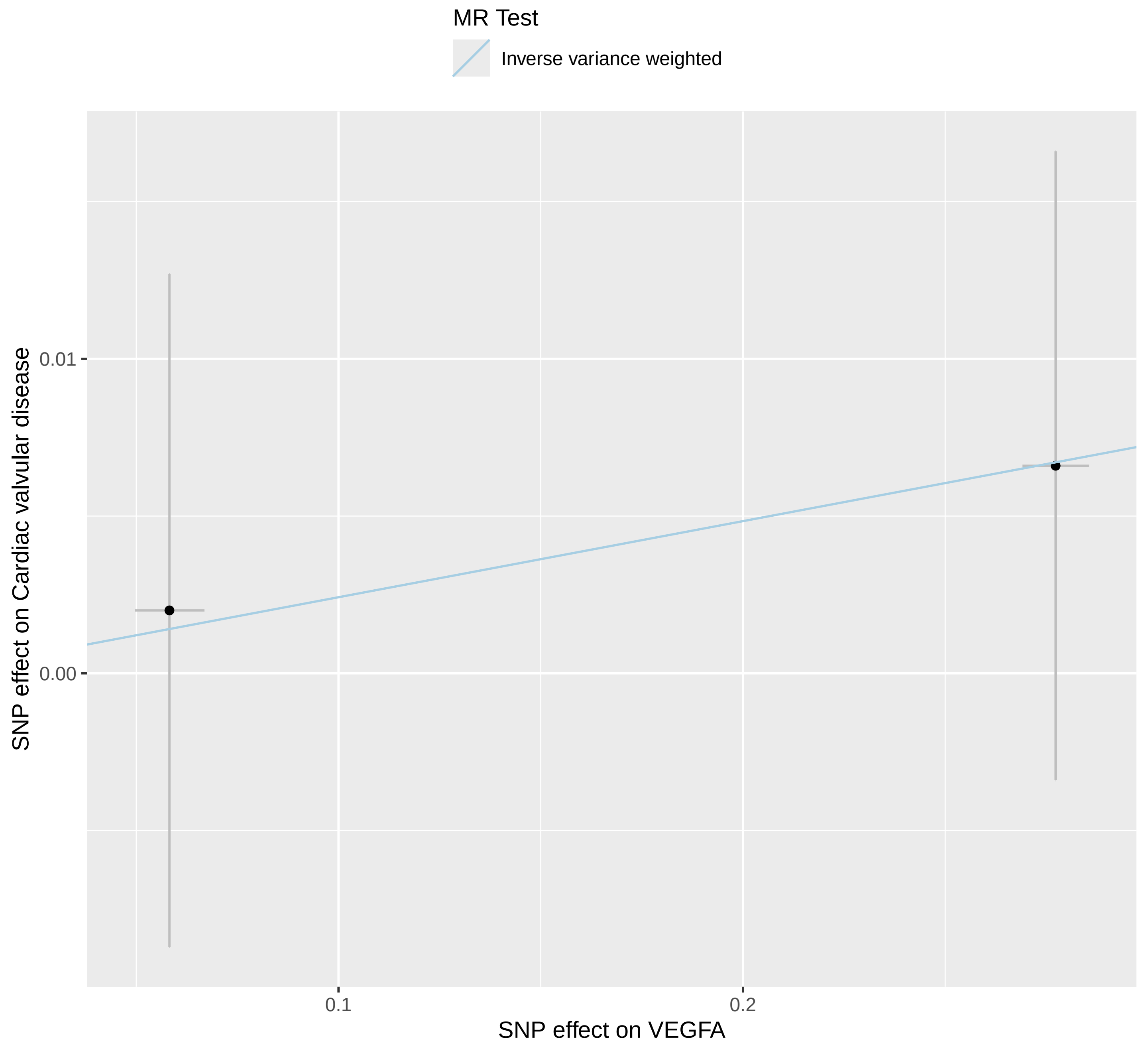
MR Test
Inverse variance weighted
SNP effect on Cardiac valvular disease
0.01
0.00
0.1
0.2
SNP effect on VEGFA

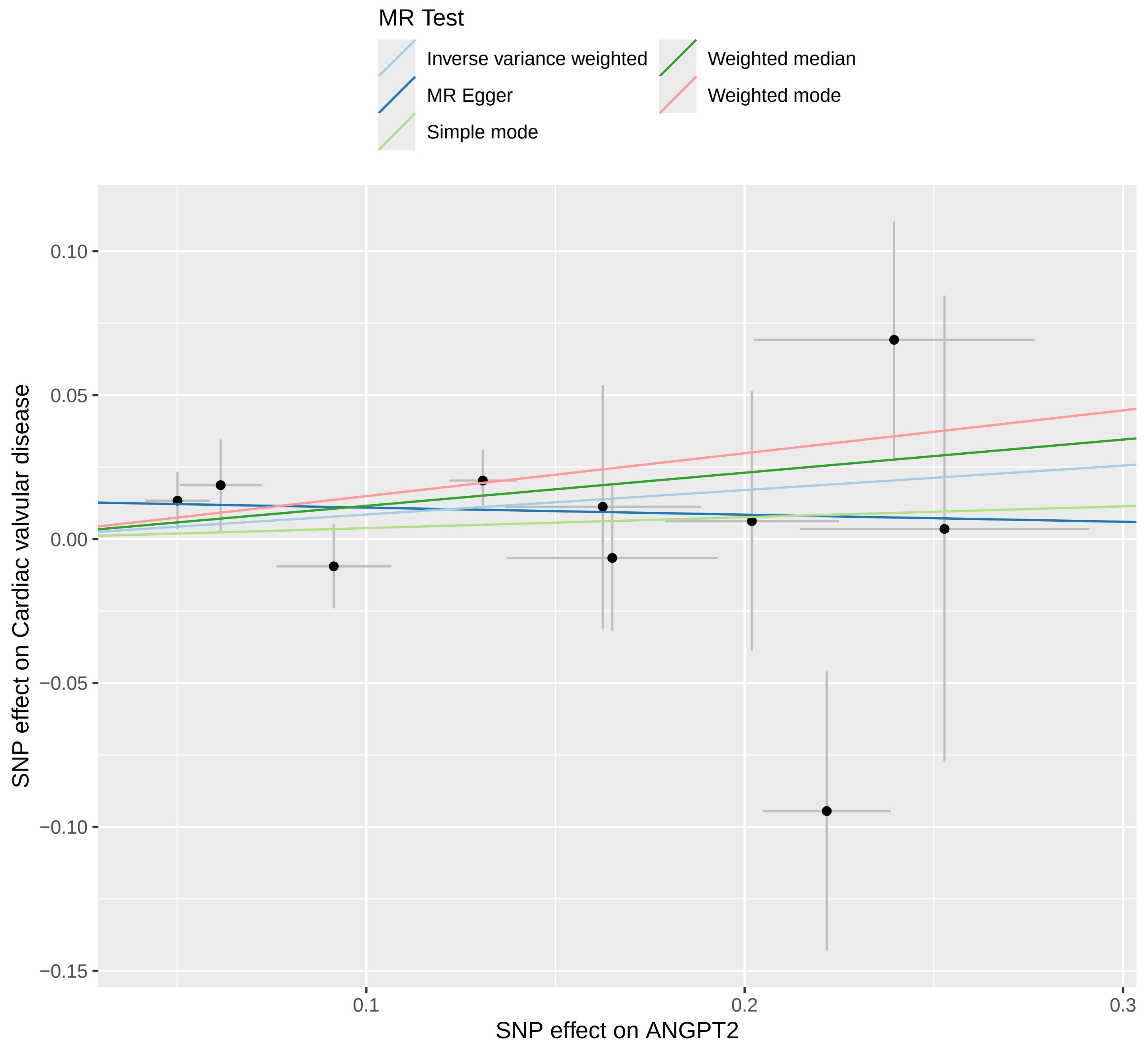

MR Test
Inverse variance weighted
MR Egger
Simple mode
Weighted median
Weighted mode
0.10
0.05
0.00
−0.05
−0.10
−0.15
SNP effect on Cardiac valvular disease
0.1
0.2
0.3
SNP effect on ANGPT2

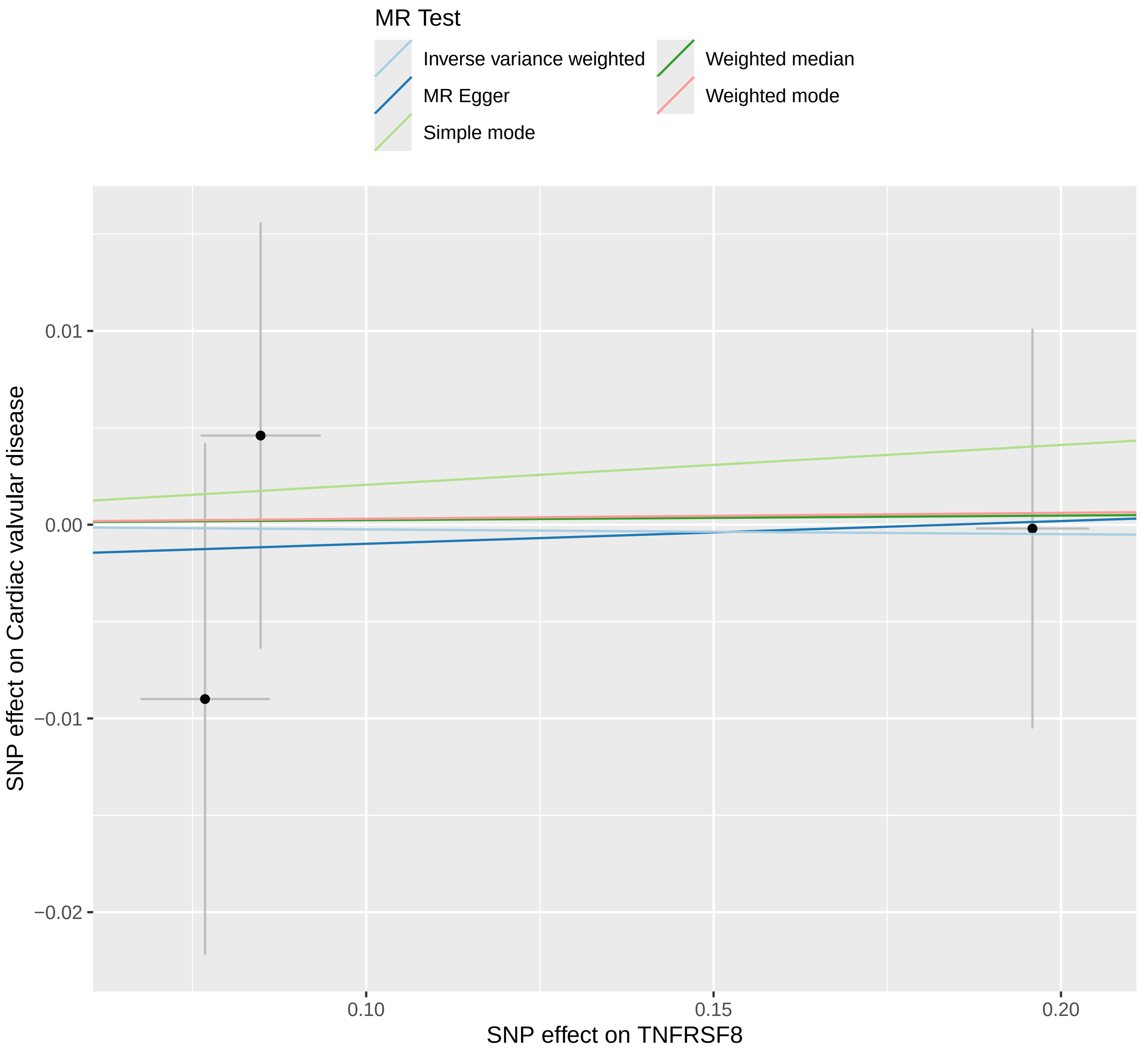
MR Test
Inverse variance weighted
MR Egger
Simple mode
Weighted median
Weighted mode
0.01
0.00
−0.01
−0.02
0.10
0.15
0.20
SNP effect on Cardiac valvular disease
SNP effect on TNFRSF8

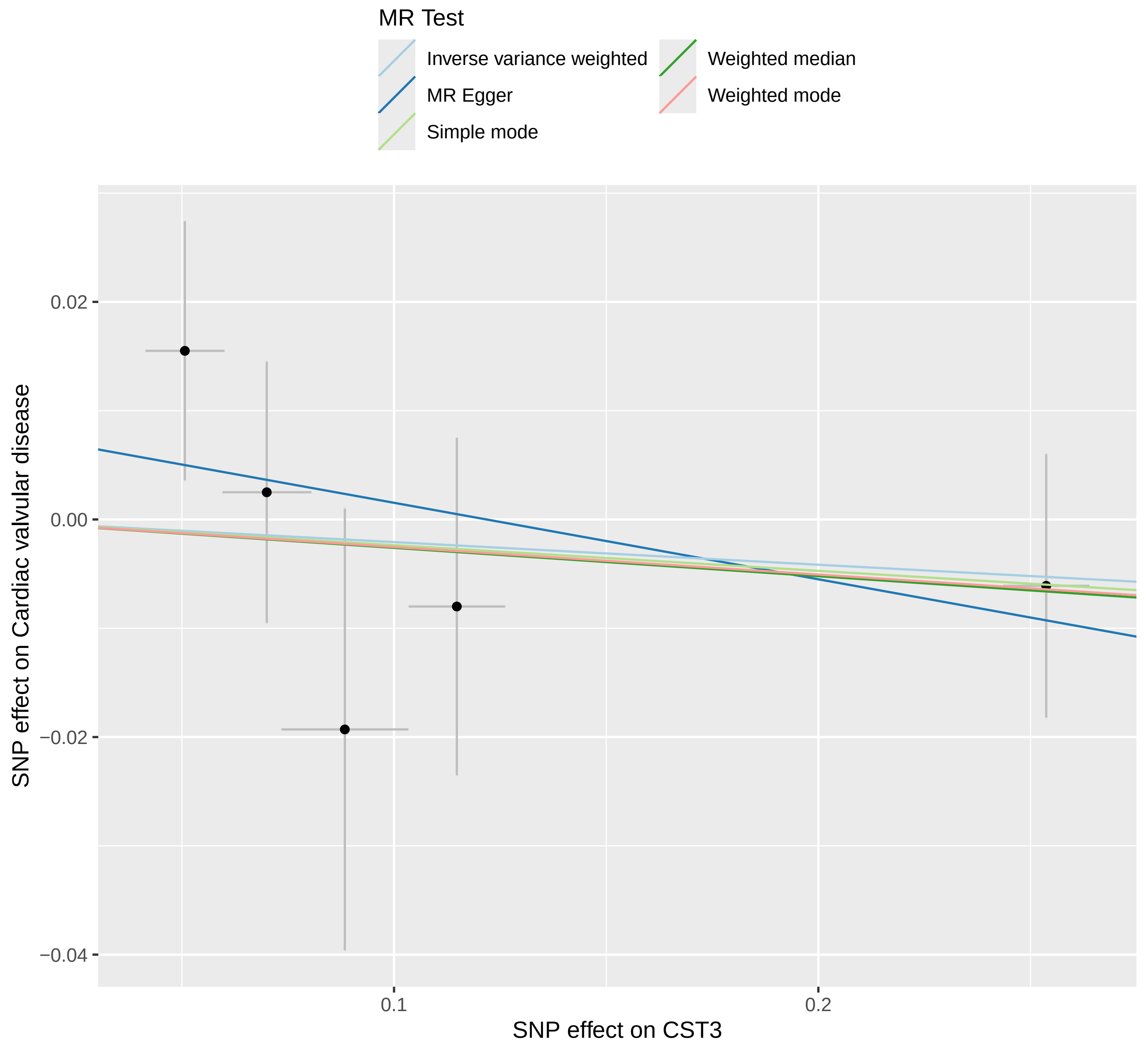
MR Test
Inverse variance weighted
MR Egger
Simple mode
Weighted median
Weighted mode
0.02
0.00
−0.02
−0.04
SNP effect on Cardiac valvular disease
0.1
0.2
SNP effect on CST3

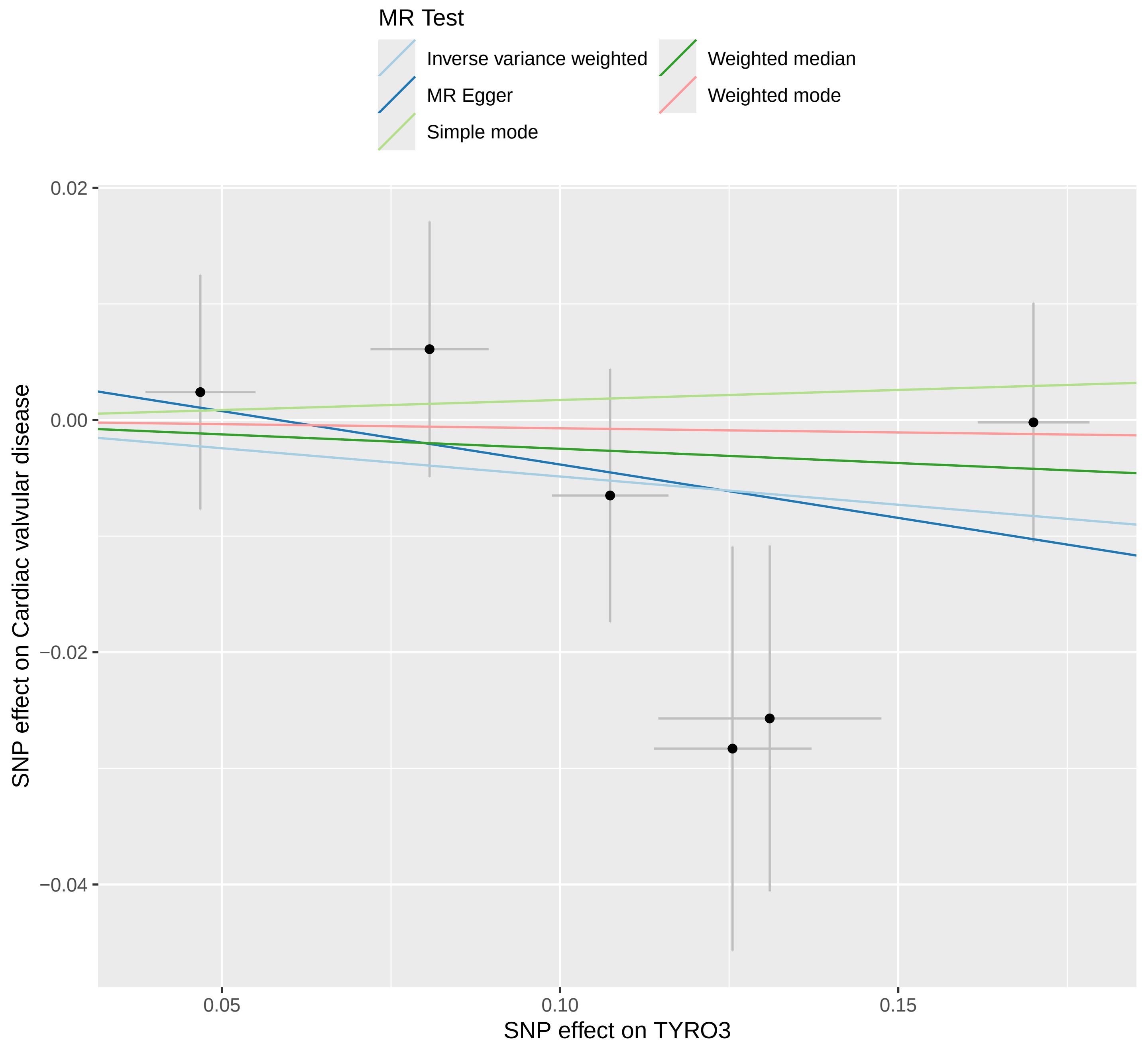
MR Test
Inverse variance weighted
MR Egger
Simple mode
Weighted median
Weighted mode
0.02
0.00
−0.02
−0.04
0.05
0.10
0.15
SNP effect on Cardiac valvular disease
SNP effect on TYRO3

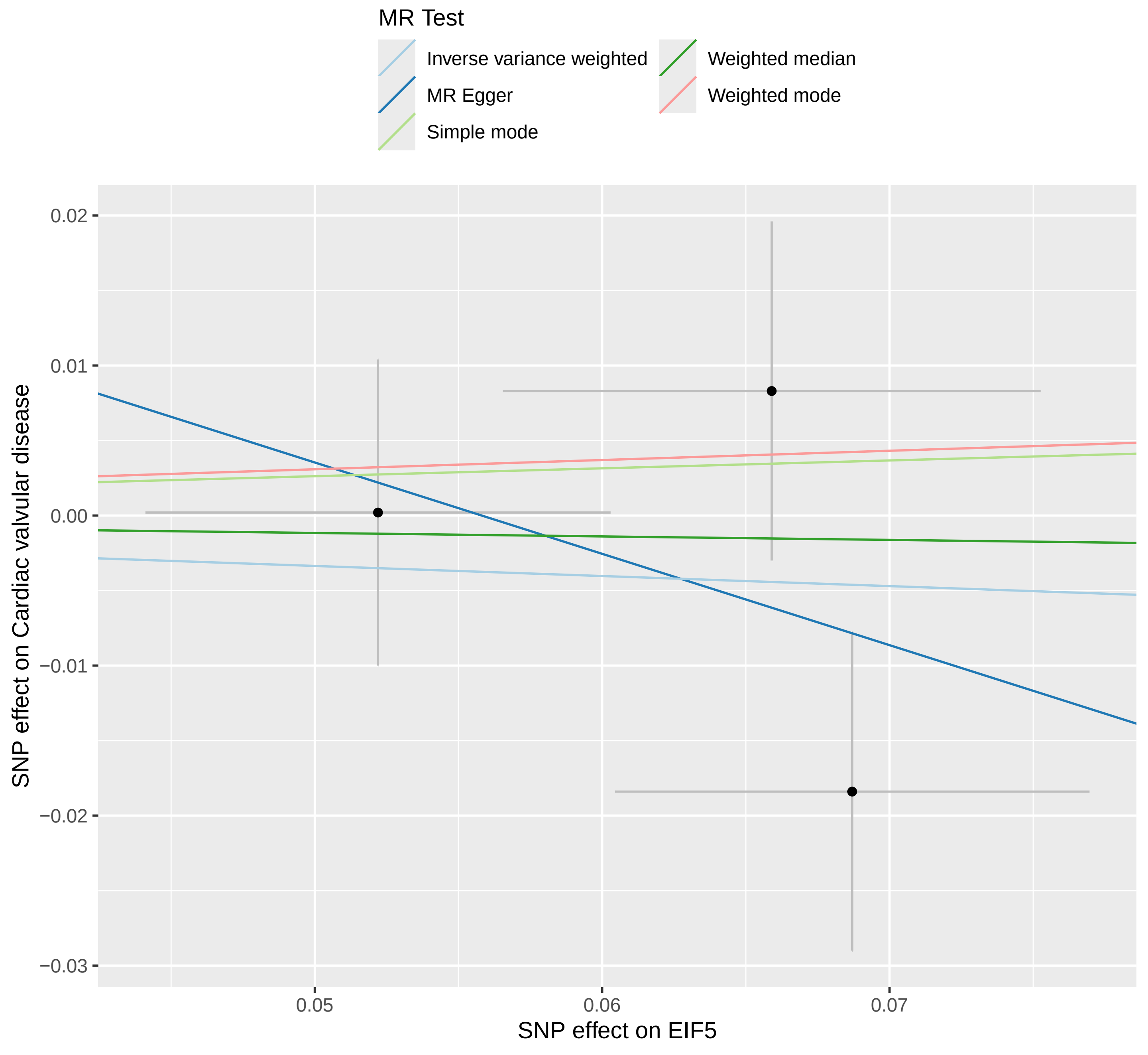
MR Test
Inverse variance weighted
MR Egger
Simple mode
Weighted median
Weighted mode
0.02
0.01
0.00
−0.01
−0.02
−0.03
SNP effect on Cardiac valvular disease
0.05
0.06
0.07
SNP effect on EIF5

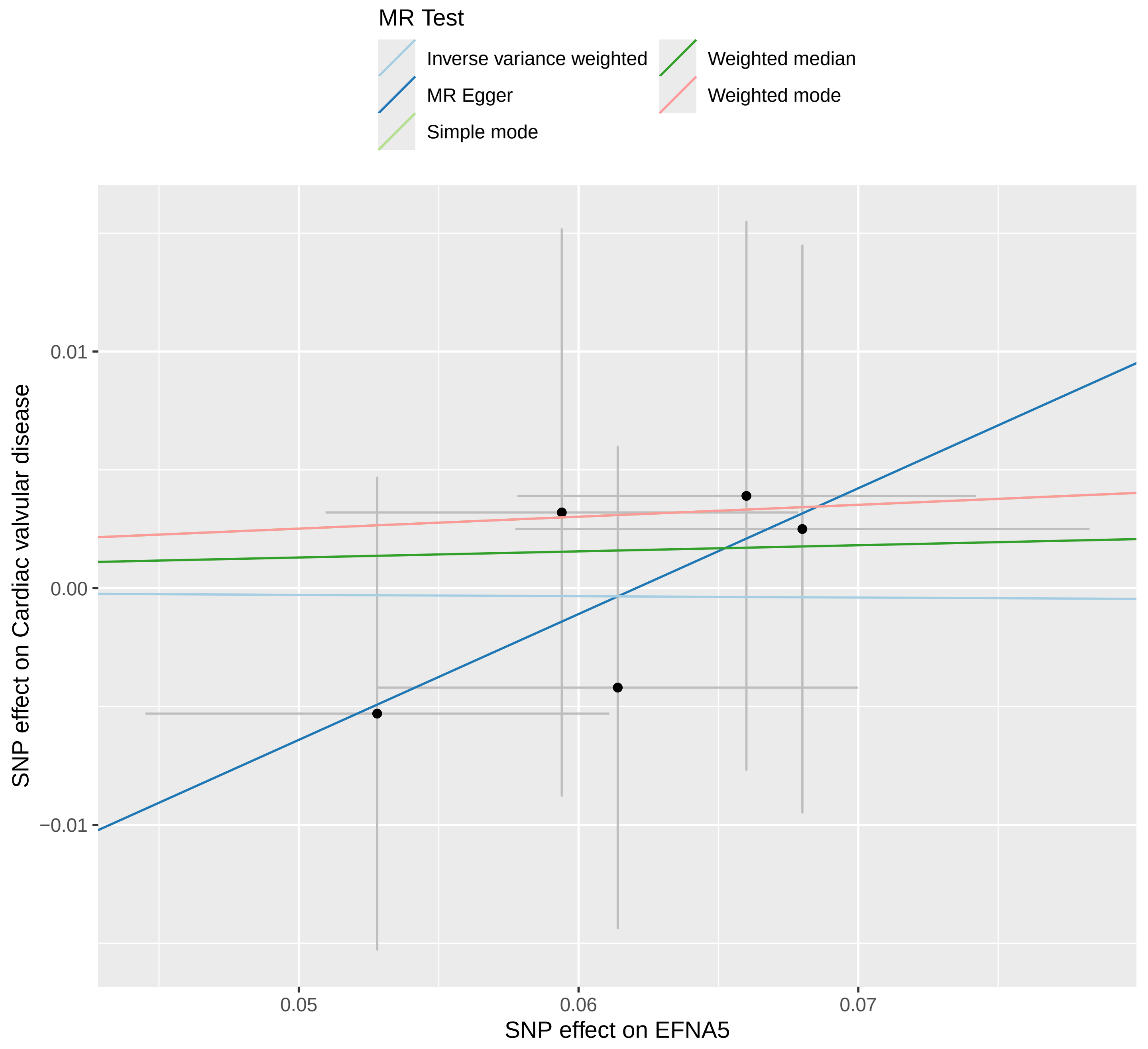

MR Test
Inverse variance weighted
MR Egger
Simple mode
Weighted median
Weighted mode
0.01
0.00
−0.01
SNP effect on Cardiac valvular disease
0.05
0.06
0.07
SNP effect on EFNA5

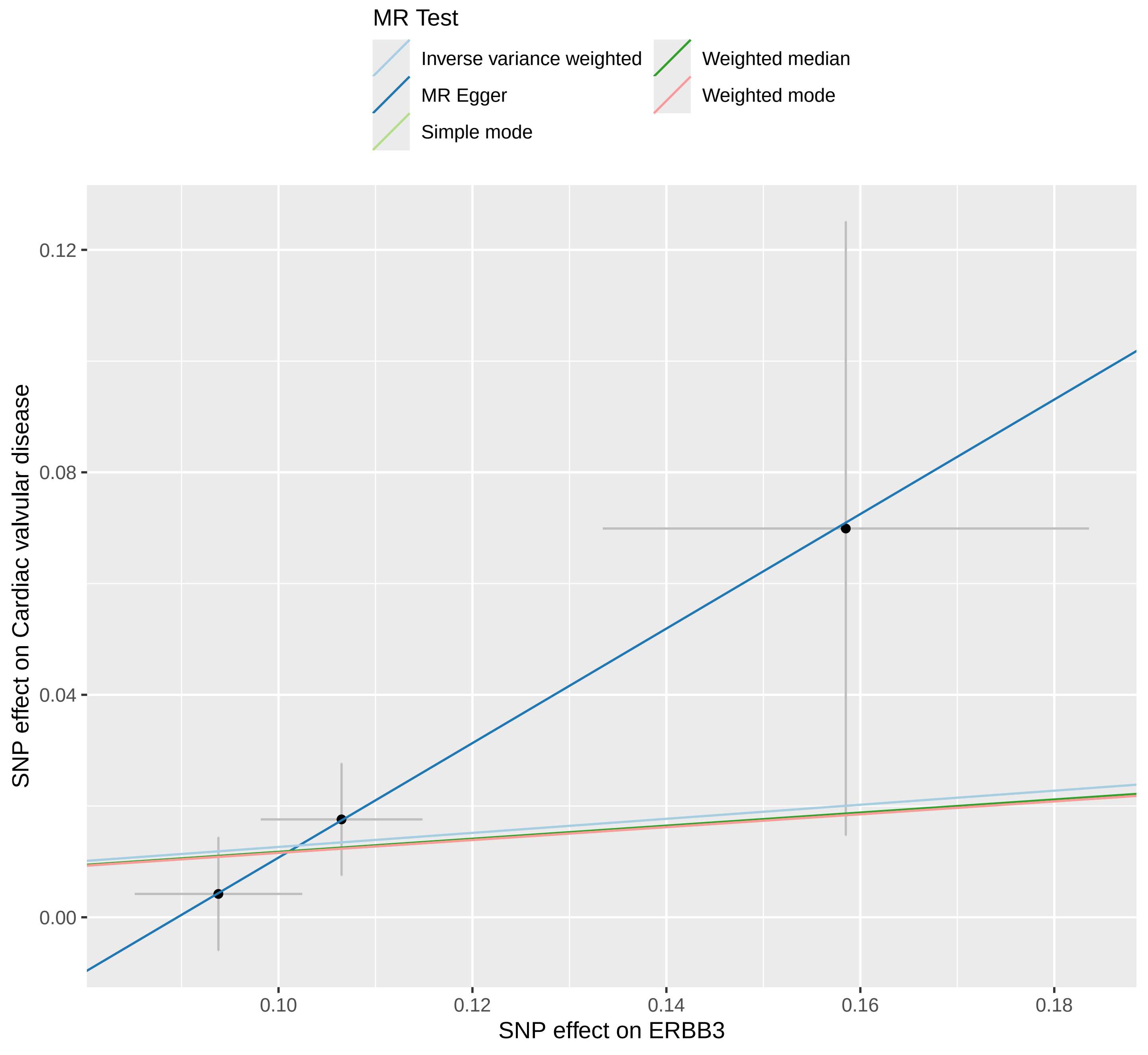

MR Test
Inverse variance weighted
MR Egger
Simple mode
Weighted median
Weighted mode
0.12
0.08
0.04
0.00
SNP effect on Cardiac valvular disease
0.10
0.12
0.14
0.16
0.18
SNP effect on ERBB3

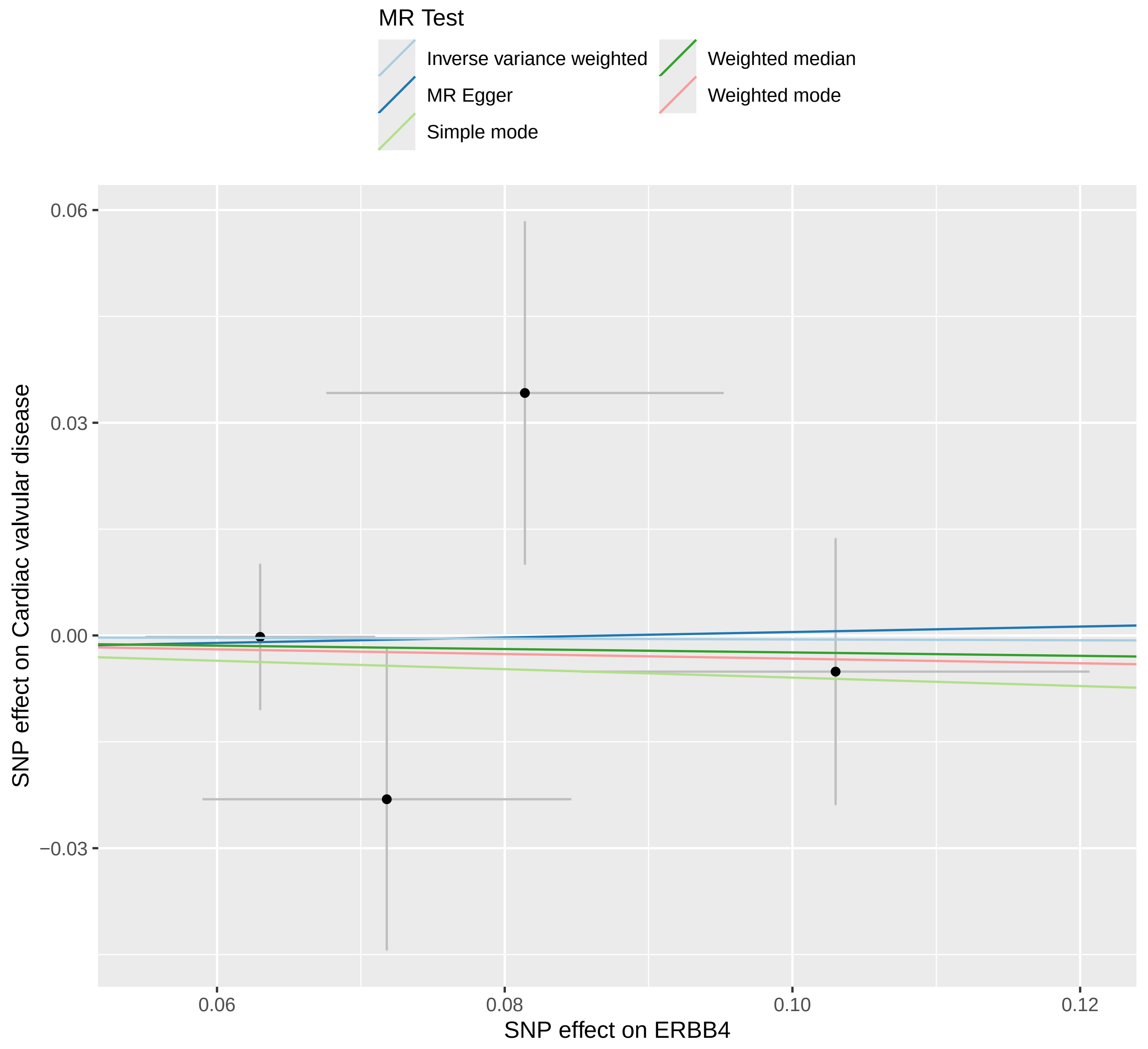

MR Test
Inverse variance weighted
MR Egger
Simple mode
Weighted median
Weighted mode
0.06
0.03
0.00
−0.03
SNP effect on Cardiac valvular disease
0.06
0.08
0.10
0.12
SNP effect on ERBB4

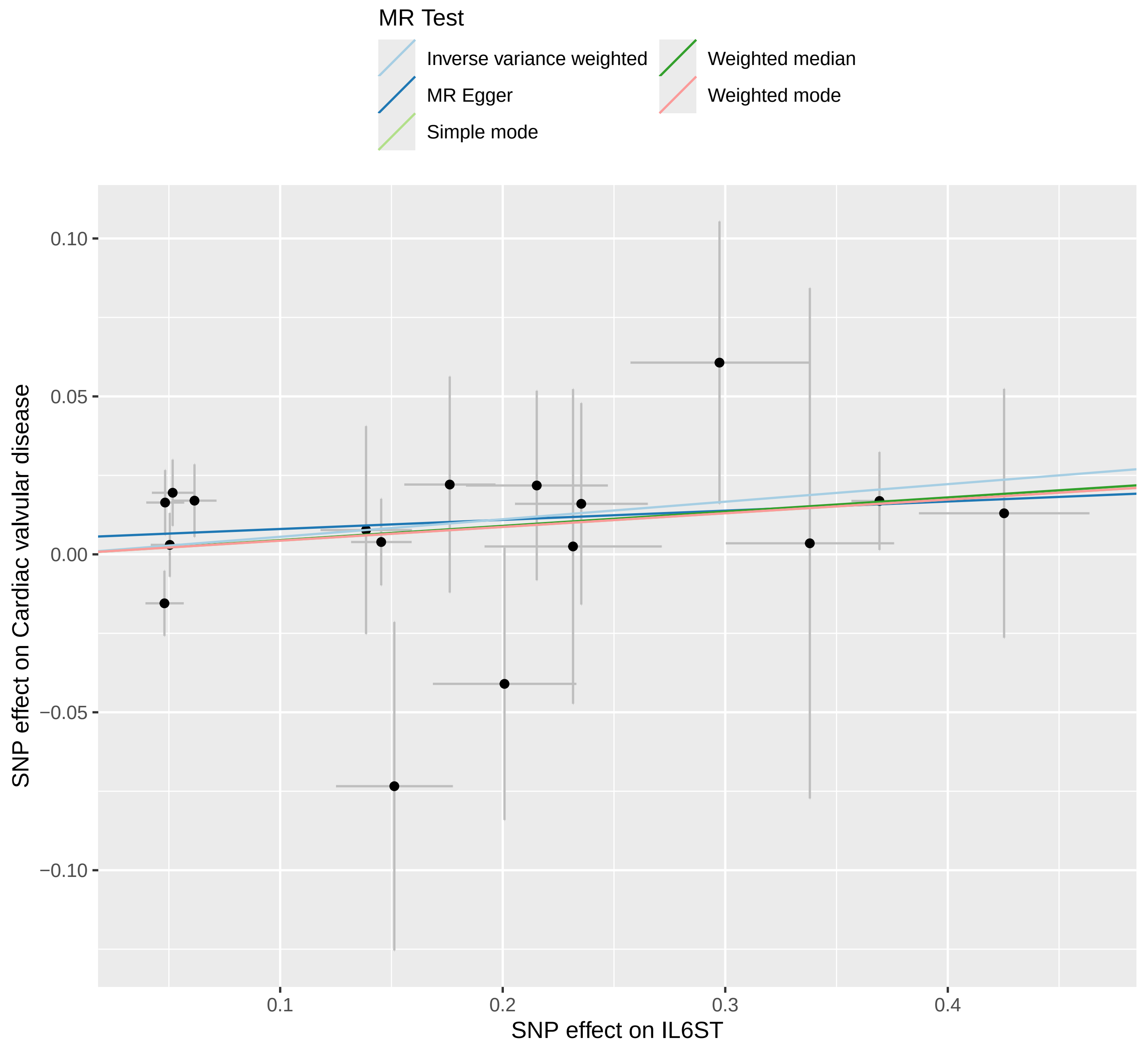

MR Test
Inverse variance weighted
MR Egger
Simple mode
Weighted median
Weighted mode
SNP effect on Cardiac valvular disease
0.10
0.05
0.00
−0.05
−0.10
0.1
0.2
0.3
0.4
SNP effect on IL6ST

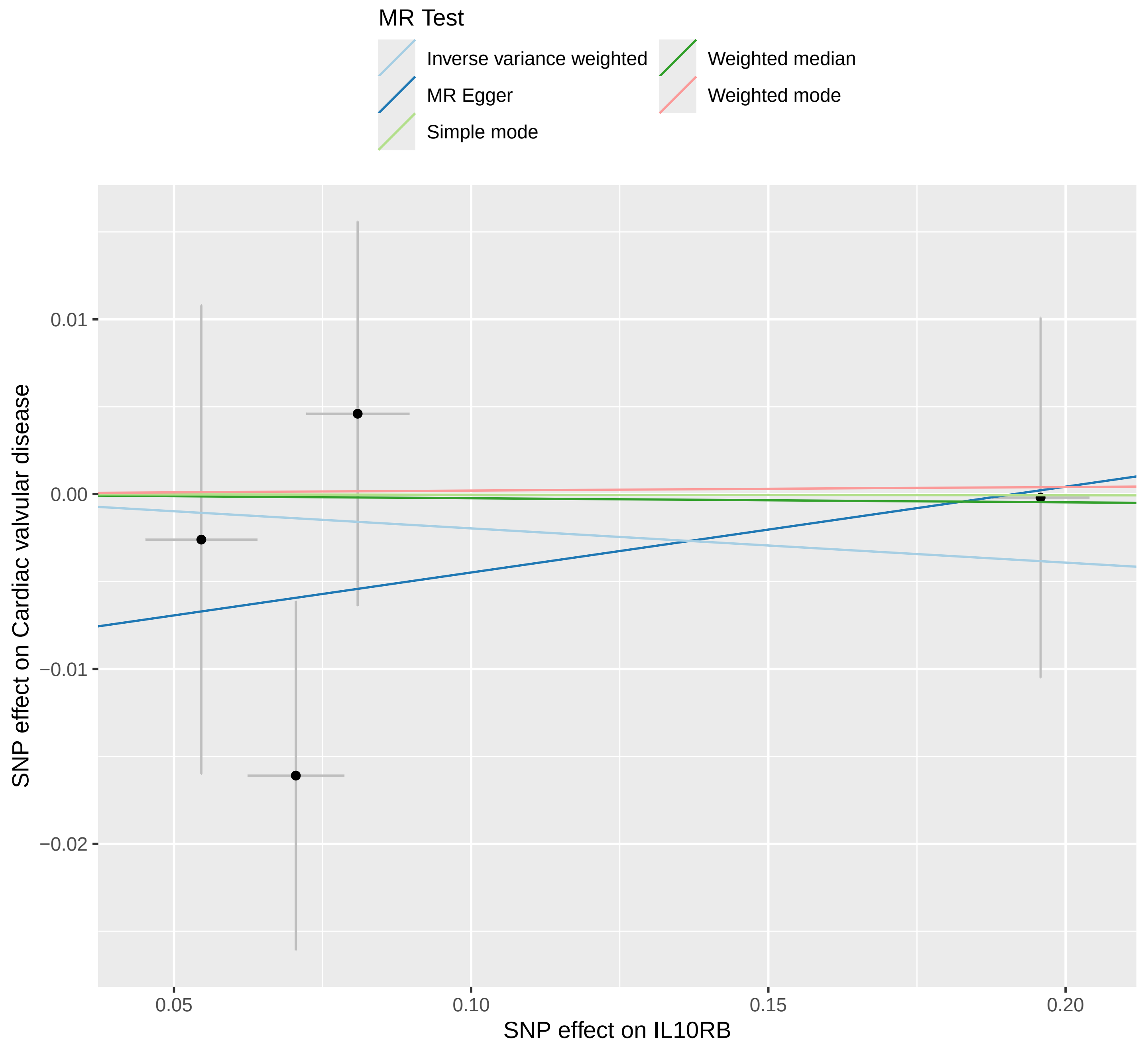

MR Test
Inverse variance weighted
MR Egger
Simple mode
Weighted median
Weighted mode
SNP effect on Cardiac valvular disease
0.01
0.00
−0.01
−0.02
0.05
0.10
0.15
0.20
SNP effect on IL10RB

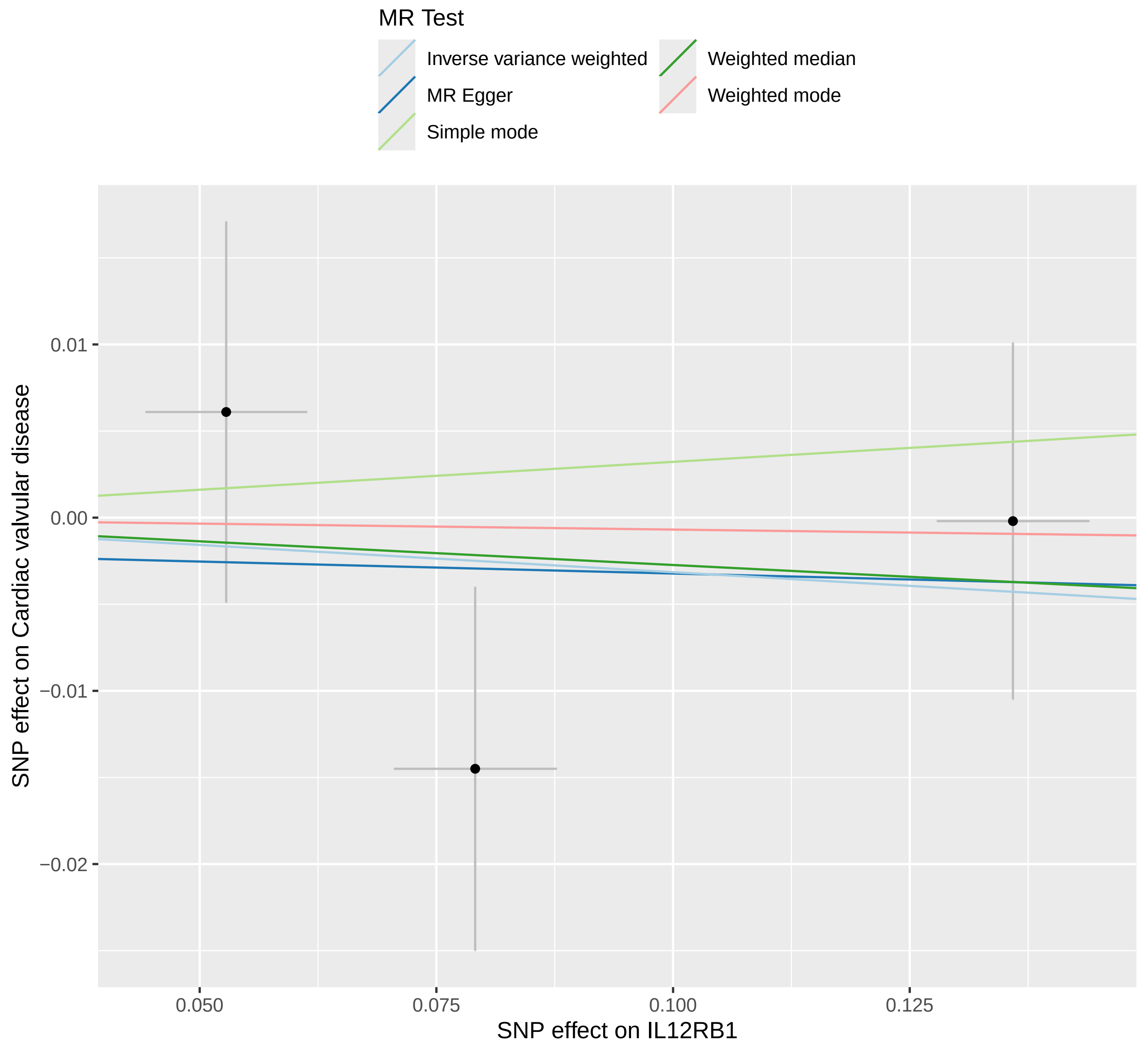

MR Test
Inverse variance weighted
MR Egger
Simple mode
Weighted median
Weighted mode
0.01
0.00
−0.01
−0.02
SNP effect on Cardiac valvular disease
0.050
0.075
0.100
0.125
SNP effect on IL12RB1

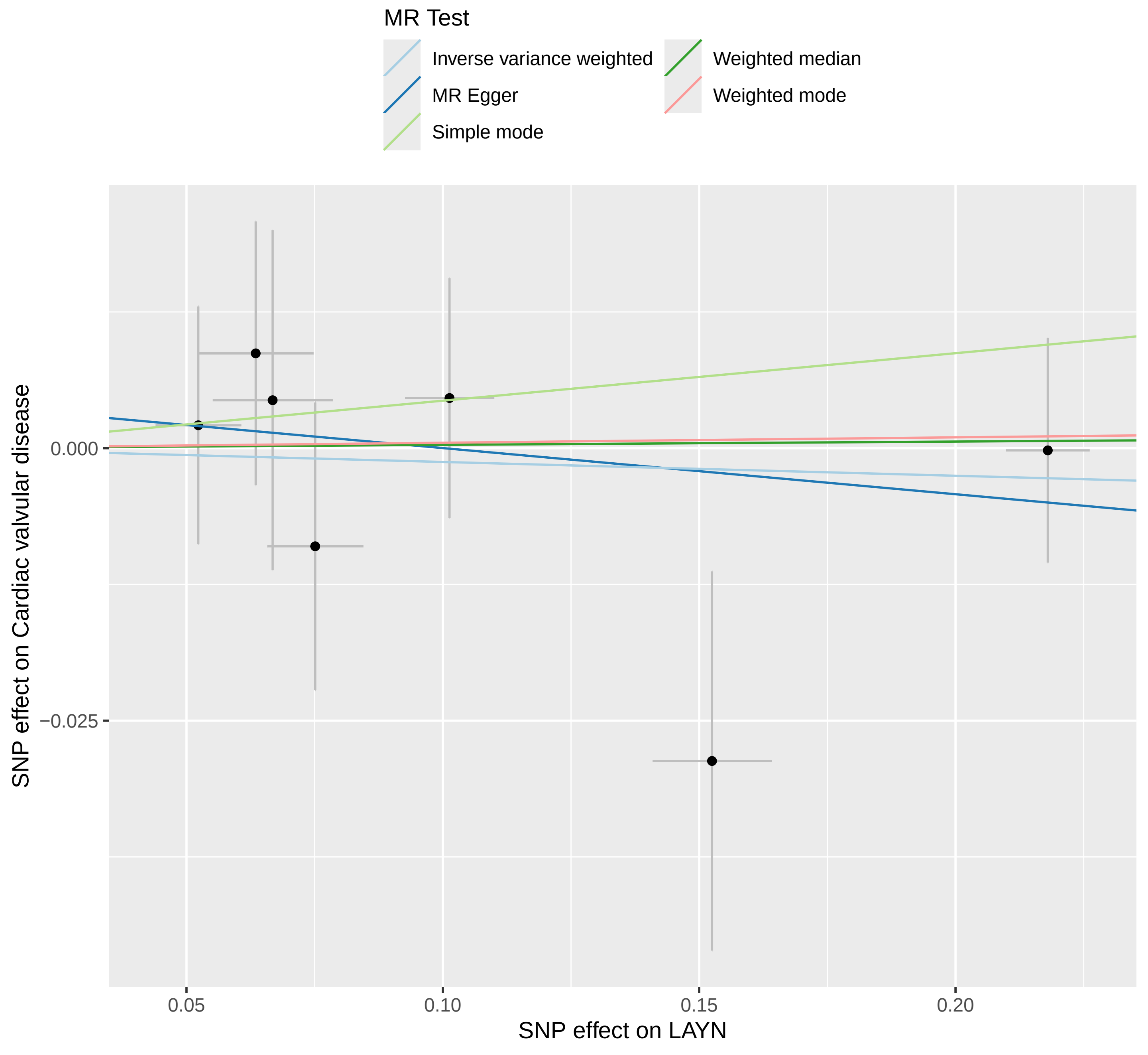
MR Test
Inverse variance weighted
MR Egger
Simple mode
Weighted median
Weighted mode
0.000
−0.025
0.05
0.10
0.15
0.20
SNP effect on LAYN
SNP effect on Cardiac valvular disease

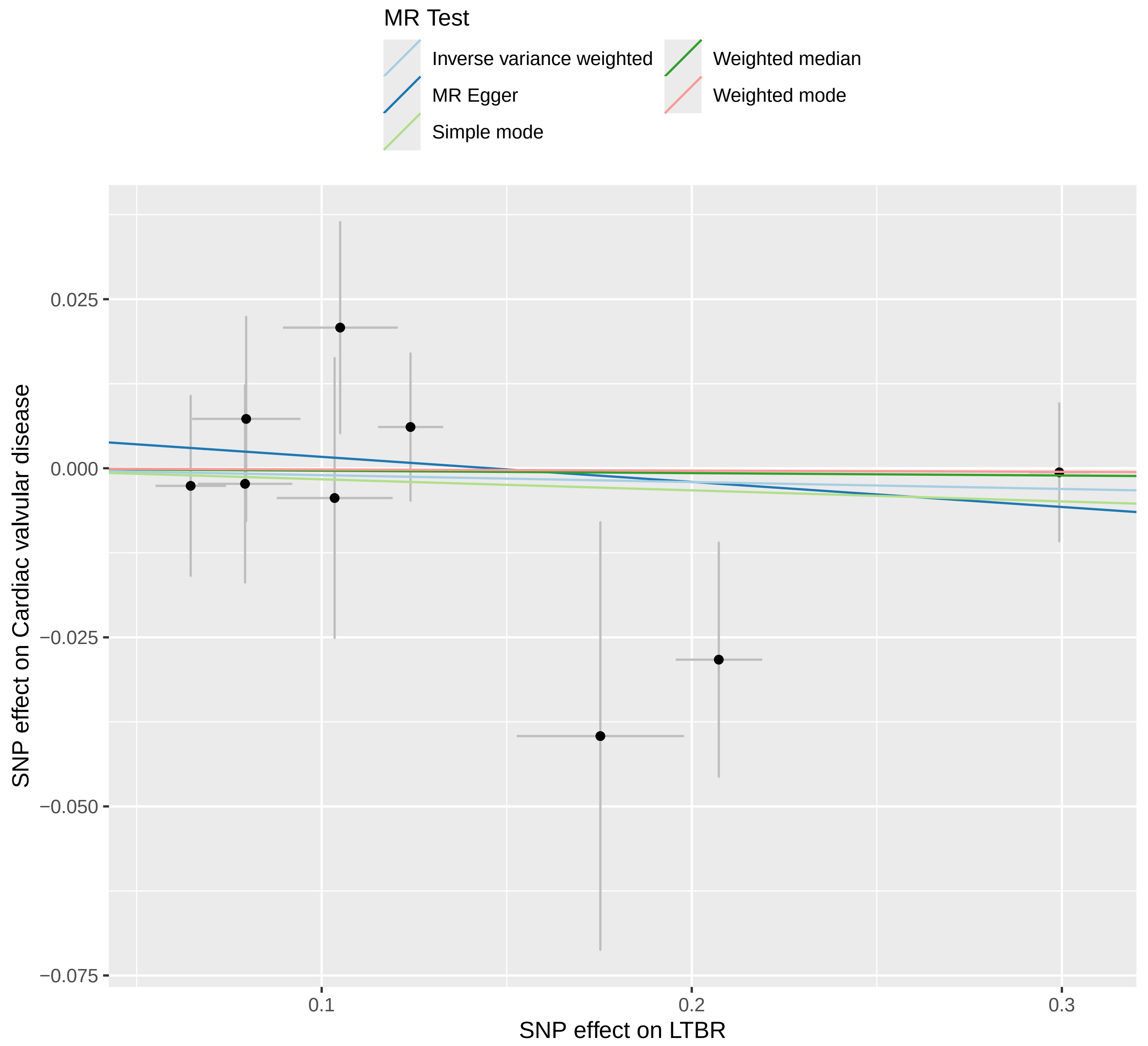
MR Test
Inverse variance weighted
MR Egger
Simple mode
Weighted median
Weighted mode
0.025
0.000
−0.025
−0.050
−0.075
0.1
0.2
0.3
SNP effect on Cardiac valvular disease
SNP effect on LTBR

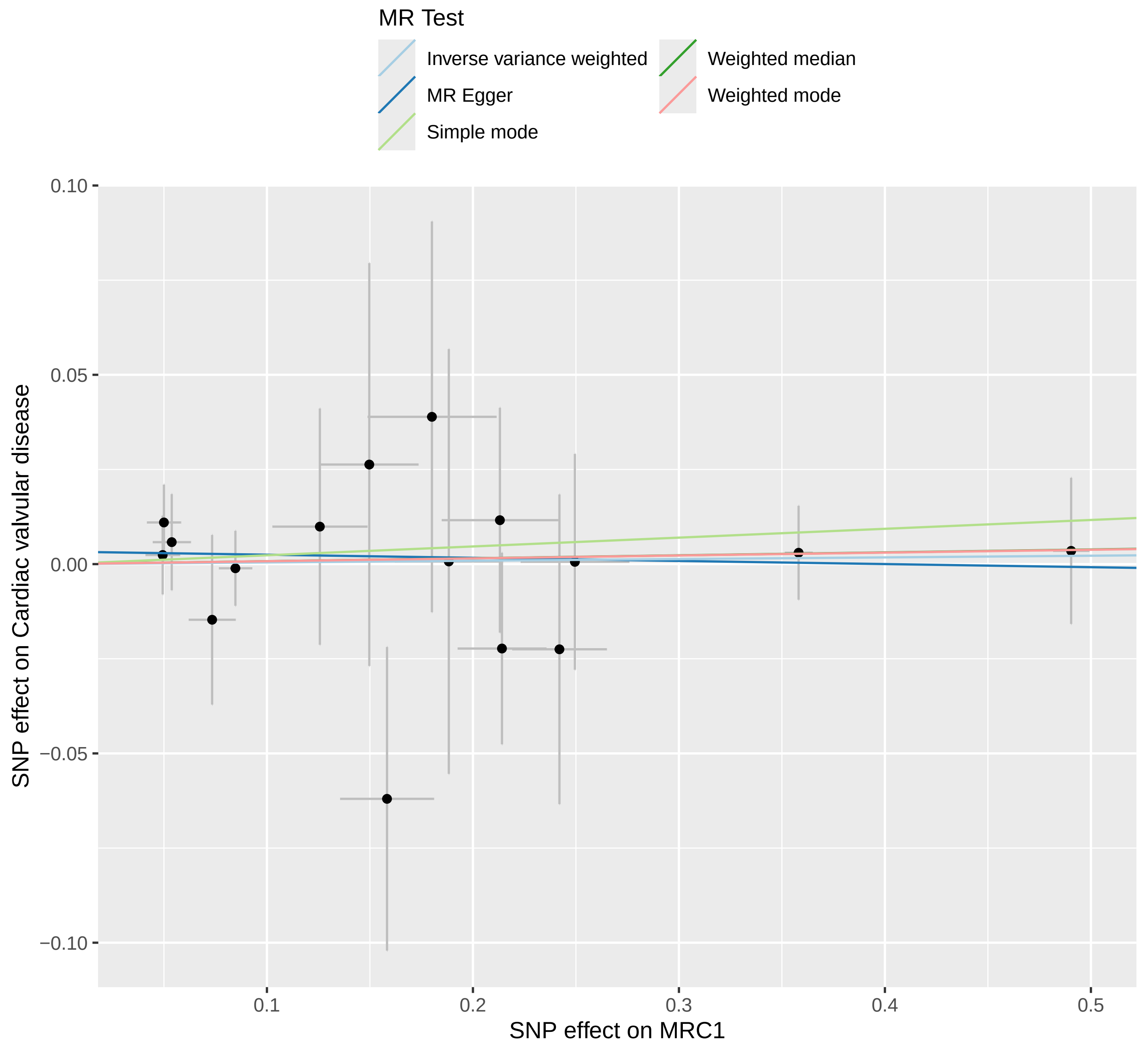

MR Test
Inverse variance weighted
MR Egger
Simple mode
Weighted median
Weighted mode
0.10
0.05
0.00
−0.05
−0.10
0.1
0.2
0.3
0.4
0.5
SNP effect on Cardiac valvular disease
SNP effect on MRC1

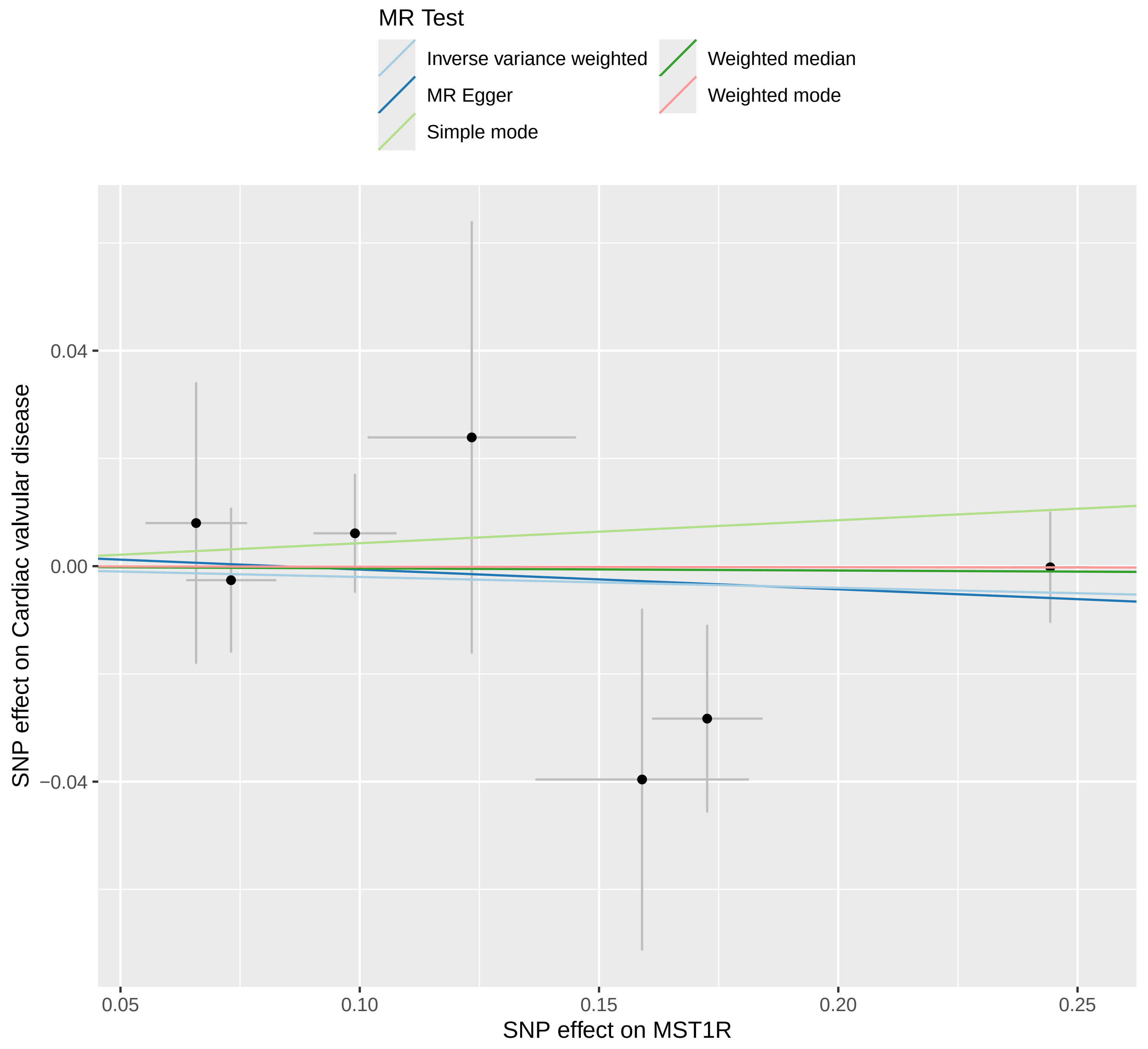
MR Test
Inverse variance weighted
MR Egger
Simple mode
Weighted median
Weighted mode
0.04
0.00
−0.04
0.05
0.10
0.15
0.20
0.25
SNP effect on Cardiac valvular disease
SNP effect on MST1R

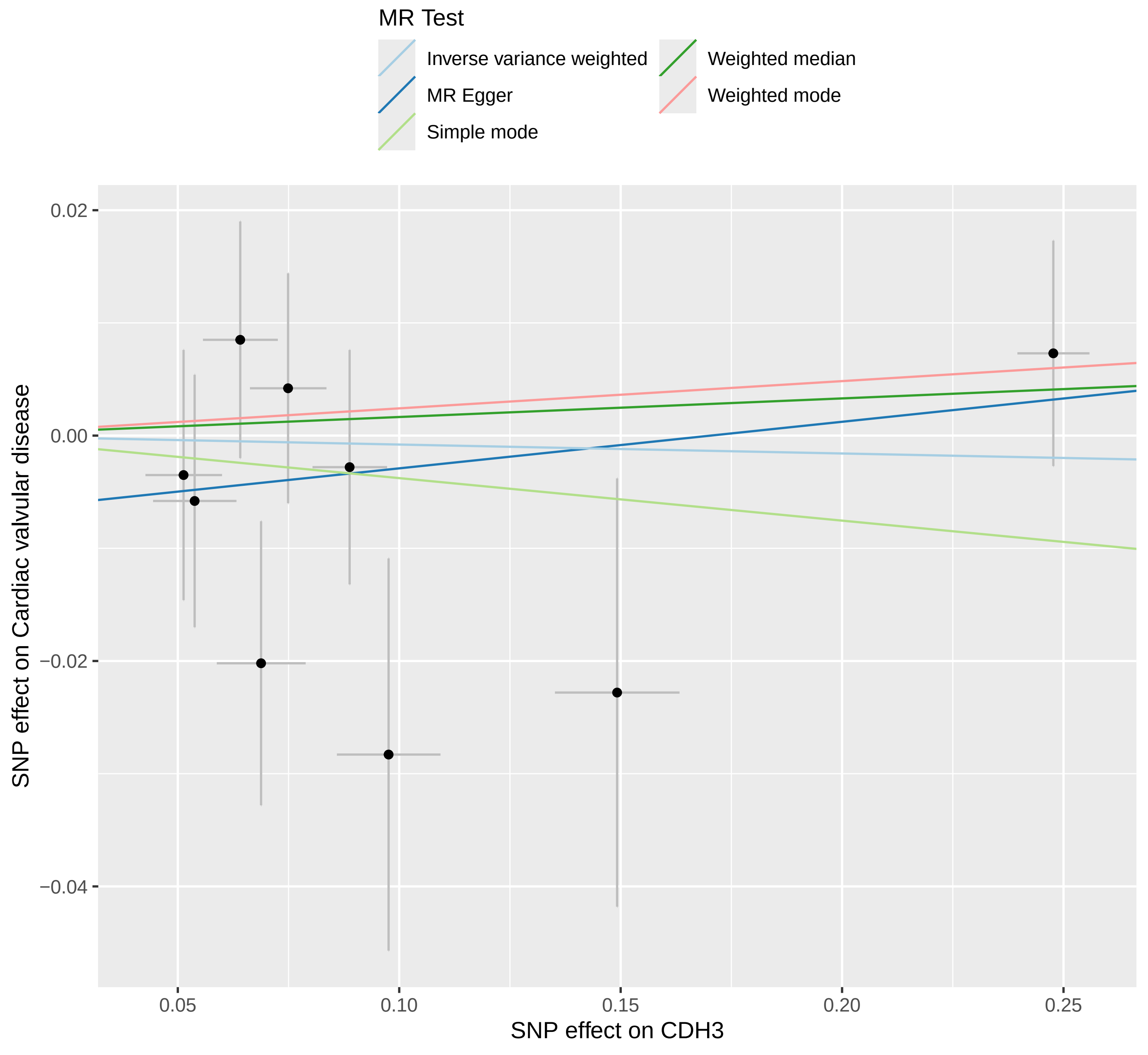

MR Test
Inverse variance weighted
MR Egger
Simple mode
Weighted median
Weighted mode
0.02
0.00
−0.02
−0.04
SNP effect on Cardiac valvular disease
0.05
0.10
0.15
0.20
0.25
SNP effect on CDH3

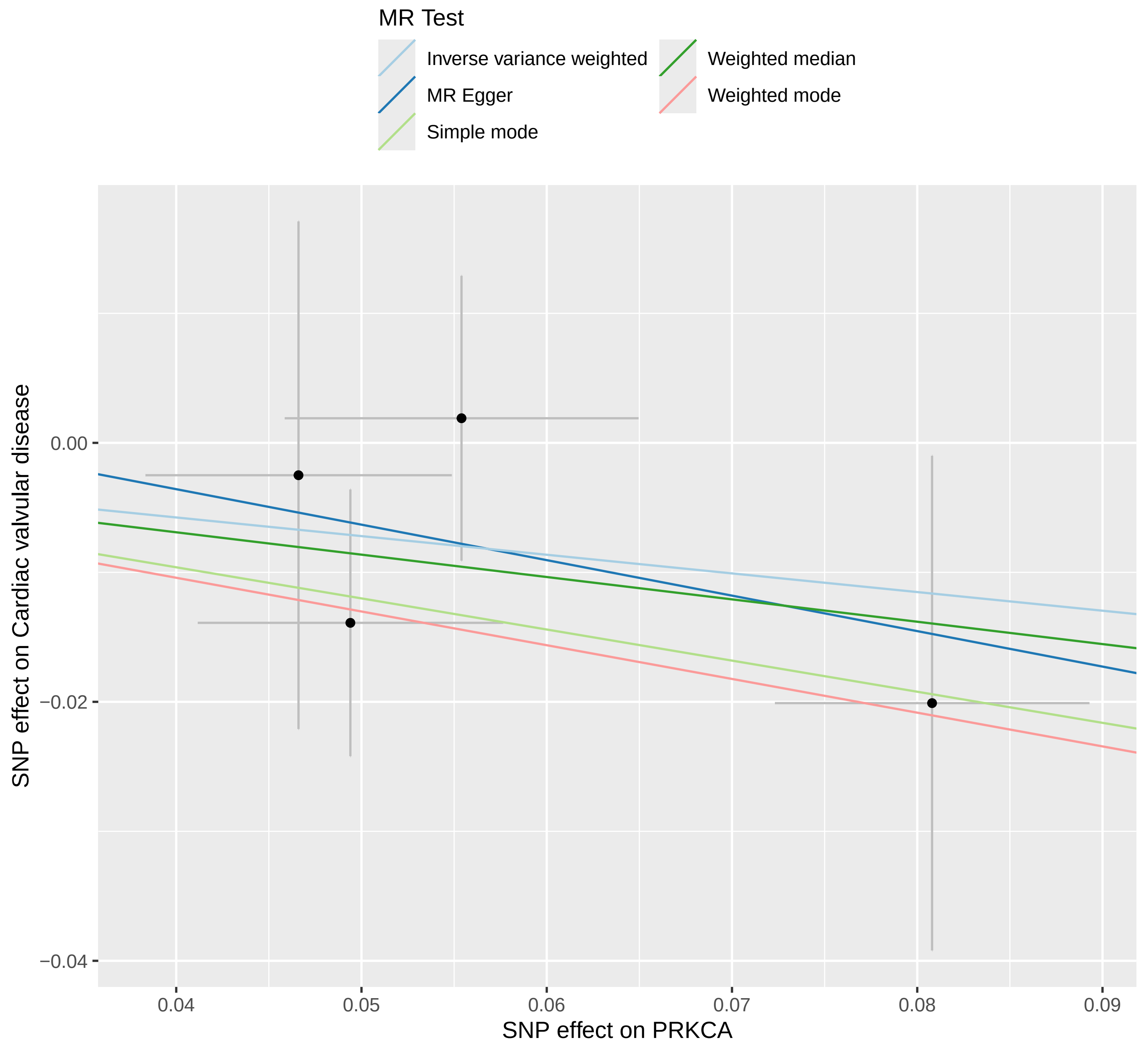
MR Test
Inverse variance weighted
MR Egger
Simple mode
Weighted median
Weighted mode
0.00
−0.02
−0.04
0.04
0.05
0.06
0.07
0.08
0.09
SNP effect on PRKCA
SNP effect on Cardiac valvular disease

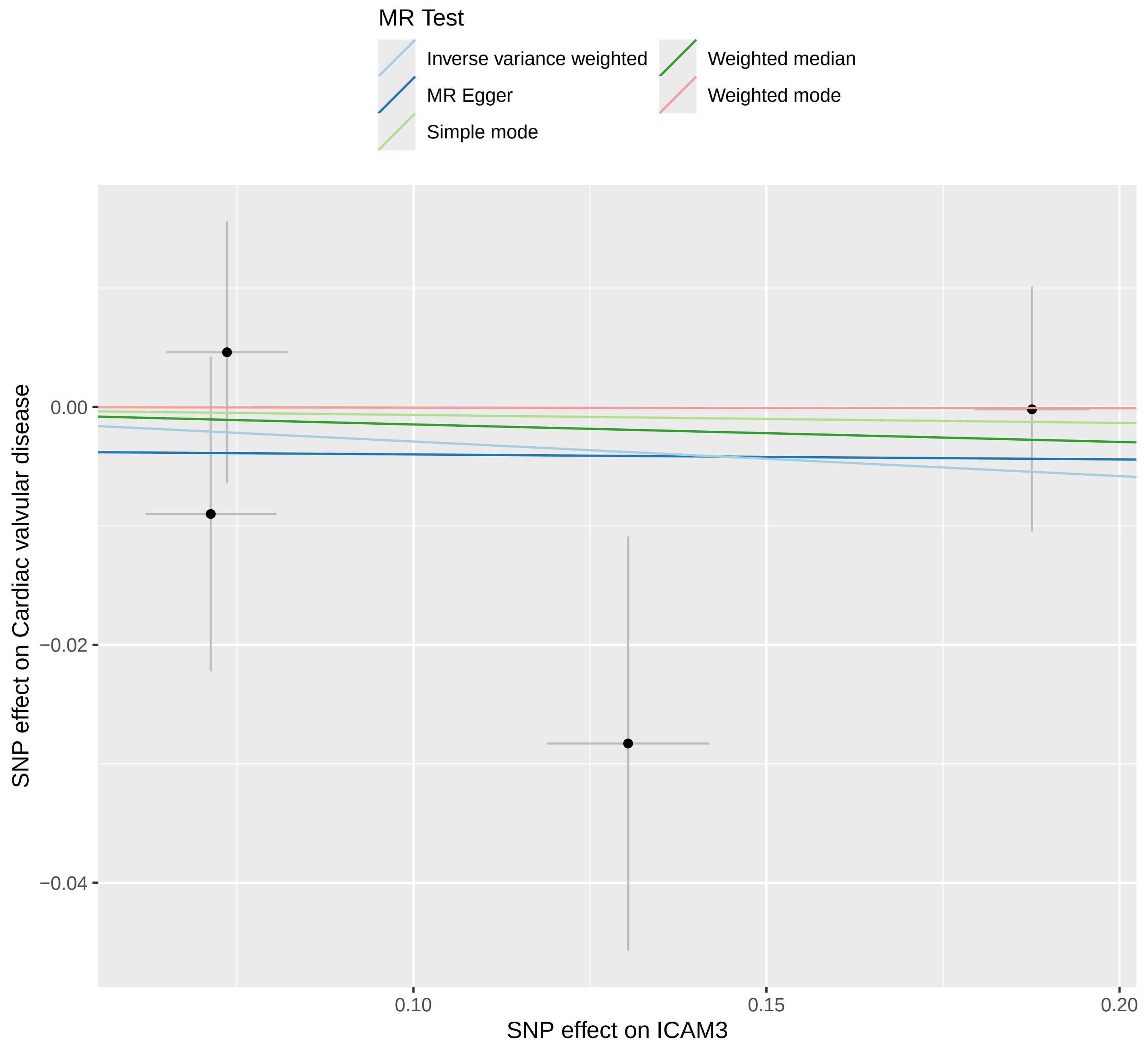
MR Test
Inverse variance weighted
MR Egger
Simple mode
Weighted median
Weighted mode
0.00
−0.02
−0.04
0.10
0.15
0.20
SNP effect on ICAM3
SNP effect on Cardiac valvular disease

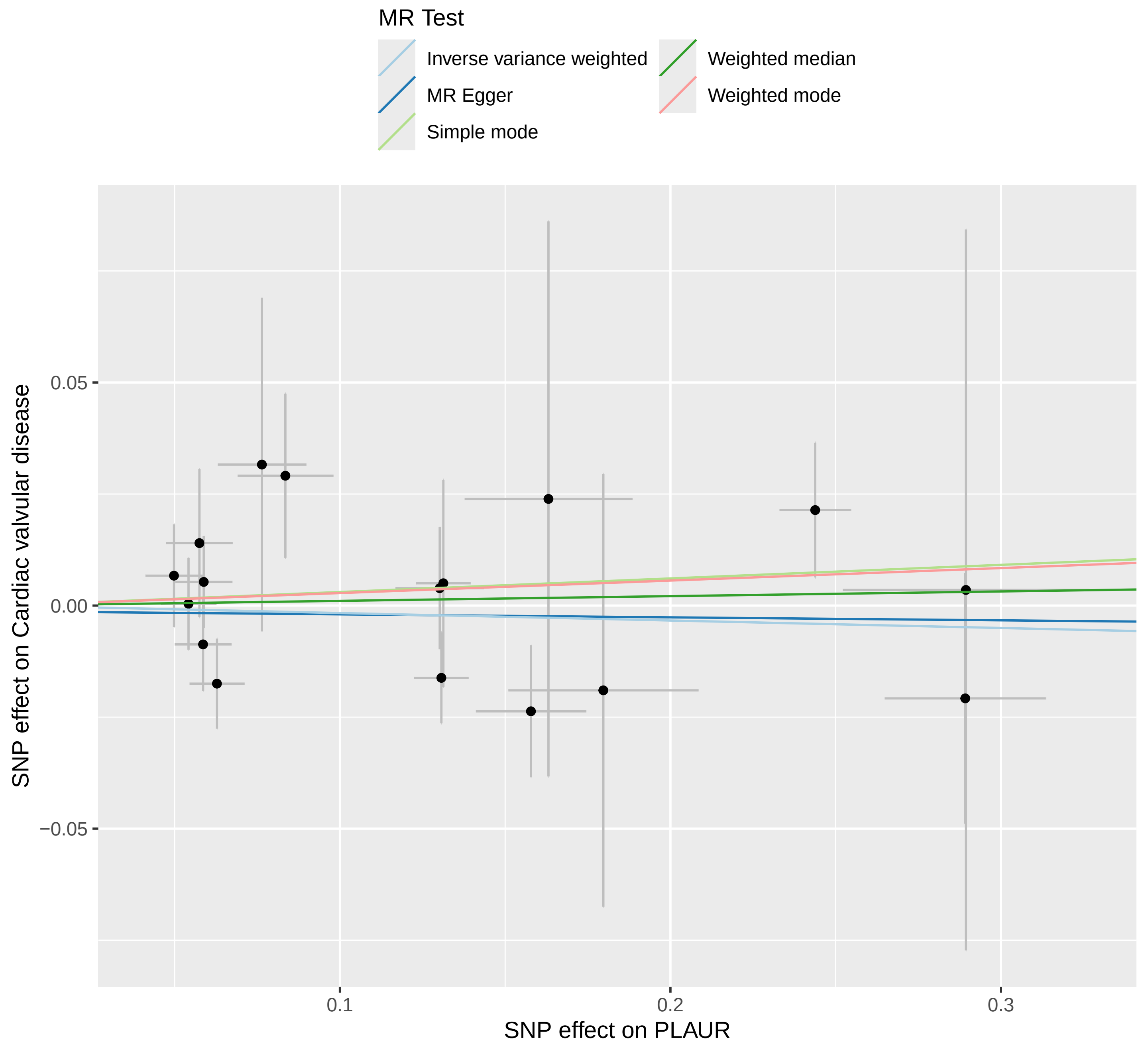
MR Test
Inverse variance weighted
MR Egger
Simple mode
Weighted median
Weighted mode
SNP effect on Cardiac valvular disease
0.05
0.00
−0.05
0.1
0.2
0.3
SNP effect on PLAUR

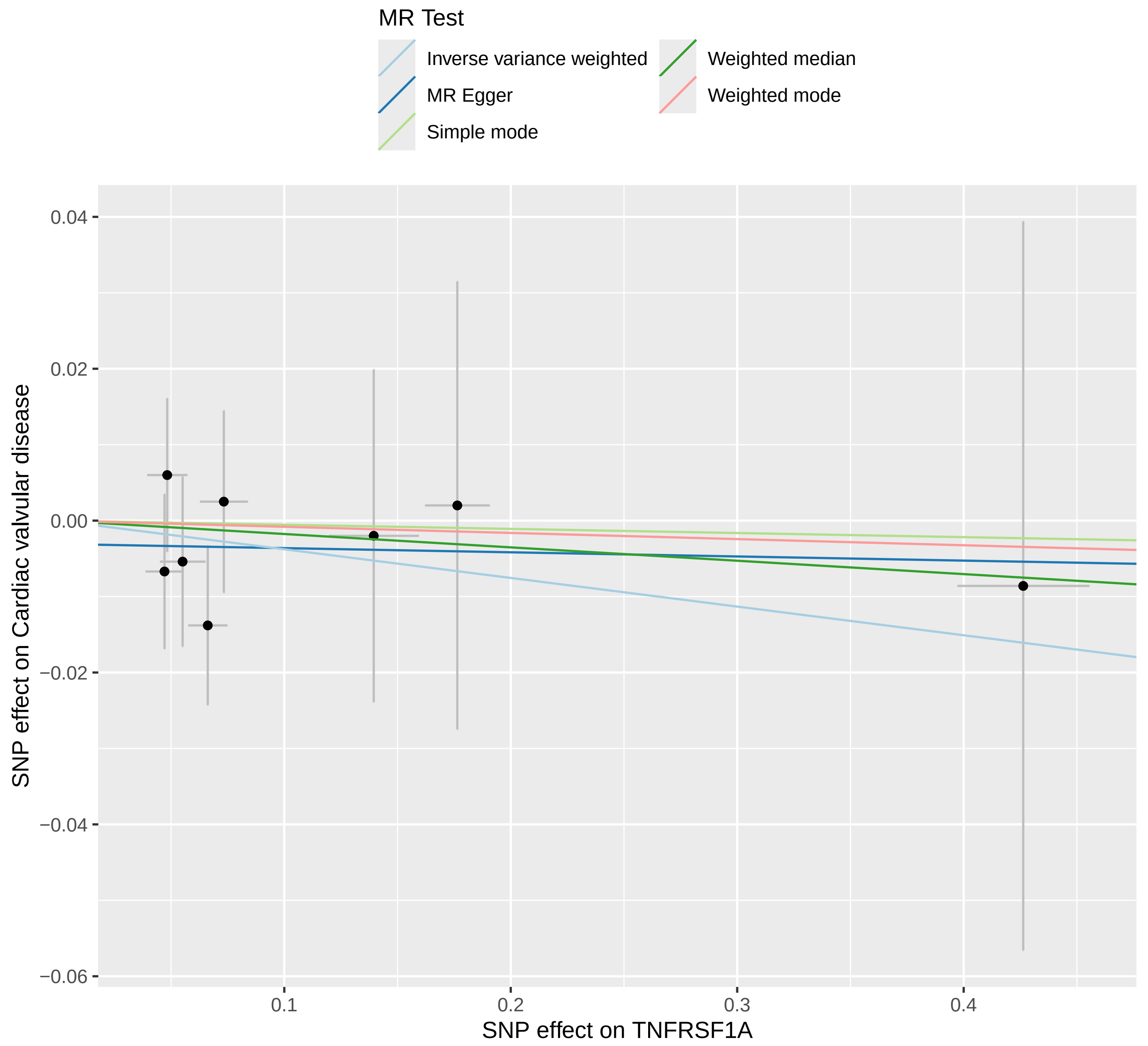

MR Test
Inverse variance weighted
MR Egger
Simple mode
Weighted median
Weighted mode
0.04
0.02
0.00
−0.02
−0.04
−0.06
SNP effect on Cardiac valvular disease
0.1
0.2
0.3
0.4
SNP effect on TNFRSF1A

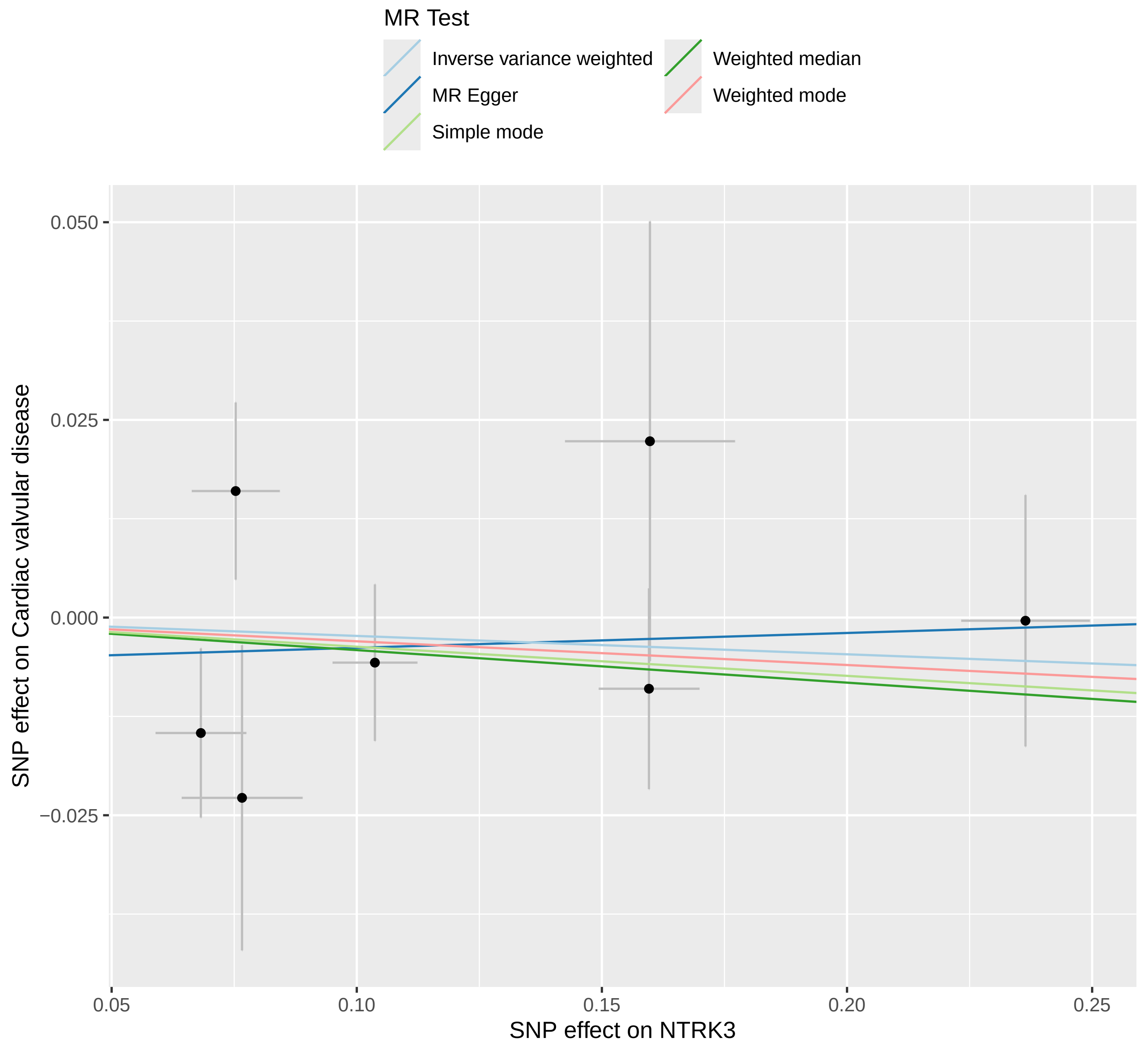
MR Test
Inverse variance weighted
MR Egger
Simple mode
Weighted median
Weighted mode
SNP effect on Cardiac valvular disease
0.050
0.025
0.000
−0.025
0.05
0.10
0.15
0.20
0.25
SNP effect on NTRK3

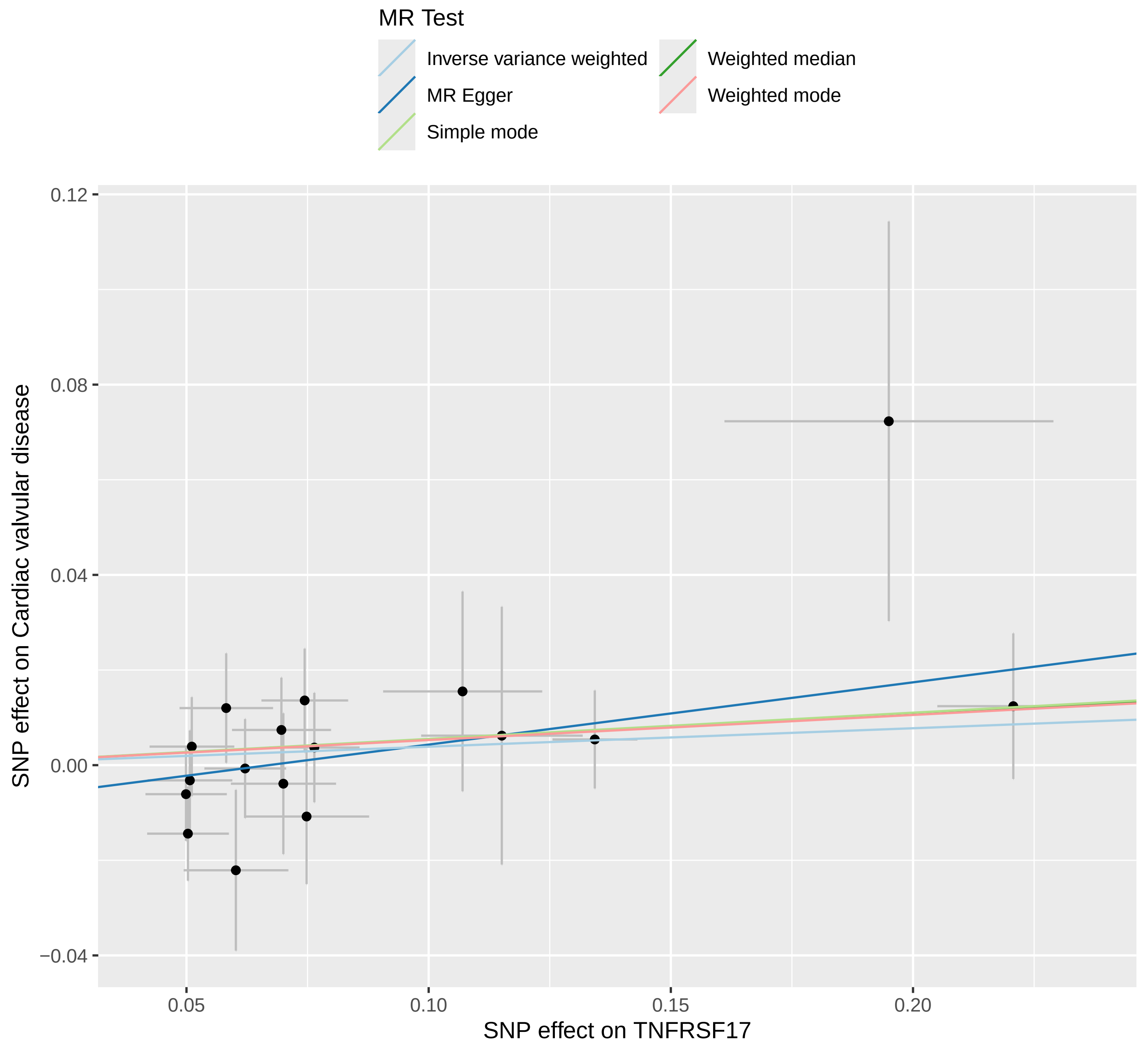
MR Test
Inverse variance weighted
MR Egger
Simple mode
Weighted median
Weighted mode
0.12
0.08
0.04
0.00
−0.04
0.05
0.10
0.15
0.20
SNP effect on Cardiac valvular disease
SNP effect on TNFRSF17

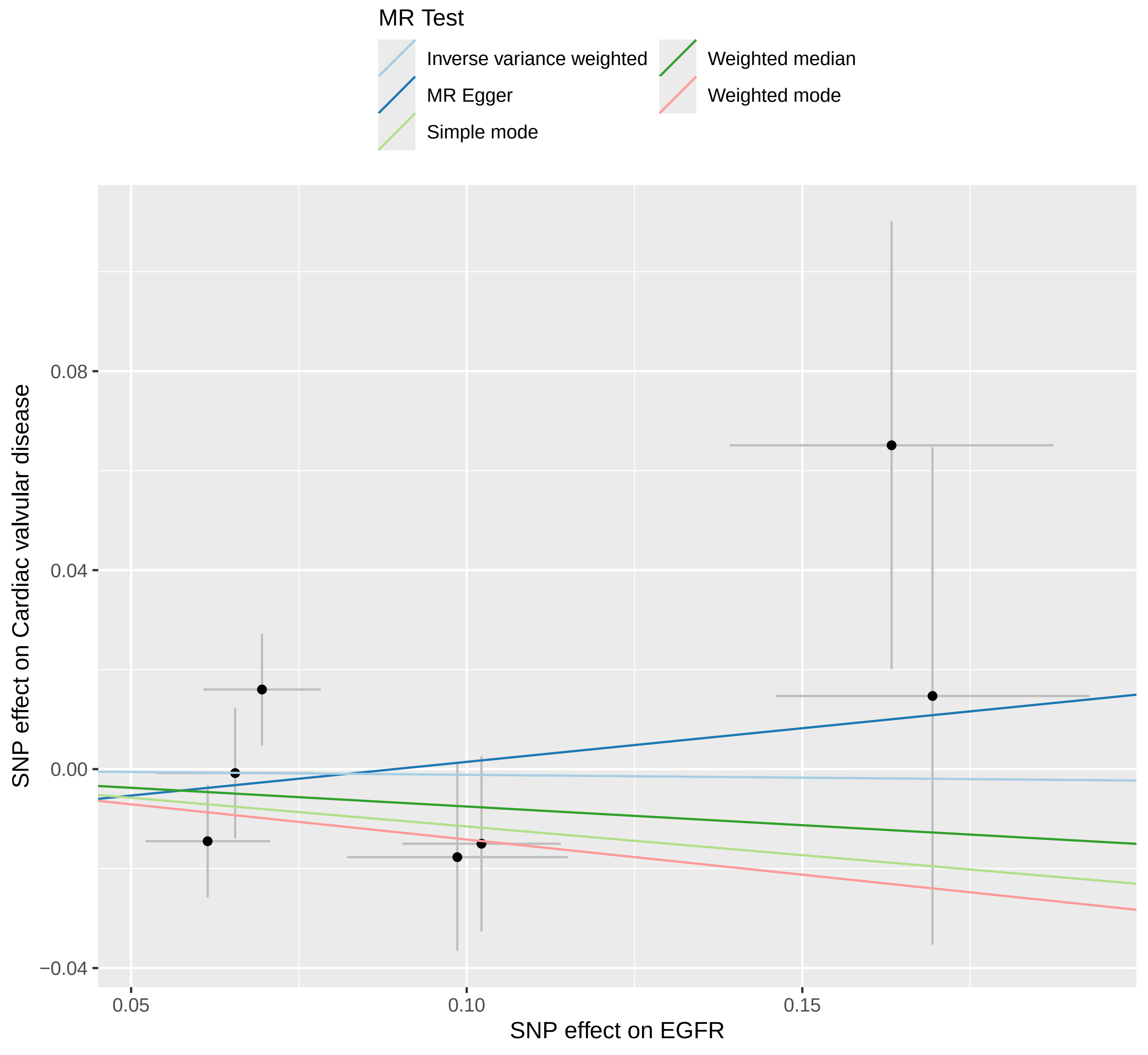
MR Test
Inverse variance weighted
MR Egger
Simple mode
Weighted median
Weighted mode
0.08
0.04
0.00
−0.04
0.05
0.10
0.15
SNP effect on Cardiac valvular disease
SNP effect on EGFR

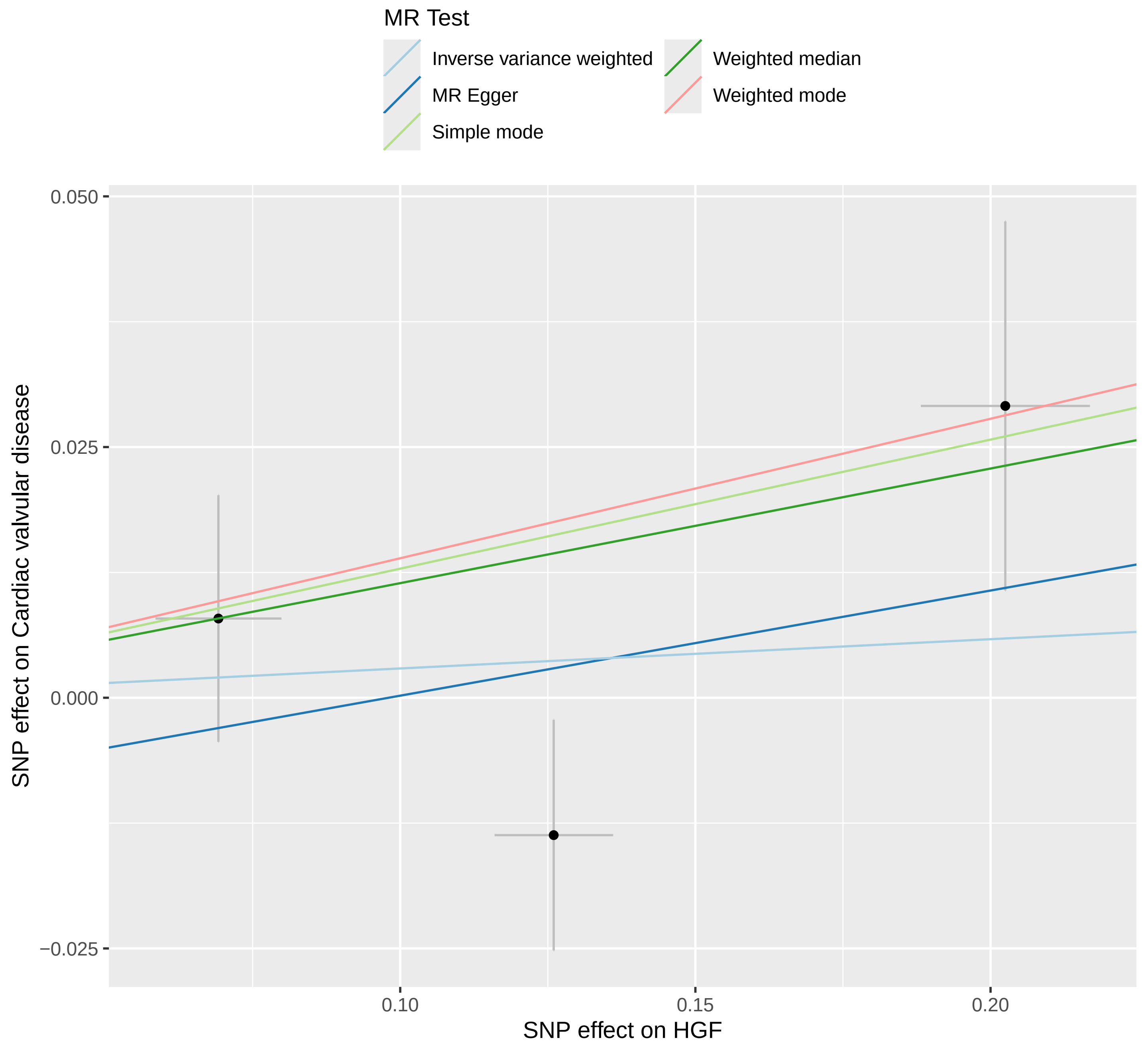
MR Test
Inverse variance weighted
MR Egger
Simple mode
Weighted median
Weighted mode
0.050
0.025
0.000
−0.025
0.10
0.15
0.20
SNP effect on Cardiac valvular disease
SNP effect on HGF

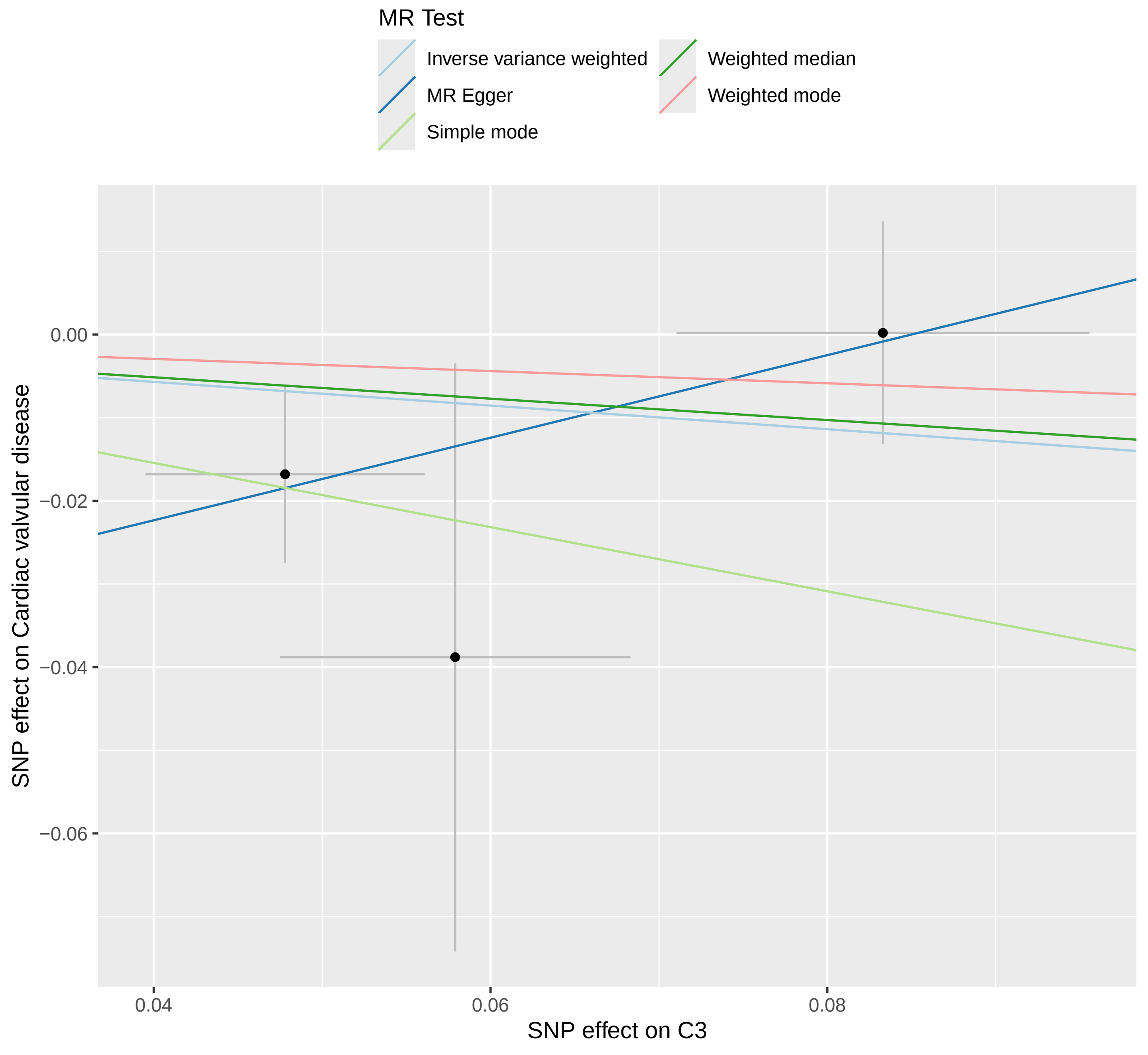

MR Test
Inverse variance weighted
MR Egger
Simple mode
Weighted median
Weighted mode
0.00
−0.02
−0.04
−0.06
SNP effect on Cardiac valvular disease
0.04
0.06
0.08
SNP effect on C3

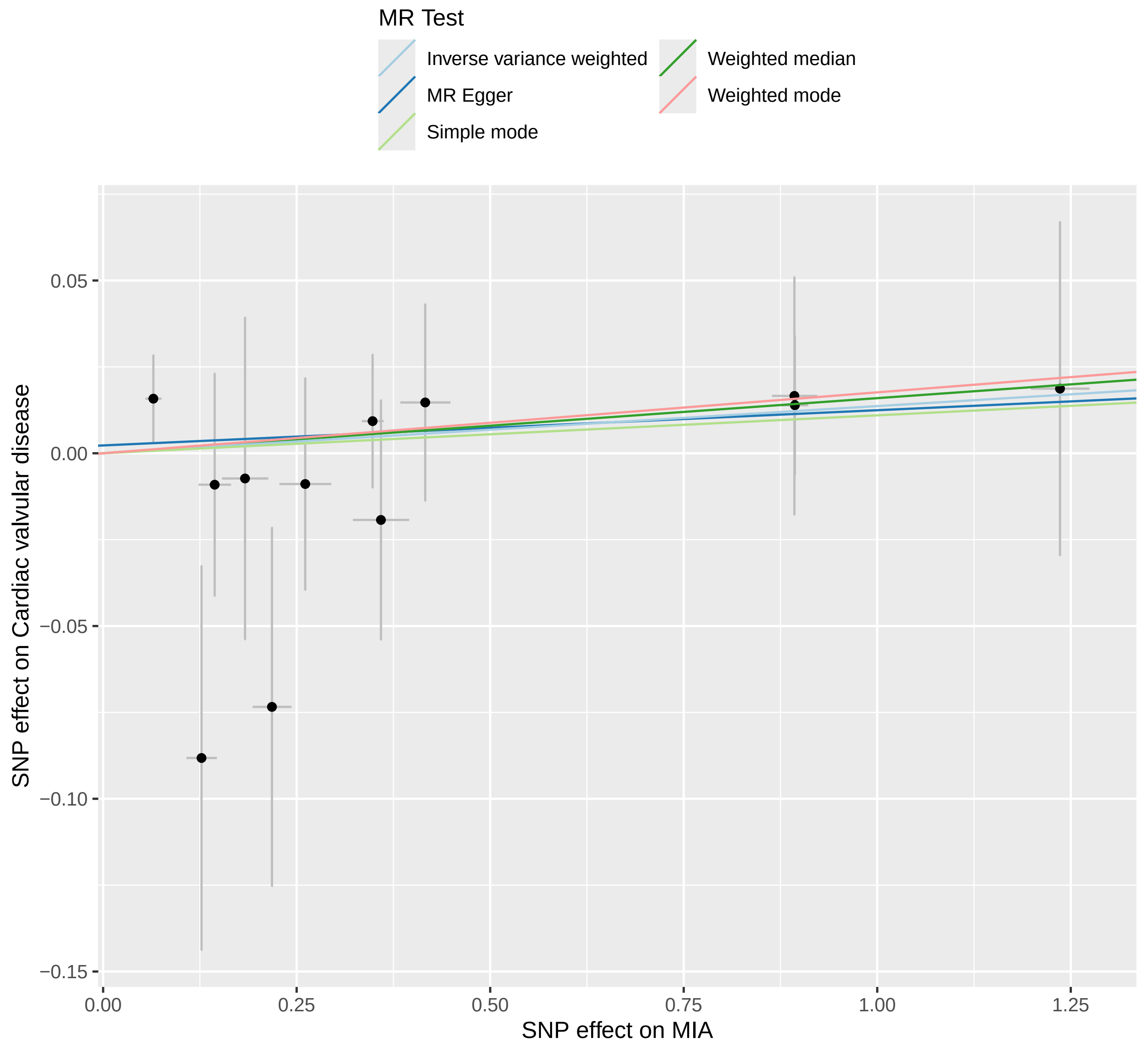
MR Test
Inverse variance weighted
MR Egger
Simple mode
Weighted median
Weighted mode
SNP effect on Cardiac valvular disease
0.05
0.00
−0.05
−0.10
−0.15
0.00
0.25
0.50
0.75
1.00
1.25
SNP effect on MIA

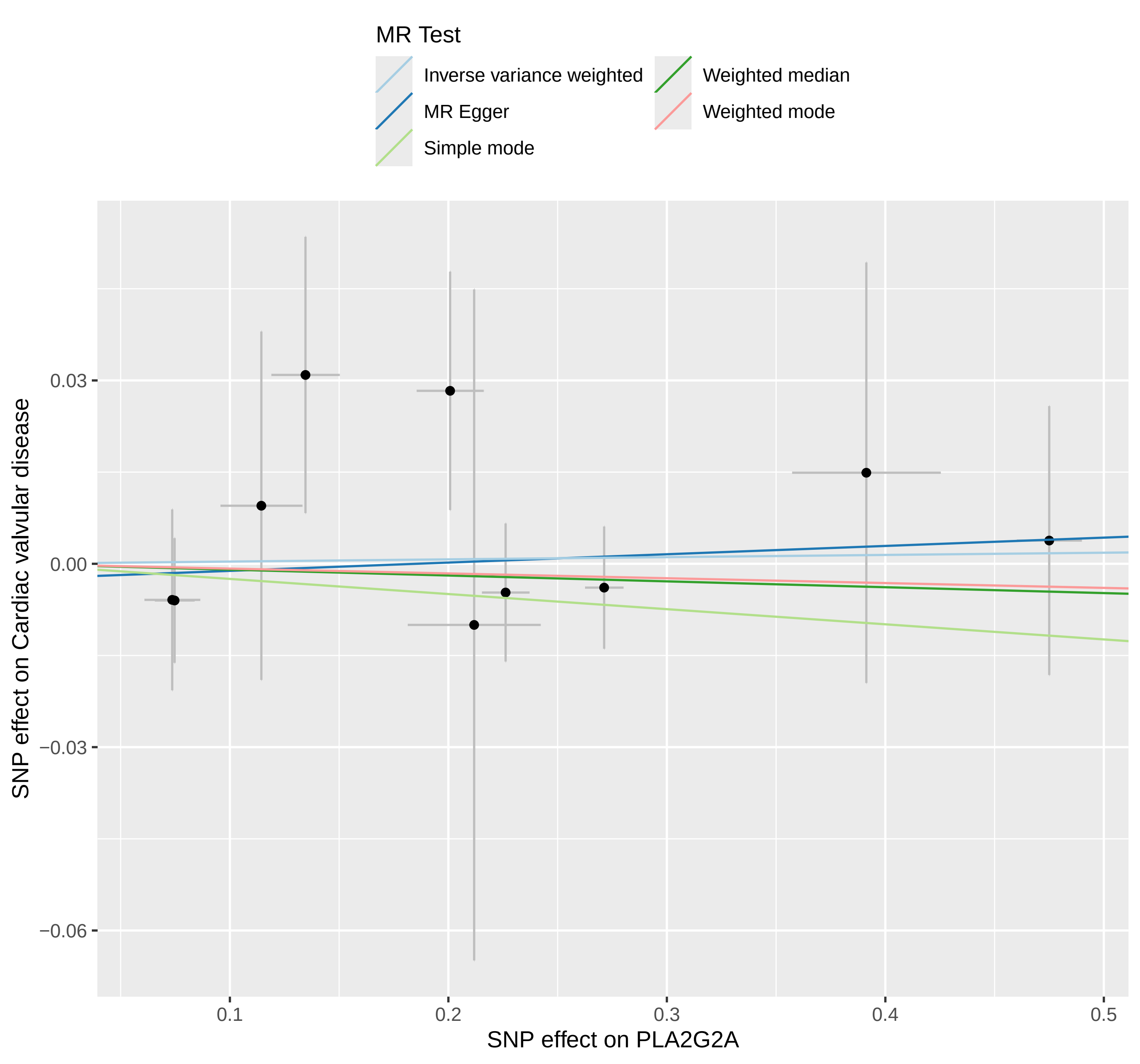
MR Test
Inverse variance weighted
MR Egger
Simple mode
Weighted median
Weighted mode
0.03
0.00
−0.03
−0.06
0.1
0.2
0.3
0.4
0.5
SNP effect on Cardiac valvular disease
SNP effect on PLA2G2A

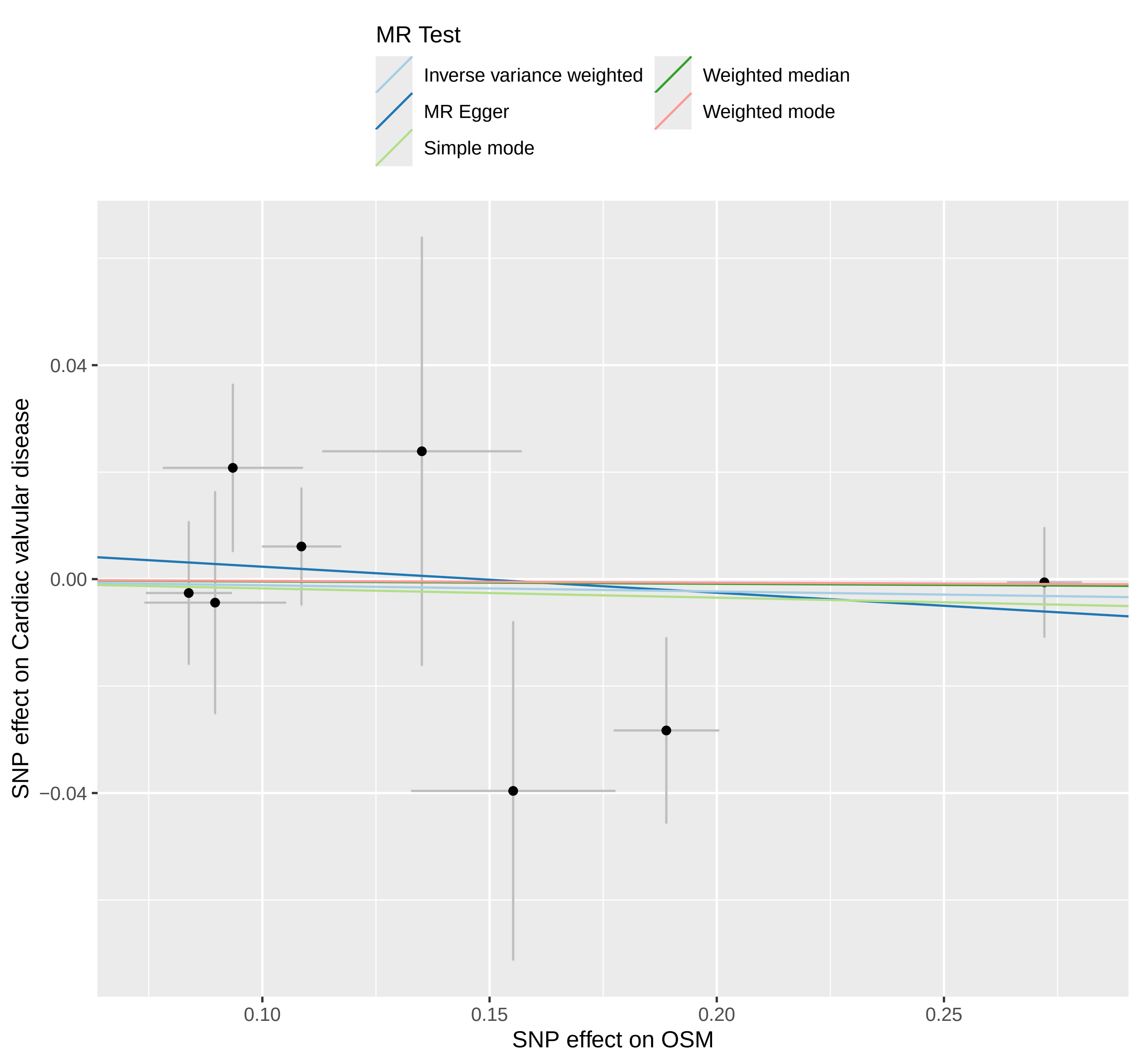
MR Test
Inverse variance weighted
MR Egger
Simple mode
Weighted median
Weighted mode
0.04
0.00
−0.04
0.10
0.15
0.20
0.25
SNP effect on OSM
SNP effect on Cardiac valvular disease

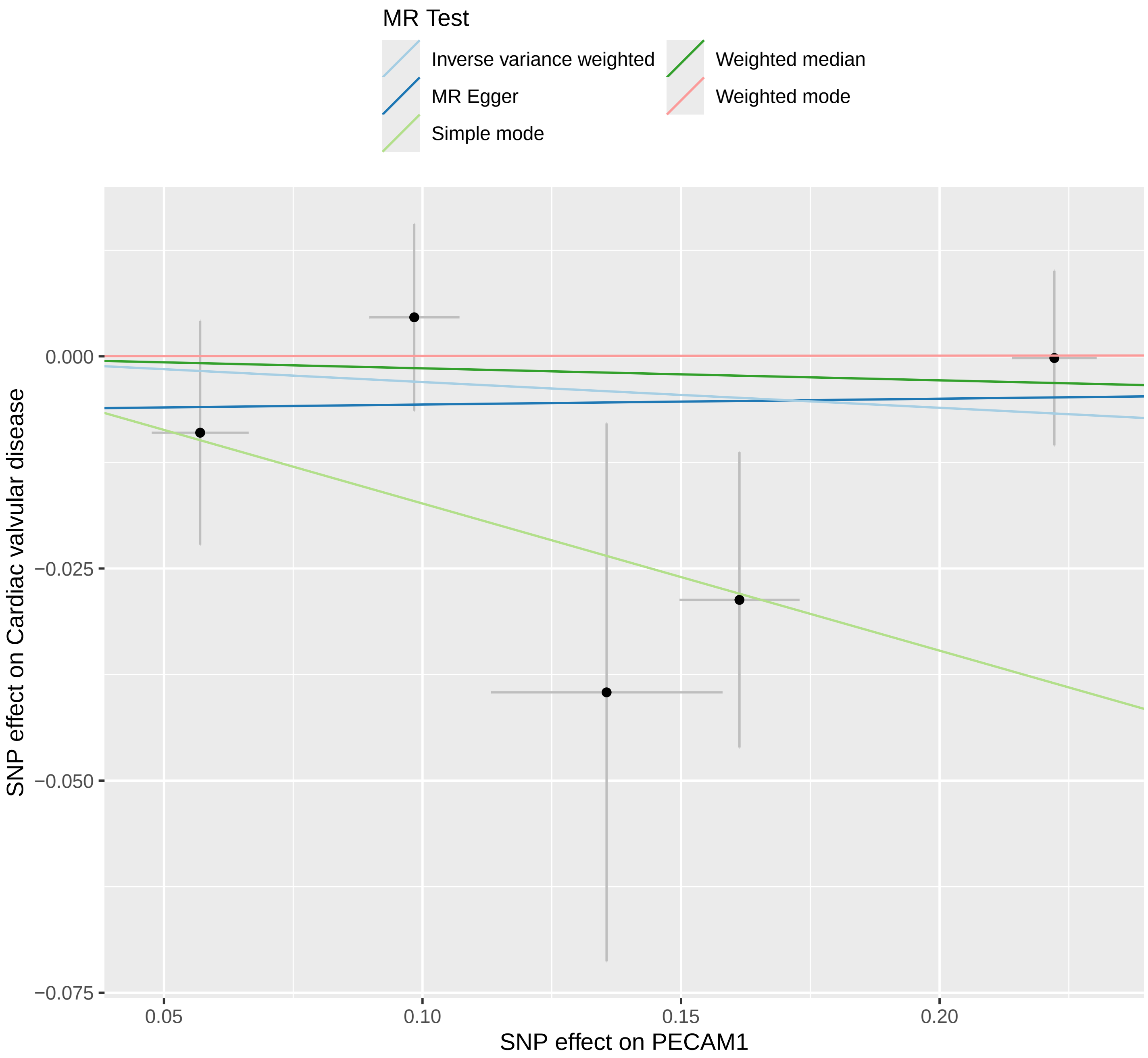

MR Test
Inverse variance weighted
MR Egger
Simple mode
Weighted median
Weighted mode
0.000
−0.025
−0.050
−0.075
SNP effect on Cardiac valvular disease
0.05
0.10
0.15
0.20
SNP effect on PECAM1

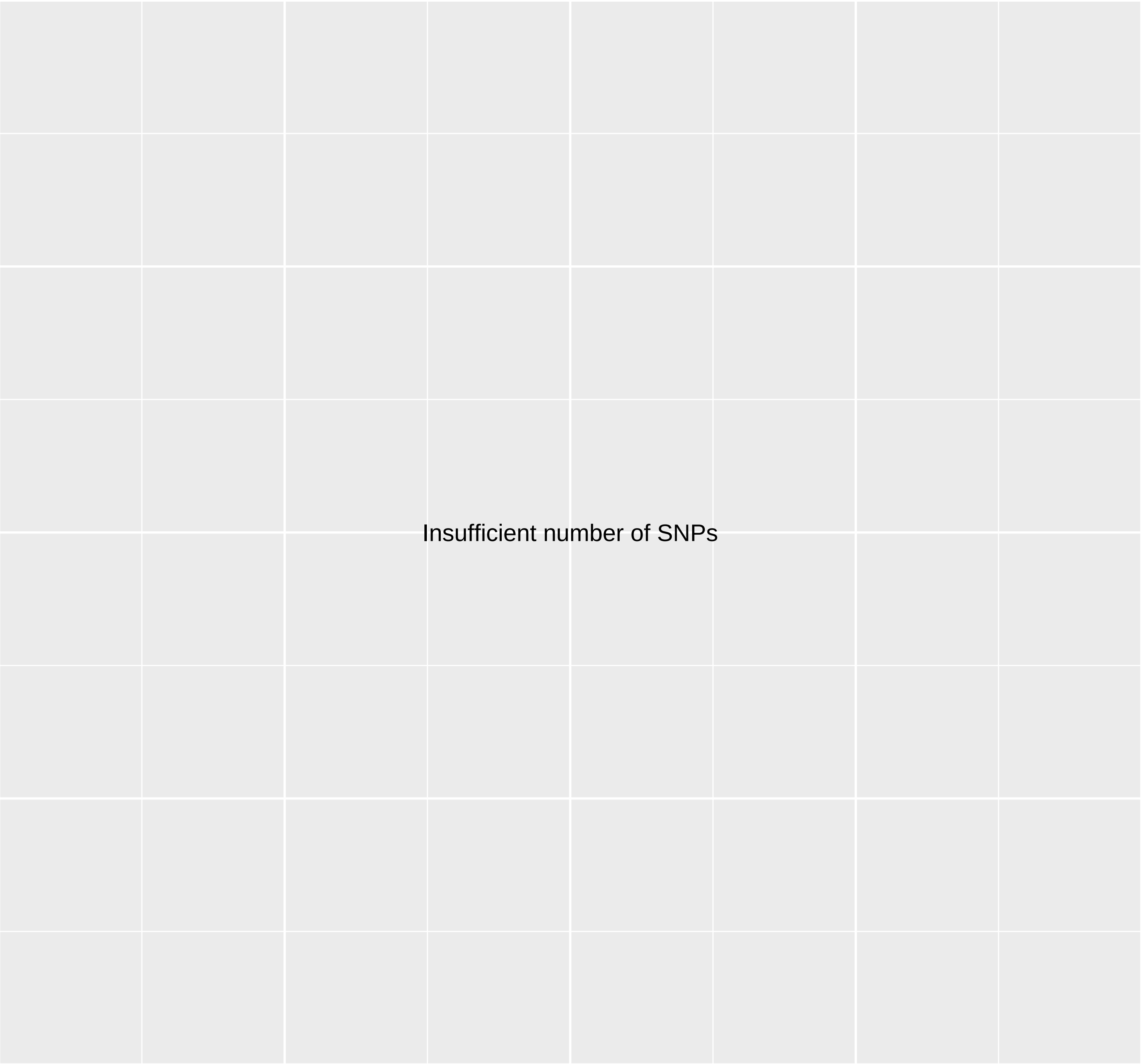
Insufficient number of SNPs

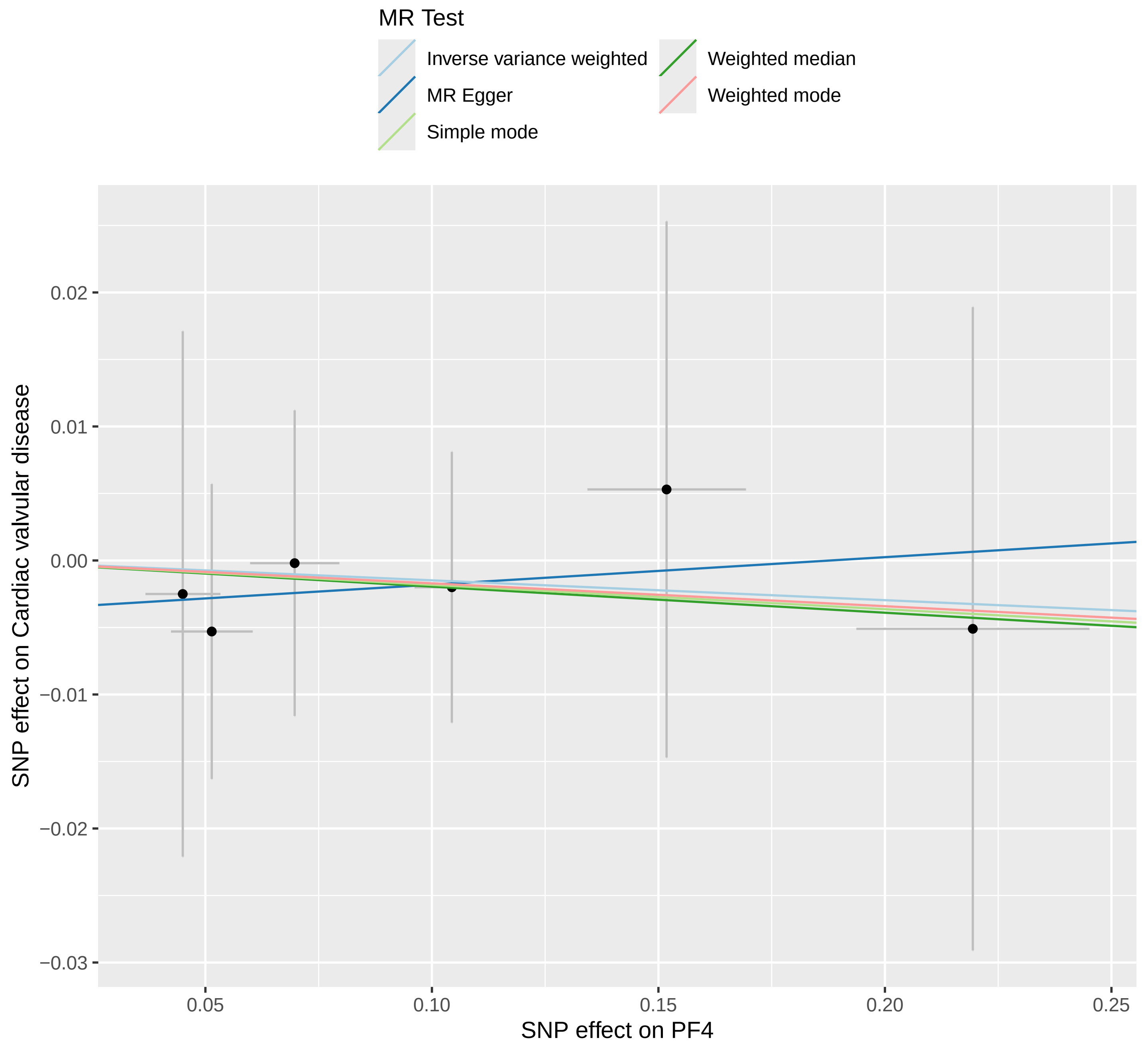

MR Test
Inverse variance weighted
MR Egger
Simple mode
Weighted median
Weighted mode
0.02
0.01
0.00
−0.01
−0.02
−0.03
SNP effect on Cardiac valvular disease
0.05
0.10
0.15
0.20
0.25
SNP effect on PF4

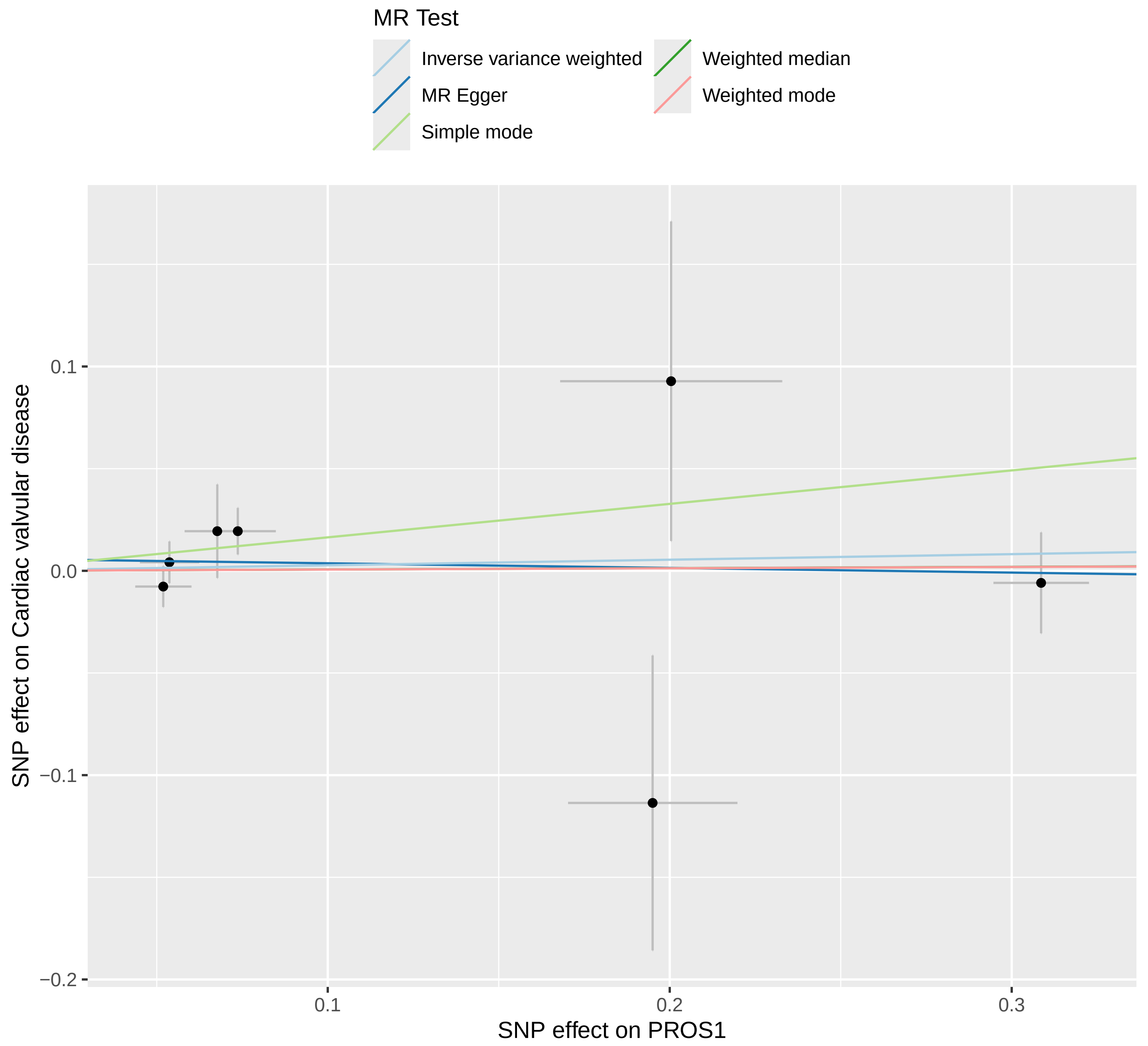
MR Test
Inverse variance weighted
MR Egger
Simple mode
Weighted median
Weighted mode
0.1
0.0
−0.1
−0.2
SNP effect on Cardiac valvular disease
0.1
0.2
0.3
SNP effect on PROS1

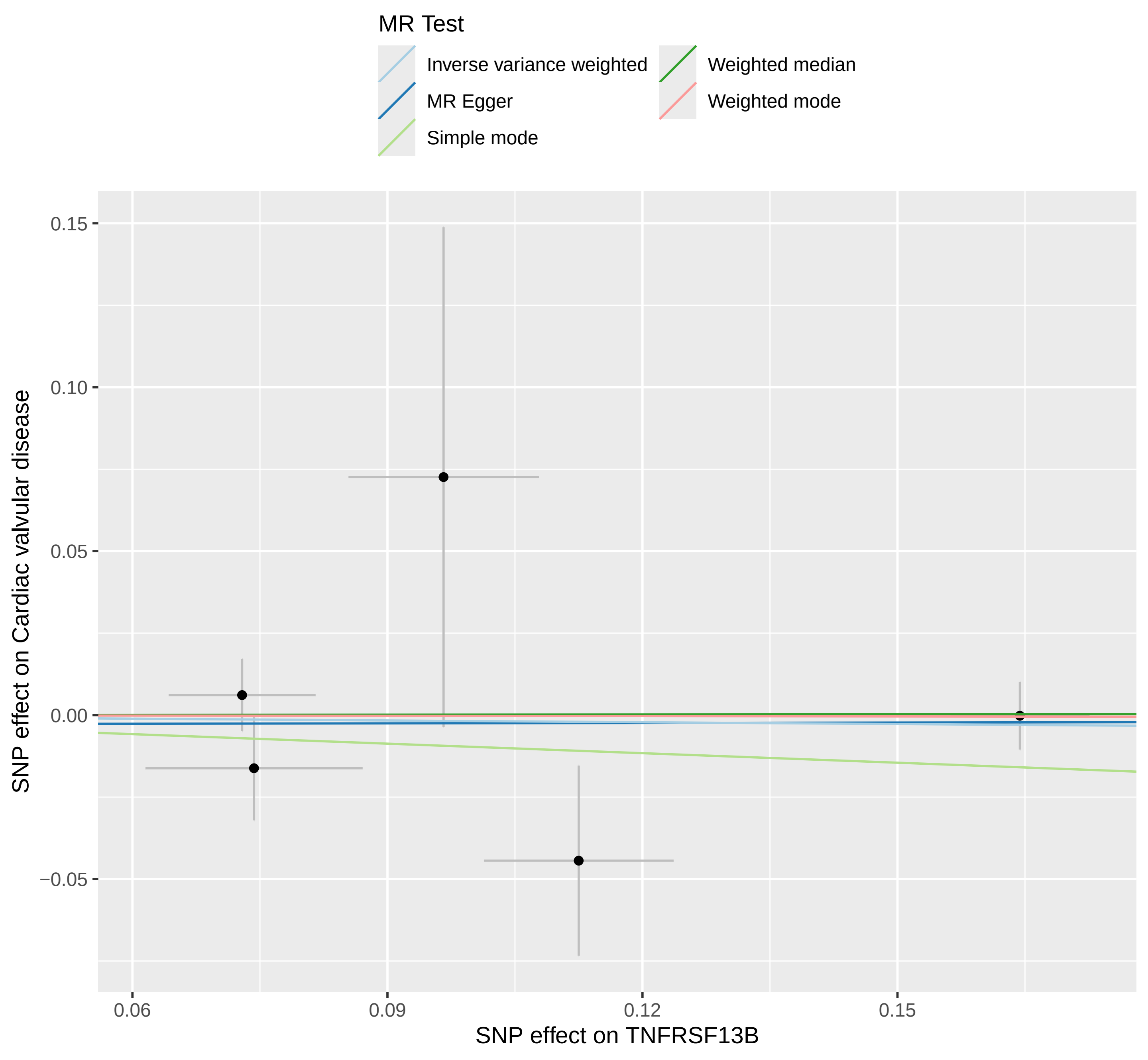

MR Test
Inverse variance weighted
MR Egger
Simple mode
Weighted median
Weighted mode
0.15
0.10
0.05
0.00
−0.05
0.06
0.09
0.12
0.15
SNP effect on TNFRSF13B
SNP effect on Cardiac valvular disease

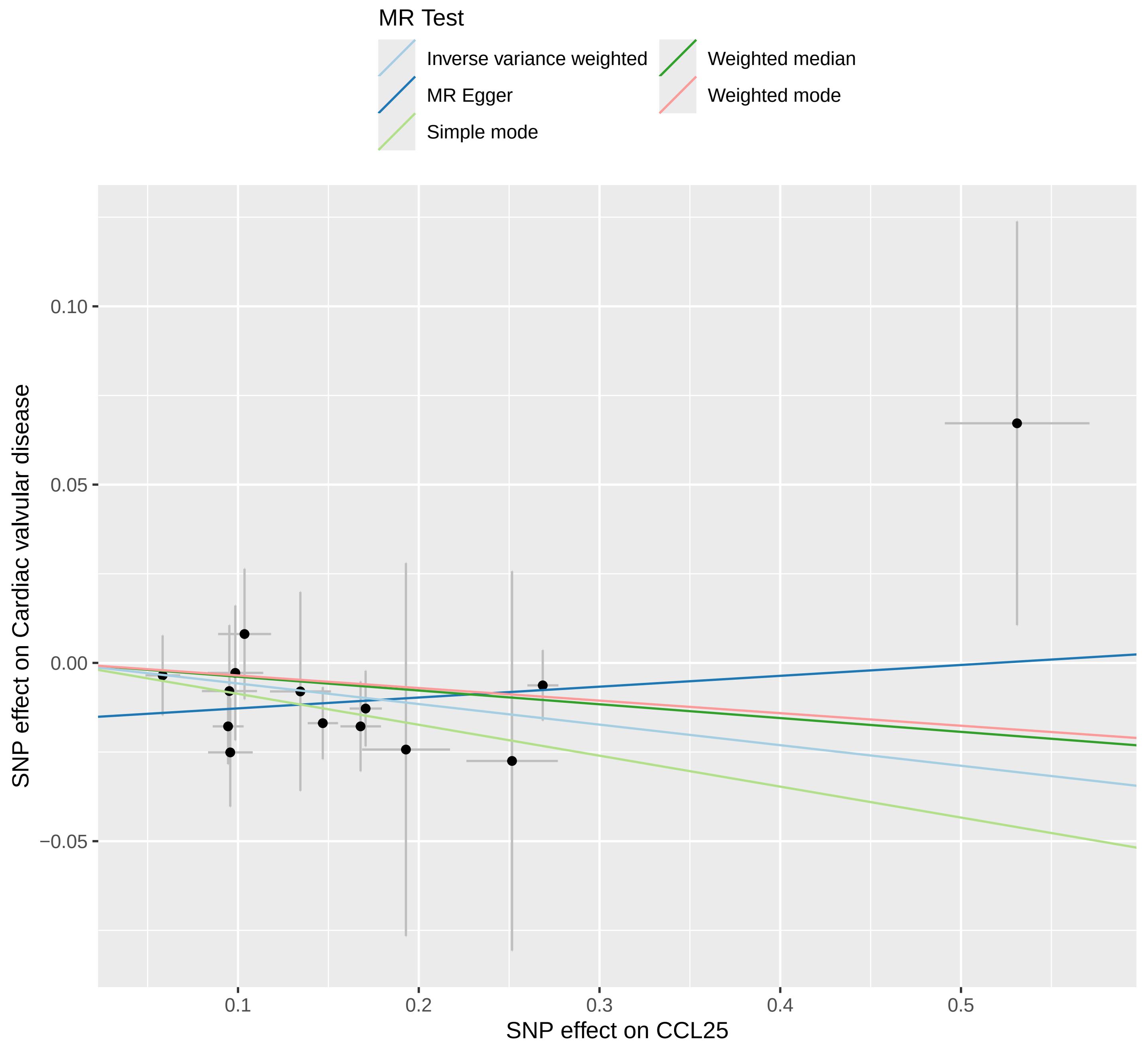

MR Test
Inverse variance weighted
MR Egger
Simple mode
Weighted median
Weighted mode
0.10
0.05
0.00
−0.05
SNP effect on Cardiac valvular disease
0.1
0.2
0.3
0.4
0.5
SNP effect on CCL25

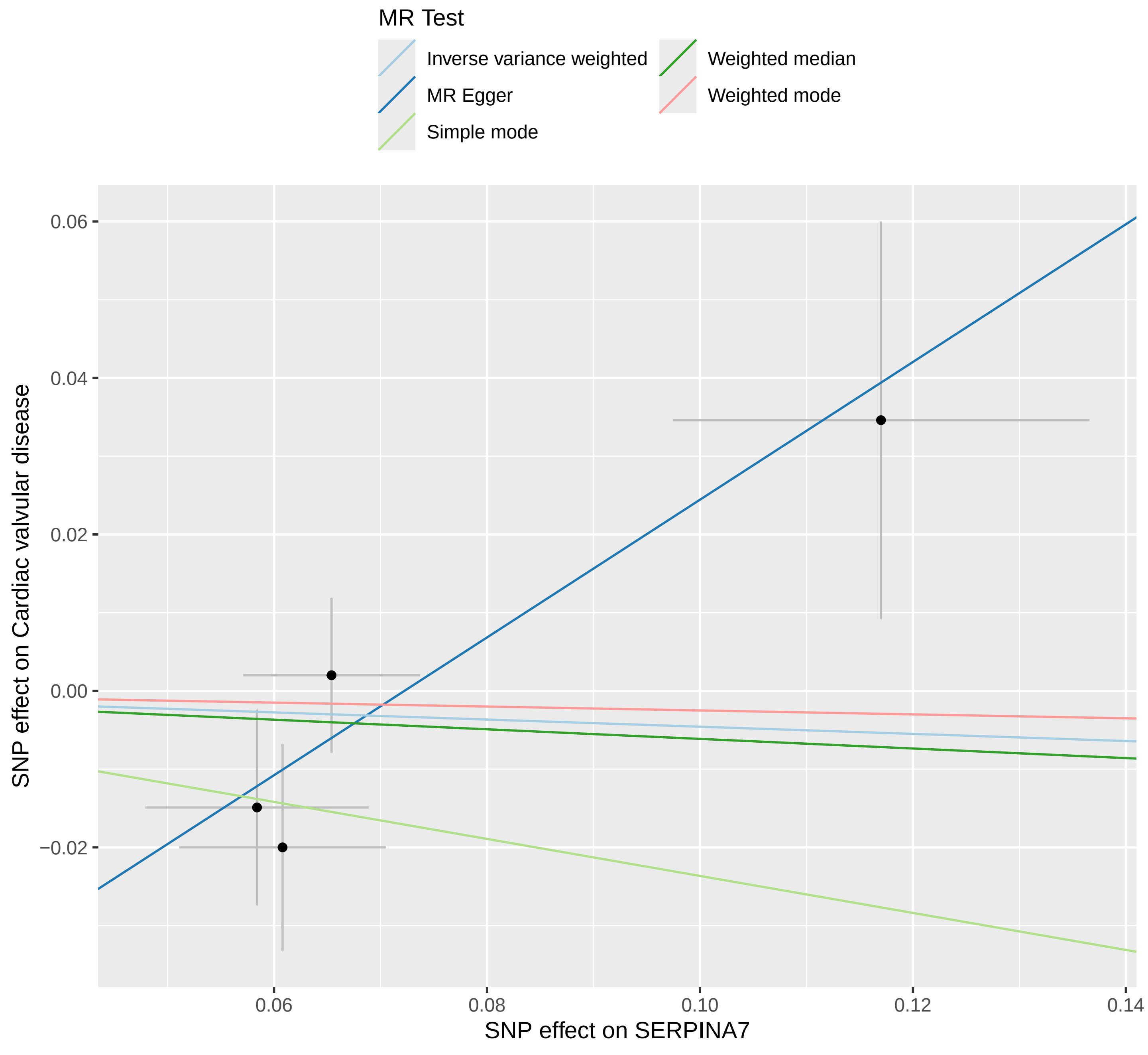
MR Test
Inverse variance weighted
MR Egger
Simple mode
Weighted median
Weighted mode
0.06
0.04
0.02
0.00
−0.02
SNP effect on Cardiac valvular disease
0.06
0.08
0.10
0.12
0.14
SNP effect on SERPINA7

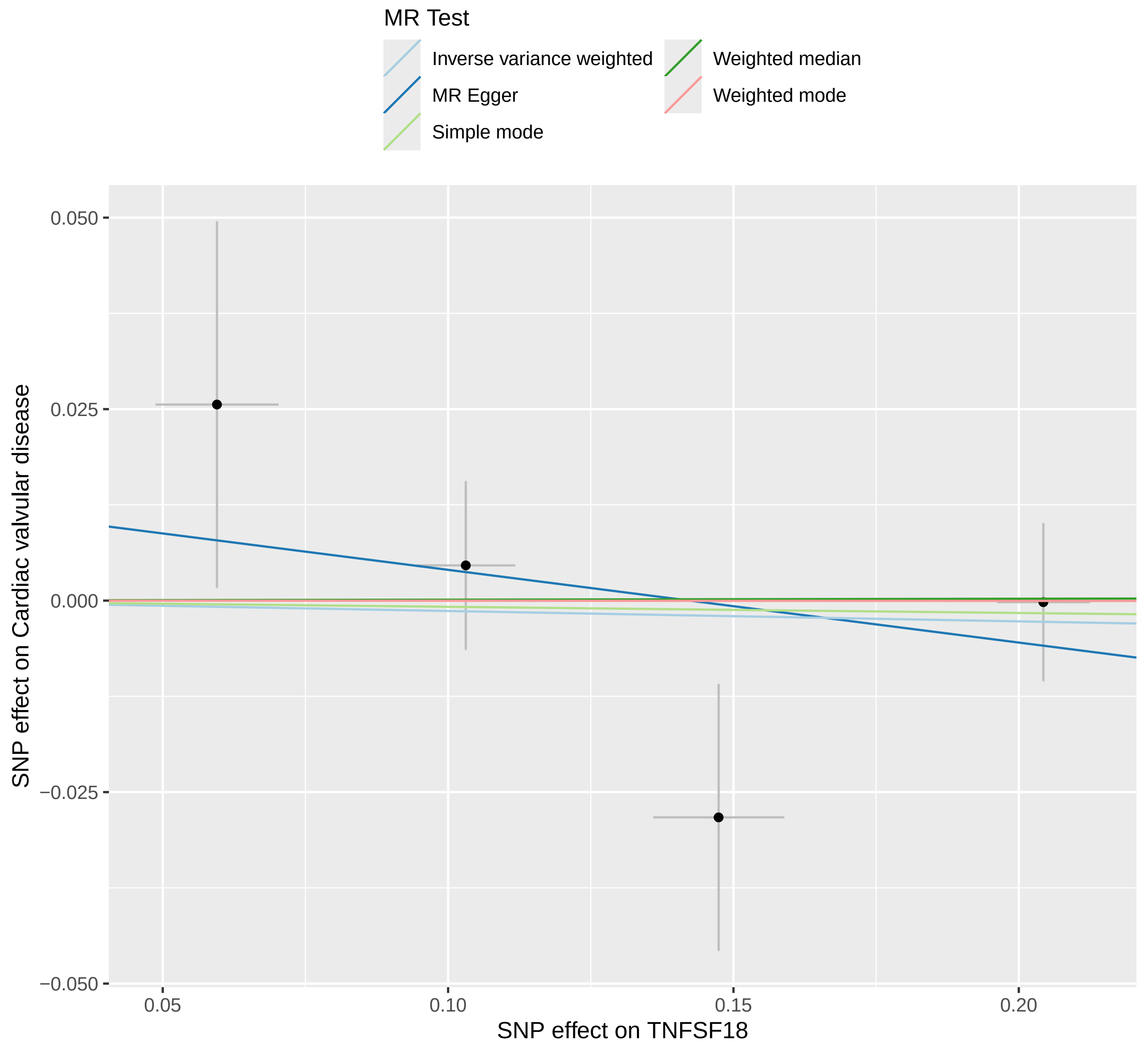

MR Test
Inverse variance weighted
MR Egger
Simple mode
Weighted median
Weighted mode
0.050
0.025
0.000
−0.025
−0.050
SNP effect on Cardiac valvular disease
0.05
0.10
0.15
0.20
SNP effect on TNFSF18

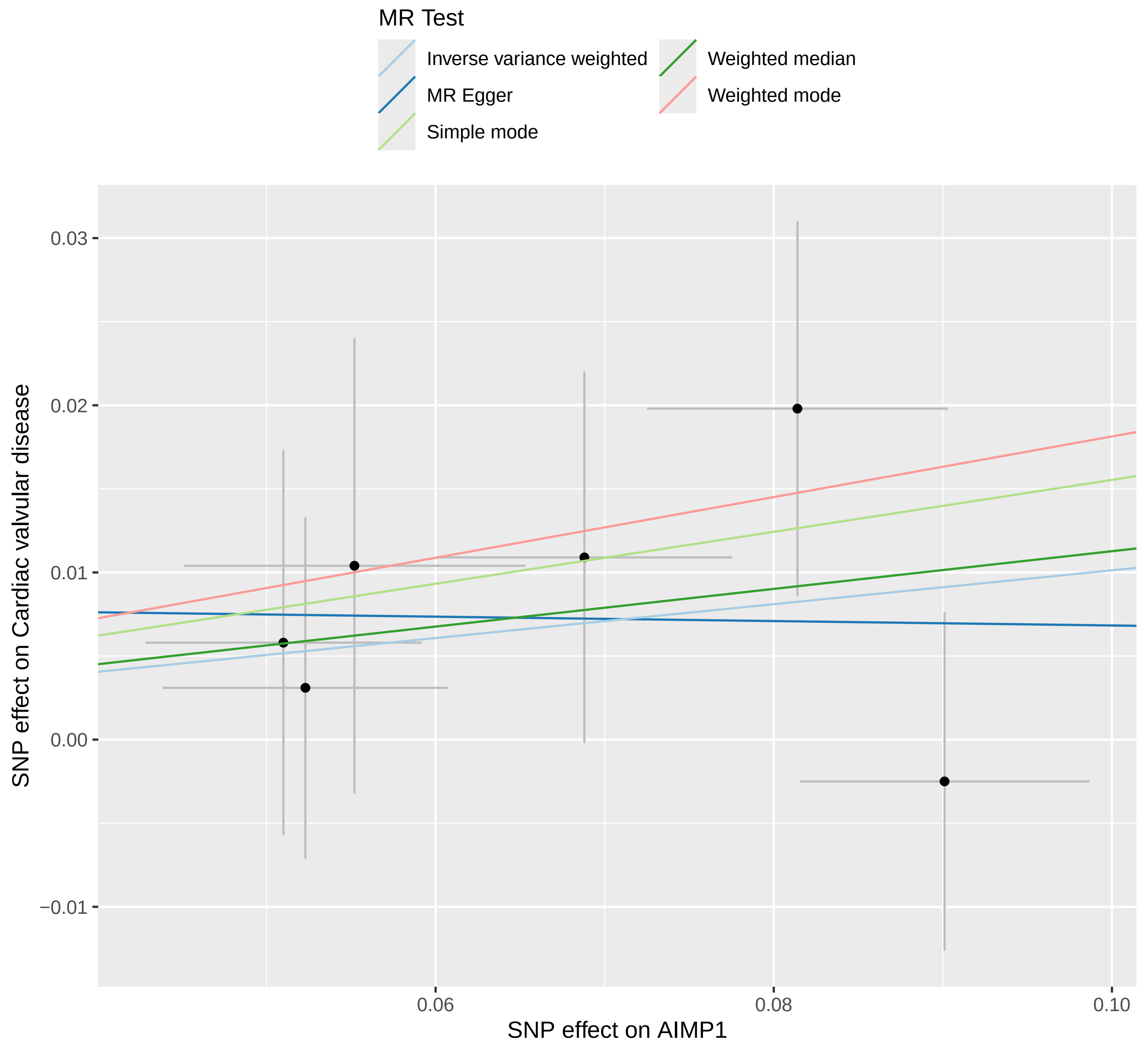

MR Test
Inverse variance weighted
MR Egger
Simple mode
Weighted median
Weighted mode
SNP effect on Cardiac valvular disease
0.03
0.02
0.01
0.00
−0.01
0.06
0.08
0.10
SNP effect on AIMP1

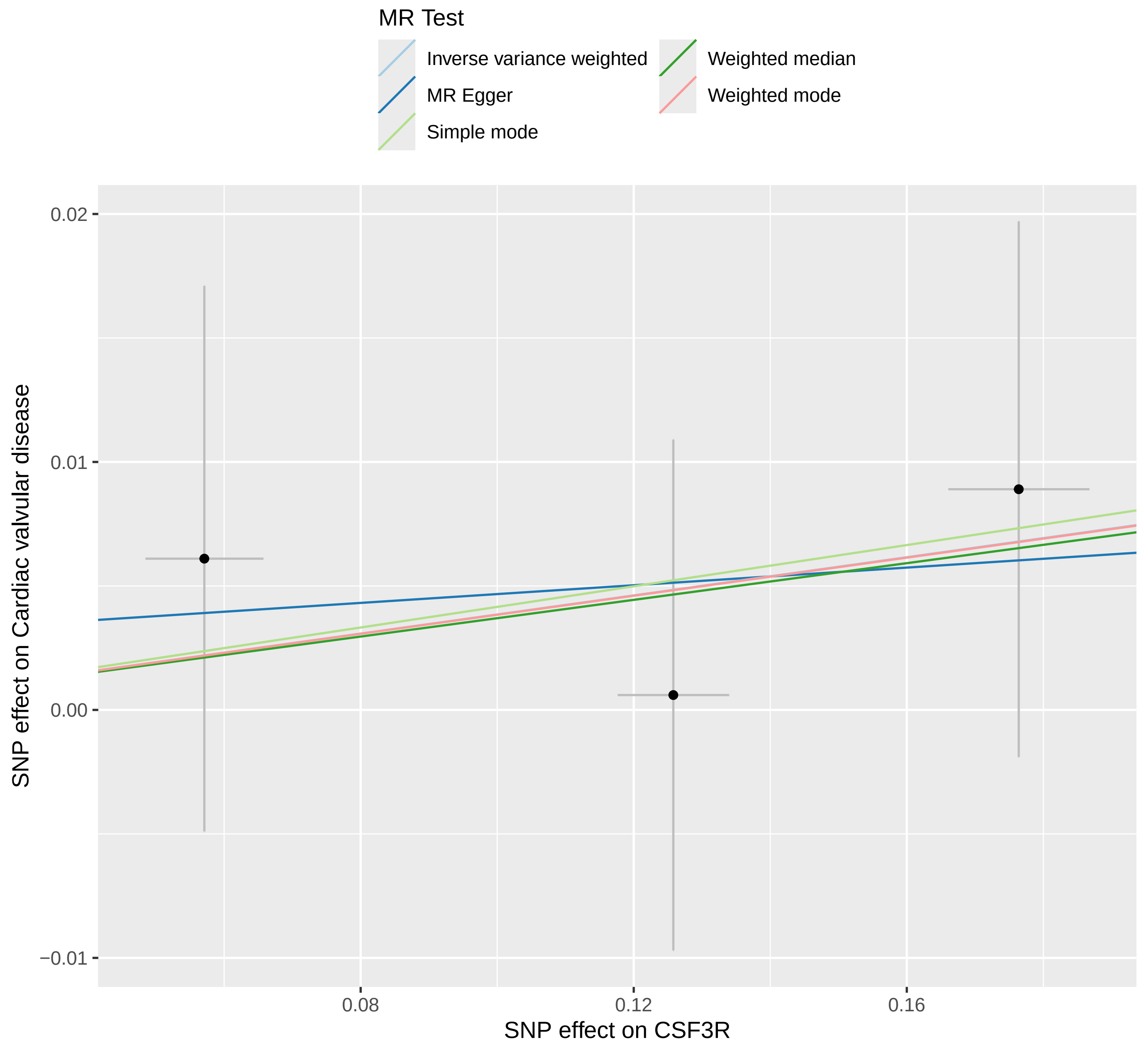

MR Test
Inverse variance weighted
MR Egger
Simple mode
Weighted median
Weighted mode
0.02
0.01
0.00
−0.01
0.08
0.12
0.16
SNP effect on Cardiac valvular disease
SNP effect on CSF3R

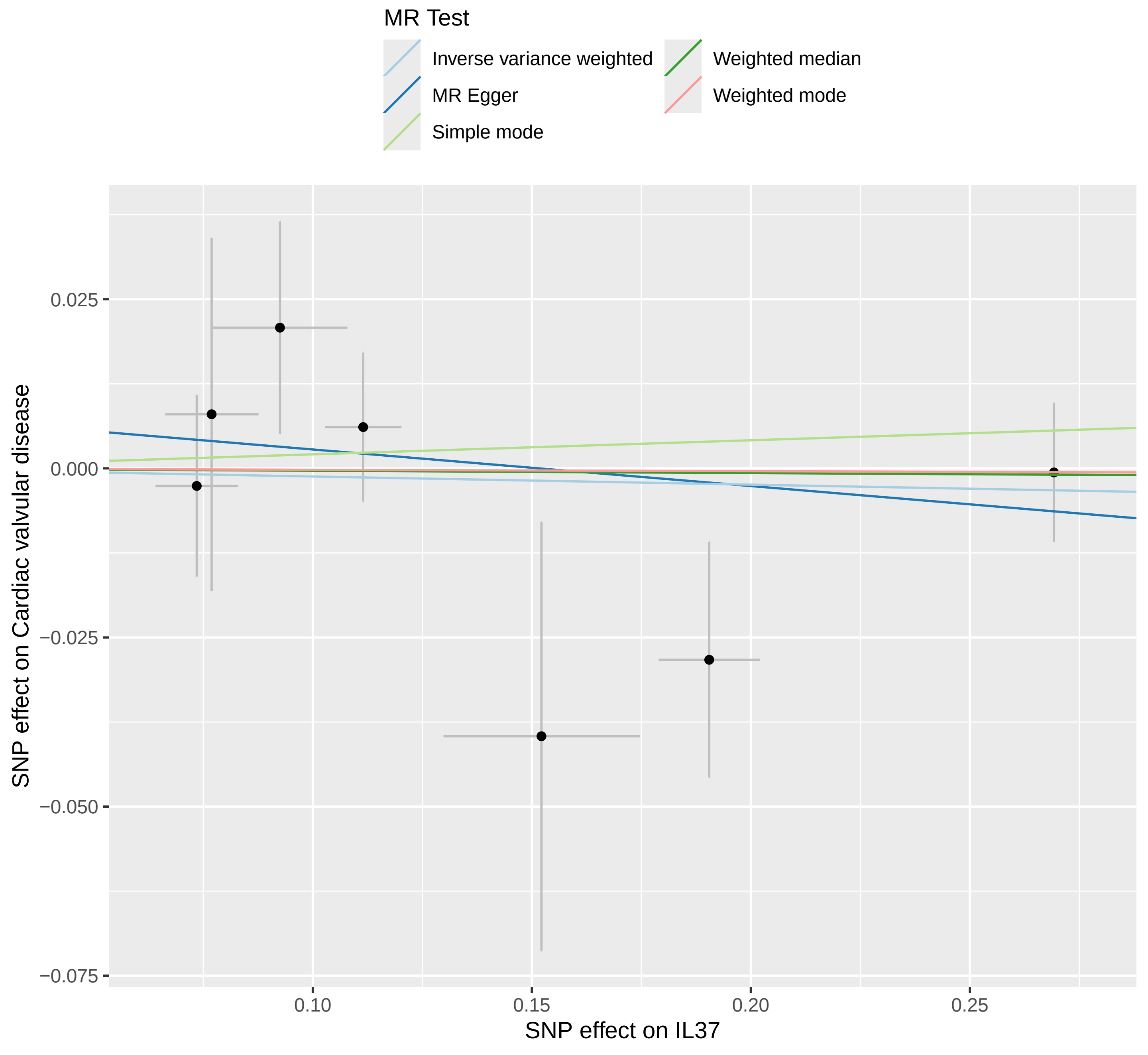
MR Test
Inverse variance weighted
MR Egger
Simple mode
Weighted median
Weighted mode
0.025
0.000
−0.025
−0.050
−0.075
0.10
0.15
0.20
0.25
SNP effect on Cardiac valvular disease
SNP effect on IL37

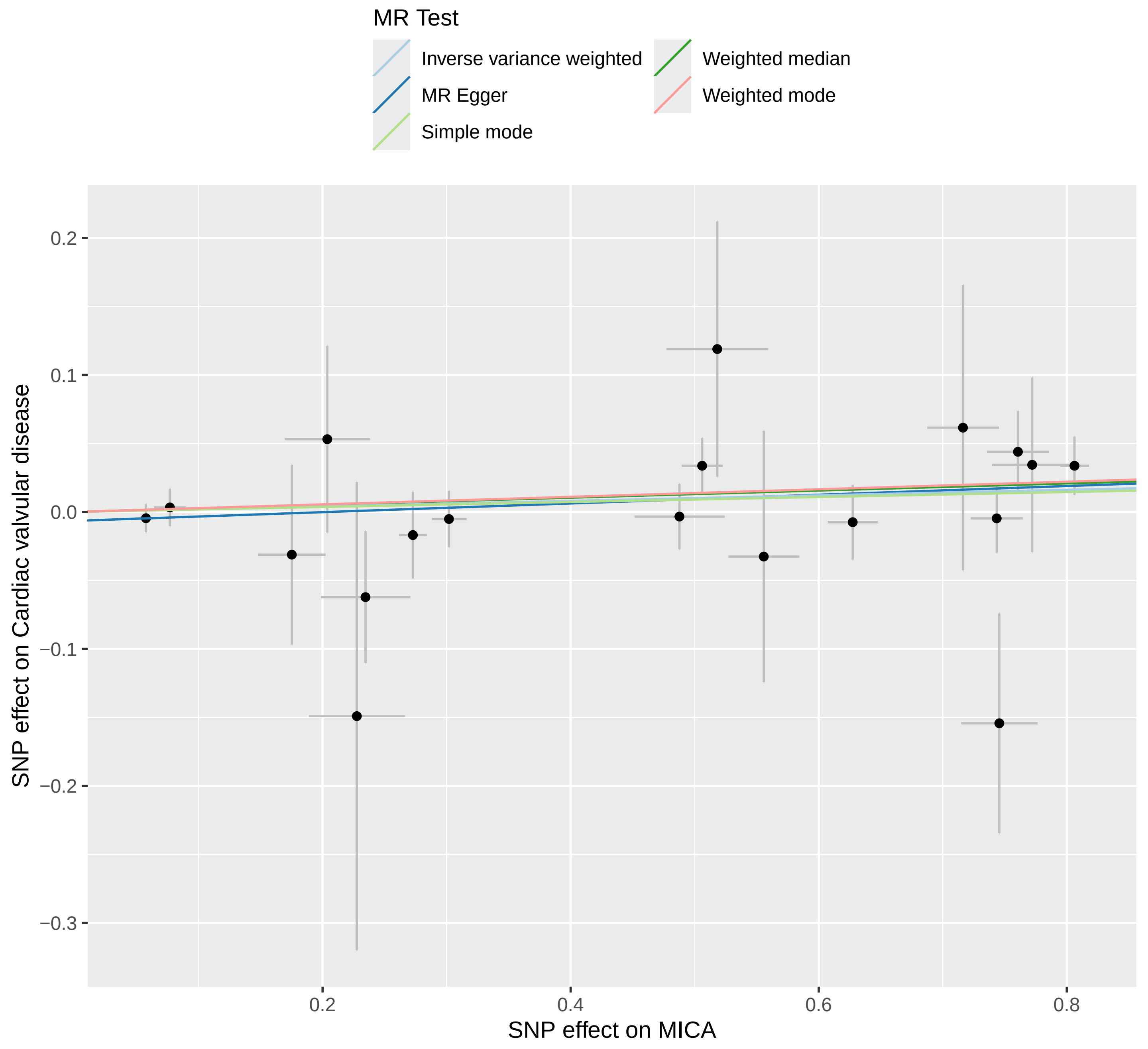
MR Test
Inverse variance weighted
MR Egger
Simple mode
Weighted median
Weighted mode
SNP effect on Cardiac valvular disease
SNP effect on MICA
0.2
0.1
0.0
−0.1
−0.2
−0.3
0.2
0.4
0.6
0.8

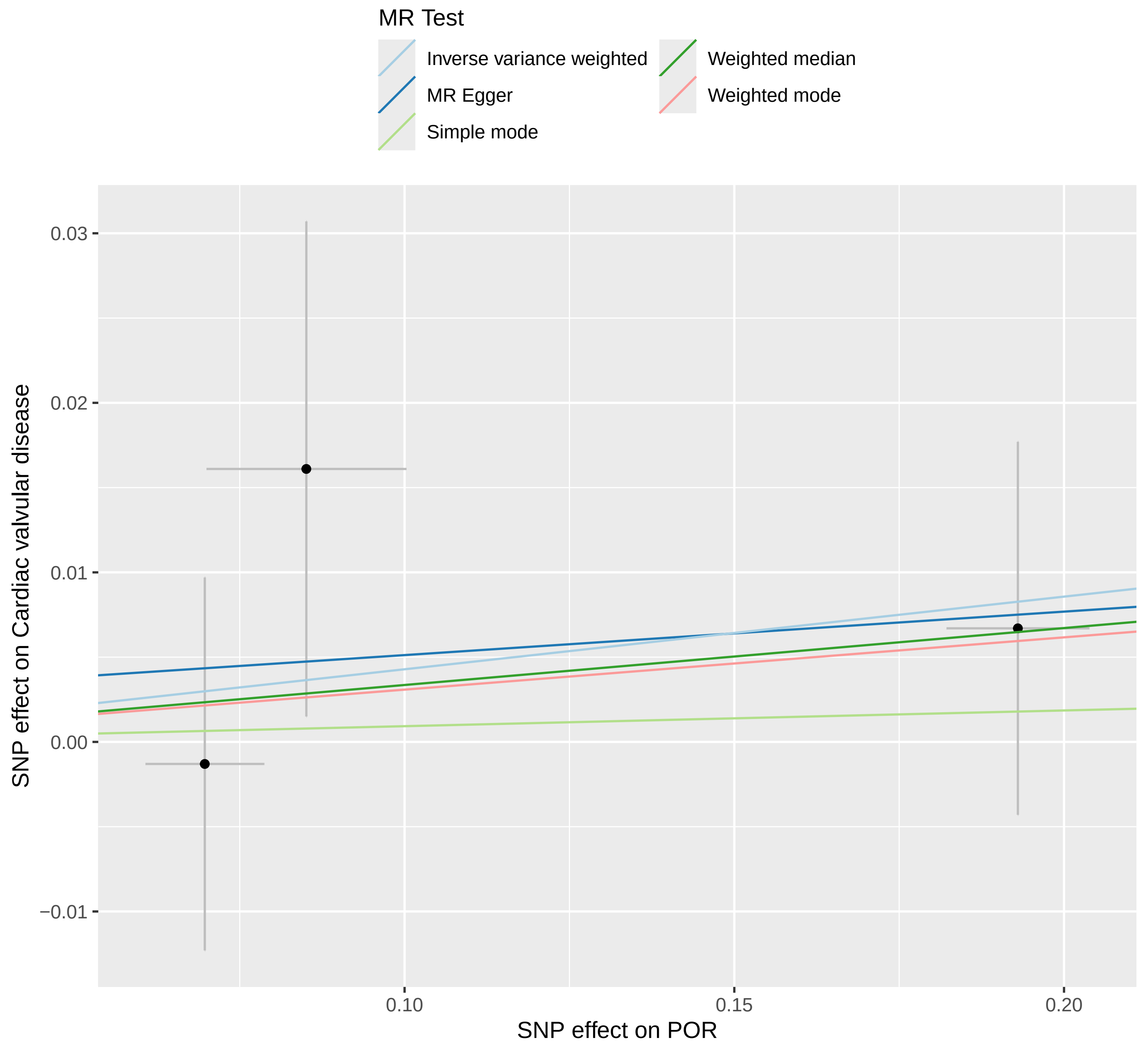
MR Test
Inverse variance weighted
MR Egger
Simple mode
Weighted median
Weighted mode
0.03
0.02
0.01
0.00
−0.01
SNP effect on Cardiac valvular disease
0.10
0.15
0.20
SNP effect on POR

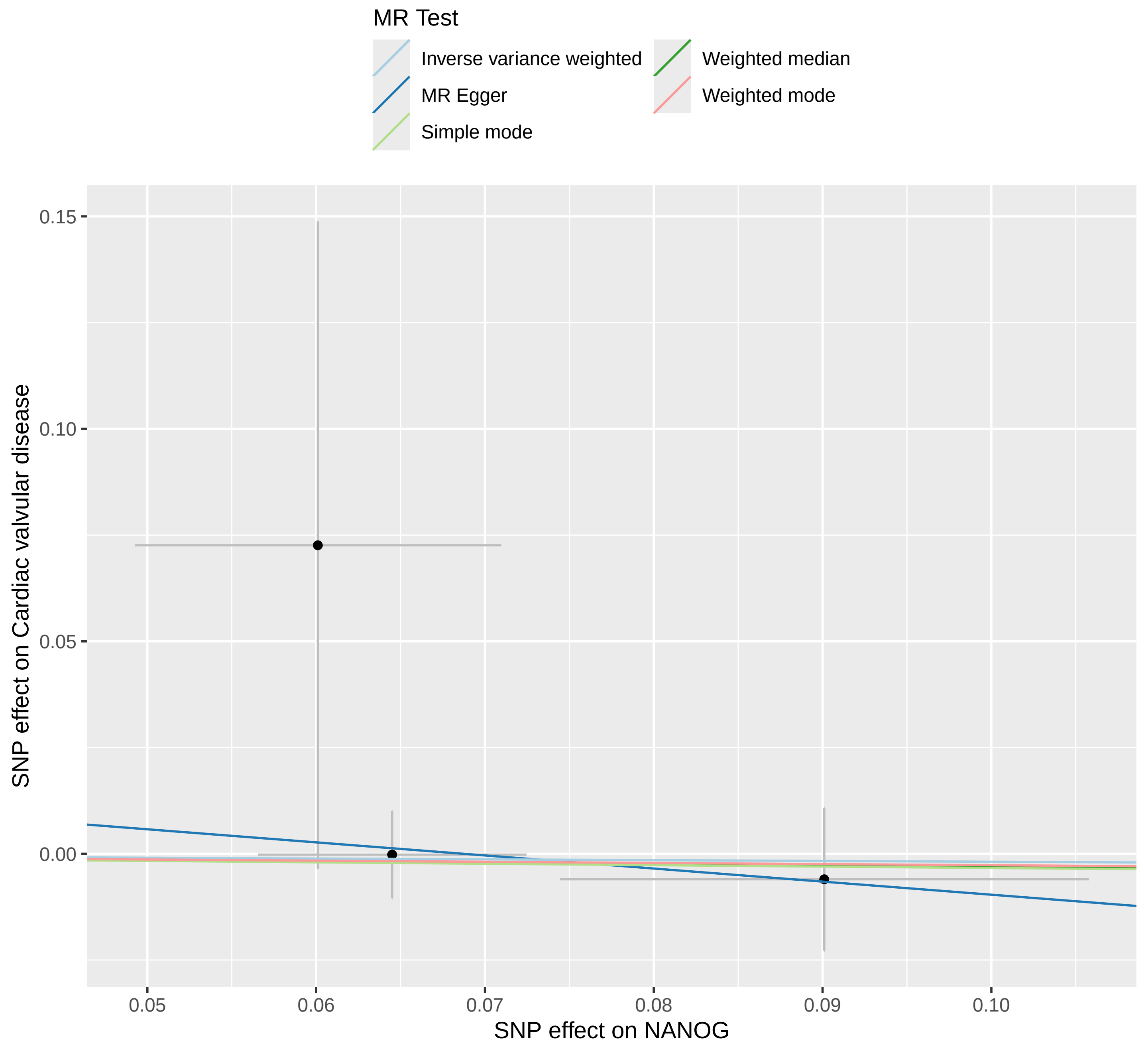

MR Test
Inverse variance weighted
MR Egger
Simple mode
Weighted median
Weighted mode
0.15
0.10
0.05
0.00
SNP effect on Cardiac valvular disease
0.05
0.06
0.07
0.08
0.09
0.10
SNP effect on NANOG

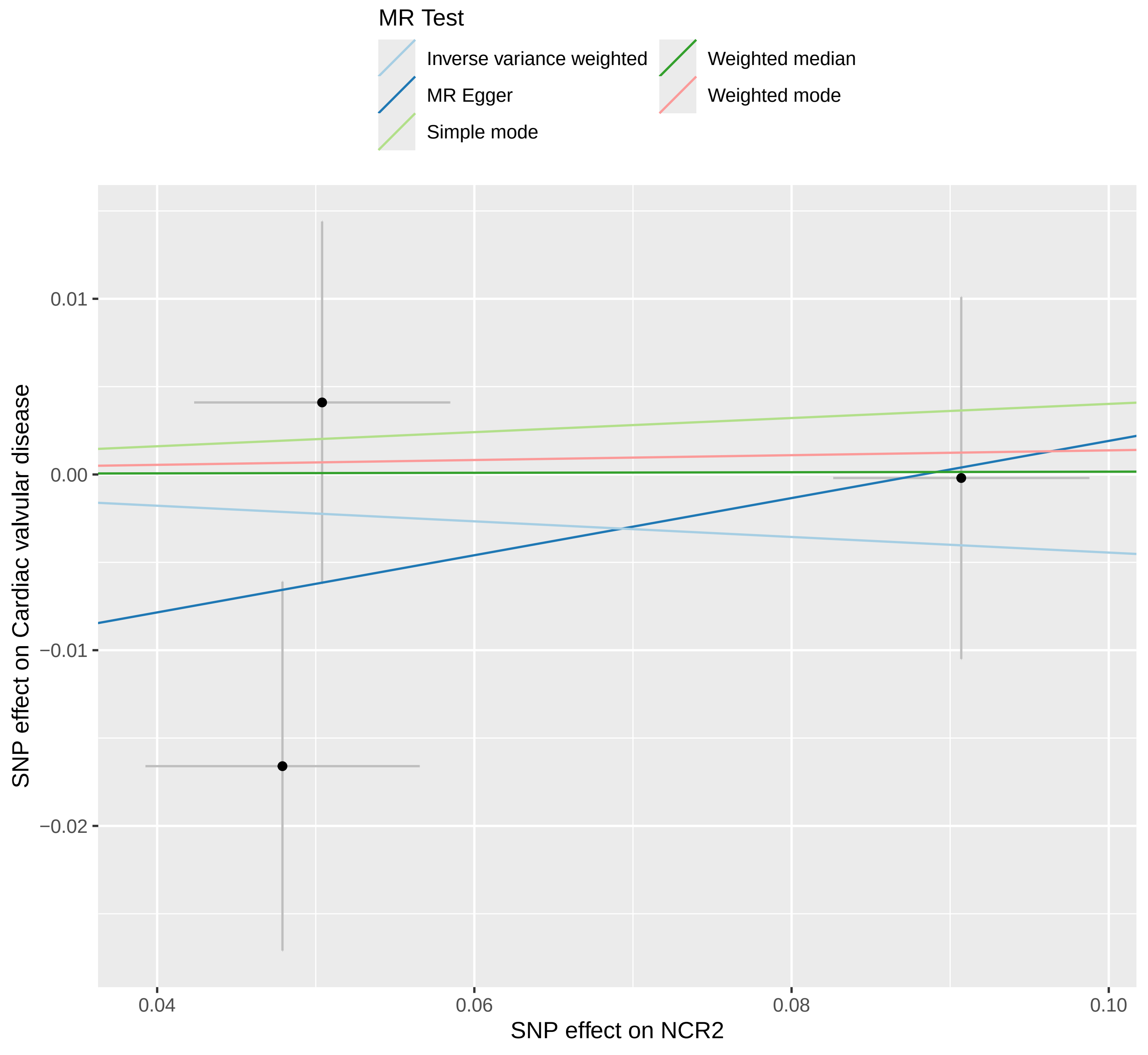
MR Test
Inverse variance weighted
MR Egger
Simple mode
Weighted median
Weighted mode
0.01
0.00
−0.01
−0.02
0.04
0.06
0.08
0.10
SNP effect on Cardiac valvular disease
SNP effect on NCR2

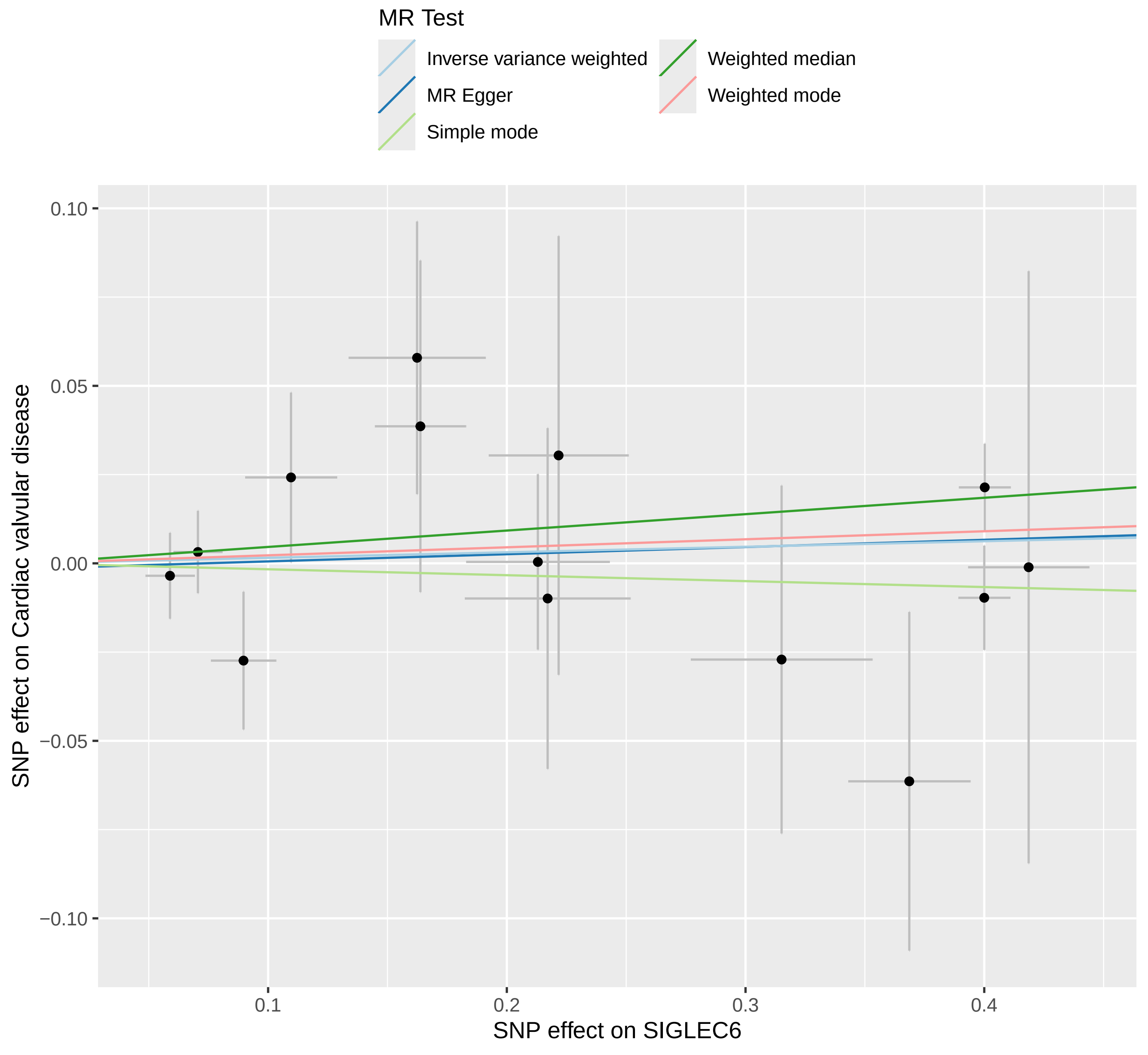
MR Test
Inverse variance weighted
MR Egger
Simple mode
Weighted median
Weighted mode
0.10
0.05
0.00
−0.05
−0.10
0.1
0.2
0.3
0.4
SNP effect on Cardiac valvular disease
SNP effect on SIGLEC6

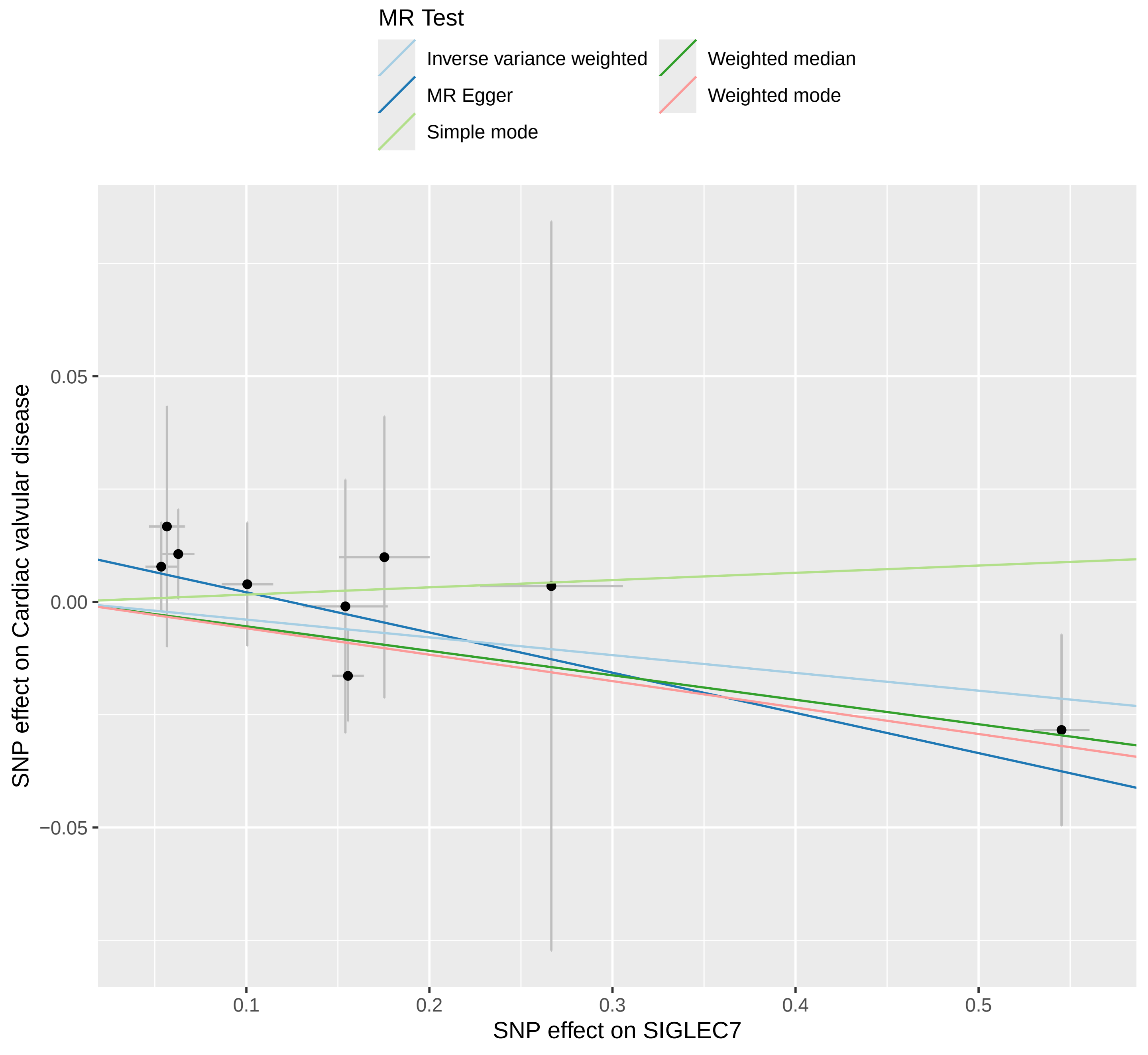

MR Test
Inverse variance weighted
MR Egger
Simple mode
Weighted median
Weighted mode
0.05
0.00
−0.05
0.1
0.2
0.3
0.4
0.5
SNP effect on Cardiac valvular disease
SNP effect on SIGLEC7

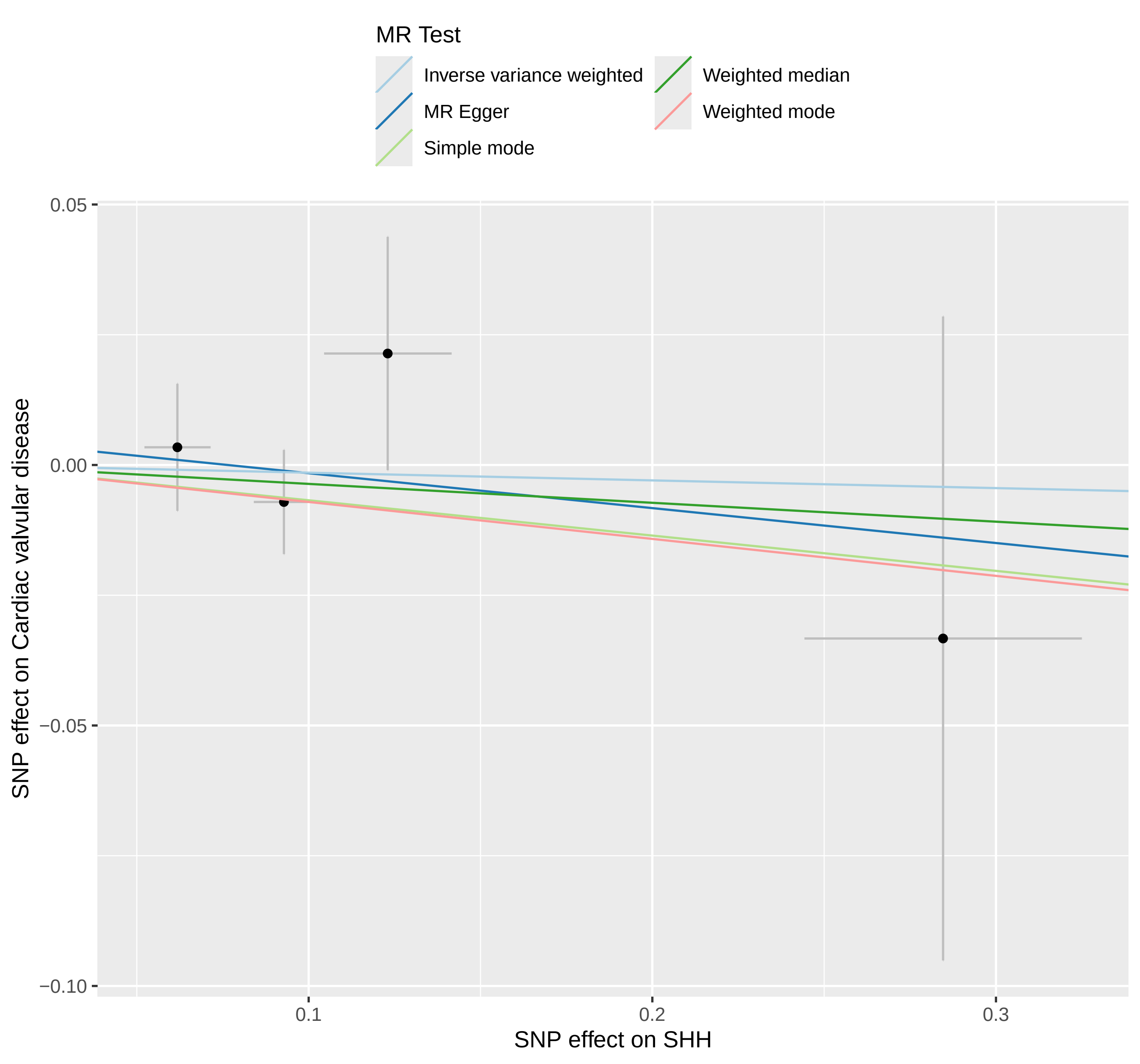

MR Test
Inverse variance weighted
MR Egger
Simple mode
Weighted median
Weighted mode
0.05
0.00
−0.05
−0.10
SNP effect on Cardiac valvular disease
0.1
0.2
0.3
SNP effect on SHH

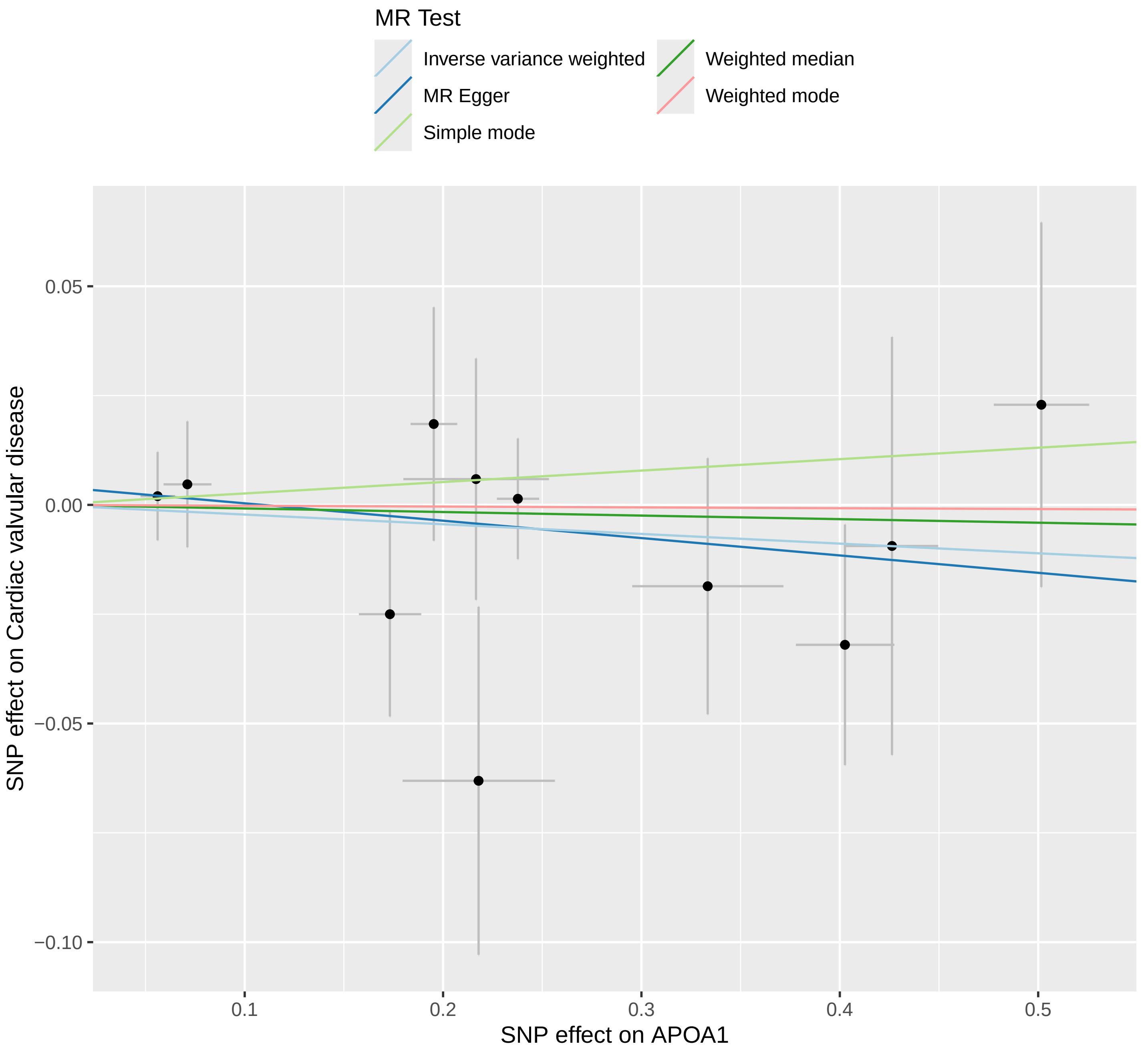
MR Test
Inverse variance weighted
MR Egger
Simple mode
Weighted median
Weighted mode
0.05
0.00
−0.05
−0.10
SNP effect on Cardiac valvular disease
0.1
0.2
0.3
0.4
0.5
SNP effect on APOA1

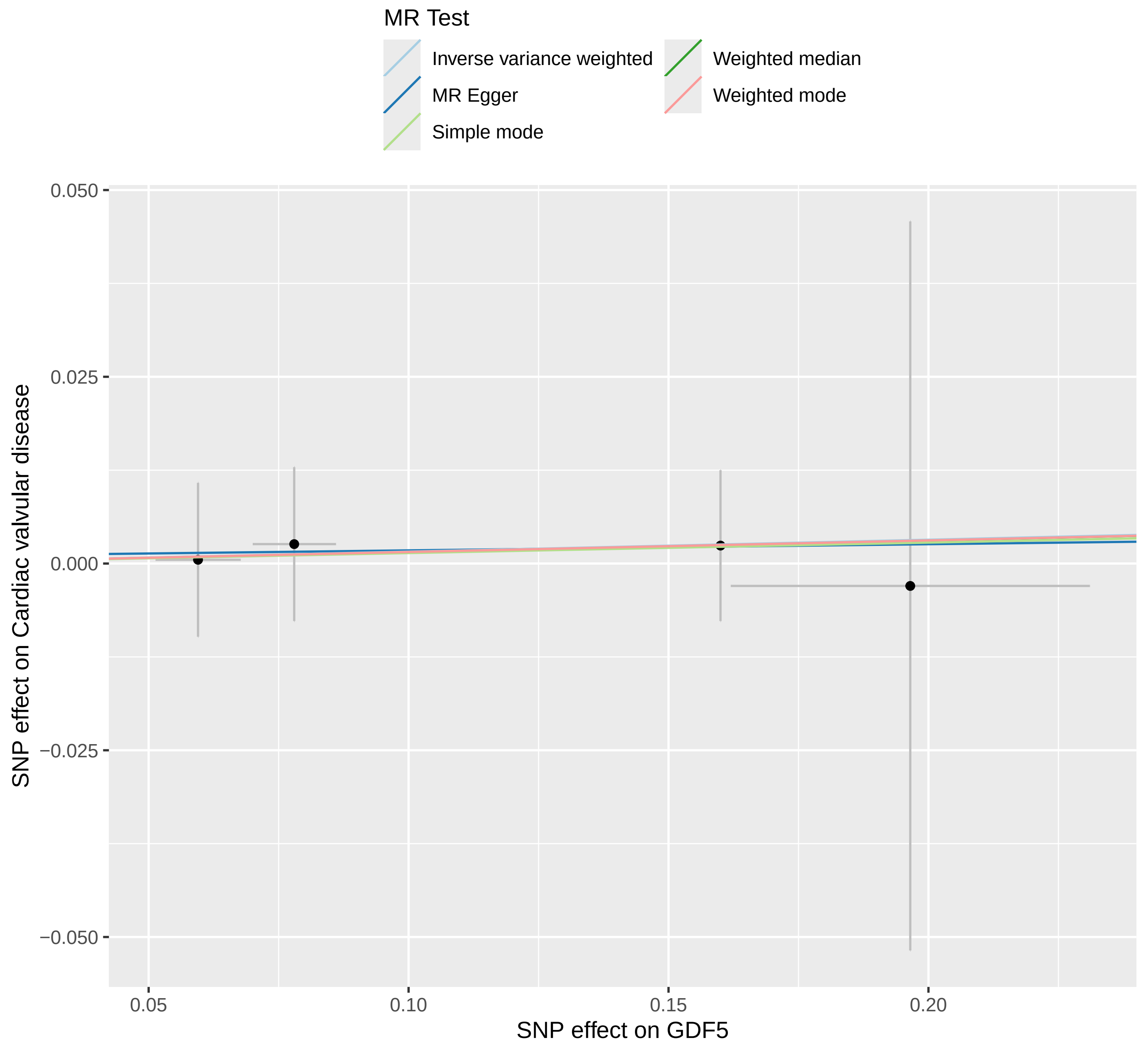
MR Test
Inverse variance weighted
MR Egger
Simple mode
Weighted median
Weighted mode
0.050
0.025
0.000
−0.025
−0.050
SNP effect on Cardiac valvular disease
0.05
0.10
0.15
0.20
SNP effect on GDF5

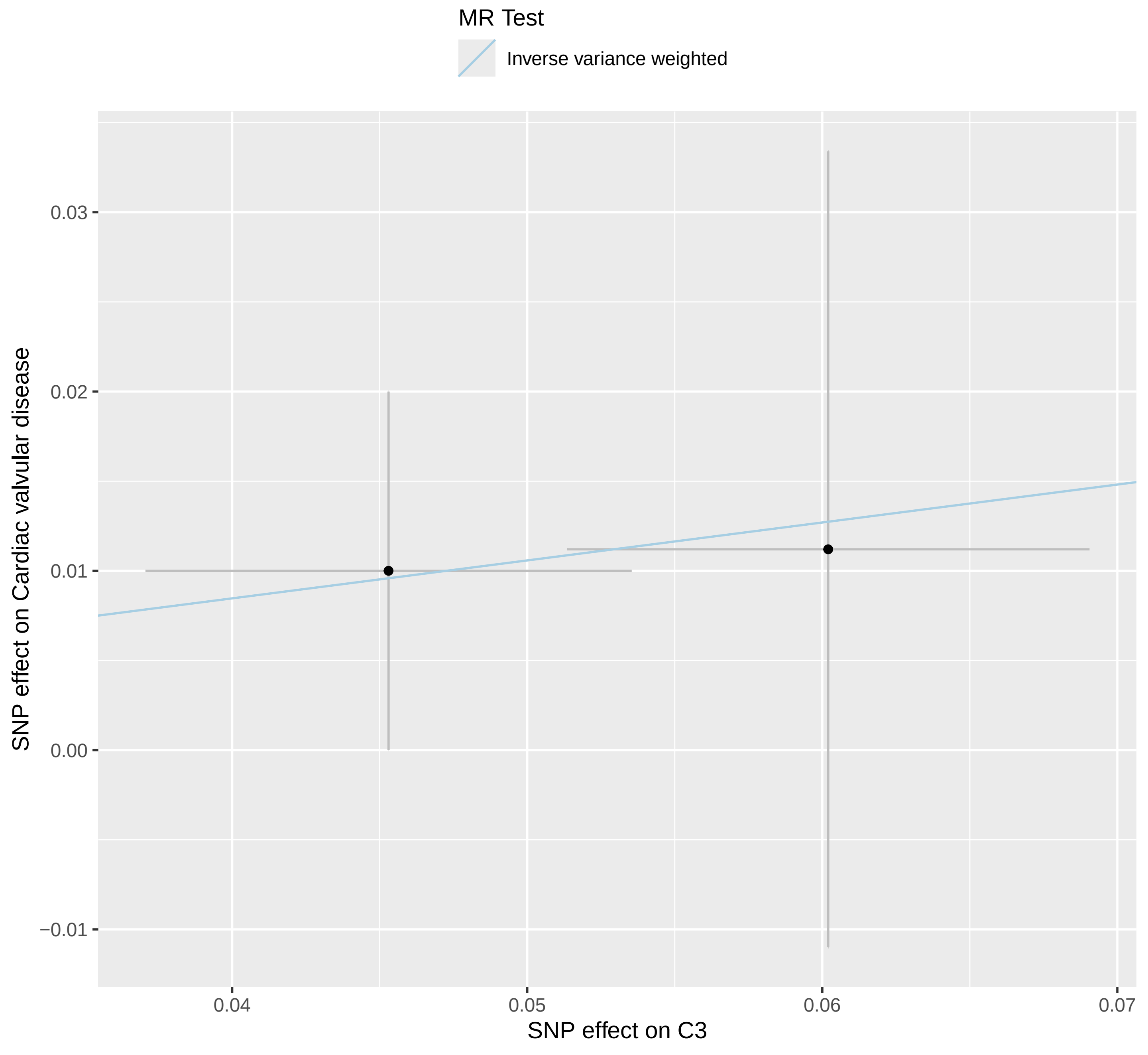
MR Test
Inverse variance weighted
SNP effect on Cardiac valvular disease
0.03
0.02
0.01
0.00
−0.01
0.04
0.05
0.06
0.07
SNP effect on C3

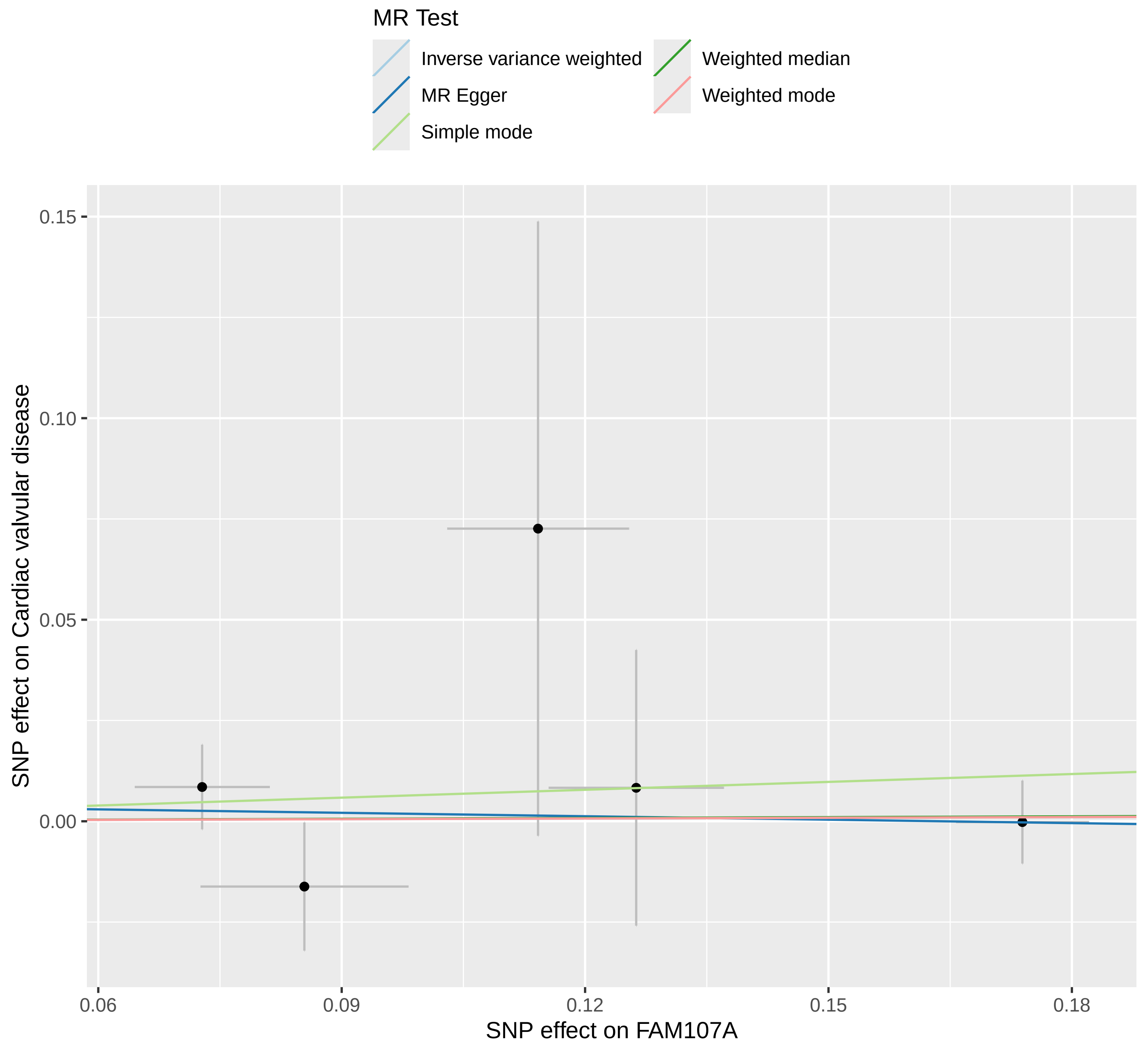
MR Test
Inverse variance weighted
MR Egger
Simple mode
Weighted median
Weighted mode
0.15
0.10
0.05
0.00
0.06
0.09
0.12
0.15
0.18
SNP effect on Cardiac valvular disease
SNP effect on FAM107A

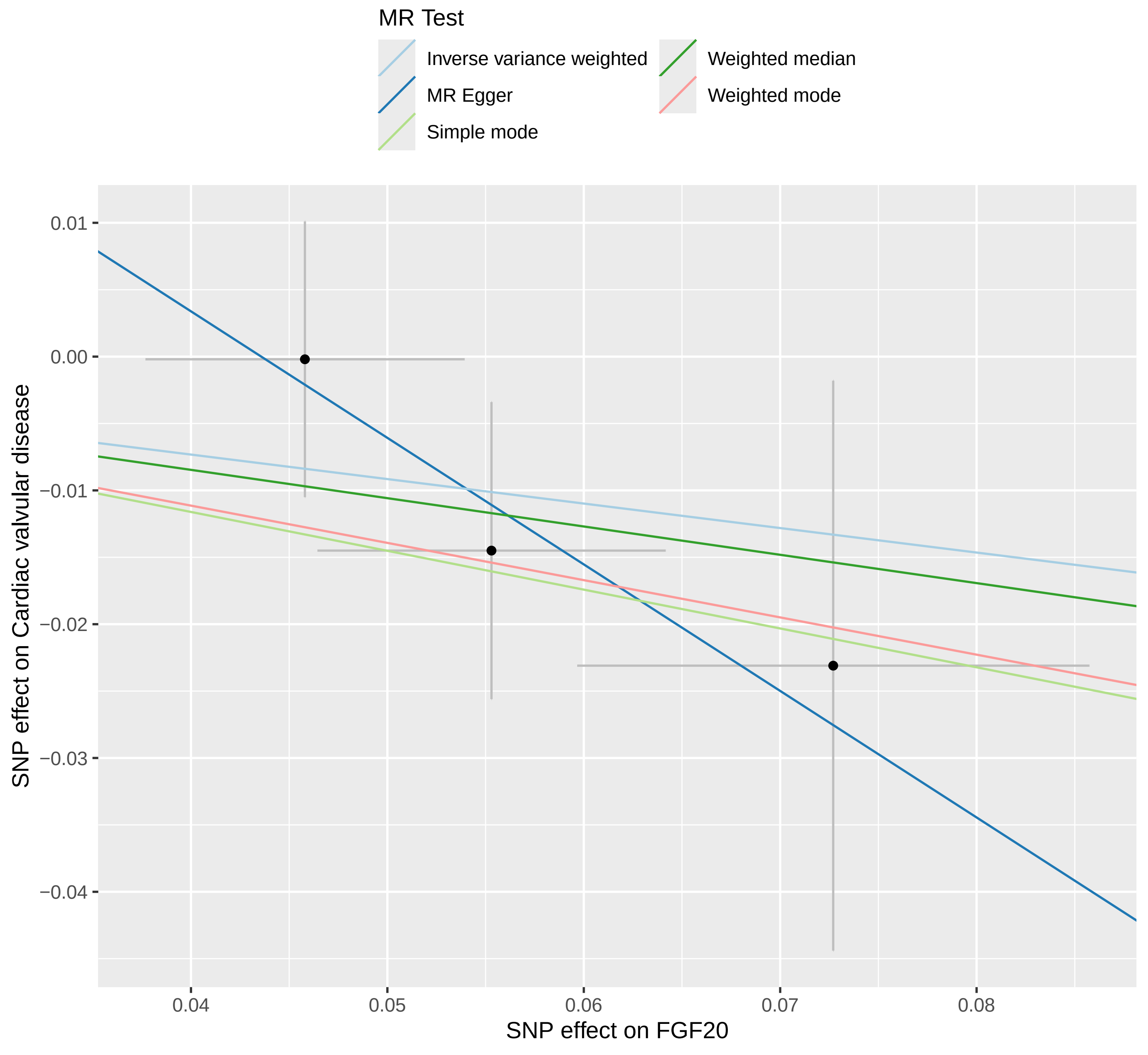

MR Test
Inverse variance weighted
MR Egger
Simple mode
Weighted median
Weighted mode
0.01
0.00
−0.01
−0.02
−0.03
−0.04
0.04
0.05
0.06
0.07
0.08
SNP effect on Cardiac valvular disease
SNP effect on FGF20

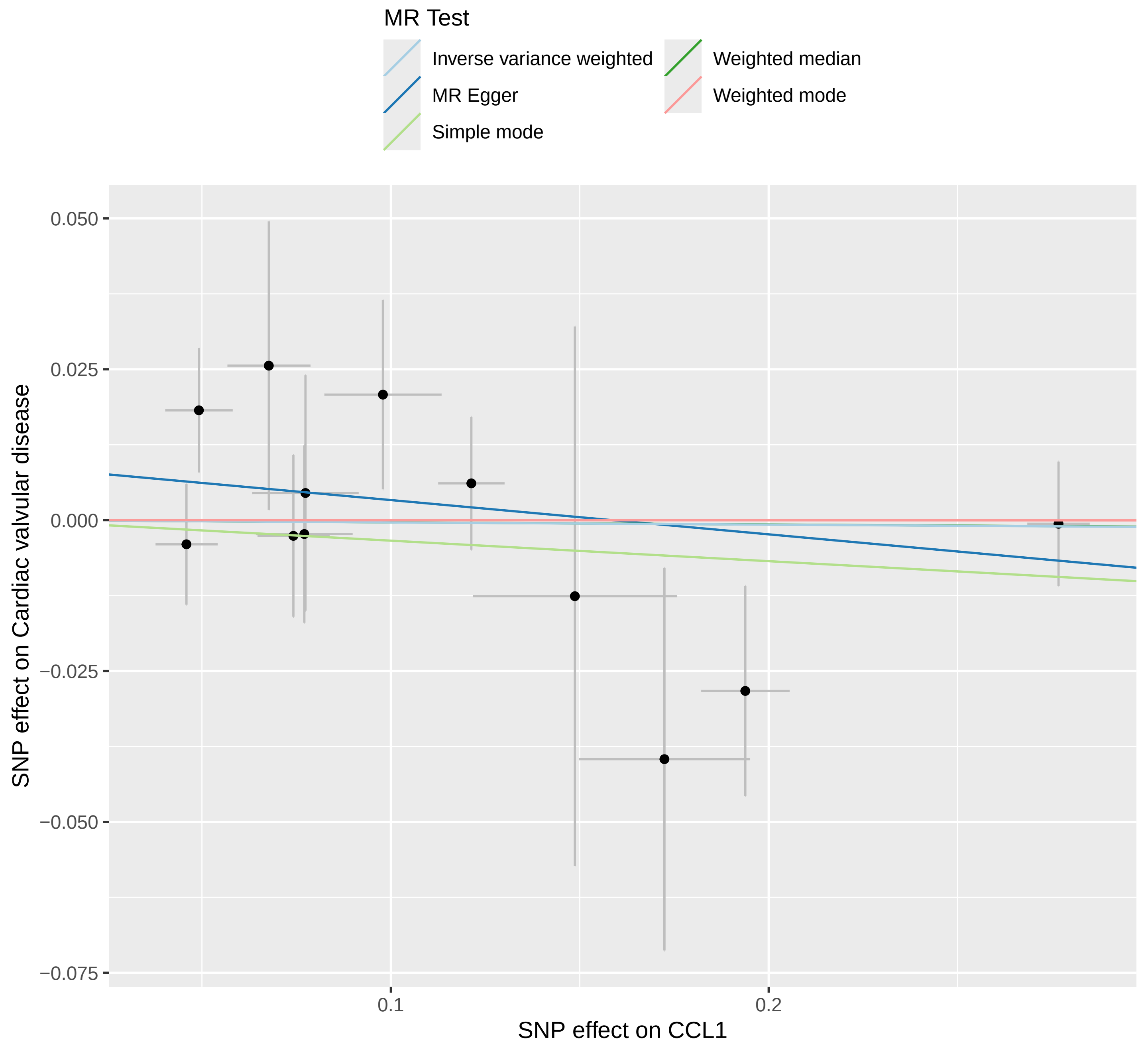

MR Test
Inverse variance weighted
MR Egger
Simple mode
Weighted median
Weighted mode
0.050
0.025
0.000
−0.025
−0.050
−0.075
SNP effect on Cardiac valvular disease
0.1
0.2
SNP effect on CCL1

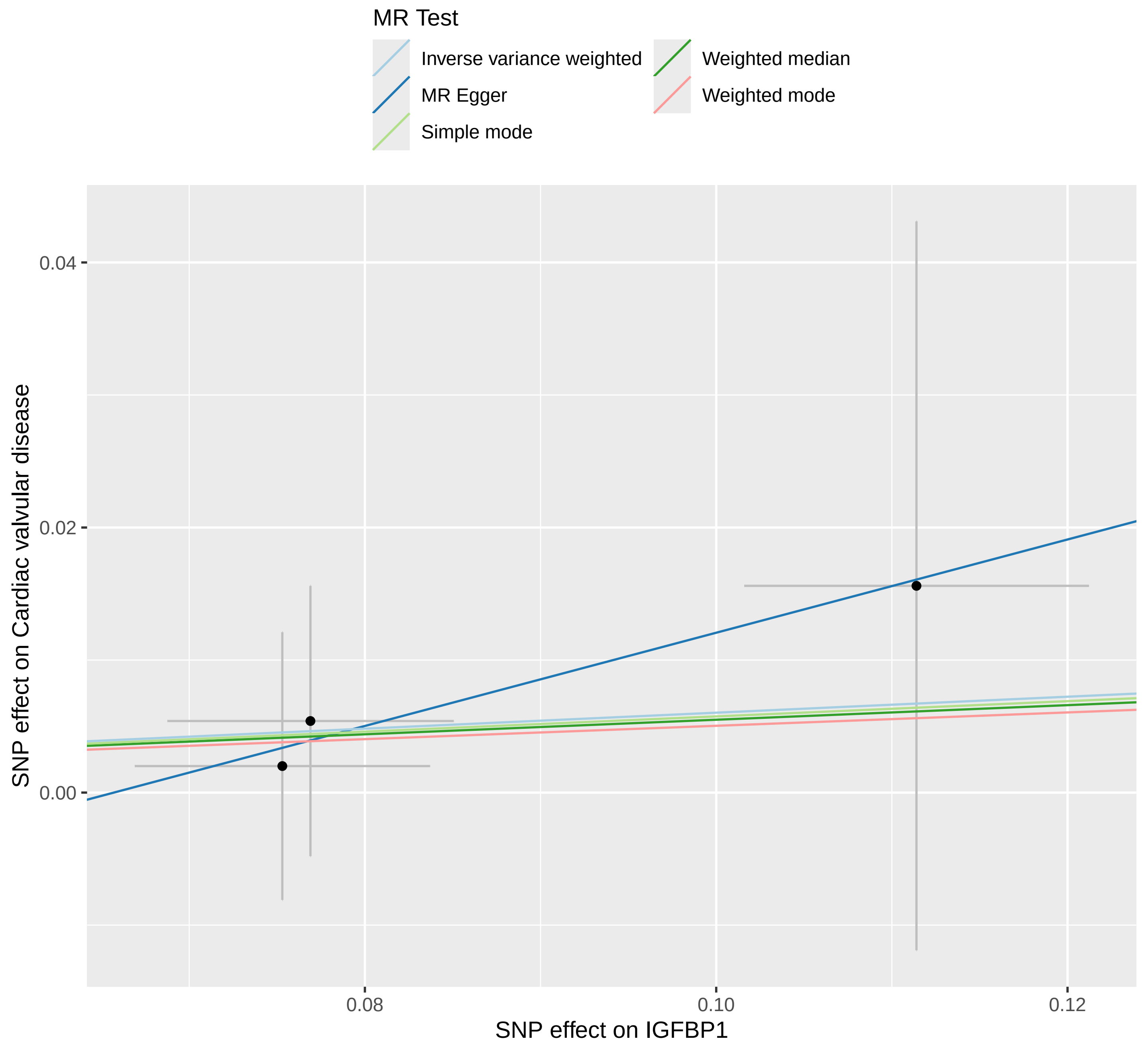
MR Test
Inverse variance weighted
MR Egger
Simple mode
Weighted median
Weighted mode
0.04
0.02
0.00
0.08
0.10
0.12
SNP effect on Cardiac valvular disease
SNP effect on IGFBP1

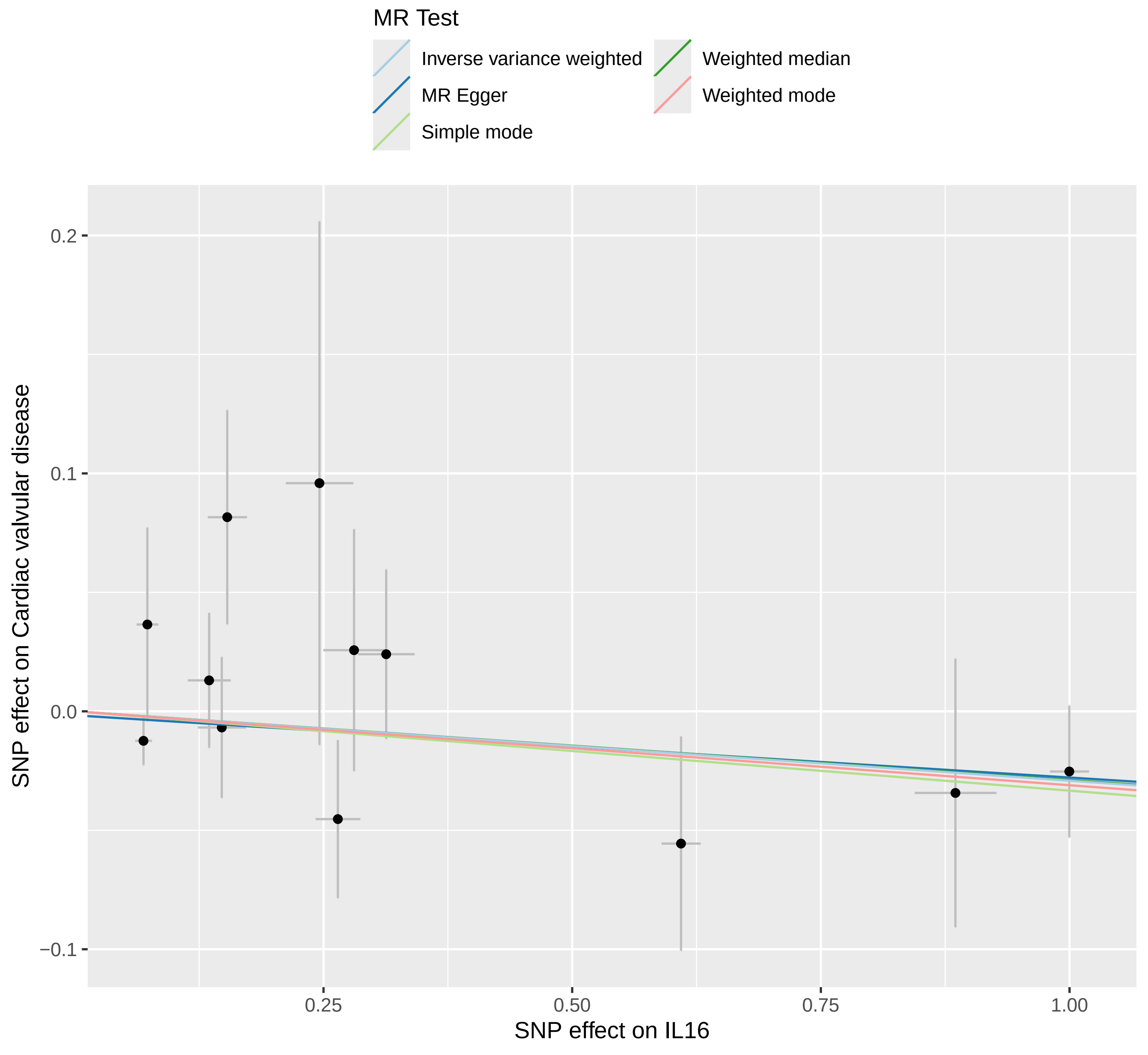
MR Test
Inverse variance weighted
MR Egger
Simple mode
Weighted median
Weighted mode
SNP effect on Cardiac valvular disease
0.2
0.1
0.0
−0.1
0.25
0.50
0.75
1.00
SNP effect on IL16

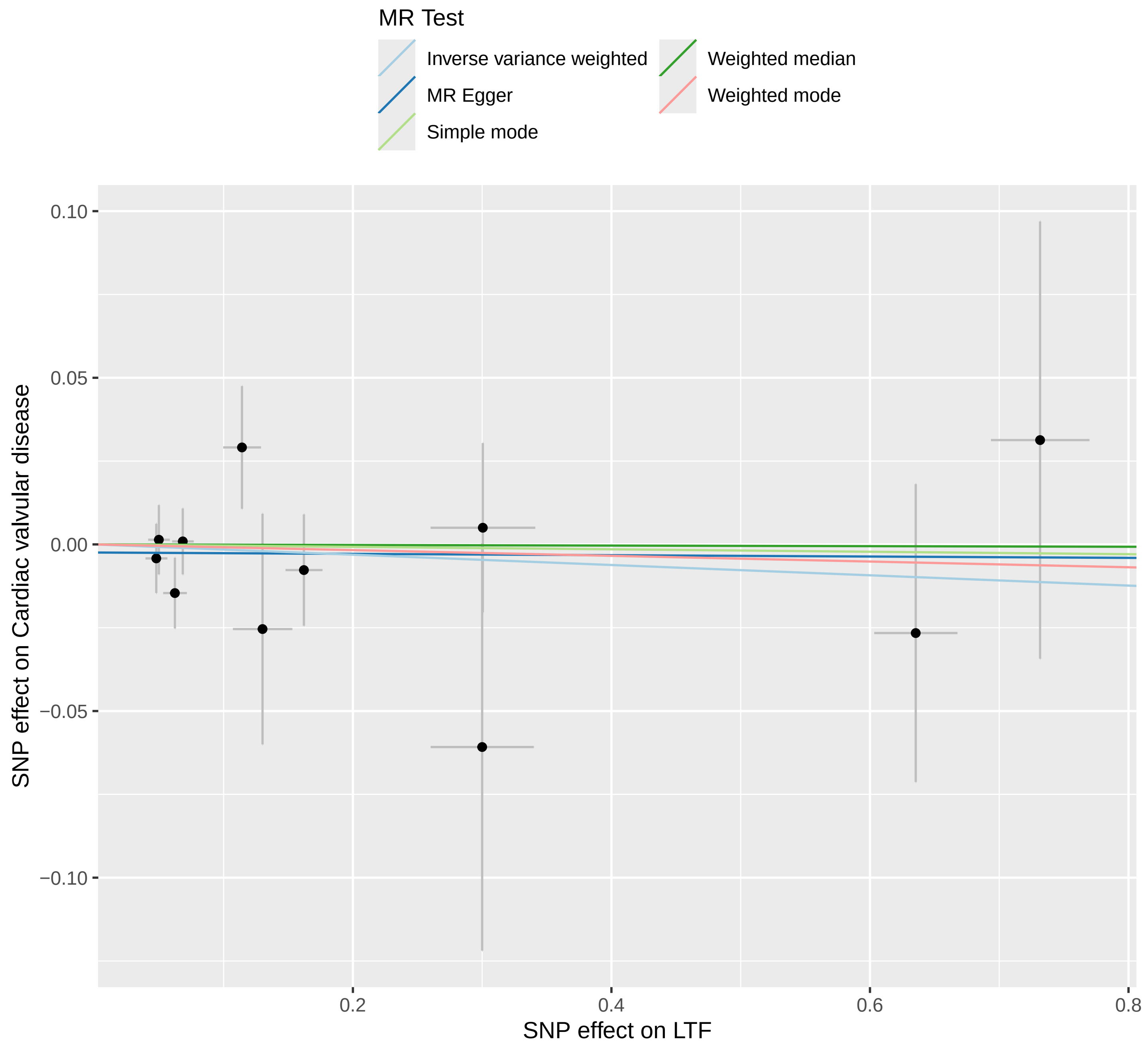
MR Test
Inverse variance weighted
MR Egger
Simple mode
Weighted median
Weighted mode
0.10
0.05
0.00
−0.05
−0.10
SNP effect on Cardiac valvular disease
0.2
0.4
0.6
0.8
SNP effect on LTF

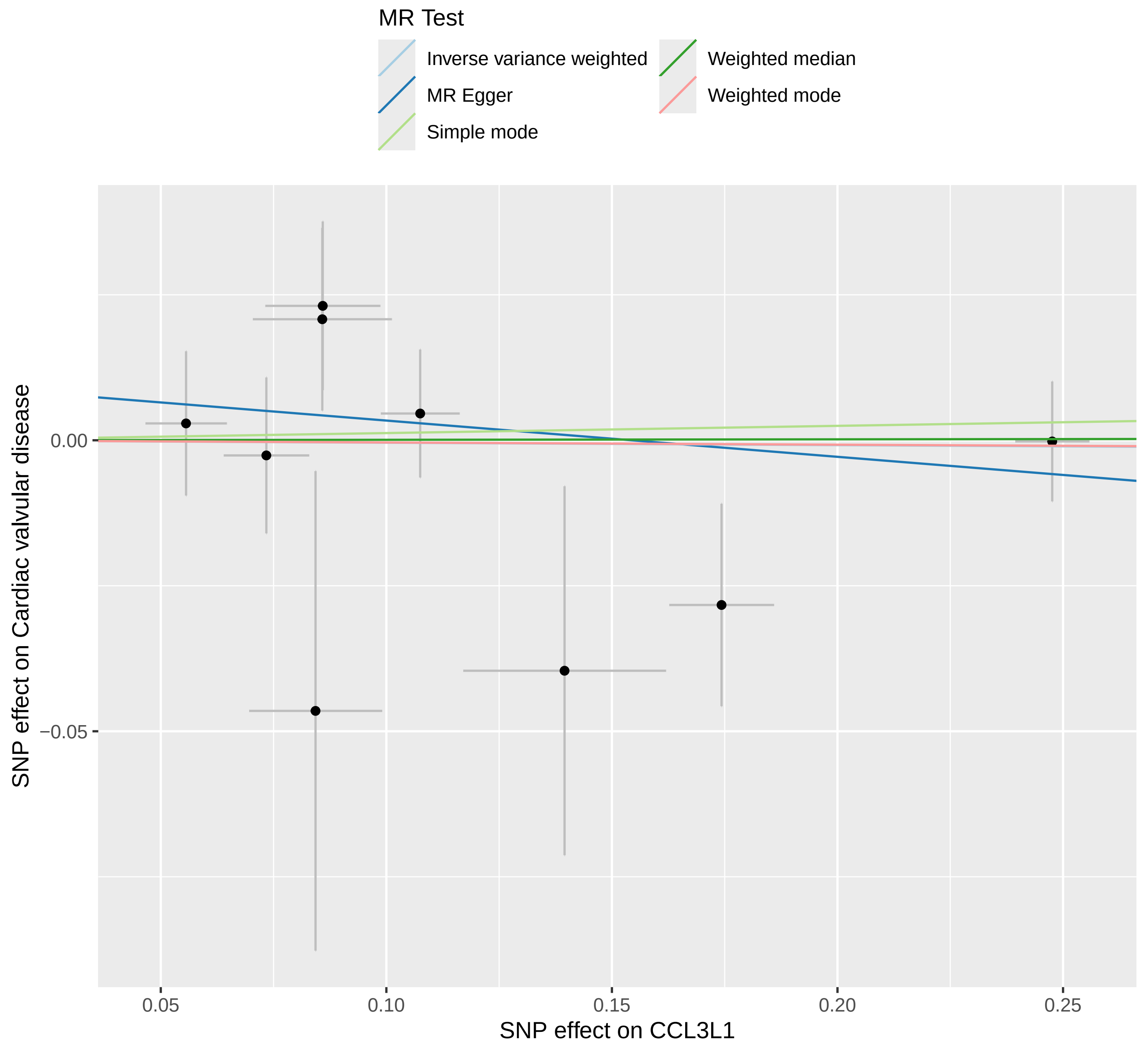
MR Test
Inverse variance weighted
MR Egger
Simple mode
Weighted median
Weighted mode
SNP effect on Cardiac valvular disease
0.00
−0.05
0.05
0.10
0.15
0.20
0.25
SNP effect on CCL3L1

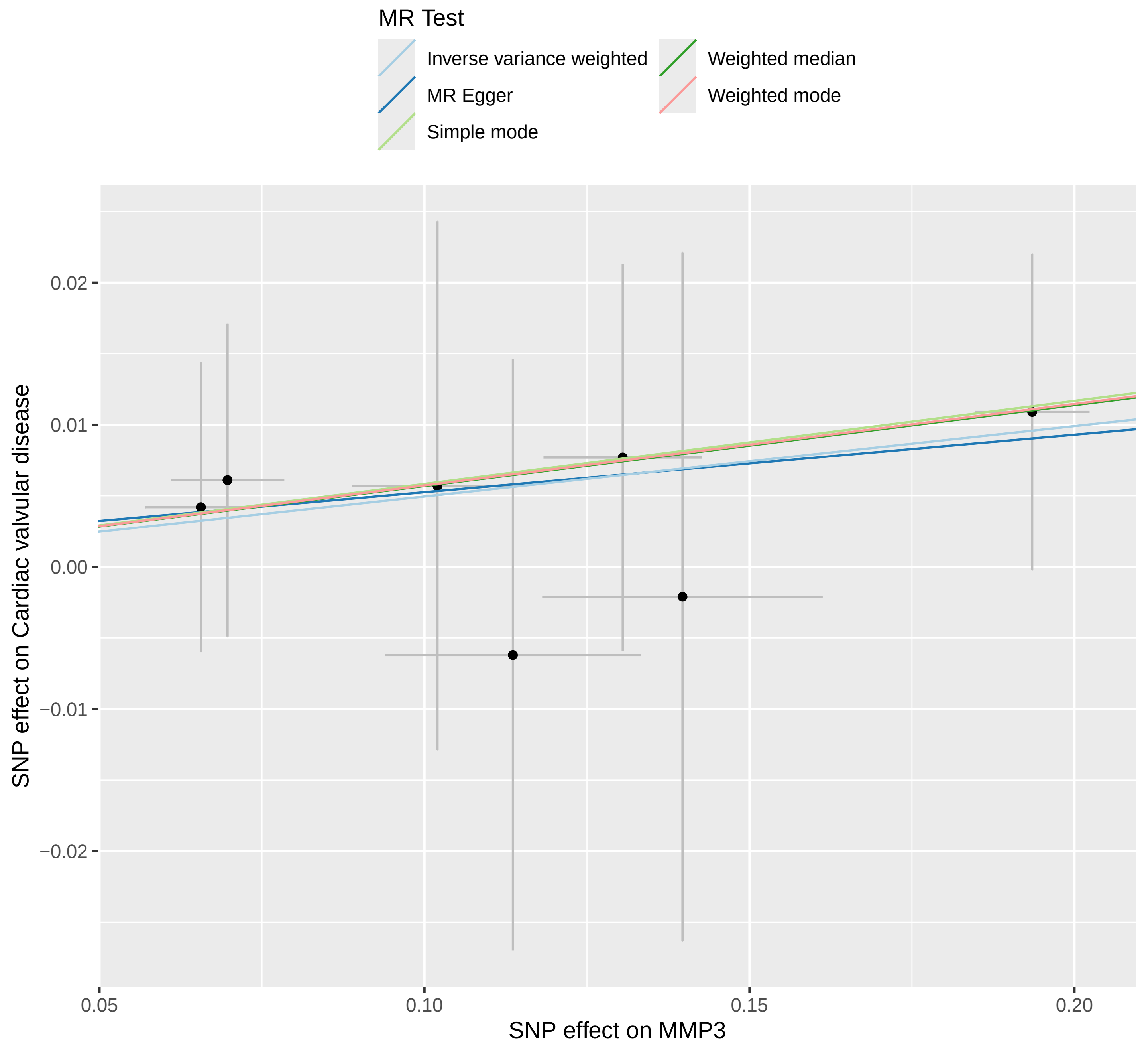

MR Test
Inverse variance weighted
MR Egger
Simple mode
Weighted median
Weighted mode
0.02
0.01
0.00
−0.01
−0.02
SNP effect on Cardiac valvular disease
0.05
0.10
0.15
0.20
SNP effect on MMP3

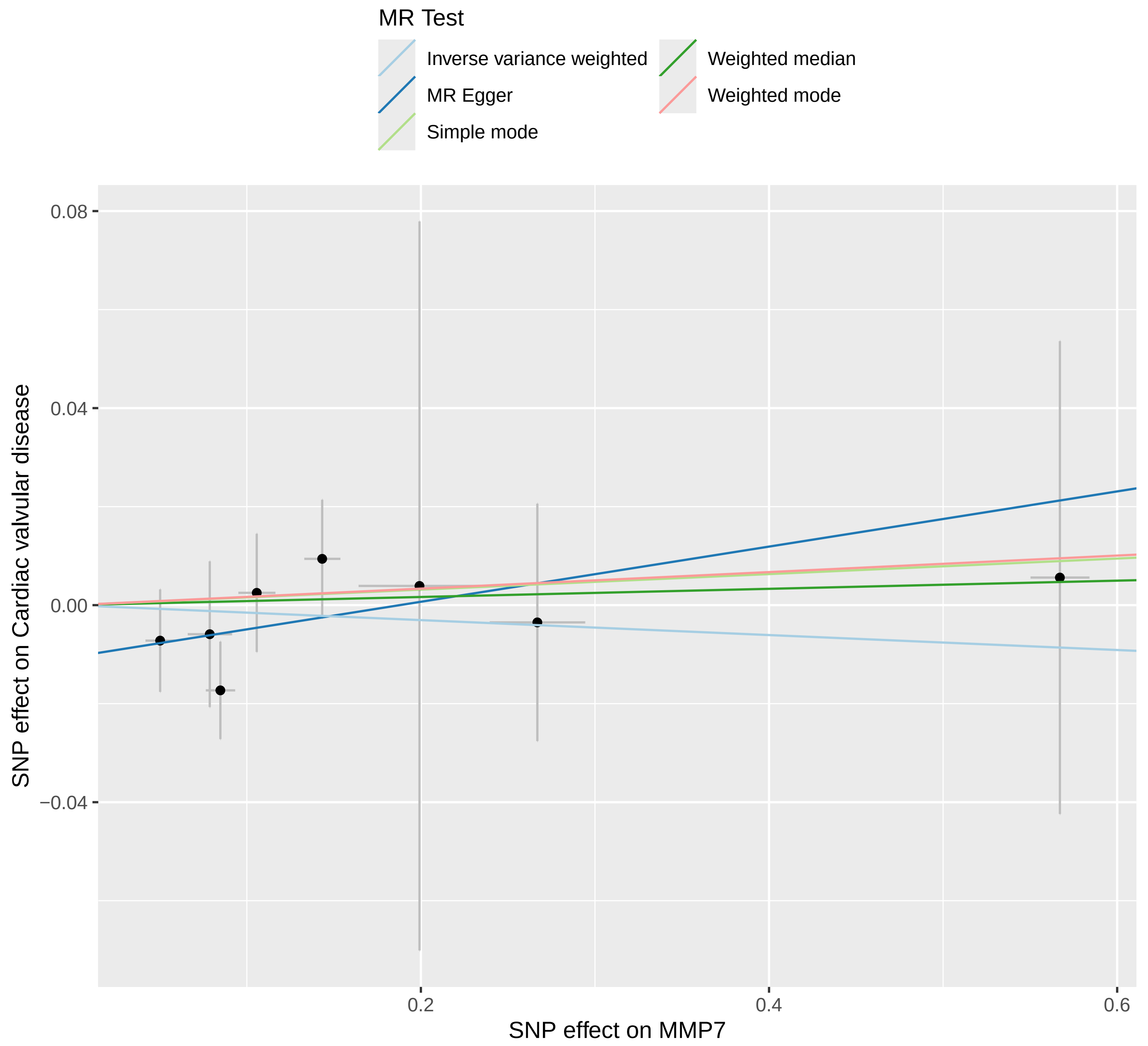
MR Test
Inverse variance weighted
MR Egger
Simple mode
Weighted median
Weighted mode
0.08
0.04
0.00
−0.04
0.2
0.4
0.6
SNP effect on Cardiac valvular disease
SNP effect on MMP7

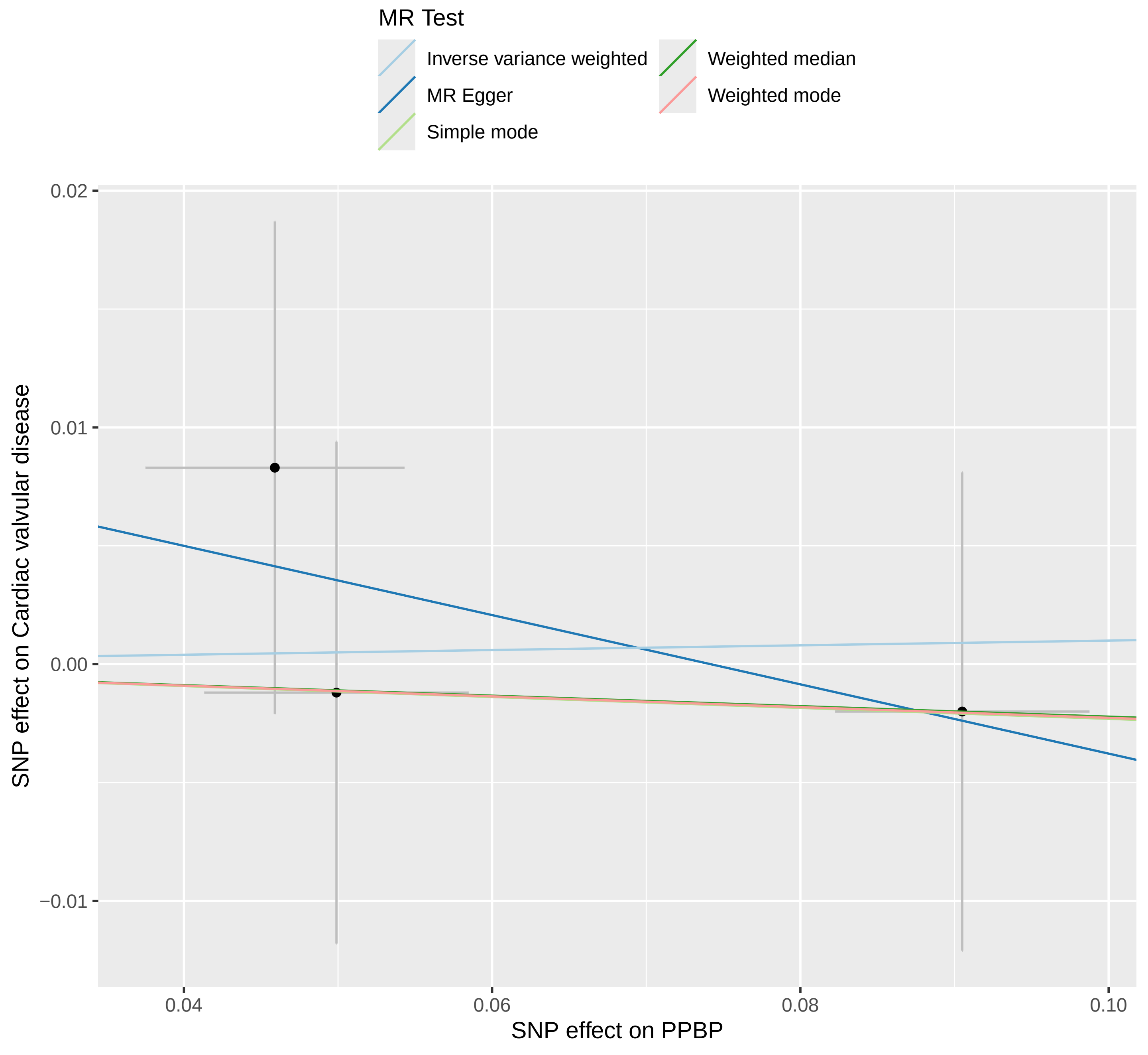
MR Test
Inverse variance weighted
MR Egger
Simple mode
Weighted median
Weighted mode
0.02
0.01
0.00
−0.01
SNP effect on Cardiac valvular disease
0.04
0.06
0.08
0.10
SNP effect on PPBP

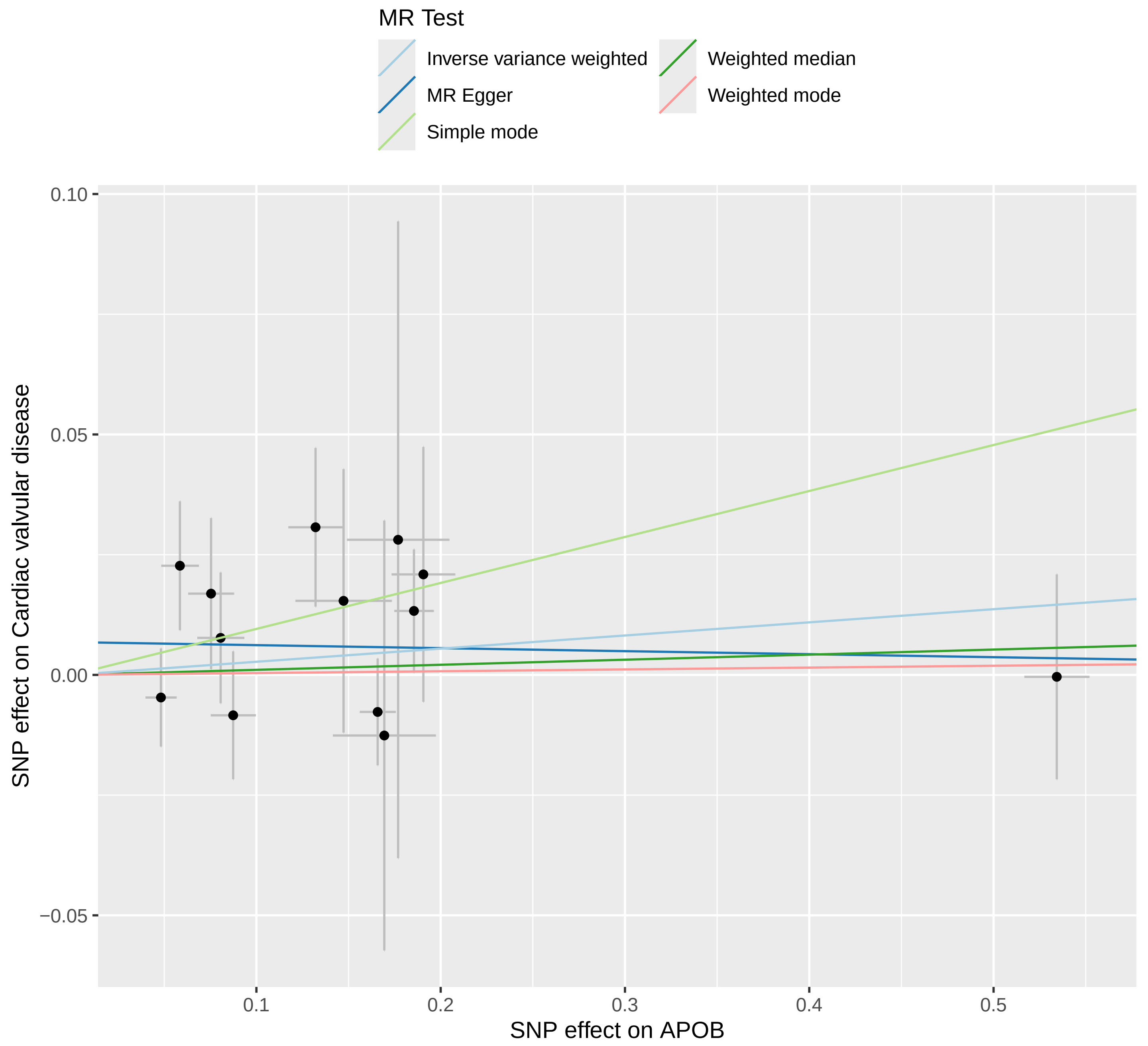

MR Test
Inverse variance weighted
MR Egger
Simple mode
Weighted median
Weighted mode
0.10
0.05
0.00
−0.05
0.1
0.2
0.3
0.4
0.5
SNP effect on Cardiac valvular disease
SNP effect on APOB

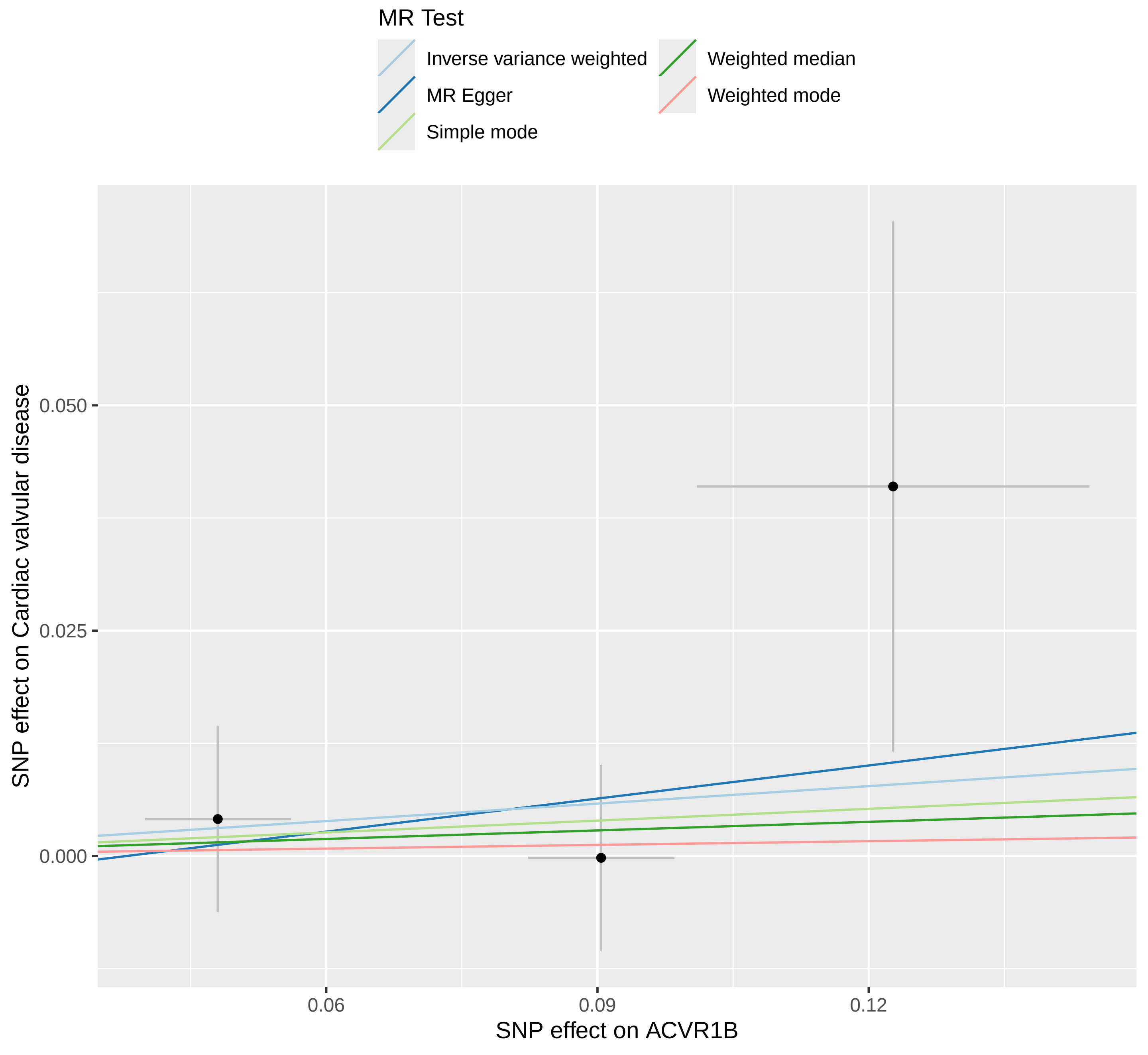

MR Test
Inverse variance weighted
MR Egger
Simple mode
Weighted median
Weighted mode
0.050
0.025
0.000
0.06
0.09
0.12
SNP effect on Cardiac valvular disease
SNP effect on ACVR1B

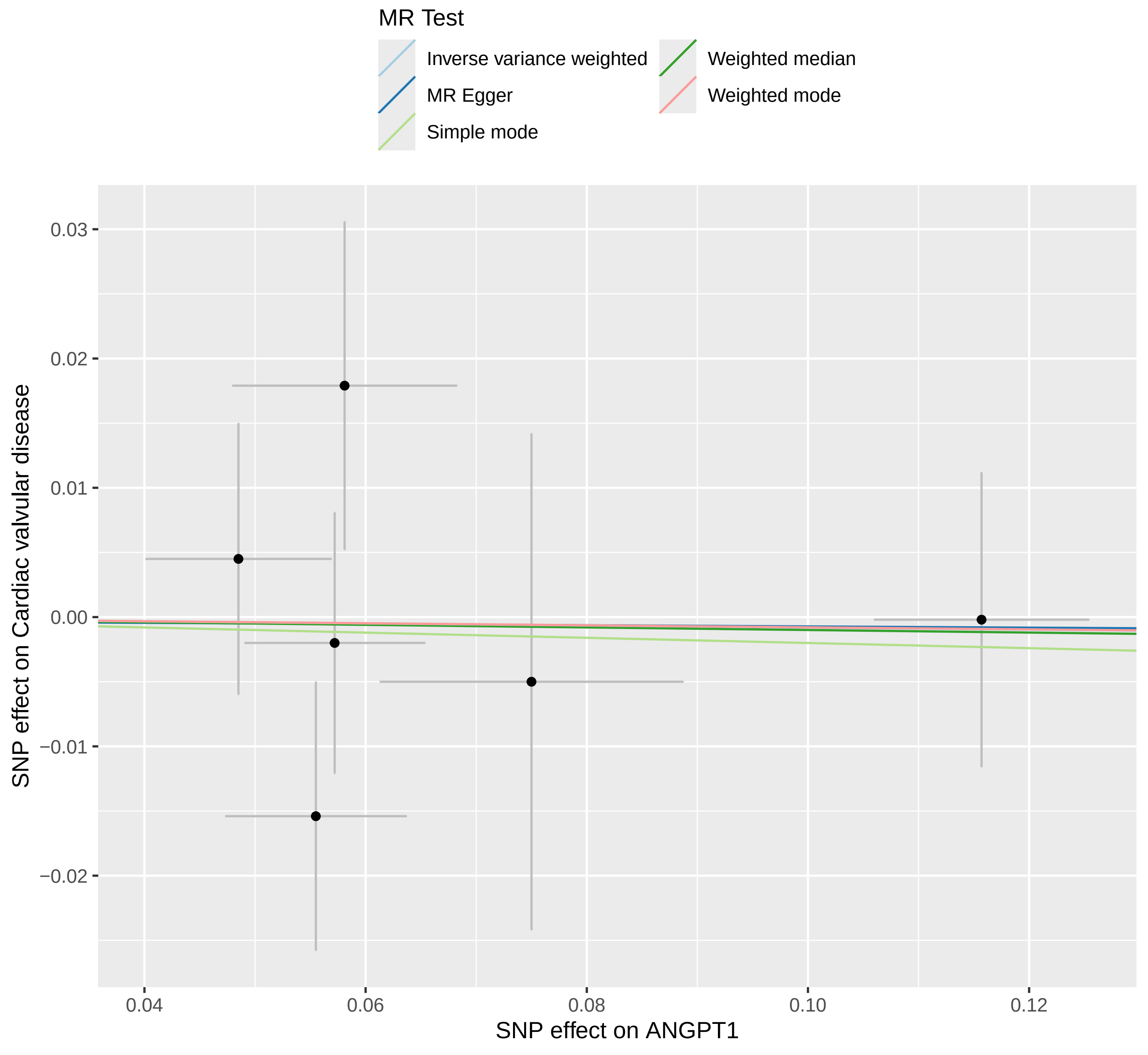
MR Test
Inverse variance weighted
MR Egger
Simple mode
Weighted median
Weighted mode
0.03
0.02
0.01
0.00
−0.01
−0.02
SNP effect on Cardiac valvular disease
0.04
0.06
0.08
0.10
0.12
SNP effect on ANGPT1

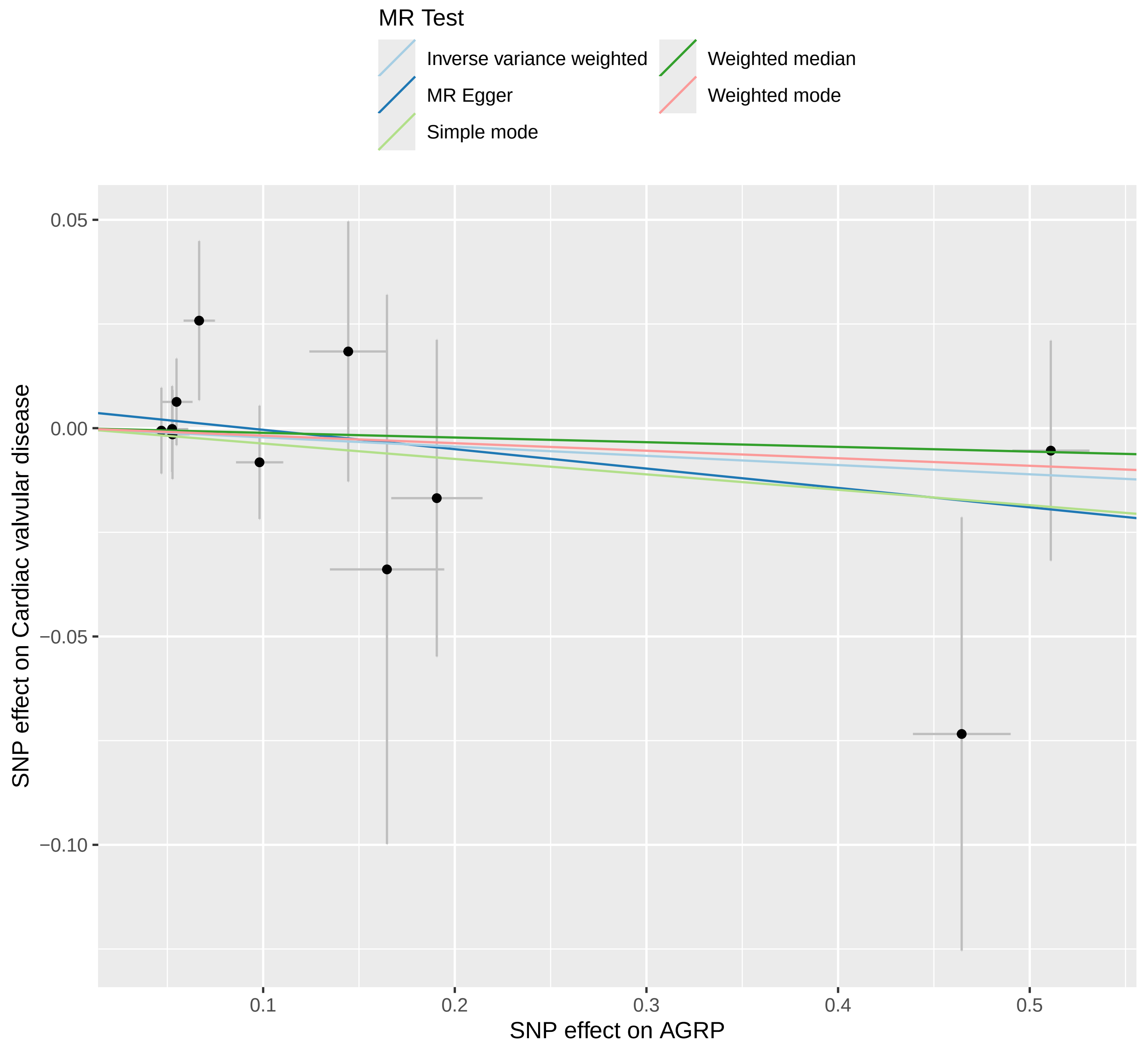
MR Test
Inverse variance weighted
MR Egger
Simple mode
Weighted median
Weighted mode
0.05
0.00
−0.05
−0.10
0.1
0.2
0.3
0.4
0.5
SNP effect on AGRP
SNP effect on Cardiac valvular disease

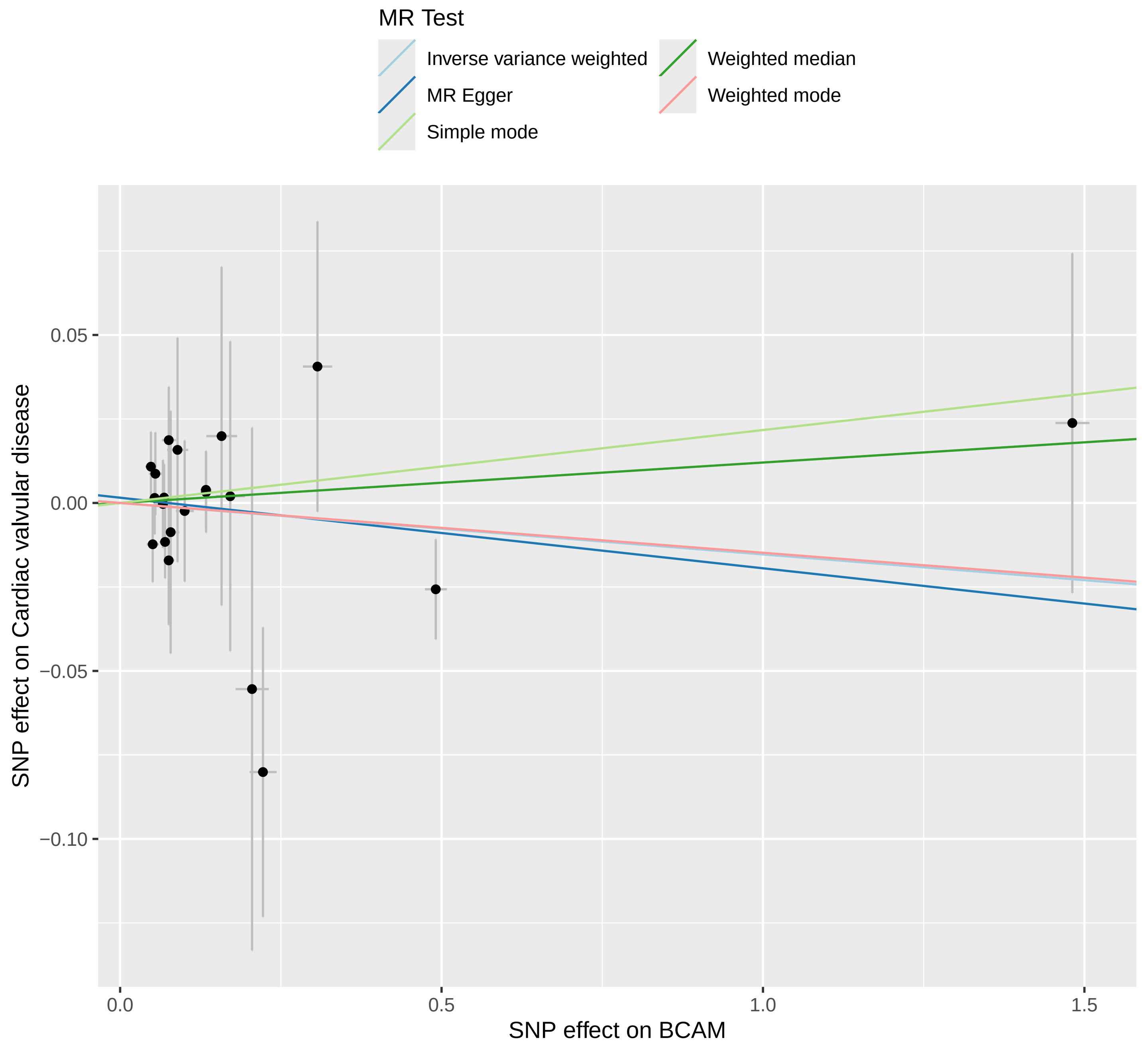
MR Test
Inverse variance weighted
MR Egger
Simple mode
Weighted median
Weighted mode
SNP effect on Cardiac valvular disease
0.05
0.00
−0.05
−0.10
0.0
0.5
1.0
1.5
SNP effect on BCAM

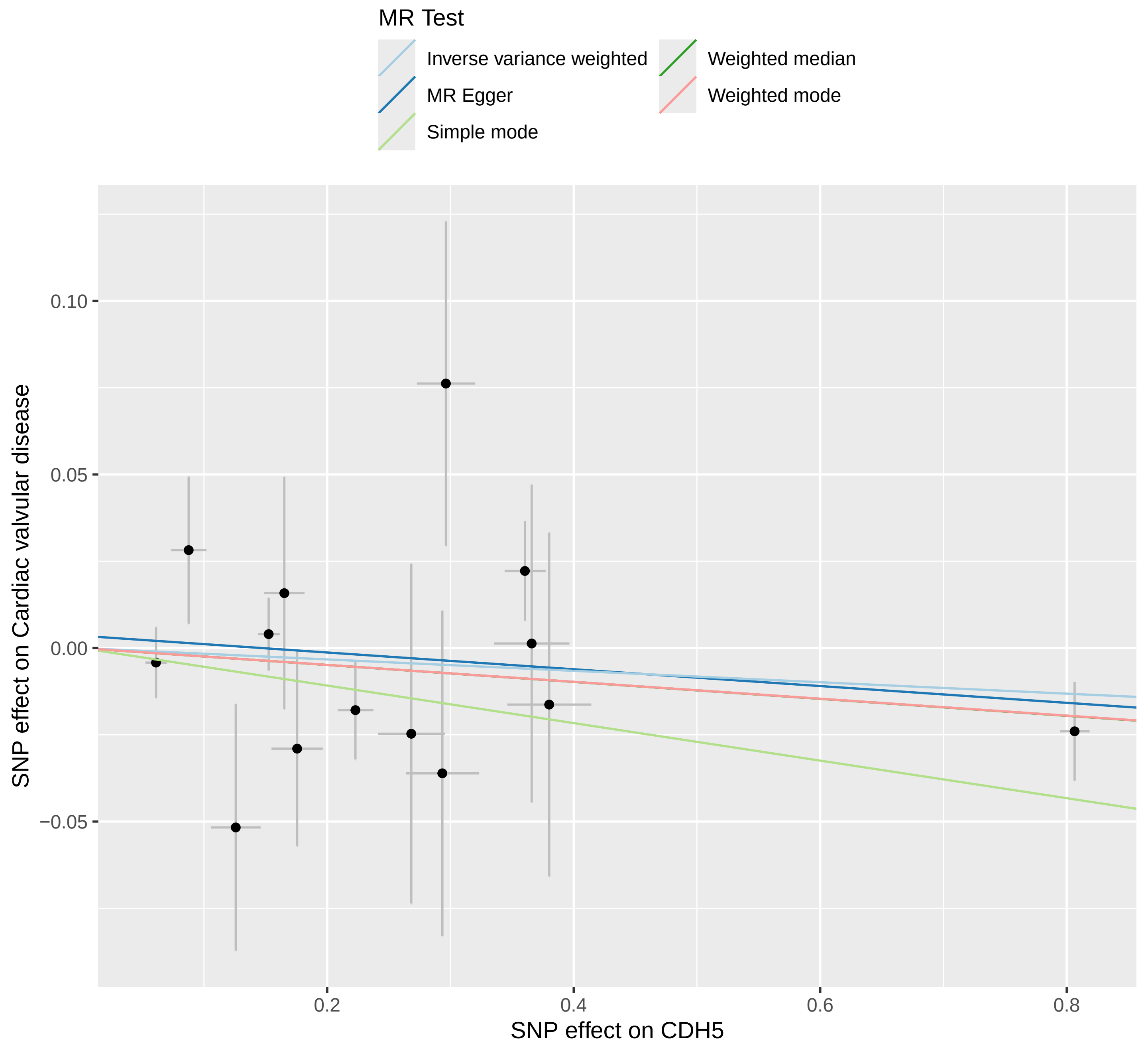
MR Test
Inverse variance weighted
MR Egger
Simple mode
Weighted median
Weighted mode
0.10
0.05
0.00
−0.05
SNP effect on Cardiac valvular disease
0.2
0.4
0.6
0.8
SNP effect on CDH5

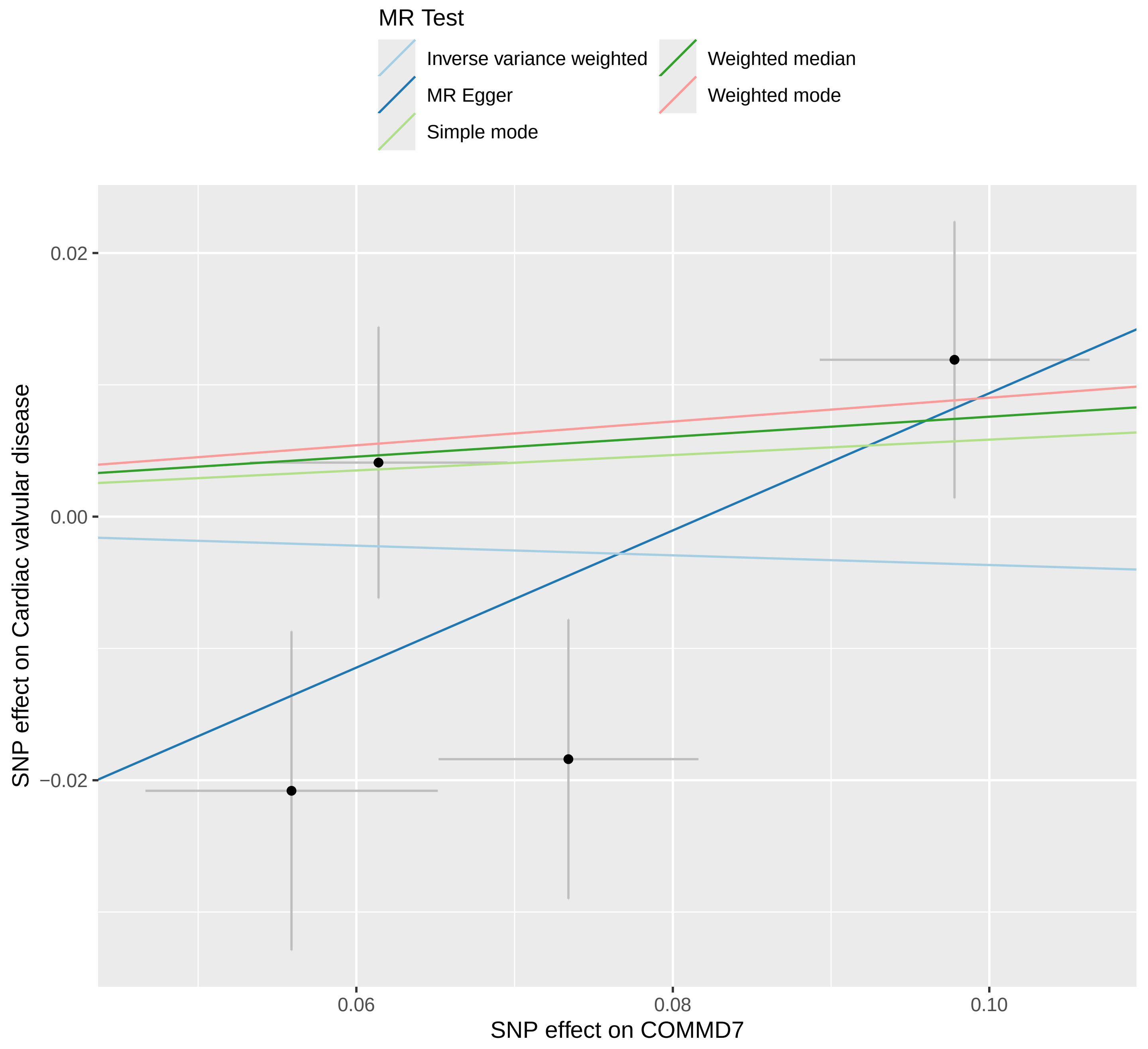

MR Test
Inverse variance weighted
MR Egger
Simple mode
Weighted median
Weighted mode
0.02
0.00
−0.02
0.06
0.08
0.10
SNP effect on Cardiac valvular disease
SNP effect on COMMD7

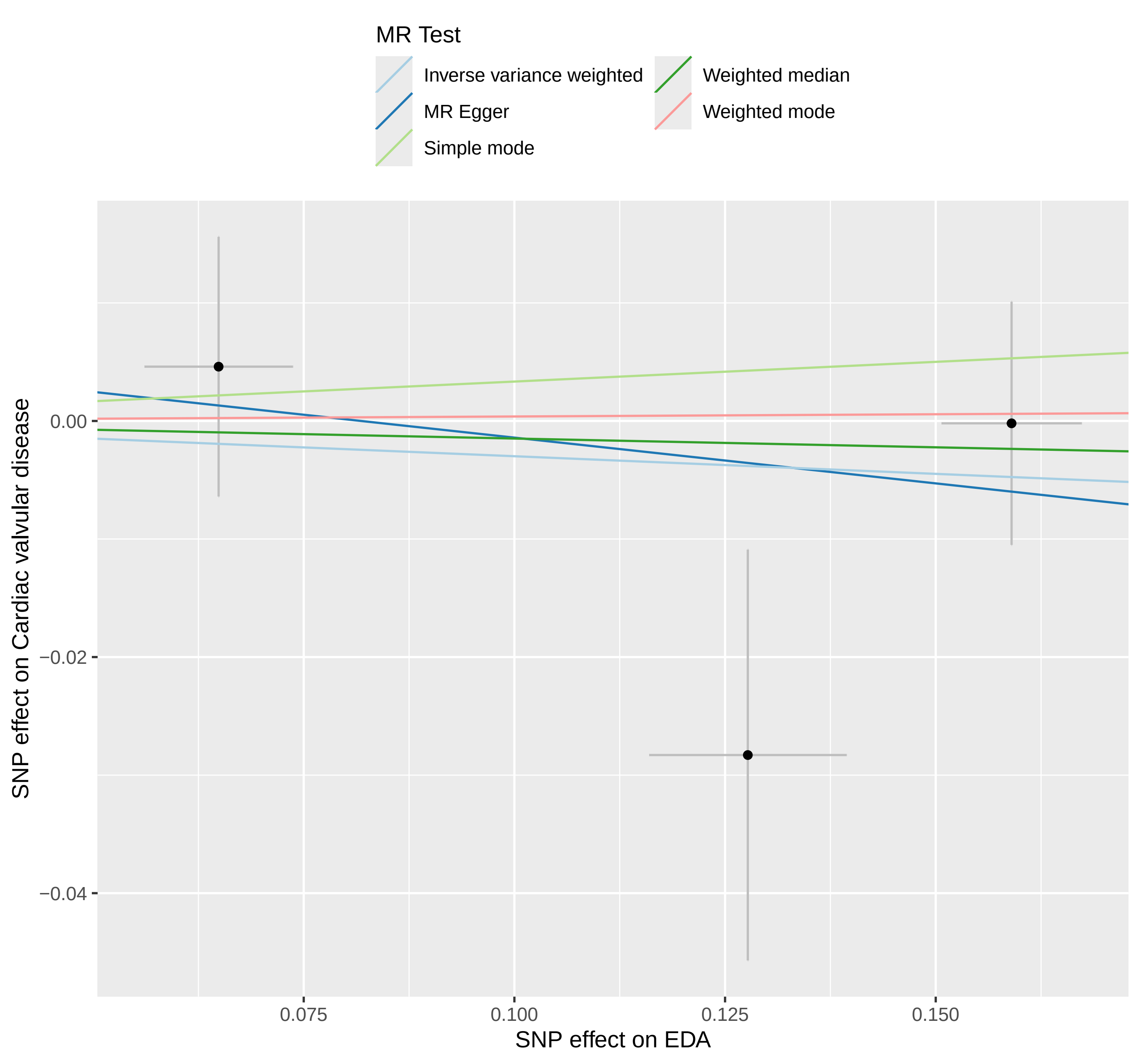
MR Test
Inverse variance weighted
MR Egger
Simple mode
Weighted median
Weighted mode
SNP effect on Cardiac valvular disease
0.00
−0.02
−0.04
0.075
0.100
0.125
0.150
SNP effect on EDA

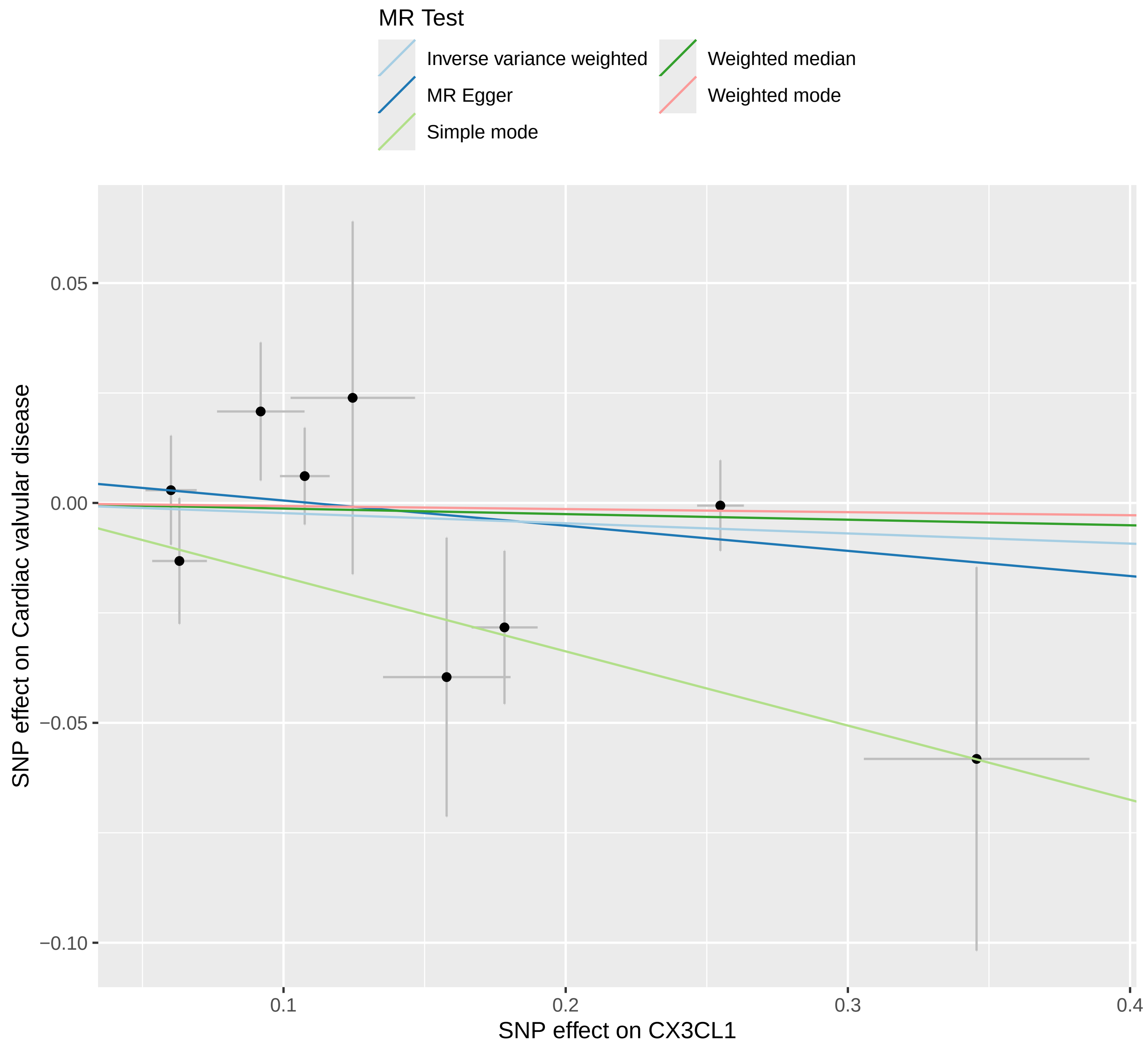
MR Test
Inverse variance weighted
MR Egger
Simple mode
Weighted median
Weighted mode
0.05
0.00
−0.05
−0.10
0.1
0.2
0.3
0.4
SNP effect on Cardiac valvular disease
SNP effect on CX3CL1

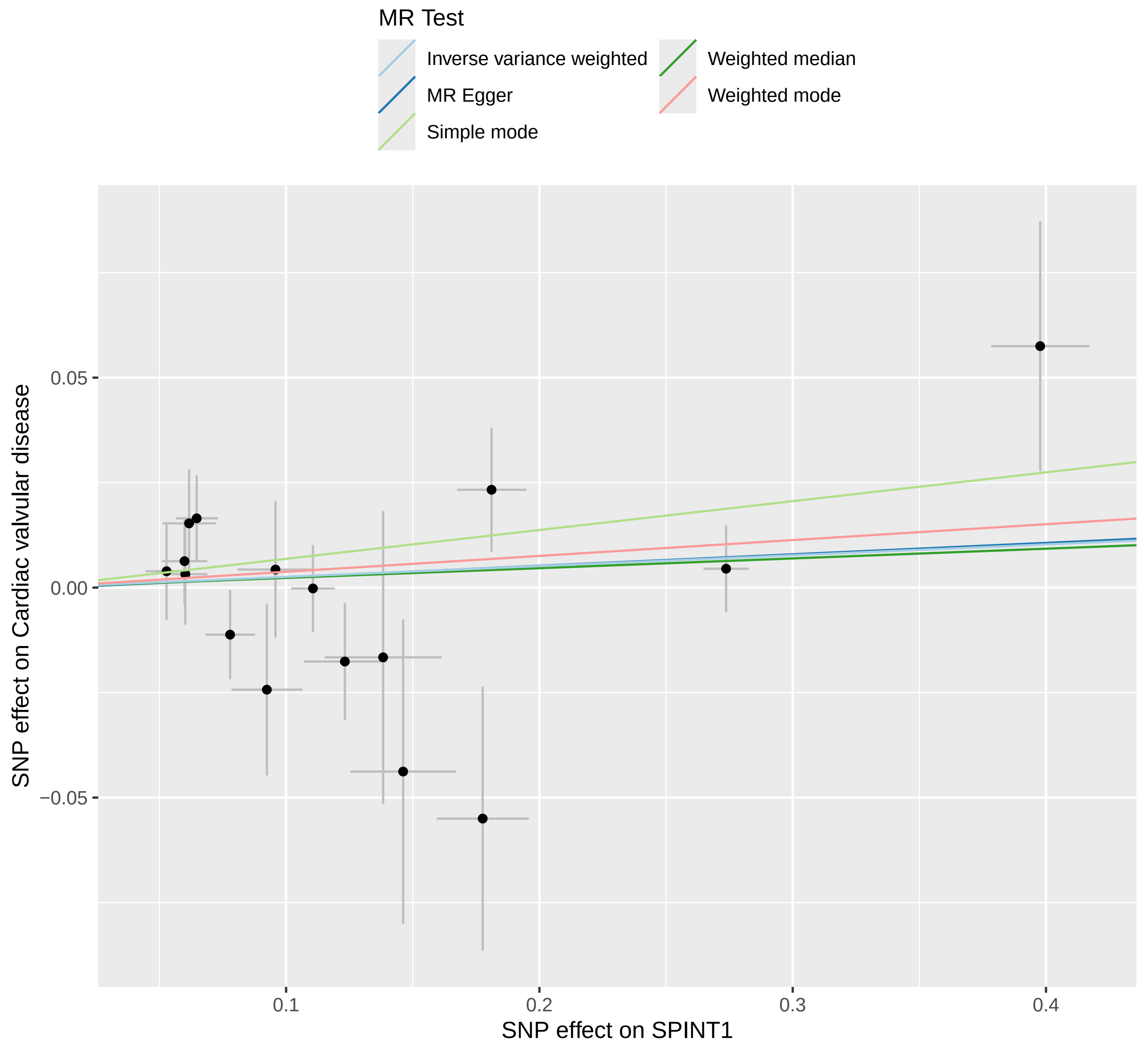
MR Test
Inverse variance weighted
MR Egger
Simple mode
Weighted median
Weighted mode
0.05
0.00
−0.05
SNP effect on Cardiac valvular disease
0.1
0.2
0.3
0.4
SNP effect on SPINT1

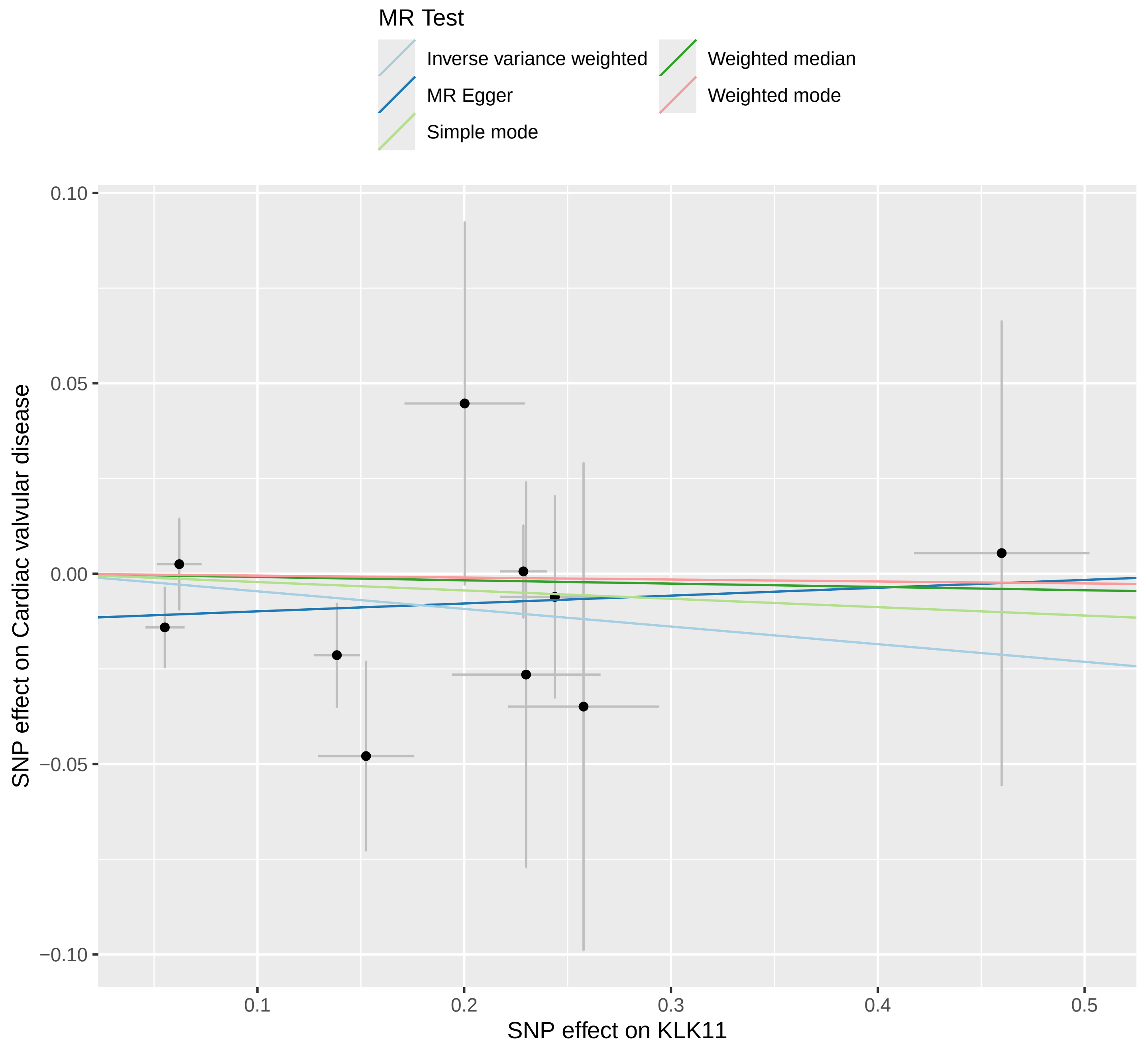

MR Test
Inverse variance weighted
MR Egger
Simple mode
Weighted median
Weighted mode
0.10
0.05
0.00
−0.05
−0.10
SNP effect on Cardiac valvular disease
0.1
0.2
0.3
0.4
0.5
SNP effect on KLK11

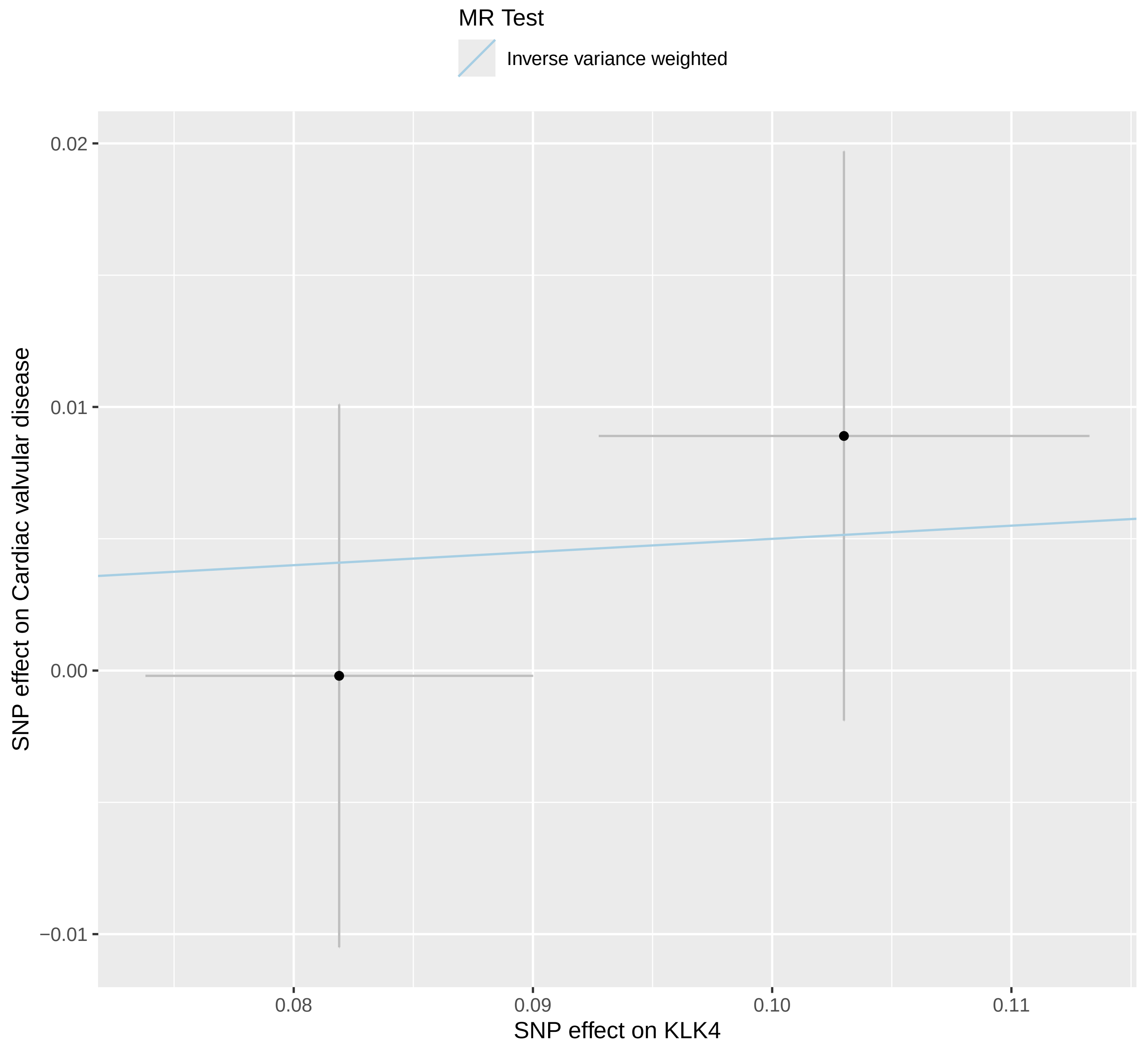
MR Test
Inverse variance weighted
0.02
0.01
0.00
−0.01
SNP effect on Cardiac valvular disease
0.08
0.09
0.10
0.11
SNP effect on KLK4

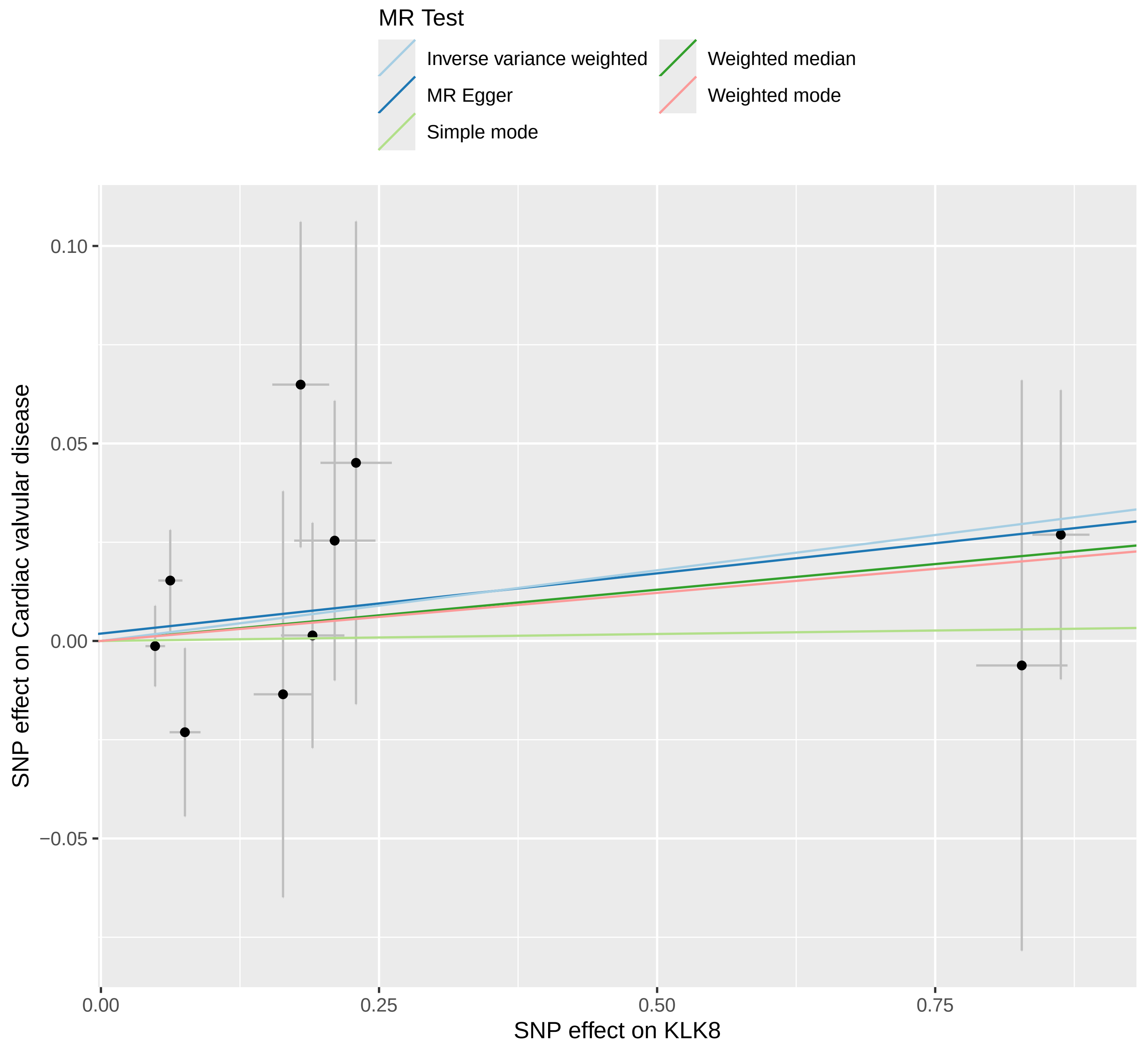

MR Test
Inverse variance weighted
MR Egger
Simple mode
Weighted median
Weighted mode
0.10
0.05
0.00
−0.05
0.00
0.25
0.50
0.75
SNP effect on Cardiac valvular disease
SNP effect on KLK8

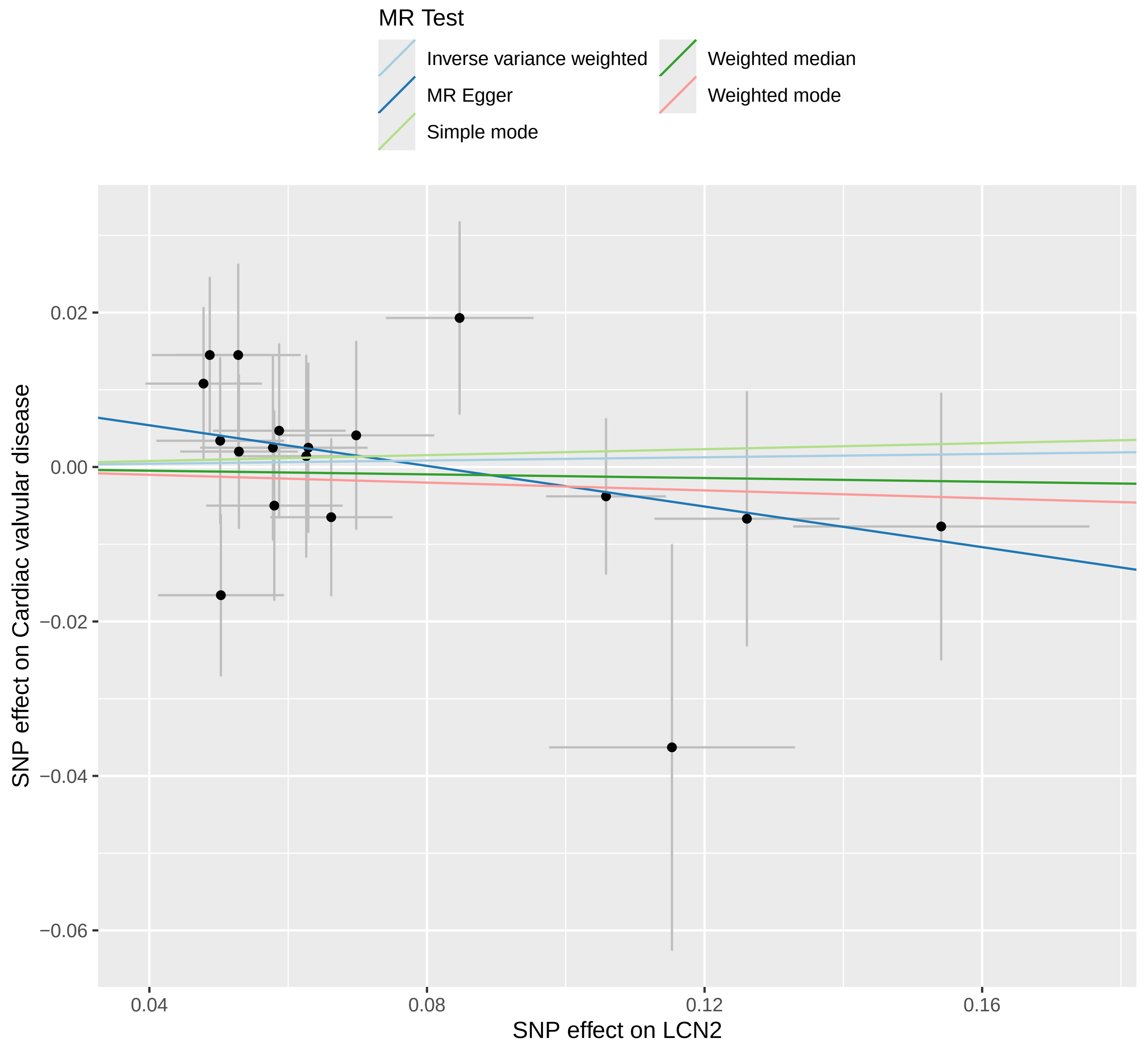
MR Test
Inverse variance weighted
MR Egger
Simple mode
Weighted median
Weighted mode
0.02
0.00
−0.02
−0.04
−0.06
0.04
0.08
0.12
0.16
SNP effect on Cardiac valvular disease
SNP effect on LCN2

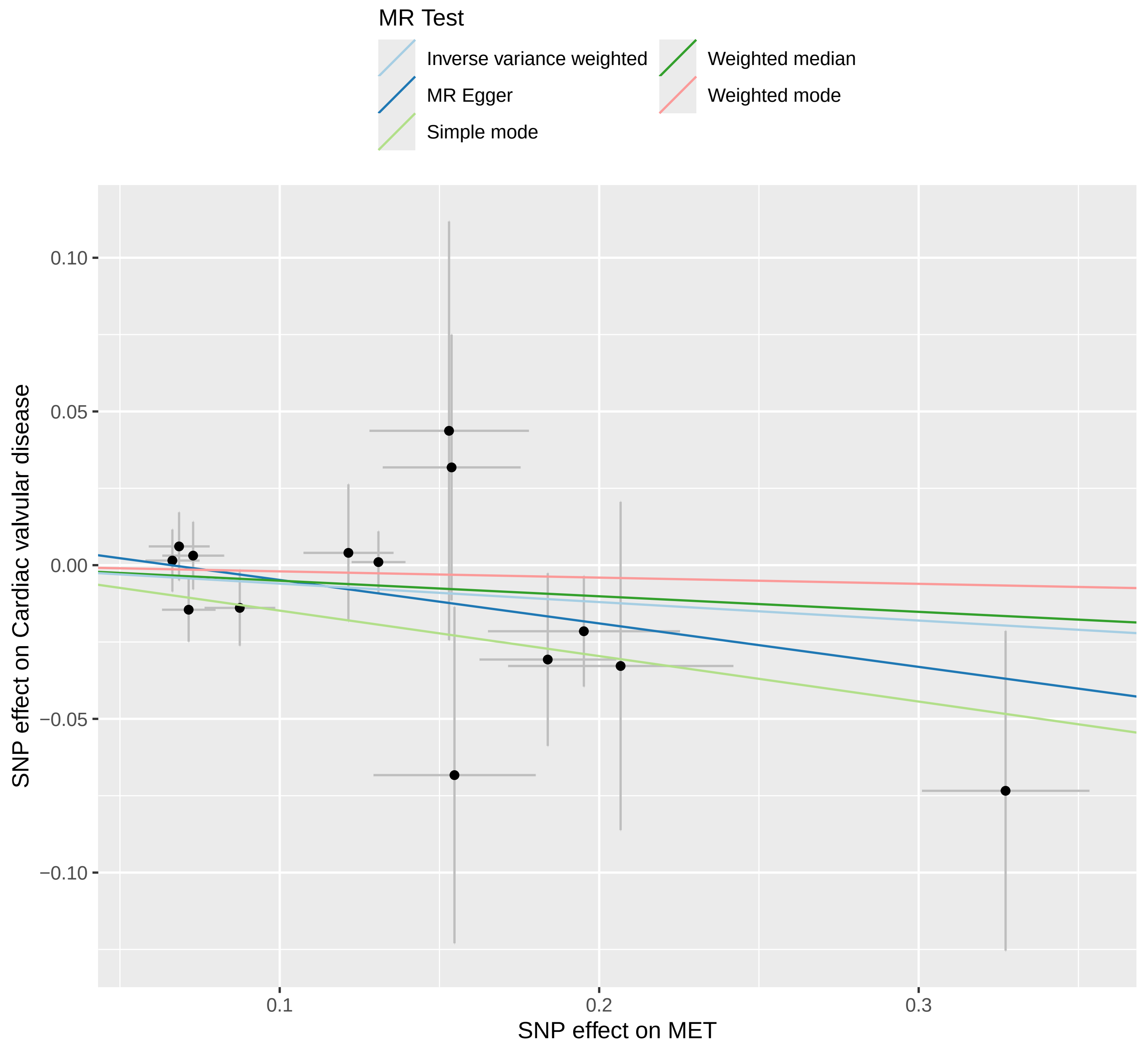

MR Test
Inverse variance weighted
MR Egger
Simple mode
Weighted median
Weighted mode
0.10
0.05
0.00
−0.05
−0.10
SNP effect on Cardiac valvular disease
0.1
0.2
0.3
SNP effect on MET

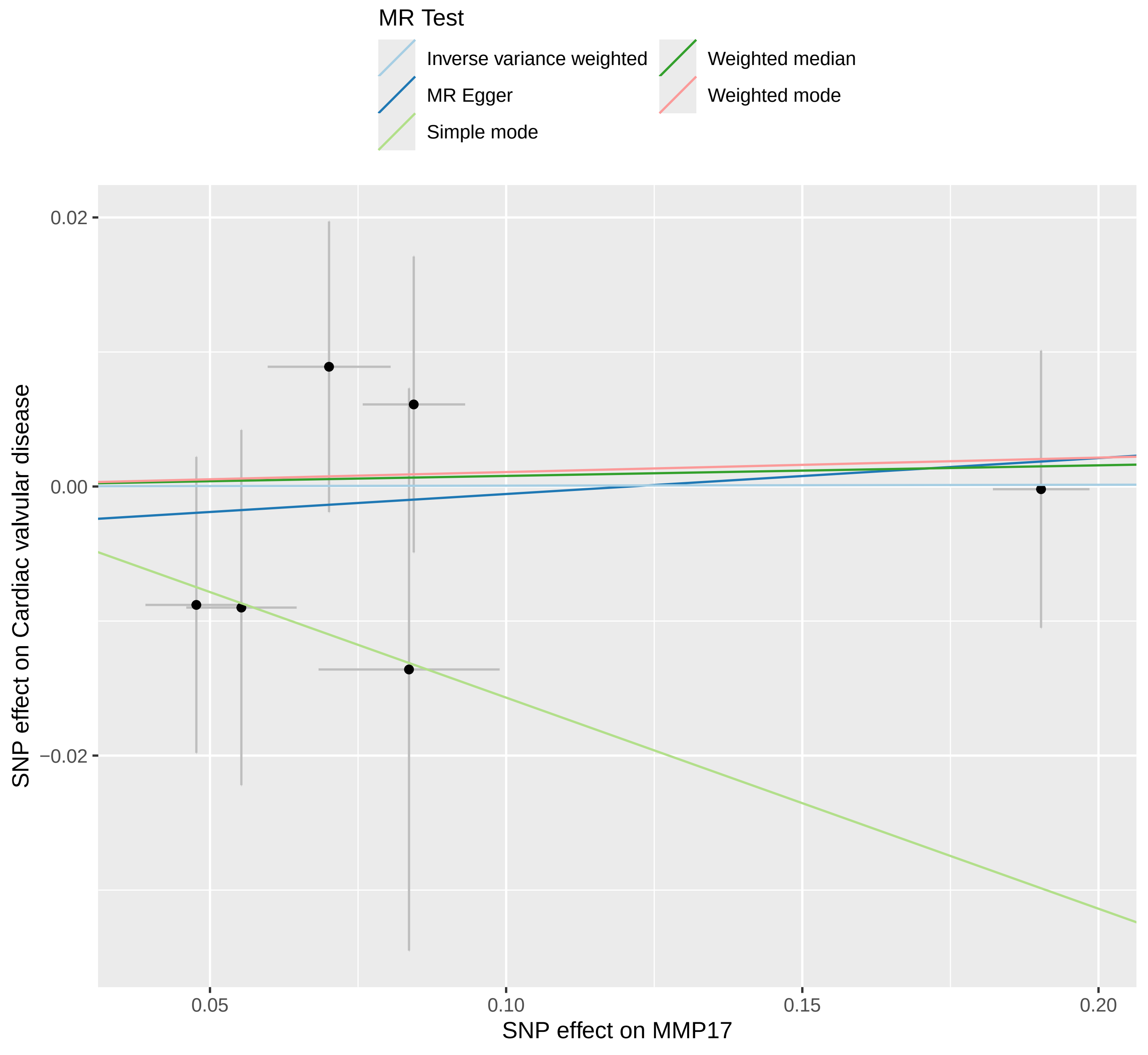
MR Test
Inverse variance weighted
MR Egger
Simple mode
Weighted median
Weighted mode
0.02
0.00
−0.02
0.05
0.10
0.15
0.20
SNP effect on Cardiac valvular disease
SNP effect on MMP17

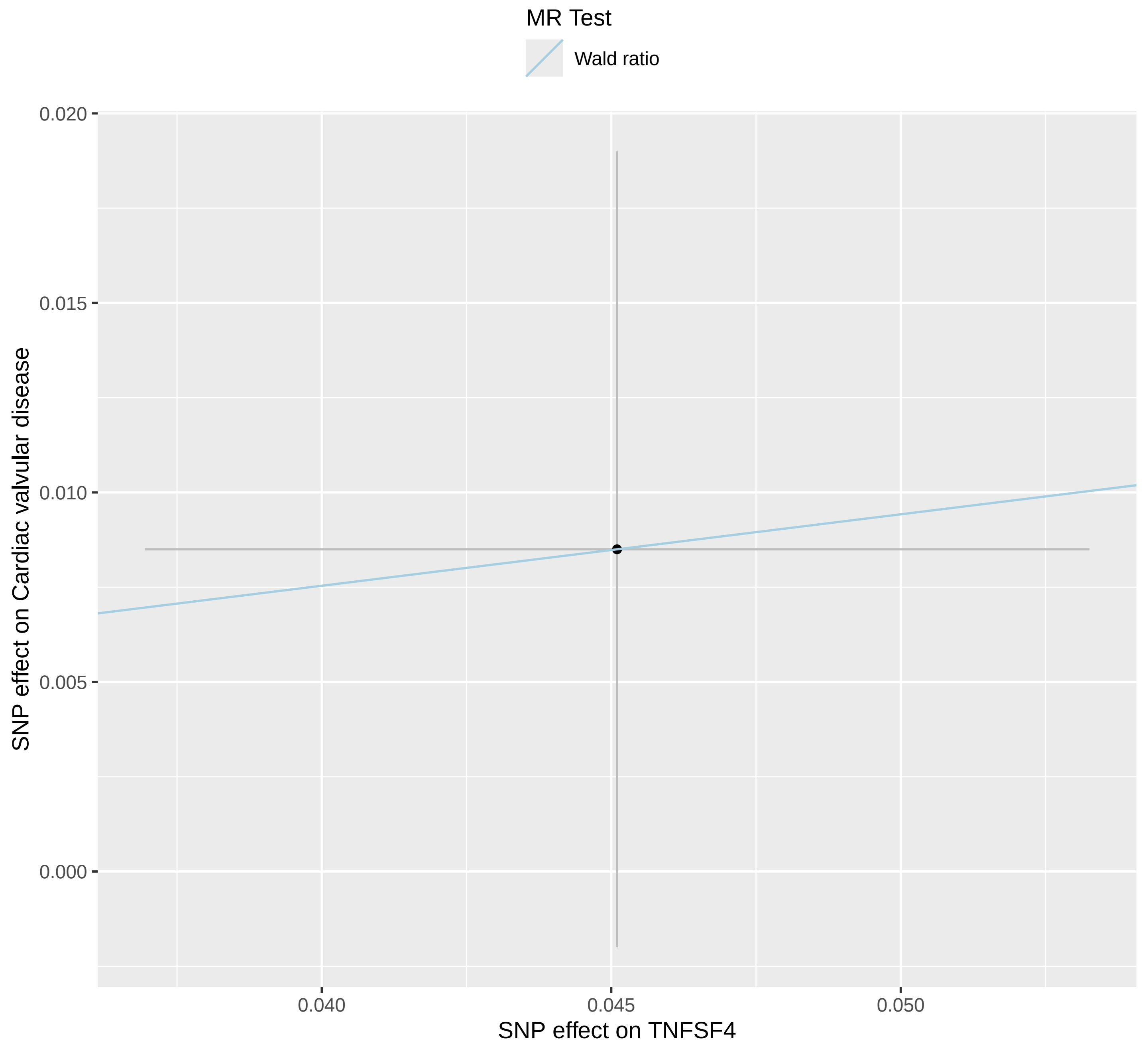
MR Test
Wald ratio
0.020
0.015
0.010
0.005
0.000
SNP effect on Cardiac valvular disease
0.040
0.045
0.050
SNP effect on TNFSF4

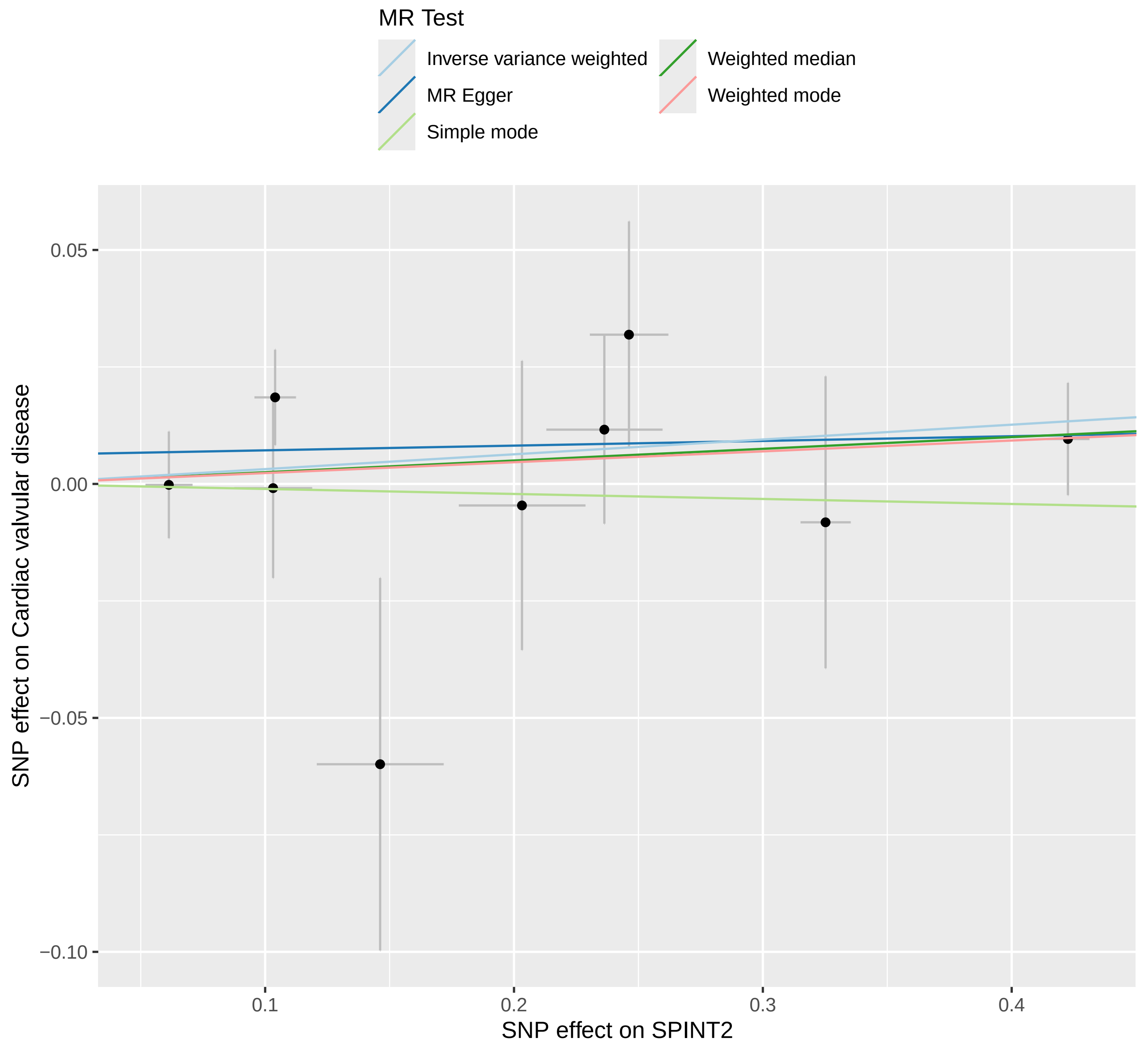

MR Test
Inverse variance weighted
MR Egger
Simple mode
Weighted median
Weighted mode
0.05
0.00
−0.05
−0.10
0.1
0.2
0.3
0.4
SNP effect on Cardiac valvular disease
SNP effect on SPINT2

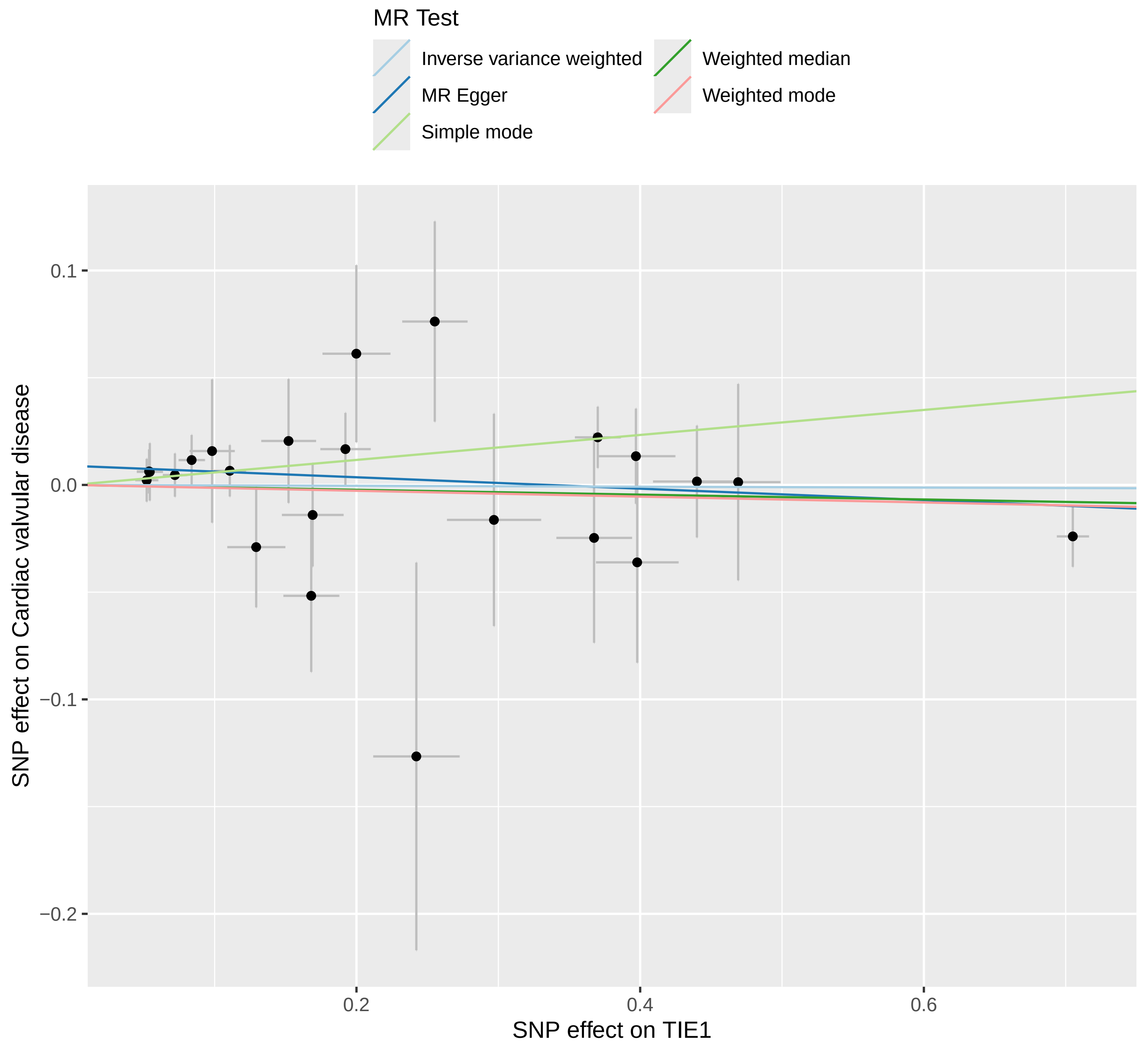

MR Test
Inverse variance weighted
MR Egger
Simple mode
Weighted median
Weighted mode
0.1
0.0
−0.1
−0.2
0.2
0.4
0.6
SNP effect on Cardiac valvular disease
SNP effect on TIE1

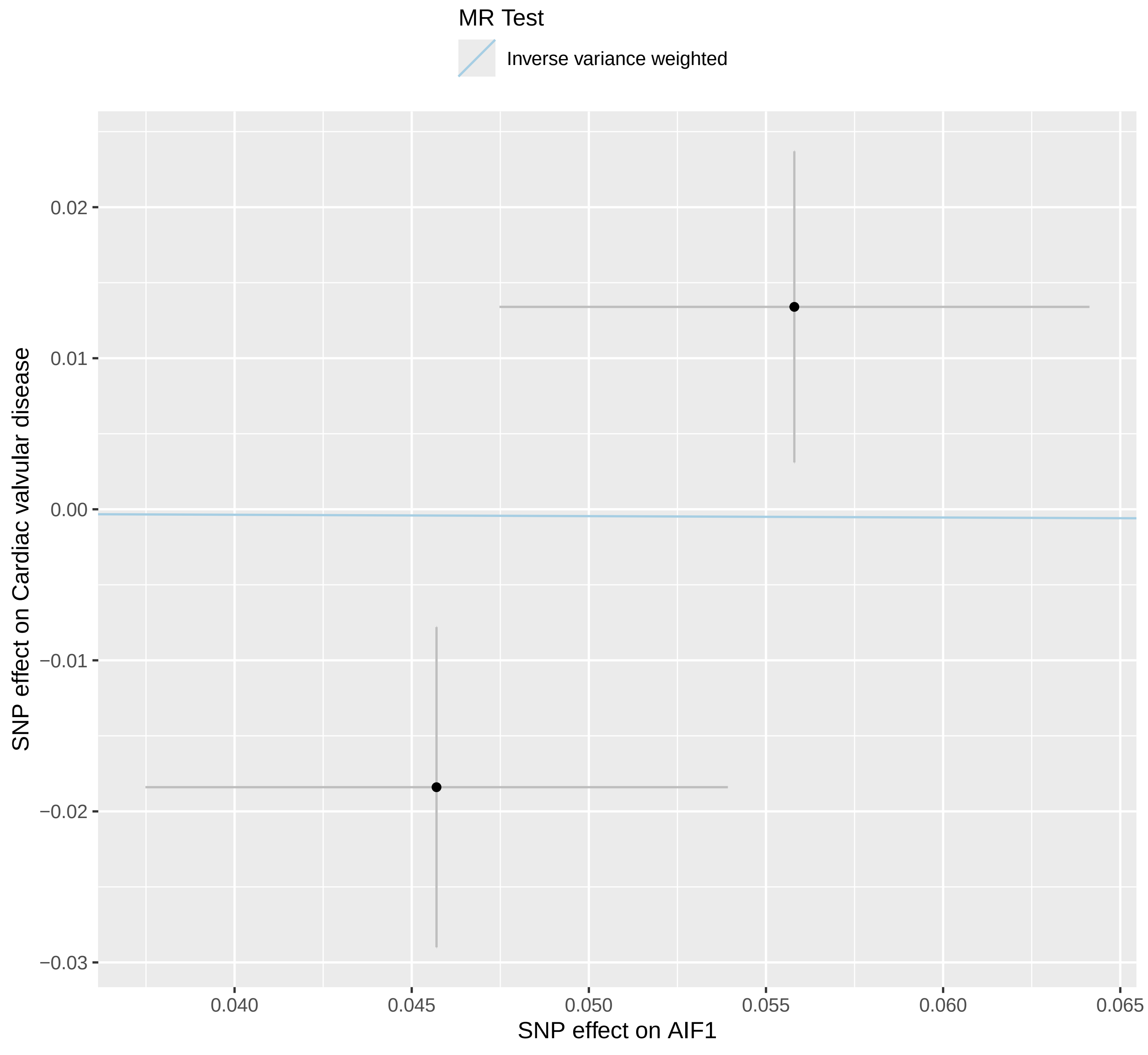
MR Test
Inverse variance weighted
SNP effect on Cardiac valvular disease
0.02
0.01
0.00
−0.01
−0.02
−0.03
0.040
0.045
0.050
0.055
0.060
0.065
SNP effect on AIF1

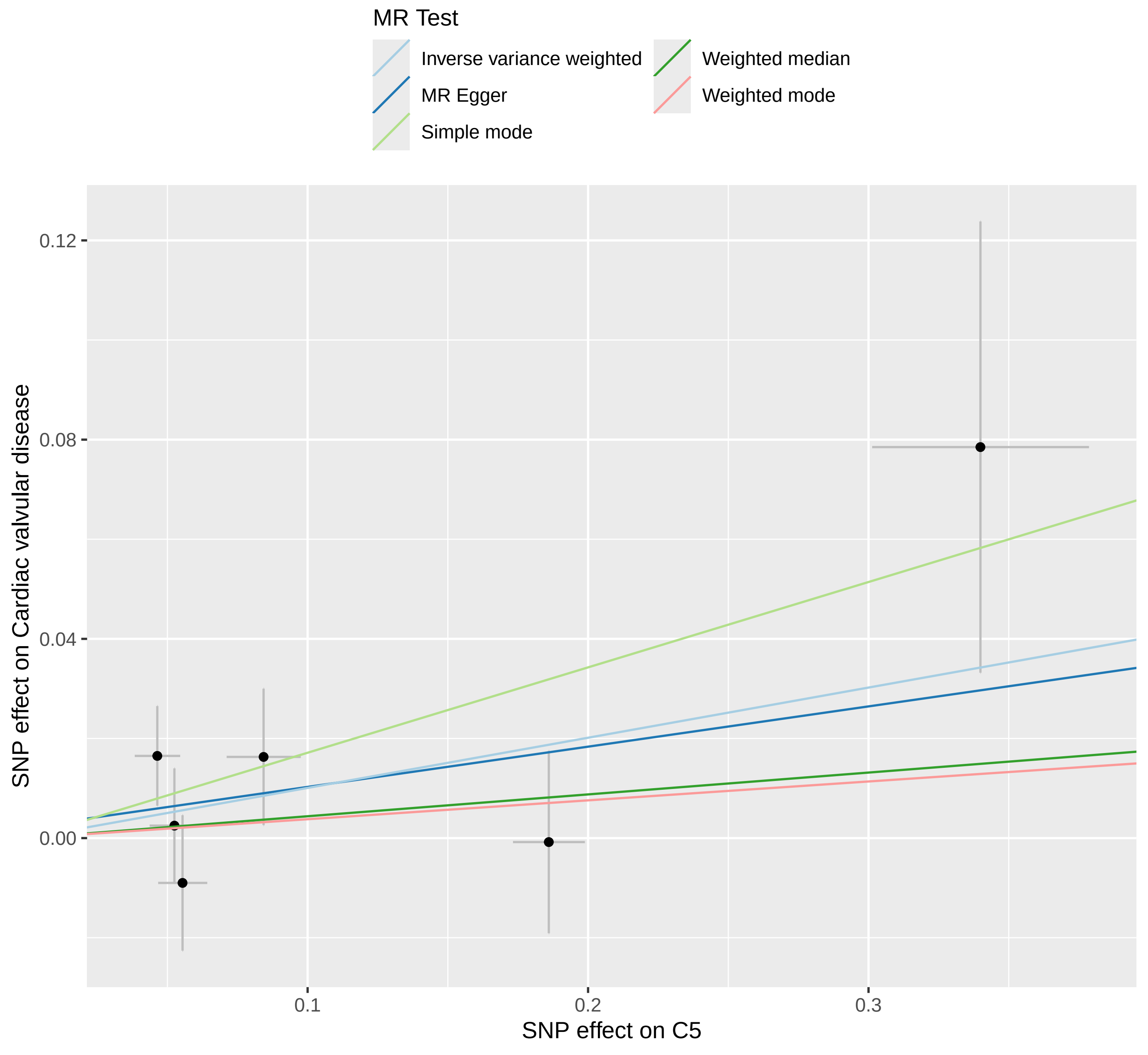

MR Test
Inverse variance weighted
MR Egger
Simple mode
Weighted median
Weighted mode
0.12
0.08
0.04
0.00
SNP effect on Cardiac valvular disease
0.1
0.2
0.3
SNP effect on C5

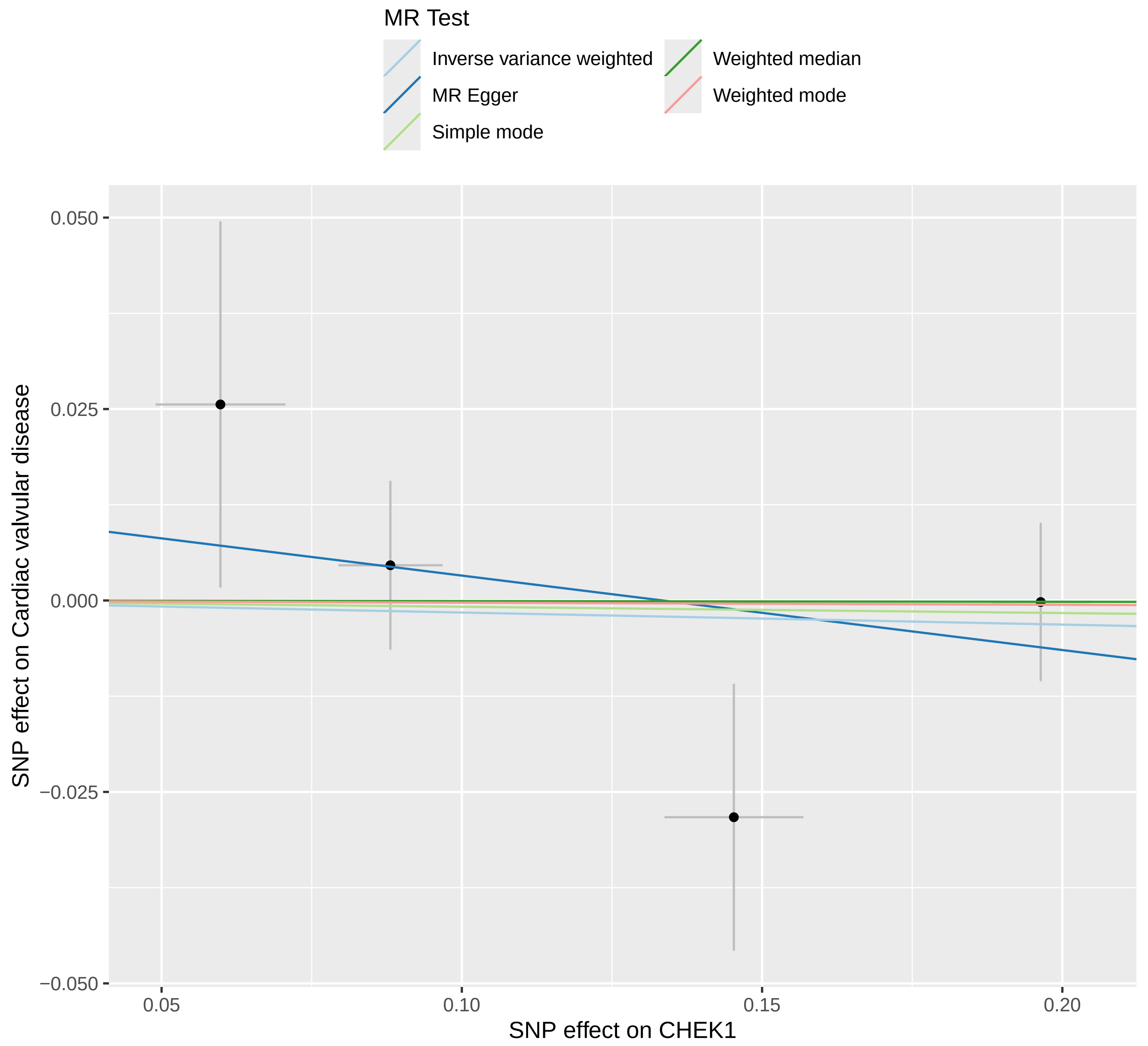
MR Test
Inverse variance weighted
MR Egger
Simple mode
Weighted median
Weighted mode
SNP effect on Cardiac valvular disease
0.050
0.025
0.000
−0.025
−0.050
0.05
0.10
0.15
0.20
SNP effect on CHEK1

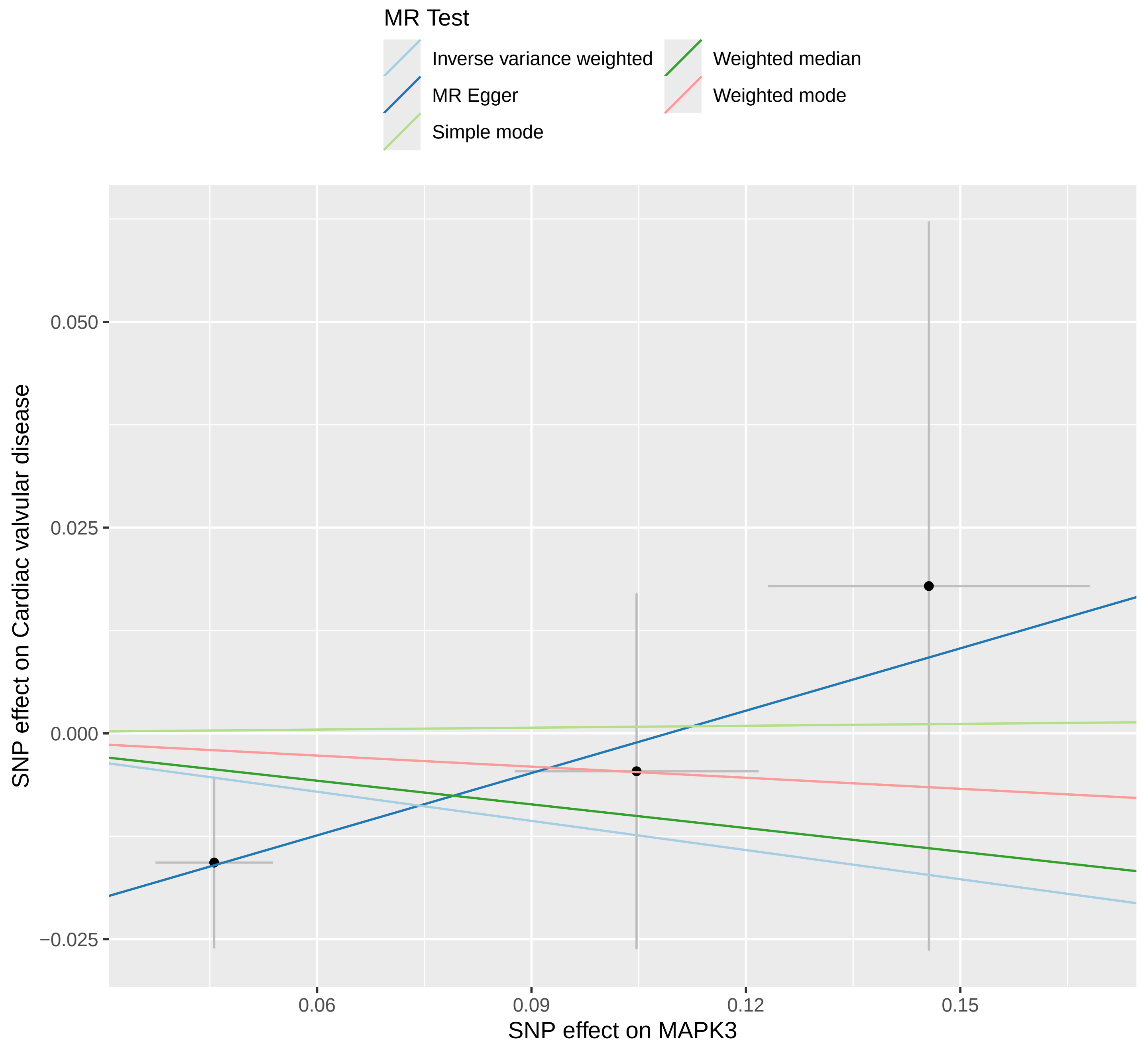
MR Test
Inverse variance weighted
MR Egger
Simple mode
Weighted median
Weighted mode
0.050
0.025
0.000
−0.025
0.06
0.09
0.12
0.15
SNP effect on Cardiac valvular disease
SNP effect on MAPK3

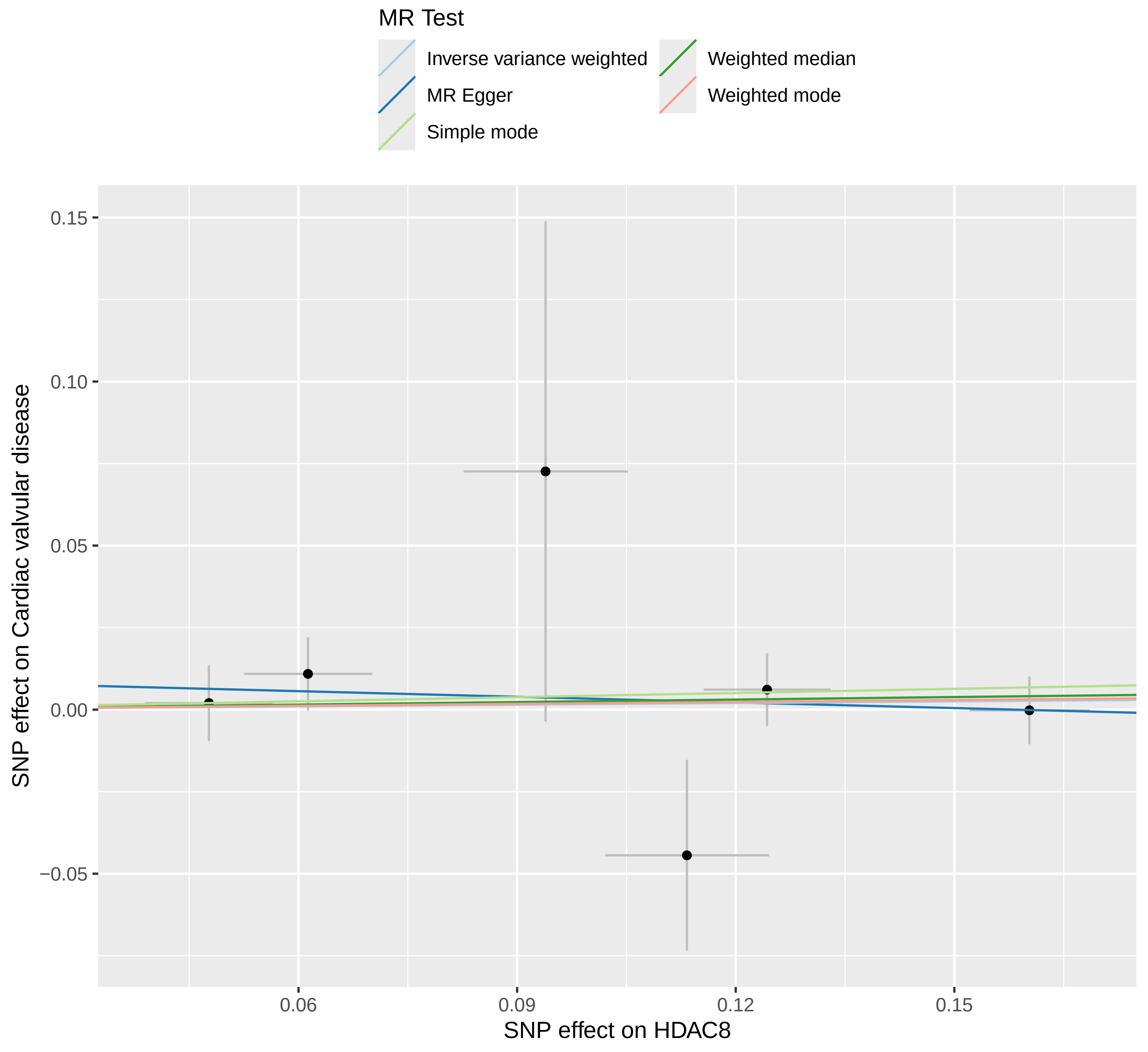
MR Test
Inverse variance weighted
MR Egger
Simple mode
Weighted median
Weighted mode
0.15
0.10
0.05
0.00
−0.05
0.06
0.09
0.12
0.15
SNP effect on Cardiac valvular disease
SNP effect on HDAC8

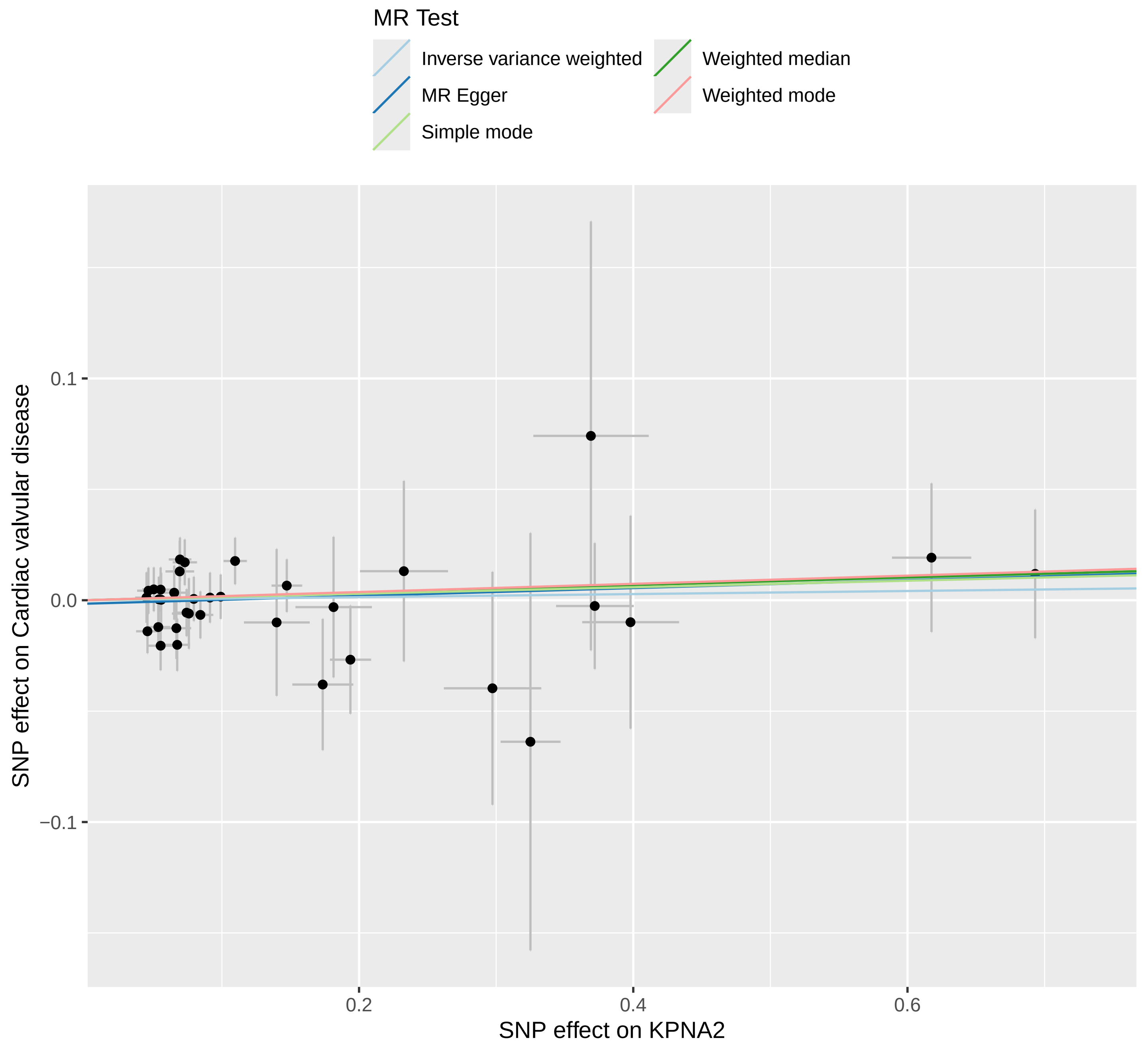
MR Test
Inverse variance weighted
MR Egger
Simple mode
Weighted median
Weighted mode
SNP effect on Cardiac valvular disease
0.1
0.0
−0.1
0.2
0.4
0.6
SNP effect on KPNA2

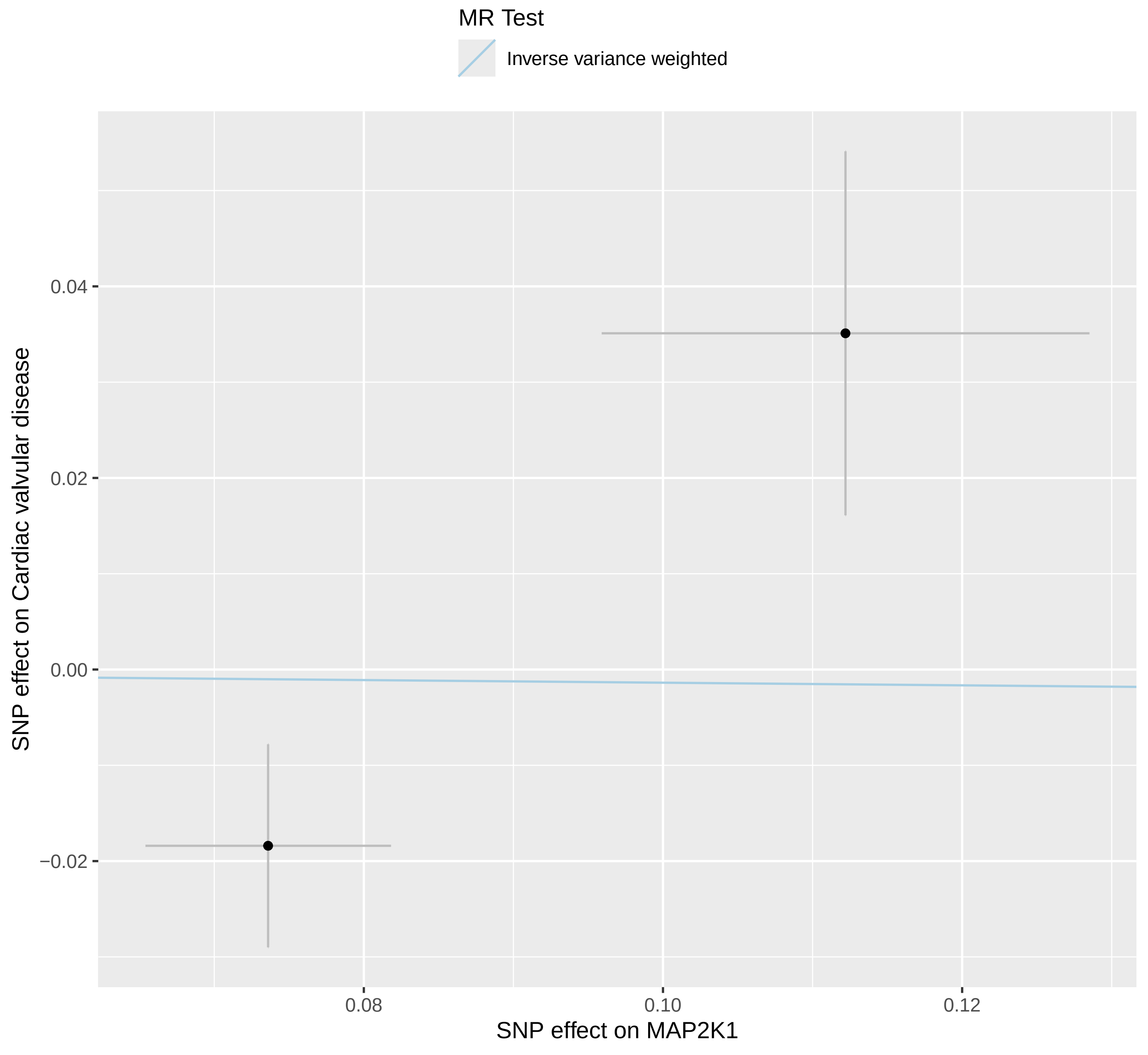

MR Test
Inverse variance weighted
0.04
0.02
0.00
−0.02
0.08
0.10
0.12
SNP effect on Cardiac valvular disease
SNP effect on MAP2K1

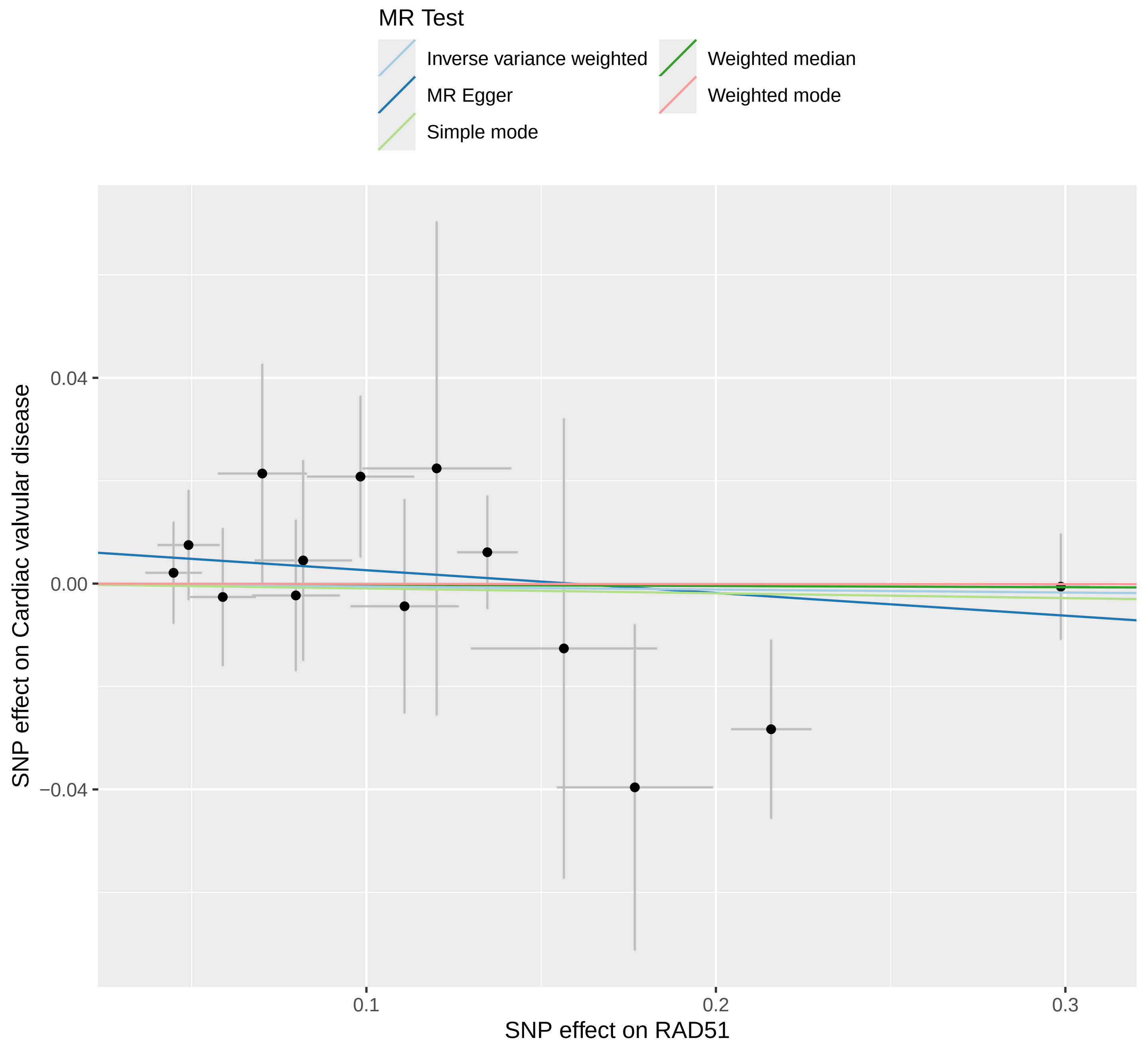
MR Test
Inverse variance weighted
MR Egger
Simple mode
Weighted median
Weighted mode
0.04
0.00
−0.04
SNP effect on Cardiac valvular disease
0.1
0.2
0.3
SNP effect on RAD51

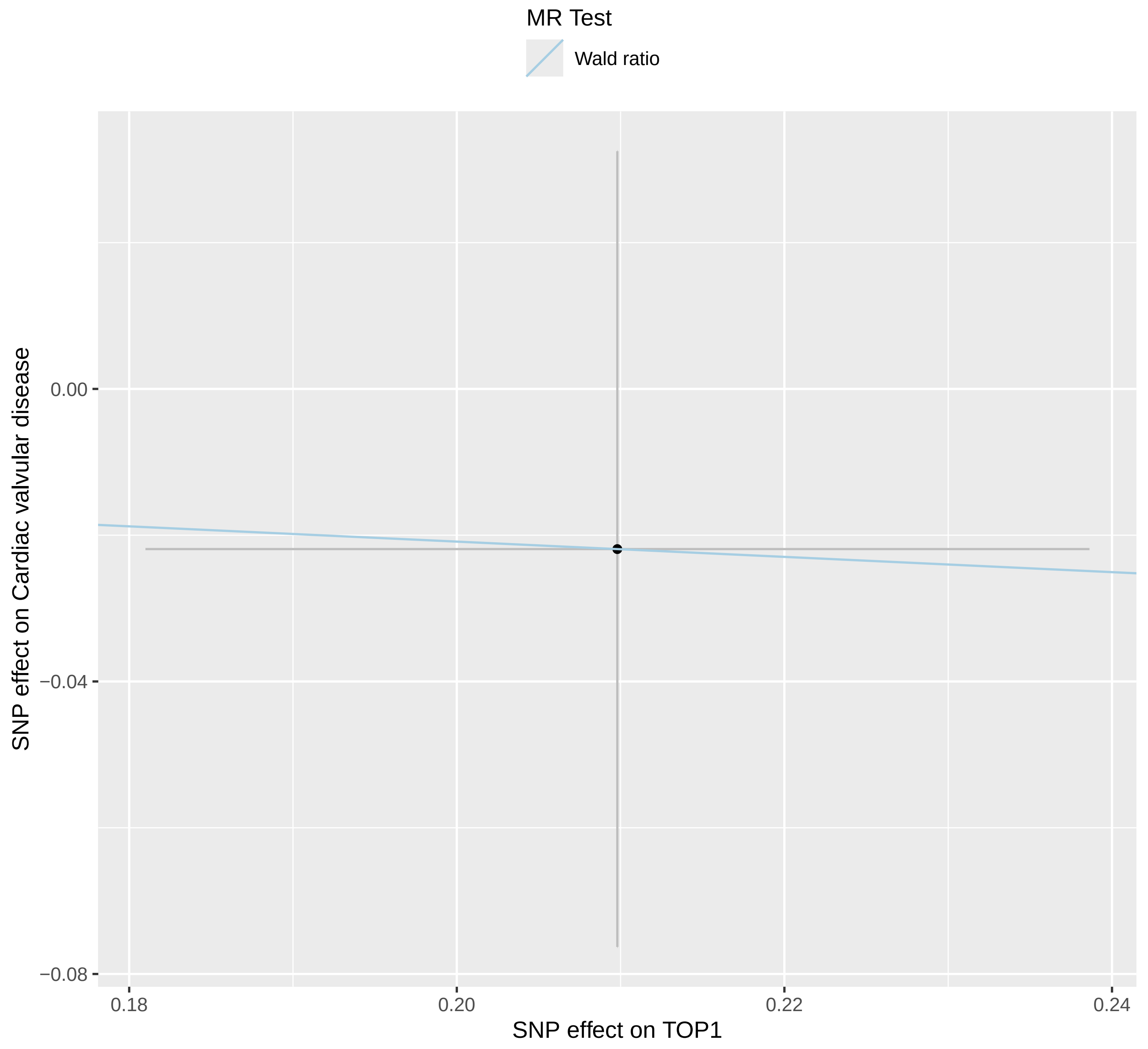

MR Test
Wald ratio
SNP effect on Cardiac valvular disease
0.00
−0.04
−0.08
0.18
0.20
0.22
0.24
SNP effect on TOP1

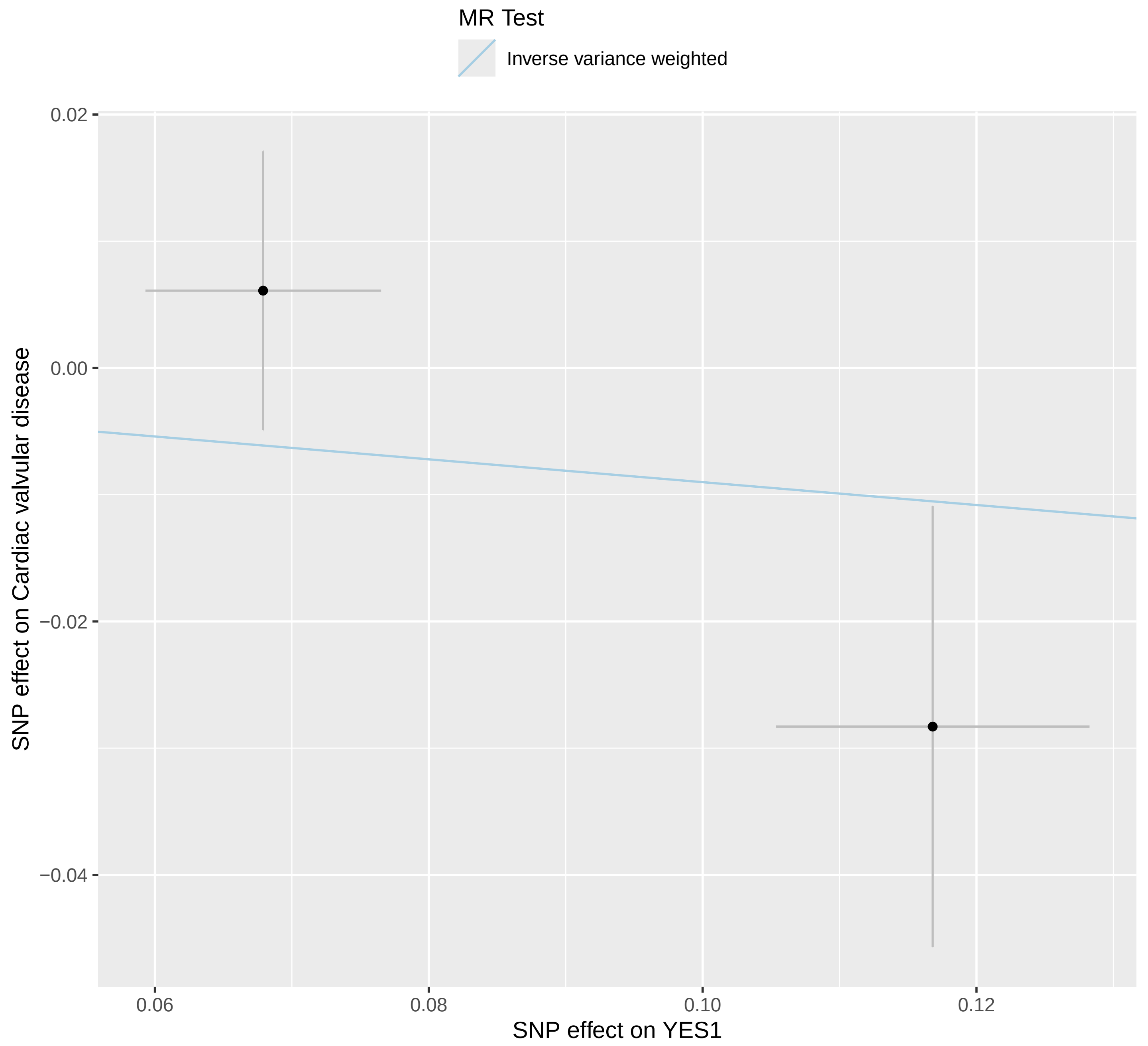
MR Test
Inverse variance weighted
0.02
0.00
−0.02
−0.04
0.06
0.08
0.10
0.12
SNP effect on YES1
SNP effect on Cardiac valvular disease

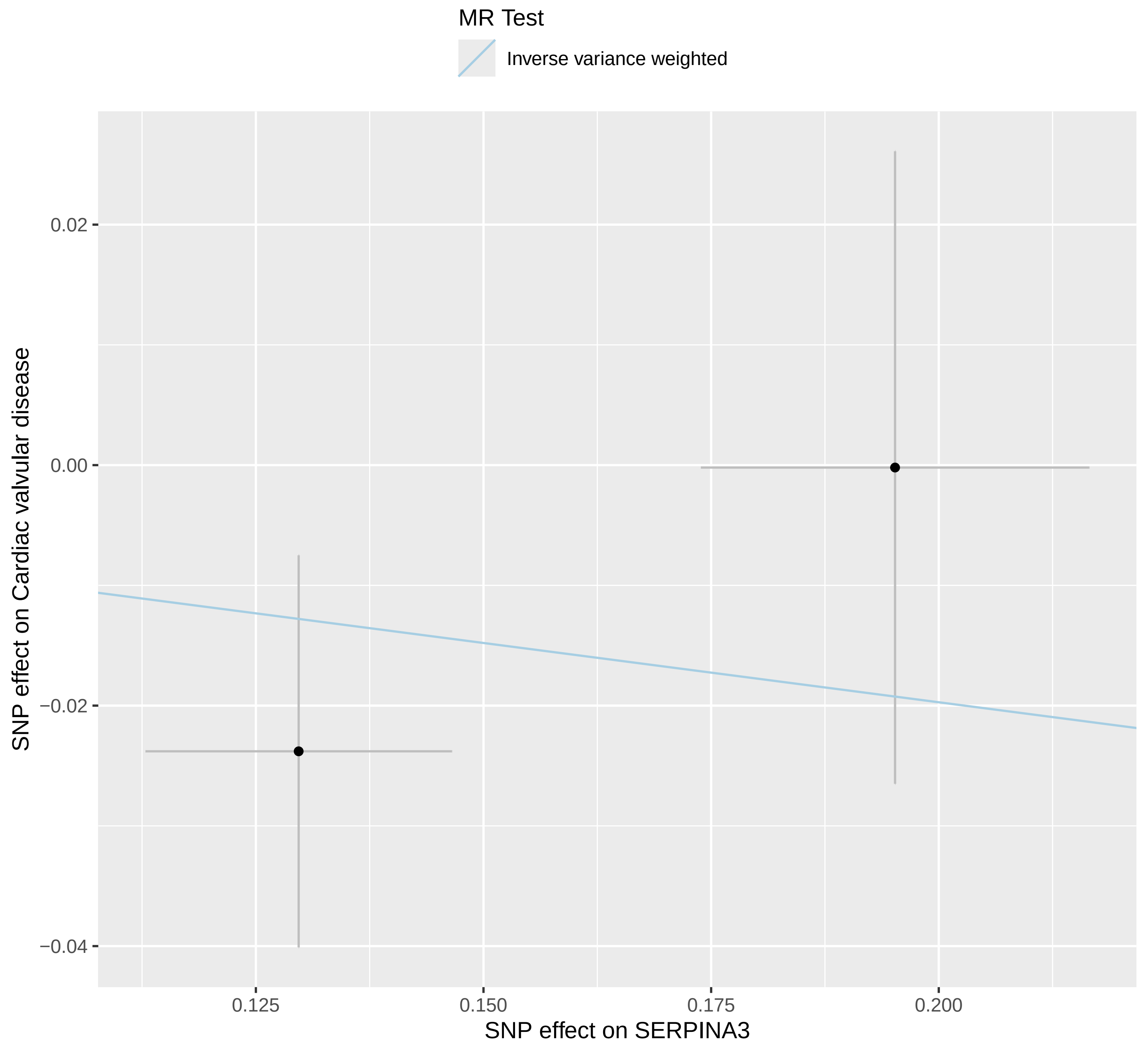
MR Test
Inverse variance weighted
0.02
0.00
−0.02
−0.04
SNP effect on Cardiac valvular disease
0.125
0.150
0.175
0.200
SNP effect on SERPINA3

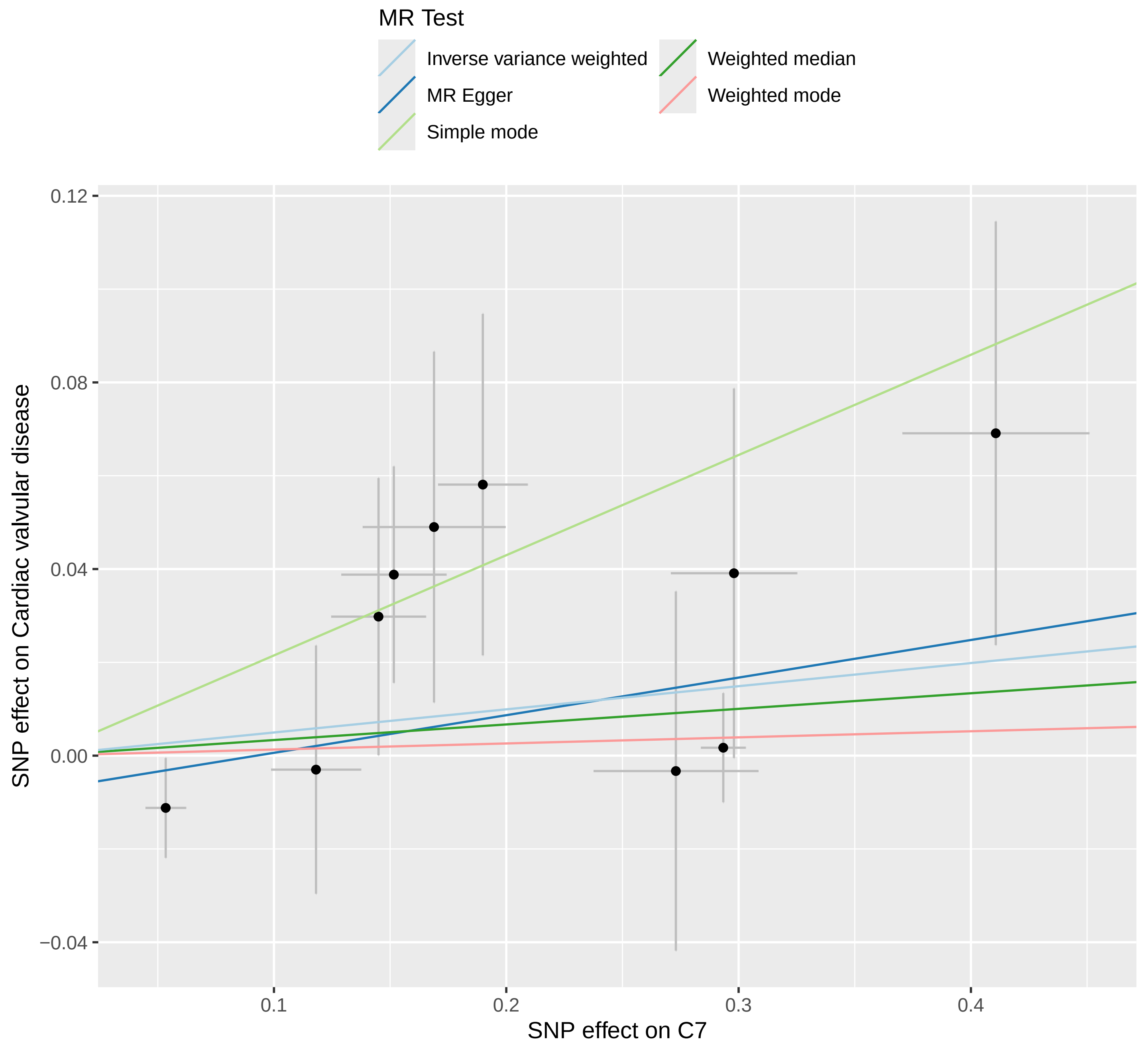

MR Test
Inverse variance weighted
MR Egger
Simple mode
Weighted median
Weighted mode
0.12
0.08
0.04
0.00
−0.04
SNP effect on Cardiac valvular disease
0.1
0.2
0.3
0.4
SNP effect on C7

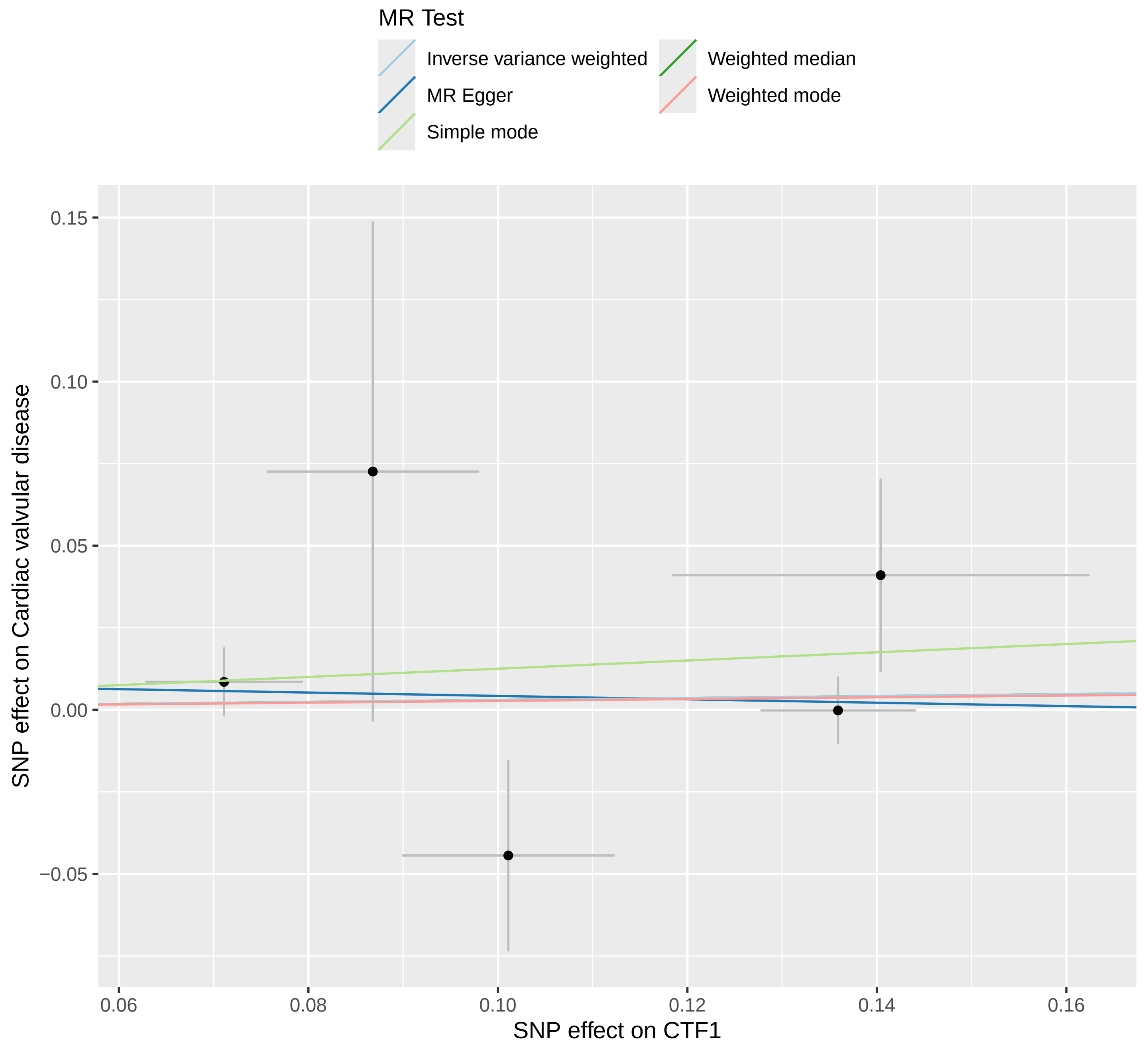

MR Test
Inverse variance weighted
MR Egger
Simple mode
Weighted median
Weighted mode
0.15
0.10
0.05
0.00
−0.05
SNP effect on Cardiac valvular disease
0.06
0.08
0.10
0.12
0.14
0.16
SNP effect on CTF1

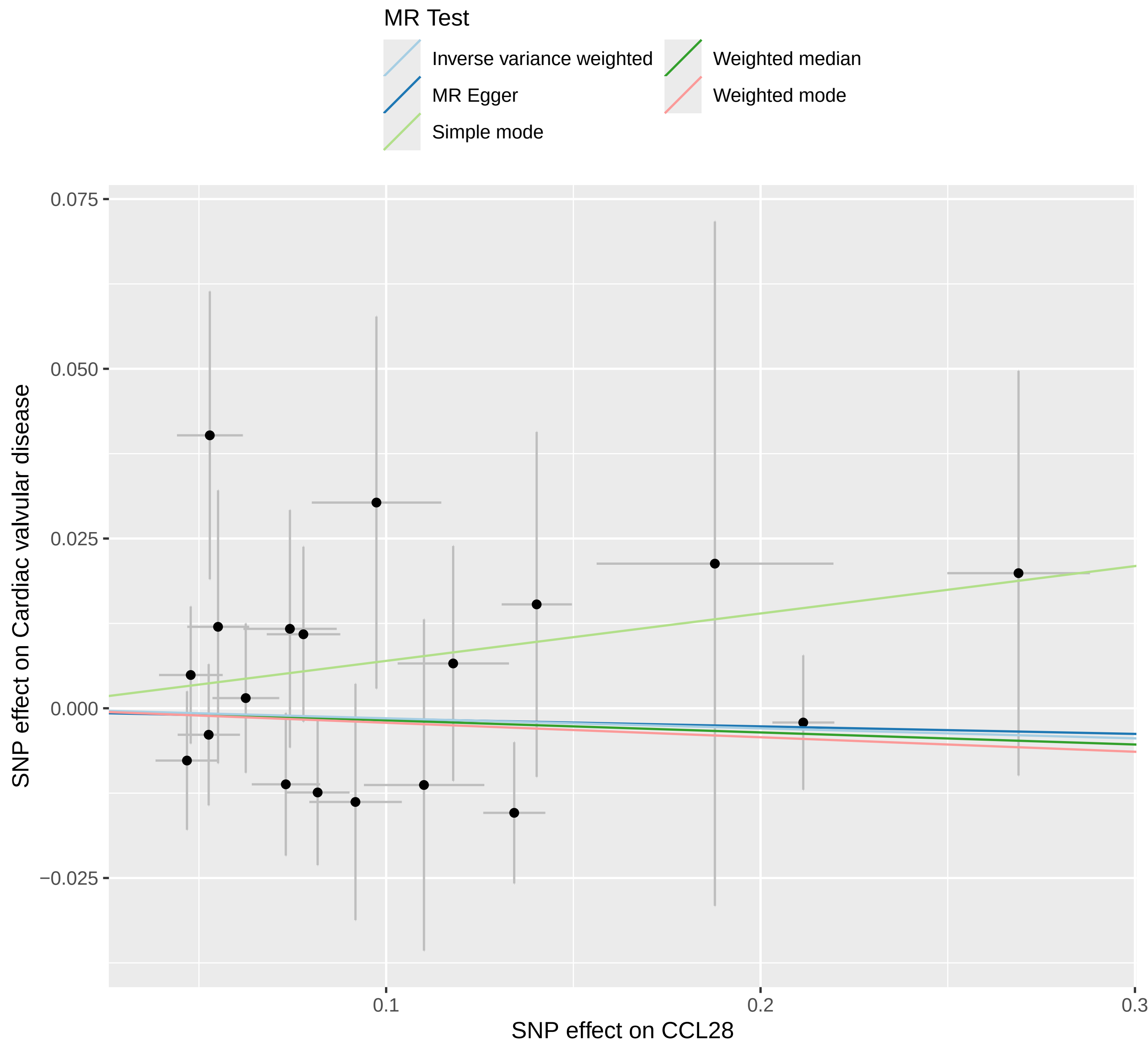
MR Test
Inverse variance weighted
MR Egger
Simple mode
Weighted median
Weighted mode
0.075
0.050
0.025
0.000
−0.025
SNP effect on Cardiac valvular disease
0.1
0.2
0.3
SNP effect on CCL28

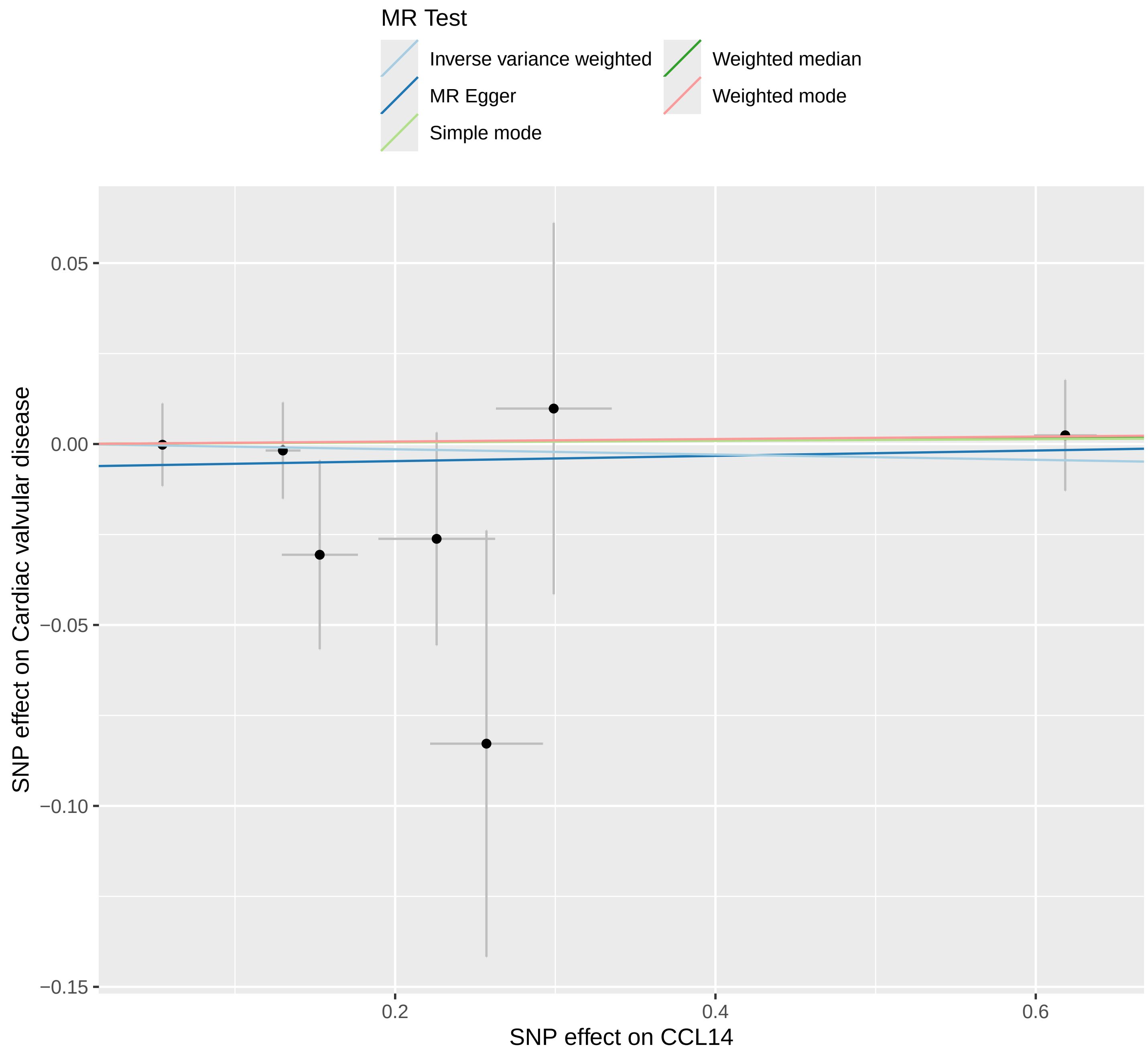
MR Test
Inverse variance weighted
MR Egger
Simple mode
Weighted median
Weighted mode
0.05
0.00
−0.05
−0.10
−0.15
0.2
0.4
0.6
SNP effect on Cardiac valvular disease
SNP effect on CCL14

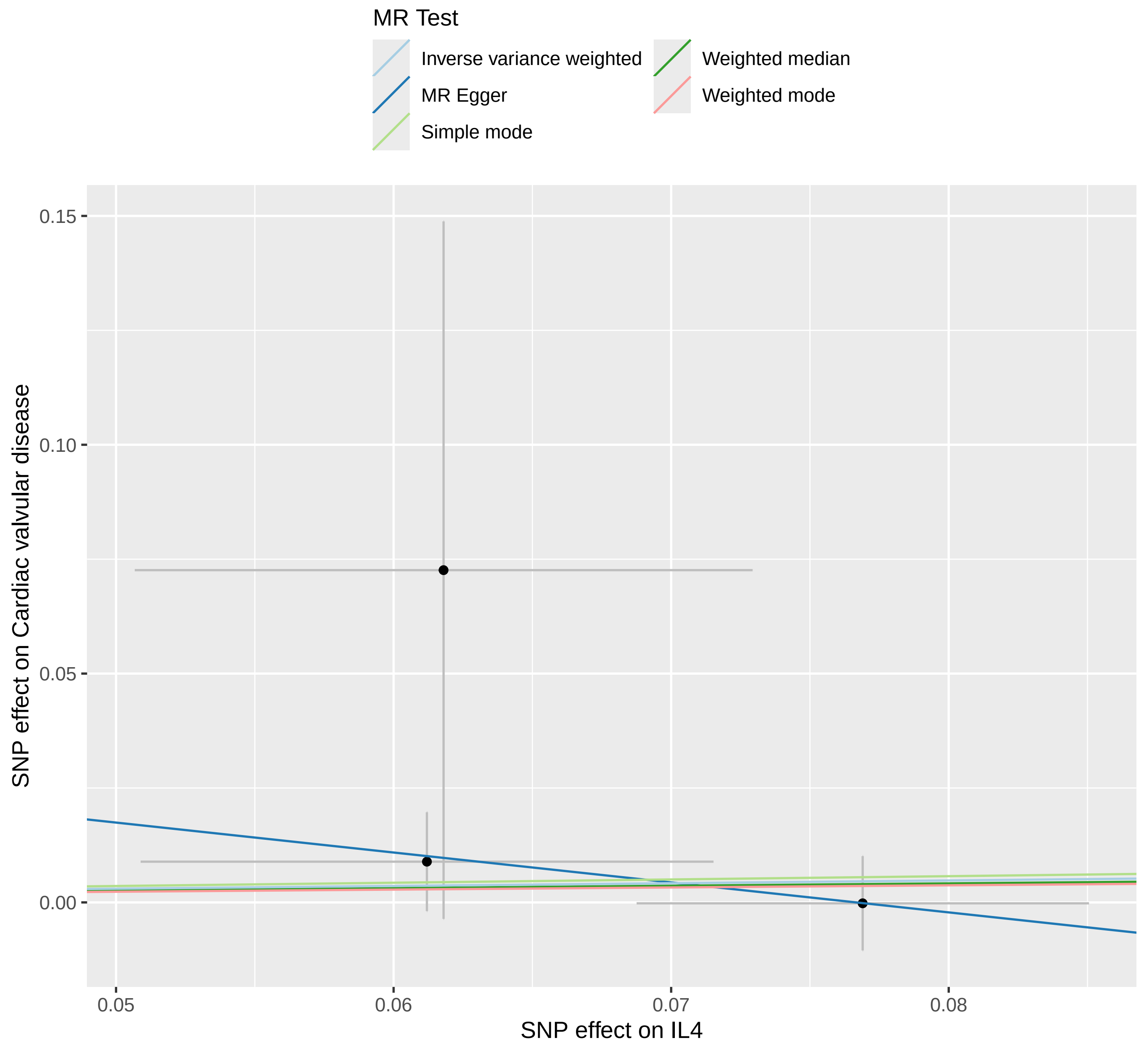
MR Test
Inverse variance weighted
MR Egger
Simple mode
Weighted median
Weighted mode
SNP effect on Cardiac valvular disease
0.15
0.10
0.05
0.00
0.05
0.06
0.07
0.08
SNP effect on IL4

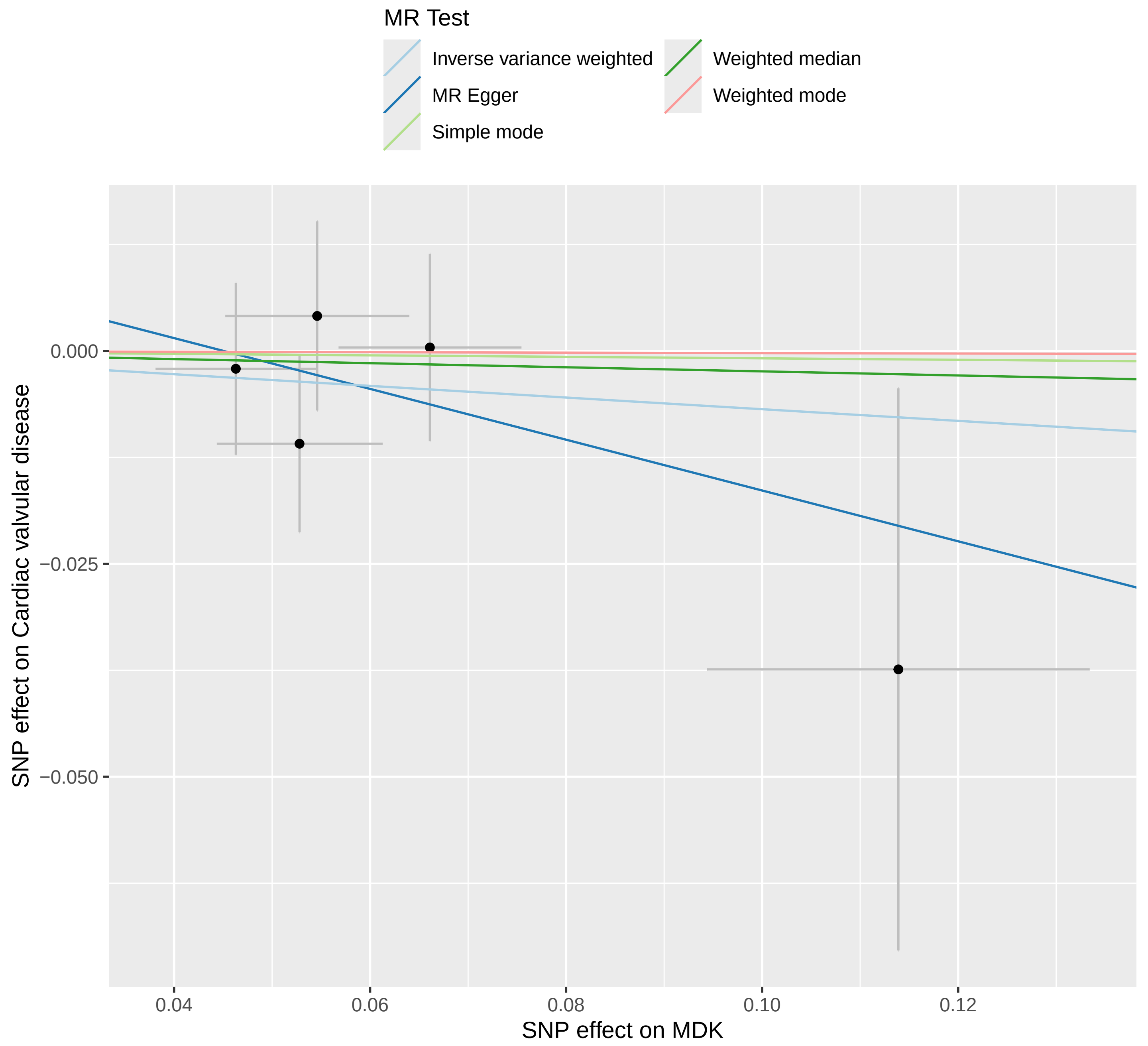

MR Test
Inverse variance weighted
MR Egger
Simple mode
Weighted median
Weighted mode
0.000
−0.025
−0.050
0.04
0.06
0.08
0.10
0.12
SNP effect on Cardiac valvular disease
SNP effect on MDK

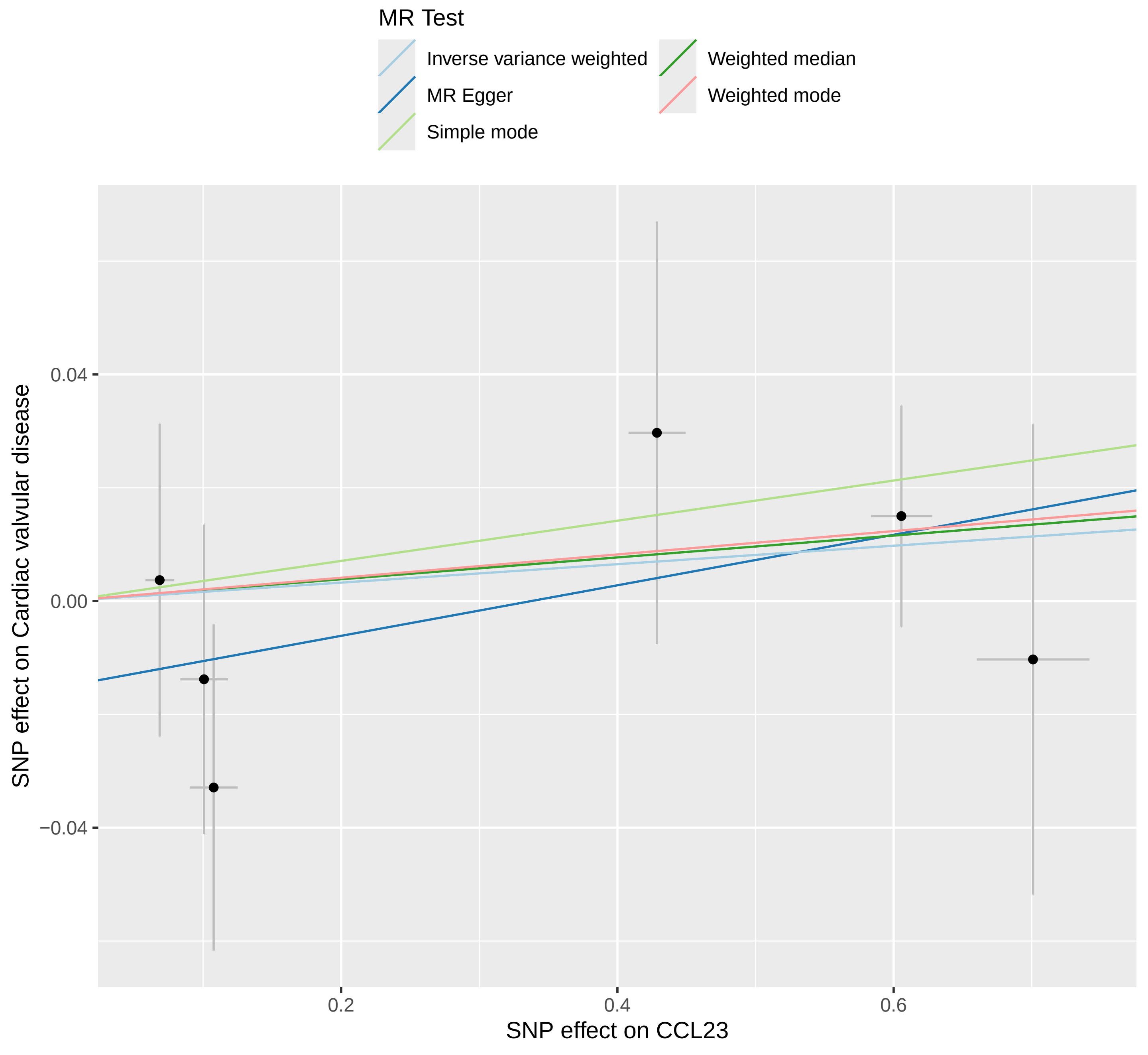
MR Test
Inverse variance weighted
MR Egger
Simple mode
Weighted median
Weighted mode
0.04
0.00
−0.04
0.2
0.4
0.6
SNP effect on Cardiac valvular disease
SNP effect on CCL23

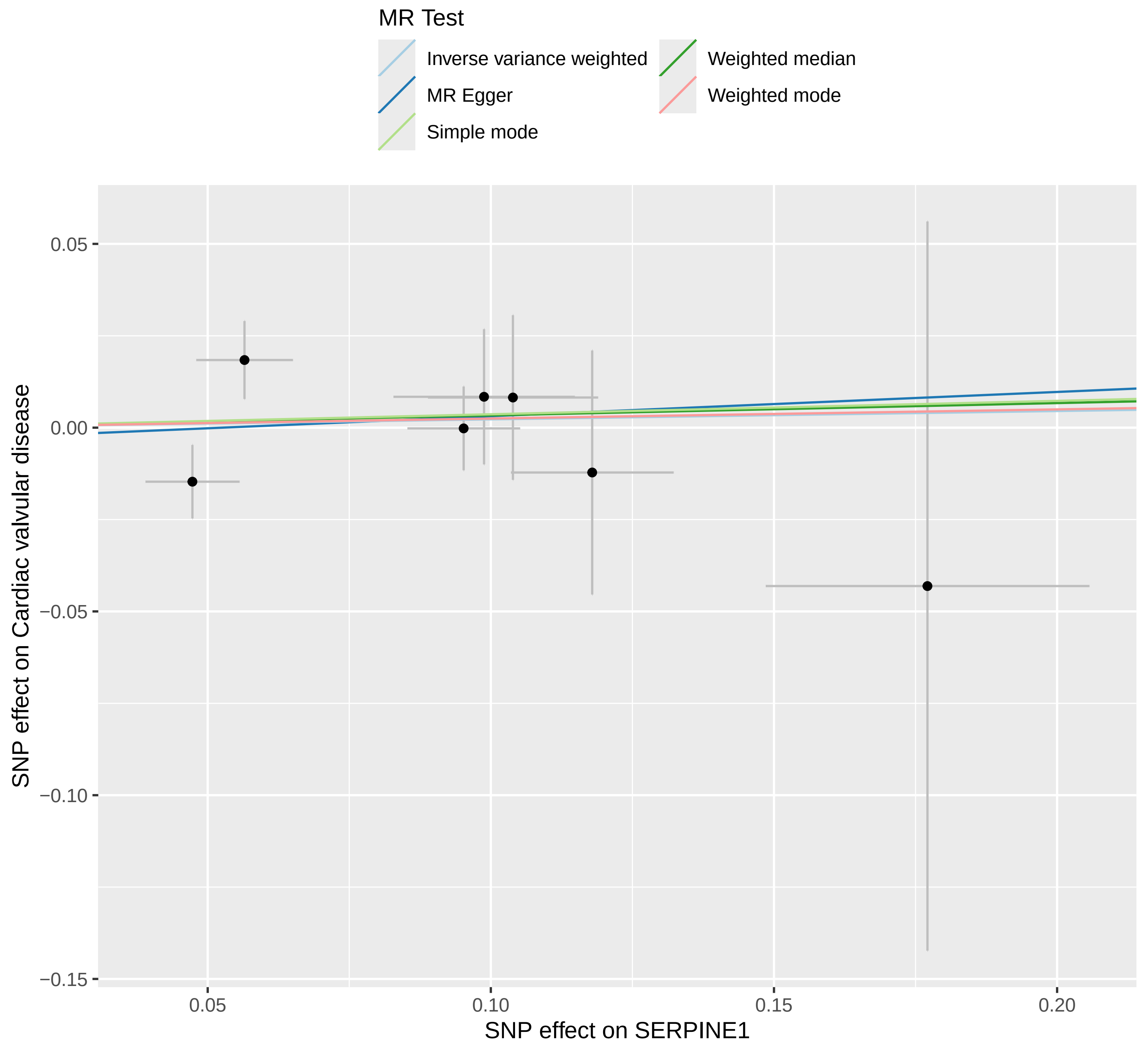
MR Test
Inverse variance weighted
MR Egger
Simple mode
Weighted median
Weighted mode
0.05
0.00
−0.05
−0.10
−0.15
SNP effect on Cardiac valvular disease
0.05
0.10
0.15
0.20
SNP effect on SERPINE1

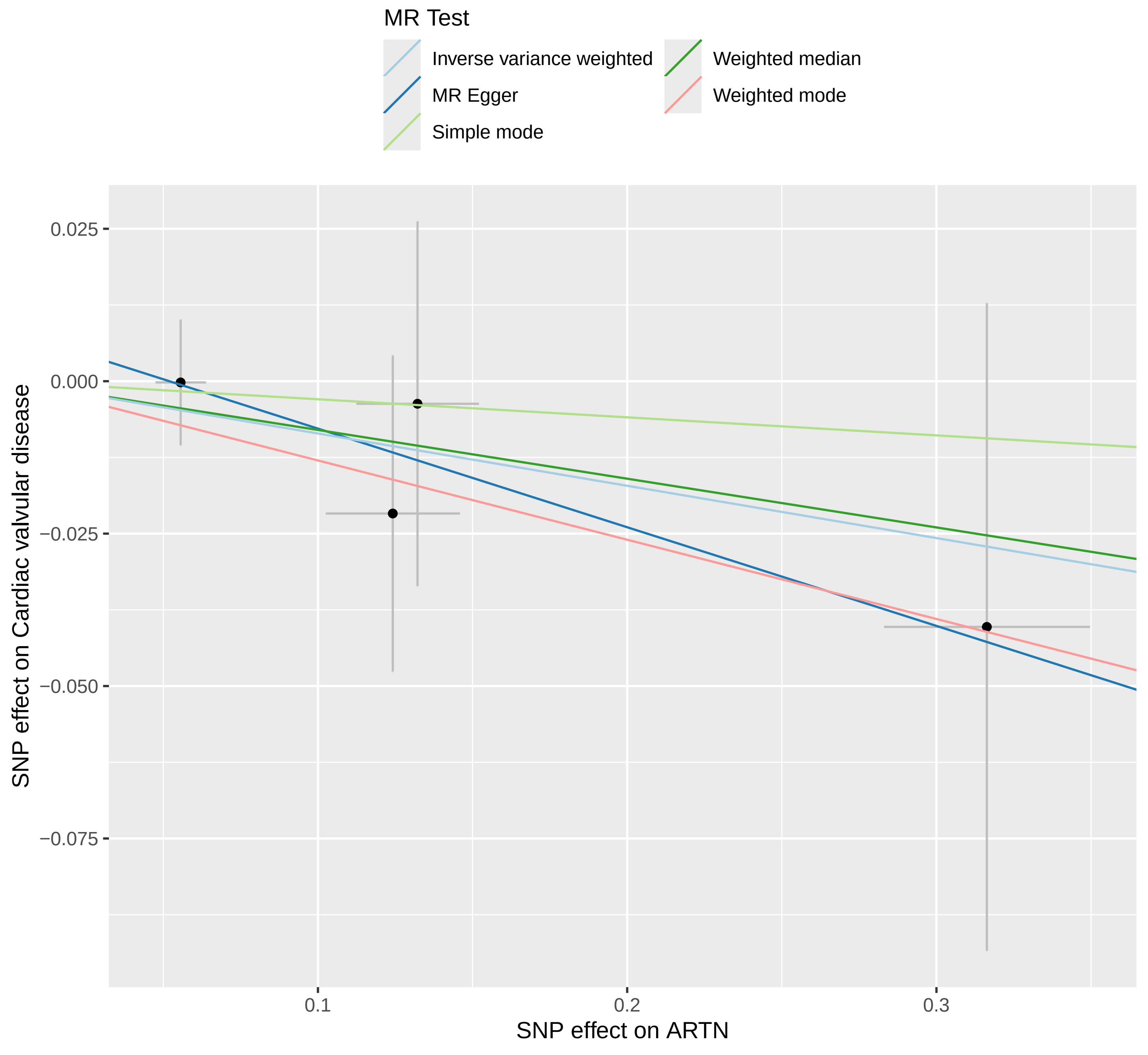
MR Test
Inverse variance weighted
MR Egger
Simple mode
Weighted median
Weighted mode
0.025
0.000
−0.025
−0.050
−0.075
SNP effect on Cardiac valvular disease
0.1
0.2
0.3
SNP effect on ARTN

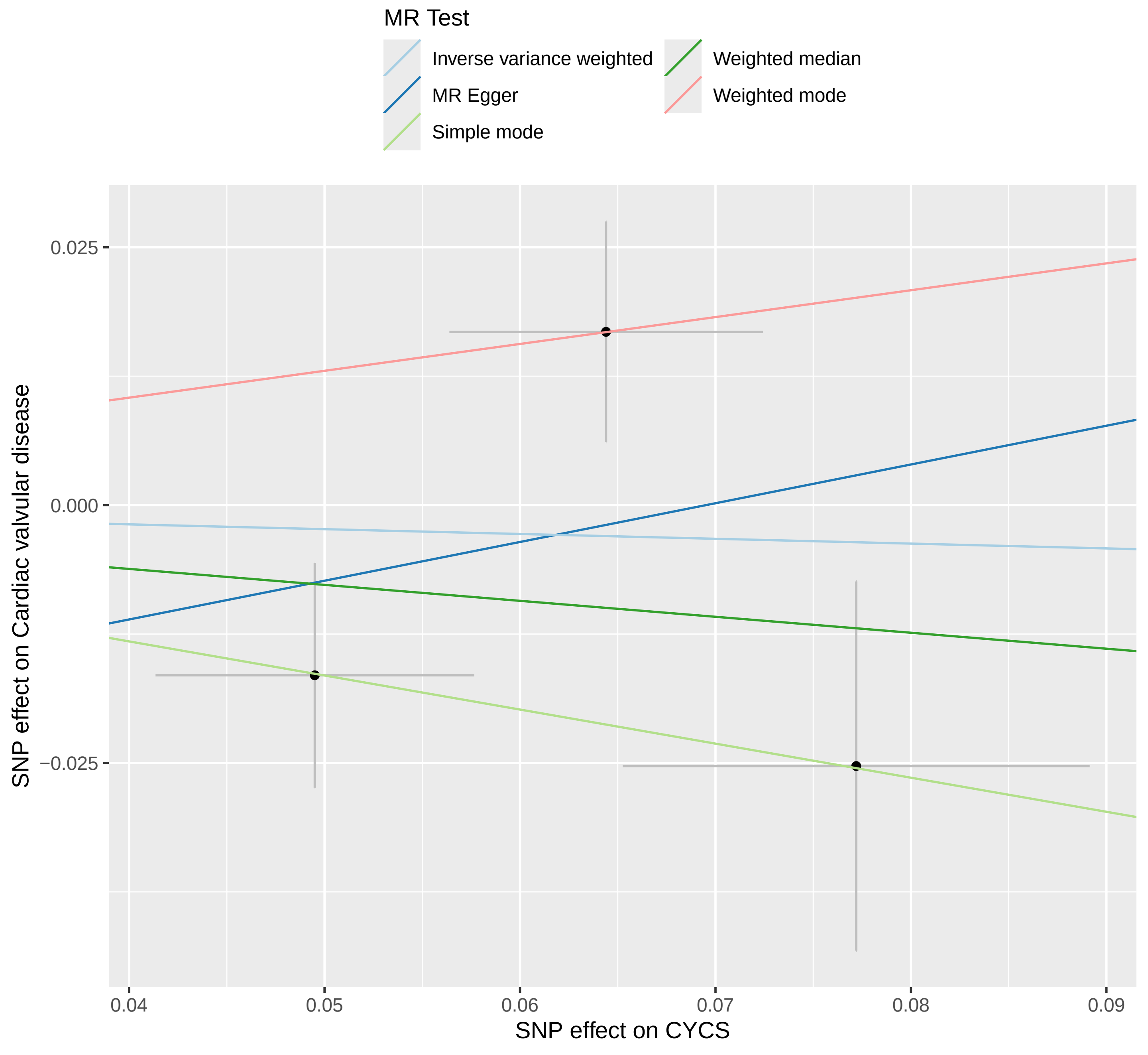

MR Test
Inverse variance weighted
MR Egger
Simple mode
Weighted median
Weighted mode
0.025
0.000
−0.025
SNP effect on Cardiac valvular disease
0.04
0.05
0.06
0.07
0.08
0.09
SNP effect on CYCS

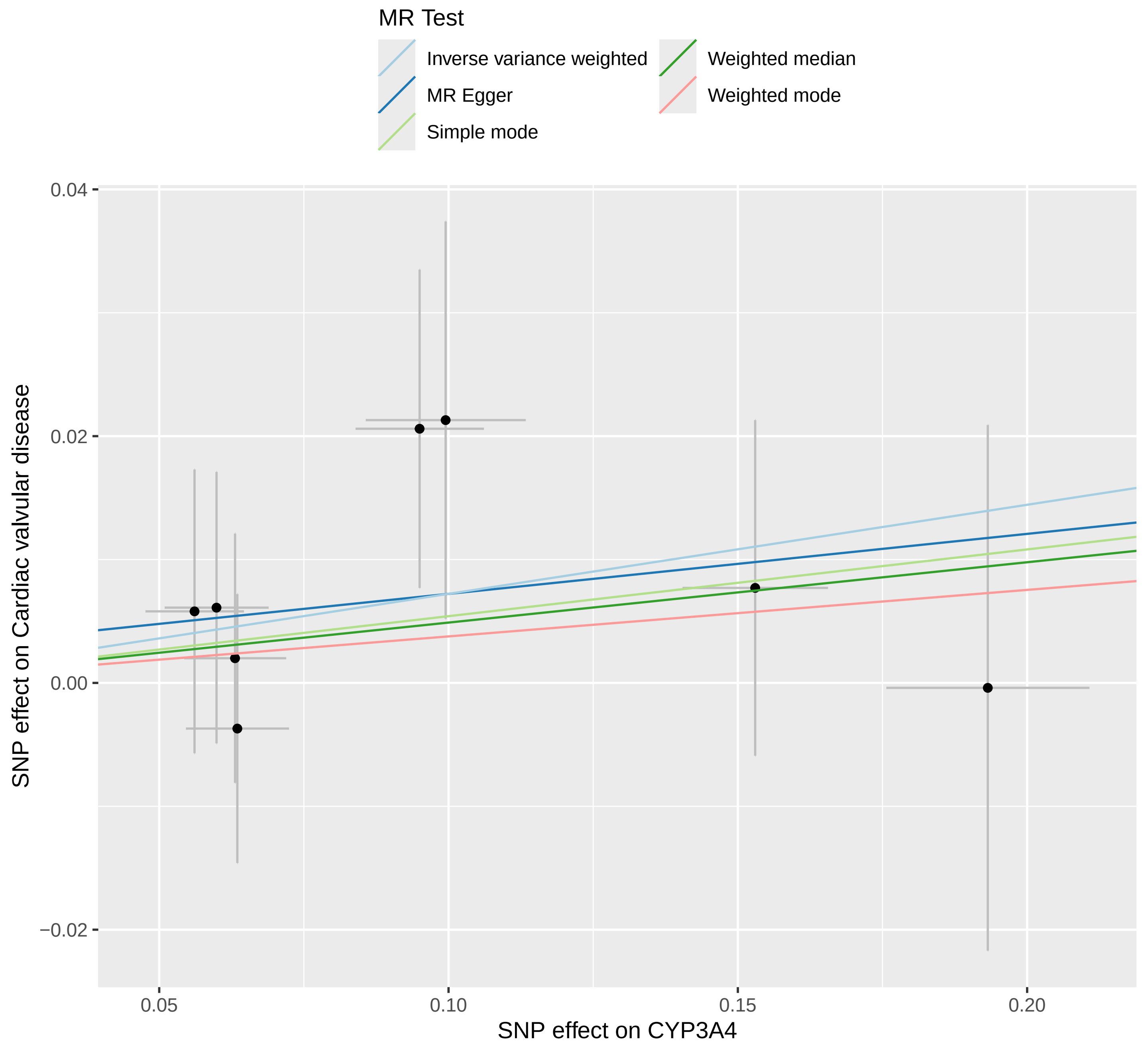
MR Test
Inverse variance weighted
MR Egger
Simple mode
Weighted median
Weighted mode
0.04
0.02
0.00
−0.02
0.05
0.10
0.15
0.20
SNP effect on Cardiac valvular disease
SNP effect on CYP3A4

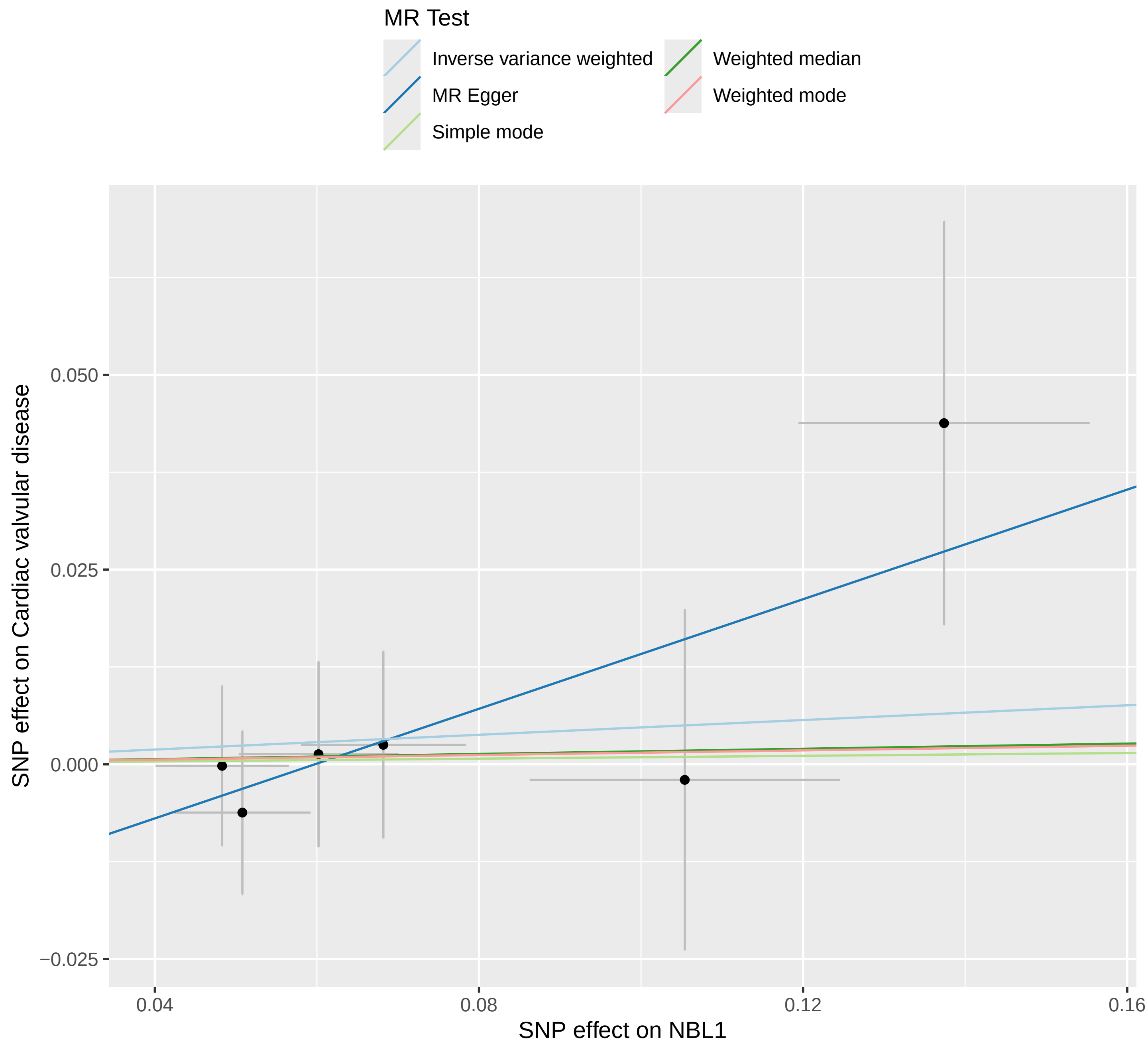
MR Test
Inverse variance weighted
MR Egger
Simple mode
Weighted median
Weighted mode
0.050
0.025
0.000
−0.025
0.04
0.08
0.12
0.16
SNP effect on Cardiac valvular disease
SNP effect on NBL1

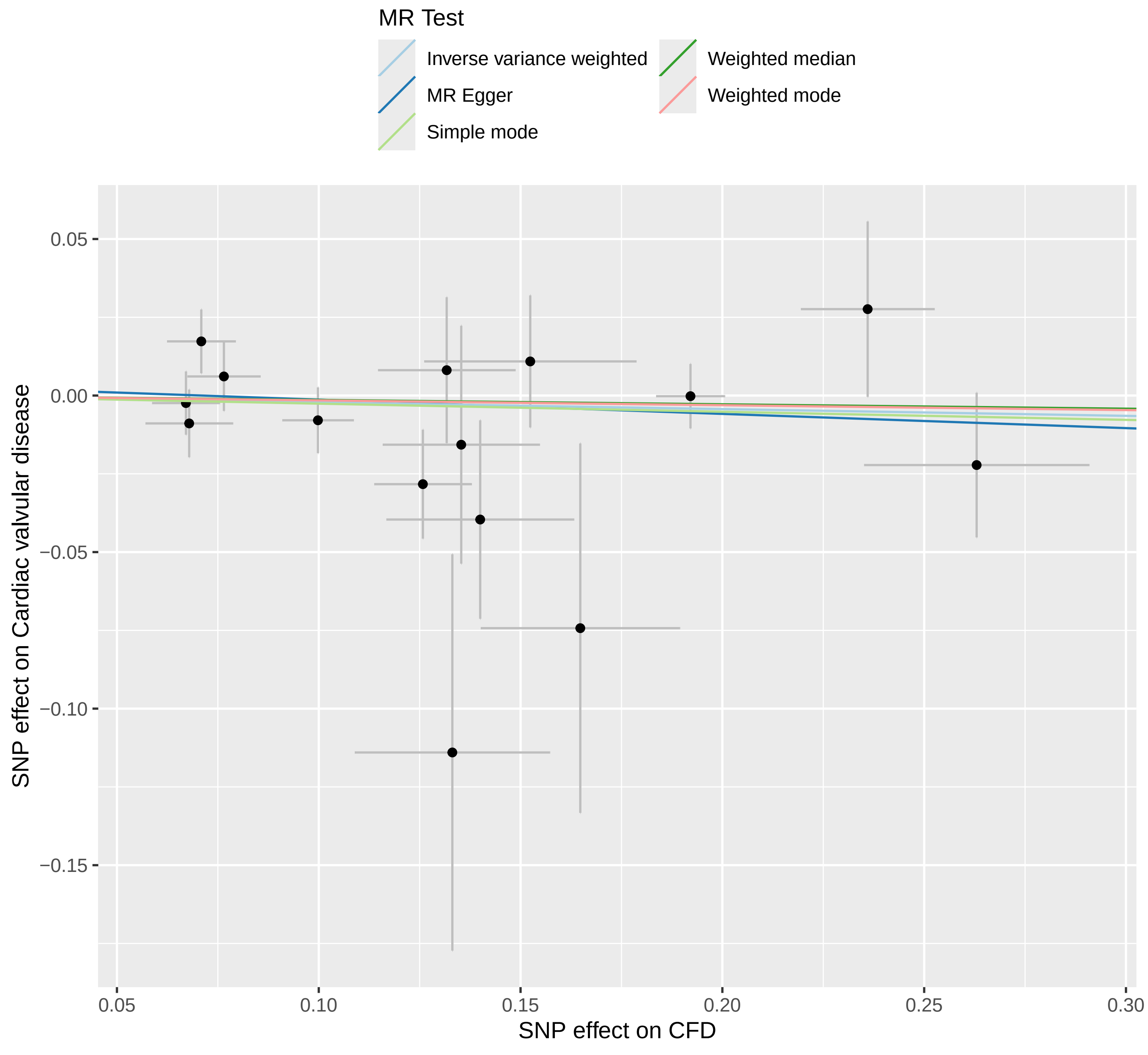
MR Test
Inverse variance weighted
MR Egger
Simple mode
Weighted median
Weighted mode
SNP effect on Cardiac valvular disease
0.05
0.00
−0.05
−0.10
−0.15
0.05
0.10
0.15
0.20
0.25
0.30
SNP effect on CFD

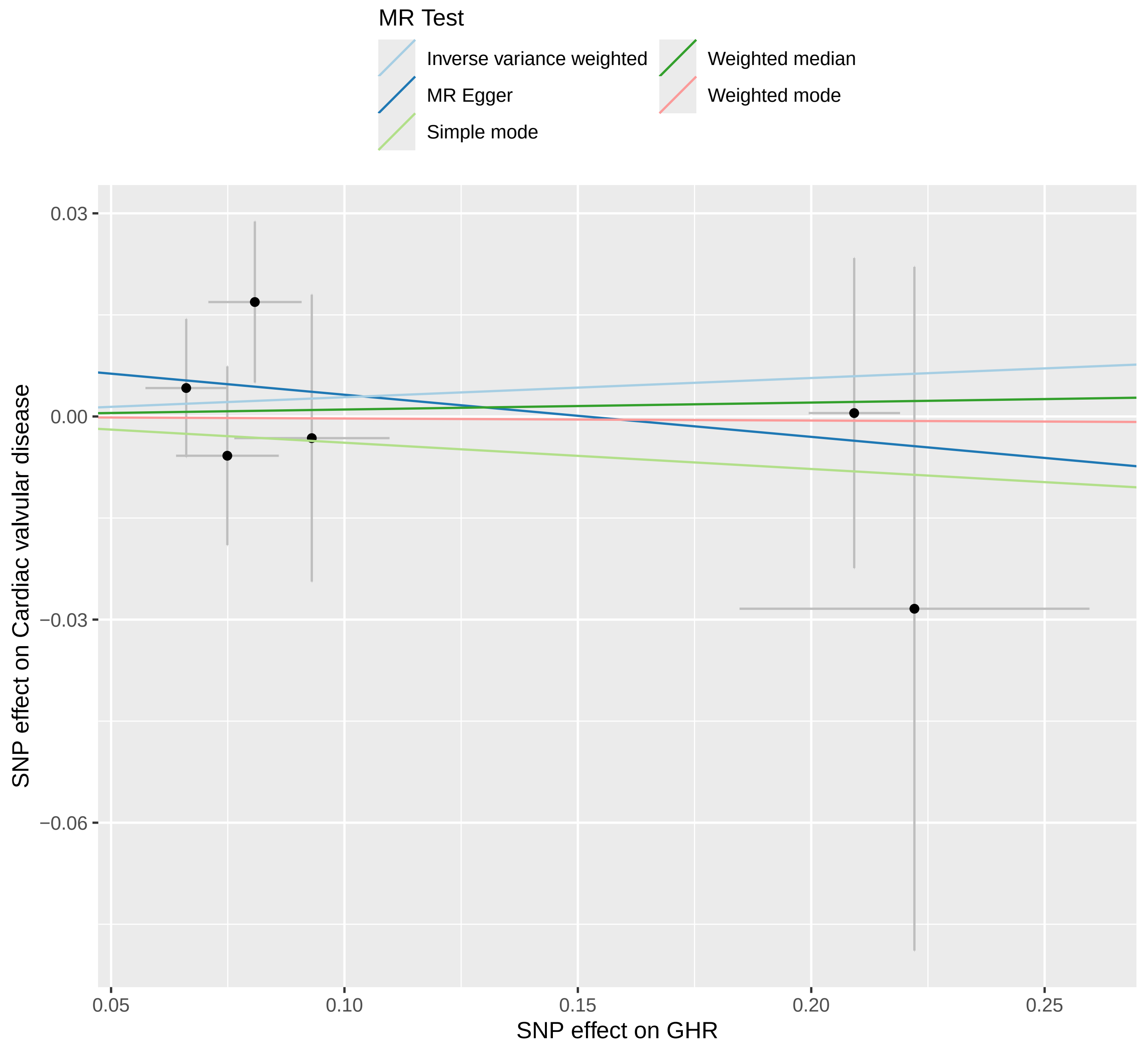

MR Test
Inverse variance weighted
MR Egger
Simple mode
Weighted median
Weighted mode
0.03
0.00
−0.03
−0.06
SNP effect on Cardiac valvular disease
0.05
0.10
0.15
0.20
0.25
SNP effect on GHR

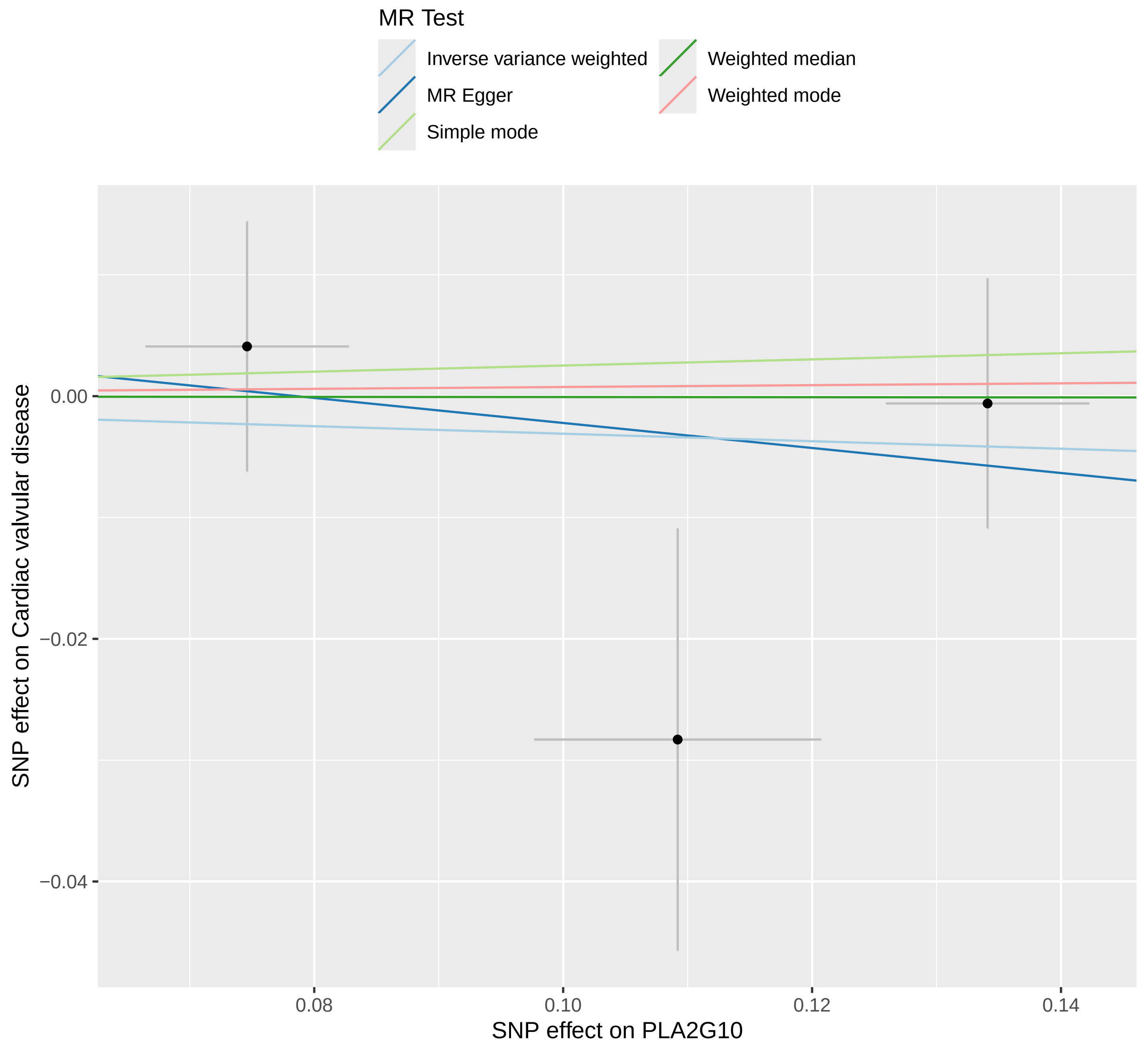
MR Test
Inverse variance weighted
MR Egger
Simple mode
Weighted median
Weighted mode
SNP effect on Cardiac valvular disease
0.00
−0.02
−0.04
0.08
0.10
0.12
0.14
SNP effect on PLA2G10

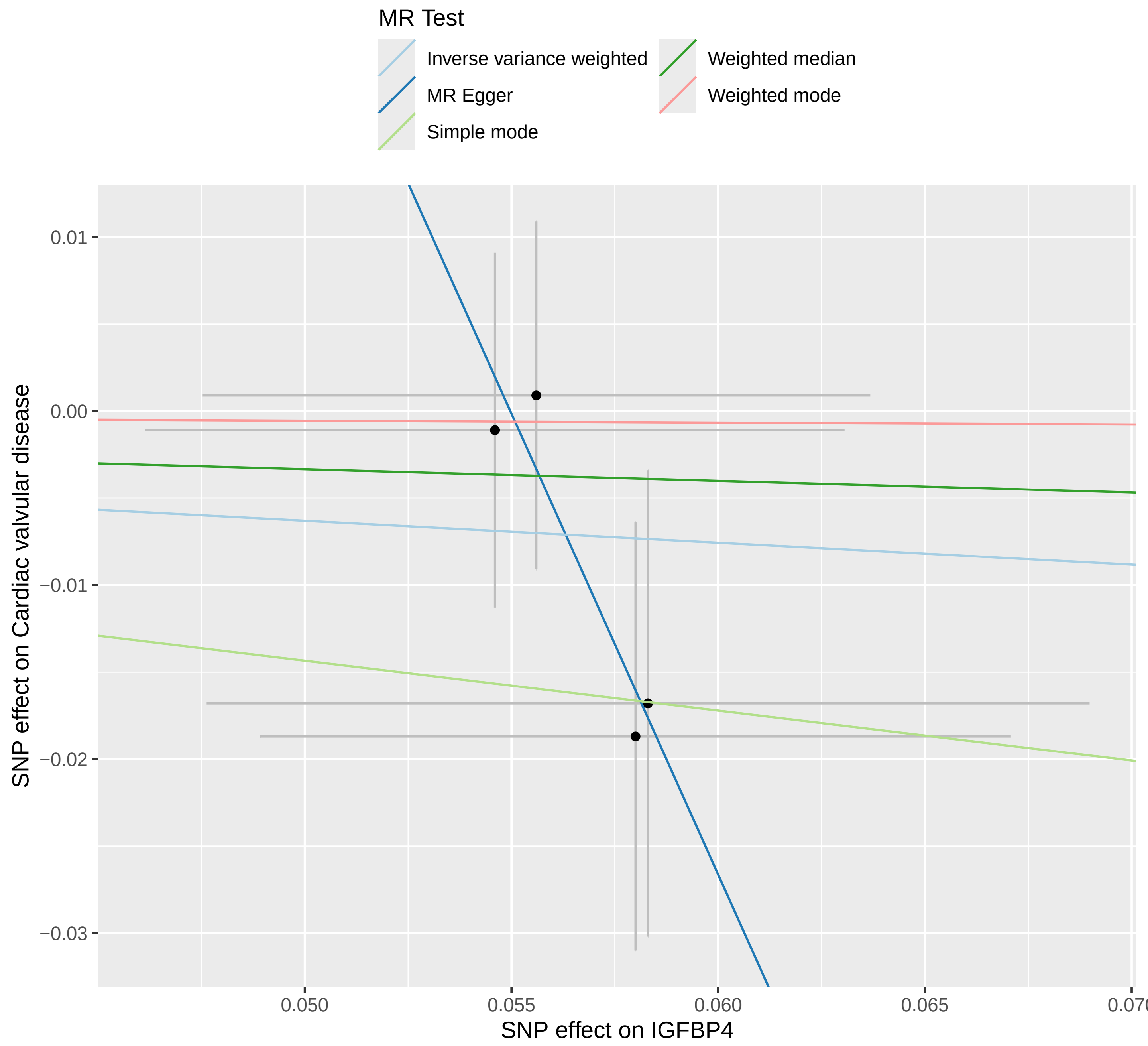
MR Test
Inverse variance weighted
MR Egger
Simple mode
Weighted median
Weighted mode
0.01
0.00
−0.01
−0.02
−0.03
SNP effect on Cardiac valvular disease
0.050
0.055
0.060
0.065
0.070
SNP effect on IGFBP4

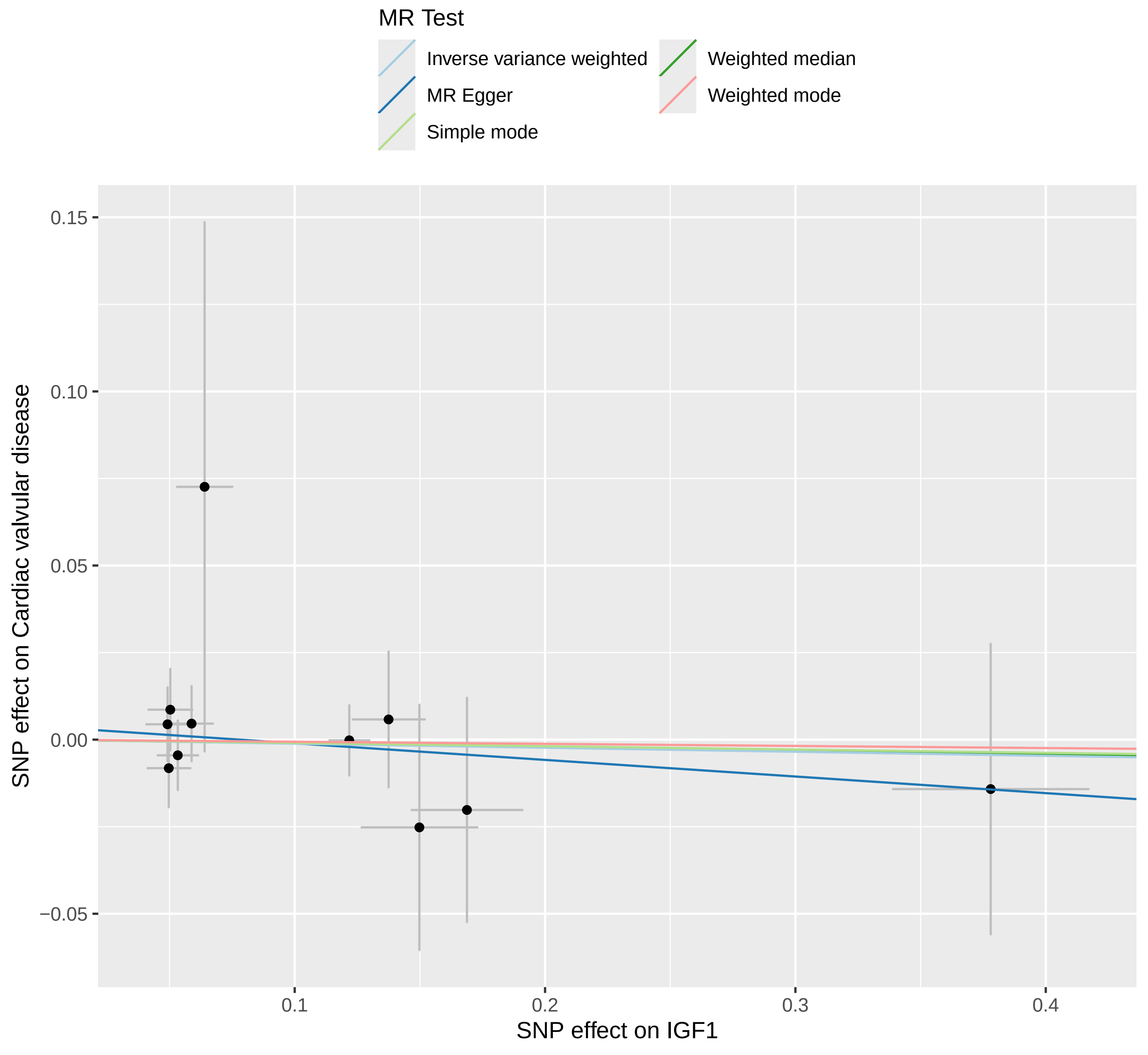
MR Test
Inverse variance weighted
MR Egger
Simple mode
Weighted median
Weighted mode
0.15
0.10
0.05
0.00
−0.05
SNP effect on Cardiac valvular disease
0.1
0.2
0.3
0.4
SNP effect on IGF1

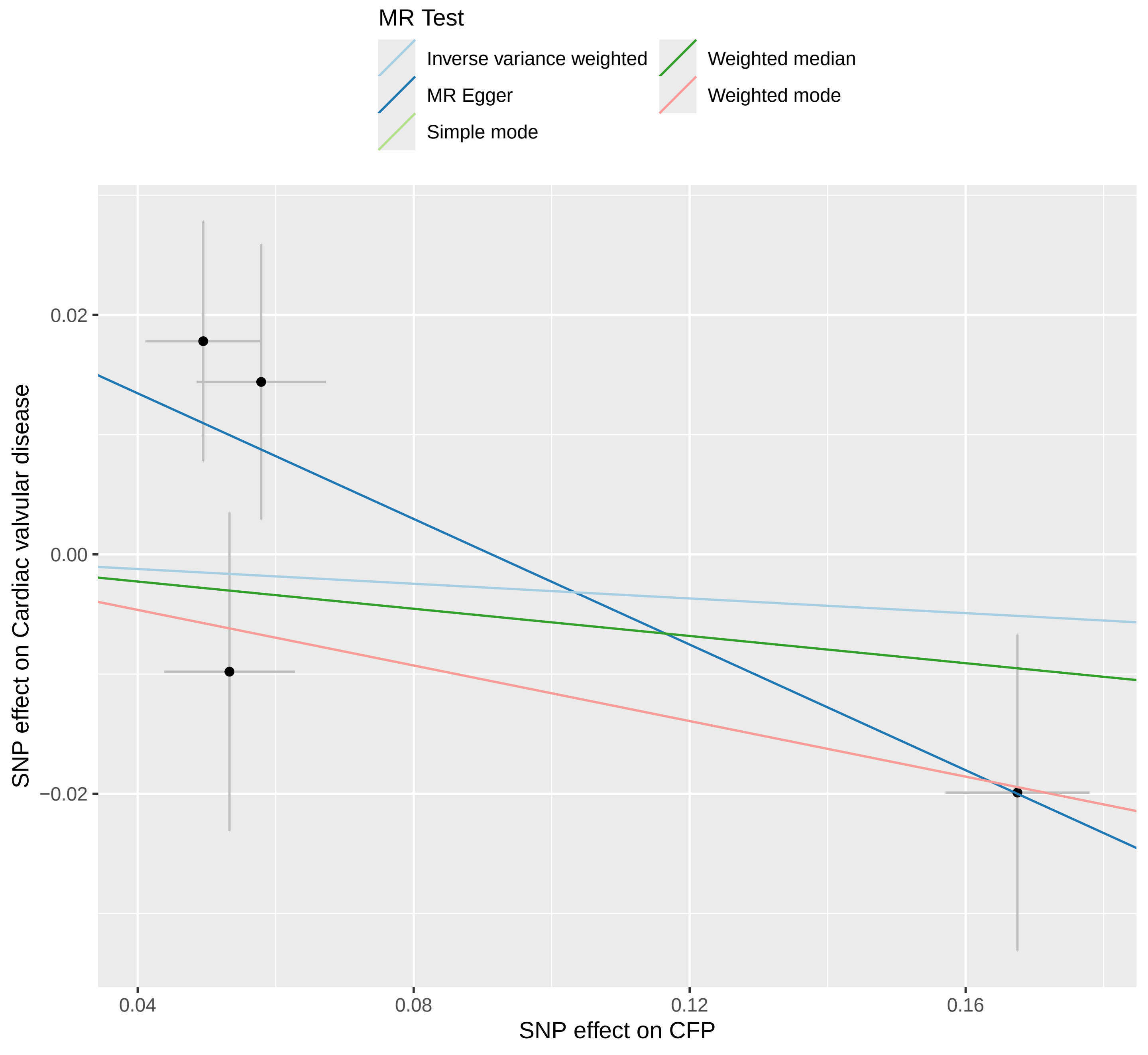
MR Test
Inverse variance weighted
MR Egger
Simple mode
Weighted median
Weighted mode
0.02
0.00
−0.02
0.04
0.08
0.12
0.16
SNP effect on Cardiac valvular disease
SNP effect on CFP

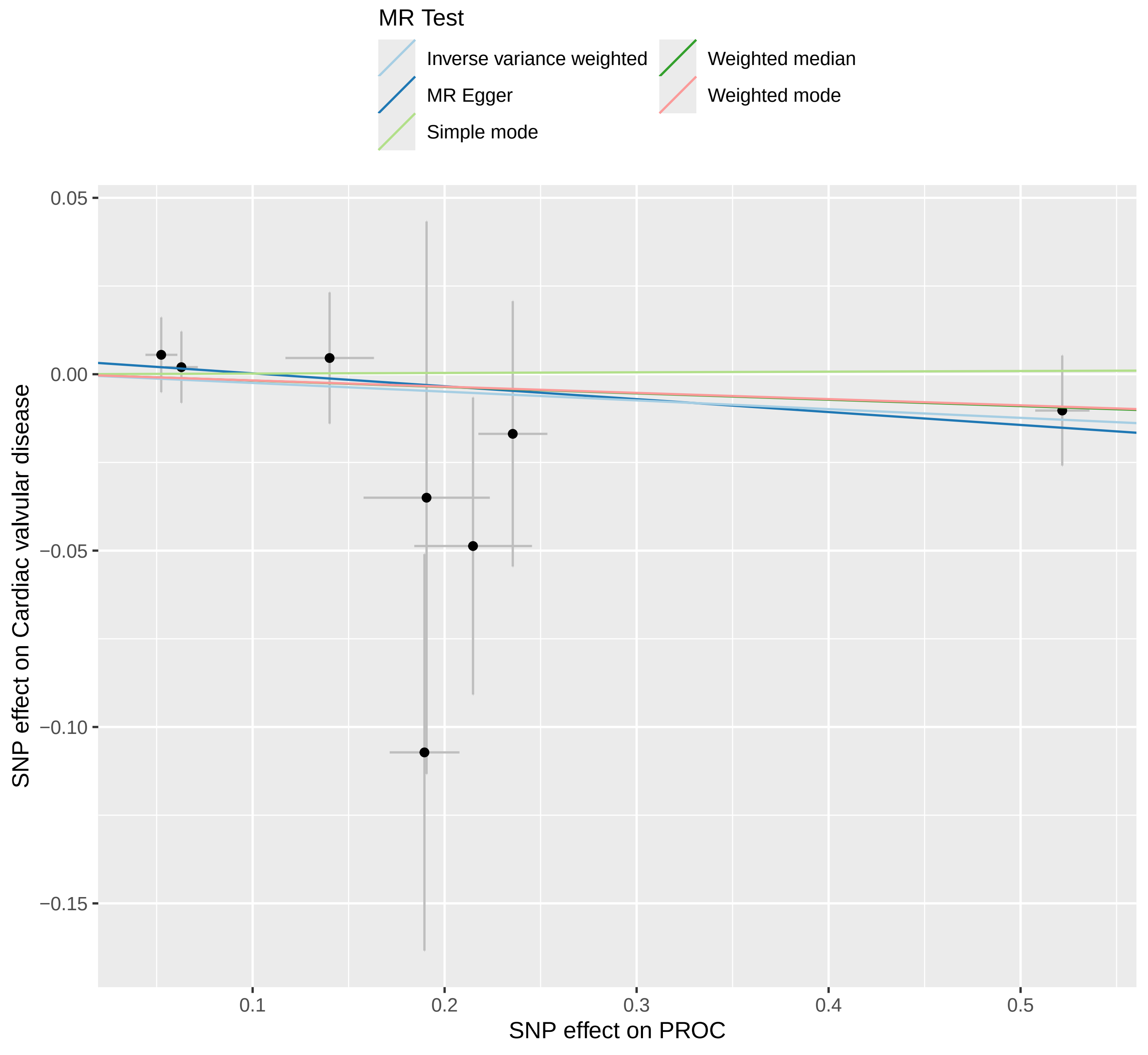
MR Test
Inverse variance weighted
MR Egger
Simple mode
Weighted median
Weighted mode
0.05
0.00
−0.05
−0.10
−0.15
0.1
0.2
0.3
0.4
0.5
SNP effect on PROC
SNP effect on Cardiac valvular disease

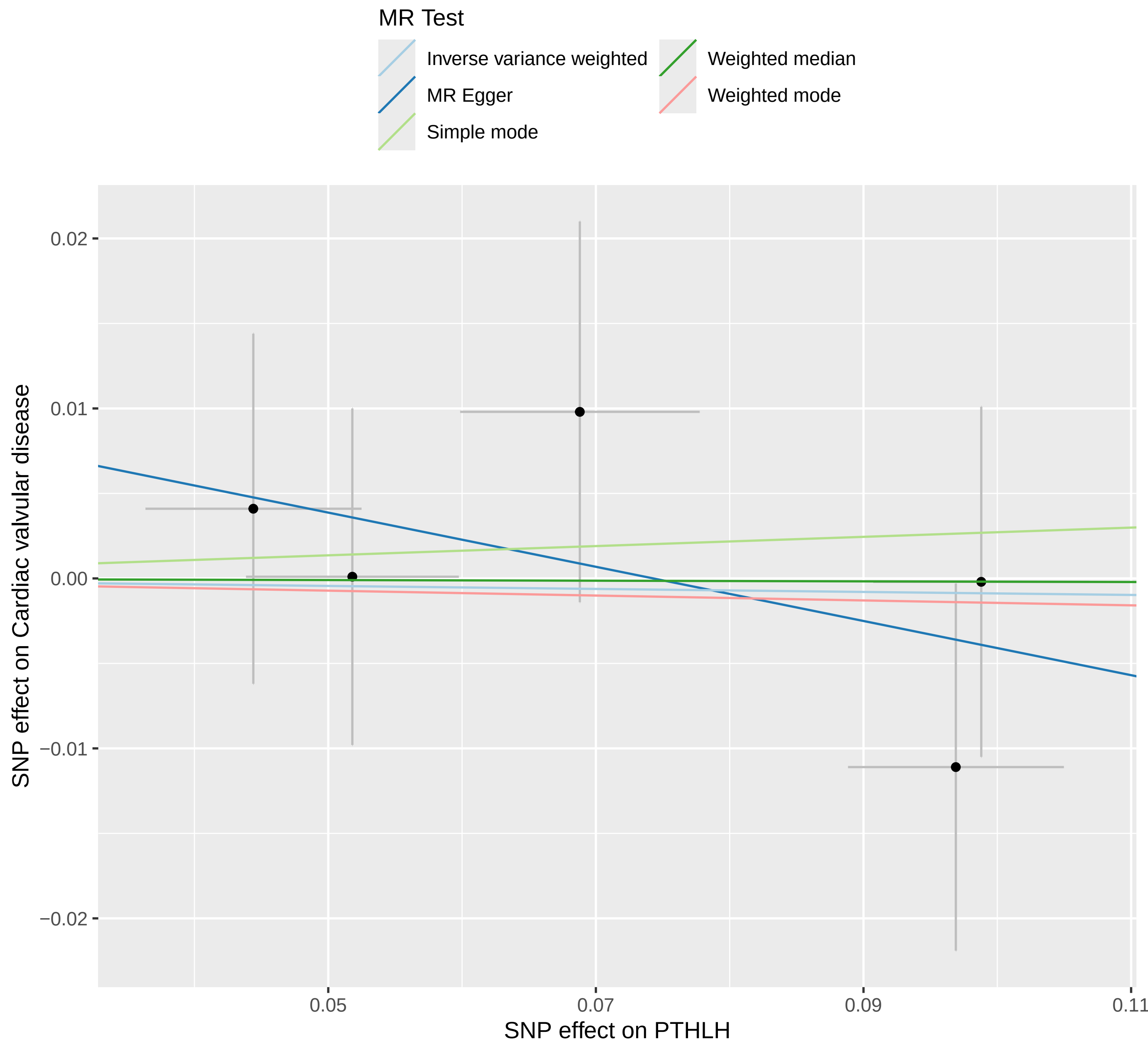
MR Test
Inverse variance weighted
MR Egger
Simple mode
Weighted median
Weighted mode
0.02
0.01
0.00
−0.01
−0.02
SNP effect on Cardiac valvular disease
0.05
0.07
0.09
0.11
SNP effect on PTHLH

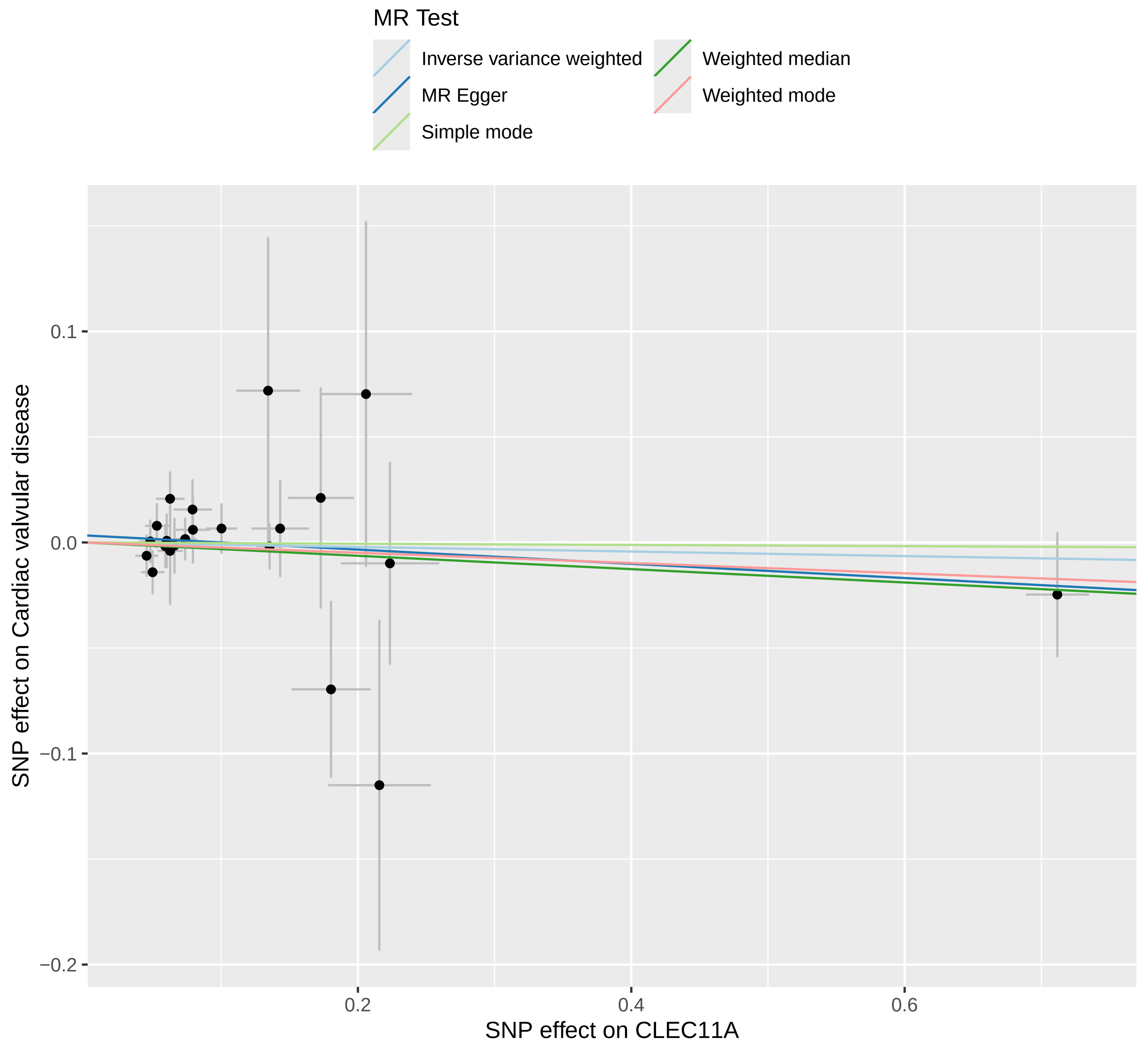
MR Test
Inverse variance weighted
MR Egger
Simple mode
Weighted median
Weighted mode
SNP effect on Cardiac valvular disease
0.1
0.0
−0.1
−0.2
0.2
0.4
0.6
SNP effect on CLEC11A

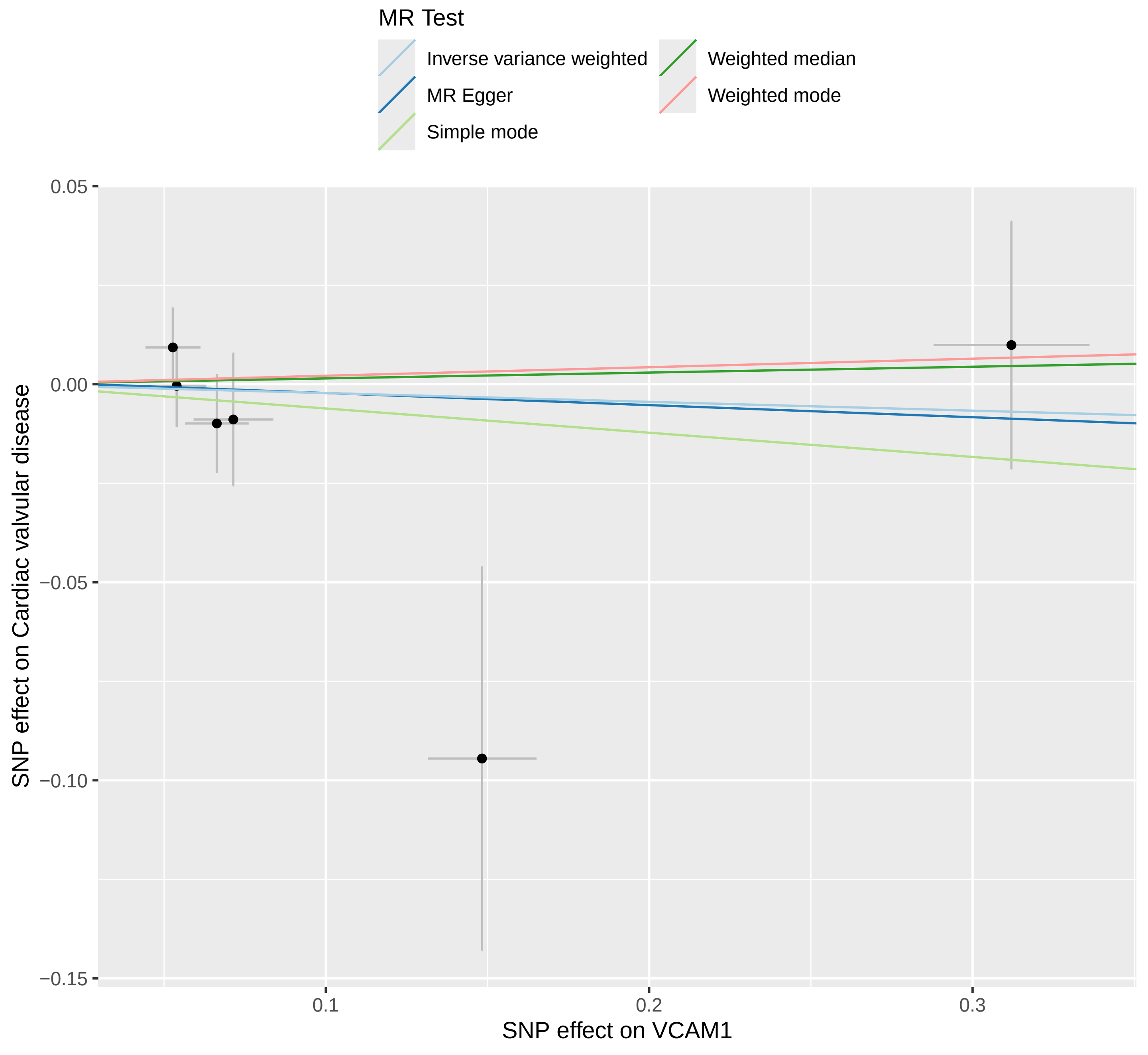
MR Test
Inverse variance weighted
MR Egger
Simple mode
Weighted median
Weighted mode
0.05
0.00
−0.05
−0.10
−0.15
0.1
0.2
0.3
SNP effect on Cardiac valvular disease
SNP effect on VCAM1

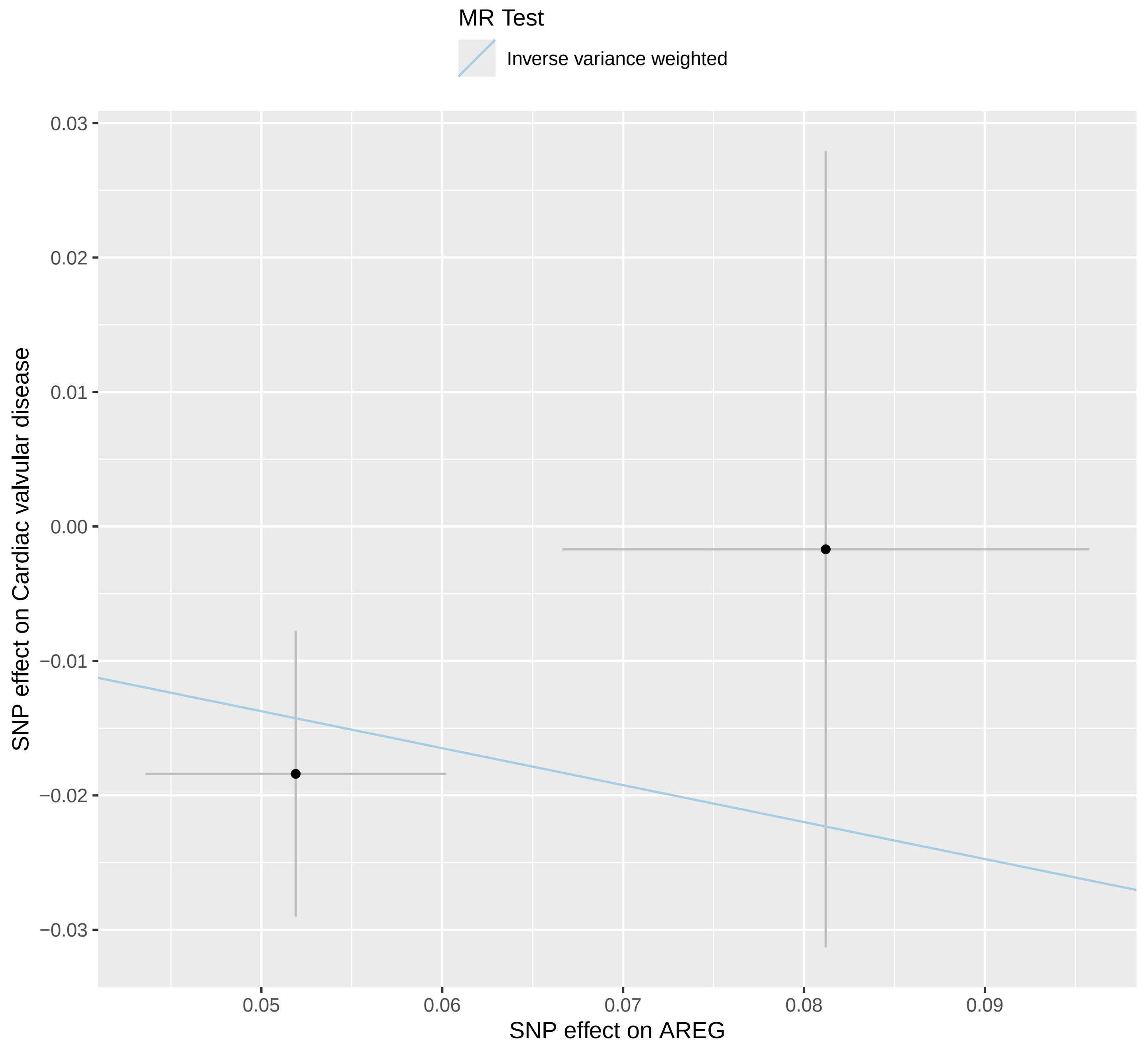
MR Test
Inverse variance weighted
0.03
0.02
0.01
0.00
−0.01
−0.02
−0.03
SNP effect on Cardiac valvular disease
0.05
0.06
0.07
0.08
0.09
SNP effect on AREG

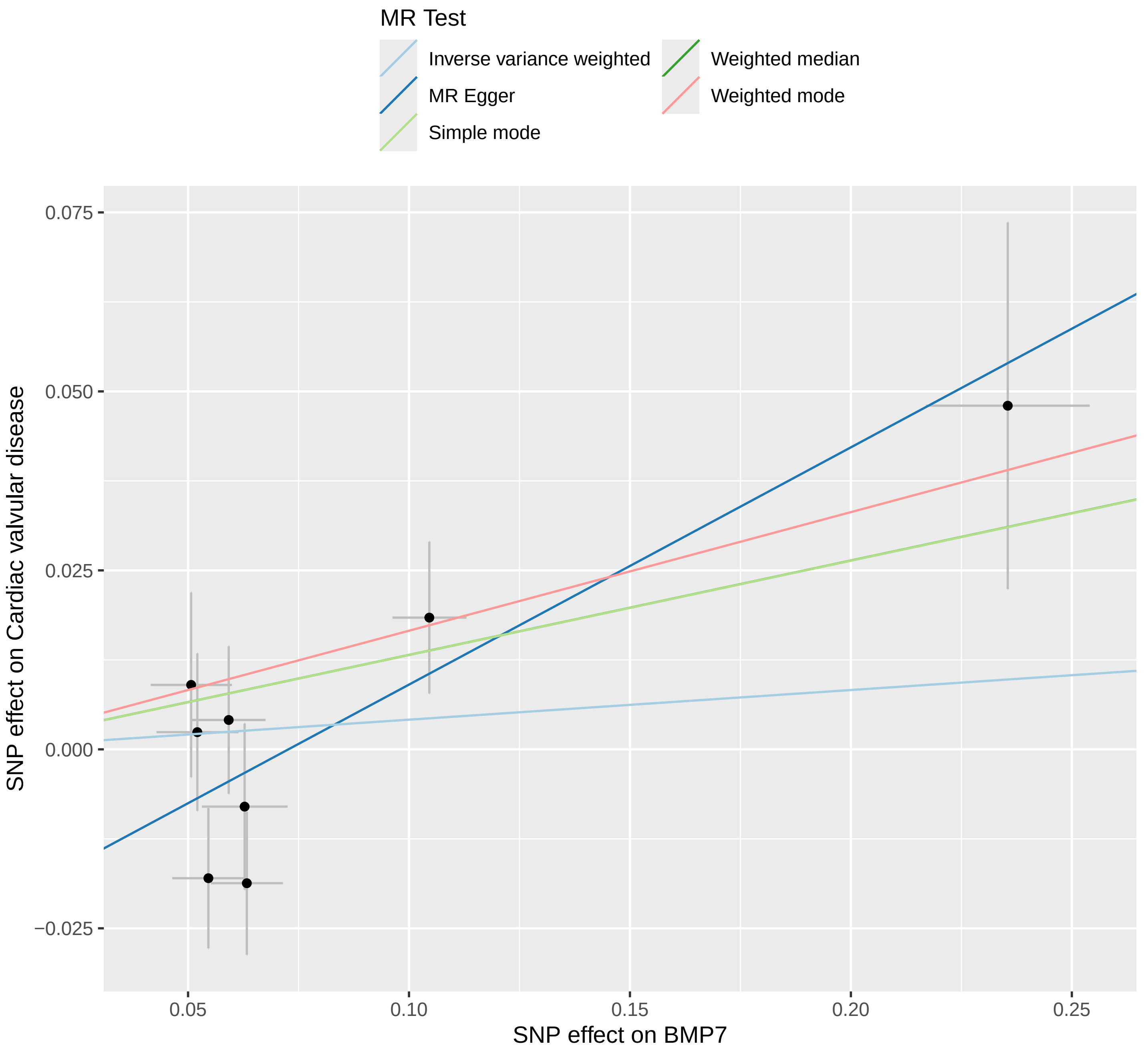
MR Test
Inverse variance weighted
MR Egger
Simple mode
Weighted median
Weighted mode
0.075
0.050
0.025
0.000
−0.025
0.05
0.10
0.15
0.20
0.25
SNP effect on Cardiac valvular disease
SNP effect on BMP7

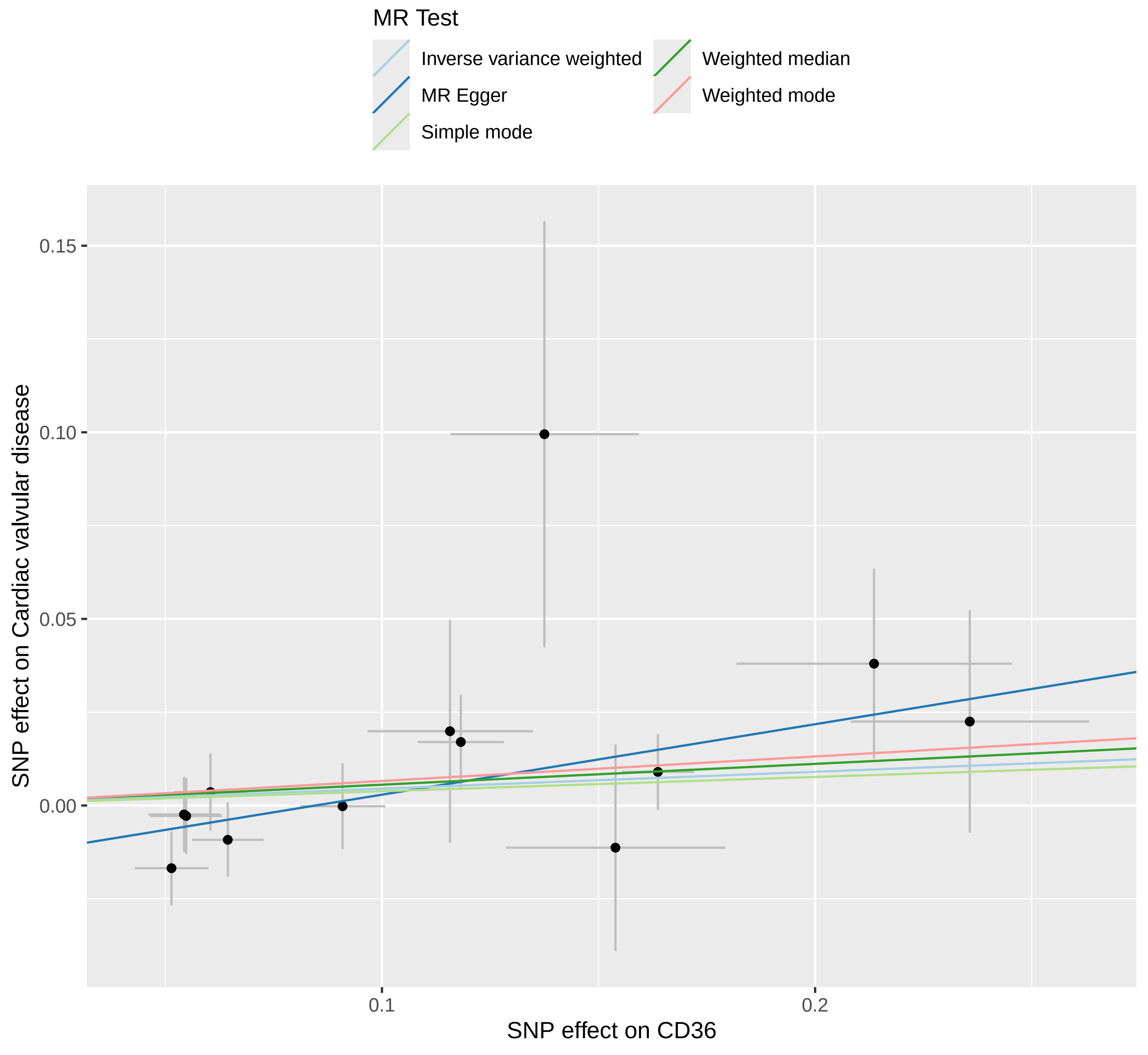
MR Test
Inverse variance weighted
MR Egger
Simple mode
Weighted median
Weighted mode
0.15
0.10
0.05
0.00
0.1
0.2
SNP effect on Cardiac valvular disease
SNP effect on CD36

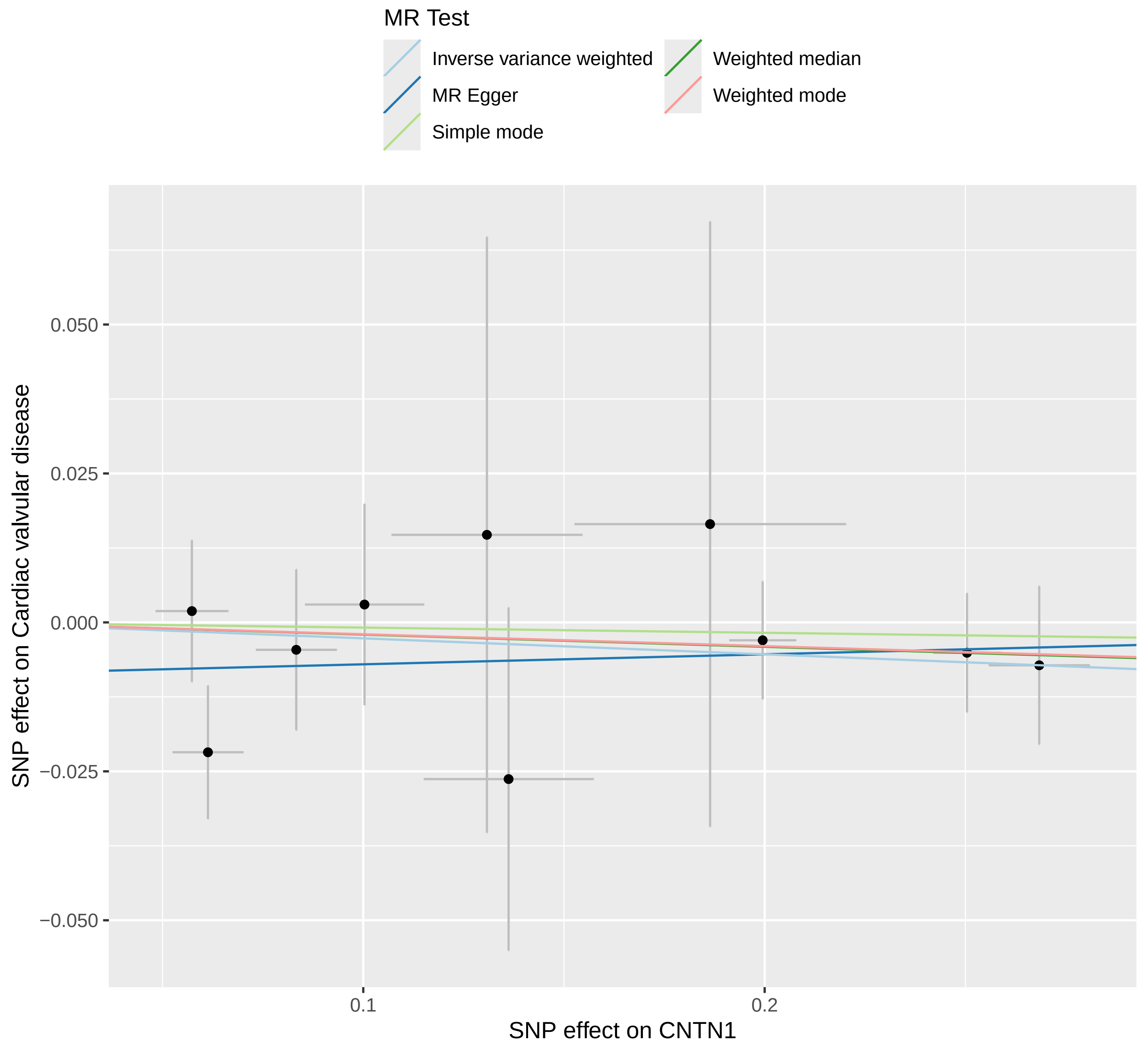

MR Test
Inverse variance weighted
MR Egger
Simple mode
Weighted median
Weighted mode
0.050
0.025
0.000
−0.025
−0.050
0.1
0.2
SNP effect on CNTN1
SNP effect on Cardiac valvular disease

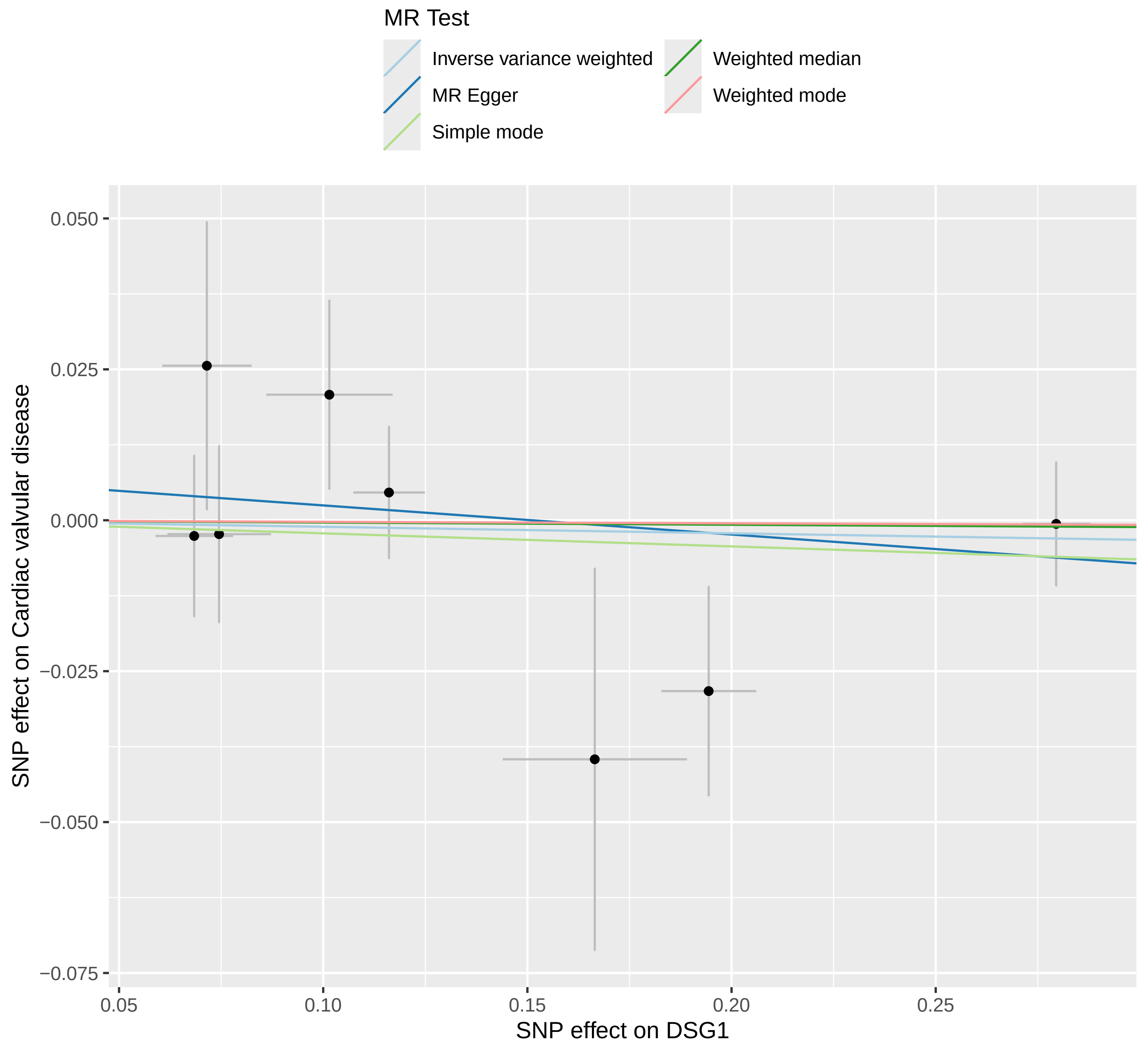

MR Test
Inverse variance weighted
MR Egger
Simple mode
Weighted median
Weighted mode
0.050
0.025
0.000
−0.025
−0.050
−0.075
SNP effect on Cardiac valvular disease
0.05
0.10
0.15
0.20
0.25
SNP effect on DSG1

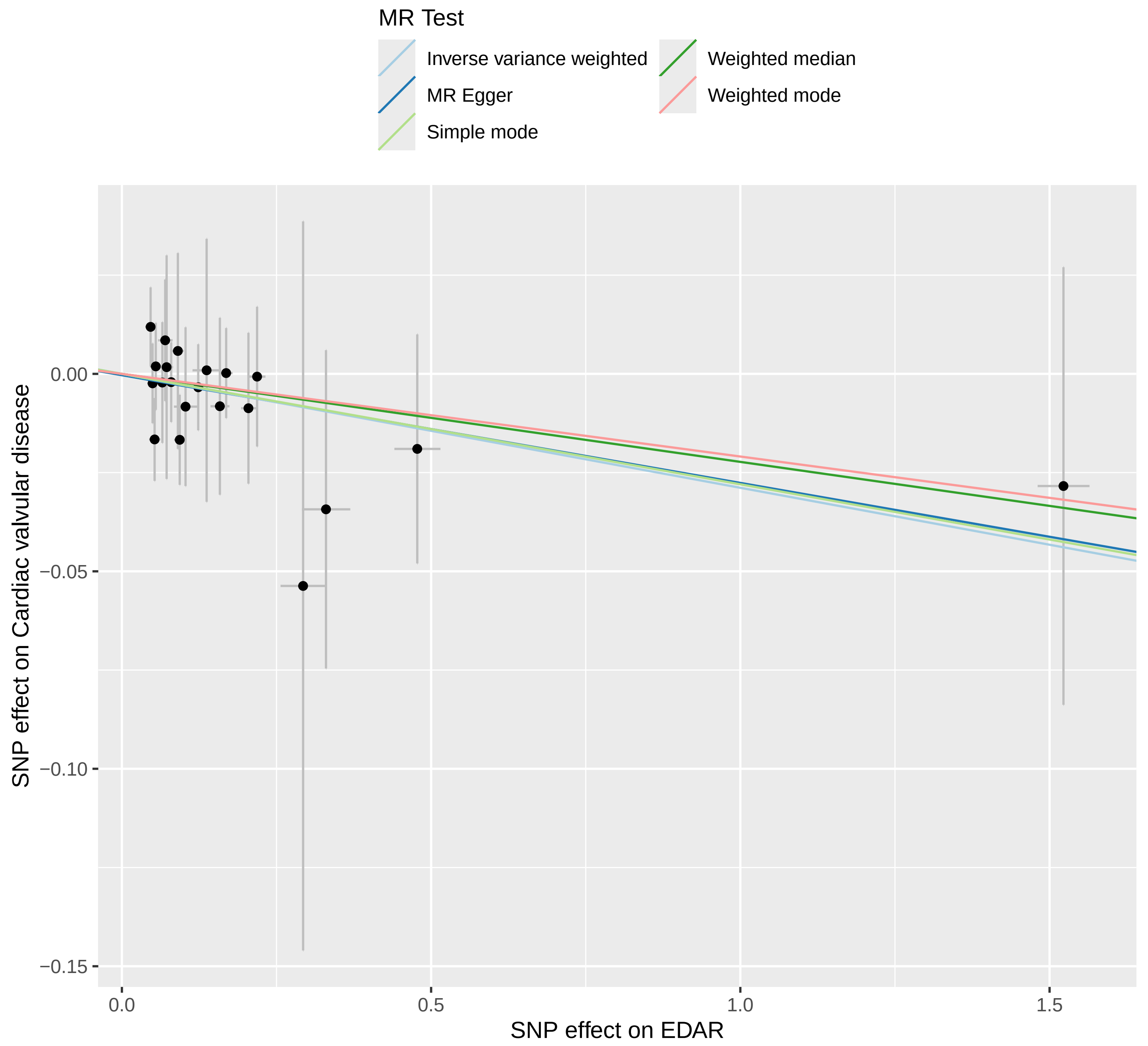

MR Test
Inverse variance weighted
MR Egger
Simple mode
Weighted median
Weighted mode
SNP effect on Cardiac valvular disease
0.00
−0.05
−0.10
−0.15
0.0
0.5
1.0
1.5
SNP effect on EDAR

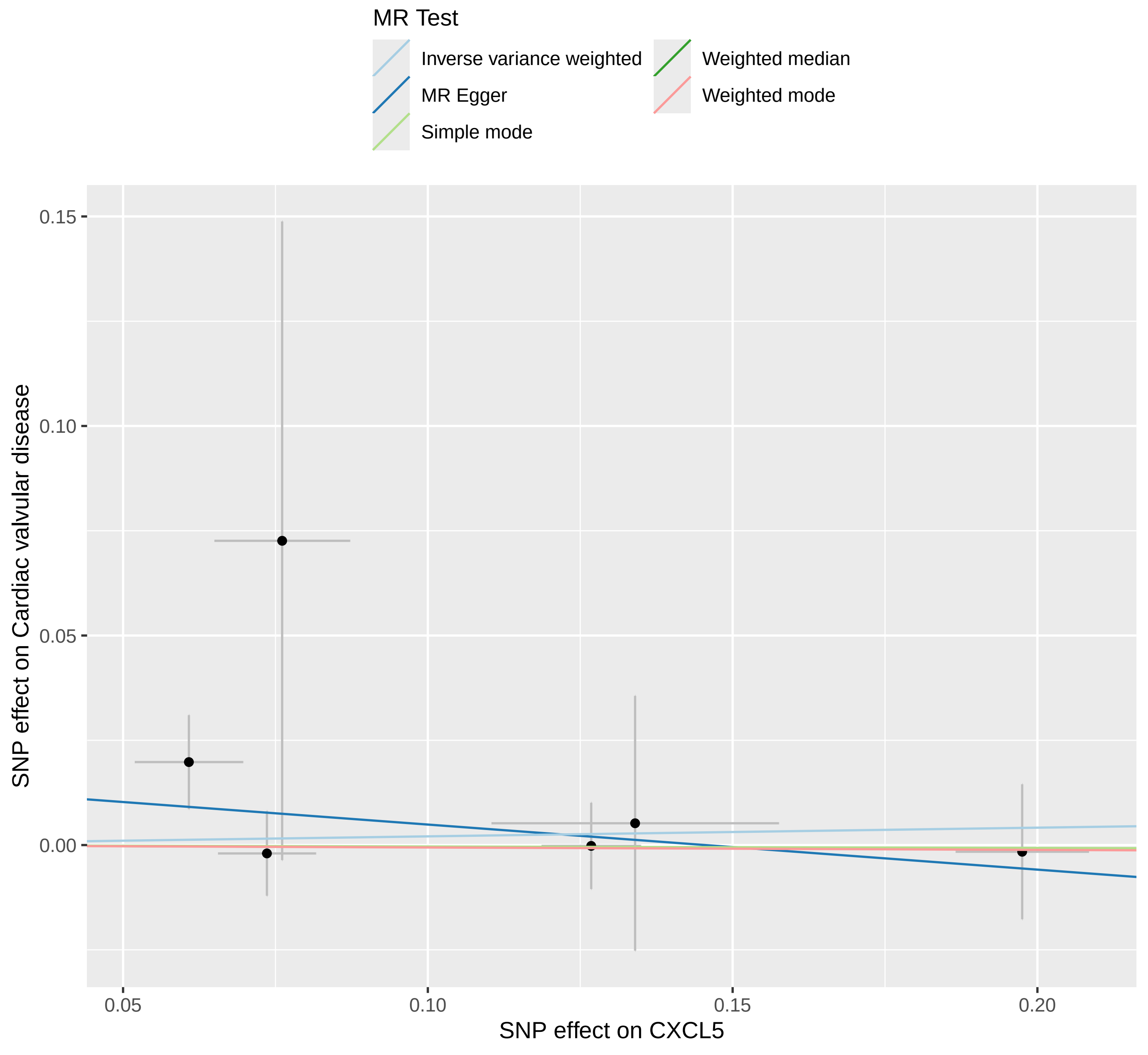
MR Test
Inverse variance weighted
MR Egger
Simple mode
Weighted median
Weighted mode
0.15
0.10
0.05
0.00
SNP effect on Cardiac valvular disease
0.05
0.10
0.15
0.20
SNP effect on CXCL5

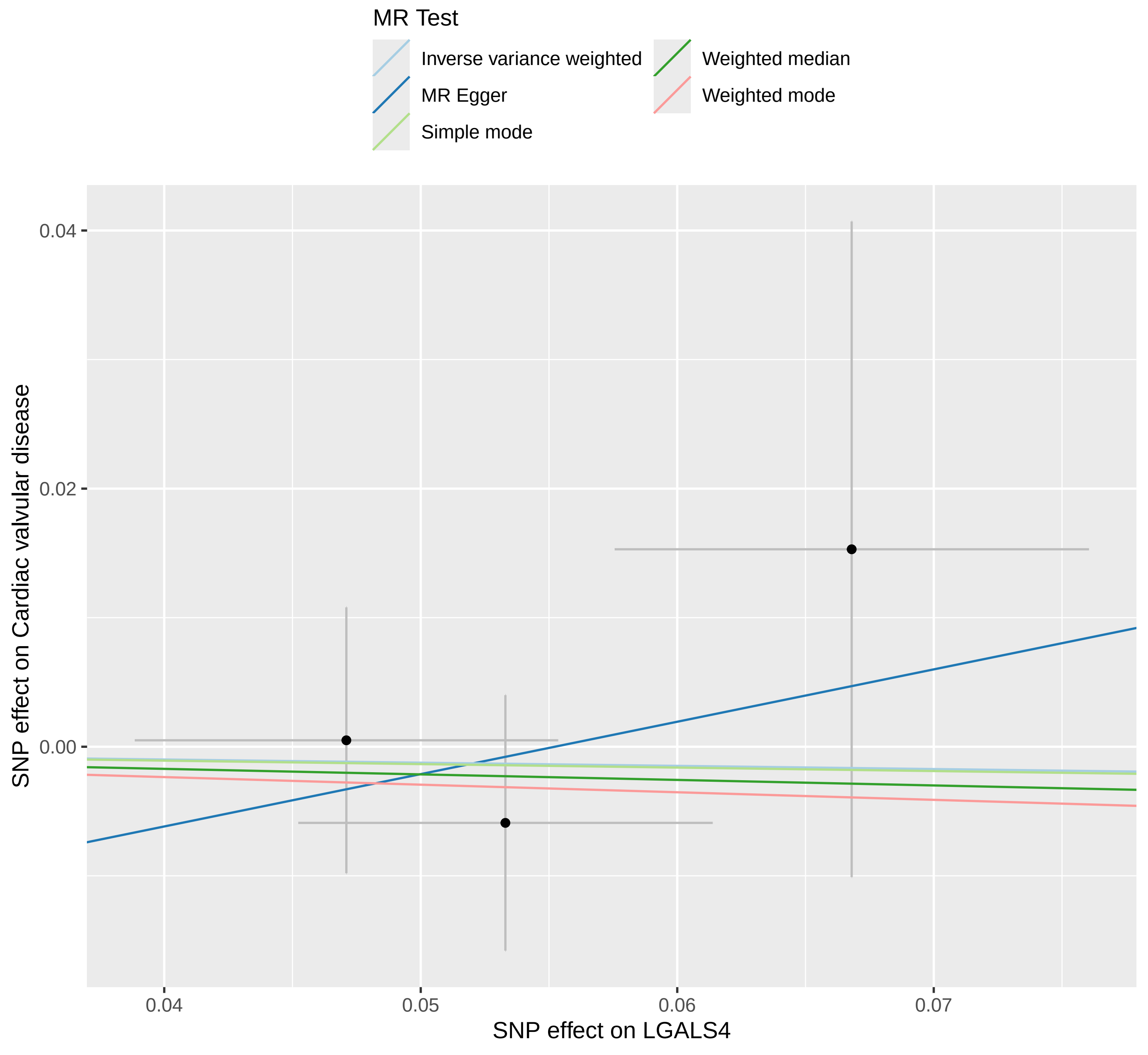
MR Test
Inverse variance weighted
MR Egger
Simple mode
Weighted median
Weighted mode
0.04
0.02
0.00
0.04
0.05
0.06
0.07
SNP effect on Cardiac valvular disease
SNP effect on LGALS4

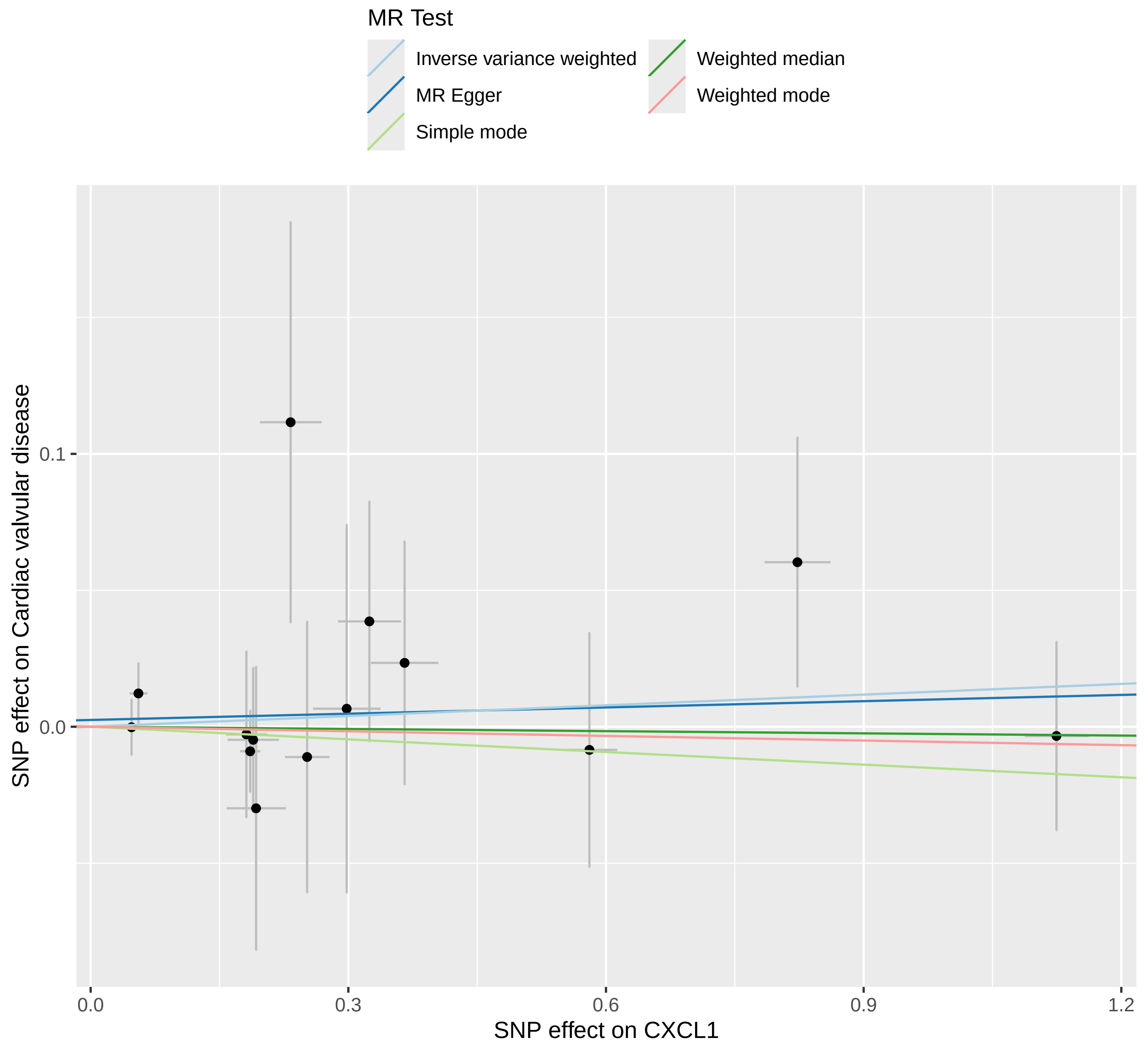
MR Test
Inverse variance weighted
MR Egger
Simple mode
Weighted median
Weighted mode
SNP effect on Cardiac valvular disease
0.1
0.0
0.0
0.3
0.6
0.9
1.2
SNP effect on CXCL1

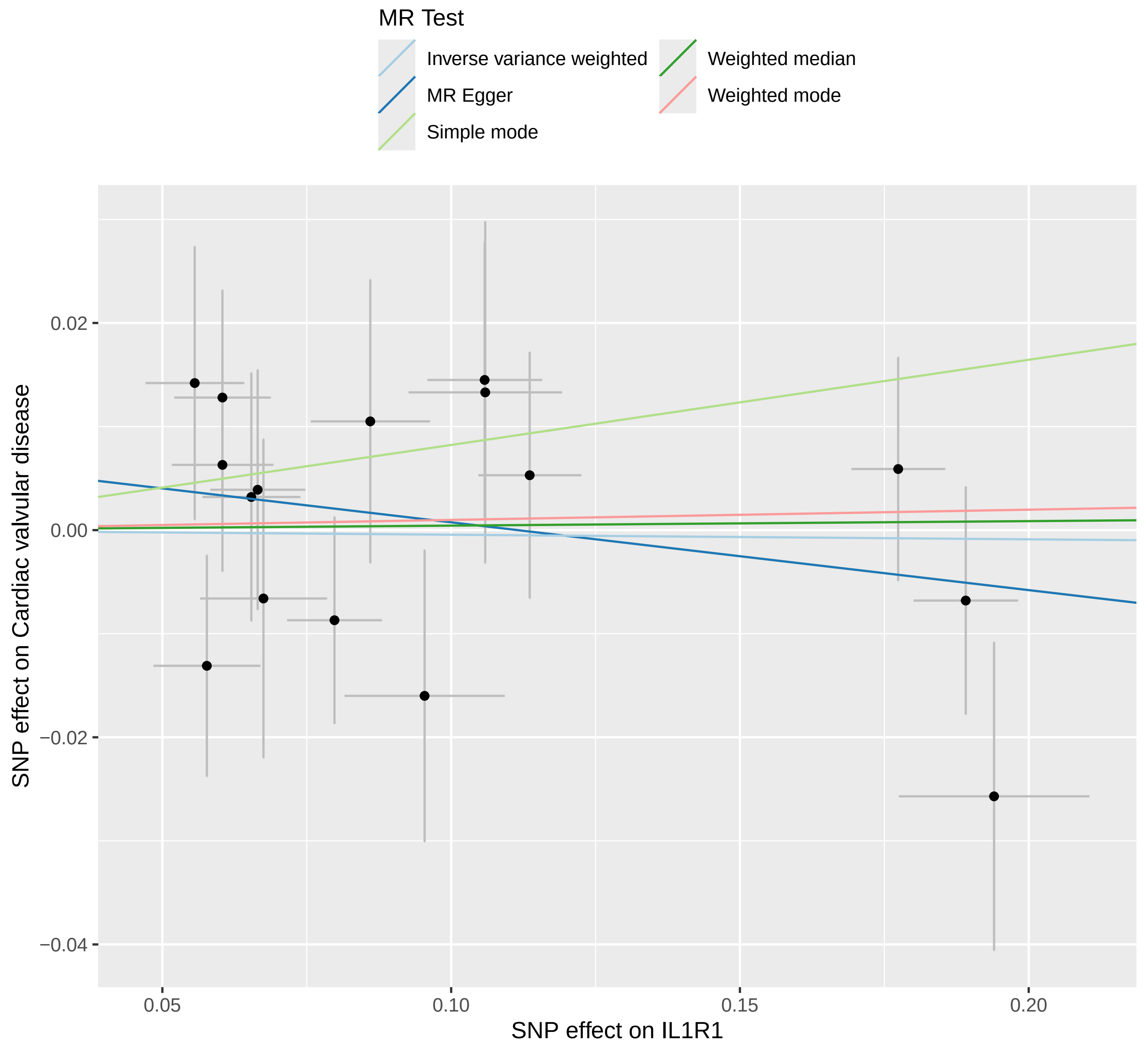
MR Test
Inverse variance weighted
MR Egger
Simple mode
Weighted median
Weighted mode
SNP effect on Cardiac valvular disease
0.02
0.00
−0.02
−0.04
0.05
0.10
0.15
0.20
SNP effect on IL1R1

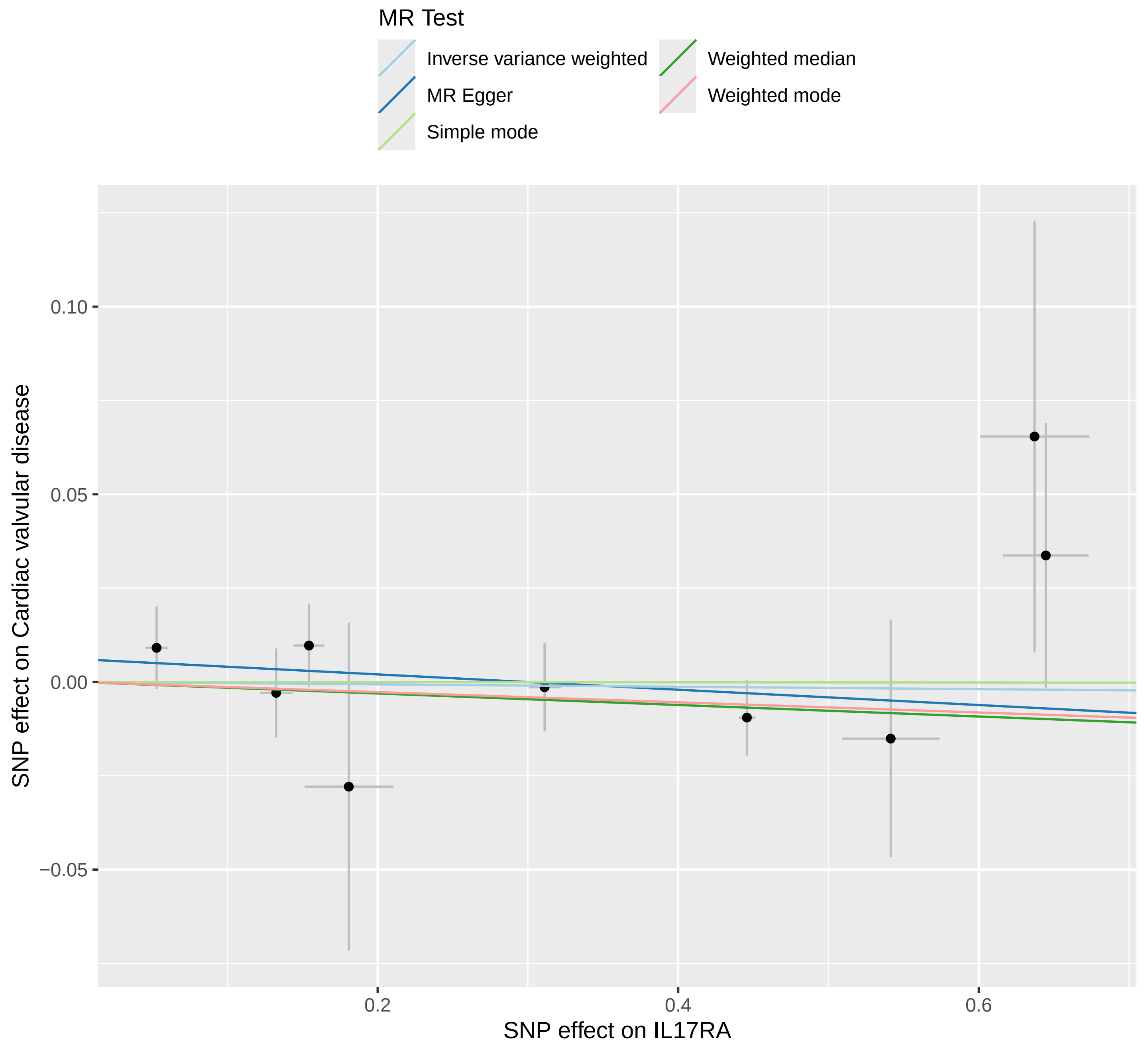

MR Test
Inverse variance weighted
MR Egger
Simple mode
Weighted median
Weighted mode
0.10
0.05
0.00
−0.05
0.2
0.4
0.6
SNP effect on Cardiac valvular disease
SNP effect on IL17RA

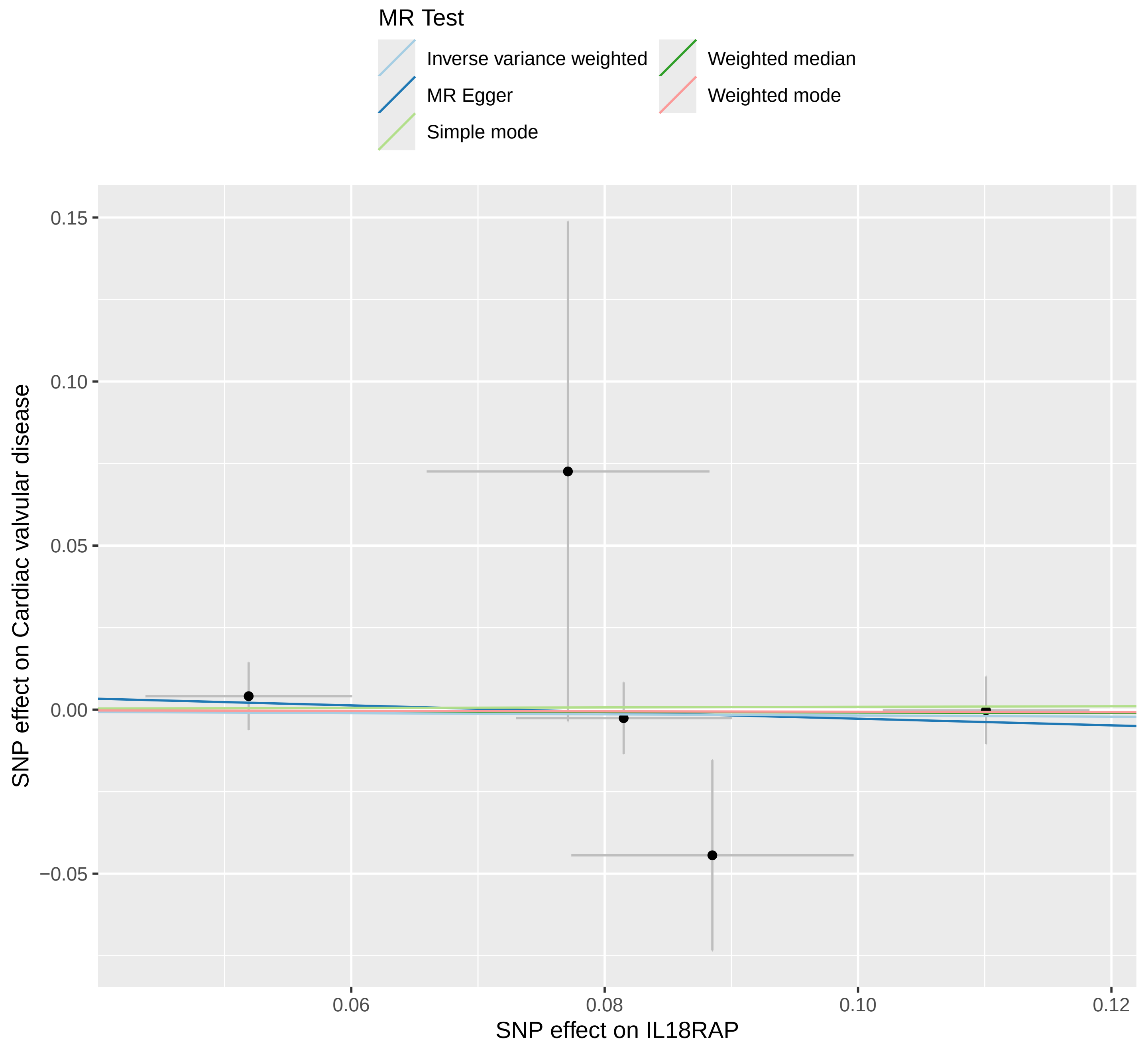

MR Test
Inverse variance weighted
MR Egger
Simple mode
Weighted median
Weighted mode
0.15
0.10
0.05
0.00
−0.05
0.06
0.08
0.10
0.12
SNP effect on Cardiac valvular disease
SNP effect on IL18RAP

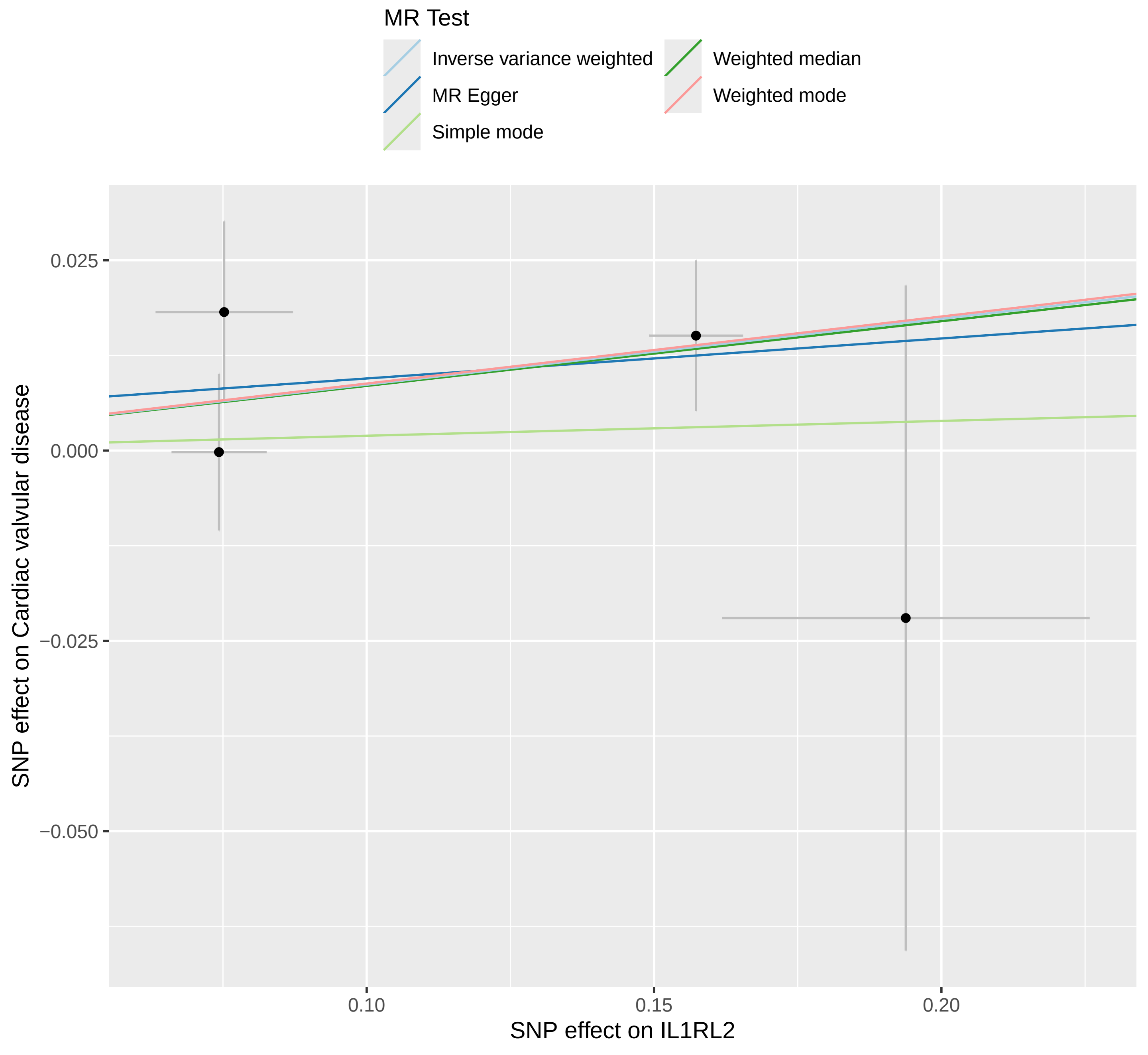
MR Test
Inverse variance weighted
MR Egger
Simple mode
Weighted median
Weighted mode
0.025
0.000
−0.025
−0.050
0.10
0.15
0.20
SNP effect on IL1RL2
SNP effect on Cardiac valvular disease

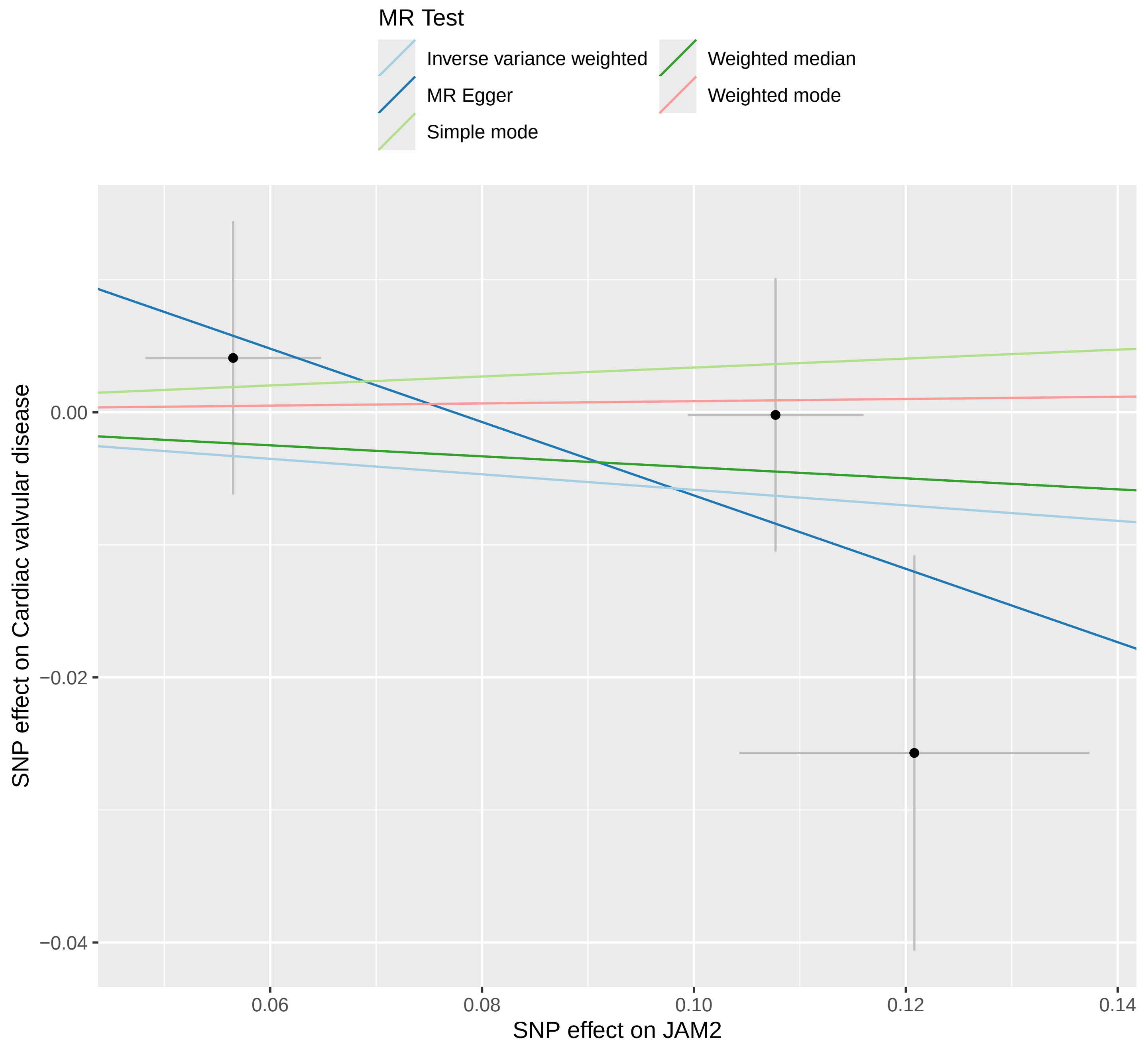

MR Test
Inverse variance weighted
MR Egger
Simple mode
Weighted median
Weighted mode
0.00
−0.02
−0.04
SNP effect on Cardiac valvular disease
0.06
0.08
0.10
0.12
0.14
SNP effect on JAM2

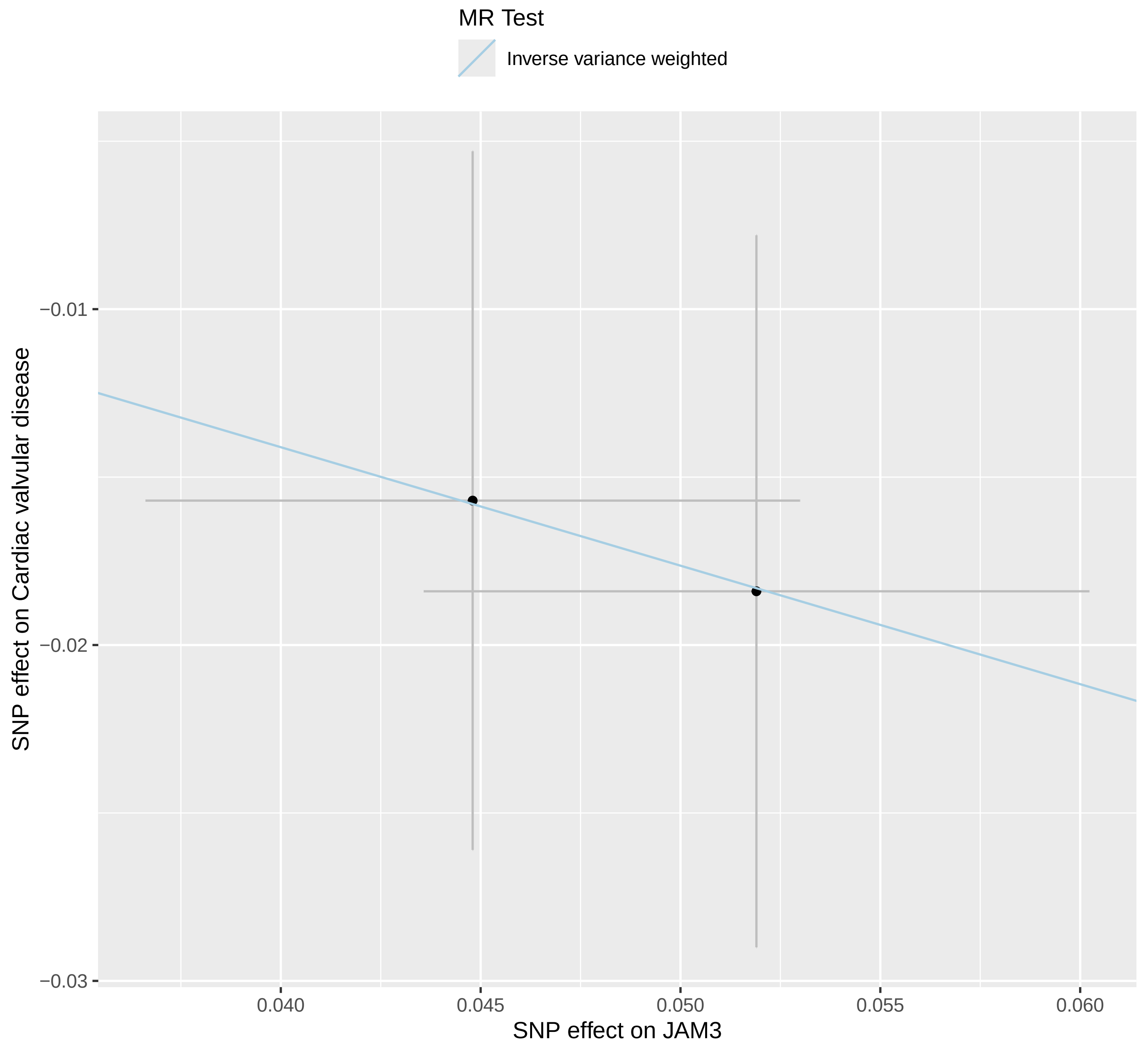
MR Test
Inverse variance weighted
SNP effect on Cardiac valvular disease
−0.01
−0.02
−0.03
0.040
0.045
0.050
0.055
0.060
SNP effect on JAM3

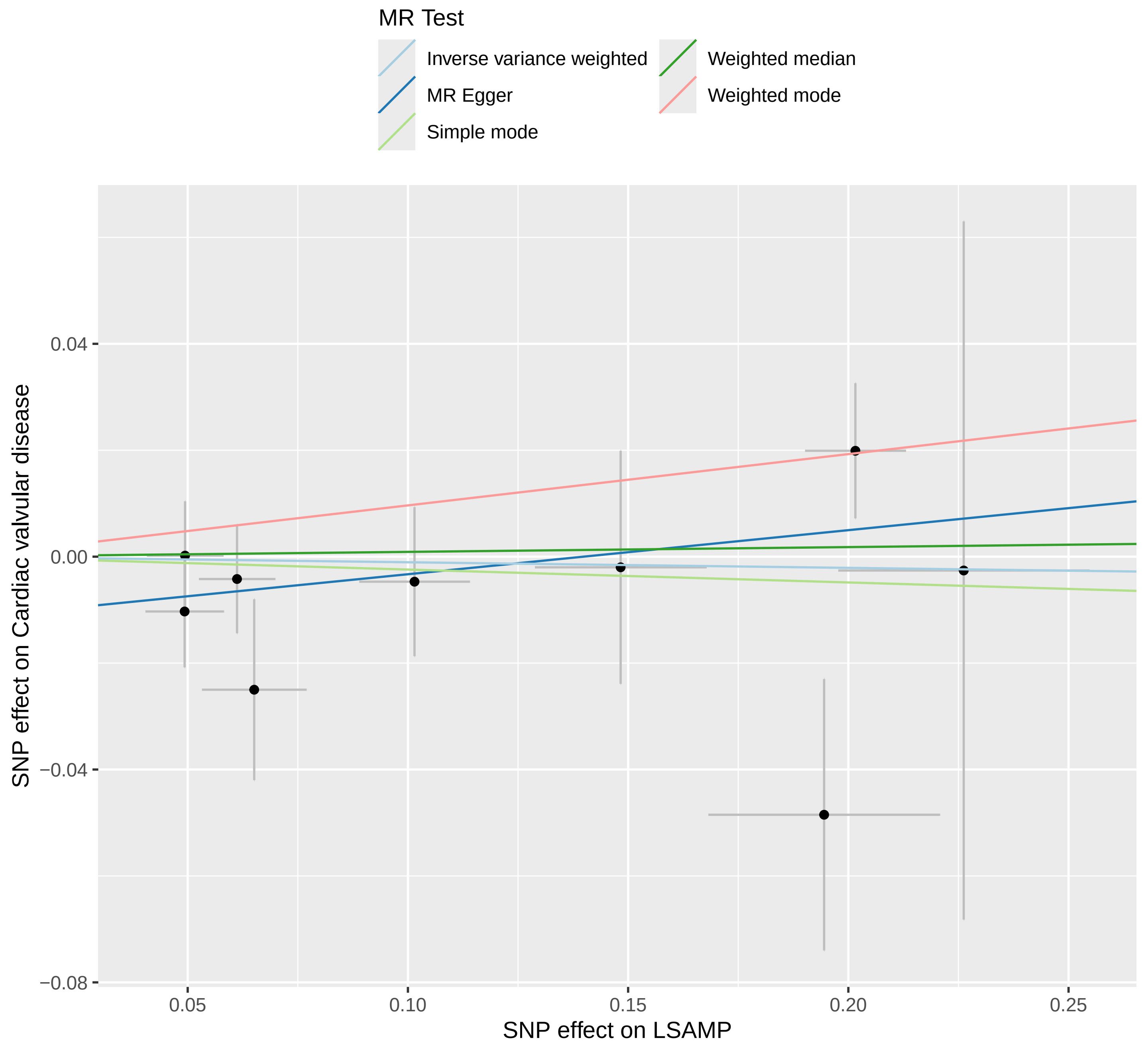

MR Test
Inverse variance weighted
MR Egger
Simple mode
Weighted median
Weighted mode
0.04
0.00
−0.04
−0.08
SNP effect on Cardiac valvular disease
0.05
0.10
0.15
0.20
0.25
SNP effect on LSAMP

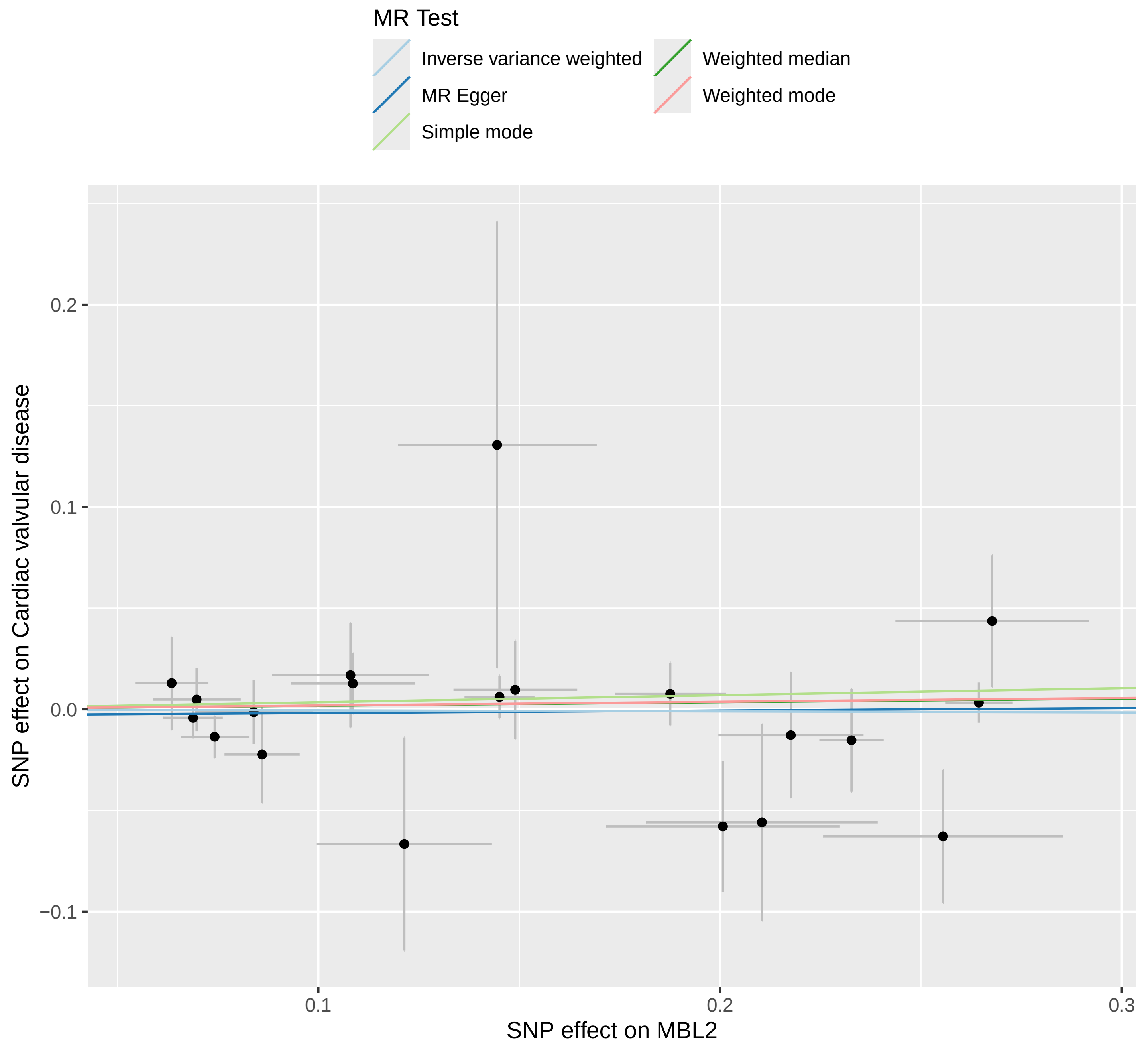

MR Test
Inverse variance weighted
MR Egger
Simple mode
Weighted median
Weighted mode
0.2
0.1
0.0
−0.1
0.1
0.2
0.3
SNP effect on Cardiac valvular disease
SNP effect on MBL2

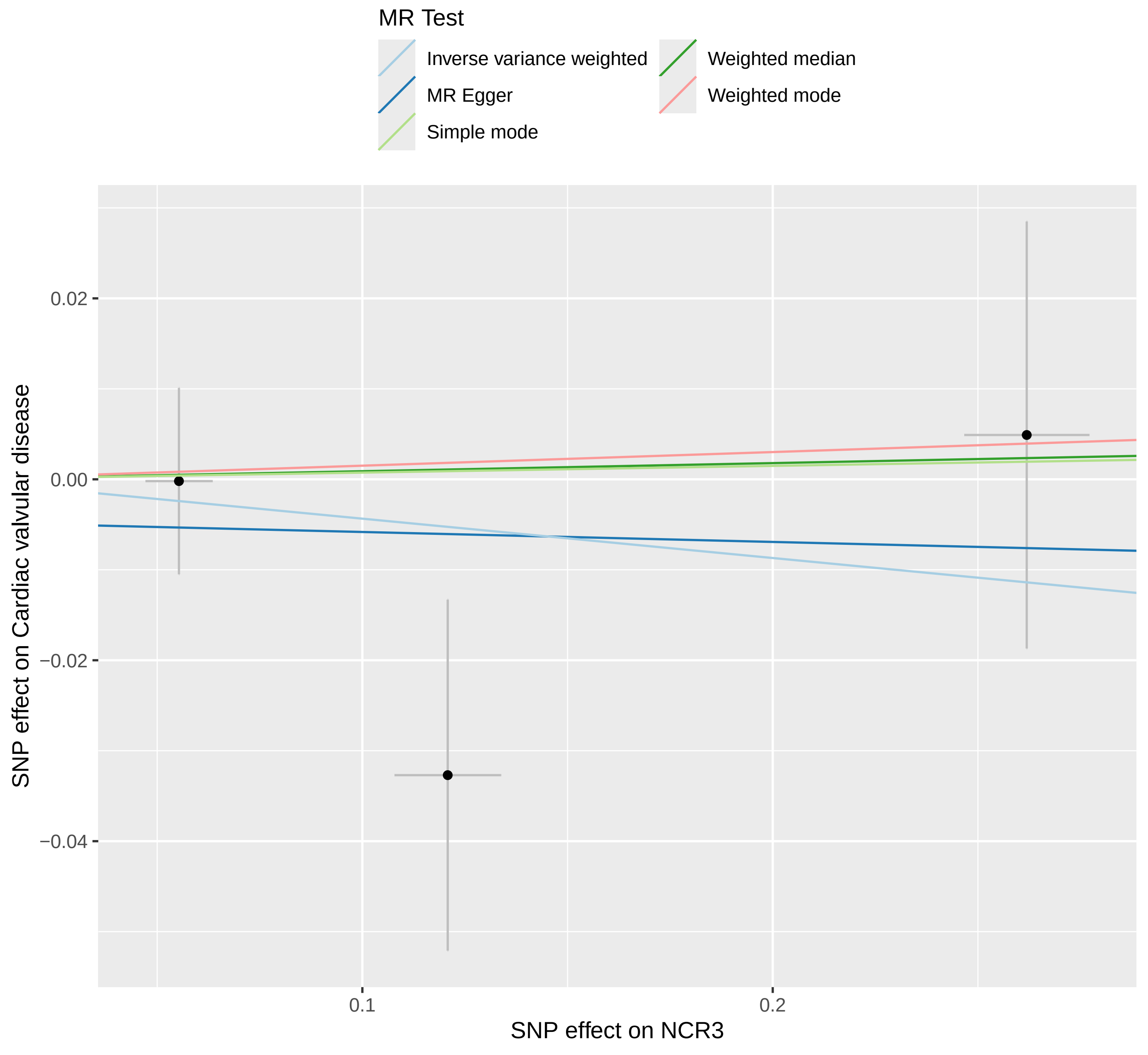
MR Test
Inverse variance weighted
MR Egger
Simple mode
Weighted median
Weighted mode
0.02
0.00
−0.02
−0.04
SNP effect on Cardiac valvular disease
0.1
0.2
SNP effect on NCR3

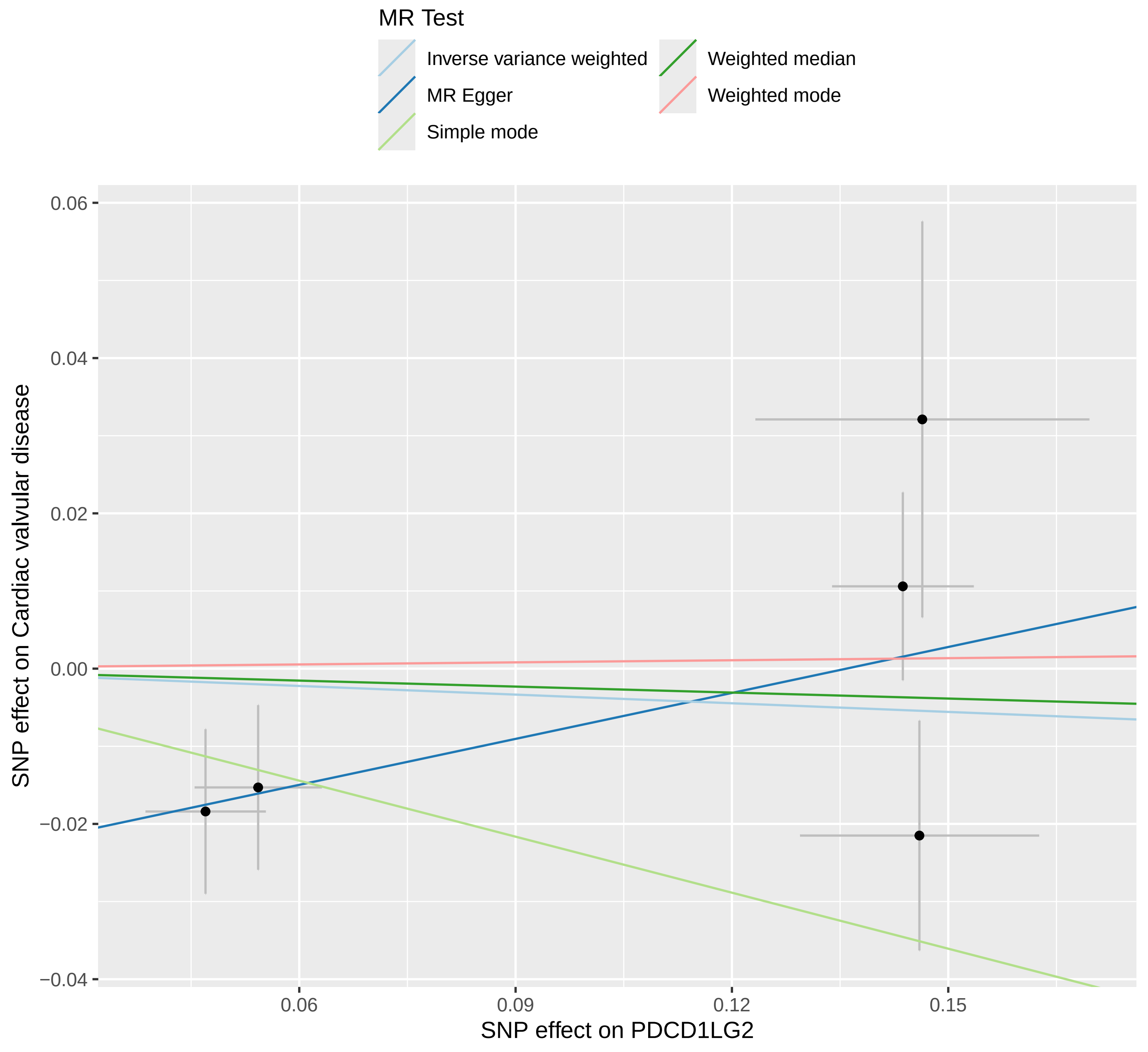
MR Test
Inverse variance weighted
MR Egger
Simple mode
Weighted median
Weighted mode
0.06
0.04
0.02
0.00
−0.02
−0.04
SNP effect on Cardiac valvular disease
0.06
0.09
0.12
0.15
SNP effect on PDCD1LG2

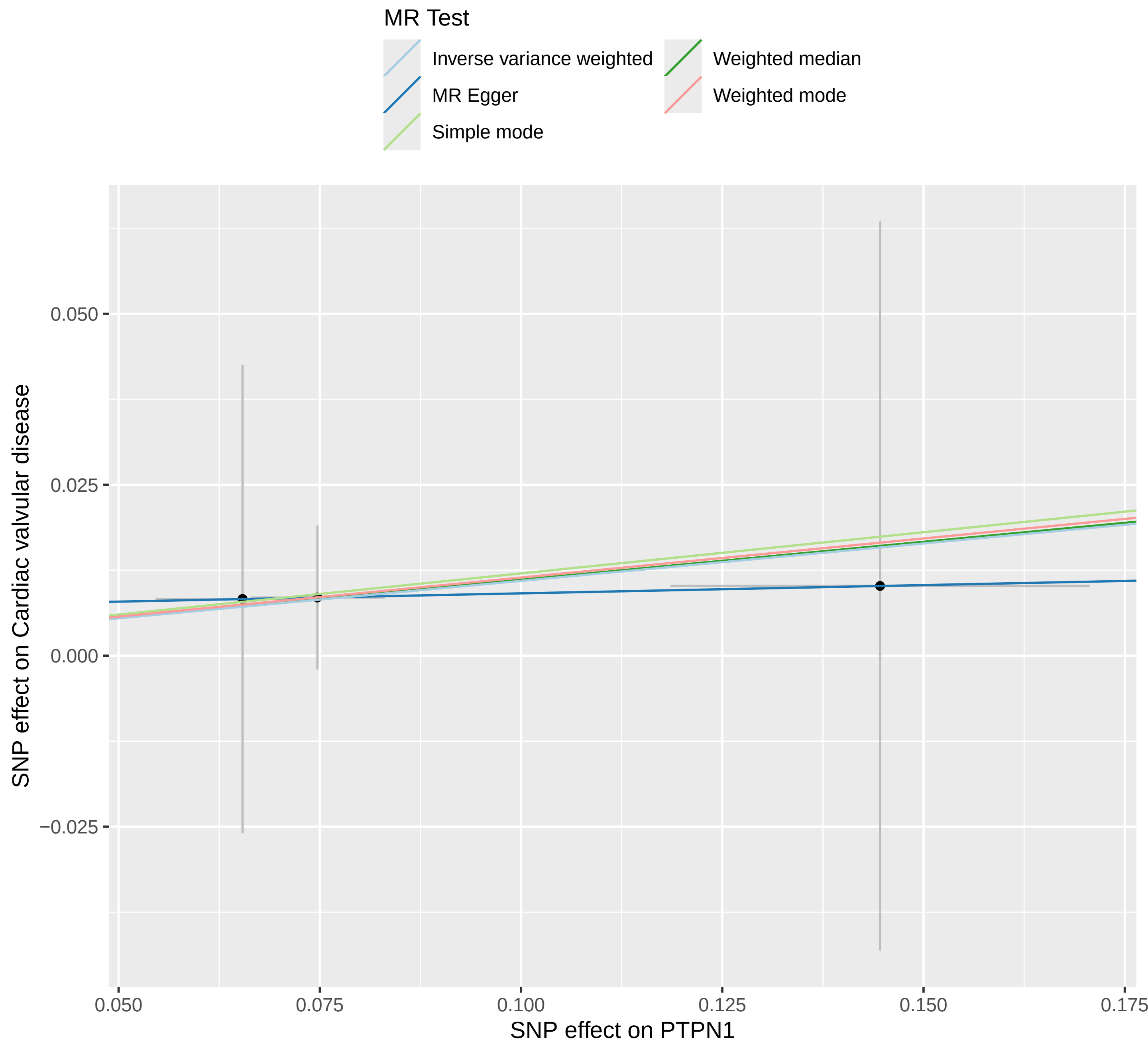

MR Test
Inverse variance weighted
MR Egger
Simple mode
Weighted median
Weighted mode
0.050
0.025
0.000
−0.025
SNP effect on Cardiac valvular disease
0.050
0.075
0.100
0.125
0.150
0.175
SNP effect on PTPN1

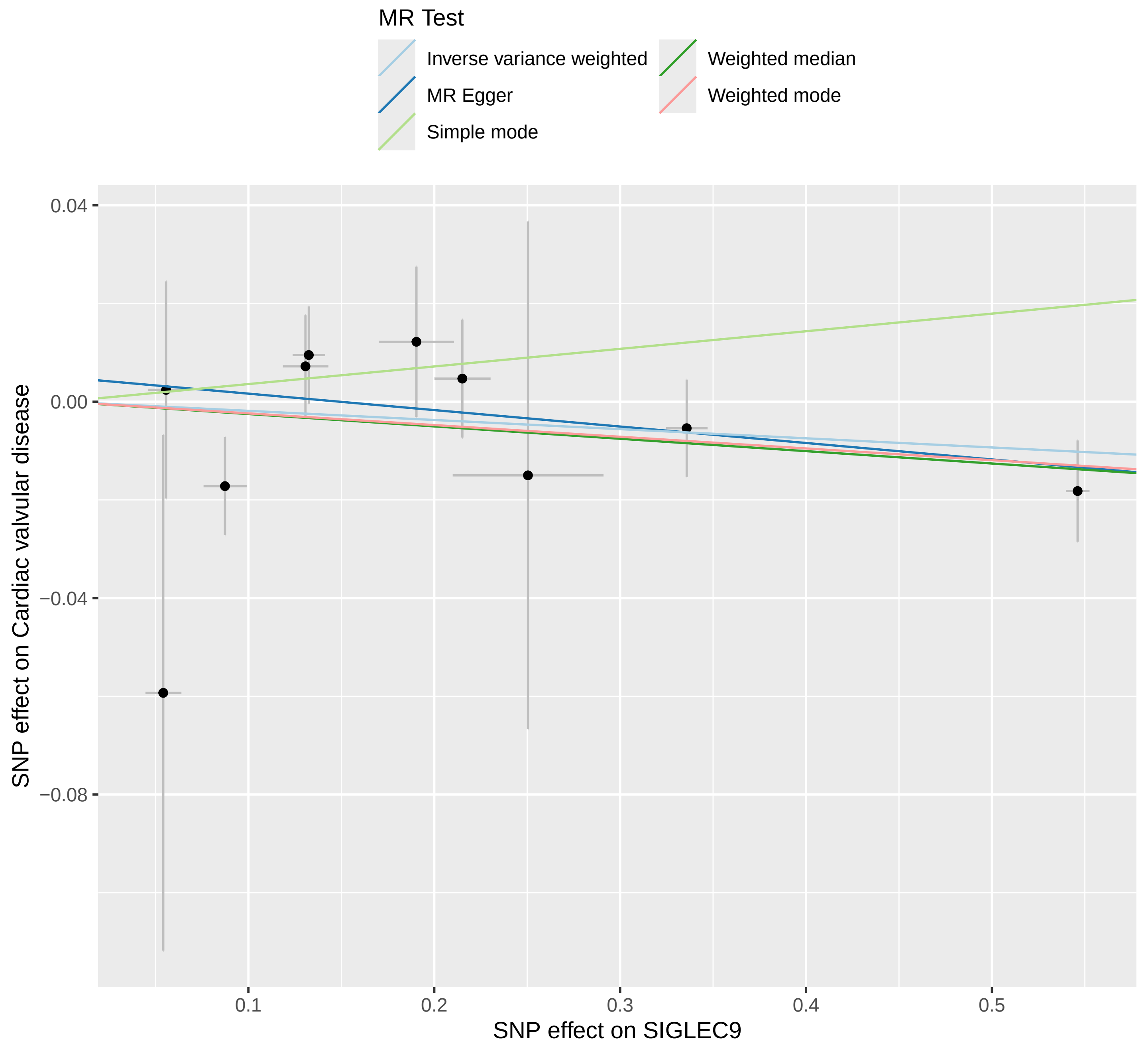

MR Test
Inverse variance weighted
MR Egger
Simple mode
Weighted median
Weighted mode
0.04
0.00
−0.04
−0.08
0.1
0.2
0.3
0.4
0.5
SNP effect on Cardiac valvular disease
SNP effect on SIGLEC9

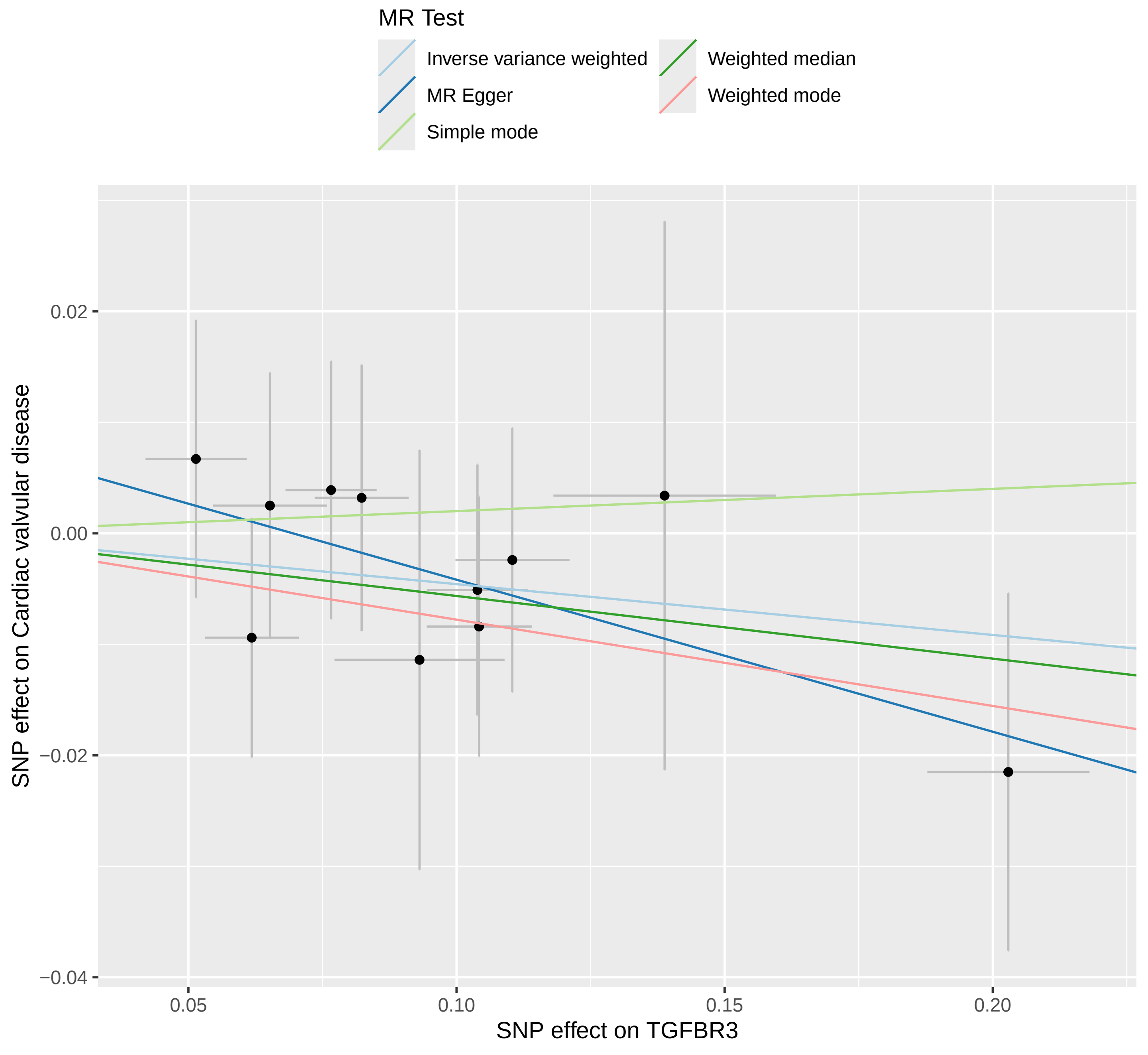
MR Test
Inverse variance weighted
MR Egger
Simple mode
Weighted median
Weighted mode
0.02
0.00
−0.02
−0.04
0.05
0.10
0.15
0.20
SNP effect on Cardiac valvular disease
SNP effect on TGFBR3

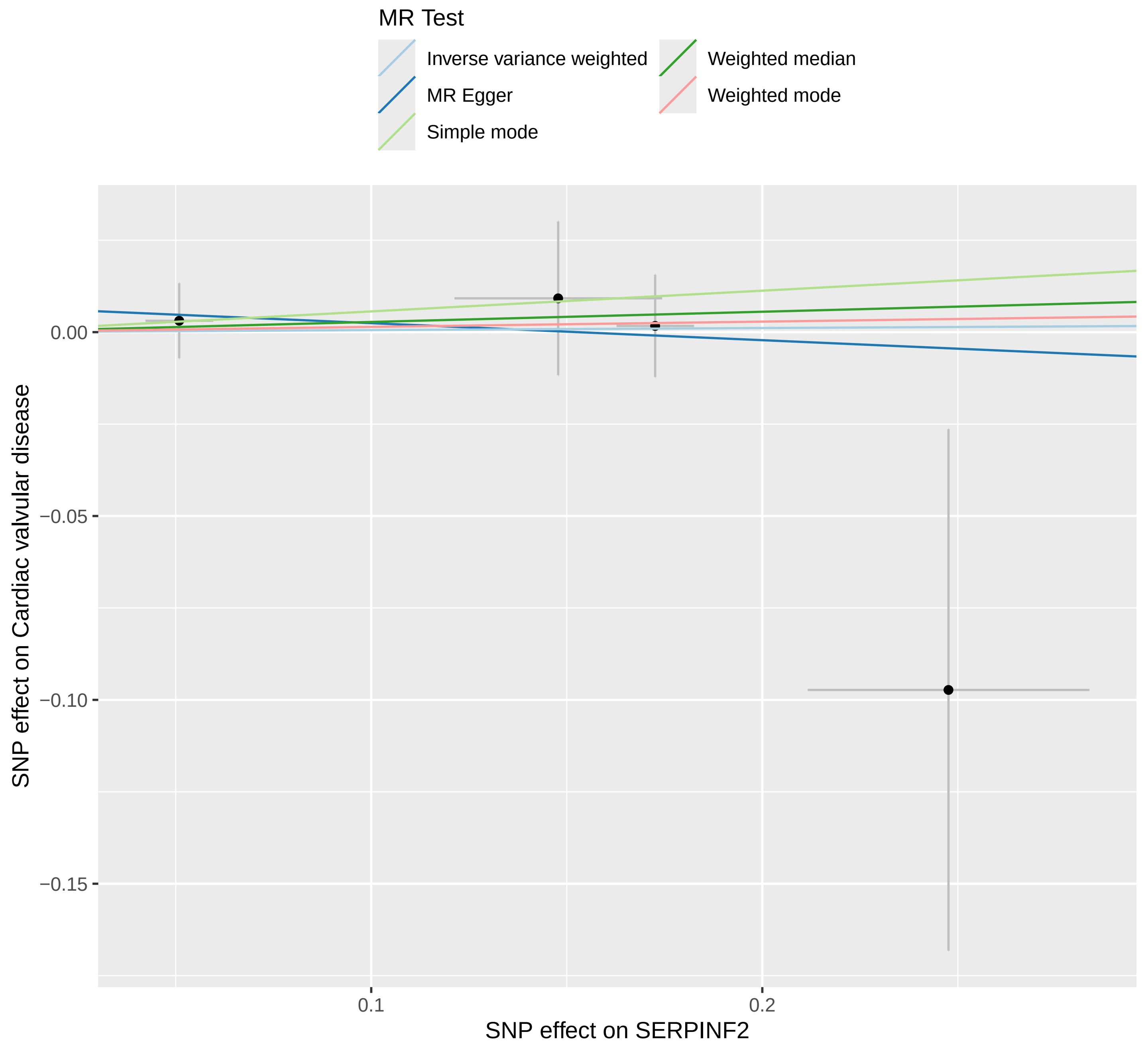

MR Test
Inverse variance weighted
MR Egger
Simple mode
Weighted median
Weighted mode
0.00
−0.05
−0.10
−0.15
0.1
0.2
SNP effect on SERPINF2
SNP effect on Cardiac valvular disease

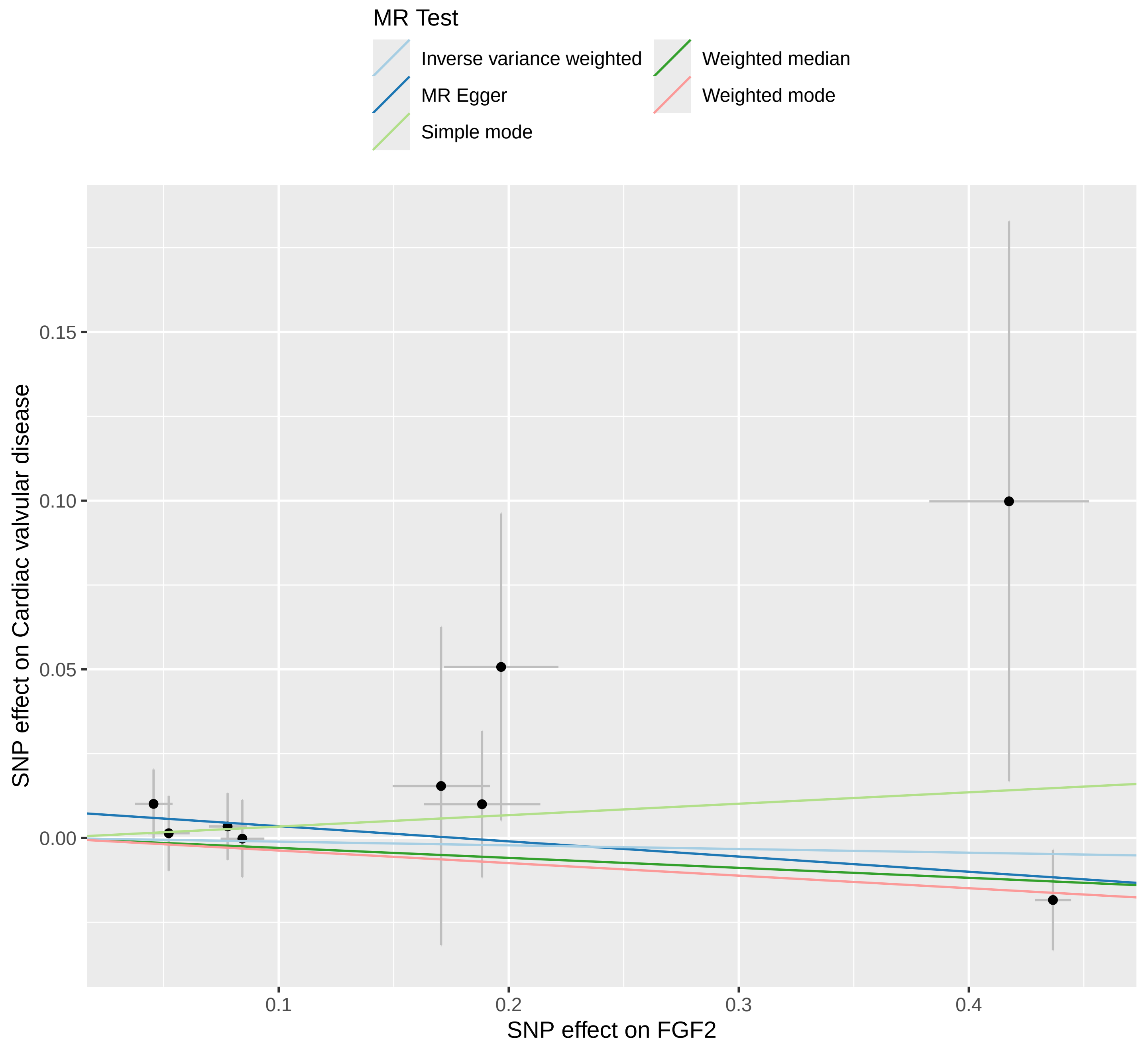

MR Test
Inverse variance weighted
MR Egger
Simple mode
Weighted median
Weighted mode
0.15
0.10
0.05
0.00
SNP effect on Cardiac valvular disease
0.1
0.2
0.3
0.4
SNP effect on FGF2

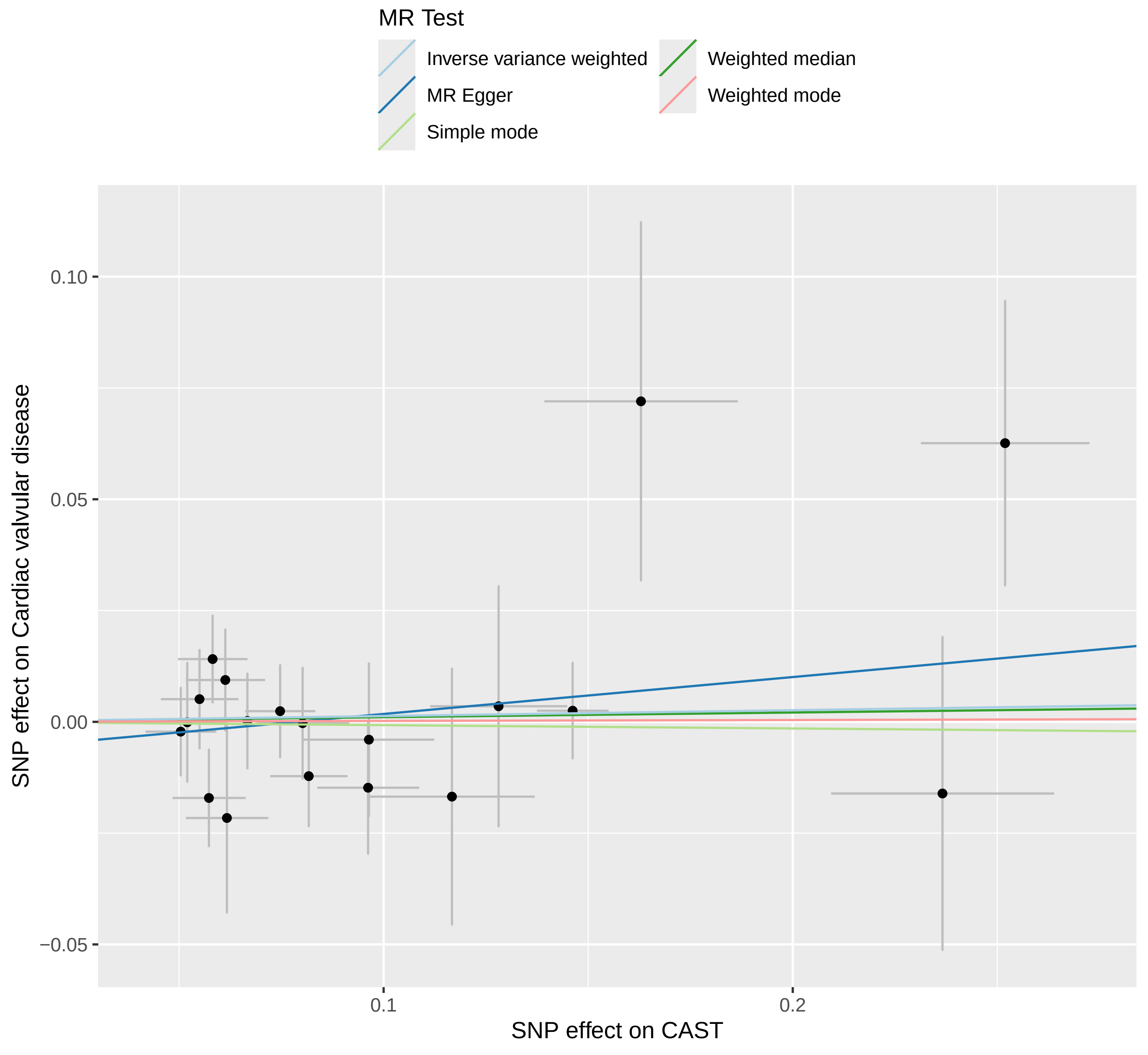

MR Test
Inverse variance weighted
MR Egger
Simple mode
Weighted median
Weighted mode
SNP effect on Cardiac valvular disease
0.10
0.05
0.00
−0.05
0.1
0.2
SNP effect on CAST

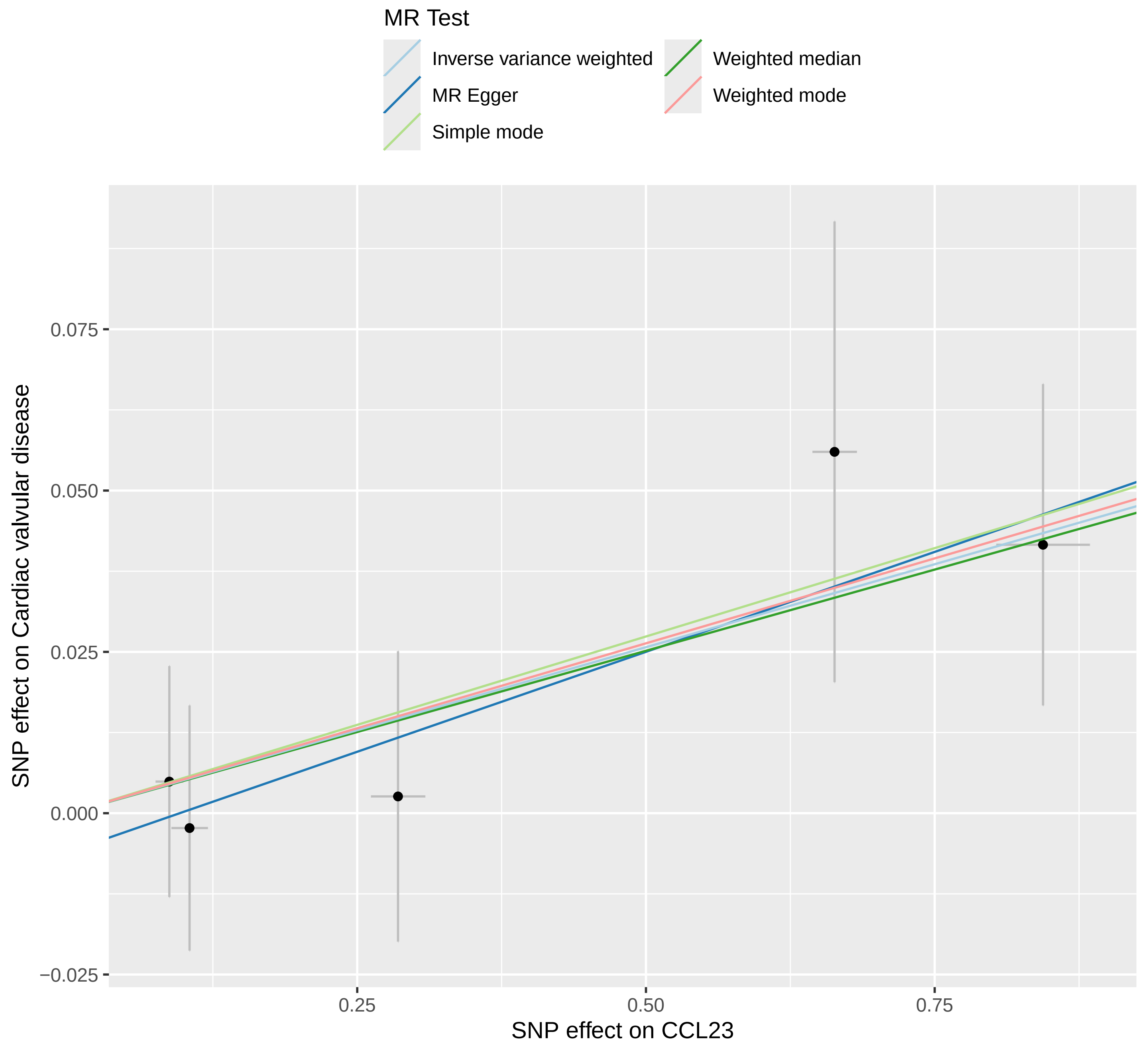
MR Test
Inverse variance weighted
MR Egger
Simple mode
Weighted median
Weighted mode
0.075
0.050
0.025
0.000
−0.025
SNP effect on Cardiac valvular disease
0.25
0.50
0.75
SNP effect on CCL23

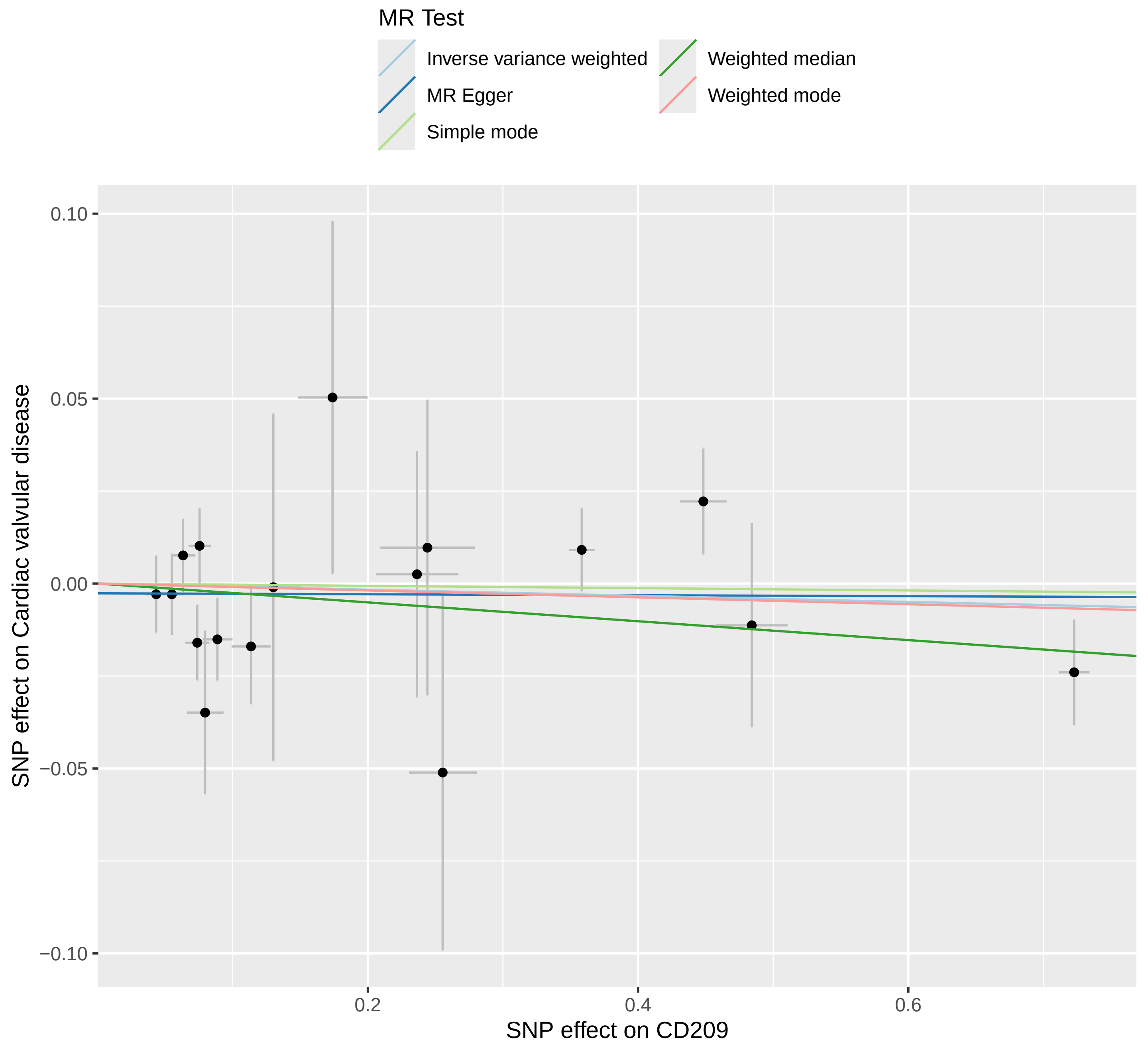

MR Test
Inverse variance weighted
MR Egger
Simple mode
Weighted median
Weighted mode
0.10
0.05
0.00
−0.05
−0.10
SNP effect on Cardiac valvular disease
0.2
0.4
0.6
SNP effect on CD209

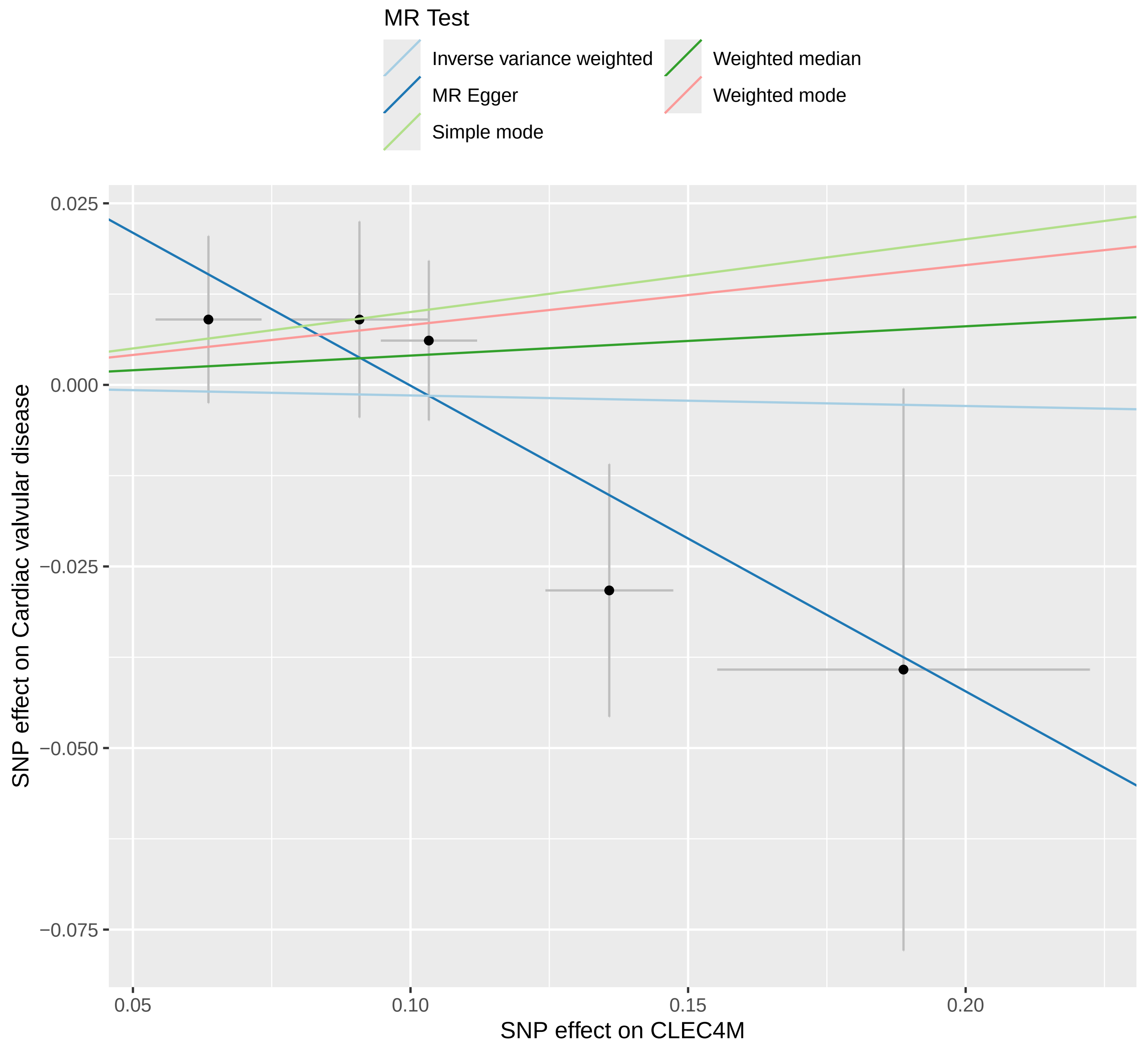

MR Test
Inverse variance weighted
MR Egger
Simple mode
Weighted median
Weighted mode
0.025
0.000
−0.025
−0.050
−0.075
SNP effect on Cardiac valvular disease
0.05
0.10
0.15
0.20
SNP effect on CLEC4M

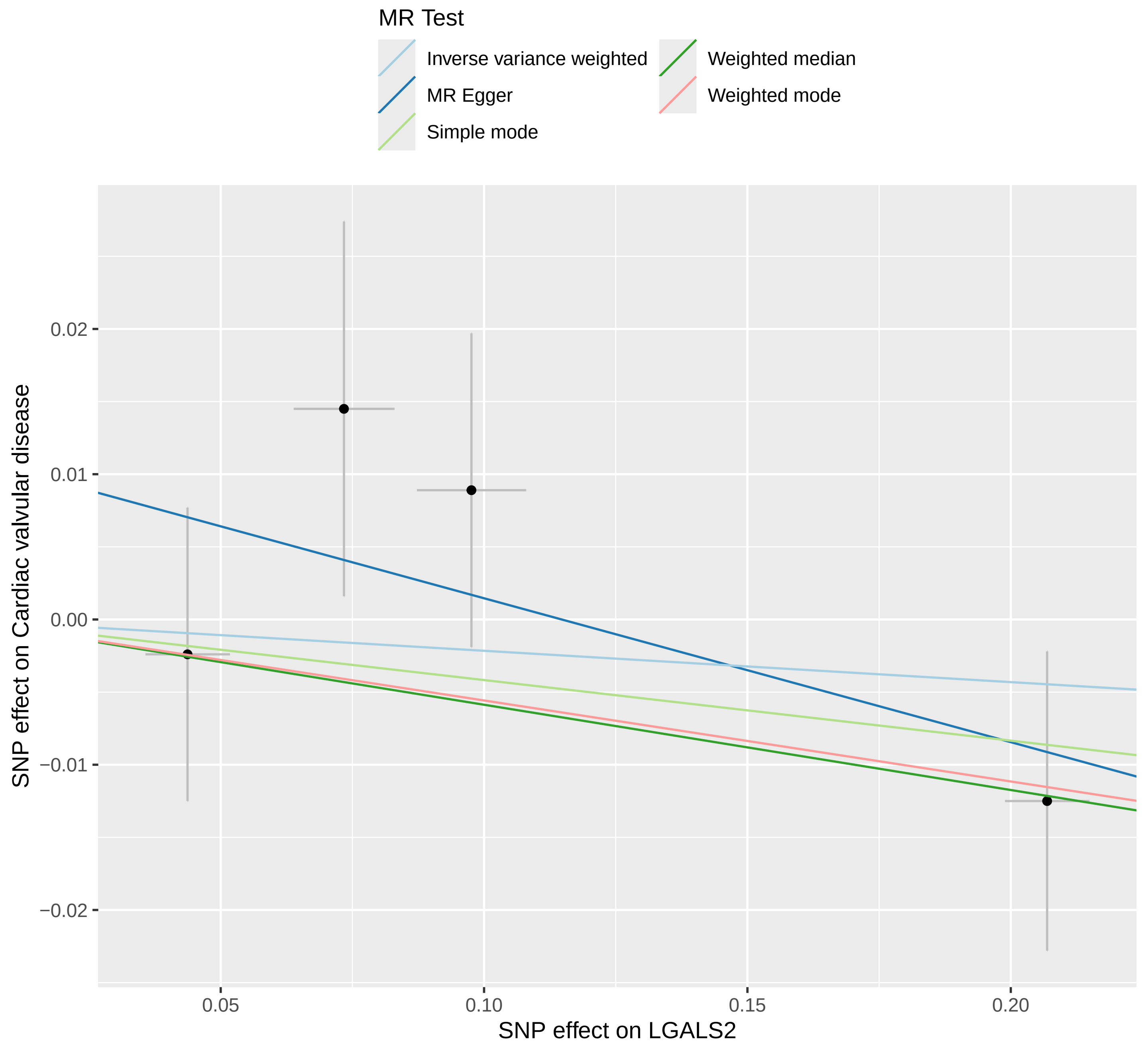
MR Test
Inverse variance weighted
MR Egger
Simple mode
Weighted median
Weighted mode
0.02
0.01
0.00
−0.01
−0.02
SNP effect on Cardiac valvular disease
0.05
0.10
0.15
0.20
SNP effect on LGALS2

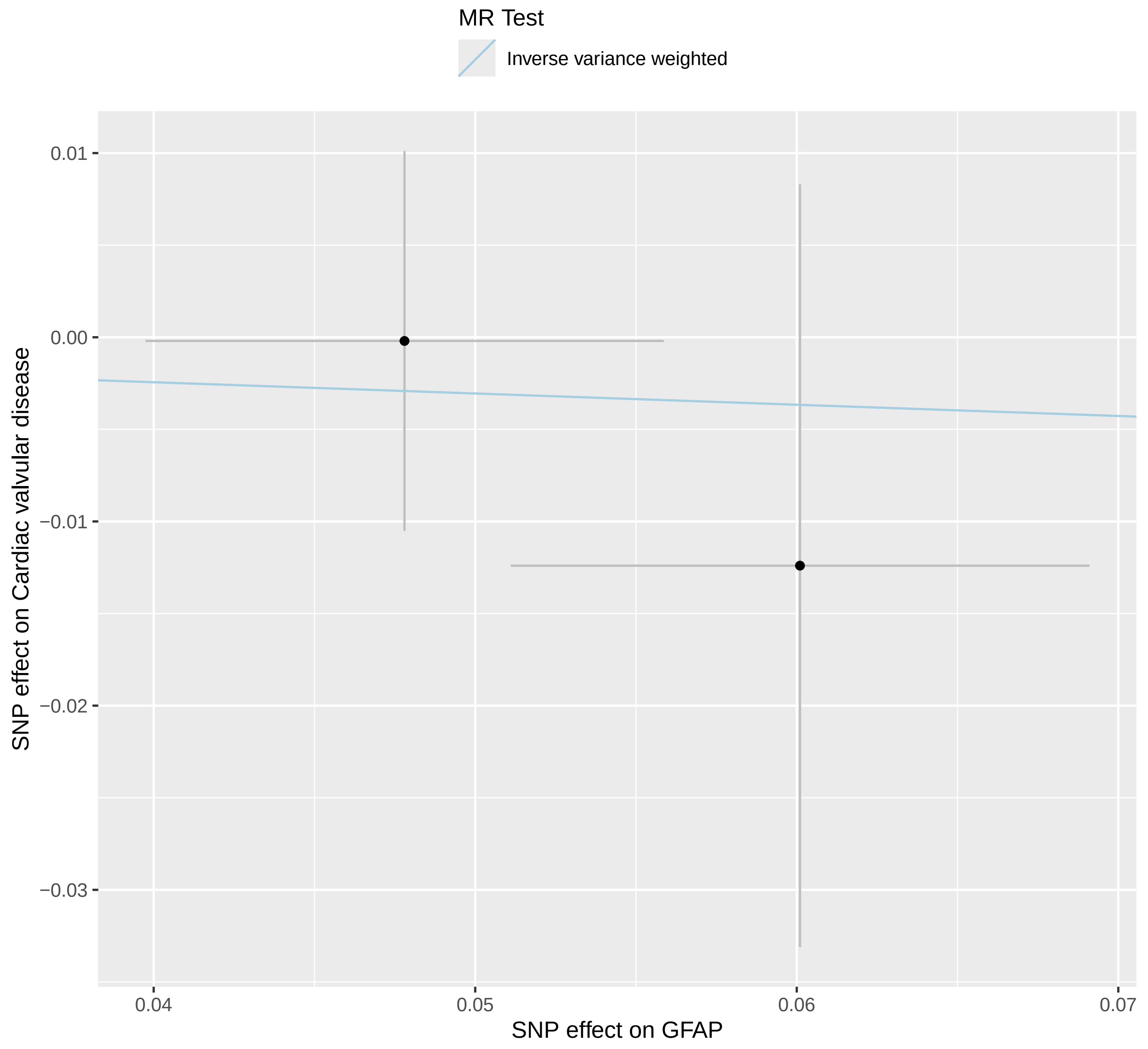
MR Test
Inverse variance weighted
0.01
0.00
−0.01
−0.02
−0.03
SNP effect on Cardiac valvular disease
0.04
0.05
0.06
0.07
SNP effect on GFAP

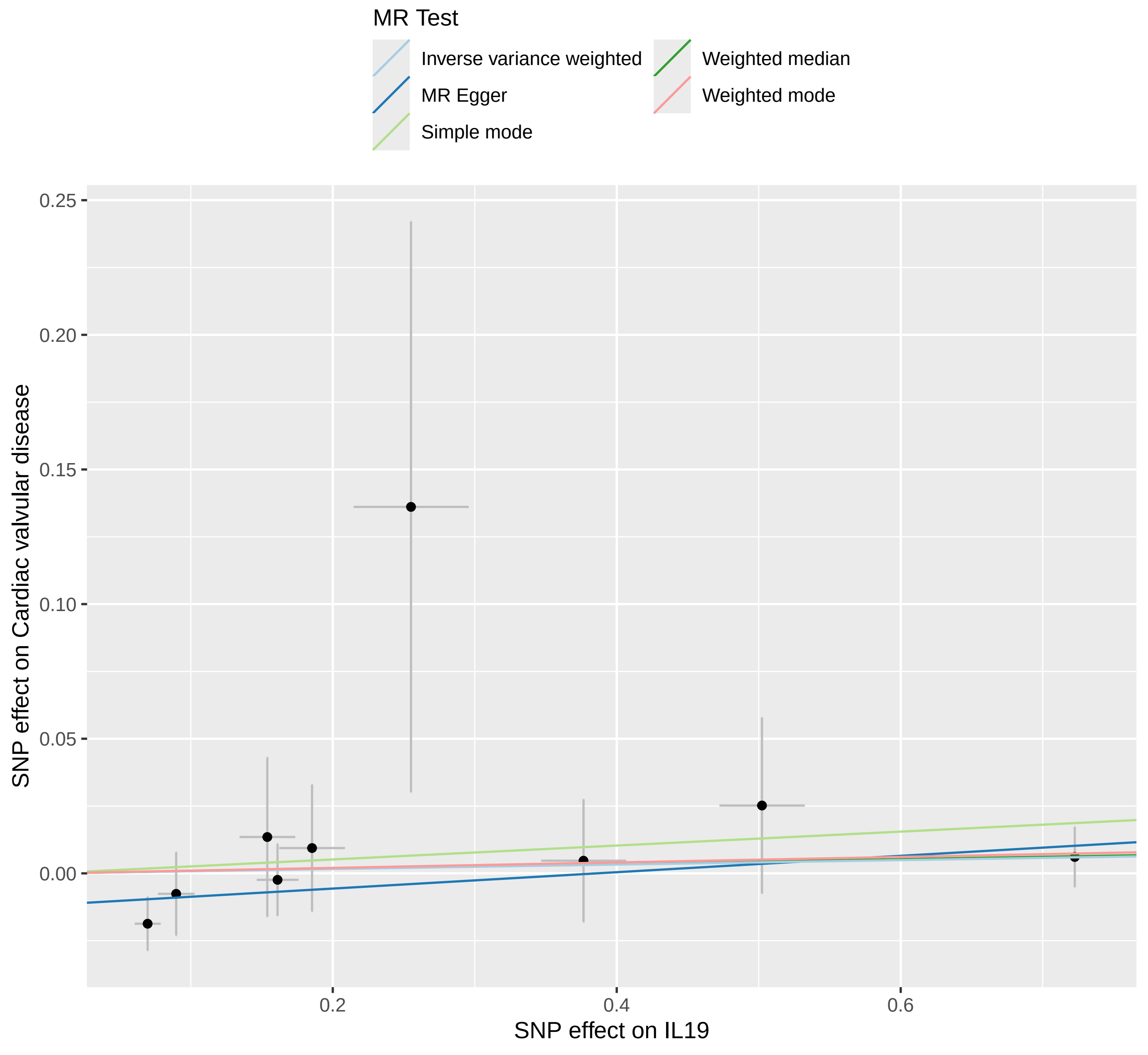
MR Test
Inverse variance weighted
MR Egger
Simple mode
Weighted median
Weighted mode
0.25
0.20
0.15
0.10
0.05
0.00
SNP effect on Cardiac valvular disease
0.2
0.4
0.6
SNP effect on IL19

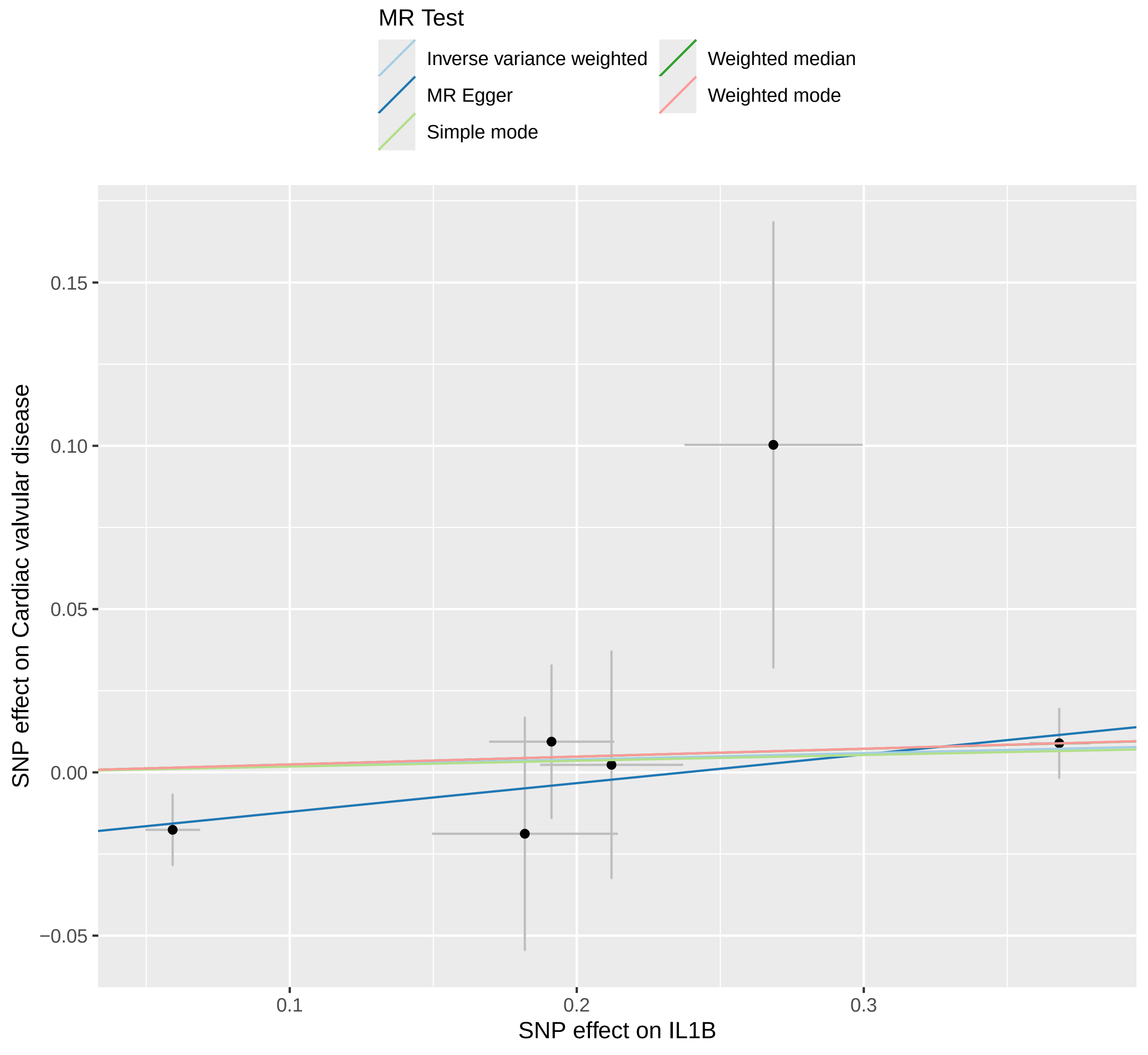

MR Test
Inverse variance weighted
MR Egger
Simple mode
Weighted median
Weighted mode
SNP effect on Cardiac valvular disease
0.15
0.10
0.05
0.00
−0.05
0.1
0.2
0.3
SNP effect on IL1B

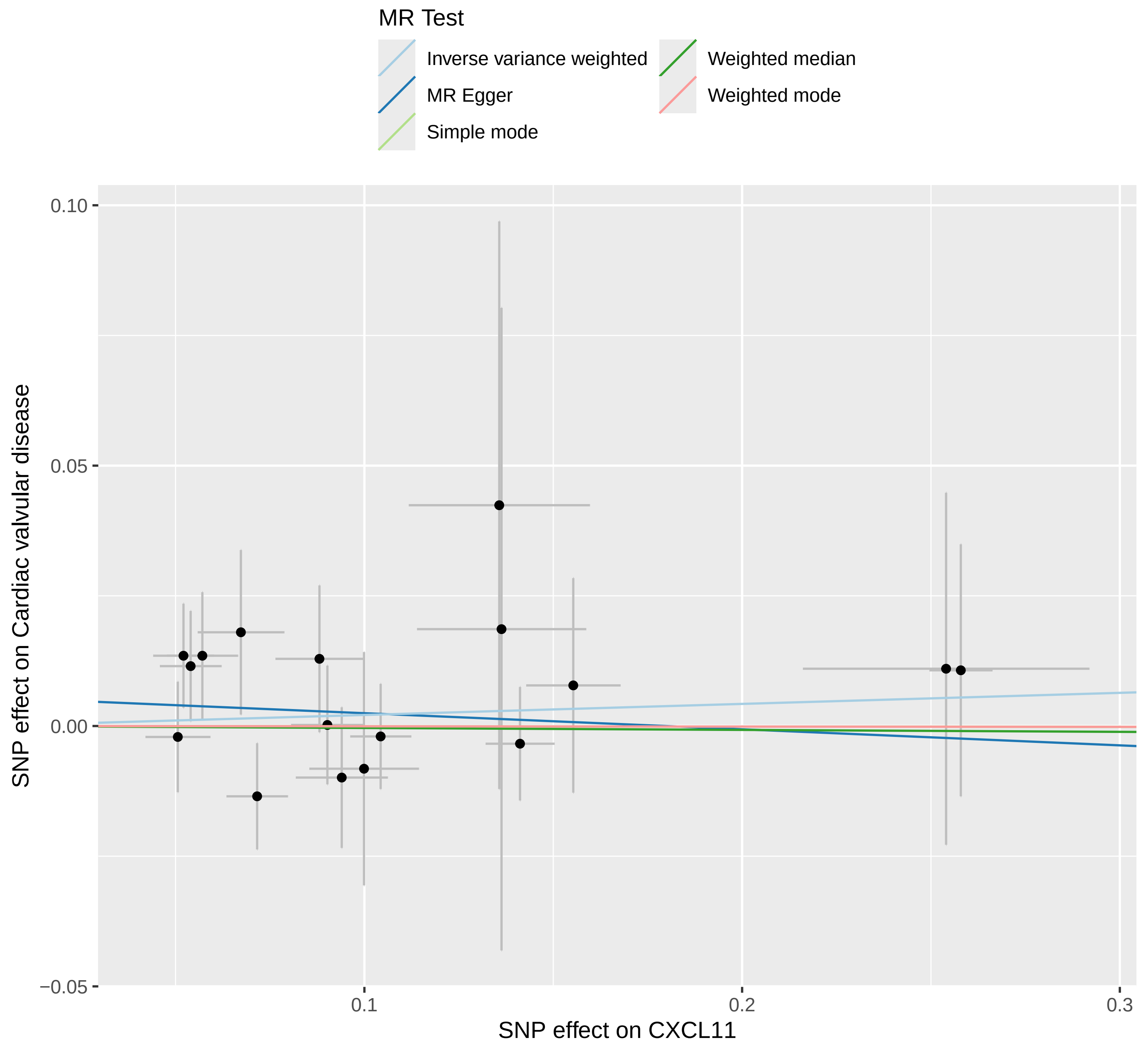

MR Test
Inverse variance weighted
MR Egger
Simple mode
Weighted median
Weighted mode
SNP effect on Cardiac valvular disease
0.10
0.05
0.00
−0.05
0.1
0.2
0.3
SNP effect on CXCL11

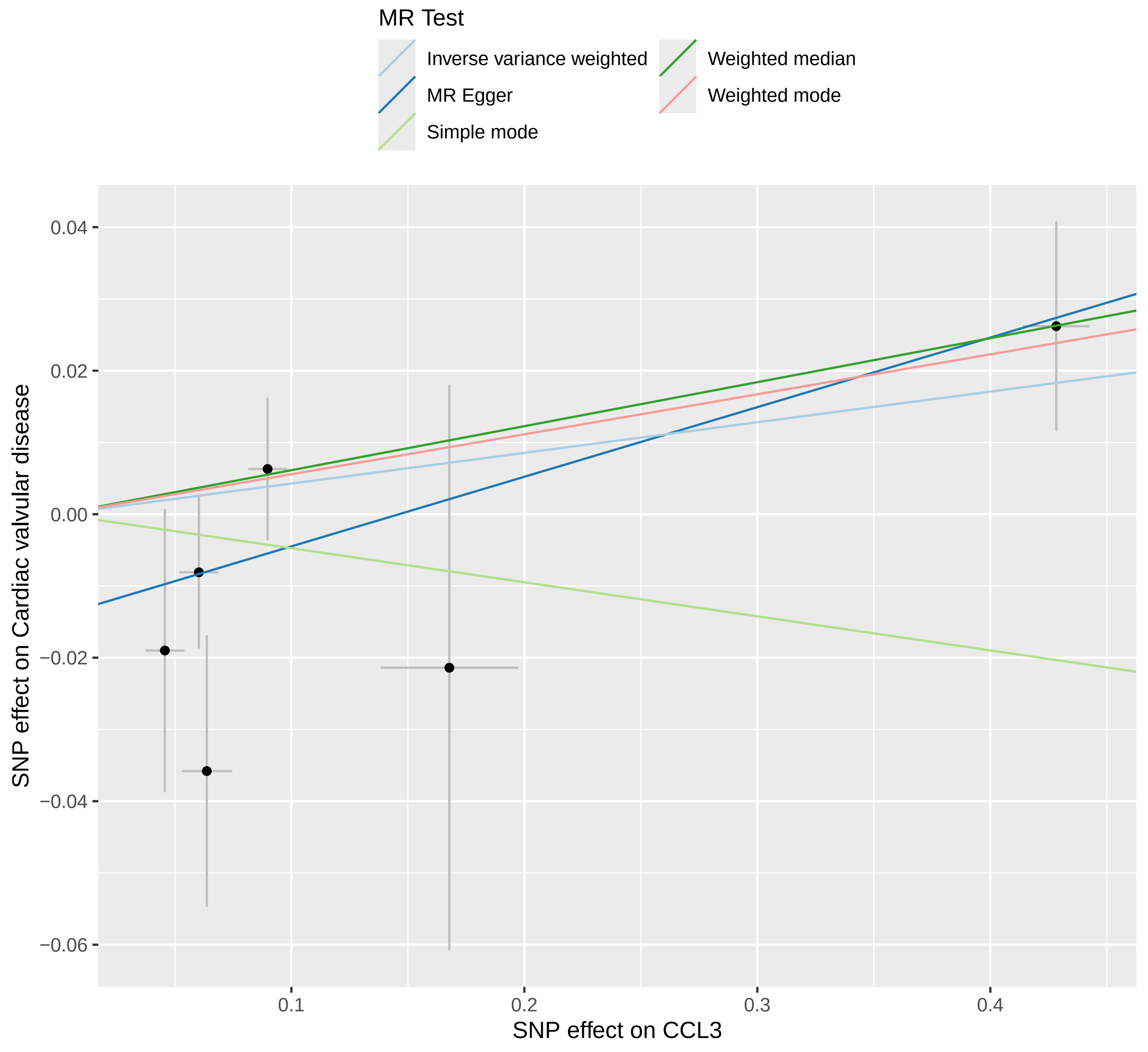

MR Test
Inverse variance weighted
MR Egger
Simple mode
Weighted median
Weighted mode
0.04
0.02
0.00
−0.02
−0.04
−0.06
SNP effect on Cardiac valvular disease
0.1
0.2
0.3
0.4
SNP effect on CCL3

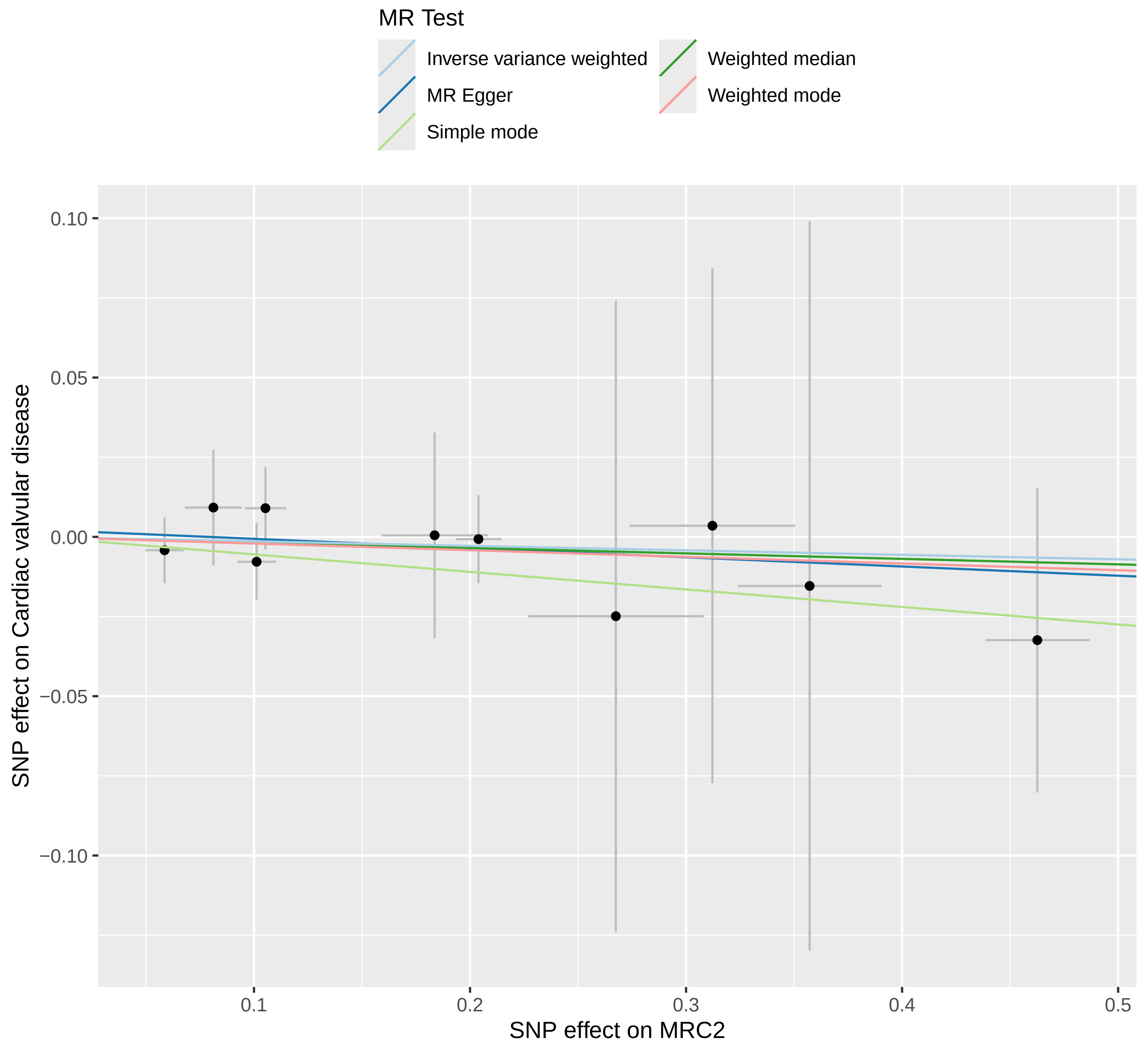
MR Test
Inverse variance weighted
MR Egger
Simple mode
Weighted median
Weighted mode
0.10
0.05
0.00
−0.05
−0.10
SNP effect on Cardiac valvular disease
0.1
0.2
0.3
0.4
0.5
SNP effect on MRC2

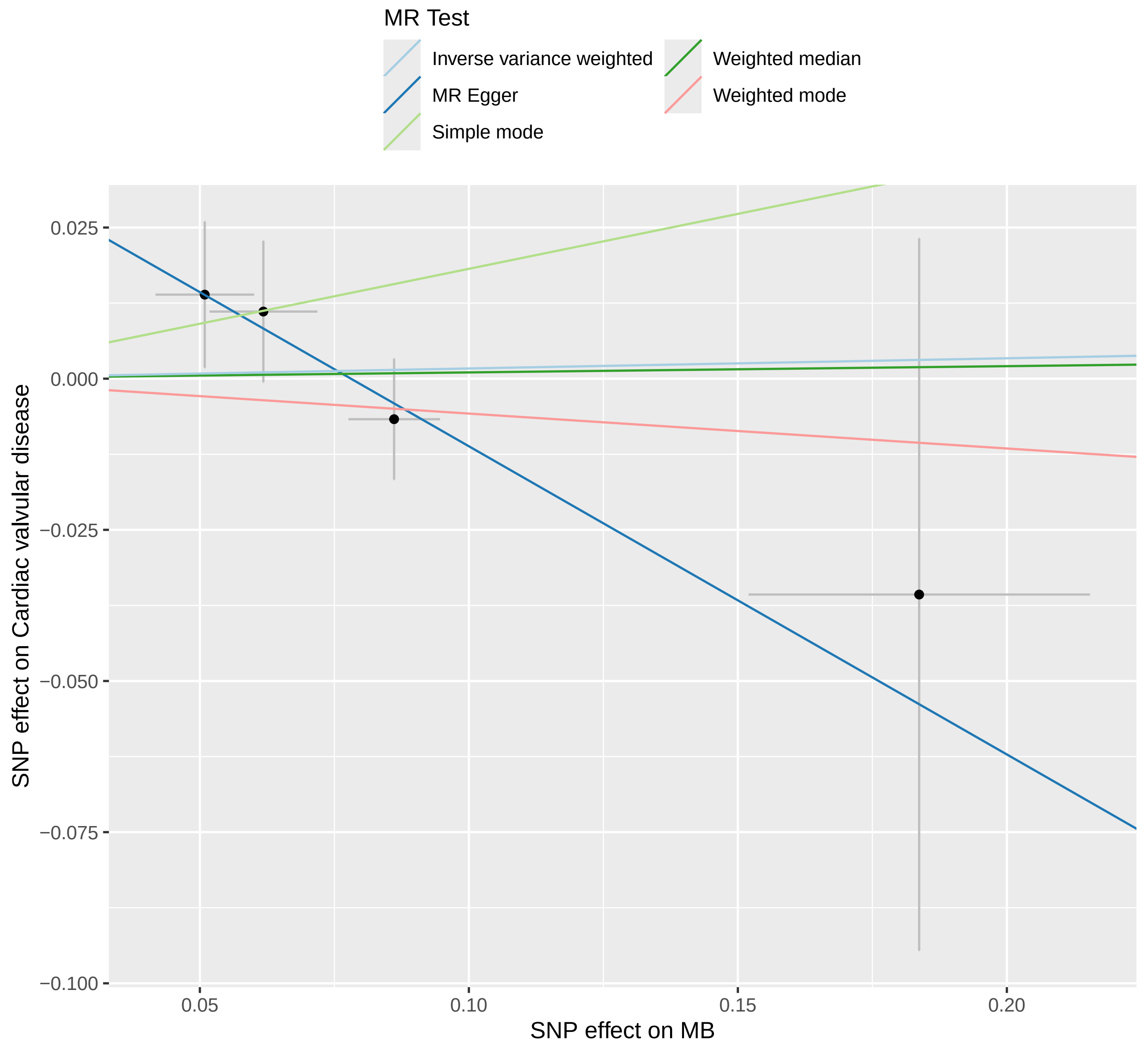
MR Test
Inverse variance weighted
MR Egger
Simple mode
Weighted median
Weighted mode
0.025
0.000
−0.025
−0.050
−0.075
−0.100
SNP effect on Cardiac valvular disease
0.05
0.10
0.15
0.20
SNP effect on MB

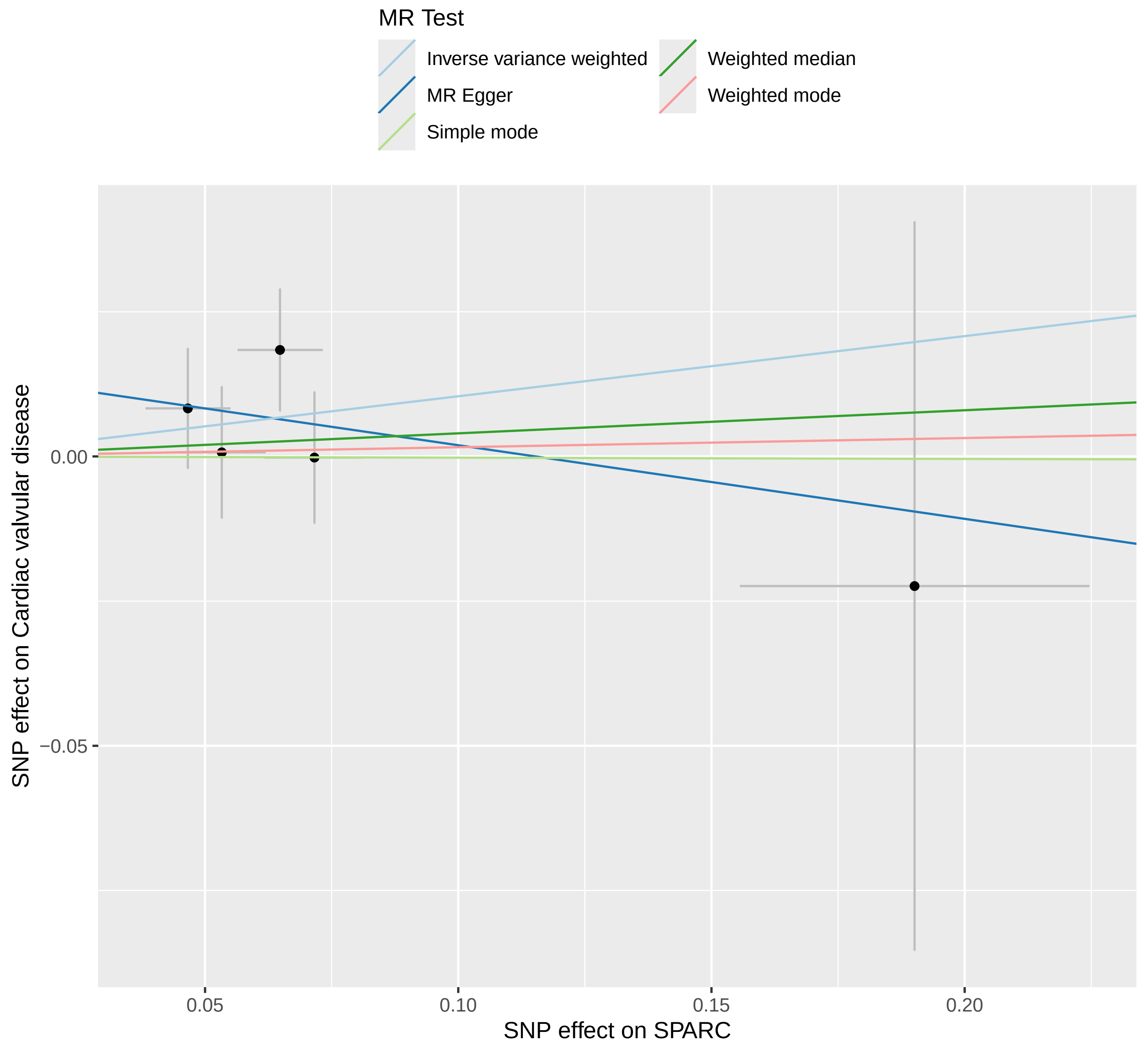

MR Test
Inverse variance weighted
MR Egger
Simple mode
Weighted median
Weighted mode
SNP effect on Cardiac valvular disease
0.00
−0.05
0.05
0.10
0.15
0.20
SNP effect on SPARC

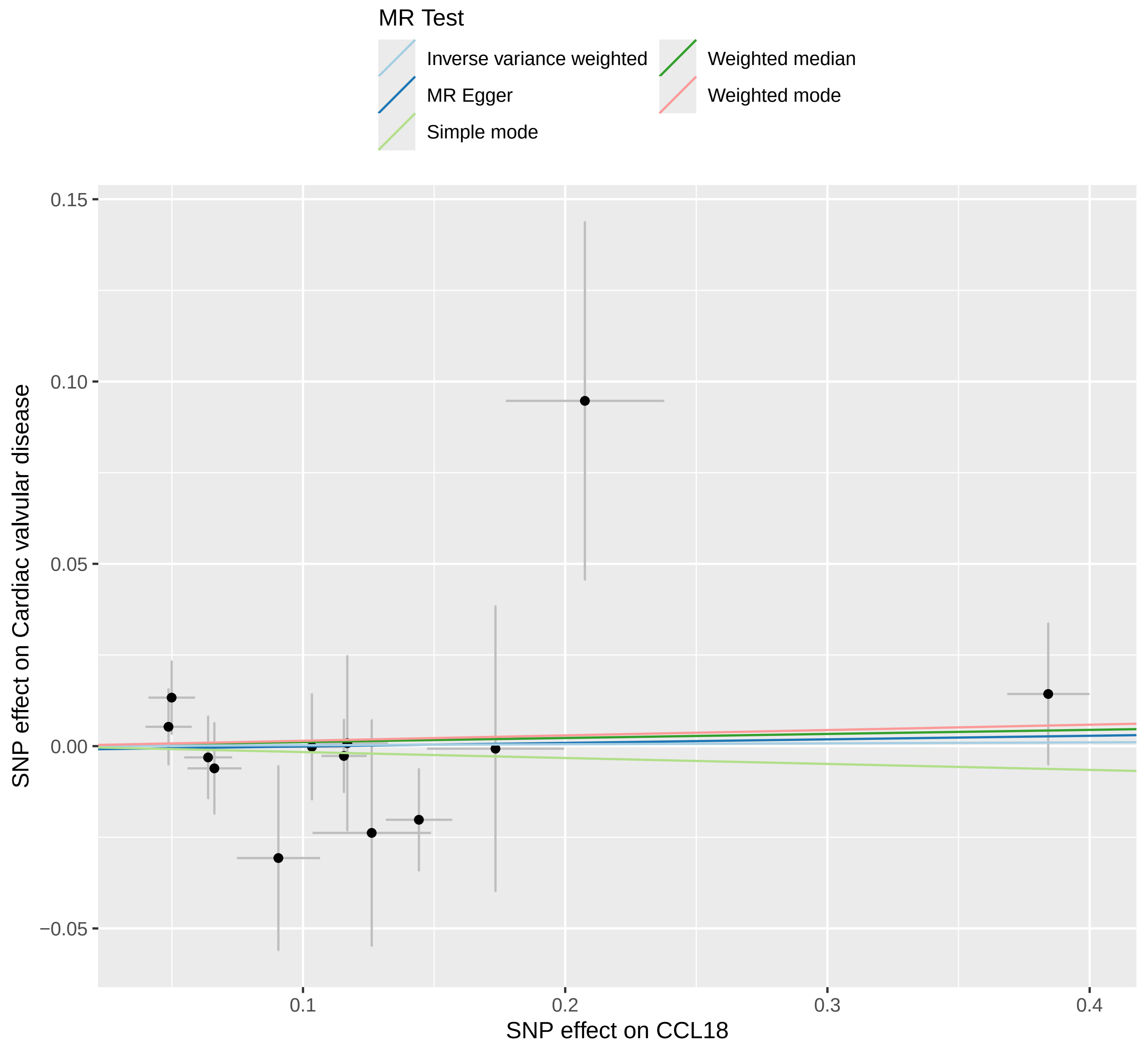
MR Test
Inverse variance weighted
MR Egger
Simple mode
Weighted median
Weighted mode
0.15
0.10
0.05
0.00
−0.05
0.1
0.2
0.3
0.4
SNP effect on Cardiac valvular disease
SNP effect on CCL18

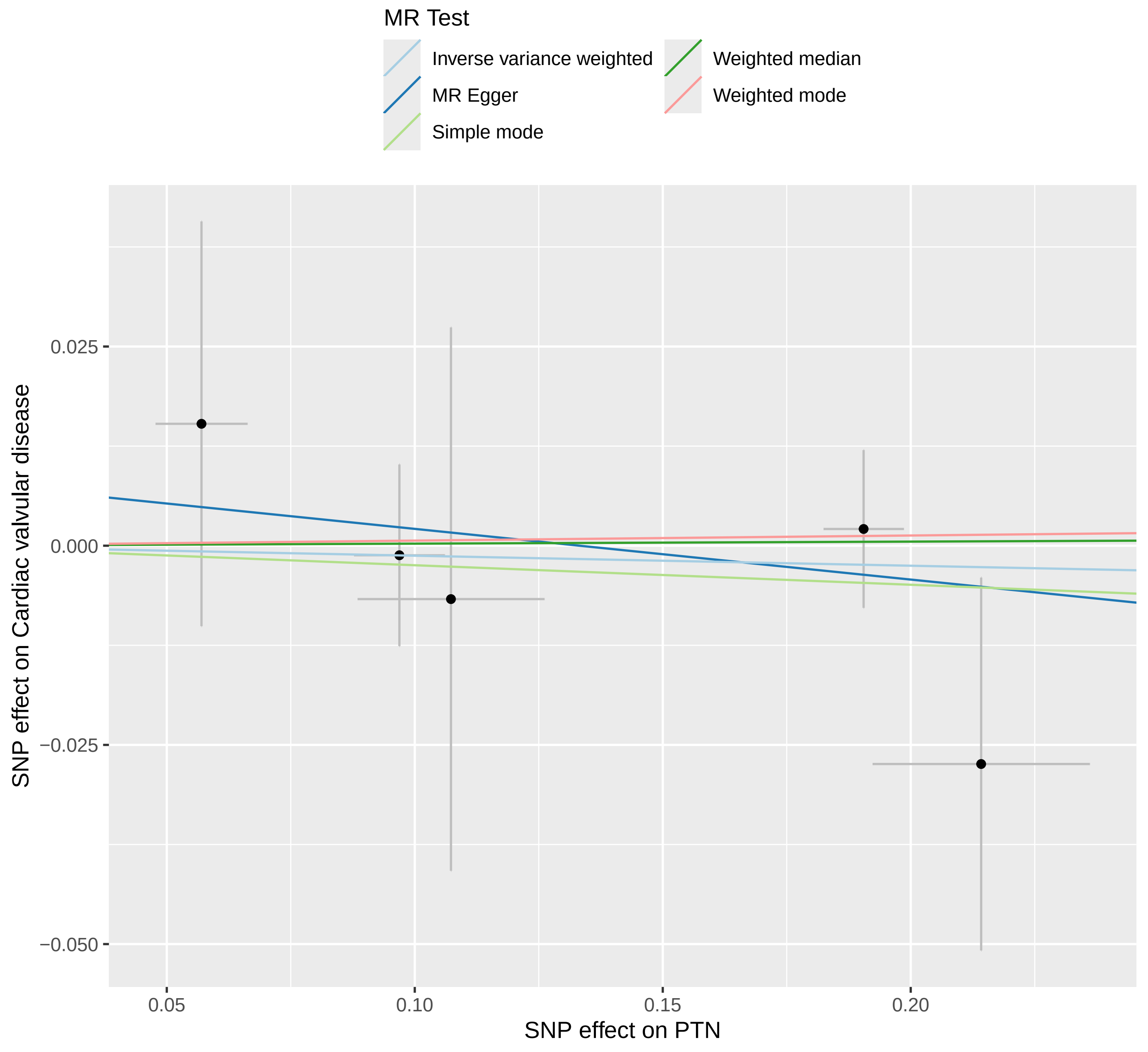
MR Test
Inverse variance weighted
MR Egger
Simple mode
Weighted median
Weighted mode
0.025
0.000
−0.025
−0.050
SNP effect on Cardiac valvular disease
0.05
0.10
0.15
0.20
SNP effect on PTN

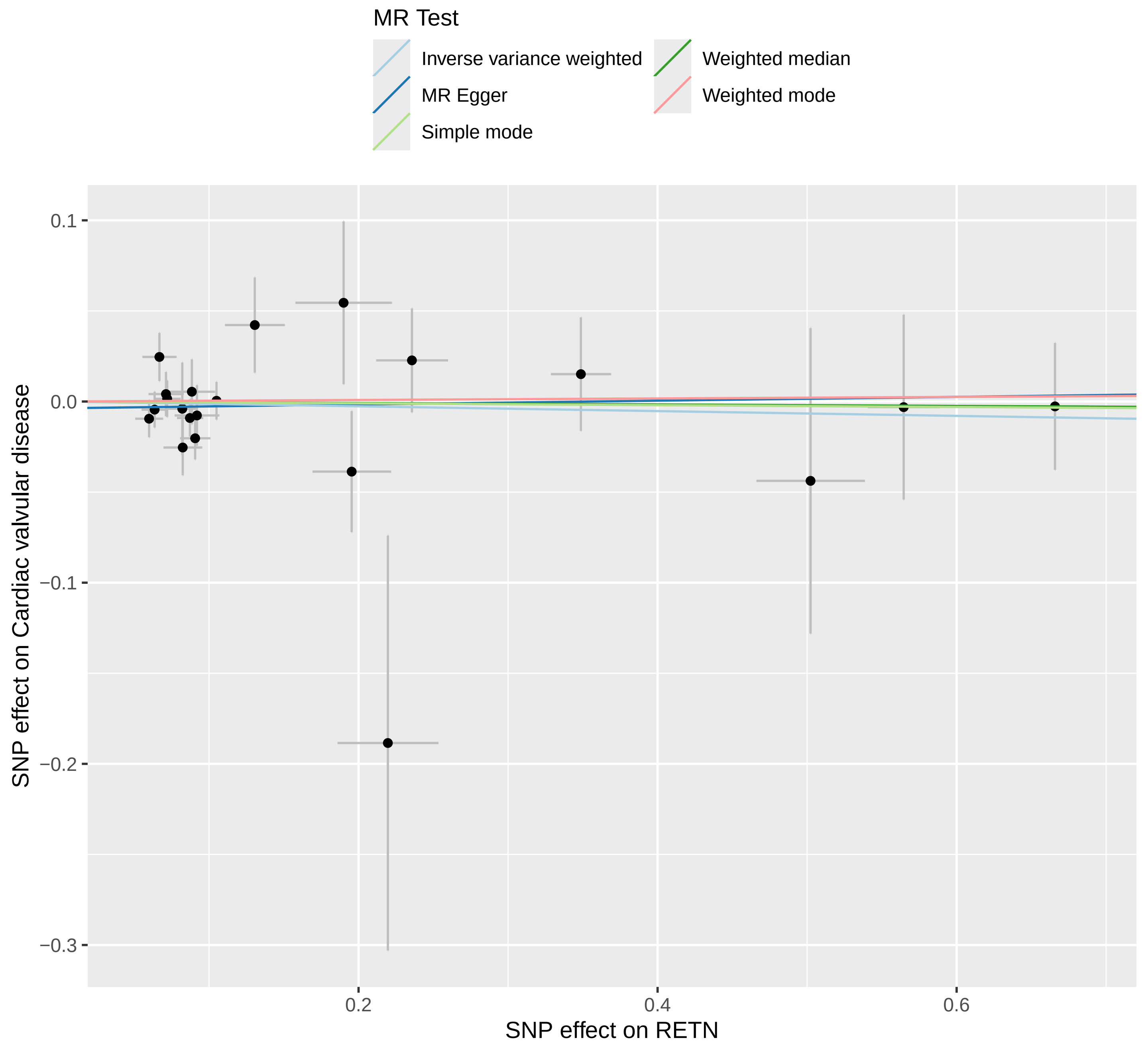
MR Test
Inverse variance weighted
MR Egger
Simple mode
Weighted median
Weighted mode
0.1
0.0
−0.1
−0.2
−0.3
SNP effect on Cardiac valvular disease
0.2
0.4
0.6
SNP effect on RETN

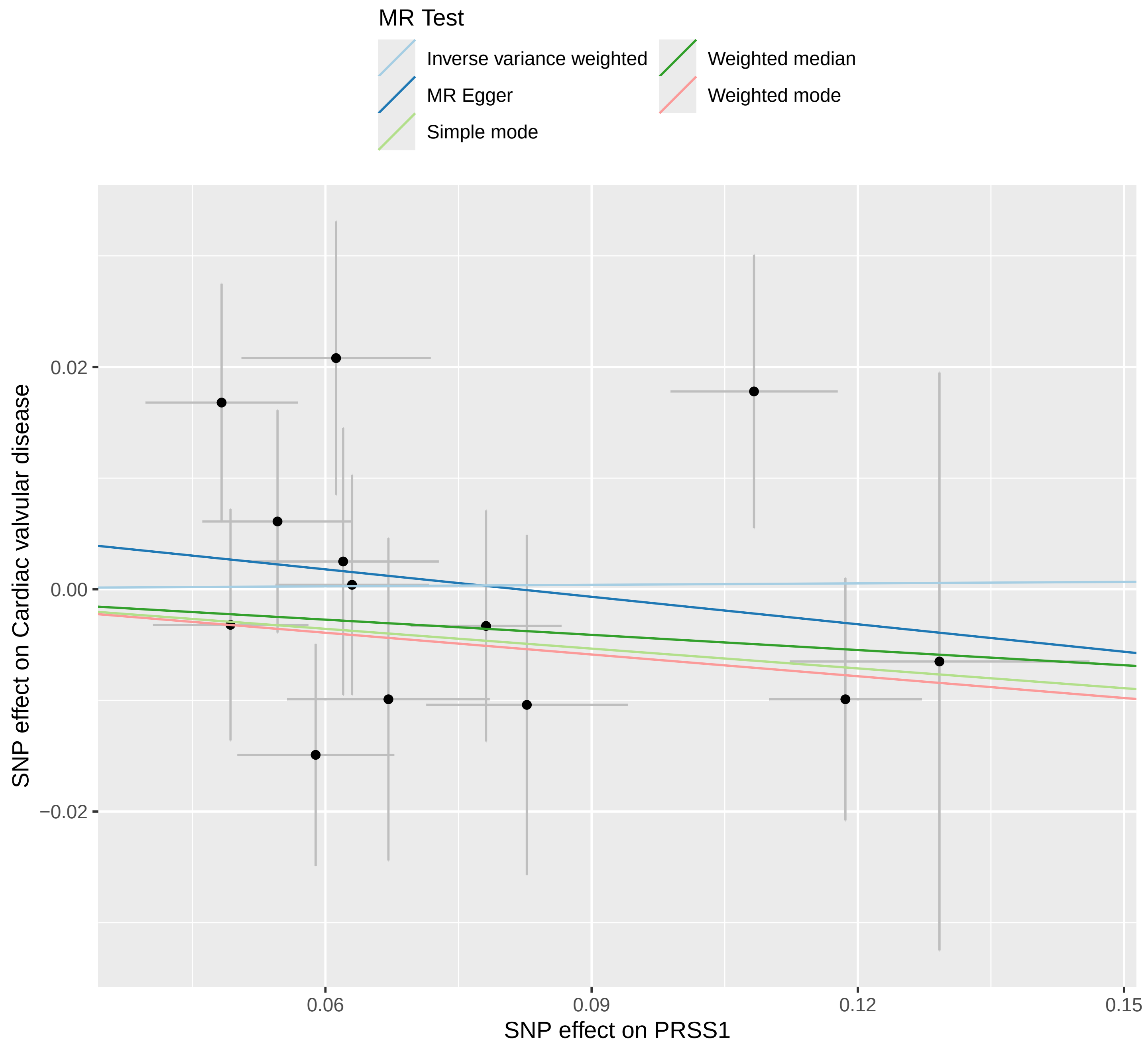
MR Test
Inverse variance weighted
MR Egger
Simple mode
Weighted median
Weighted mode
0.02
0.00
−0.02
0.06
0.09
0.12
0.15
SNP effect on PRSS1
SNP effect on Cardiac valvular disease

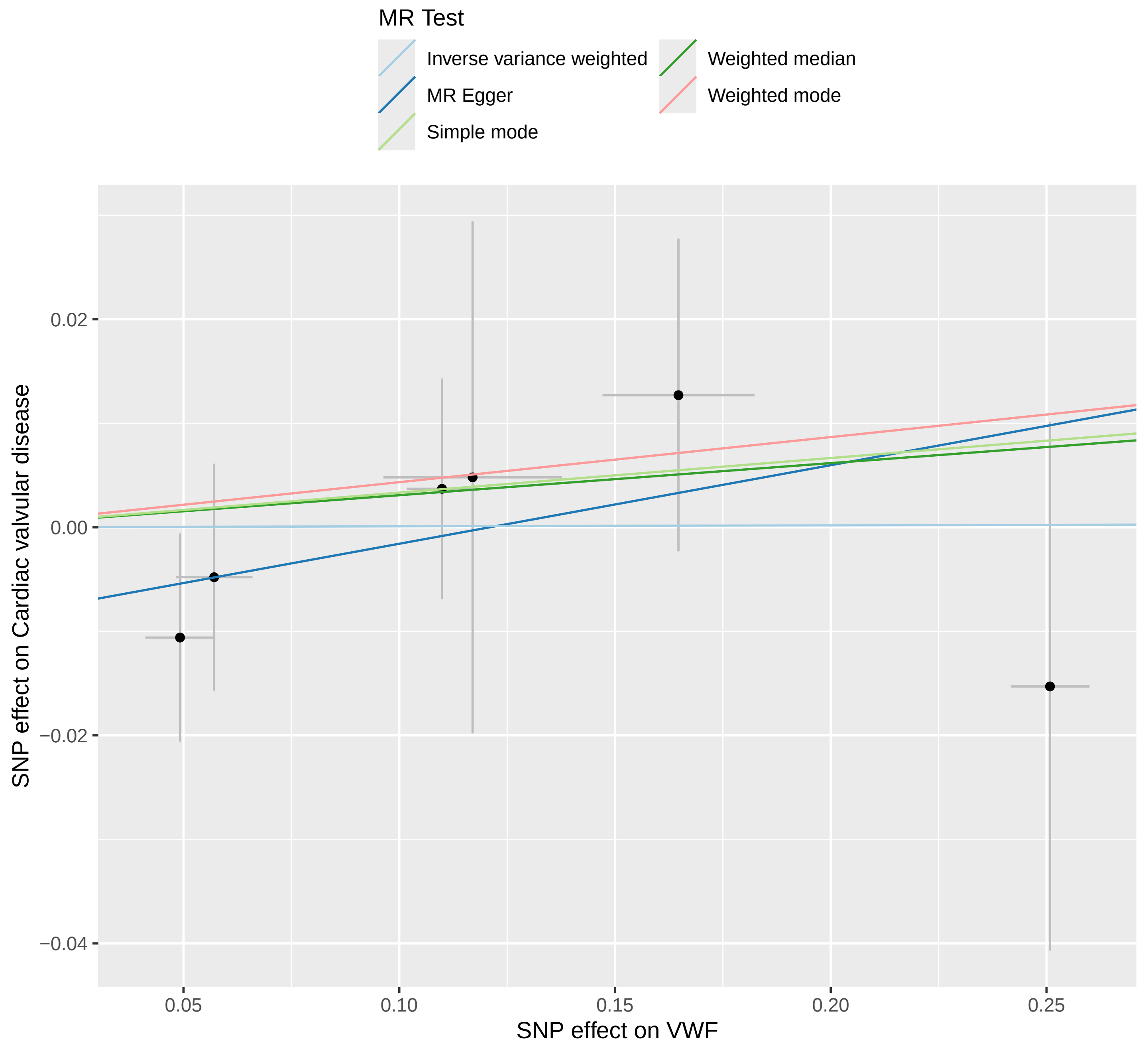
MR Test
Inverse variance weighted
MR Egger
Simple mode
Weighted median
Weighted mode
0.02
0.00
−0.02
−0.04
0.05
0.10
0.15
0.20
0.25
SNP effect on VWF
SNP effect on Cardiac valvular disease

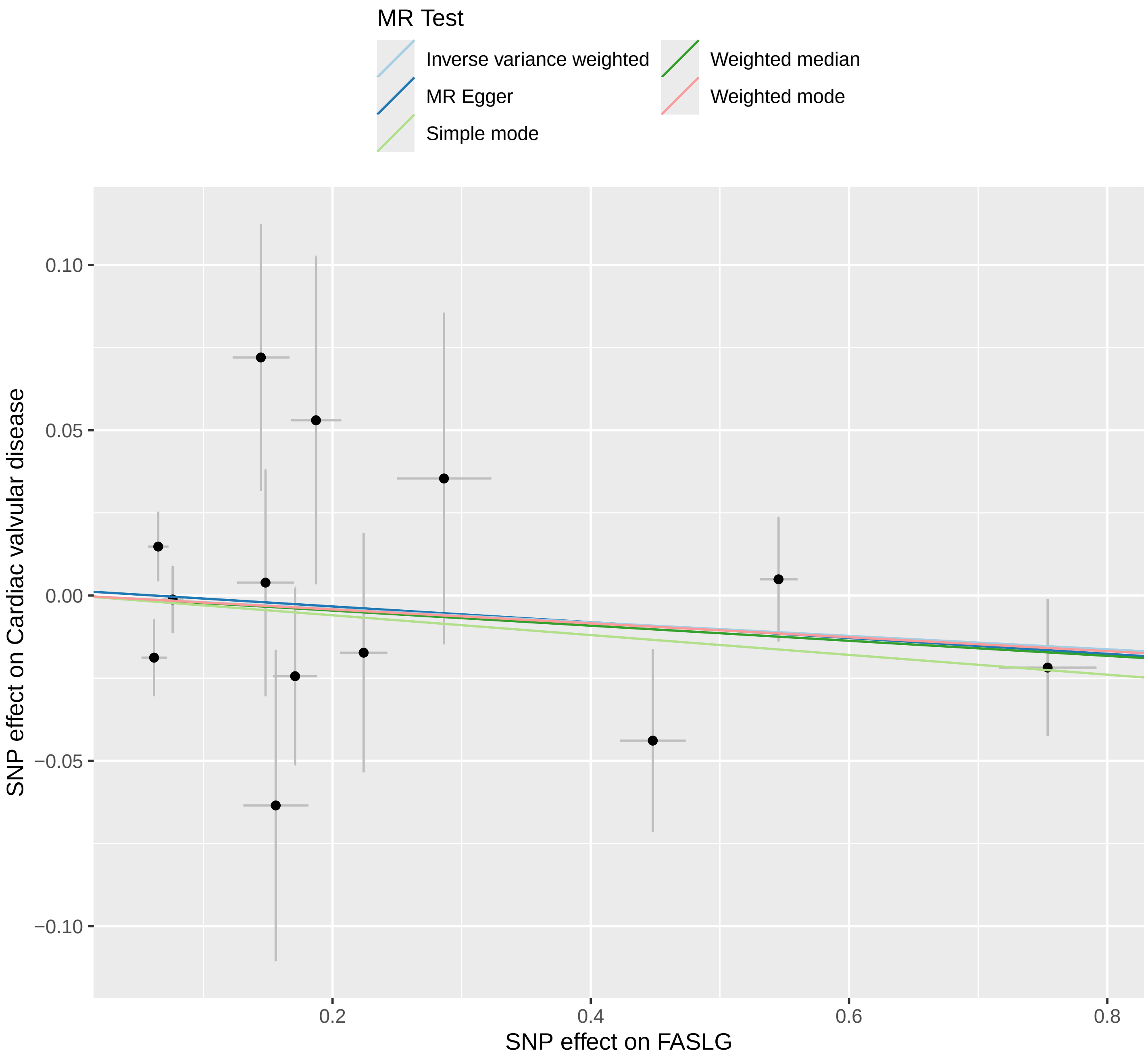

MR Test
Inverse variance weighted
MR Egger
Simple mode
Weighted median
Weighted mode
0.10
0.05
0.00
−0.05
−0.10
SNP effect on Cardiac valvular disease
0.2
0.4
0.6
0.8
SNP effect on FASLG

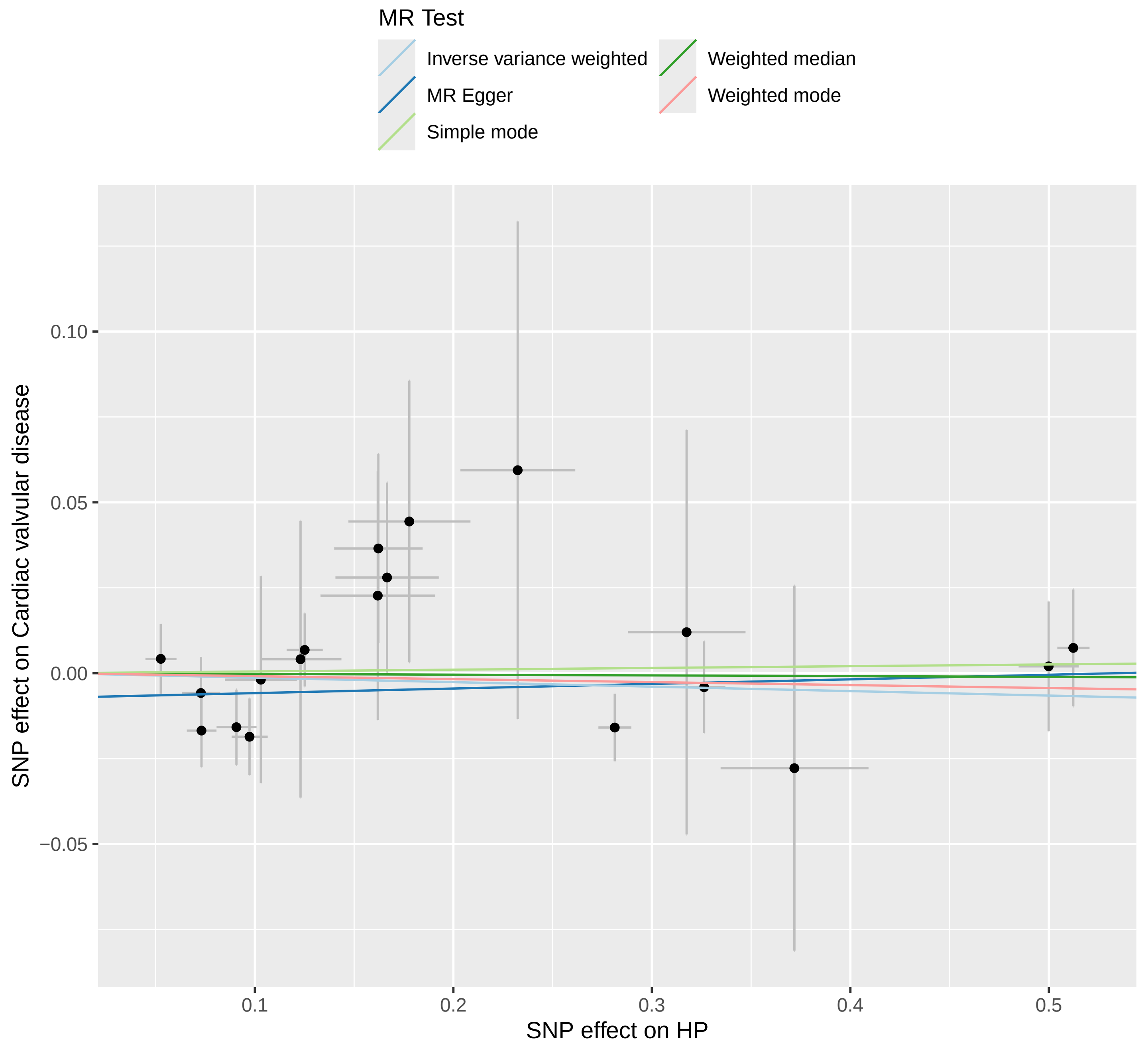
MR Test
Inverse variance weighted
MR Egger
Simple mode
Weighted median
Weighted mode
SNP effect on Cardiac valvular disease
0.10
0.05
0.00
−0.05
0.1
0.2
0.3
0.4
0.5
SNP effect on HP

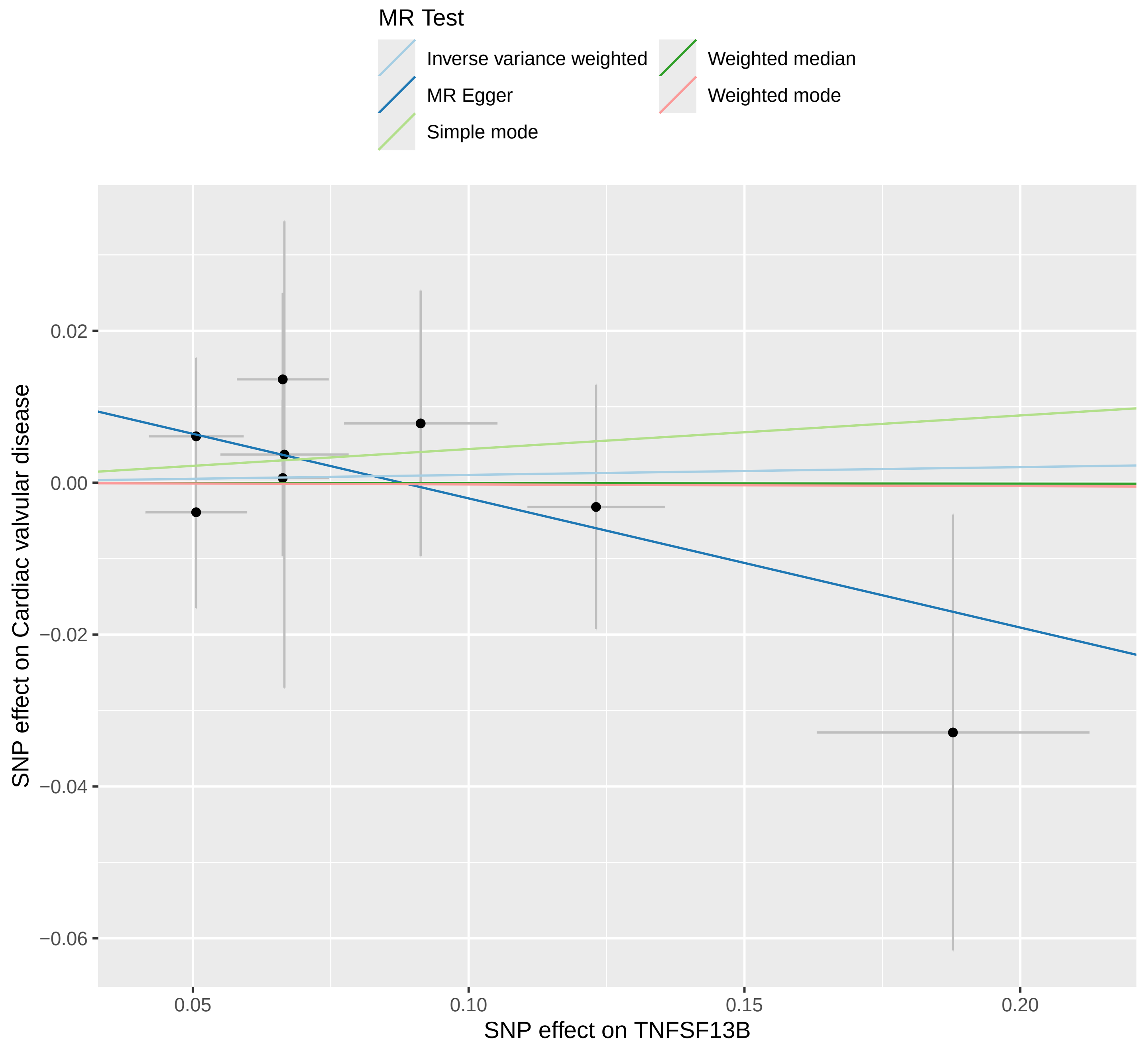
MR Test
Inverse variance weighted
MR Egger
Simple mode
Weighted median
Weighted mode
0.02
0.00
−0.02
−0.04
−0.06
SNP effect on Cardiac valvular disease
0.05
0.10
0.15
0.20
SNP effect on TNFSF13B

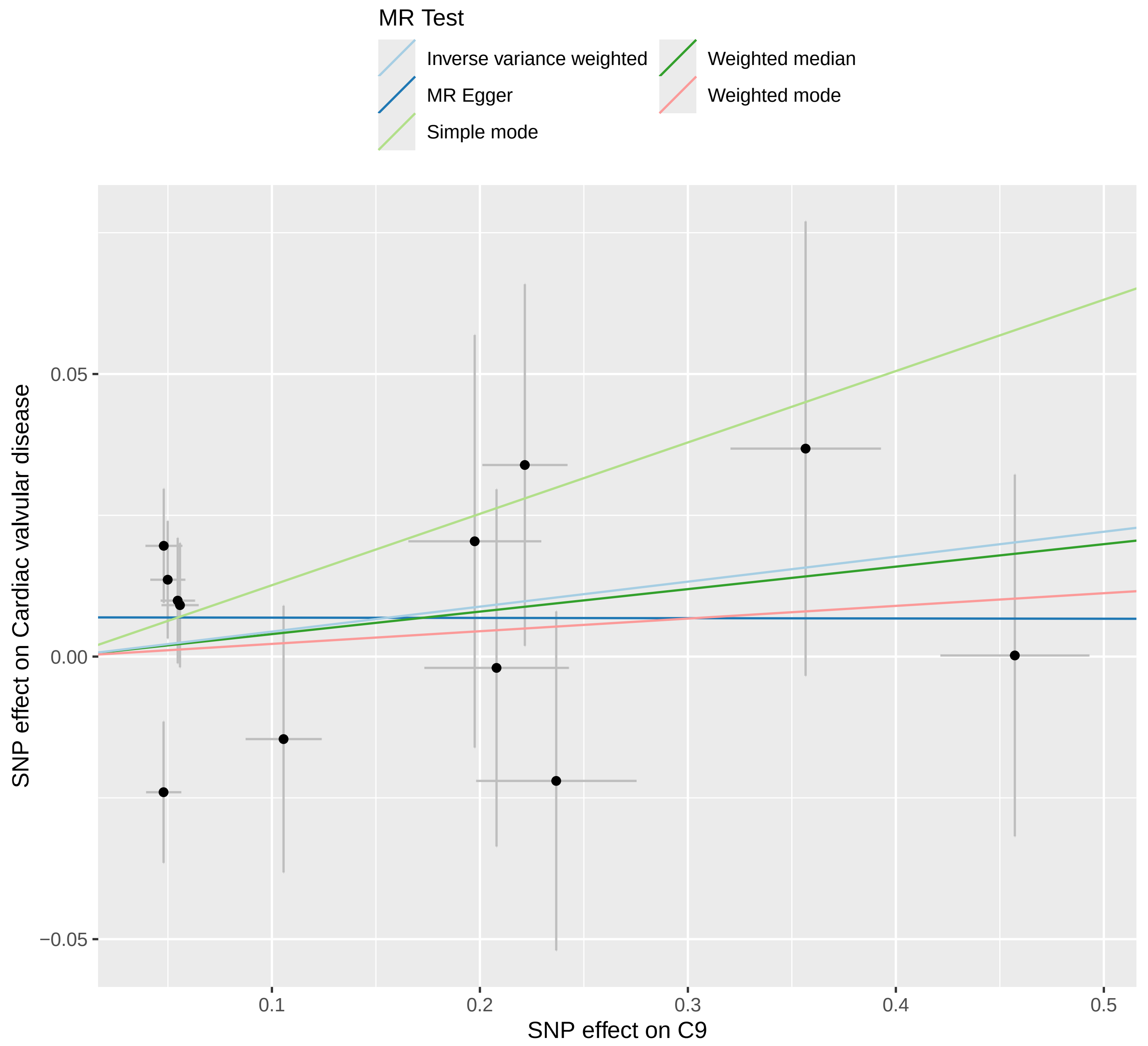

MR Test
Inverse variance weighted
MR Egger
Simple mode
Weighted median
Weighted mode
0.05
0.00
−0.05
SNP effect on Cardiac valvular disease
0.1
0.2
0.3
0.4
0.5
SNP effect on C9

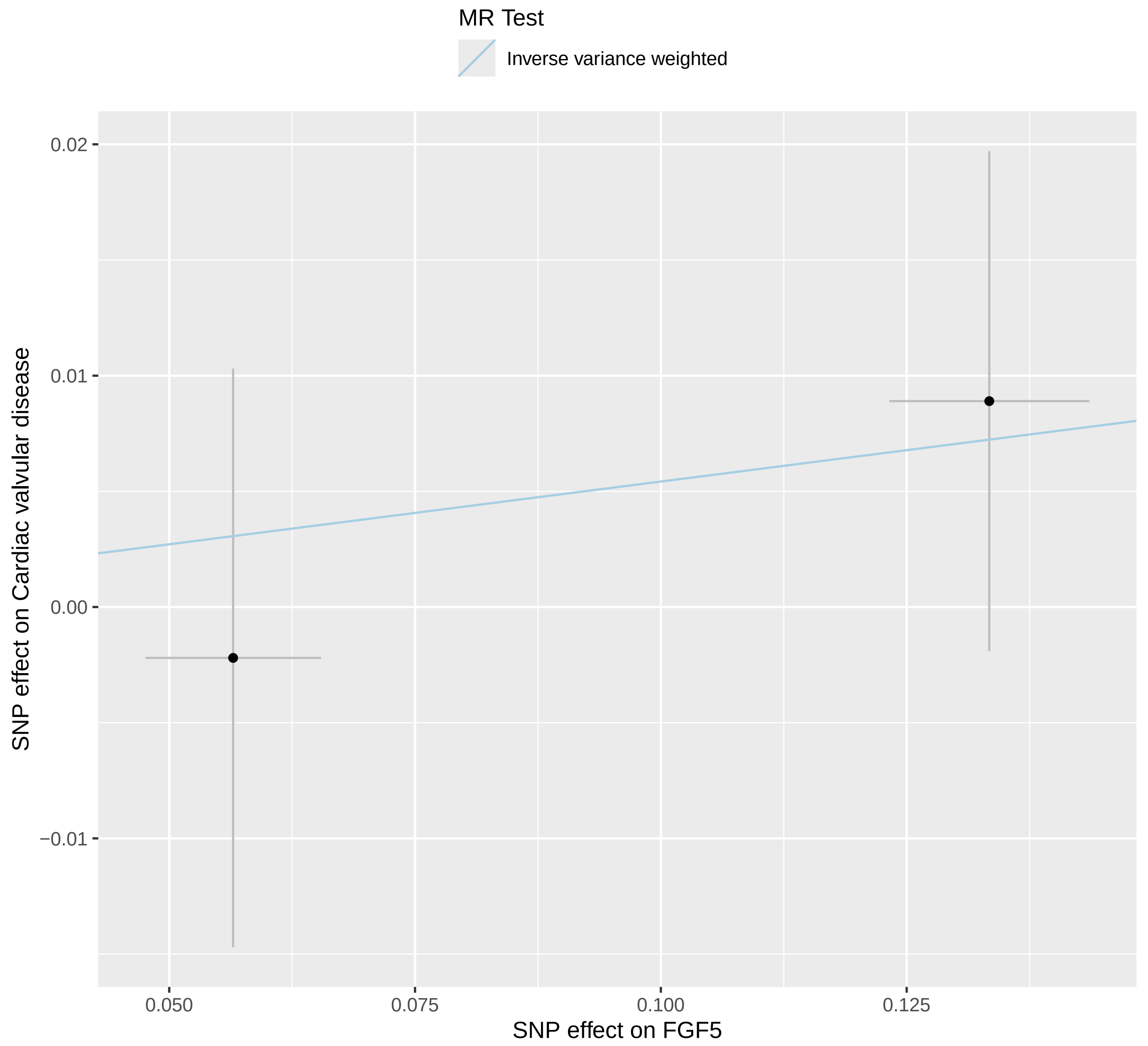

MR Test
Inverse variance weighted
0.02
0.01
0.00
−0.01
0.050
0.075
0.100
0.125
SNP effect on FGF5
SNP effect on Cardiac valvular disease

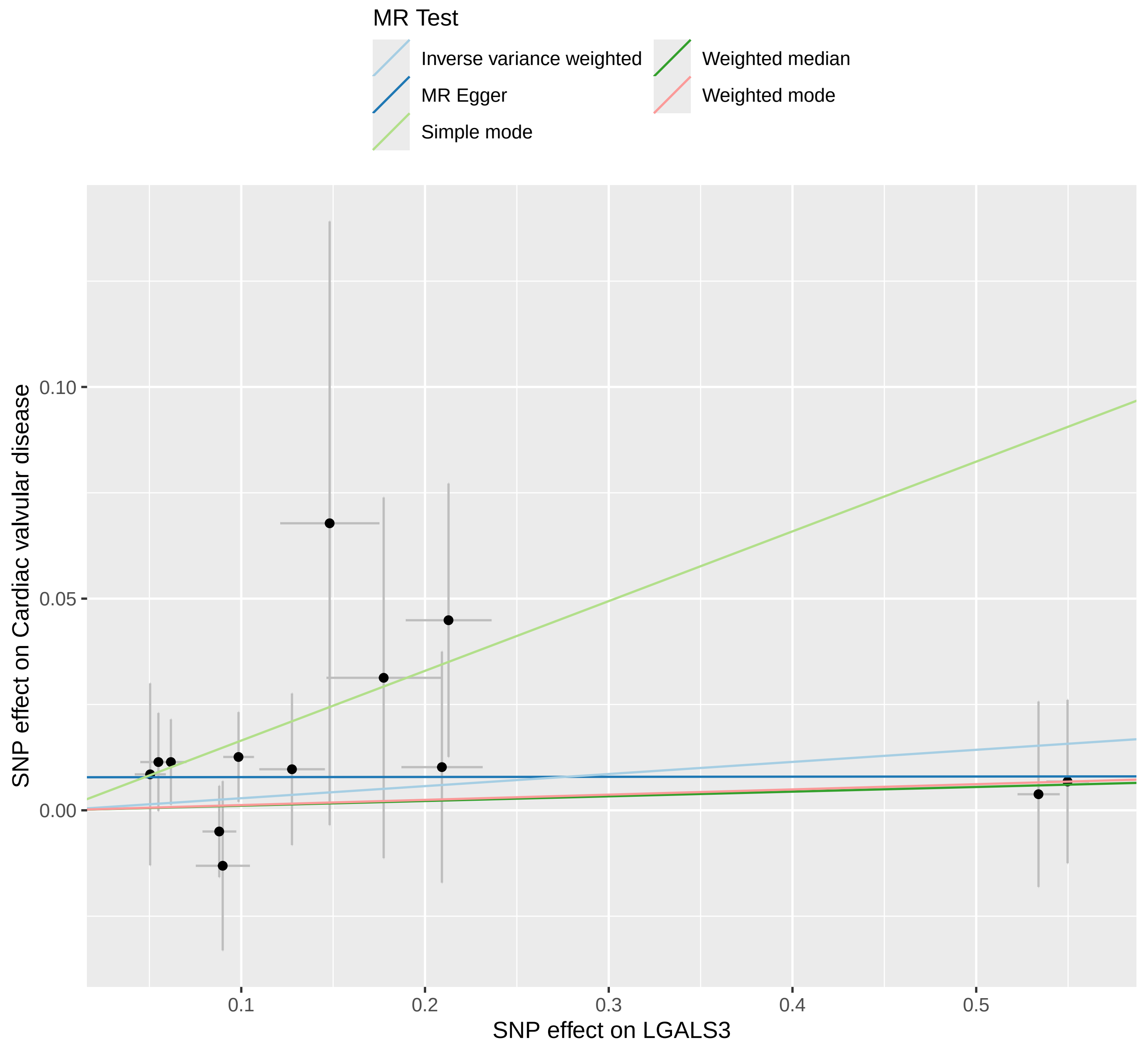

MR Test
Inverse variance weighted
MR Egger
Simple mode
Weighted median
Weighted mode
0.10
0.05
0.00
SNP effect on Cardiac valvular disease
0.1
0.2
0.3
0.4
0.5
SNP effect on LGALS3

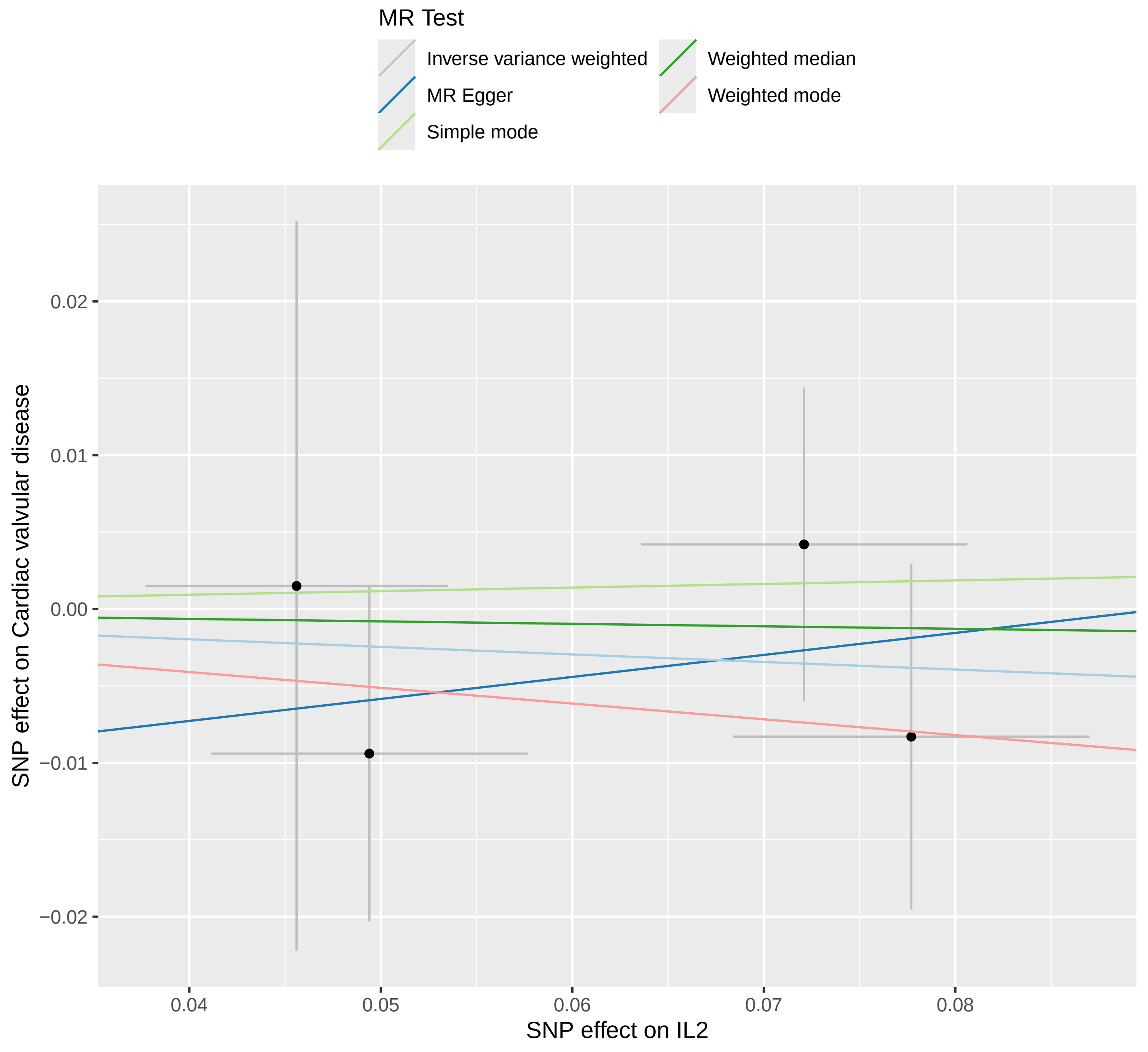
MR Test
Inverse variance weighted
MR Egger
Simple mode
Weighted median
Weighted mode
SNP effect on Cardiac valvular disease
0.02
0.01
0.00
−0.01
−0.02
0.04
0.05
0.06
0.07
0.08
SNP effect on IL2

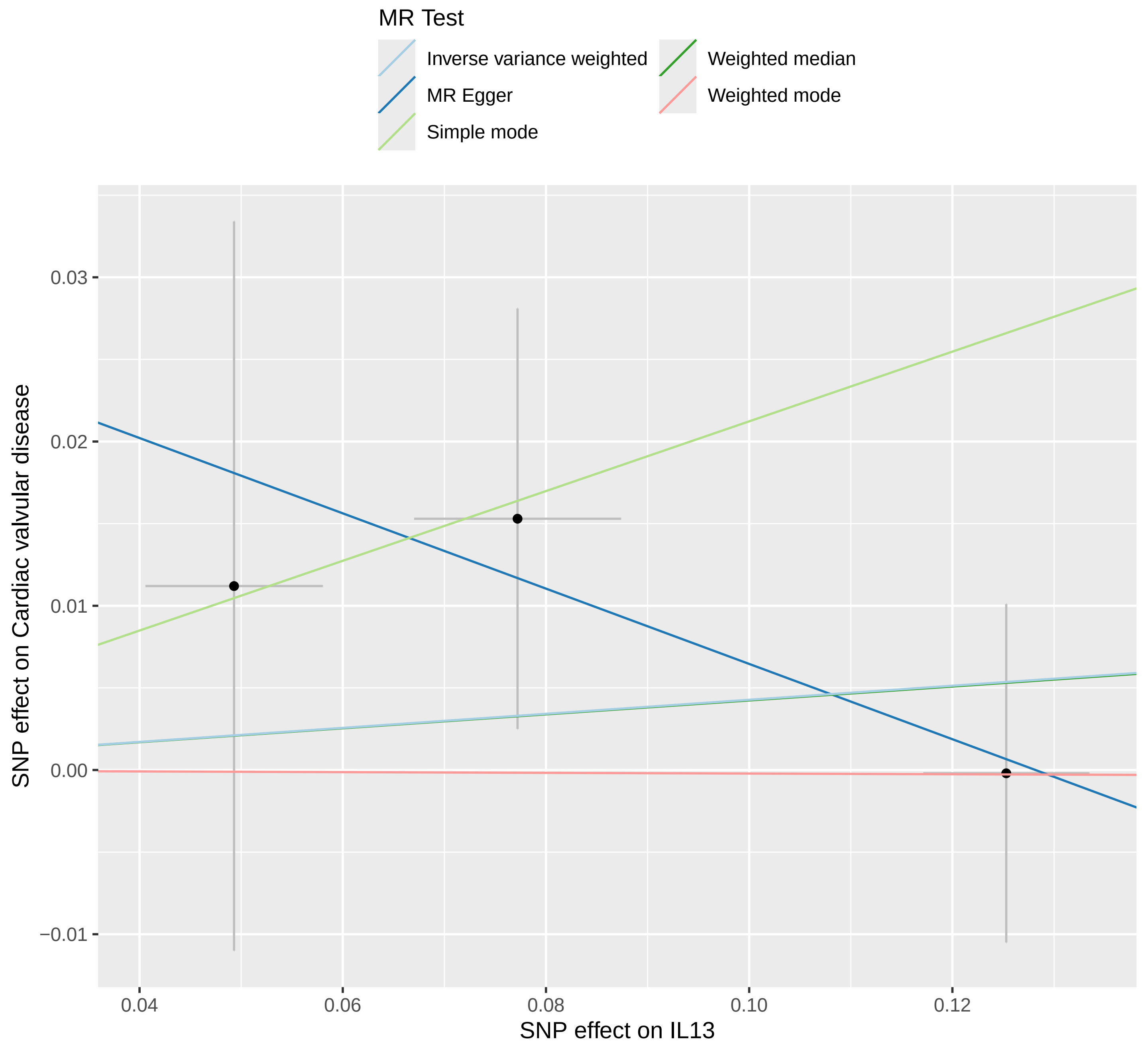
MR Test
Inverse variance weighted
MR Egger
Simple mode
Weighted median
Weighted mode
0.03
0.02
0.01
0.00
−0.01
0.04
0.06
0.08
0.10
0.12
SNP effect on IL13
SNP effect on Cardiac valvular disease

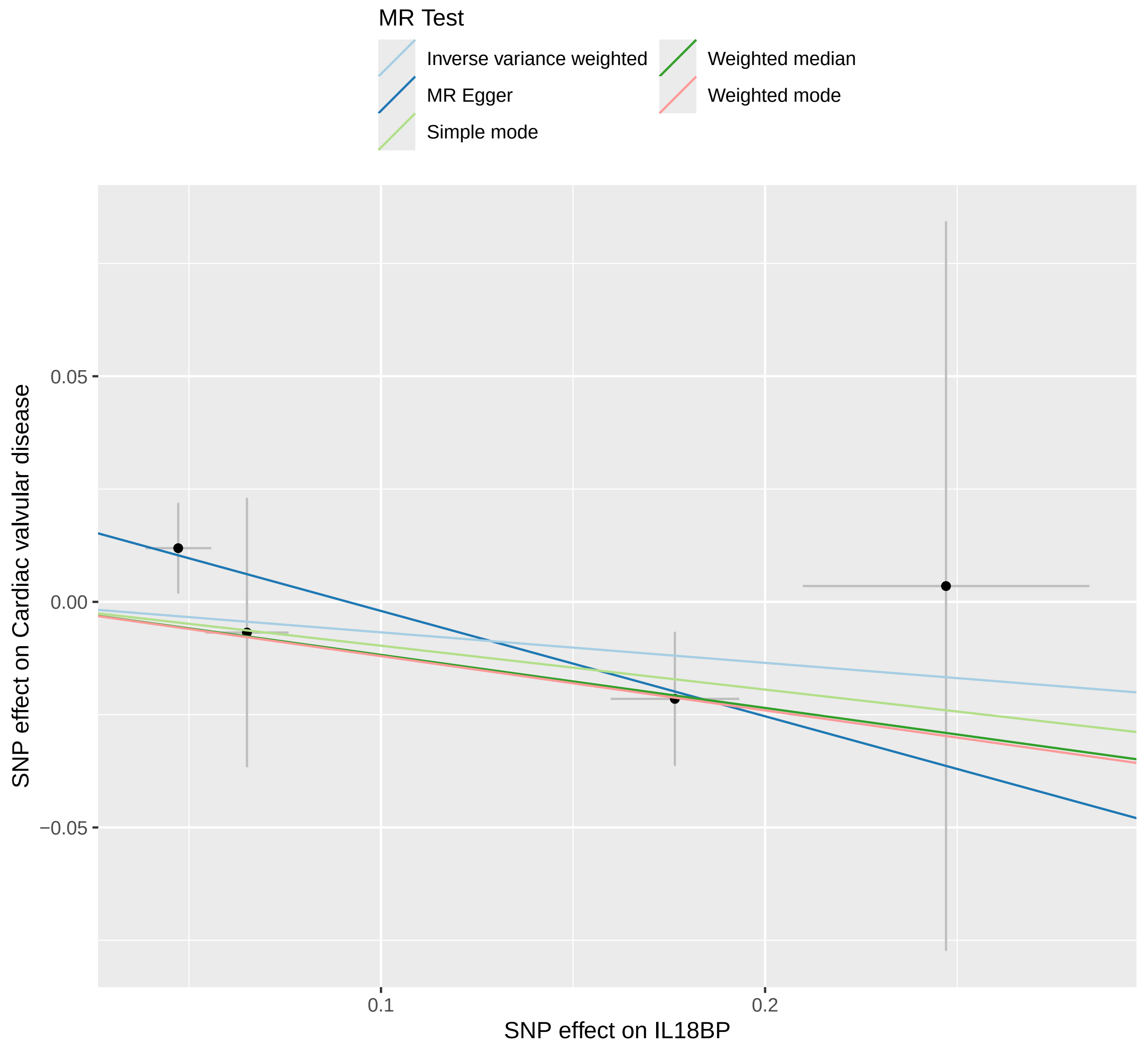

MR Test
Inverse variance weighted
MR Egger
Simple mode
Weighted median
Weighted mode
0.05
0.00
−0.05
0.1
0.2
SNP effect on Cardiac valvular disease
SNP effect on IL18BP

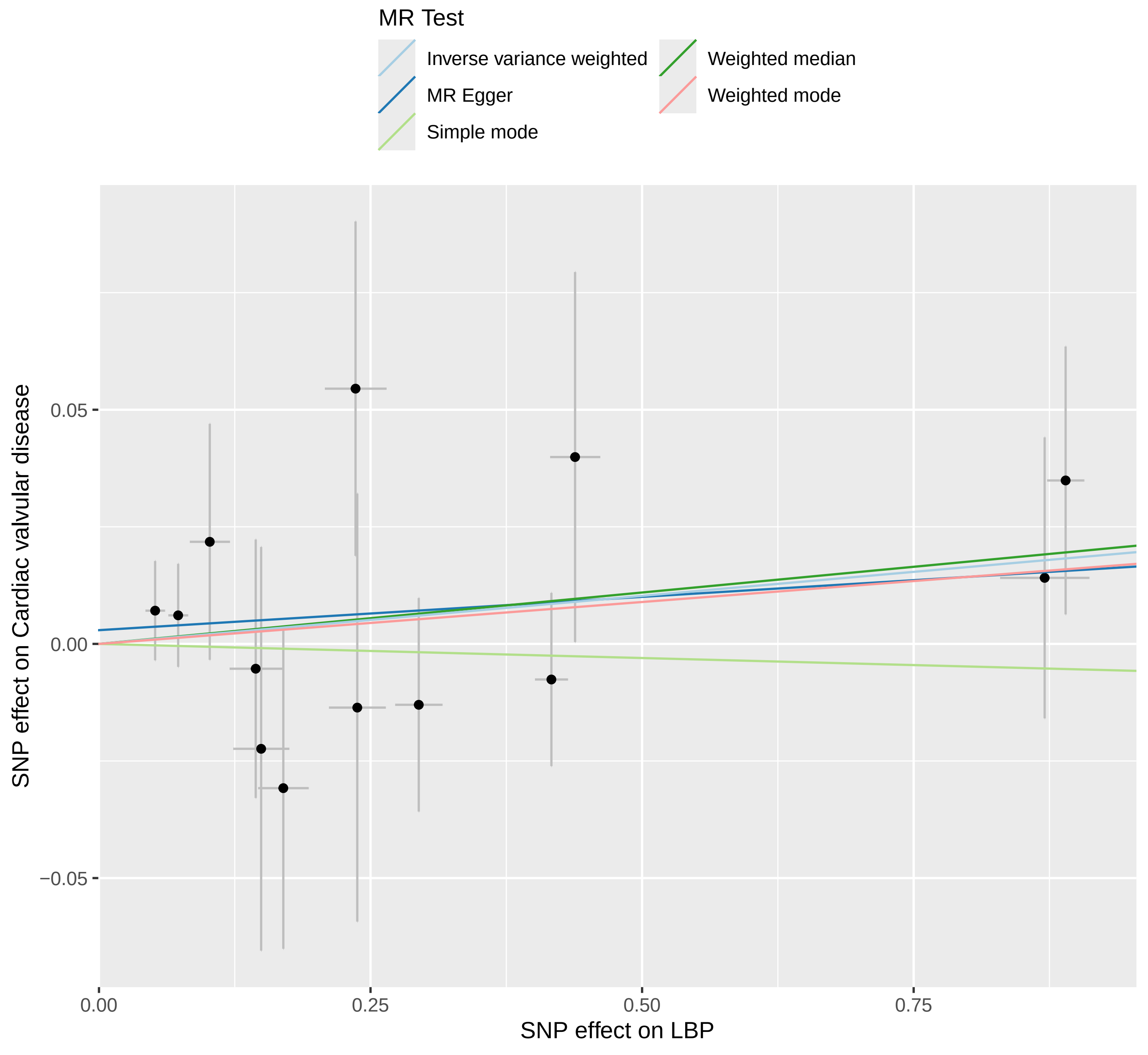
MR Test
Inverse variance weighted
MR Egger
Simple mode
Weighted median
Weighted mode
SNP effect on Cardiac valvular disease
0.05
0.00
−0.05
0.00
0.25
0.50
0.75
SNP effect on LBP

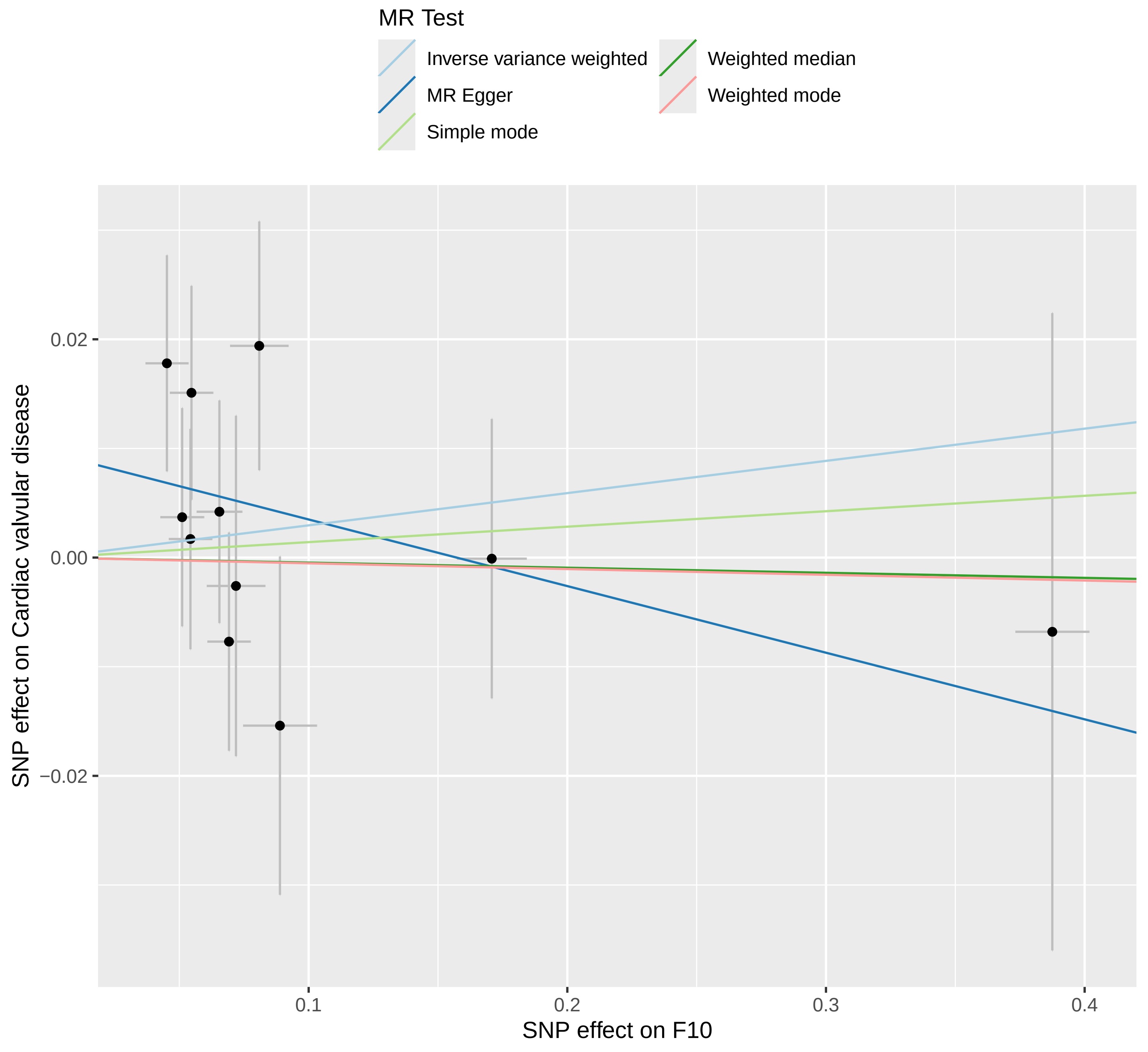
MR Test
Inverse variance weighted
MR Egger
Simple mode
Weighted median
Weighted mode
0.02
0.00
−0.02
0.1
0.2
0.3
0.4
SNP effect on Cardiac valvular disease
SNP effect on F10

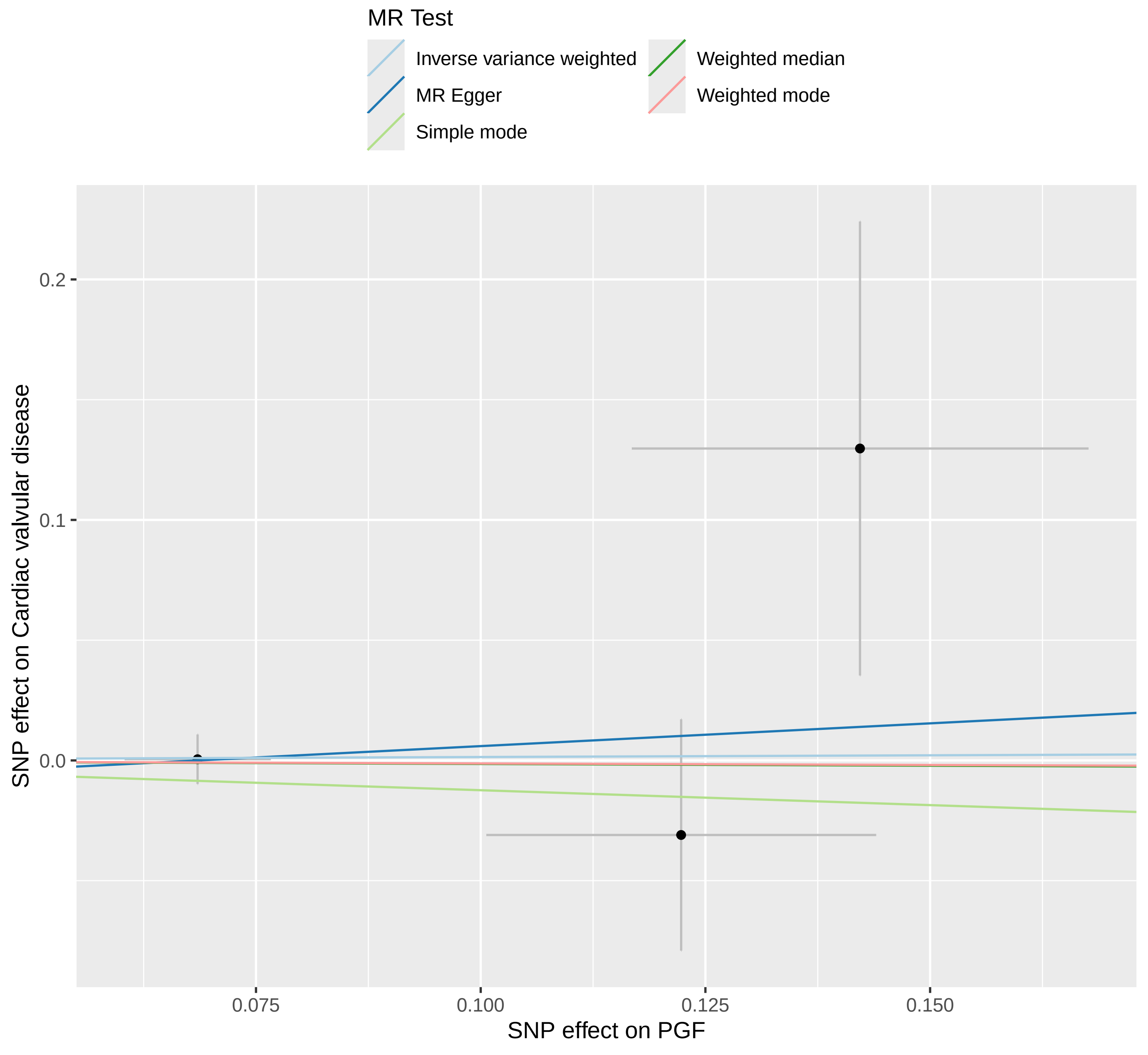
MR Test
Inverse variance weighted
MR Egger
Simple mode
Weighted median
Weighted mode
SNP effect on Cardiac valvular disease
0.2
0.1
0.0
0.075
0.100
0.125
0.150
SNP effect on PGF

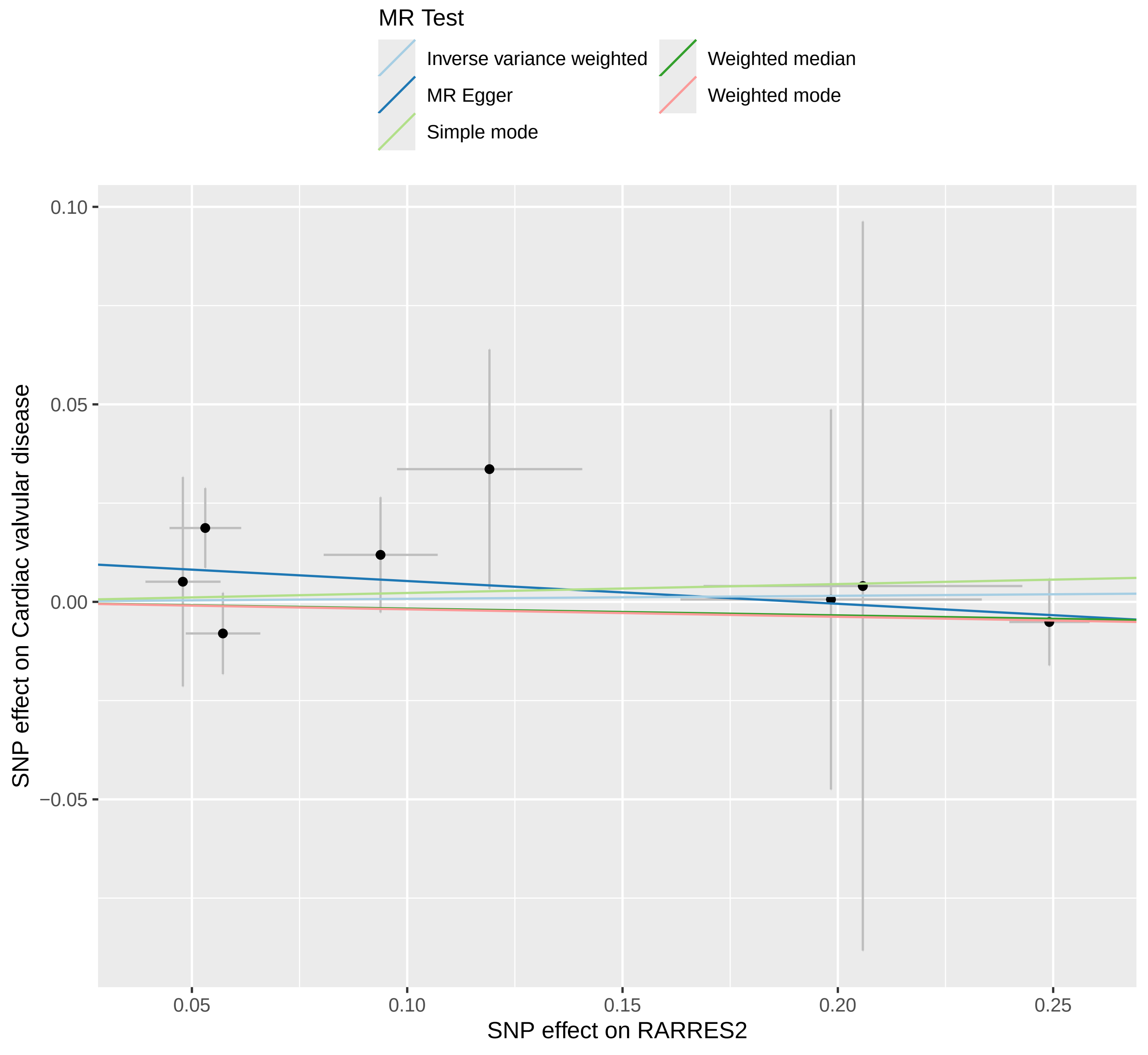

MR Test
Inverse variance weighted
MR Egger
Simple mode
Weighted median
Weighted mode
0.10
0.05
0.00
−0.05
SNP effect on Cardiac valvular disease
0.05
0.10
0.15
0.20
0.25
SNP effect on RARRES2

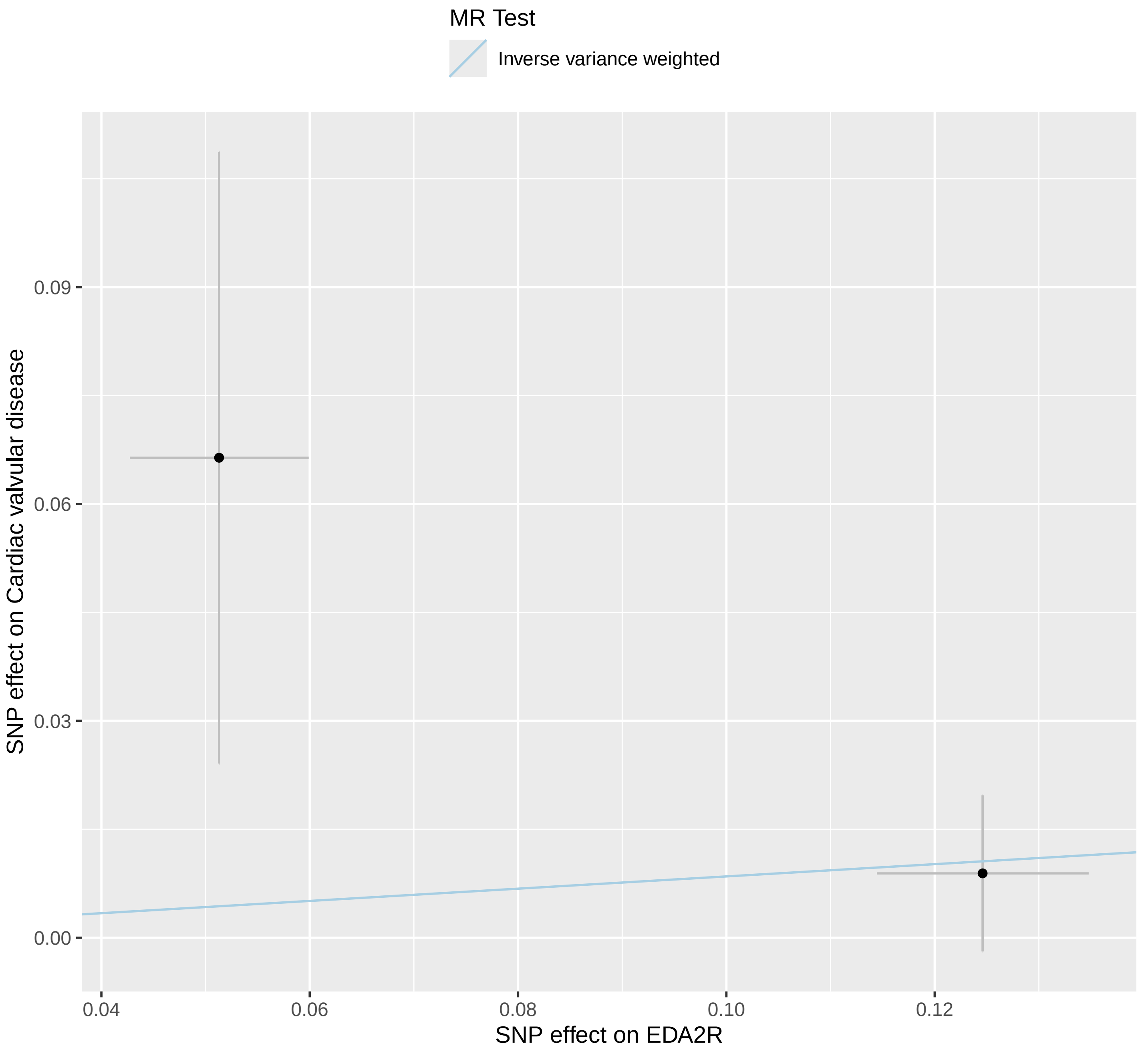
MR Test
Inverse variance weighted
0.09
0.06
0.03
0.00
SNP effect on Cardiac valvular disease
0.04
0.06
0.08
0.10
0.12
SNP effect on EDA2R

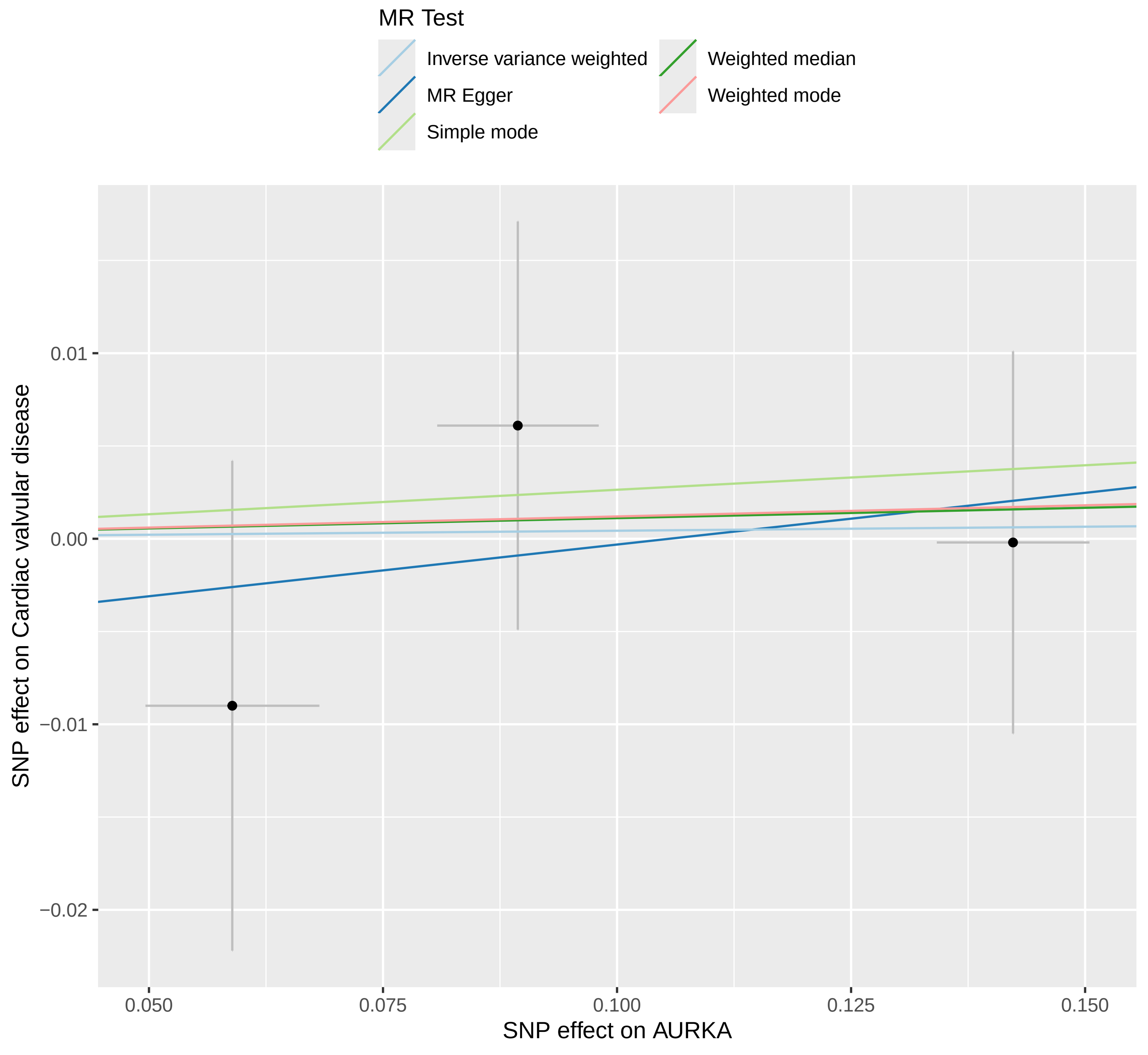

MR Test
Inverse variance weighted
MR Egger
Simple mode
Weighted median
Weighted mode
SNP effect on Cardiac valvular disease
0.01
0.00
−0.01
−0.02
0.050
0.075
0.100
0.125
0.150
SNP effect on AURKA

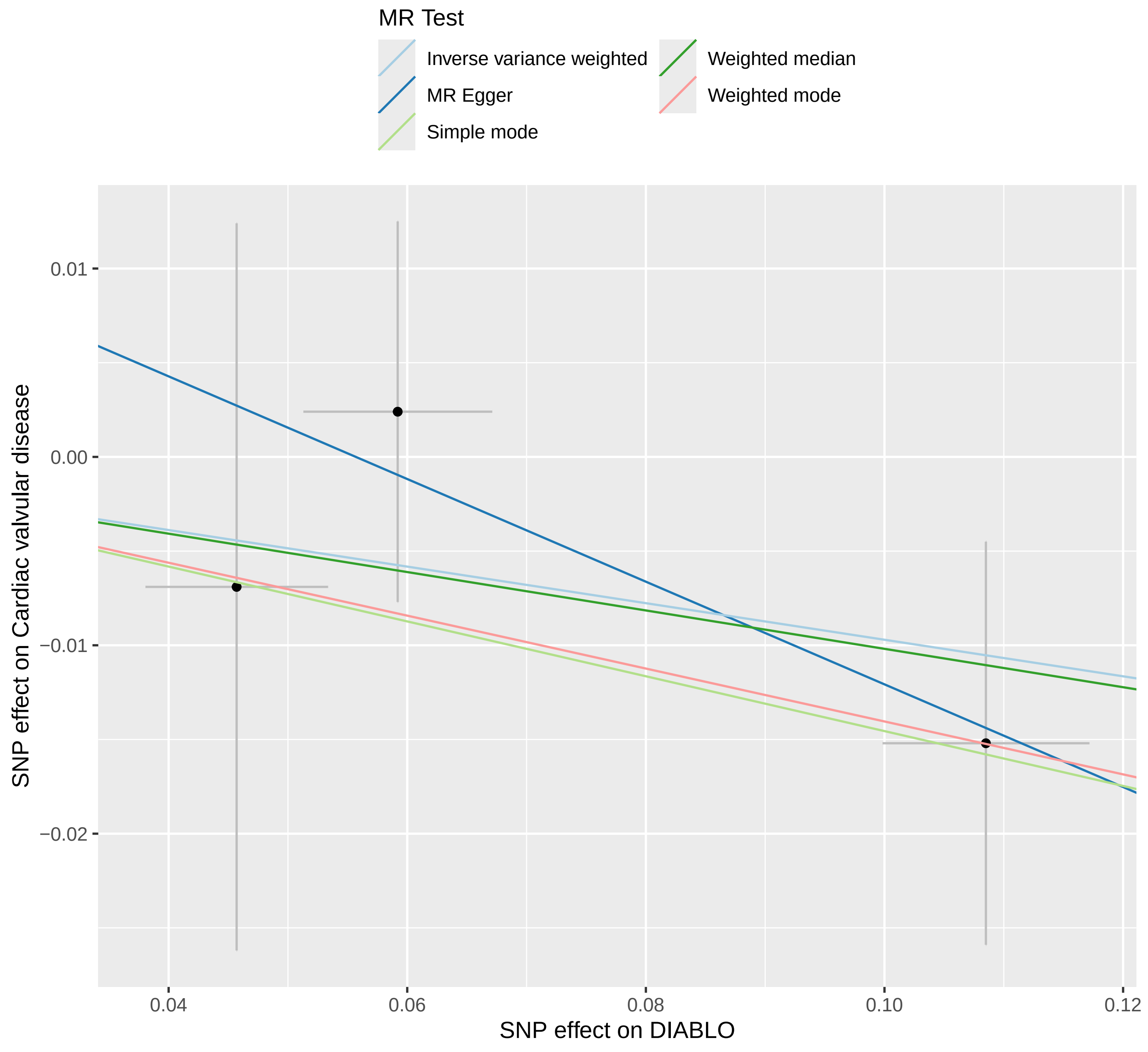
MR Test
Inverse variance weighted
MR Egger
Simple mode
Weighted median
Weighted mode
0.01
0.00
−0.01
−0.02
0.04
0.06
0.08
0.10
0.12
SNP effect on DIABLO
SNP effect on Cardiac valvular disease

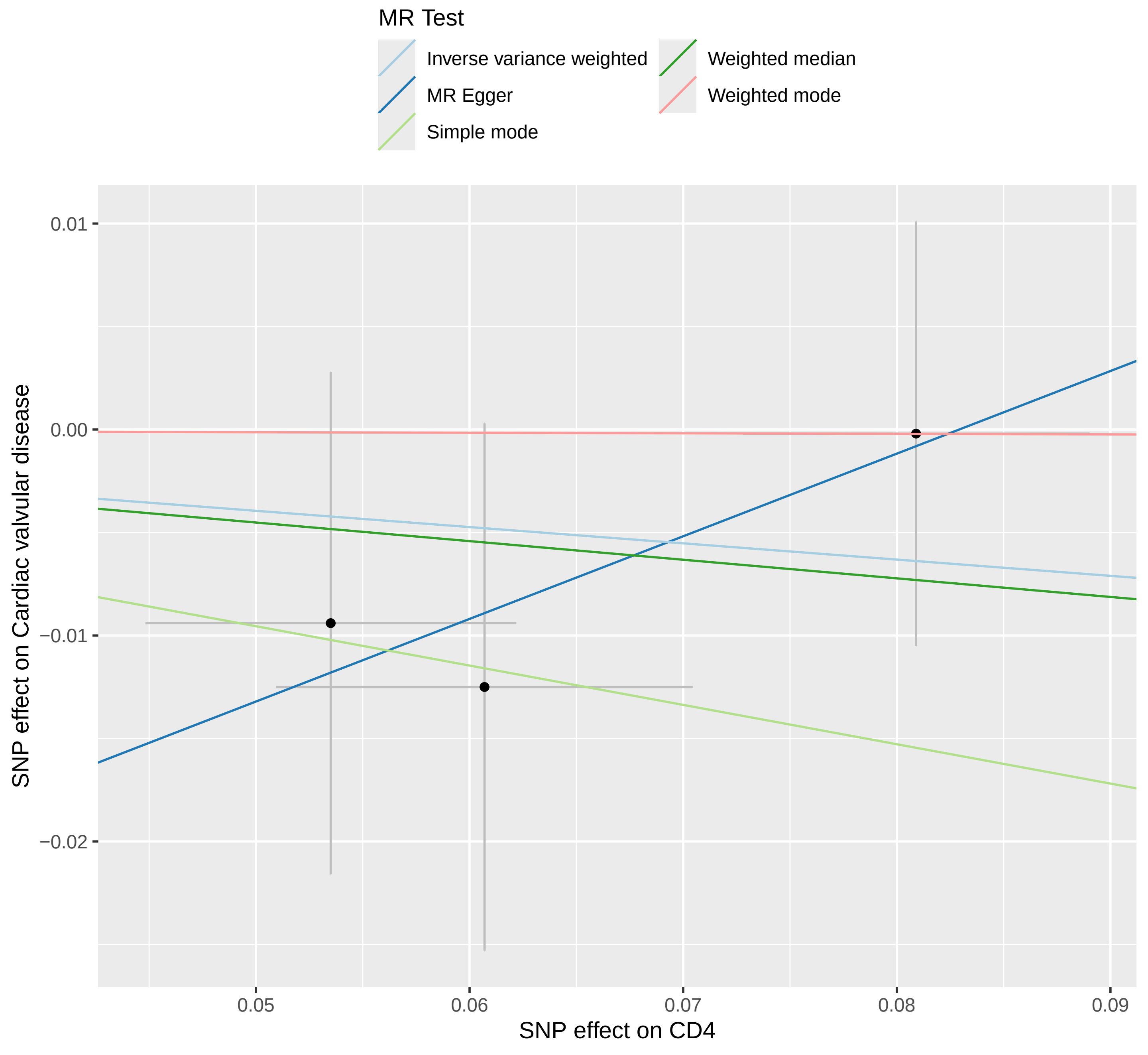

MR Test
Inverse variance weighted
MR Egger
Simple mode
Weighted median
Weighted mode
0.01
0.00
−0.01
−0.02
0.05
0.06
0.07
0.08
0.09
SNP effect on CD4
SNP effect on Cardiac valvular disease

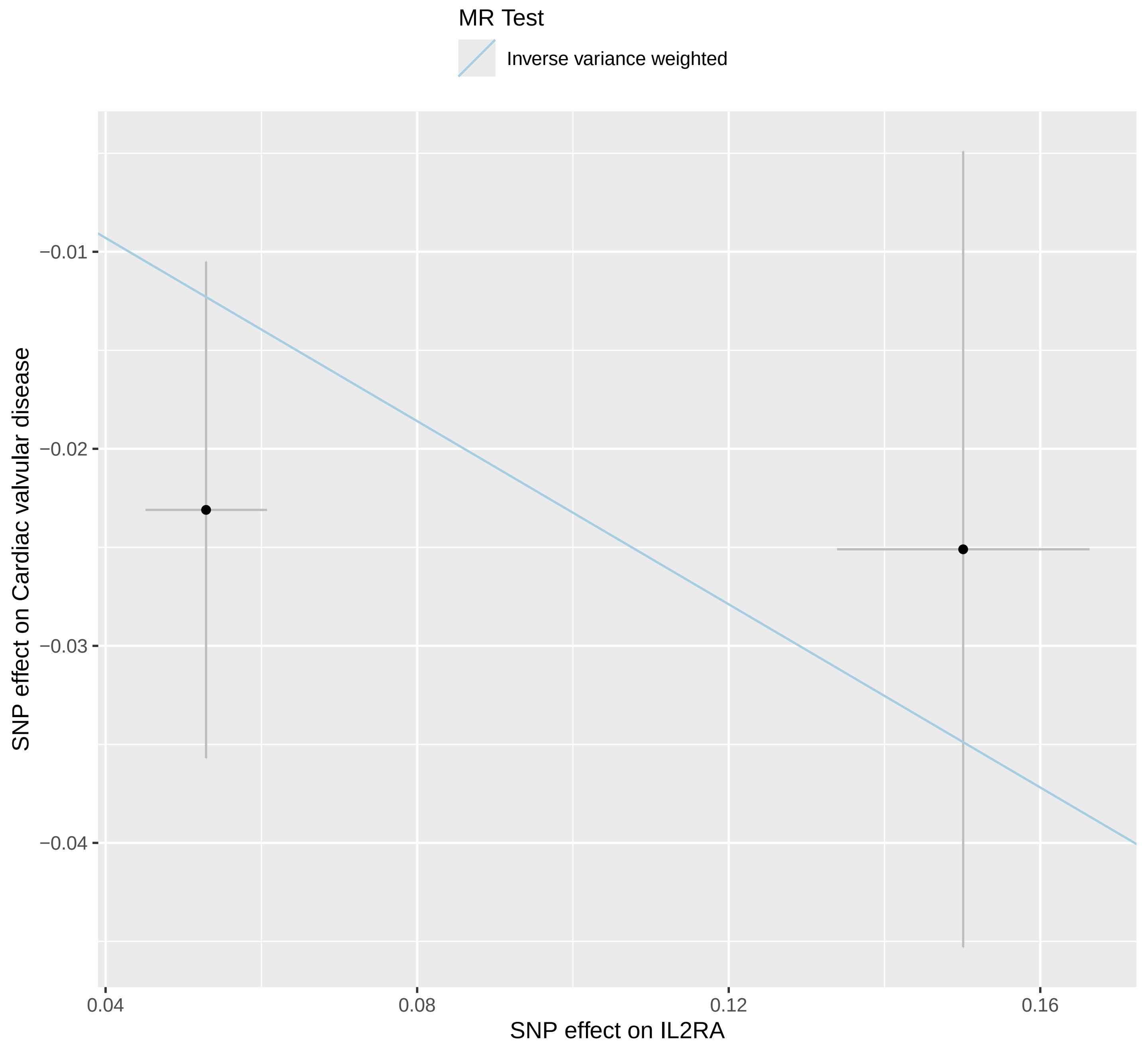
MR Test
Inverse variance weighted
SNP effect on Cardiac valvular disease
−0.01
−0.02
−0.03
−0.04
0.04
0.08
0.12
0.16
SNP effect on IL2RA

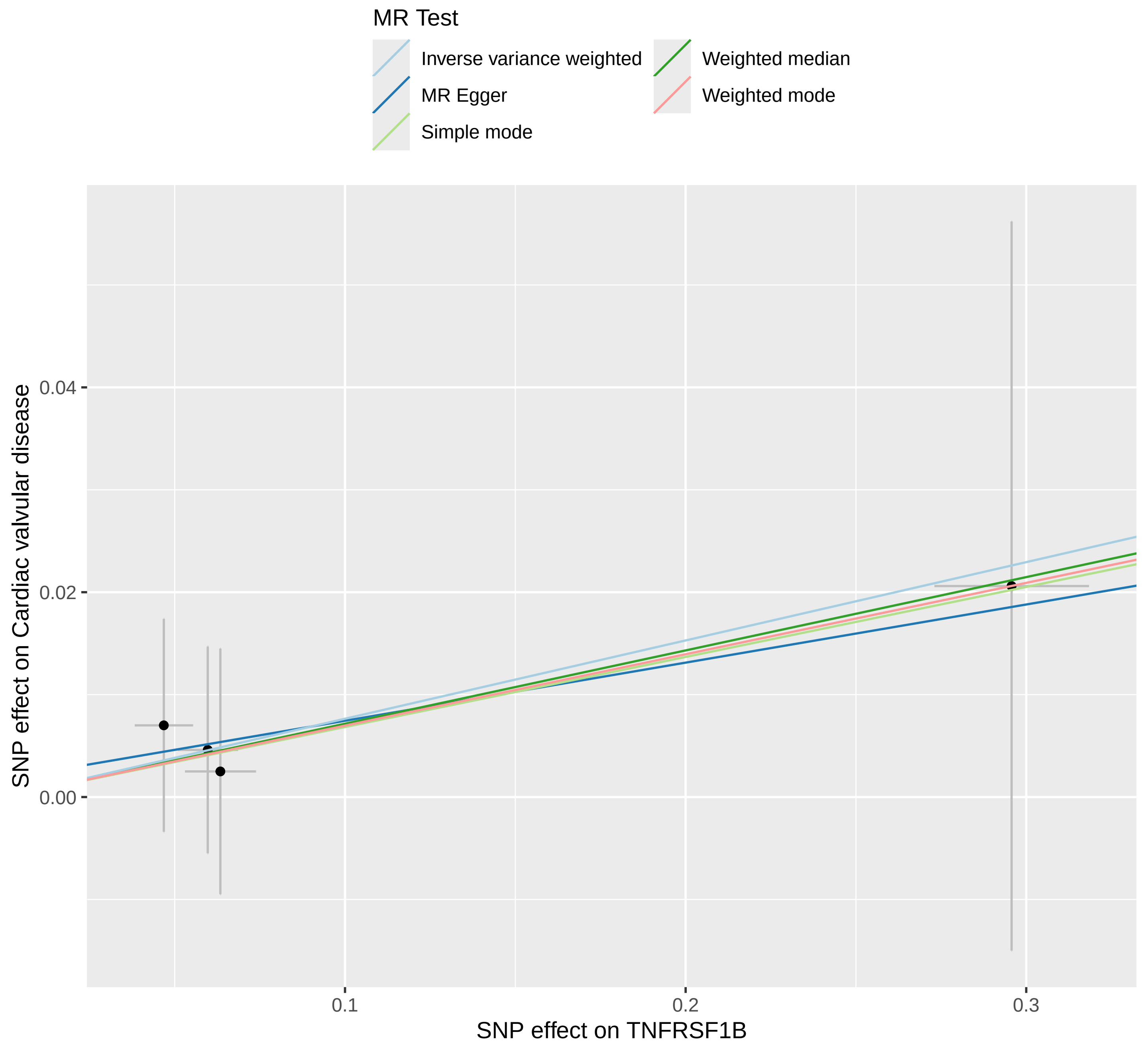

MR Test
Inverse variance weighted
MR Egger
Simple mode
Weighted median
Weighted mode
0.04
0.02
0.00
0.1
0.2
0.3
SNP effect on Cardiac valvular disease
SNP effect on TNFRSF1B

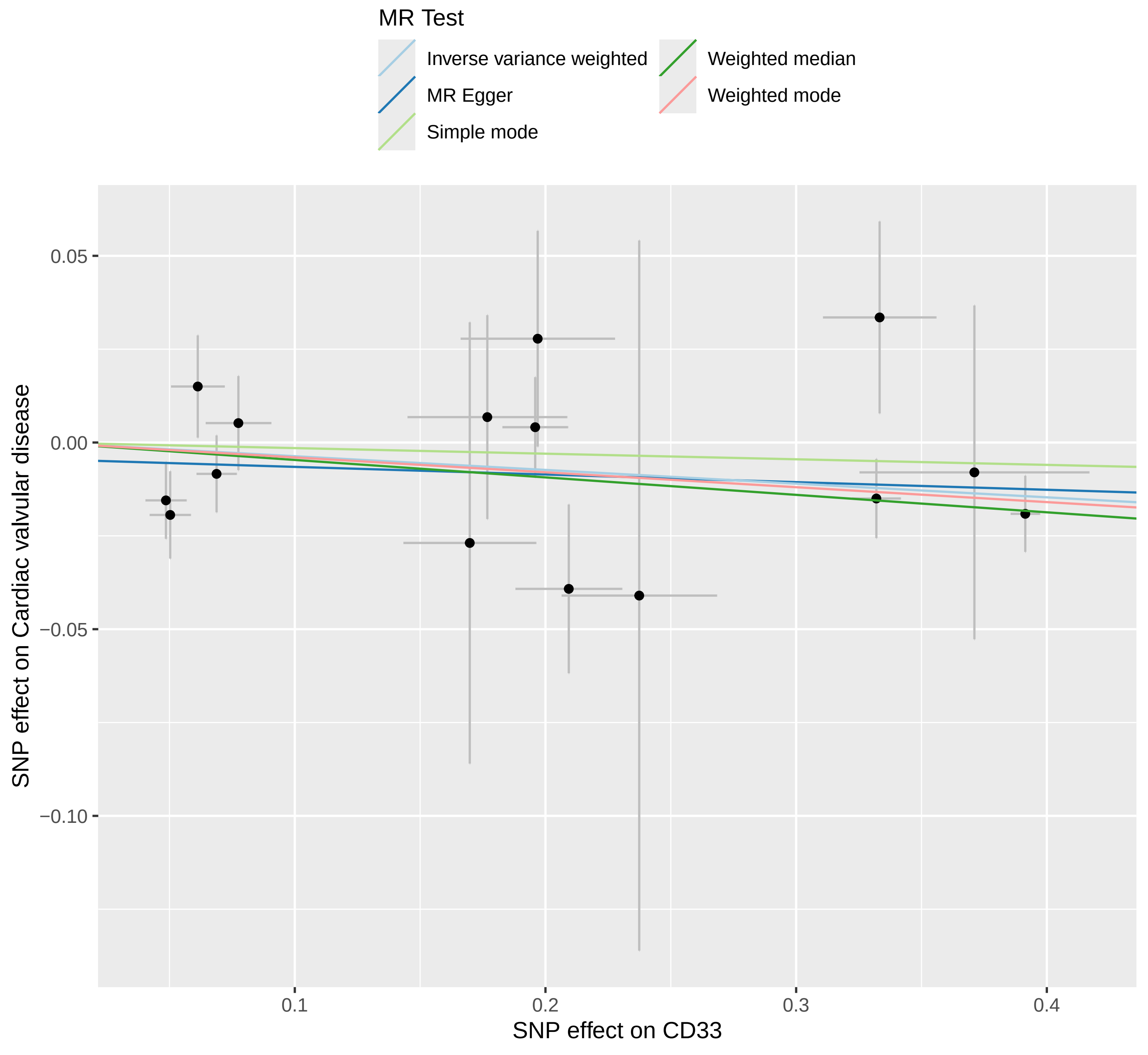
MR Test
Inverse variance weighted
MR Egger
Simple mode
Weighted median
Weighted mode
SNP effect on Cardiac valvular disease
0.05
0.00
−0.05
−0.10
0.1
0.2
0.3
0.4
SNP effect on CD33

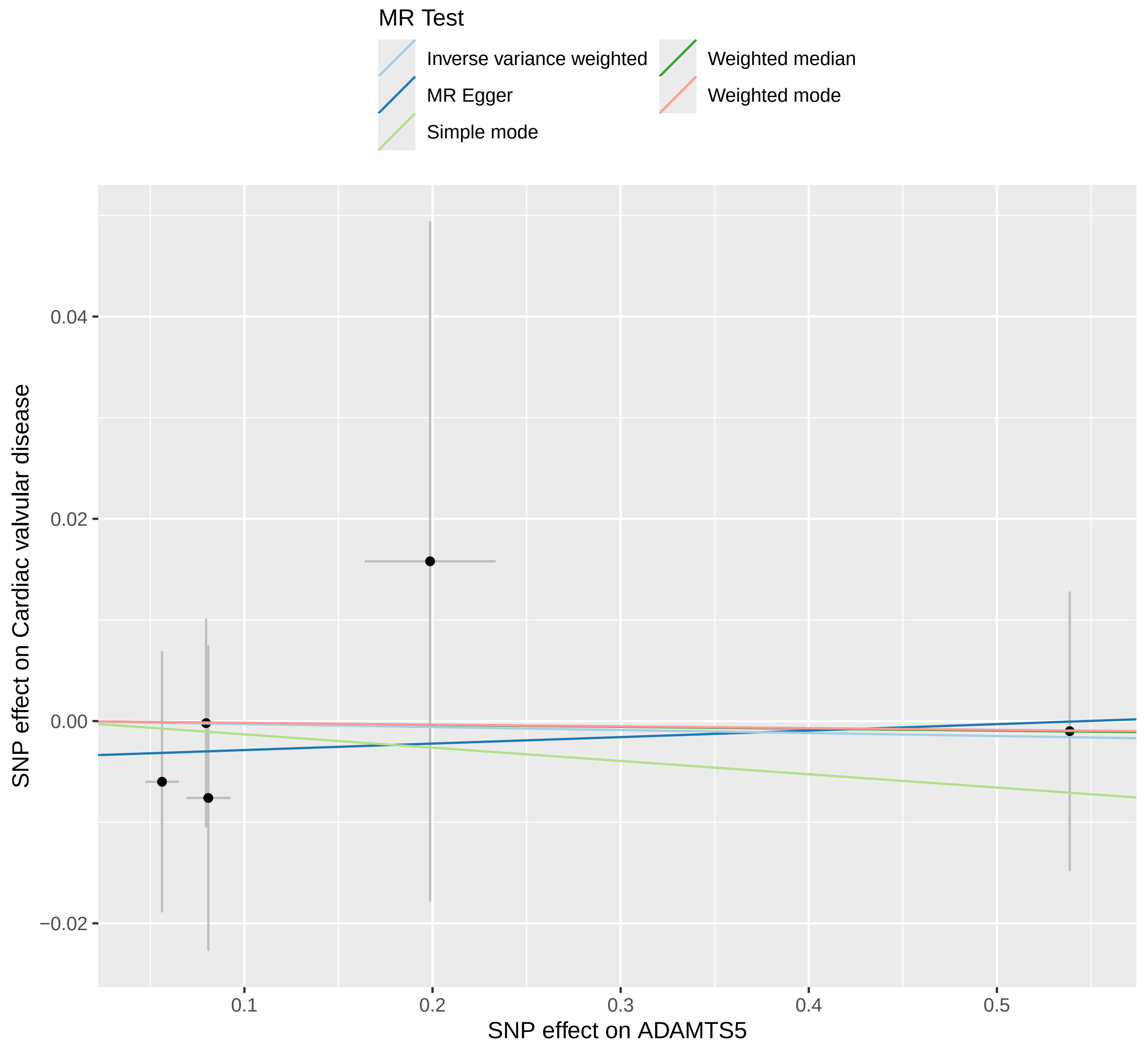
MR Test
Inverse variance weighted
MR Egger
Simple mode
Weighted median
Weighted mode
SNP effect on Cardiac valvular disease
0.04
0.02
0.00
−0.02
0.1
0.2
0.3
0.4
0.5
SNP effect on ADAMTS5

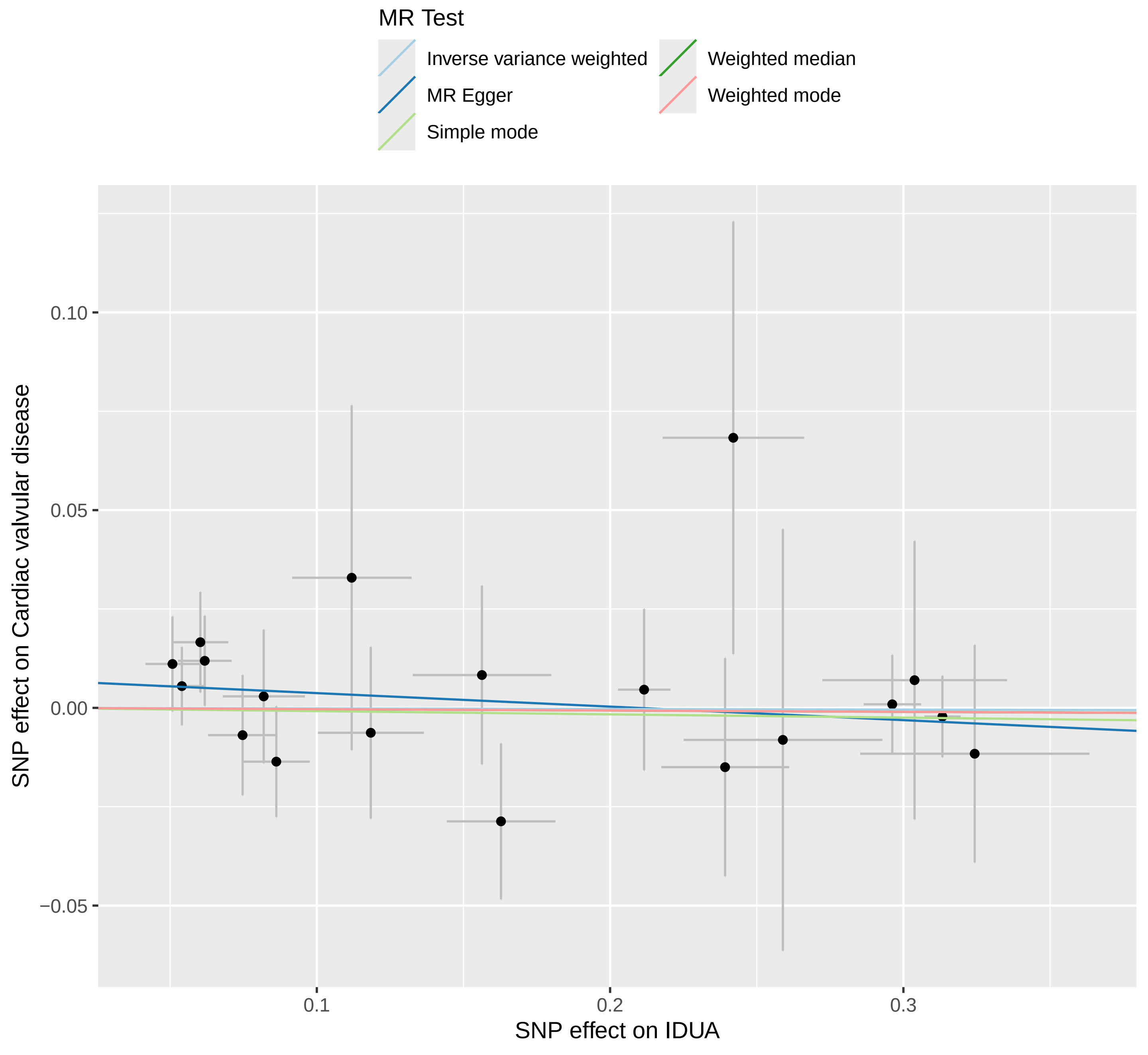

MR Test
Inverse variance weighted
MR Egger
Simple mode
Weighted median
Weighted mode
SNP effect on Cardiac valvular disease
0.10
0.05
0.00
−0.05
0.1
0.2
0.3
SNP effect on IDUA

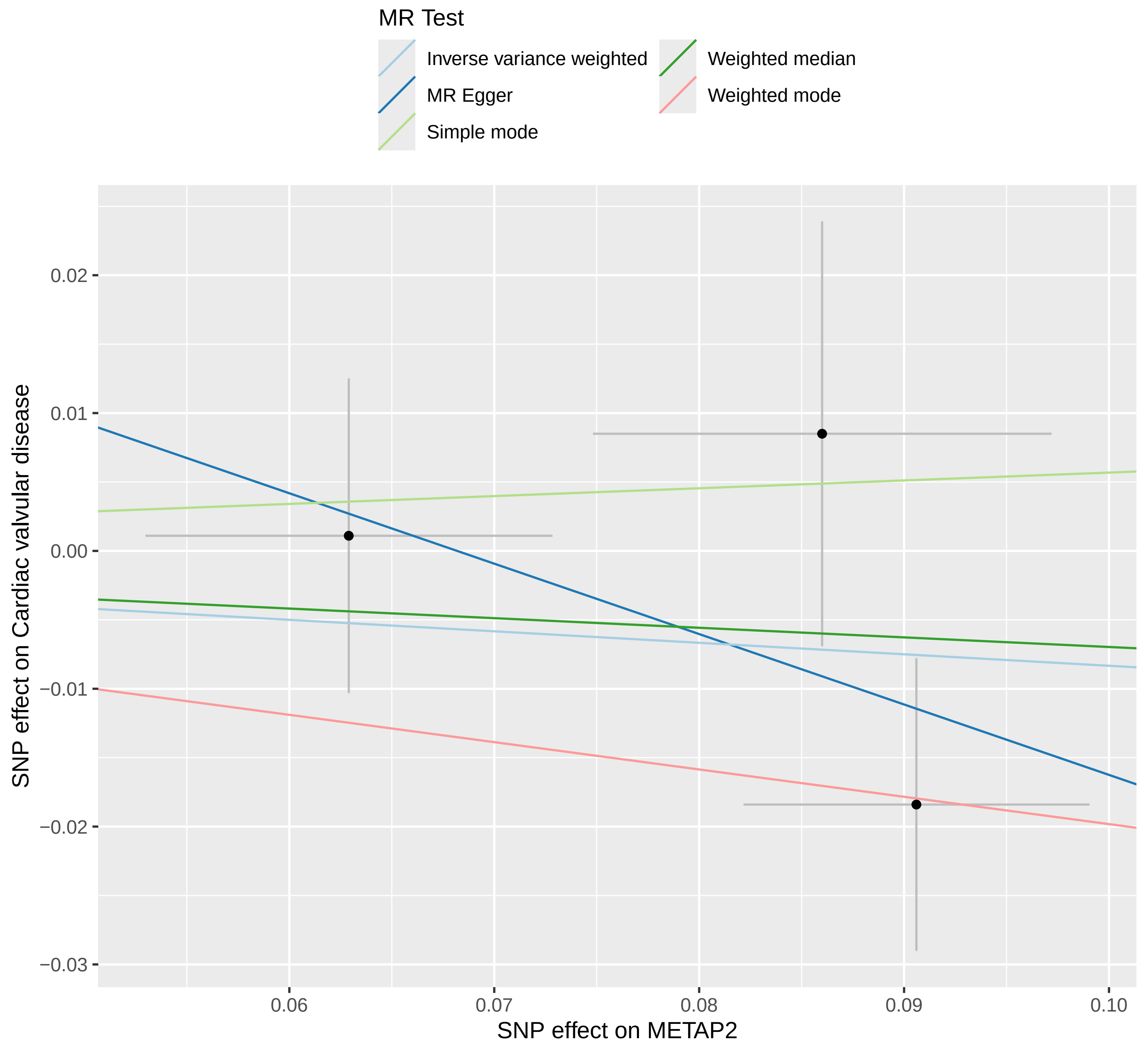

MR Test
Inverse variance weighted
MR Egger
Simple mode
Weighted median
Weighted mode
SNP effect on Cardiac valvular disease
0.02
0.01
0.00
−0.01
−0.02
−0.03
0.06
0.07
0.08
0.09
0.10
SNP effect on METAP2

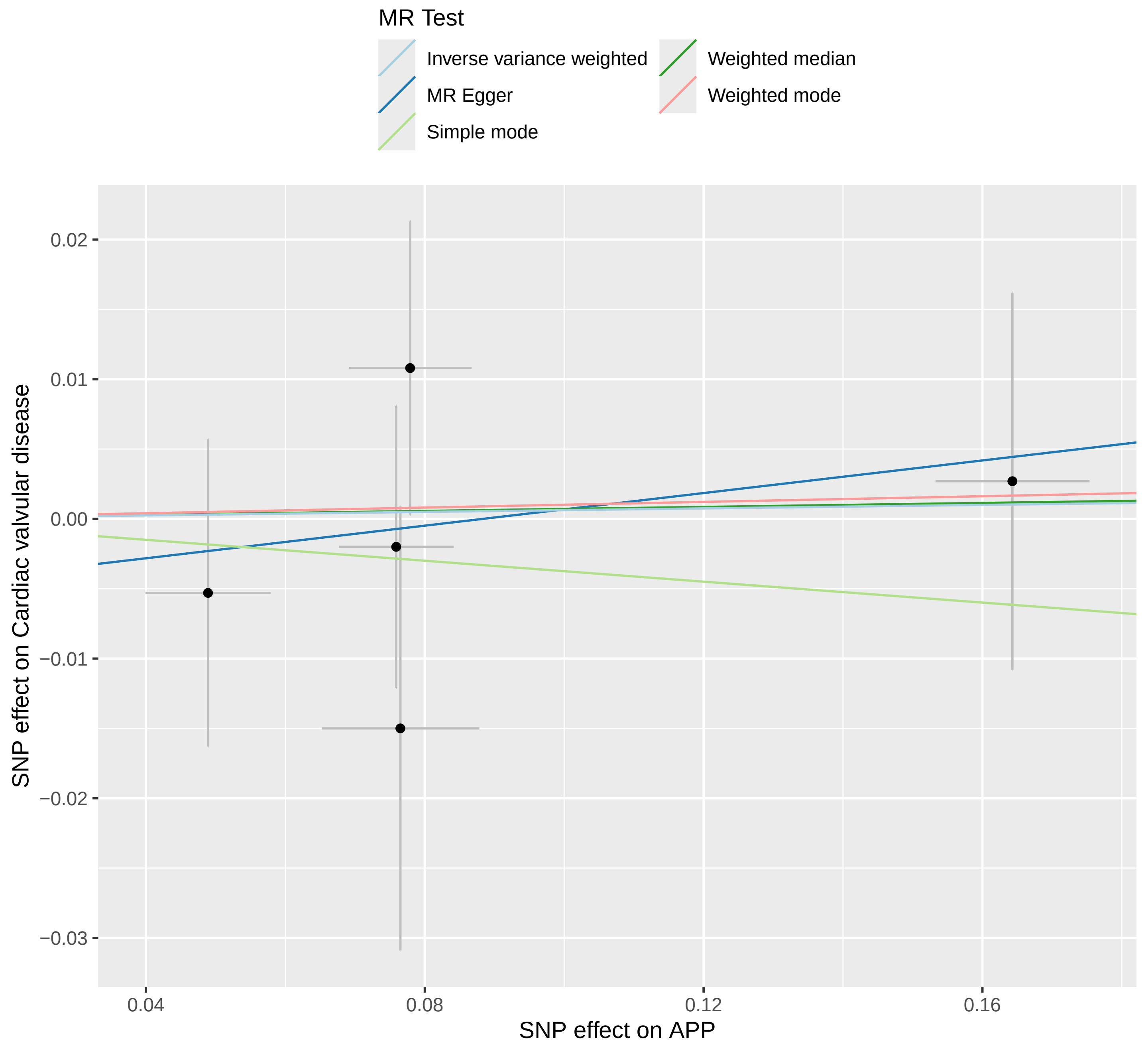

MR Test
Inverse variance weighted
MR Egger
Simple mode
Weighted median
Weighted mode
0.02
0.01
0.00
−0.01
−0.02
−0.03
0.04
0.08
0.12
0.16
SNP effect on Cardiac valvular disease
SNP effect on APP

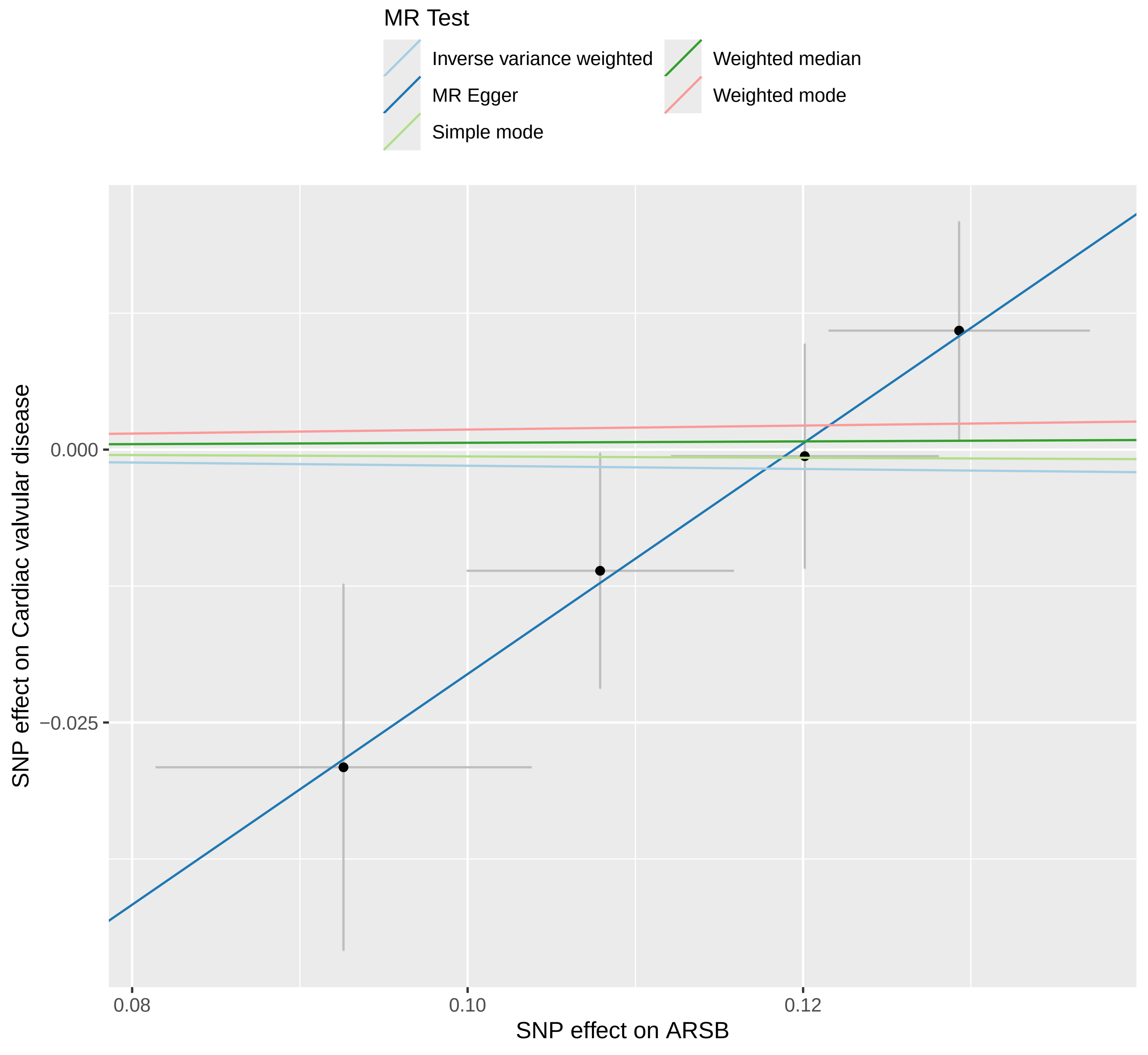
MR Test
Inverse variance weighted
MR Egger
Simple mode
Weighted median
Weighted mode
0.000
−0.025
0.08
0.10
0.12
SNP effect on ARSB
SNP effect on Cardiac valvular disease

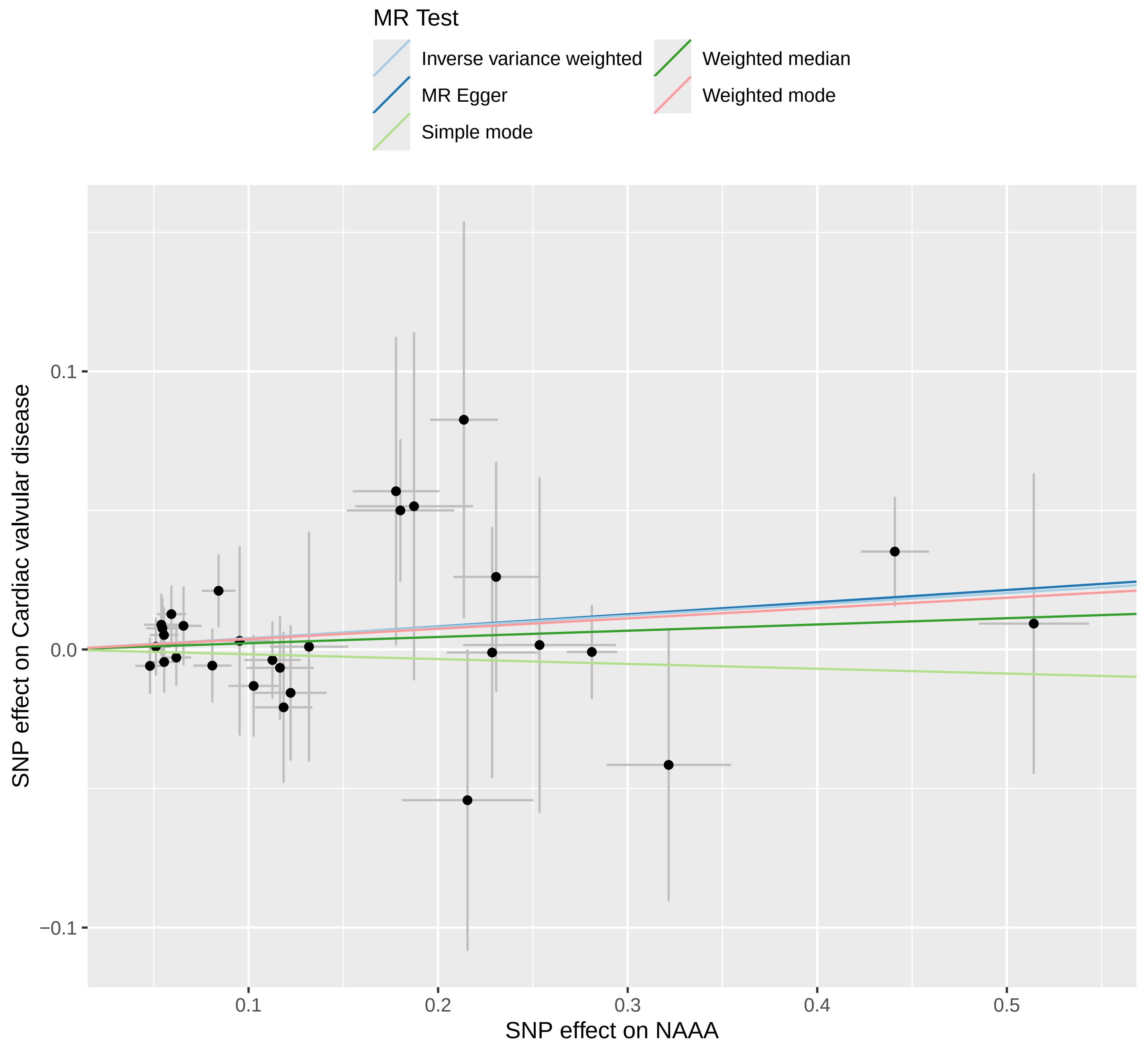
MR Test
Inverse variance weighted
MR Egger
Simple mode
Weighted median
Weighted mode
0.1
0.0
−0.1
0.1
0.2
0.3
0.4
0.5
SNP effect on NAAA
SNP effect on Cardiac valvular disease

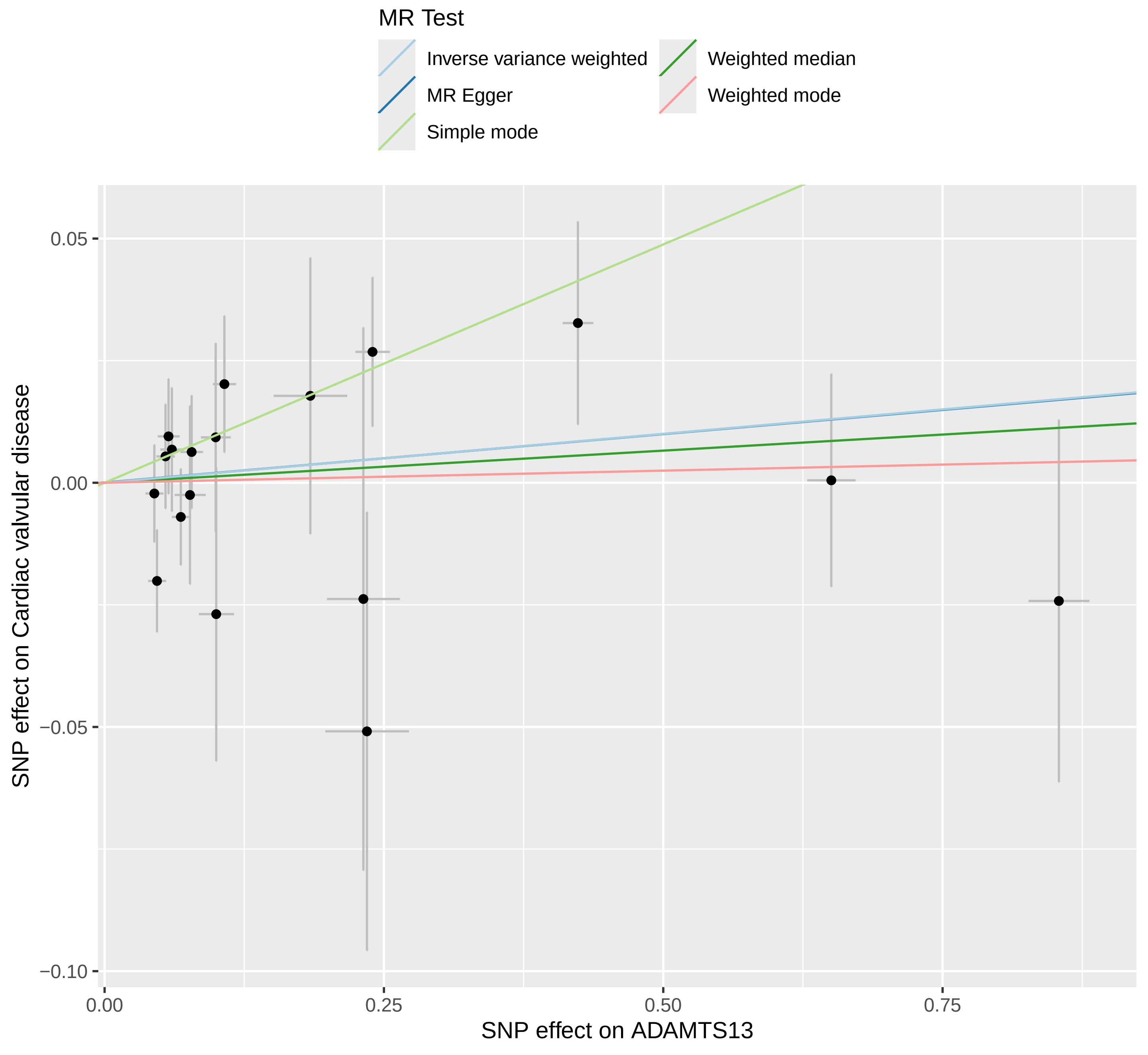
MR Test
Inverse variance weighted
MR Egger
Simple mode
Weighted median
Weighted mode
0.05
0.00
−0.05
−0.10
0.00
0.25
0.50
0.75
SNP effect on Cardiac valvular disease
SNP effect on ADAMTS13

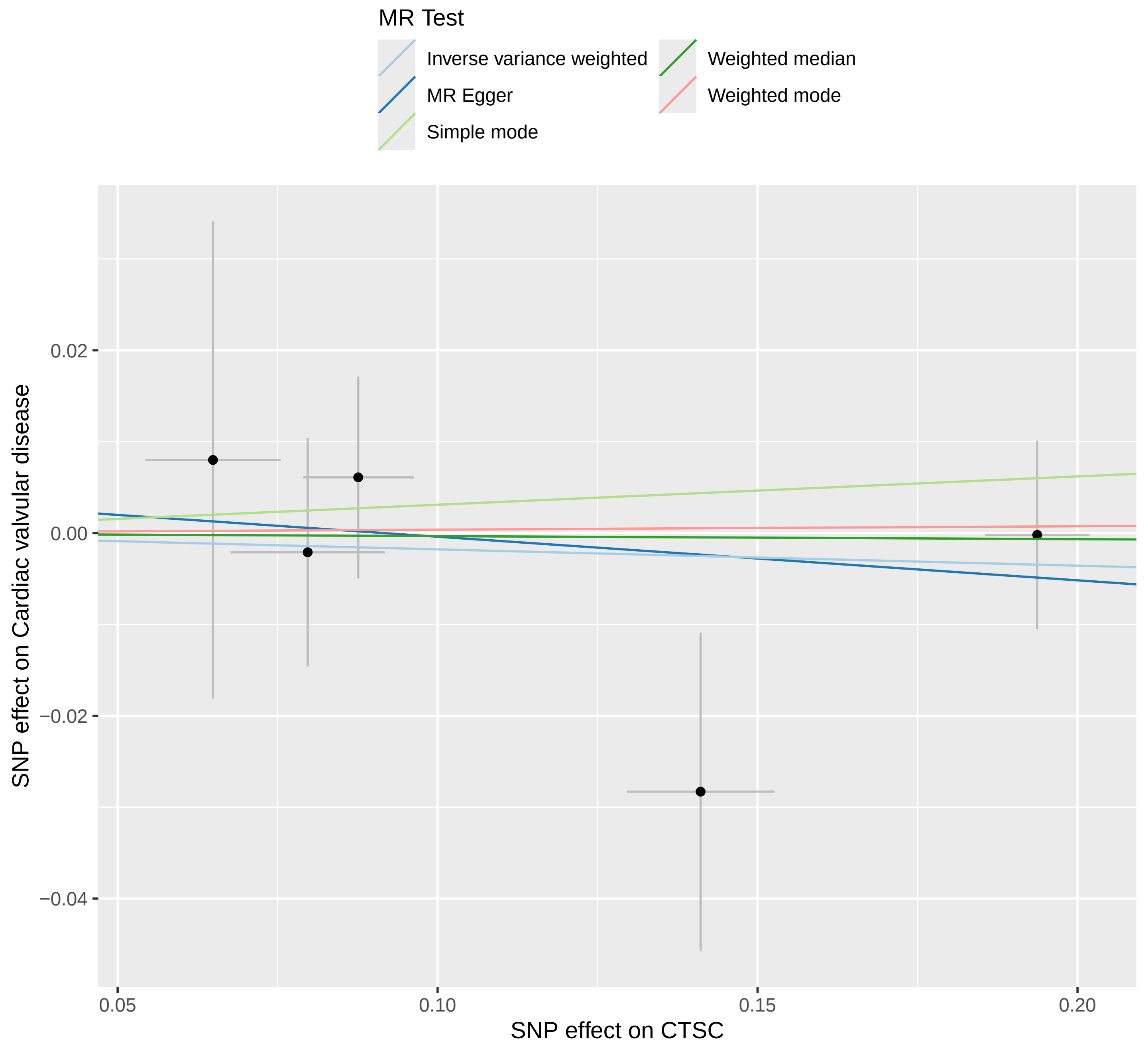
MR Test
Inverse variance weighted
MR Egger
Simple mode
Weighted median
Weighted mode
0.02
0.00
−0.02
−0.04
SNP effect on Cardiac valvular disease
0.05
0.10
0.15
0.20
SNP effect on CTSC

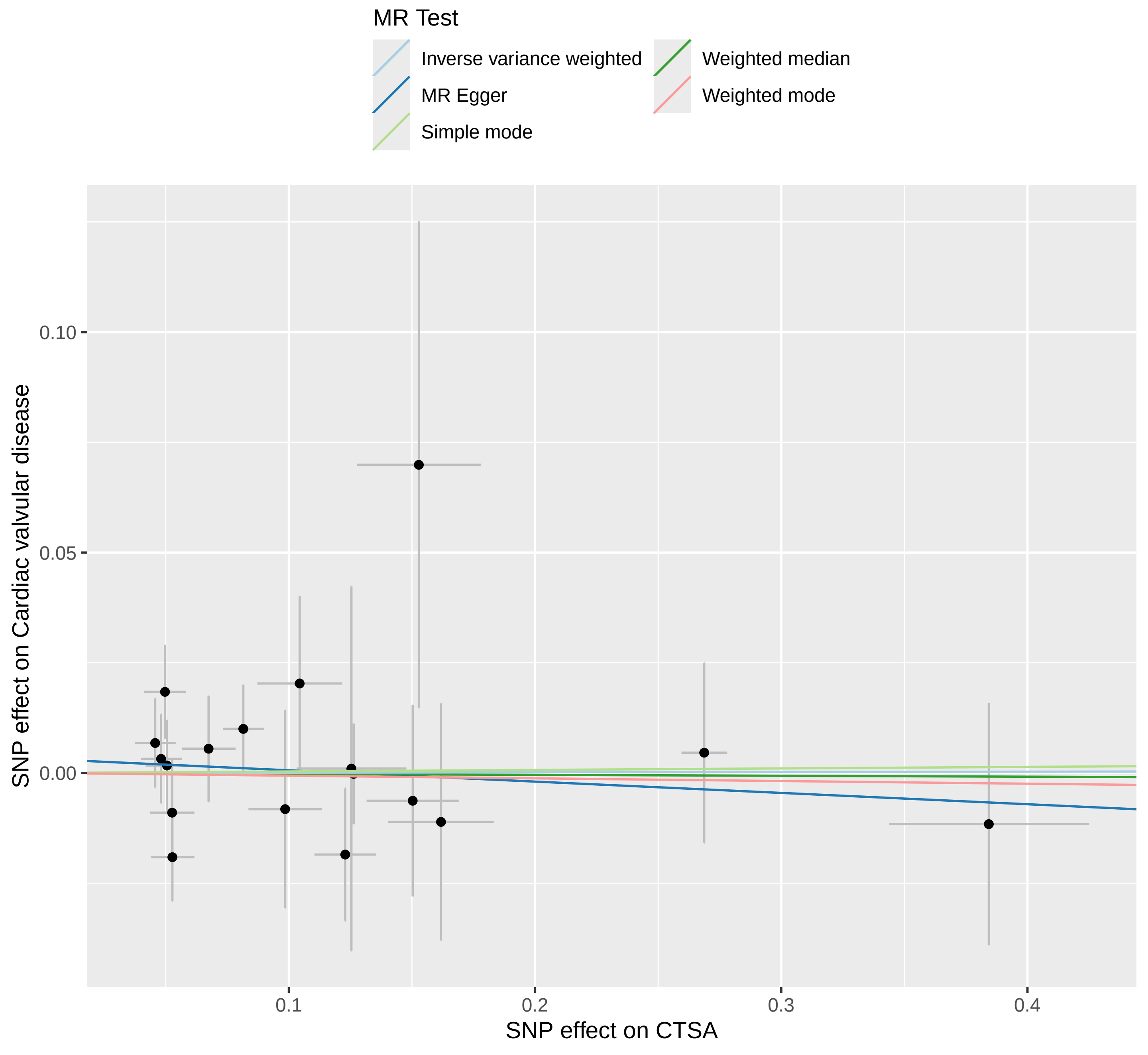
MR Test
Inverse variance weighted
MR Egger
Simple mode
Weighted median
Weighted mode
0.10
0.05
0.00
SNP effect on Cardiac valvular disease
0.1
0.2
0.3
0.4
SNP effect on CTSA

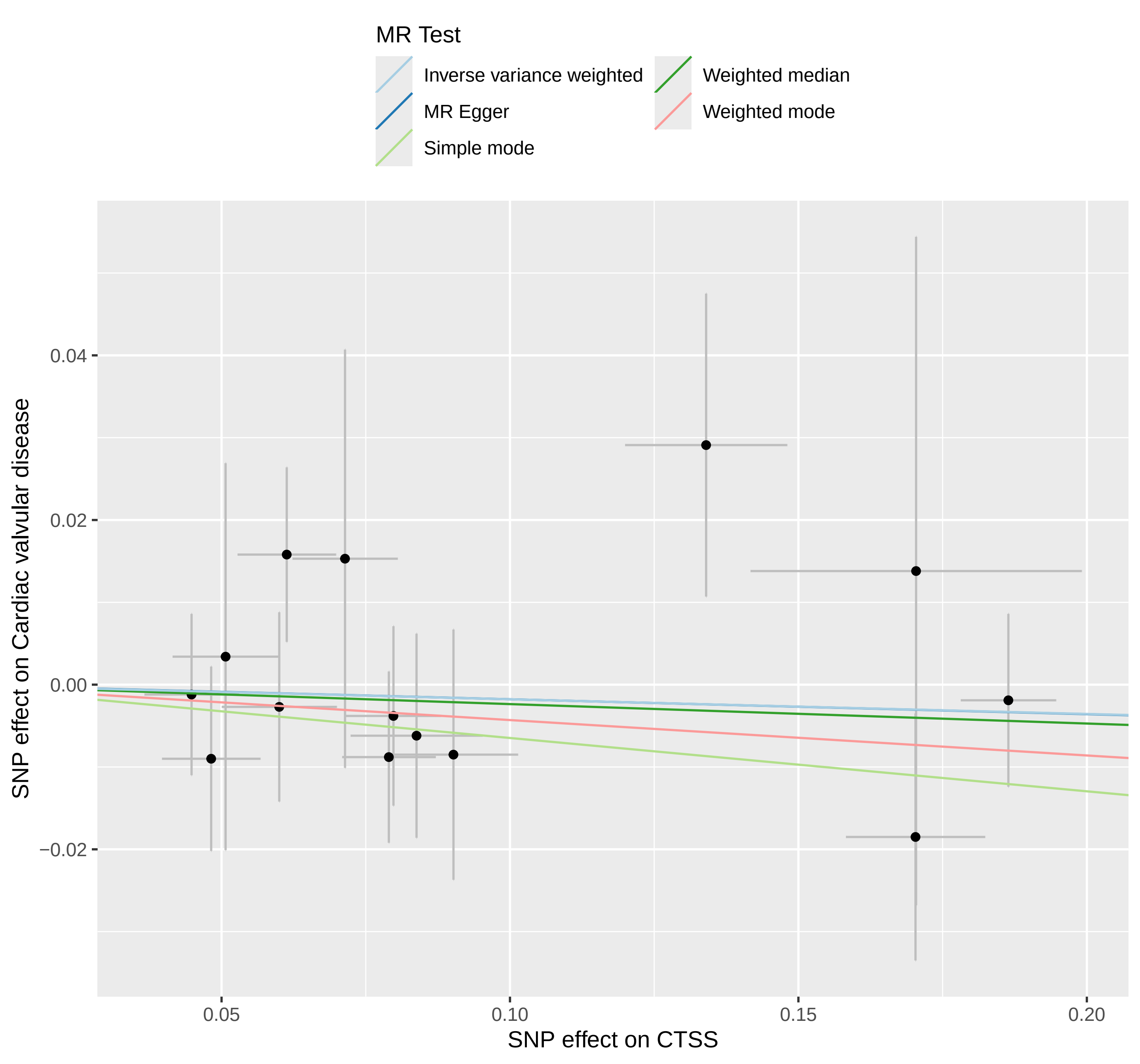
MR Test
Inverse variance weighted
MR Egger
Simple mode
Weighted median
Weighted mode
0.04
0.02
0.00
−0.02
SNP effect on Cardiac valvular disease
0.05
0.10
0.15
0.20
SNP effect on CTSS

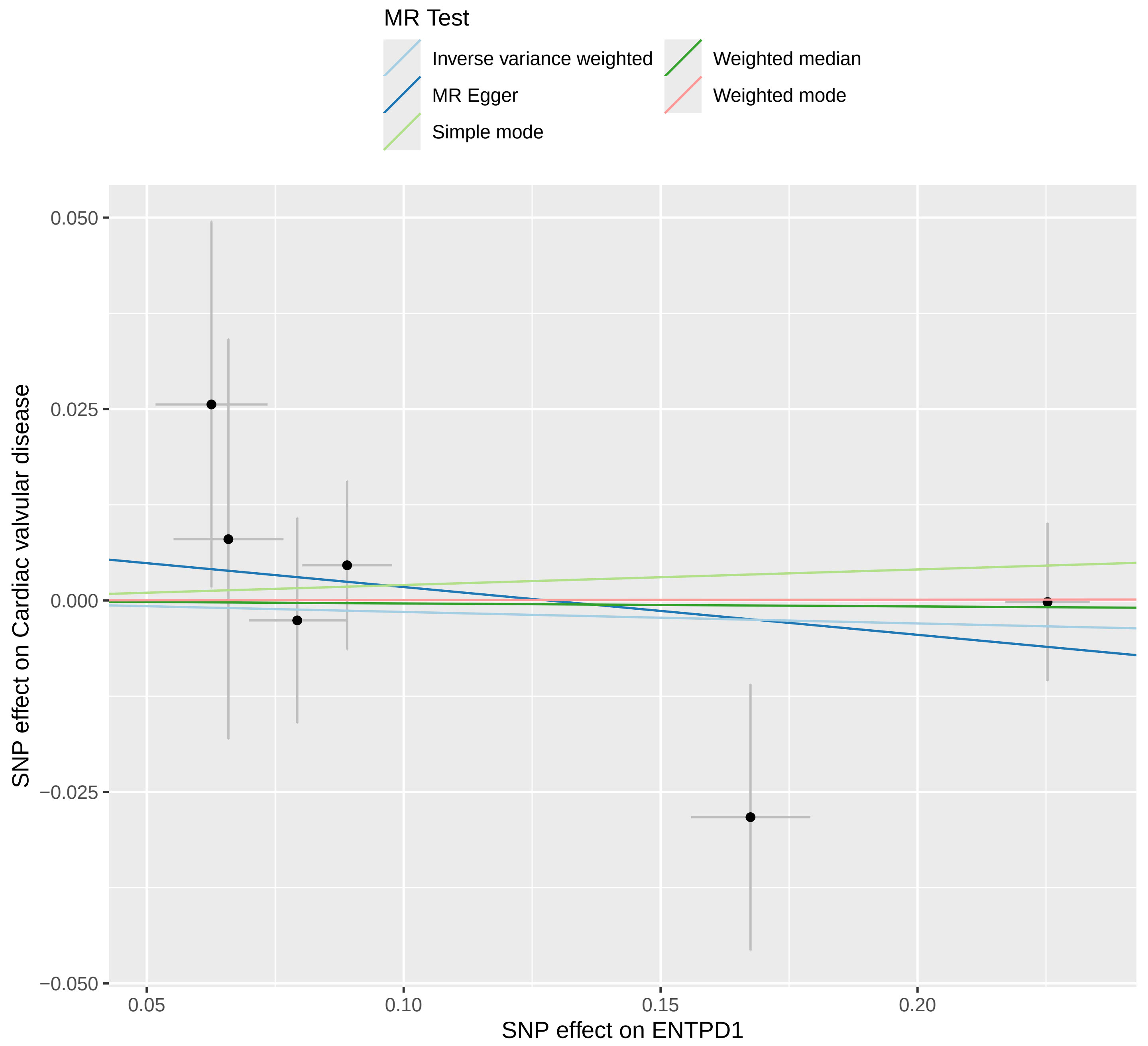

MR Test
Inverse variance weighted
MR Egger
Simple mode
Weighted median
Weighted mode
0.050
0.025
0.000
−0.025
−0.050
0.05
0.10
0.15
0.20
SNP effect on ENTPD1
SNP effect on Cardiac valvular disease

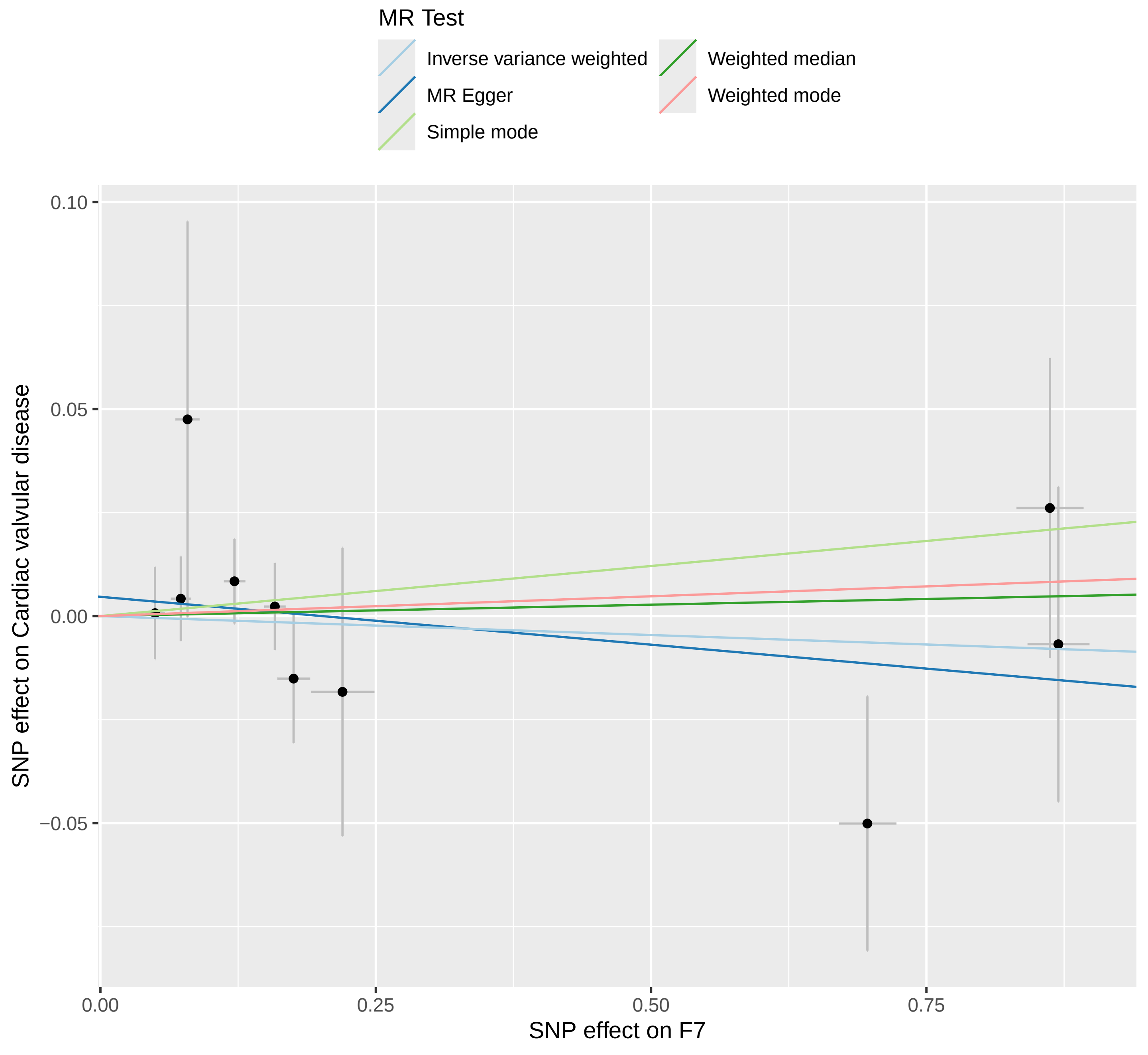
MR Test
Inverse variance weighted
MR Egger
Simple mode
Weighted median
Weighted mode
0.10
0.05
0.00
−0.05
0.00
0.25
0.50
0.75
SNP effect on Cardiac valvular disease
SNP effect on F7

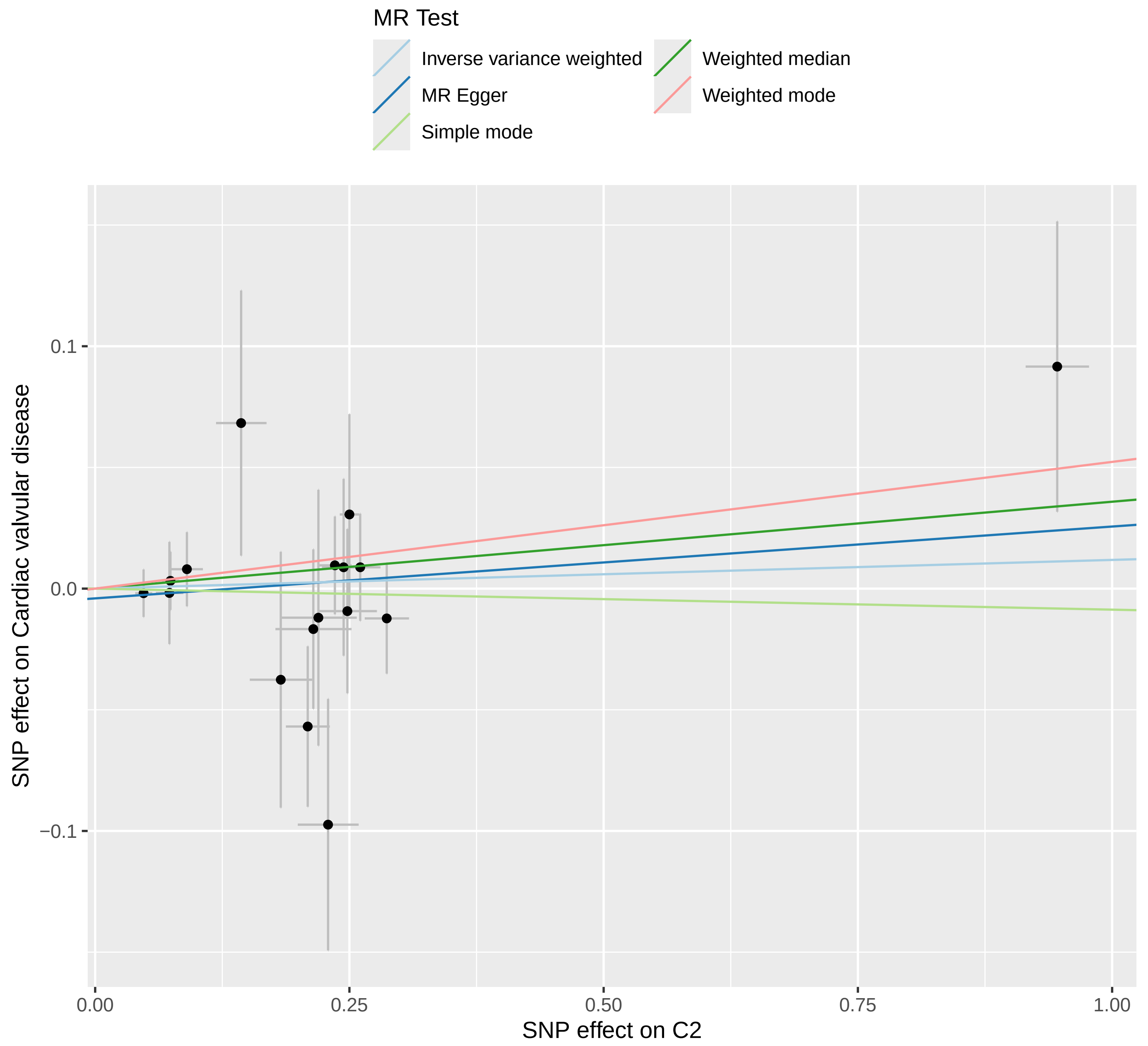
MR Test
Inverse variance weighted
MR Egger
Simple mode
Weighted median
Weighted mode
SNP effect on Cardiac valvular disease
0.1
0.0
−0.1
0.00
0.25
0.50
0.75
1.00
SNP effect on C2

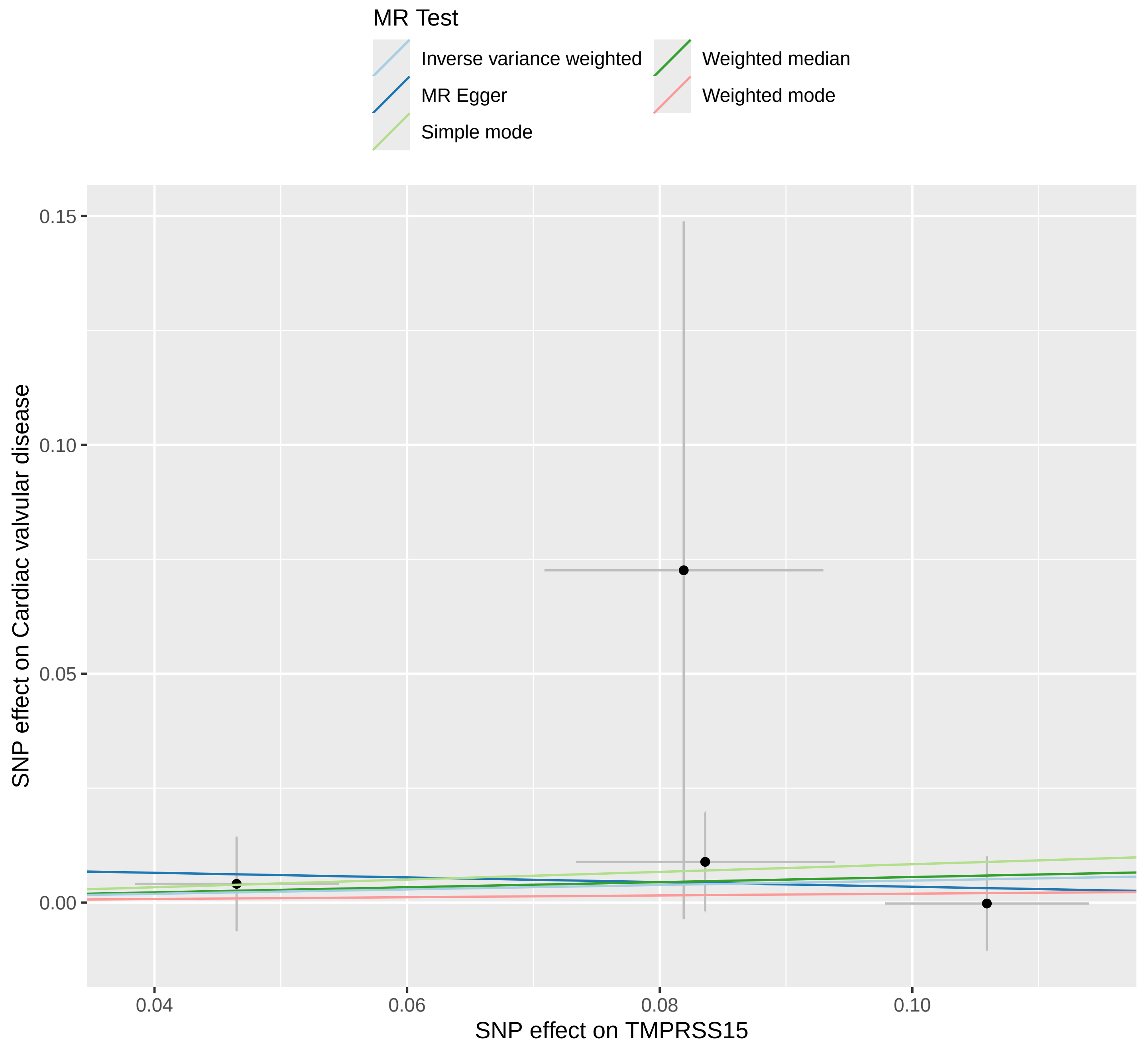
MR Test
Inverse variance weighted
MR Egger
Simple mode
Weighted median
Weighted mode
0.15
0.10
0.05
0.00
0.04
0.06
0.08
0.10
SNP effect on Cardiac valvular disease
SNP effect on TMPRSS15

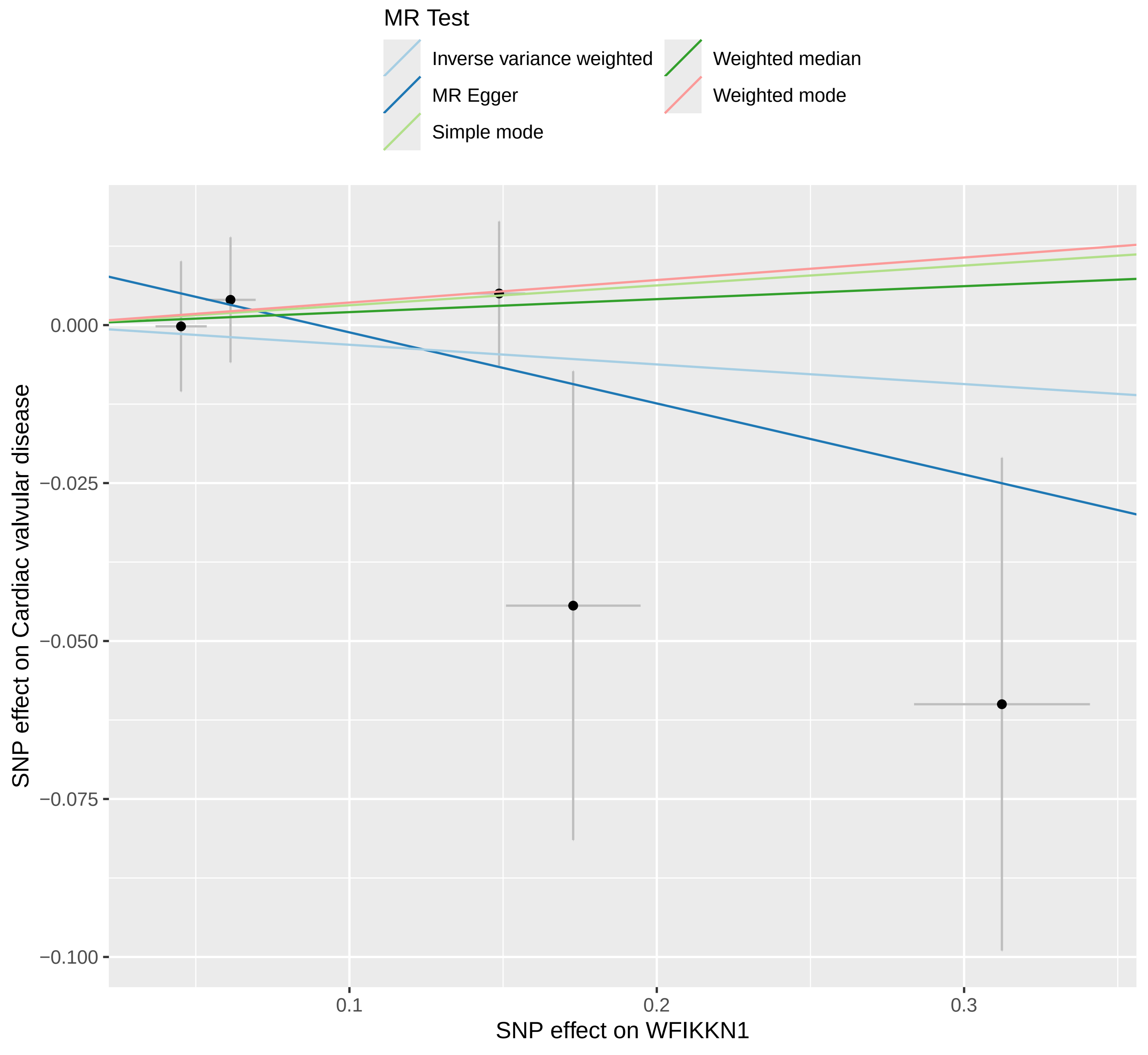

MR Test
Inverse variance weighted
MR Egger
Simple mode
Weighted median
Weighted mode
SNP effect on Cardiac valvular disease
0.000
−0.025
−0.050
−0.075
−0.100
0.1
0.2
0.3
SNP effect on WFIKKN1

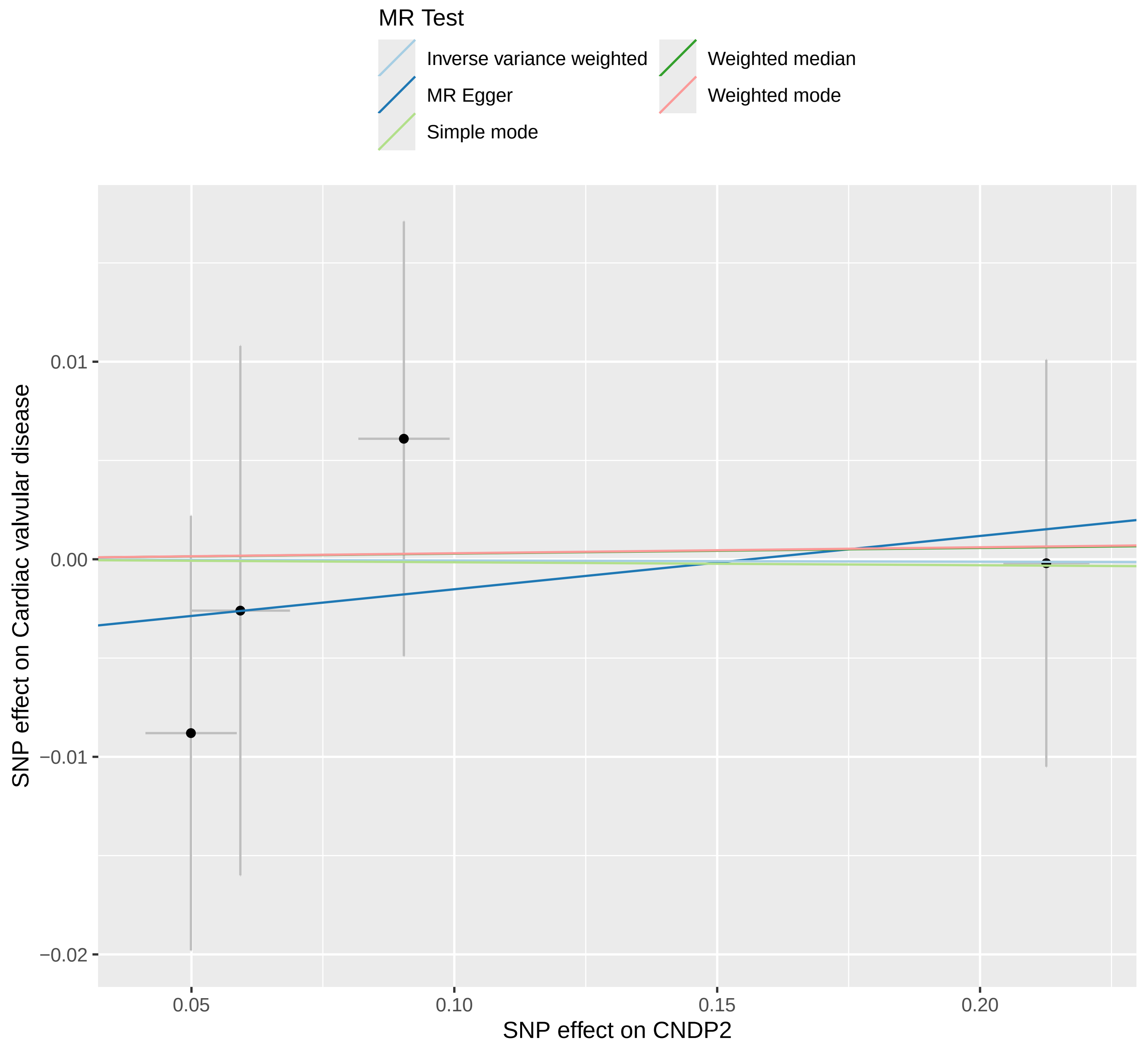
MR Test
Inverse variance weighted
MR Egger
Simple mode
Weighted median
Weighted mode
0.01
0.00
−0.01
−0.02
0.05
0.10
0.15
0.20
SNP effect on Cardiac valvular disease
SNP effect on CNDP2

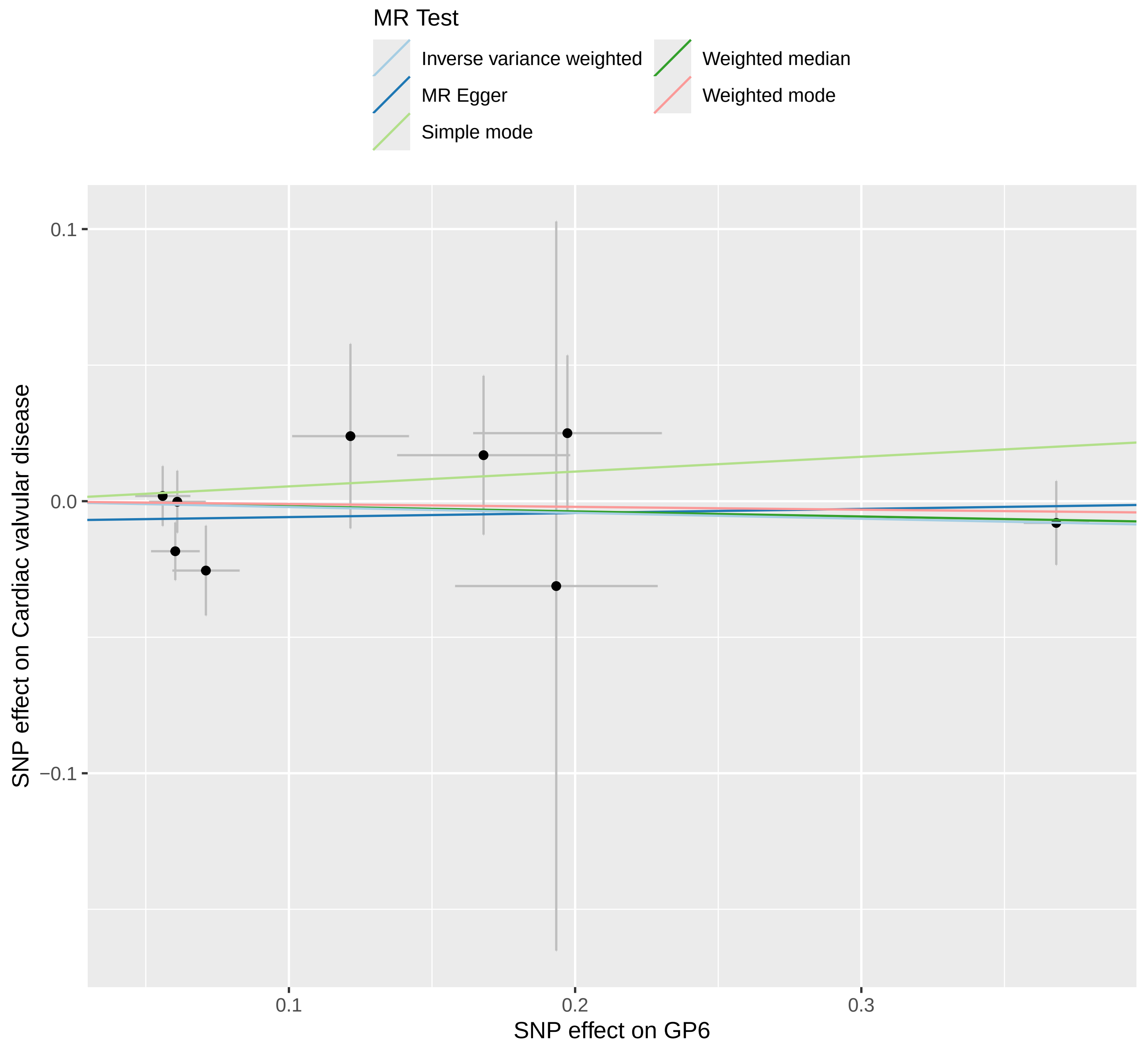
MR Test
Inverse variance weighted
MR Egger
Simple mode
Weighted median
Weighted mode
0.1
0.0
−0.1
SNP effect on Cardiac valvular disease
0.1
0.2
0.3
SNP effect on GP6

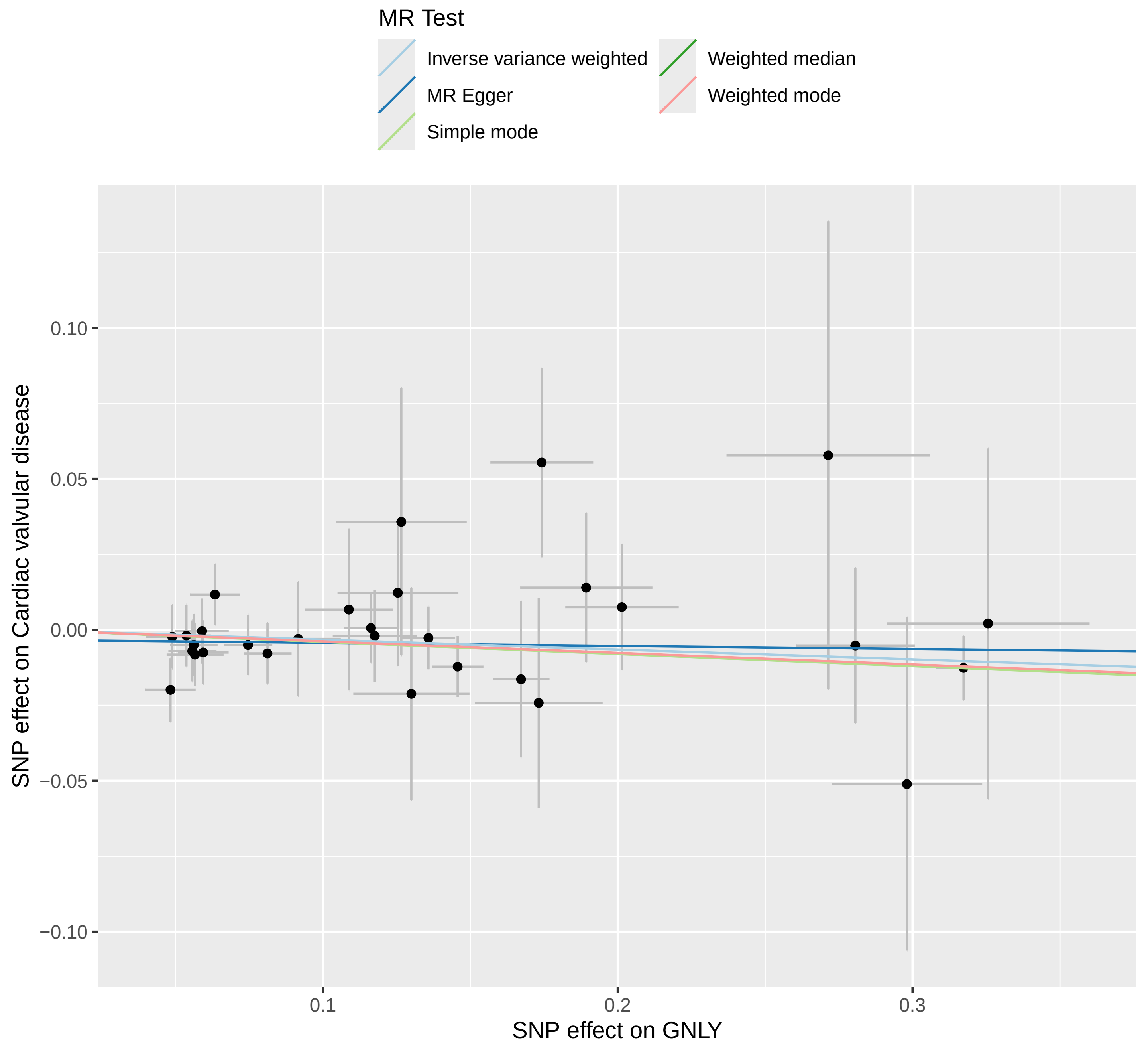
MR Test
Inverse variance weighted
MR Egger
Simple mode
Weighted median
Weighted mode
0.10
0.05
0.00
−0.05
−0.10
0.1
0.2
0.3
SNP effect on Cardiac valvular disease
SNP effect on GNLY

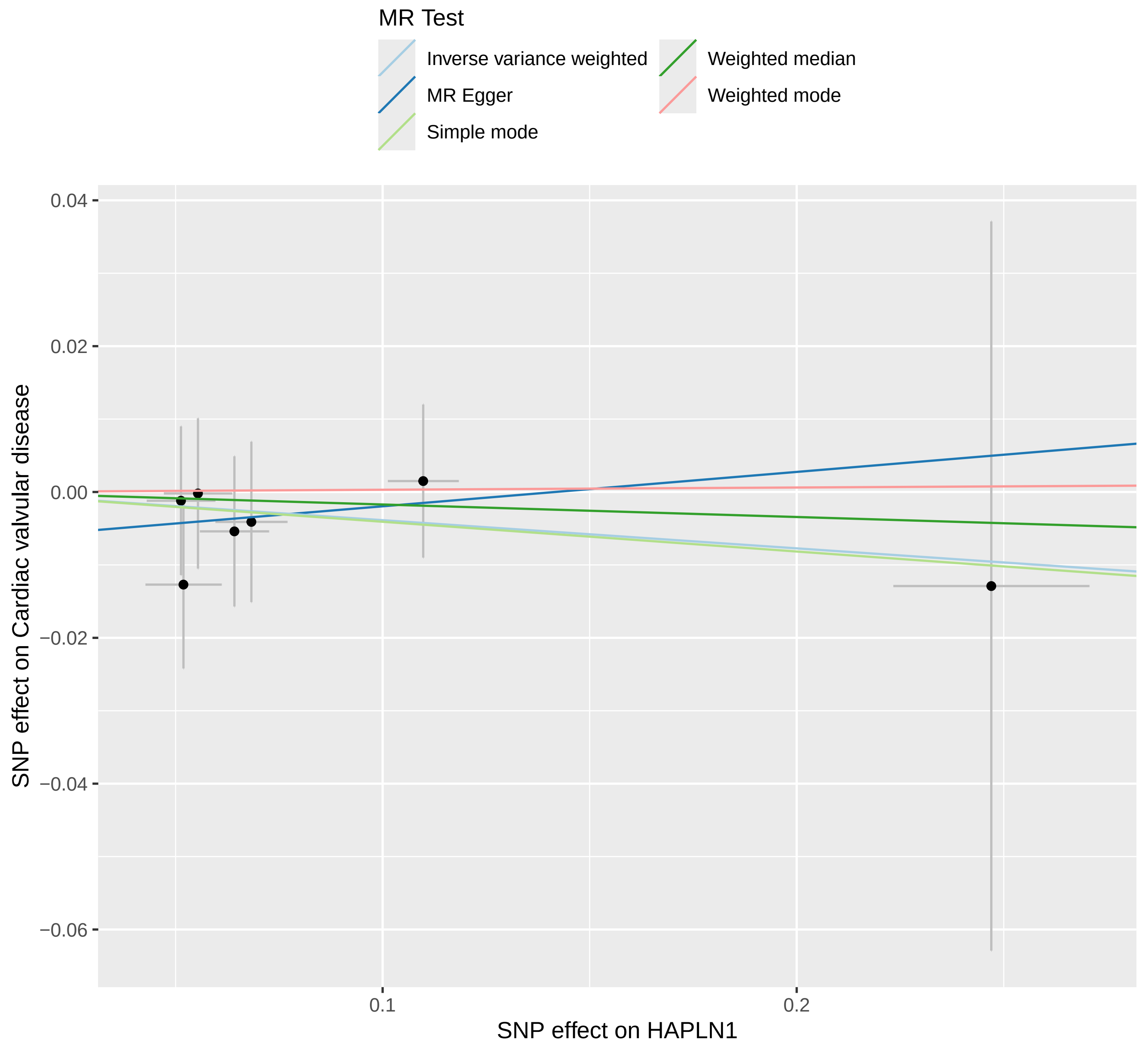
MR Test
Inverse variance weighted
MR Egger
Simple mode
Weighted median
Weighted mode
0.04
0.02
0.00
−0.02
−0.04
−0.06
SNP effect on Cardiac valvular disease
0.1
0.2
SNP effect on HAPLN1

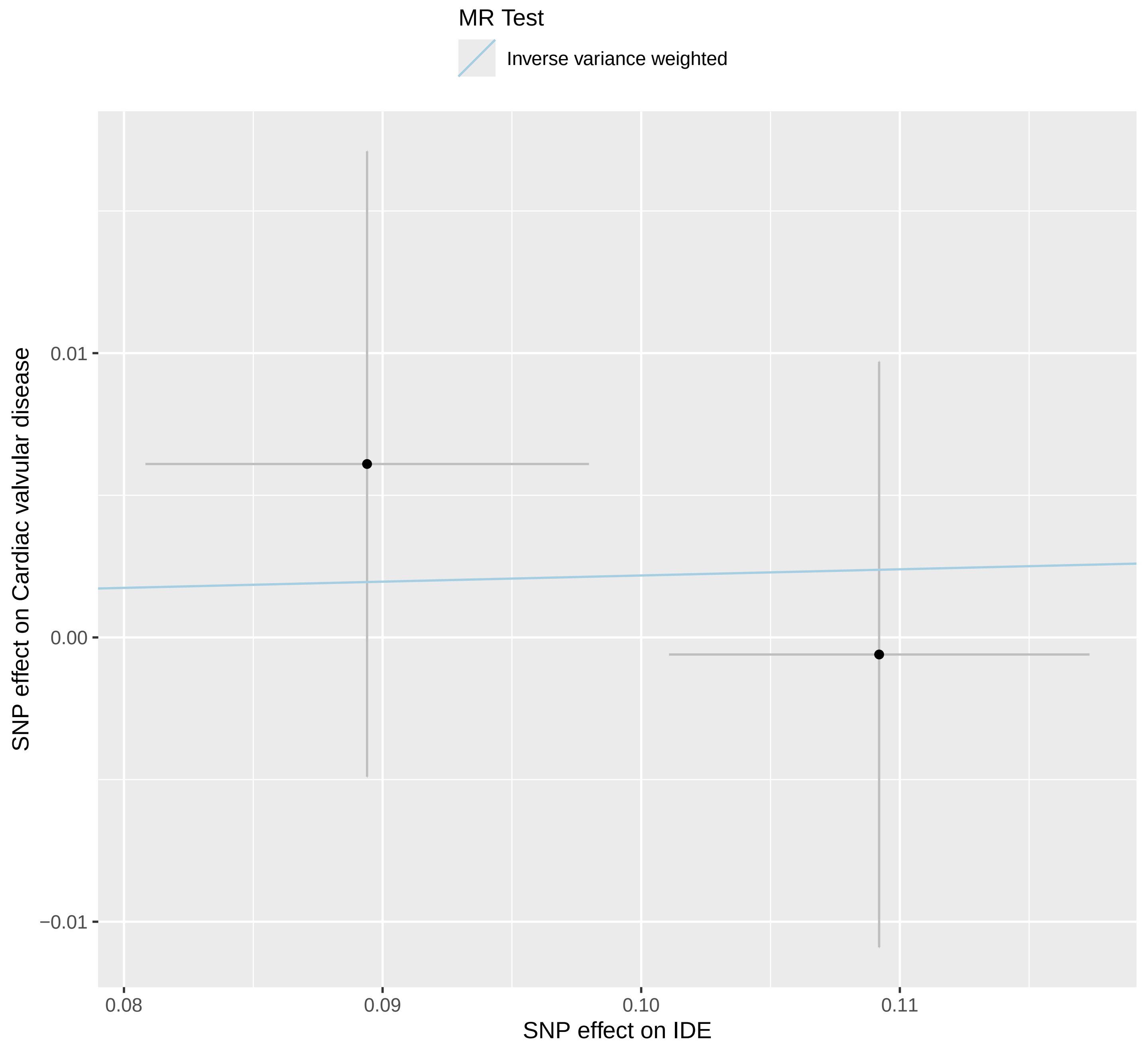

MR Test
Inverse variance weighted
SNP effect on Cardiac valvular disease
0.01
0.00
−0.01
0.08
0.09
0.10
0.11
SNP effect on IDE

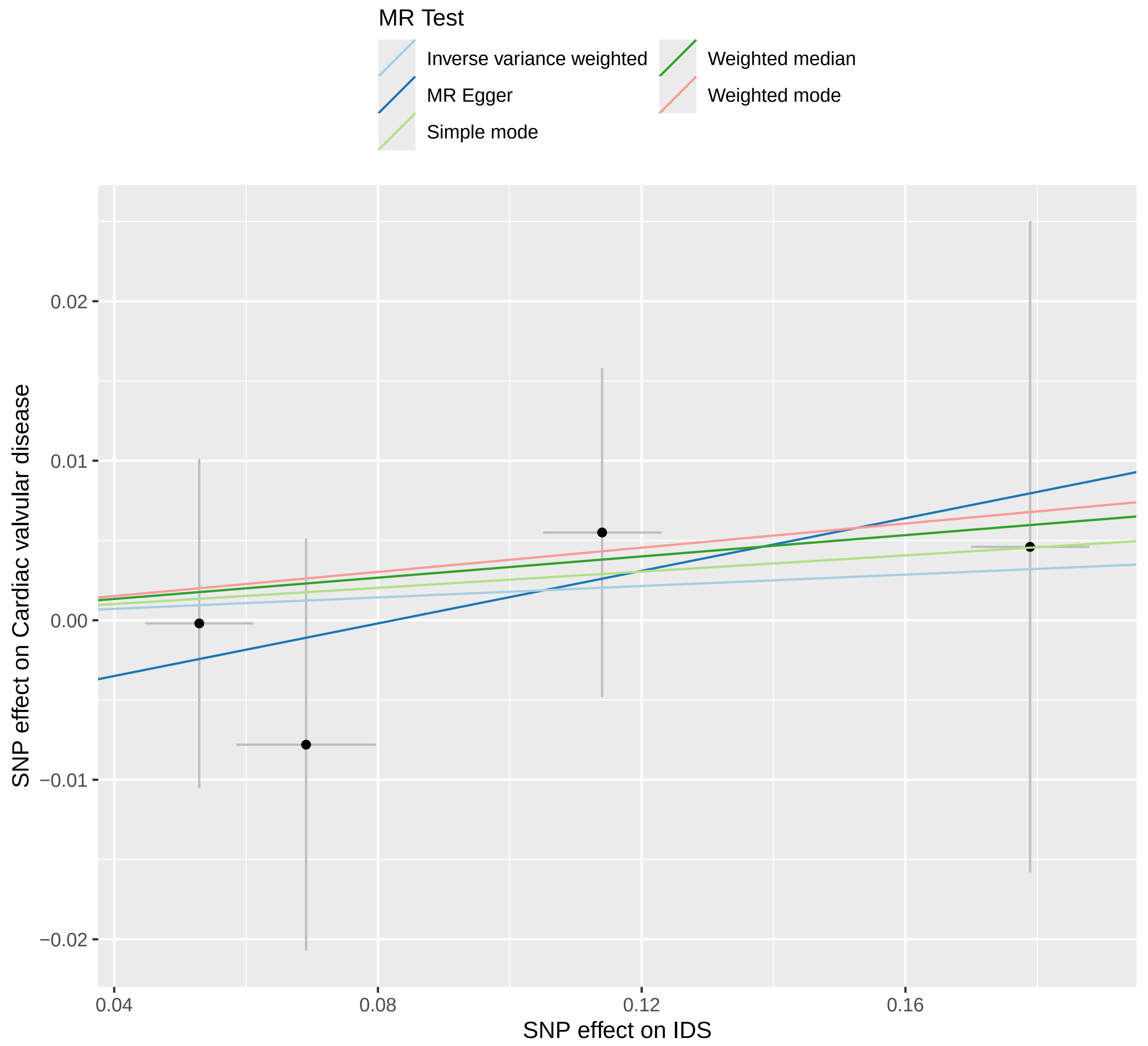
MR Test
Inverse variance weighted
MR Egger
Simple mode
Weighted median
Weighted mode
SNP effect on Cardiac valvular disease
0.02
0.01
0.00
−0.01
−0.02
0.04
0.08
0.12
0.16
SNP effect on IDS

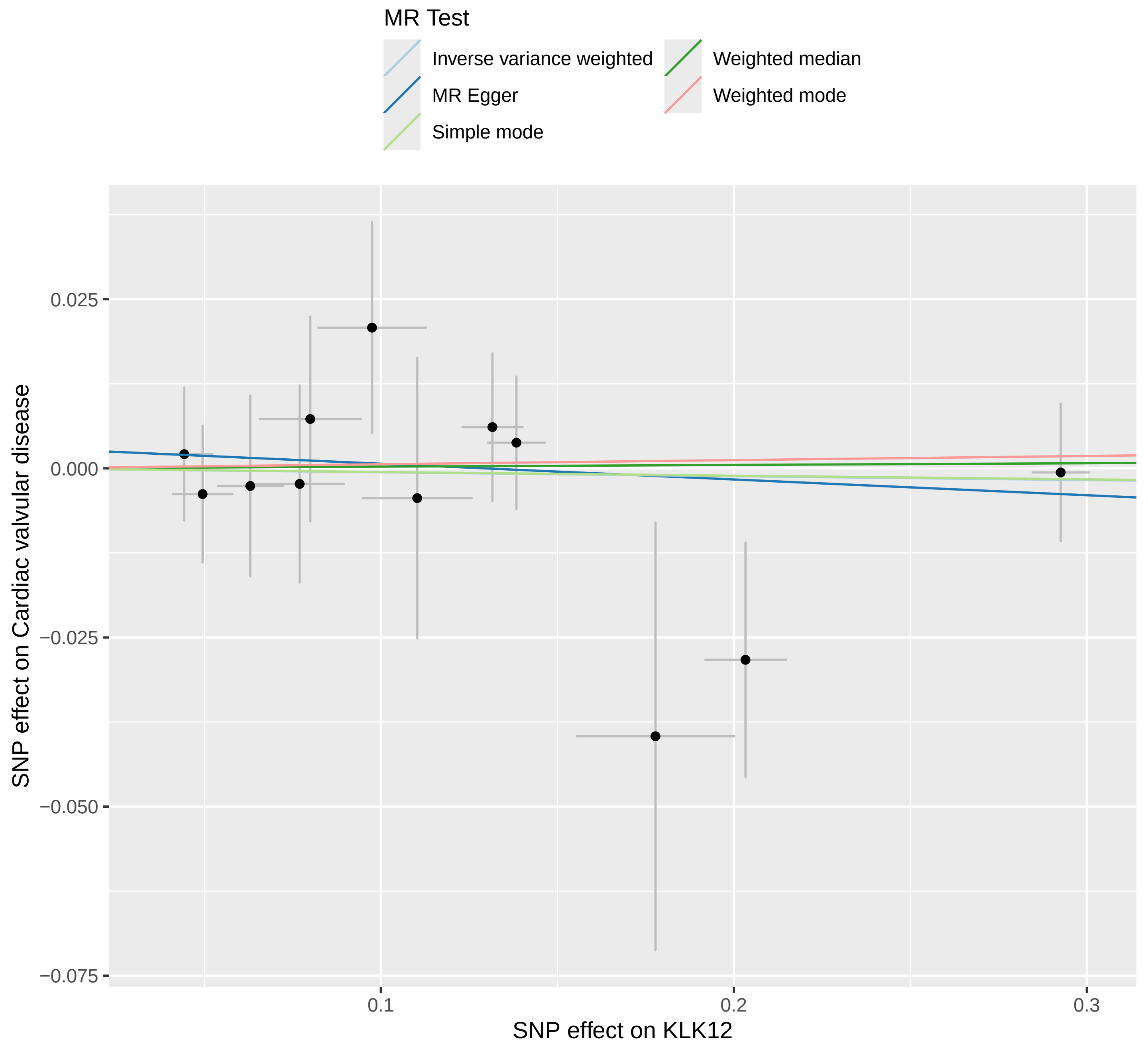
MR Test
Inverse variance weighted
MR Egger
Simple mode
Weighted median
Weighted mode
0.025
0.000
−0.025
−0.050
−0.075
0.1
0.2
0.3
SNP effect on Cardiac valvular disease
SNP effect on KLK12

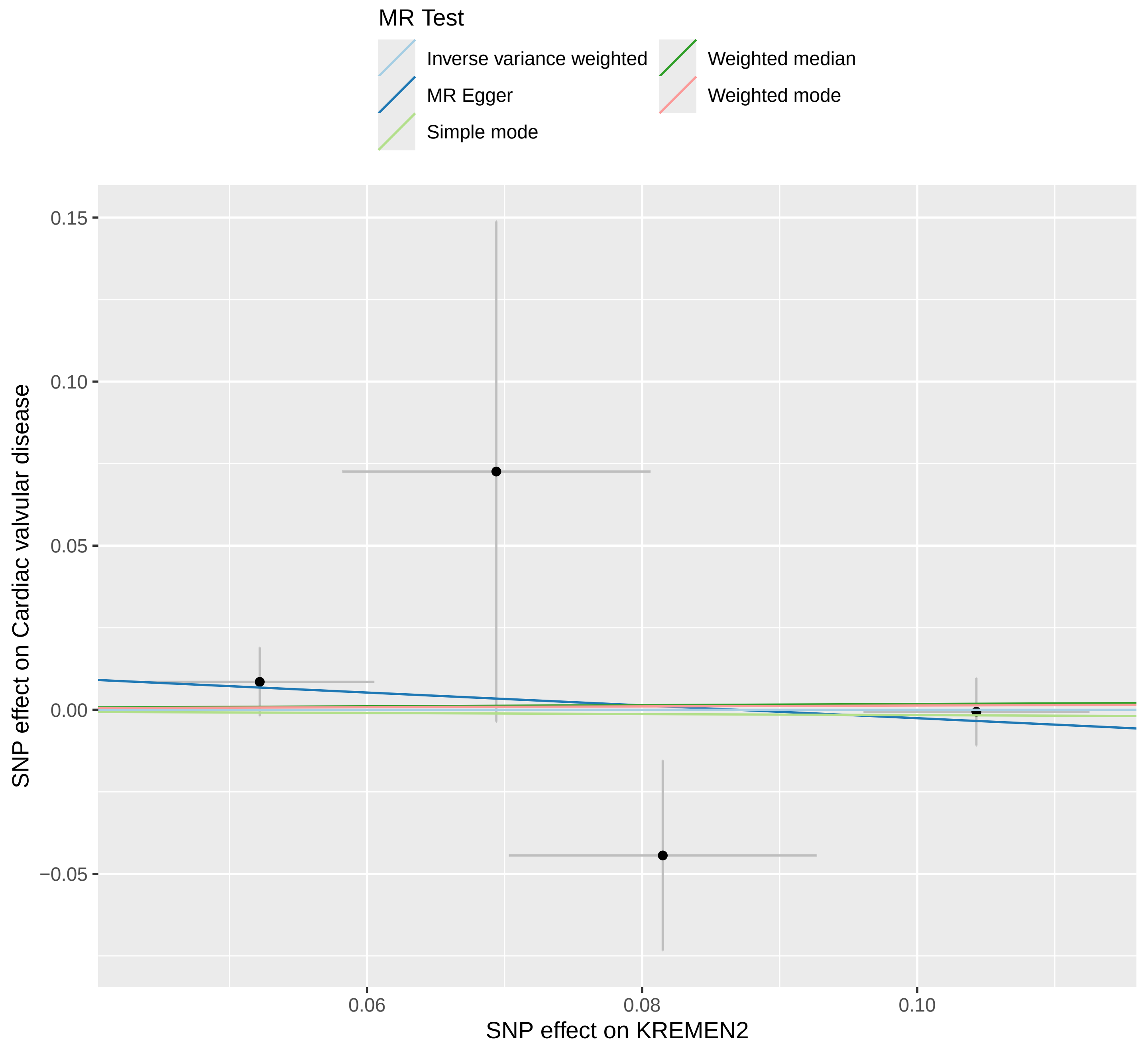
MR Test
Inverse variance weighted
MR Egger
Simple mode
Weighted median
Weighted mode
0.15
0.10
0.05
0.00
−0.05
0.06
0.08
0.10
SNP effect on Cardiac valvular disease
SNP effect on KREMEN2

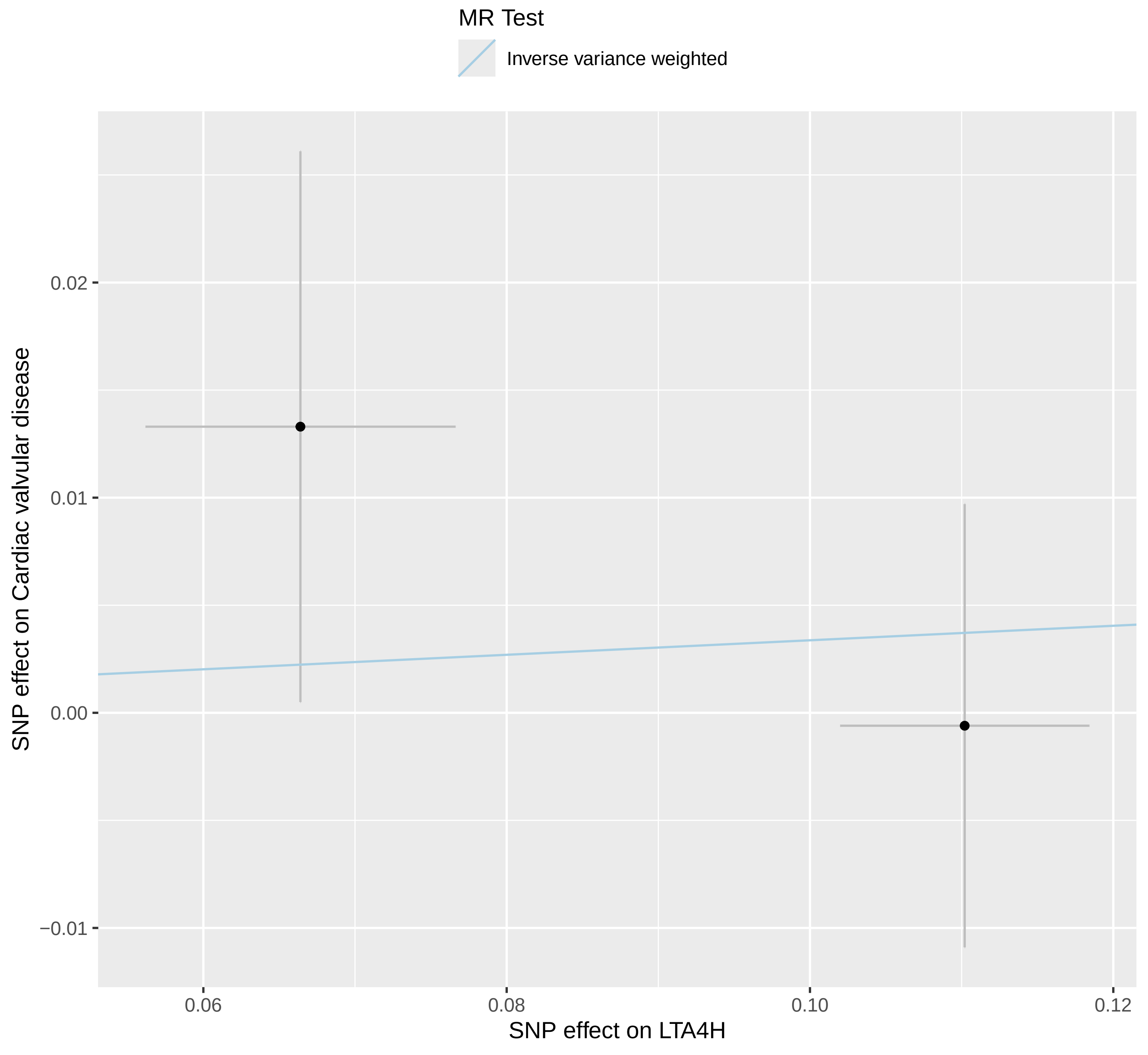
MR Test
Inverse variance weighted
0.02
0.01
0.00
−0.01
0.06
0.08
0.10
0.12
SNP effect on Cardiac valvular disease
SNP effect on LTA4H

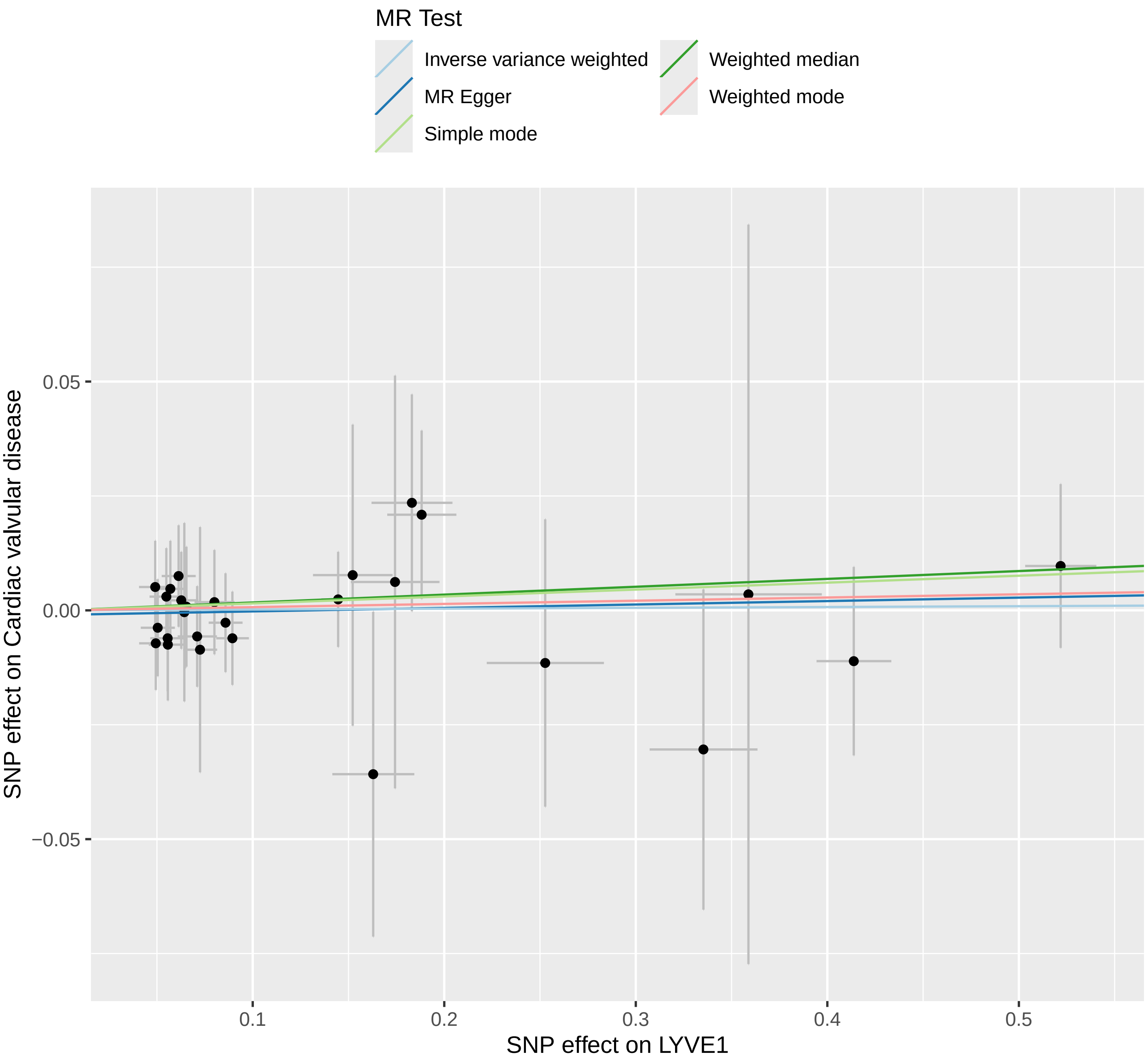

MR Test
Inverse variance weighted
MR Egger
Simple mode
Weighted median
Weighted mode
0.05
0.00
−0.05
0.1
0.2
0.3
0.4
0.5
SNP effect on Cardiac valvular disease
SNP effect on LYVE1

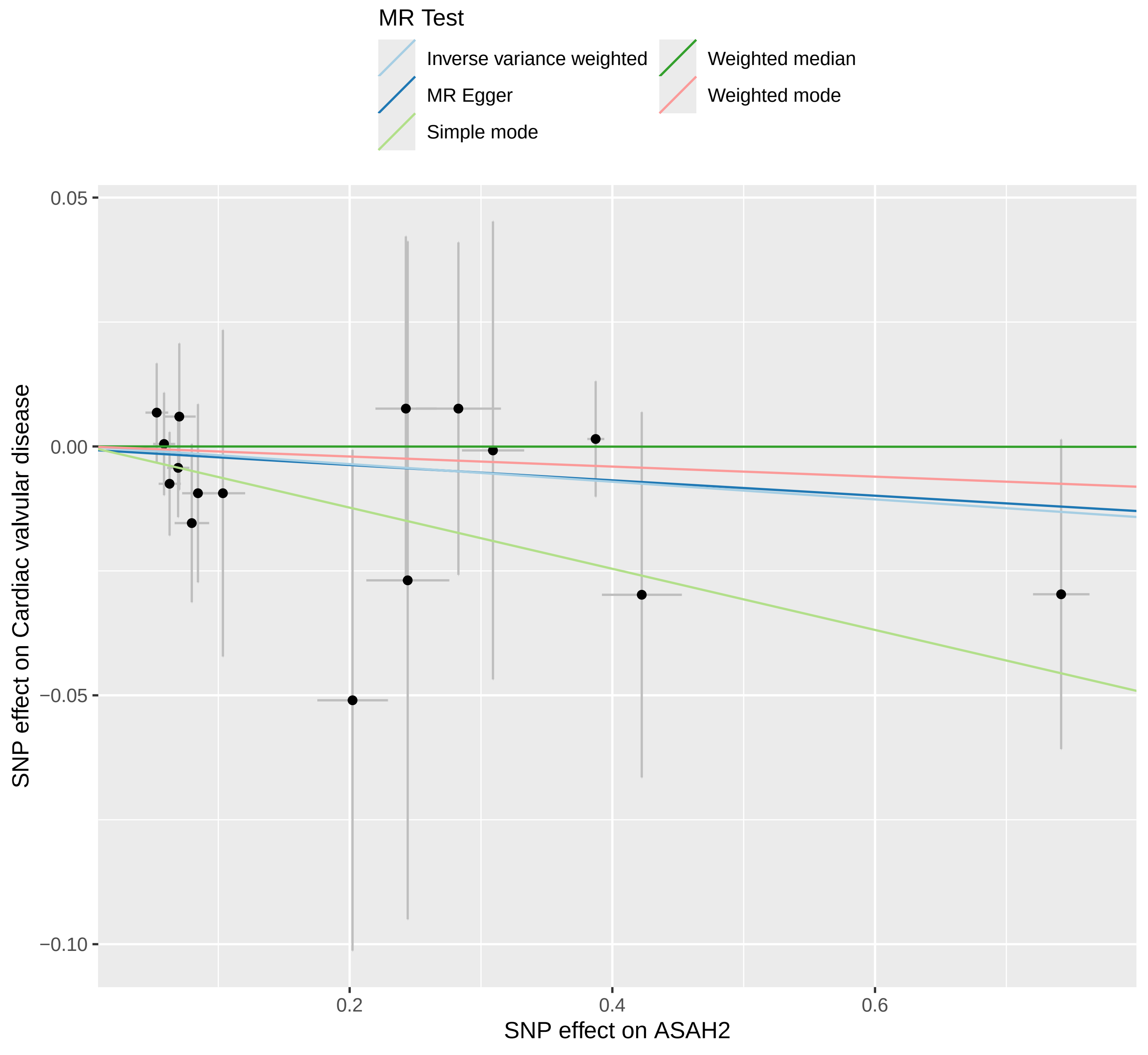
MR Test
Inverse variance weighted
MR Egger
Simple mode
Weighted median
Weighted mode
0.05
0.00
−0.05
−0.10
0.2
0.4
0.6
SNP effect on Cardiac valvular disease
SNP effect on ASAH2

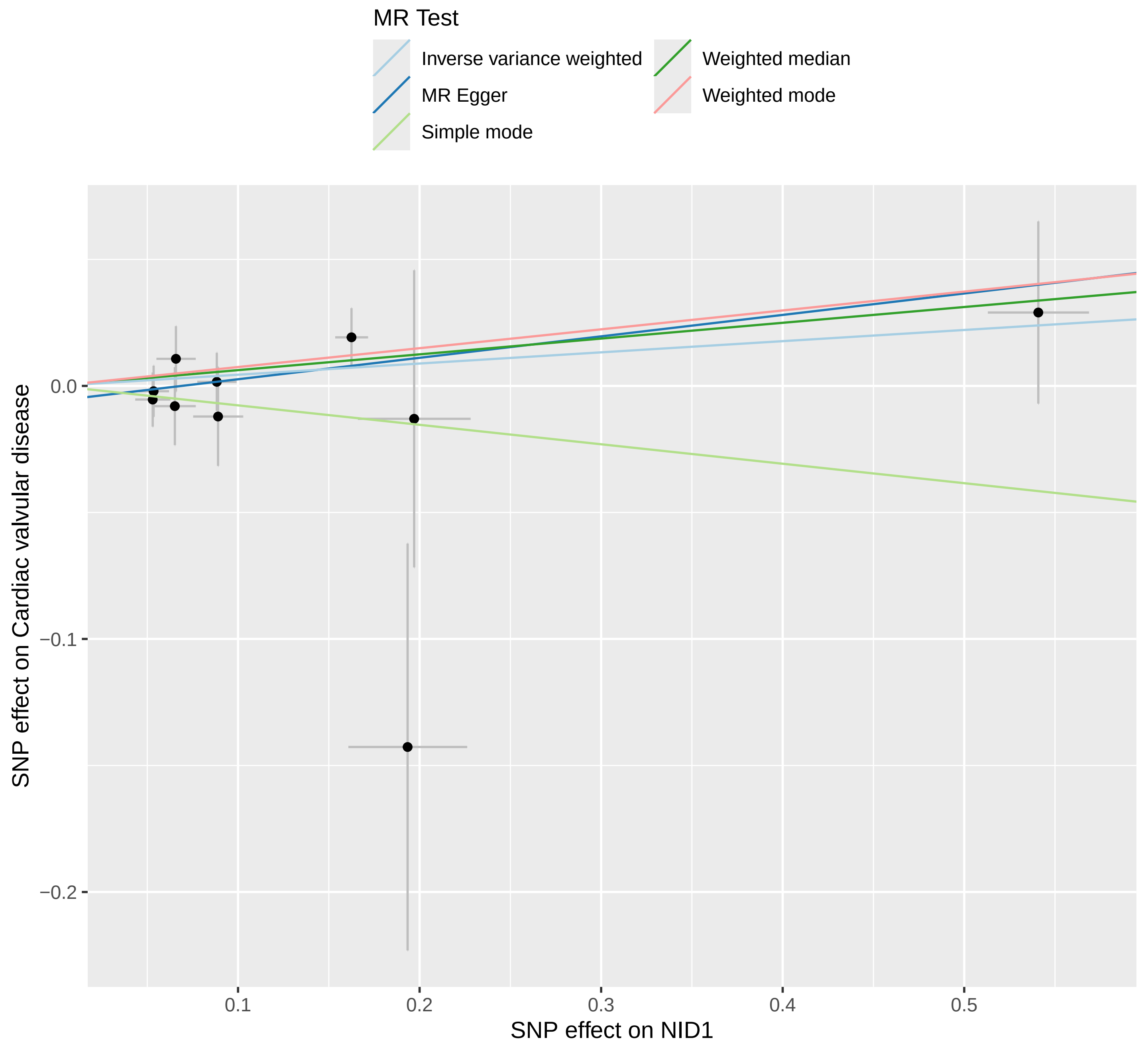

MR Test
Inverse variance weighted
MR Egger
Simple mode
Weighted median
Weighted mode
SNP effect on Cardiac valvular disease
0.0
−0.1
−0.2
0.1
0.2
0.3
0.4
0.5
SNP effect on NID1

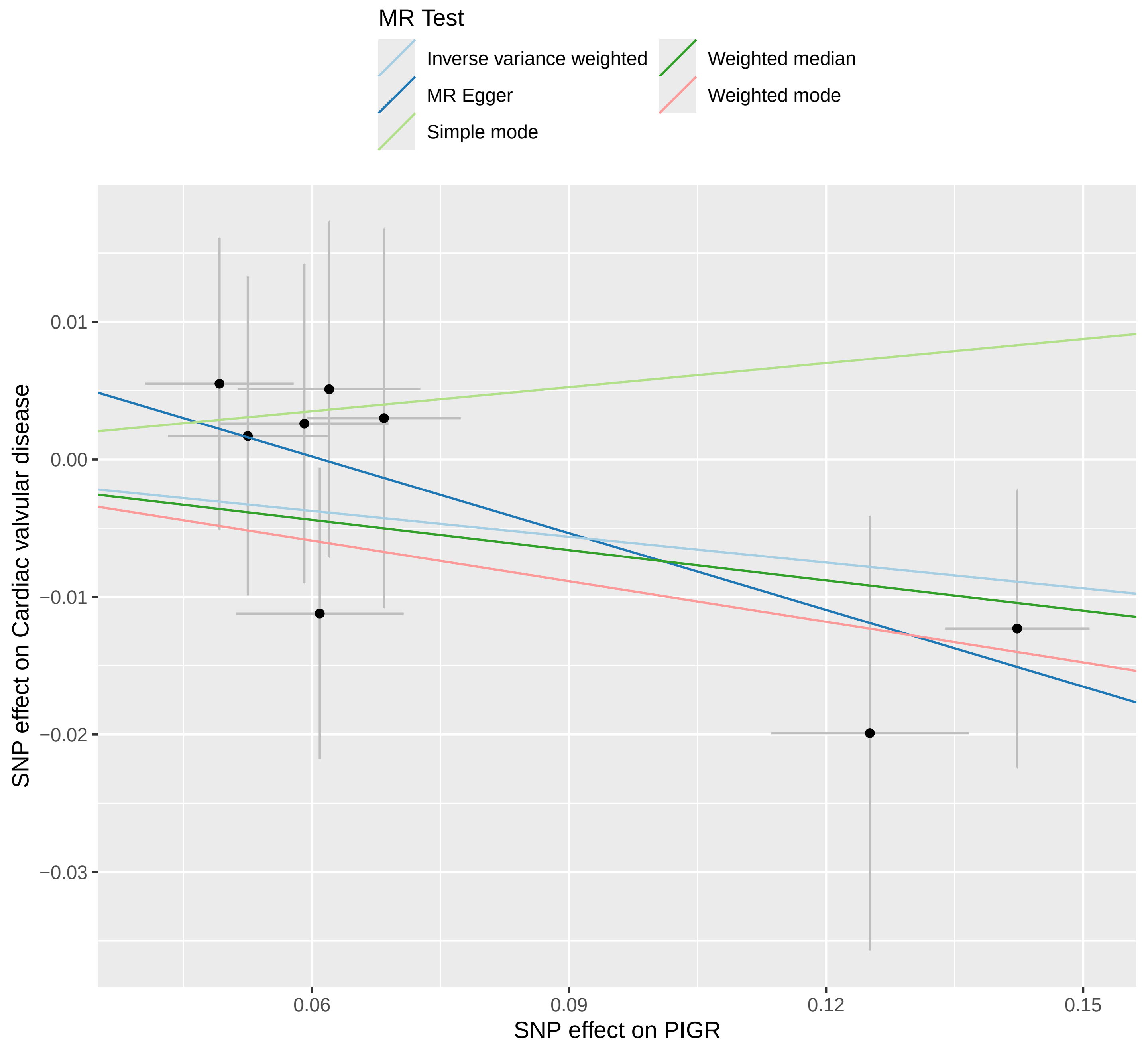
MR Test
Inverse variance weighted
MR Egger
Simple mode
Weighted median
Weighted mode
0.01
0.00
−0.01
−0.02
−0.03
SNP effect on Cardiac valvular disease
0.06
0.09
0.12
0.15
SNP effect on PIGR

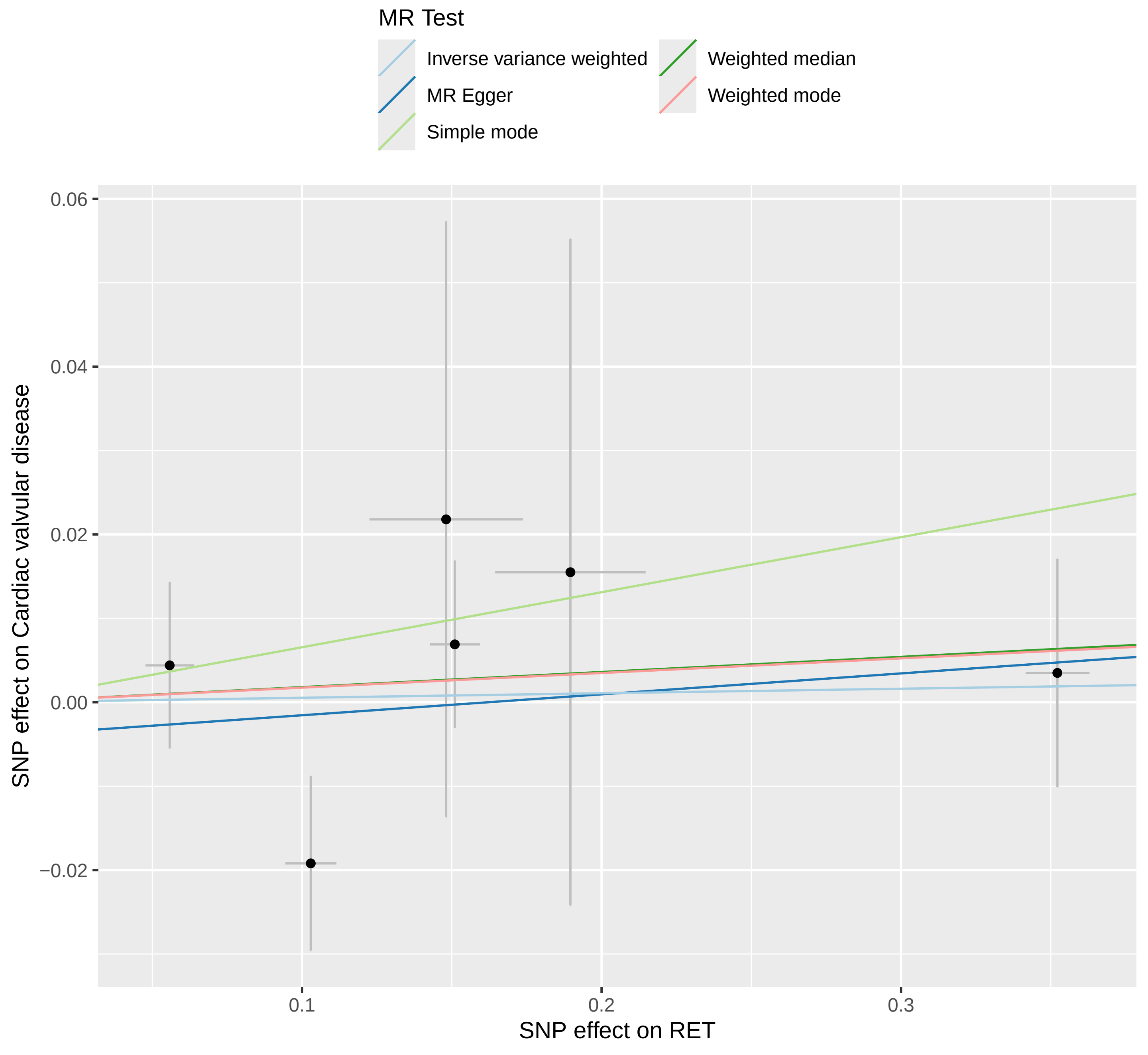
MR Test
Inverse variance weighted
MR Egger
Simple mode
Weighted median
Weighted mode
0.06
0.04
0.02
0.00
−0.02
0.1
0.2
0.3
SNP effect on Cardiac valvular disease
SNP effect on RET

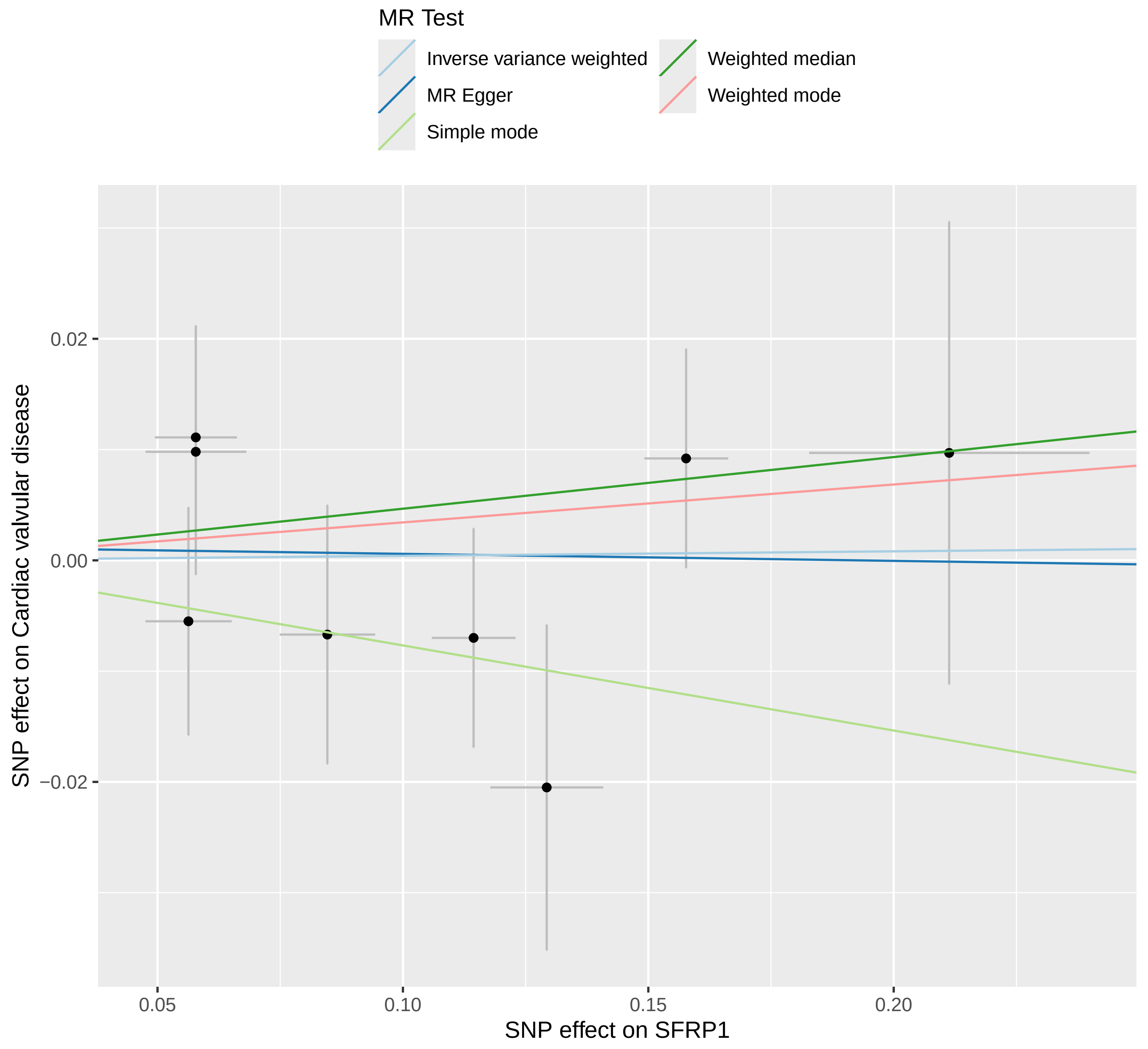
MR Test
Inverse variance weighted
MR Egger
Simple mode
Weighted median
Weighted mode
0.02
0.00
−0.02
SNP effect on Cardiac valvular disease
0.05
0.10
0.15
0.20
SNP effect on SFRP1

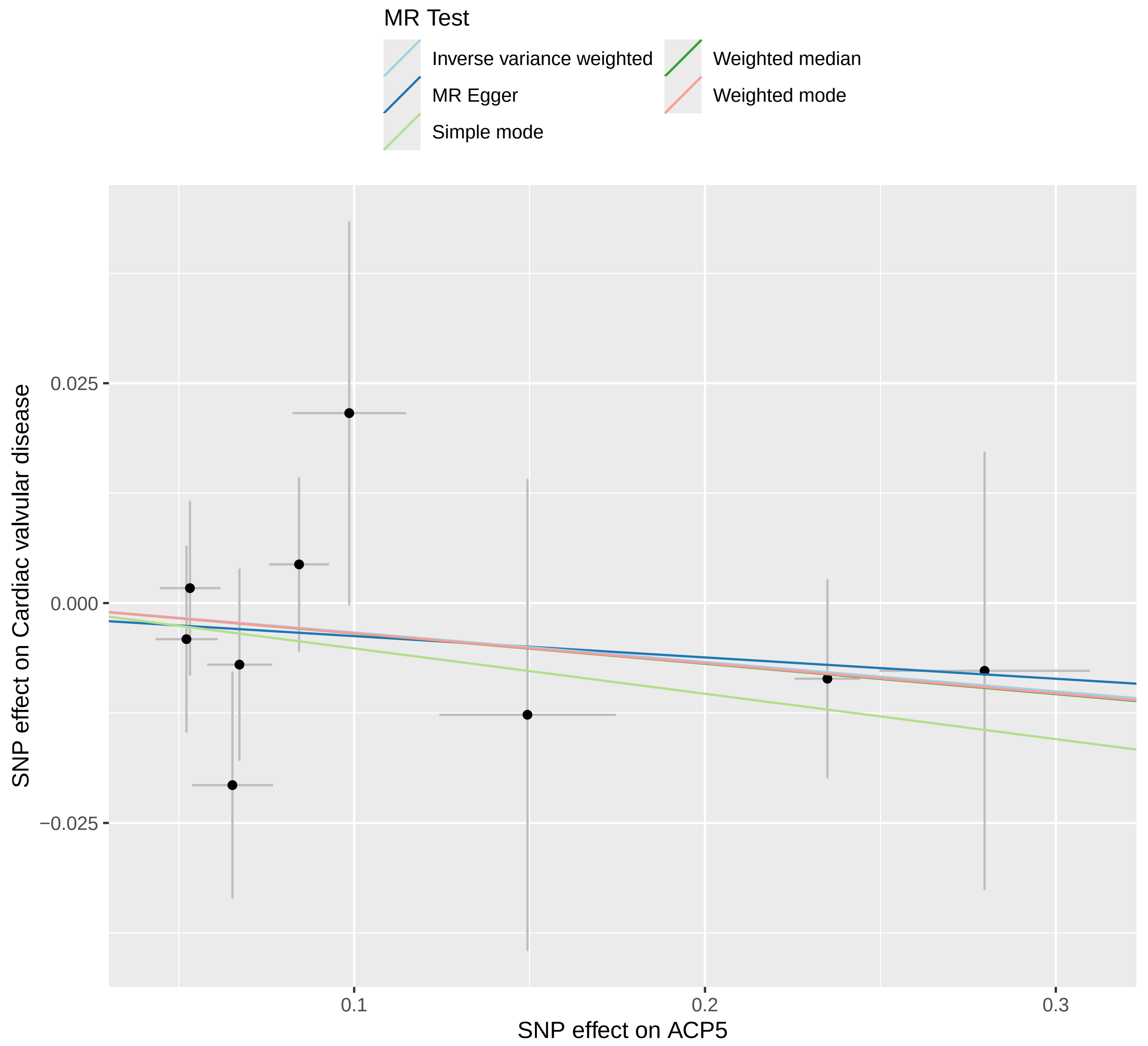
MR Test
Inverse variance weighted
MR Egger
Simple mode
Weighted median
Weighted mode
0.025
0.000
−0.025
0.1
0.2
0.3
SNP effect on Cardiac valvular disease
SNP effect on ACP5

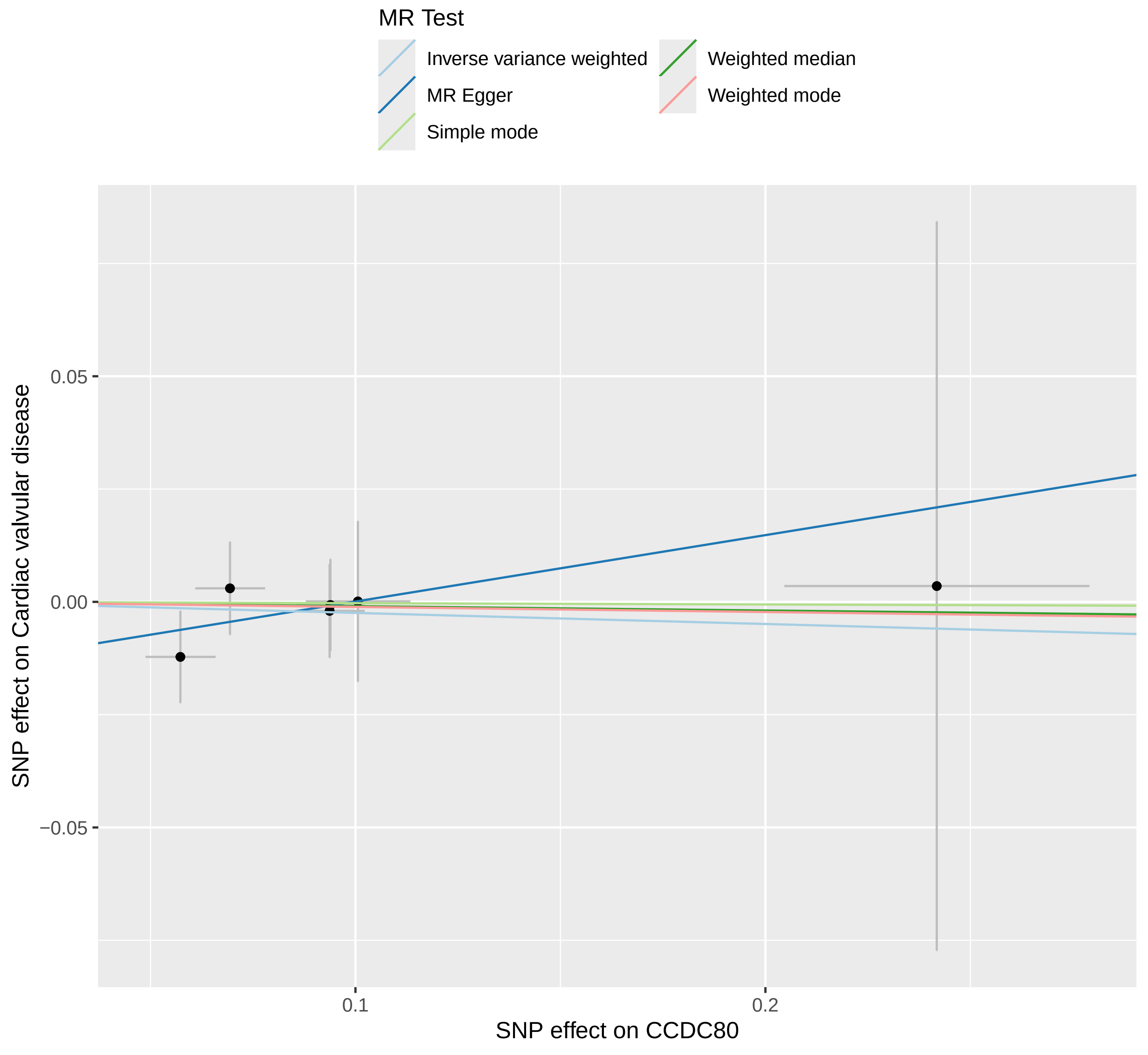

MR Test
Inverse variance weighted
MR Egger
Simple mode
Weighted median
Weighted mode
0.05
0.00
−0.05
0.1
0.2
SNP effect on Cardiac valvular disease
SNP effect on CCDC80

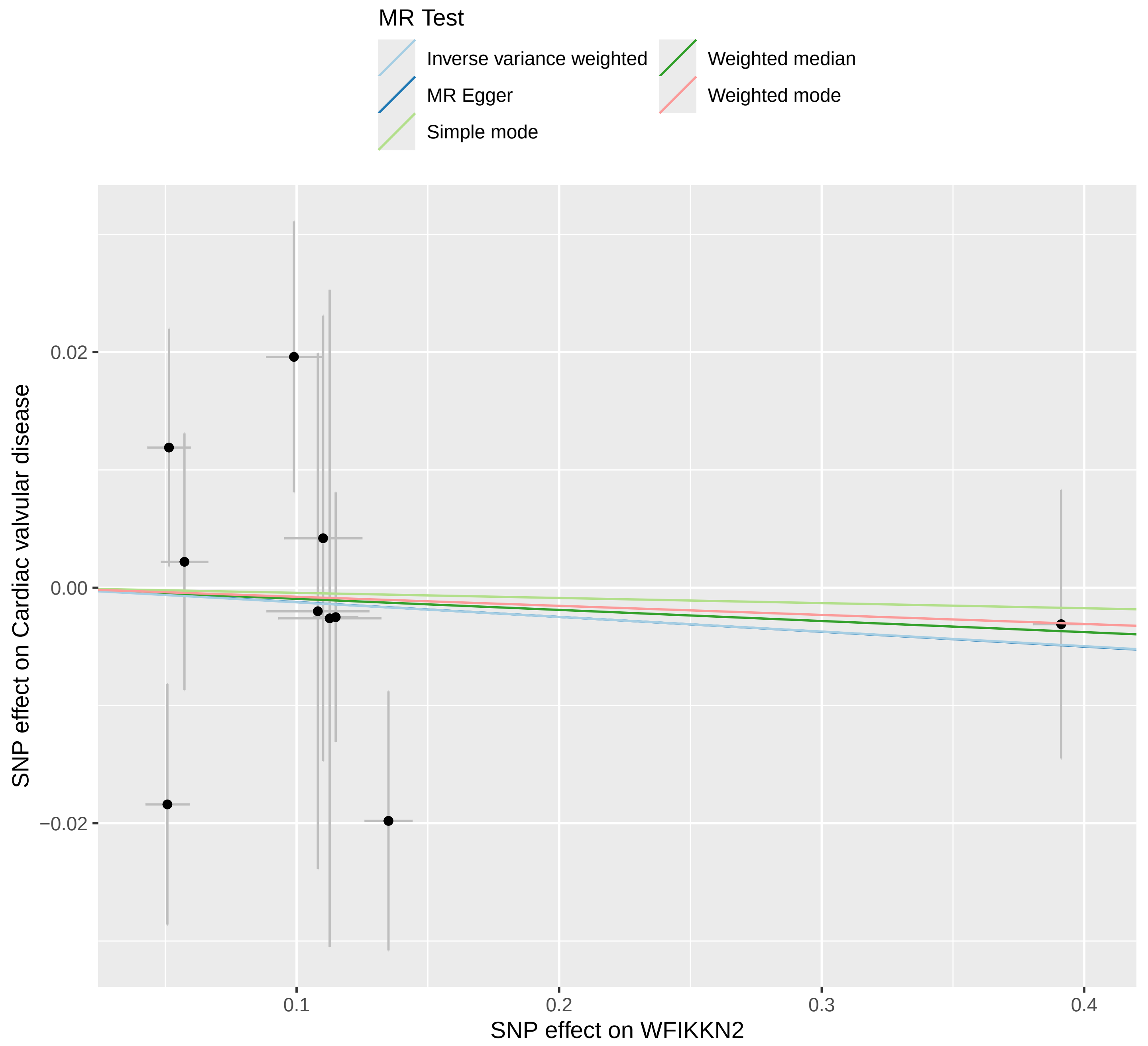
MR Test
Inverse variance weighted
MR Egger
Simple mode
Weighted median
Weighted mode
0.02
0.00
−0.02
SNP effect on Cardiac valvular disease
0.1
0.2
0.3
0.4
SNP effect on WFIKKN2

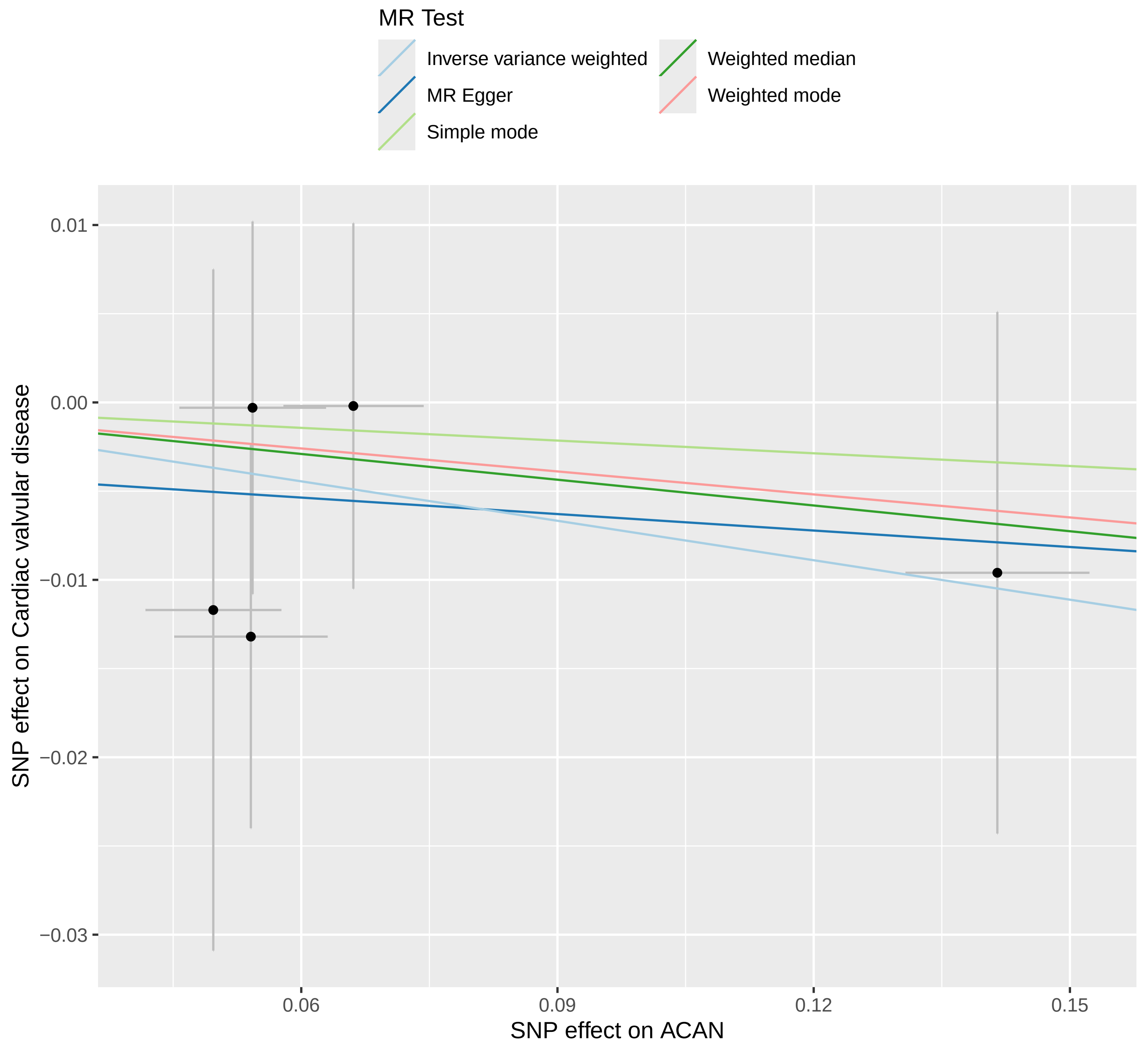
MR Test
Inverse variance weighted
MR Egger
Simple mode
Weighted median
Weighted mode
0.01
0.00
−0.01
−0.02
−0.03
SNP effect on Cardiac valvular disease
0.06
0.09
0.12
0.15
SNP effect on ACAN

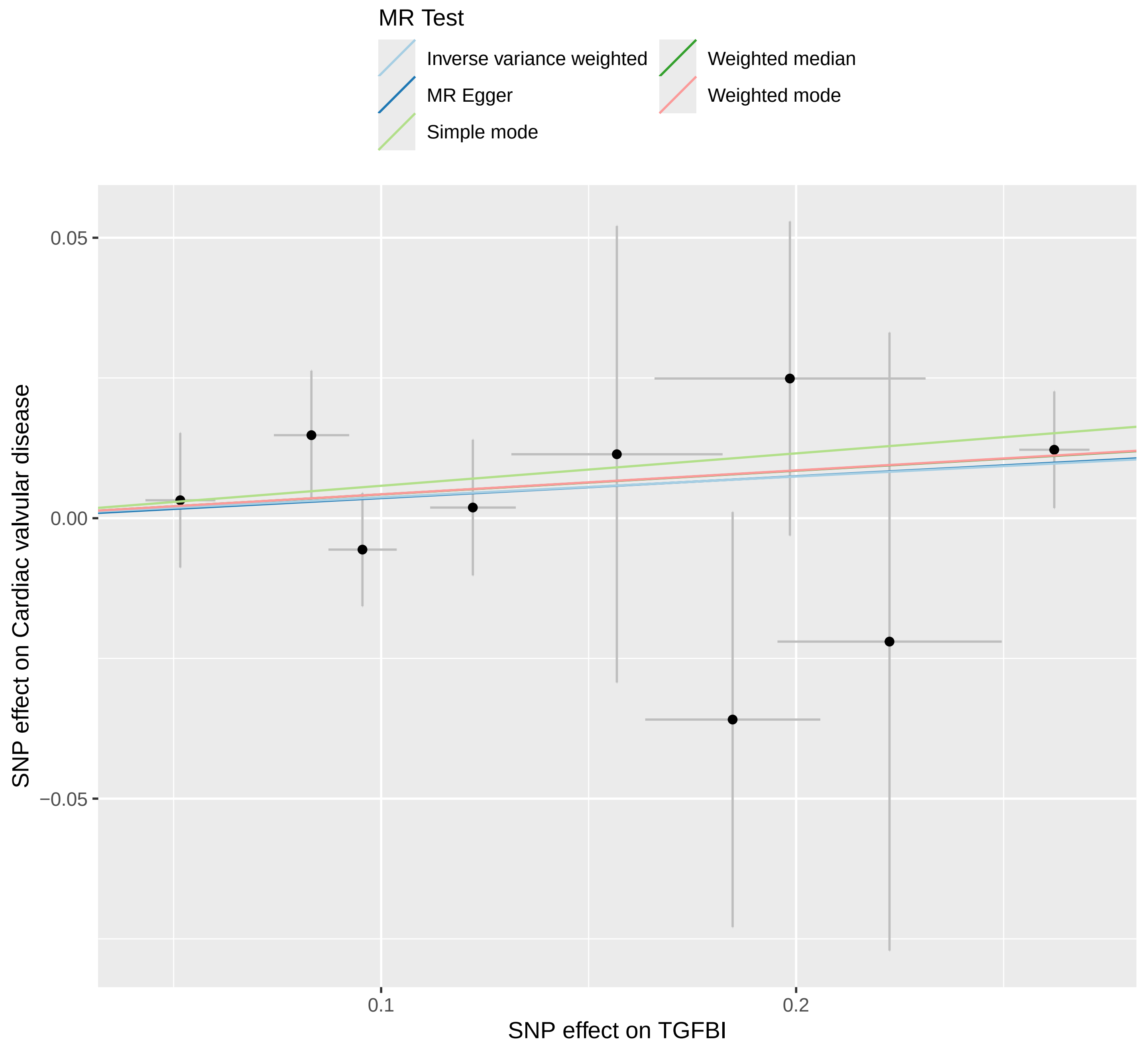
MR Test
Inverse variance weighted
MR Egger
Simple mode
Weighted median
Weighted mode
0.05
0.00
−0.05
0.1
0.2
SNP effect on Cardiac valvular disease
SNP effect on TGFBI

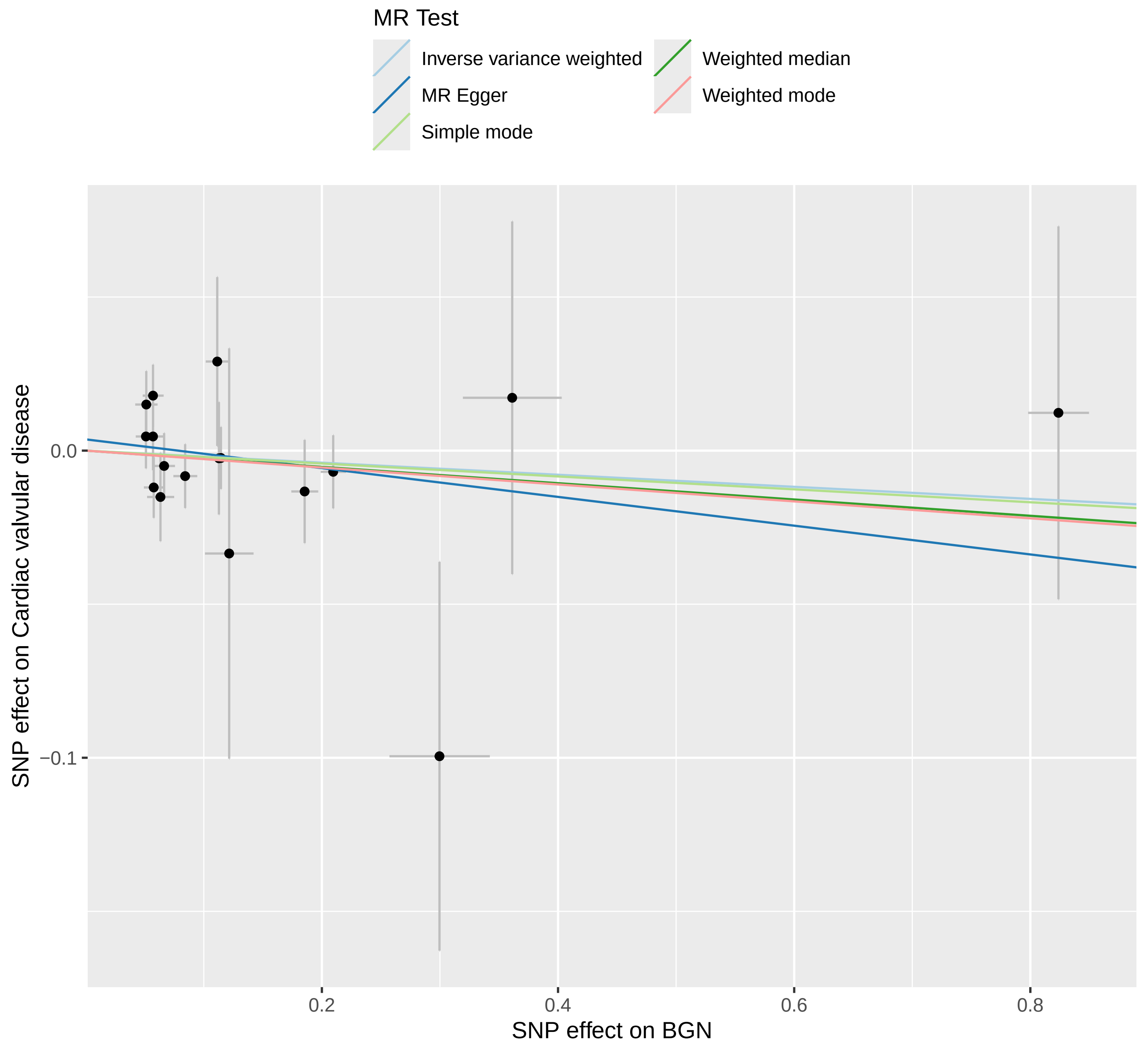
MR Test
Inverse variance weighted
MR Egger
Simple mode
Weighted median
Weighted mode
SNP effect on Cardiac valvular disease
0.0
−0.1
0.2
0.4
0.6
0.8
SNP effect on BGN

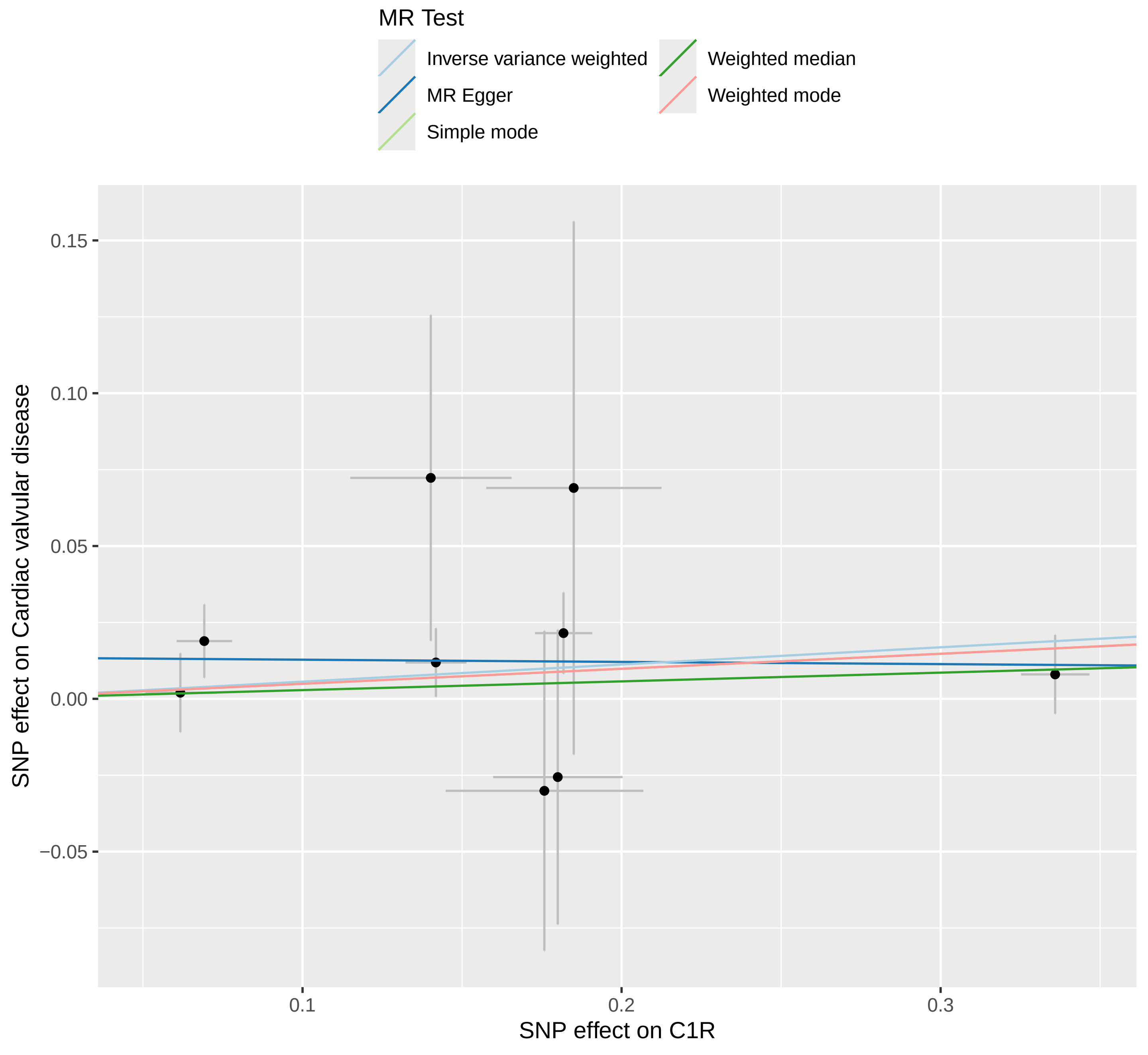
MR Test
Inverse variance weighted
MR Egger
Simple mode
Weighted median
Weighted mode
0.15
0.10
0.05
0.00
−0.05
SNP effect on Cardiac valvular disease
0.1
0.2
0.3
SNP effect on C1R

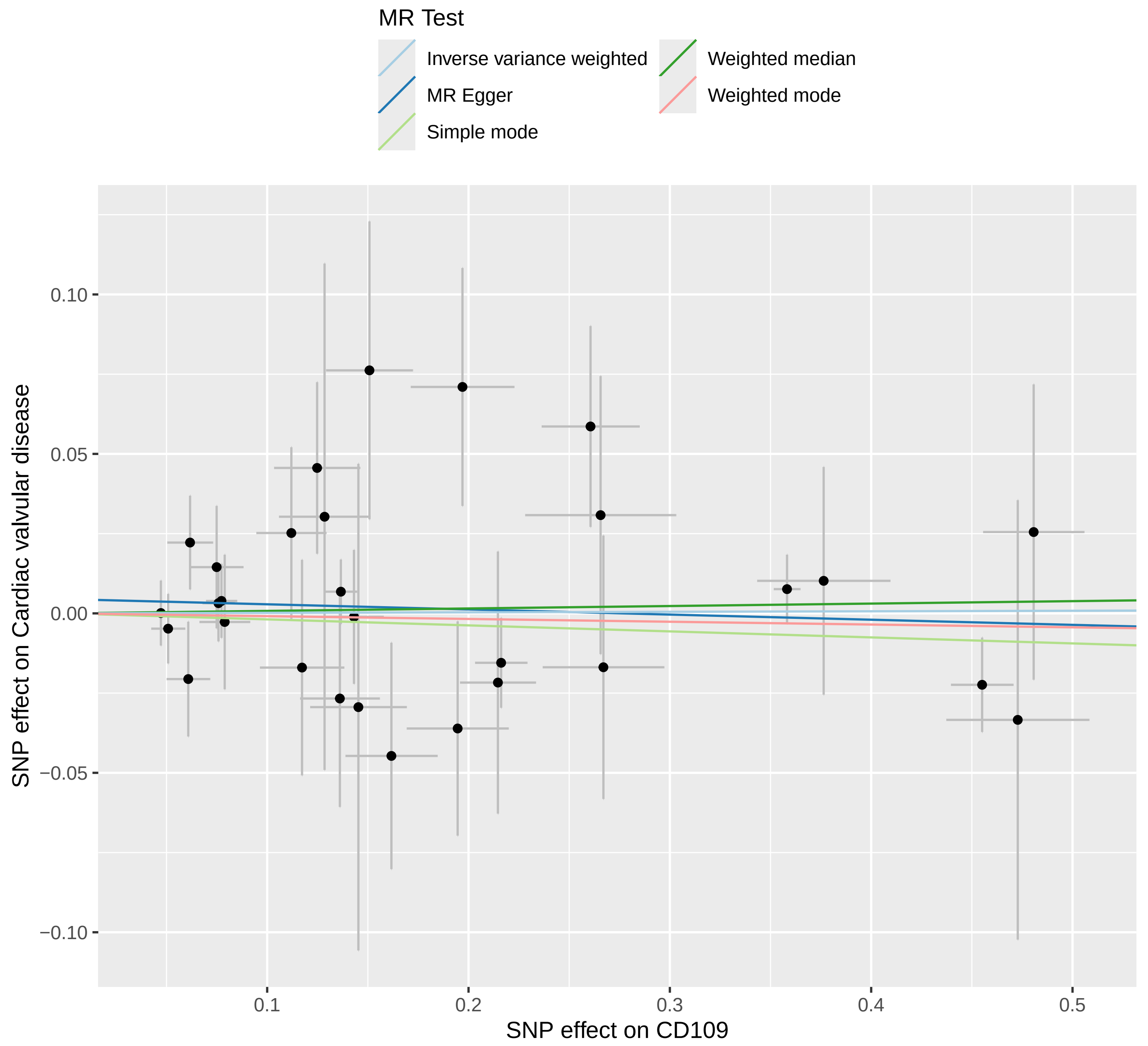

MR Test
Inverse variance weighted
MR Egger
Simple mode
Weighted median
Weighted mode
SNP effect on Cardiac valvular disease
0.10
0.05
0.00
−0.05
−0.10
0.1
0.2
0.3
0.4
0.5
SNP effect on CD109

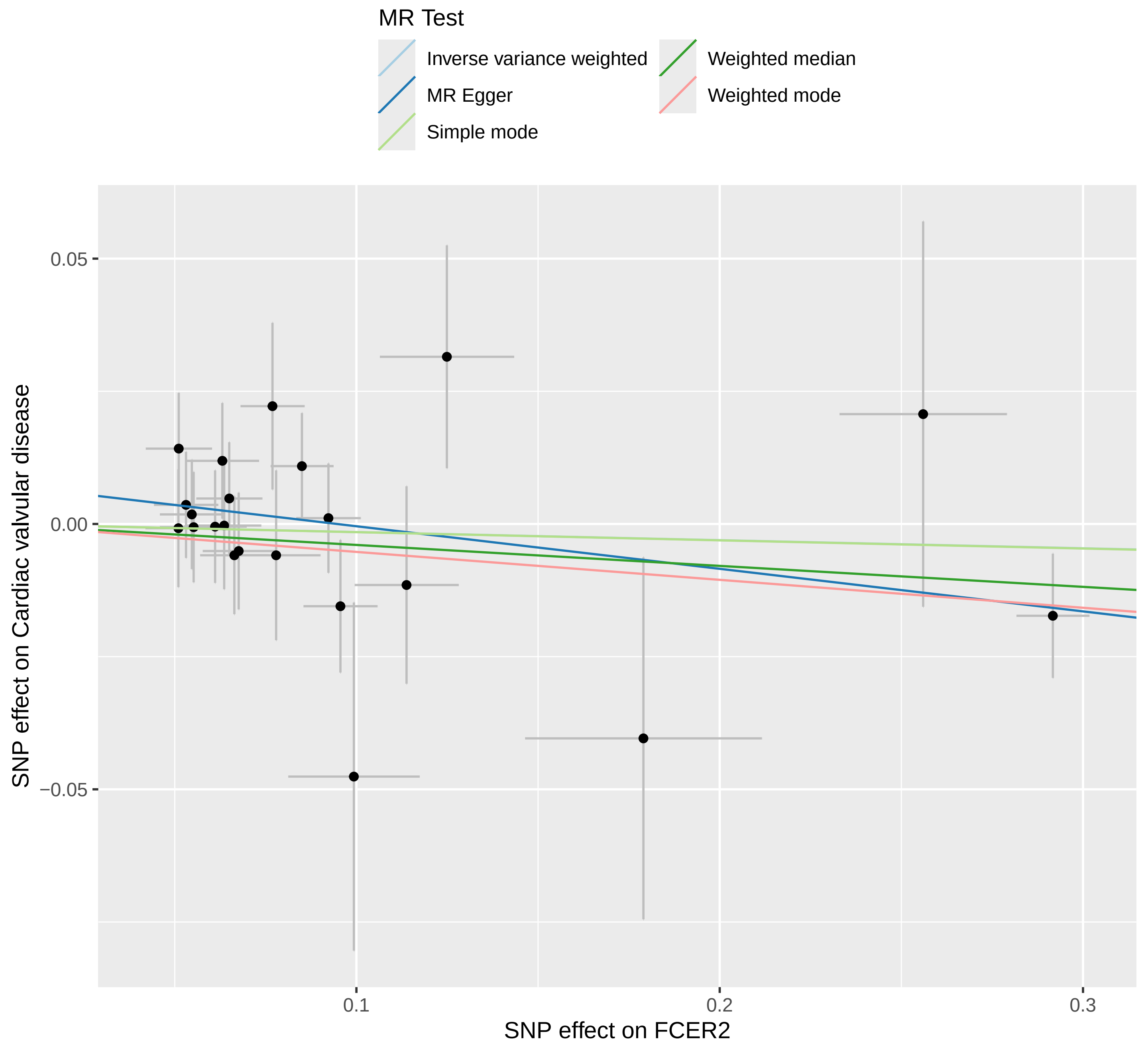

MR Test
Inverse variance weighted
MR Egger
Simple mode
Weighted median
Weighted mode
0.05
0.00
−0.05
0.1
0.2
0.3
SNP effect on Cardiac valvular disease
SNP effect on FCER2

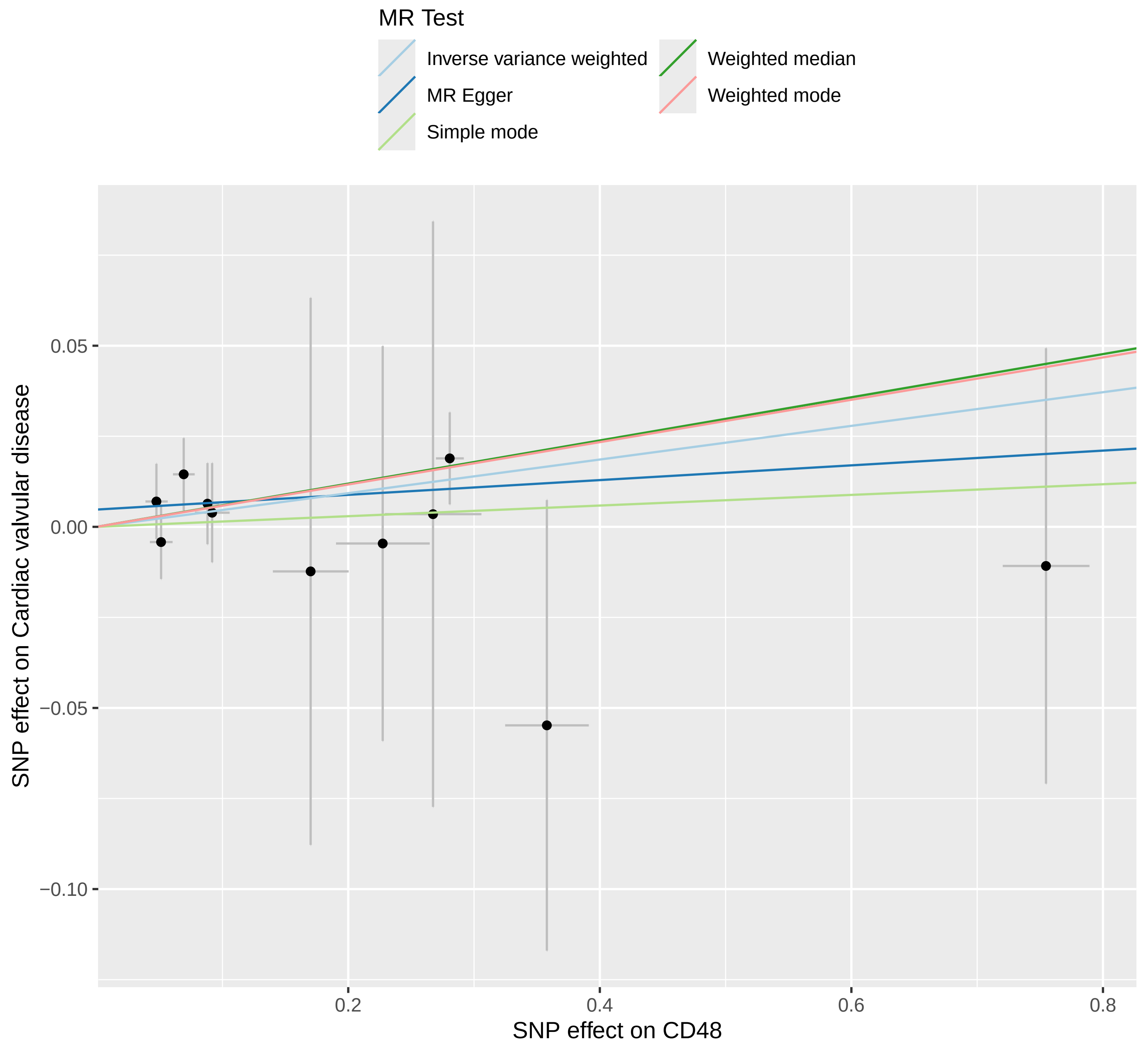

MR Test
Inverse variance weighted
MR Egger
Simple mode
Weighted median
Weighted mode
0.05
0.00
−0.05
−0.10
0.2
0.4
0.6
0.8
SNP effect on Cardiac valvular disease
SNP effect on CD48

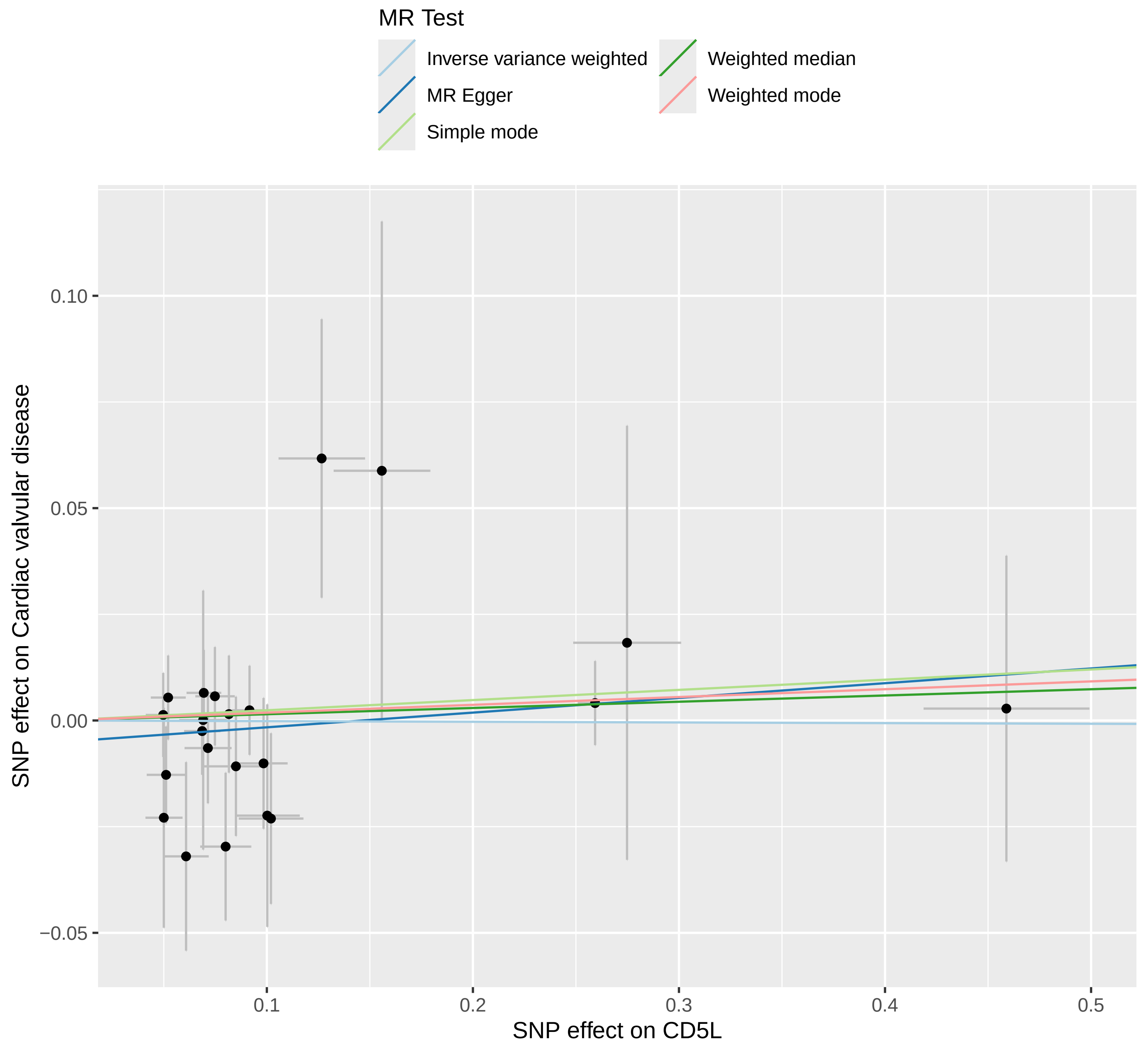

MR Test
Inverse variance weighted
MR Egger
Simple mode
Weighted median
Weighted mode
SNP effect on Cardiac valvular disease
0.10
0.05
0.00
−0.05
0.1
0.2
0.3
0.4
0.5
SNP effect on CD5L

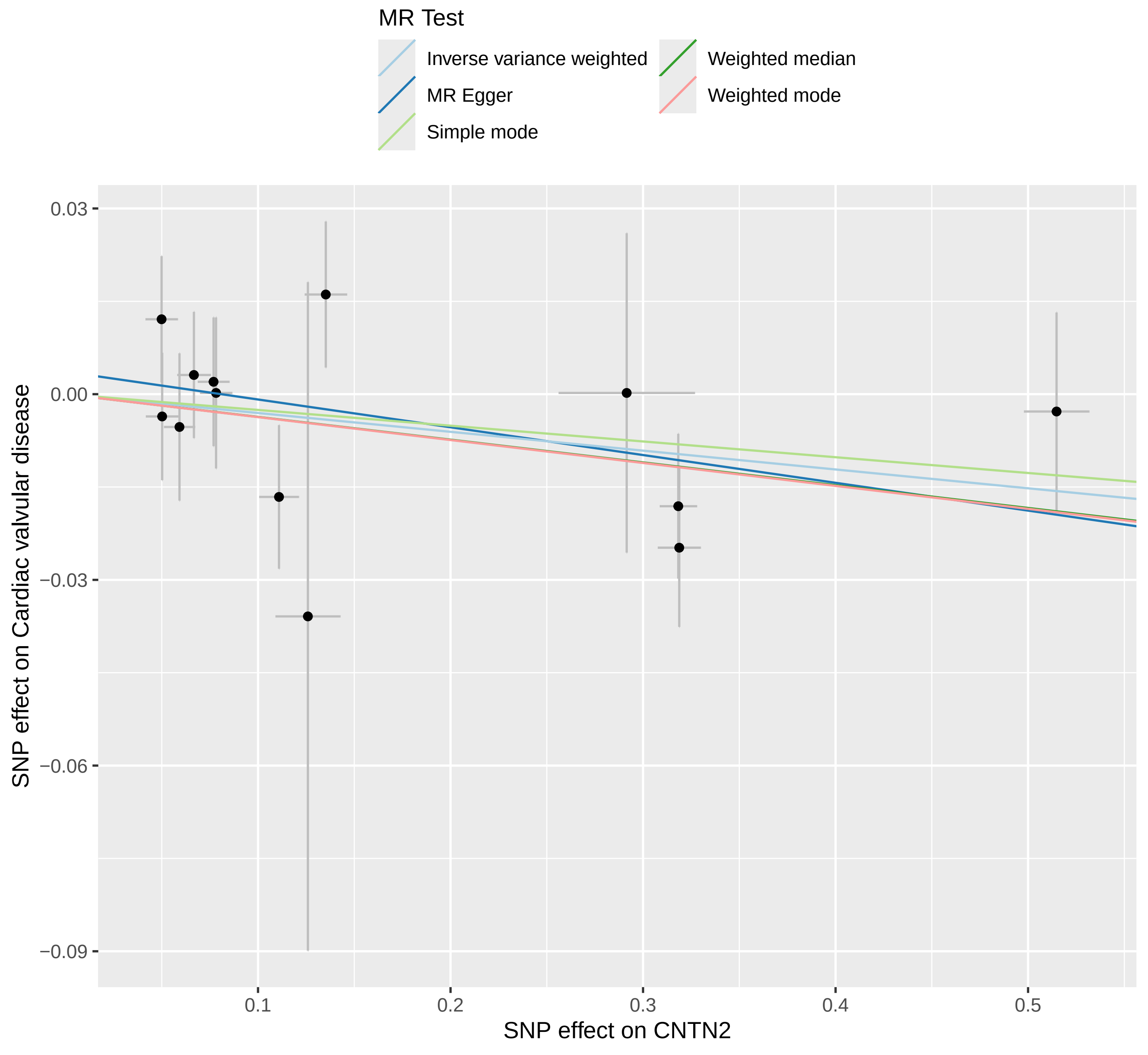
MR Test
Inverse variance weighted
MR Egger
Simple mode
Weighted median
Weighted mode
0.03
0.00
−0.03
−0.06
−0.09
0.1
0.2
0.3
0.4
0.5
SNP effect on Cardiac valvular disease
SNP effect on CNTN2

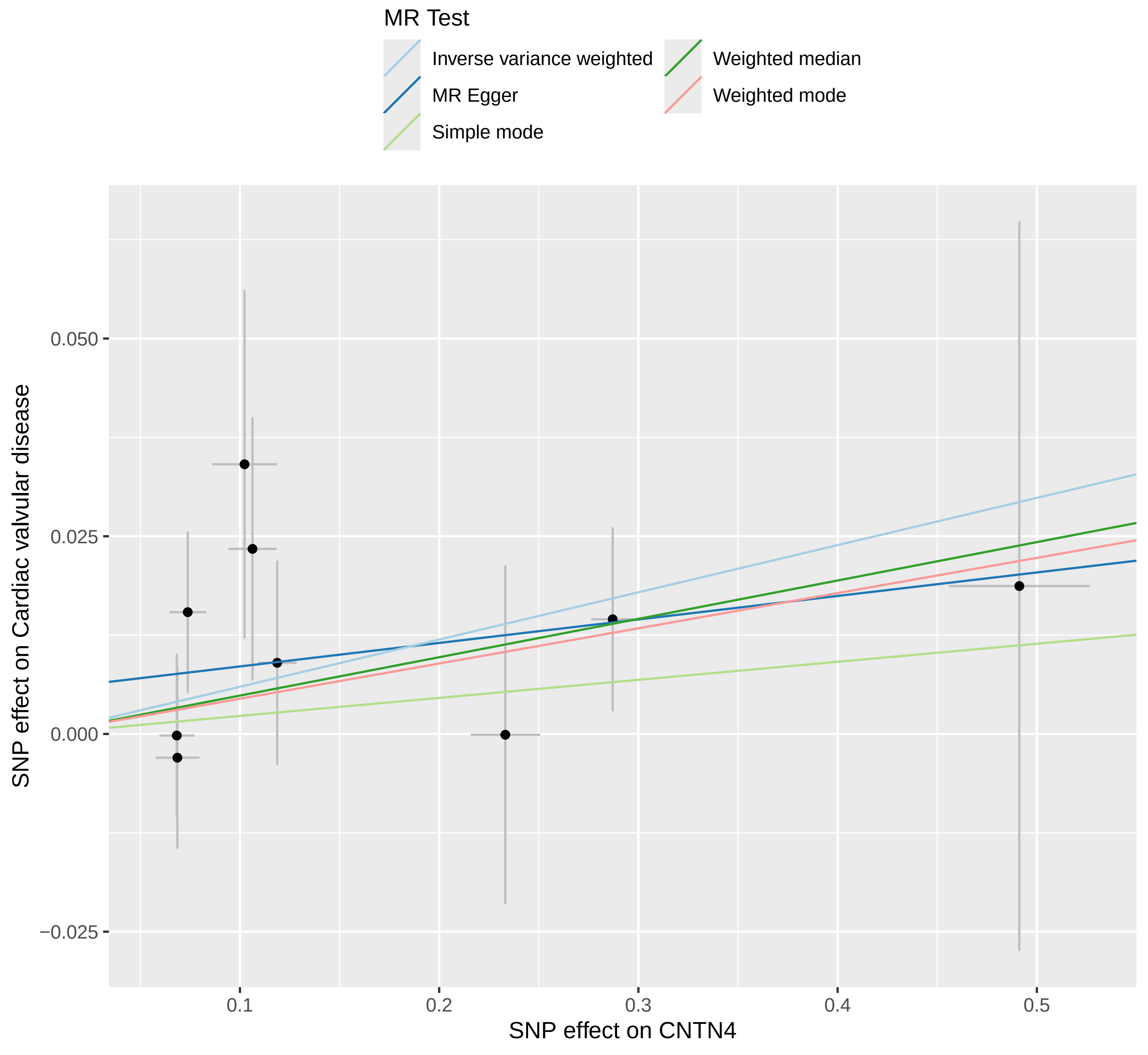
MR Test
Inverse variance weighted
MR Egger
Simple mode
Weighted median
Weighted mode
SNP effect on Cardiac valvular disease
0.050
0.025
0.000
−0.025
0.1
0.2
0.3
0.4
0.5
SNP effect on CNTN4

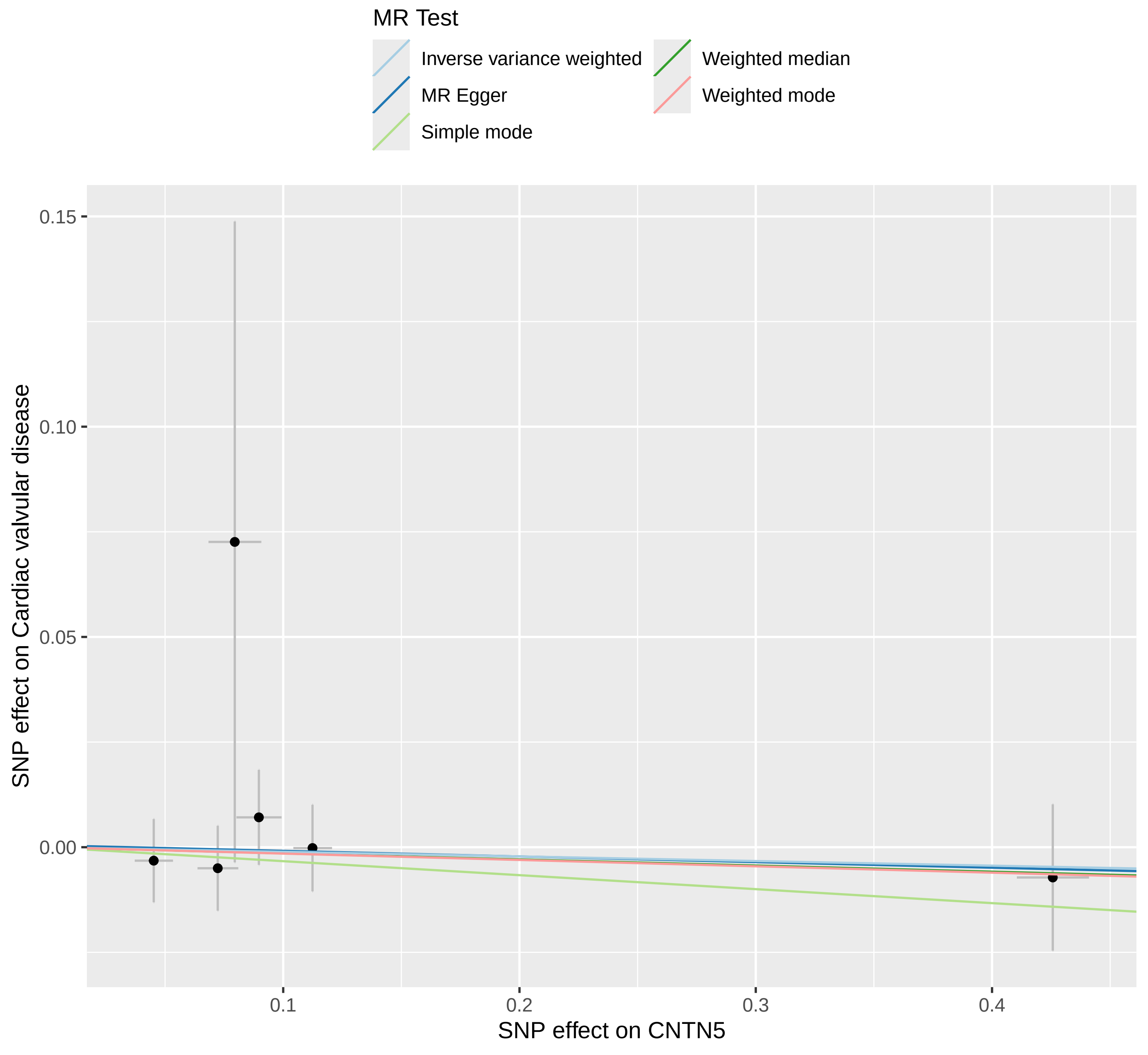
MR Test
Inverse variance weighted
MR Egger
Simple mode
Weighted median
Weighted mode
0.15
0.10
0.05
0.00
SNP effect on Cardiac valvular disease
0.1
0.2
0.3
0.4
SNP effect on CNTN5

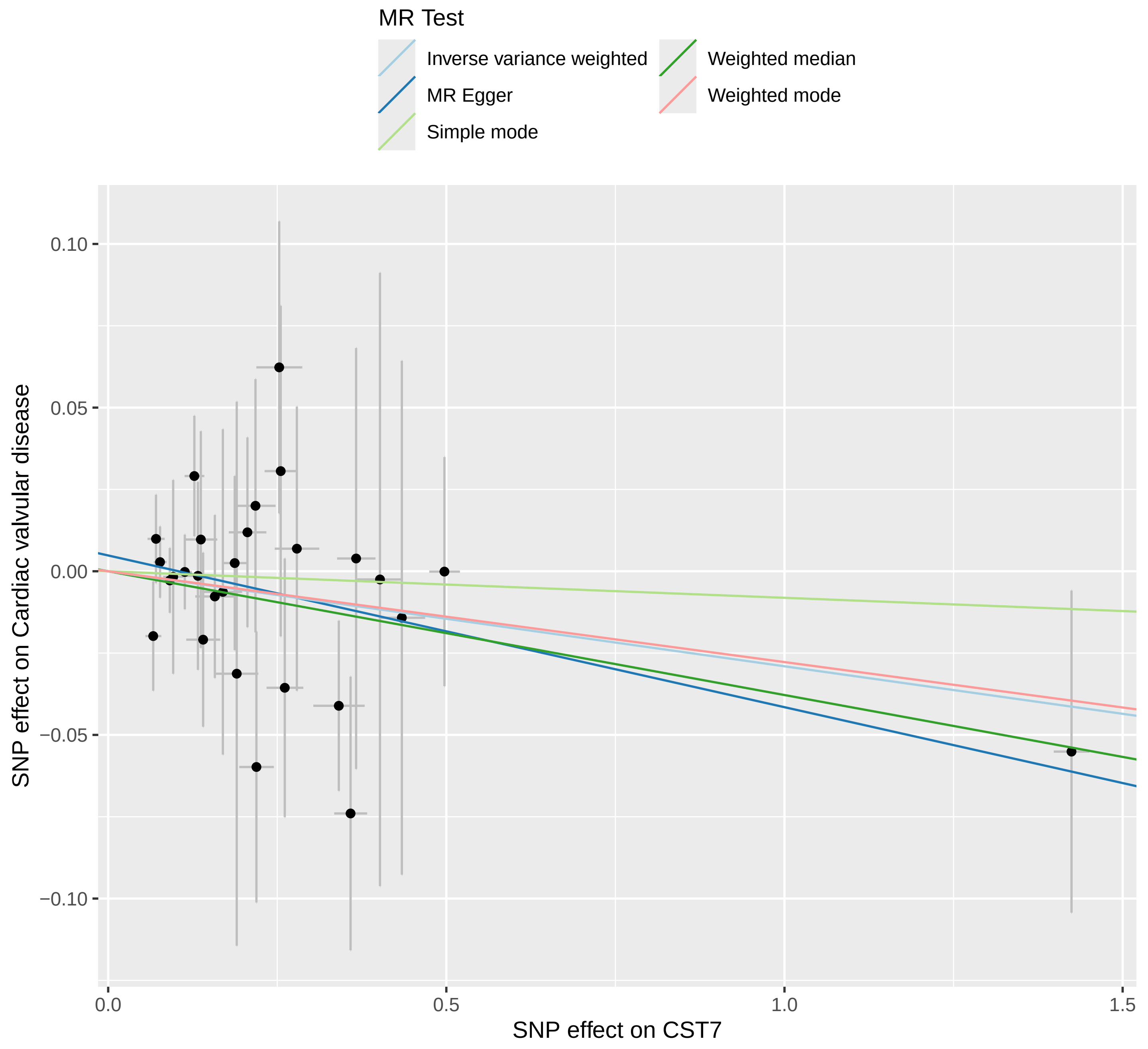

MR Test
Inverse variance weighted
MR Egger
Simple mode
Weighted median
Weighted mode
0.10
0.05
0.00
−0.05
−0.10
0.0
0.5
1.0
1.5
SNP effect on Cardiac valvular disease
SNP effect on CST7

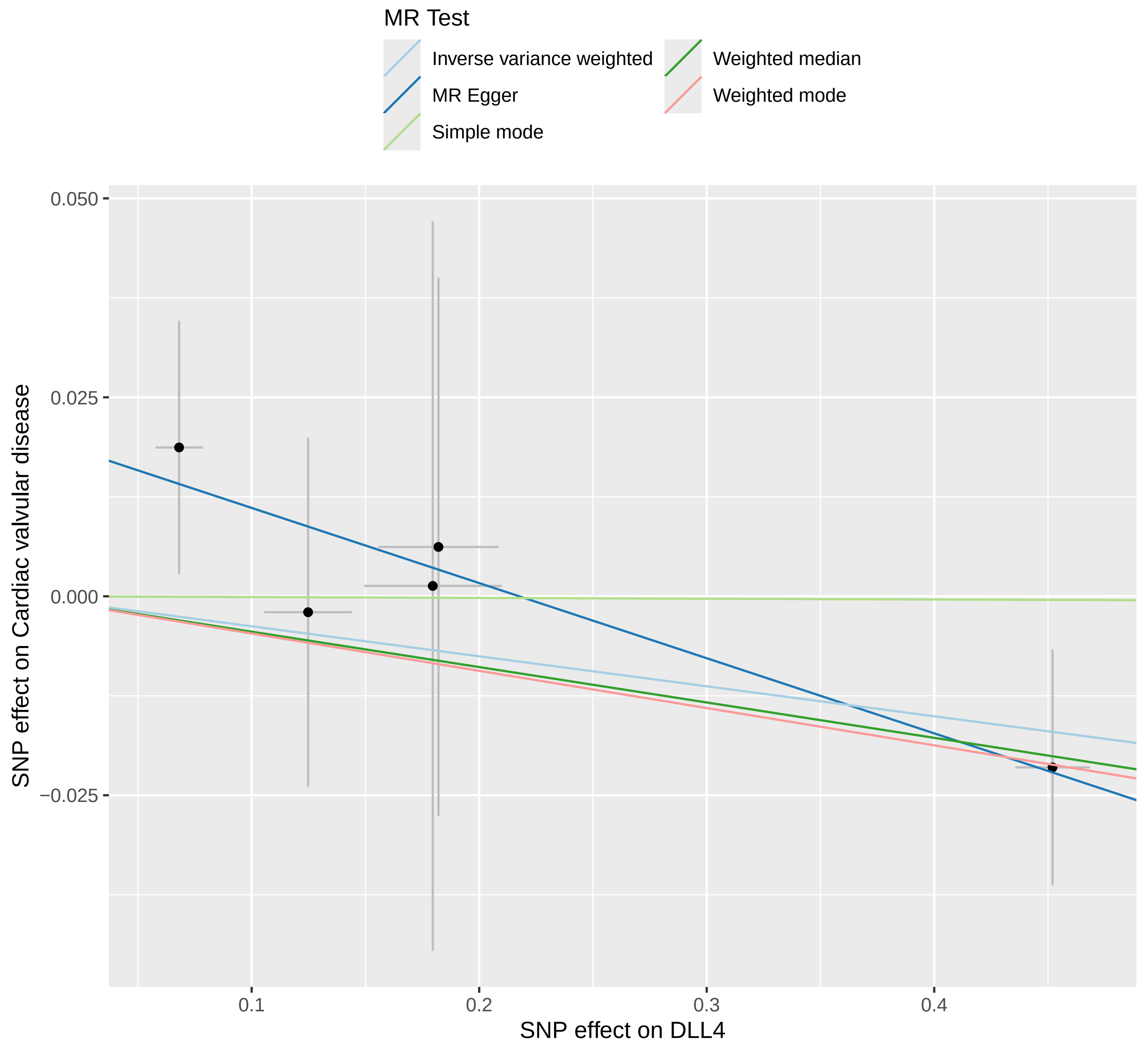
MR Test
Inverse variance weighted
MR Egger
Simple mode
Weighted median
Weighted mode
0.050
0.025
0.000
−0.025
0.1
0.2
0.3
0.4
SNP effect on Cardiac valvular disease
SNP effect on DLL4

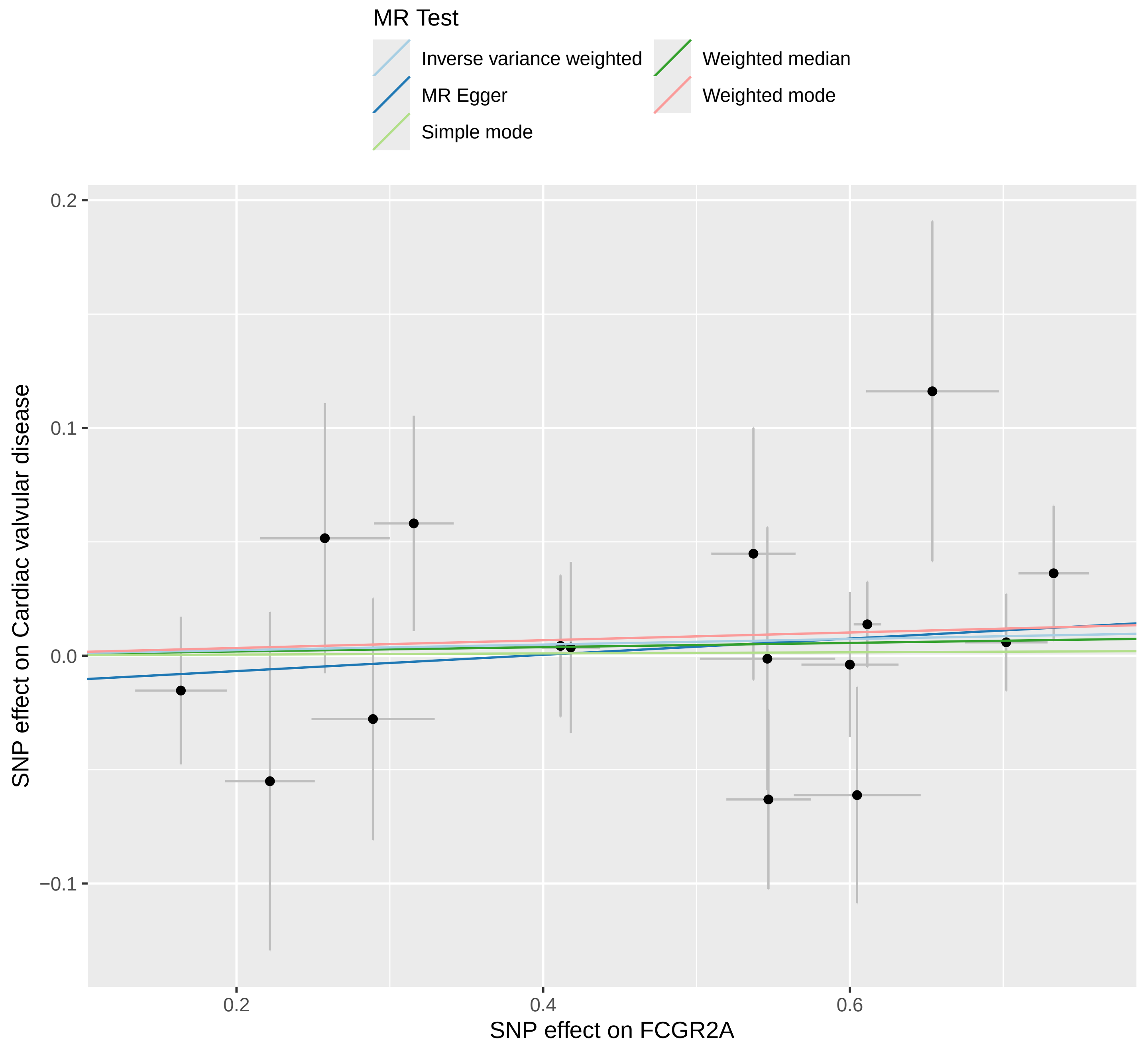

MR Test
Inverse variance weighted
MR Egger
Simple mode
Weighted median
Weighted mode
0.2
0.1
0.0
−0.1
SNP effect on Cardiac valvular disease
0.2
0.4
0.6
SNP effect on FCGR2A

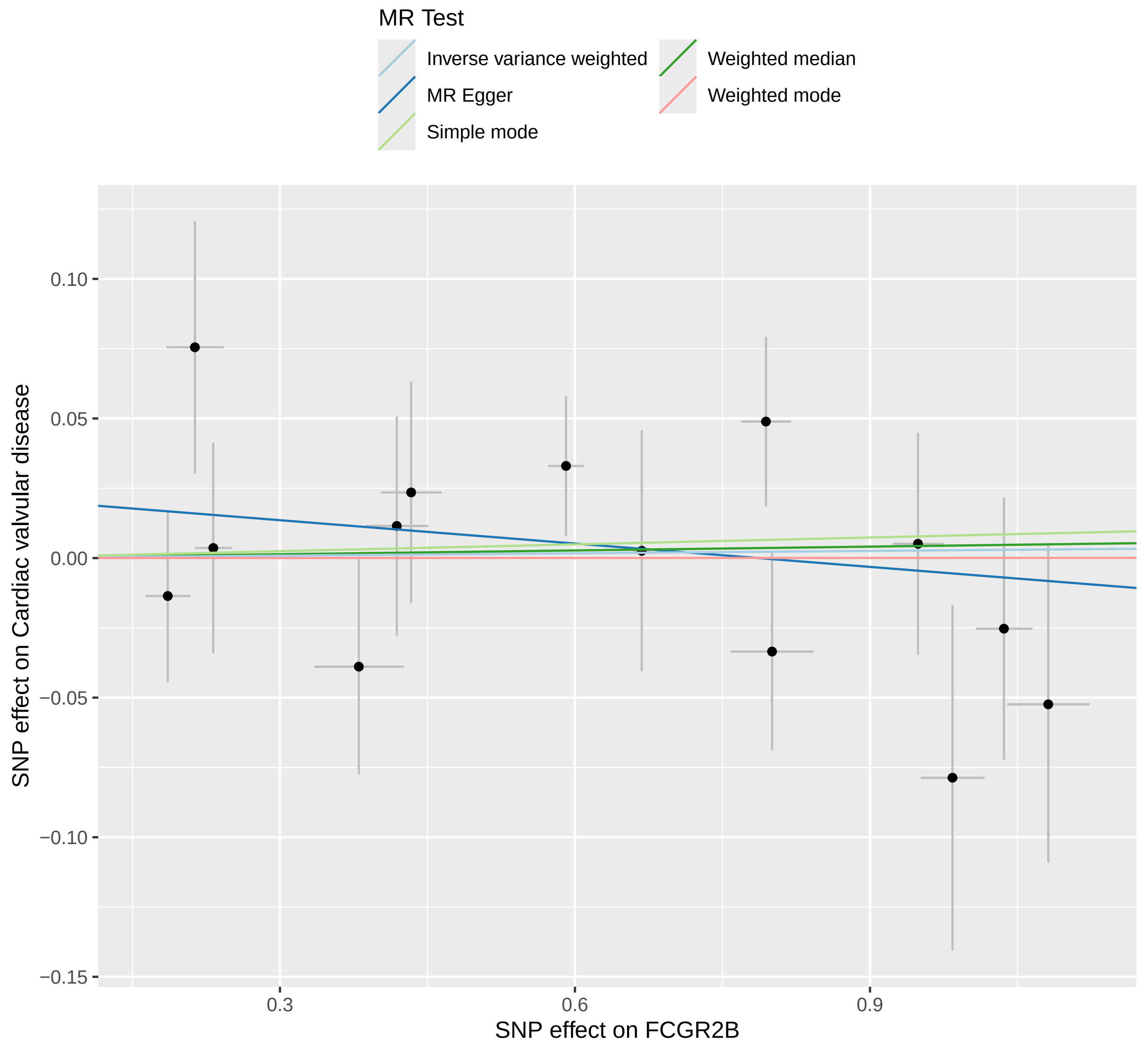
MR Test
Inverse variance weighted
MR Egger
Simple mode
Weighted median
Weighted mode
0.10
0.05
0.00
−0.05
−0.10
−0.15
SNP effect on Cardiac valvular disease
0.3
0.6
0.9
SNP effect on FCGR2B

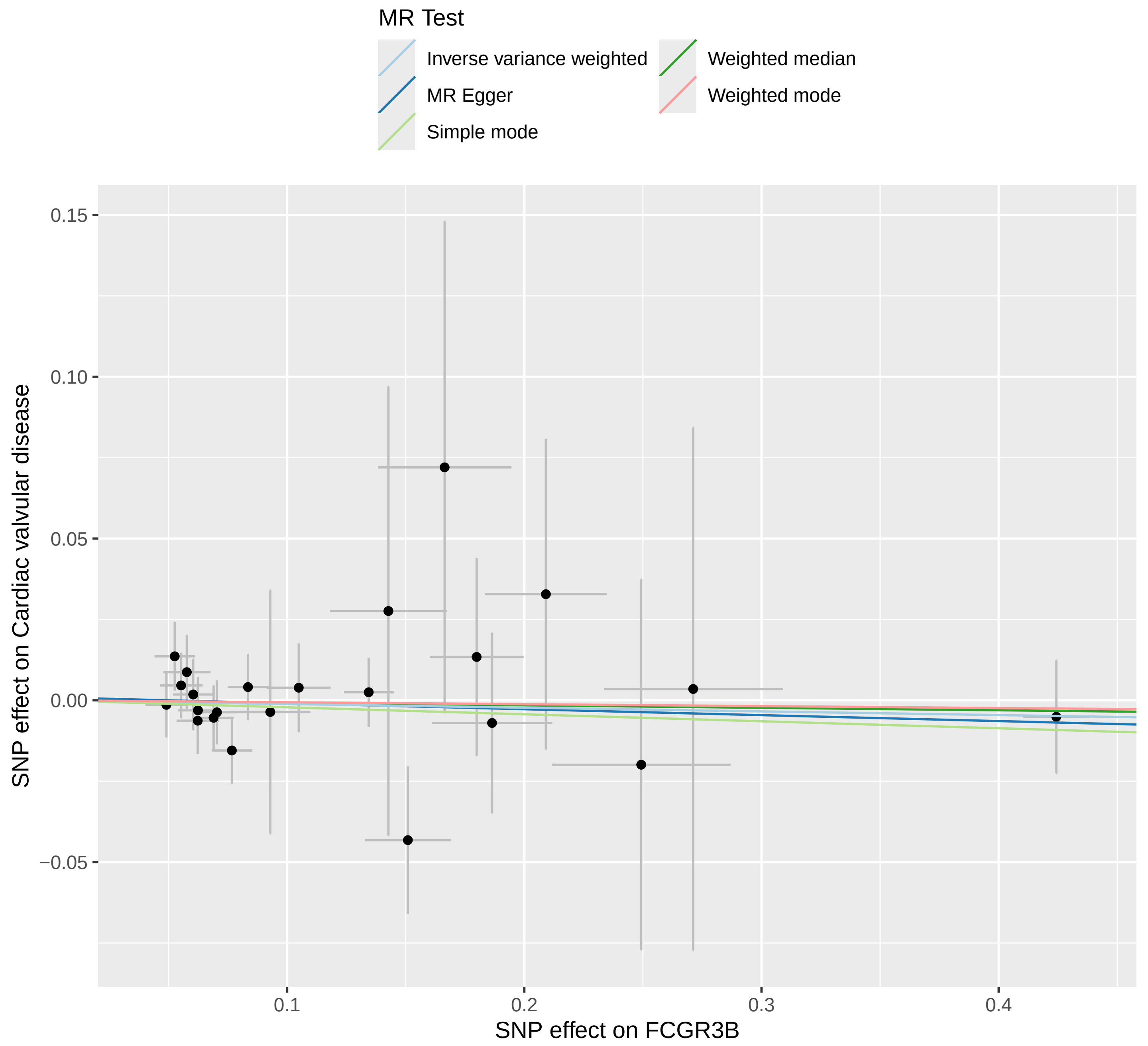
MR Test
Inverse variance weighted
MR Egger
Simple mode
Weighted median
Weighted mode
0.15
0.10
0.05
0.00
−0.05
0.1
0.2
0.3
0.4
SNP effect on Cardiac valvular disease
SNP effect on FCGR3B

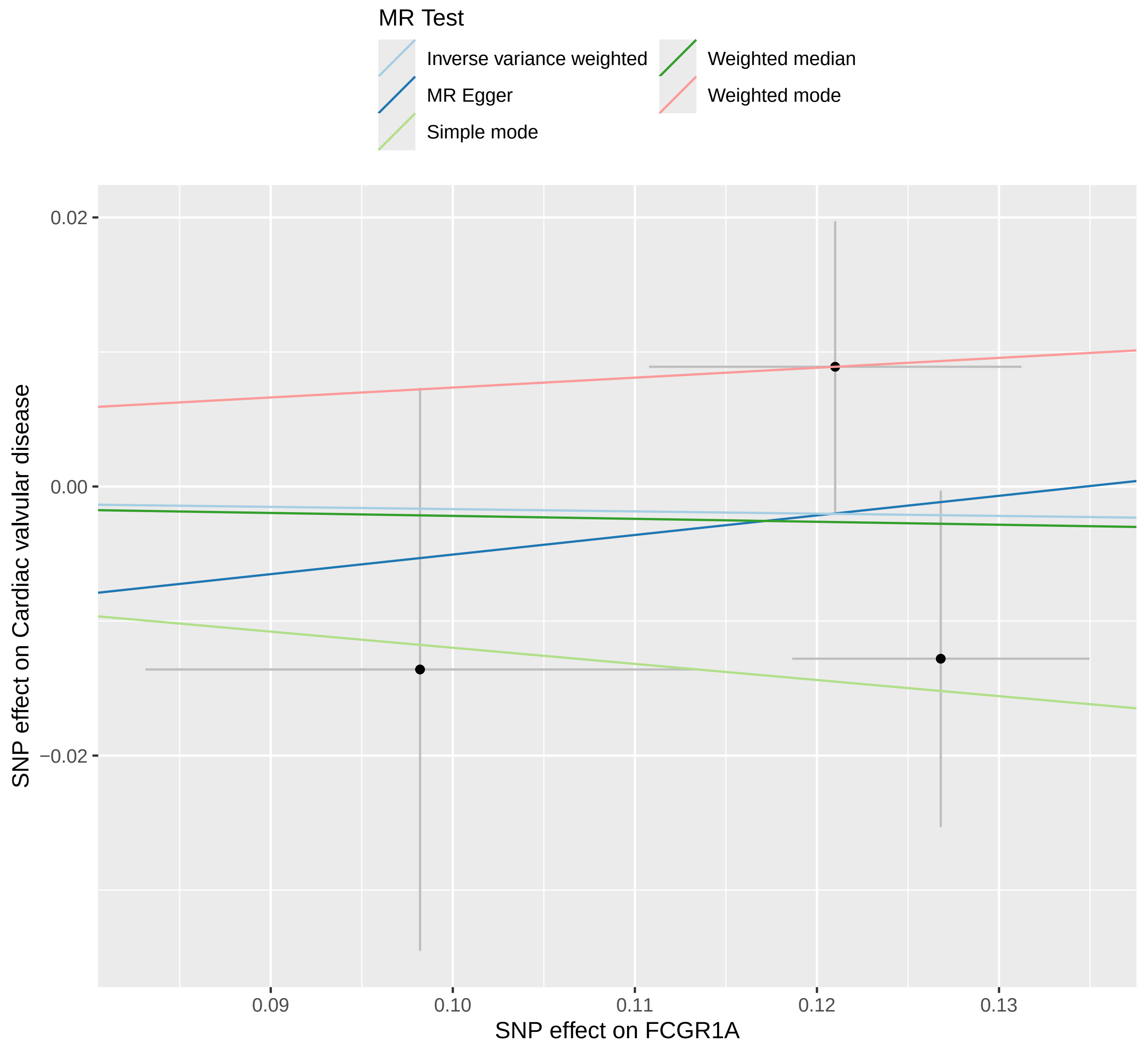
MR Test
Inverse variance weighted
MR Egger
Simple mode
Weighted median
Weighted mode
0.02
0.00
−0.02
0.09
0.10
0.11
0.12
0.13
SNP effect on Cardiac valvular disease
SNP effect on FCGR1A

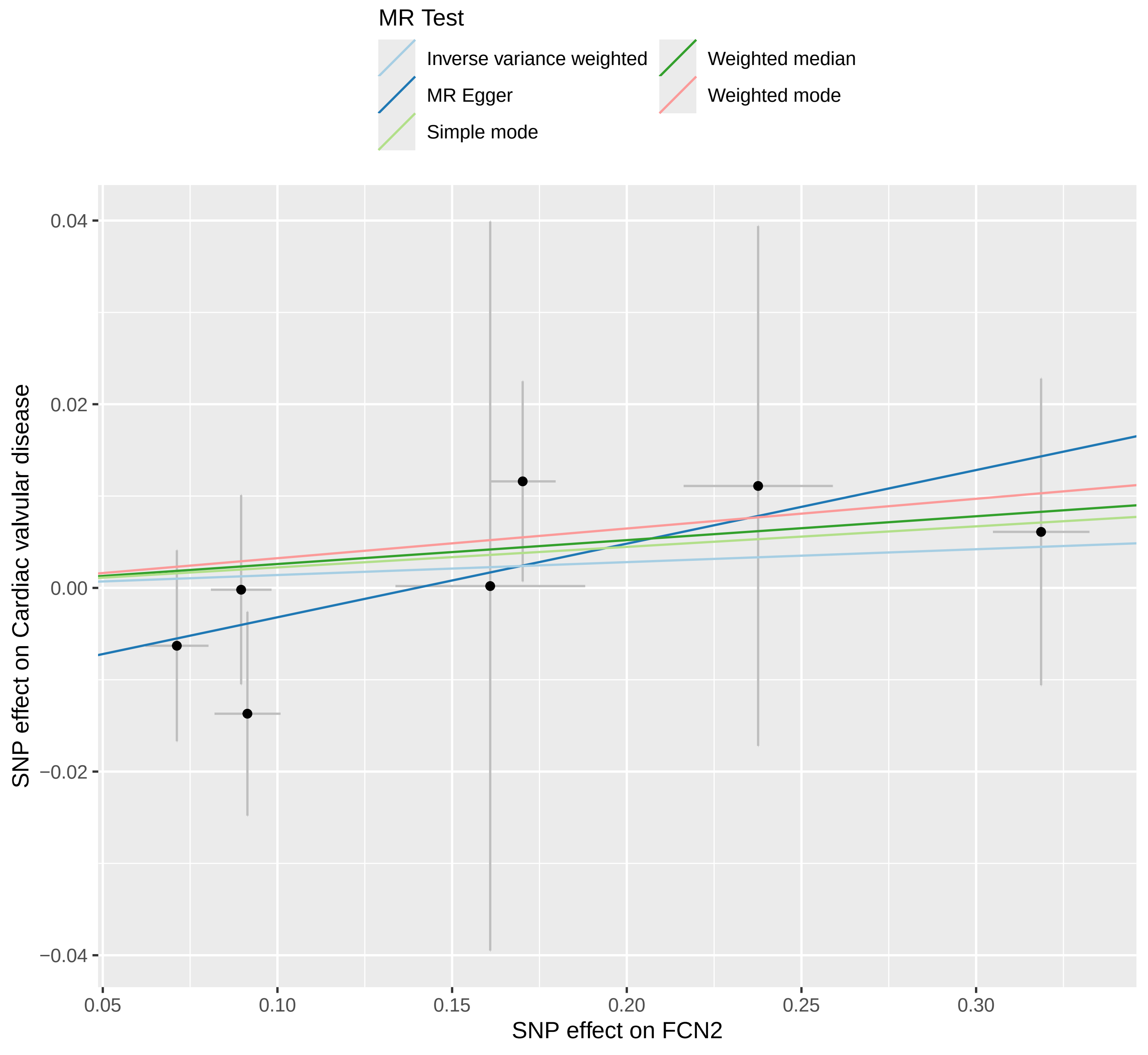

MR Test
Inverse variance weighted
MR Egger
Simple mode
Weighted median
Weighted mode
SNP effect on Cardiac valvular disease
SNP effect on FCN2
0.04
0.02
0.00
−0.02
−0.04
0.05
0.10
0.15
0.20
0.25
0.30

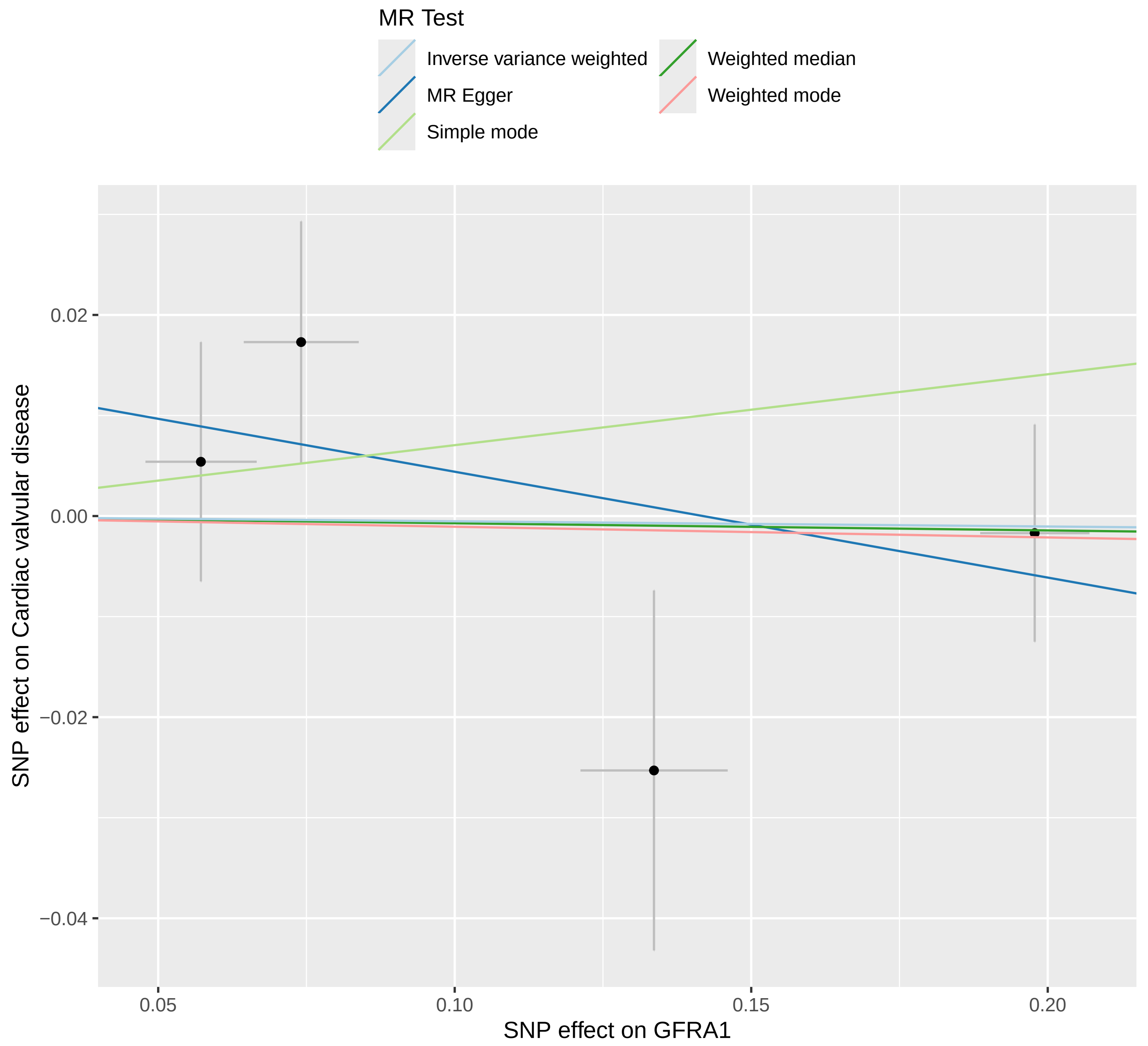
MR Test
Inverse variance weighted
MR Egger
Simple mode
Weighted median
Weighted mode
0.02
0.00
−0.02
−0.04
0.05
0.10
0.15
0.20
SNP effect on Cardiac valvular disease
SNP effect on GFRA1

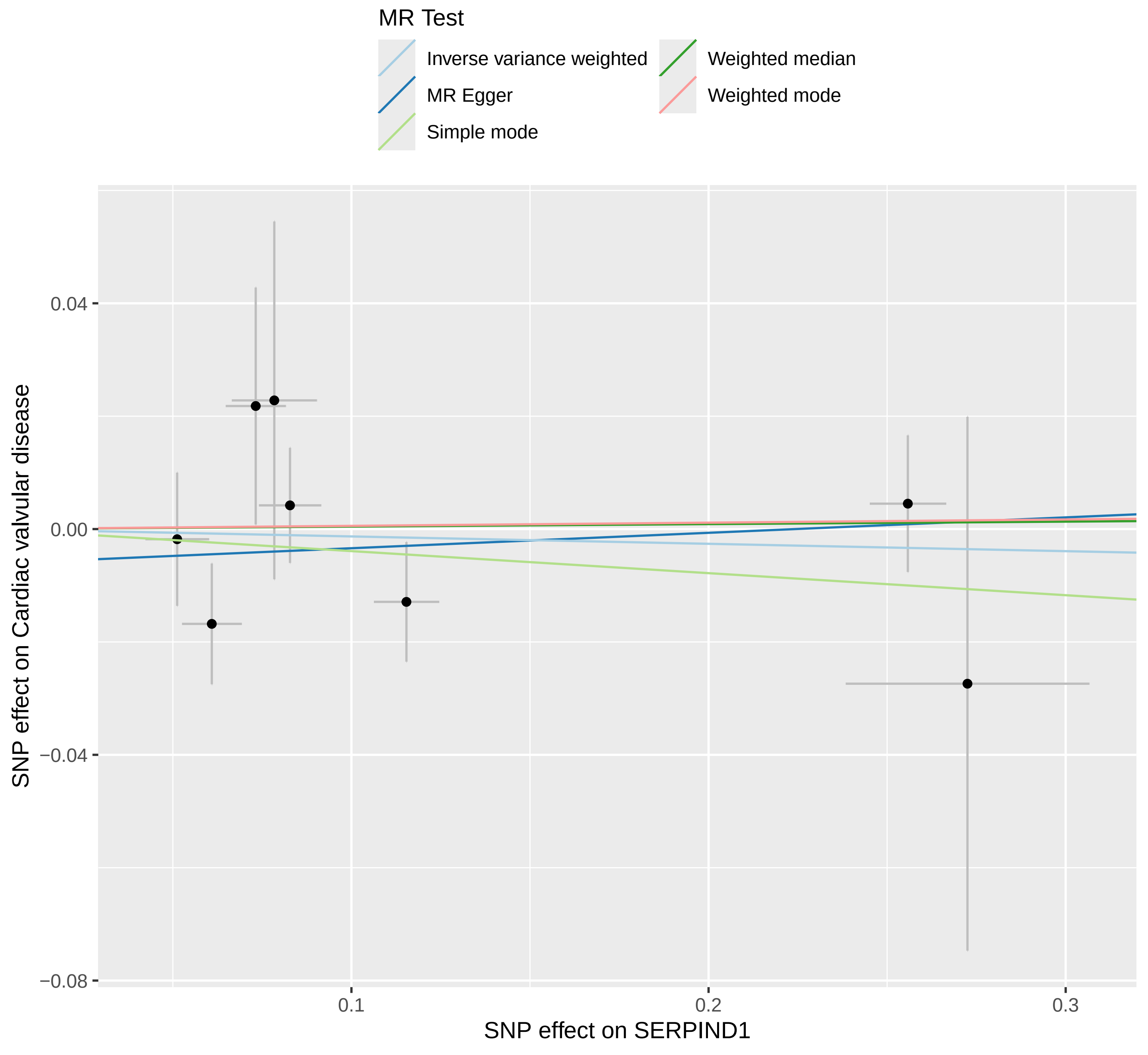
MR Test
Inverse variance weighted
MR Egger
Simple mode
Weighted median
Weighted mode
0.04
0.00
−0.04
−0.08
SNP effect on Cardiac valvular disease
0.1
0.2
0.3
SNP effect on SERPIND1

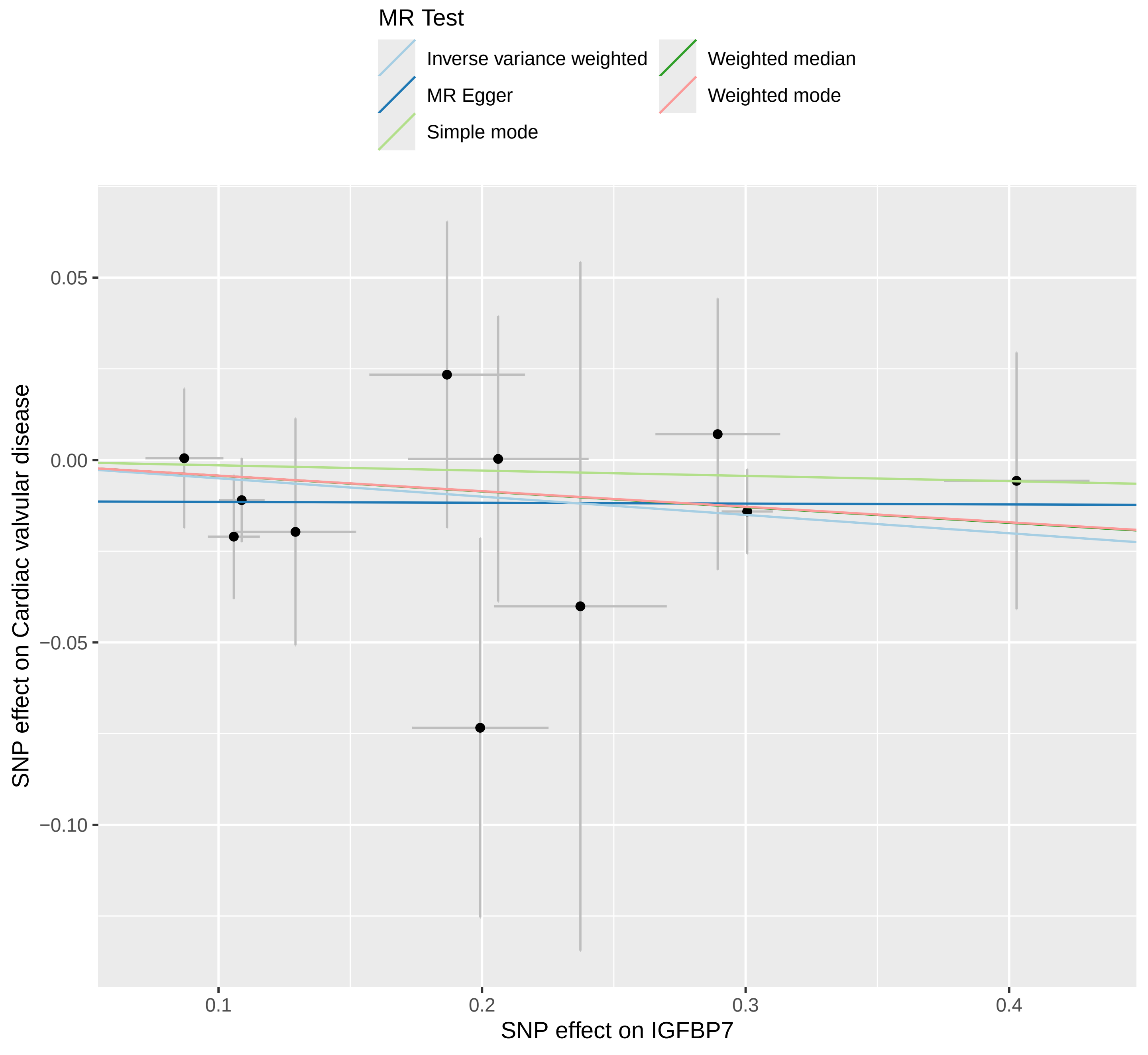

MR Test
Inverse variance weighted
MR Egger
Simple mode
Weighted median
Weighted mode
0.05
0.00
−0.05
−0.10
0.1
0.2
0.3
0.4
SNP effect on Cardiac valvular disease
SNP effect on IGFBP7

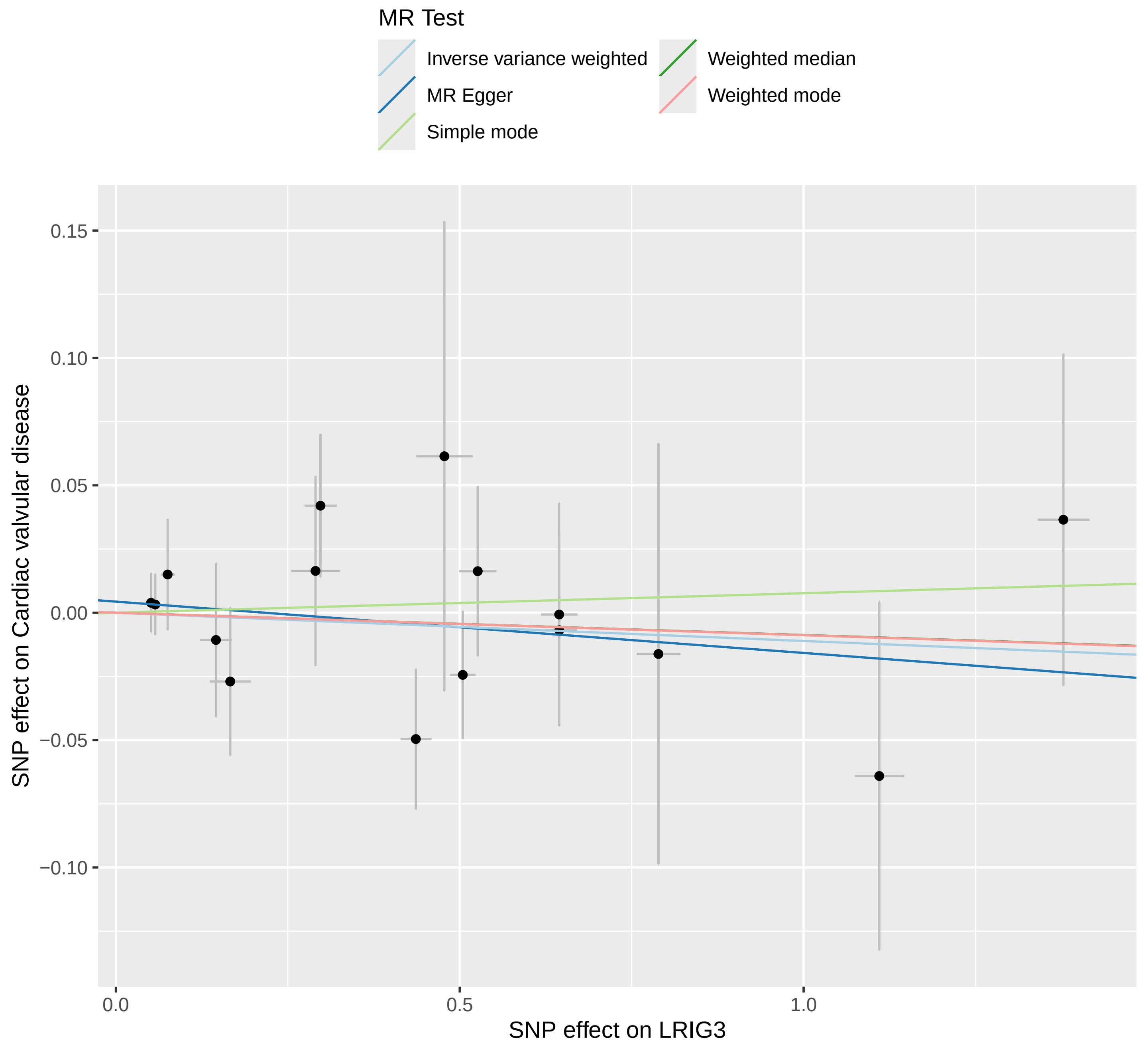
MR Test
Inverse variance weighted
MR Egger
Simple mode
Weighted median
Weighted mode
SNP effect on Cardiac valvular disease
0.15
0.10
0.05
0.00
−0.05
−0.10
0.0
0.5
1.0
SNP effect on LRIG3

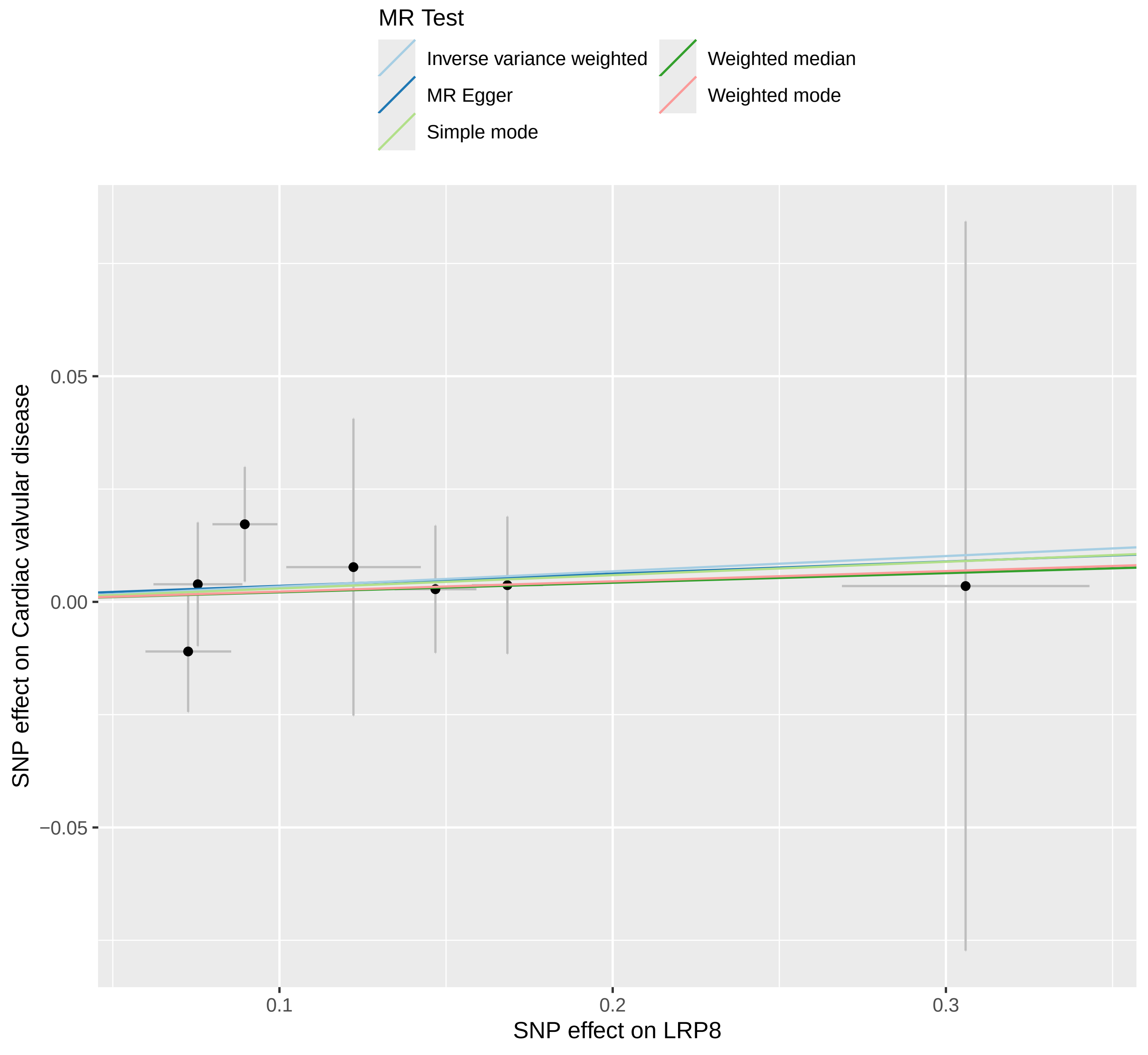
MR Test
Inverse variance weighted
MR Egger
Simple mode
Weighted median
Weighted mode
0.05
0.00
−0.05
0.1
0.2
0.3
SNP effect on Cardiac valvular disease
SNP effect on LRP8

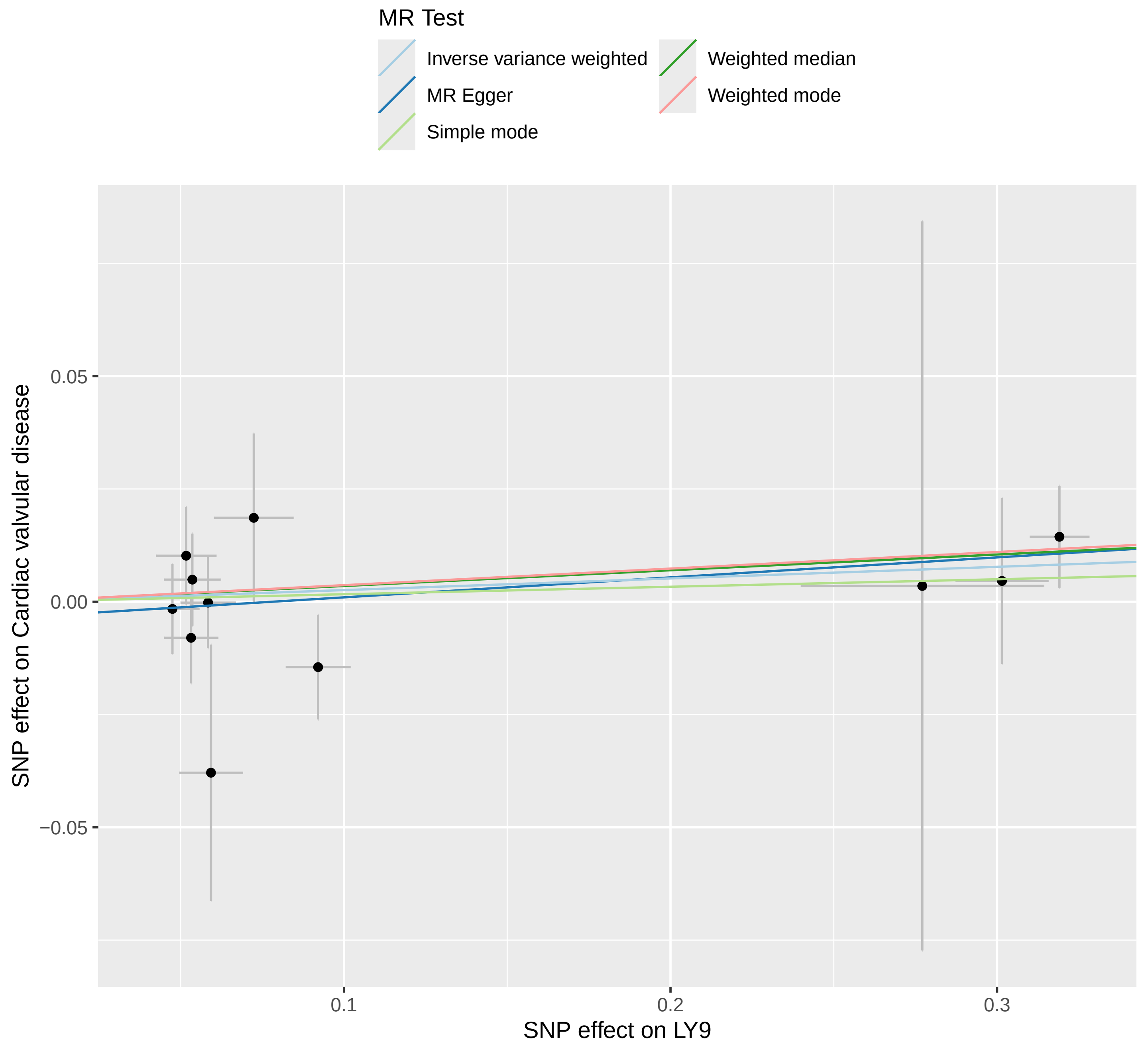

MR Test
Inverse variance weighted
MR Egger
Simple mode
Weighted median
Weighted mode
0.05
0.00
−0.05
SNP effect on Cardiac valvular disease
0.1
0.2
0.3
SNP effect on LY9

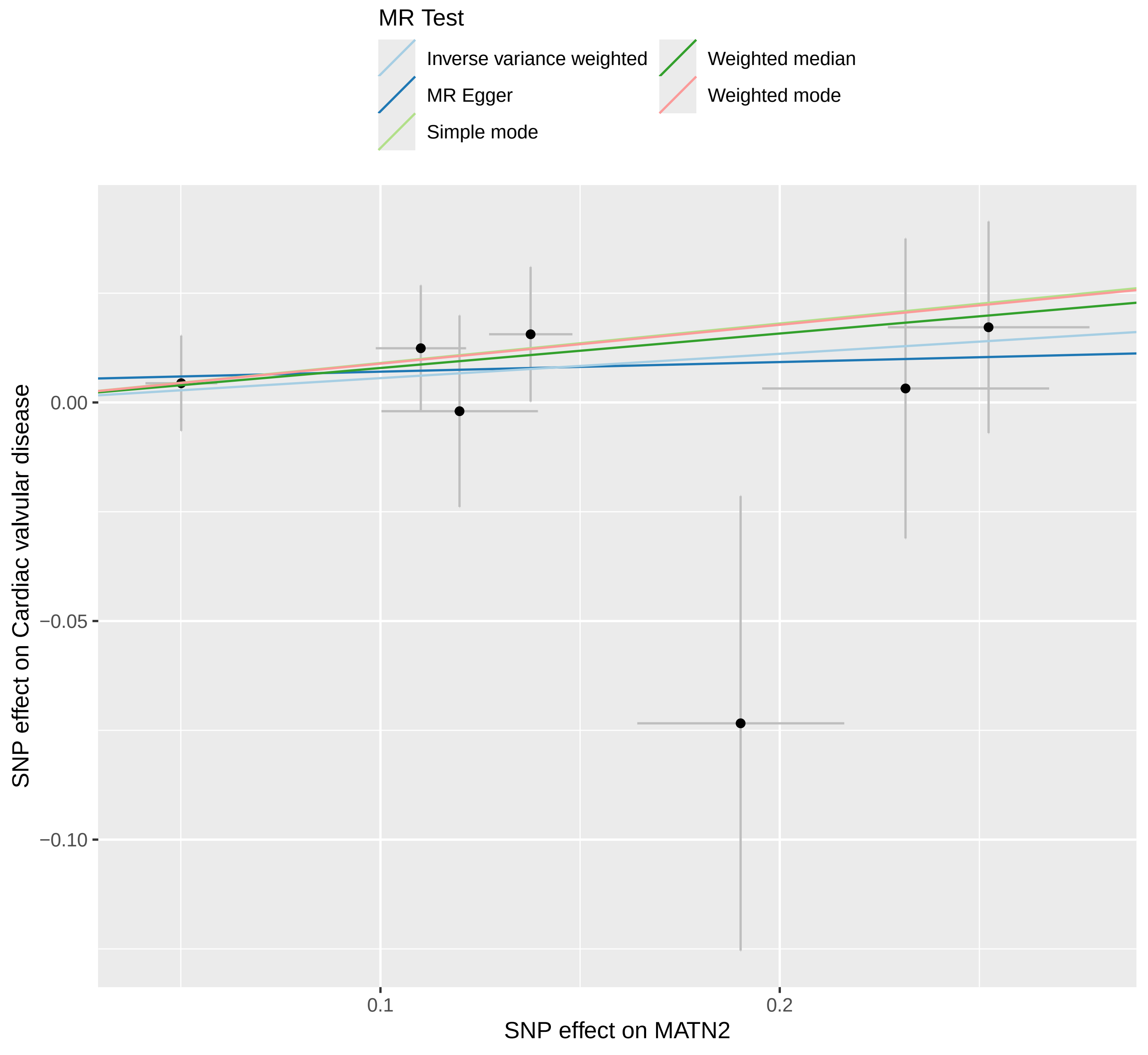

MR Test
Inverse variance weighted
MR Egger
Simple mode
Weighted median
Weighted mode
SNP effect on Cardiac valvular disease
0.00
−0.05
−0.10
0.1
0.2
SNP effect on MATN2

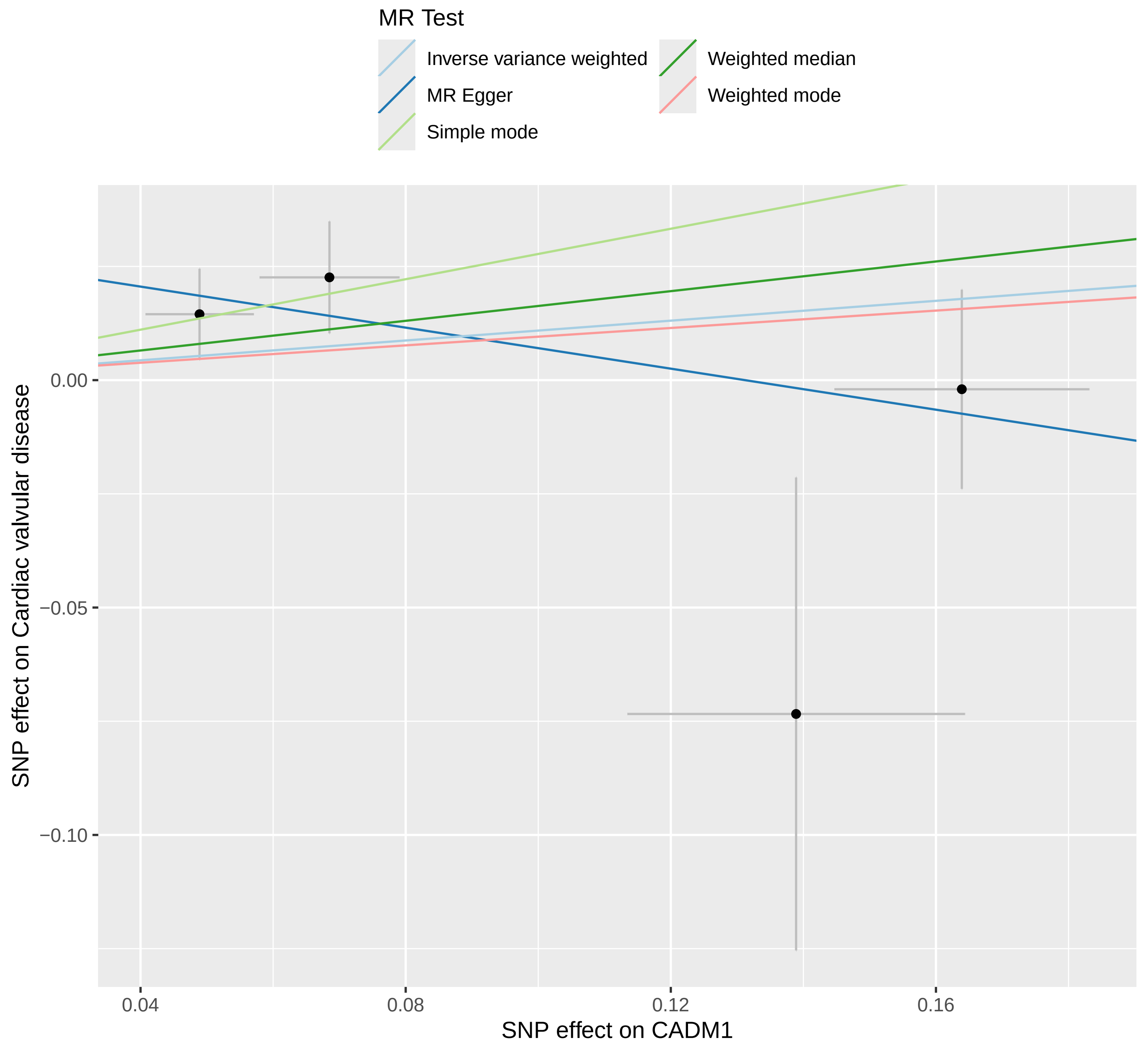

MR Test
Inverse variance weighted
MR Egger
Simple mode
Weighted median
Weighted mode
0.00
−0.05
−0.10
0.04
0.08
0.12
0.16
SNP effect on Cardiac valvular disease
SNP effect on CADM1

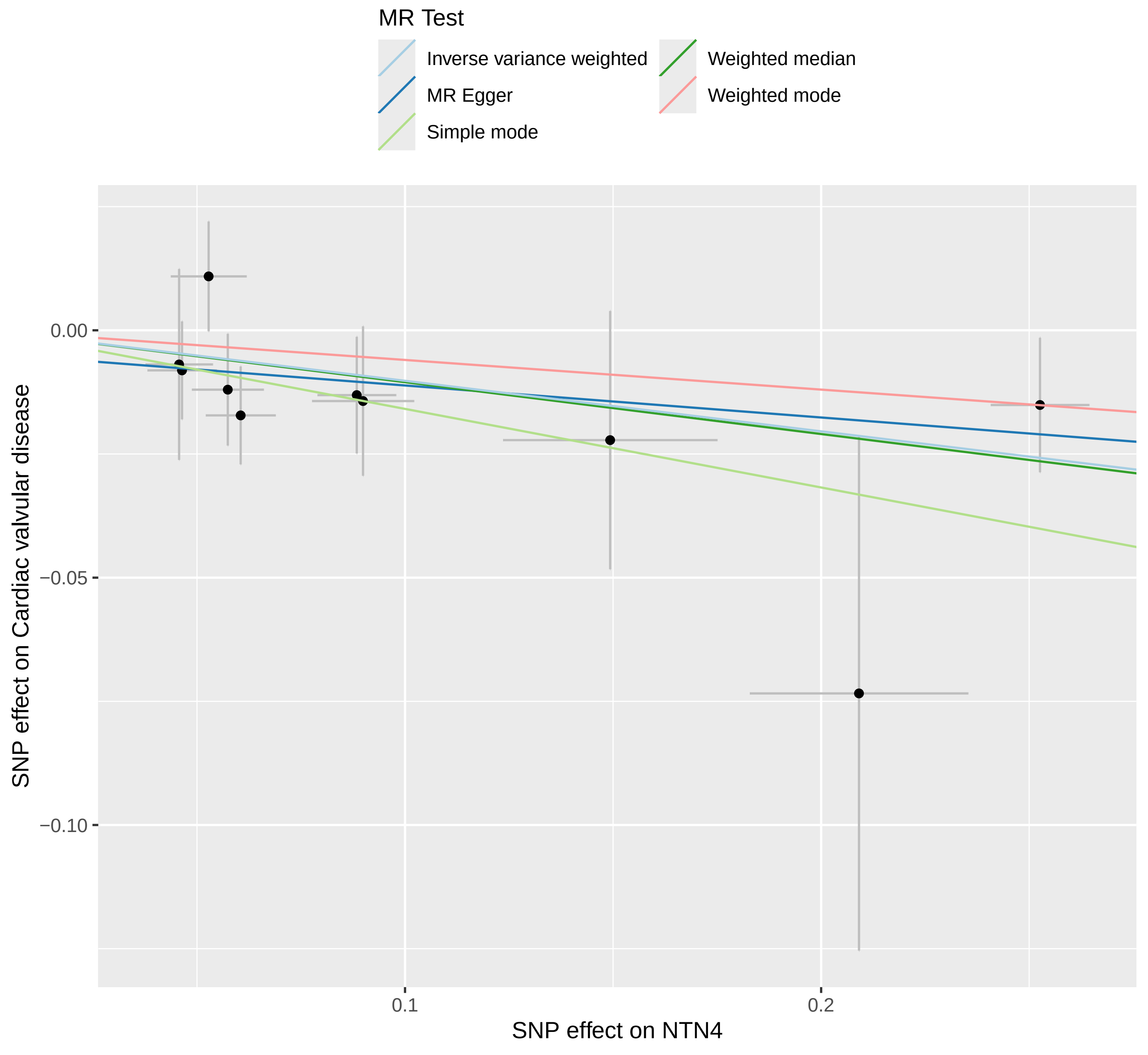

MR Test
Inverse variance weighted
MR Egger
Simple mode
Weighted median
Weighted mode
SNP effect on Cardiac valvular disease
0.00
−0.05
−0.10
0.1
0.2
SNP effect on NTN4

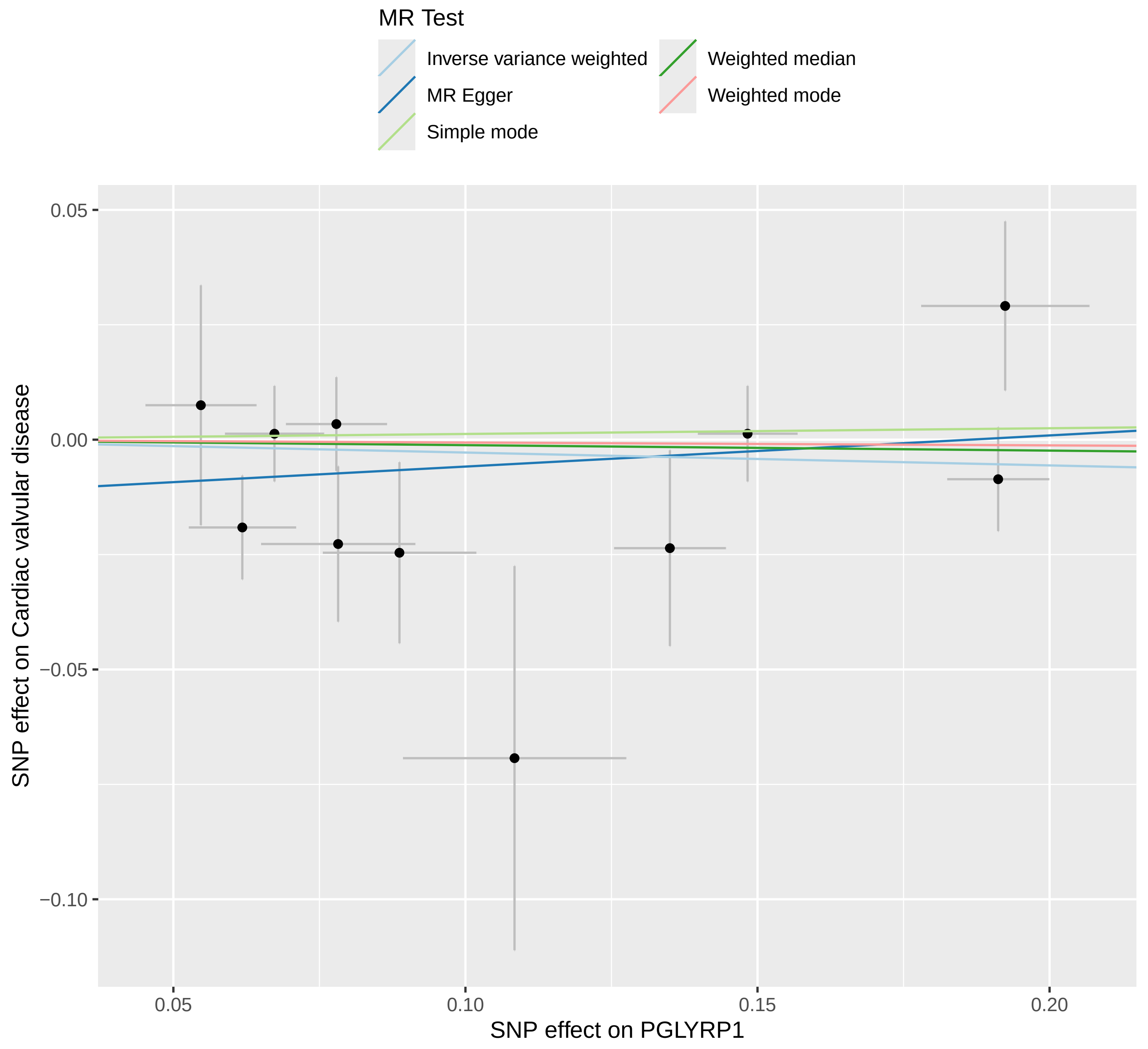
MR Test
Inverse variance weighted
MR Egger
Simple mode
Weighted median
Weighted mode
0.05
0.00
−0.05
−0.10
0.05
0.10
0.15
0.20
SNP effect on PGLYRP1
SNP effect on Cardiac valvular disease

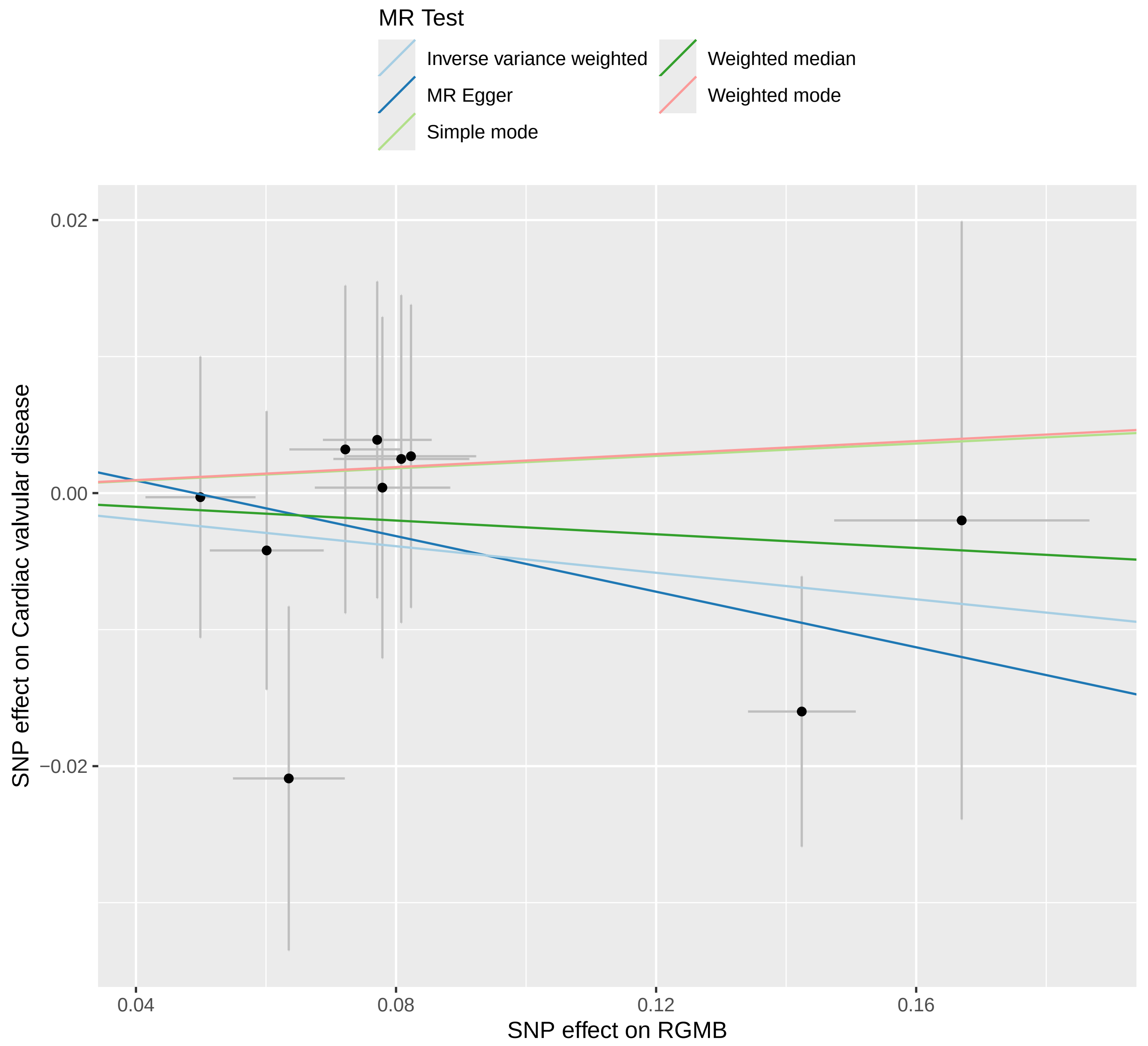
MR Test
Inverse variance weighted
MR Egger
Simple mode
Weighted median
Weighted mode
0.02
0.00
−0.02
0.04
0.08
0.12
0.16
SNP effect on Cardiac valvular disease
SNP effect on RGMB

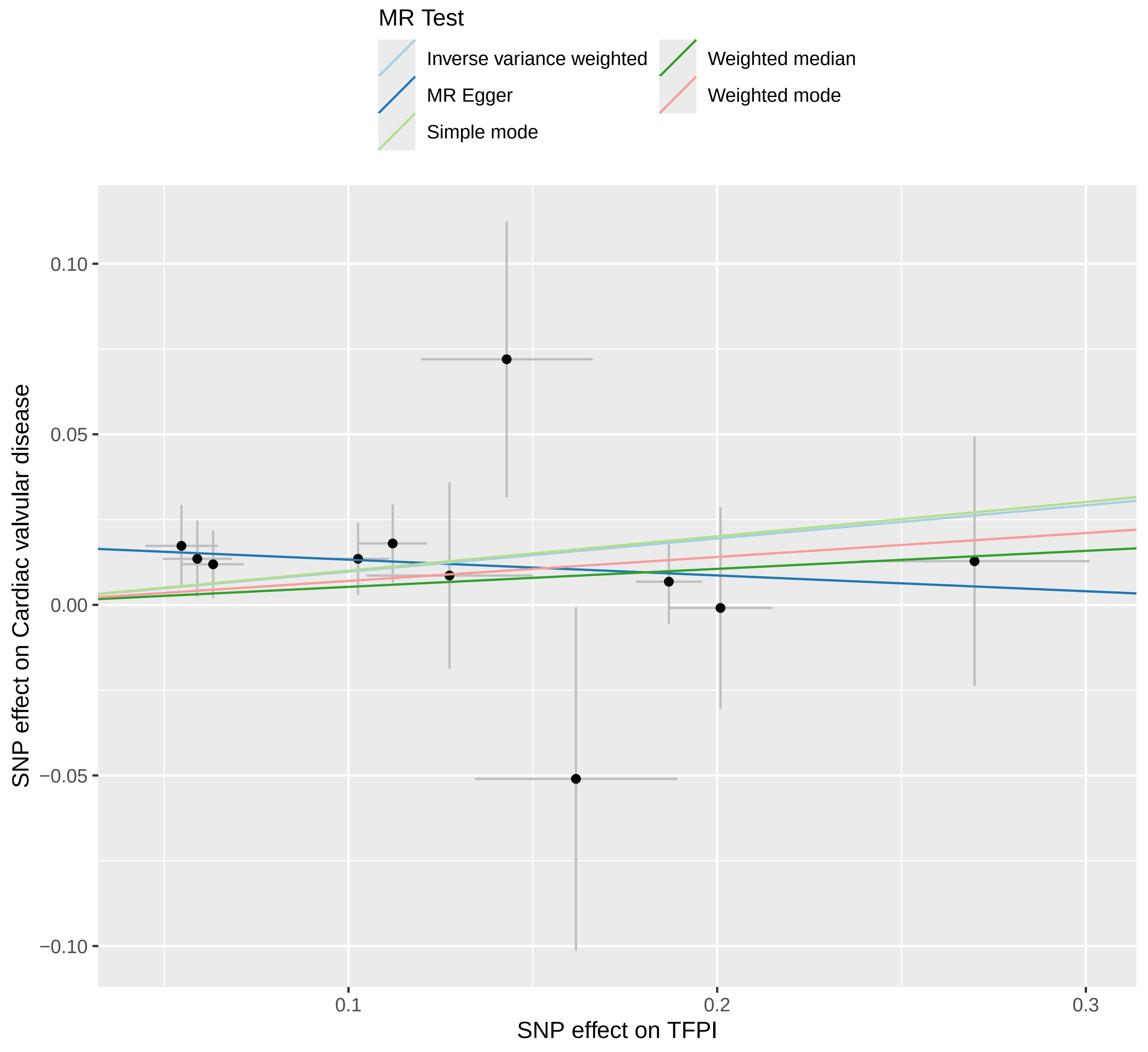

MR Test
Inverse variance weighted
MR Egger
Simple mode
Weighted median
Weighted mode
0.10
0.05
0.00
−0.05
−0.10
SNP effect on Cardiac valvular disease
0.1
0.2
0.3
SNP effect on TFPI

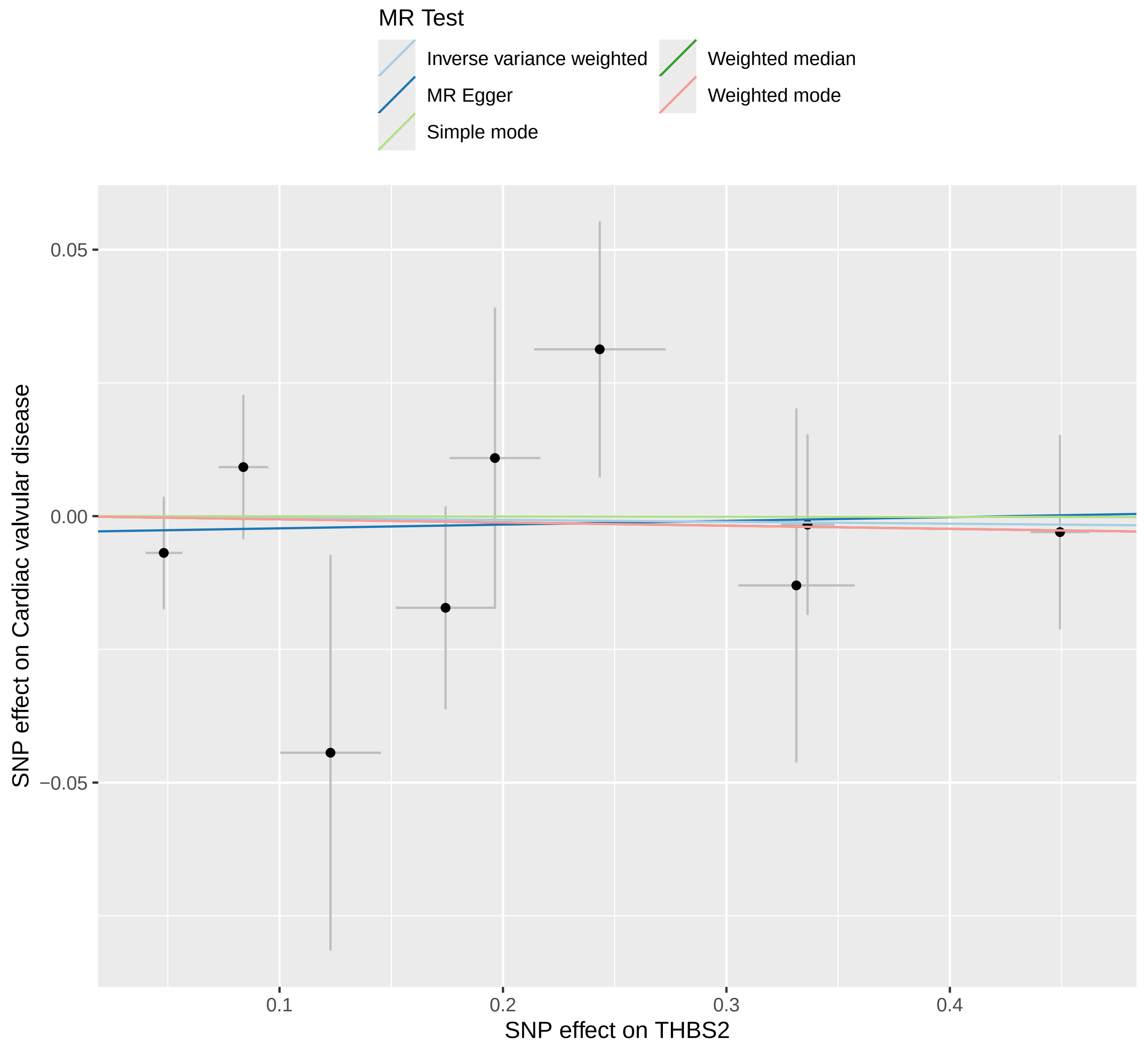
MR Test
Inverse variance weighted
MR Egger
Simple mode
Weighted median
Weighted mode
0.05
0.00
−0.05
0.1
0.2
0.3
0.4
SNP effect on Cardiac valvular disease
SNP effect on THBS2

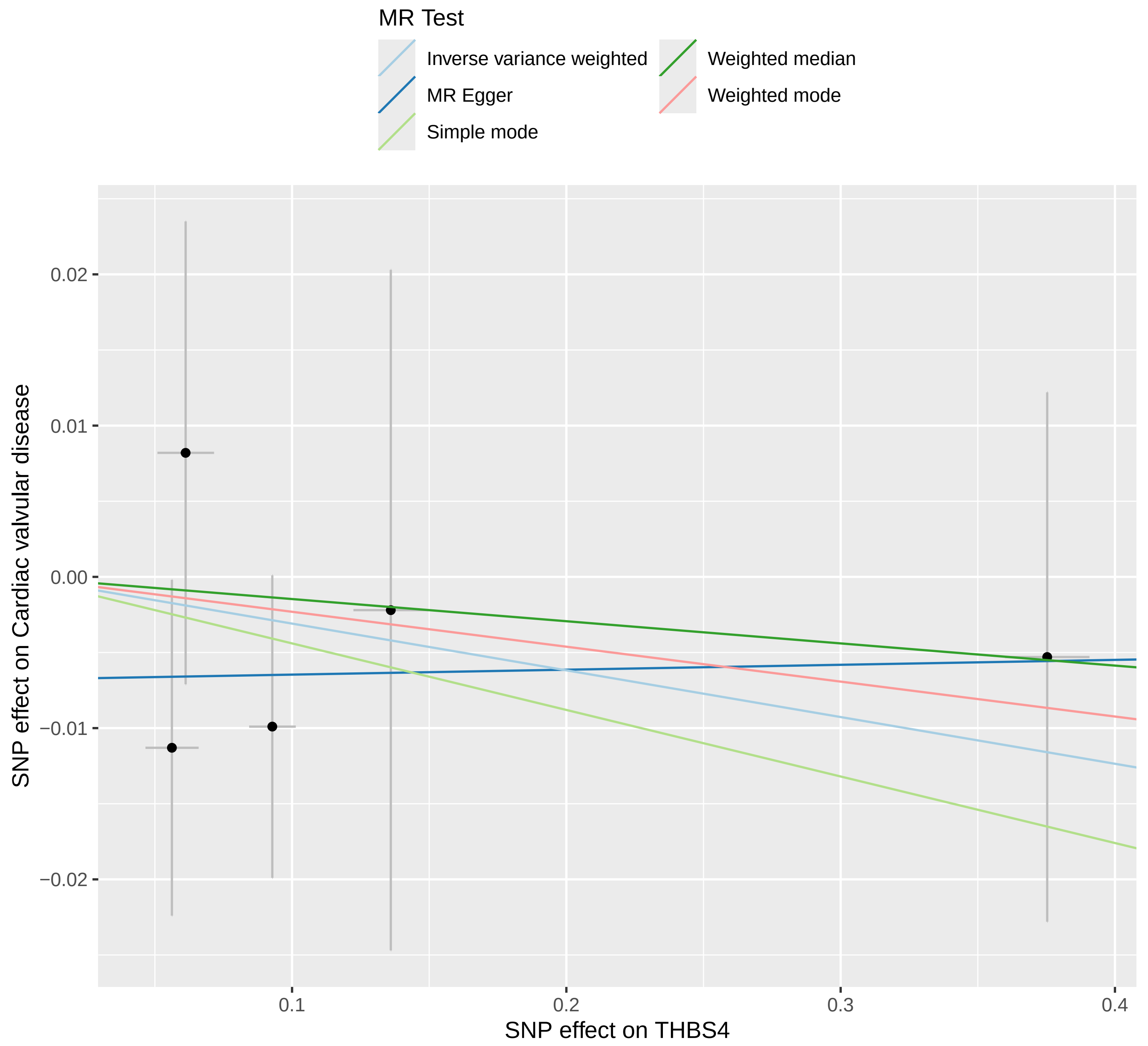

MR Test
Inverse variance weighted
MR Egger
Simple mode
Weighted median
Weighted mode
0.02
0.01
0.00
−0.01
−0.02
SNP effect on Cardiac valvular disease
0.1
0.2
0.3
0.4
SNP effect on THBS4

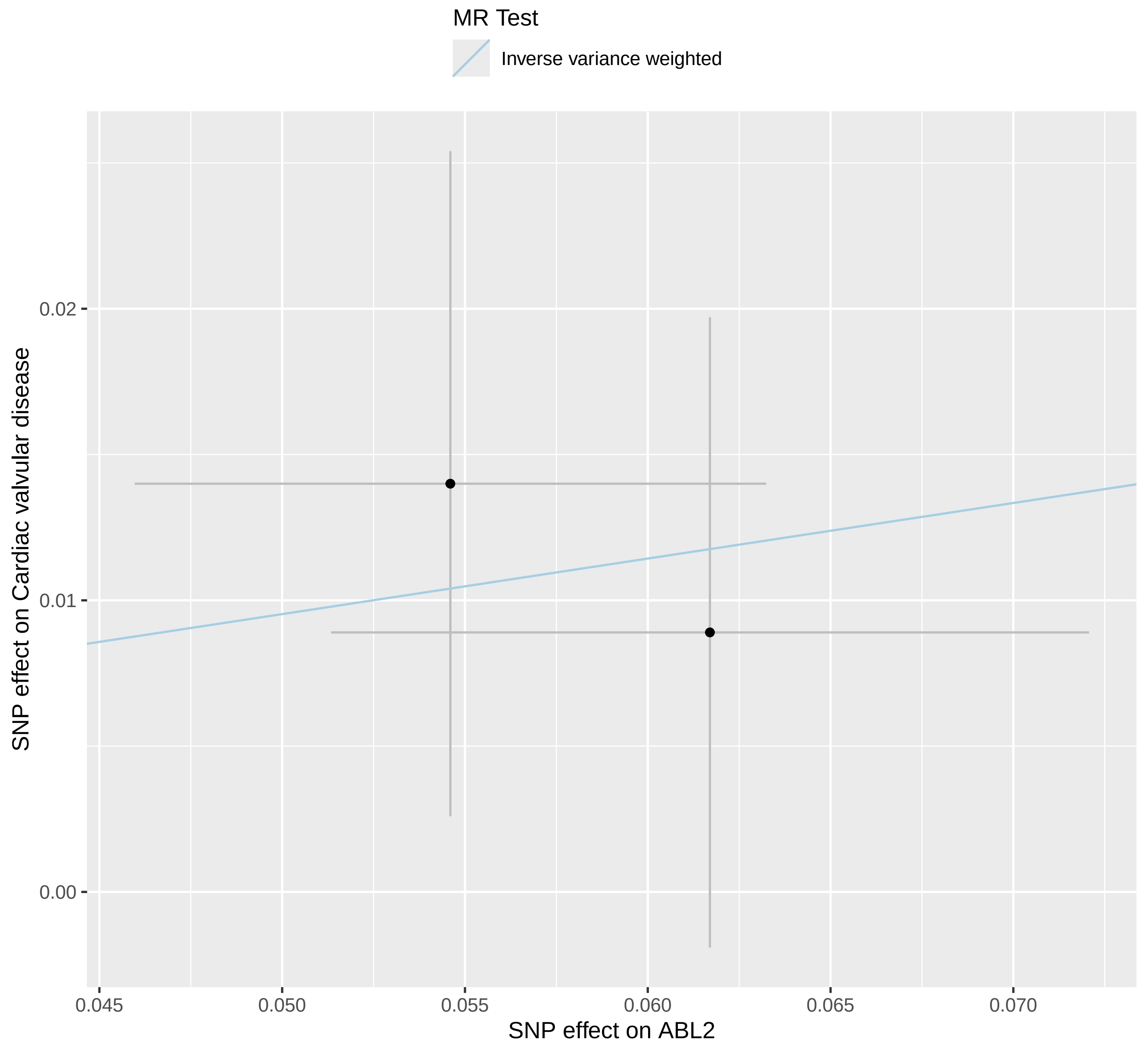
MR Test
Inverse variance weighted
SNP effect on Cardiac valvular disease
0.02
0.01
0.00
0.045
0.050
0.055
0.060
0.065
0.070
SNP effect on ABL2

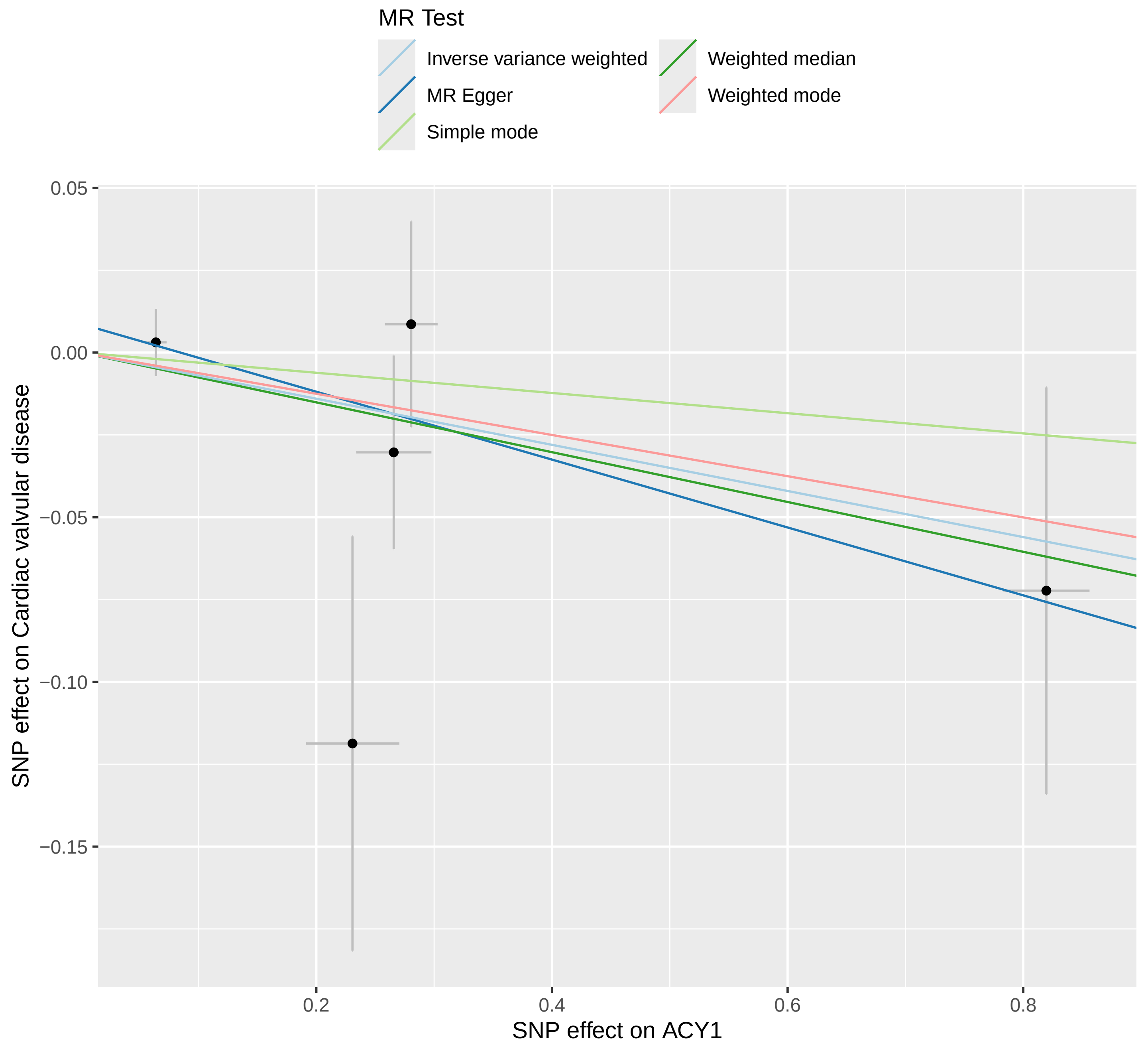
MR Test
Inverse variance weighted
MR Egger
Simple mode
Weighted median
Weighted mode
0.05
0.00
−0.05
−0.10
−0.15
0.2
0.4
0.6
0.8
SNP effect on Cardiac valvular disease
SNP effect on ACY1

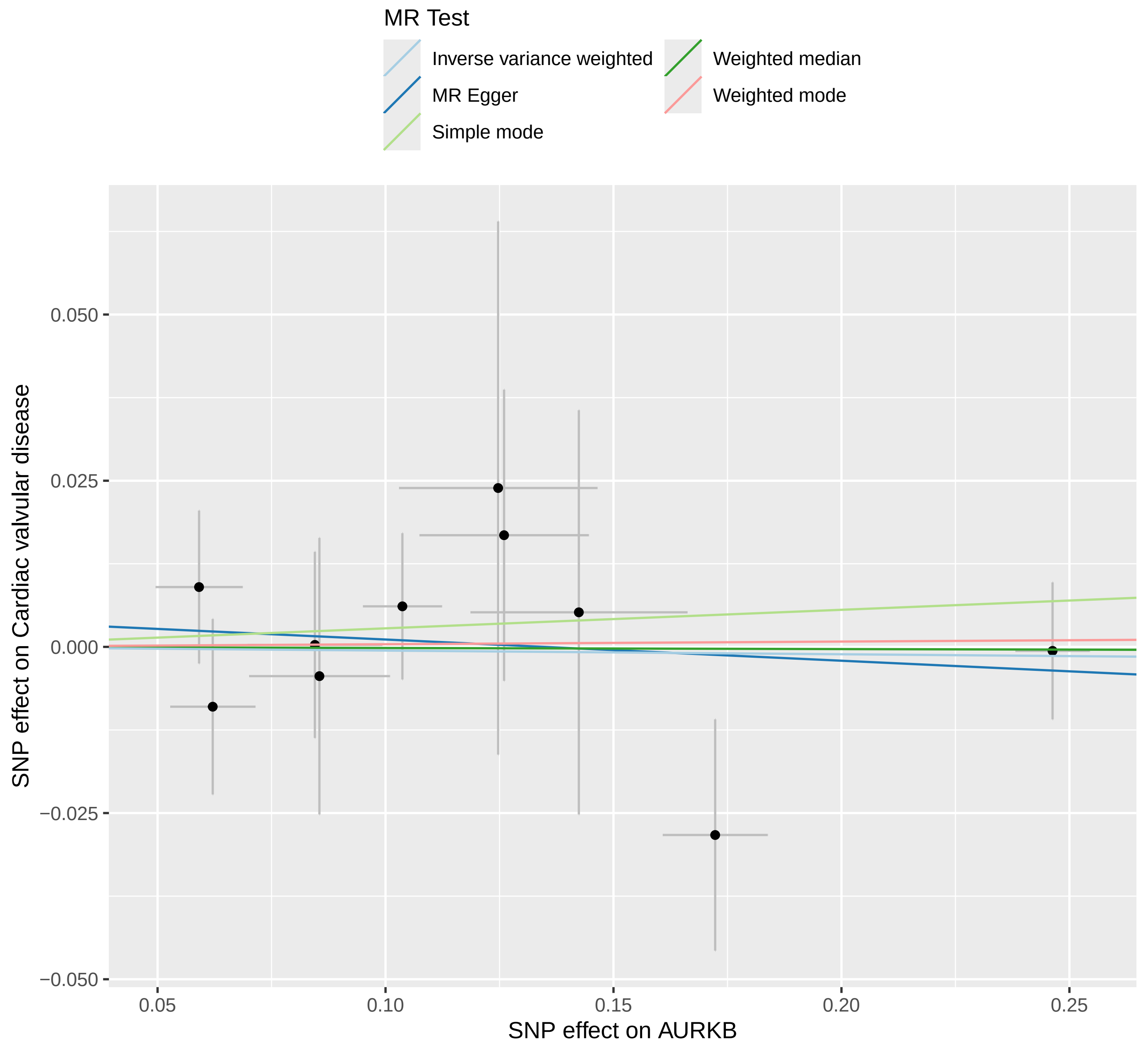
MR Test
Inverse variance weighted
MR Egger
Simple mode
Weighted median
Weighted mode
SNP effect on Cardiac valvular disease
0.050
0.025
0.000
−0.025
−0.050
0.05
0.10
0.15
0.20
0.25
SNP effect on AURKB

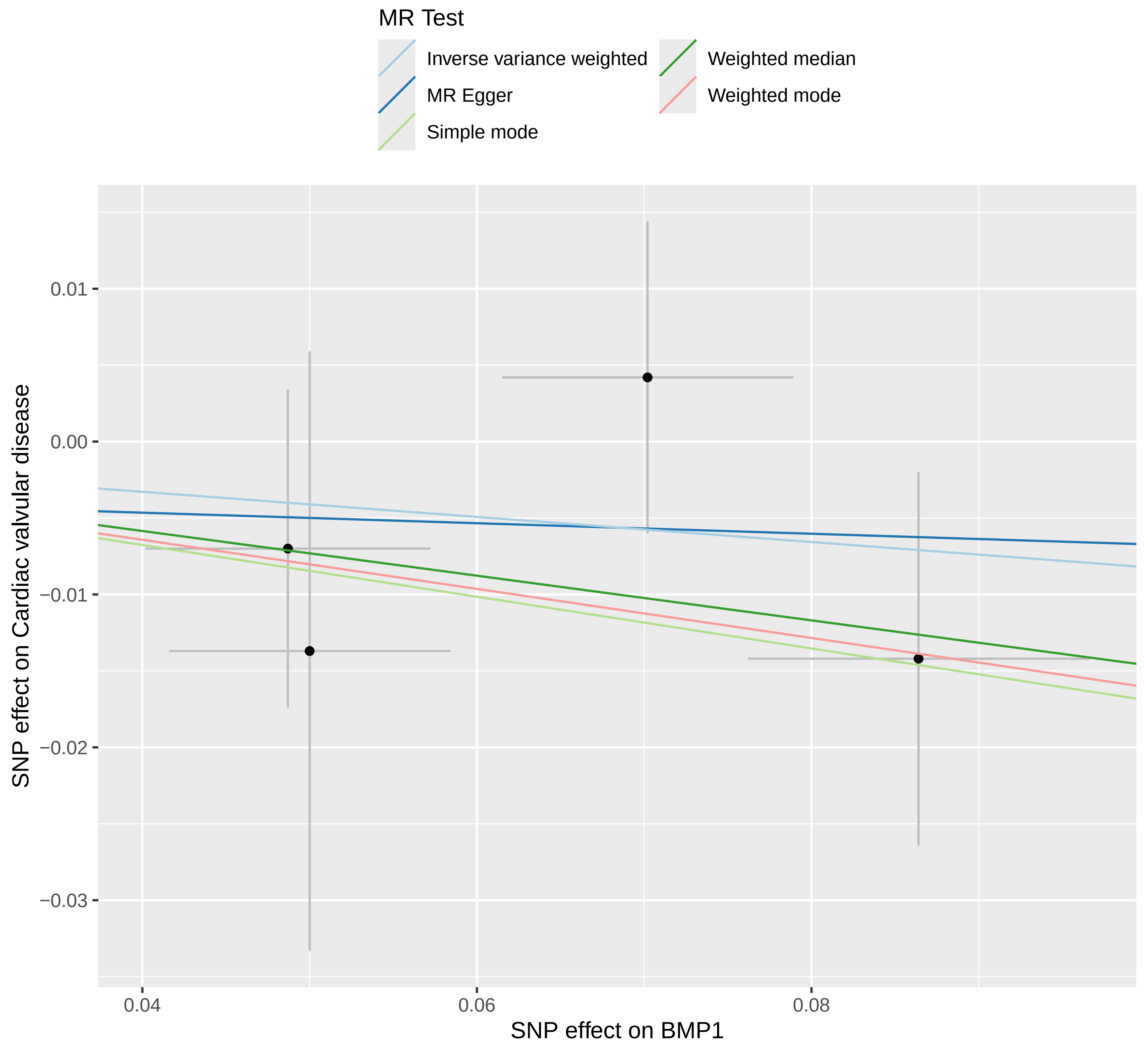
MR Test
Inverse variance weighted
MR Egger
Simple mode
Weighted median
Weighted mode
0.01
0.00
−0.01
−0.02
−0.03
0.04
0.06
0.08
SNP effect on Cardiac valvular disease
SNP effect on BMP1

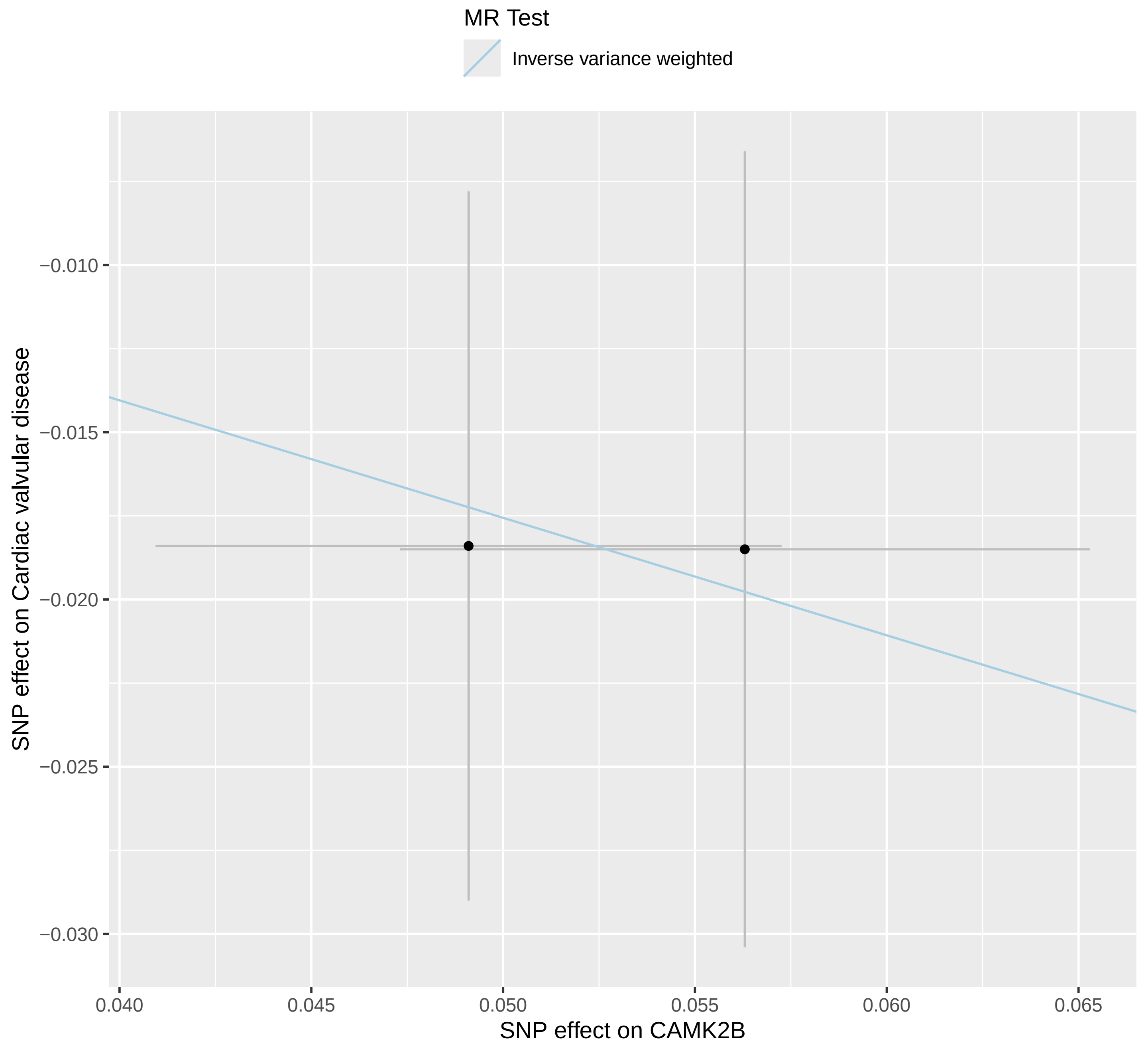
MR Test
Inverse variance weighted
SNP effect on Cardiac valvular disease
−0.010
−0.015
−0.020
−0.025
−0.030
0.040
0.045
0.050
0.055
0.060
0.065
SNP effect on CAMK2B

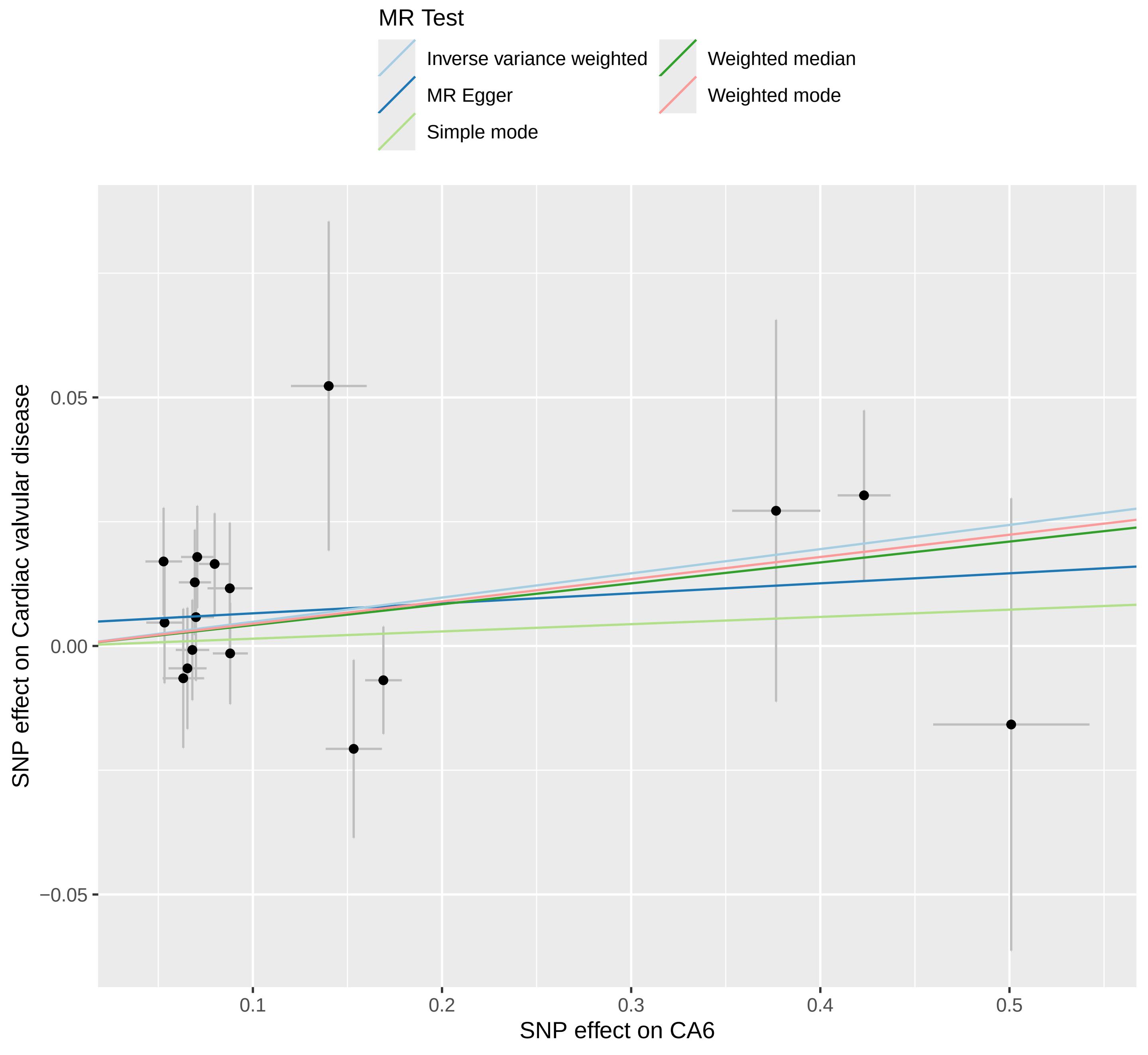
MR Test
Inverse variance weighted
MR Egger
Simple mode
Weighted median
Weighted mode
0.05
0.00
−0.05
0.1
0.2
0.3
0.4
0.5
SNP effect on Cardiac valvular disease
SNP effect on CA6

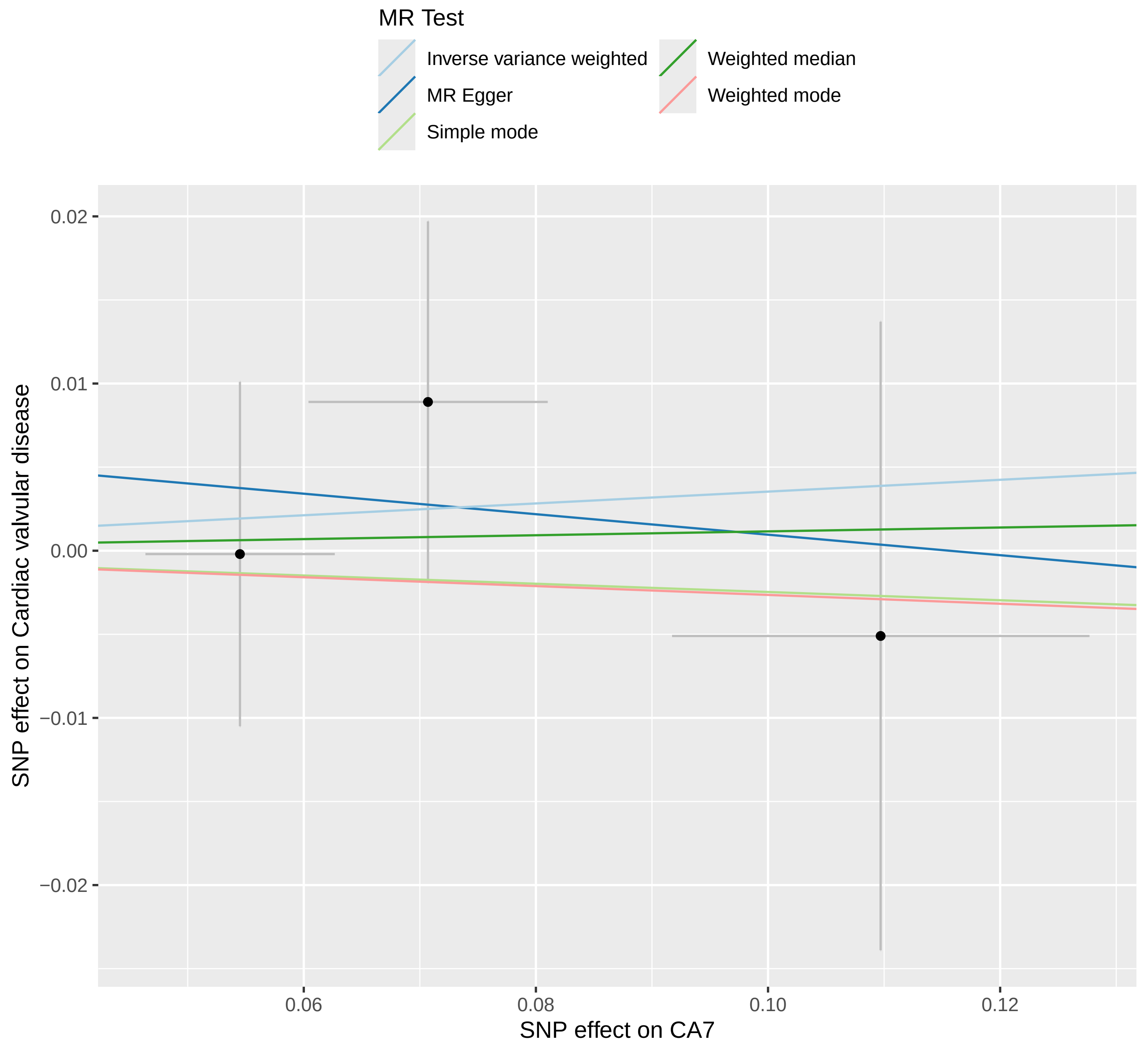
MR Test
Inverse variance weighted
MR Egger
Simple mode
Weighted median
Weighted mode
SNP effect on Cardiac valvular disease
0.02
0.01
0.00
−0.01
−0.02
0.06
0.08
0.10
0.12
SNP effect on CA7

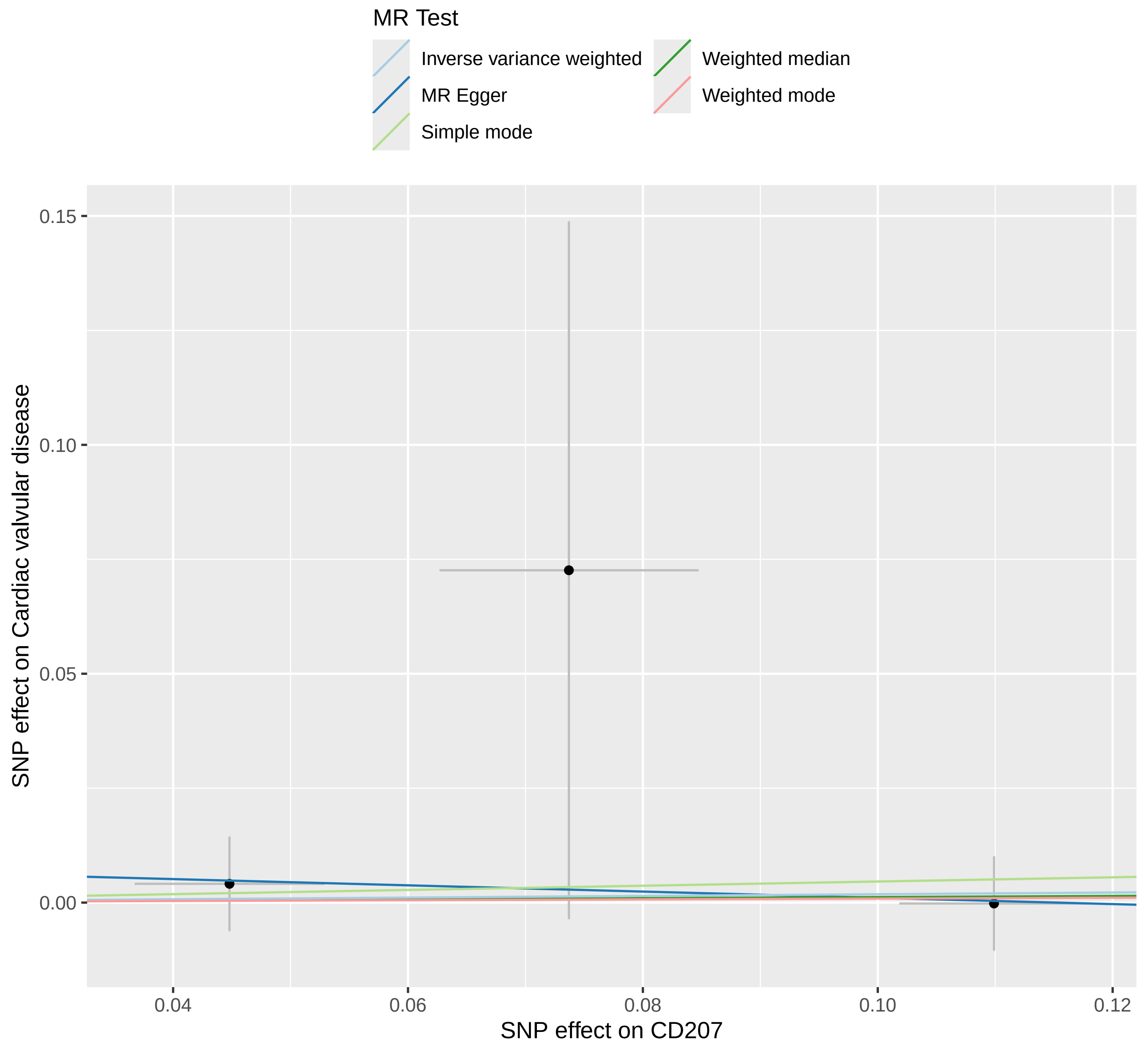
MR Test
Inverse variance weighted
MR Egger
Simple mode
Weighted median
Weighted mode
SNP effect on Cardiac valvular disease
0.15
0.10
0.05
0.00
0.04
0.06
0.08
0.10
0.12
SNP effect on CD207

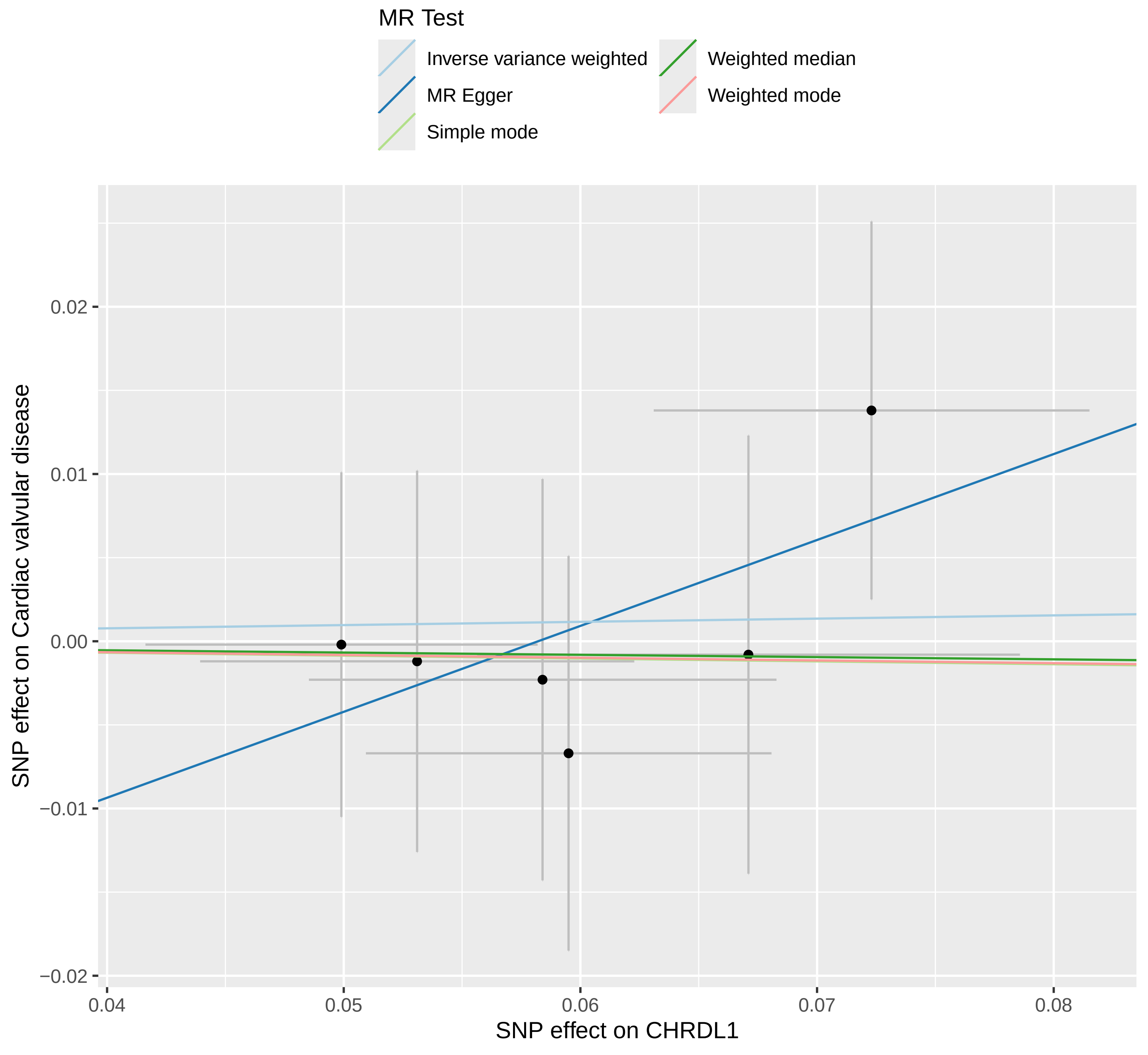
MR Test
Inverse variance weighted
MR Egger
Simple mode
Weighted median
Weighted mode
SNP effect on Cardiac valvular disease
0.02
0.01
0.00
−0.01
−0.02
0.04
0.05
0.06
0.07
0.08
SNP effect on CHRDL1

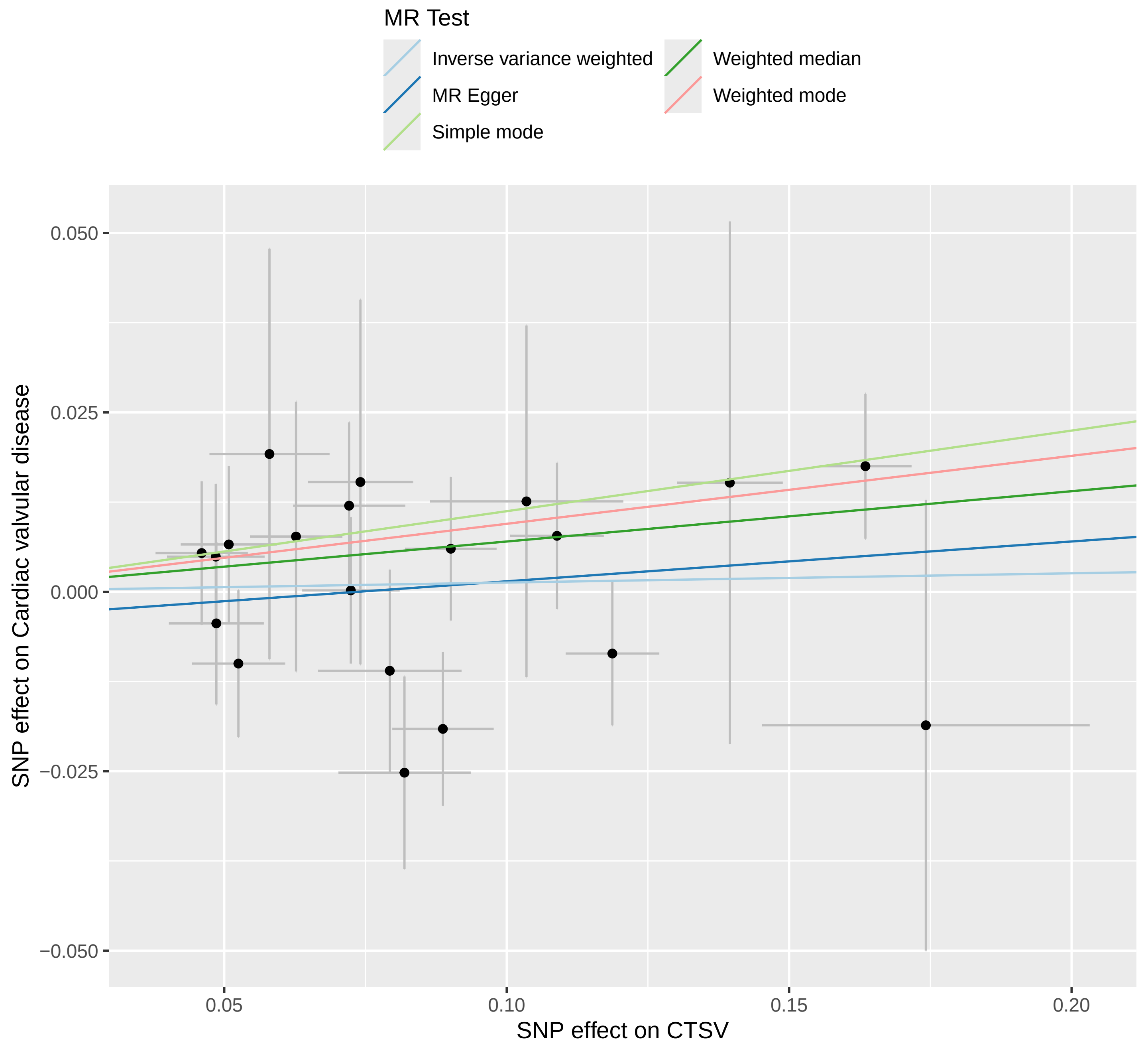
MR Test
Inverse variance weighted
MR Egger
Simple mode
Weighted median
Weighted mode
SNP effect on Cardiac valvular disease
0.050
0.025
0.000
−0.025
−0.050
0.05
0.10
0.15
0.20
SNP effect on CTSV

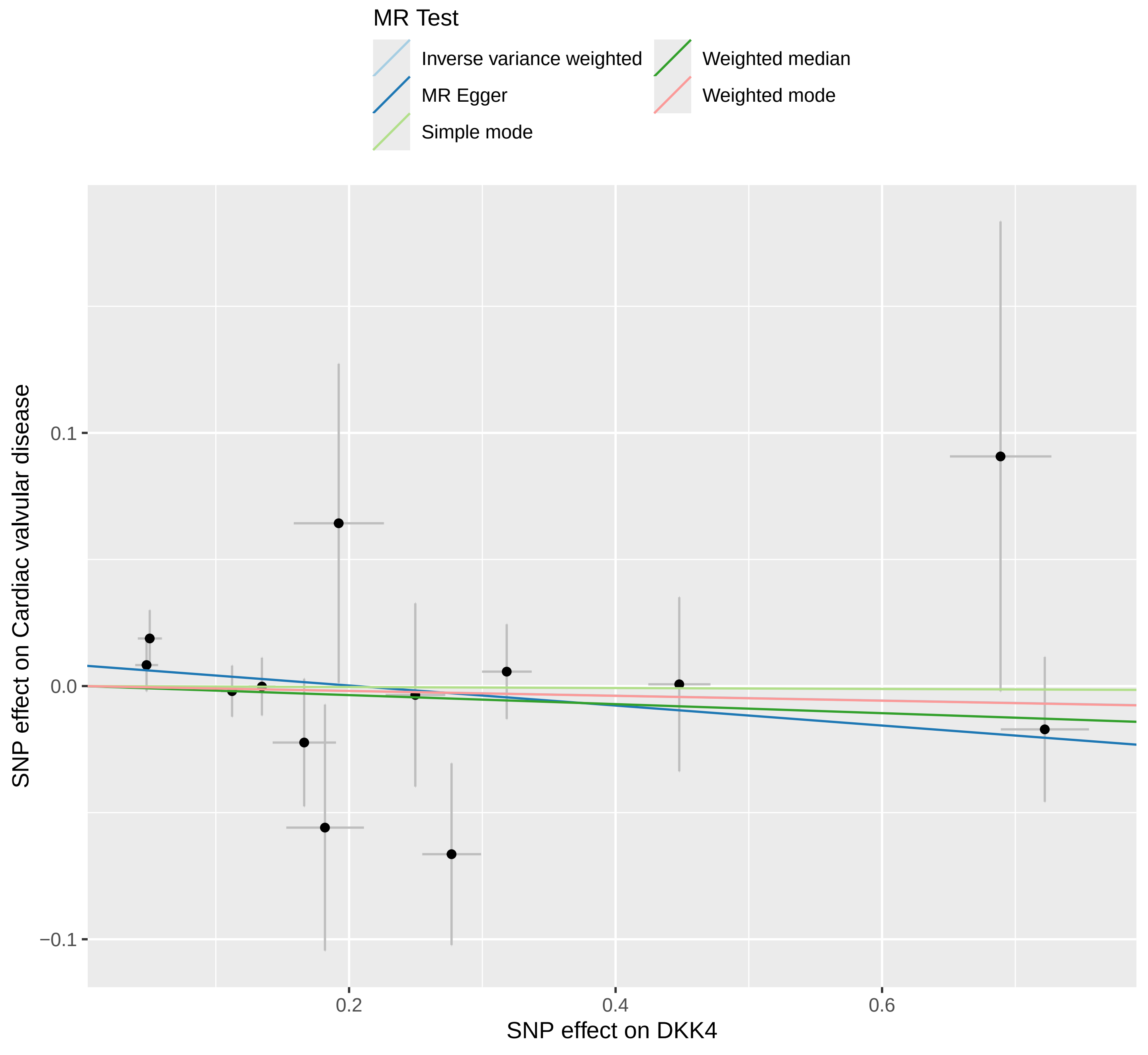

MR Test
Inverse variance weighted
MR Egger
Simple mode
Weighted median
Weighted mode
0.1
0.0
−0.1
0.2
0.4
0.6
SNP effect on Cardiac valvular disease
SNP effect on DKK4

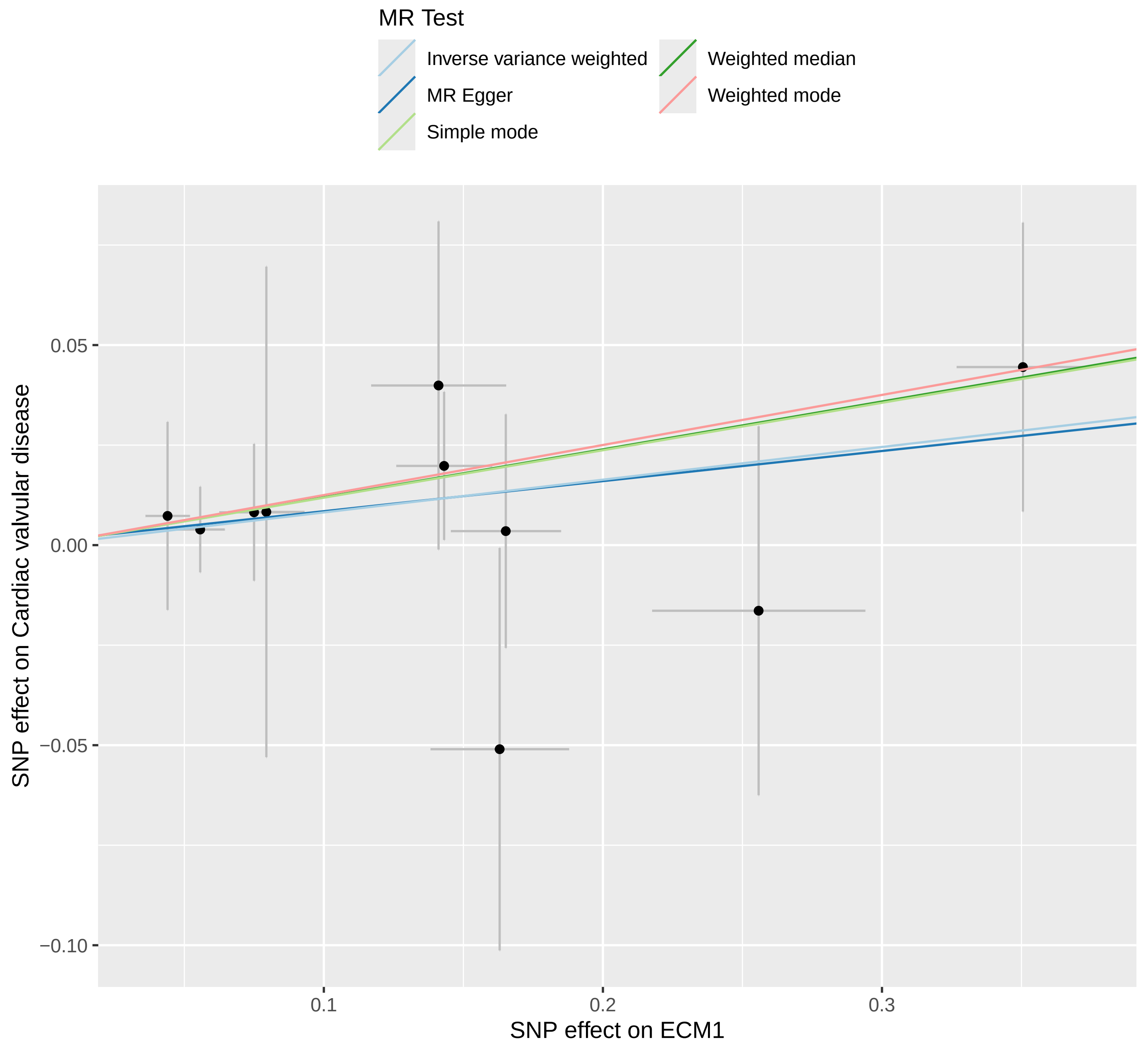
MR Test
Inverse variance weighted
MR Egger
Simple mode
Weighted median
Weighted mode
0.05
0.00
−0.05
−0.10
0.1
0.2
0.3
SNP effect on Cardiac valvular disease
SNP effect on ECM1

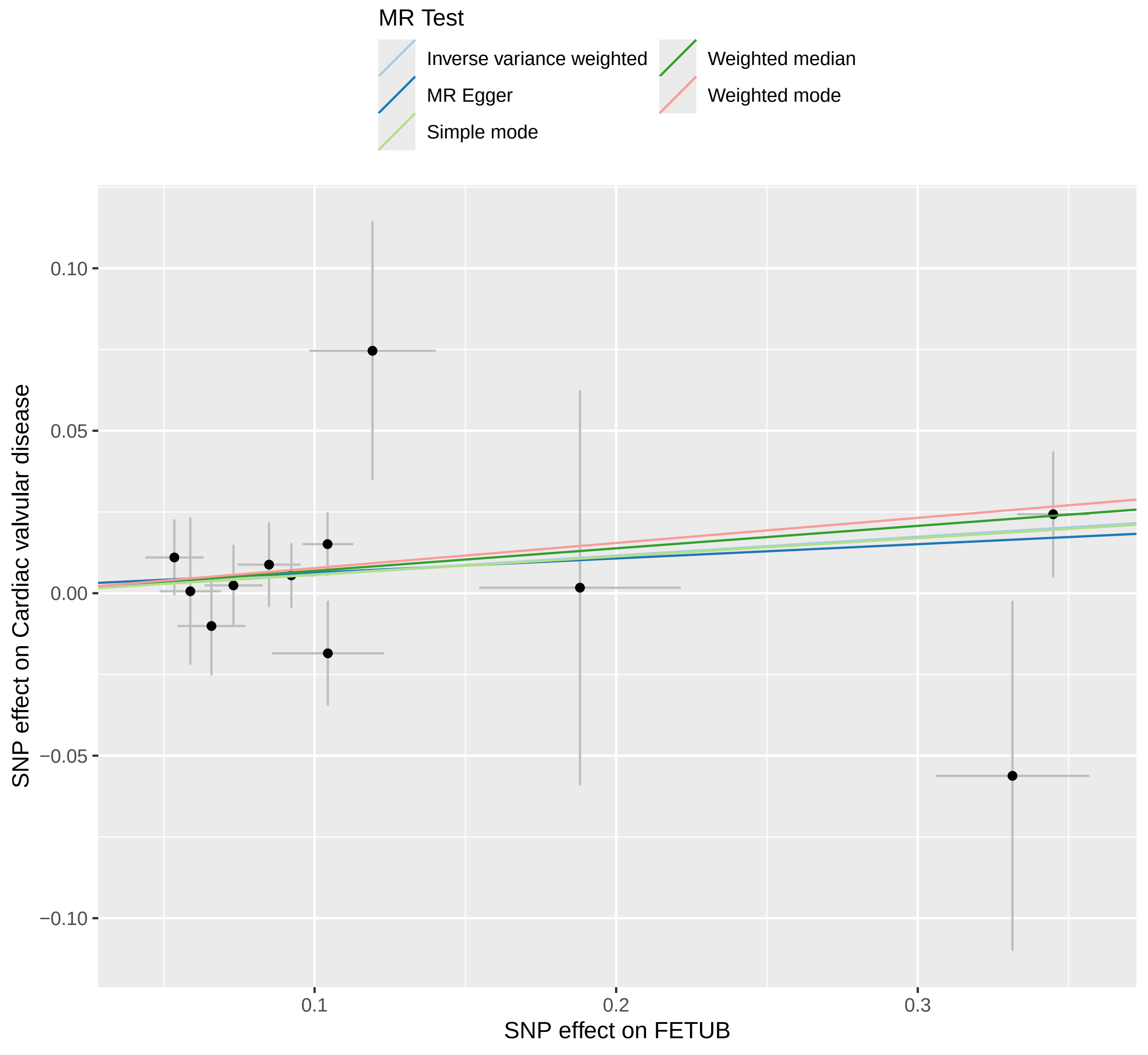
MR Test
Inverse variance weighted
MR Egger
Simple mode
Weighted median
Weighted mode
0.10
0.05
0.00
−0.05
−0.10
0.1
0.2
0.3
SNP effect on Cardiac valvular disease
SNP effect on FETUB

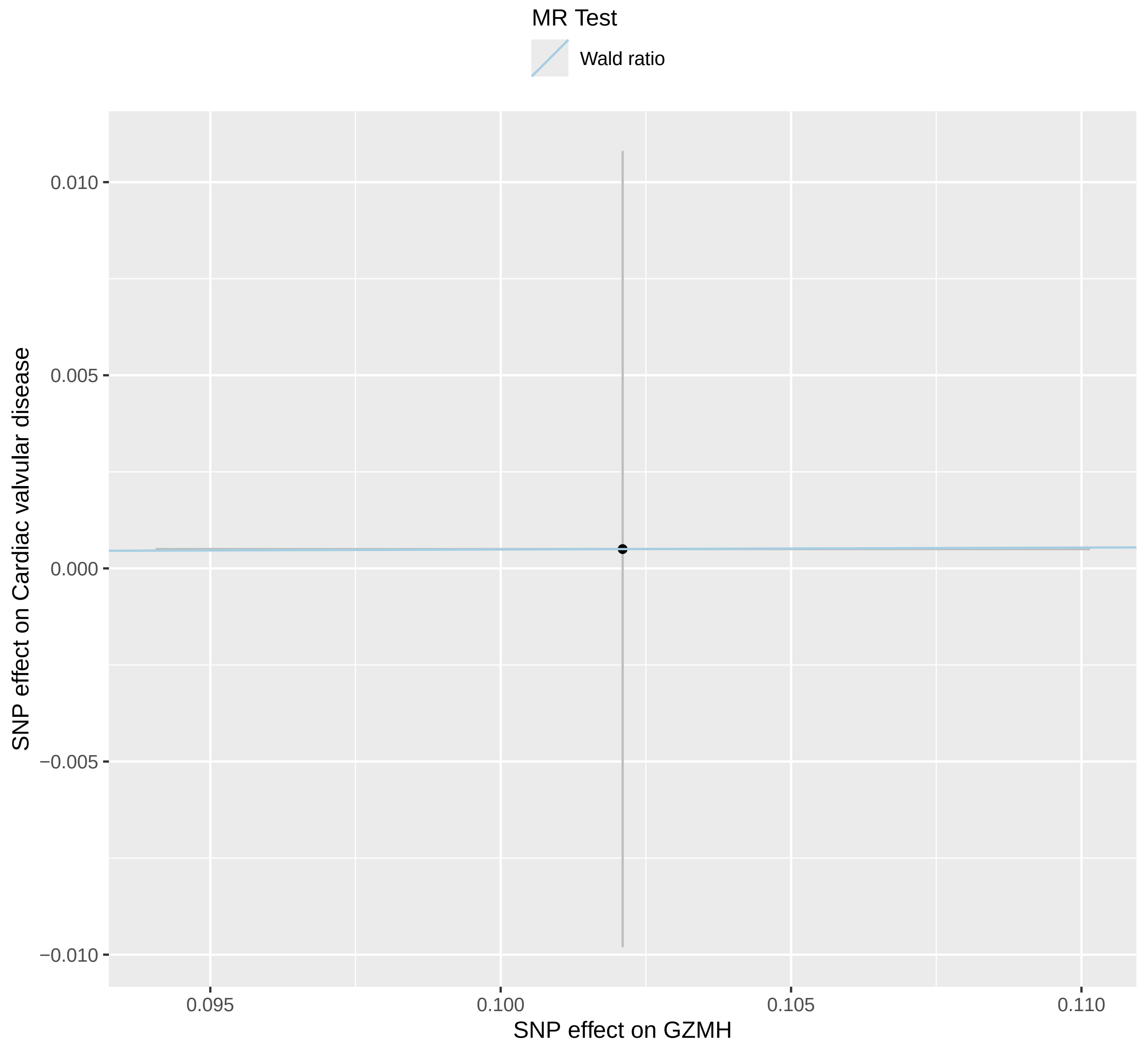
MR Test
Wald ratio
0.010
0.005
0.000
−0.005
−0.010
0.095
0.100
0.105
0.110
SNP effect on Cardiac valvular disease
SNP effect on GZMH

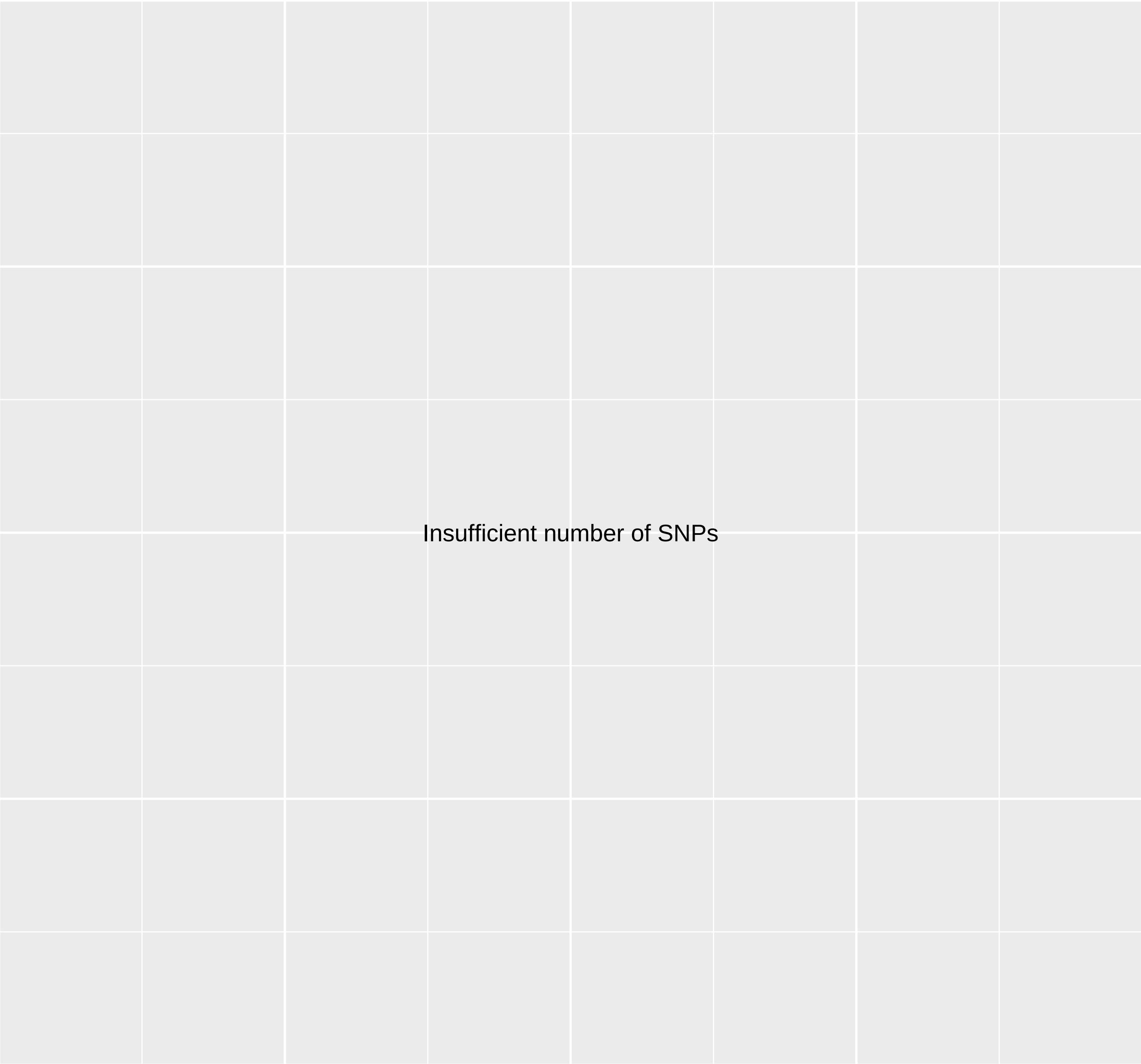
Insufficient number of SNPs

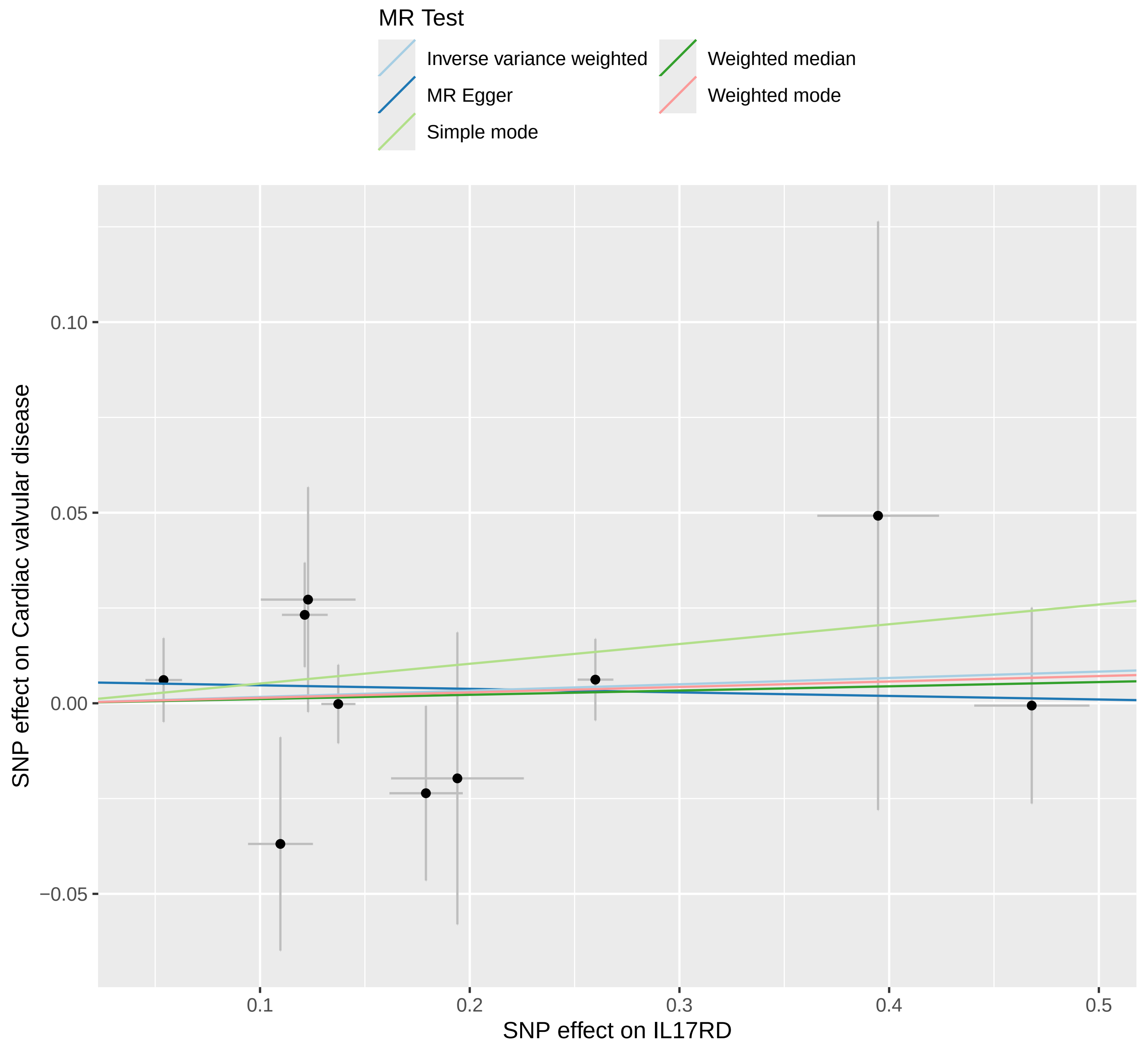

MR Test
Inverse variance weighted
MR Egger
Simple mode
Weighted median
Weighted mode
0.10
0.05
0.00
−0.05
0.1
0.2
0.3
0.4
0.5
SNP effect on Cardiac valvular disease
SNP effect on IL17RD

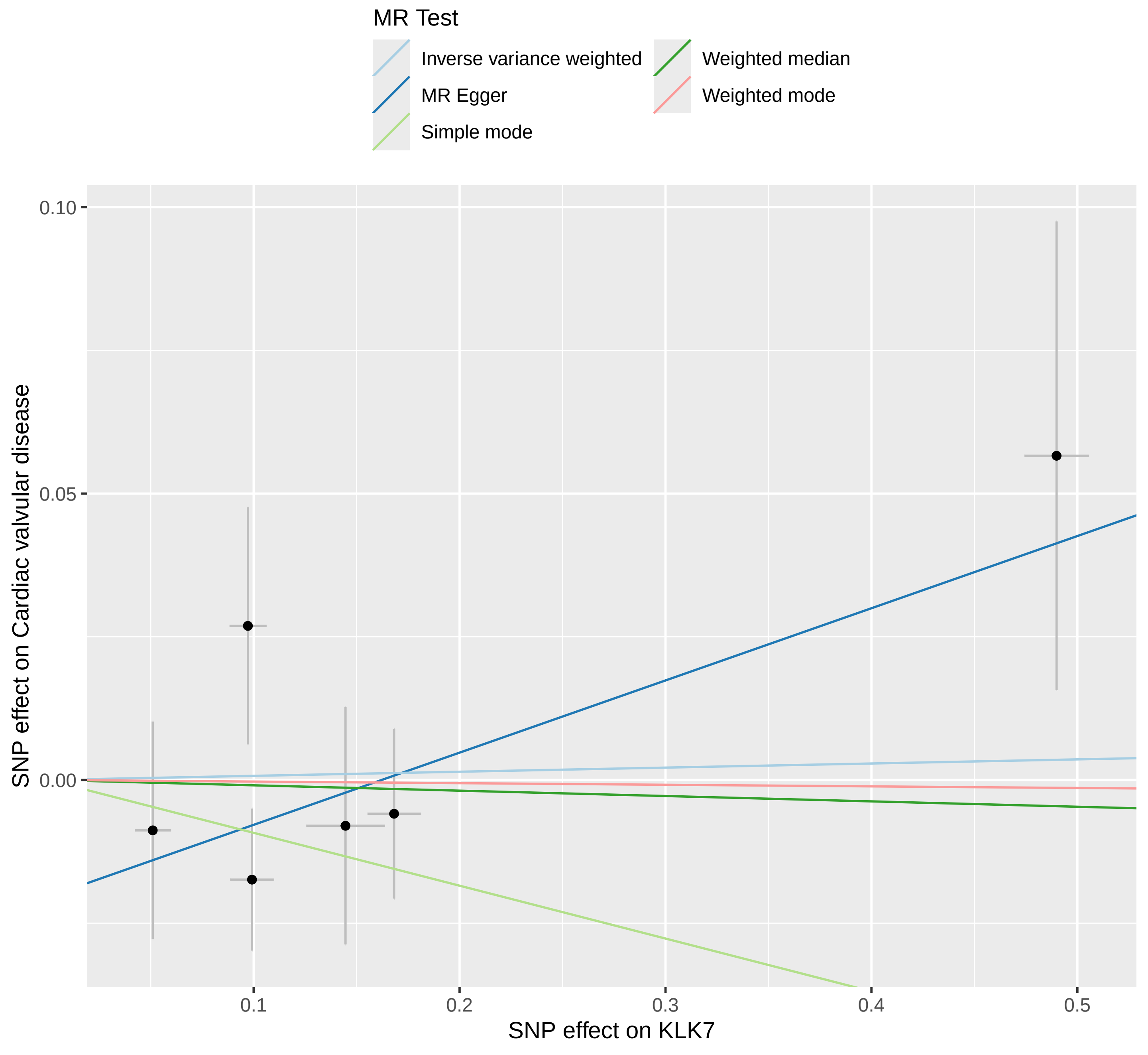
MR Test
Inverse variance weighted
MR Egger
Simple mode
Weighted median
Weighted mode
0.10
0.05
0.00
SNP effect on Cardiac valvular disease
0.1
0.2
0.3
0.4
0.5
SNP effect on KLK7

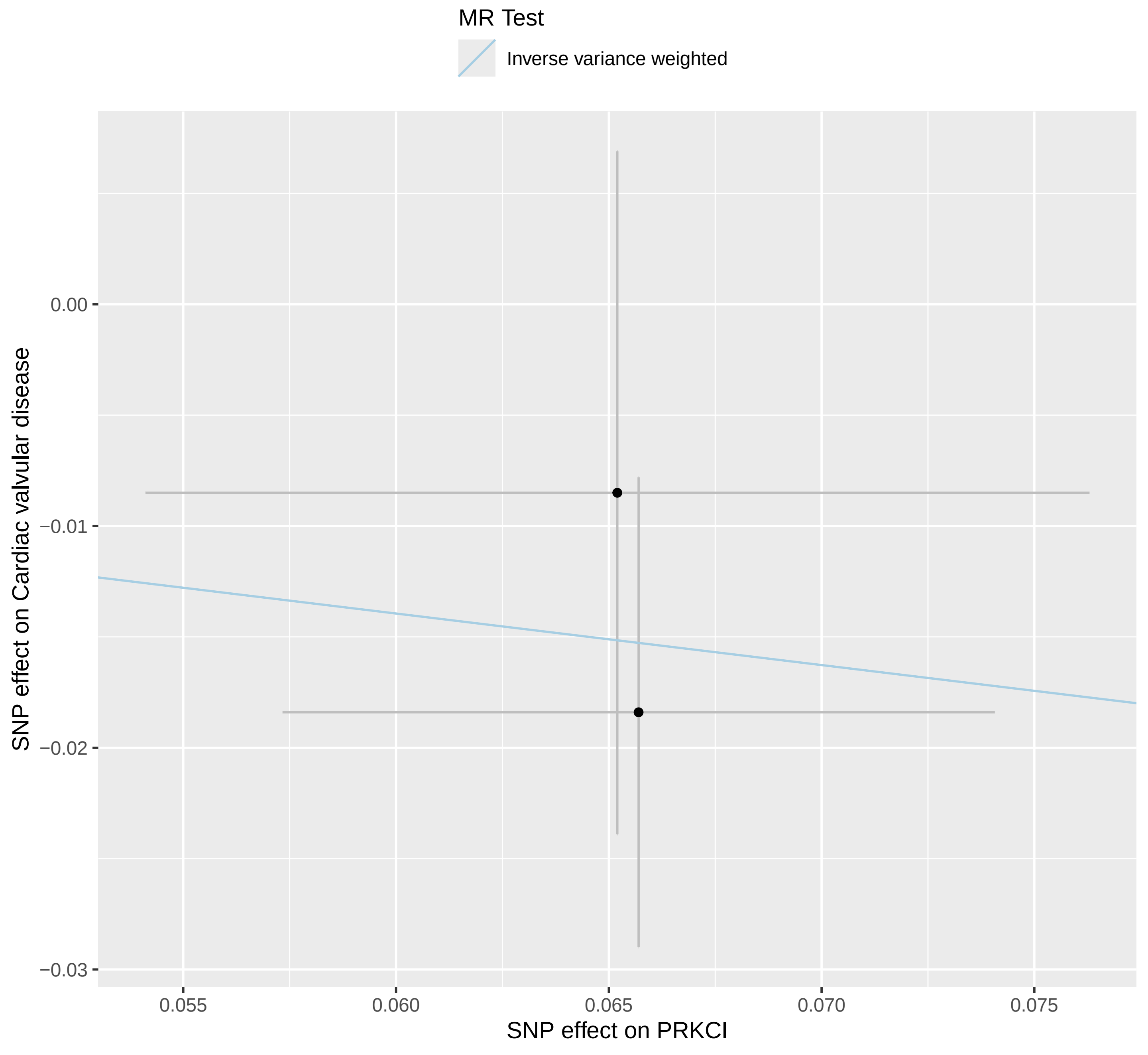
MR Test
Inverse variance weighted
0.00
−0.01
−0.02
−0.03
0.055
0.060
0.065
0.070
0.075
SNP effect on PRKCI
SNP effect on Cardiac valvular disease

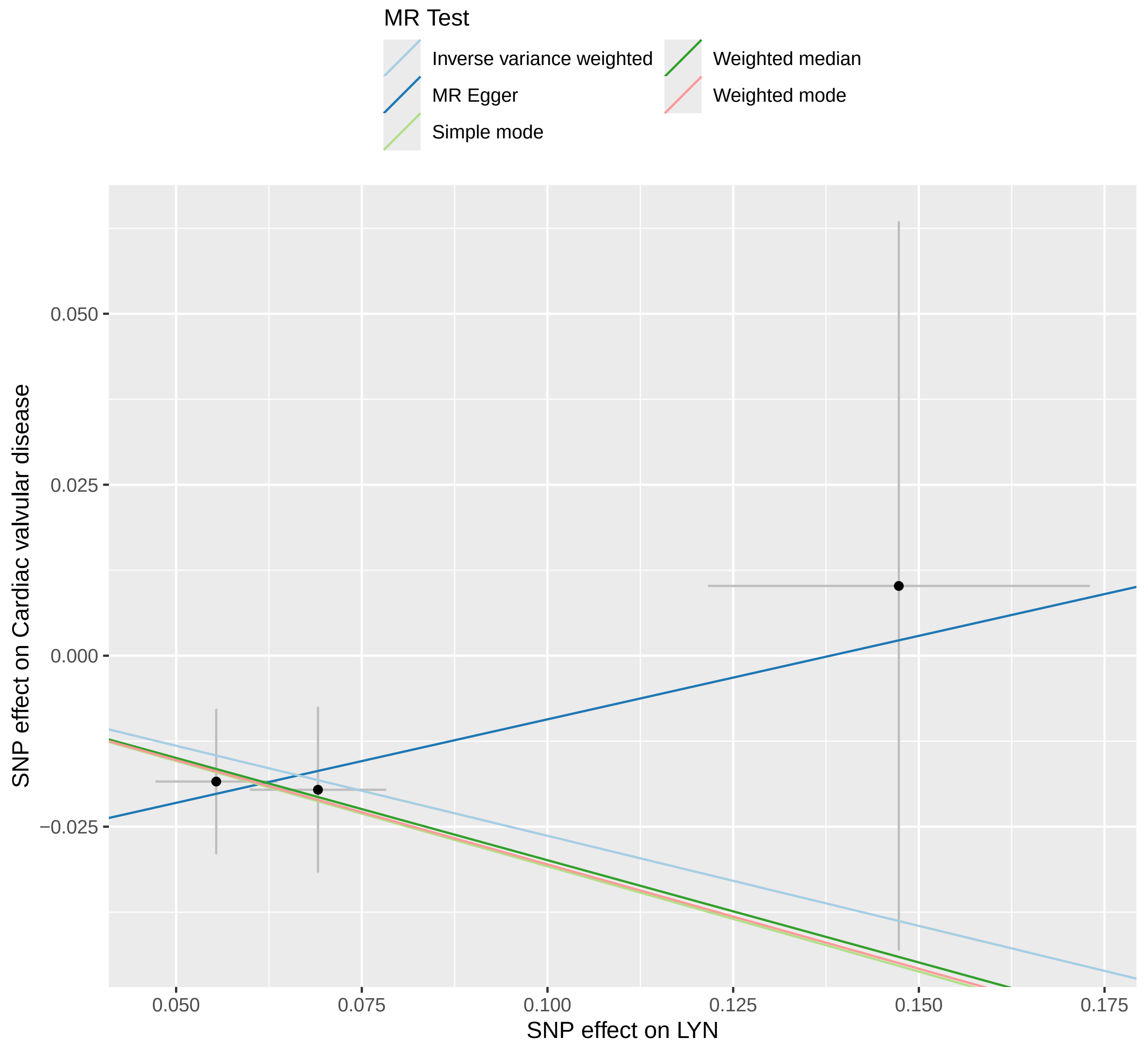
MR Test
Inverse variance weighted
MR Egger
Simple mode
Weighted median
Weighted mode
SNP effect on Cardiac valvular disease
0.050
0.025
0.000
−0.025
0.050
0.075
0.100
0.125
0.150
0.175
SNP effect on LYN

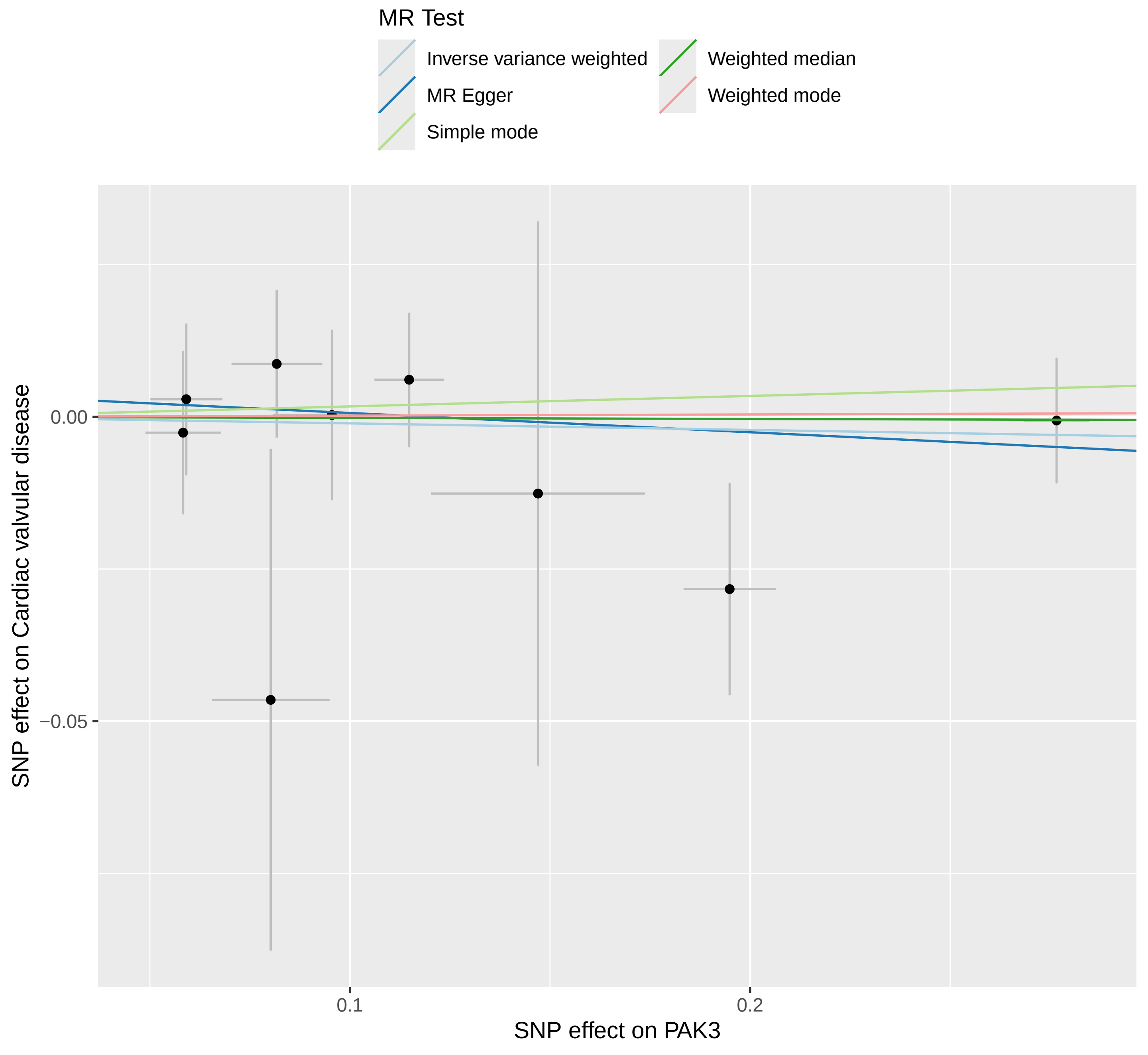
MR Test
Inverse variance weighted
MR Egger
Simple mode
Weighted median
Weighted mode
0.00
−0.05
0.1
0.2
SNP effect on PAK3
SNP effect on Cardiac valvular disease

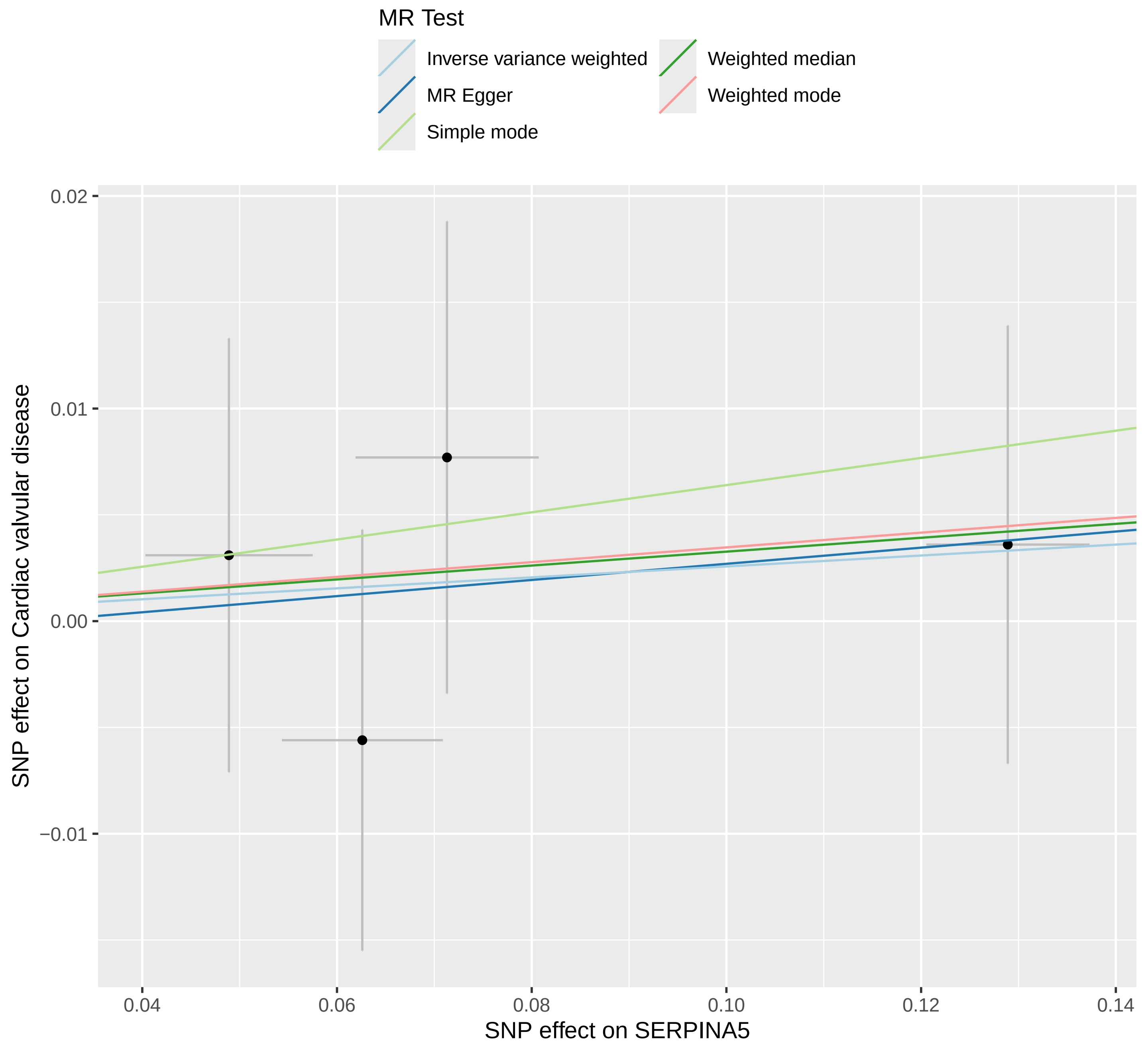
MR Test
Inverse variance weighted
MR Egger
Simple mode
Weighted median
Weighted mode
0.02
0.01
0.00
−0.01
SNP effect on Cardiac valvular disease
0.04
0.06
0.08
0.10
0.12
0.14
SNP effect on SERPINA5

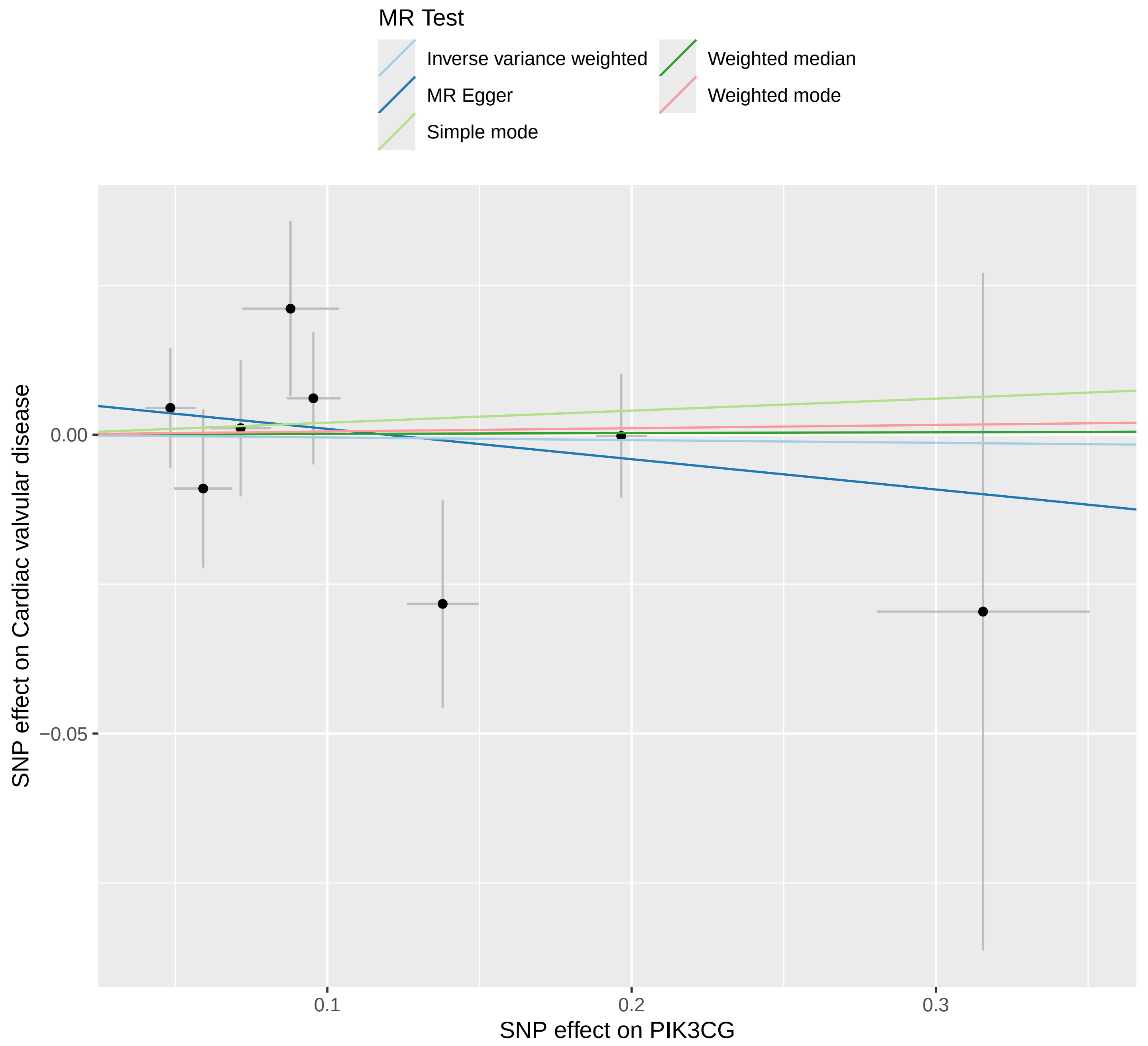

MR Test
Inverse variance weighted
MR Egger
Simple mode
Weighted median
Weighted mode
0.00
−0.05
0.1
0.2
0.3
SNP effect on Cardiac valvular disease
SNP effect on PIK3CG

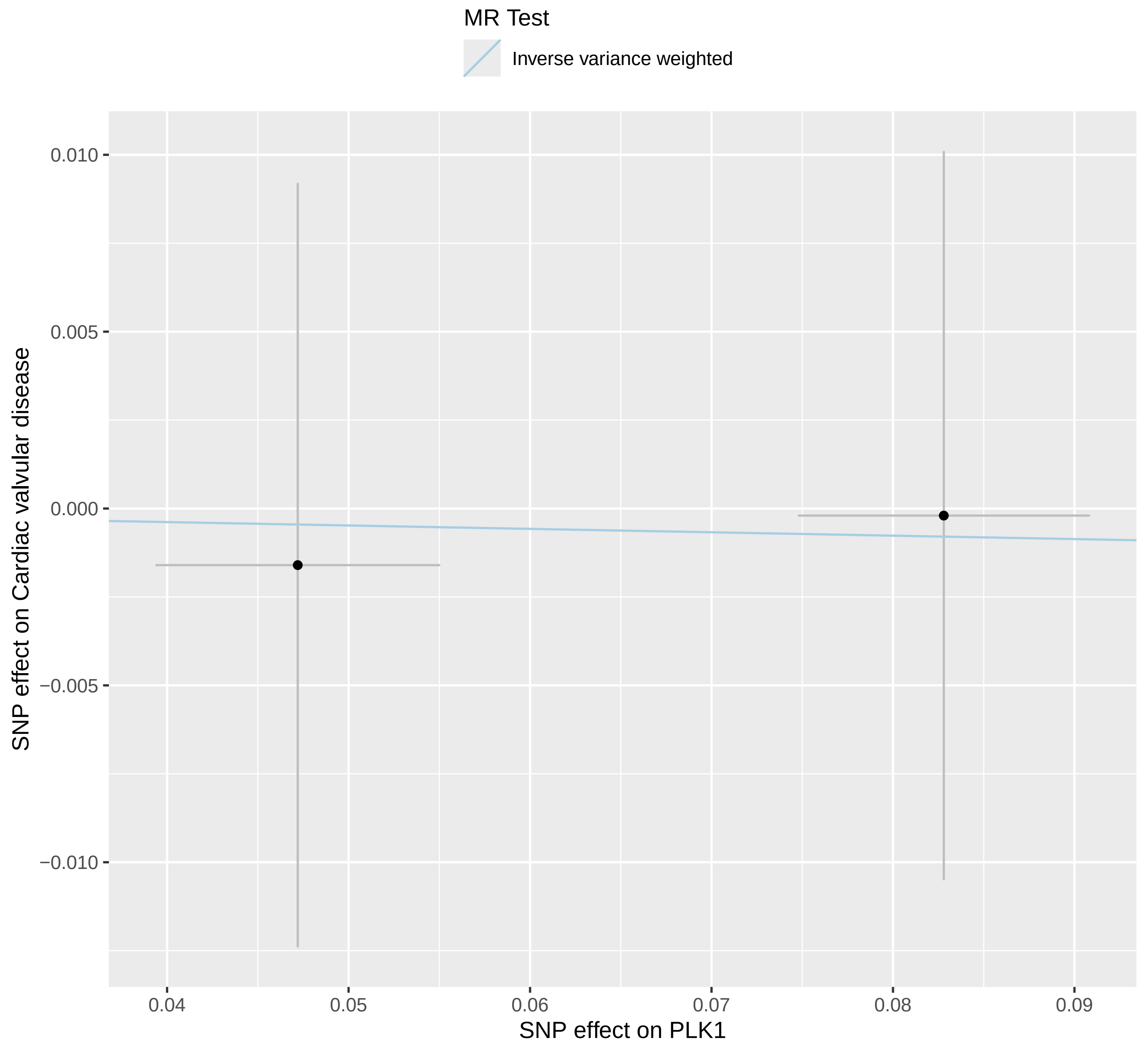

MR Test
Inverse variance weighted
0.010
0.005
0.000
−0.005
−0.010
0.04
0.05
0.06
0.07
0.08
0.09
SNP effect on PLK1
SNP effect on Cardiac valvular disease

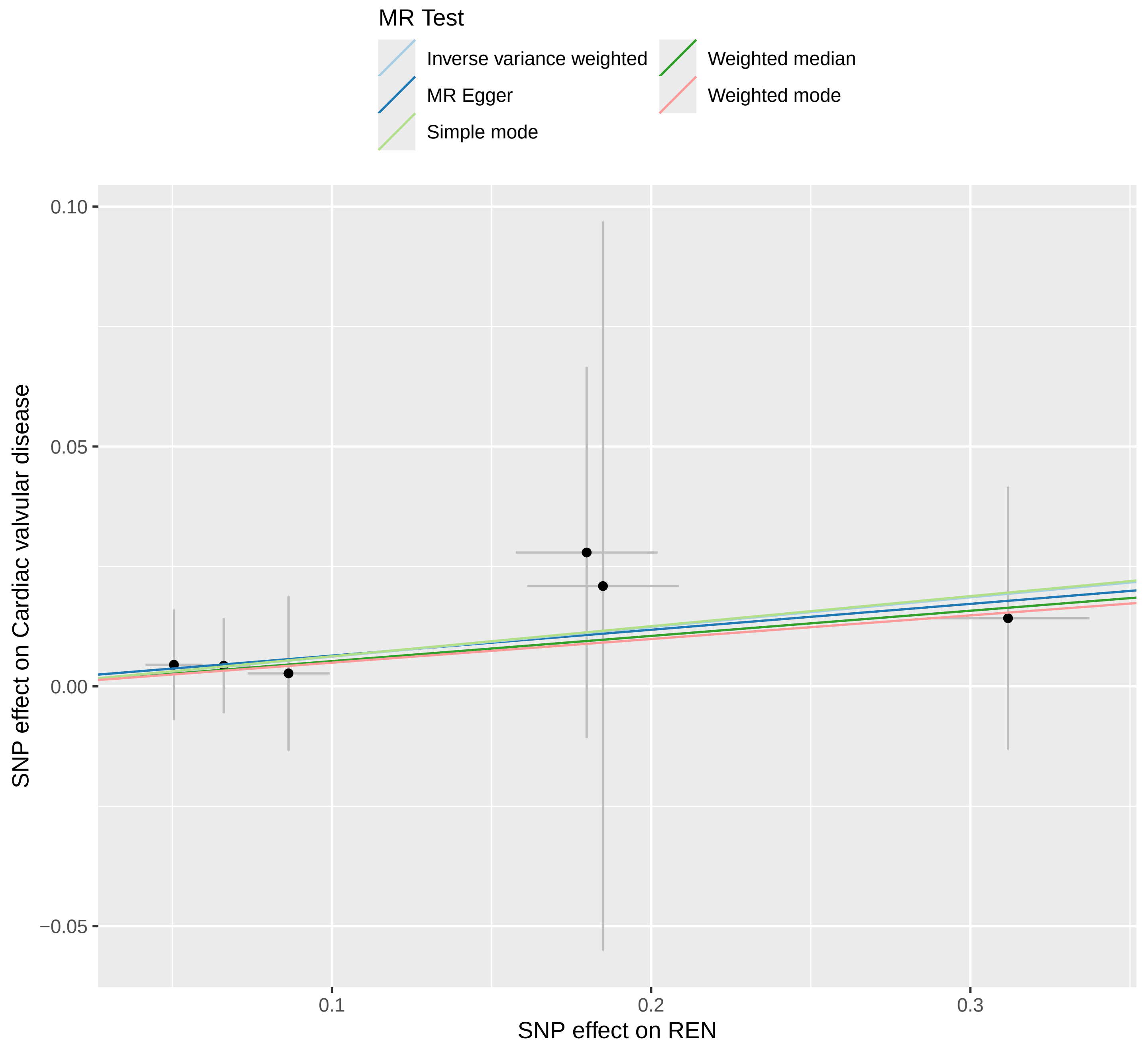
MR Test
Inverse variance weighted
MR Egger
Simple mode
Weighted median
Weighted mode
0.10
0.05
0.00
−0.05
0.1
0.2
0.3
SNP effect on Cardiac valvular disease
SNP effect on REN

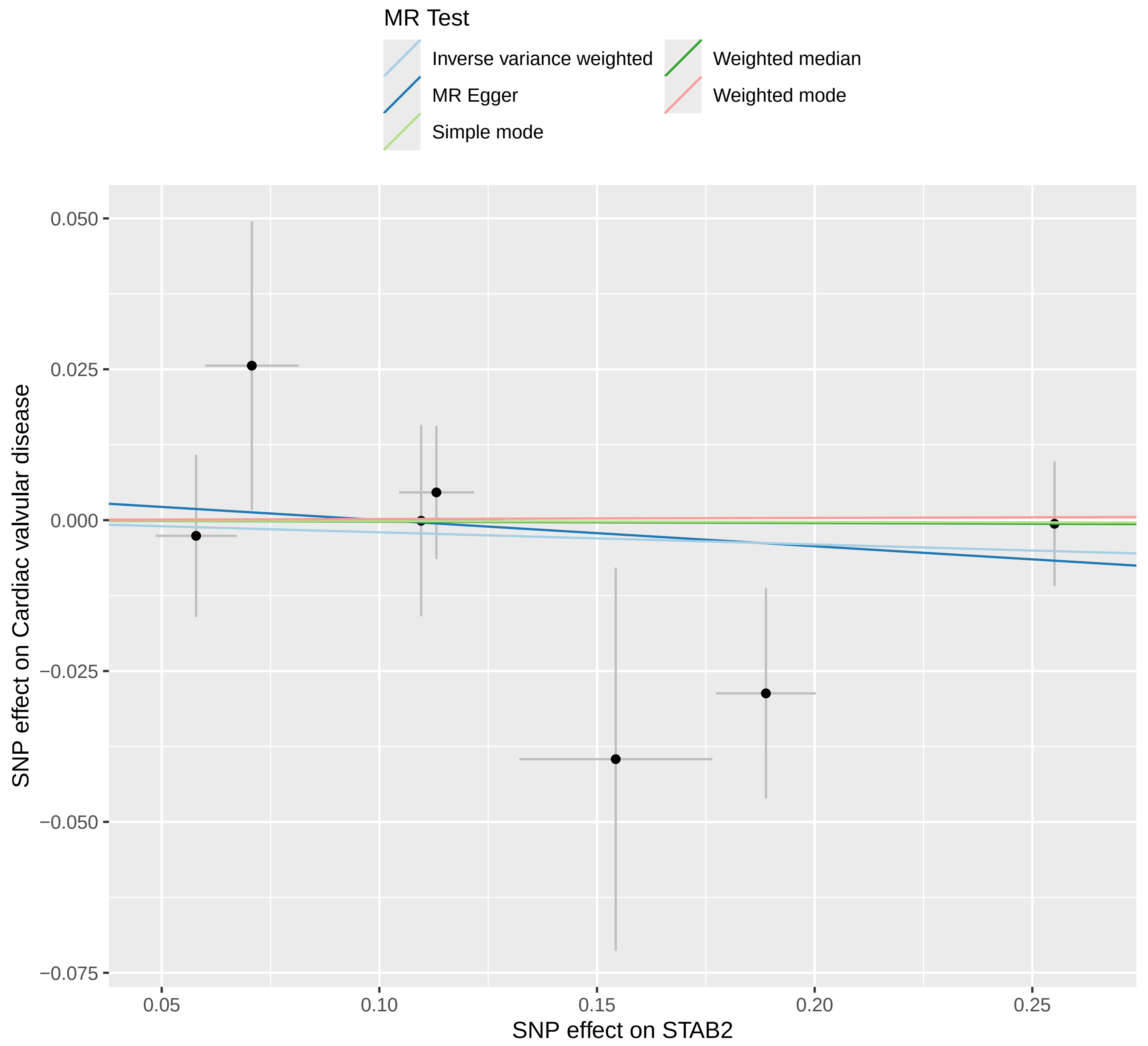
MR Test
Inverse variance weighted
MR Egger
Simple mode
Weighted median
Weighted mode
0.050
0.025
0.000
−0.025
−0.050
−0.075
SNP effect on Cardiac valvular disease
0.05
0.10
0.15
0.20
0.25
SNP effect on STAB2

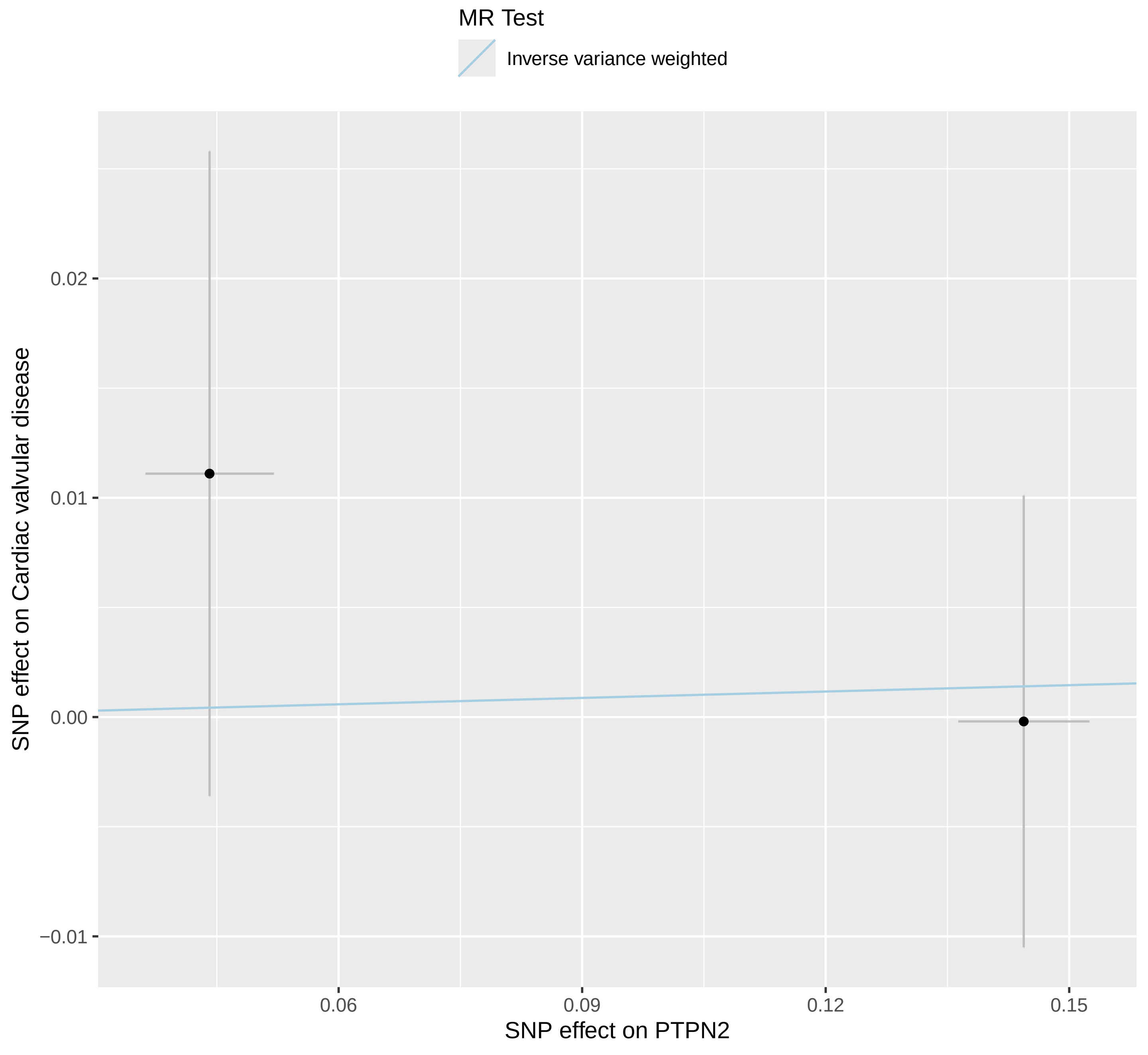

MR Test
Inverse variance weighted
0.02
0.01
0.00
−0.01
0.06
0.09
0.12
0.15
SNP effect on Cardiac valvular disease
SNP effect on PTPN2

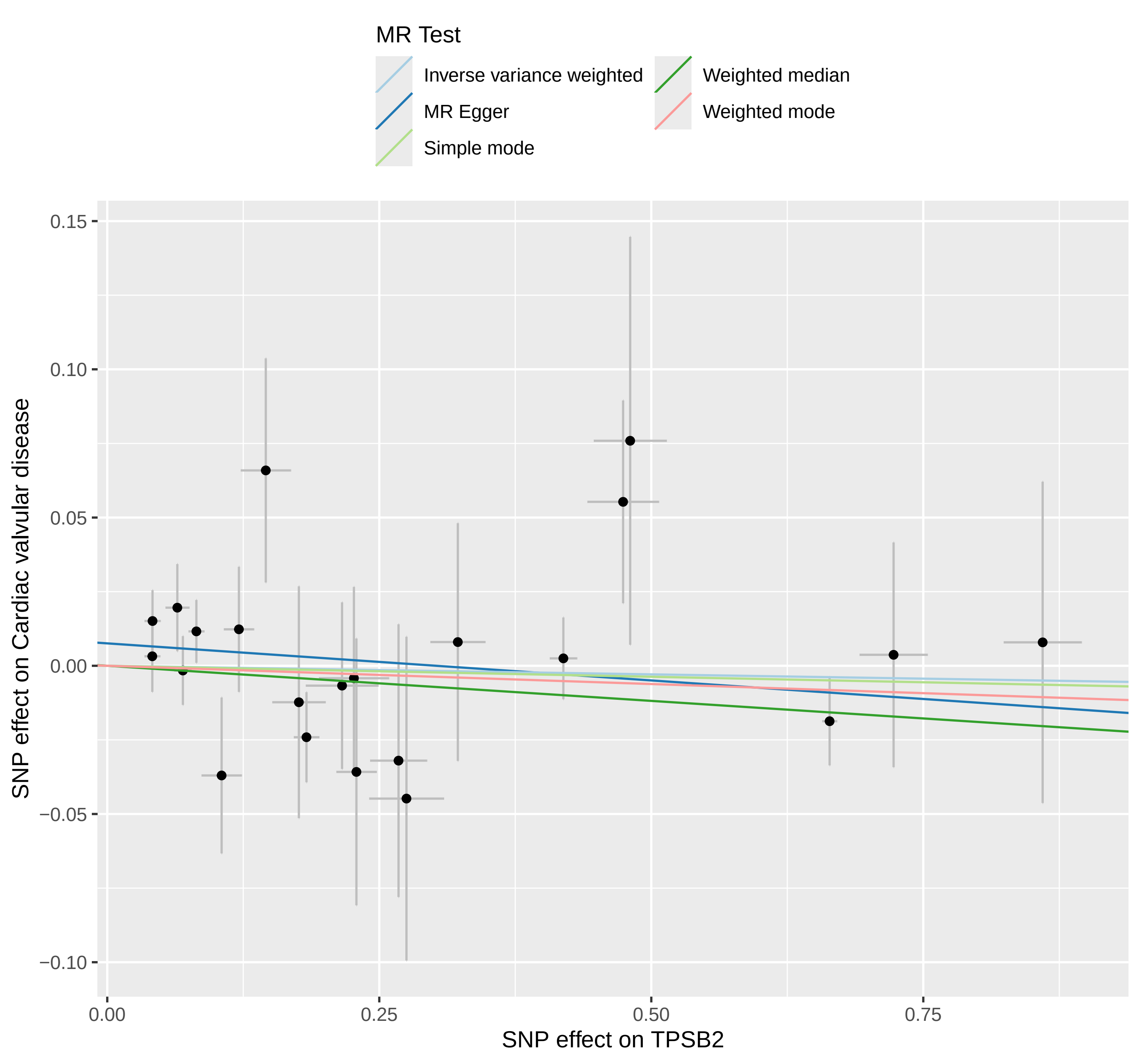
MR Test
Inverse variance weighted
MR Egger
Simple mode
Weighted median
Weighted mode
0.15
0.10
0.05
0.00
−0.05
−0.10
0.00
0.25
0.50
0.75
SNP effect on Cardiac valvular disease
SNP effect on TPSB2

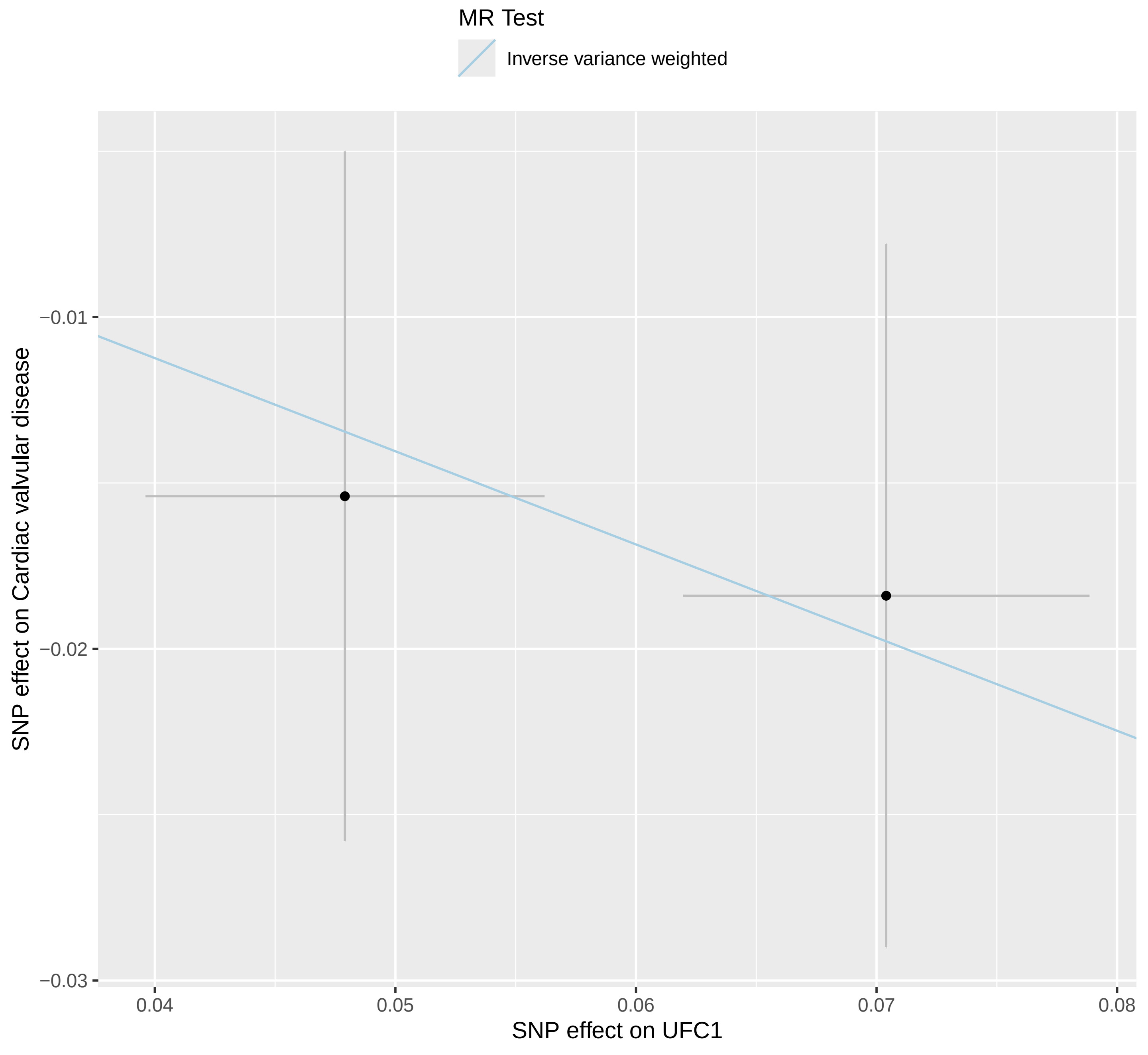
MR Test
Inverse variance weighted
−0.01
−0.02
−0.03
0.04
0.05
0.06
0.07
0.08
SNP effect on UFC1
SNP effect on Cardiac valvular disease

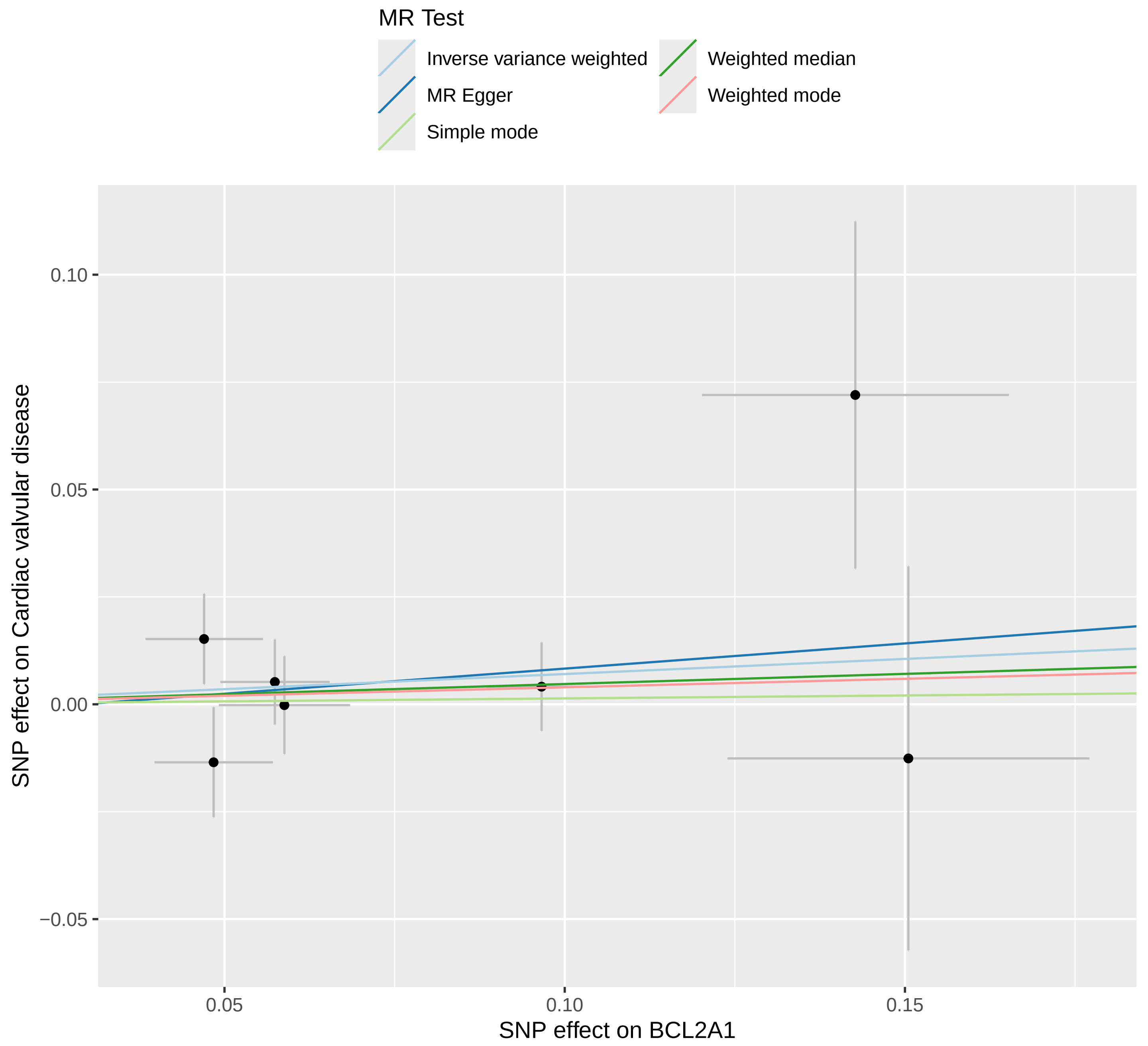
MR Test
Inverse variance weighted
MR Egger
Simple mode
Weighted median
Weighted mode
SNP effect on Cardiac valvular disease
0.10
0.05
0.00
−0.05
0.05
0.10
0.15
SNP effect on BCL2A1

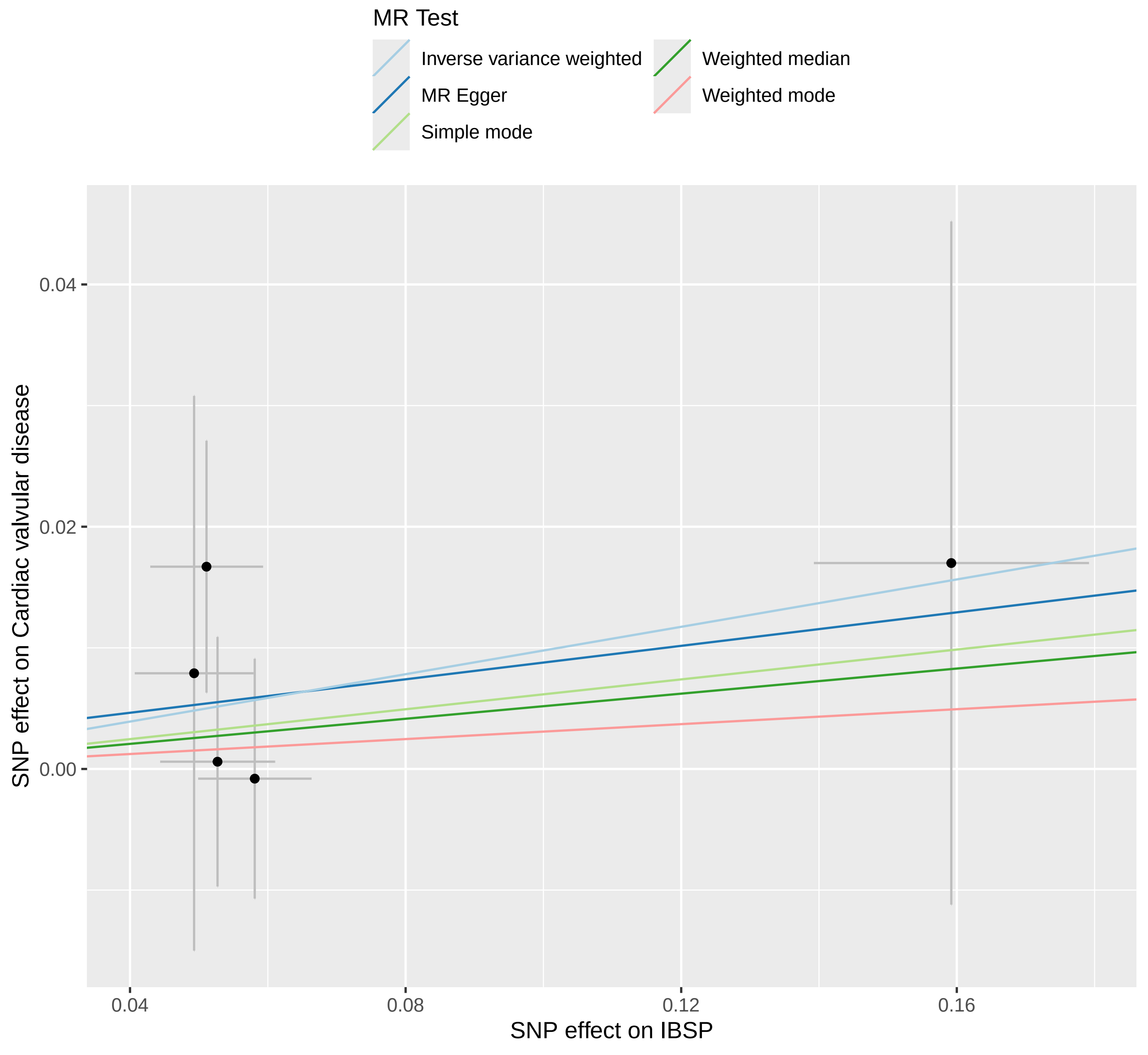
MR Test
Inverse variance weighted
MR Egger
Simple mode
Weighted median
Weighted mode
0.04
0.02
0.00
0.04
0.08
0.12
0.16
SNP effect on Cardiac valvular disease
SNP effect on IBSP

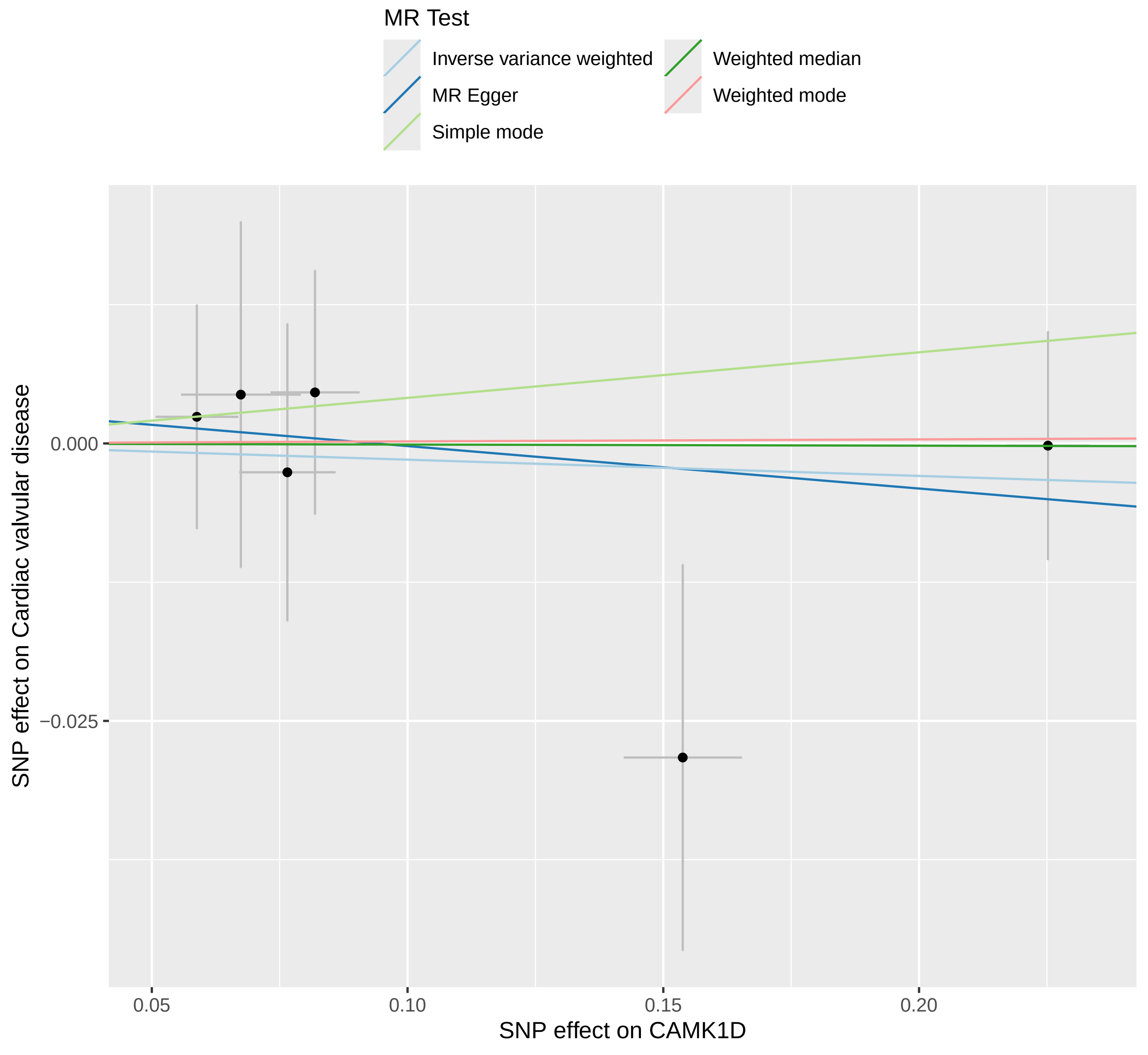

MR Test
Inverse variance weighted
MR Egger
Simple mode
Weighted median
Weighted mode
SNP effect on Cardiac valvular disease
0.000
−0.025
0.05
0.10
0.15
0.20
SNP effect on CAMK1D

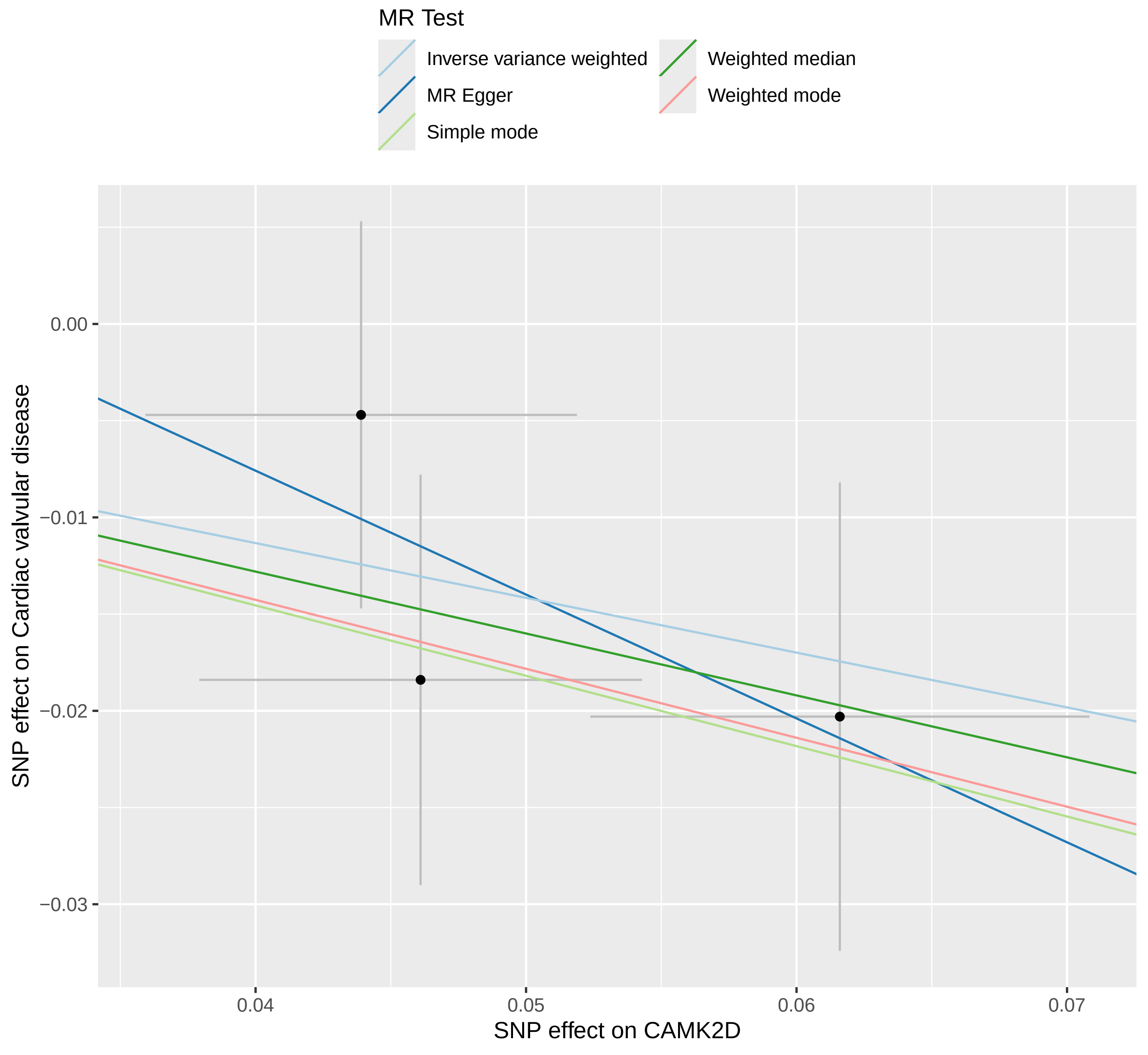
MR Test
Inverse variance weighted
MR Egger
Simple mode
Weighted median
Weighted mode
SNP effect on Cardiac valvular disease
0.00
−0.01
−0.02
−0.03
0.04
0.05
0.06
0.07
SNP effect on CAMK2D

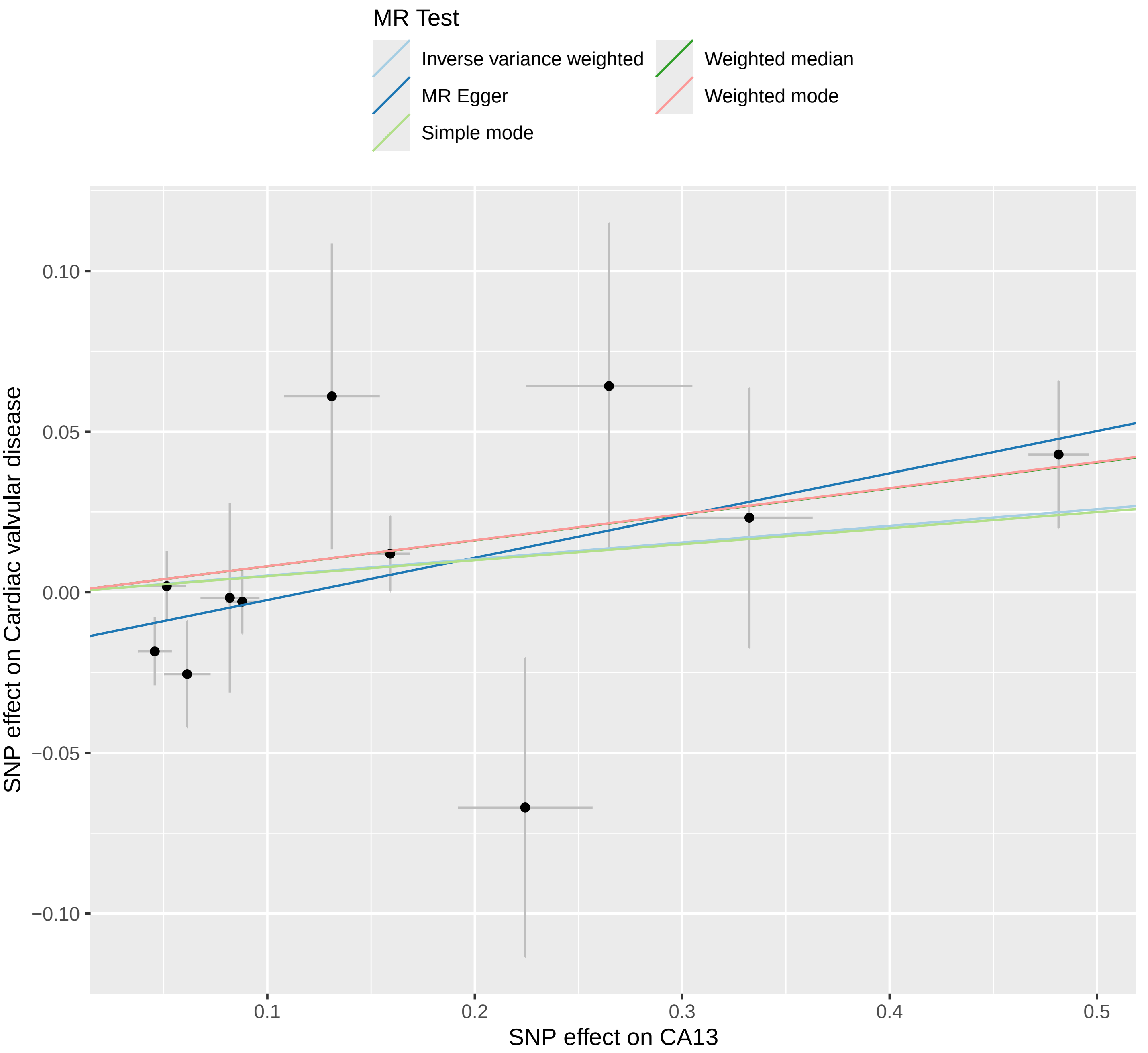
MR Test
Inverse variance weighted
MR Egger
Simple mode
Weighted median
Weighted mode
0.10
0.05
0.00
−0.05
−0.10
SNP effect on Cardiac valvular disease
0.1
0.2
0.3
0.4
0.5
SNP effect on CA13

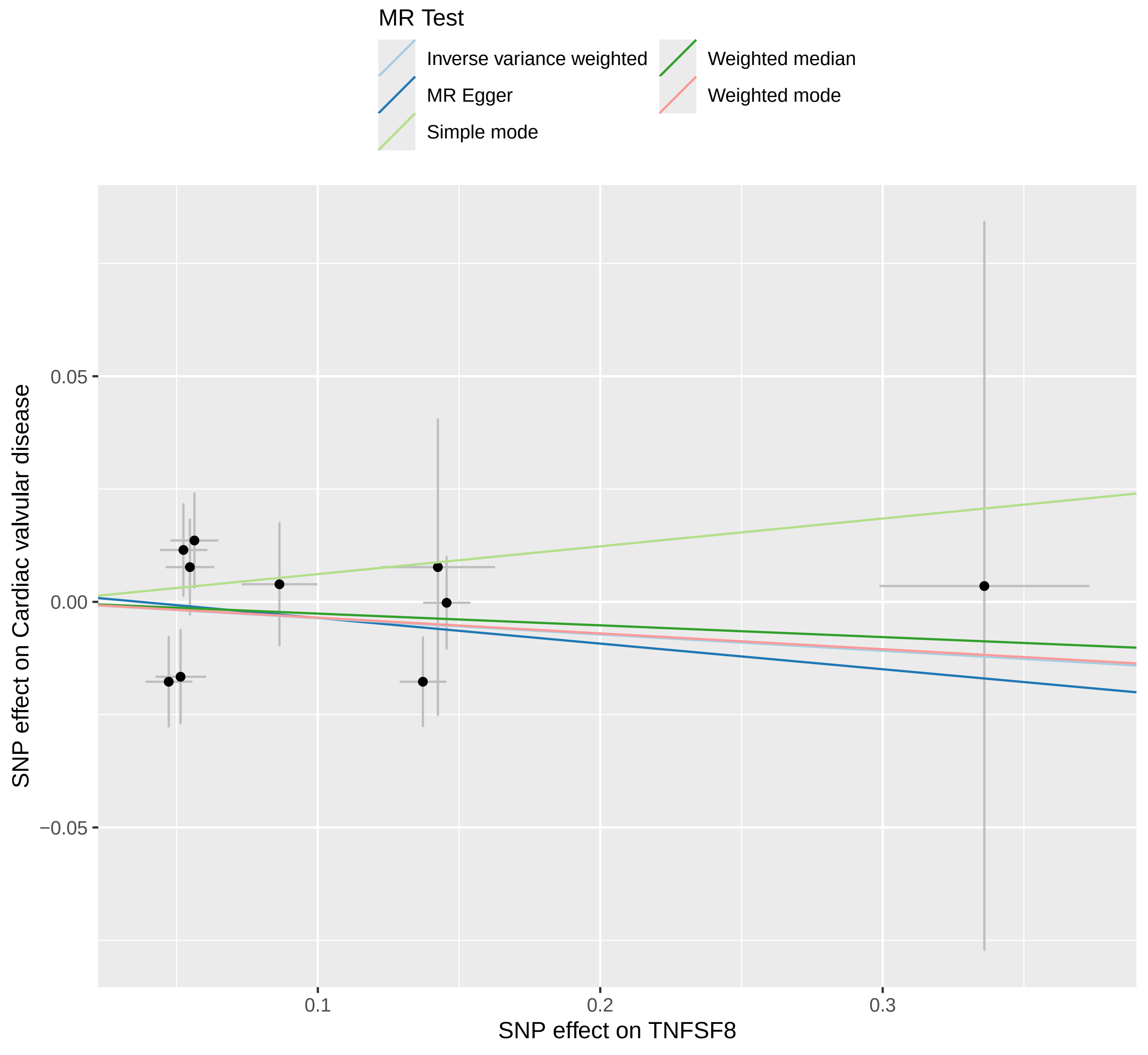

MR Test
Inverse variance weighted
MR Egger
Simple mode
Weighted median
Weighted mode
0.05
0.00
−0.05
0.1
0.2
0.3
SNP effect on Cardiac valvular disease
SNP effect on TNFSF8

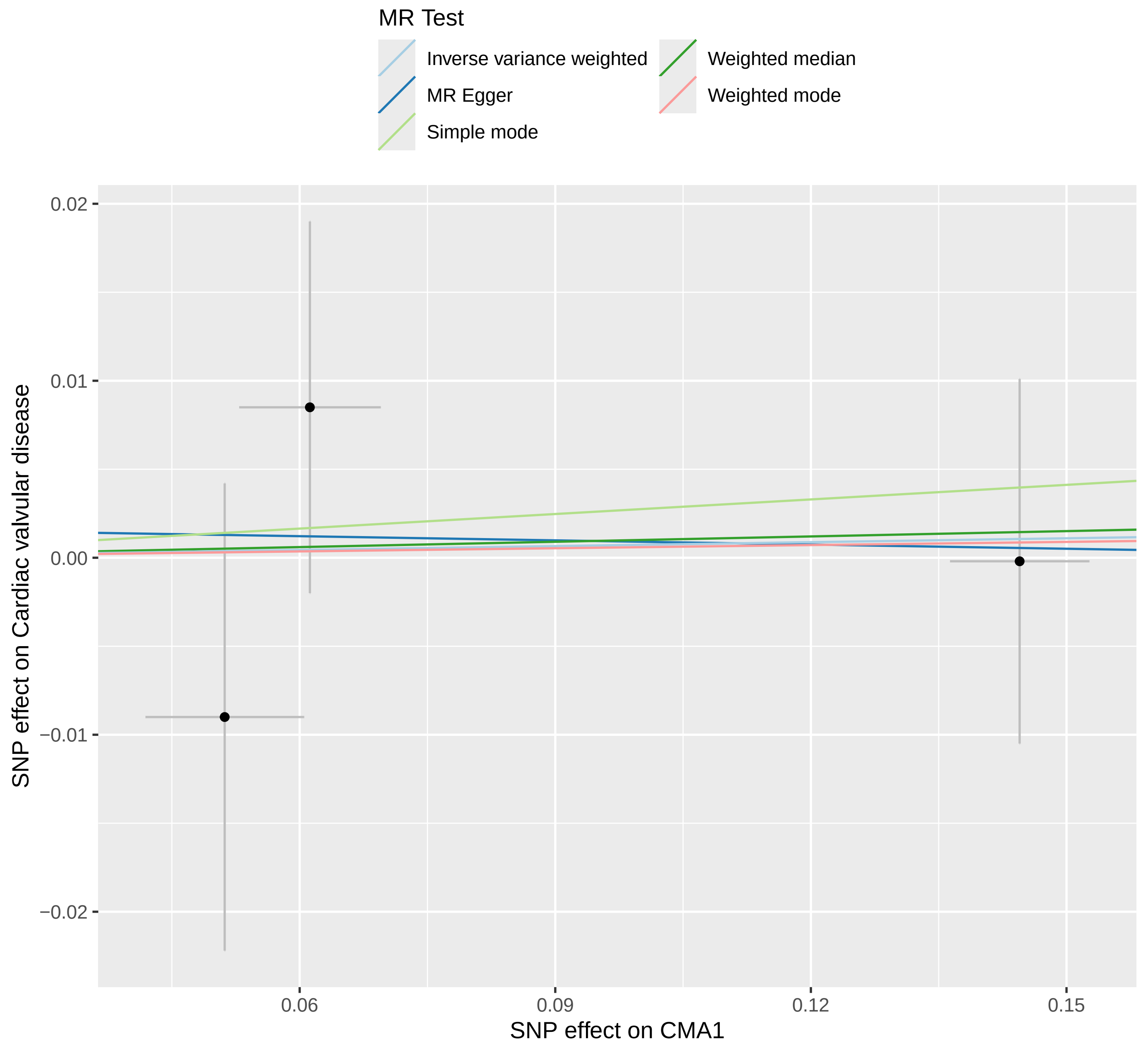
MR Test
Inverse variance weighted
MR Egger
Simple mode
Weighted median
Weighted mode
0.02
0.01
0.00
−0.01
−0.02
SNP effect on Cardiac valvular disease
0.06
0.09
0.12
0.15
SNP effect on CMA1

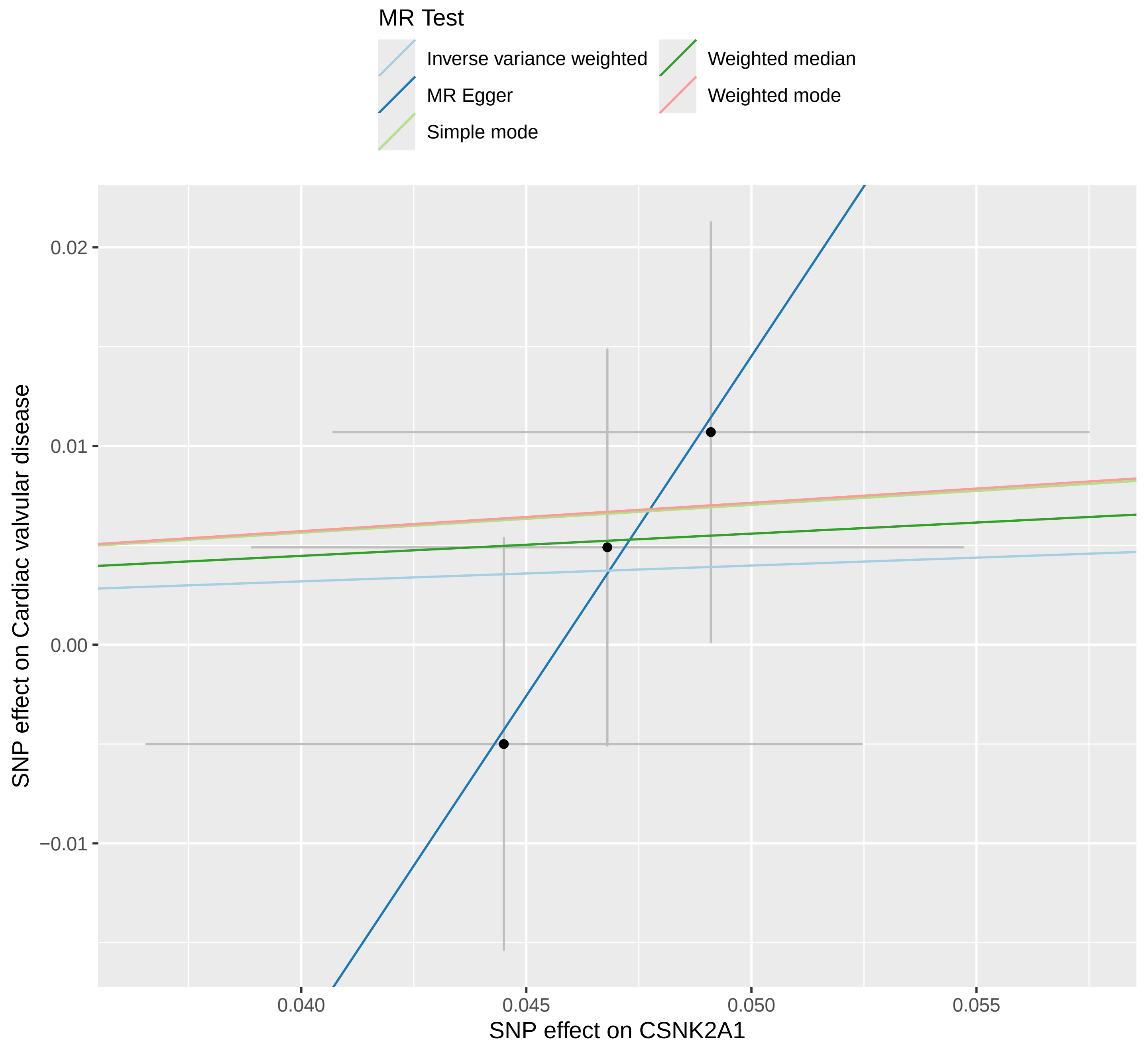
MR Test
Inverse variance weighted
MR Egger
Simple mode
Weighted median
Weighted mode
0.02
0.01
0.00
−0.01
SNP effect on Cardiac valvular disease
0.040
0.045
0.050
0.055
SNP effect on CSNK2A1

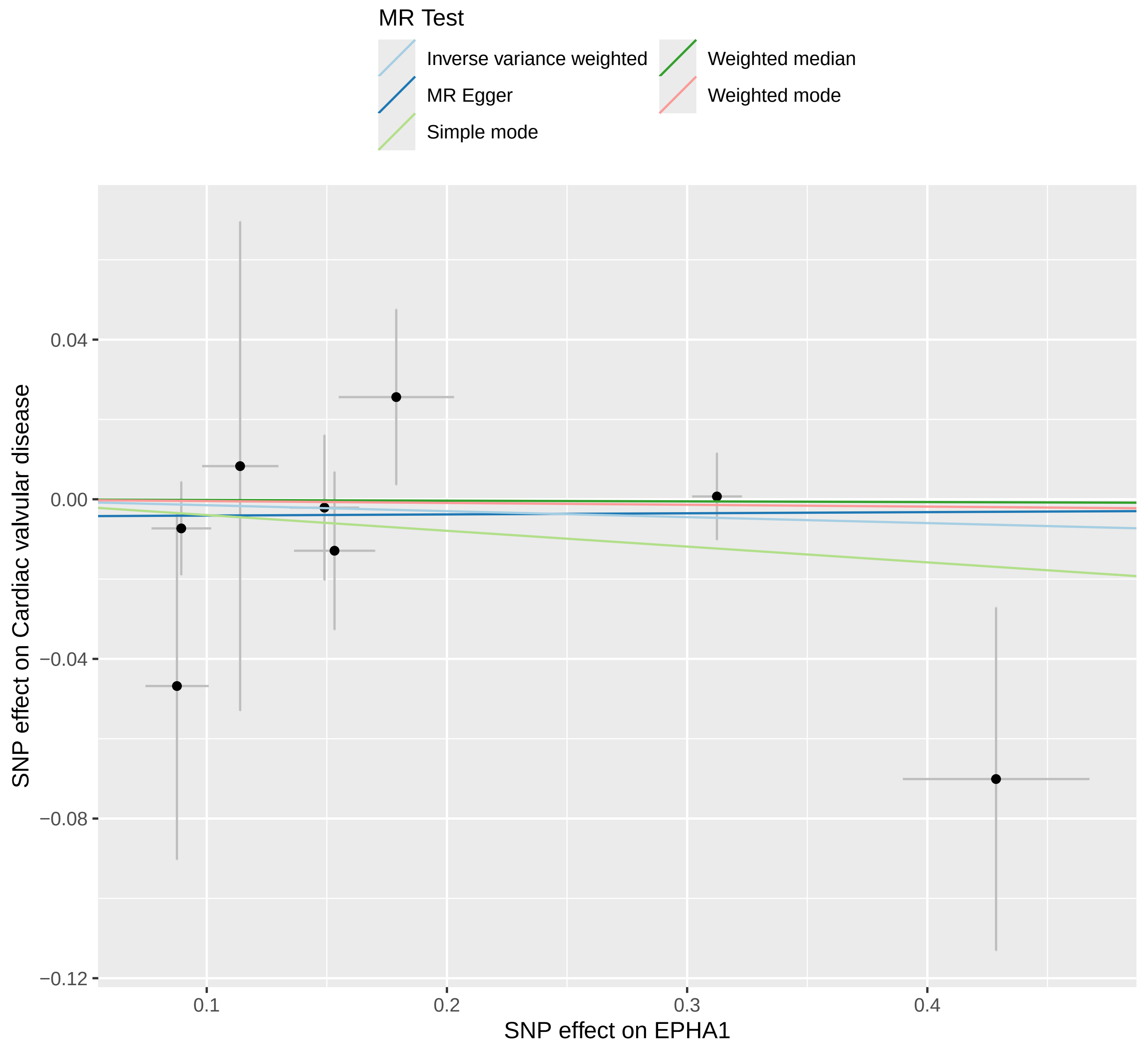
MR Test
Inverse variance weighted
MR Egger
Simple mode
Weighted median
Weighted mode
0.04
0.00
−0.04
−0.08
−0.12
SNP effect on Cardiac valvular disease
0.1
0.2
0.3
0.4
SNP effect on EPHA1

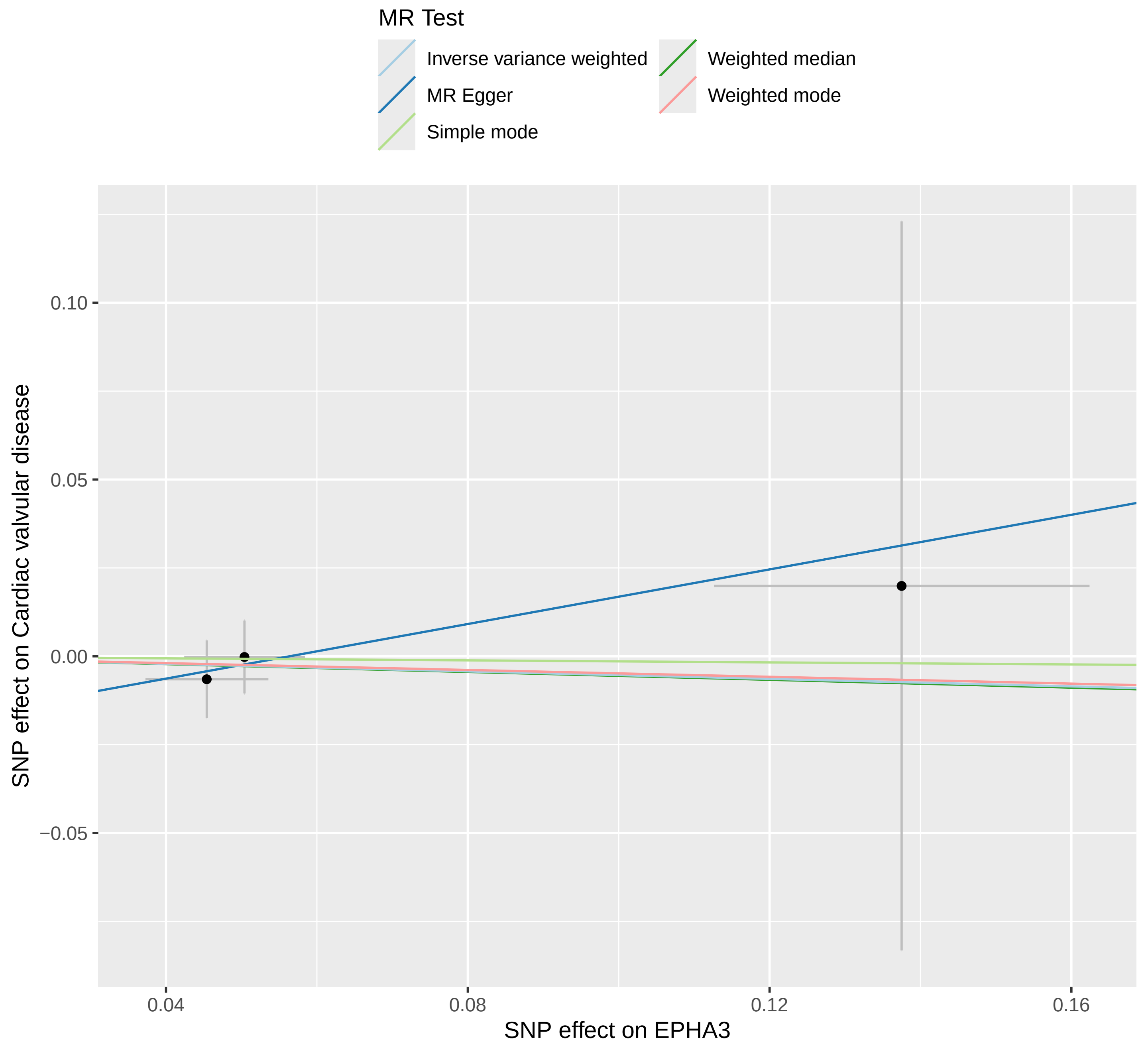
MR Test
Inverse variance weighted
MR Egger
Simple mode
Weighted median
Weighted mode
0.10
0.05
0.00
−0.05
0.04
0.08
0.12
0.16
SNP effect on Cardiac valvular disease
SNP effect on EPHA3

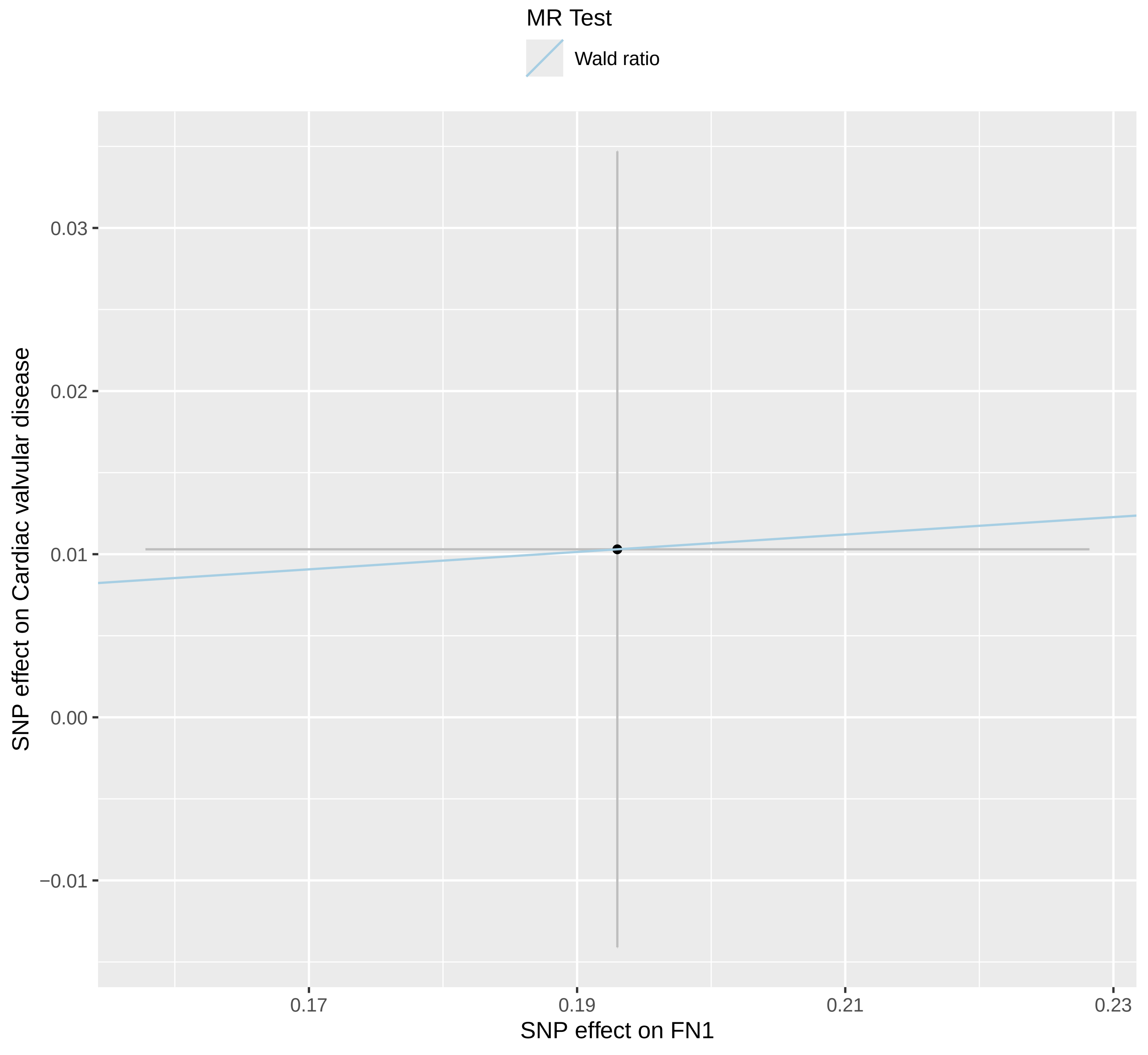

MR Test
Wald ratio
SNP effect on Cardiac valvular disease
0.03
0.02
0.01
0.00
−0.01
0.17
0.19
0.21
0.23
SNP effect on FN1

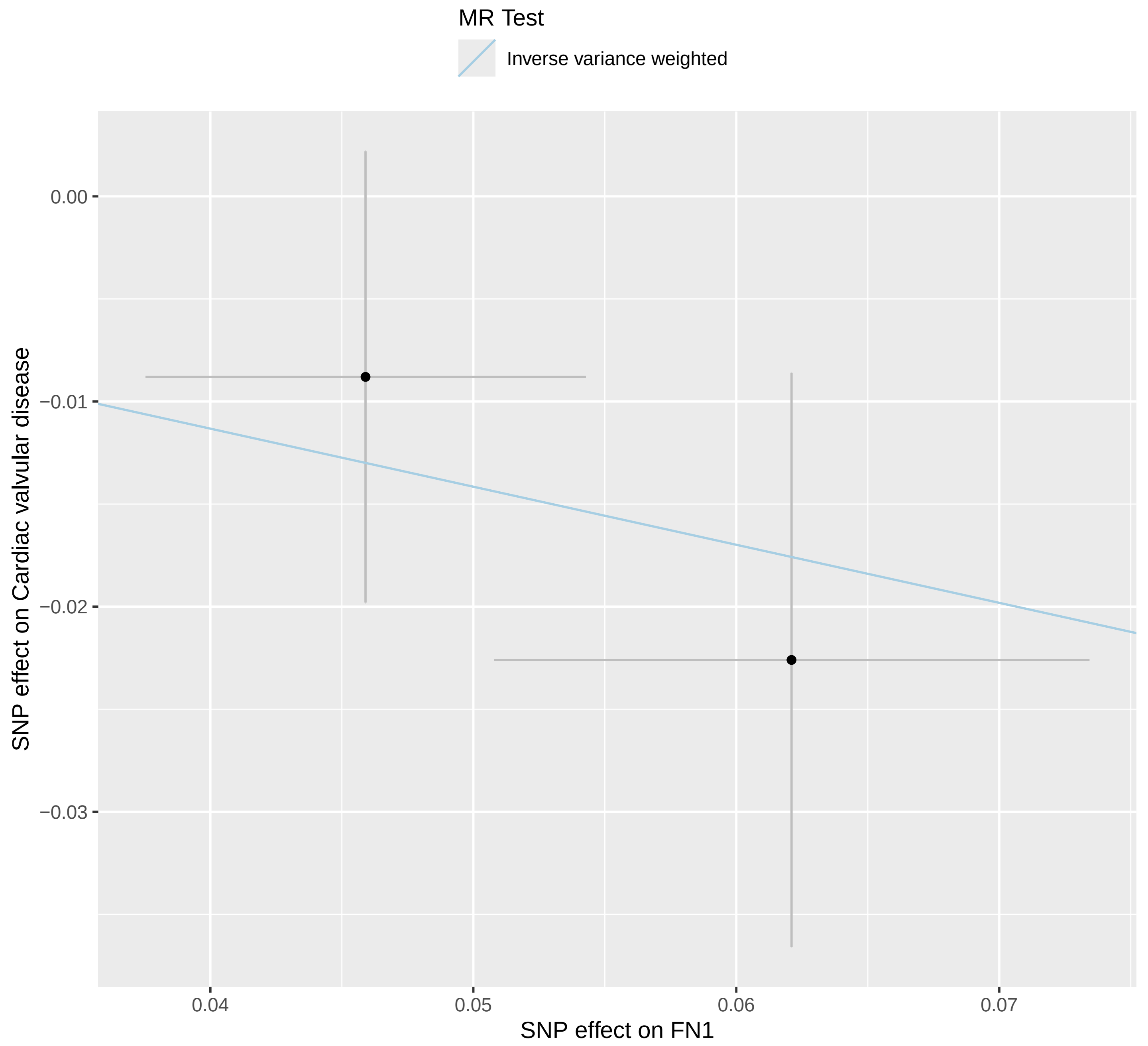
MR Test
Inverse variance weighted
0.00
−0.01
−0.02
−0.03
SNP effect on Cardiac valvular disease
0.04
0.05
0.06
0.07
SNP effect on FN1

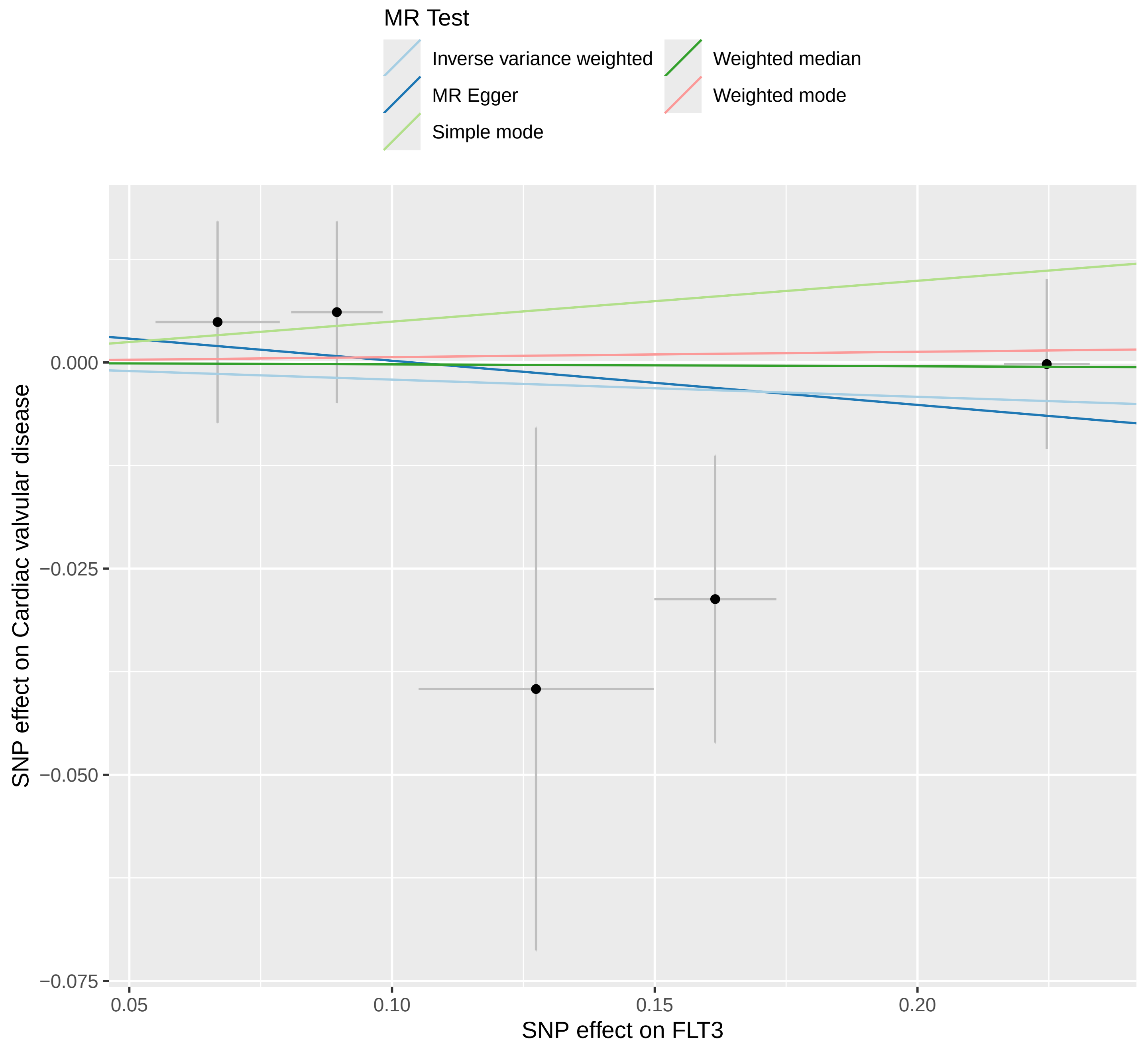
MR Test
Inverse variance weighted
MR Egger
Simple mode
Weighted median
Weighted mode
0.000
−0.025
−0.050
−0.075
SNP effect on Cardiac valvular disease
0.05
0.10
0.15
0.20
SNP effect on FLT3

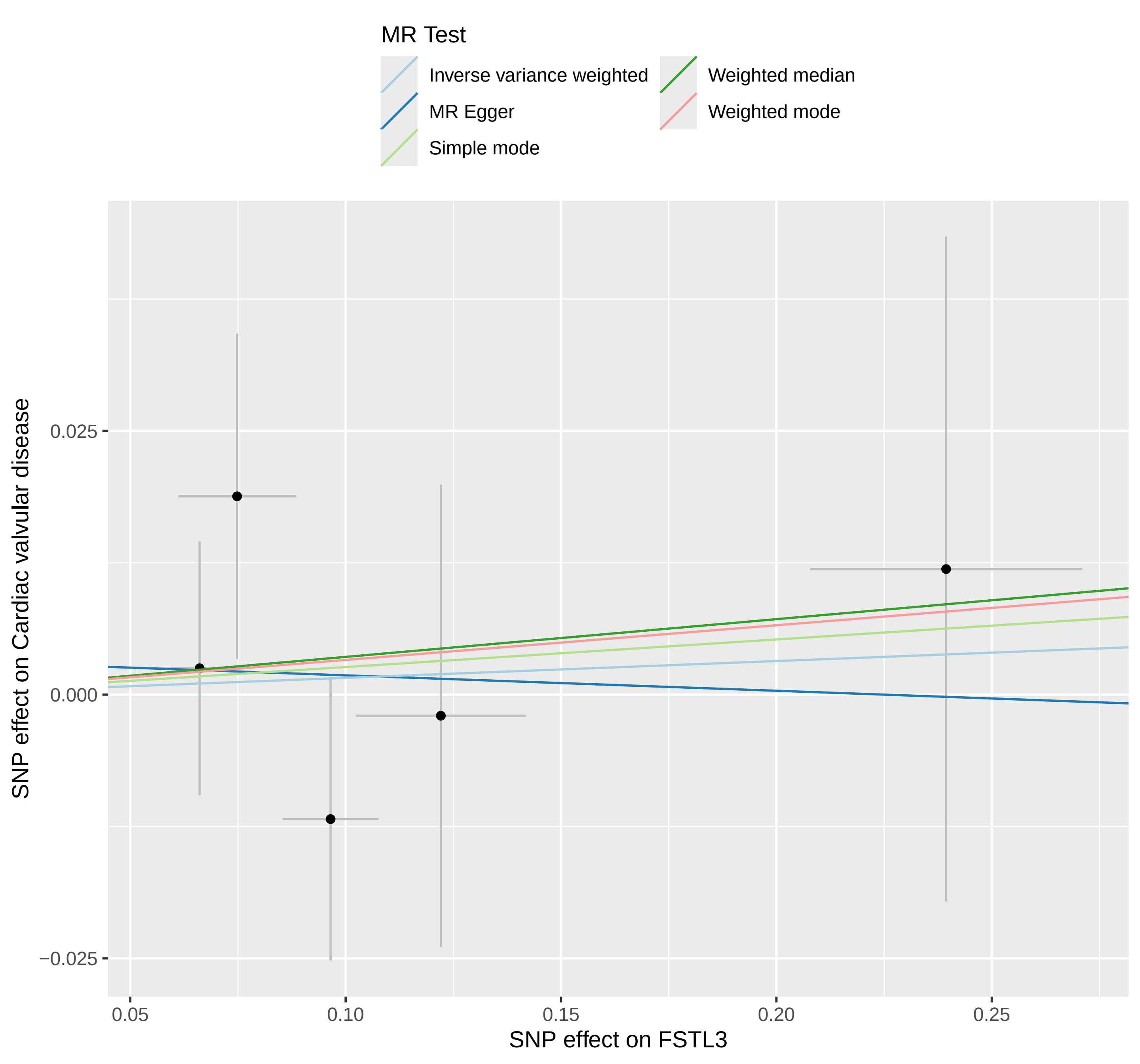
MR Test
Inverse variance weighted
MR Egger
Simple mode
Weighted median
Weighted mode
0.025
0.000
−0.025
0.05
0.10
0.15
0.20
0.25
SNP effect on Cardiac valvular disease
SNP effect on FSTL3

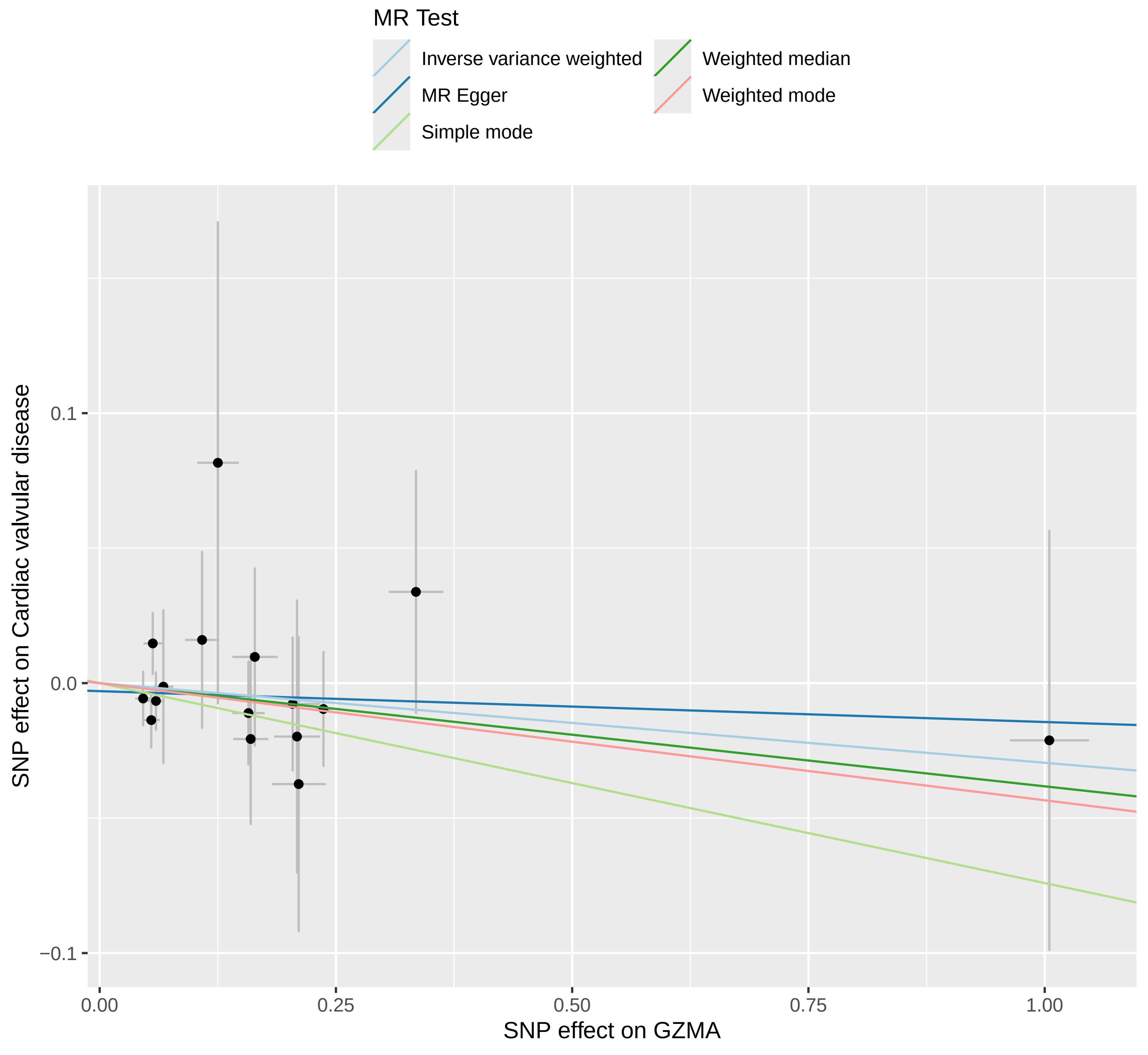
MR Test
Inverse variance weighted
MR Egger
Simple mode
Weighted median
Weighted mode
SNP effect on Cardiac valvular disease
0.1
0.0
−0.1
0.00
0.25
0.50
0.75
1.00
SNP effect on GZMA

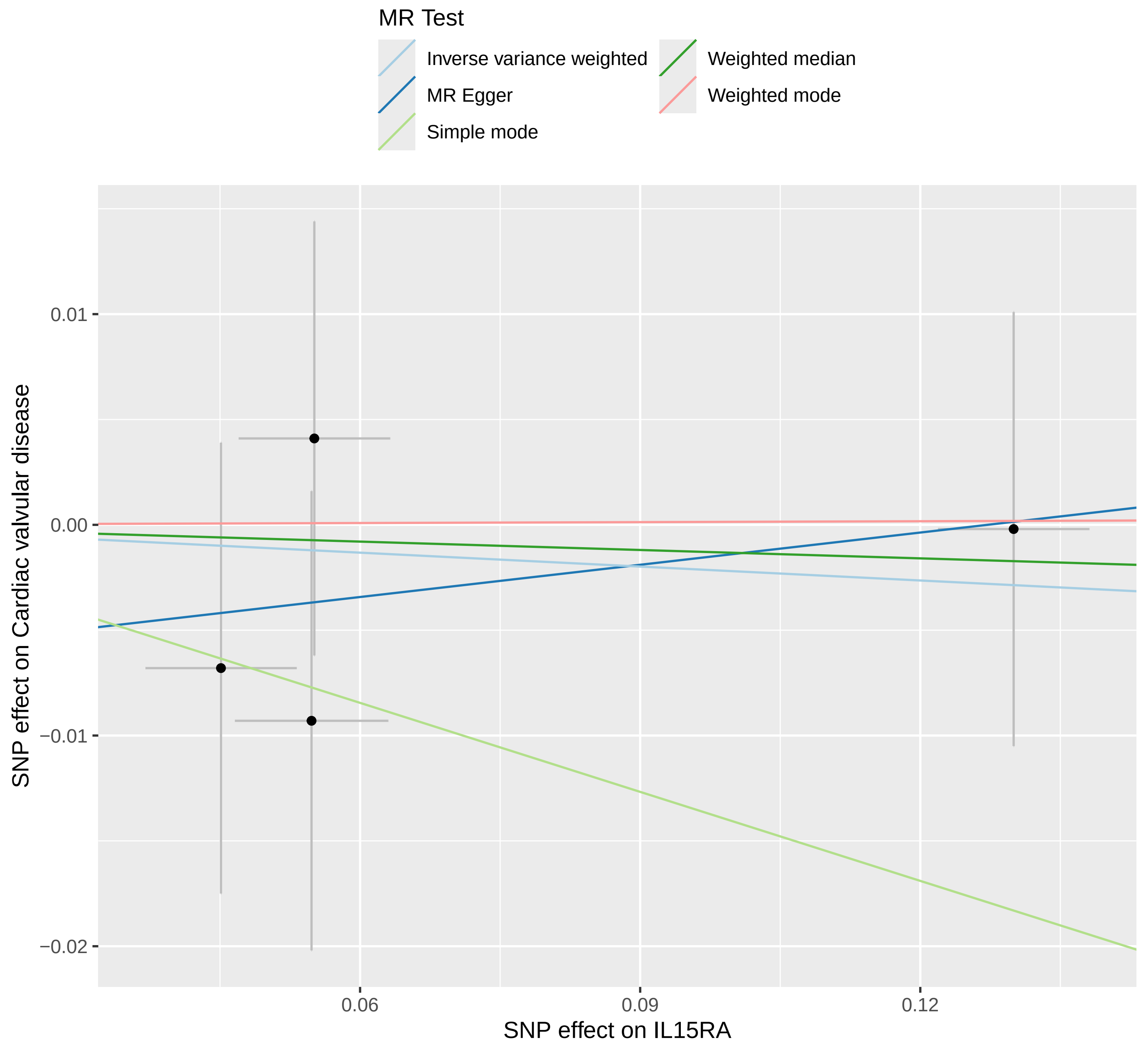

MR Test
Inverse variance weighted
MR Egger
Simple mode
Weighted median
Weighted mode
0.01
0.00
−0.01
−0.02
0.06
0.09
0.12
SNP effect on Cardiac valvular disease
SNP effect on IL15RA

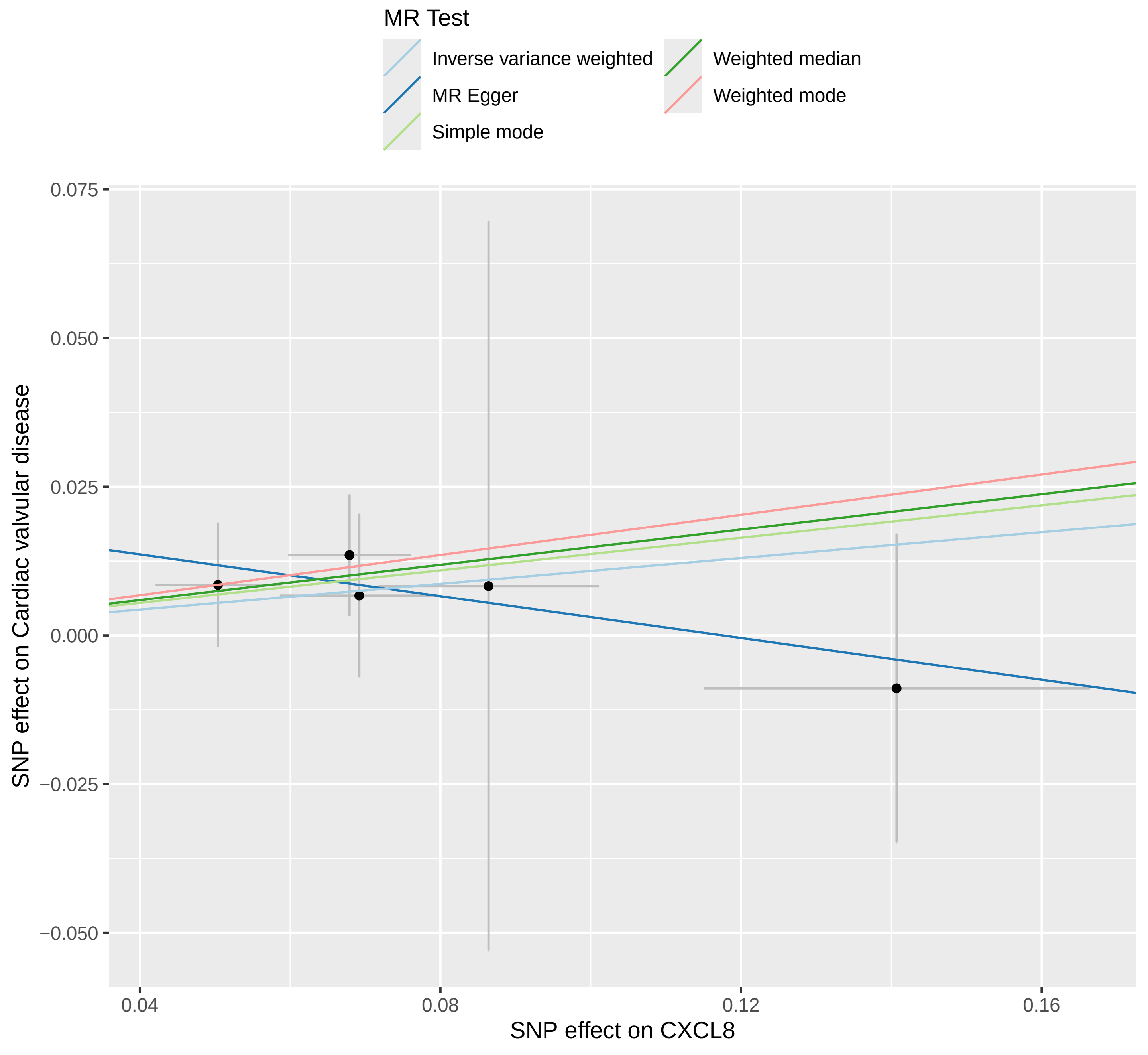
MR Test
Inverse variance weighted
MR Egger
Simple mode
Weighted median
Weighted mode
0.075
0.050
0.025
0.000
−0.025
−0.050
SNP effect on Cardiac valvular disease
0.04
0.08
0.12
0.16
SNP effect on CXCL8

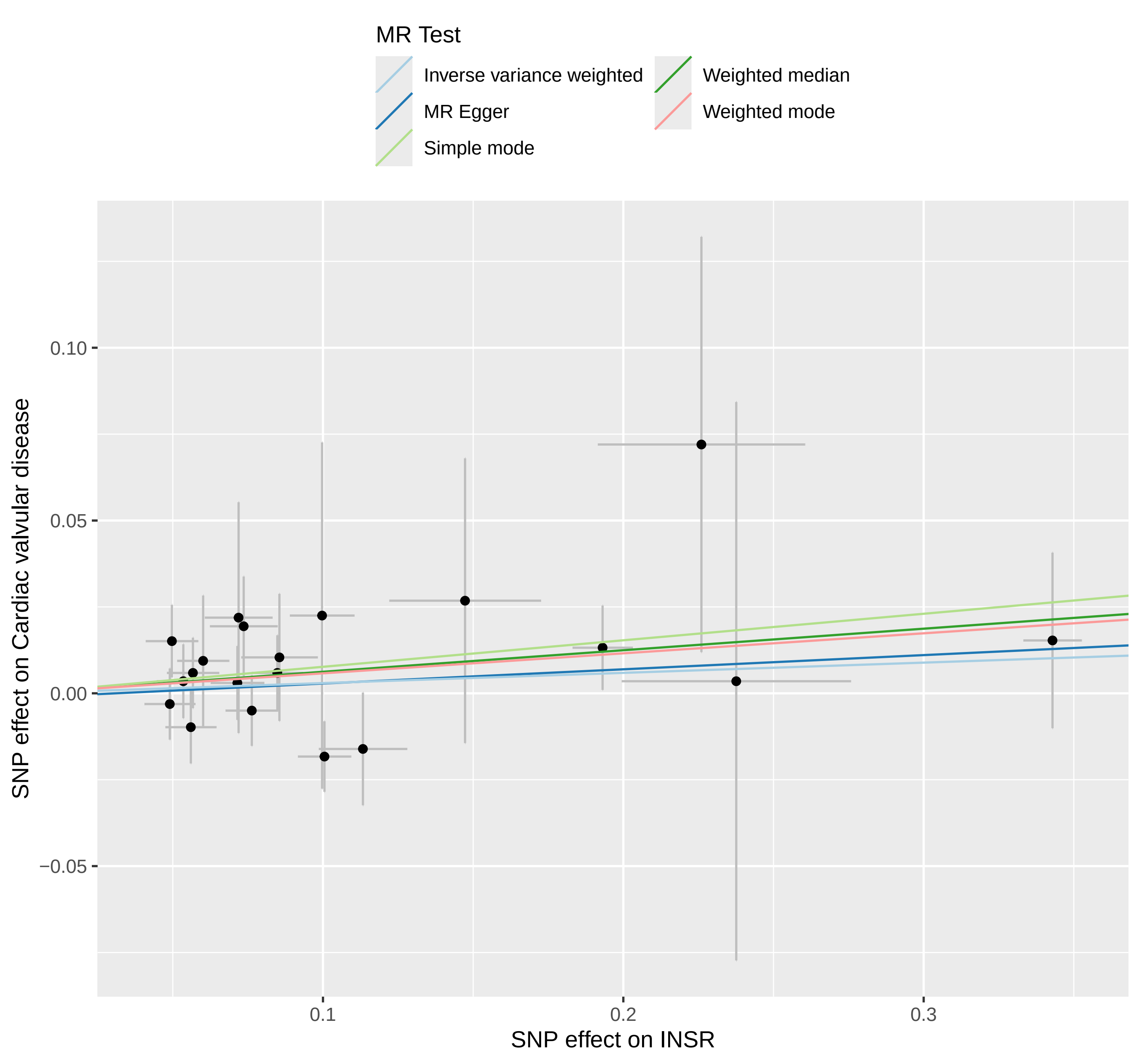
MR Test
Inverse variance weighted
MR Egger
Simple mode
Weighted median
Weighted mode
0.10
0.05
0.00
−0.05
SNP effect on Cardiac valvular disease
0.1
0.2
0.3
SNP effect on INSR

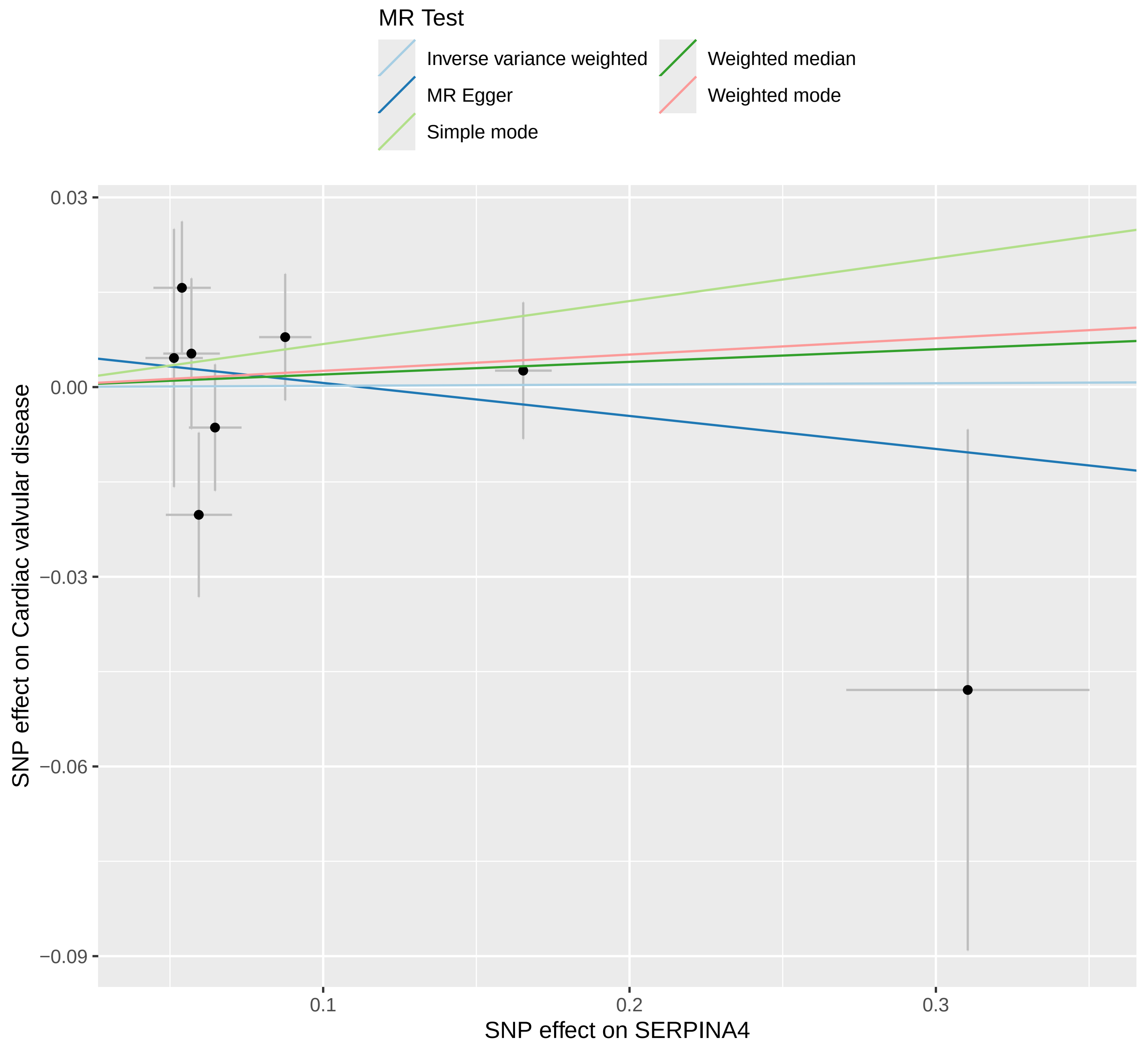
MR Test
Inverse variance weighted
MR Egger
Simple mode
Weighted median
Weighted mode
0.03
0.00
−0.03
−0.06
−0.09
SNP effect on Cardiac valvular disease
0.1
0.2
0.3
SNP effect on SERPINA4

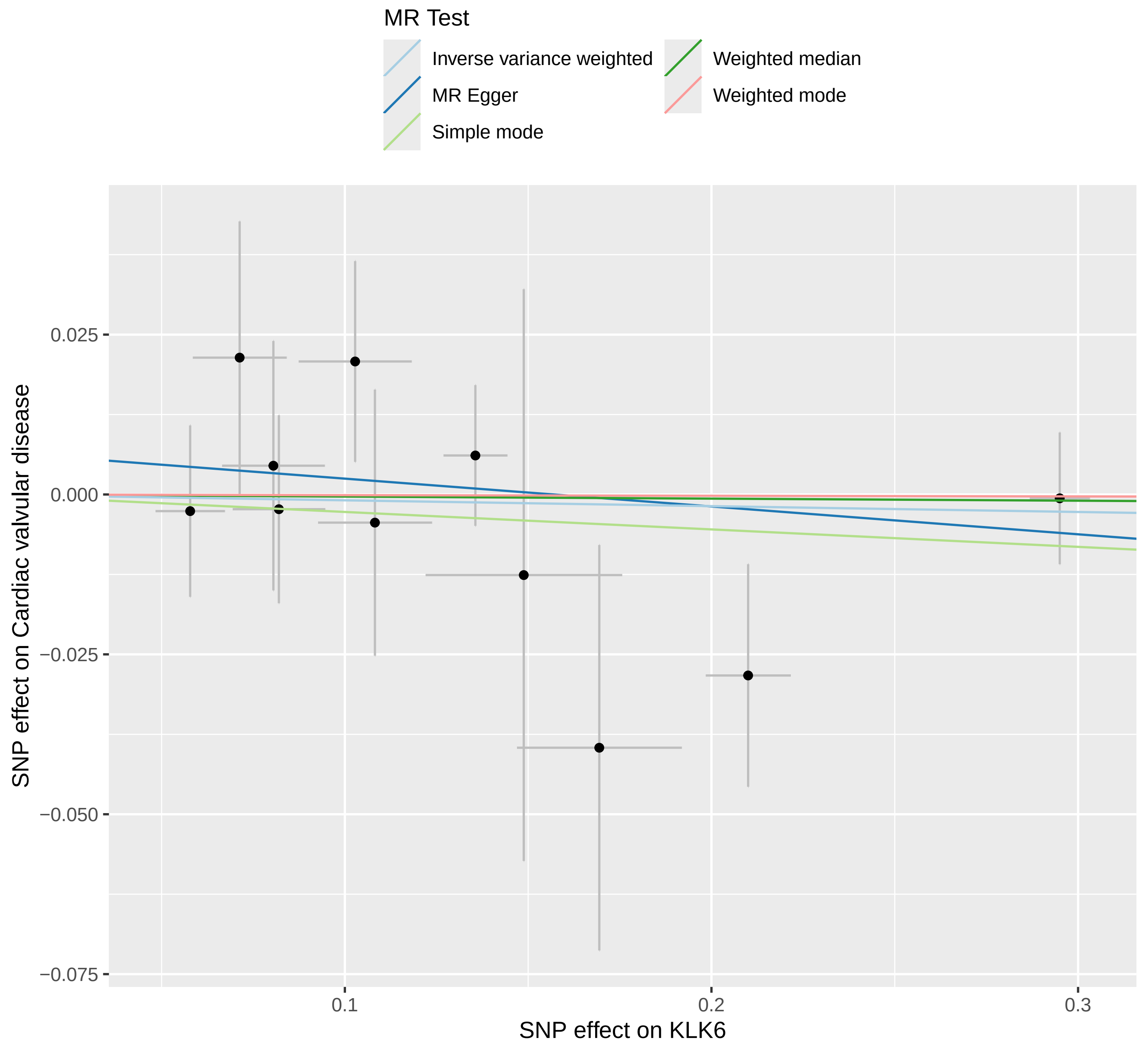
MR Test
Inverse variance weighted
MR Egger
Simple mode
Weighted median
Weighted mode
0.025
0.000
−0.025
−0.050
−0.075
SNP effect on Cardiac valvular disease
0.1
0.2
0.3
SNP effect on KLK6

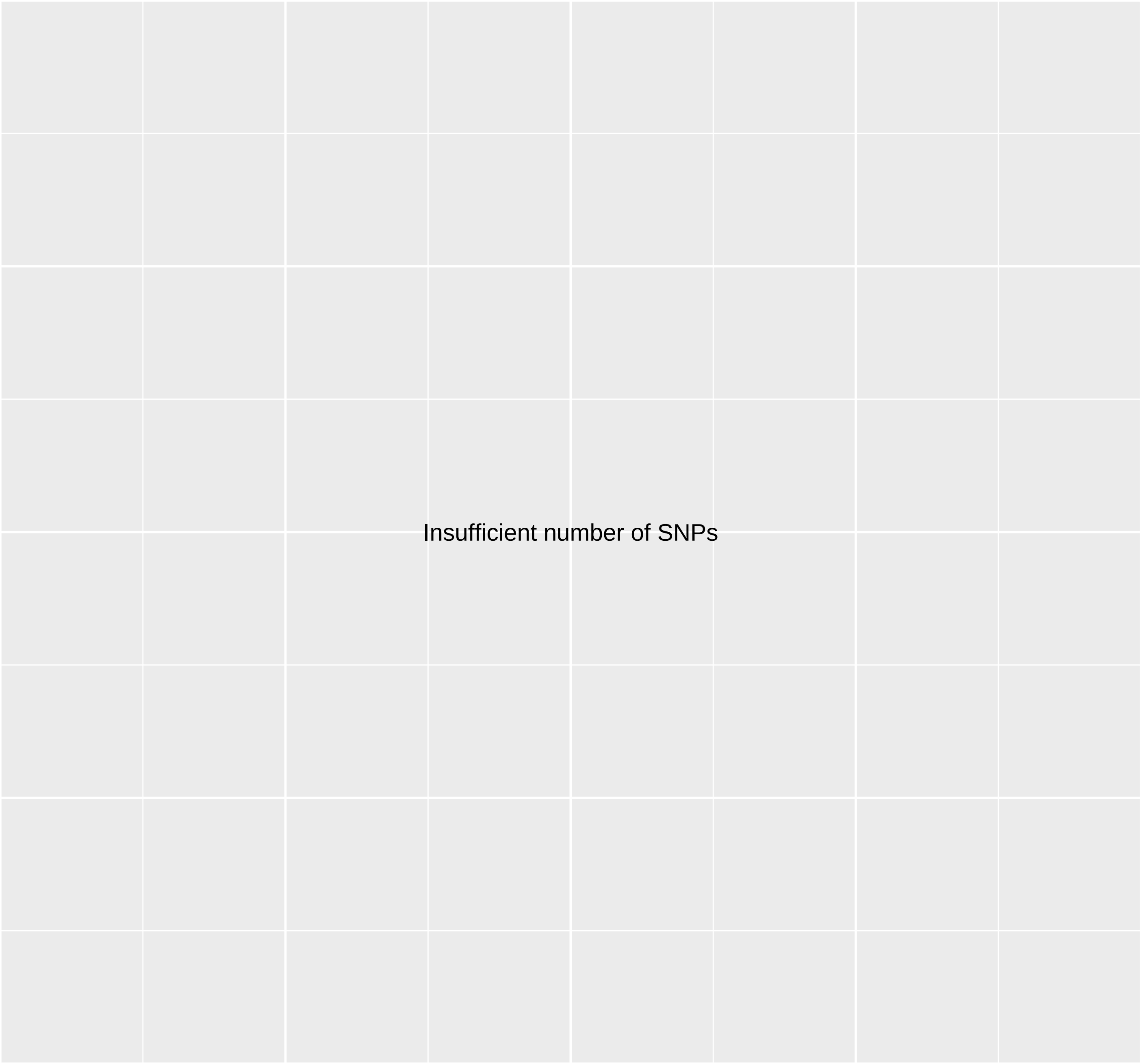
Insufficient number of SNPs

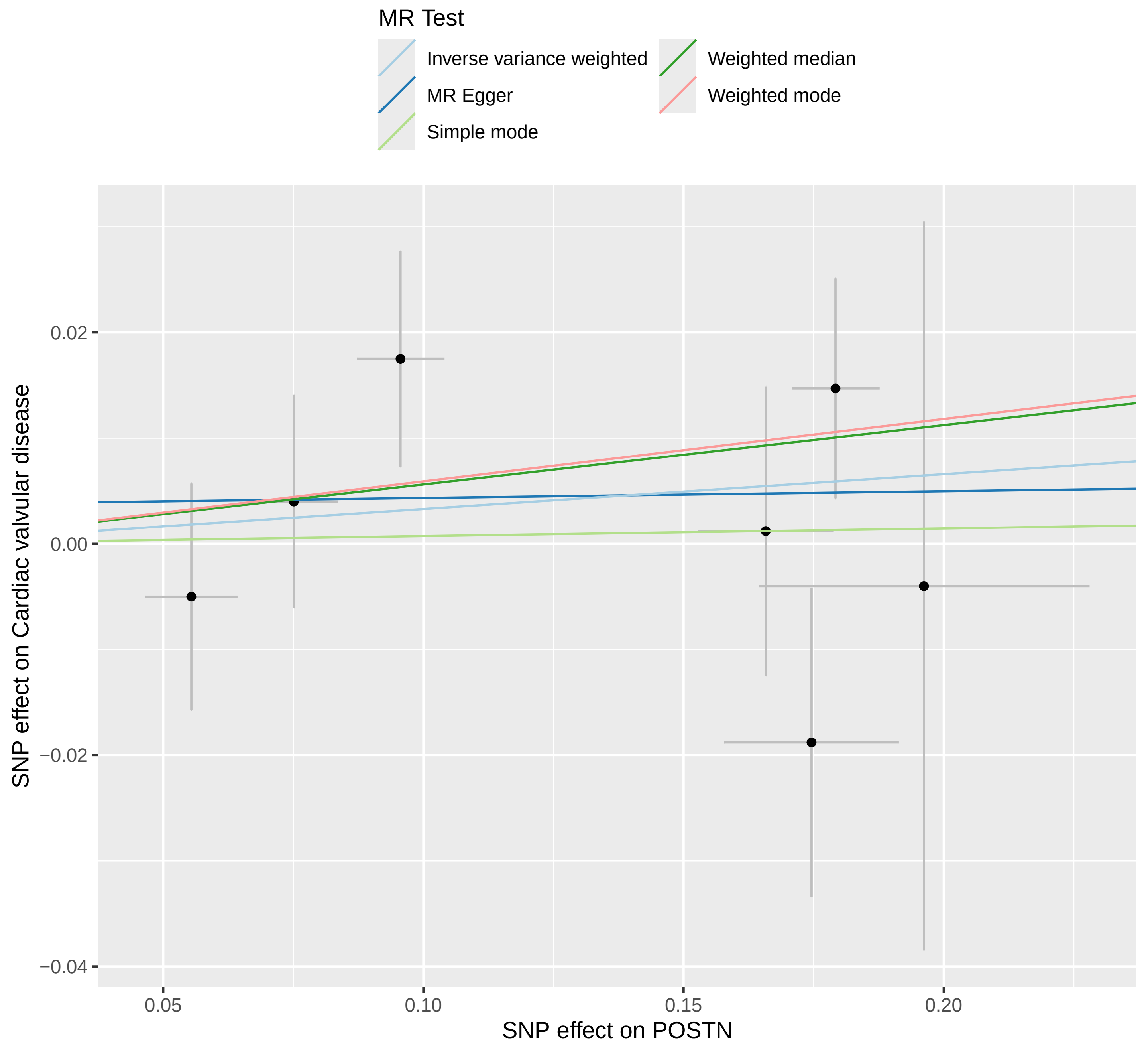
MR Test
Inverse variance weighted
MR Egger
Simple mode
Weighted median
Weighted mode
0.02
0.00
−0.02
−0.04
SNP effect on Cardiac valvular disease
0.05
0.10
0.15
0.20
SNP effect on POSTN

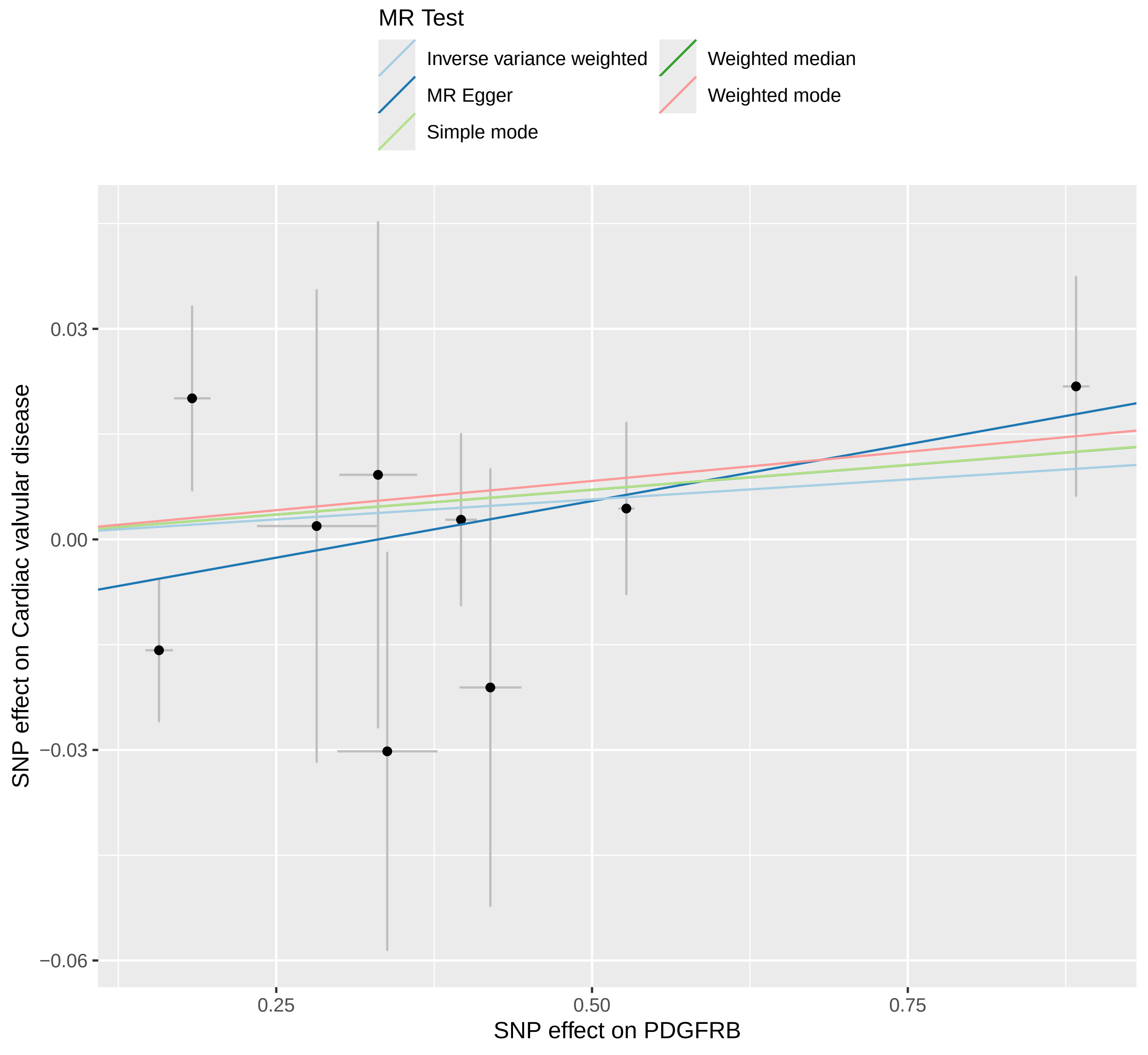
MR Test
Inverse variance weighted
MR Egger
Simple mode
Weighted median
Weighted mode
0.03
0.00
−0.03
−0.06
0.25
0.50
0.75
SNP effect on Cardiac valvular disease
SNP effect on PDGFRB

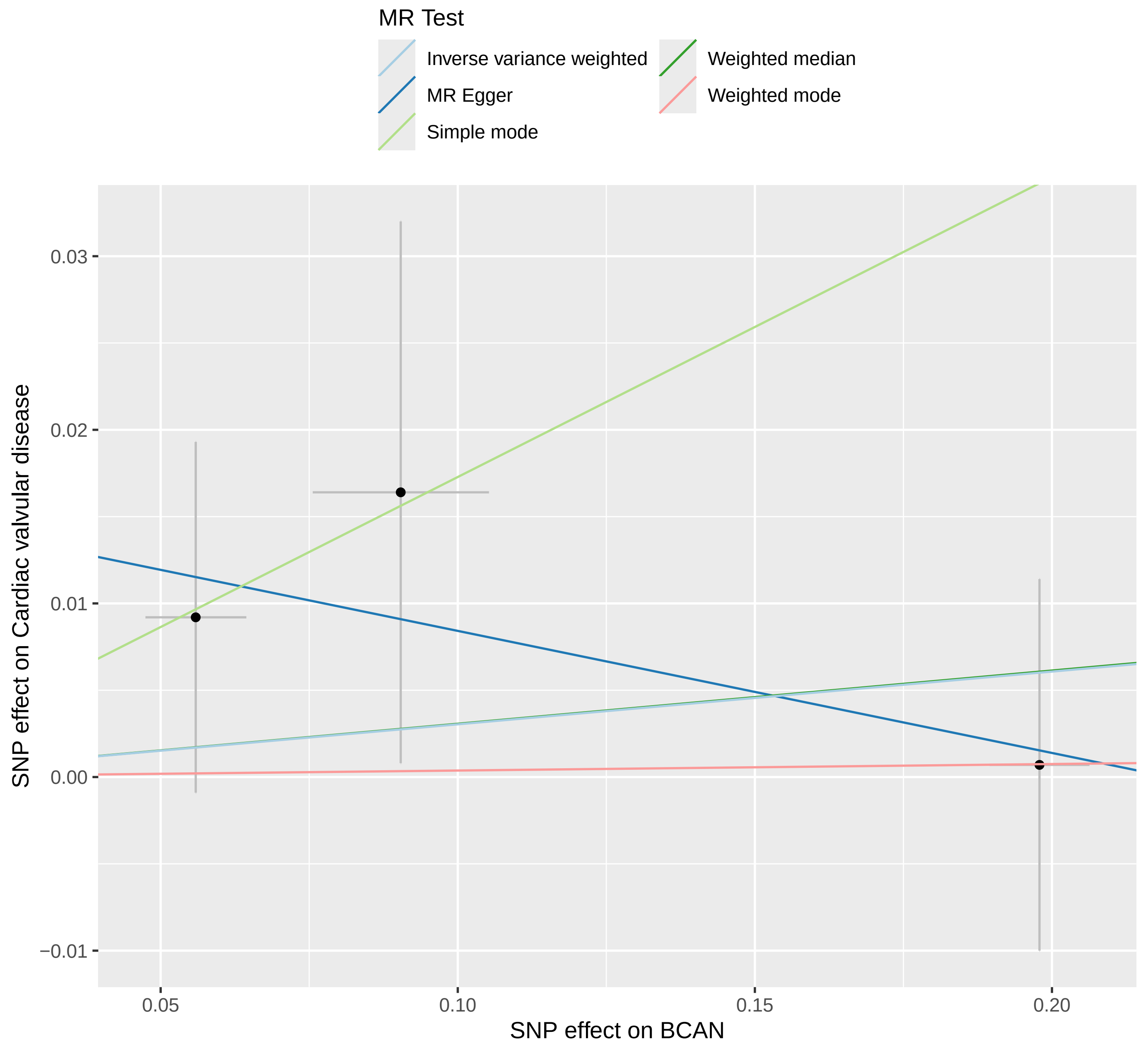

MR Test
Inverse variance weighted
MR Egger
Simple mode
Weighted median
Weighted mode
0.03
0.02
0.01
0.00
−0.01
0.05
0.10
0.15
0.20
SNP effect on Cardiac valvular disease
SNP effect on BCAN

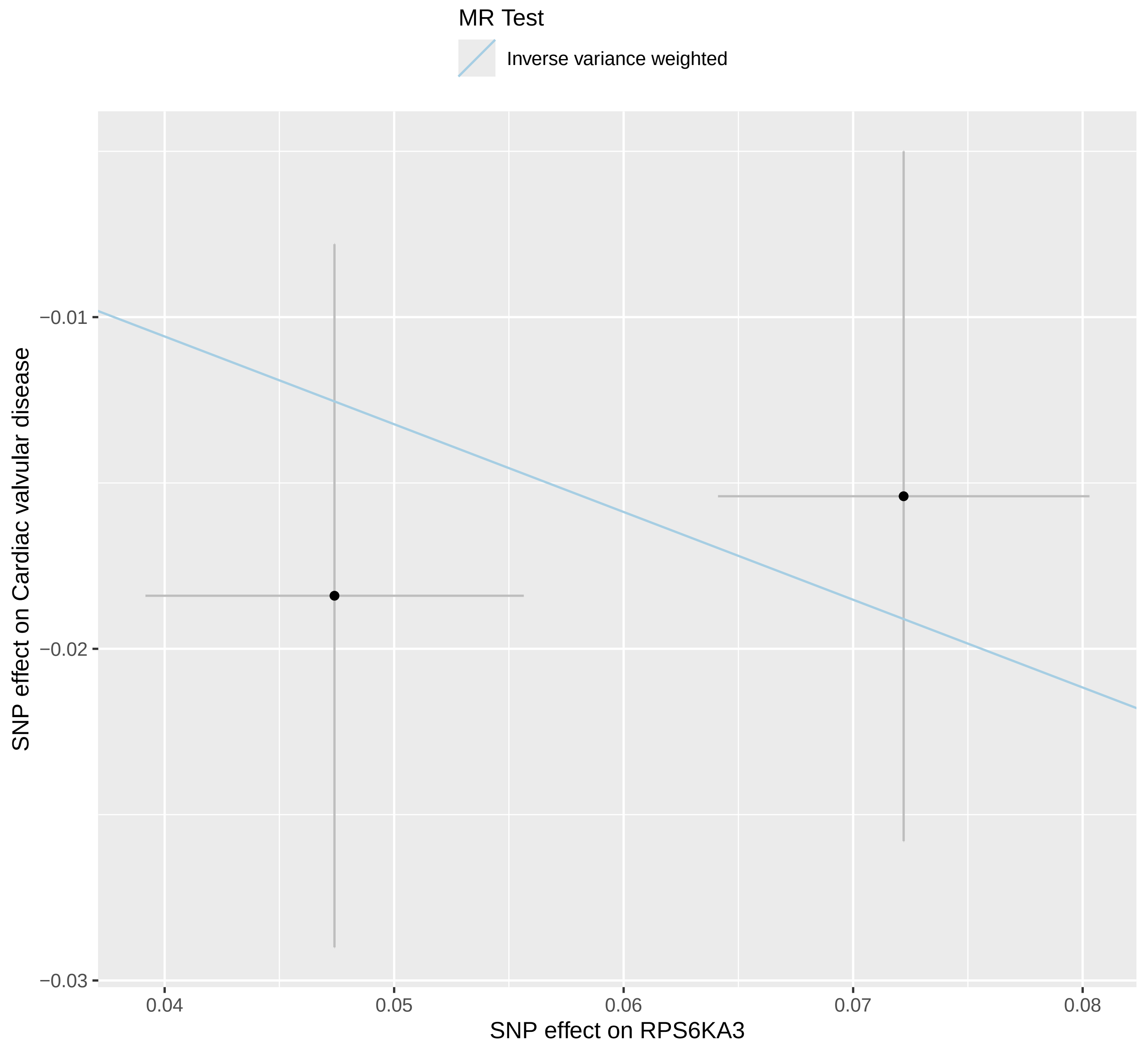
MR Test
Inverse variance weighted
SNP effect on Cardiac valvular disease
−0.01
−0.02
−0.03
0.04
0.05
0.06
0.07
0.08
SNP effect on RPS6KA3

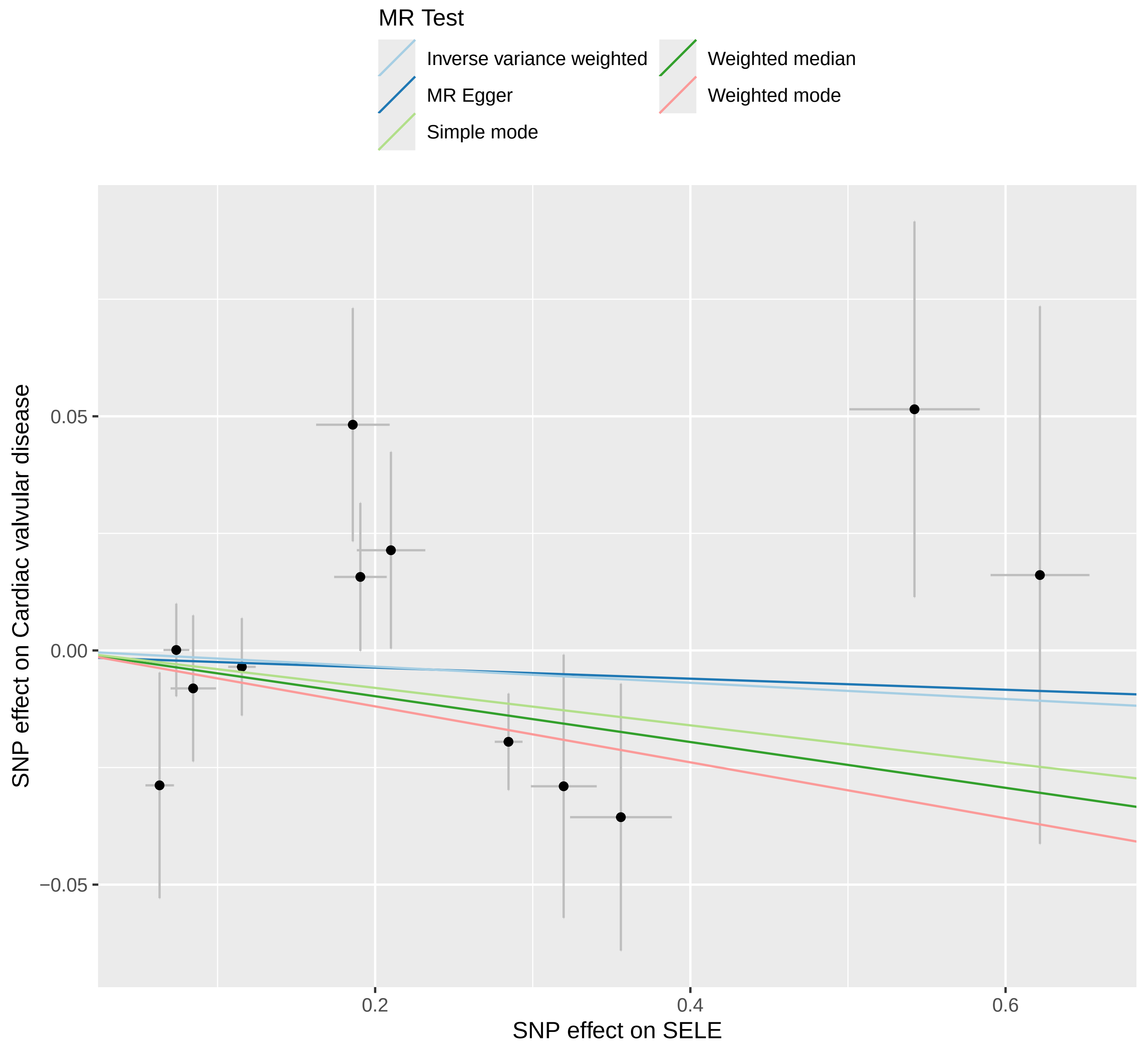
MR Test
Inverse variance weighted
MR Egger
Simple mode
Weighted median
Weighted mode
0.05
0.00
−0.05
0.2
0.4
0.6
SNP effect on Cardiac valvular disease
SNP effect on SELE

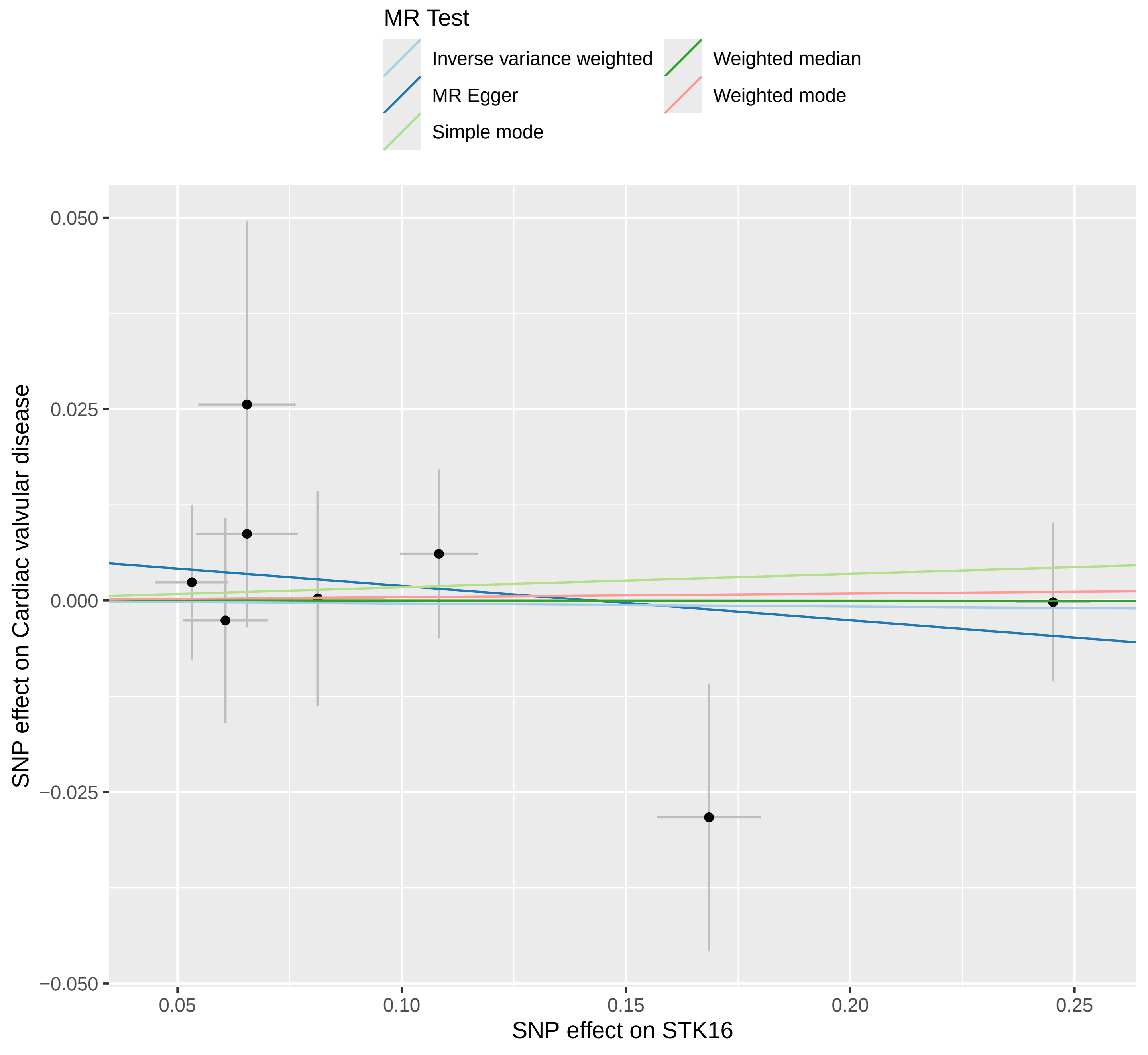
MR Test
Inverse variance weighted
MR Egger
Simple mode
Weighted median
Weighted mode
SNP effect on Cardiac valvular disease
0.050
0.025
0.000
−0.025
−0.050
0.05
0.10
0.15
0.20
0.25
SNP effect on STK16

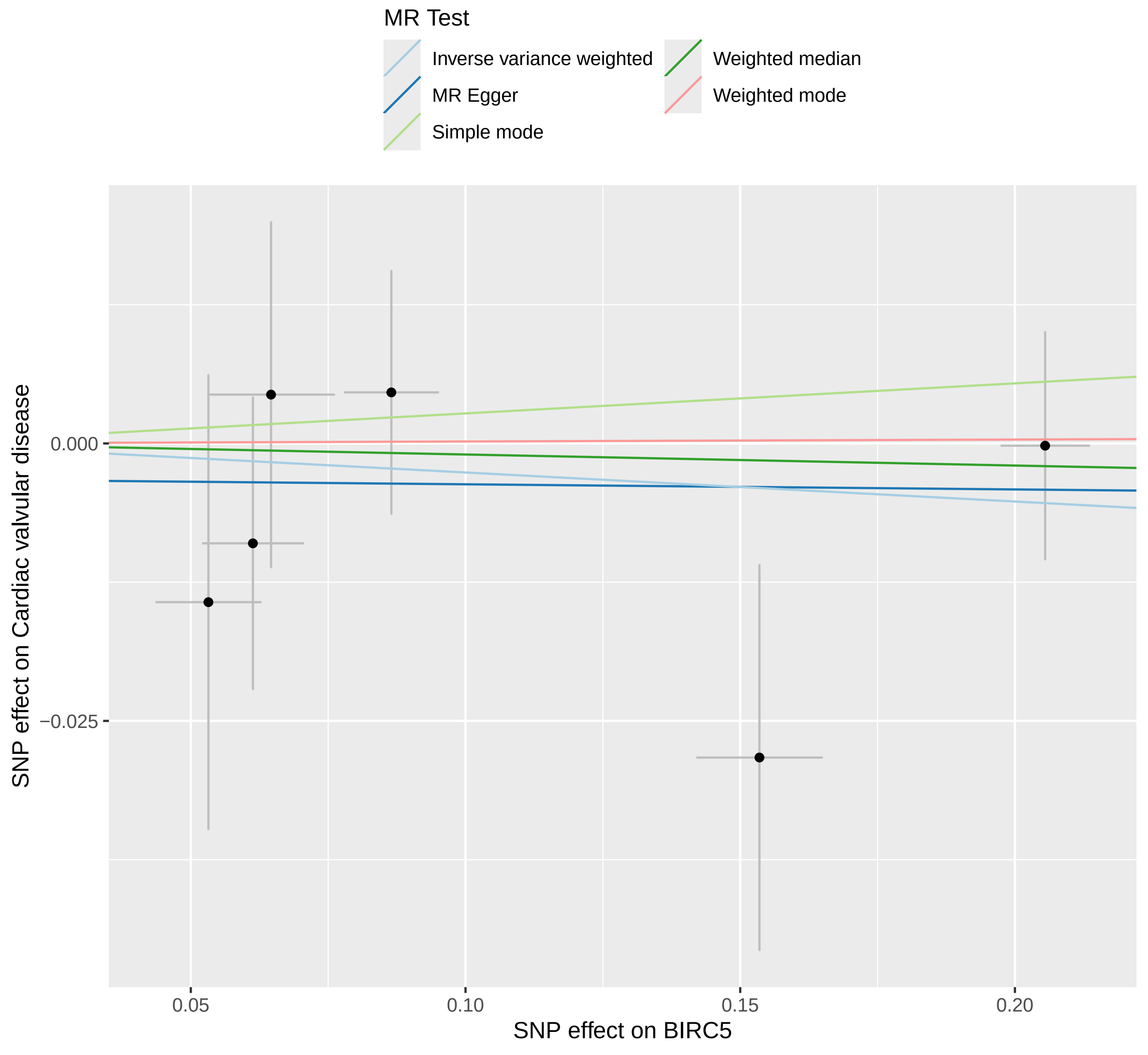

MR Test
Inverse variance weighted
MR Egger
Simple mode
Weighted median
Weighted mode
SNP effect on Cardiac valvular disease
0.000
−0.025
0.05
0.10
0.15
0.20
SNP effect on BIRC5

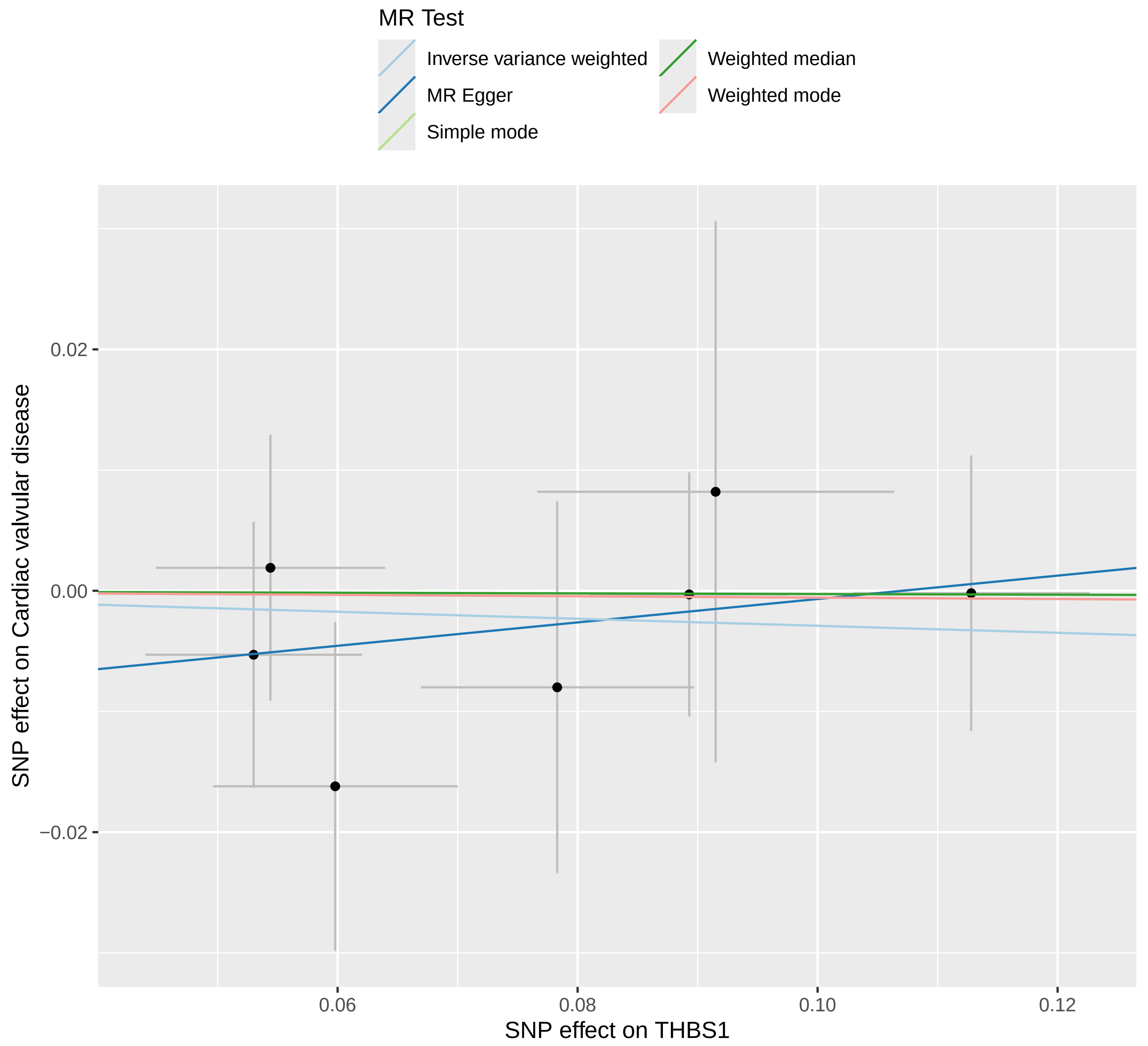

MR Test
Inverse variance weighted
MR Egger
Simple mode
Weighted median
Weighted mode
0.02
0.00
−0.02
0.06
0.08
0.10
0.12
SNP effect on Cardiac valvular disease
SNP effect on THBS1

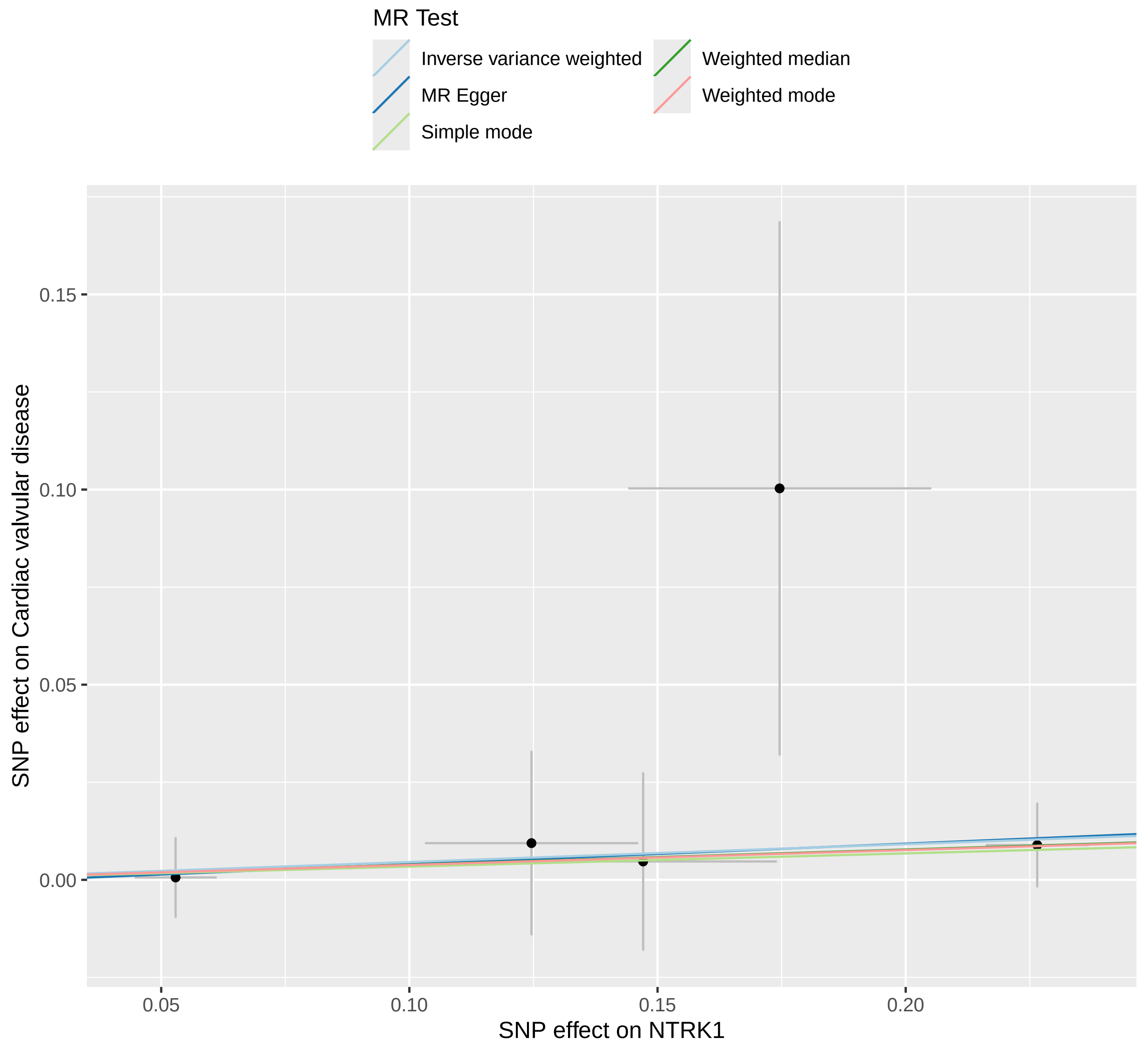
MR Test
Inverse variance weighted
MR Egger
Simple mode
Weighted median
Weighted mode
0.15
0.10
0.05
0.00
SNP effect on Cardiac valvular disease
0.05
0.10
0.15
0.20
SNP effect on NTRK1

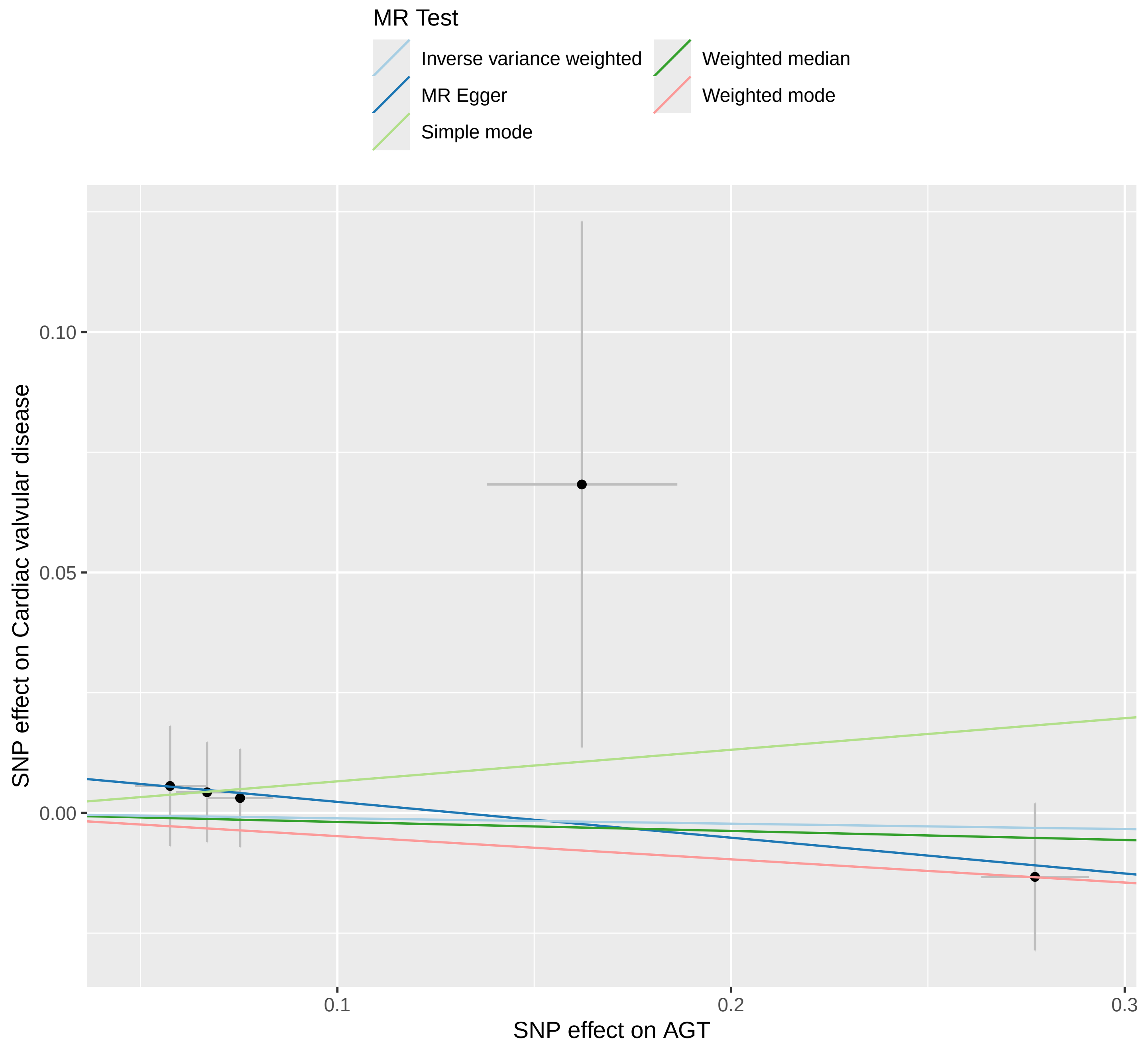
MR Test
Inverse variance weighted
MR Egger
Simple mode
Weighted median
Weighted mode
0.10
0.05
0.00
SNP effect on Cardiac valvular disease
0.1
0.2
0.3
SNP effect on AGT

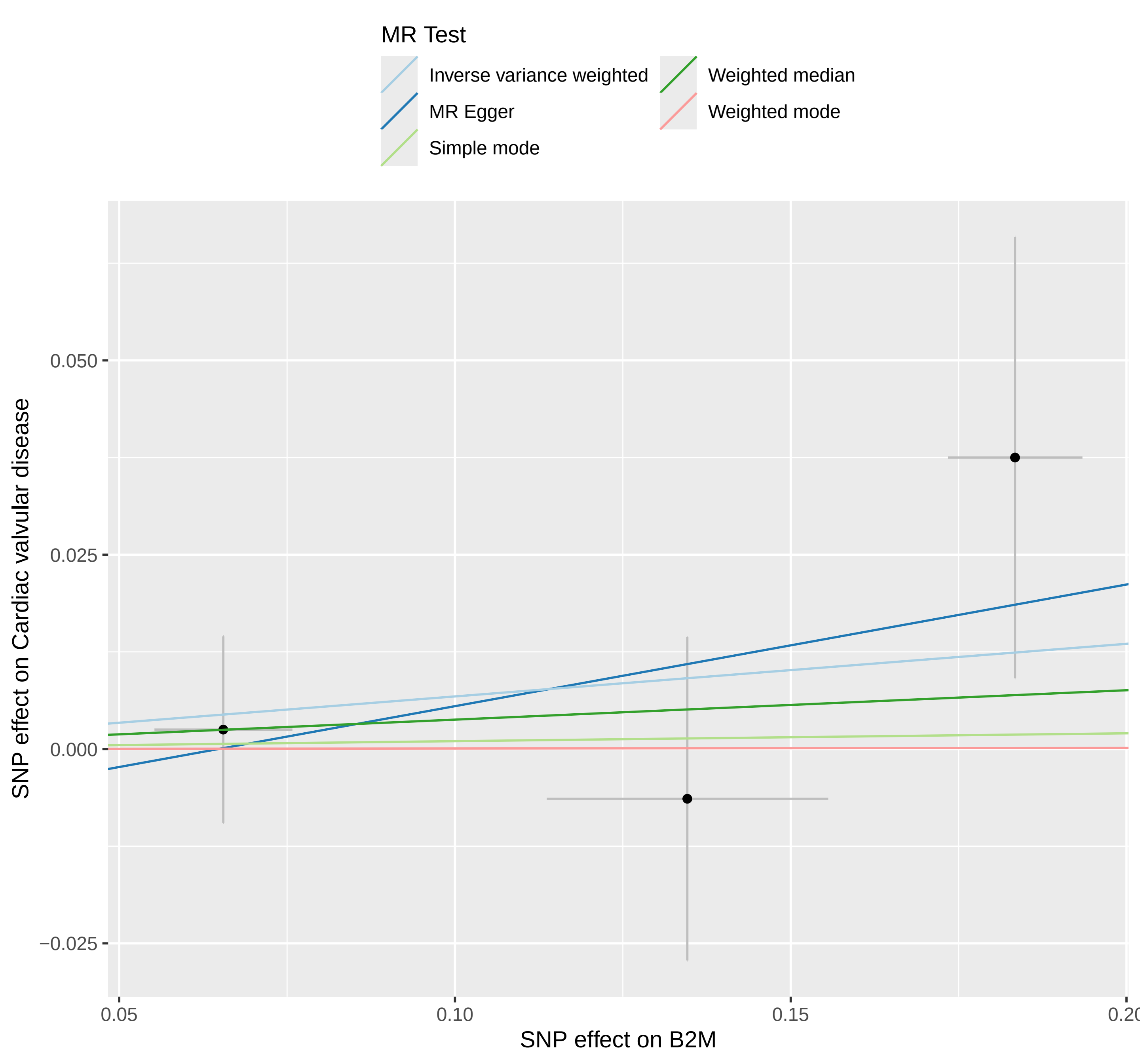

MR Test
Inverse variance weighted
MR Egger
Simple mode
Weighted median
Weighted mode
0.050
0.025
0.000
−0.025
0.05
0.10
0.15
0.20
SNP effect on B2M
SNP effect on Cardiac valvular disease

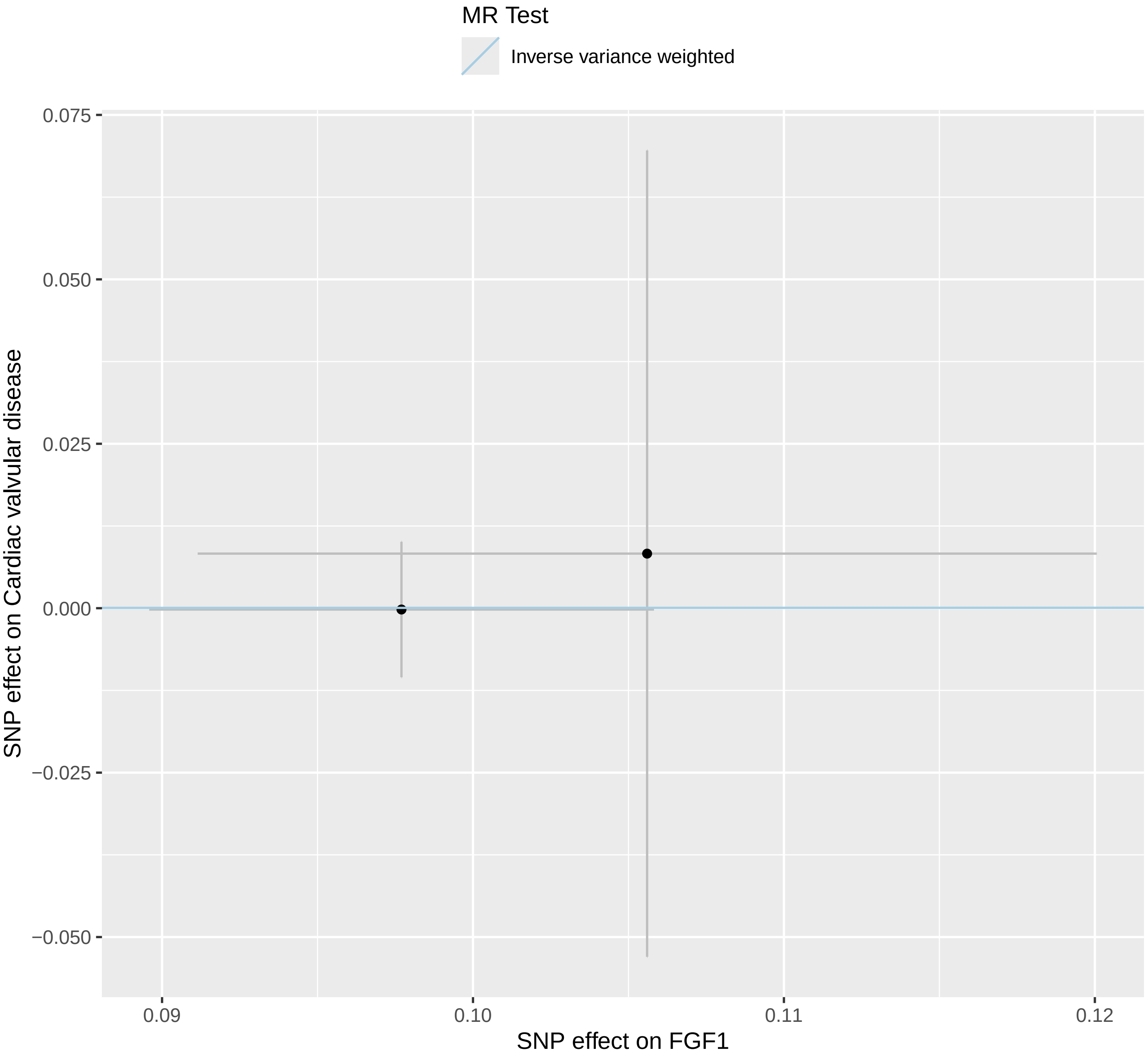

MR Test
Inverse variance weighted
0.075
0.050
0.025
0.000
−0.025
−0.050
SNP effect on Cardiac valvular disease
0.09
0.10
0.11
0.12
SNP effect on FGF1

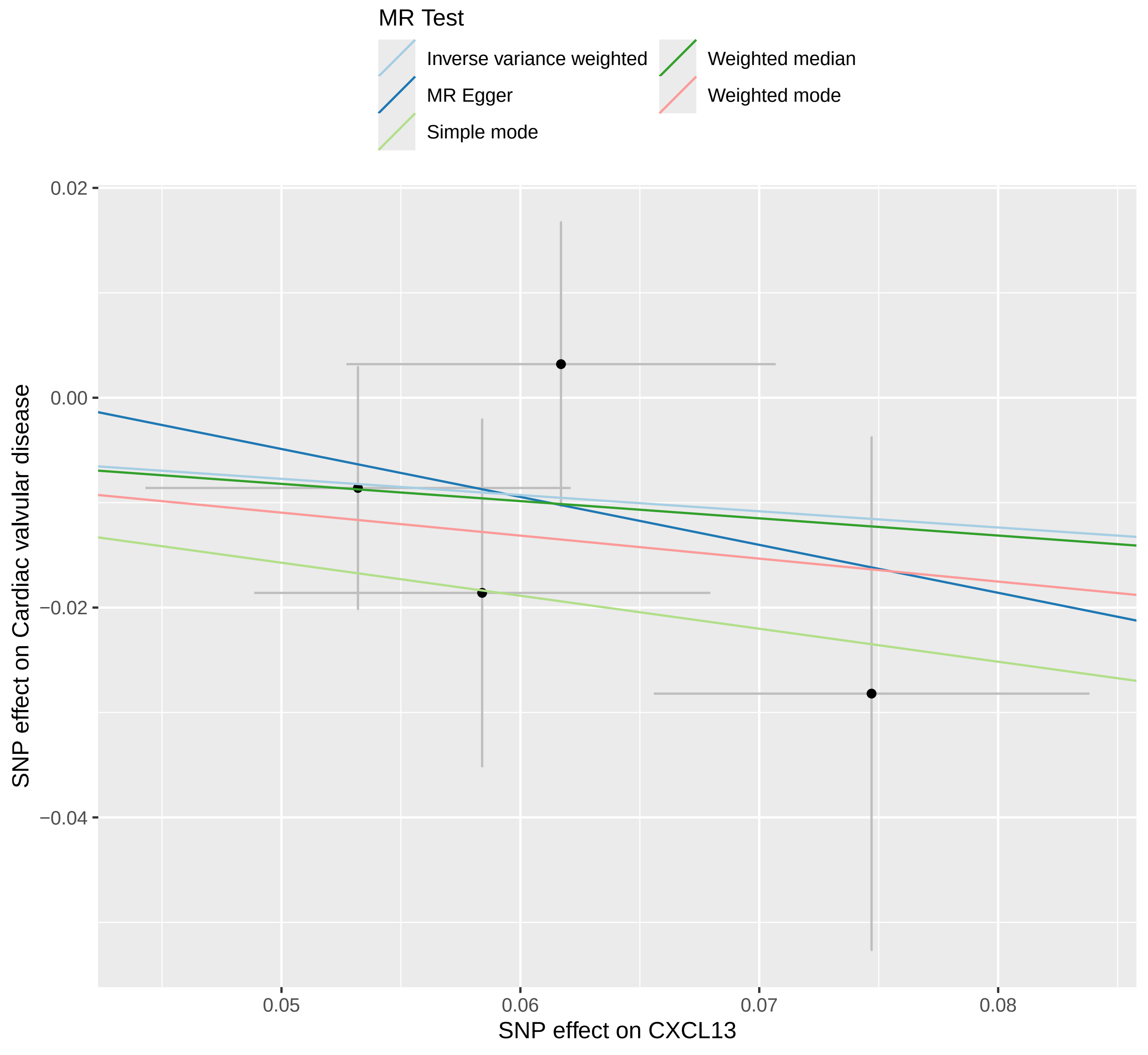
MR Test
Inverse variance weighted
MR Egger
Simple mode
Weighted median
Weighted mode
0.02
0.00
−0.02
−0.04
SNP effect on Cardiac valvular disease
0.05
0.06
0.07
0.08
SNP effect on CXCL13

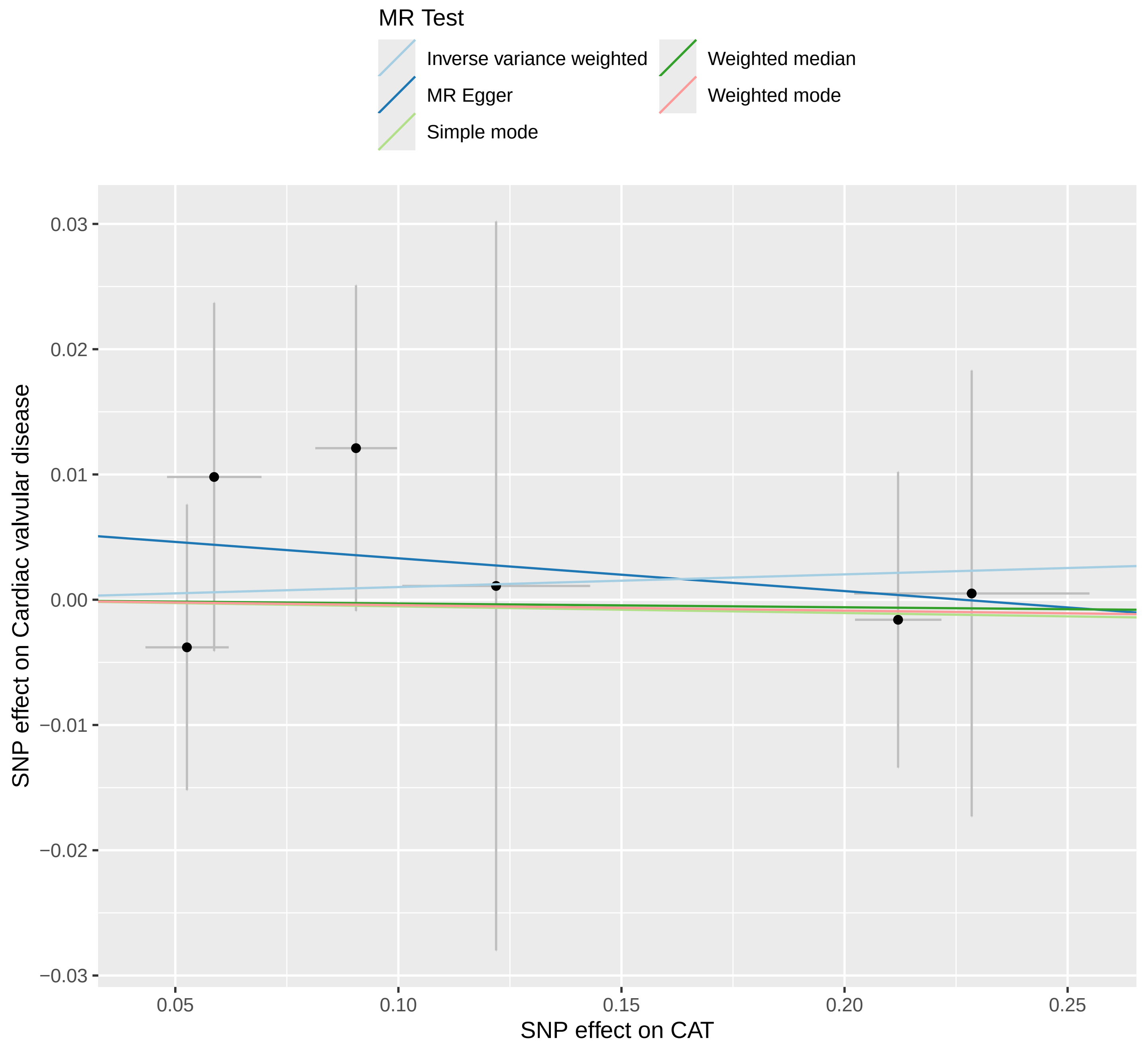
MR Test
Inverse variance weighted
MR Egger
Simple mode
Weighted median
Weighted mode
0.03
0.02
0.01
0.00
−0.01
−0.02
−0.03
SNP effect on Cardiac valvular disease
0.05
0.10
0.15
0.20
0.25
SNP effect on CAT

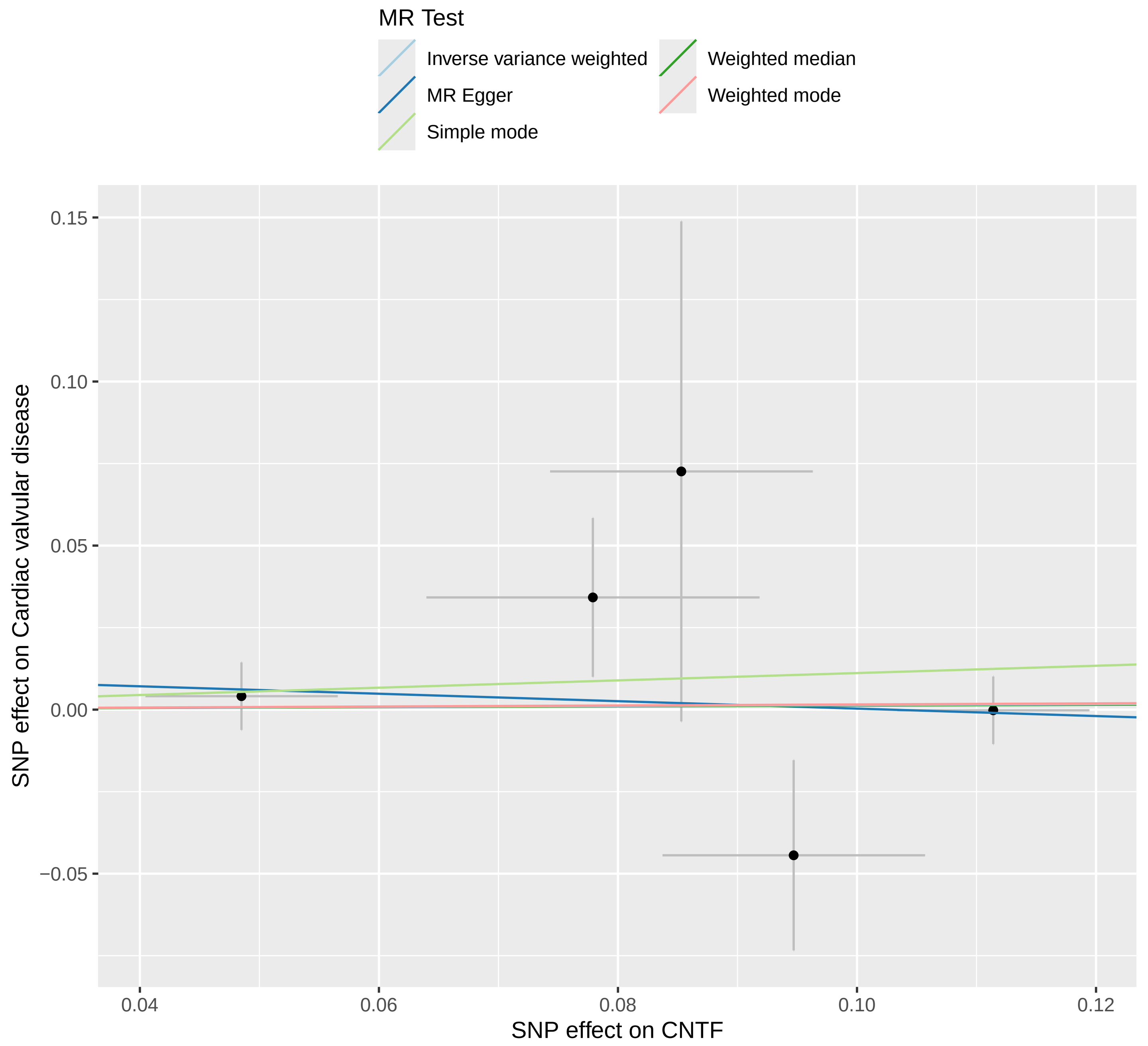
MR Test
Inverse variance weighted
MR Egger
Simple mode
Weighted median
Weighted mode
0.15
0.10
0.05
0.00
−0.05
SNP effect on Cardiac valvular disease
0.04
0.06
0.08
0.10
0.12
SNP effect on CNTF

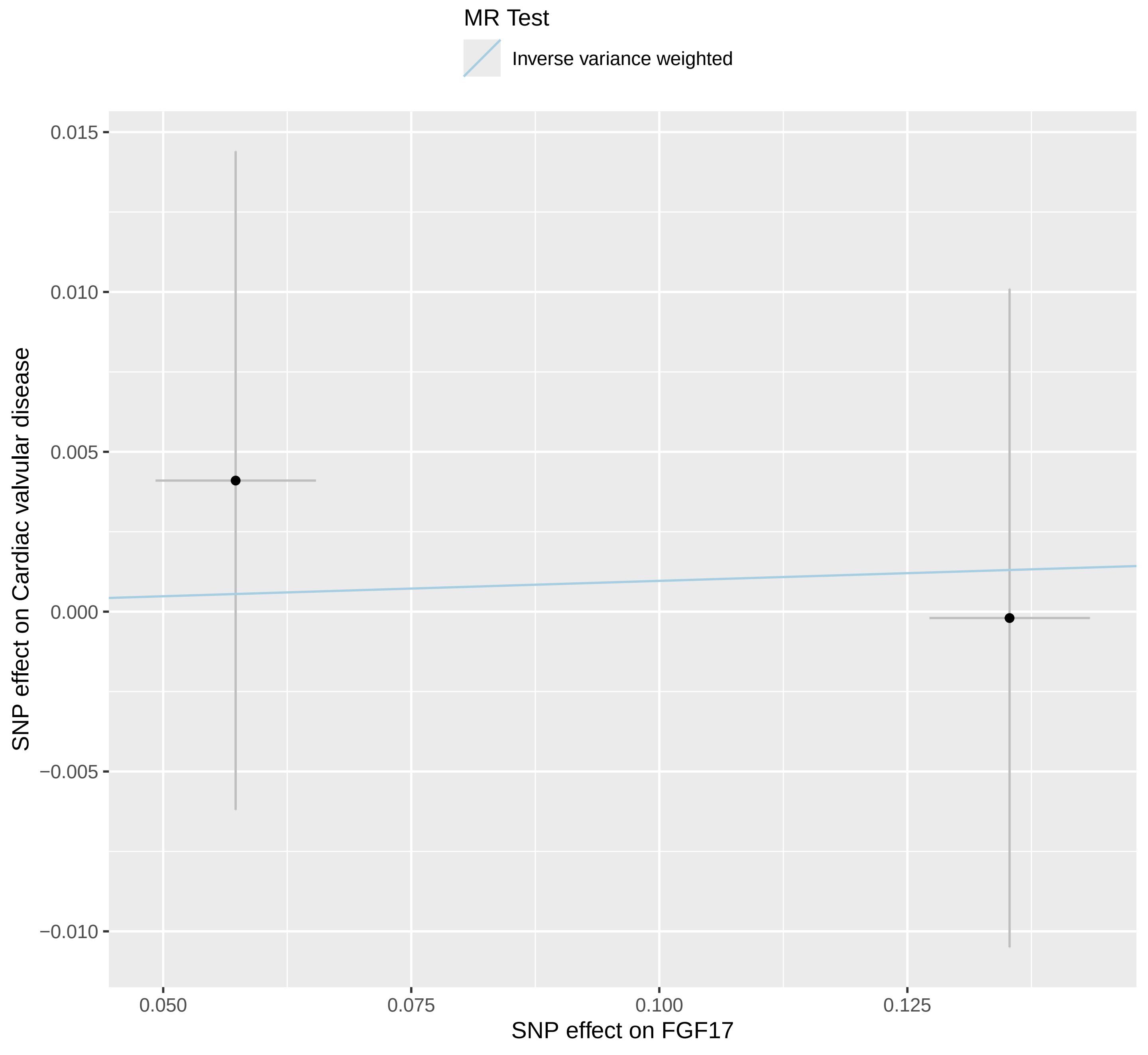

MR Test
Inverse variance weighted
0.015
0.010
0.005
0.000
−0.005
−0.010
SNP effect on Cardiac valvular disease
0.050
0.075
0.100
0.125
SNP effect on FGF17

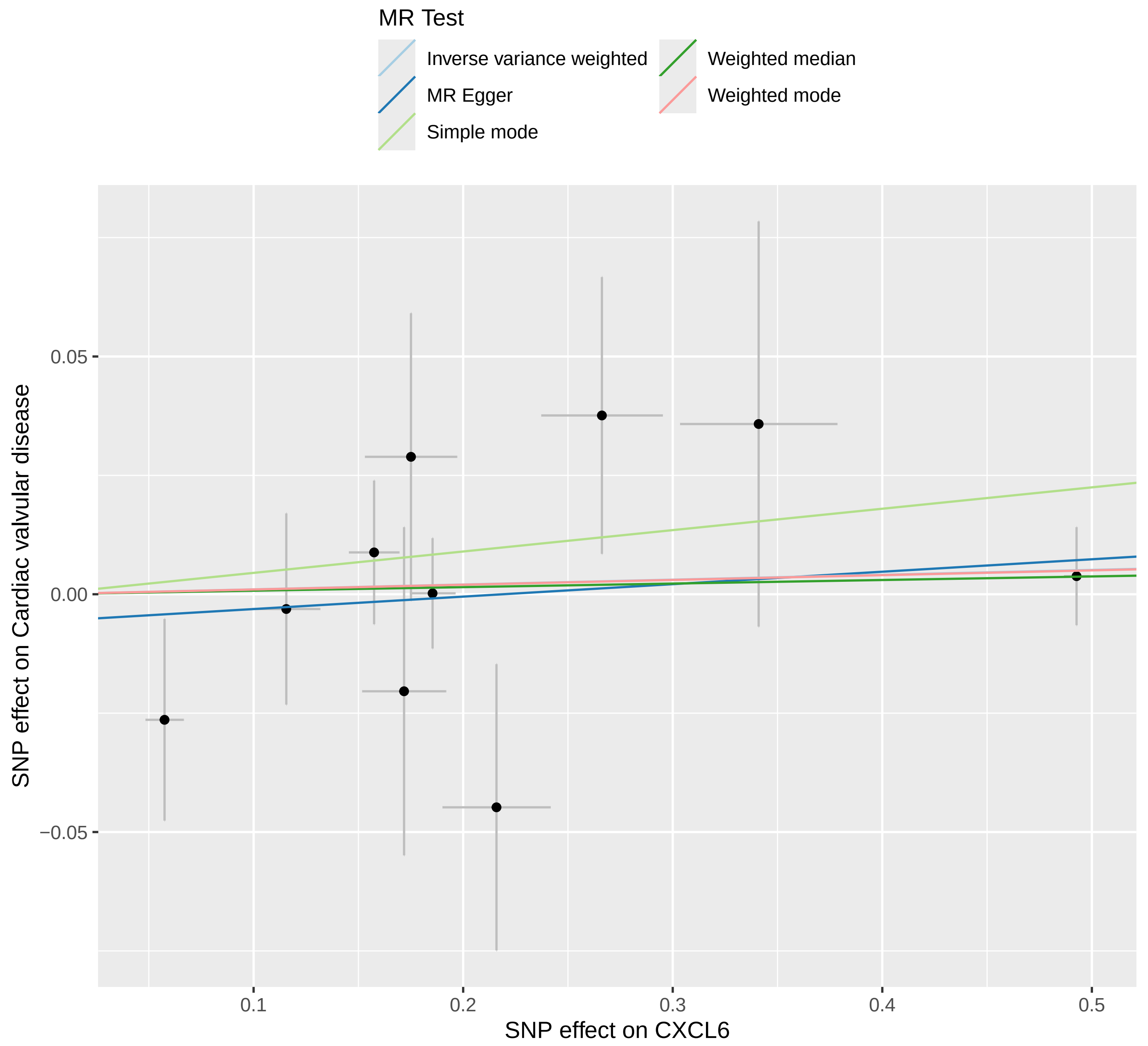
MR Test
Inverse variance weighted
MR Egger
Simple mode
Weighted median
Weighted mode
0.05
0.00
−0.05
0.1
0.2
0.3
0.4
0.5
SNP effect on Cardiac valvular disease
SNP effect on CXCL6

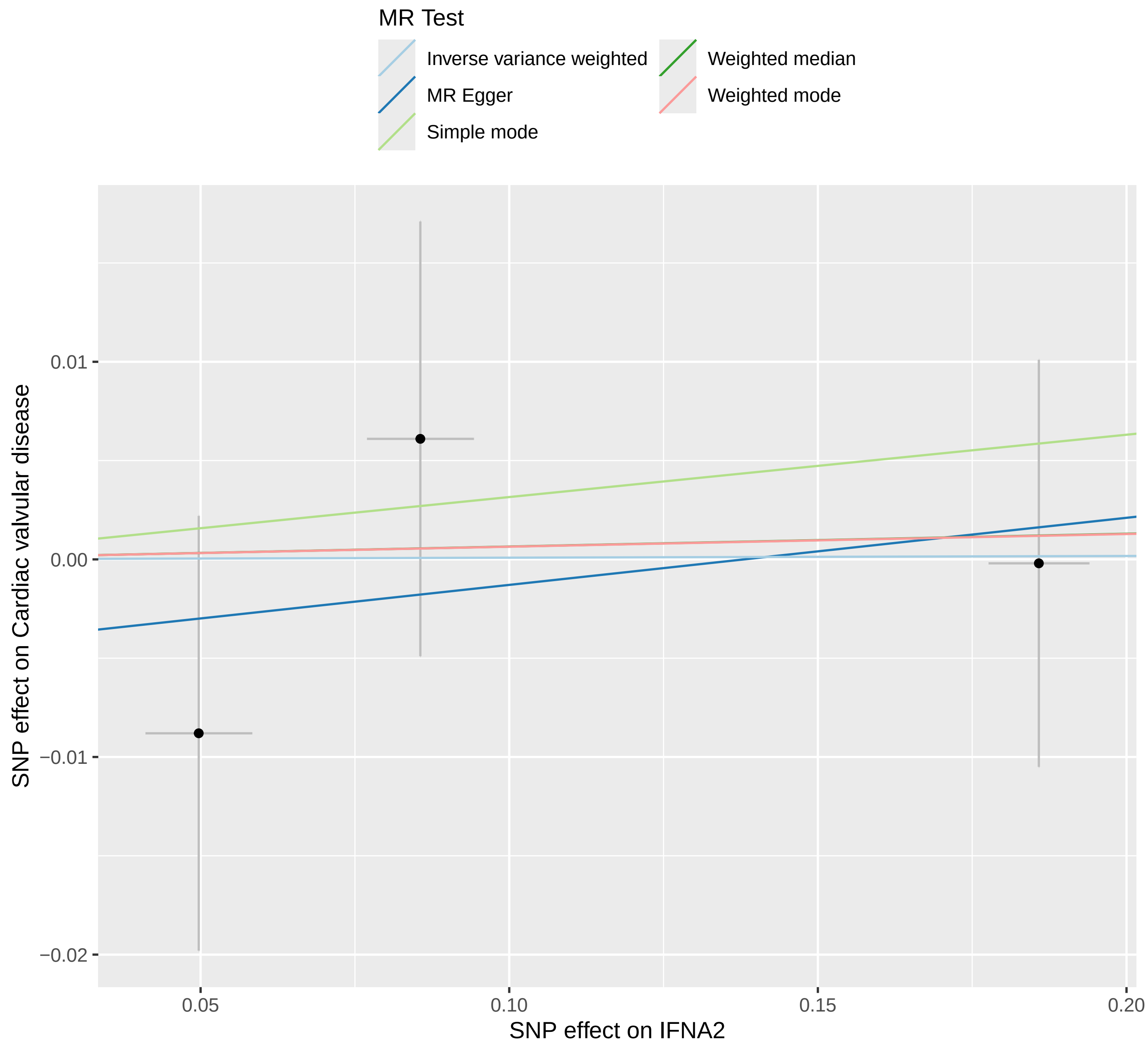
MR Test
Inverse variance weighted
MR Egger
Simple mode
Weighted median
Weighted mode
0.01
0.00
−0.01
−0.02
SNP effect on Cardiac valvular disease
0.05
0.10
0.15
0.20
SNP effect on IFNA2

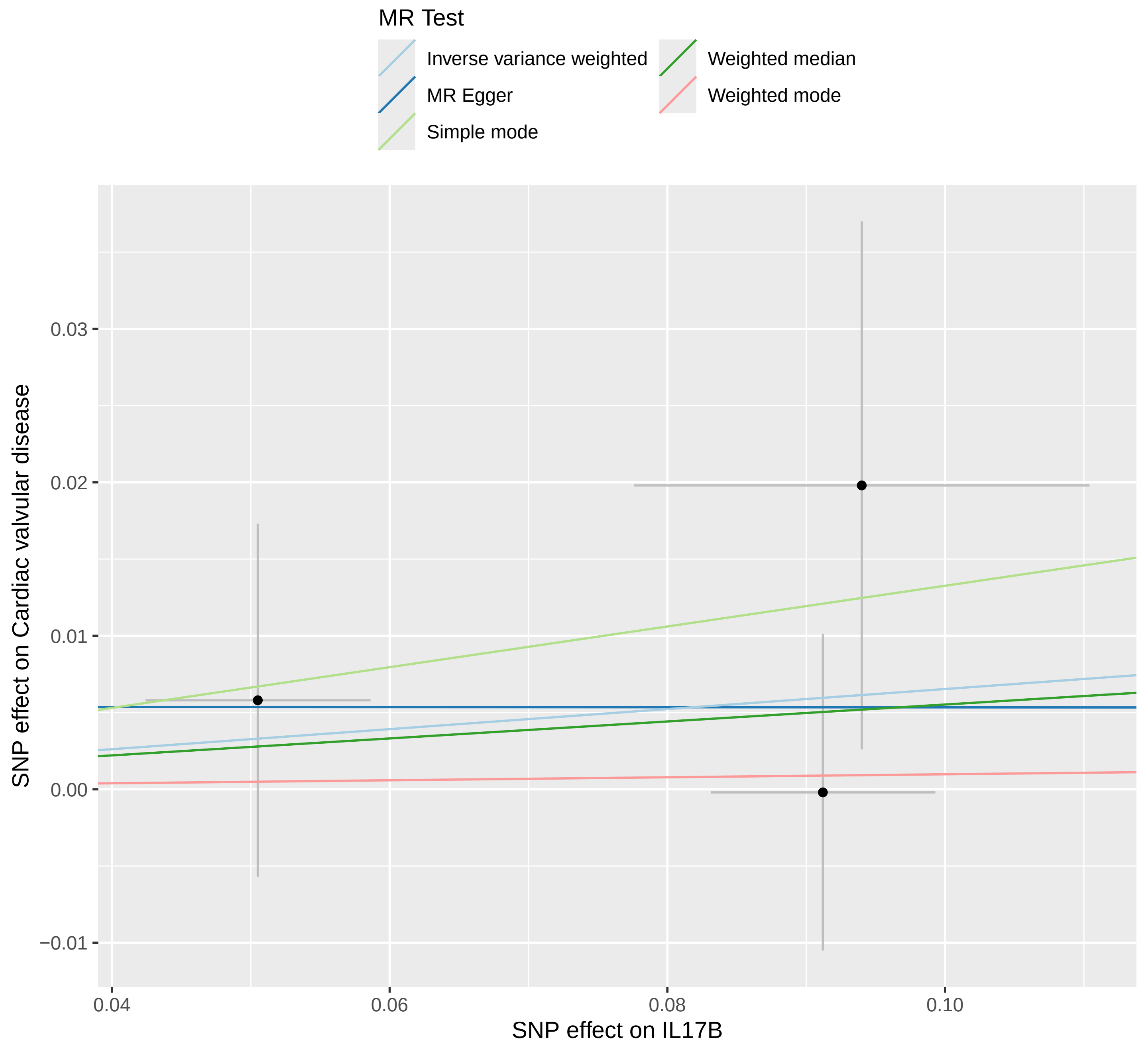

MR Test
Inverse variance weighted
MR Egger
Simple mode
Weighted median
Weighted mode
SNP effect on Cardiac valvular disease
0.03
0.02
0.01
0.00
−0.01
0.04
0.06
0.08
0.10
SNP effect on IL17B

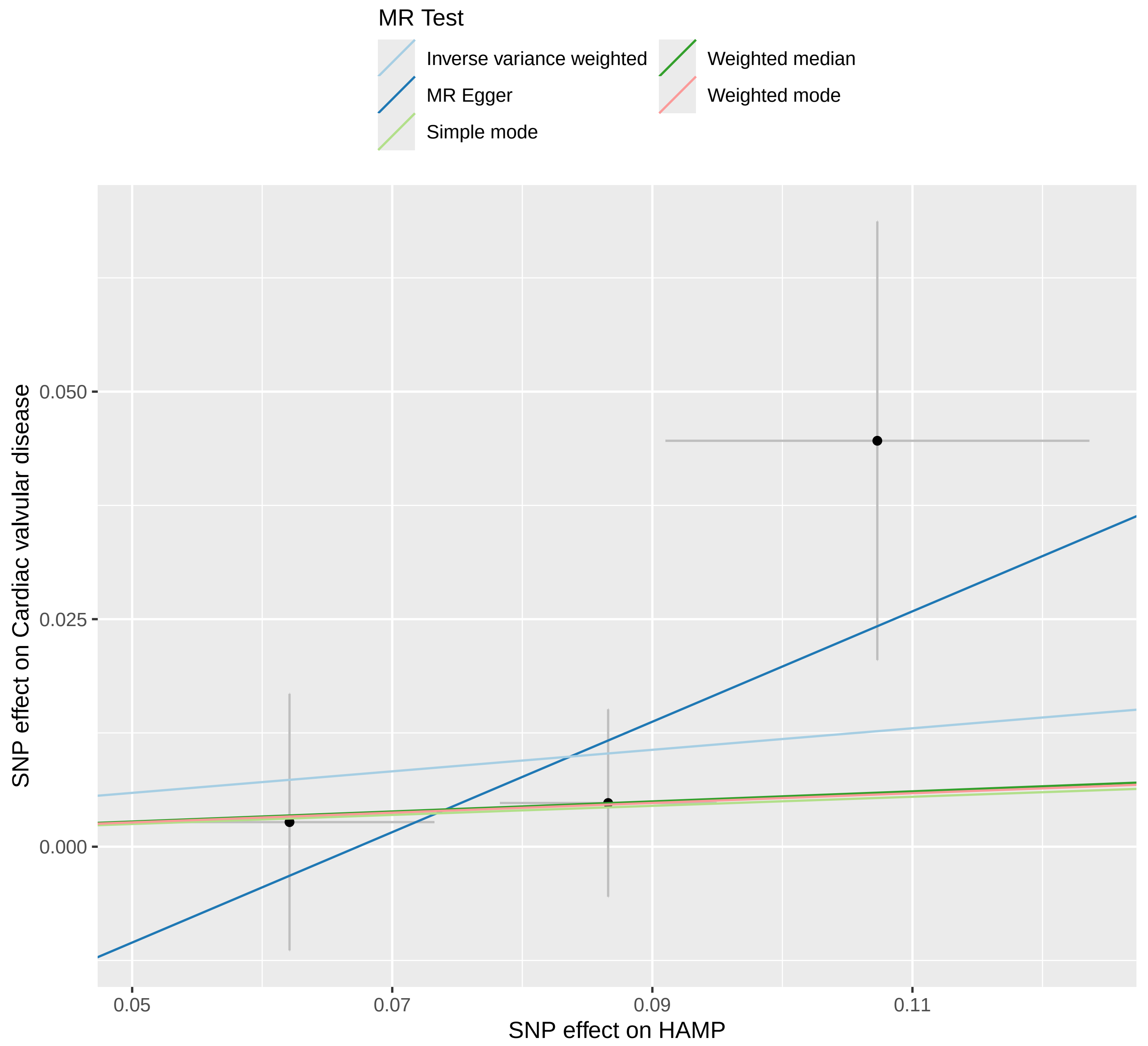
MR Test
Inverse variance weighted
MR Egger
Simple mode
Weighted median
Weighted mode
SNP effect on Cardiac valvular disease
0.050
0.025
0.000
0.05
0.07
0.09
0.11
SNP effect on HAMP

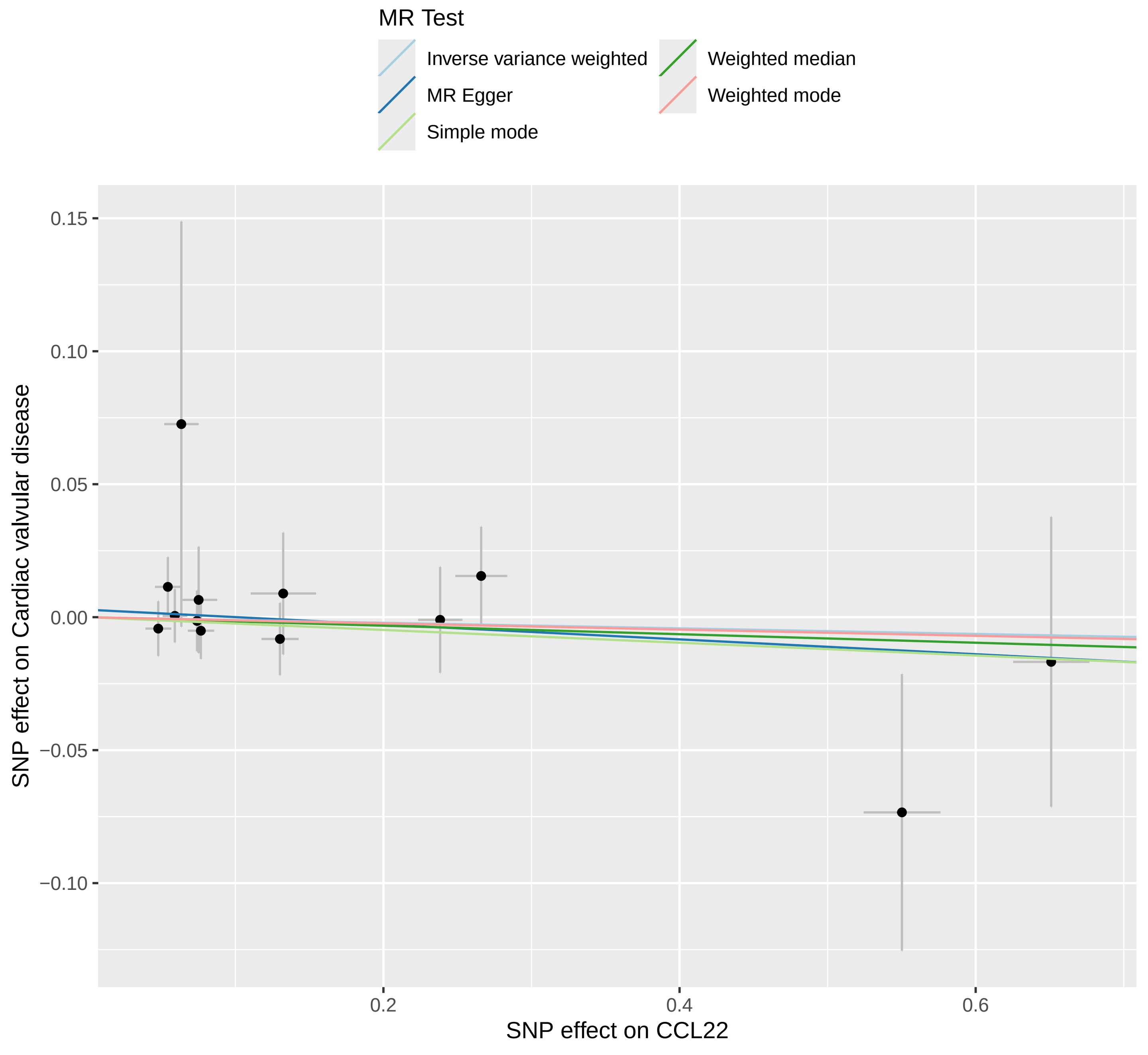
MR Test
Inverse variance weighted
MR Egger
Simple mode
Weighted median
Weighted mode
0.15
0.10
0.05
0.00
−0.05
−0.10
0.2
0.4
0.6
SNP effect on Cardiac valvular disease
SNP effect on CCL22

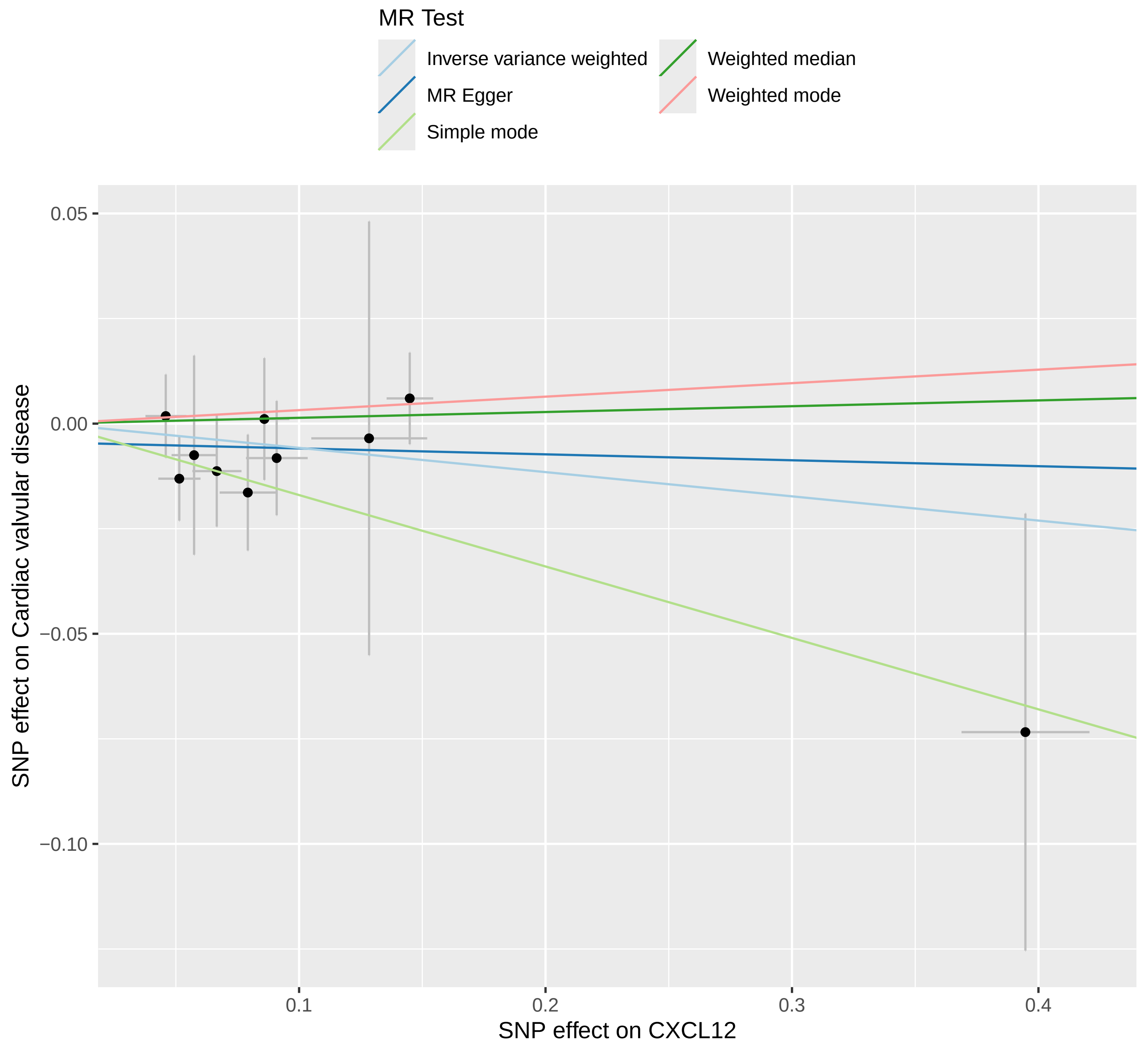

MR Test
Inverse variance weighted
MR Egger
Simple mode
Weighted median
Weighted mode
0.05
0.00
−0.05
−0.10
0.1
0.2
0.3
0.4
SNP effect on Cardiac valvular disease
SNP effect on CXCL12

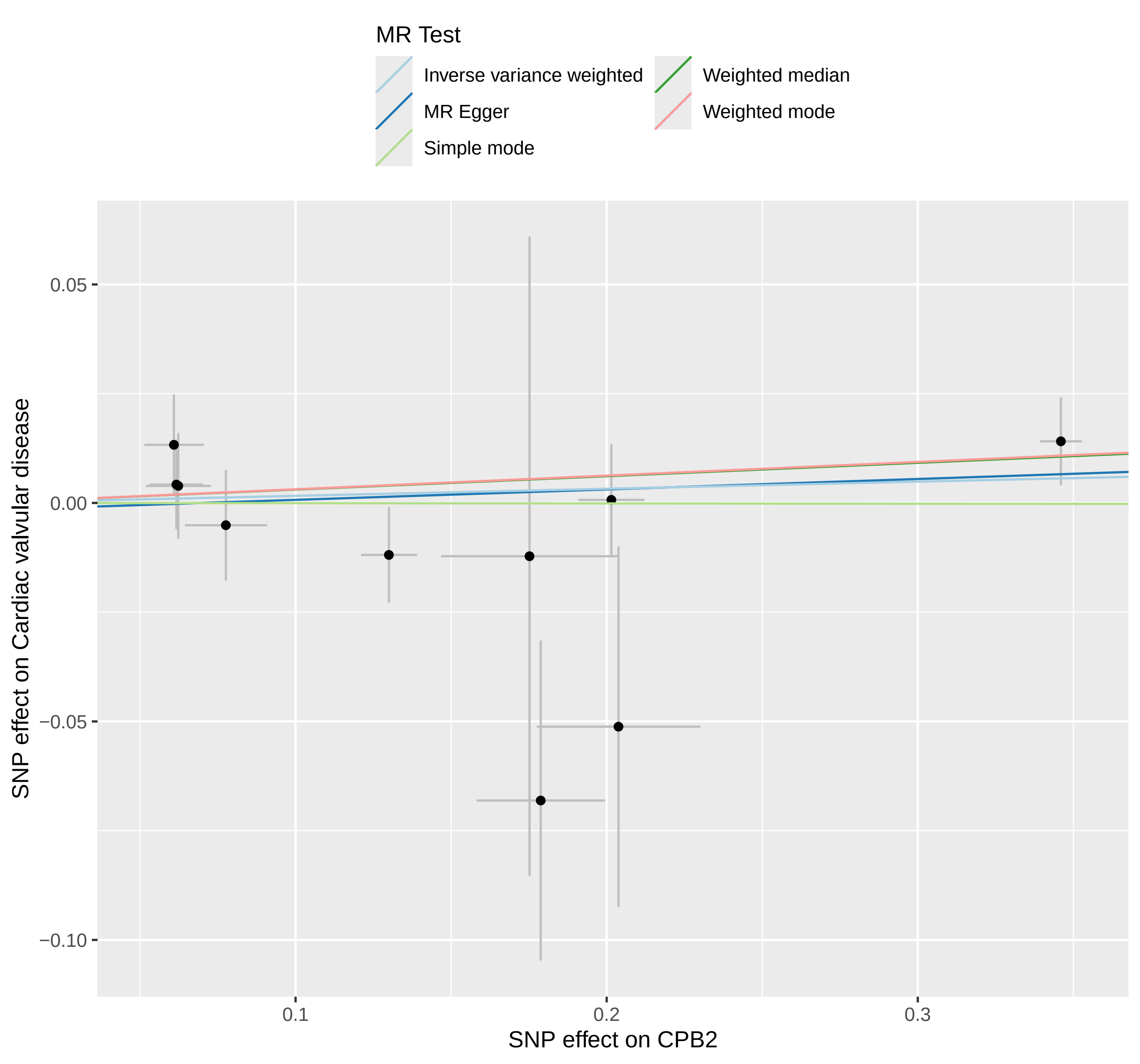
MR Test
Inverse variance weighted
MR Egger
Simple mode
Weighted median
Weighted mode
0.05
0.00
−0.05
−0.10
0.1
0.2
0.3
SNP effect on Cardiac valvular disease
SNP effect on CPB2

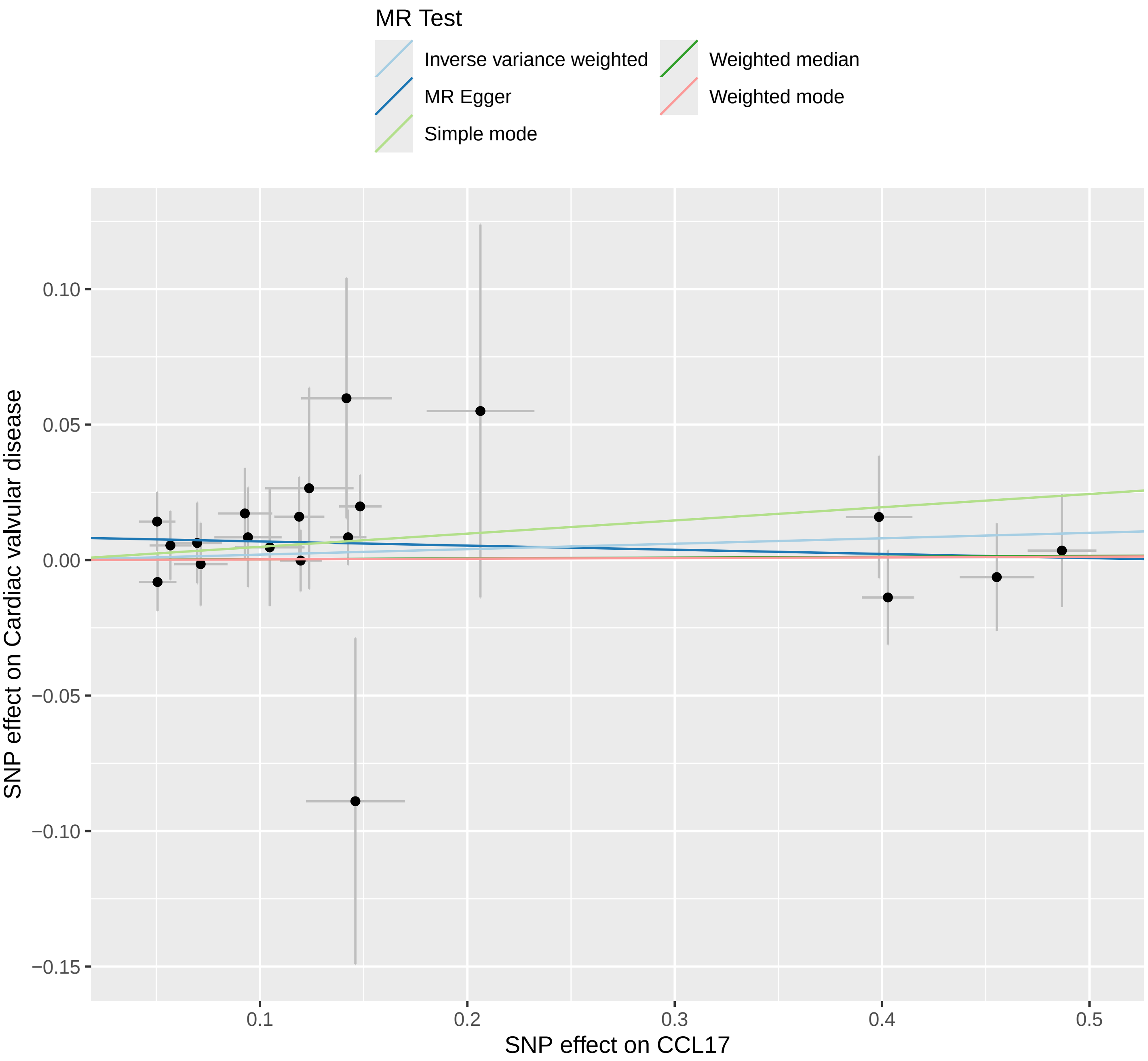
MR Test
Inverse variance weighted
MR Egger
Simple mode
Weighted median
Weighted mode
0.10
0.05
0.00
−0.05
−0.10
−0.15
0.1
0.2
0.3
0.4
0.5
SNP effect on Cardiac valvular disease
SNP effect on CCL17

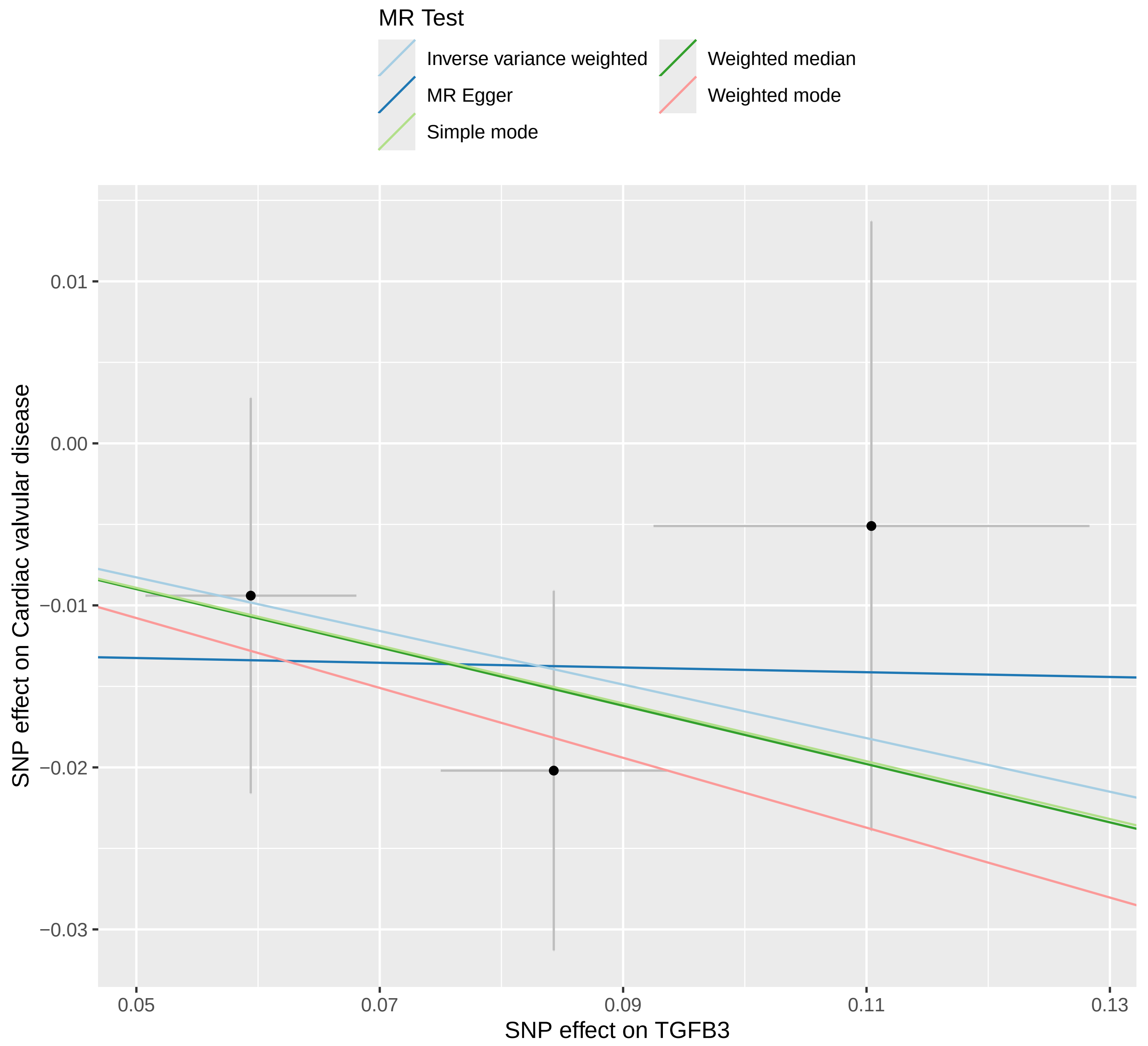
MR Test
Inverse variance weighted
MR Egger
Simple mode
Weighted median
Weighted mode
0.01
0.00
−0.01
−0.02
−0.03
0.05
0.07
0.09
0.11
0.13
SNP effect on Cardiac valvular disease
SNP effect on TGFB3

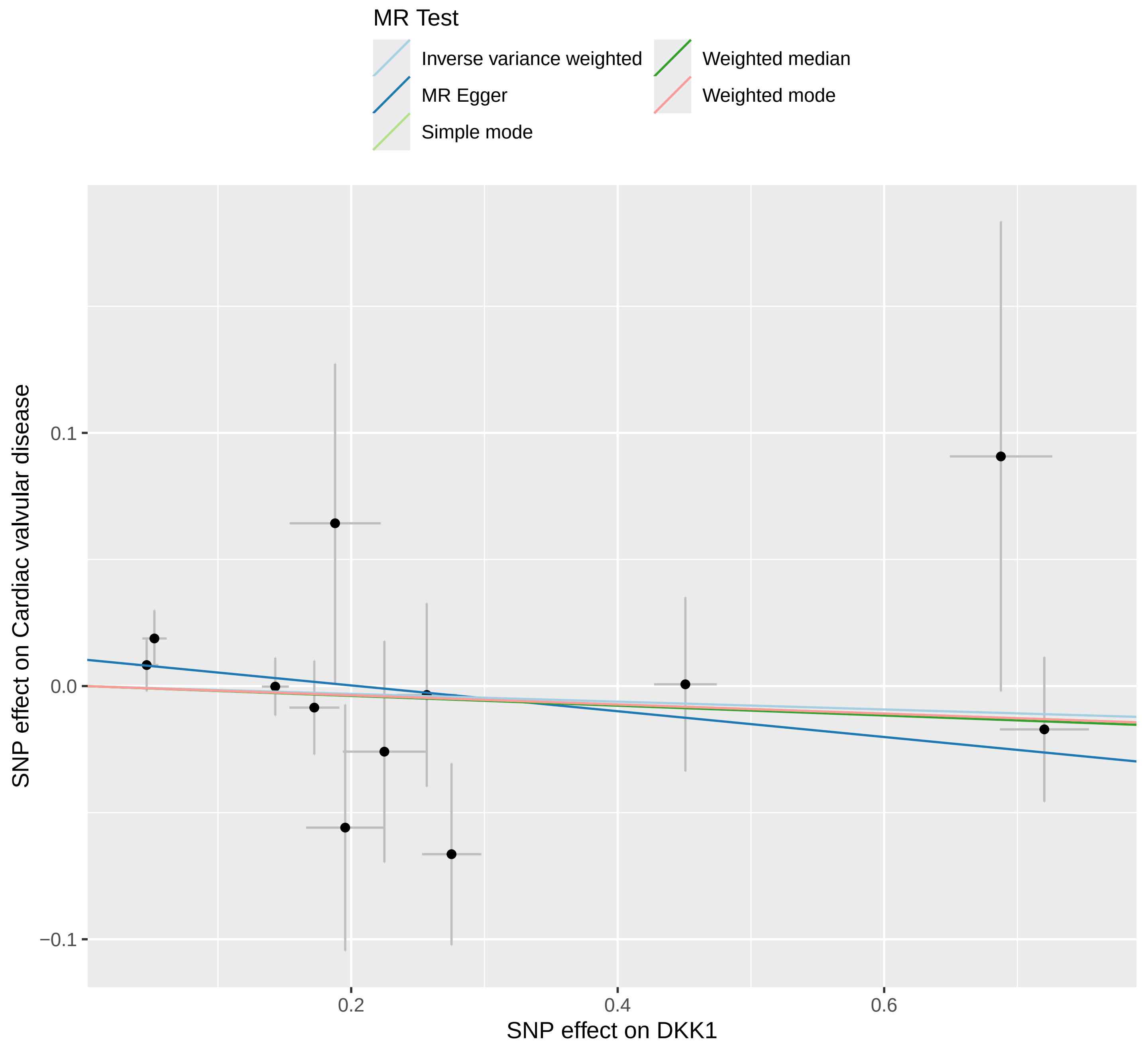

MR Test
Inverse variance weighted
MR Egger
Simple mode
Weighted median
Weighted mode
0.1
0.0
−0.1
0.2
0.4
0.6
SNP effect on DKK1
SNP effect on Cardiac valvular disease

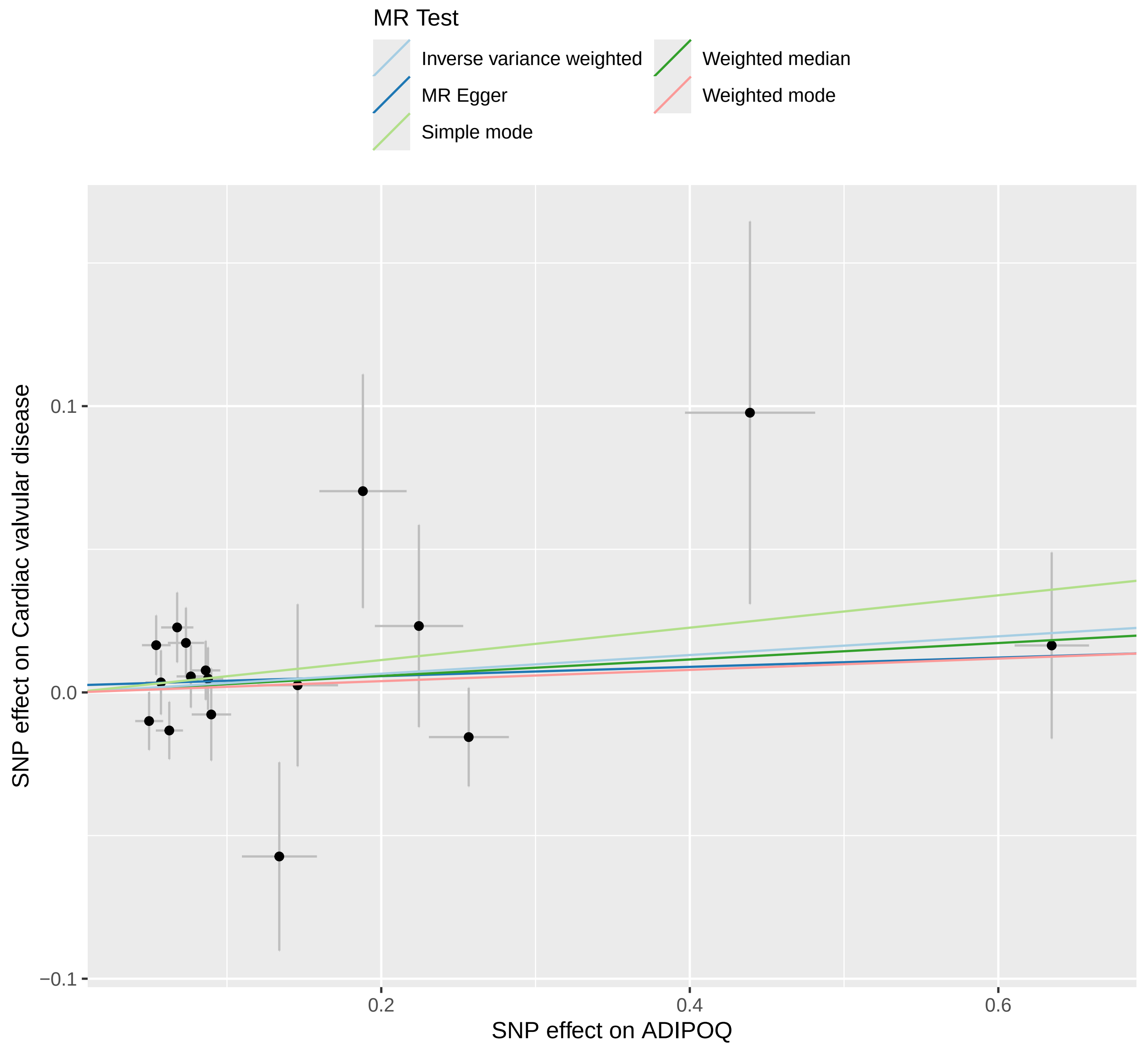
MR Test
Inverse variance weighted
MR Egger
Simple mode
Weighted median
Weighted mode
SNP effect on Cardiac valvular disease
0.1
0.0
−0.1
0.2
0.4
0.6
SNP effect on ADIPOQ

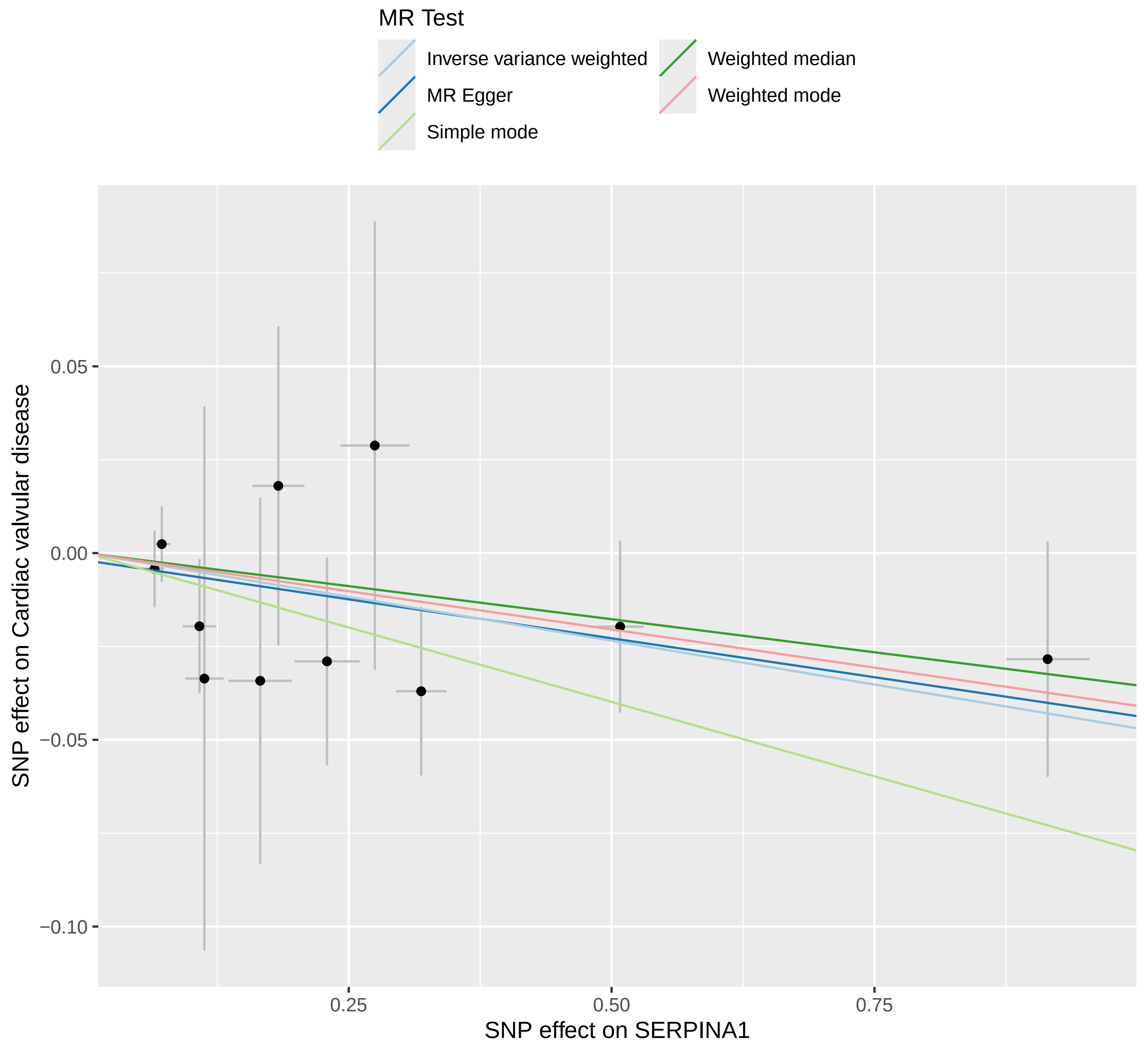
MR Test
Inverse variance weighted
MR Egger
Simple mode
Weighted median
Weighted mode
0.05
0.00
−0.05
−0.10
SNP effect on Cardiac valvular disease
0.25
0.50
0.75
SNP effect on SERPINA1

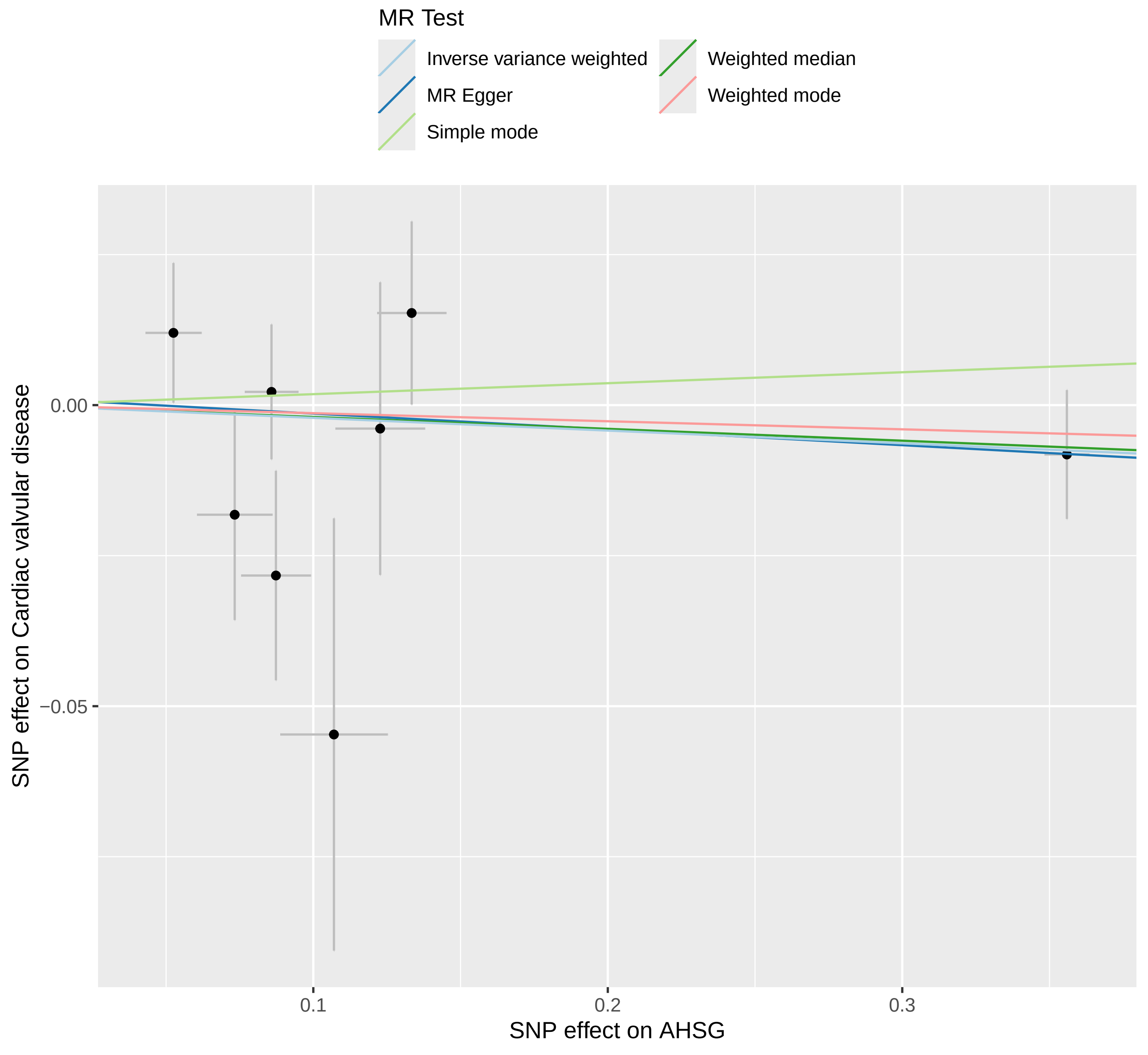
MR Test
Inverse variance weighted
MR Egger
Simple mode
Weighted median
Weighted mode
SNP effect on Cardiac valvular disease
0.00
−0.05
0.1
0.2
0.3
SNP effect on AHSG

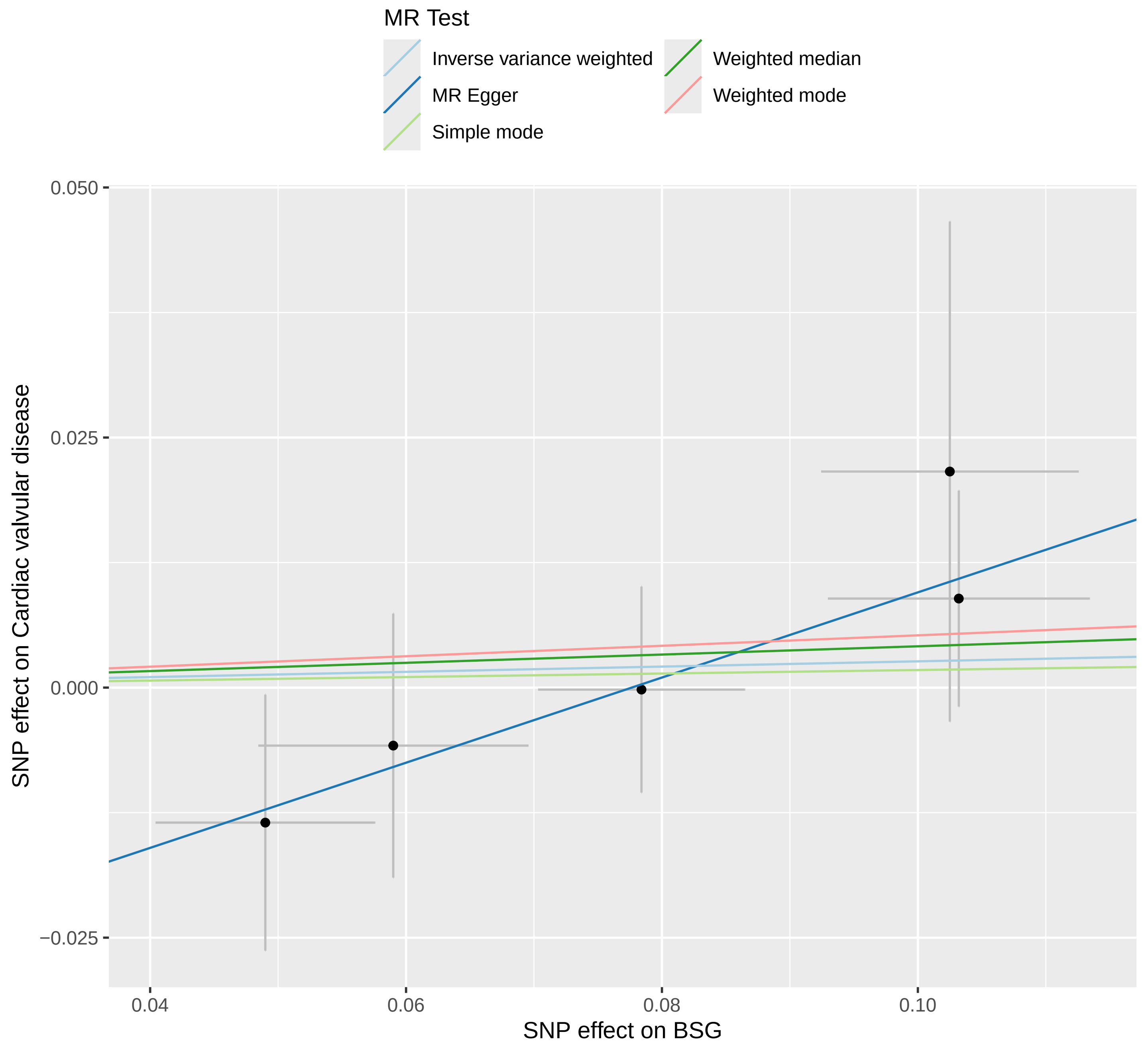
MR Test
Inverse variance weighted
MR Egger
Simple mode
Weighted median
Weighted mode
0.050
0.025
0.000
−0.025
SNP effect on Cardiac valvular disease
0.04
0.06
0.08
0.10
SNP effect on BSG

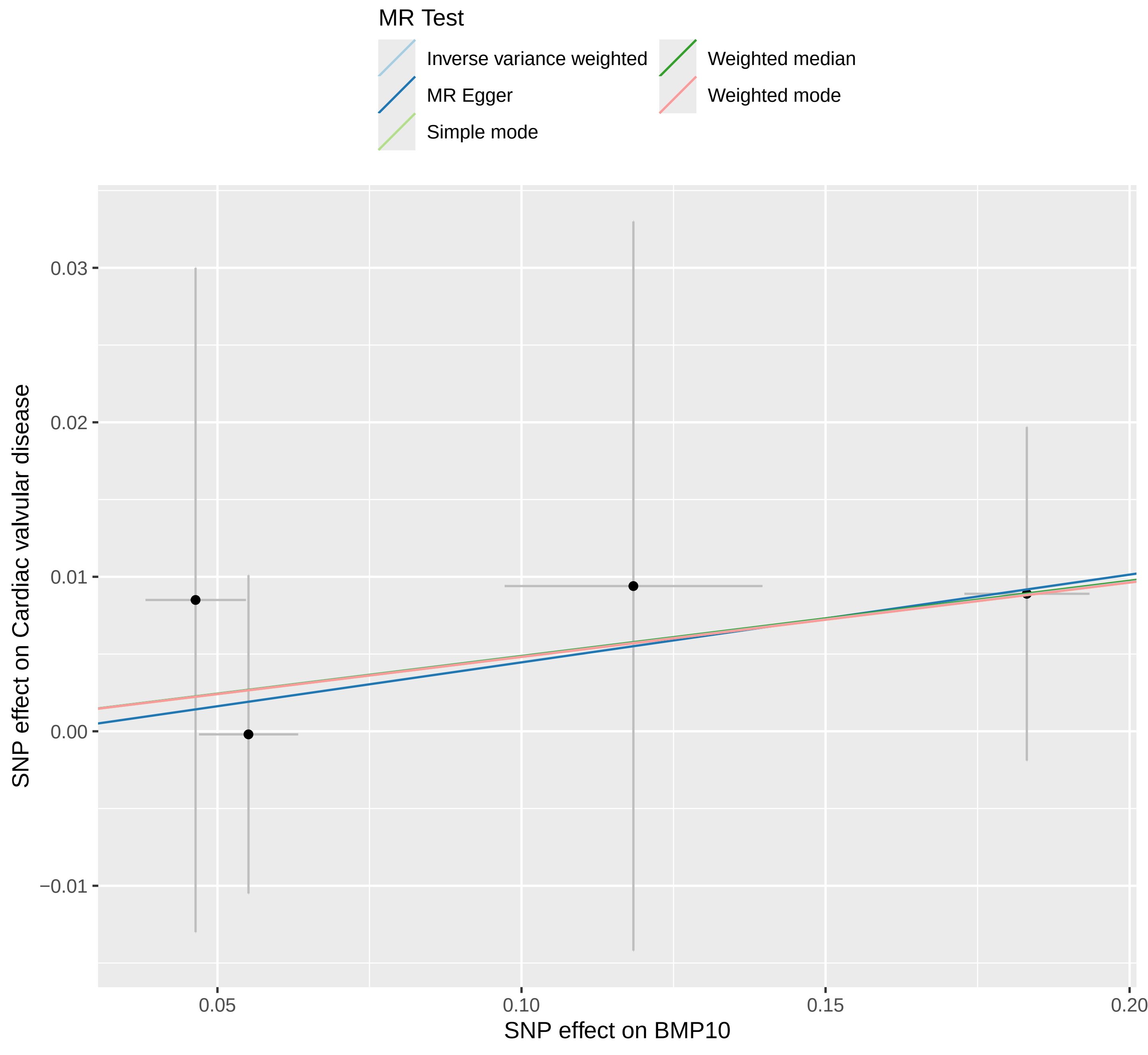
MR Test
Inverse variance weighted
MR Egger
Simple mode
Weighted median
Weighted mode
0.03
0.02
0.01
0.00
−0.01
0.05
0.10
0.15
0.20
SNP effect on Cardiac valvular disease
SNP effect on BMP10

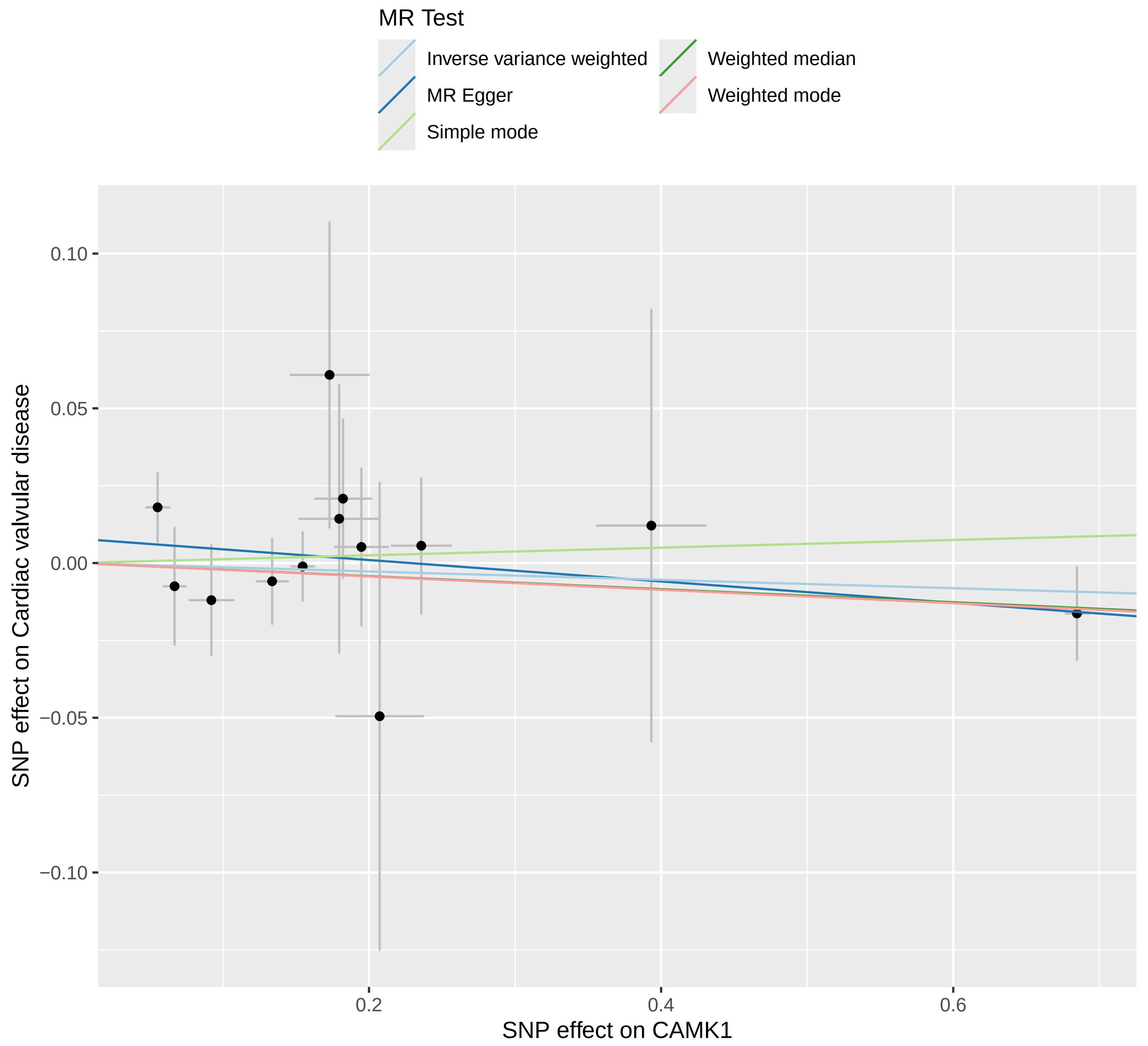
MR Test
Inverse variance weighted
MR Egger
Simple mode
Weighted median
Weighted mode
0.10
0.05
0.00
−0.05
−0.10
SNP effect on Cardiac valvular disease
0.2
0.4
0.6
SNP effect on CAMK1

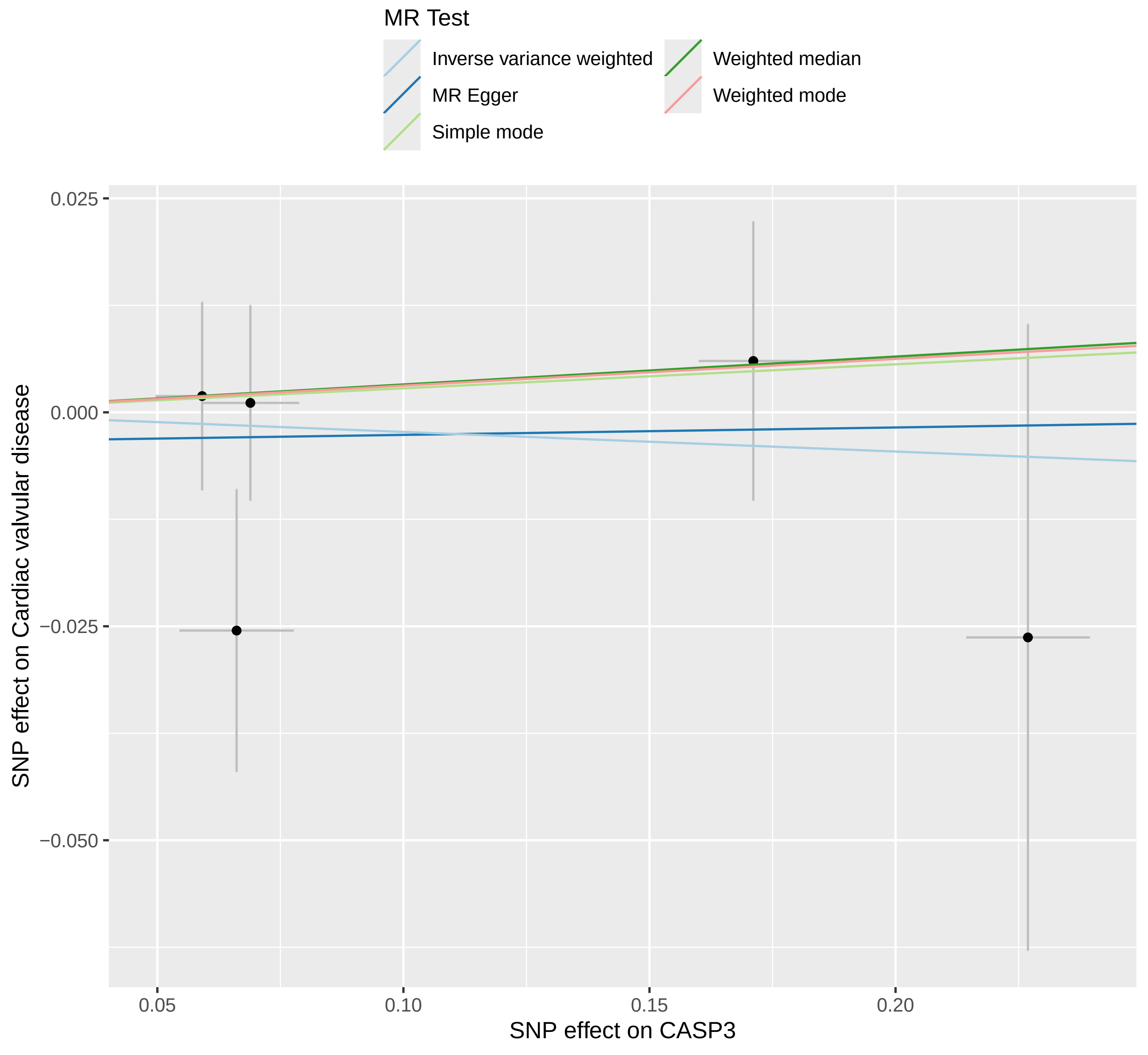
MR Test
Inverse variance weighted
MR Egger
Simple mode
Weighted median
Weighted mode
0.025
0.000
−0.025
−0.050
0.05
0.10
0.15
0.20
SNP effect on Cardiac valvular disease
SNP effect on CASP3

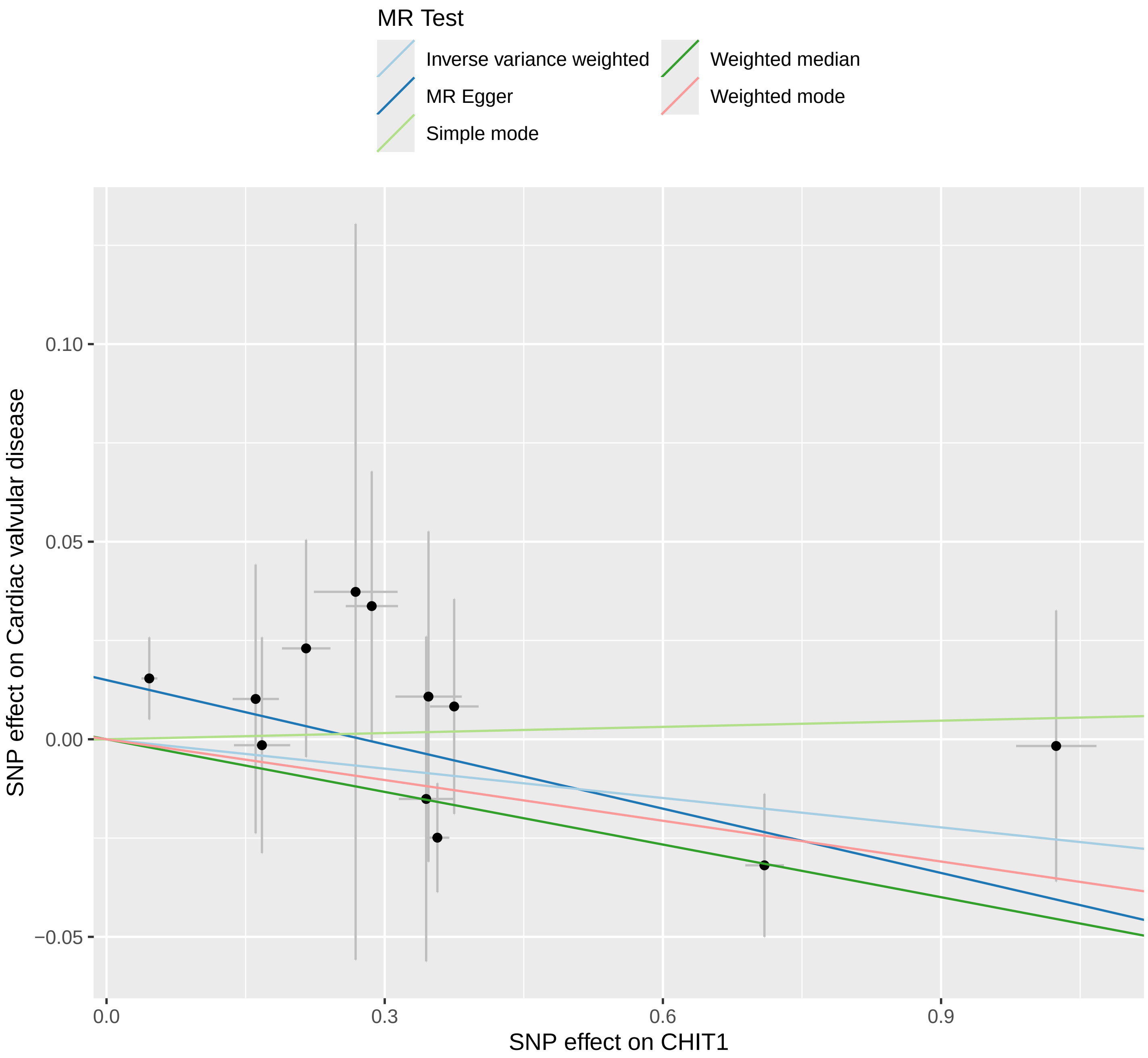

MR Test
Inverse variance weighted
MR Egger
Simple mode
Weighted median
Weighted mode
SNP effect on Cardiac valvular disease
0.10
0.05
0.00
−0.05
0.0
0.3
0.6
0.9
SNP effect on CHIT1

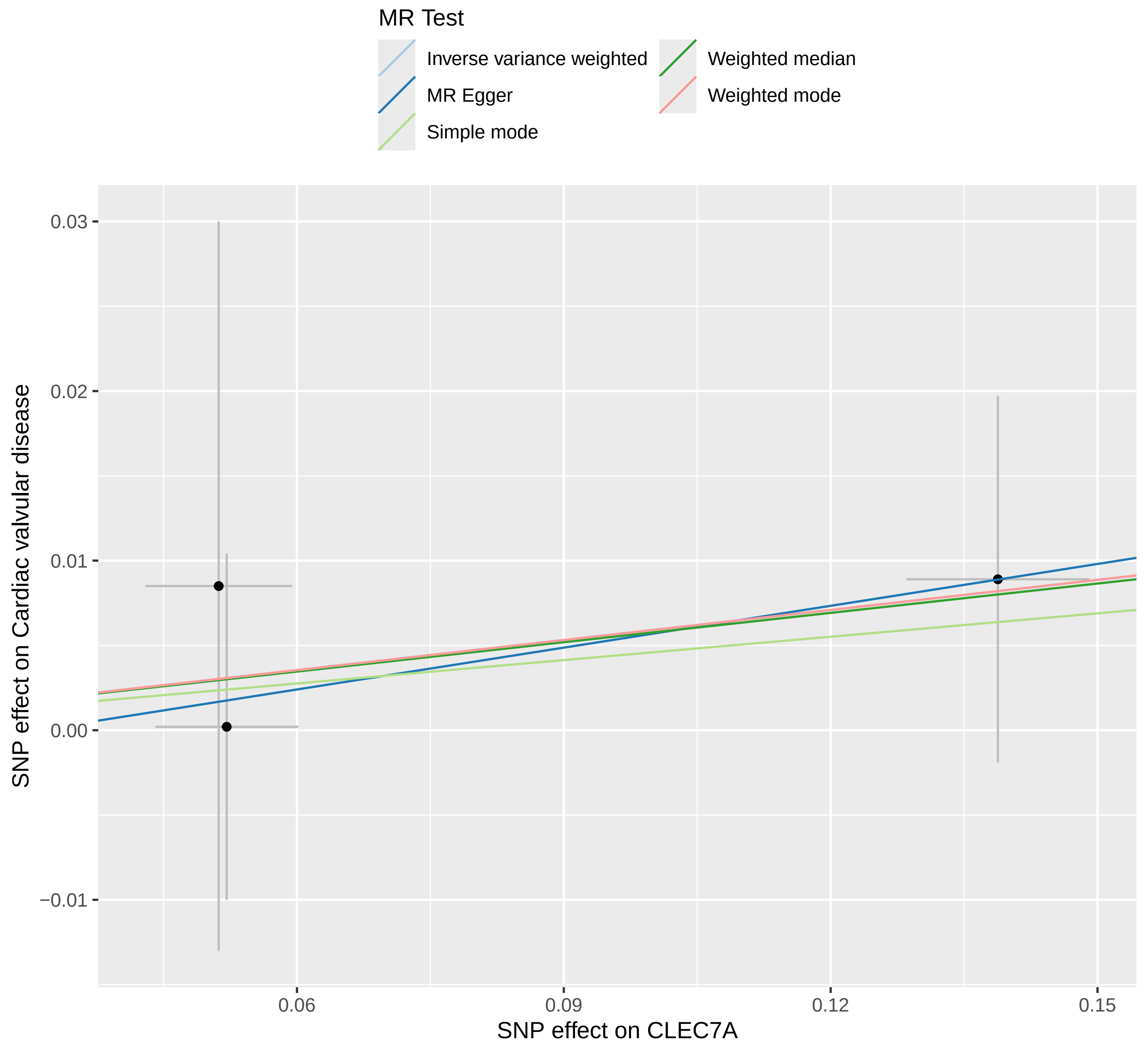

MR Test
Inverse variance weighted
MR Egger
Simple mode
Weighted median
Weighted mode
SNP effect on Cardiac valvular disease
0.03
0.02
0.01
0.00
−0.01
0.06
0.09
0.12
0.15
SNP effect on CLEC7A

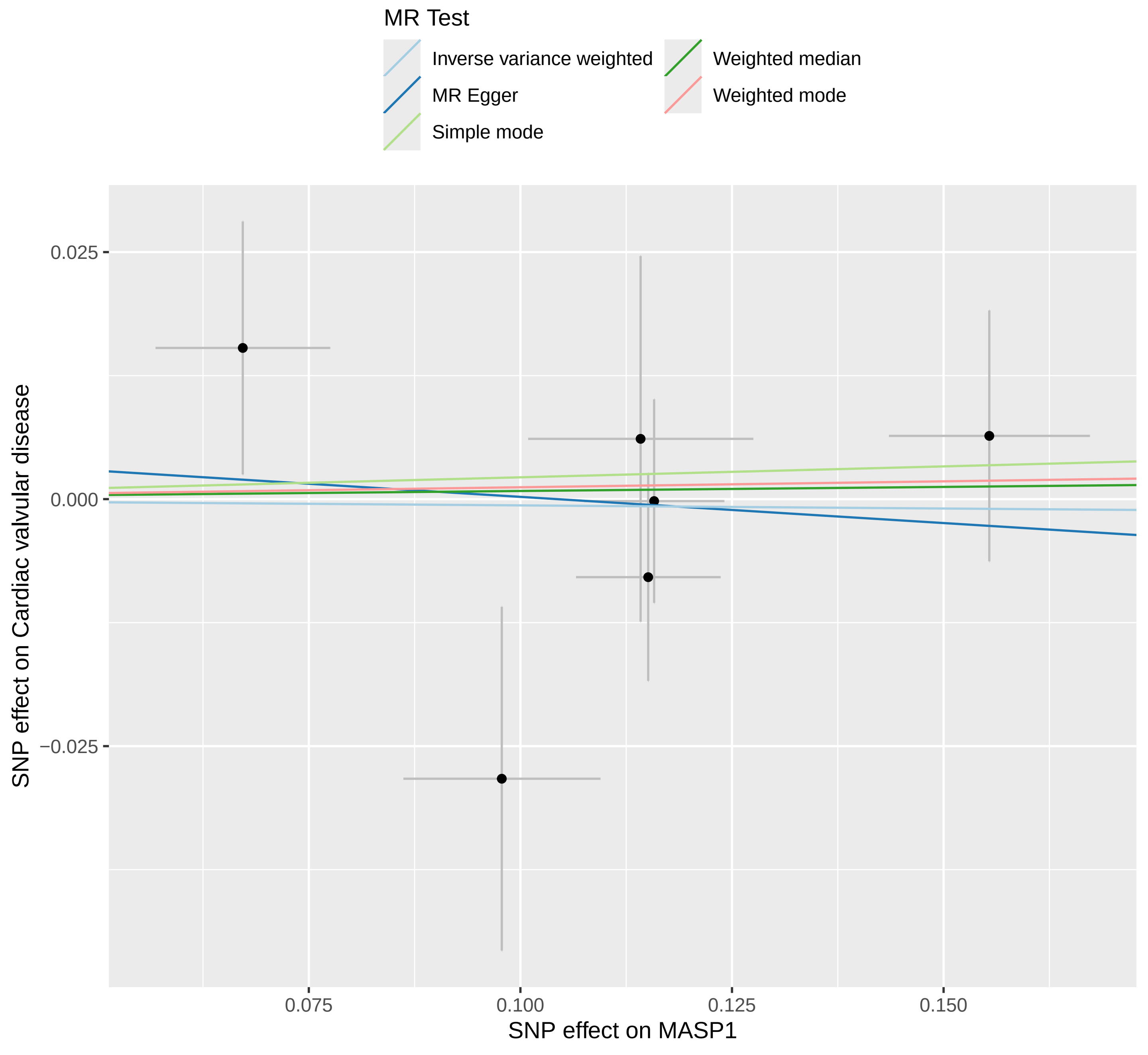
MR Test
Inverse variance weighted
MR Egger
Simple mode
Weighted median
Weighted mode
0.025
0.000
−0.025
0.075
0.100
0.125
0.150
SNP effect on Cardiac valvular disease
SNP effect on MASP1

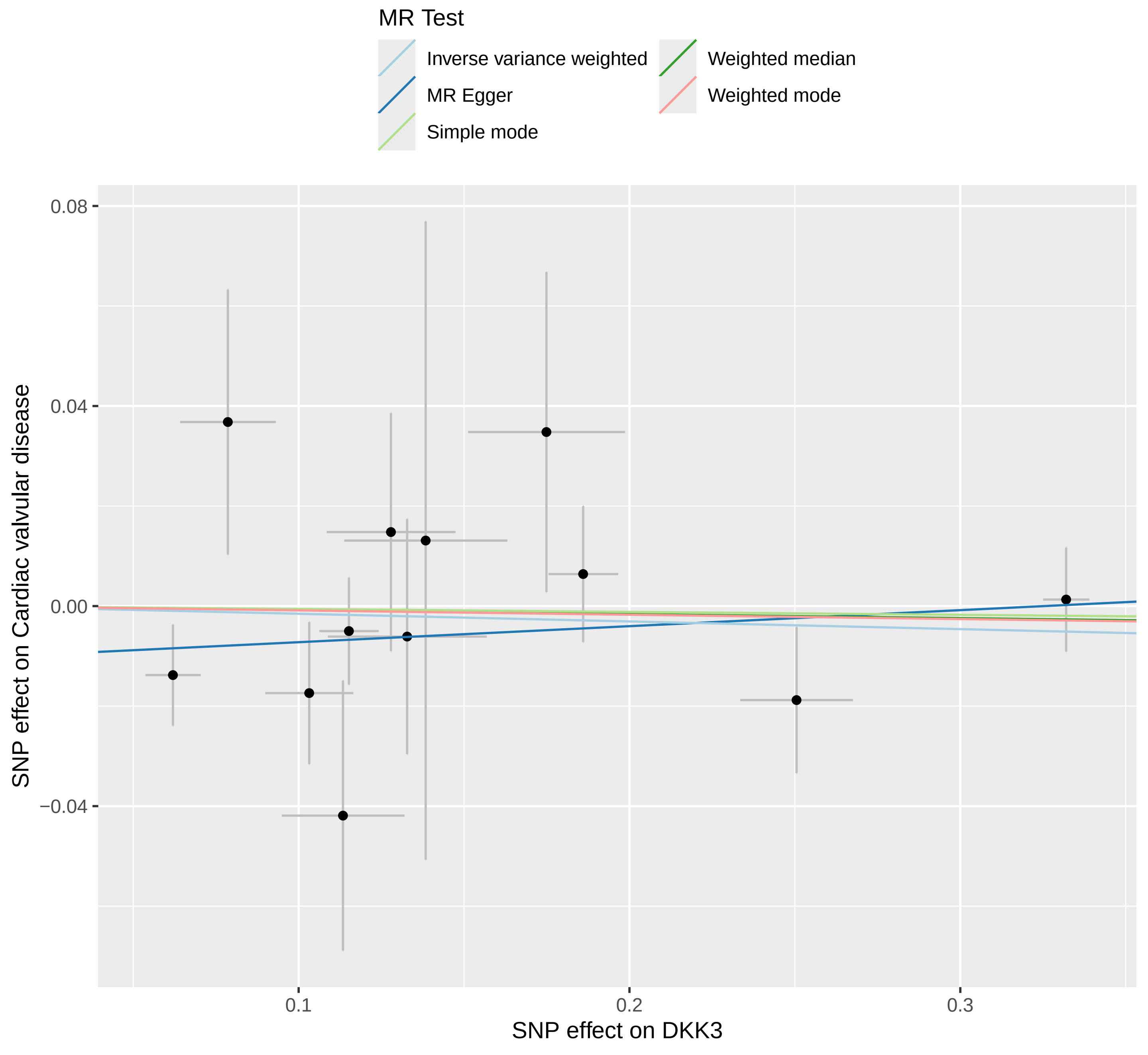

MR Test
Inverse variance weighted
MR Egger
Simple mode
Weighted median
Weighted mode
0.08
0.04
0.00
−0.04
SNP effect on Cardiac valvular disease
0.1
0.2
0.3
SNP effect on DKK3

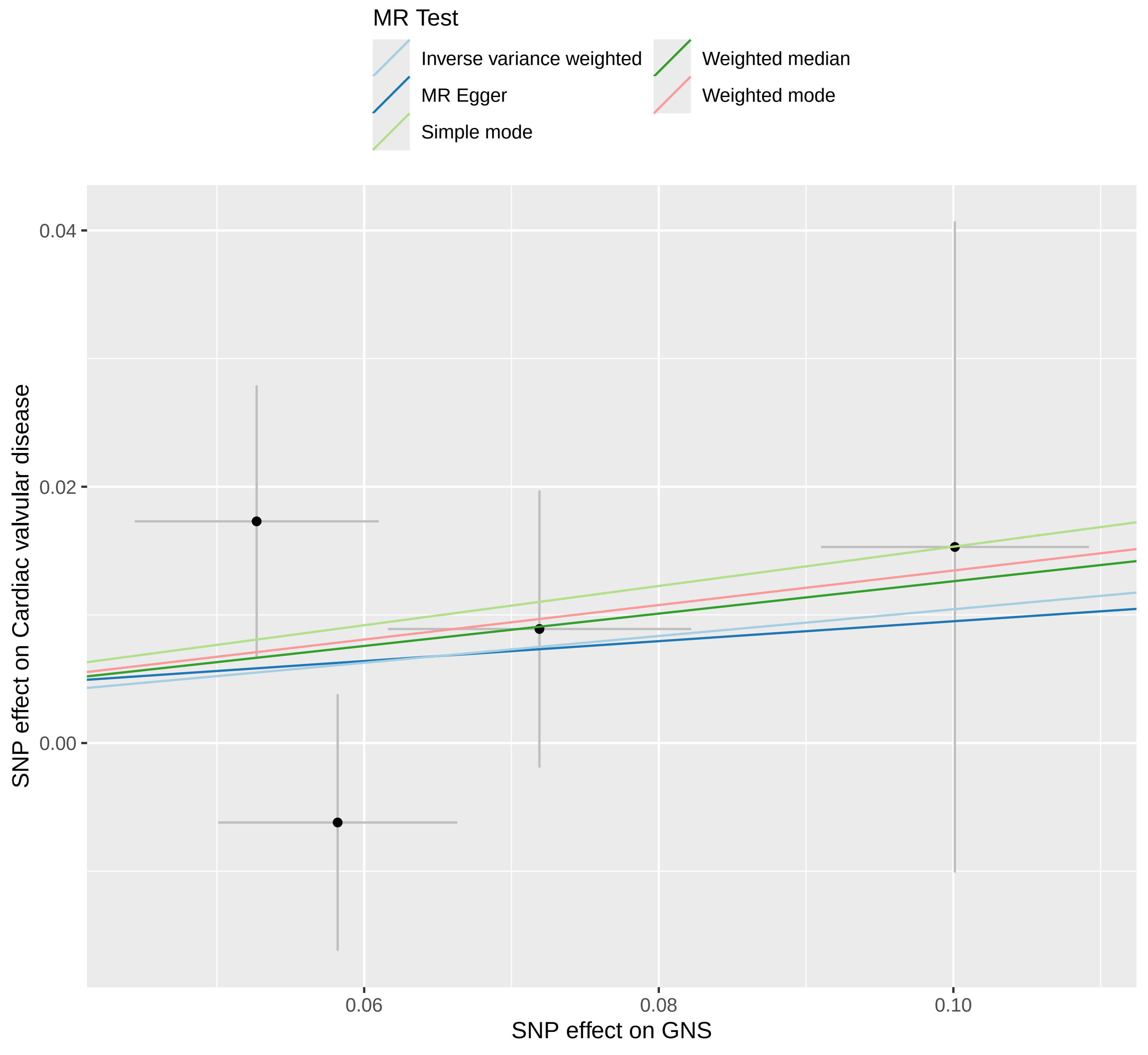
MR Test
Inverse variance weighted
MR Egger
Simple mode
Weighted median
Weighted mode
0.04
0.02
0.00
SNP effect on Cardiac valvular disease
0.06
0.08
0.10
SNP effect on GNS

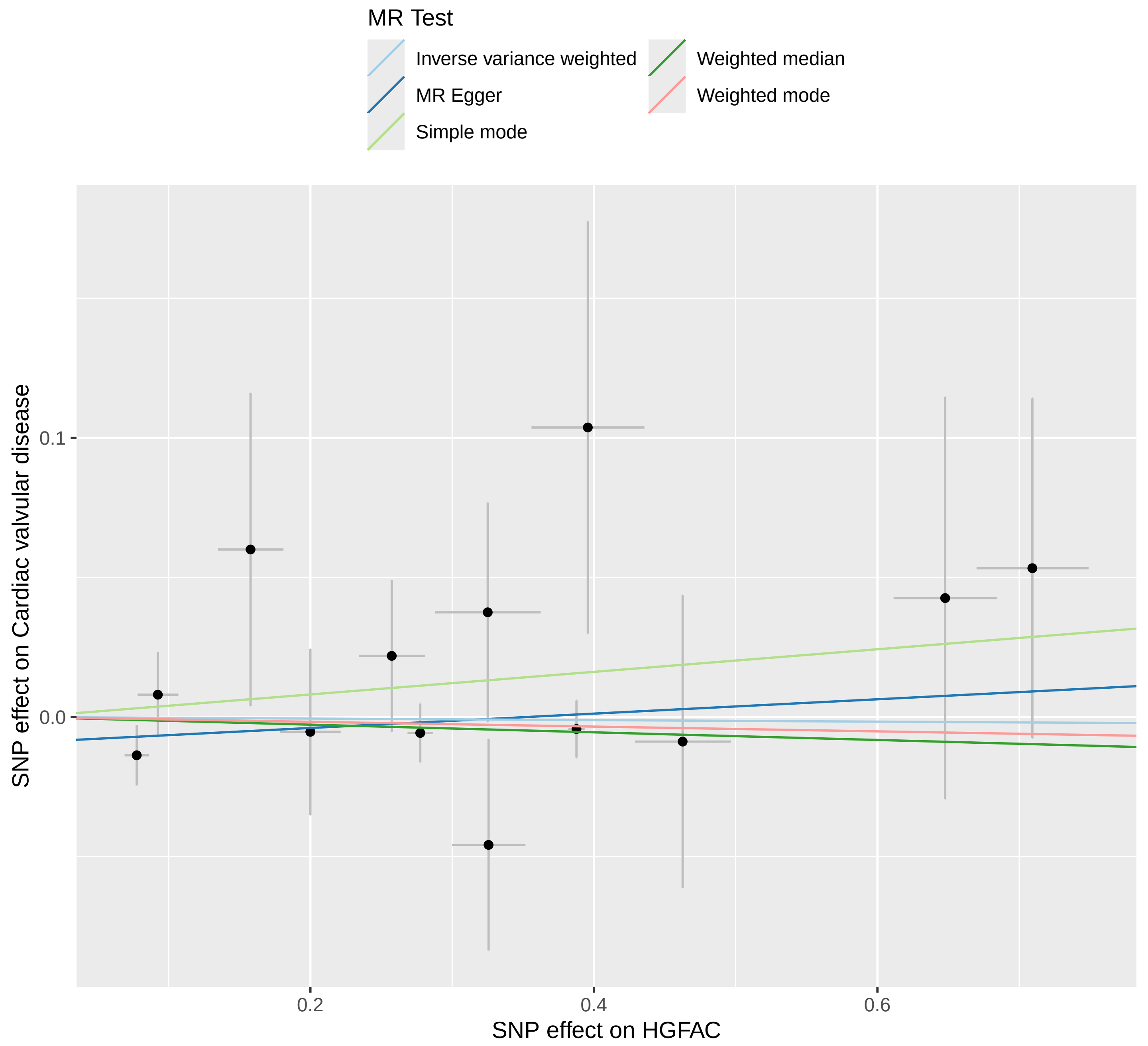
MR Test
Inverse variance weighted
MR Egger
Simple mode
Weighted median
Weighted mode
0.1
0.0
0.2
0.4
0.6
SNP effect on HGFAC
SNP effect on Cardiac valvular disease

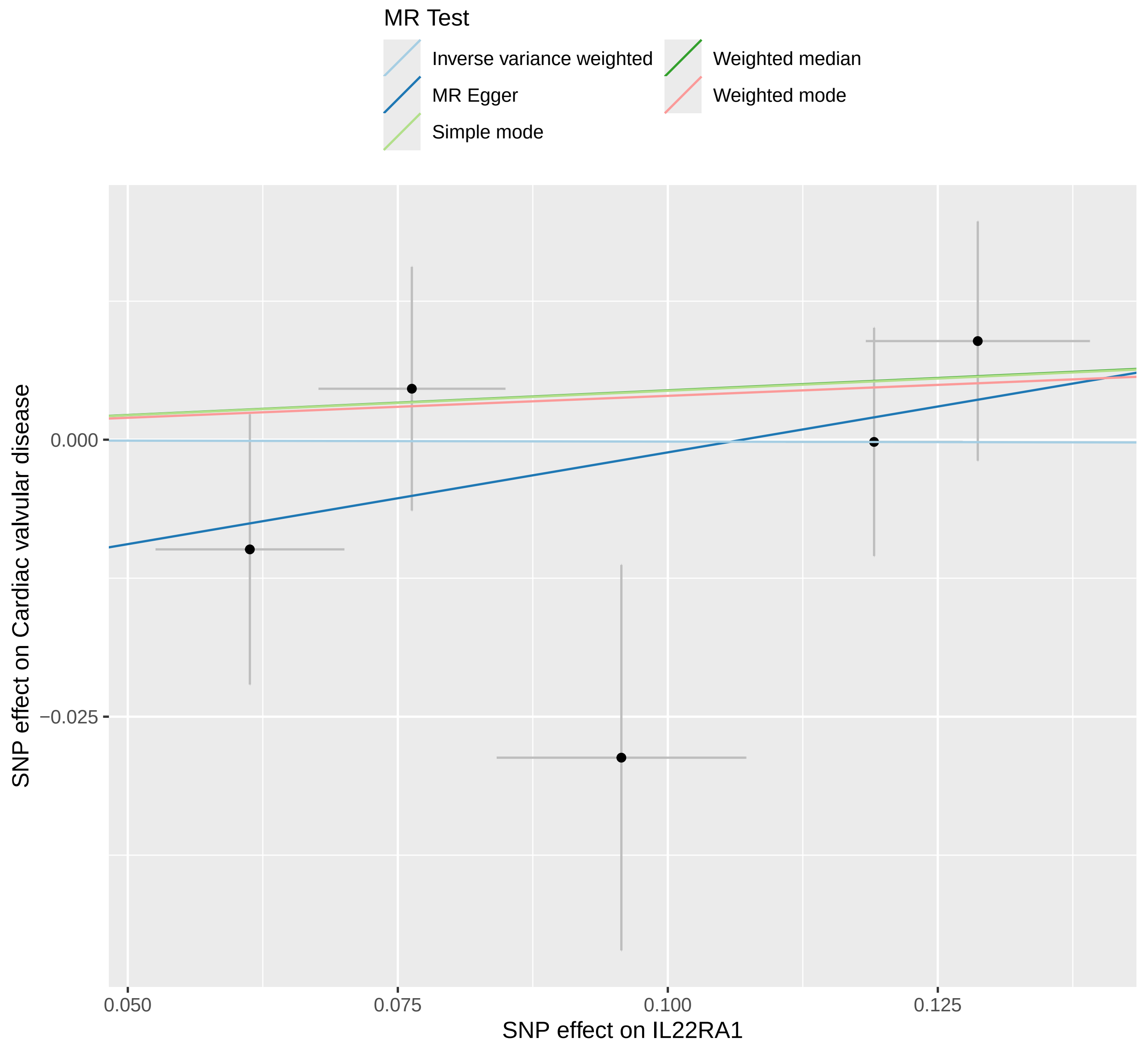
MR Test
Inverse variance weighted
MR Egger
Simple mode
Weighted median
Weighted mode
SNP effect on Cardiac valvular disease
0.000
−0.025
0.050
0.075
0.100
0.125
SNP effect on IL22RA1

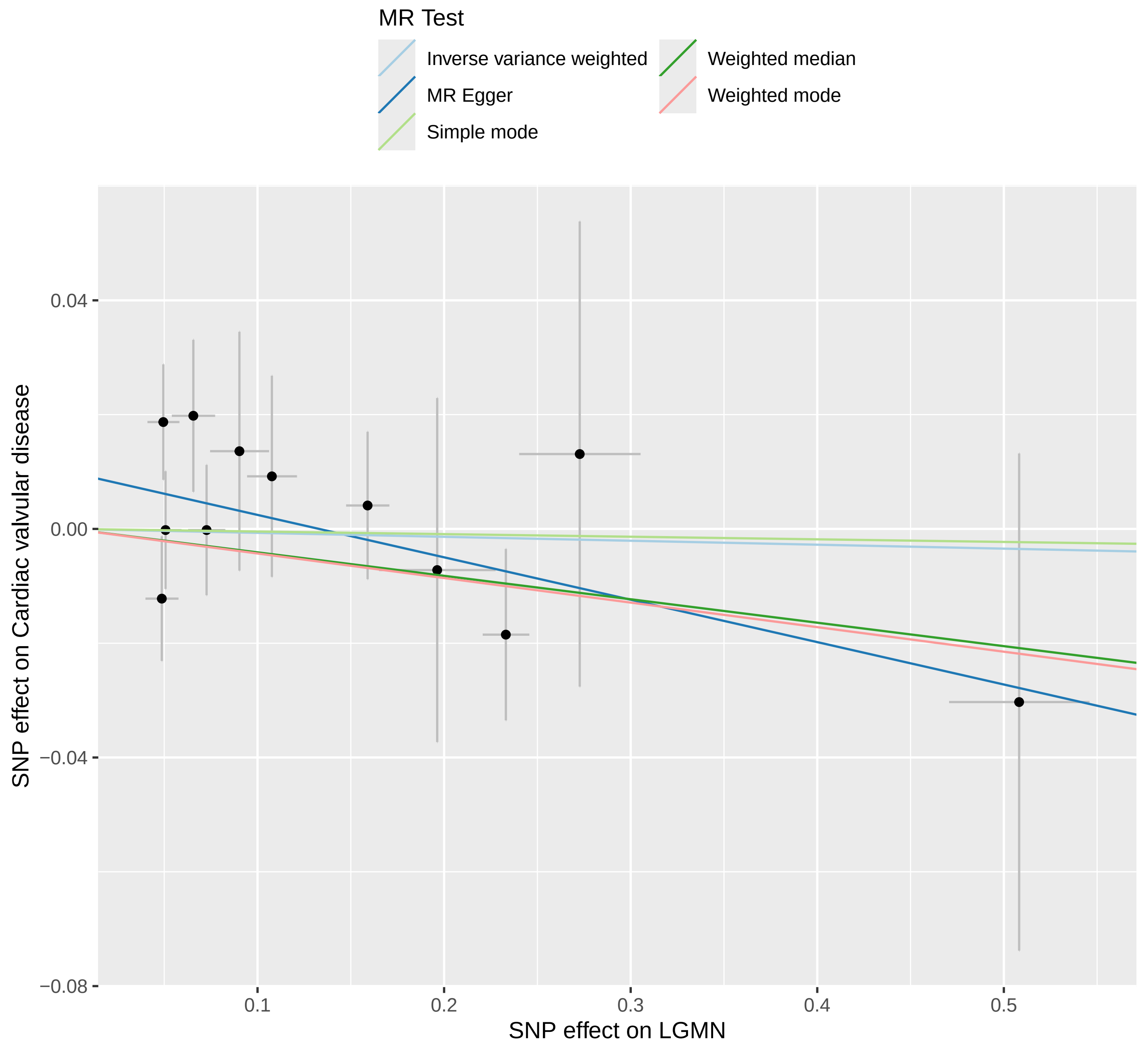

MR Test
Inverse variance weighted
MR Egger
Simple mode
Weighted median
Weighted mode
0.04
0.00
−0.04
−0.08
0.1
0.2
0.3
0.4
0.5
SNP effect on Cardiac valvular disease
SNP effect on LGMN

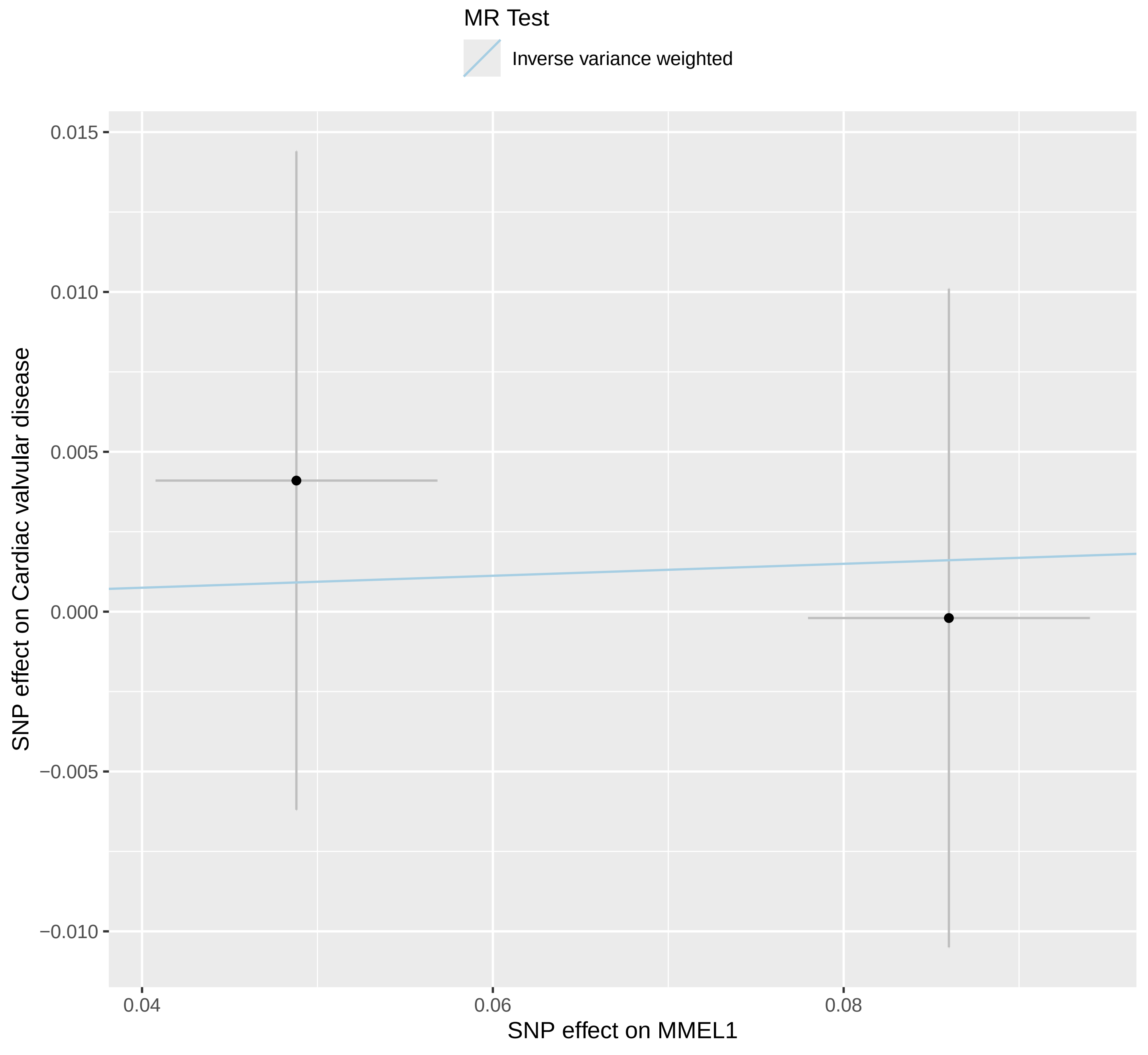

MR Test
Inverse variance weighted
SNP effect on Cardiac valvular disease
0.015
0.010
0.005
0.000
−0.005
−0.010
0.04
0.06
0.08
SNP effect on MMEL1

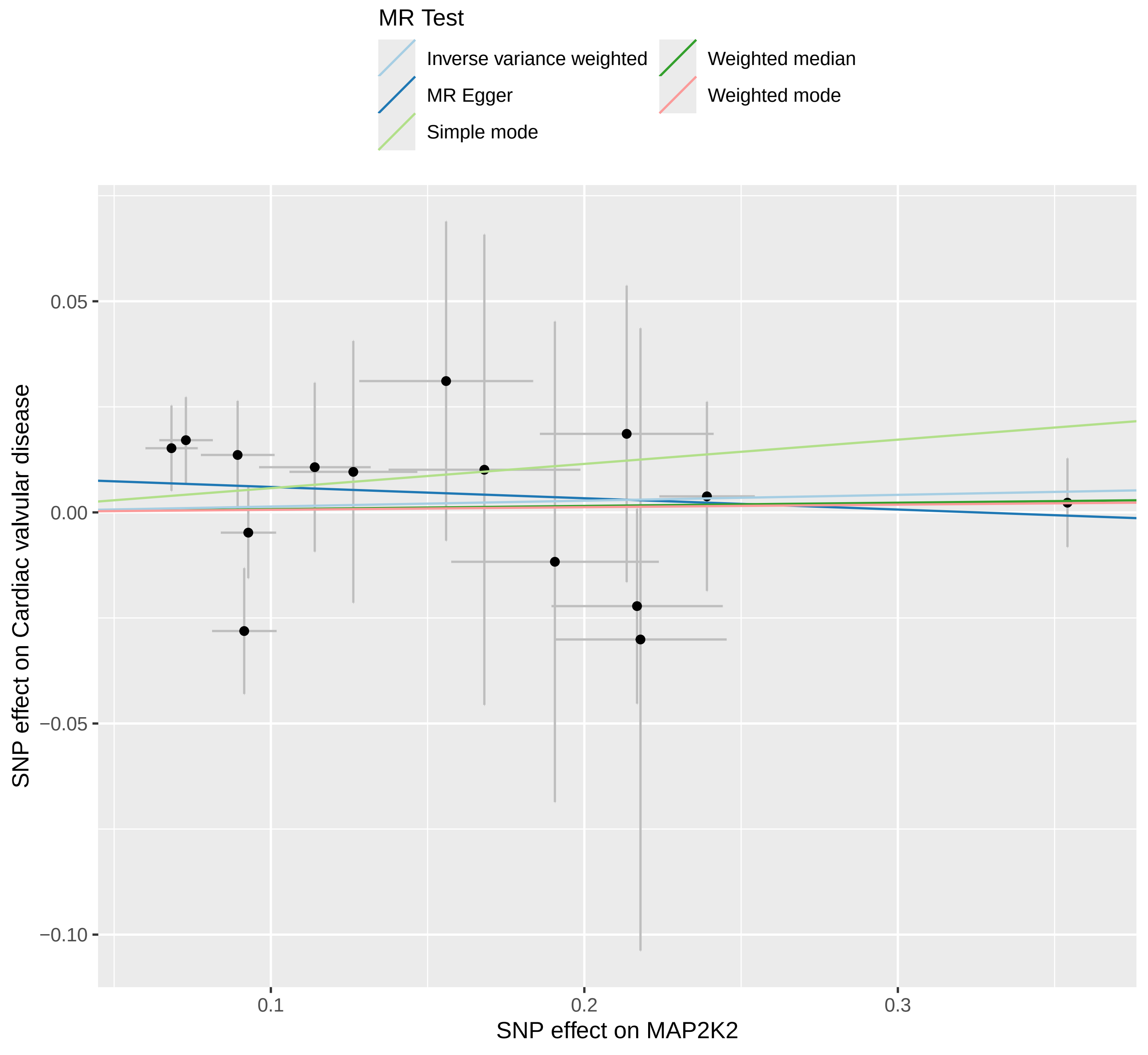

MR Test
Inverse variance weighted
MR Egger
Simple mode
Weighted median
Weighted mode
0.05
0.00
−0.05
−0.10
0.1
0.2
0.3
SNP effect on Cardiac valvular disease
SNP effect on MAP2K2

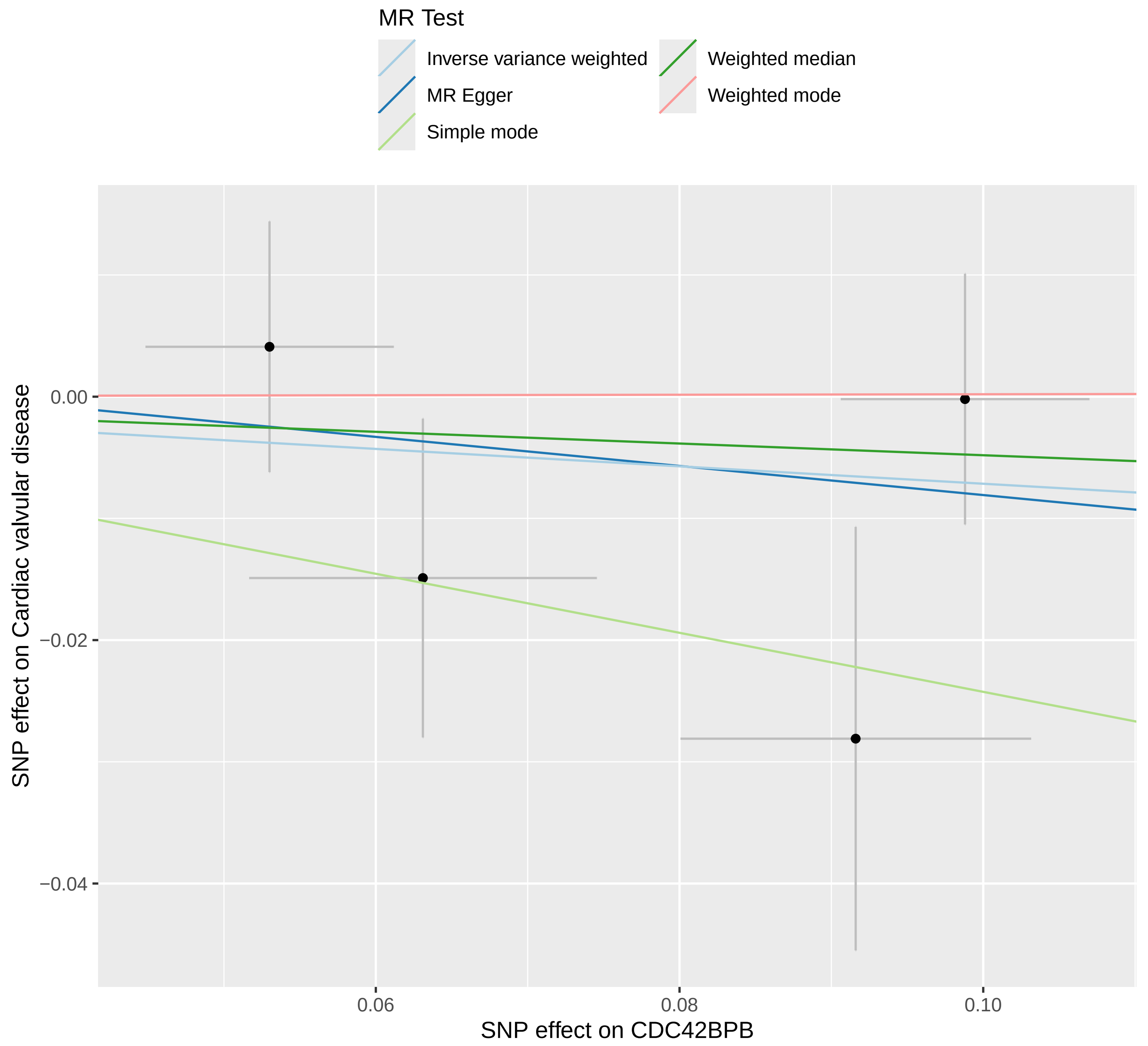
MR Test
Inverse variance weighted
MR Egger
Simple mode
Weighted median
Weighted mode
SNP effect on Cardiac valvular disease
0.00
−0.02
−0.04
0.06
0.08
0.10
SNP effect on CDC42BPB

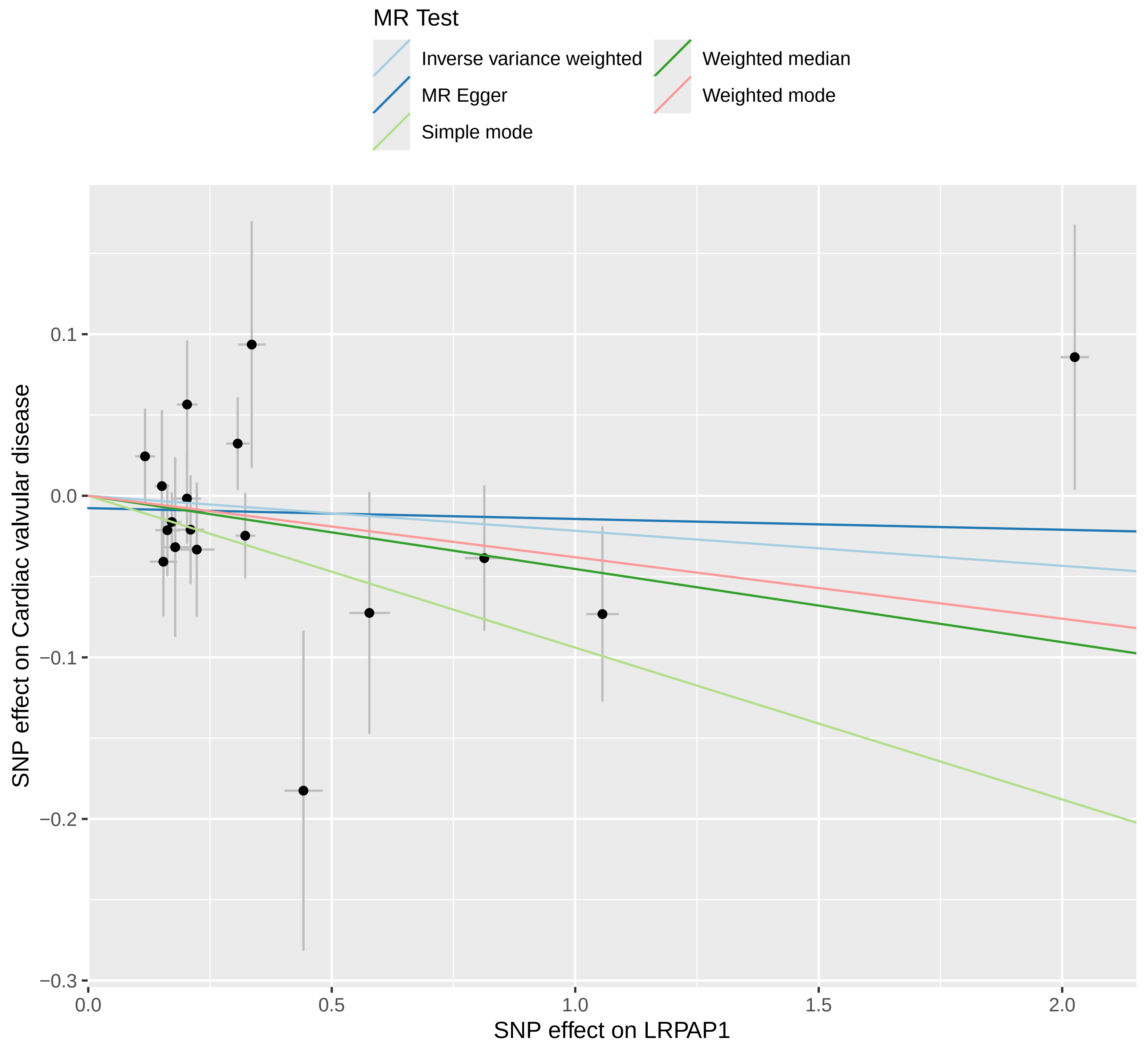

MR Test
Inverse variance weighted
MR Egger
Simple mode
Weighted median
Weighted mode
SNP effect on Cardiac valvular disease
0.1
0.0
−0.1
−0.2
−0.3
0.0
0.5
1.0
1.5
2.0
SNP effect on LRPAP1

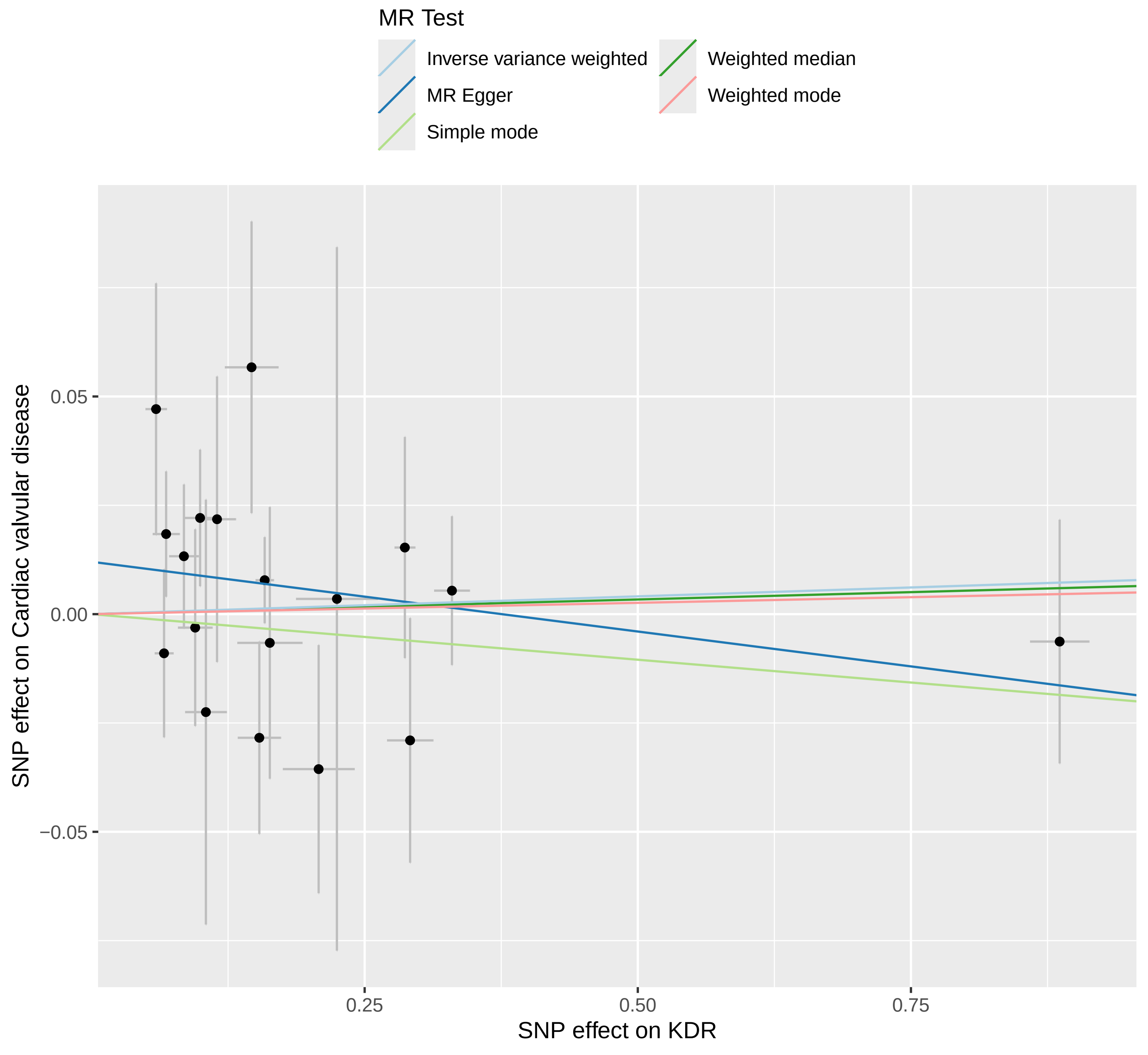
MR Test
Inverse variance weighted
MR Egger
Simple mode
Weighted median
Weighted mode
SNP effect on Cardiac valvular disease
0.05
0.00
−0.05
0.25
0.50
0.75
SNP effect on KDR

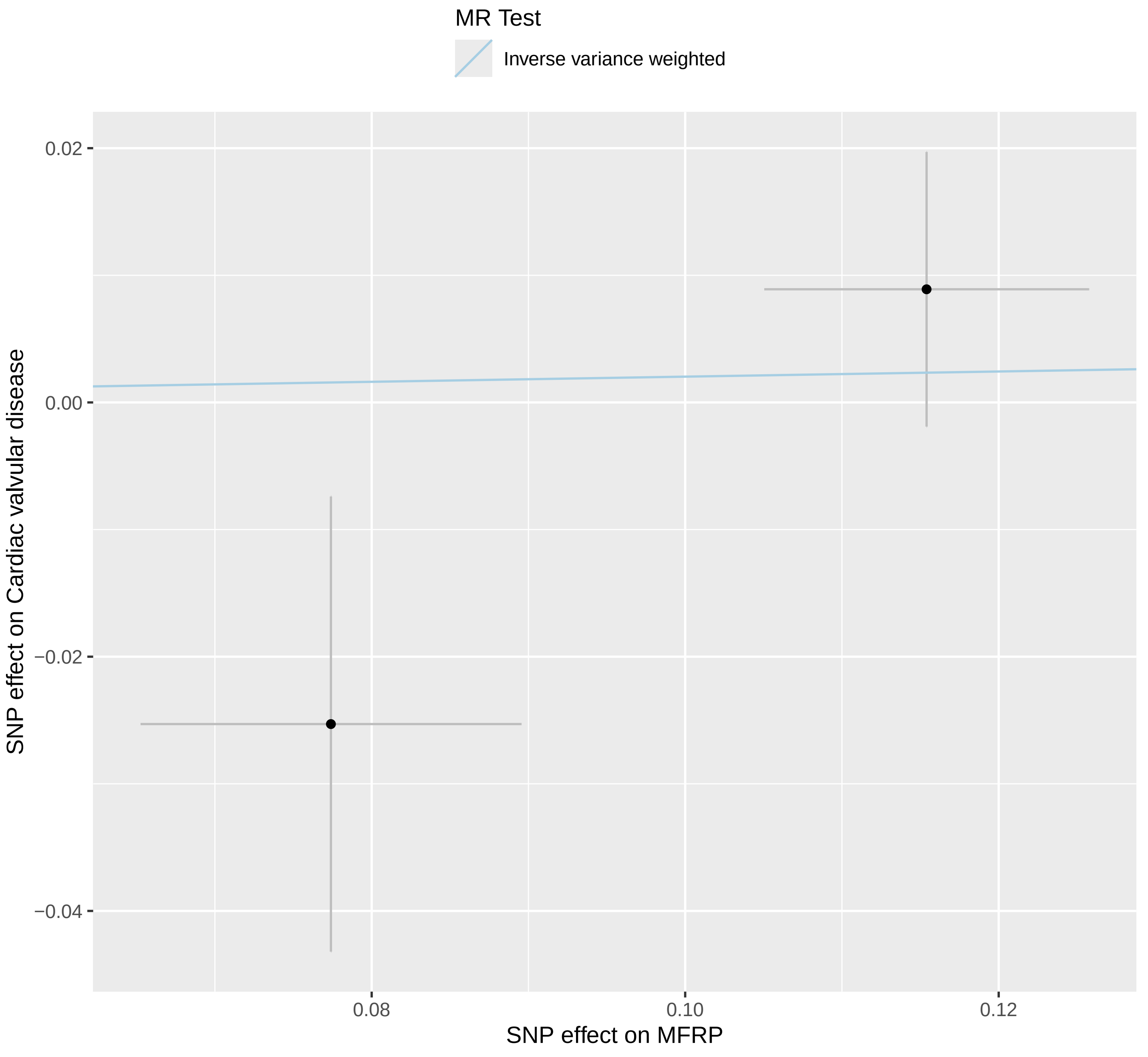
MR Test
Inverse variance weighted
0.02
0.00
−0.02
−0.04
0.08
0.10
0.12
SNP effect on MFRP
SNP effect on Cardiac valvular disease

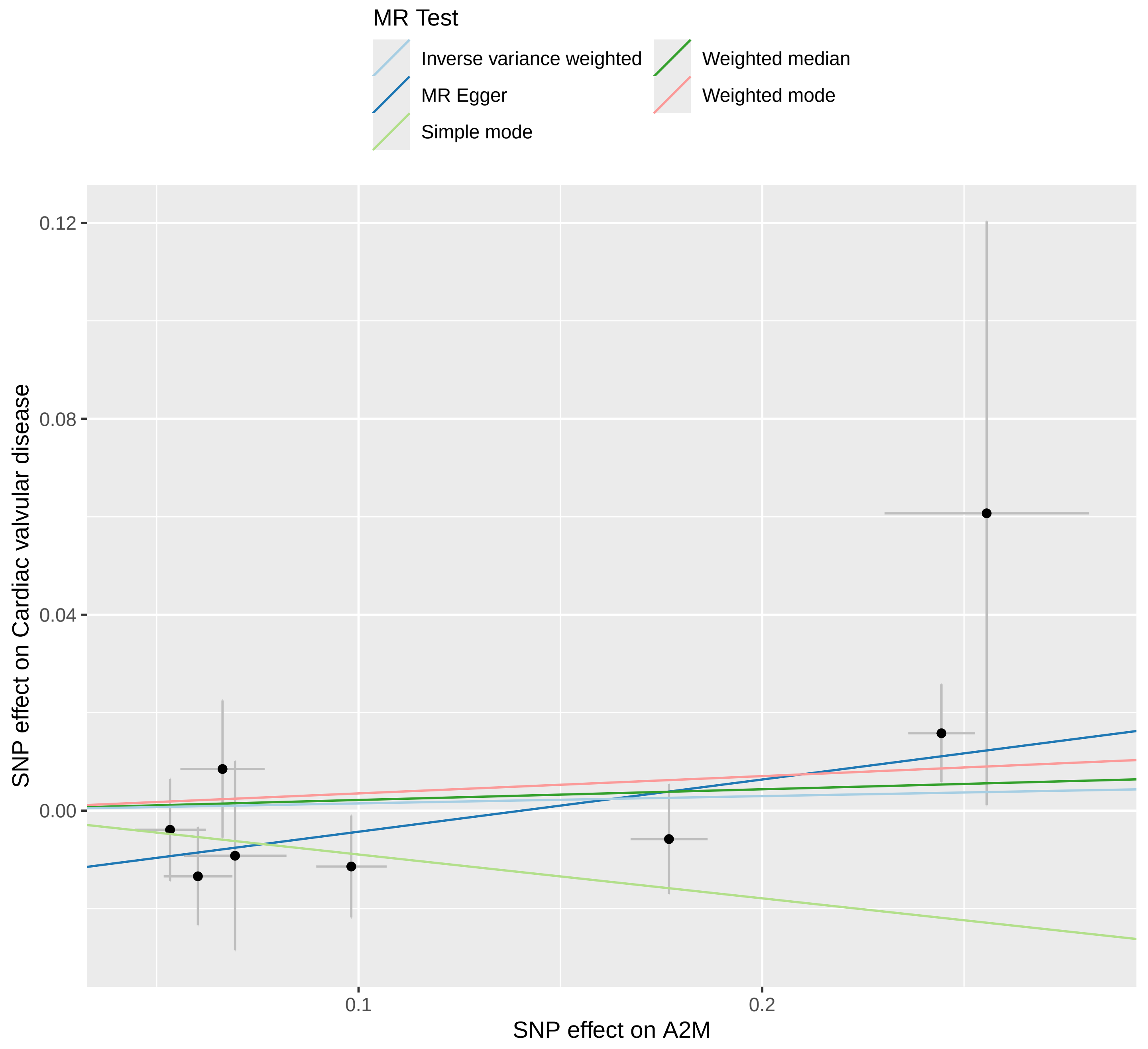
MR Test
Inverse variance weighted
MR Egger
Simple mode
Weighted median
Weighted mode
0.12
0.08
0.04
0.00
0.1
0.2
SNP effect on Cardiac valvular disease
SNP effect on A2M

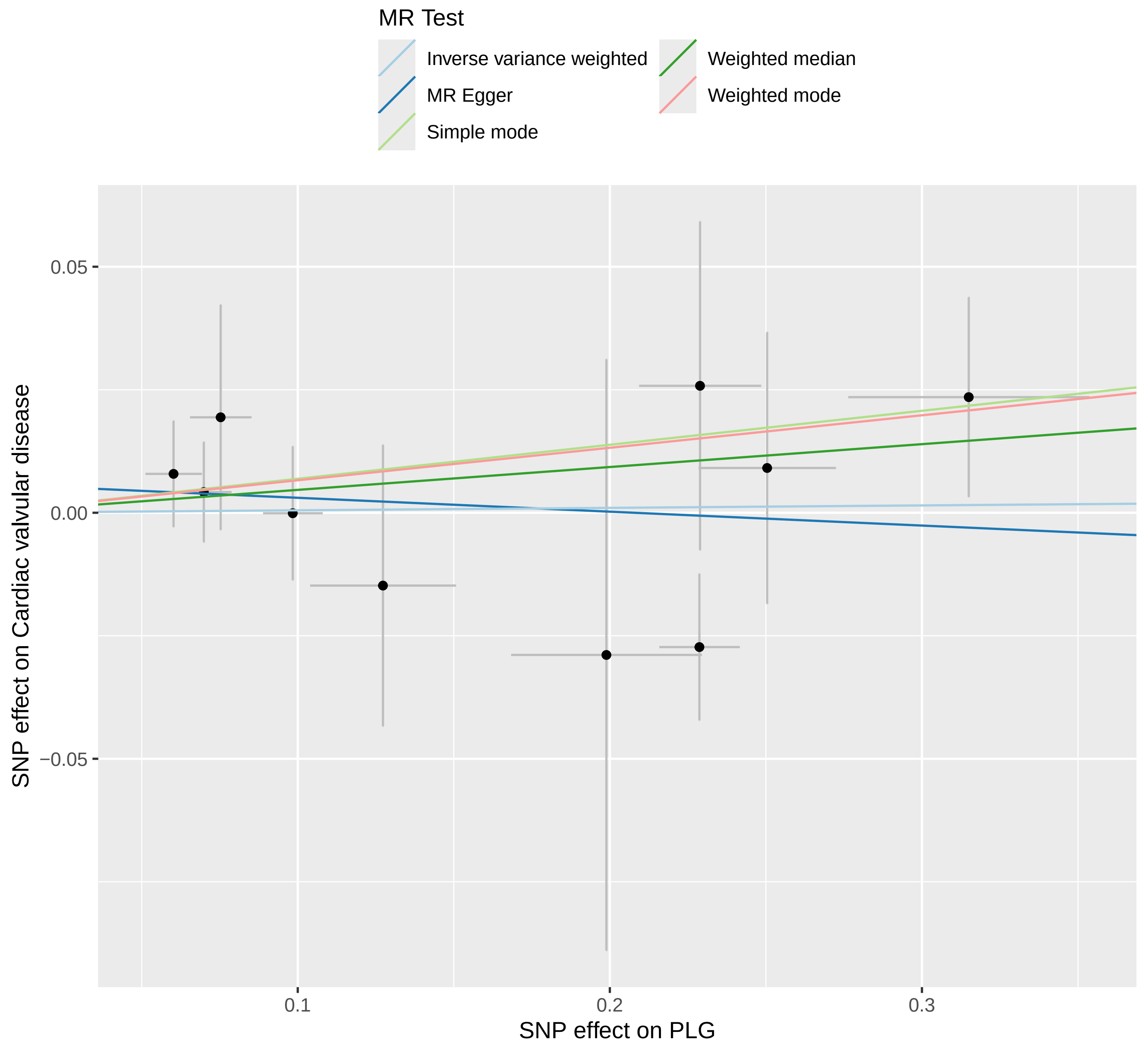
MR Test
Inverse variance weighted
MR Egger
Simple mode
Weighted median
Weighted mode
SNP effect on Cardiac valvular disease
0.05
0.00
−0.05
0.1
0.2
0.3
SNP effect on PLG

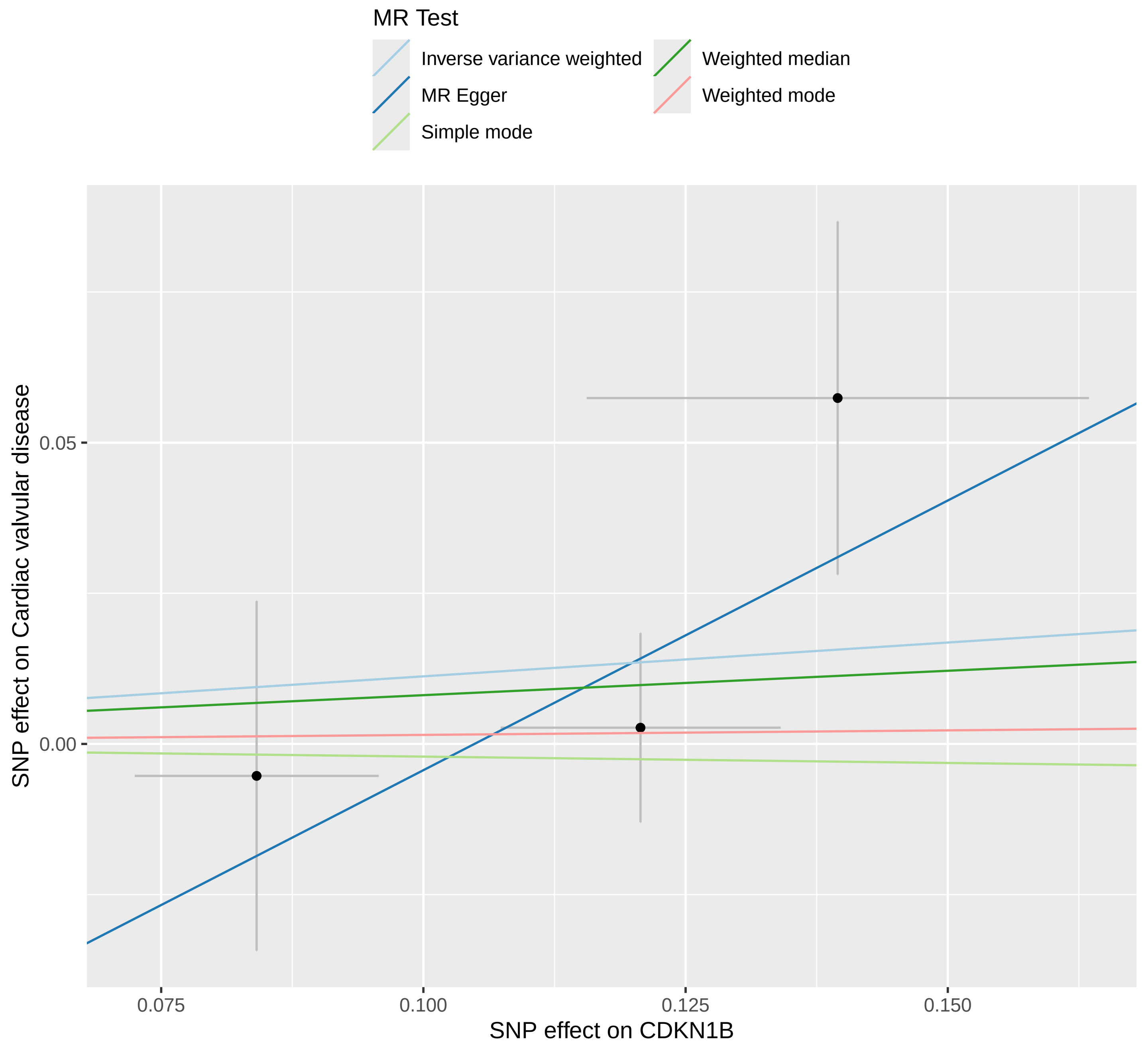
MR Test
Inverse variance weighted
MR Egger
Simple mode
Weighted median
Weighted mode
0.05
0.00
0.075
0.100
0.125
0.150
SNP effect on CDKN1B
SNP effect on Cardiac valvular disease

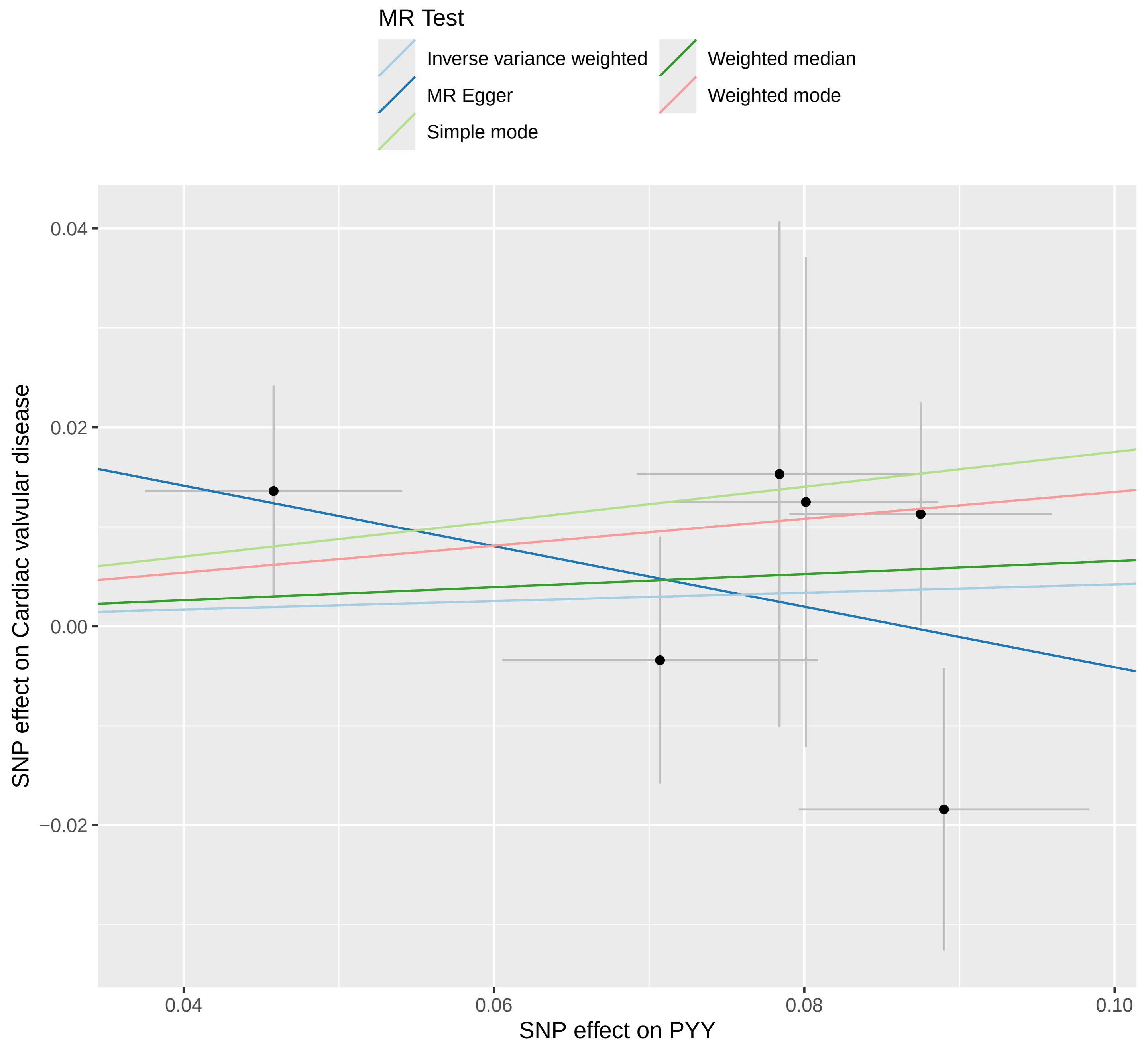
MR Test
Inverse variance weighted
MR Egger
Simple mode
Weighted median
Weighted mode
0.04
0.02
0.00
−0.02
0.04
0.06
0.08
0.10
SNP effect on PYY
SNP effect on Cardiac valvular disease

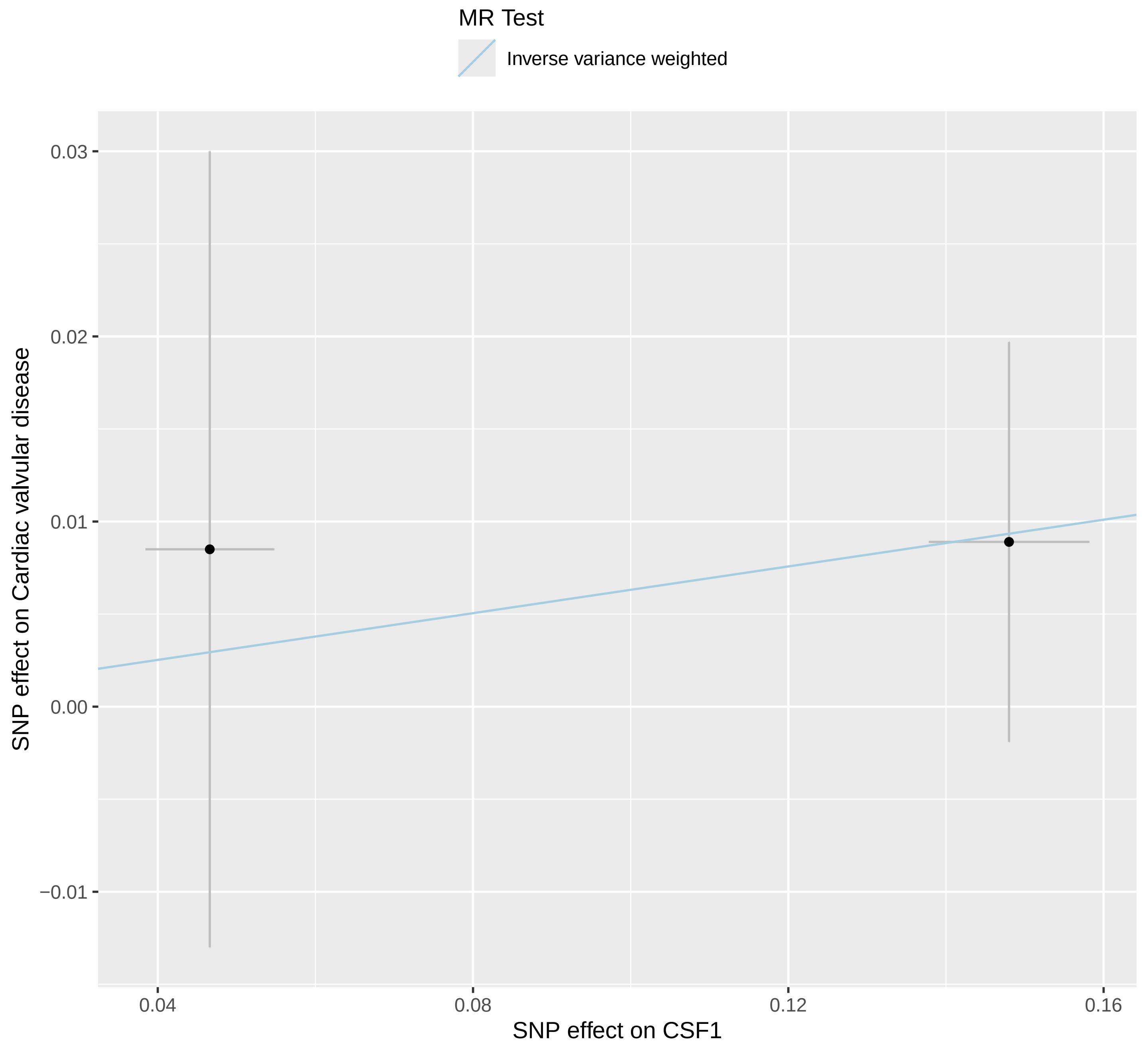

MR Test
Inverse variance weighted
0.03
0.02
0.01
0.00
−0.01
0.04
0.08
0.12
0.16
SNP effect on Cardiac valvular disease
SNP effect on CSF1

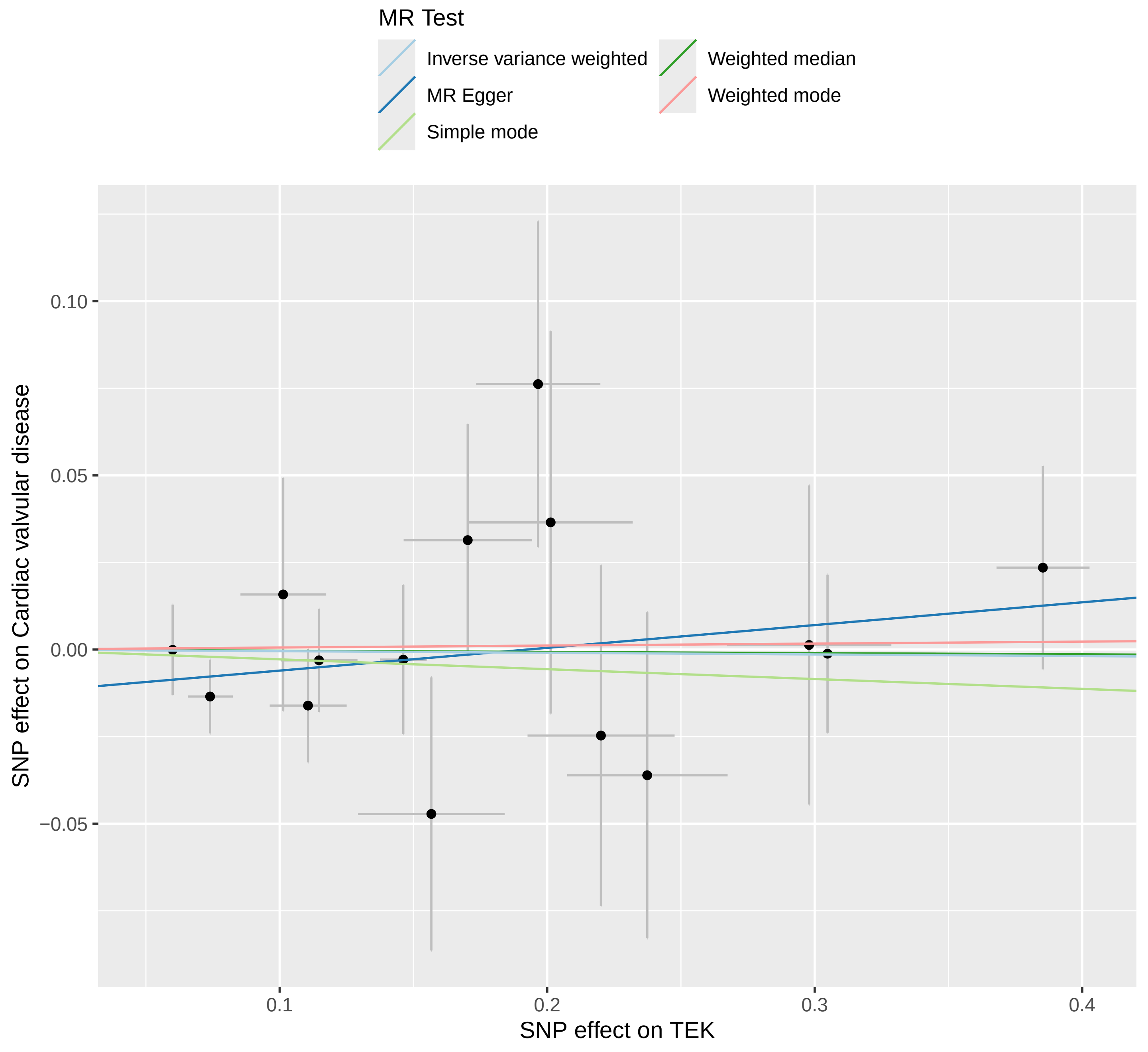
MR Test
Inverse variance weighted
MR Egger
Simple mode
Weighted median
Weighted mode
0.10
0.05
0.00
−0.05
SNP effect on Cardiac valvular disease
0.1
0.2
0.3
0.4
SNP effect on TEK

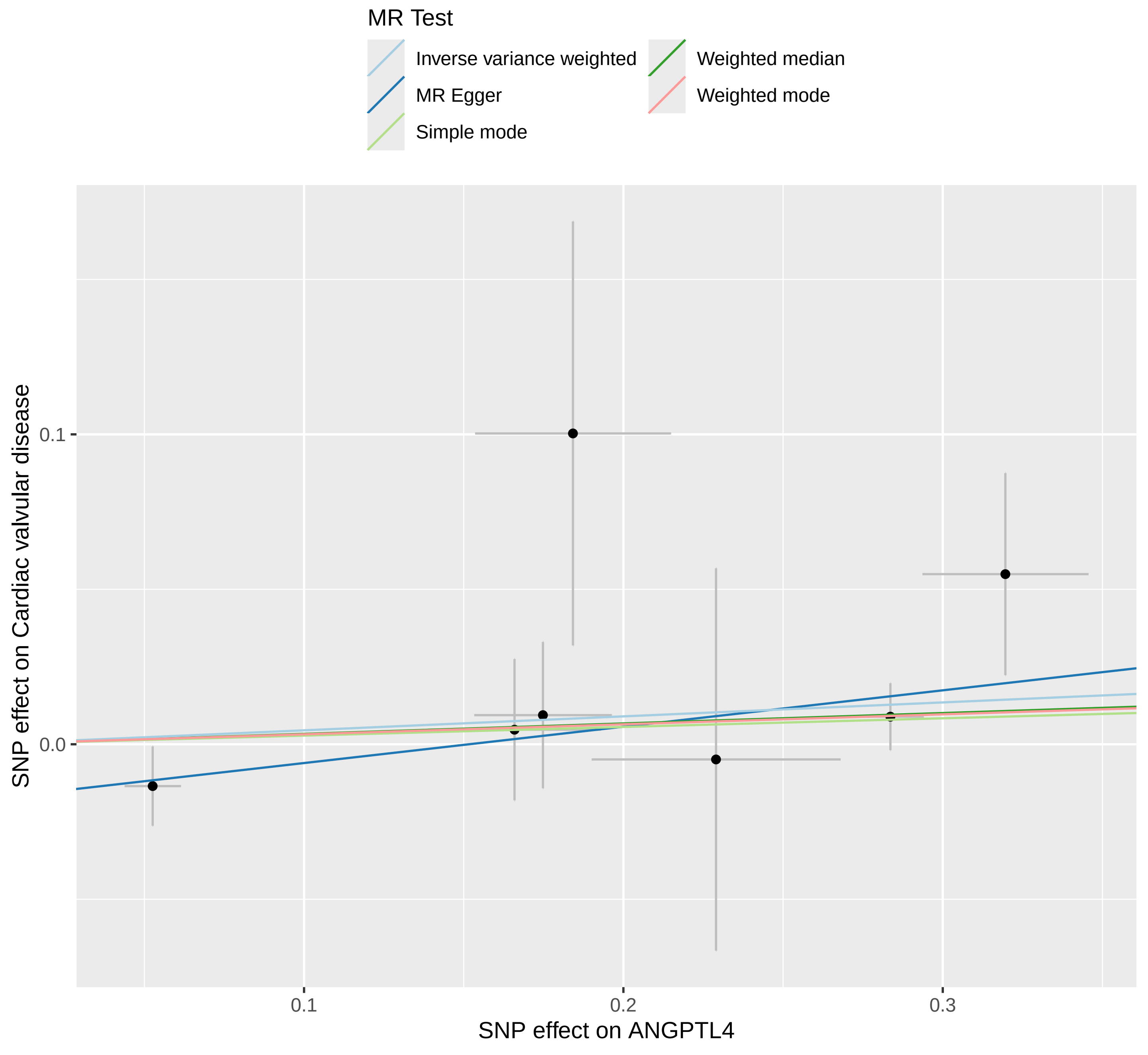
MR Test
Inverse variance weighted
MR Egger
Simple mode
Weighted median
Weighted mode
SNP effect on Cardiac valvular disease
0.1
0.0
0.1
0.2
0.3
SNP effect on ANGPTL4

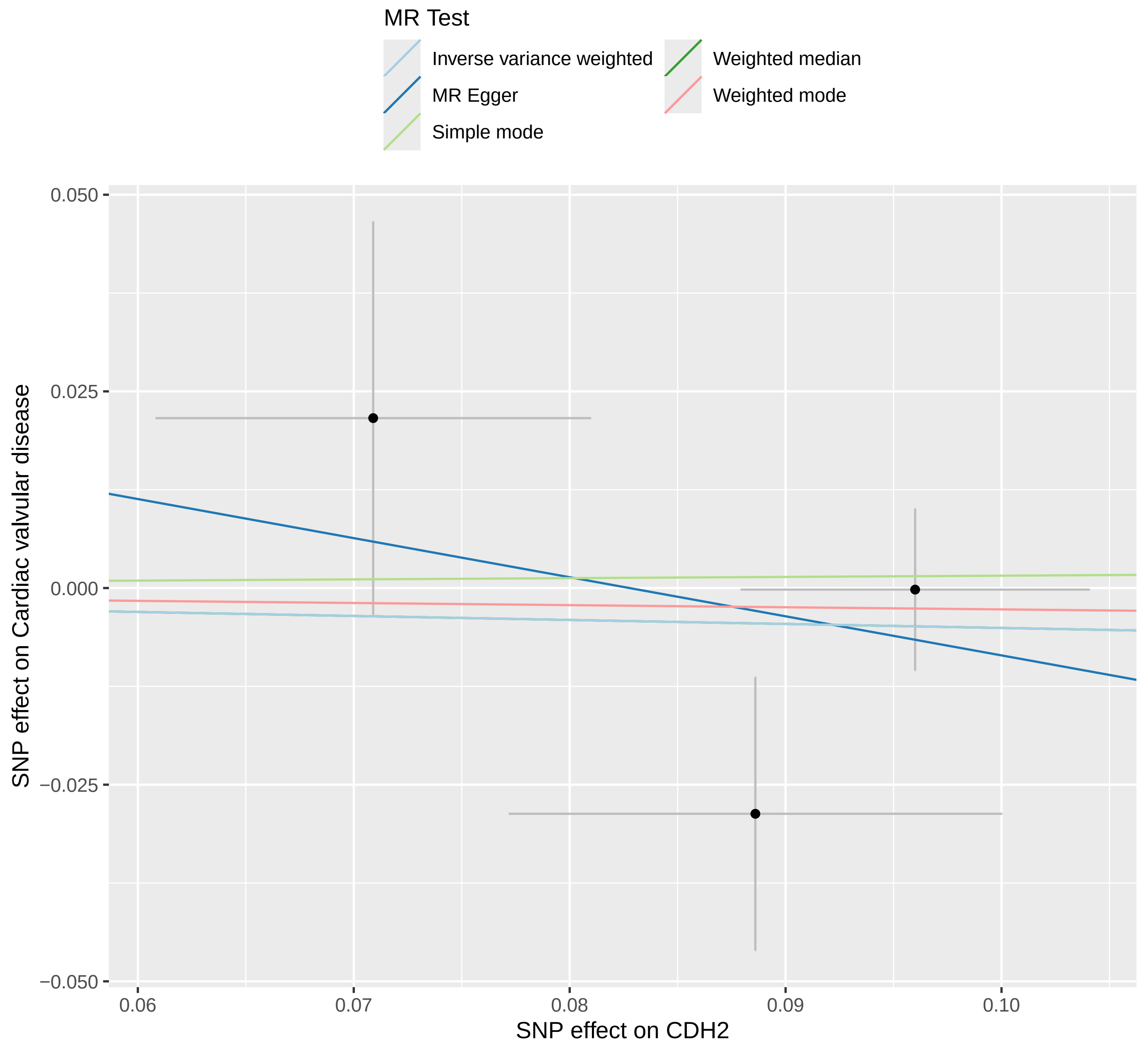

MR Test
Inverse variance weighted
MR Egger
Simple mode
Weighted median
Weighted mode
0.050
0.025
0.000
−0.025
−0.050
SNP effect on Cardiac valvular disease
0.06
0.07
0.08
0.09
0.10
SNP effect on CDH2

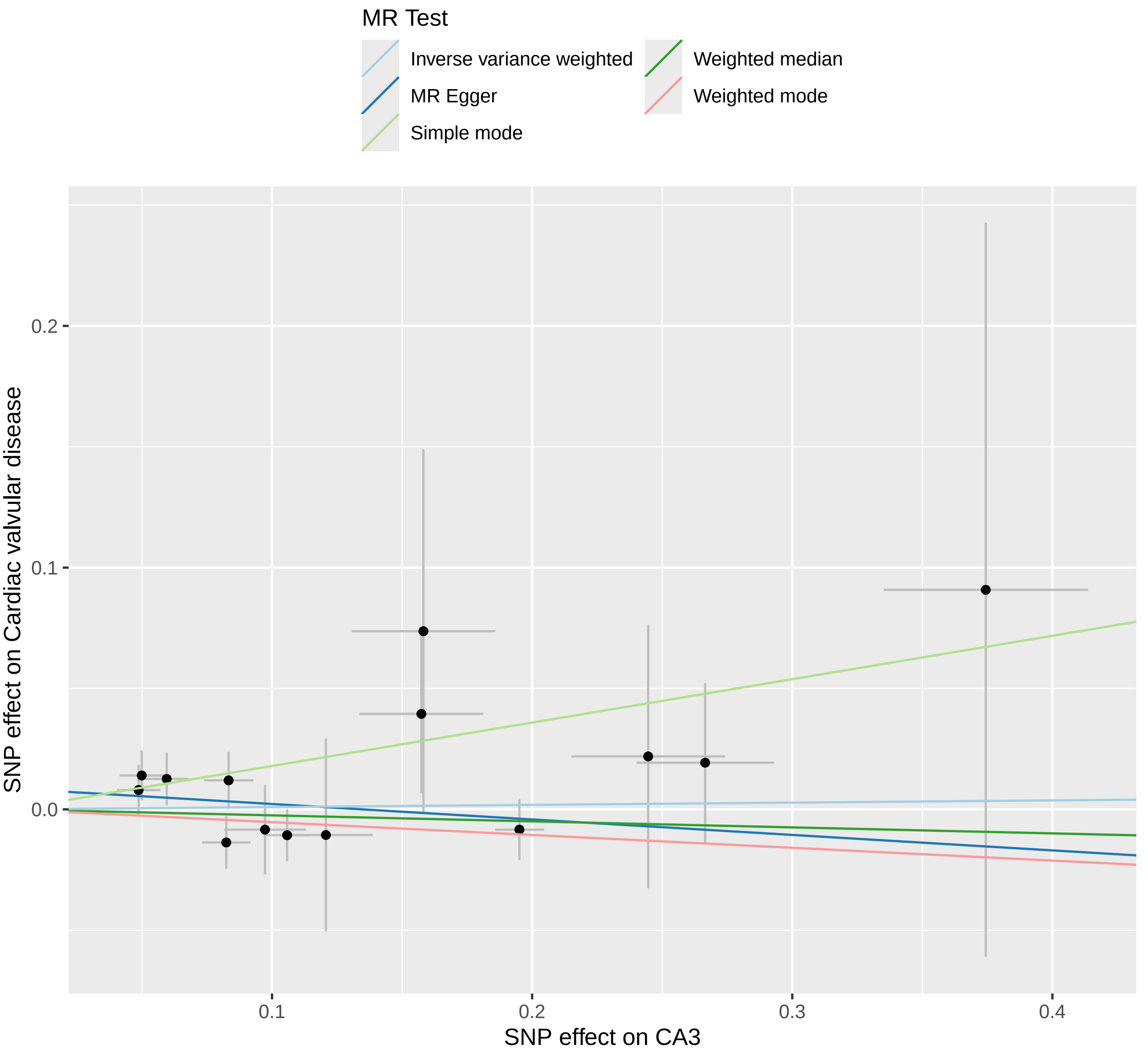

MR Test
Inverse variance weighted
MR Egger
Simple mode
Weighted median
Weighted mode
SNP effect on Cardiac valvular disease
0.2
0.1
0.0
0.1
0.2
0.3
0.4
SNP effect on CA3

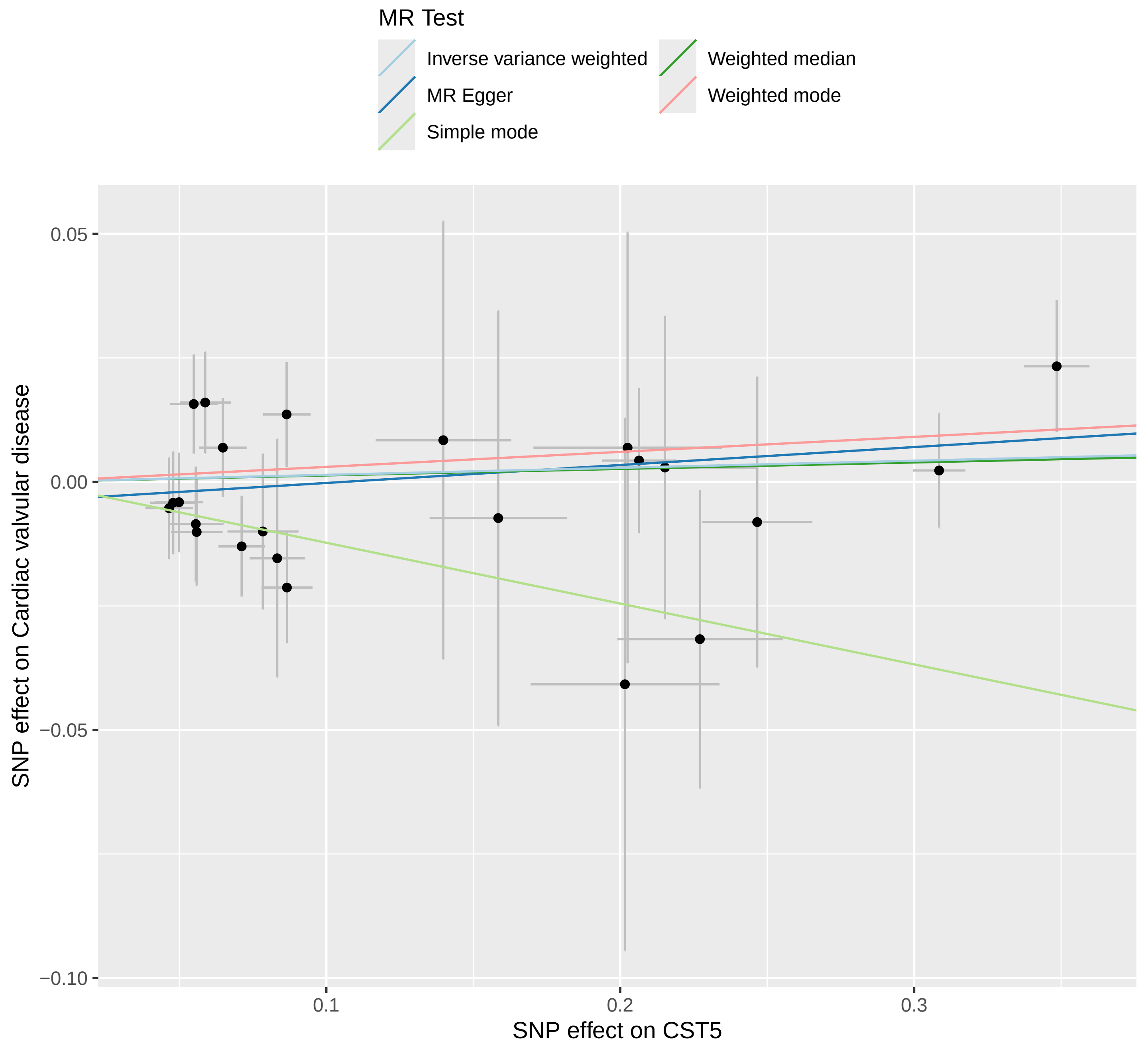

MR Test
Inverse variance weighted
MR Egger
Simple mode
Weighted median
Weighted mode
0.05
0.00
−0.05
−0.10
0.1
0.2
0.3
SNP effect on Cardiac valvular disease
SNP effect on CST5

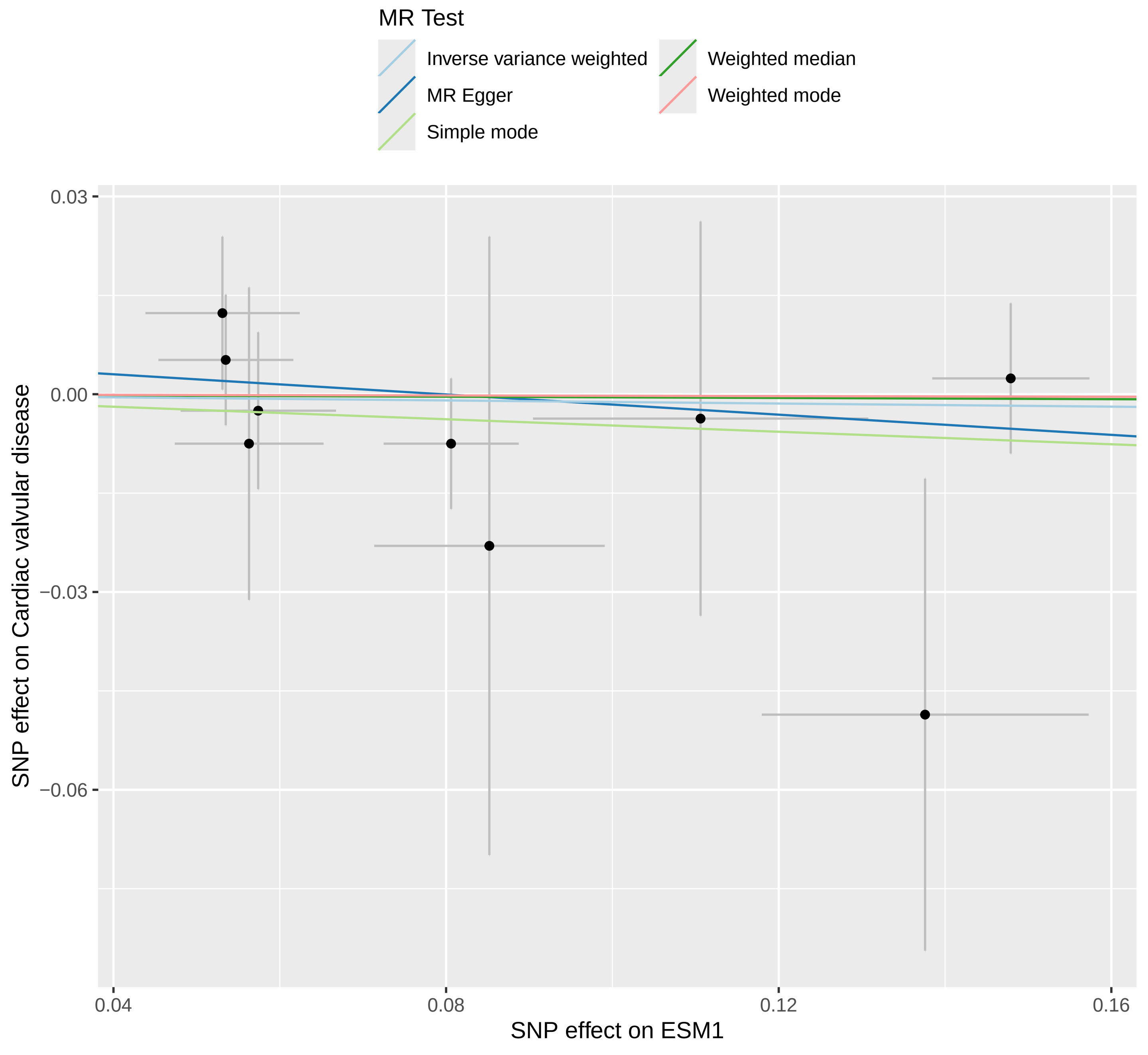

MR Test
Inverse variance weighted
MR Egger
Simple mode
Weighted median
Weighted mode
0.03
0.00
−0.03
−0.06
SNP effect on Cardiac valvular disease
0.04
0.08
0.12
0.16
SNP effect on ESM1

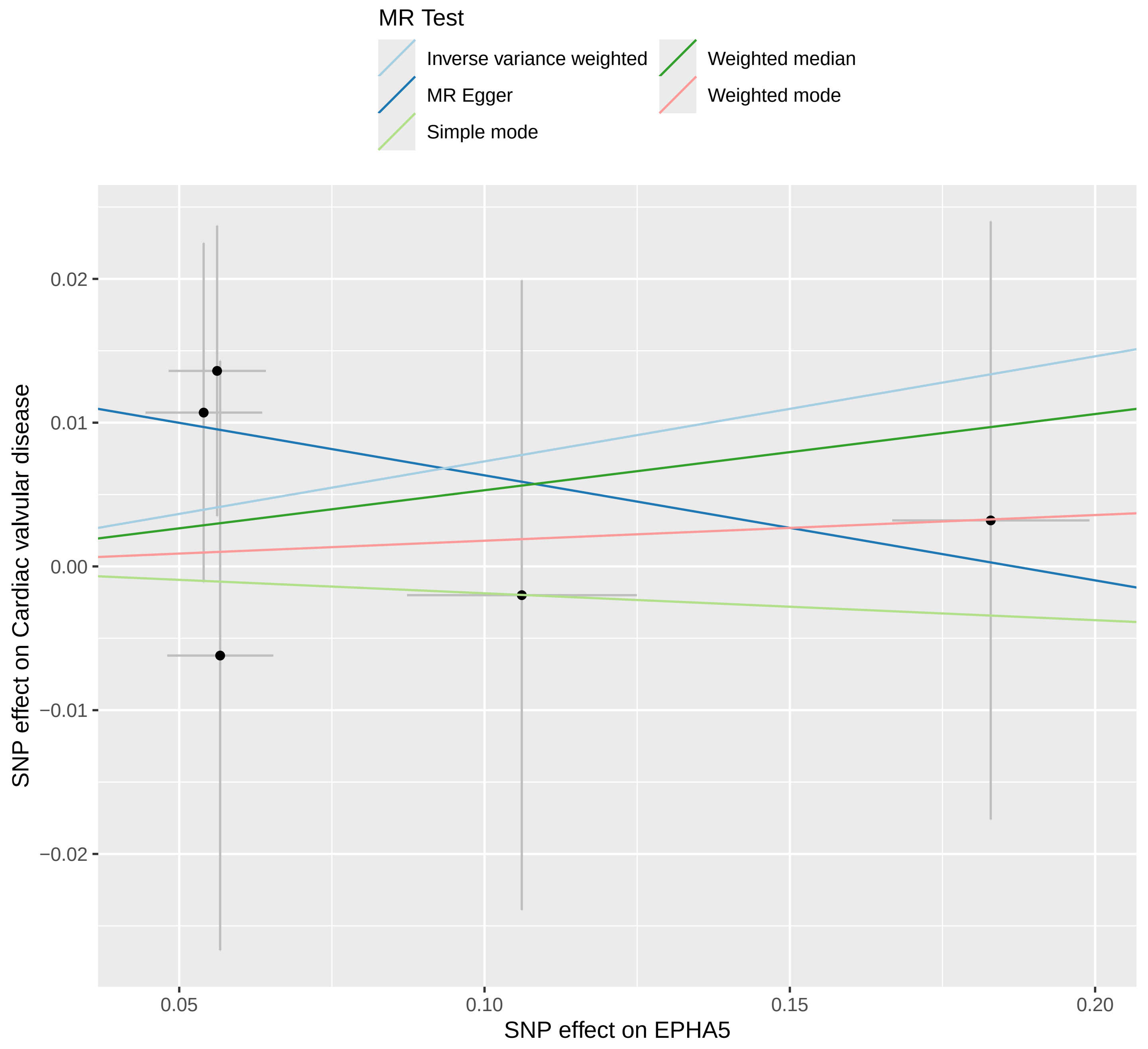
MR Test
Inverse variance weighted
MR Egger
Simple mode
Weighted median
Weighted mode
0.02
0.01
0.00
−0.01
−0.02
SNP effect on Cardiac valvular disease
0.05
0.10
0.15
0.20
SNP effect on EPHA5

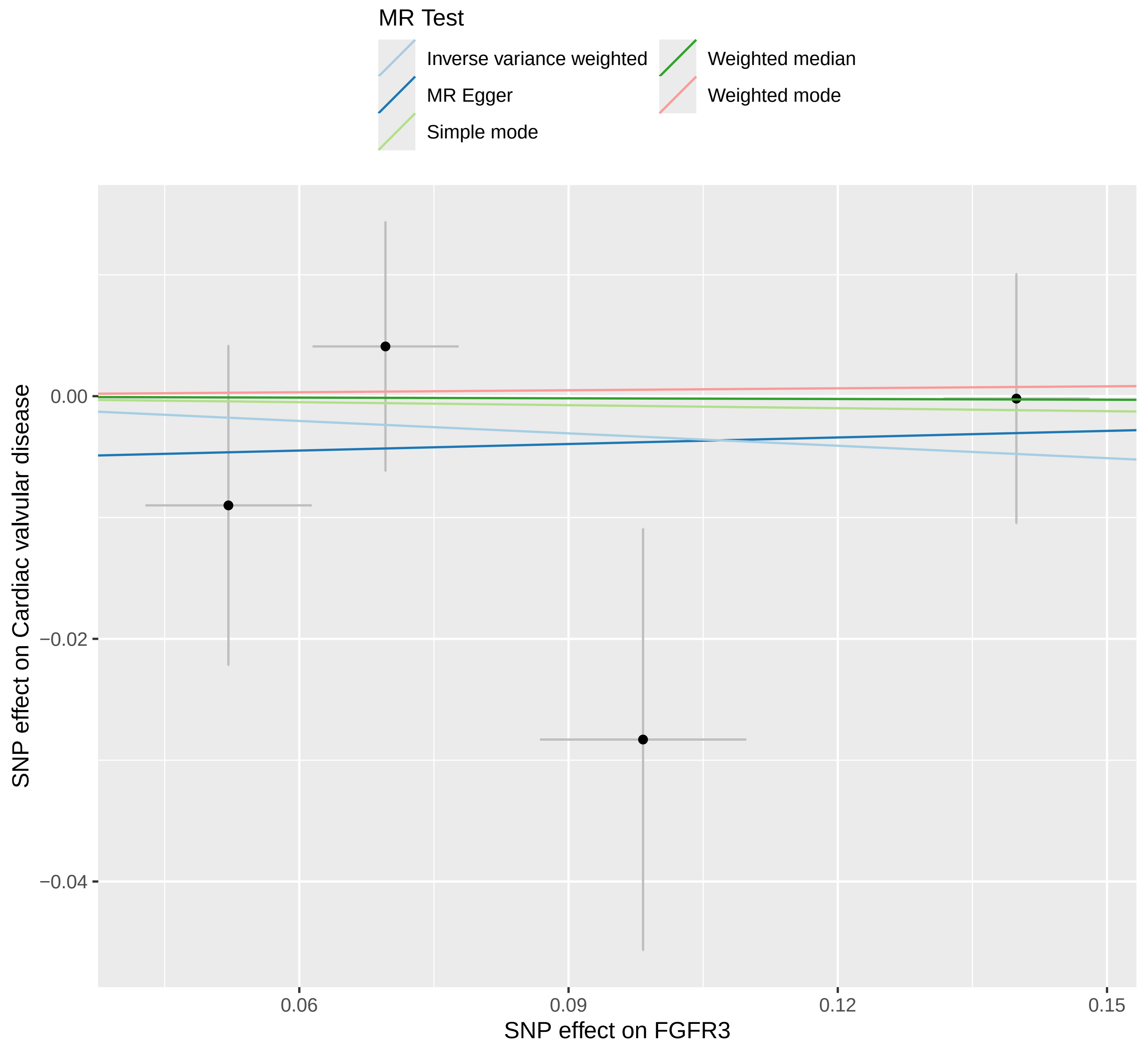
MR Test
Inverse variance weighted
MR Egger
Simple mode
Weighted median
Weighted mode
SNP effect on Cardiac valvular disease
0.00
−0.02
−0.04
0.06
0.09
0.12
0.15
SNP effect on FGFR3

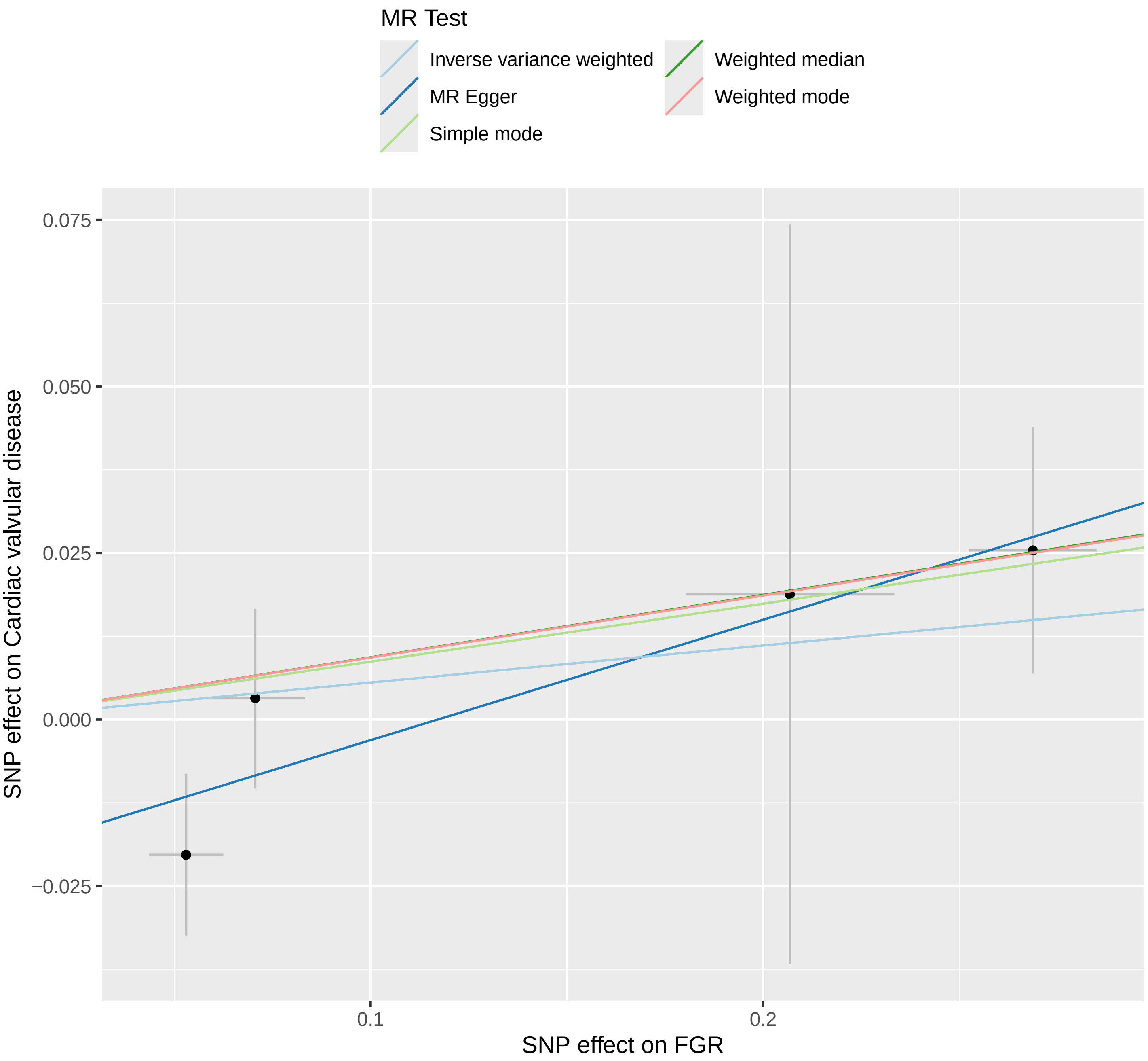
MR Test
Inverse variance weighted
MR Egger
Simple mode
Weighted median
Weighted mode
0.075
0.050
0.025
0.000
−0.025
0.1
0.2
SNP effect on Cardiac valvular disease
SNP effect on FGR

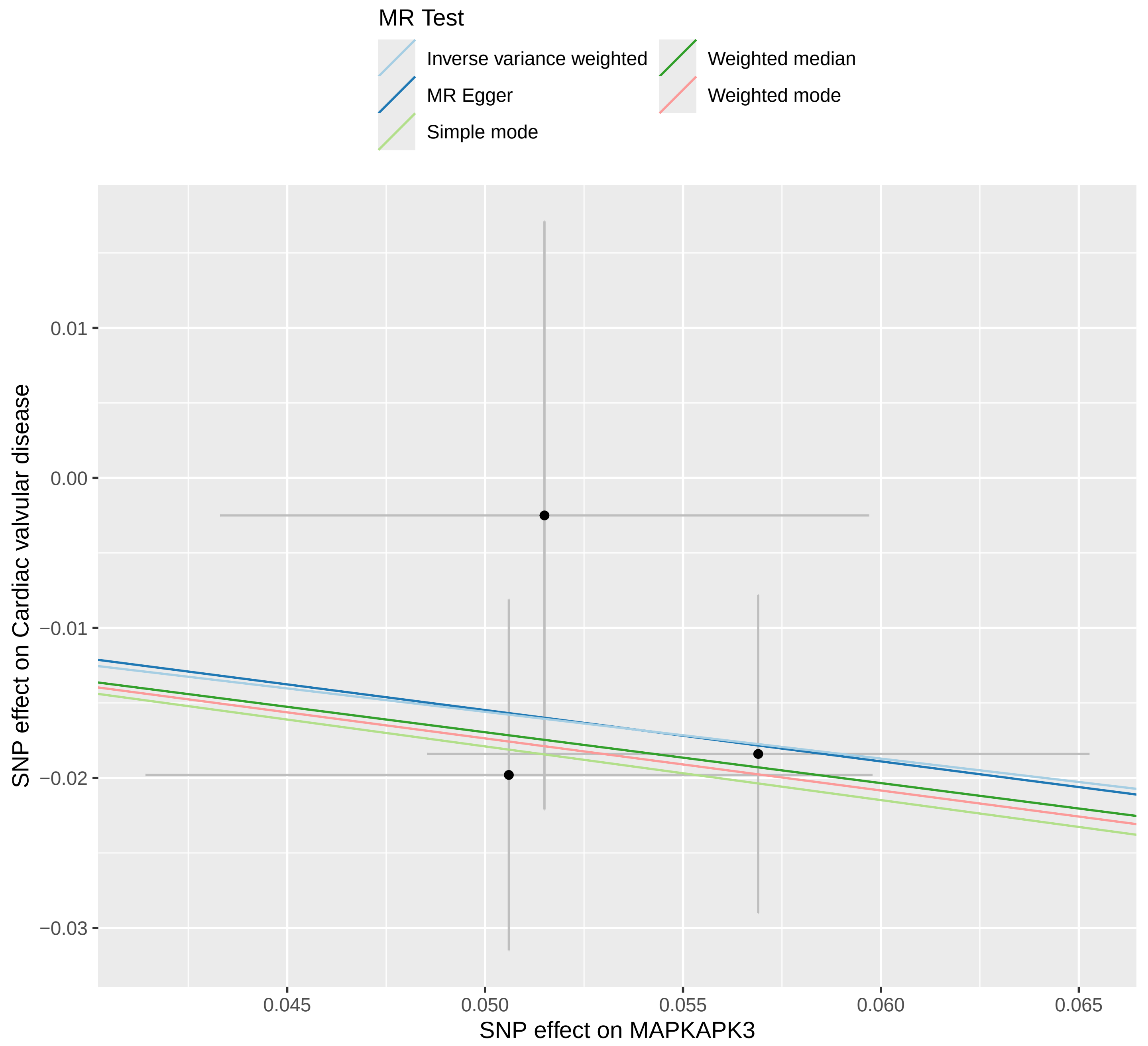

MR Test
Inverse variance weighted
MR Egger
Simple mode
Weighted median
Weighted mode
0.01
0.00
−0.01
−0.02
−0.03
0.045
0.050
0.055
0.060
0.065
SNP effect on MAPKAPK3
SNP effect on Cardiac valvular disease

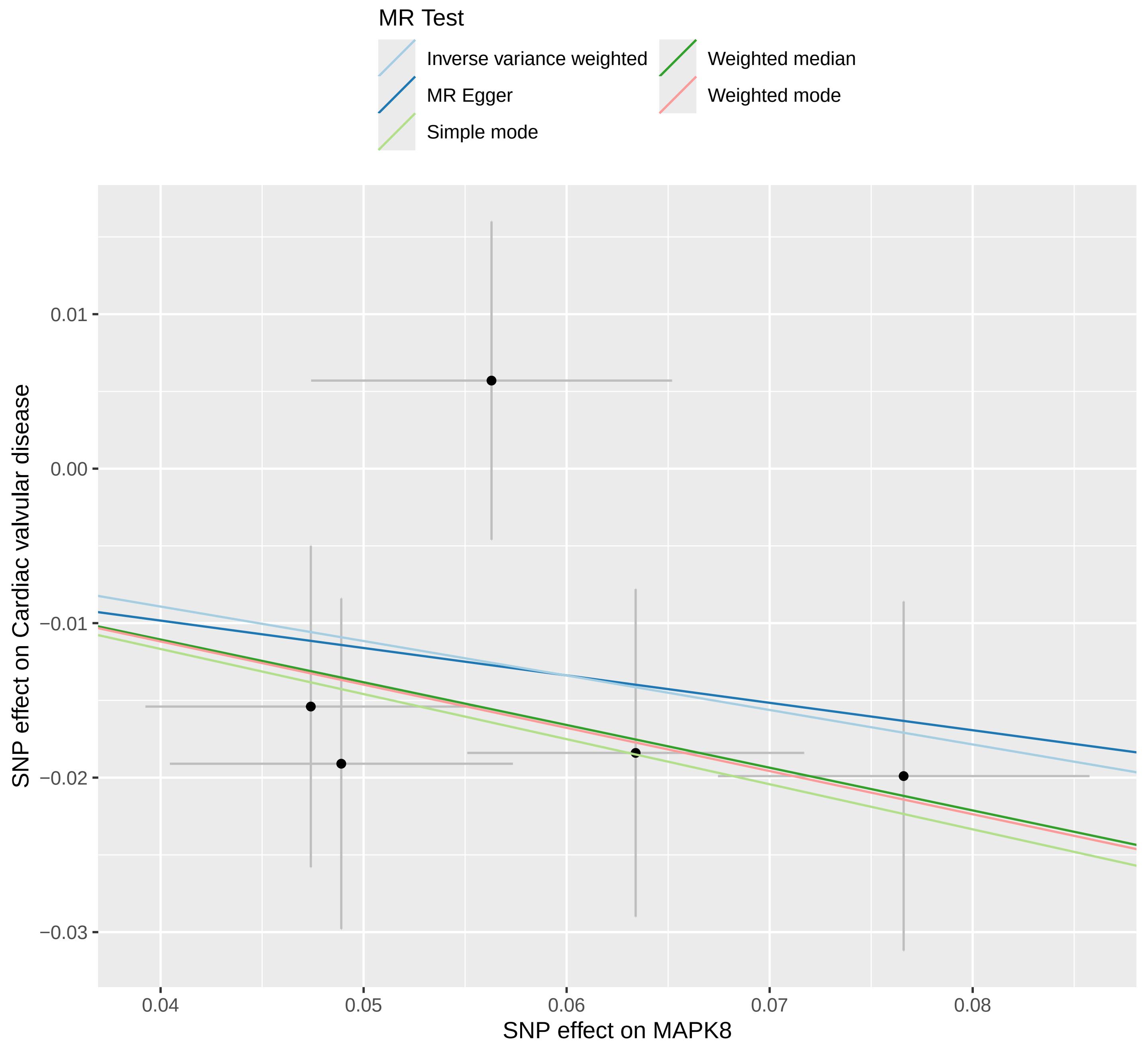

MR Test
Inverse variance weighted
MR Egger
Simple mode
Weighted median
Weighted mode
0.01
0.00
−0.01
−0.02
−0.03
SNP effect on Cardiac valvular disease
0.04
0.05
0.06
0.07
0.08
SNP effect on MAPK8

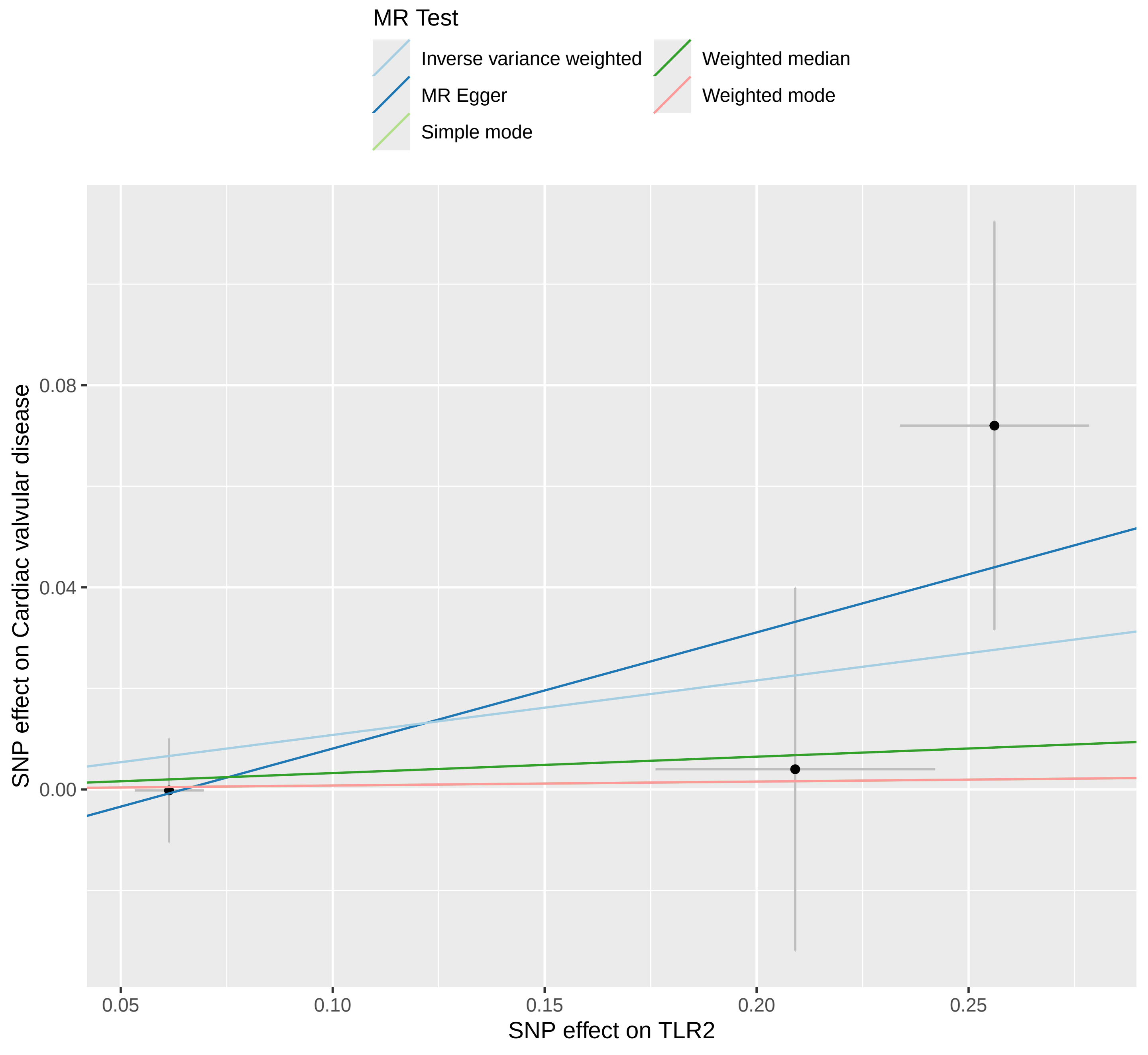
MR Test
Inverse variance weighted
MR Egger
Simple mode
Weighted median
Weighted mode
0.08
0.04
0.00
SNP effect on Cardiac valvular disease
0.05
0.10
0.15
0.20
0.25
SNP effect on TLR2

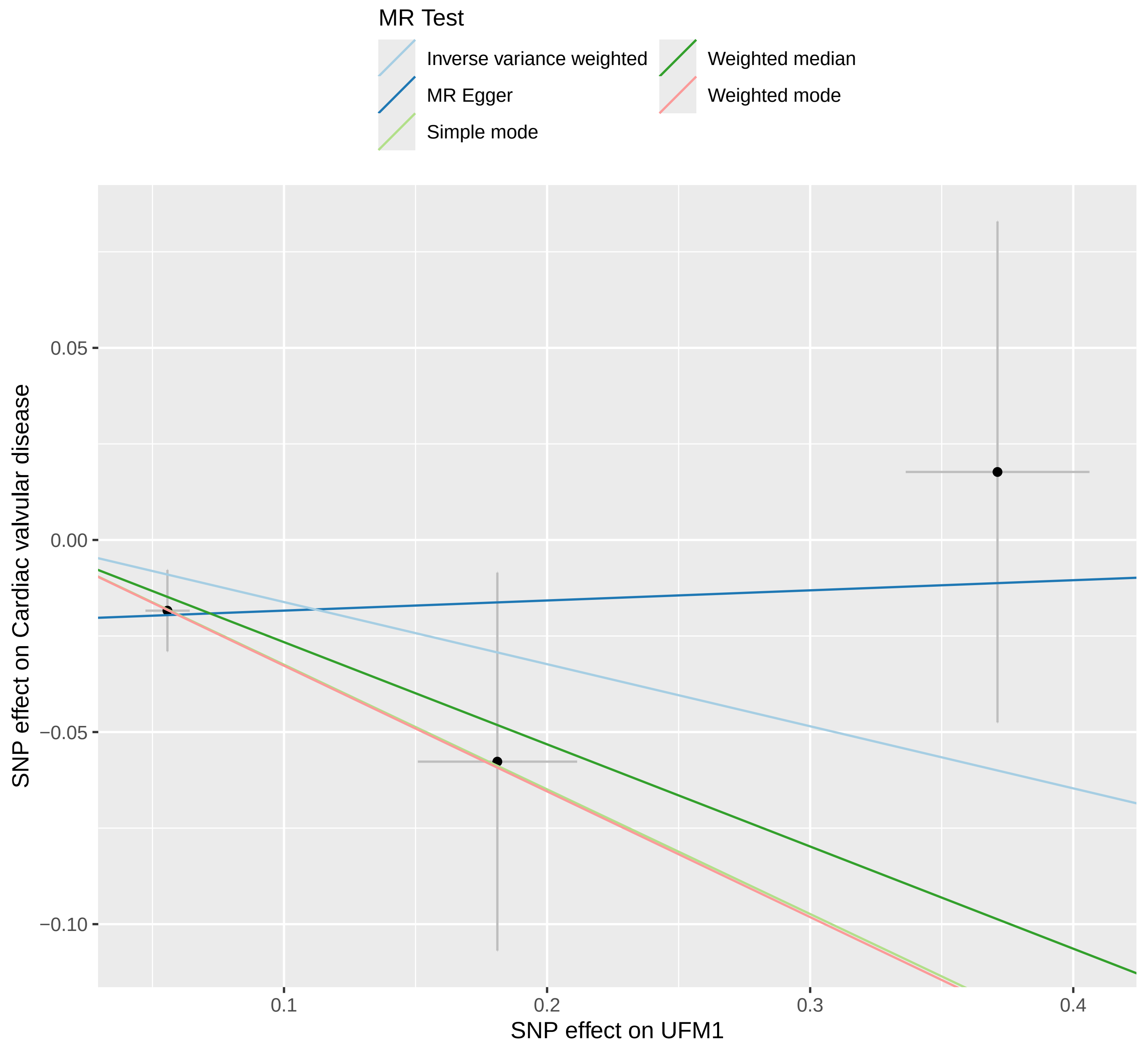

MR Test
Inverse variance weighted
MR Egger
Simple mode
Weighted median
Weighted mode
0.05
0.00
−0.05
−0.10
0.1
0.2
0.3
0.4
SNP effect on Cardiac valvular disease
SNP effect on UFM1

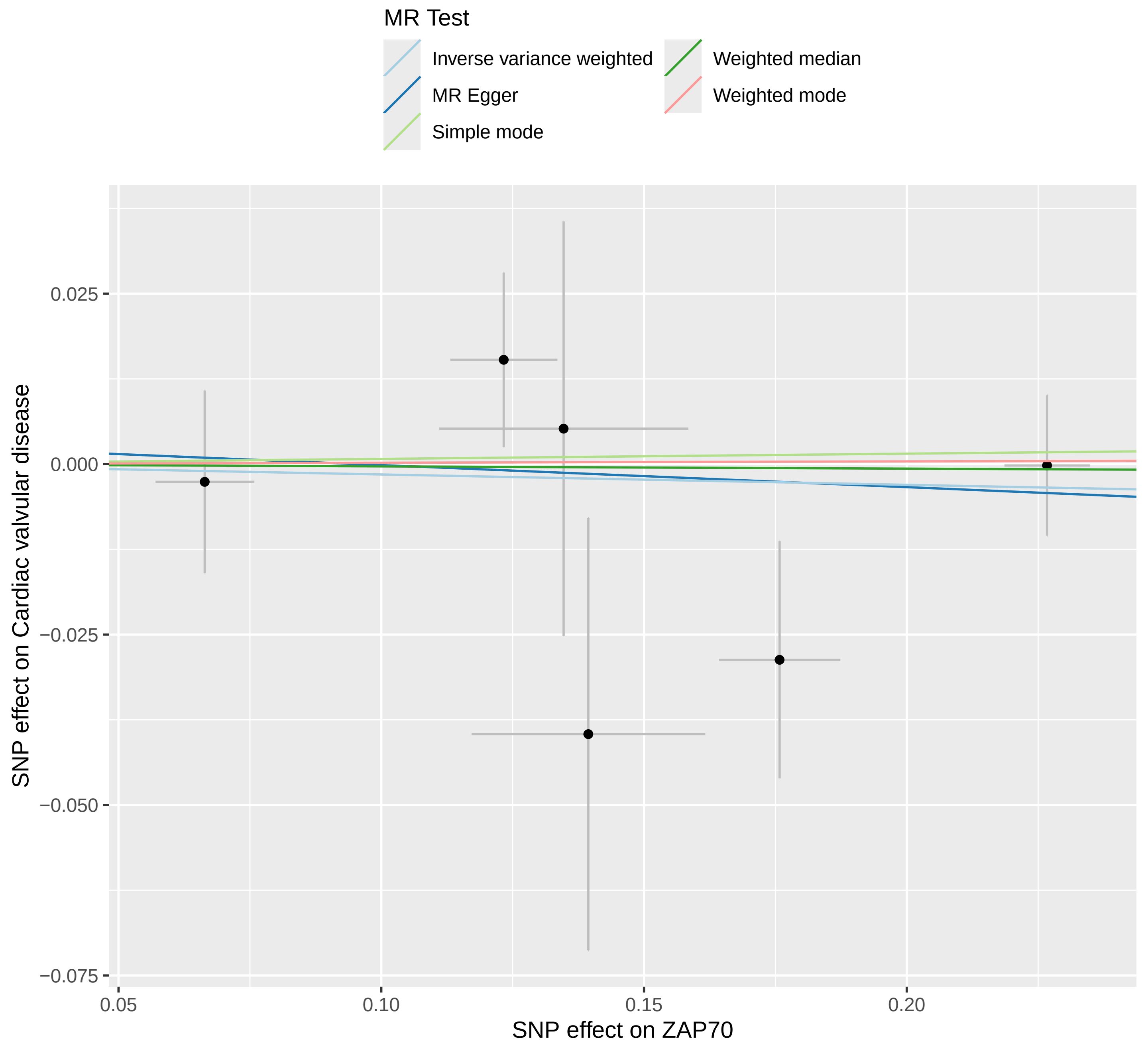
MR Test
Inverse variance weighted
MR Egger
Simple mode
Weighted median
Weighted mode
SNP effect on Cardiac valvular disease
0.025
0.000
−0.025
−0.050
−0.075
0.05
0.10
0.15
0.20
SNP effect on ZAP70

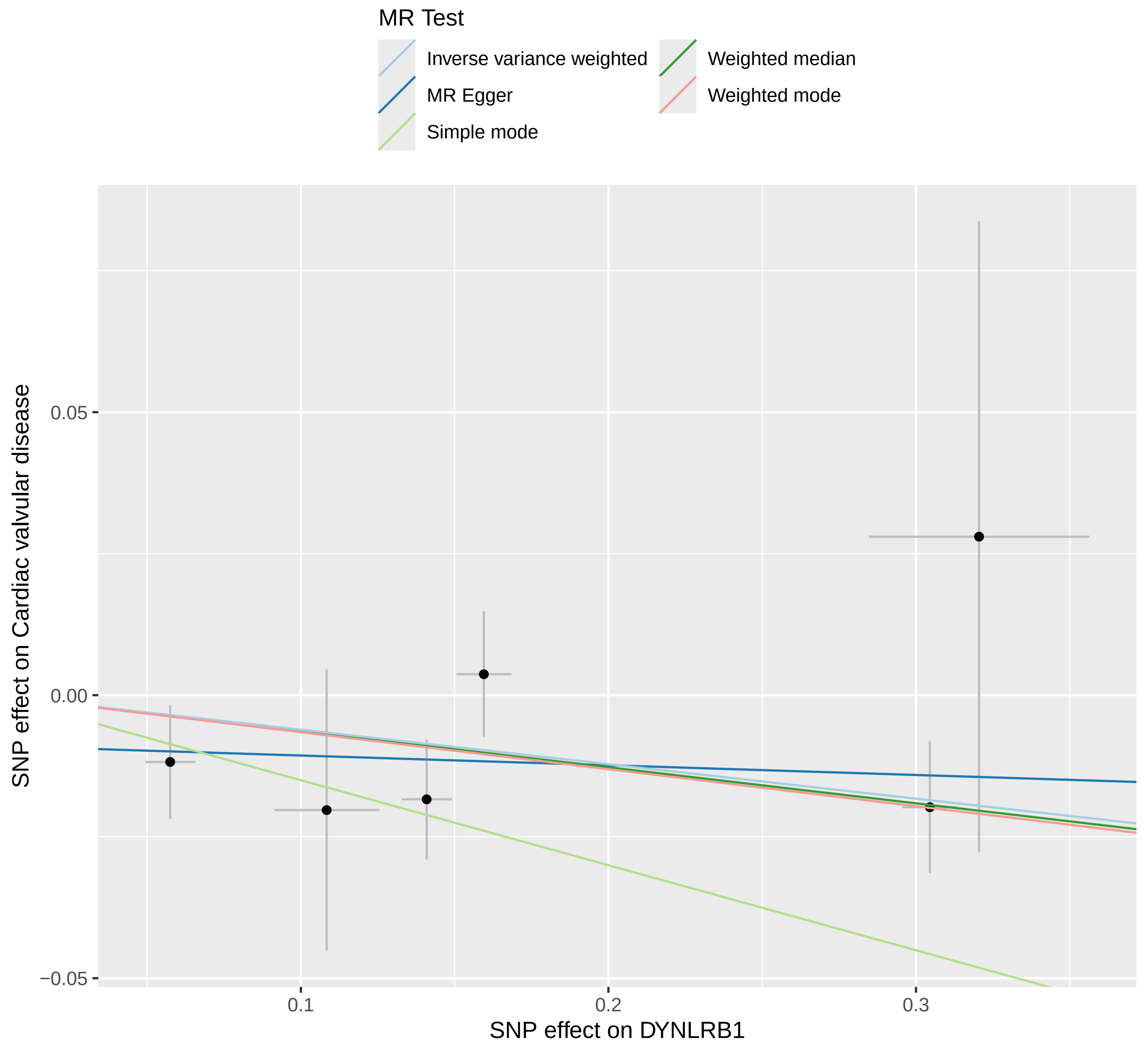
MR Test
Inverse variance weighted
MR Egger
Simple mode
Weighted median
Weighted mode
0.05
0.00
−0.05
0.1
0.2
0.3
SNP effect on Cardiac valvular disease
SNP effect on DYNLRB1

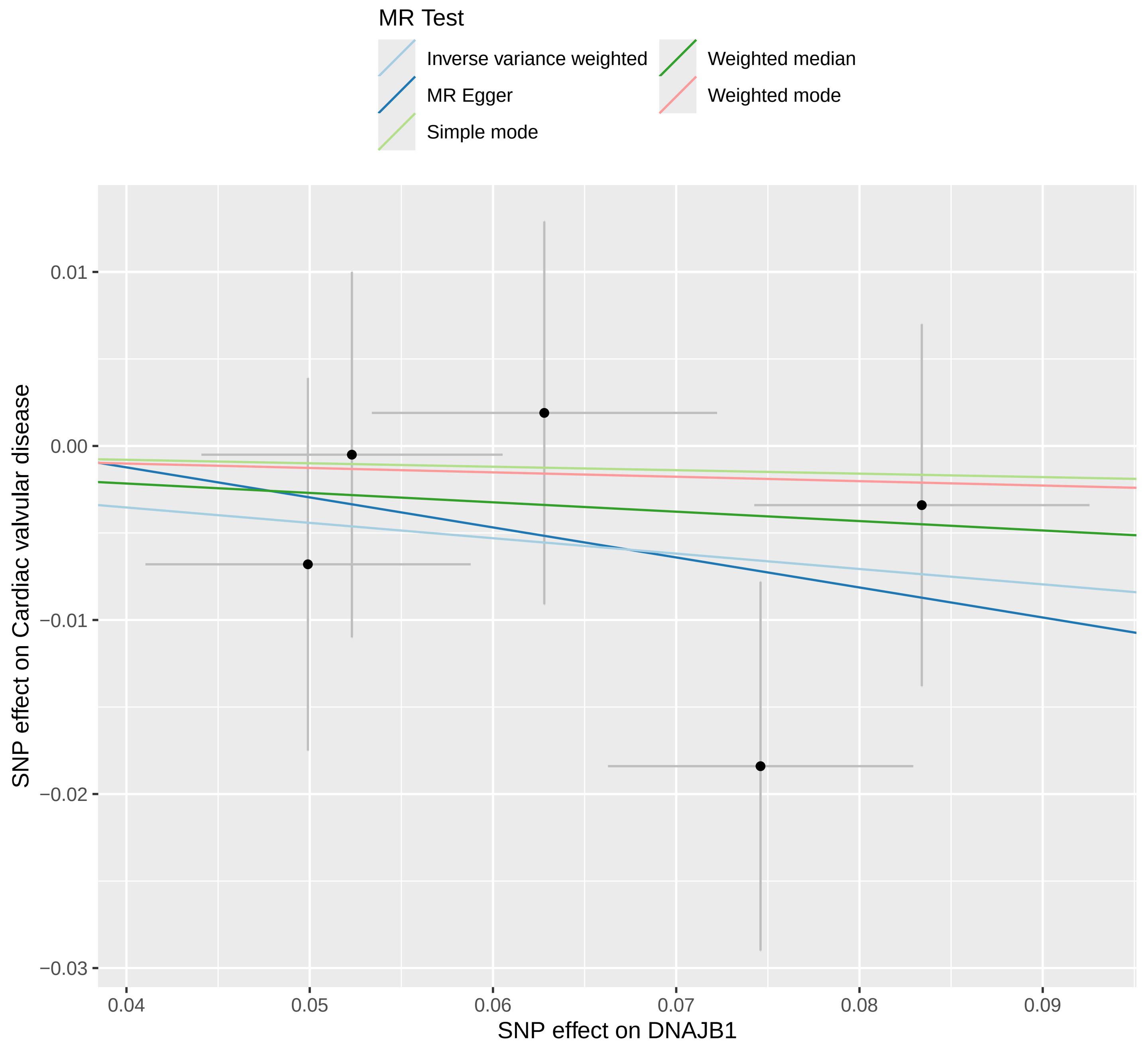
MR Test
Inverse variance weighted
MR Egger
Simple mode
Weighted median
Weighted mode
0.01
0.00
−0.01
−0.02
−0.03
SNP effect on Cardiac valvular disease
0.04
0.05
0.06
0.07
0.08
0.09
SNP effect on DNAJB1

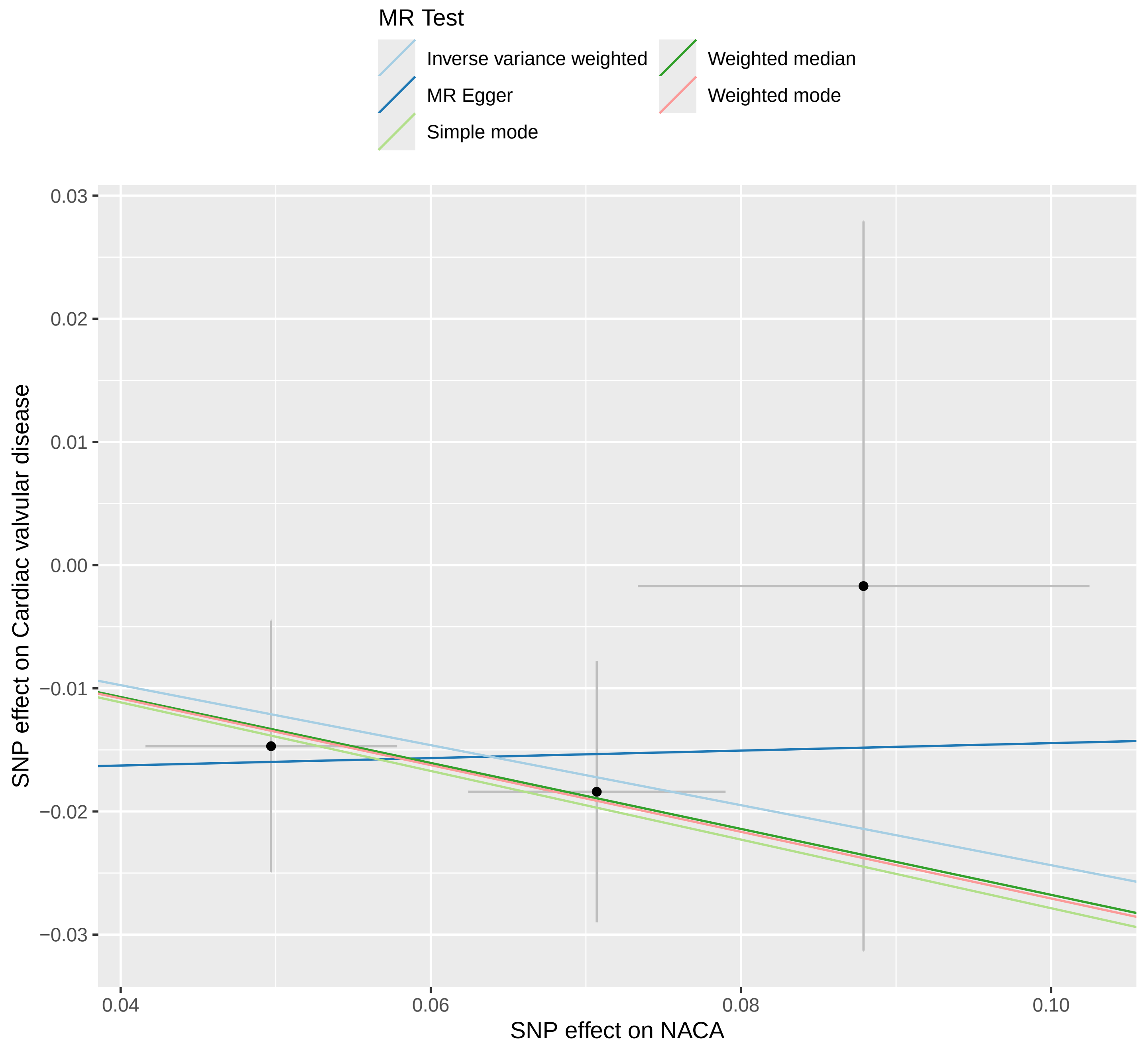
MR Test
Inverse variance weighted
MR Egger
Simple mode
Weighted median
Weighted mode
SNP effect on Cardiac valvular disease
0.03
0.02
0.01
0.00
−0.01
−0.02
−0.03
0.04
0.06
0.08
0.10
SNP effect on NACA

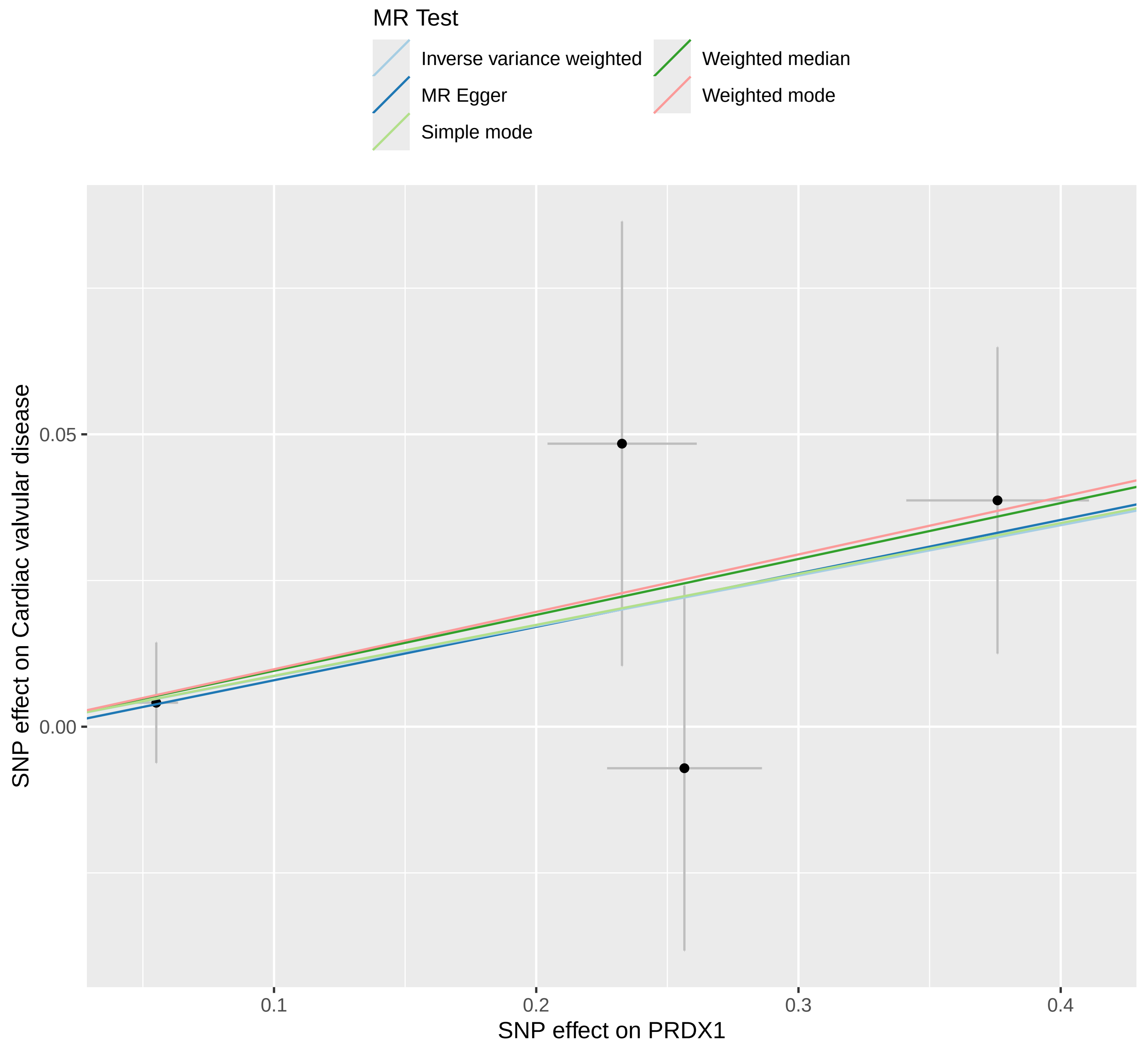

MR Test
Inverse variance weighted
MR Egger
Simple mode
Weighted median
Weighted mode
SNP effect on Cardiac valvular disease
0.05
0.00
0.1
0.2
0.3
0.4
SNP effect on PRDX1

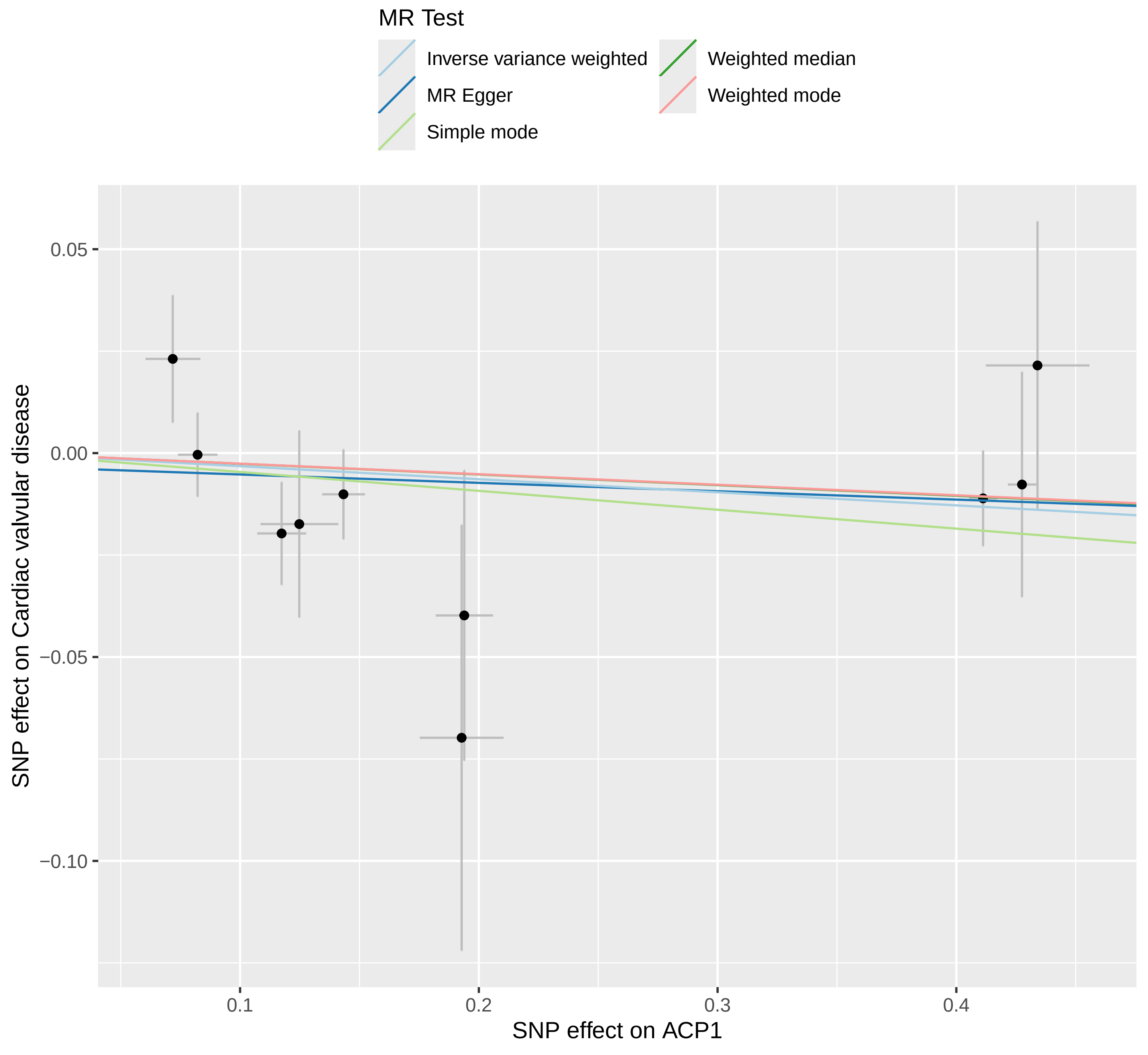

MR Test
Inverse variance weighted
MR Egger
Simple mode
Weighted median
Weighted mode
0.05
0.00
−0.05
−0.10
SNP effect on Cardiac valvular disease
0.1
0.2
0.3
0.4
SNP effect on ACP1

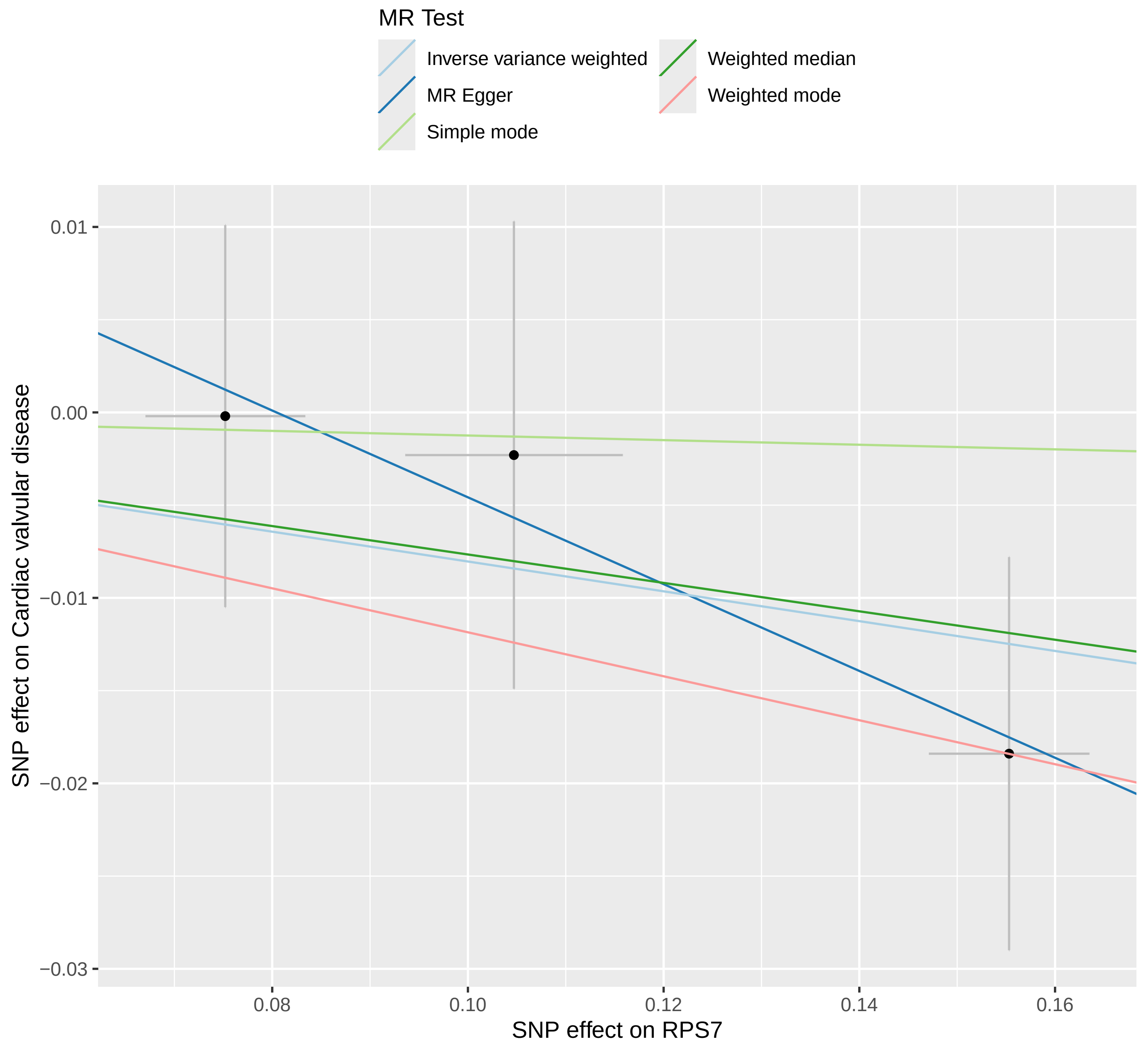
MR Test
Inverse variance weighted
MR Egger
Simple mode
Weighted median
Weighted mode
0.01
0.00
−0.01
−0.02
−0.03
SNP effect on Cardiac valvular disease
0.08
0.10
0.12
0.14
0.16
SNP effect on RPS7

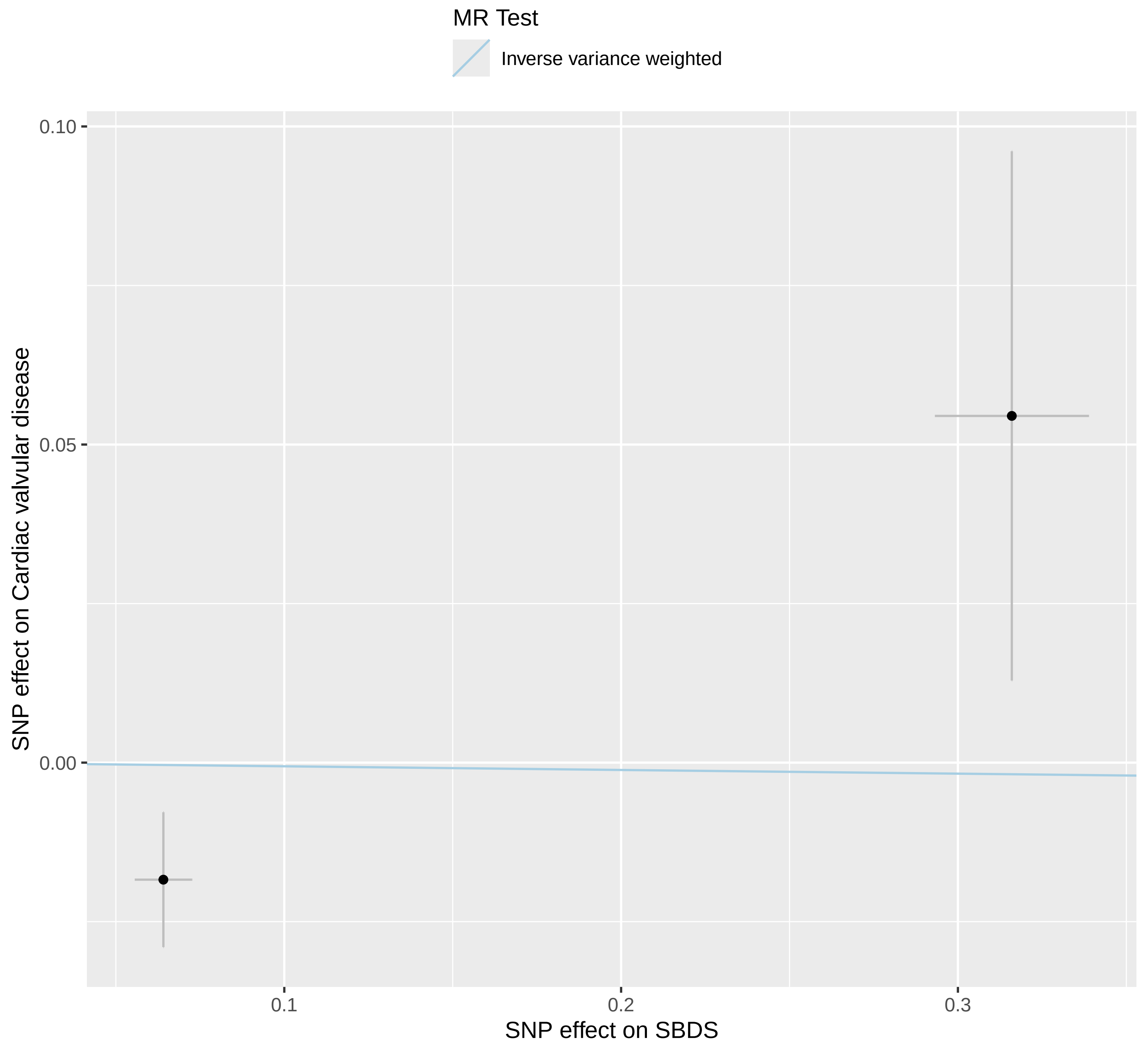

MR Test
Inverse variance weighted
0.10
0.05
0.00
SNP effect on Cardiac valvular disease
0.1
0.2
0.3
SNP effect on SBDS

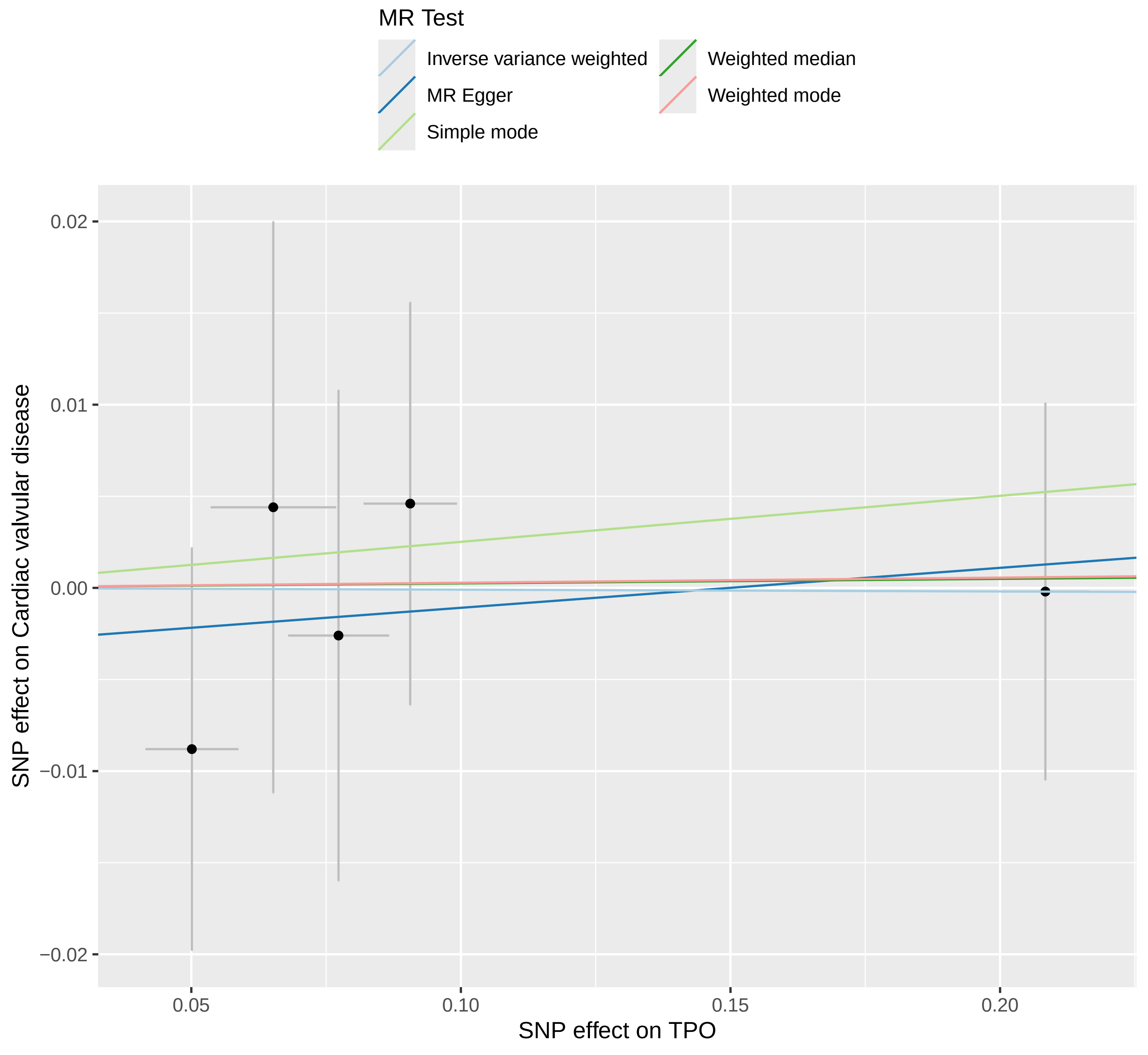
MR Test
Inverse variance weighted
MR Egger
Simple mode
Weighted median
Weighted mode
0.02
0.01
0.00
−0.01
−0.02
0.05
0.10
0.15
0.20
SNP effect on Cardiac valvular disease
SNP effect on TPO

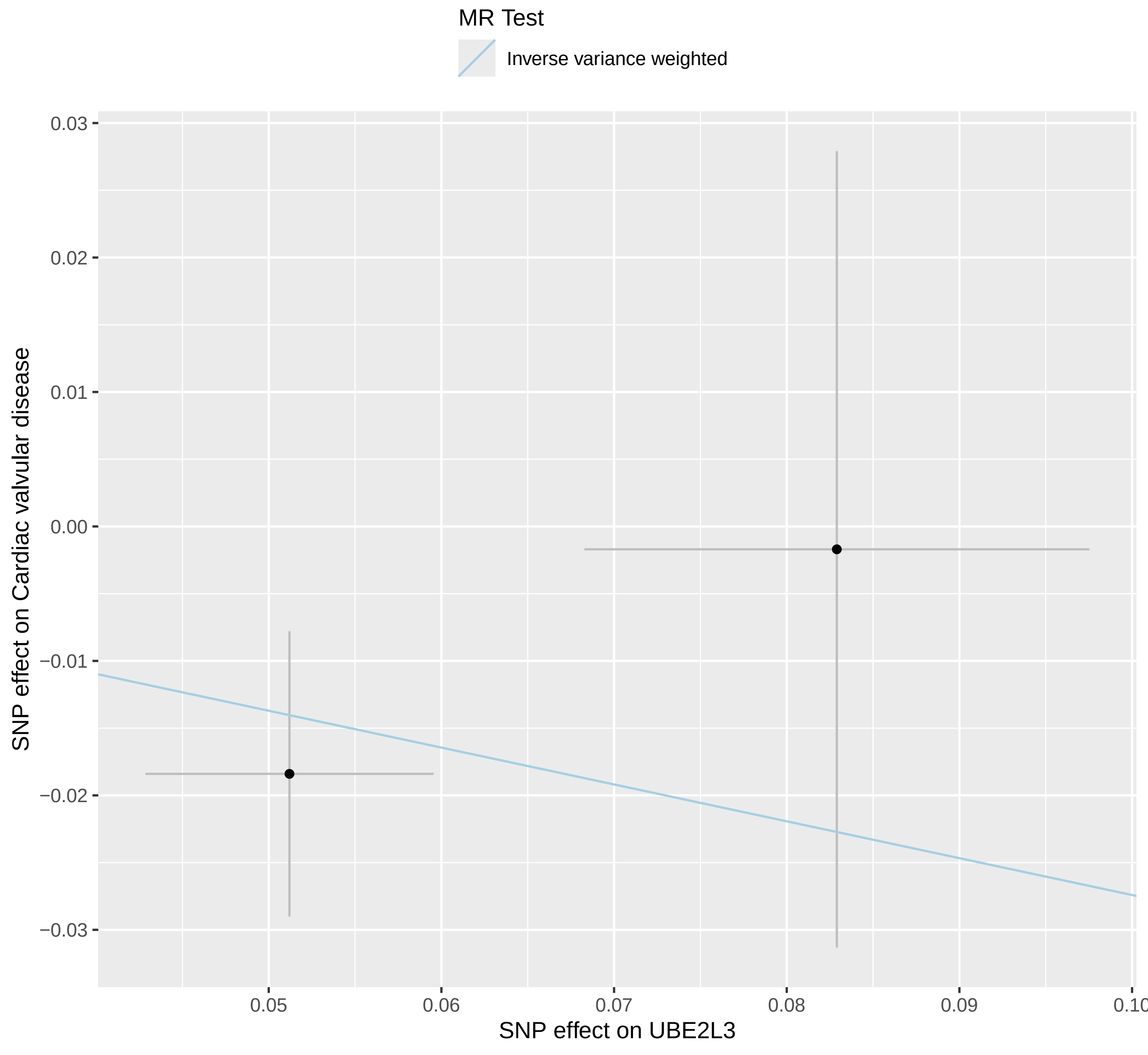
MR Test
Inverse variance weighted
0.03
0.02
0.01
0.00
−0.01
−0.02
−0.03
0.05
0.06
0.07
0.08
0.09
0.10
SNP effect on Cardiac valvular disease
SNP effect on UBE2L3

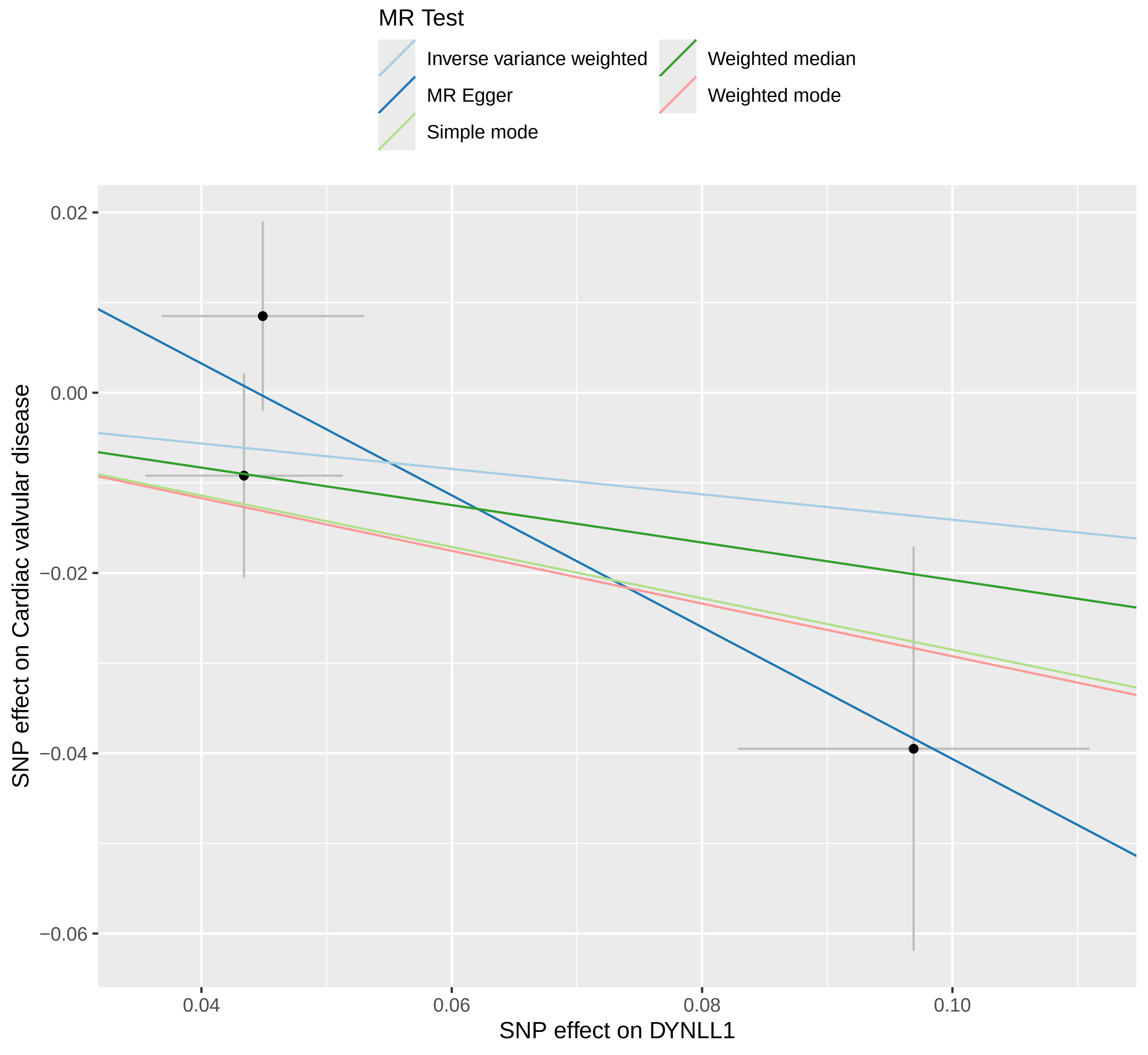
MR Test
Inverse variance weighted
MR Egger
Simple mode
Weighted median
Weighted mode
0.02
0.00
−0.02
−0.04
−0.06
SNP effect on Cardiac valvular disease
0.04
0.06
0.08
0.10
SNP effect on DYNLL1

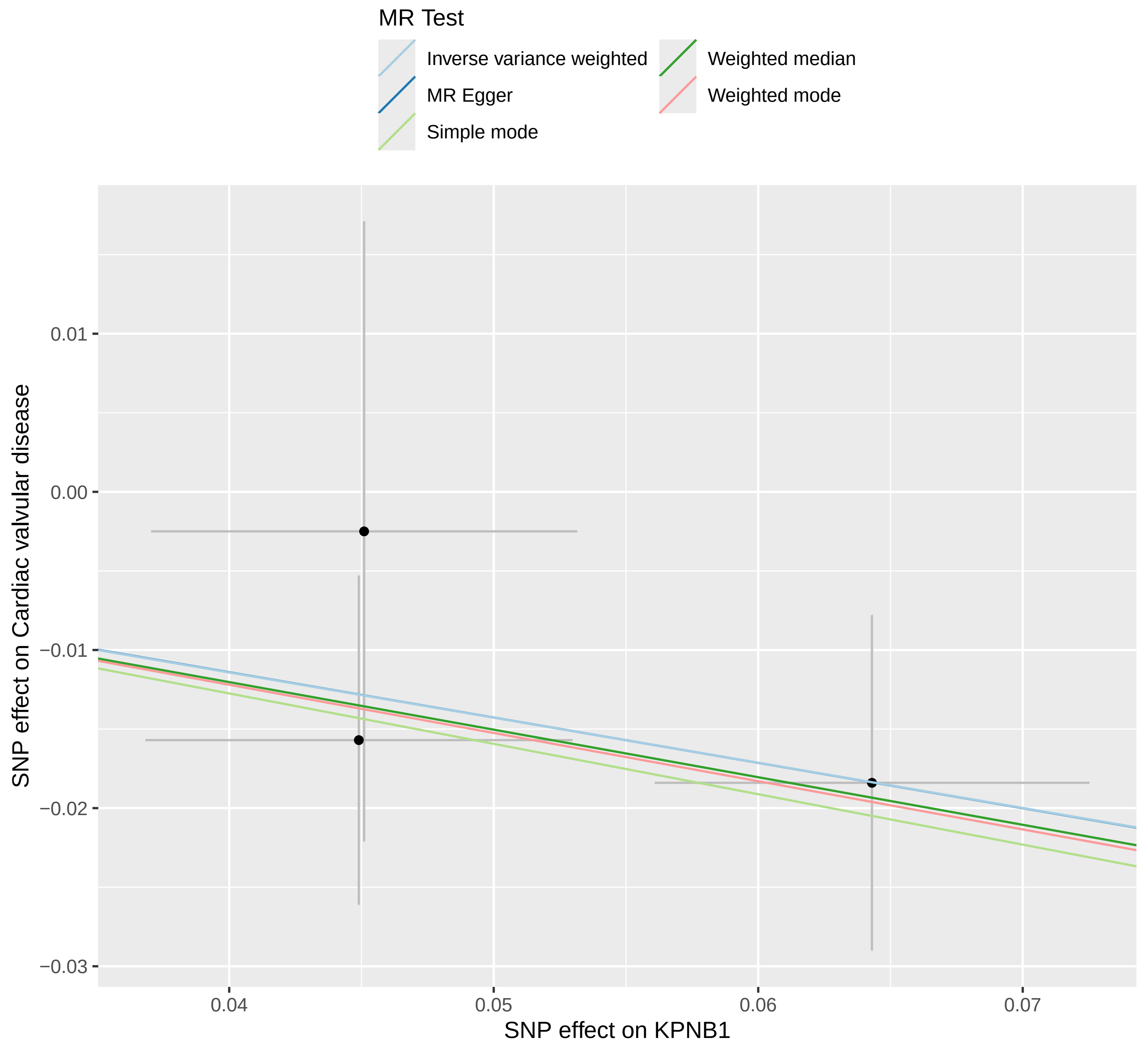
MR Test
Inverse variance weighted
MR Egger
Simple mode
Weighted median
Weighted mode
0.01
0.00
−0.01
−0.02
−0.03
0.04
0.05
0.06
0.07
SNP effect on Cardiac valvular disease
SNP effect on KPNB1

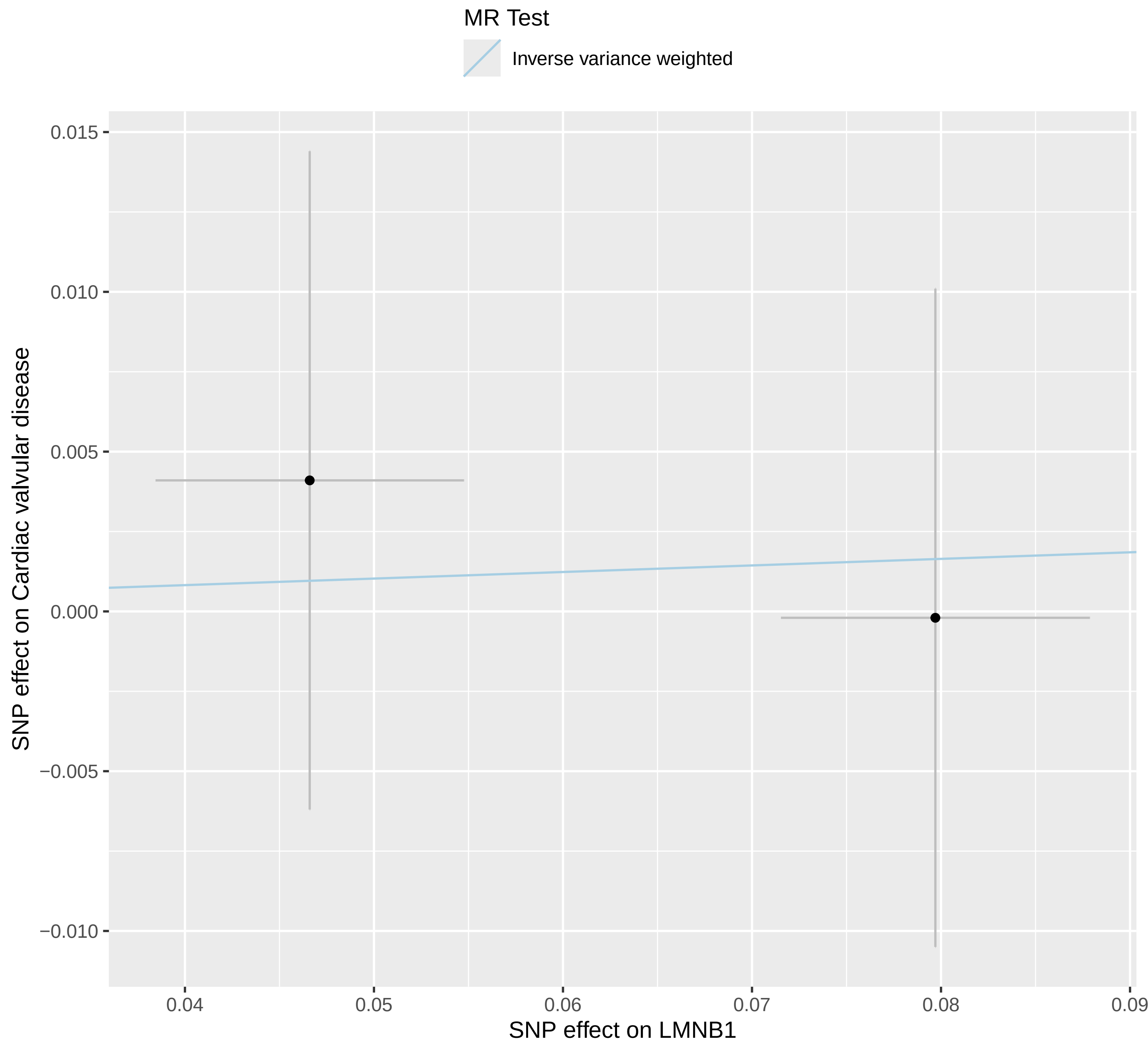
MR Test
Inverse variance weighted
0.015
0.010
0.005
0.000
−0.005
−0.010
SNP effect on Cardiac valvular disease
0.04
0.05
0.06
0.07
0.08
0.09
SNP effect on LMNB1

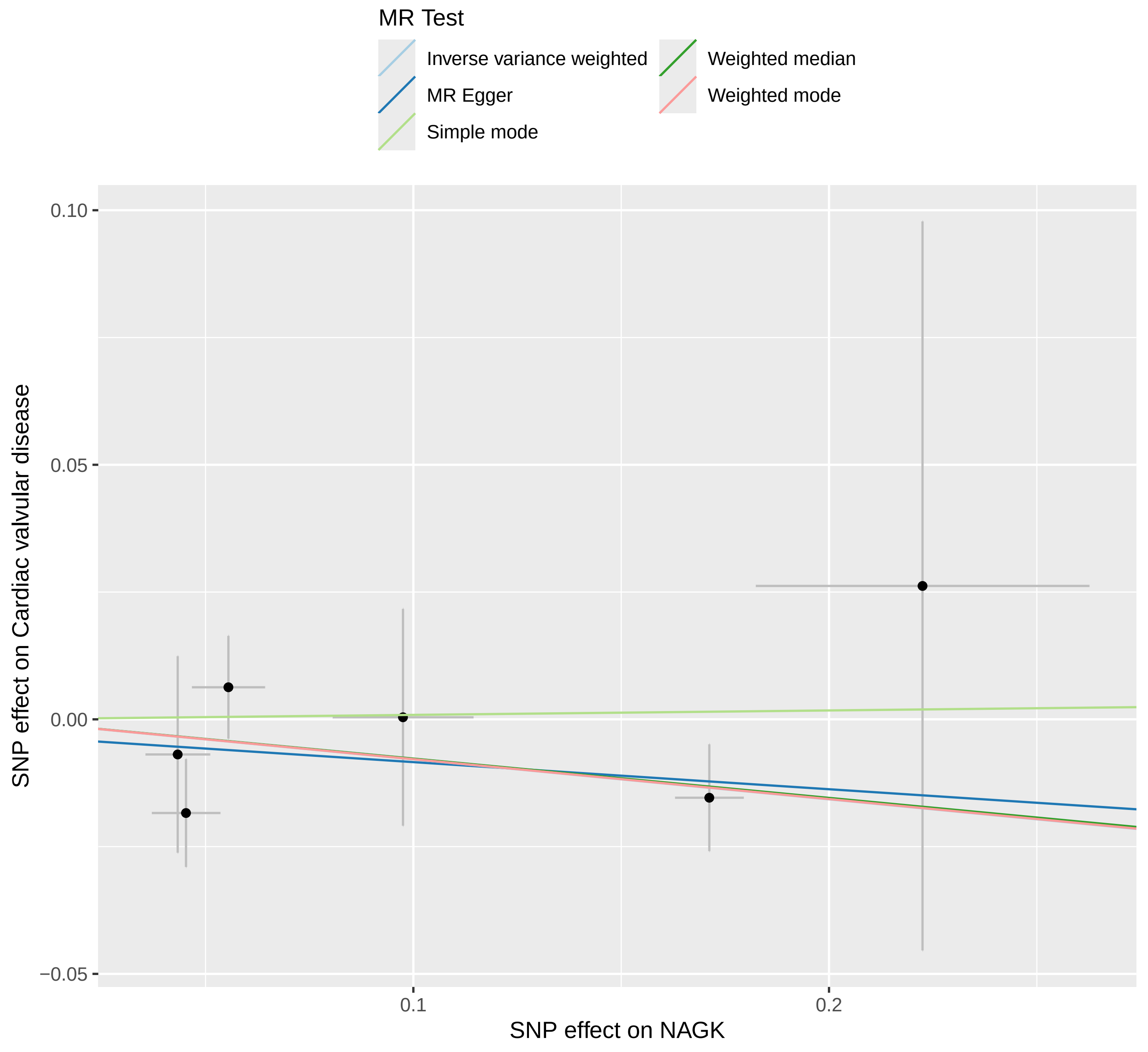
MR Test
Inverse variance weighted
MR Egger
Simple mode
Weighted median
Weighted mode
0.10
0.05
0.00
−0.05
0.1
0.2
SNP effect on Cardiac valvular disease
SNP effect on NAGK

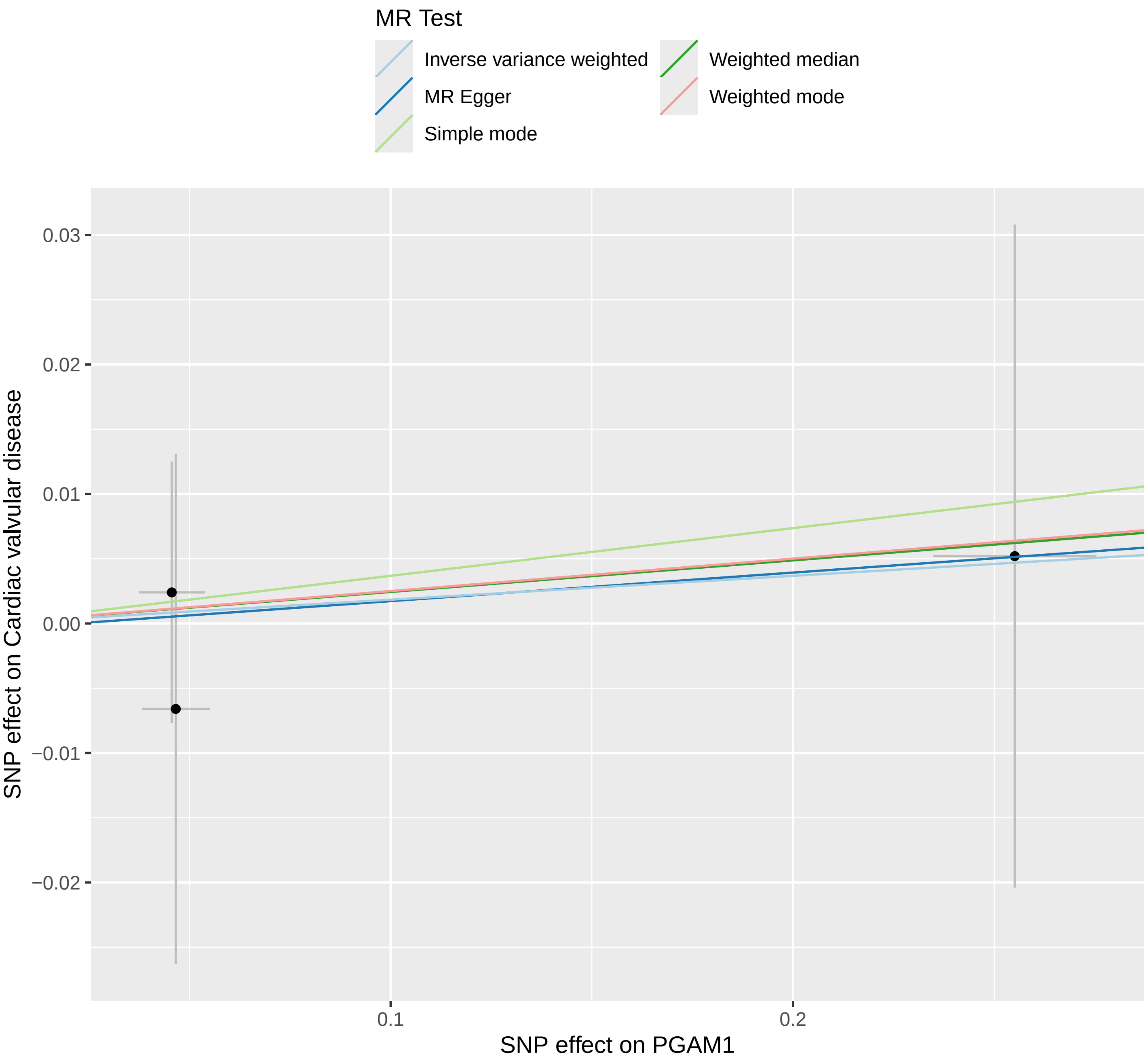
MR Test
Inverse variance weighted
MR Egger
Simple mode
Weighted median
Weighted mode
0.03
0.02
0.01
0.00
−0.01
−0.02
SNP effect on Cardiac valvular disease
0.1
0.2
SNP effect on PGAM1

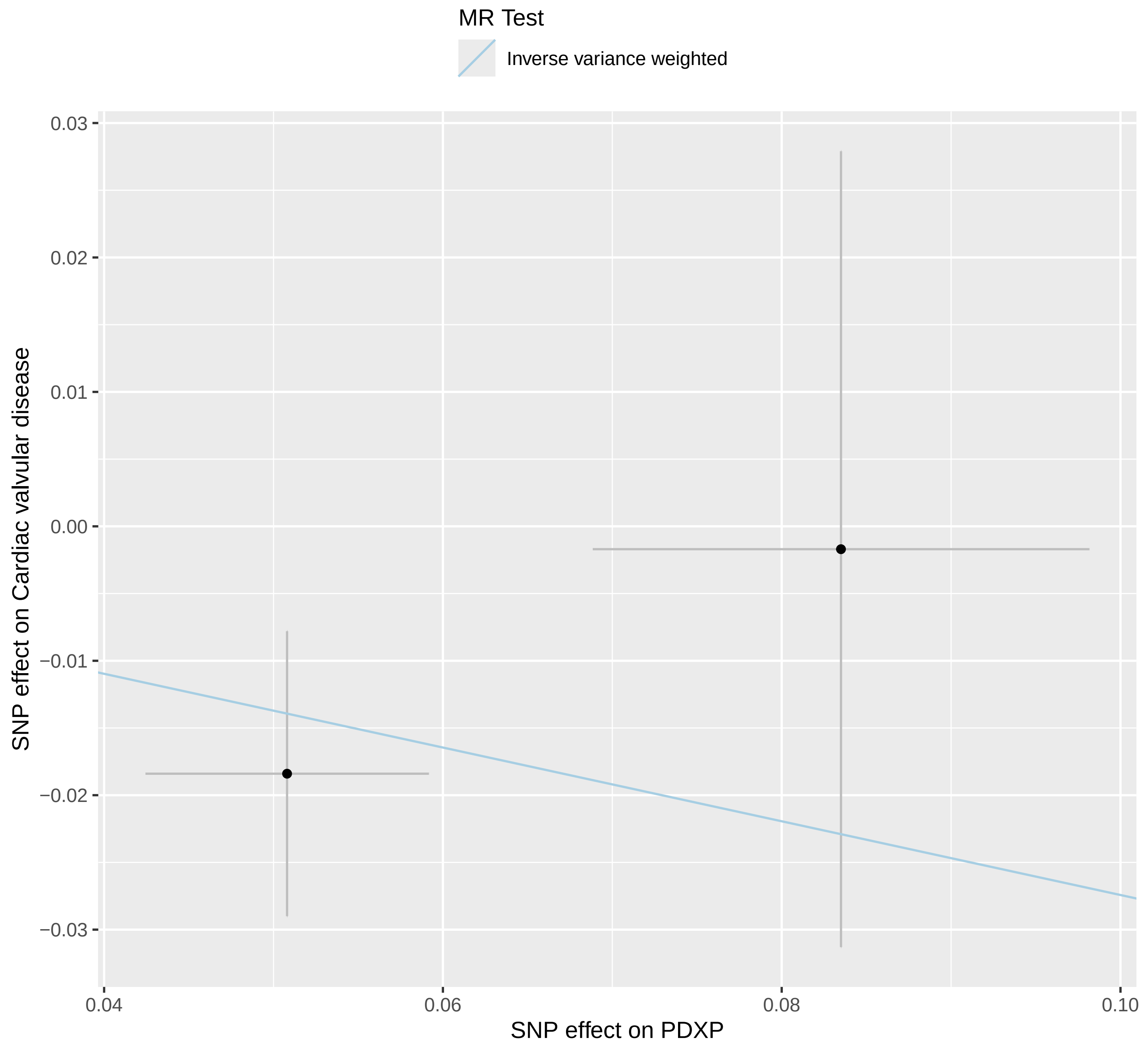

MR Test
Inverse variance weighted
SNP effect on Cardiac valvular disease
0.03
0.02
0.01
0.00
−0.01
−0.02
−0.03
0.04
0.06
0.08
0.10
SNP effect on PDXP

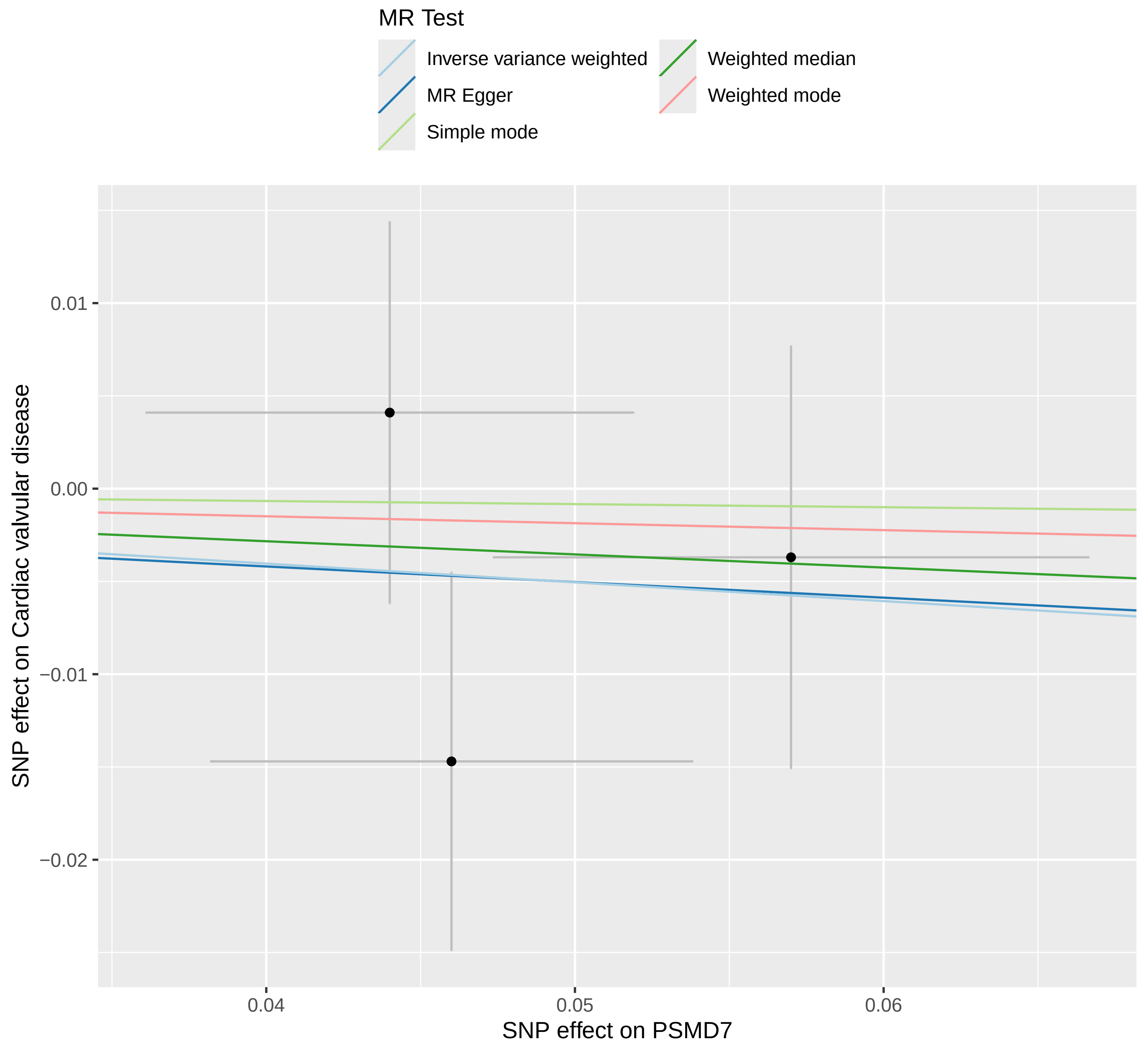
MR Test
Inverse variance weighted
MR Egger
Simple mode
Weighted median
Weighted mode
0.01
0.00
−0.01
−0.02
SNP effect on Cardiac valvular disease
0.04
0.05
0.06
SNP effect on PSMD7

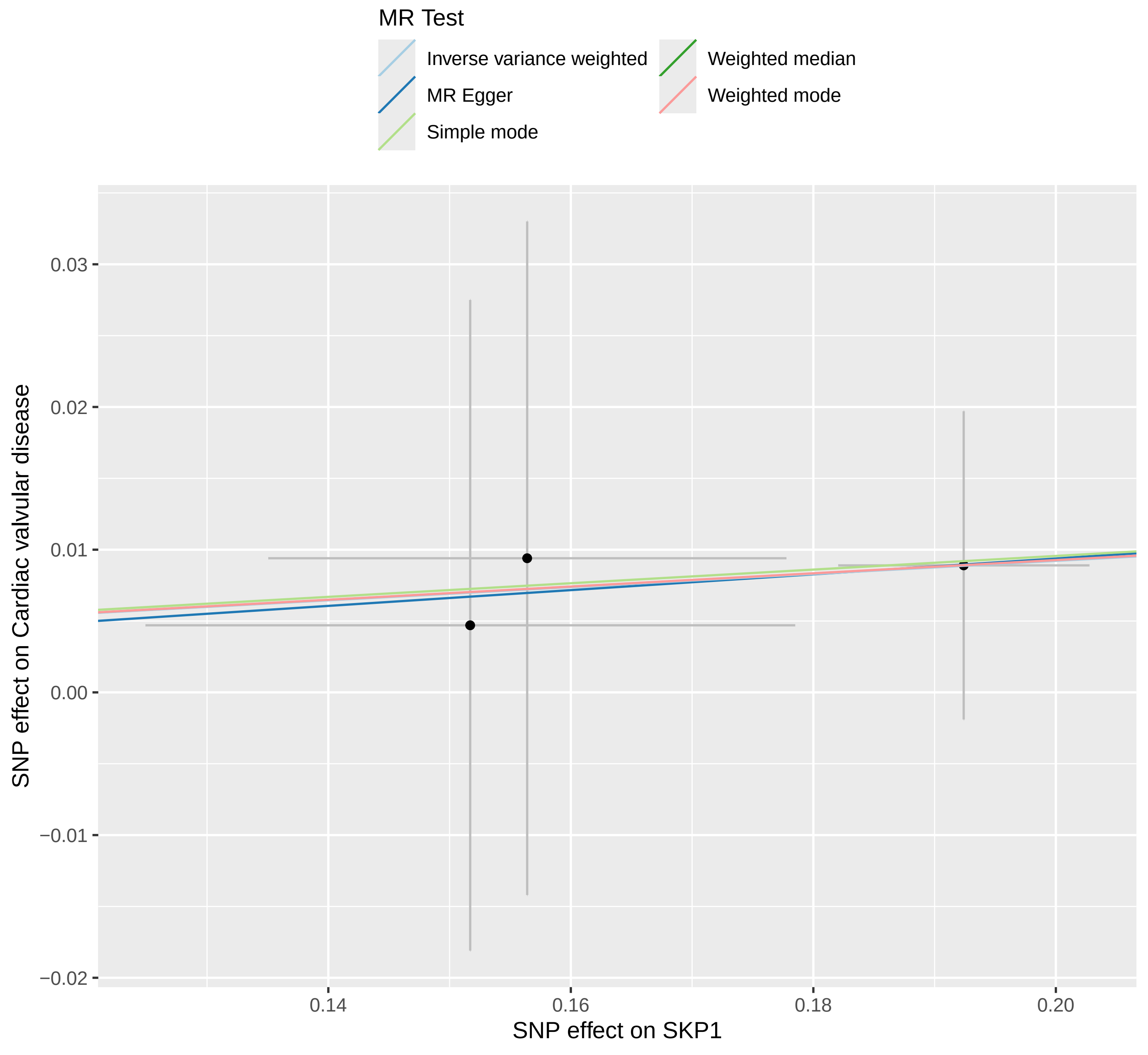
MR Test
Inverse variance weighted
MR Egger
Simple mode
Weighted median
Weighted mode
0.03
0.02
0.01
0.00
−0.01
−0.02
SNP effect on Cardiac valvular disease
0.14
0.16
0.18
0.20
SNP effect on SKP1

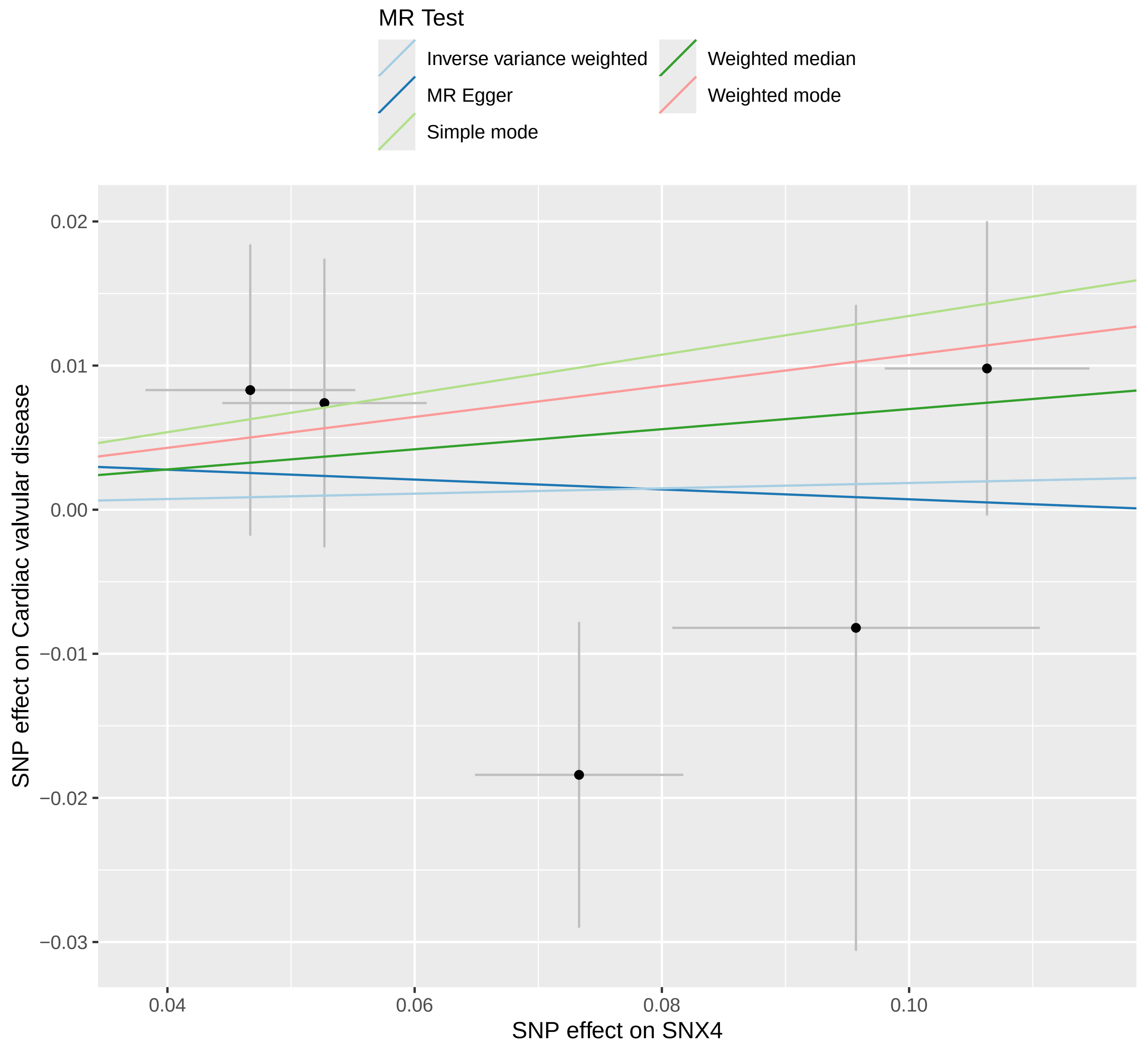
MR Test
Inverse variance weighted
MR Egger
Simple mode
Weighted median
Weighted mode
0.02
0.01
0.00
−0.01
−0.02
−0.03
SNP effect on Cardiac valvular disease
0.04
0.06
0.08
0.10
SNP effect on SNX4

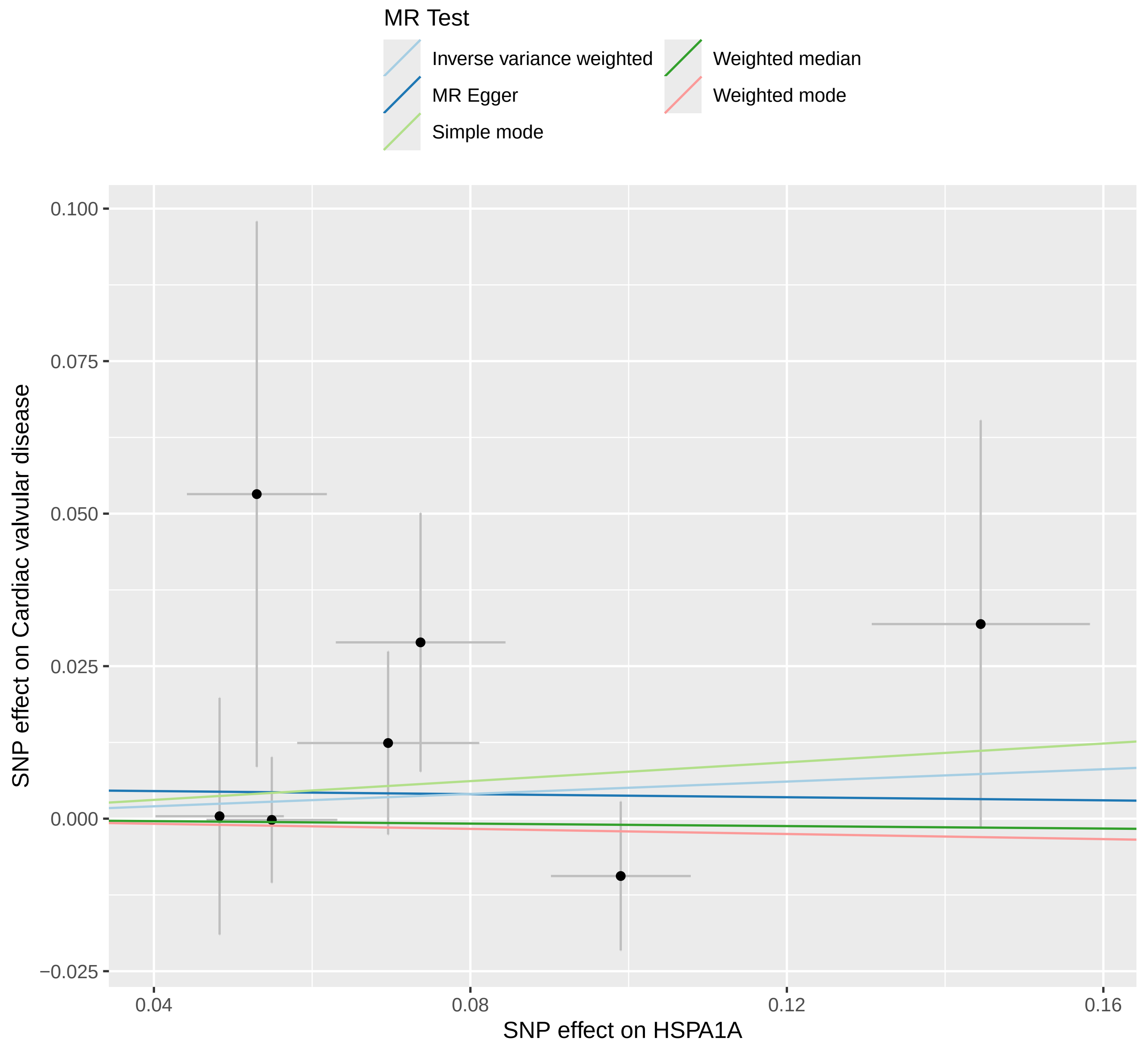
MR Test
Inverse variance weighted
MR Egger
Simple mode
Weighted median
Weighted mode
0.100
0.075
0.050
0.025
0.000
−0.025
0.04
0.08
0.12
0.16
SNP effect on Cardiac valvular disease
SNP effect on HSPA1A

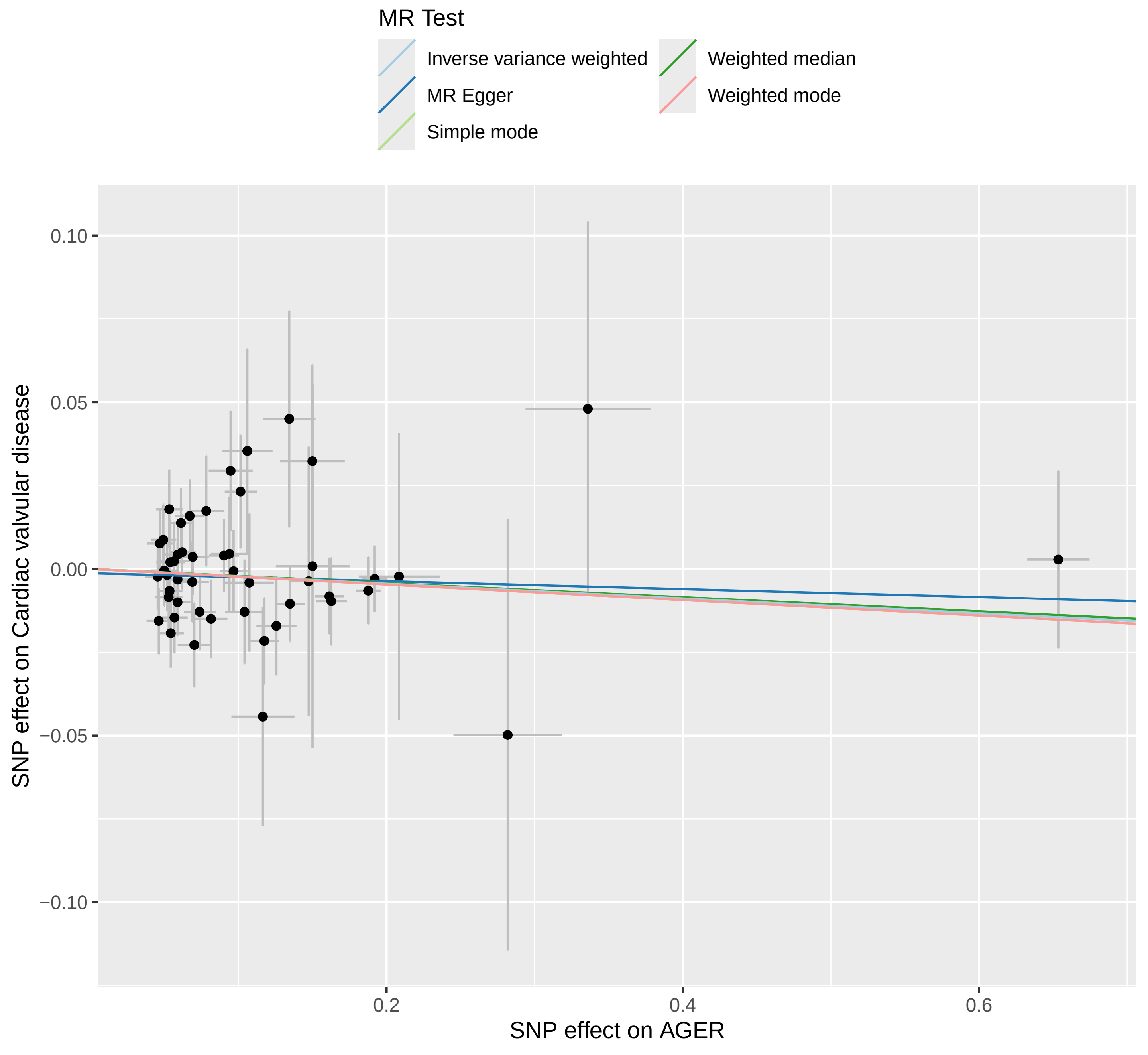

MR Test
Inverse variance weighted
MR Egger
Simple mode
Weighted median
Weighted mode
SNP effect on Cardiac valvular disease
0.10
0.05
0.00
−0.05
−0.10
SNP effect on AGER
0.2
0.4
0.6

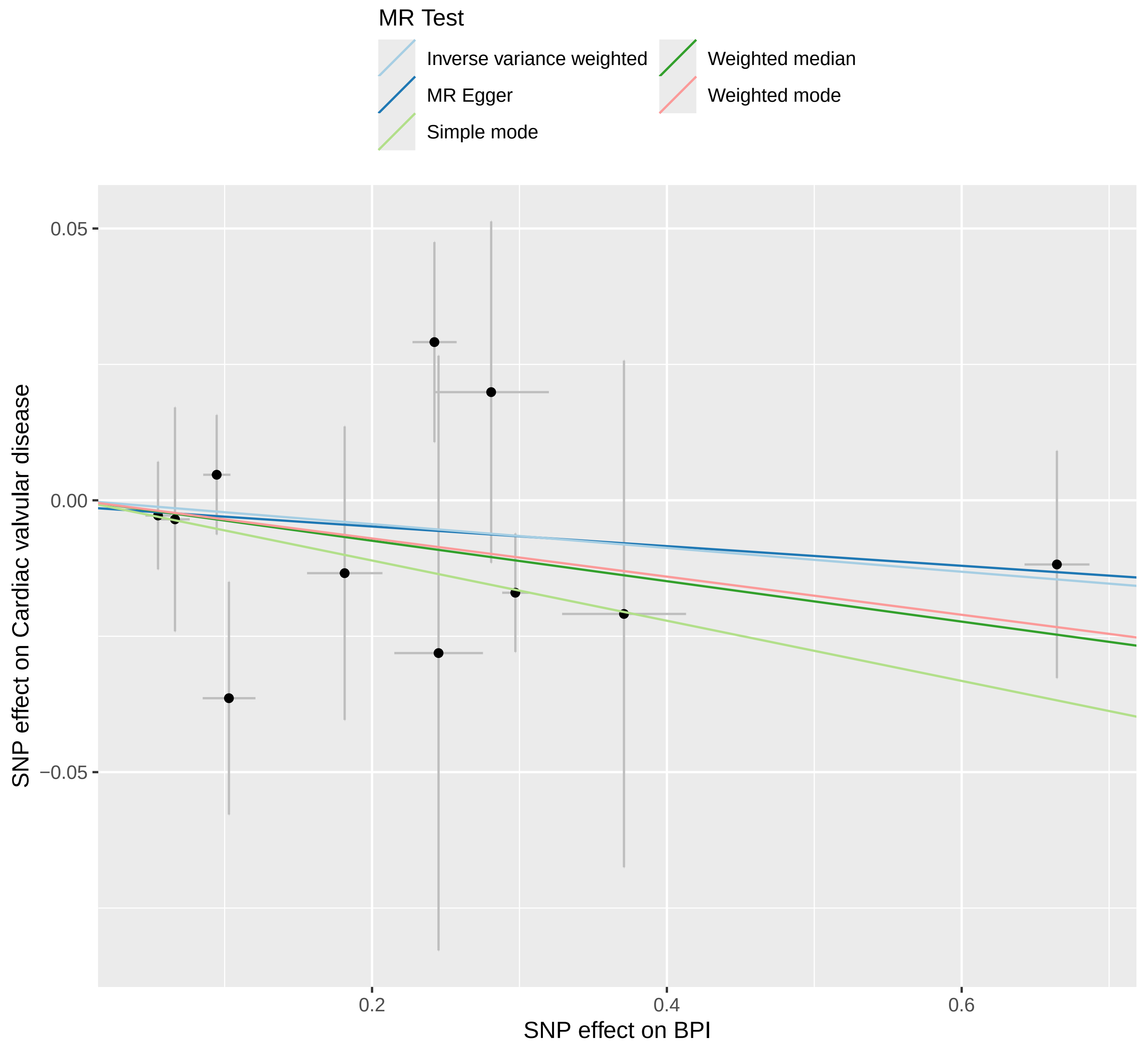
MR Test
Inverse variance weighted
MR Egger
Simple mode
Weighted median
Weighted mode
0.05
0.00
−0.05
0.2
0.4
0.6
SNP effect on BPI
SNP effect on Cardiac valvular disease

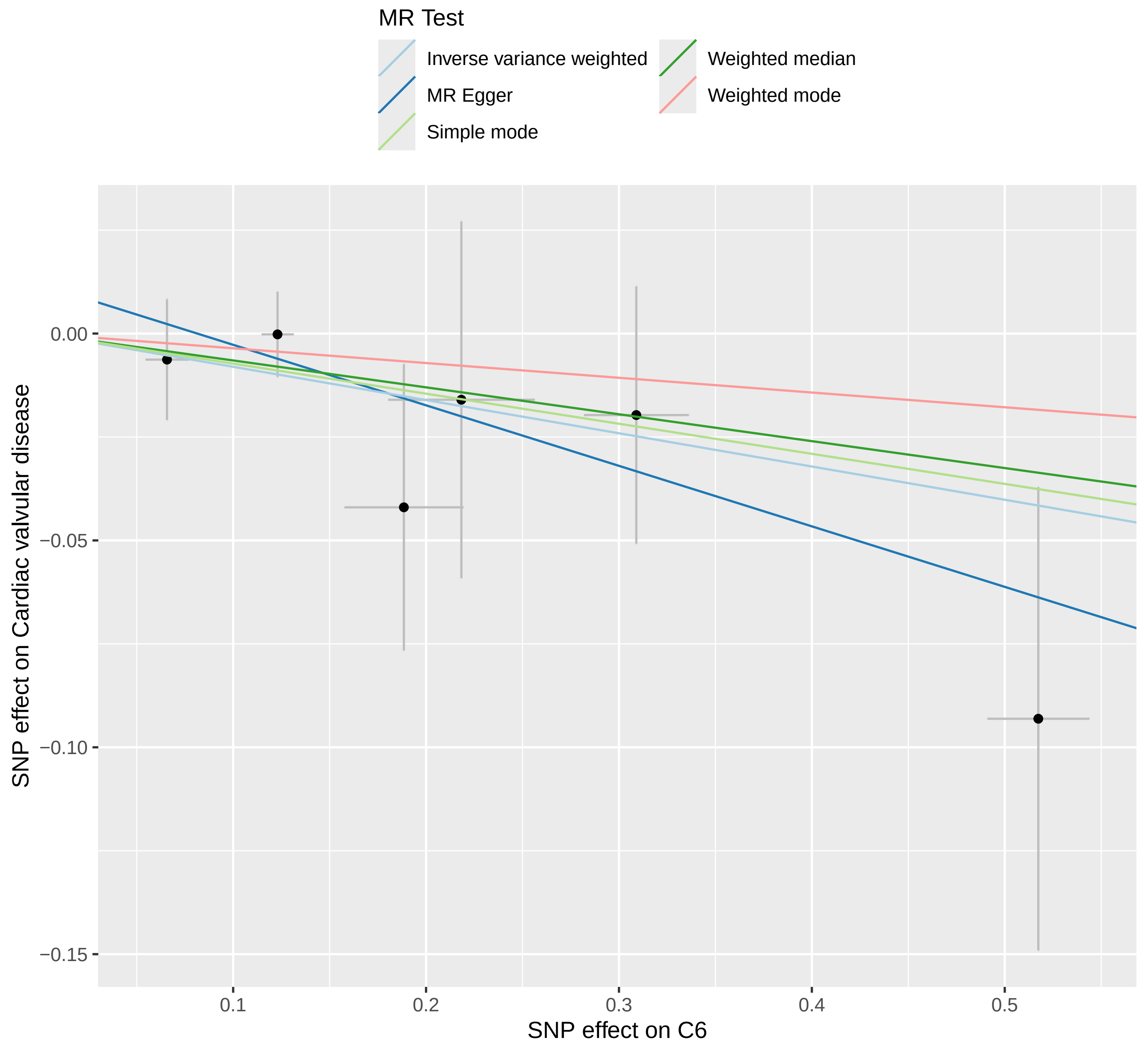
MR Test
Inverse variance weighted
MR Egger
Simple mode
Weighted median
Weighted mode
0.00
−0.05
−0.10
−0.15
SNP effect on Cardiac valvular disease
0.1
0.2
0.3
0.4
0.5
SNP effect on C6

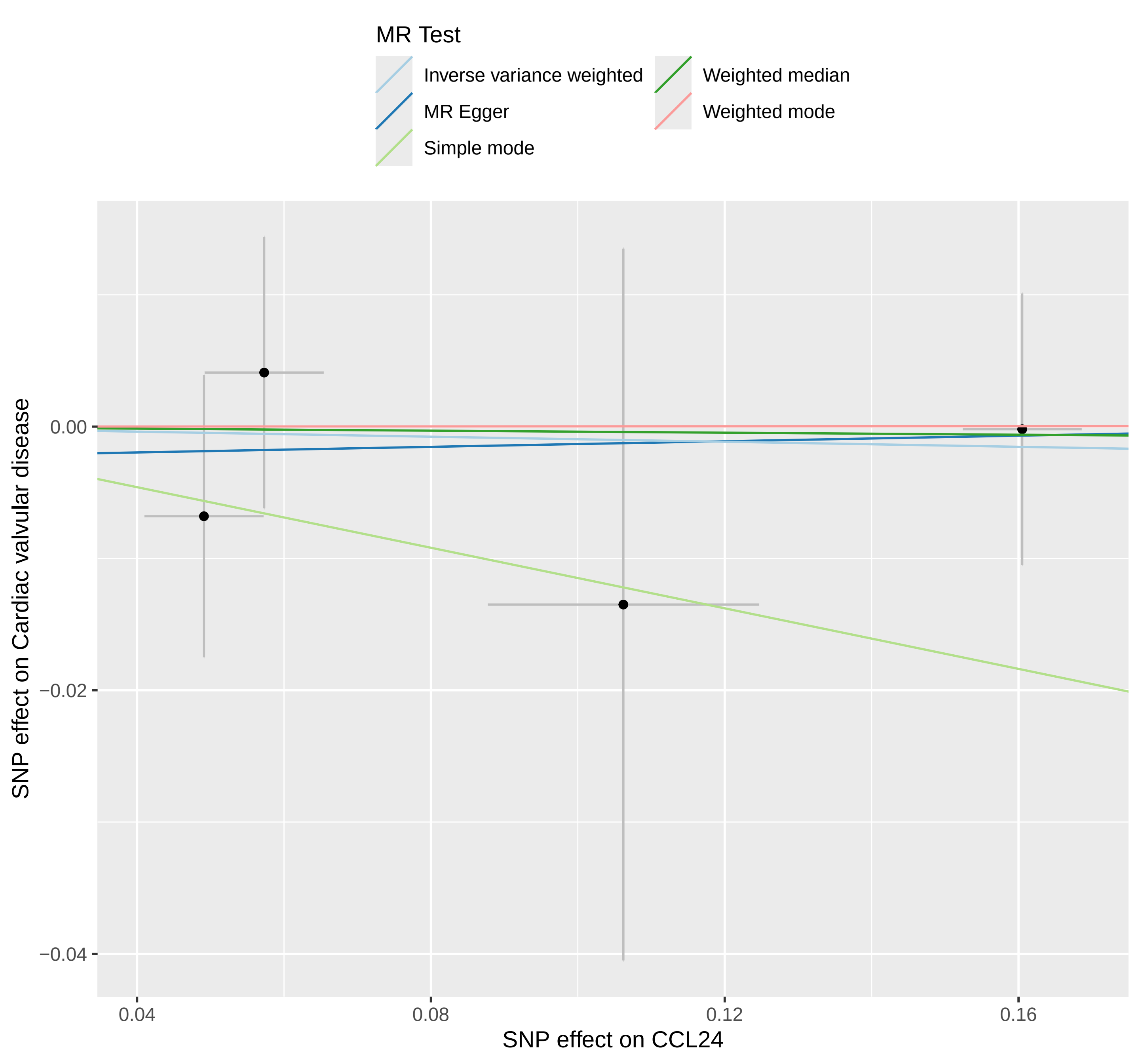

MR Test
Inverse variance weighted
MR Egger
Simple mode
Weighted median
Weighted mode
0.00
−0.02
−0.04
0.04
0.08
0.12
0.16
SNP effect on Cardiac valvular disease
SNP effect on CCL24

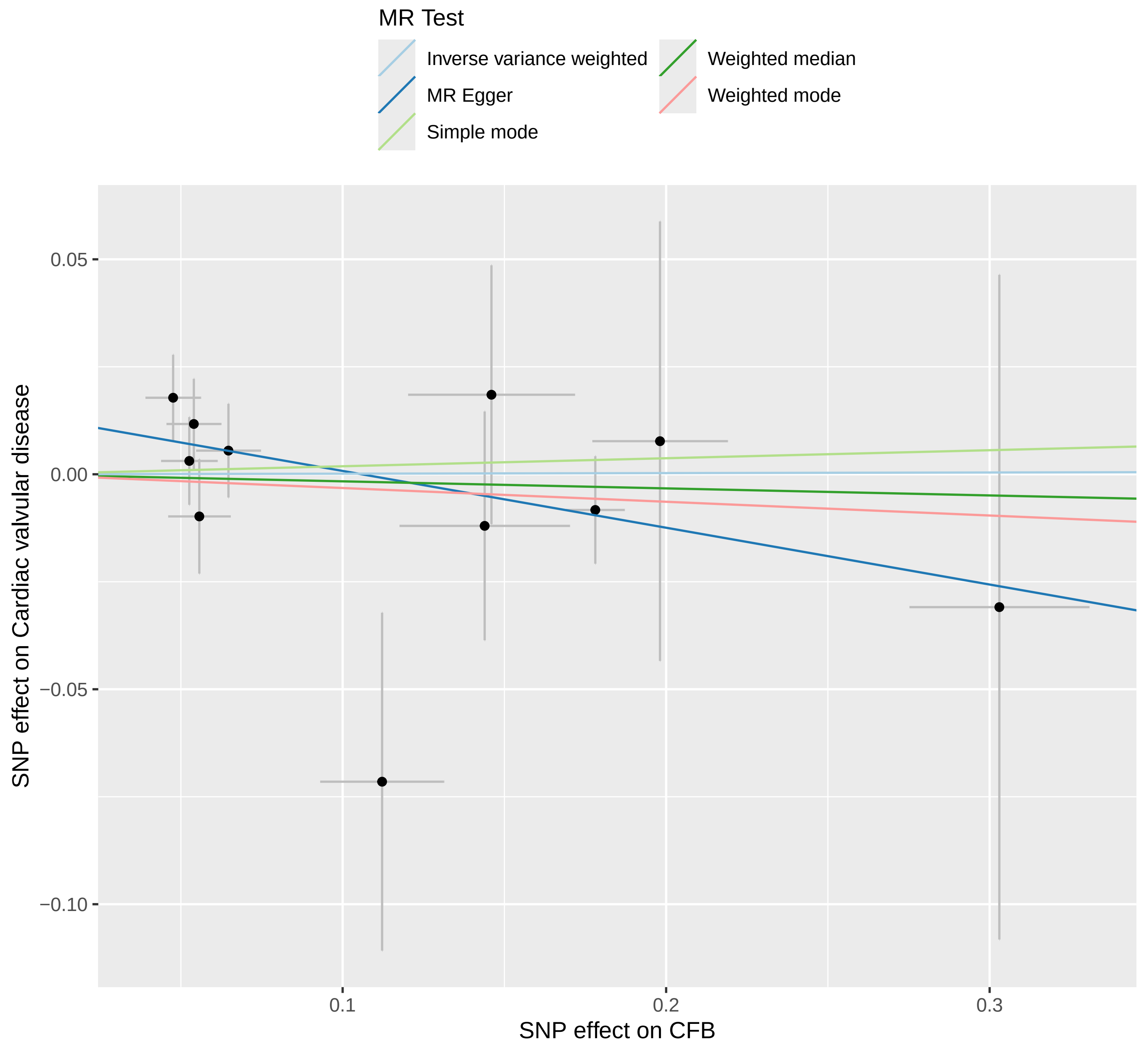

MR Test
Inverse variance weighted
MR Egger
Simple mode
Weighted median
Weighted mode
0.05
0.00
−0.05
−0.10
0.1
0.2
0.3
SNP effect on Cardiac valvular disease
SNP effect on CFB

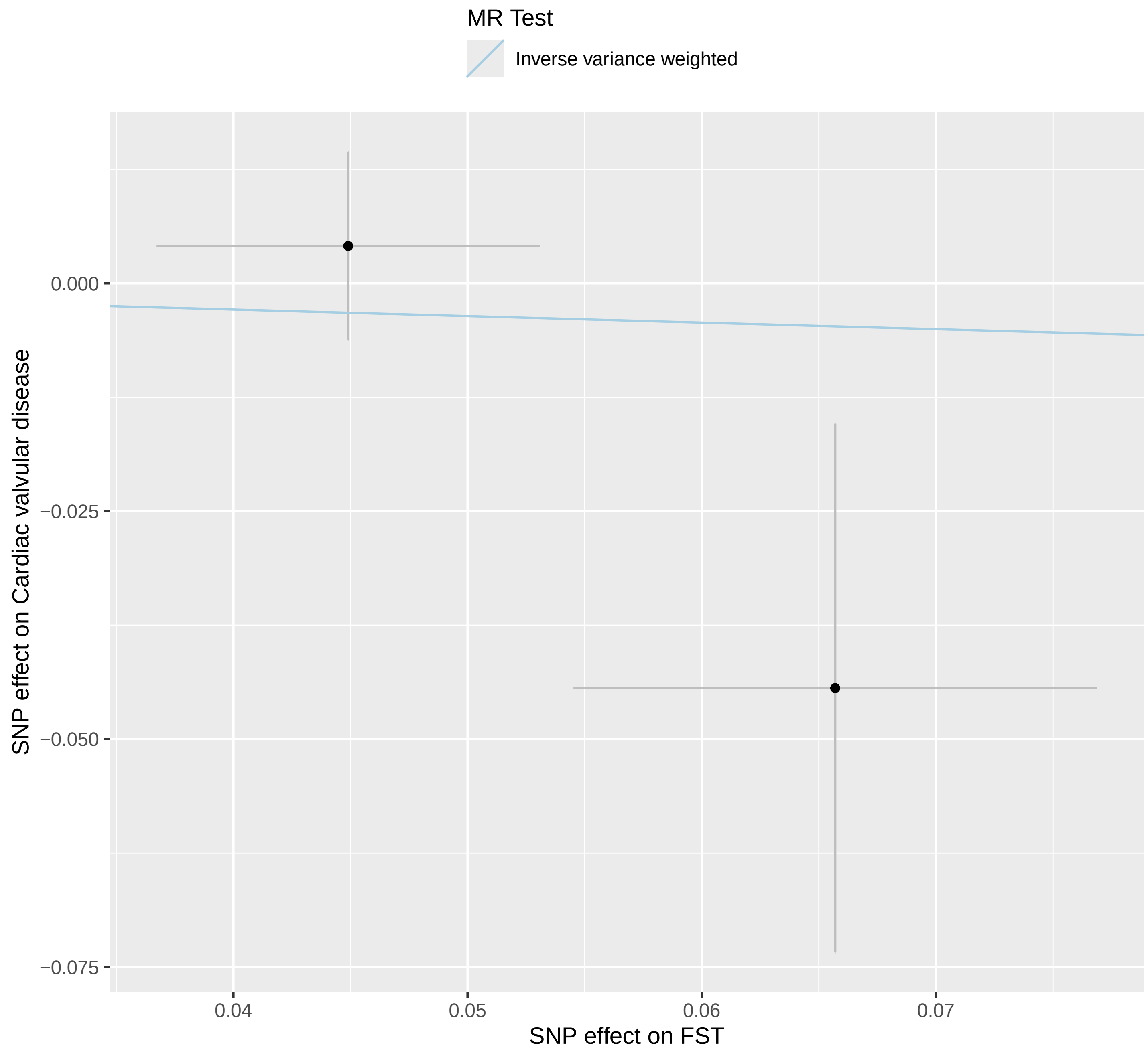
MR Test
Inverse variance weighted
0.000
−0.025
−0.050
−0.075
0.04
0.05
0.06
0.07
SNP effect on FST
SNP effect on Cardiac valvular disease

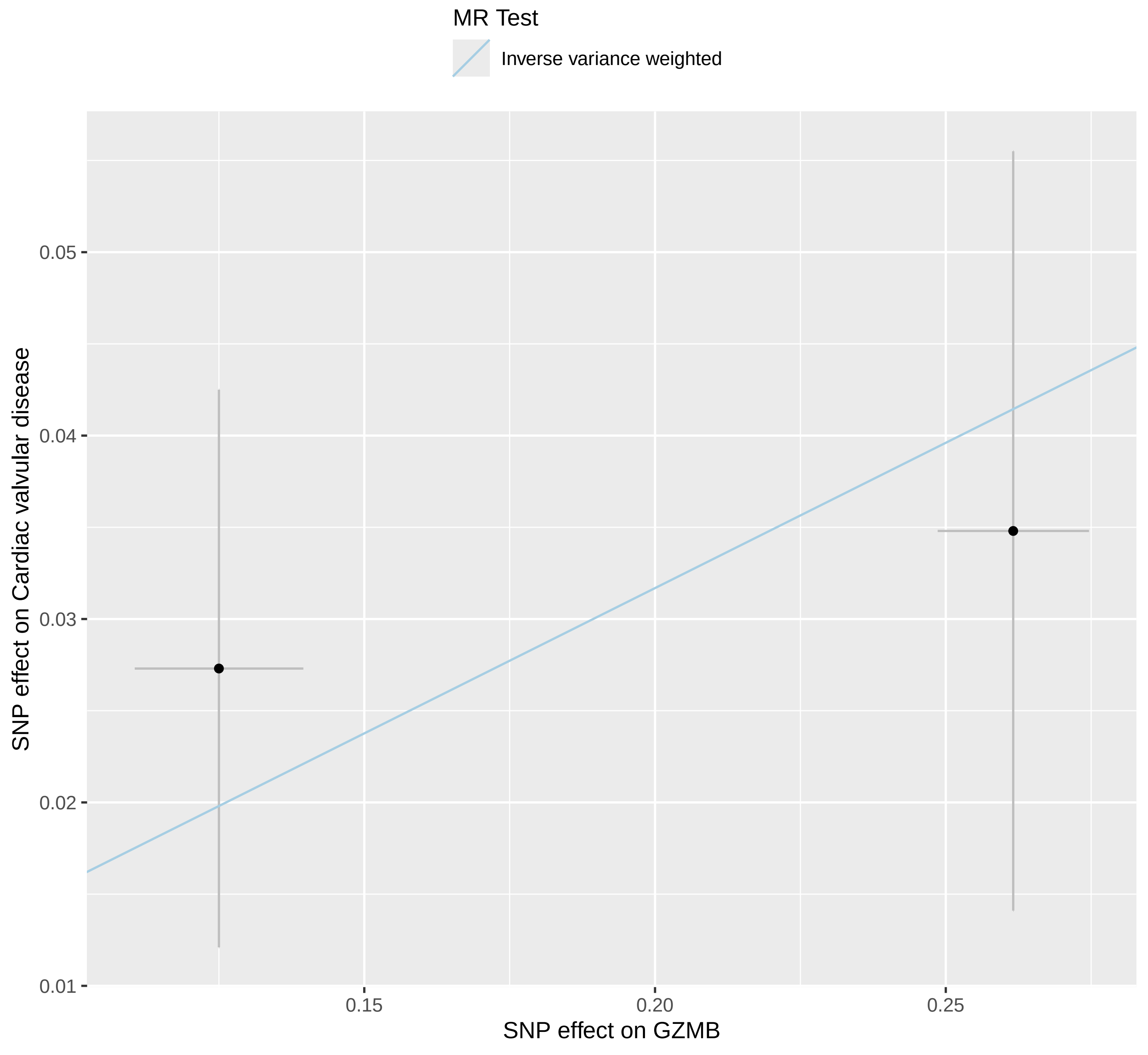

MR Test
Inverse variance weighted
SNP effect on Cardiac valvular disease
0.05
0.04
0.03
0.02
0.01
0.15
0.20
0.25
SNP effect on GZMB

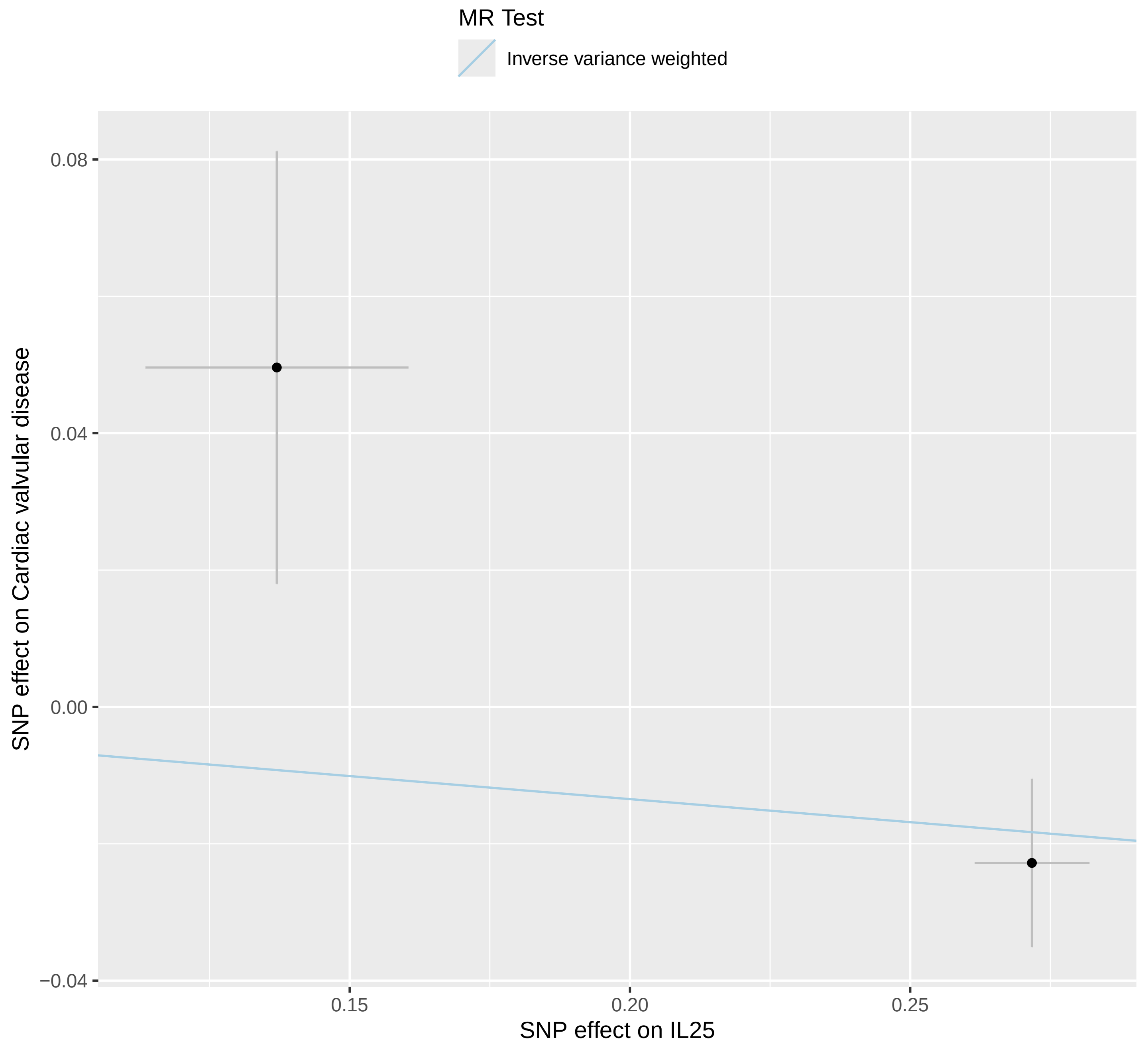

MR Test
Inverse variance weighted
SNP effect on Cardiac valvular disease
0.08
0.04
0.00
−0.04
0.15
0.20
0.25
SNP effect on IL25

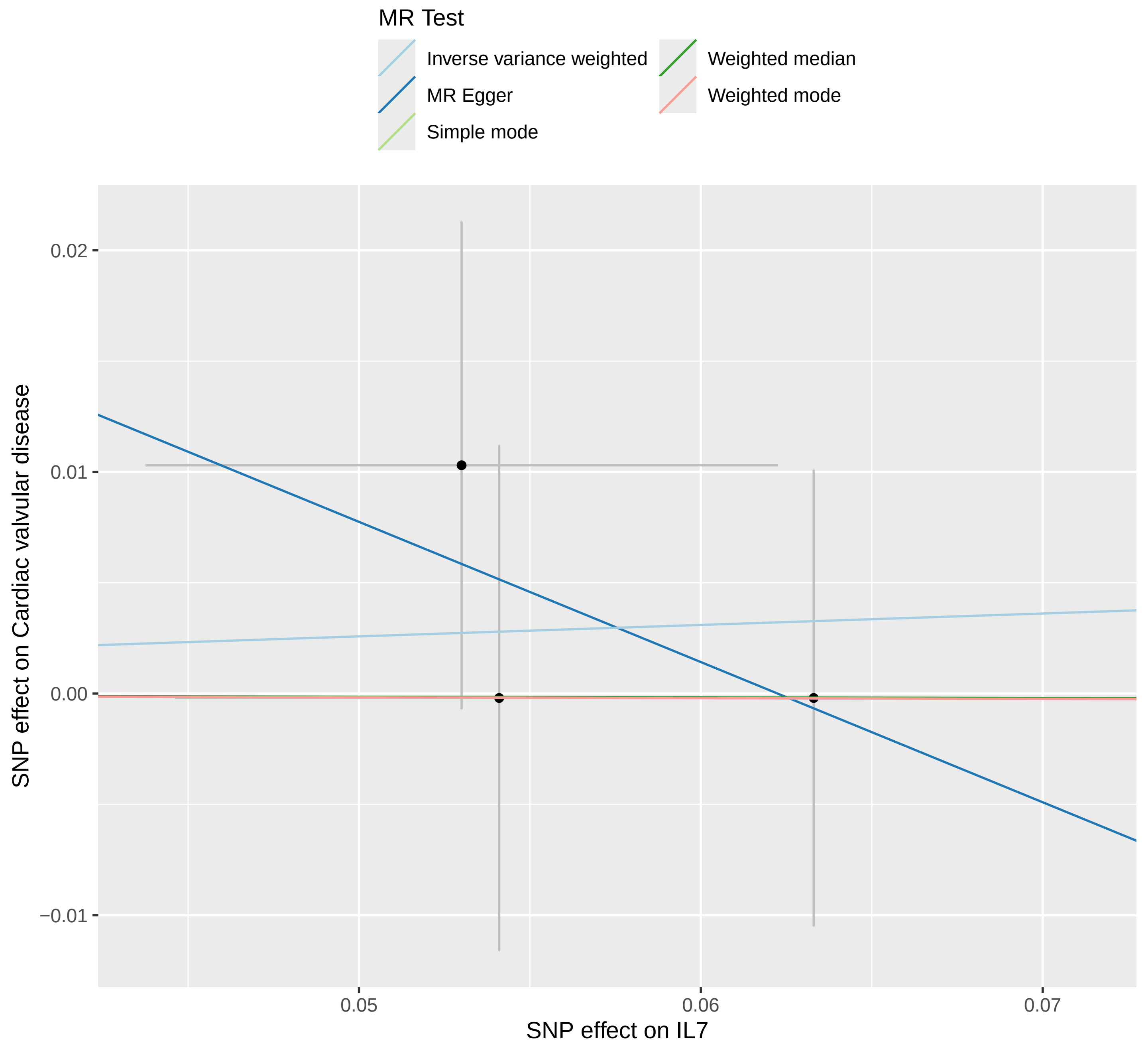
MR Test
Inverse variance weighted
MR Egger
Simple mode
Weighted median
Weighted mode
0.02
0.01
0.00
−0.01
SNP effect on Cardiac valvular disease
0.05
0.06
0.07
SNP effect on IL7

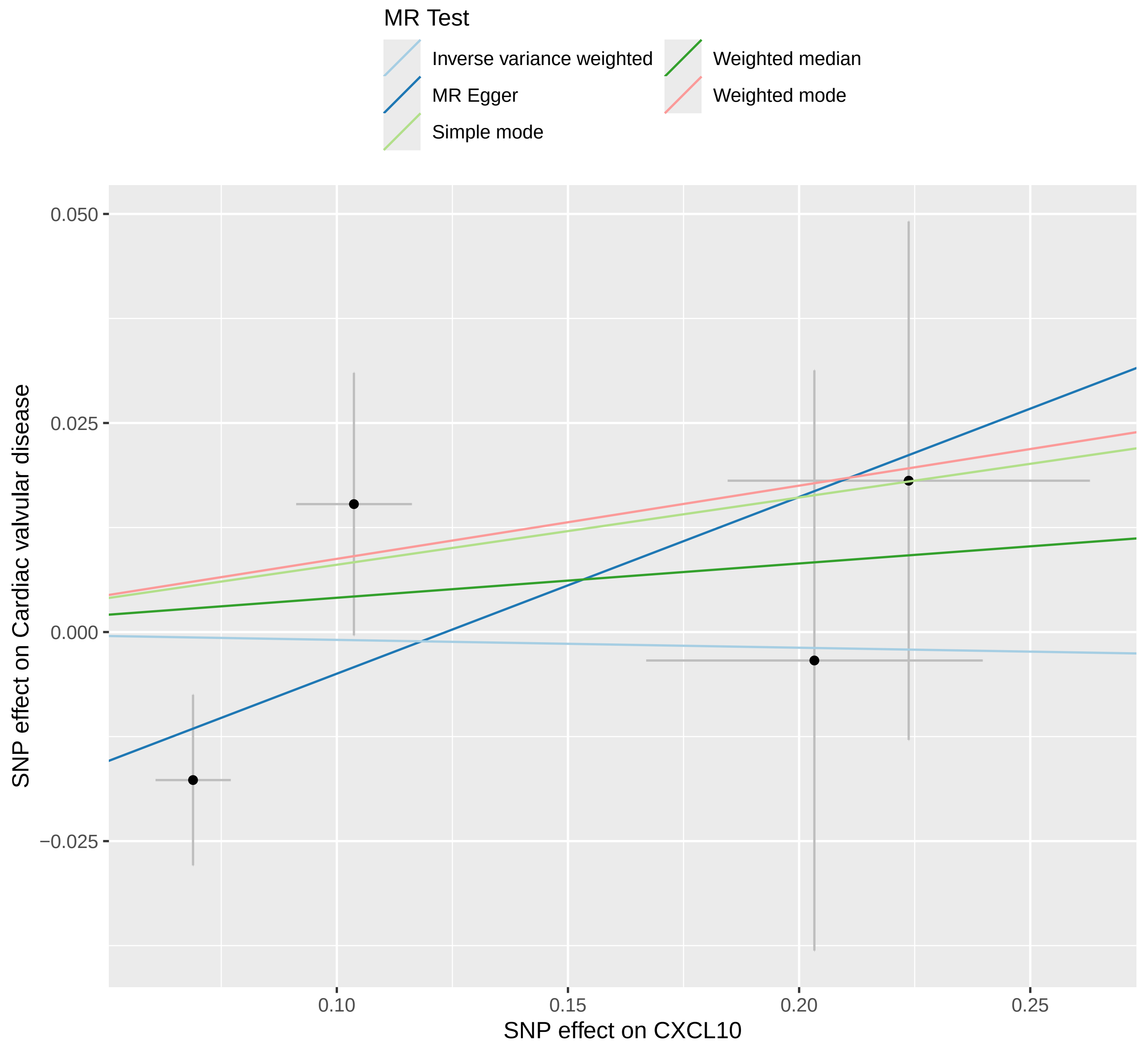
MR Test
Inverse variance weighted
MR Egger
Simple mode
Weighted median
Weighted mode
0.050
0.025
0.000
−0.025
0.10
0.15
0.20
0.25
SNP effect on CXCL10
SNP effect on Cardiac valvular disease

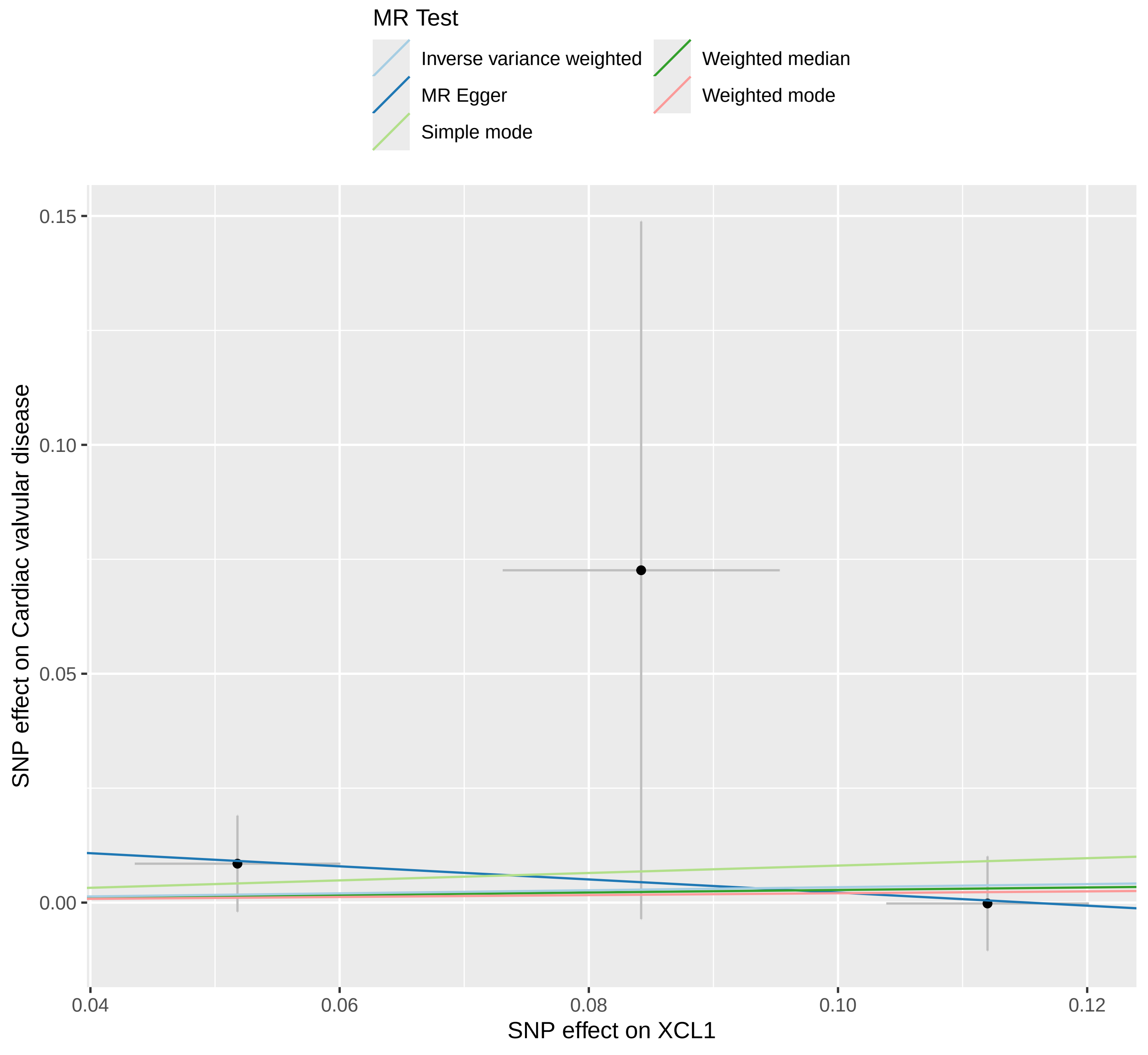
MR Test
Inverse variance weighted
MR Egger
Simple mode
Weighted median
Weighted mode
0.15
0.10
0.05
0.00
SNP effect on Cardiac valvular disease
0.04
0.06
0.08
0.10
0.12
SNP effect on XCL1

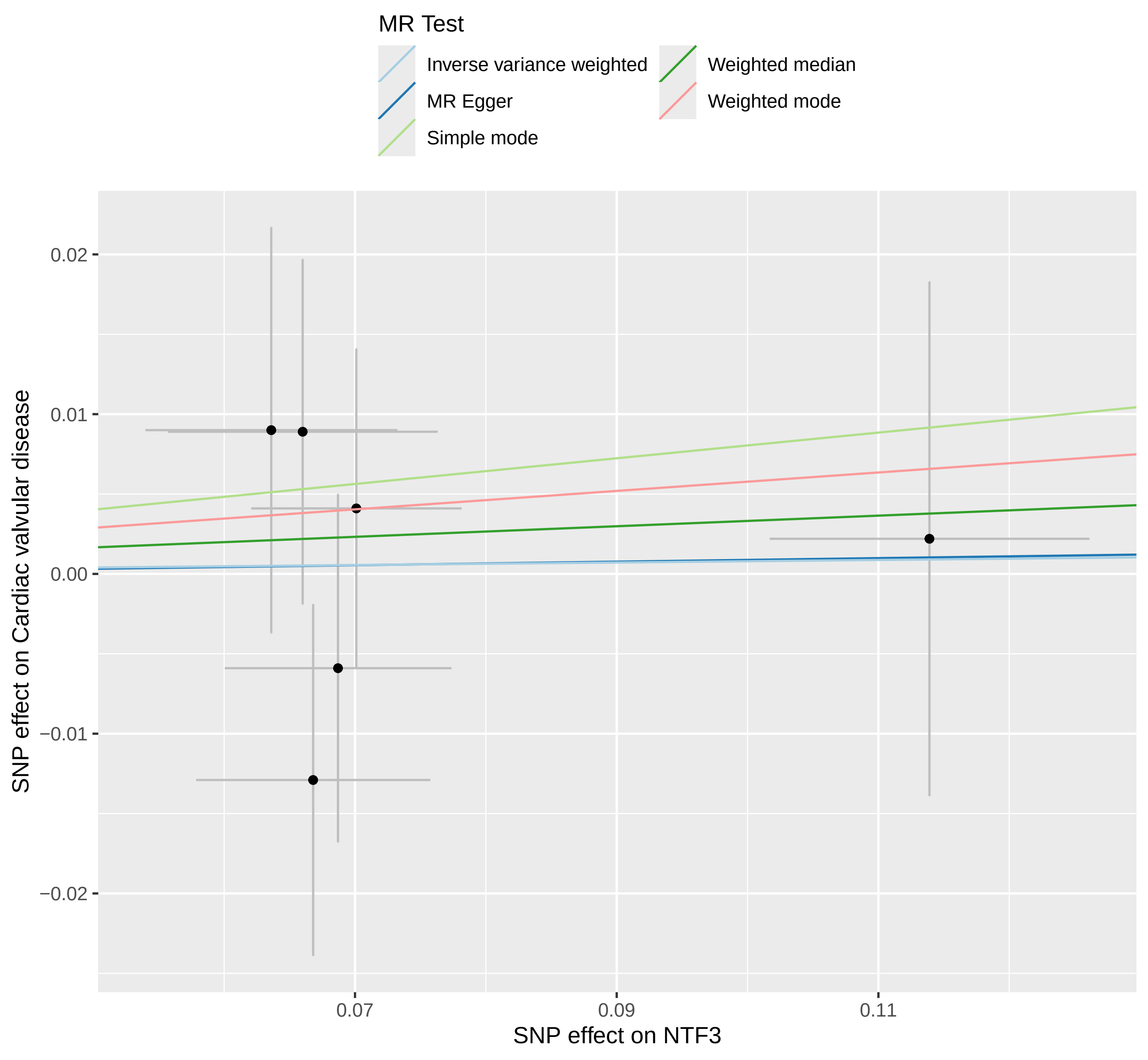
MR Test
Inverse variance weighted
MR Egger
Simple mode
Weighted median
Weighted mode
0.02
0.01
0.00
−0.01
−0.02
SNP effect on Cardiac valvular disease
0.07
0.09
0.11
SNP effect on NTF3

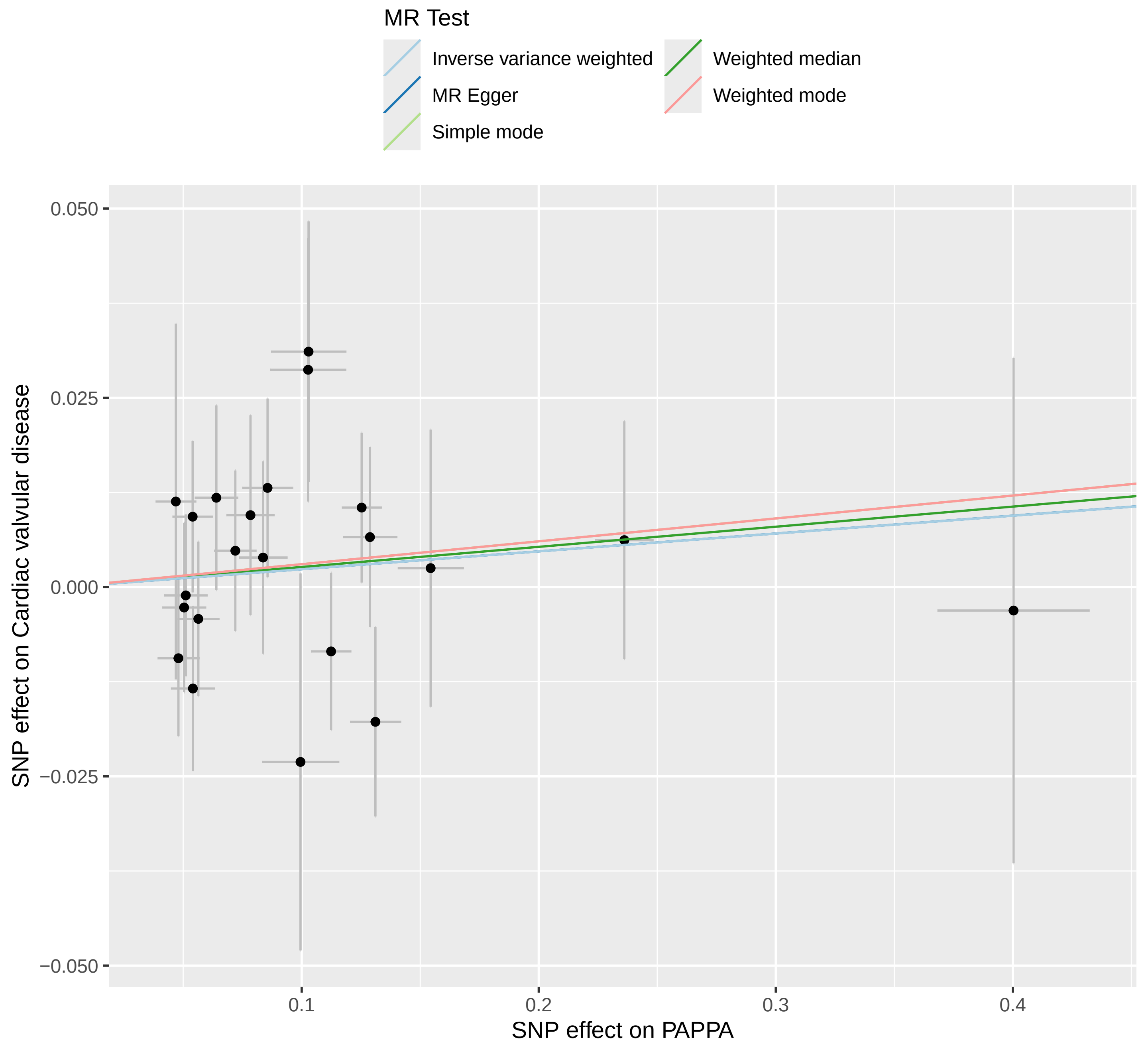
MR Test
Inverse variance weighted
MR Egger
Simple mode
Weighted median
Weighted mode
0.050
0.025
0.000
−0.025
−0.050
SNP effect on Cardiac valvular disease
0.1
0.2
0.3
0.4
SNP effect on PAPPA

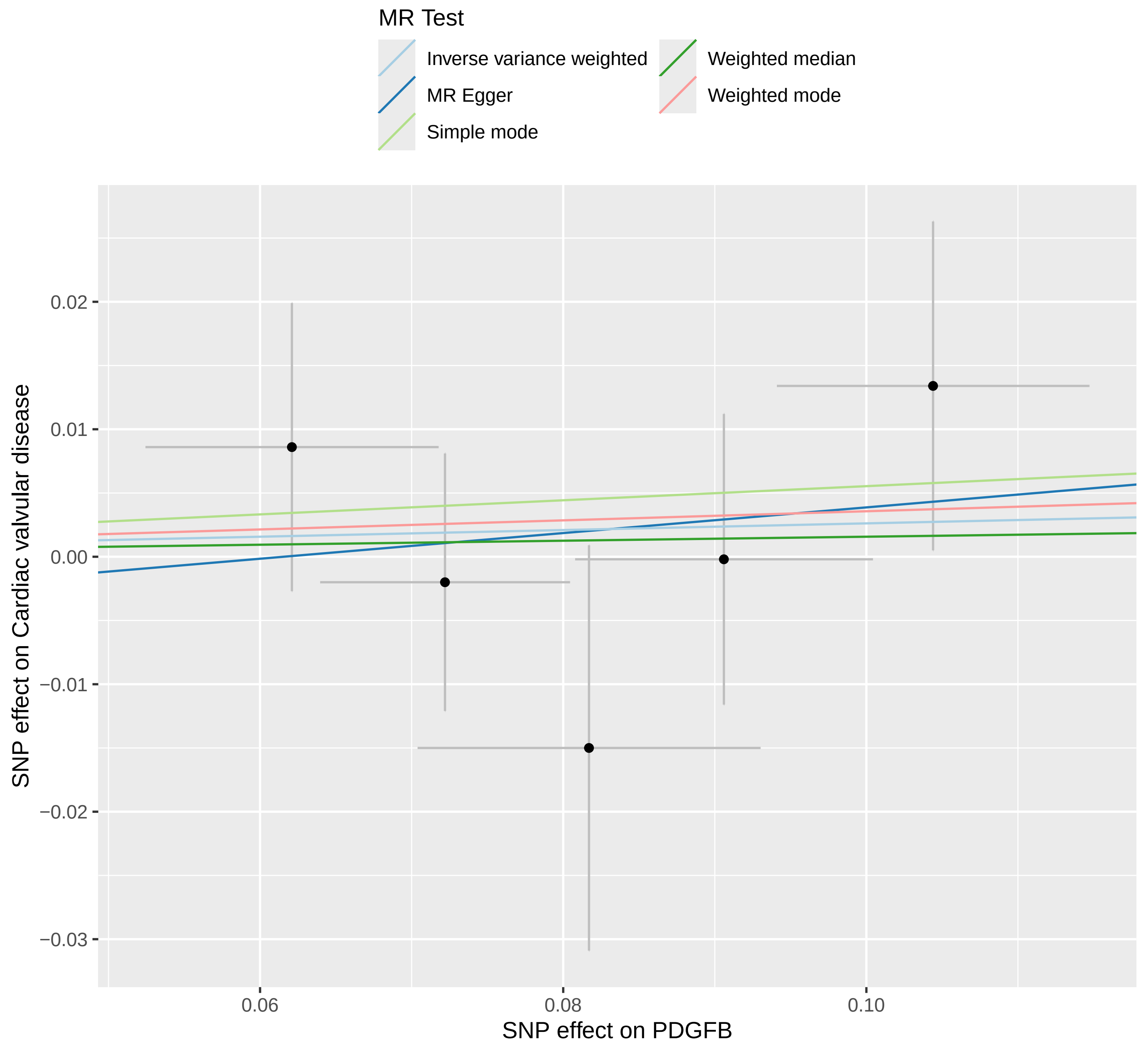
MR Test
Inverse variance weighted
MR Egger
Simple mode
Weighted median
Weighted mode
0.02
0.01
0.00
−0.01
−0.02
−0.03
SNP effect on Cardiac valvular disease
0.06
0.08
0.10
SNP effect on PDGFB

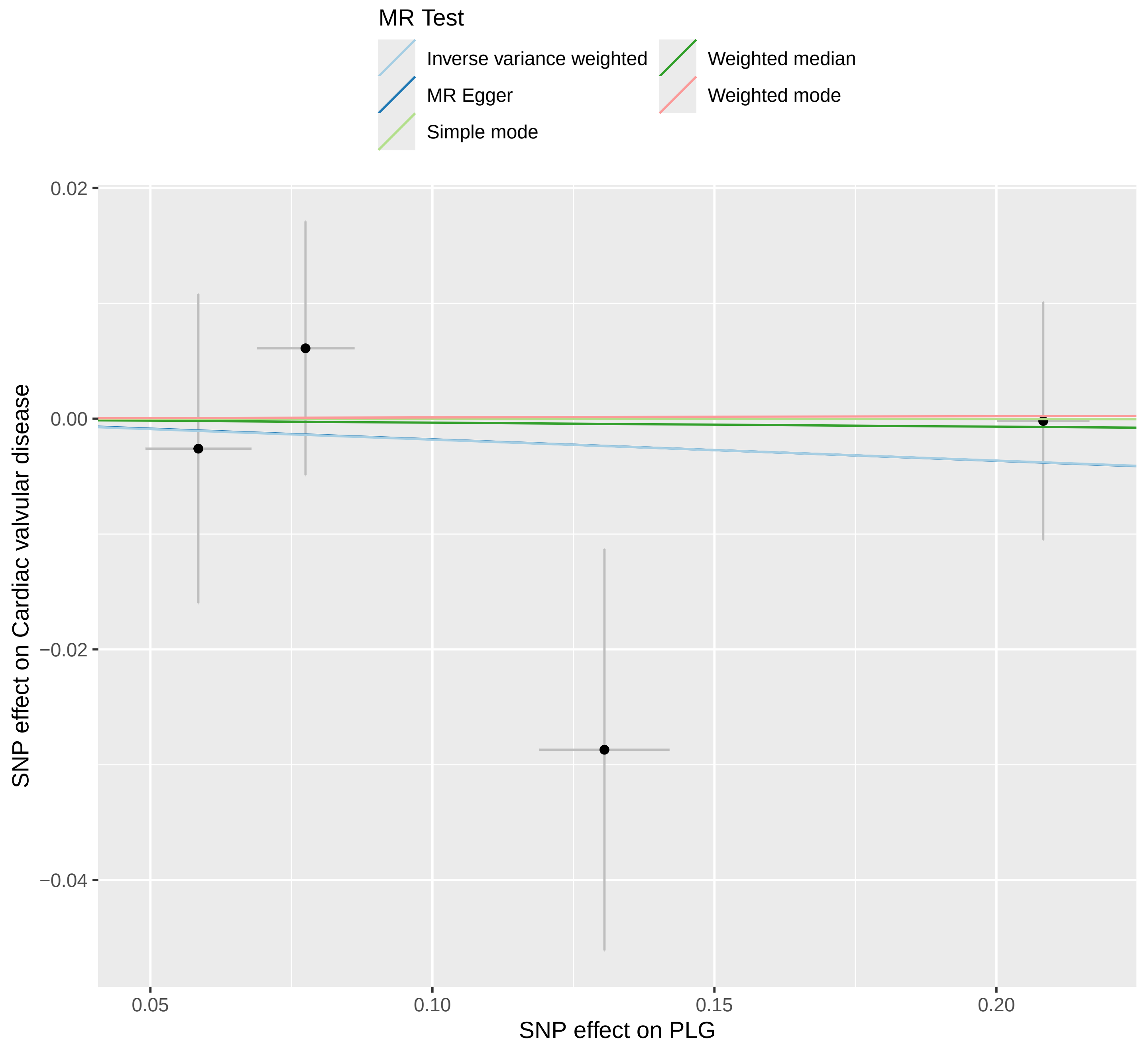
MR Test
Inverse variance weighted
MR Egger
Simple mode
Weighted median
Weighted mode
0.02
0.00
−0.02
−0.04
SNP effect on Cardiac valvular disease
0.05
0.10
0.15
0.20
SNP effect on PLG

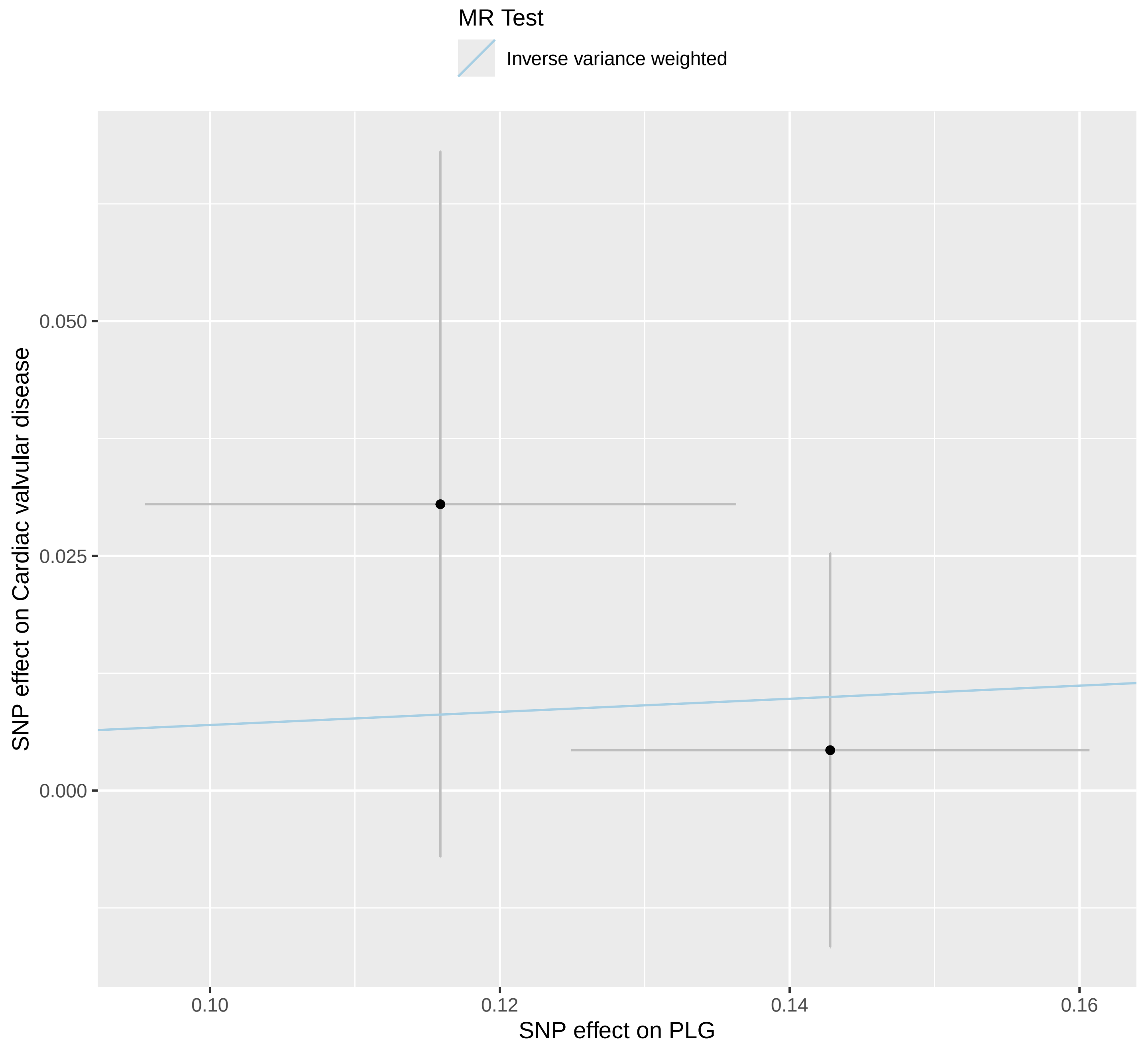
MR Test
Inverse variance weighted
0.050
0.025
0.000
0.10
0.12
0.14
0.16
SNP effect on PLG
SNP effect on Cardiac valvular disease

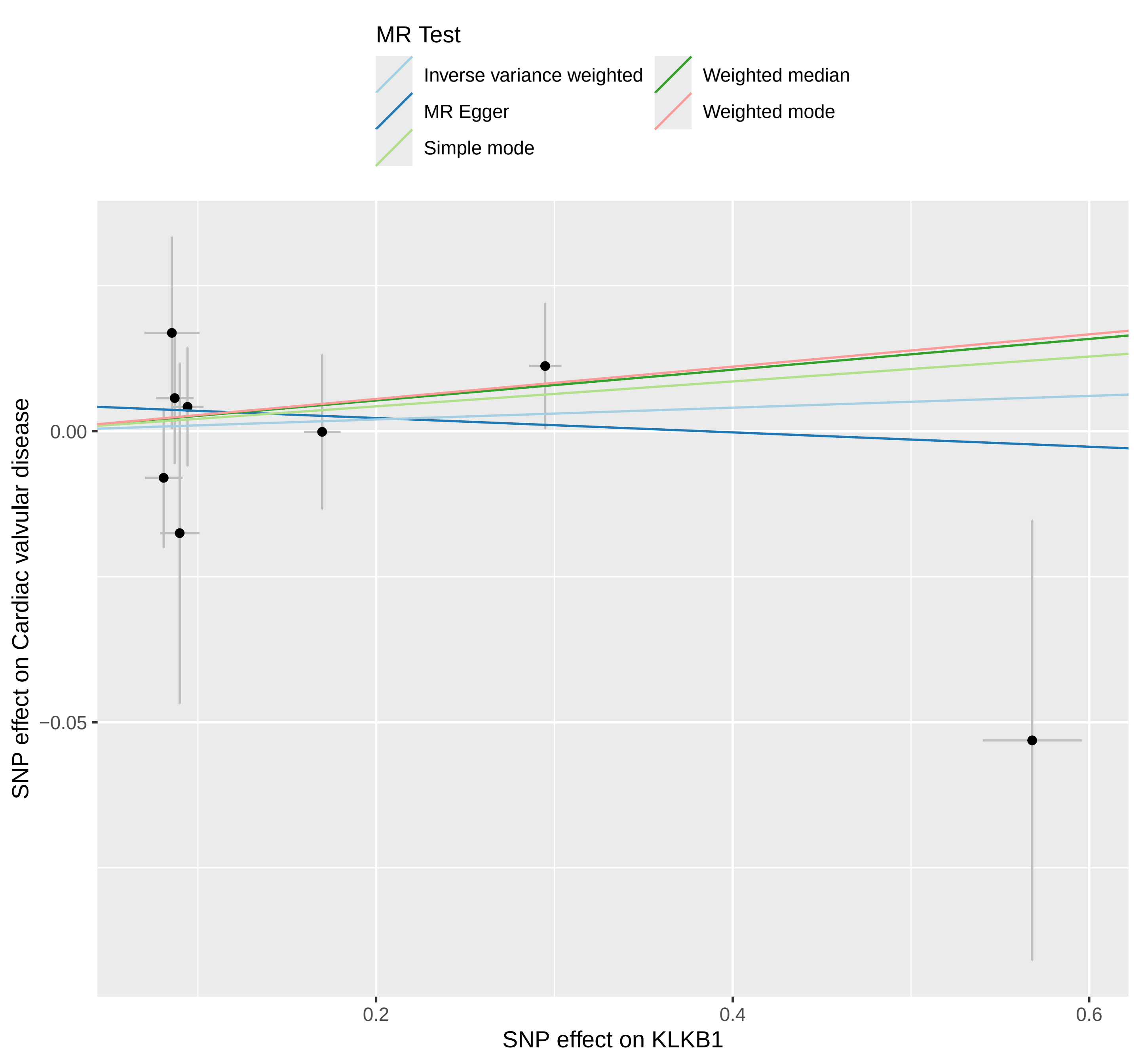
MR Test
Inverse variance weighted
MR Egger
Simple mode
Weighted median
Weighted mode
0.00
−0.05
0.2
0.4
0.6
SNP effect on KLKB1
SNP effect on Cardiac valvular disease

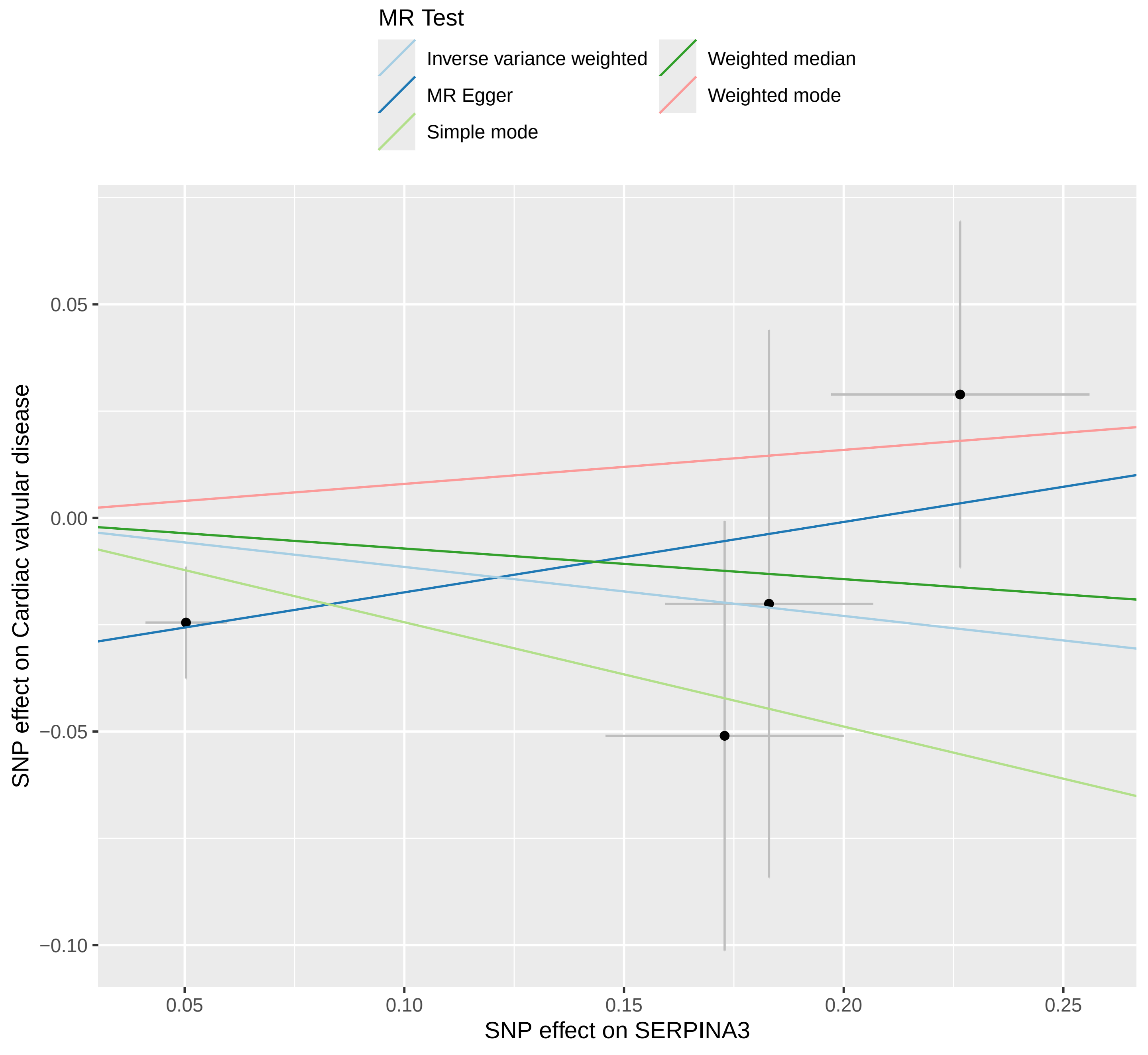

MR Test
Inverse variance weighted
MR Egger
Simple mode
Weighted median
Weighted mode
0.05
0.00
−0.05
−0.10
SNP effect on Cardiac valvular disease
0.05
0.10
0.15
0.20
0.25
SNP effect on SERPINA3

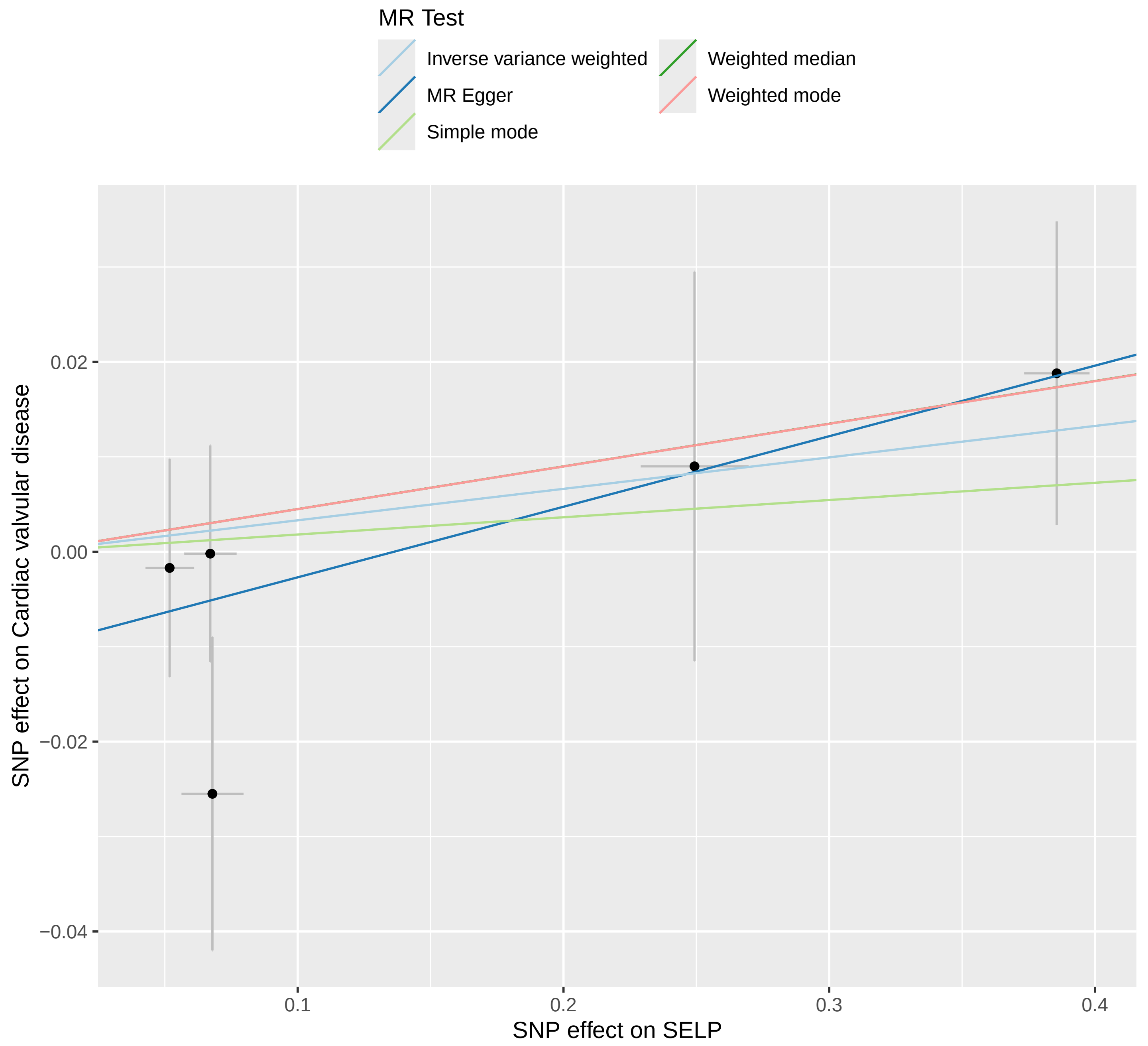
MR Test
Inverse variance weighted
MR Egger
Simple mode
Weighted median
Weighted mode
SNP effect on Cardiac valvular disease
0.02
0.00
−0.02
−0.04
0.1
0.2
0.3
0.4
SNP effect on SELP

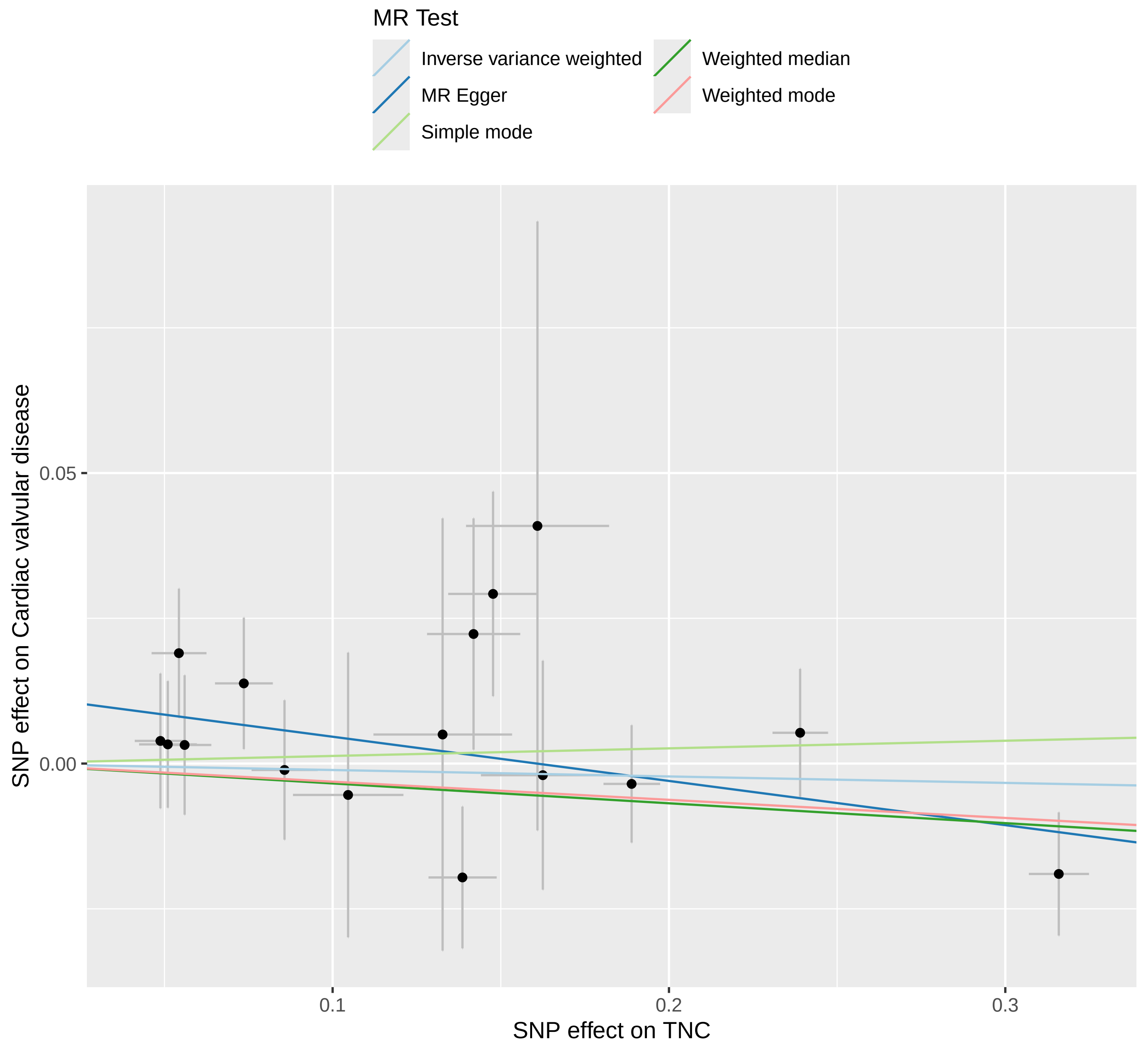
MR Test
Inverse variance weighted
Weighted median
MR Egger
Weighted mode
Simple mode
SNP effect on Cardiac valvular disease
0.05
0.00
0.1
0.2
0.3
SNP effect on TNC

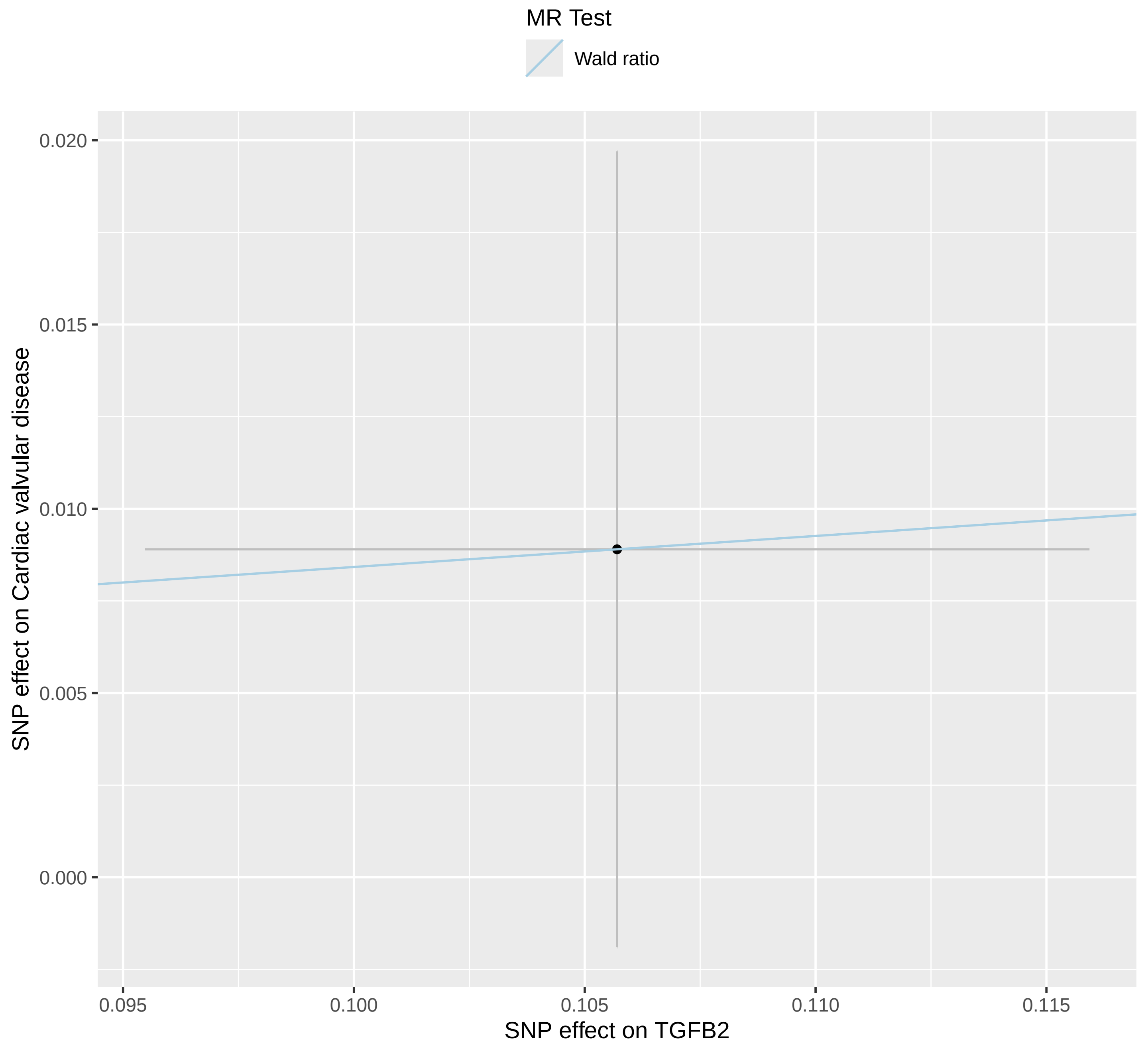
MR Test
Wald ratio
0.020
0.015
0.010
0.005
0.000
SNP effect on Cardiac valvular disease
0.095
0.100
0.105
0.110
0.115
SNP effect on TGFB2

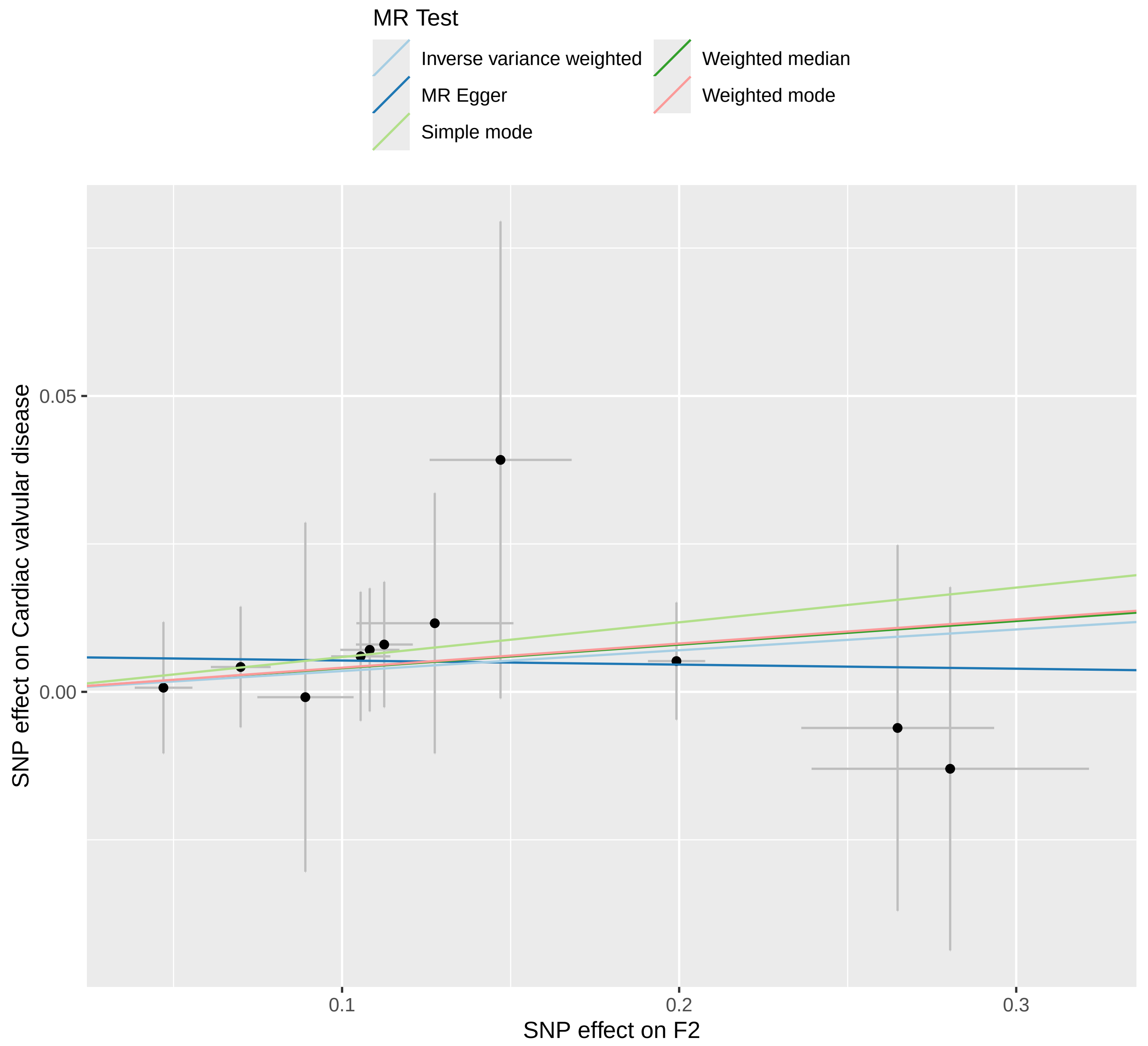
MR Test
Inverse variance weighted
MR Egger
Simple mode
Weighted median
Weighted mode
SNP effect on Cardiac valvular disease
0.05
0.00
0.1
0.2
0.3
SNP effect on F2

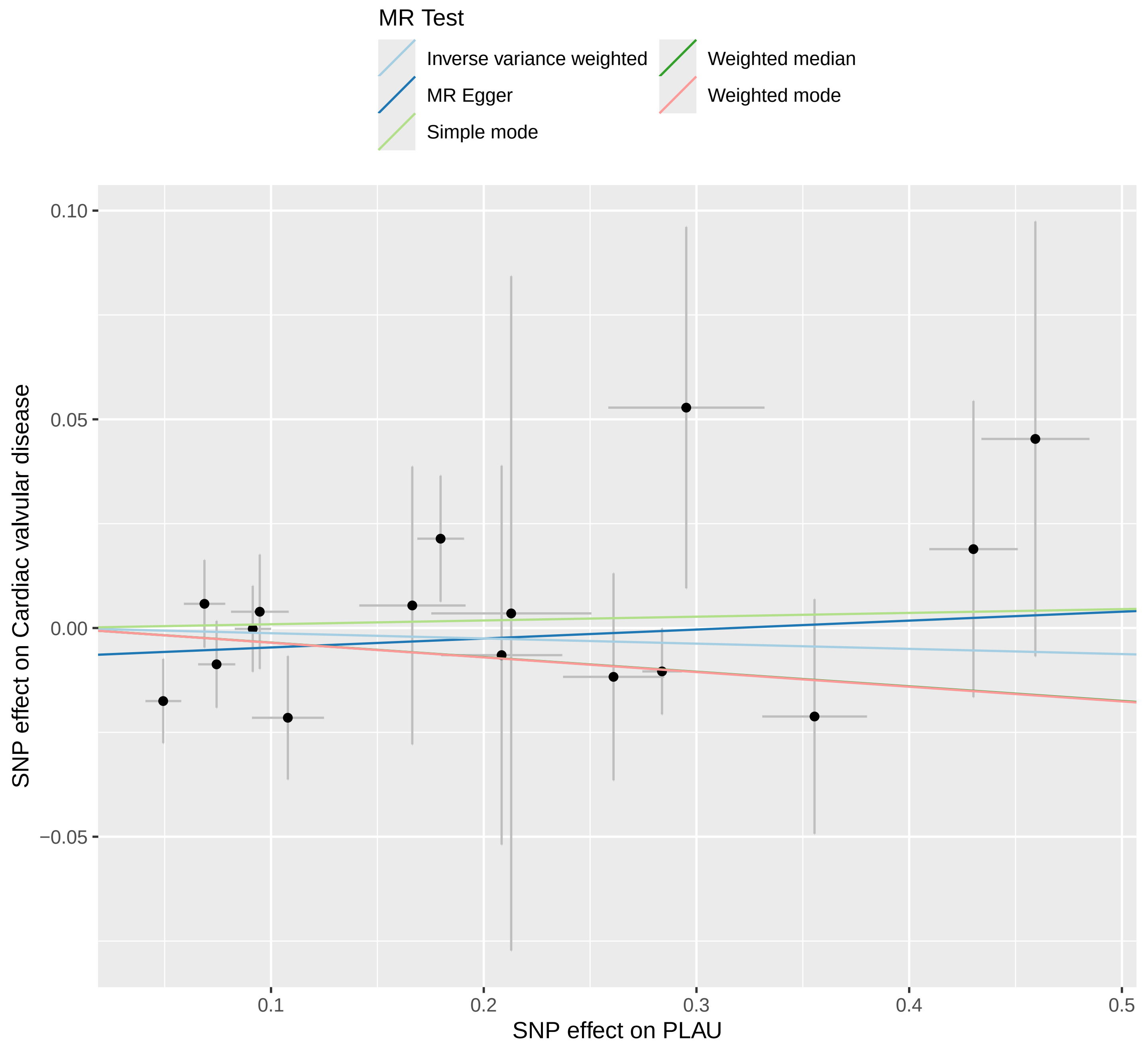

MR Test
Inverse variance weighted
MR Egger
Simple mode
Weighted median
Weighted mode
0.10
0.05
0.00
−0.05
0.1
0.2
0.3
0.4
0.5
SNP effect on Cardiac valvular disease
SNP effect on PLAU

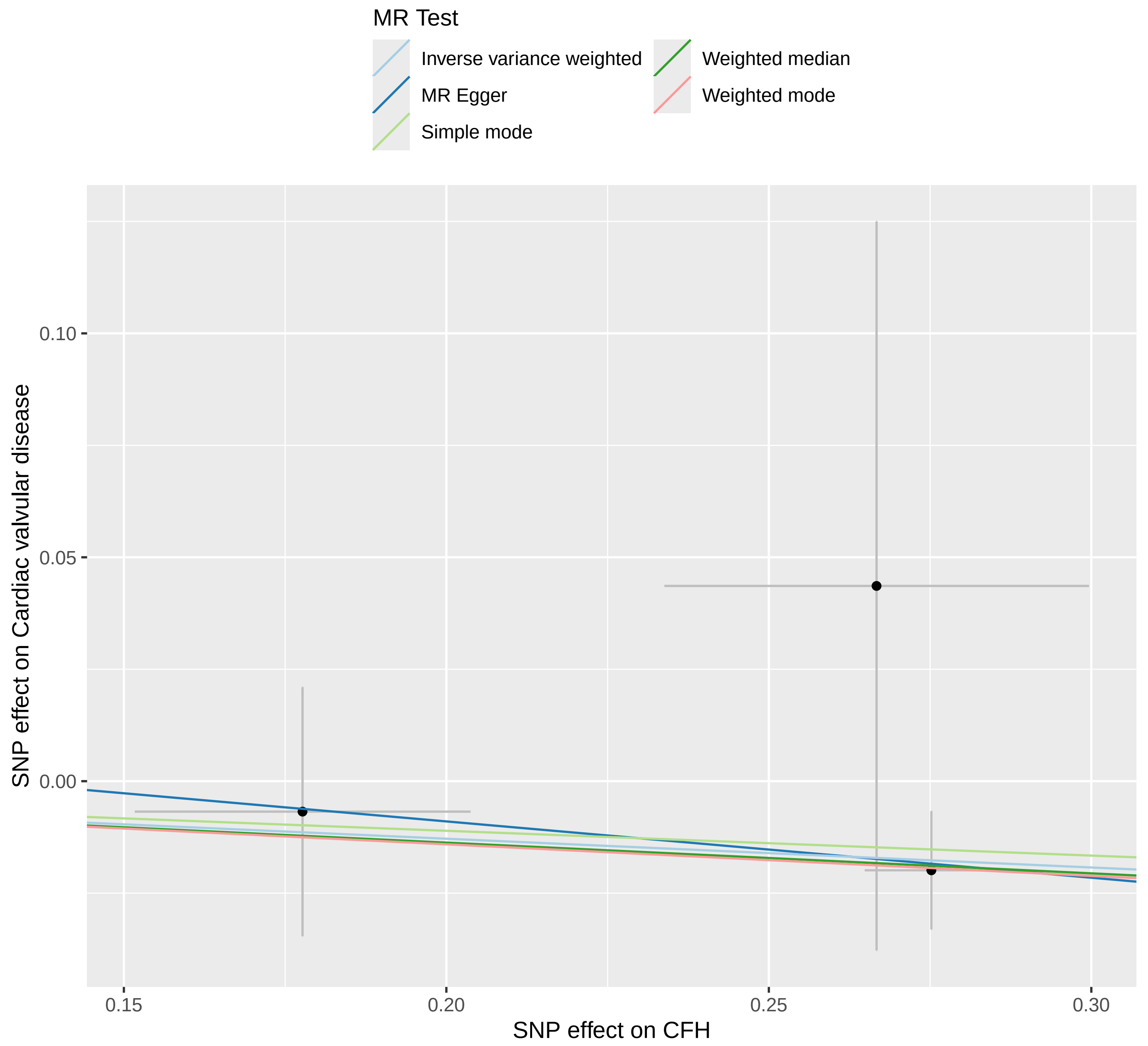
MR Test
Inverse variance weighted
MR Egger
Simple mode
Weighted median
Weighted mode
0.10
0.05
0.00
SNP effect on Cardiac valvular disease
0.15
0.20
0.25
0.30
SNP effect on CFH

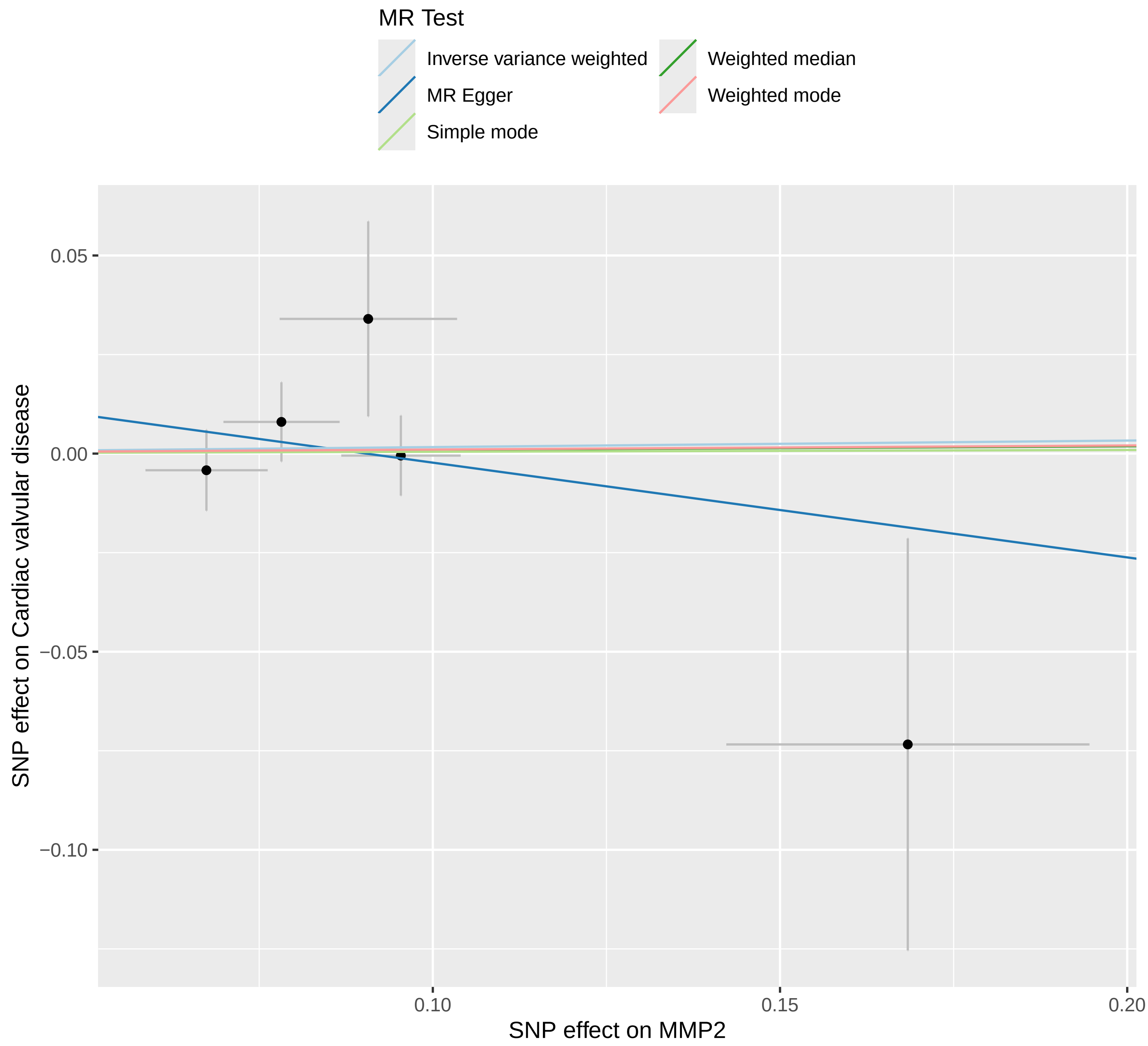
MR Test
Inverse variance weighted
MR Egger
Simple mode
Weighted median
Weighted mode
0.05
0.00
−0.05
−0.10
SNP effect on Cardiac valvular disease
0.10
0.15
0.20
SNP effect on MMP2

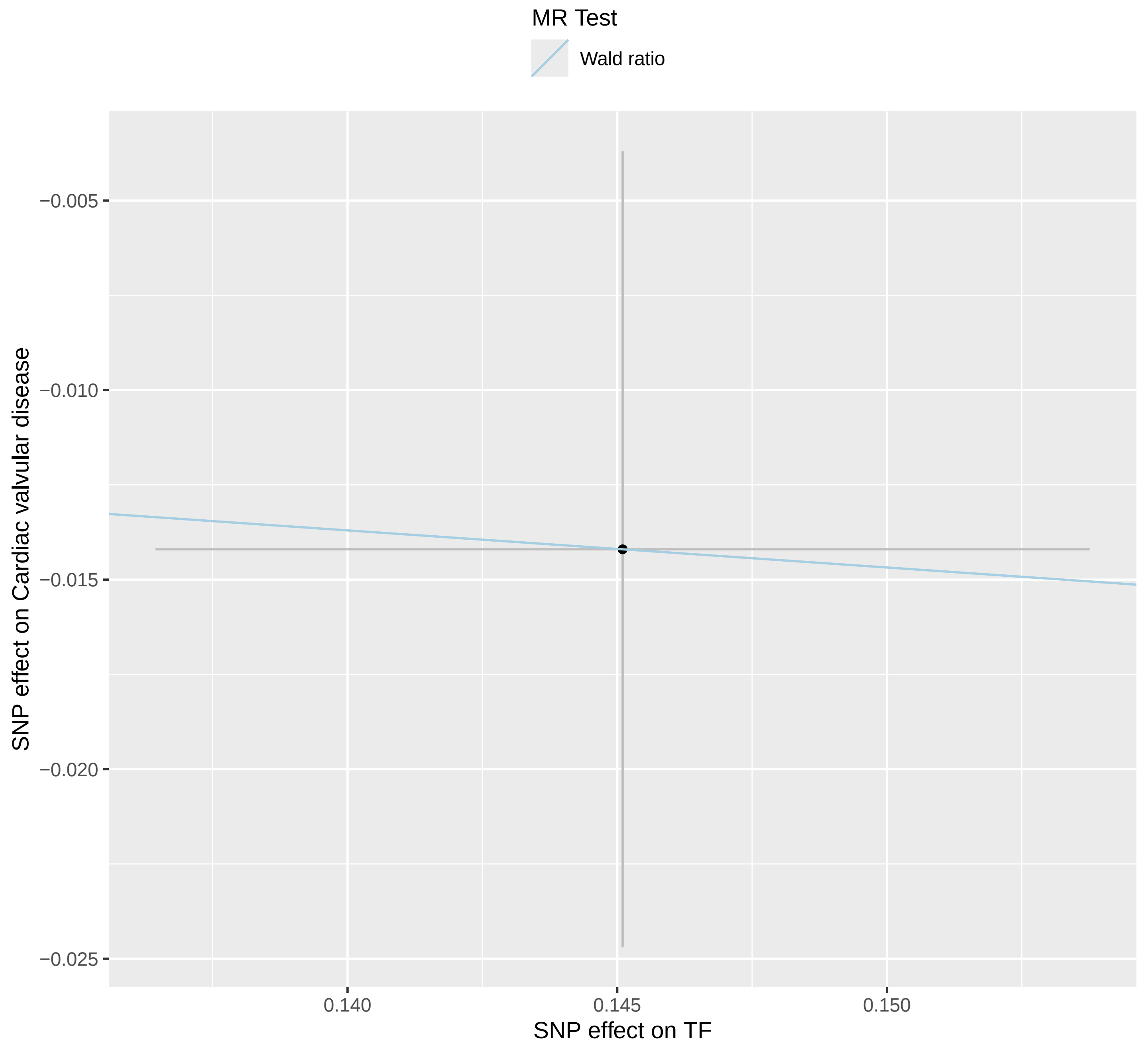
MR Test
Wald ratio
−0.005
−0.010
−0.015
−0.020
−0.025
SNP effect on Cardiac valvular disease
0.140
0.145
0.150
SNP effect on TF

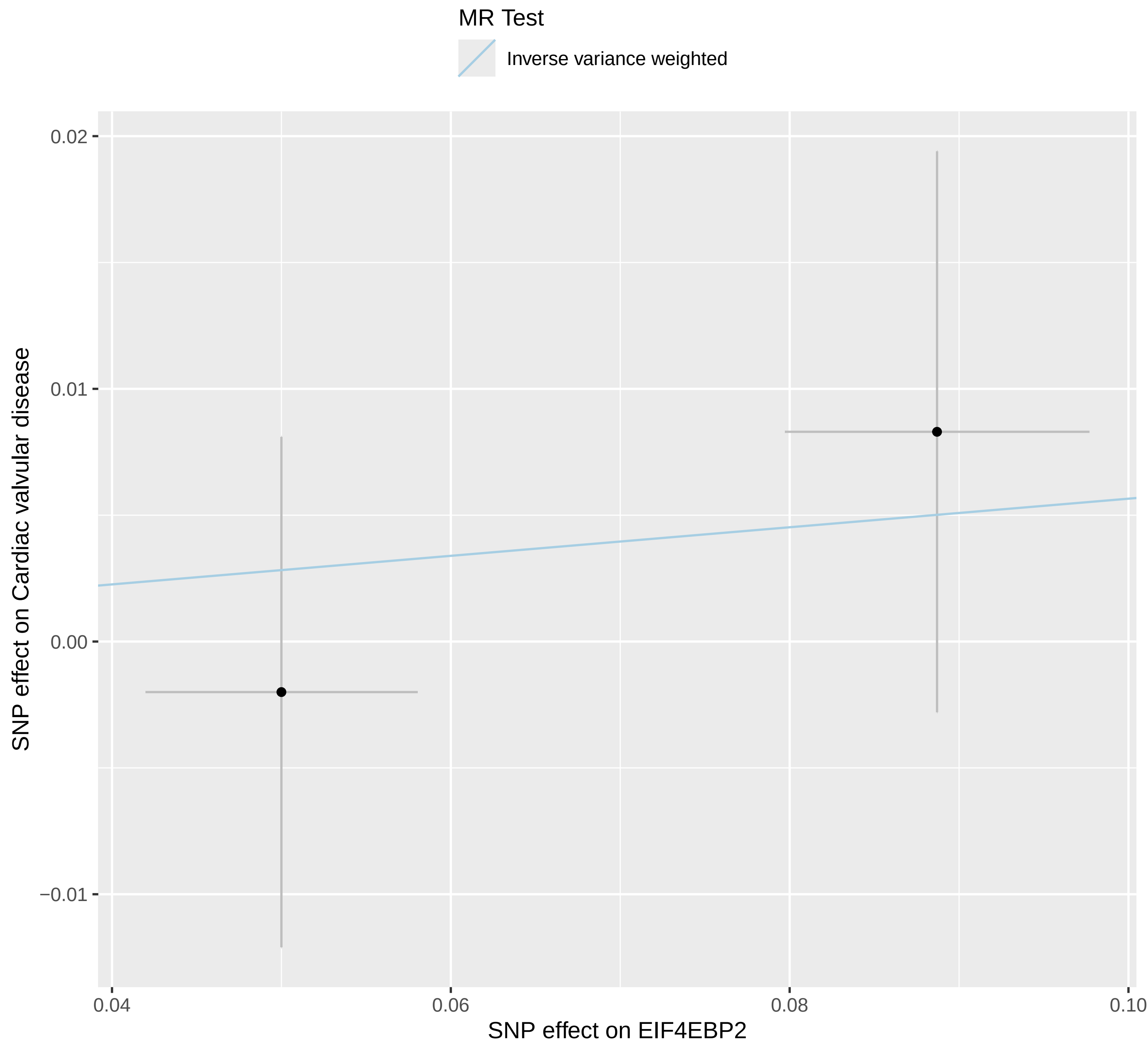

MR Test
Inverse variance weighted
0.02
0.01
0.00
−0.01
0.04
0.06
0.08
0.10
SNP effect on Cardiac valvular disease
SNP effect on EIF4EBP2

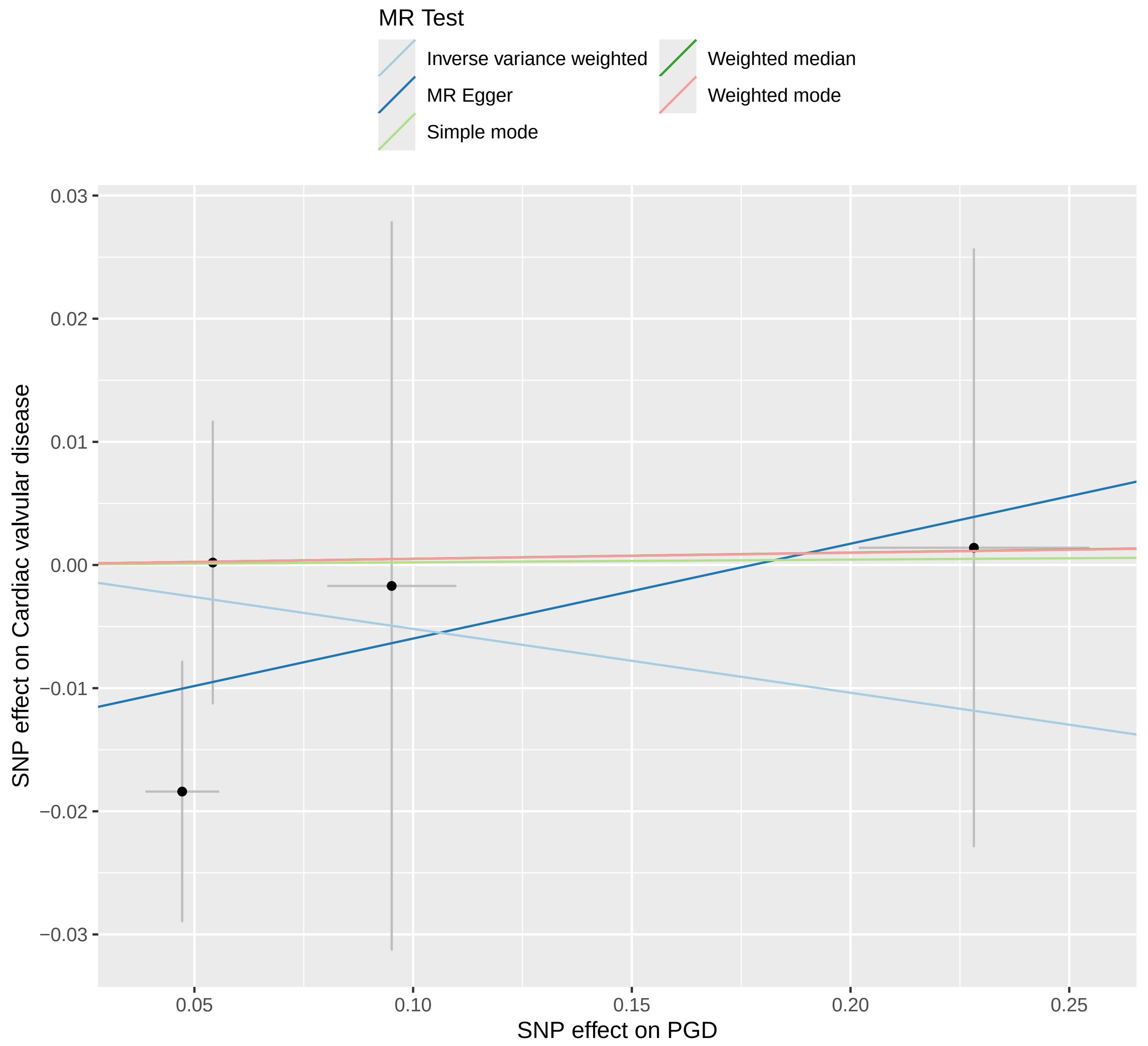
MR Test
Inverse variance weighted
MR Egger
Simple mode
Weighted median
Weighted mode
0.03
0.02
0.01
0.00
−0.01
−0.02
−0.03
0.05
0.10
0.15
0.20
0.25
SNP effect on Cardiac valvular disease
SNP effect on PGD

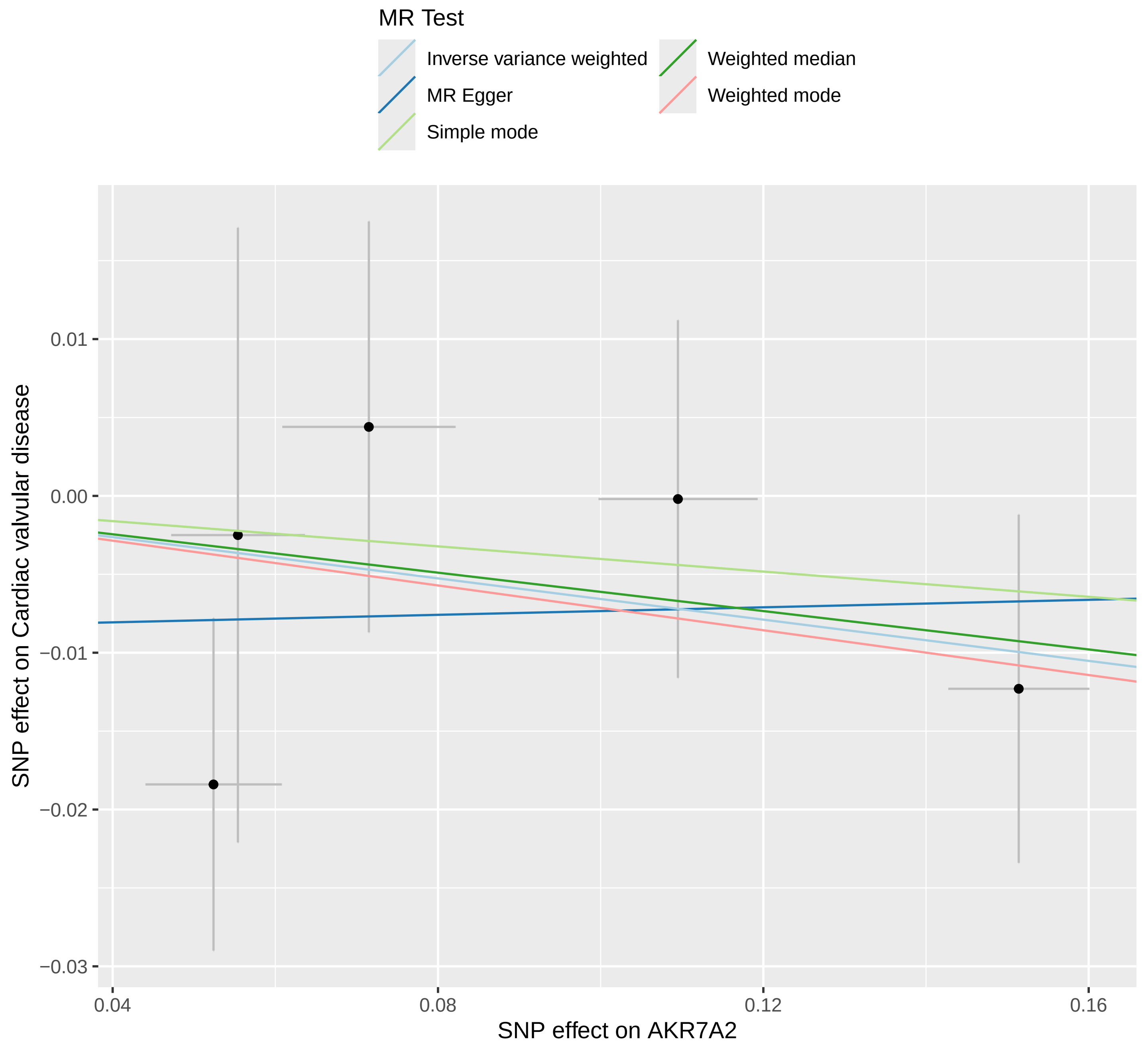
MR Test
Inverse variance weighted
MR Egger
Simple mode
Weighted median
Weighted mode
0.01
0.00
−0.01
−0.02
−0.03
0.04
0.08
0.12
0.16
SNP effect on AKR7A2
SNP effect on Cardiac valvular disease

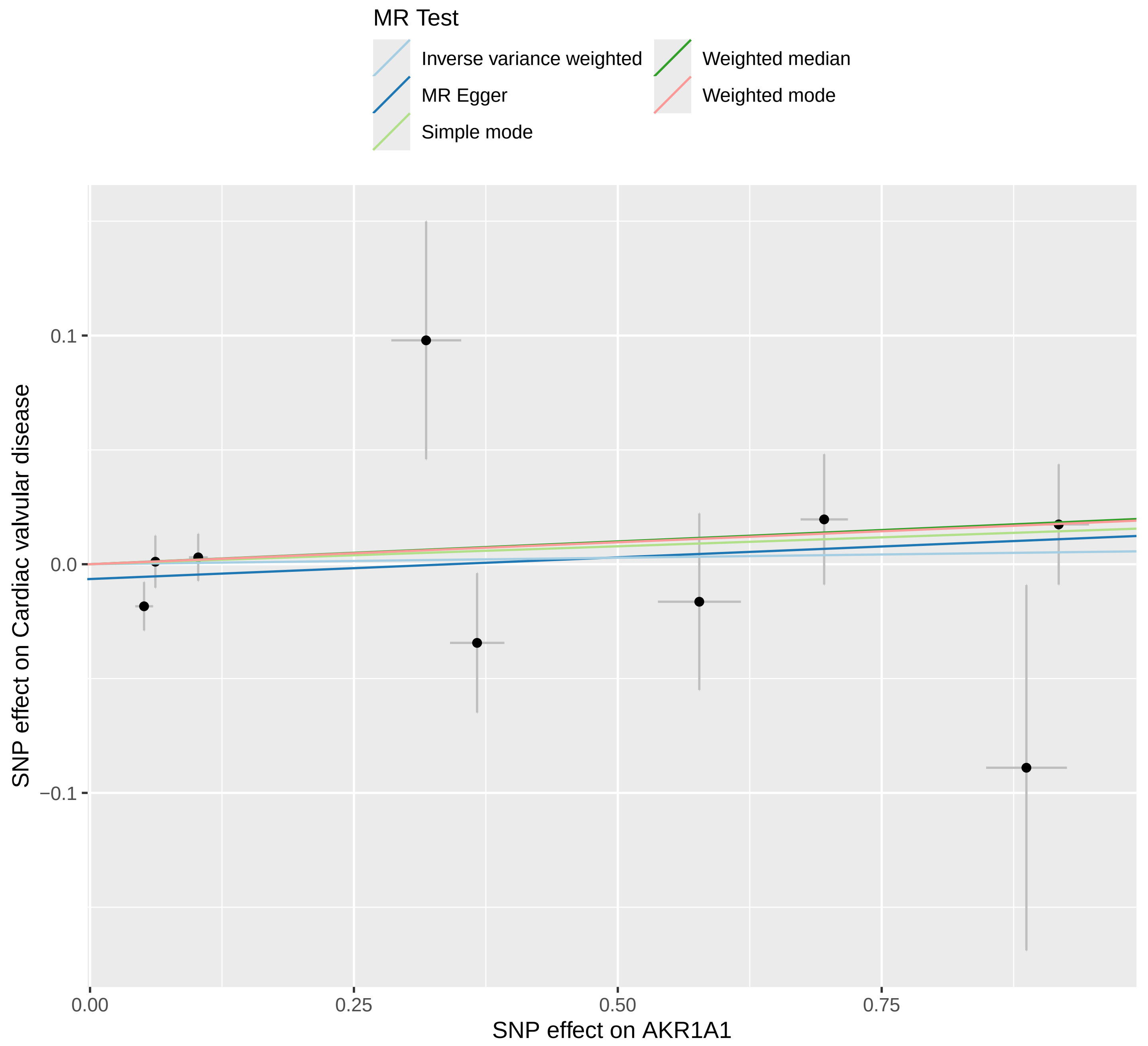
MR Test
Inverse variance weighted
MR Egger
Simple mode
Weighted median
Weighted mode
0.1
0.0
−0.1
0.00
0.25
0.50
0.75
SNP effect on Cardiac valvular disease
SNP effect on AKR1A1

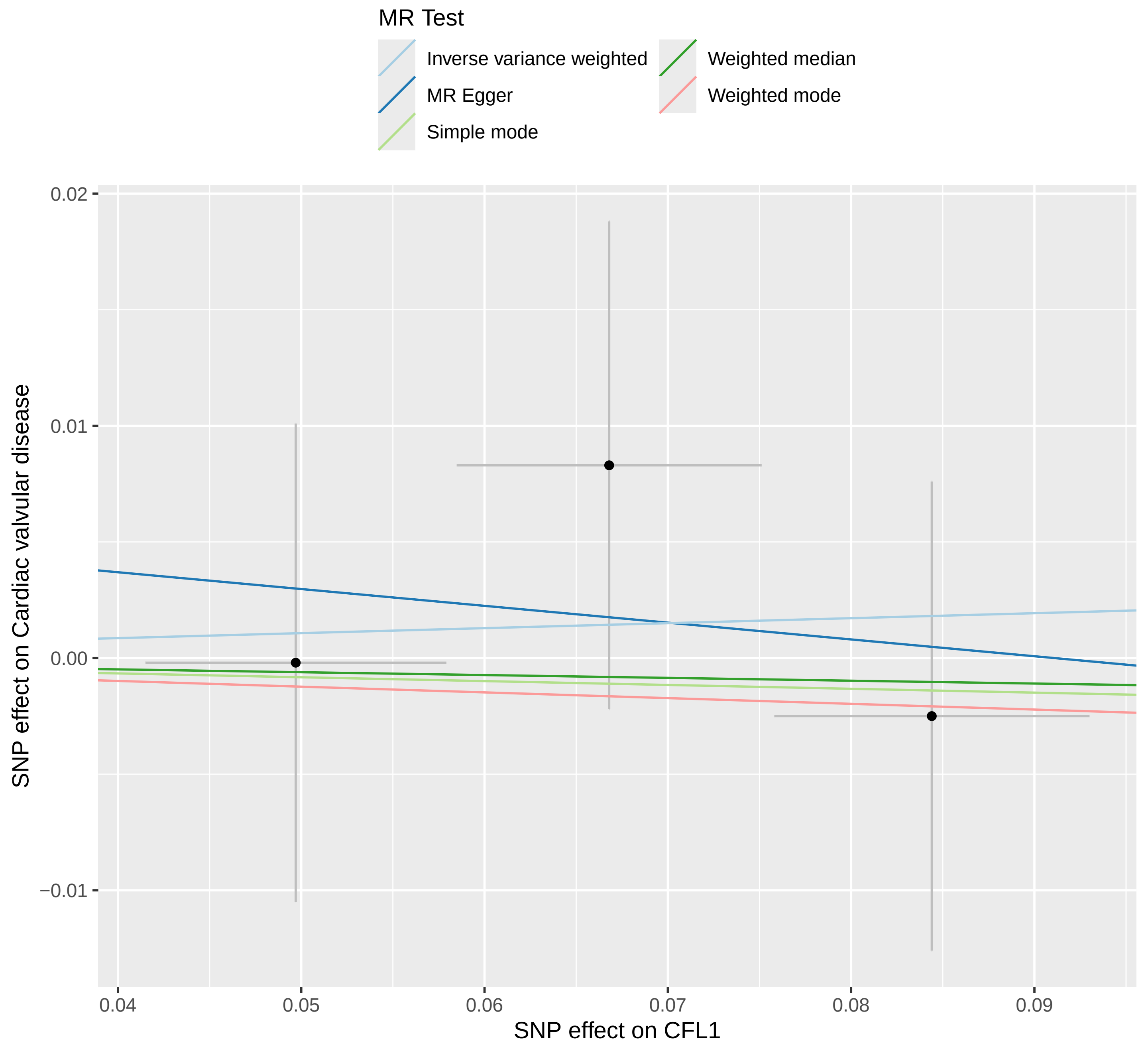

MR Test
Inverse variance weighted
MR Egger
Simple mode
Weighted median
Weighted mode
0.02
0.01
0.00
−0.01
0.04
0.05
0.06
0.07
0.08
0.09
SNP effect on Cardiac valvular disease
SNP effect on CFL1

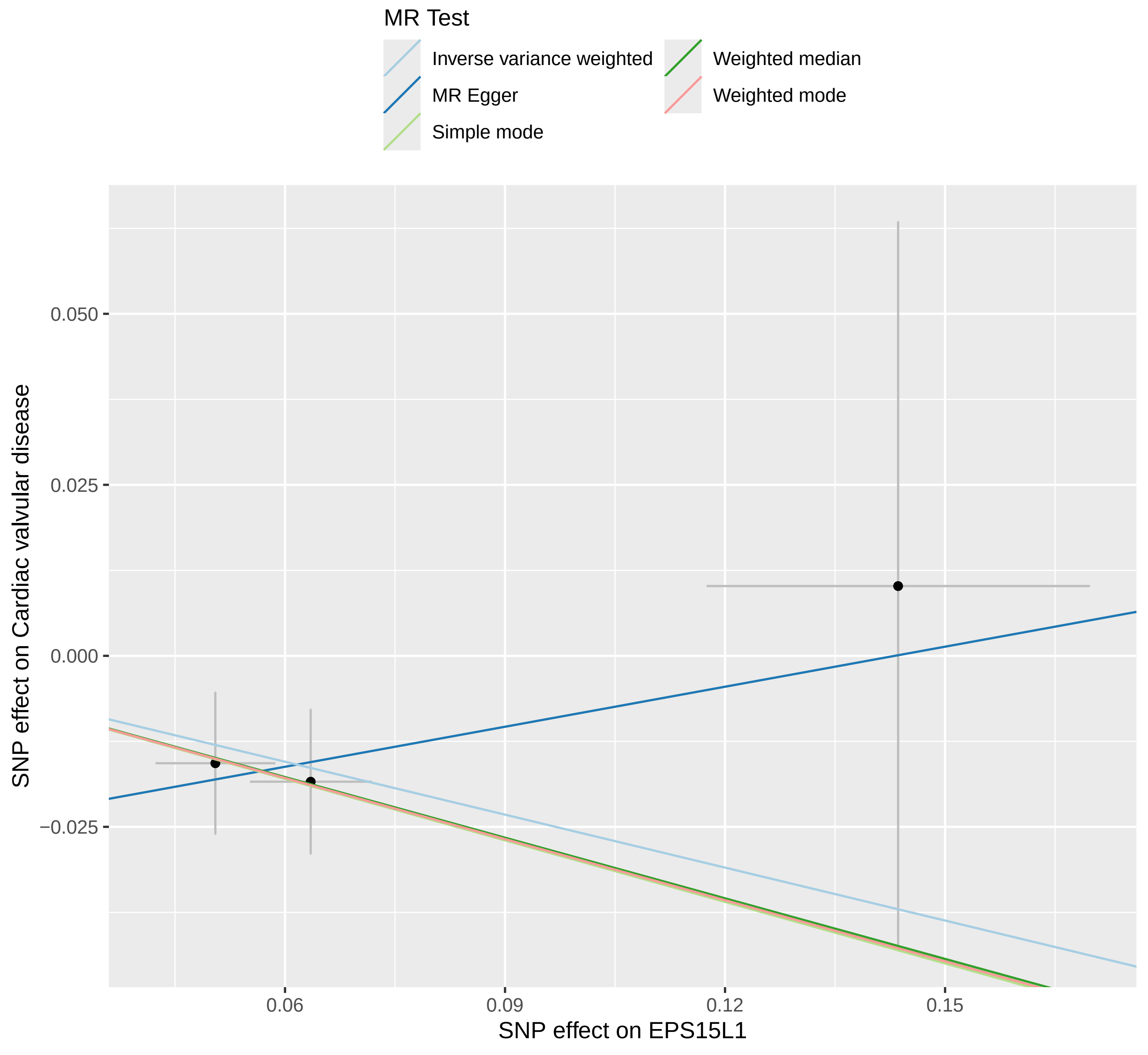

MR Test
Inverse variance weighted
MR Egger
Simple mode
Weighted median
Weighted mode
0.050
0.025
0.000
−0.025
SNP effect on Cardiac valvular disease
0.06
0.09
0.12
0.15
SNP effect on EPS15L1

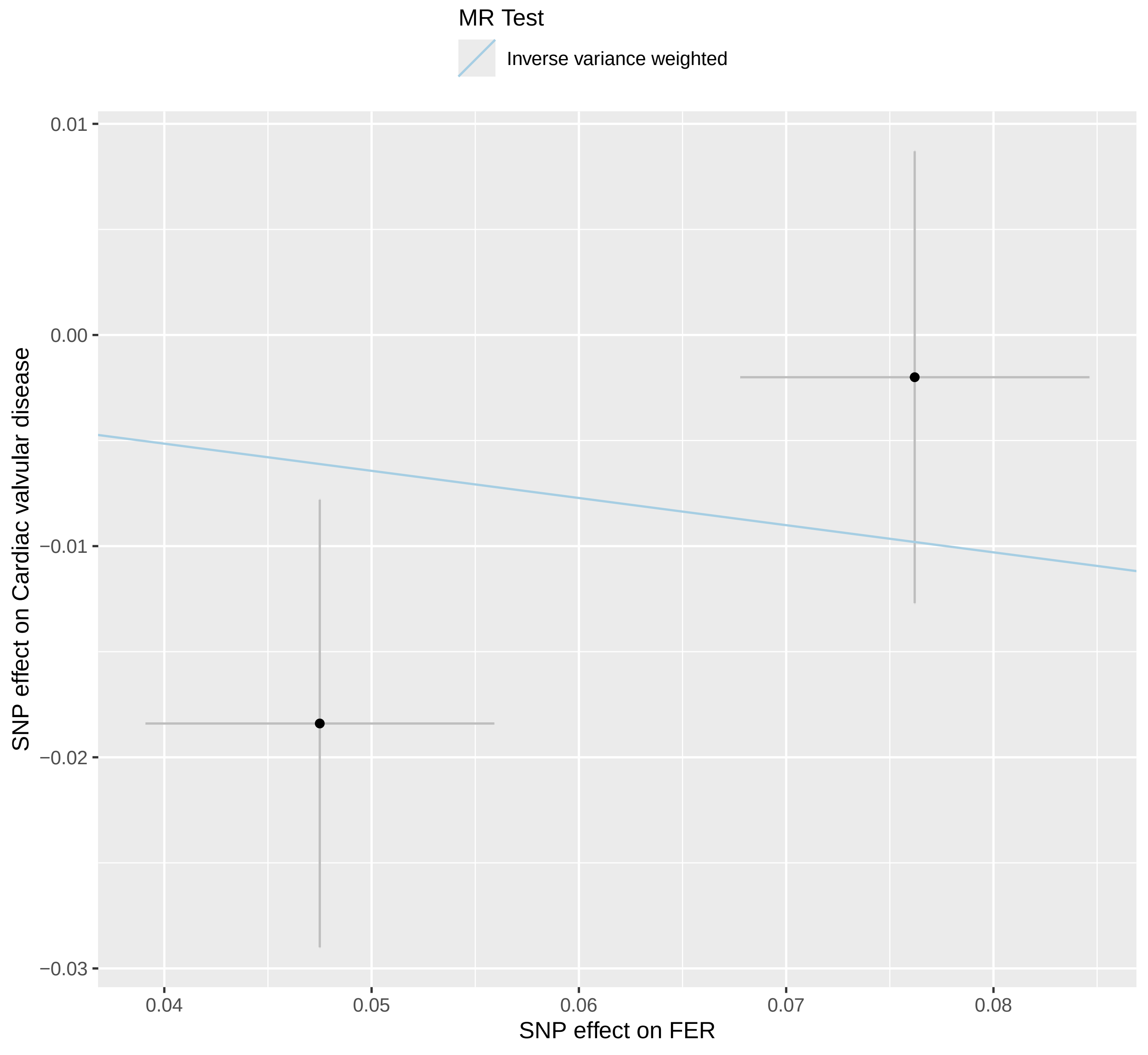

MR Test
Inverse variance weighted
0.01
0.00
−0.01
−0.02
−0.03
SNP effect on Cardiac valvular disease
0.04
0.05
0.06
0.07
0.08
SNP effect on FER

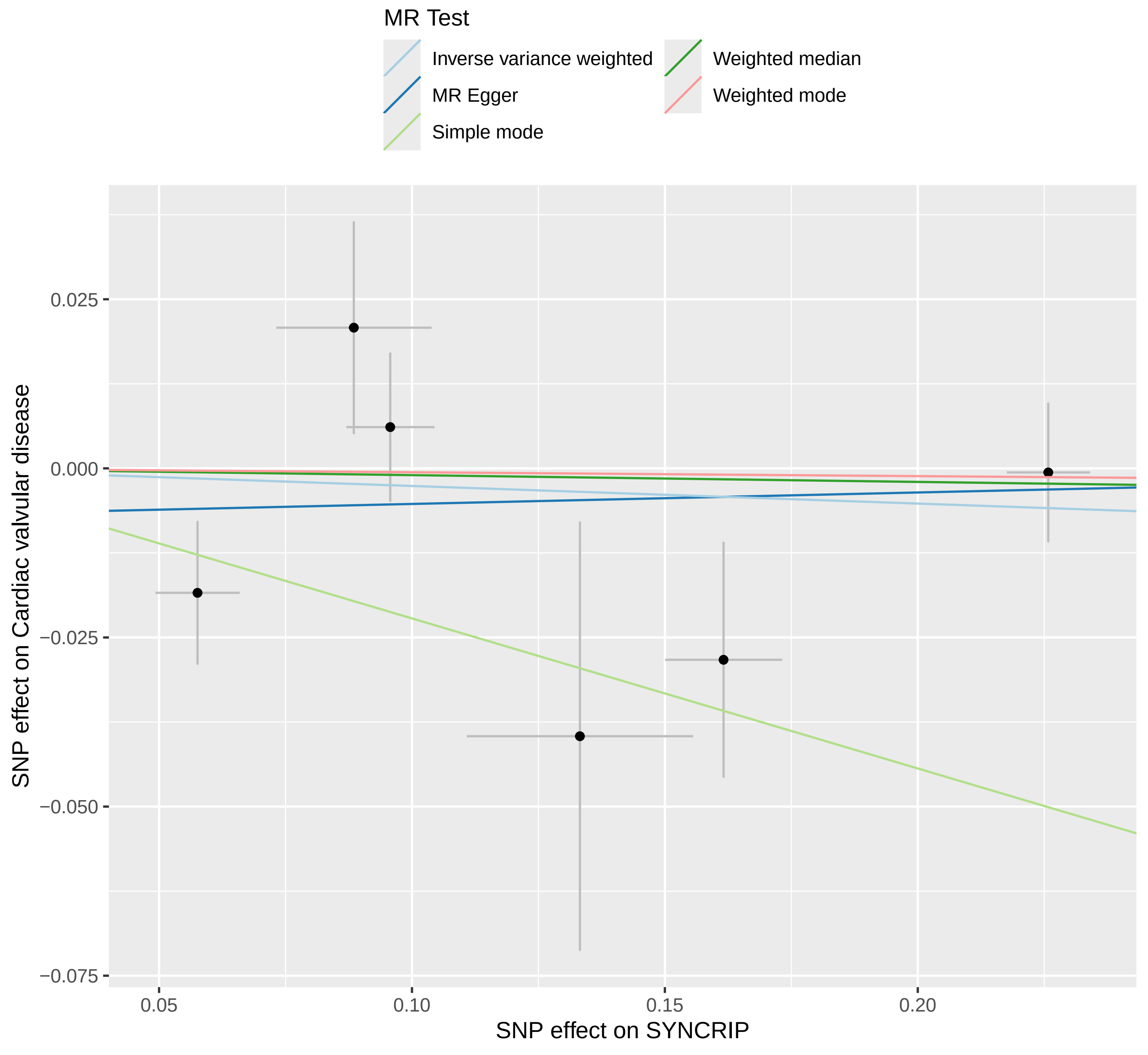

MR Test
Inverse variance weighted
MR Egger
Simple mode
Weighted median
Weighted mode
0.025
0.000
−0.025
−0.050
−0.075
0.05
0.10
0.15
0.20
SNP effect on Cardiac valvular disease
SNP effect on SYNCRIP

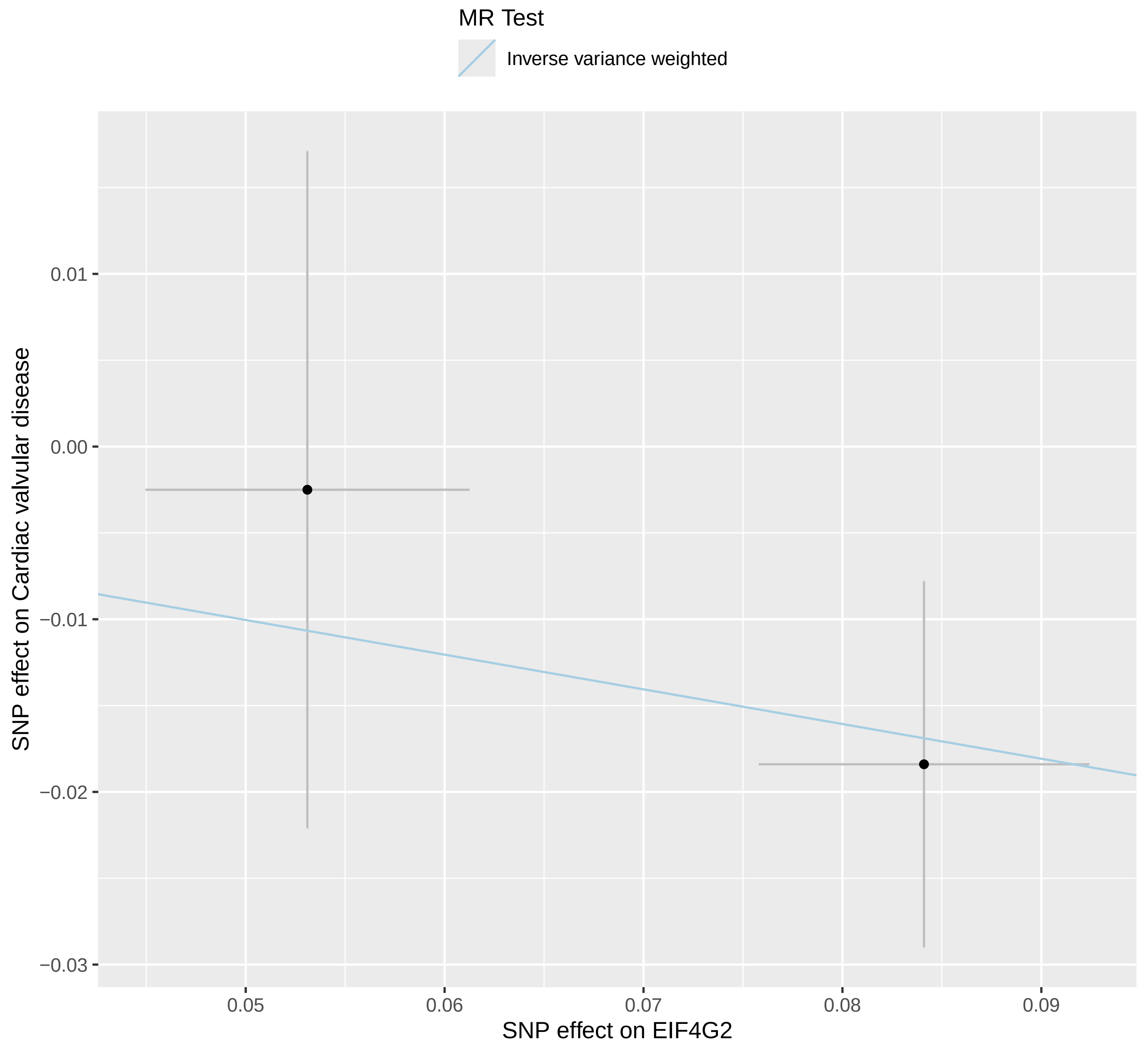
MR Test
Inverse variance weighted
0.01
0.00
−0.01
−0.02
−0.03
0.05
0.06
0.07
0.08
0.09
SNP effect on EIF4G2
SNP effect on Cardiac valvular disease

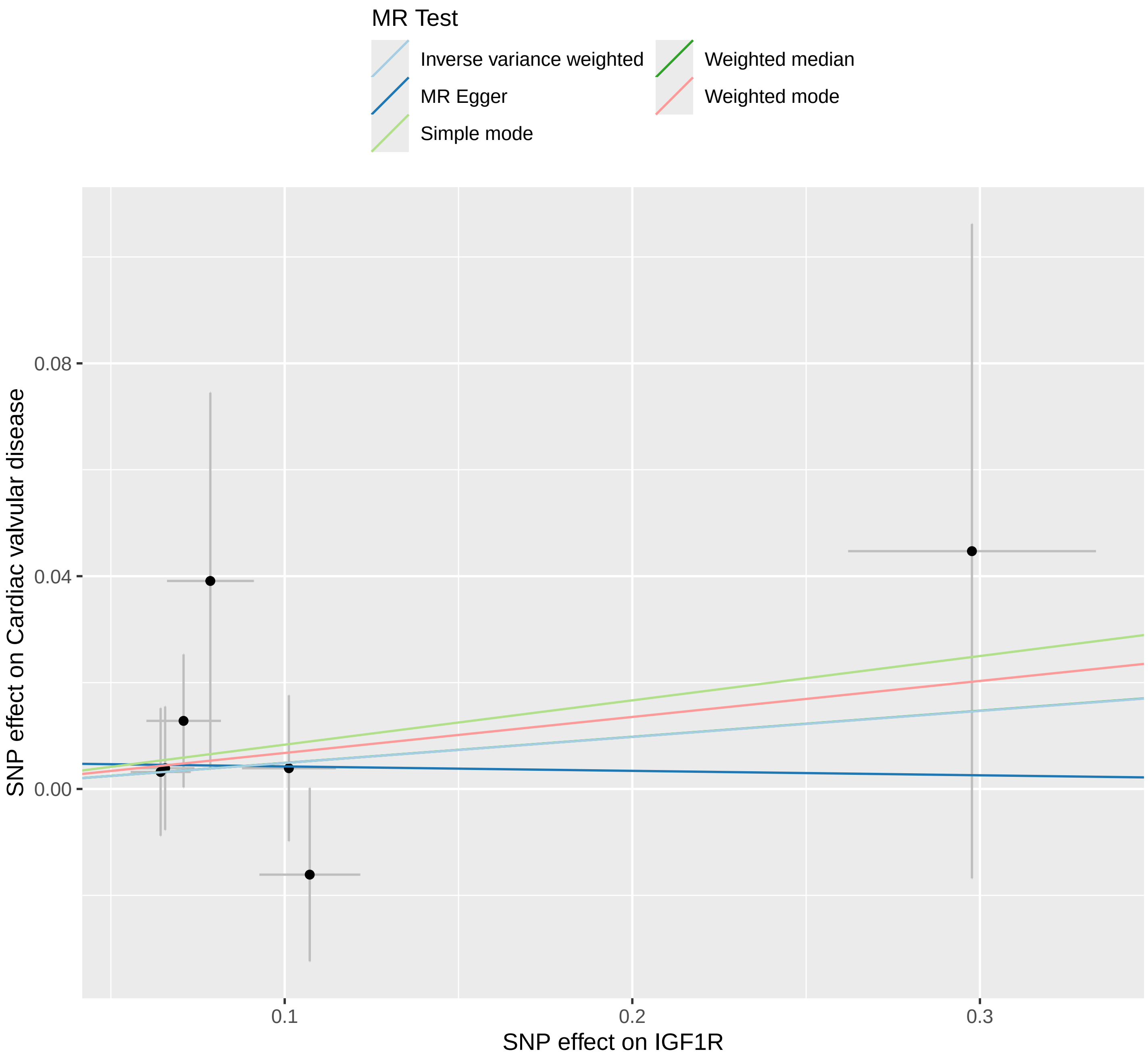

MR Test
Inverse variance weighted
MR Egger
Simple mode
Weighted median
Weighted mode
0.08
0.04
0.00
SNP effect on Cardiac valvular disease
0.1
0.2
0.3
SNP effect on IGF1R

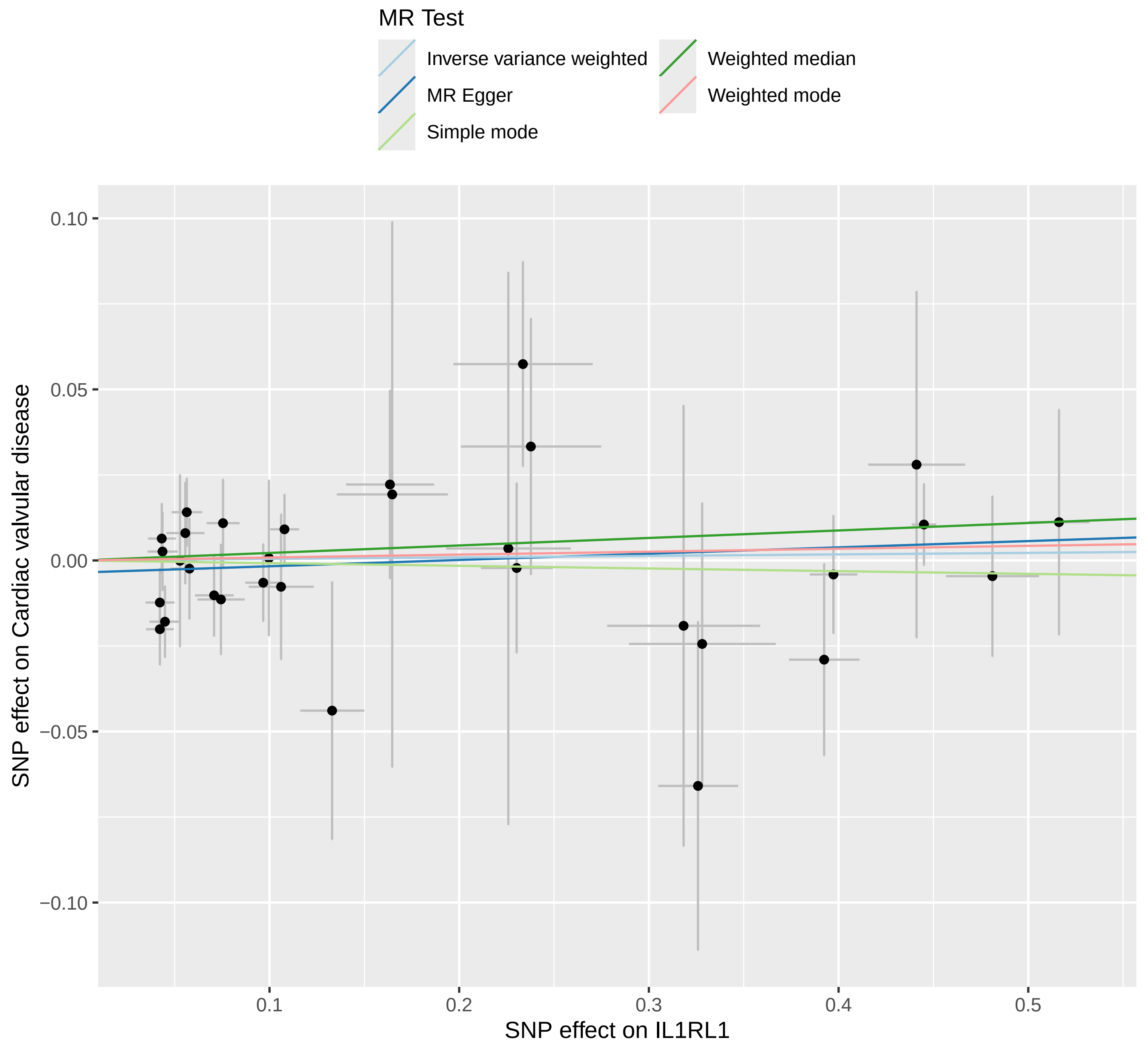
MR Test
Inverse variance weighted
MR Egger
Simple mode
Weighted median
Weighted mode
0.10
0.05
0.00
−0.05
−0.10
0.1
0.2
0.3
0.4
0.5
SNP effect on Cardiac valvular disease
SNP effect on IL1RL1

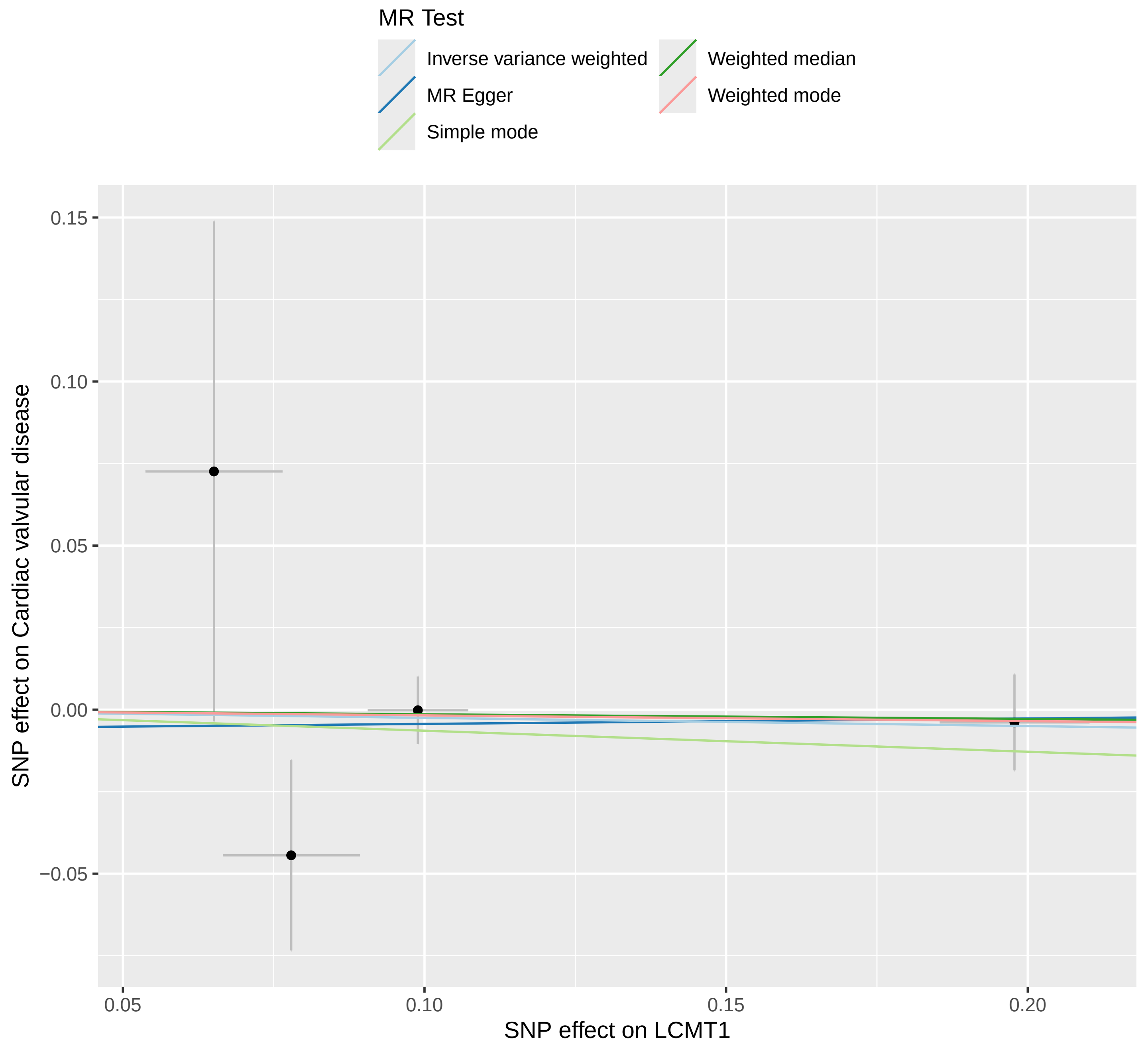

MR Test
Inverse variance weighted
MR Egger
Simple mode
Weighted median
Weighted mode
0.15
0.10
0.05
0.00
−0.05
SNP effect on Cardiac valvular disease
0.05
0.10
0.15
0.20
SNP effect on LCMT1

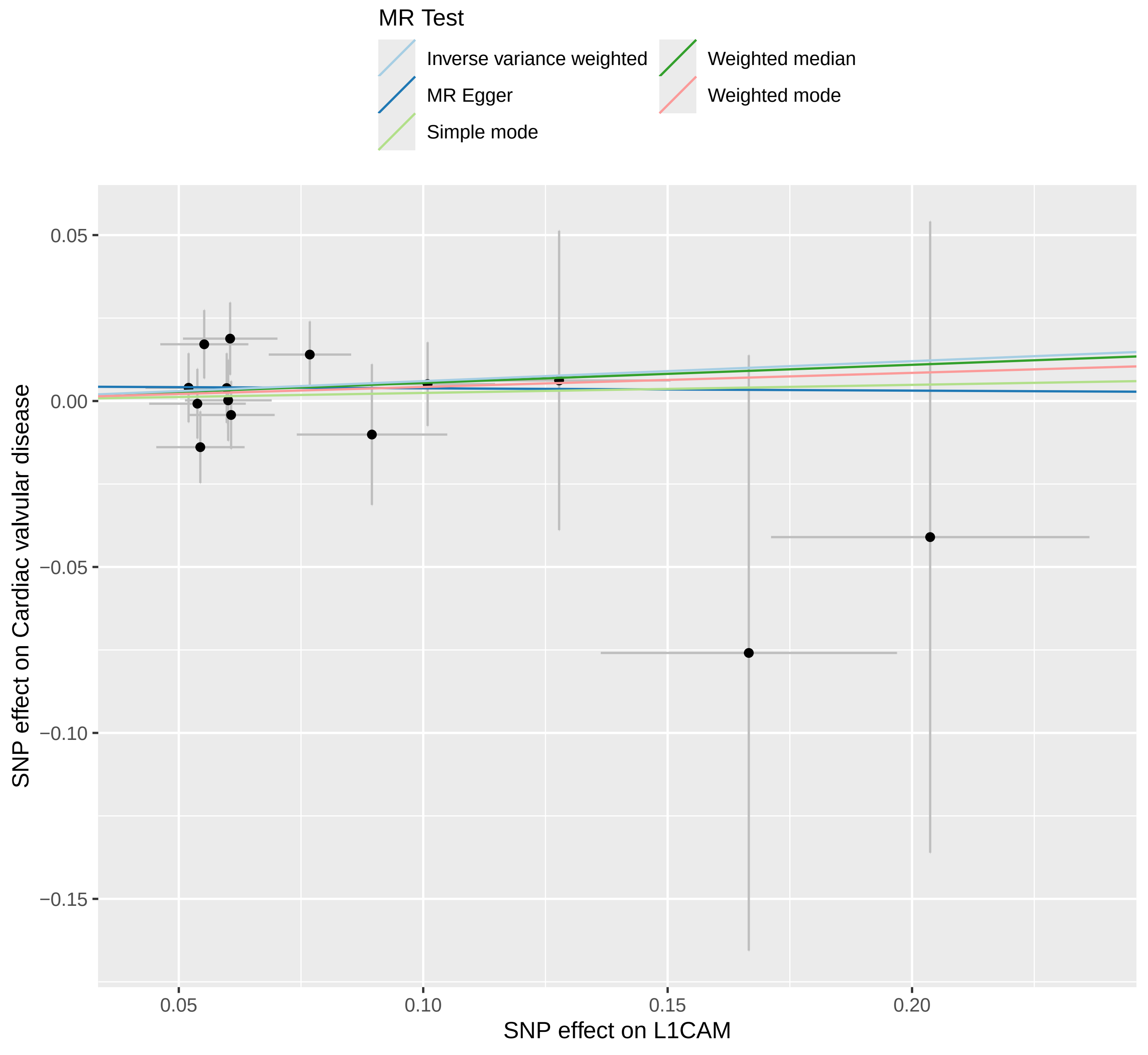
MR Test
Inverse variance weighted
MR Egger
Simple mode
Weighted median
Weighted mode
0.05
0.00
−0.05
−0.10
−0.15
0.05
0.10
0.15
0.20
SNP effect on L1CAM
SNP effect on Cardiac valvular disease

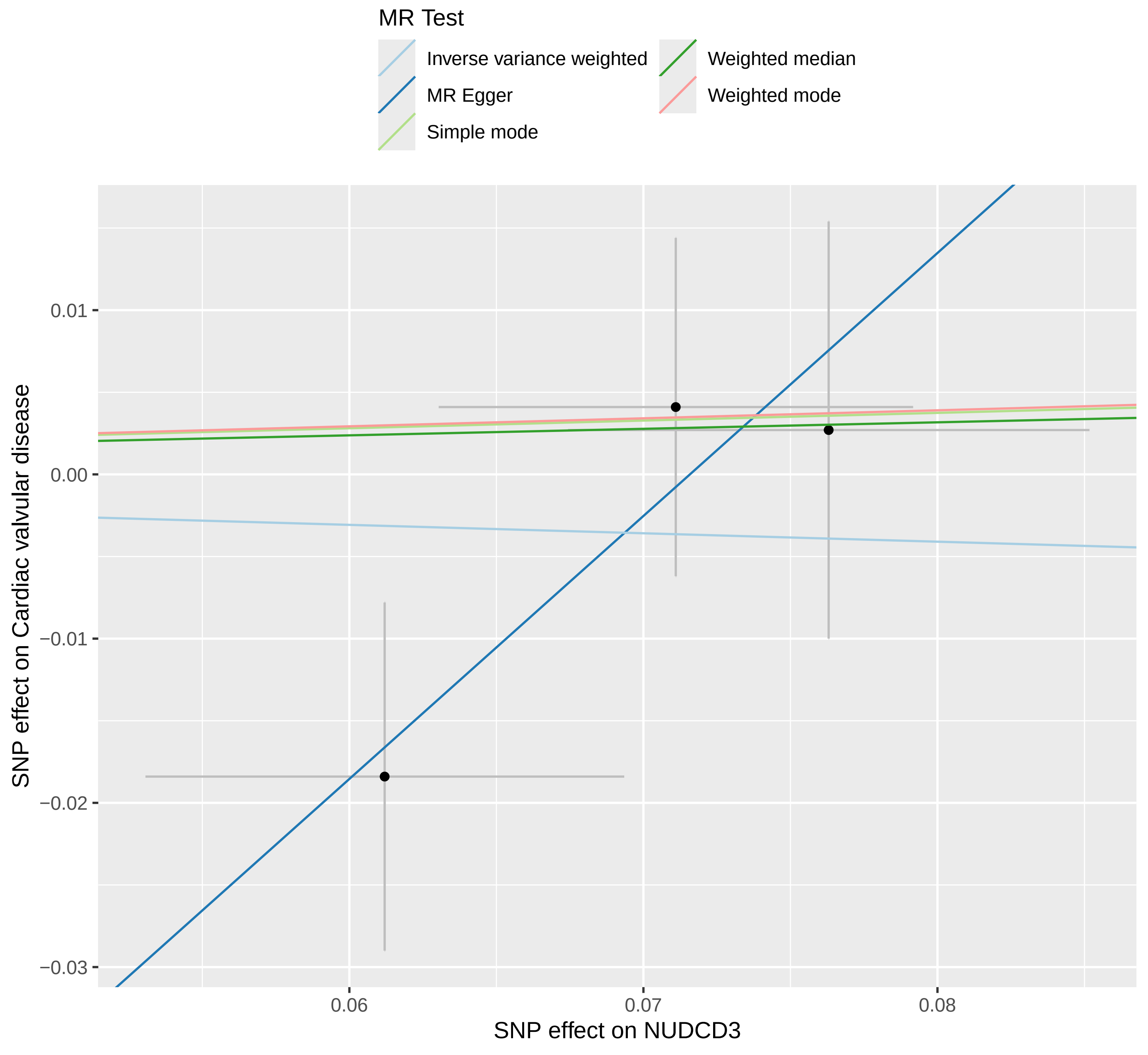
MR Test
Inverse variance weighted
MR Egger
Simple mode
Weighted median
Weighted mode
0.01
0.00
−0.01
−0.02
−0.03
SNP effect on Cardiac valvular disease
0.06
0.07
0.08
SNP effect on NUDCD3

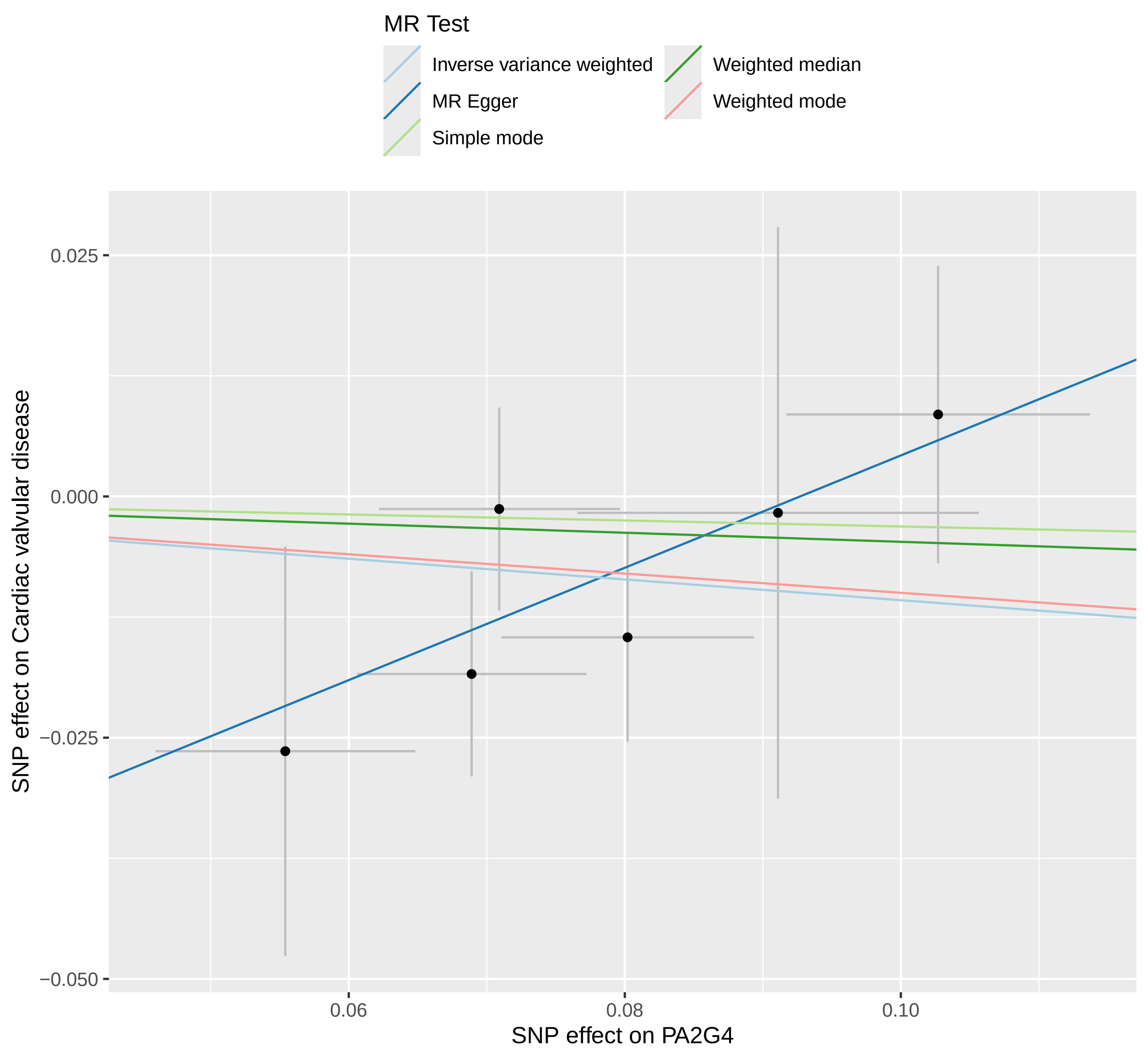

MR Test
Inverse variance weighted
MR Egger
Simple mode
Weighted median
Weighted mode
0.025
0.000
−0.025
−0.050
0.06
0.08
0.10
SNP effect on Cardiac valvular disease
SNP effect on PA2G4

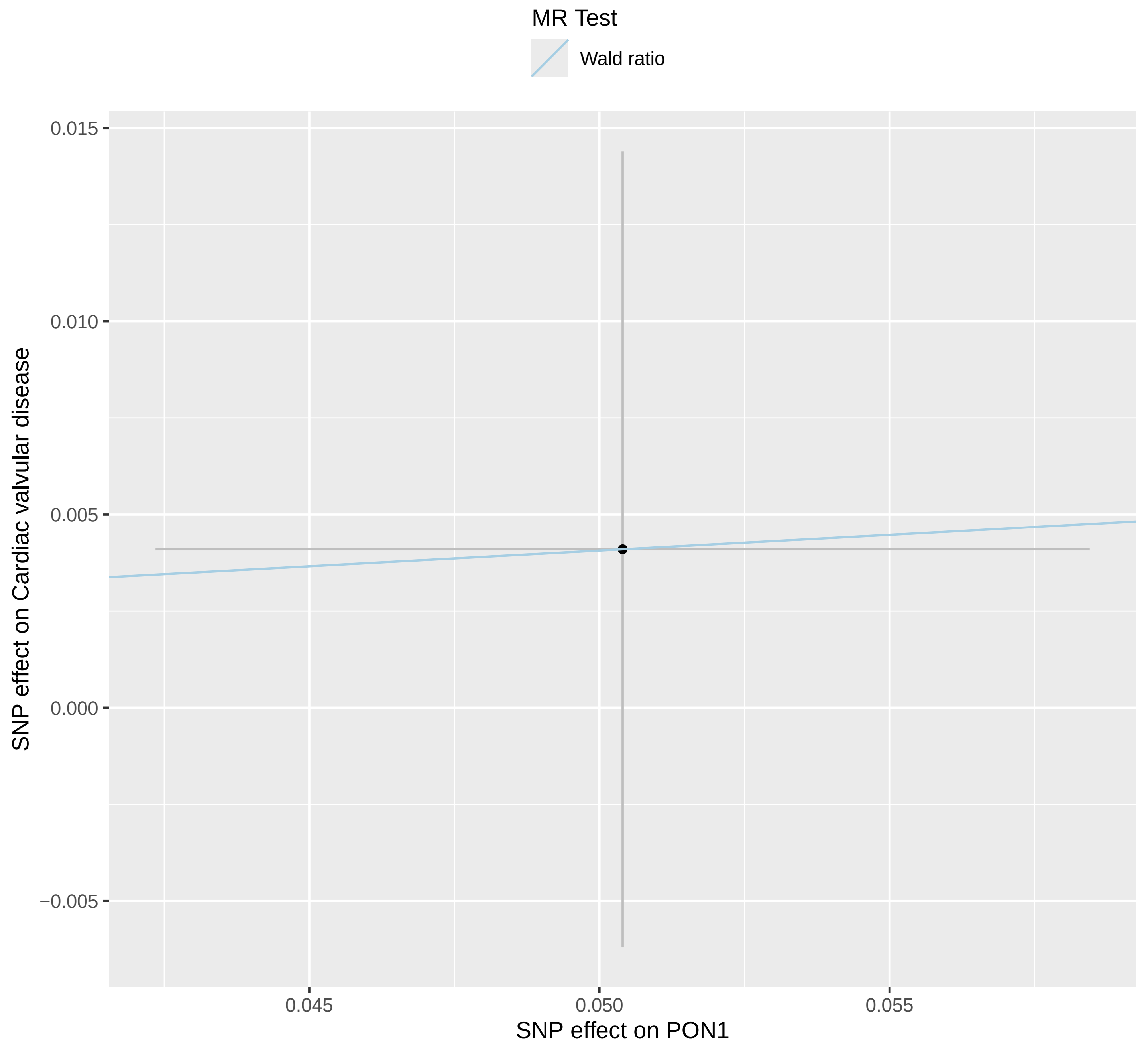

MR Test
Wald ratio
0.015
0.010
0.005
0.000
−0.005
SNP effect on Cardiac valvular disease
0.045
0.050
0.055
SNP effect on PON1

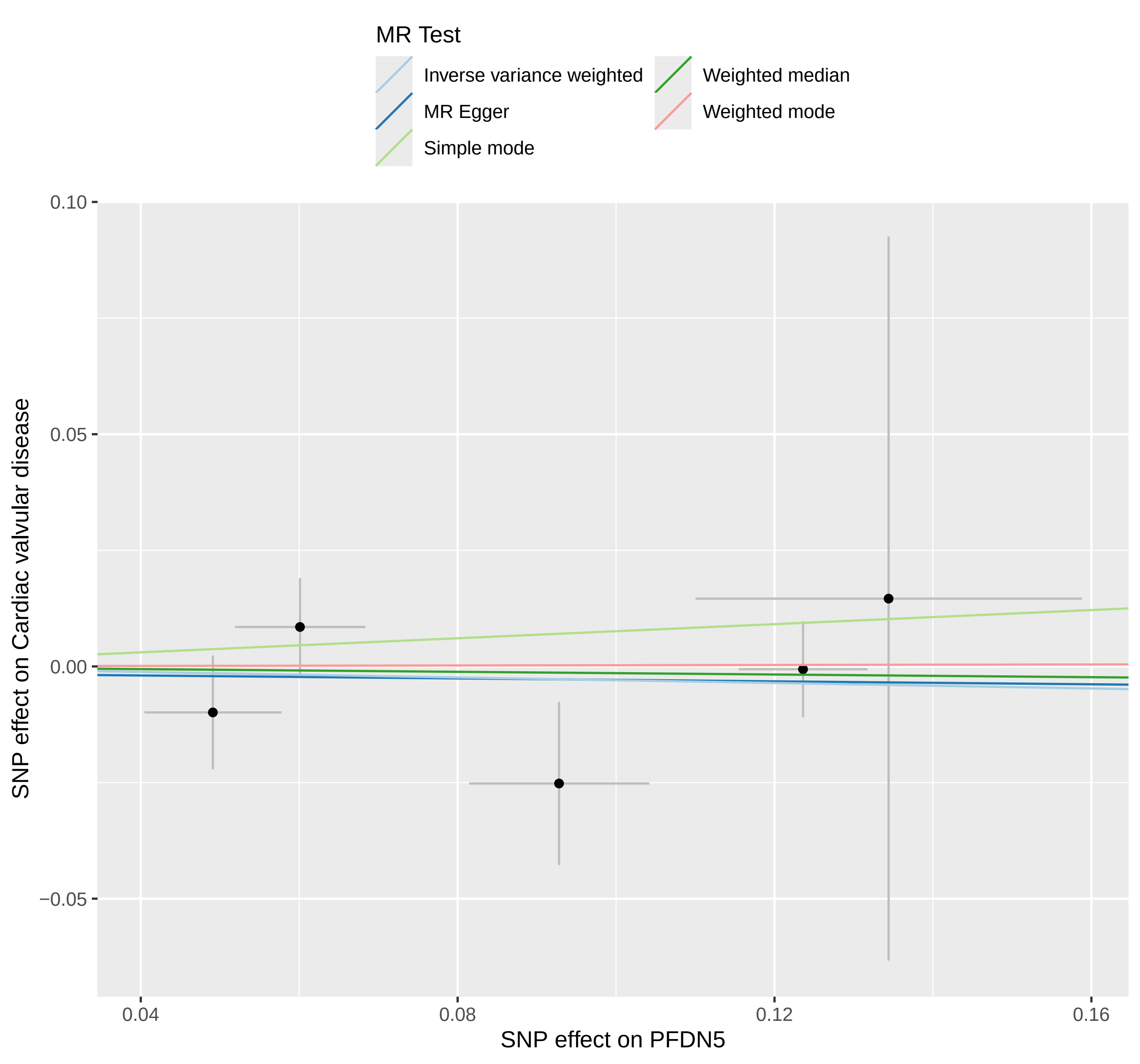

MR Test
Inverse variance weighted
MR Egger
Simple mode
Weighted median
Weighted mode
0.10
0.05
0.00
−0.05
0.04
0.08
0.12
0.16
SNP effect on PFDN5
SNP effect on Cardiac valvular disease

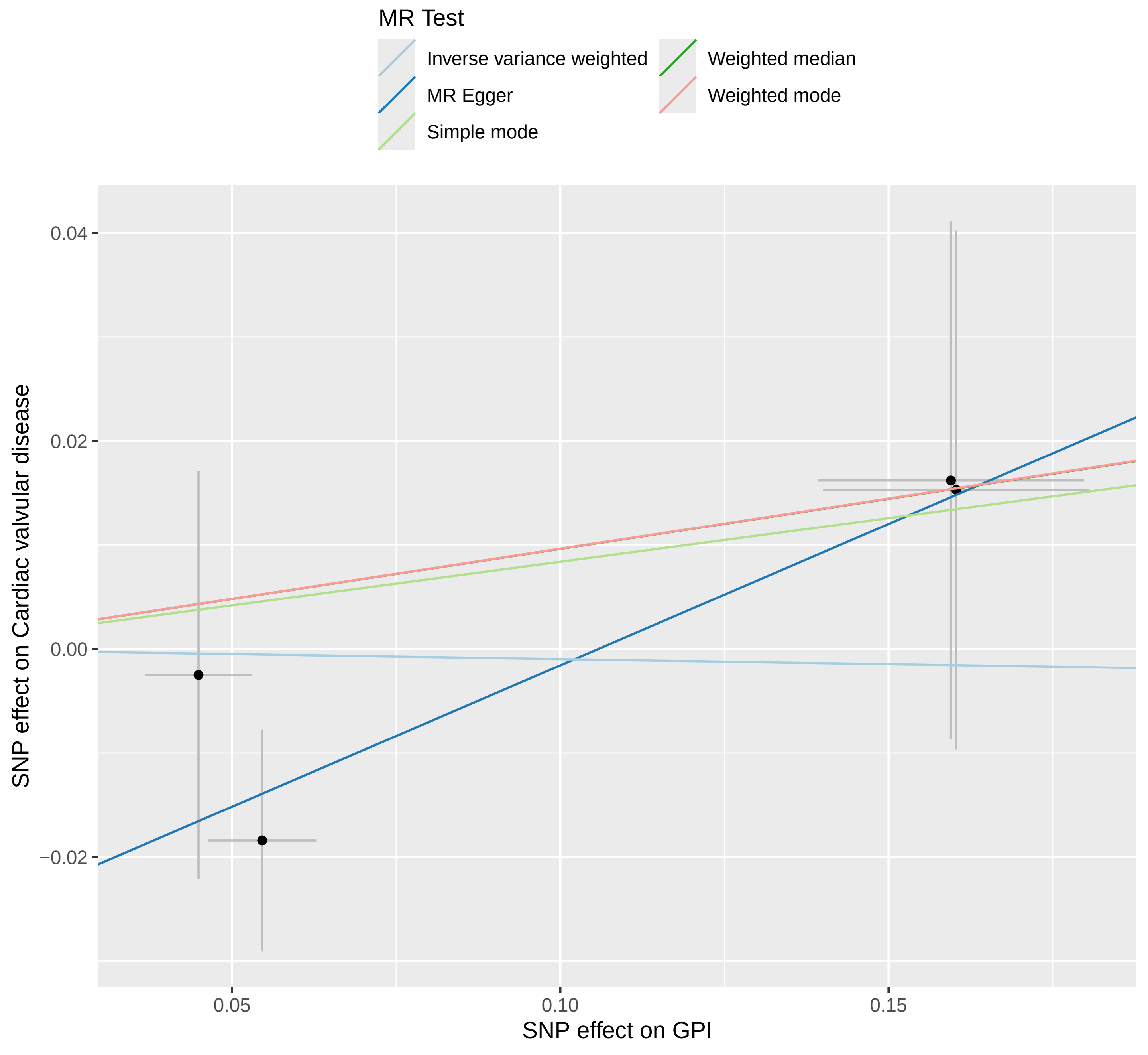
MR Test
Inverse variance weighted
MR Egger
Simple mode
Weighted median
Weighted mode
0.04
0.02
0.00
−0.02
0.05
0.10
0.15
SNP effect on Cardiac valvular disease
SNP effect on GPI

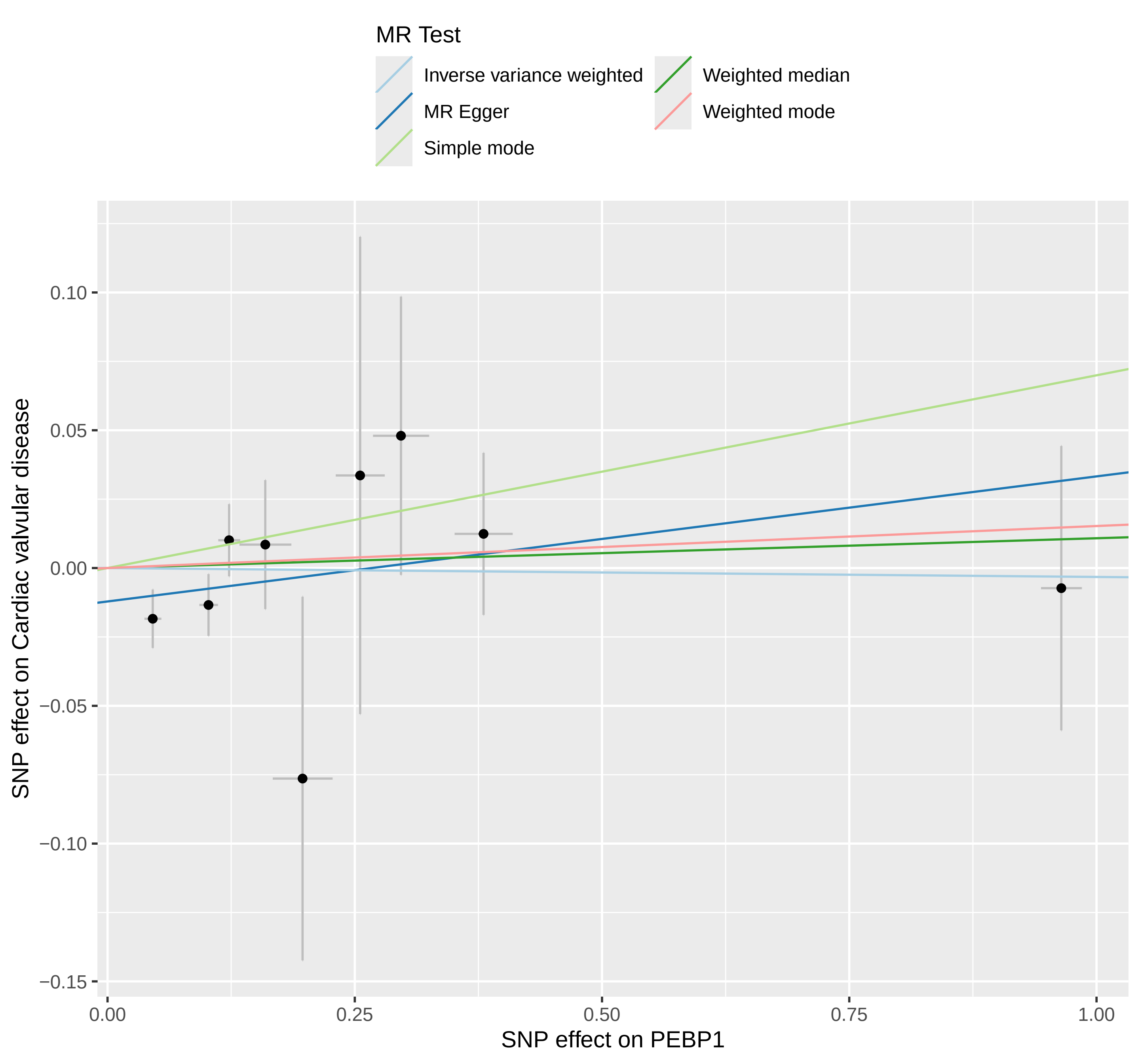

MR Test
Inverse variance weighted
MR Egger
Simple mode
Weighted median
Weighted mode
0.10
0.05
0.00
−0.05
−0.10
−0.15
SNP effect on Cardiac valvular disease
0.00
0.25
0.50
0.75
1.00
SNP effect on PEBP1

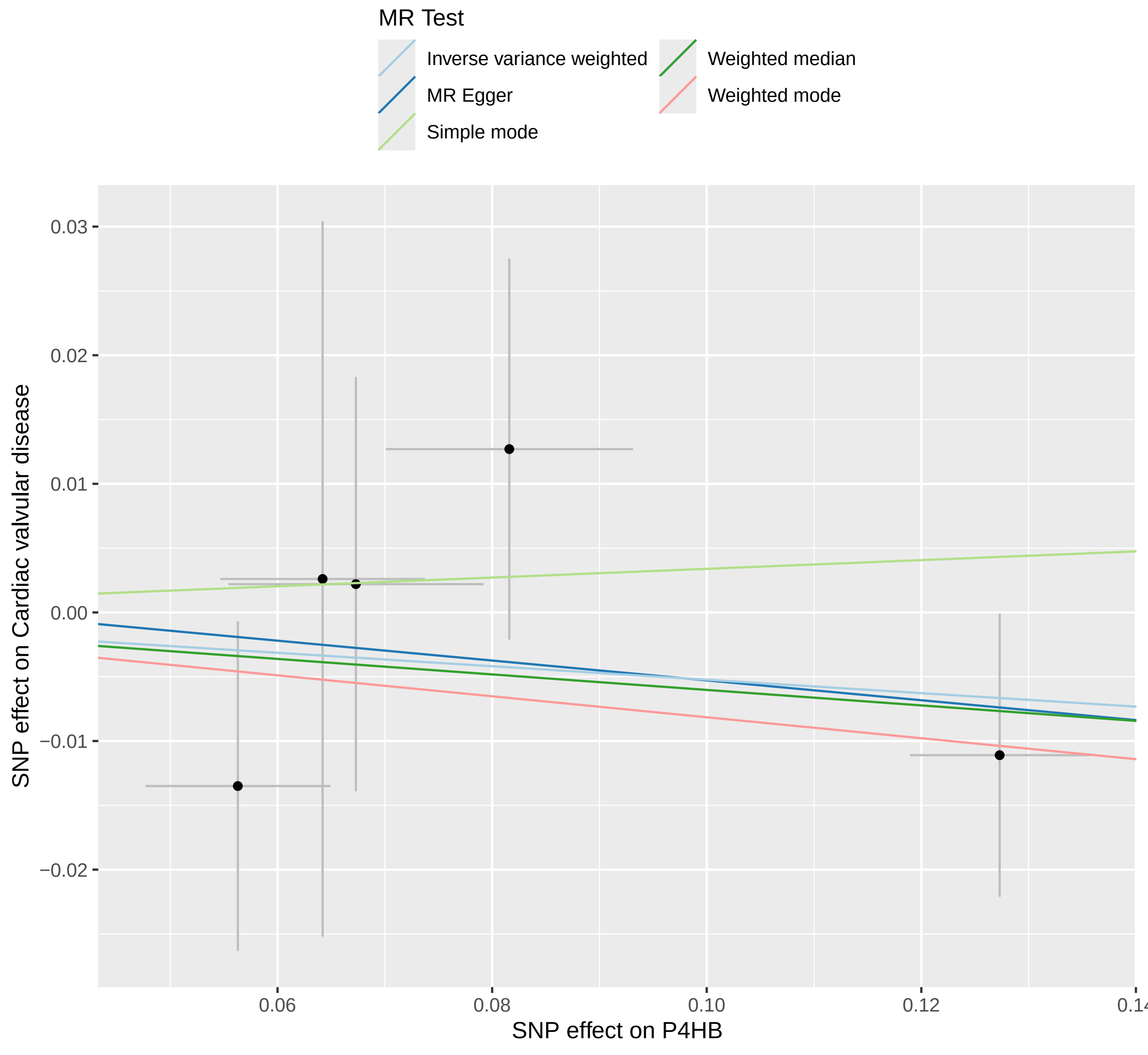
MR Test
Inverse variance weighted
MR Egger
Simple mode
Weighted median
Weighted mode
0.03
0.02
0.01
0.00
−0.01
−0.02
SNP effect on Cardiac valvular disease
0.06
0.08
0.10
0.12
SNP effect on P4HB

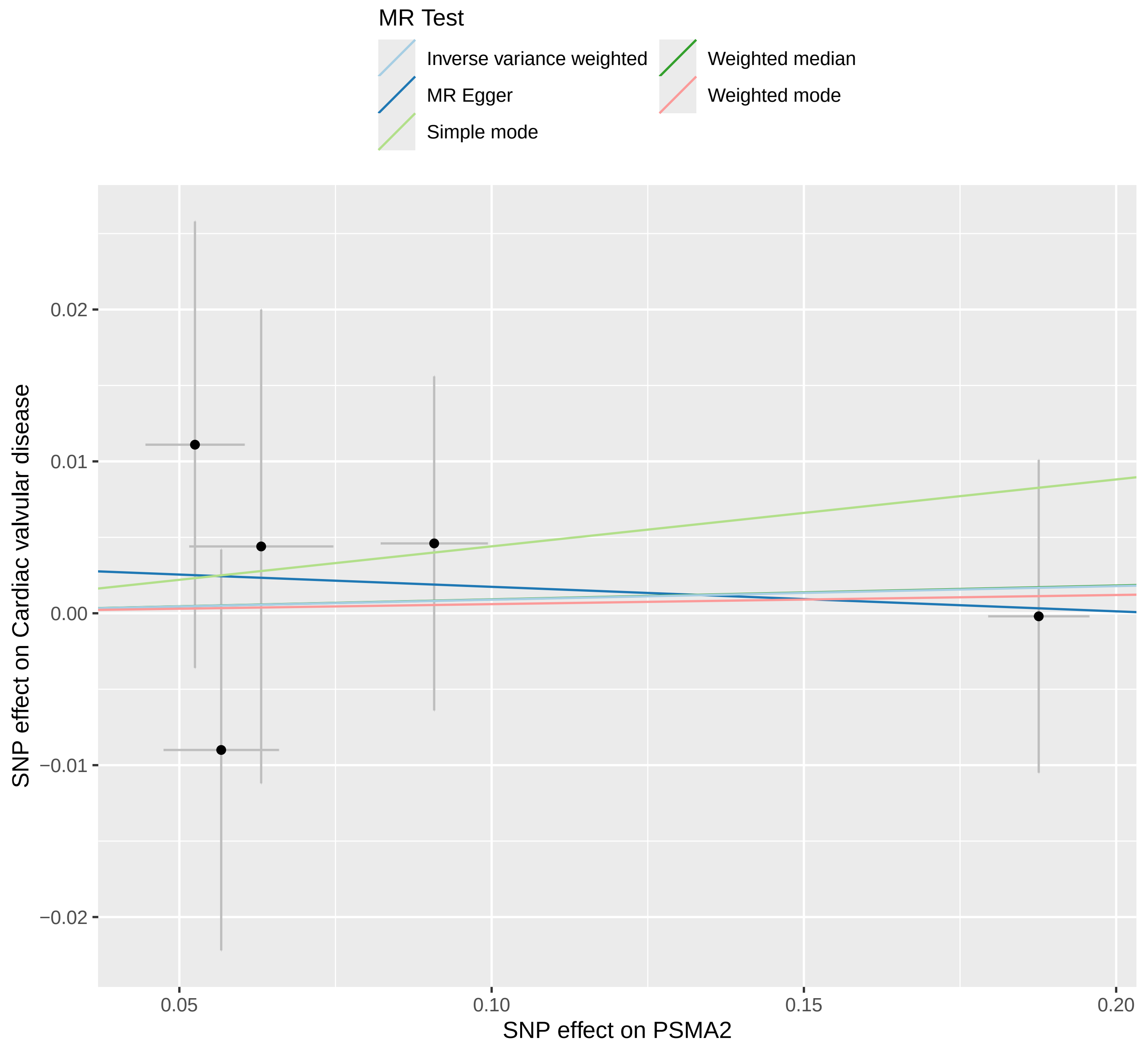
MR Test
Inverse variance weighted
MR Egger
Simple mode
Weighted median
Weighted mode
0.02
0.01
0.00
−0.01
−0.02
SNP effect on Cardiac valvular disease
0.05
0.10
0.15
0.20
SNP effect on PSMA2

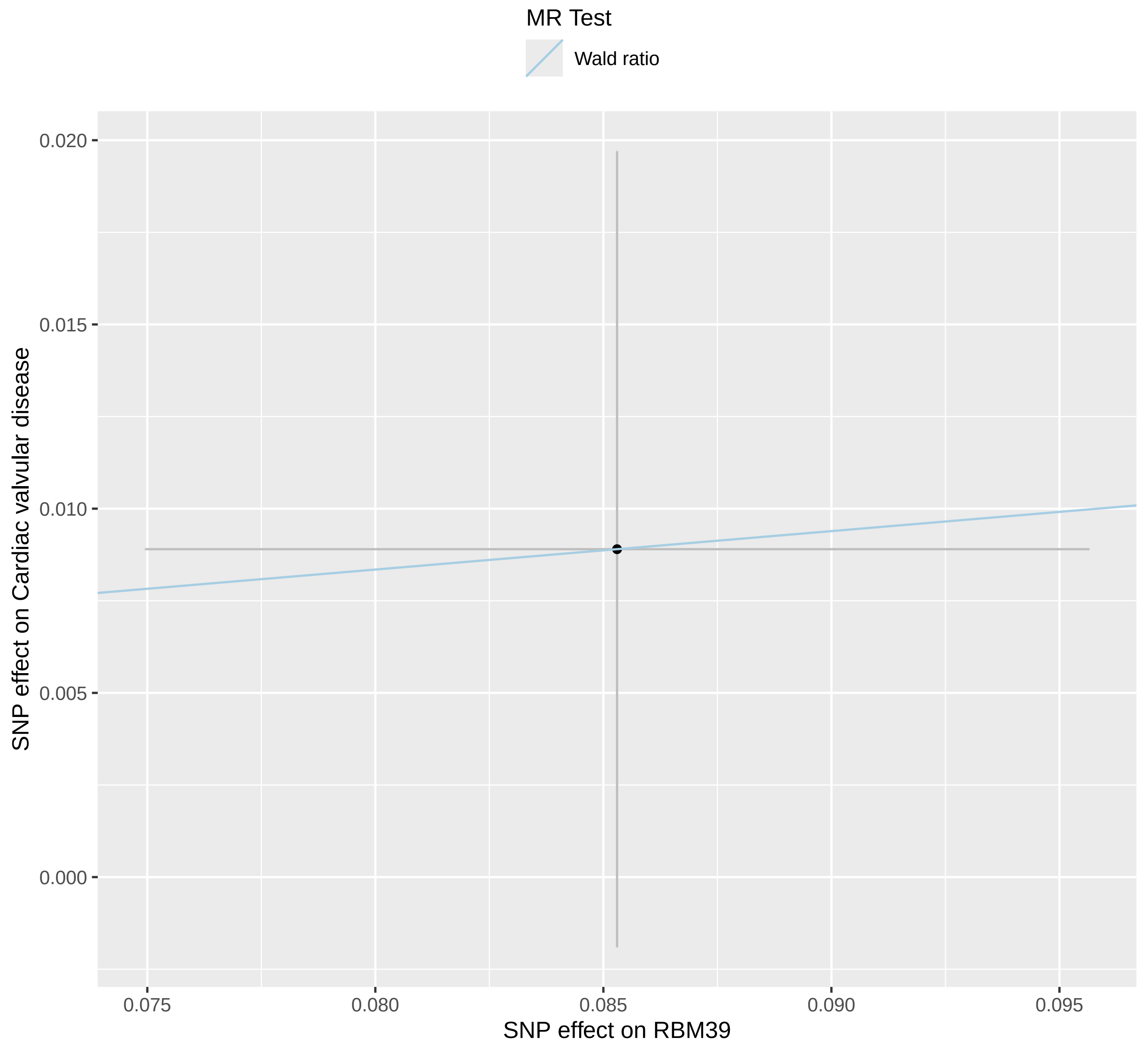

MR Test
Wald ratio
0.020
0.015
0.010
0.005
0.000
SNP effect on Cardiac valvular disease
0.075
0.080
0.085
0.090
0.095
SNP effect on RBM39

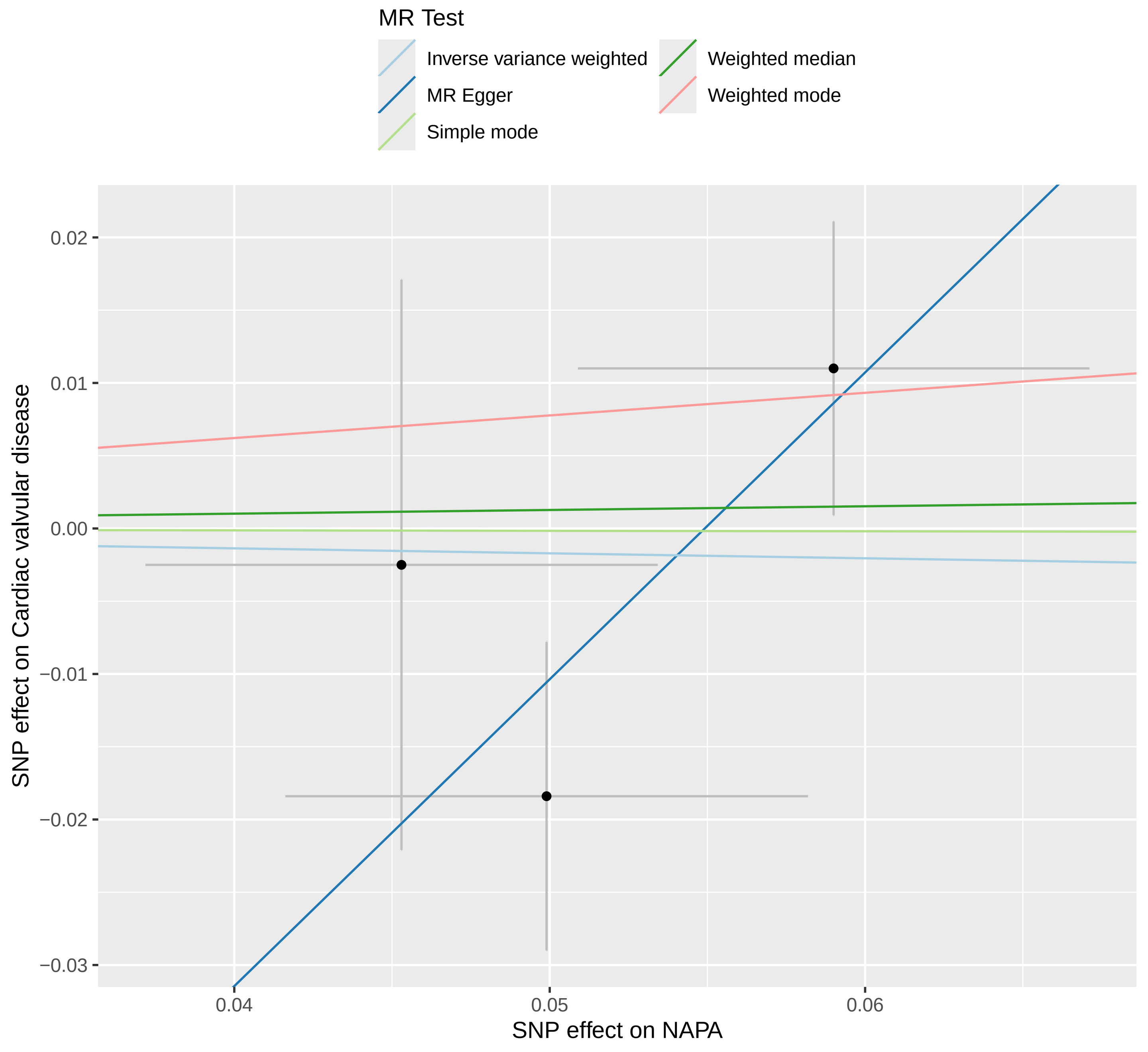
MR Test
Inverse variance weighted
MR Egger
Simple mode
Weighted median
Weighted mode
0.02
0.01
0.00
−0.01
−0.02
−0.03
SNP effect on Cardiac valvular disease
0.04
0.05
0.06
SNP effect on NAPA

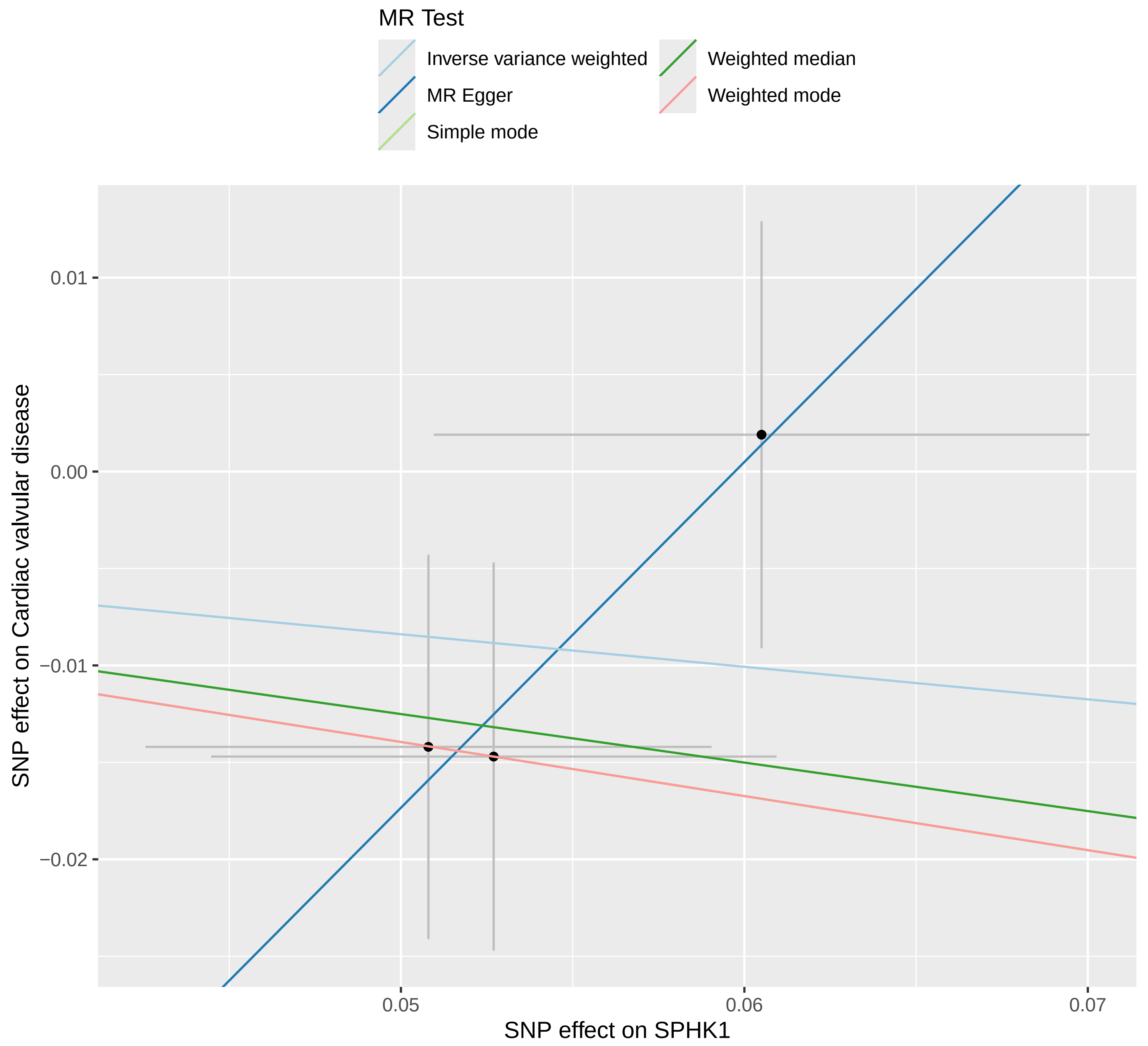
MR Test
Inverse variance weighted
MR Egger
Simple mode
Weighted median
Weighted mode
SNP effect on Cardiac valvular disease
0.01
0.00
−0.01
−0.02
0.05
0.06
0.07
SNP effect on SPHK1

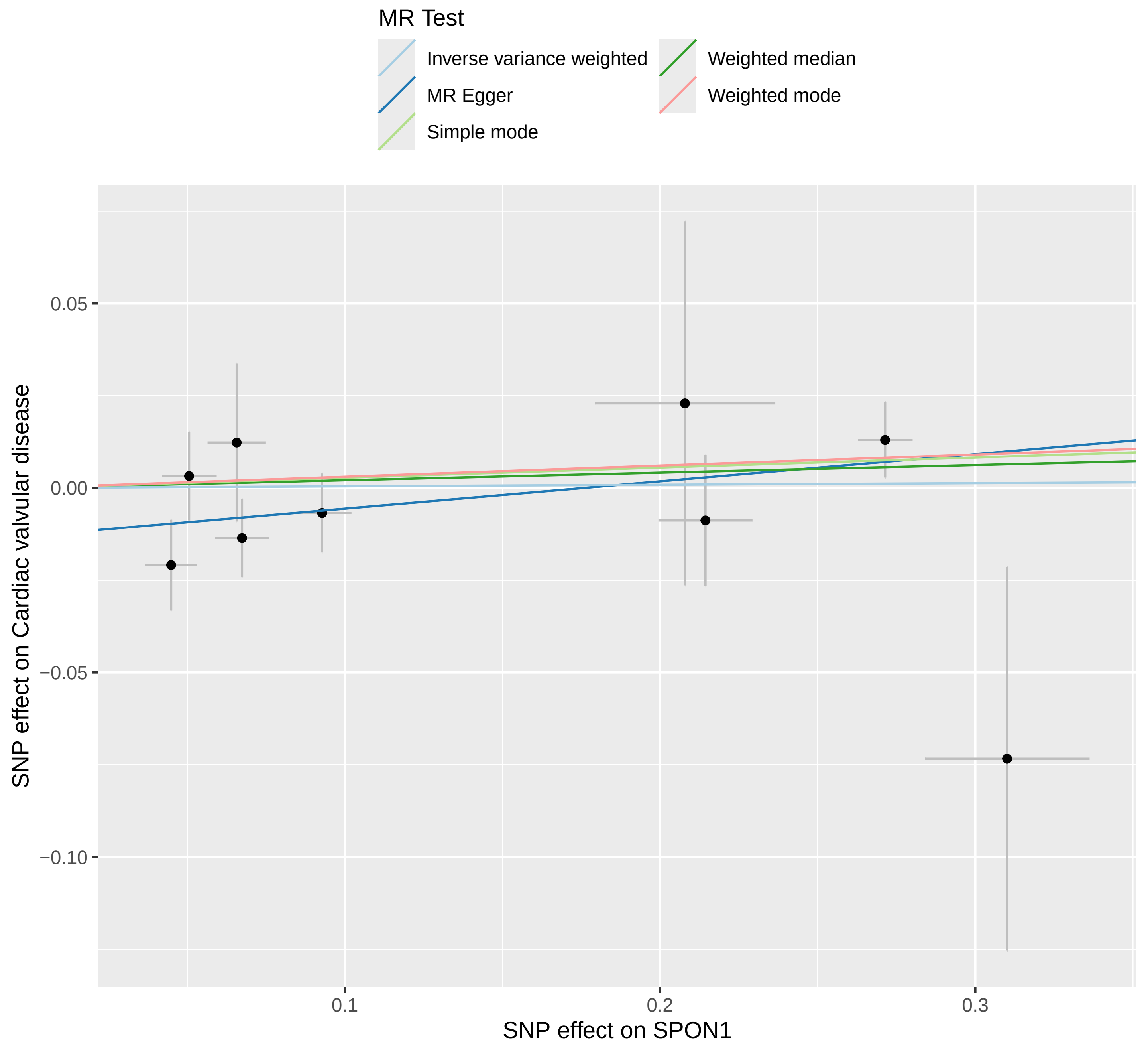
MR Test
Inverse variance weighted
MR Egger
Simple mode
Weighted median
Weighted mode
0.05
0.00
−0.05
−0.10
0.1
0.2
0.3
SNP effect on Cardiac valvular disease
SNP effect on SPON1

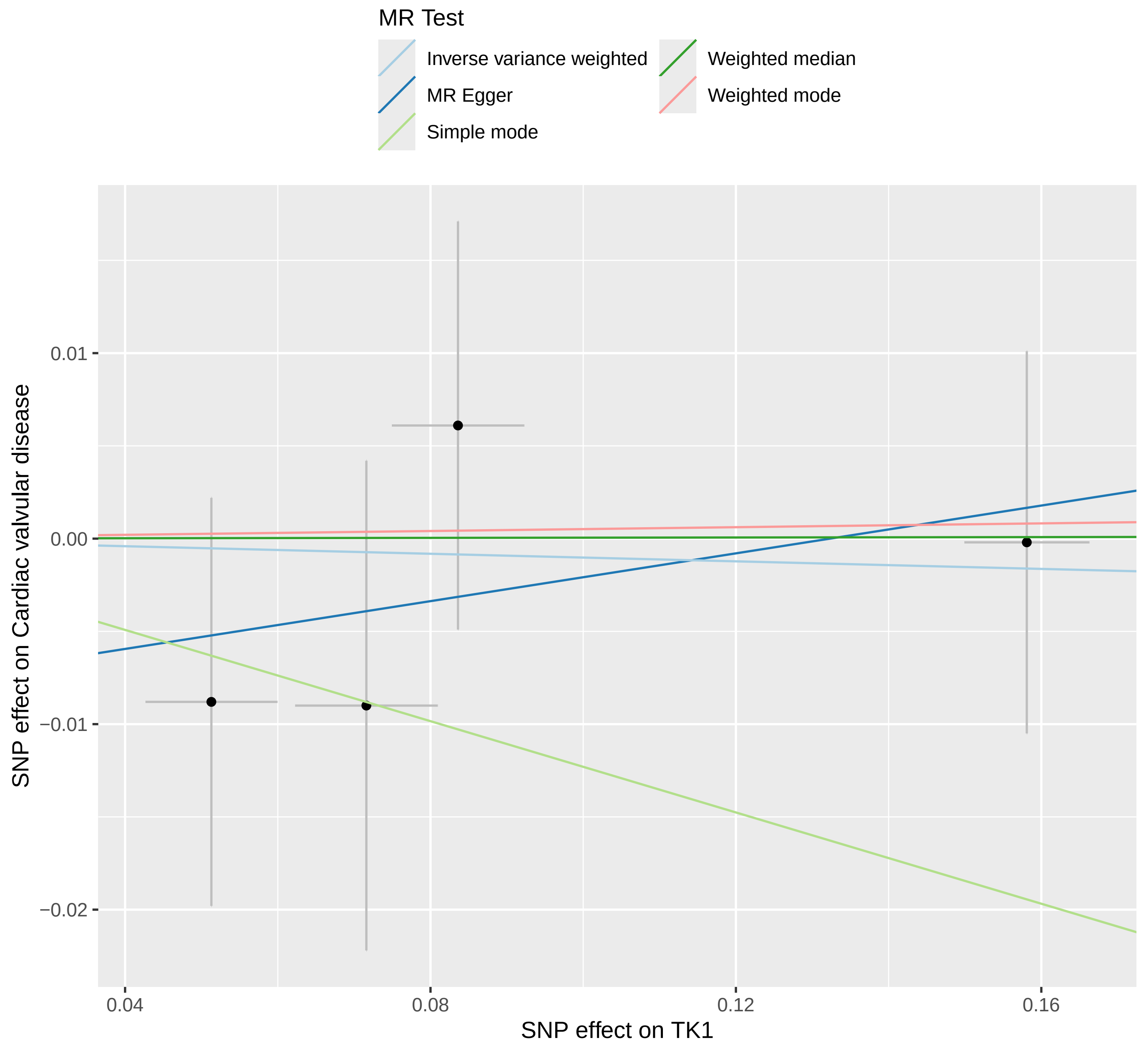

MR Test
Inverse variance weighted
MR Egger
Simple mode
Weighted median
Weighted mode
0.01
0.00
−0.01
−0.02
SNP effect on Cardiac valvular disease
0.04
0.08
0.12
0.16
SNP effect on TK1

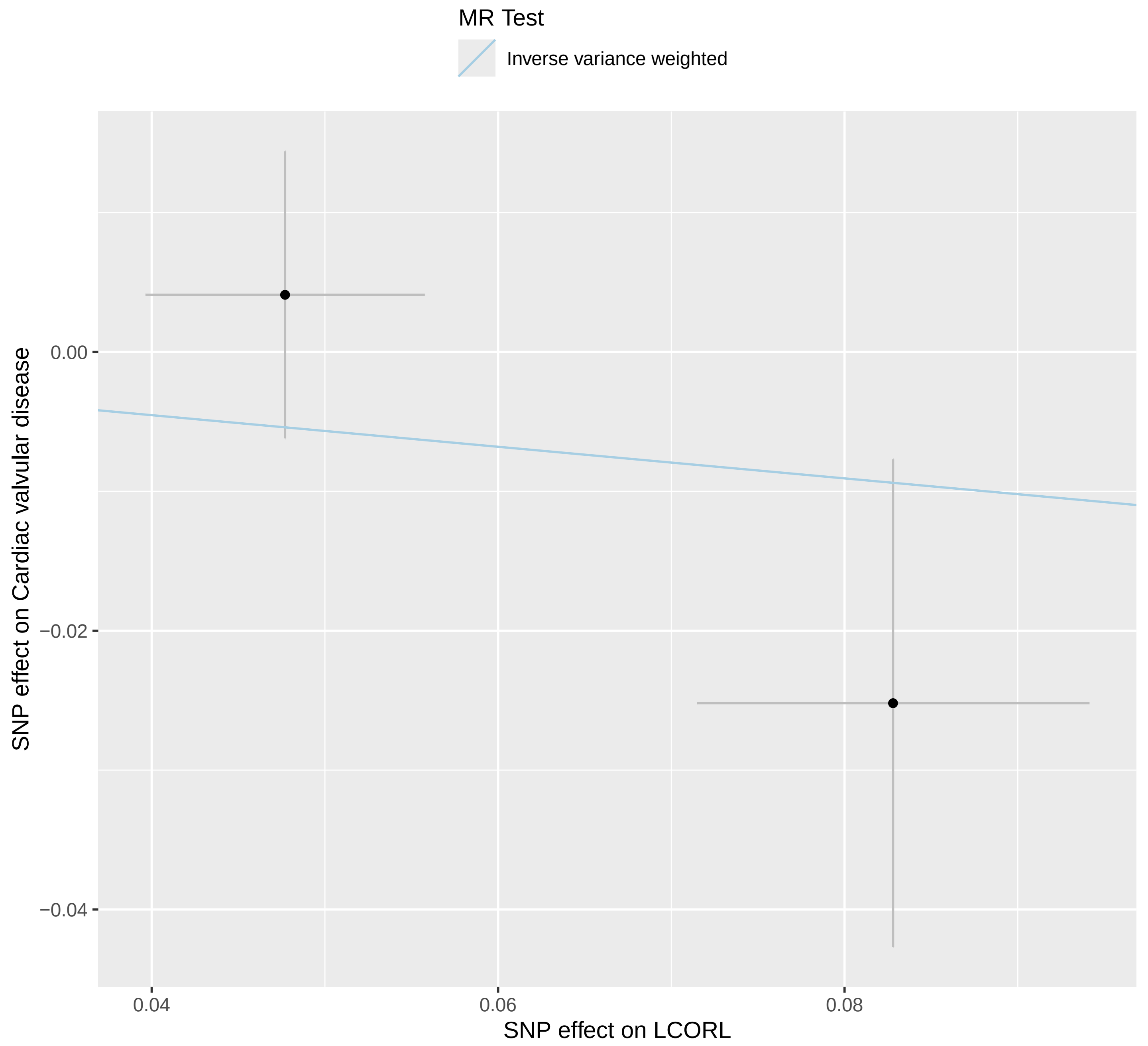
MR Test
Inverse variance weighted
SNP effect on Cardiac valvular disease
0.00
−0.02
−0.04
0.04
0.06
0.08
SNP effect on LCORL

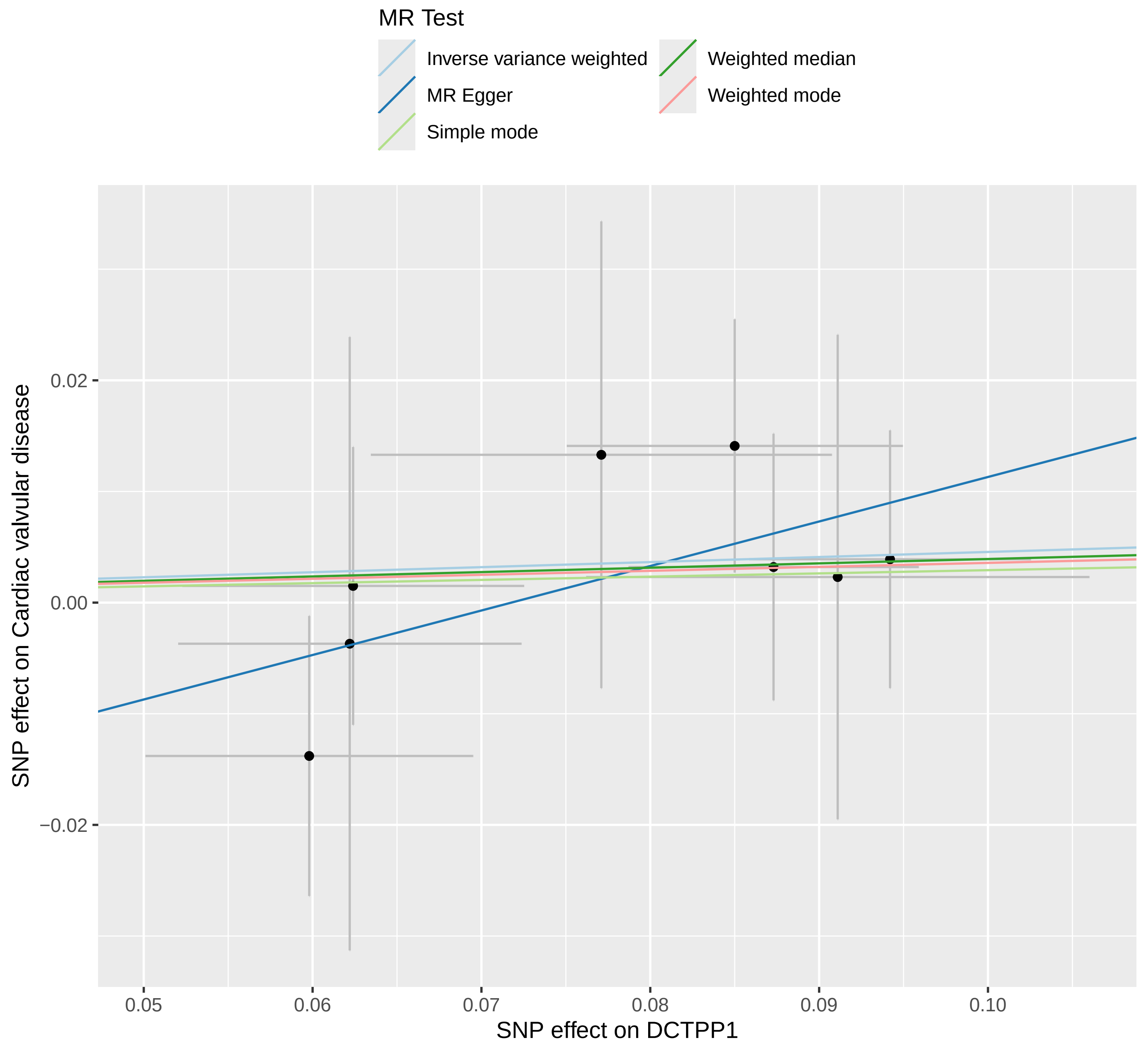

MR Test
Inverse variance weighted
MR Egger
Simple mode
Weighted median
Weighted mode
0.02
0.00
−0.02
0.05
0.06
0.07
0.08
0.09
0.10
SNP effect on DCTPP1
SNP effect on Cardiac valvular disease

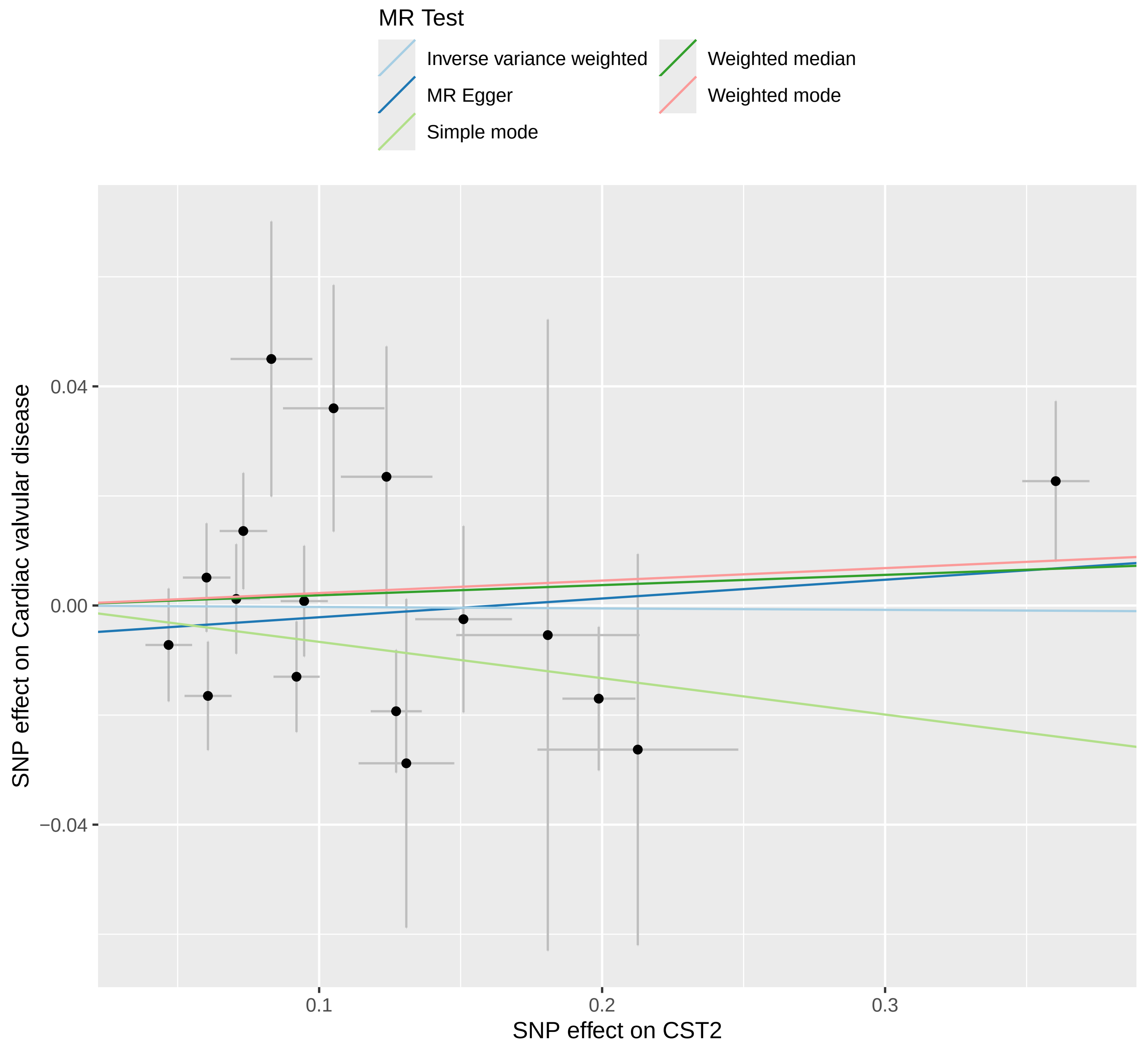

MR Test
Inverse variance weighted
MR Egger
Simple mode
Weighted median
Weighted mode
0.04
0.00
−0.04
0.1
0.2
0.3
SNP effect on Cardiac valvular disease
SNP effect on CST2

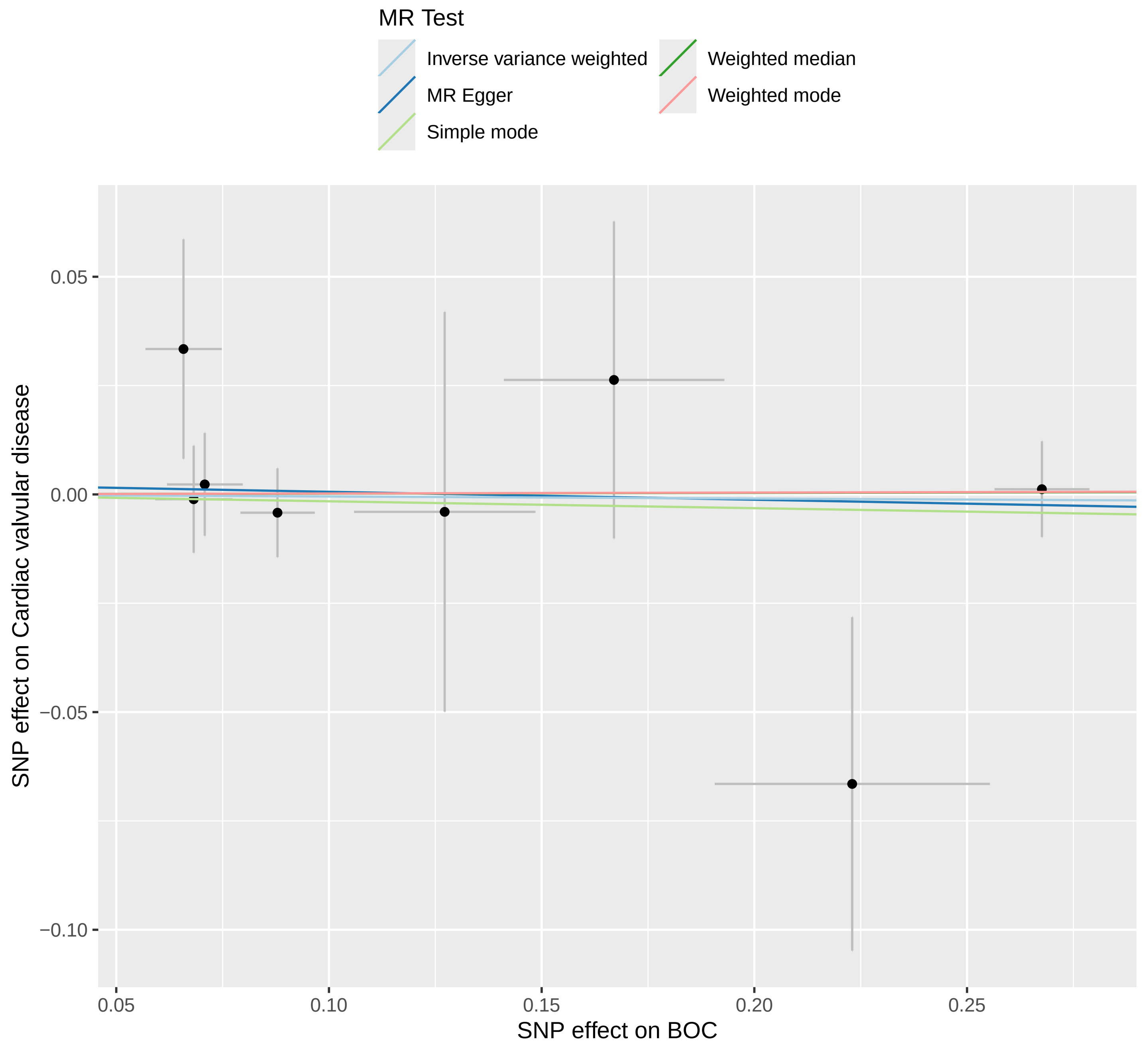

MR Test
Inverse variance weighted
MR Egger
Simple mode
Weighted median
Weighted mode
0.05
0.00
−0.05
−0.10
SNP effect on Cardiac valvular disease
0.05
0.10
0.15
0.20
0.25
SNP effect on BOC

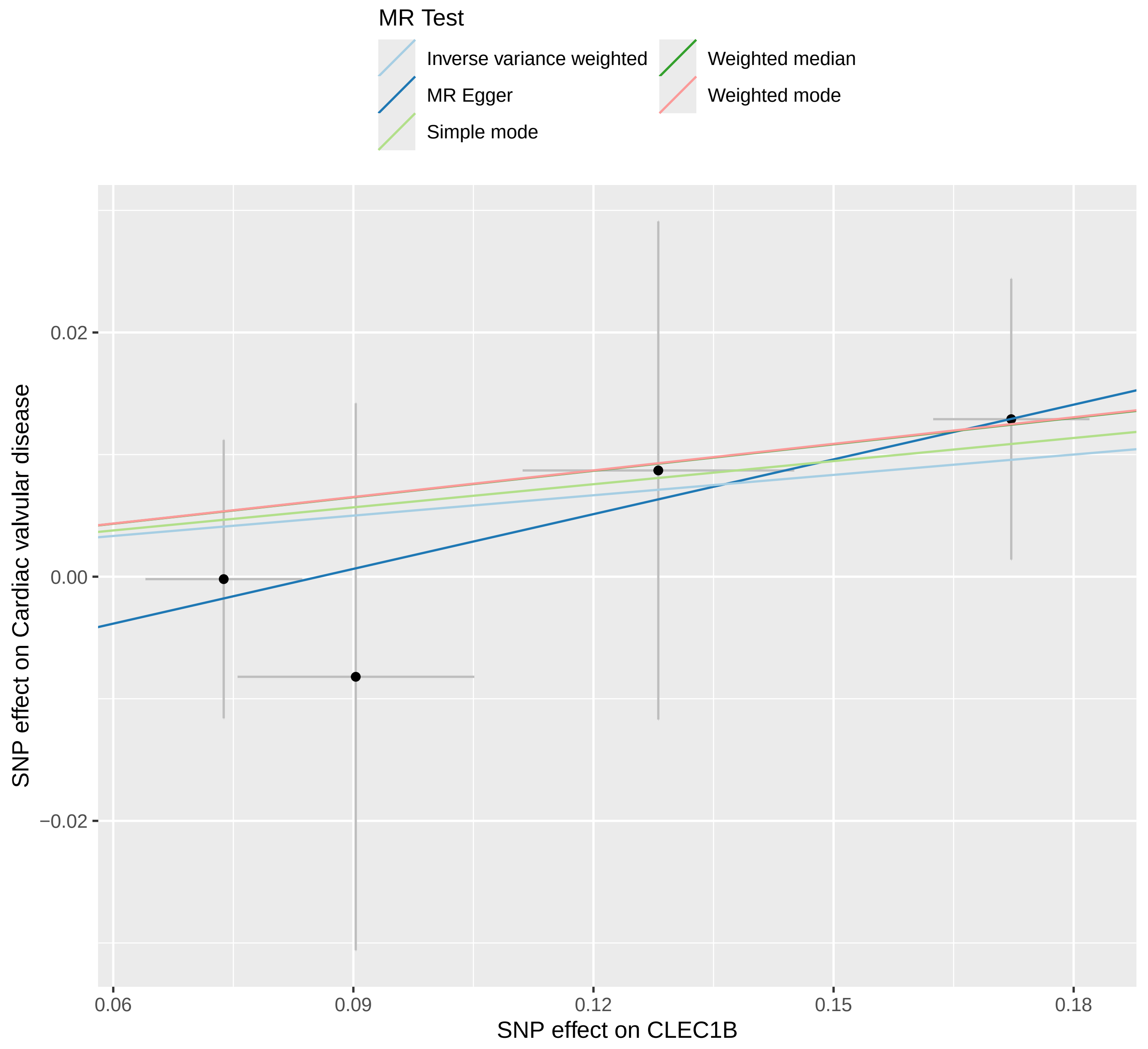
MR Test
Inverse variance weighted
MR Egger
Simple mode
Weighted median
Weighted mode
0.02
0.00
−0.02
0.06
0.09
0.12
0.15
0.18
SNP effect on Cardiac valvular disease
SNP effect on CLEC1B

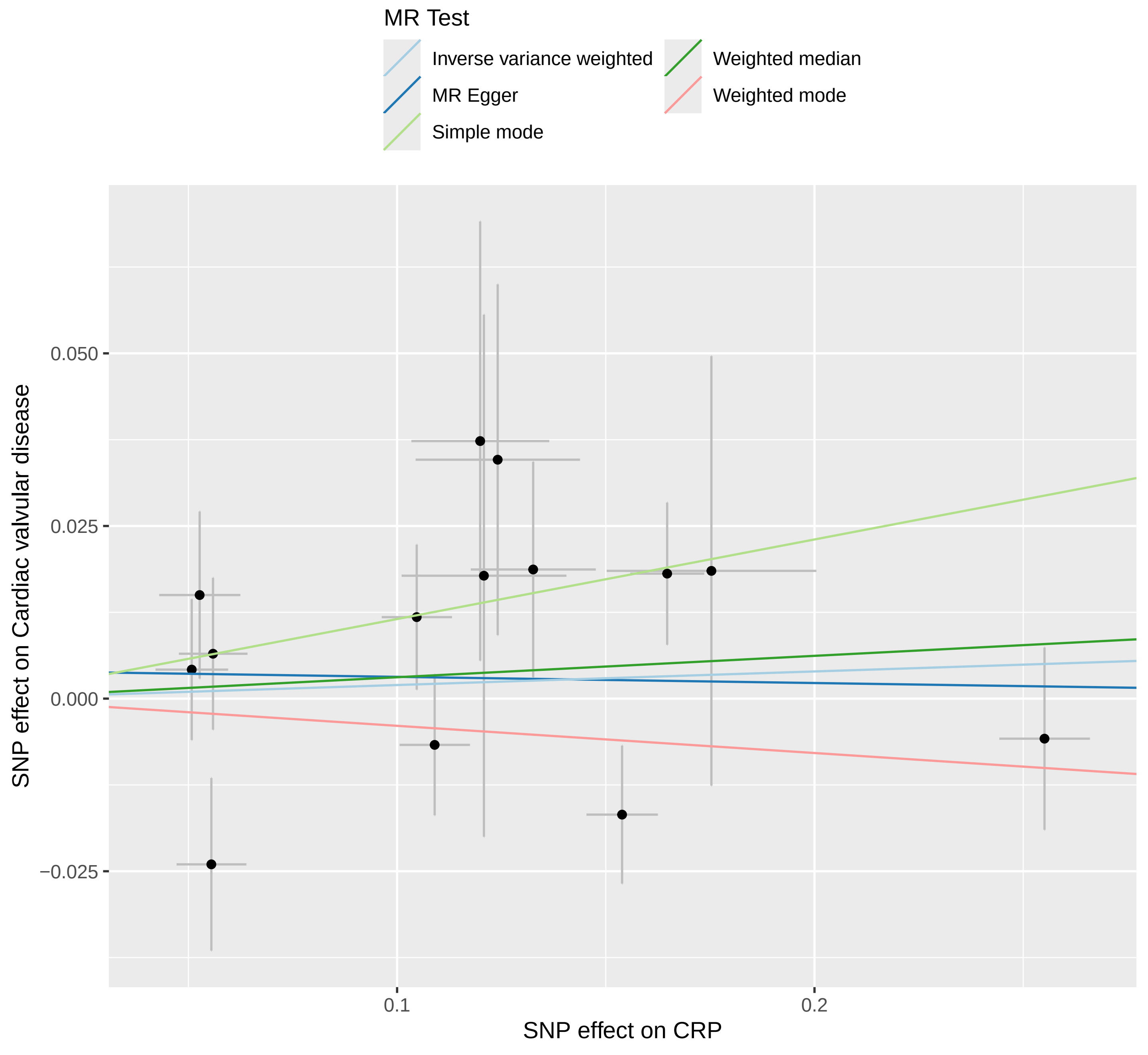
MR Test
Inverse variance weighted
MR Egger
Simple mode
Weighted median
Weighted mode
SNP effect on Cardiac valvular disease
0.050
0.025
0.000
−0.025
0.1
0.2
SNP effect on CRP

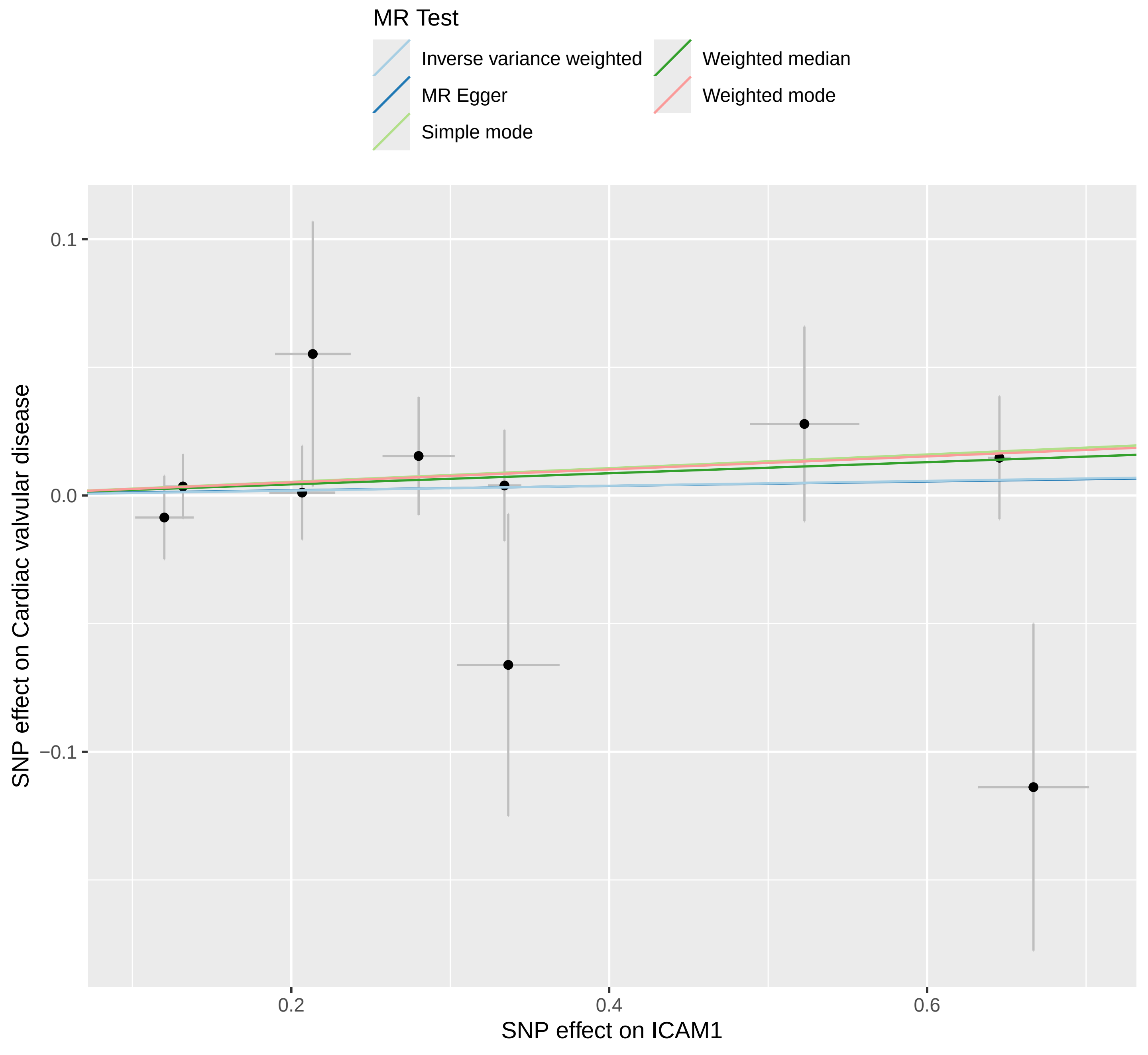
MR Test
Inverse variance weighted
MR Egger
Simple mode
Weighted median
Weighted mode
0.1
0.0
−0.1
0.2
0.4
0.6
SNP effect on Cardiac valvular disease
SNP effect on ICAM1

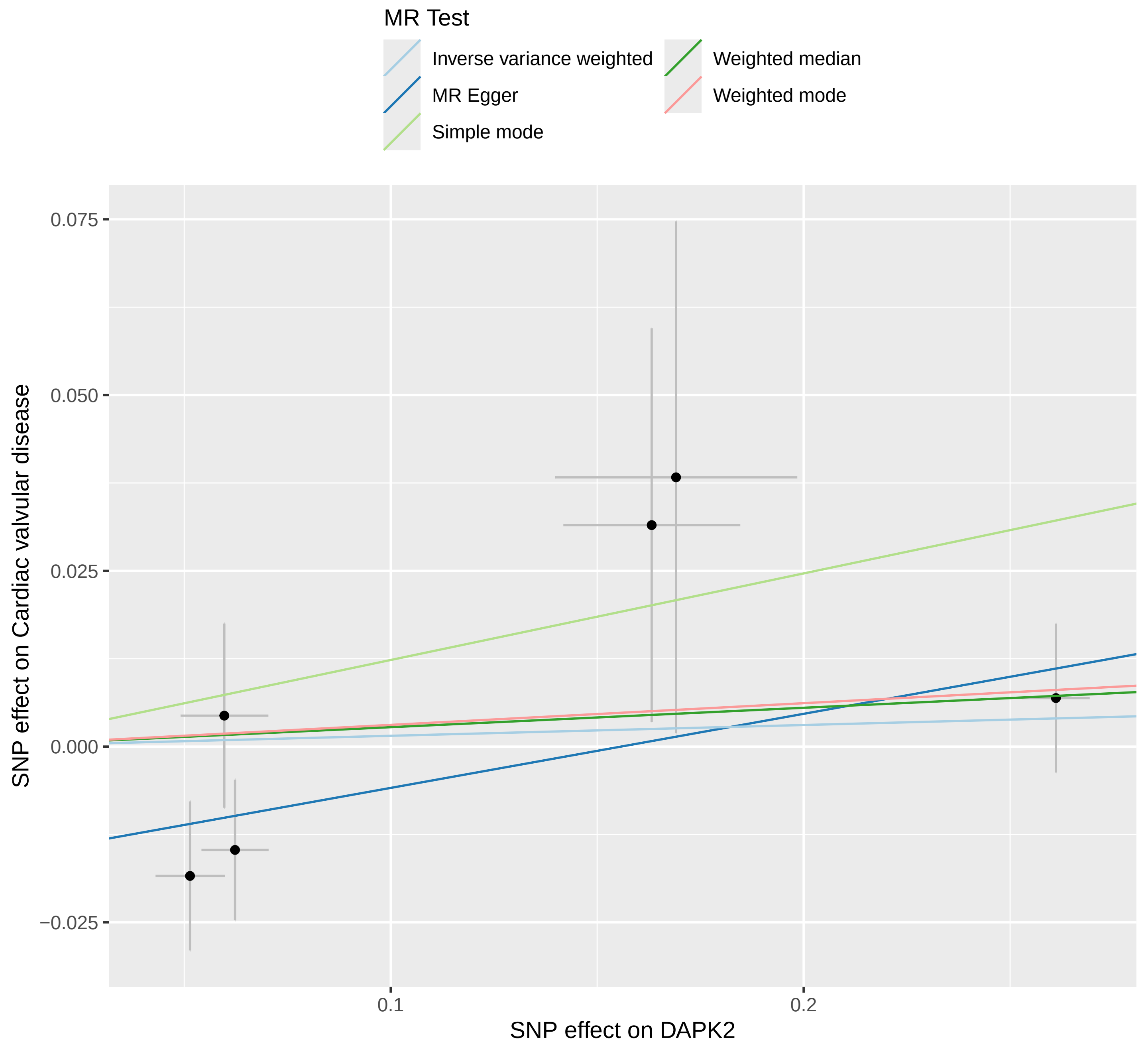

MR Test
Inverse variance weighted
MR Egger
Simple mode
Weighted median
Weighted mode
0.075
0.050
0.025
0.000
−0.025
0.1
0.2
SNP effect on Cardiac valvular disease
SNP effect on DAPK2

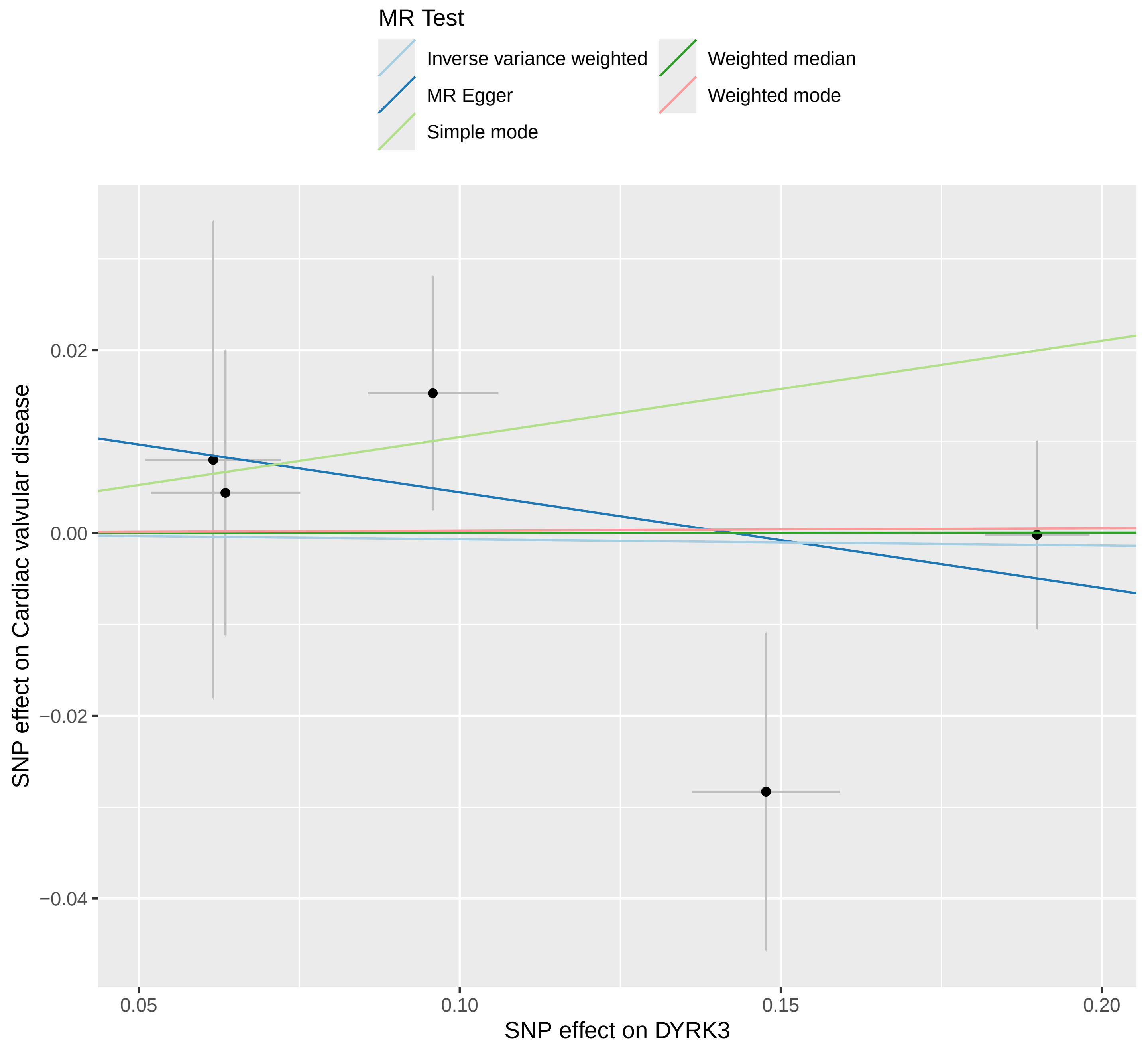

MR Test
Inverse variance weighted
MR Egger
Simple mode
Weighted median
Weighted mode
SNP effect on Cardiac valvular disease
0.02
0.00
−0.02
−0.04
0.05
0.10
0.15
0.20
SNP effect on DYRK3

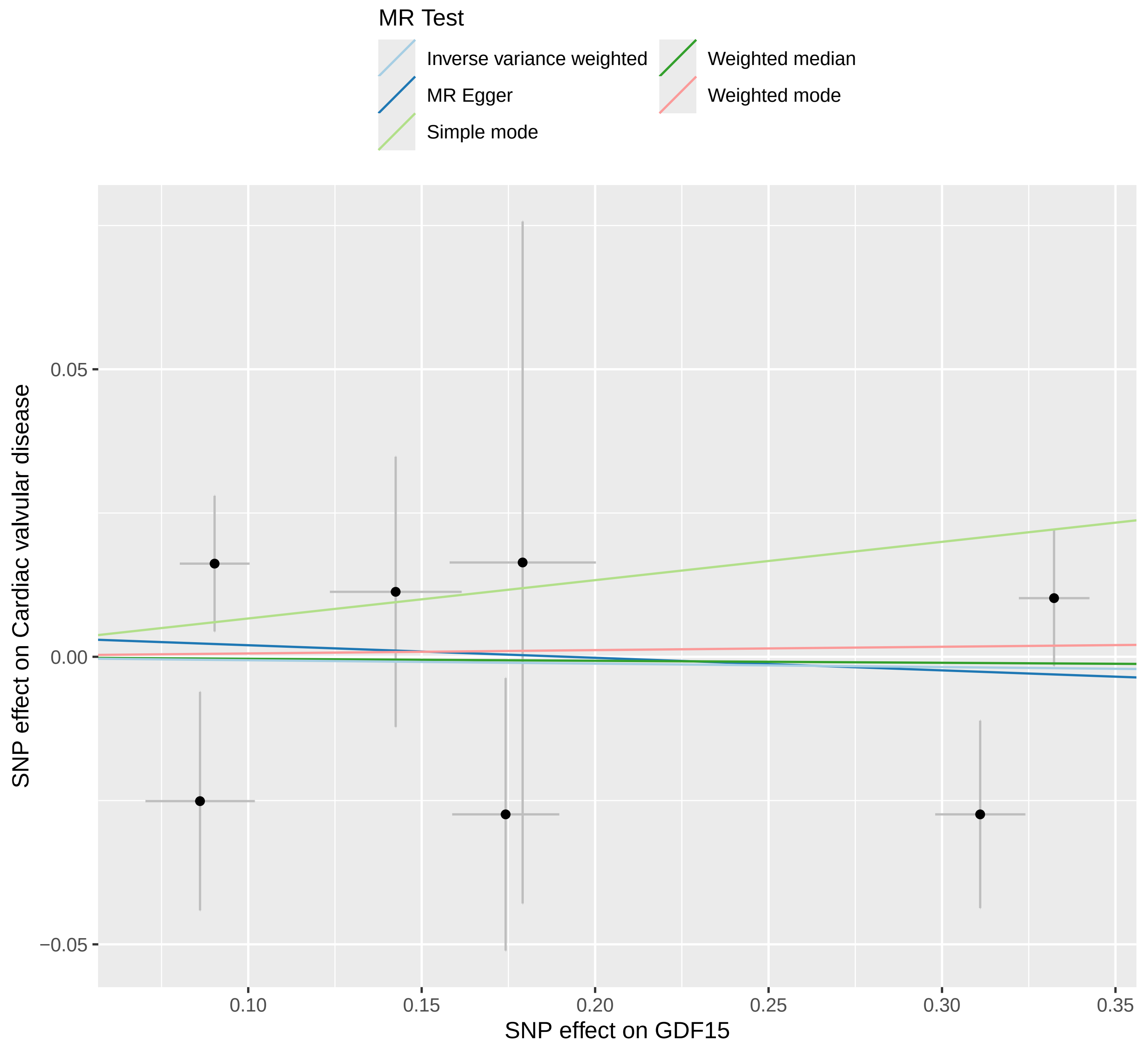
MR Test
Inverse variance weighted
MR Egger
Simple mode
Weighted median
Weighted mode
SNP effect on Cardiac valvular disease
0.05
0.00
−0.05
0.10
0.15
0.20
0.25
0.30
0.35
SNP effect on GDF15

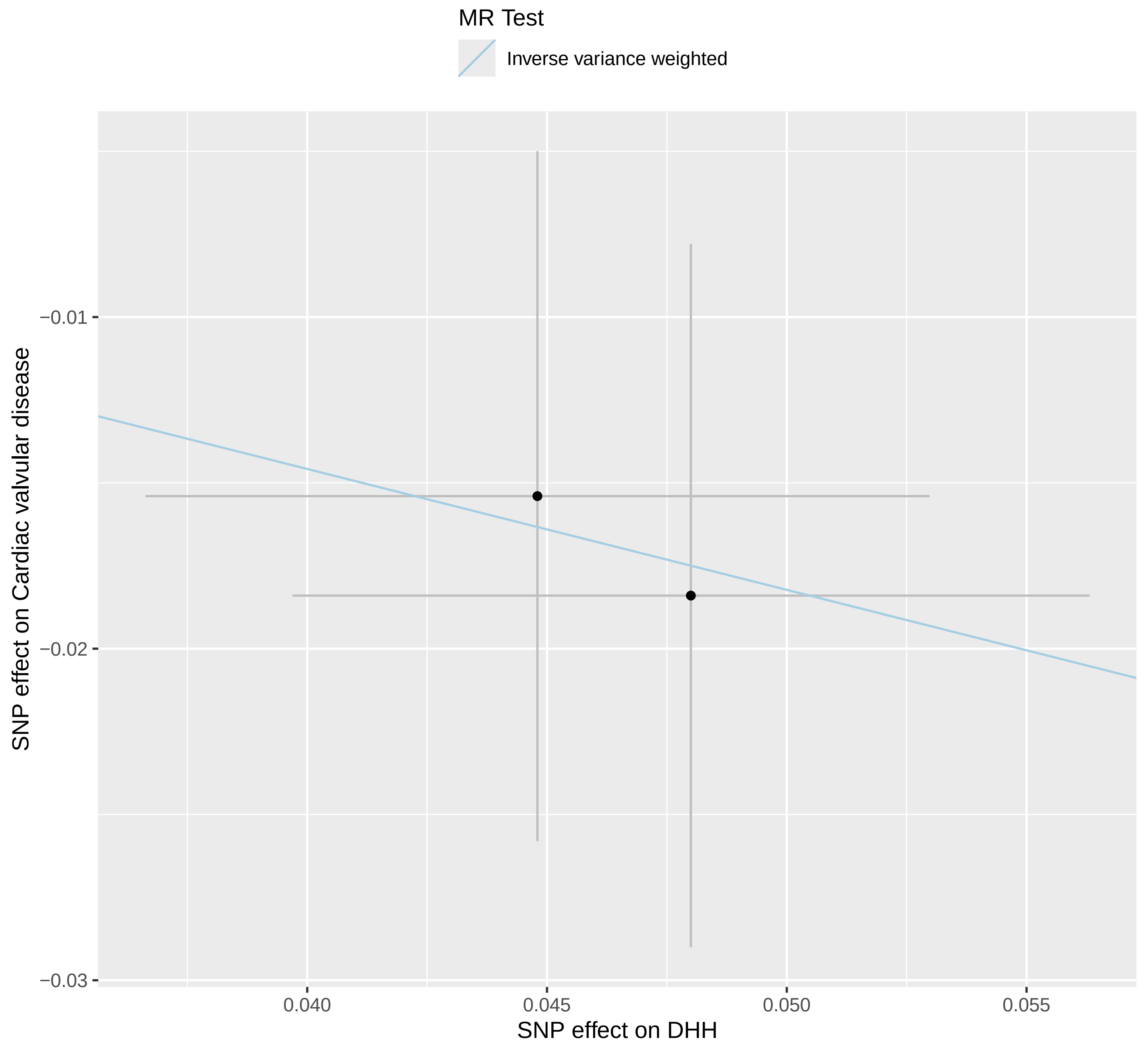
MR Test
Inverse variance weighted
−0.01
−0.02
−0.03
0.040
0.045
0.050
0.055
SNP effect on DHH
SNP effect on Cardiac valvular disease

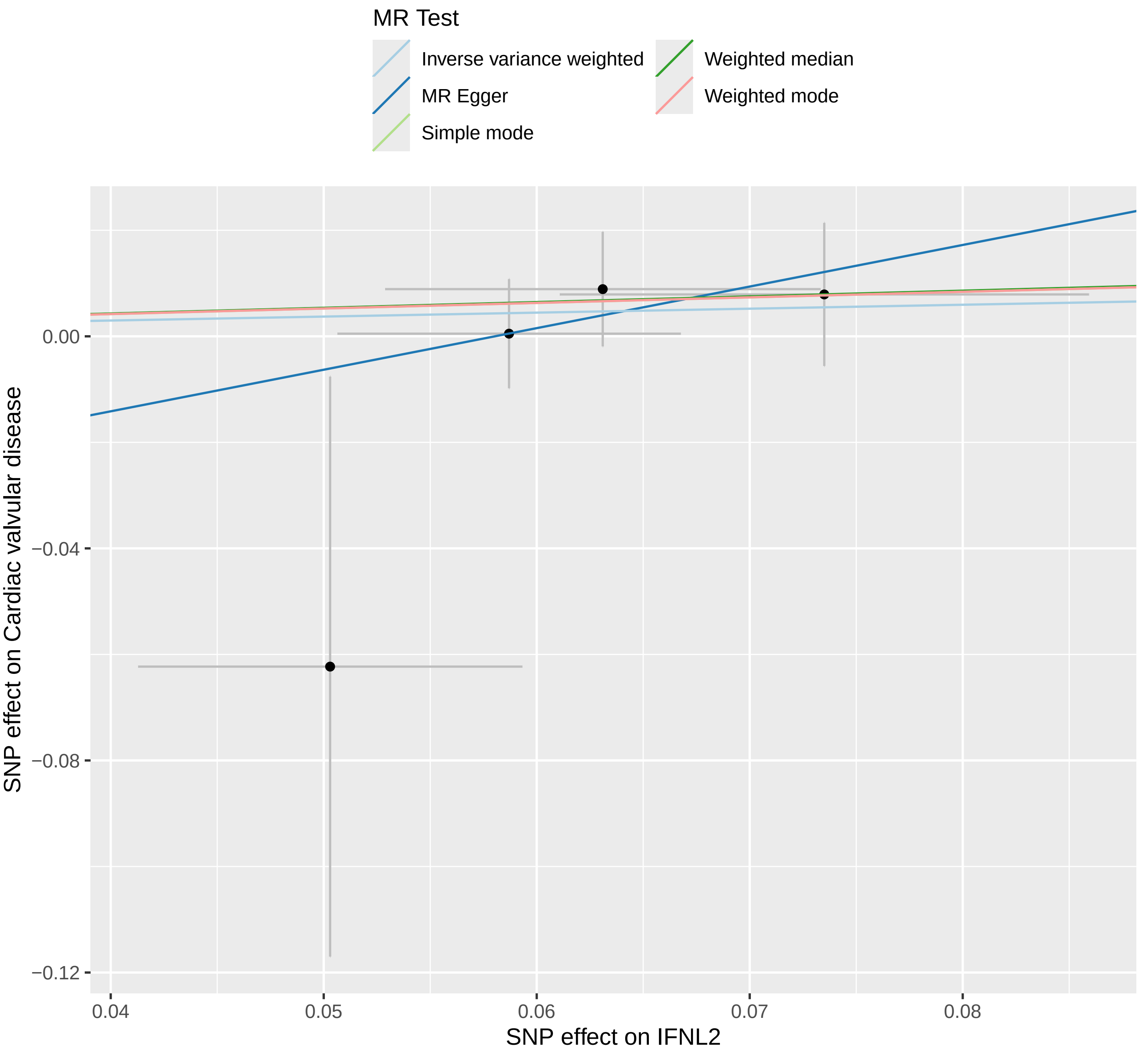

MR Test
Inverse variance weighted
MR Egger
Simple mode
Weighted median
Weighted mode
SNP effect on Cardiac valvular disease
0.00
−0.04
−0.08
−0.12
0.04
0.05
0.06
0.07
0.08
SNP effect on IFNL2

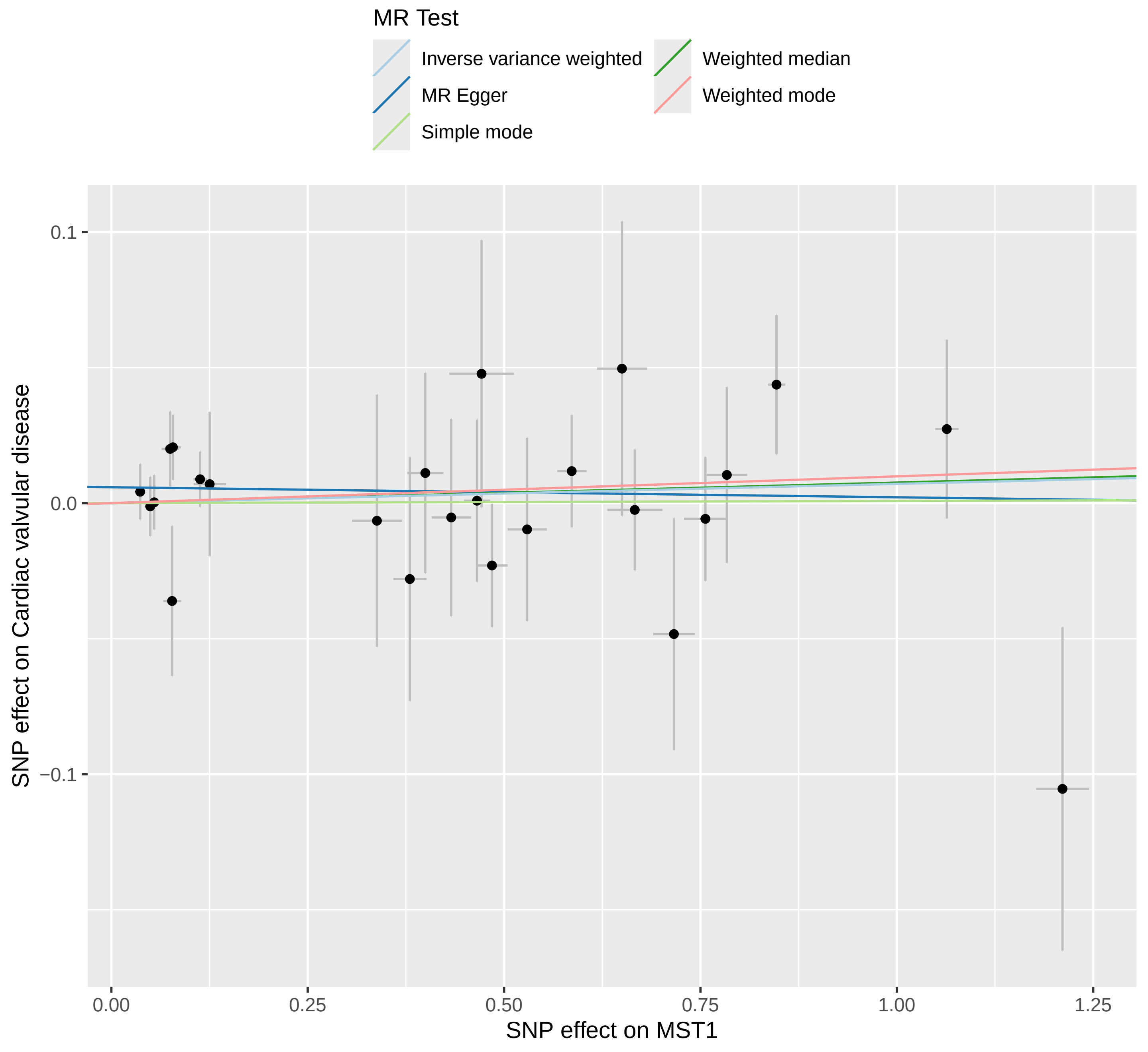
MR Test
Inverse variance weighted
MR Egger
Simple mode
Weighted median
Weighted mode
0.1
0.0
−0.1
0.00
0.25
0.50
0.75
1.00
1.25
SNP effect on MST1
SNP effect on Cardiac valvular disease

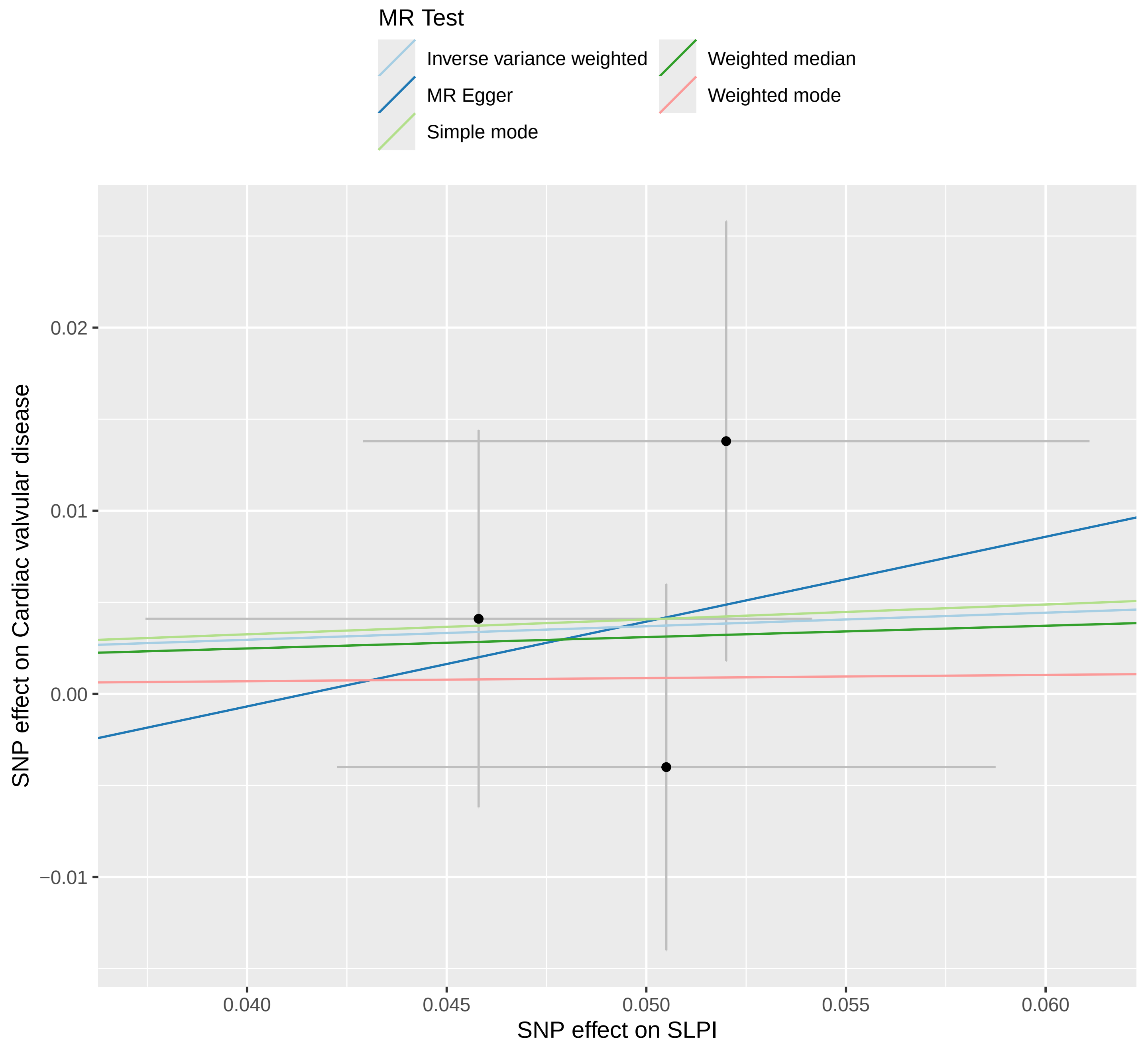
MR Test
Inverse variance weighted
MR Egger
Simple mode
Weighted median
Weighted mode
0.02
0.01
0.00
−0.01
SNP effect on Cardiac valvular disease
0.040
0.045
0.050
0.055
0.060
SNP effect on SLPI

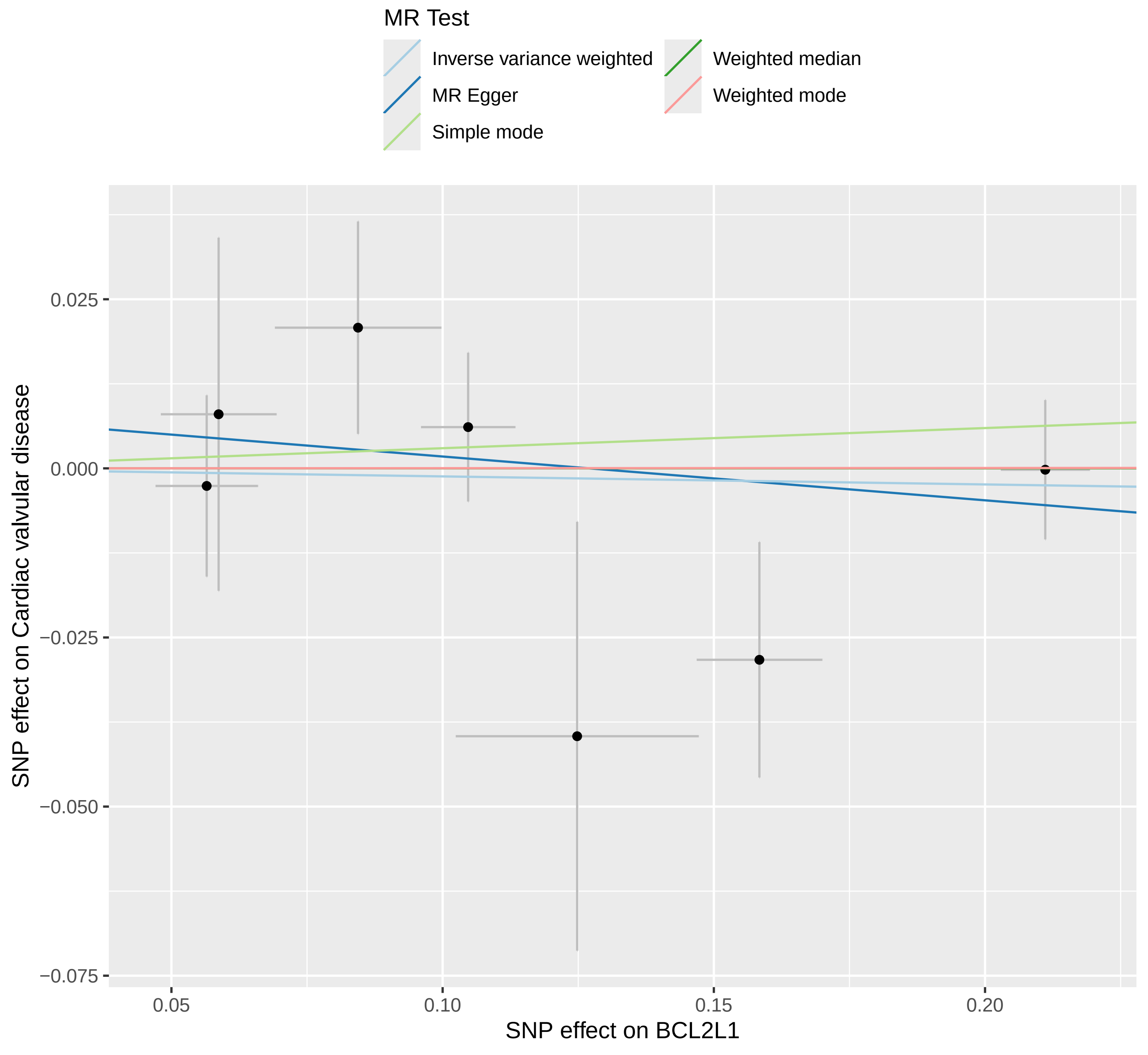
MR Test
Inverse variance weighted
MR Egger
Simple mode
Weighted median
Weighted mode
0.025
0.000
−0.025
−0.050
−0.075
SNP effect on Cardiac valvular disease
0.05
0.10
0.15
0.20
SNP effect on BCL2L1

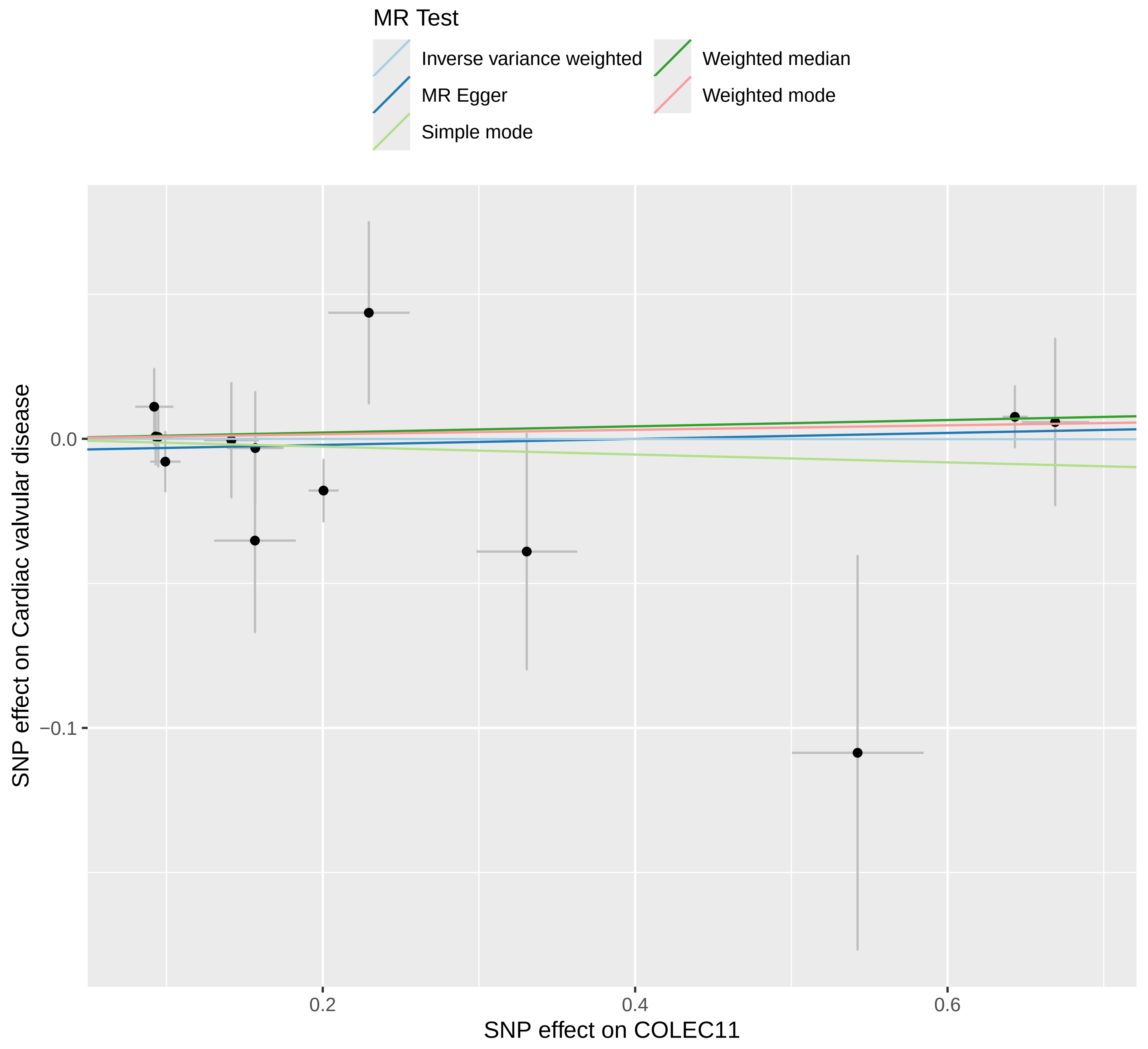

MR Test
Inverse variance weighted
MR Egger
Simple mode
Weighted median
Weighted mode
SNP effect on Cardiac valvular disease
0.0
−0.1
0.2
0.4
0.6
SNP effect on COLEC11

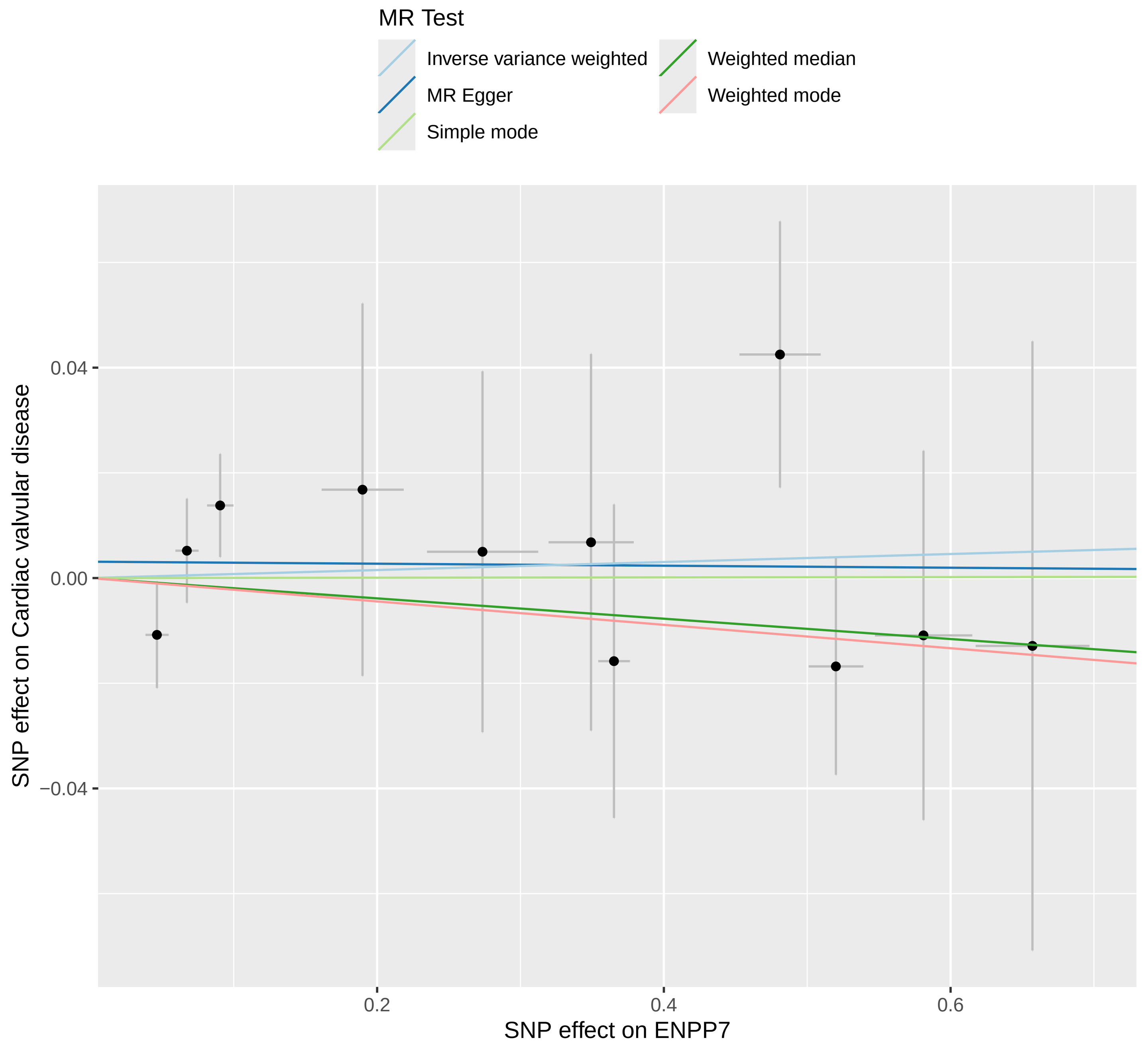
MR Test
Inverse variance weighted
MR Egger
Simple mode
Weighted median
Weighted mode
0.04
0.00
−0.04
0.2
0.4
0.6
SNP effect on Cardiac valvular disease
SNP effect on ENPP7

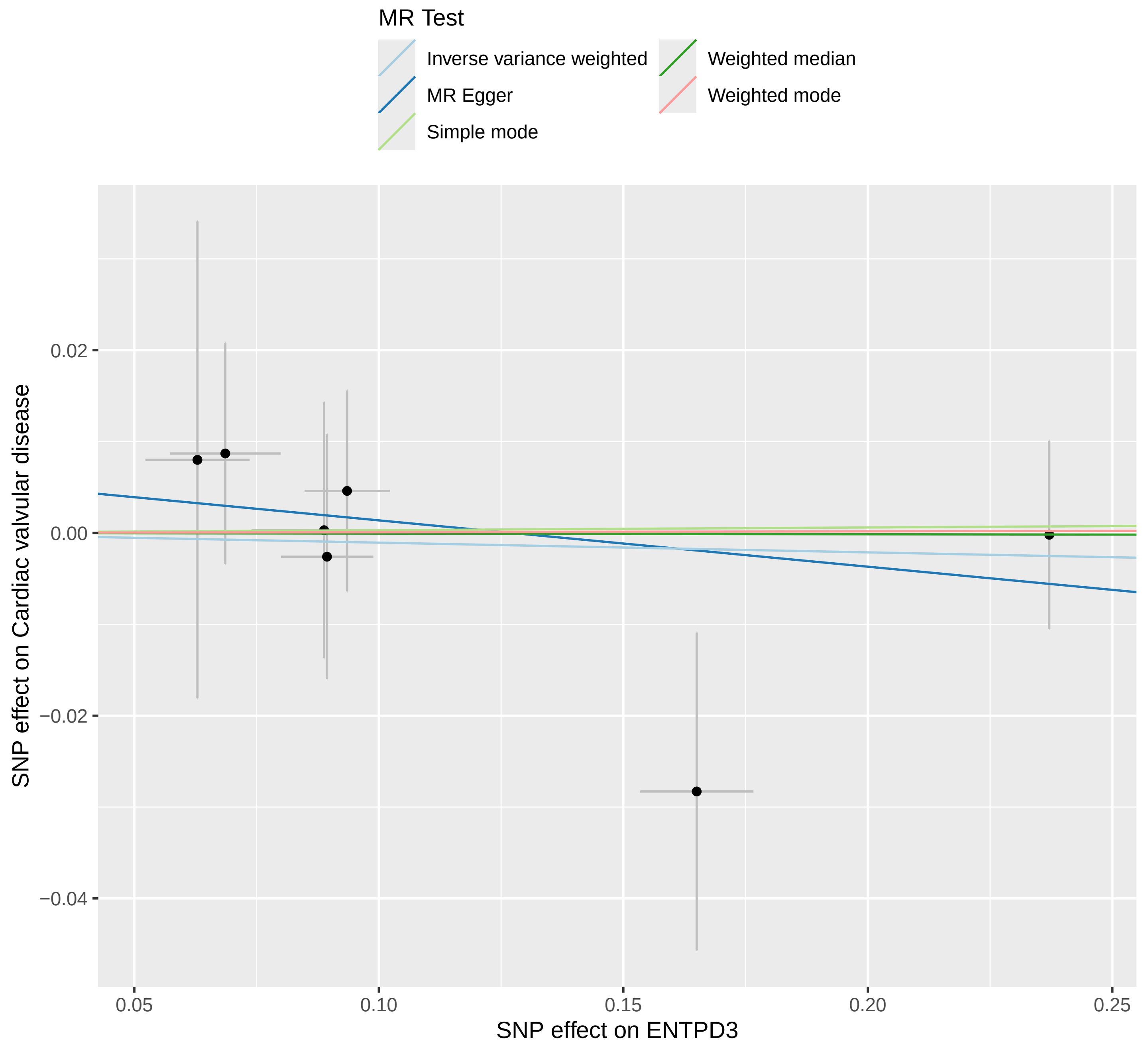

MR Test
Inverse variance weighted
MR Egger
Simple mode
Weighted median
Weighted mode
0.02
0.00
−0.02
−0.04
SNP effect on Cardiac valvular disease
0.05
0.10
0.15
0.20
0.25
SNP effect on ENTPD3

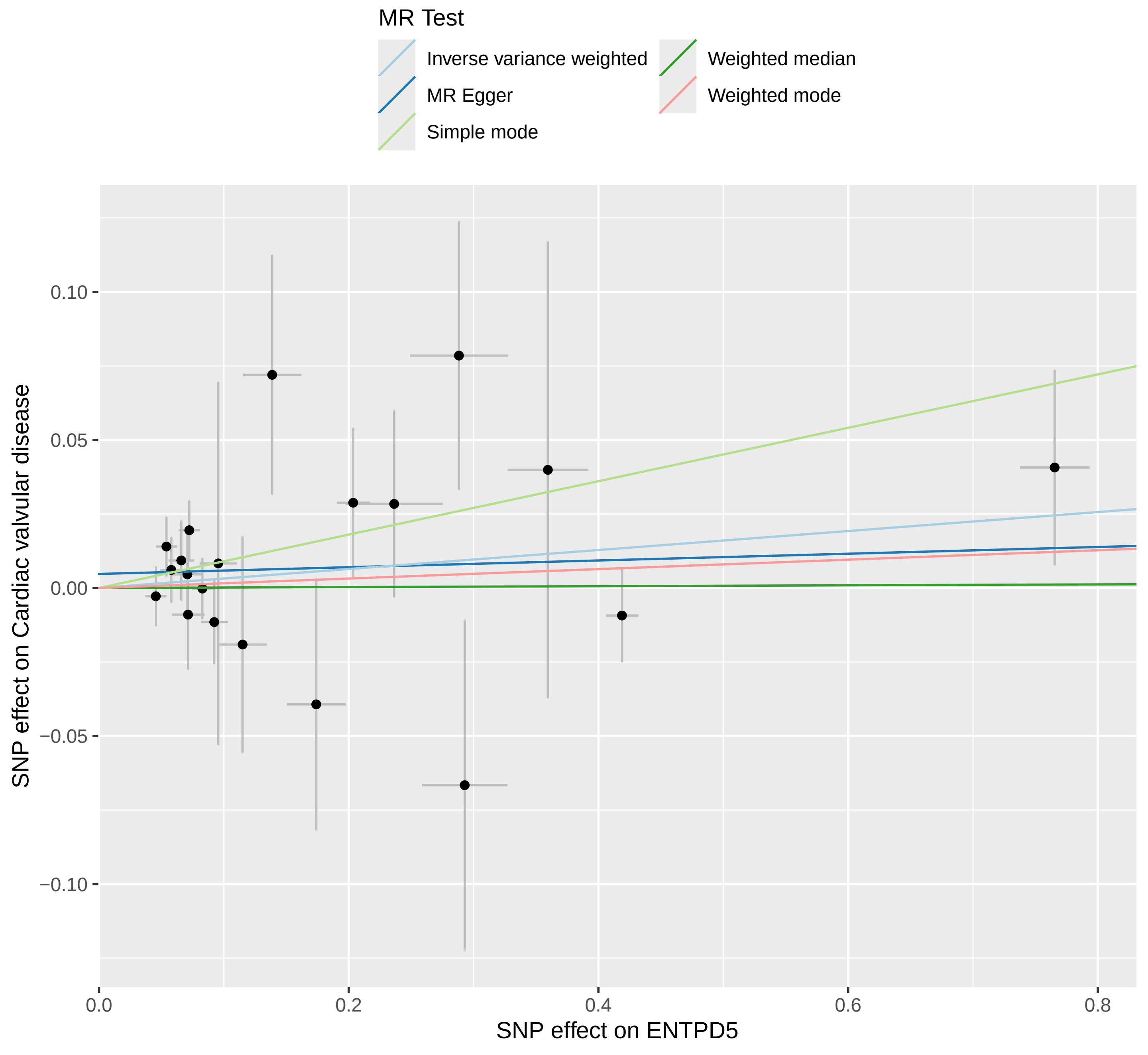
MR Test
Inverse variance weighted
MR Egger
Simple mode
Weighted median
Weighted mode
SNP effect on Cardiac valvular disease
0.10
0.05
0.00
−0.05
−0.10
0.0
0.2
0.4
0.6
0.8
SNP effect on ENTPD5

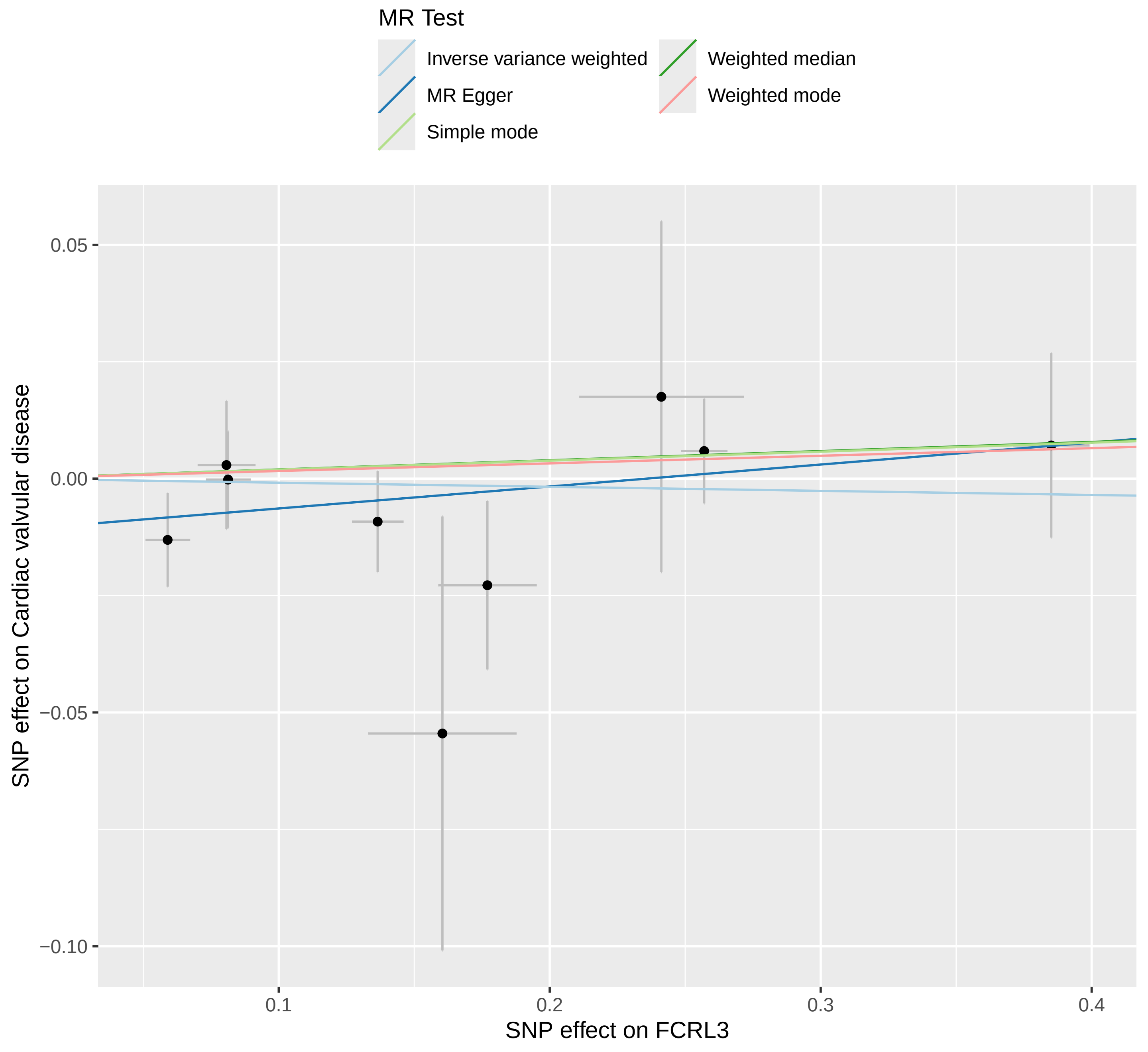
MR Test
Inverse variance weighted
MR Egger
Simple mode
Weighted median
Weighted mode
SNP effect on Cardiac valvular disease
0.05
0.00
−0.05
−0.10
0.1
0.2
0.3
0.4
SNP effect on FCRL3

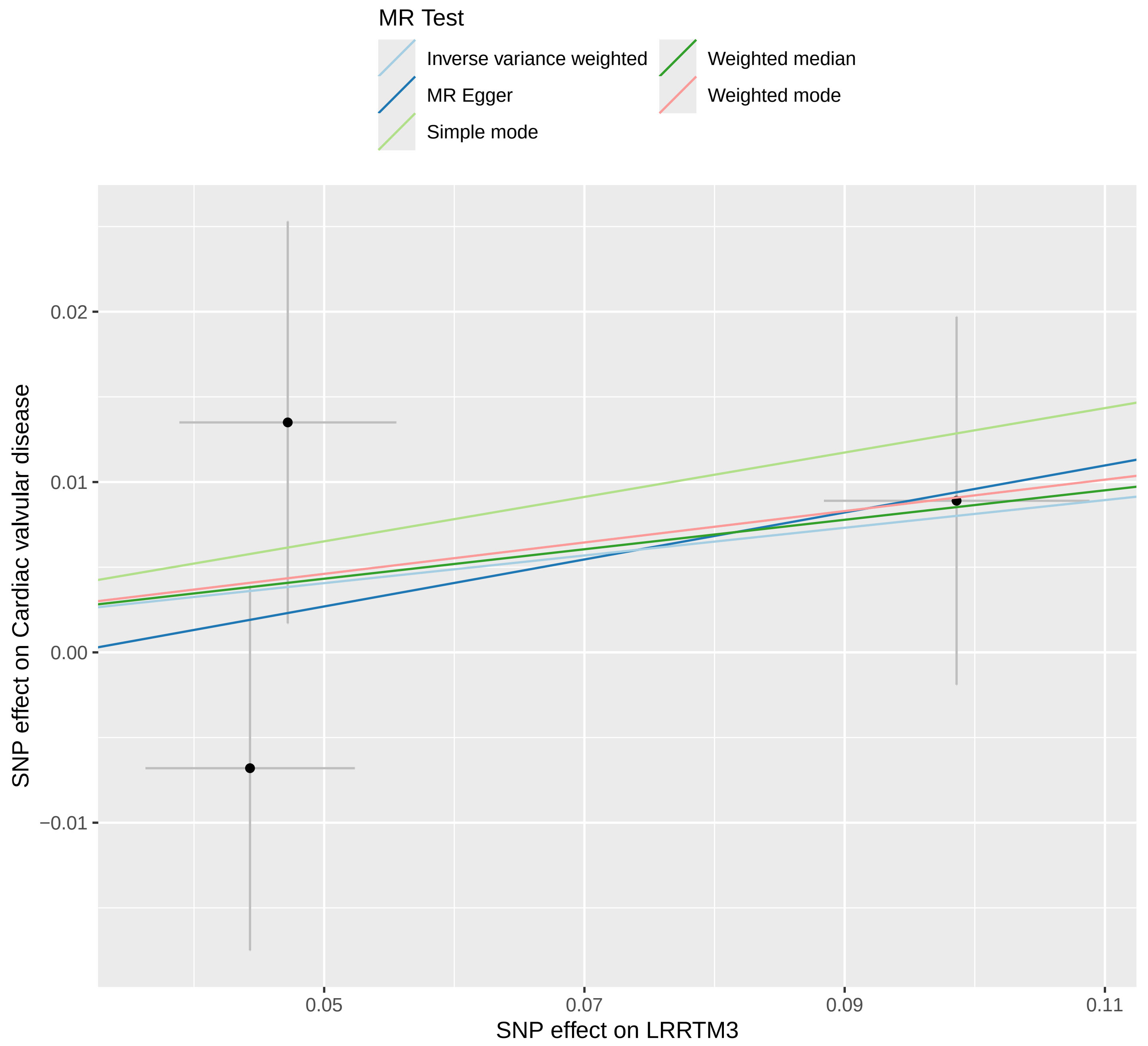

MR Test
Inverse variance weighted
MR Egger
Simple mode
Weighted median
Weighted mode
0.02
0.01
0.00
−0.01
0.05
0.07
0.09
0.11
SNP effect on Cardiac valvular disease
SNP effect on LRRTM3

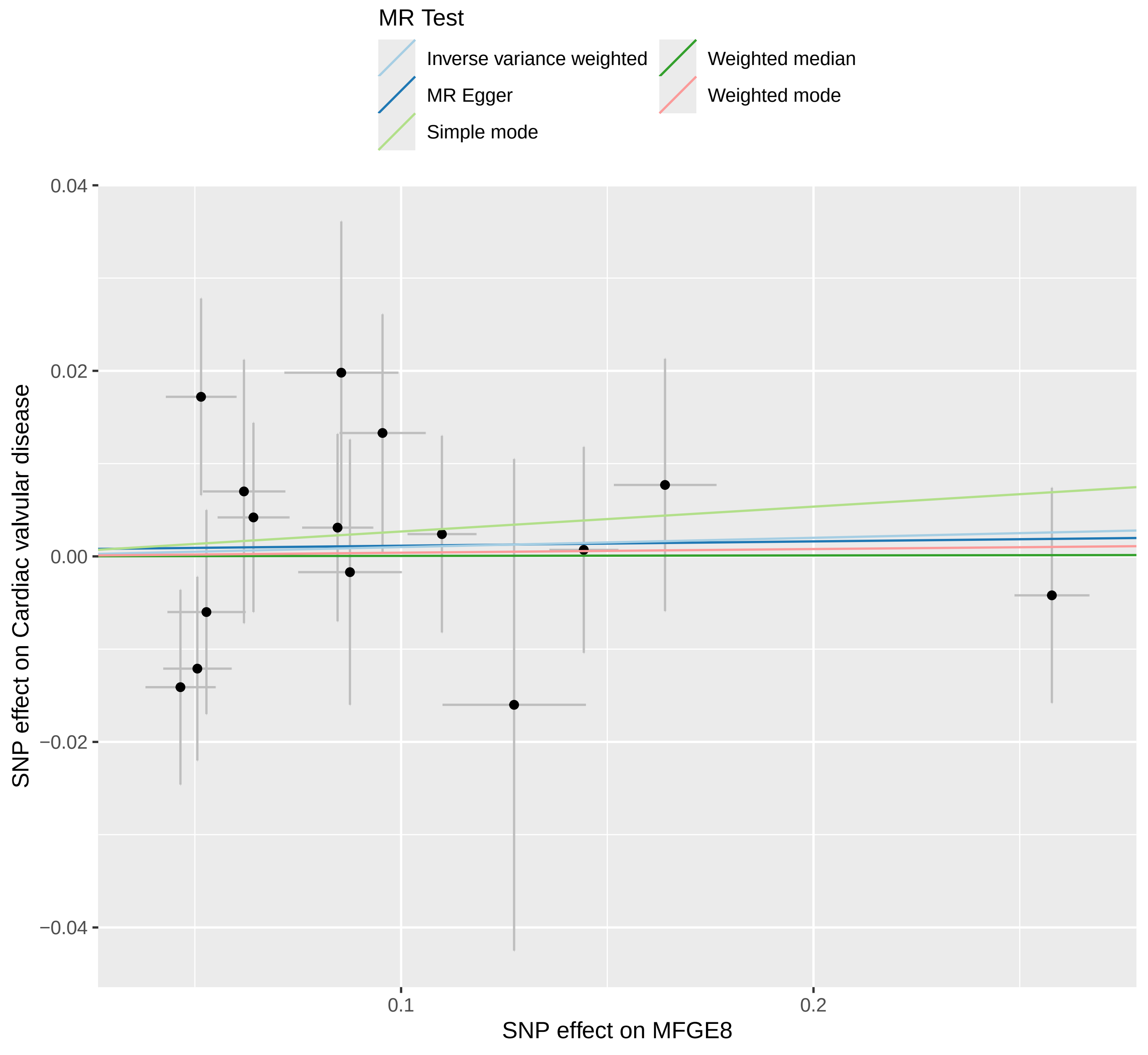
MR Test
Inverse variance weighted
MR Egger
Simple mode
Weighted median
Weighted mode
0.04
0.02
0.00
−0.02
−0.04
0.1
0.2
SNP effect on MFGE8
SNP effect on Cardiac valvular disease

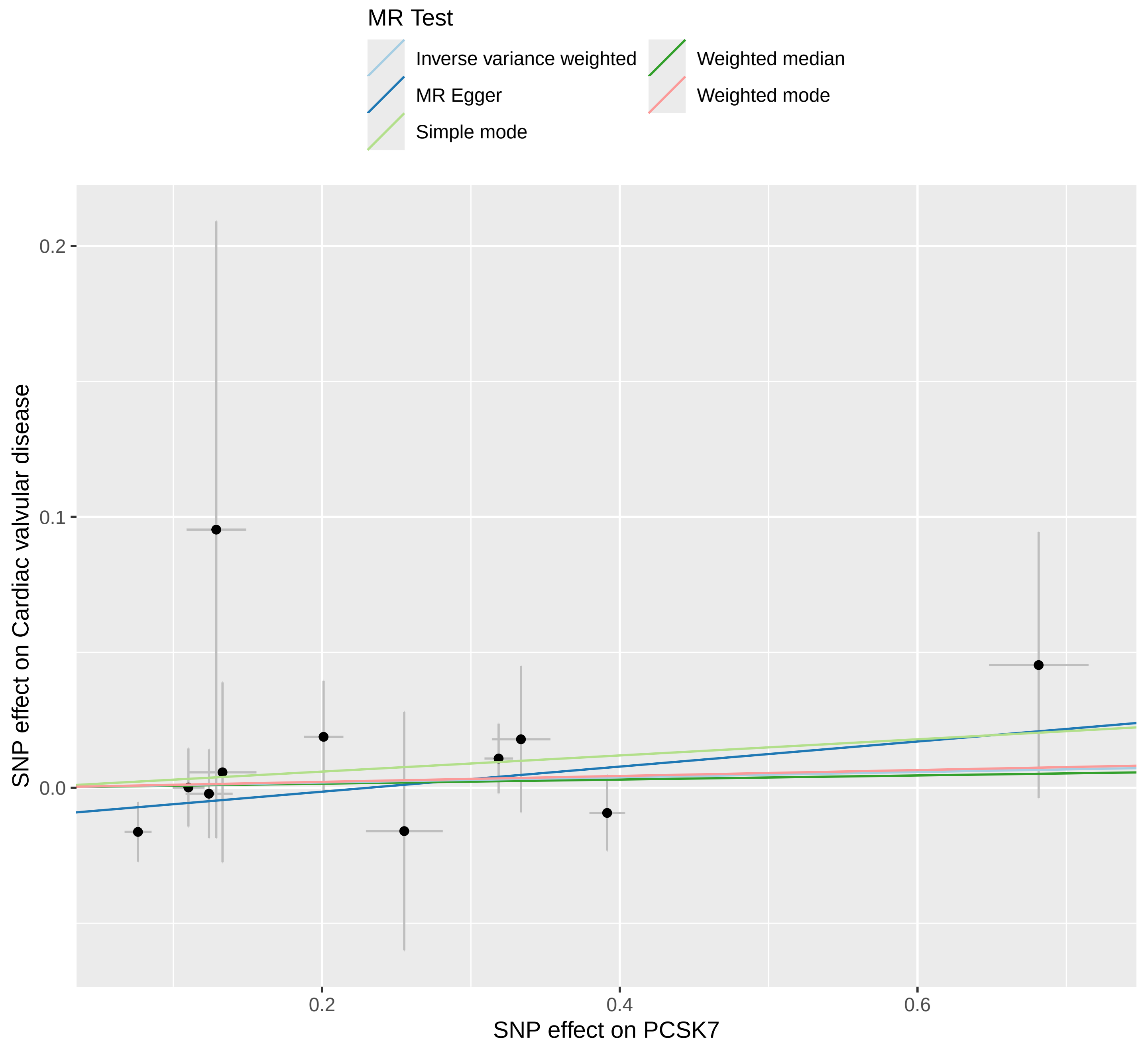

MR Test
Inverse variance weighted
MR Egger
Simple mode
Weighted median
Weighted mode
0.2
0.1
0.0
0.2
0.4
0.6
SNP effect on Cardiac valvular disease
SNP effect on PCSK7

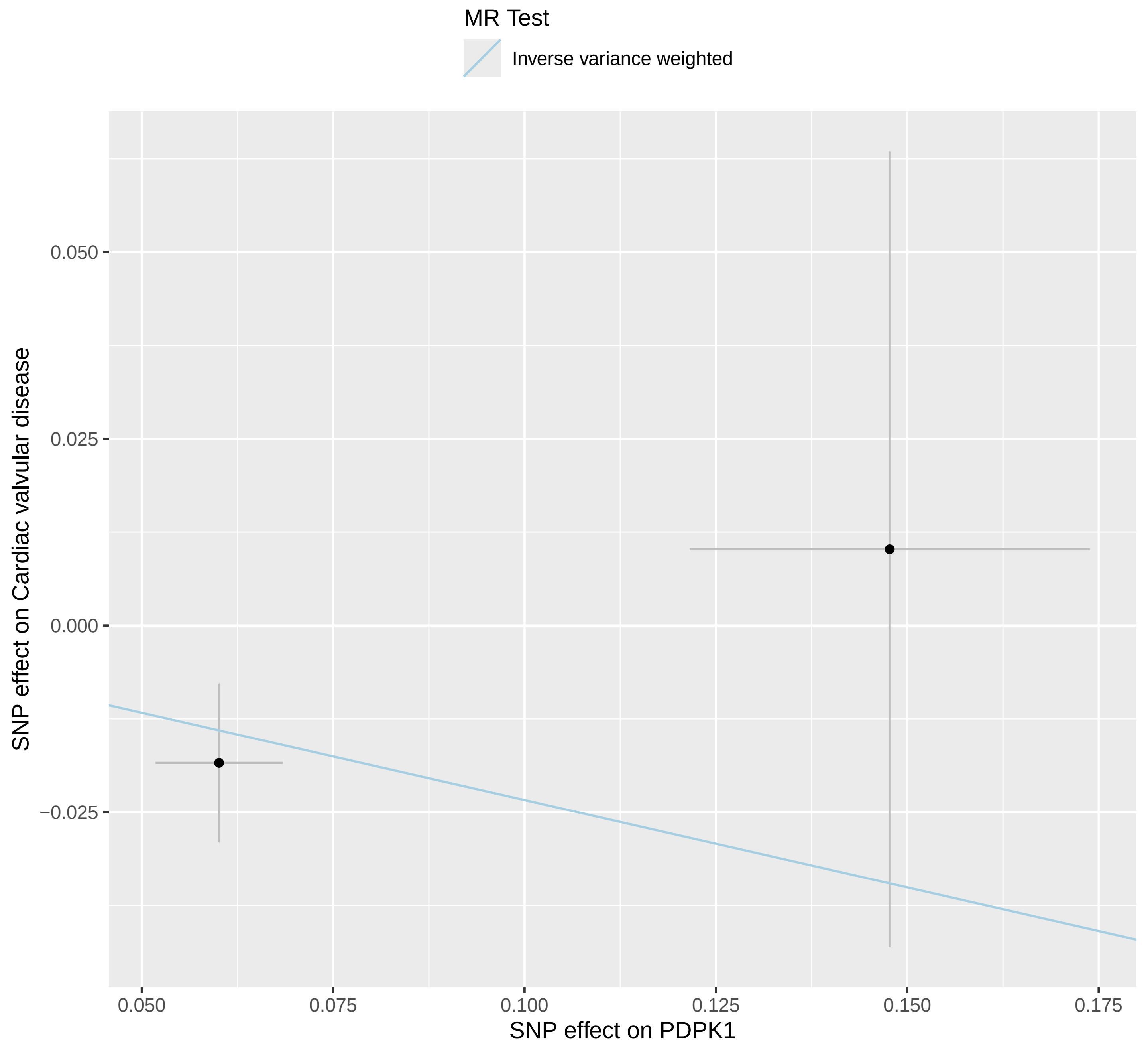

MR Test
Inverse variance weighted
SNP effect on Cardiac valvular disease
0.050
0.025
0.000
−0.025
0.050
0.075
0.100
0.125
0.150
0.175
SNP effect on PDPK1

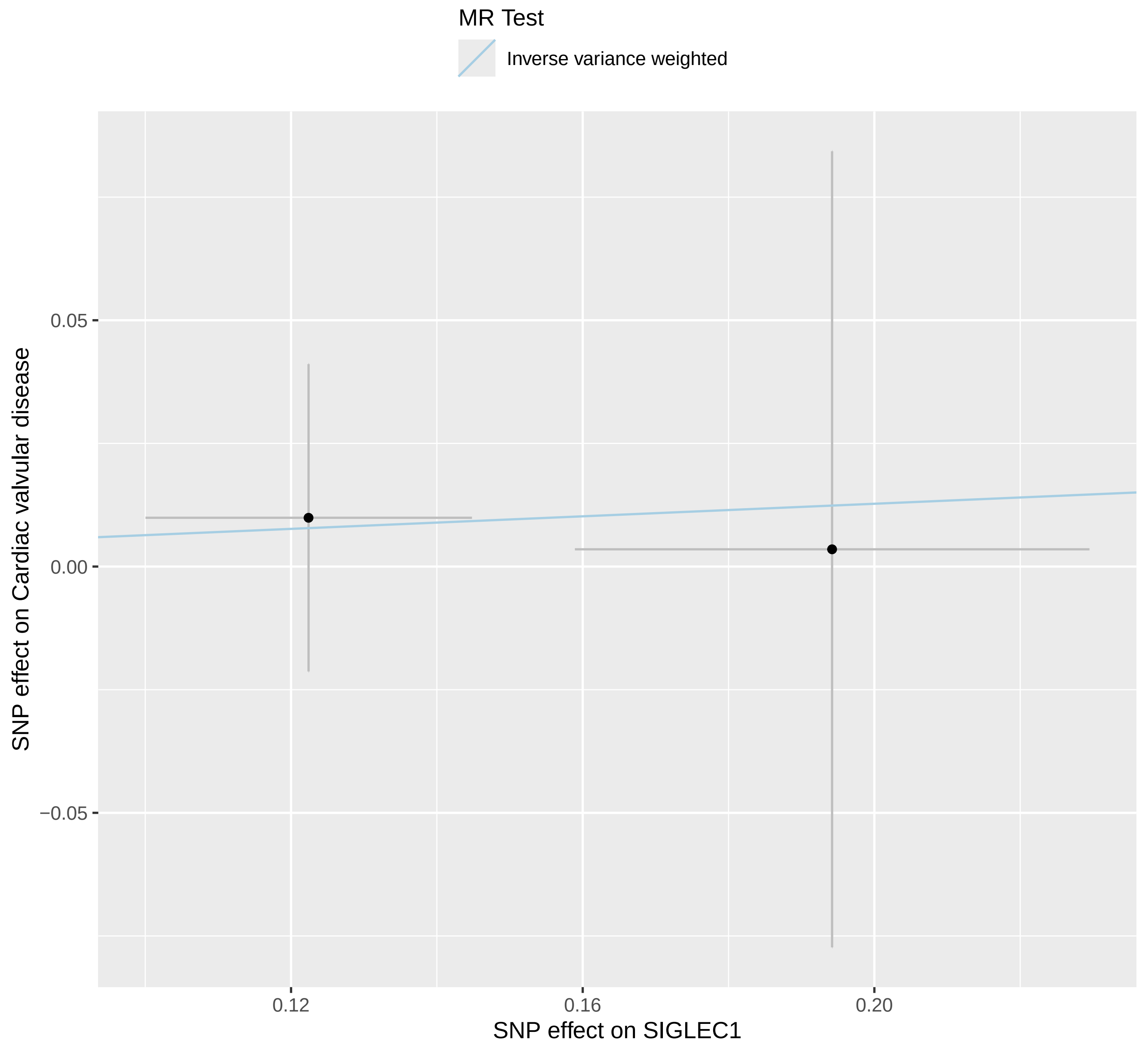

MR Test
Inverse variance weighted
SNP effect on Cardiac valvular disease
0.05
0.00
−0.05
0.12
0.16
0.20
SNP effect on SIGLEC1

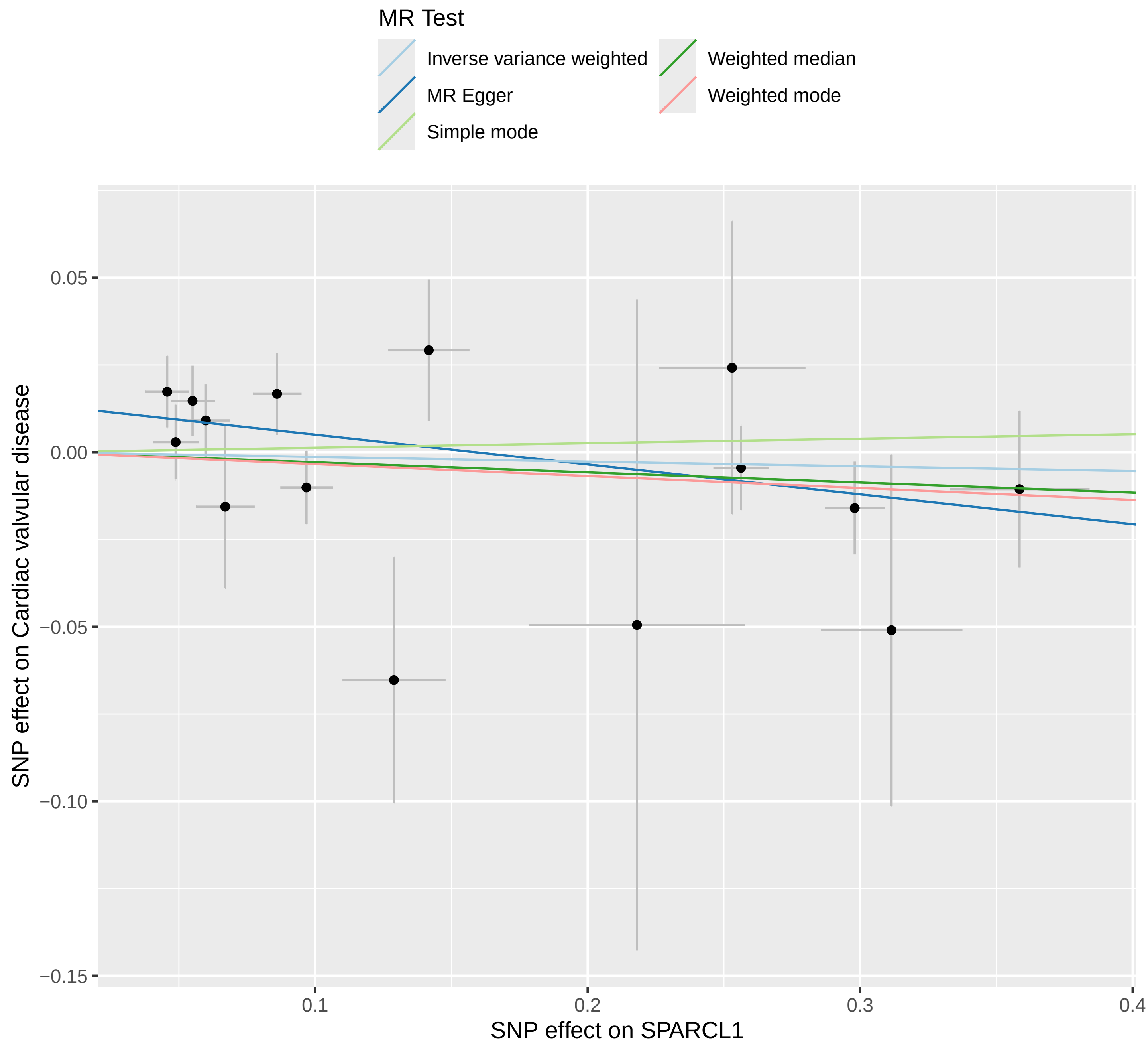

MR Test
Inverse variance weighted
MR Egger
Simple mode
Weighted median
Weighted mode
0.05
0.00
−0.05
−0.10
−0.15
0.1
0.2
0.3
0.4
SNP effect on Cardiac valvular disease
SNP effect on SPARCL1

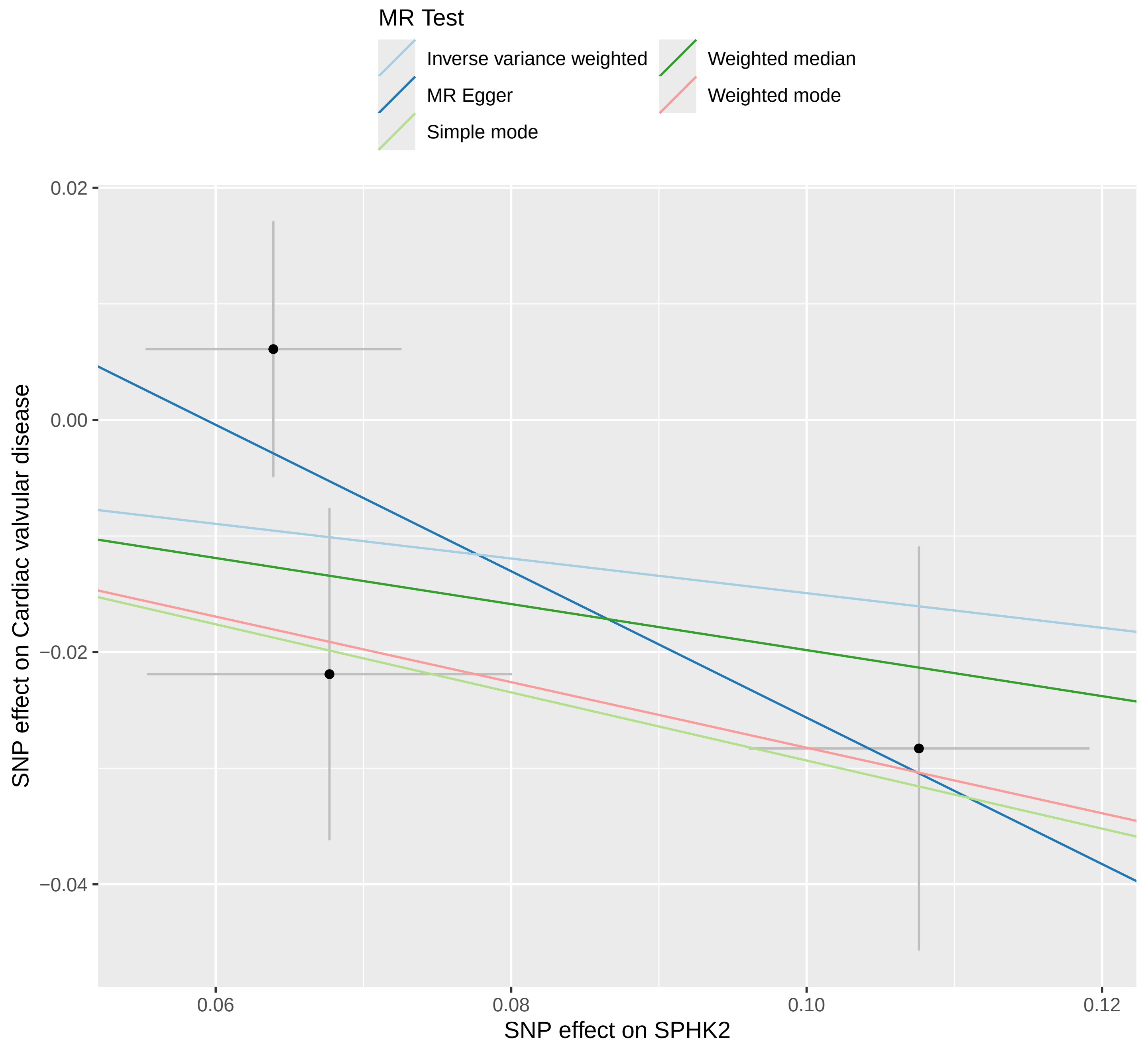

MR Test
Inverse variance weighted
MR Egger
Simple mode
Weighted median
Weighted mode
0.02
0.00
−0.02
−0.04
SNP effect on Cardiac valvular disease
0.06
0.08
0.10
0.12
SNP effect on SPHK2

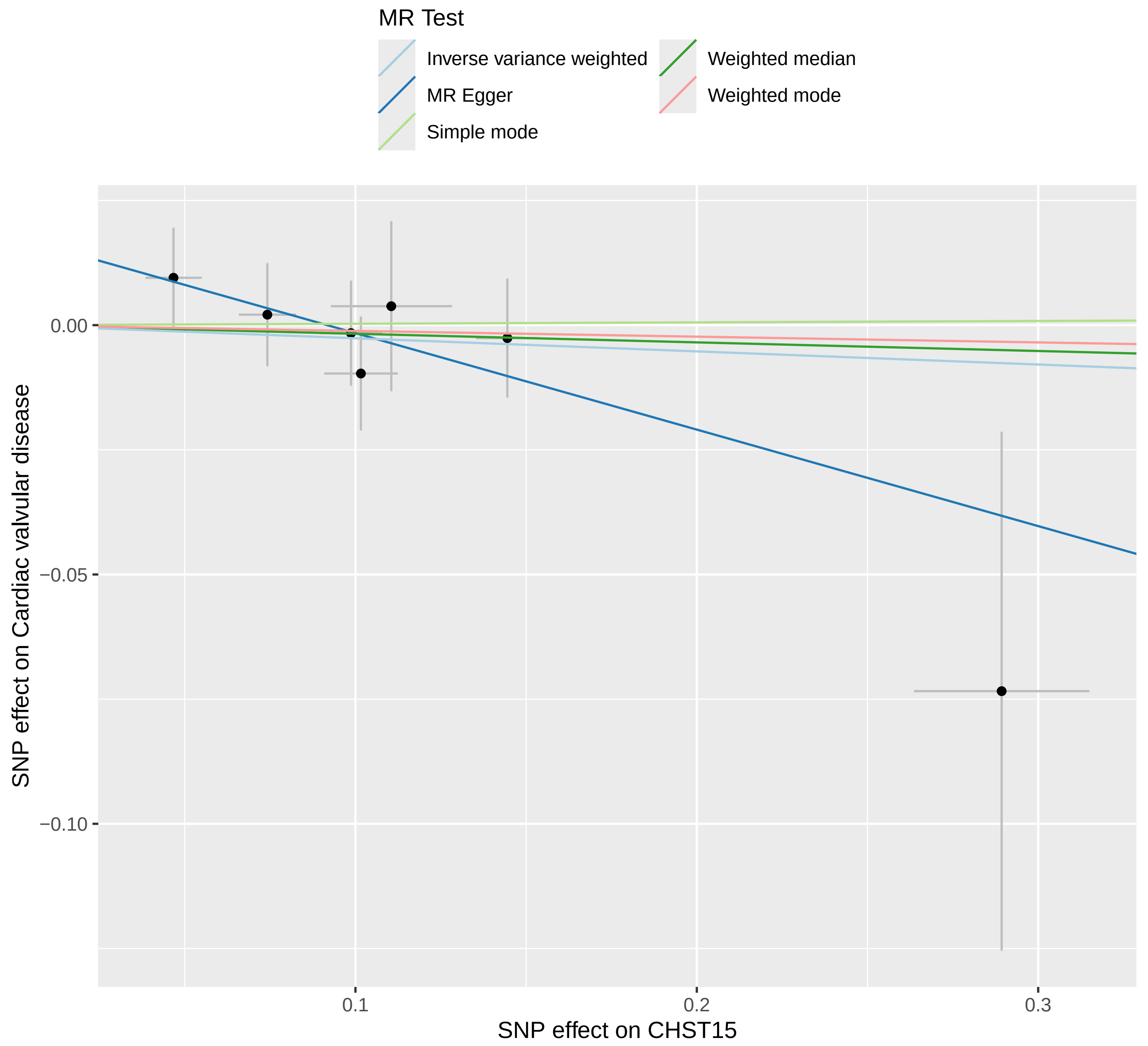
MR Test
Inverse variance weighted
MR Egger
Simple mode
Weighted median
Weighted mode
0.00
−0.05
−0.10
0.1
0.2
0.3
SNP effect on Cardiac valvular disease
SNP effect on CHST15

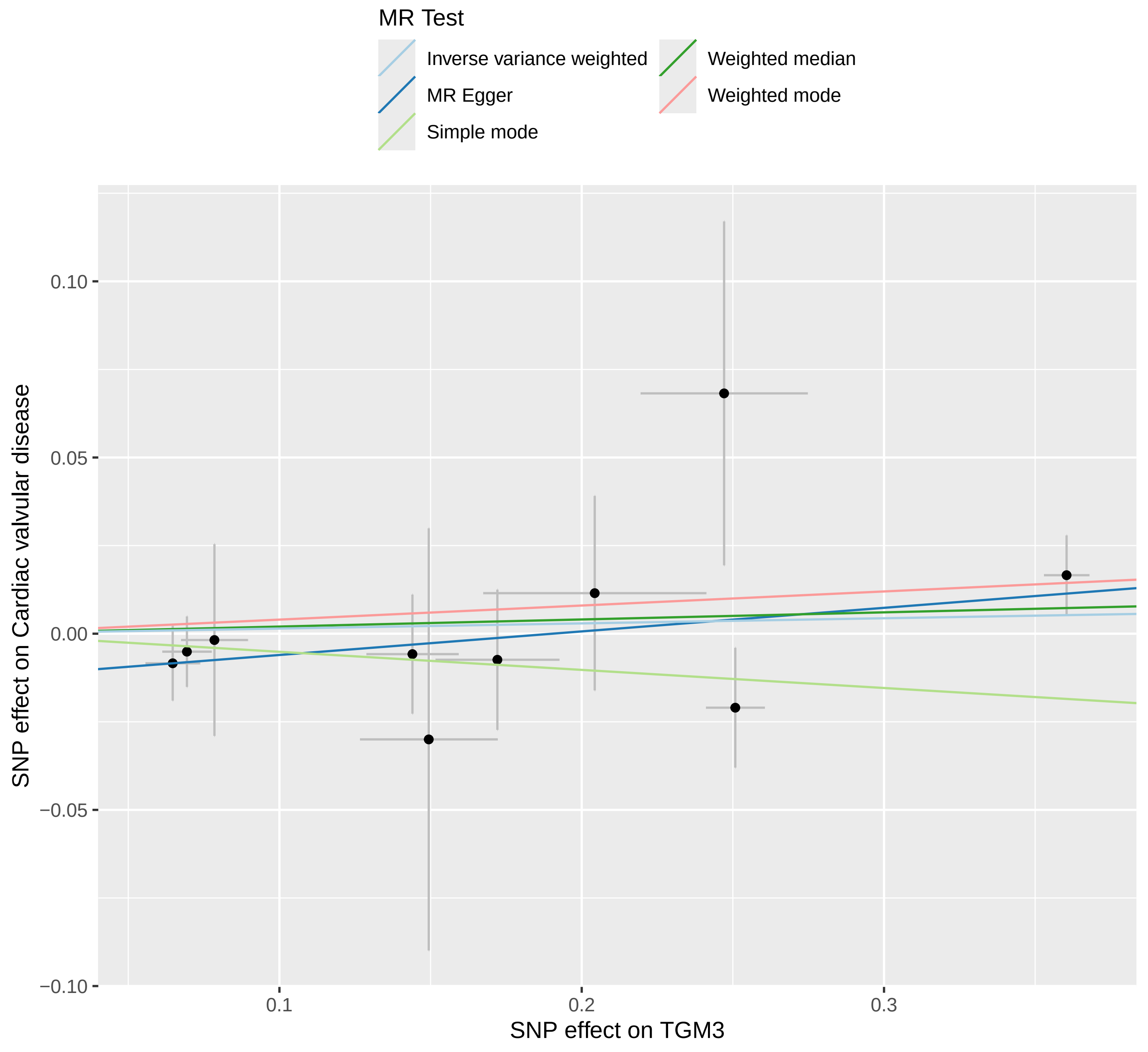

MR Test
Inverse variance weighted
MR Egger
Simple mode
Weighted median
Weighted mode
0.10
0.05
0.00
−0.05
−0.10
0.1
0.2
0.3
SNP effect on Cardiac valvular disease
SNP effect on TGM3

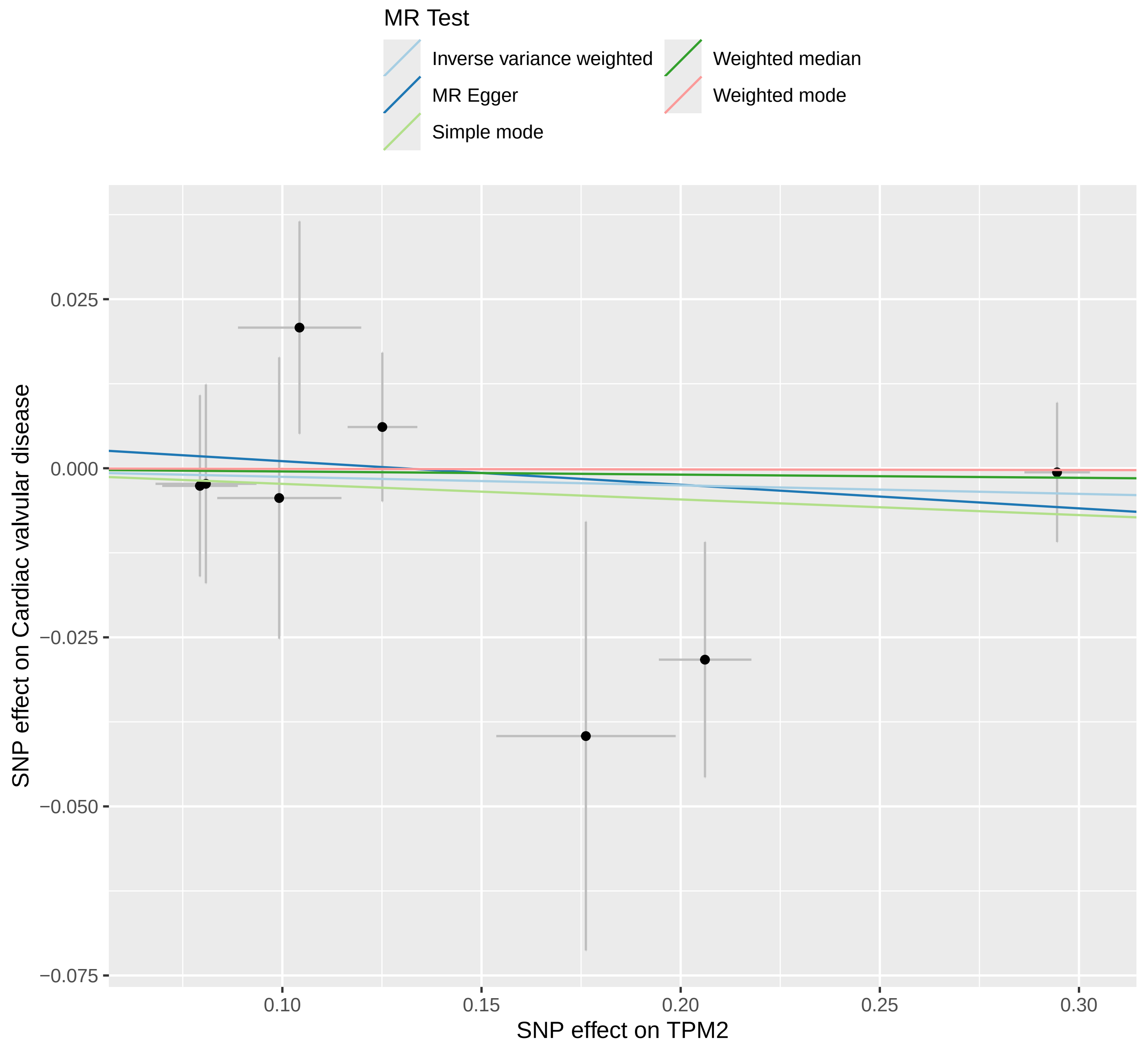
MR Test
Inverse variance weighted
MR Egger
Simple mode
Weighted median
Weighted mode
0.025
0.000
−0.025
−0.050
−0.075
SNP effect on Cardiac valvular disease
0.10
0.15
0.20
0.25
0.30
SNP effect on TPM2

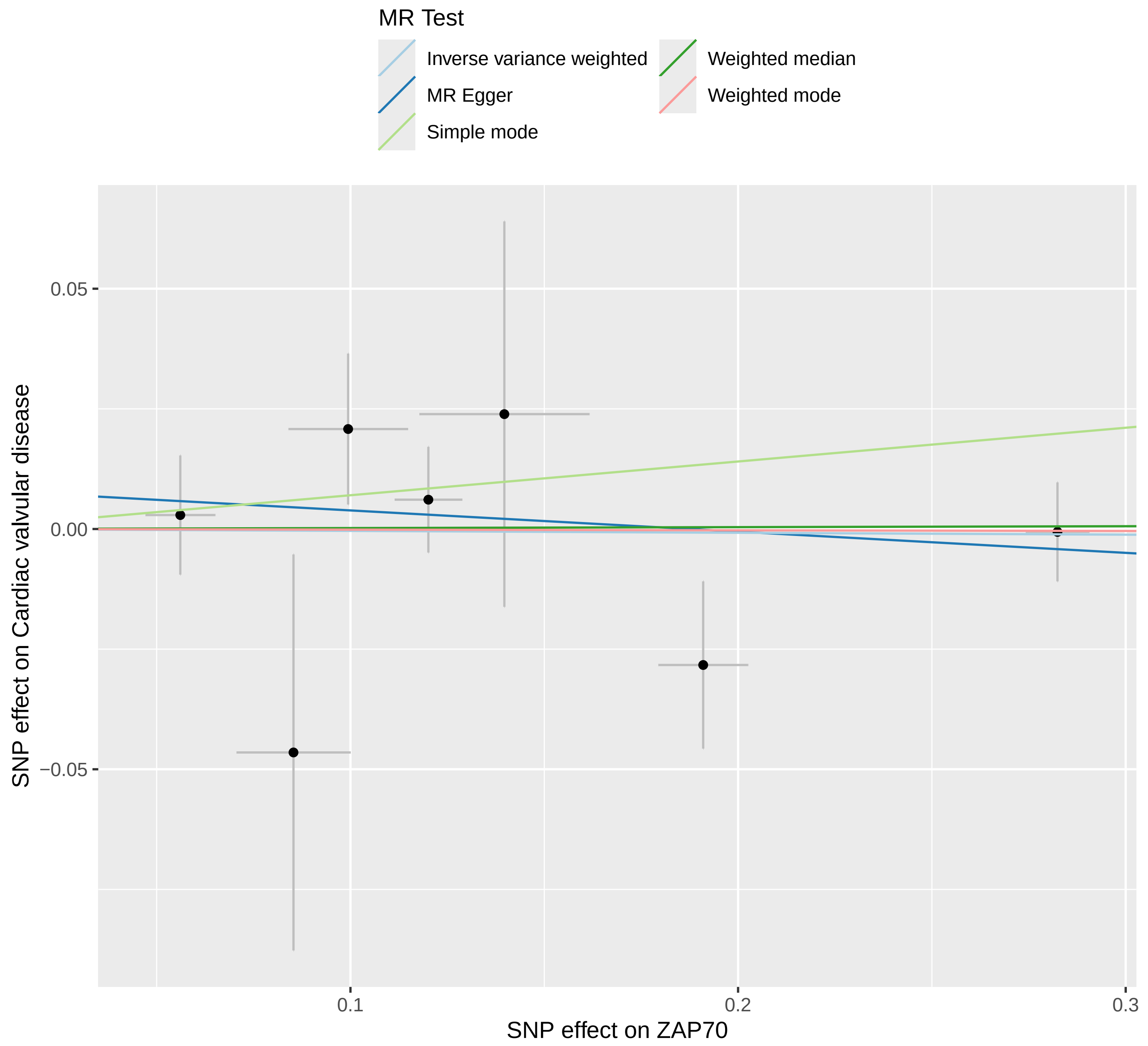
MR Test
Inverse variance weighted
MR Egger
Simple mode
Weighted median
Weighted mode
0.05
0.00
−0.05
SNP effect on Cardiac valvular disease
0.1
0.2
0.3
SNP effect on ZAP70

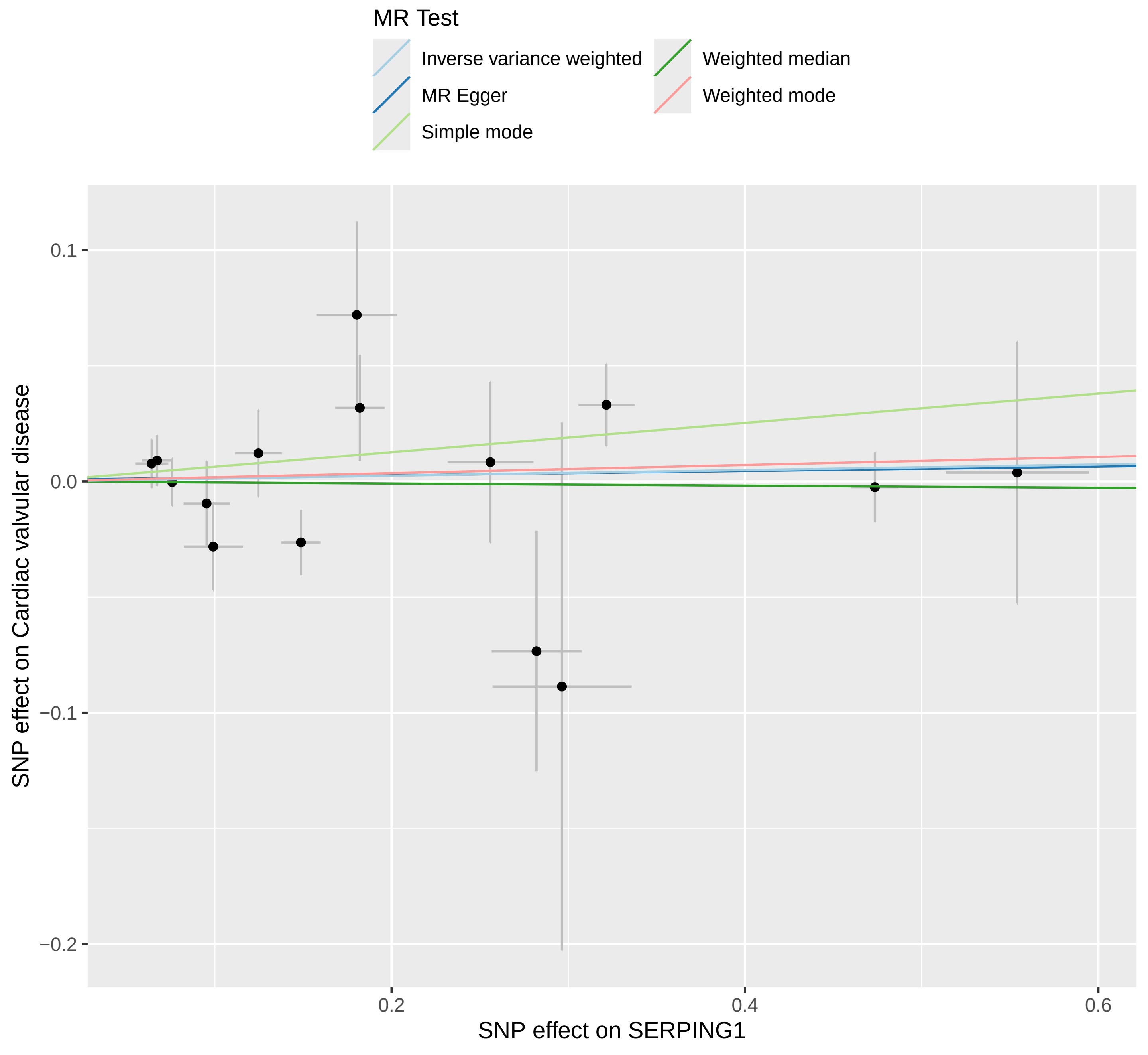
MR Test
Inverse variance weighted
MR Egger
Simple mode
Weighted median
Weighted mode
SNP effect on Cardiac valvular disease
0.1
0.0
−0.1
−0.2
0.2
0.4
0.6
SNP effect on SERPING1

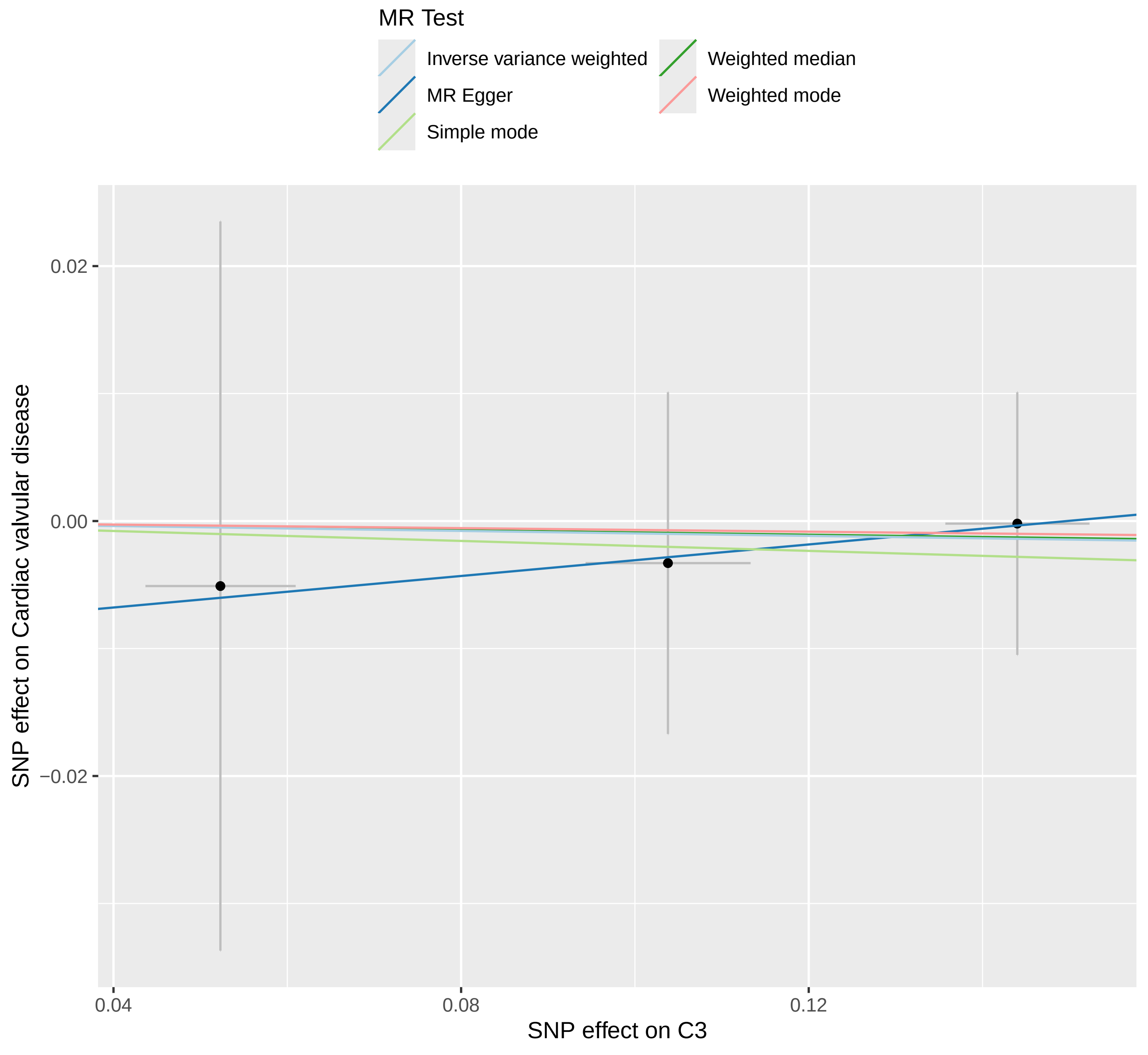

MR Test
Inverse variance weighted
MR Egger
Simple mode
Weighted median
Weighted mode
0.02
0.00
−0.02
SNP effect on Cardiac valvular disease
0.04
0.08
0.12
SNP effect on C3

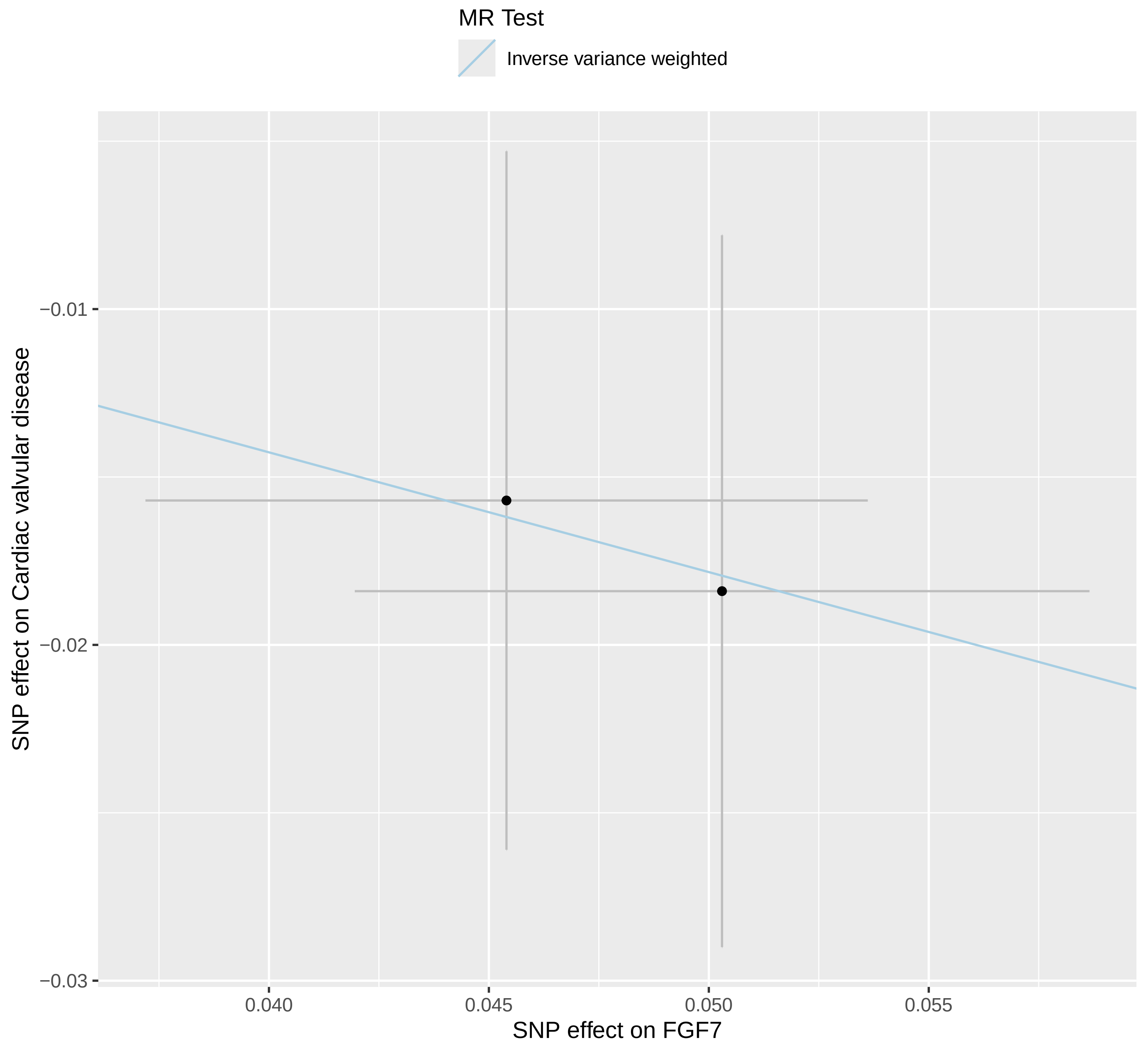
MR Test
Inverse variance weighted
−0.01
−0.02
−0.03
0.040
0.045
0.050
0.055
SNP effect on Cardiac valvular disease
SNP effect on FGF7

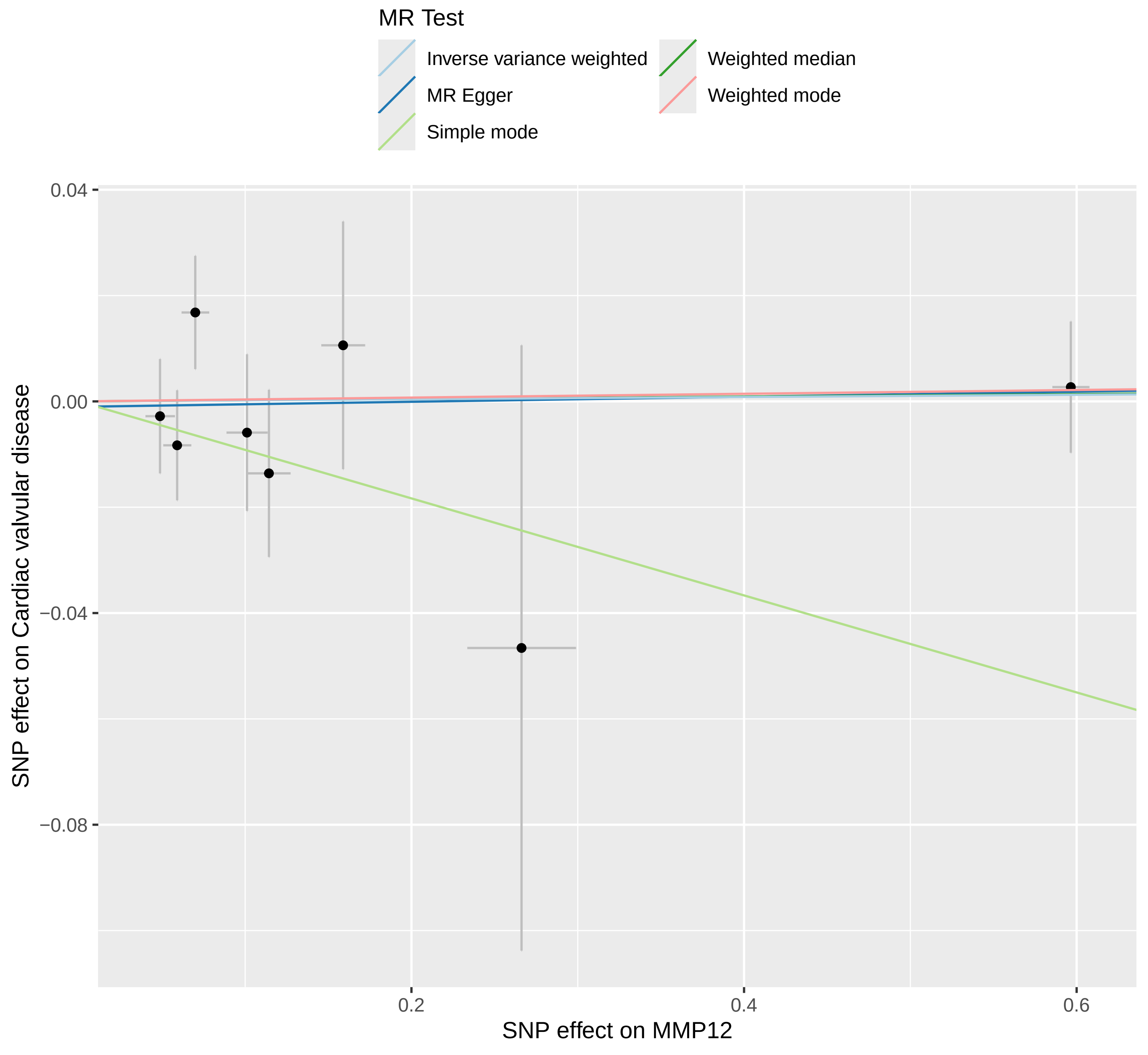

MR Test
Inverse variance weighted
MR Egger
Simple mode
Weighted median
Weighted mode
0.04
0.00
−0.04
−0.08
0.2
0.4
0.6
SNP effect on MMP12
SNP effect on Cardiac valvular disease

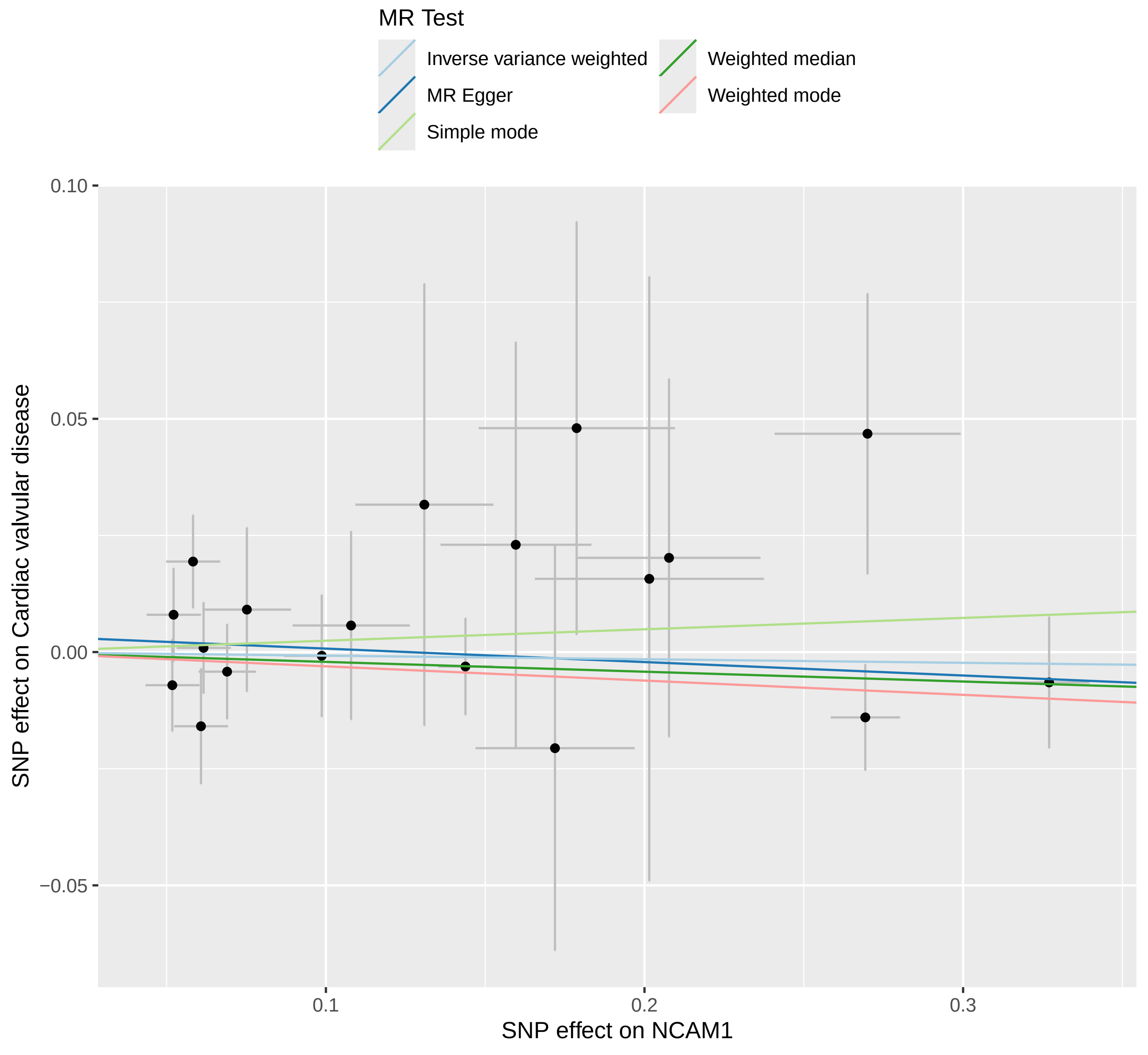

MR Test
Inverse variance weighted
MR Egger
Simple mode
Weighted median
Weighted mode
0.10
0.05
0.00
−0.05
0.1
0.2
0.3
SNP effect on NCAM1
SNP effect on Cardiac valvular disease

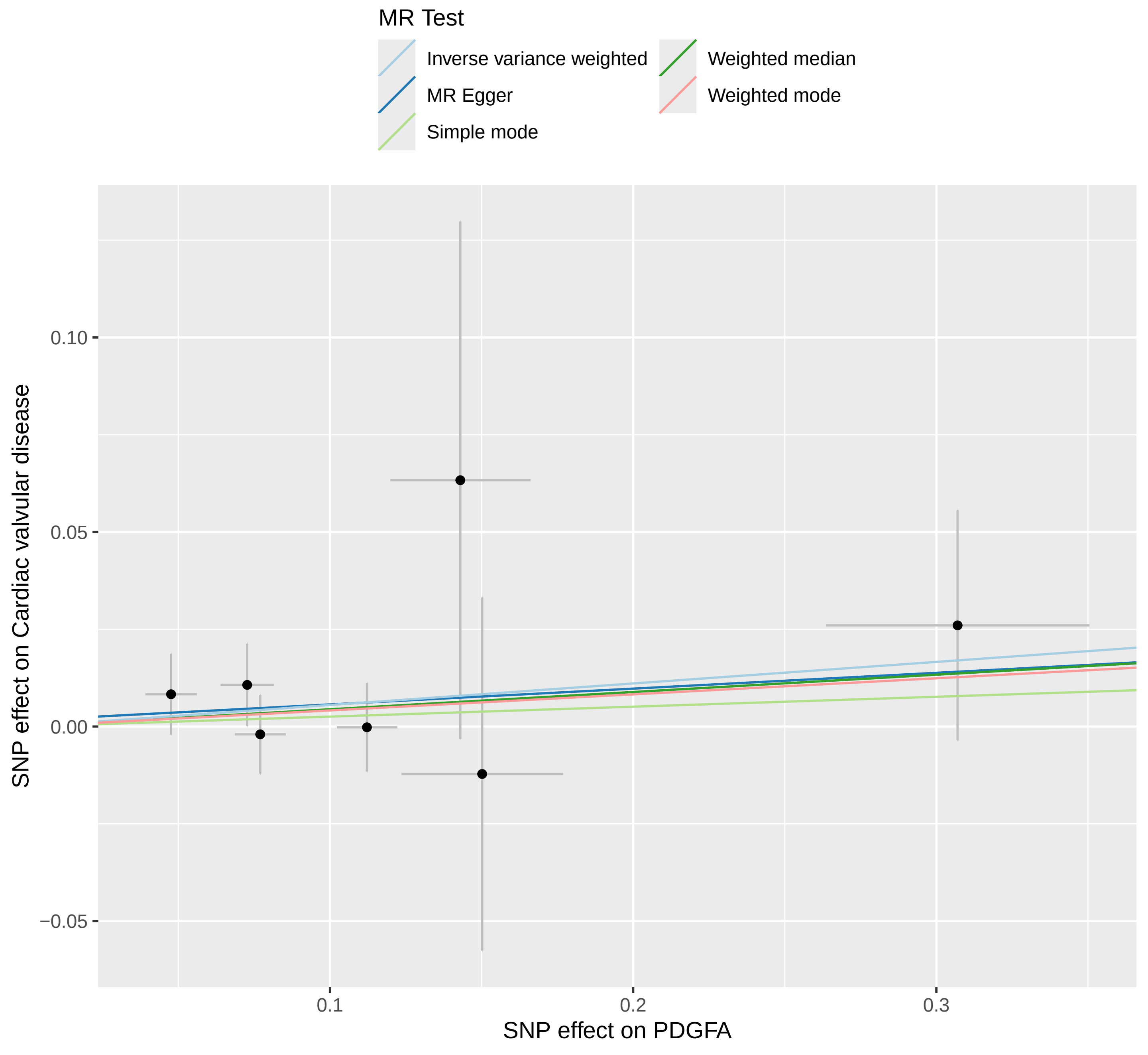
MR Test
Inverse variance weighted
MR Egger
Simple mode
Weighted median
Weighted mode
0.10
0.05
0.00
−0.05
0.1
0.2
0.3
SNP effect on Cardiac valvular disease
SNP effect on PDGFA

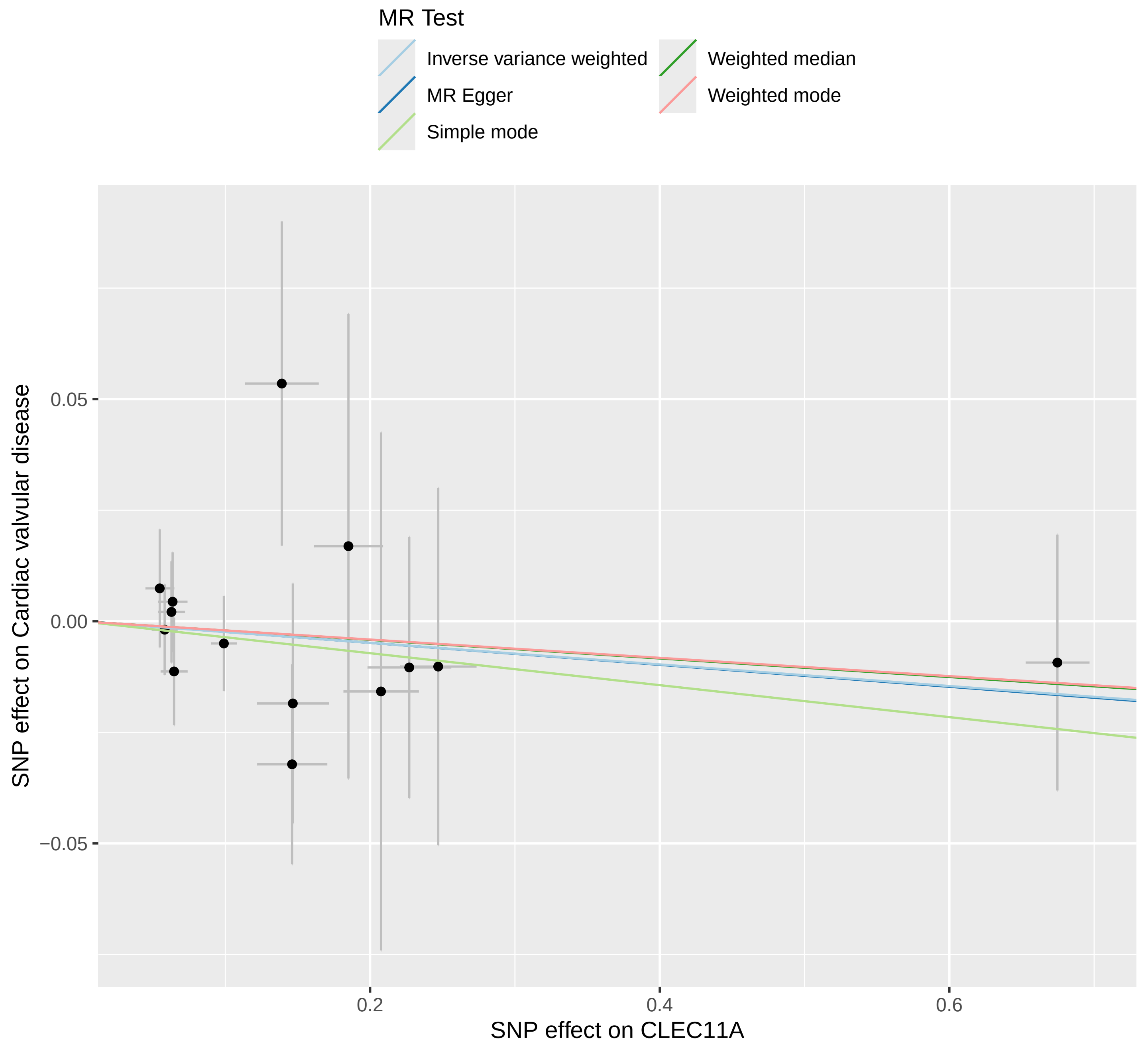

MR Test
Inverse variance weighted
MR Egger
Simple mode
Weighted median
Weighted mode
0.05
0.00
−0.05
SNP effect on Cardiac valvular disease
0.2
0.4
0.6
SNP effect on CLEC11A

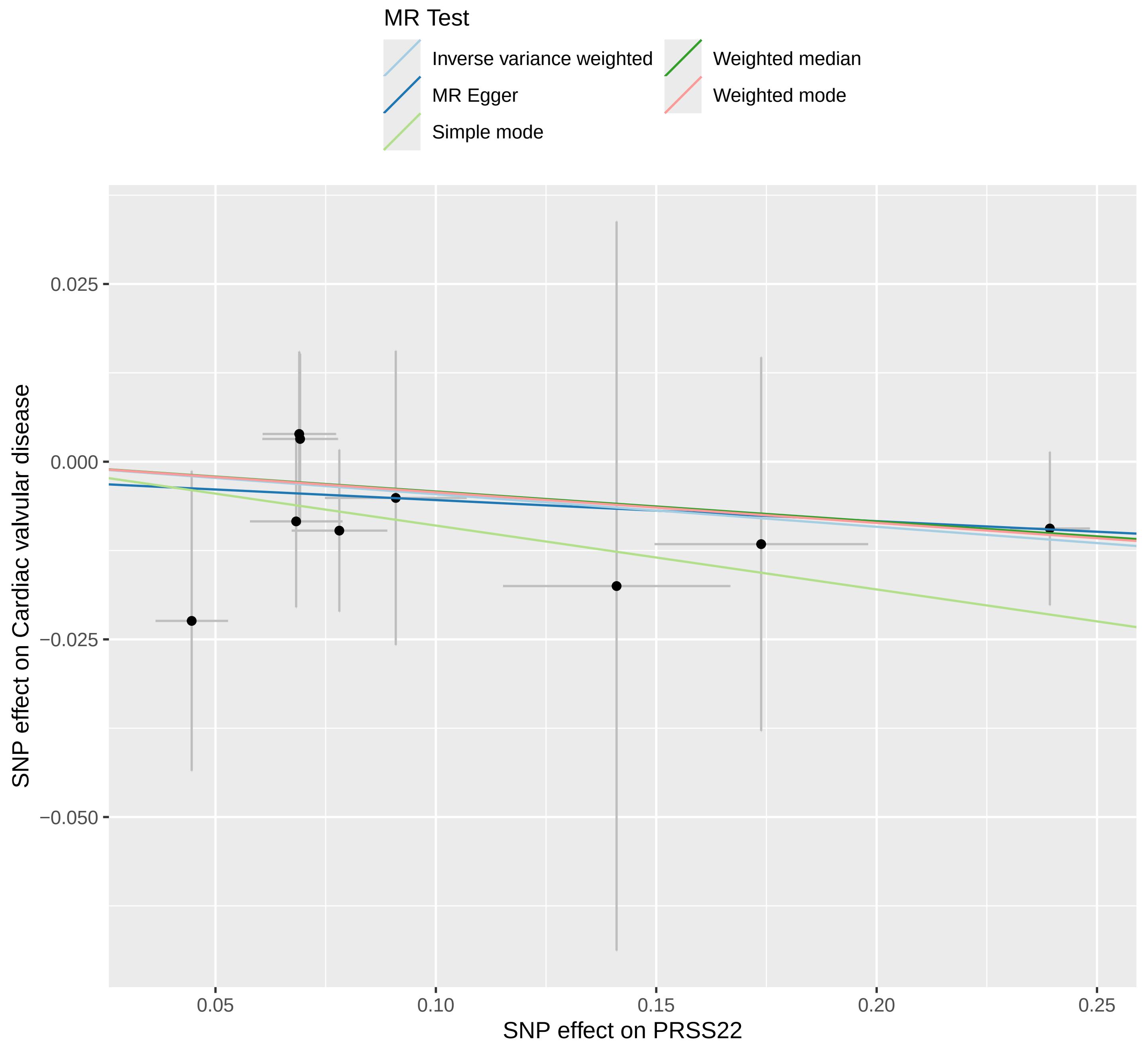
MR Test
Inverse variance weighted
MR Egger
Simple mode
Weighted median
Weighted mode
0.025
0.000
−0.025
−0.050
SNP effect on Cardiac valvular disease
0.05
0.10
0.15
0.20
0.25
SNP effect on PRSS22

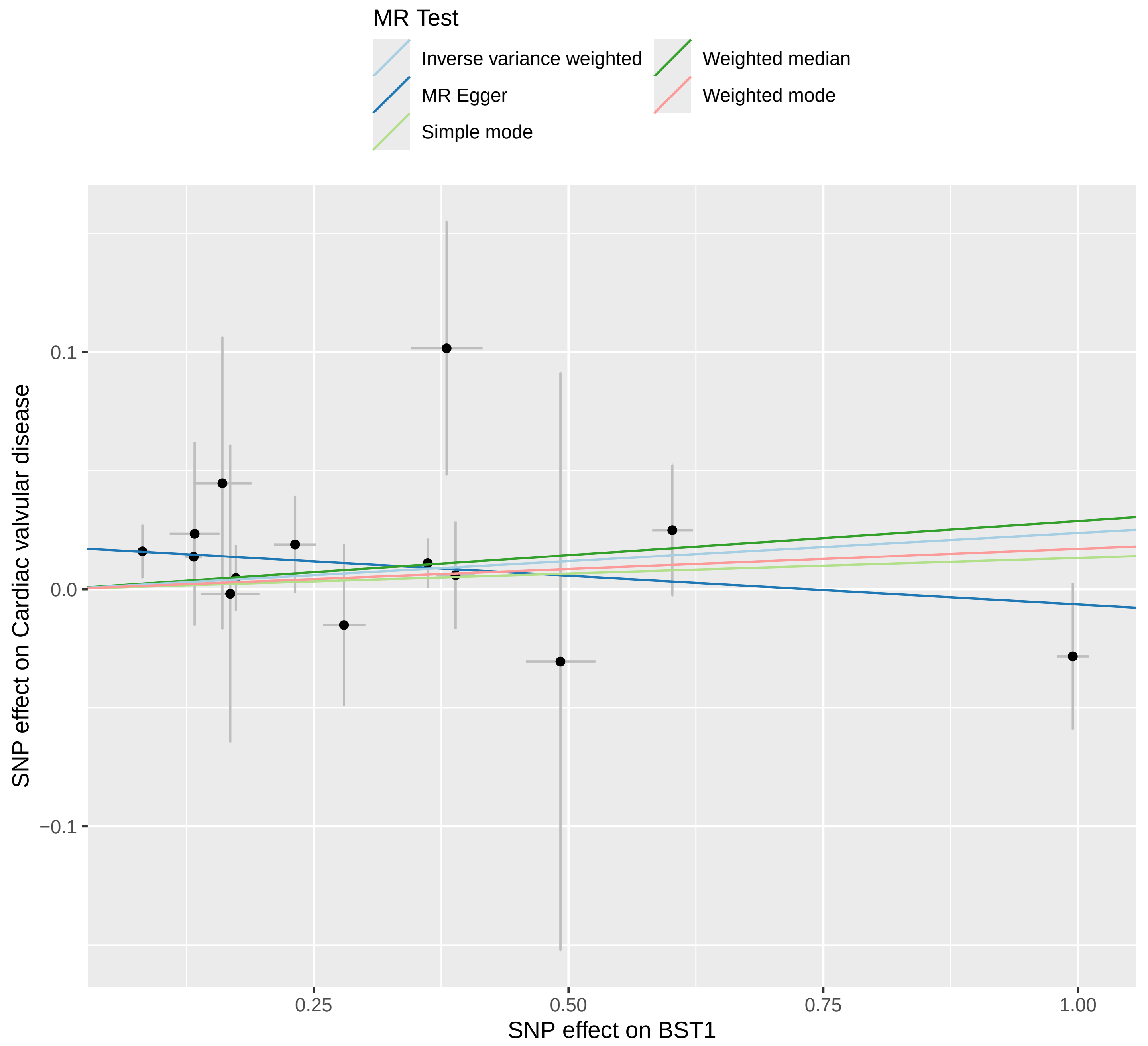

MR Test
Inverse variance weighted
MR Egger
Simple mode
Weighted median
Weighted mode
SNP effect on Cardiac valvular disease
0.1
0.0
−0.1
0.25
0.50
0.75
1.00
SNP effect on BST1

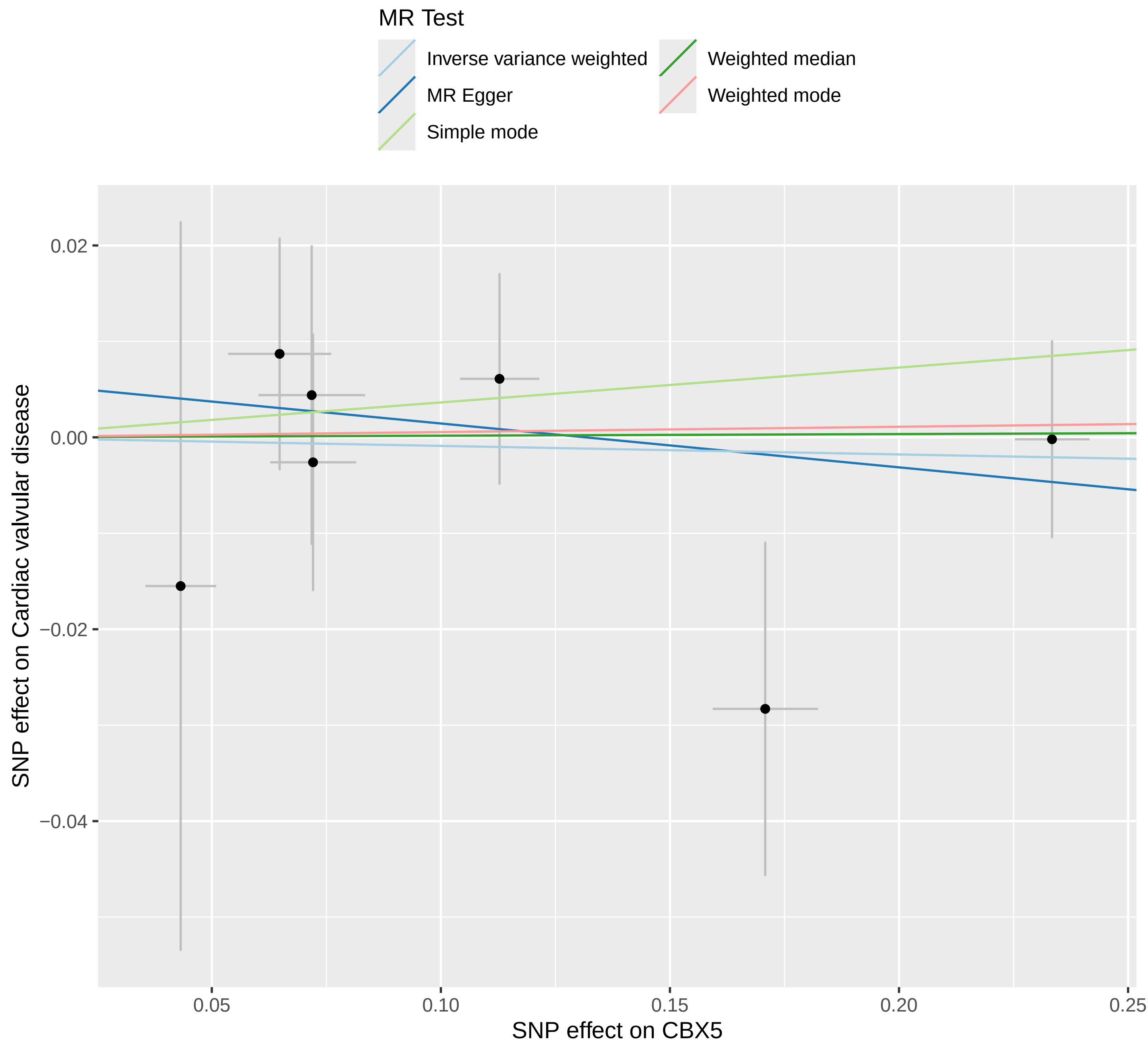
MR Test
Inverse variance weighted
MR Egger
Simple mode
Weighted median
Weighted mode
SNP effect on Cardiac valvular disease
0.02
0.00
−0.02
−0.04
0.05
0.10
0.15
0.20
0.25
SNP effect on CBX5

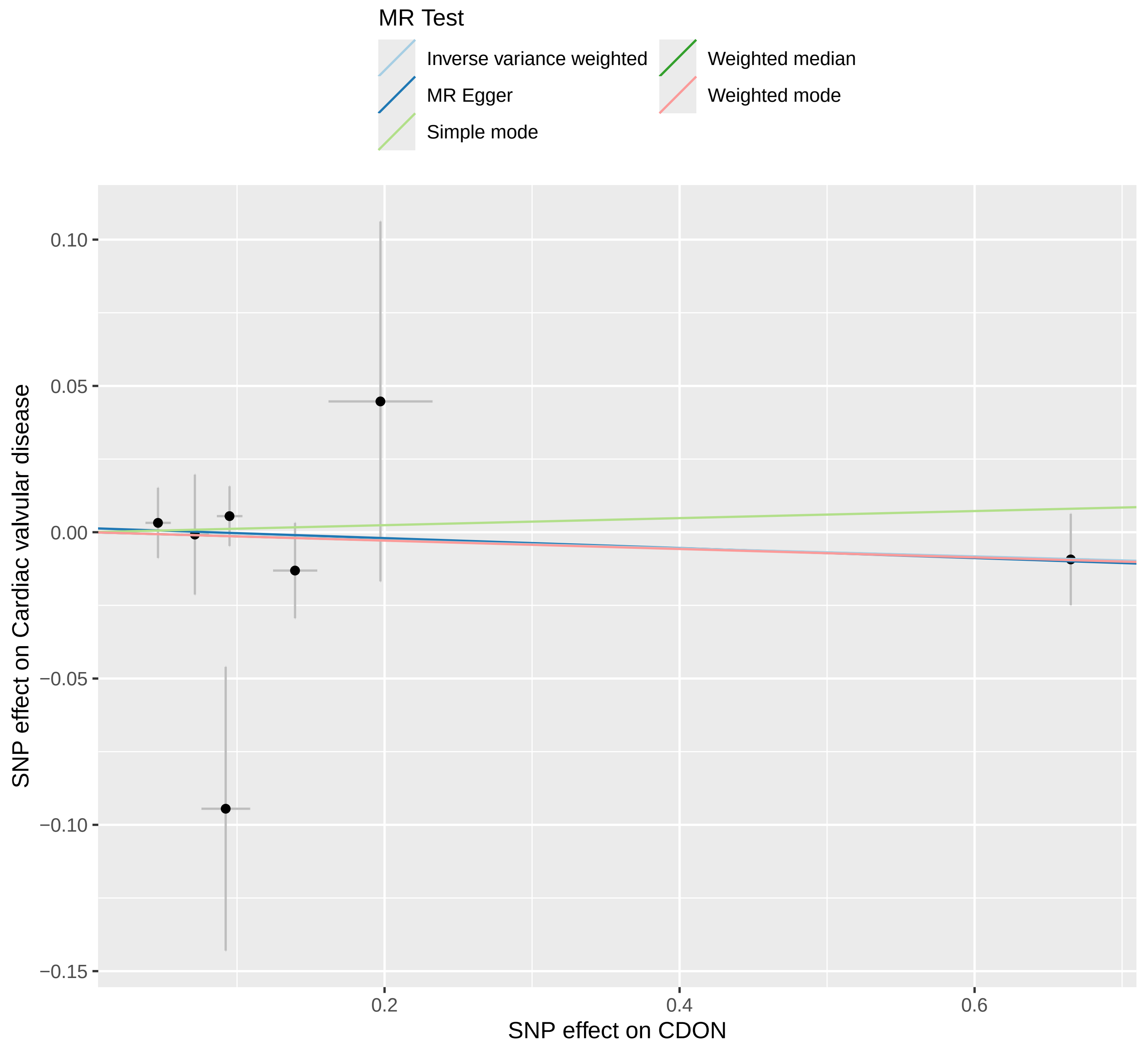
MR Test
Inverse variance weighted
MR Egger
Simple mode
Weighted median
Weighted mode
0.10
0.05
0.00
−0.05
−0.10
−0.15
SNP effect on Cardiac valvular disease
0.2
0.4
0.6
SNP effect on CDON

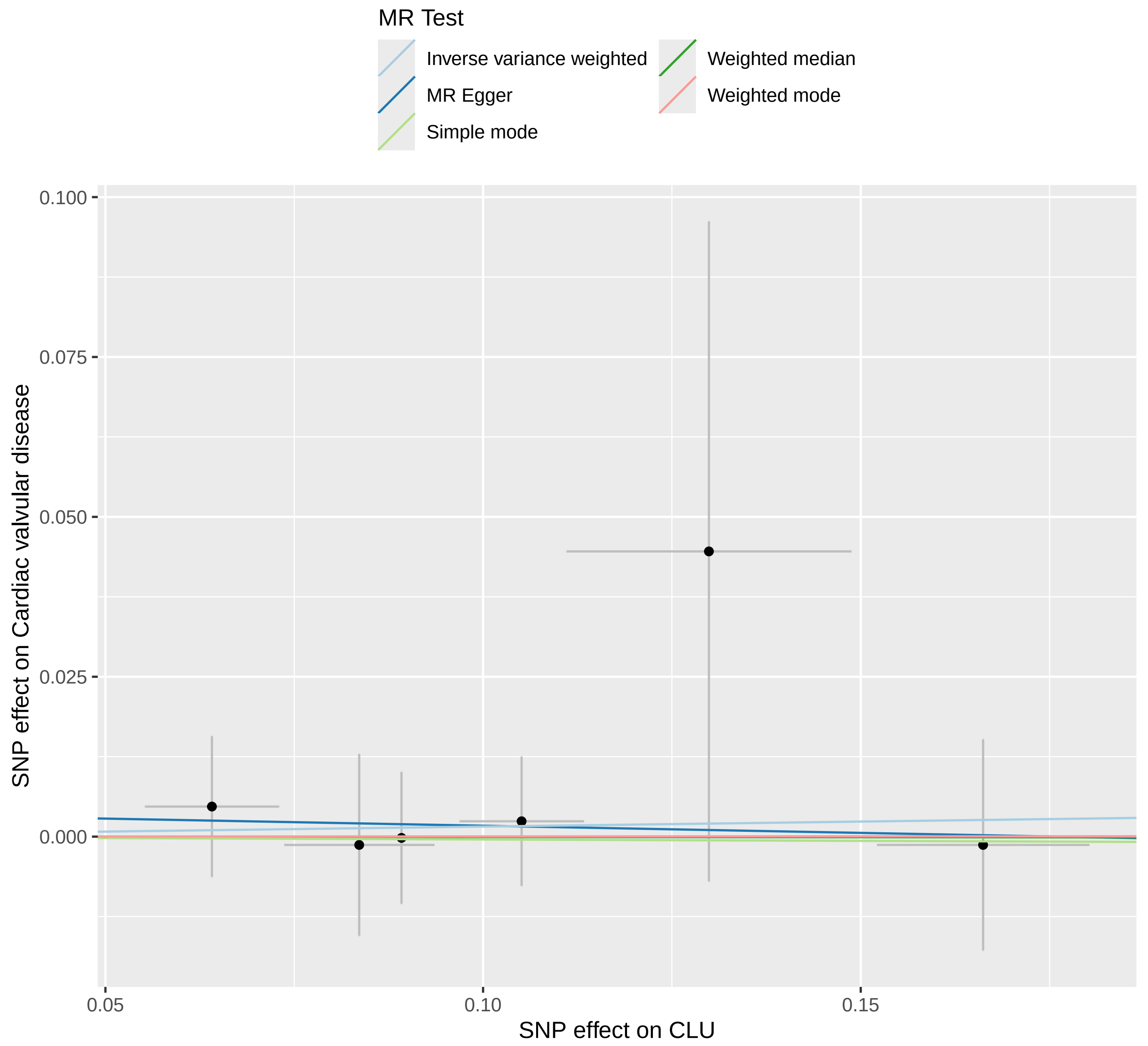

MR Test
Inverse variance weighted
MR Egger
Simple mode
Weighted median
Weighted mode
0.100
0.075
0.050
0.025
0.000
SNP effect on Cardiac valvular disease
0.05
0.10
0.15
SNP effect on CLU

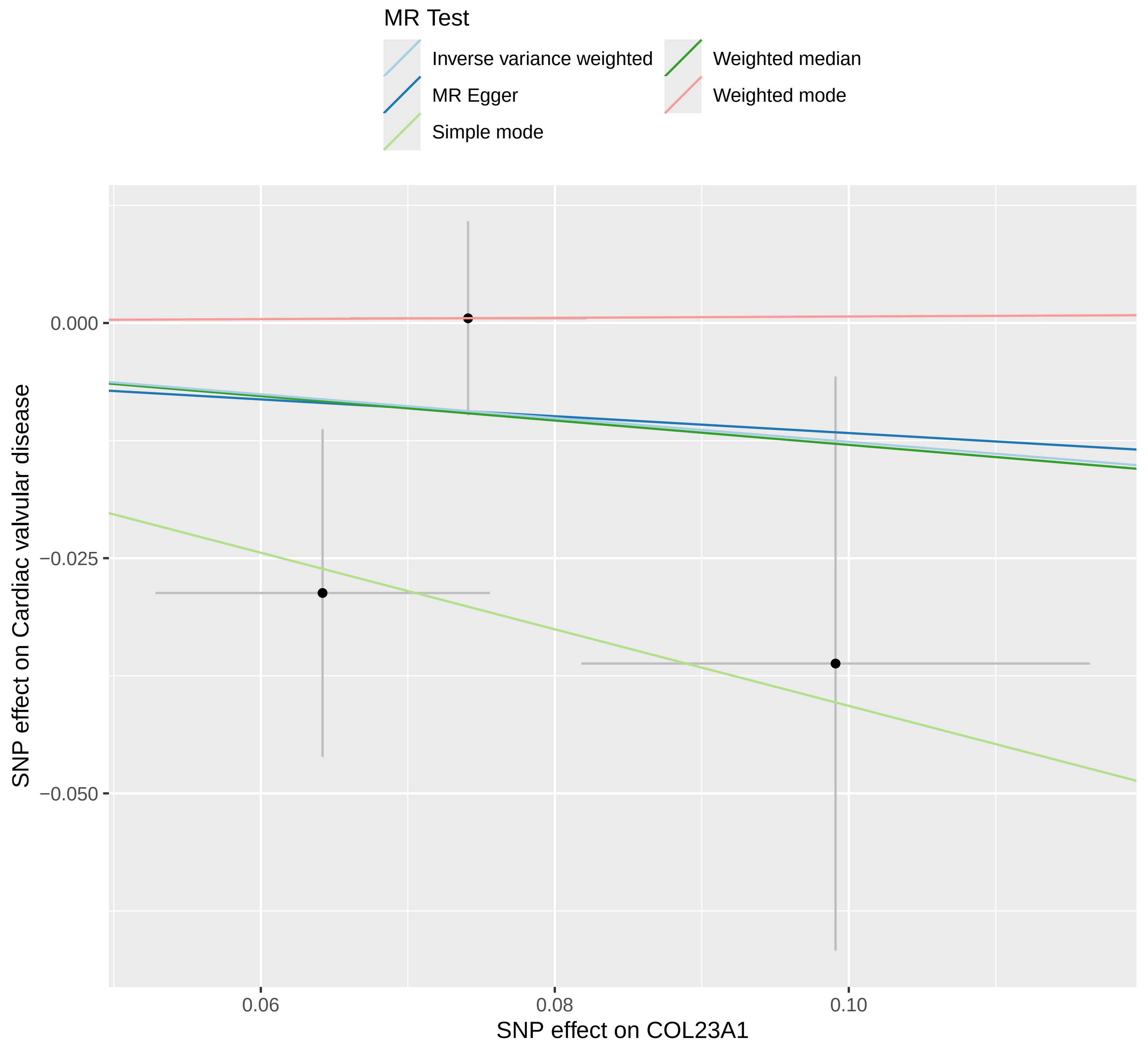

MR Test
Inverse variance weighted
MR Egger
Simple mode
Weighted median
Weighted mode
0.000
−0.025
−0.050
0.06
0.08
0.10
SNP effect on Cardiac valvular disease
SNP effect on COL23A1

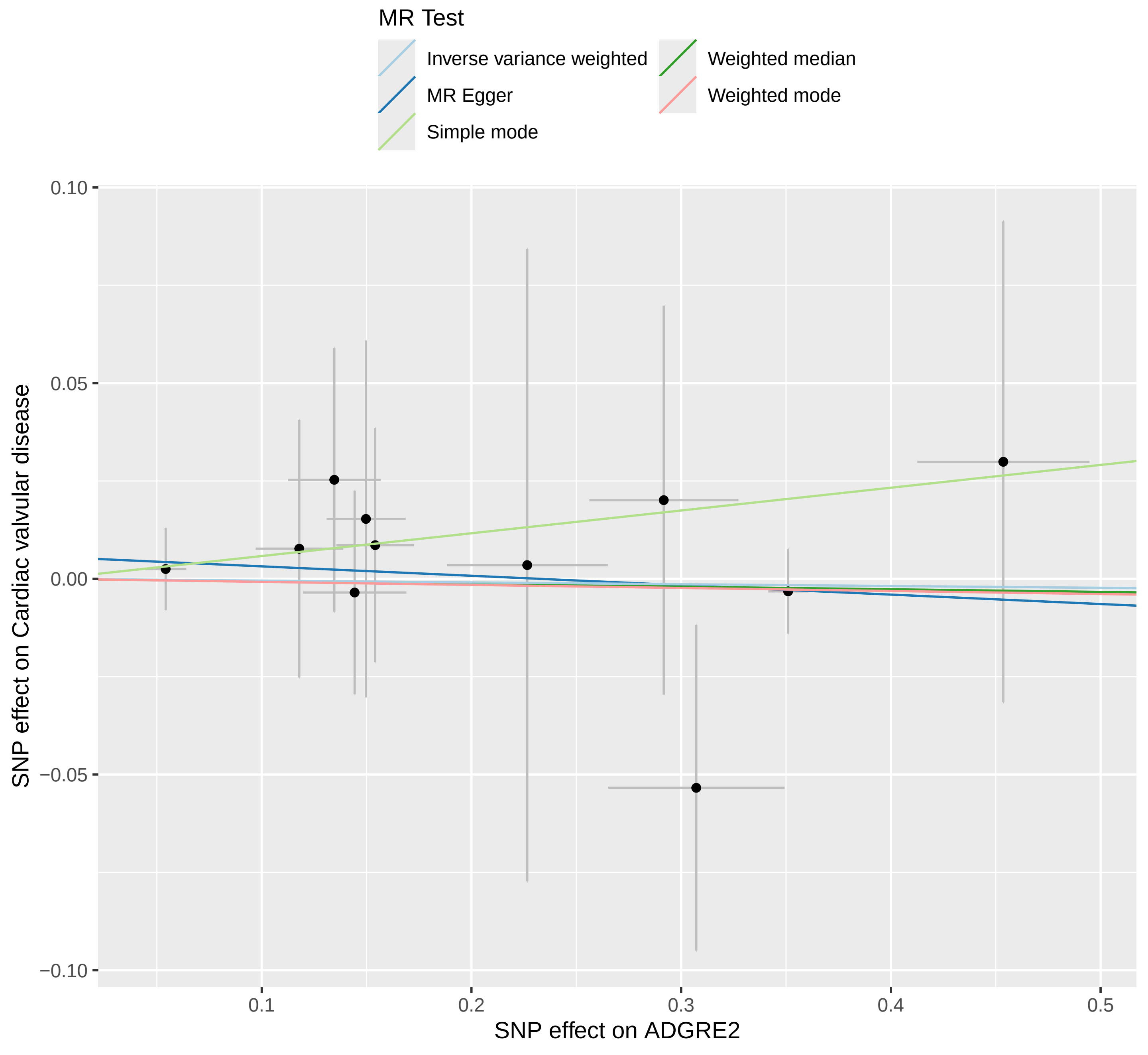
MR Test
Inverse variance weighted
MR Egger
Simple mode
Weighted median
Weighted mode
0.10
0.05
0.00
−0.05
−0.10
0.1
0.2
0.3
0.4
0.5
SNP effect on ADGRE2
SNP effect on Cardiac valvular disease

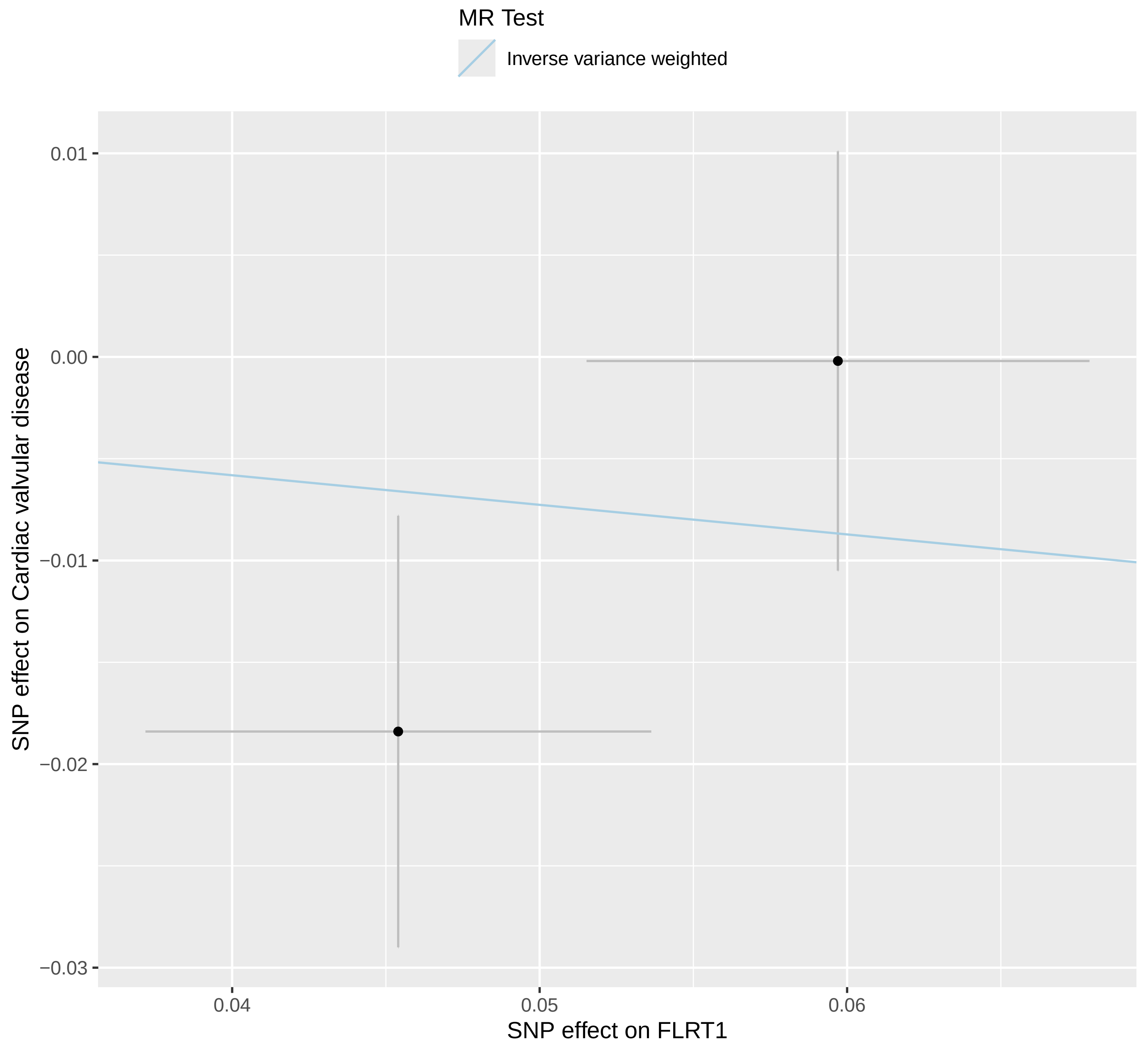
MR Test
Inverse variance weighted
0.01
0.00
−0.01
−0.02
−0.03
0.04
0.05
0.06
SNP effect on Cardiac valvular disease
SNP effect on FLRT1

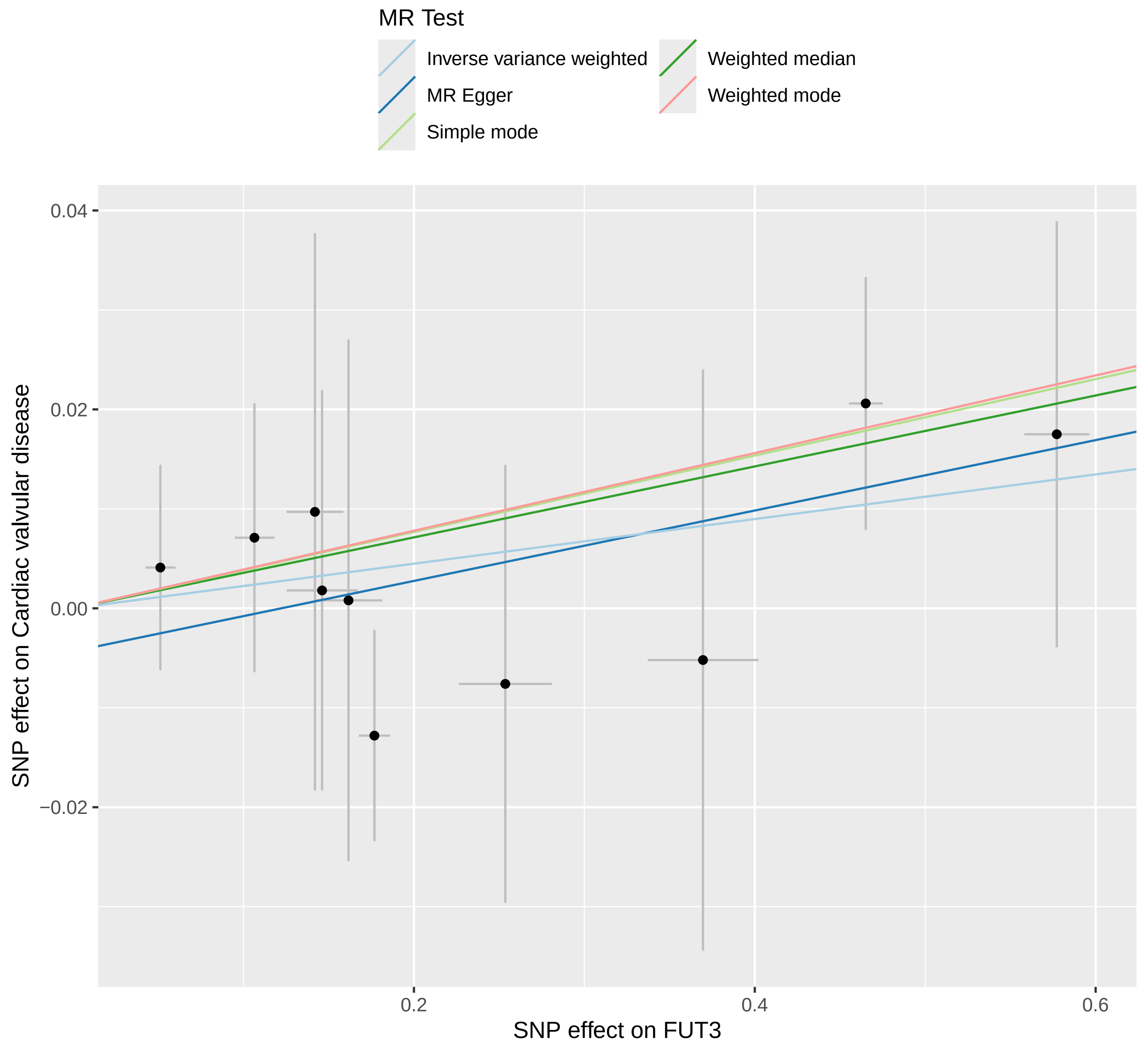
MR Test
Inverse variance weighted
MR Egger
Simple mode
Weighted median
Weighted mode
0.04
0.02
0.00
−0.02
SNP effect on Cardiac valvular disease
0.2
0.4
0.6
SNP effect on FUT3

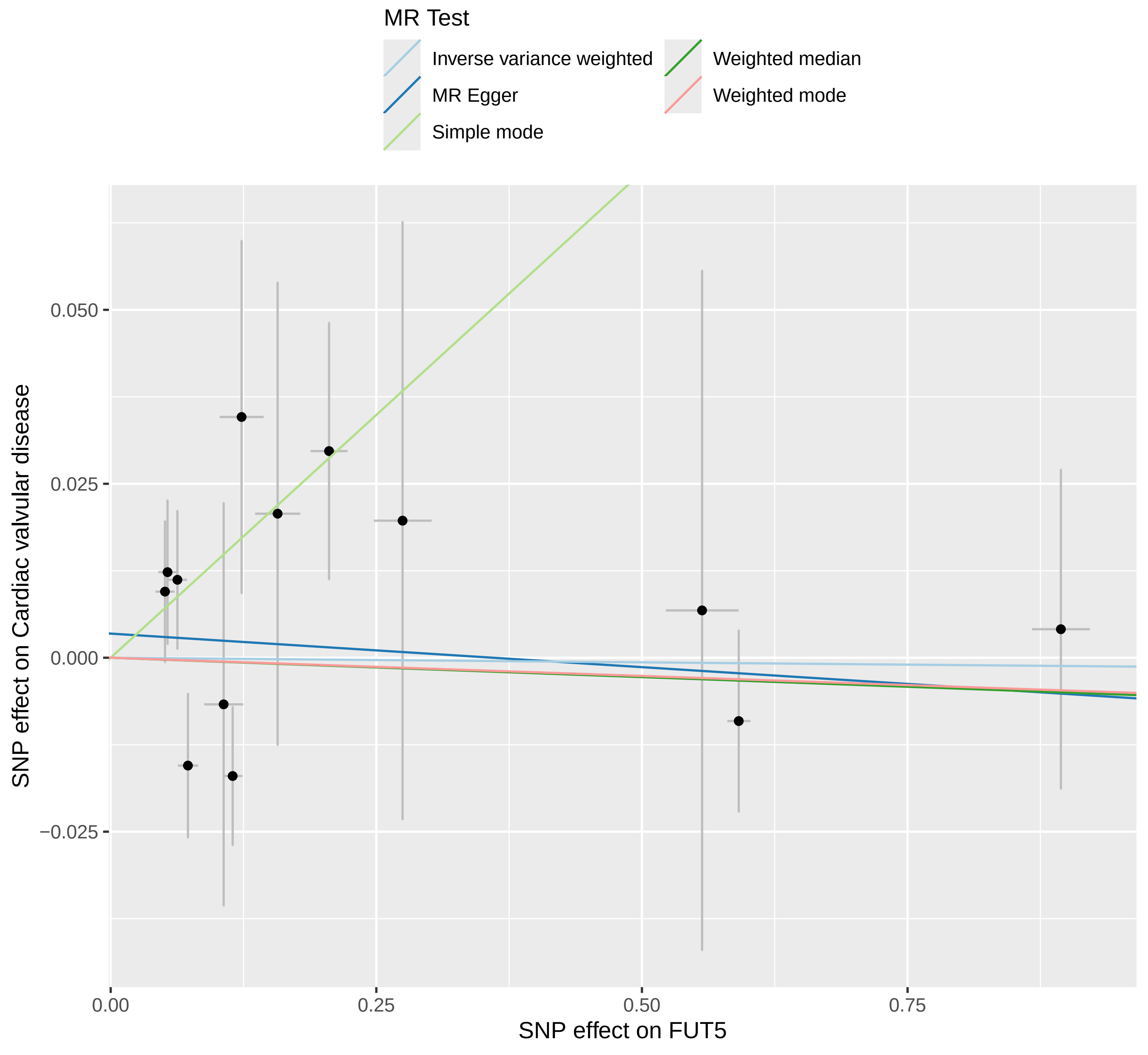
MR Test
Inverse variance weighted
MR Egger
Simple mode
Weighted median
Weighted mode
SNP effect on Cardiac valvular disease
0.050
0.025
0.000
−0.025
0.00
0.25
0.50
0.75
SNP effect on FUT5

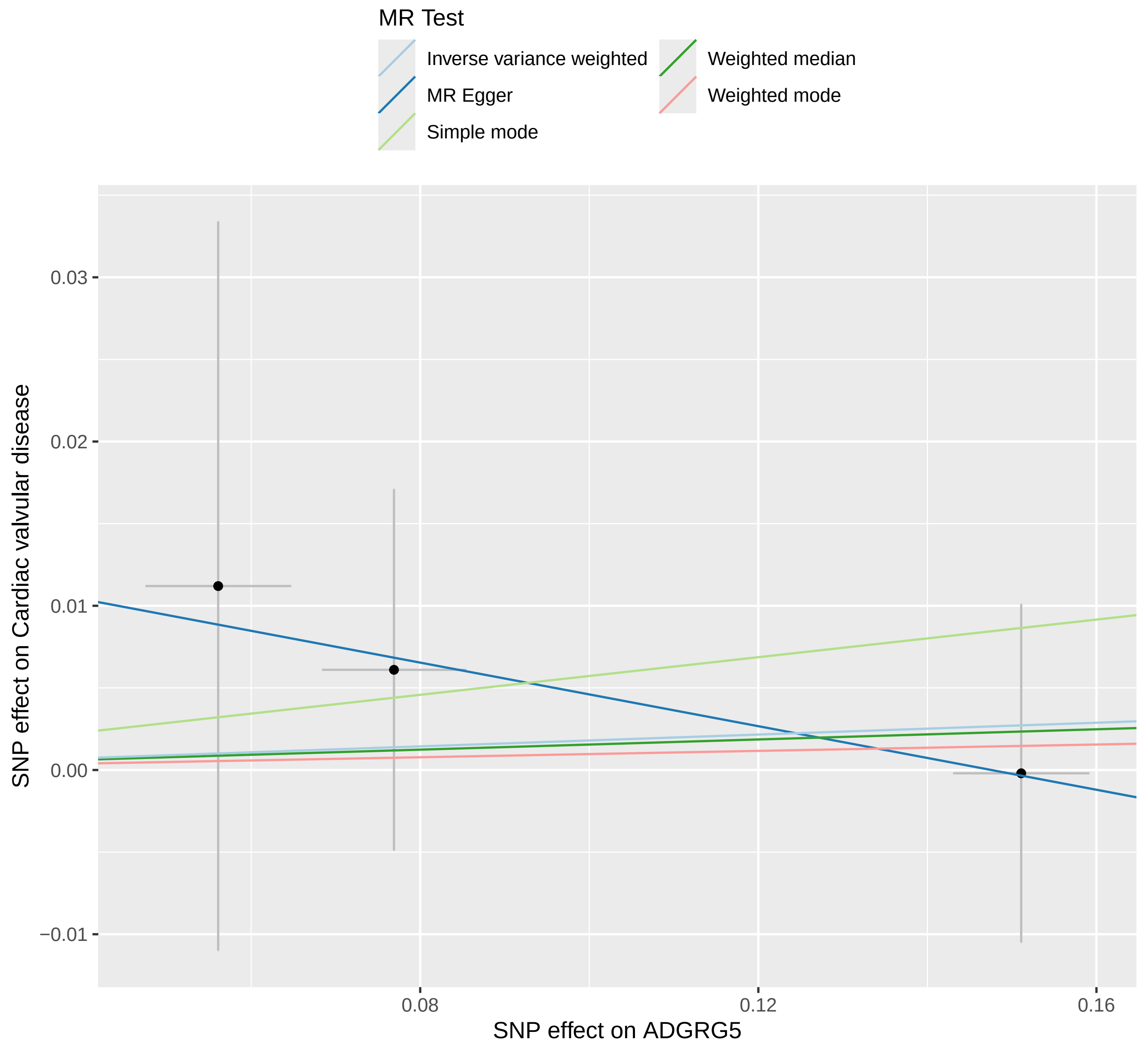
MR Test
Inverse variance weighted
MR Egger
Simple mode
Weighted median
Weighted mode
0.03
0.02
0.01
0.00
−0.01
SNP effect on Cardiac valvular disease
0.08
0.12
0.16
SNP effect on ADGRG5

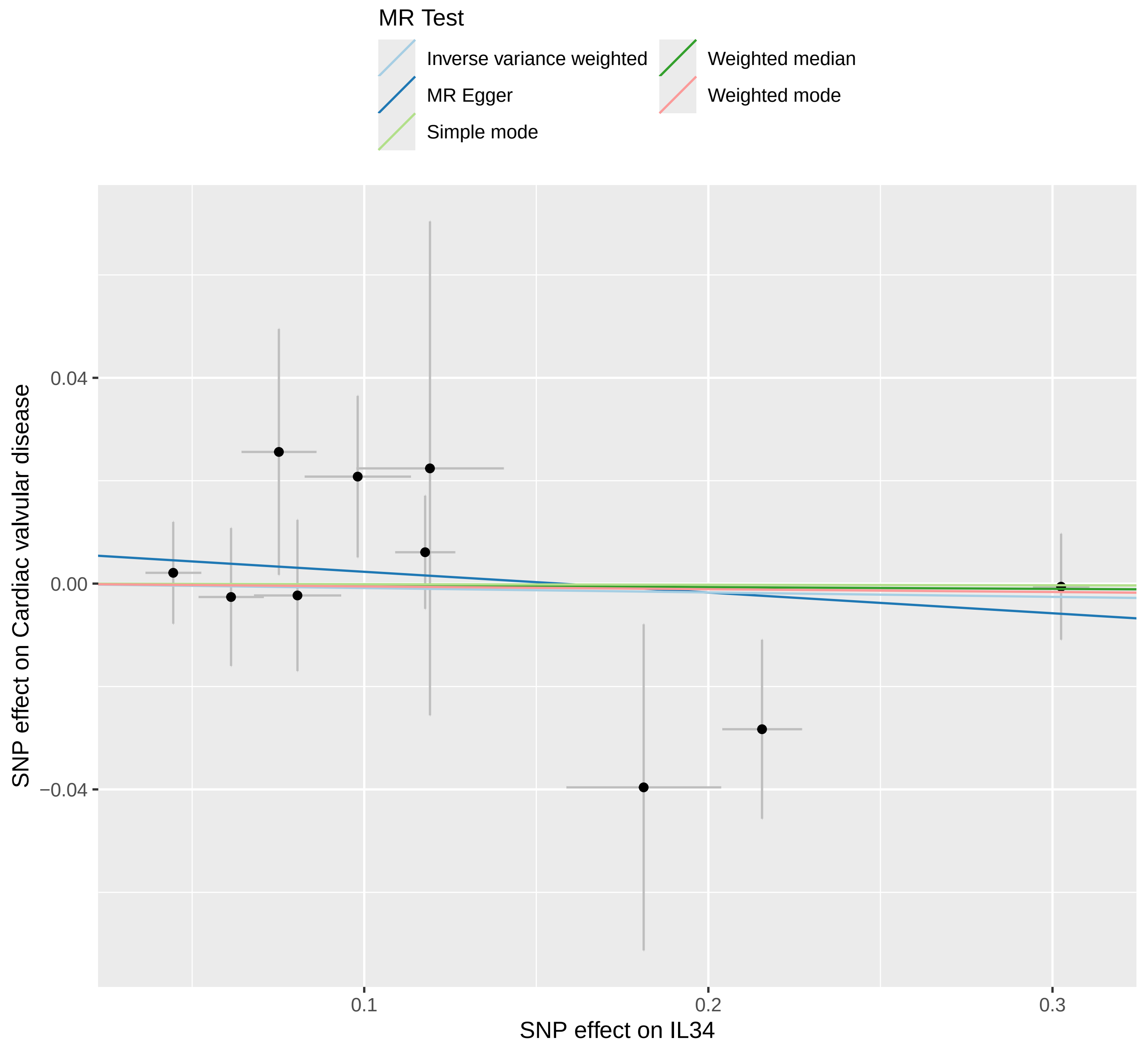

MR Test
Inverse variance weighted
MR Egger
Simple mode
Weighted median
Weighted mode
0.04
0.00
−0.04
0.1
0.2
0.3
SNP effect on Cardiac valvular disease
SNP effect on IL34

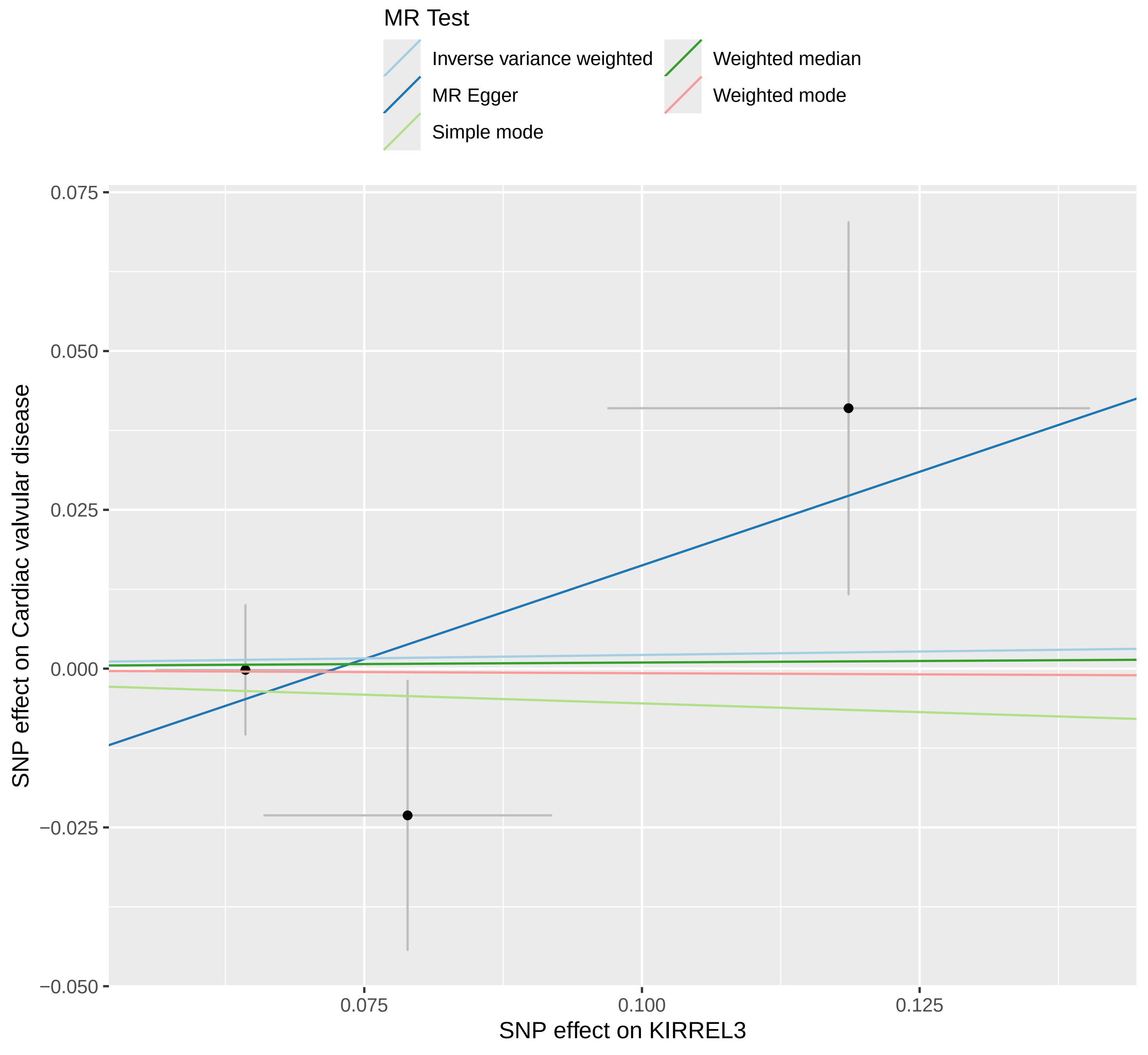
MR Test
Inverse variance weighted
MR Egger
Simple mode
Weighted median
Weighted mode
0.075
0.050
0.025
0.000
−0.025
−0.050
SNP effect on Cardiac valvular disease
0.075
0.100
0.125
SNP effect on KIRREL3

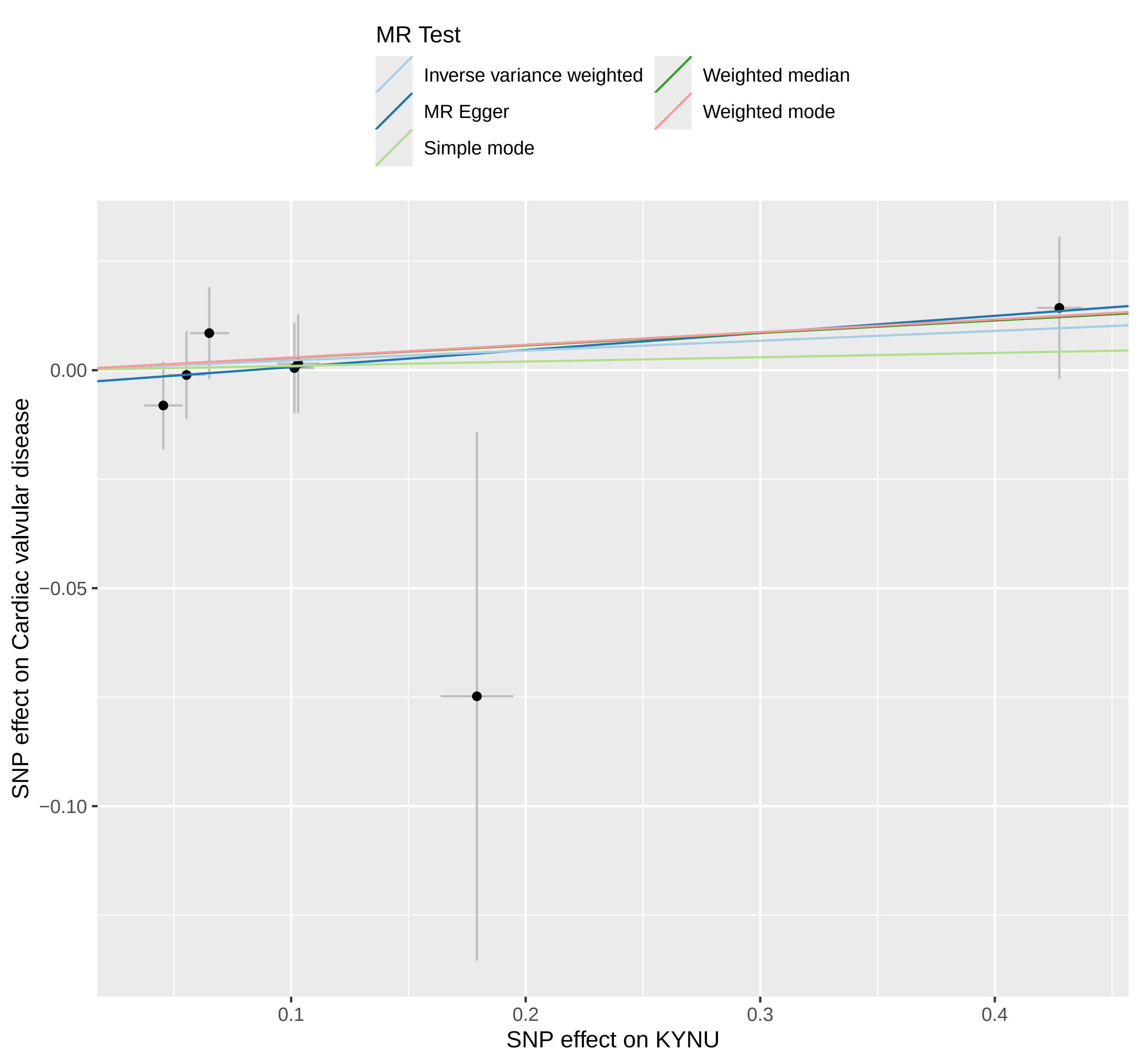

MR Test
Inverse variance weighted
MR Egger
Simple mode
Weighted median
Weighted mode
0.00
−0.05
−0.10
0.1
0.2
0.3
0.4
SNP effect on KYNU
SNP effect on Cardiac valvular disease

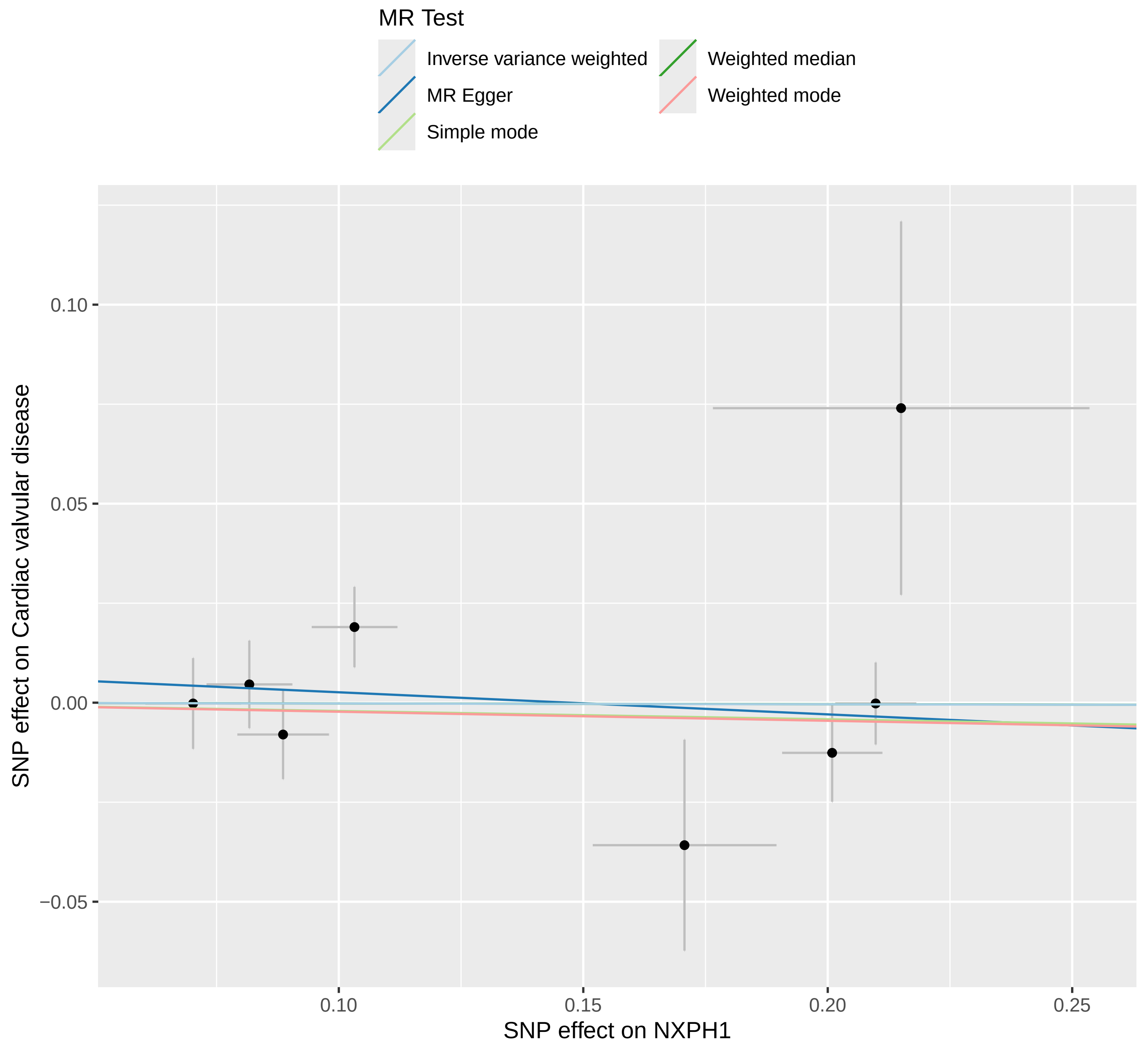

MR Test
Inverse variance weighted
MR Egger
Simple mode
Weighted median
Weighted mode
0.10
0.05
0.00
−0.05
0.10
0.15
0.20
0.25
SNP effect on Cardiac valvular disease
SNP effect on NXPH1

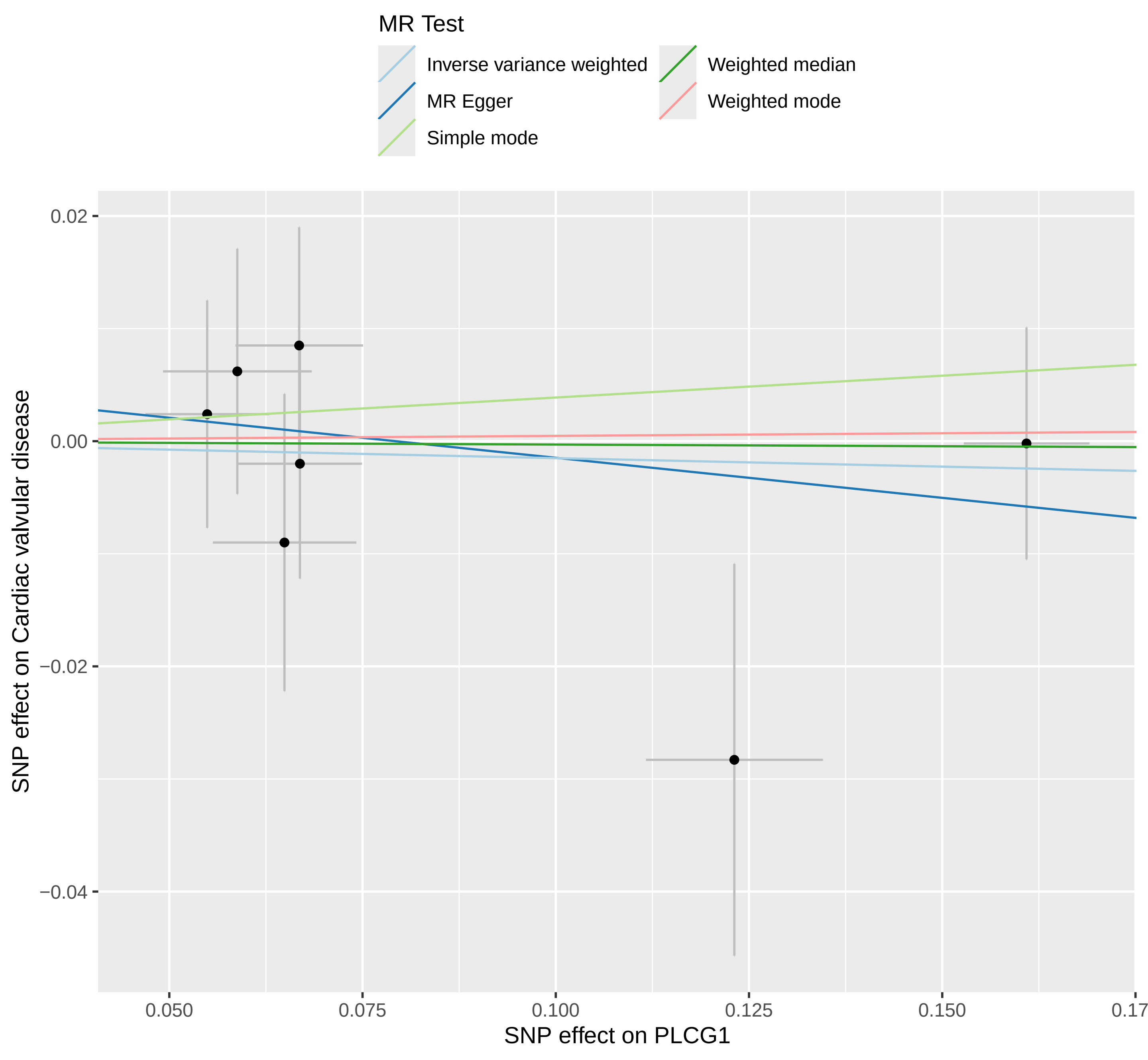

MR Test
Inverse variance weighted
MR Egger
Simple mode
Weighted median
Weighted mode
0.02
0.00
−0.02
−0.04
0.050
0.075
0.100
0.125
0.150
0.17
SNP effect on PLCG1
SNP effect on Cardiac valvular disease

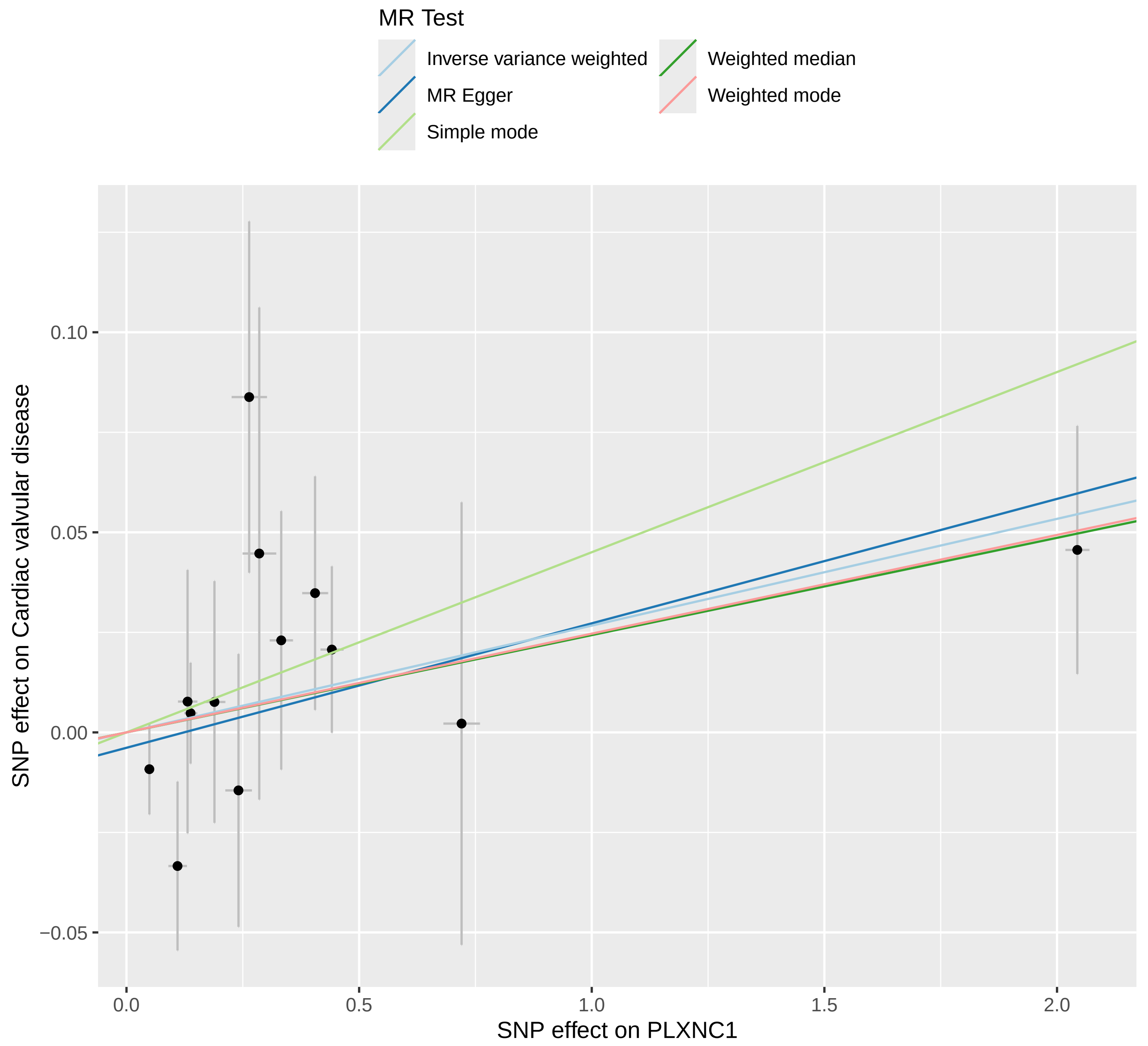

MR Test
Inverse variance weighted
MR Egger
Simple mode
Weighted median
Weighted mode
0.10
0.05
0.00
−0.05
0.0
0.5
1.0
1.5
2.0
SNP effect on PLXNC1
SNP effect on Cardiac valvular disease

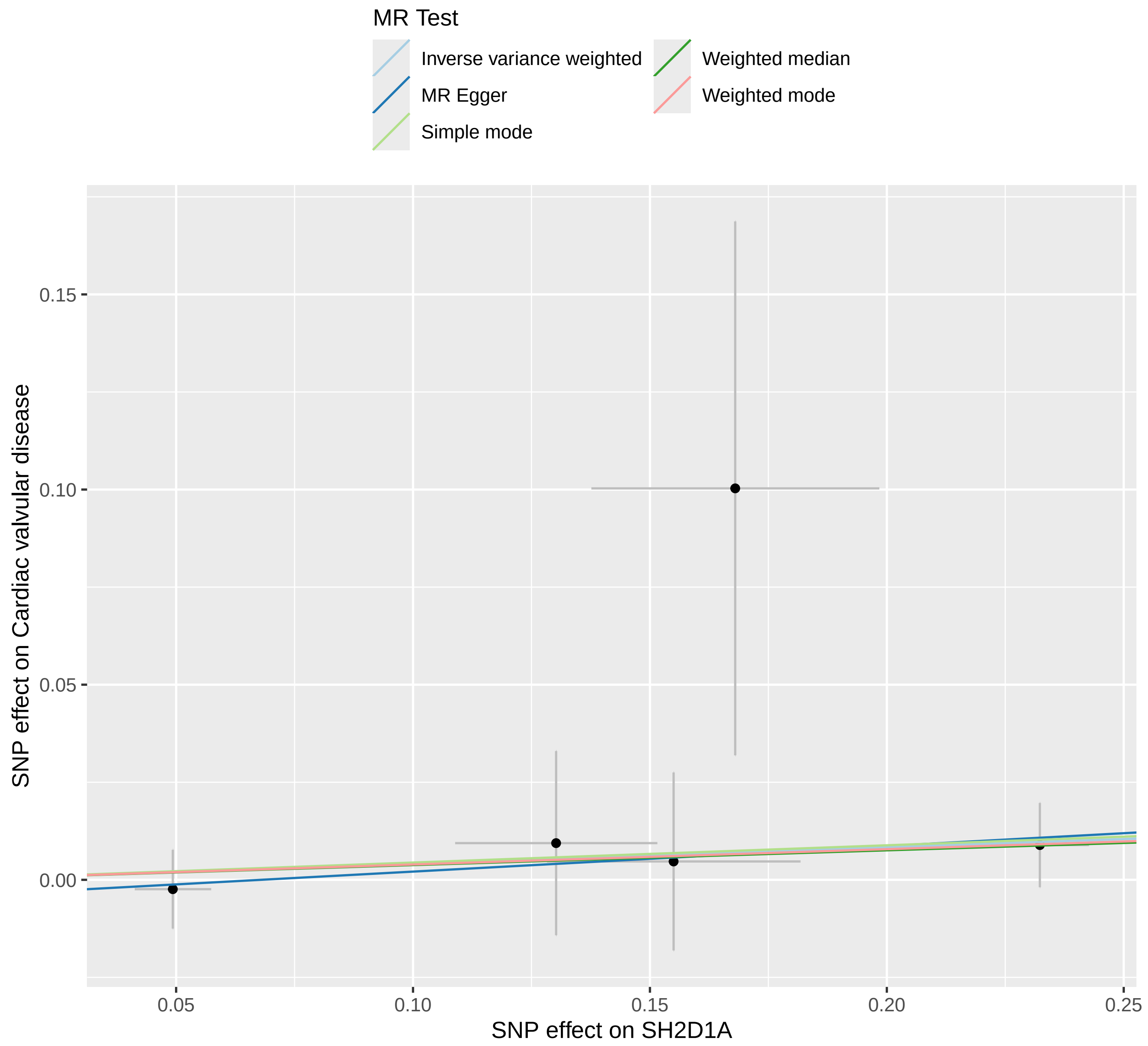
MR Test
Inverse variance weighted
MR Egger
Simple mode
Weighted median
Weighted mode
SNP effect on Cardiac valvular disease
0.15
0.10
0.05
0.00
0.05
0.10
0.15
0.20
0.25
SNP effect on SH2D1A

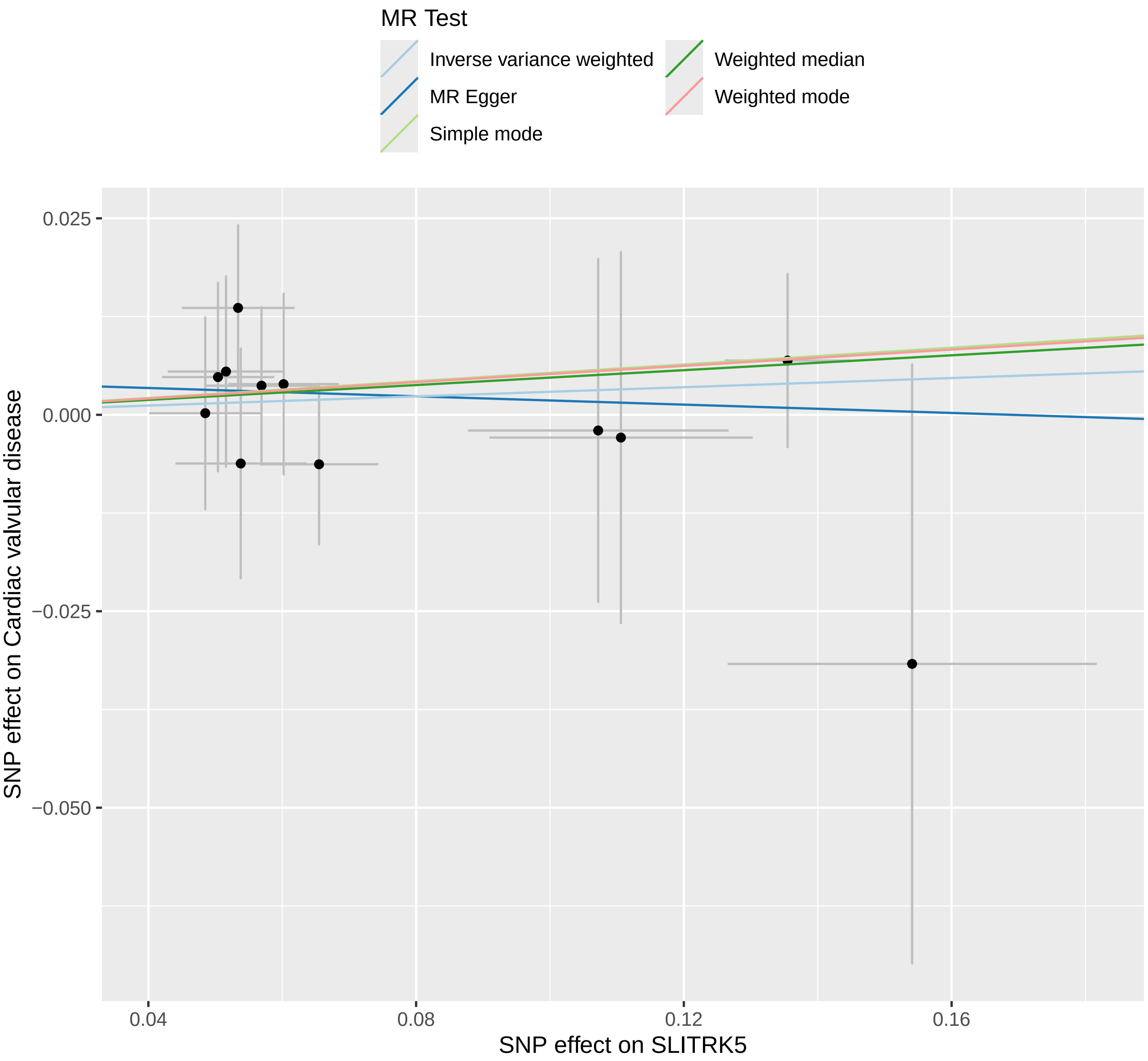

MR Test
Inverse variance weighted
MR Egger
Simple mode
Weighted median
Weighted mode
0.025
0.000
−0.025
−0.050
SNP effect on Cardiac valvular disease
0.04
0.08
0.12
0.16
SNP effect on SLITRK5

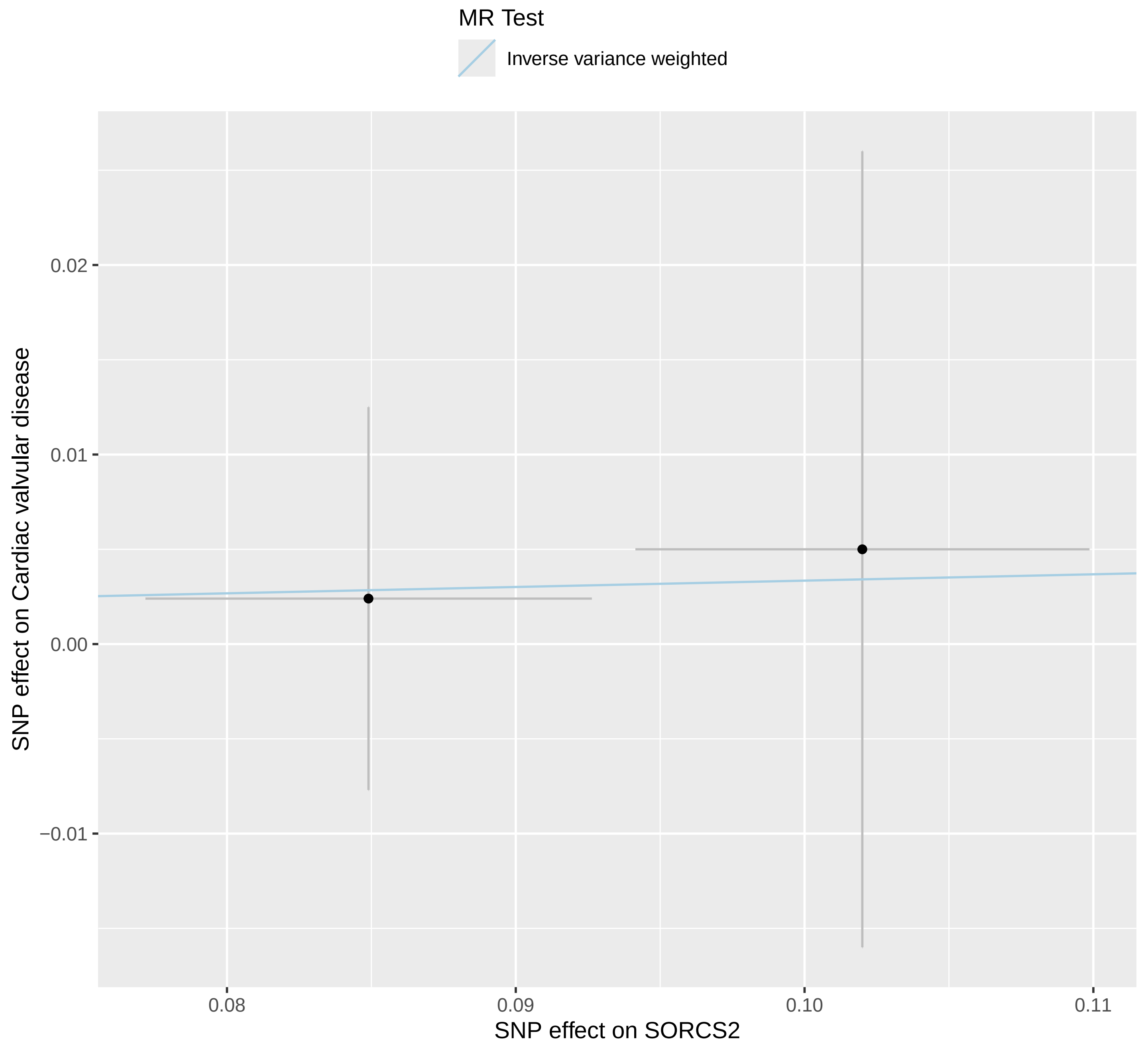

MR Test
Inverse variance weighted
0.02
0.01
0.00
−0.01
0.08
0.09
0.10
0.11
SNP effect on Cardiac valvular disease
SNP effect on SORCS2

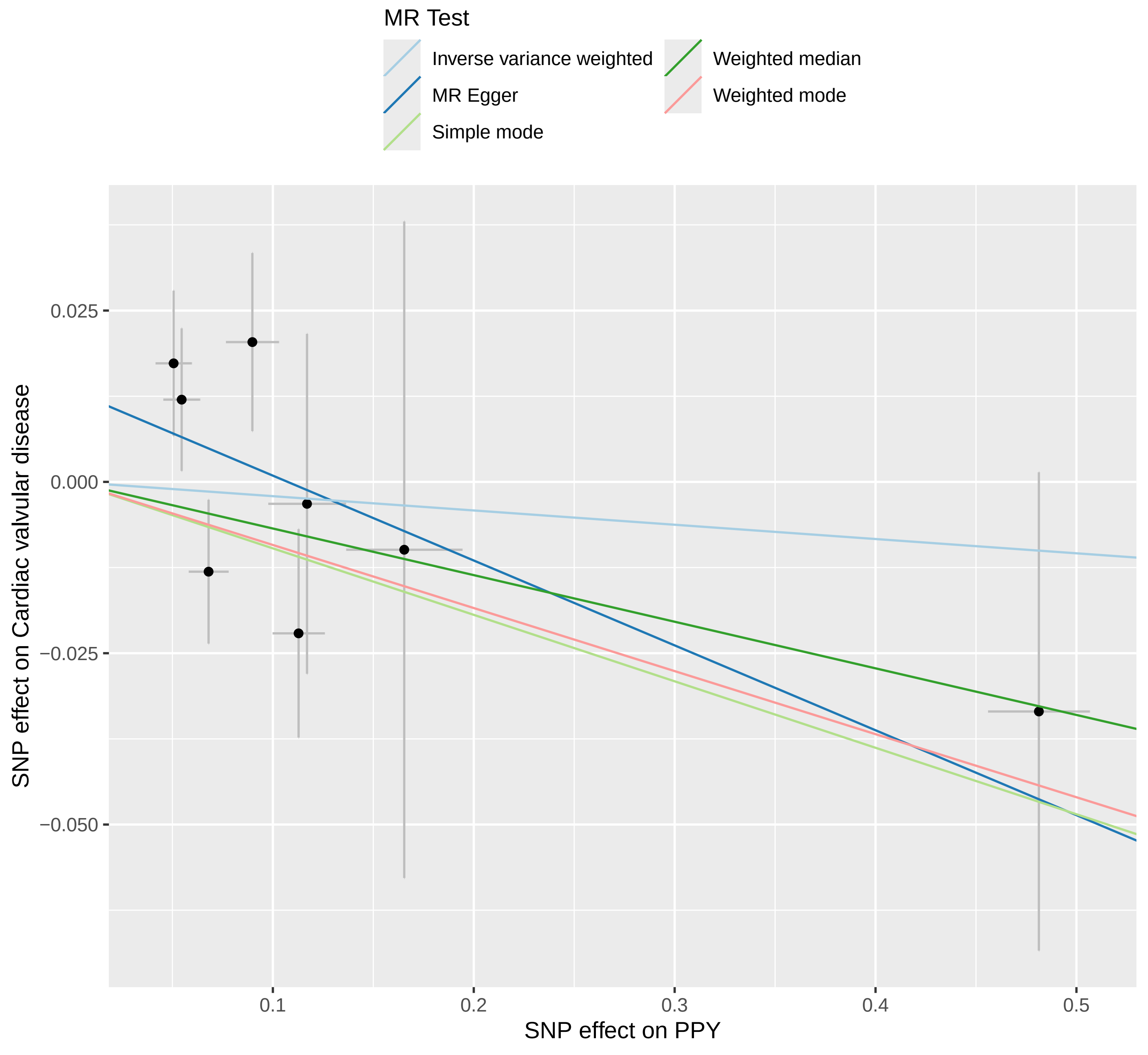
MR Test
Inverse variance weighted
MR Egger
Simple mode
Weighted median
Weighted mode
0.025
0.000
−0.025
−0.050
0.1
0.2
0.3
0.4
0.5
SNP effect on Cardiac valvular disease
SNP effect on PPY

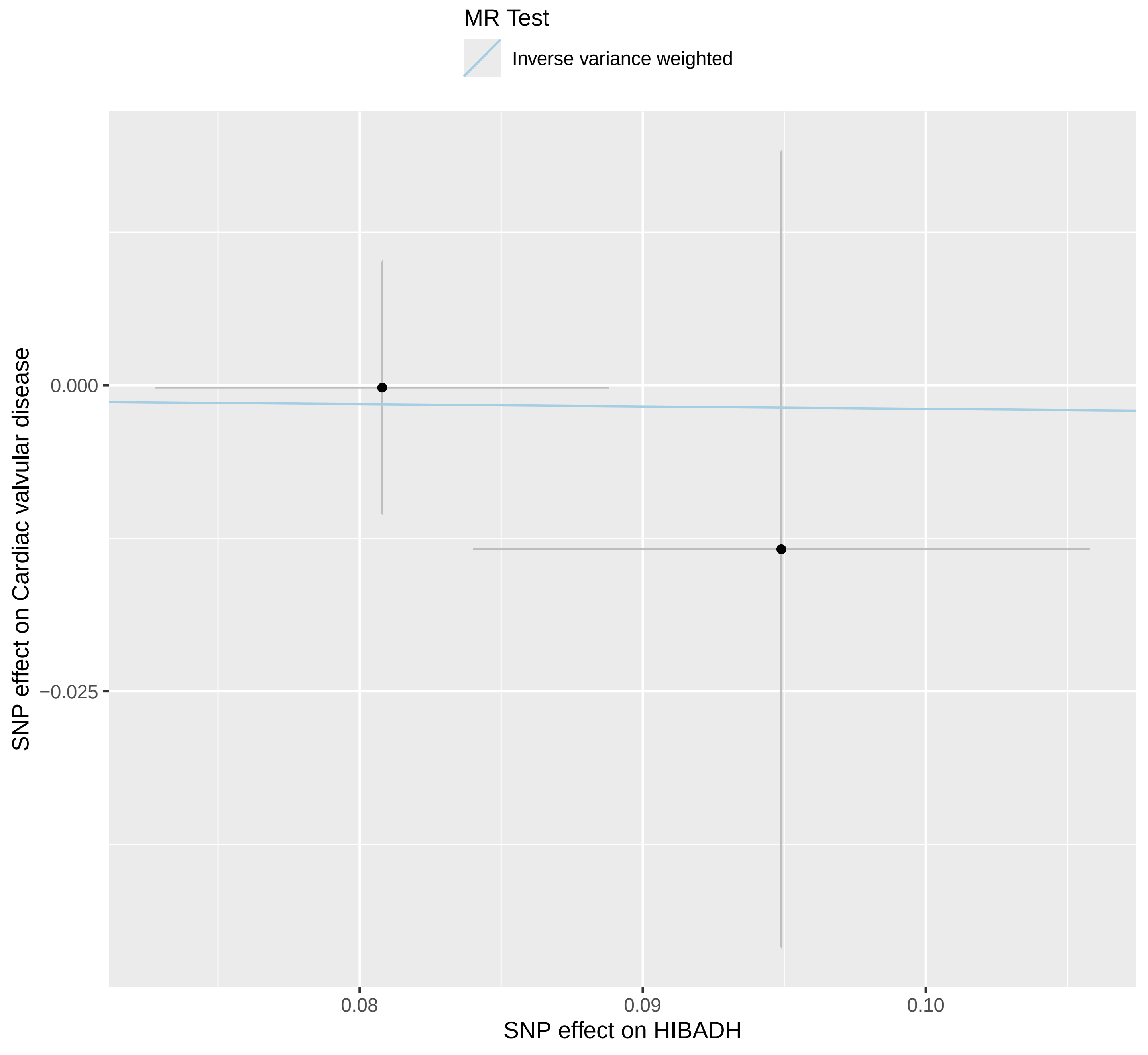
MR Test
Inverse variance weighted
0.000
−0.025
0.08
0.09
0.10
SNP effect on HIBADH
SNP effect on Cardiac valvular disease

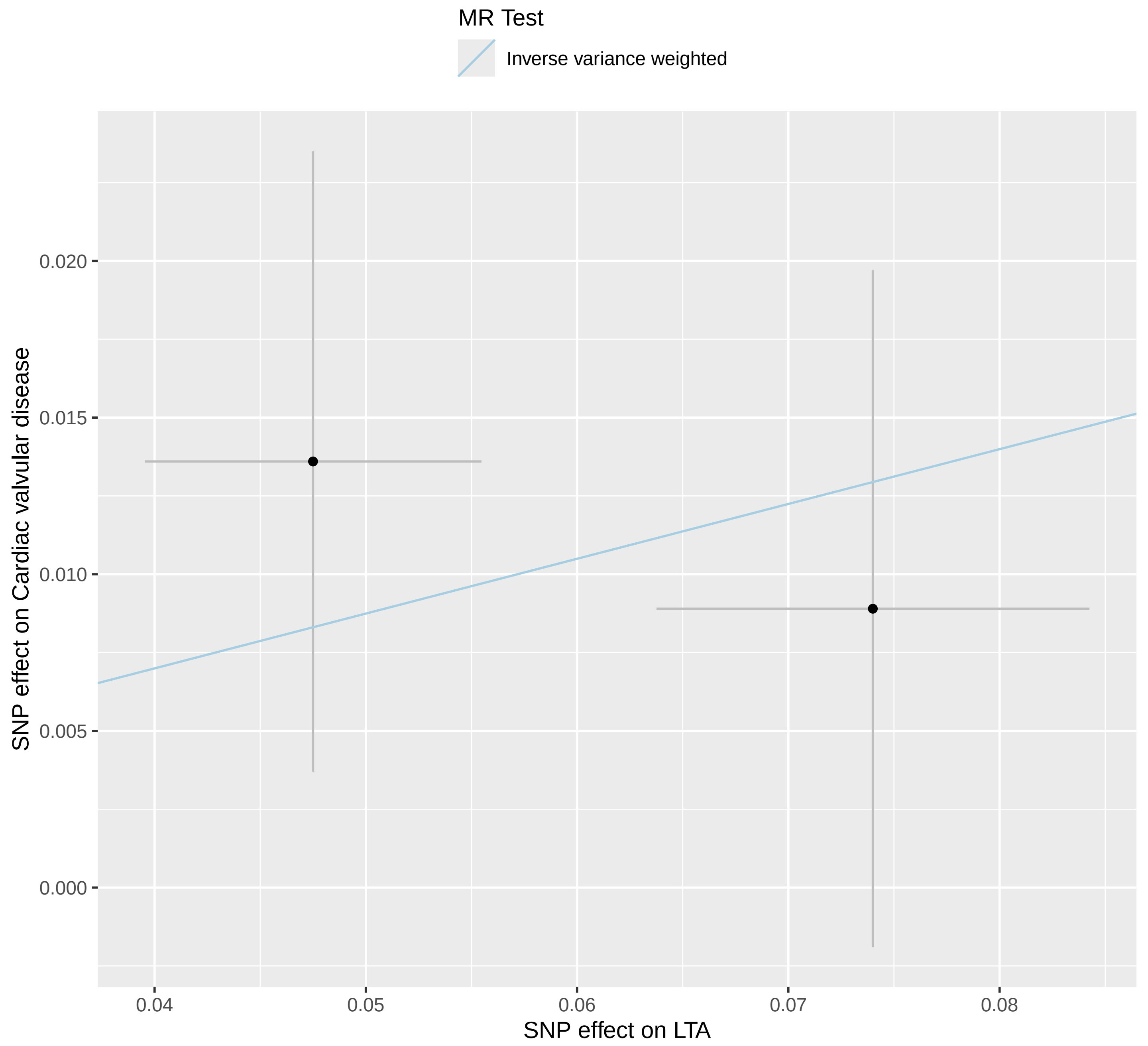
MR Test
Inverse variance weighted
0.020
0.015
0.010
0.005
0.000
0.04
0.05
0.06
0.07
0.08
SNP effect on Cardiac valvular disease
SNP effect on LTA

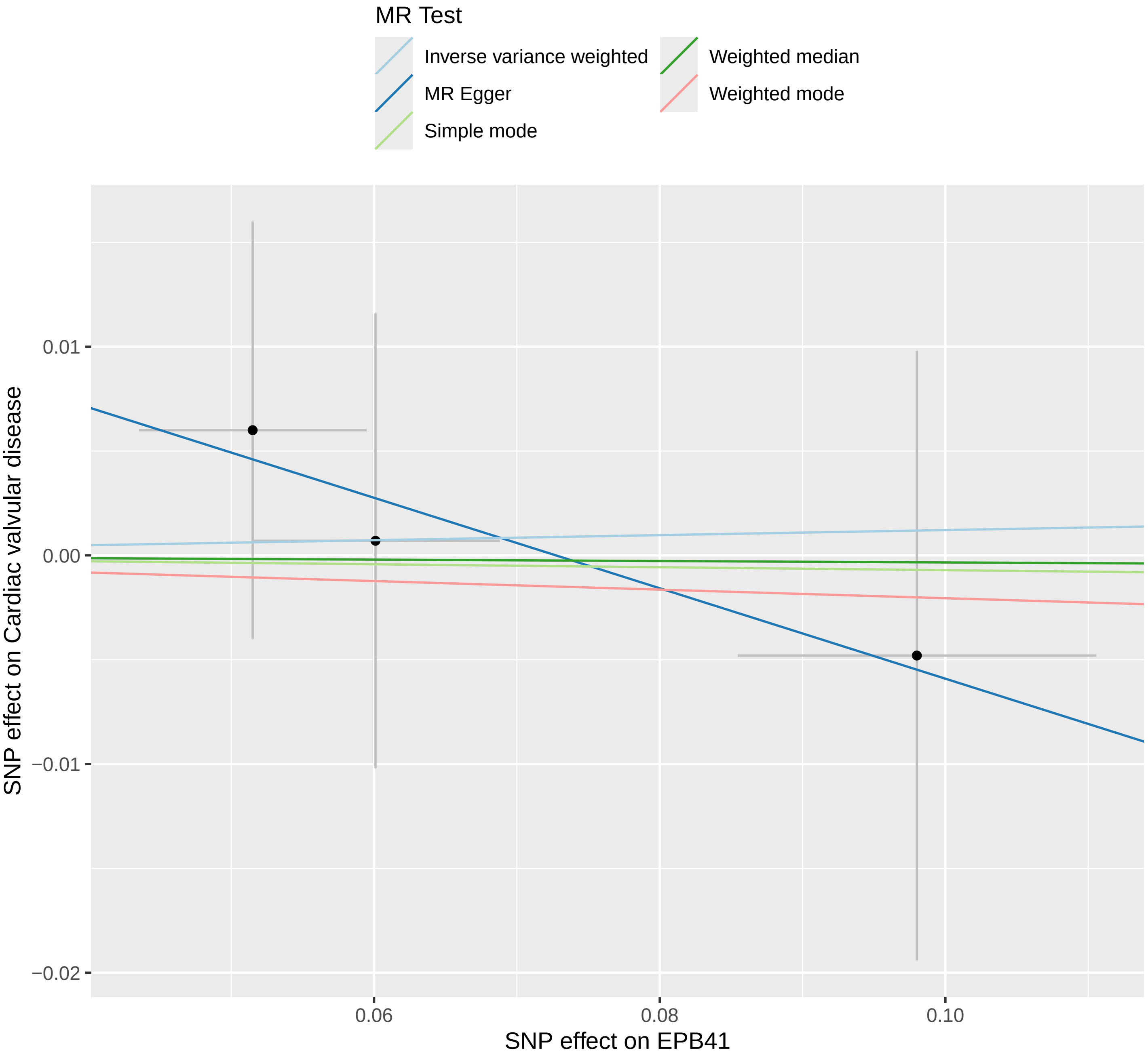

MR Test
Inverse variance weighted
MR Egger
Simple mode
Weighted median
Weighted mode
0.01
0.00
−0.01
−0.02
0.06
0.08
0.10
SNP effect on Cardiac valvular disease
SNP effect on EPB41

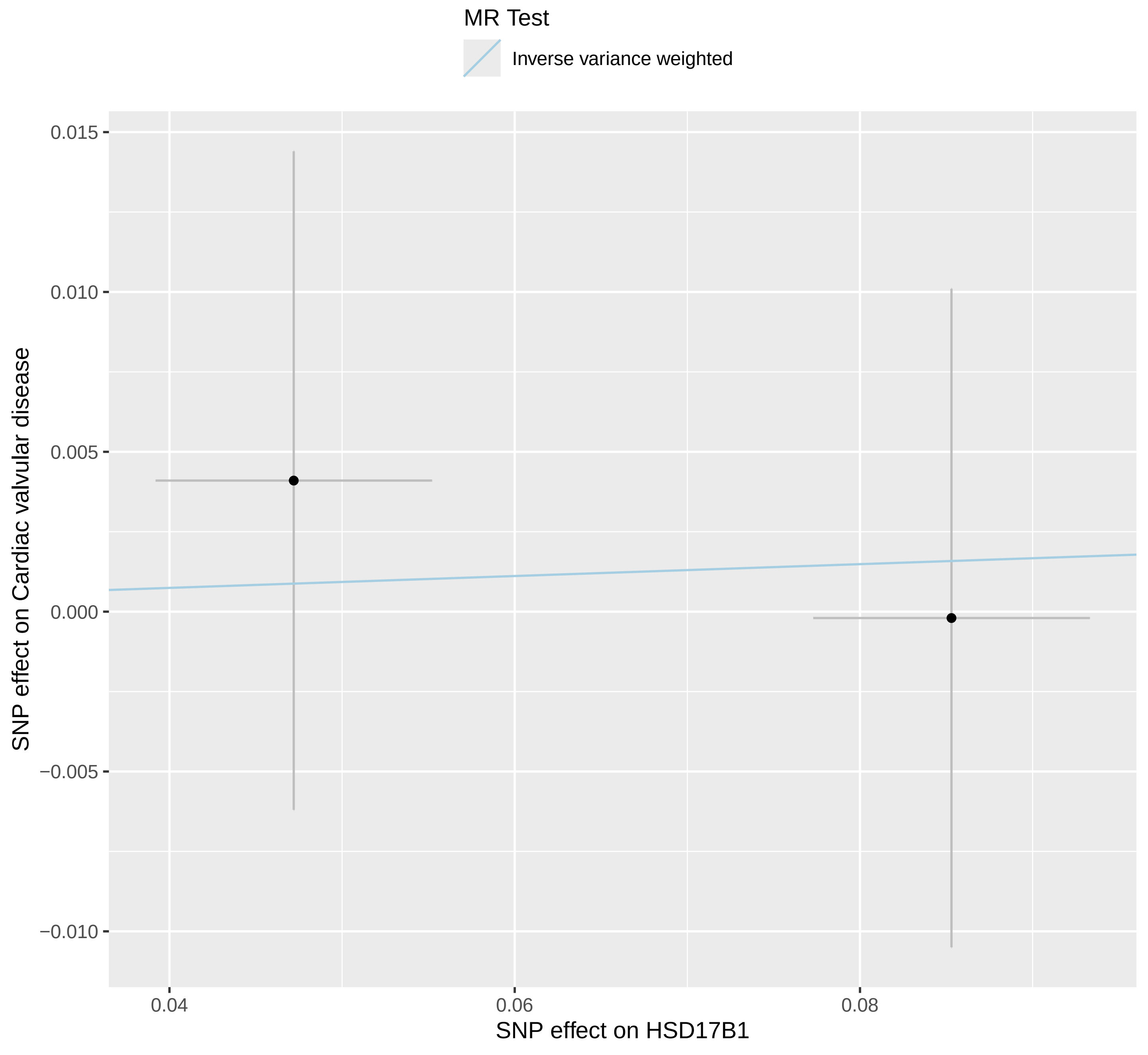
MR Test
Inverse variance weighted
0.015
0.010
0.005
0.000
−0.005
−0.010
0.04
0.06
0.08
SNP effect on Cardiac valvular disease
SNP effect on HSD17B1

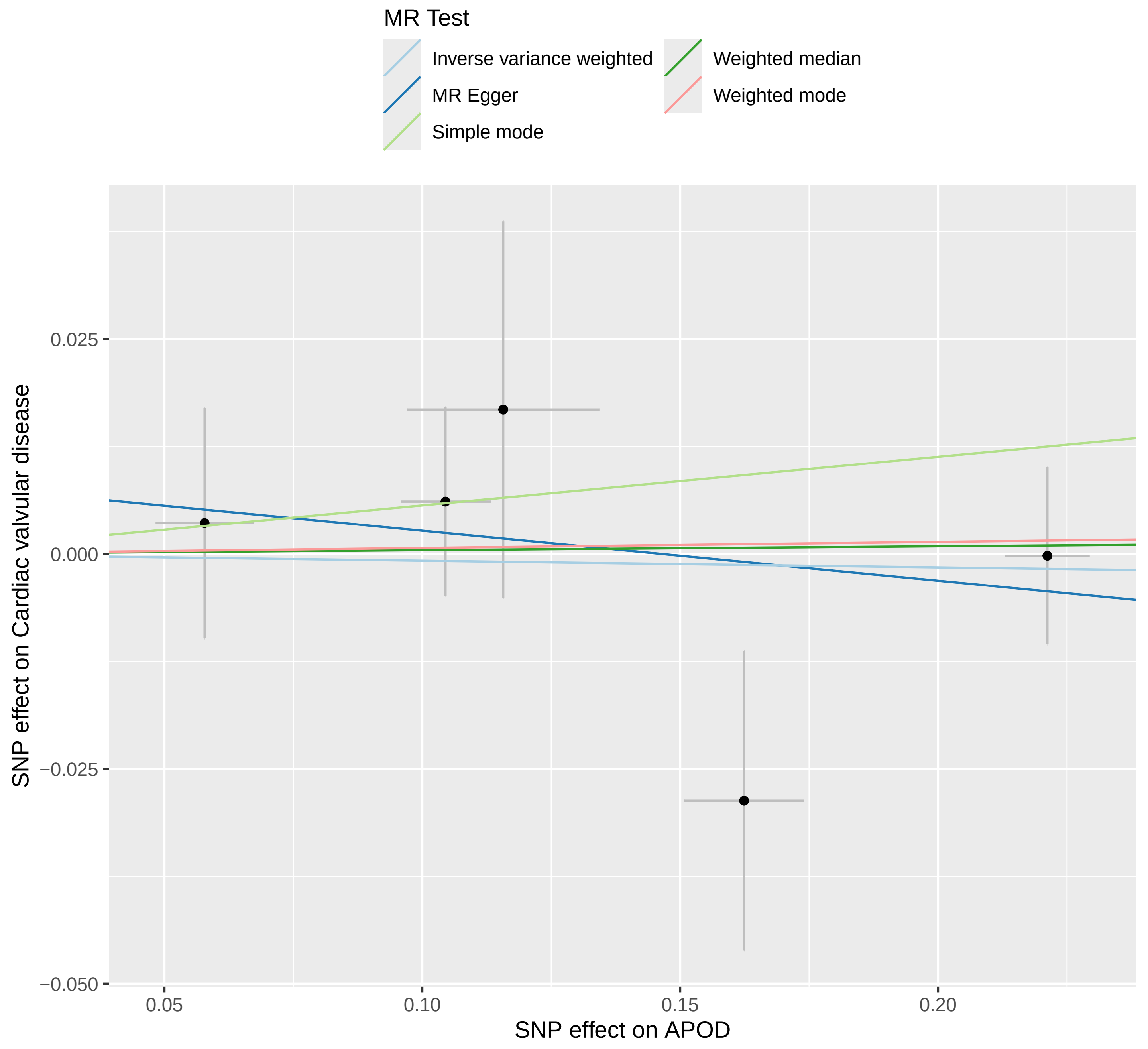
MR Test
Inverse variance weighted
MR Egger
Simple mode
Weighted median
Weighted mode
0.025
0.000
−0.025
−0.050
0.05
0.10
0.15
0.20
SNP effect on APOD
SNP effect on Cardiac valvular disease

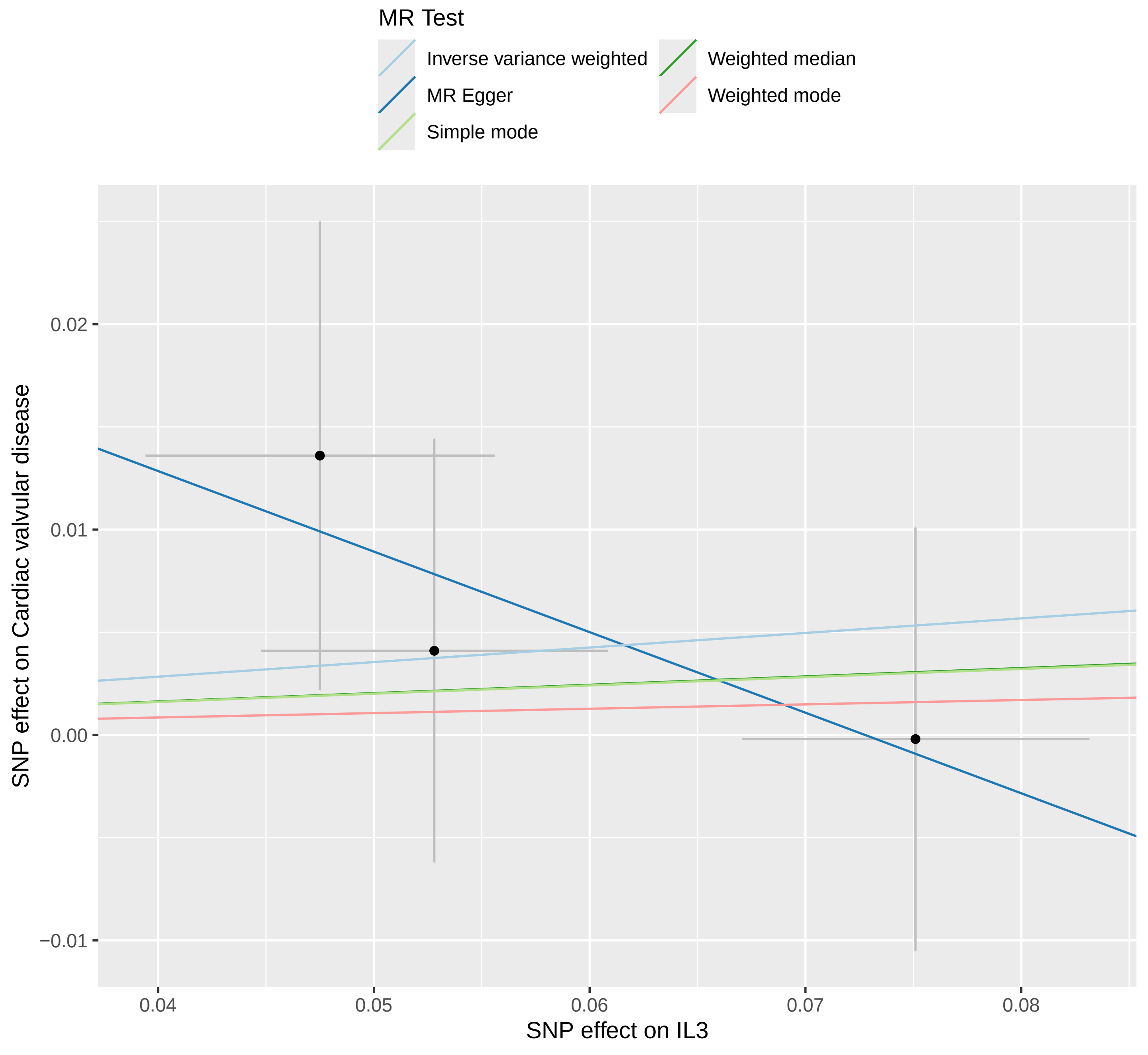

MR Test
Inverse variance weighted
MR Egger
Simple mode
Weighted median
Weighted mode
0.02
0.01
0.00
−0.01
0.04
0.05
0.06
0.07
0.08
SNP effect on Cardiac valvular disease
SNP effect on IL3

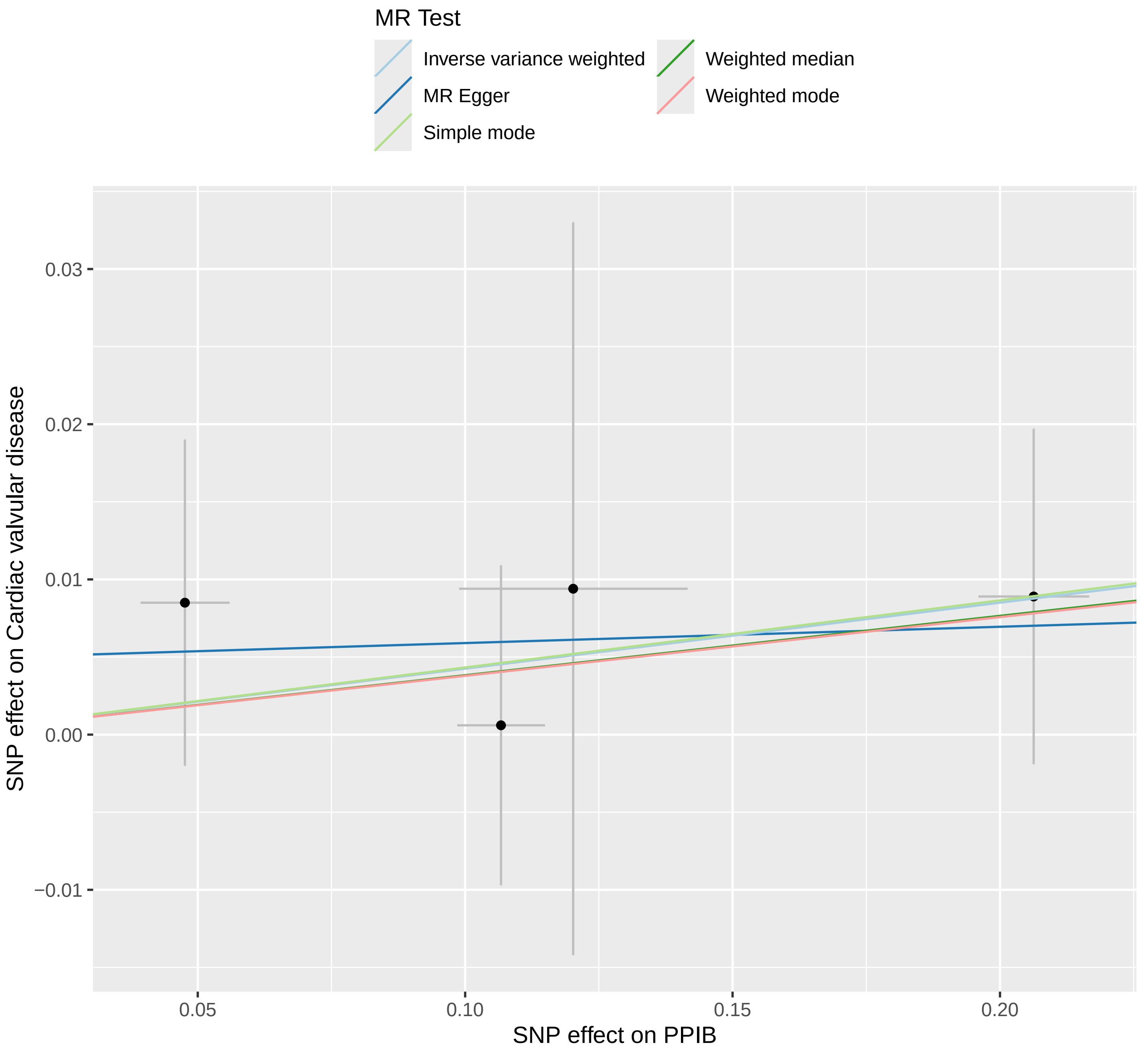
MR Test
Inverse variance weighted
MR Egger
Simple mode
Weighted median
Weighted mode
0.03
0.02
0.01
0.00
−0.01
SNP effect on Cardiac valvular disease
0.05
0.10
0.15
0.20
SNP effect on PPIB

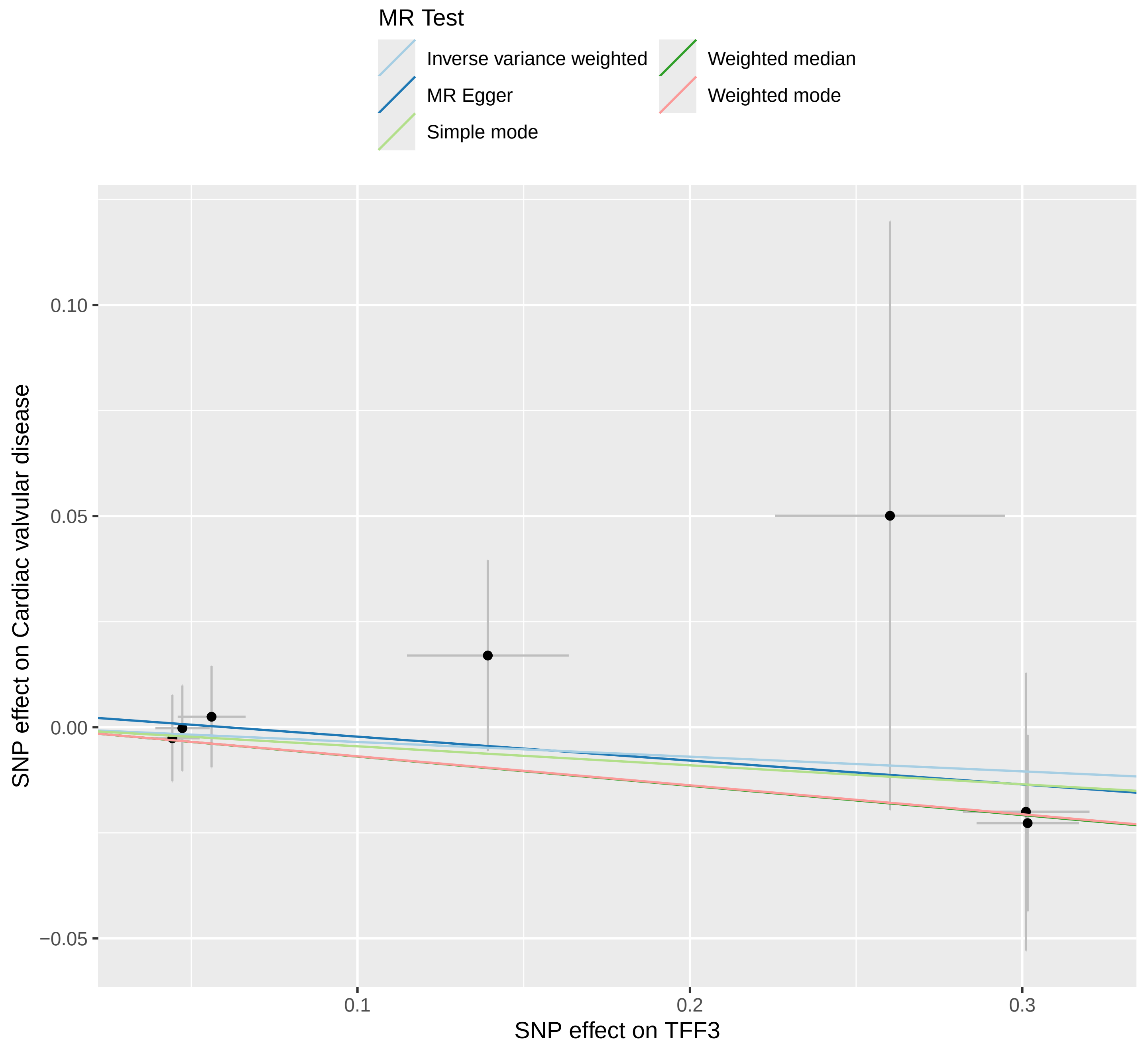
MR Test
Inverse variance weighted
MR Egger
Simple mode
Weighted median
Weighted mode
0.10
0.05
0.00
−0.05
0.1
0.2
0.3
SNP effect on Cardiac valvular disease
SNP effect on TFF3

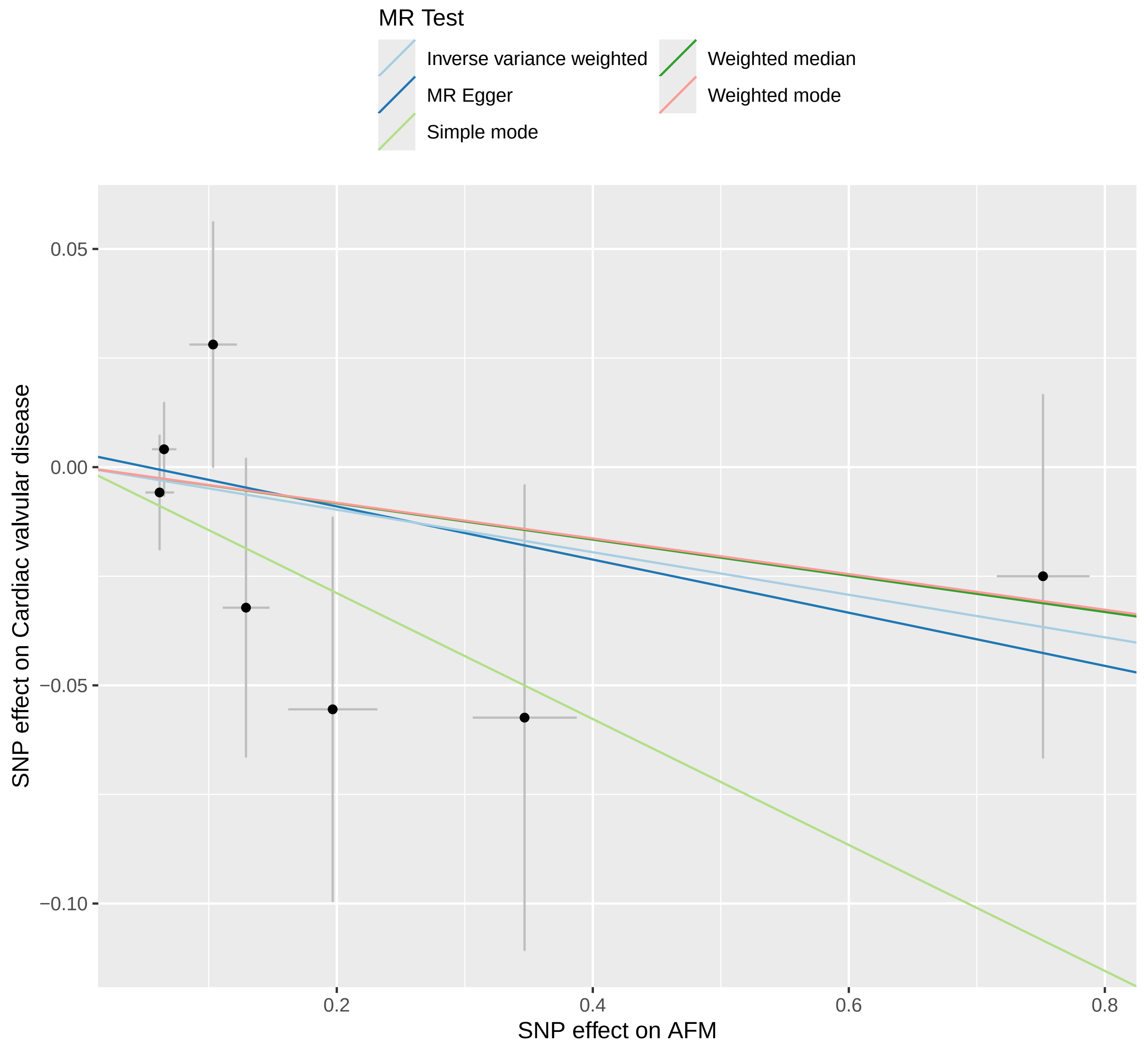
MR Test
Inverse variance weighted
MR Egger
Simple mode
Weighted median
Weighted mode
0.05
0.00
−0.05
−0.10
SNP effect on Cardiac valvular disease
0.2
0.4
0.6
0.8
SNP effect on AFM

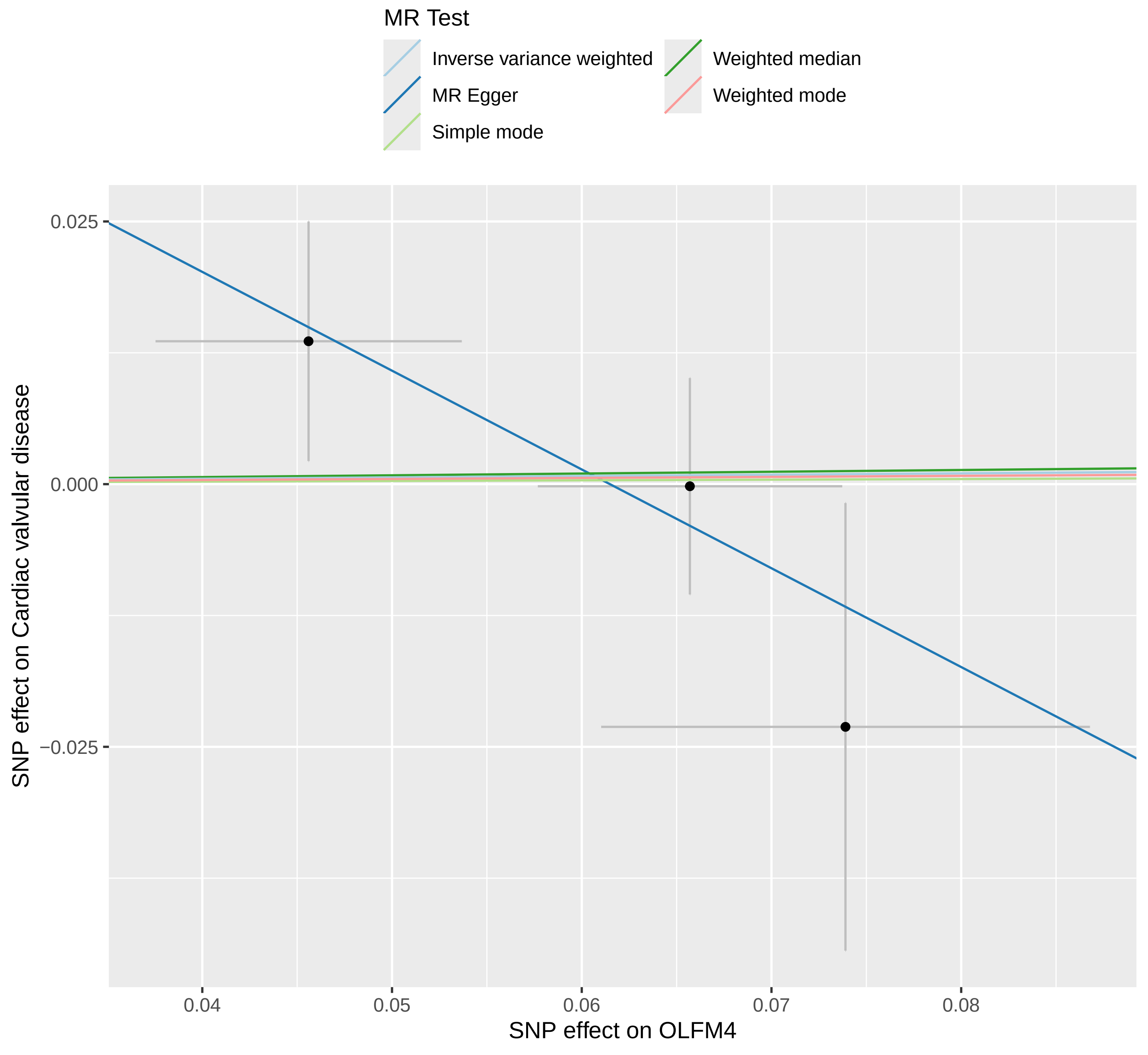
MR Test
Inverse variance weighted
MR Egger
Simple mode
Weighted median
Weighted mode
0.025
0.000
−0.025
0.04
0.05
0.06
0.07
0.08
SNP effect on Cardiac valvular disease
SNP effect on OLFM4

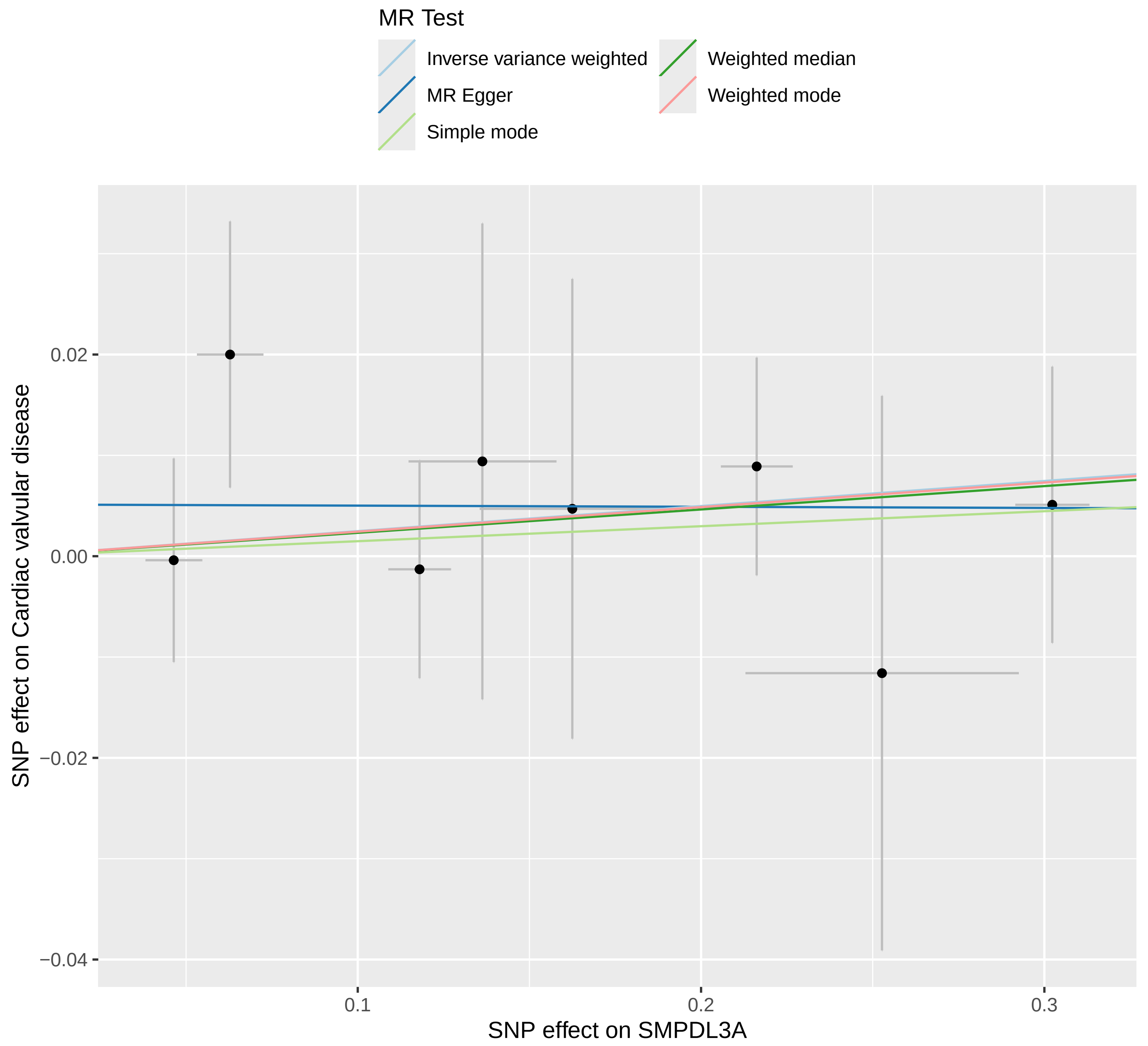

MR Test
Inverse variance weighted
MR Egger
Simple mode
Weighted median
Weighted mode
0.02
0.00
−0.02
−0.04
SNP effect on Cardiac valvular disease
0.1
0.2
0.3
SNP effect on SMPDL3A

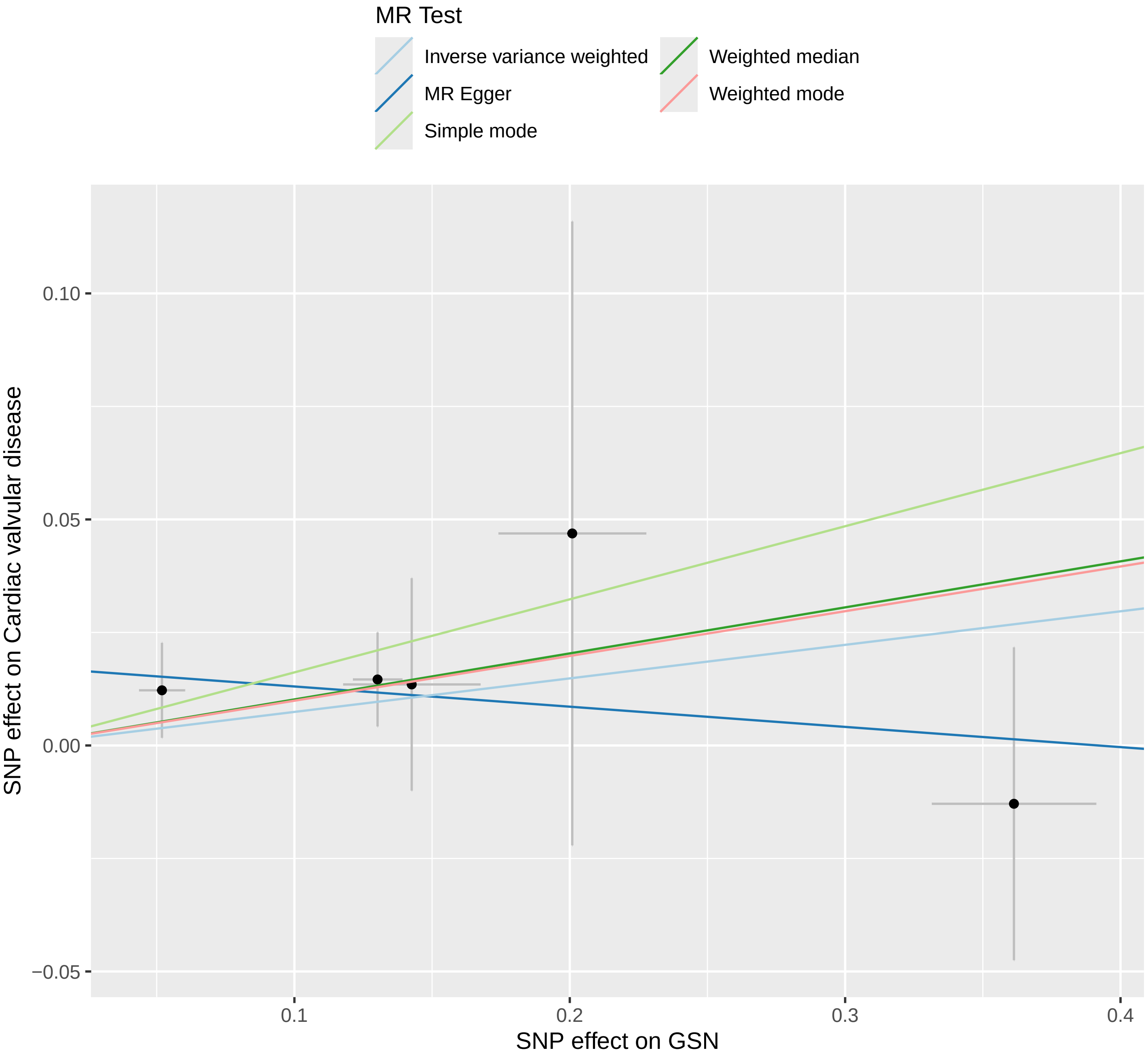
MR Test
Inverse variance weighted
MR Egger
Simple mode
Weighted median
Weighted mode
0.10
0.05
0.00
−0.05
SNP effect on Cardiac valvular disease
0.1
0.2
0.3
0.4
SNP effect on GSN

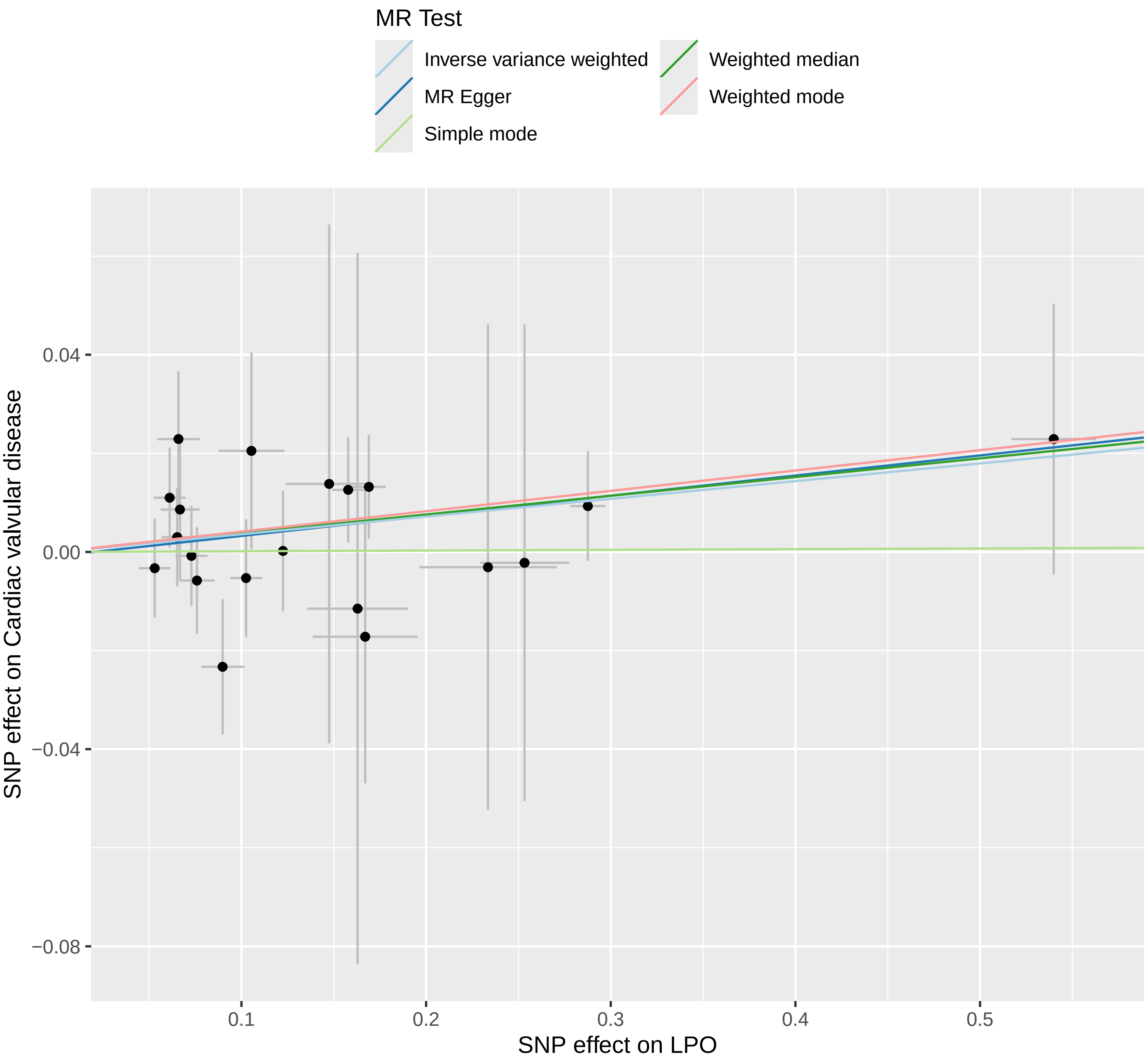

MR Test
Inverse variance weighted
MR Egger
Simple mode
Weighted median
Weighted mode
SNP effect on Cardiac valvular disease
0.04
0.00
−0.04
−0.08
0.1
0.2
0.3
0.4
0.5
SNP effect on LPO

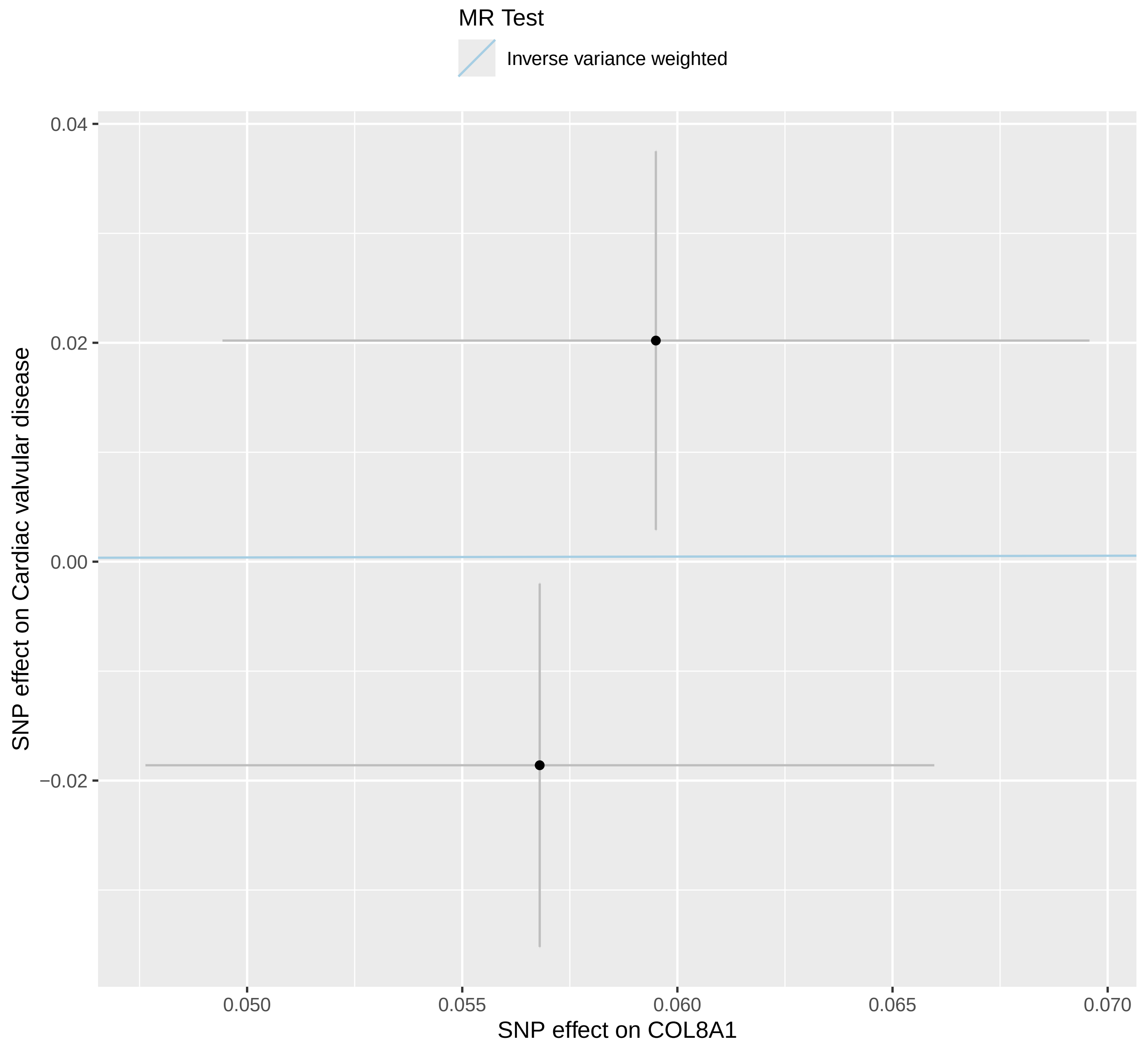
MR Test
Inverse variance weighted
0.04
0.02
0.00
−0.02
SNP effect on Cardiac valvular disease
0.050
0.055
0.060
0.065
0.070
SNP effect on COL8A1

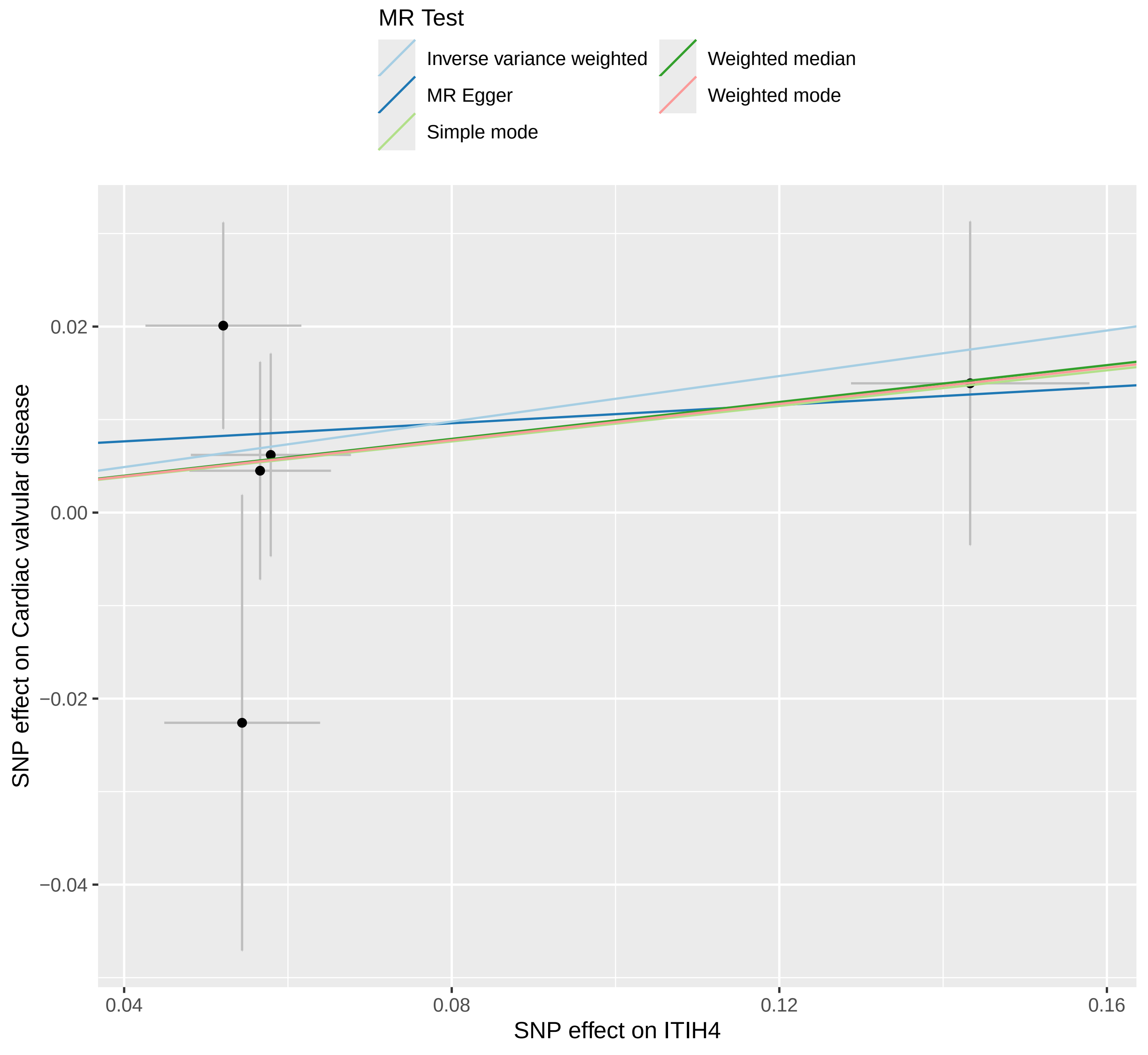
MR Test
Inverse variance weighted
MR Egger
Simple mode
Weighted median
Weighted mode
0.02
0.00
−0.02
−0.04
0.04
0.08
0.12
0.16
SNP effect on Cardiac valvular disease
SNP effect on ITIH4

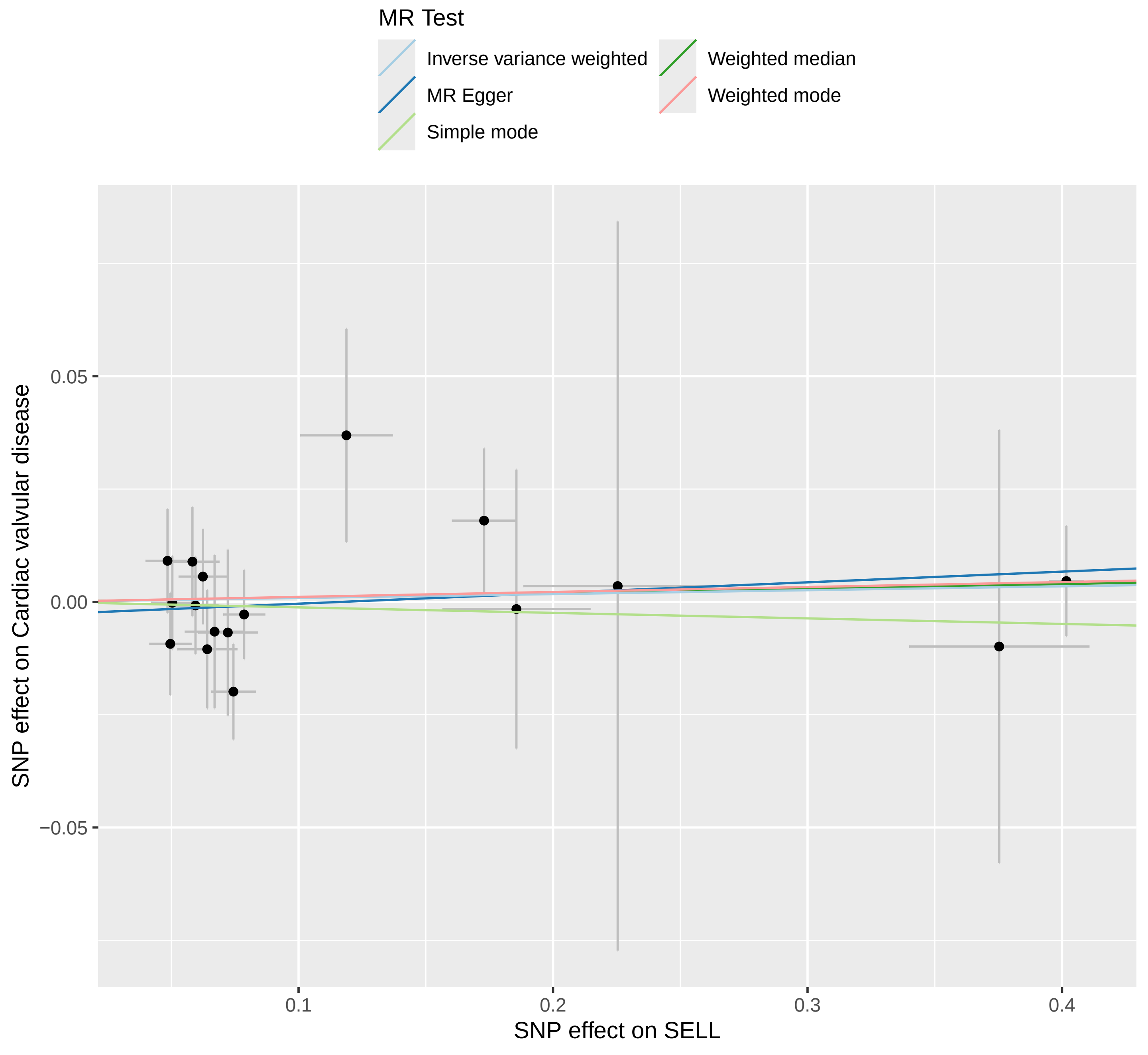

MR Test
Inverse variance weighted
MR Egger
Simple mode
Weighted median
Weighted mode
0.05
0.00
−0.05
0.1
0.2
0.3
0.4
SNP effect on Cardiac valvular disease
SNP effect on SELL

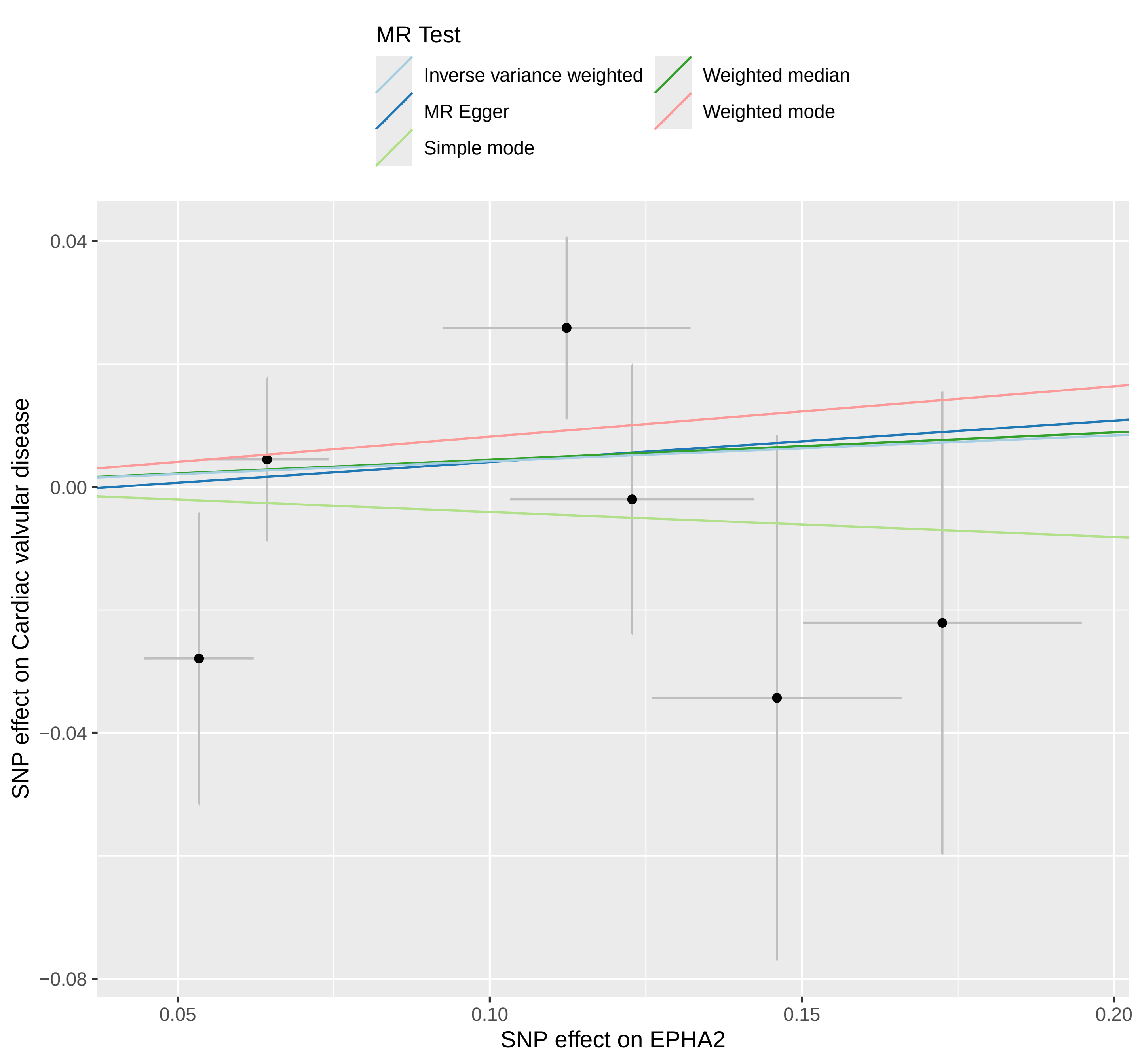

MR Test
Inverse variance weighted
MR Egger
Simple mode
Weighted median
Weighted mode
0.04
0.00
−0.04
−0.08
0.05
0.10
0.15
0.20
SNP effect on EPHA2
SNP effect on Cardiac valvular disease

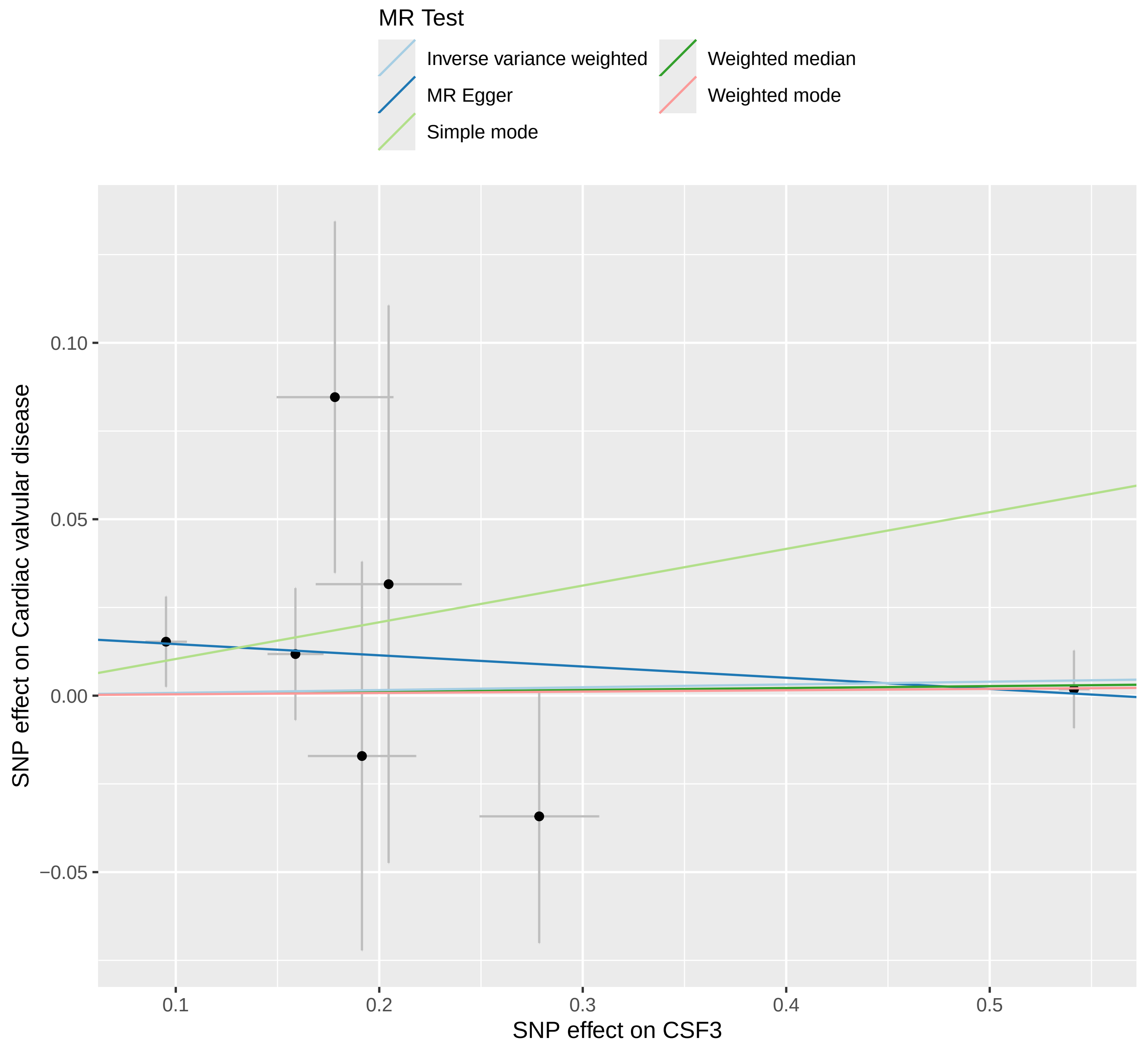
MR Test
Inverse variance weighted
MR Egger
Simple mode
Weighted median
Weighted mode
0.10
0.05
0.00
−0.05
SNP effect on Cardiac valvular disease
0.1
0.2
0.3
0.4
0.5
SNP effect on CSF3

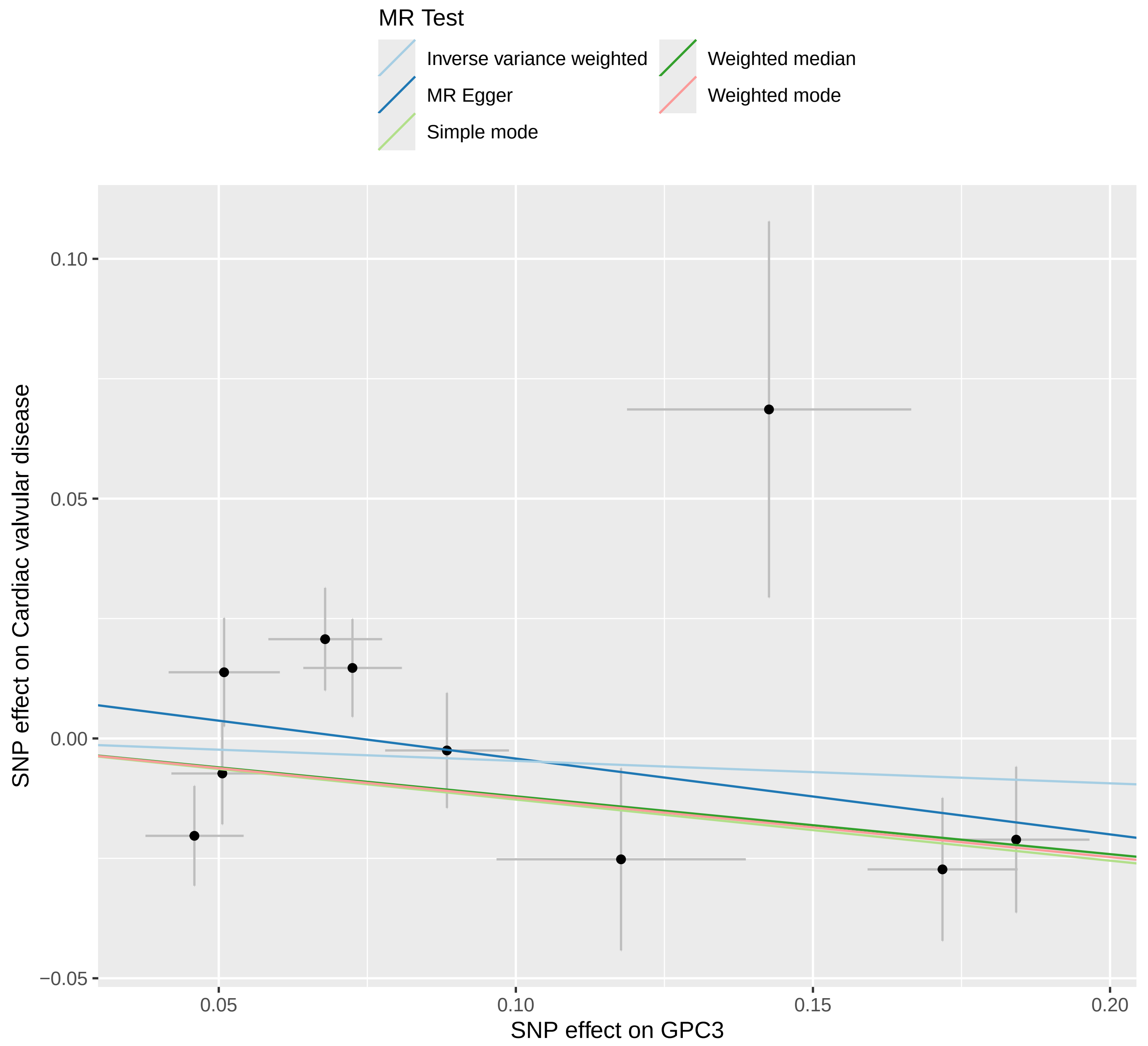
MR Test
Inverse variance weighted
MR Egger
Simple mode
Weighted median
Weighted mode
SNP effect on Cardiac valvular disease
0.10
0.05
0.00
−0.05
0.05
0.10
0.15
0.20
SNP effect on GPC3

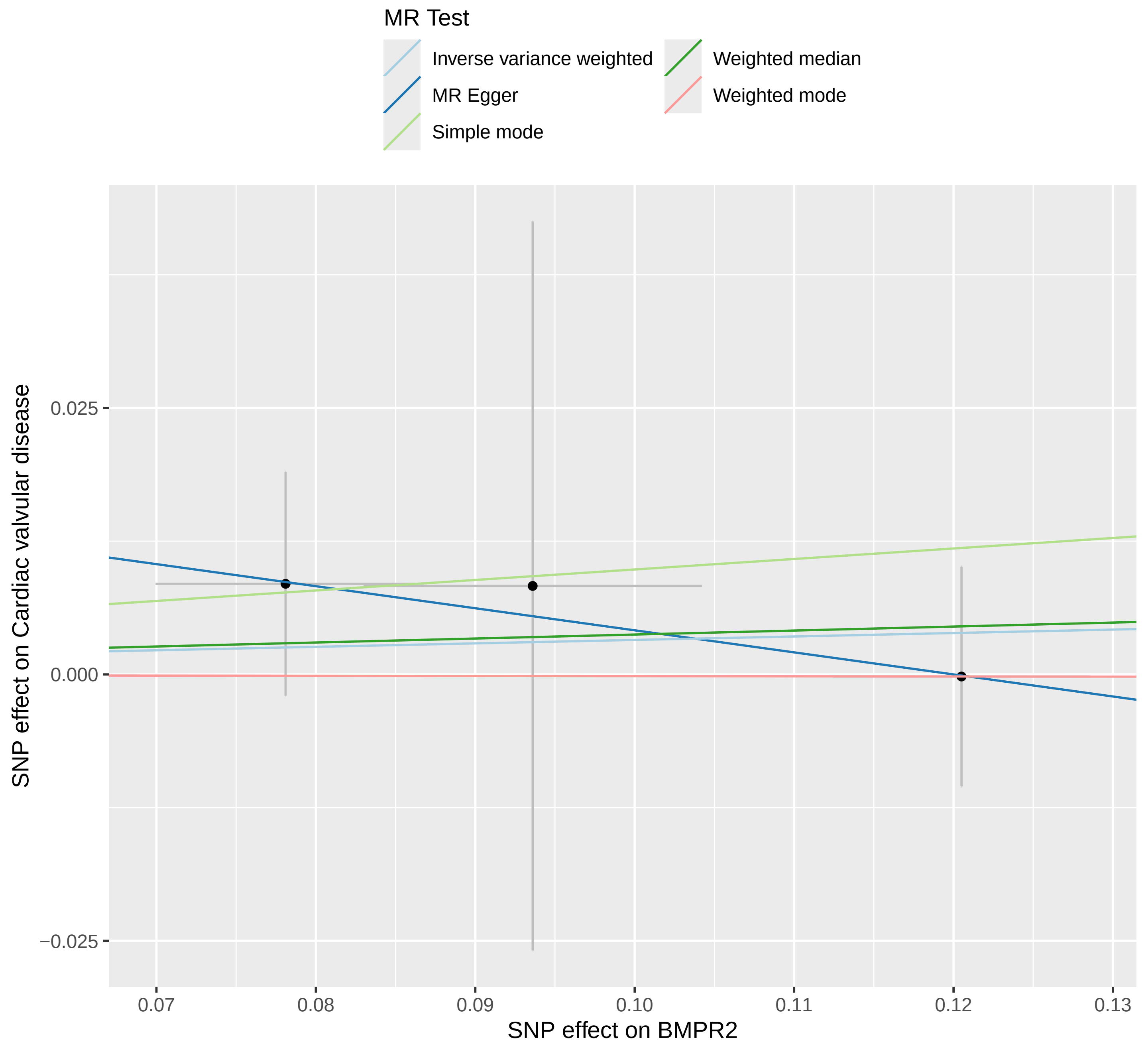

MR Test
Inverse variance weighted
MR Egger
Simple mode
Weighted median
Weighted mode
SNP effect on Cardiac valvular disease
0.025
0.000
−0.025
0.07
0.08
0.09
0.10
0.11
0.12
0.13
SNP effect on BMPR2

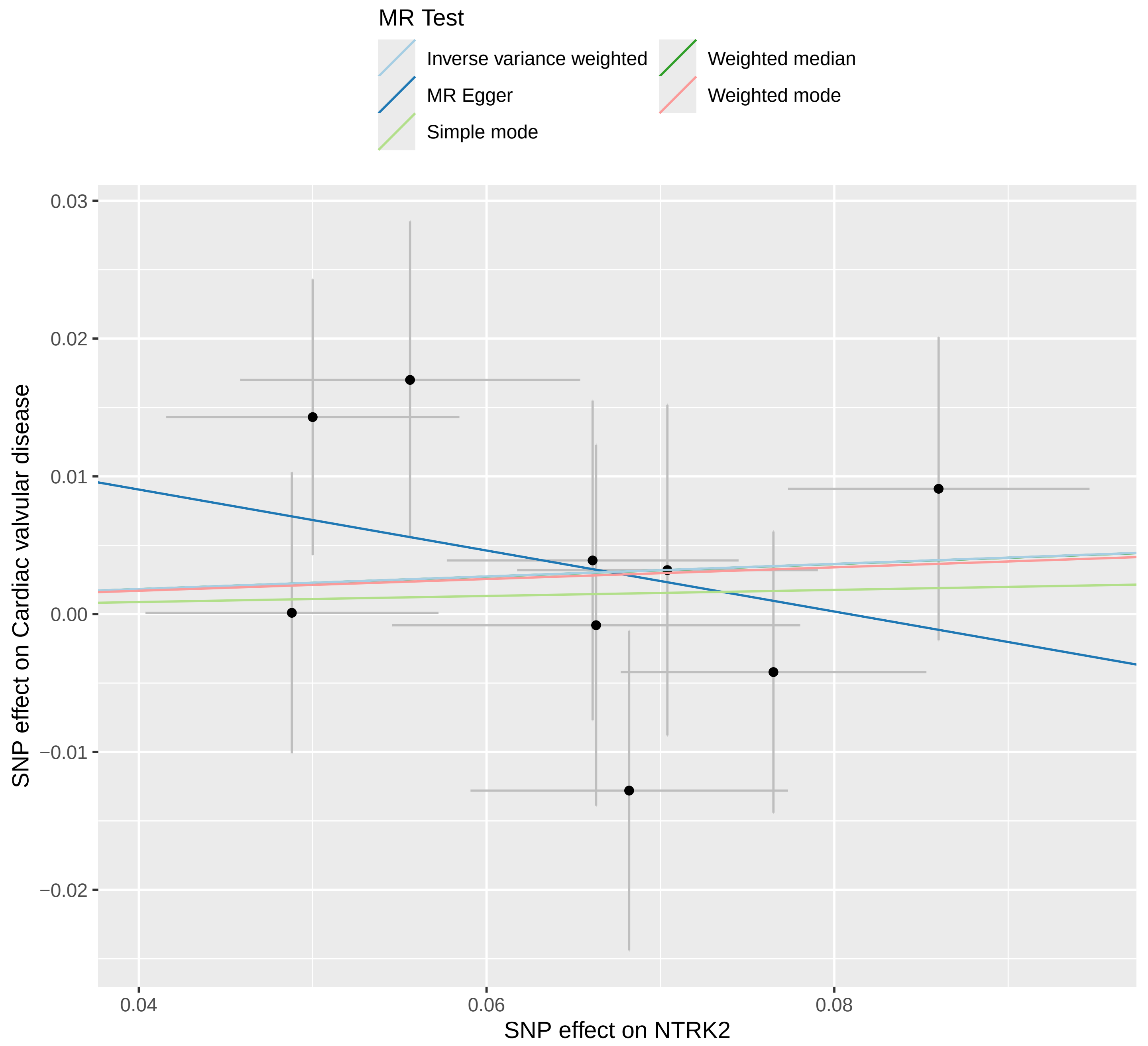

MR Test
Inverse variance weighted
MR Egger
Simple mode
Weighted median
Weighted mode
0.03
0.02
0.01
0.00
−0.01
−0.02
SNP effect on Cardiac valvular disease
0.04
0.06
0.08
SNP effect on NTRK2

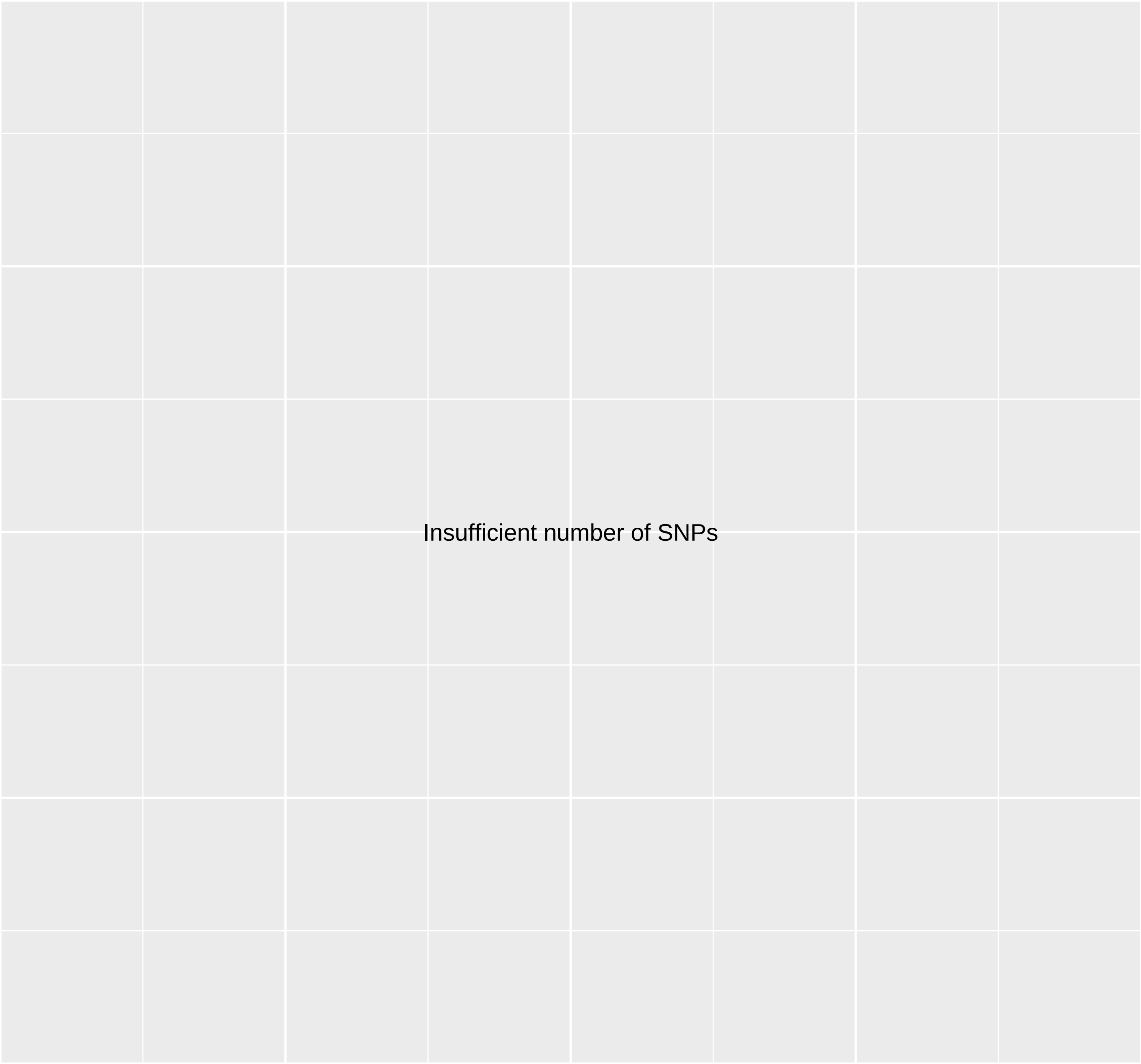
Insufficient number of SNPs

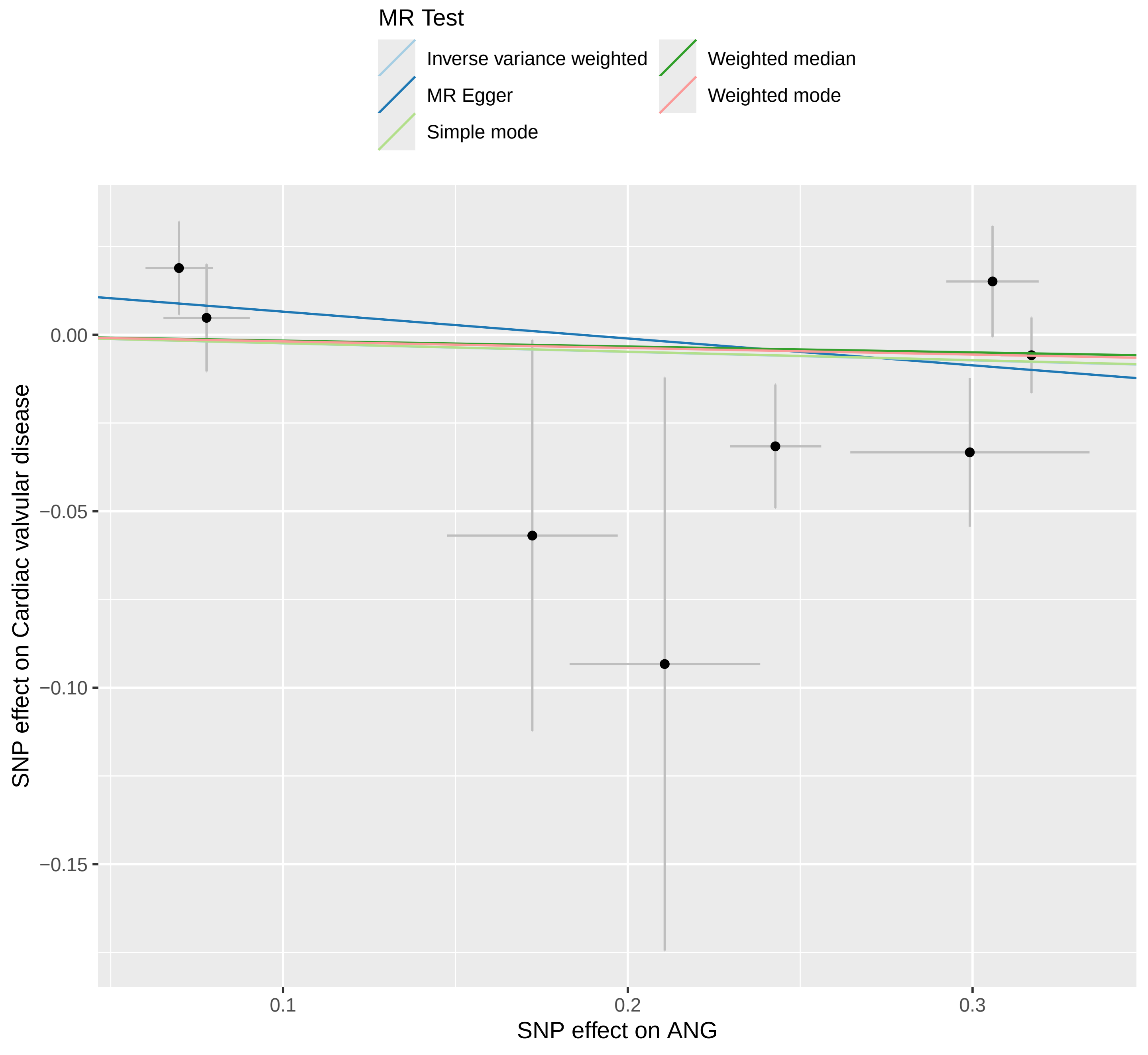
MR Test
Inverse variance weighted
MR Egger
Simple mode
Weighted median
Weighted mode
0.00
−0.05
−0.10
−0.15
0.1
0.2
0.3
SNP effect on Cardiac valvular disease
SNP effect on ANG

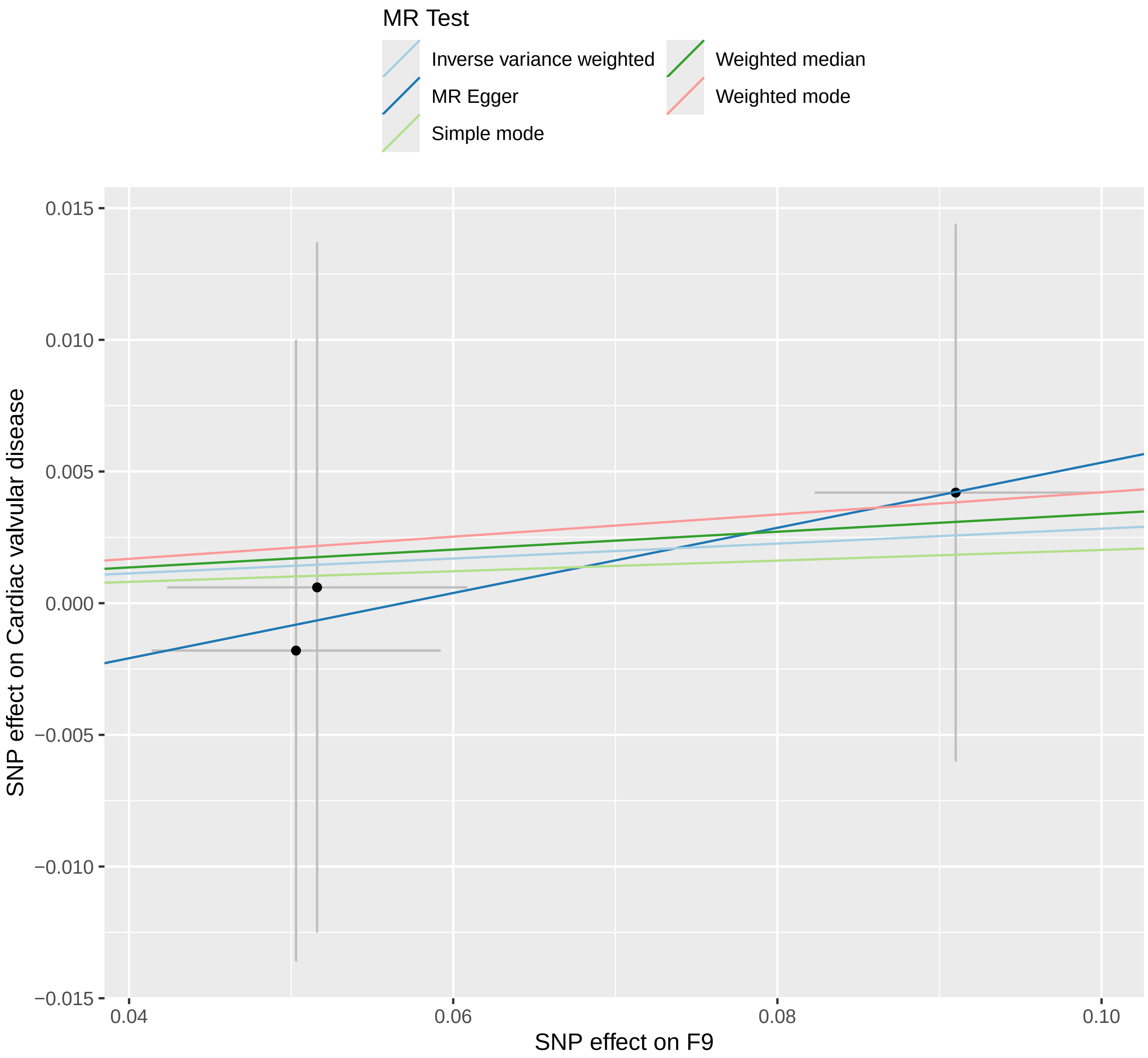
MR Test
Inverse variance weighted
MR Egger
Simple mode
Weighted median
Weighted mode
0.015
0.010
0.005
0.000
−0.005
−0.010
−0.015
0.04
0.06
0.08
0.10
SNP effect on F9
SNP effect on Cardiac valvular disease

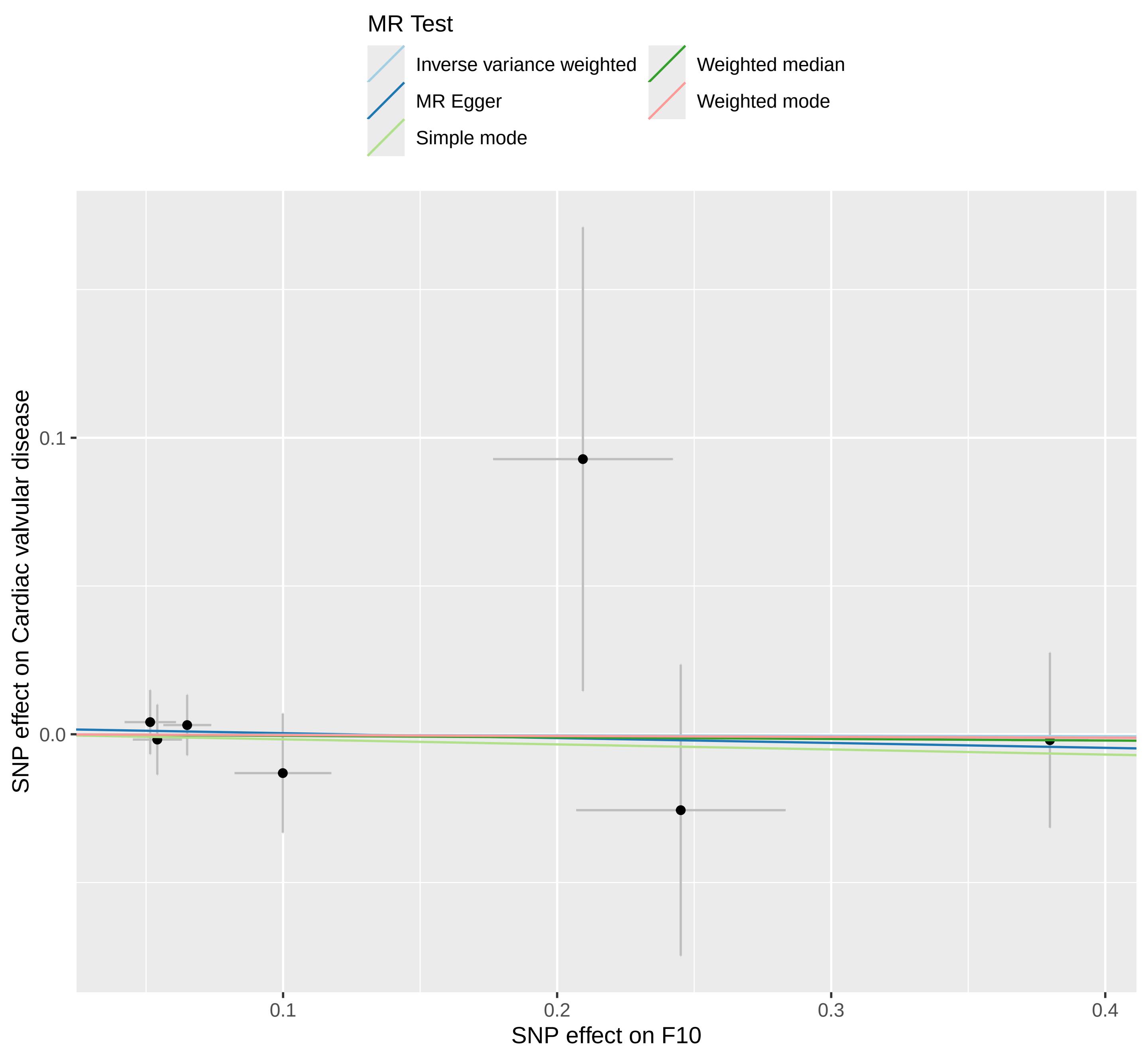

MR Test
Inverse variance weighted
MR Egger
Simple mode
Weighted median
Weighted mode
SNP effect on Cardiac valvular disease
0.1
0.0
0.1
0.2
0.3
0.4
SNP effect on F10

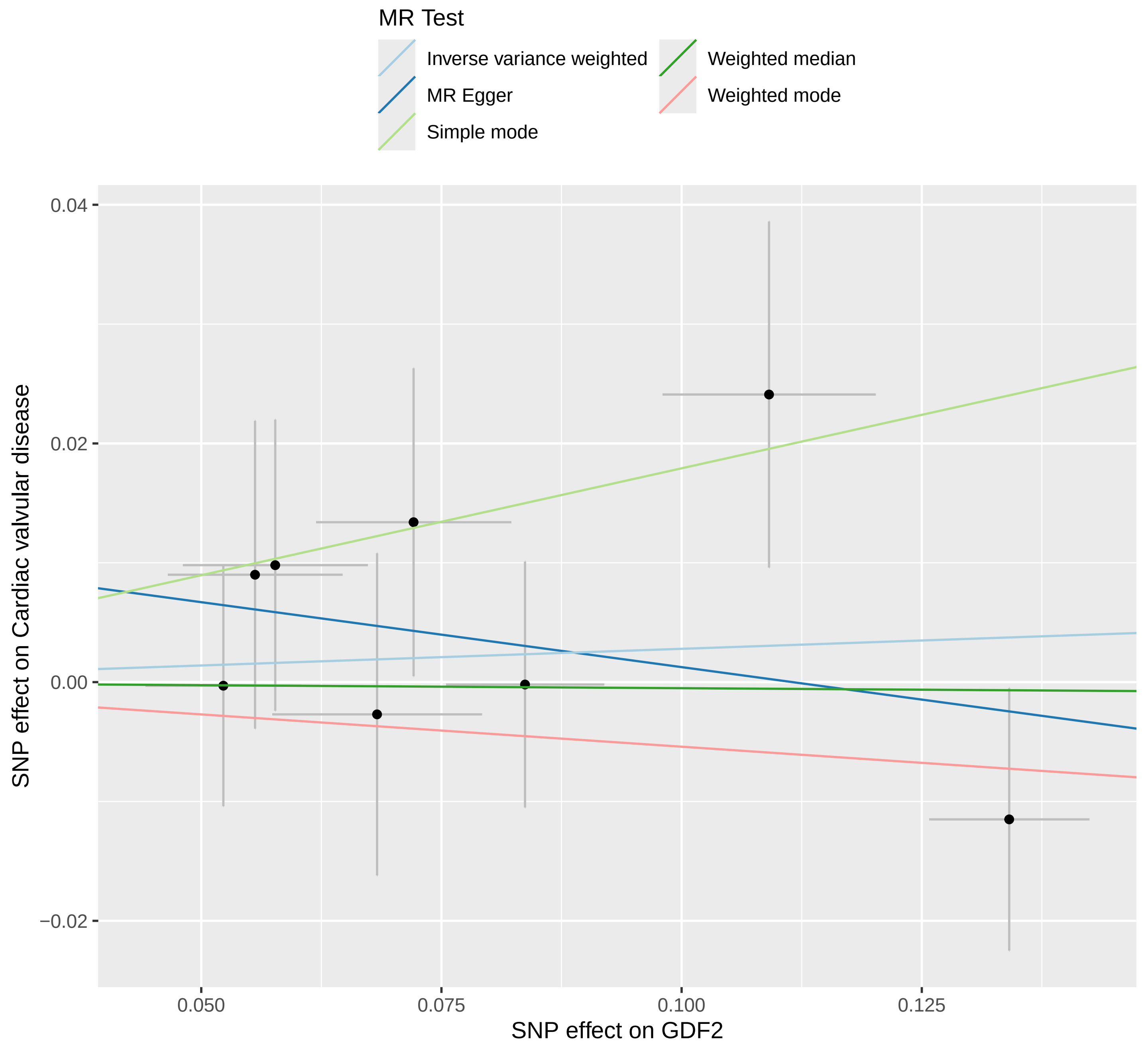
MR Test
Inverse variance weighted
MR Egger
Simple mode
Weighted median
Weighted mode
0.04
0.02
0.00
−0.02
SNP effect on Cardiac valvular disease
0.050
0.075
0.100
0.125
SNP effect on GDF2

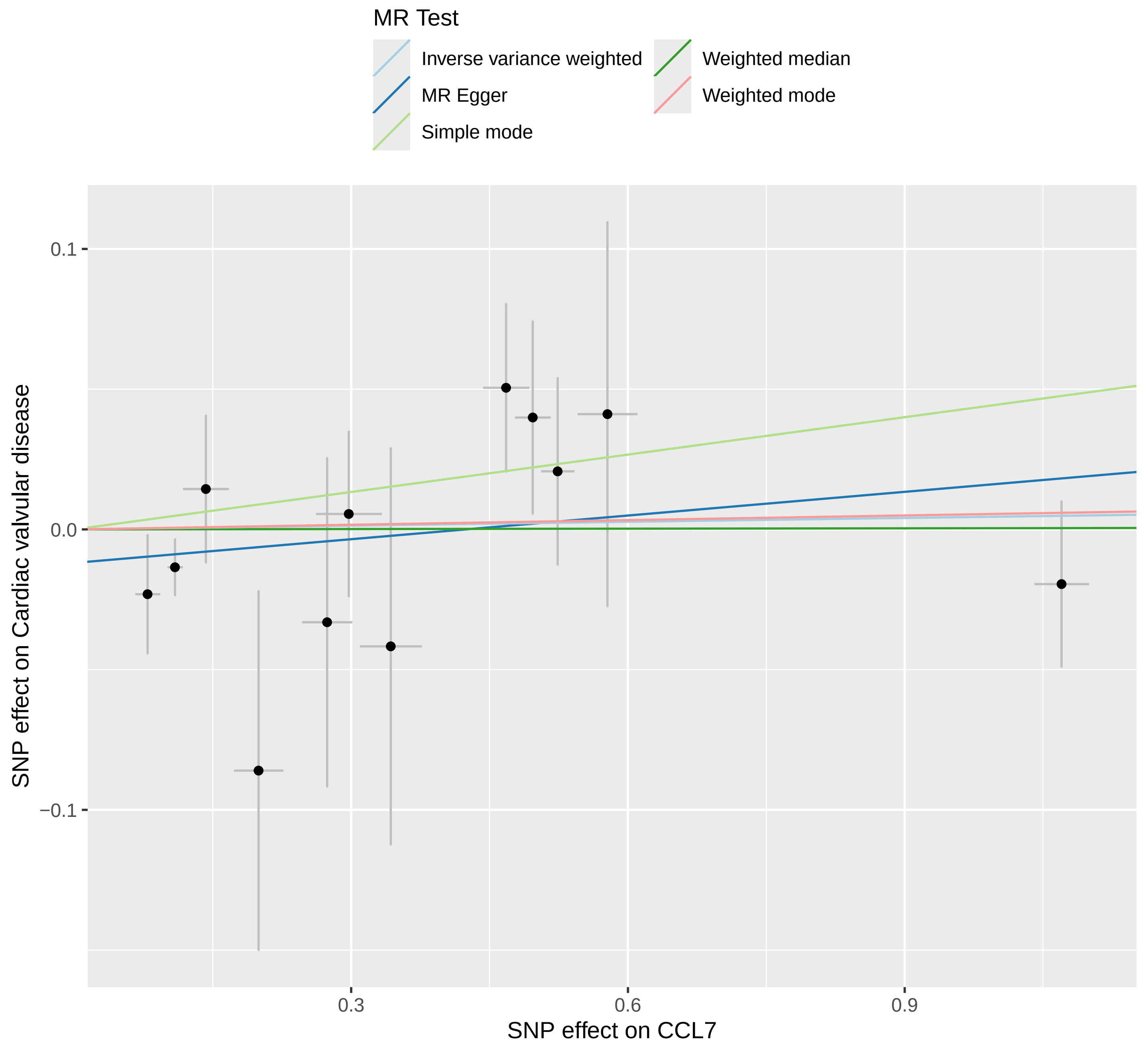

MR Test
Inverse variance weighted
MR Egger
Simple mode
Weighted median
Weighted mode
0.1
0.0
−0.1
0.3
0.6
0.9
SNP effect on CCL7
SNP effect on Cardiac valvular disease

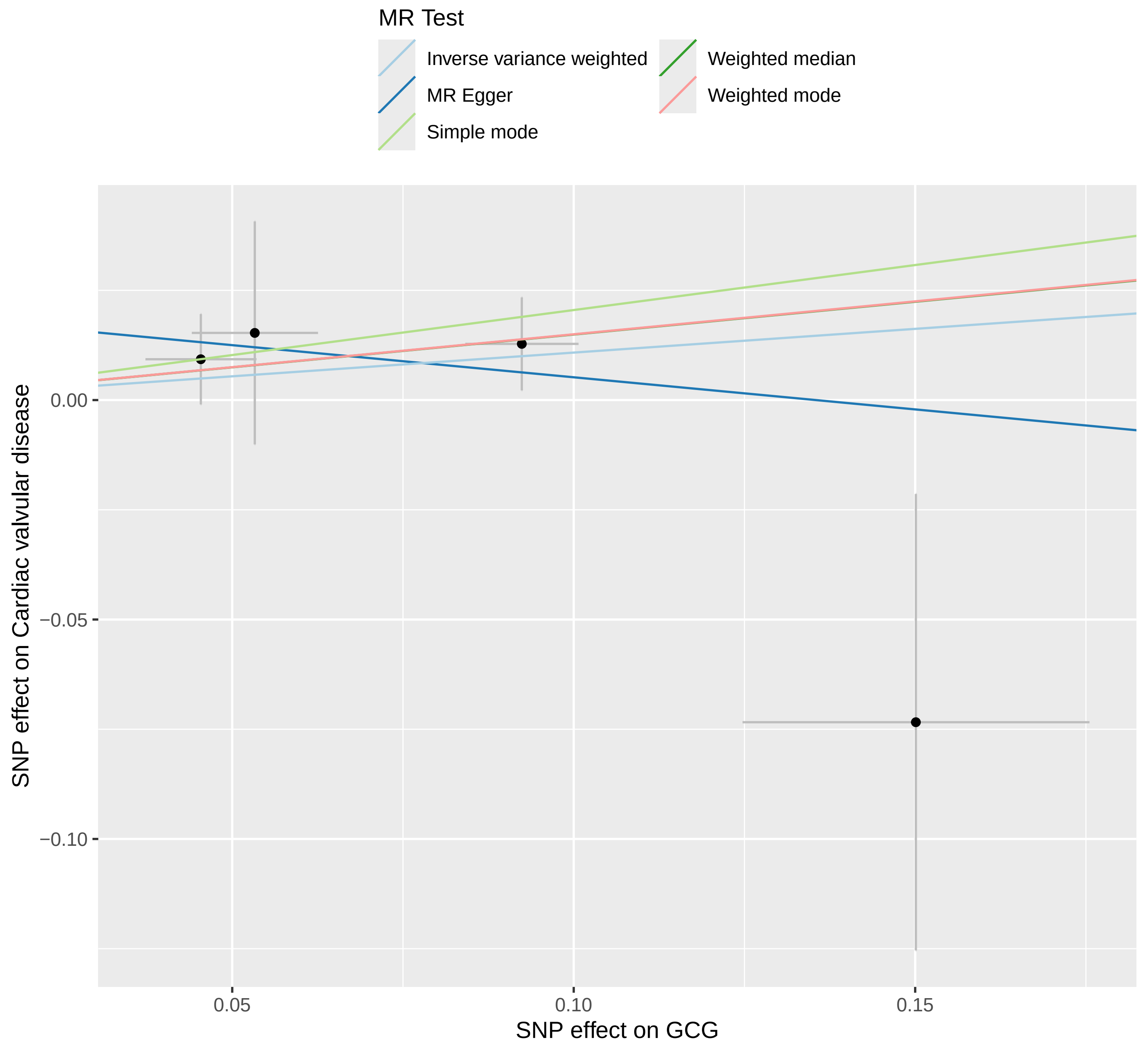
MR Test
Inverse variance weighted
MR Egger
Simple mode
Weighted median
Weighted mode
SNP effect on Cardiac valvular disease
0.00
−0.05
−0.10
0.05
0.10
0.15
SNP effect on GCG

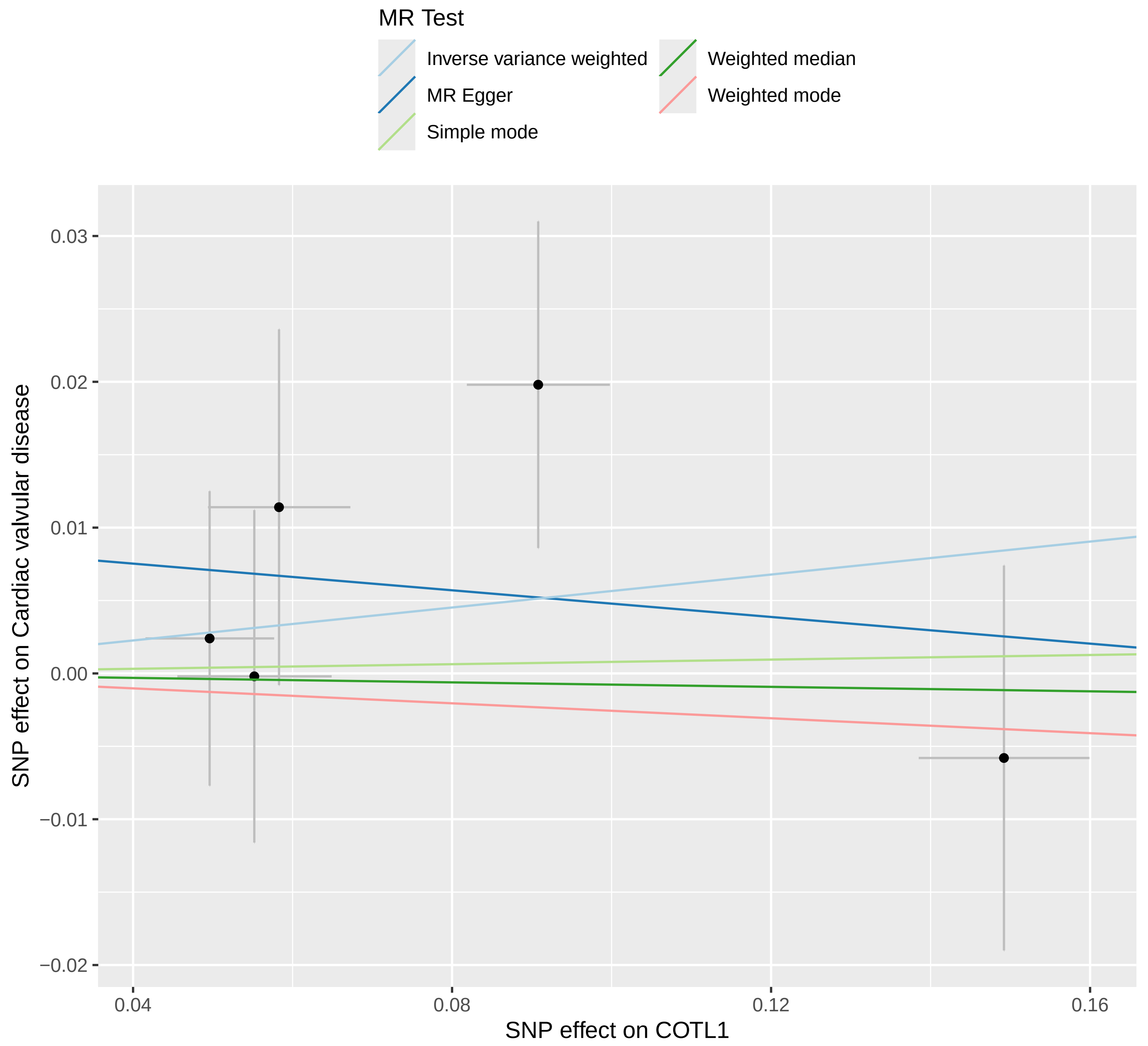
MR Test
Inverse variance weighted
MR Egger
Simple mode
Weighted median
Weighted mode
0.03
0.02
0.01
0.00
−0.01
−0.02
SNP effect on Cardiac valvular disease
0.04
0.08
0.12
0.16
SNP effect on COTL1

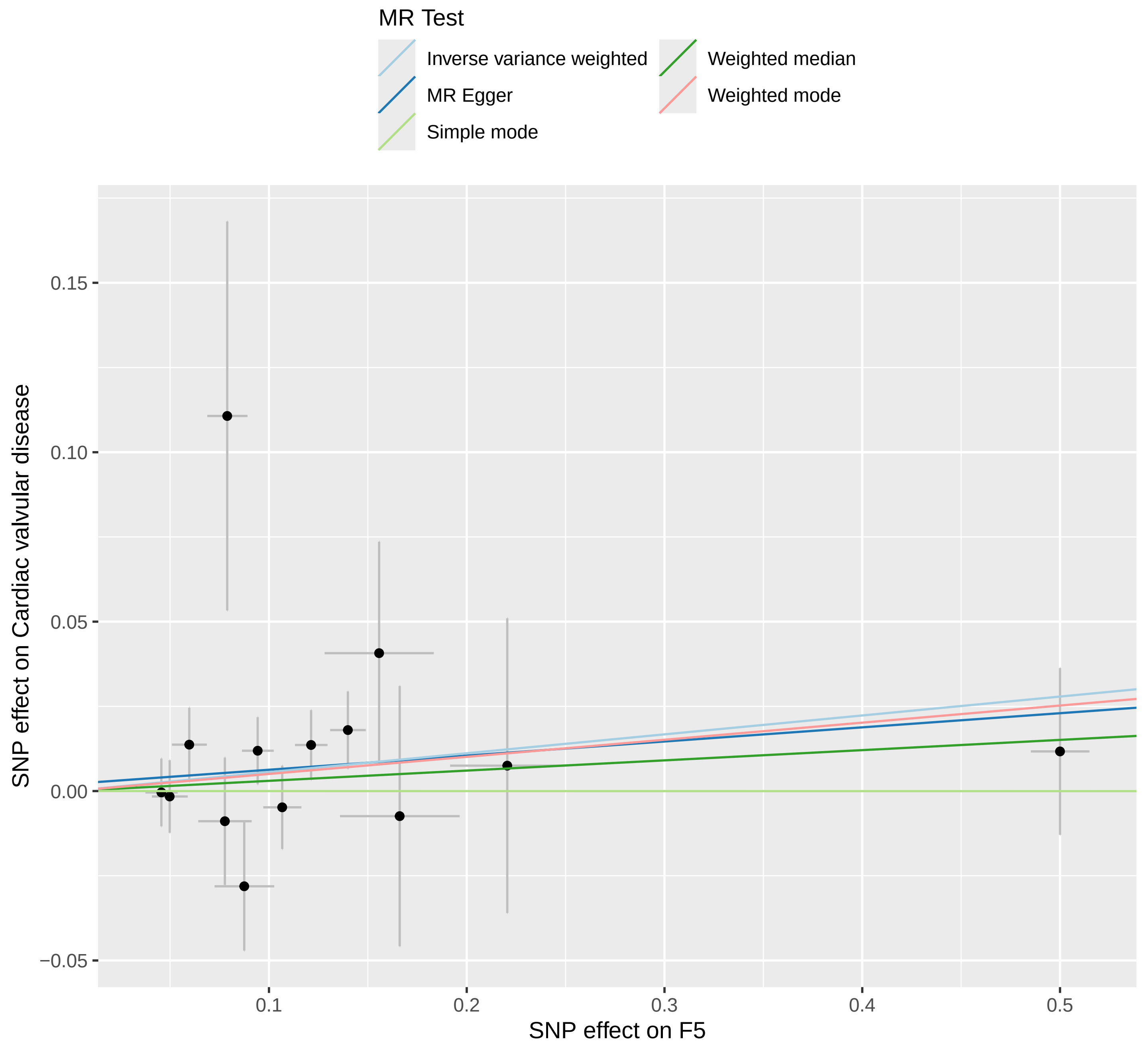
MR Test
Inverse variance weighted
MR Egger
Simple mode
Weighted median
Weighted mode
0.15
0.10
0.05
0.00
−0.05
0.1
0.2
0.3
0.4
0.5
SNP effect on Cardiac valvular disease
SNP effect on F5

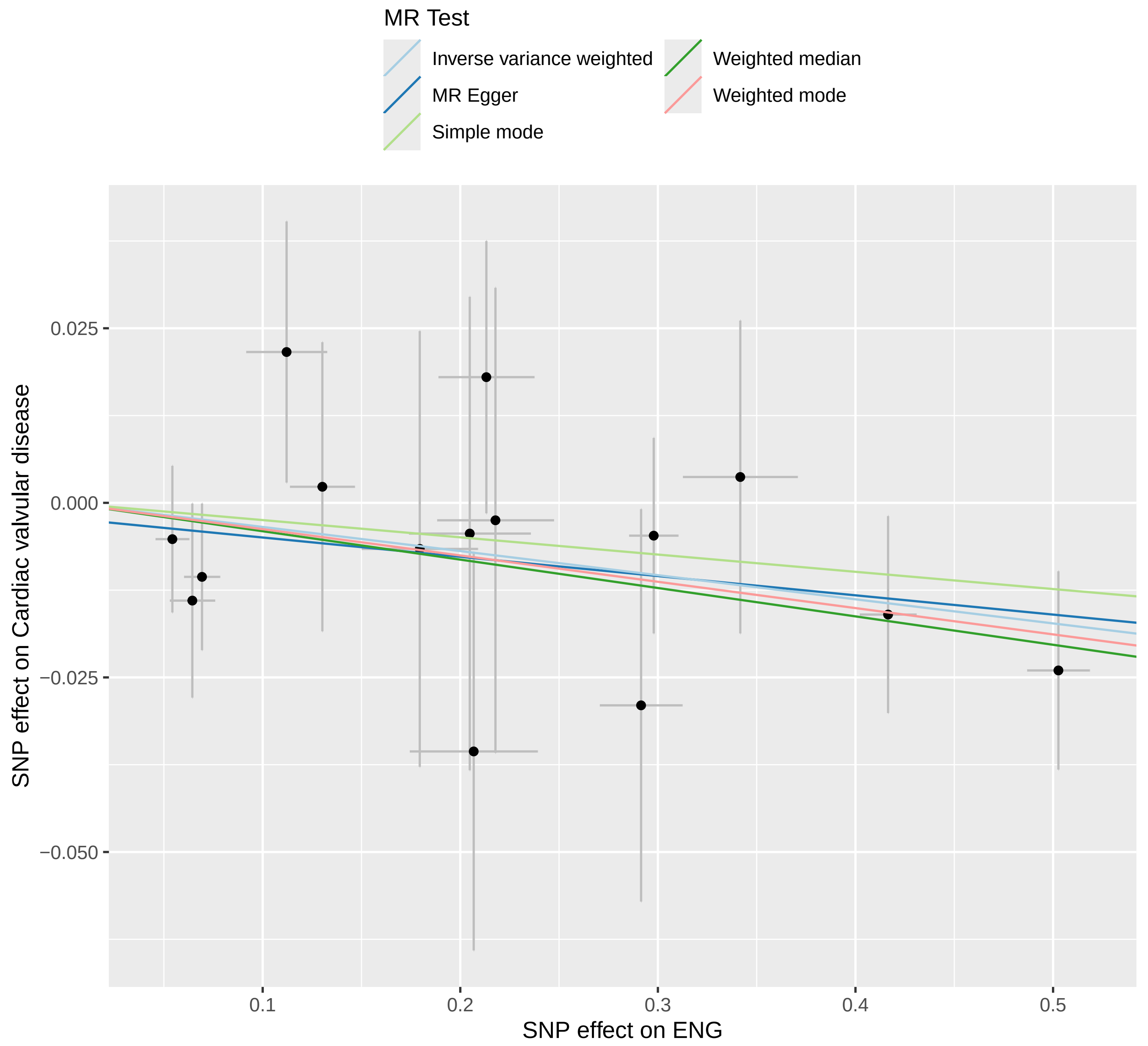
MR Test
Inverse variance weighted
MR Egger
Simple mode
Weighted median
Weighted mode
0.025
0.000
−0.025
−0.050
SNP effect on Cardiac valvular disease
0.1
0.2
0.3
0.4
0.5
SNP effect on ENG

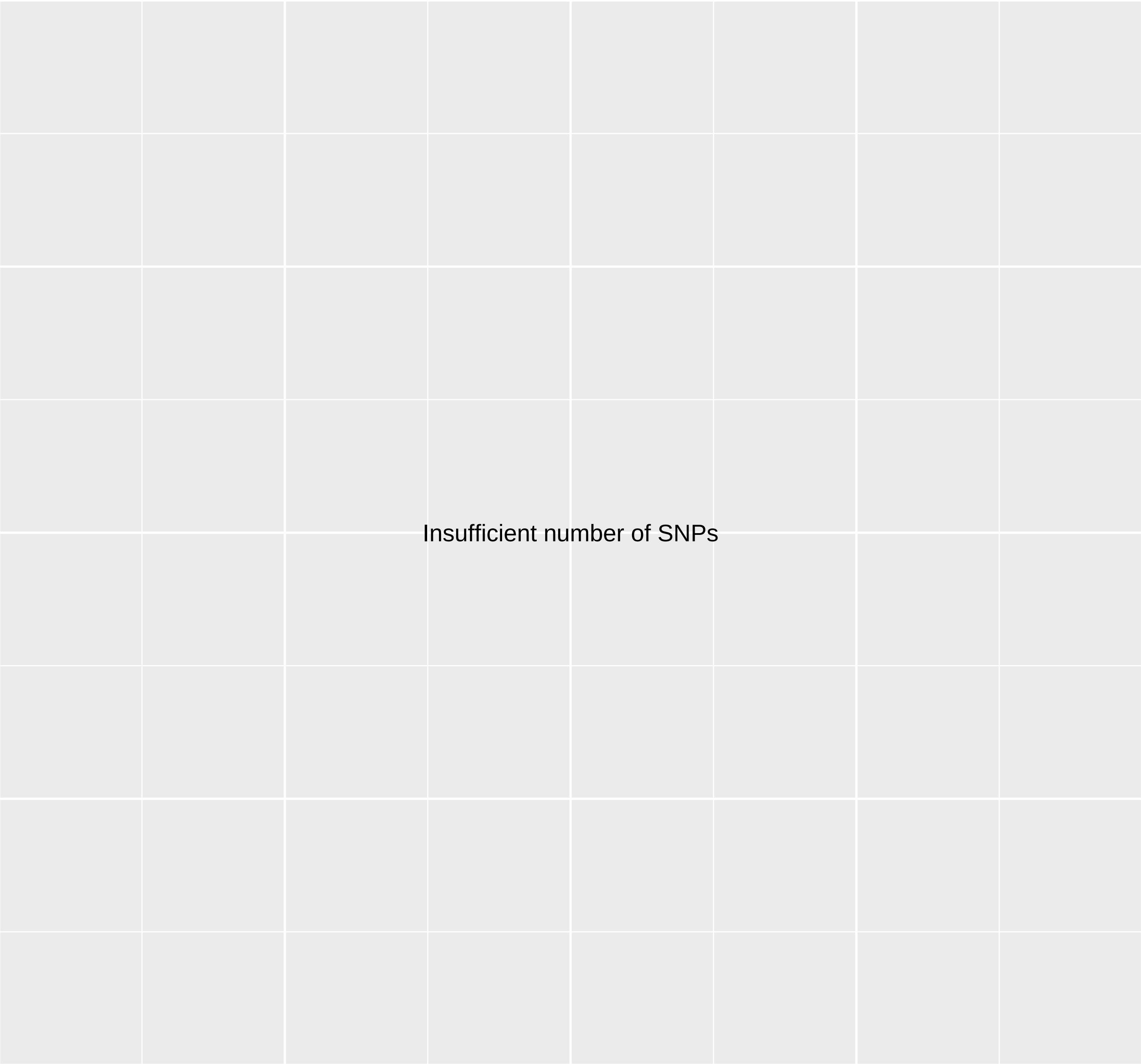
Insufficient number of SNPs

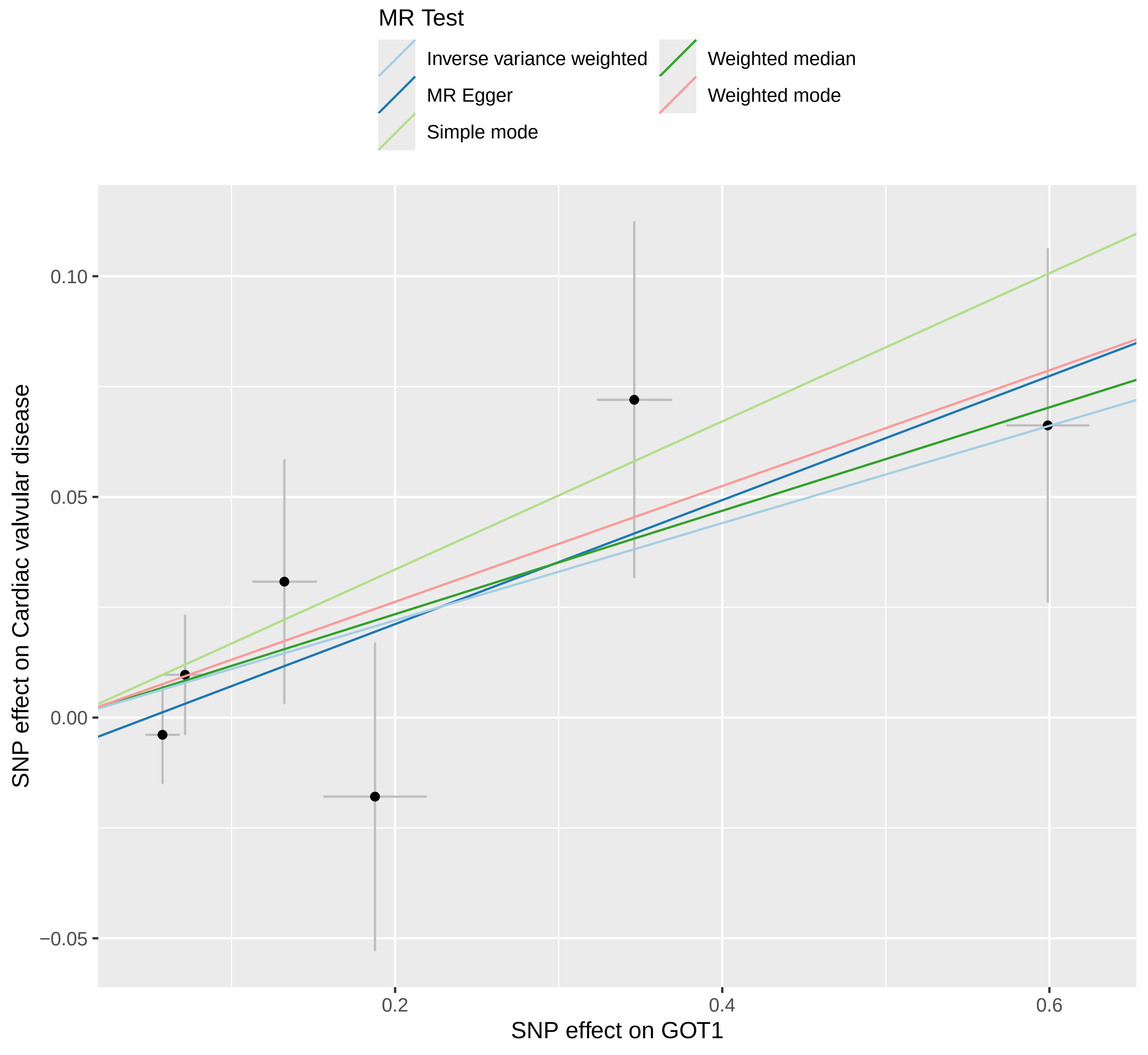
MR Test
Inverse variance weighted
MR Egger
Simple mode
Weighted median
Weighted mode
0.10
0.05
0.00
−0.05
0.2
0.4
0.6
SNP effect on Cardiac valvular disease
SNP effect on GOT1

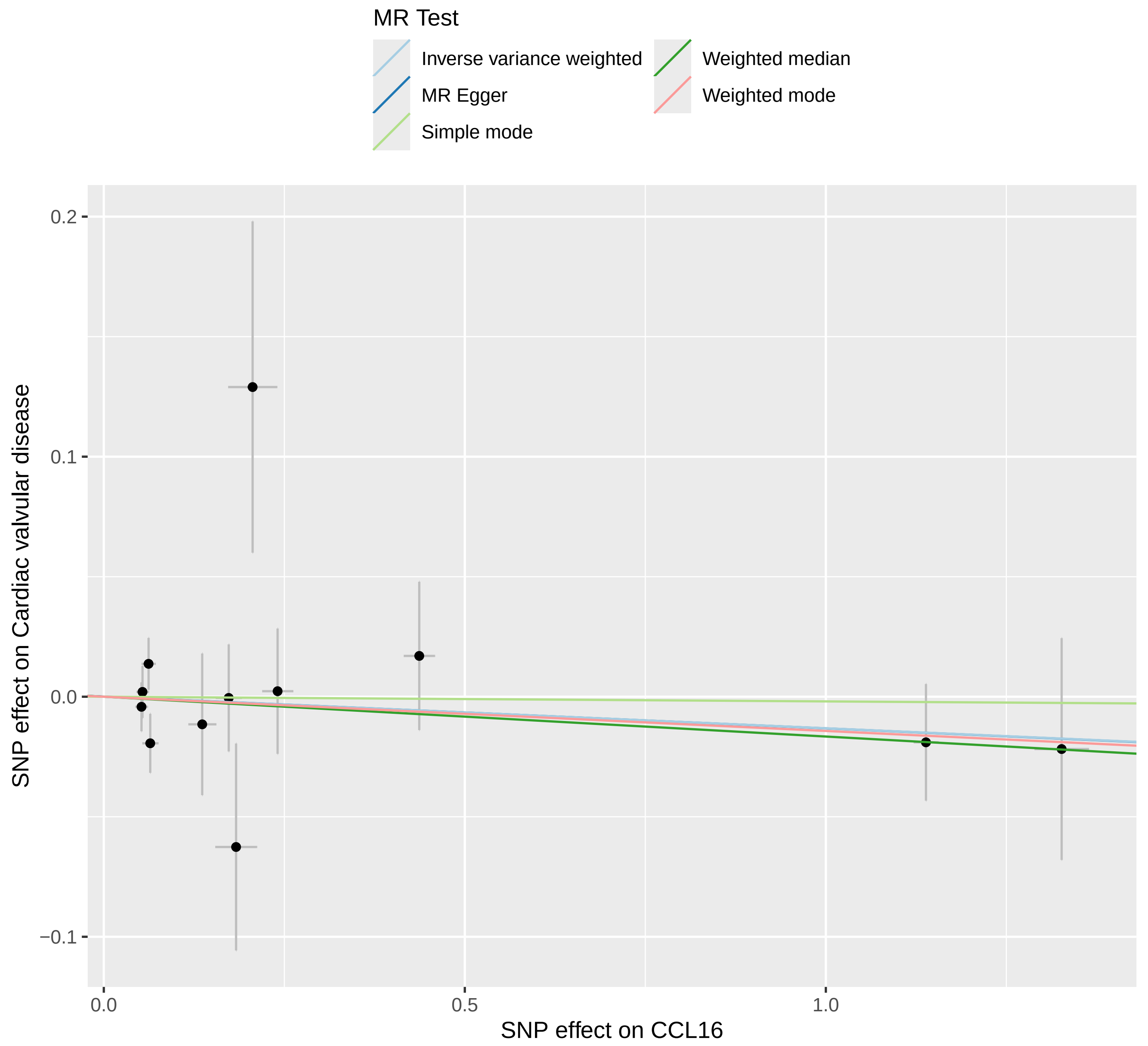
MR Test
Inverse variance weighted
MR Egger
Simple mode
Weighted median
Weighted mode
0.2
0.1
0.0
−0.1
SNP effect on Cardiac valvular disease
0.0
0.5
1.0
SNP effect on CCL16

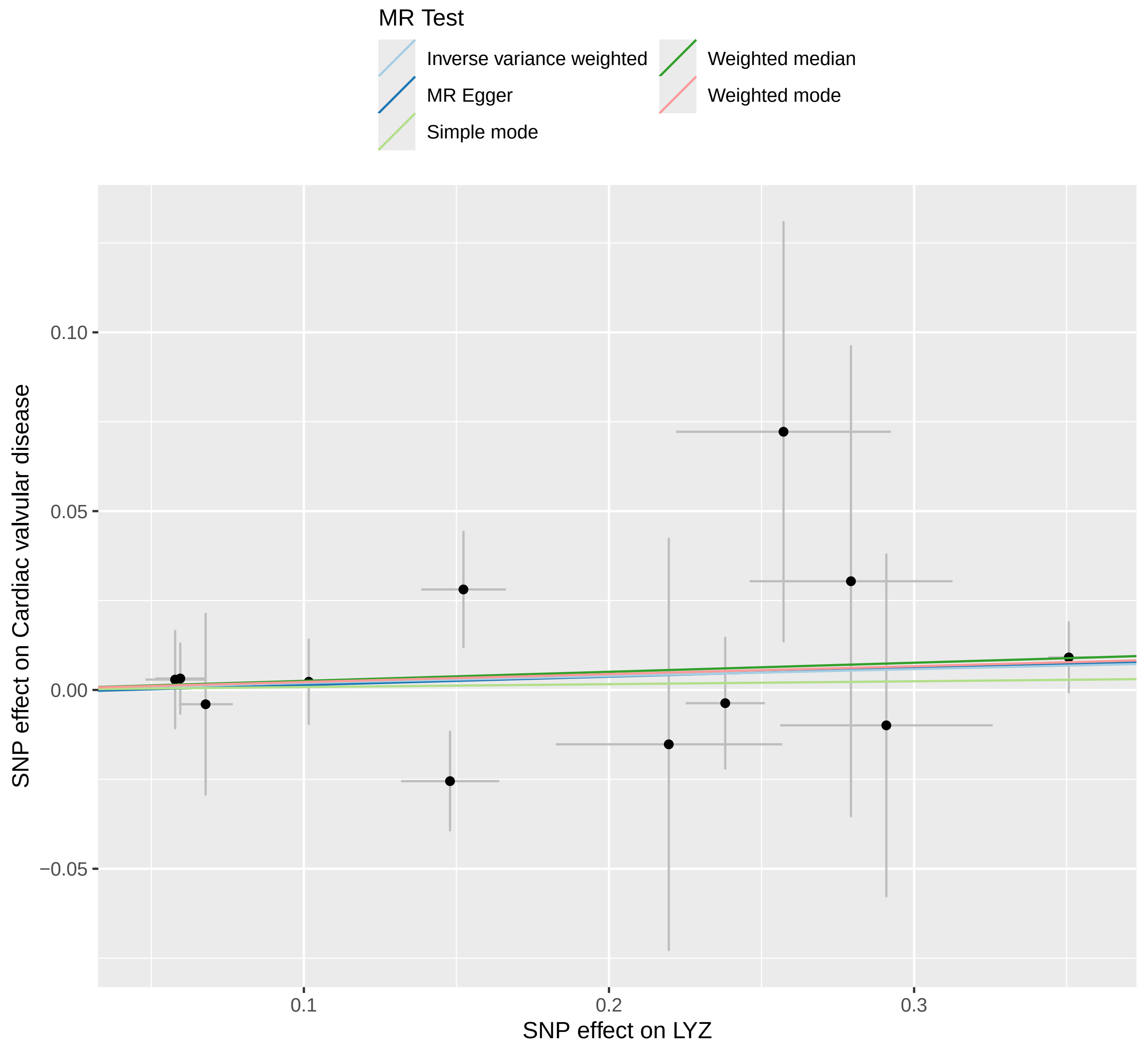
MR Test
Inverse variance weighted
MR Egger
Simple mode
Weighted median
Weighted mode
0.10
0.05
0.00
−0.05
SNP effect on Cardiac valvular disease
0.1
0.2
0.3
SNP effect on LYZ

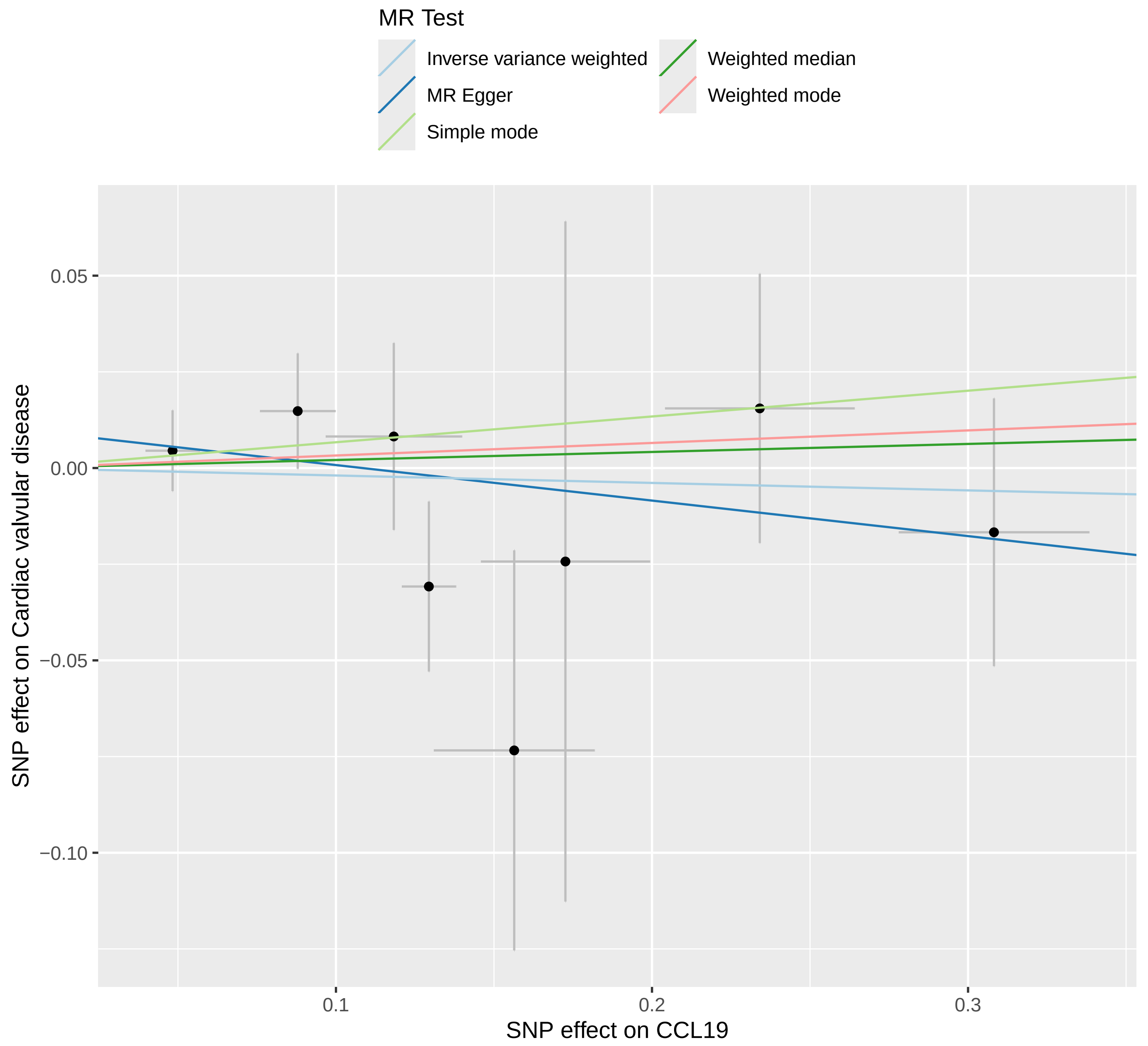
MR Test
Inverse variance weighted
MR Egger
Simple mode
Weighted median
Weighted mode
0.05
0.00
−0.05
−0.10
SNP effect on Cardiac valvular disease
0.1
0.2
0.3
SNP effect on CCL19

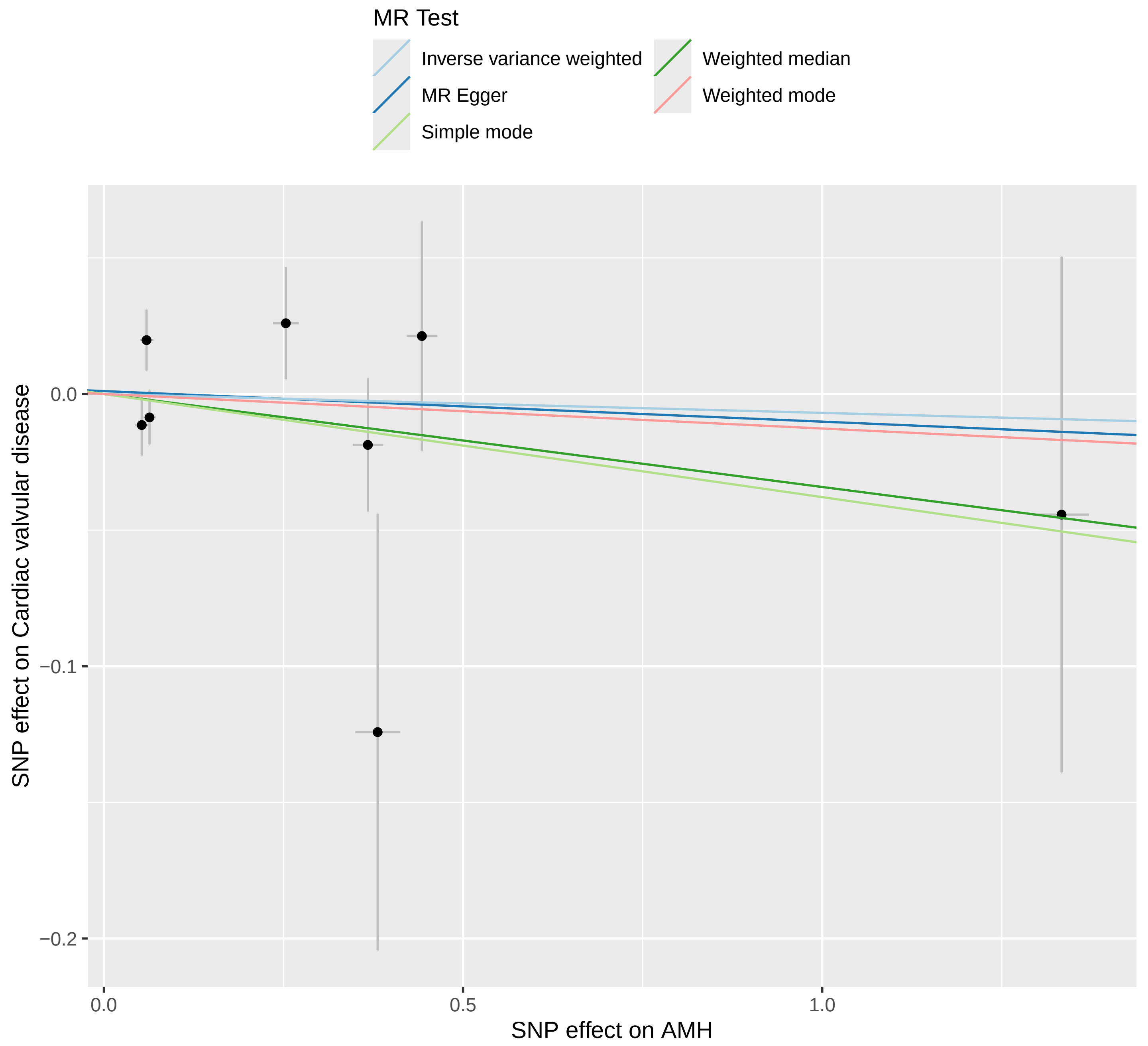
MR Test
Inverse variance weighted
MR Egger
Simple mode
Weighted median
Weighted mode
0.0
−0.1
−0.2
SNP effect on Cardiac valvular disease
0.0
0.5
1.0
SNP effect on AMH

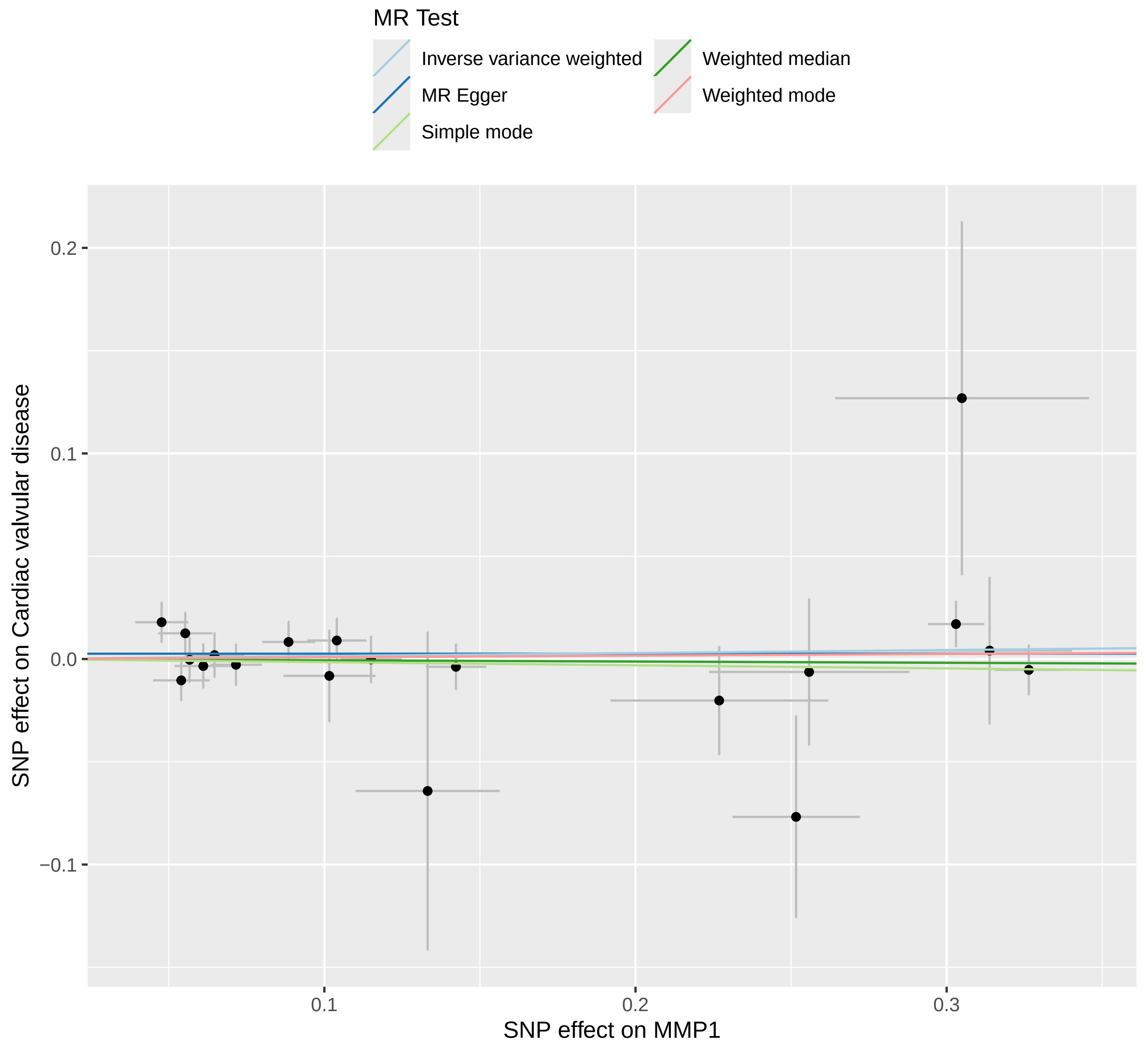
MR Test
Inverse variance weighted
MR Egger
Simple mode
Weighted median
Weighted mode
0.2
0.1
0.0
−0.1
0.1
0.2
0.3
SNP effect on Cardiac valvular disease
SNP effect on MMP1

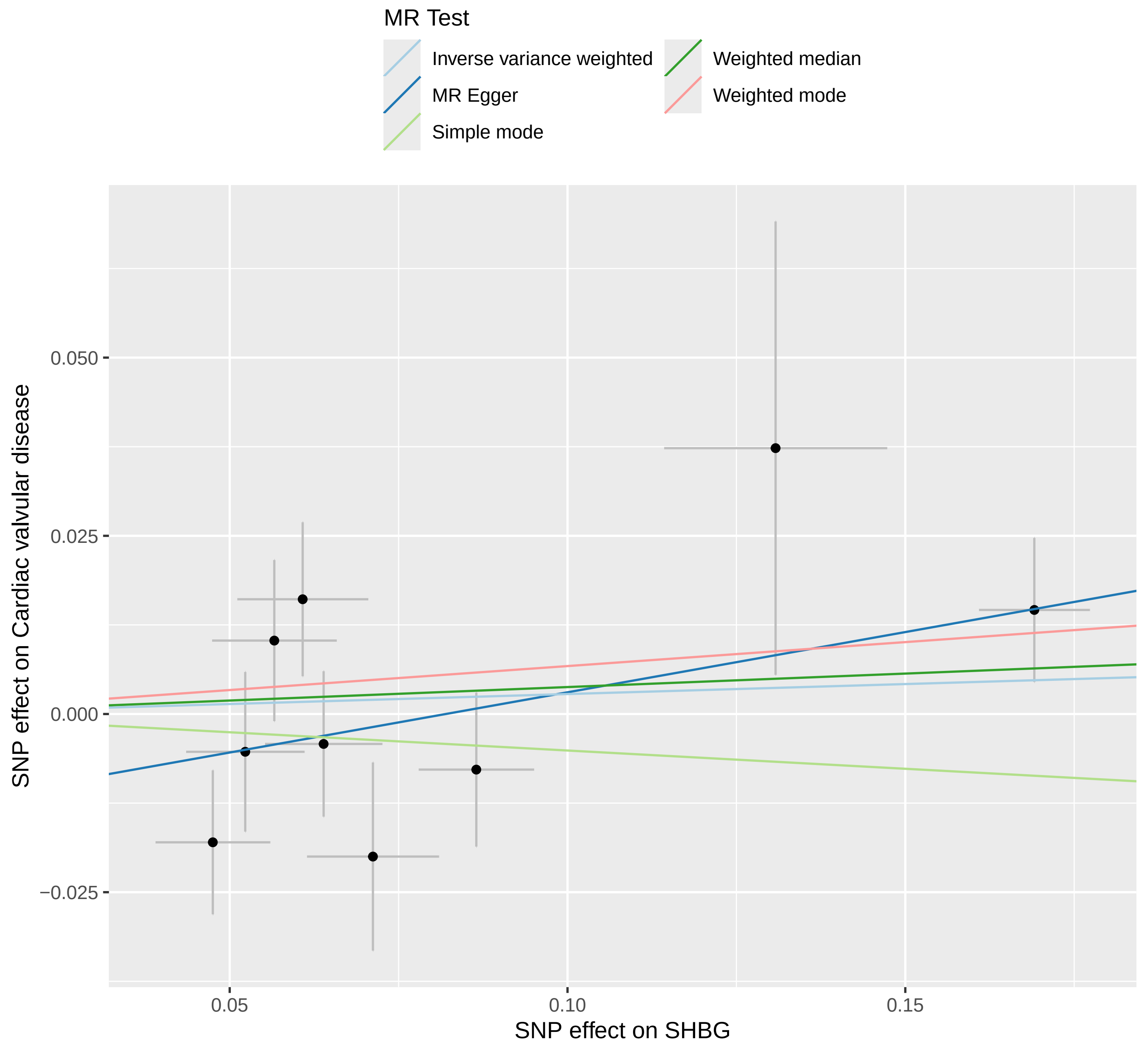
MR Test
Inverse variance weighted
MR Egger
Simple mode
Weighted median
Weighted mode
0.050
0.025
0.000
−0.025
0.05
0.10
0.15
SNP effect on Cardiac valvular disease
SNP effect on SHBG

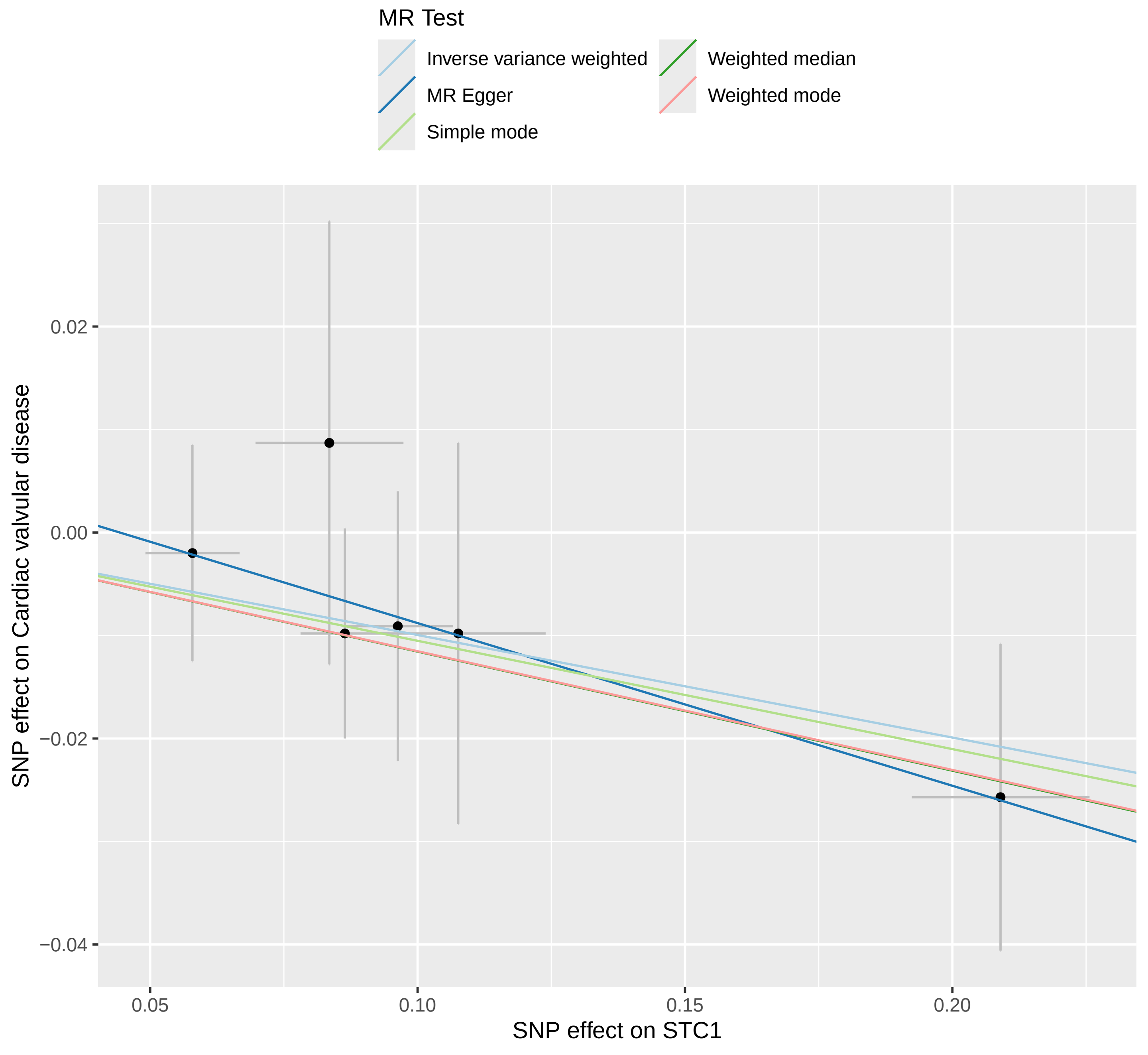
MR Test
Inverse variance weighted
MR Egger
Simple mode
Weighted median
Weighted mode
0.02
0.00
−0.02
−0.04
SNP effect on Cardiac valvular disease
0.05
0.10
0.15
0.20
SNP effect on STC1

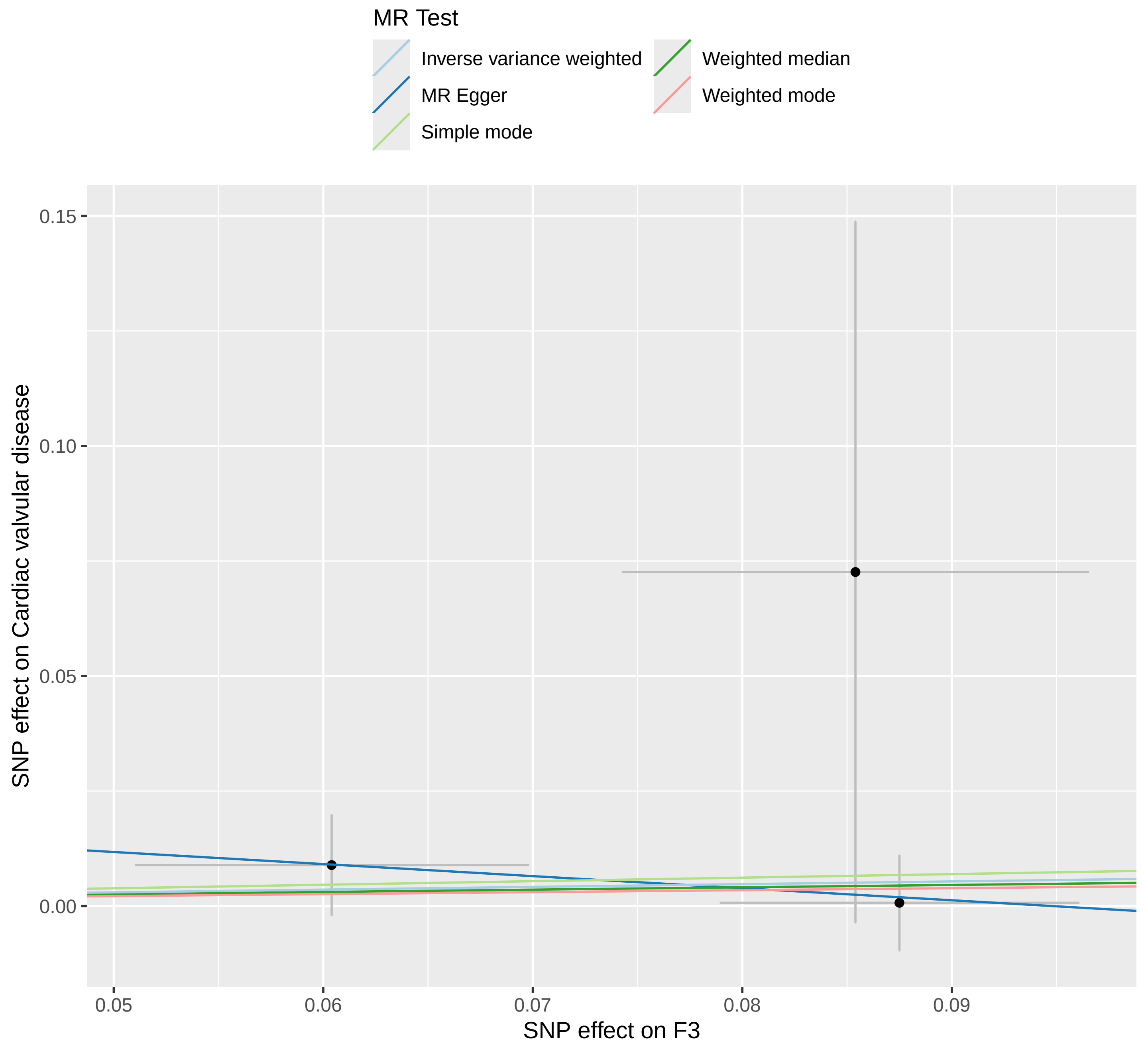

MR Test
Inverse variance weighted
MR Egger
Simple mode
Weighted median
Weighted mode
0.15
0.10
0.05
0.00
SNP effect on Cardiac valvular disease
0.05
0.06
0.07
0.08
0.09
SNP effect on F3

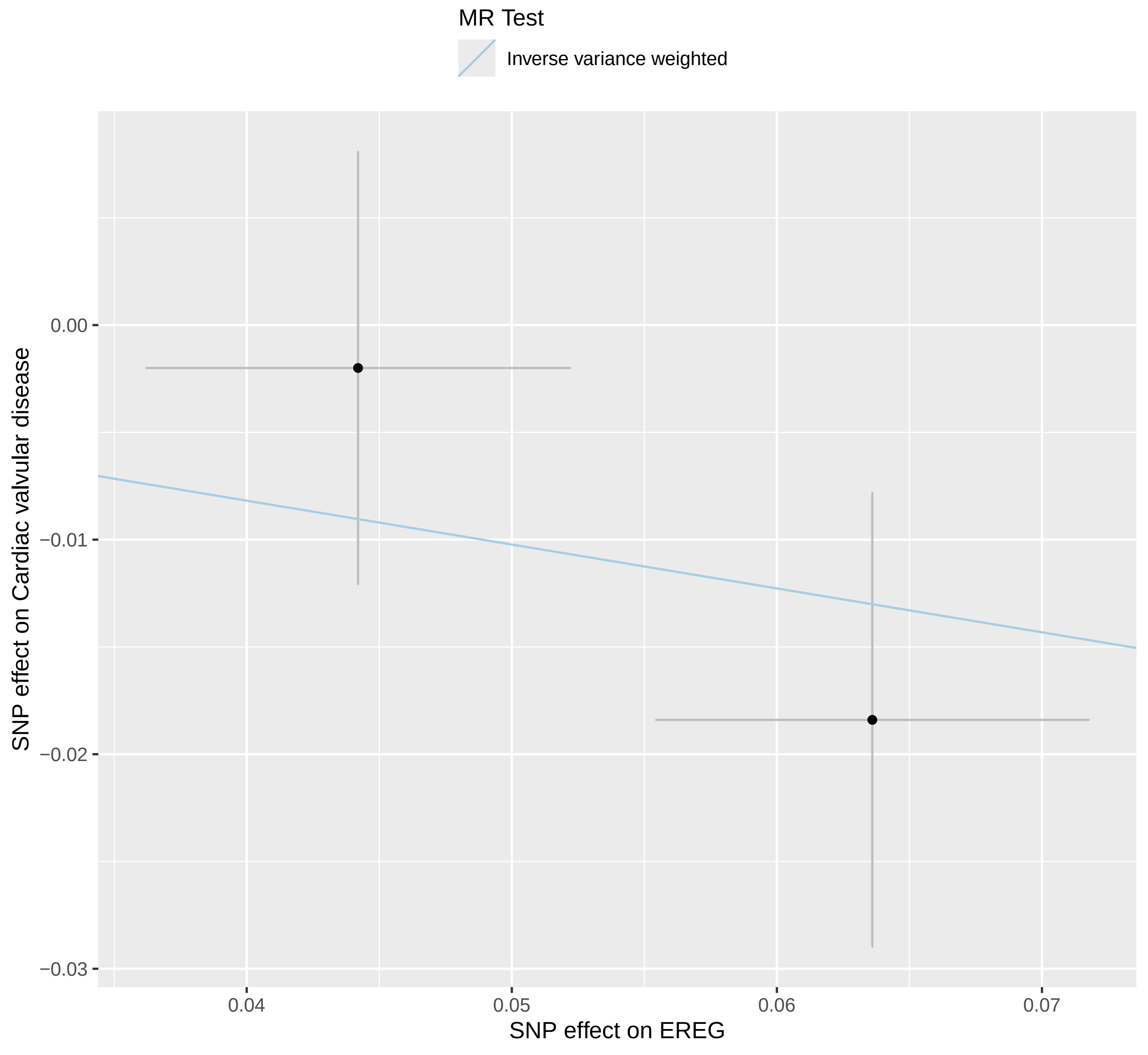

MR Test
Inverse variance weighted
0.00
−0.01
−0.02
−0.03
SNP effect on Cardiac valvular disease
0.04
0.05
0.06
0.07
SNP effect on EREG

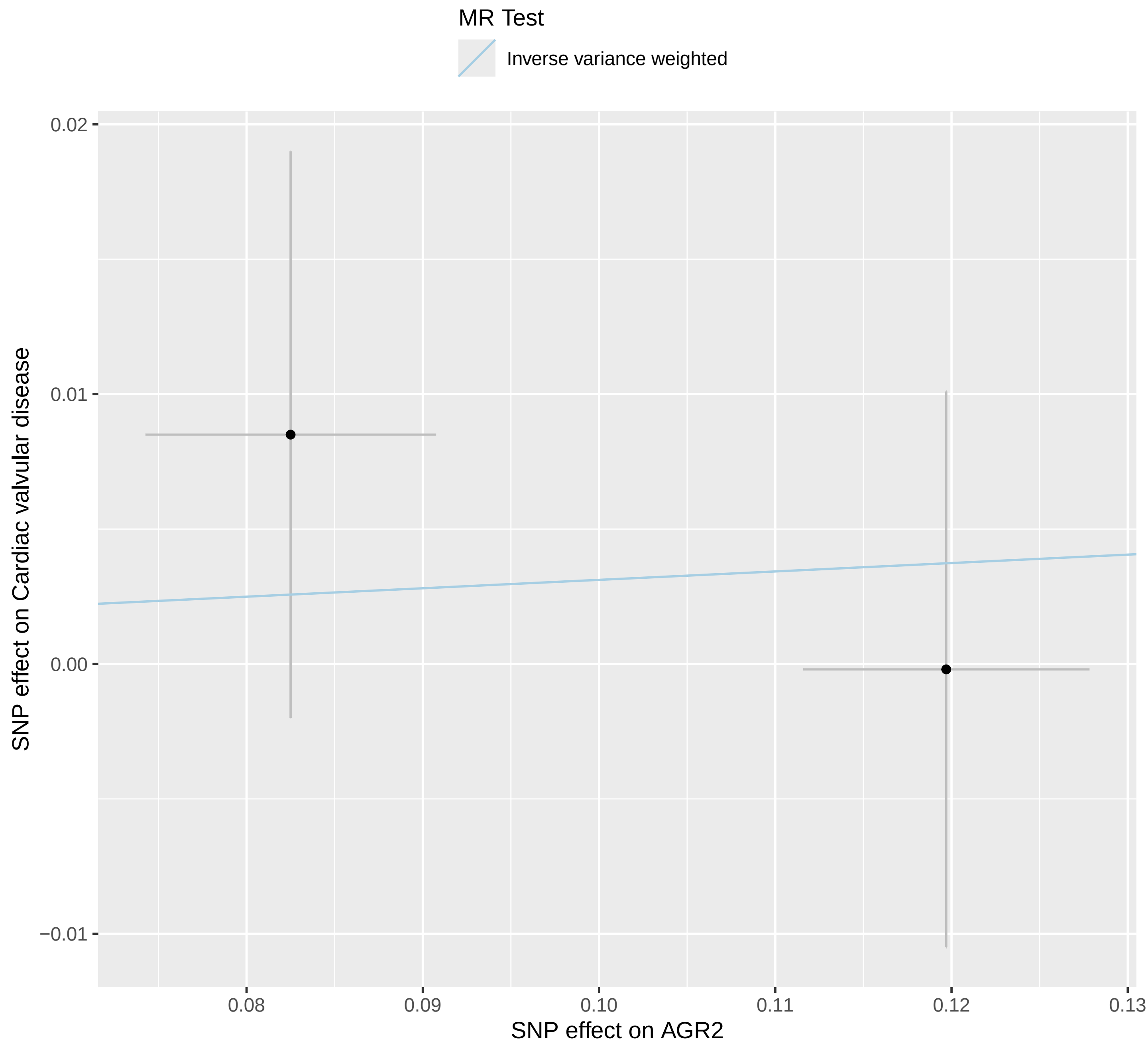
MR Test
Inverse variance weighted
0.02
0.01
0.00
−0.01
SNP effect on Cardiac valvular disease
0.08
0.09
0.10
0.11
0.12
0.13
SNP effect on AGR2

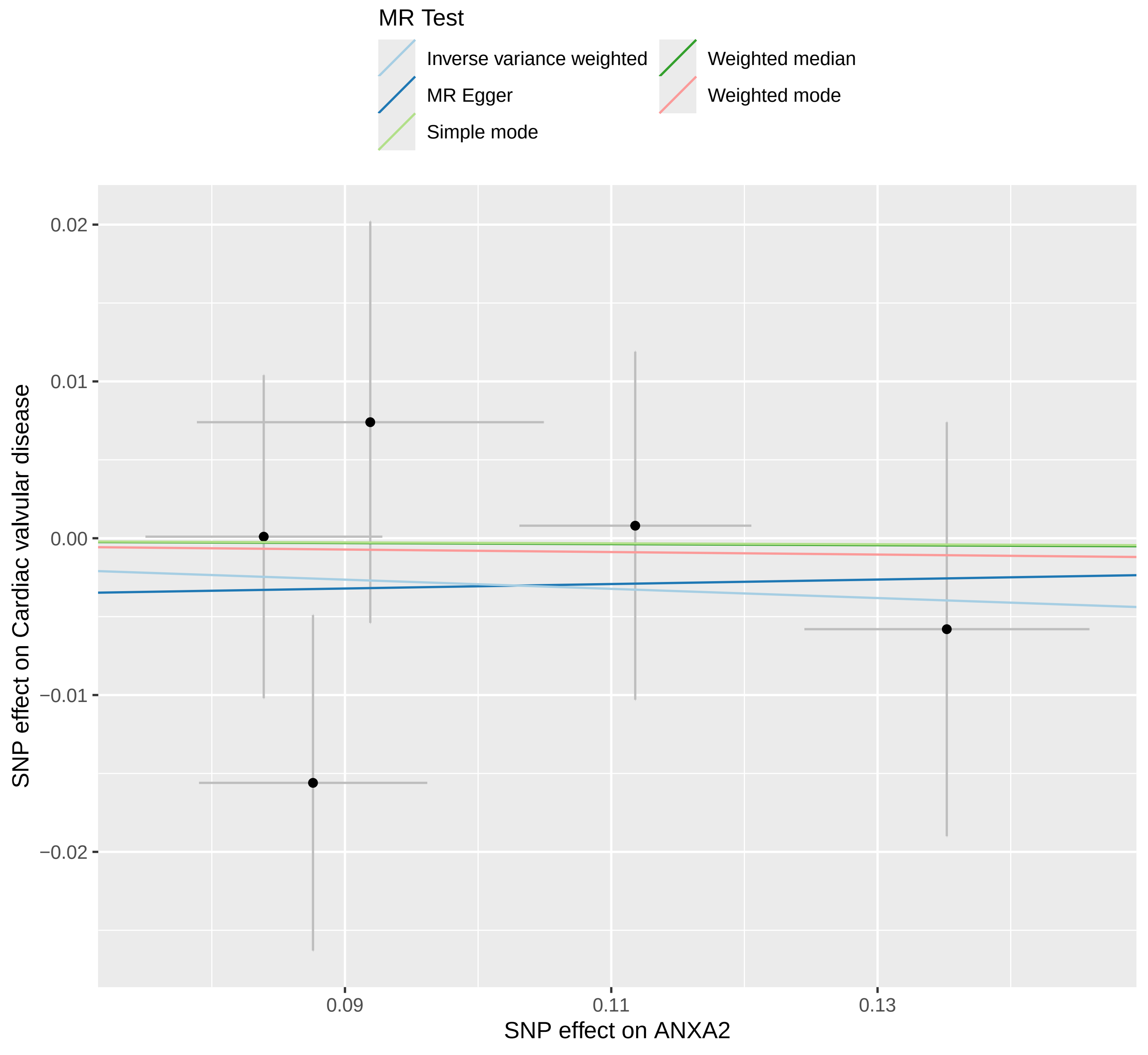
MR Test
Inverse variance weighted
MR Egger
Simple mode
Weighted median
Weighted mode
0.02
0.01
0.00
−0.01
−0.02
SNP effect on Cardiac valvular disease
0.09
0.11
0.13
SNP effect on ANXA2

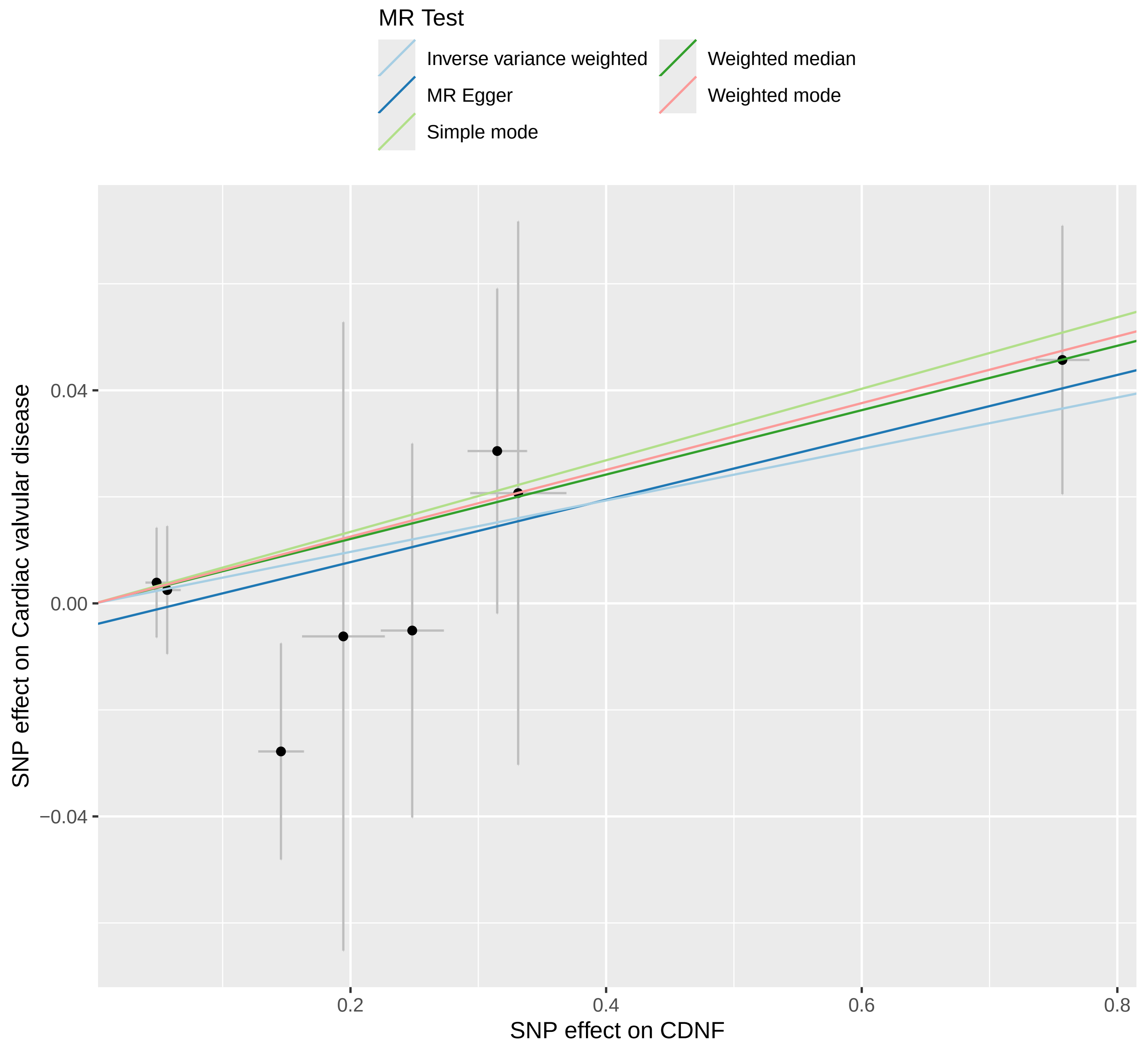
MR Test
Inverse variance weighted
MR Egger
Simple mode
Weighted median
Weighted mode
SNP effect on Cardiac valvular disease
0.04
0.00
−0.04
0.2
0.4
0.6
0.8
SNP effect on CDNF

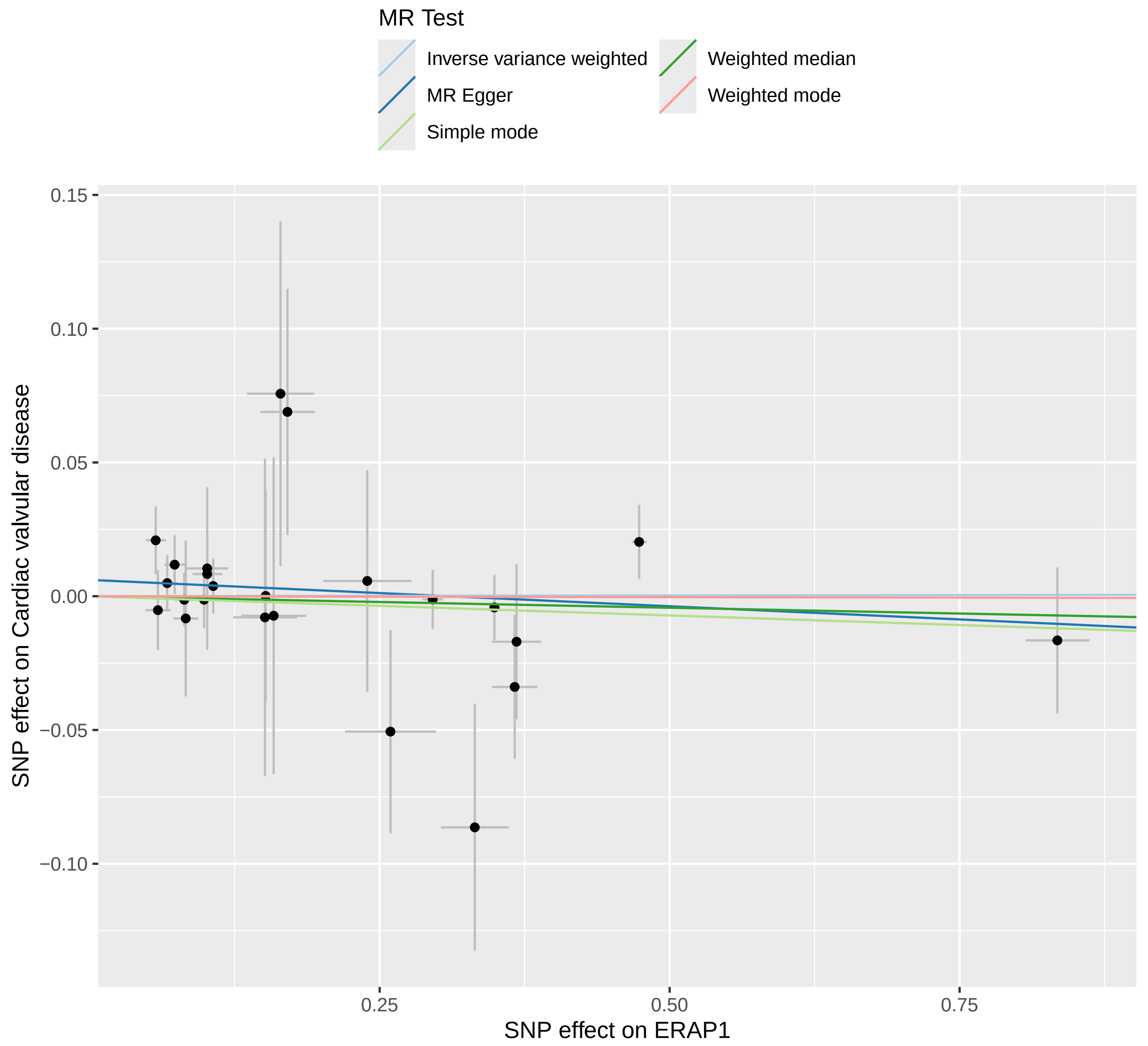

MR Test
Inverse variance weighted
MR Egger
Simple mode
Weighted median
Weighted mode
0.15
0.10
0.05
0.00
−0.05
−0.10
SNP effect on Cardiac valvular disease
0.25
0.50
0.75
SNP effect on ERAP1

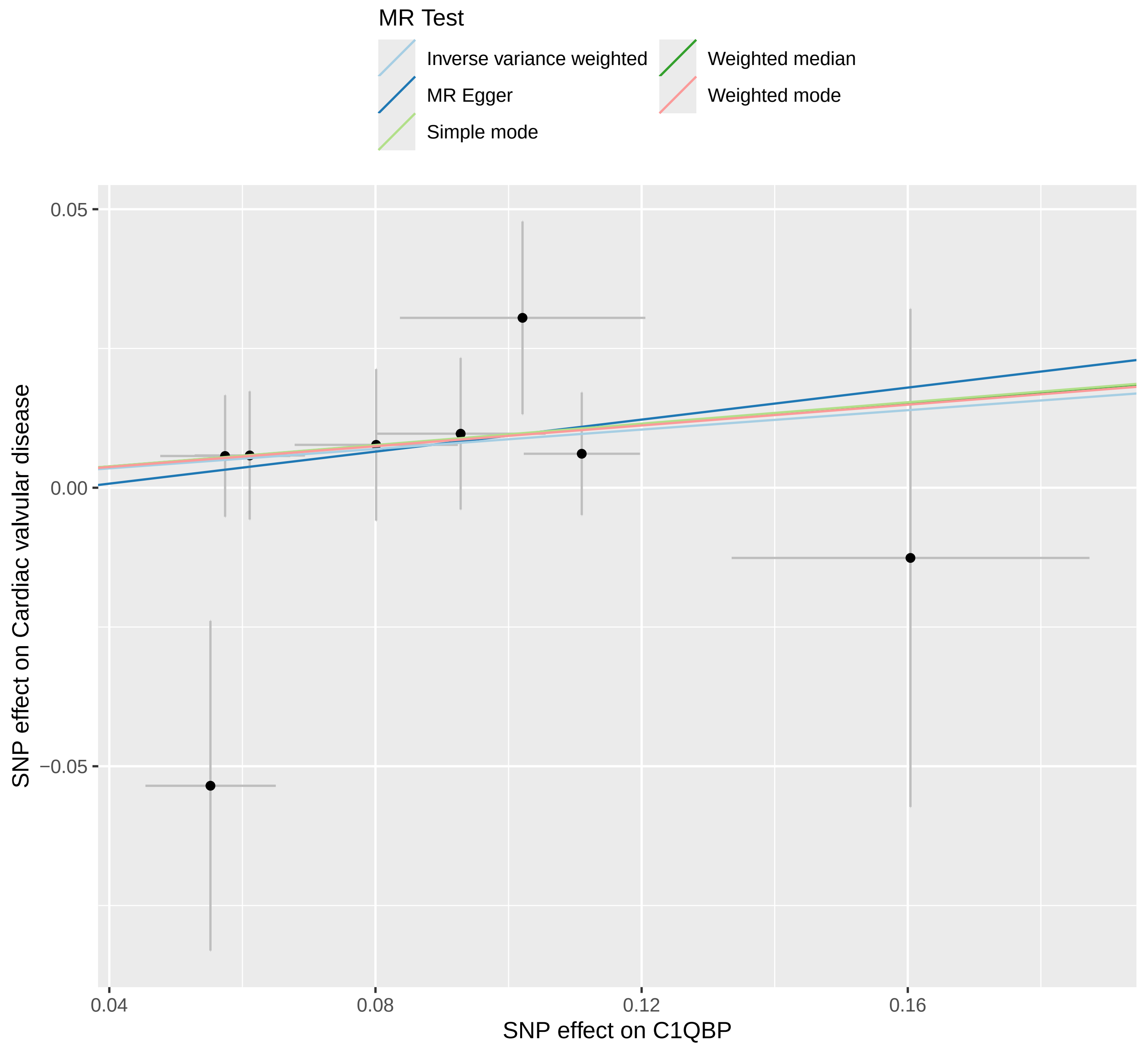
MR Test
Inverse variance weighted
MR Egger
Simple mode
Weighted median
Weighted mode
0.05
0.00
−0.05
SNP effect on Cardiac valvular disease
0.04
0.08
0.12
0.16
SNP effect on C1QBP

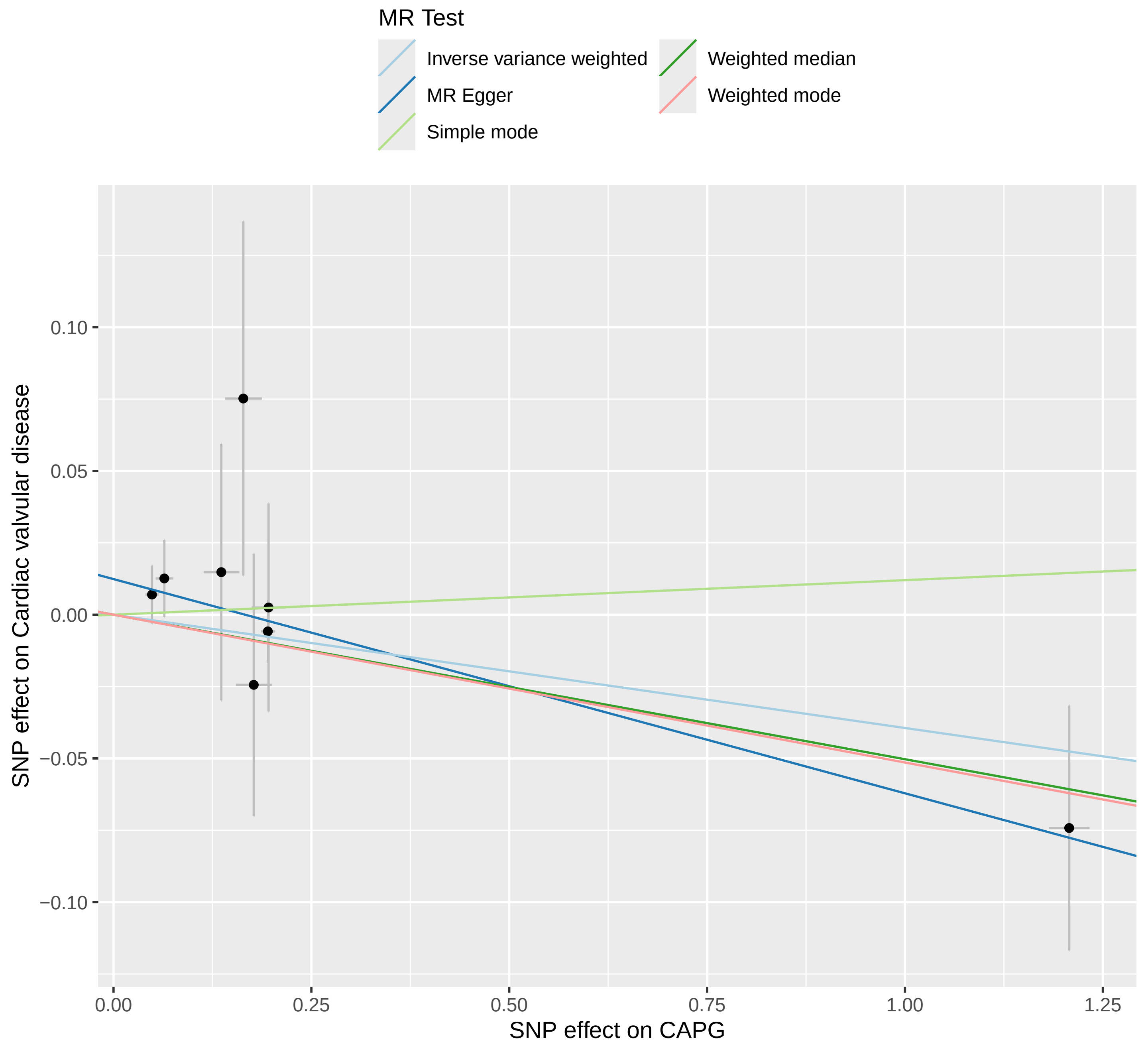

MR Test
Inverse variance weighted
MR Egger
Simple mode
Weighted median
Weighted mode
0.10
0.05
0.00
−0.05
−0.10
SNP effect on Cardiac valvular disease
0.00
0.25
0.50
0.75
1.00
1.25
SNP effect on CAPG

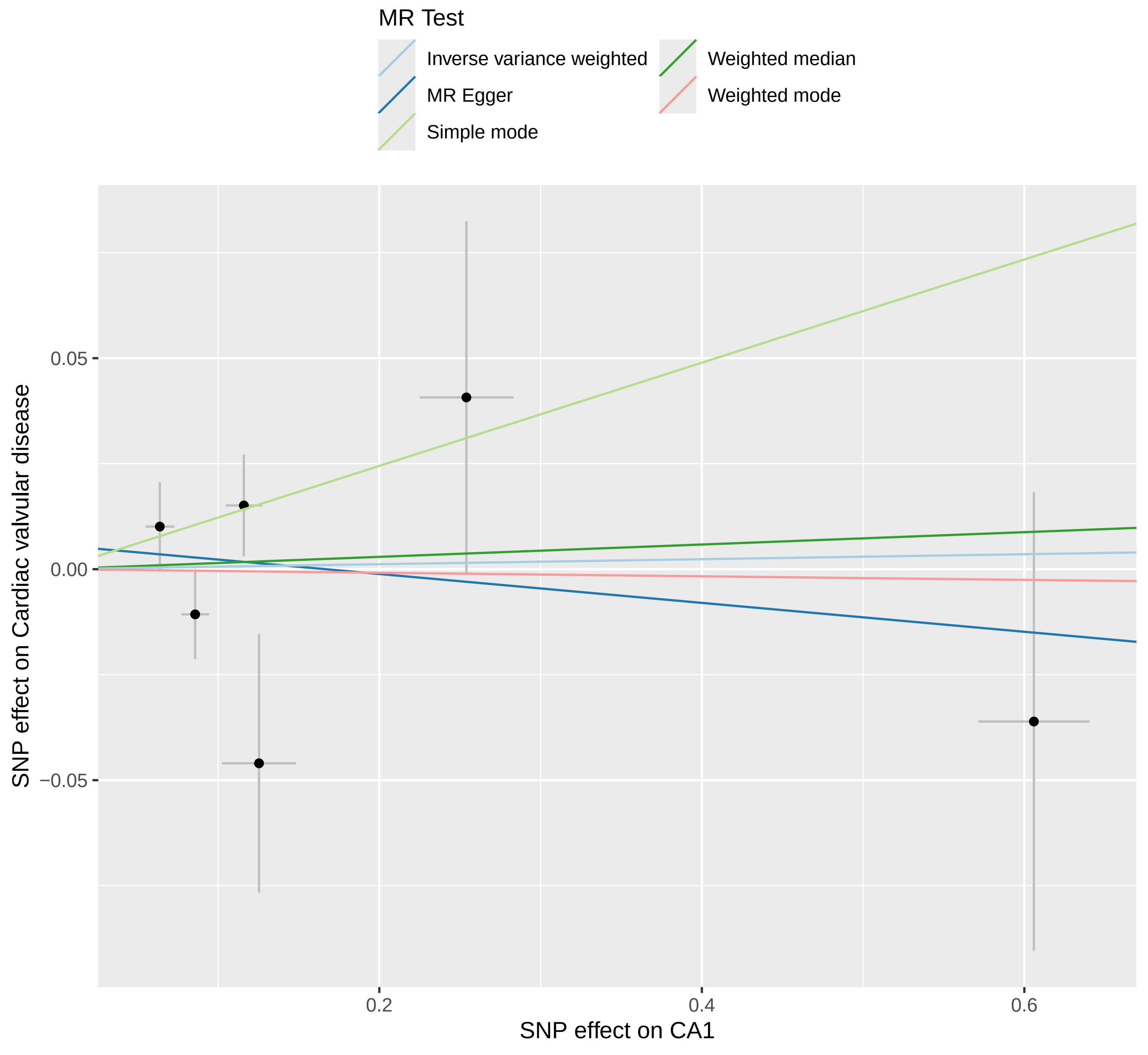
MR Test
Inverse variance weighted
MR Egger
Simple mode
Weighted median
Weighted mode
0.05
0.00
−0.05
0.2
0.4
0.6
SNP effect on Cardiac valvular disease
SNP effect on CA1

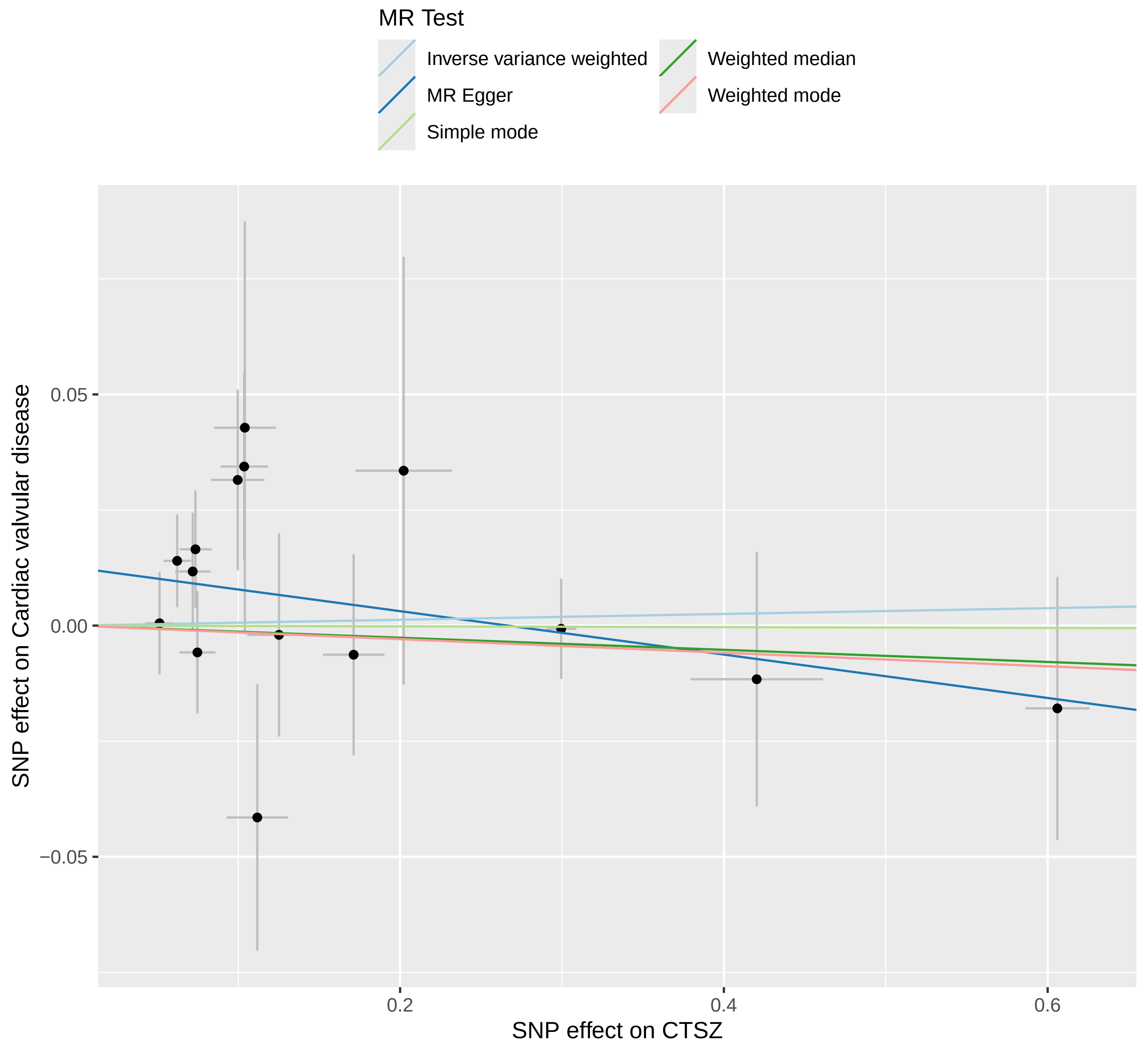
MR Test
Inverse variance weighted
MR Egger
Simple mode
Weighted median
Weighted mode
SNP effect on Cardiac valvular disease
0.05
0.00
−0.05
0.2
0.4
0.6
SNP effect on CTSZ

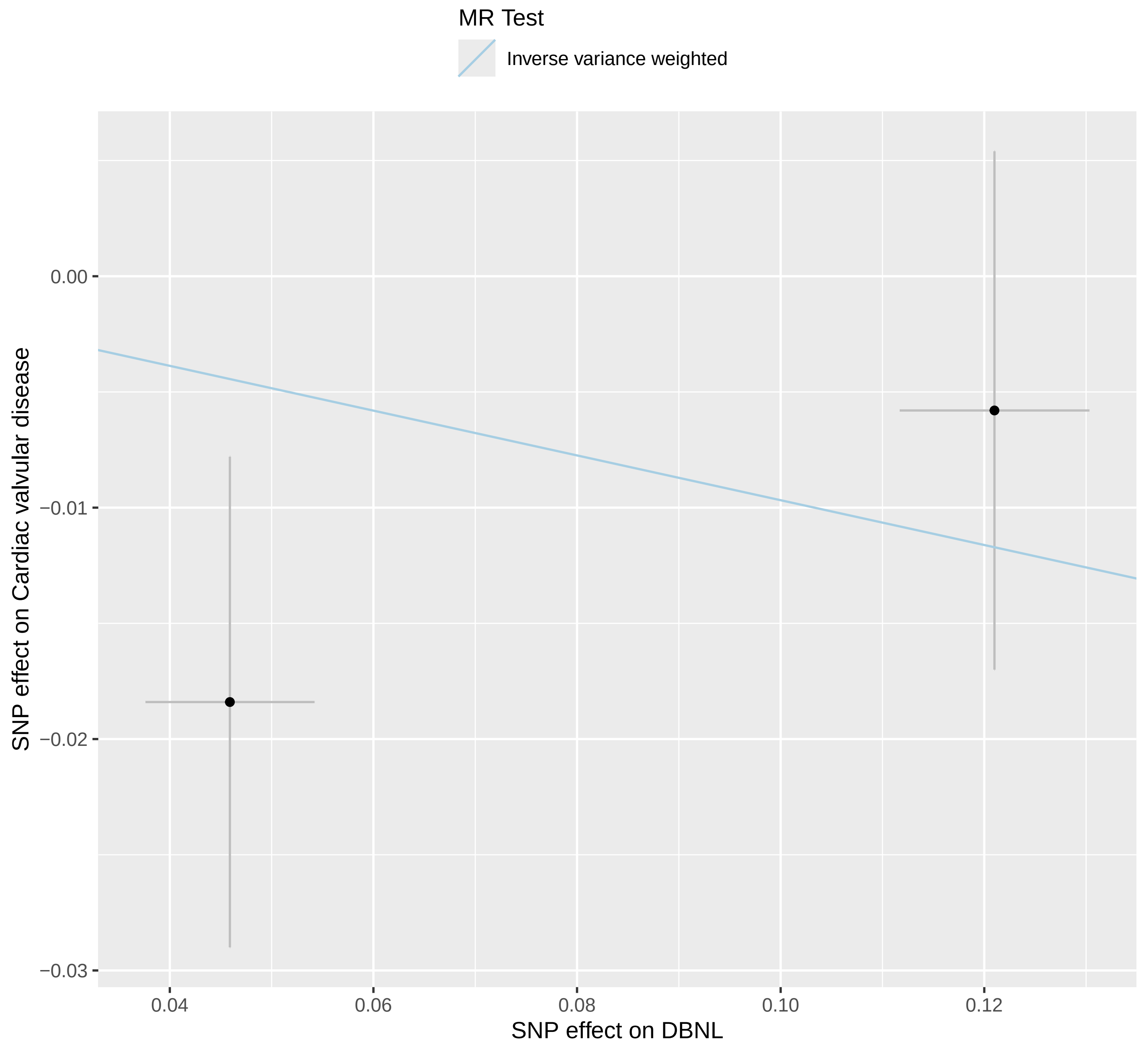

MR Test
Inverse variance weighted
0.00
−0.01
−0.02
−0.03
SNP effect on Cardiac valvular disease
0.04
0.06
0.08
0.10
0.12
SNP effect on DBNL

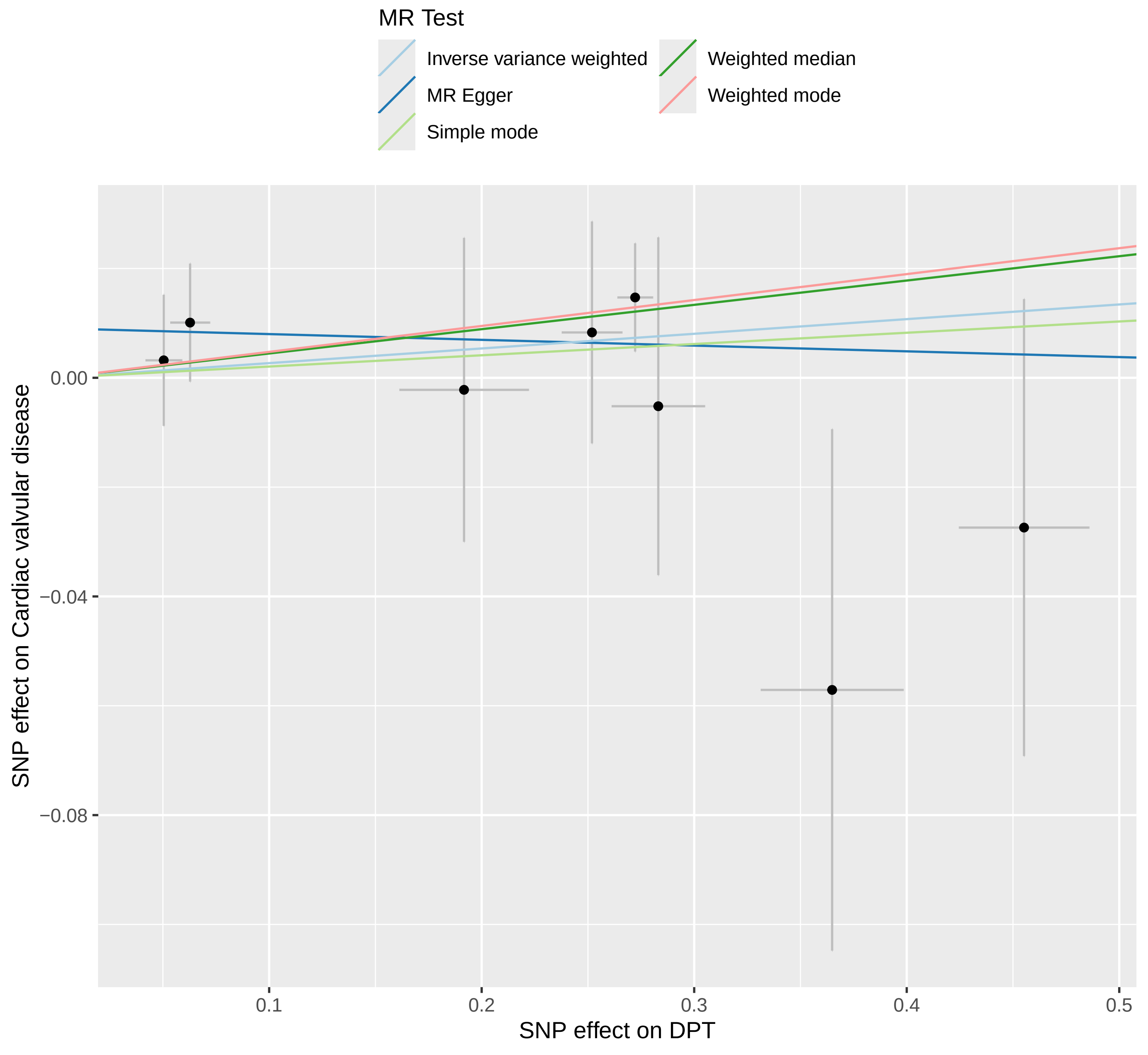
MR Test
Inverse variance weighted
MR Egger
Simple mode
Weighted median
Weighted mode
SNP effect on Cardiac valvular disease
0.00
−0.04
−0.08
0.1
0.2
0.3
0.4
0.5
SNP effect on DPT

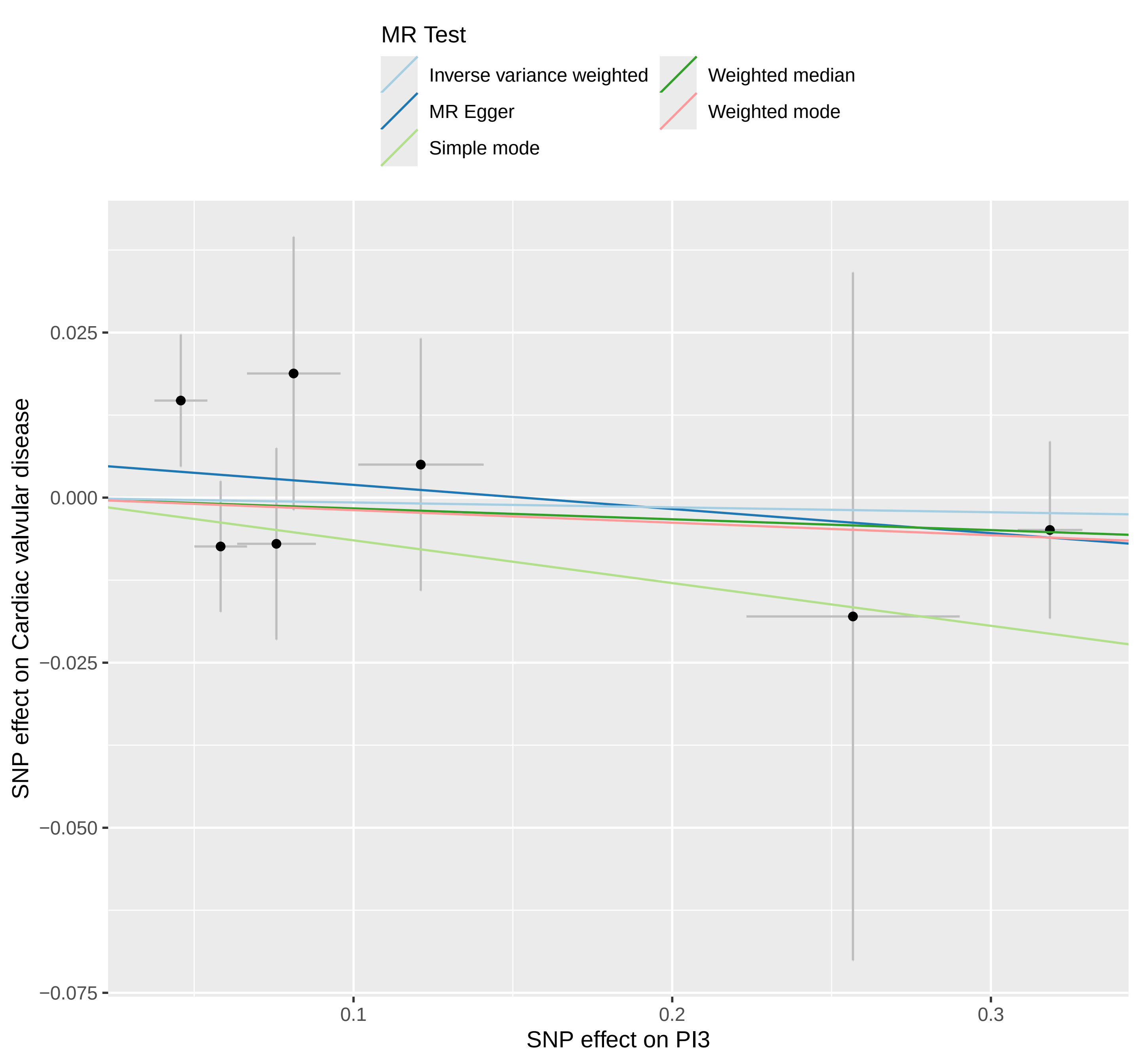
MR Test
Inverse variance weighted
MR Egger
Simple mode
Weighted median
Weighted mode
0.025
0.000
−0.025
−0.050
−0.075
0.1
0.2
0.3
SNP effect on PI3
SNP effect on Cardiac valvular disease

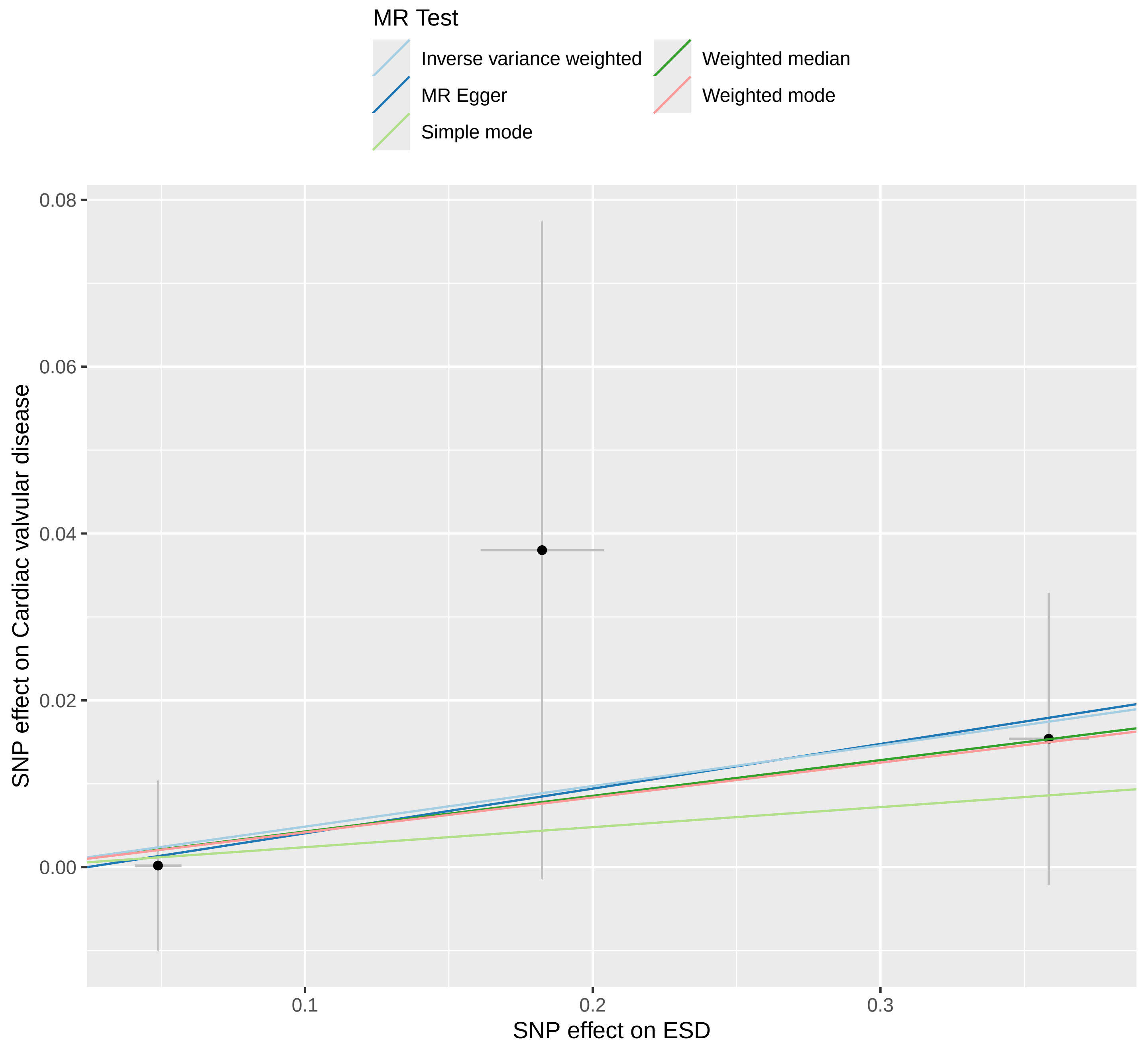

MR Test
Inverse variance weighted
MR Egger
Simple mode
Weighted median
Weighted mode
0.08
0.06
0.04
0.02
0.00
0.1
0.2
0.3
SNP effect on Cardiac valvular disease
SNP effect on ESD

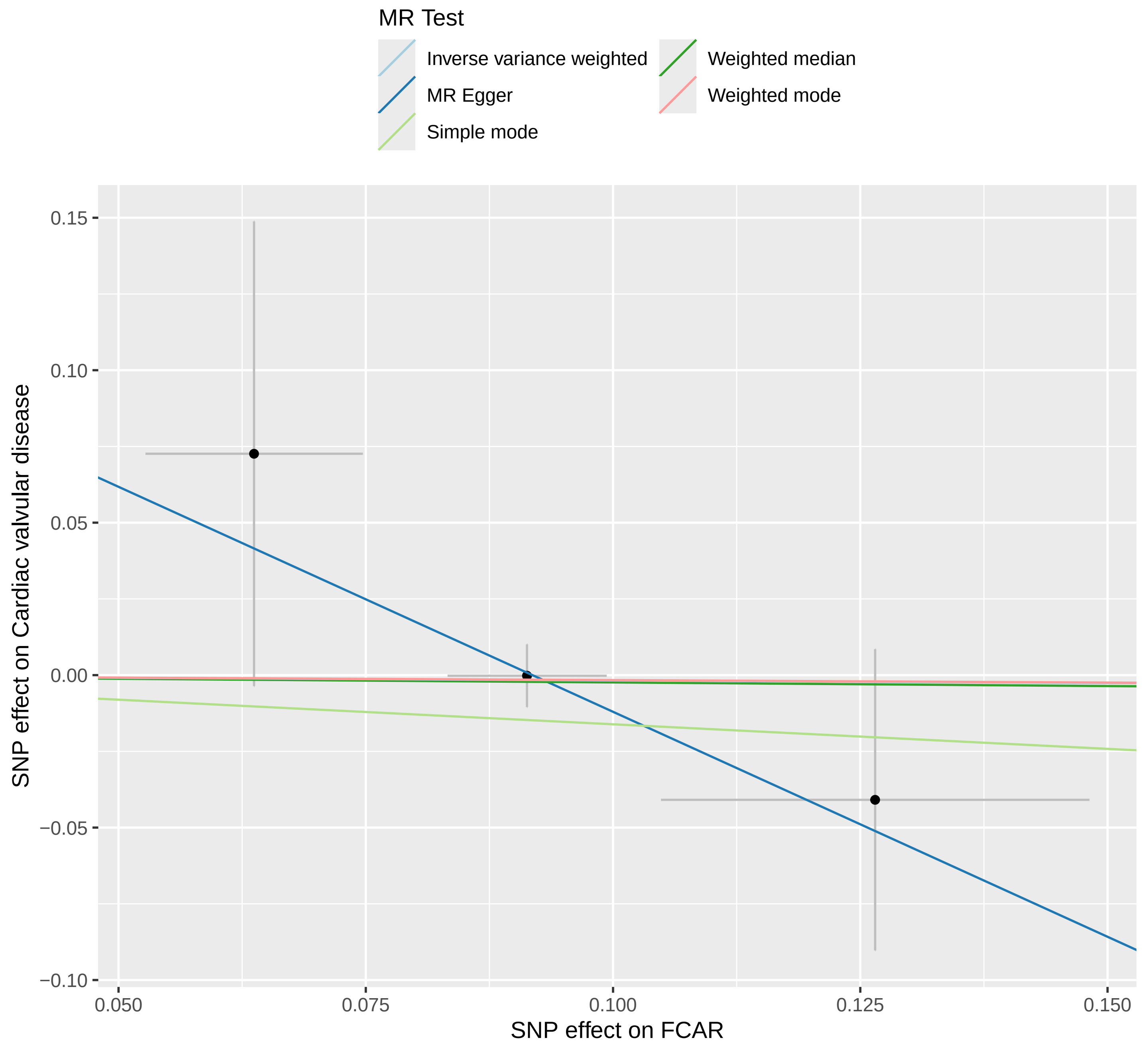
MR Test
Inverse variance weighted
MR Egger
Simple mode
Weighted median
Weighted mode
SNP effect on Cardiac valvular disease
0.15
0.10
0.05
0.00
−0.05
−0.10
0.050
0.075
0.100
0.125
0.150
SNP effect on FCAR

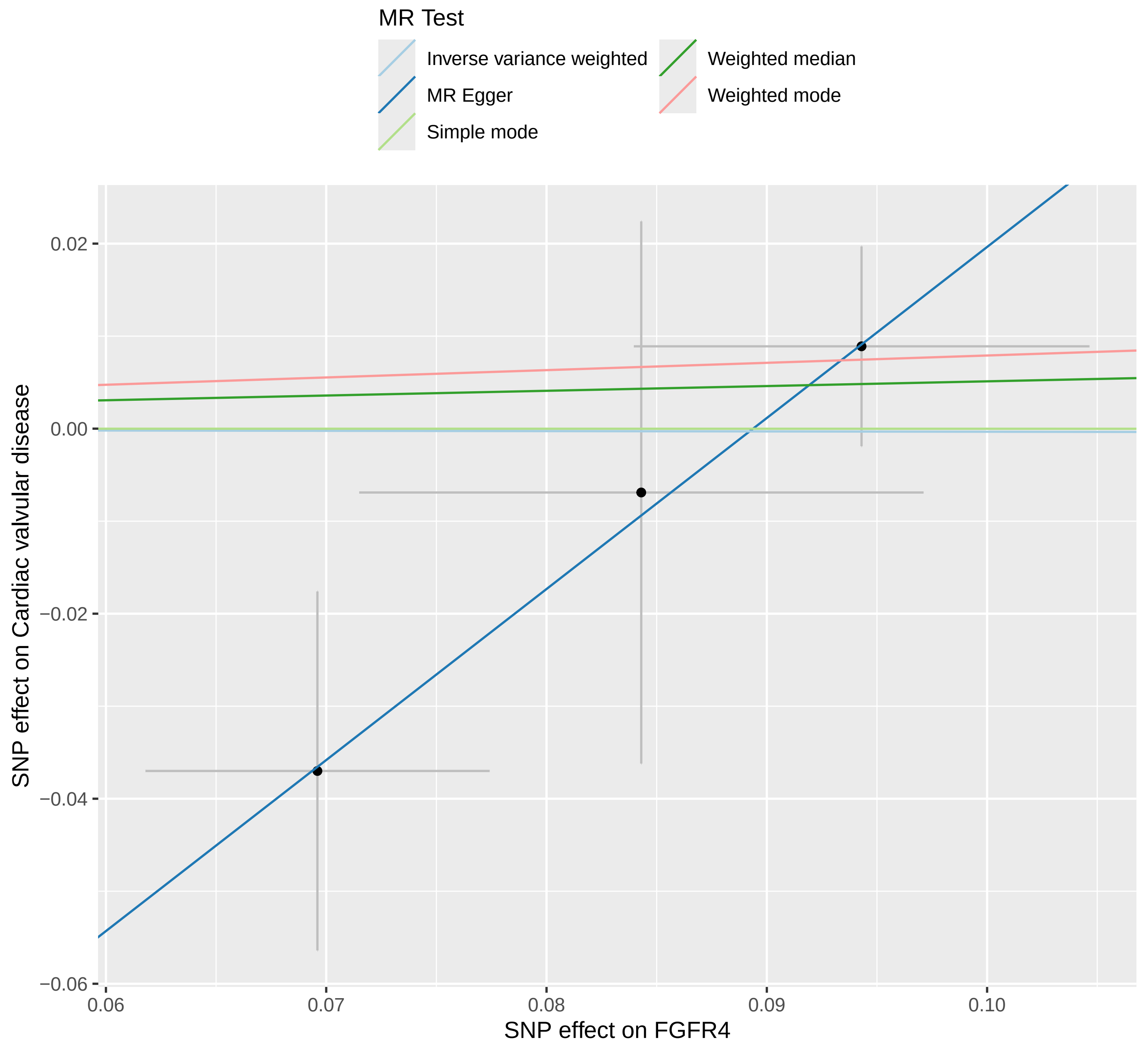
MR Test
Inverse variance weighted
MR Egger
Simple mode
Weighted median
Weighted mode
0.02
0.00
−0.02
−0.04
−0.06
0.06
0.07
0.08
0.09
0.10
SNP effect on Cardiac valvular disease
SNP effect on FGFR4

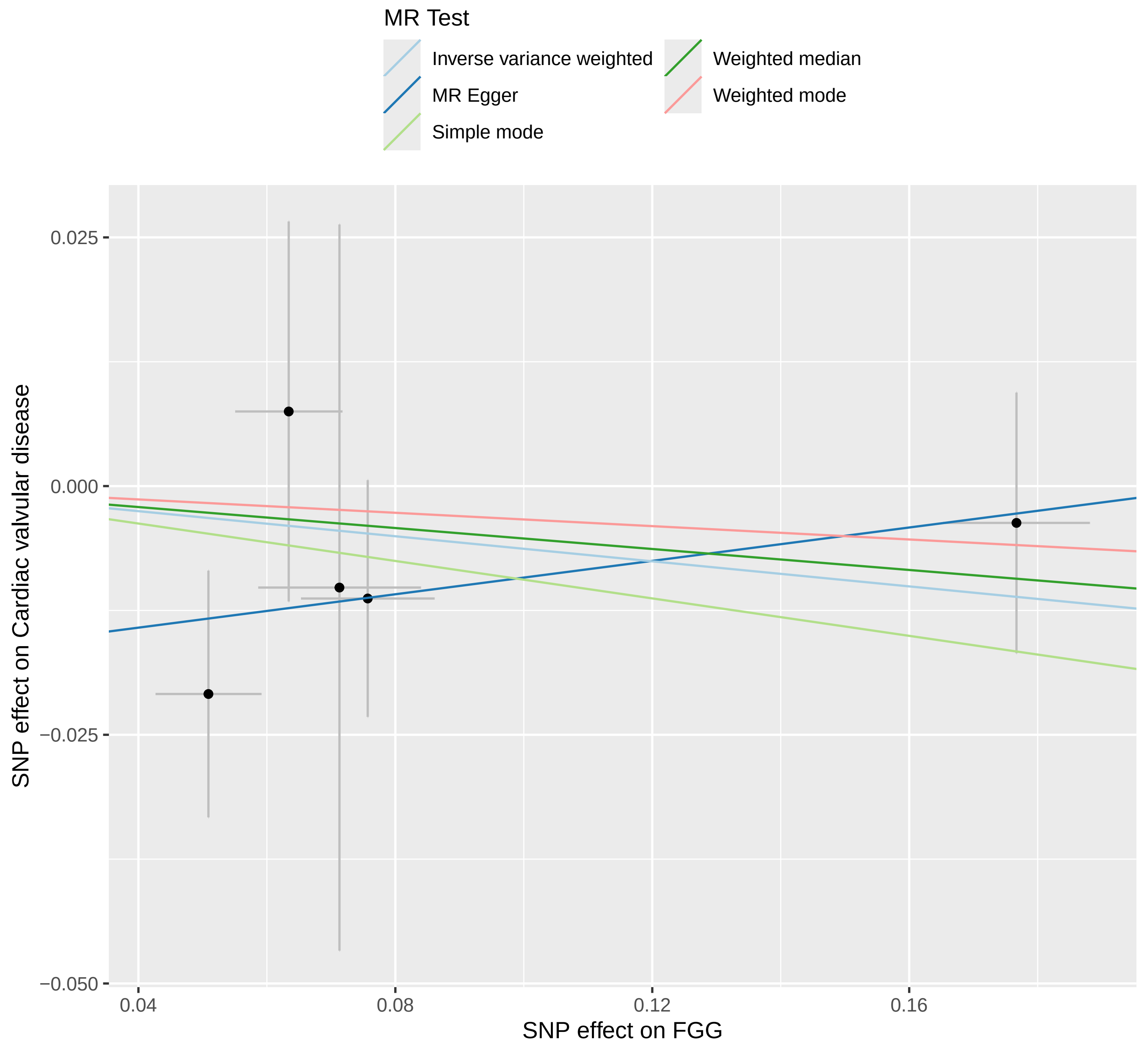
MR Test
Inverse variance weighted
MR Egger
Simple mode
Weighted median
Weighted mode
0.025
0.000
−0.025
−0.050
0.04
0.08
0.12
0.16
SNP effect on Cardiac valvular disease
SNP effect on FGG

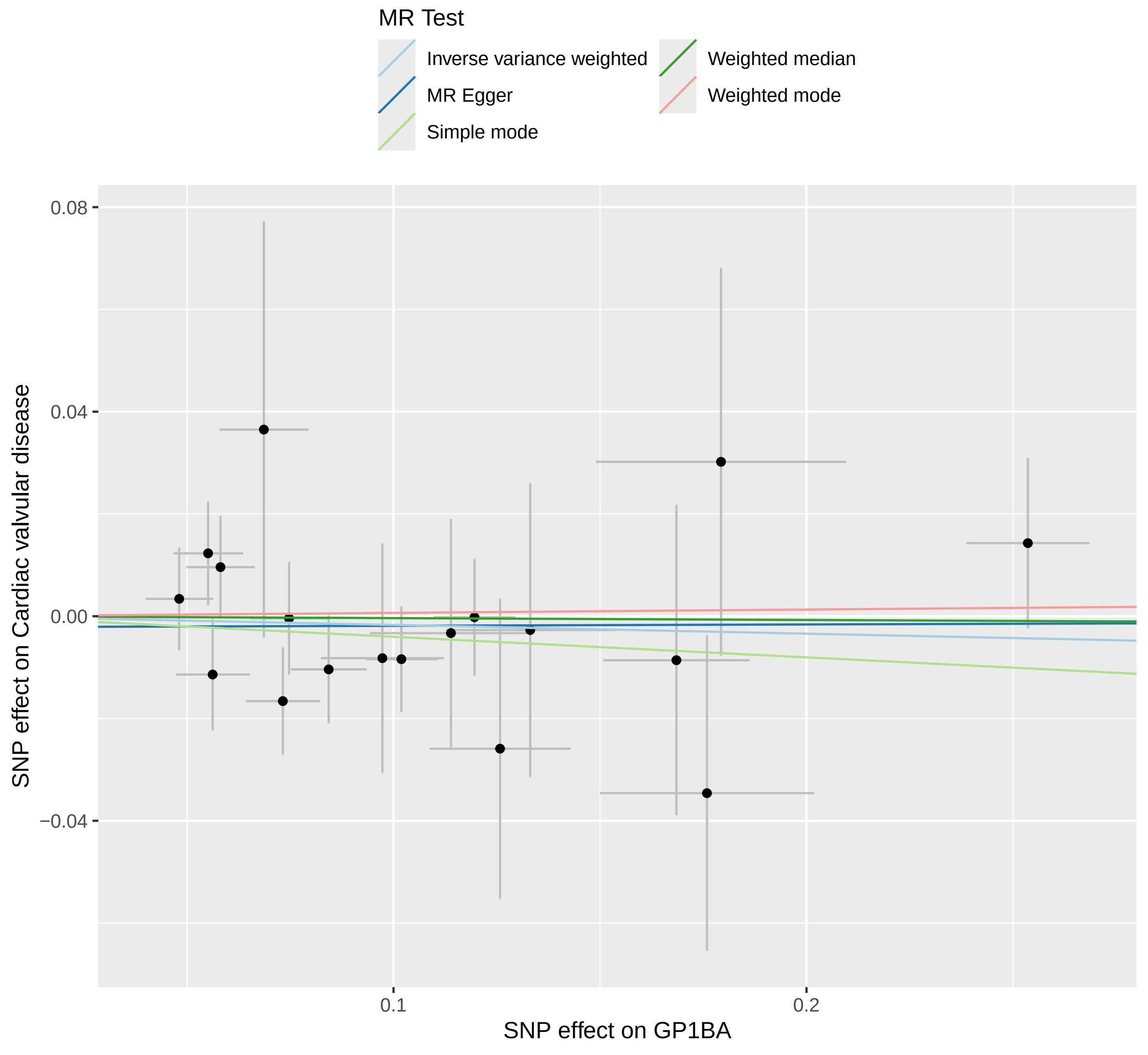
MR Test
Inverse variance weighted
MR Egger
Simple mode
Weighted median
Weighted mode
0.08
0.04
0.00
−0.04
SNP effect on Cardiac valvular disease
0.1
0.2
SNP effect on GP1BA

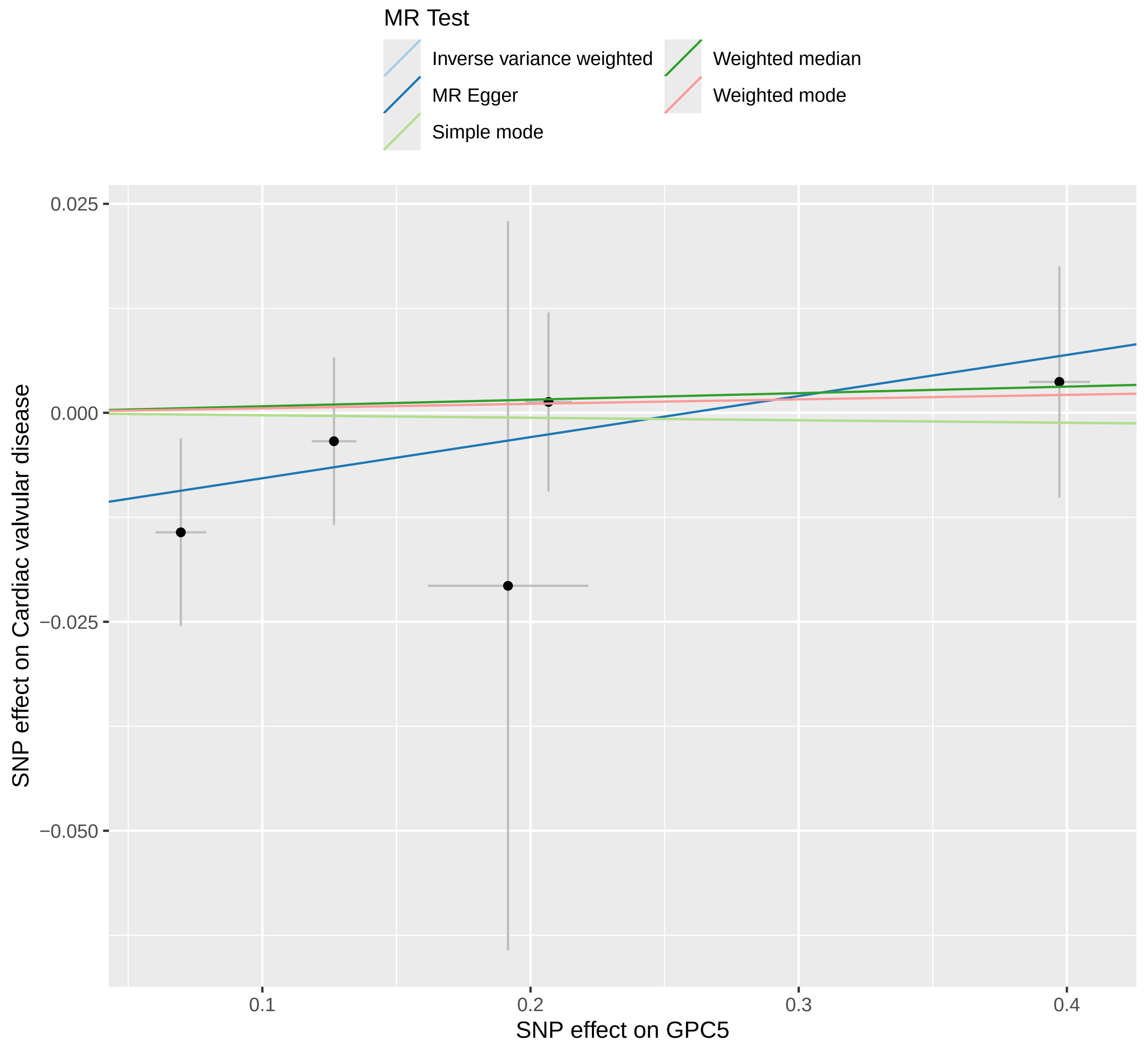

MR Test
Inverse variance weighted
MR Egger
Simple mode
Weighted median
Weighted mode
0.025
0.000
−0.025
−0.050
SNP effect on Cardiac valvular disease
0.1
0.2
0.3
0.4
SNP effect on GPC5

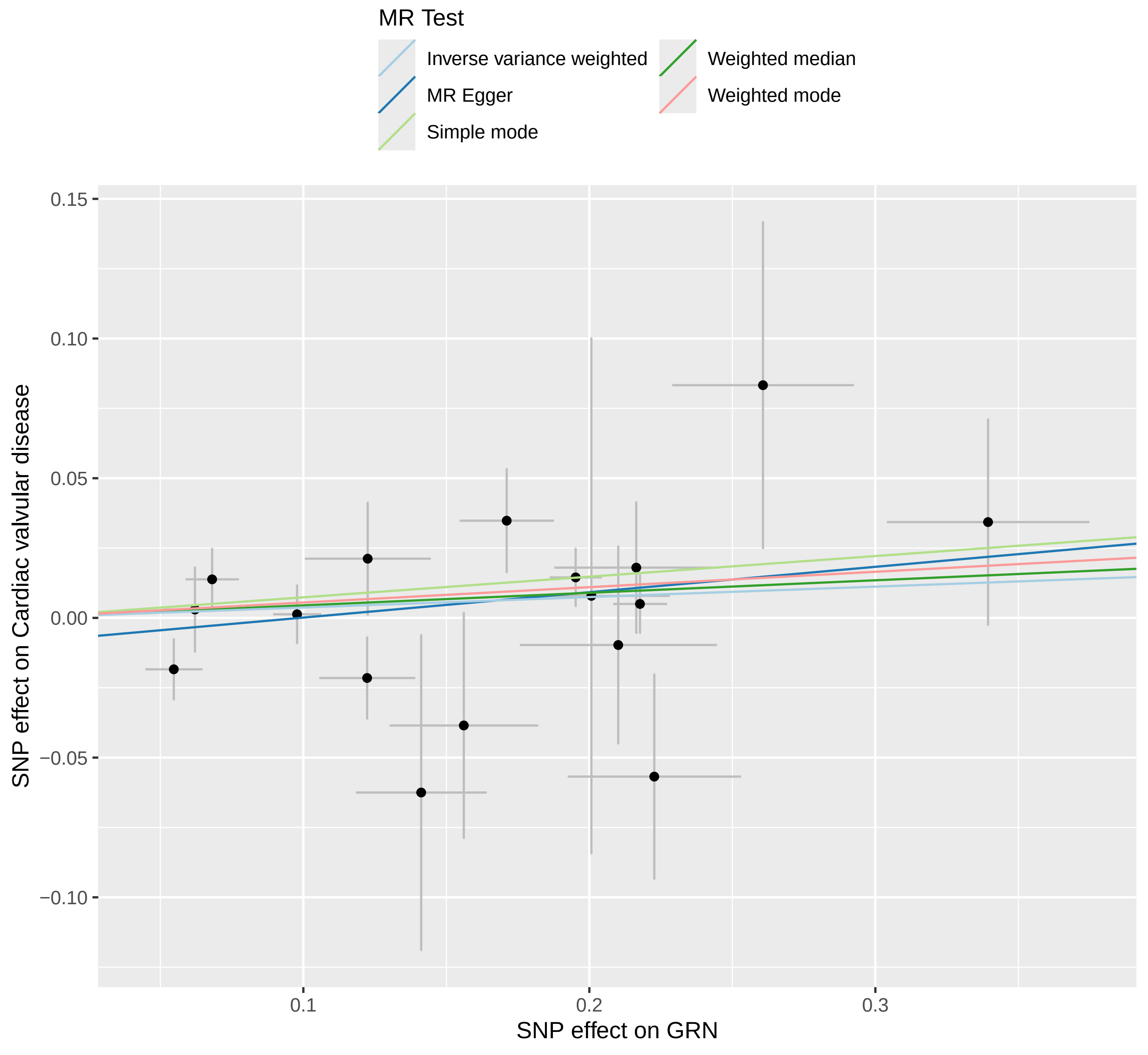
MR Test
Inverse variance weighted
MR Egger
Simple mode
Weighted median
Weighted mode
0.15
0.10
0.05
0.00
−0.05
−0.10
SNP effect on Cardiac valvular disease
0.1
0.2
0.3
SNP effect on GRN

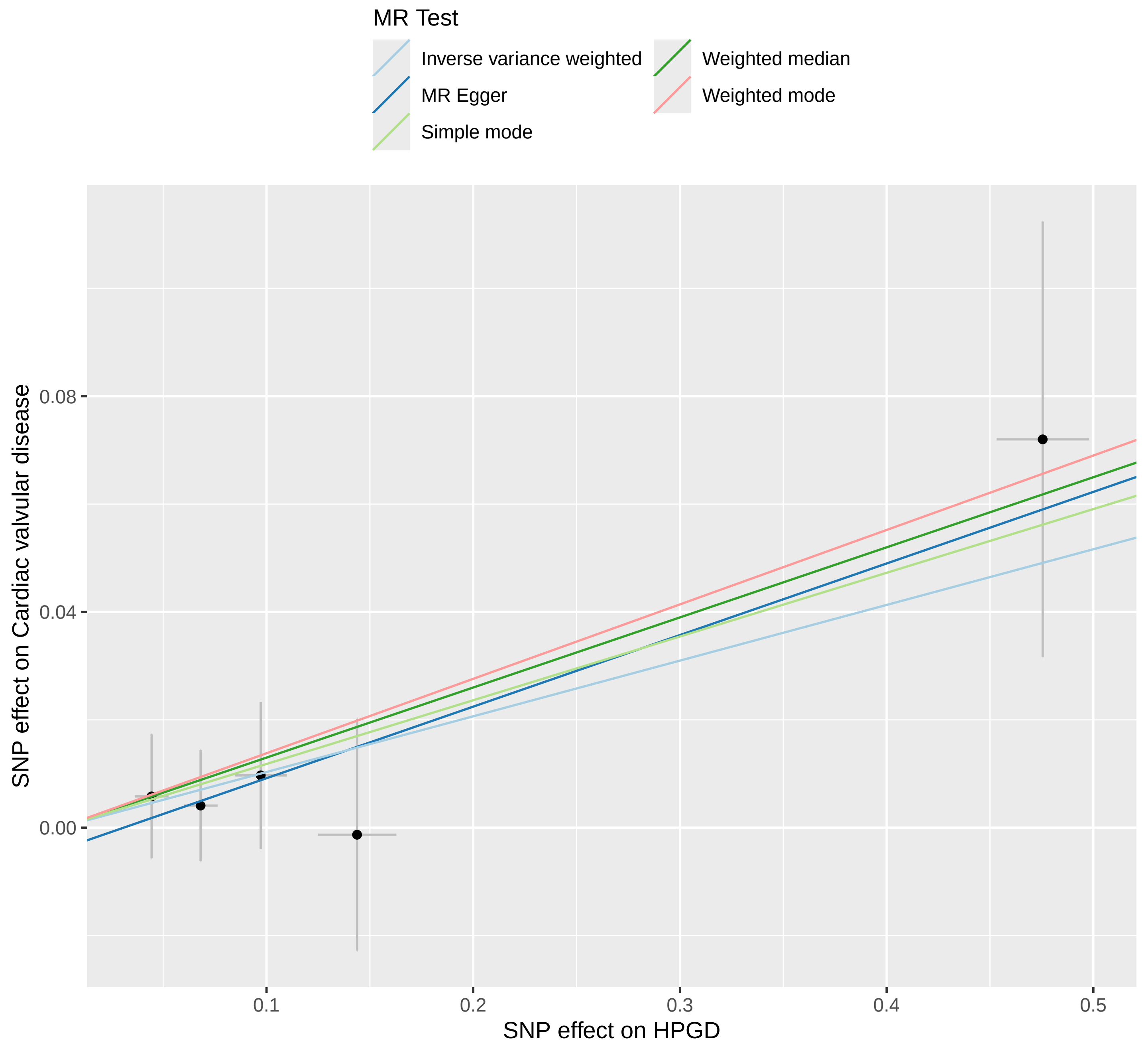
MR Test
Inverse variance weighted
MR Egger
Simple mode
Weighted median
Weighted mode
0.08
0.04
0.00
0.1
0.2
0.3
0.4
0.5
SNP effect on HPGD
SNP effect on Cardiac valvular disease

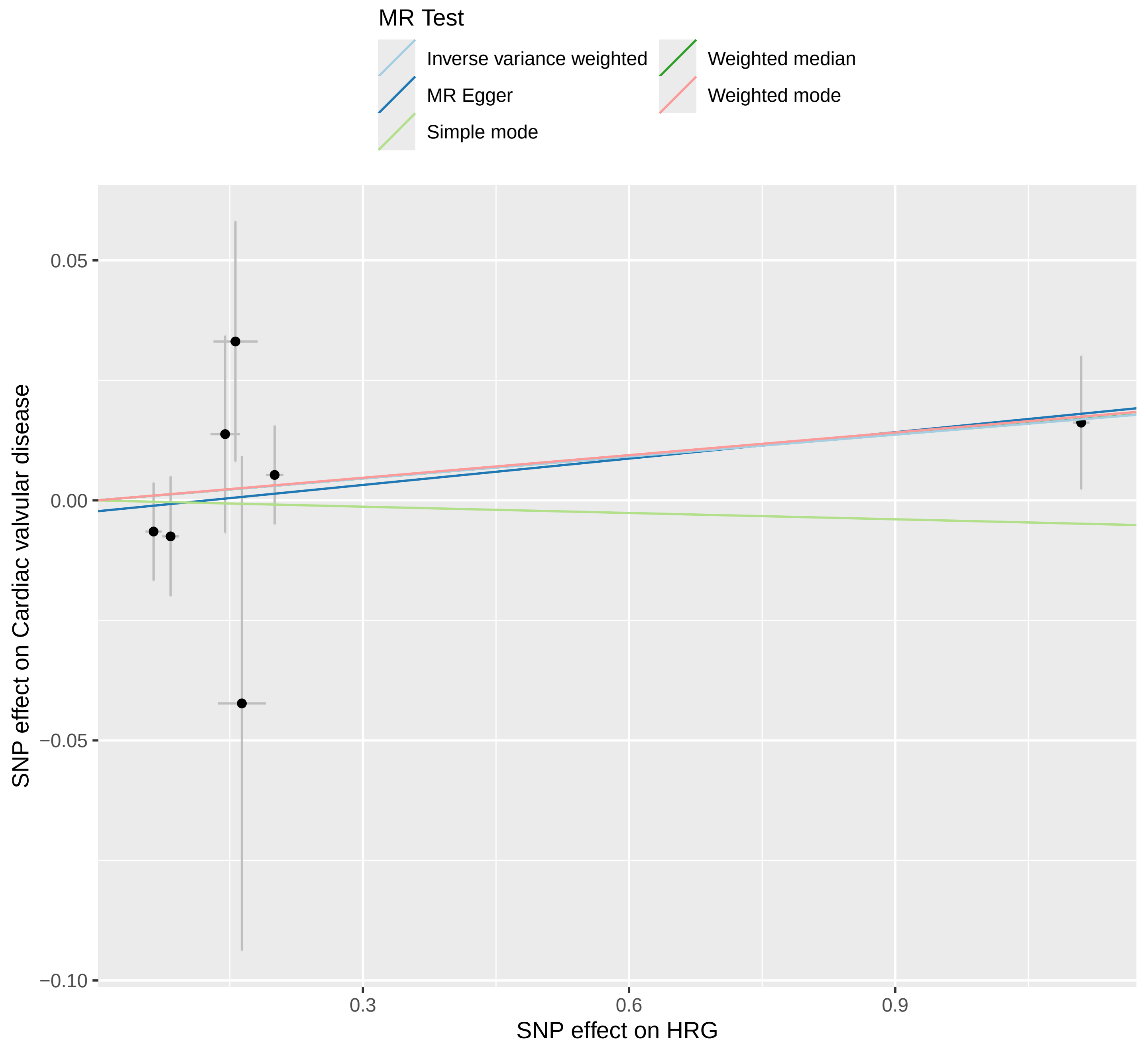
MR Test
Inverse variance weighted
MR Egger
Simple mode
Weighted median
Weighted mode
0.05
0.00
−0.05
−0.10
0.3
0.6
0.9
SNP effect on Cardiac valvular disease
SNP effect on HRG

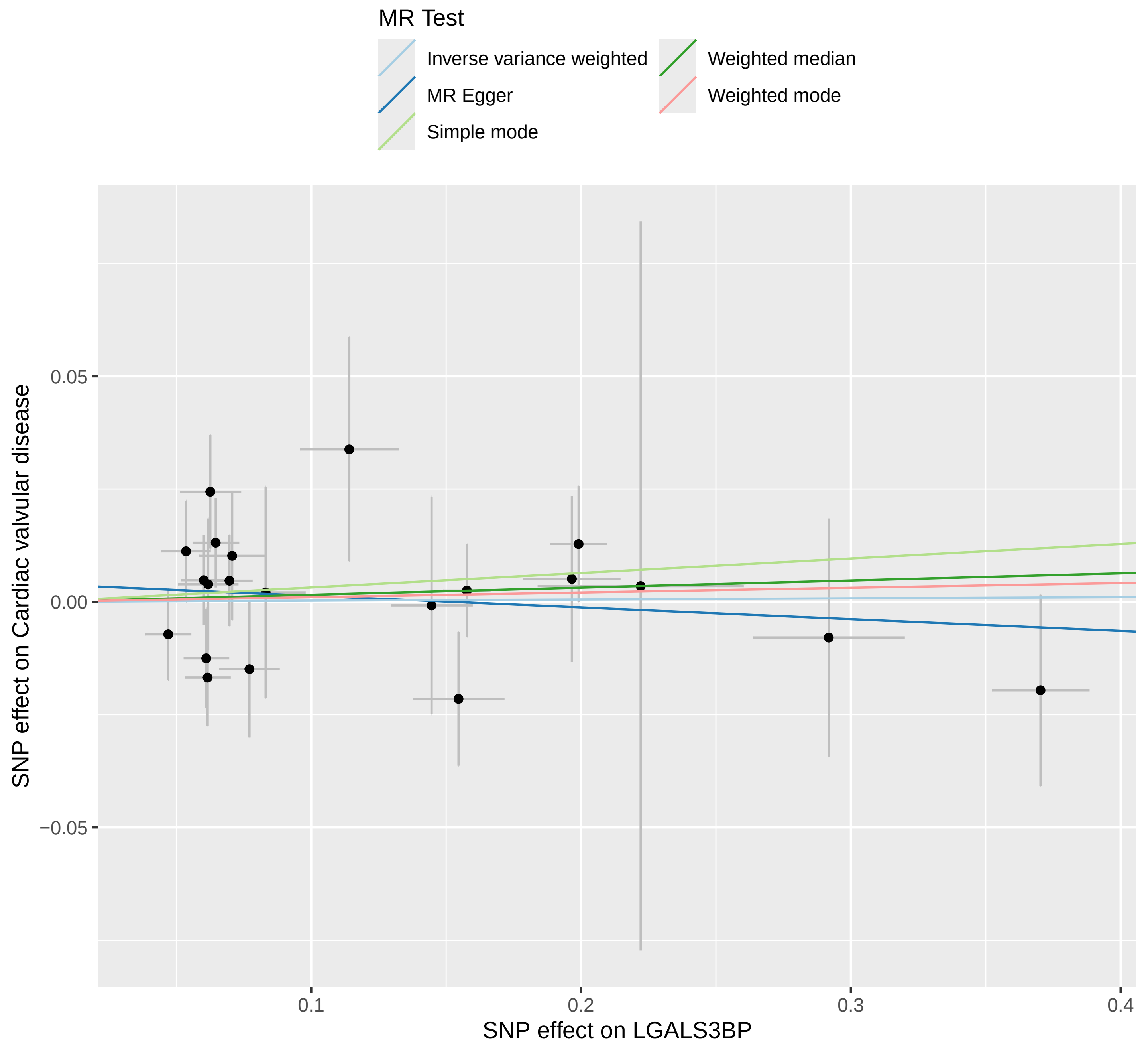
MR Test
Inverse variance weighted
MR Egger
Simple mode
Weighted median
Weighted mode
0.05
0.00
−0.05
SNP effect on Cardiac valvular disease
0.1
0.2
0.3
0.4
SNP effect on LGALS3BP

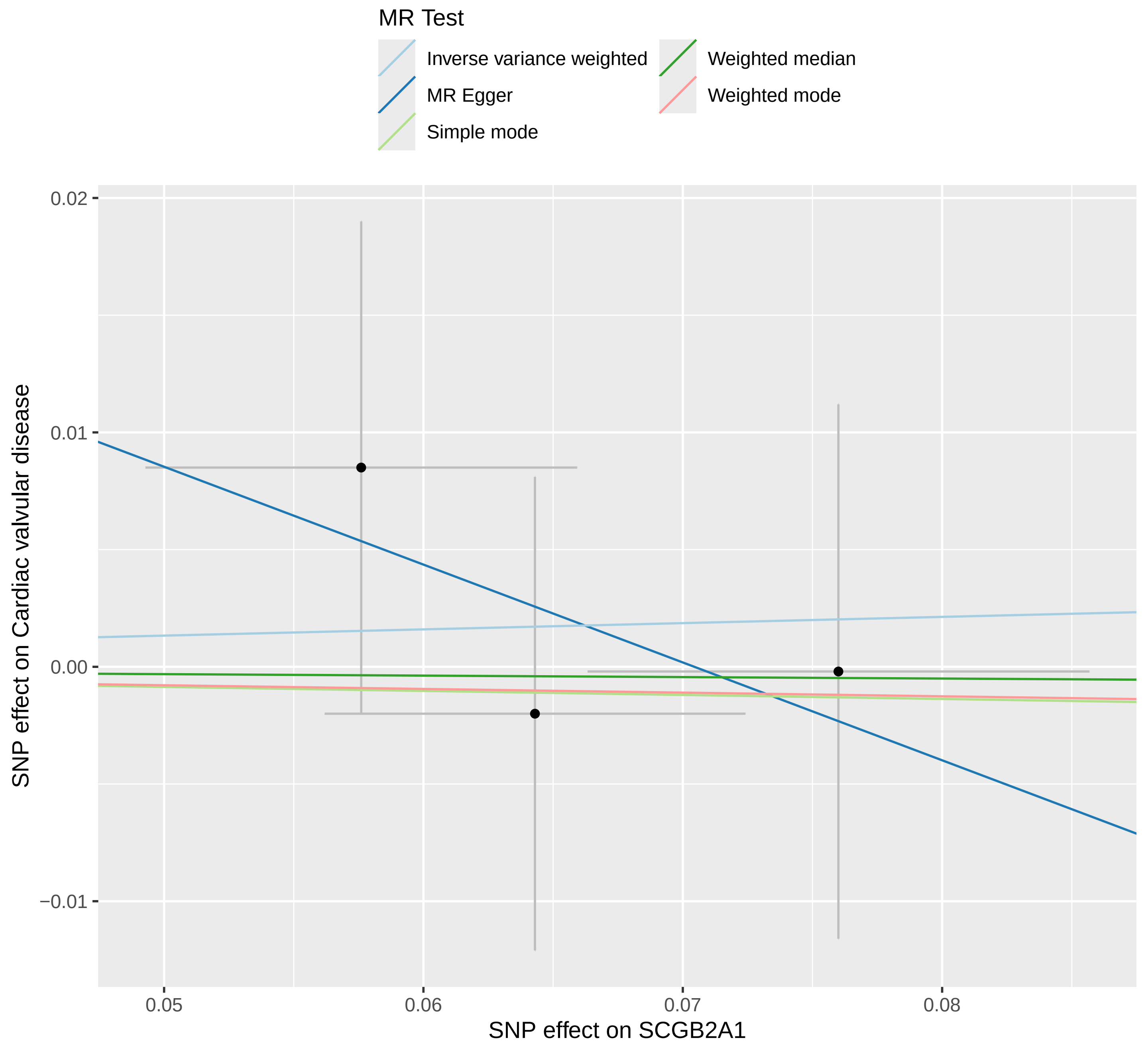

MR Test
Inverse variance weighted
MR Egger
Simple mode
Weighted median
Weighted mode
0.02
0.01
0.00
−0.01
0.05
0.06
0.07
0.08
SNP effect on Cardiac valvular disease
SNP effect on SCGB2A1

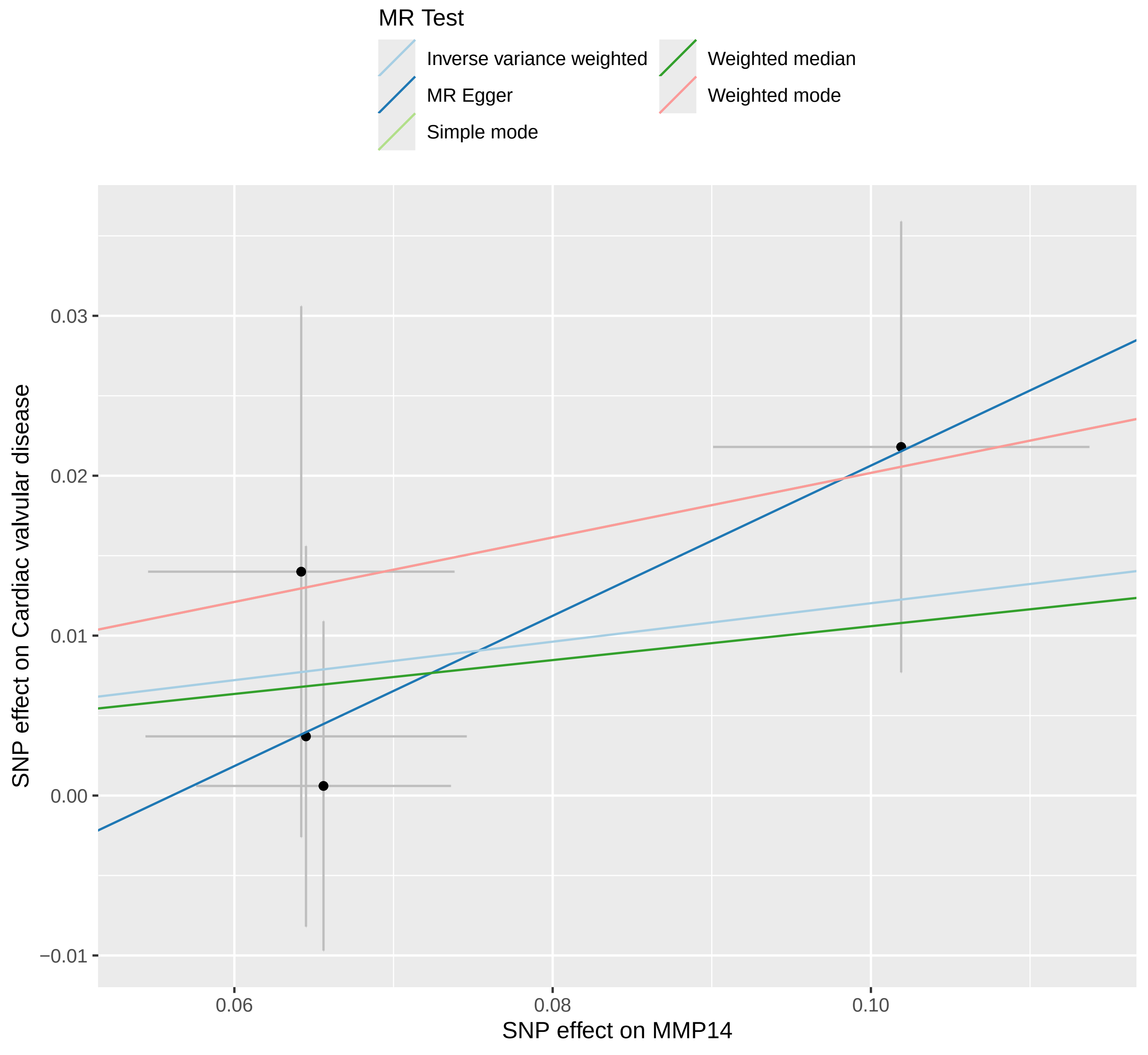
MR Test
Inverse variance weighted
MR Egger
Simple mode
Weighted median
Weighted mode
SNP effect on Cardiac valvular disease
0.03
0.02
0.01
0.00
−0.01
0.06
0.08
0.10
SNP effect on MMP14

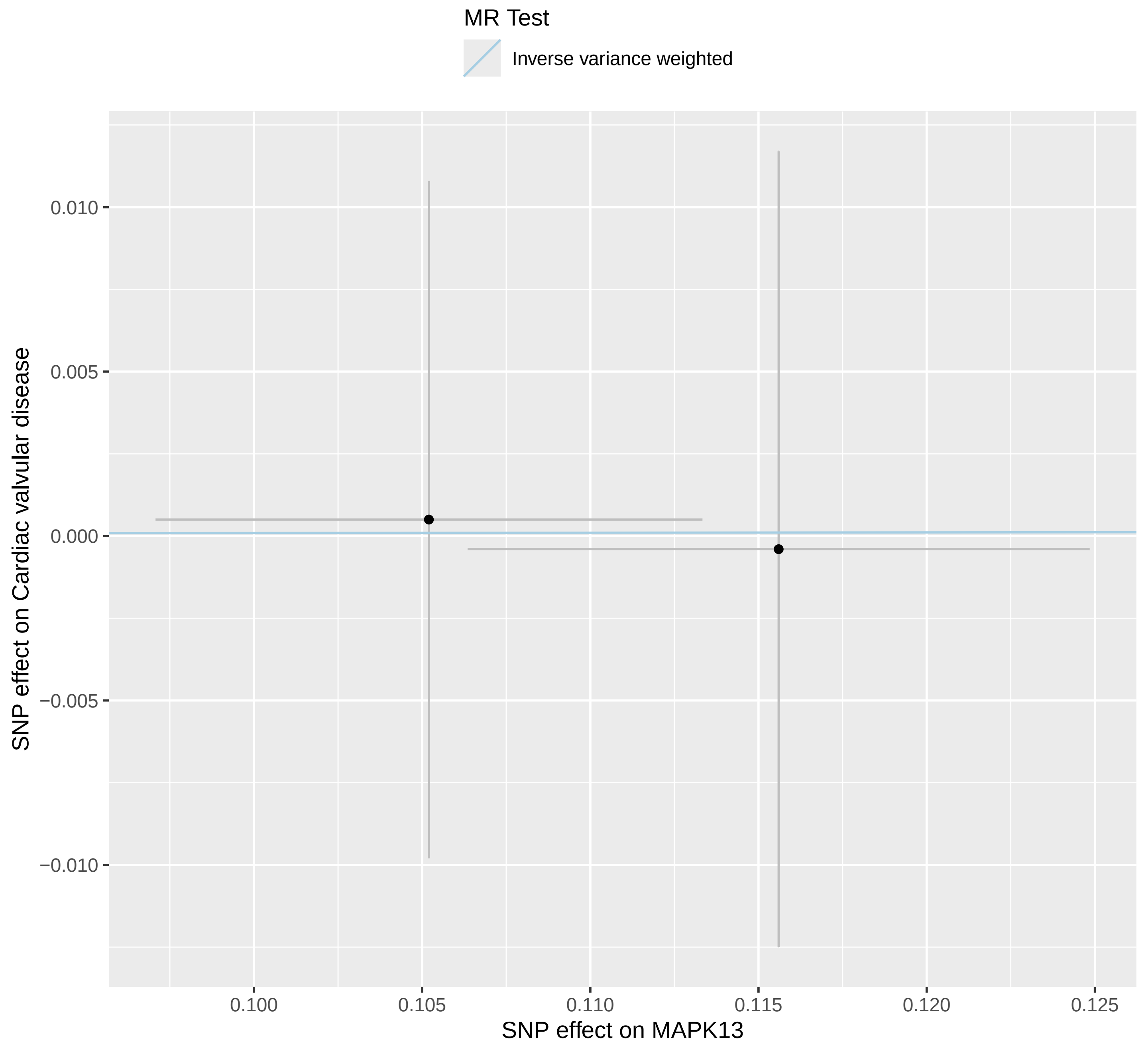
MR Test
Inverse variance weighted
SNP effect on Cardiac valvular disease
0.010
0.005
0.000
−0.005
−0.010
0.100
0.105
0.110
0.115
0.120
0.125
SNP effect on MAPK13

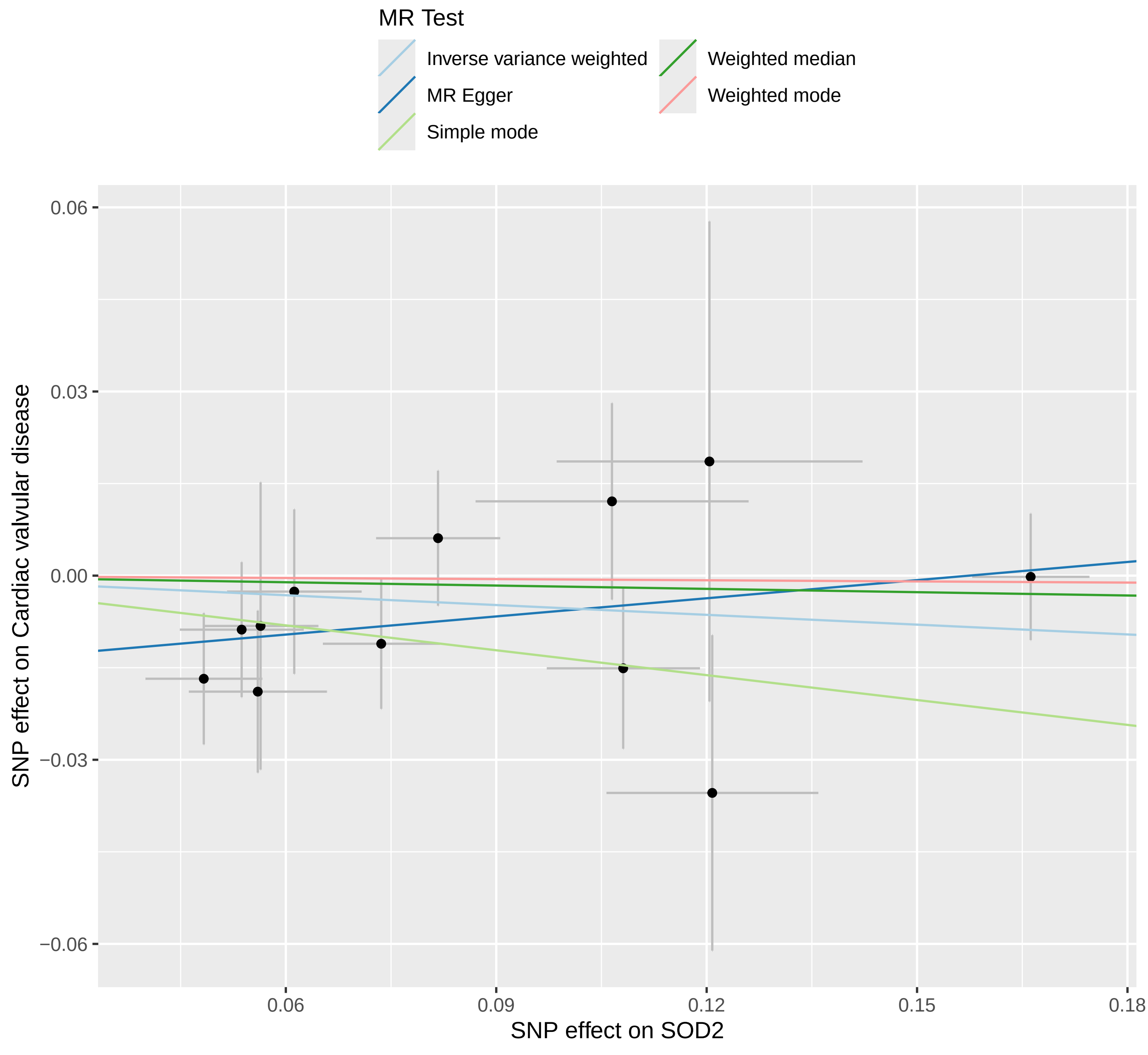
MR Test
Inverse variance weighted
MR Egger
Simple mode
Weighted median
Weighted mode
0.06
0.03
0.00
−0.03
−0.06
0.06
0.09
0.12
0.15
0.18
SNP effect on Cardiac valvular disease
SNP effect on SOD2

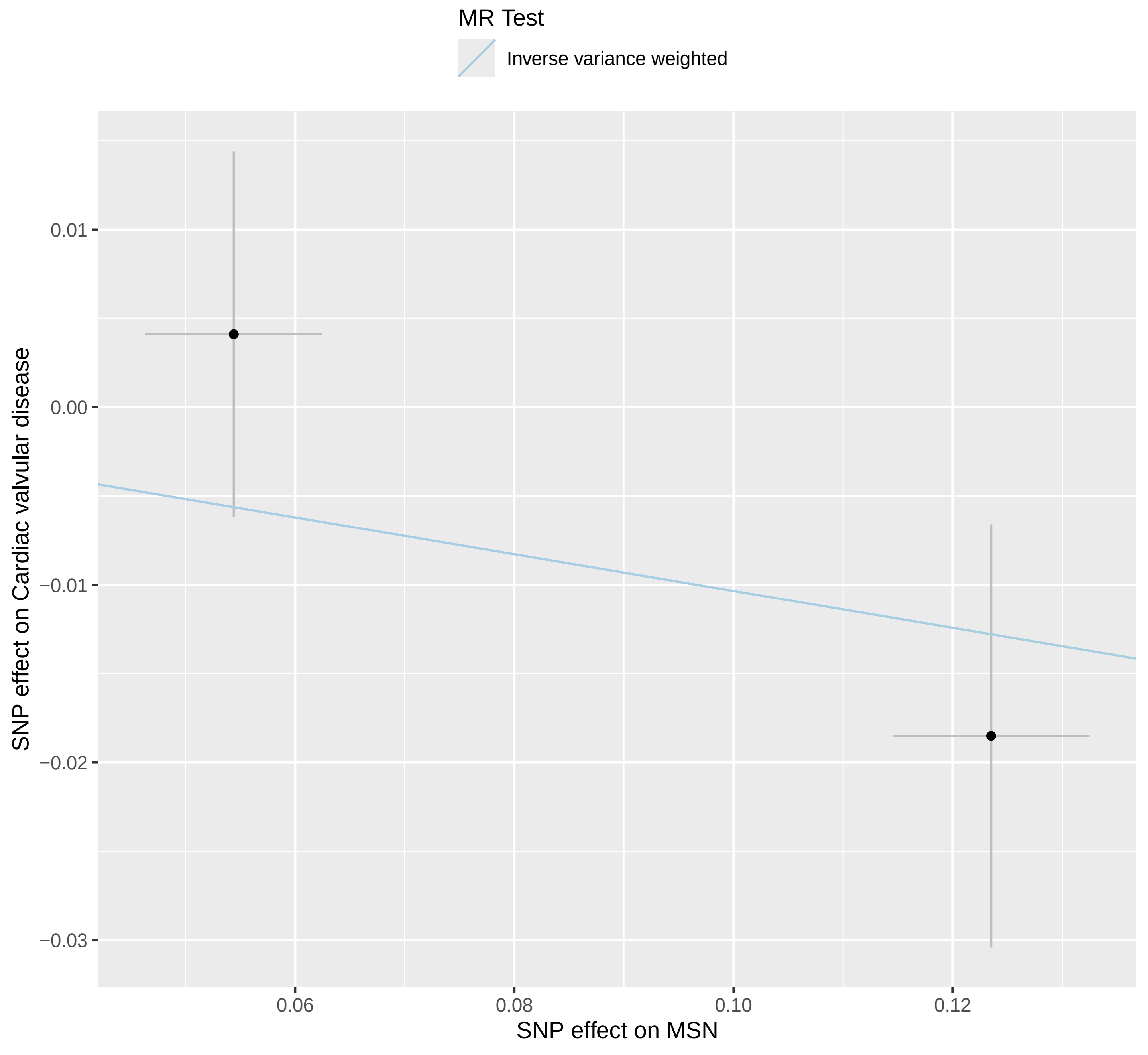
MR Test
Inverse variance weighted
0.01
0.00
−0.01
−0.02
−0.03
SNP effect on Cardiac valvular disease
0.06
0.08
0.10
0.12
SNP effect on MSN

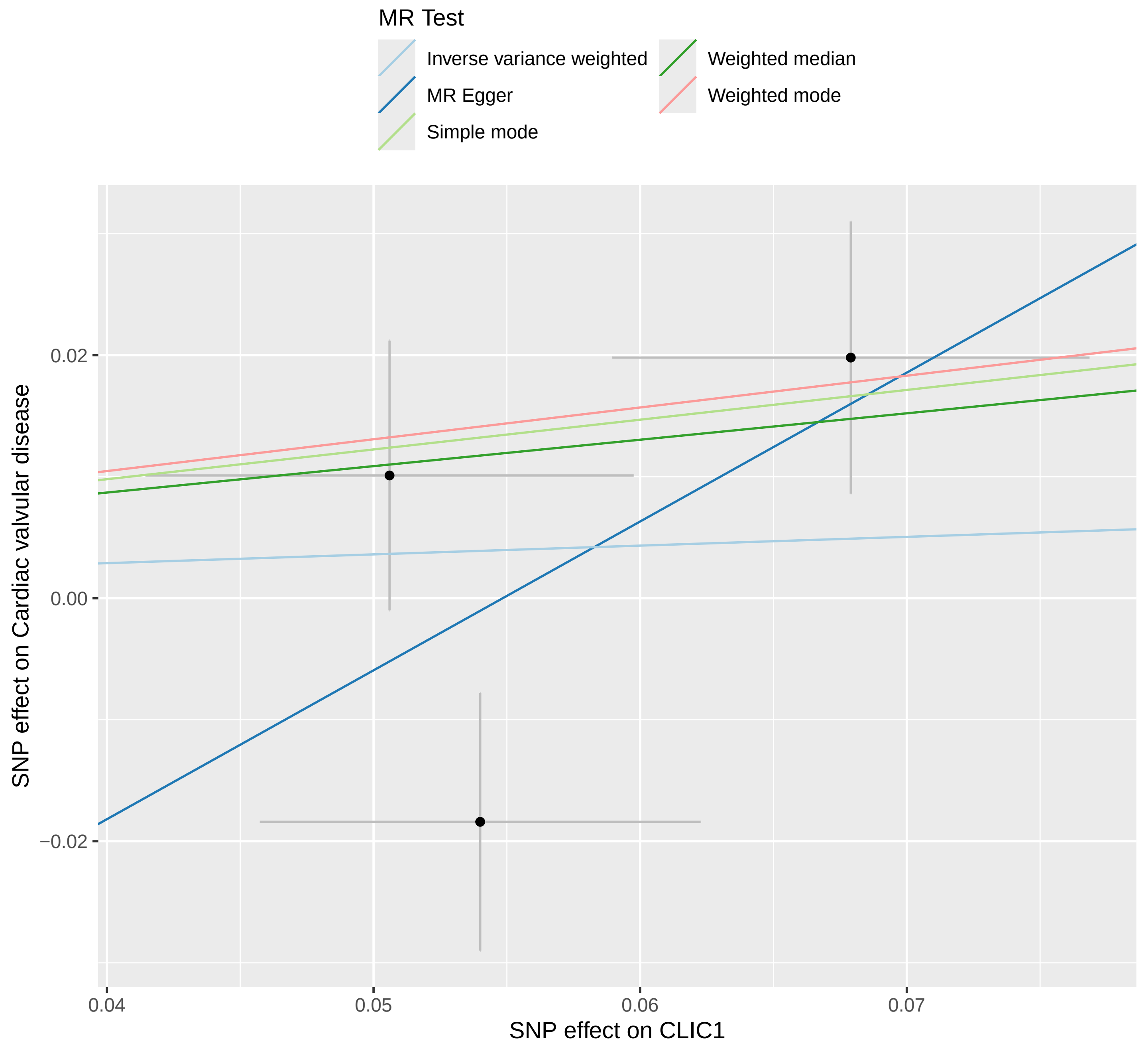

MR Test
Inverse variance weighted
MR Egger
Simple mode
Weighted median
Weighted mode
0.02
0.00
−0.02
0.04
0.05
0.06
0.07
SNP effect on Cardiac valvular disease
SNP effect on CLIC1

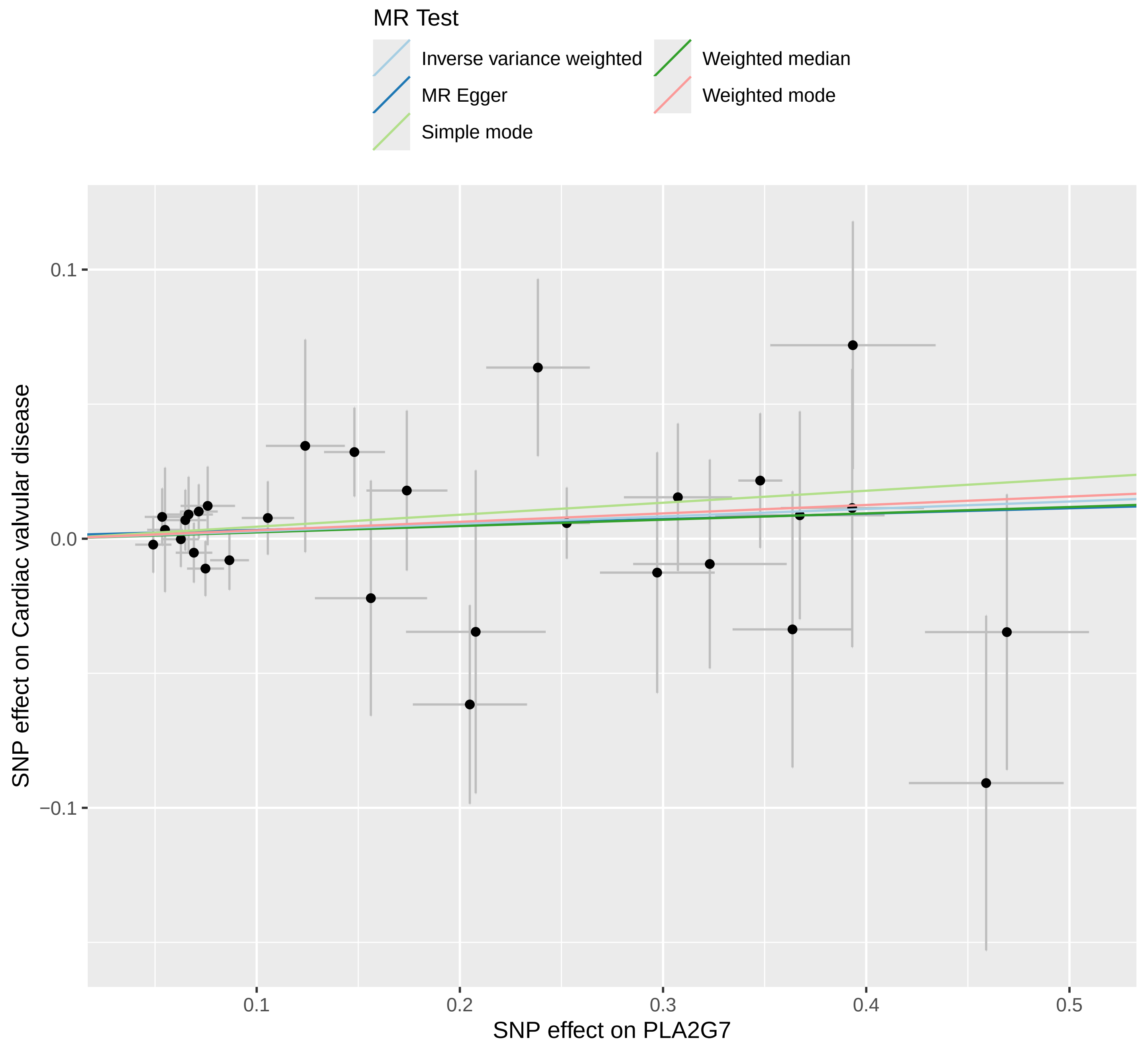

MR Test
Inverse variance weighted
MR Egger
Simple mode
Weighted median
Weighted mode
0.1
0.0
−0.1
SNP effect on Cardiac valvular disease
0.1
0.2
0.3
0.4
0.5
SNP effect on PLA2G7

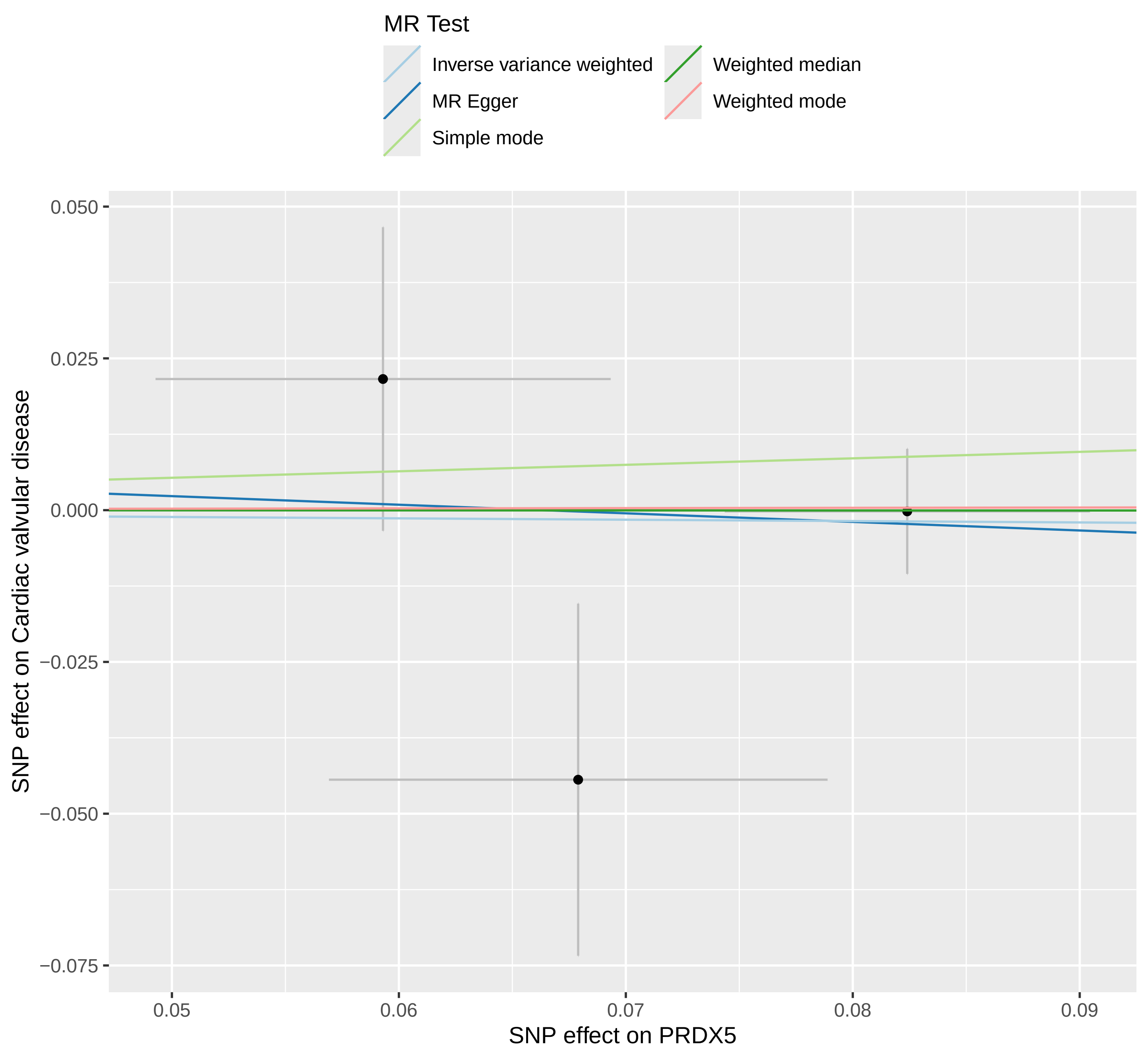
MR Test
Inverse variance weighted
MR Egger
Simple mode
Weighted median
Weighted mode
0.050
0.025
0.000
−0.025
−0.050
−0.075
0.05
0.06
0.07
0.08
0.09
SNP effect on Cardiac valvular disease
SNP effect on PRDX5

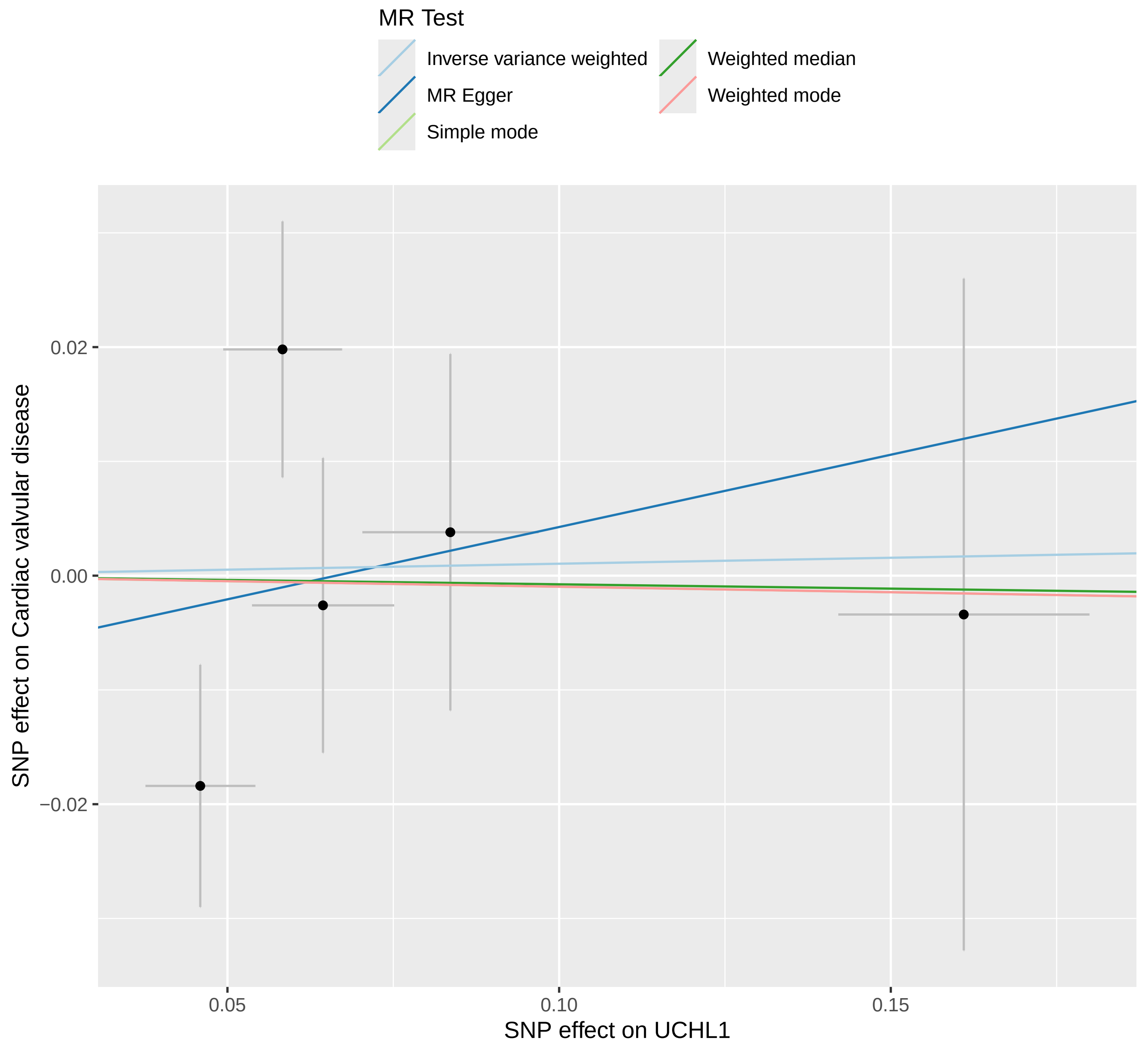

MR Test
Inverse variance weighted
MR Egger
Simple mode
Weighted median
Weighted mode
0.02
0.00
−0.02
0.05
0.10
0.15
SNP effect on UCHL1
SNP effect on Cardiac valvular disease

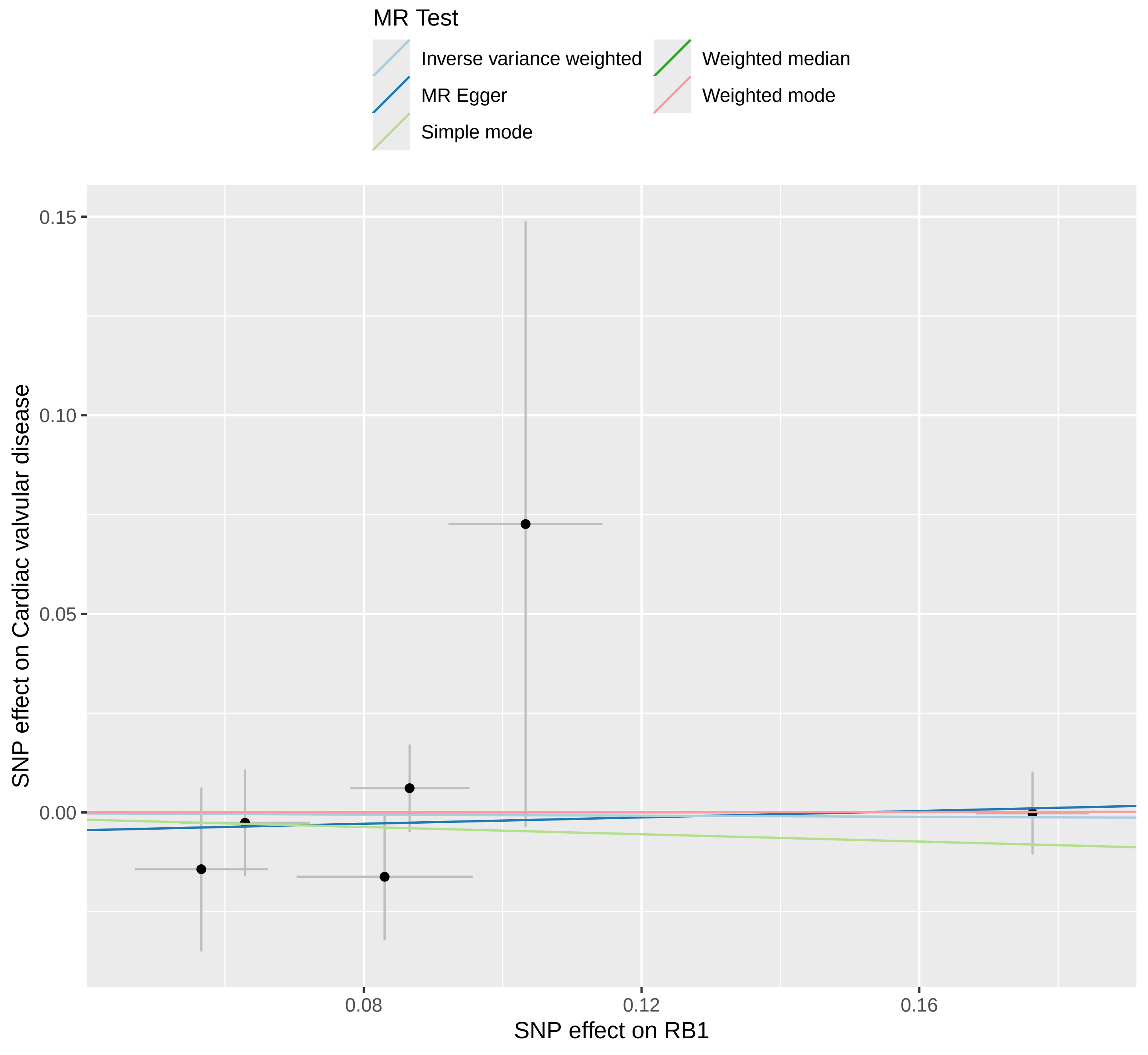
MR Test
Inverse variance weighted
MR Egger
Simple mode
Weighted median
Weighted mode
0.15
0.10
0.05
0.00
SNP effect on Cardiac valvular disease
0.08
0.12
0.16
SNP effect on RB1

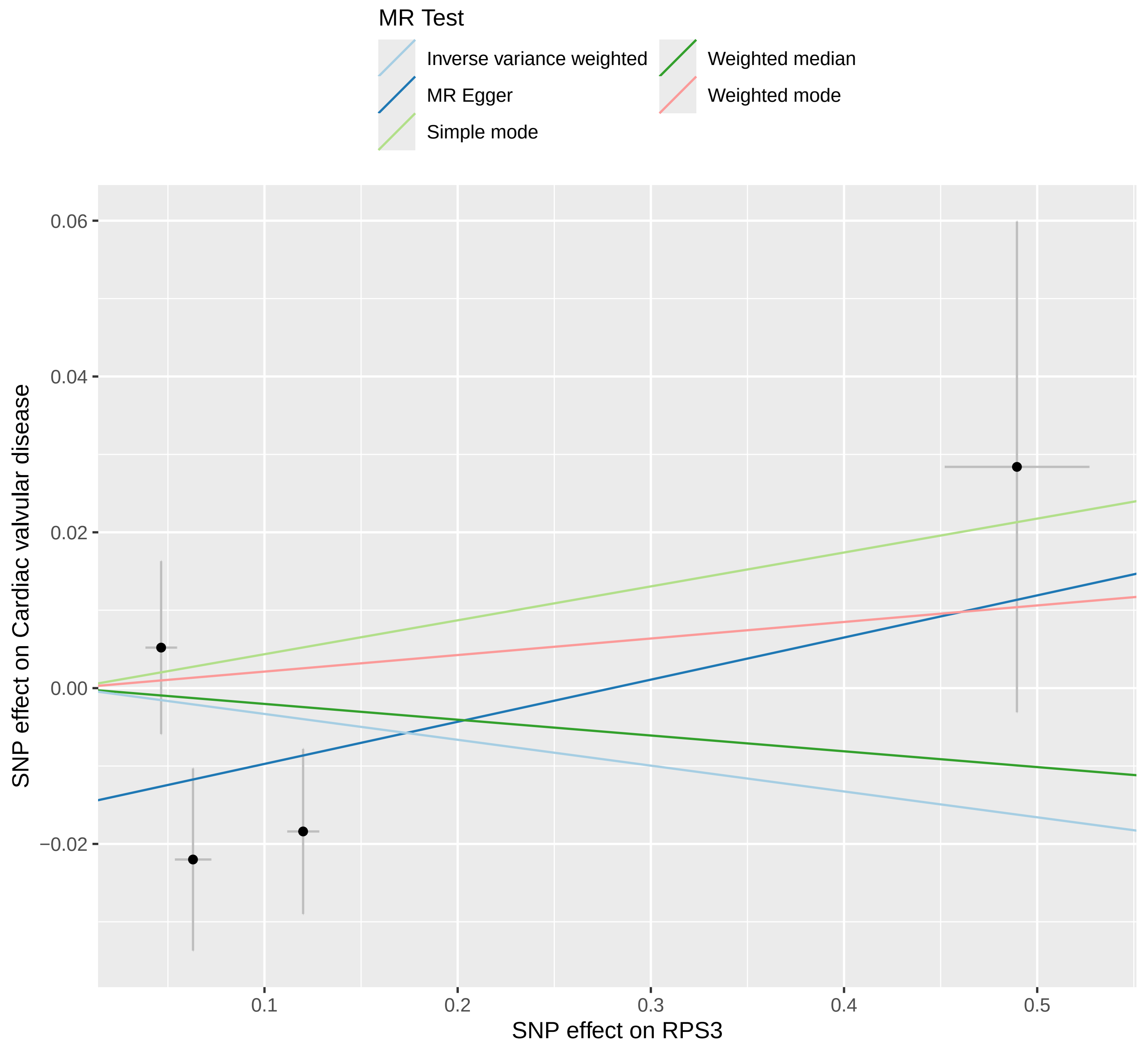
MR Test
Inverse variance weighted
MR Egger
Simple mode
Weighted median
Weighted mode
0.06
0.04
0.02
0.00
−0.02
0.1
0.2
0.3
0.4
0.5
SNP effect on Cardiac valvular disease
SNP effect on RPS3

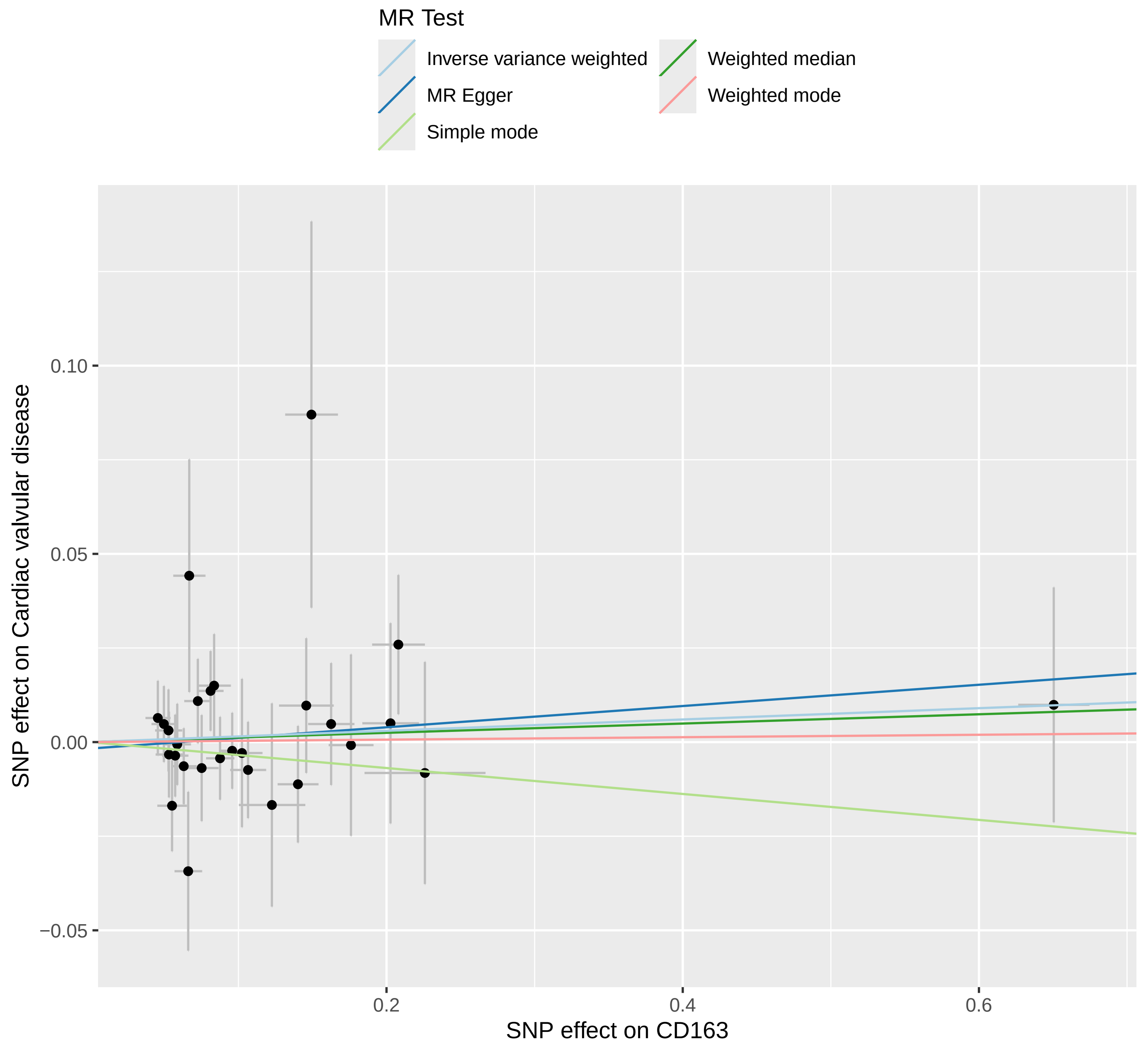

MR Test
Inverse variance weighted
MR Egger
Simple mode
Weighted median
Weighted mode
0.10
0.05
0.00
−0.05
SNP effect on Cardiac valvular disease
0.2
0.4
0.6
SNP effect on CD163

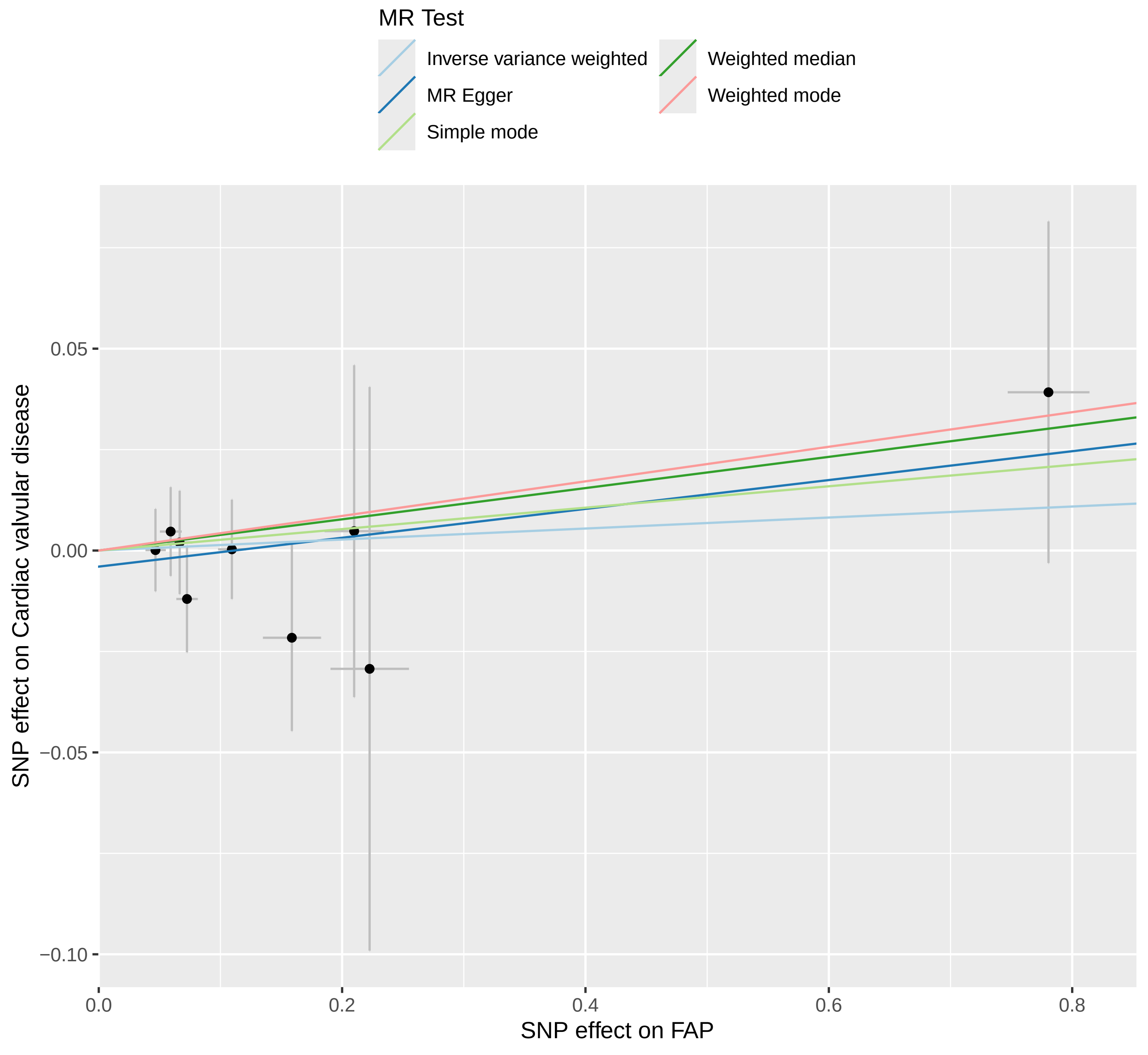
MR Test
Inverse variance weighted
MR Egger
Simple mode
Weighted median
Weighted mode
SNP effect on Cardiac valvular disease
0.05
0.00
−0.05
−0.10
0.0
0.2
0.4
0.6
0.8
SNP effect on FAP

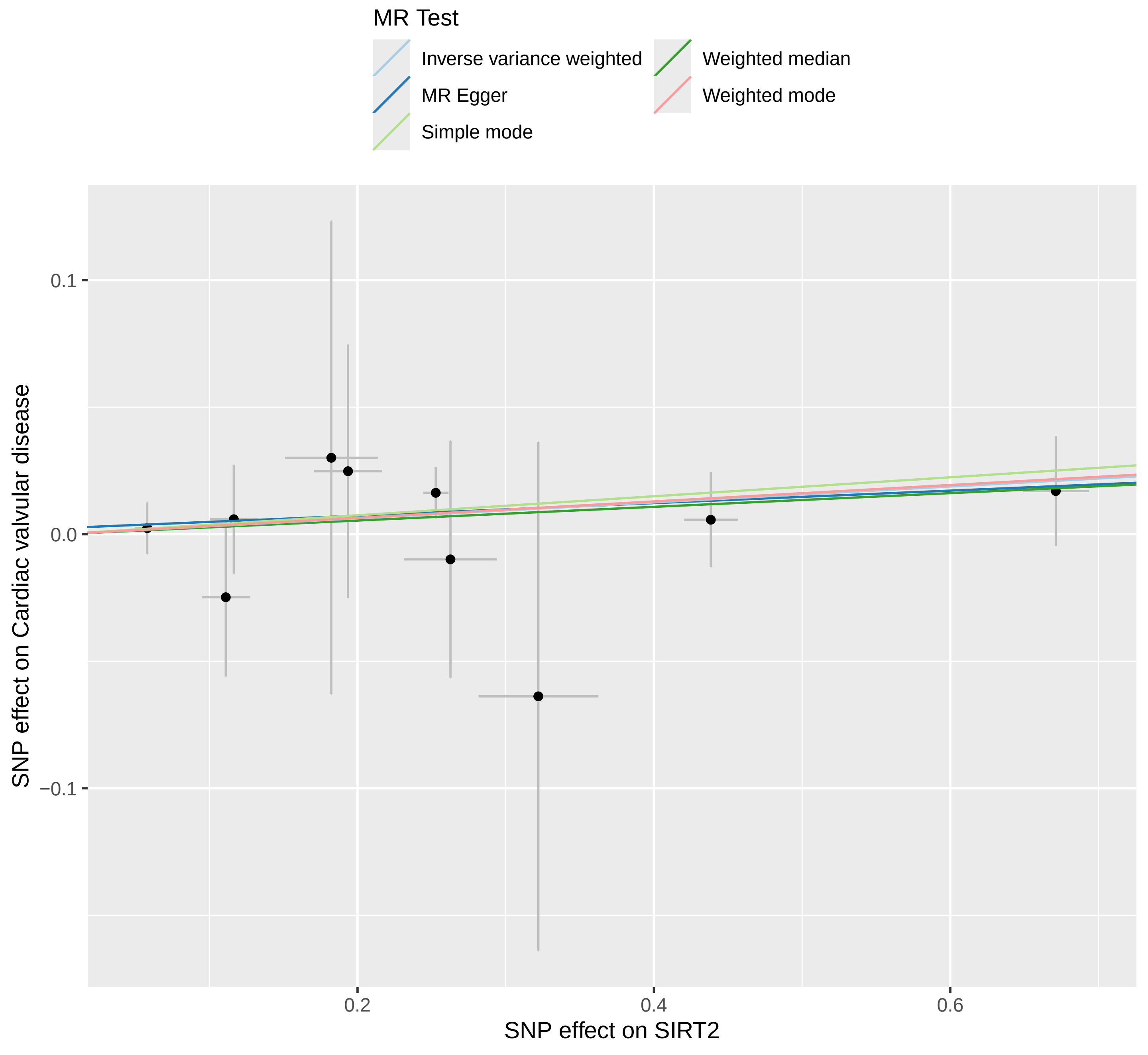
MR Test
Inverse variance weighted
MR Egger
Simple mode
Weighted median
Weighted mode
0.1
0.0
−0.1
SNP effect on Cardiac valvular disease
0.2
0.4
0.6
SNP effect on SIRT2

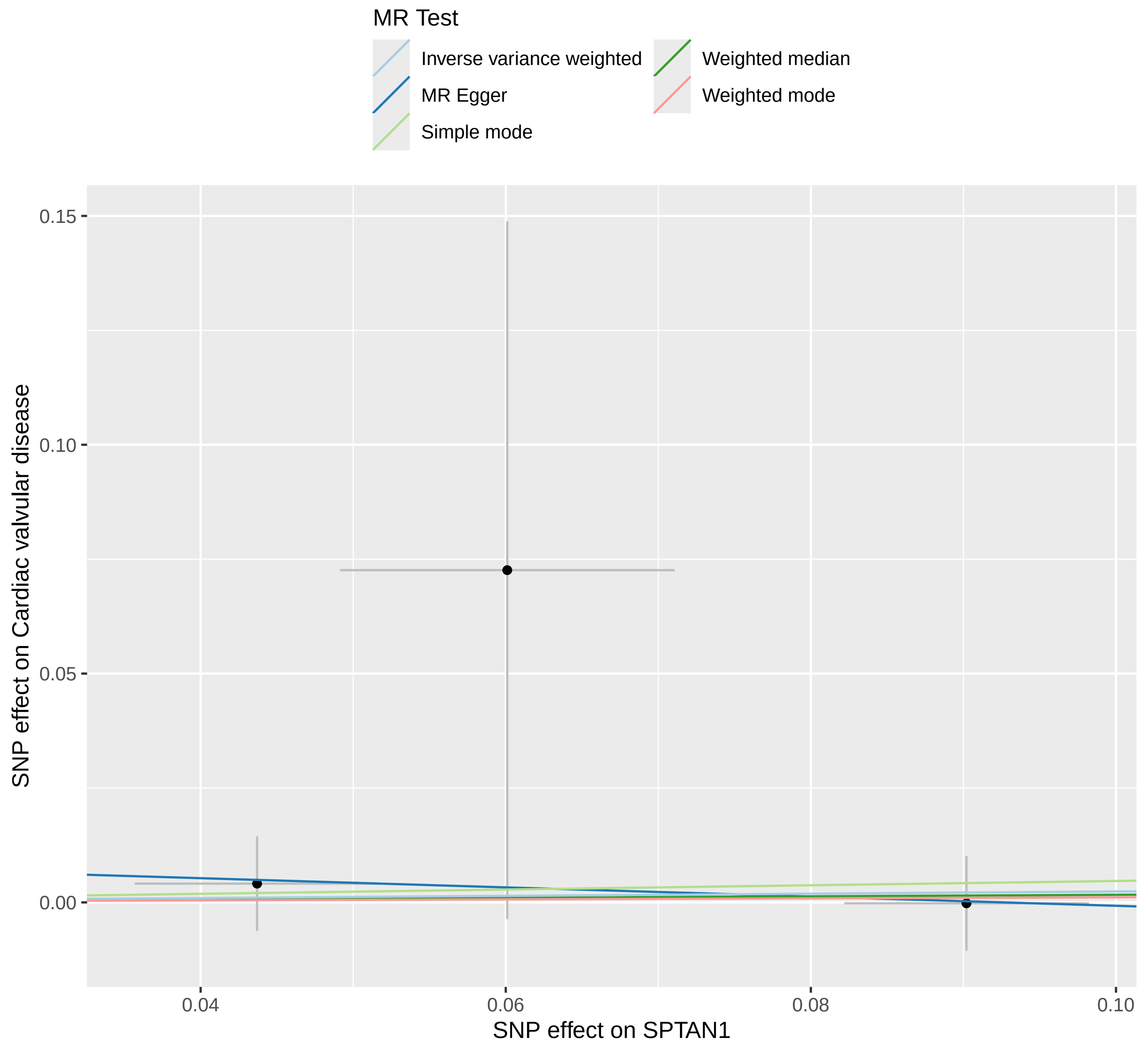
MR Test
Inverse variance weighted
MR Egger
Simple mode
Weighted median
Weighted mode
0.15
0.10
0.05
0.00
SNP effect on Cardiac valvular disease
0.04
0.06
0.08
0.10
SNP effect on SPTAN1

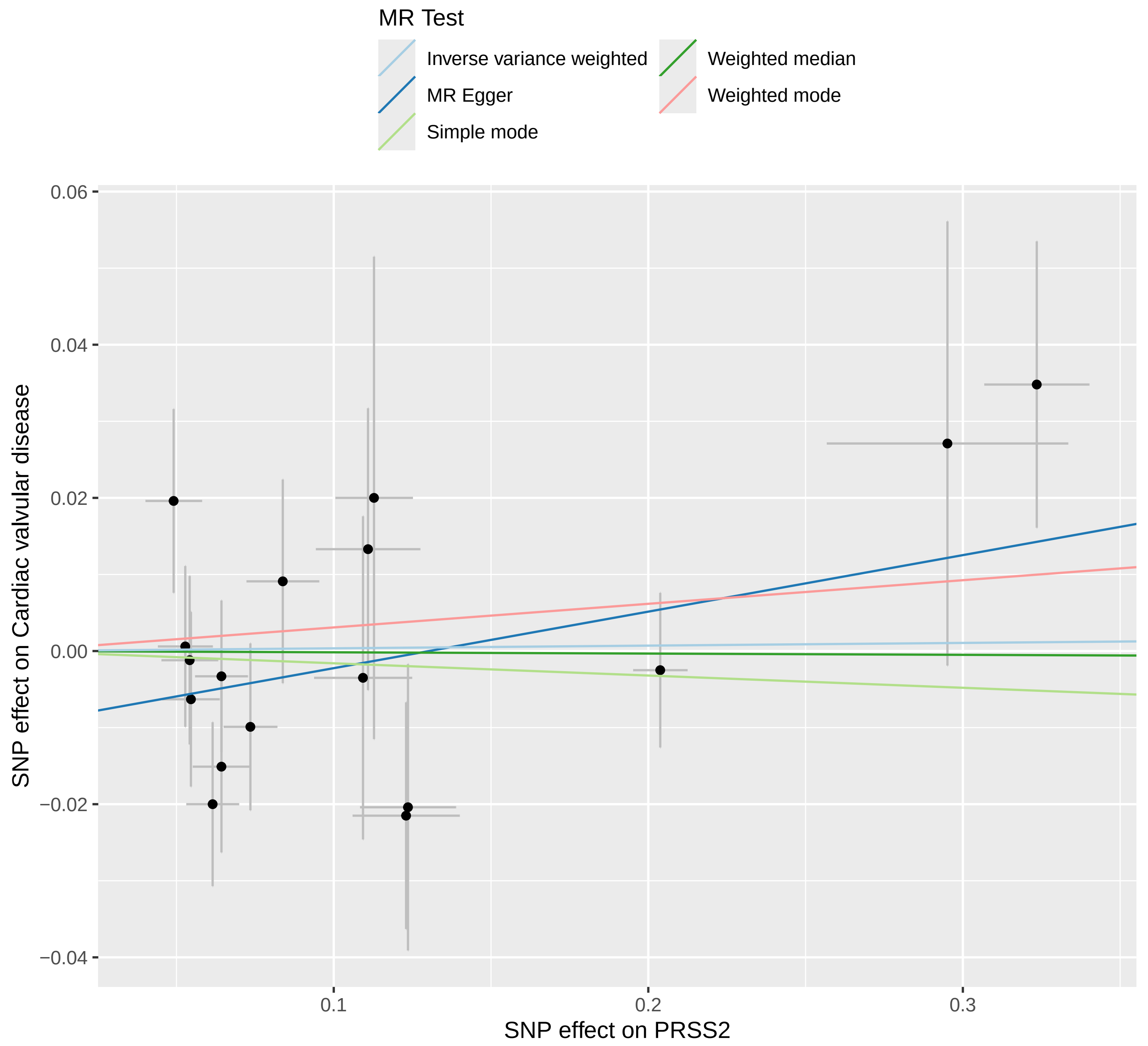

MR Test
Inverse variance weighted
MR Egger
Simple mode
Weighted median
Weighted mode
0.06
0.04
0.02
0.00
−0.02
−0.04
SNP effect on Cardiac valvular disease
0.1
0.2
0.3
SNP effect on PRSS2

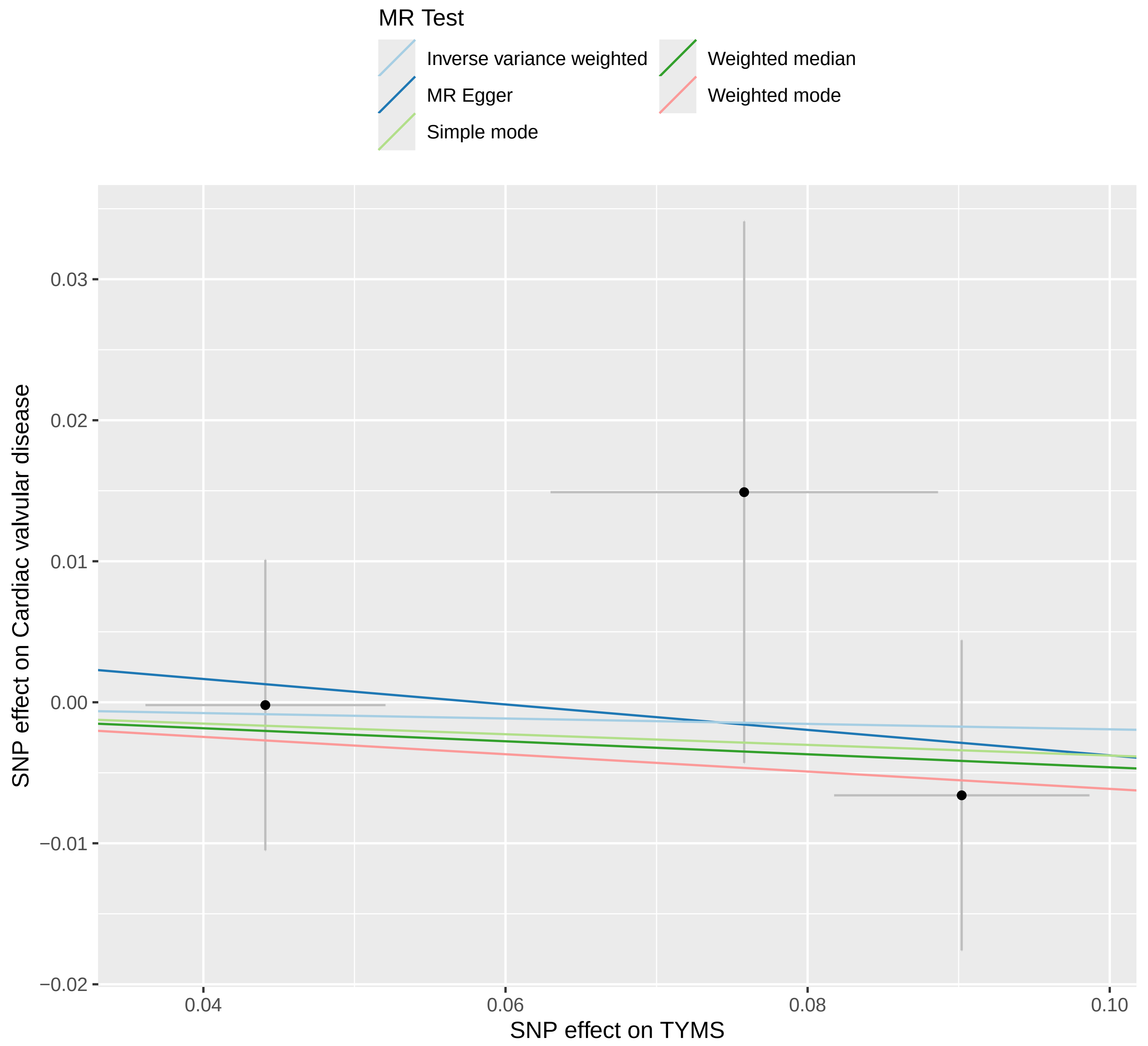

MR Test
Inverse variance weighted
MR Egger
Simple mode
Weighted median
Weighted mode
0.03
0.02
0.01
0.00
−0.01
−0.02
SNP effect on Cardiac valvular disease
0.04
0.06
0.08
0.10
SNP effect on TYMS

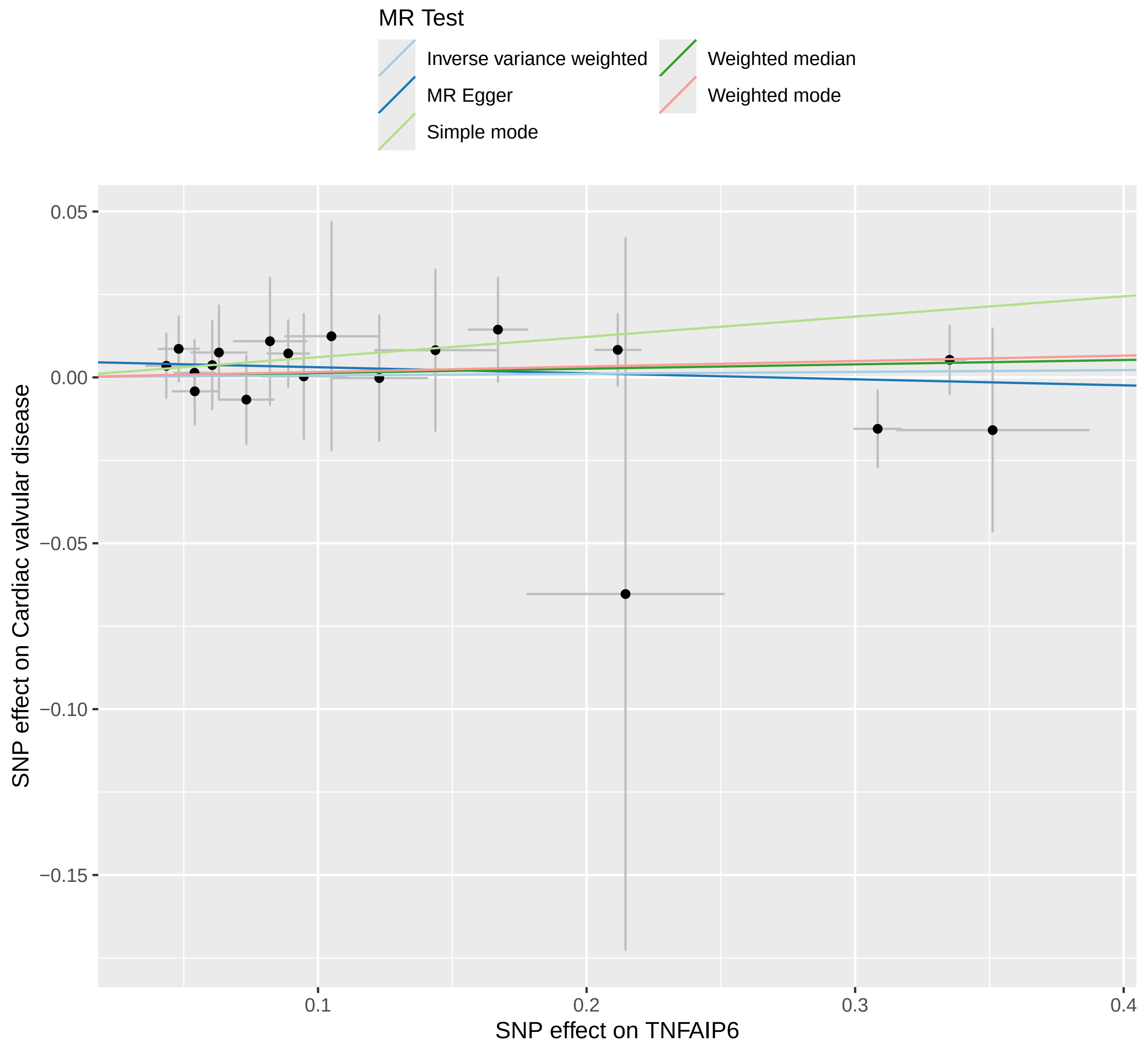
MR Test
Inverse variance weighted
MR Egger
Simple mode
Weighted median
Weighted mode
0.05
0.00
−0.05
−0.10
−0.15
SNP effect on Cardiac valvular disease
0.1
0.2
0.3
0.4
SNP effect on TNFAIP6

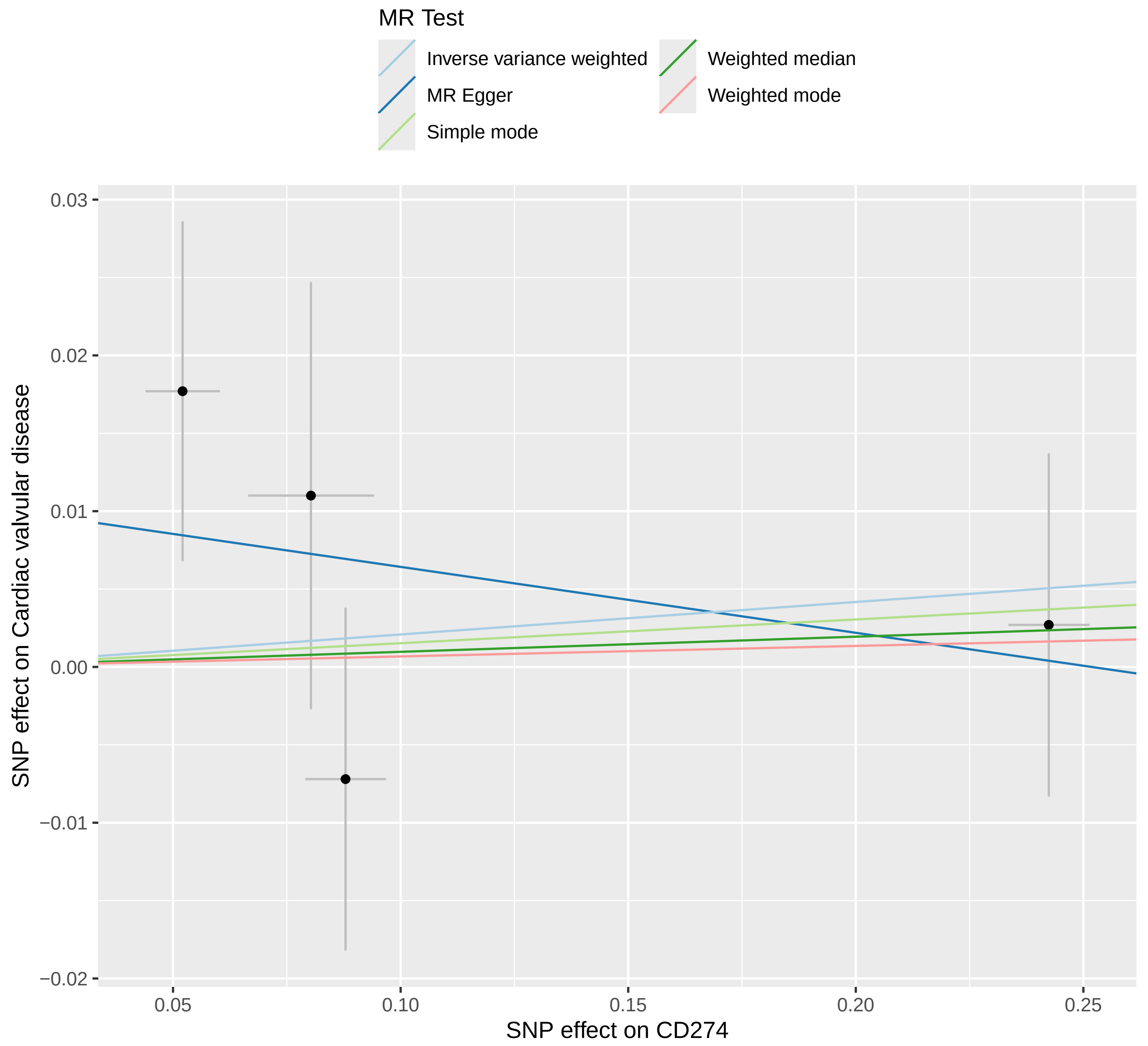
MR Test
Inverse variance weighted
MR Egger
Simple mode
Weighted median
Weighted mode
0.03
0.02
0.01
0.00
−0.01
−0.02
0.05
0.10
0.15
0.20
0.25
SNP effect on Cardiac valvular disease
SNP effect on CD274

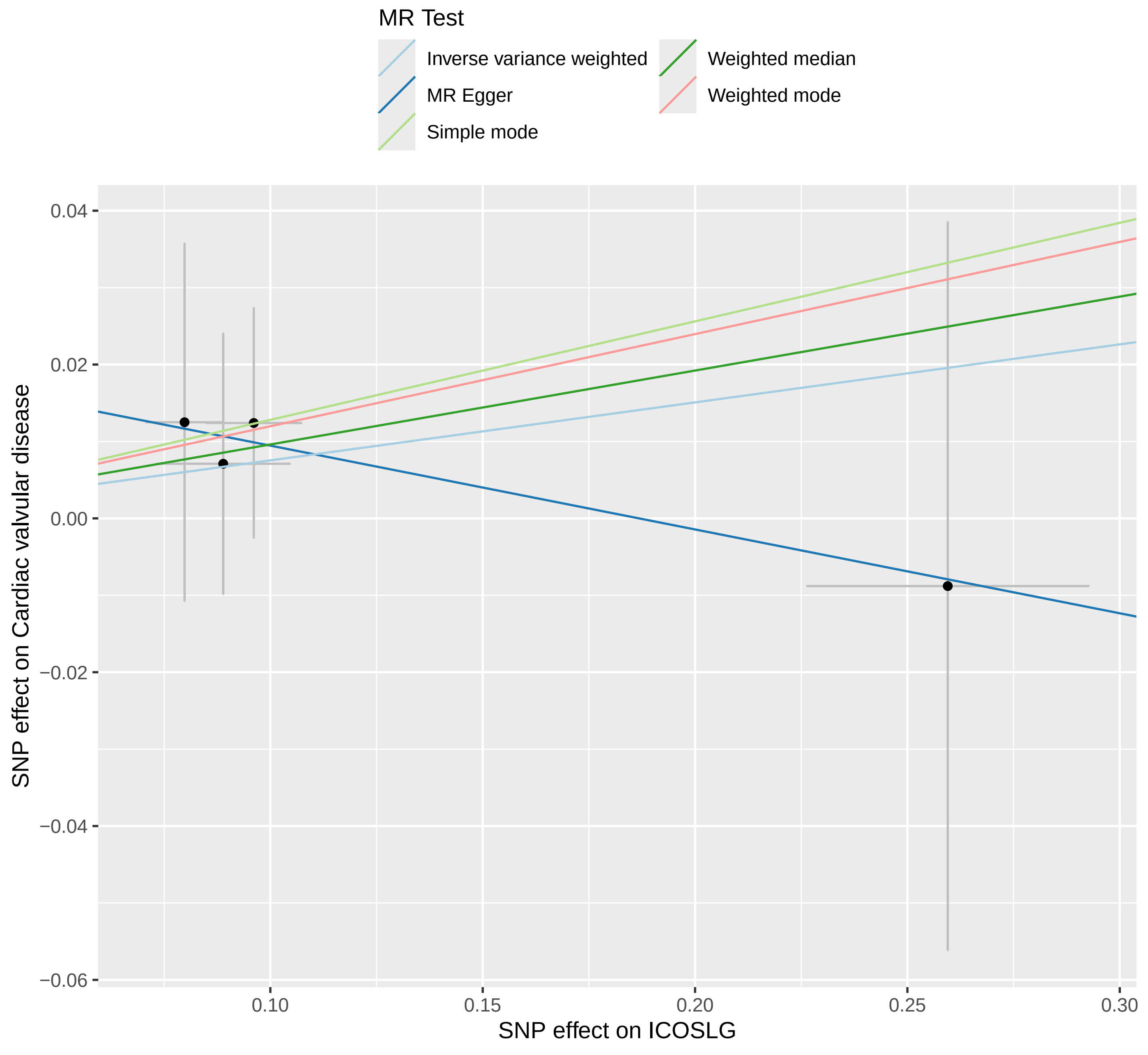
MR Test
Inverse variance weighted
MR Egger
Simple mode
Weighted median
Weighted mode
0.04
0.02
0.00
−0.02
−0.04
−0.06
SNP effect on Cardiac valvular disease
0.10
0.15
0.20
0.25
0.30
SNP effect on ICOSLG

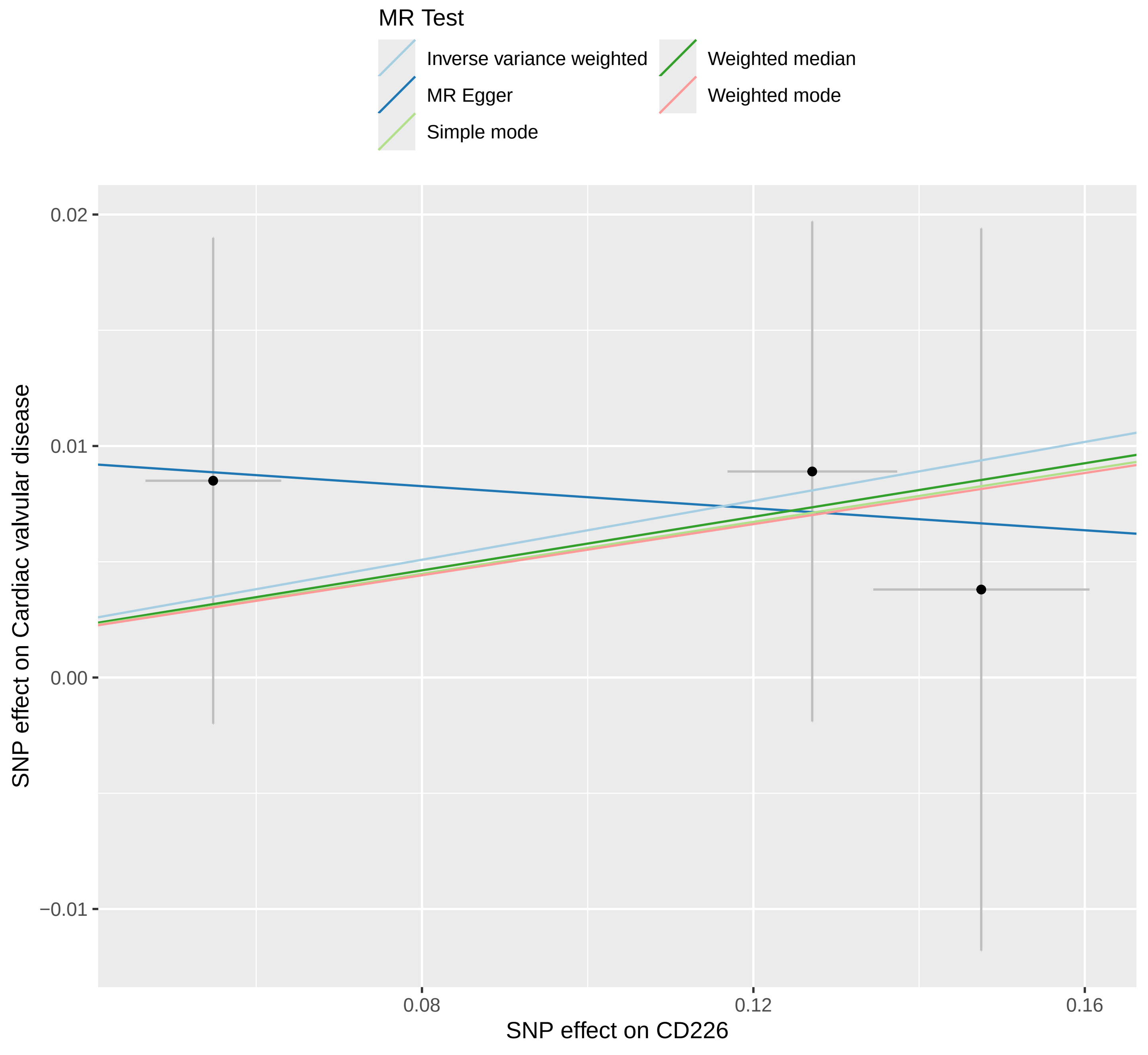

MR Test
Inverse variance weighted
MR Egger
Simple mode
Weighted median
Weighted mode
0.02
0.01
0.00
−0.01
SNP effect on Cardiac valvular disease
0.08
0.12
0.16
SNP effect on CD226

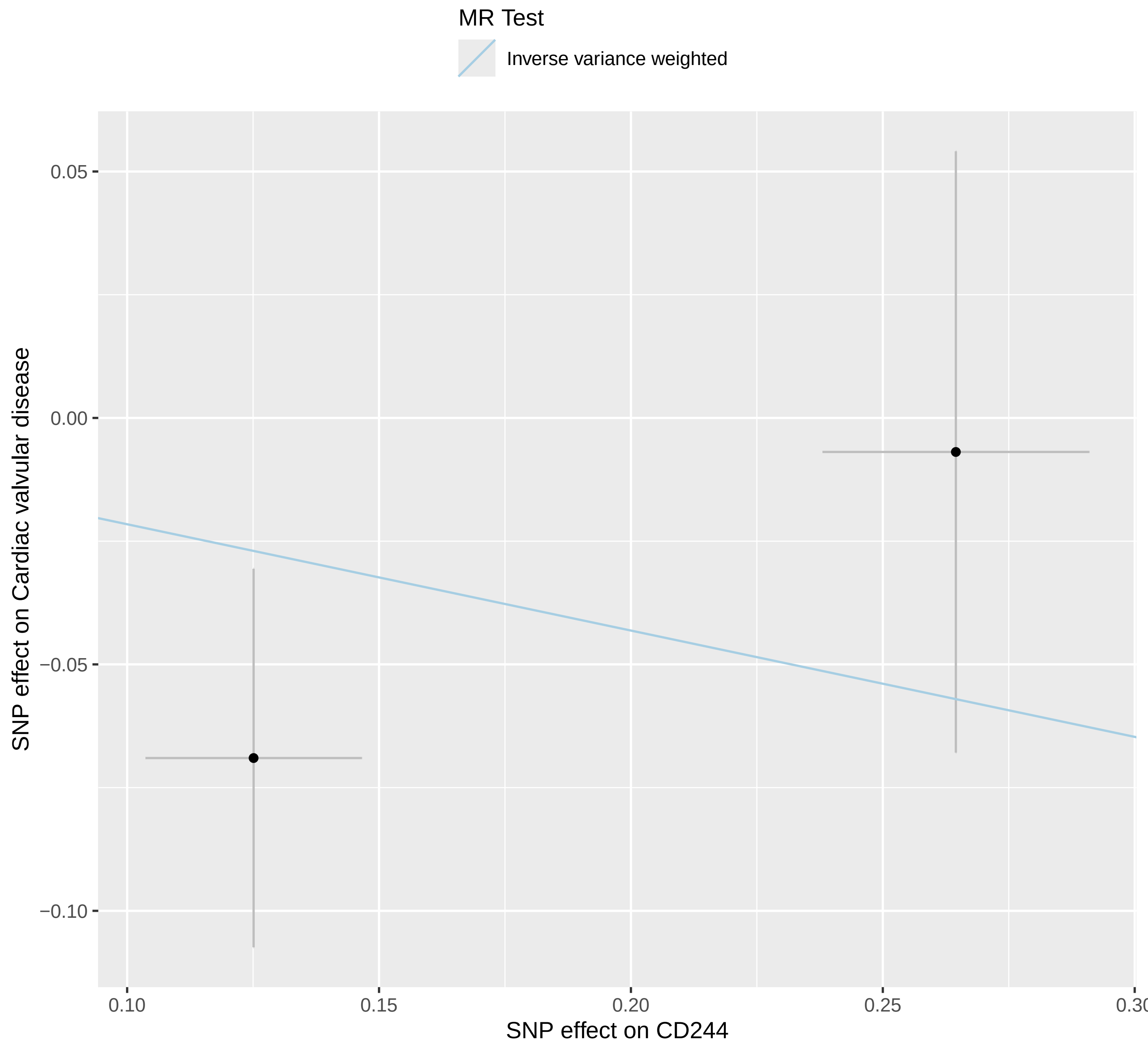
MR Test
Inverse variance weighted
0.05
0.00
−0.05
−0.10
0.10
0.15
0.20
0.25
0.30
SNP effect on CD244
SNP effect on Cardiac valvular disease

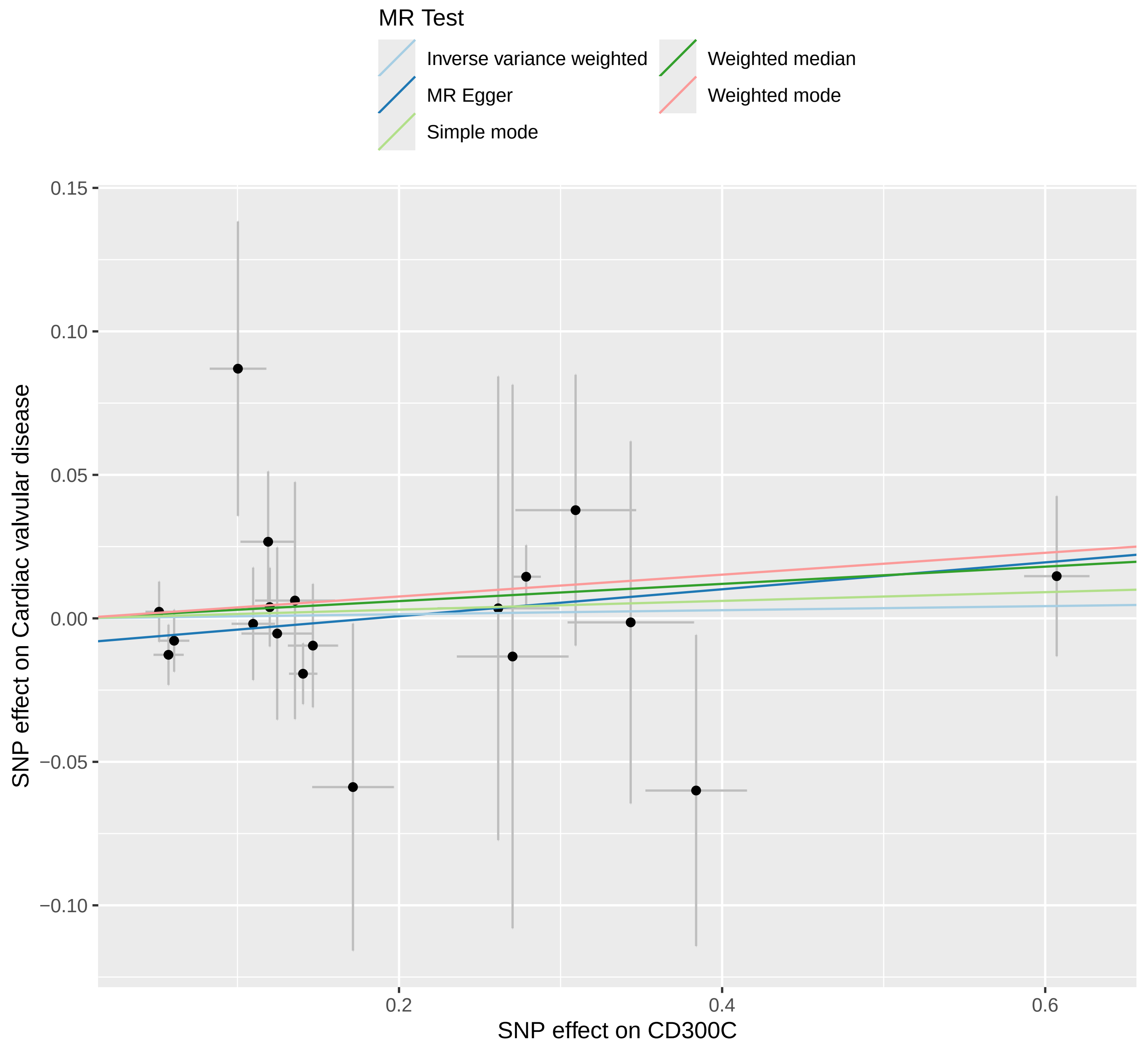

MR Test
Inverse variance weighted
MR Egger
Simple mode
Weighted median
Weighted mode
0.15
0.10
0.05
0.00
−0.05
−0.10
0.2
0.4
0.6
SNP effect on Cardiac valvular disease
SNP effect on CD300C

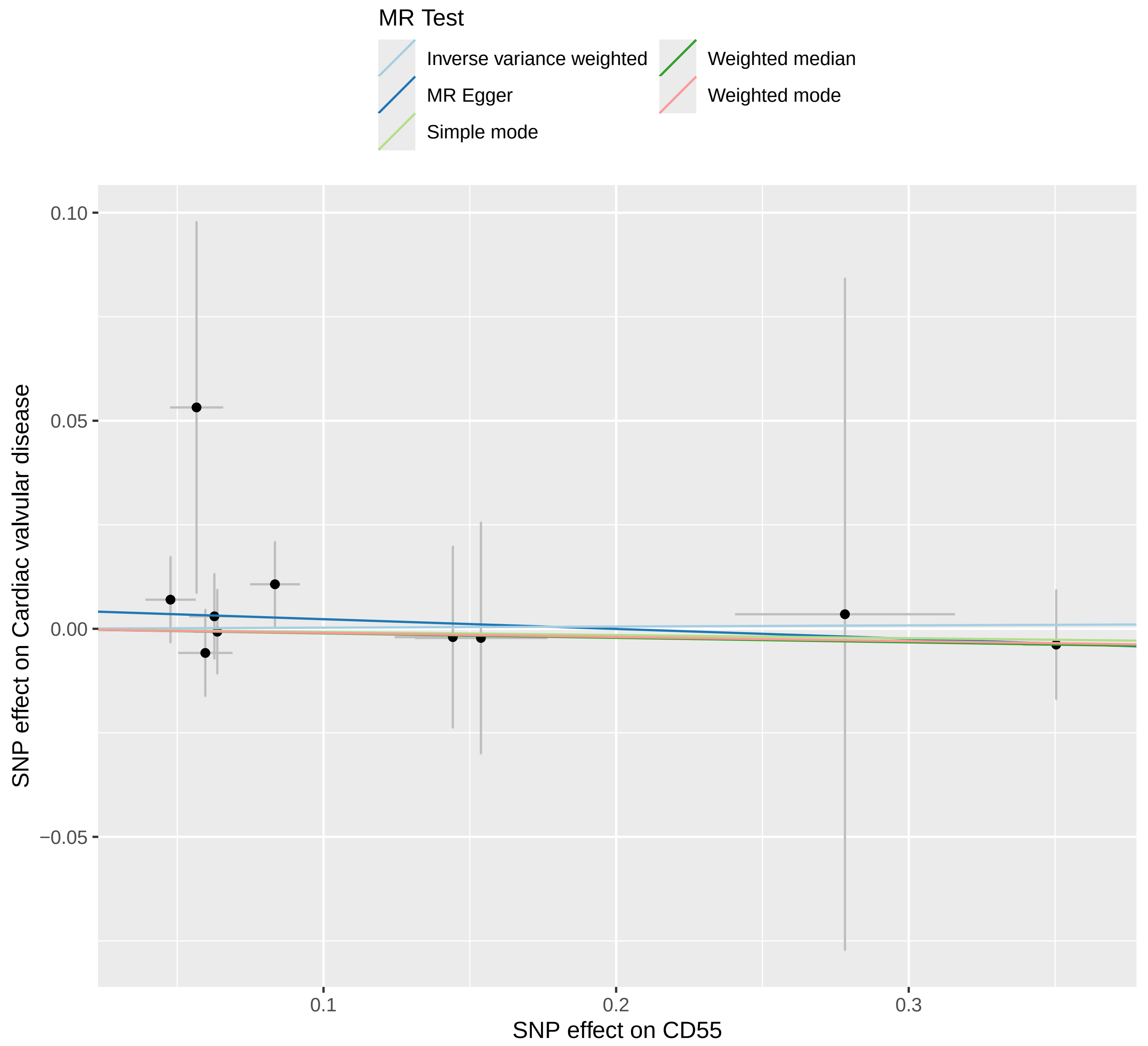
MR Test
Inverse variance weighted
MR Egger
Simple mode
Weighted median
Weighted mode
0.10
0.05
0.00
−0.05
0.1
0.2
0.3
SNP effect on Cardiac valvular disease
SNP effect on CD55

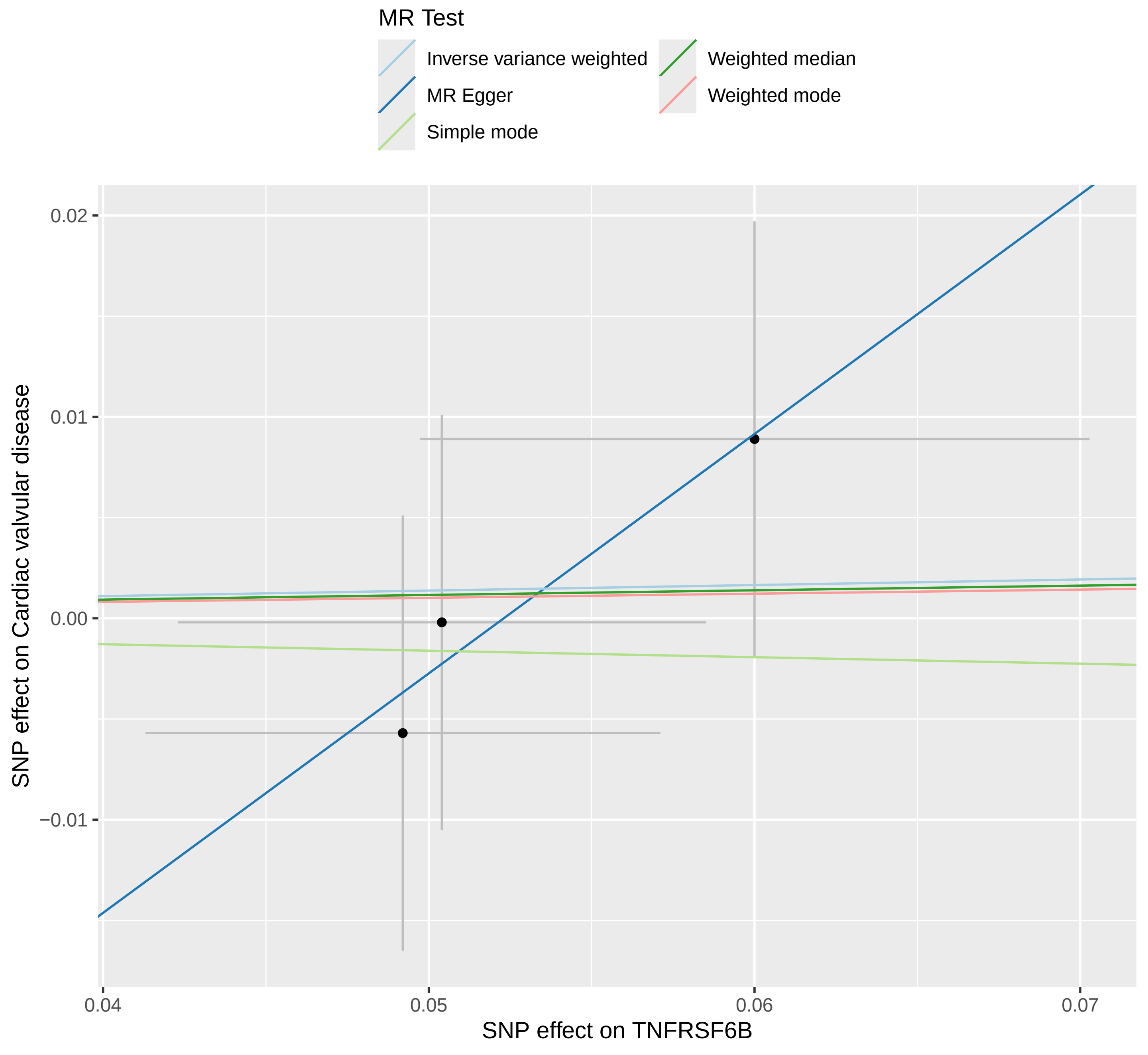
MR Test
Inverse variance weighted
MR Egger
Simple mode
Weighted median
Weighted mode
0.02
0.01
0.00
−0.01
SNP effect on Cardiac valvular disease
0.04
0.05
0.06
0.07
SNP effect on TNFRSF6B

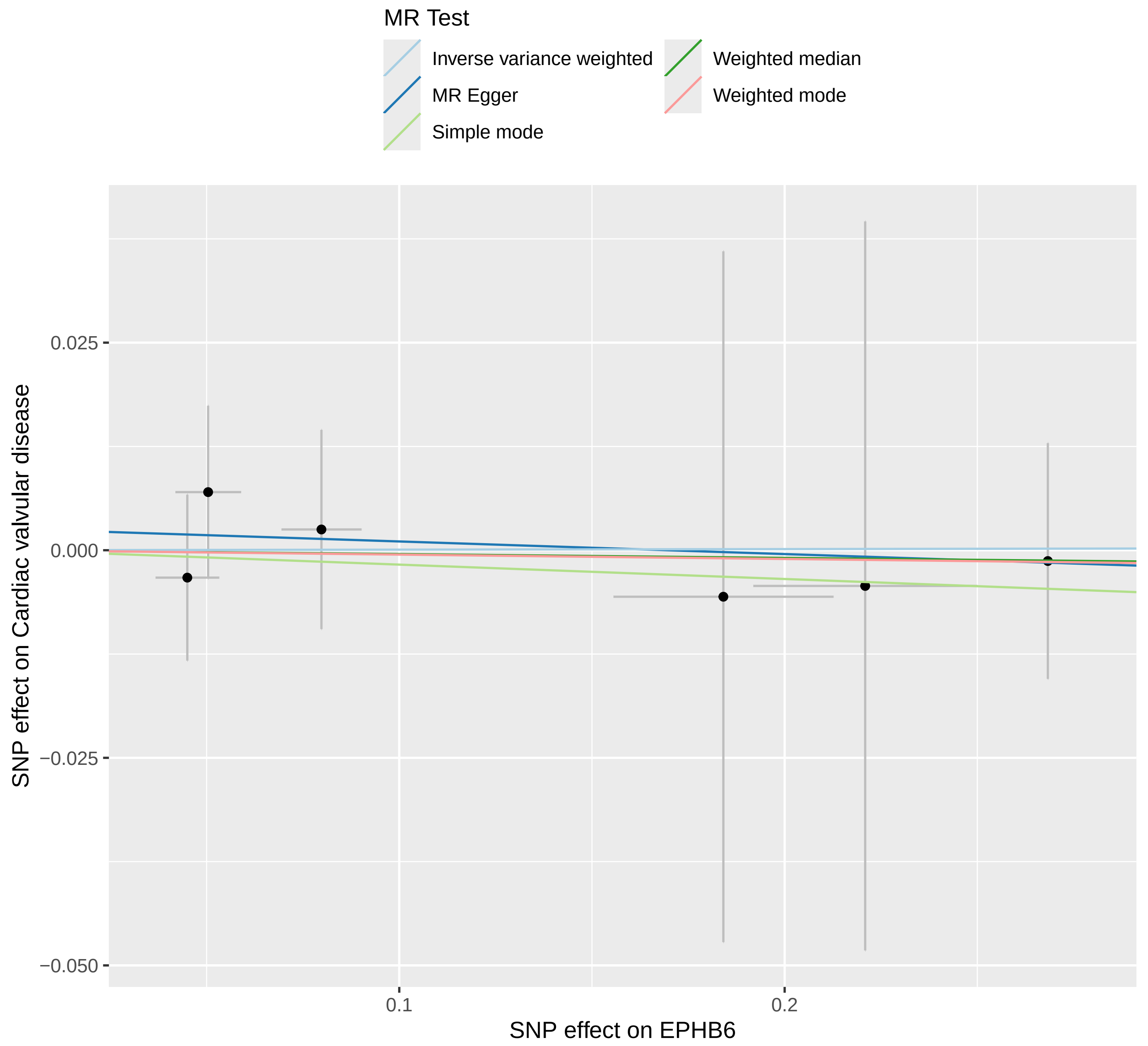
MR Test
Inverse variance weighted
MR Egger
Simple mode
Weighted median
Weighted mode
0.025
0.000
−0.025
−0.050
SNP effect on Cardiac valvular disease
0.1
0.2
SNP effect on EPHB6

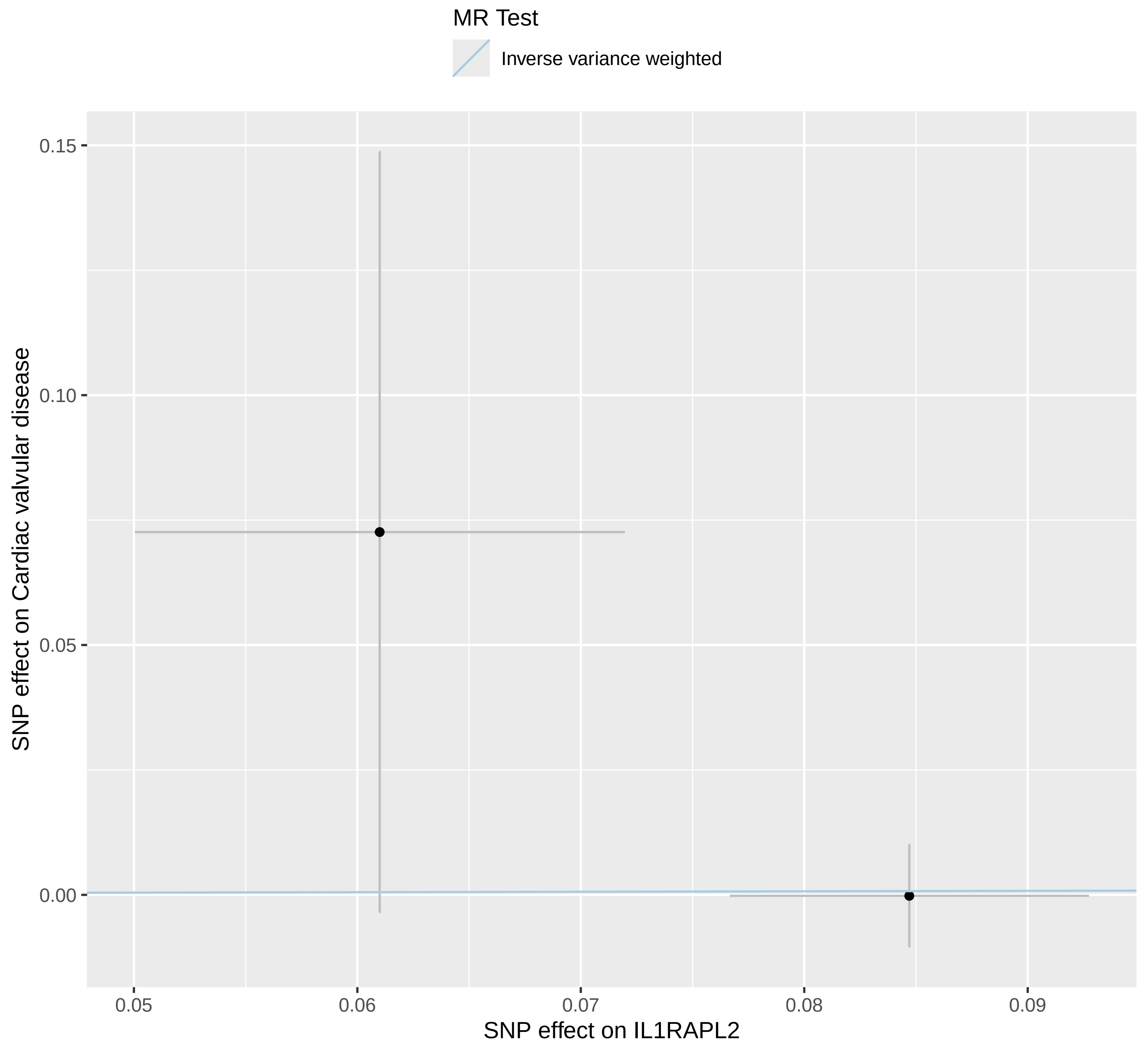

MR Test
Inverse variance weighted
0.15
0.10
0.05
0.00
SNP effect on Cardiac valvular disease
0.05
0.06
0.07
0.08
0.09
SNP effect on IL1RAPL2

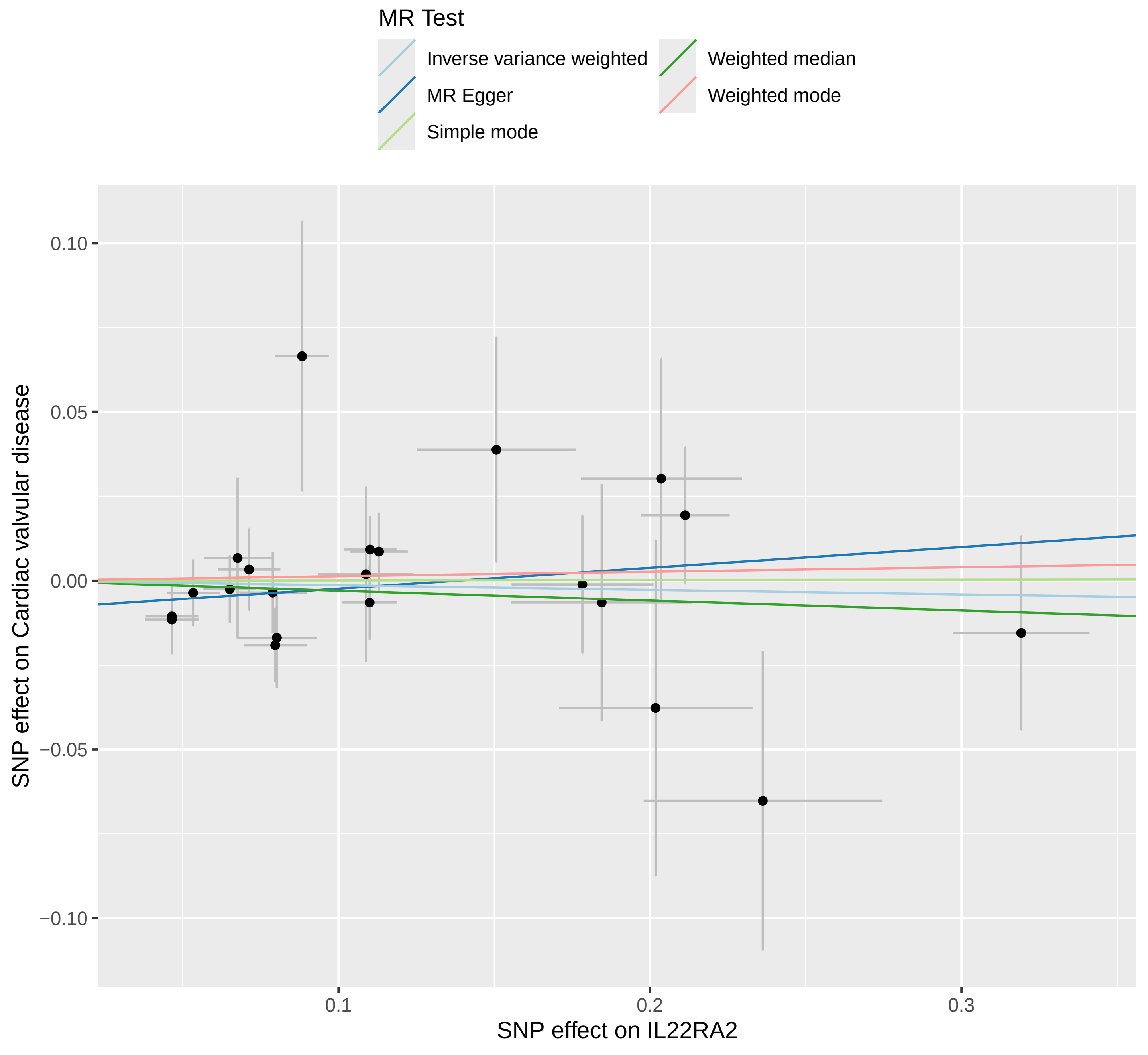

MR Test
Inverse variance weighted
MR Egger
Simple mode
Weighted median
Weighted mode
0.10
0.05
0.00
−0.05
−0.10
0.1
0.2
0.3
SNP effect on Cardiac valvular disease
SNP effect on IL22RA2

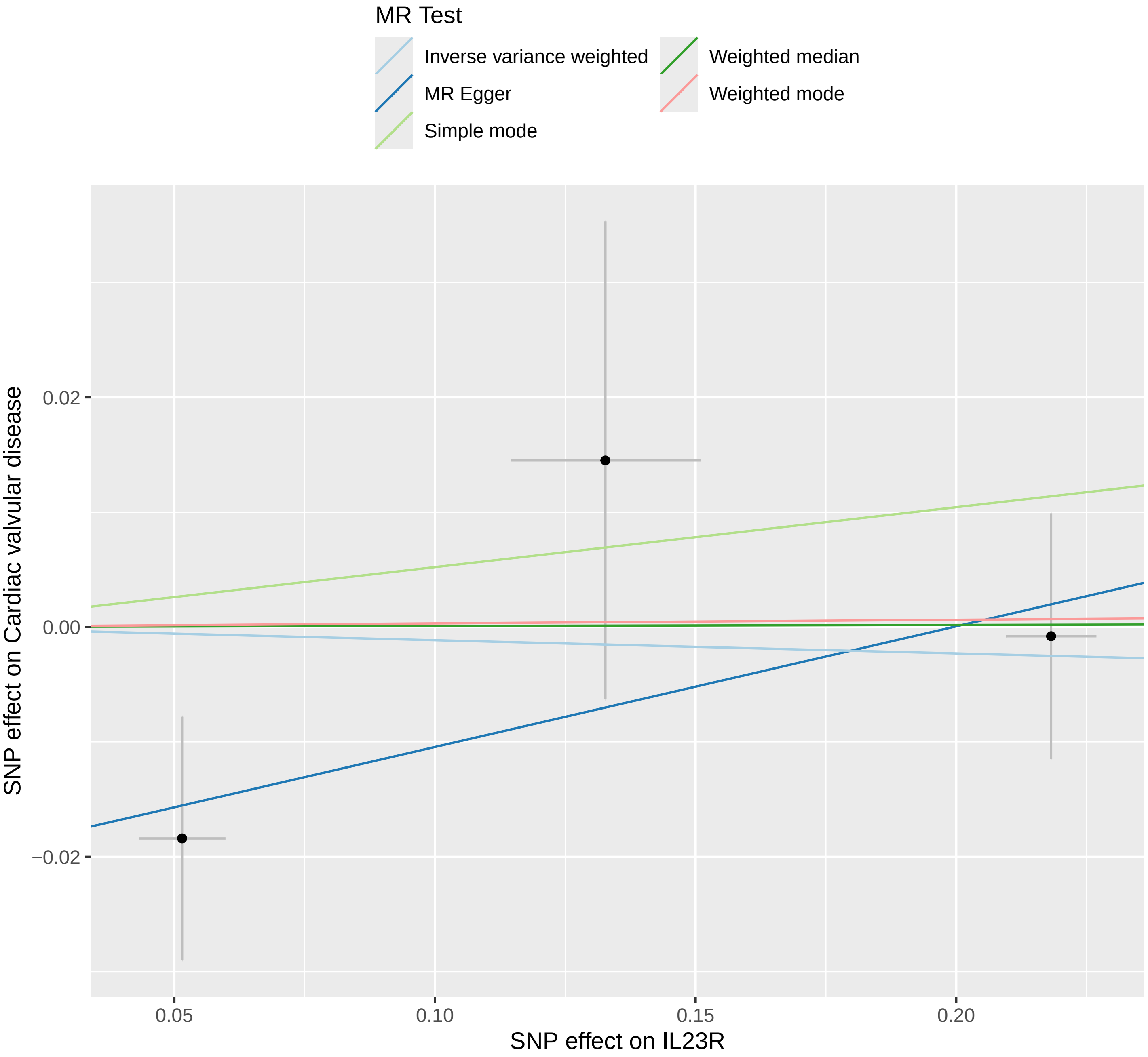
MR Test
Inverse variance weighted
MR Egger
Simple mode
Weighted median
Weighted mode
0.02
0.00
−0.02
0.05
0.10
0.15
0.20
SNP effect on IL23R
SNP effect on Cardiac valvular disease

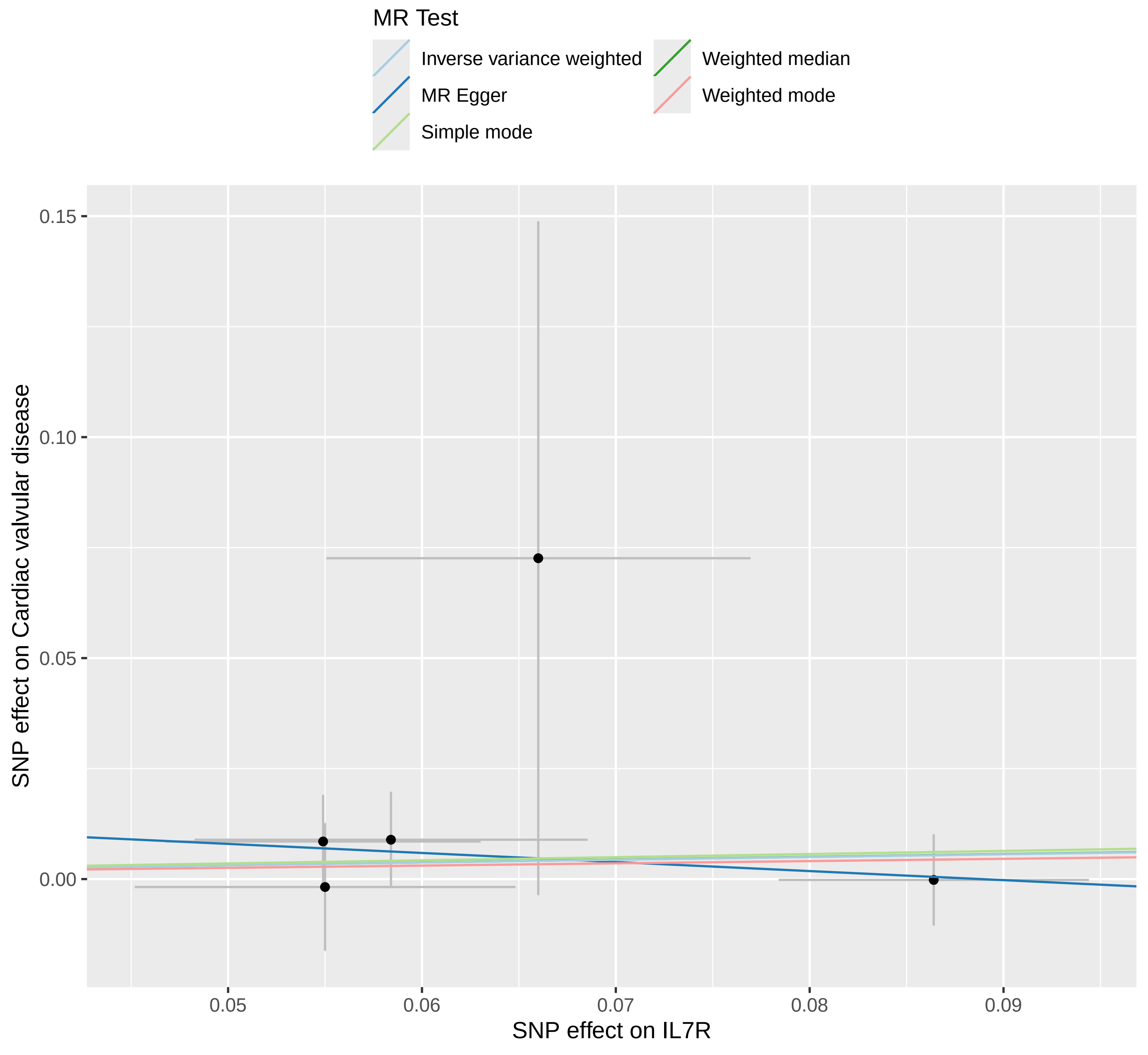

MR Test
Inverse variance weighted
MR Egger
Simple mode
Weighted median
Weighted mode
SNP effect on Cardiac valvular disease
0.15
0.10
0.05
0.00
0.05
0.06
0.07
0.08
0.09
SNP effect on IL7R

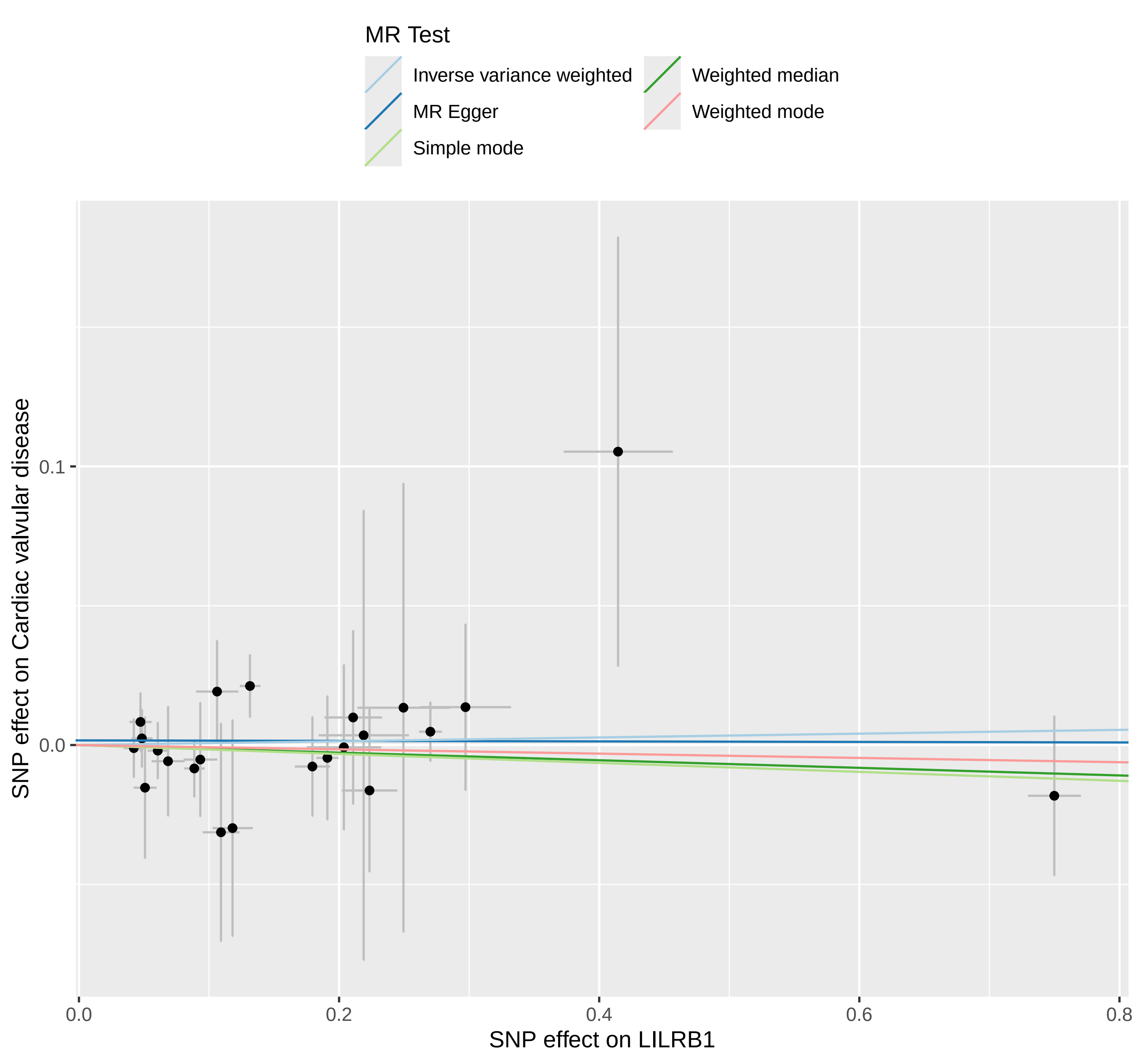
MR Test
Inverse variance weighted
MR Egger
Simple mode
Weighted median
Weighted mode
SNP effect on Cardiac valvular disease
0.1
0.0
0.0
0.2
0.4
0.6
0.8
SNP effect on LILRB1

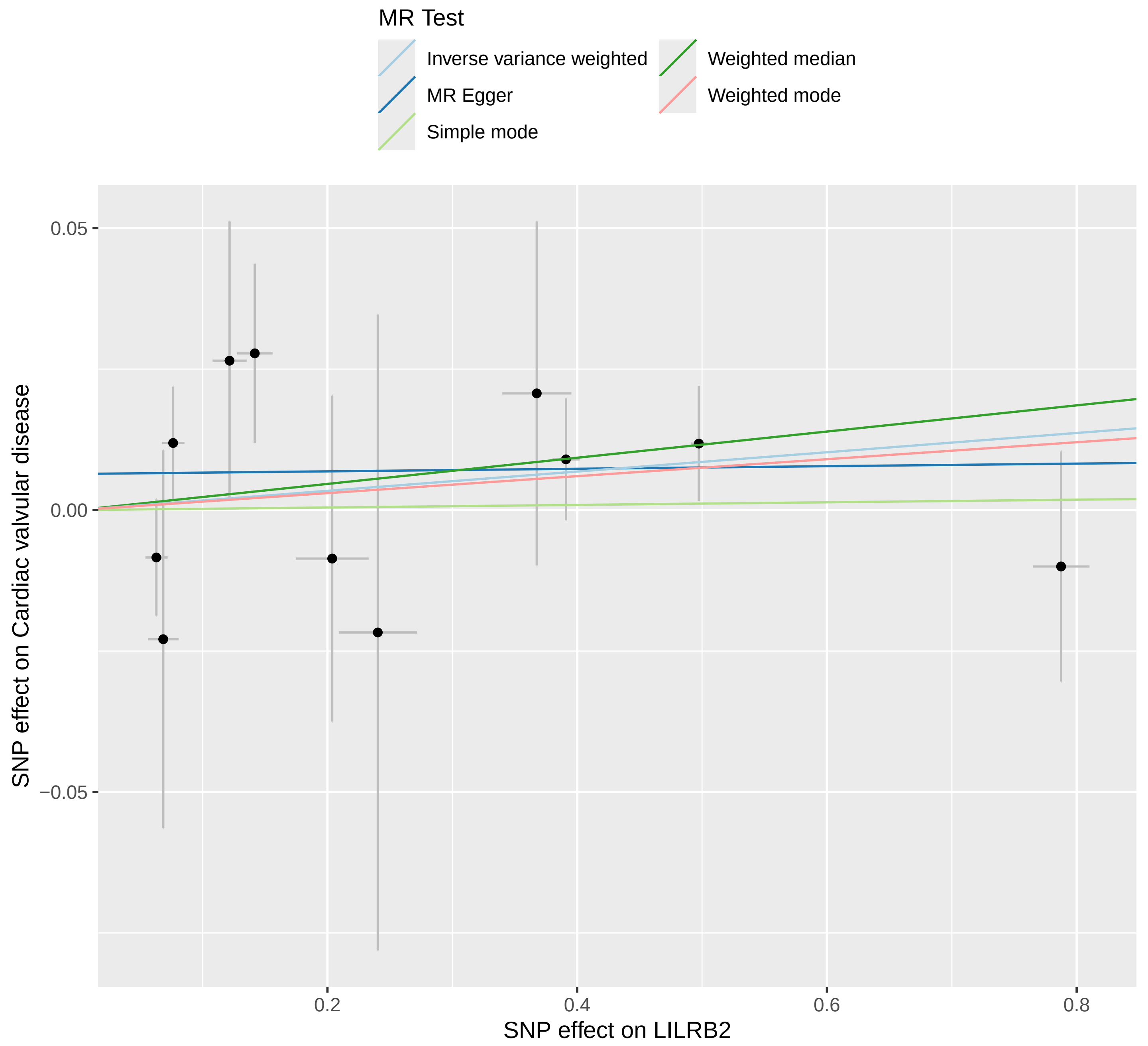
MR Test
Inverse variance weighted
MR Egger
Simple mode
Weighted median
Weighted mode
0.05
0.00
−0.05
0.2
0.4
0.6
0.8
SNP effect on Cardiac valvular disease
SNP effect on LILRB2

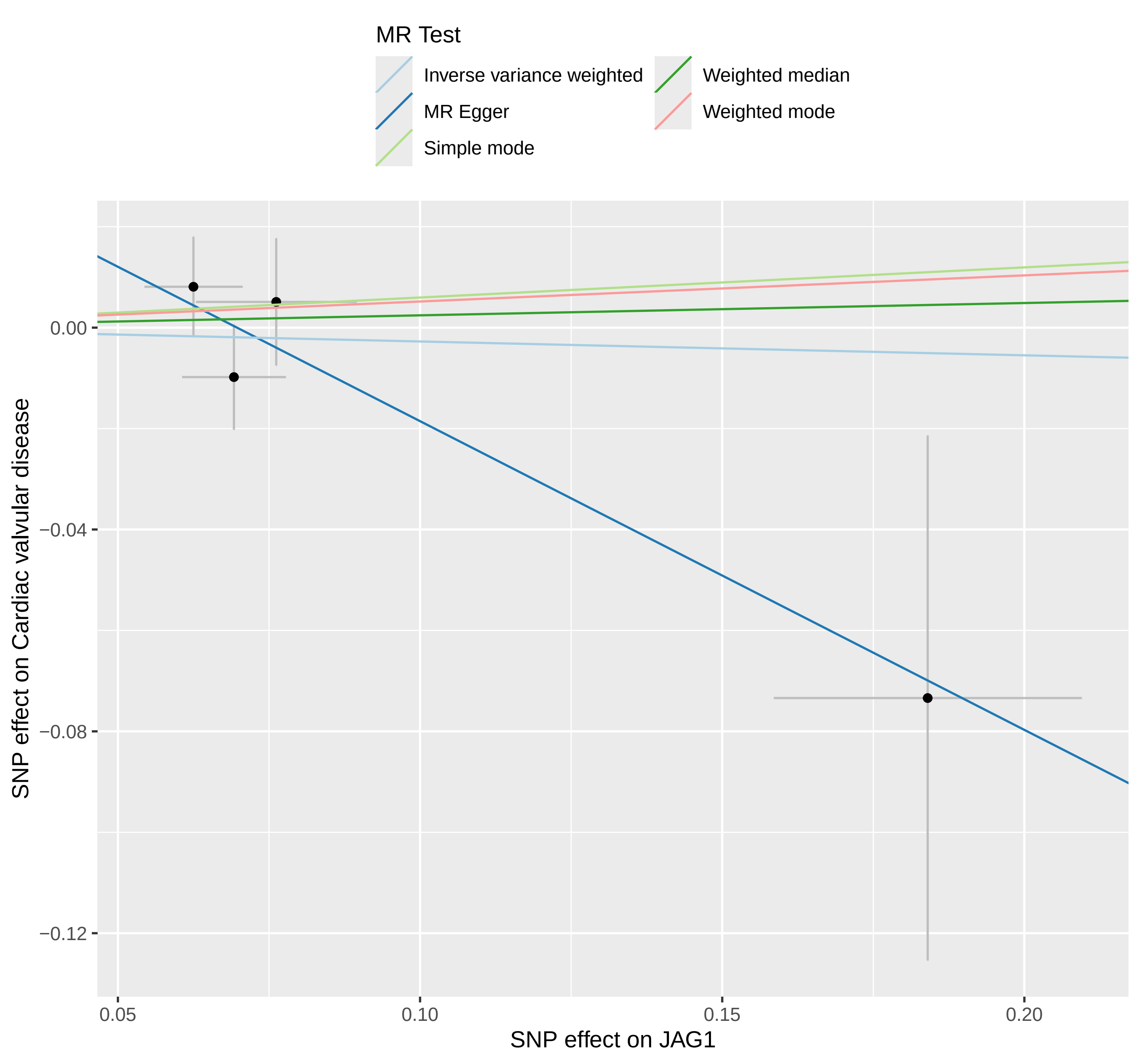
MR Test
Inverse variance weighted
MR Egger
Simple mode
Weighted median
Weighted mode
0.00
−0.04
−0.08
−0.12
0.05
0.10
0.15
0.20
SNP effect on JAG1
SNP effect on Cardiac valvular disease

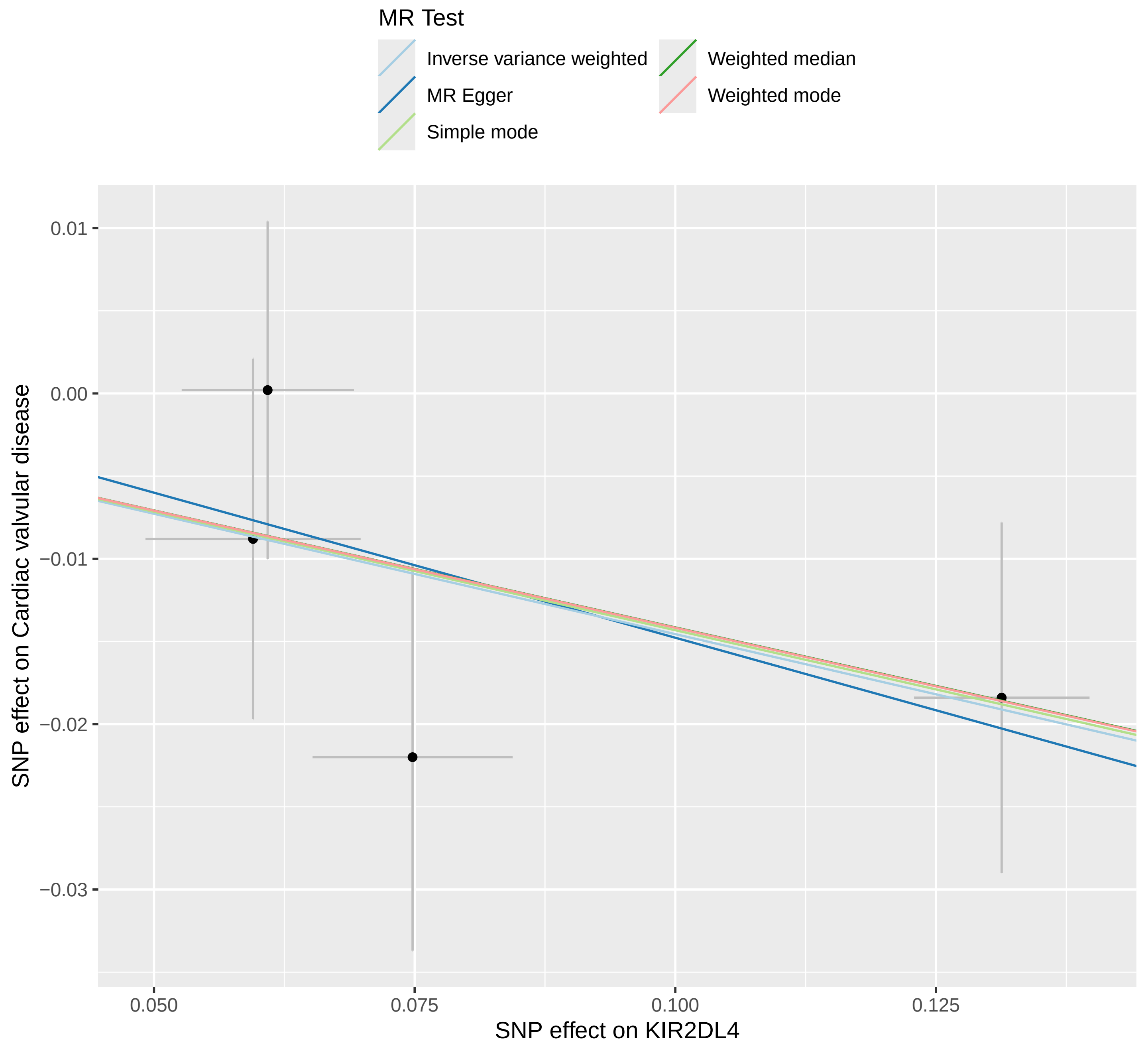

MR Test
Inverse variance weighted
MR Egger
Simple mode
Weighted median
Weighted mode
0.01
0.00
−0.01
−0.02
−0.03
SNP effect on Cardiac valvular disease
0.050
0.075
0.100
0.125
SNP effect on KIR2DL4

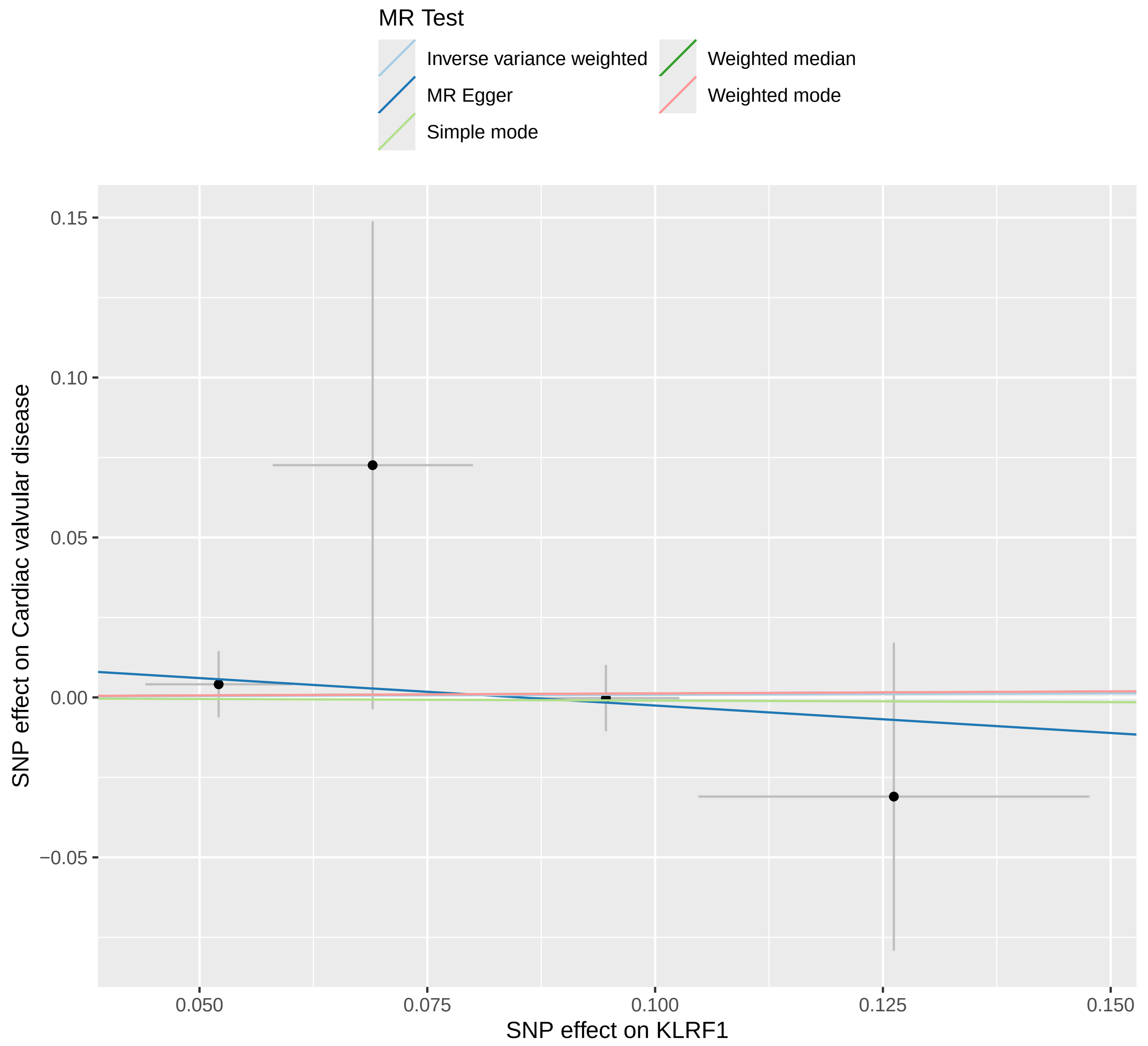

MR Test
Inverse variance weighted
MR Egger
Simple mode
Weighted median
Weighted mode
0.15
0.10
0.05
0.00
−0.05
0.050
0.075
0.100
0.125
0.150
SNP effect on Cardiac valvular disease
SNP effect on KLRF1

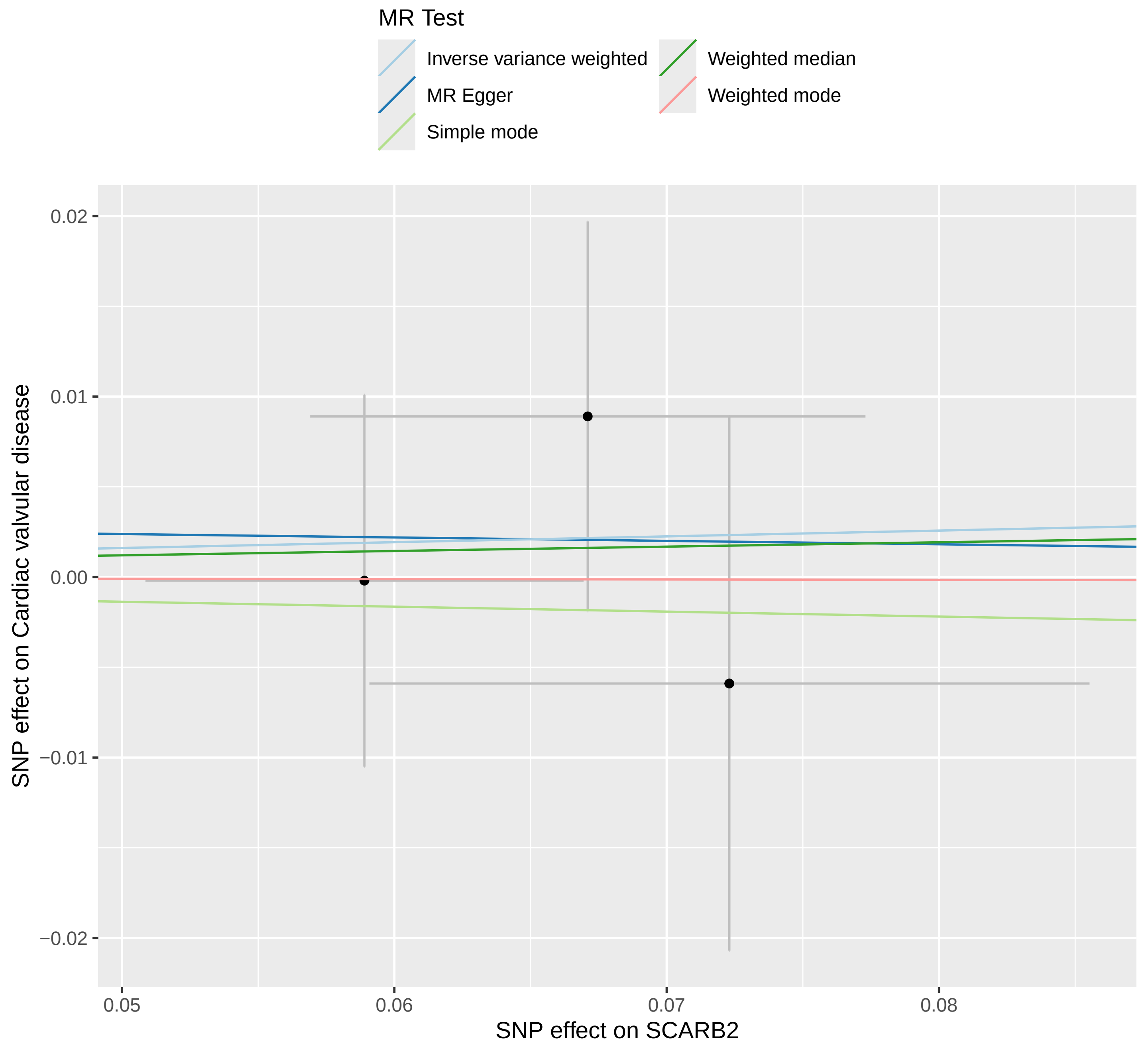
MR Test
Inverse variance weighted
MR Egger
Simple mode
Weighted median
Weighted mode
0.02
0.01
0.00
−0.01
−0.02
SNP effect on Cardiac valvular disease
0.05
0.06
0.07
0.08
SNP effect on SCARB2

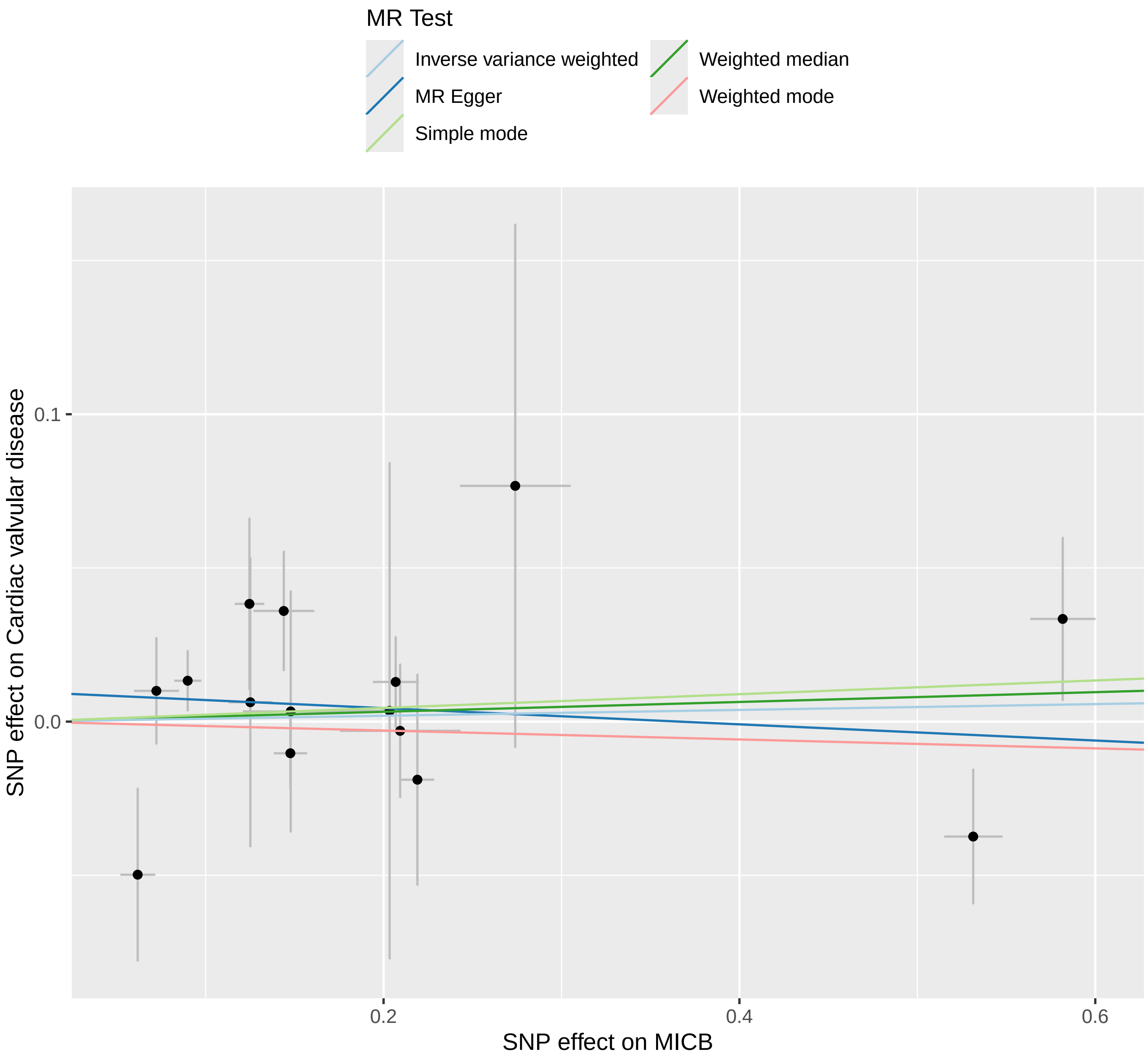
MR Test
Inverse variance weighted
MR Egger
Simple mode
Weighted median
Weighted mode
SNP effect on Cardiac valvular disease
0.1
0.0
0.2
0.4
0.6
SNP effect on MICB

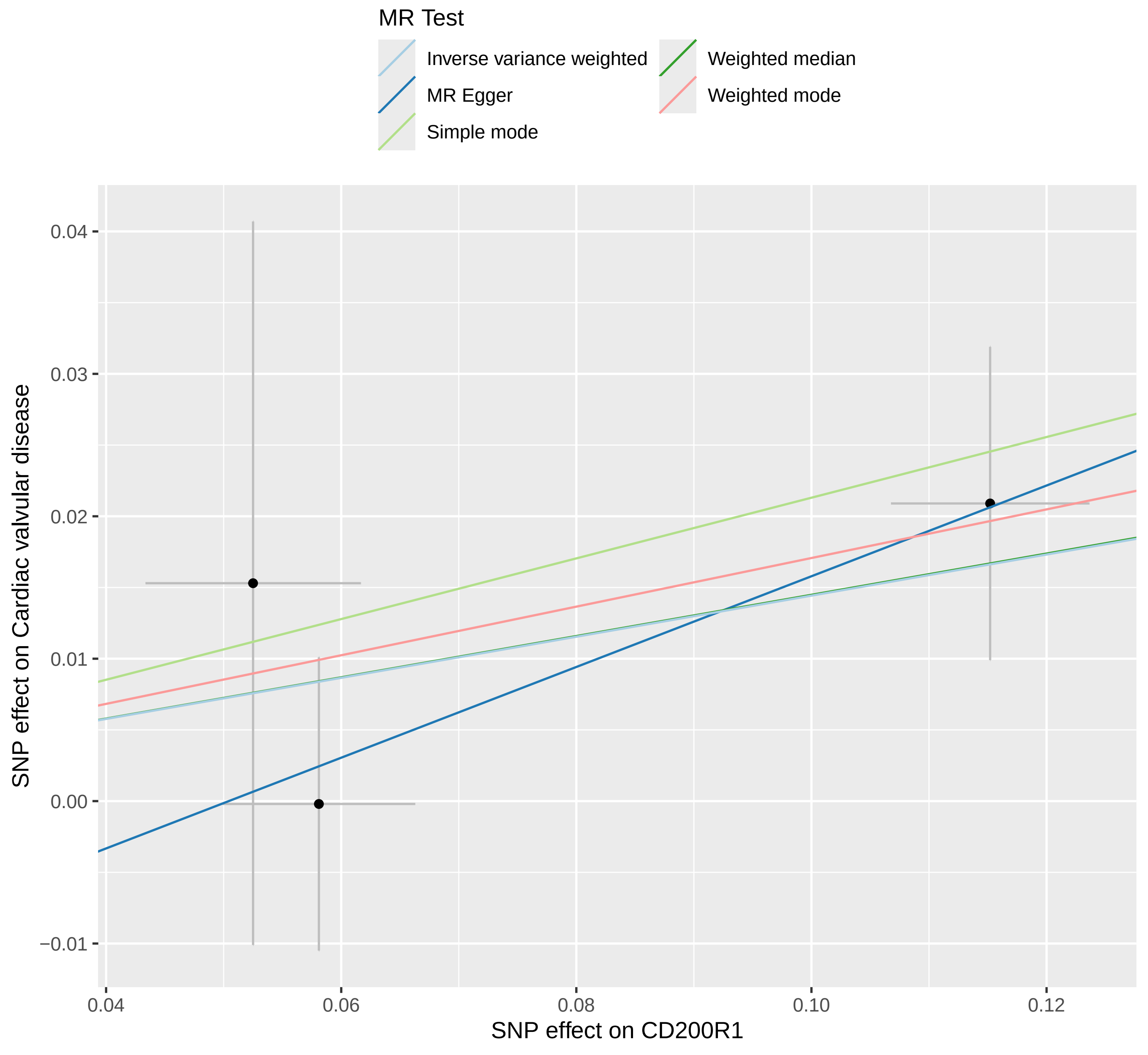
MR Test
Inverse variance weighted
MR Egger
Simple mode
Weighted median
Weighted mode
SNP effect on Cardiac valvular disease
0.04
0.03
0.02
0.01
0.00
−0.01
0.04
0.06
0.08
0.10
0.12
SNP effect on CD200R1

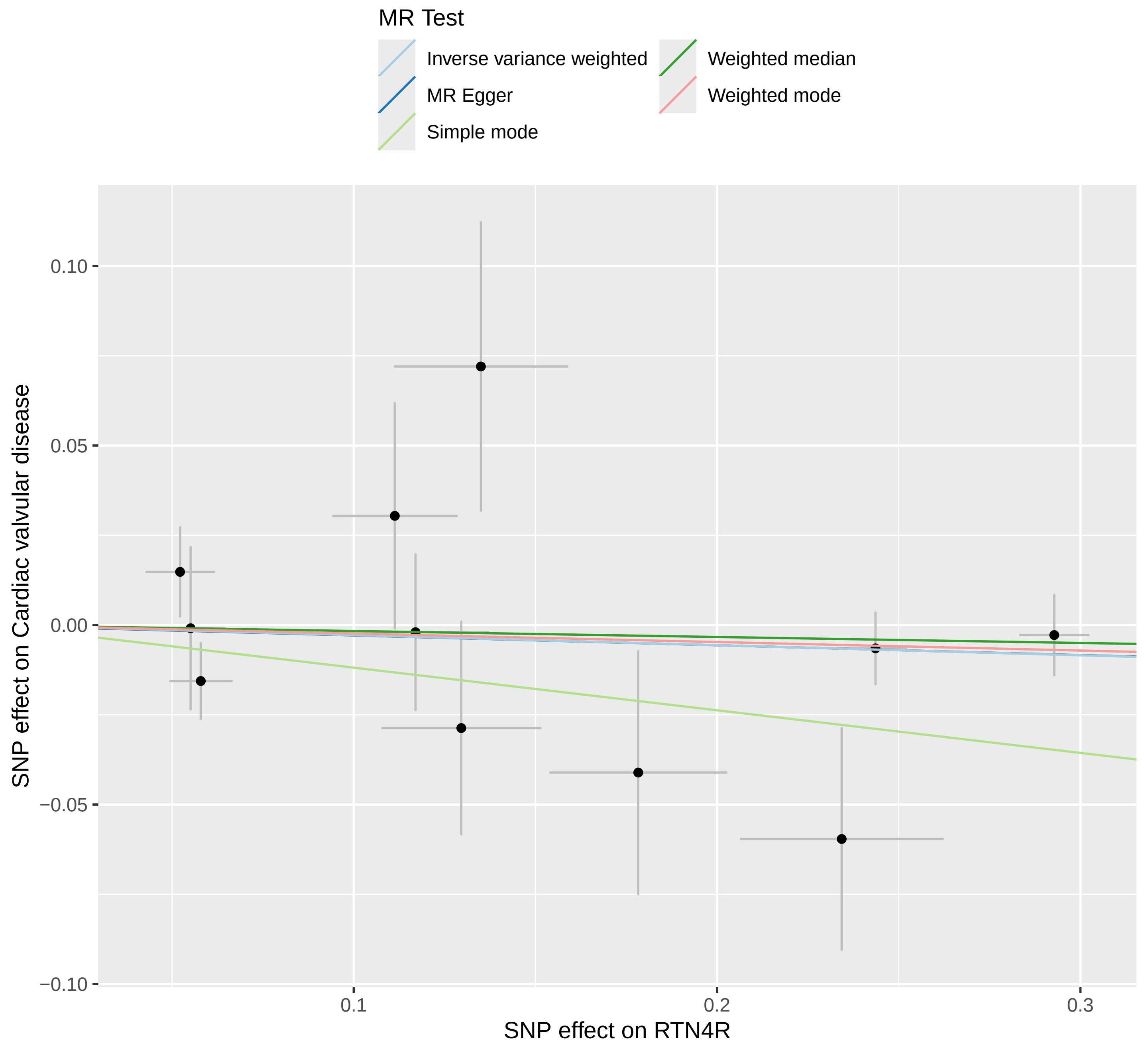

MR Test
Inverse variance weighted
MR Egger
Simple mode
Weighted median
Weighted mode
0.10
0.05
0.00
−0.05
−0.10
SNP effect on Cardiac valvular disease
0.1
0.2
0.3
SNP effect on RTN4R

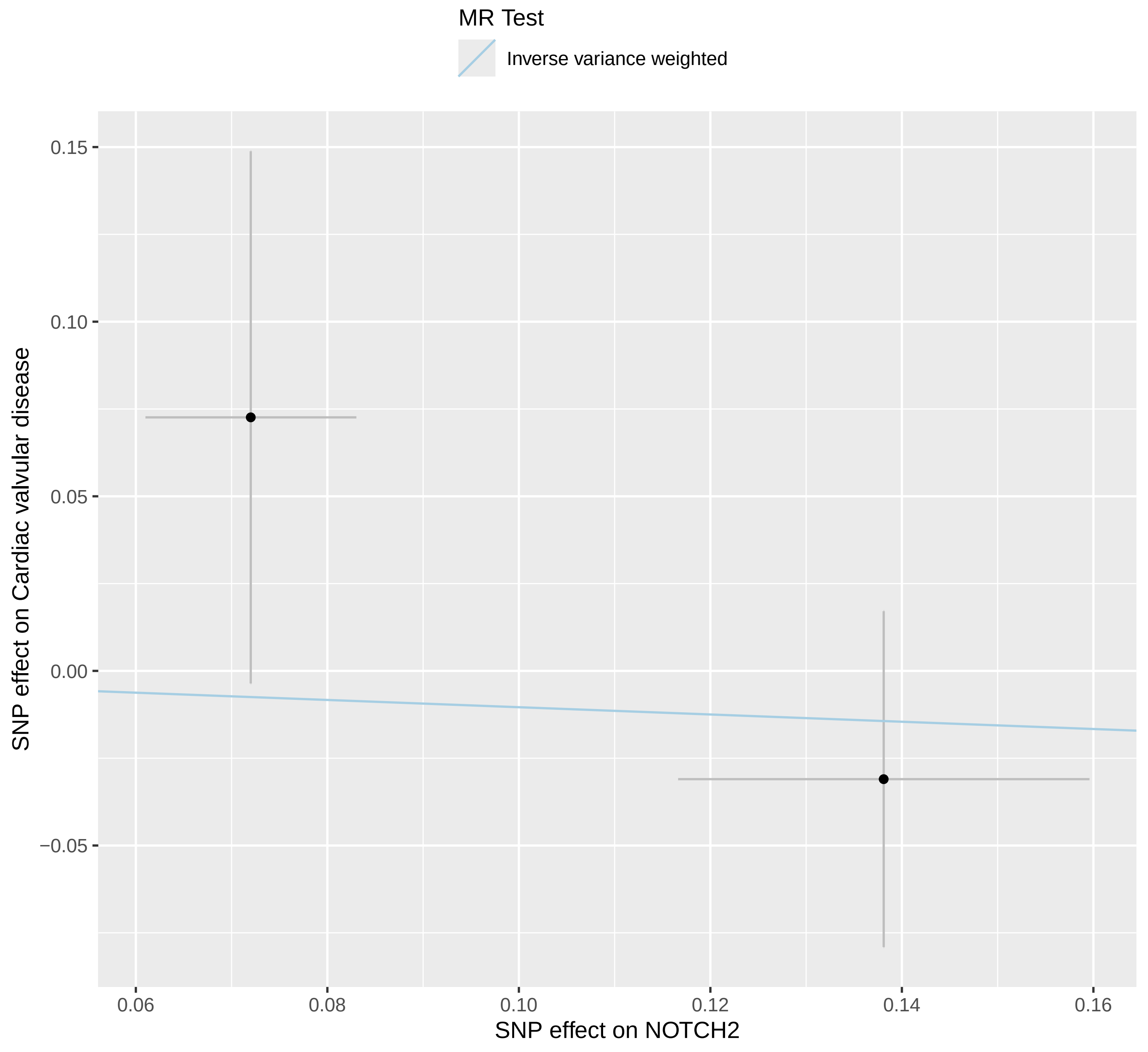
MR Test
Inverse variance weighted
0.15
0.10
0.05
0.00
−0.05
SNP effect on Cardiac valvular disease
0.06
0.08
0.10
0.12
0.14
0.16
SNP effect on NOTCH2

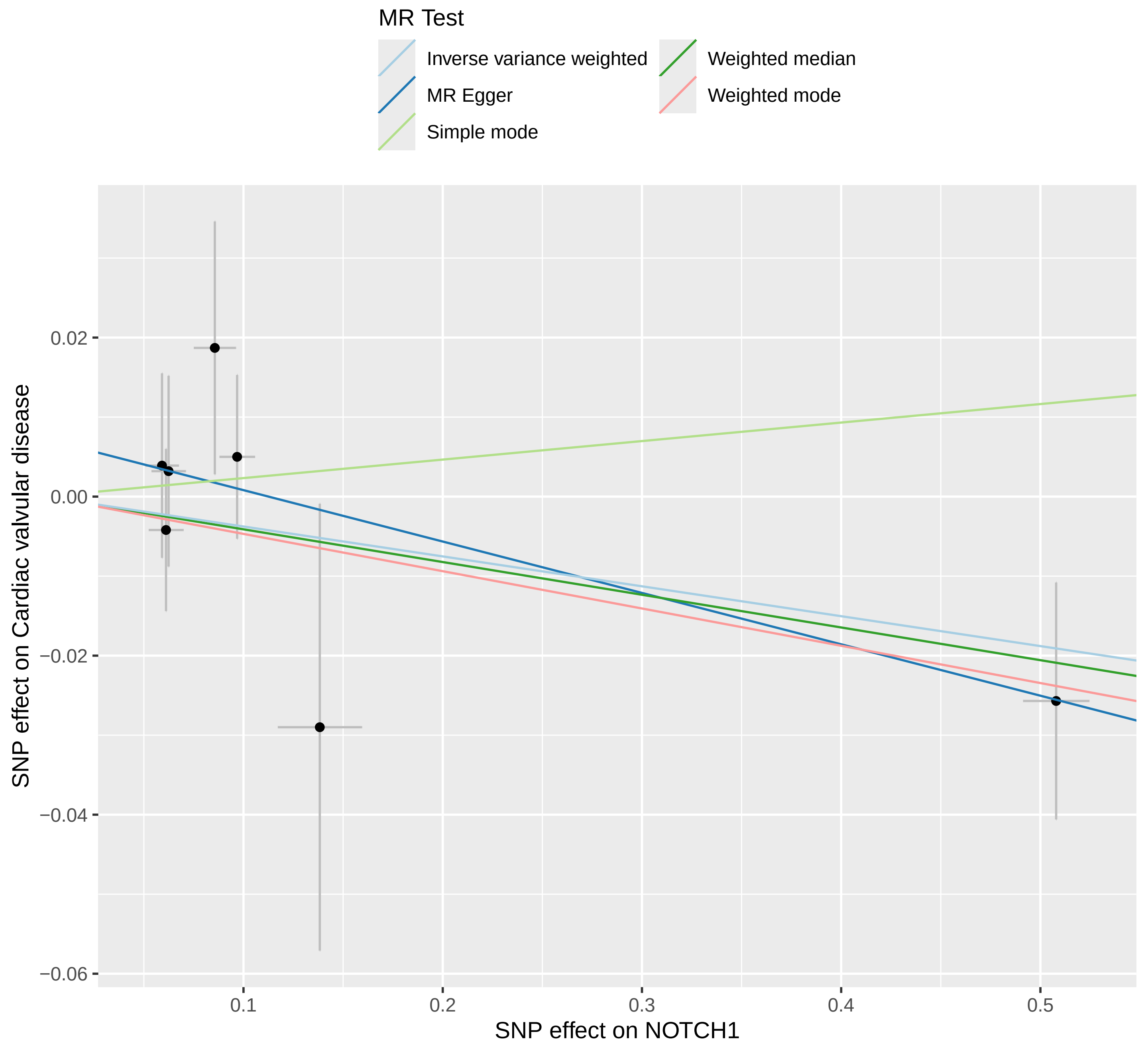
MR Test
Inverse variance weighted
MR Egger
Simple mode
Weighted median
Weighted mode
0.02
0.00
−0.02
−0.04
−0.06
SNP effect on Cardiac valvular disease
0.1
0.2
0.3
0.4
0.5
SNP effect on NOTCH1

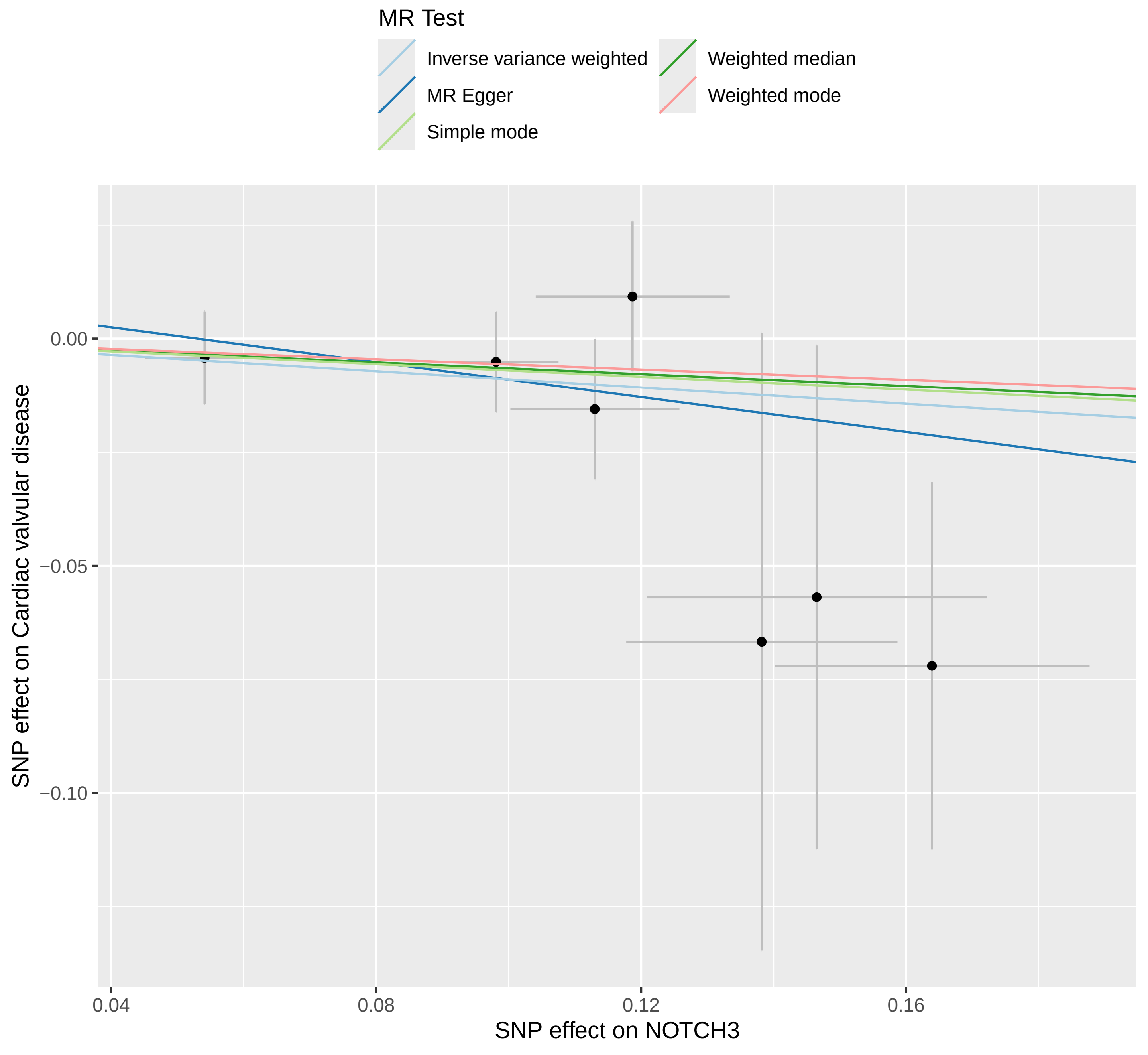

MR Test
Inverse variance weighted
MR Egger
Simple mode
Weighted median
Weighted mode
0.00
−0.05
−0.10
0.04
0.08
0.12
0.16
SNP effect on Cardiac valvular disease
SNP effect on NOTCH3

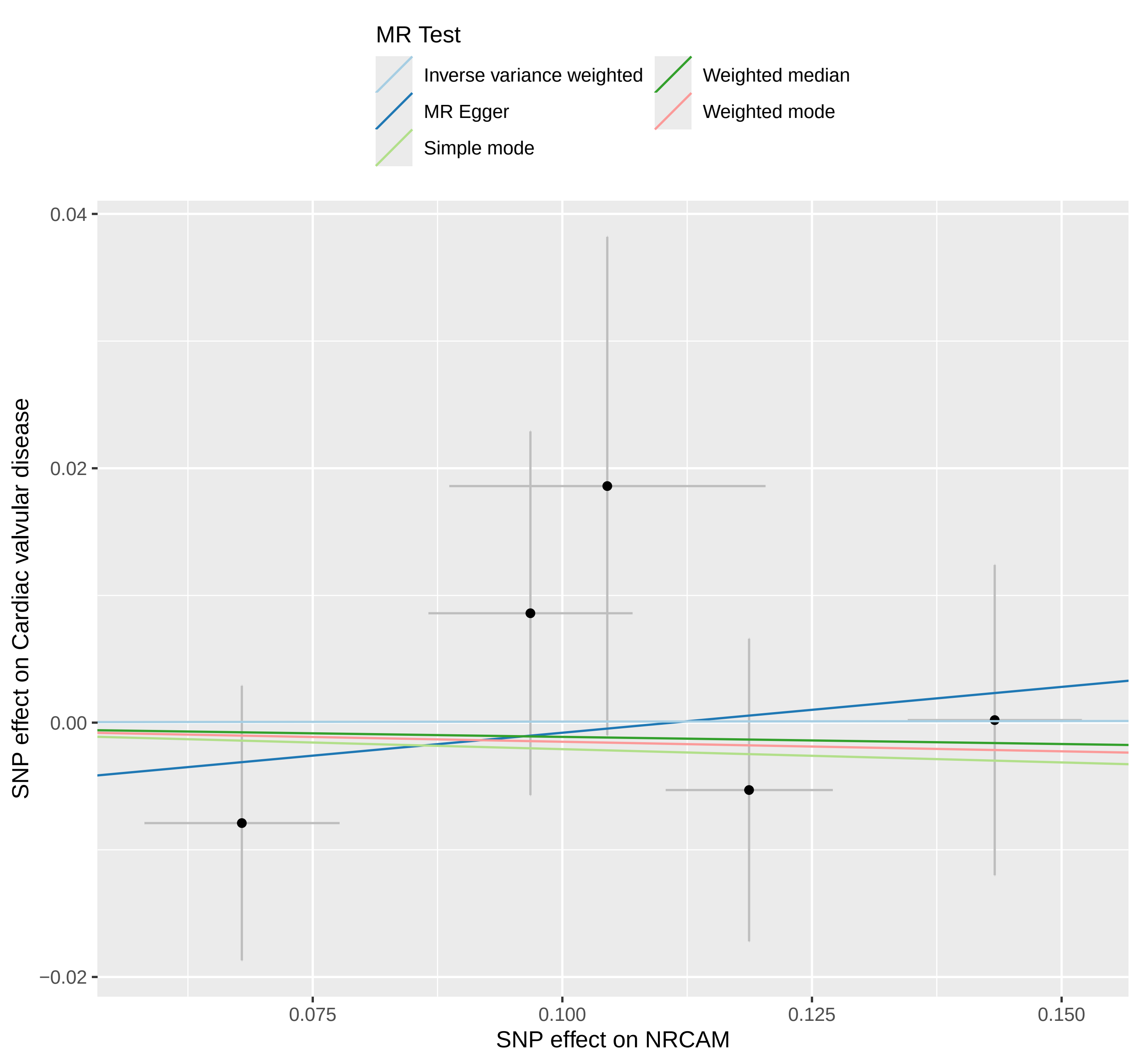
MR Test
Inverse variance weighted
MR Egger
Simple mode
Weighted median
Weighted mode
0.04
0.02
0.00
−0.02
0.075
0.100
0.125
0.150
SNP effect on NRCAM
SNP effect on Cardiac valvular disease

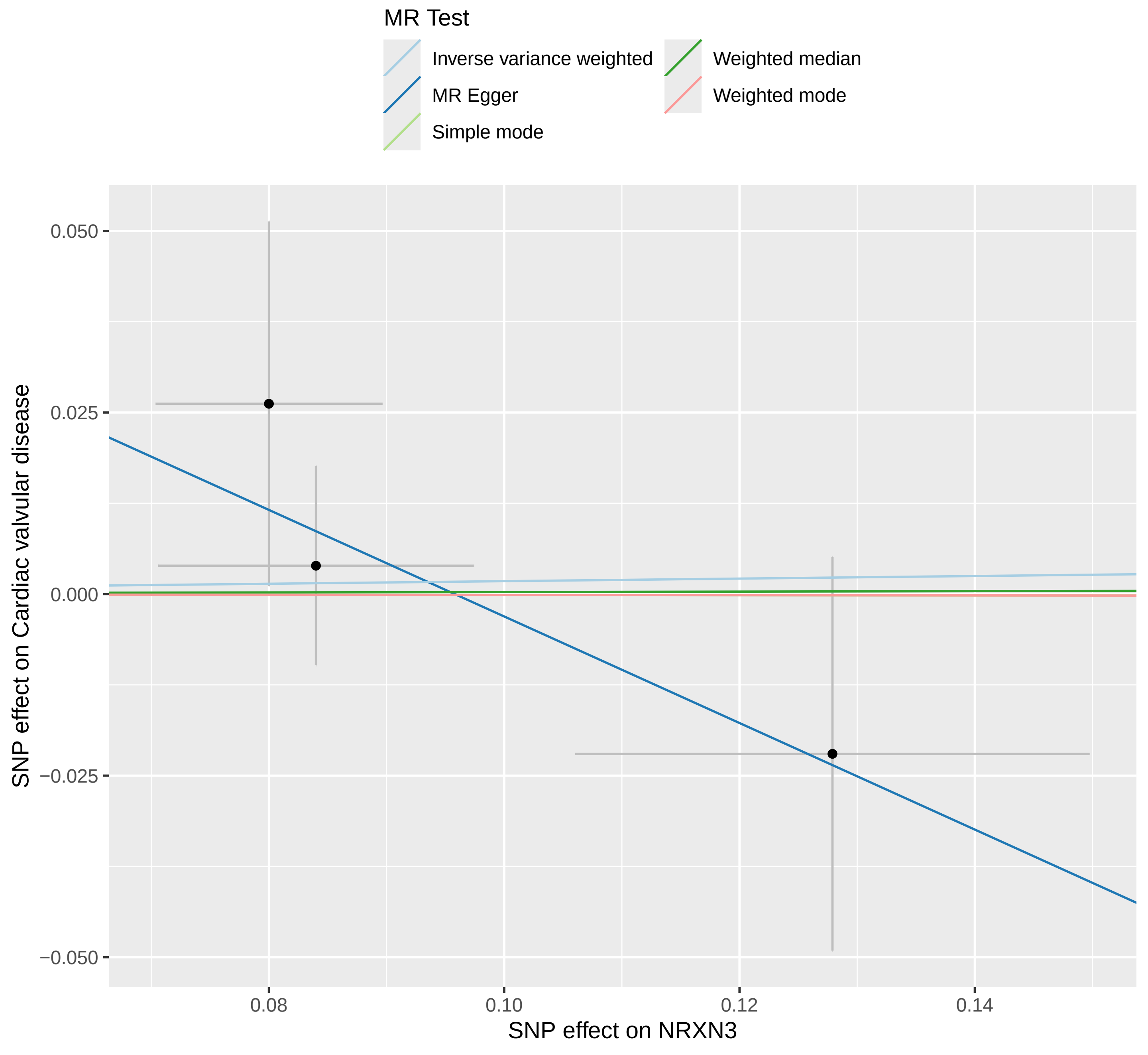

MR Test
Inverse variance weighted
MR Egger
Simple mode
Weighted median
Weighted mode
0.050
0.025
0.000
−0.025
−0.050
0.08
0.10
0.12
0.14
SNP effect on NRXN3
SNP effect on Cardiac valvular disease

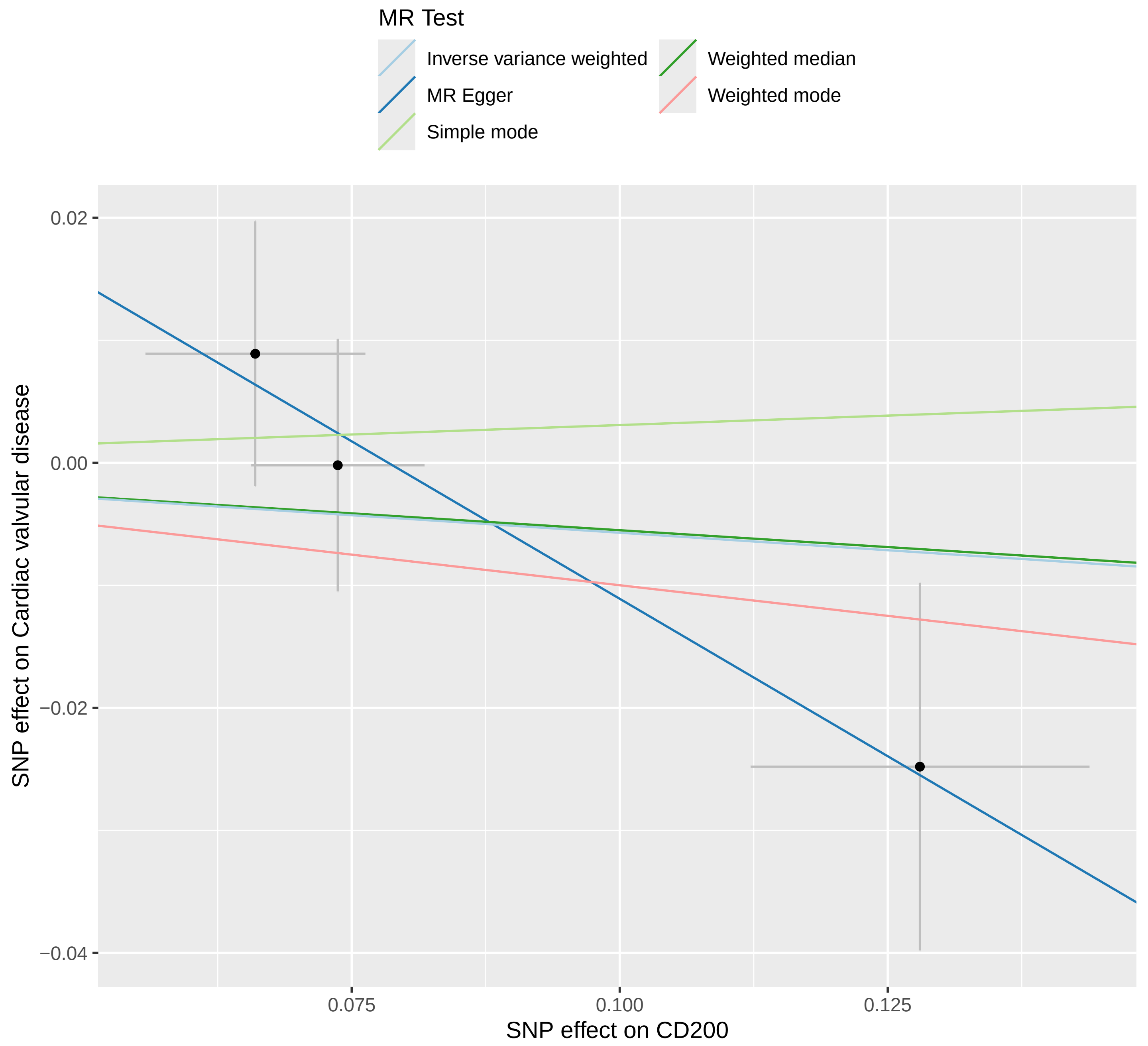
MR Test
Inverse variance weighted
MR Egger
Simple mode
Weighted median
Weighted mode
0.02
0.00
−0.02
−0.04
SNP effect on Cardiac valvular disease
0.075
0.100
0.125
SNP effect on CD200

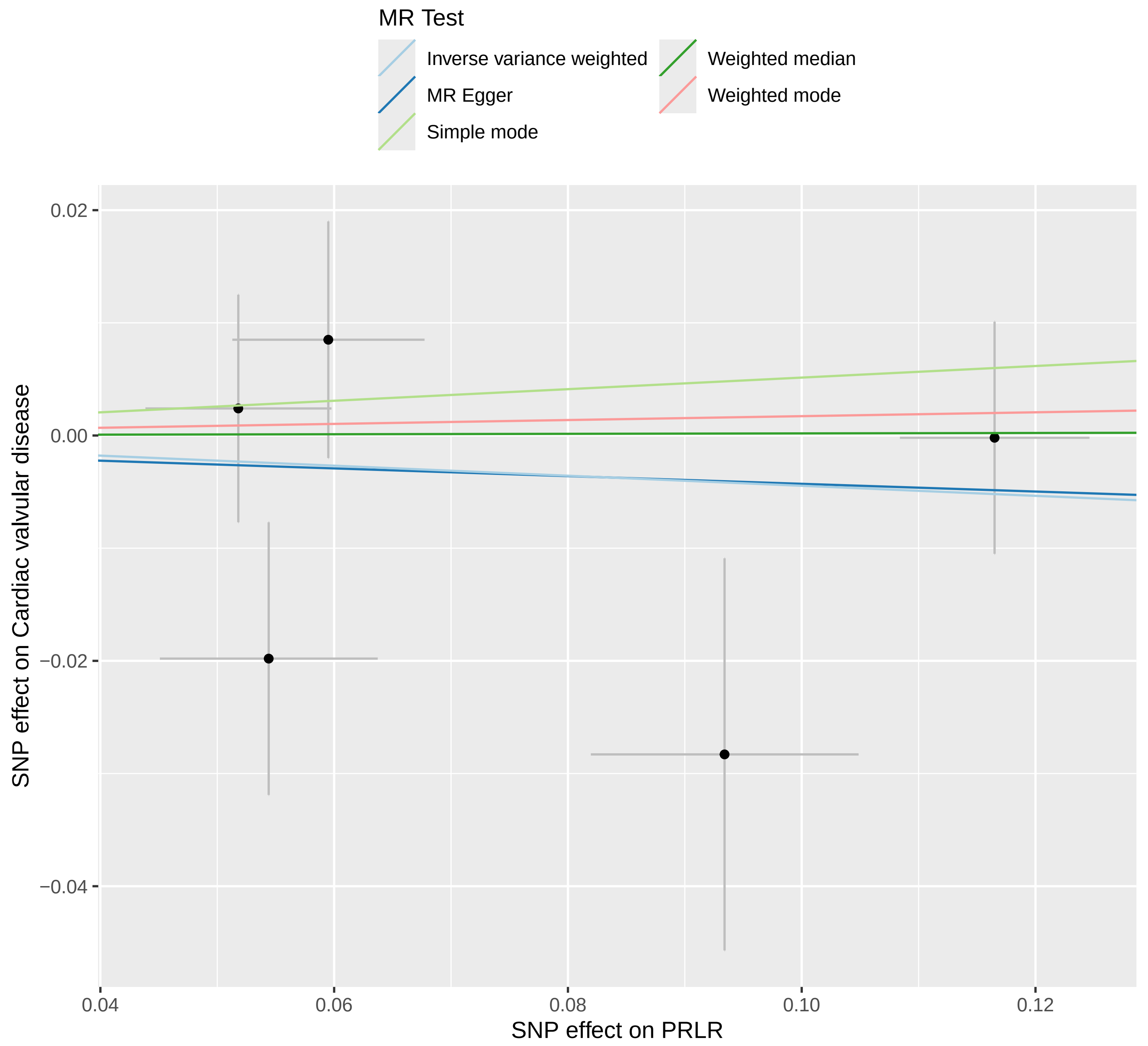
MR Test
Inverse variance weighted
MR Egger
Simple mode
Weighted median
Weighted mode
0.02
0.00
−0.02
−0.04
SNP effect on Cardiac valvular disease
0.04
0.06
0.08
0.10
0.12
SNP effect on PRLR

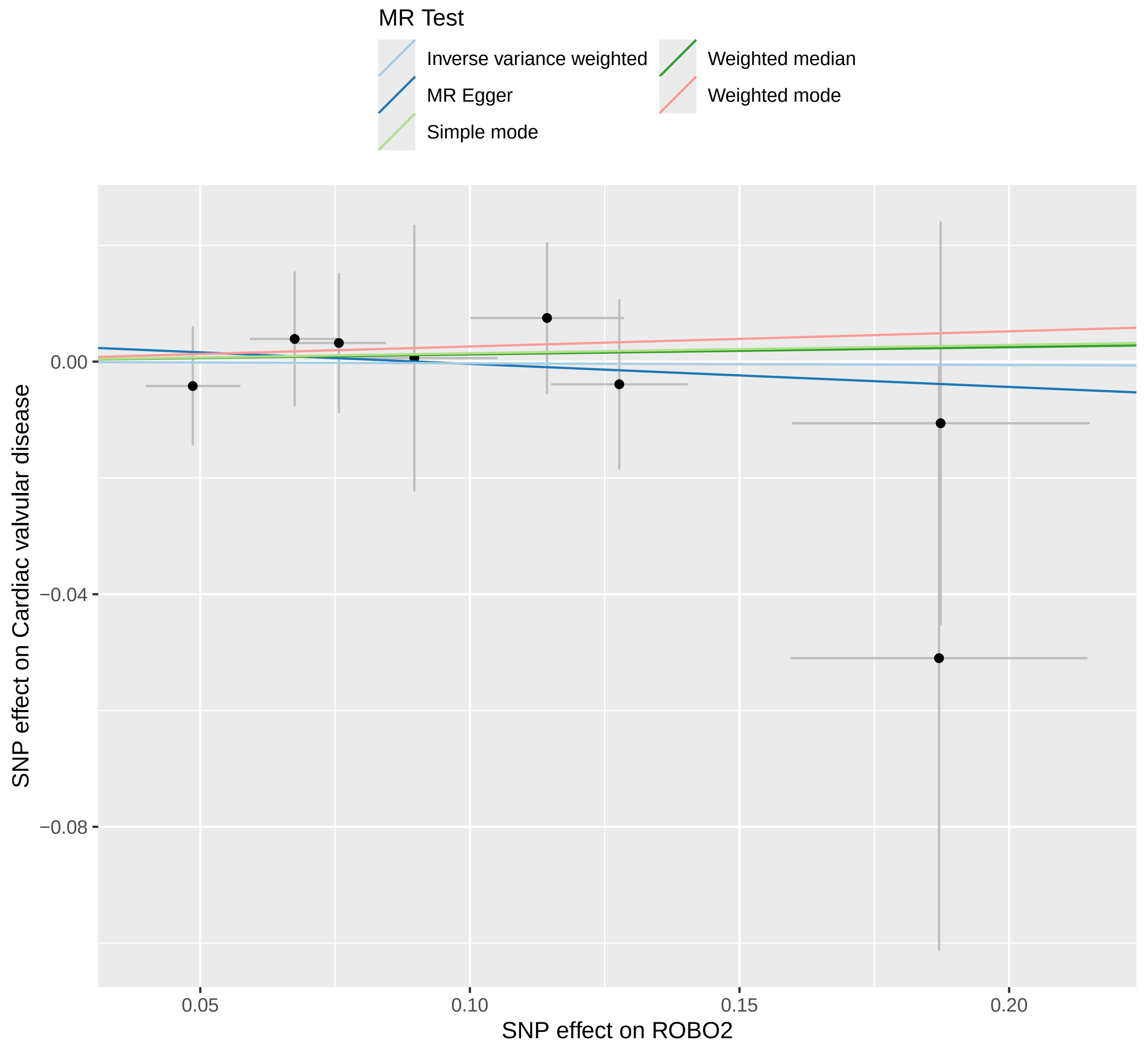
MR Test
Inverse variance weighted
MR Egger
Simple mode
Weighted median
Weighted mode
0.00
−0.04
−0.08
0.05
0.10
0.15
0.20
SNP effect on ROBO2
SNP effect on Cardiac valvular disease

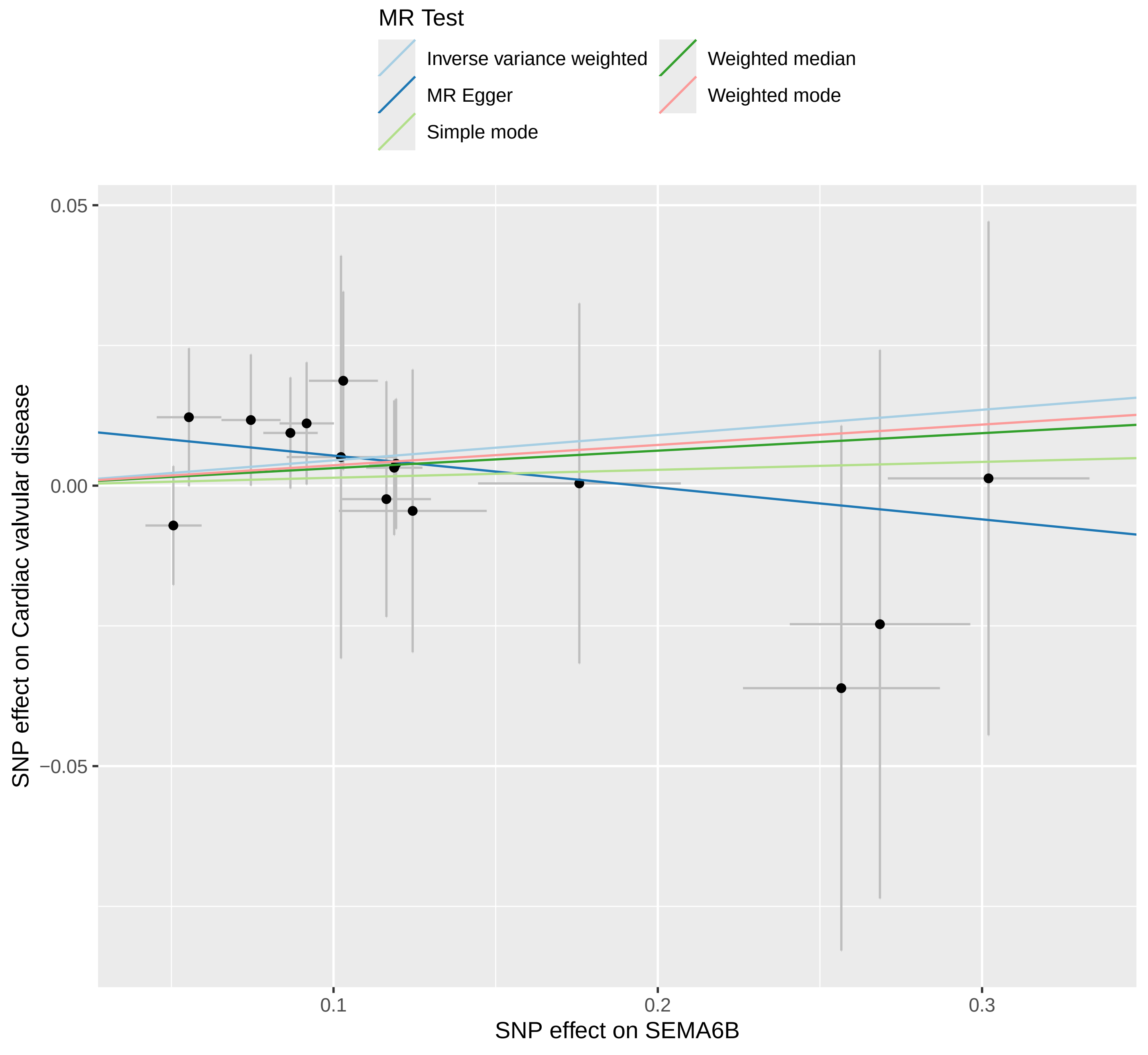

MR Test
Inverse variance weighted
MR Egger
Simple mode
Weighted median
Weighted mode
0.05
0.00
−0.05
0.1
0.2
0.3
SNP effect on Cardiac valvular disease
SNP effect on SEMA6B

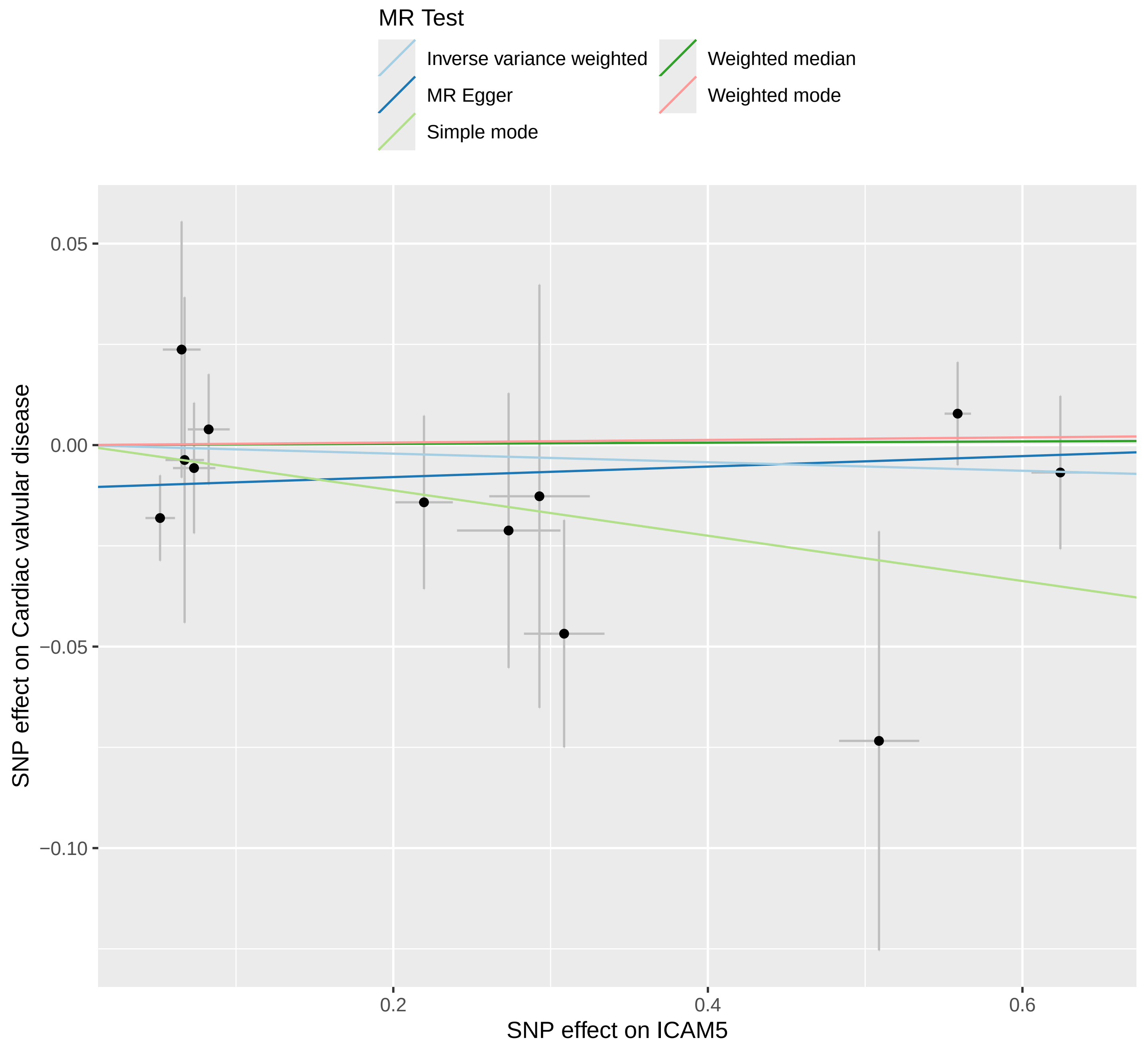
MR Test
Inverse variance weighted
MR Egger
Simple mode
Weighted median
Weighted mode
0.05
0.00
−0.05
−0.10
SNP effect on Cardiac valvular disease
0.2
0.4
0.6
SNP effect on ICAM5

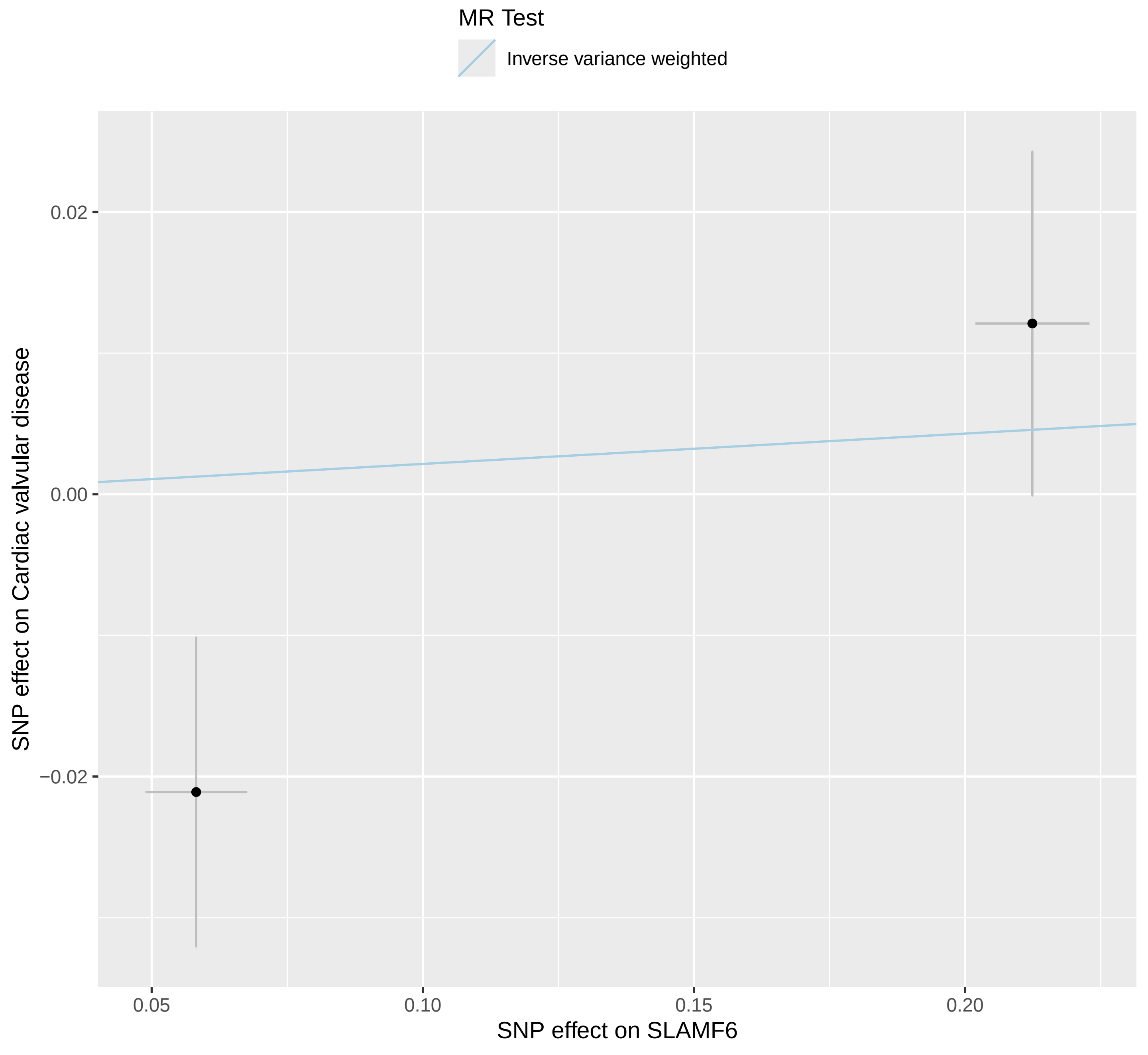
MR Test
Inverse variance weighted
0.02
0.00
−0.02
0.05
0.10
0.15
0.20
SNP effect on Cardiac valvular disease
SNP effect on SLAMF6

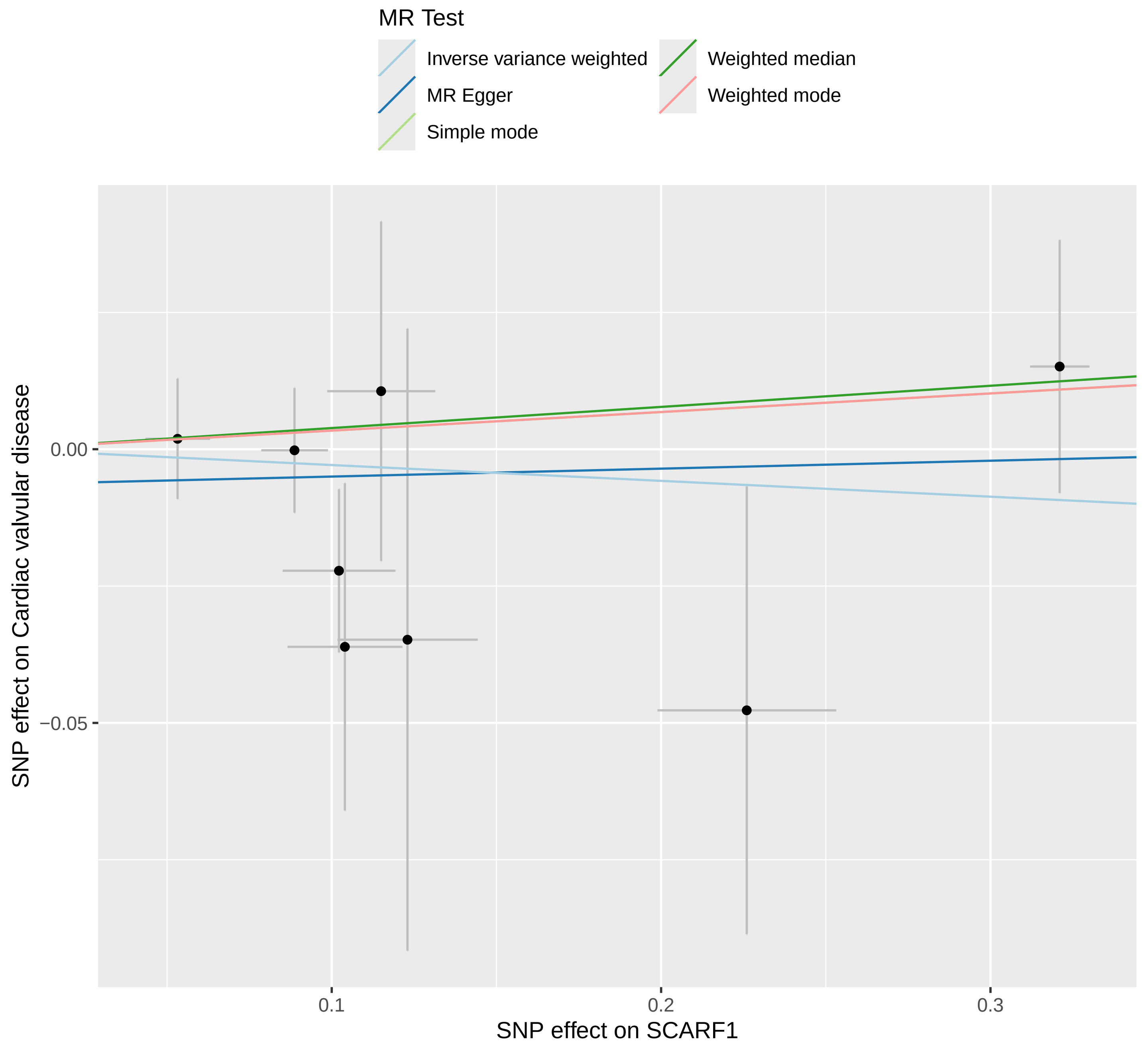

MR Test
Inverse variance weighted
MR Egger
Simple mode
Weighted median
Weighted mode
0.00
−0.05
0.1
0.2
0.3
SNP effect on Cardiac valvular disease
SNP effect on SCARF1

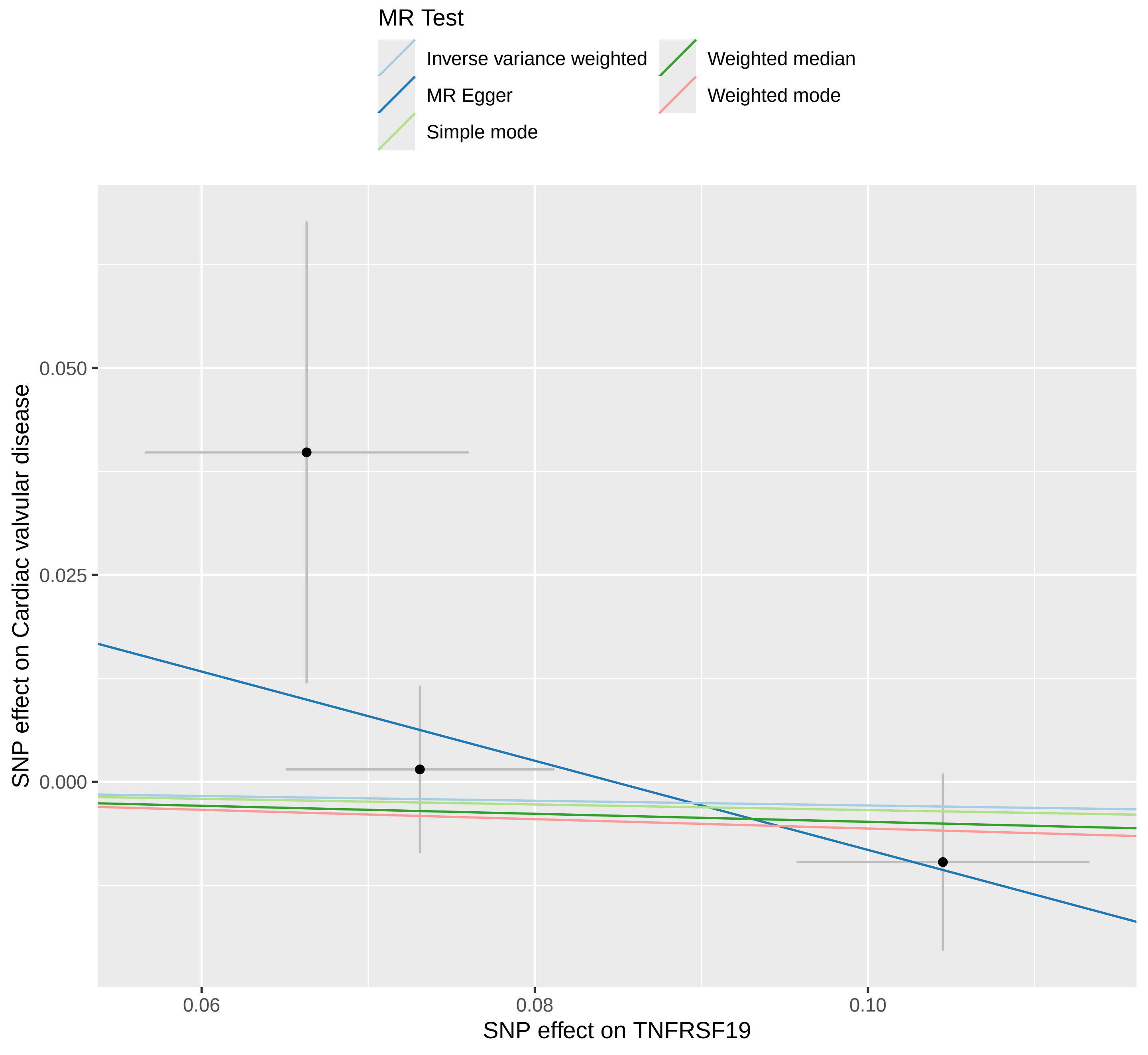

MR Test
Inverse variance weighted
MR Egger
Simple mode
Weighted median
Weighted mode
0.050
0.025
0.000
SNP effect on Cardiac valvular disease
0.06
0.08
0.10
SNP effect on TNFRSF19

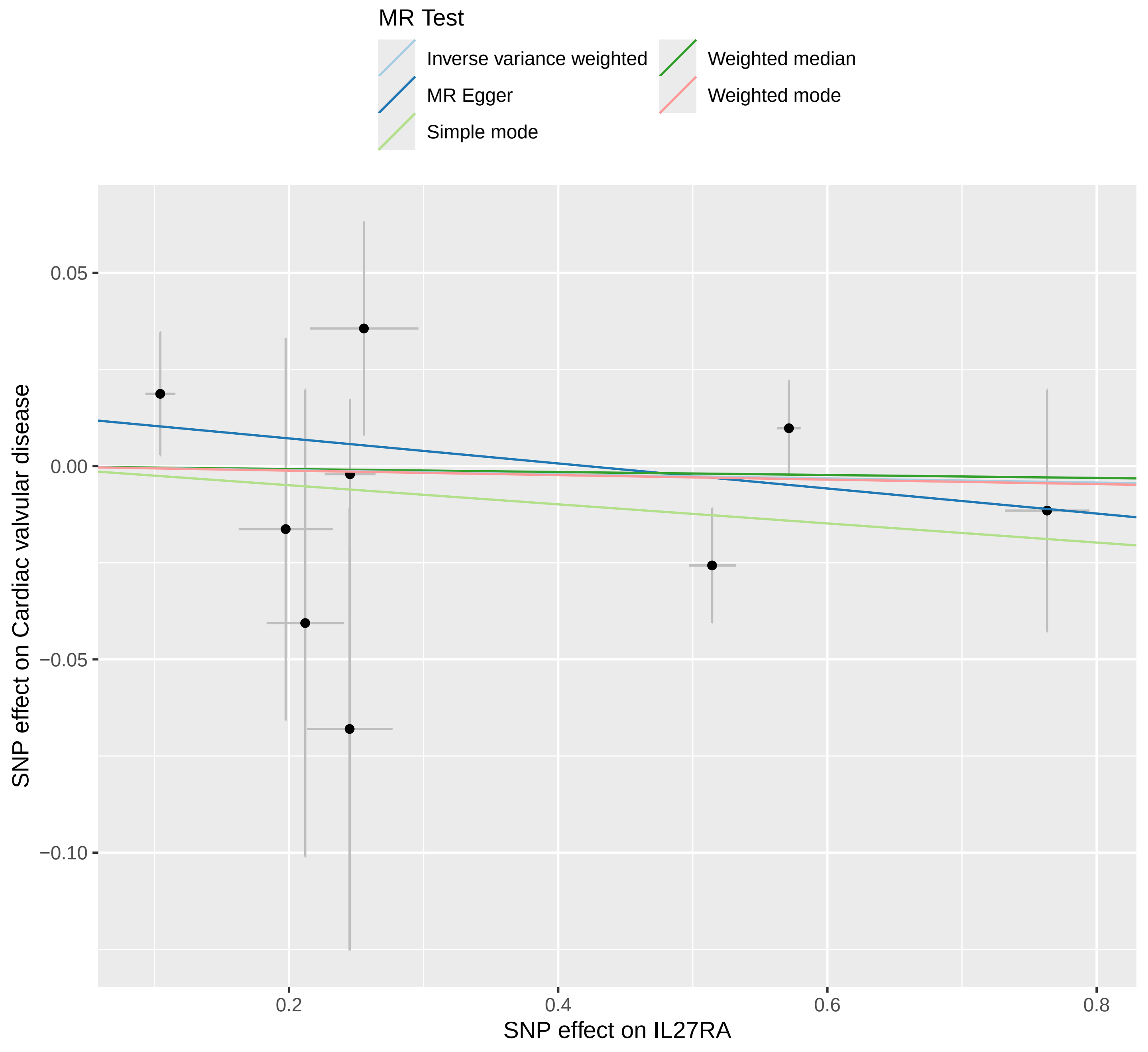
MR Test
Inverse variance weighted
MR Egger
Simple mode
Weighted median
Weighted mode
0.05
0.00
−0.05
−0.10
0.2
0.4
0.6
0.8
SNP effect on Cardiac valvular disease
SNP effect on IL27RA

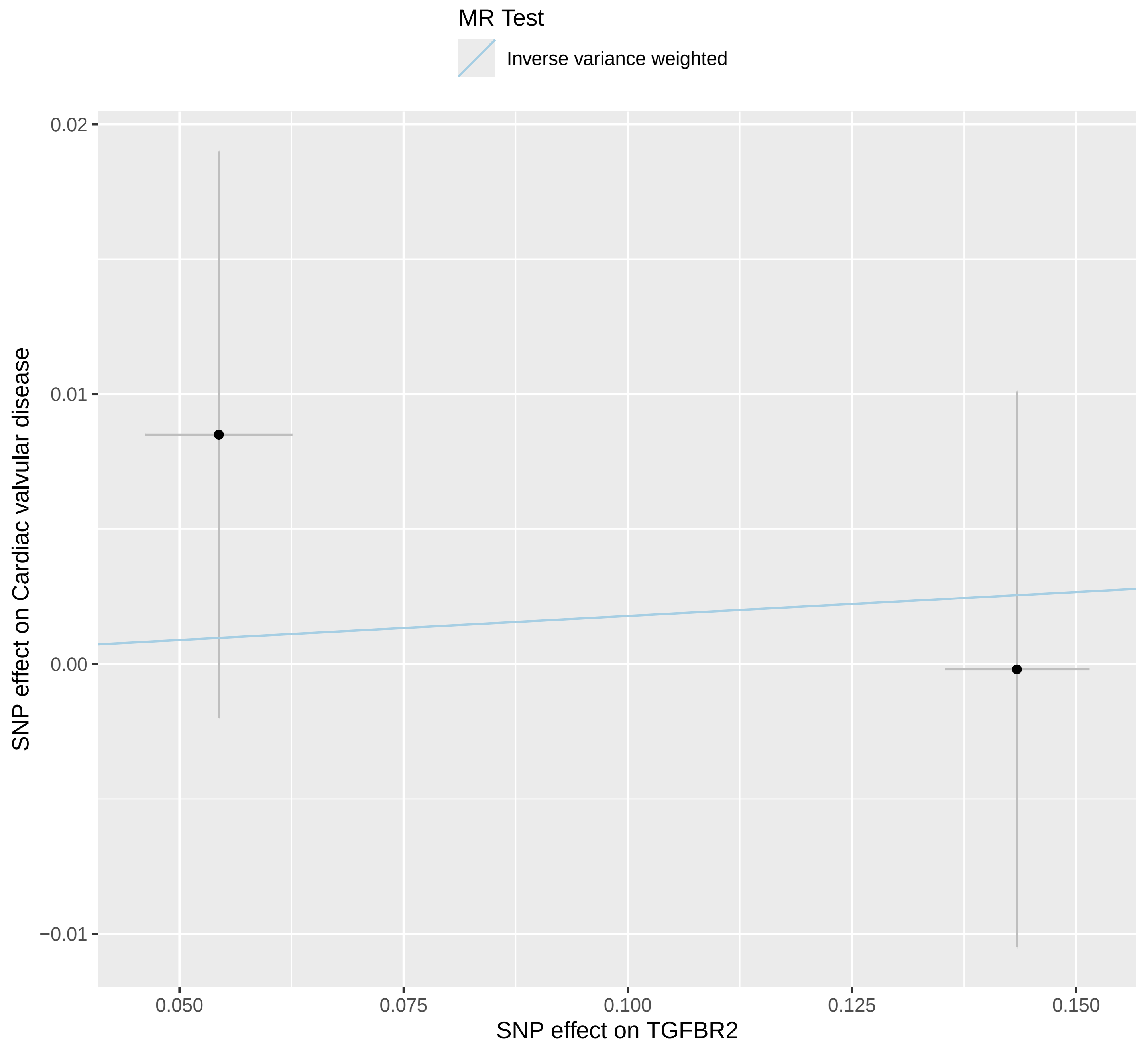

MR Test
Inverse variance weighted
0.02
0.01
0.00
−0.01
SNP effect on Cardiac valvular disease
0.050
0.075
0.100
0.125
0.150
SNP effect on TGFBR2

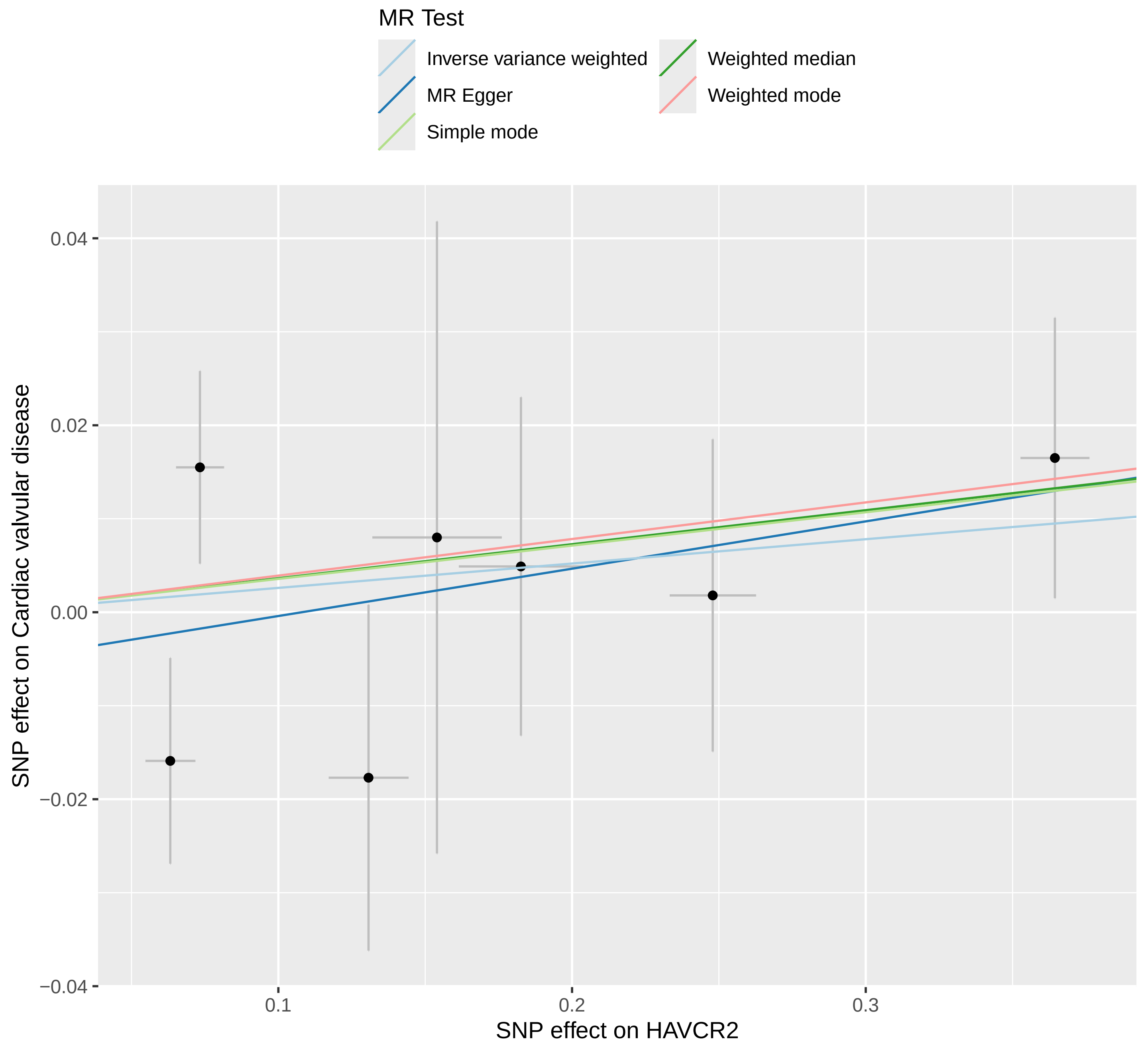
MR Test
Inverse variance weighted
MR Egger
Simple mode
Weighted median
Weighted mode
0.04
0.02
0.00
−0.02
−0.04
0.1
0.2
0.3
SNP effect on Cardiac valvular disease
SNP effect on HAVCR2

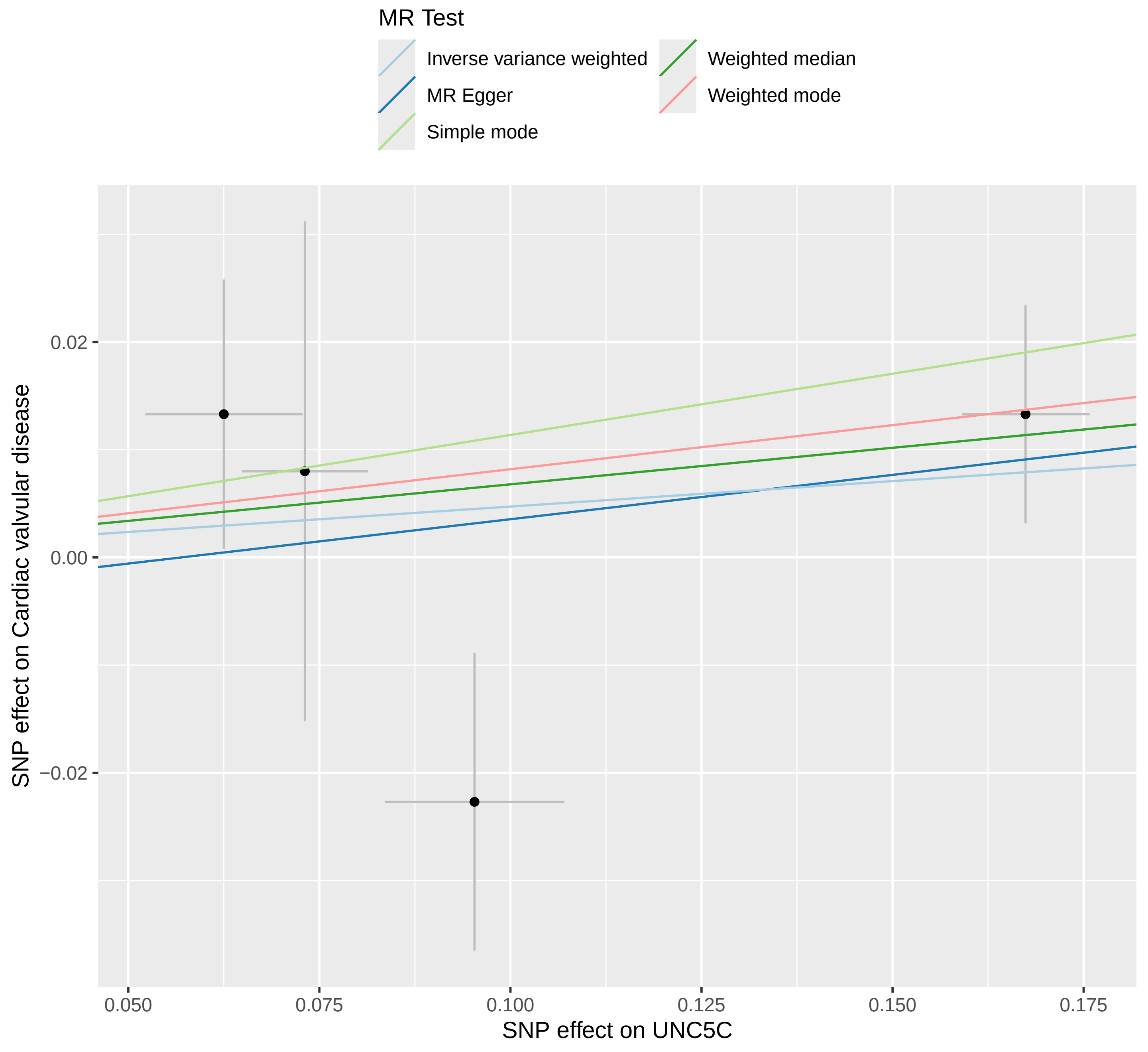
MR Test
Inverse variance weighted
MR Egger
Simple mode
Weighted median
Weighted mode
0.02
0.00
−0.02
0.050
0.075
0.100
0.125
0.150
0.175
SNP effect on UNC5C
SNP effect on Cardiac valvular disease

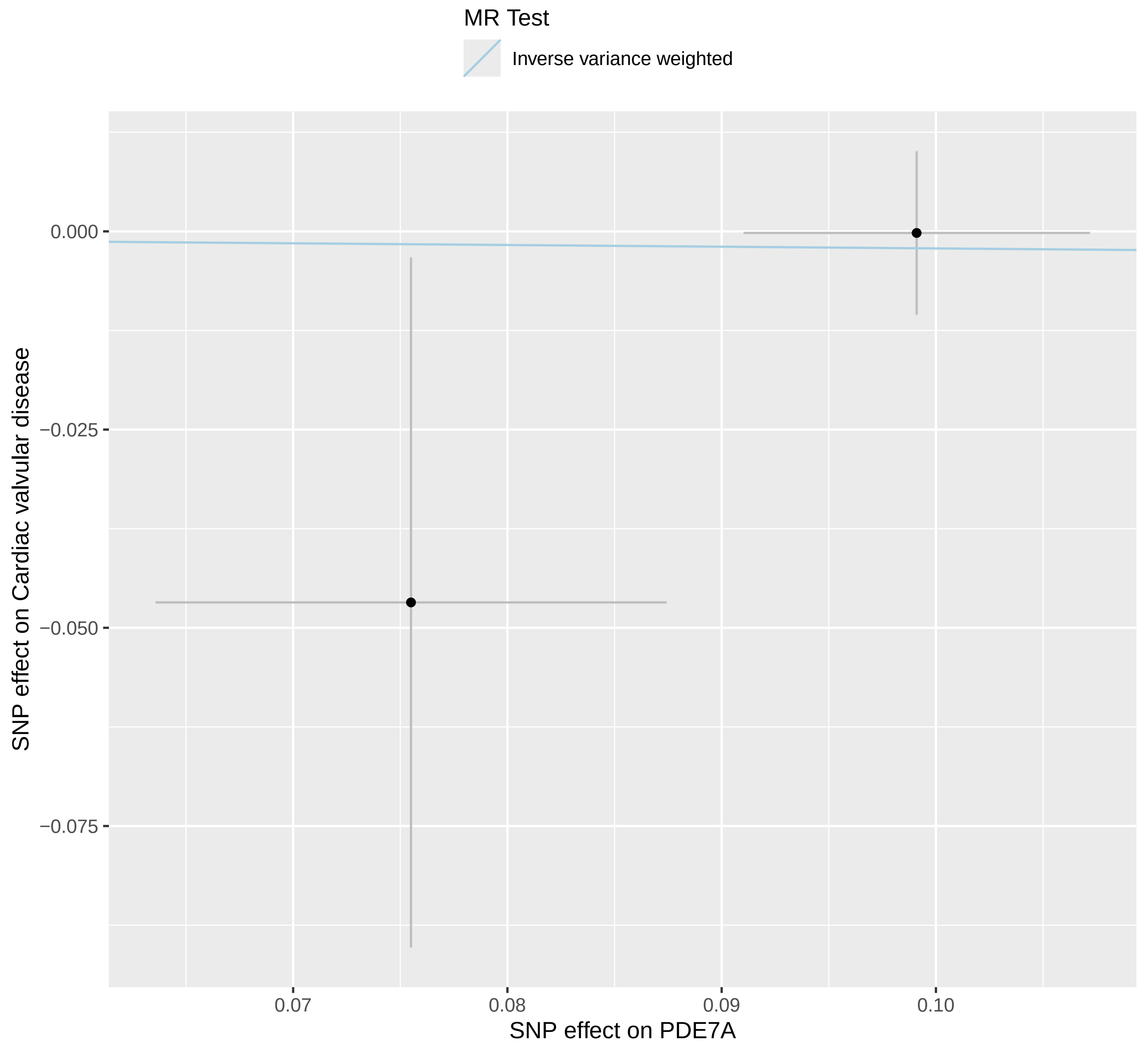

MR Test
Inverse variance weighted
0.000
−0.025
−0.050
−0.075
0.07
0.08
0.09
0.10
SNP effect on PDE7A
SNP effect on Cardiac valvular disease

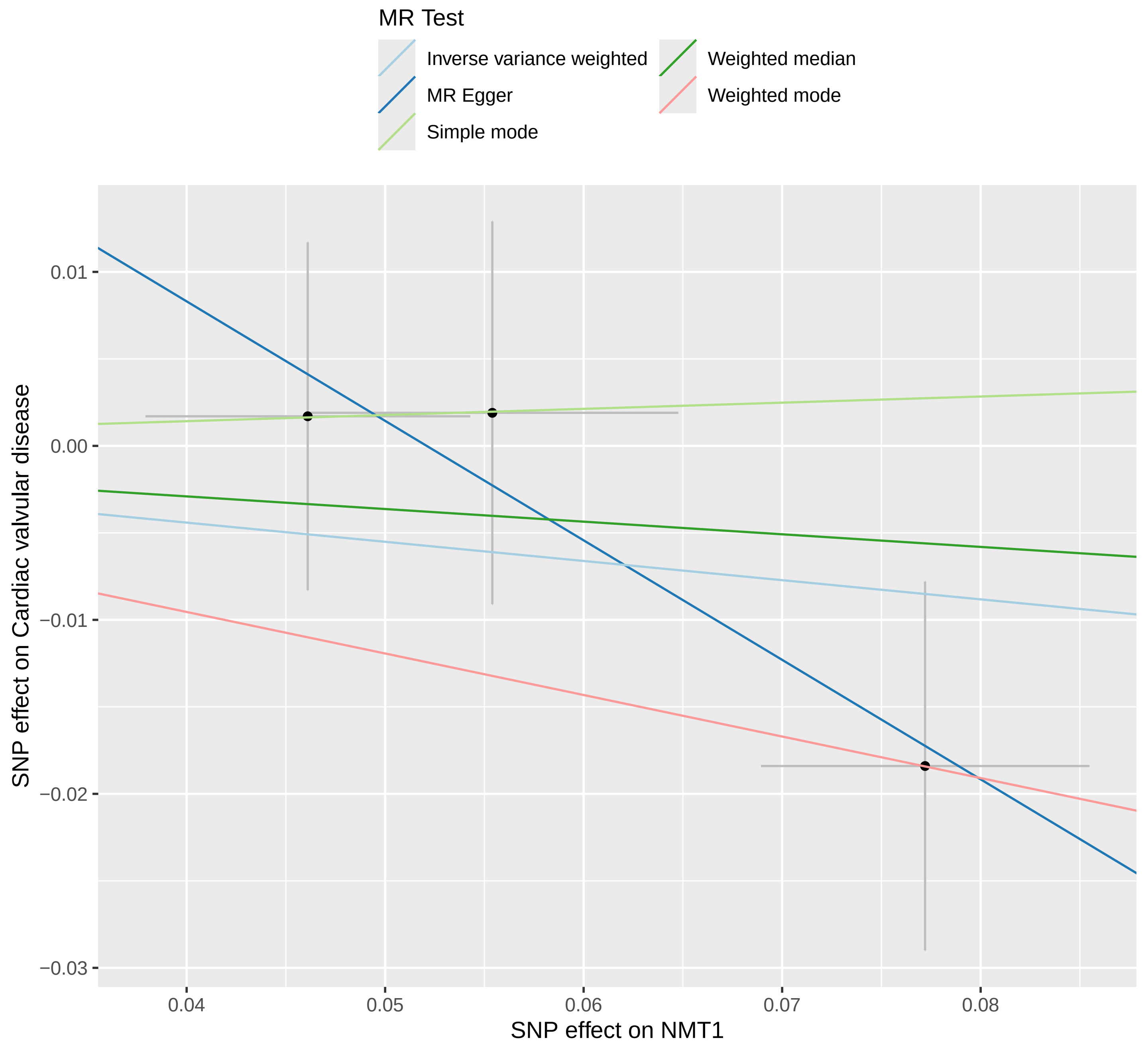
MR Test
Inverse variance weighted
MR Egger
Simple mode
Weighted median
Weighted mode
SNP effect on Cardiac valvular disease
0.01
0.00
−0.01
−0.02
−0.03
0.04
0.05
0.06
0.07
0.08
SNP effect on NMT1

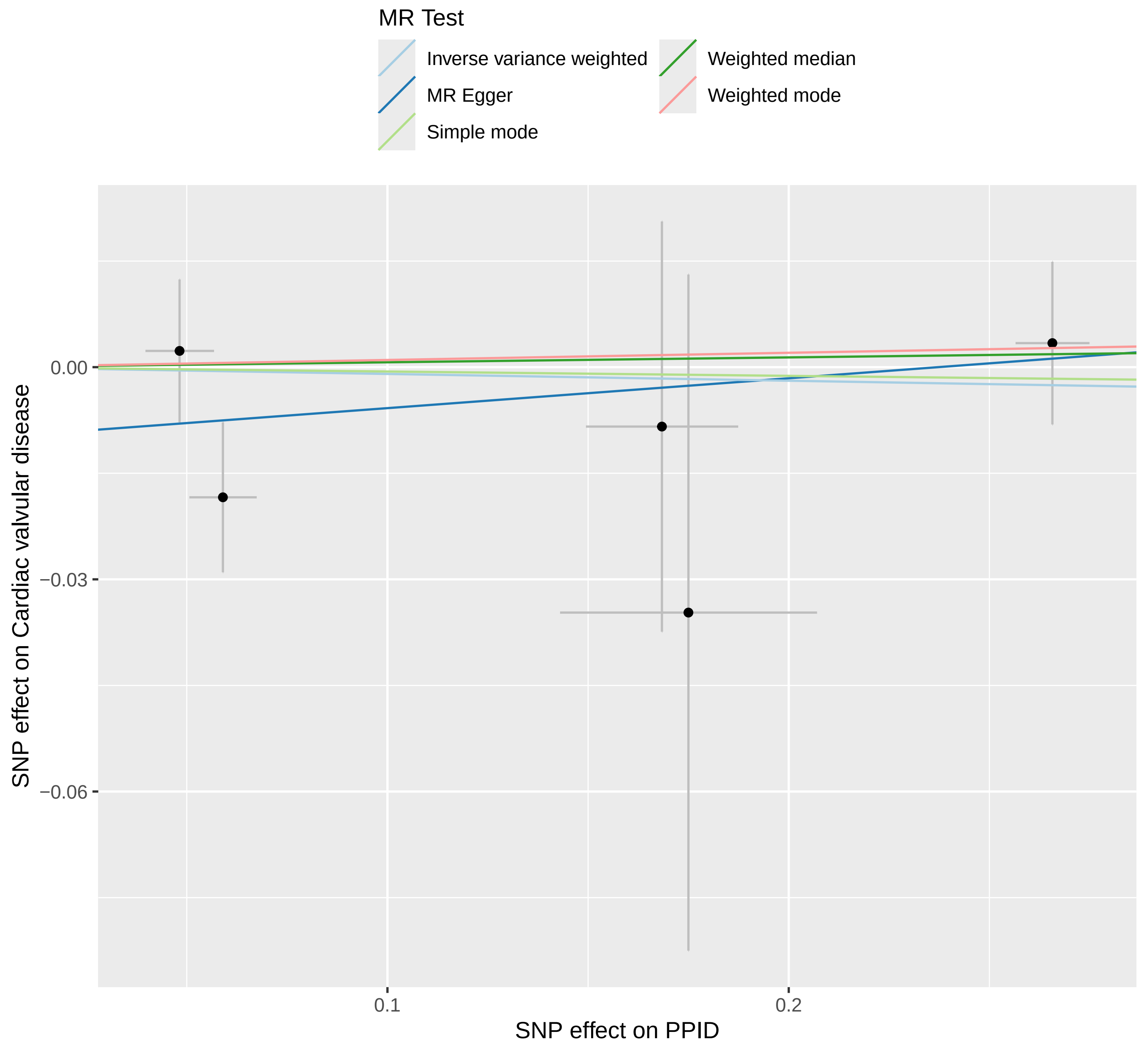
MR Test
Inverse variance weighted
MR Egger
Simple mode
Weighted median
Weighted mode
0.00
−0.03
−0.06
0.1
0.2
SNP effect on Cardiac valvular disease
SNP effect on PPID

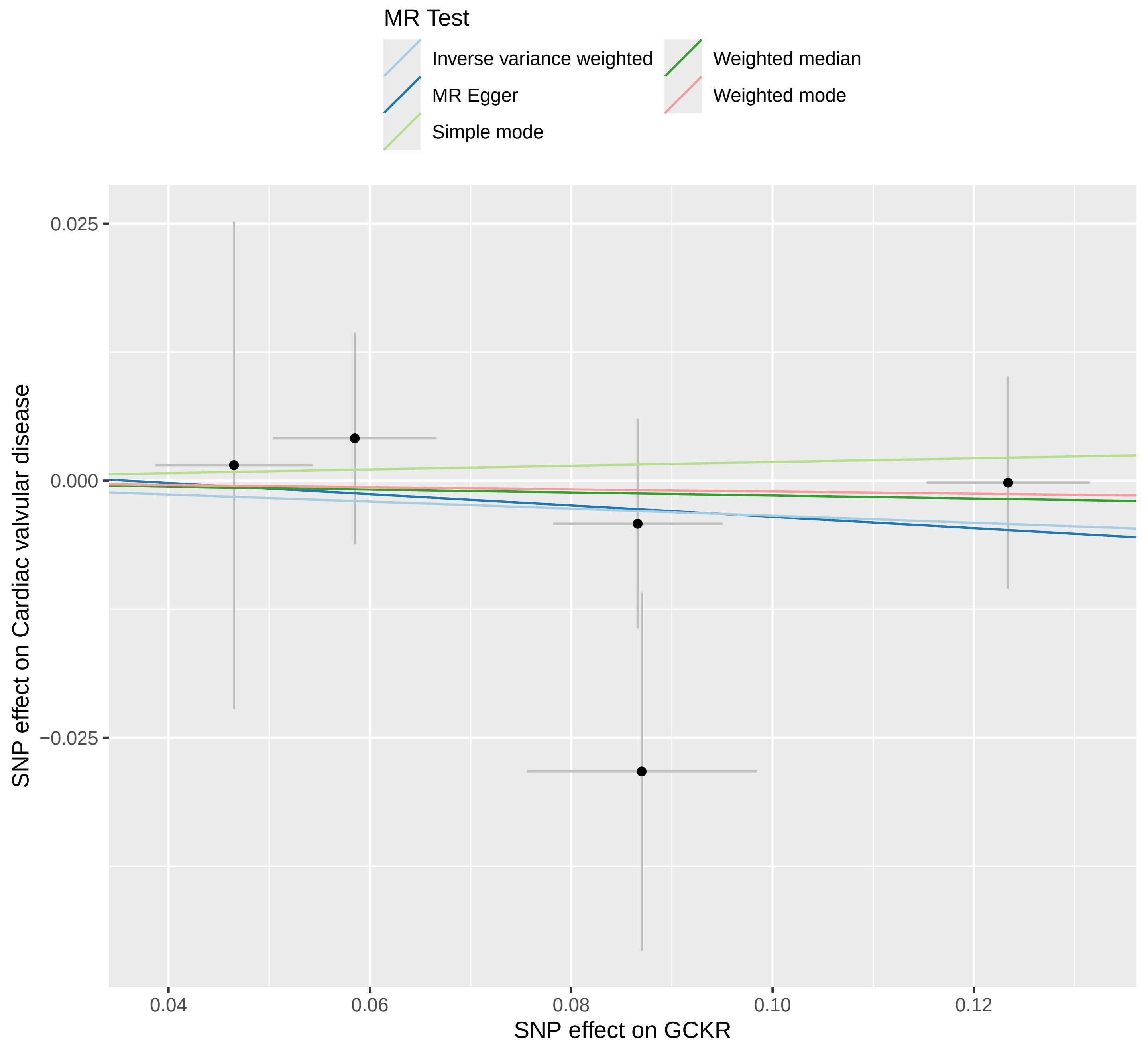
MR Test
Inverse variance weighted
MR Egger
Simple mode
Weighted median
Weighted mode
0.025
0.000
−0.025
0.04
0.06
0.08
0.10
0.12
SNP effect on GCKR
SNP effect on Cardiac valvular disease

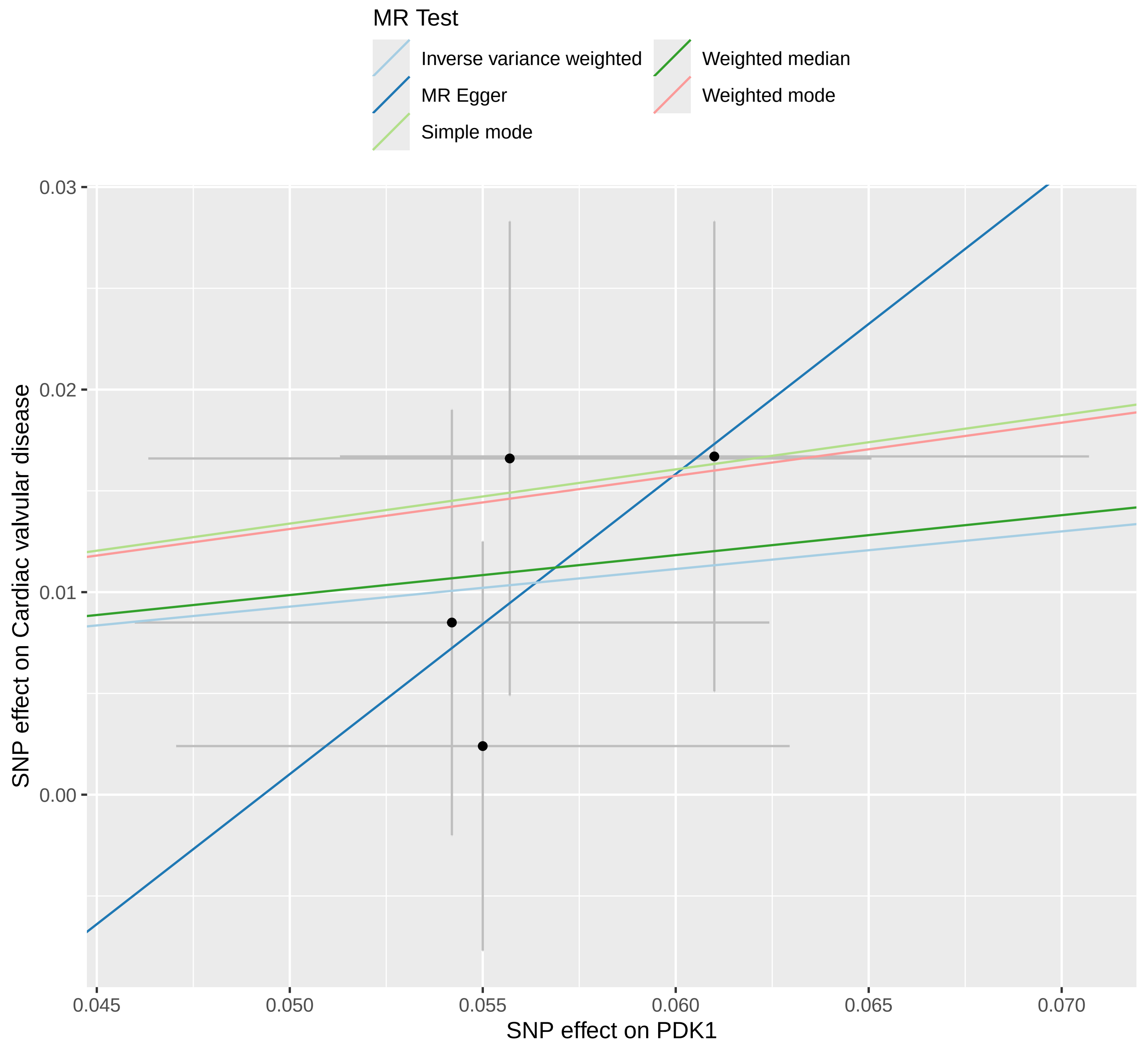
MR Test
Inverse variance weighted
MR Egger
Simple mode
Weighted median
Weighted mode
0.03
0.02
0.01
0.00
SNP effect on Cardiac valvular disease
0.045
0.050
0.055
0.060
0.065
0.070
SNP effect on PDK1

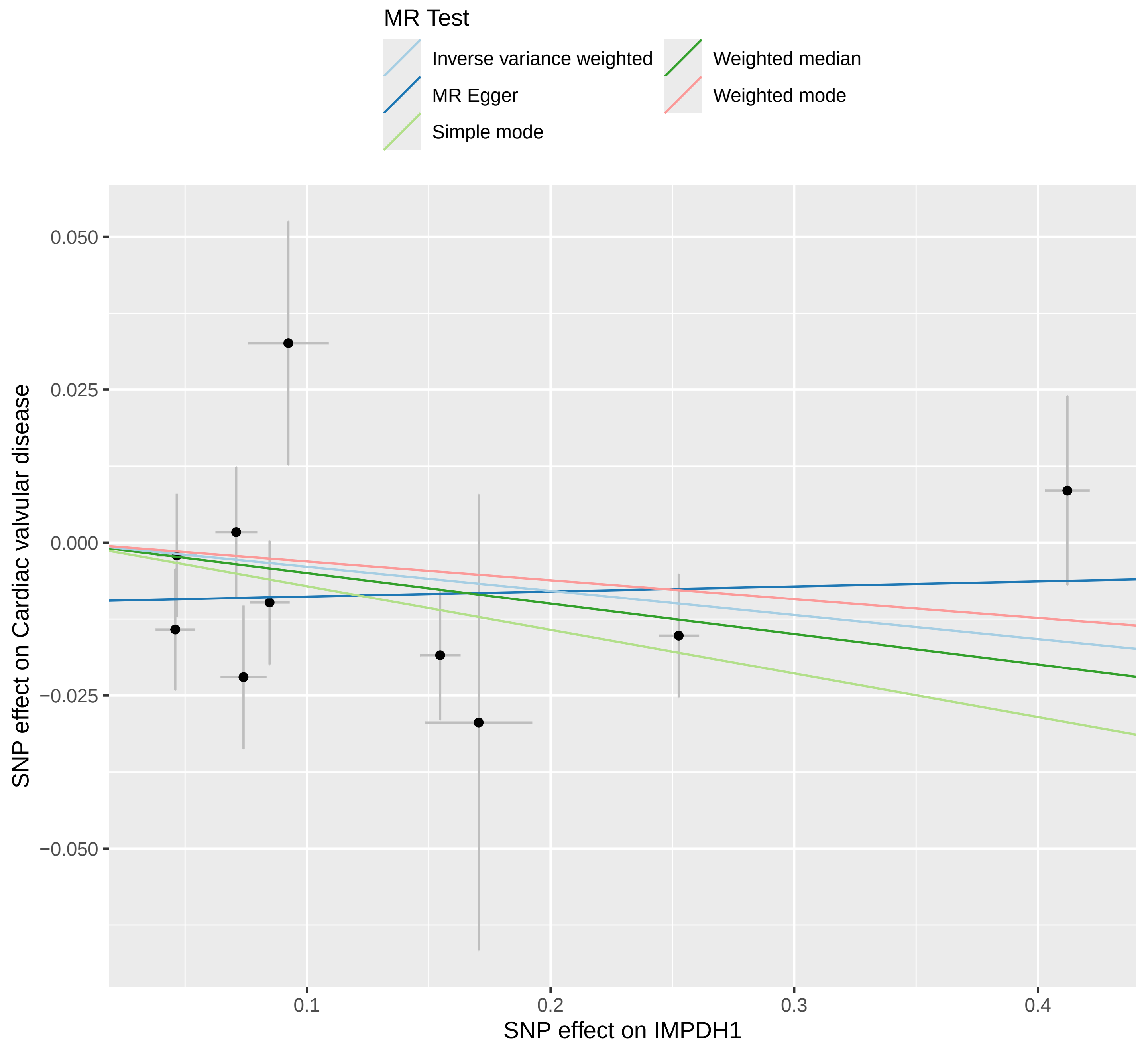
MR Test
Inverse variance weighted
MR Egger
Simple mode
Weighted median
Weighted mode
0.050
0.025
0.000
−0.025
−0.050
0.1
0.2
0.3
0.4
SNP effect on Cardiac valvular disease
SNP effect on IMPDH1

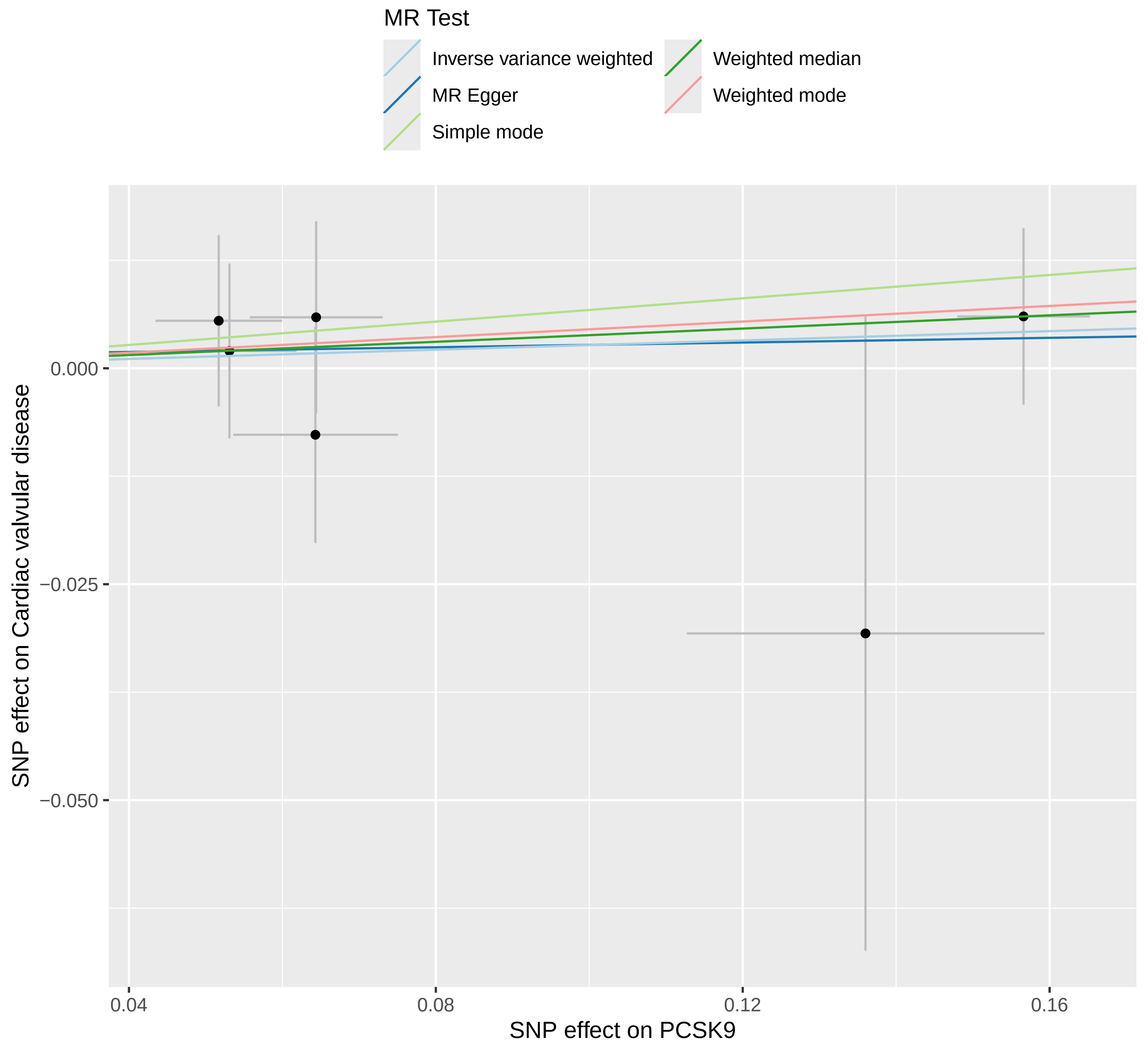
MR Test
Inverse variance weighted
MR Egger
Simple mode
Weighted median
Weighted mode
0.000
−0.025
−0.050
0.04
0.08
0.12
0.16
SNP effect on PCSK9
SNP effect on Cardiac valvular disease

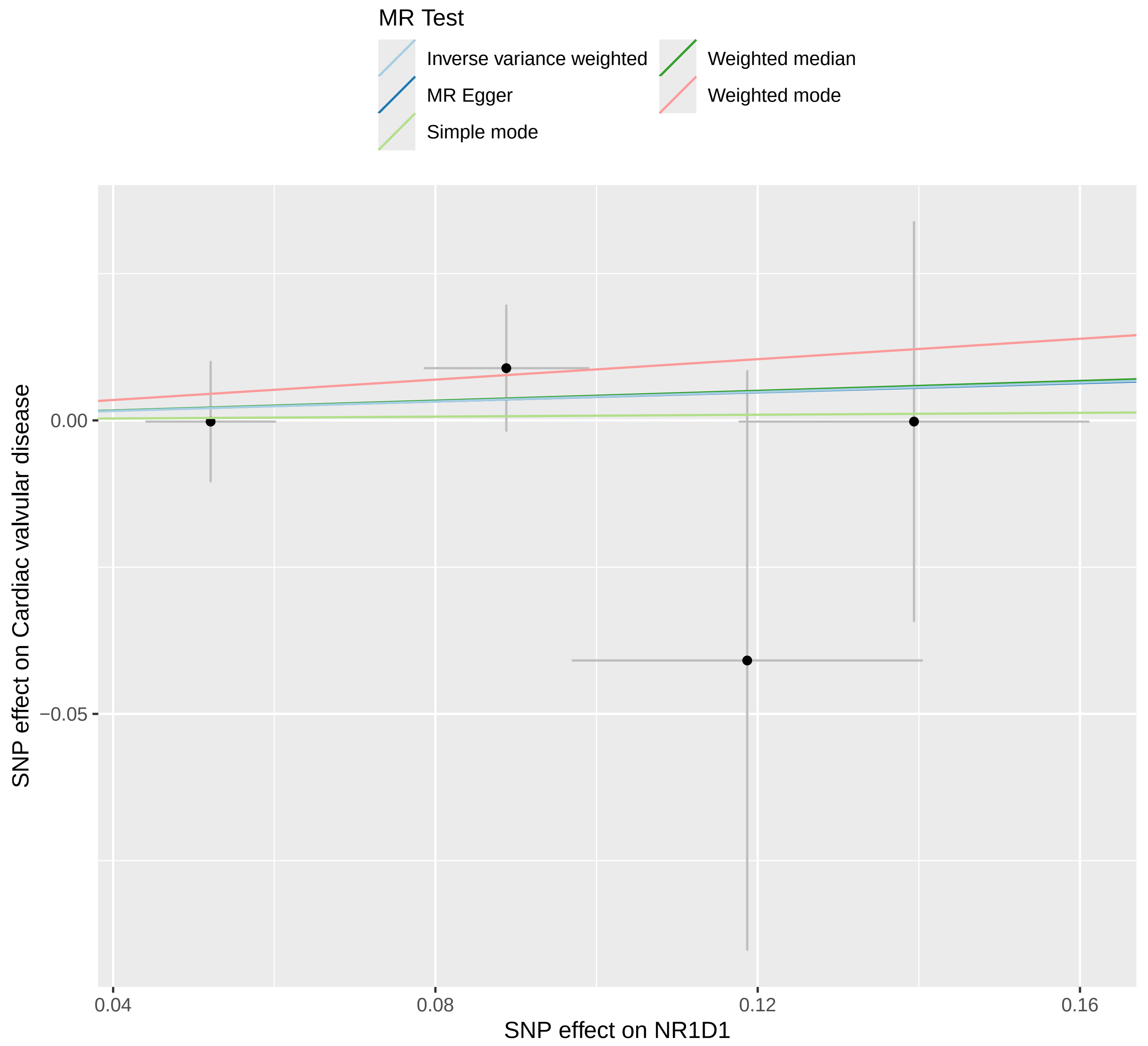
MR Test
Inverse variance weighted
MR Egger
Simple mode
Weighted median
Weighted mode
0.00
−0.05
0.04
0.08
0.12
0.16
SNP effect on NR1D1
SNP effect on Cardiac valvular disease

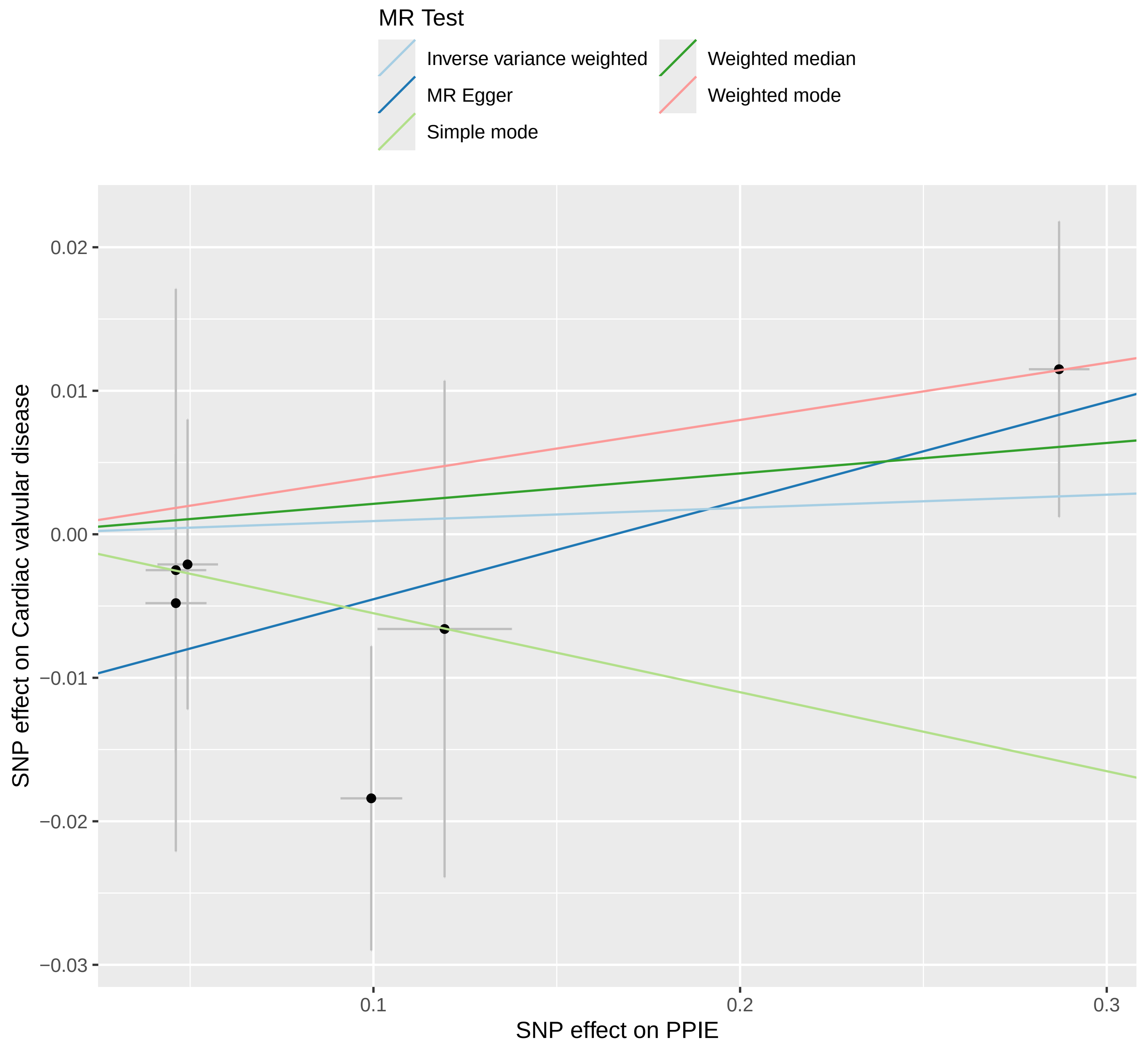
MR Test
Inverse variance weighted
MR Egger
Simple mode
Weighted median
Weighted mode
SNP effect on Cardiac valvular disease
0.02
0.01
0.00
−0.01
−0.02
−0.03
0.1
0.2
0.3
SNP effect on PPIE

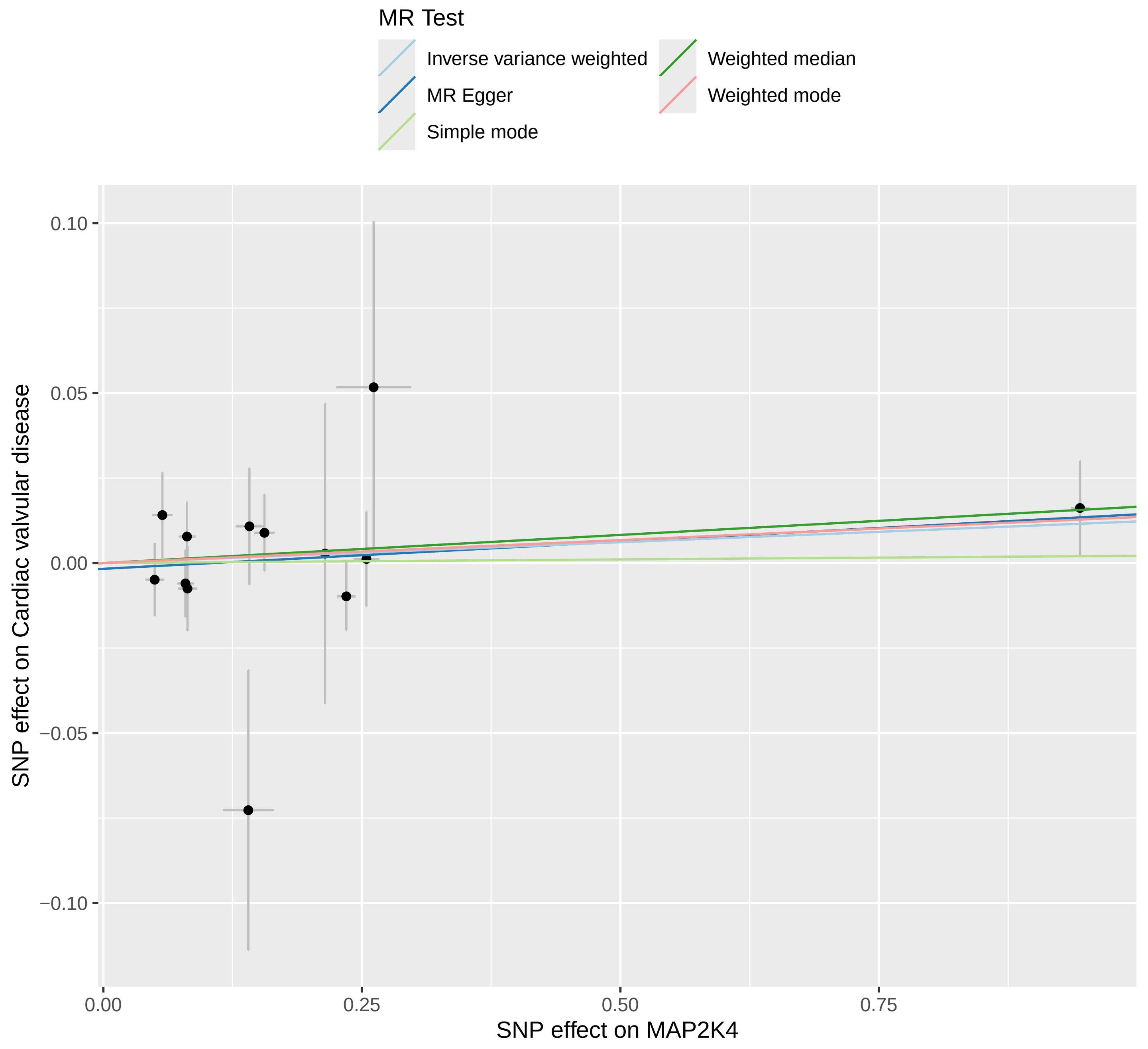
MR Test
Inverse variance weighted
MR Egger
Simple mode
Weighted median
Weighted mode
0.10
0.05
0.00
−0.05
−0.10
SNP effect on Cardiac valvular disease
0.00
0.25
0.50
0.75
SNP effect on MAP2K4

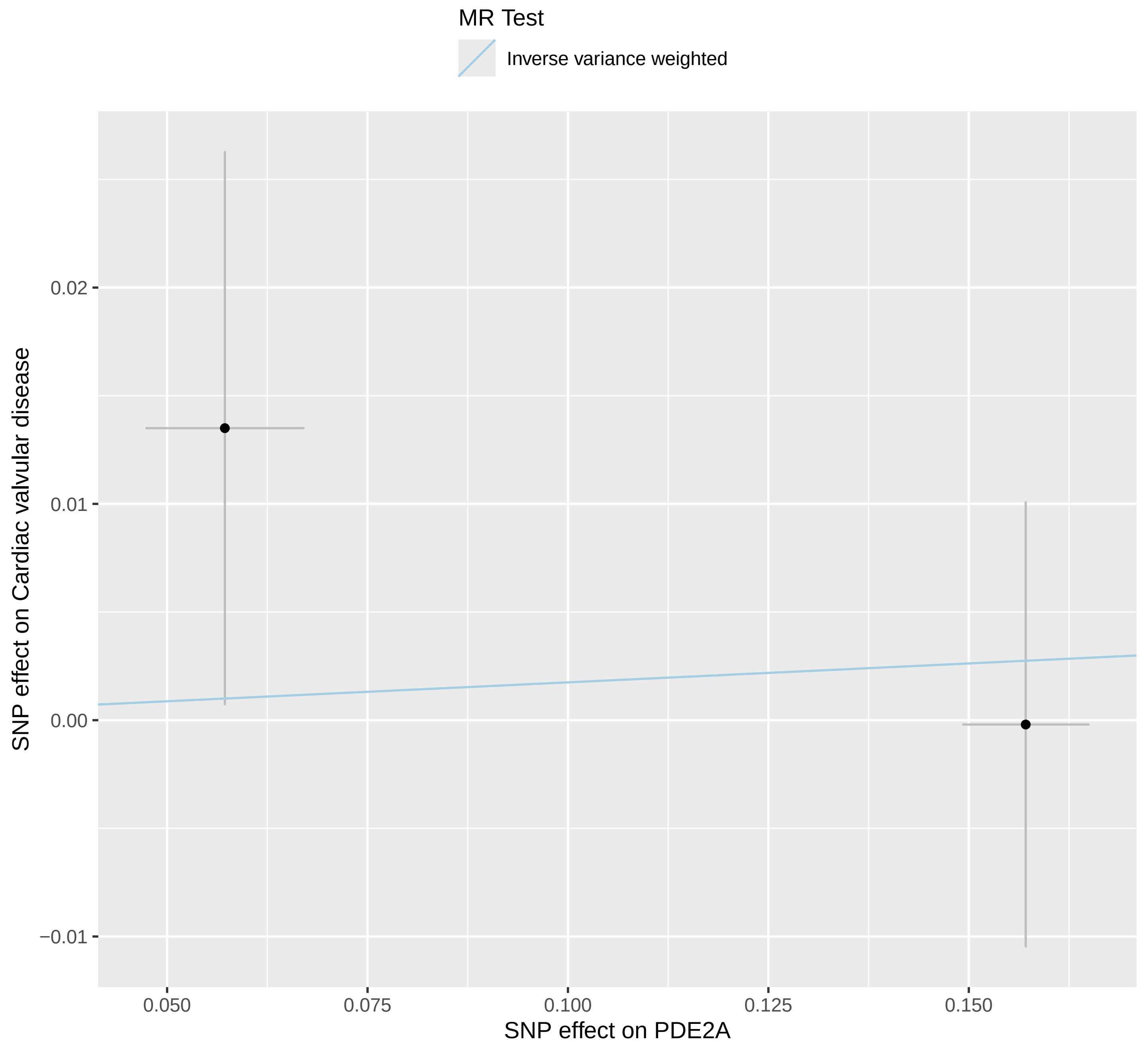
MR Test
Inverse variance weighted
SNP effect on Cardiac valvular disease
0.02
0.01
0.00
−0.01
0.050
0.075
0.100
0.125
0.150
SNP effect on PDE2A

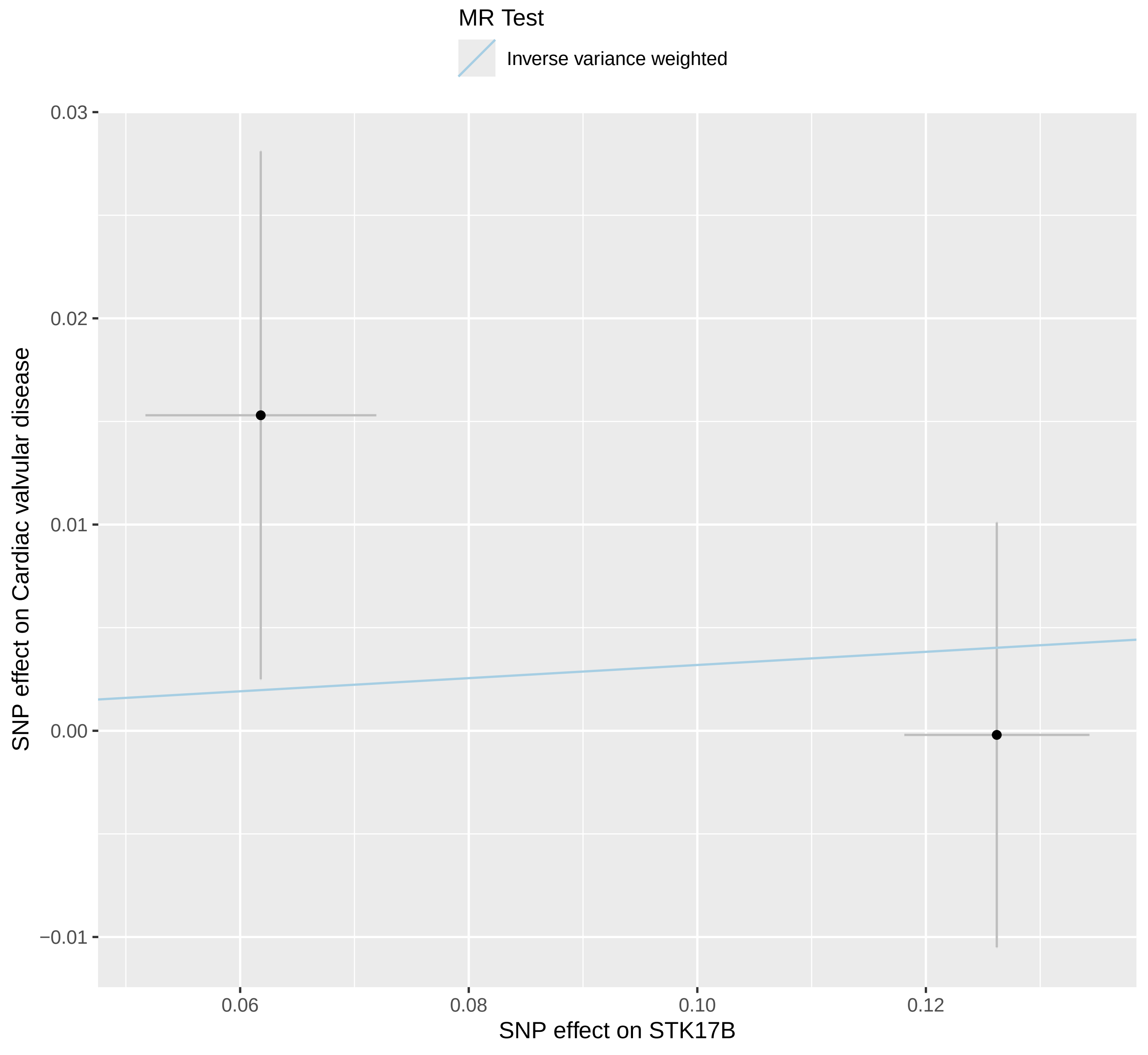
MR Test
Inverse variance weighted
0.03
0.02
0.01
0.00
−0.01
0.06
0.08
0.10
0.12
SNP effect on STK17B
SNP effect on Cardiac valvular disease

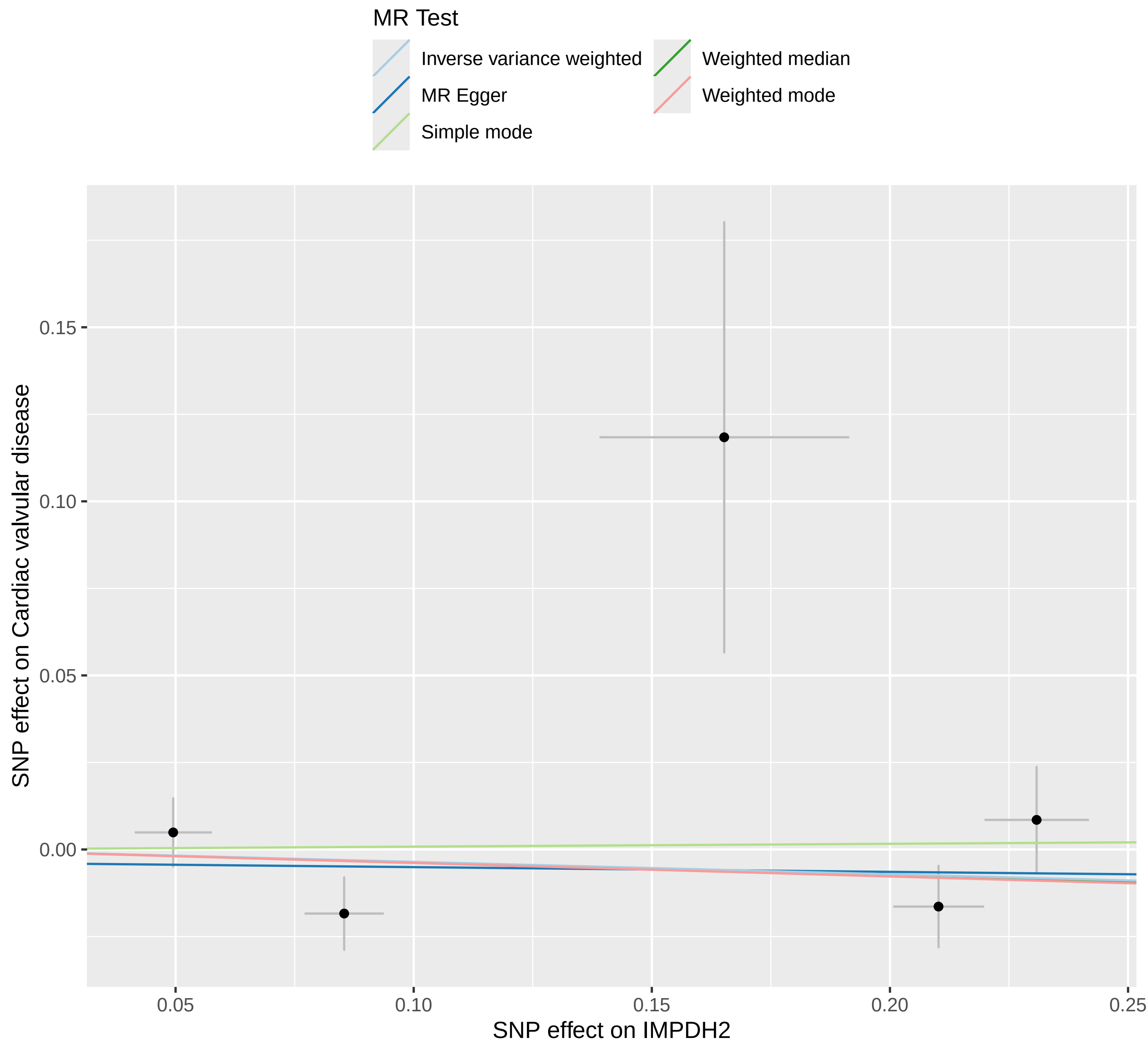
MR Test
Inverse variance weighted
MR Egger
Simple mode
Weighted median
Weighted mode
SNP effect on Cardiac valvular disease
0.15
0.10
0.05
0.00
0.05
0.10
0.15
0.20
0.25
SNP effect on IMPDH2

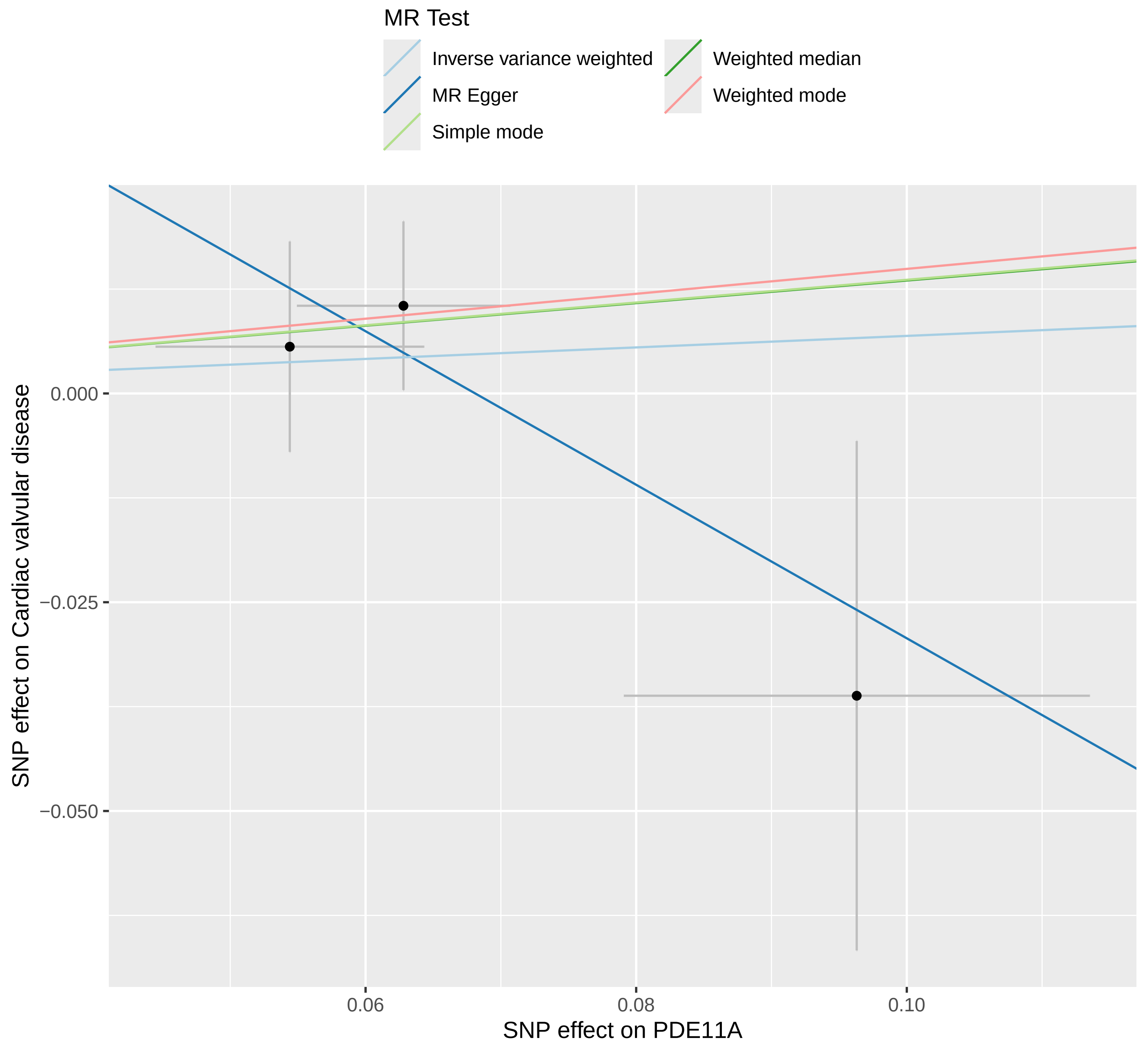
MR Test
Inverse variance weighted
MR Egger
Simple mode
Weighted median
Weighted mode
SNP effect on Cardiac valvular disease
0.000
−0.025
−0.050
0.06
0.08
0.10
SNP effect on PDE11A

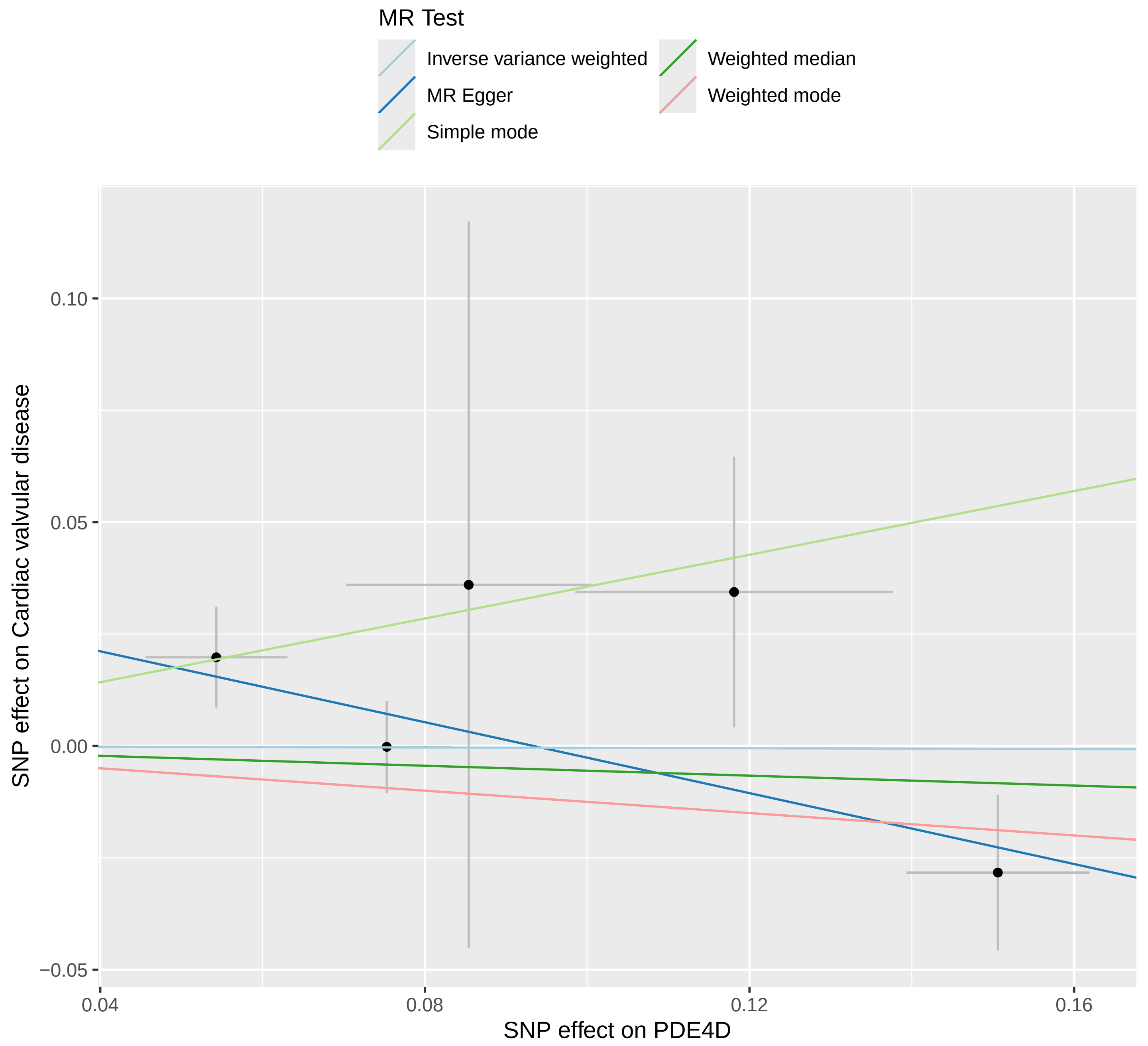

MR Test
Inverse variance weighted
MR Egger
Simple mode
Weighted median
Weighted mode
SNP effect on Cardiac valvular disease
0.10
0.05
0.00
−0.05
0.04
0.08
0.12
0.16
SNP effect on PDE4D

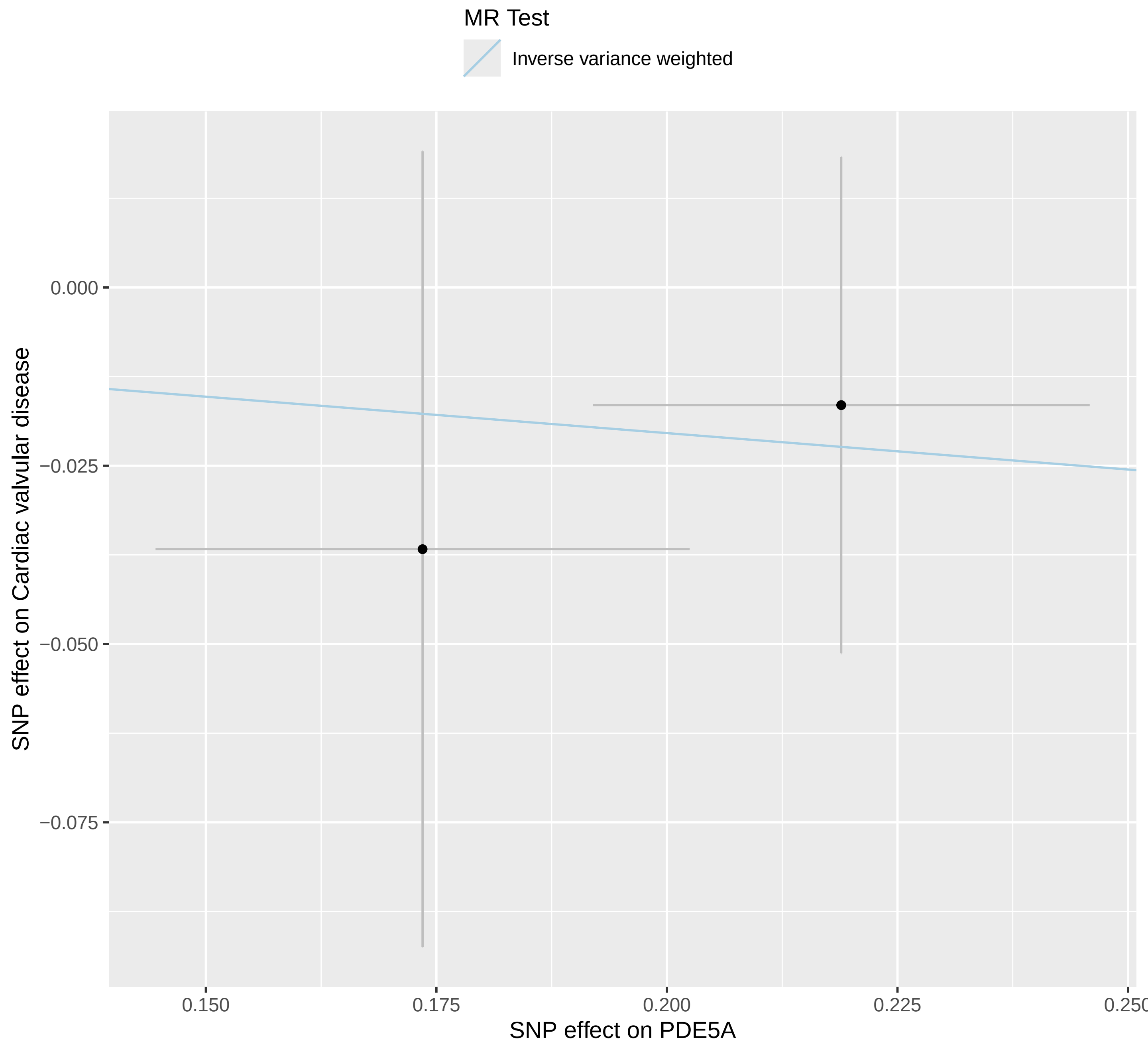

MR Test
Inverse variance weighted
0.000
−0.025
−0.050
−0.075
SNP effect on Cardiac valvular disease
0.150
0.175
0.200
0.225
0.250
SNP effect on PDE5A

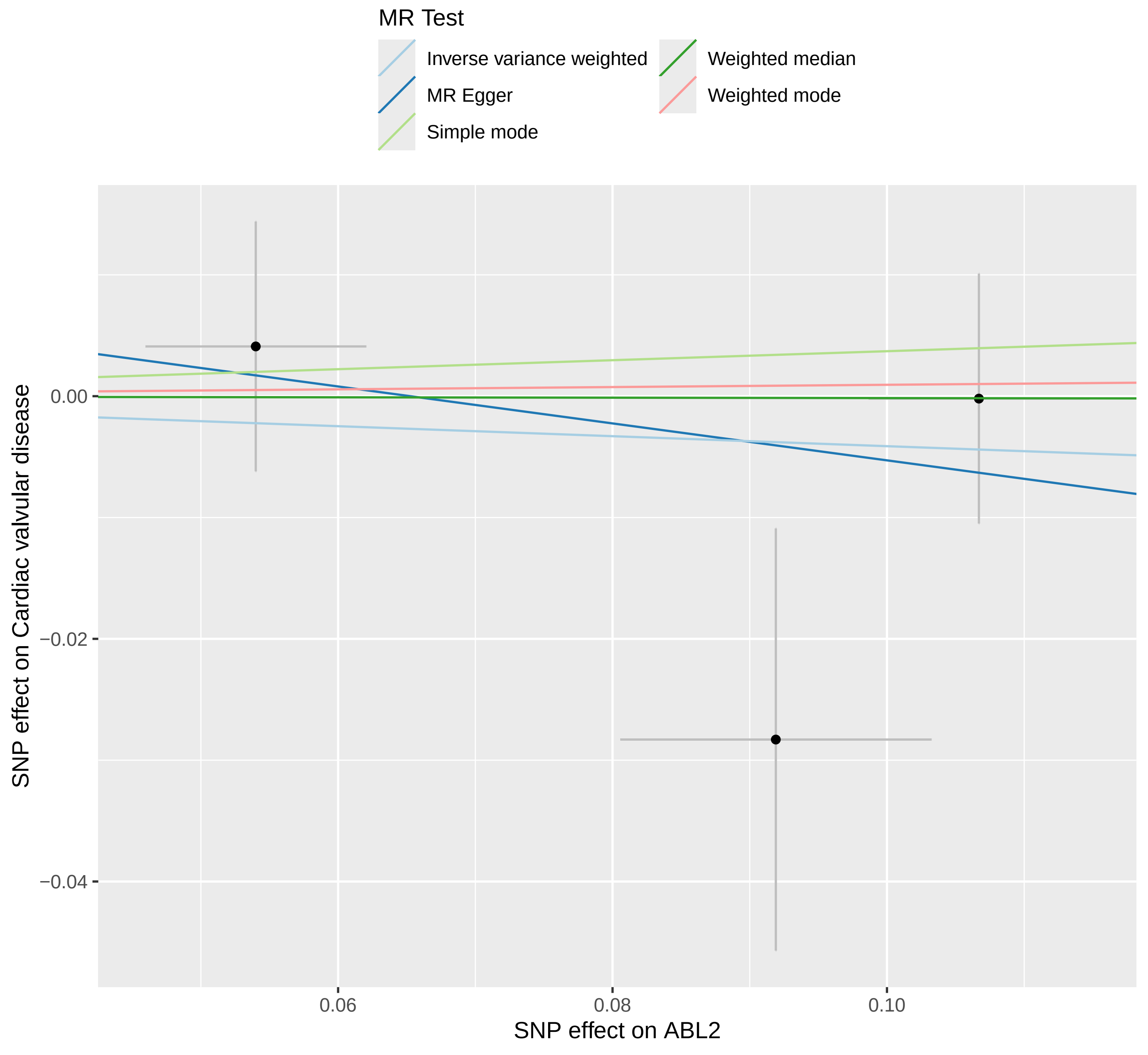
MR Test
Inverse variance weighted
MR Egger
Simple mode
Weighted median
Weighted mode
SNP effect on Cardiac valvular disease
0.00
−0.02
−0.04
0.06
0.08
0.10
SNP effect on ABL2

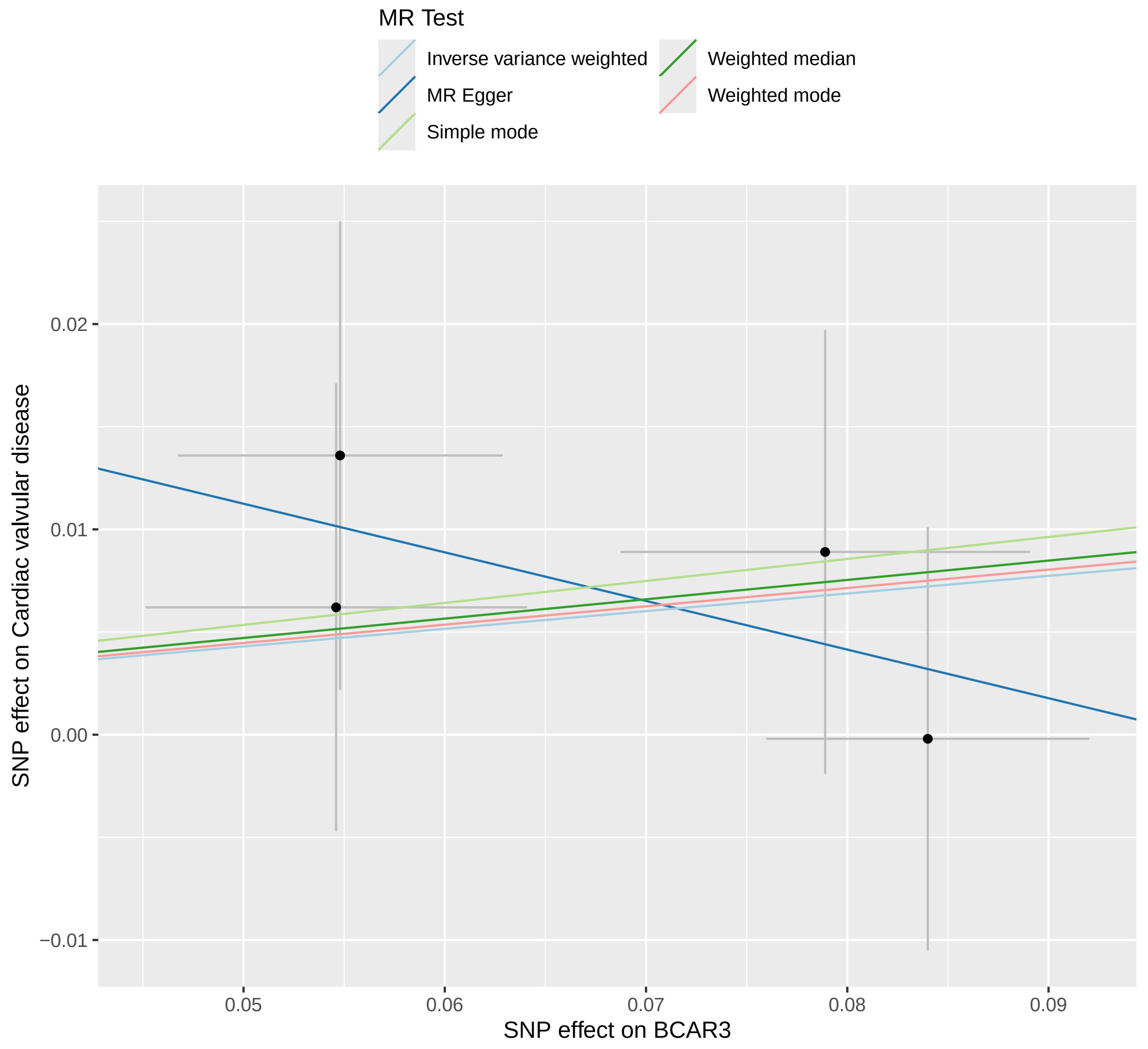

MR Test
Inverse variance weighted
MR Egger
Simple mode
Weighted median
Weighted mode
0.02
0.01
0.00
−0.01
SNP effect on Cardiac valvular disease
0.05
0.06
0.07
0.08
0.09
SNP effect on BCAR3

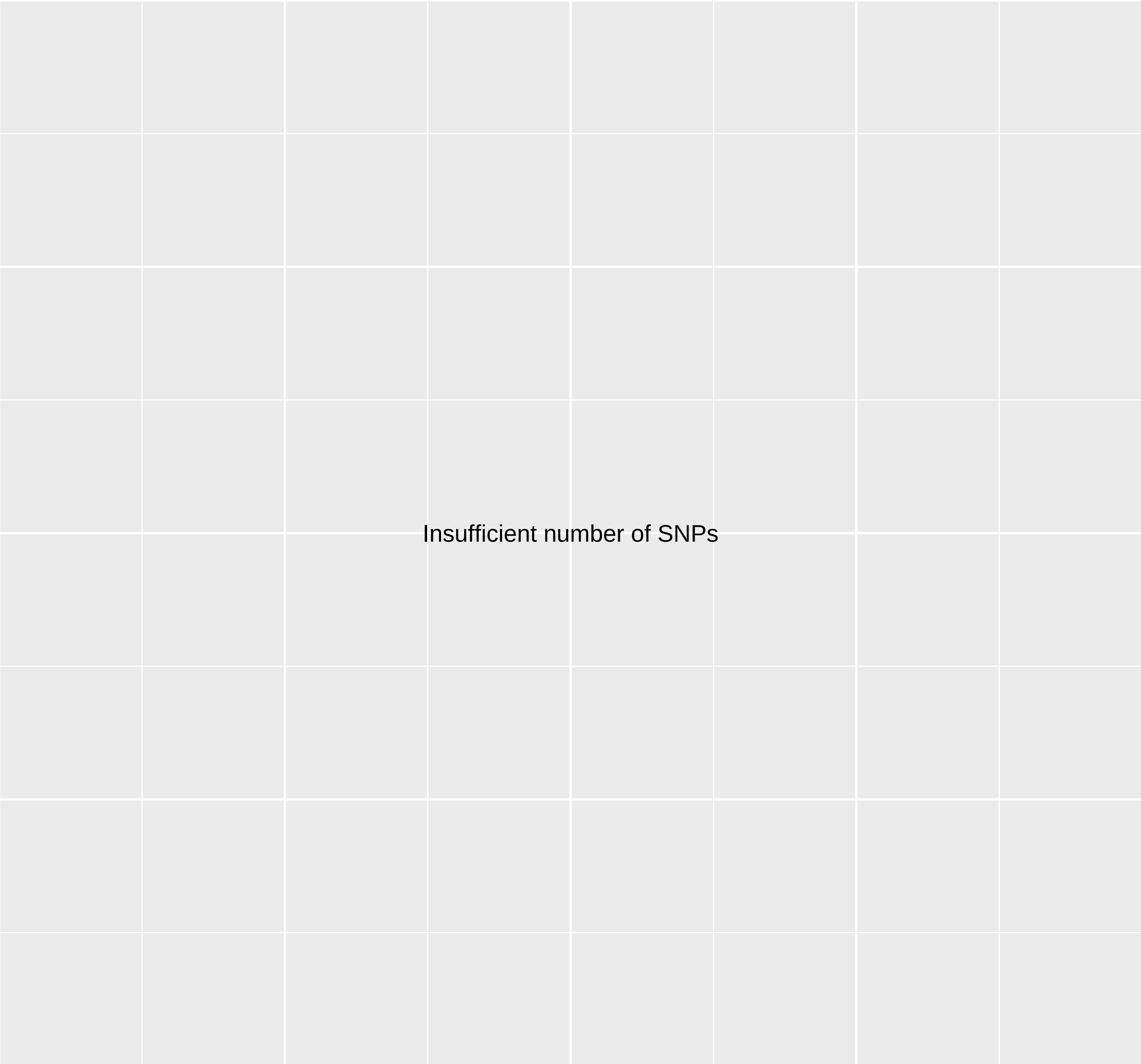
Insufficient number of SNPs

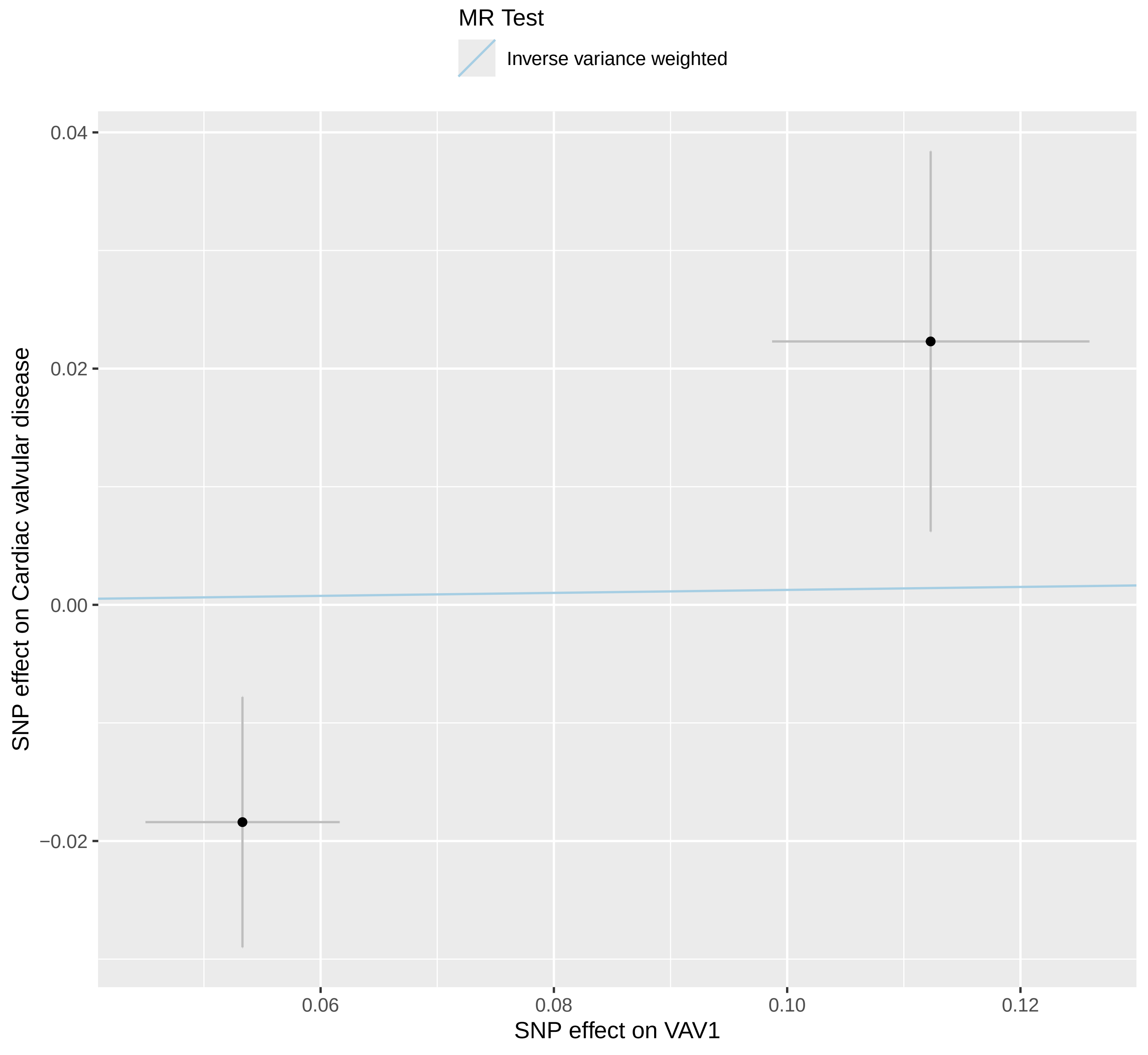

MR Test
Inverse variance weighted
0.04
0.02
0.00
−0.02
SNP effect on Cardiac valvular disease
0.06
0.08
0.10
0.12
SNP effect on VAV1

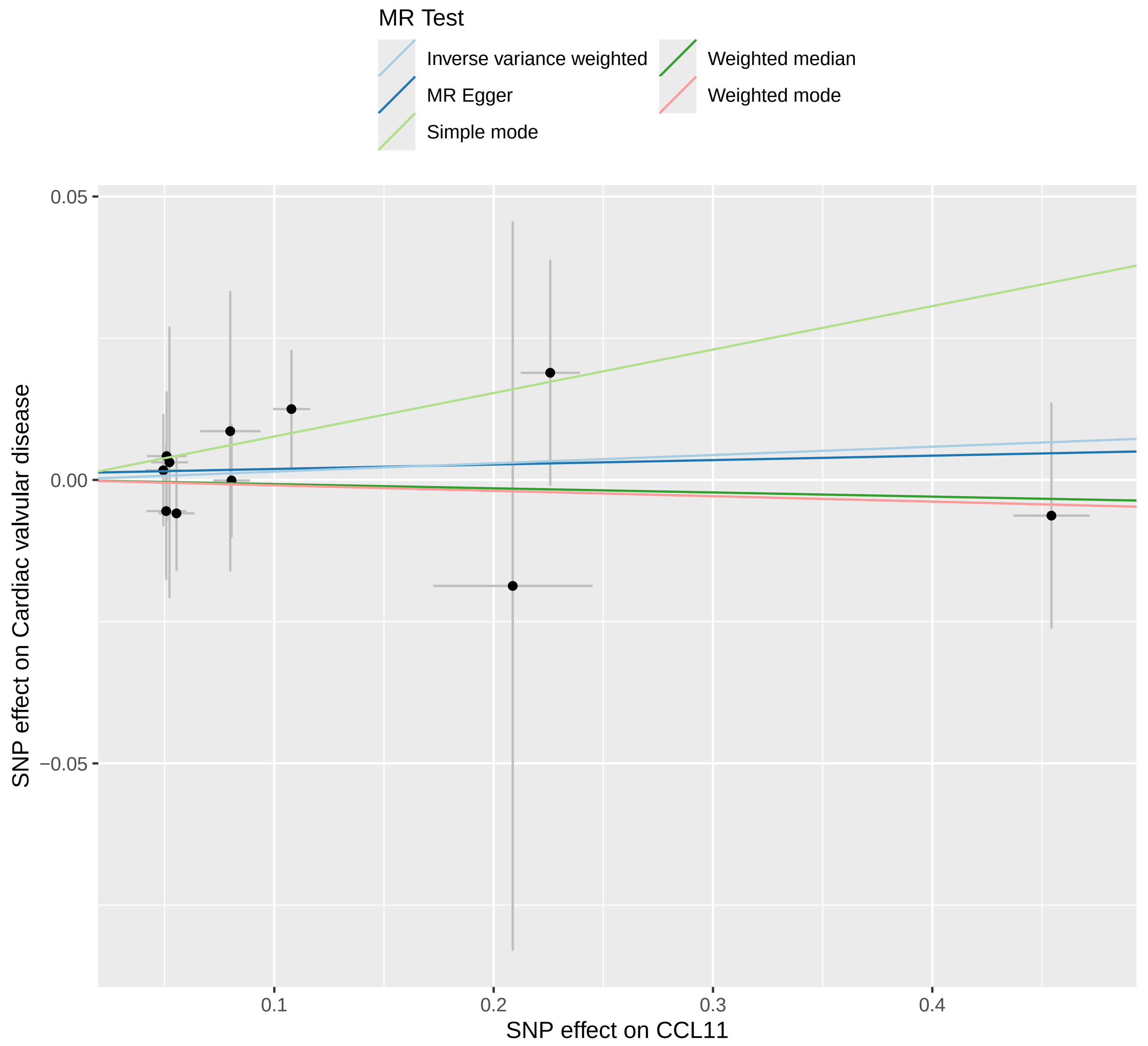
MR Test
Inverse variance weighted
MR Egger
Simple mode
Weighted median
Weighted mode
0.05
0.00
−0.05
SNP effect on Cardiac valvular disease
0.1
0.2
0.3
0.4
SNP effect on CCL11

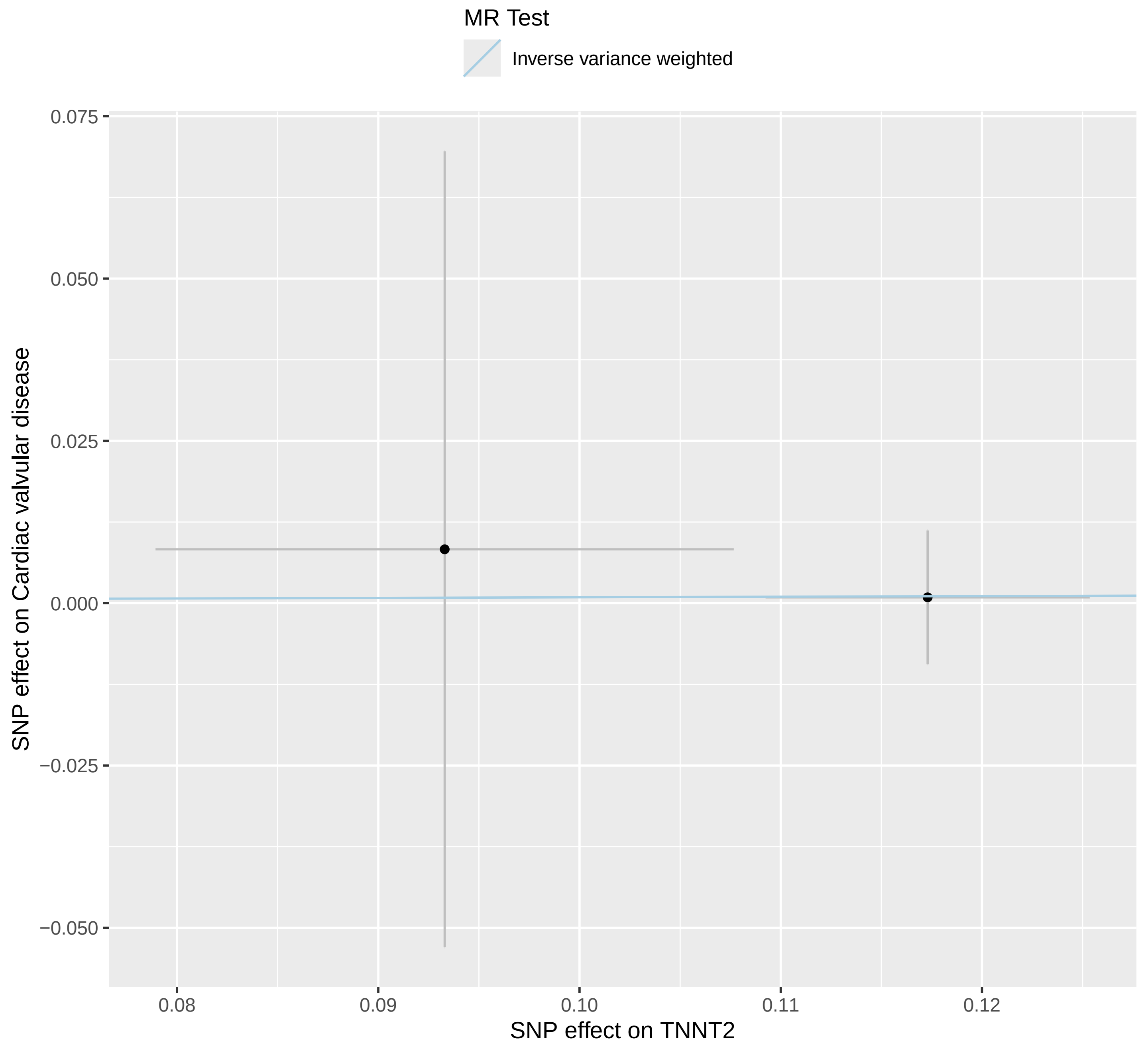
MR Test
Inverse variance weighted
0.075
0.050
0.025
0.000
−0.025
−0.050
SNP effect on Cardiac valvular disease
0.08
0.09
0.10
0.11
0.12
SNP effect on TNNT2

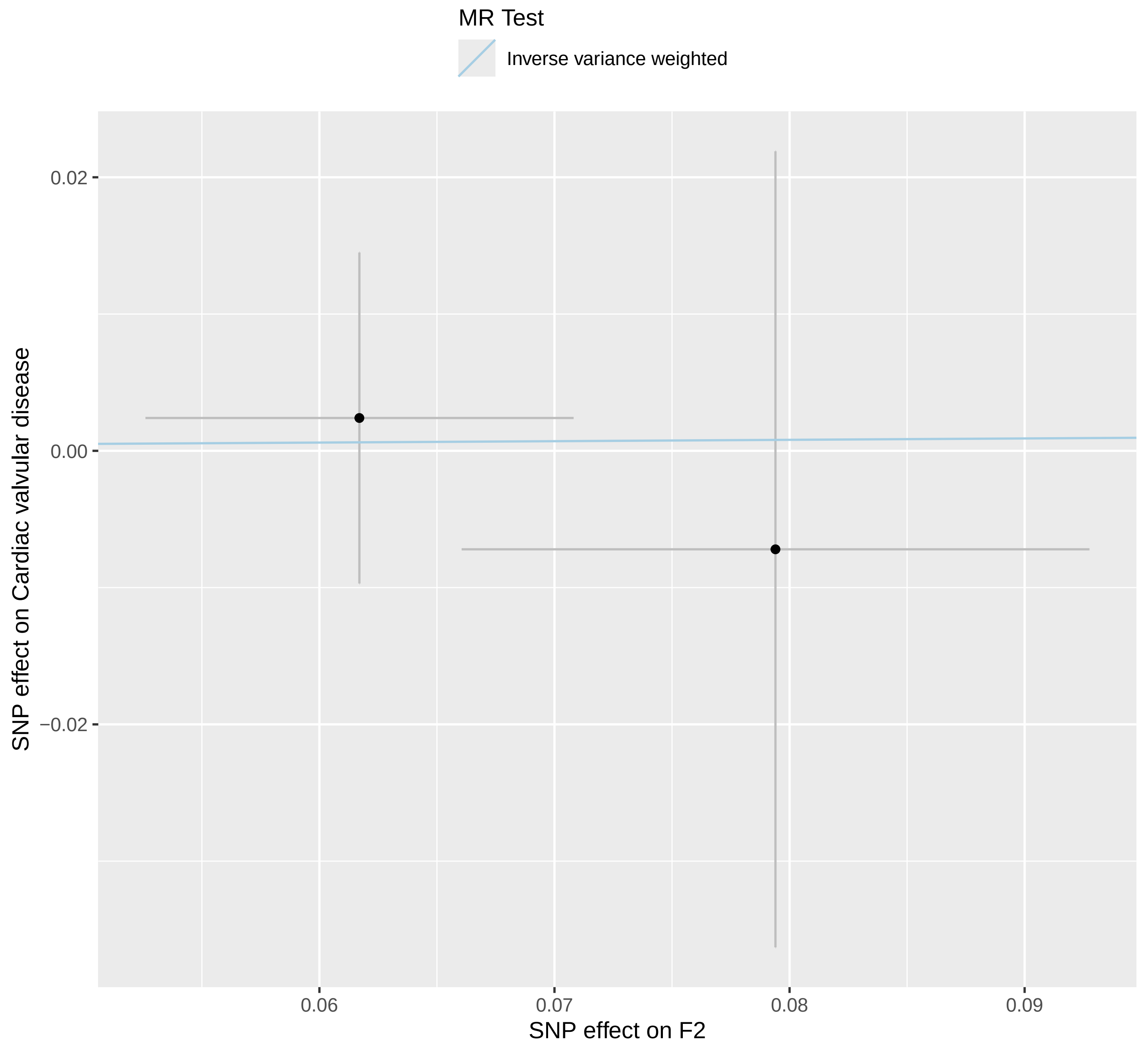
MR Test
Inverse variance weighted
SNP effect on Cardiac valvular disease
0.02
0.00
−0.02
0.06
0.07
0.08
0.09
SNP effect on F2

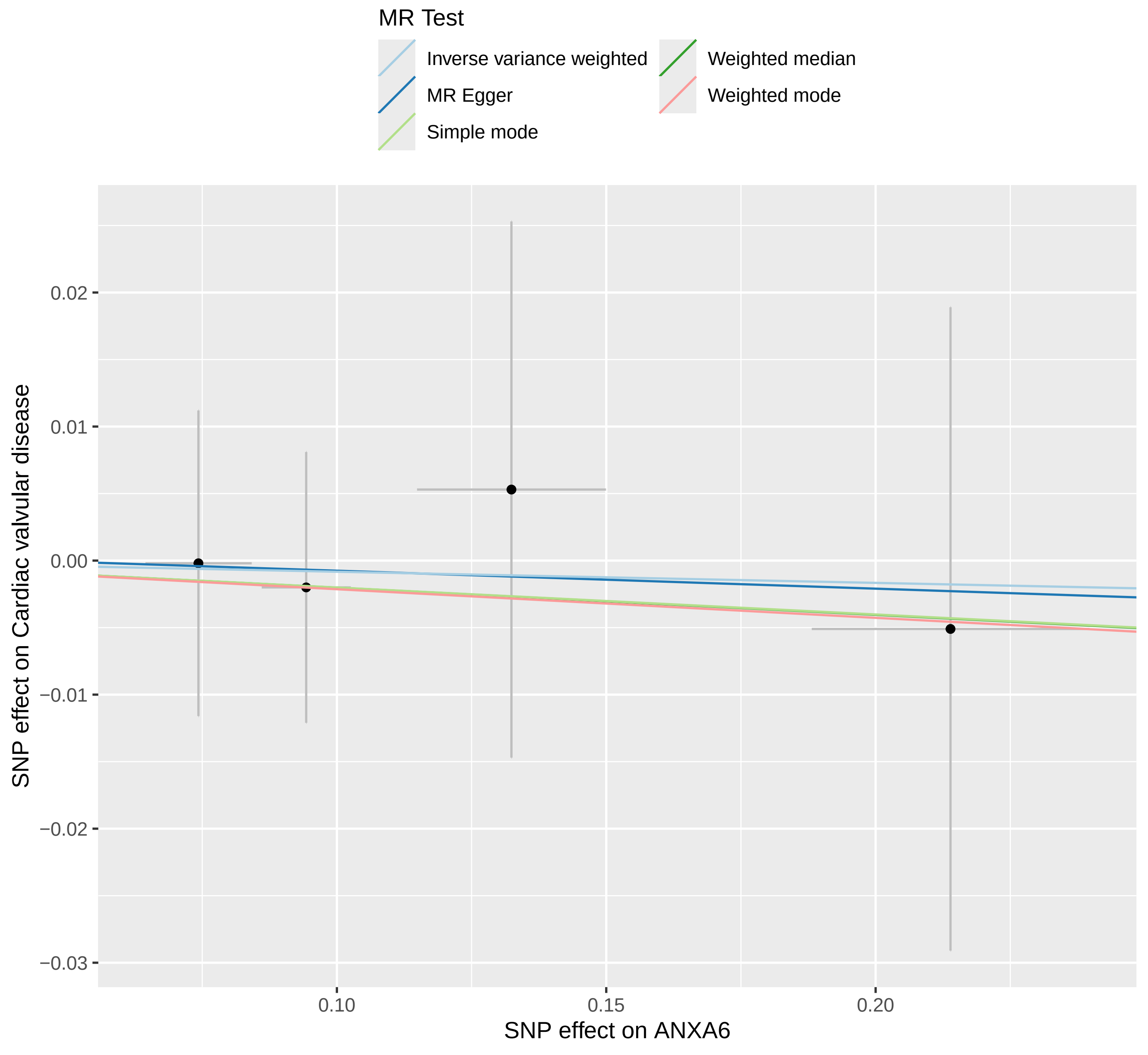
MR Test
Inverse variance weighted
MR Egger
Simple mode
Weighted median
Weighted mode
0.02
0.01
0.00
−0.01
−0.02
−0.03
SNP effect on Cardiac valvular disease
0.10
0.15
0.20
SNP effect on ANXA6

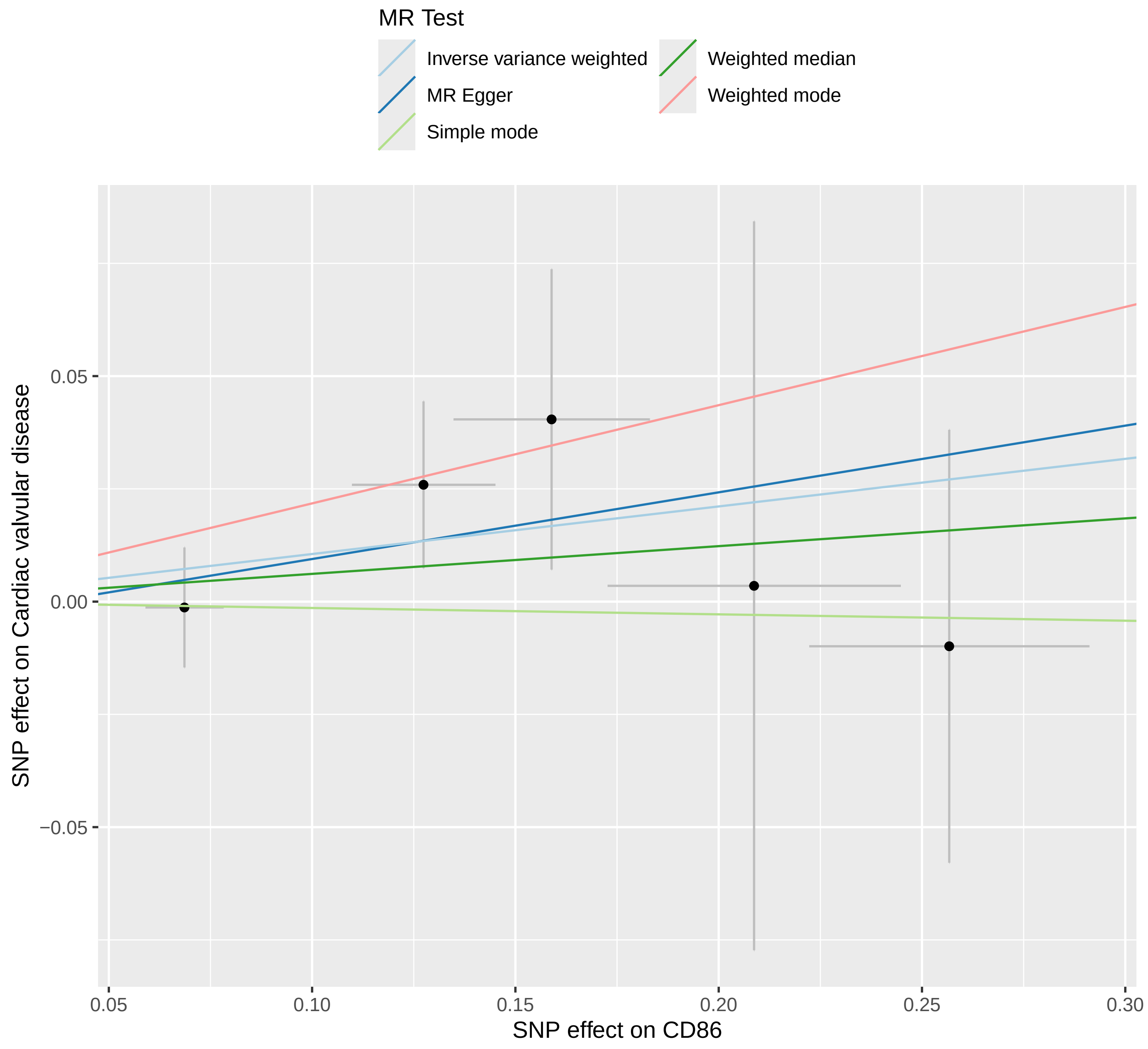

MR Test
Inverse variance weighted
MR Egger
Simple mode
Weighted median
Weighted mode
0.05
0.00
−0.05
SNP effect on Cardiac valvular disease
0.05
0.10
0.15
0.20
0.25
0.30
SNP effect on CD86

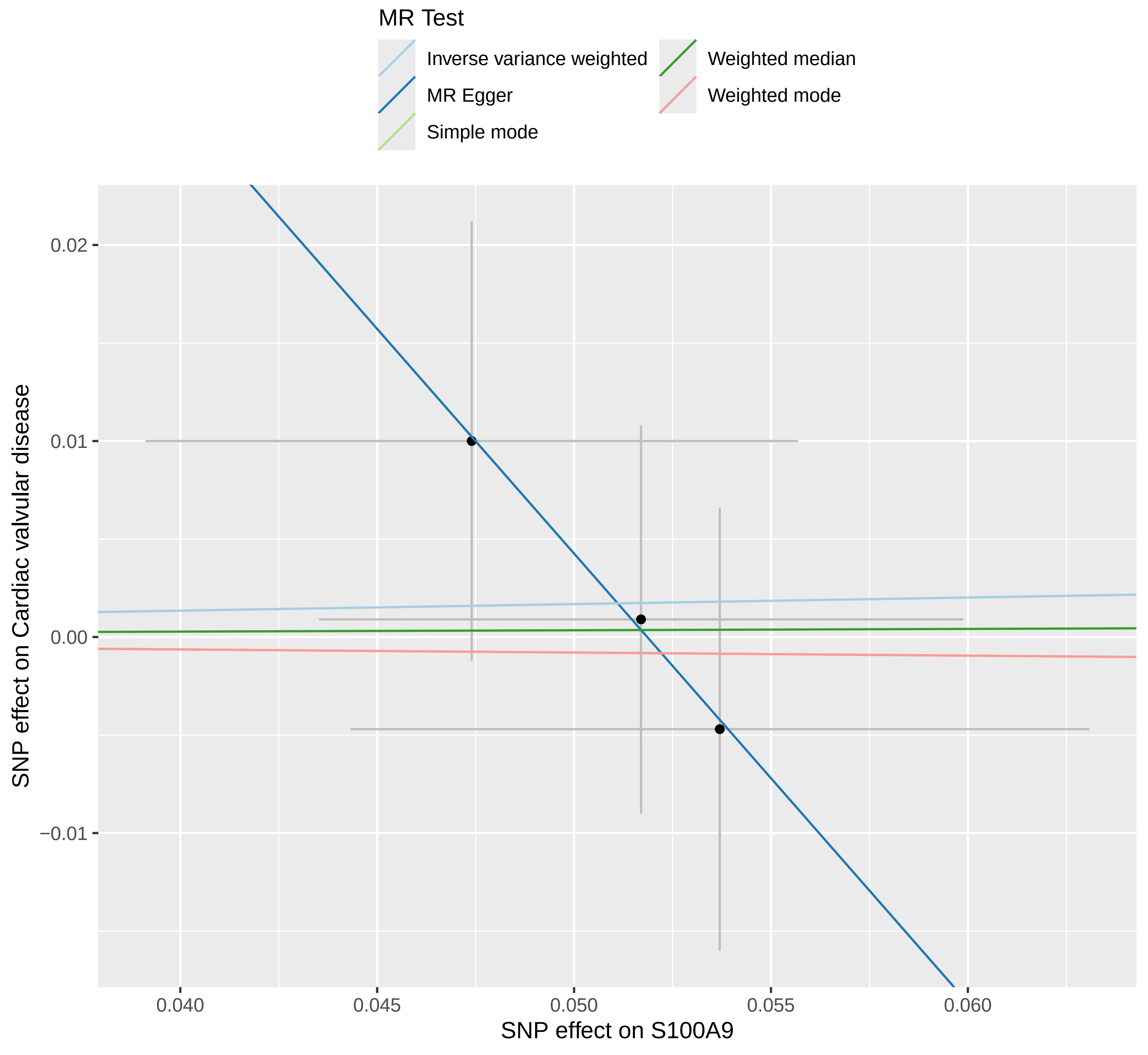
MR Test
Inverse variance weighted
MR Egger
Simple mode
Weighted median
Weighted mode
0.02
0.01
0.00
−0.01
0.040
0.045
0.050
0.055
0.060
SNP effect on S100A9
SNP effect on Cardiac valvular disease

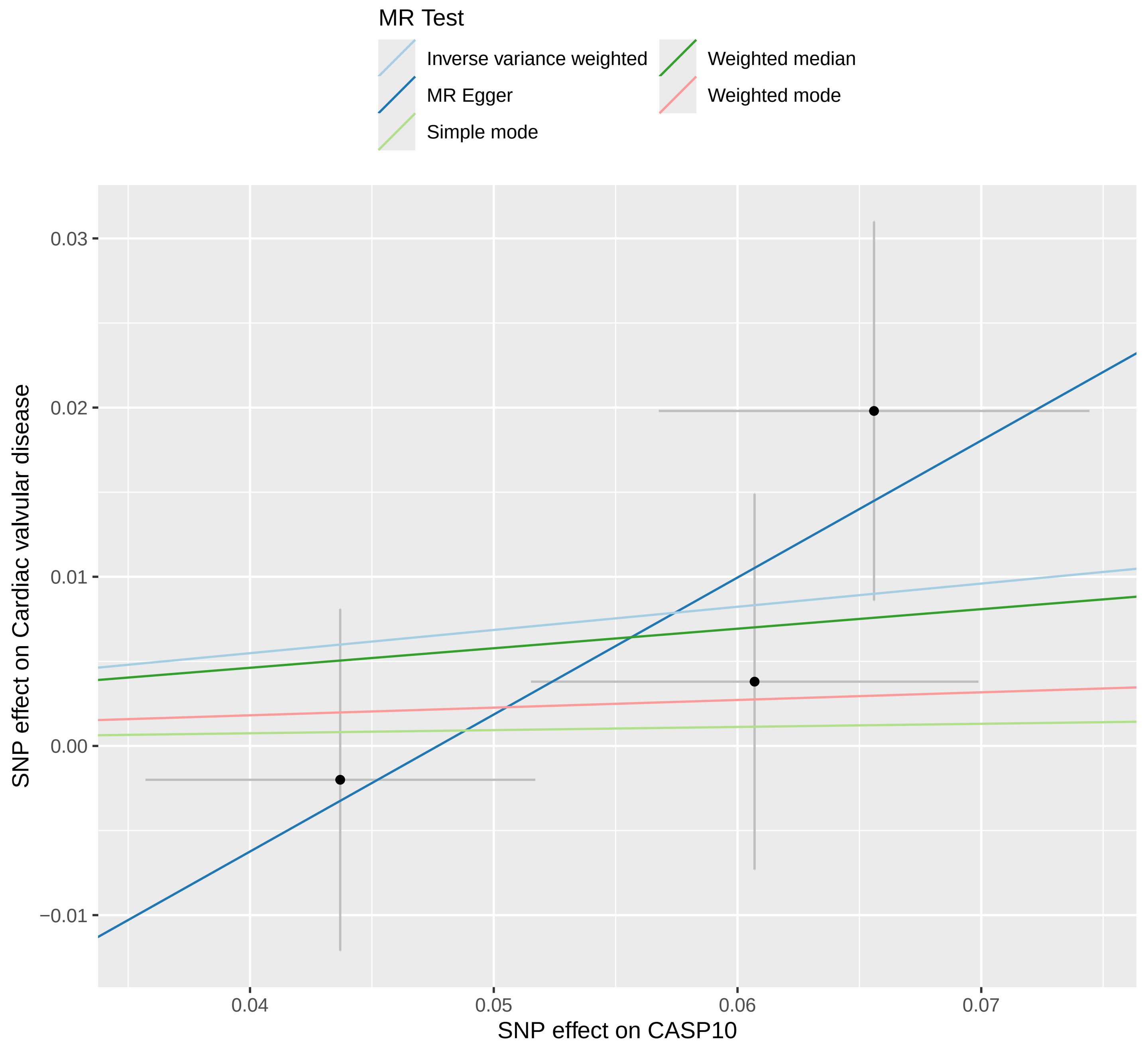
MR Test
Inverse variance weighted
MR Egger
Simple mode
Weighted median
Weighted mode
0.03
0.02
0.01
0.00
−0.01
0.04
0.05
0.06
0.07
SNP effect on Cardiac valvular disease
SNP effect on CASP10

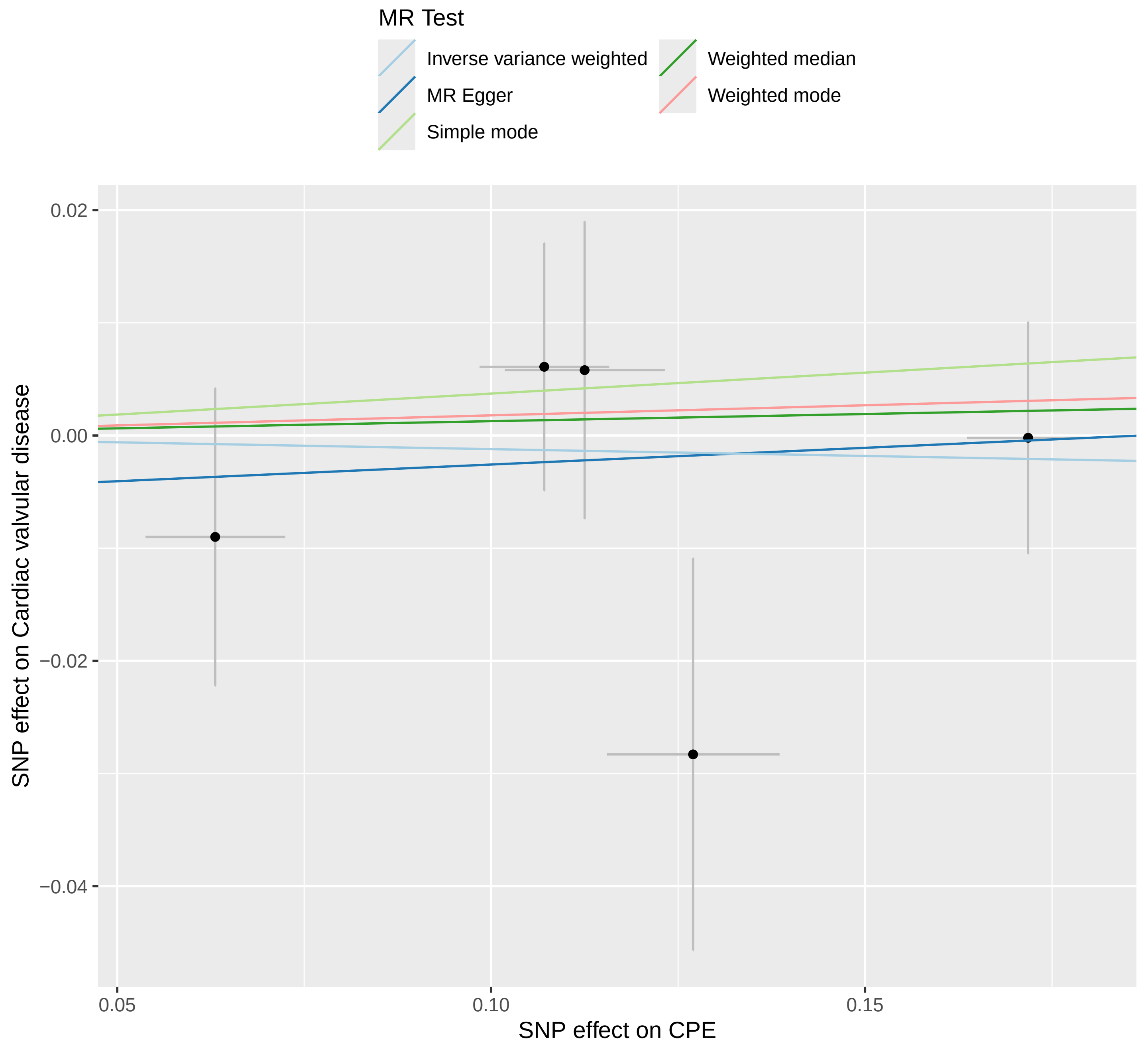
MR Test
Inverse variance weighted
MR Egger
Simple mode
Weighted median
Weighted mode
0.02
0.00
−0.02
−0.04
0.05
0.10
0.15
SNP effect on Cardiac valvular disease
SNP effect on CPE

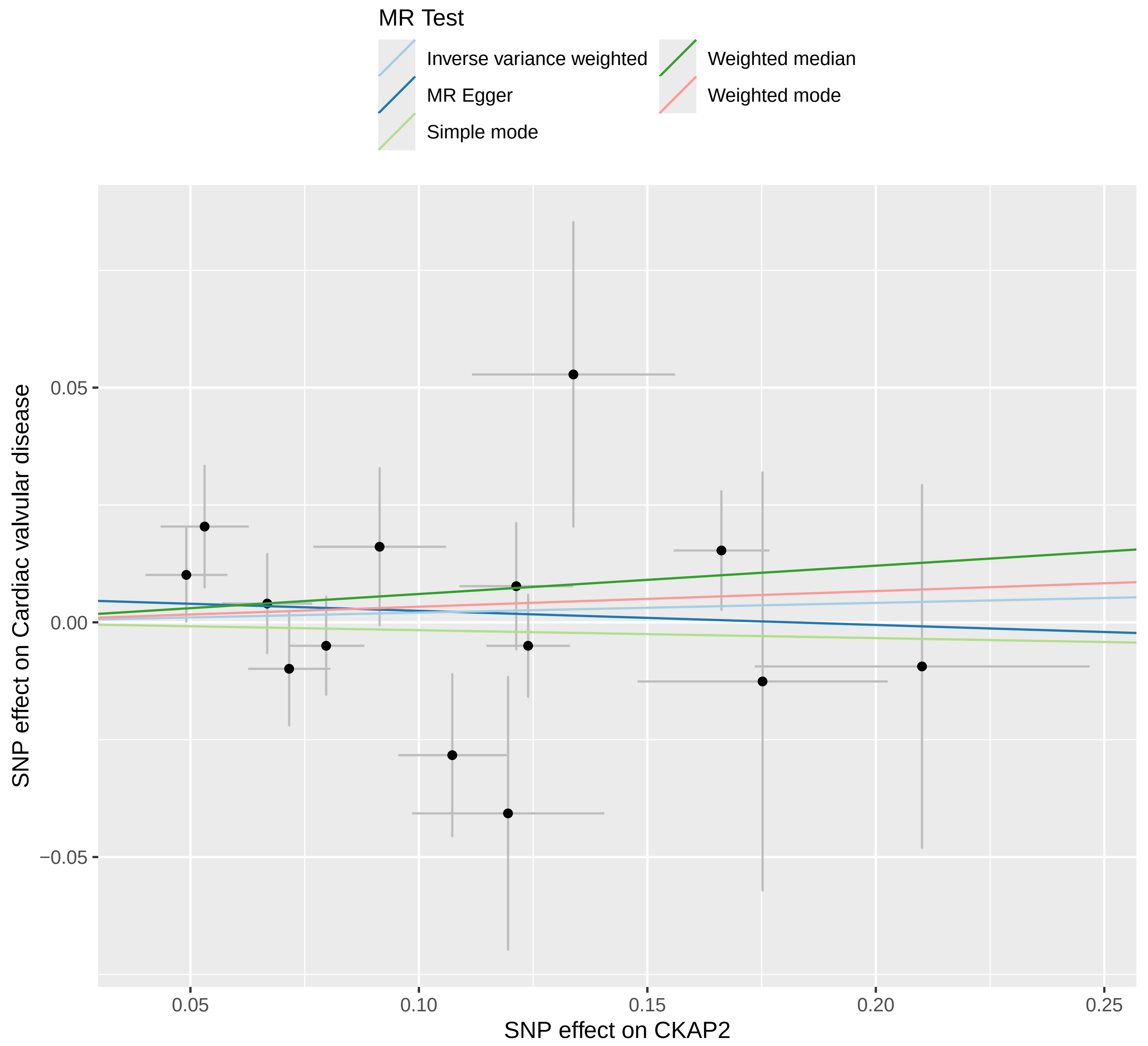

MR Test
Inverse variance weighted
MR Egger
Simple mode
Weighted median
Weighted mode
SNP effect on Cardiac valvular disease
0.05
0.00
−0.05
0.05
0.10
0.15
0.20
0.25
SNP effect on CKAP2

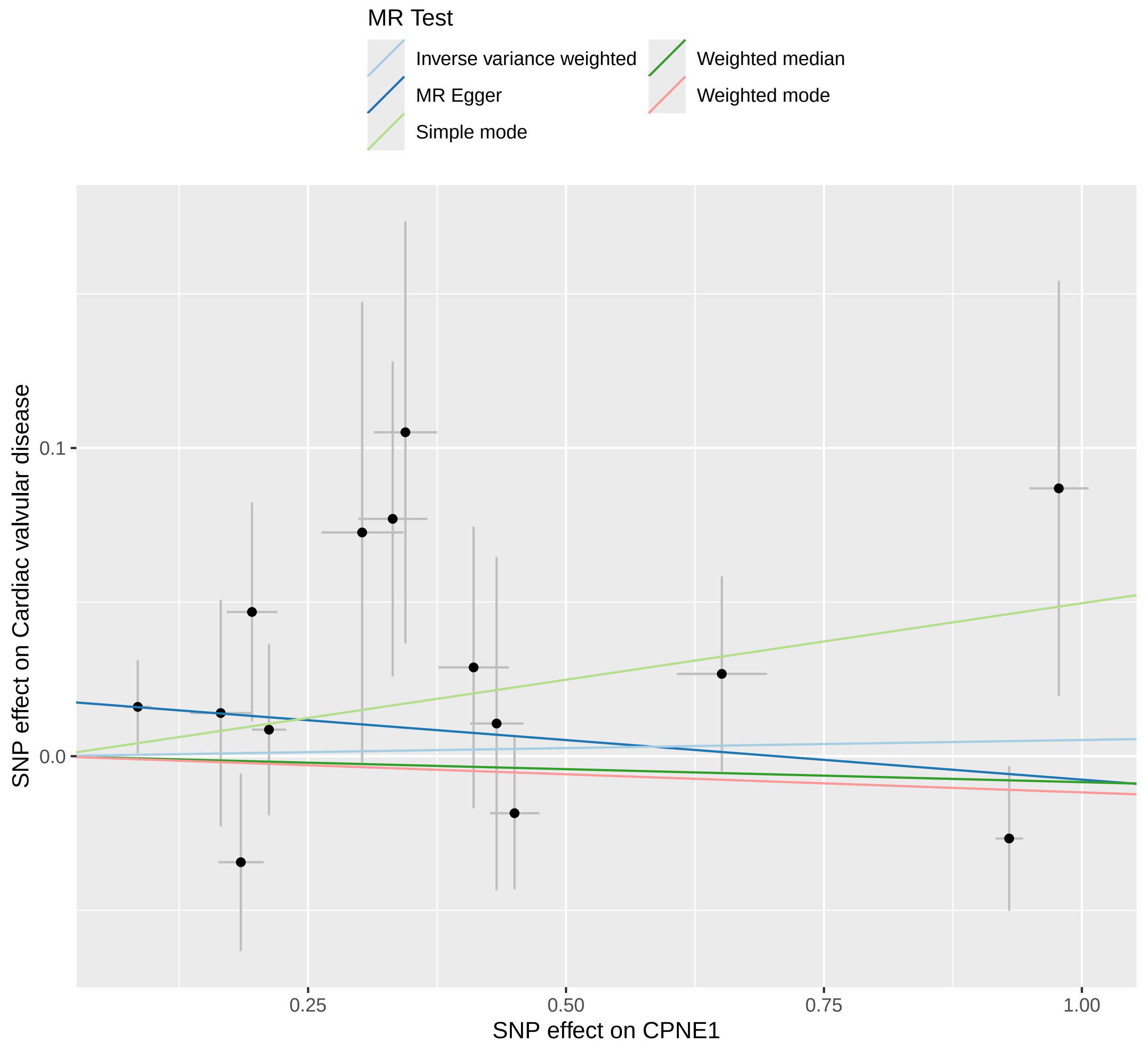

MR Test
Inverse variance weighted
MR Egger
Simple mode
Weighted median
Weighted mode
SNP effect on Cardiac valvular disease
0.1
0.0
0.25
0.50
0.75
1.00
SNP effect on CPNE1

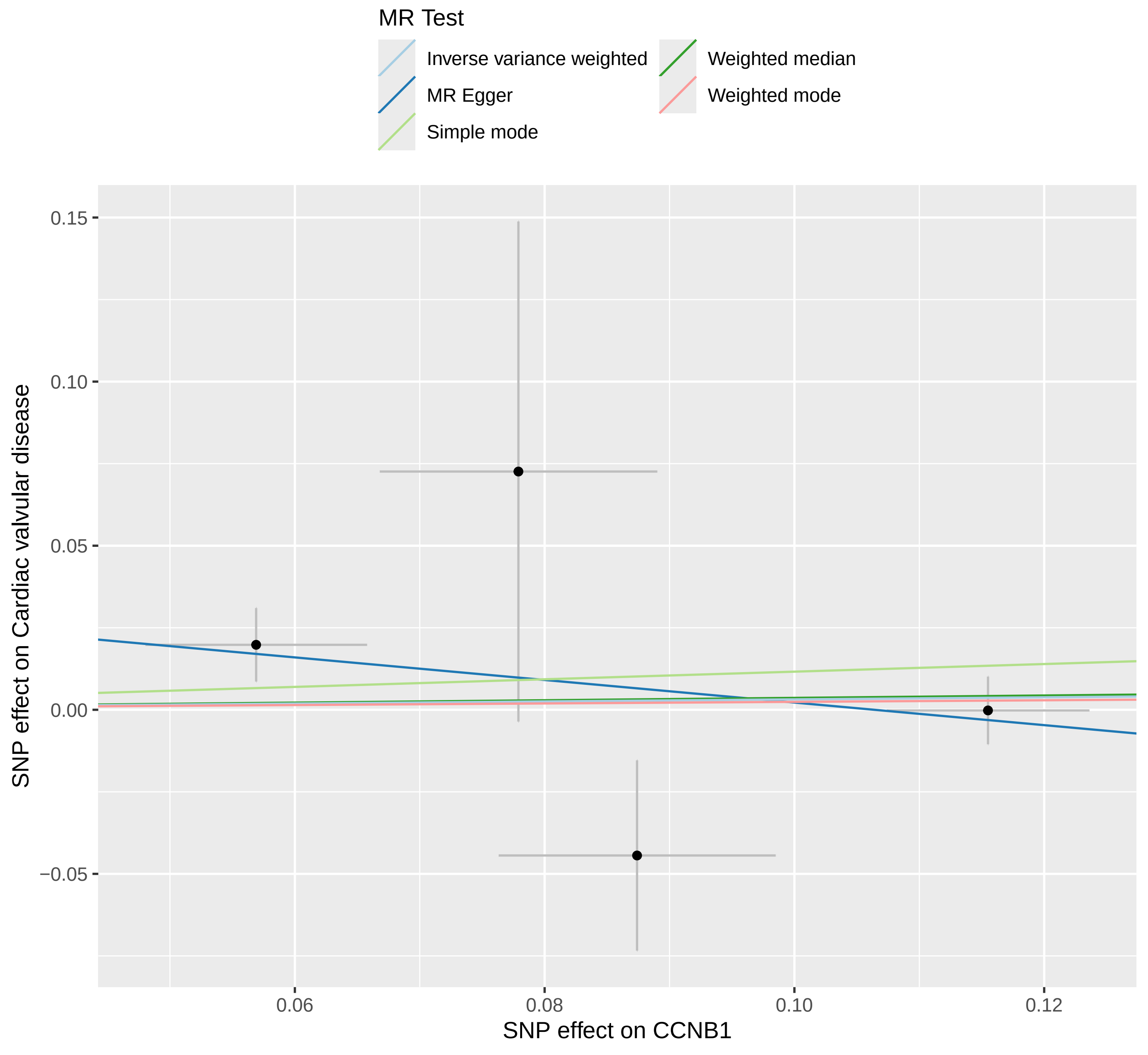

MR Test
Inverse variance weighted
MR Egger
Simple mode
Weighted median
Weighted mode
0.15
0.10
0.05
0.00
−0.05
0.06
0.08
0.10
0.12
SNP effect on Cardiac valvular disease
SNP effect on CCNB1

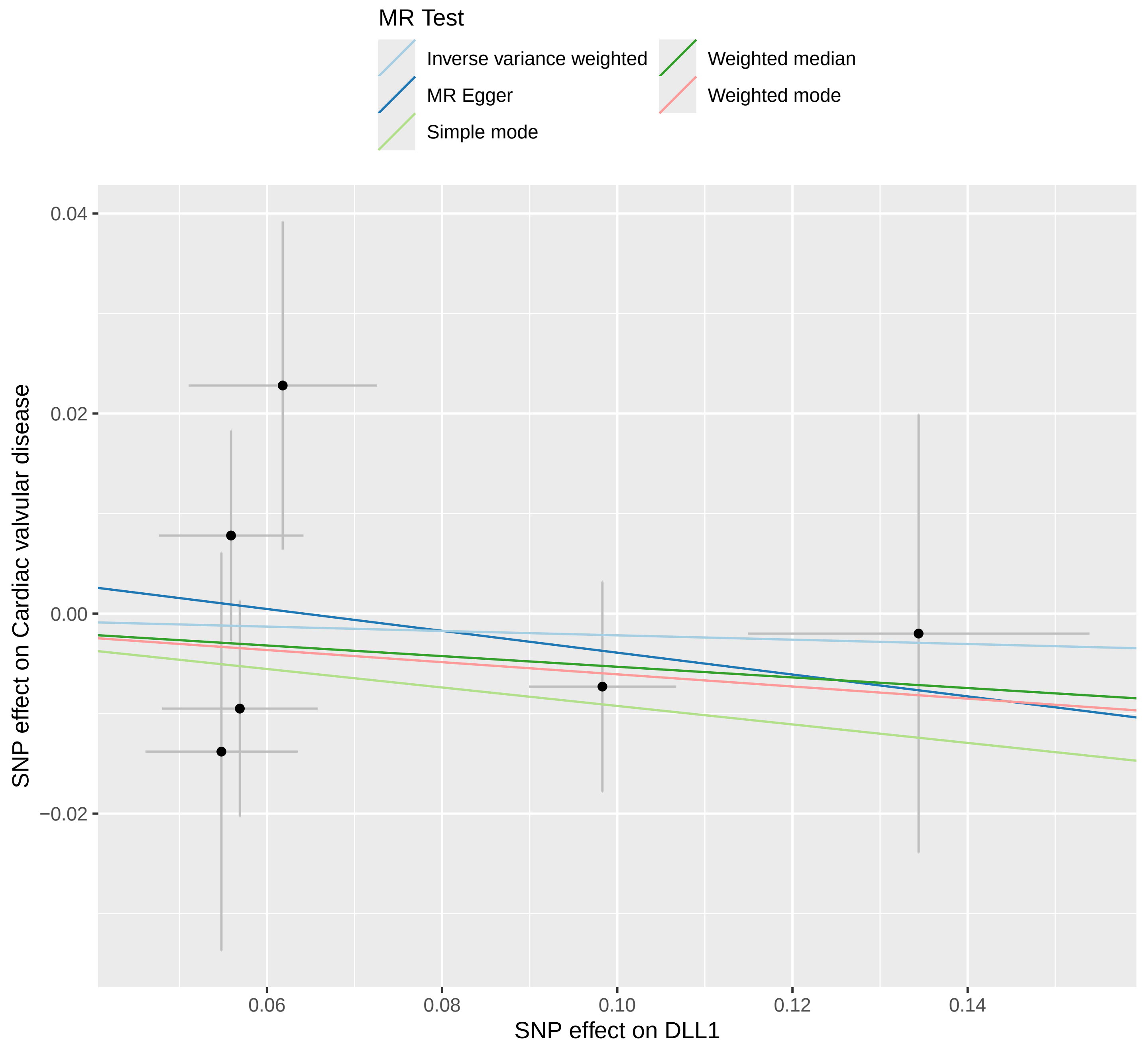
MR Test
Inverse variance weighted
MR Egger
Simple mode
Weighted median
Weighted mode
0.04
0.02
0.00
−0.02
SNP effect on Cardiac valvular disease
0.06
0.08
0.10
0.12
0.14
SNP effect on DLL1

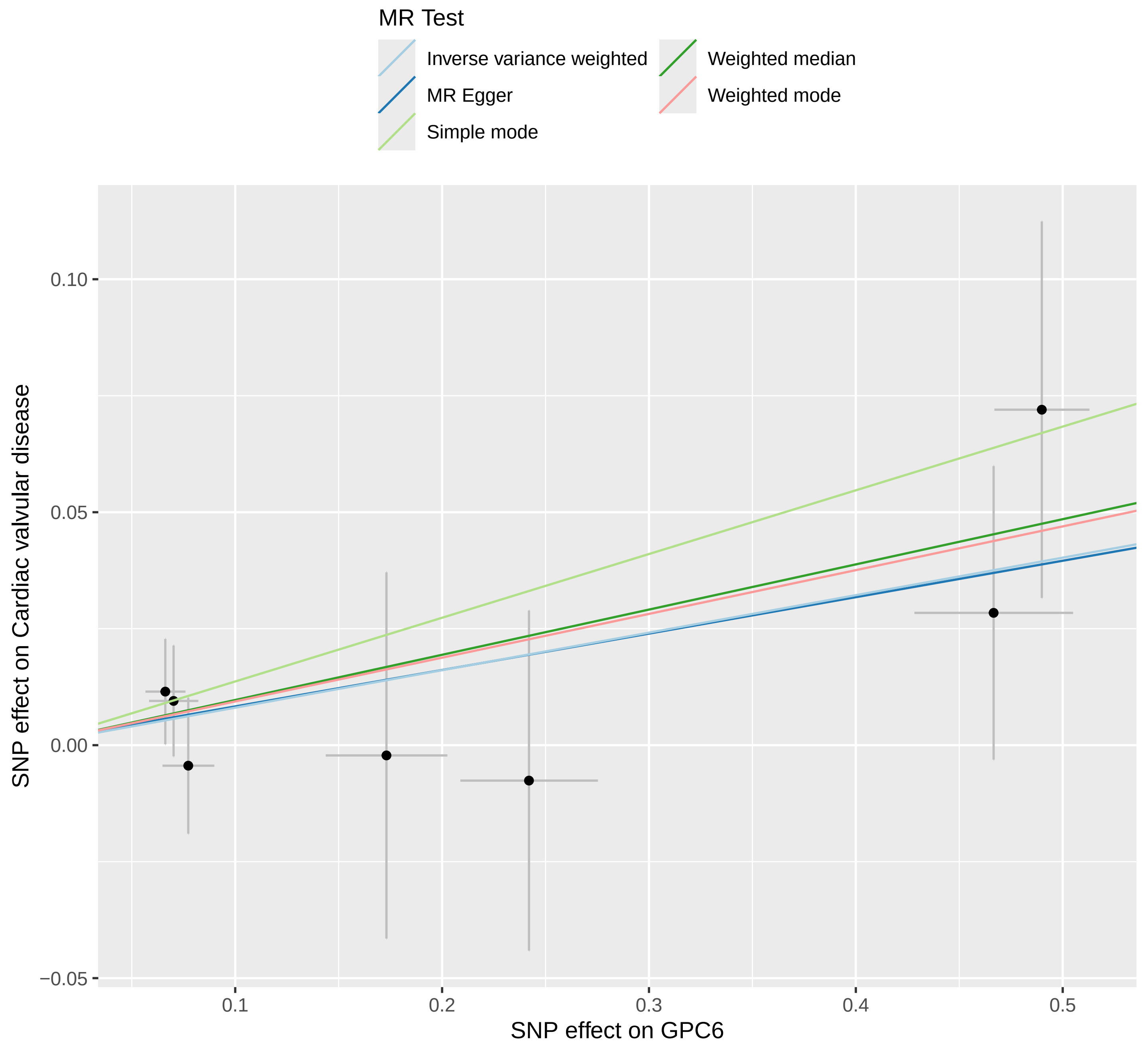

MR Test
Inverse variance weighted
MR Egger
Simple mode
Weighted median
Weighted mode
0.10
0.05
0.00
−0.05
0.1
0.2
0.3
0.4
0.5
SNP effect on Cardiac valvular disease
SNP effect on GPC6

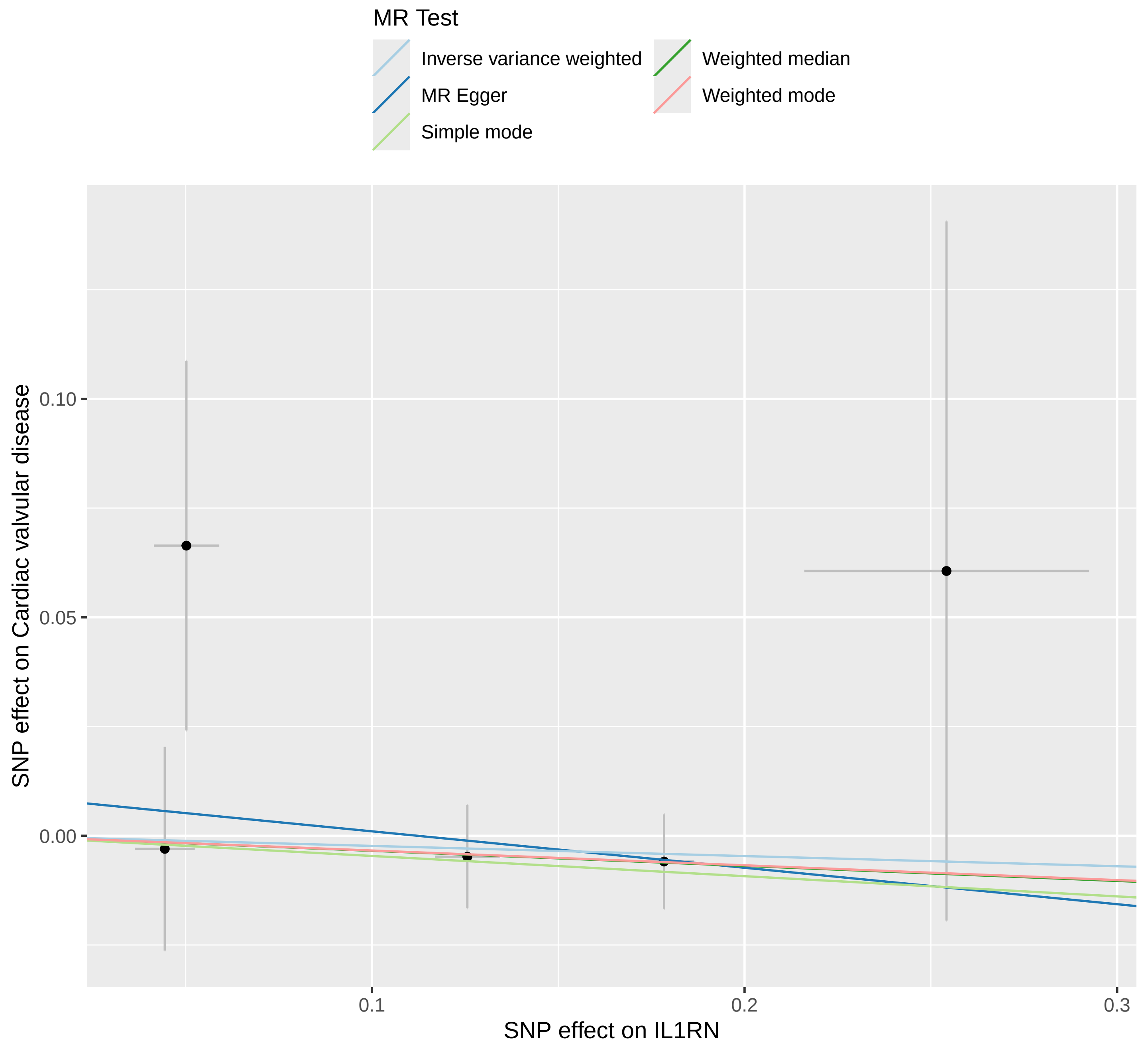
MR Test
Inverse variance weighted
MR Egger
Simple mode
Weighted median
Weighted mode
SNP effect on Cardiac valvular disease
0.10
0.05
0.00
0.1
0.2
0.3
SNP effect on IL1RN

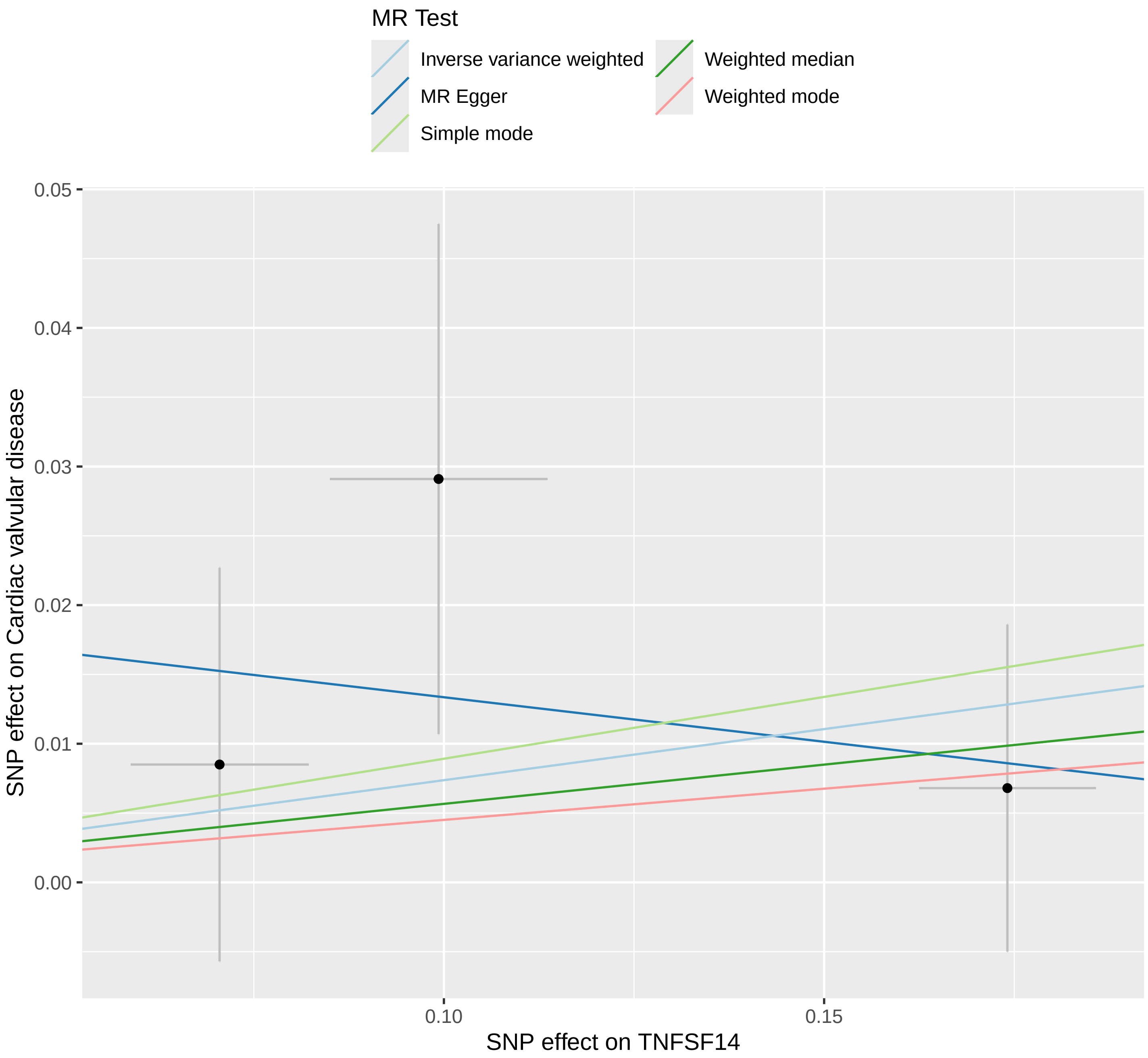

MR Test
Inverse variance weighted
MR Egger
Simple mode
Weighted median
Weighted mode
0.05
0.04
0.03
0.02
0.01
0.00
SNP effect on Cardiac valvular disease
0.10
0.15
SNP effect on TNFSF14

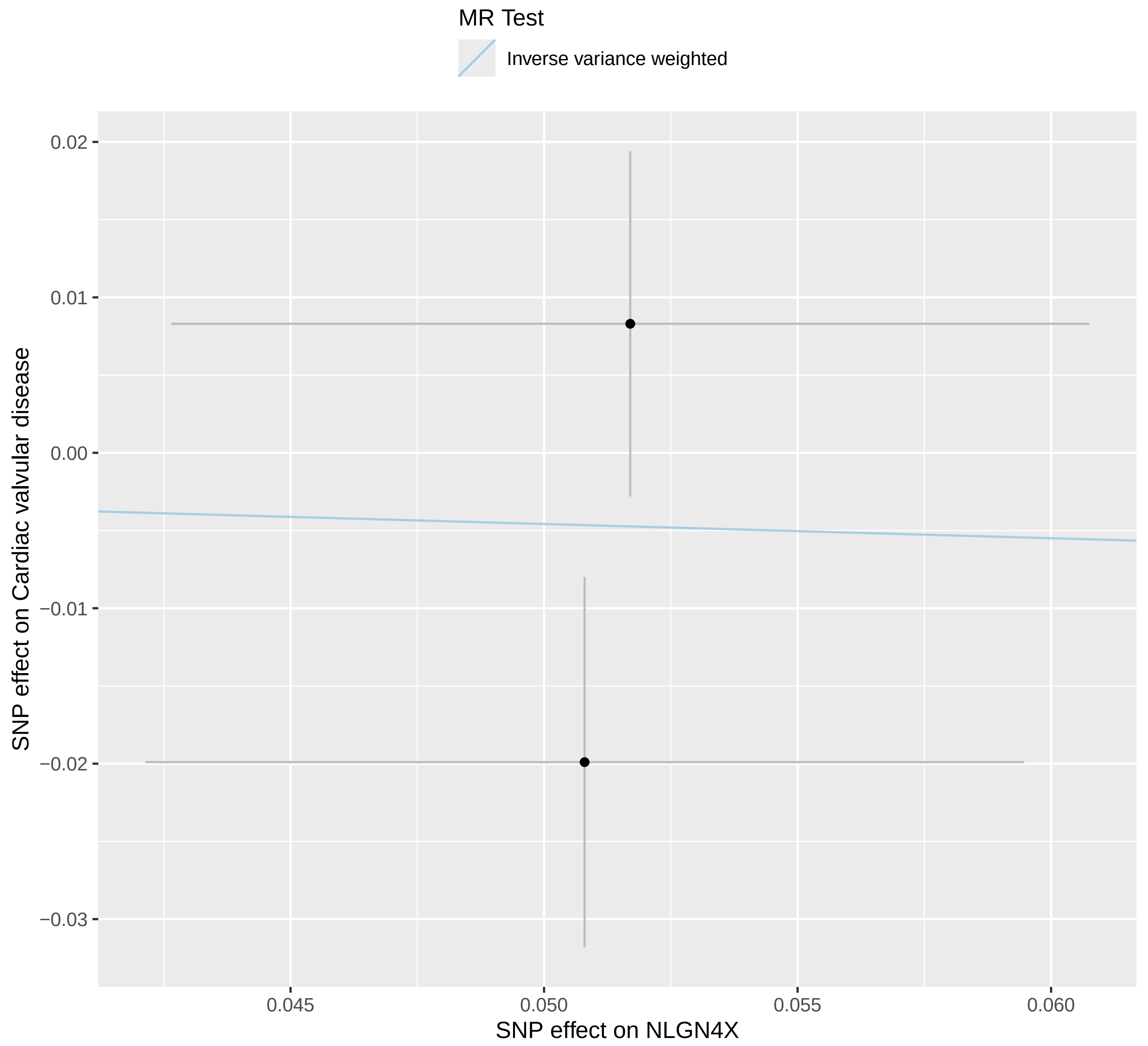
MR Test
Inverse variance weighted
0.02
0.01
0.00
−0.01
−0.02
−0.03
0.045
0.050
0.055
0.060
SNP effect on Cardiac valvular disease
SNP effect on NLGN4X

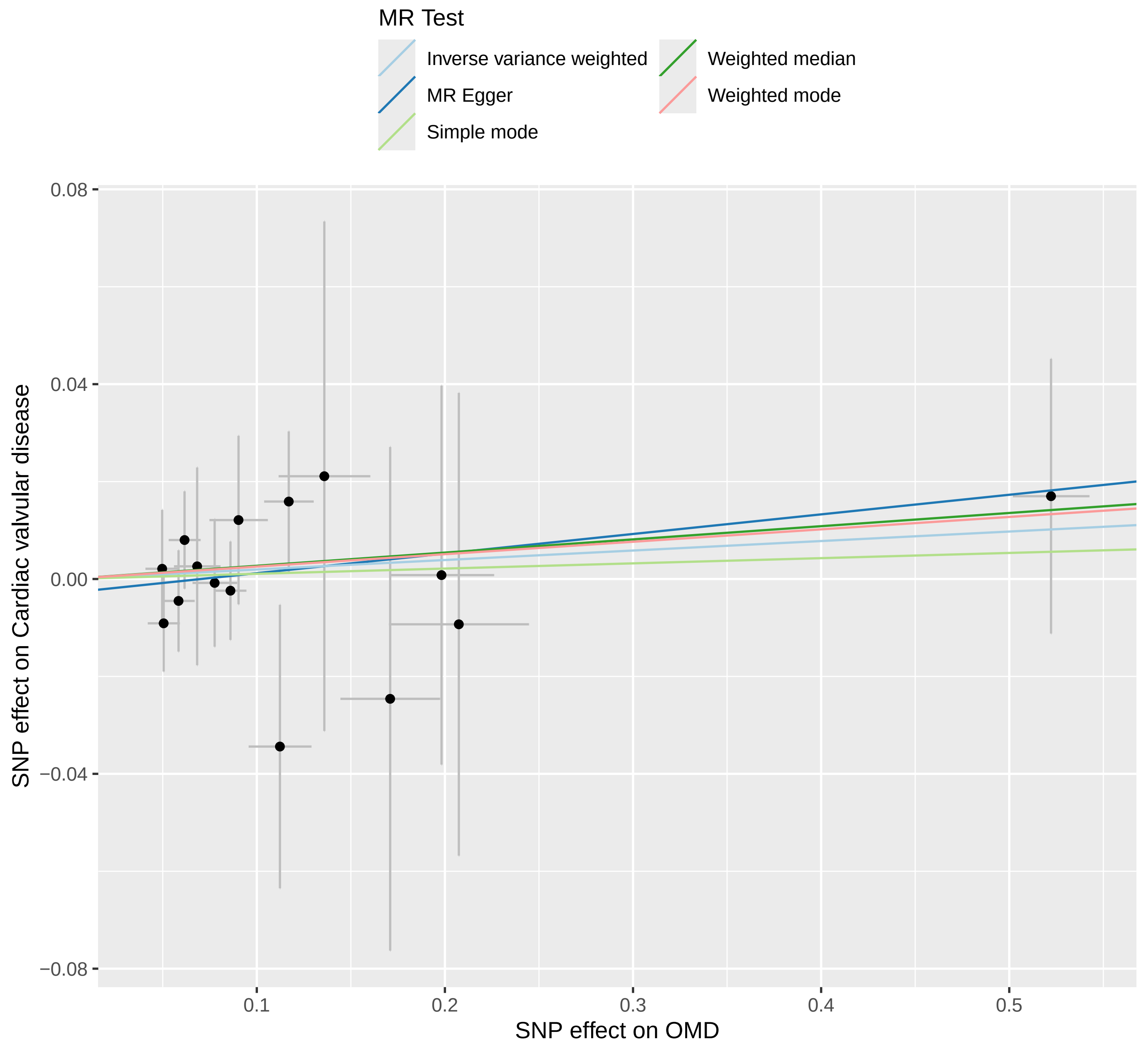

MR Test
Inverse variance weighted
MR Egger
Simple mode
Weighted median
Weighted mode
0.08
0.04
0.00
−0.04
−0.08
0.1
0.2
0.3
0.4
0.5
SNP effect on Cardiac valvular disease
SNP effect on OMD

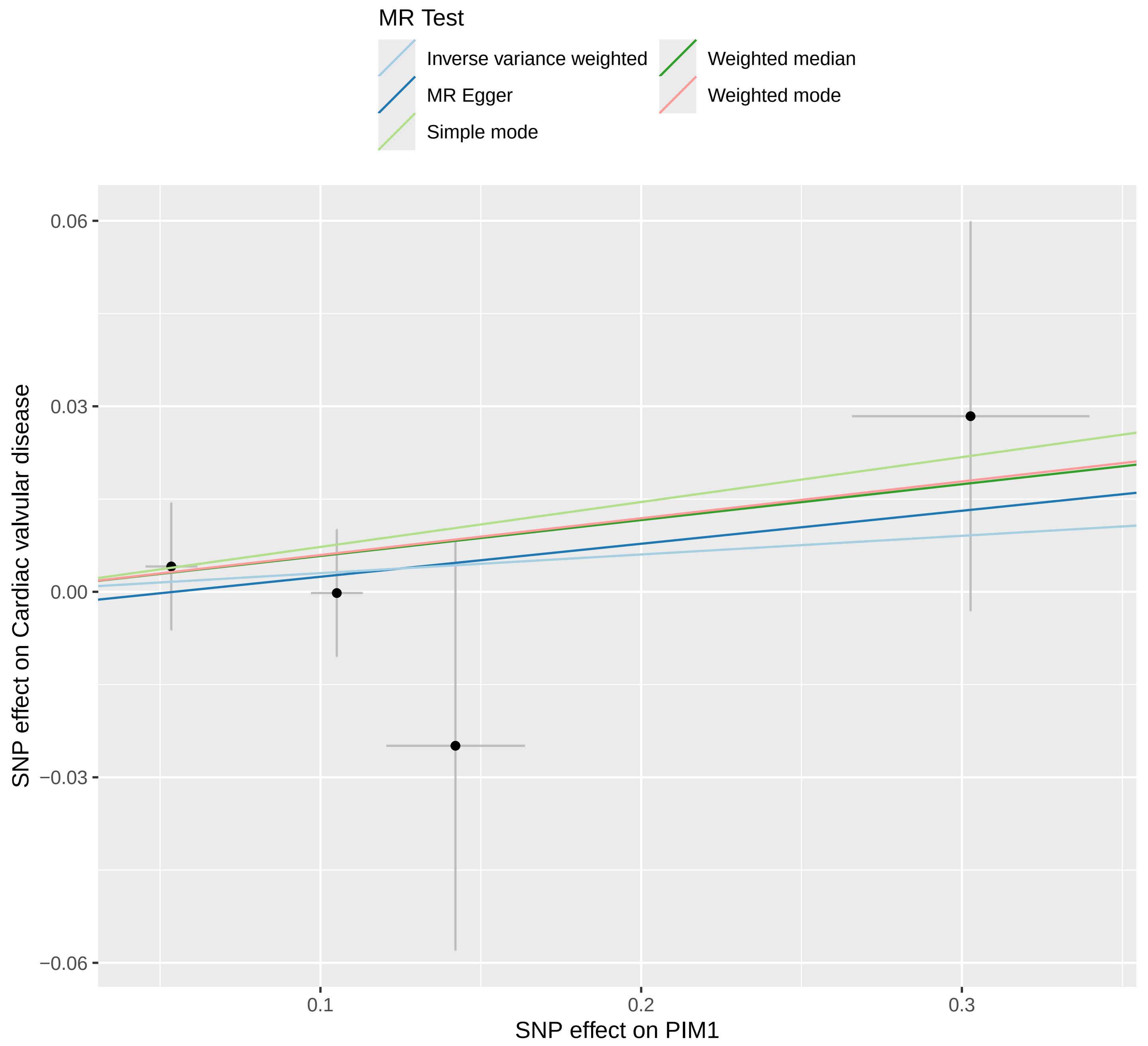
MR Test
Inverse variance weighted
MR Egger
Simple mode
Weighted median
Weighted mode
0.06
0.03
0.00
−0.03
−0.06
0.1
0.2
0.3
SNP effect on Cardiac valvular disease
SNP effect on PIM1

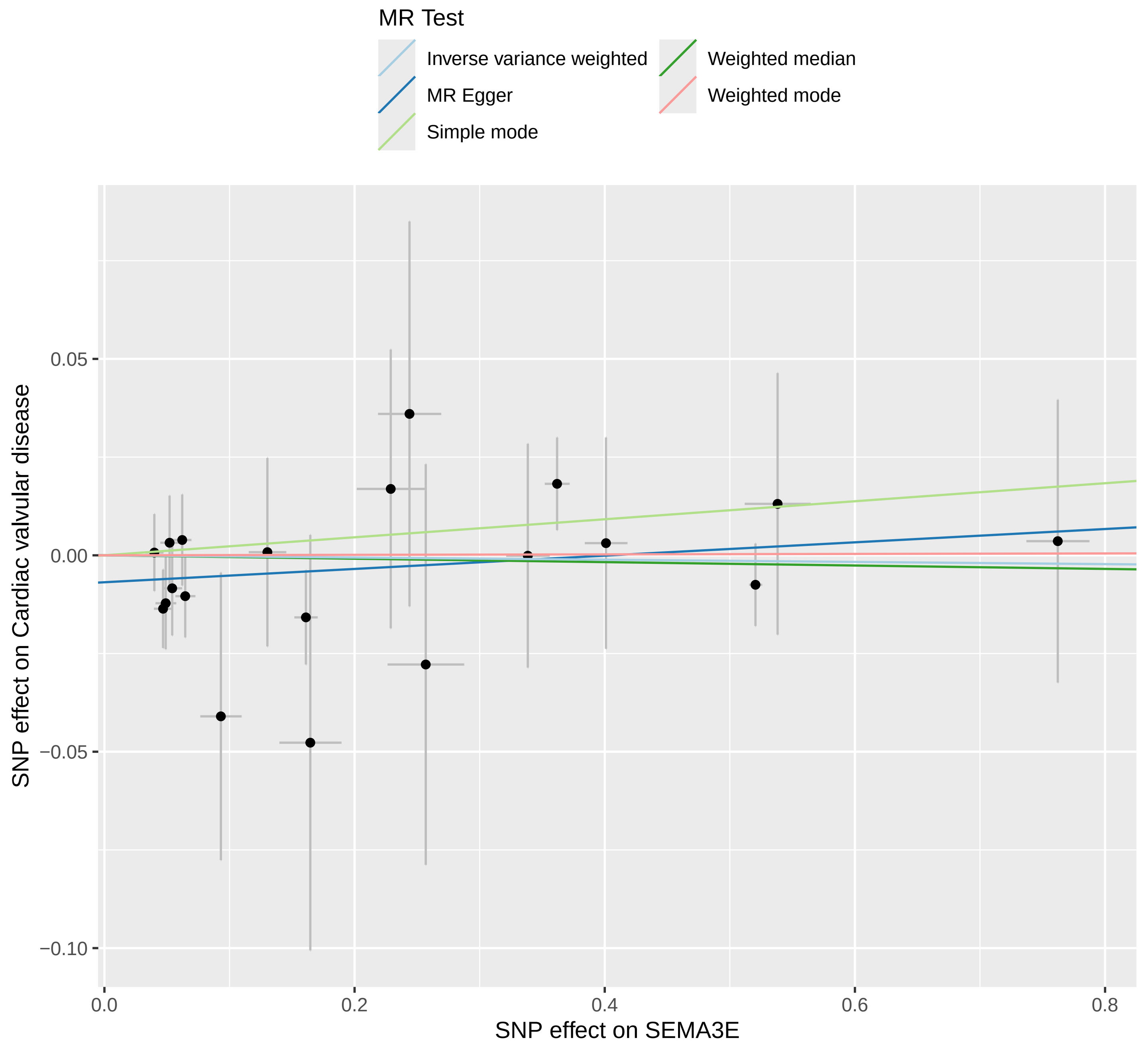
MR Test
Inverse variance weighted
MR Egger
Simple mode
Weighted median
Weighted mode
0.05
0.00
−0.05
−0.10
SNP effect on Cardiac valvular disease
0.0
0.2
0.4
0.6
0.8
SNP effect on SEMA3E

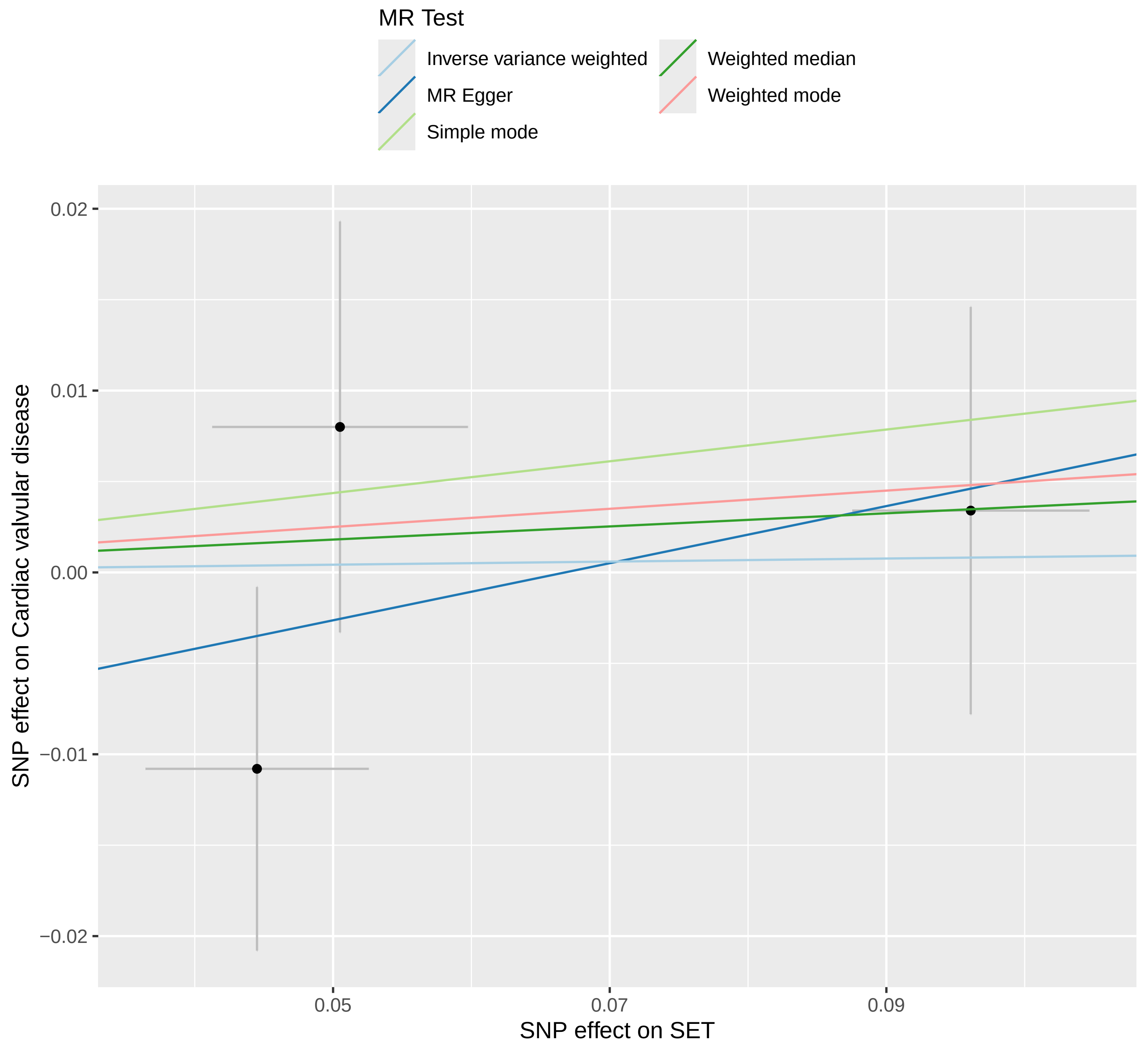
MR Test
Inverse variance weighted
MR Egger
Simple mode
Weighted median
Weighted mode
0.02
0.01
0.00
−0.01
−0.02
SNP effect on Cardiac valvular disease
0.05
0.07
0.09
SNP effect on SET

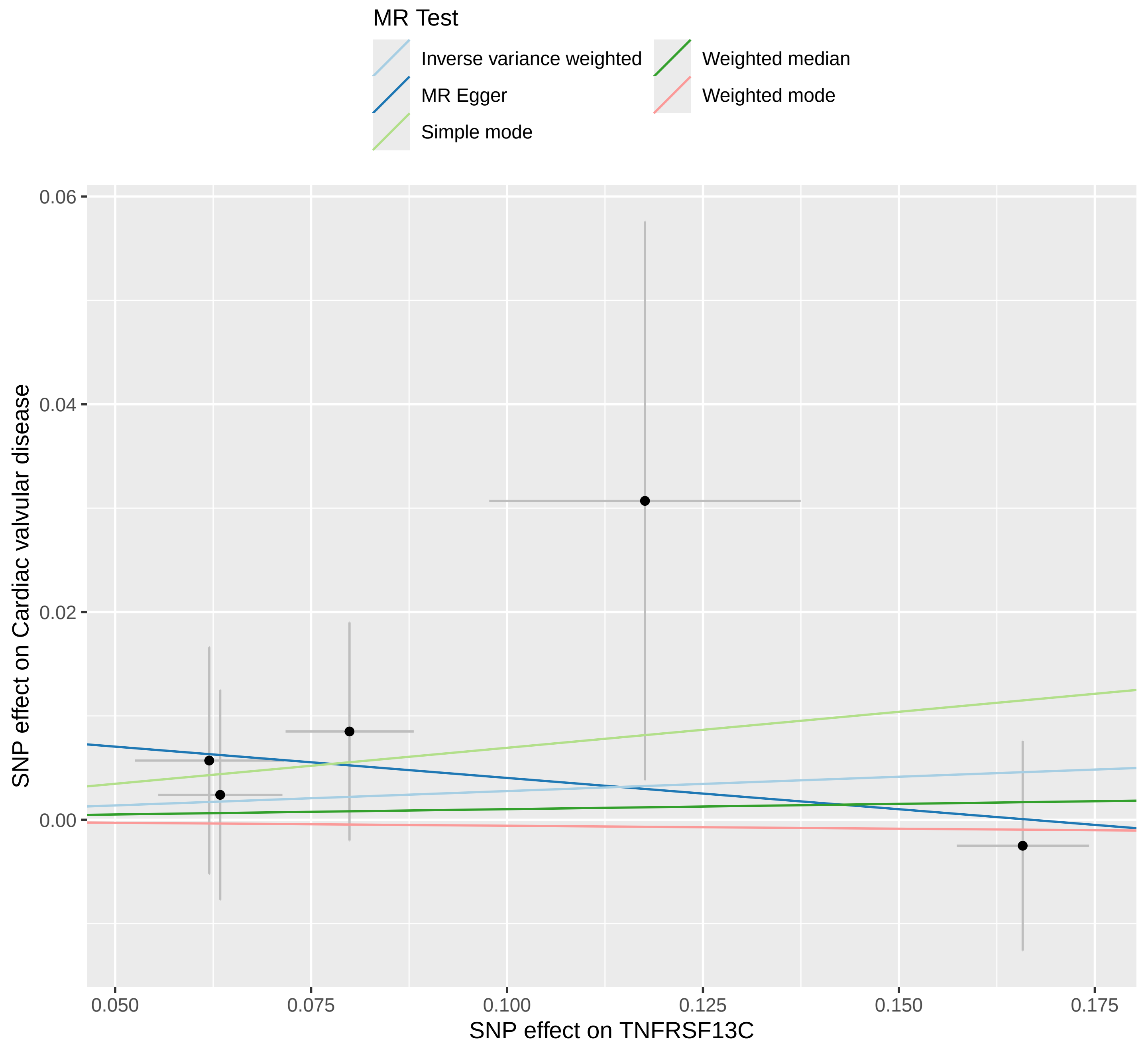

MR Test
Inverse variance weighted
MR Egger
Simple mode
Weighted median
Weighted mode
0.06
0.04
0.02
0.00
0.050
0.075
0.100
0.125
0.150
0.175
SNP effect on Cardiac valvular disease
SNP effect on TNFRSF13C

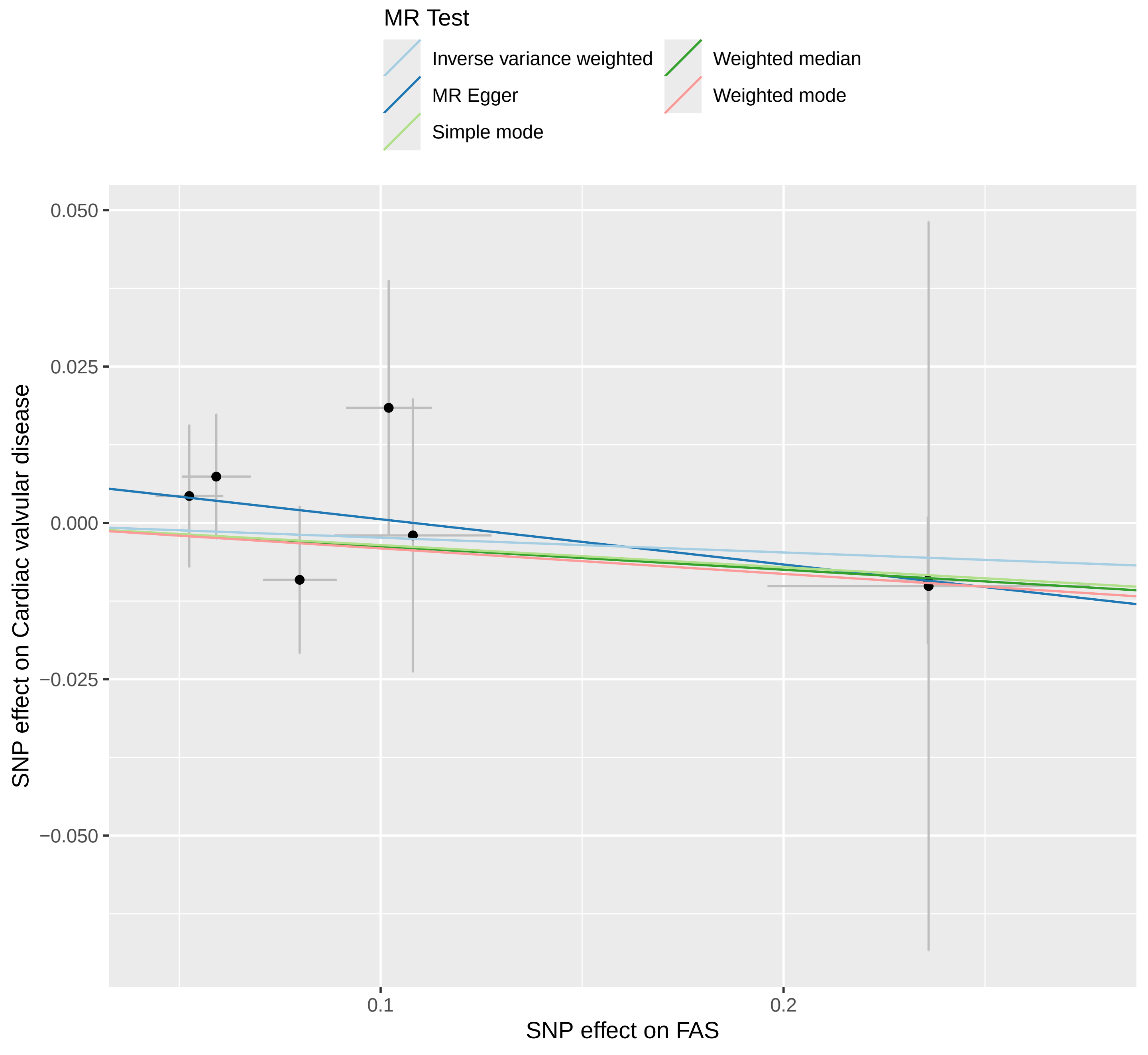

MR Test
Inverse variance weighted
MR Egger
Simple mode
Weighted median
Weighted mode
0.050
0.025
0.000
−0.025
−0.050
0.1
0.2
SNP effect on FAS
SNP effect on Cardiac valvular disease

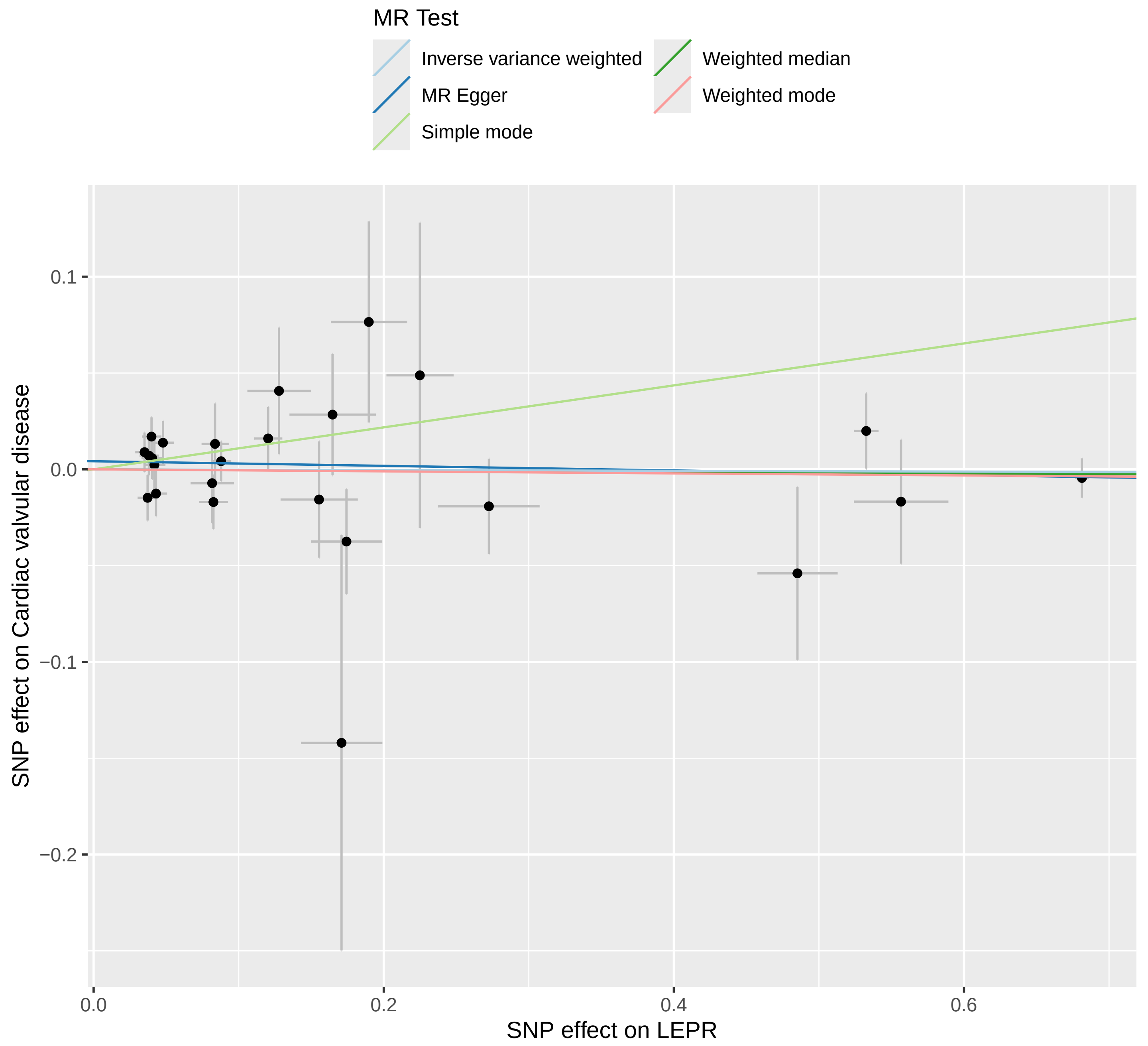
MR Test
Inverse variance weighted
MR Egger
Simple mode
Weighted median
Weighted mode
0.1
0.0
−0.1
−0.2
SNP effect on Cardiac valvular disease
0.0
0.2
0.4
0.6
SNP effect on LEPR

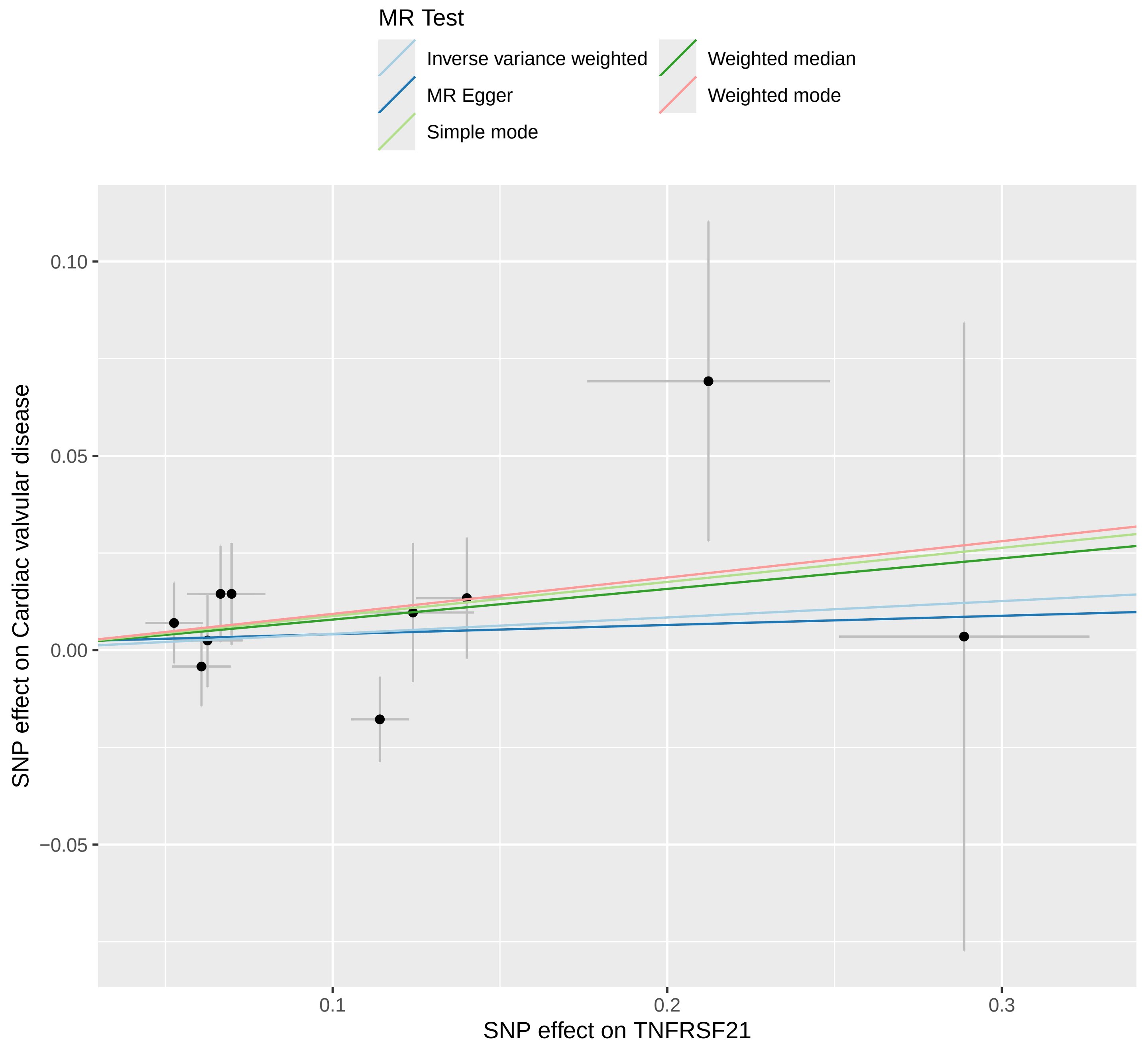
MR Test
Inverse variance weighted
MR Egger
Simple mode
Weighted median
Weighted mode
0.10
0.05
0.00
−0.05
0.1
0.2
0.3
SNP effect on Cardiac valvular disease
SNP effect on TNFRSF21

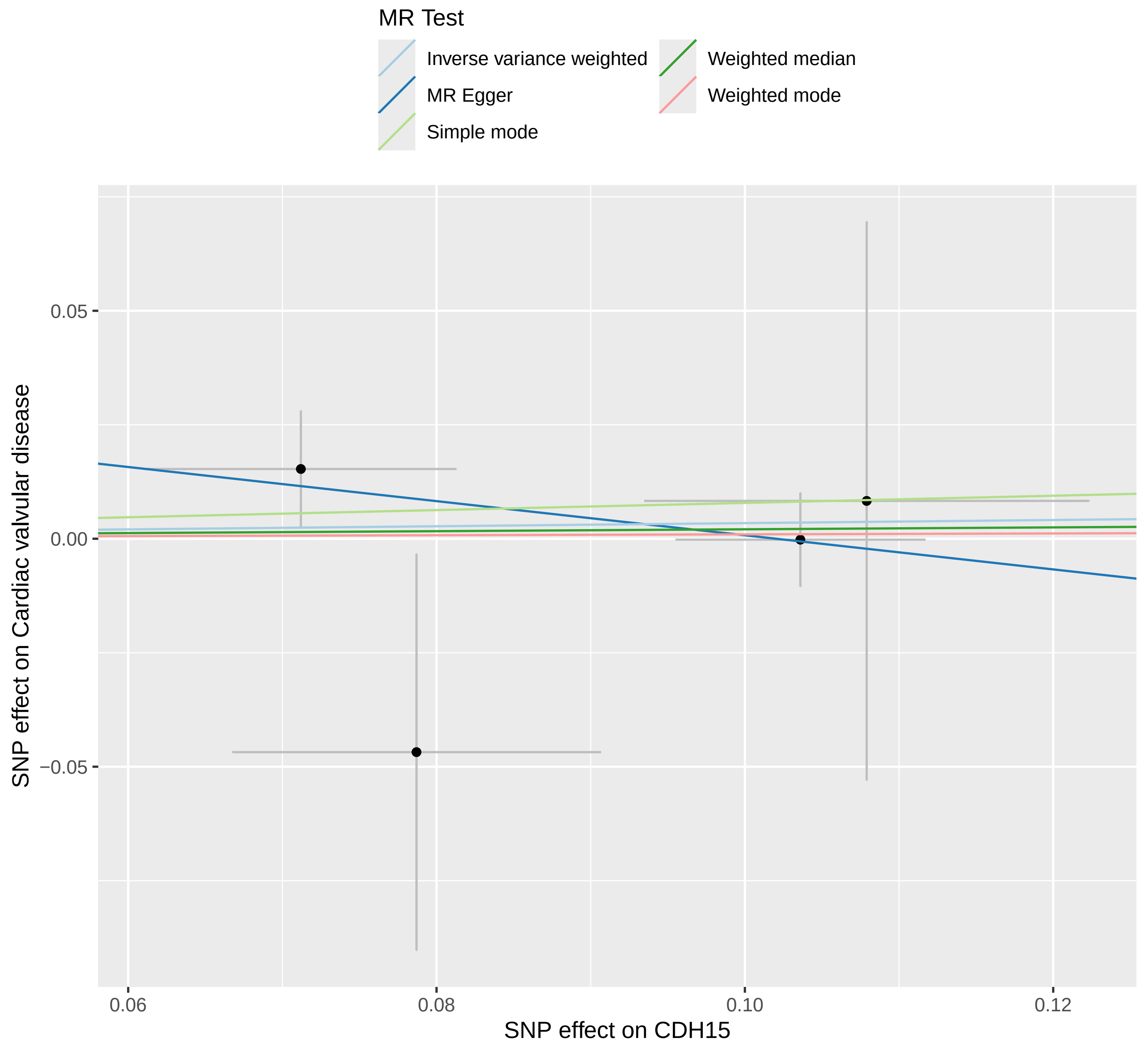

MR Test
Inverse variance weighted
MR Egger
Simple mode
Weighted median
Weighted mode
0.05
0.00
−0.05
0.06
0.08
0.10
0.12
SNP effect on CDH15
SNP effect on Cardiac valvular disease

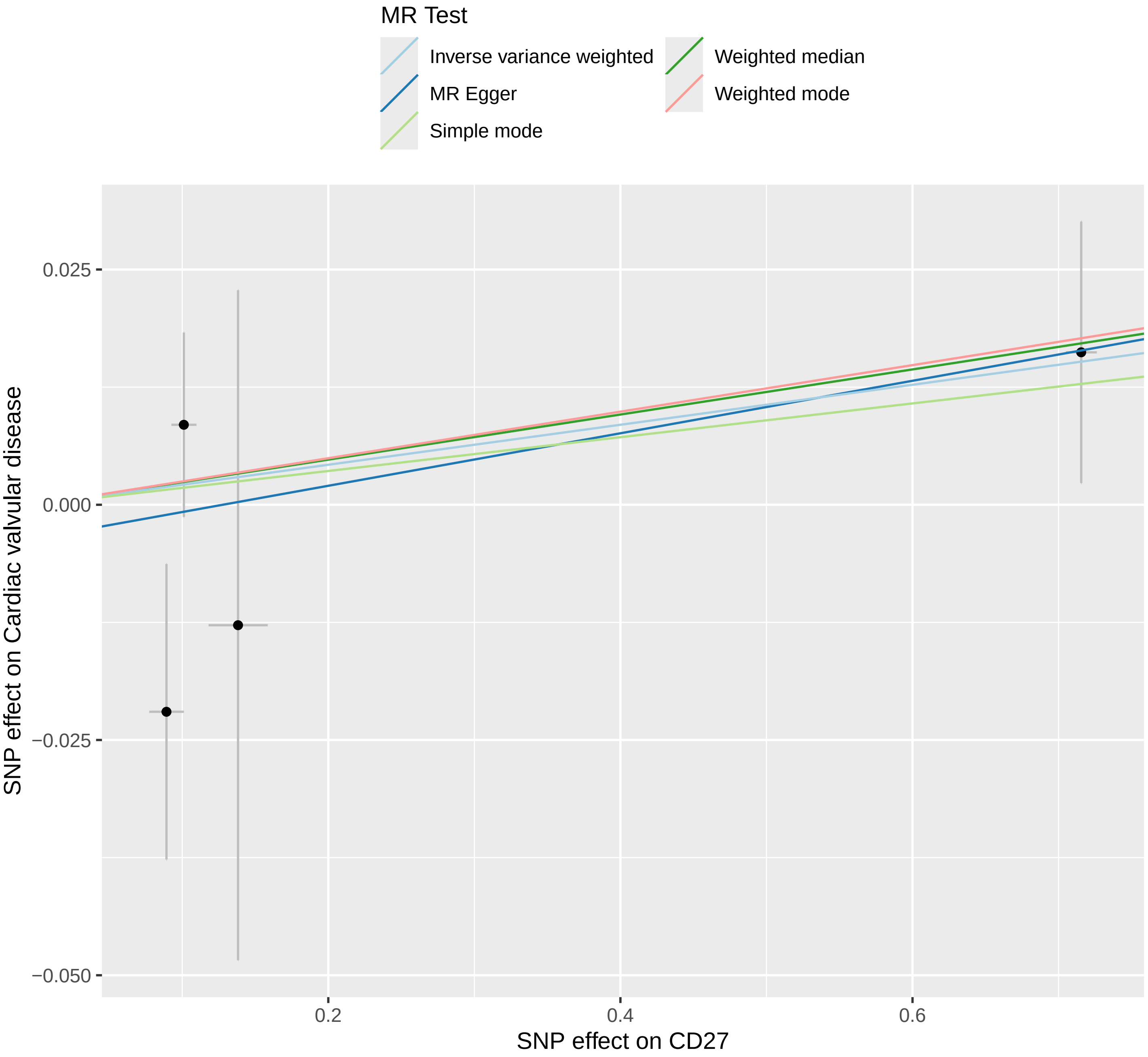

MR Test
Inverse variance weighted
MR Egger
Simple mode
Weighted median
Weighted mode
0.025
0.000
−0.025
−0.050
0.2
0.4
0.6
SNP effect on CD27
SNP effect on Cardiac valvular disease

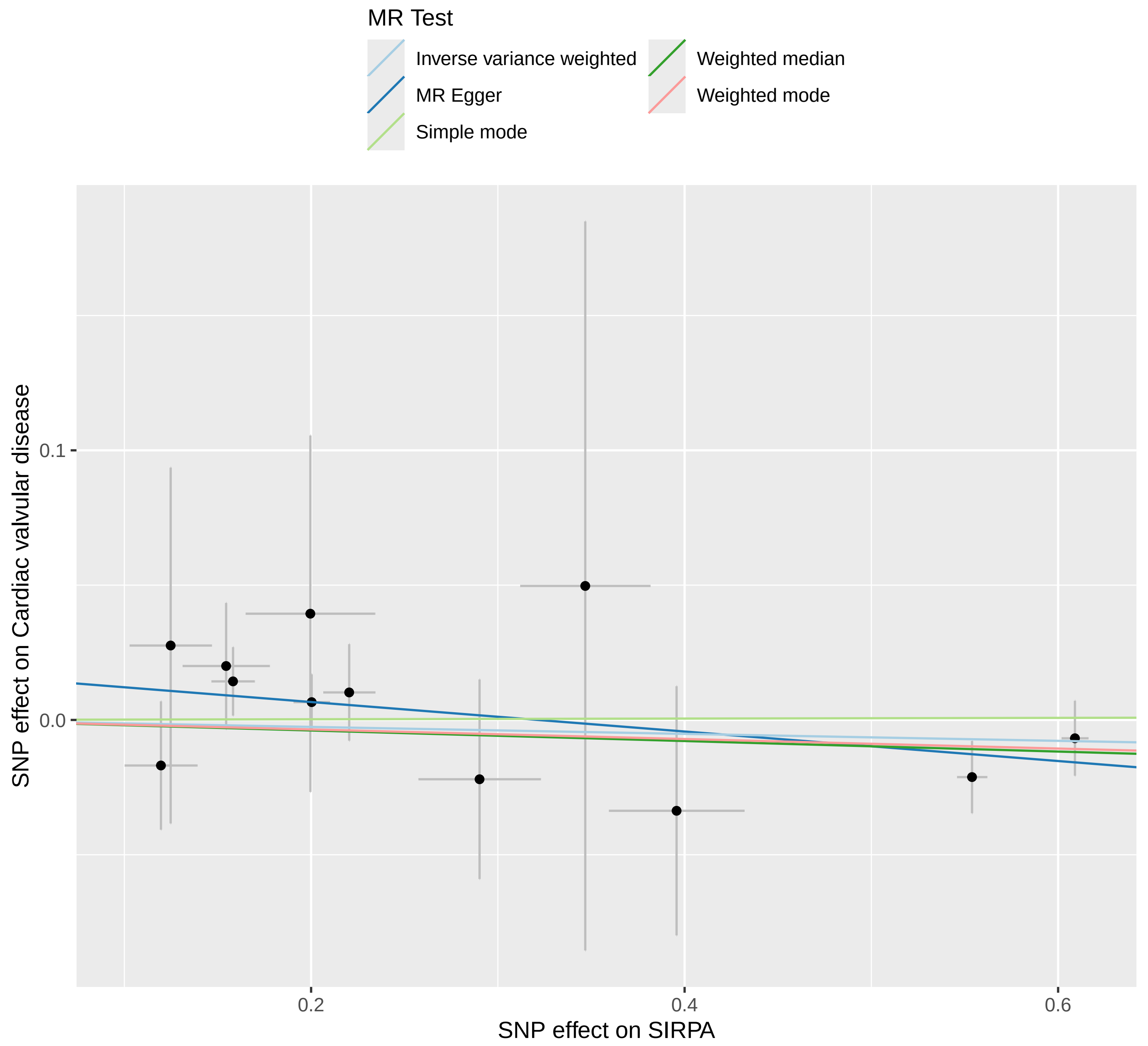
MR Test
Inverse variance weighted
MR Egger
Simple mode
Weighted median
Weighted mode
SNP effect on Cardiac valvular disease
0.1
0.0
0.2
0.4
0.6
SNP effect on SIRPA

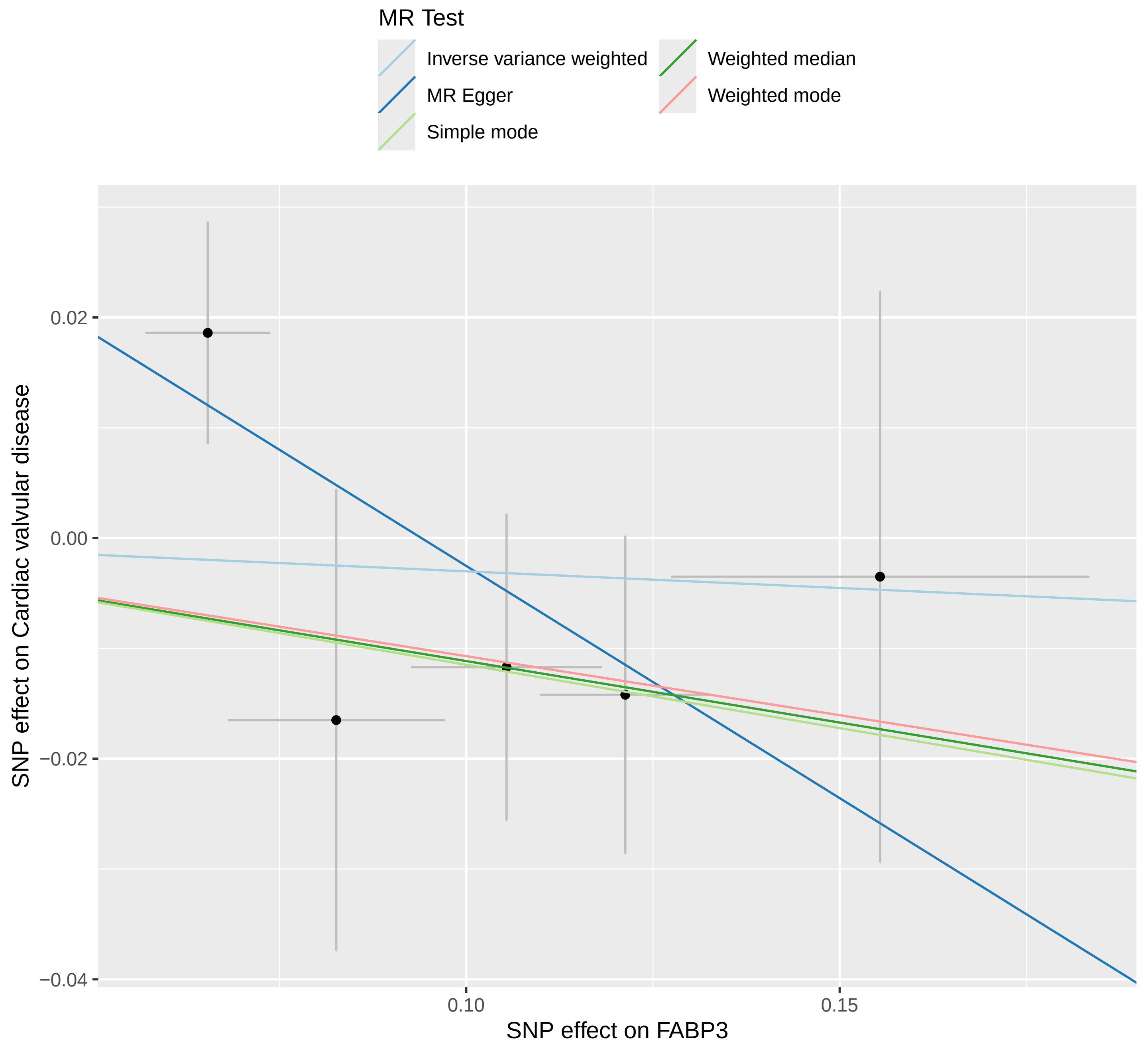

MR Test
Inverse variance weighted
MR Egger
Simple mode
Weighted median
Weighted mode
0.02
0.00
−0.02
−0.04
0.10
0.15
SNP effect on Cardiac valvular disease
SNP effect on FABP3

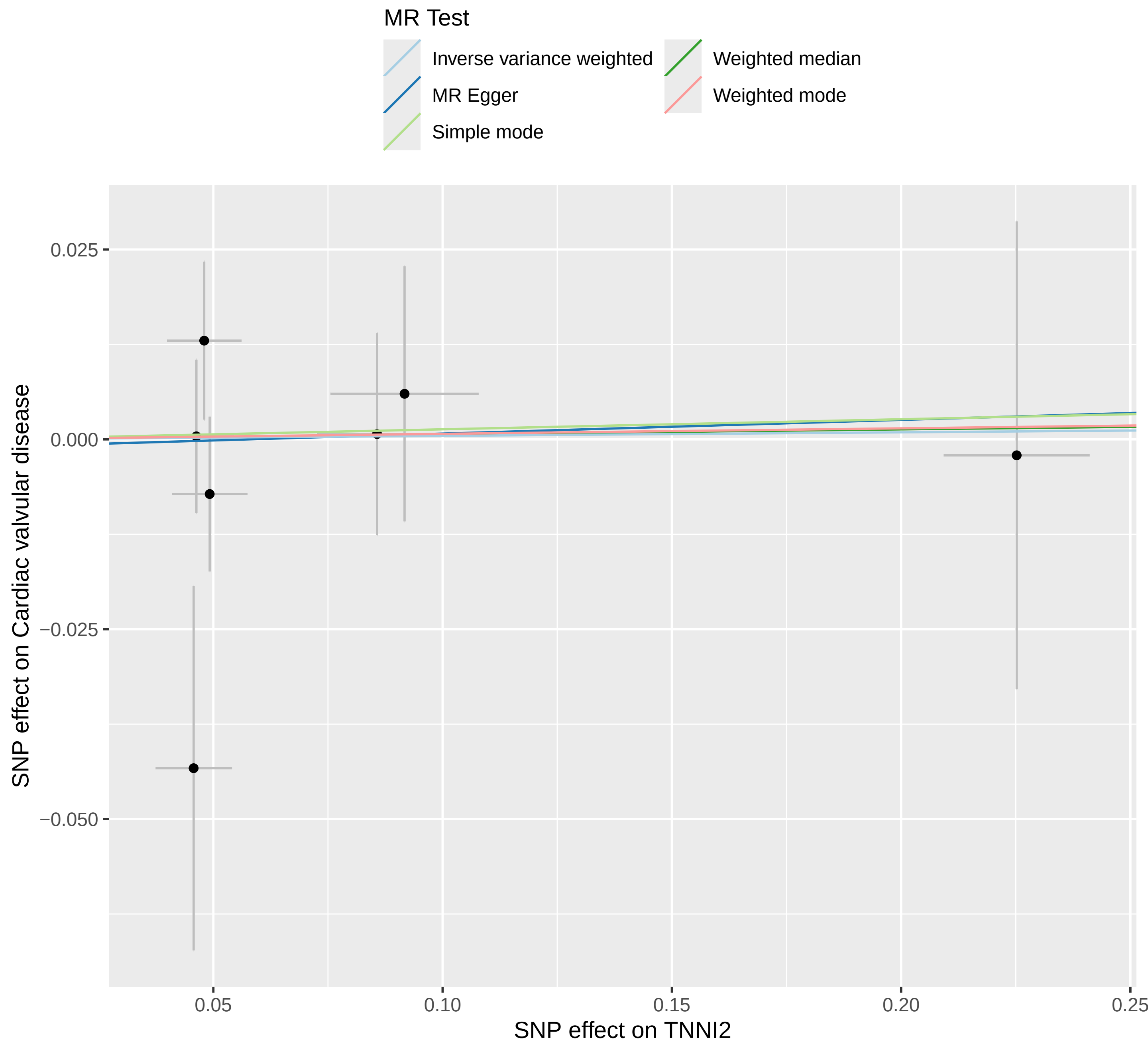
MR Test
Inverse variance weighted
MR Egger
Simple mode
Weighted median
Weighted mode
0.025
0.000
−0.025
−0.050
SNP effect on Cardiac valvular disease
0.05
0.10
0.15
0.20
0.25
SNP effect on TNNI2

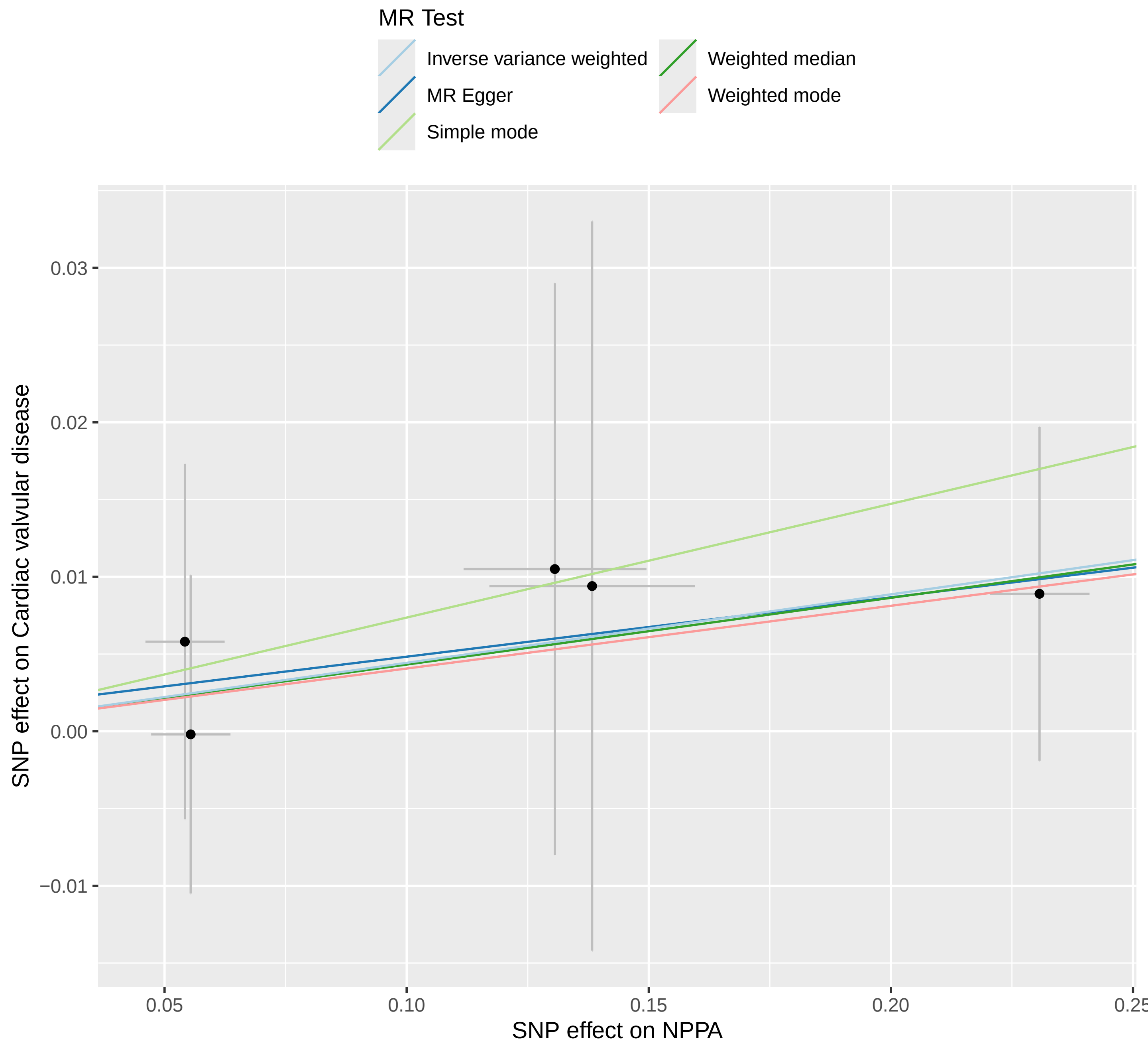

MR Test
Inverse variance weighted
MR Egger
Simple mode
Weighted median
Weighted mode
0.03
0.02
0.01
0.00
−0.01
SNP effect on Cardiac valvular disease
0.05
0.10
0.15
0.20
0.25
SNP effect on NPPA

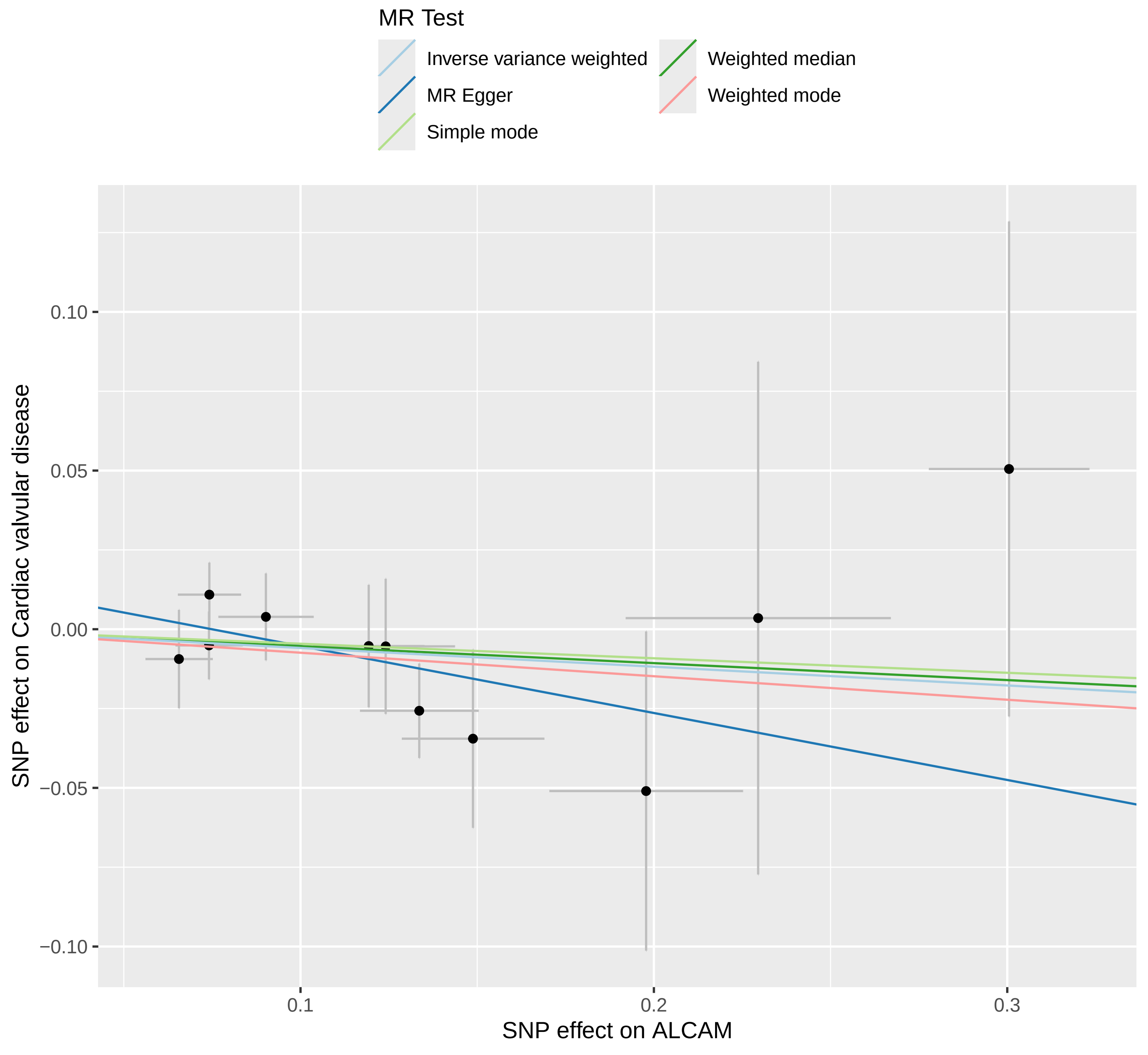
MR Test
Inverse variance weighted
MR Egger
Simple mode
Weighted median
Weighted mode
SNP effect on Cardiac valvular disease
0.10
0.05
0.00
−0.05
−0.10
0.1
0.2
0.3
SNP effect on ALCAM

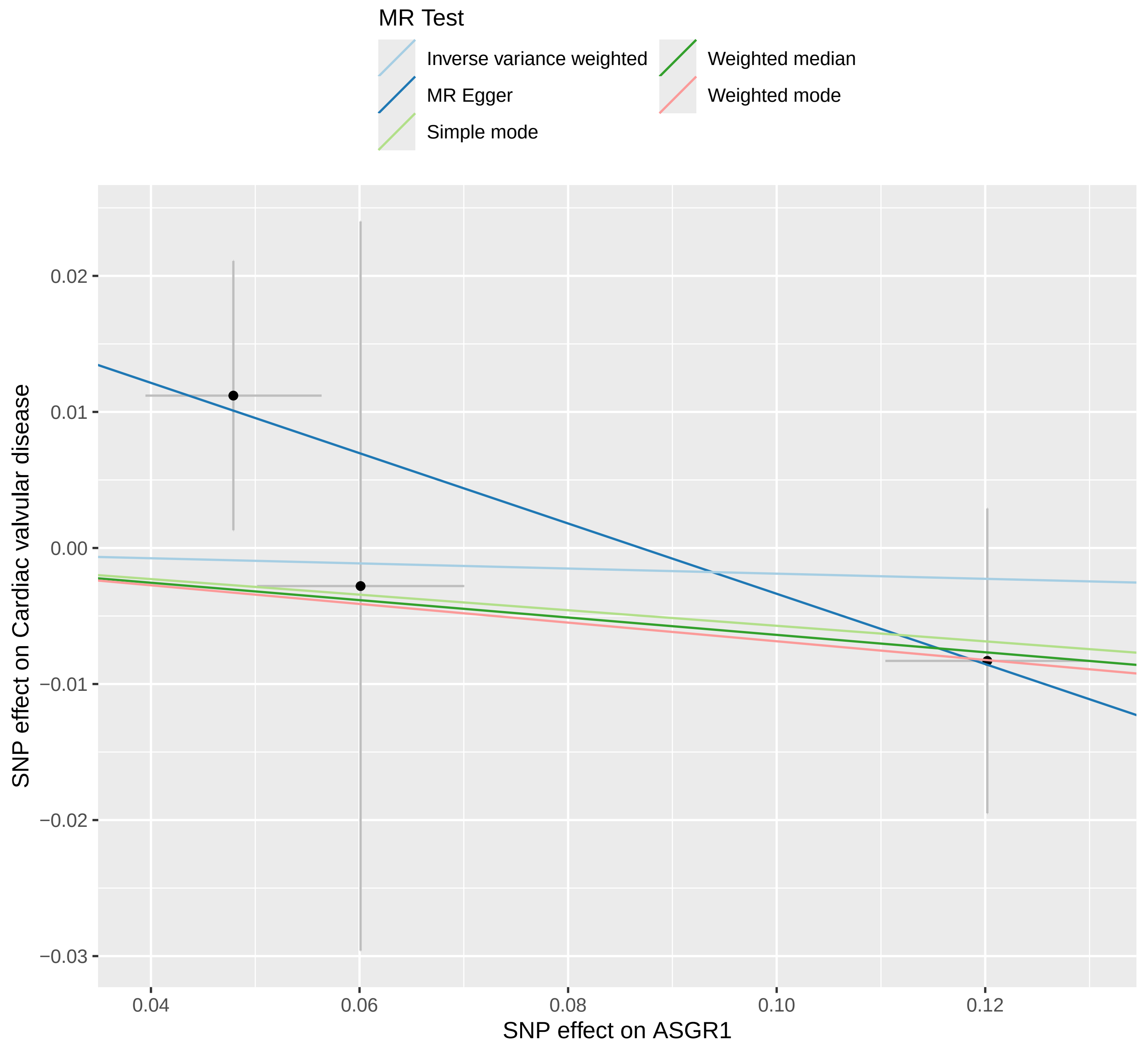

MR Test
Inverse variance weighted
MR Egger
Simple mode
Weighted median
Weighted mode
0.02
0.01
0.00
−0.01
−0.02
−0.03
SNP effect on Cardiac valvular disease
0.04
0.06
0.08
0.10
0.12
SNP effect on ASGR1

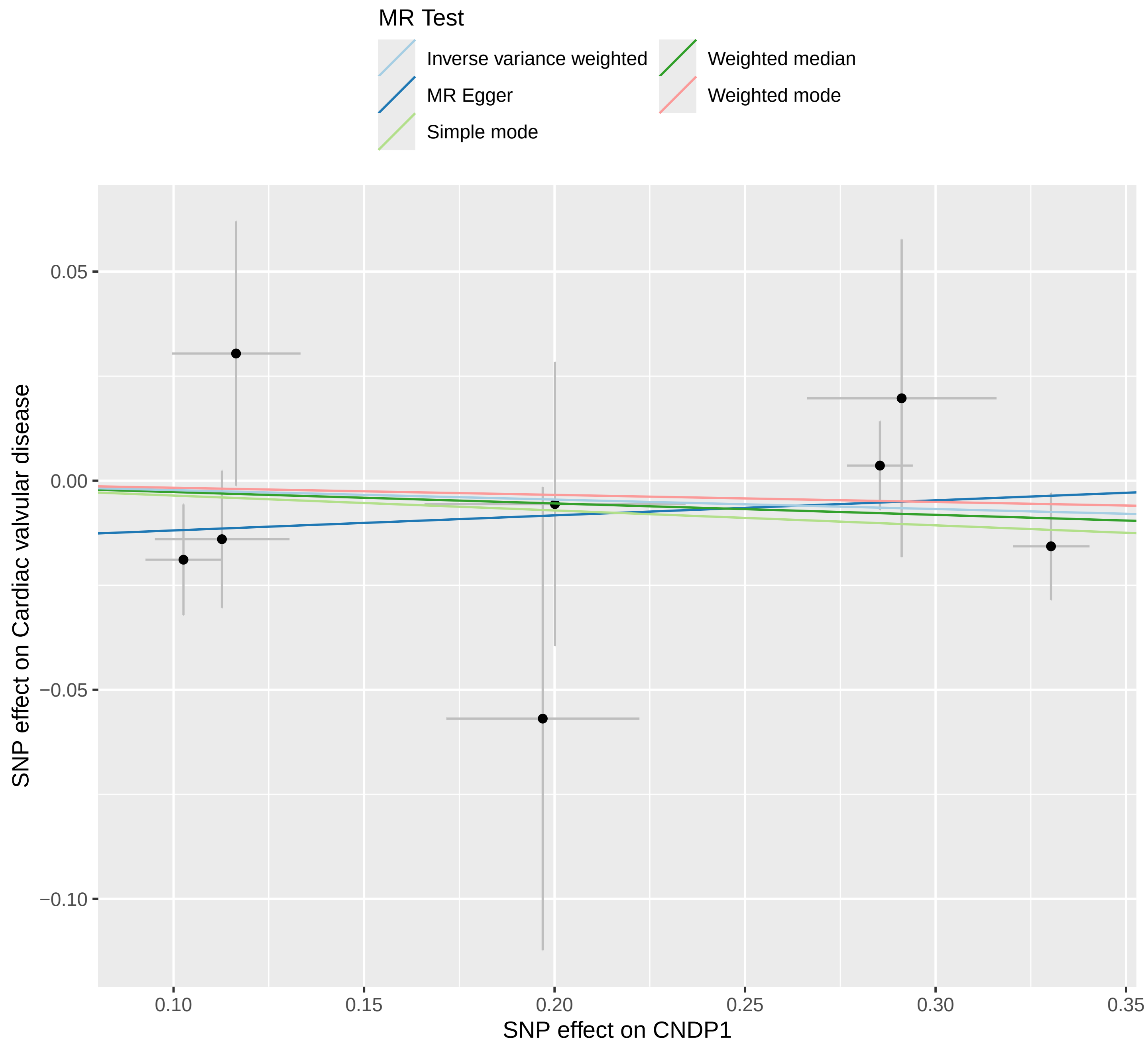

MR Test
Inverse variance weighted
MR Egger
Simple mode
Weighted median
Weighted mode
0.05
0.00
−0.05
−0.10
0.10
0.15
0.20
0.25
0.30
0.35
SNP effect on CNDP1
SNP effect on Cardiac valvular disease

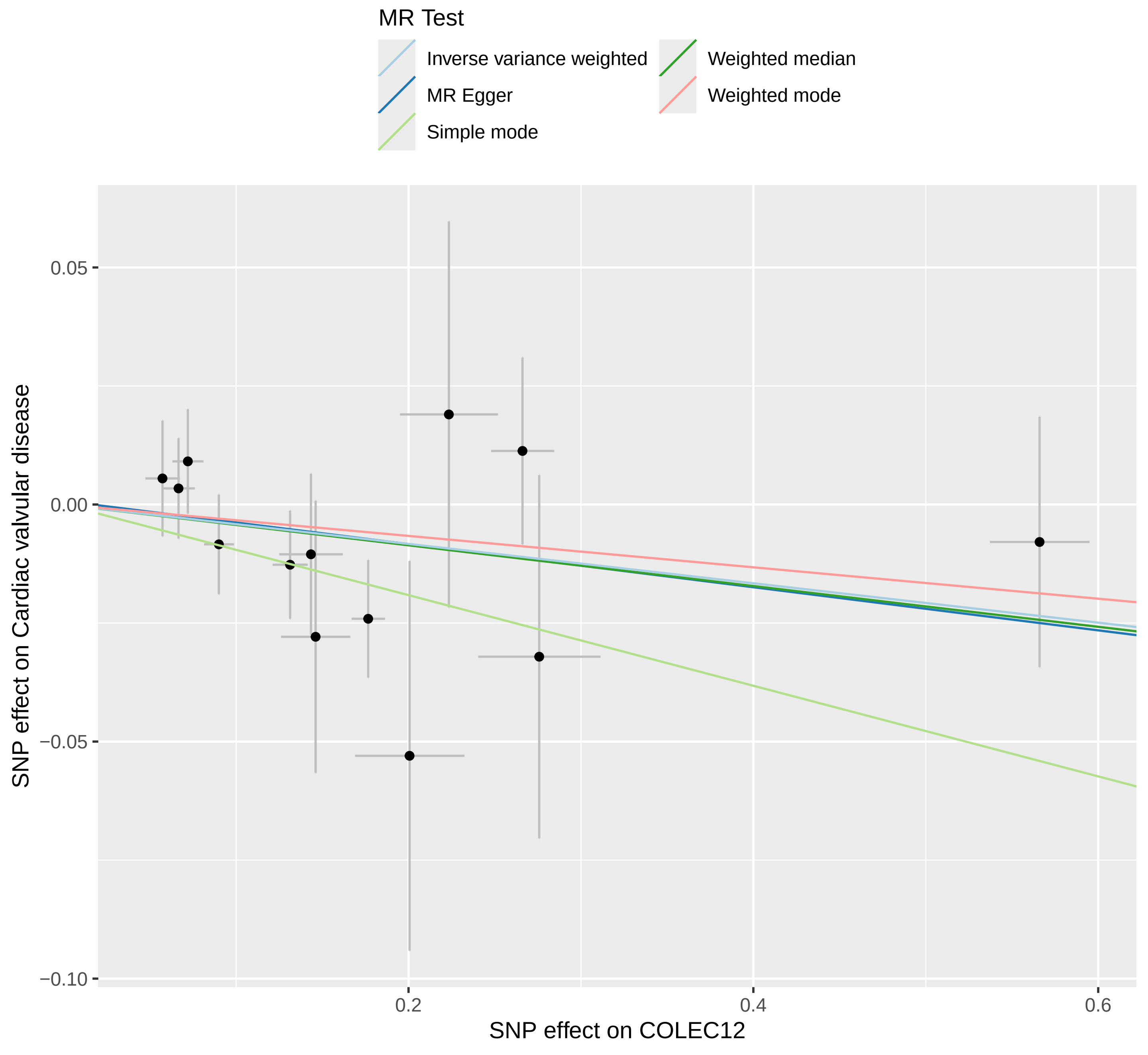

MR Test
Inverse variance weighted
MR Egger
Simple mode
Weighted median
Weighted mode
0.05
0.00
−0.05
−0.10
0.2
0.4
0.6
SNP effect on Cardiac valvular disease
SNP effect on COLEC12

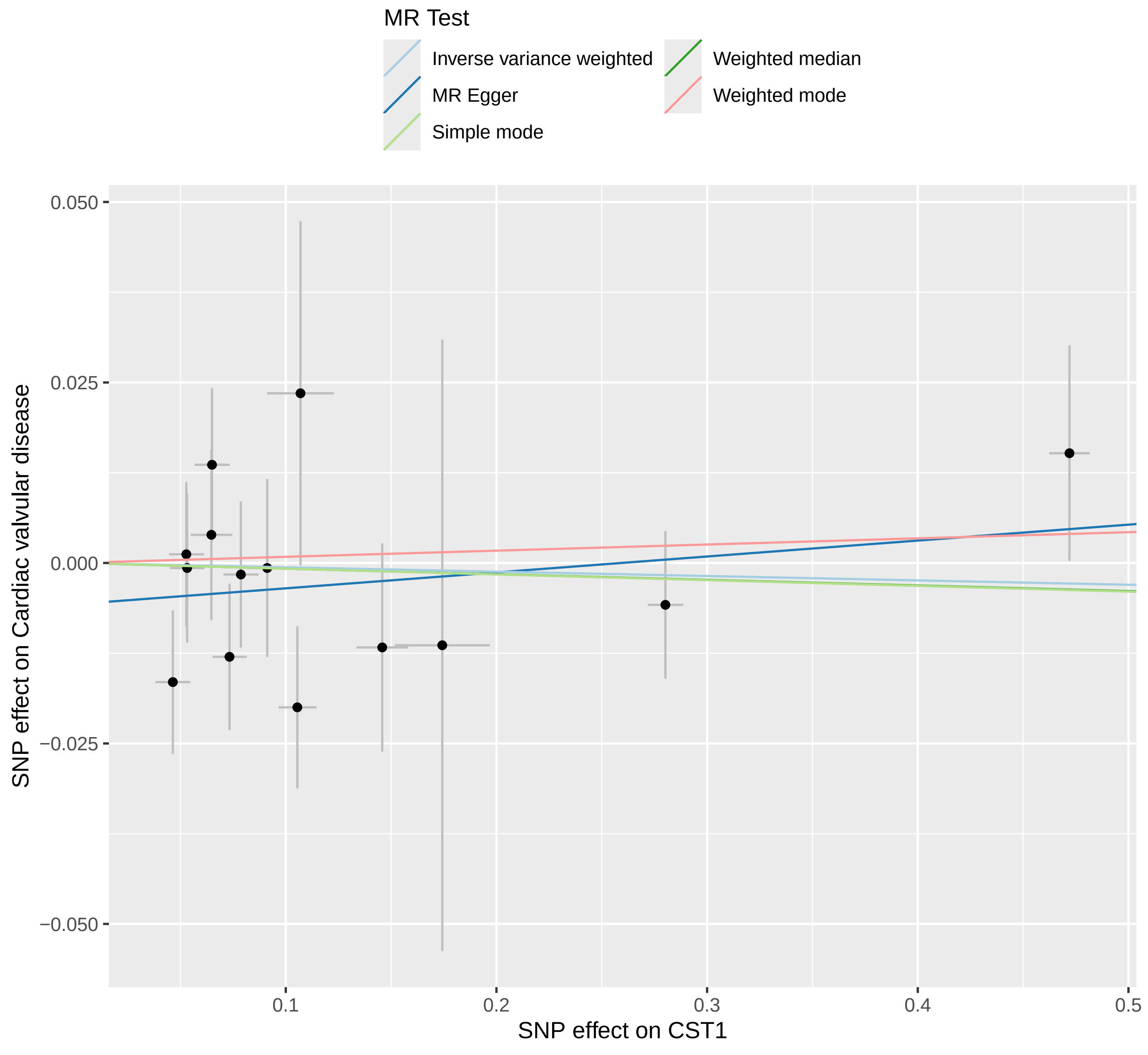
MR Test
Inverse variance weighted
MR Egger
Simple mode
Weighted median
Weighted mode
0.050
0.025
0.000
−0.025
−0.050
SNP effect on Cardiac valvular disease
0.1
0.2
0.3
0.4
0.5
SNP effect on CST1

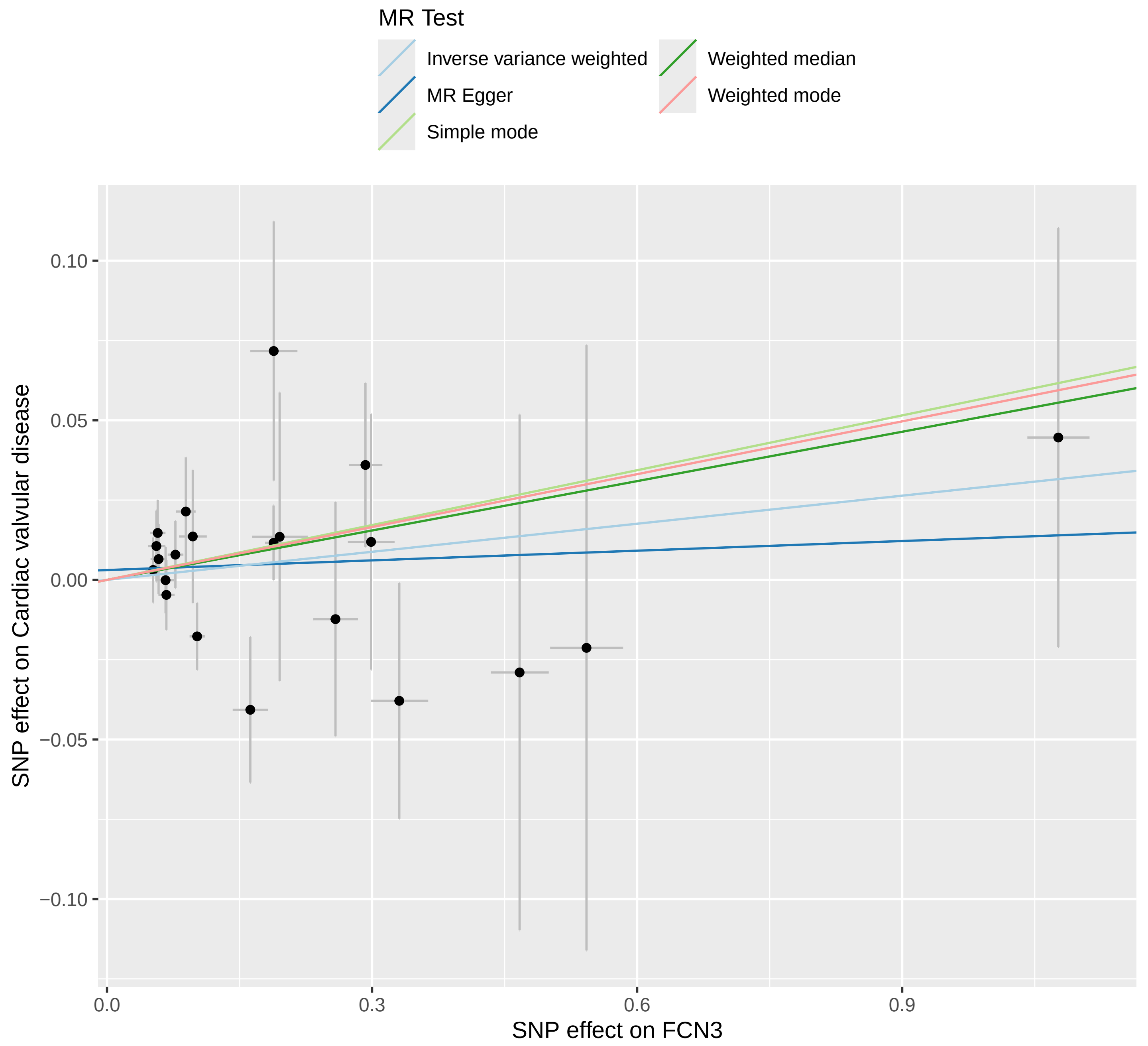

MR Test
Inverse variance weighted
MR Egger
Simple mode
Weighted median
Weighted mode
0.10
0.05
0.00
−0.05
−0.10
SNP effect on Cardiac valvular disease
0.0
0.3
0.6
0.9
SNP effect on FCN3

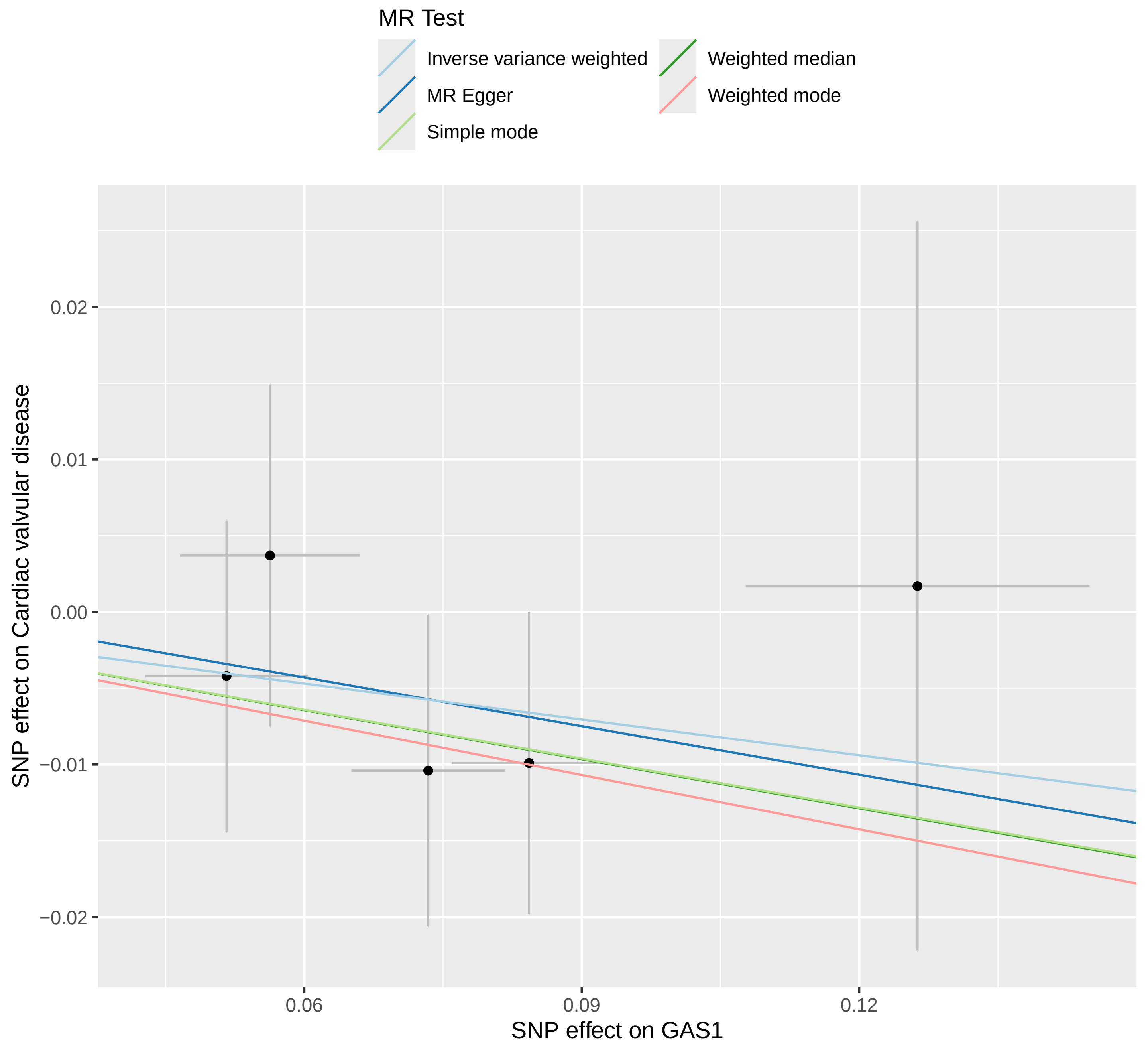
MR Test
Inverse variance weighted
MR Egger
Simple mode
Weighted median
Weighted mode
0.02
0.01
0.00
−0.01
−0.02
SNP effect on Cardiac valvular disease
0.06
0.09
0.12
SNP effect on GAS1

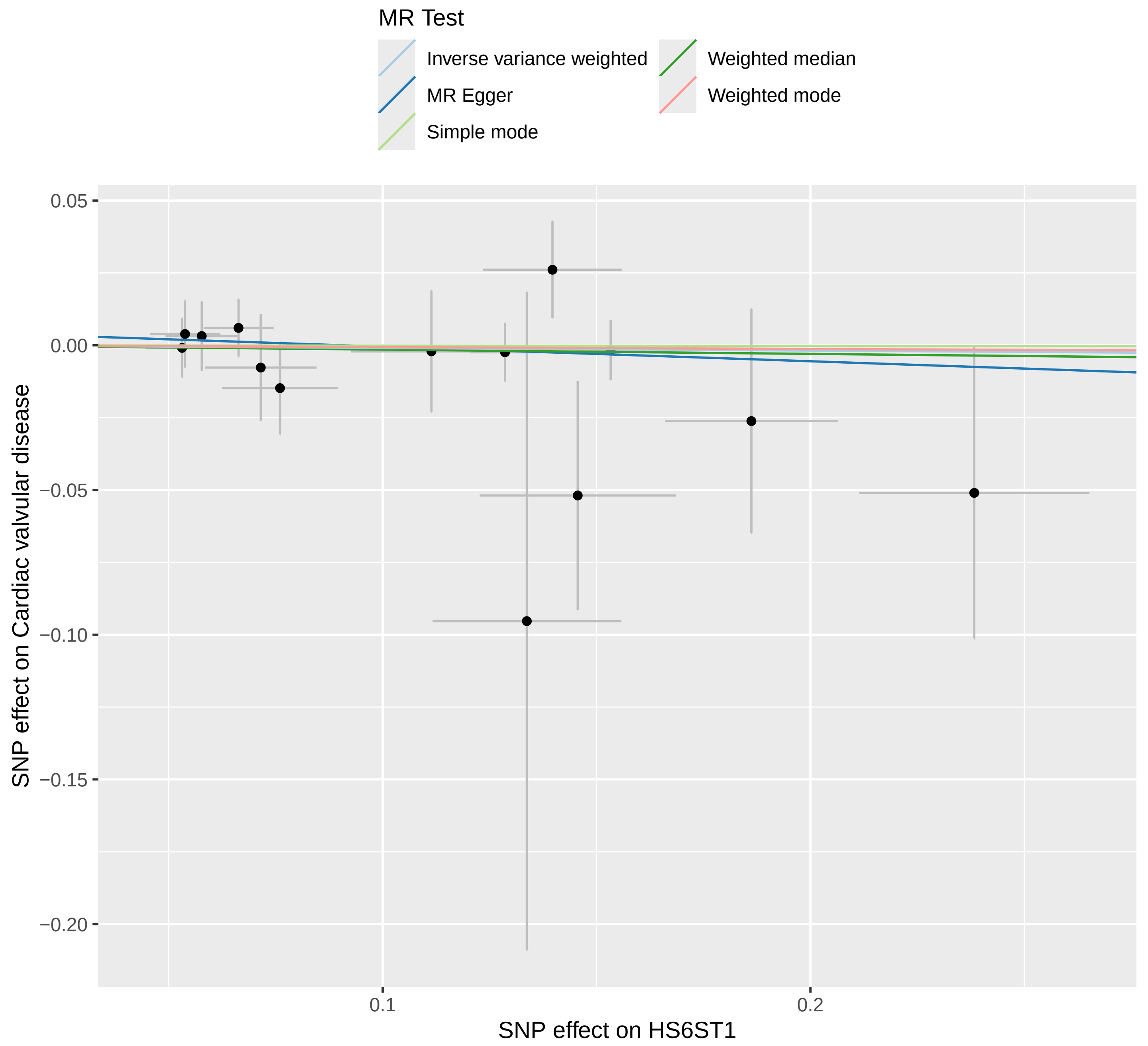

MR Test
Inverse variance weighted
MR Egger
Simple mode
Weighted median
Weighted mode
0.05
0.00
−0.05
−0.10
−0.15
−0.20
0.1
0.2
SNP effect on Cardiac valvular disease
SNP effect on HS6ST1

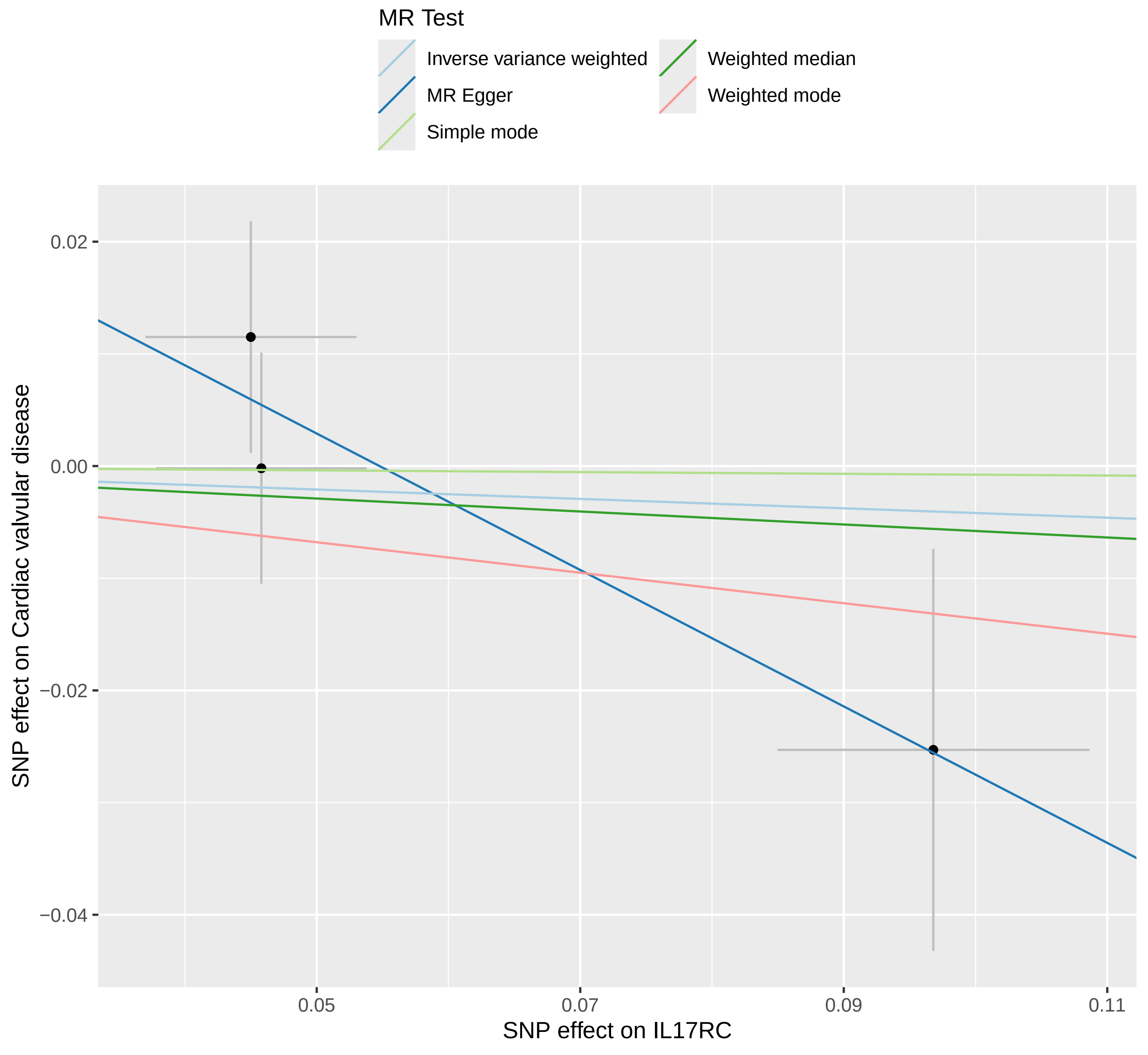

MR Test
Inverse variance weighted
MR Egger
Simple mode
Weighted median
Weighted mode
0.02
0.00
−0.02
−0.04
0.05
0.07
0.09
0.11
SNP effect on IL17RC
SNP effect on Cardiac valvular disease

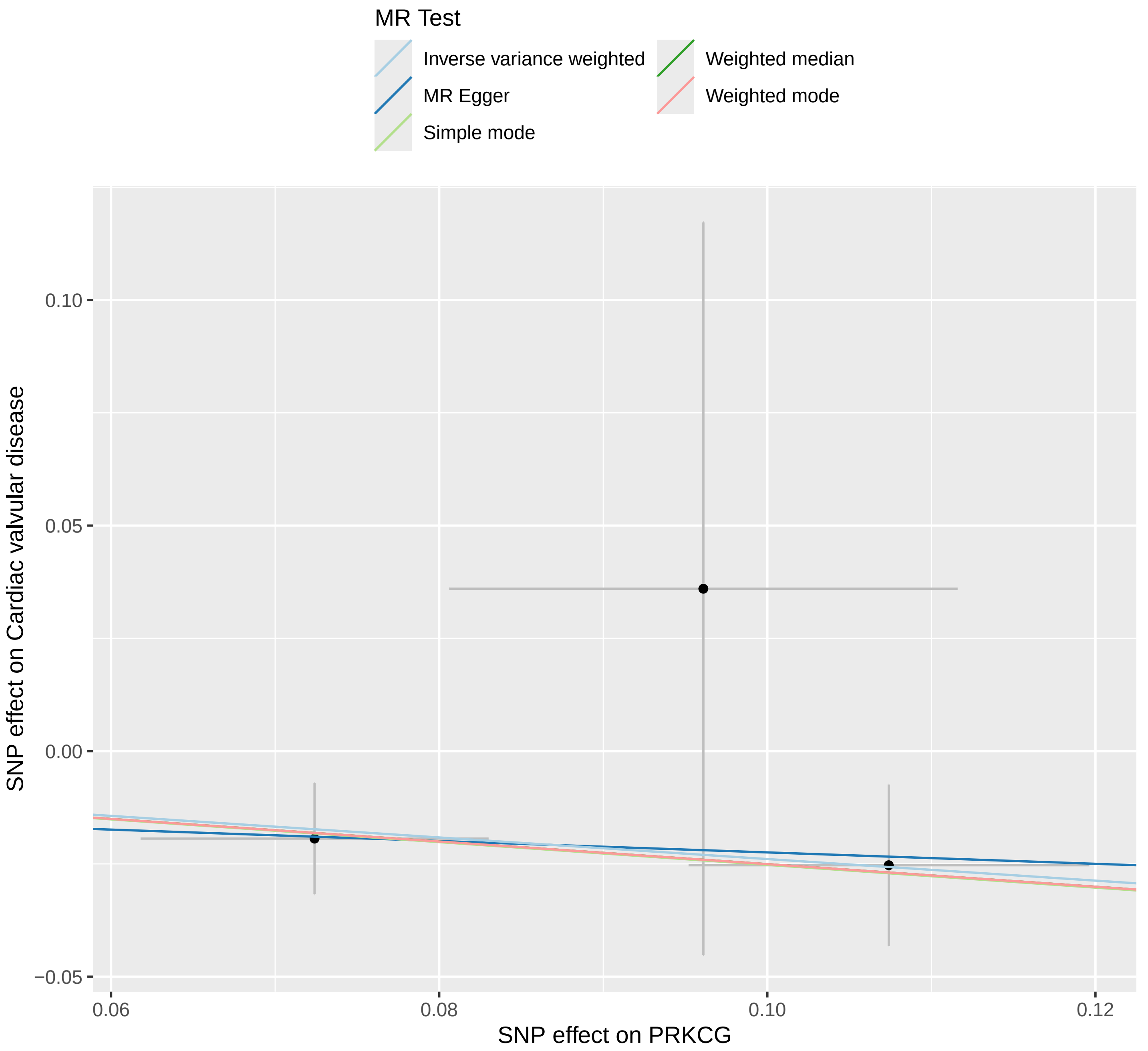

MR Test
Inverse variance weighted
MR Egger
Simple mode
Weighted median
Weighted mode
0.10
0.05
0.00
−0.05
0.06
0.08
0.10
0.12
SNP effect on Cardiac valvular disease
SNP effect on PRKCG

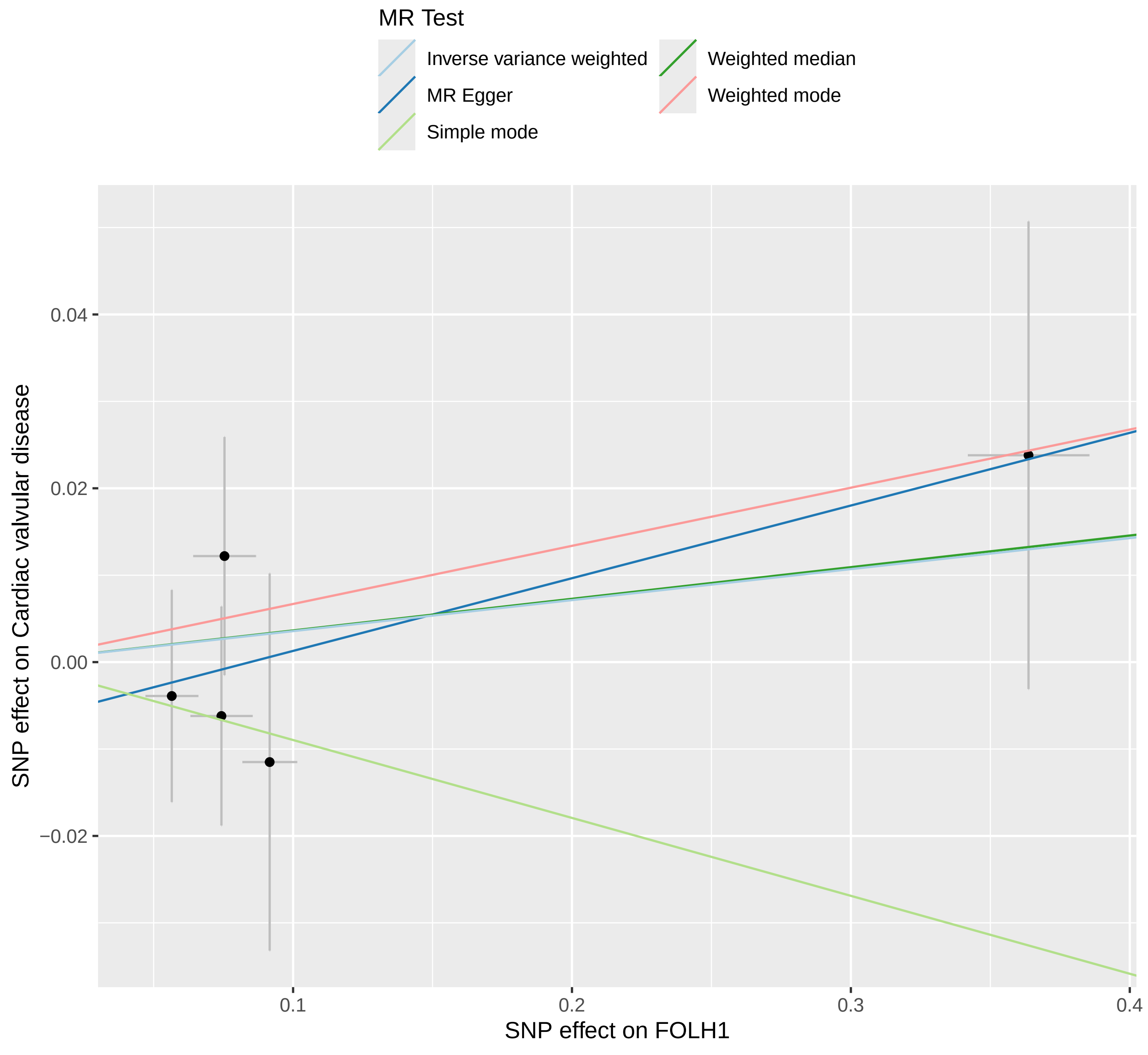

MR Test
Inverse variance weighted
MR Egger
Simple mode
Weighted median
Weighted mode
0.04
0.02
0.00
−0.02
SNP effect on Cardiac valvular disease
0.1
0.2
0.3
0.4
SNP effect on FOLH1

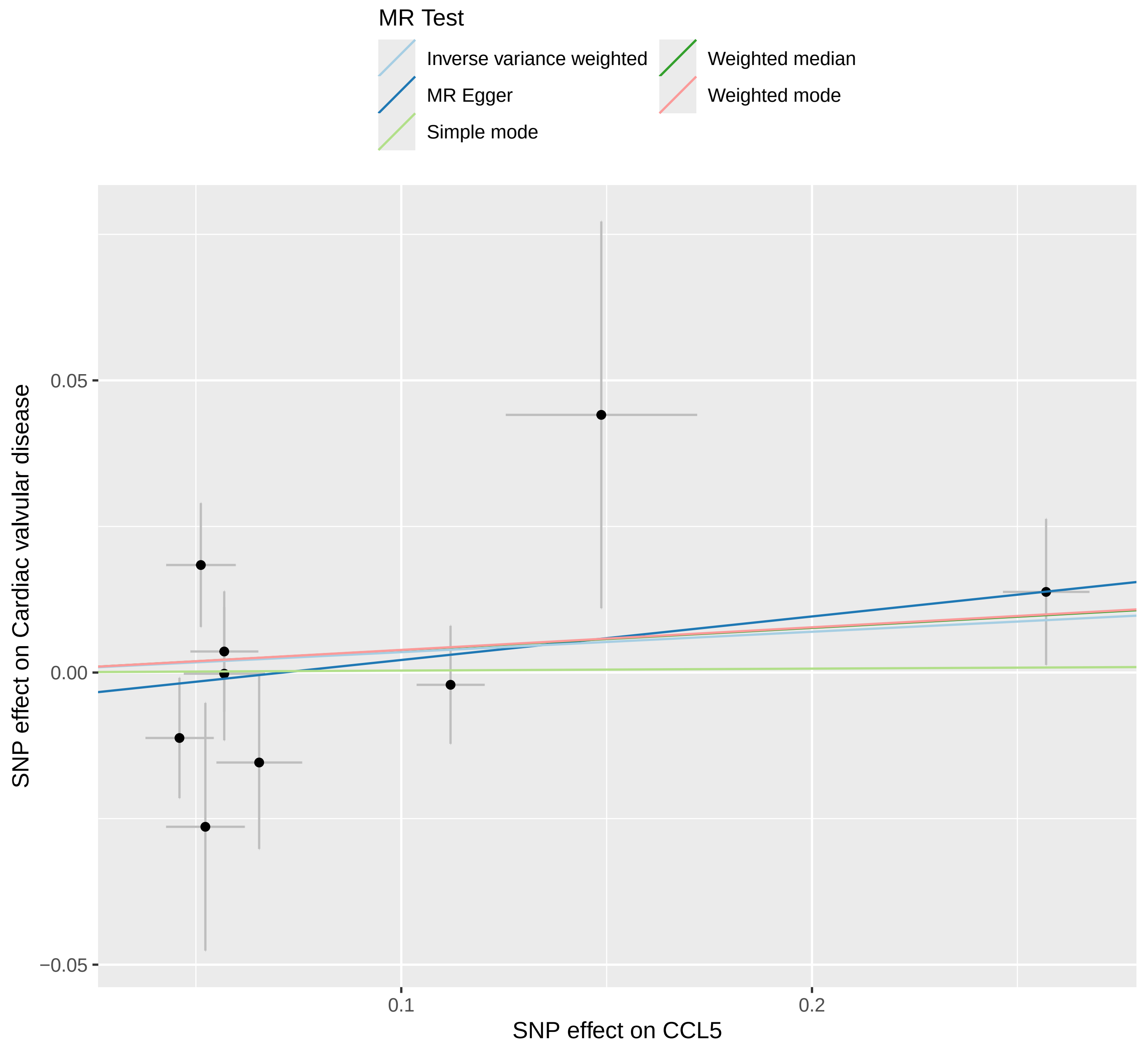
MR Test
Inverse variance weighted
MR Egger
Simple mode
Weighted median
Weighted mode
SNP effect on Cardiac valvular disease
0.05
0.00
−0.05
0.1
0.2
SNP effect on CCL5

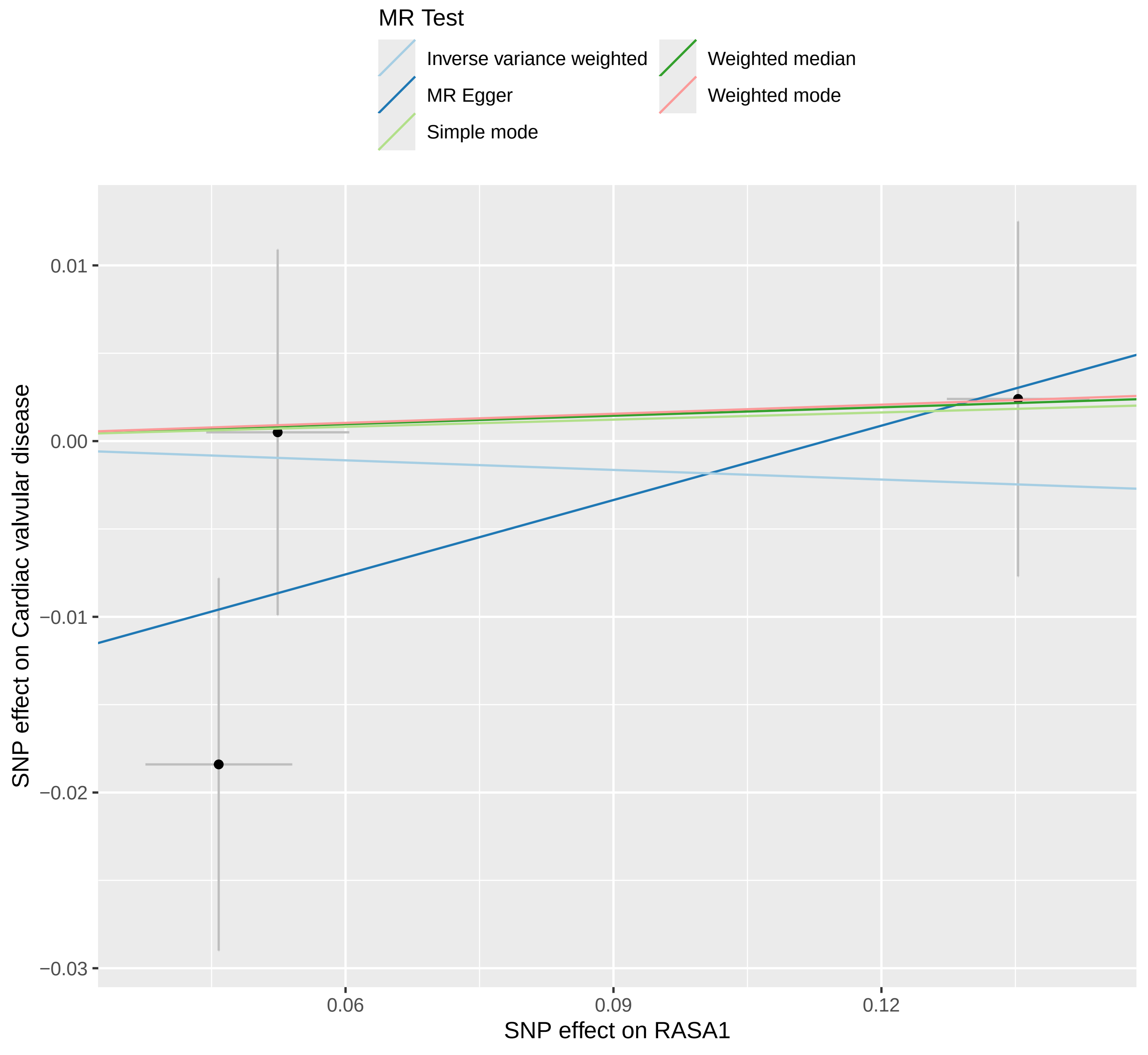

MR Test
Inverse variance weighted
MR Egger
Simple mode
Weighted median
Weighted mode
SNP effect on Cardiac valvular disease
0.01
0.00
−0.01
−0.02
−0.03
0.06
0.09
0.12
SNP effect on RASA1

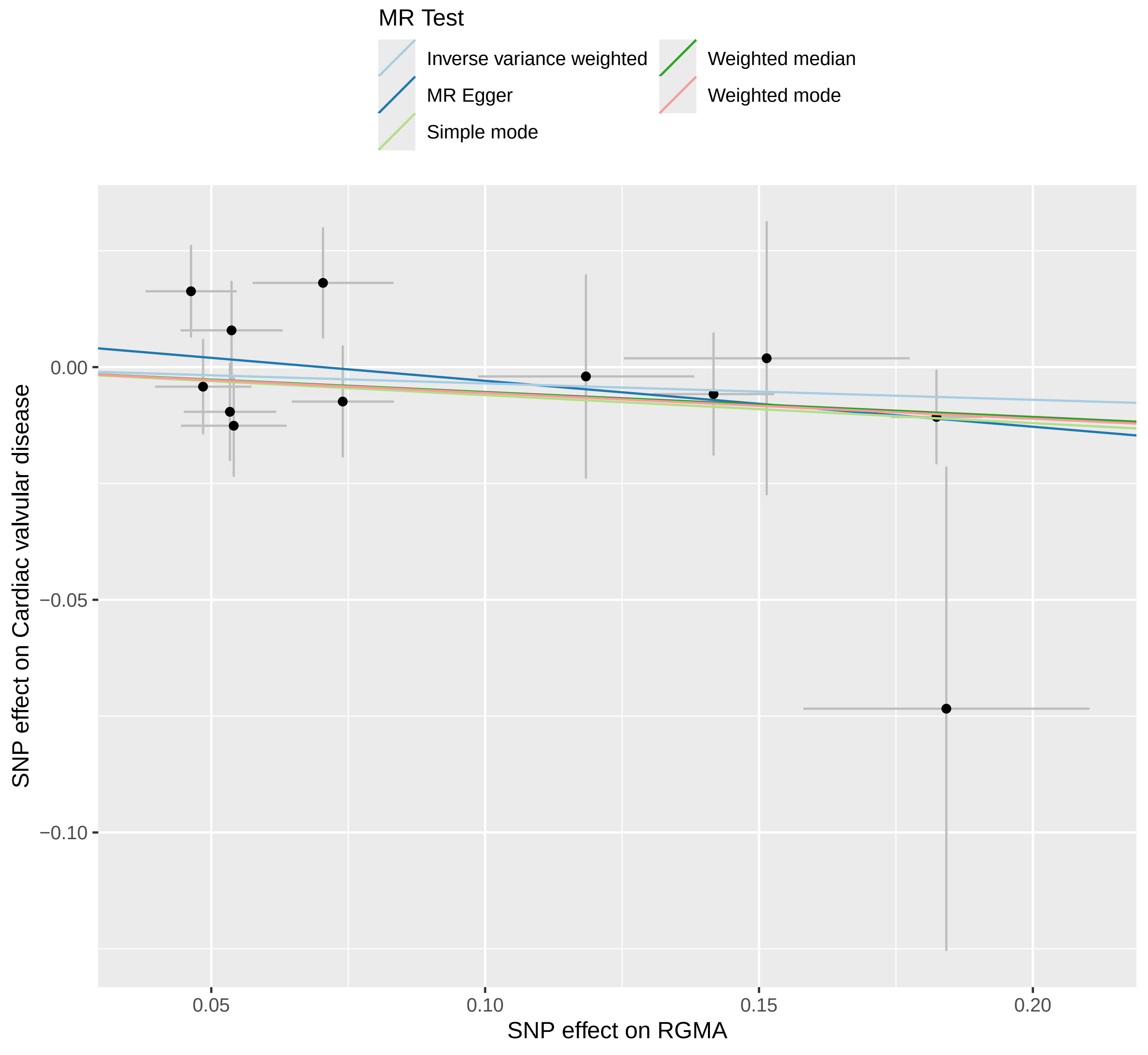
MR Test
Inverse variance weighted
MR Egger
Simple mode
Weighted median
Weighted mode
0.00
−0.05
−0.10
0.05
0.10
0.15
0.20
SNP effect on RGMA
SNP effect on Cardiac valvular disease

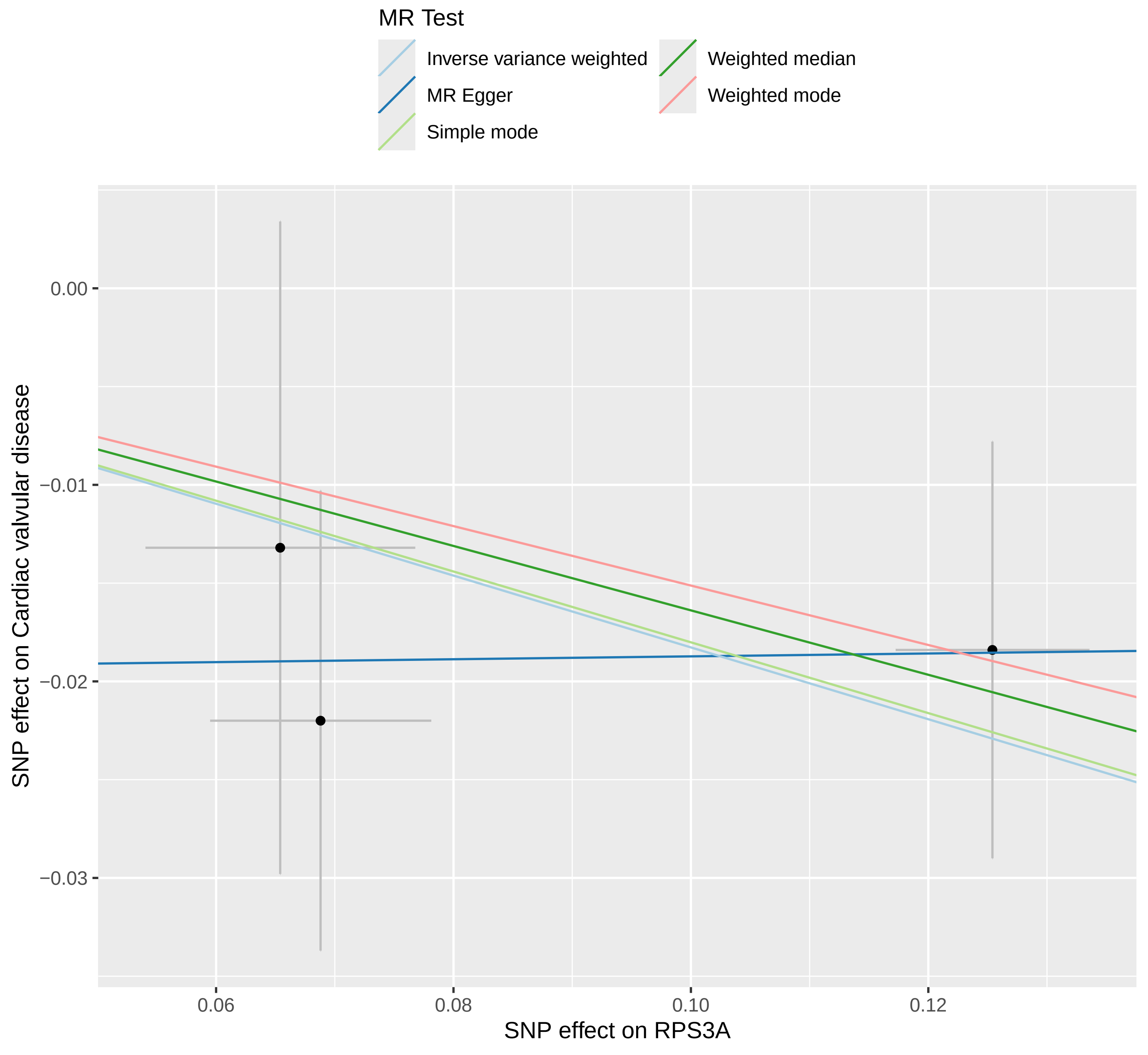
MR Test
Inverse variance weighted
MR Egger
Simple mode
Weighted median
Weighted mode
0.00
−0.01
−0.02
−0.03
0.06
0.08
0.10
0.12
SNP effect on Cardiac valvular disease
SNP effect on RPS3A

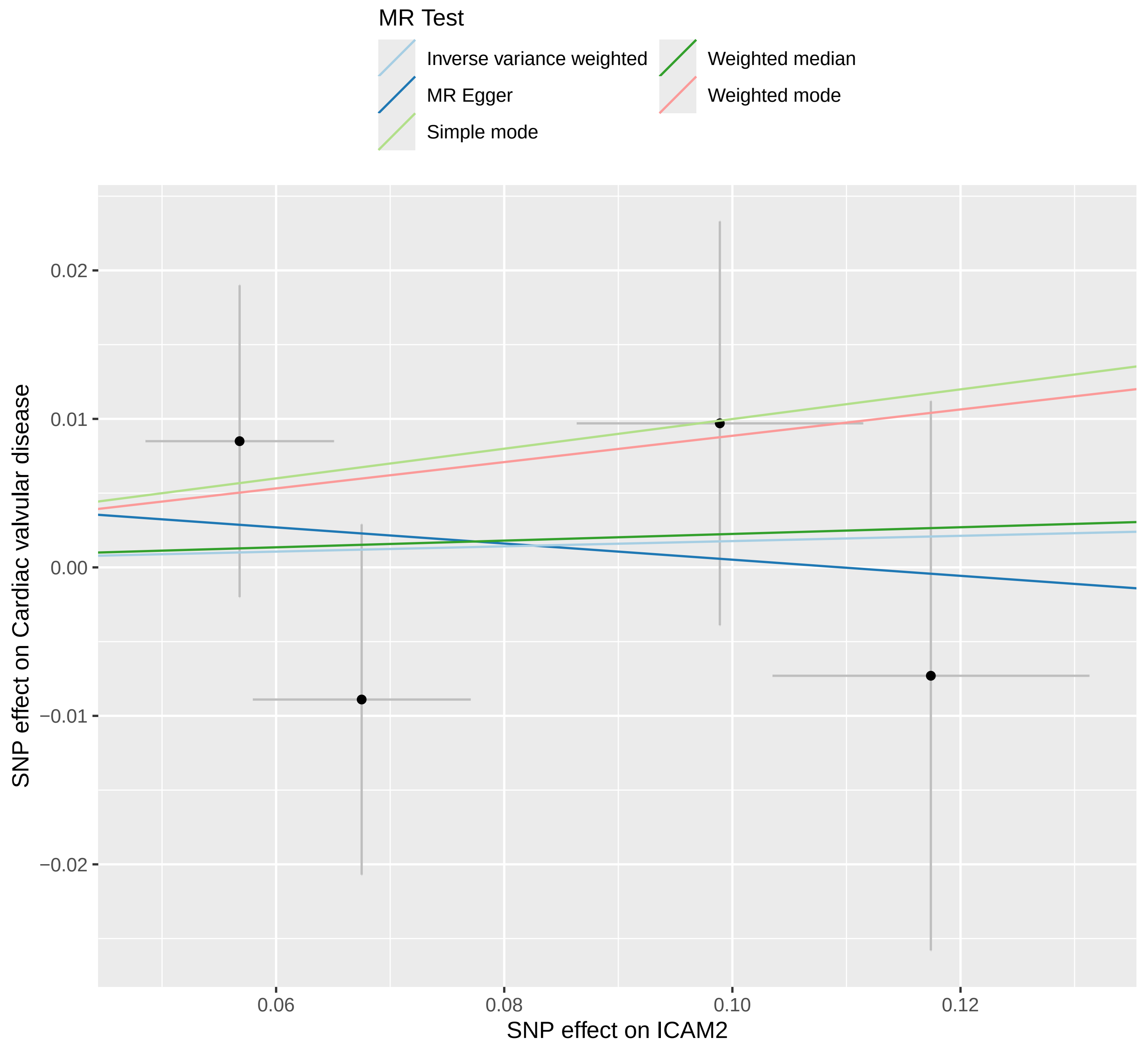
MR Test
Inverse variance weighted
MR Egger
Simple mode
Weighted median
Weighted mode
0.02
0.01
0.00
−0.01
−0.02
SNP effect on Cardiac valvular disease
0.06
0.08
0.10
0.12
SNP effect on ICAM2

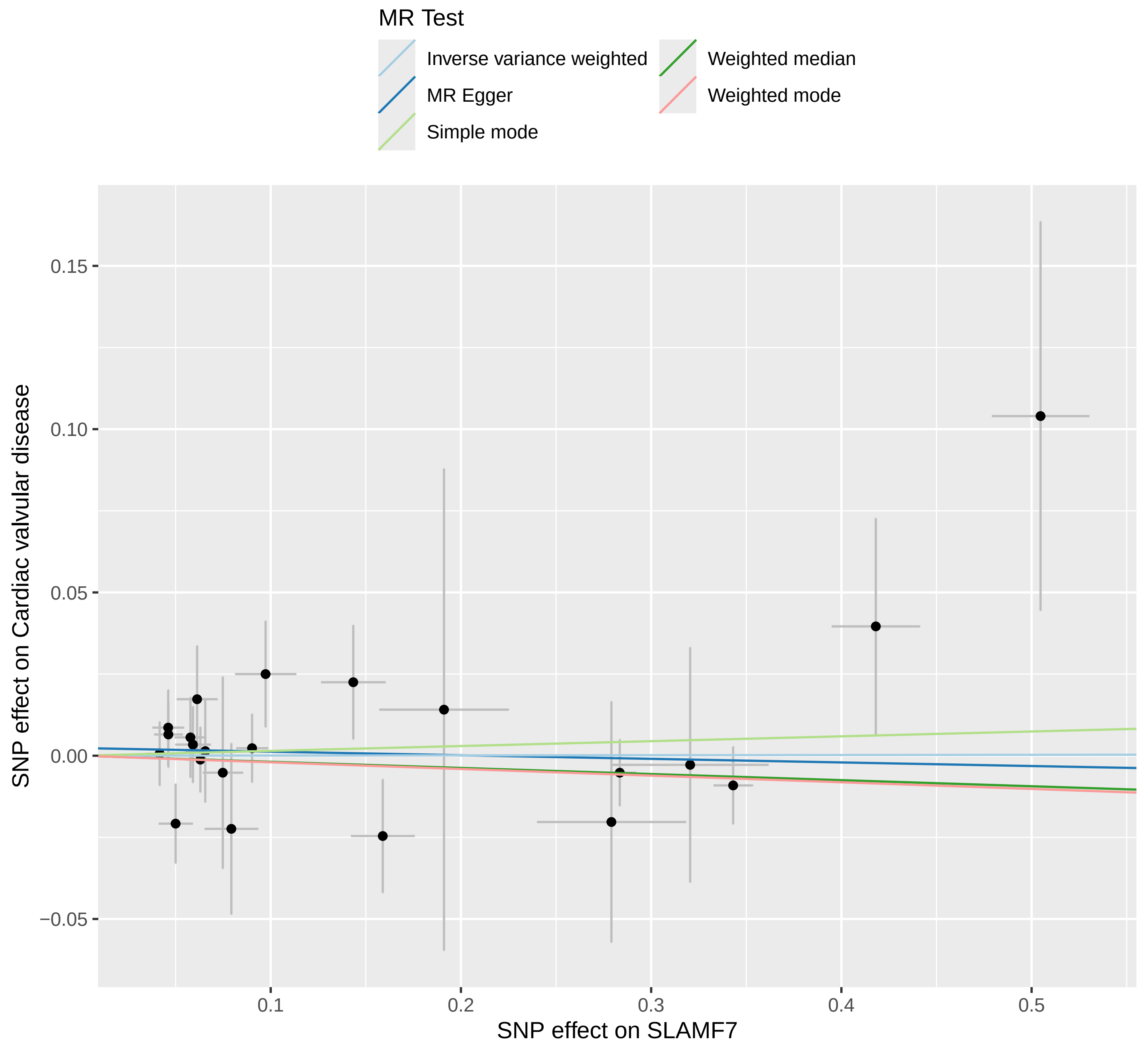
MR Test
Inverse variance weighted
MR Egger
Simple mode
Weighted median
Weighted mode
SNP effect on Cardiac valvular disease
0.15
0.10
0.05
0.00
−0.05
0.1
0.2
0.3
0.4
0.5
SNP effect on SLAMF7

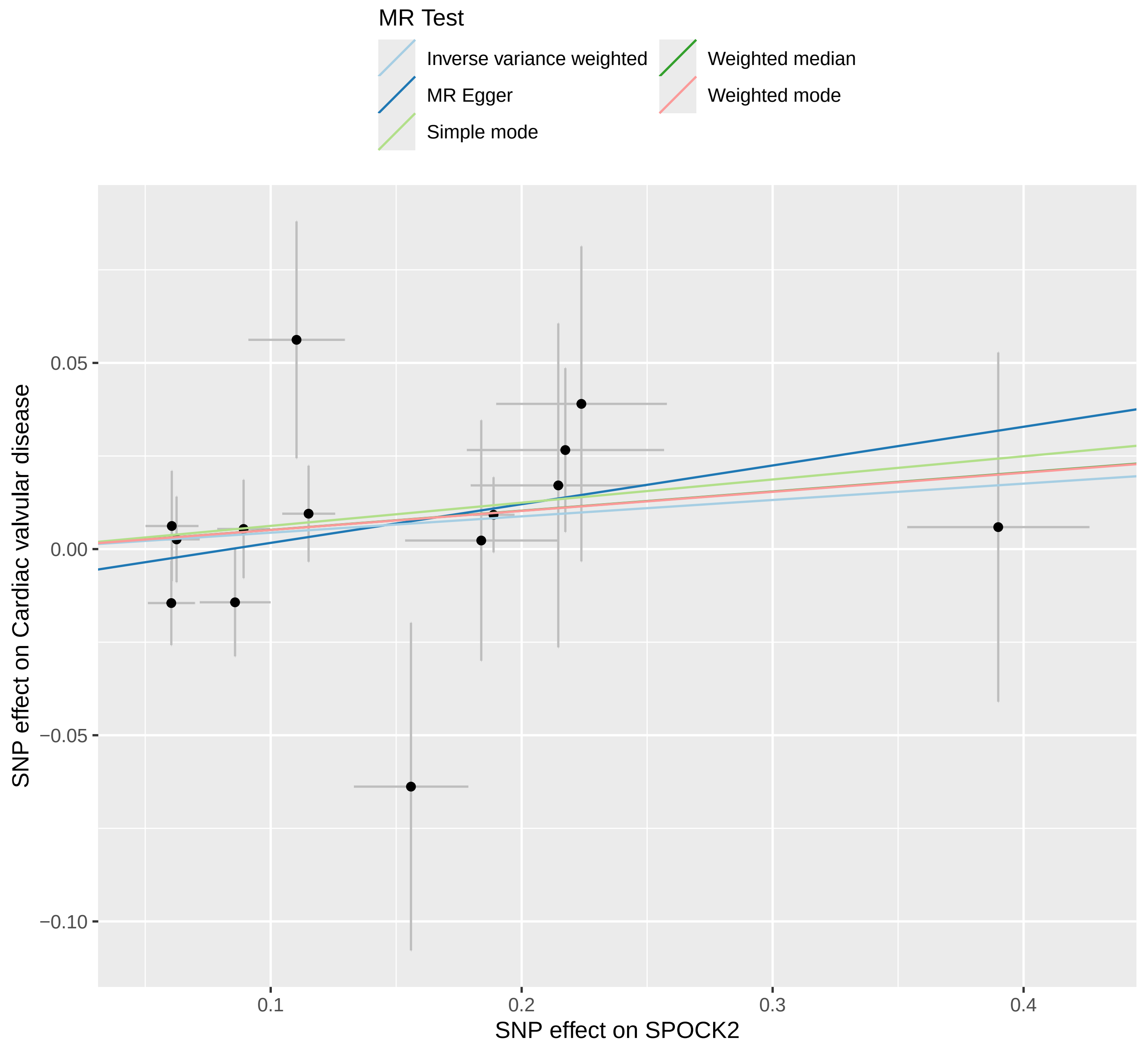
MR Test
Inverse variance weighted
MR Egger
Simple mode
Weighted median
Weighted mode
0.05
0.00
−0.05
−0.10
0.1
0.2
0.3
0.4
SNP effect on Cardiac valvular disease
SNP effect on SPOCK2

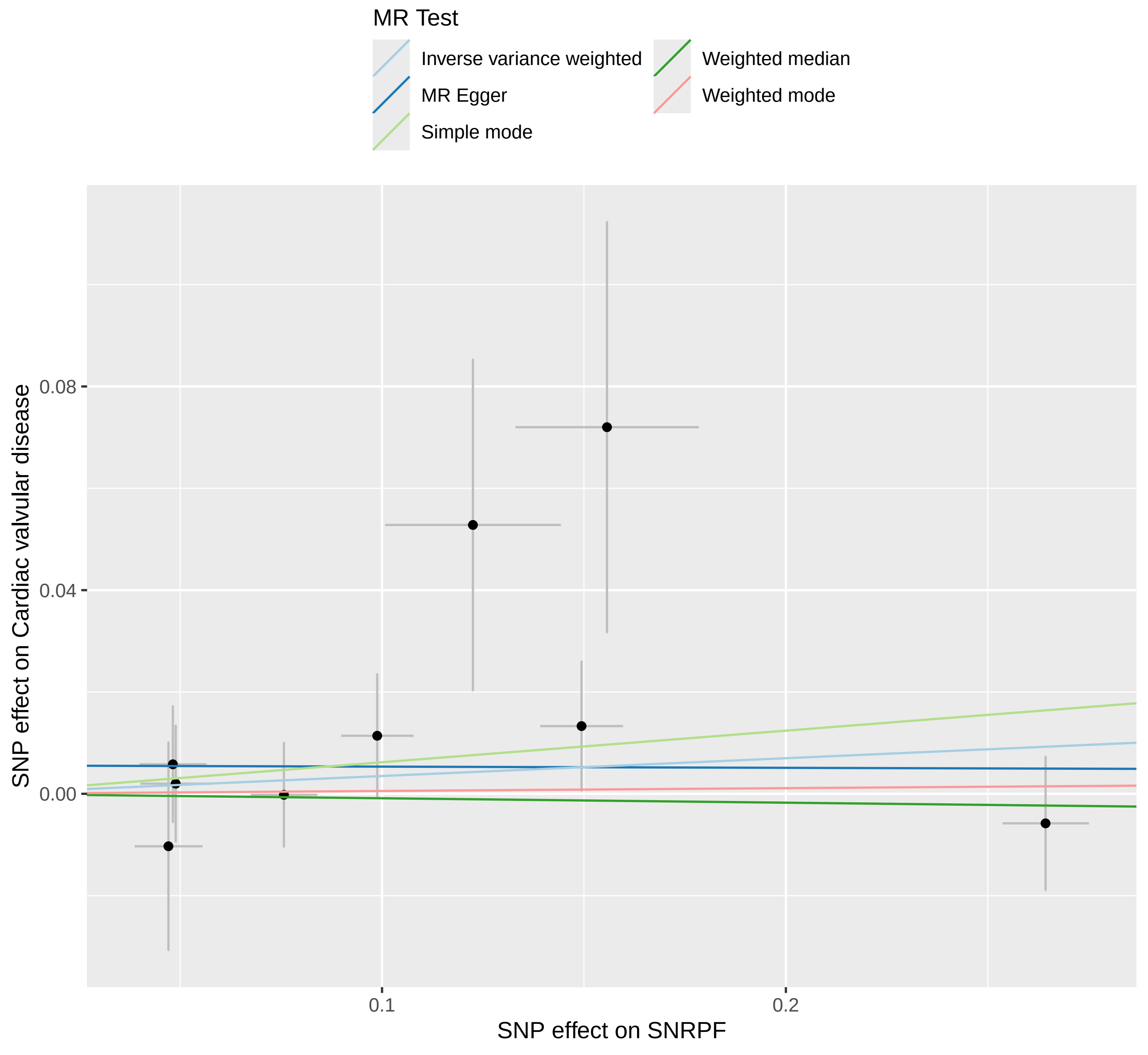
MR Test
Inverse variance weighted
MR Egger
Simple mode
Weighted median
Weighted mode
0.08
0.04
0.00
0.1
0.2
SNP effect on Cardiac valvular disease
SNP effect on SNRPF

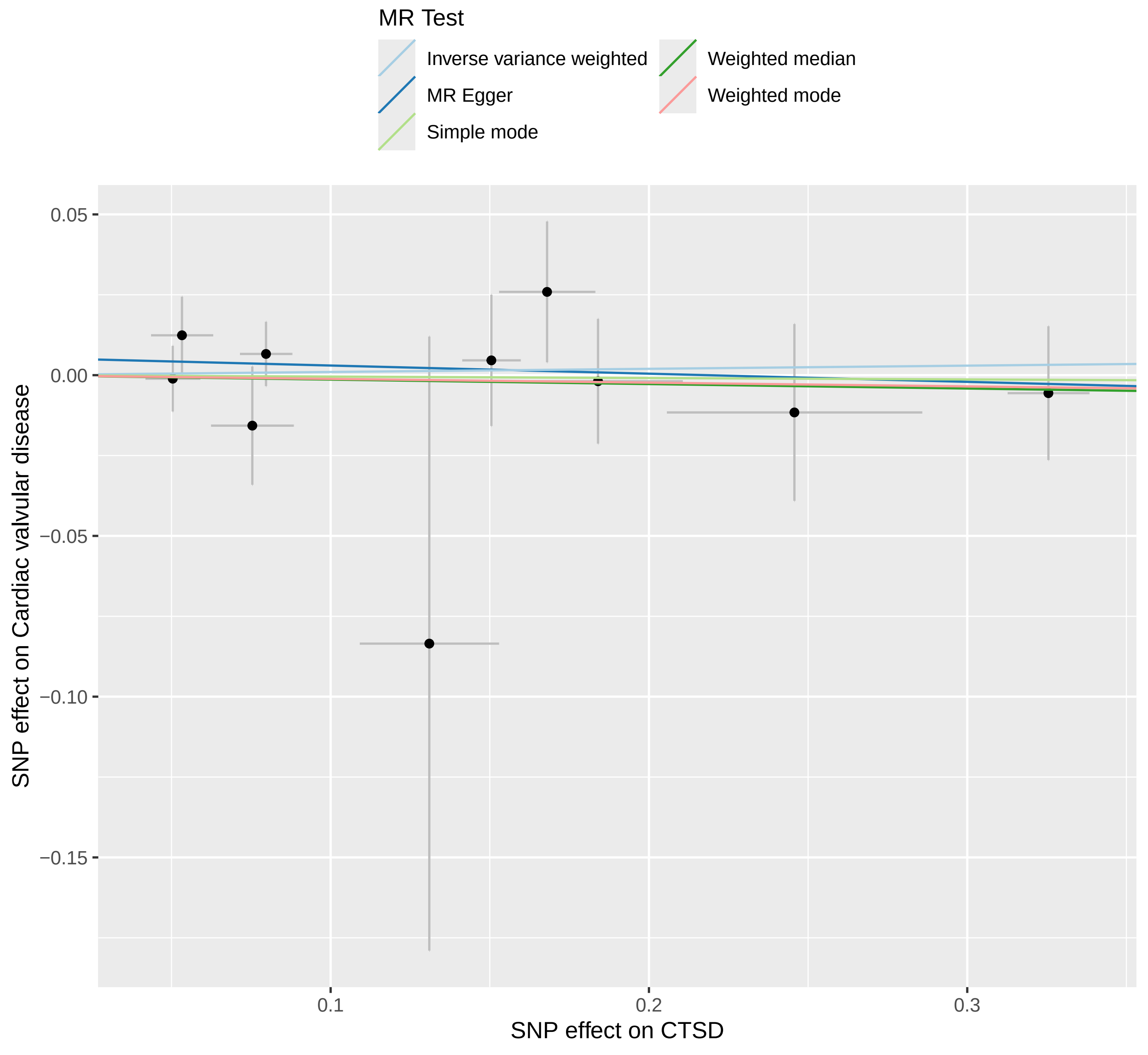

MR Test
Inverse variance weighted
MR Egger
Simple mode
Weighted median
Weighted mode
0.05
0.00
−0.05
−0.10
−0.15
SNP effect on Cardiac valvular disease
0.1
0.2
0.3
SNP effect on CTSD

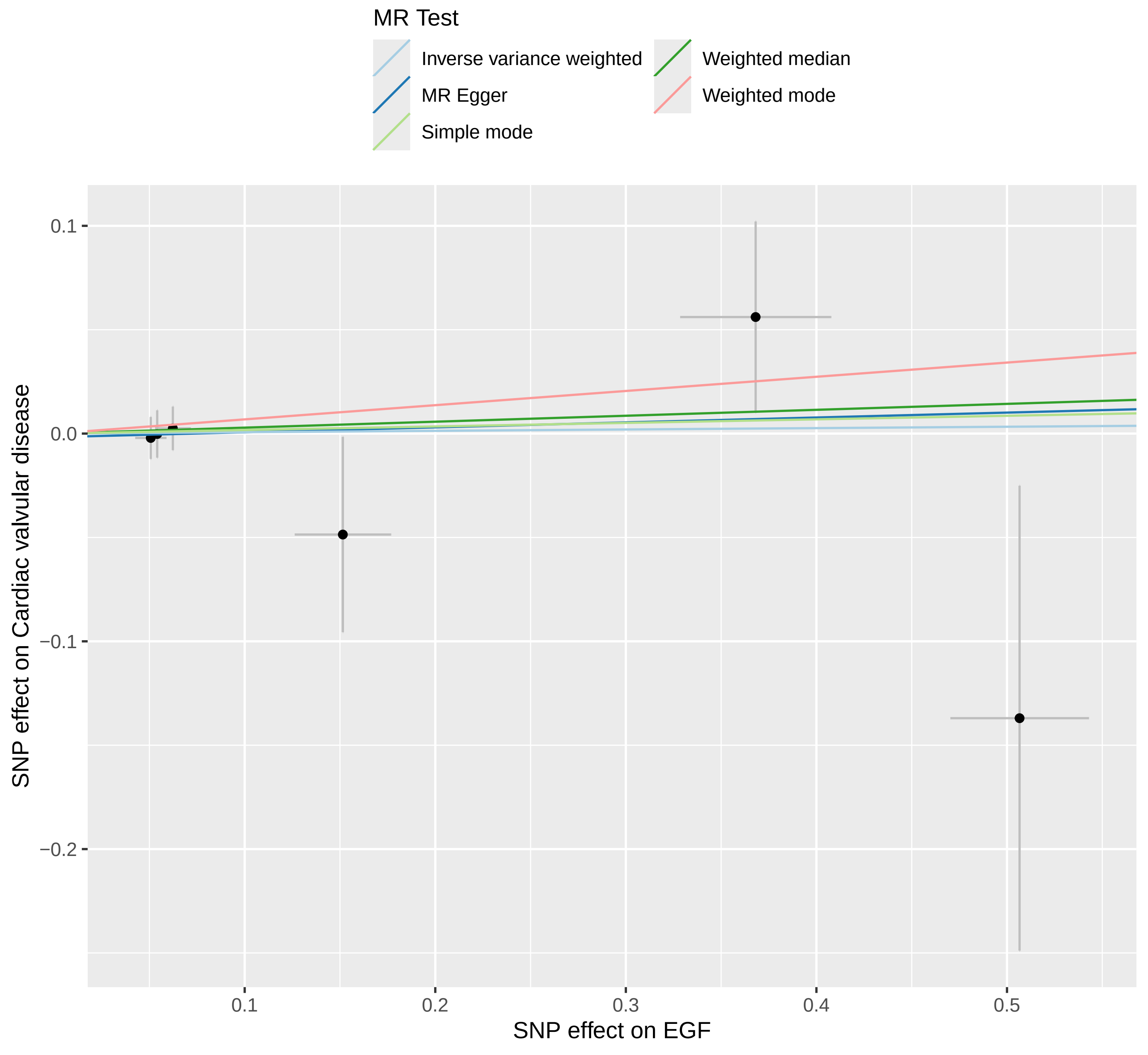
MR Test
Inverse variance weighted
MR Egger
Simple mode
Weighted median
Weighted mode
0.1
0.0
−0.1
−0.2
SNP effect on Cardiac valvular disease
0.1
0.2
0.3
0.4
0.5
SNP effect on EGF

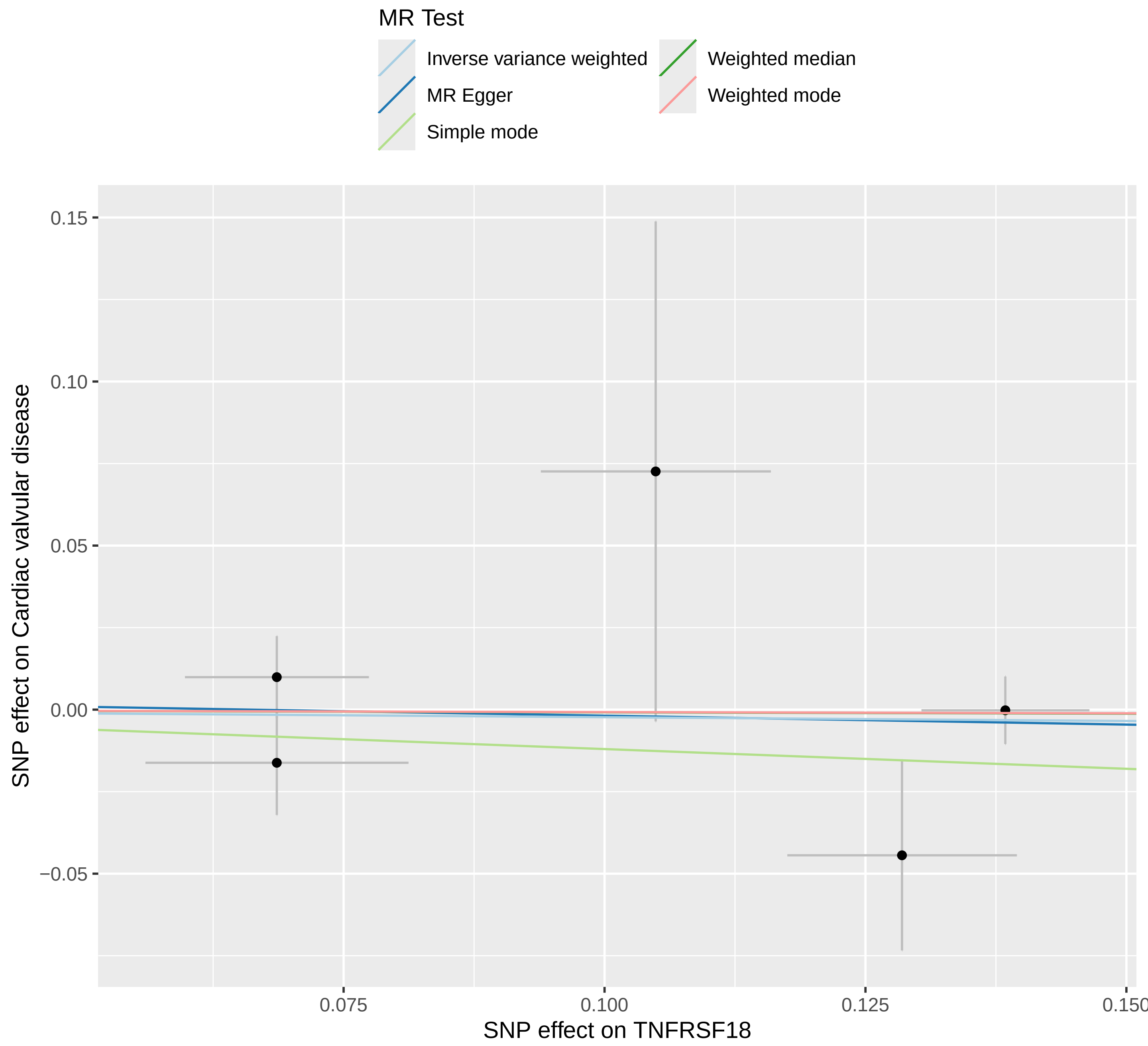
MR Test
Inverse variance weighted
MR Egger
Simple mode
Weighted median
Weighted mode
0.15
0.10
0.05
0.00
−0.05
0.075
0.100
0.125
0.150
SNP effect on Cardiac valvular disease
SNP effect on TNFRSF18

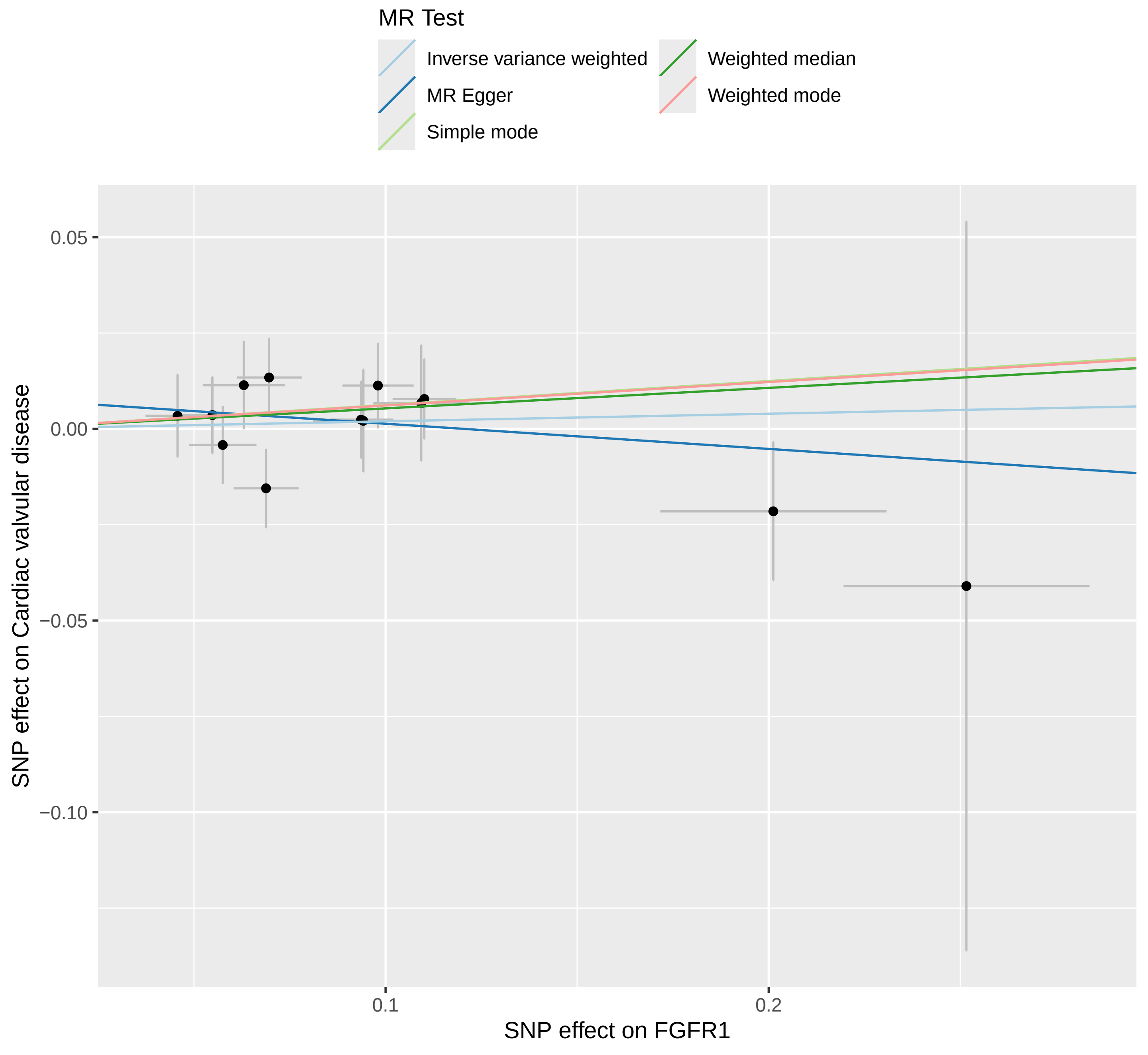

MR Test
Inverse variance weighted
MR Egger
Simple mode
Weighted median
Weighted mode
0.05
0.00
−0.05
−0.10
0.1
0.2
SNP effect on FGFR1
SNP effect on Cardiac valvular disease

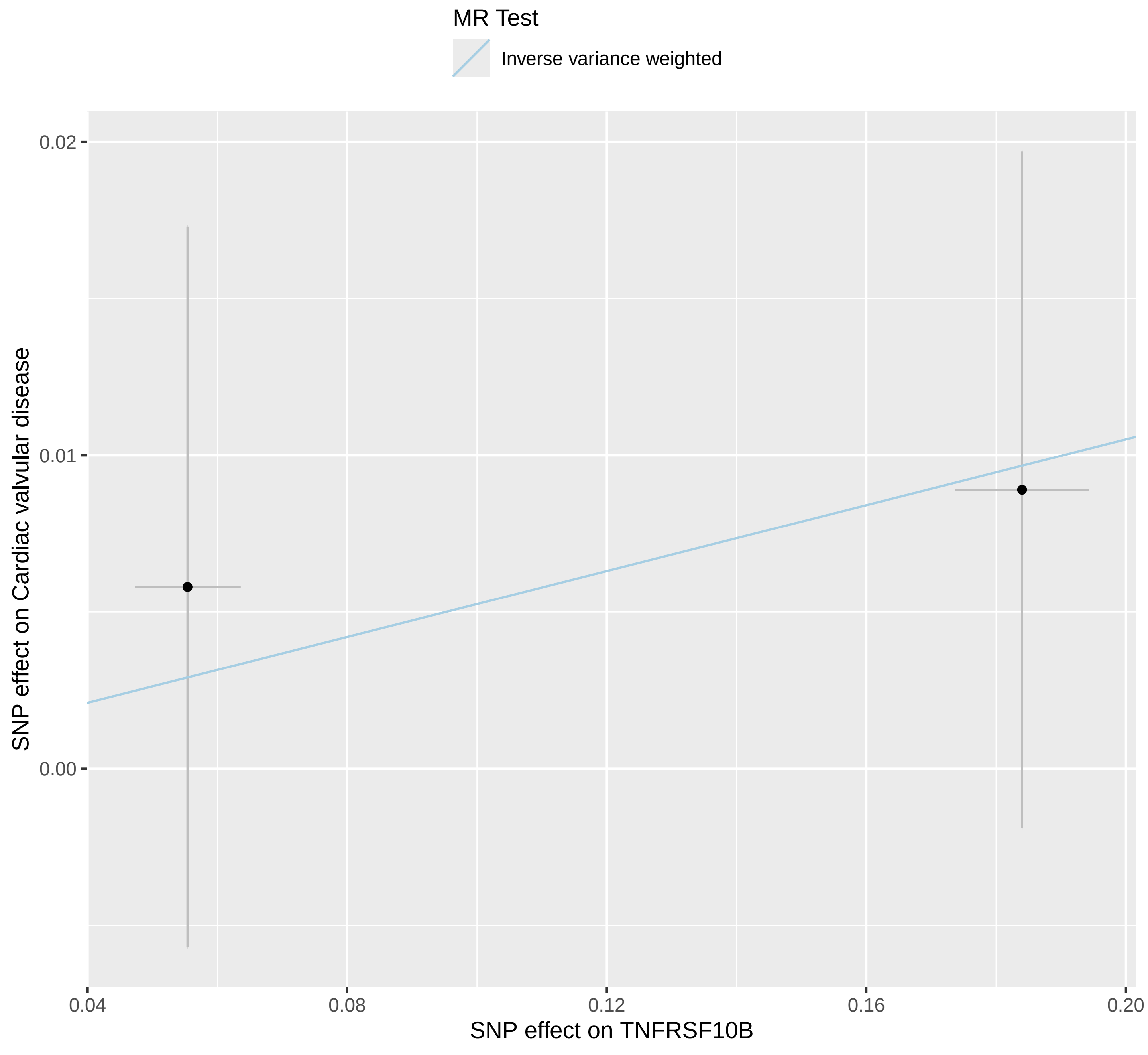
MR Test
Inverse variance weighted
0.02
0.01
0.00
0.04
0.08
0.12
0.16
0.20
SNP effect on TNFRSF10B
SNP effect on Cardiac valvular disease

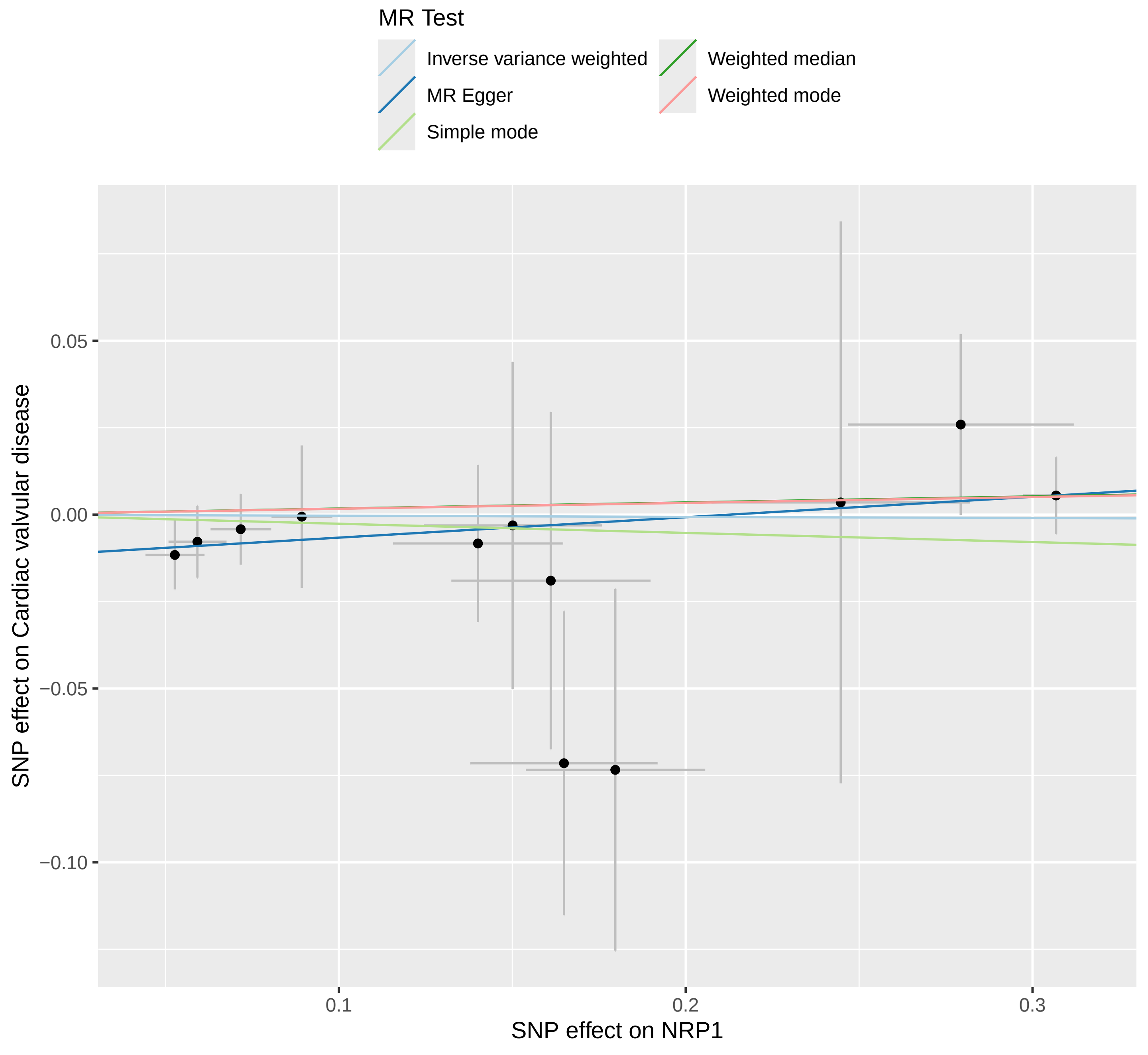
MR Test
Inverse variance weighted
MR Egger
Simple mode
Weighted median
Weighted mode
0.05
0.00
−0.05
−0.10
0.1
0.2
0.3
SNP effect on Cardiac valvular disease
SNP effect on NRP1

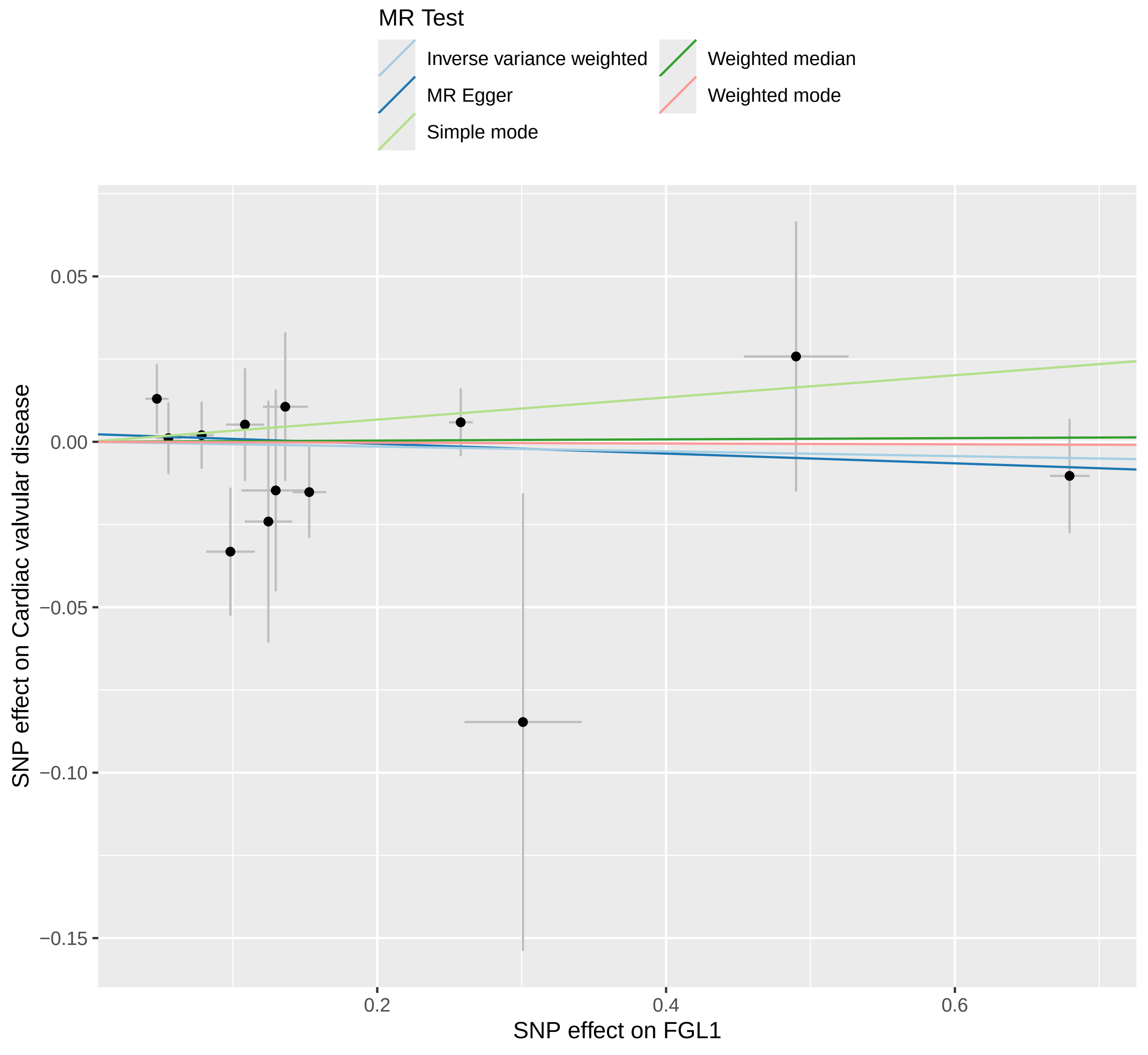
MR Test
Inverse variance weighted
MR Egger
Simple mode
Weighted median
Weighted mode
0.05
0.00
−0.05
−0.10
−0.15
0.2
0.4
0.6
SNP effect on Cardiac valvular disease
SNP effect on FGL1

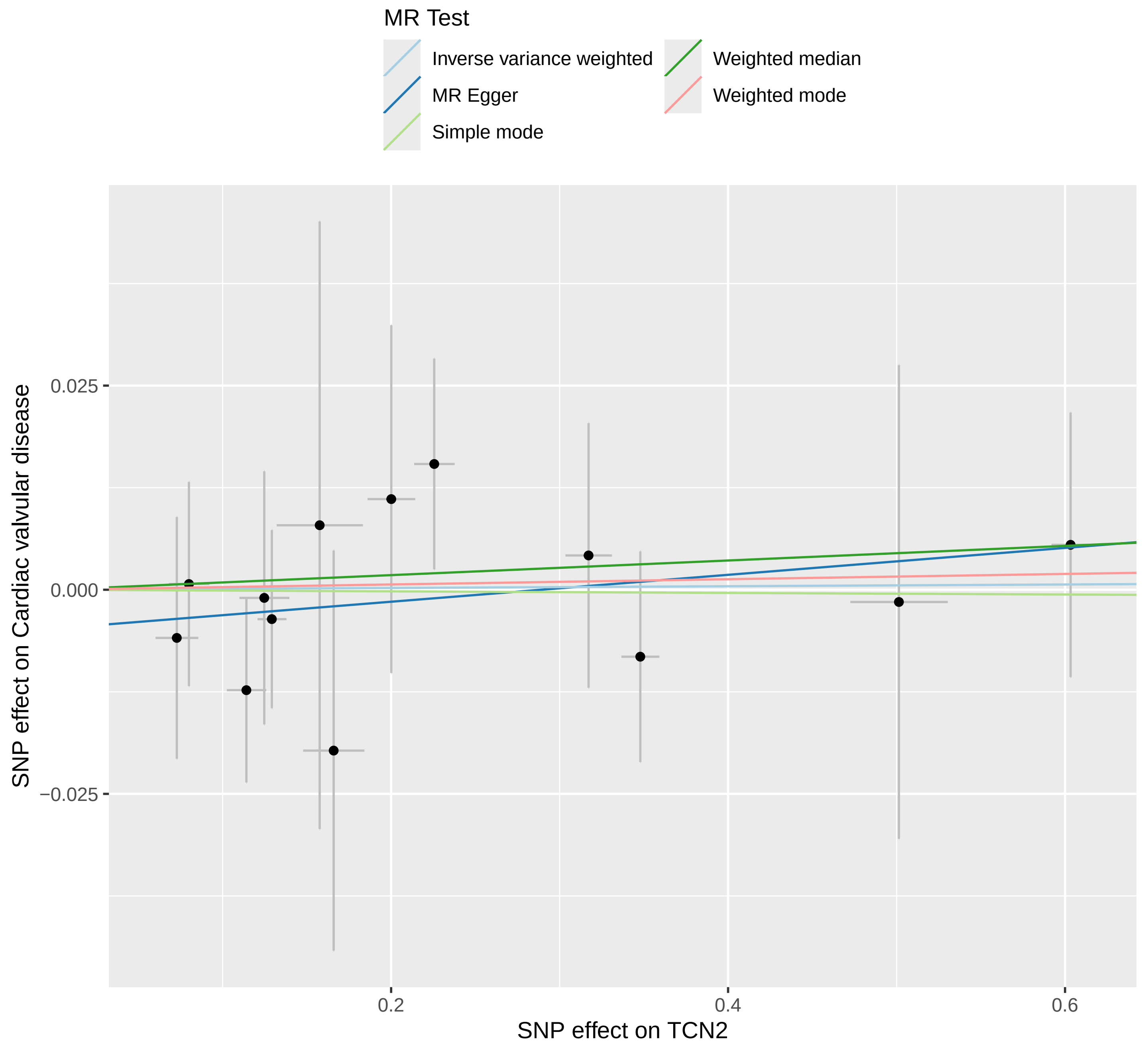

MR Test
Inverse variance weighted
MR Egger
Simple mode
Weighted median
Weighted mode
0.025
0.000
−0.025
0.2
0.4
0.6
SNP effect on Cardiac valvular disease
SNP effect on TCN2

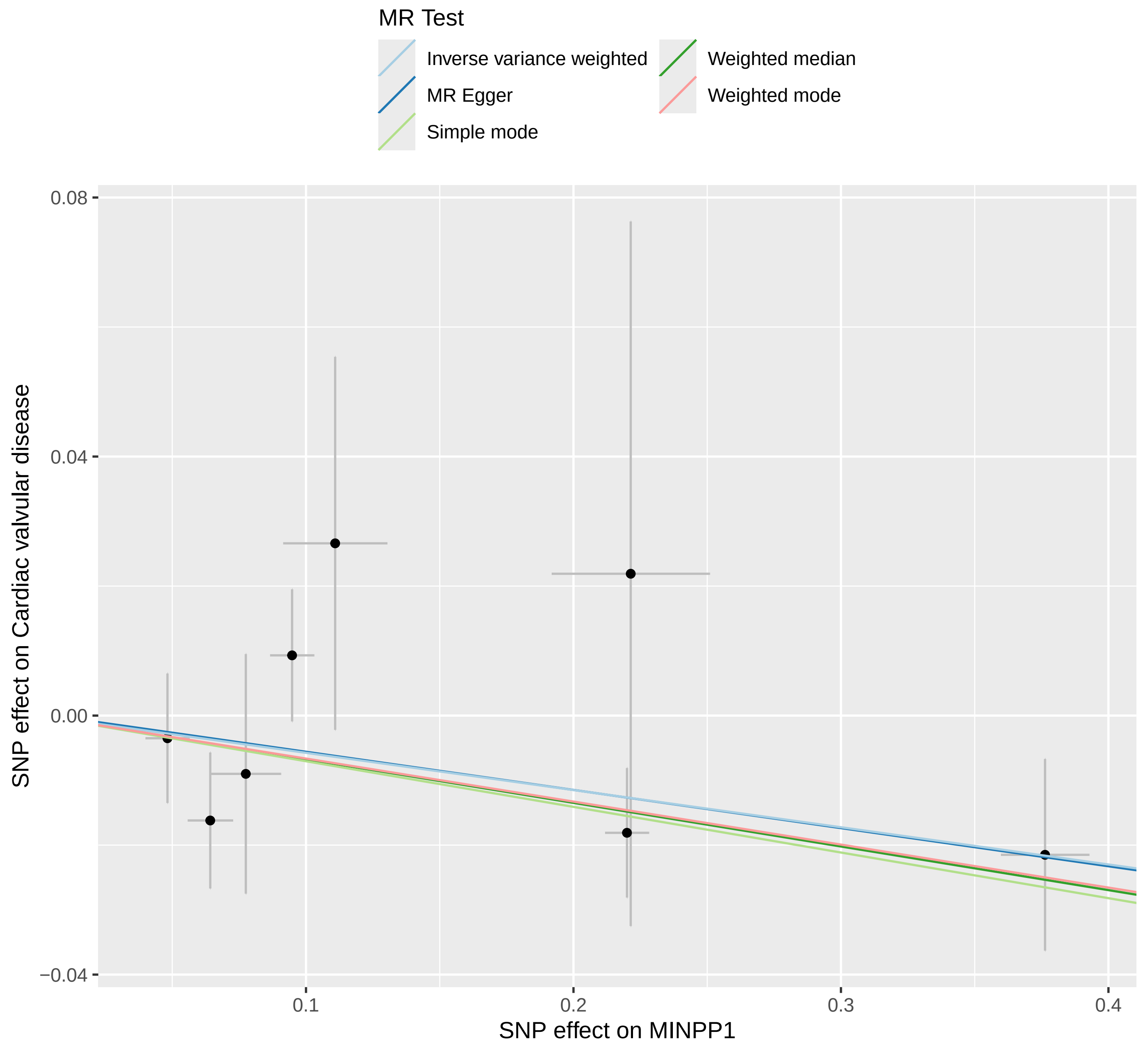

MR Test
Inverse variance weighted
MR Egger
Simple mode
Weighted median
Weighted mode
0.08
0.04
0.00
−0.04
0.1
0.2
0.3
0.4
SNP effect on Cardiac valvular disease
SNP effect on MINPP1

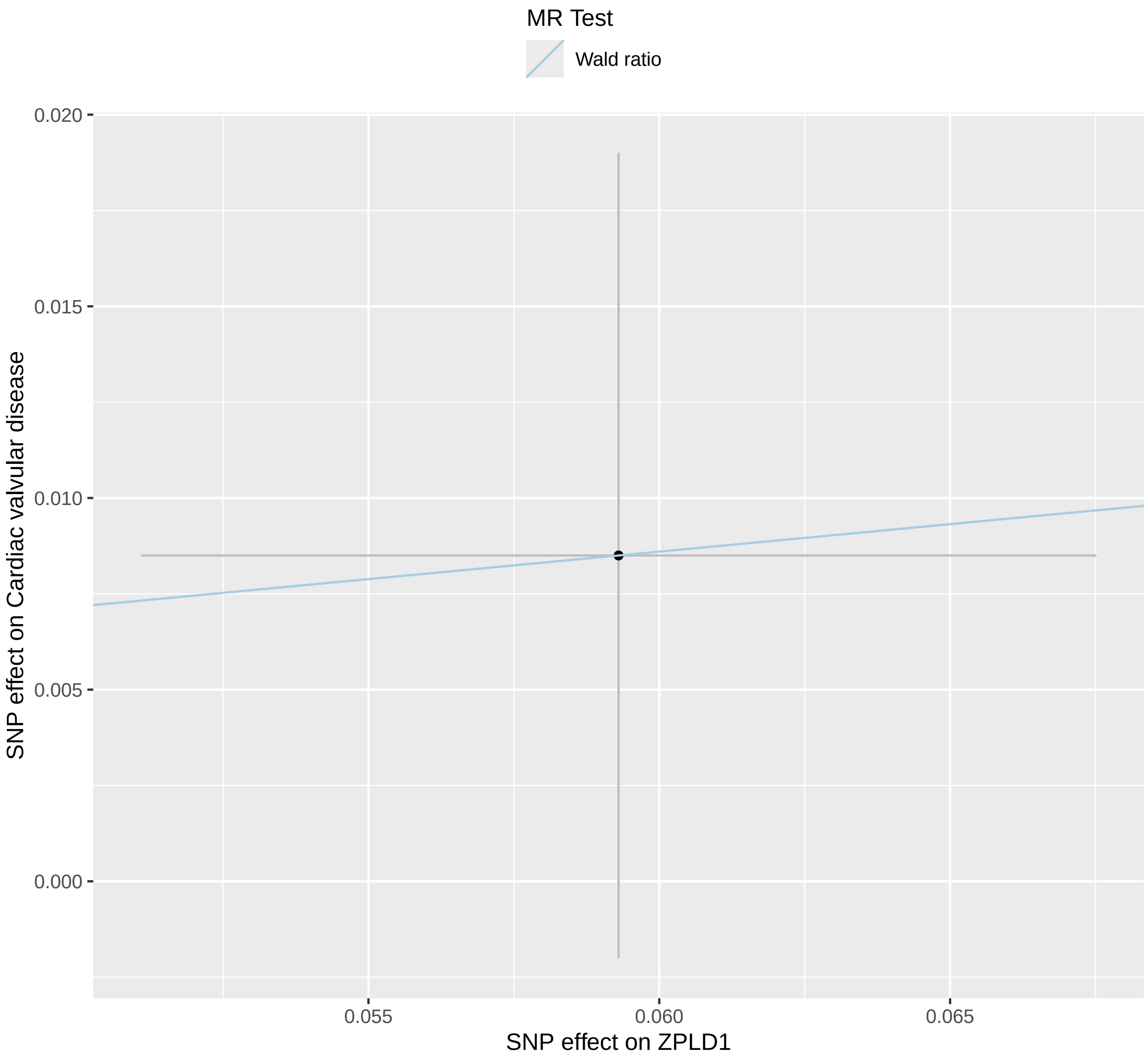
MR Test
Wald ratio
0.020
0.015
0.010
0.005
0.000
0.055
0.060
0.065
SNP effect on Cardiac valvular disease
SNP effect on ZPLD1

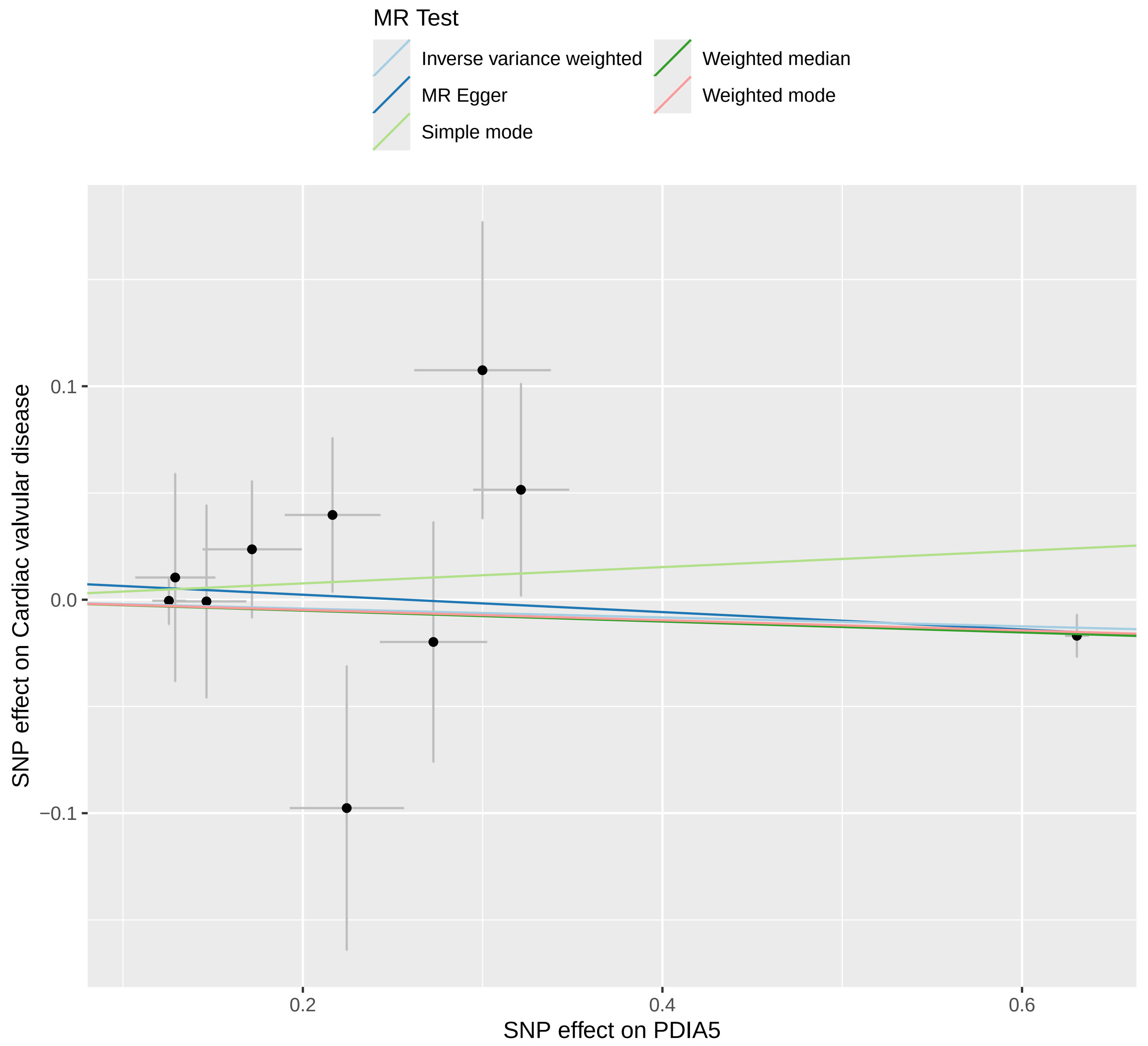
MR Test
Inverse variance weighted
MR Egger
Simple mode
Weighted median
Weighted mode
0.1
0.0
−0.1
0.2
0.4
0.6
SNP effect on Cardiac valvular disease
SNP effect on PDIA5

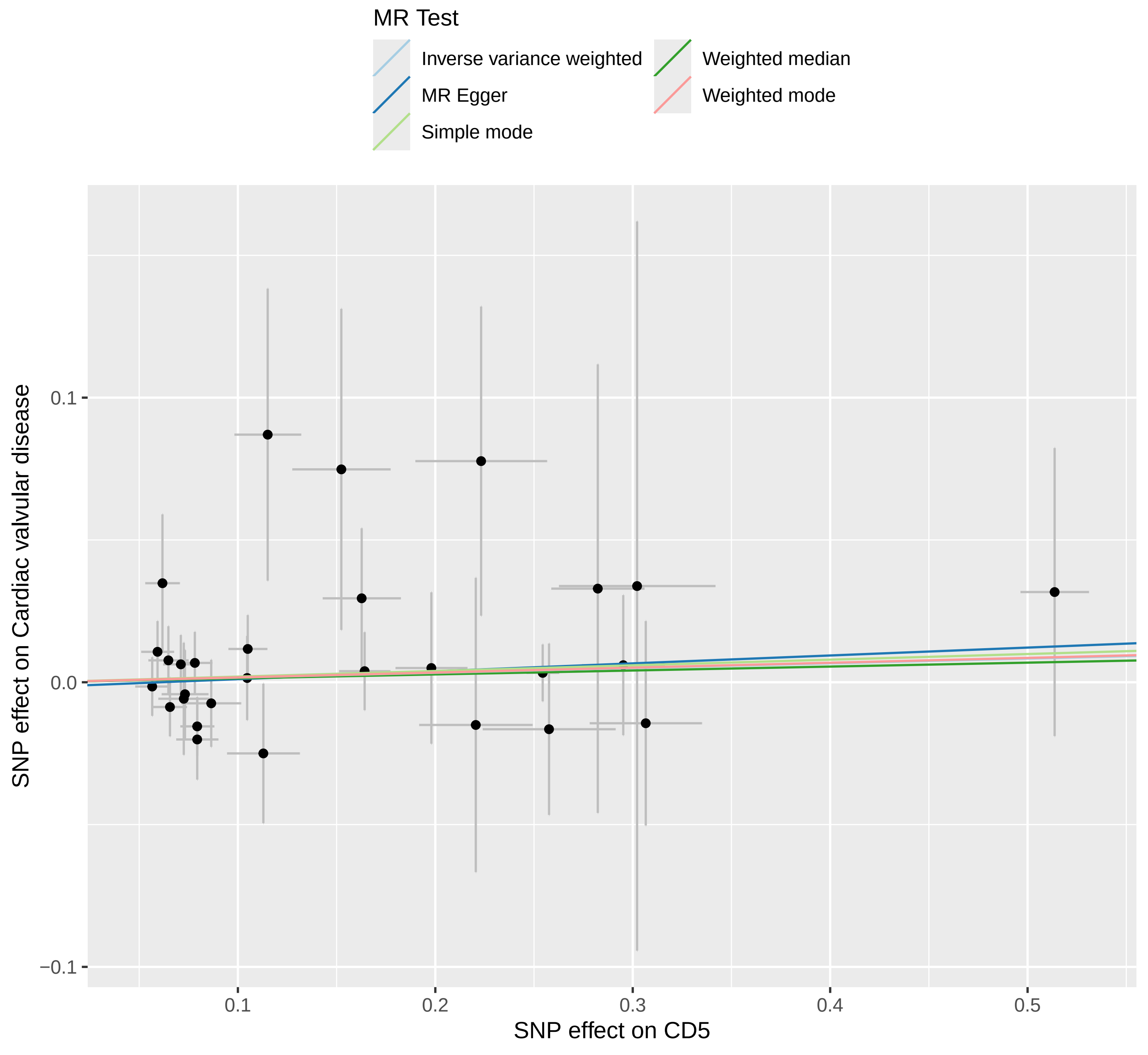
MR Test
Inverse variance weighted
MR Egger
Simple mode
Weighted median
Weighted mode
SNP effect on Cardiac valvular disease
0.1
0.0
−0.1
0.1
0.2
0.3
0.4
0.5
SNP effect on CD5

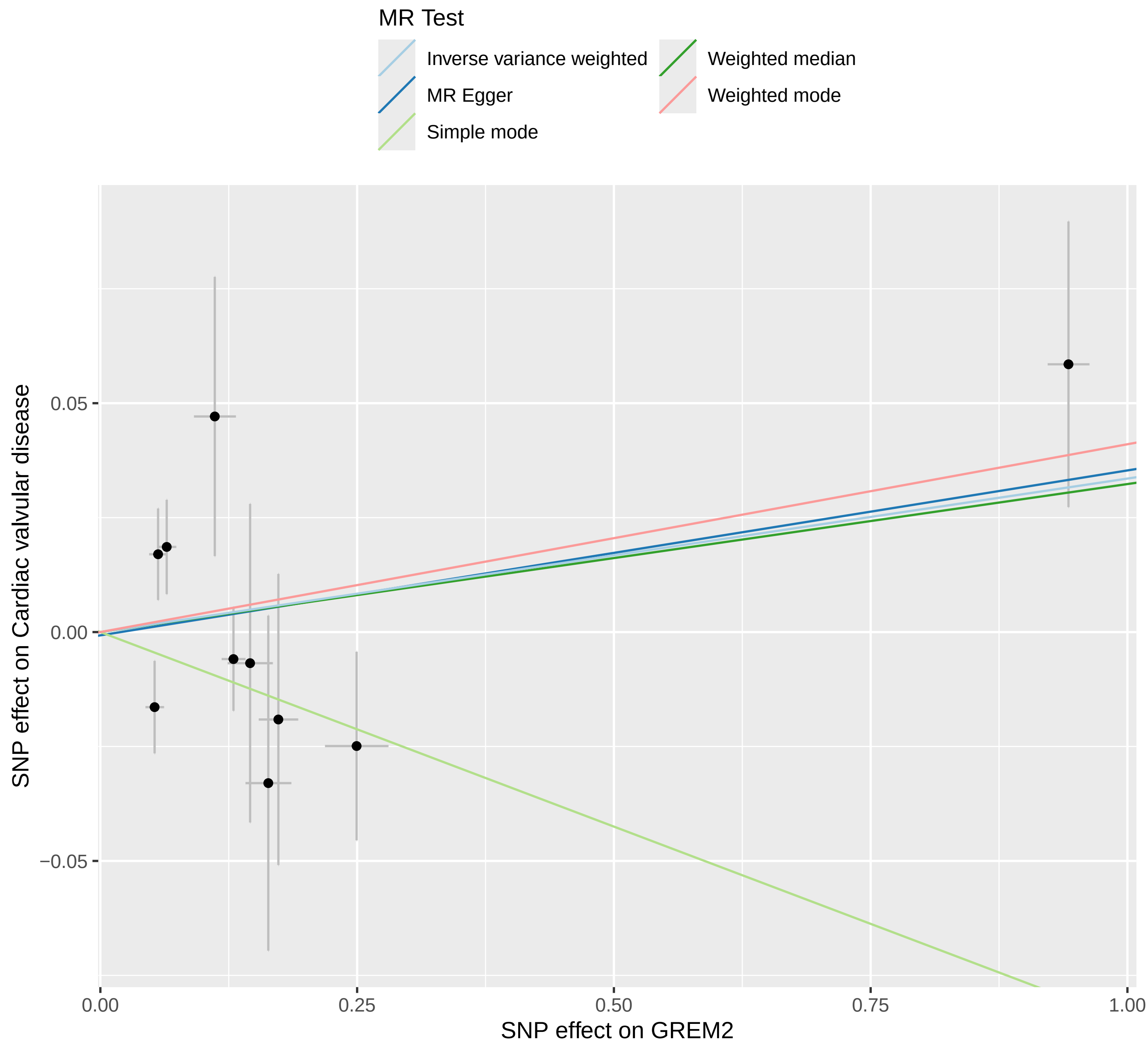
MR Test
Inverse variance weighted
MR Egger
Simple mode
Weighted median
Weighted mode
SNP effect on Cardiac valvular disease
0.05
0.00
−0.05
0.00
0.25
0.50
0.75
1.00
SNP effect on GREM2

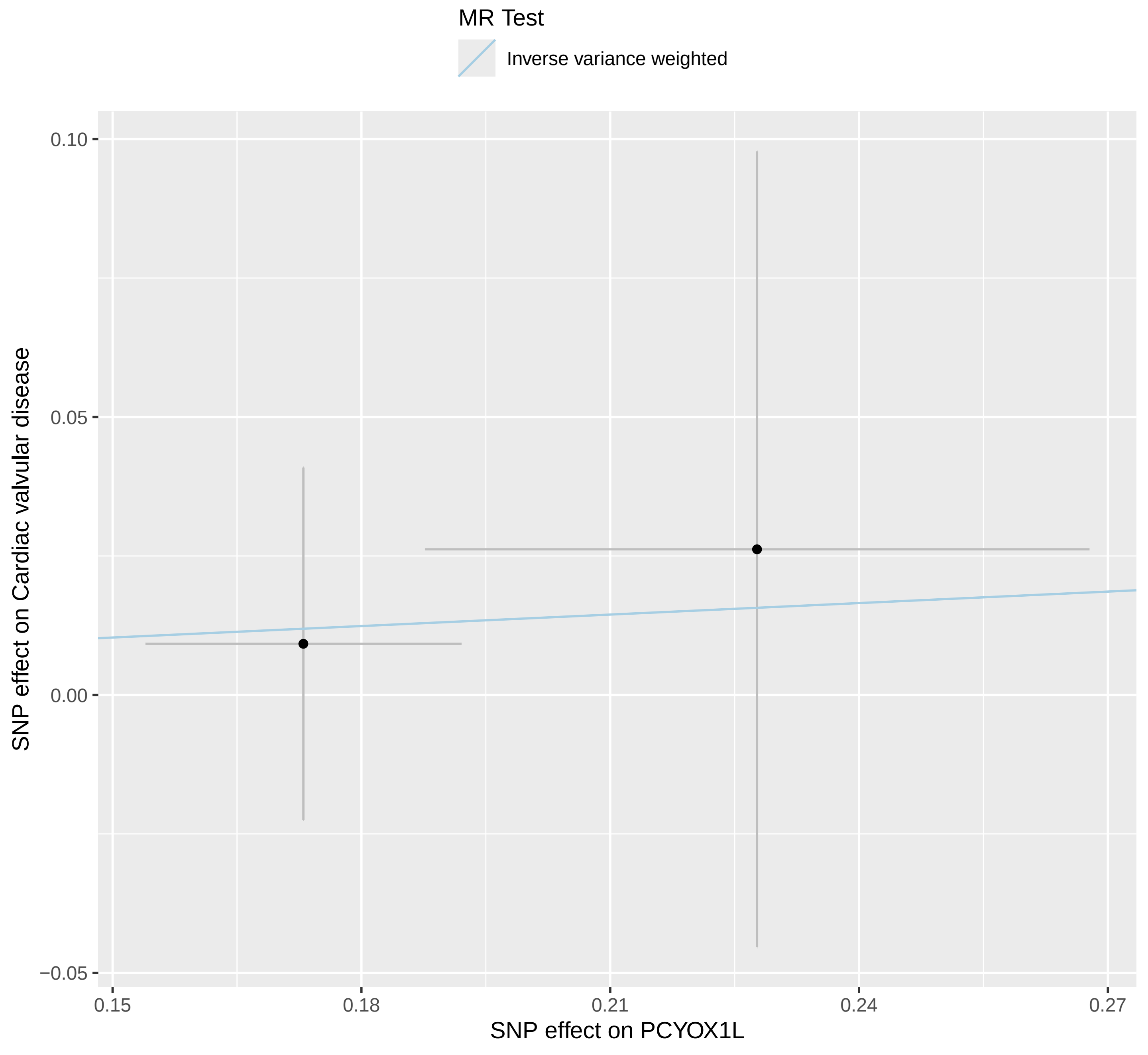

MR Test
Inverse variance weighted
0.10
0.05
0.00
−0.05
0.15
0.18
0.21
0.24
0.27
SNP effect on Cardiac valvular disease
SNP effect on PCYOX1L

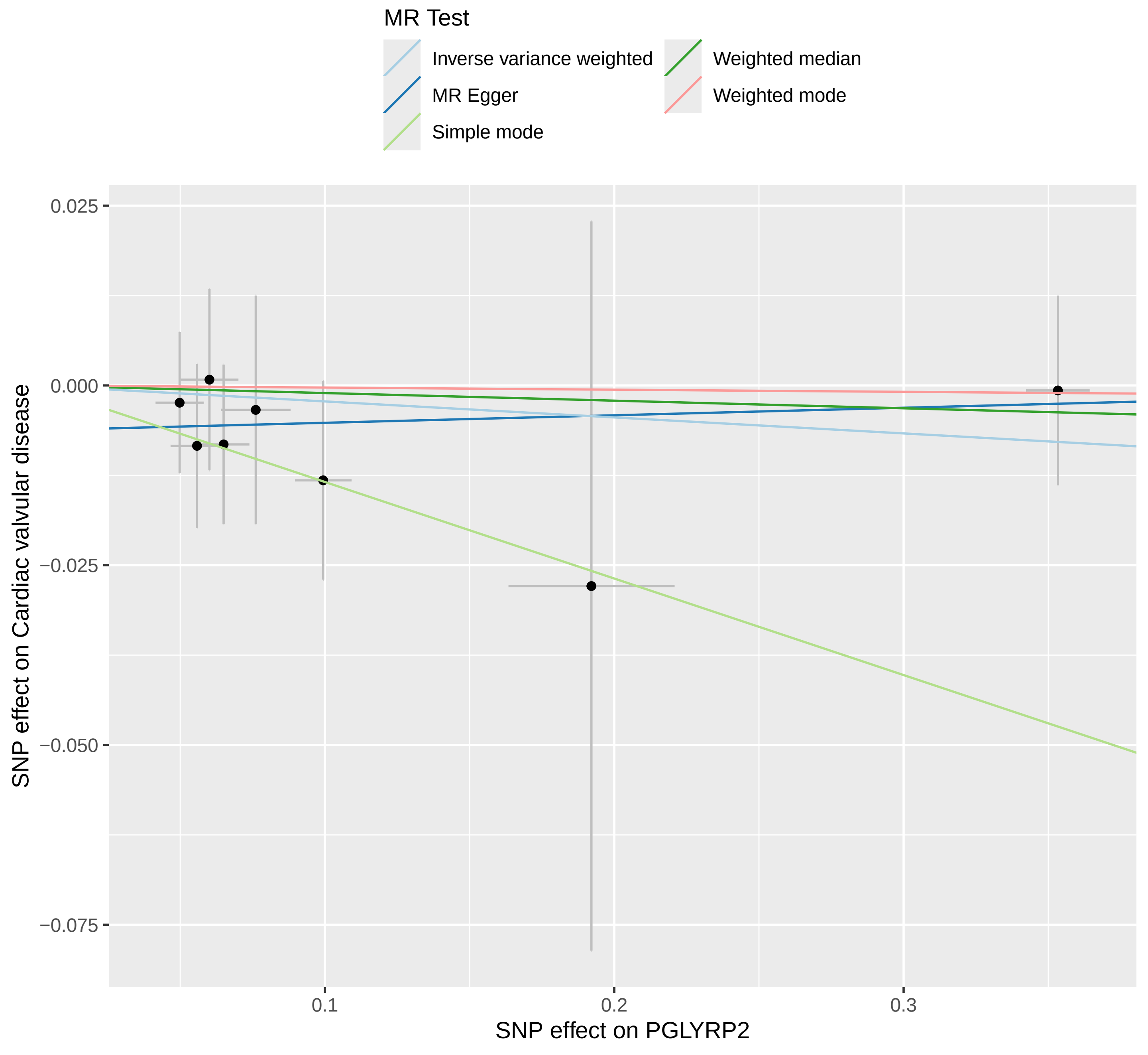
MR Test
Inverse variance weighted
MR Egger
Simple mode
Weighted median
Weighted mode
0.025
0.000
−0.025
−0.050
−0.075
0.1
0.2
0.3
SNP effect on Cardiac valvular disease
SNP effect on PGLYRP2

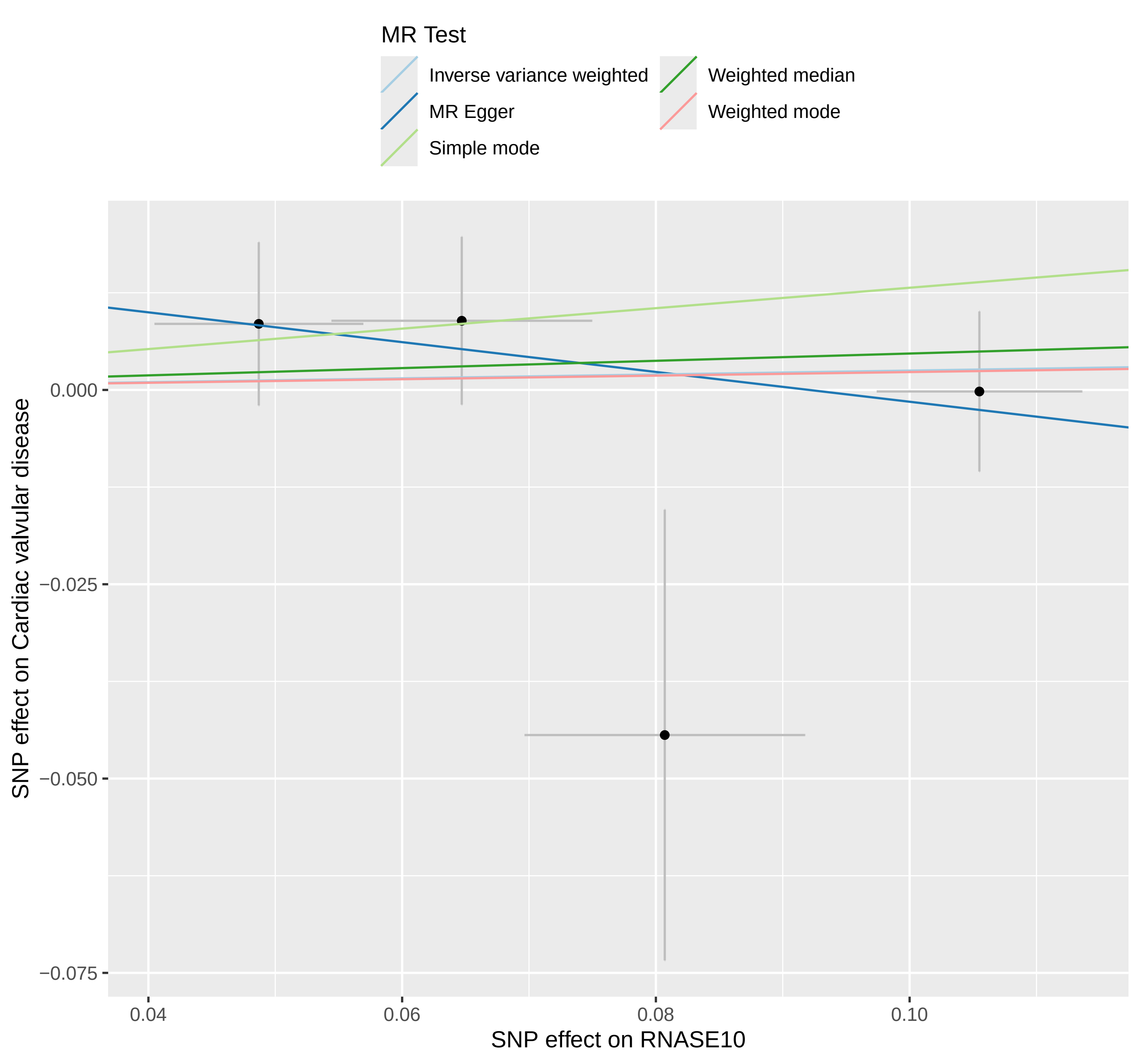

MR Test
Inverse variance weighted
MR Egger
Simple mode
Weighted median
Weighted mode
0.000
−0.025
−0.050
−0.075
0.04
0.06
0.08
0.10
SNP effect on Cardiac valvular disease
SNP effect on RNASE10

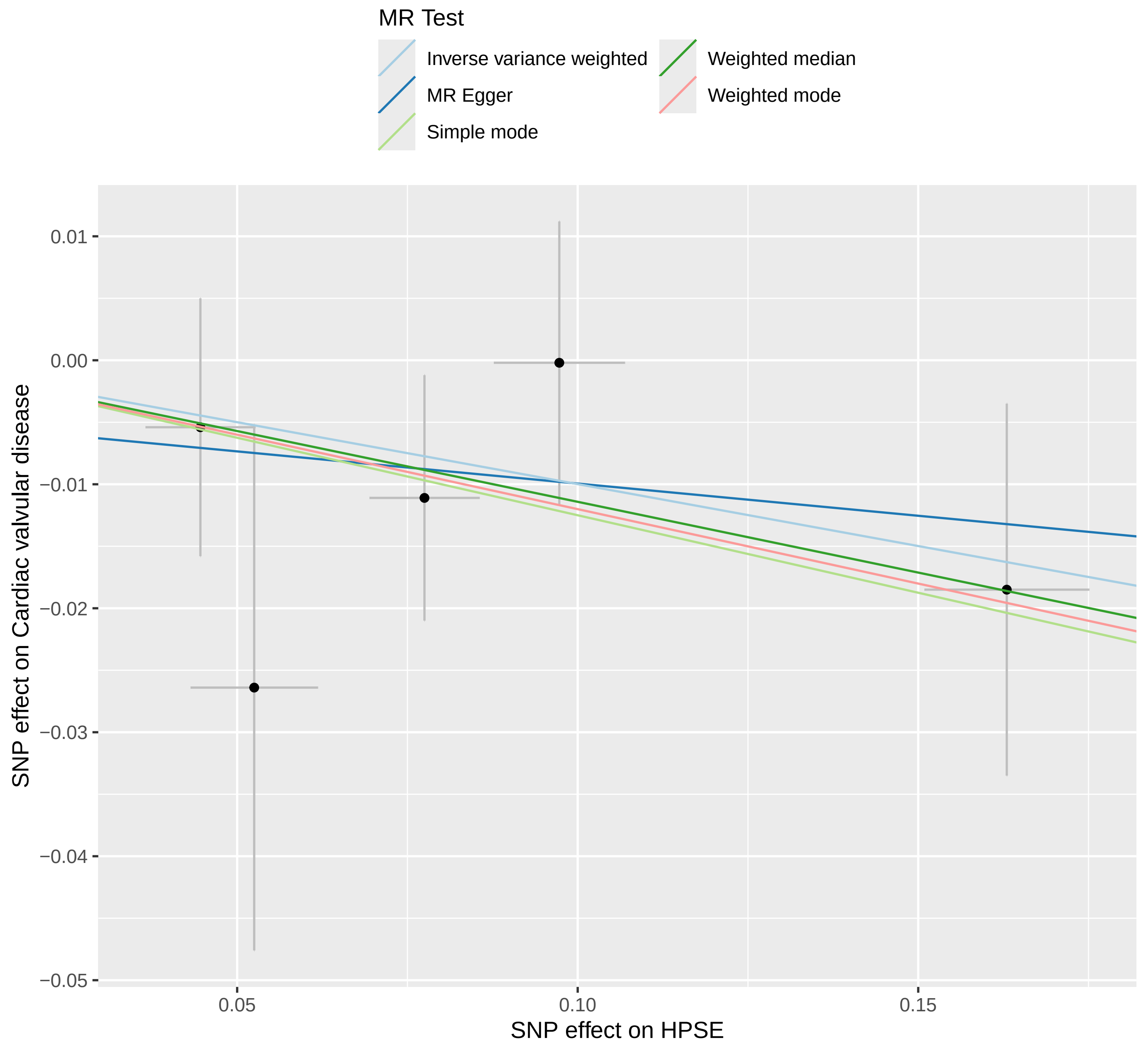

MR Test
Inverse variance weighted
MR Egger
Simple mode
Weighted median
Weighted mode
0.01
0.00
−0.01
−0.02
−0.03
−0.04
−0.05
0.05
0.10
0.15
SNP effect on Cardiac valvular disease
SNP effect on HPSE

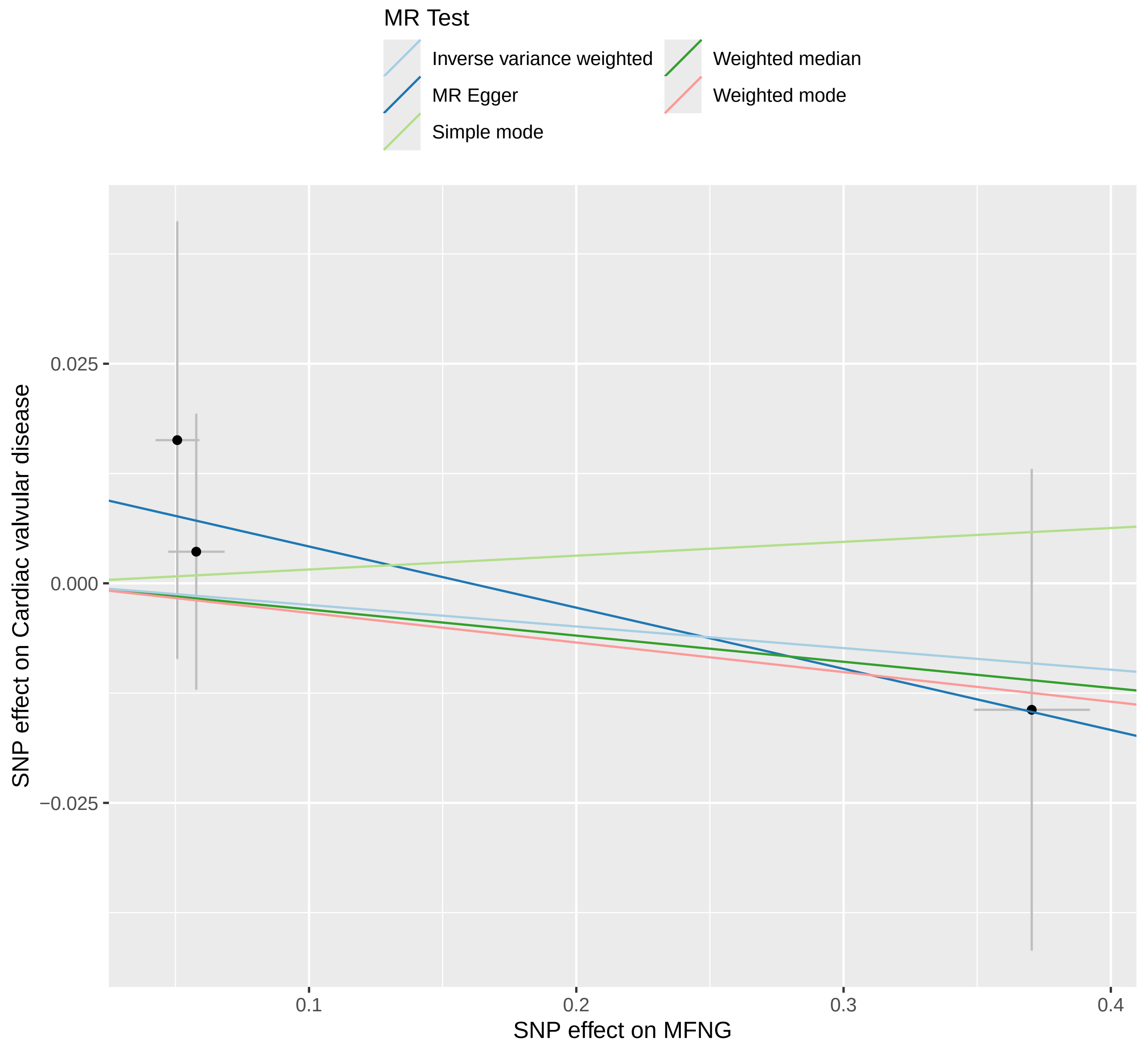

MR Test
Inverse variance weighted
MR Egger
Simple mode
Weighted median
Weighted mode
0.025
0.000
−0.025
SNP effect on Cardiac valvular disease
0.1
0.2
0.3
0.4
SNP effect on MFNG

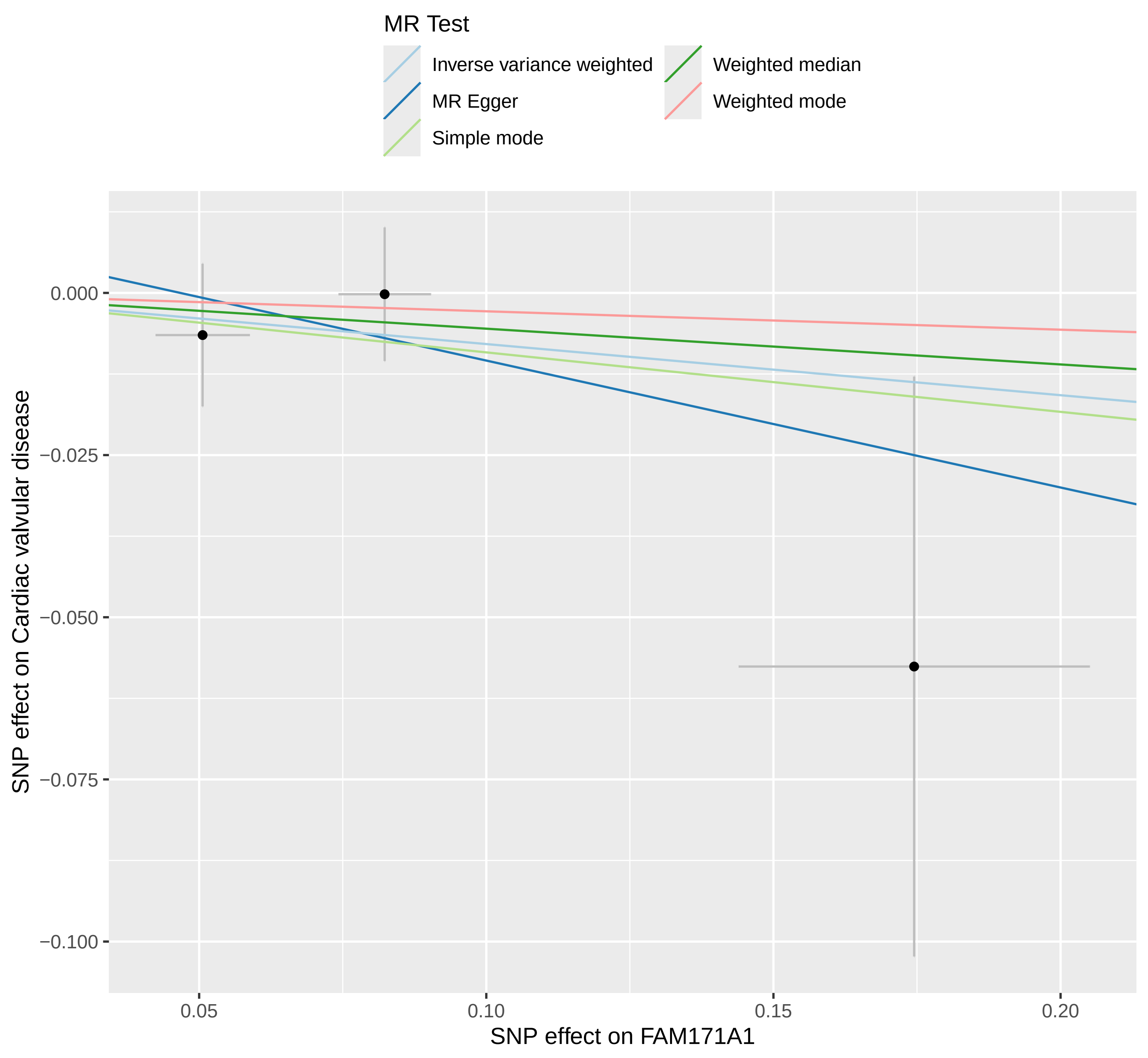

MR Test
Inverse variance weighted
MR Egger
Simple mode
Weighted median
Weighted mode
0.000
−0.025
−0.050
−0.075
−0.100
SNP effect on Cardiac valvular disease
0.05
0.10
0.15
0.20
SNP effect on FAM171A1

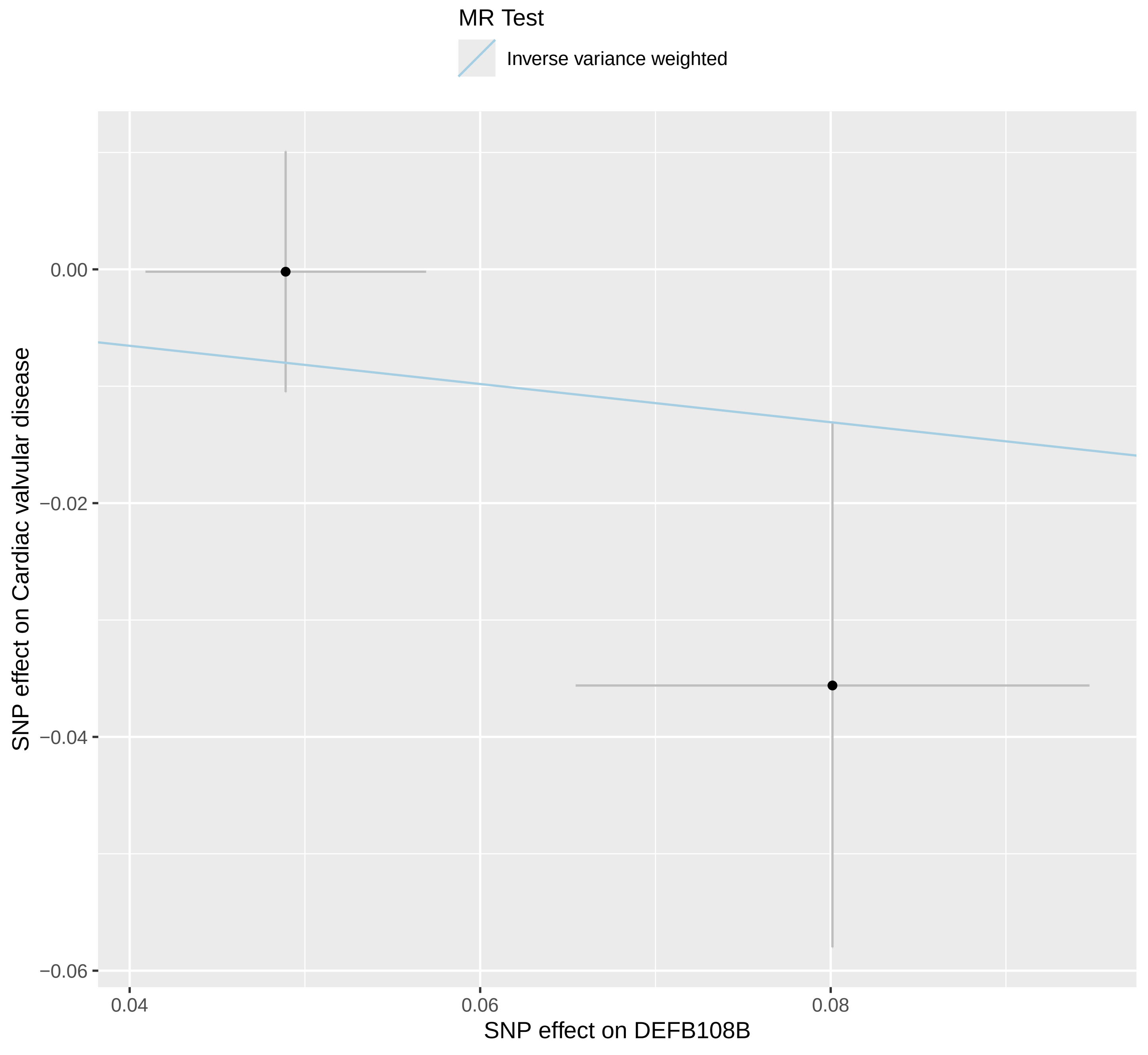

MR Test
Inverse variance weighted
0.00
−0.02
−0.04
−0.06
0.04
0.06
0.08
SNP effect on Cardiac valvular disease
SNP effect on DEFB108B

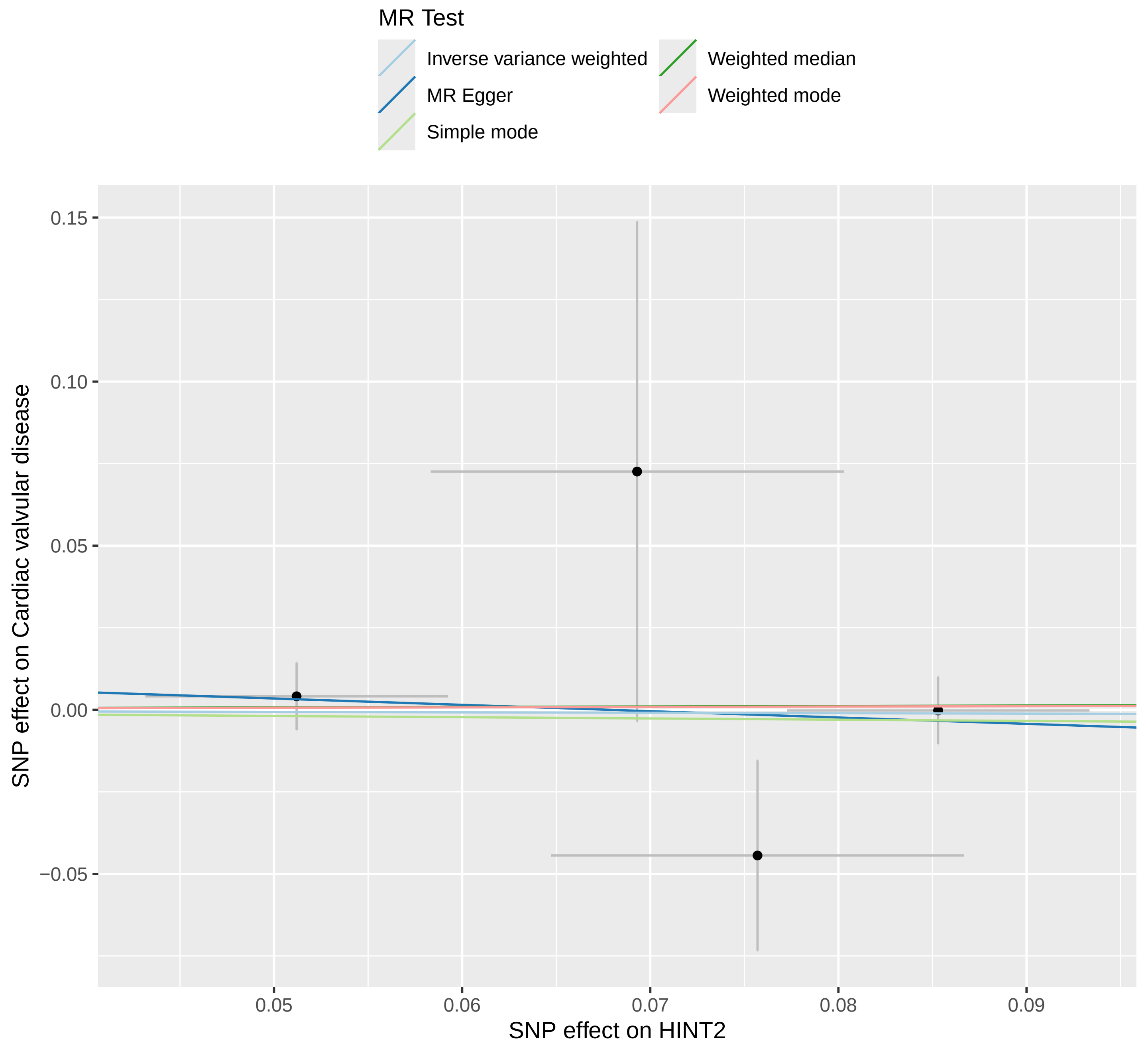

MR Test
Inverse variance weighted
MR Egger
Simple mode
Weighted median
Weighted mode
0.15
0.10
0.05
0.00
−0.05
0.05
0.06
0.07
0.08
0.09
SNP effect on Cardiac valvular disease
SNP effect on HINT2

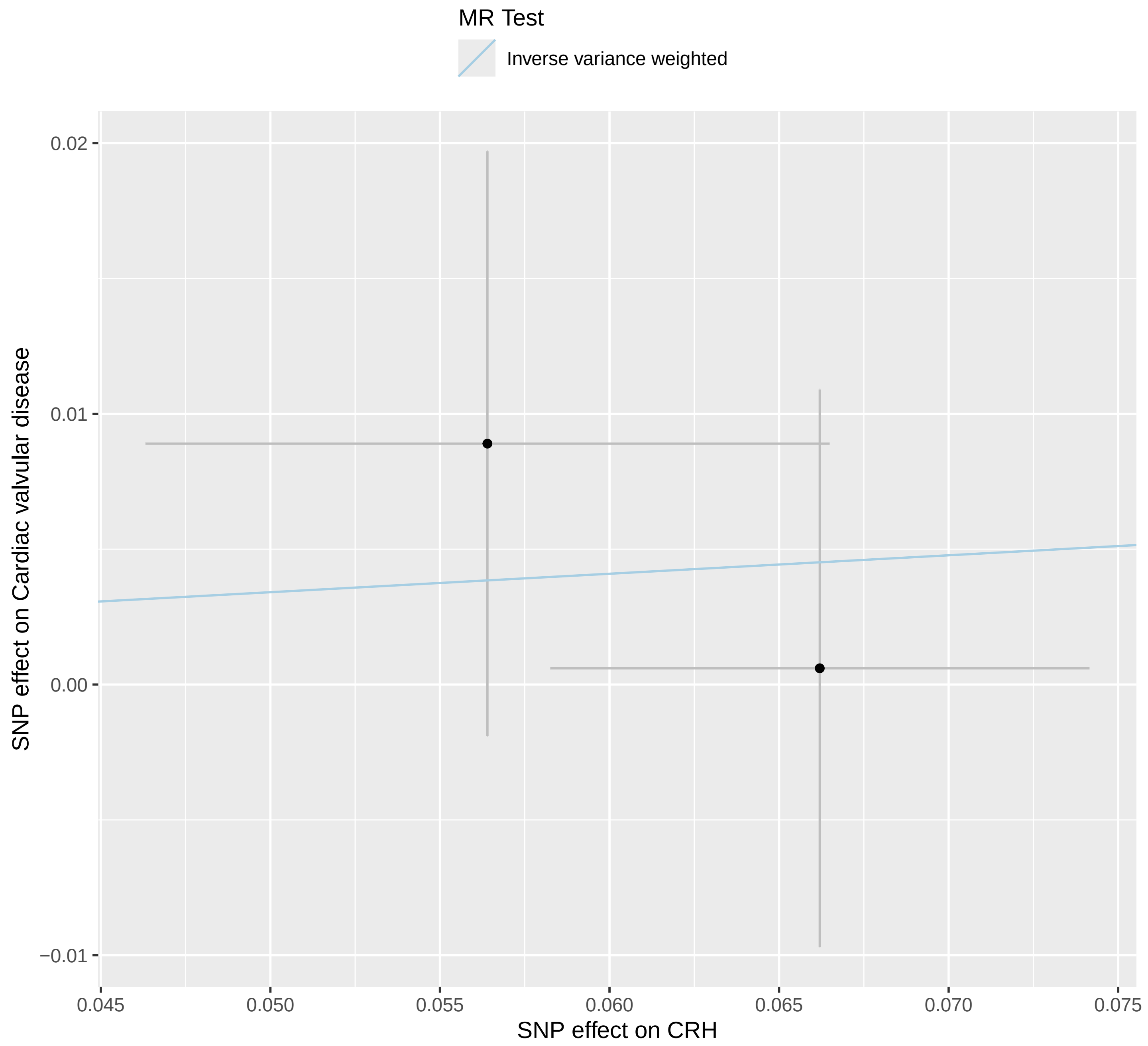
MR Test
Inverse variance weighted
0.02
0.01
0.00
−0.01
0.045
0.050
0.055
0.060
0.065
0.070
0.075
SNP effect on Cardiac valvular disease
SNP effect on CRH

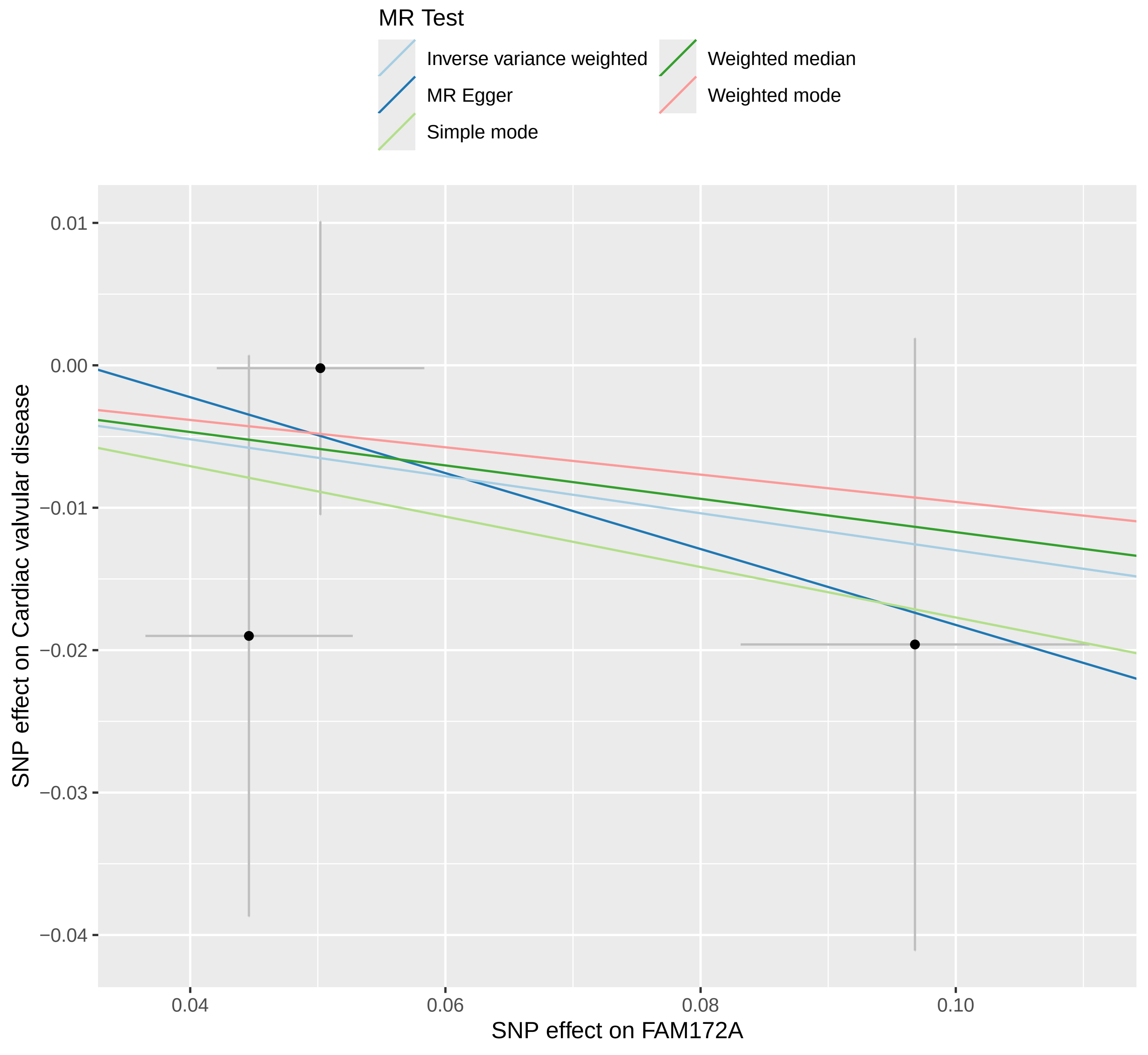

MR Test
Inverse variance weighted
MR Egger
Simple mode
Weighted median
Weighted mode
0.01
0.00
−0.01
−0.02
−0.03
−0.04
SNP effect on Cardiac valvular disease
0.04
0.06
0.08
0.10
SNP effect on FAM172A

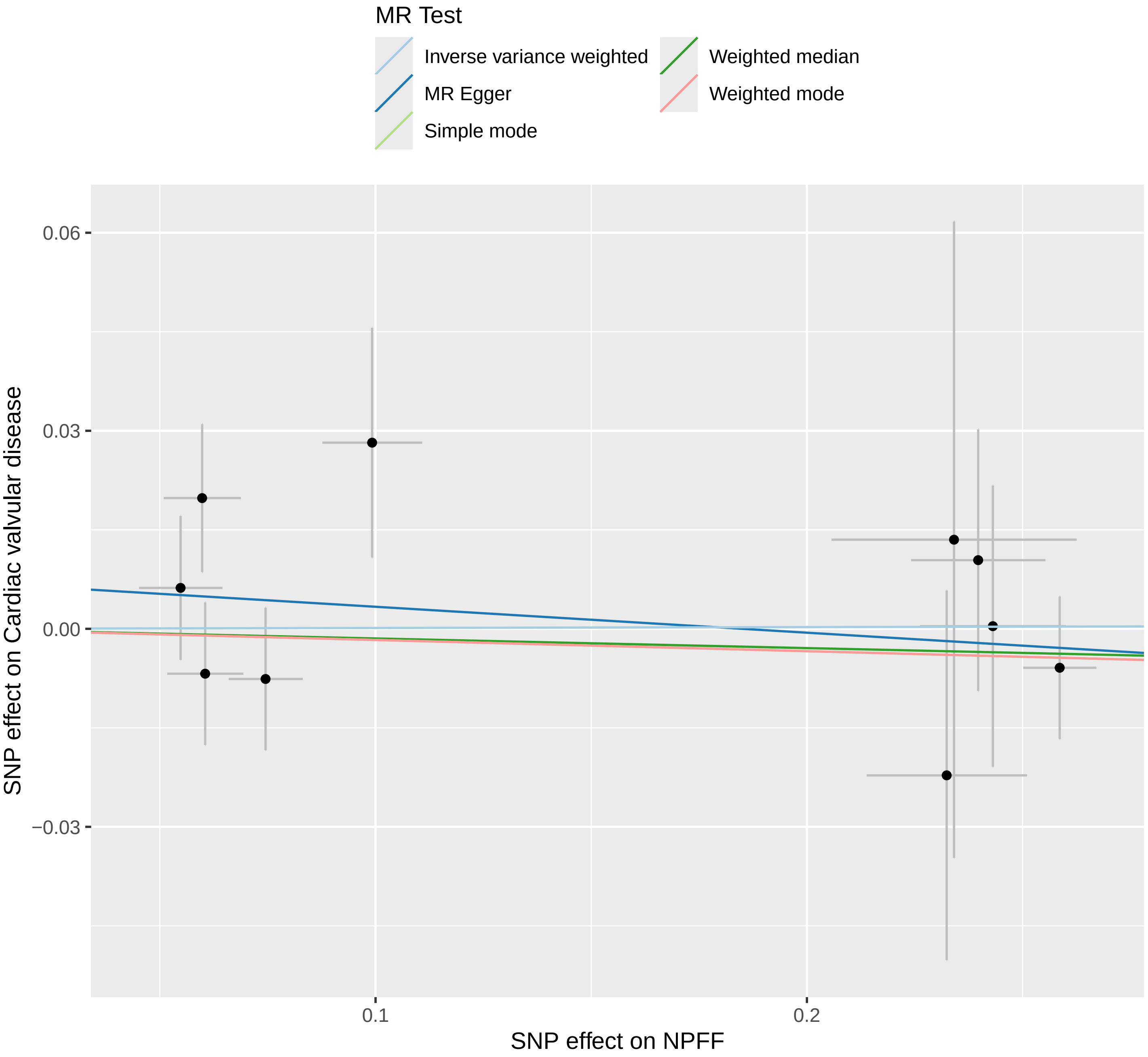
MR Test
Inverse variance weighted
MR Egger
Simple mode
Weighted median
Weighted mode
0.06
0.03
0.00
−0.03
0.1
0.2
SNP effect on NPFF
SNP effect on Cardiac valvular disease

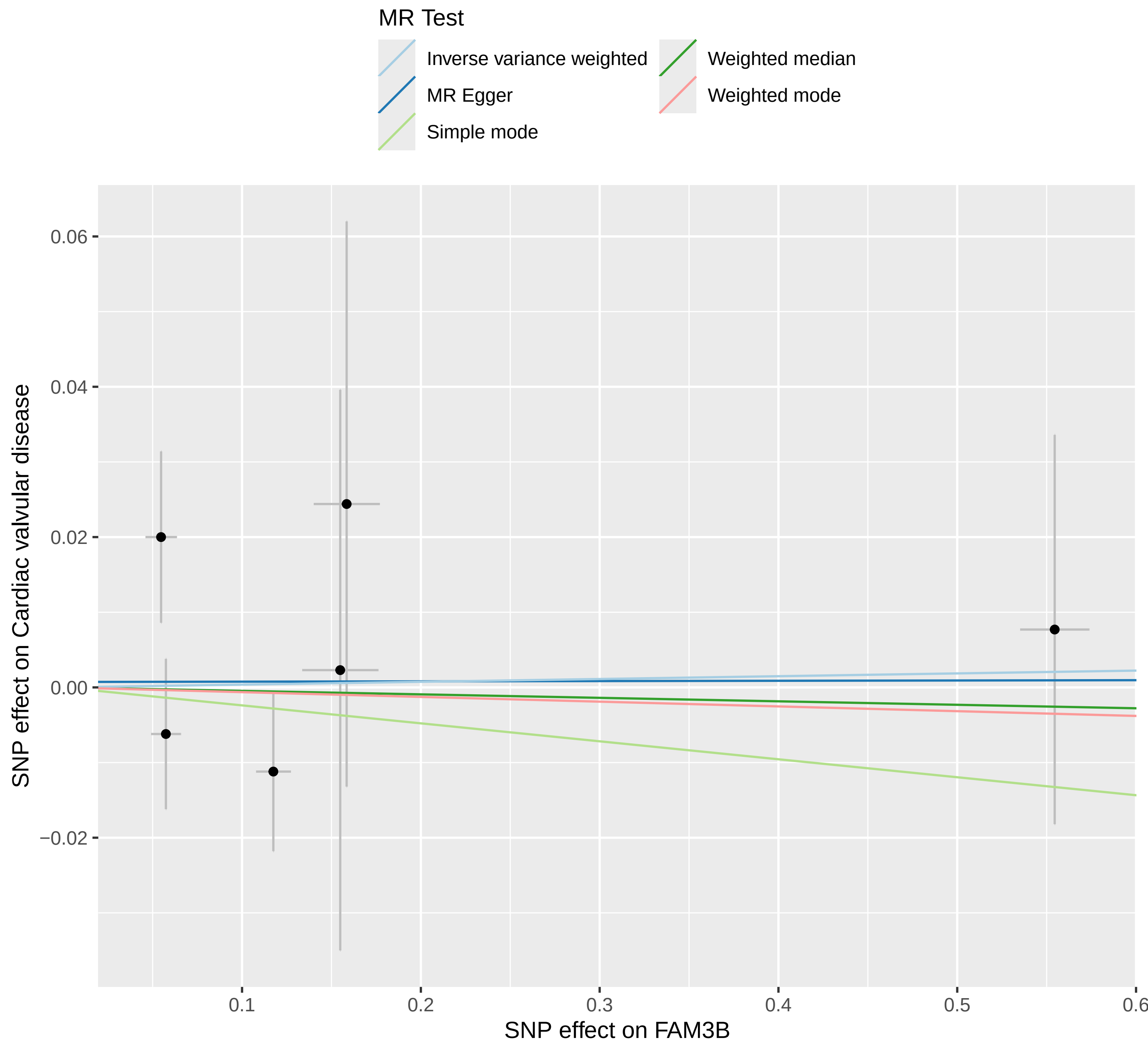
MR Test
Inverse variance weighted
MR Egger
Simple mode
Weighted median
Weighted mode
0.06
0.04
0.02
0.00
−0.02
0.1
0.2
0.3
0.4
0.5
0.6
SNP effect on FAM3B
SNP effect on Cardiac valvular disease

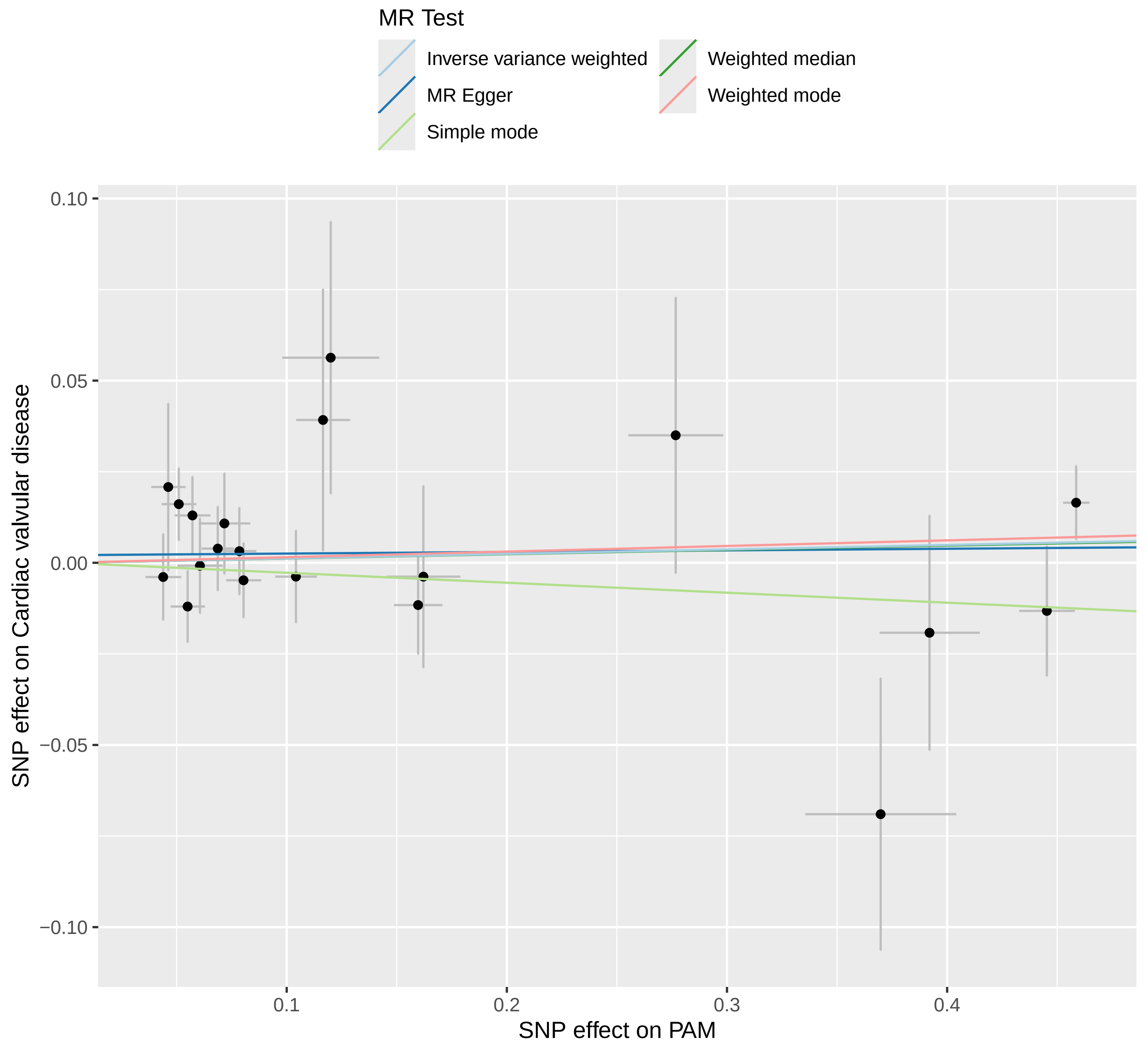
MR Test
Inverse variance weighted
MR Egger
Simple mode
Weighted median
Weighted mode
0.10
0.05
0.00
−0.05
−0.10
0.1
0.2
0.3
0.4
SNP effect on Cardiac valvular disease
SNP effect on PAM

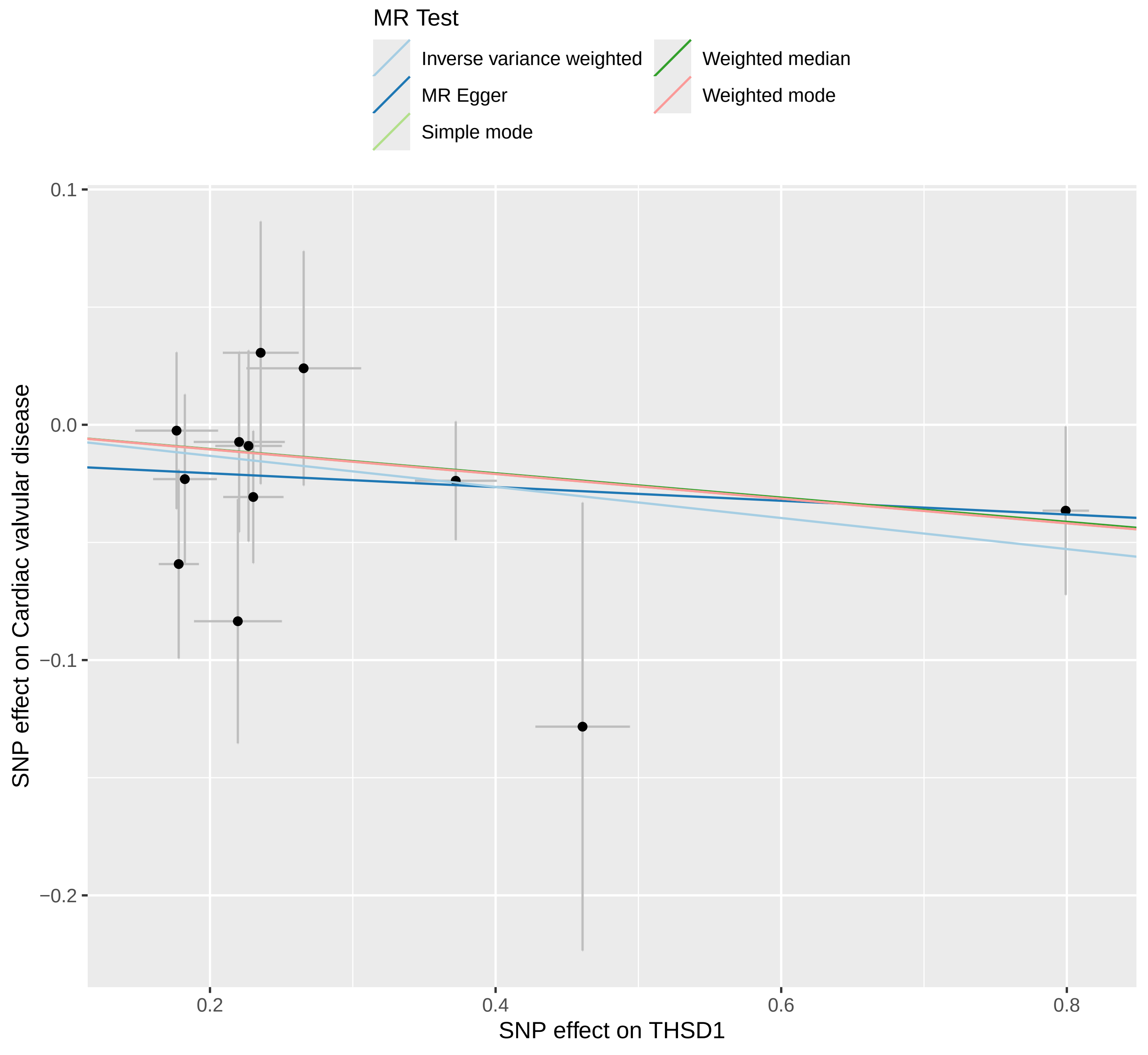
MR Test
Inverse variance weighted
MR Egger
Simple mode
Weighted median
Weighted mode
0.1
0.0
−0.1
−0.2
0.2
0.4
0.6
0.8
SNP effect on Cardiac valvular disease
SNP effect on THSD1

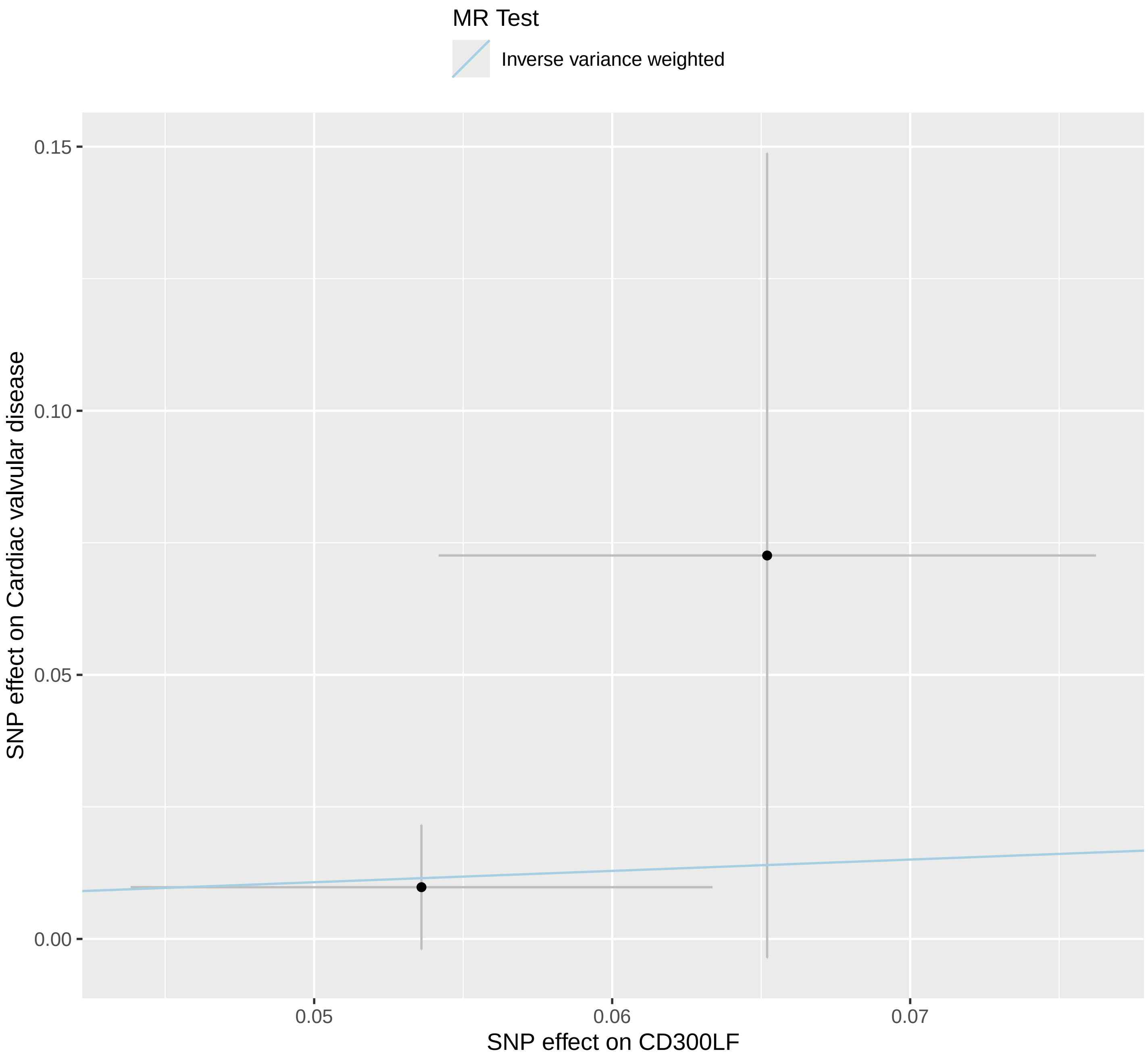

MR Test
Inverse variance weighted
0.15
0.10
0.05
0.00
SNP effect on Cardiac valvular disease
0.05
0.06
0.07
SNP effect on CD300LF

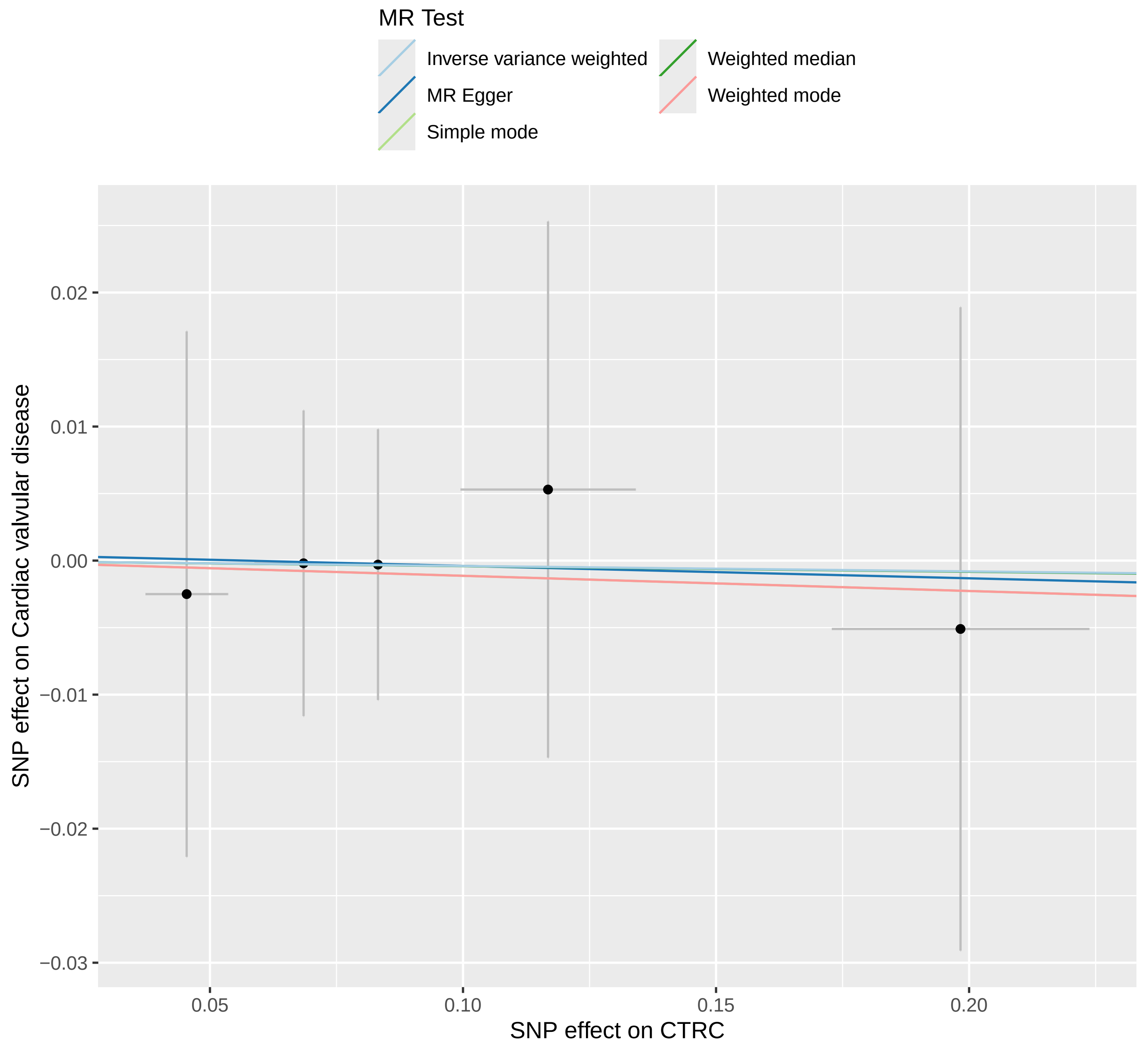
MR Test
Inverse variance weighted
MR Egger
Simple mode
Weighted median
Weighted mode
0.02
0.01
0.00
−0.01
−0.02
−0.03
SNP effect on Cardiac valvular disease
0.05
0.10
0.15
0.20
SNP effect on CTRC

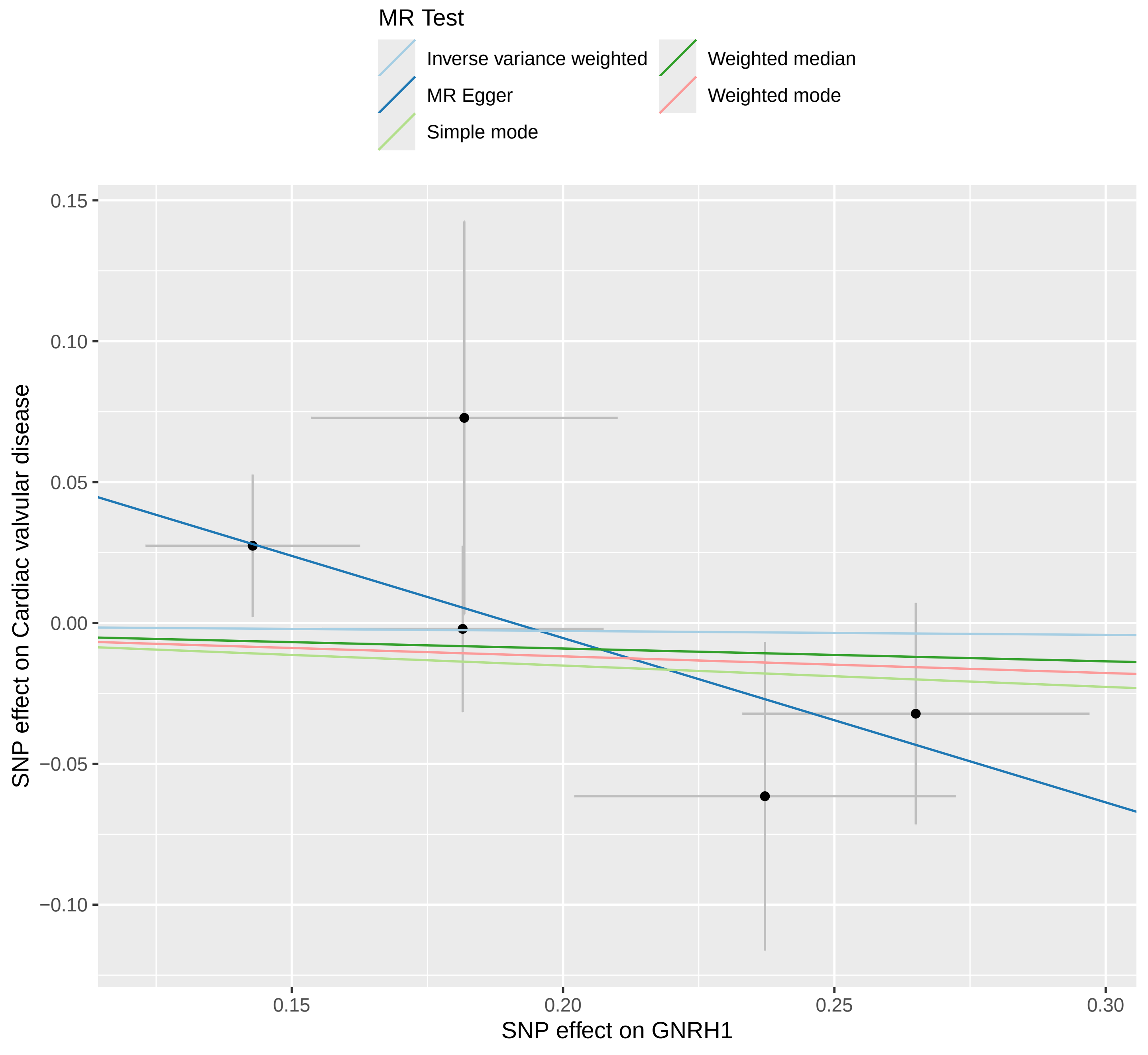

MR Test
Inverse variance weighted
MR Egger
Simple mode
Weighted median
Weighted mode
0.15
0.10
0.05
0.00
−0.05
−0.10
SNP effect on Cardiac valvular disease
0.15
0.20
0.25
0.30
SNP effect on GNRH1

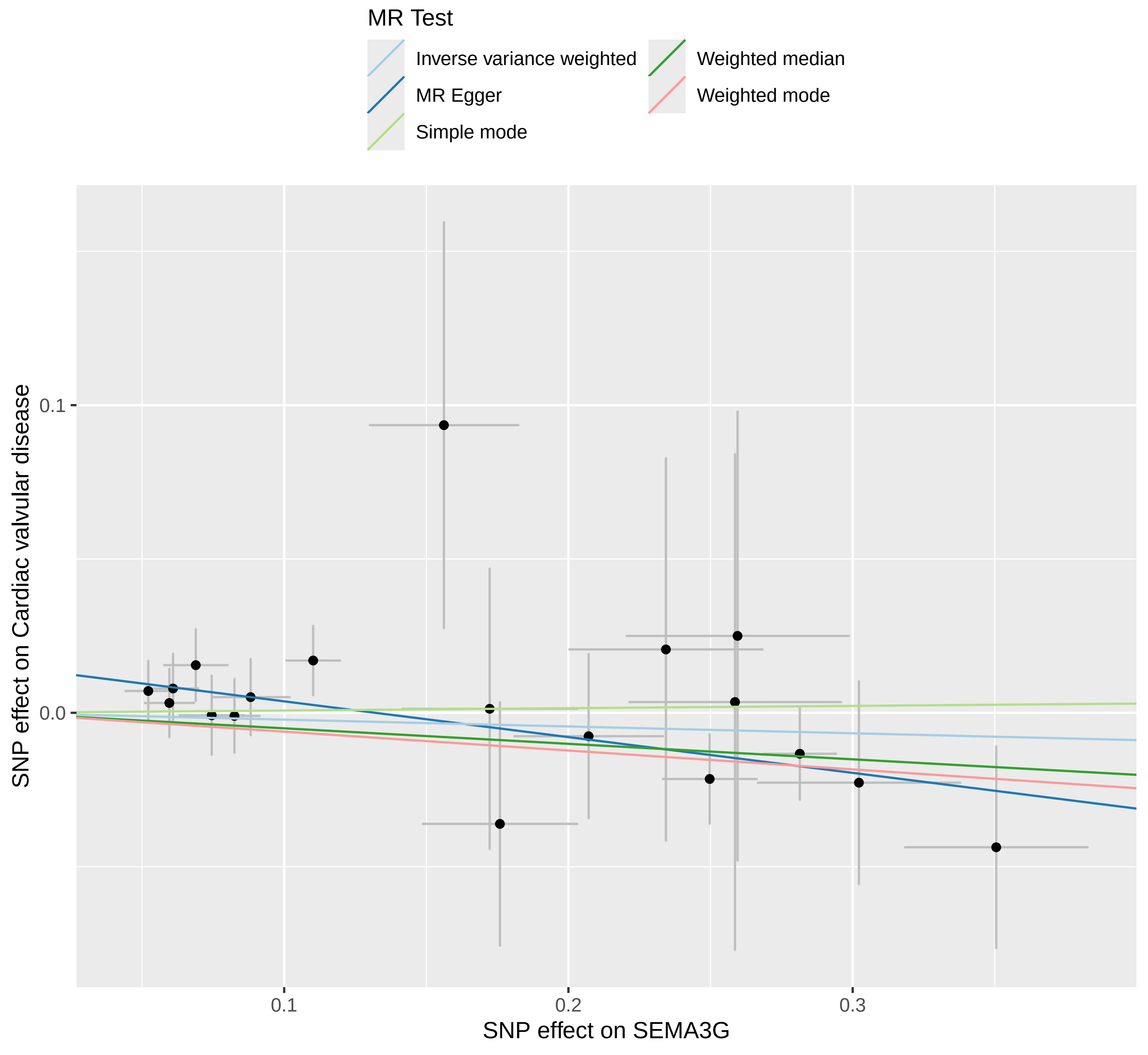
MR Test
Inverse variance weighted
MR Egger
Simple mode
Weighted median
Weighted mode
0.1
0.0
SNP effect on Cardiac valvular disease
0.1
0.2
0.3
SNP effect on SEMA3G

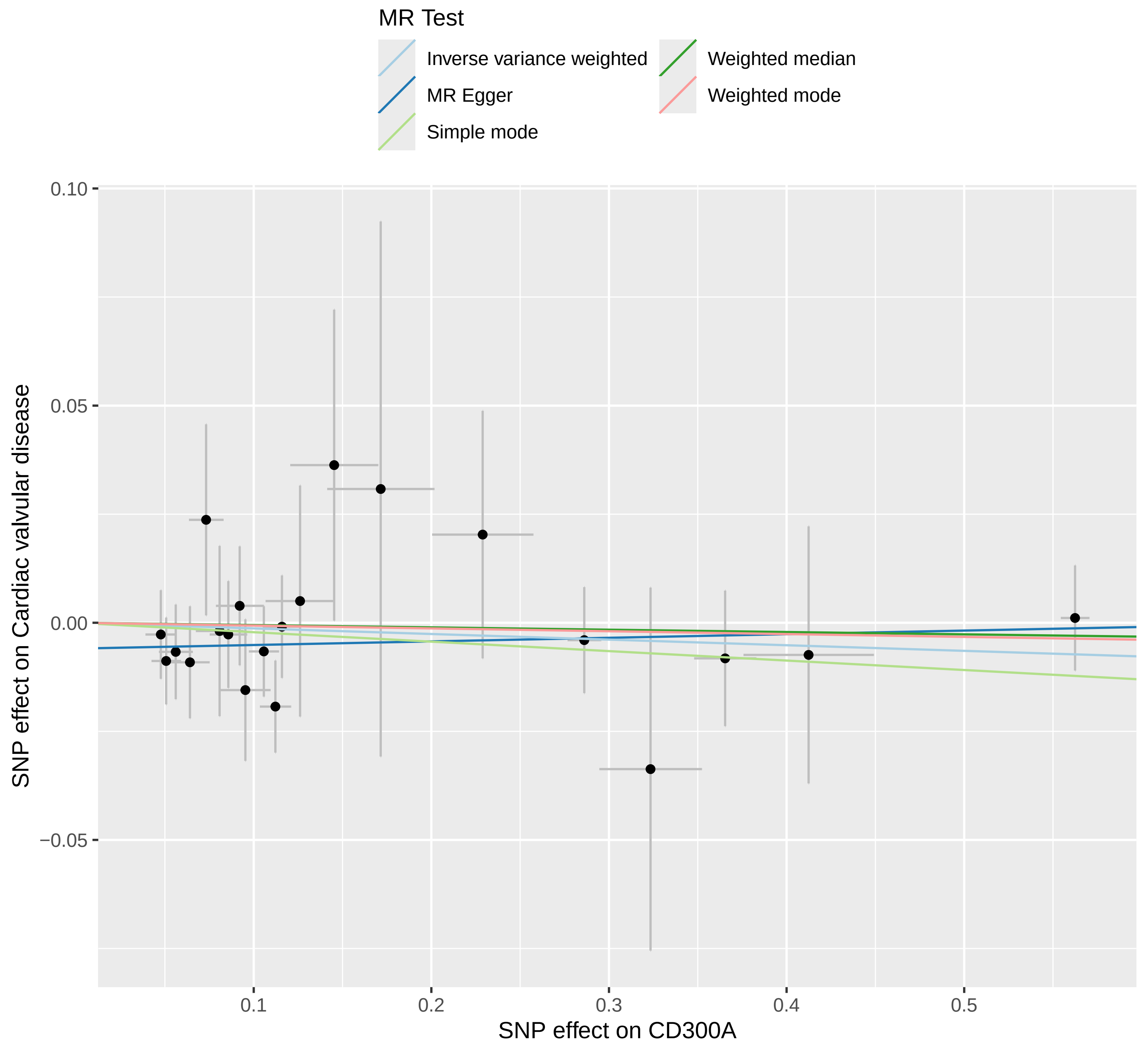

MR Test
Inverse variance weighted
MR Egger
Simple mode
Weighted median
Weighted mode
0.10
0.05
0.00
−0.05
0.1
0.2
0.3
0.4
0.5
SNP effect on Cardiac valvular disease
SNP effect on CD300A

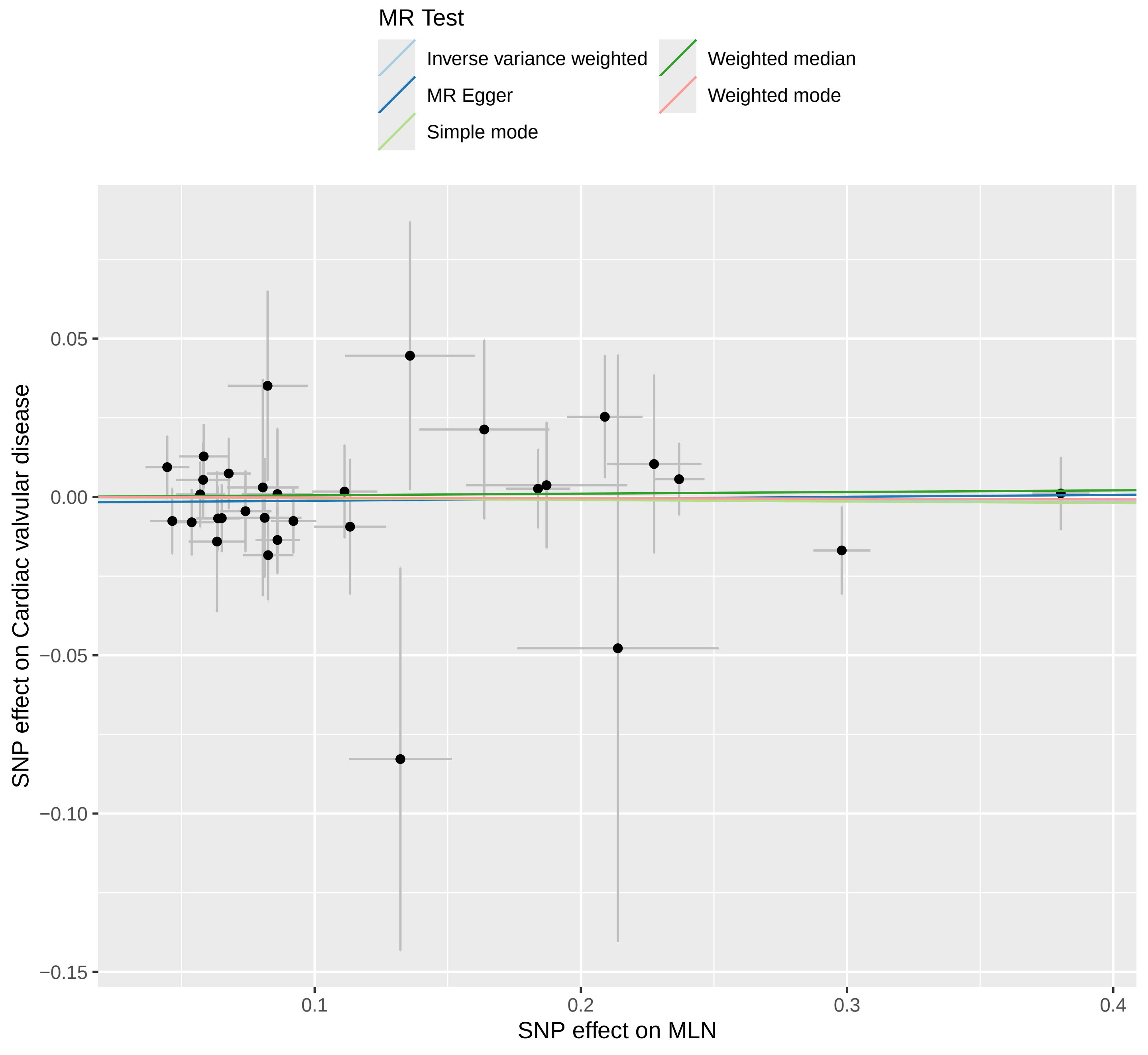

MR Test
Inverse variance weighted
MR Egger
Simple mode
Weighted median
Weighted mode
0.05
0.00
−0.05
−0.10
−0.15
SNP effect on Cardiac valvular disease
0.1
0.2
0.3
0.4
SNP effect on MLN

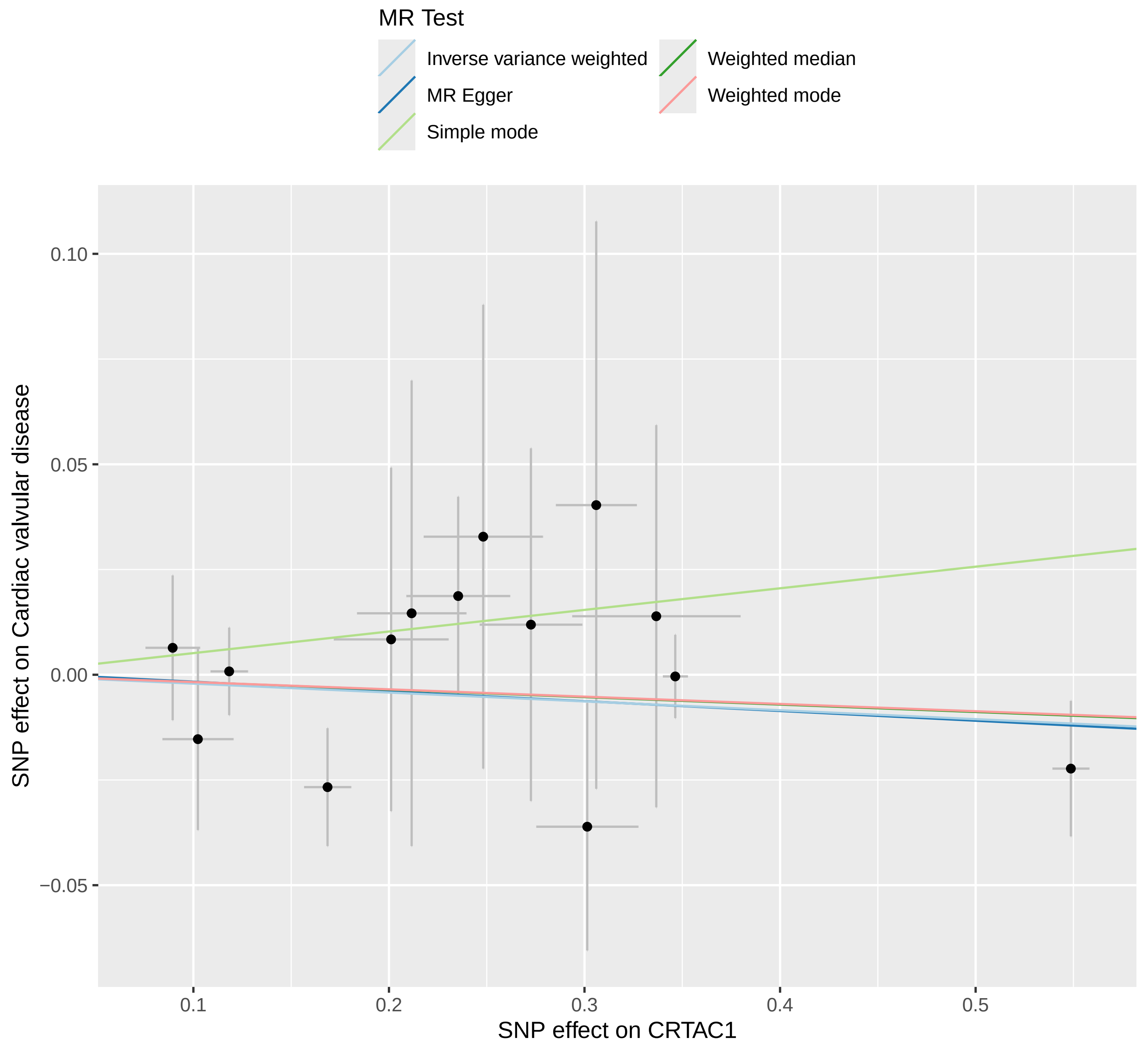
MR Test
Inverse variance weighted
MR Egger
Simple mode
Weighted median
Weighted mode
0.10
0.05
0.00
−0.05
SNP effect on Cardiac valvular disease
0.1
0.2
0.3
0.4
0.5
SNP effect on CRTAC1

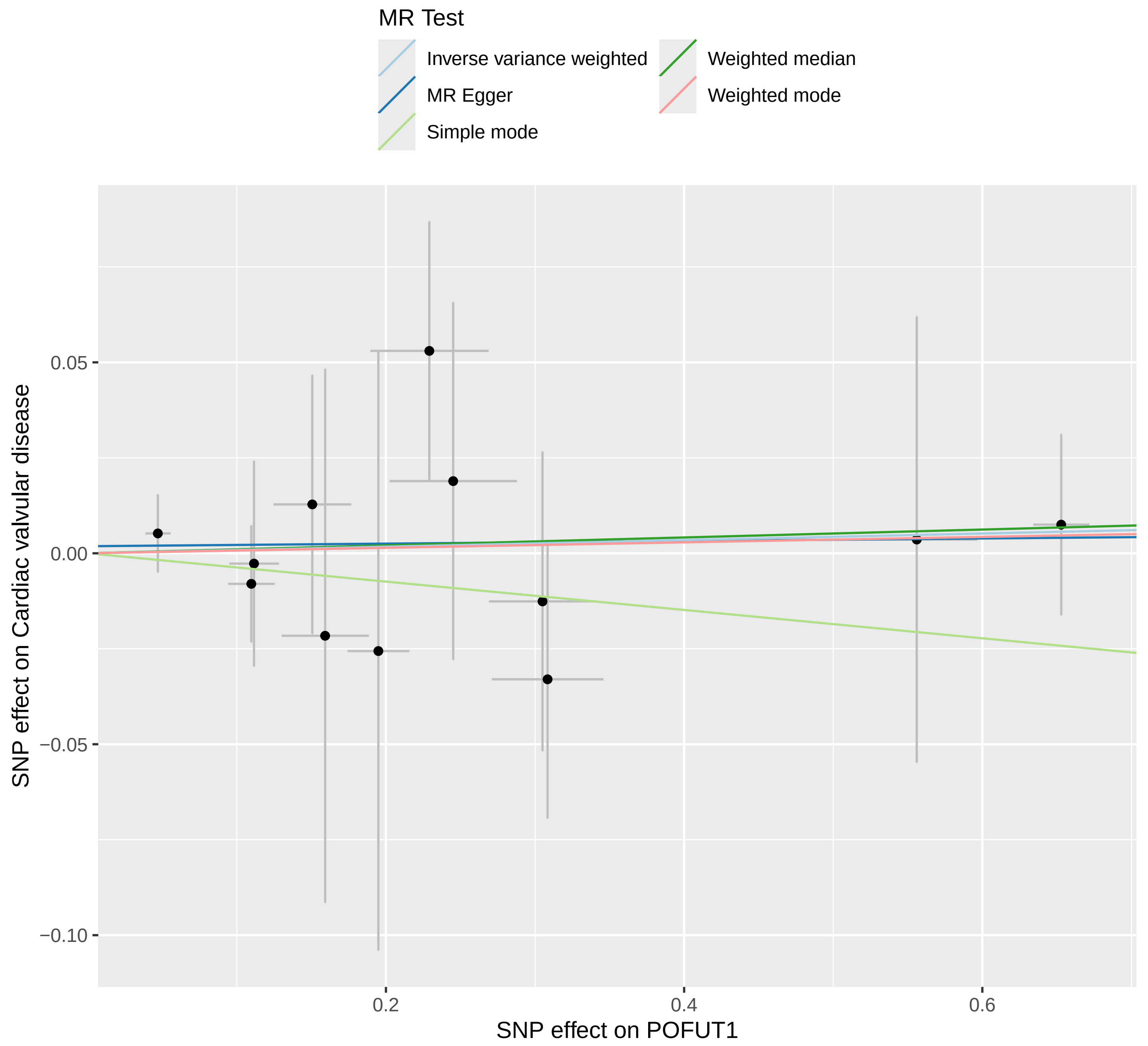
MR Test
Inverse variance weighted
MR Egger
Simple mode
Weighted median
Weighted mode
SNP effect on Cardiac valvular disease
0.05
0.00
−0.05
−0.10
0.2
0.4
0.6
SNP effect on POFUT1

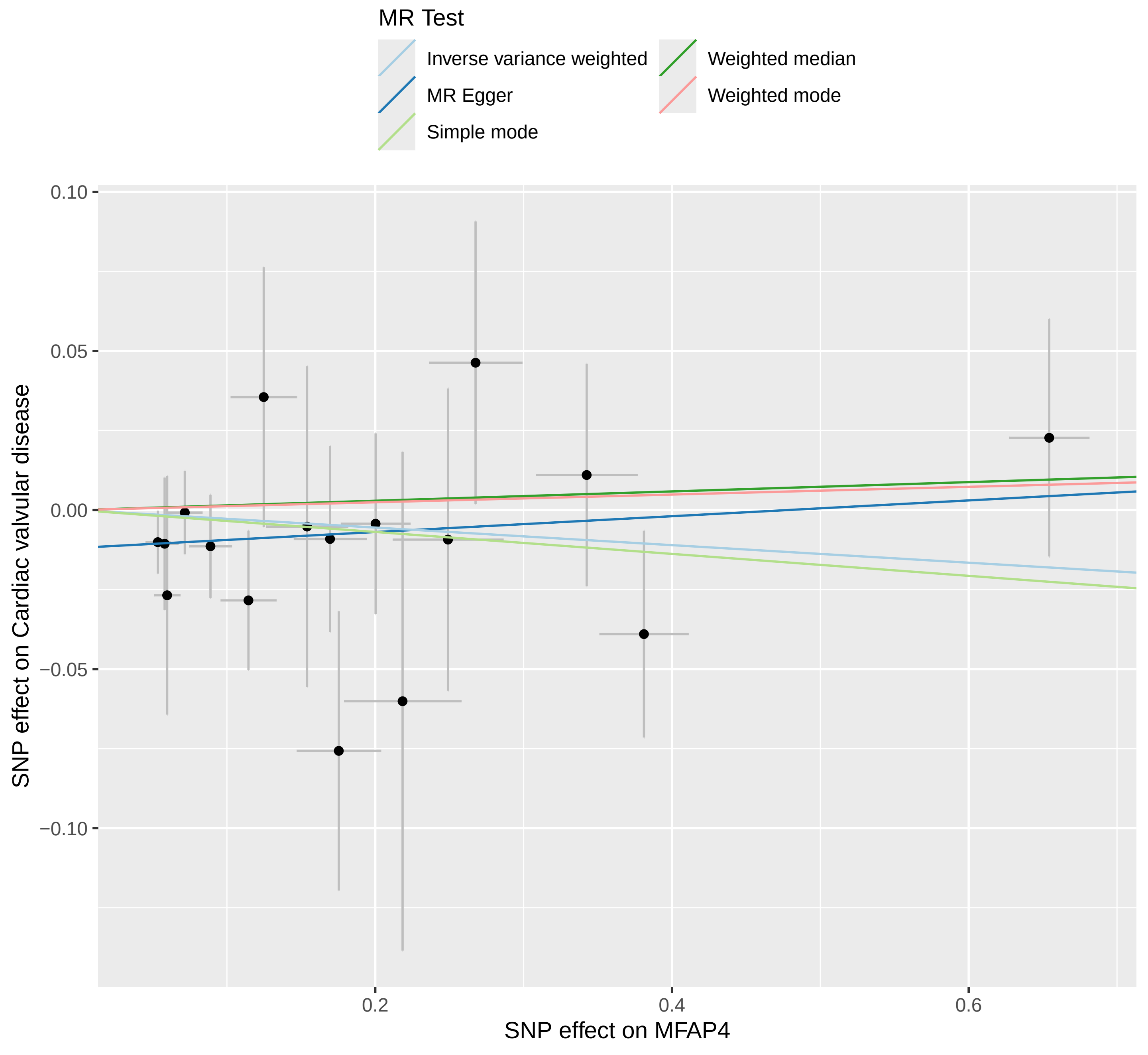
MR Test
Inverse variance weighted
MR Egger
Simple mode
Weighted median
Weighted mode
0.10
0.05
0.00
−0.05
−0.10
SNP effect on Cardiac valvular disease
0.2
0.4
0.6
SNP effect on MFAP4

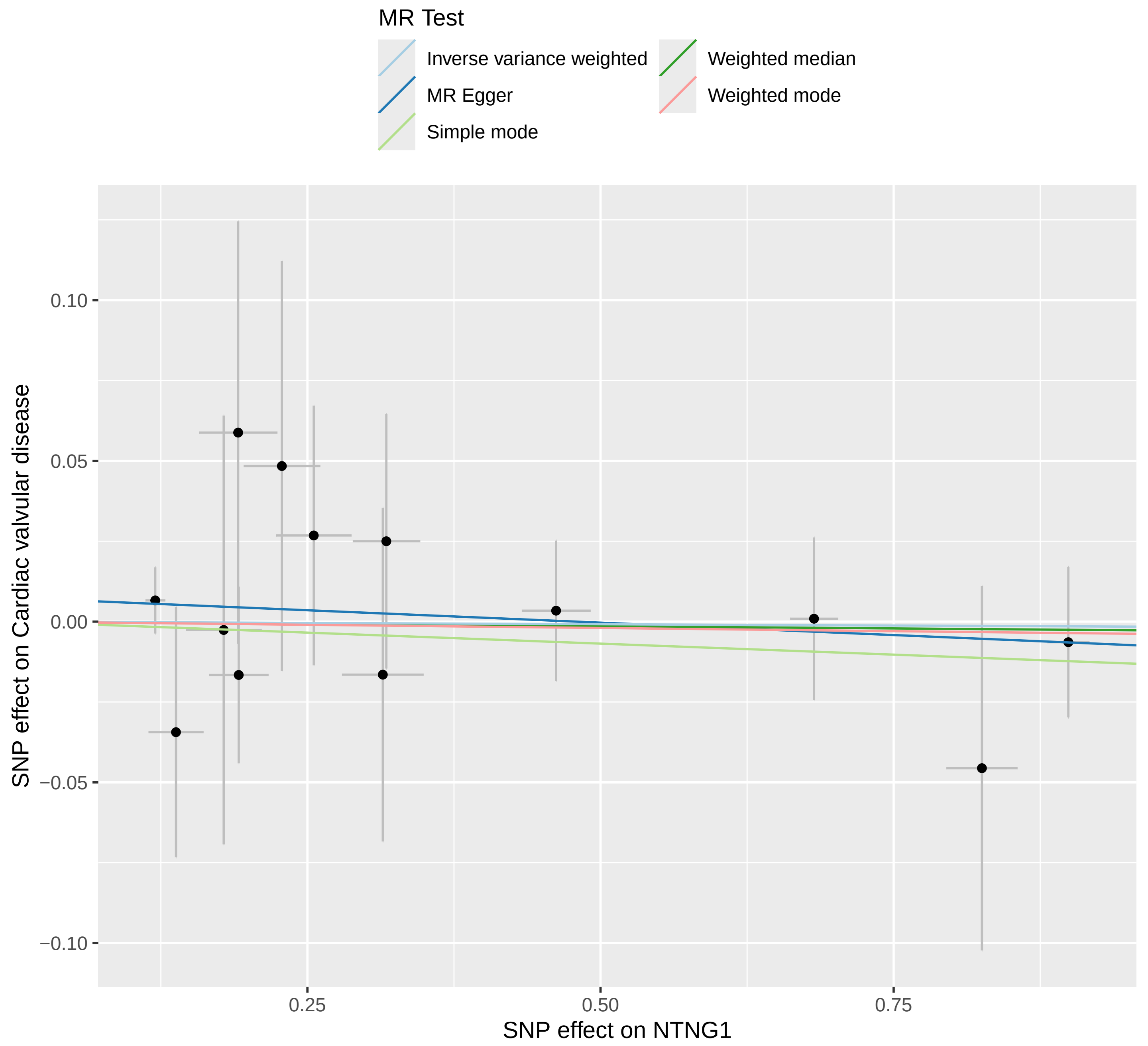
MR Test
Inverse variance weighted
MR Egger
Simple mode
Weighted median
Weighted mode
0.10
0.05
0.00
−0.05
−0.10
SNP effect on Cardiac valvular disease
0.25
0.50
0.75
SNP effect on NTNG1

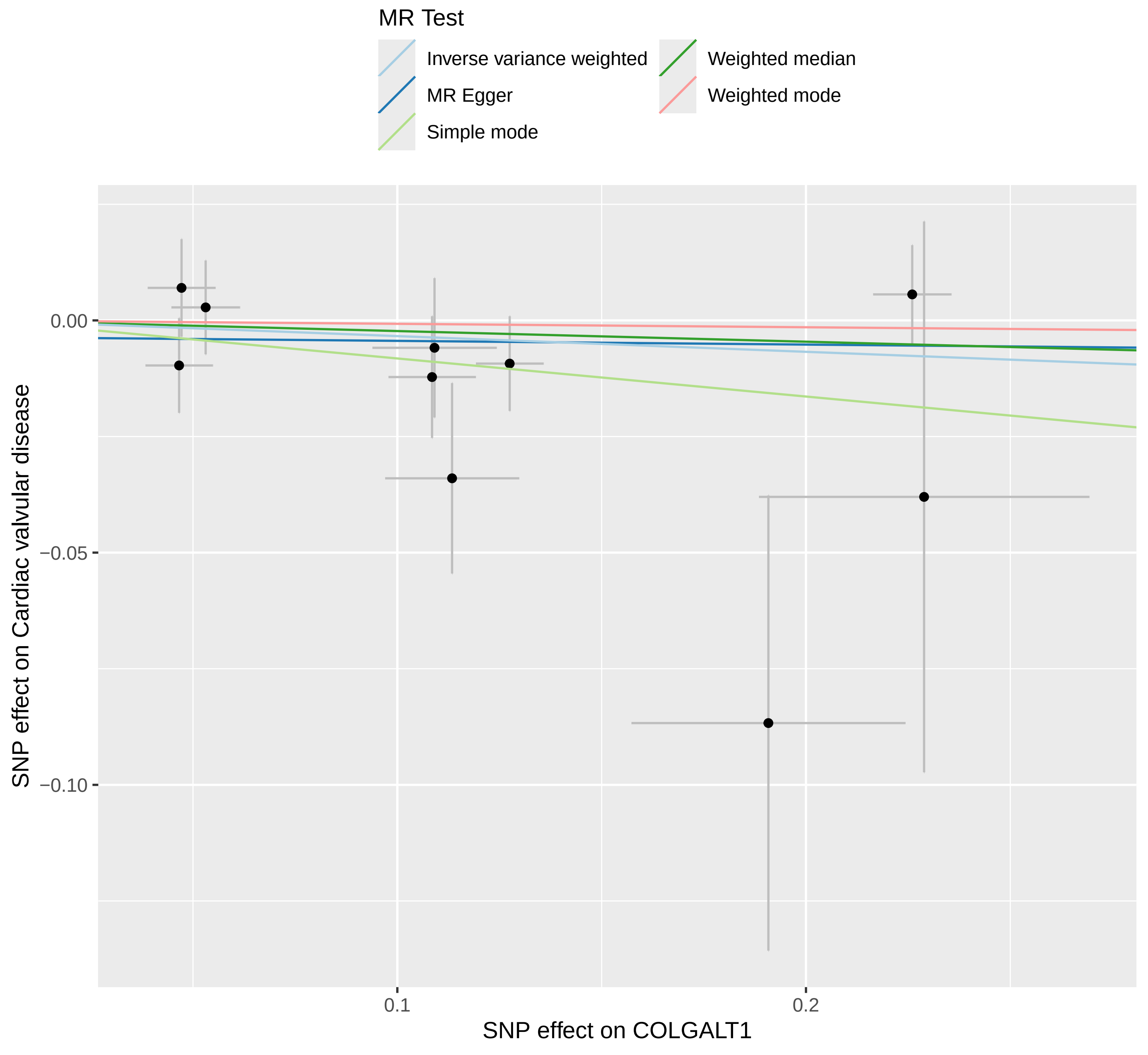

MR Test
Inverse variance weighted
MR Egger
Simple mode
Weighted median
Weighted mode
0.00
−0.05
−0.10
0.1
0.2
SNP effect on Cardiac valvular disease
SNP effect on COLGALT1

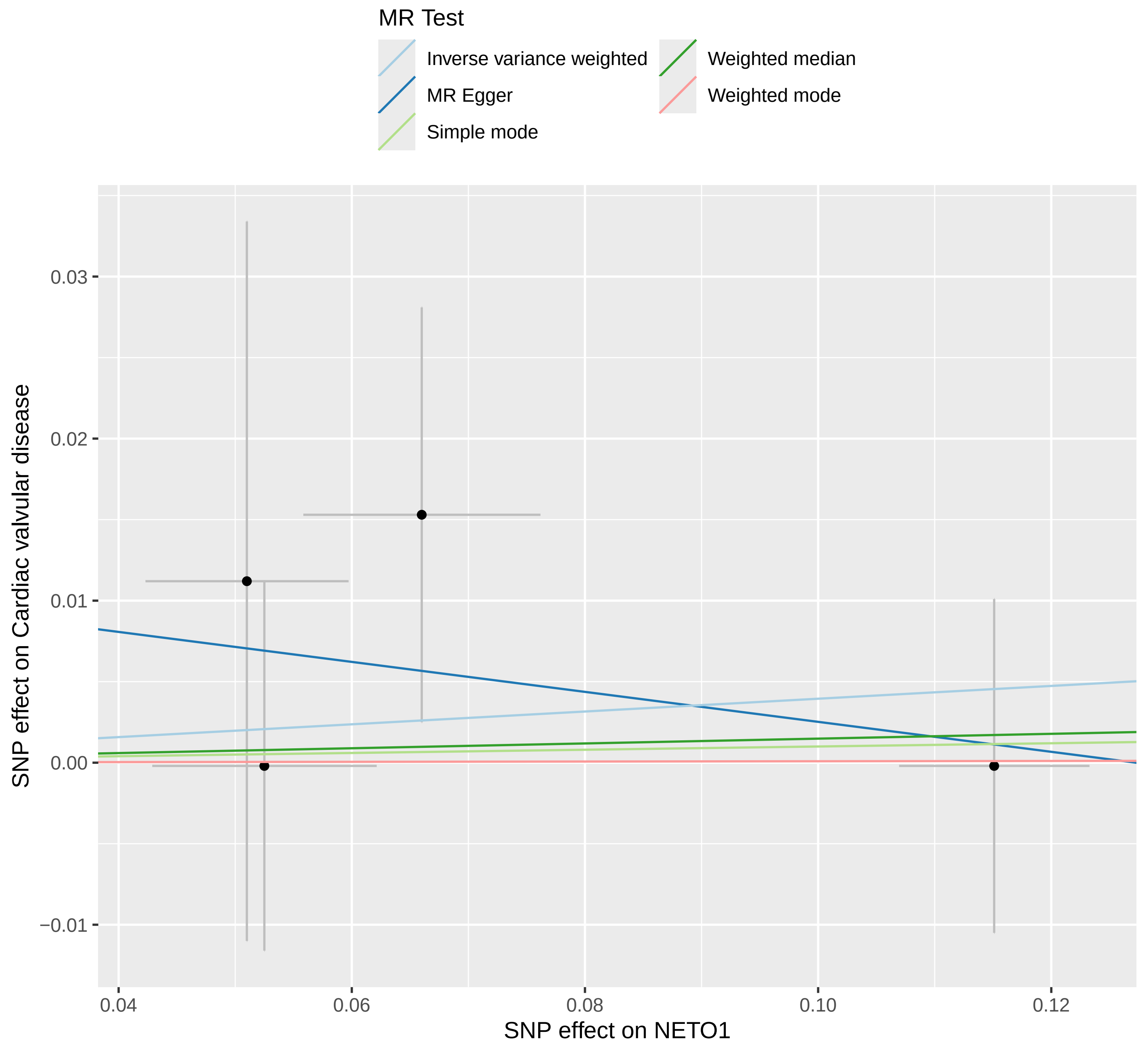

MR Test
Inverse variance weighted
MR Egger
Simple mode
Weighted median
Weighted mode
0.03
0.02
0.01
0.00
−0.01
0.04
0.06
0.08
0.10
0.12
SNP effect on Cardiac valvular disease
SNP effect on NETO1

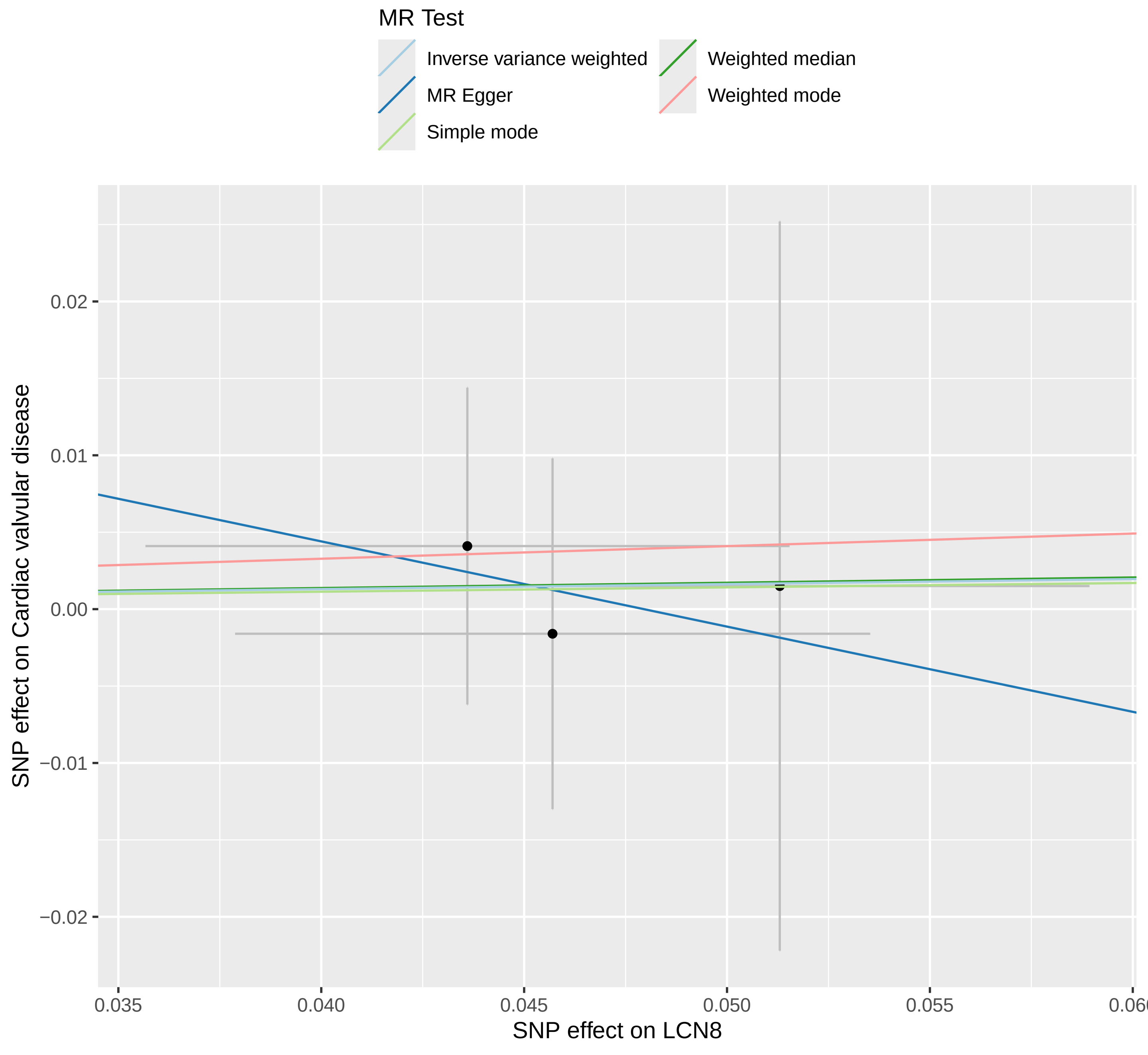

MR Test
Inverse variance weighted
MR Egger
Simple mode
Weighted median
Weighted mode
SNP effect on Cardiac valvular disease
0.02
0.01
0.00
−0.01
−0.02
0.035
0.040
0.045
0.050
0.055
SNP effect on LCN8

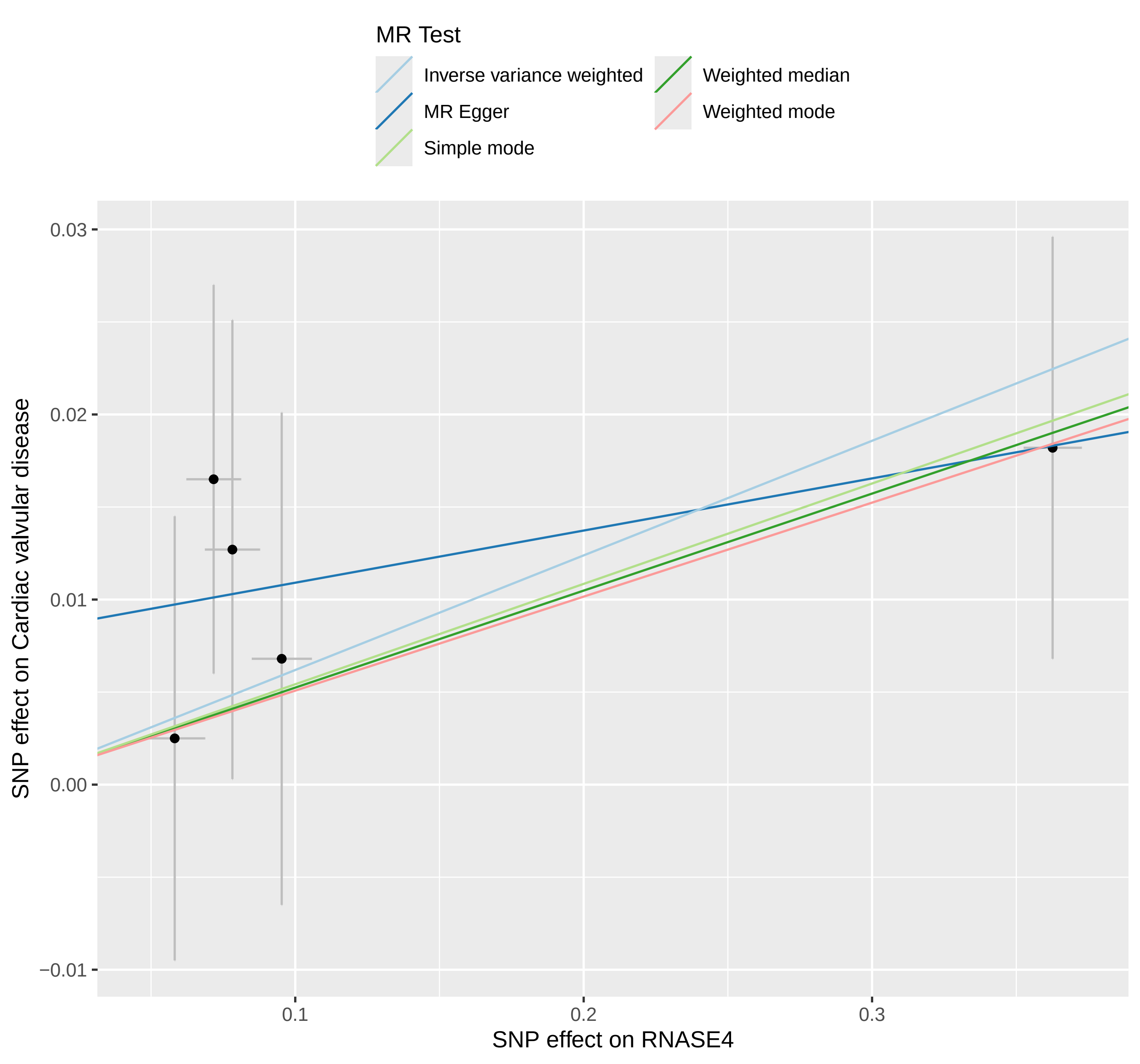

MR Test
Inverse variance weighted
MR Egger
Simple mode
Weighted median
Weighted mode
0.03
0.02
0.01
0.00
−0.01
0.1
0.2
0.3
SNP effect on Cardiac valvular disease
SNP effect on RNASE4

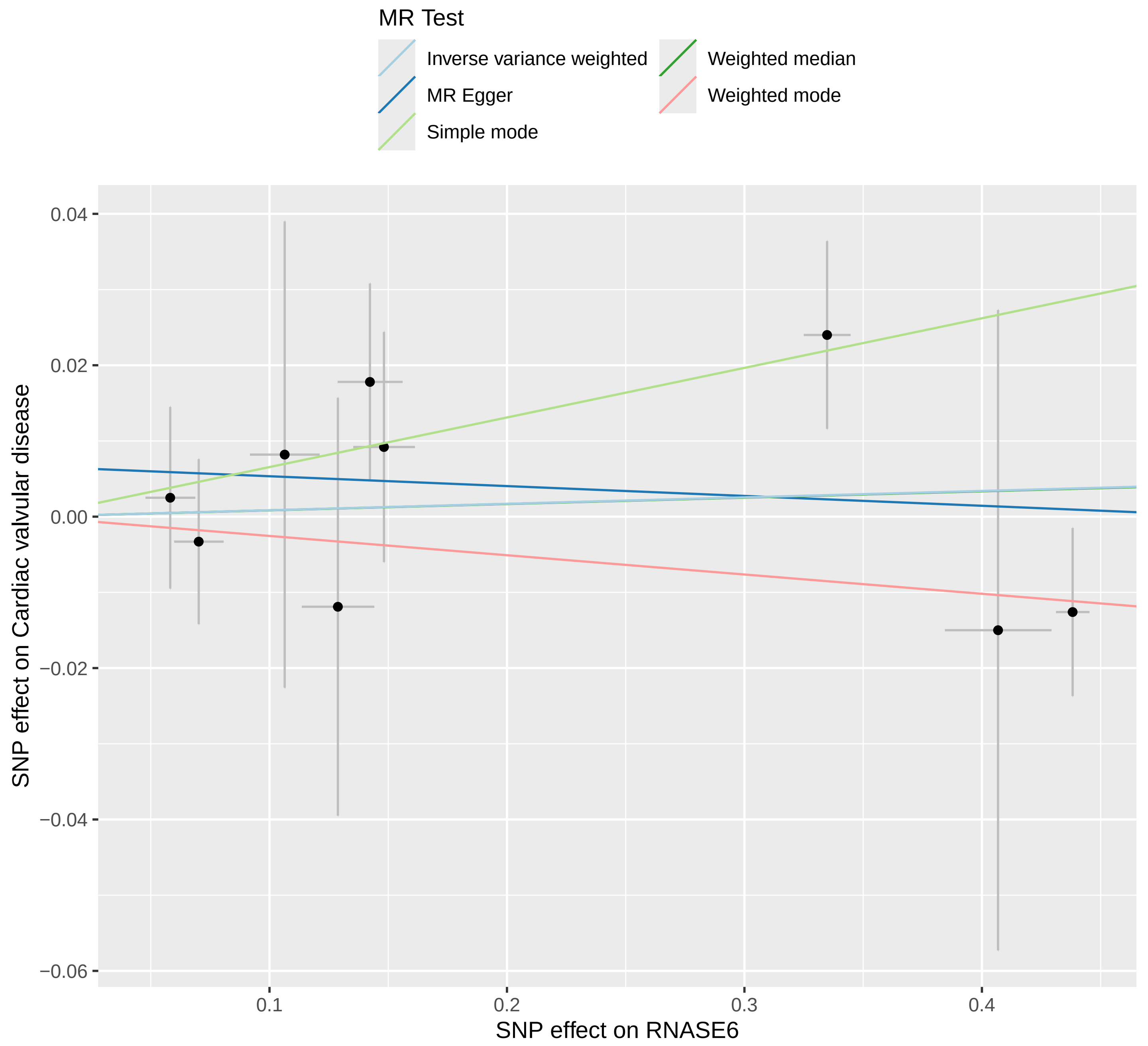
MR Test
Inverse variance weighted
MR Egger
Simple mode
Weighted median
Weighted mode
0.04
0.02
0.00
−0.02
−0.04
−0.06
SNP effect on Cardiac valvular disease
0.1
0.2
0.3
0.4
SNP effect on RNASE6

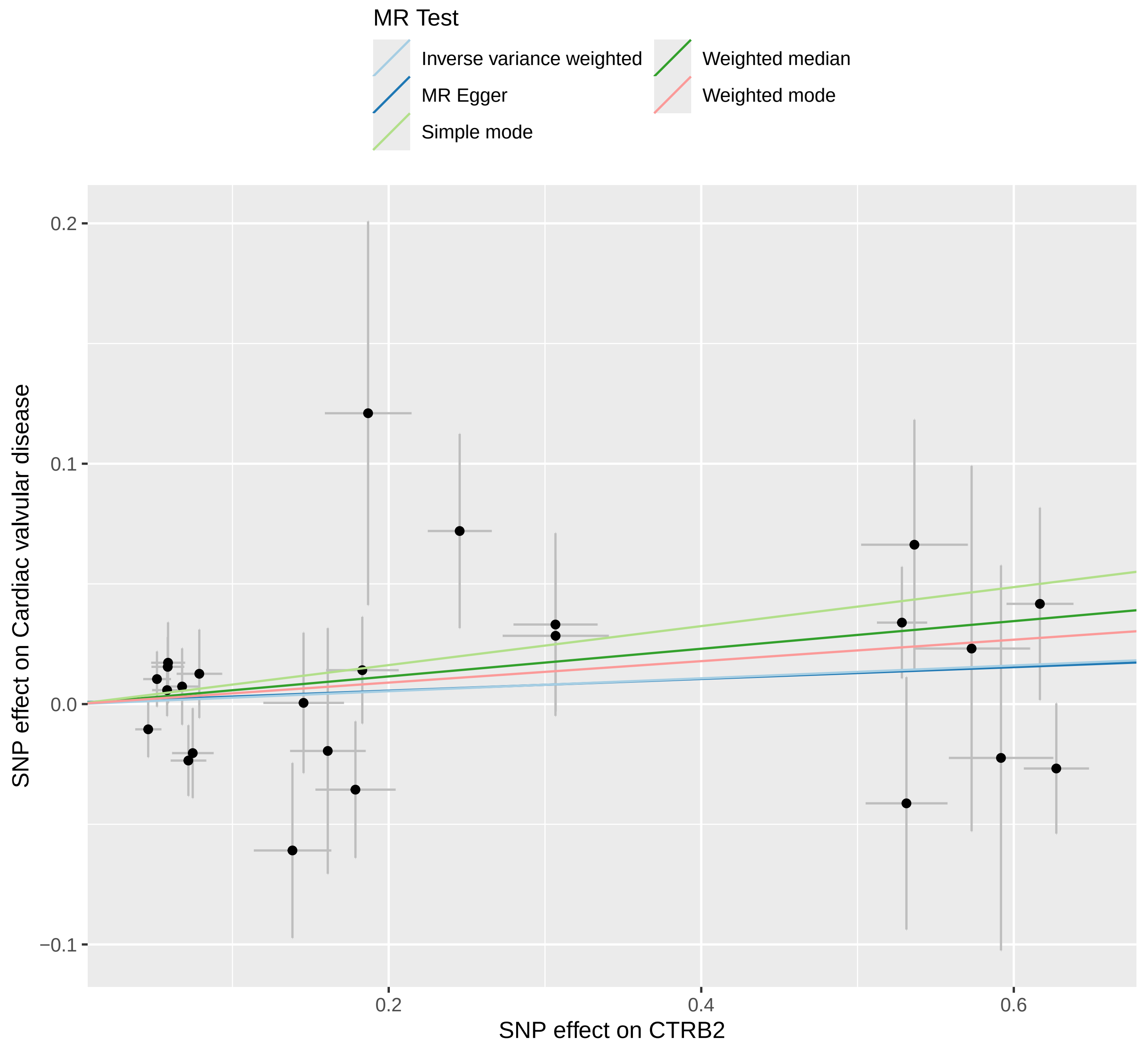
MR Test
Inverse variance weighted
MR Egger
Simple mode
Weighted median
Weighted mode
0.2
0.1
0.0
−0.1
0.2
0.4
0.6
SNP effect on Cardiac valvular disease
SNP effect on CTRB2

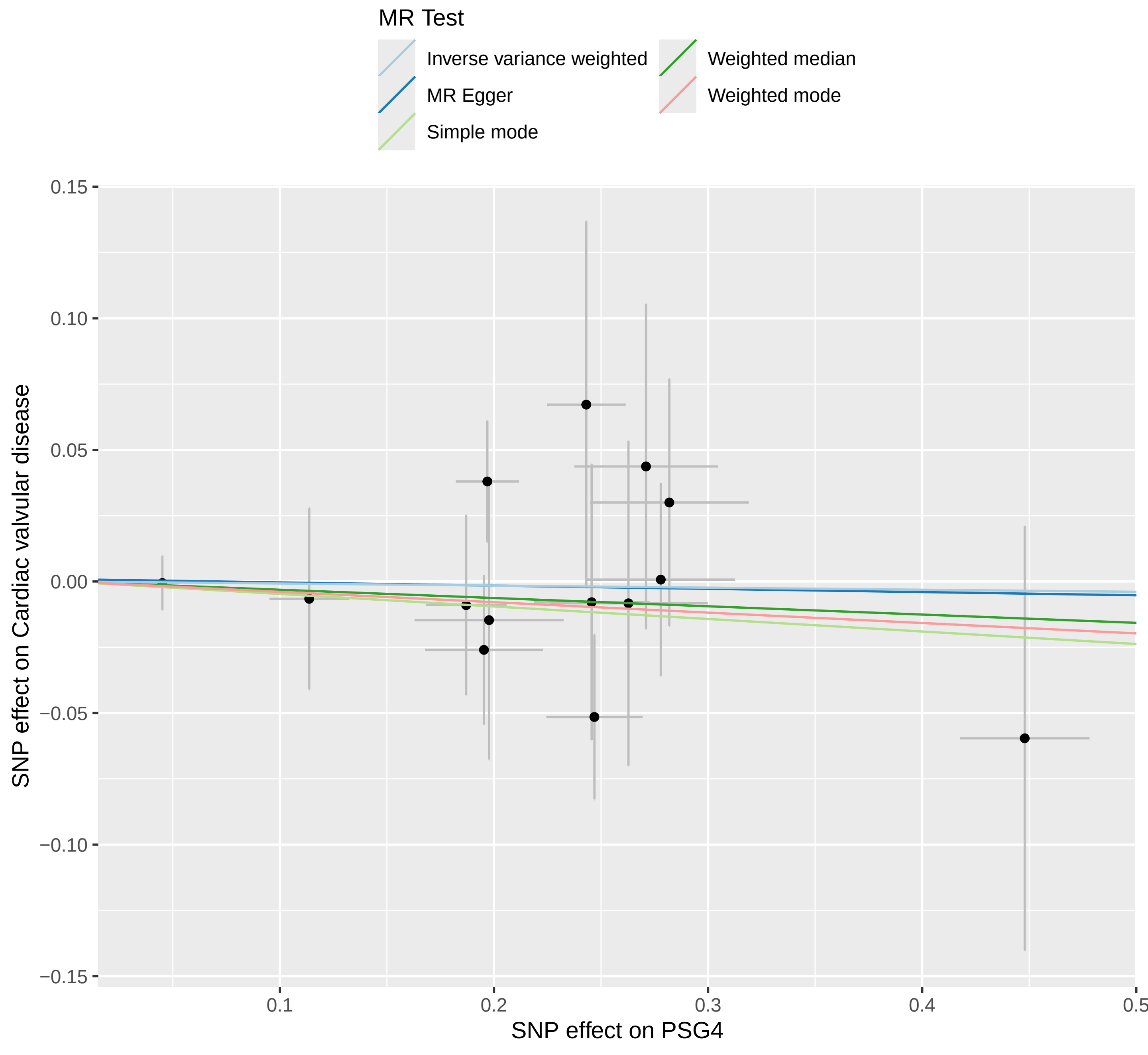

MR Test
Inverse variance weighted
MR Egger
Simple mode
Weighted median
Weighted mode
0.15
0.10
0.05
0.00
−0.05
−0.10
−0.15
0.1
0.2
0.3
0.4
0.5
SNP effect on Cardiac valvular disease
SNP effect on PSG4

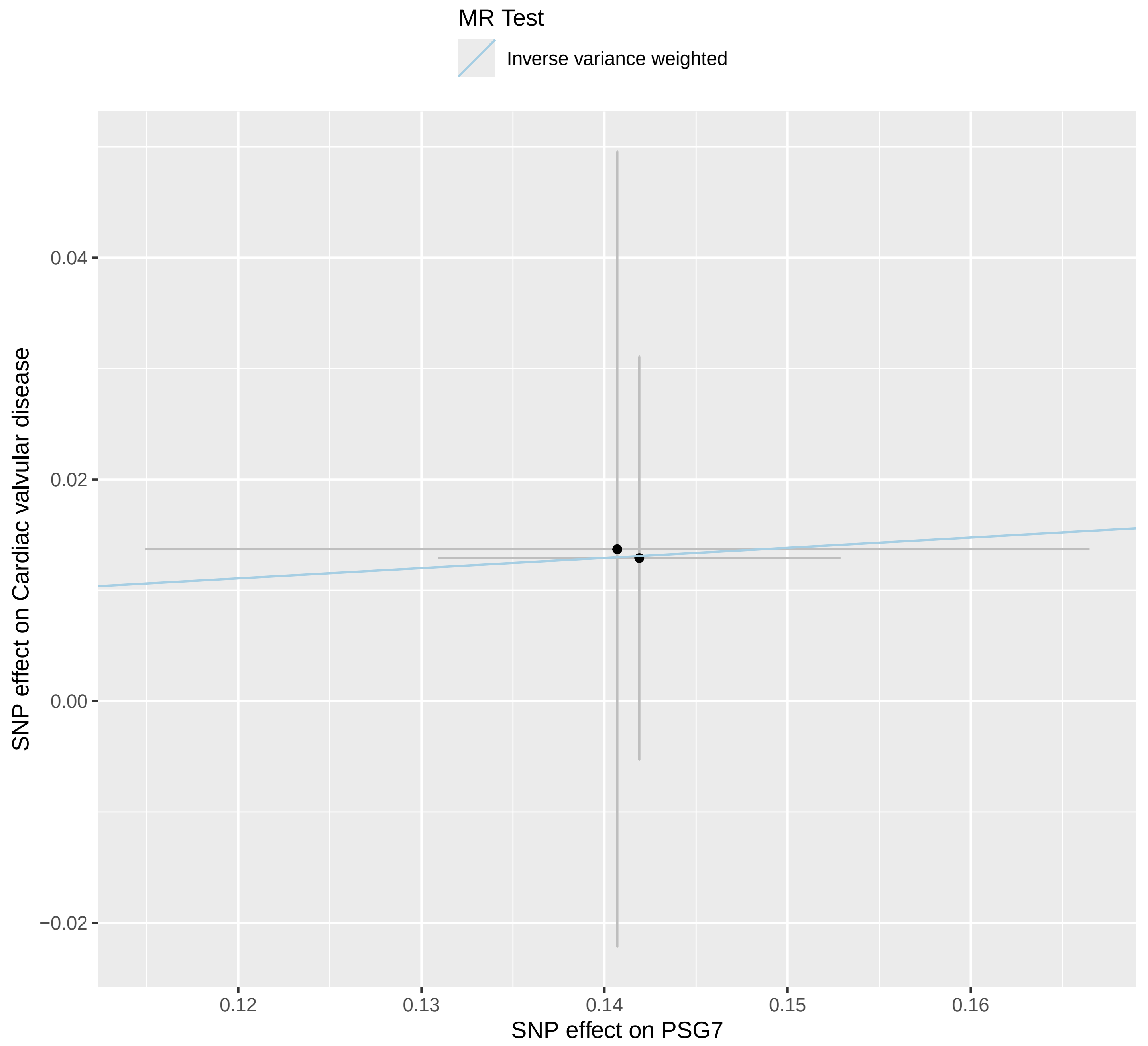
MR Test
Inverse variance weighted
SNP effect on Cardiac valvular disease
0.04
0.02
0.00
−0.02
0.12
0.13
0.14
0.15
0.16
SNP effect on PSG7

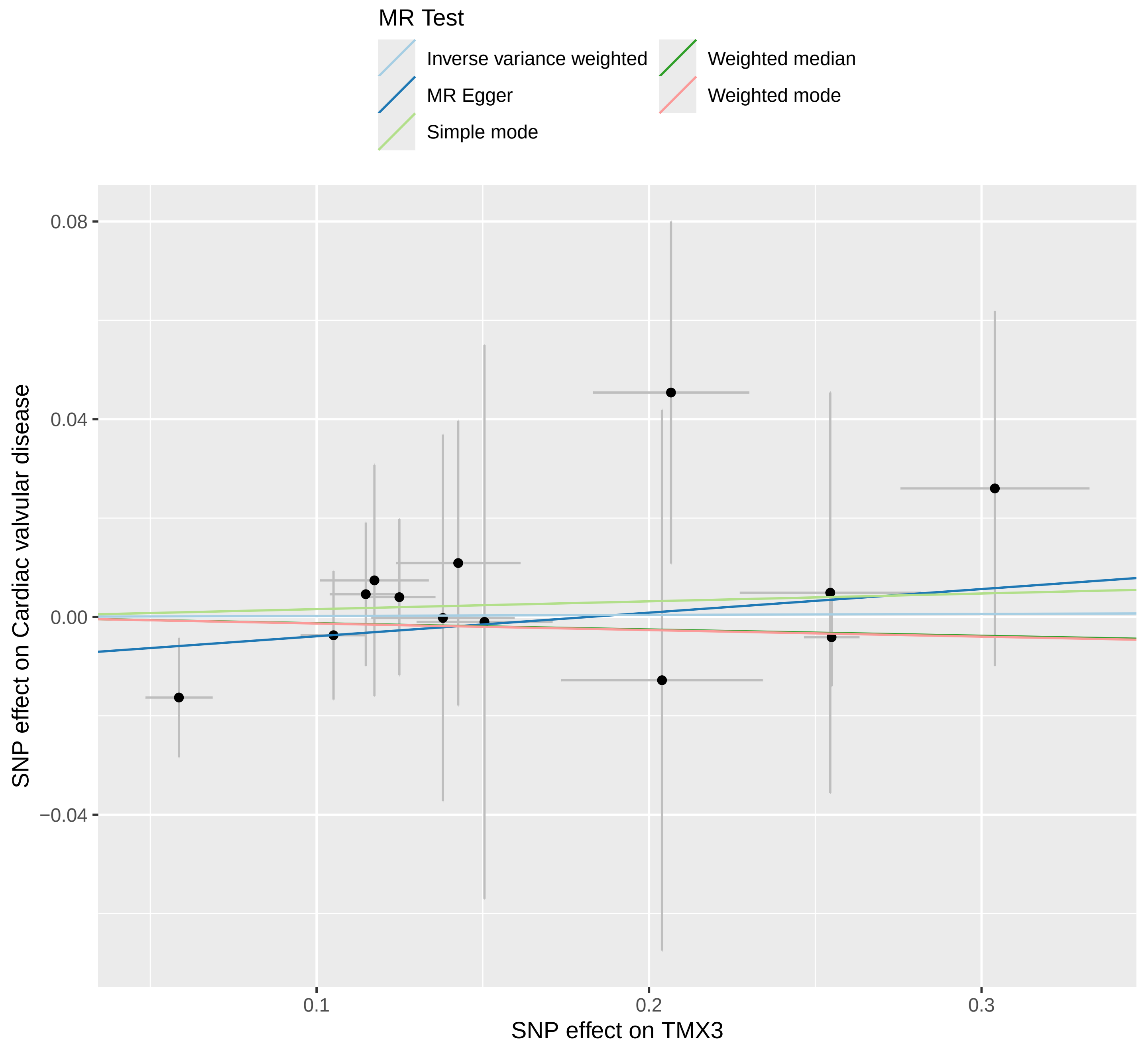
MR Test
Inverse variance weighted
MR Egger
Simple mode
Weighted median
Weighted mode
0.08
0.04
0.00
−0.04
SNP effect on Cardiac valvular disease
0.1
0.2
0.3
SNP effect on TMX3

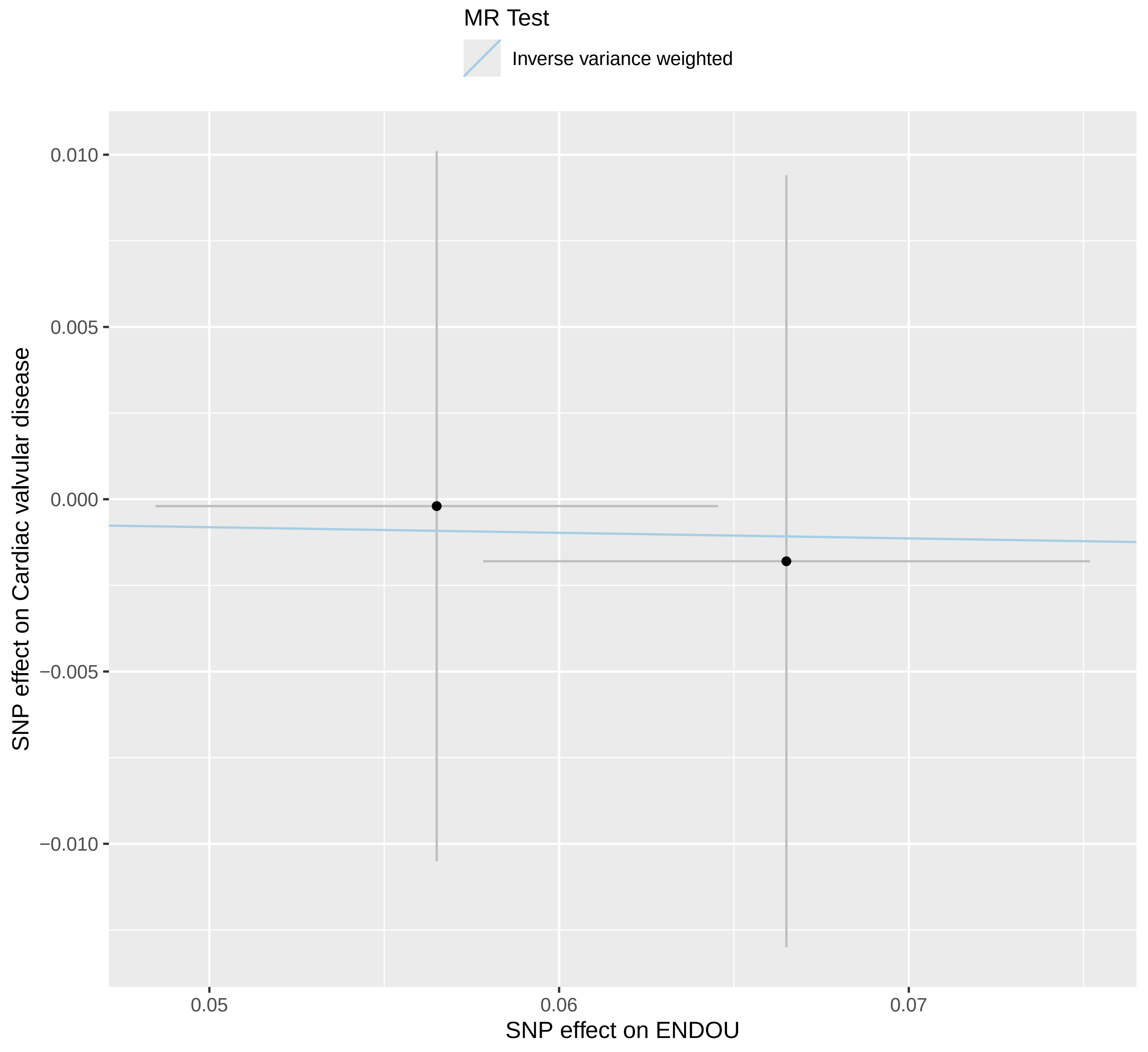
MR Test
Inverse variance weighted
0.010
0.005
0.000
−0.005
−0.010
0.05
0.06
0.07
SNP effect on ENDOU
SNP effect on Cardiac valvular disease

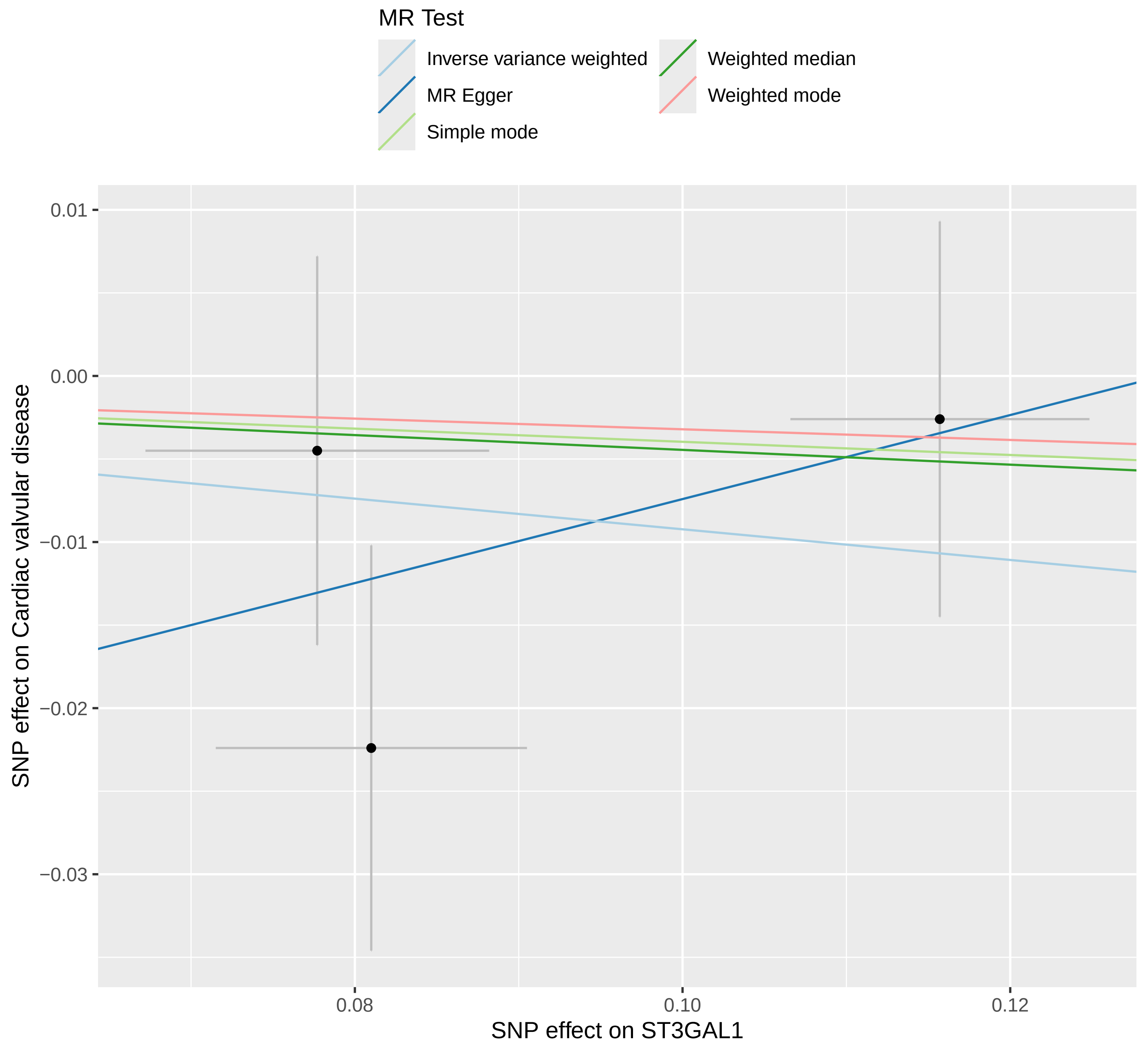
MR Test
Inverse variance weighted
MR Egger
Simple mode
Weighted median
Weighted mode
0.01
0.00
−0.01
−0.02
−0.03
SNP effect on Cardiac valvular disease
0.08
0.10
0.12
SNP effect on ST3GAL1

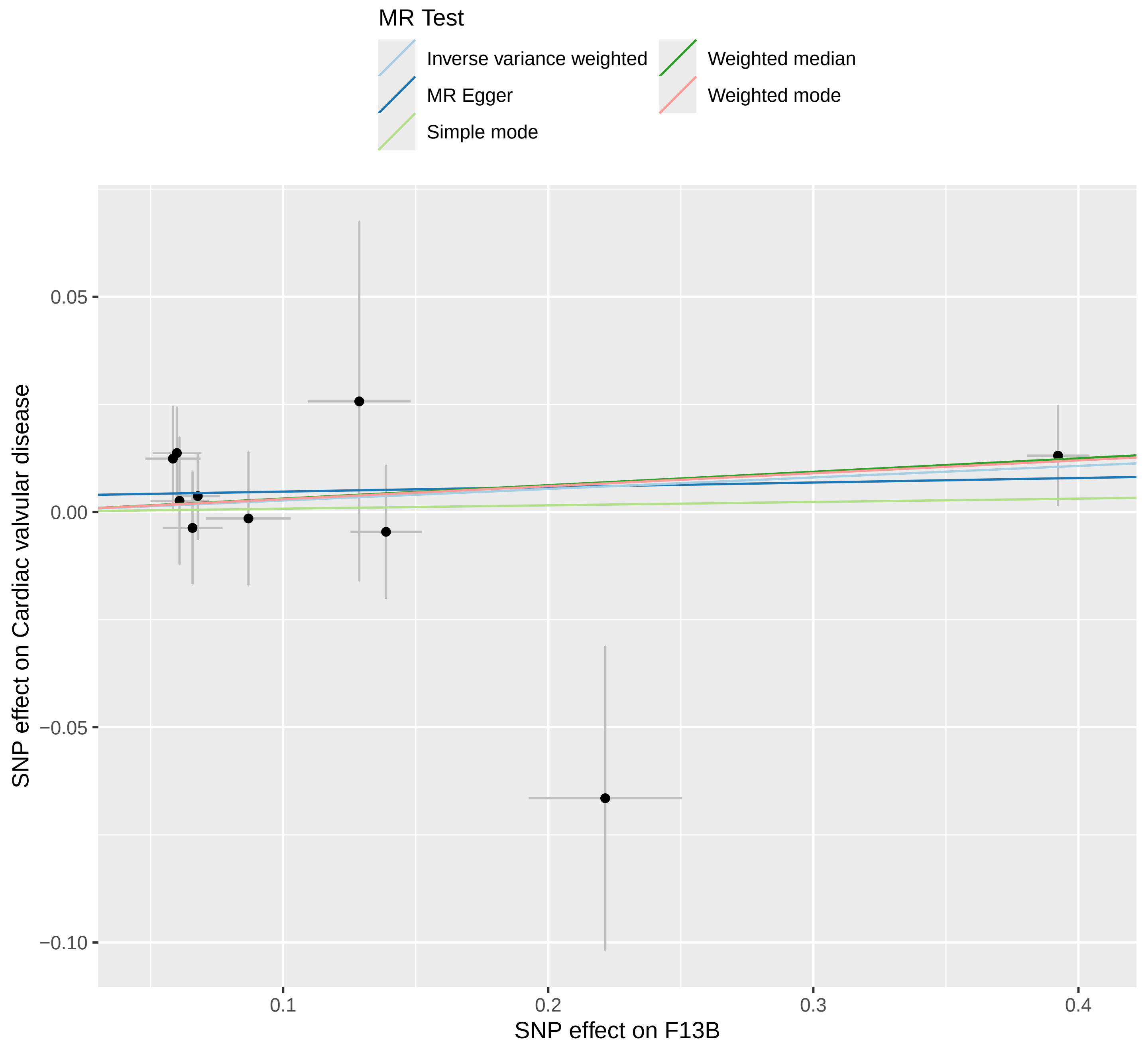
MR Test
Inverse variance weighted
MR Egger
Simple mode
Weighted median
Weighted mode
0.05
0.00
−0.05
−0.10
0.1
0.2
0.3
0.4
SNP effect on Cardiac valvular disease
SNP effect on F13B

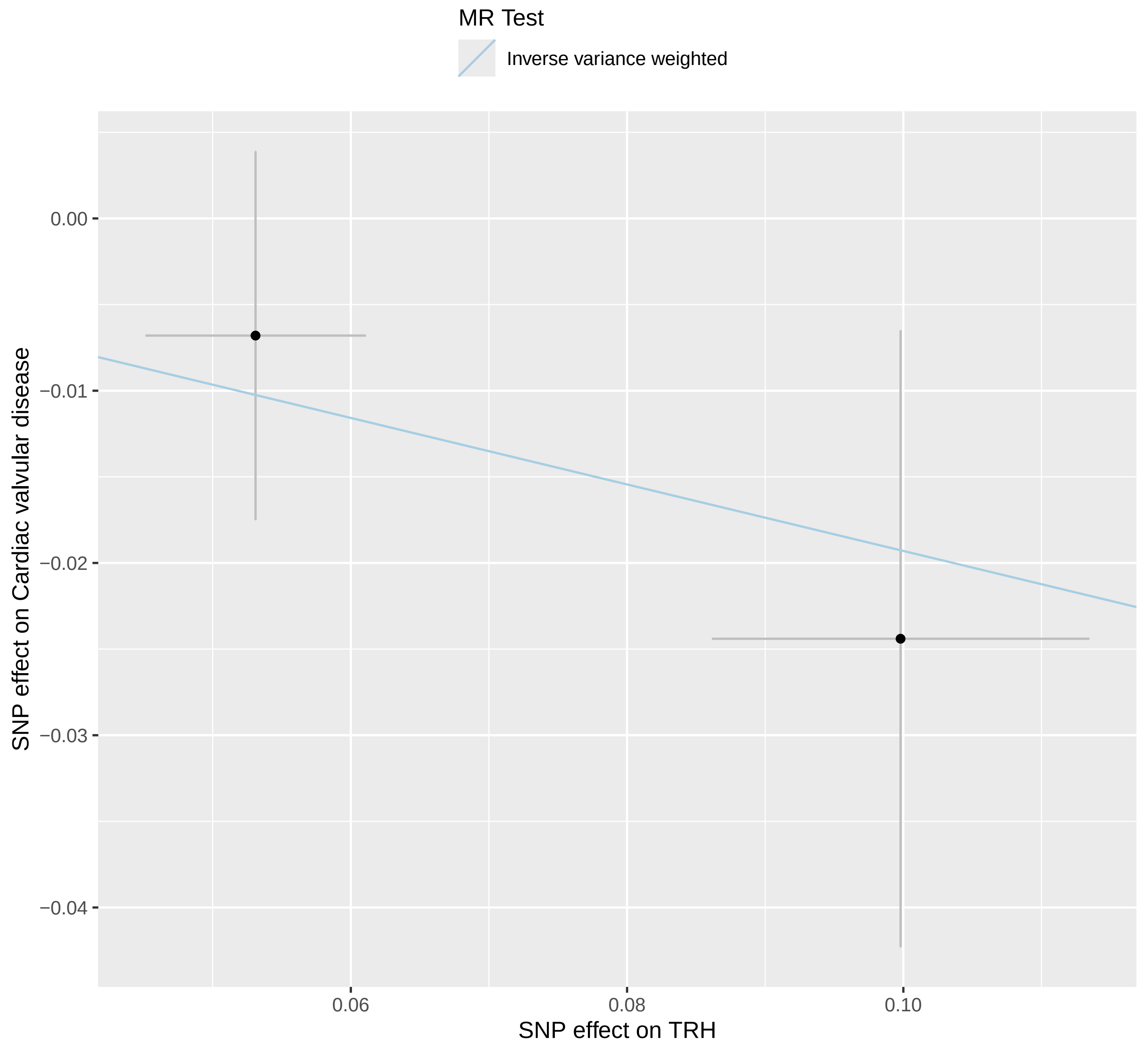
MR Test
Inverse variance weighted
0.00
−0.01
−0.02
−0.03
−0.04
SNP effect on Cardiac valvular disease
0.06
0.08
0.10
SNP effect on TRH

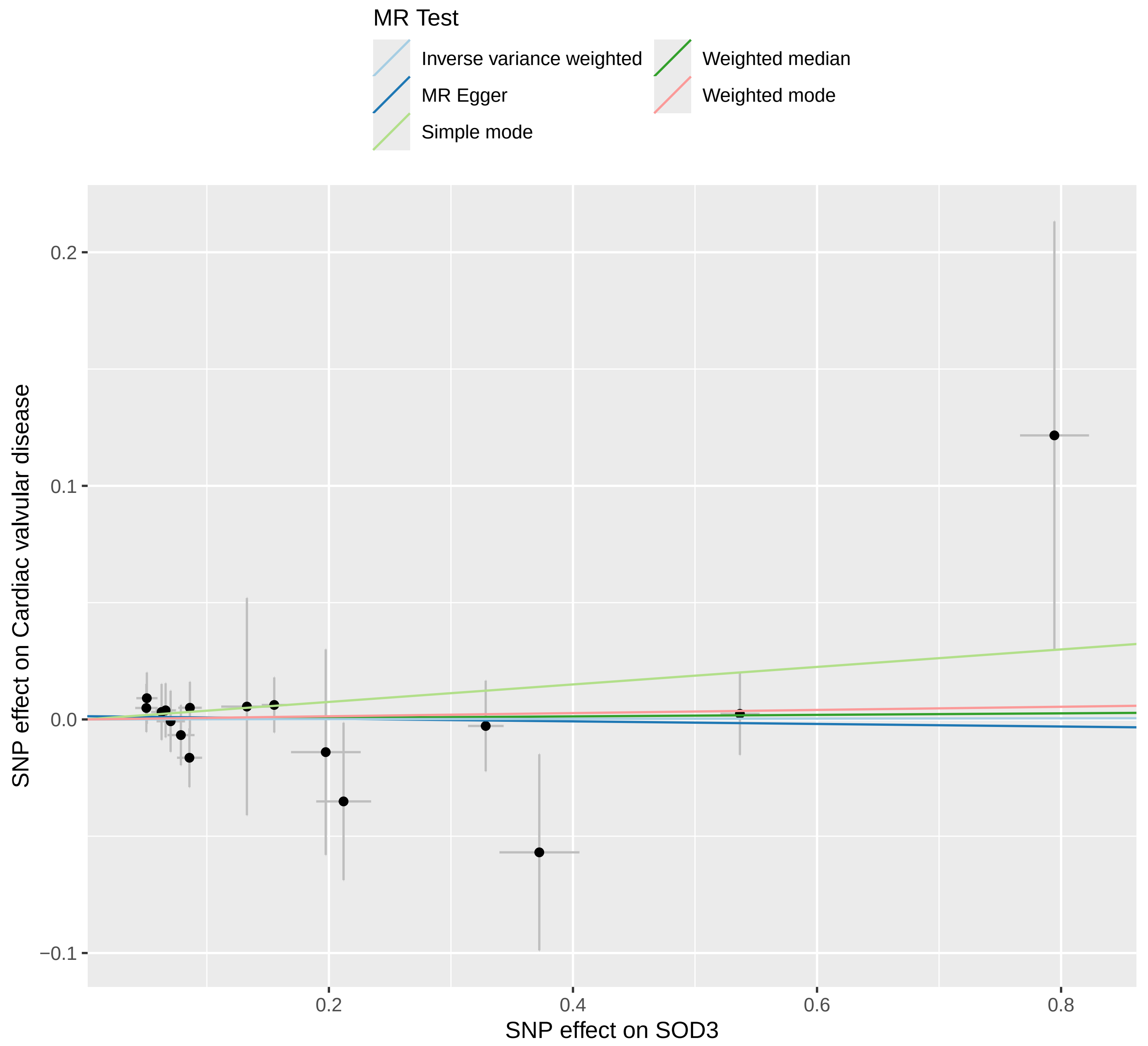

MR Test
Inverse variance weighted
MR Egger
Simple mode
Weighted median
Weighted mode
0.2
0.1
0.0
−0.1
0.2
0.4
0.6
0.8
SNP effect on Cardiac valvular disease
SNP effect on SOD3

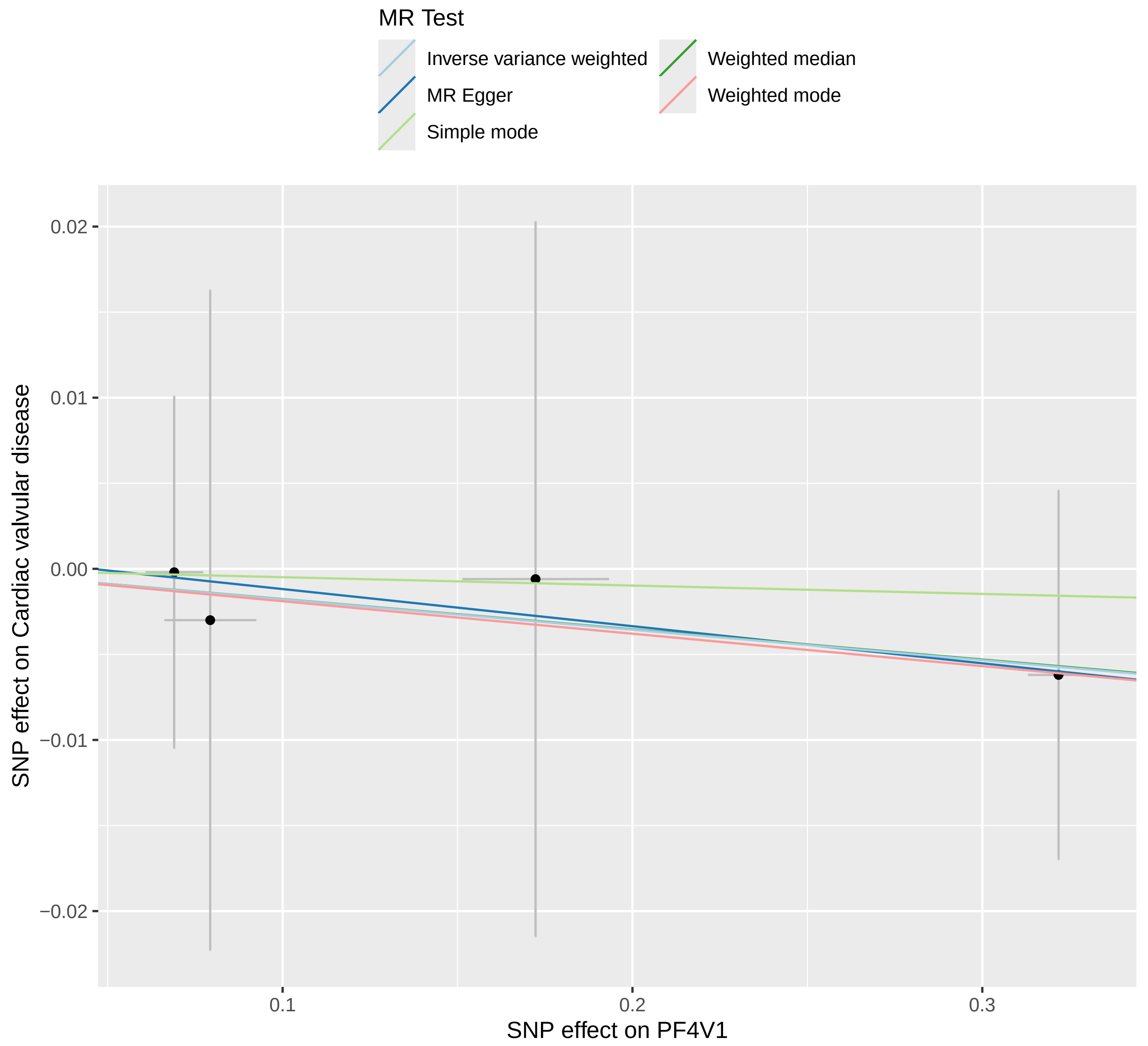
MR Test
Inverse variance weighted
MR Egger
Simple mode
Weighted median
Weighted mode
0.02
0.01
0.00
−0.01
−0.02
SNP effect on Cardiac valvular disease
0.1
0.2
0.3
SNP effect on PF4V1

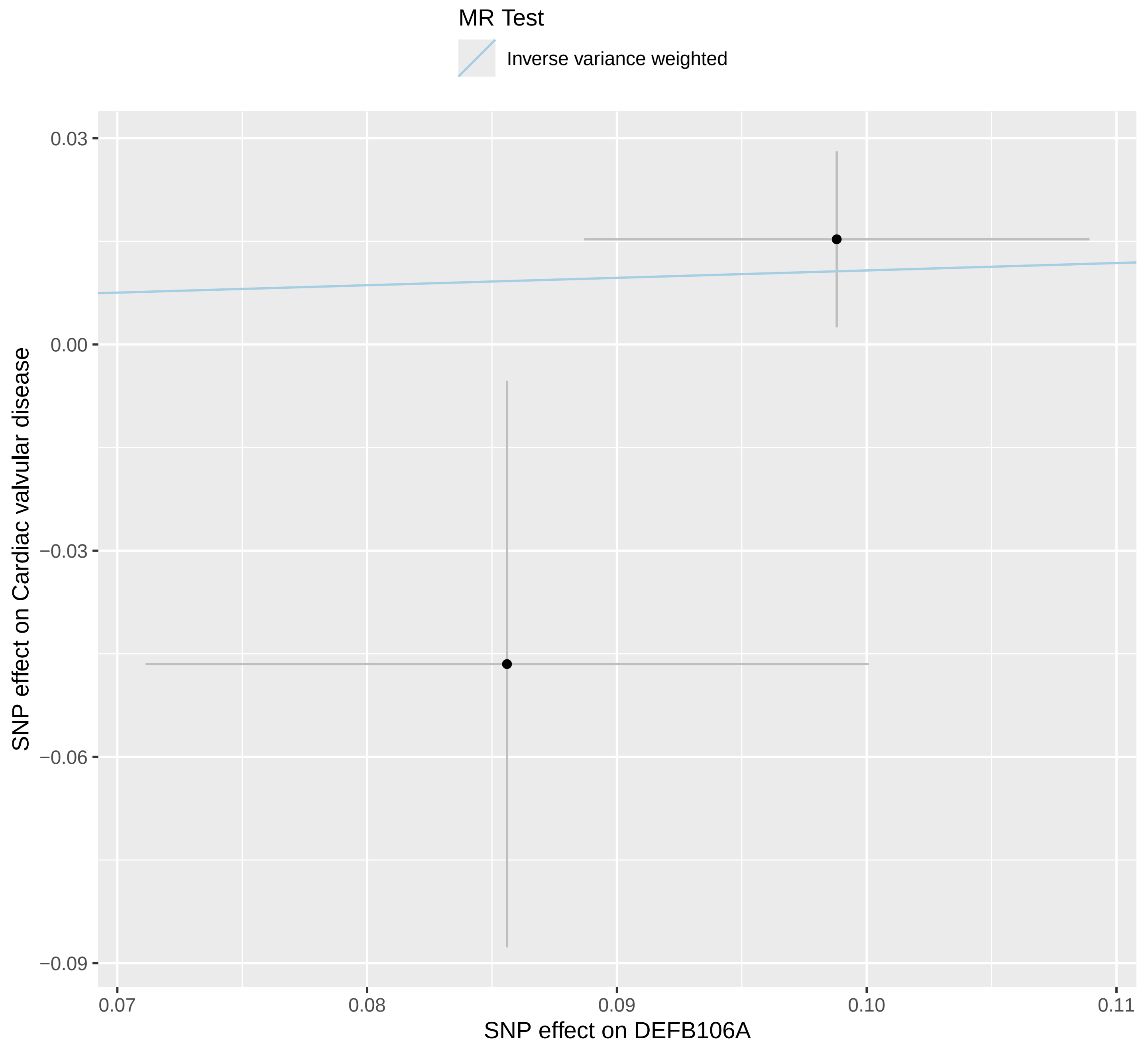
MR Test
Inverse variance weighted
0.03
0.00
−0.03
−0.06
−0.09
0.07
0.08
0.09
0.10
0.11
SNP effect on Cardiac valvular disease
SNP effect on DEFB106A

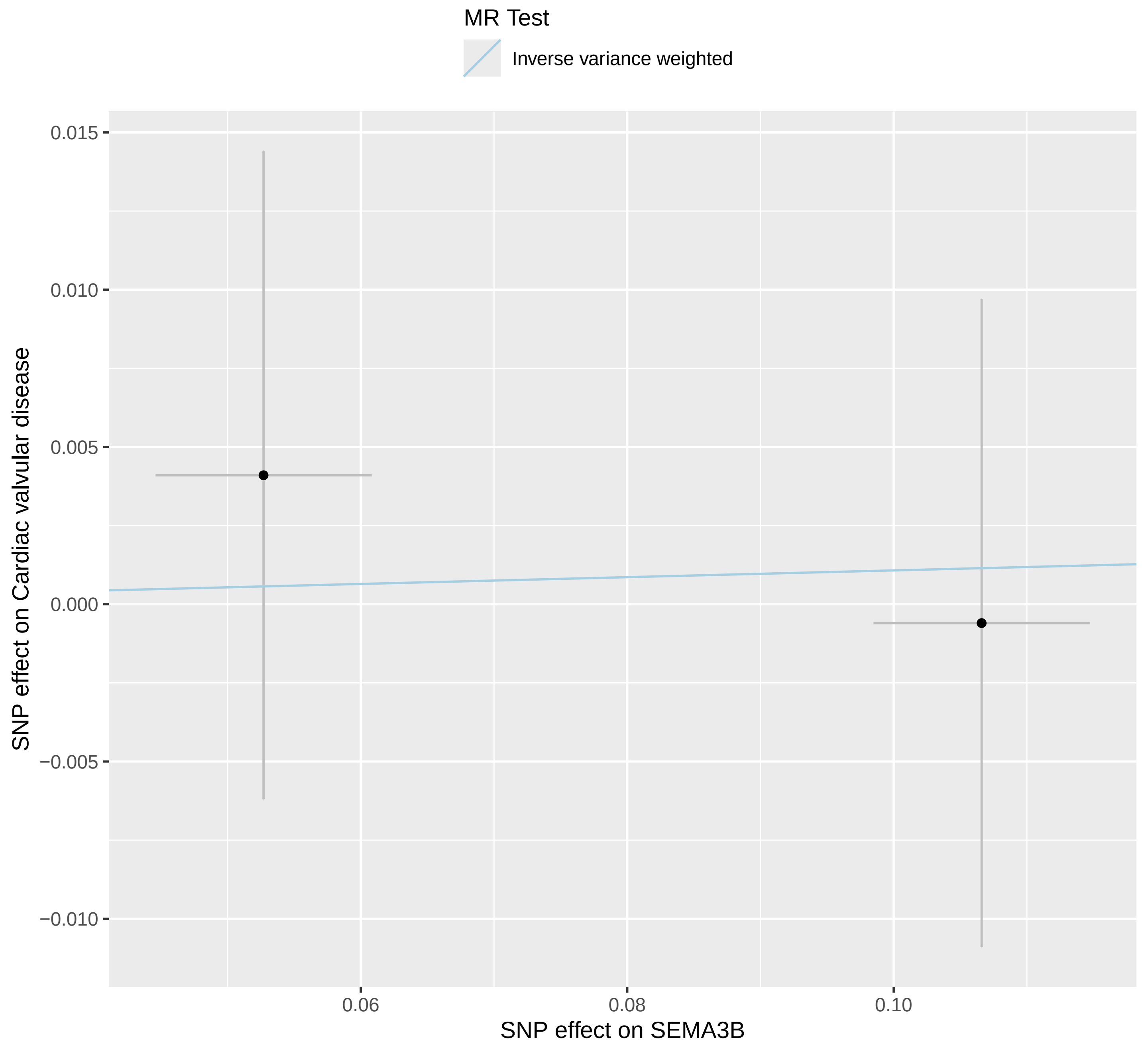

MR Test
Inverse variance weighted
0.015
0.010
0.005
0.000
−0.005
−0.010
0.06
0.08
0.10
SNP effect on SEMA3B
SNP effect on Cardiac valvular disease

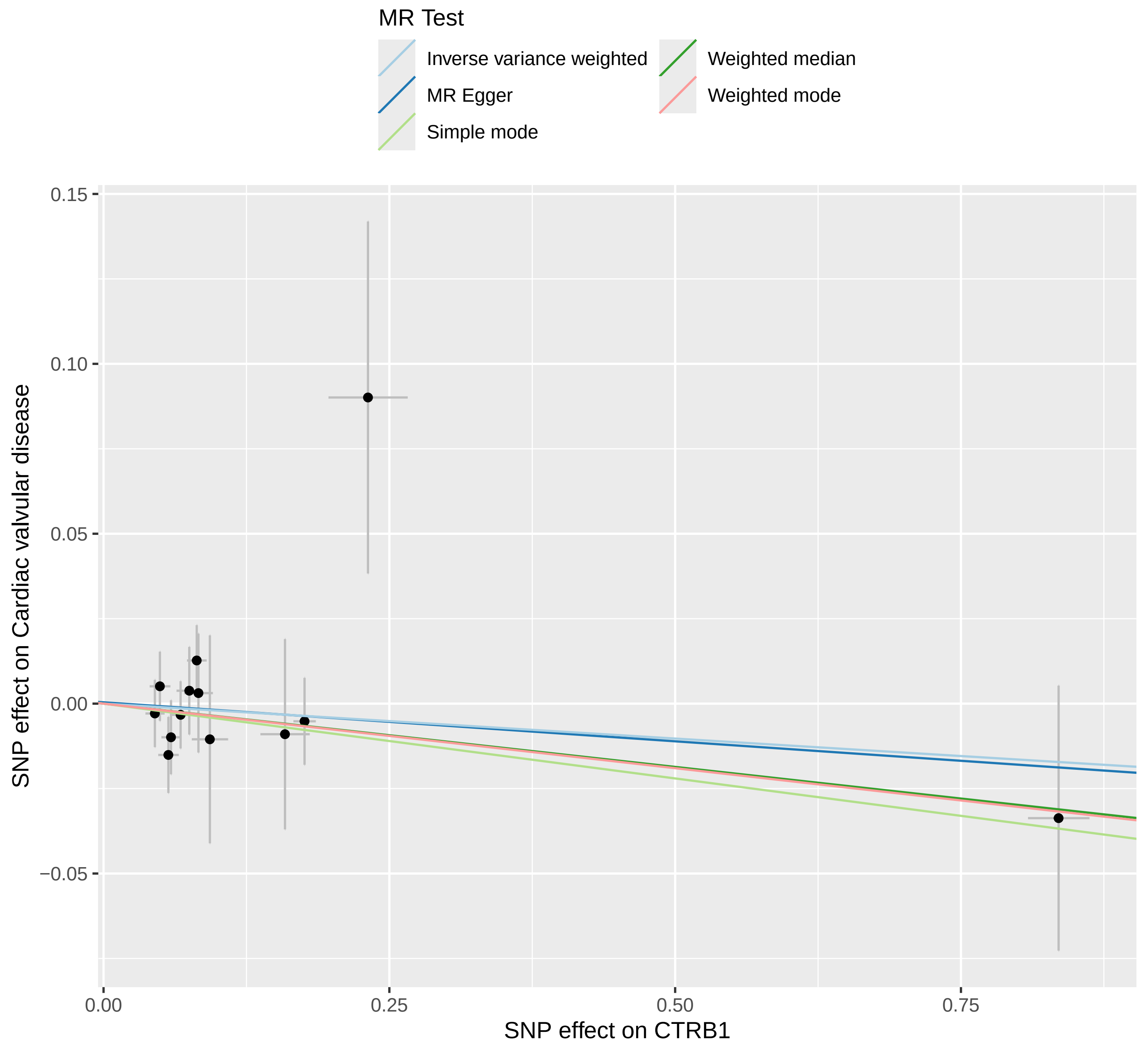
MR Test
Inverse variance weighted
MR Egger
Simple mode
Weighted median
Weighted mode
0.15
0.10
0.05
0.00
−0.05
SNP effect on Cardiac valvular disease
0.00
0.25
0.50
0.75
SNP effect on CTRB1

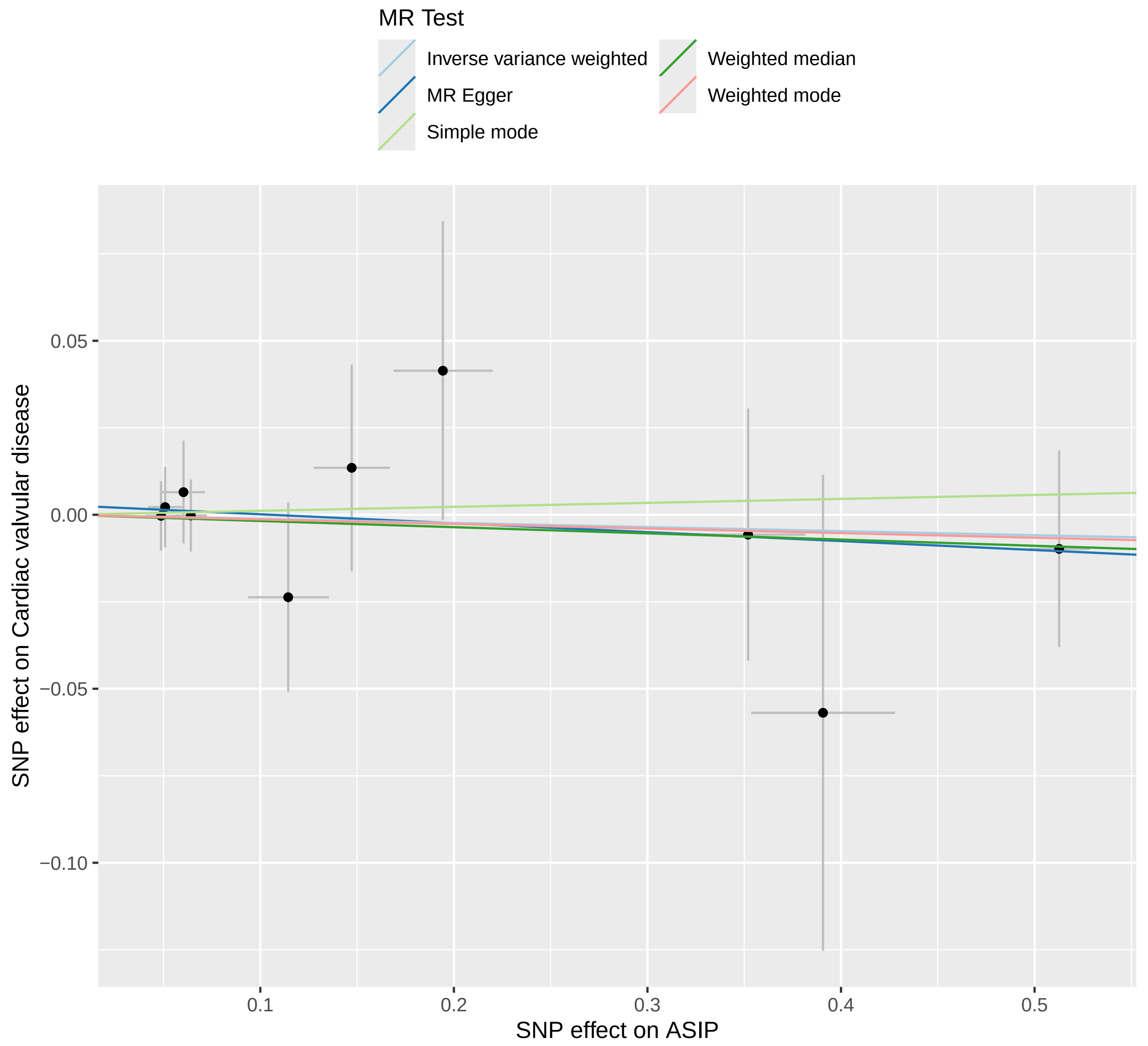

MR Test
Inverse variance weighted
MR Egger
Simple mode
Weighted median
Weighted mode
0.05
0.00
−0.05
−0.10
0.1
0.2
0.3
0.4
0.5
SNP effect on Cardiac valvular disease
SNP effect on ASIP

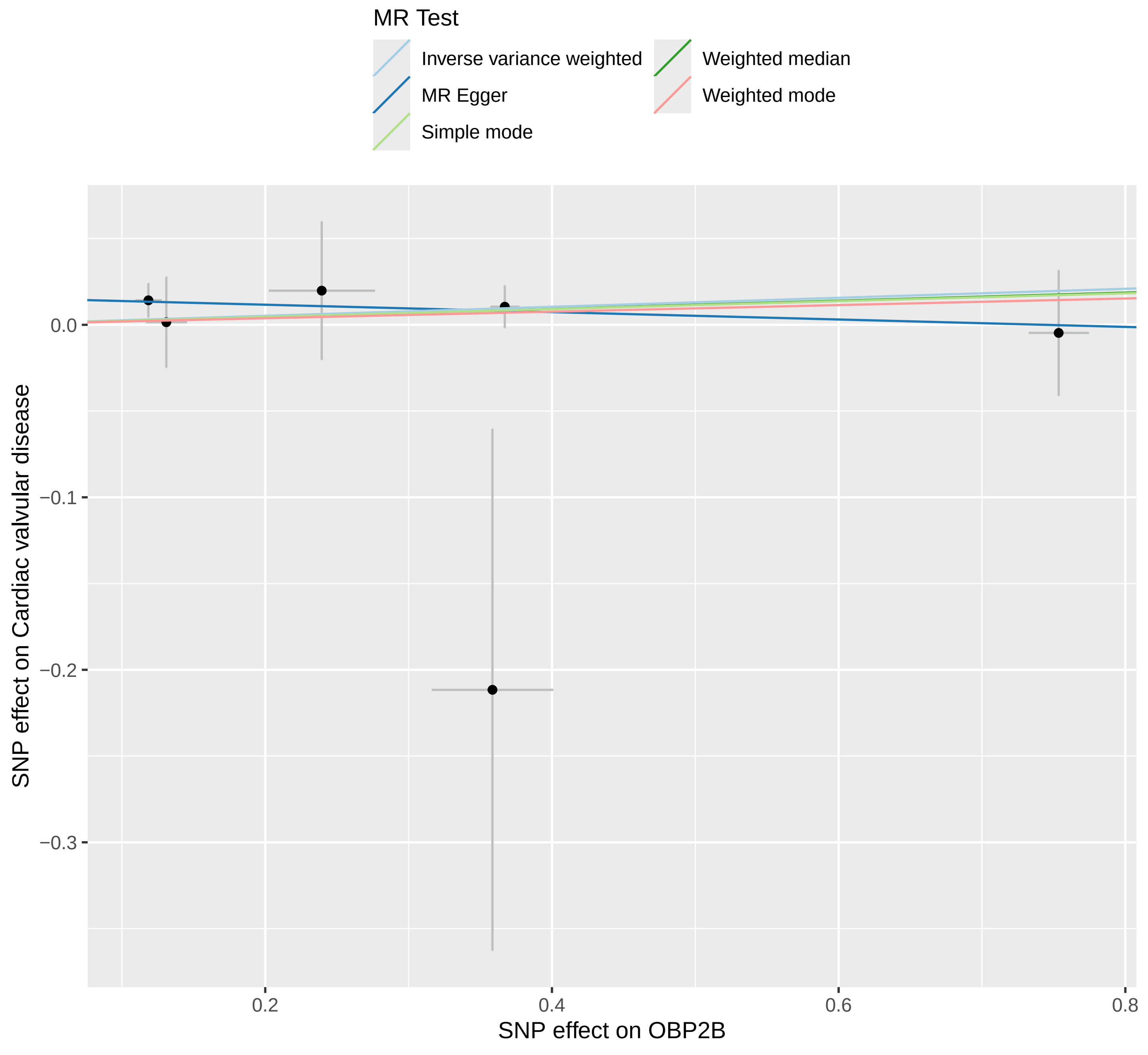
MR Test
Inverse variance weighted
MR Egger
Simple mode
Weighted median
Weighted mode
SNP effect on Cardiac valvular disease
0.0
−0.1
−0.2
−0.3
0.2
0.4
0.6
0.8
SNP effect on OBP2B

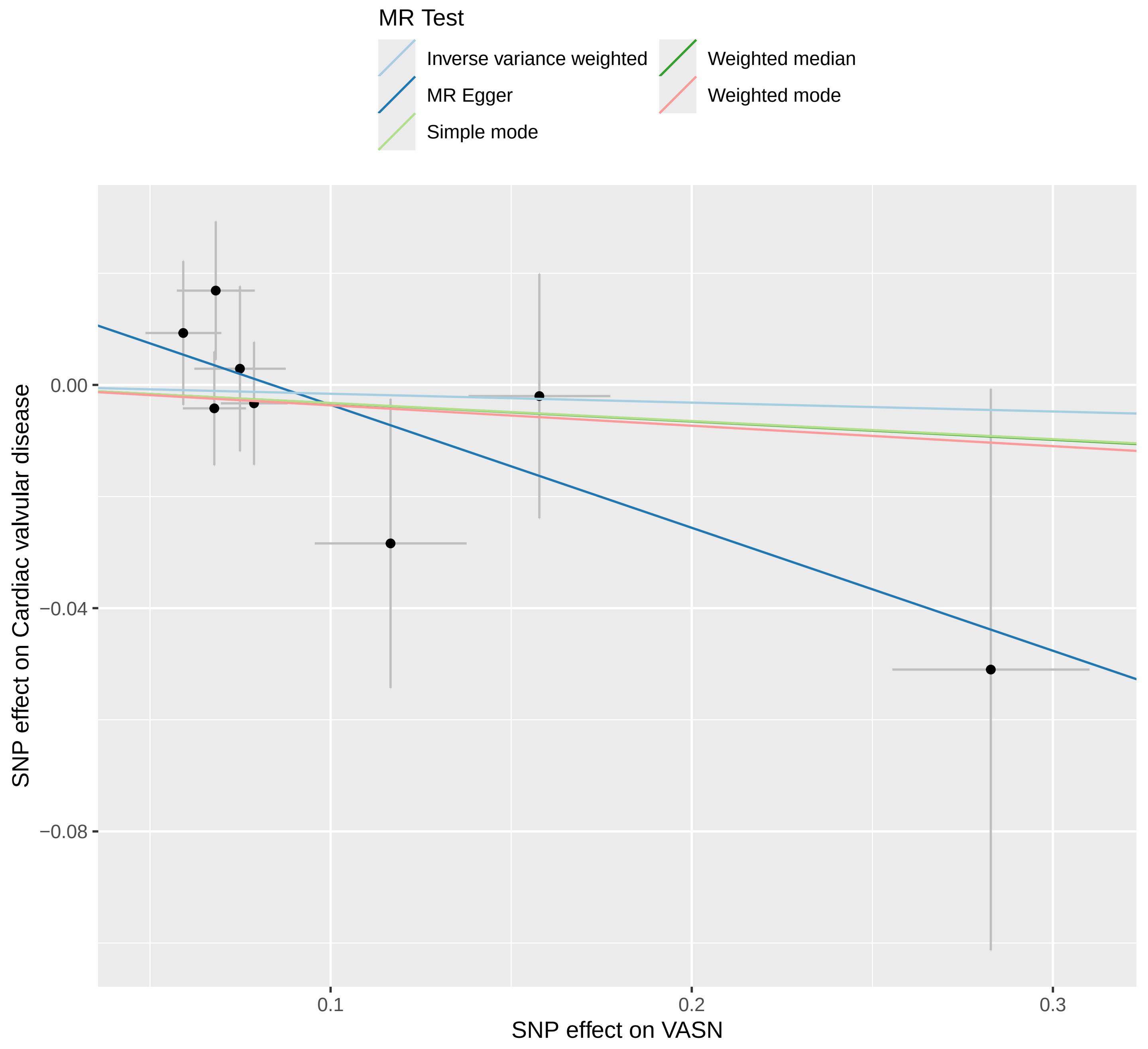

MR Test
Inverse variance weighted
MR Egger
Simple mode
Weighted median
Weighted mode
0.00
−0.04
−0.08
0.1
0.2
0.3
SNP effect on Cardiac valvular disease
SNP effect on VASN

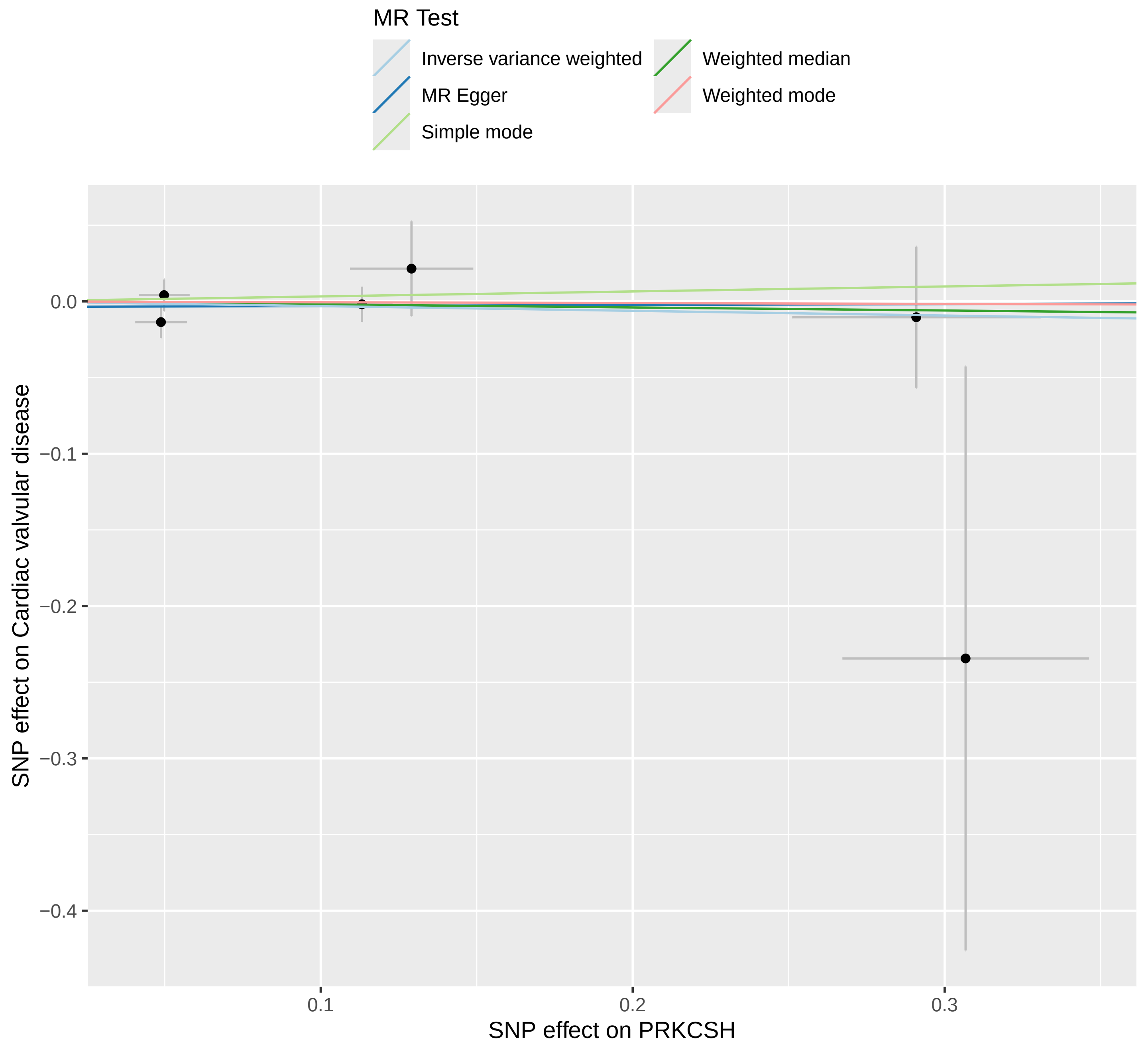
MR Test
Inverse variance weighted
MR Egger
Simple mode
Weighted median
Weighted mode
0.0
−0.1
−0.2
−0.3
−0.4
SNP effect on Cardiac valvular disease
0.1
0.2
0.3
SNP effect on PRKCSH

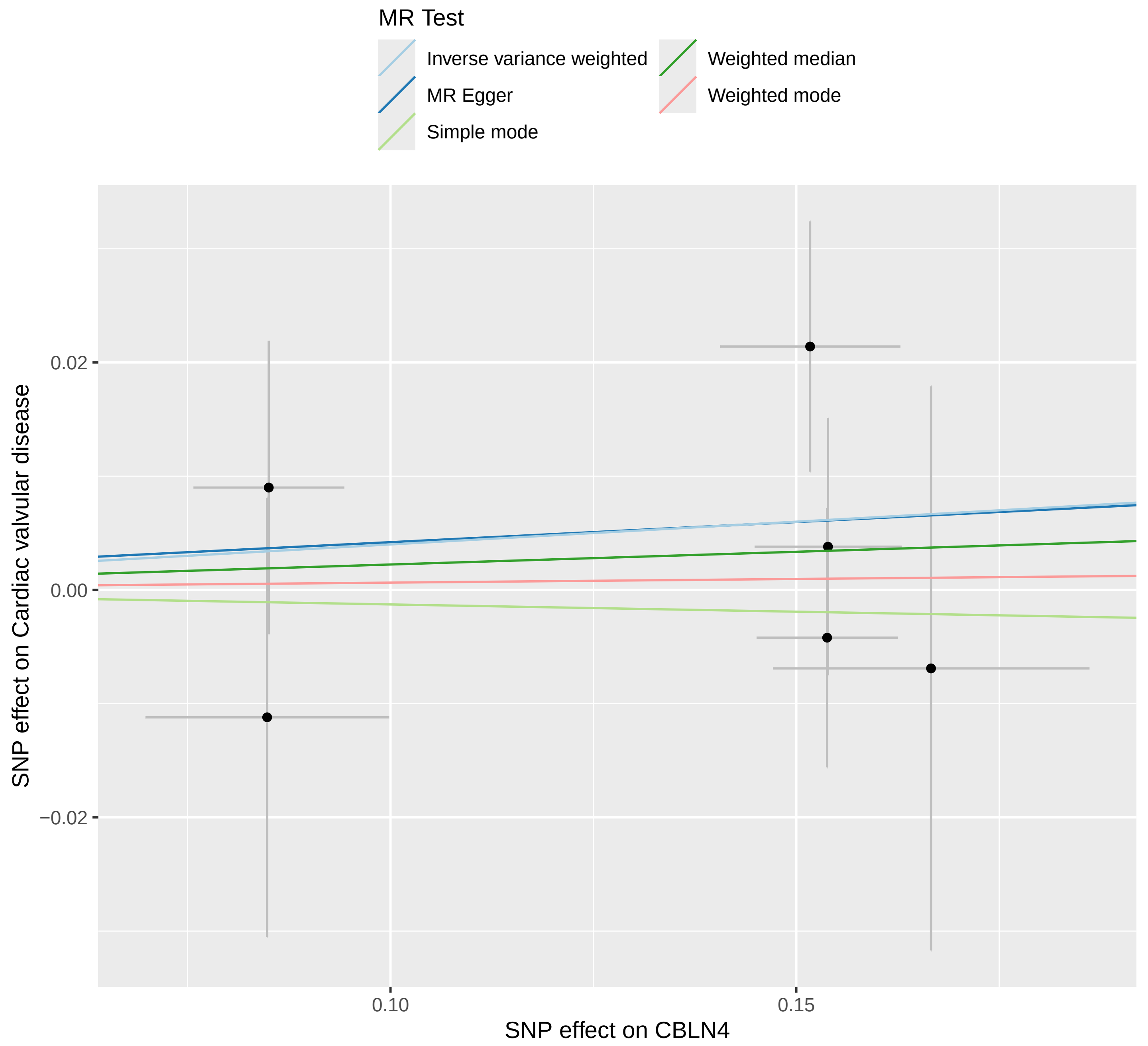
MR Test
Inverse variance weighted
MR Egger
Simple mode
Weighted median
Weighted mode
0.02
0.00
−0.02
0.10
0.15
SNP effect on Cardiac valvular disease
SNP effect on CBLN4

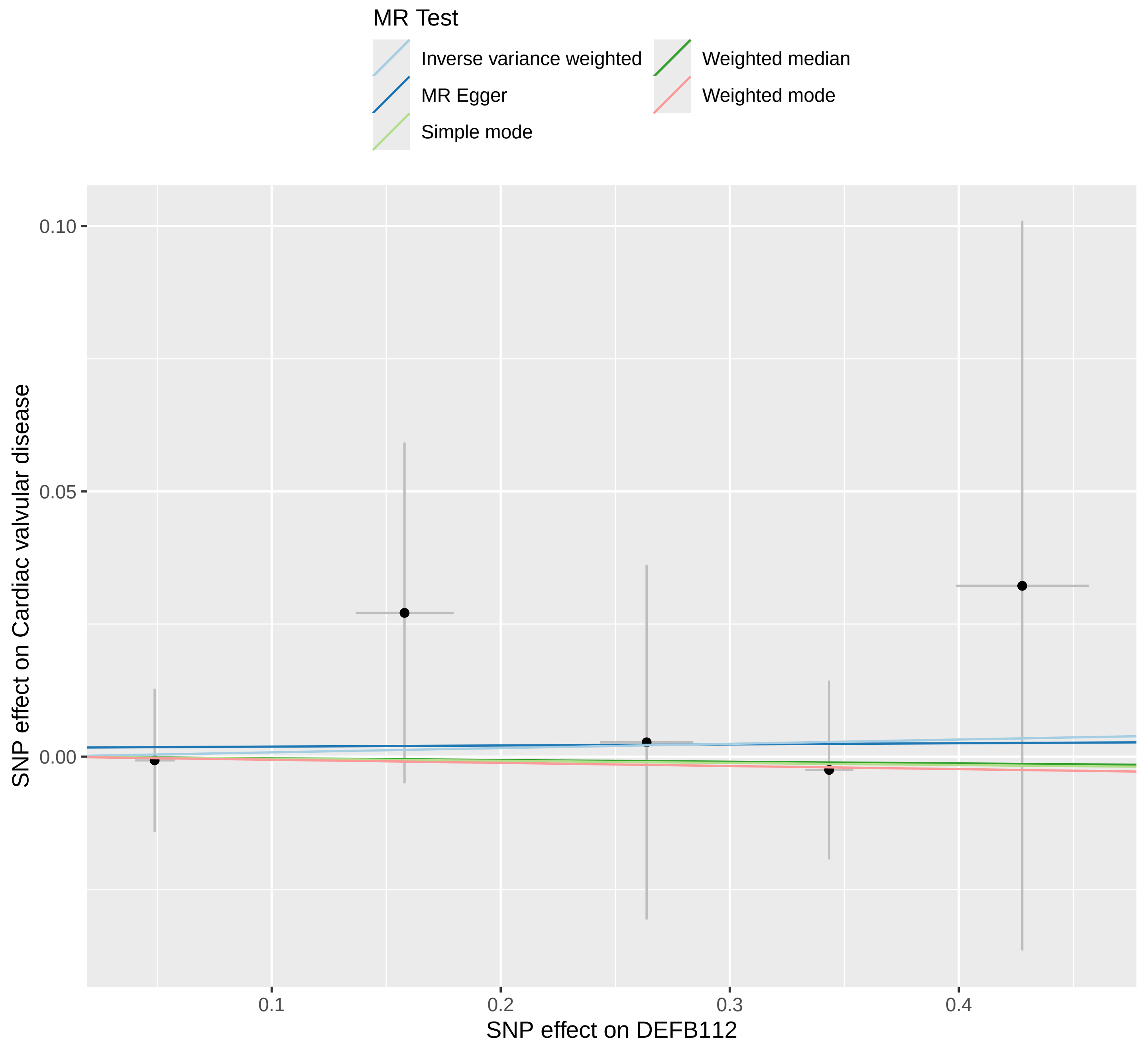
MR Test
Inverse variance weighted
MR Egger
Simple mode
Weighted median
Weighted mode
0.10
0.05
0.00
0.1
0.2
0.3
0.4
SNP effect on Cardiac valvular disease
SNP effect on DEFB112

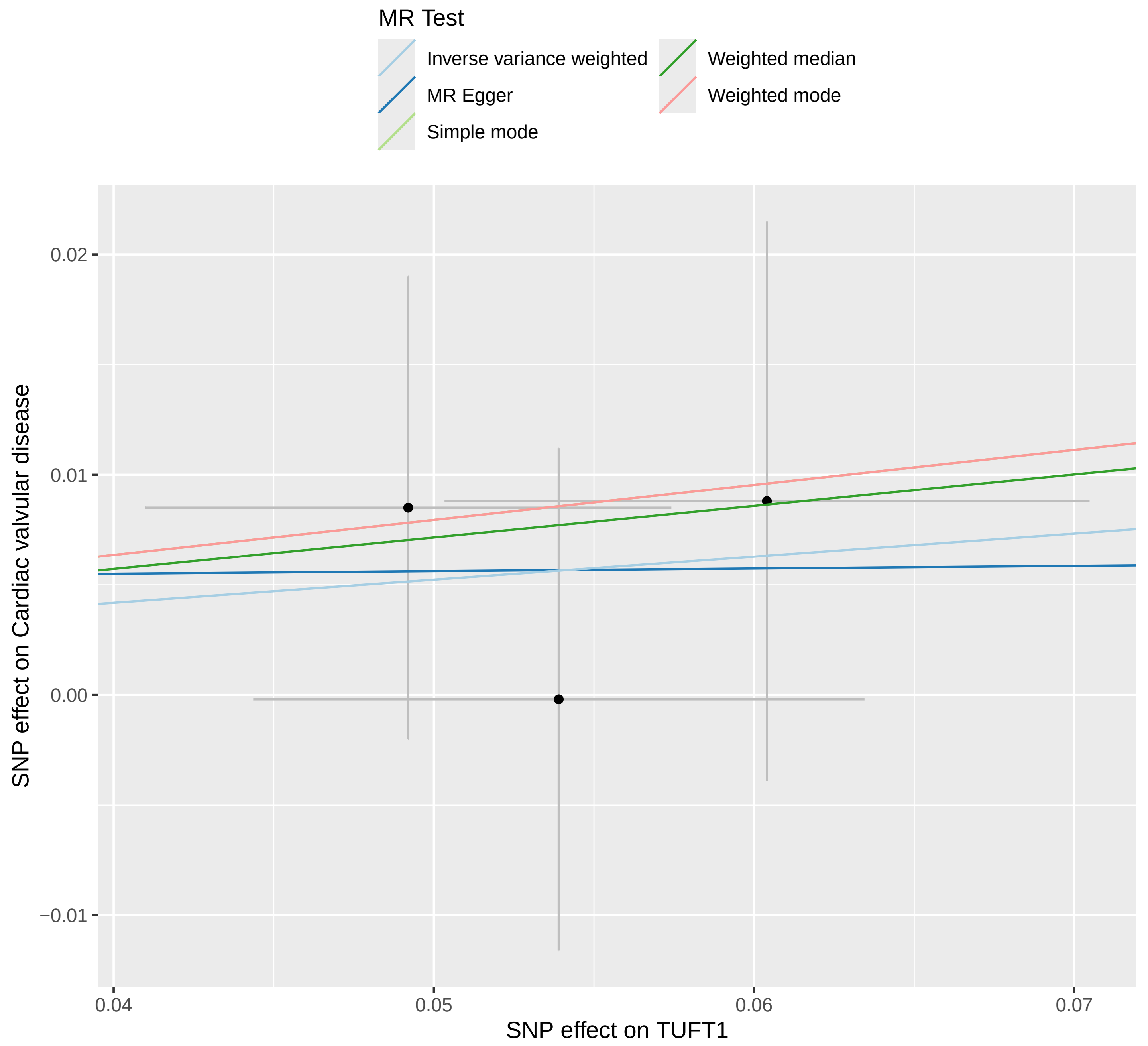

MR Test
Inverse variance weighted
MR Egger
Simple mode
Weighted median
Weighted mode
0.02
0.01
0.00
−0.01
0.04
0.05
0.06
0.07
SNP effect on Cardiac valvular disease
SNP effect on TUFT1

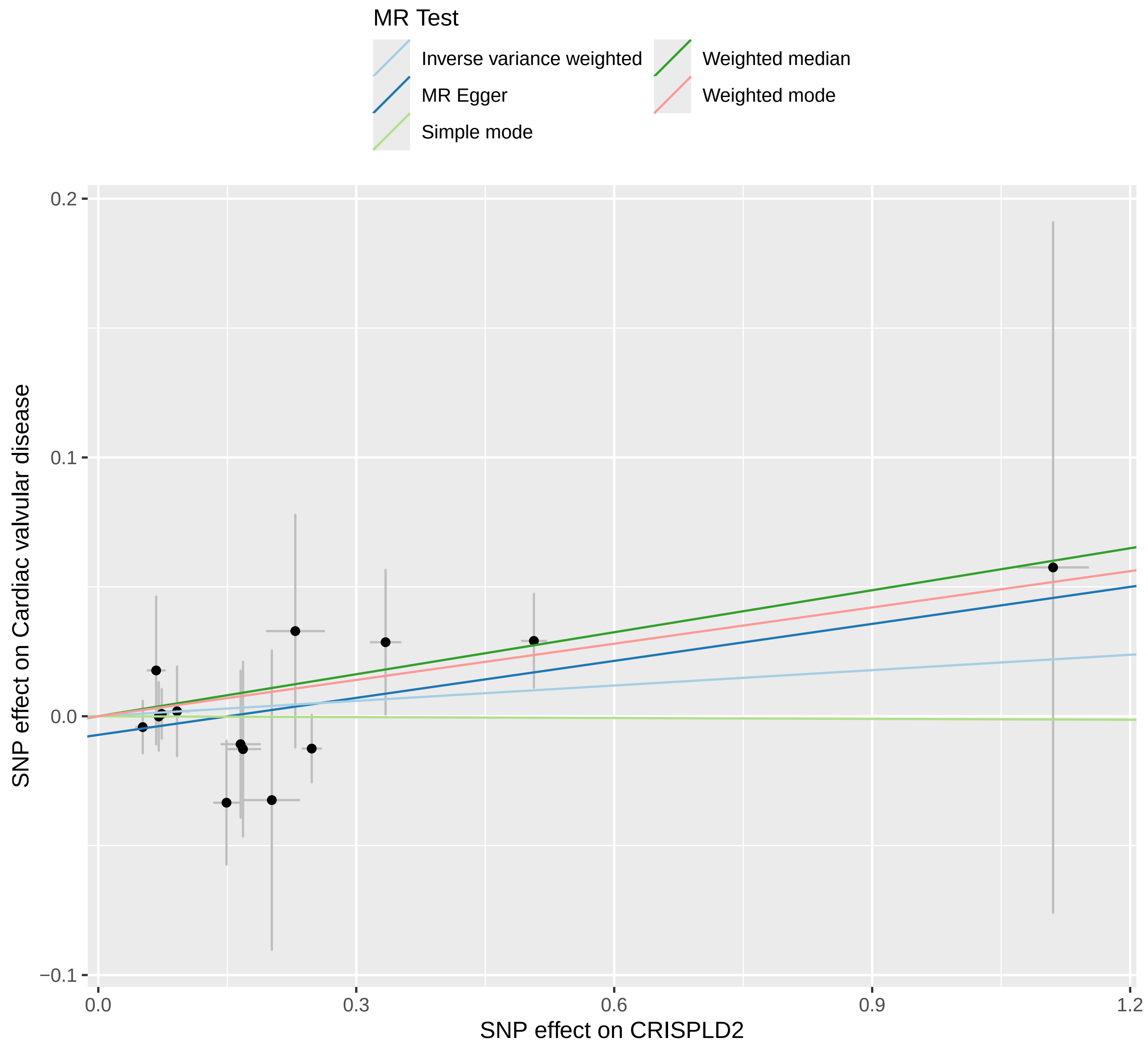

MR Test
Inverse variance weighted
MR Egger
Simple mode
Weighted median
Weighted mode
0.2
0.1
0.0
−0.1
0.0
0.3
0.6
0.9
1.2
SNP effect on Cardiac valvular disease
SNP effect on CRISPLD2

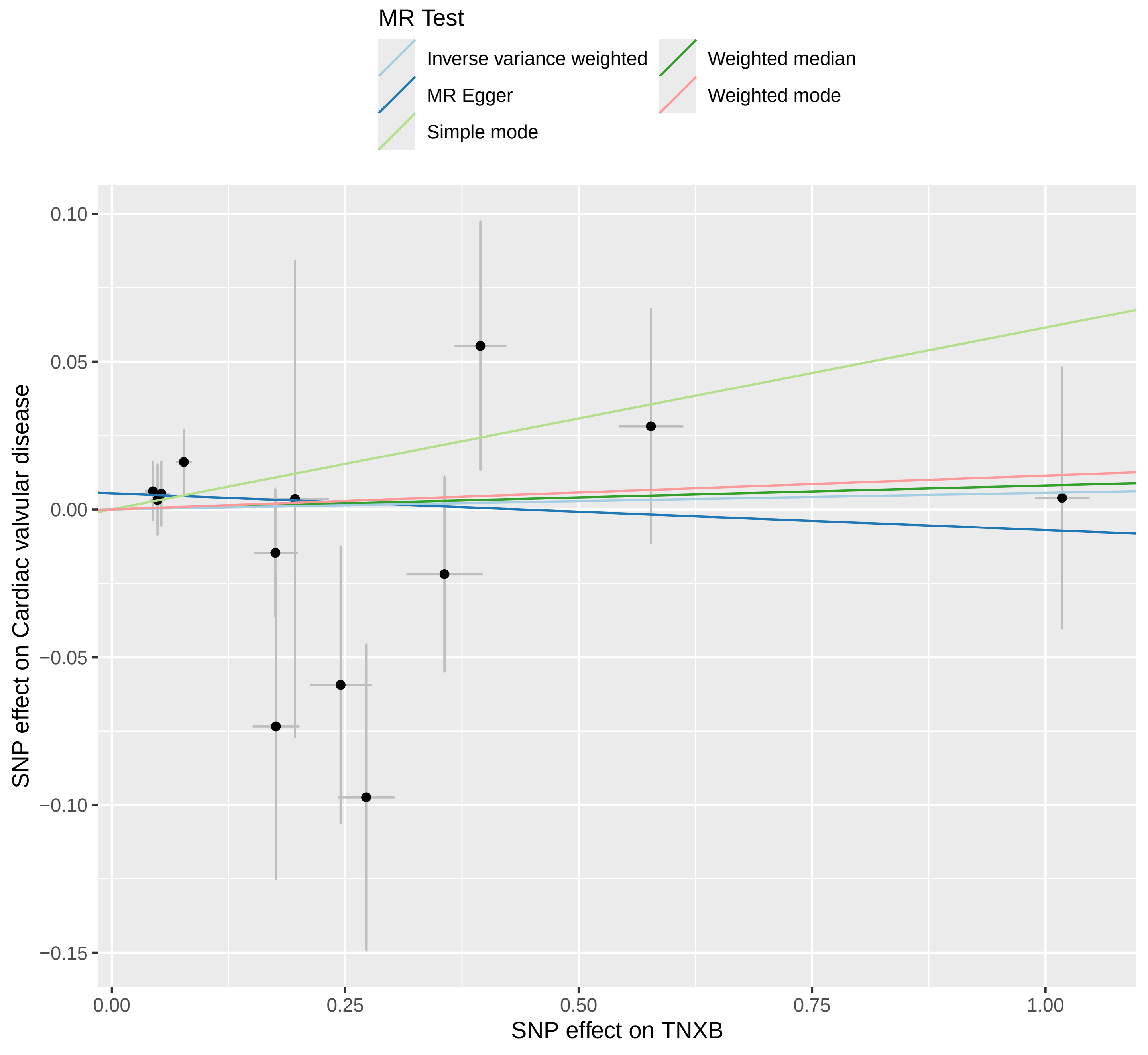
MR Test
Inverse variance weighted
MR Egger
Simple mode
Weighted median
Weighted mode
0.10
0.05
0.00
−0.05
−0.10
−0.15
0.00
0.25
0.50
0.75
1.00
SNP effect on Cardiac valvular disease
SNP effect on TNXB

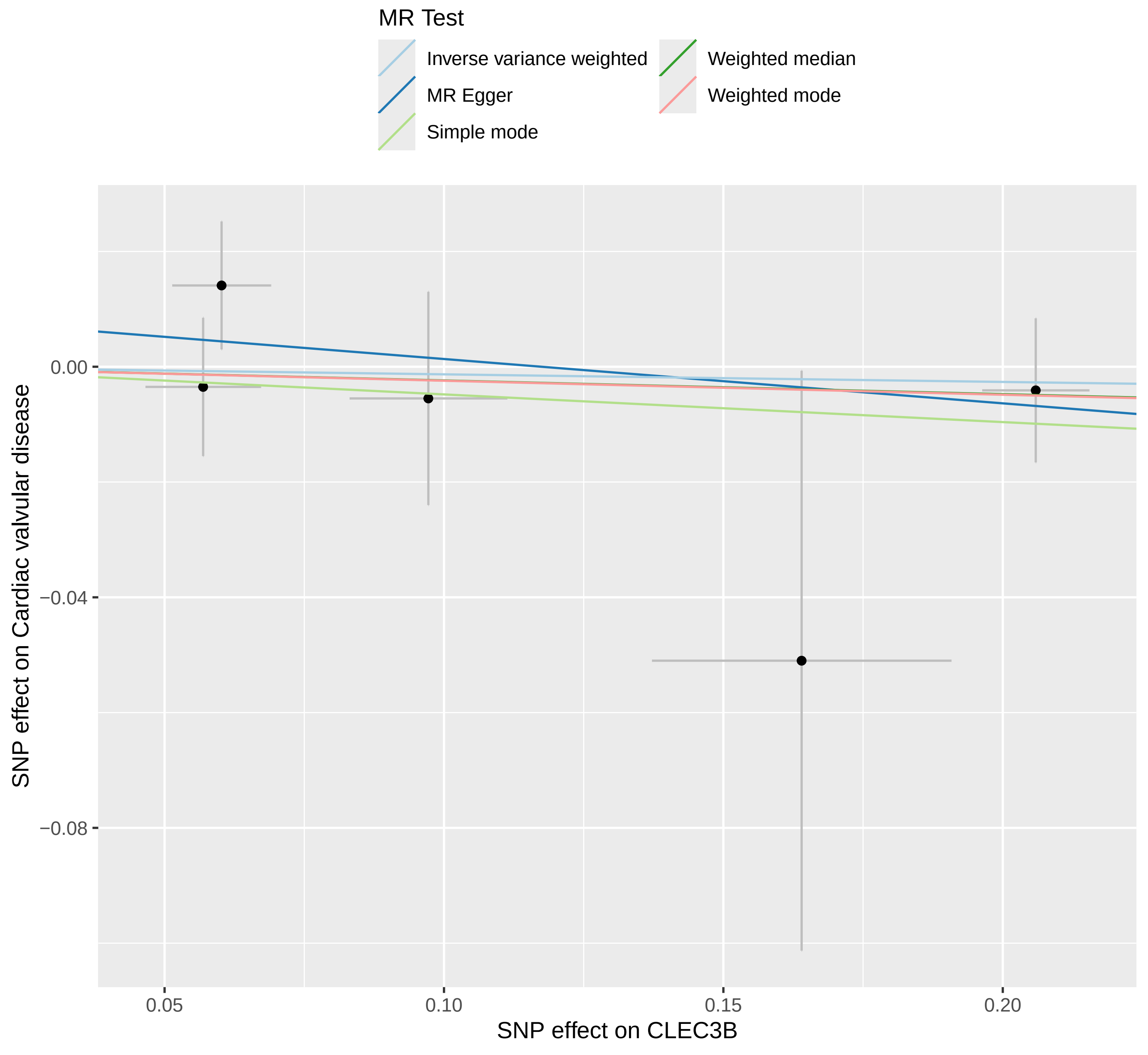

MR Test
Inverse variance weighted
MR Egger
Simple mode
Weighted median
Weighted mode
0.00
−0.04
−0.08
0.05
0.10
0.15
0.20
SNP effect on CLEC3B
SNP effect on Cardiac valvular disease

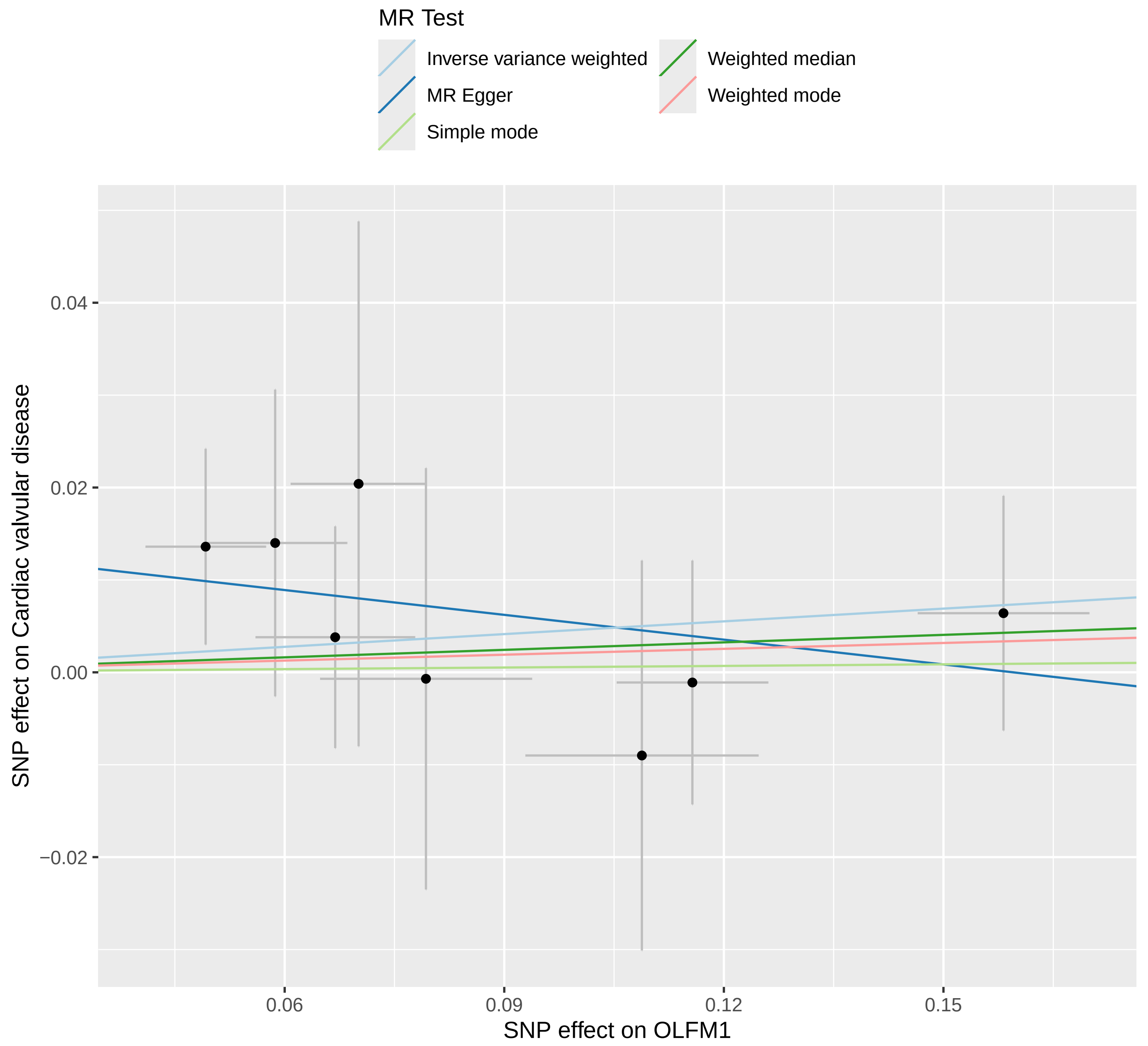
MR Test
Inverse variance weighted
MR Egger
Simple mode
Weighted median
Weighted mode
SNP effect on Cardiac valvular disease
0.04
0.02
0.00
−0.02
0.06
0.09
0.12
0.15
SNP effect on OLFM1

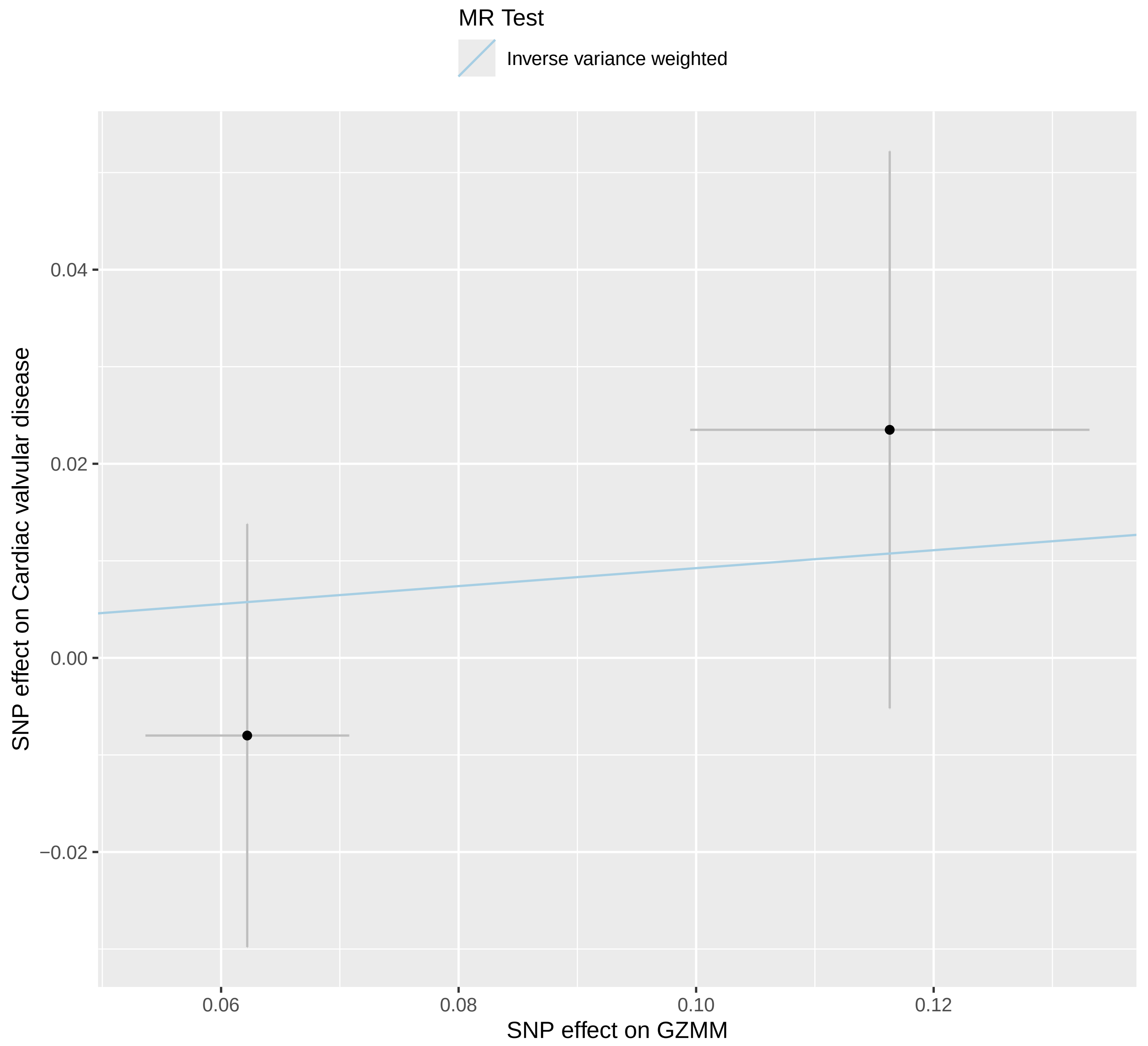

MR Test
Inverse variance weighted
0.04
0.02
0.00
−0.02
0.06
0.08
0.10
0.12
SNP effect on Cardiac valvular disease
SNP effect on GZMM

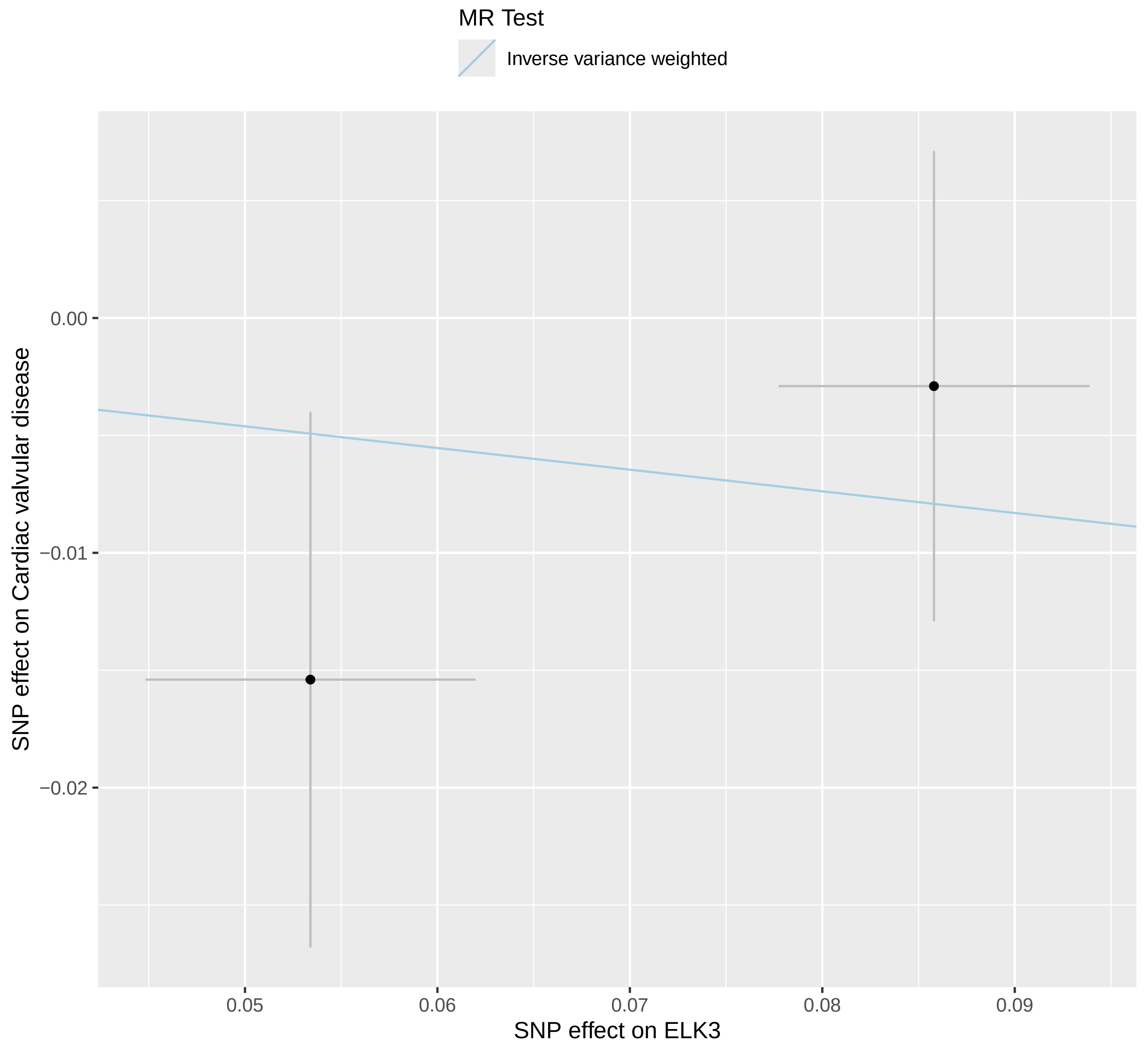

MR Test
Inverse variance weighted
0.00
−0.01
−0.02
SNP effect on Cardiac valvular disease
0.05
0.06
0.07
0.08
0.09
SNP effect on ELK3

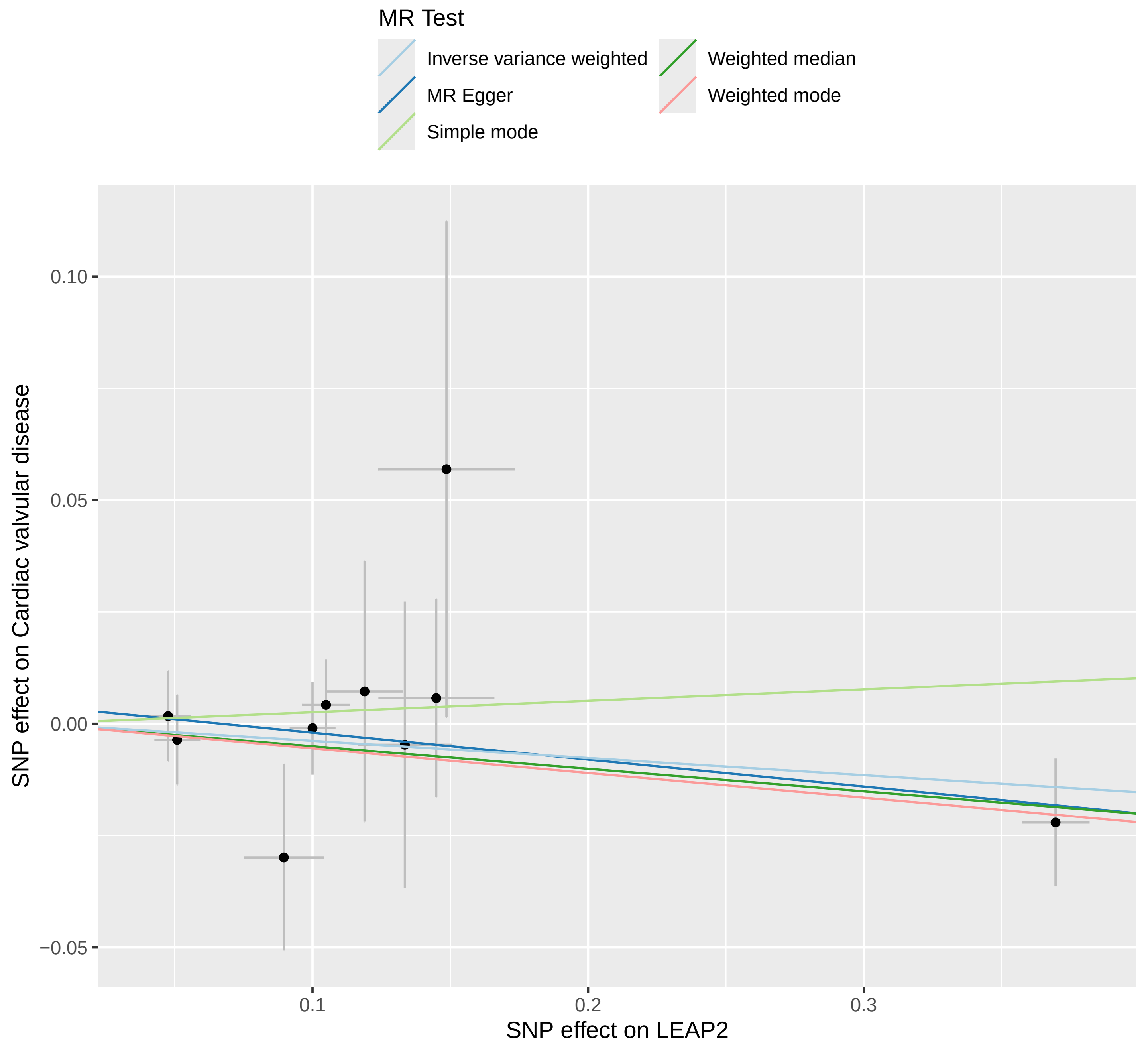
MR Test
Inverse variance weighted
MR Egger
Simple mode
Weighted median
Weighted mode
0.10
0.05
0.00
−0.05
0.1
0.2
0.3
SNP effect on Cardiac valvular disease
SNP effect on LEAP2

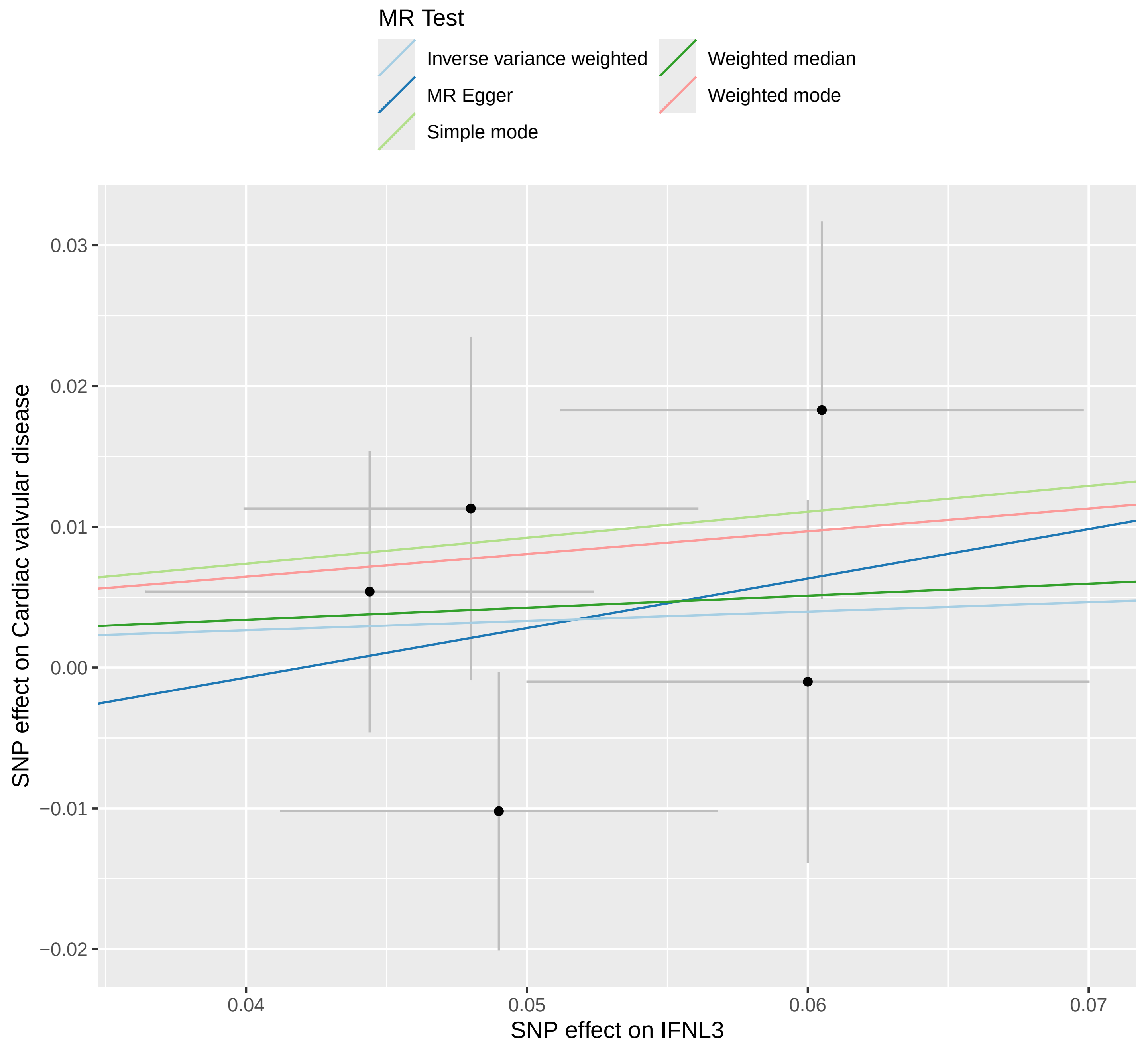

MR Test
Inverse variance weighted
MR Egger
Simple mode
Weighted median
Weighted mode
0.03
0.02
0.01
0.00
−0.01
−0.02
SNP effect on Cardiac valvular disease
0.04
0.05
0.06
0.07
SNP effect on IFNL3

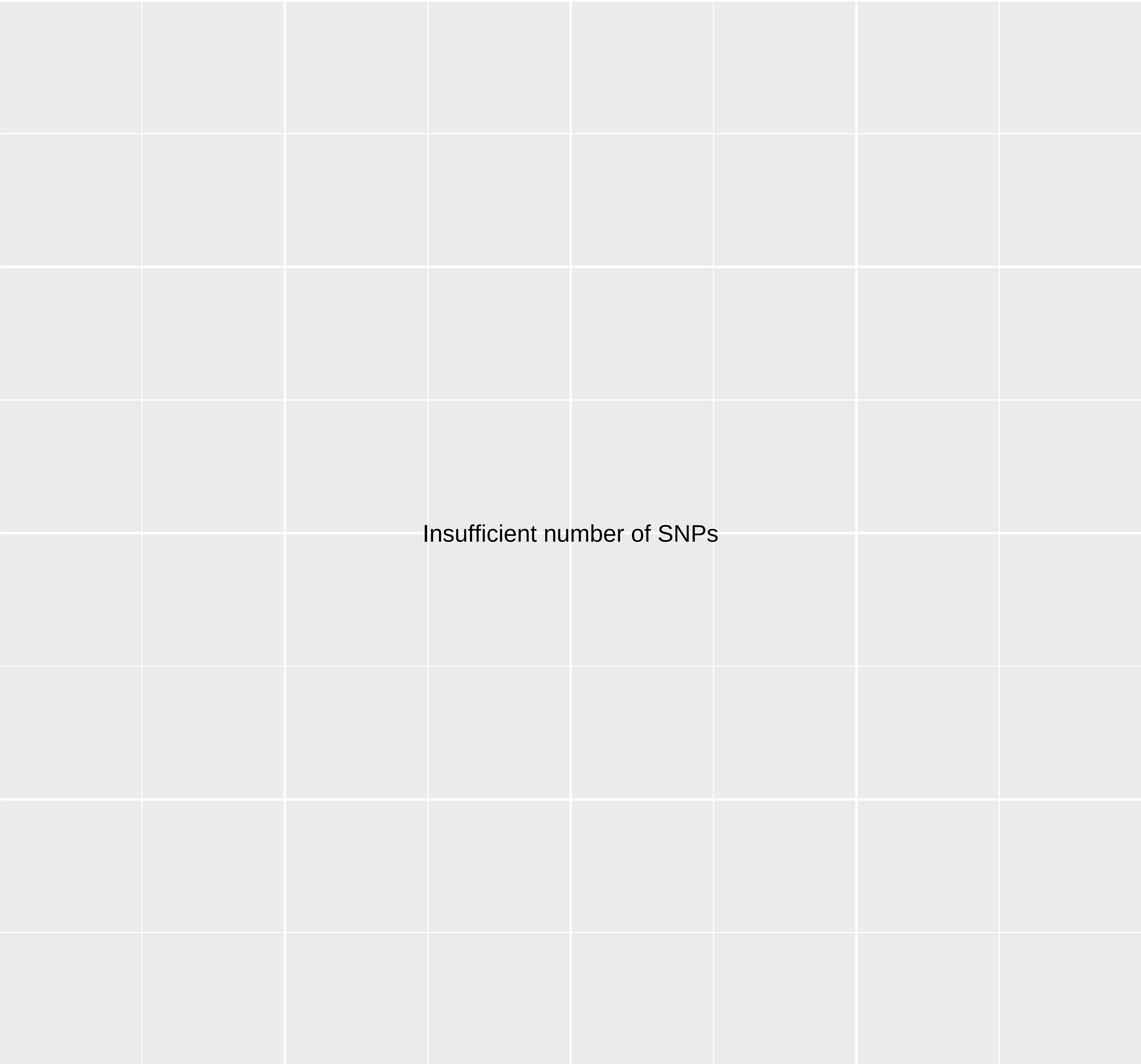
Insufficient number of SNPs

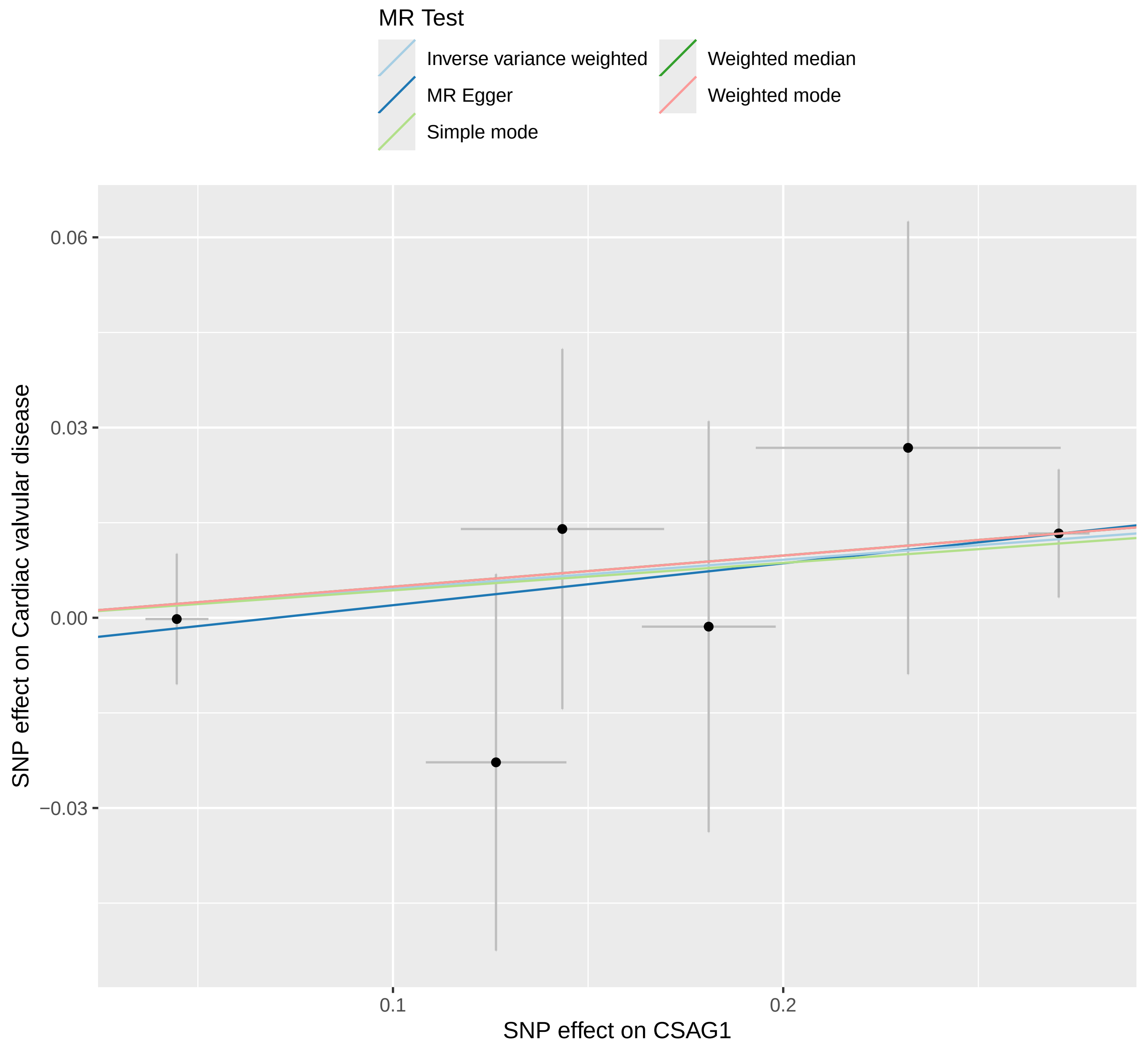
MR Test
Inverse variance weighted
MR Egger
Simple mode
Weighted median
Weighted mode
0.06
0.03
0.00
−0.03
0.1
0.2
SNP effect on Cardiac valvular disease
SNP effect on CSAG1

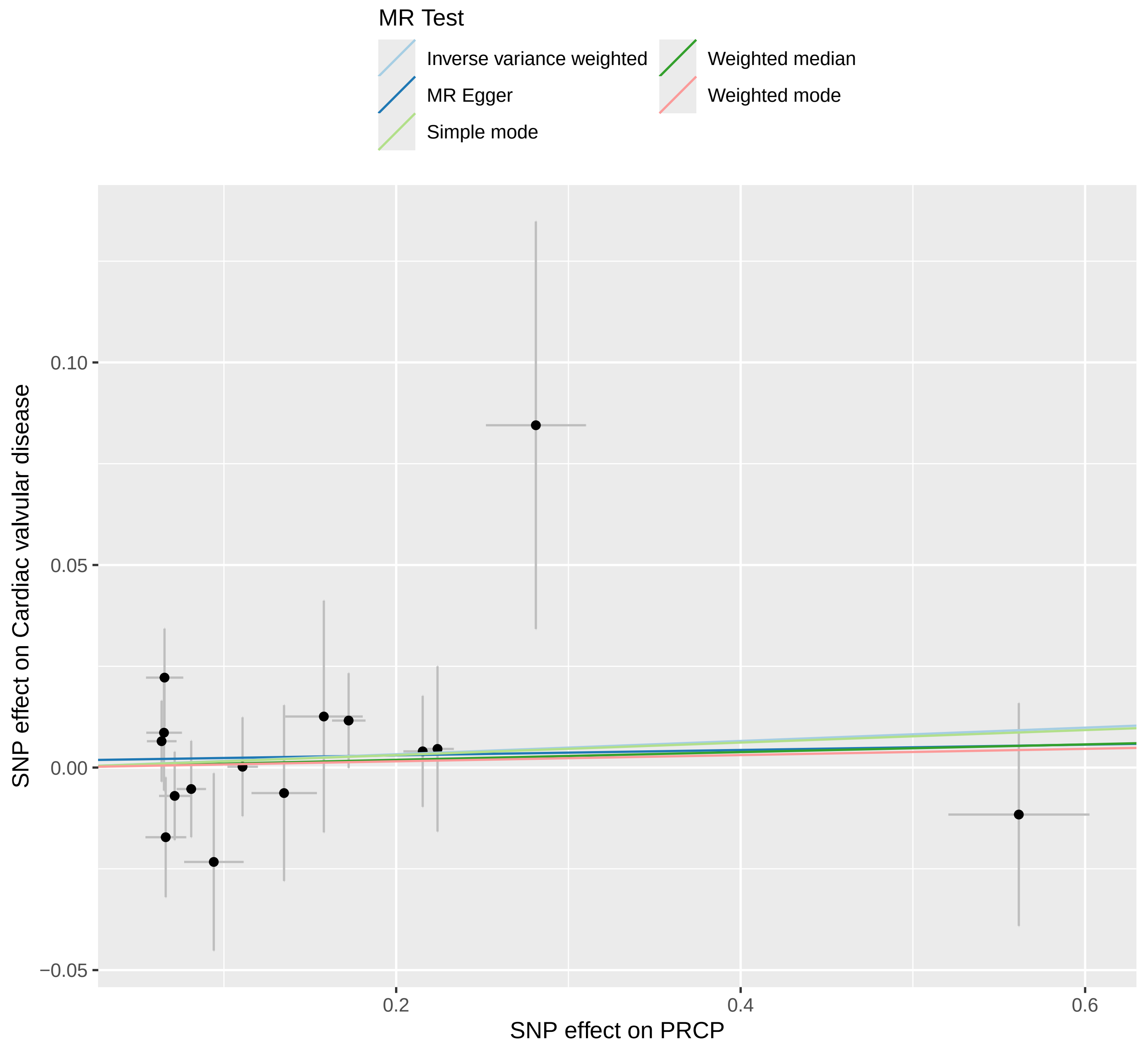
MR Test
Inverse variance weighted
MR Egger
Simple mode
Weighted median
Weighted mode
SNP effect on Cardiac valvular disease
0.10
0.05
0.00
−0.05
0.2
0.4
0.6
SNP effect on PRCP

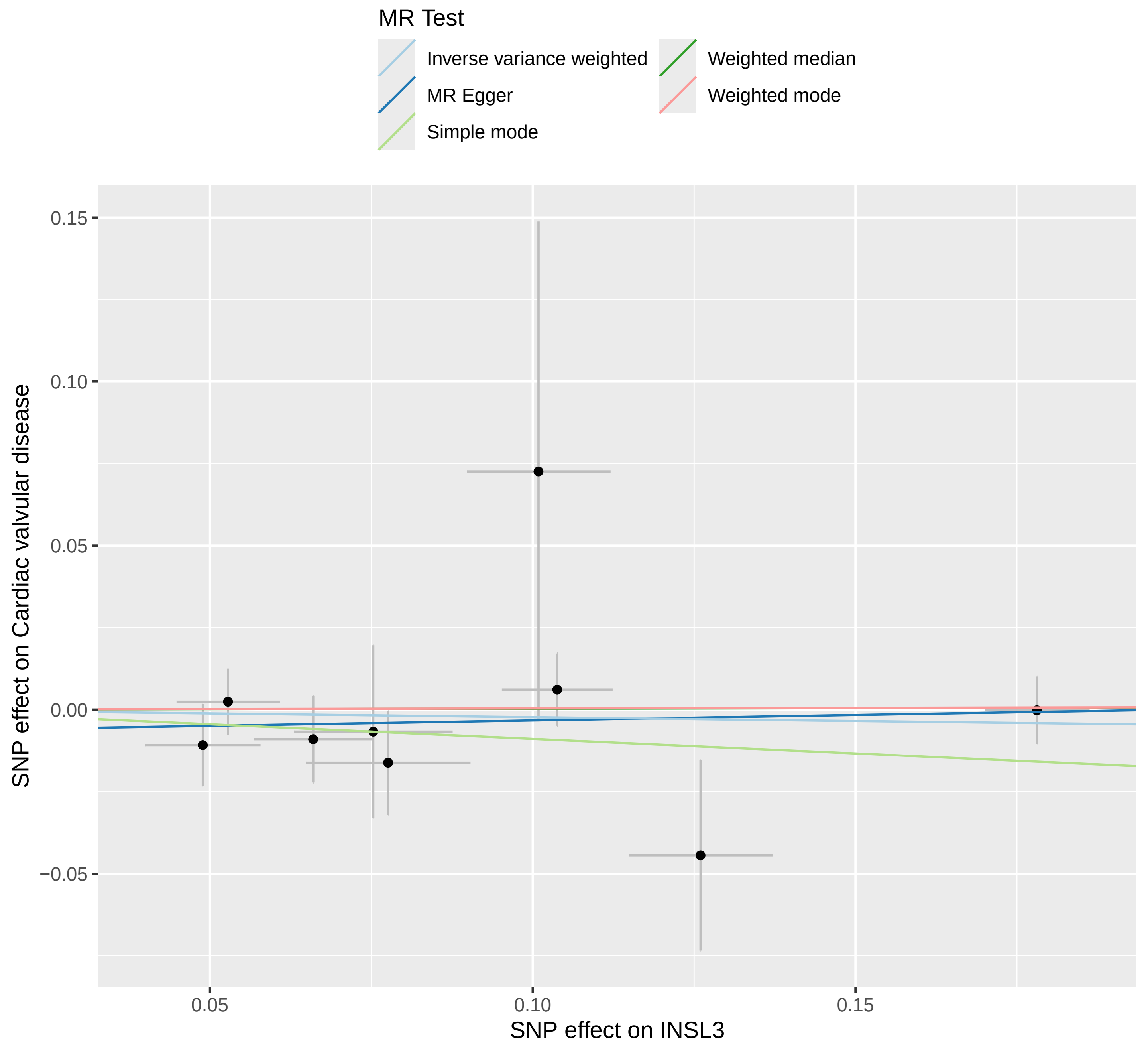
MR Test
Inverse variance weighted
MR Egger
Simple mode
Weighted median
Weighted mode
0.15
0.10
0.05
0.00
−0.05
SNP effect on Cardiac valvular disease
0.05
0.10
0.15
SNP effect on INSL3

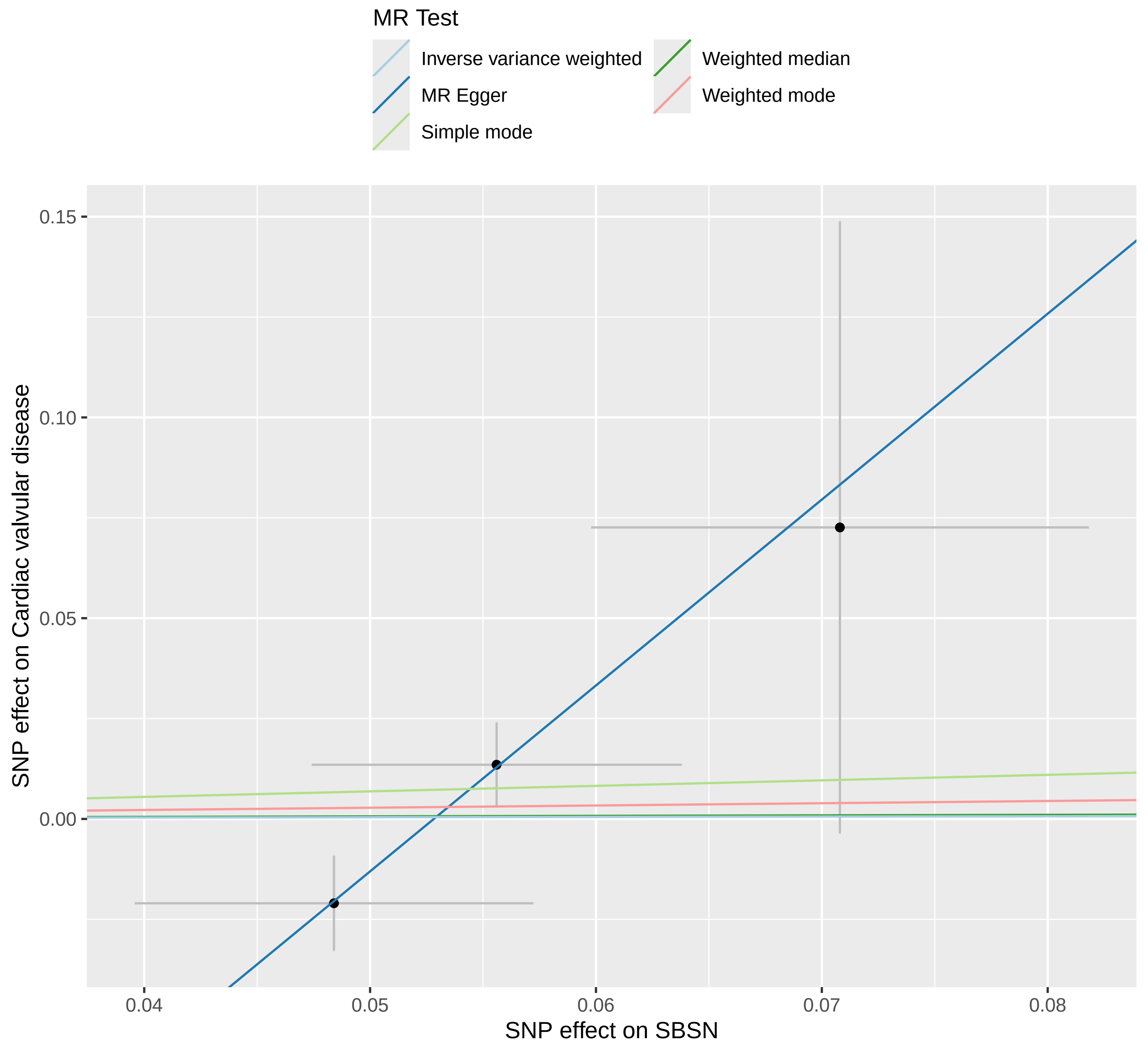

MR Test
Inverse variance weighted
MR Egger
Simple mode
Weighted median
Weighted mode
0.15
0.10
0.05
0.00
0.04
0.05
0.06
0.07
0.08
SNP effect on Cardiac valvular disease
SNP effect on SBSN

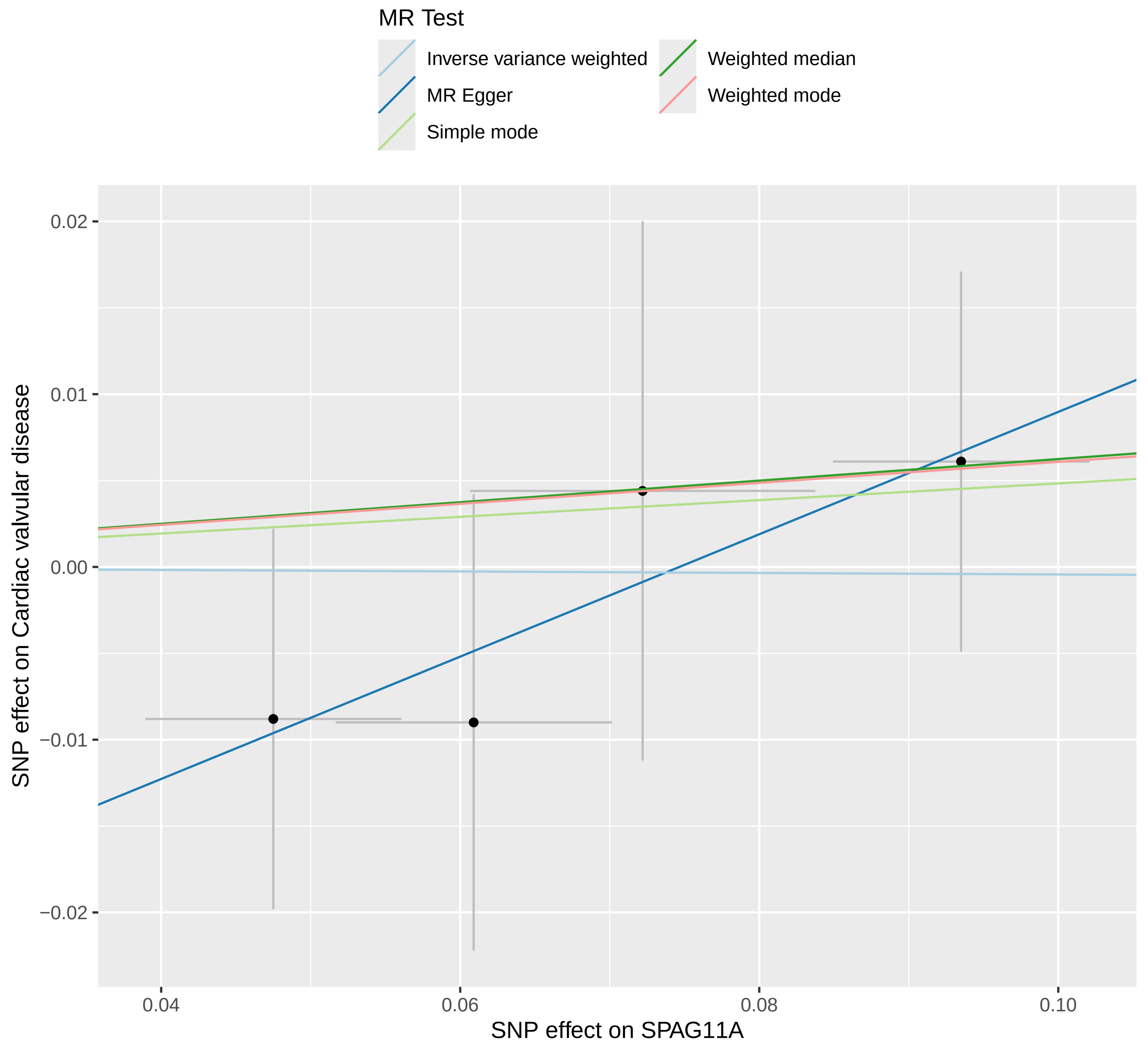
MR Test
Inverse variance weighted
MR Egger
Simple mode
Weighted median
Weighted mode
0.02
0.01
0.00
−0.01
−0.02
SNP effect on Cardiac valvular disease
0.04
0.06
0.08
0.10
SNP effect on SPAG11A

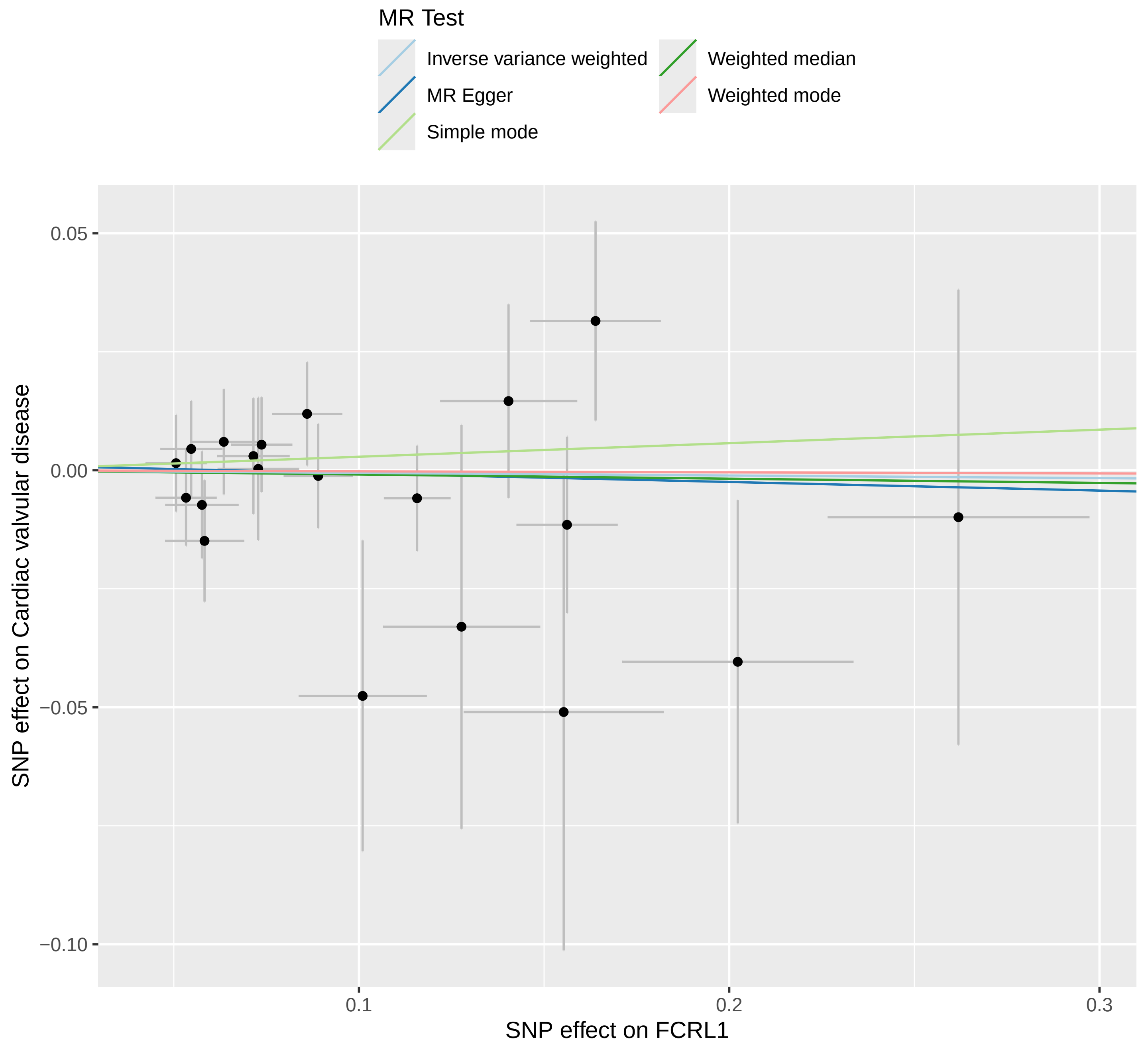
MR Test
Inverse variance weighted
MR Egger
Simple mode
Weighted median
Weighted mode
0.05
0.00
−0.05
−0.10
0.1
0.2
0.3
SNP effect on Cardiac valvular disease
SNP effect on FCRL1

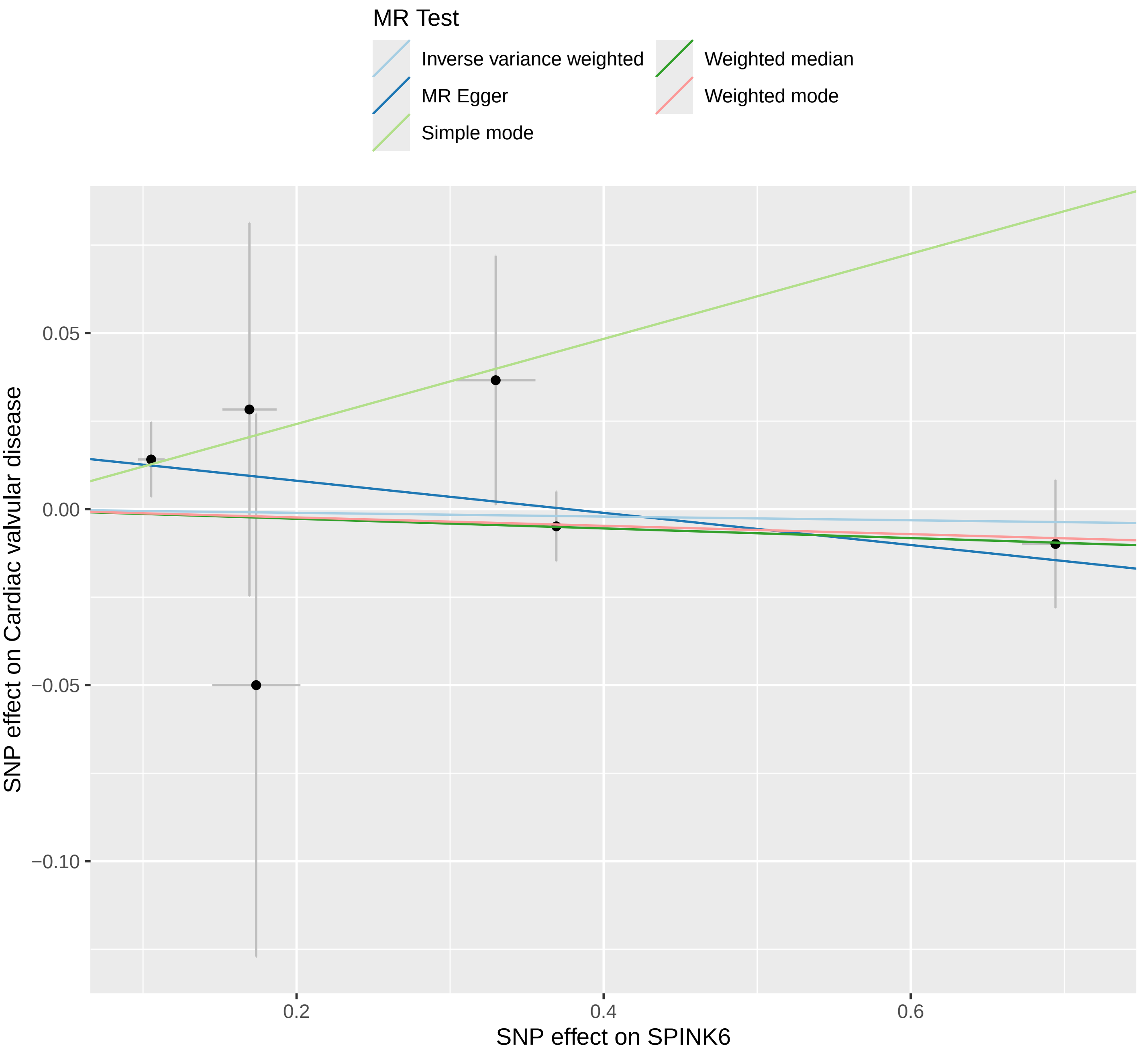
MR Test
Inverse variance weighted
MR Egger
Simple mode
Weighted median
Weighted mode
0.05
0.00
−0.05
−0.10
0.2
0.4
0.6
SNP effect on Cardiac valvular disease
SNP effect on SPINK6

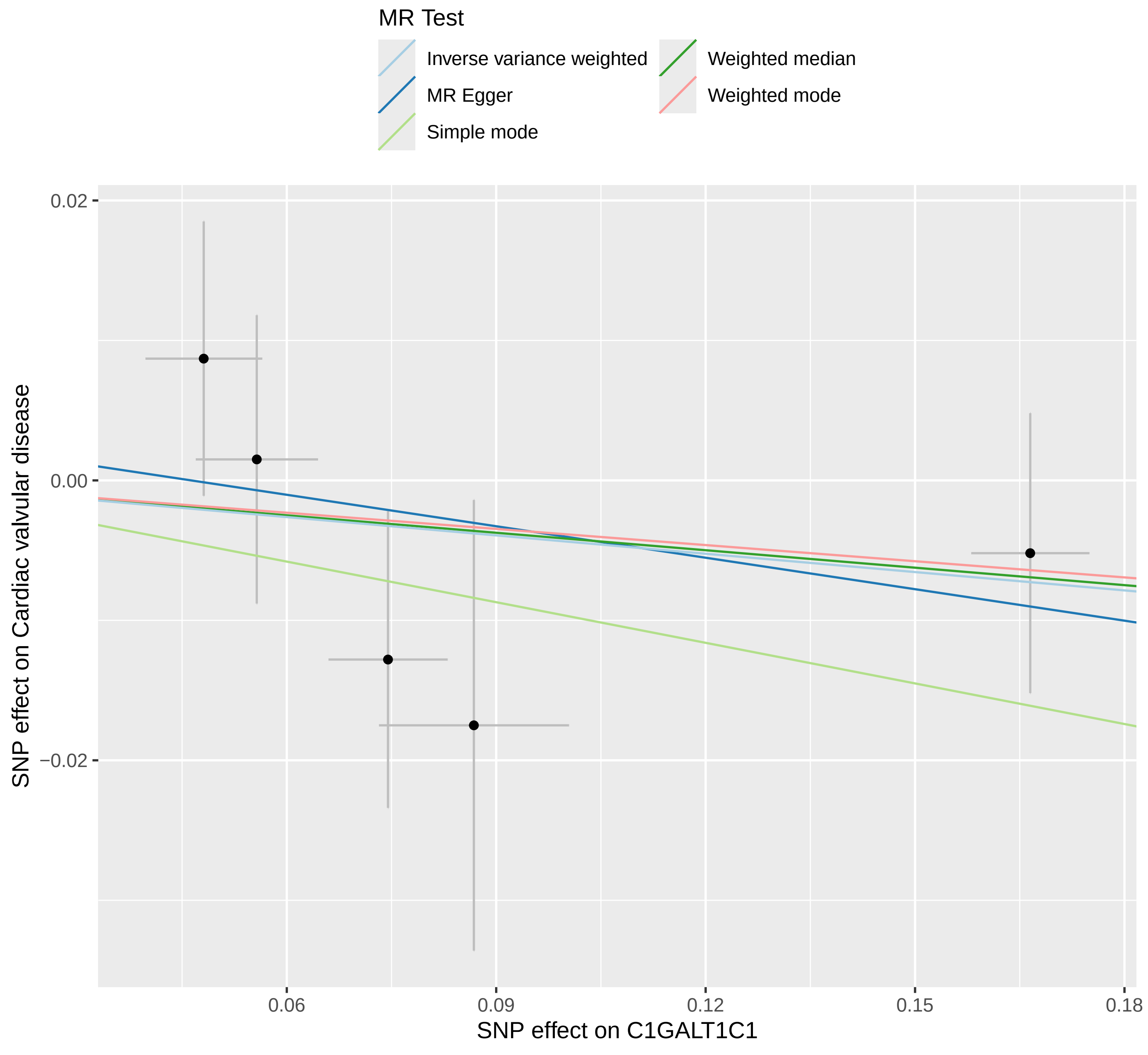
MR Test
Inverse variance weighted
MR Egger
Simple mode
Weighted median
Weighted mode
0.02
0.00
−0.02
0.06
0.09
0.12
0.15
0.18
SNP effect on C1GALT1C1
SNP effect on Cardiac valvular disease

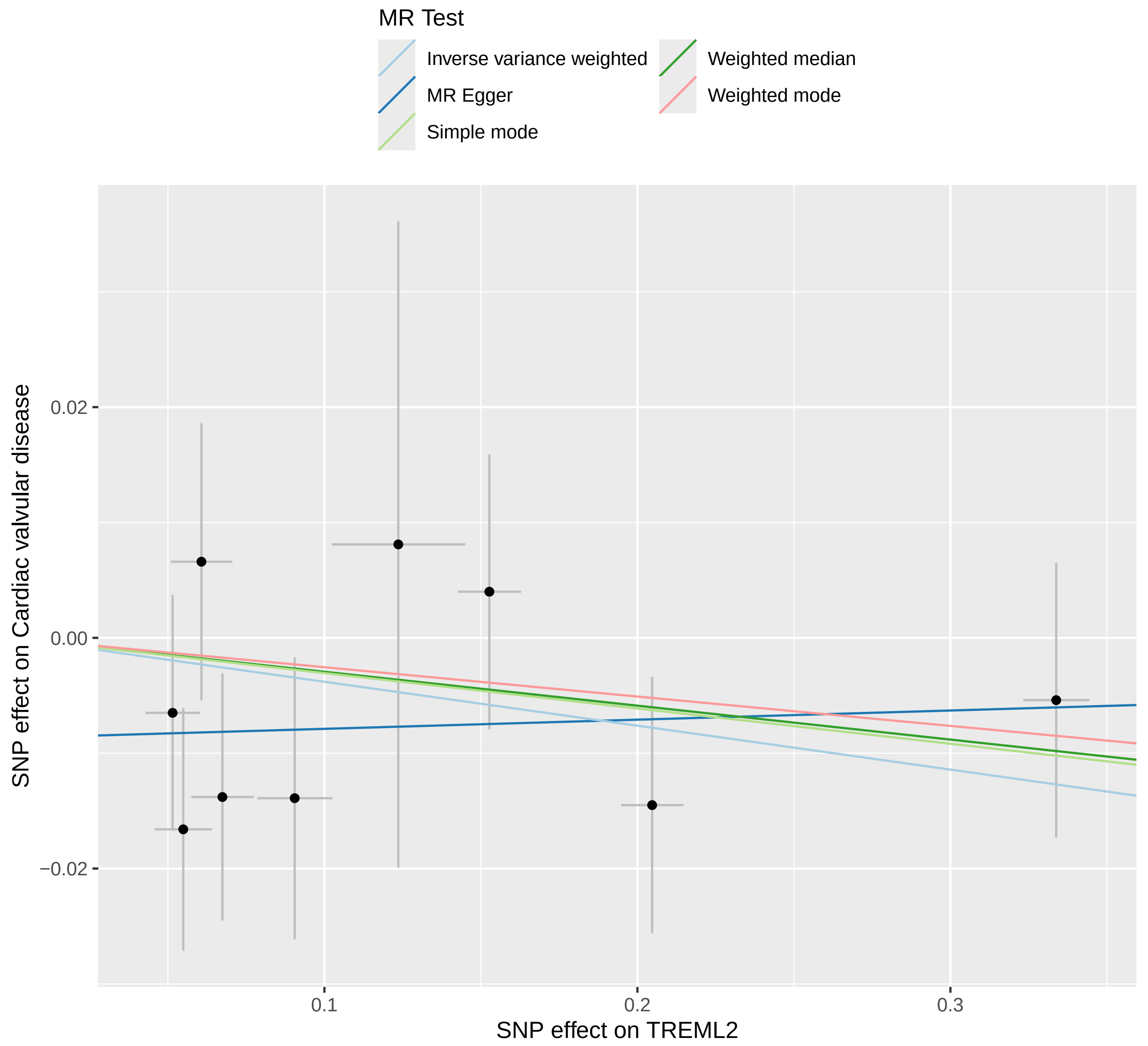

MR Test
Inverse variance weighted
MR Egger
Simple mode
Weighted median
Weighted mode
SNP effect on Cardiac valvular disease
0.02
0.00
−0.02
0.1
0.2
0.3
SNP effect on TREML2

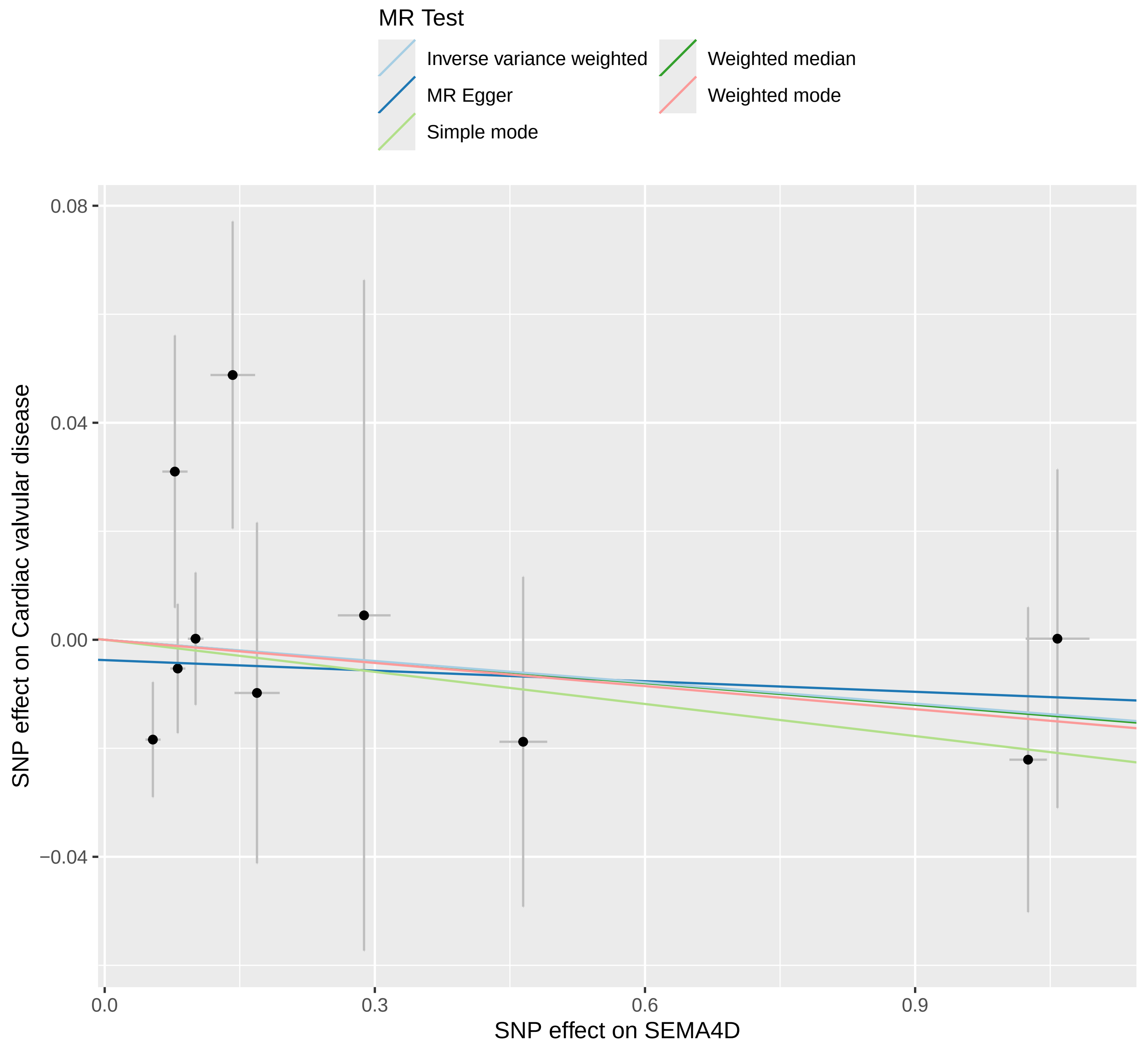
MR Test
Inverse variance weighted
MR Egger
Simple mode
Weighted median
Weighted mode
0.08
0.04
0.00
−0.04
0.0
0.3
0.6
0.9
SNP effect on Cardiac valvular disease
SNP effect on SEMA4D

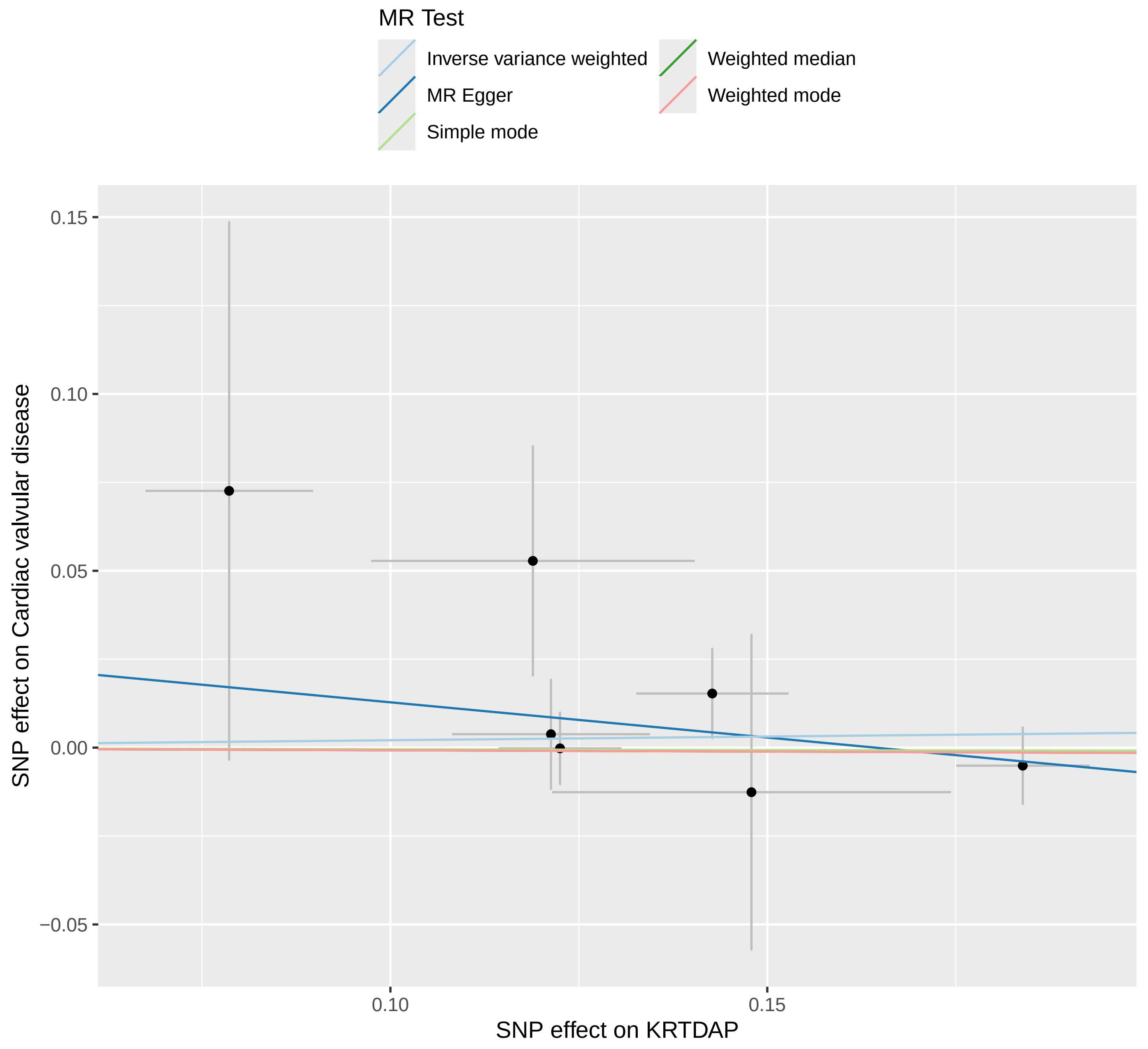

MR Test
Inverse variance weighted
MR Egger
Simple mode
Weighted median
Weighted mode
0.15
0.10
0.05
0.00
−0.05
SNP effect on Cardiac valvular disease
0.10
0.15
SNP effect on KRTDAP

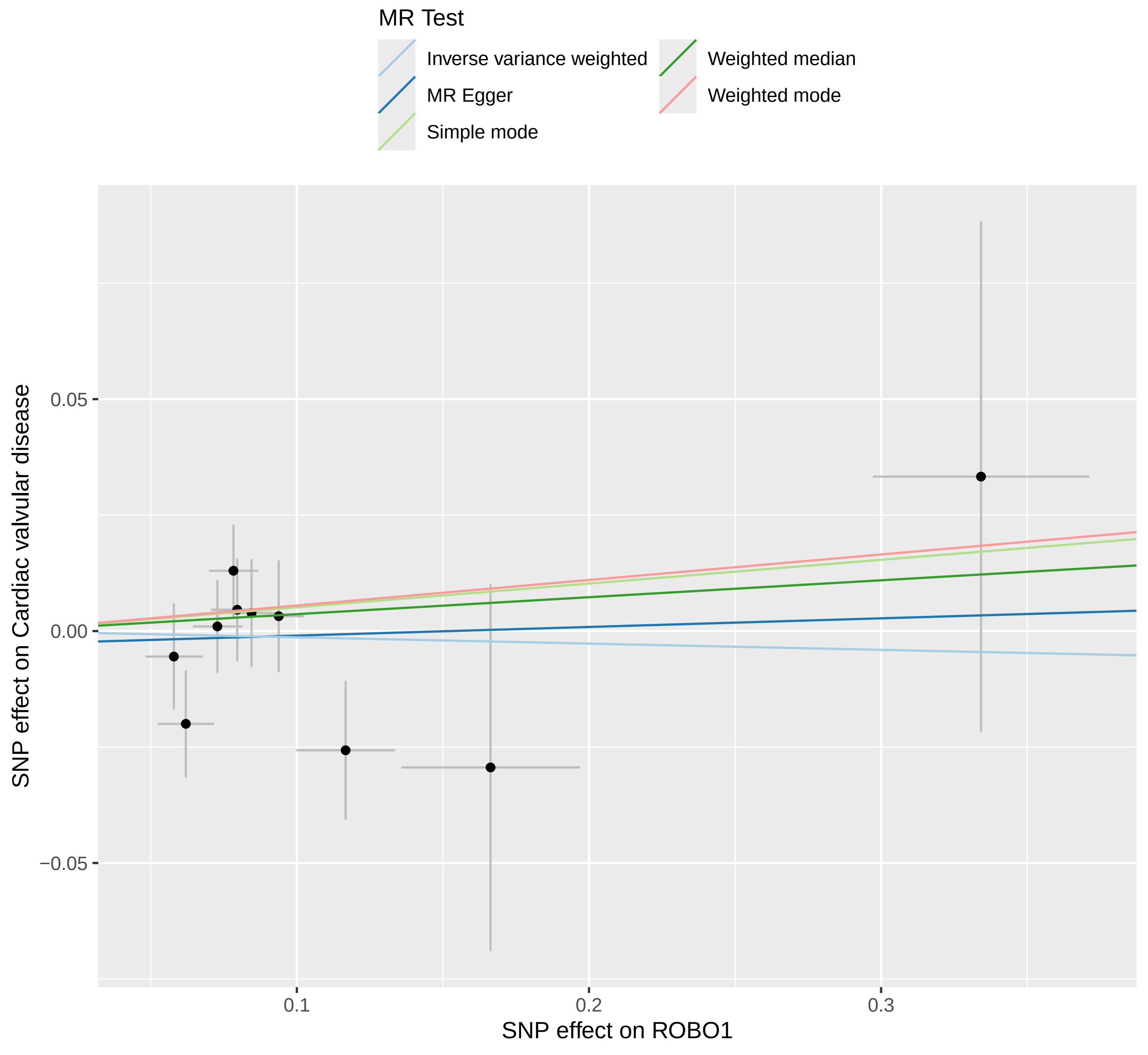

MR Test
Inverse variance weighted
MR Egger
Simple mode
Weighted median
Weighted mode
SNP effect on Cardiac valvular disease
0.05
0.00
−0.05
0.1
0.2
0.3
SNP effect on ROBO1

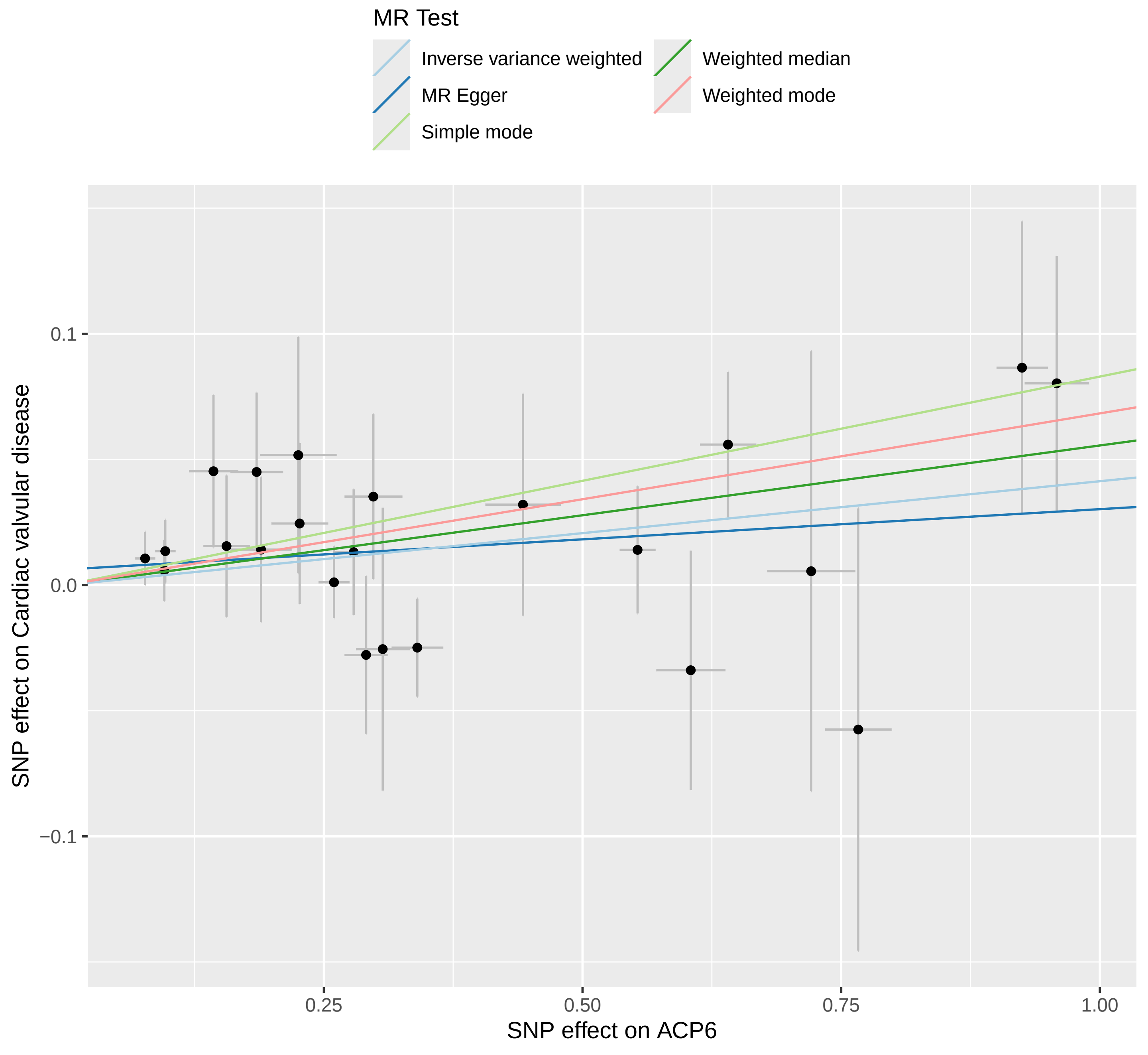
MR Test
Inverse variance weighted
MR Egger
Simple mode
Weighted median
Weighted mode
SNP effect on Cardiac valvular disease
0.1
0.0
−0.1
0.25
0.50
0.75
1.00
SNP effect on ACP6

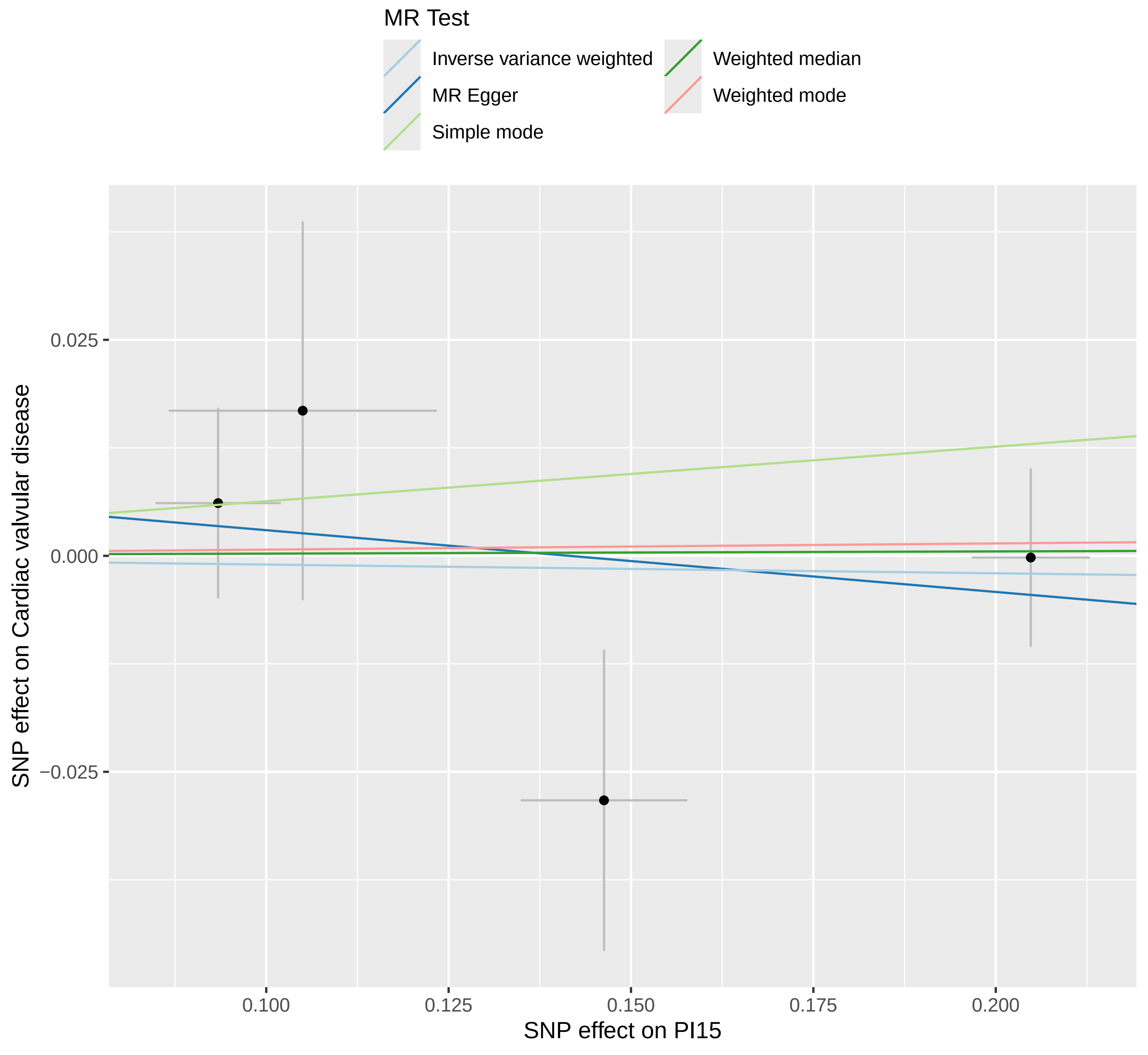

MR Test
Inverse variance weighted
MR Egger
Simple mode
Weighted median
Weighted mode
0.025
0.000
−0.025
0.100
0.125
0.150
0.175
0.200
SNP effect on PI15
SNP effect on Cardiac valvular disease

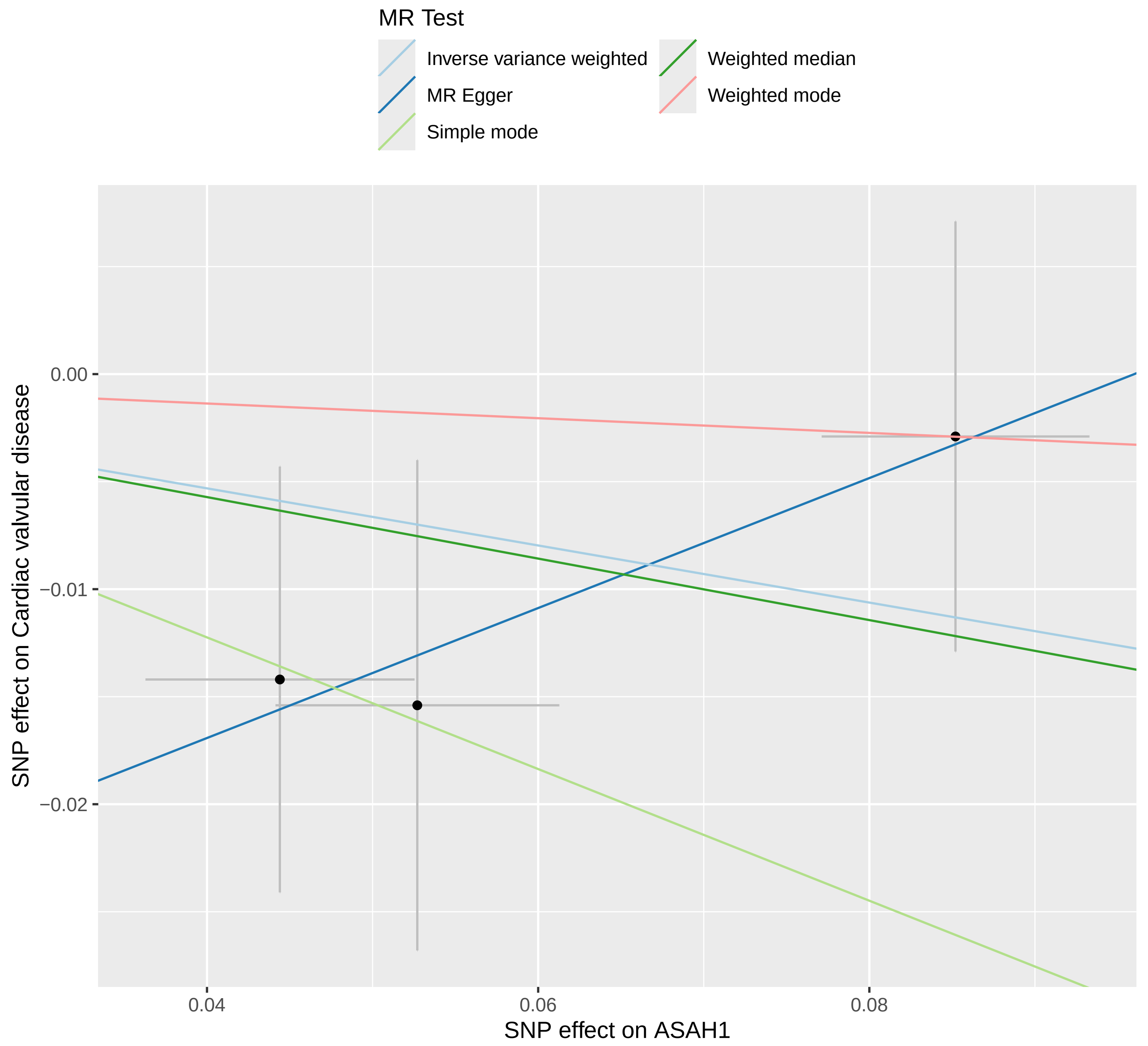
MR Test
Inverse variance weighted
MR Egger
Simple mode
Weighted median
Weighted mode
0.00
−0.01
−0.02
0.04
0.06
0.08
SNP effect on Cardiac valvular disease
SNP effect on ASAH1

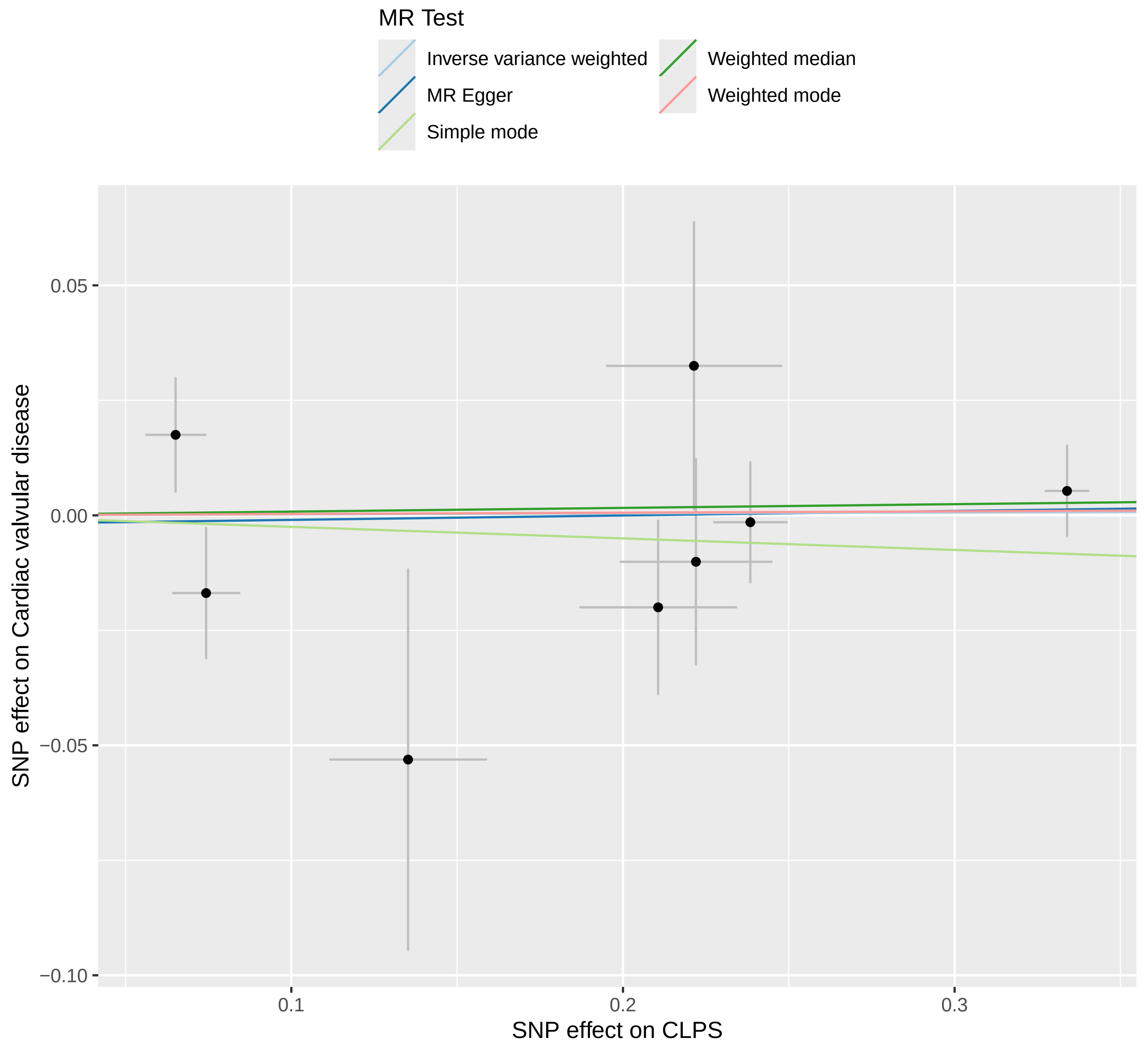

MR Test
Inverse variance weighted
MR Egger
Simple mode
Weighted median
Weighted mode
0.05
0.00
−0.05
−0.10
0.1
0.2
0.3
SNP effect on Cardiac valvular disease
SNP effect on CLPS

Insufficient number of SNPs

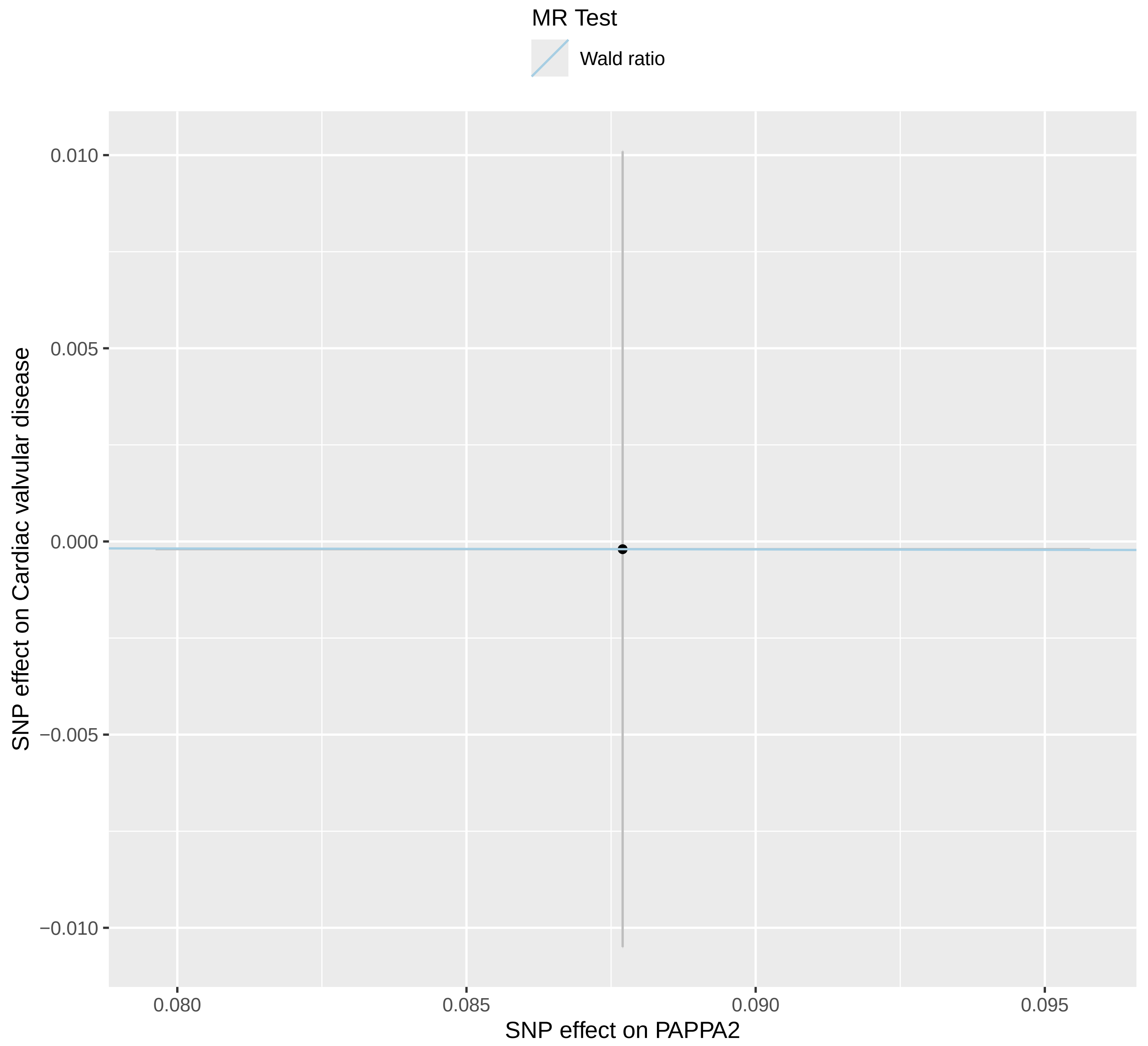

MR Test
Wald ratio
0.010
0.005
0.000
−0.005
−0.010
SNP effect on Cardiac valvular disease
0.080
0.085
0.090
0.095
SNP effect on PAPPA2

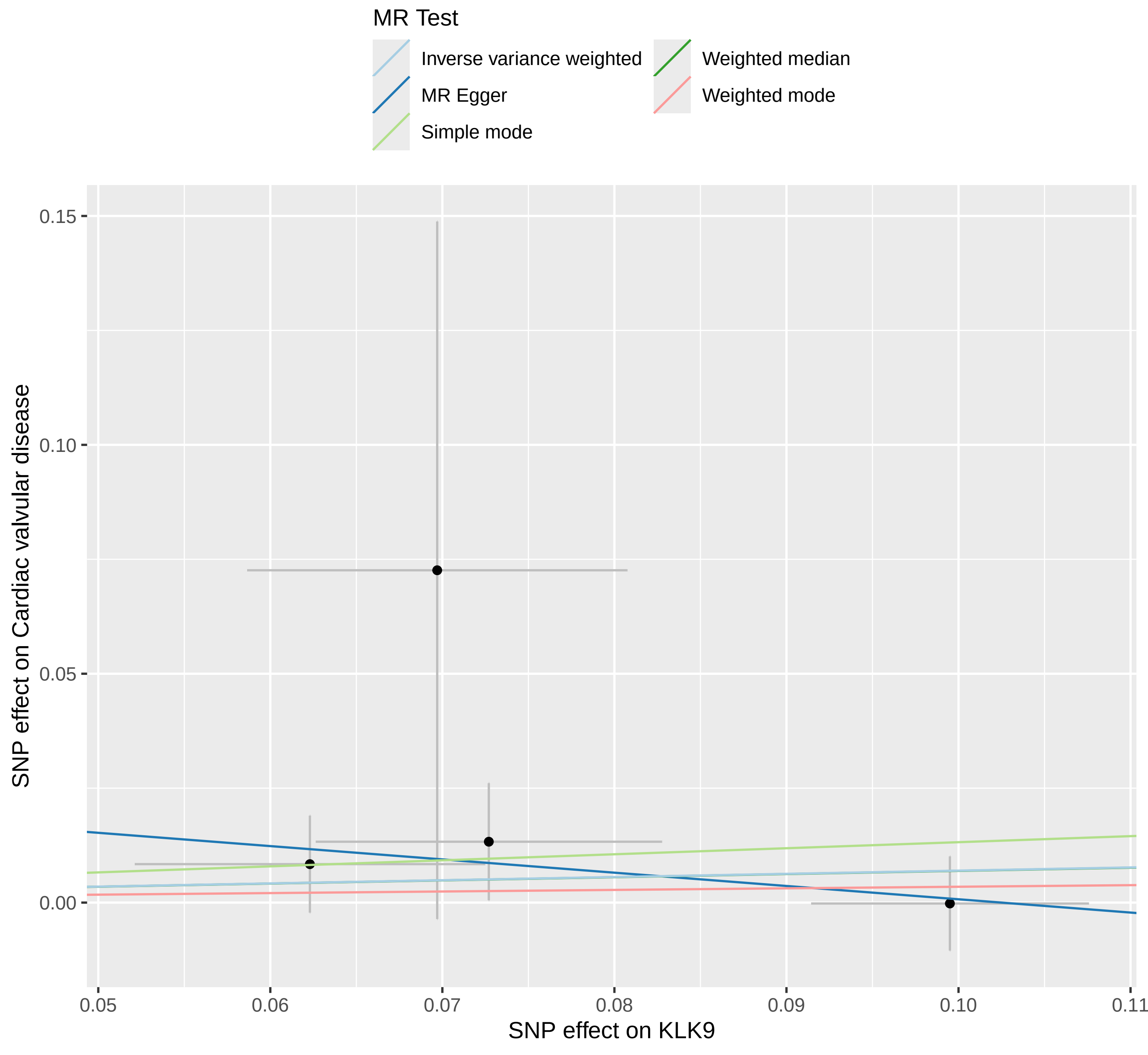
MR Test
Inverse variance weighted
MR Egger
Simple mode
Weighted median
Weighted mode
0.15
0.10
0.05
0.00
SNP effect on Cardiac valvular disease
0.05
0.06
0.07
0.08
0.09
0.10
0.11
SNP effect on KLK9

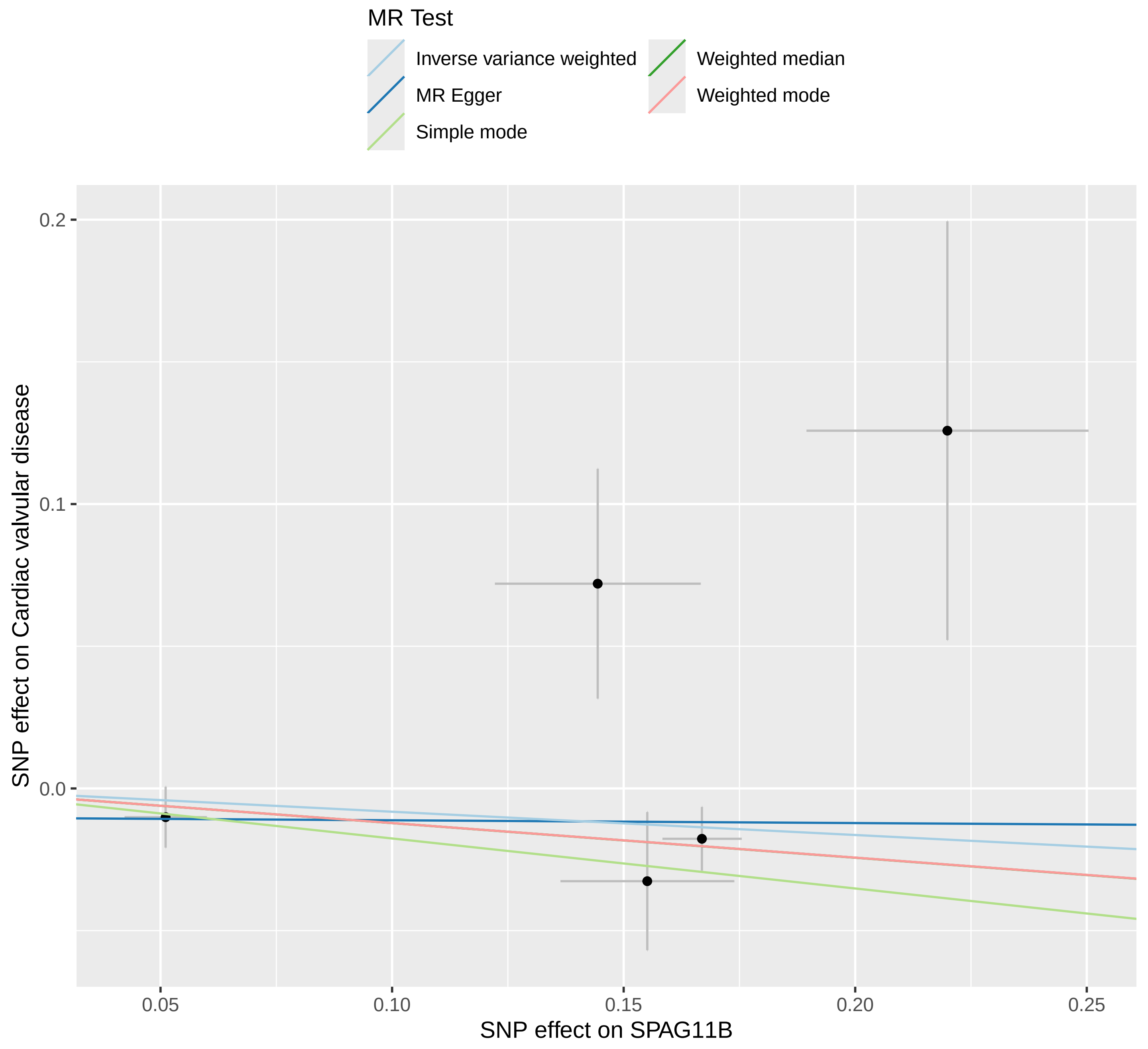
MR Test
Inverse variance weighted
MR Egger
Simple mode
Weighted median
Weighted mode
0.2
0.1
0.0
0.05
0.10
0.15
0.20
0.25
SNP effect on Cardiac valvular disease
SNP effect on SPAG11B

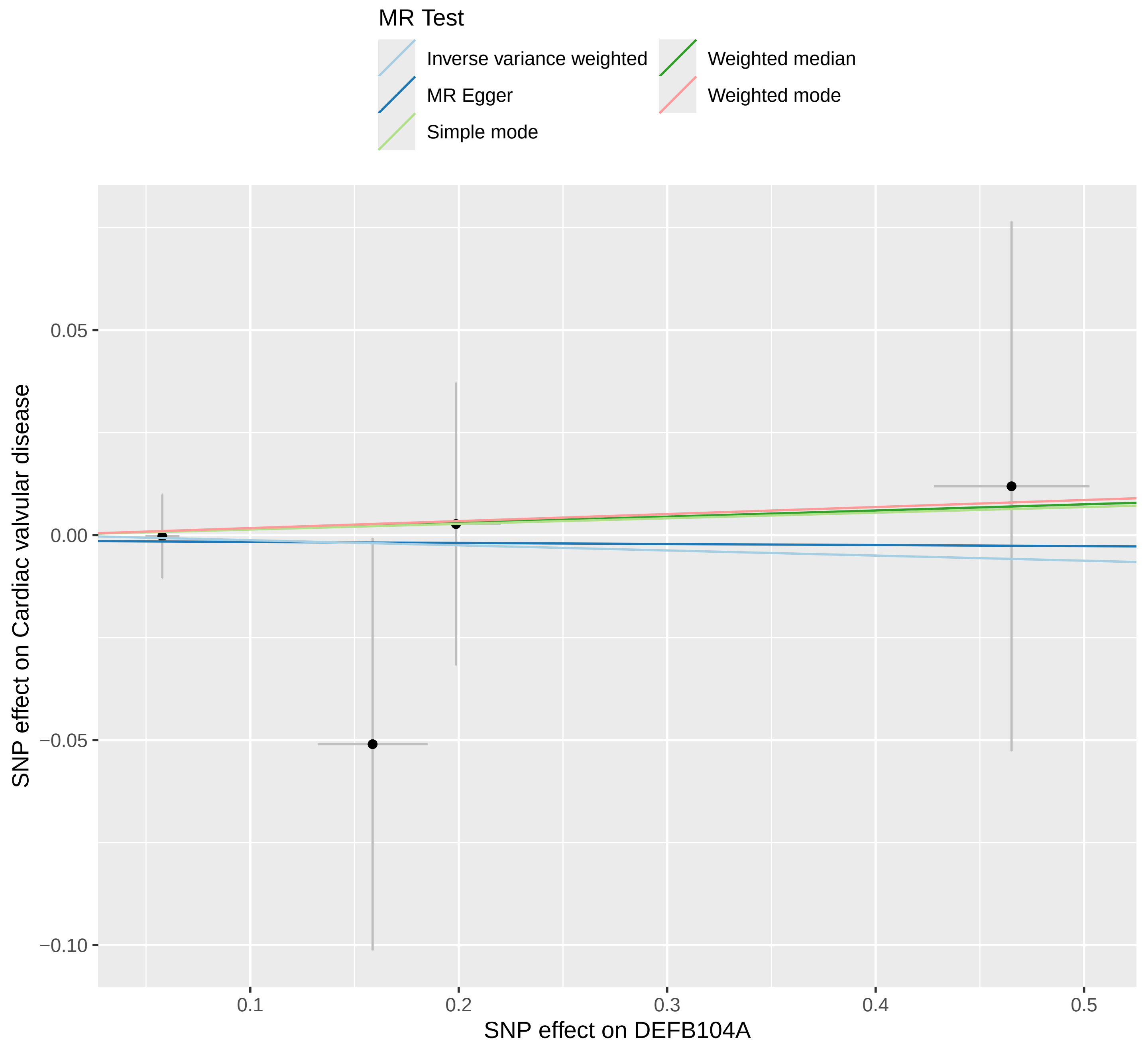
MR Test
Inverse variance weighted
MR Egger
Simple mode
Weighted median
Weighted mode
SNP effect on Cardiac valvular disease
0.05
0.00
−0.05
−0.10
0.1
0.2
0.3
0.4
0.5
SNP effect on DEFB104A

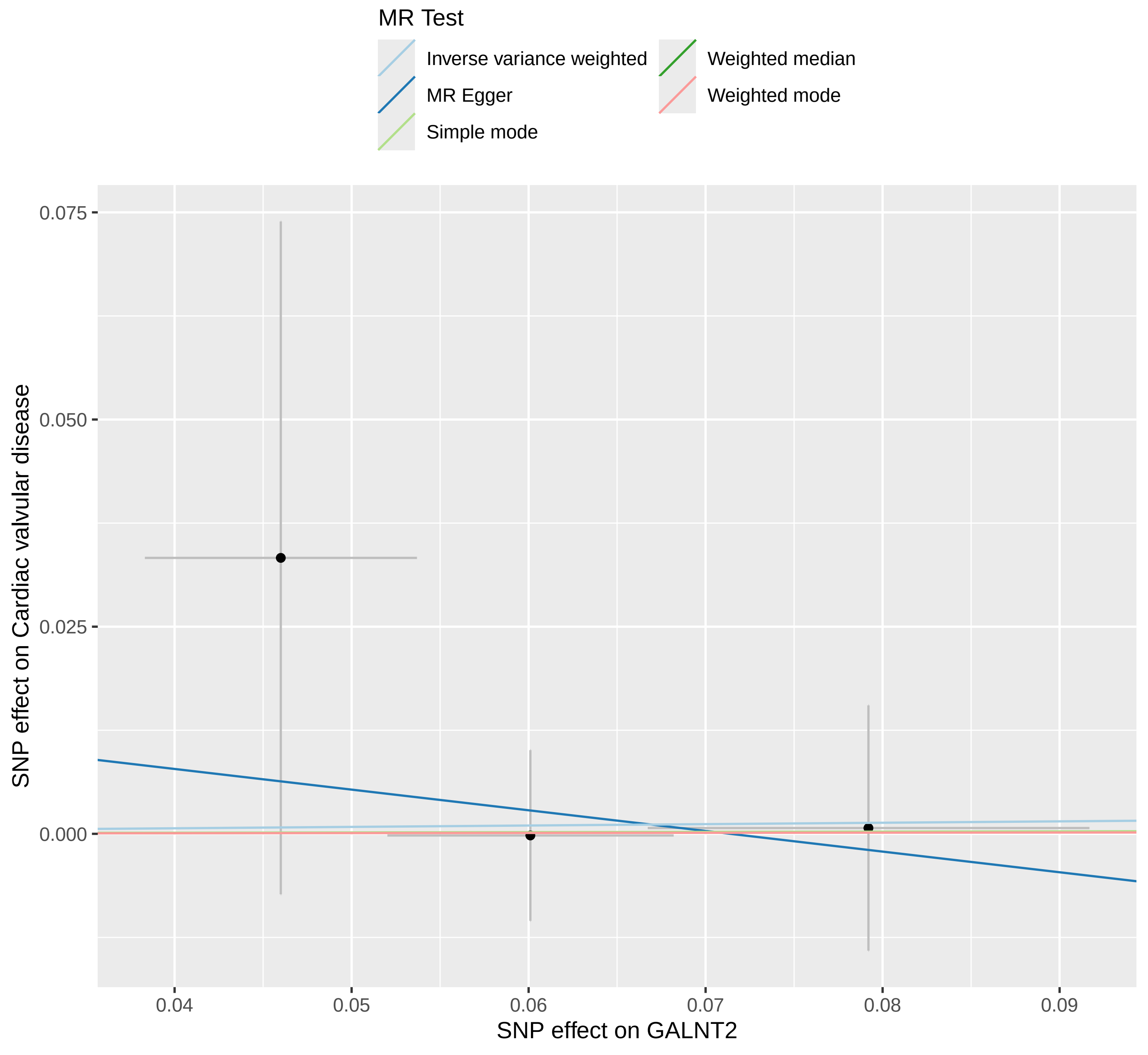

MR Test
Inverse variance weighted
MR Egger
Simple mode
Weighted median
Weighted mode
0.075
0.050
0.025
0.000
SNP effect on Cardiac valvular disease
0.04
0.05
0.06
0.07
0.08
0.09
SNP effect on GALNT2

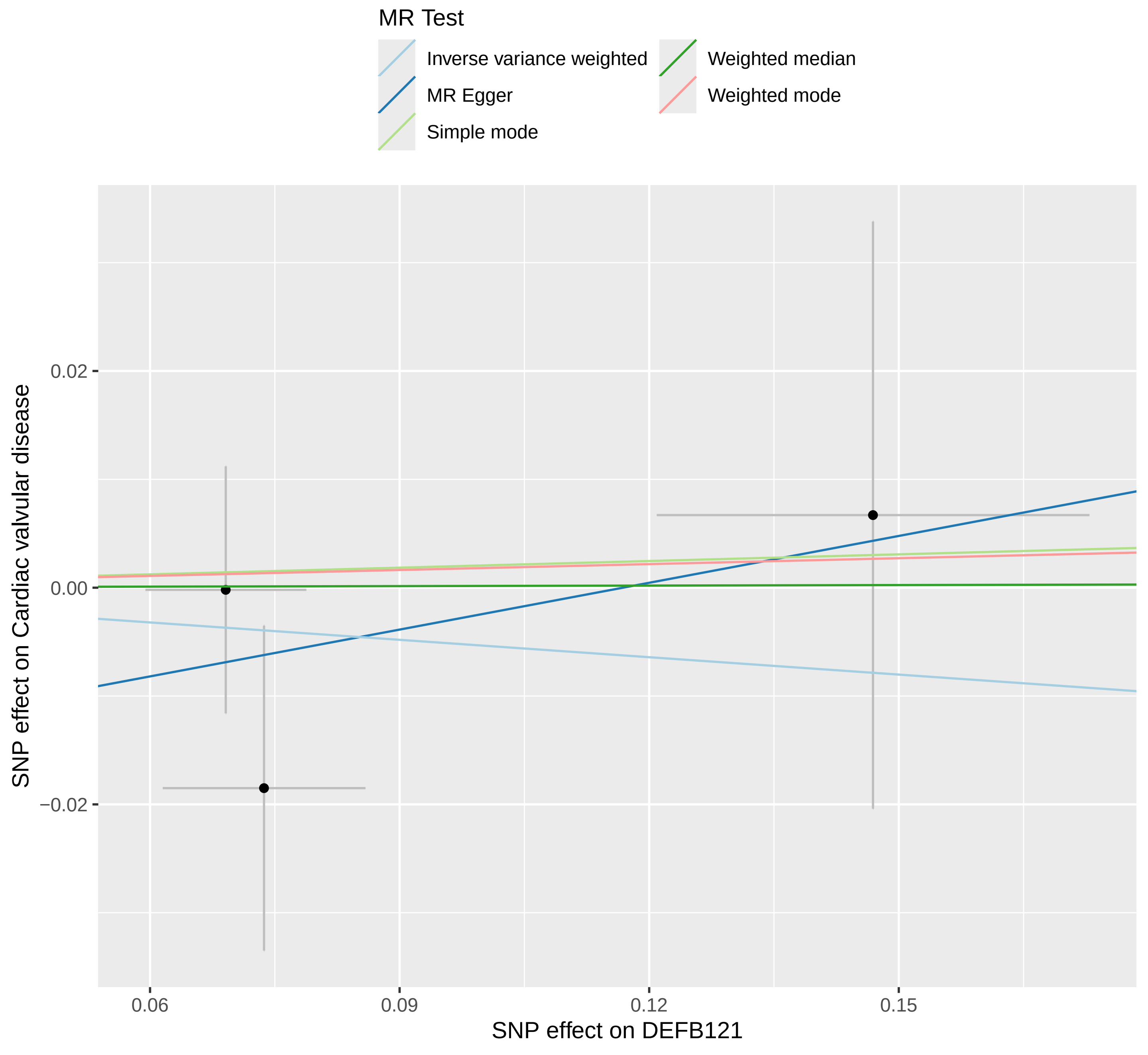
MR Test
Inverse variance weighted
MR Egger
Simple mode
Weighted median
Weighted mode
0.02
0.00
−0.02
0.06
0.09
0.12
0.15
SNP effect on Cardiac valvular disease
SNP effect on DEFB121

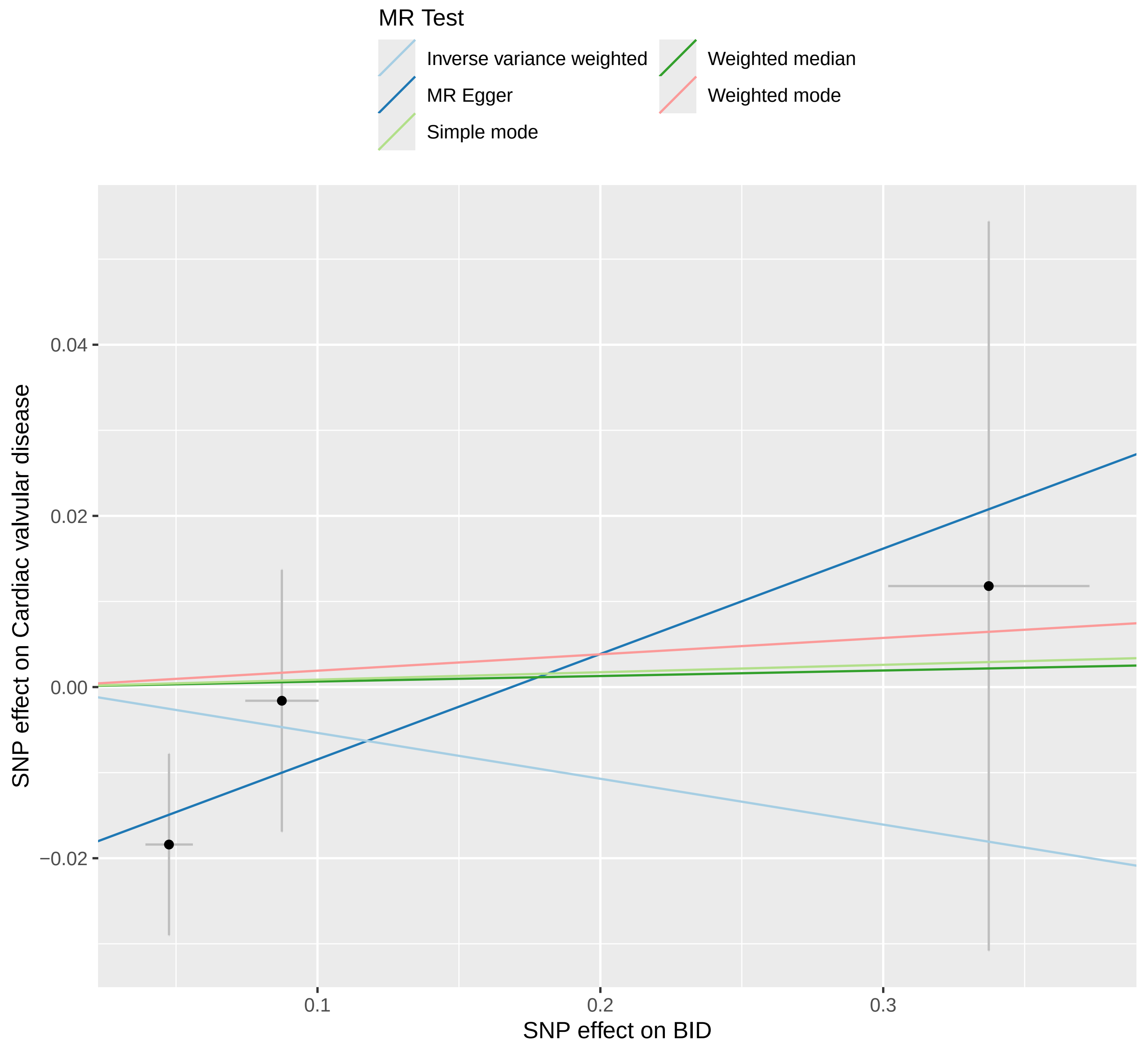
MR Test
Inverse variance weighted
MR Egger
Simple mode
Weighted median
Weighted mode
0.04
0.02
0.00
−0.02
SNP effect on Cardiac valvular disease
0.1
0.2
0.3
SNP effect on BID

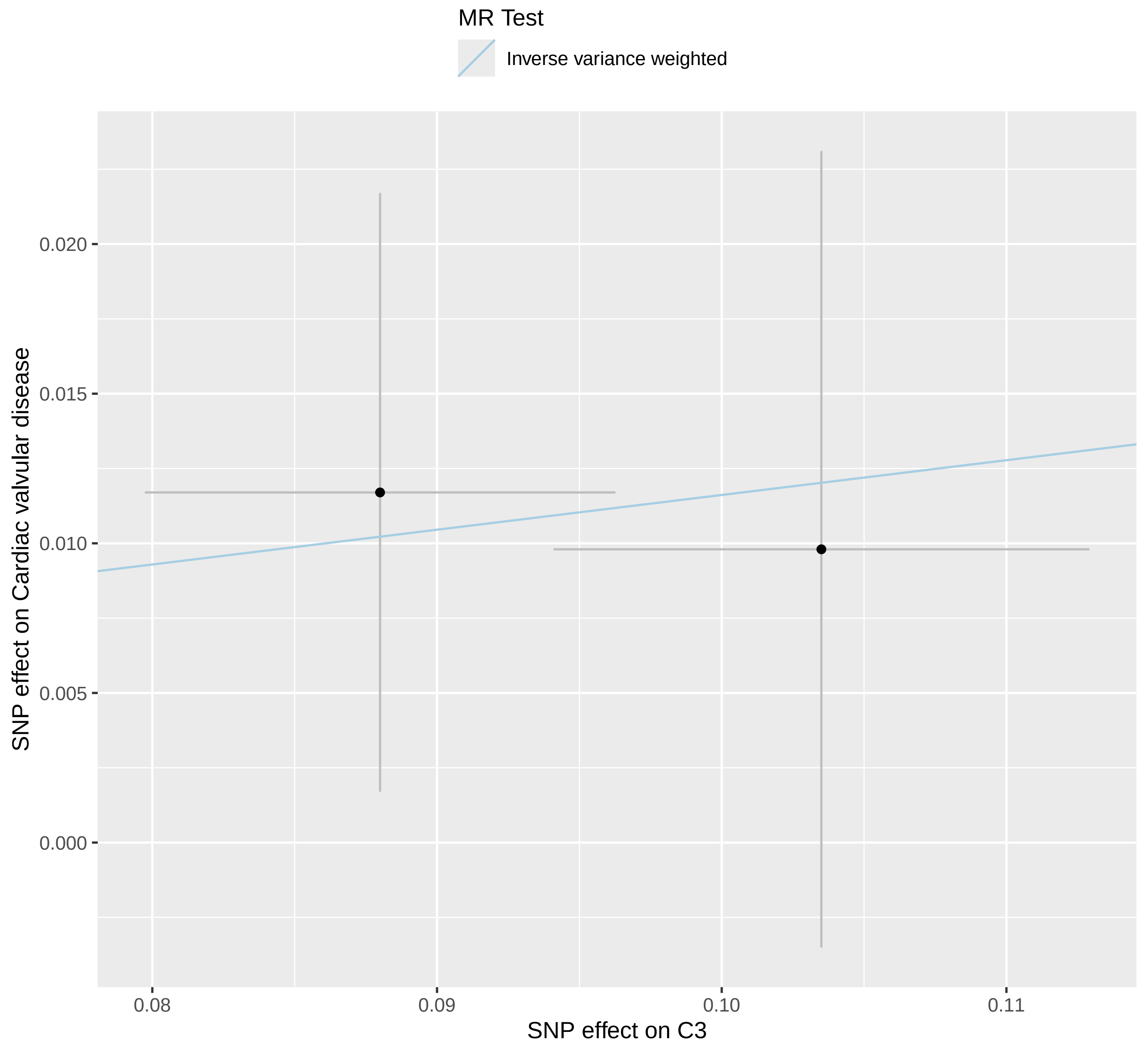

MR Test
Inverse variance weighted
SNP effect on Cardiac valvular disease
0.020
0.015
0.010
0.005
0.000
0.08
0.09
0.10
0.11
SNP effect on C3

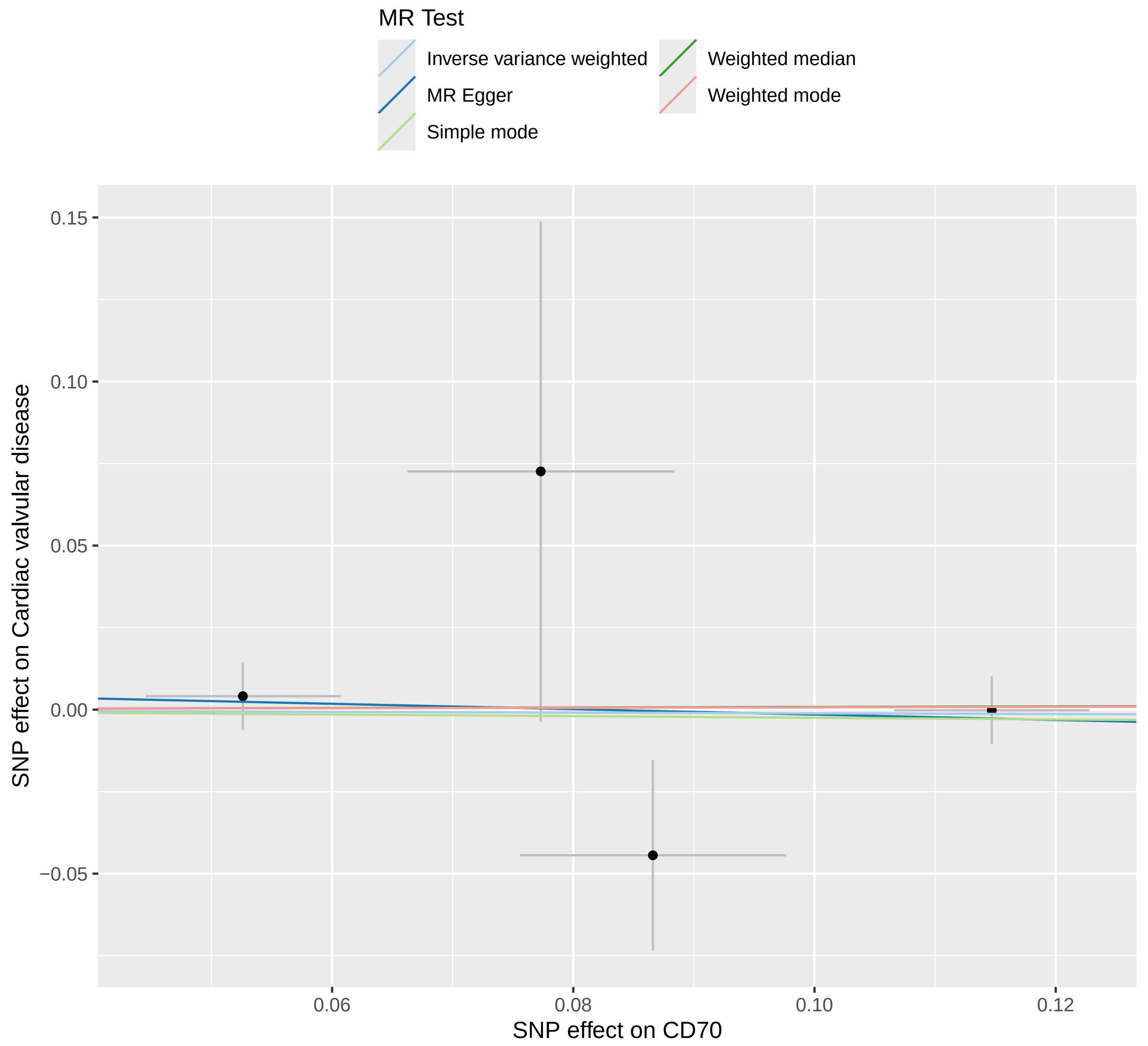
MR Test
Inverse variance weighted
MR Egger
Simple mode
Weighted median
Weighted mode
0.15
0.10
0.05
0.00
−0.05
SNP effect on Cardiac valvular disease
0.06
0.08
0.10
0.12
SNP effect on CD70

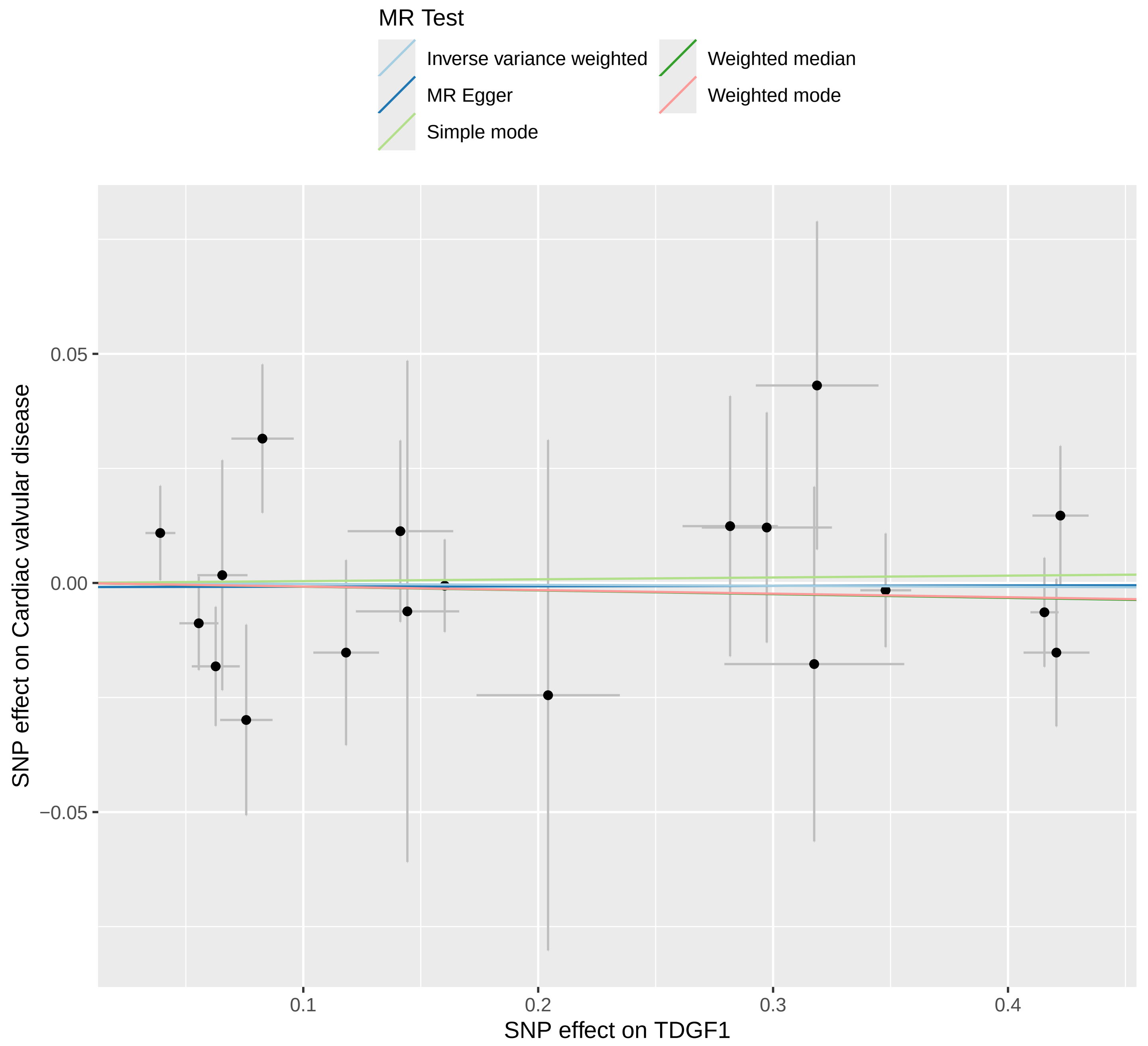

MR Test
Inverse variance weighted
MR Egger
Simple mode
Weighted median
Weighted mode
0.05
0.00
−0.05
SNP effect on Cardiac valvular disease
0.1
0.2
0.3
0.4
SNP effect on TDGF1

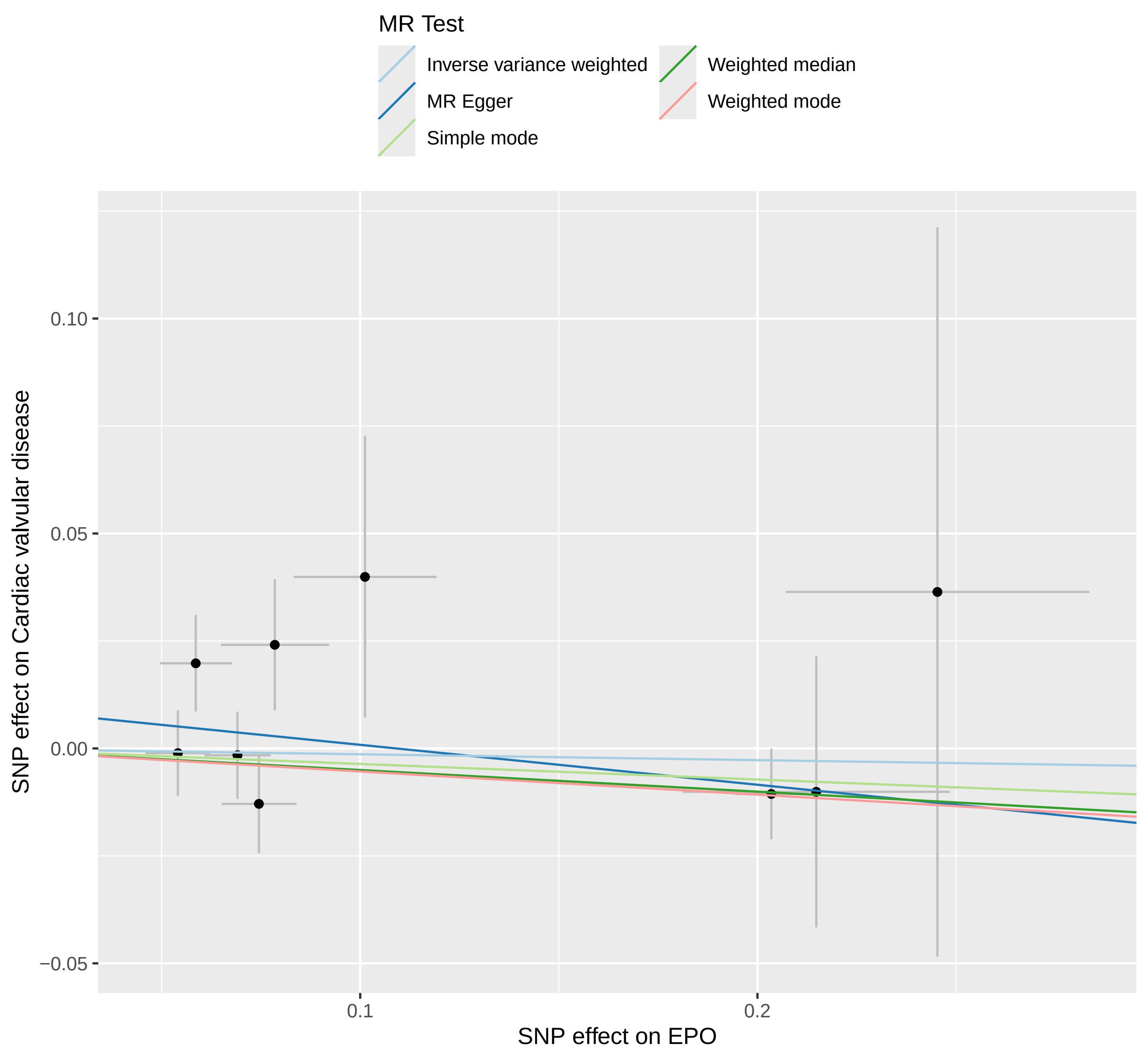

MR Test
Inverse variance weighted
MR Egger
Simple mode
Weighted median
Weighted mode
0.10
0.05
0.00
−0.05
0.1
0.2
SNP effect on Cardiac valvular disease
SNP effect on EPO

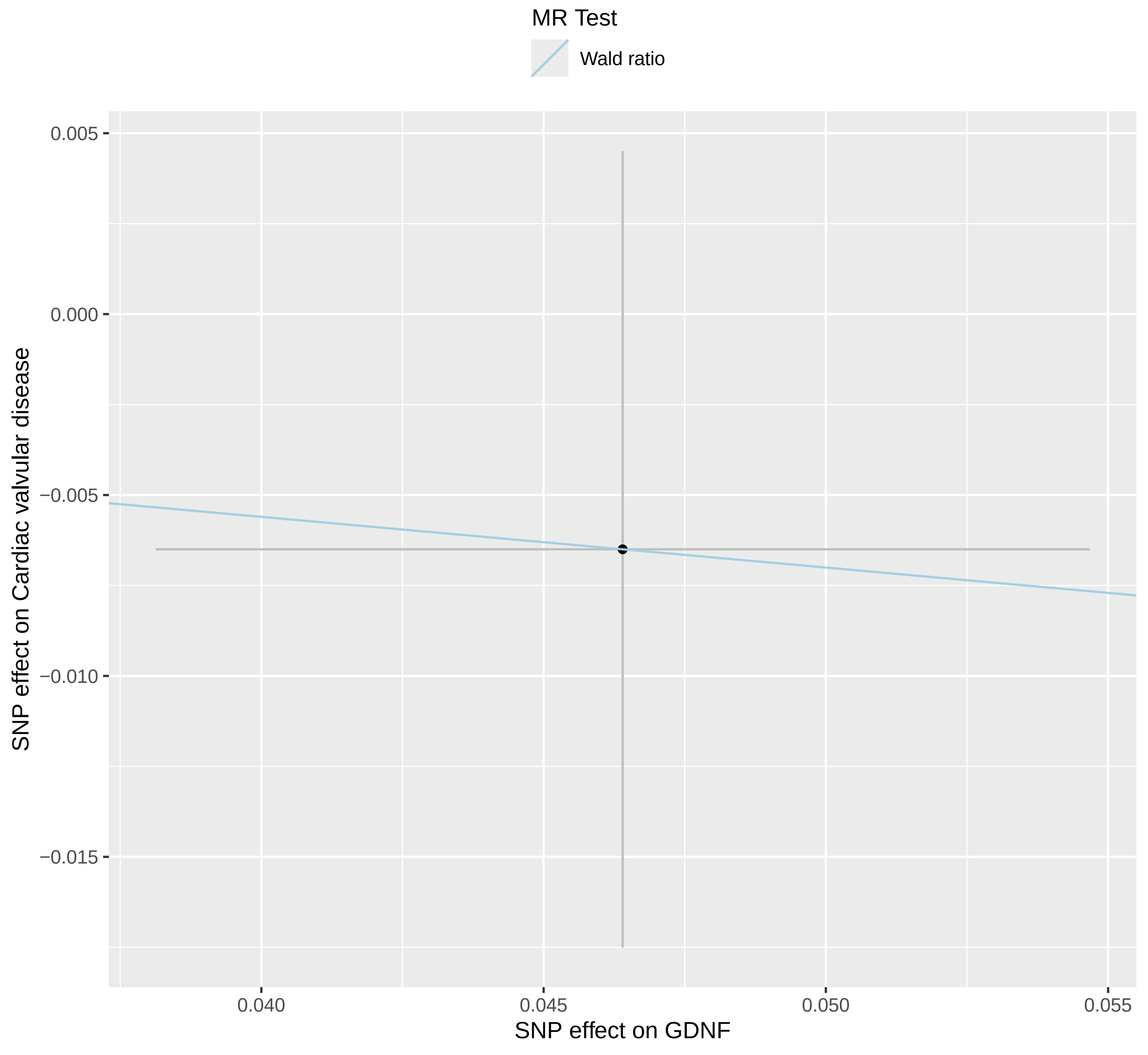

MR Test
Wald ratio
0.005
0.000
−0.005
−0.010
−0.015
SNP effect on Cardiac valvular disease
0.040
0.045
0.050
0.055
SNP effect on GDNF

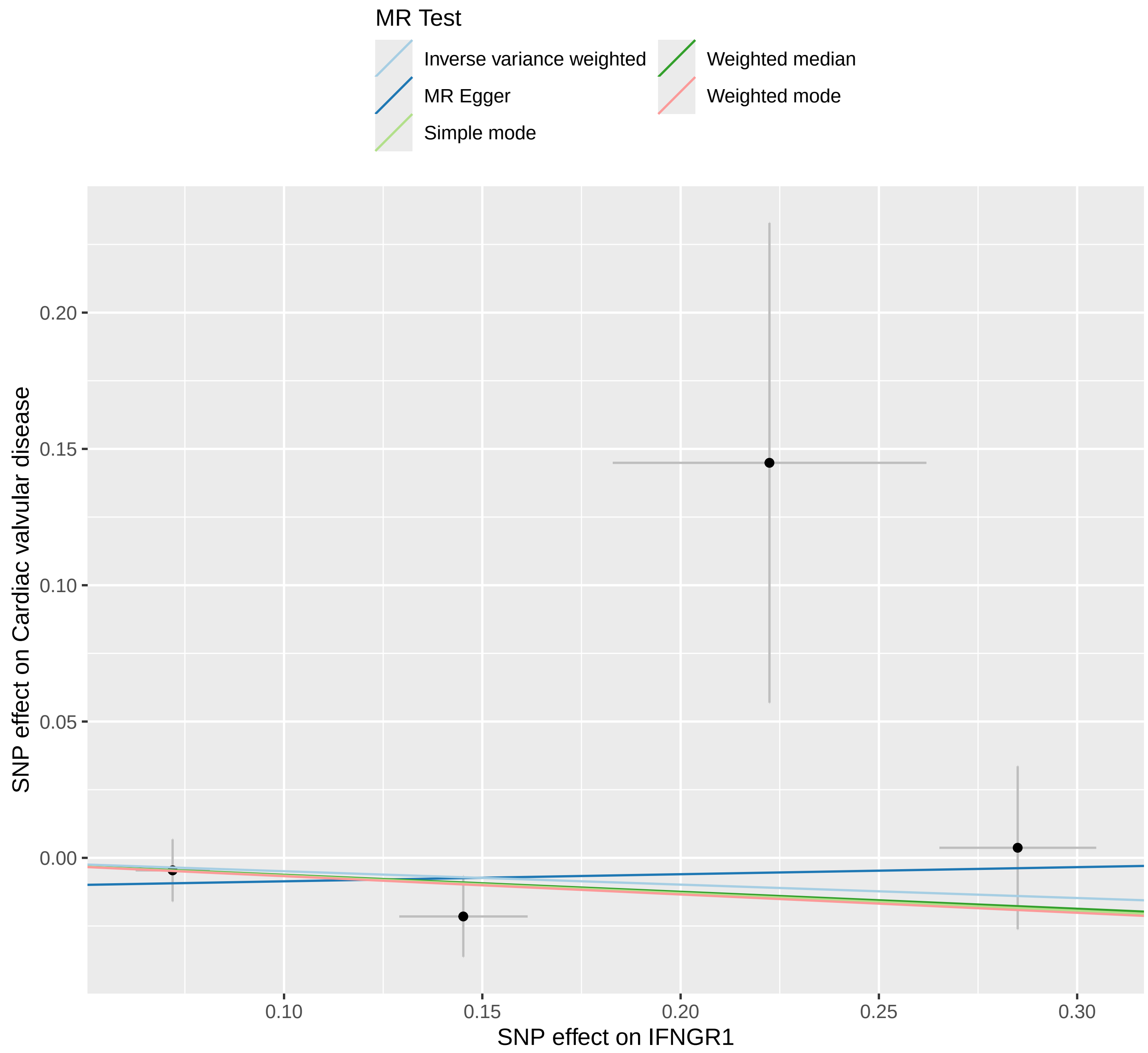

MR Test
Inverse variance weighted
MR Egger
Simple mode
Weighted median
Weighted mode
0.20
0.15
0.10
0.05
0.00
SNP effect on Cardiac valvular disease
0.10
0.15
0.20
0.25
0.30
SNP effect on IFNGR1

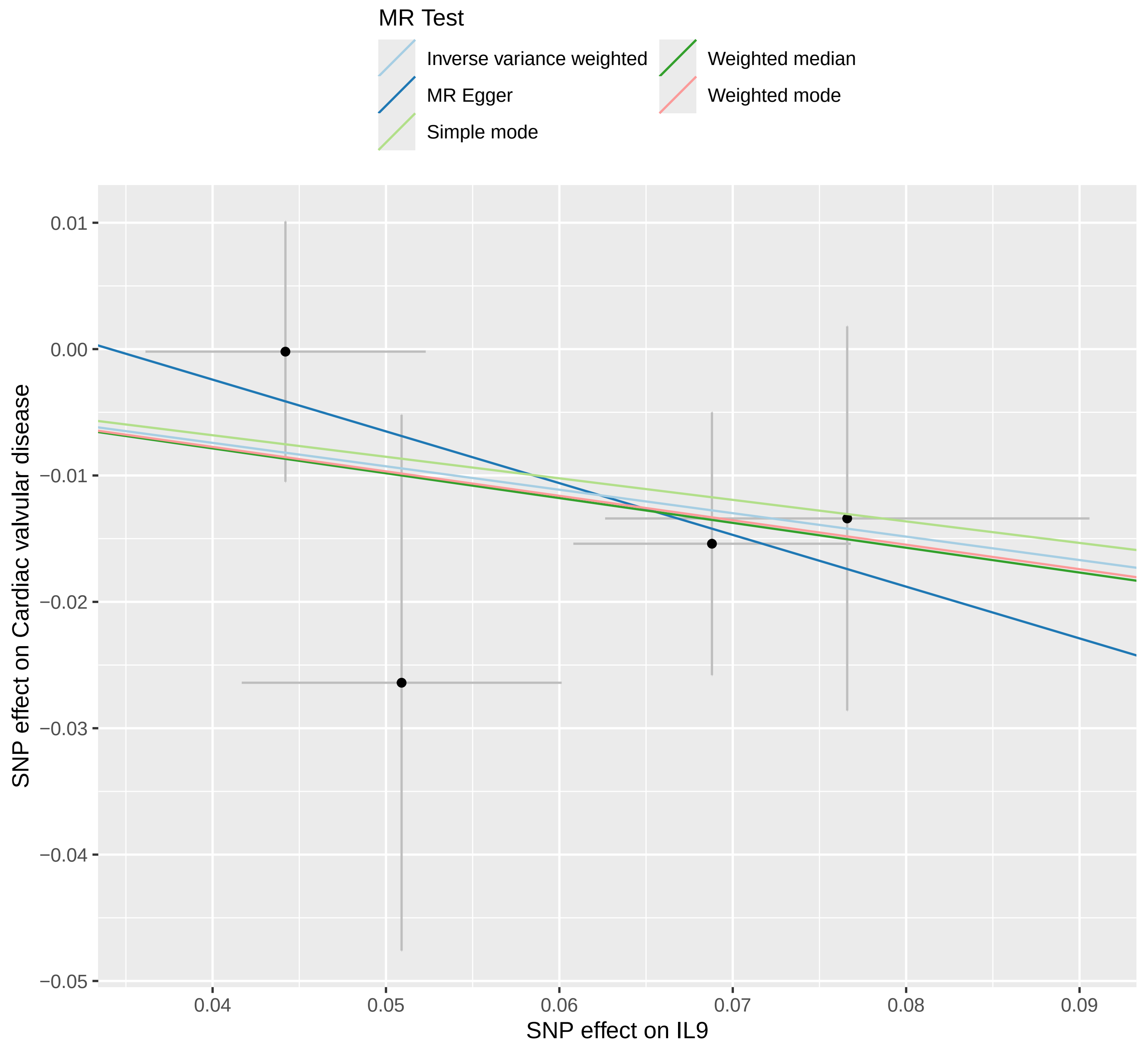
MR Test
Inverse variance weighted
MR Egger
Simple mode
Weighted median
Weighted mode
SNP effect on Cardiac valvular disease
0.01
0.00
−0.01
−0.02
−0.03
−0.04
−0.05
0.04
0.05
0.06
0.07
0.08
0.09
SNP effect on IL9

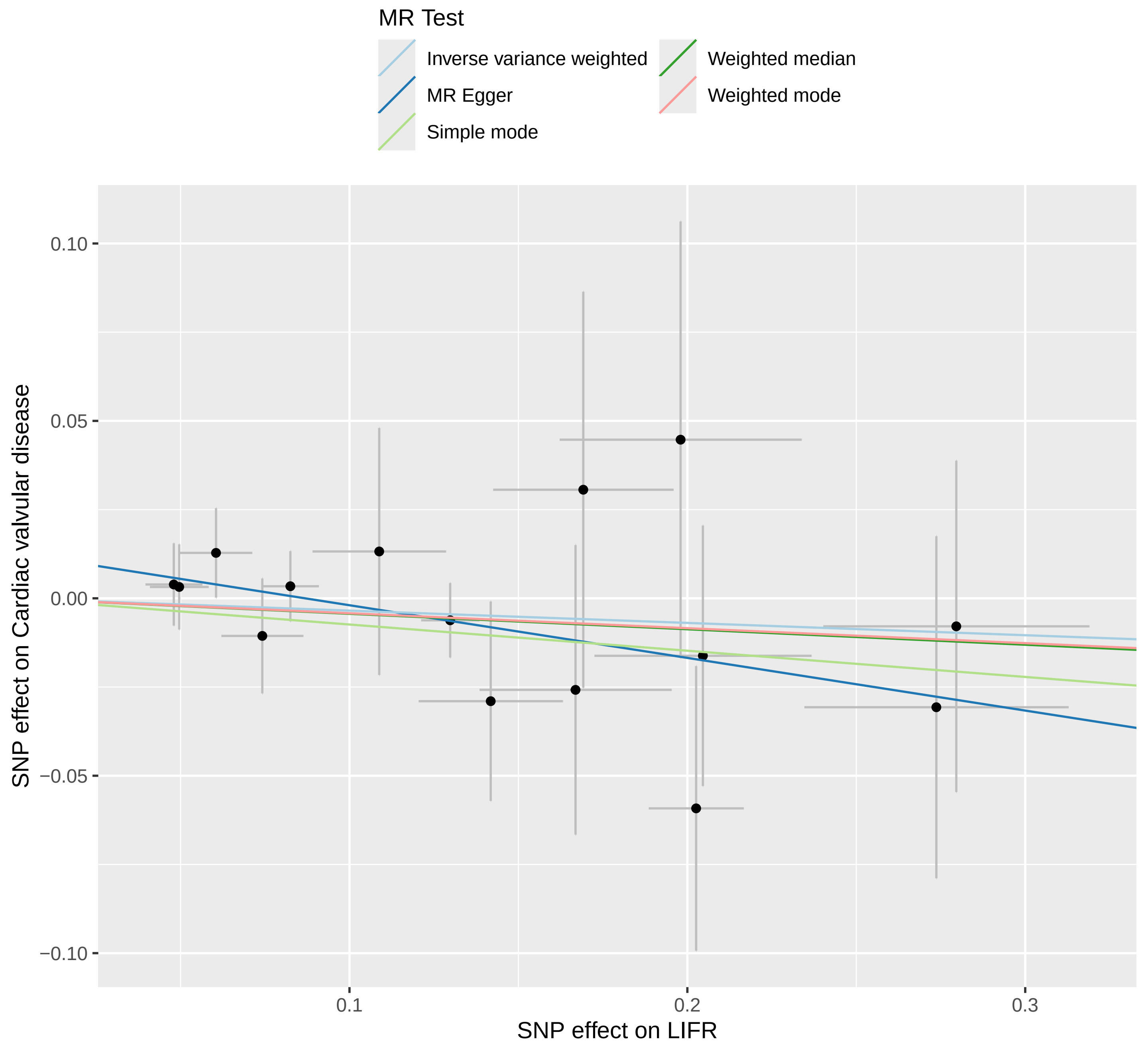
MR Test
Inverse variance weighted
MR Egger
Simple mode
Weighted median
Weighted mode
0.10
0.05
0.00
−0.05
−0.10
SNP effect on Cardiac valvular disease
0.1
0.2
0.3
SNP effect on LIFR

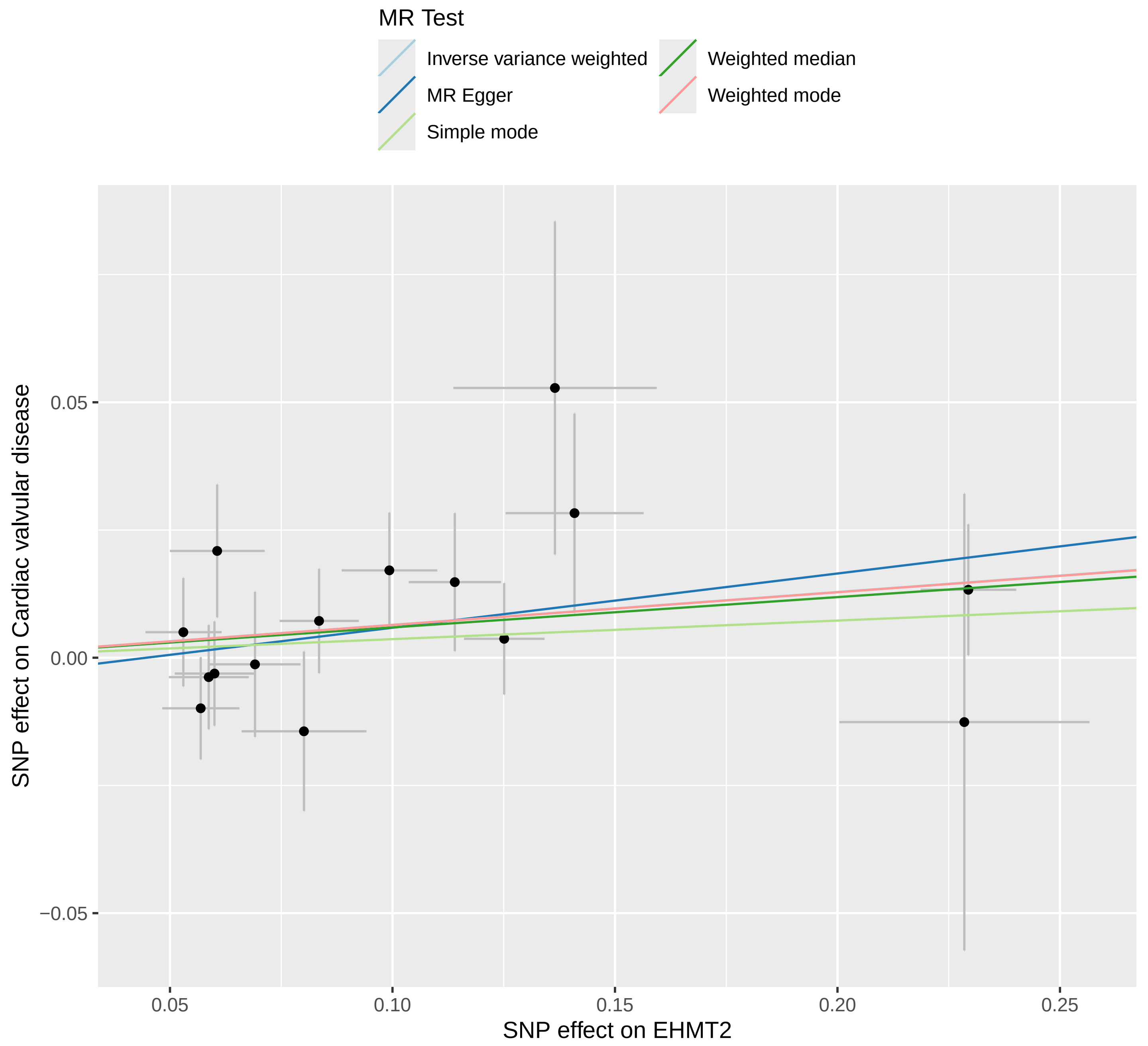

MR Test
Inverse variance weighted
MR Egger
Simple mode
Weighted median
Weighted mode
SNP effect on Cardiac valvular disease
0.05
0.00
−0.05
0.05
0.10
0.15
0.20
0.25
SNP effect on EHMT2

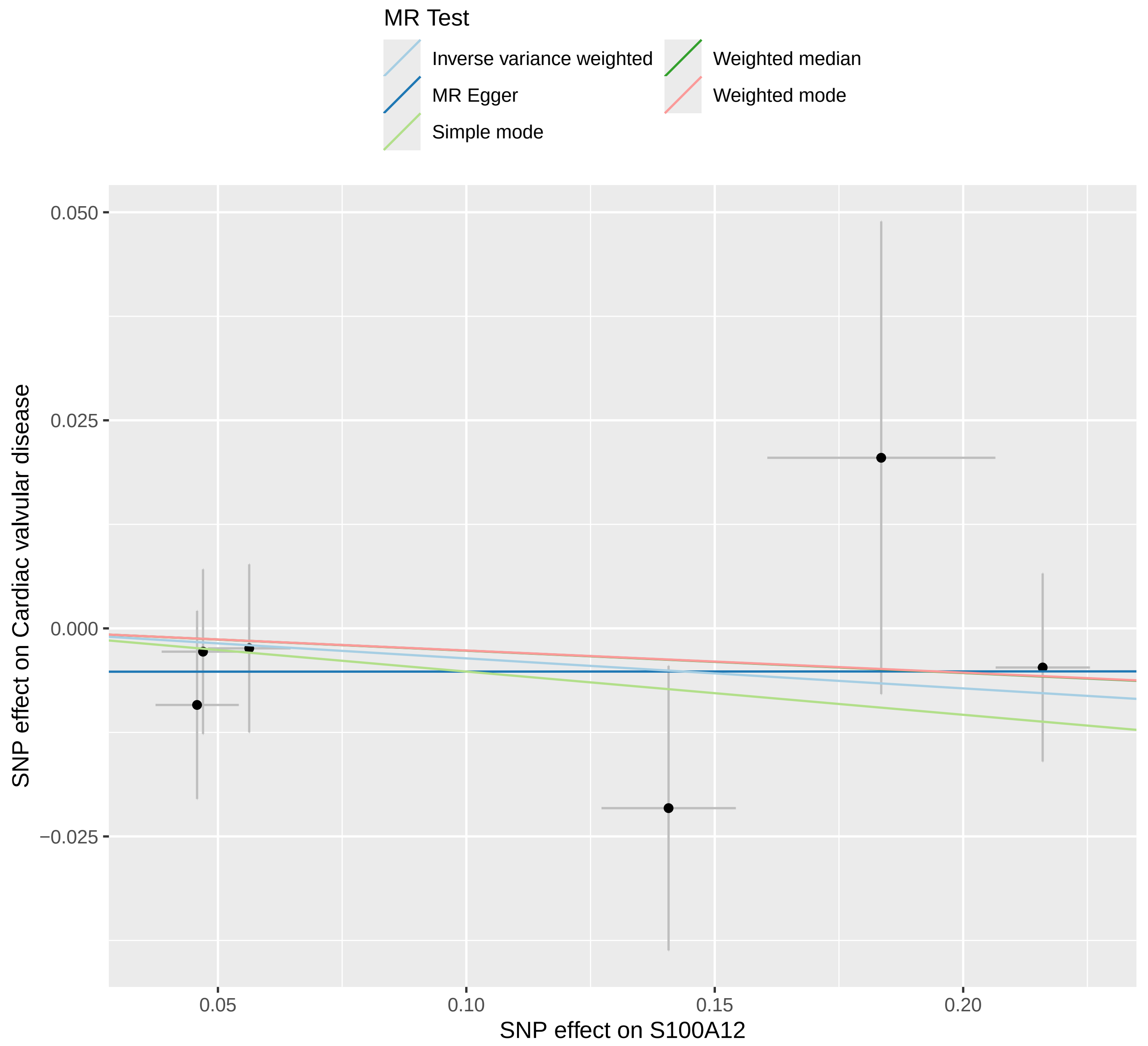
MR Test
Inverse variance weighted
MR Egger
Simple mode
Weighted median
Weighted mode
0.050
0.025
0.000
−0.025
0.05
0.10
0.15
0.20
SNP effect on Cardiac valvular disease
SNP effect on S100A12

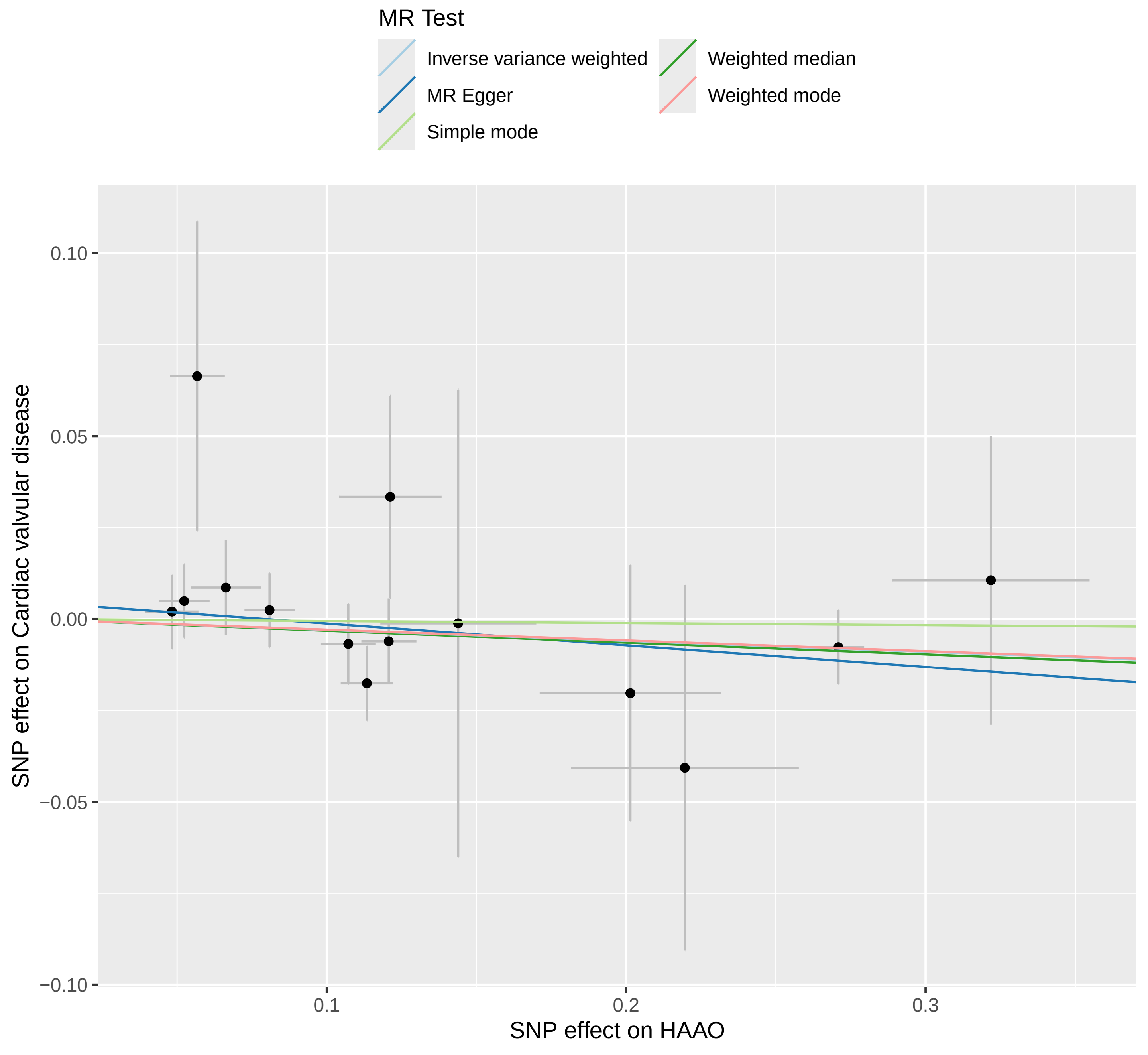

MR Test
Inverse variance weighted
MR Egger
Simple mode
Weighted median
Weighted mode
0.10
0.05
0.00
−0.05
−0.10
0.1
0.2
0.3
SNP effect on Cardiac valvular disease
SNP effect on HAAO

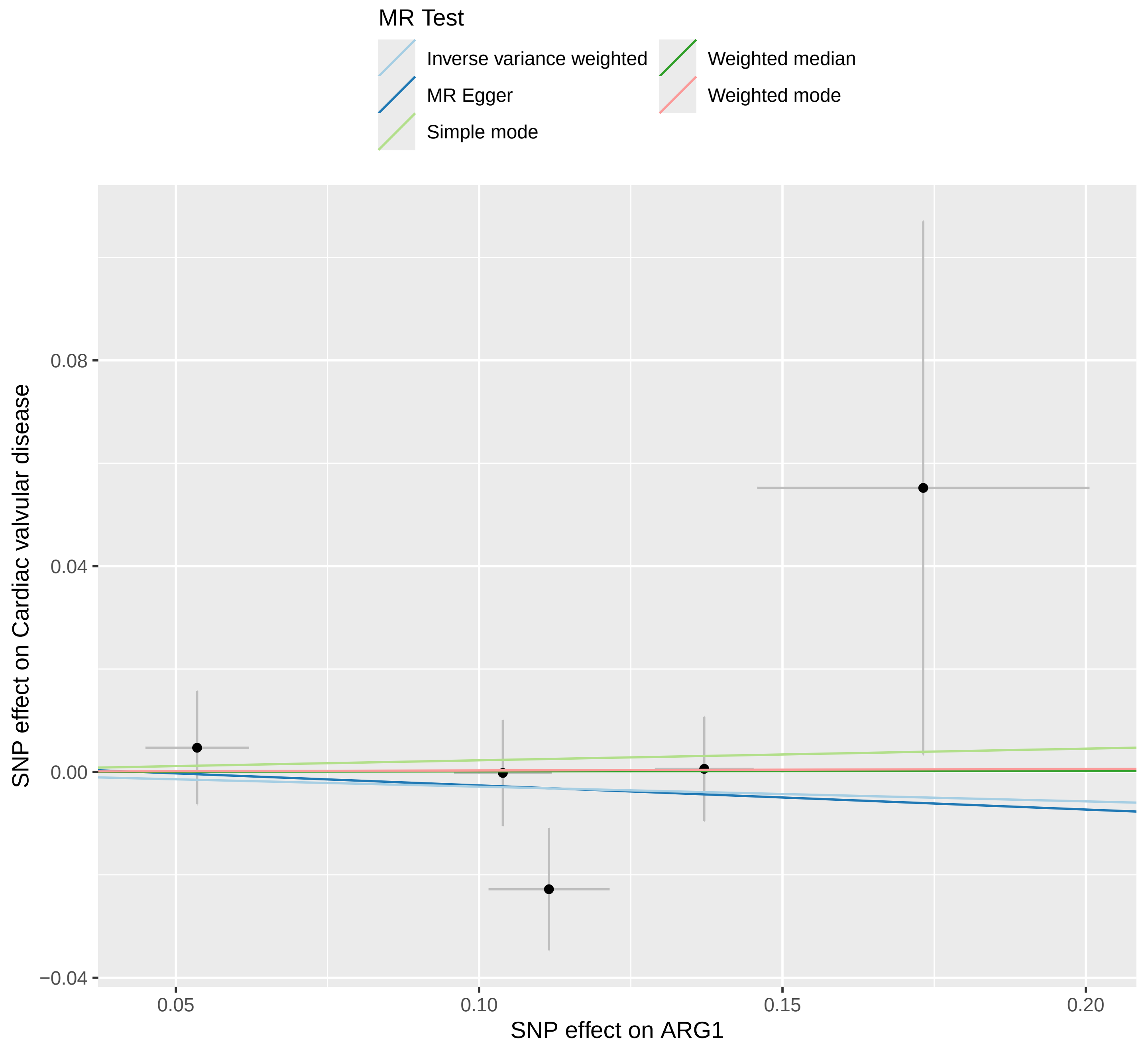
MR Test
Inverse variance weighted
MR Egger
Simple mode
Weighted median
Weighted mode
0.08
0.04
0.00
−0.04
0.05
0.10
0.15
0.20
SNP effect on Cardiac valvular disease
SNP effect on ARG1

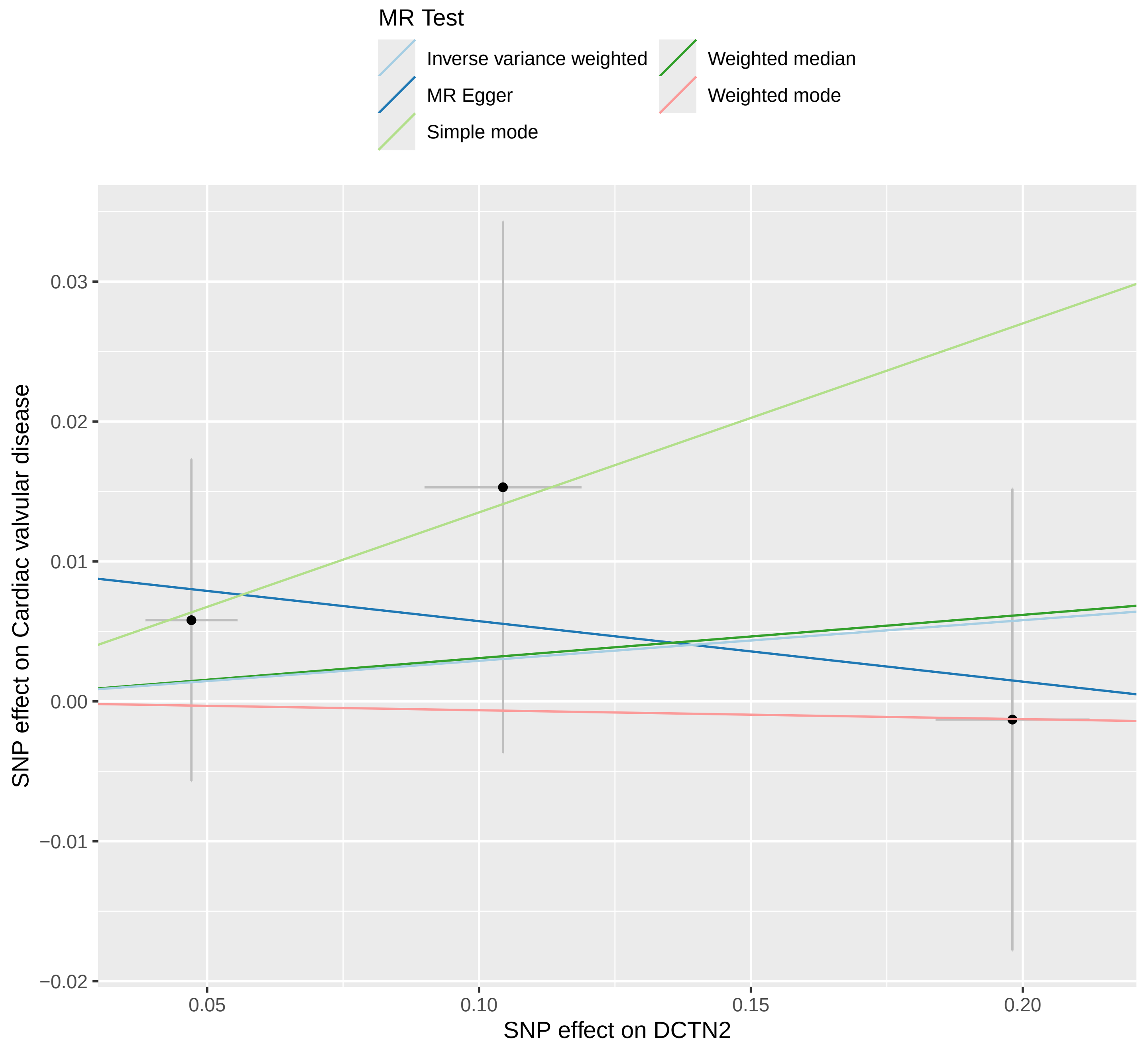

MR Test
Inverse variance weighted
MR Egger
Simple mode
Weighted median
Weighted mode
0.03
0.02
0.01
0.00
−0.01
−0.02
SNP effect on Cardiac valvular disease
0.05
0.10
0.15
0.20
SNP effect on DCTN2

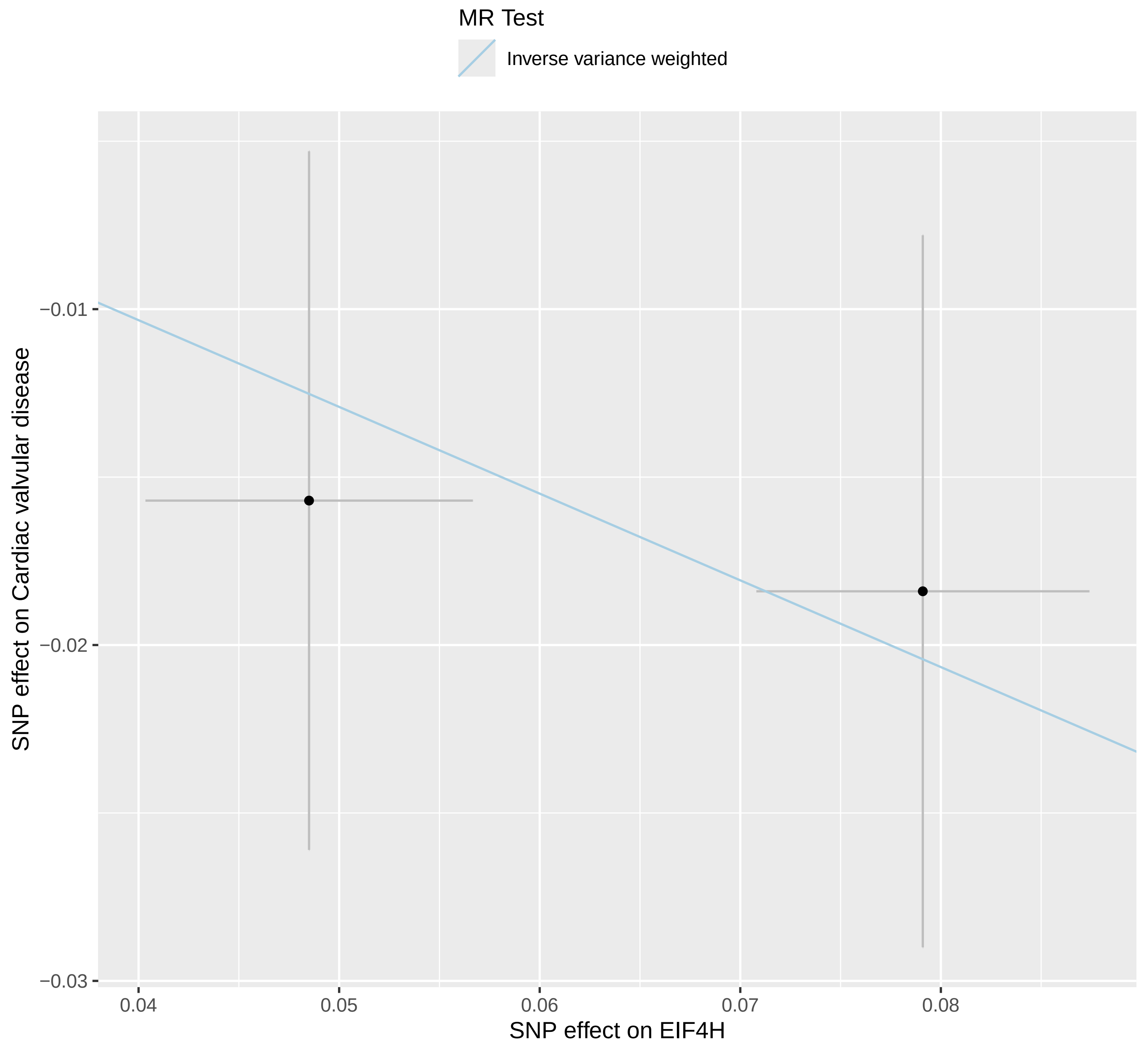

MR Test
Inverse variance weighted
−0.01
−0.02
−0.03
0.04
0.05
0.06
0.07
0.08
SNP effect on Cardiac valvular disease
SNP effect on EIF4H

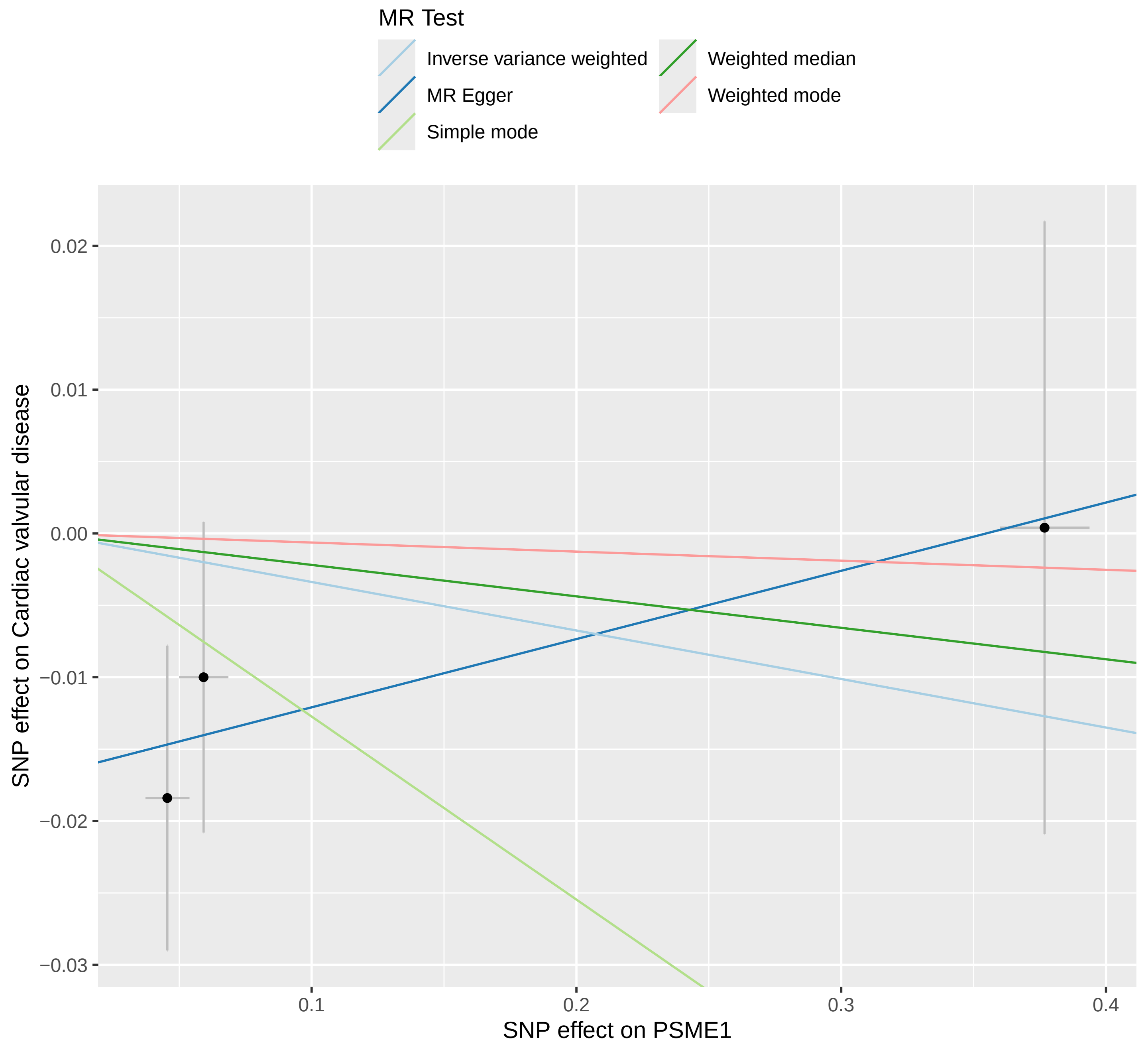

MR Test
Inverse variance weighted
MR Egger
Simple mode
Weighted median
Weighted mode
0.02
0.01
0.00
−0.01
−0.02
−0.03
SNP effect on Cardiac valvular disease
0.1
0.2
0.3
0.4
SNP effect on PSME1

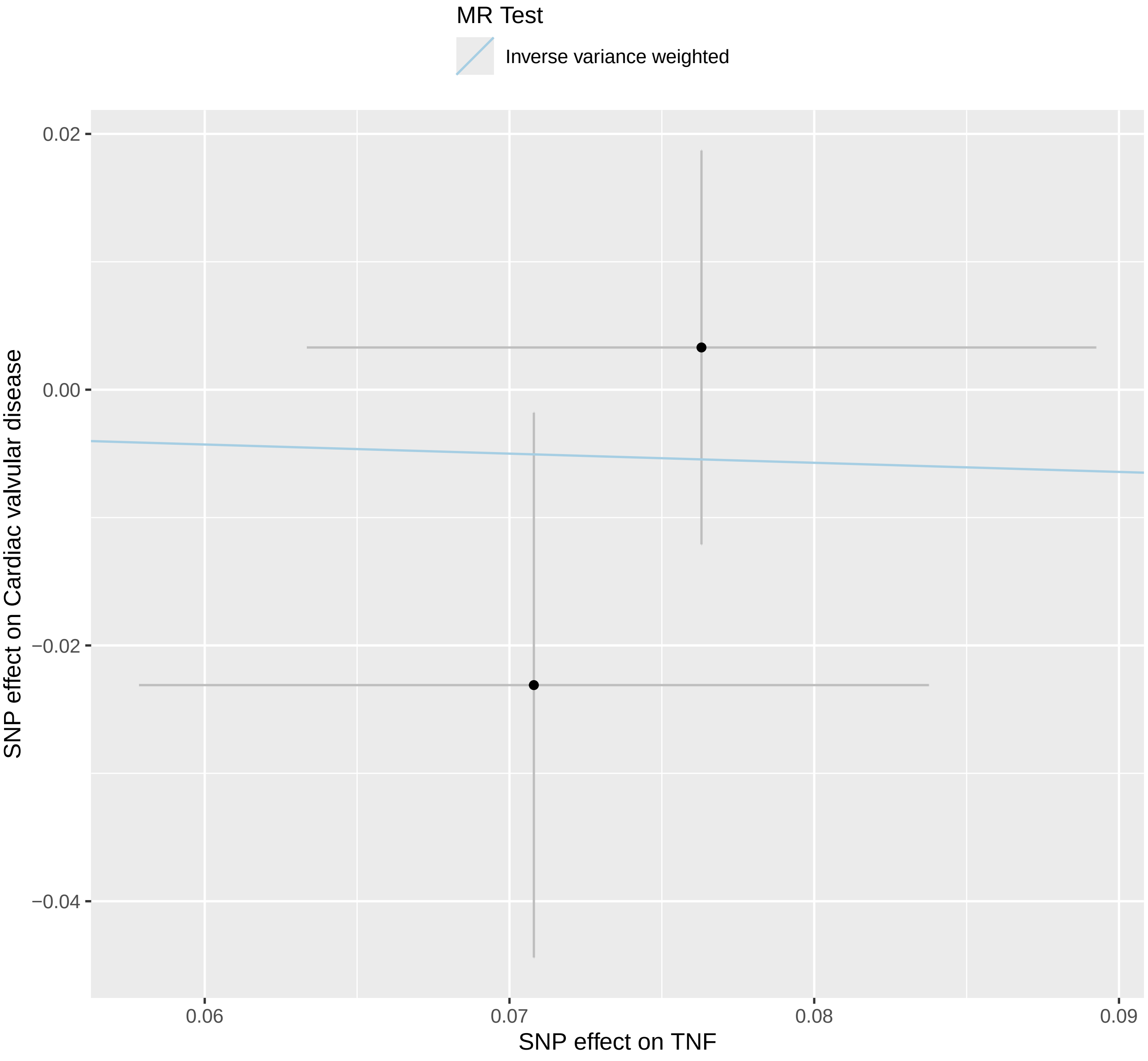
MR Test
Inverse variance weighted
0.02
0.00
−0.02
−0.04
0.06
0.07
0.08
0.09
SNP effect on Cardiac valvular disease
SNP effect on TNF

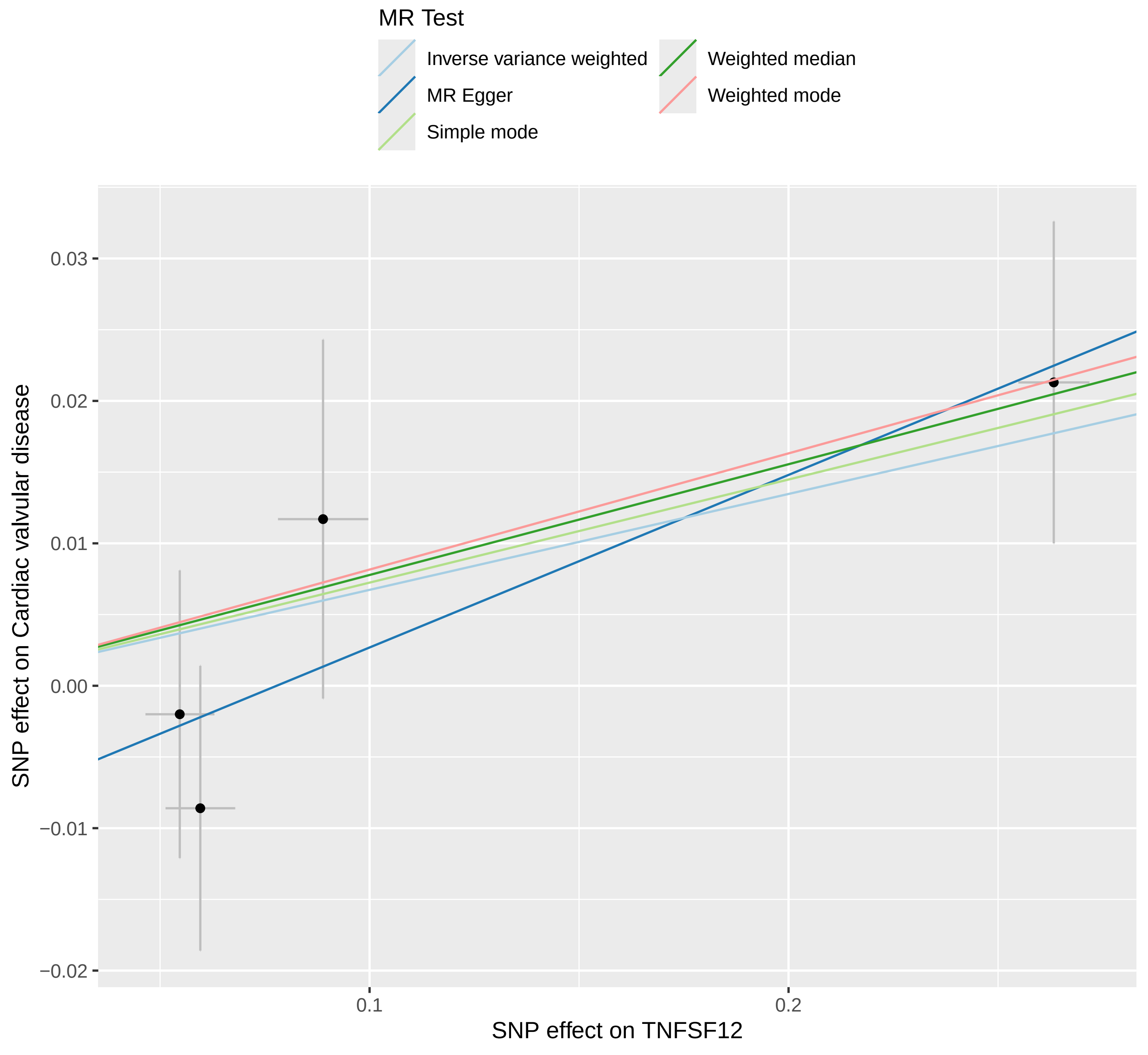
MR Test
Inverse variance weighted
MR Egger
Simple mode
Weighted median
Weighted mode
0.03
0.02
0.01
0.00
−0.01
−0.02
0.1
0.2
SNP effect on Cardiac valvular disease
SNP effect on TNFSF12

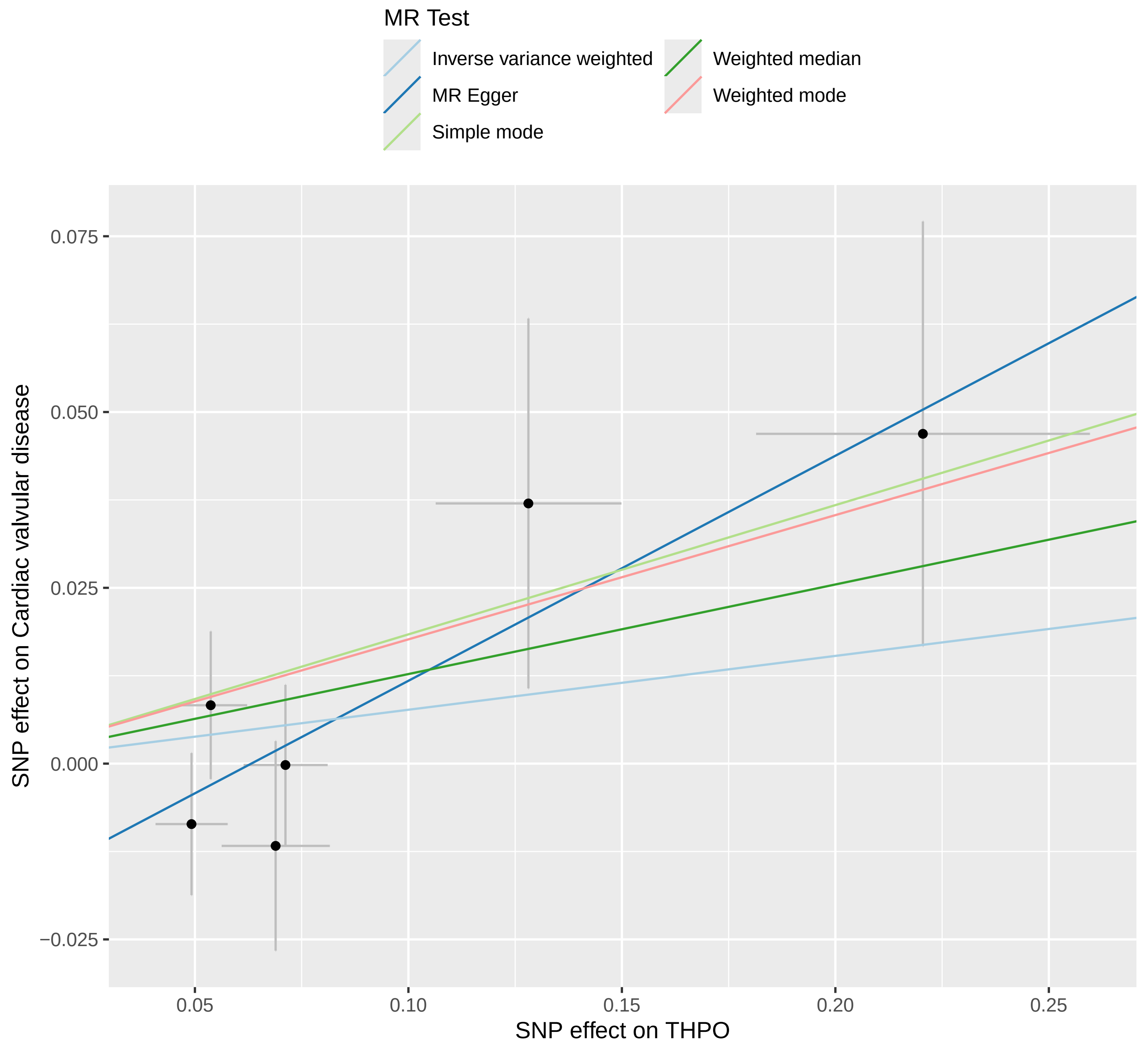

MR Test
Inverse variance weighted
MR Egger
Simple mode
Weighted median
Weighted mode
0.075
0.050
0.025
0.000
−0.025
SNP effect on Cardiac valvular disease
0.05
0.10
0.15
0.20
0.25
SNP effect on THPO

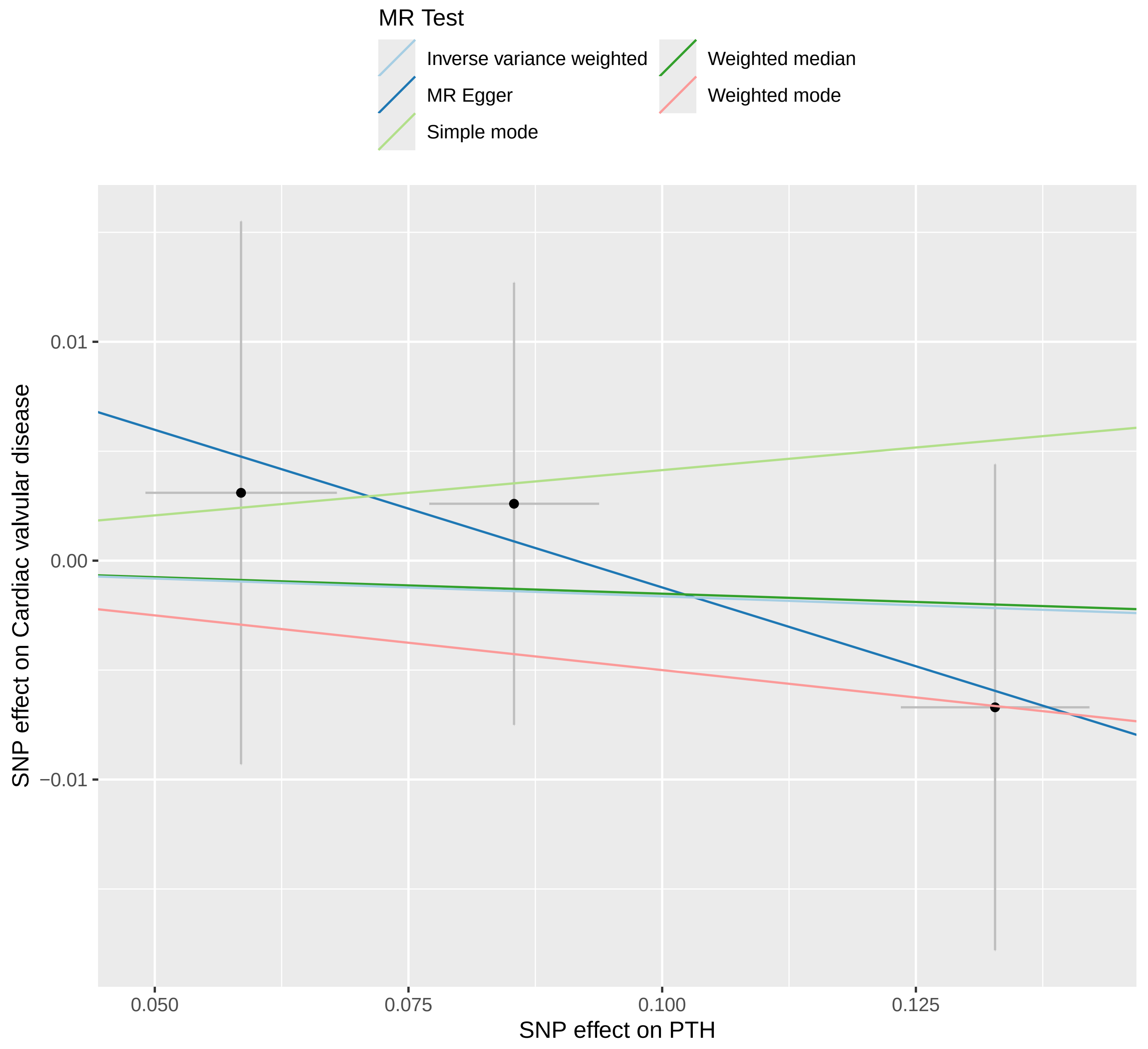

MR Test
Inverse variance weighted
MR Egger
Simple mode
Weighted median
Weighted mode
0.01
0.00
−0.01
SNP effect on Cardiac valvular disease
0.050
0.075
0.100
0.125
SNP effect on PTH

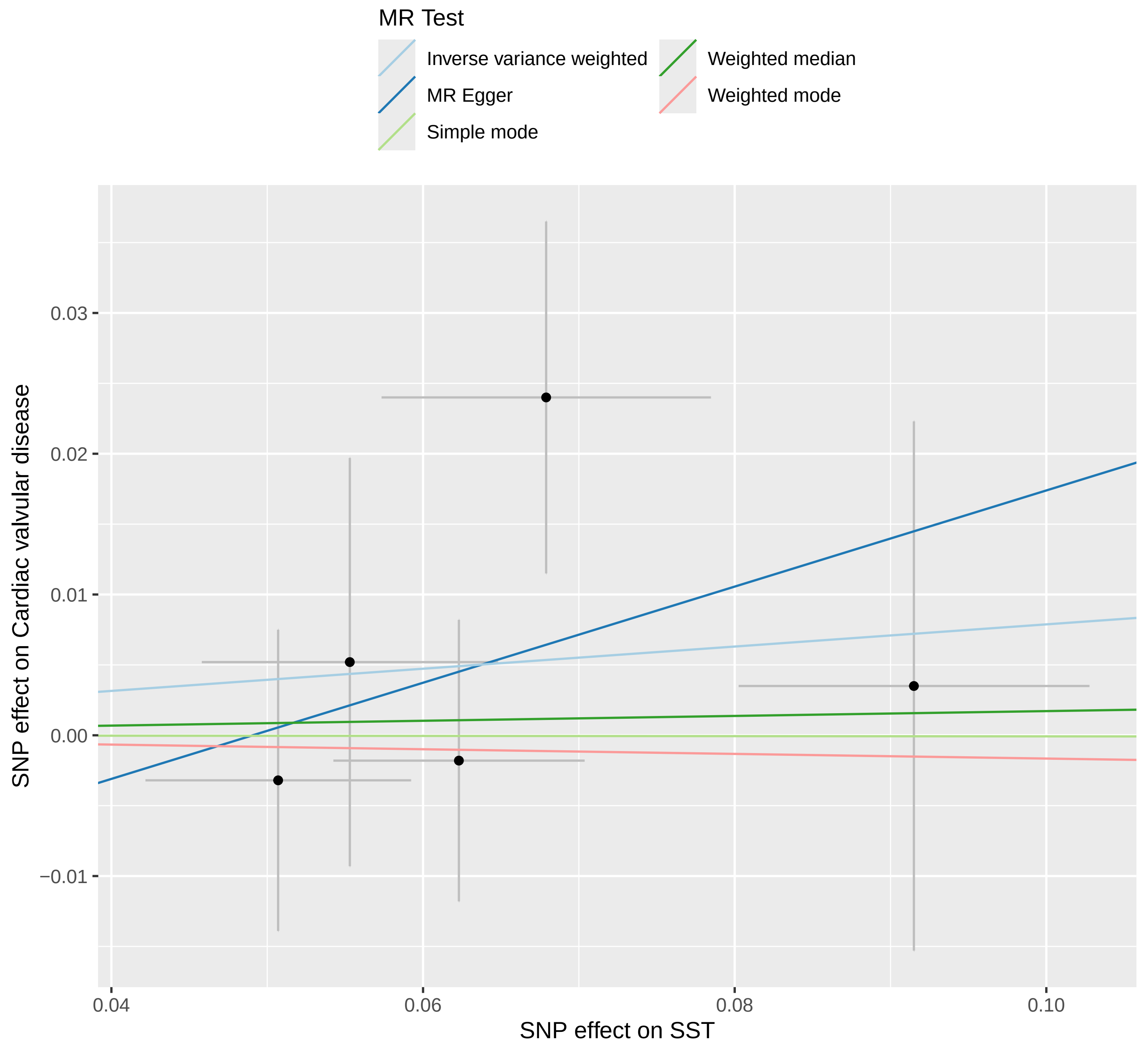
MR Test
Inverse variance weighted
MR Egger
Simple mode
Weighted median
Weighted mode
0.03
0.02
0.01
0.00
−0.01
0.04
0.06
0.08
0.10
SNP effect on Cardiac valvular disease
SNP effect on SST

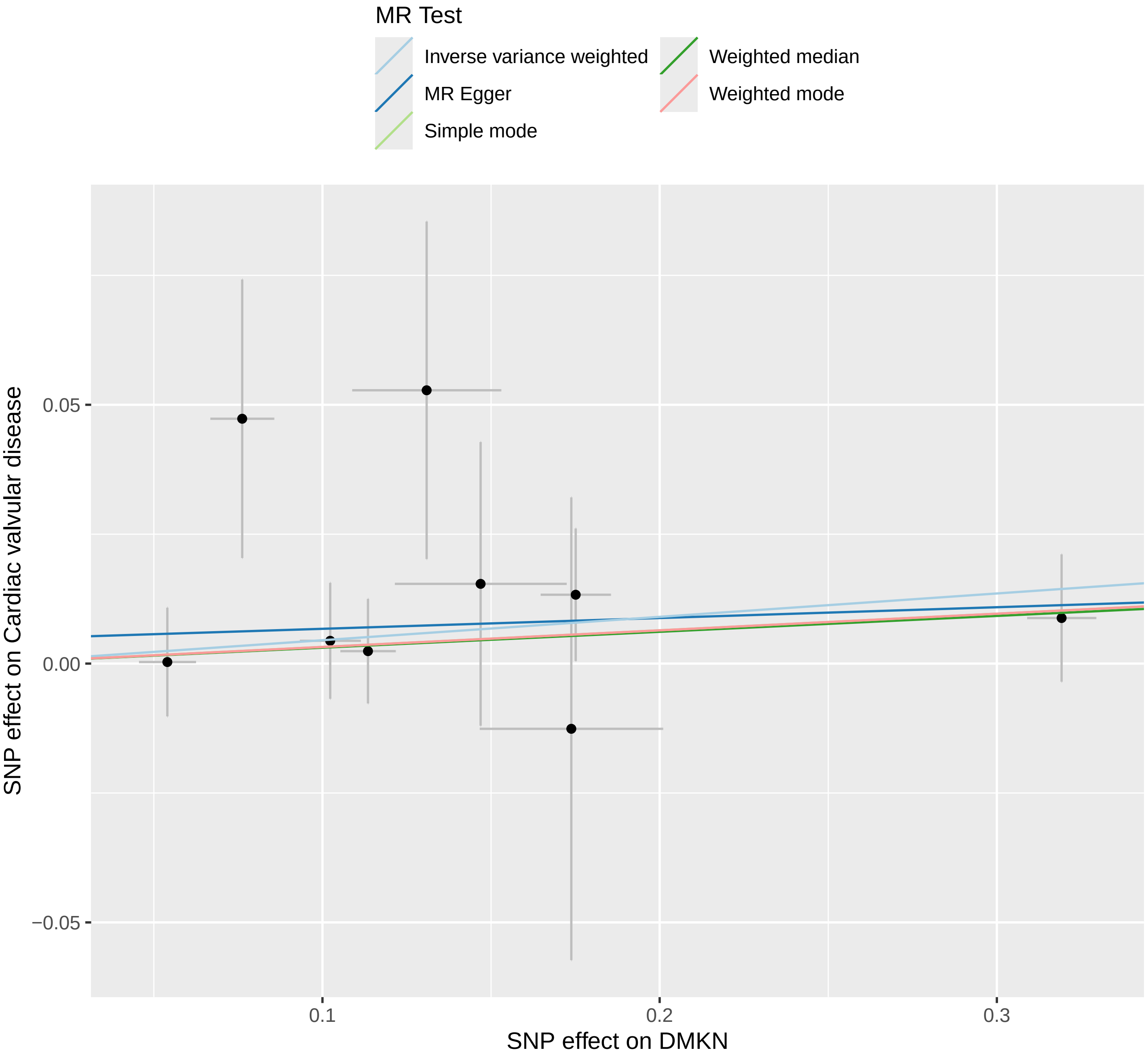

MR Test
Inverse variance weighted
MR Egger
Simple mode
Weighted median
Weighted mode
0.05
0.00
−0.05
0.1
0.2
0.3
SNP effect on Cardiac valvular disease
SNP effect on DMKN

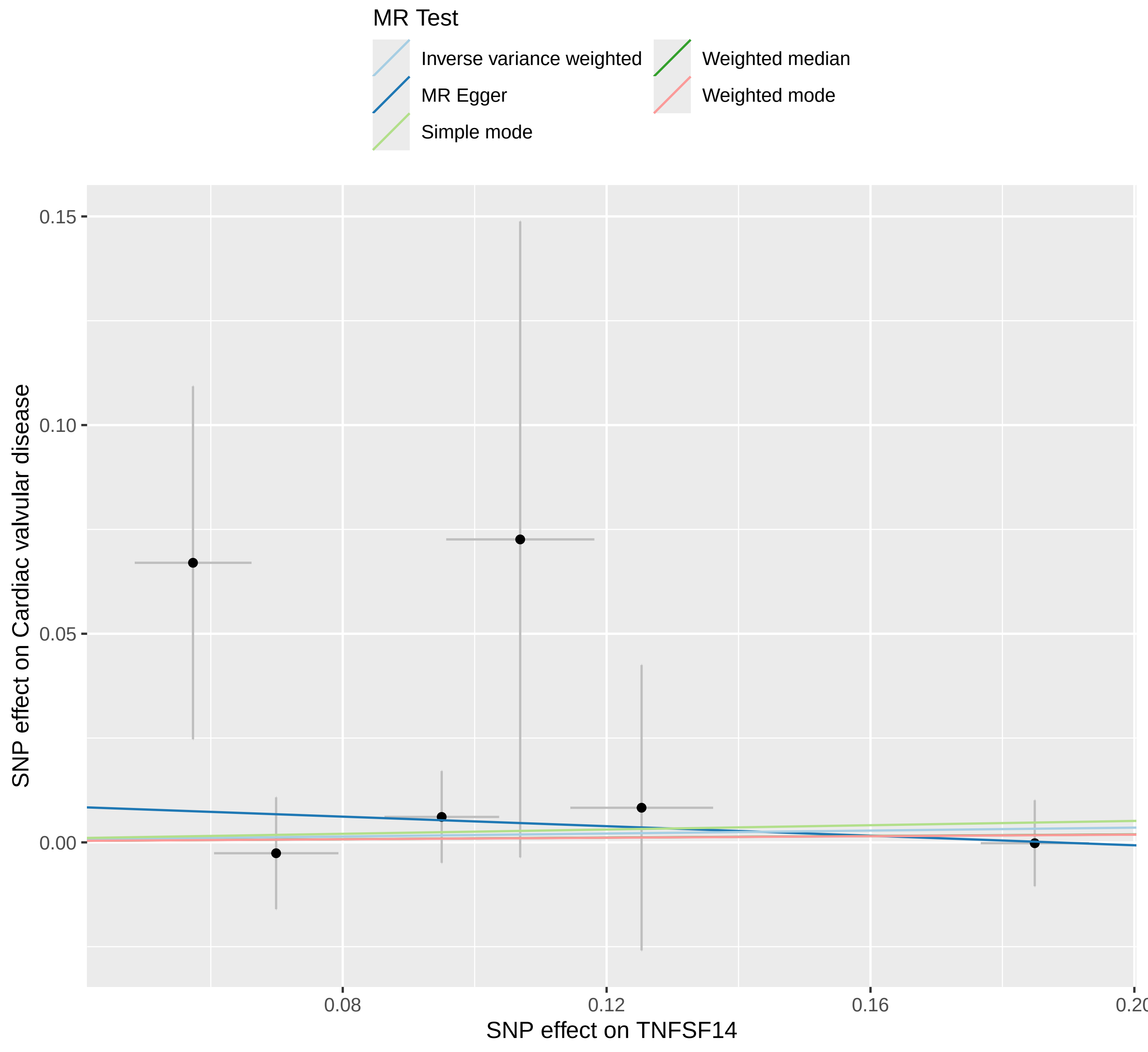
MR Test
Inverse variance weighted
MR Egger
Simple mode
Weighted median
Weighted mode
SNP effect on Cardiac valvular disease
0.15
0.10
0.05
0.00
0.08
0.12
0.16
0.20
SNP effect on TNFSF14

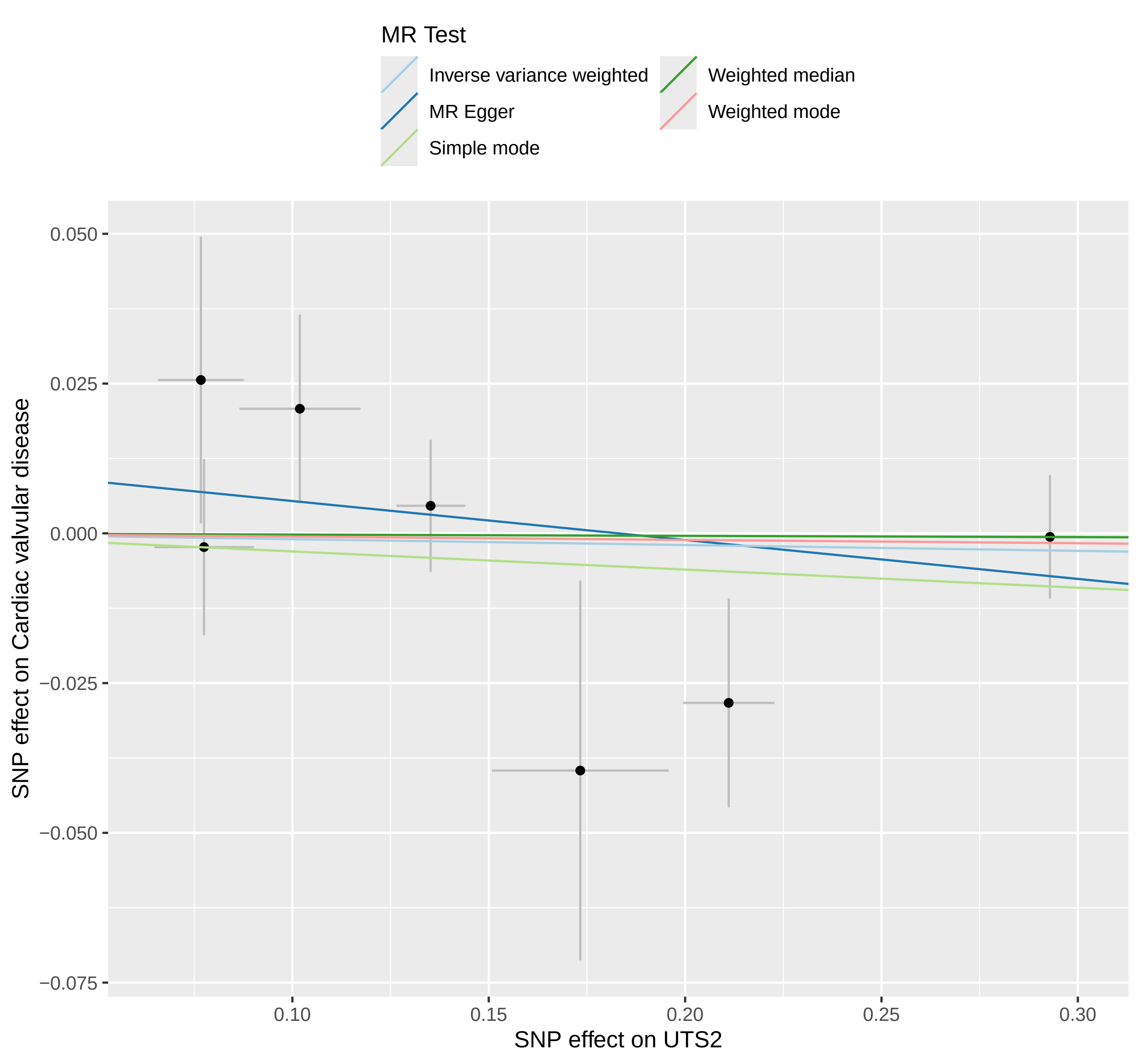

MR Test
Inverse variance weighted
MR Egger
Simple mode
Weighted median
Weighted mode
0.050
0.025
0.000
−0.025
−0.050
−0.075
0.10
0.15
0.20
0.25
0.30
SNP effect on Cardiac valvular disease
SNP effect on UTS2

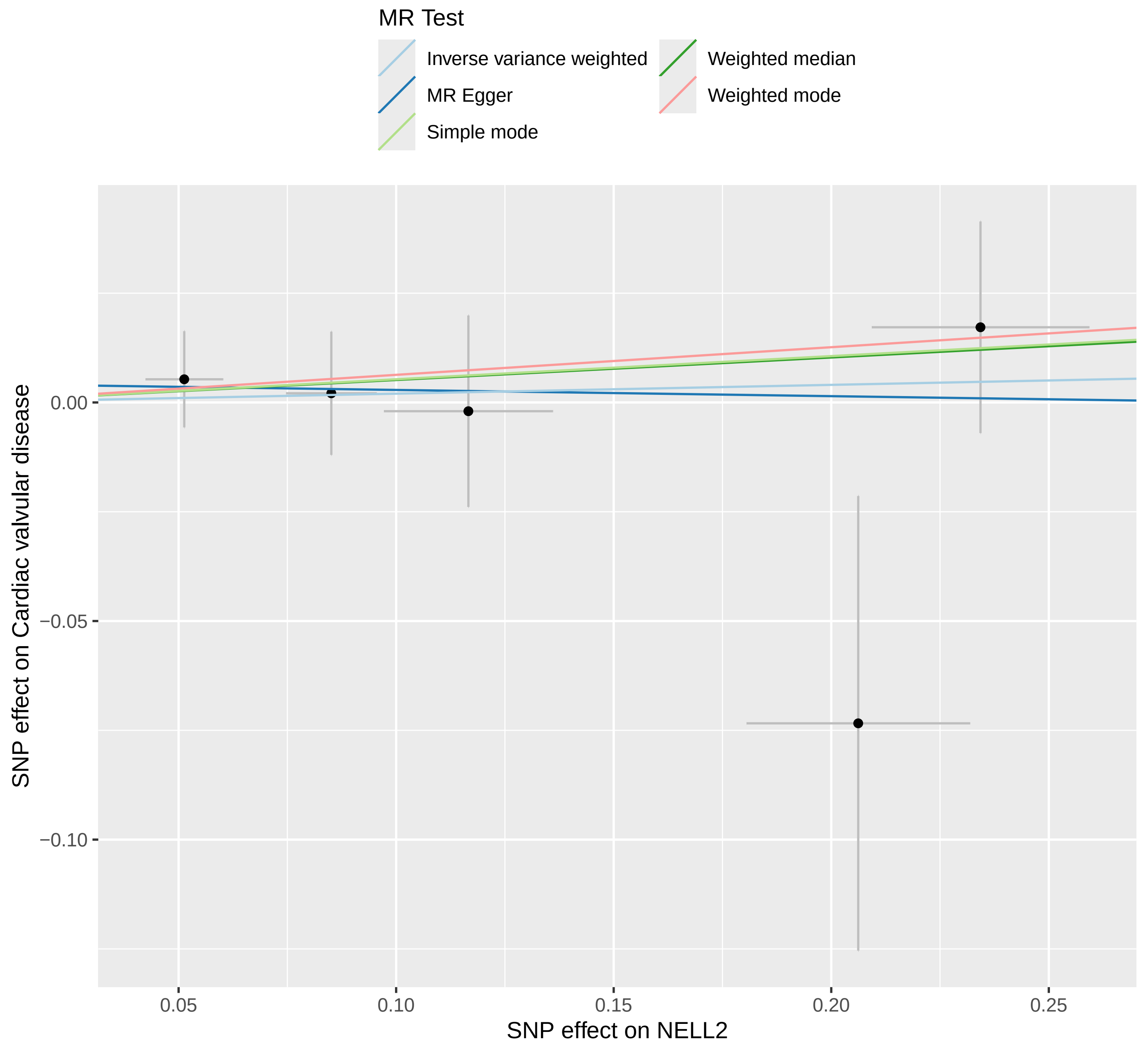

MR Test
Inverse variance weighted
MR Egger
Simple mode
Weighted median
Weighted mode
SNP effect on Cardiac valvular disease
0.00
−0.05
−0.10
0.05
0.10
0.15
0.20
0.25
SNP effect on NELL2

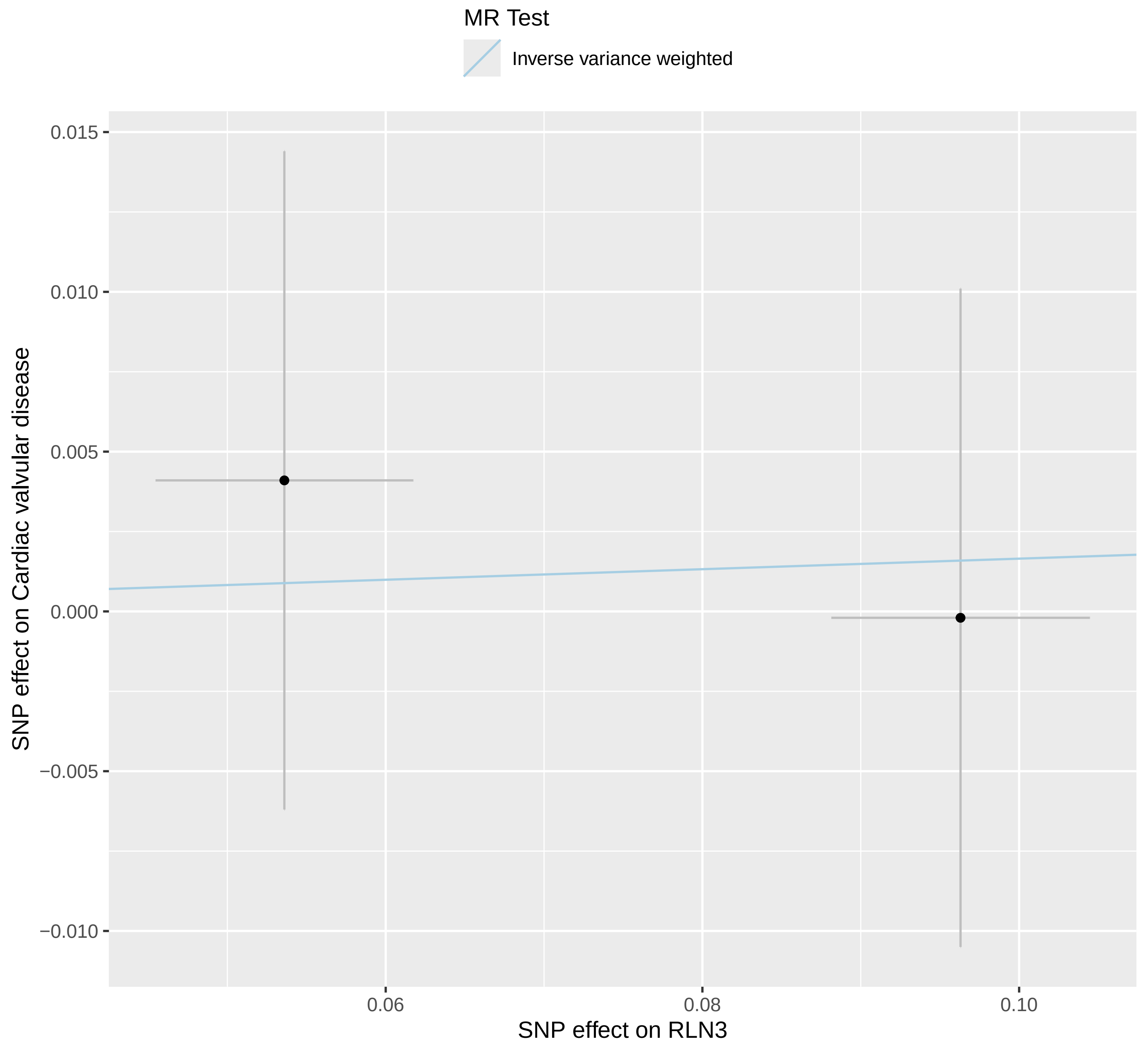

MR Test
Inverse variance weighted
0.015
0.010
0.005
0.000
−0.005
−0.010
0.06
0.08
0.10
SNP effect on RLN3
SNP effect on Cardiac valvular disease

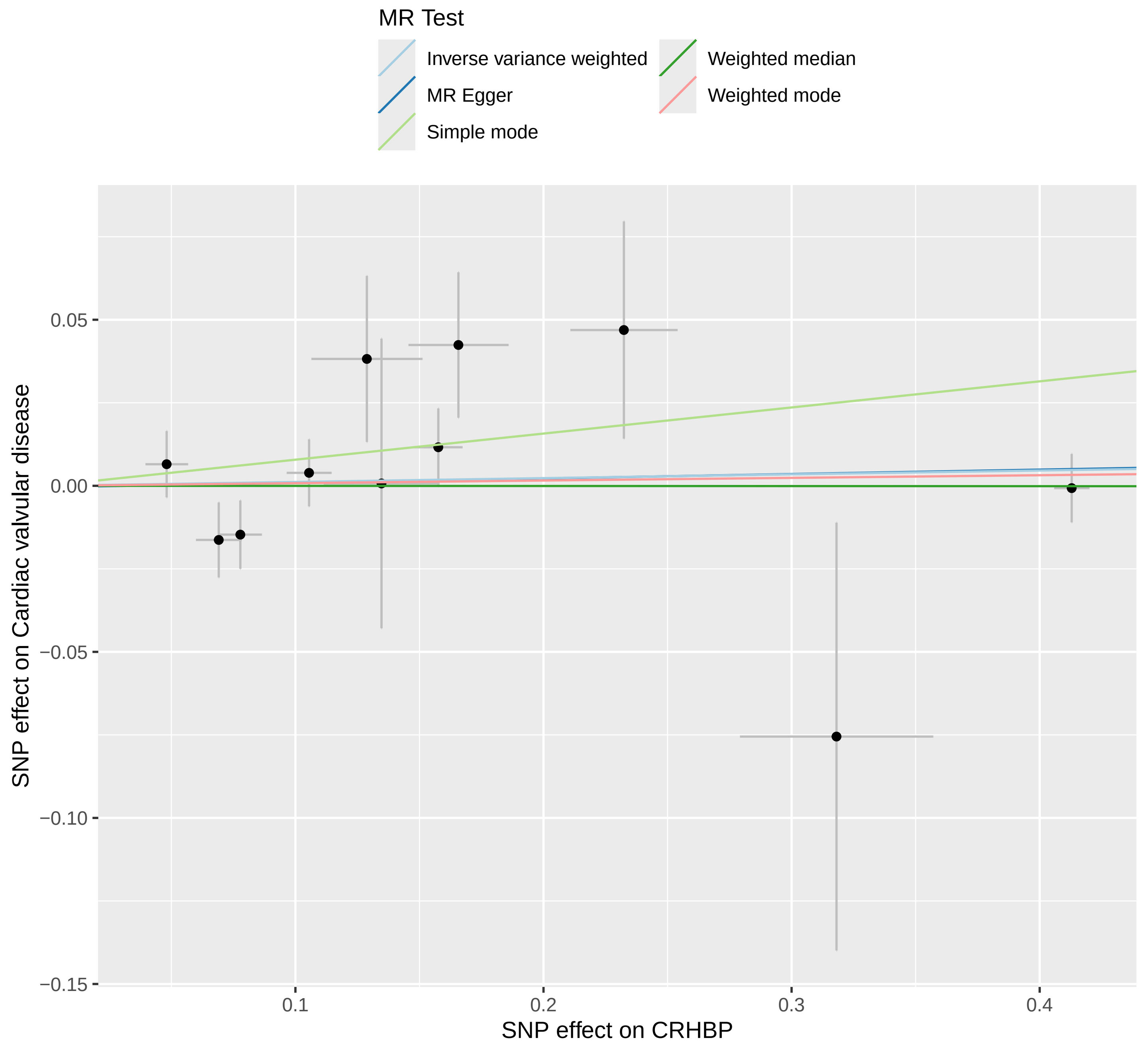
MR Test
Inverse variance weighted
MR Egger
Simple mode
Weighted median
Weighted mode
SNP effect on Cardiac valvular disease
0.05
0.00
−0.05
−0.10
−0.15
0.1
0.2
0.3
0.4
SNP effect on CRHBP

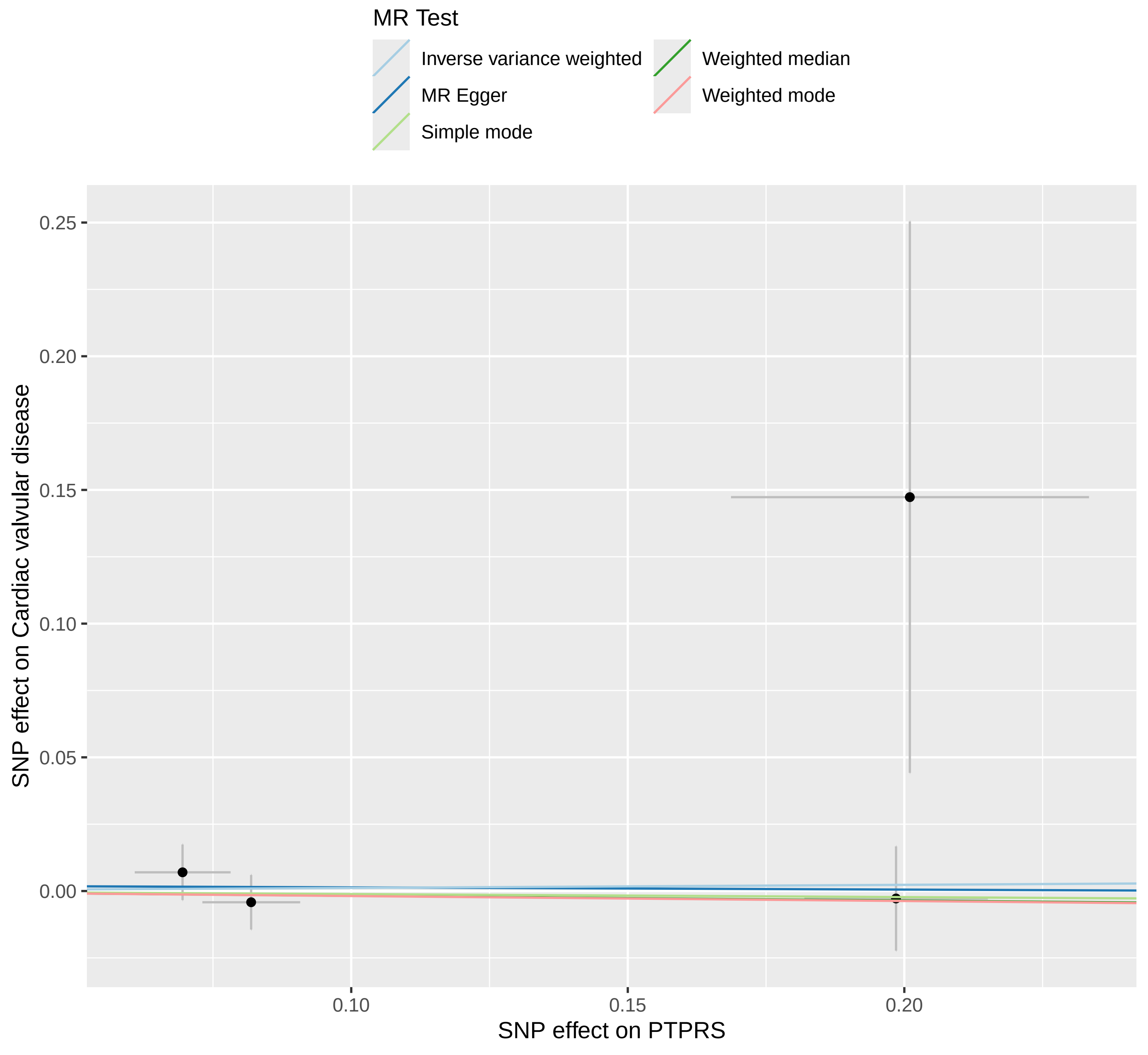

MR Test
Inverse variance weighted
MR Egger
Simple mode
Weighted median
Weighted mode
SNP effect on Cardiac valvular disease
0.25
0.20
0.15
0.10
0.05
0.00
0.10
0.15
0.20
SNP effect on PTPRS

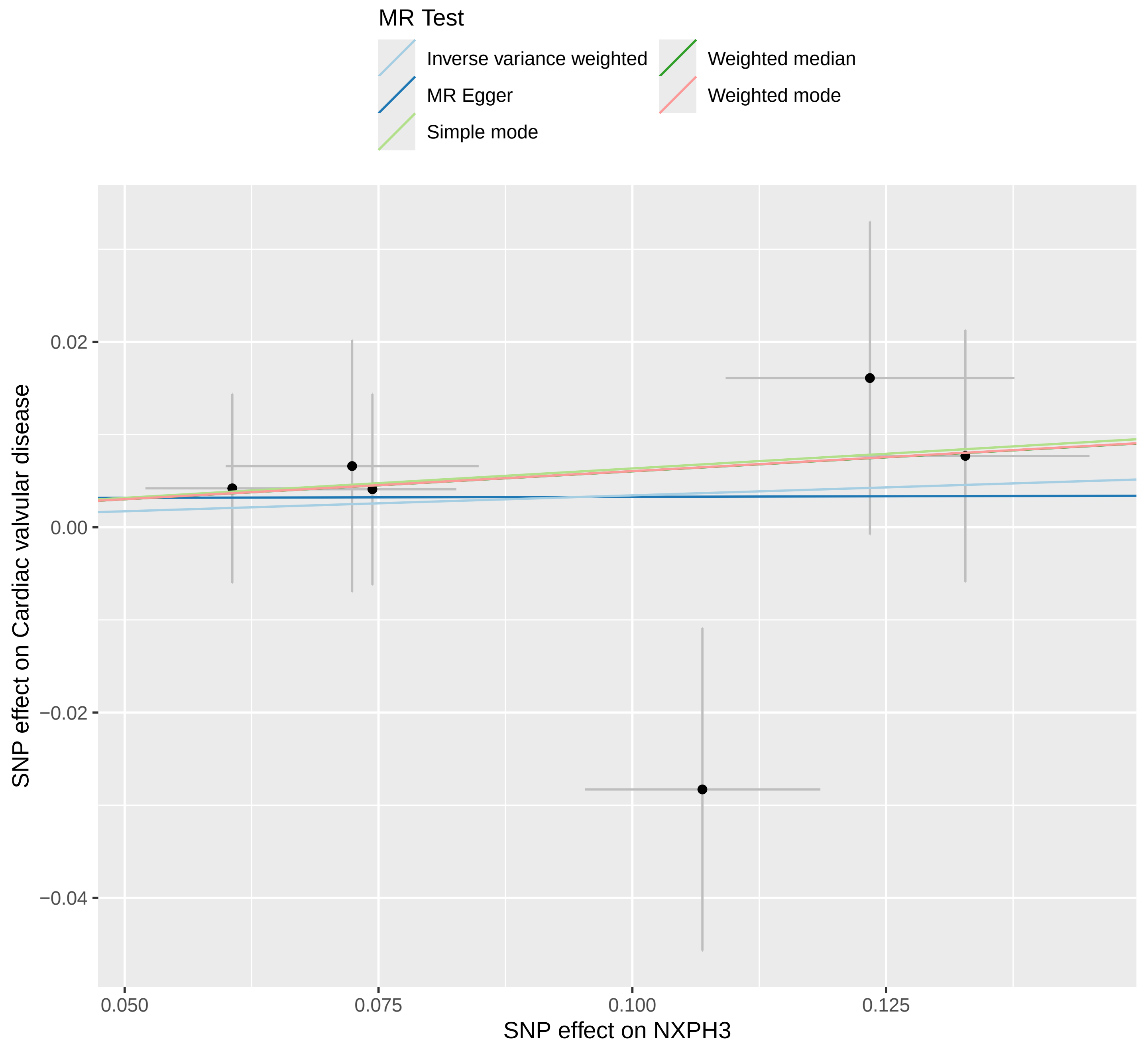
MR Test
Inverse variance weighted
MR Egger
Simple mode
Weighted median
Weighted mode
0.02
0.00
−0.02
−0.04
0.050
0.075
0.100
0.125
SNP effect on NXPH3
SNP effect on Cardiac valvular disease

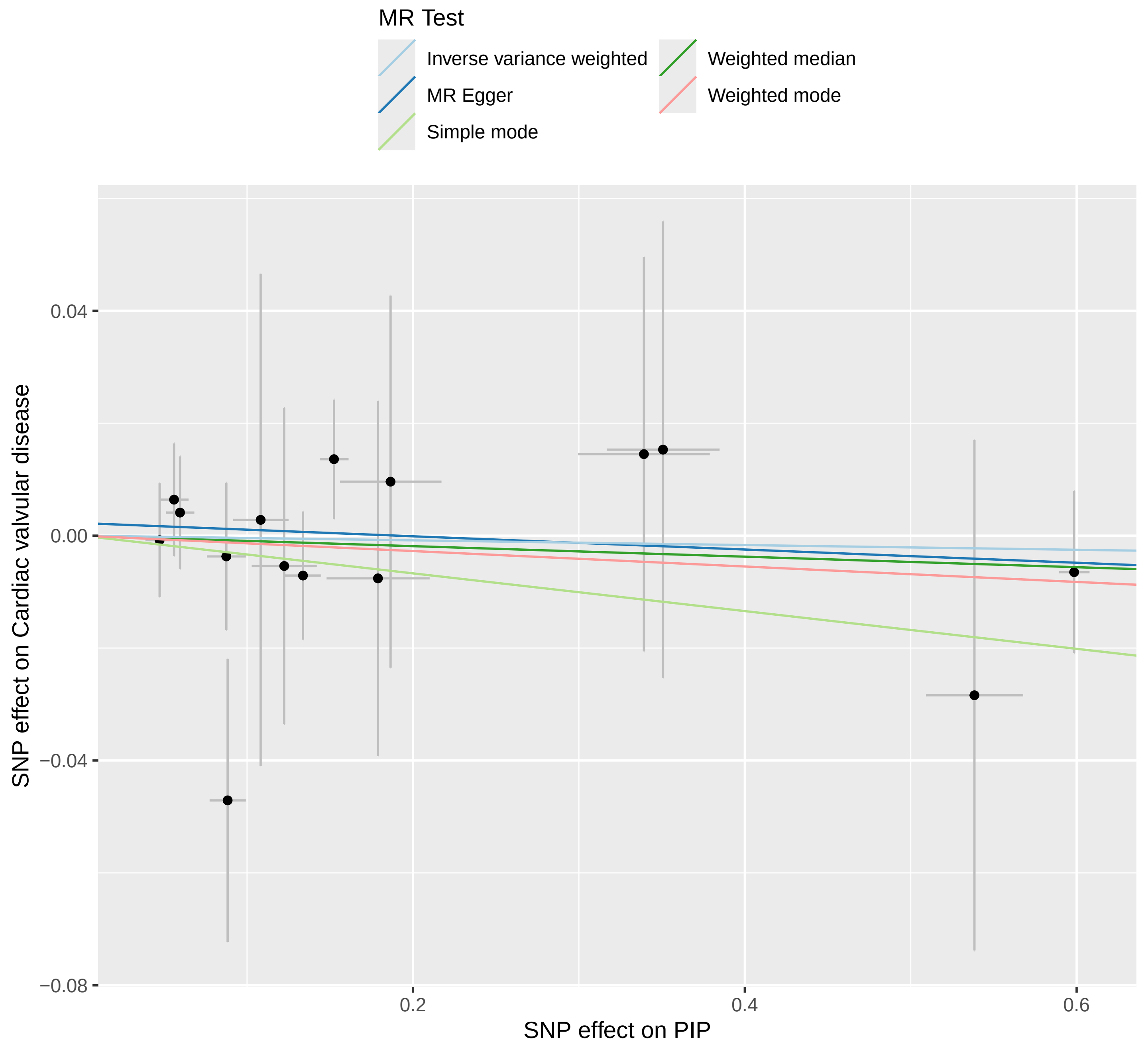

MR Test
Inverse variance weighted
MR Egger
Simple mode
Weighted median
Weighted mode
SNP effect on Cardiac valvular disease
0.04
0.00
−0.04
−0.08
0.2
0.4
0.6
SNP effect on PIP

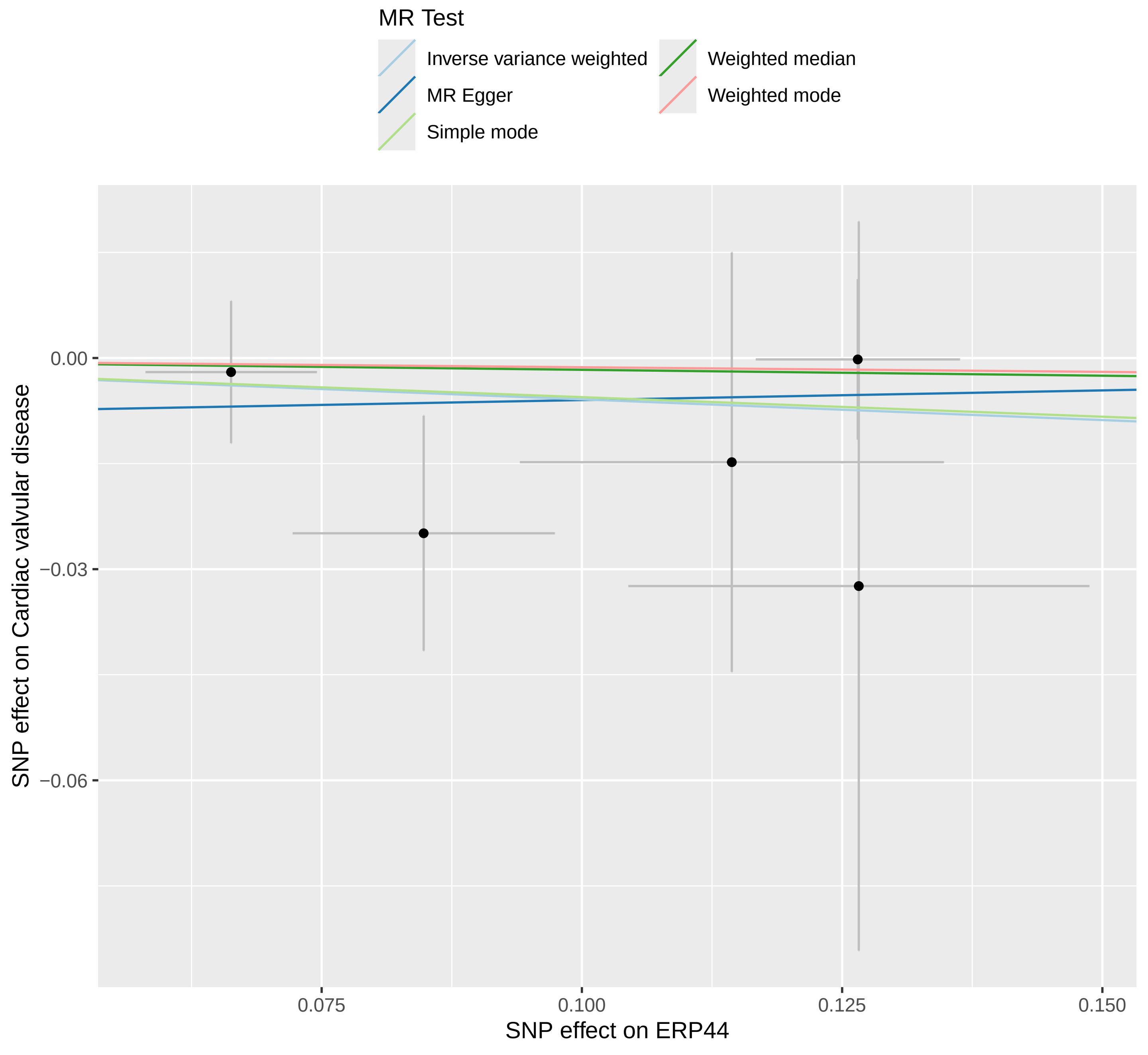
MR Test
Inverse variance weighted
MR Egger
Simple mode
Weighted median
Weighted mode
SNP effect on Cardiac valvular disease
0.00
−0.03
−0.06
0.075
0.100
0.125
0.150
SNP effect on ERP44

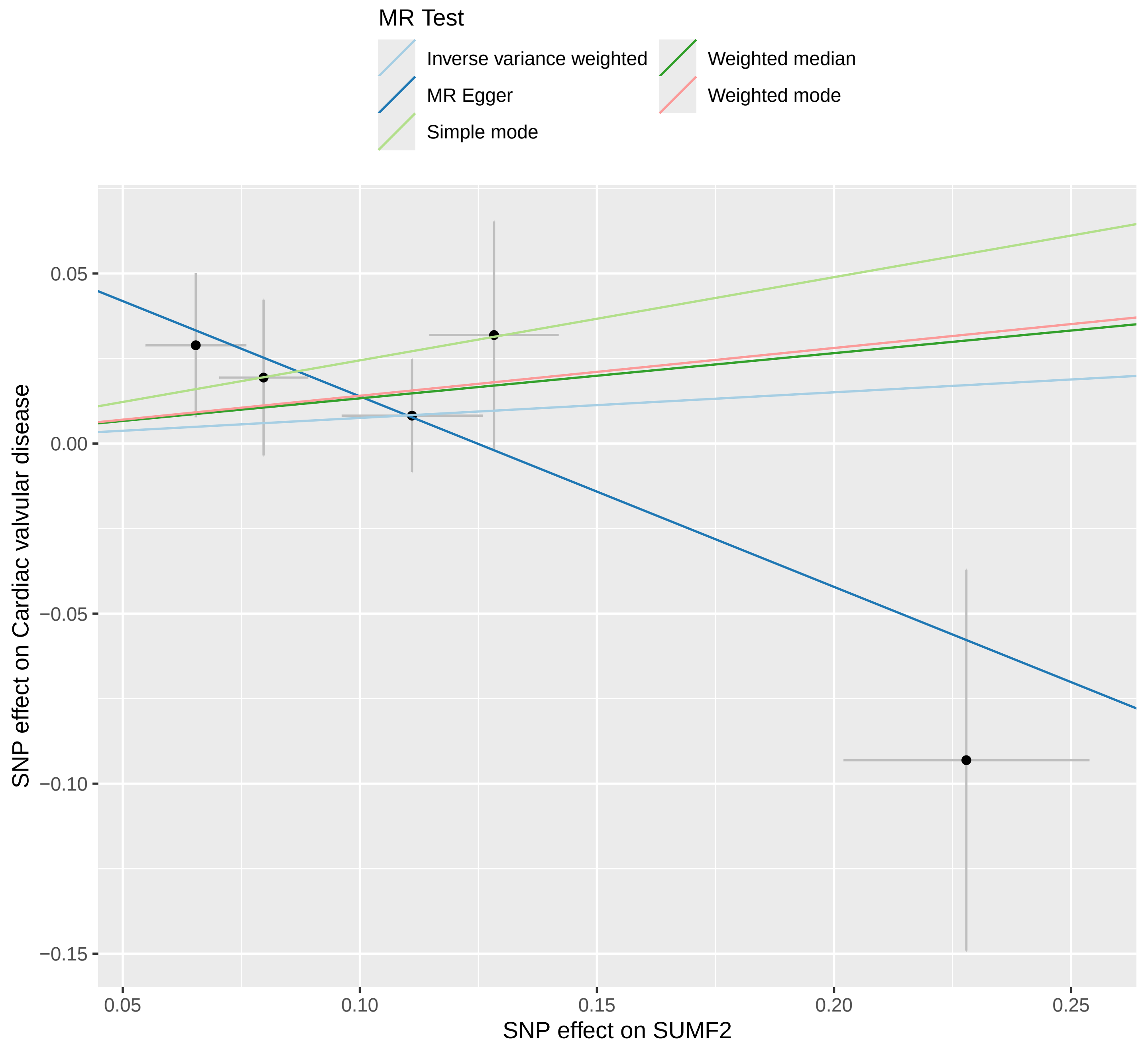
MR Test
Inverse variance weighted
MR Egger
Simple mode
Weighted median
Weighted mode
SNP effect on Cardiac valvular disease
0.05
0.00
−0.05
−0.10
−0.15
0.05
0.10
0.15
0.20
0.25
SNP effect on SUMF2

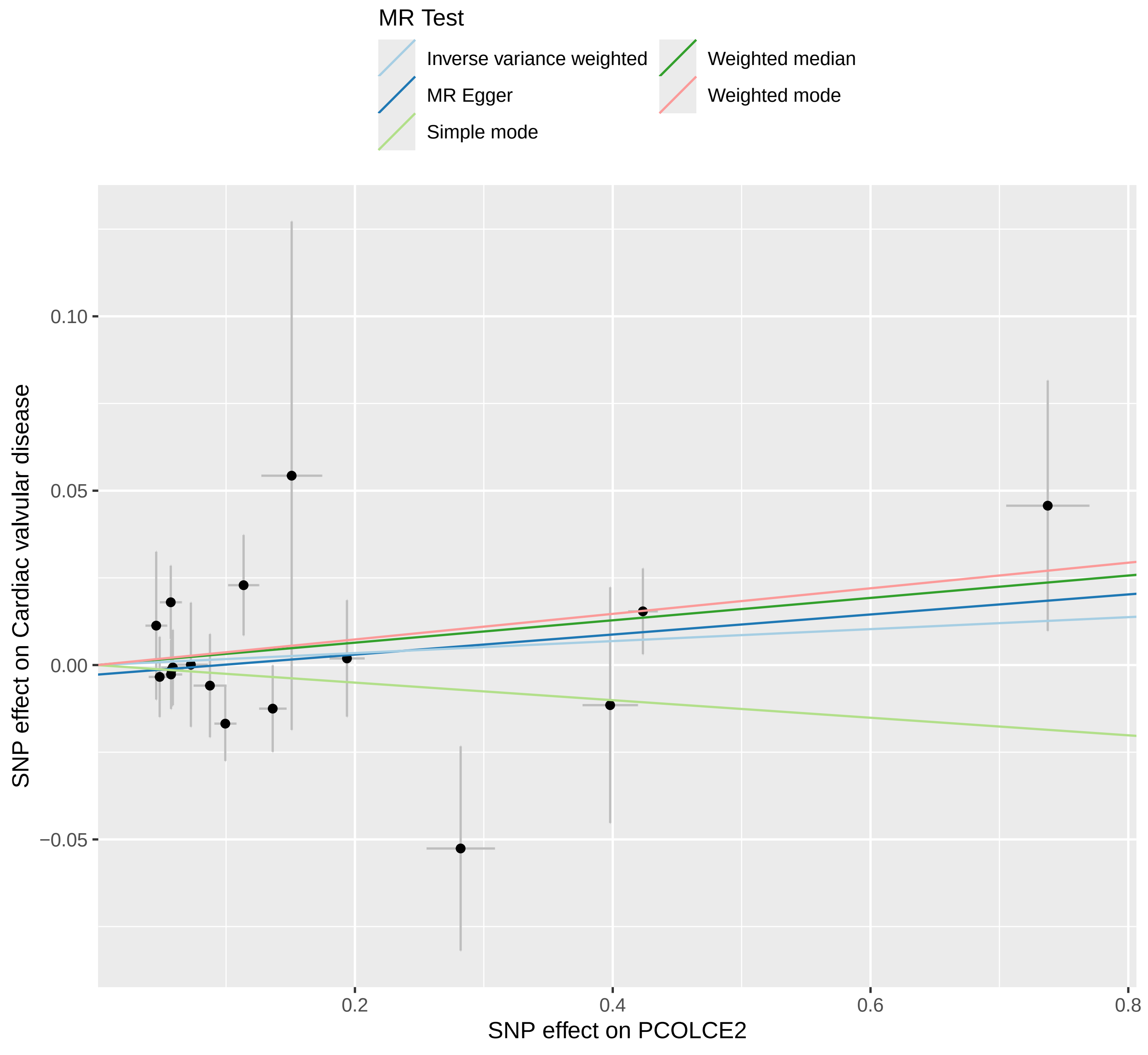

MR Test
Inverse variance weighted
MR Egger
Simple mode
Weighted median
Weighted mode
0.10
0.05
0.00
−0.05
SNP effect on Cardiac valvular disease
0.2
0.4
0.6
0.8
SNP effect on PCOLCE2

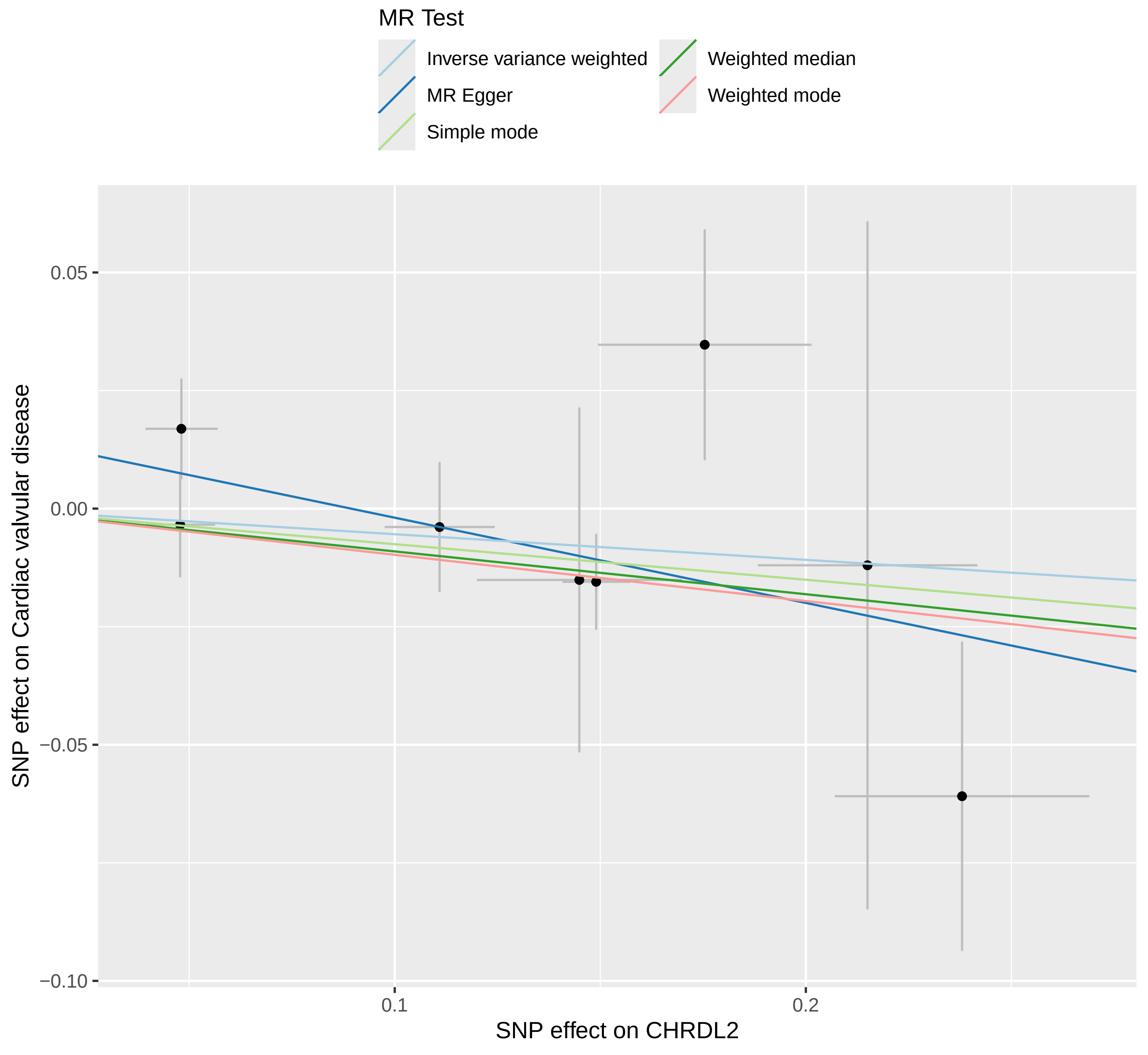
MR Test
Inverse variance weighted
MR Egger
Simple mode
Weighted median
Weighted mode
0.05
0.00
−0.05
−0.10
0.1
0.2
SNP effect on Cardiac valvular disease
SNP effect on CHRDL2

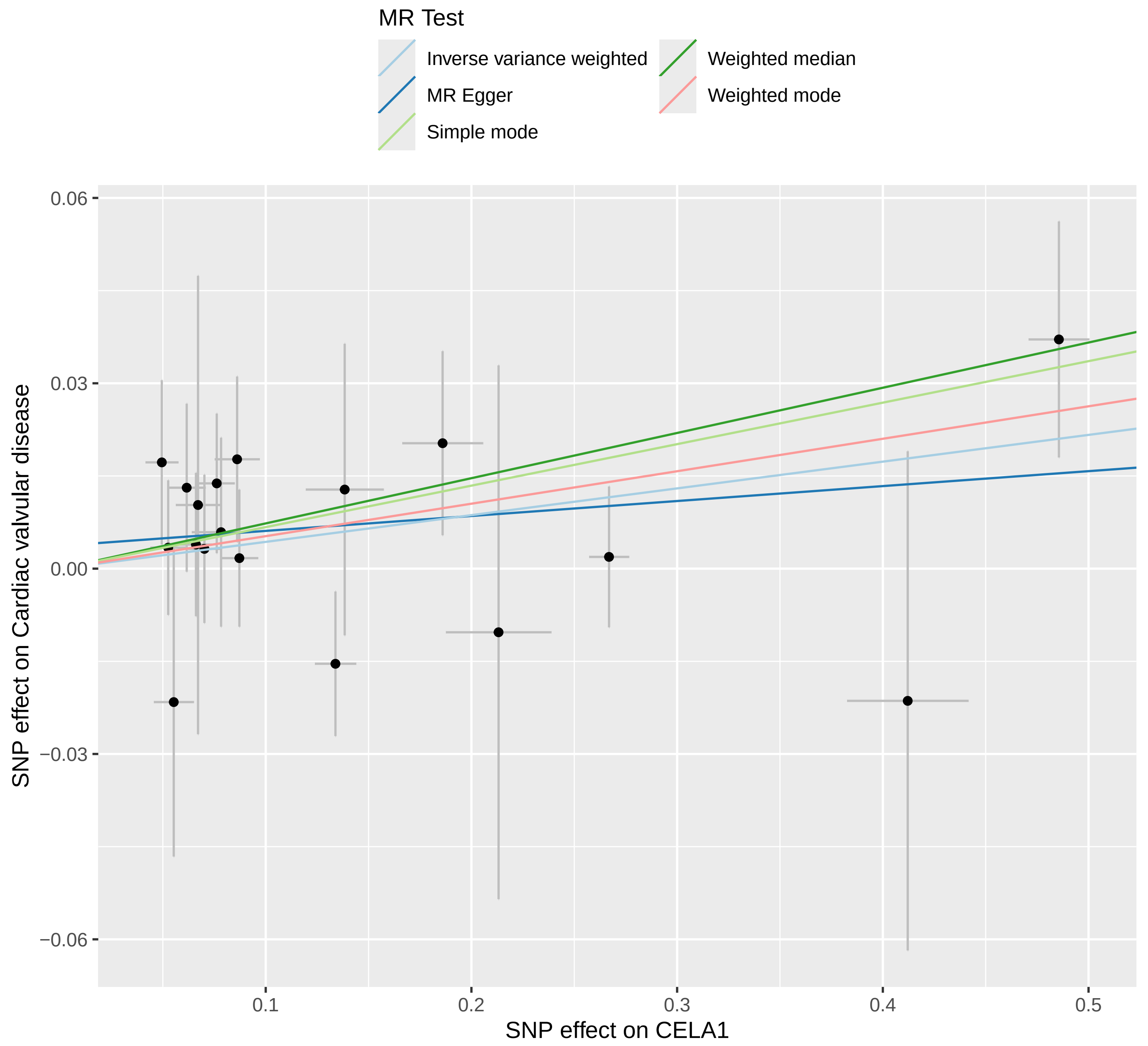

MR Test
Inverse variance weighted
MR Egger
Simple mode
Weighted median
Weighted mode
0.06
0.03
0.00
−0.03
−0.06
SNP effect on Cardiac valvular disease
0.1
0.2
0.3
0.4
0.5
SNP effect on CELA1

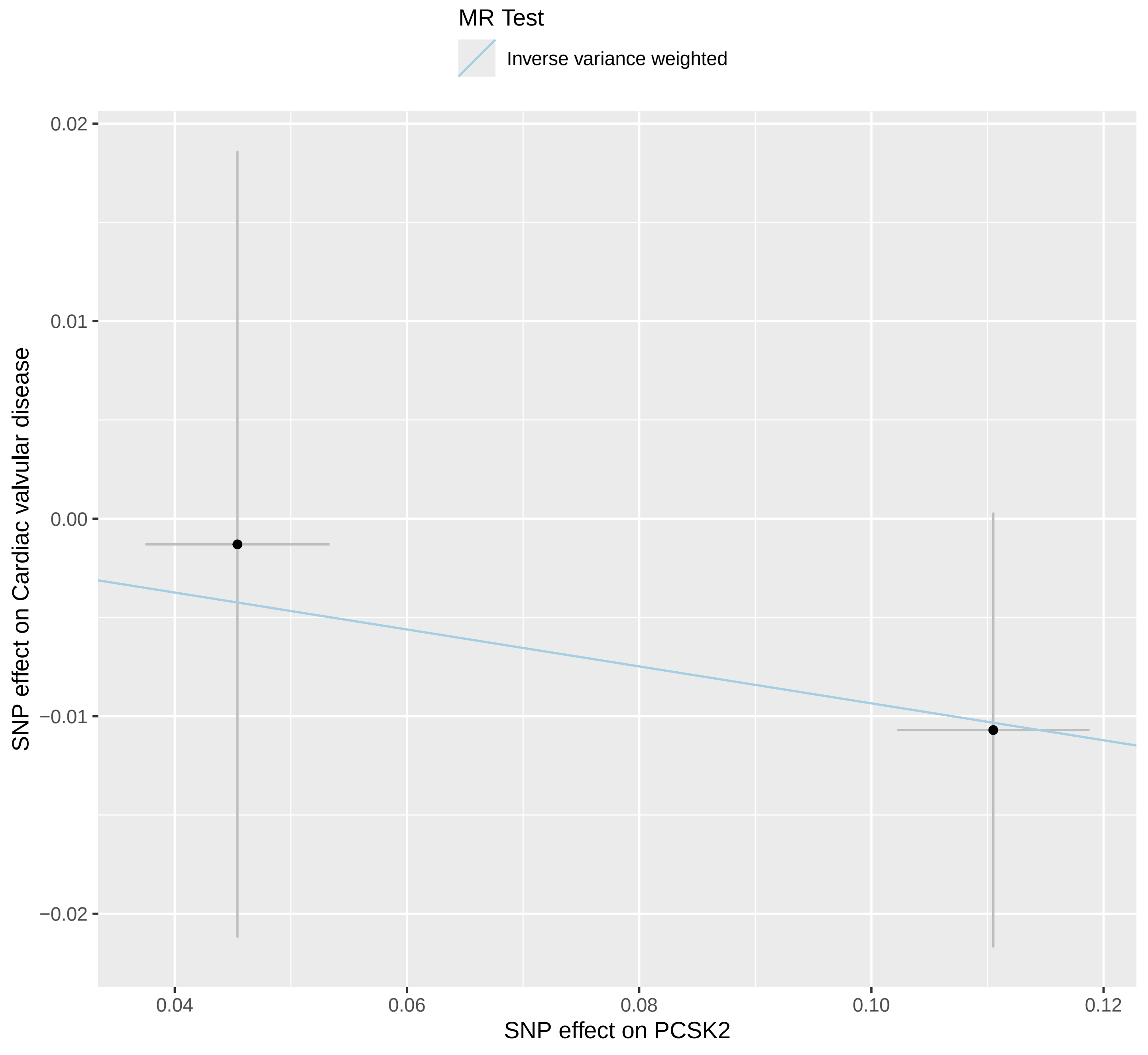

MR Test
Inverse variance weighted
0.02
0.01
0.00
−0.01
−0.02
SNP effect on Cardiac valvular disease
0.04
0.06
0.08
0.10
0.12
SNP effect on PCSK2

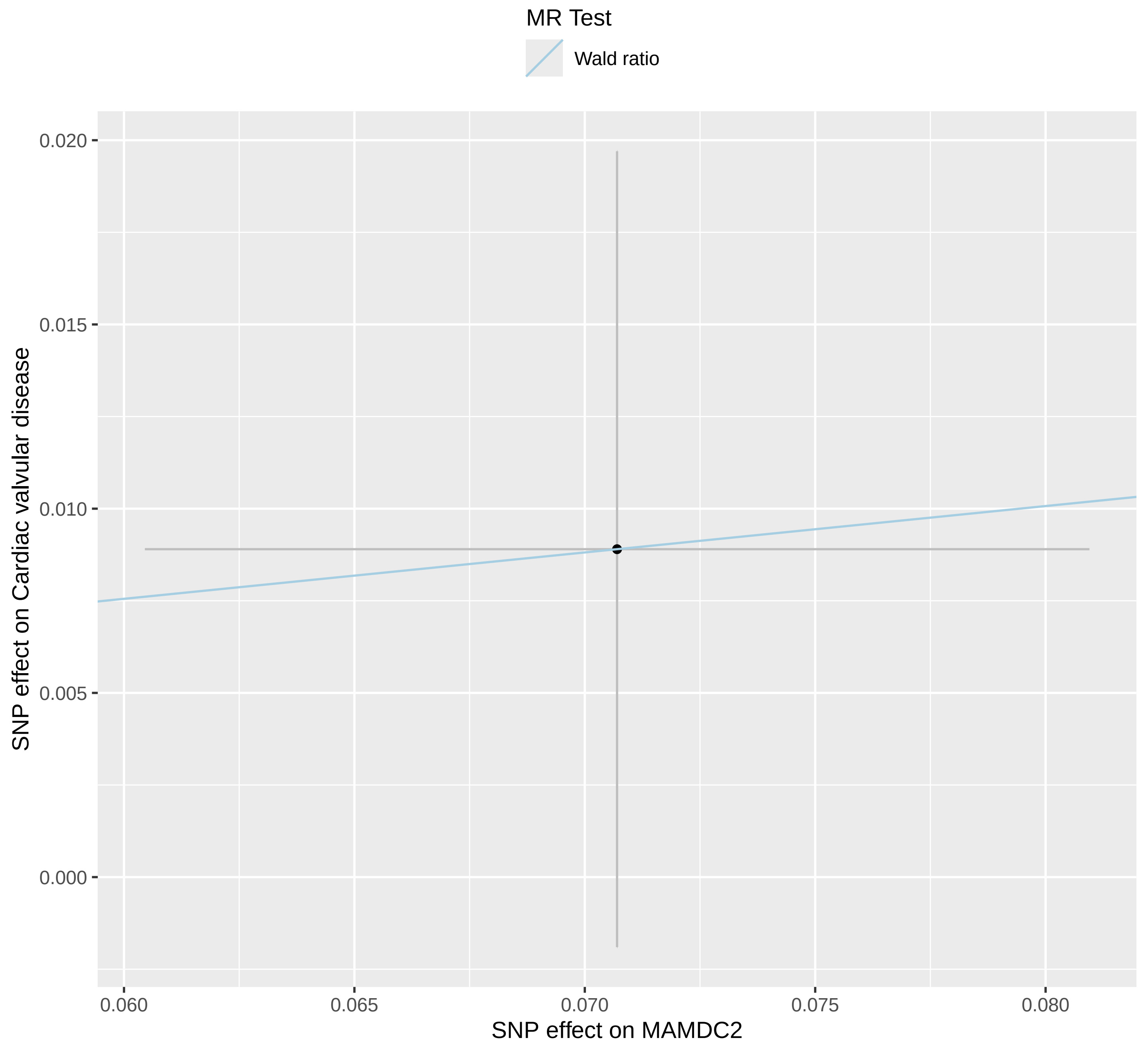

MR Test
Wald ratio
0.020
0.015
0.010
0.005
0.000
SNP effect on Cardiac valvular disease
0.060
0.065
0.070
0.075
0.080
SNP effect on MAMDC2

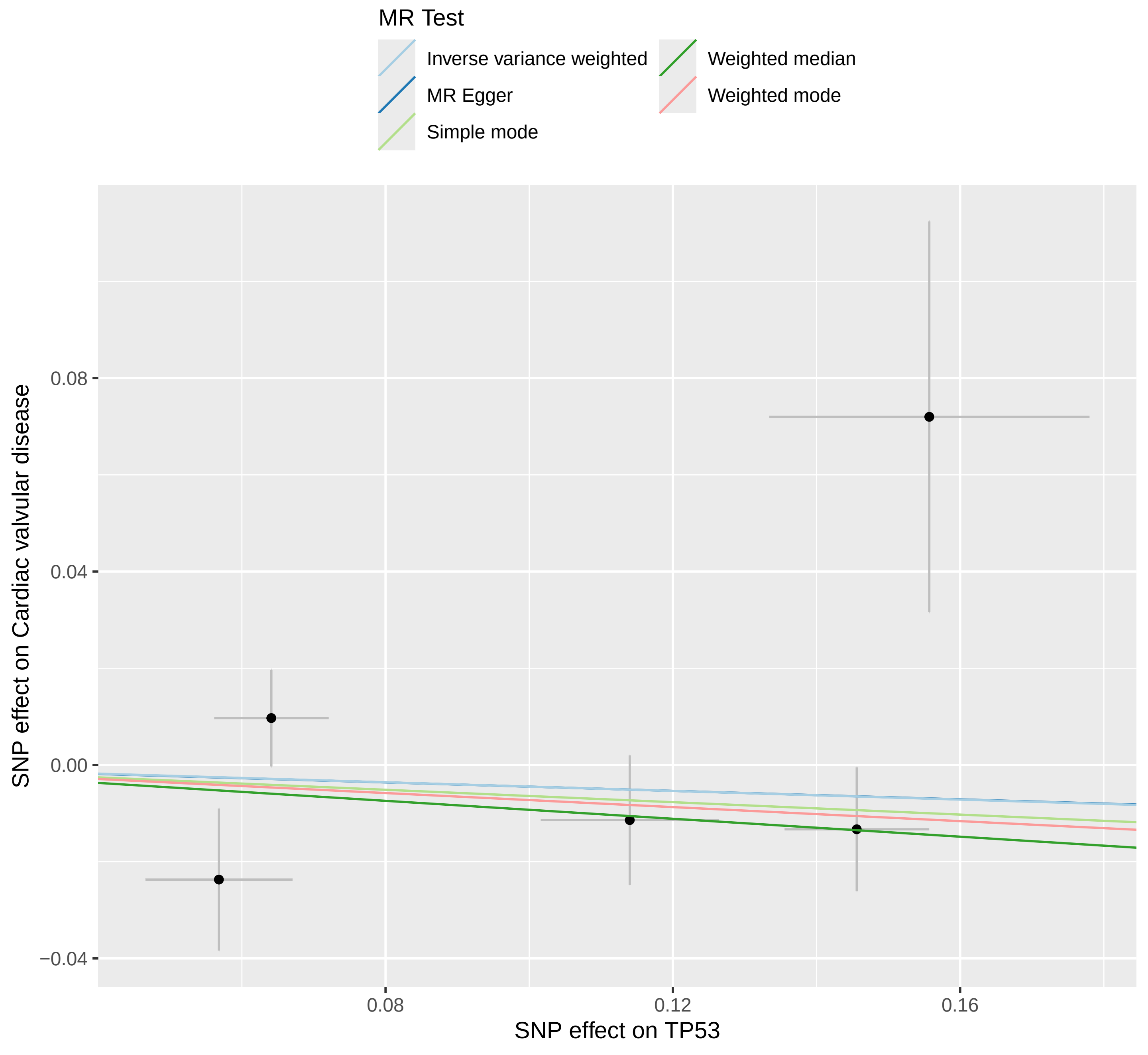

MR Test
Inverse variance weighted
MR Egger
Simple mode
Weighted median
Weighted mode
0.08
0.04
0.00
−0.04
SNP effect on Cardiac valvular disease
0.08
0.12
0.16
SNP effect on TP53

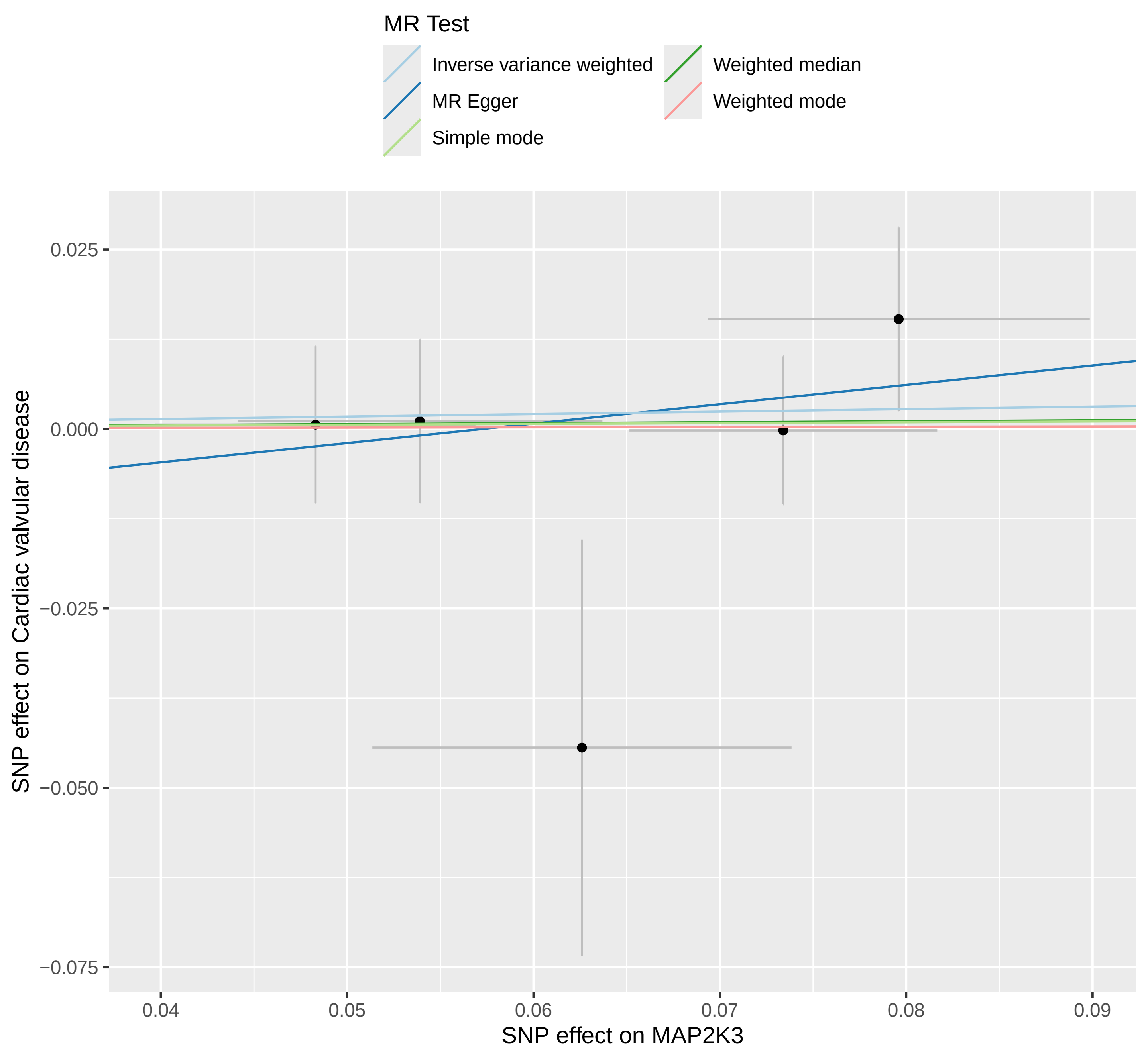
MR Test
Inverse variance weighted
MR Egger
Simple mode
Weighted median
Weighted mode
0.025
0.000
−0.025
−0.050
−0.075
SNP effect on Cardiac valvular disease
0.04
0.05
0.06
0.07
0.08
0.09
SNP effect on MAP2K3

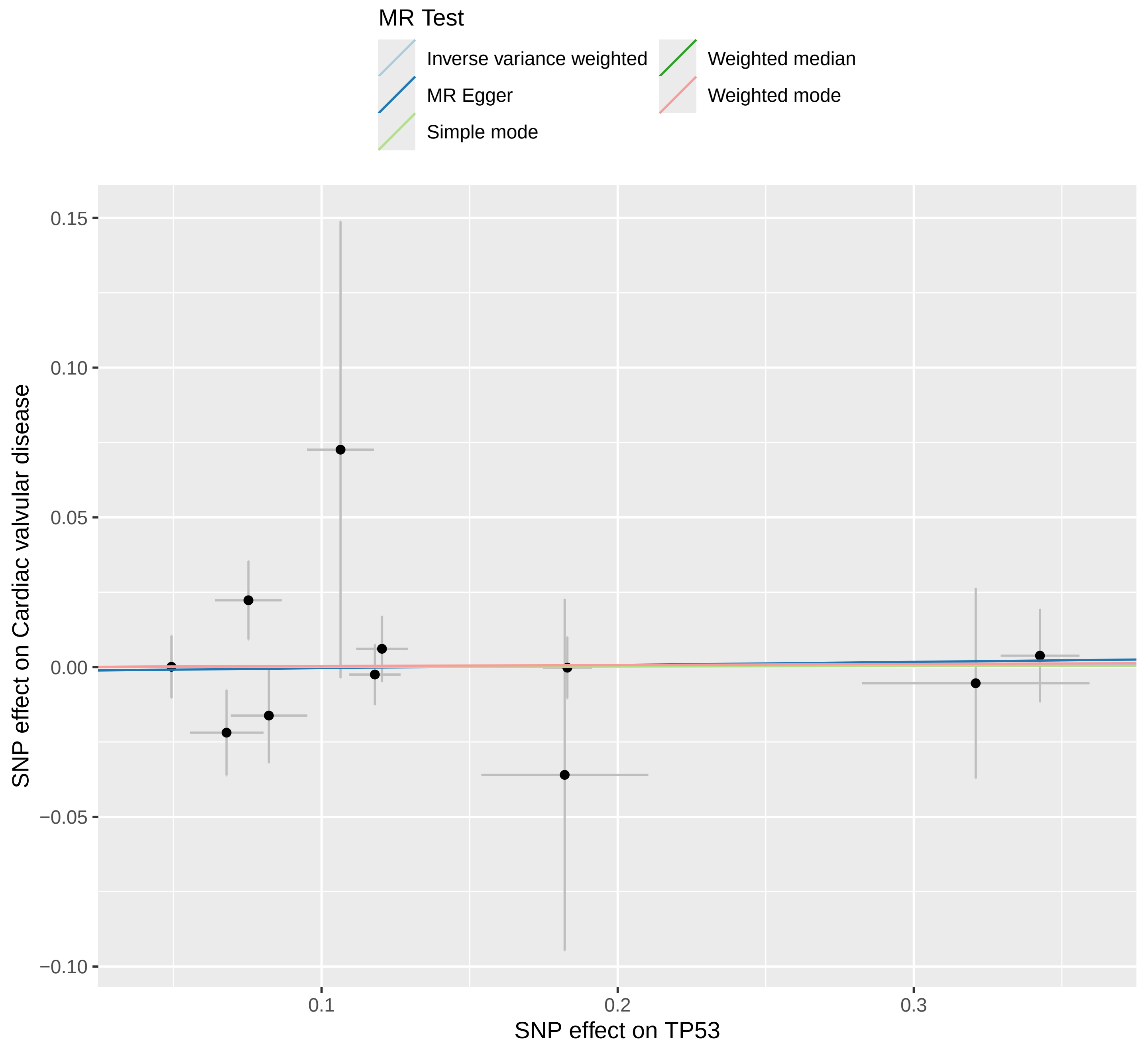

MR Test
Inverse variance weighted
MR Egger
Simple mode
Weighted median
Weighted mode
0.15
0.10
0.05
0.00
−0.05
−0.10
0.1
0.2
0.3
SNP effect on Cardiac valvular disease
SNP effect on TP53

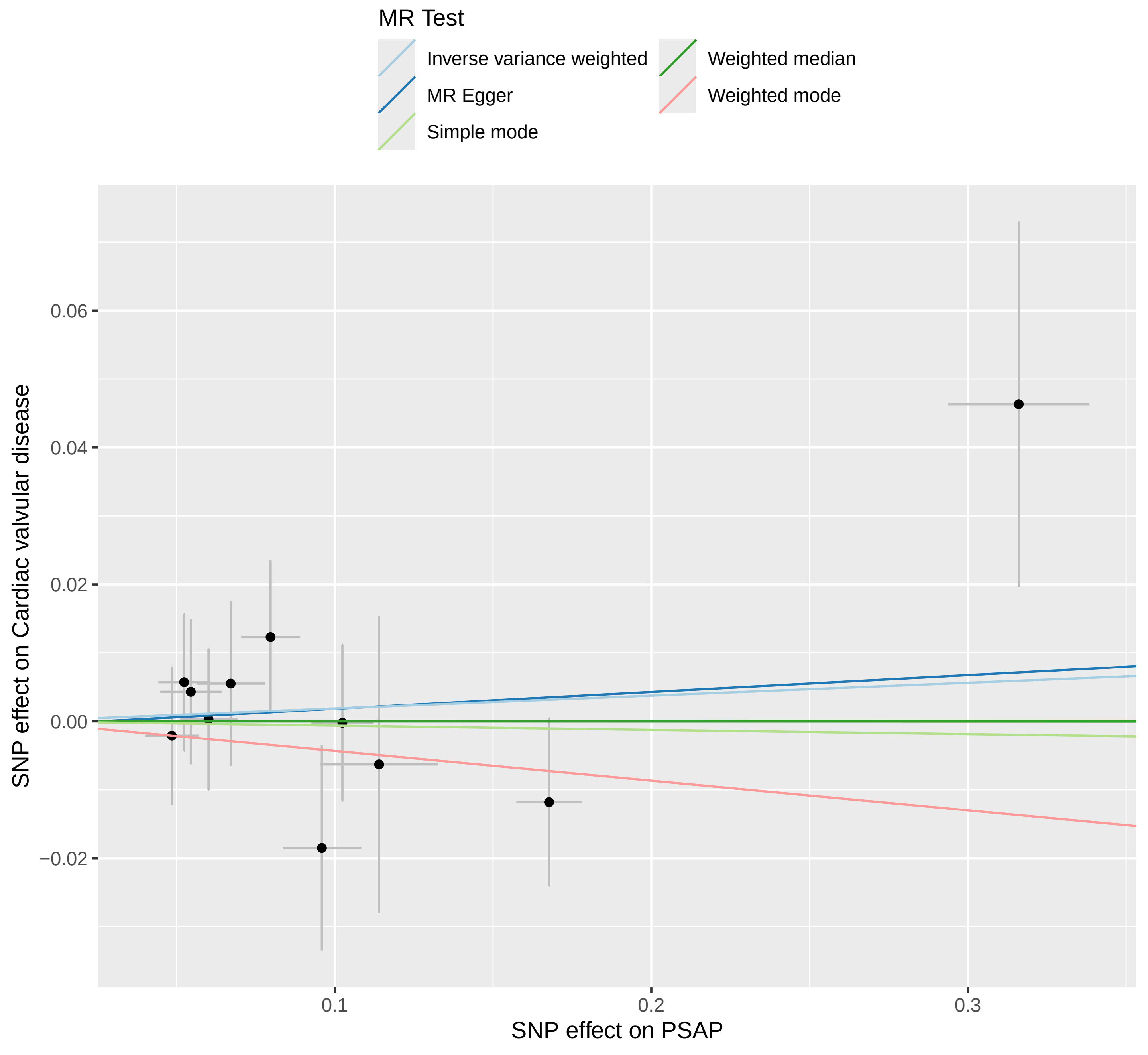
MR Test
Inverse variance weighted
MR Egger
Simple mode
Weighted median
Weighted mode
0.06
0.04
0.02
0.00
−0.02
SNP effect on Cardiac valvular disease
0.1
0.2
0.3
SNP effect on PSAP

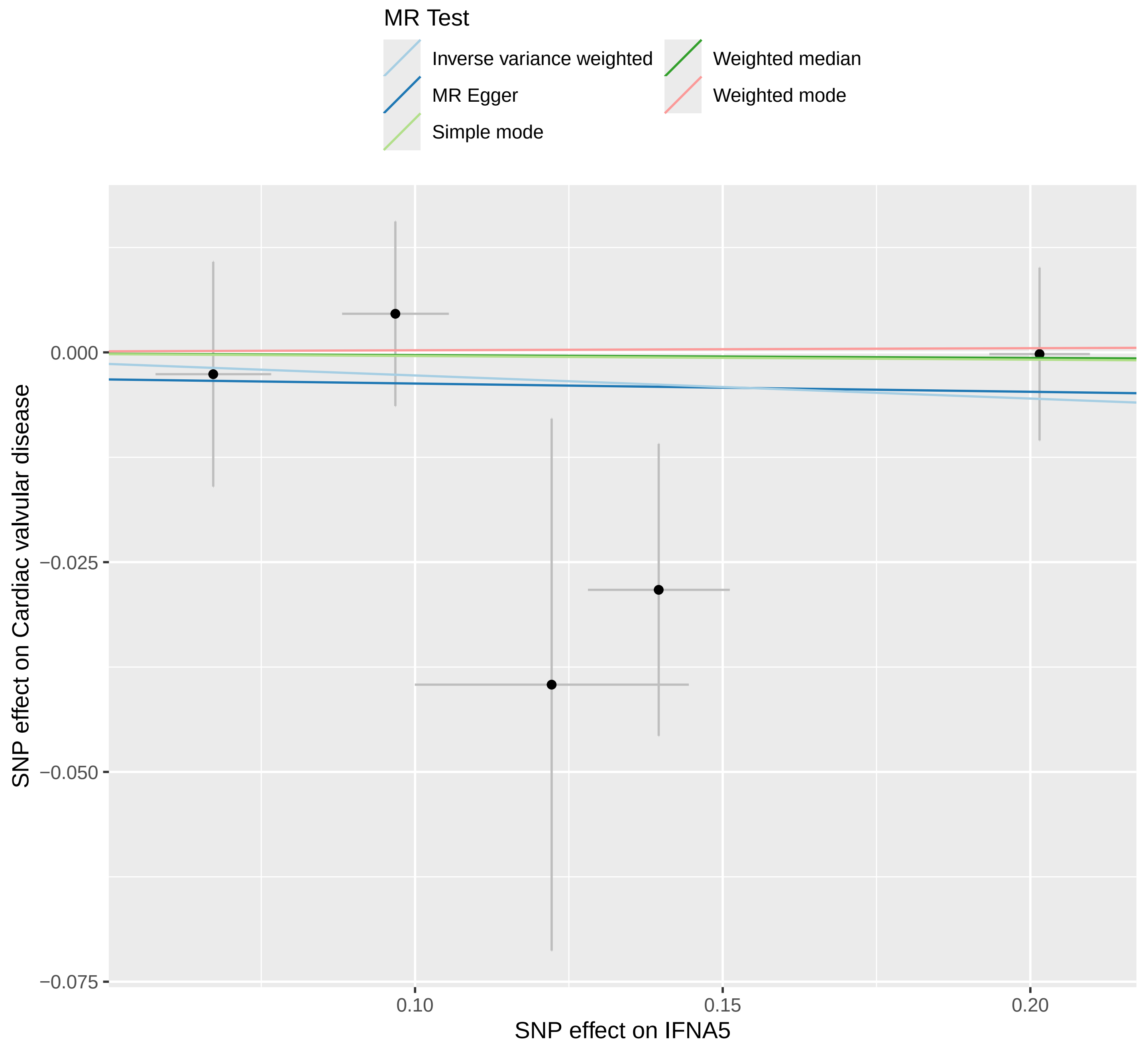
MR Test
Inverse variance weighted
MR Egger
Simple mode
Weighted median
Weighted mode
0.000
−0.025
−0.050
−0.075
0.10
0.15
0.20
SNP effect on IFNA5
SNP effect on Cardiac valvular disease

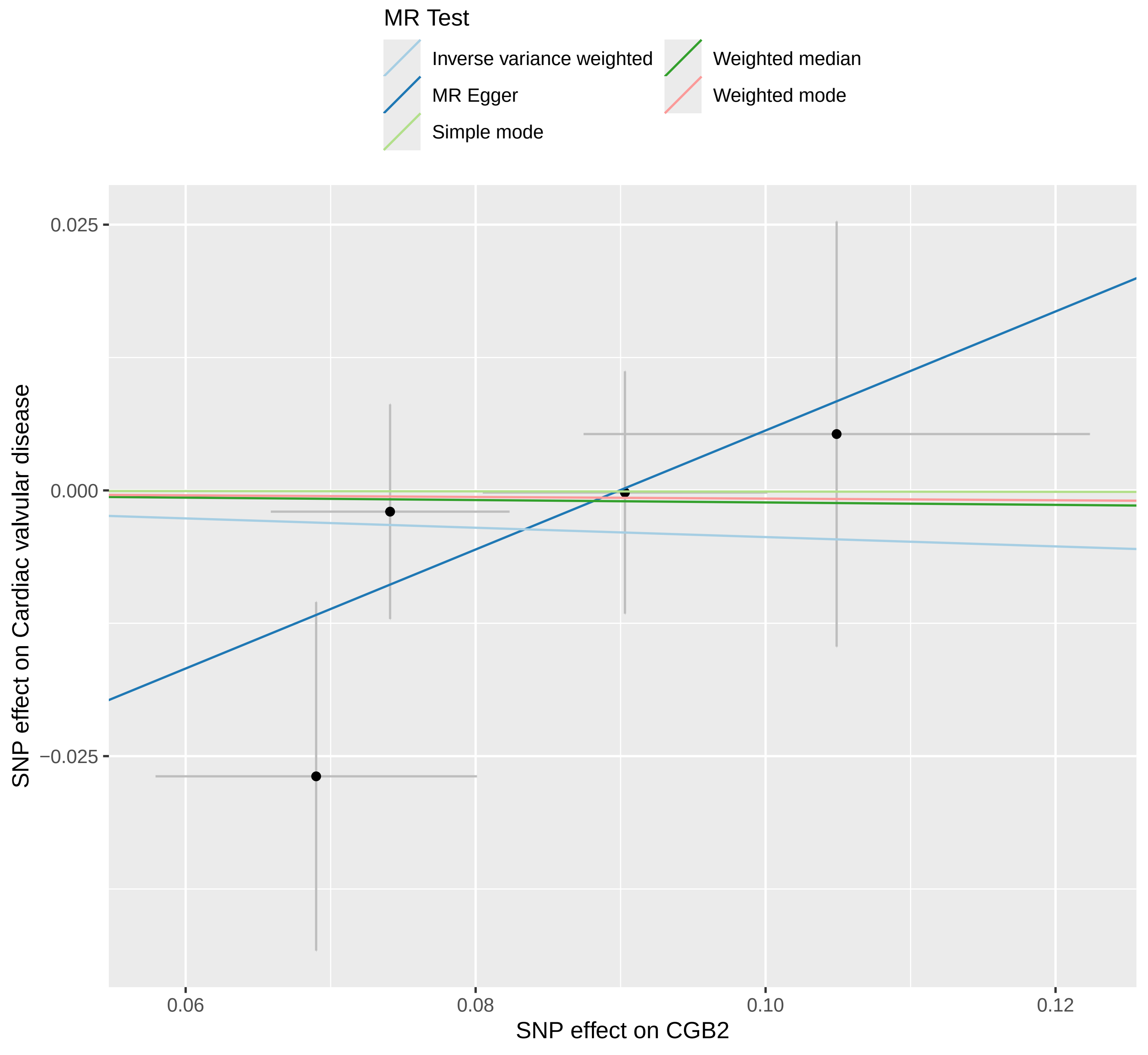

MR Test
Inverse variance weighted
MR Egger
Simple mode
Weighted median
Weighted mode
0.025
0.000
−0.025
SNP effect on Cardiac valvular disease
0.06
0.08
0.10
0.12
SNP effect on CGB2

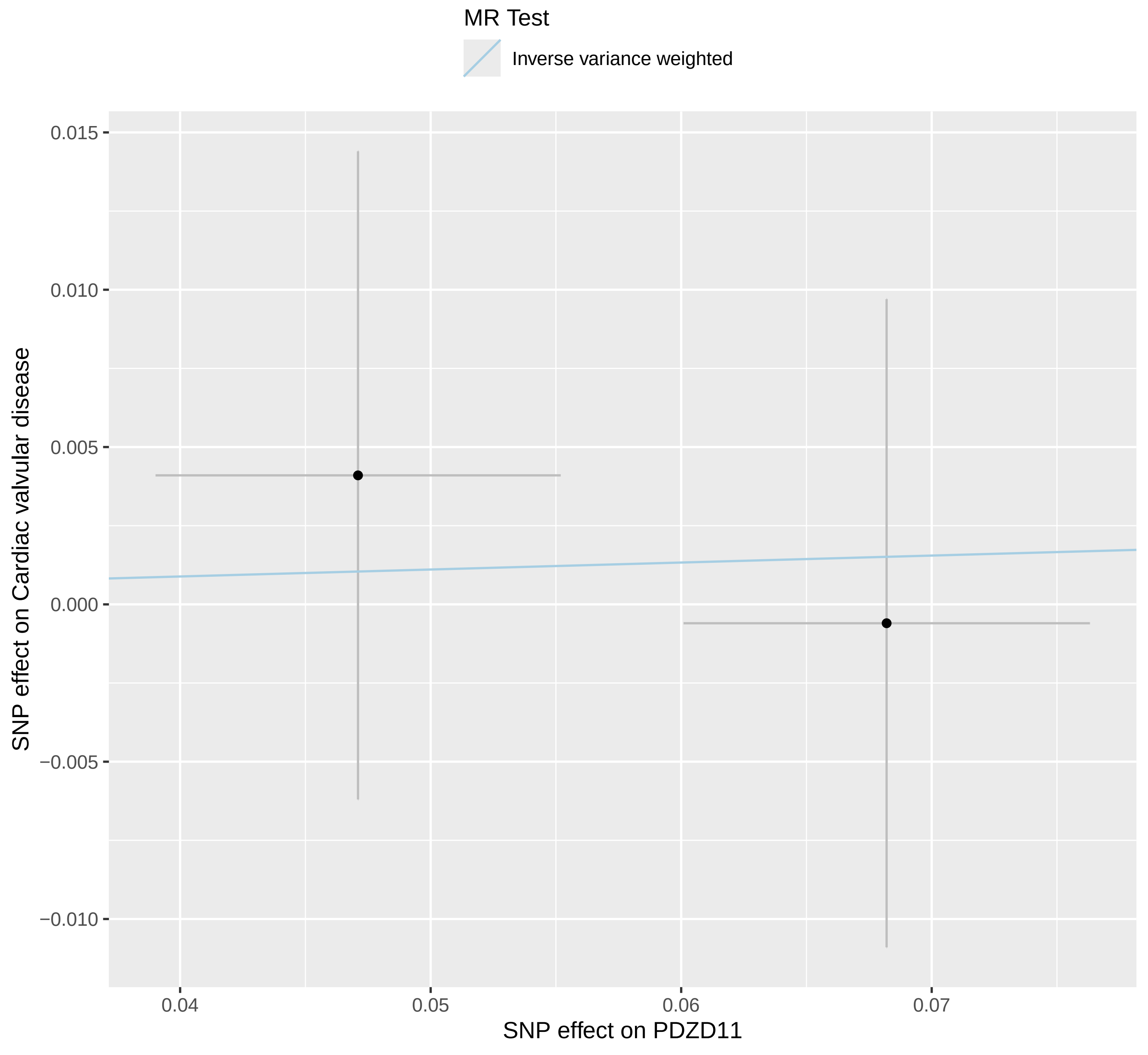
MR Test
Inverse variance weighted
0.015
0.010
0.005
0.000
−0.005
−0.010
SNP effect on Cardiac valvular disease
0.04
0.05
0.06
0.07
SNP effect on PDZD11

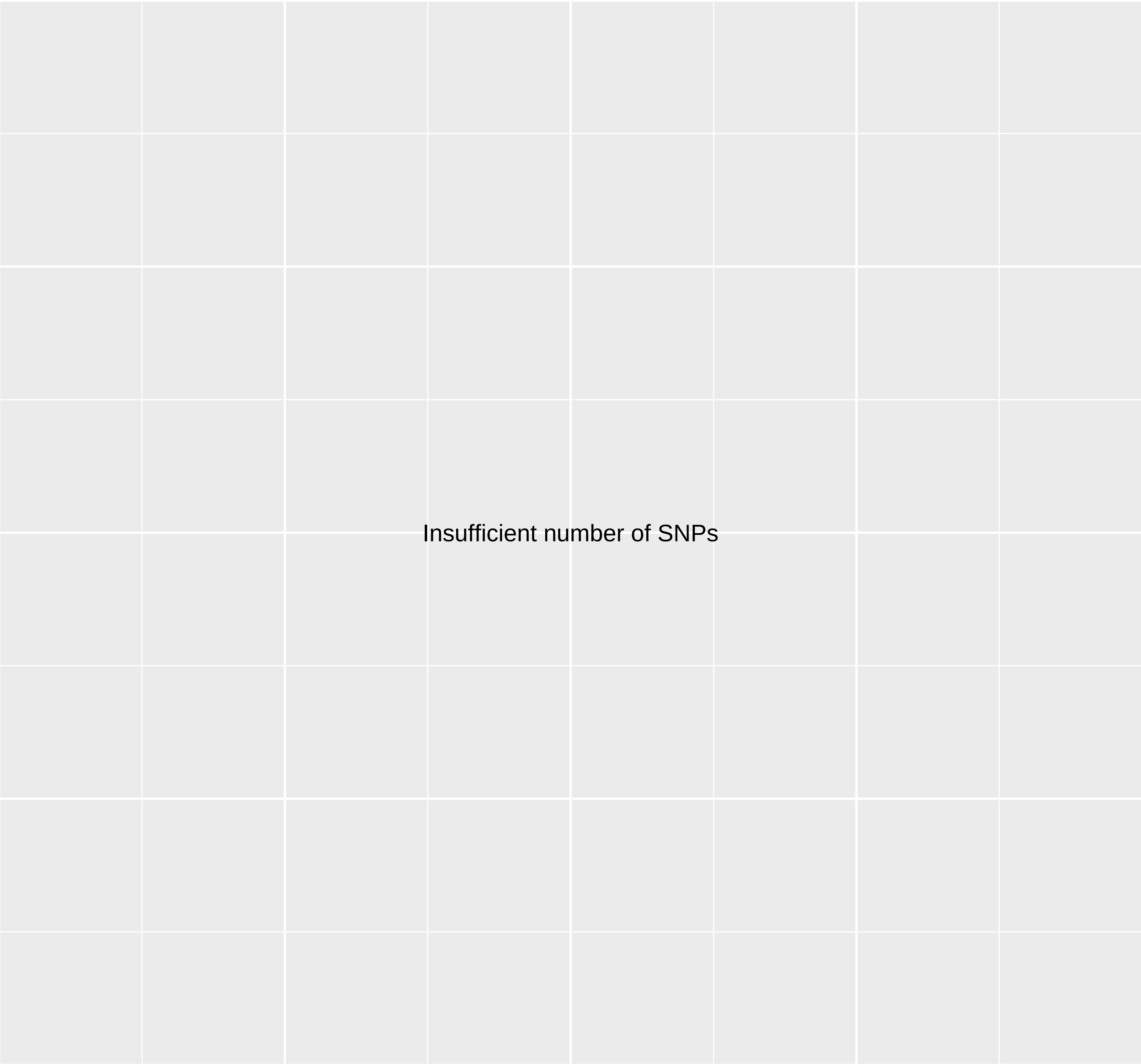
Insufficient number of SNPs

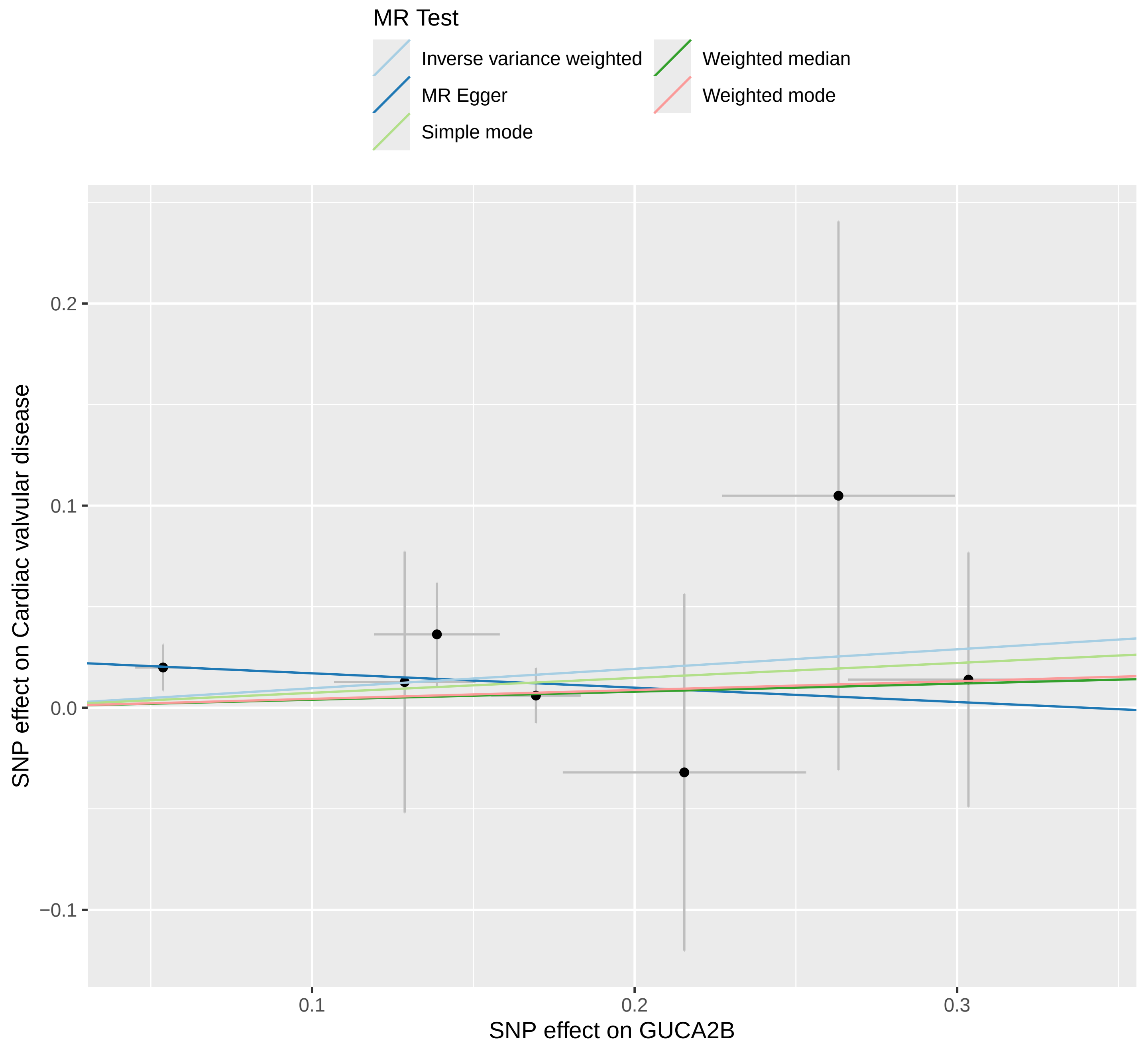
MR Test
Inverse variance weighted
MR Egger
Simple mode
Weighted median
Weighted mode
0.2
0.1
0.0
−0.1
0.1
0.2
0.3
SNP effect on Cardiac valvular disease
SNP effect on GUCA2B

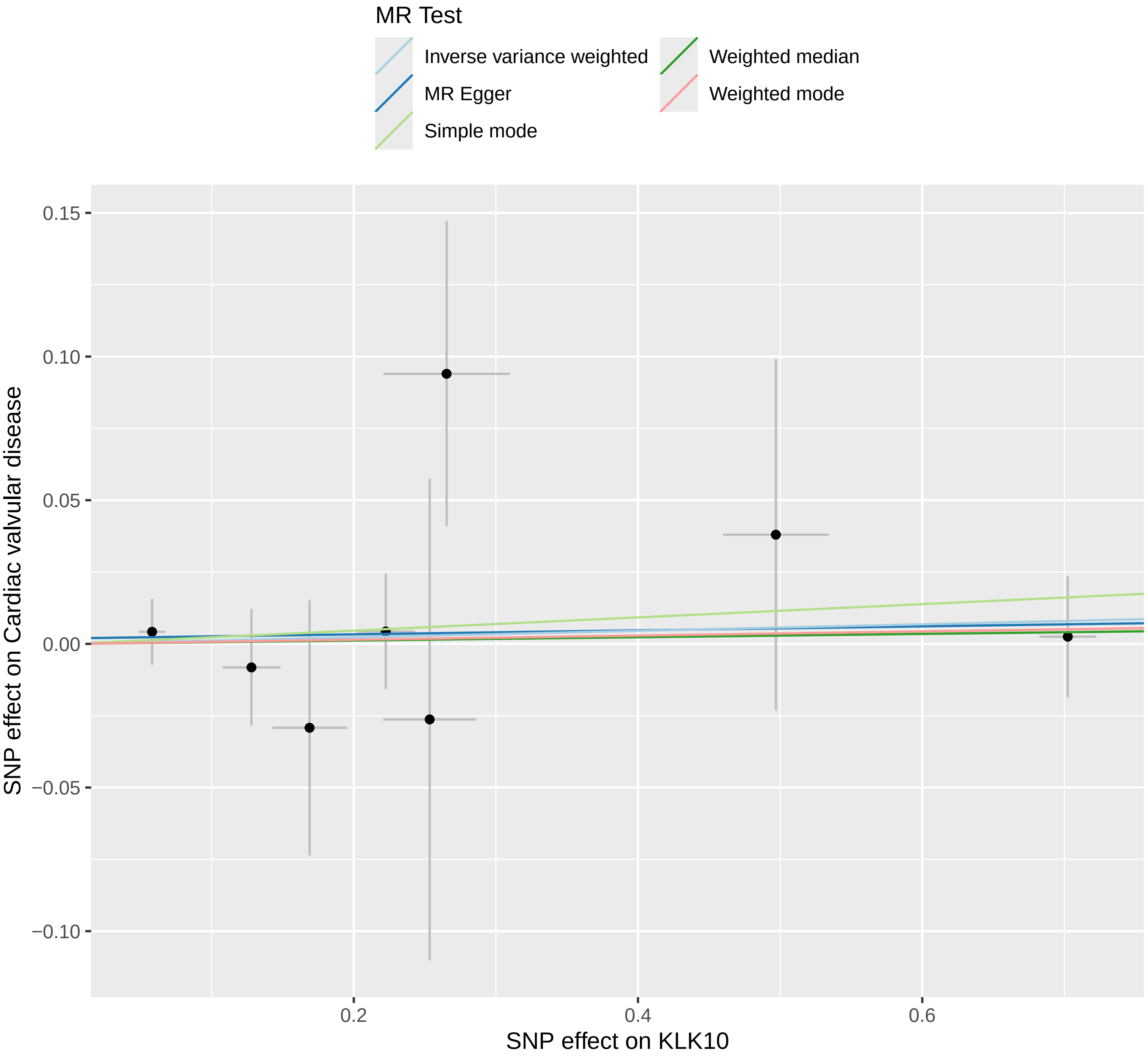
MR Test
Inverse variance weighted
MR Egger
Simple mode
Weighted median
Weighted mode
0.15
0.10
0.05
0.00
−0.05
−0.10
SNP effect on Cardiac valvular disease
0.2
0.4
0.6
SNP effect on KLK10

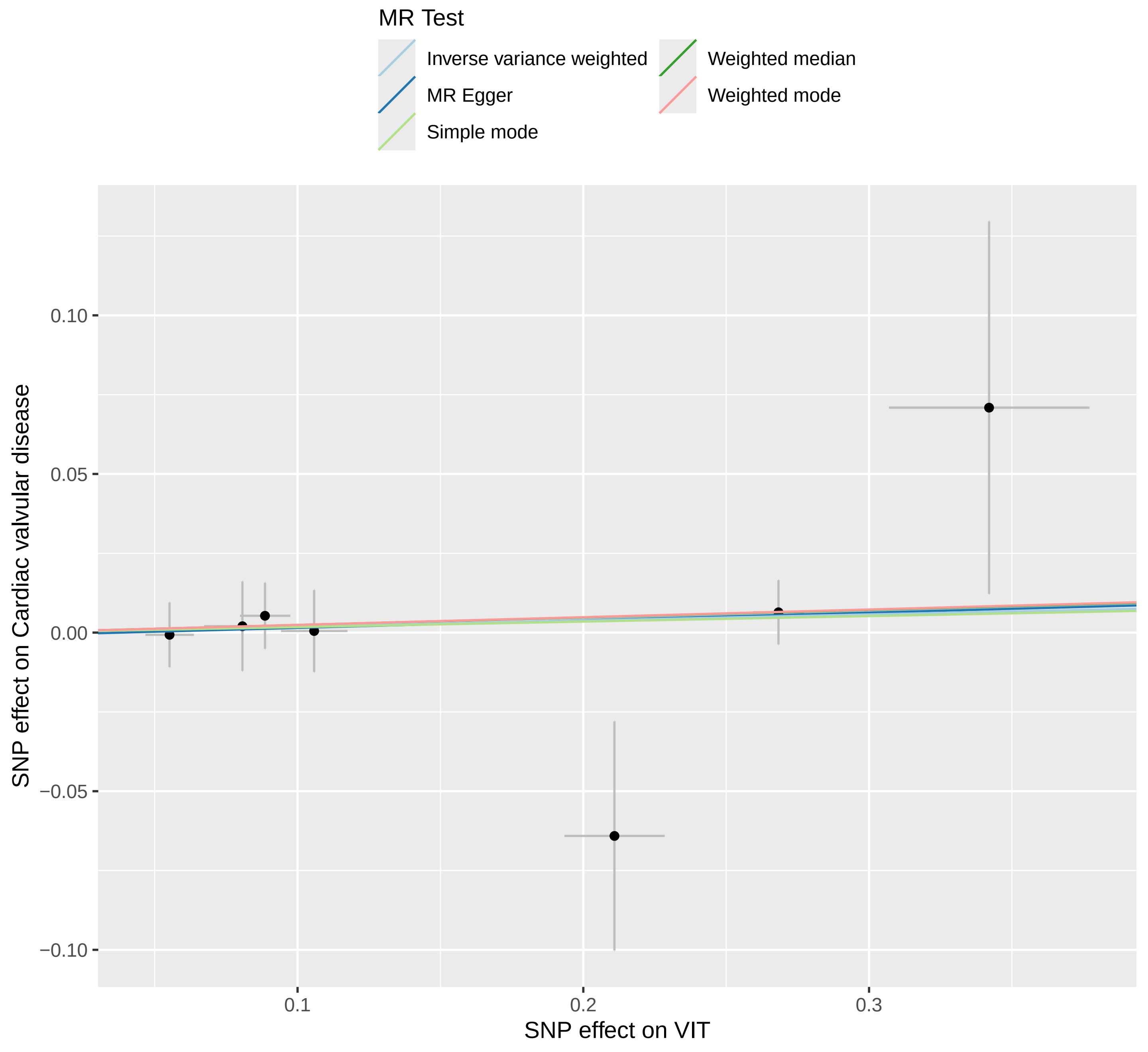
MR Test
Inverse variance weighted
MR Egger
Simple mode
Weighted median
Weighted mode
SNP effect on Cardiac valvular disease
0.10
0.05
0.00
−0.05
−0.10
0.1
0.2
0.3
SNP effect on VIT

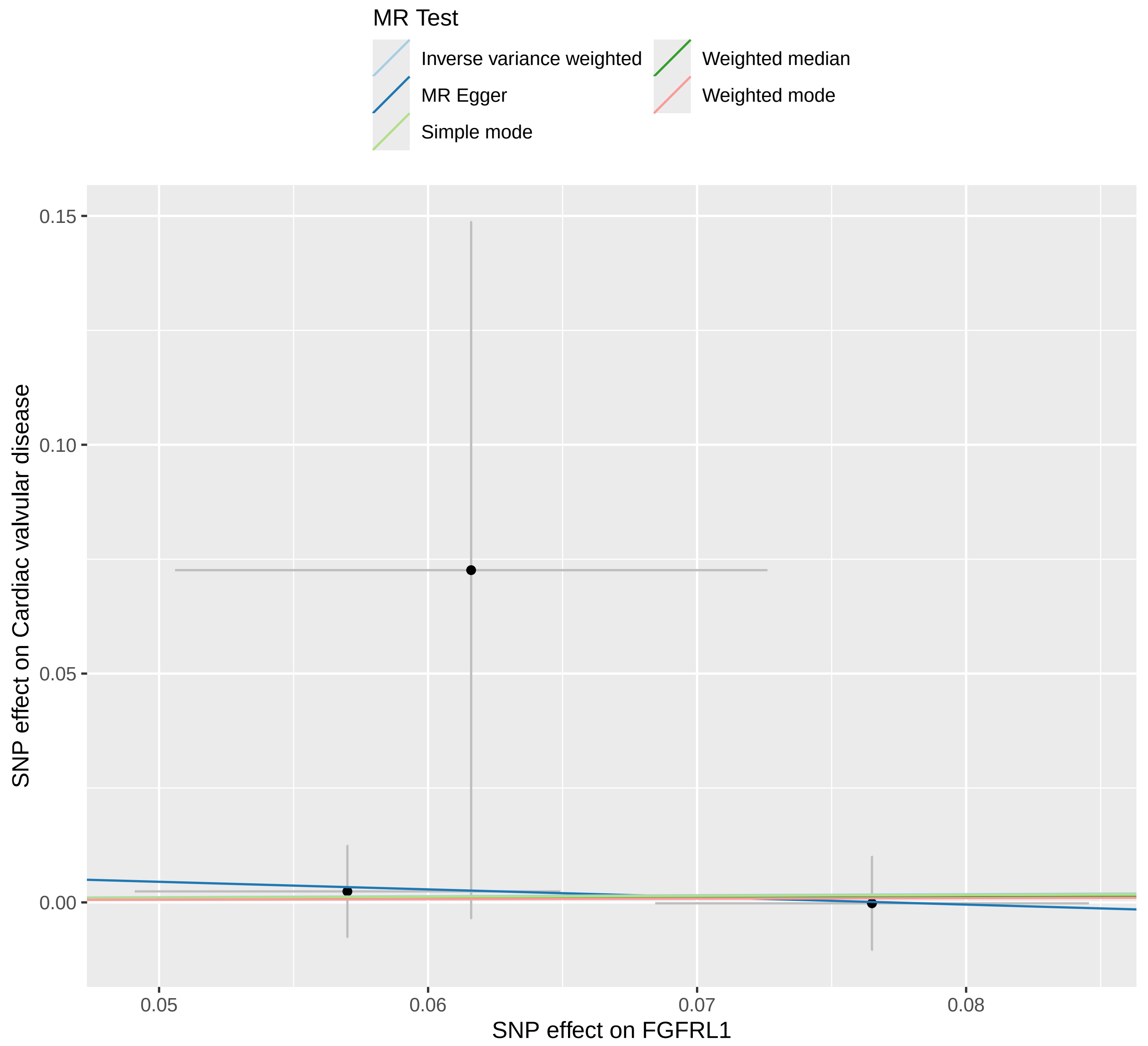

MR Test
Inverse variance weighted
MR Egger
Simple mode
Weighted median
Weighted mode
0.15
0.10
0.05
0.00
0.05
0.06
0.07
0.08
SNP effect on Cardiac valvular disease
SNP effect on FGFRL1

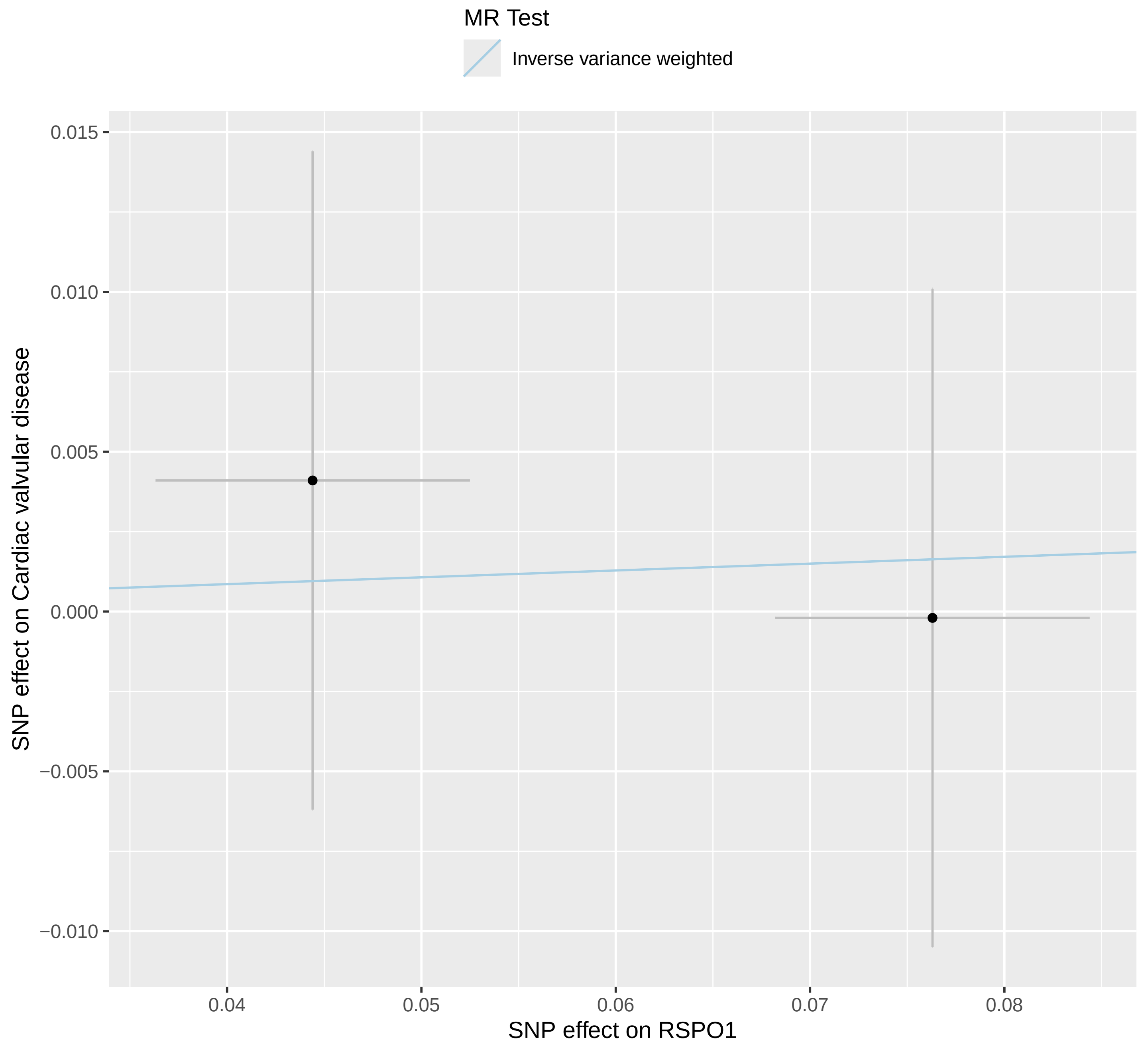
MR Test
Inverse variance weighted
SNP effect on Cardiac valvular disease
0.015
0.010
0.005
0.000
−0.005
−0.010
0.04
0.05
0.06
0.07
0.08
SNP effect on RSPO1

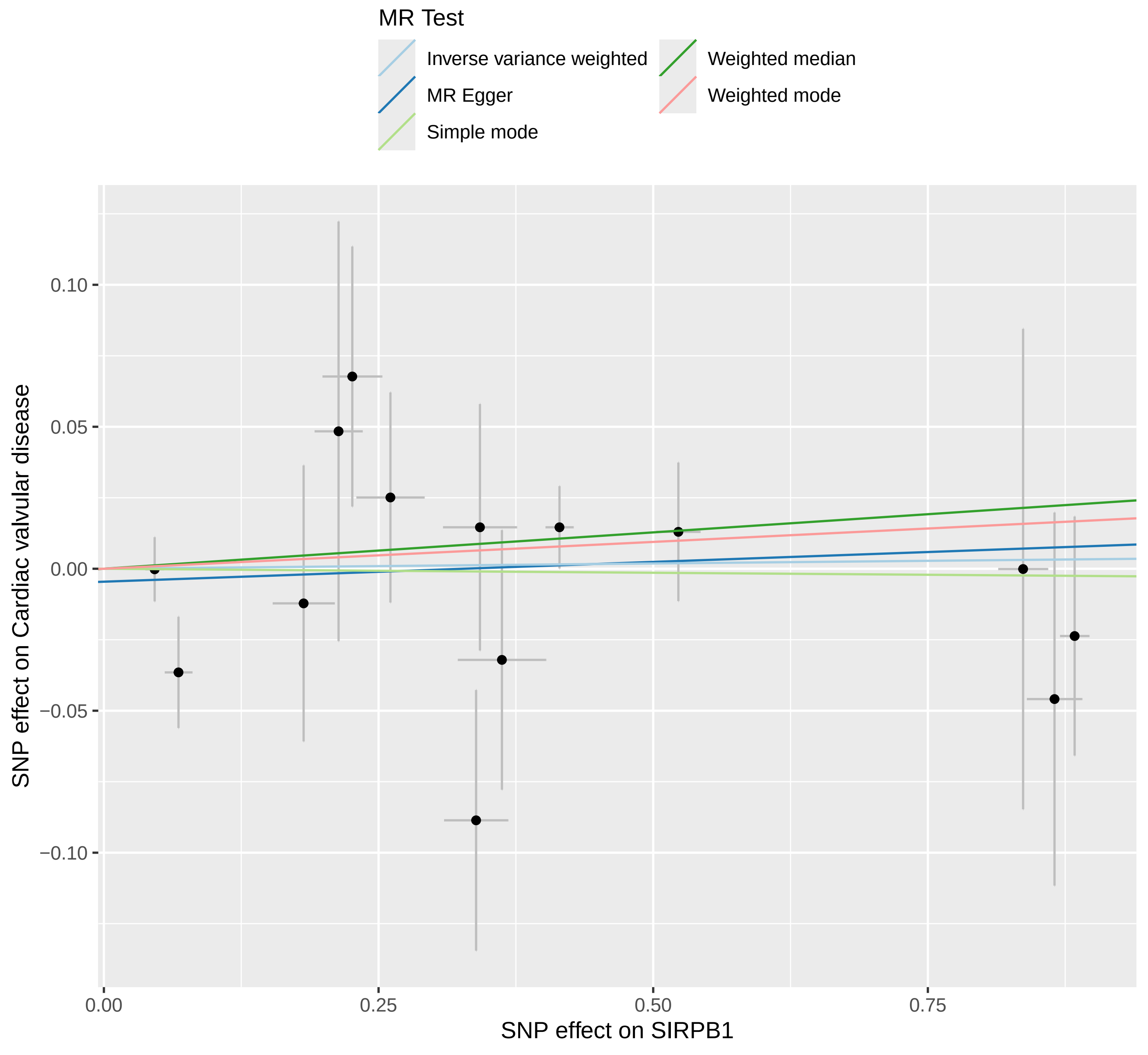
MR Test
Inverse variance weighted
MR Egger
Simple mode
Weighted median
Weighted mode
SNP effect on Cardiac valvular disease
0.10
0.05
0.00
−0.05
−0.10
0.00
0.25
0.50
0.75
SNP effect on SIRPB1

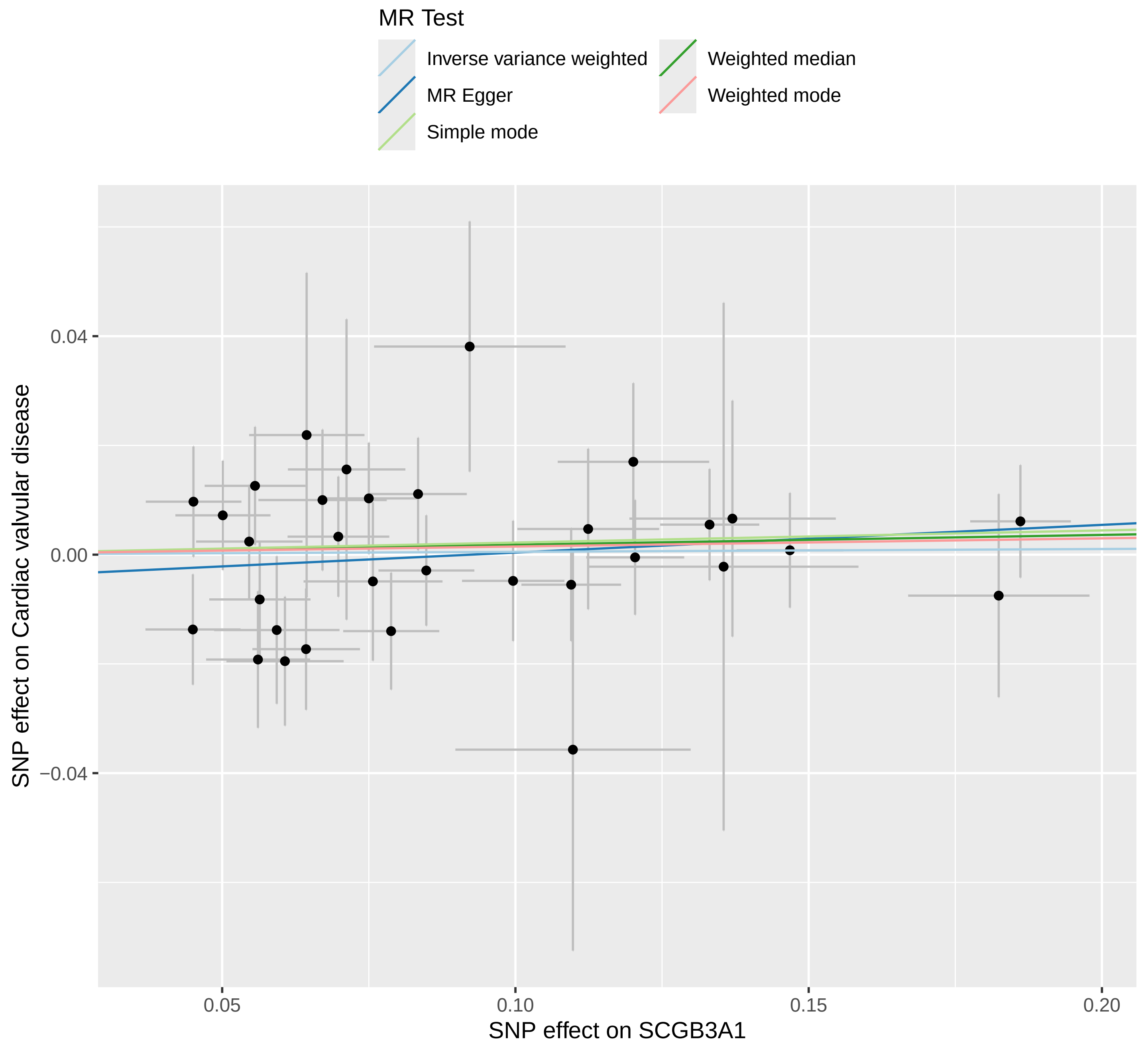

MR Test
Inverse variance weighted
MR Egger
Simple mode
Weighted median
Weighted mode
0.04
0.00
−0.04
0.05
0.10
0.15
0.20
SNP effect on SCGB3A1
SNP effect on Cardiac valvular disease

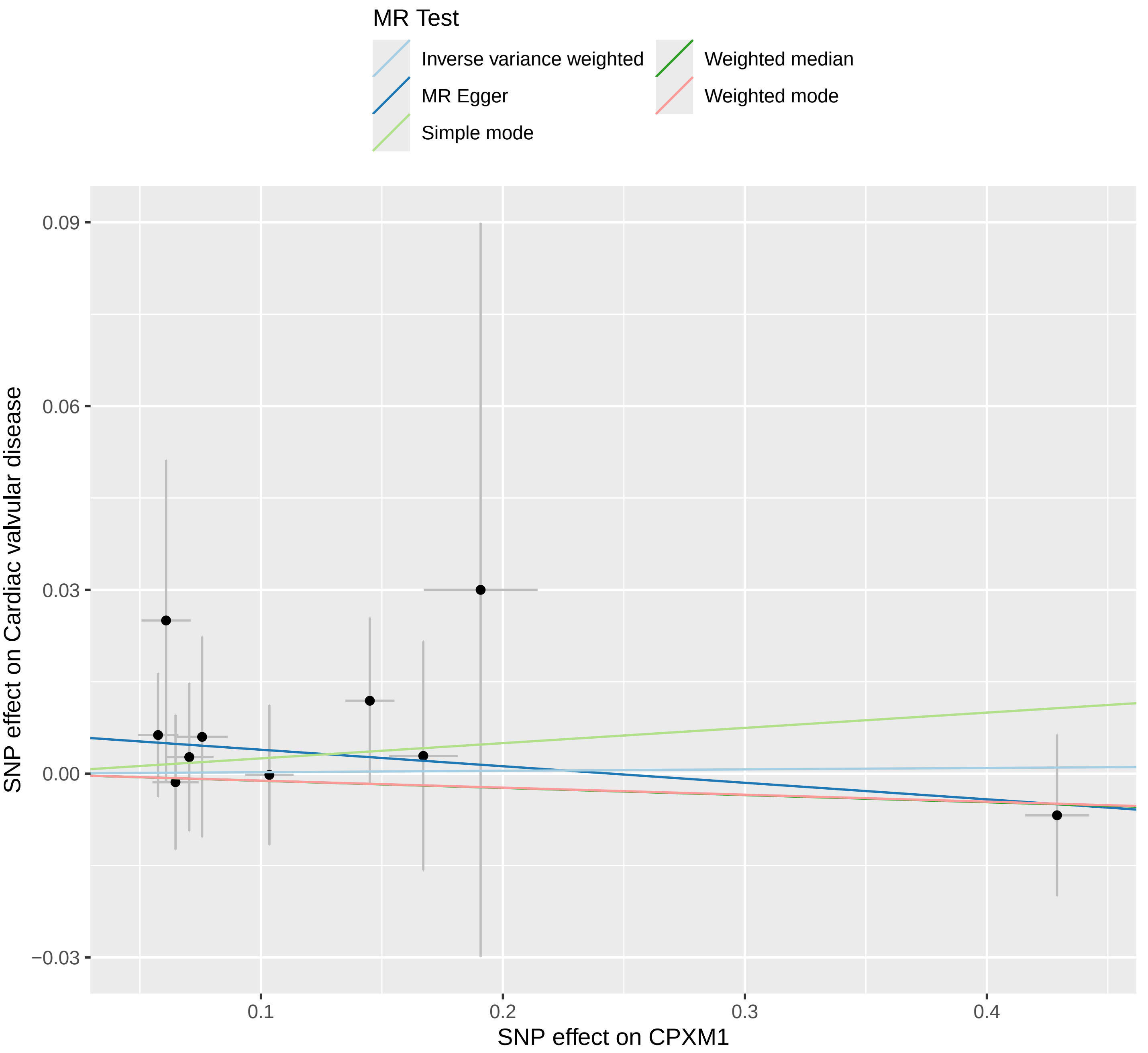
MR Test
Inverse variance weighted
MR Egger
Simple mode
Weighted median
Weighted mode
0.09
0.06
0.03
0.00
−0.03
0.1
0.2
0.3
0.4
SNP effect on Cardiac valvular disease
SNP effect on CPXM1

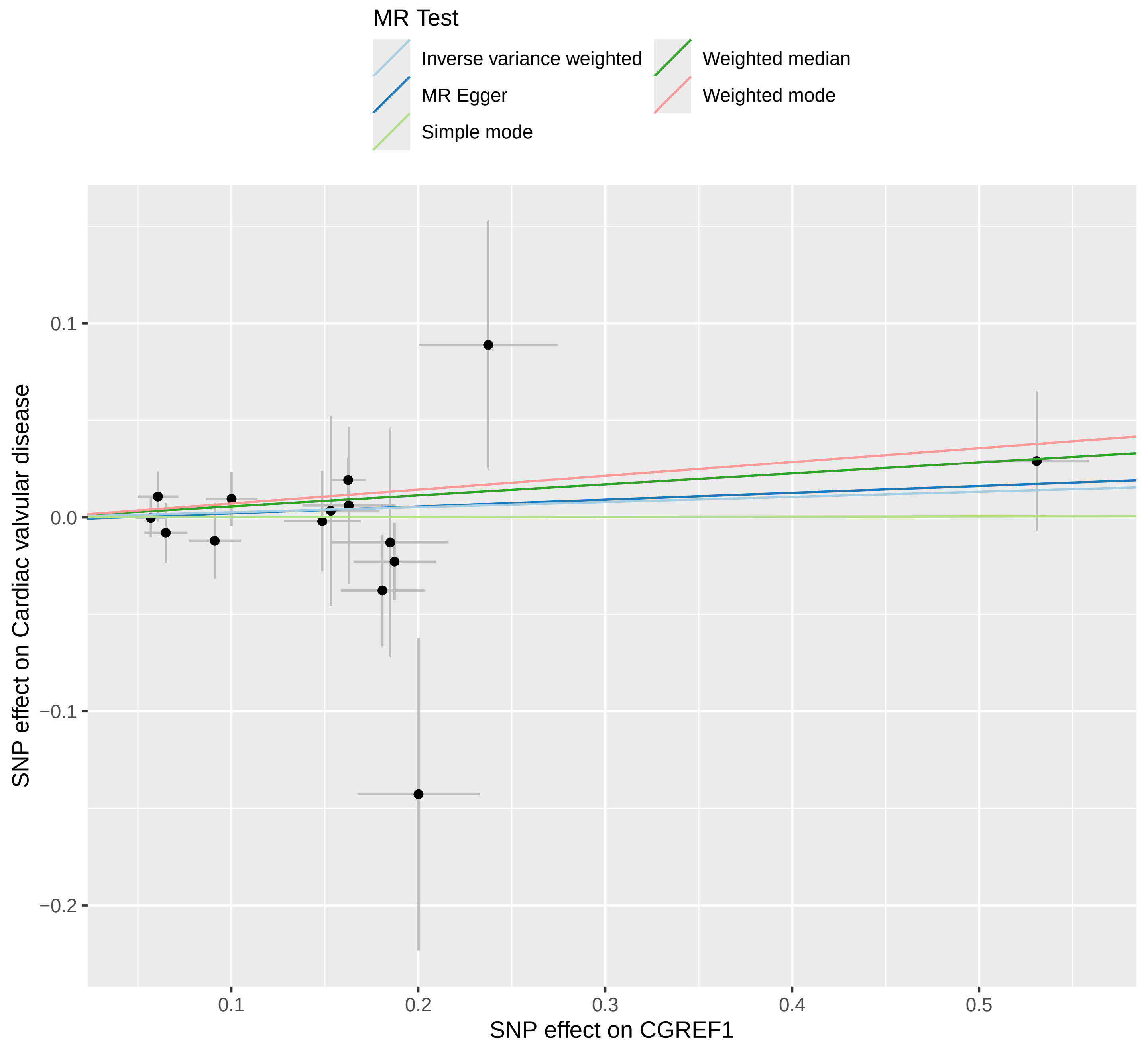
MR Test
Inverse variance weighted
MR Egger
Simple mode
Weighted median
Weighted mode
SNP effect on Cardiac valvular disease
0.1
0.0
−0.1
−0.2
0.1
0.2
0.3
0.4
0.5
SNP effect on CGREF1

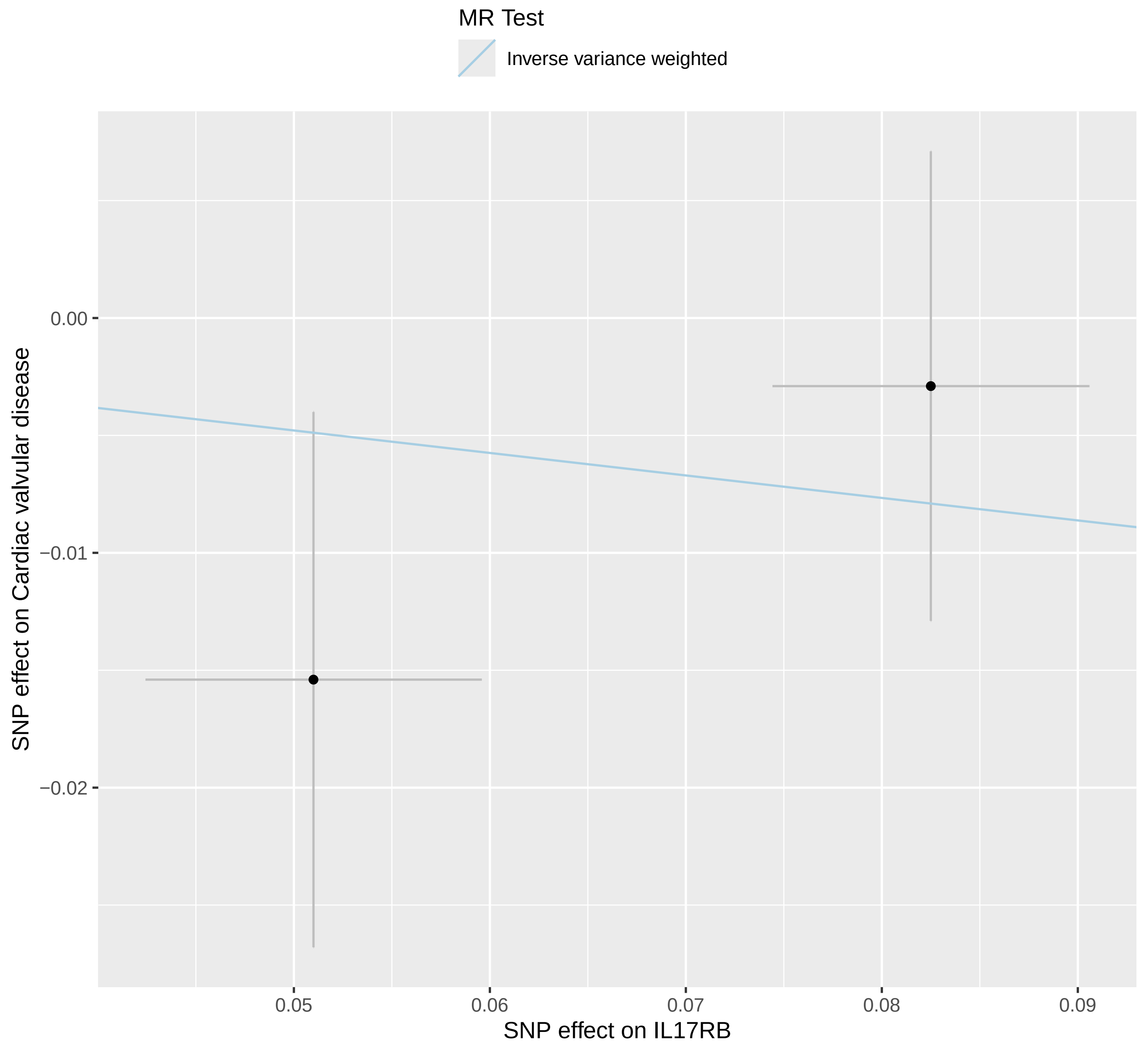
MR Test
Inverse variance weighted
0.00
−0.01
−0.02
0.05
0.06
0.07
0.08
0.09
SNP effect on IL17RB
SNP effect on Cardiac valvular disease

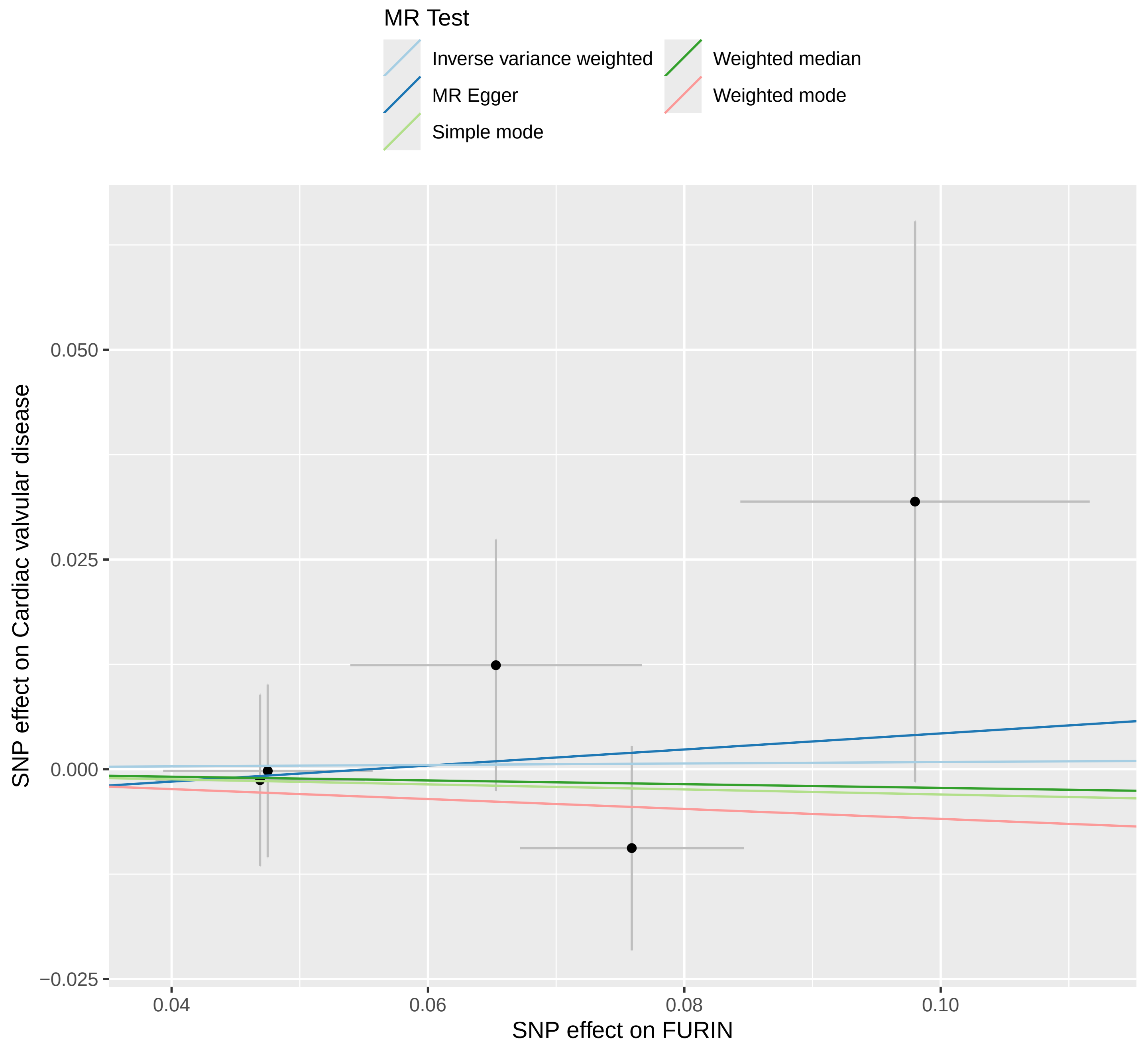

MR Test
Inverse variance weighted
MR Egger
Simple mode
Weighted median
Weighted mode
0.050
0.025
0.000
−0.025
0.04
0.06
0.08
0.10
SNP effect on Cardiac valvular disease
SNP effect on FURIN

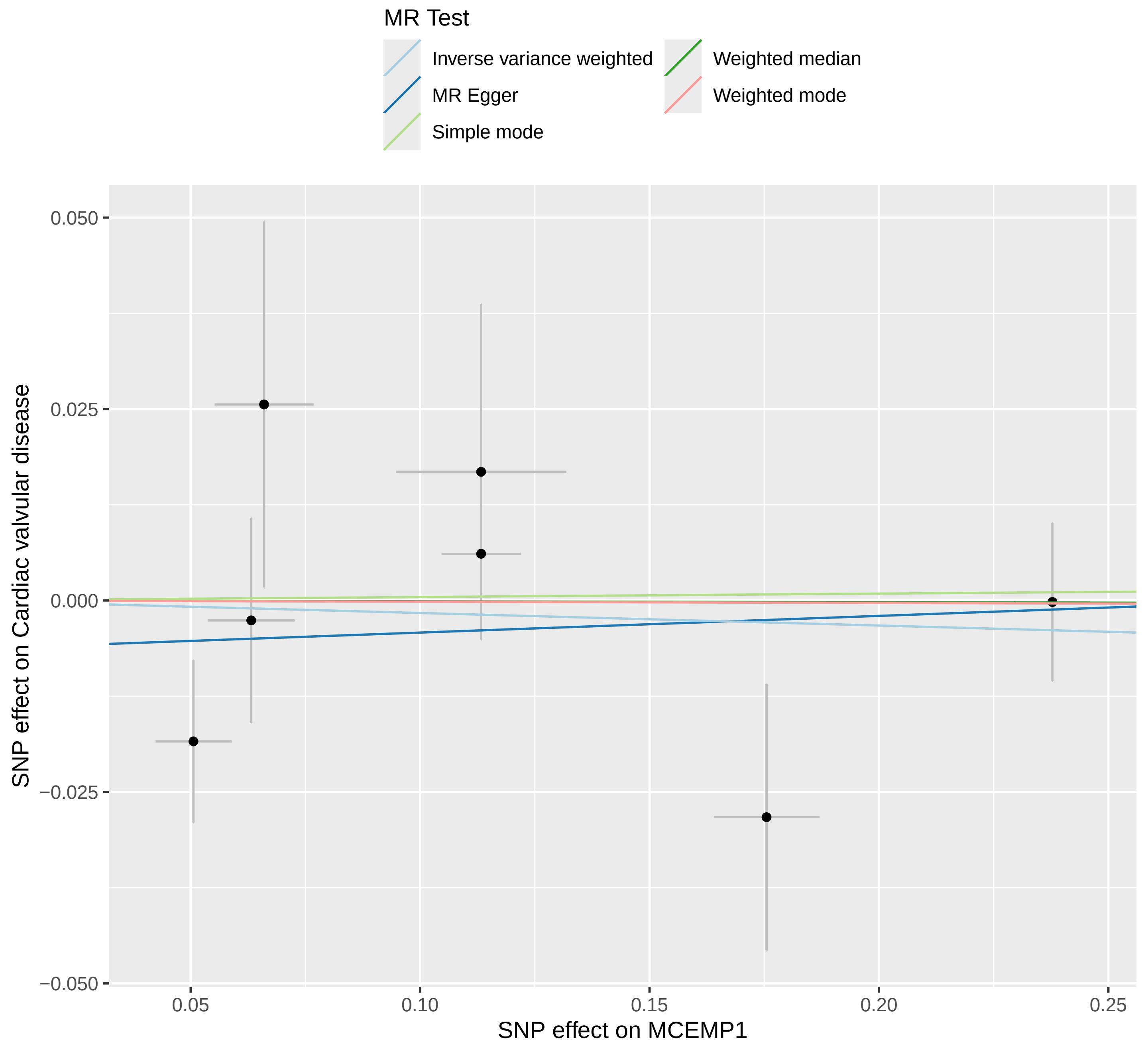

MR Test
Inverse variance weighted
MR Egger
Simple mode
Weighted median
Weighted mode
0.050
0.025
0.000
−0.025
−0.050
SNP effect on Cardiac valvular disease
0.05
0.10
0.15
0.20
0.25
SNP effect on MCEMP1

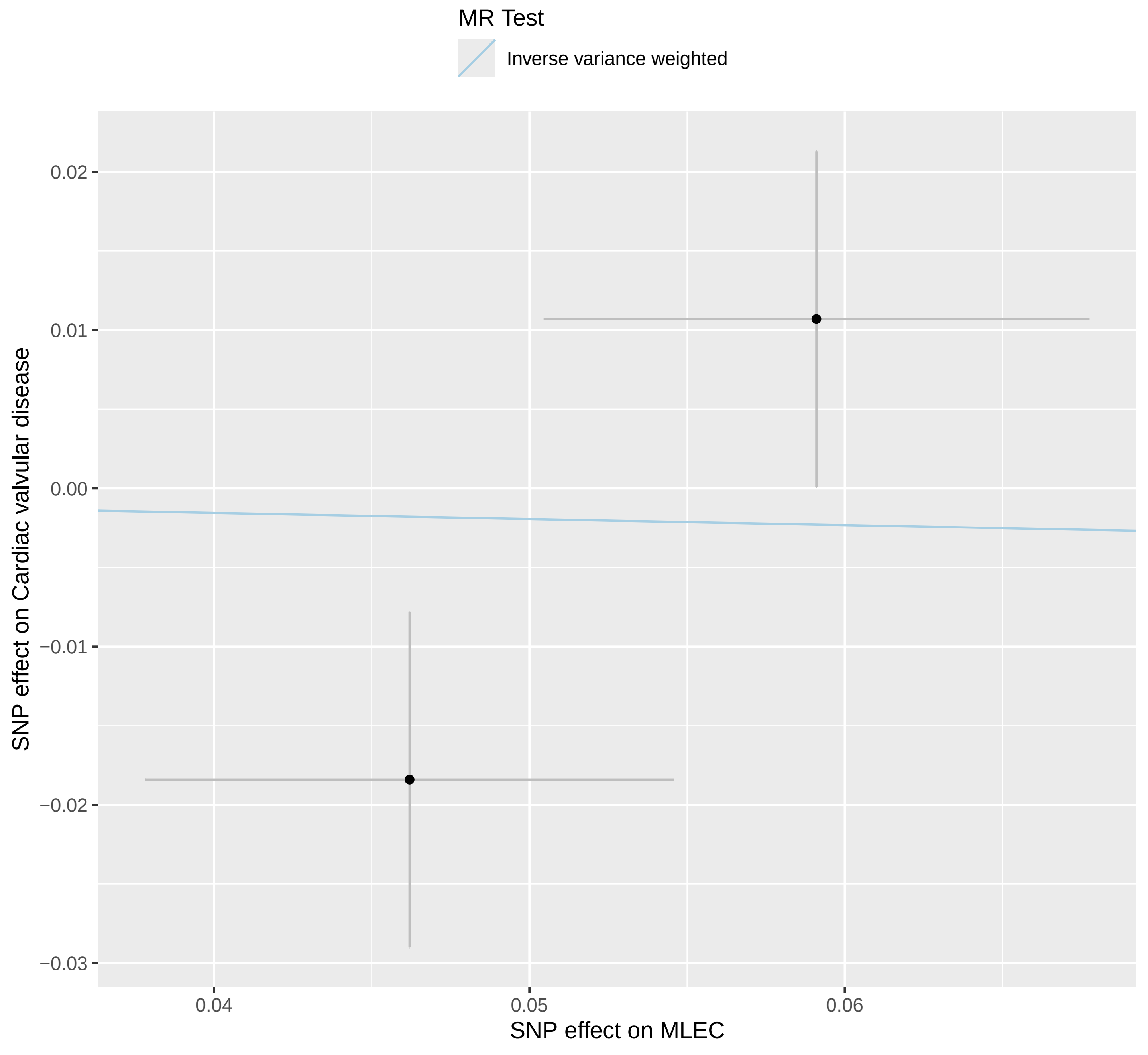
MR Test
Inverse variance weighted
0.02
0.01
0.00
−0.01
−0.02
−0.03
0.04
0.05
0.06
SNP effect on MLEC
SNP effect on Cardiac valvular disease

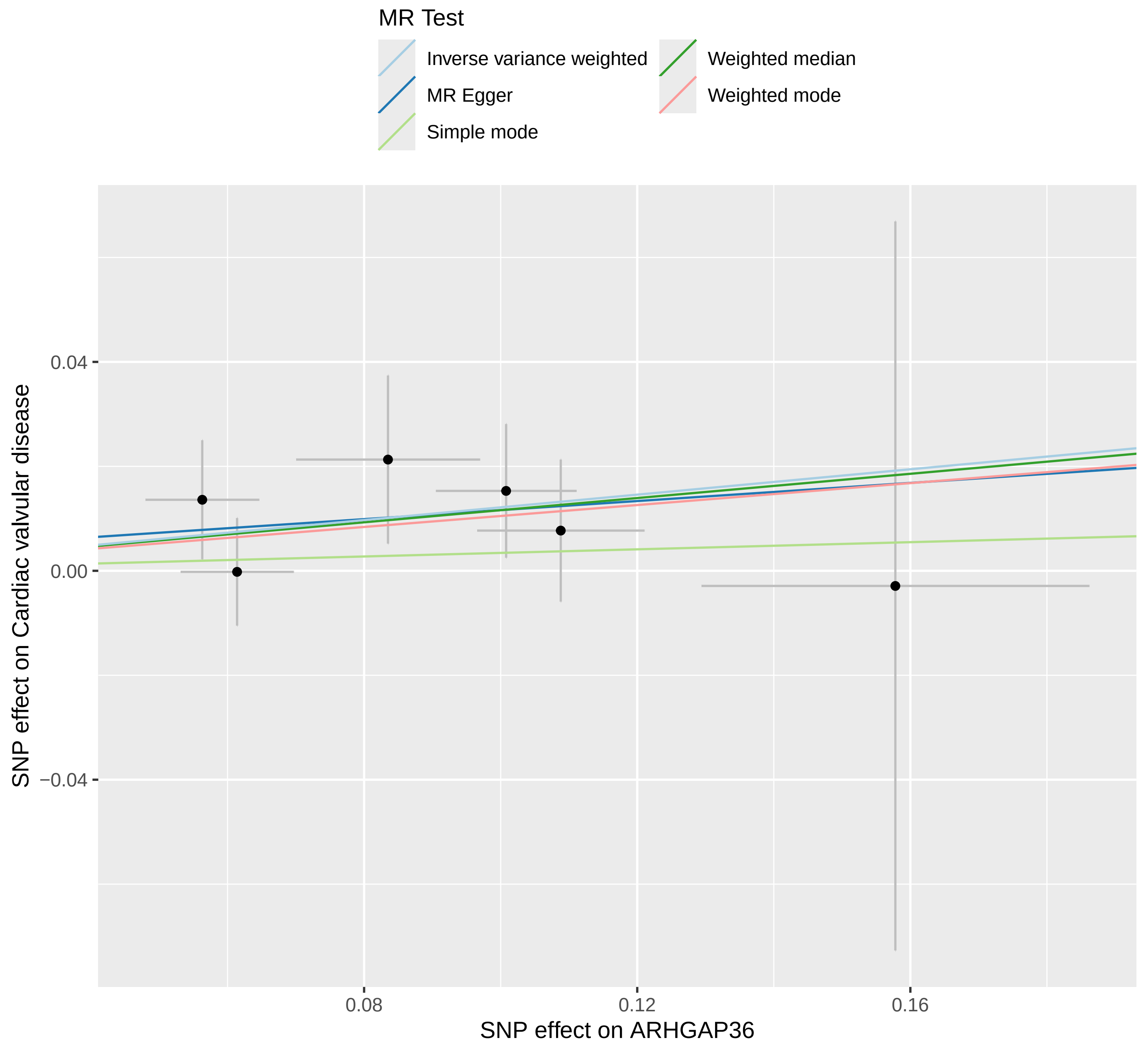
MR Test
Inverse variance weighted
MR Egger
Simple mode
Weighted median
Weighted mode
SNP effect on Cardiac valvular disease
0.04
0.00
−0.04
0.08
0.12
0.16
SNP effect on ARHGAP36

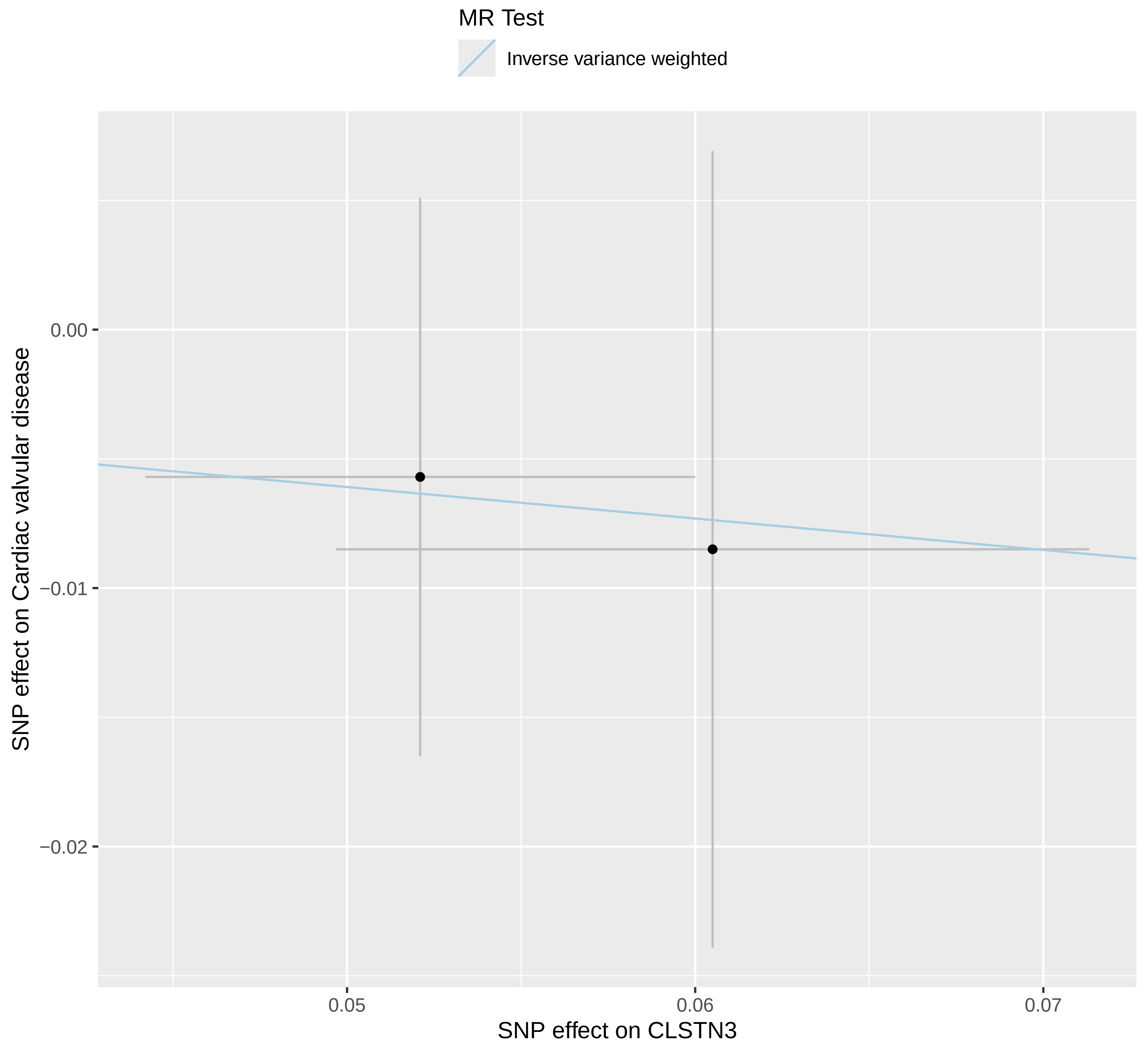
MR Test
Inverse variance weighted
0.00
−0.01
−0.02
0.05
0.06
0.07
SNP effect on Cardiac valvular disease
SNP effect on CLSTN3

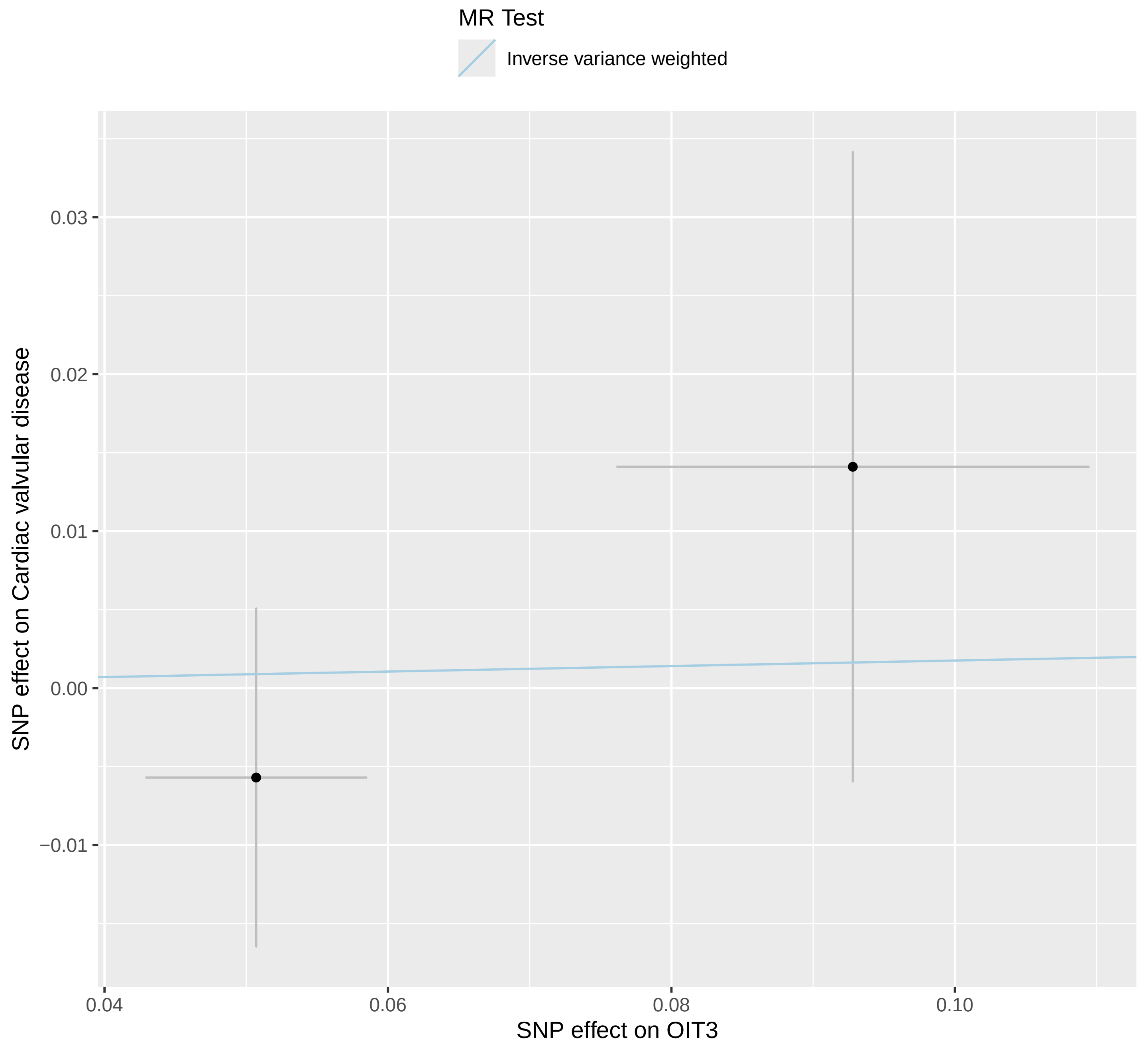
MR Test
Inverse variance weighted
0.03
0.02
0.01
0.00
−0.01
SNP effect on Cardiac valvular disease
0.04
0.06
0.08
0.10
SNP effect on OIT3

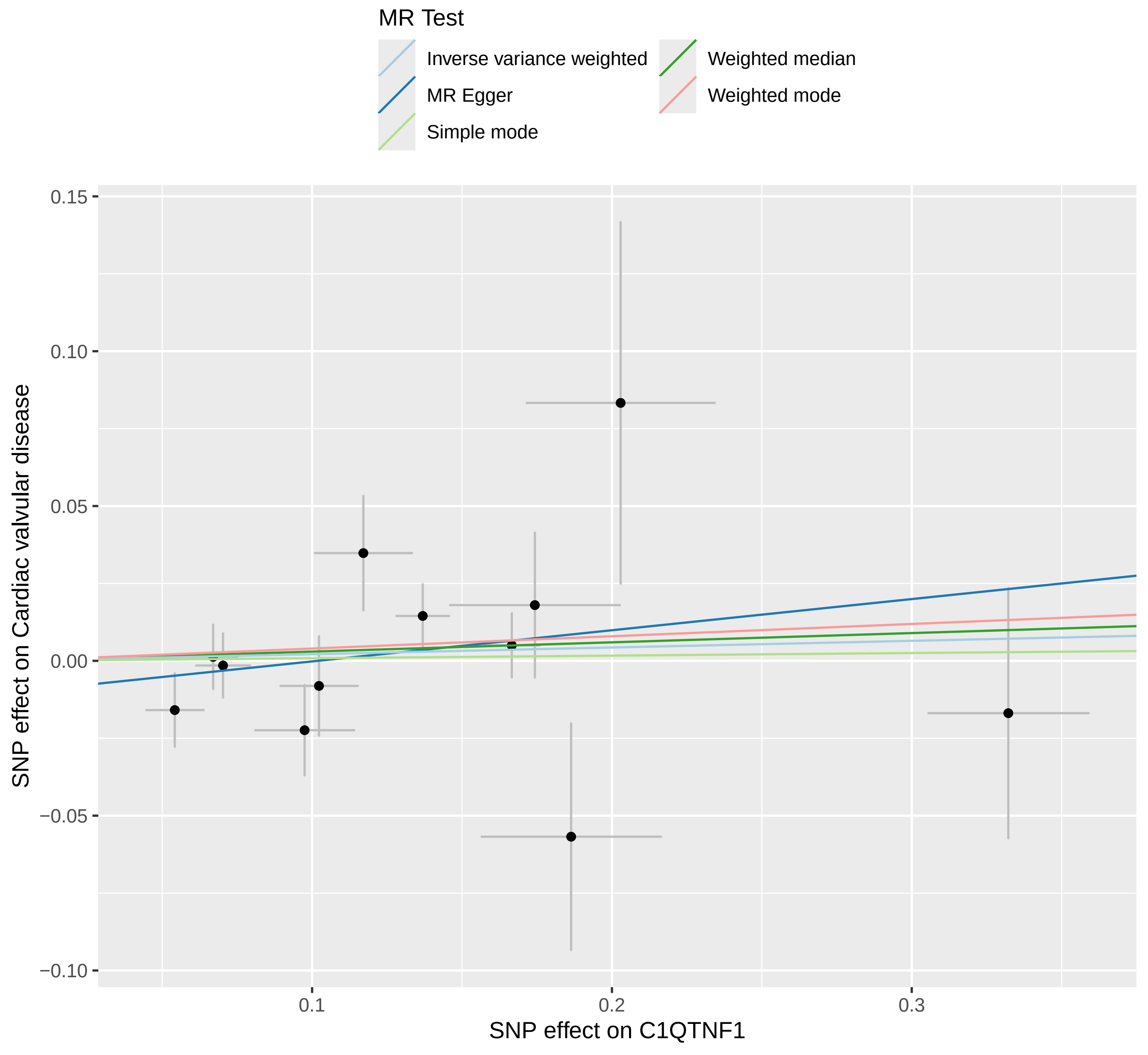
MR Test
Inverse variance weighted
MR Egger
Simple mode
Weighted median
Weighted mode
0.15
0.10
0.05
0.00
−0.05
−0.10
0.1
0.2
0.3
SNP effect on Cardiac valvular disease
SNP effect on C1QTNF1

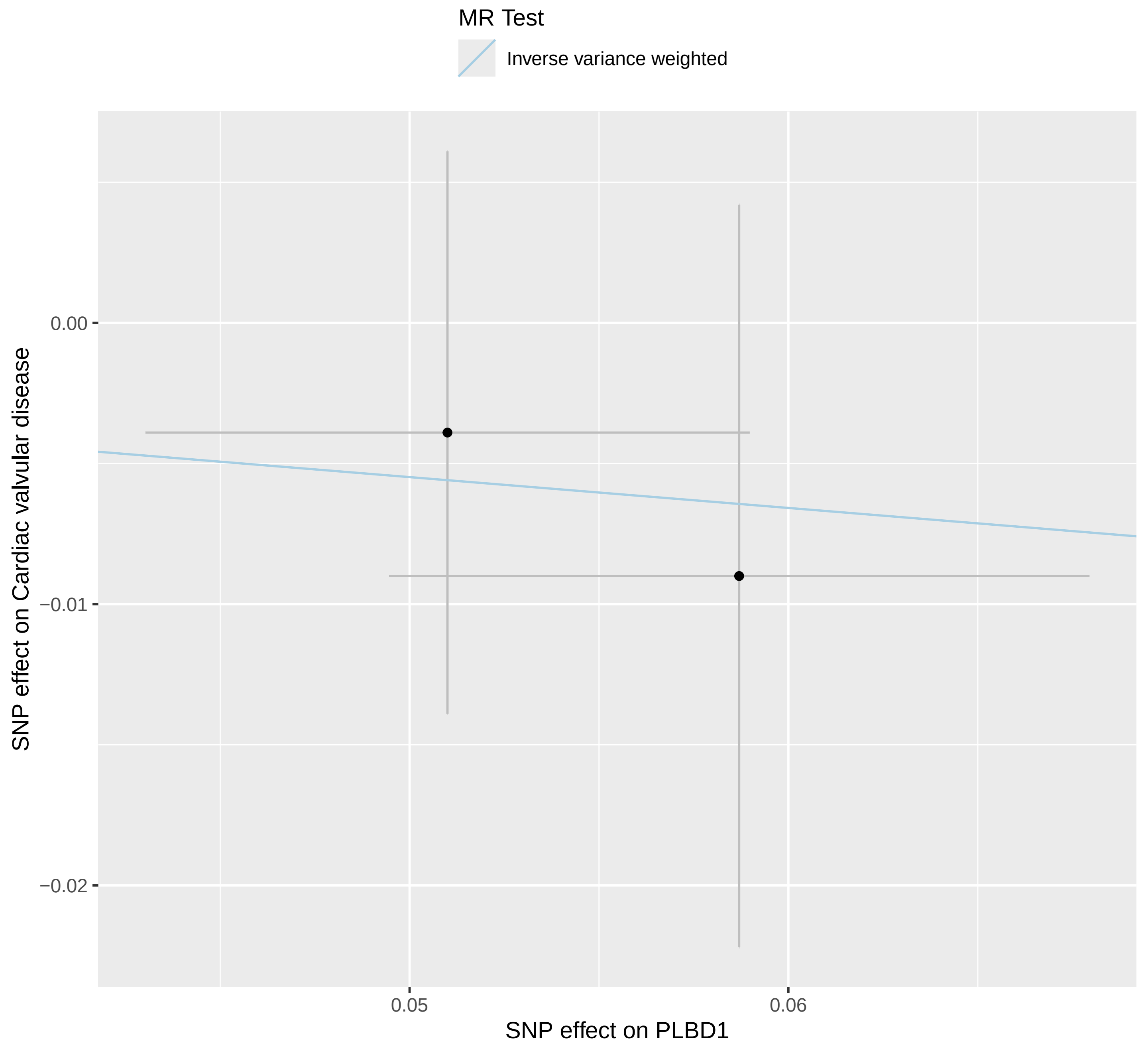
MR Test
Inverse variance weighted
0.00
−0.01
−0.02
0.05
0.06
SNP effect on PLBD1
SNP effect on Cardiac valvular disease

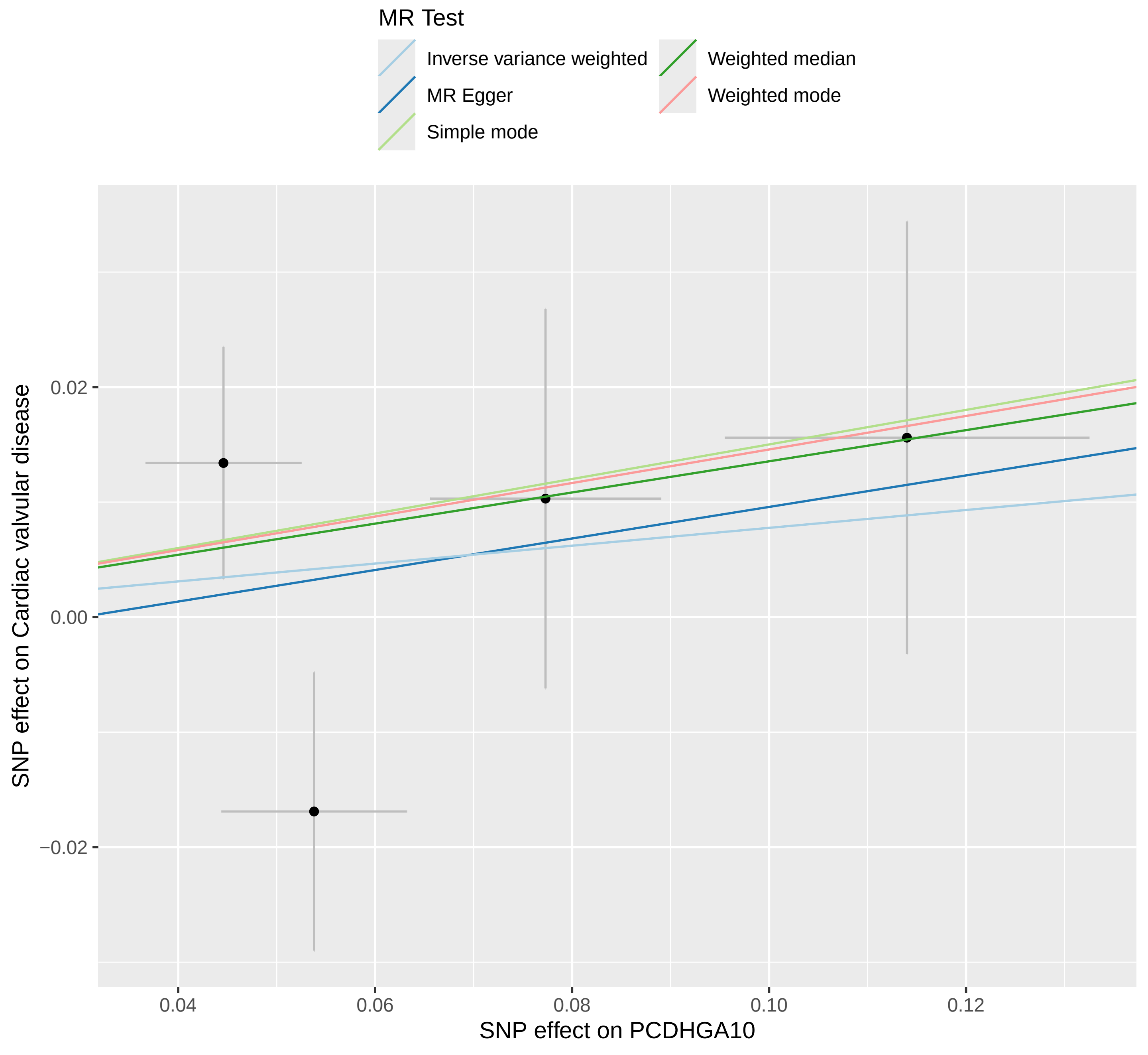
MR Test
Inverse variance weighted
MR Egger
Simple mode
Weighted median
Weighted mode
SNP effect on Cardiac valvular disease
0.02
0.00
−0.02
0.04
0.06
0.08
0.10
0.12
SNP effect on PCDHGA10

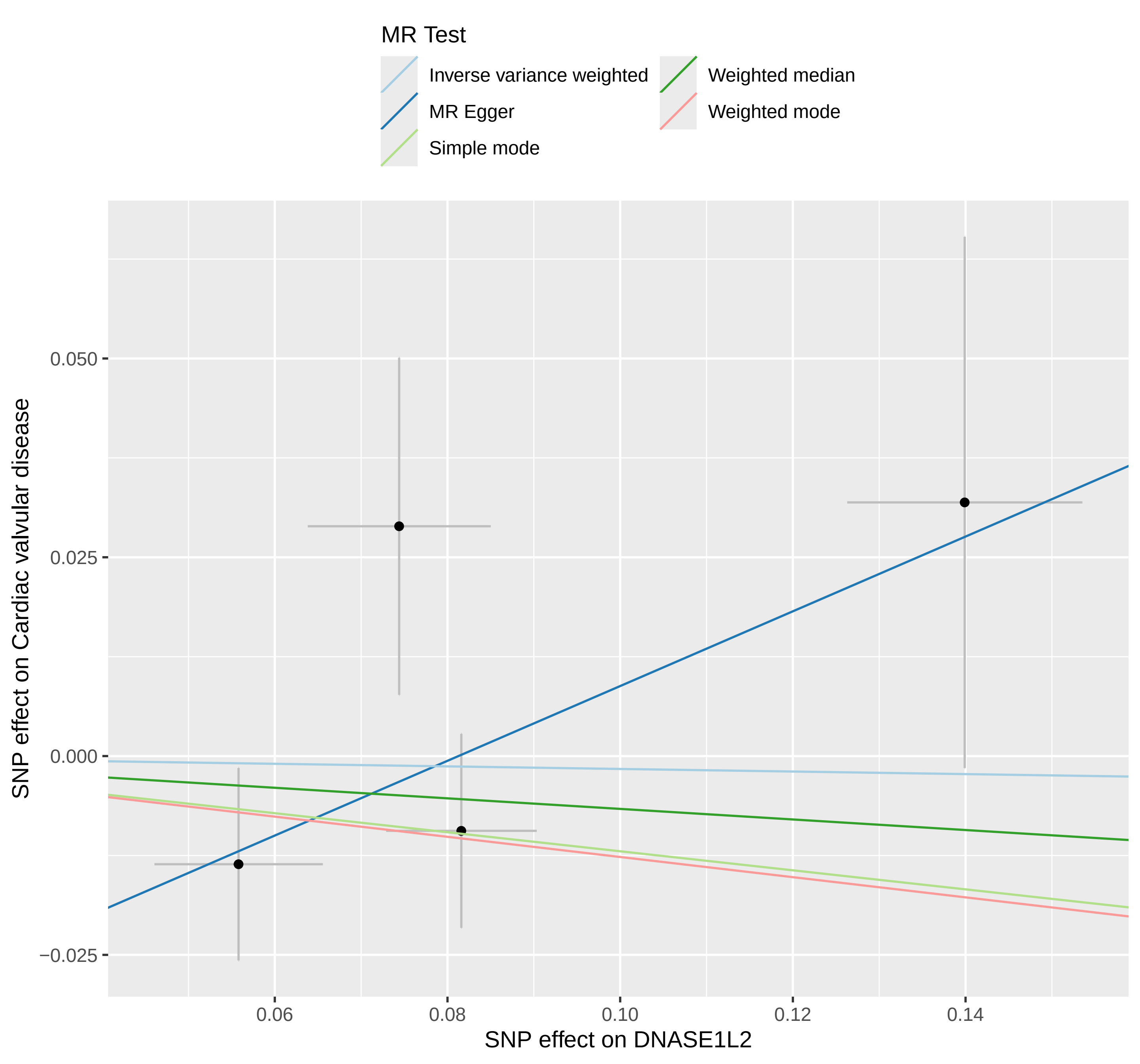
MR Test
Inverse variance weighted
MR Egger
Simple mode
Weighted median
Weighted mode
0.050
0.025
0.000
−0.025
0.06
0.08
0.10
0.12
0.14
SNP effect on Cardiac valvular disease
SNP effect on DNASE1L2

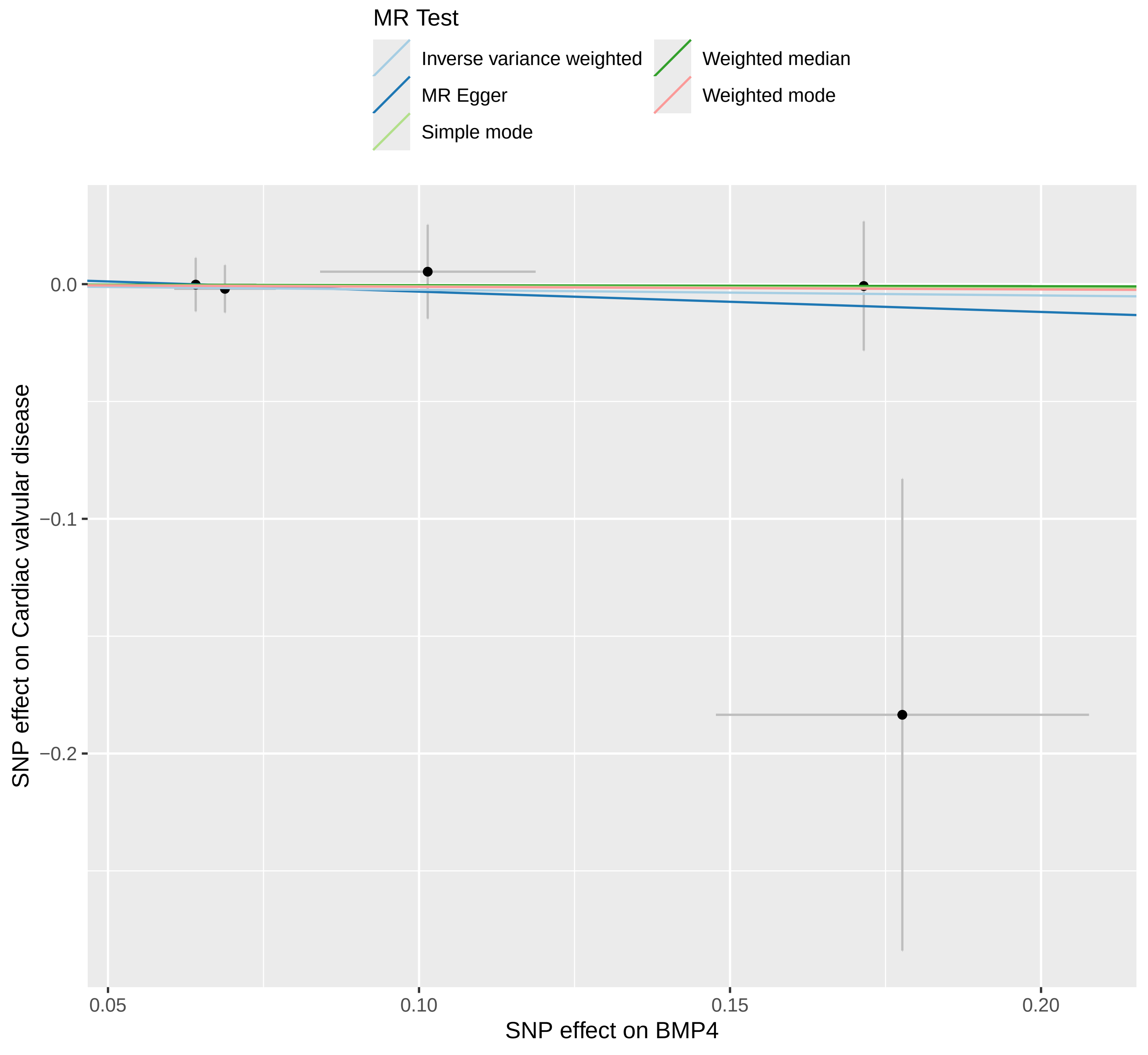

MR Test
Inverse variance weighted
MR Egger
Simple mode
Weighted median
Weighted mode
0.0
−0.1
−0.2
0.05
0.10
0.15
0.20
SNP effect on Cardiac valvular disease
SNP effect on BMP4

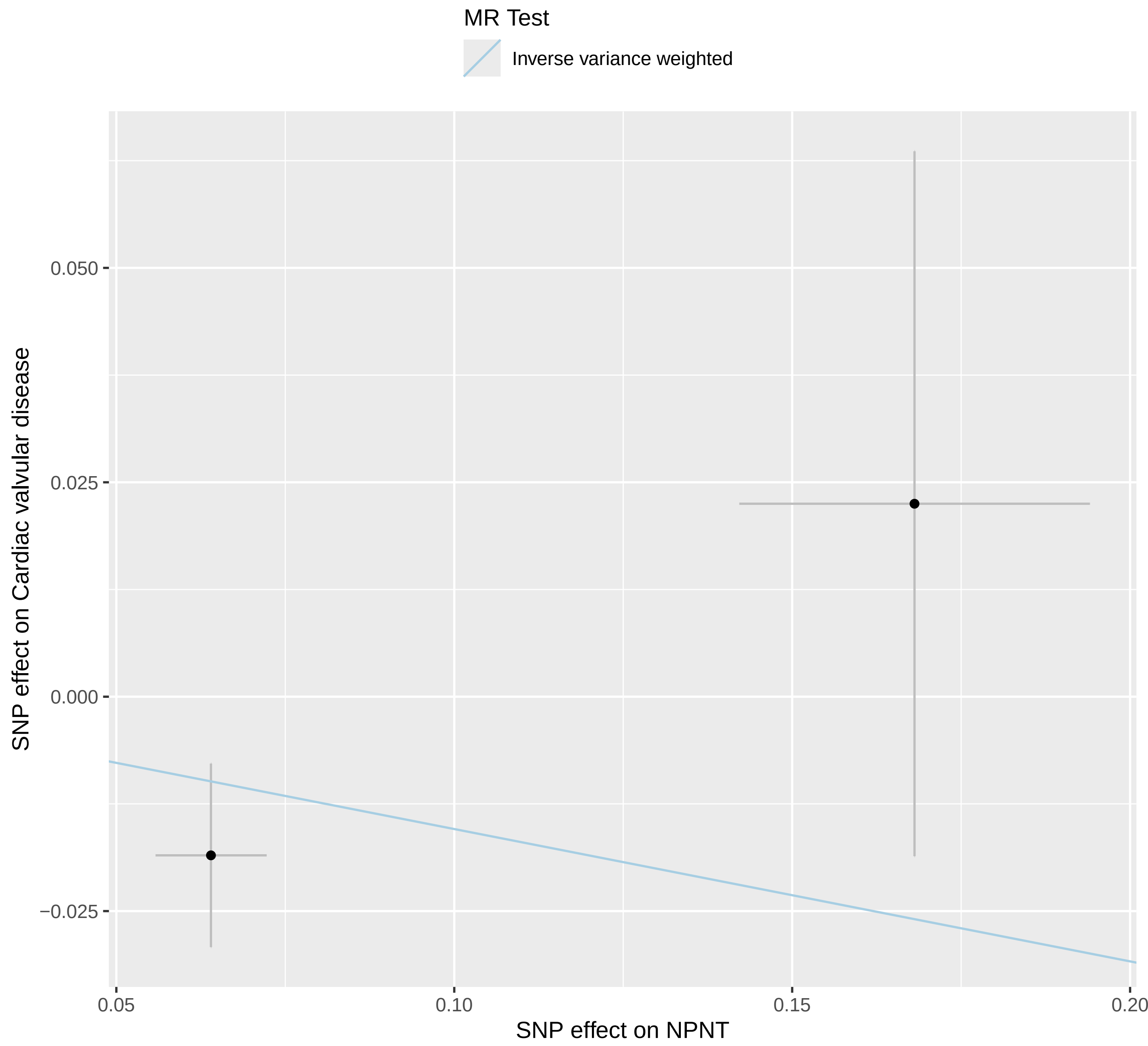

MR Test
Inverse variance weighted
0.050
0.025
0.000
−0.025
SNP effect on Cardiac valvular disease
0.05
0.10
0.15
0.20
SNP effect on NPNT

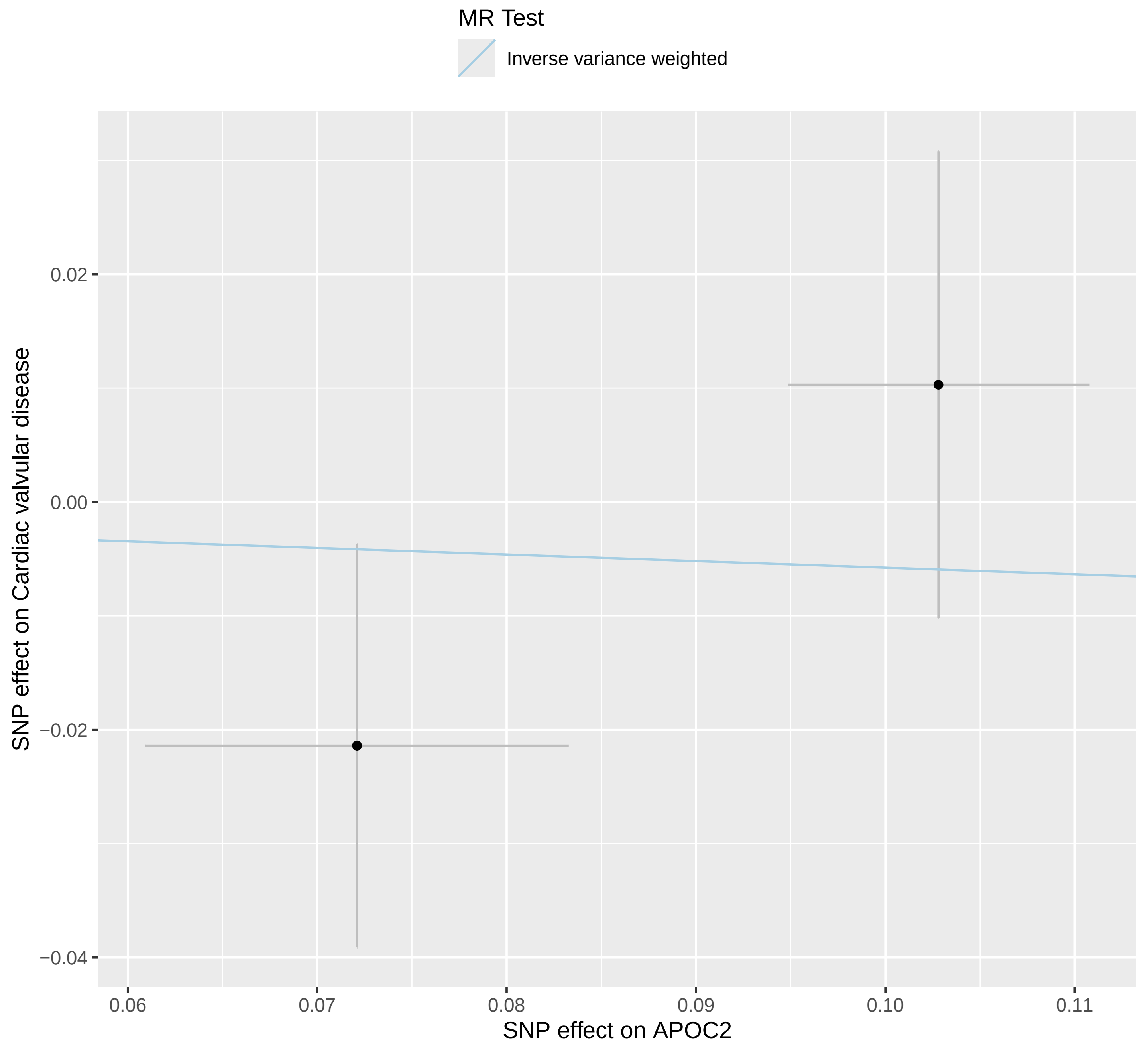

MR Test
Inverse variance weighted
SNP effect on Cardiac valvular disease
0.02
0.00
−0.02
−0.04
0.06
0.07
0.08
0.09
0.10
0.11
SNP effect on APOC2

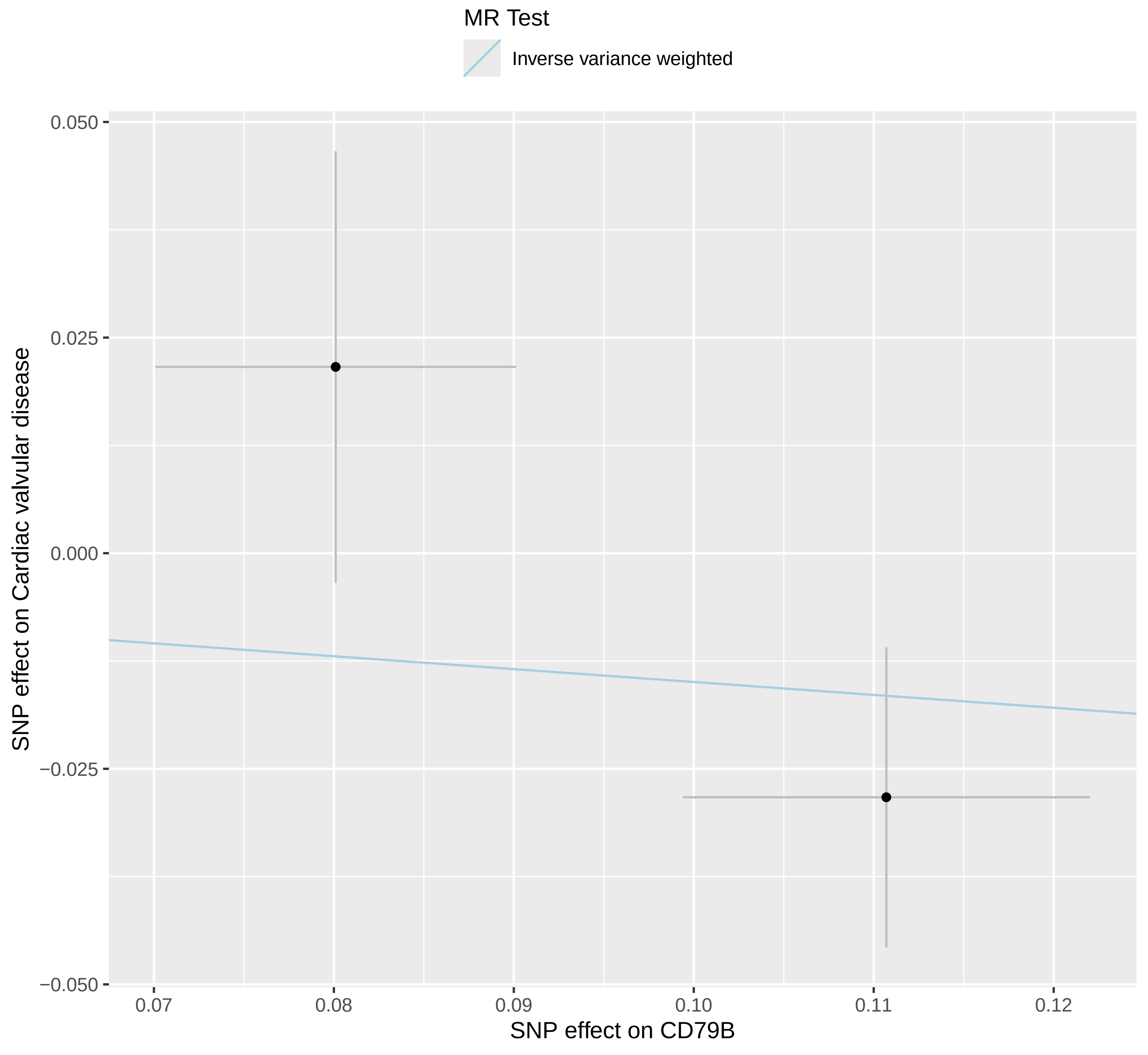
MR Test
Inverse variance weighted
SNP effect on Cardiac valvular disease
0.050
0.025
0.000
−0.025
−0.050
0.07
0.08
0.09
0.10
0.11
0.12
SNP effect on CD79B

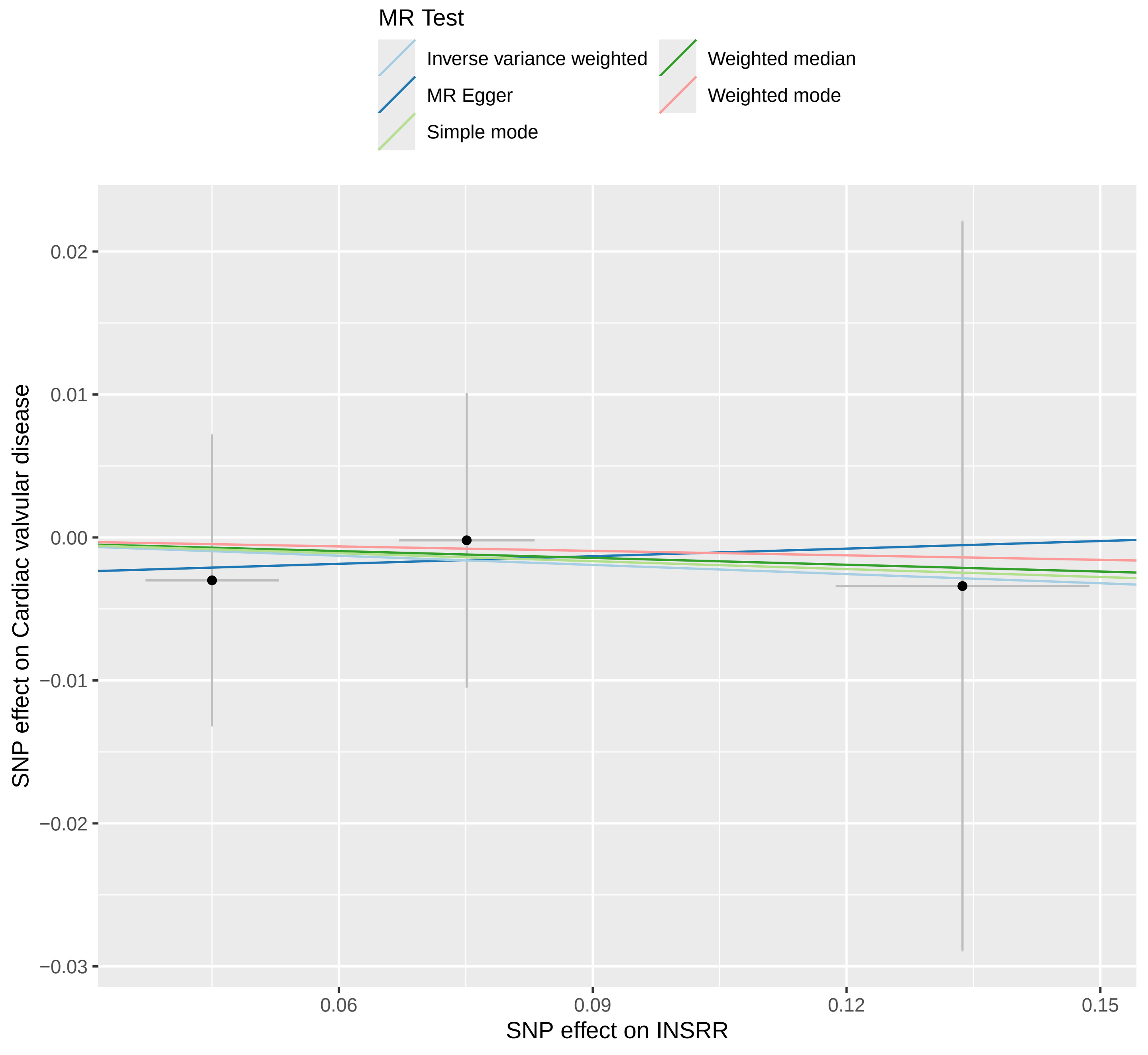
MR Test
Inverse variance weighted
MR Egger
Simple mode
Weighted median
Weighted mode
0.02
0.01
0.00
−0.01
−0.02
−0.03
SNP effect on Cardiac valvular disease
0.06
0.09
0.12
0.15
SNP effect on INSRR

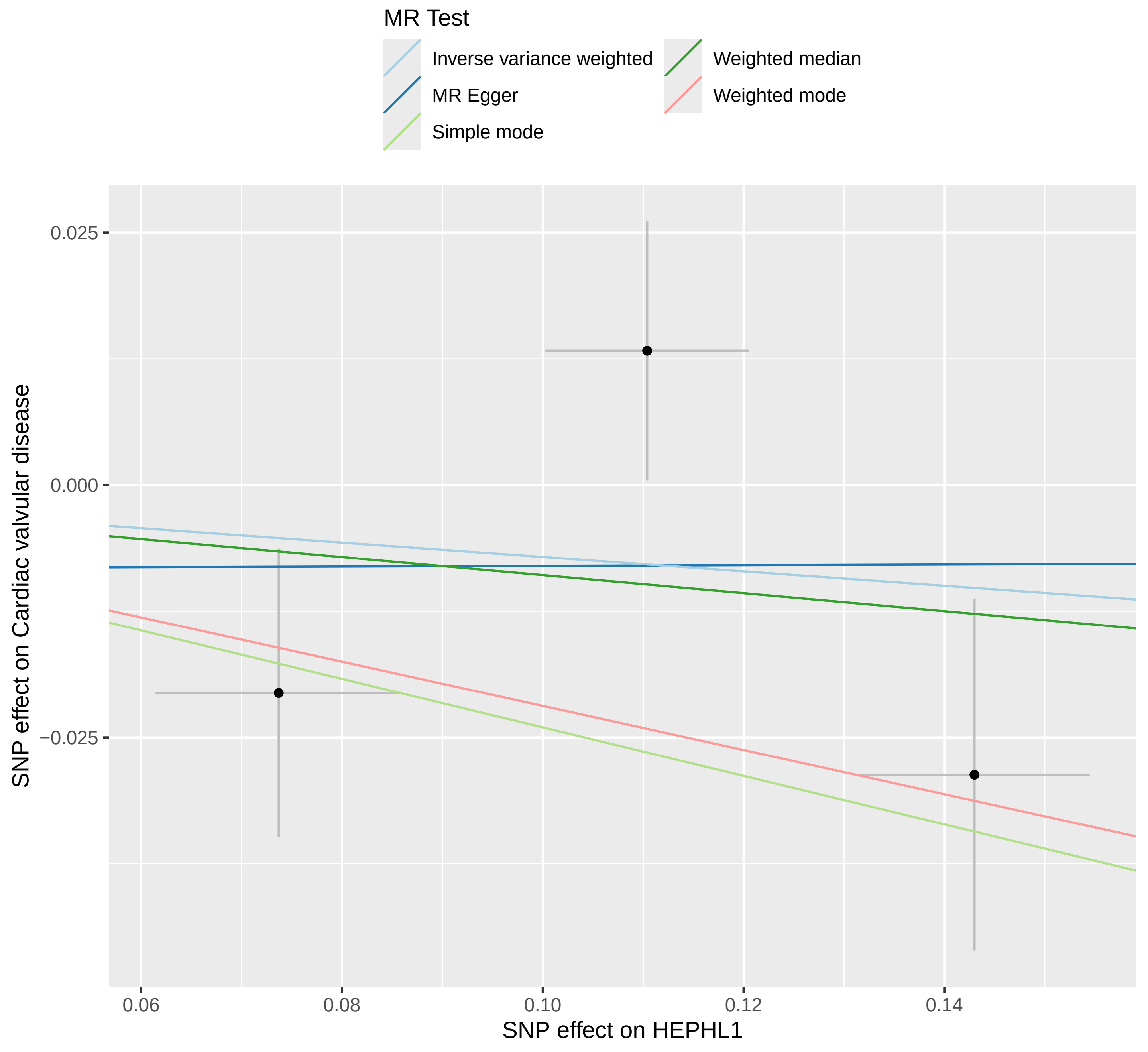

MR Test
Inverse variance weighted
MR Egger
Simple mode
Weighted median
Weighted mode
0.025
0.000
−0.025
0.06
0.08
0.10
0.12
0.14
SNP effect on Cardiac valvular disease
SNP effect on HEPHL1

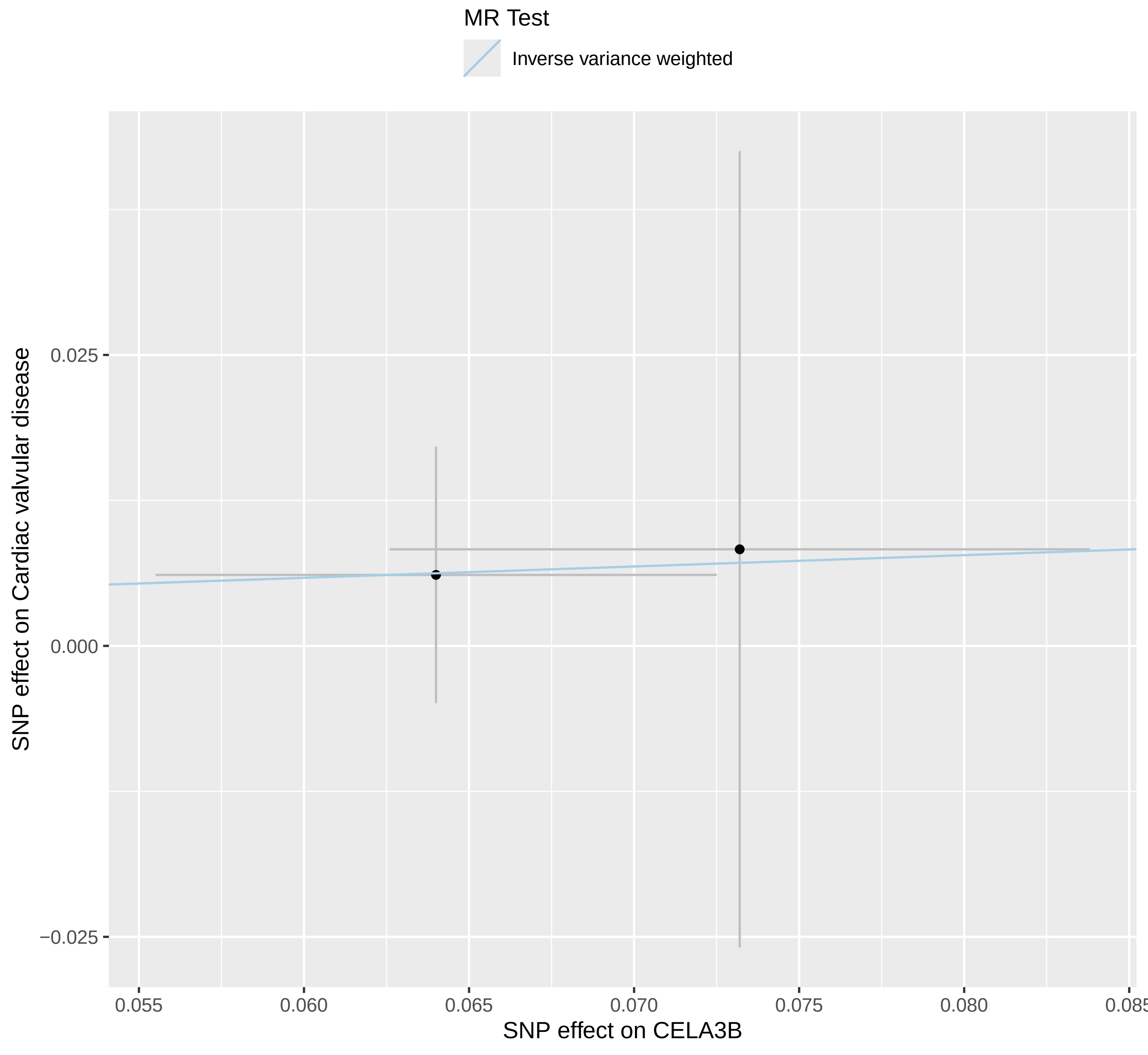
MR Test
Inverse variance weighted
0.025
0.000
−0.025
0.055
0.060
0.065
0.070
0.075
0.080
0.085
SNP effect on Cardiac valvular disease
SNP effect on CELA3B

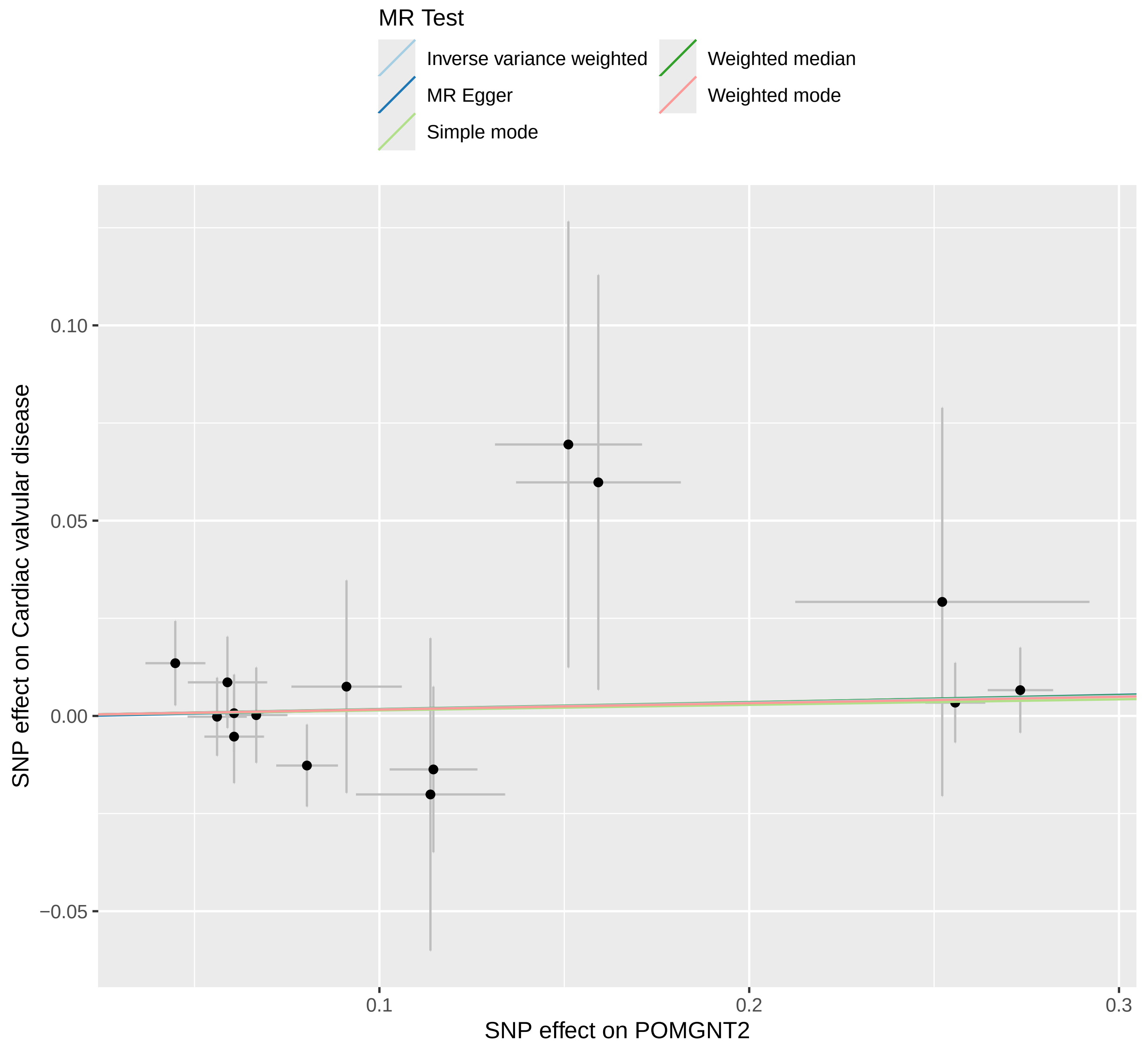

MR Test
Inverse variance weighted
MR Egger
Simple mode
Weighted median
Weighted mode
0.10
0.05
0.00
−0.05
SNP effect on Cardiac valvular disease
0.1
0.2
0.3
SNP effect on POMGNT2

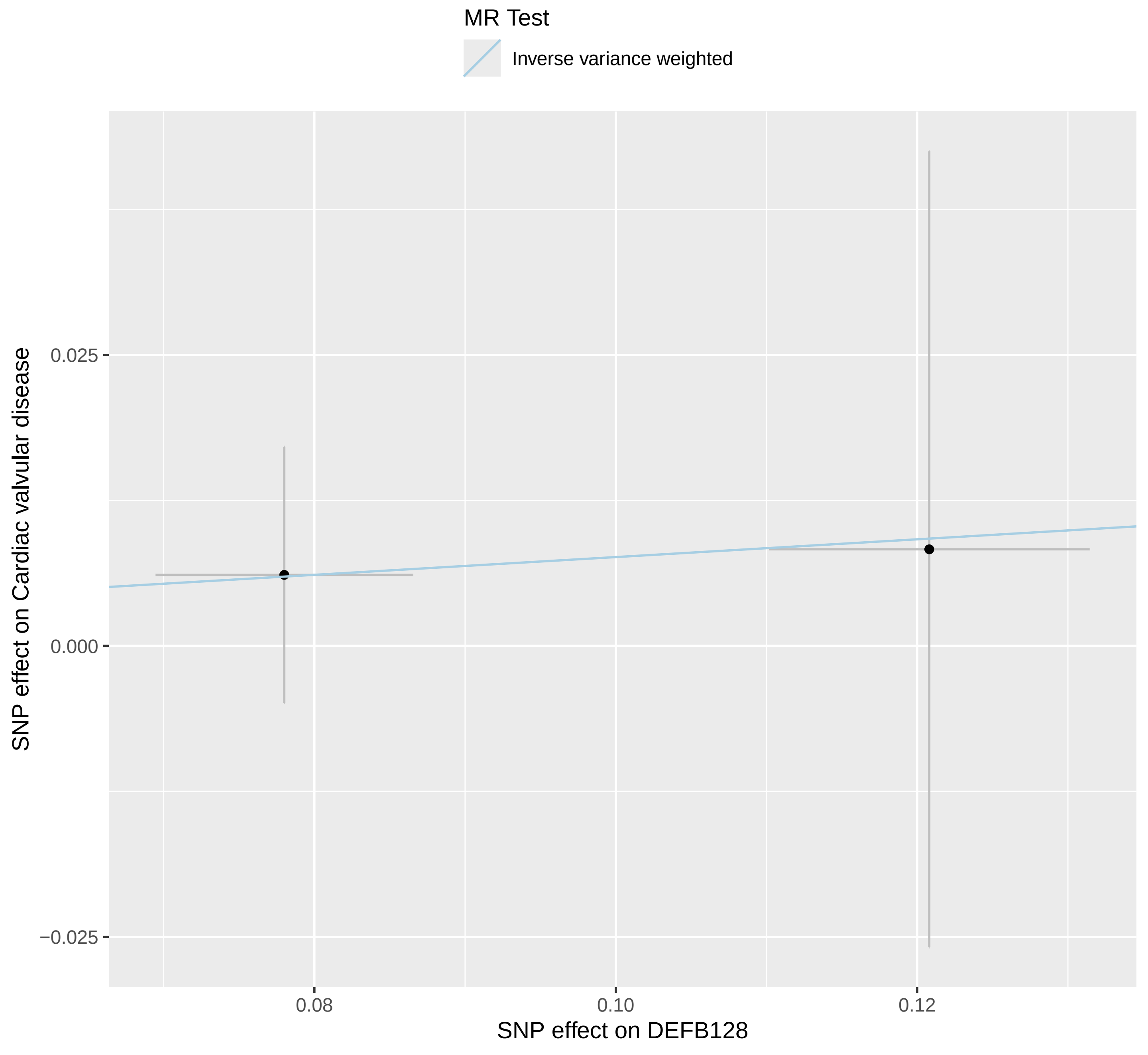

MR Test
Inverse variance weighted
0.025
0.000
−0.025
0.08
0.10
0.12
SNP effect on DEFB128
SNP effect on Cardiac valvular disease

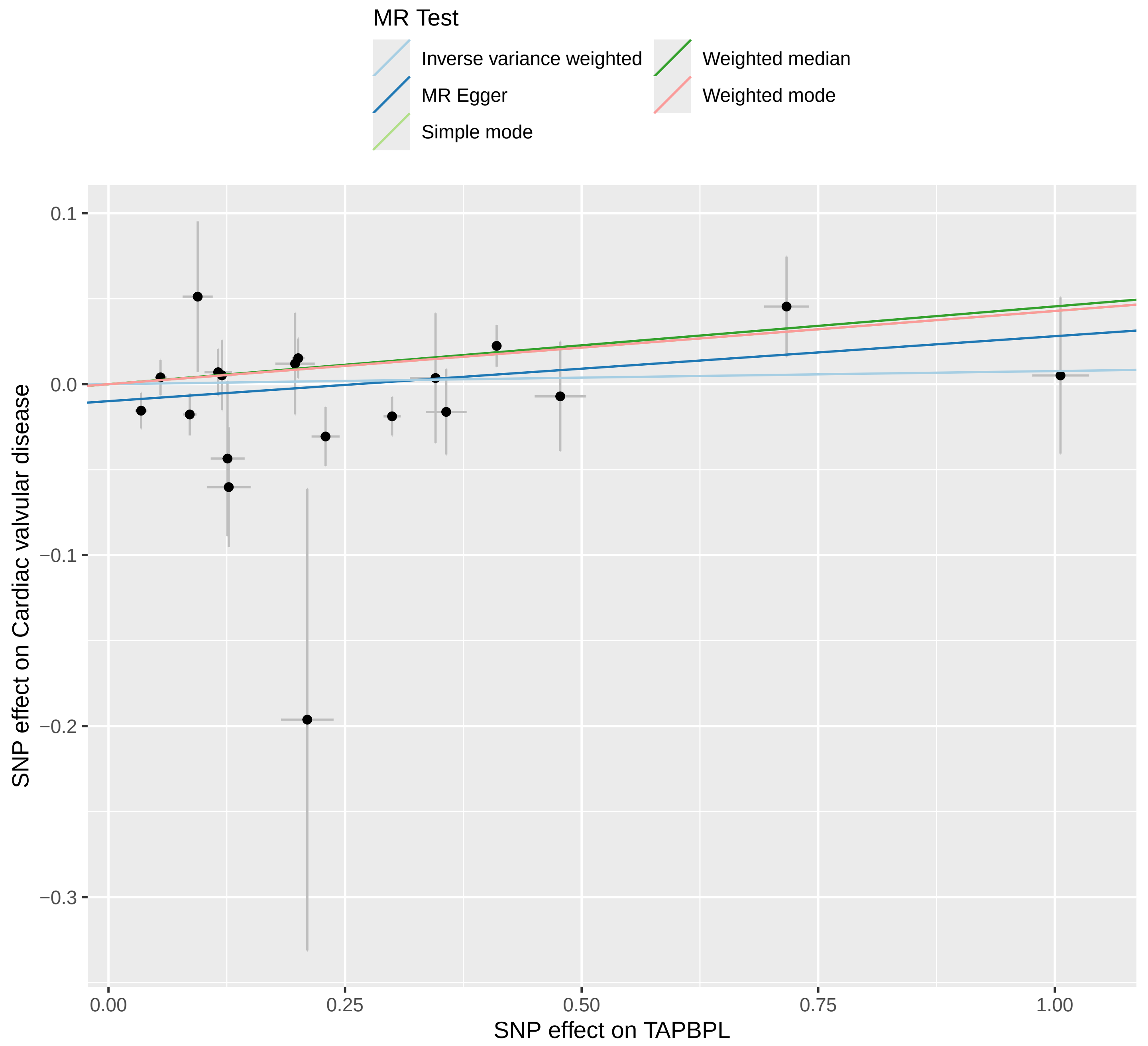

MR Test
Inverse variance weighted
MR Egger
Simple mode
Weighted median
Weighted mode
0.1
0.0
−0.1
−0.2
−0.3
0.00
0.25
0.50
0.75
1.00
SNP effect on TAPBPL
SNP effect on Cardiac valvular disease

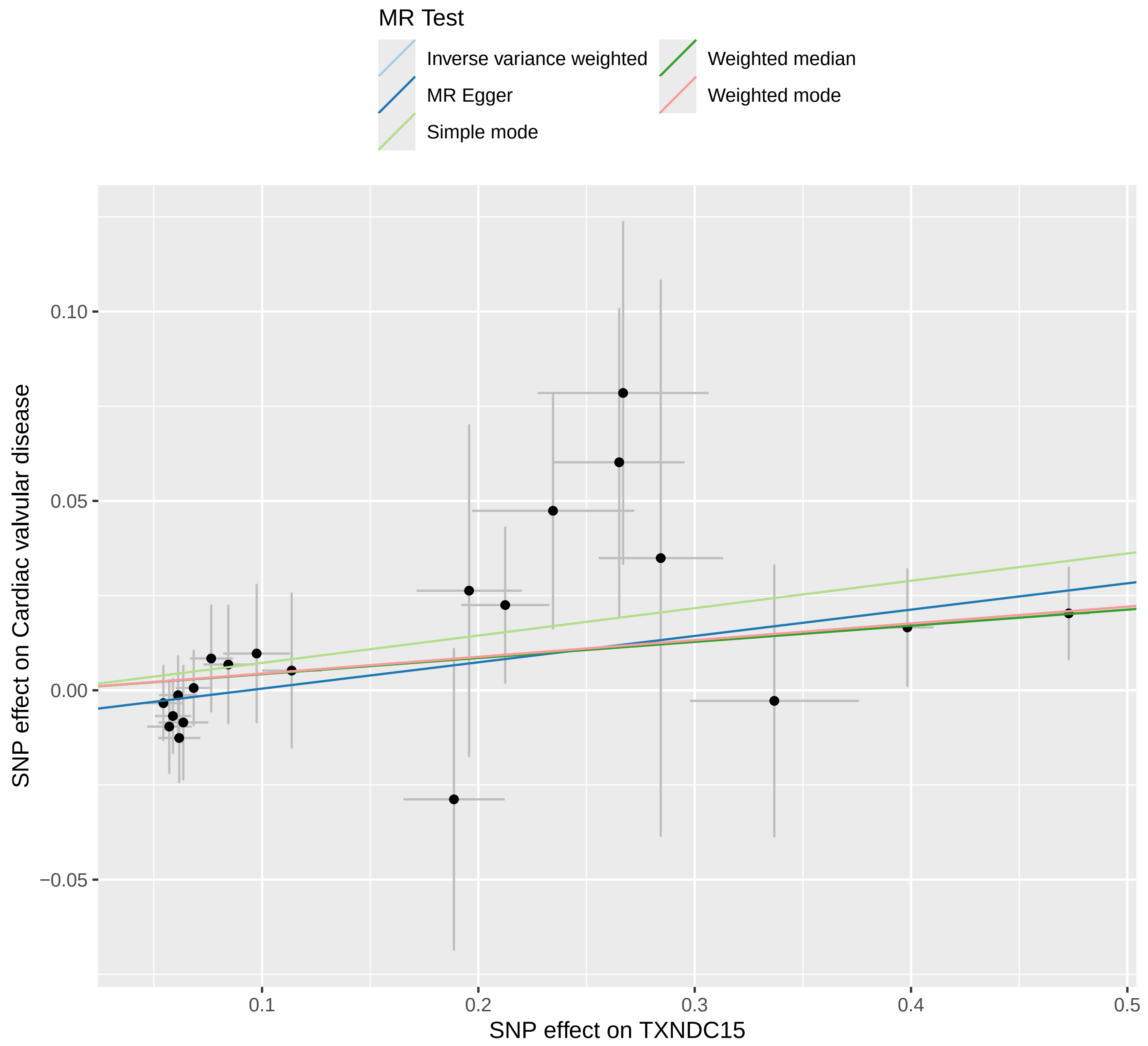
MR Test
Inverse variance weighted
MR Egger
Simple mode
Weighted median
Weighted mode
0.10
0.05
0.00
−0.05
SNP effect on Cardiac valvular disease
0.1
0.2
0.3
0.4
0.5
SNP effect on TXNDC15

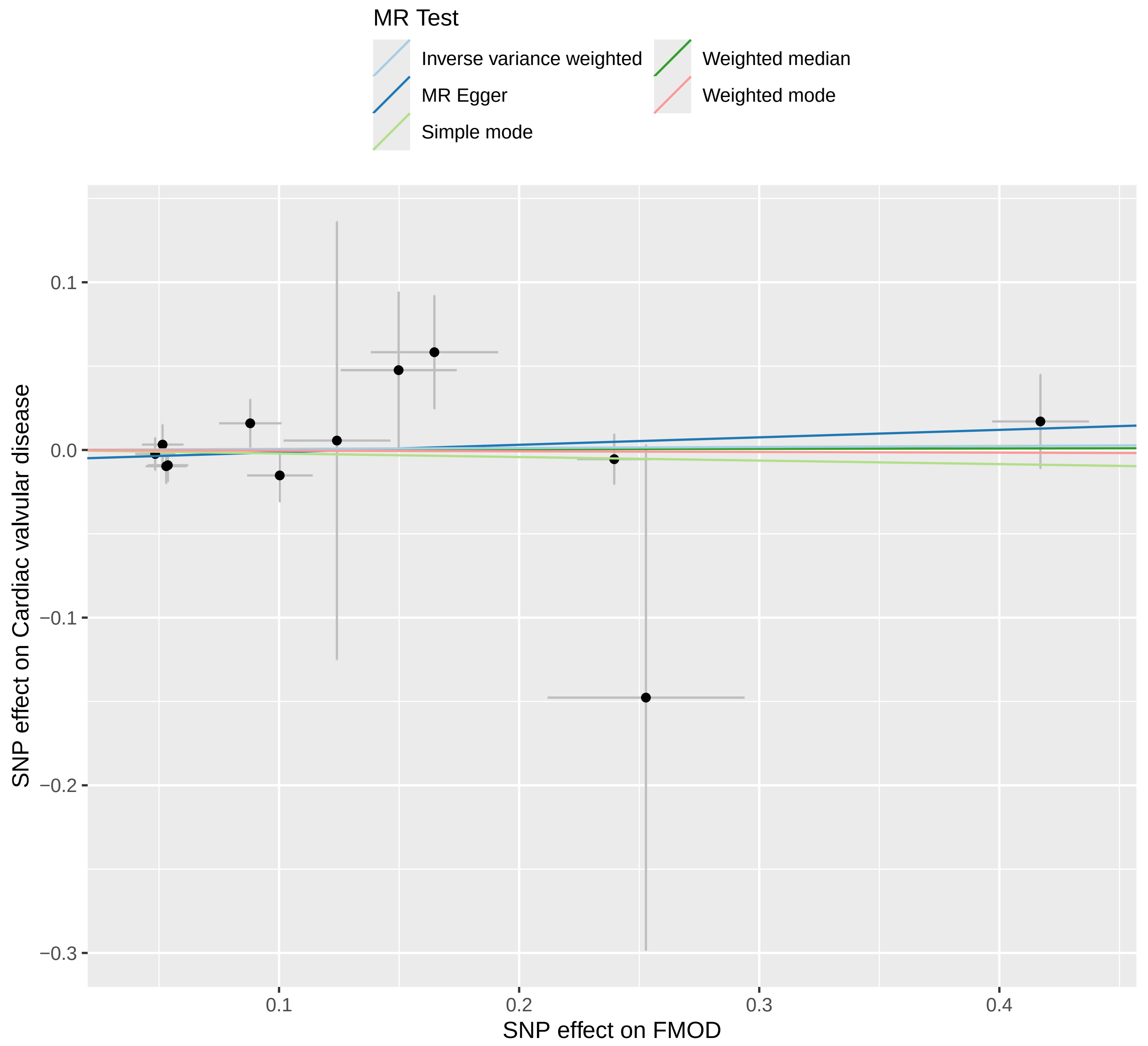
MR Test
Inverse variance weighted
MR Egger
Simple mode
Weighted median
Weighted mode
0.1
0.0
−0.1
−0.2
−0.3
SNP effect on Cardiac valvular disease
0.1
0.2
0.3
0.4
SNP effect on FMOD

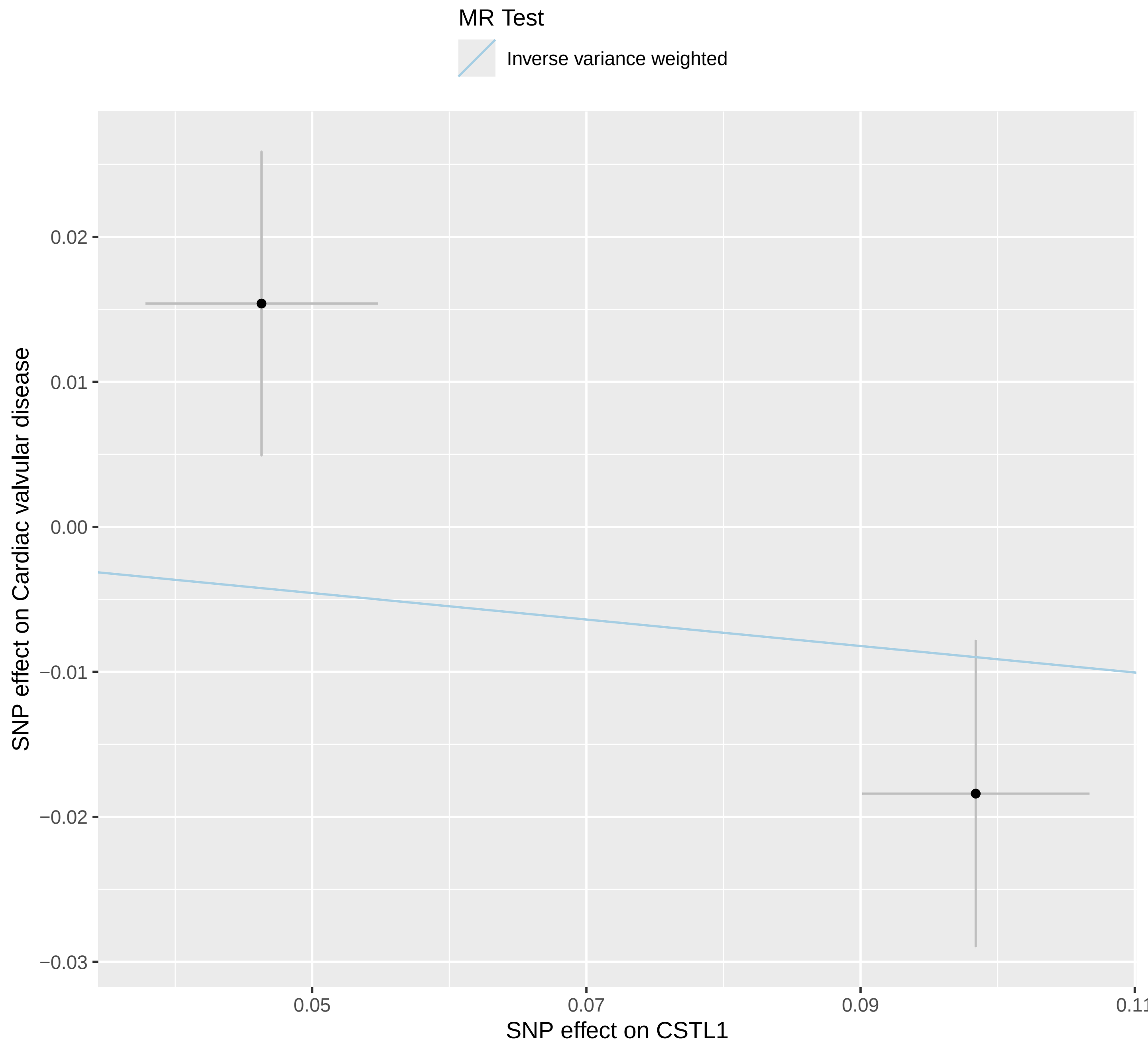
MR Test
Inverse variance weighted
0.02
0.01
0.00
−0.01
−0.02
−0.03
SNP effect on Cardiac valvular disease
0.05
0.07
0.09
0.11
SNP effect on CSTL1

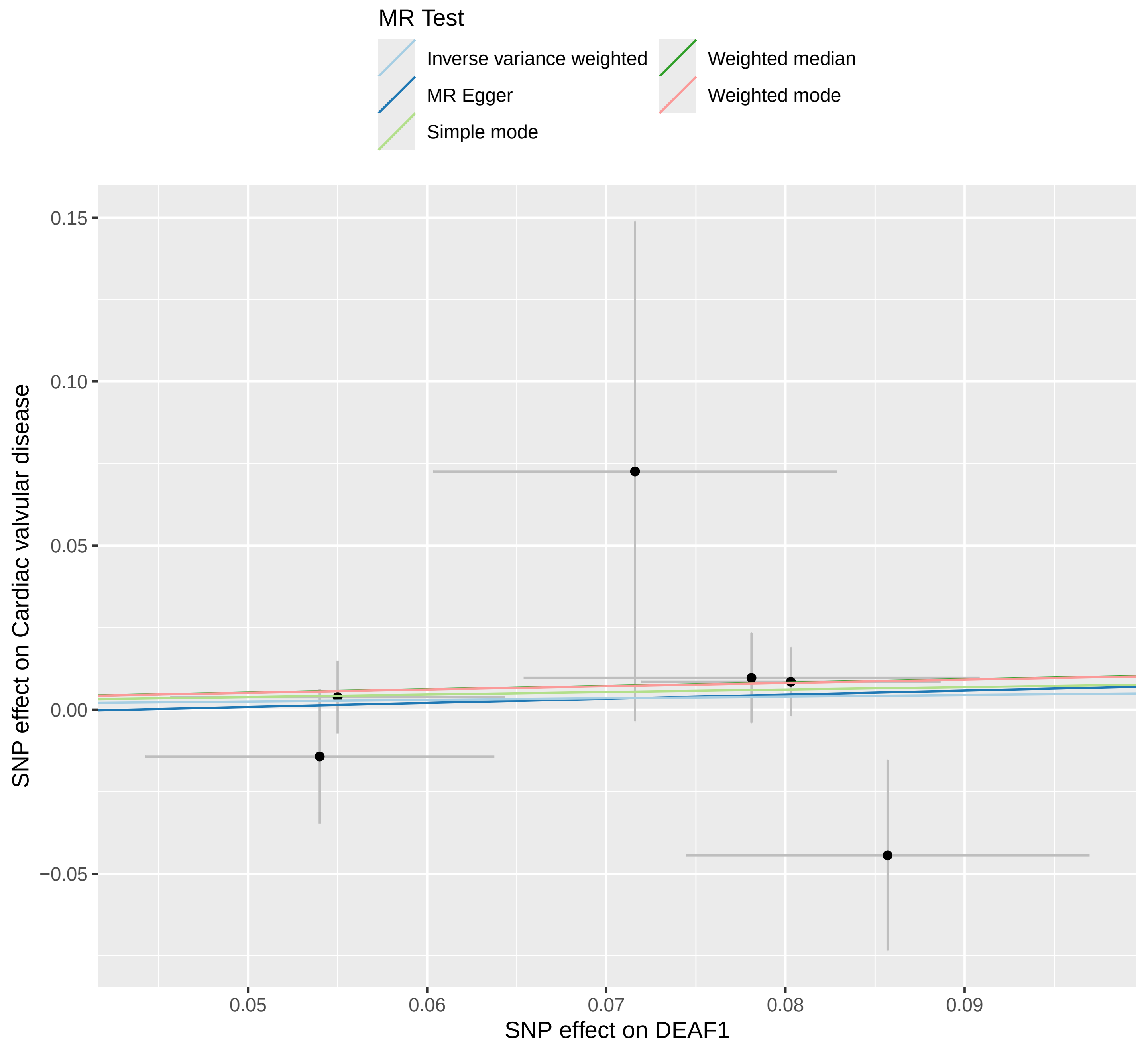

MR Test
Inverse variance weighted
MR Egger
Simple mode
Weighted median
Weighted mode
0.15
0.10
0.05
0.00
−0.05
0.05
0.06
0.07
0.08
0.09
SNP effect on Cardiac valvular disease
SNP effect on DEAF1

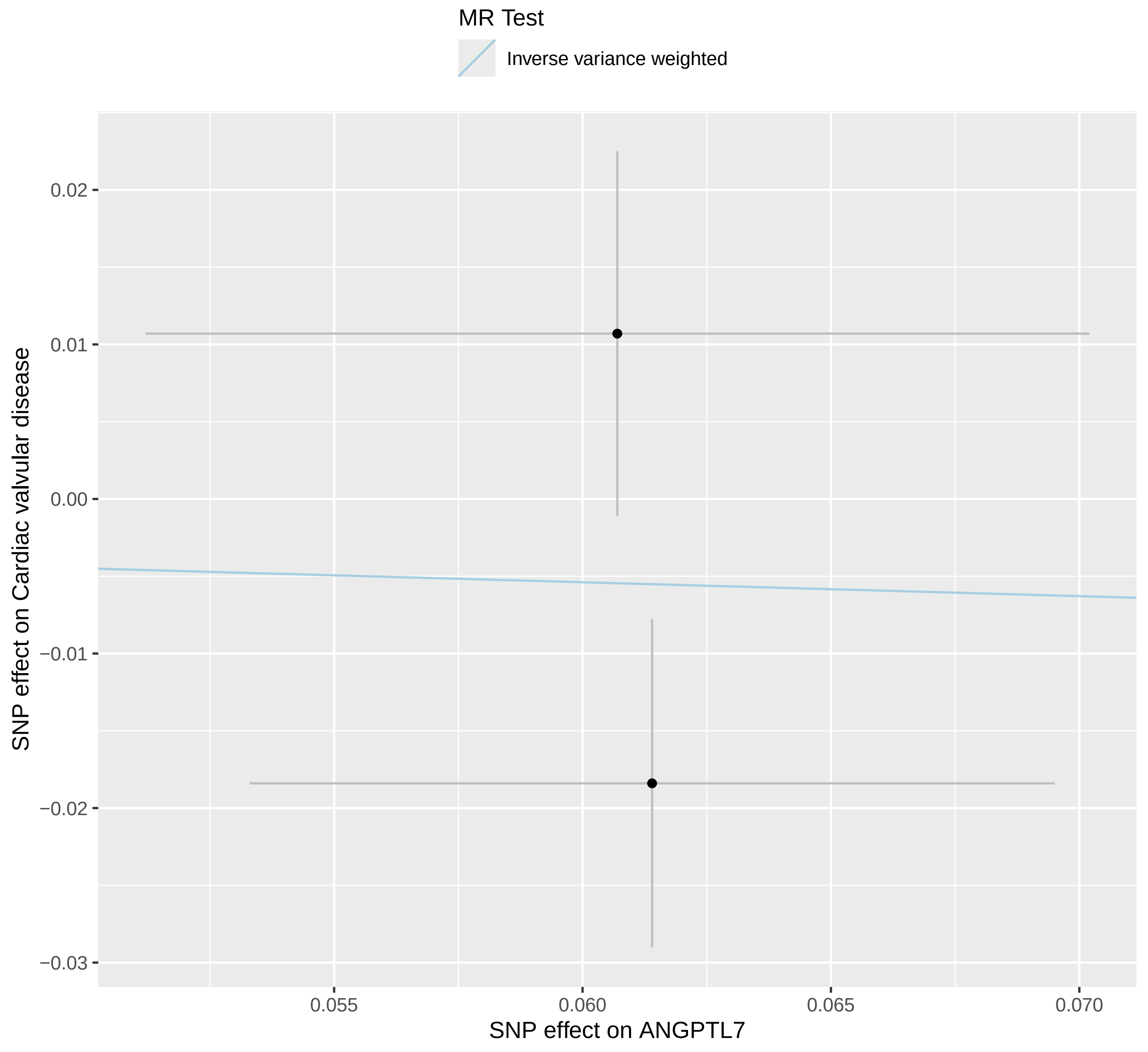
MR Test
Inverse variance weighted
0.02
0.01
0.00
−0.01
−0.02
−0.03
SNP effect on Cardiac valvular disease
0.055
0.060
0.065
0.070
SNP effect on ANGPTL7

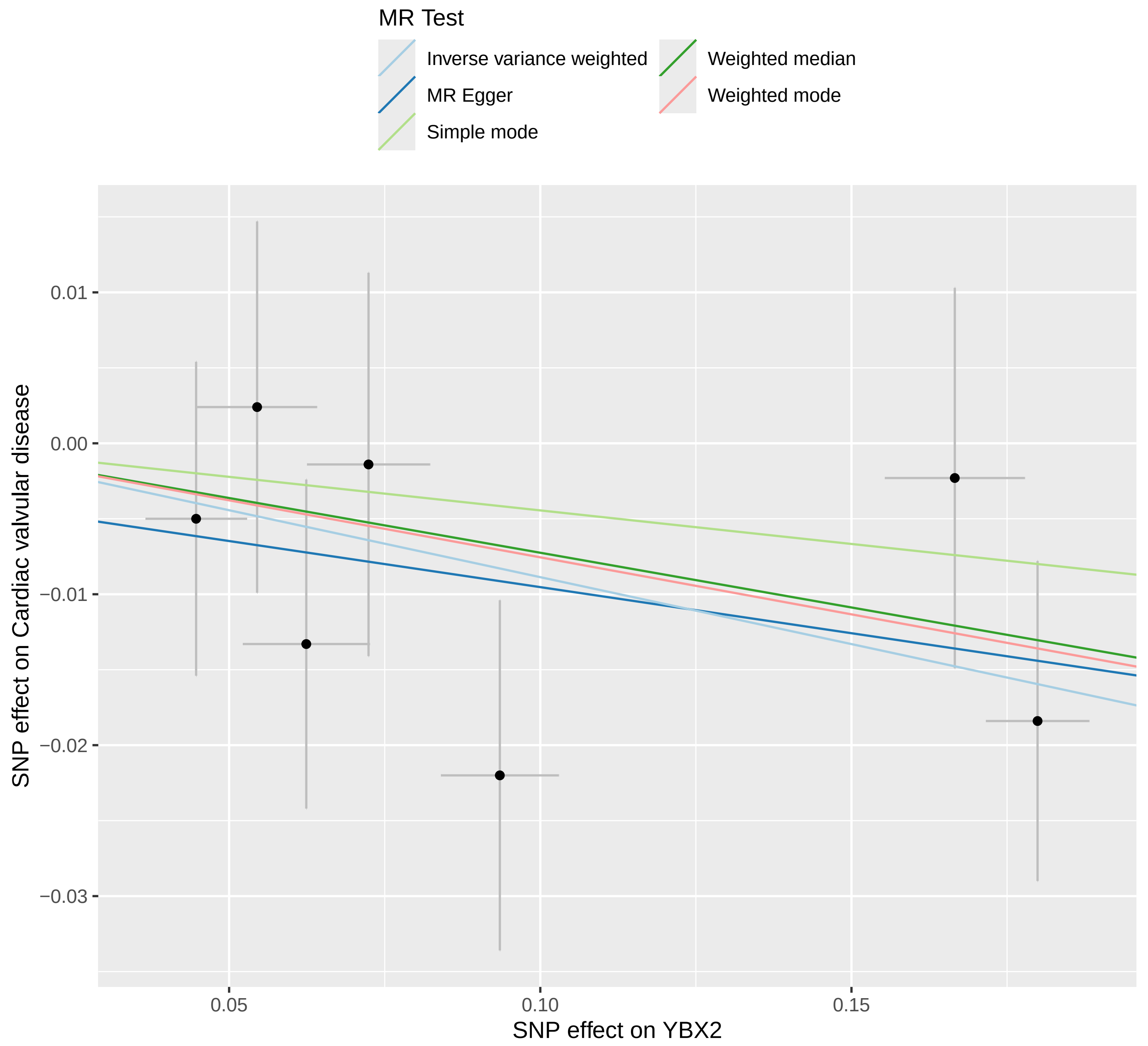

MR Test
Inverse variance weighted
MR Egger
Simple mode
Weighted median
Weighted mode
SNP effect on Cardiac valvular disease
0.01
0.00
−0.01
−0.02
−0.03
0.05
0.10
0.15
SNP effect on YBX2

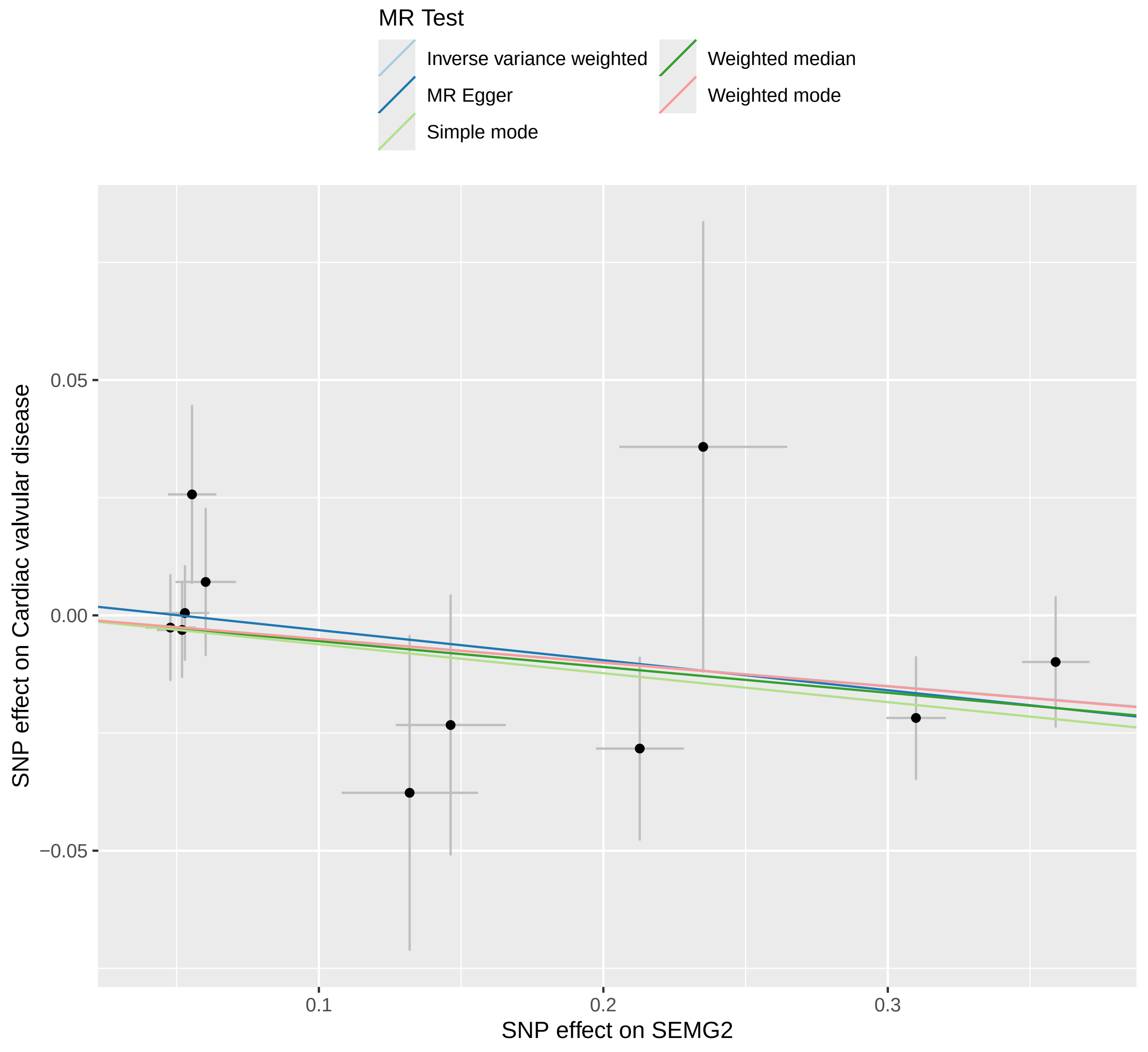
MR Test
Inverse variance weighted
MR Egger
Simple mode
Weighted median
Weighted mode
0.05
0.00
−0.05
0.1
0.2
0.3
SNP effect on Cardiac valvular disease
SNP effect on SEMG2

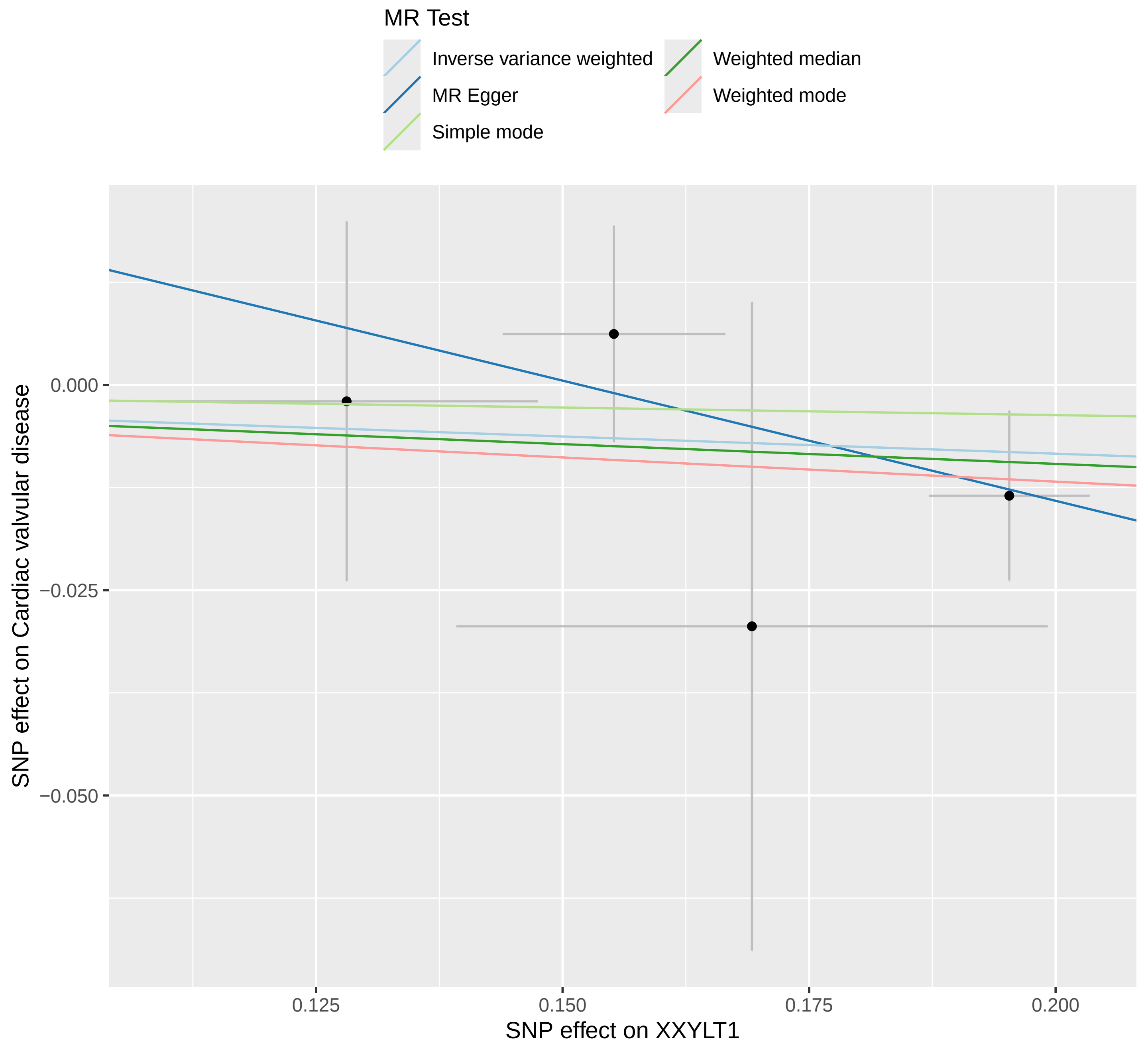

MR Test
Inverse variance weighted
MR Egger
Simple mode
Weighted median
Weighted mode
0.000
−0.025
−0.050
0.125
0.150
0.175
0.200
SNP effect on Cardiac valvular disease
SNP effect on XXYLT1

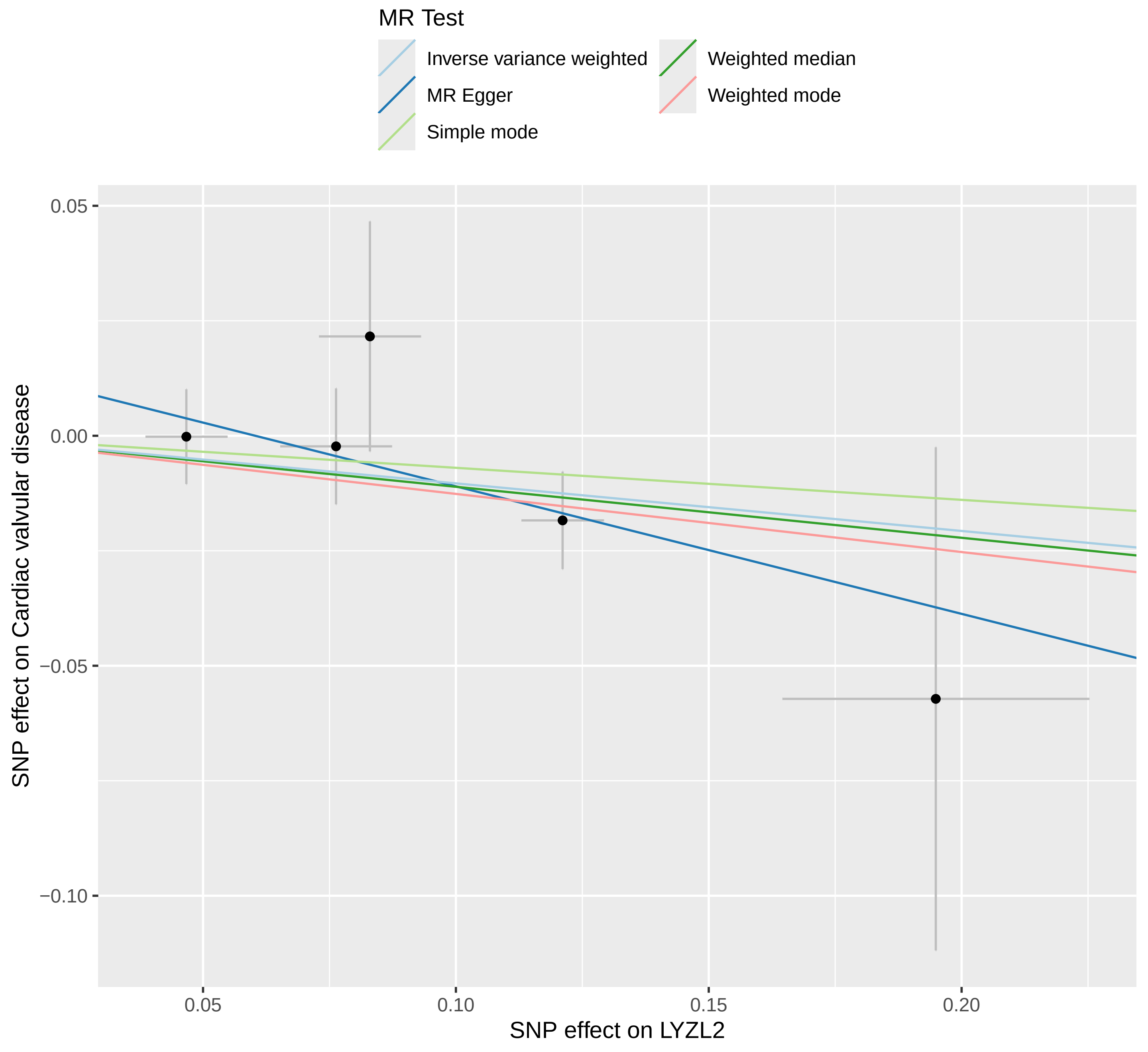
MR Test
Inverse variance weighted
MR Egger
Simple mode
Weighted median
Weighted mode
0.05
0.00
−0.05
−0.10
SNP effect on Cardiac valvular disease
0.05
0.10
0.15
0.20
SNP effect on LYZL2

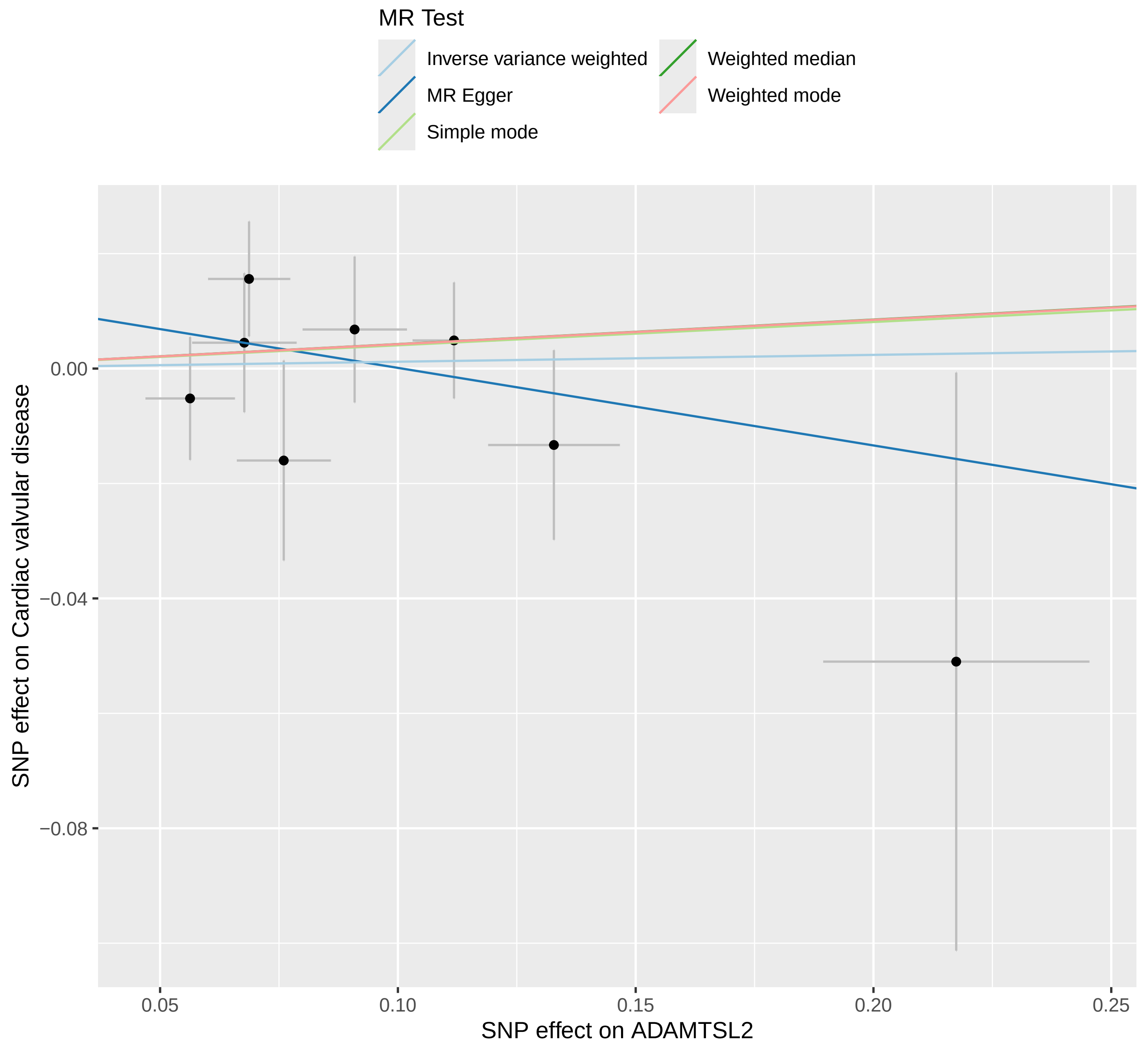

MR Test
Inverse variance weighted
MR Egger
Simple mode
Weighted median
Weighted mode
0.00
−0.04
−0.08
0.05
0.10
0.15
0.20
0.25
SNP effect on ADAMTSL2
SNP effect on Cardiac valvular disease

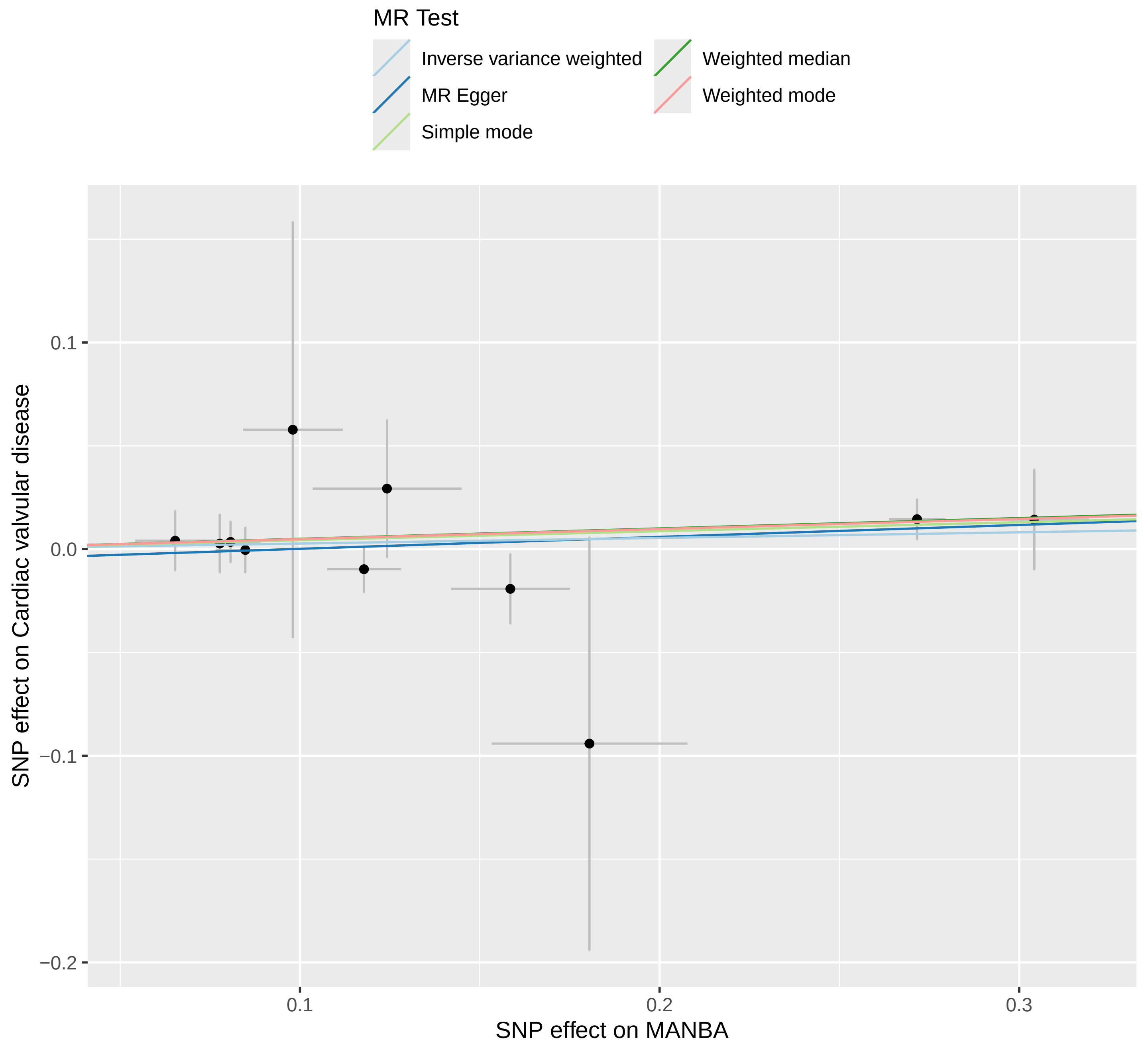
MR Test
Inverse variance weighted
MR Egger
Simple mode
Weighted median
Weighted mode
0.1
0.0
−0.1
−0.2
0.1
0.2
0.3
SNP effect on Cardiac valvular disease
SNP effect on MANBA

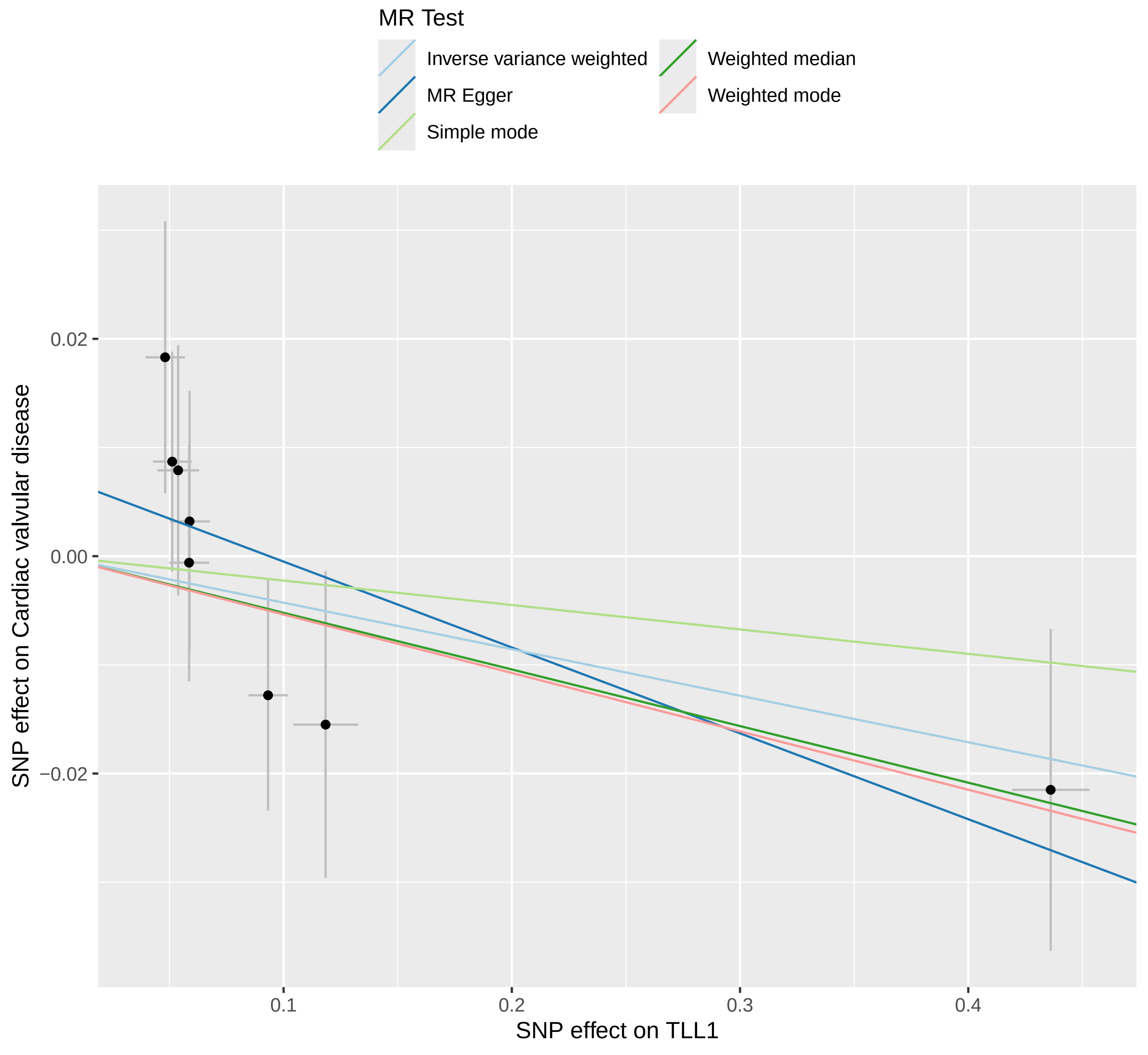

MR Test
Inverse variance weighted
MR Egger
Simple mode
Weighted median
Weighted mode
0.02
0.00
−0.02
0.1
0.2
0.3
0.4
SNP effect on Cardiac valvular disease
SNP effect on TLL1

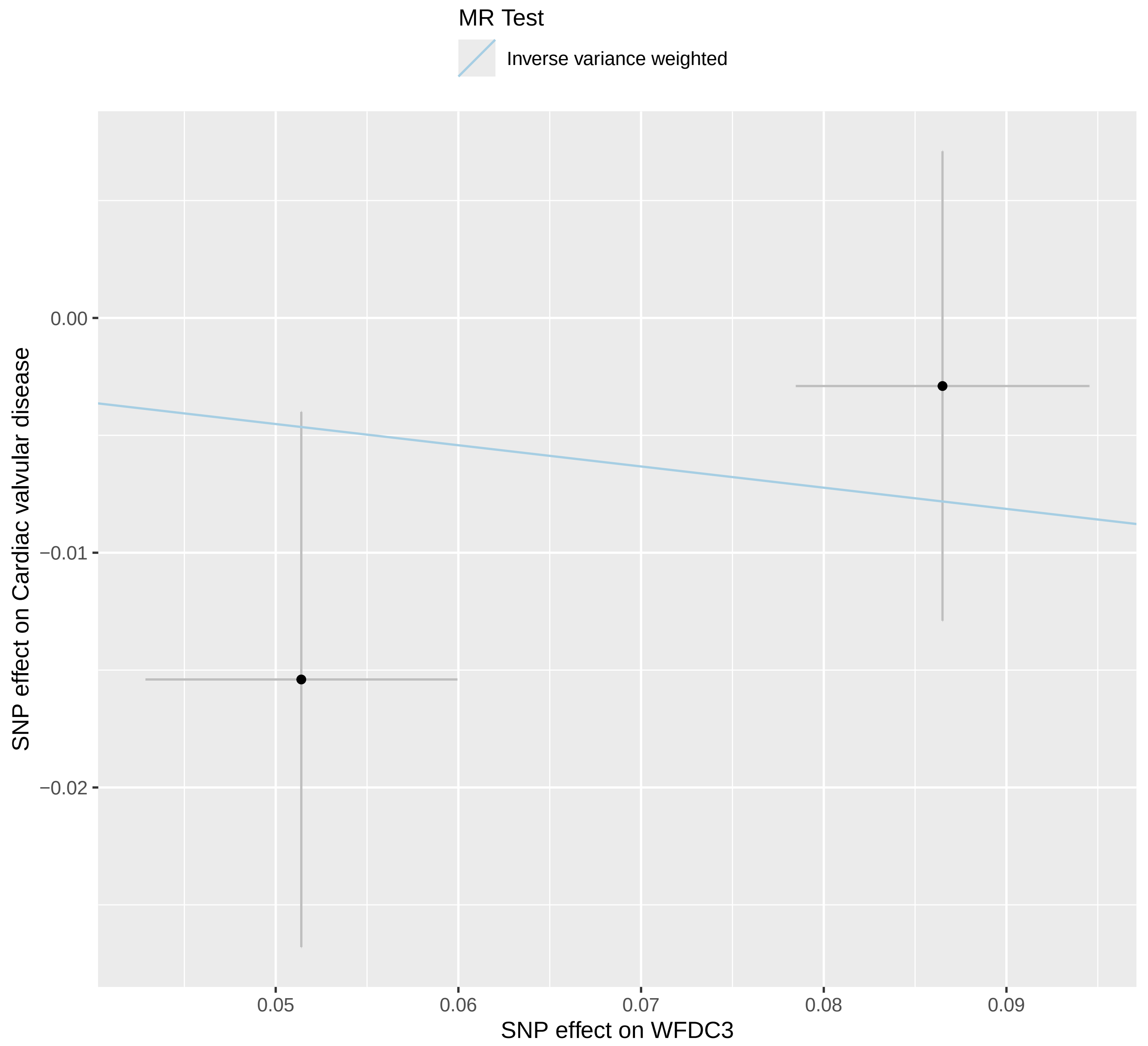

MR Test
Inverse variance weighted
0.00
−0.01
−0.02
0.05
0.06
0.07
0.08
0.09
SNP effect on WFDC3
SNP effect on Cardiac valvular disease

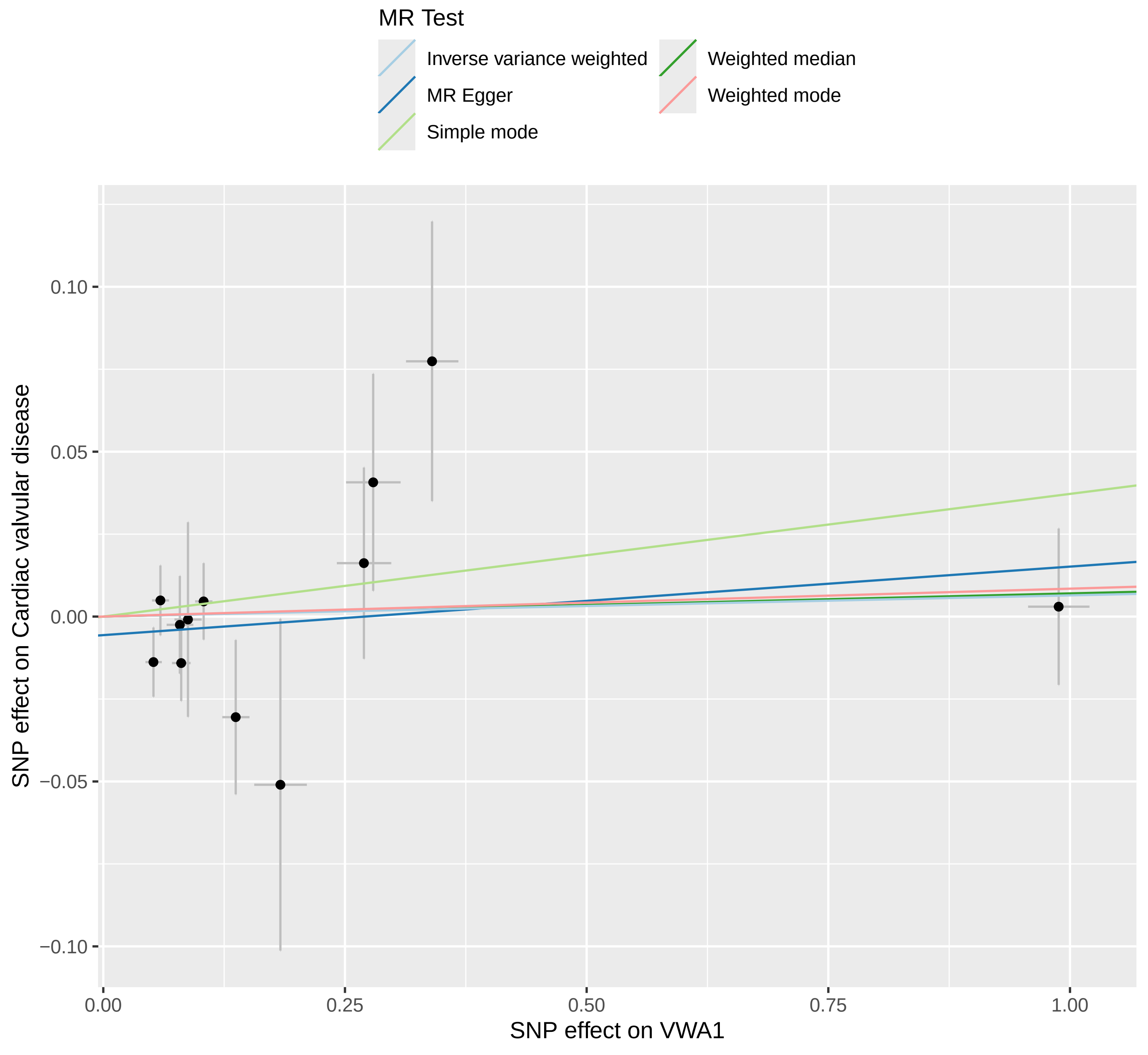
MR Test
Inverse variance weighted
MR Egger
Simple mode
Weighted median
Weighted mode
SNP effect on Cardiac valvular disease
0.10
0.05
0.00
−0.05
−0.10
0.00
0.25
0.50
0.75
1.00
SNP effect on VWA1

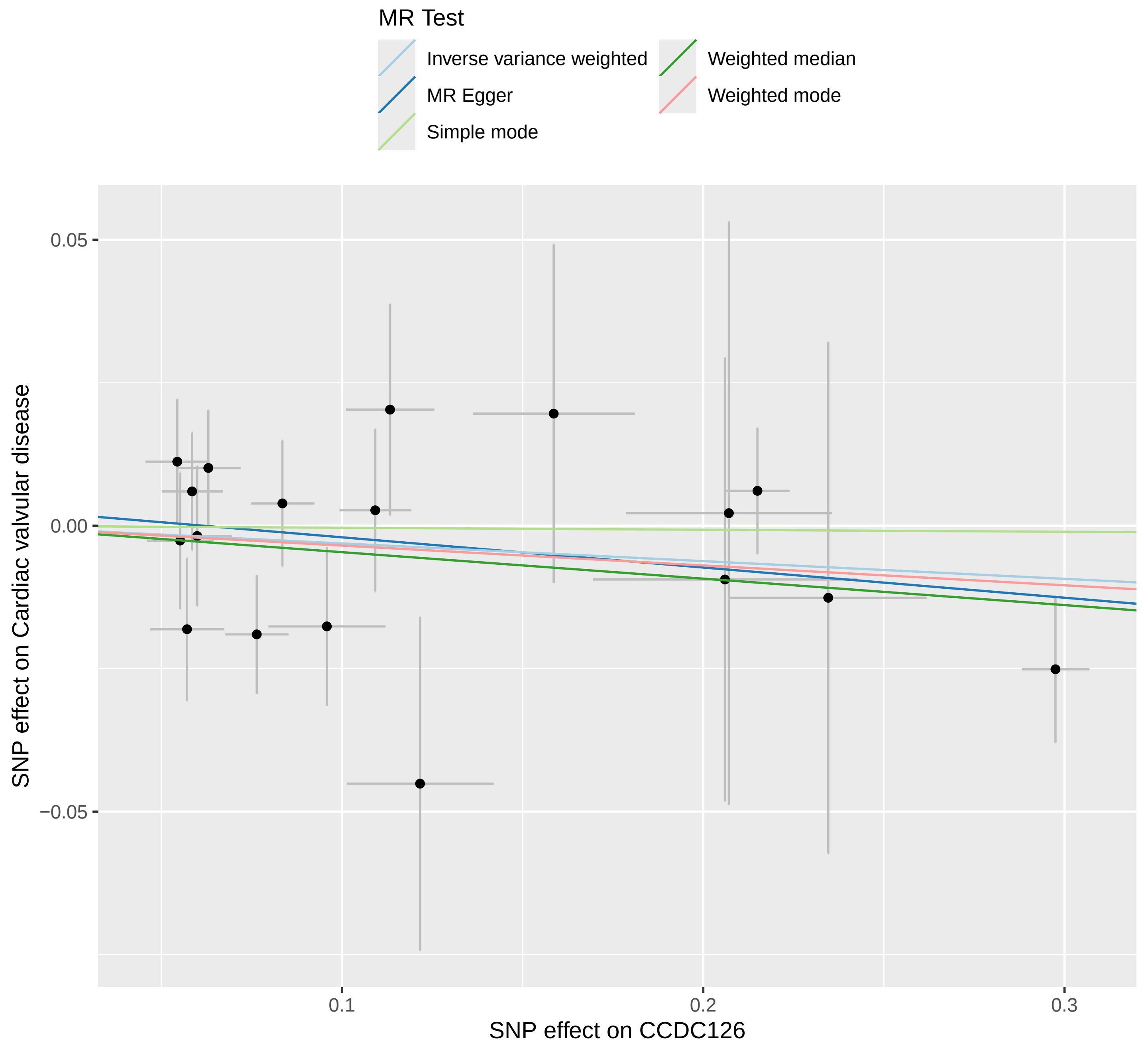

MR Test
Inverse variance weighted
MR Egger
Simple mode
Weighted median
Weighted mode
0.05
0.00
−0.05
0.1
0.2
0.3
SNP effect on Cardiac valvular disease
SNP effect on CCDC126

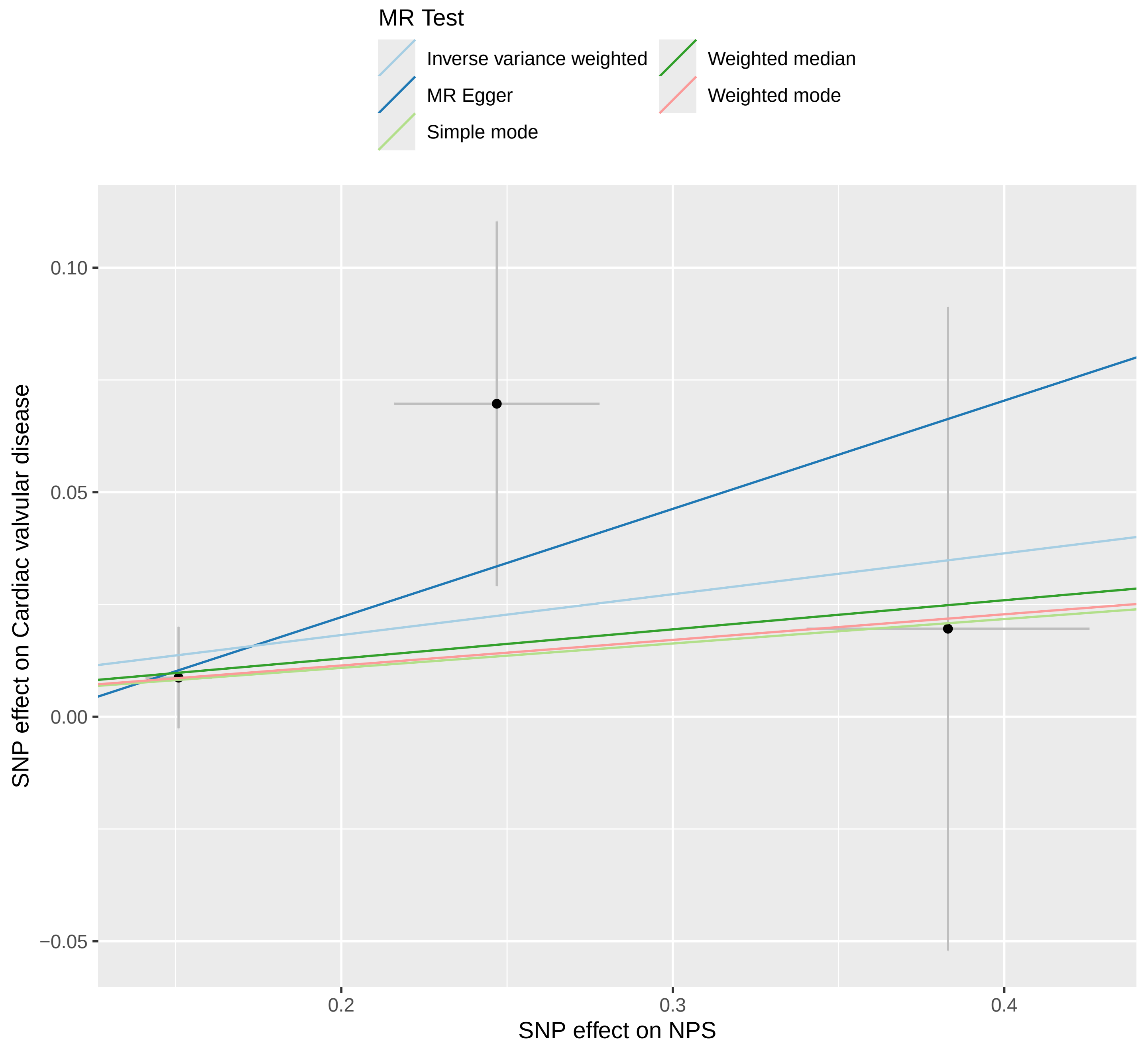
MR Test
Inverse variance weighted
MR Egger
Simple mode
Weighted median
Weighted mode
SNP effect on Cardiac valvular disease
0.10
0.05
0.00
−0.05
0.2
0.3
0.4
SNP effect on NPS

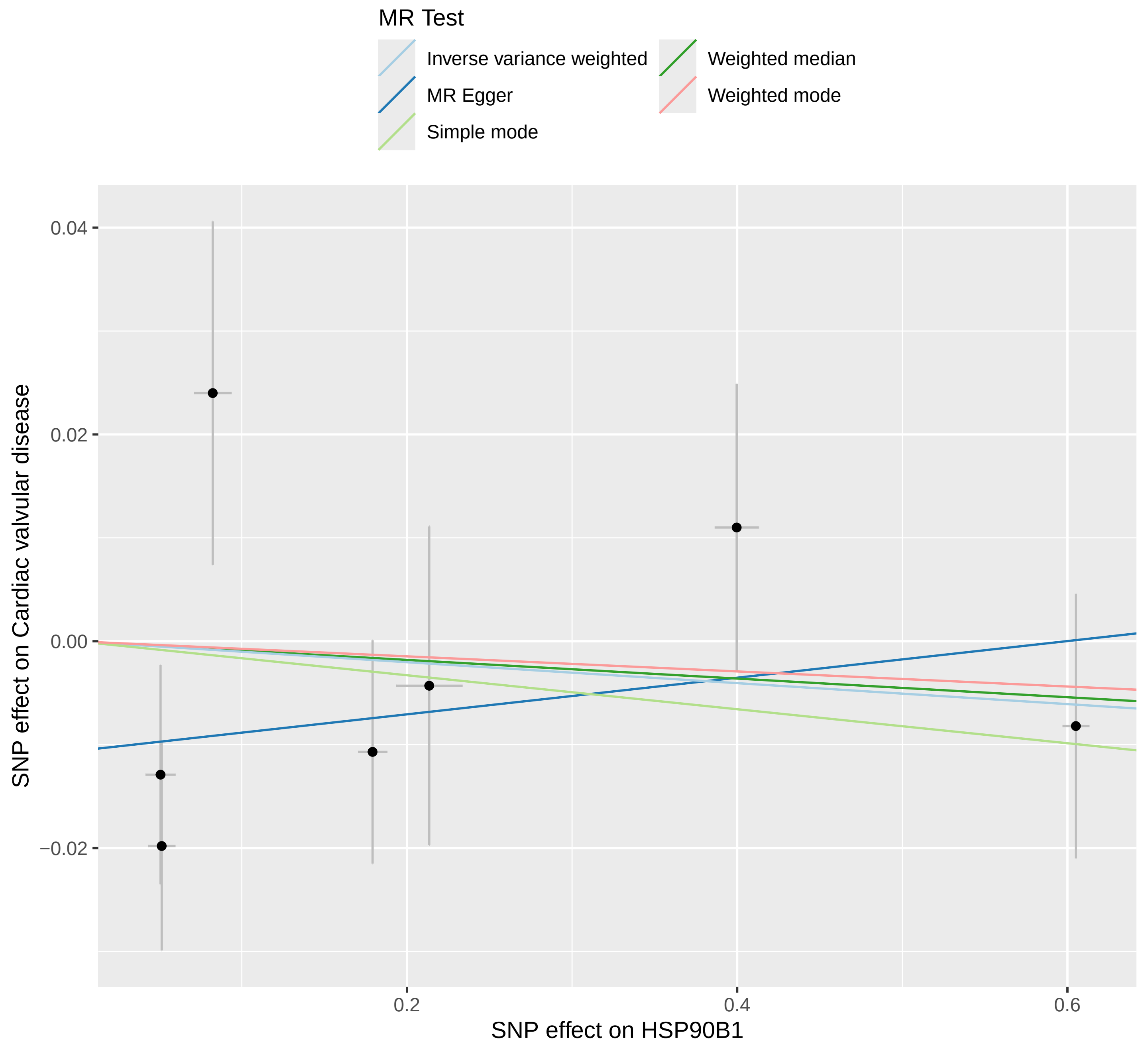
MR Test
Inverse variance weighted
MR Egger
Simple mode
Weighted median
Weighted mode
0.04
0.02
0.00
−0.02
0.2
0.4
0.6
SNP effect on Cardiac valvular disease
SNP effect on HSP90B1

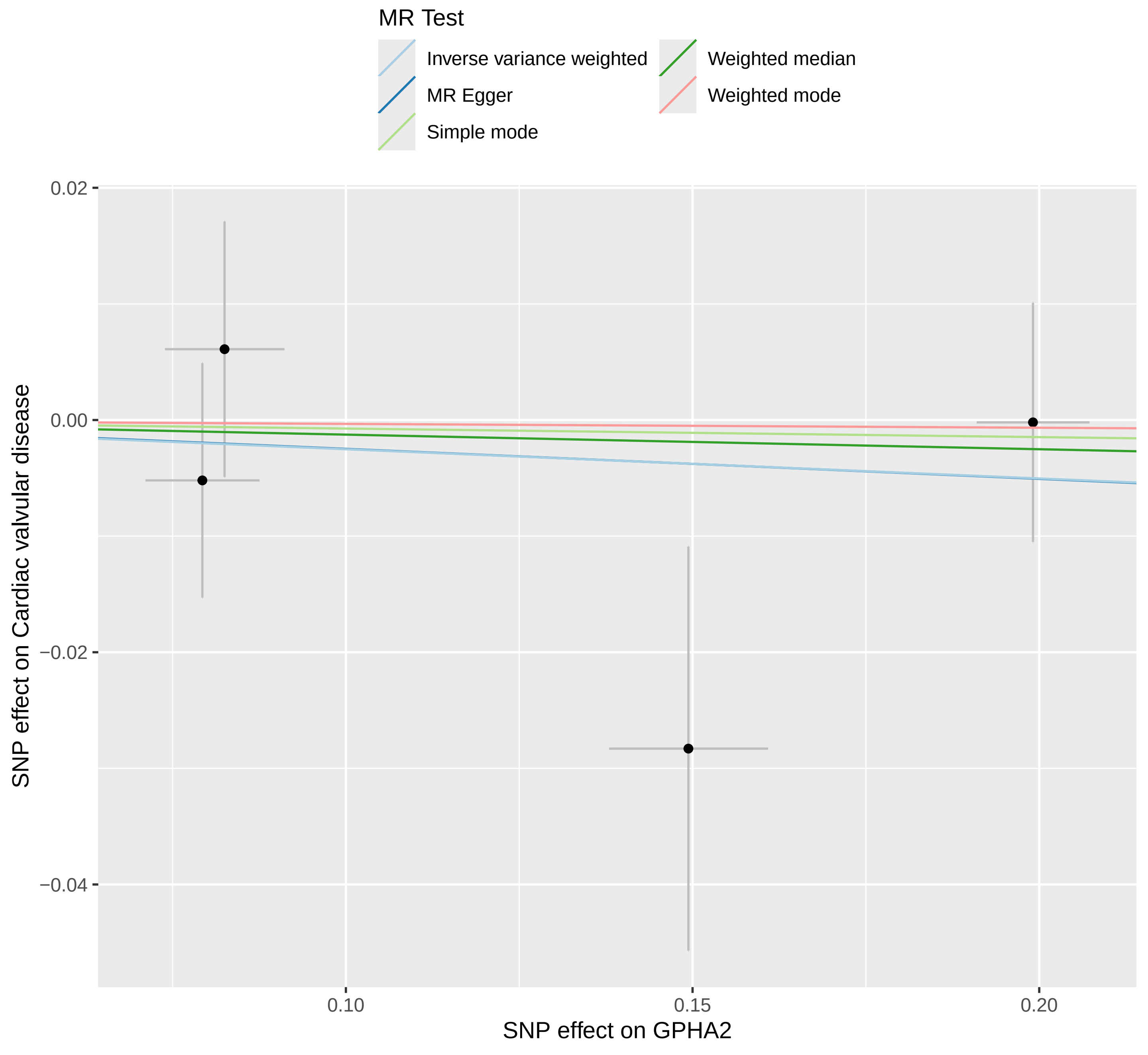
MR Test
Inverse variance weighted
MR Egger
Simple mode
Weighted median
Weighted mode
0.02
0.00
−0.02
−0.04
SNP effect on Cardiac valvular disease
0.10
0.15
0.20
SNP effect on GPHA2

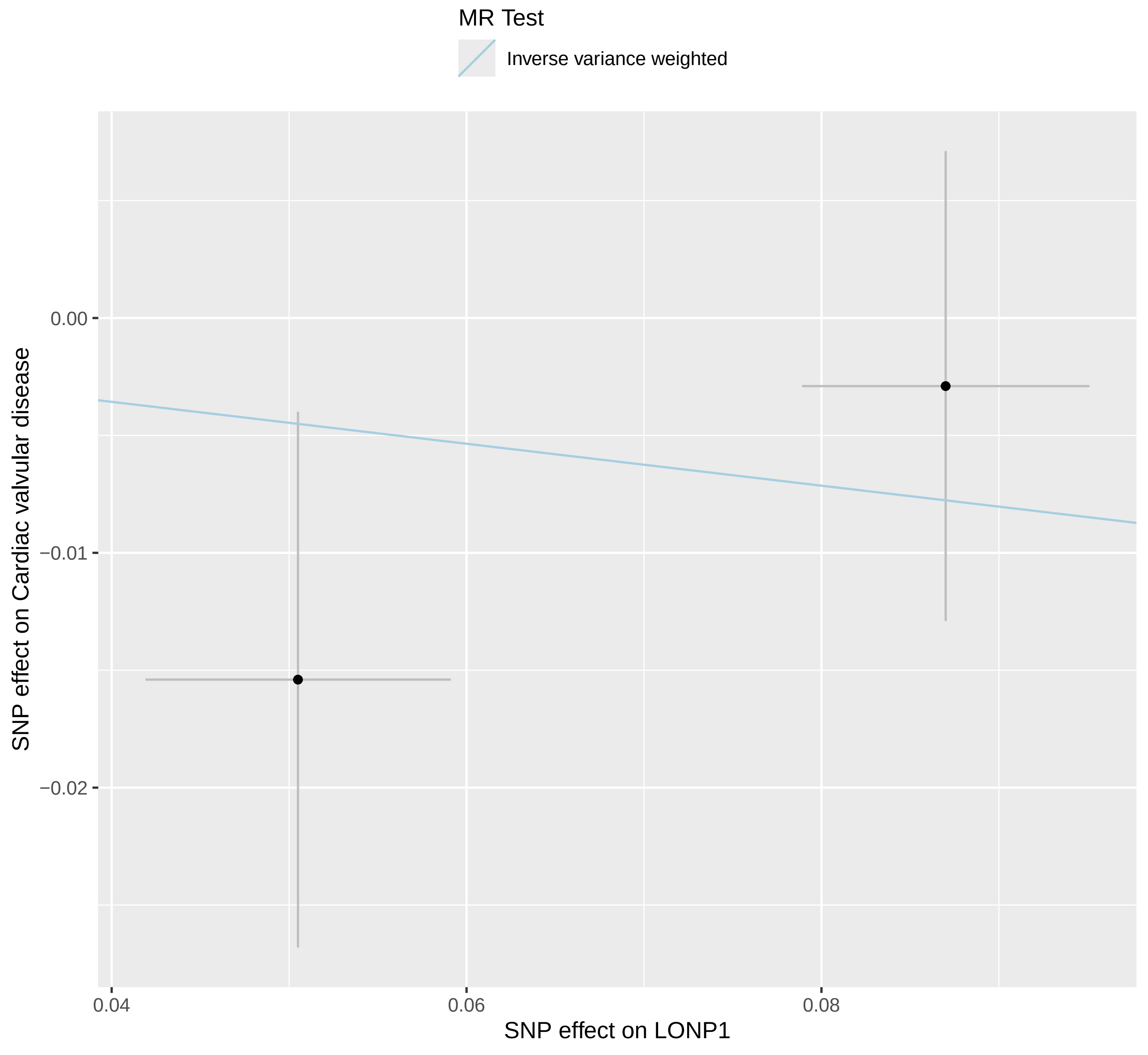

MR Test
Inverse variance weighted
0.00
−0.01
−0.02
SNP effect on Cardiac valvular disease
0.04
0.06
0.08
SNP effect on LONP1

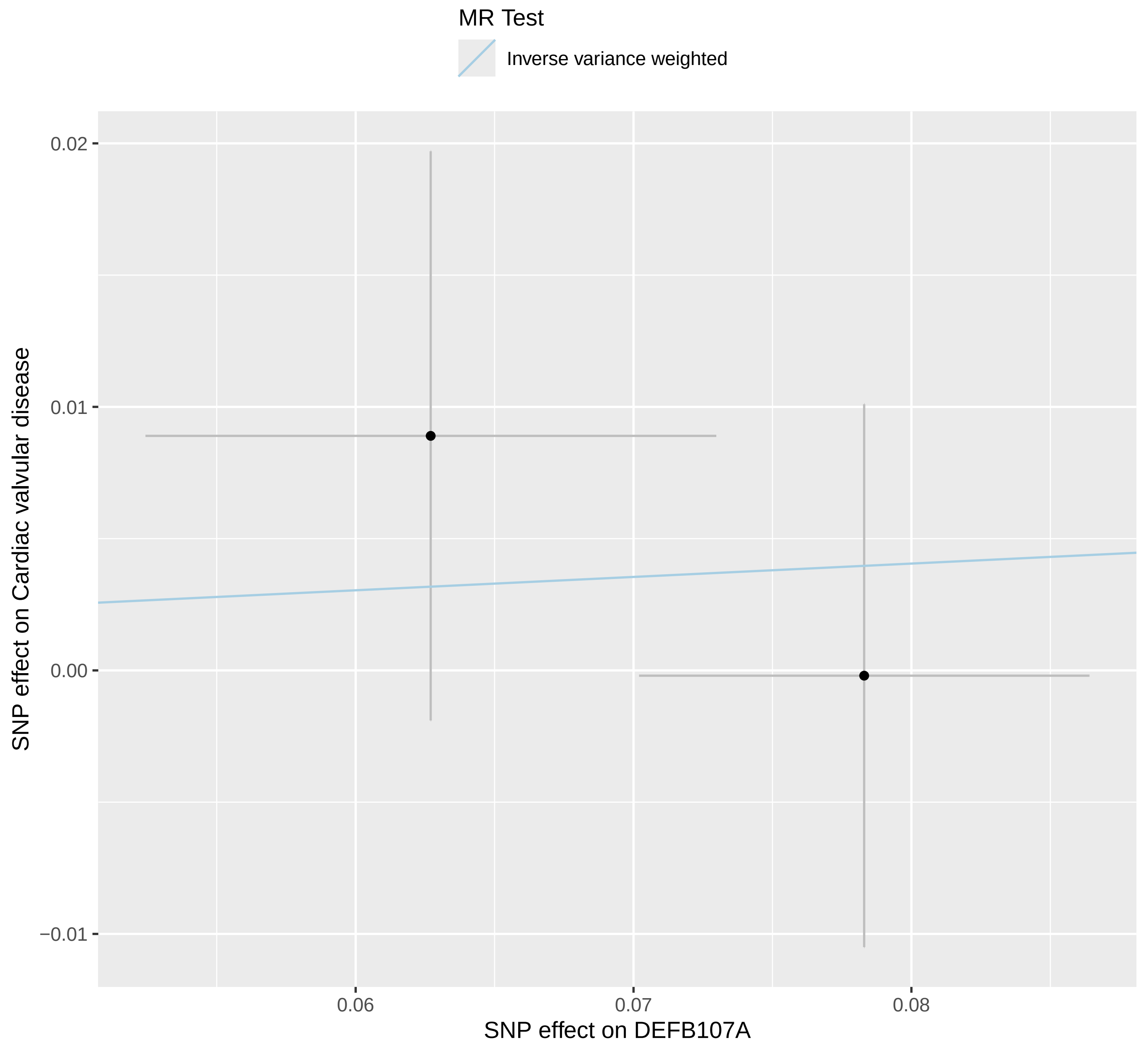

MR Test
Inverse variance weighted
0.02
0.01
0.00
−0.01
SNP effect on Cardiac valvular disease
0.06
0.07
0.08
SNP effect on DEFB107A

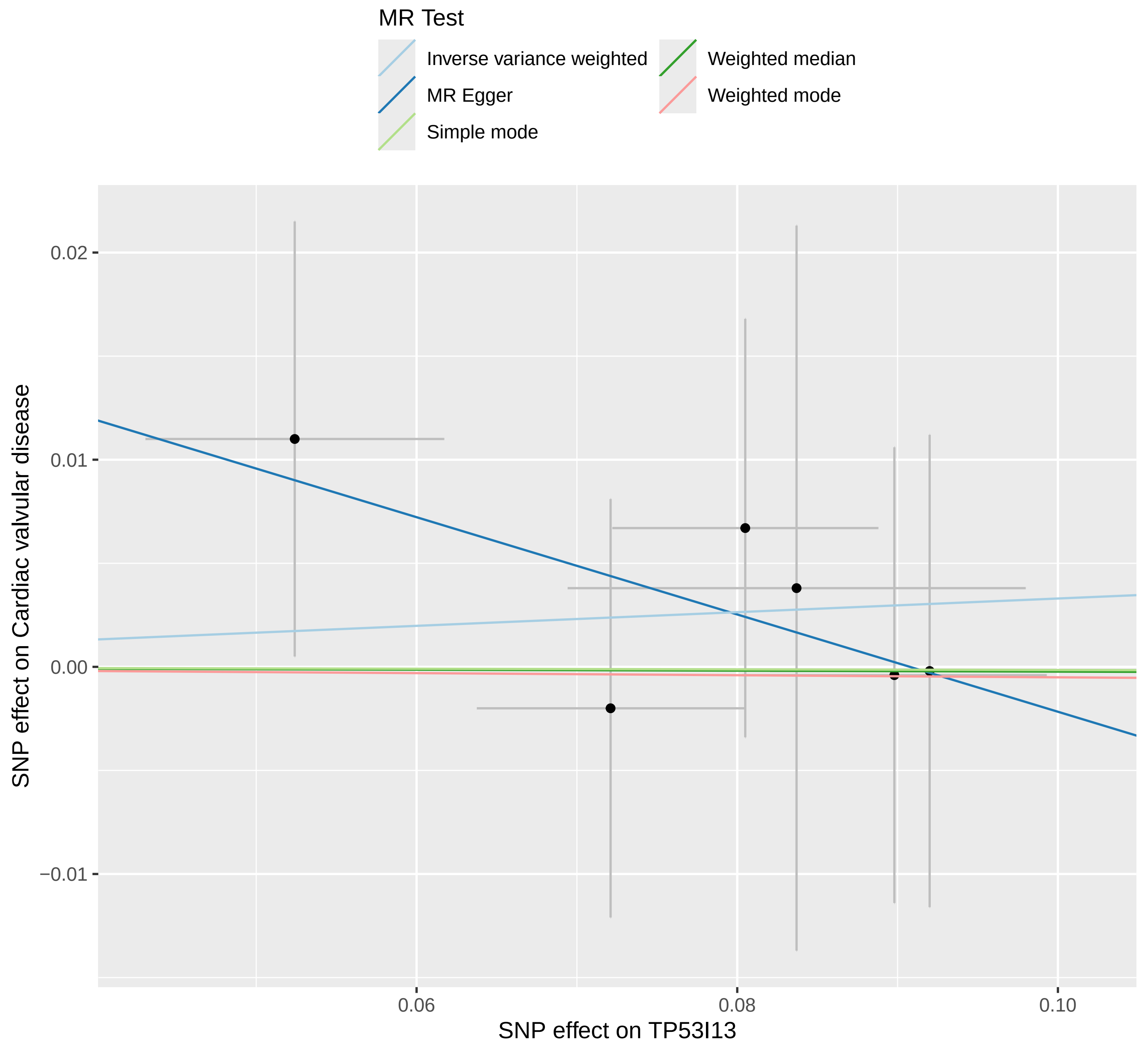
MR Test
Inverse variance weighted
MR Egger
Simple mode
Weighted median
Weighted mode
SNP effect on Cardiac valvular disease
0.02
0.01
0.00
−0.01
0.06
0.08
0.10
SNP effect on TP53I13

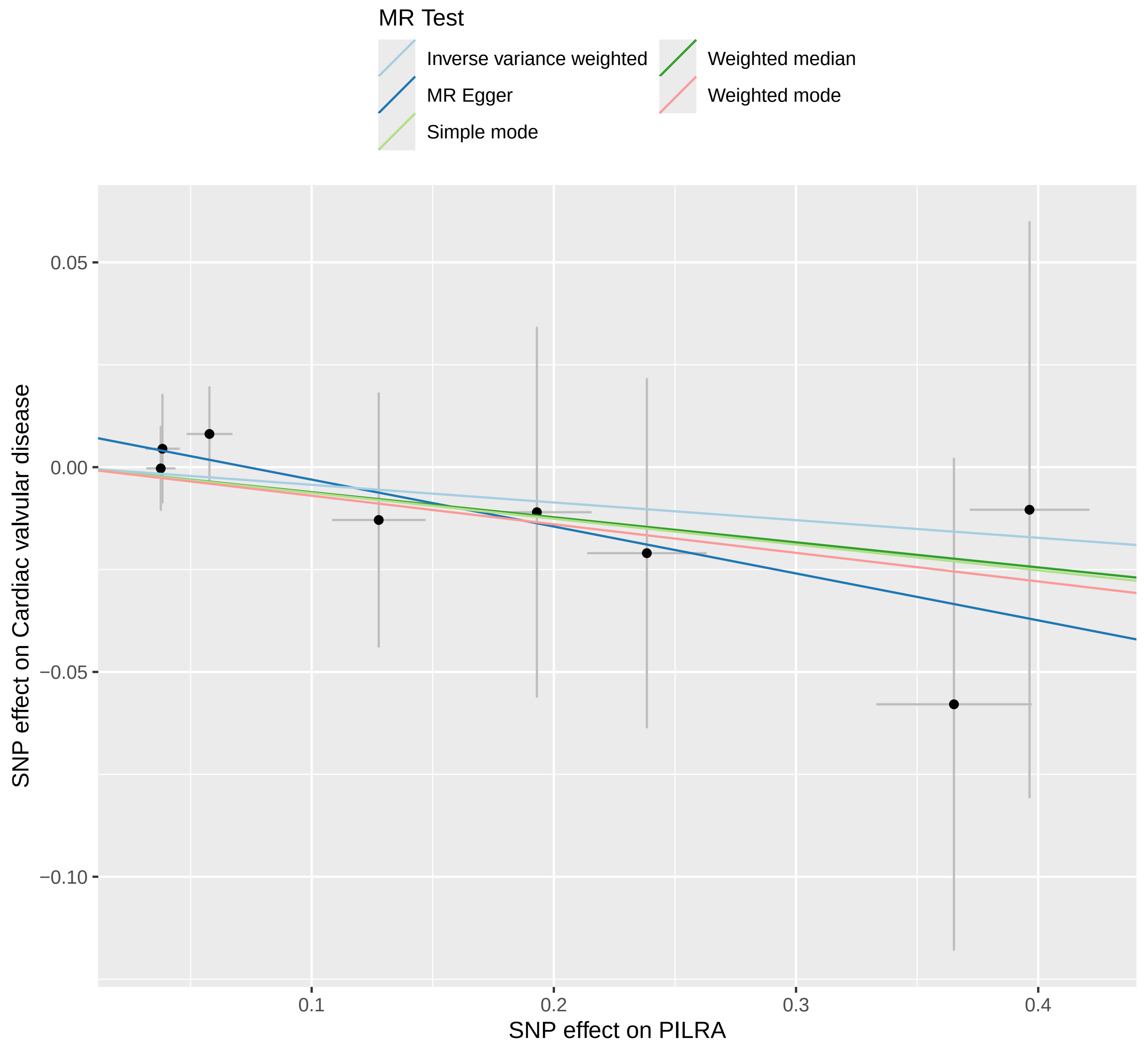
MR Test
Inverse variance weighted
MR Egger
Simple mode
Weighted median
Weighted mode
SNP effect on Cardiac valvular disease
0.05
0.00
−0.05
−0.10
0.1
0.2
0.3
0.4
SNP effect on PILRA

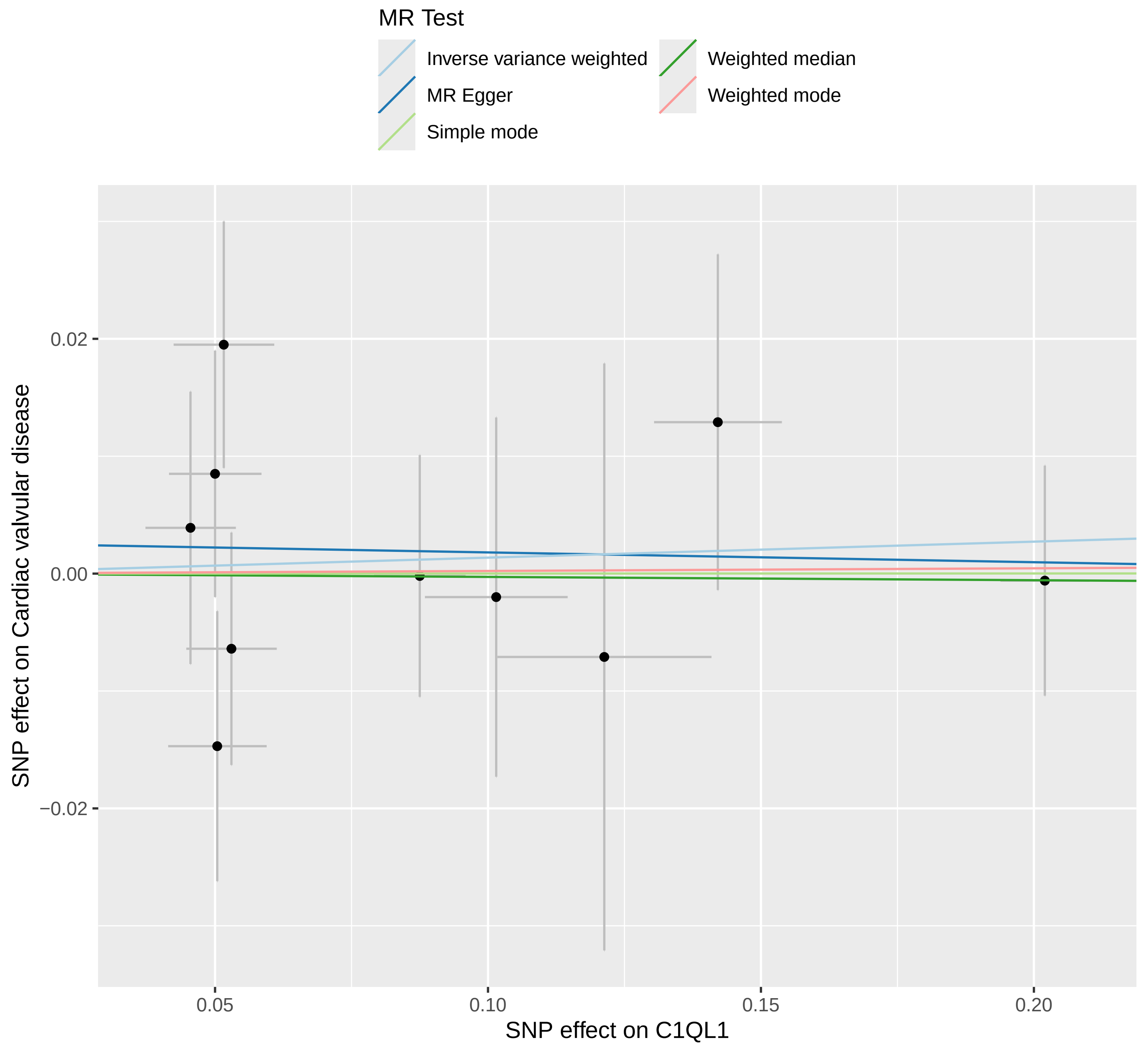
MR Test
Inverse variance weighted
MR Egger
Simple mode
Weighted median
Weighted mode
SNP effect on Cardiac valvular disease
0.02
0.00
−0.02
0.05
0.10
0.15
0.20
SNP effect on C1QL1

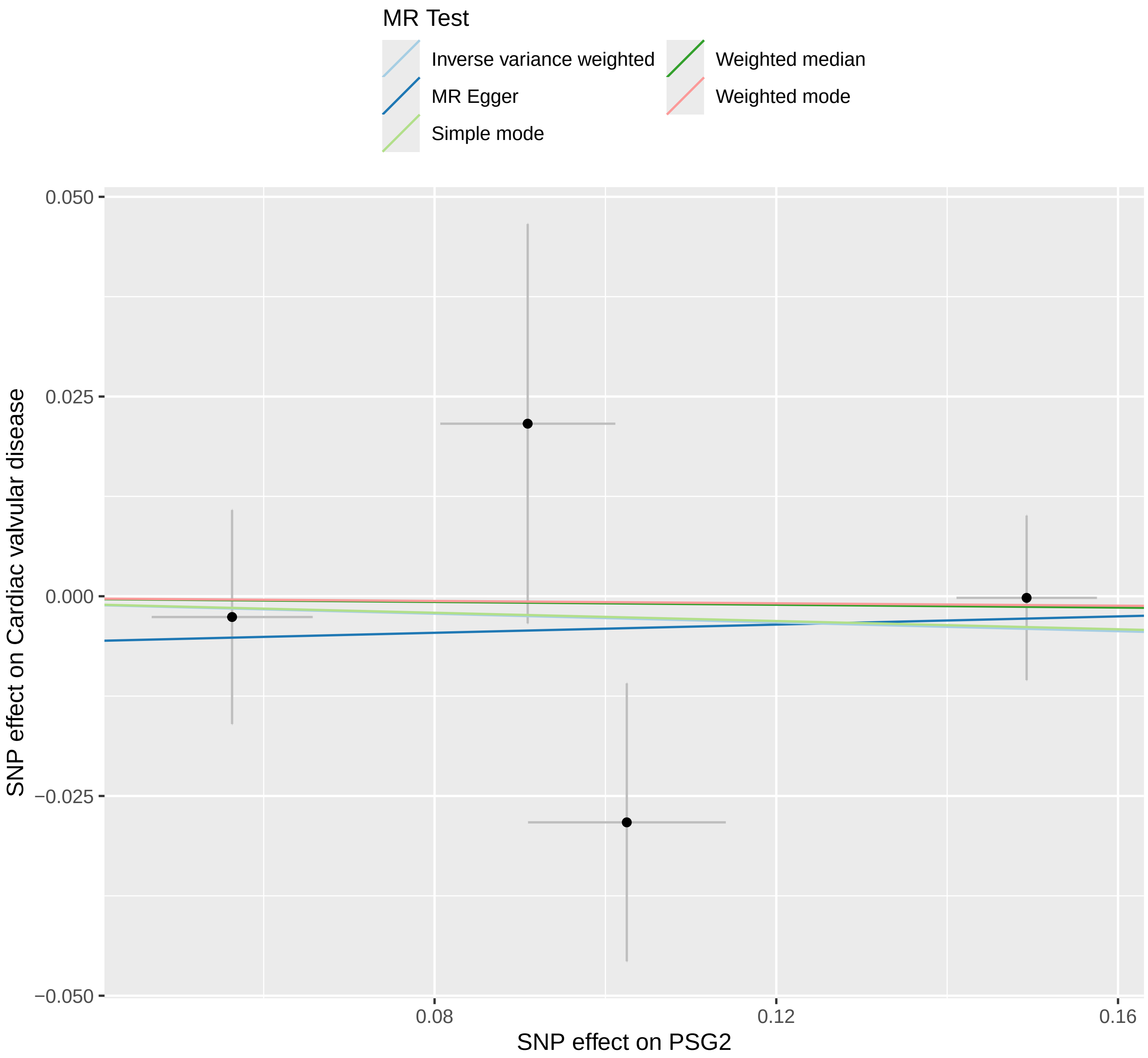

MR Test
Inverse variance weighted
MR Egger
Simple mode
Weighted median
Weighted mode
0.050
0.025
0.000
−0.025
−0.050
SNP effect on Cardiac valvular disease
0.08
0.12
0.16
SNP effect on PSG2

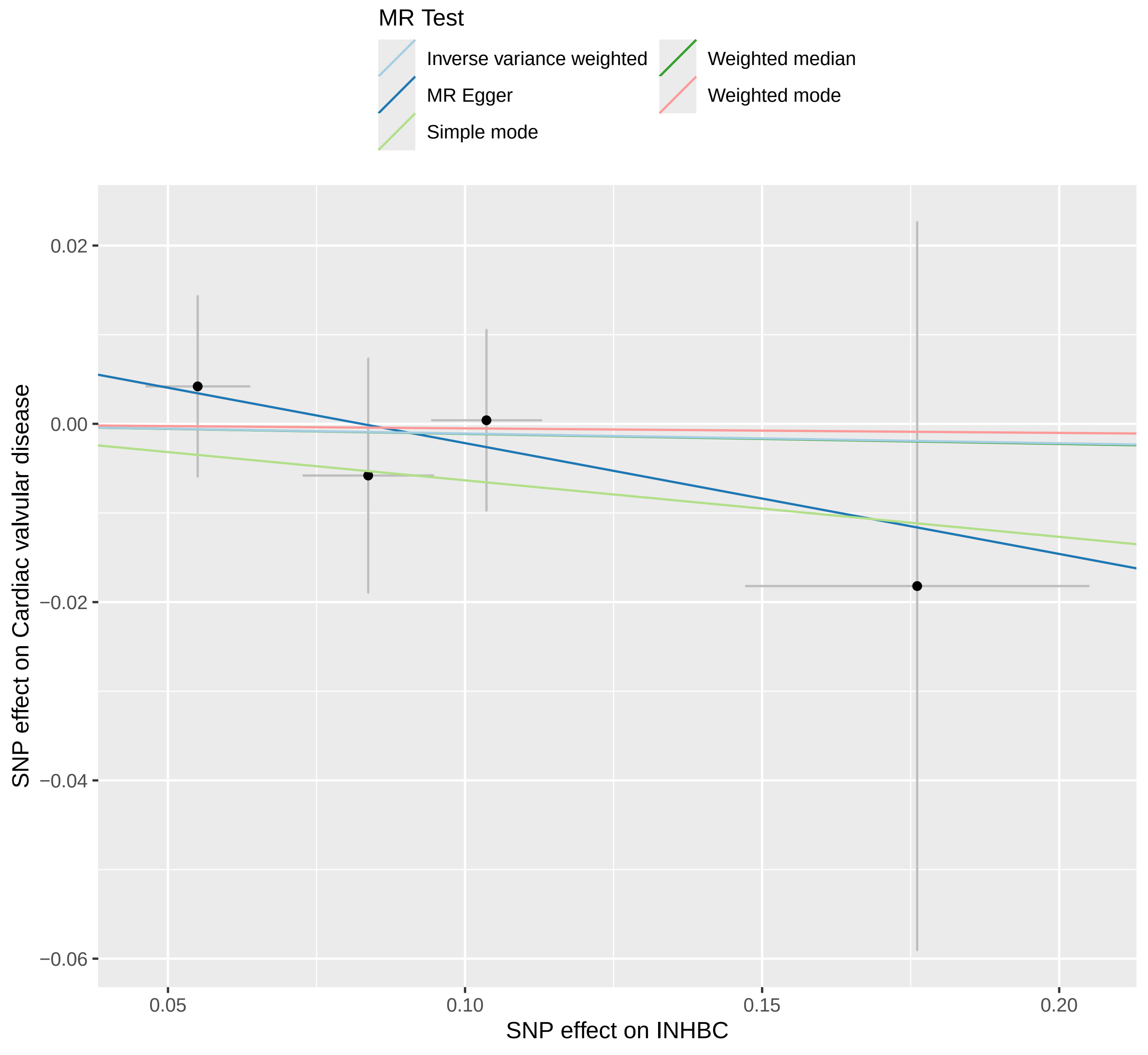
MR Test
Inverse variance weighted
MR Egger
Simple mode
Weighted median
Weighted mode
0.02
0.00
−0.02
−0.04
−0.06
0.05
0.10
0.15
0.20
SNP effect on Cardiac valvular disease
SNP effect on INHBC

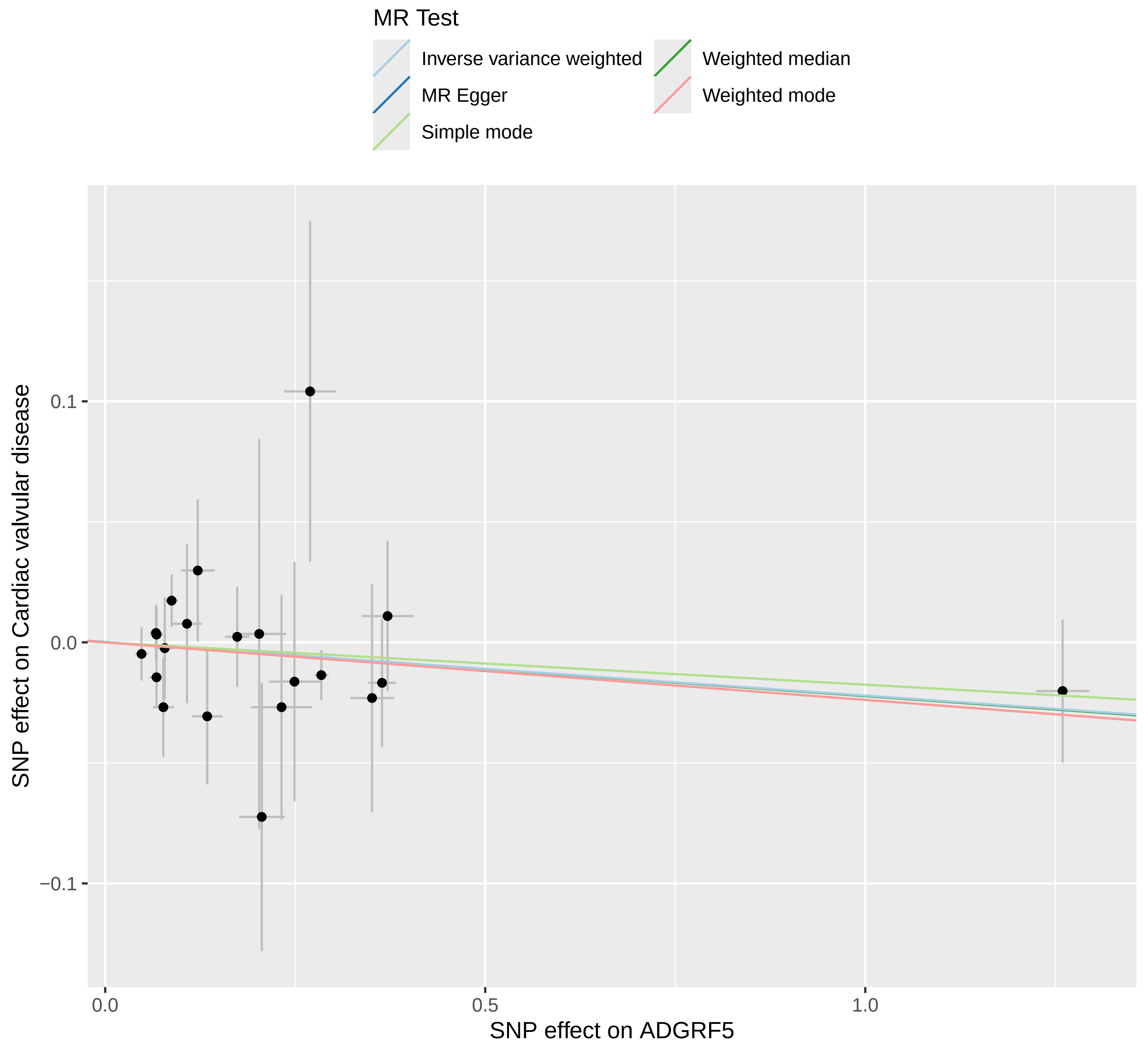

MR Test
Inverse variance weighted
MR Egger
Simple mode
Weighted median
Weighted mode
0.1
0.0
−0.1
0.0
0.5
1.0
SNP effect on ADGRF5
SNP effect on Cardiac valvular disease

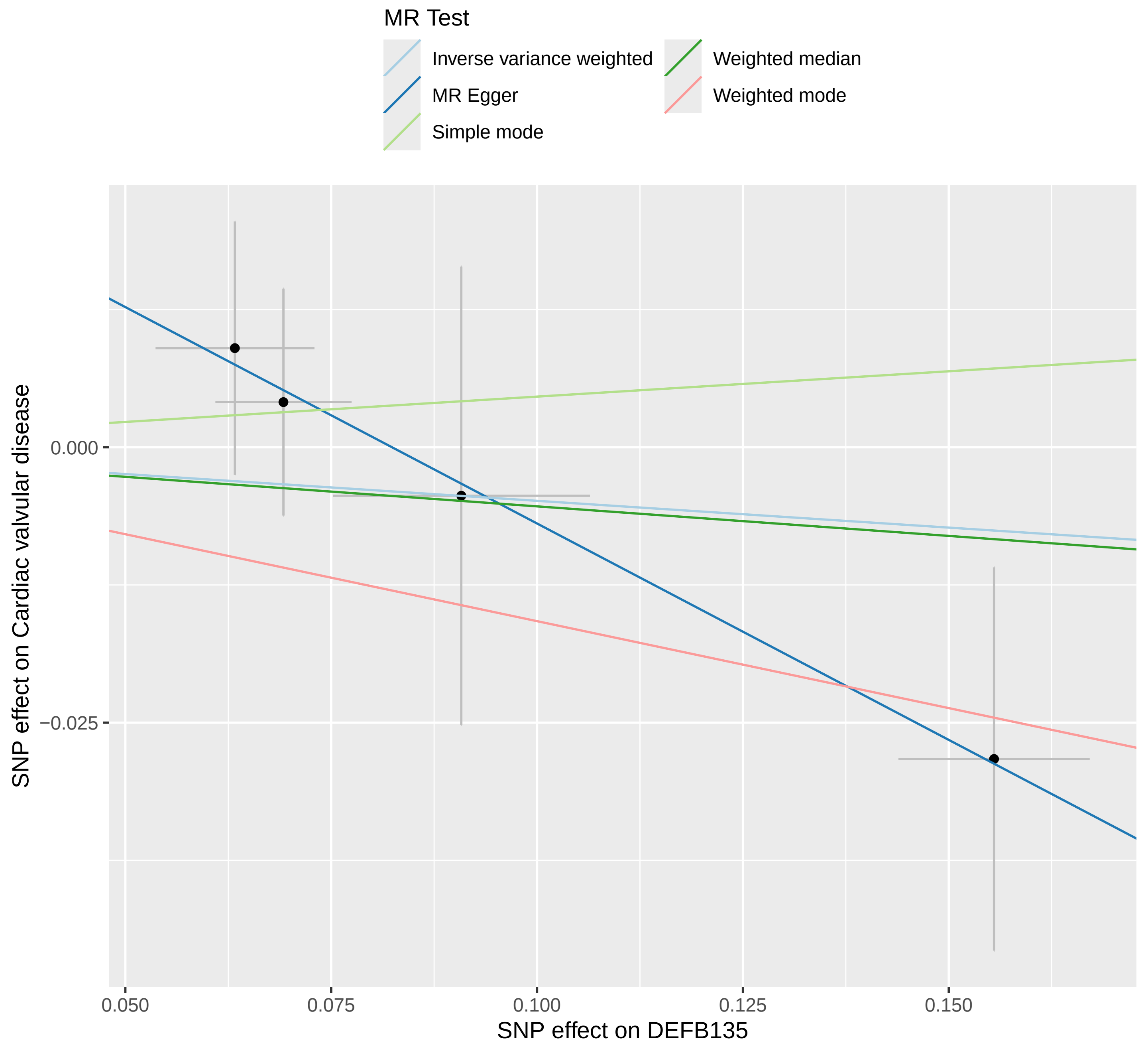

MR Test
Inverse variance weighted
MR Egger
Simple mode
Weighted median
Weighted mode
0.000
−0.025
0.050
0.075
0.100
0.125
0.150
SNP effect on Cardiac valvular disease
SNP effect on DEFB135

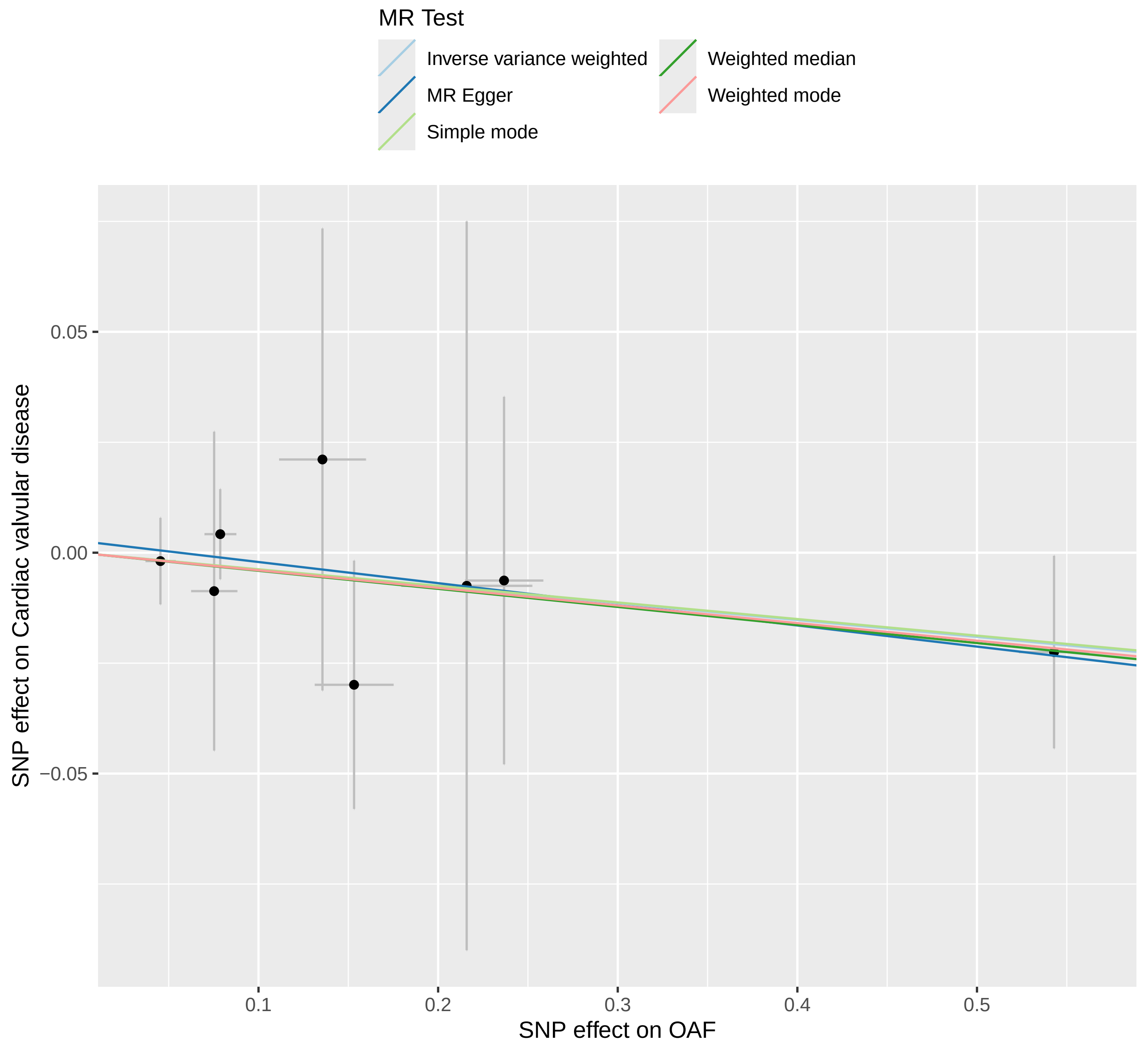

MR Test
Inverse variance weighted
MR Egger
Simple mode
Weighted median
Weighted mode
SNP effect on Cardiac valvular disease
0.05
0.00
−0.05
0.1
0.2
0.3
0.4
0.5
SNP effect on OAF

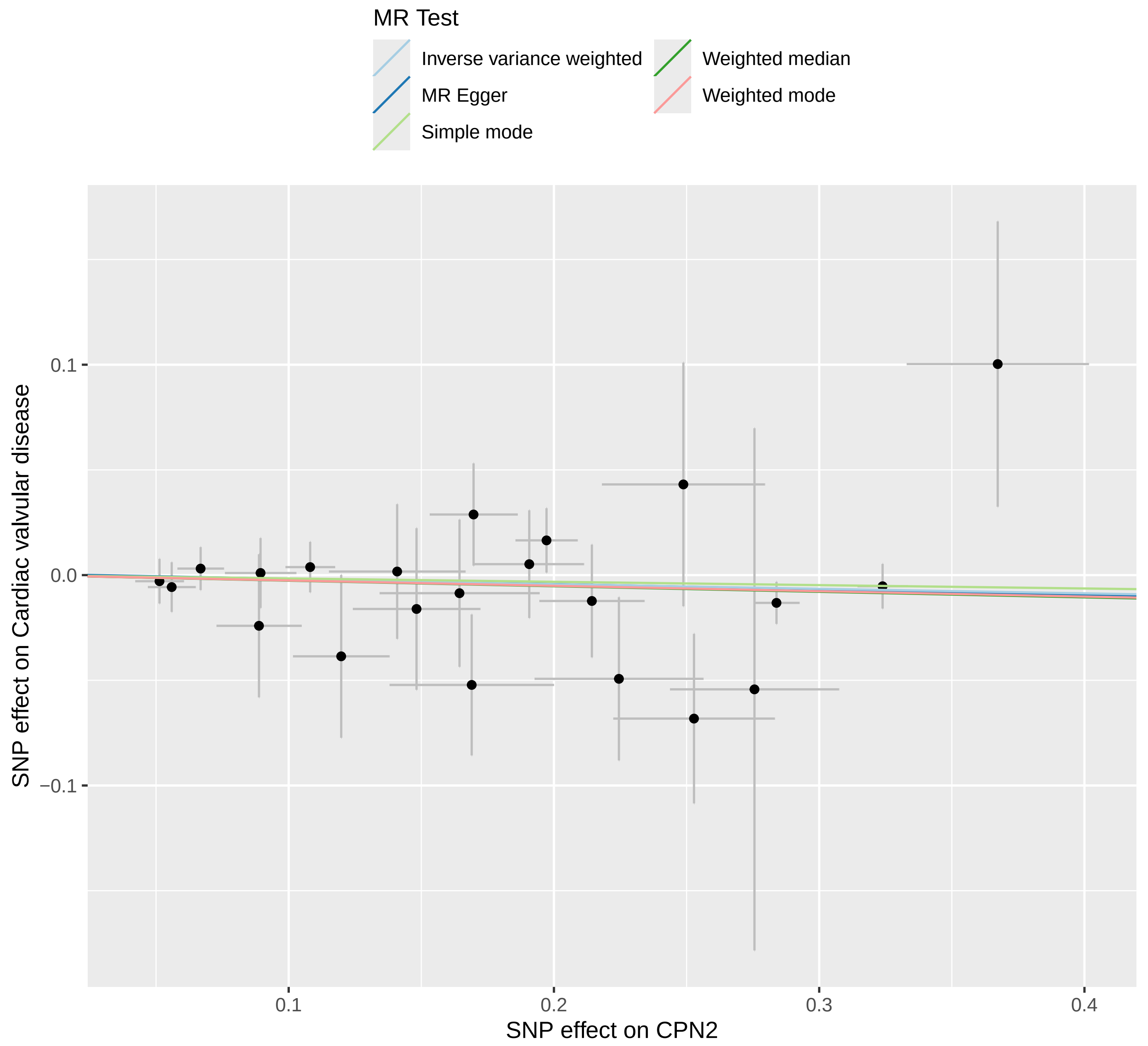
MR Test
Inverse variance weighted
MR Egger
Simple mode
Weighted median
Weighted mode
SNP effect on Cardiac valvular disease
0.1
0.0
−0.1
0.1
0.2
0.3
0.4
SNP effect on CPN2

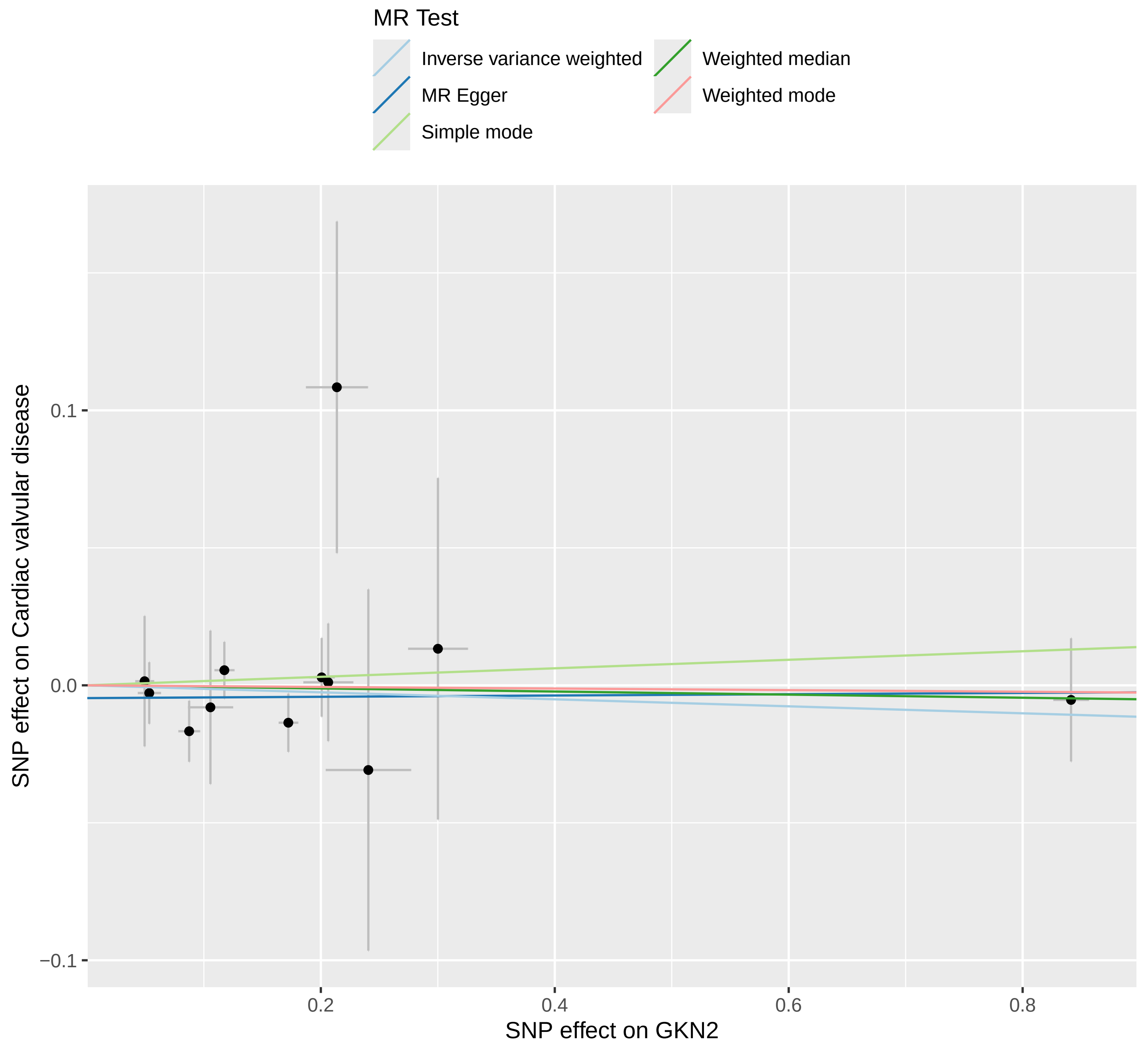

MR Test
Inverse variance weighted
MR Egger
Simple mode
Weighted median
Weighted mode
SNP effect on Cardiac valvular disease
0.1
0.0
−0.1
0.2
0.4
0.6
0.8
SNP effect on GKN2

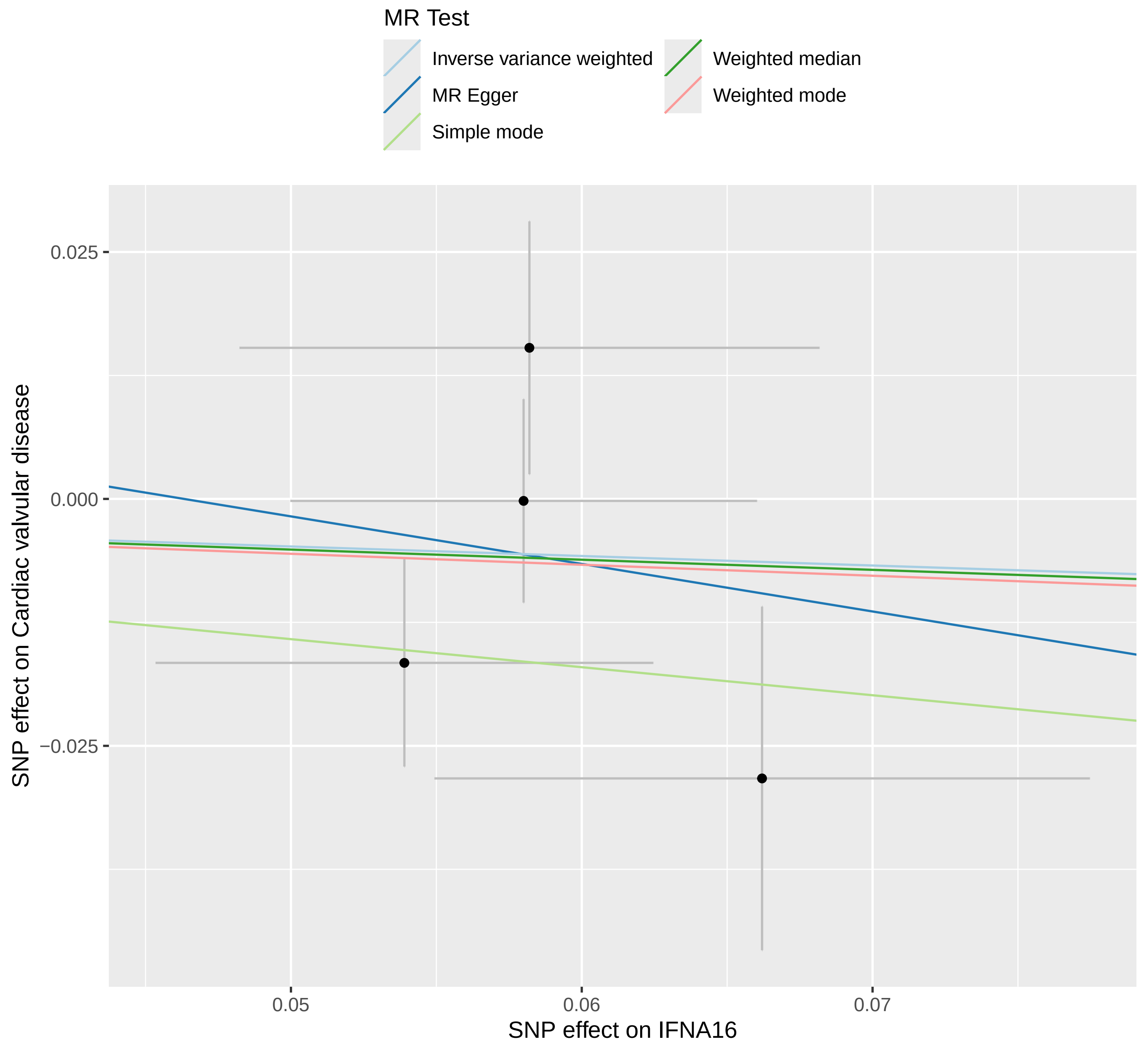
MR Test
Inverse variance weighted
MR Egger
Simple mode
Weighted median
Weighted mode
0.025
0.000
−0.025
0.05
0.06
0.07
SNP effect on Cardiac valvular disease
SNP effect on IFNA16

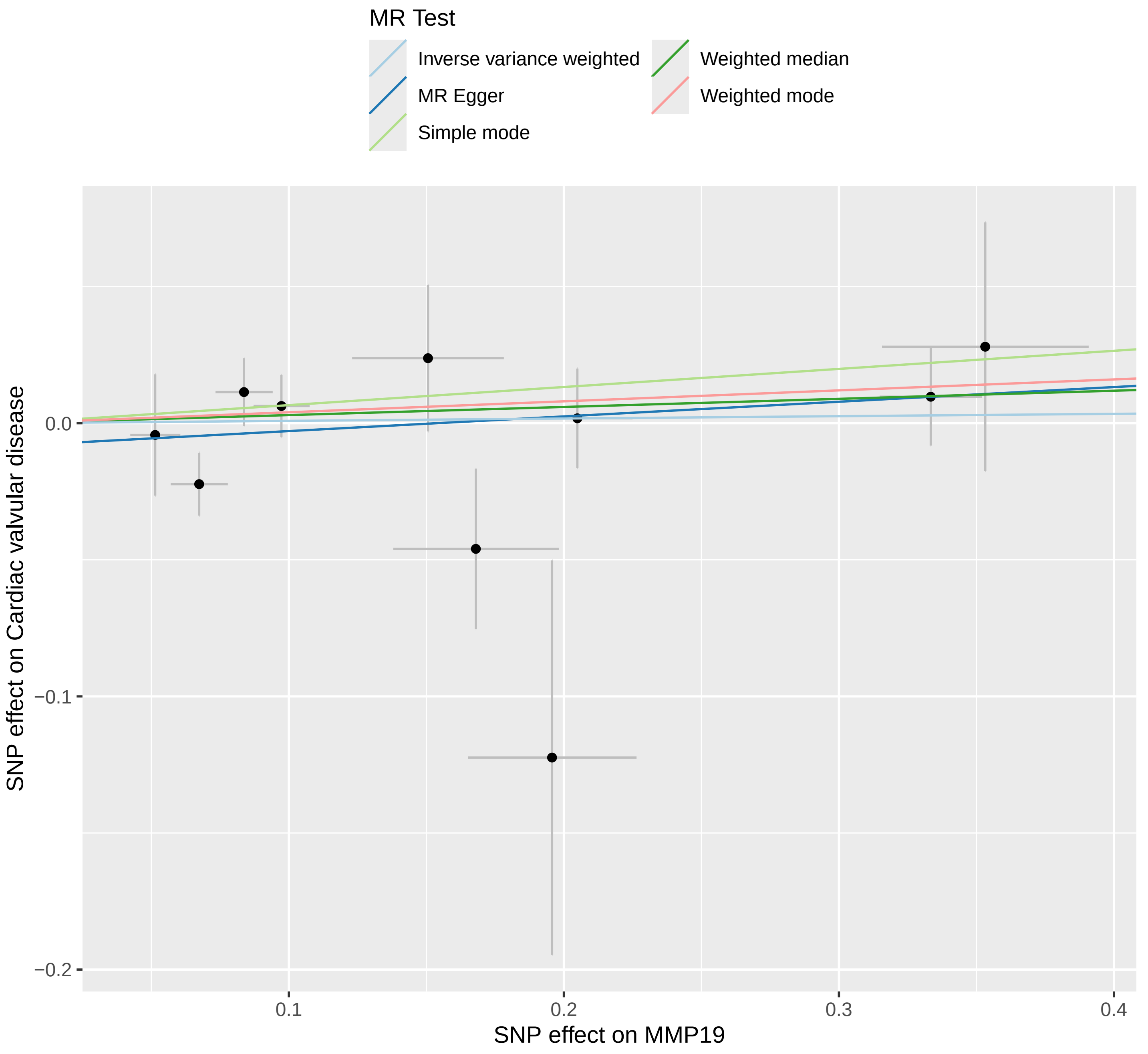

MR Test
Inverse variance weighted
MR Egger
Simple mode
Weighted median
Weighted mode
SNP effect on Cardiac valvular disease
0.0
−0.1
−0.2
0.1
0.2
0.3
0.4
SNP effect on MMP19

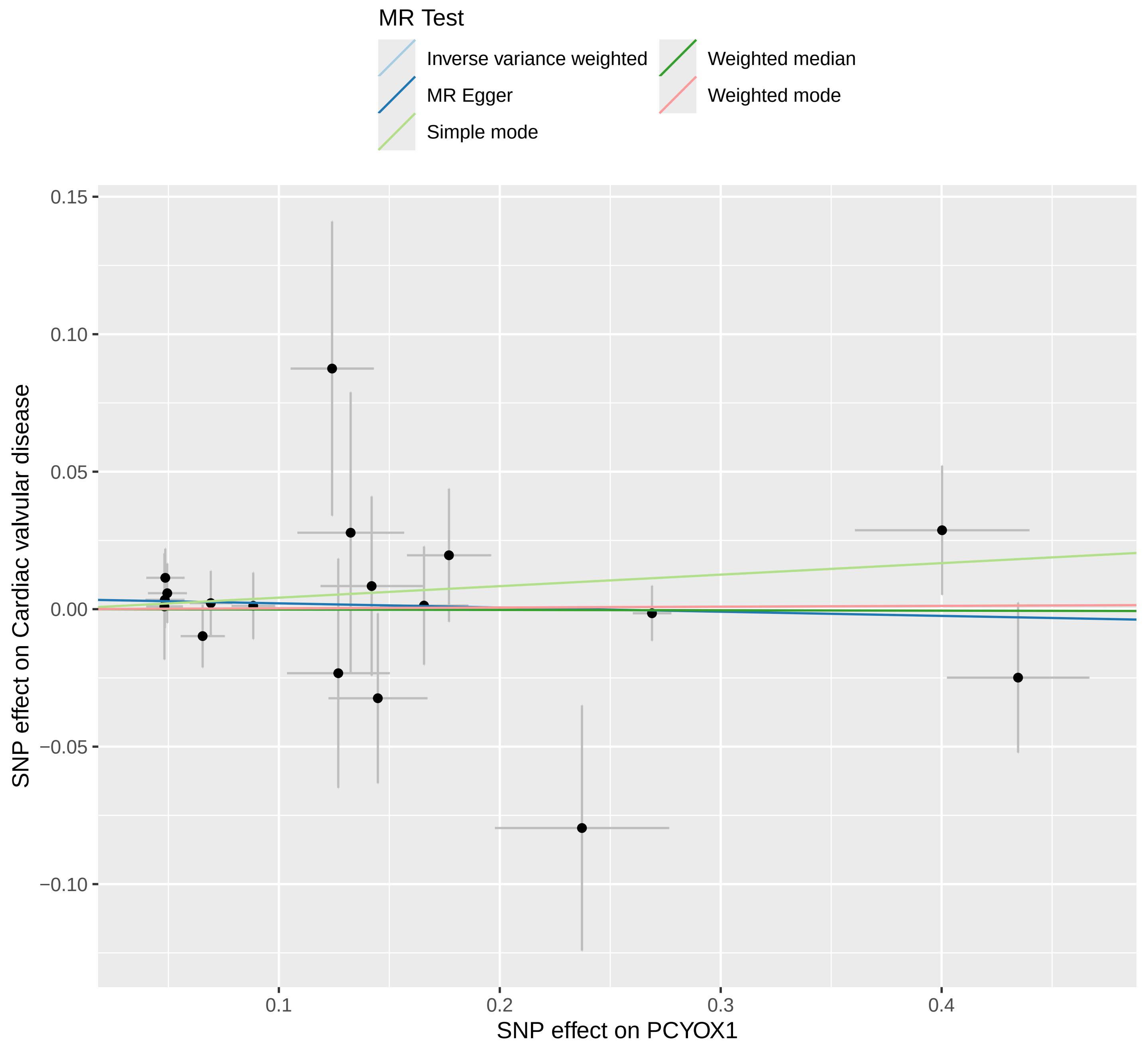
MR Test
Inverse variance weighted
MR Egger
Simple mode
Weighted median
Weighted mode
0.15
0.10
0.05
0.00
−0.05
−0.10
SNP effect on Cardiac valvular disease
0.1
0.2
0.3
0.4
SNP effect on PCYOX1

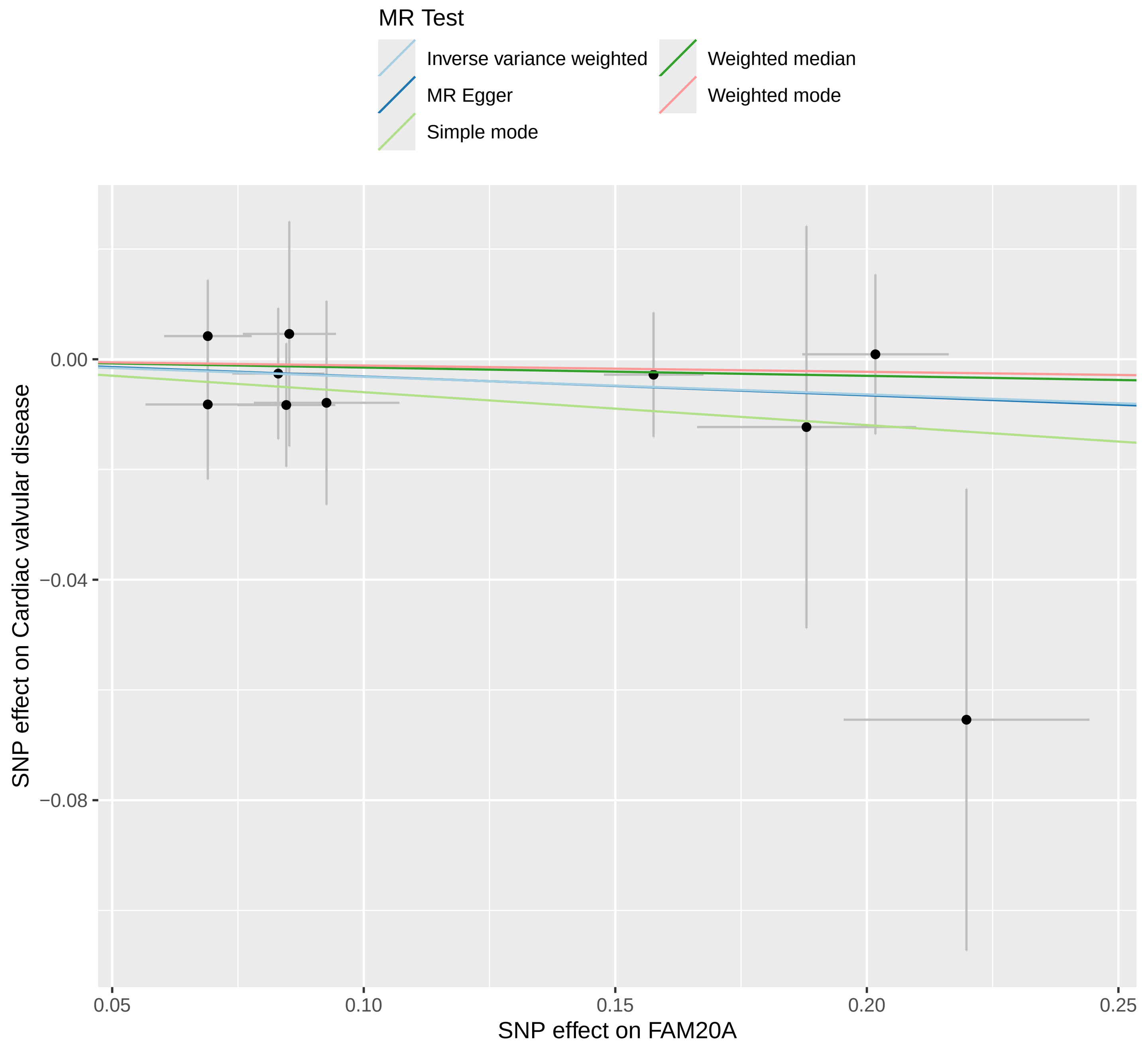
MR Test
Inverse variance weighted
MR Egger
Simple mode
Weighted median
Weighted mode
SNP effect on Cardiac valvular disease
0.00
−0.04
−0.08
0.05
0.10
0.15
0.20
0.25
SNP effect on FAM20A

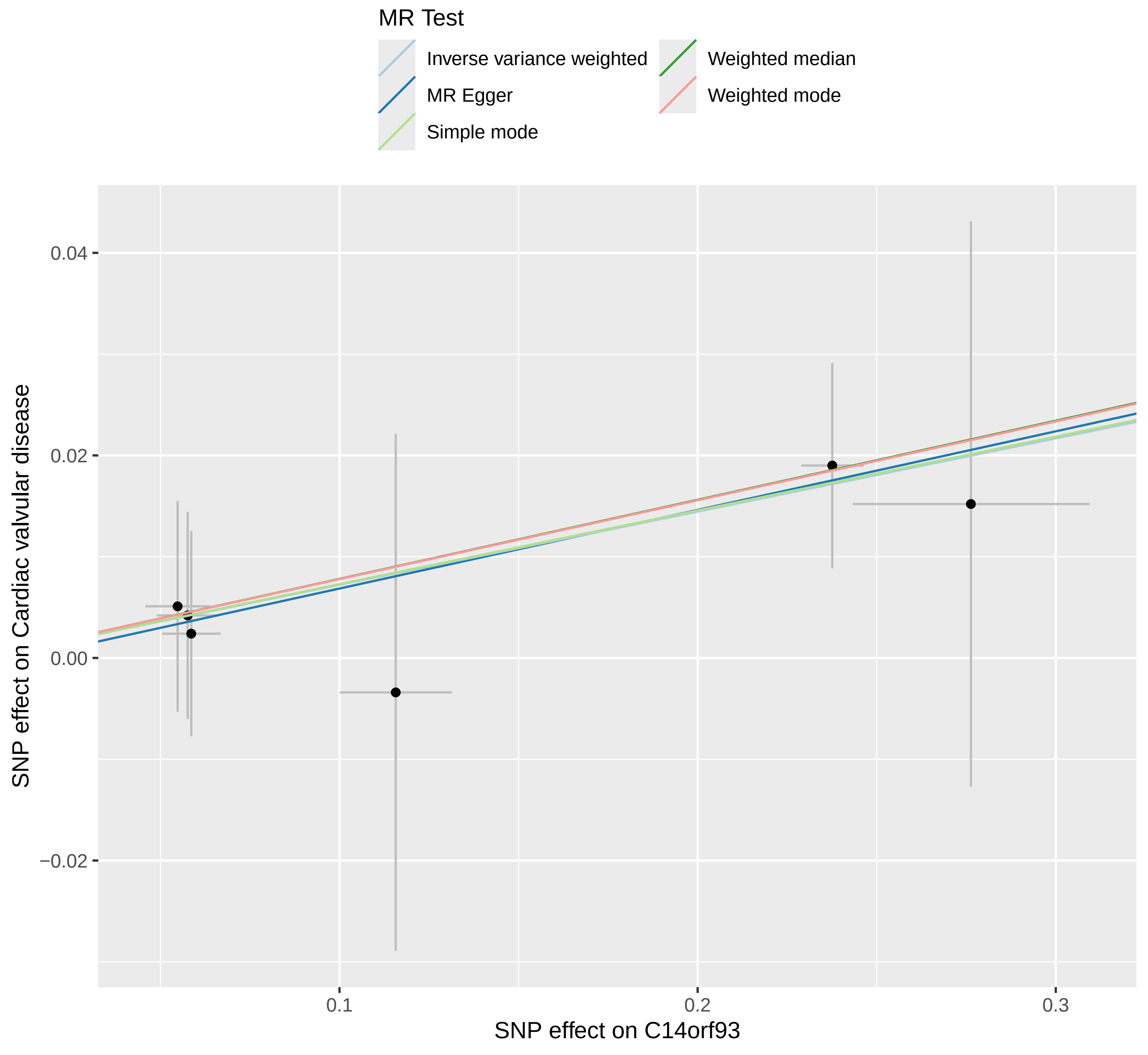

MR Test
Inverse variance weighted
MR Egger
Simple mode
Weighted median
Weighted mode
0.04
0.02
0.00
−0.02
0.1
0.2
0.3
SNP effect on Cardiac valvular disease
SNP effect on C14orf93

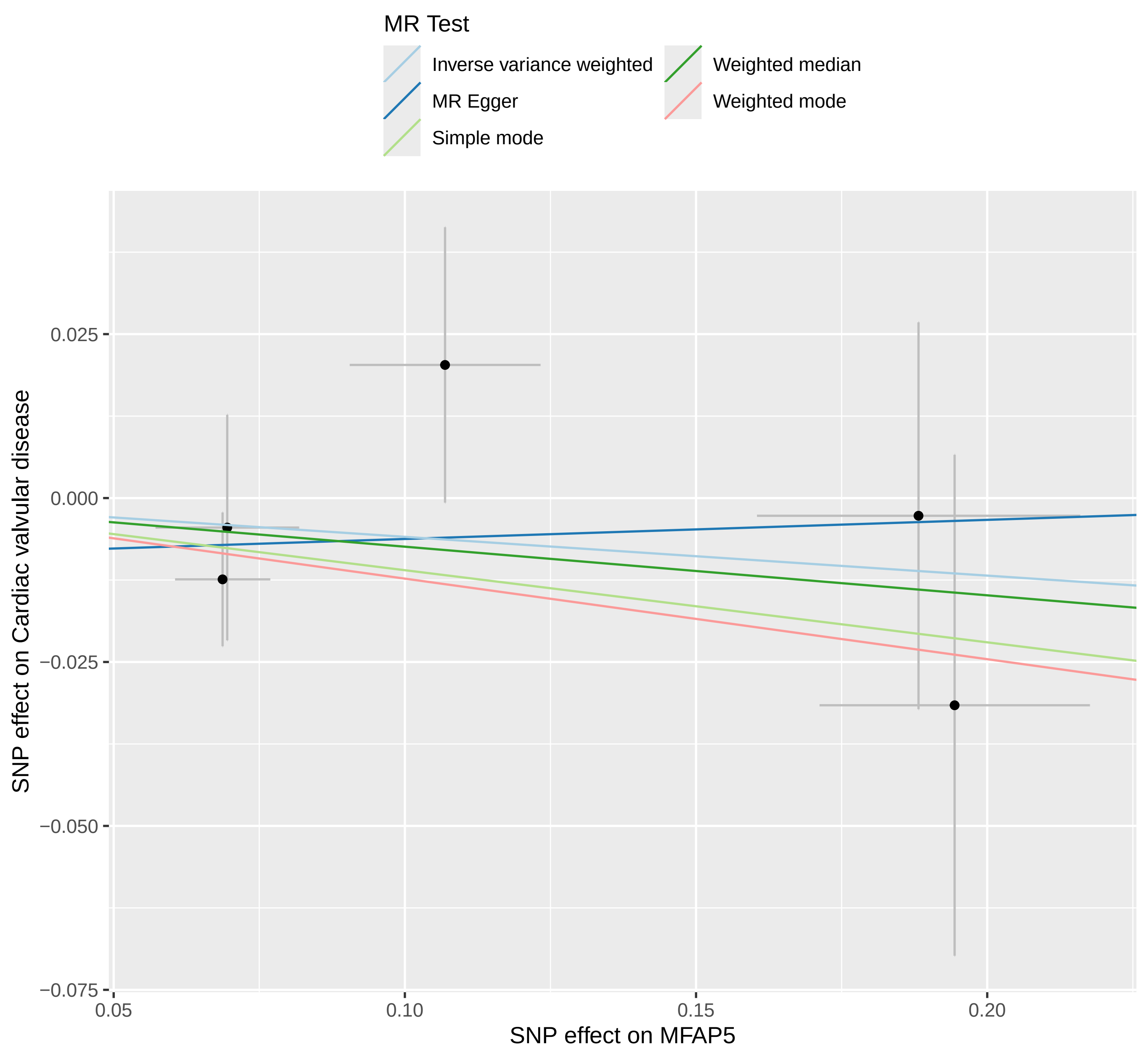

MR Test
Inverse variance weighted
MR Egger
Simple mode
Weighted median
Weighted mode
0.025
0.000
−0.025
−0.050
−0.075
0.05
0.10
0.15
0.20
SNP effect on Cardiac valvular disease
SNP effect on MFAP5

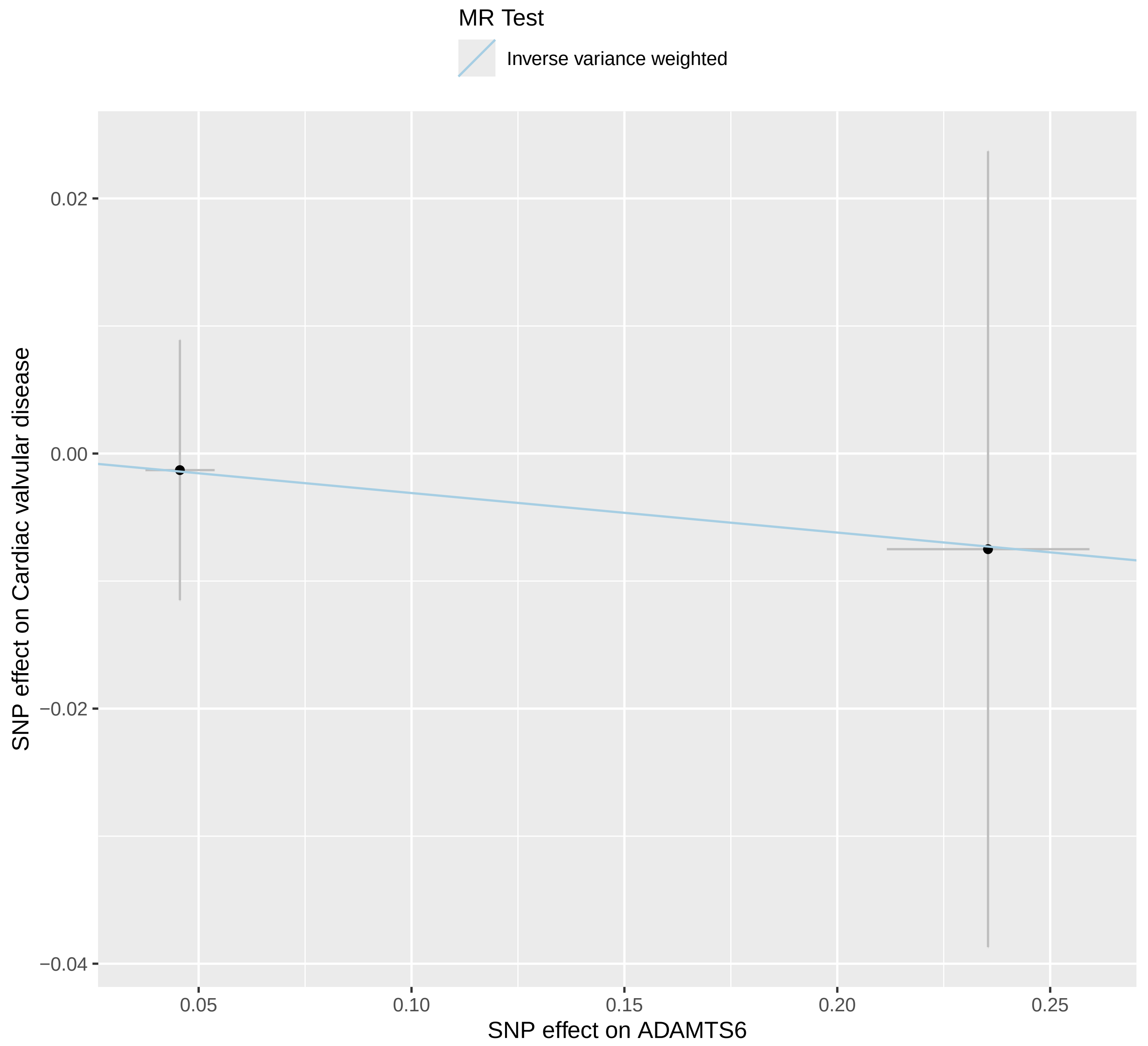

MR Test
Inverse variance weighted
SNP effect on Cardiac valvular disease
0.02
0.00
−0.02
−0.04
0.05
0.10
0.15
0.20
0.25
SNP effect on ADAMTS6

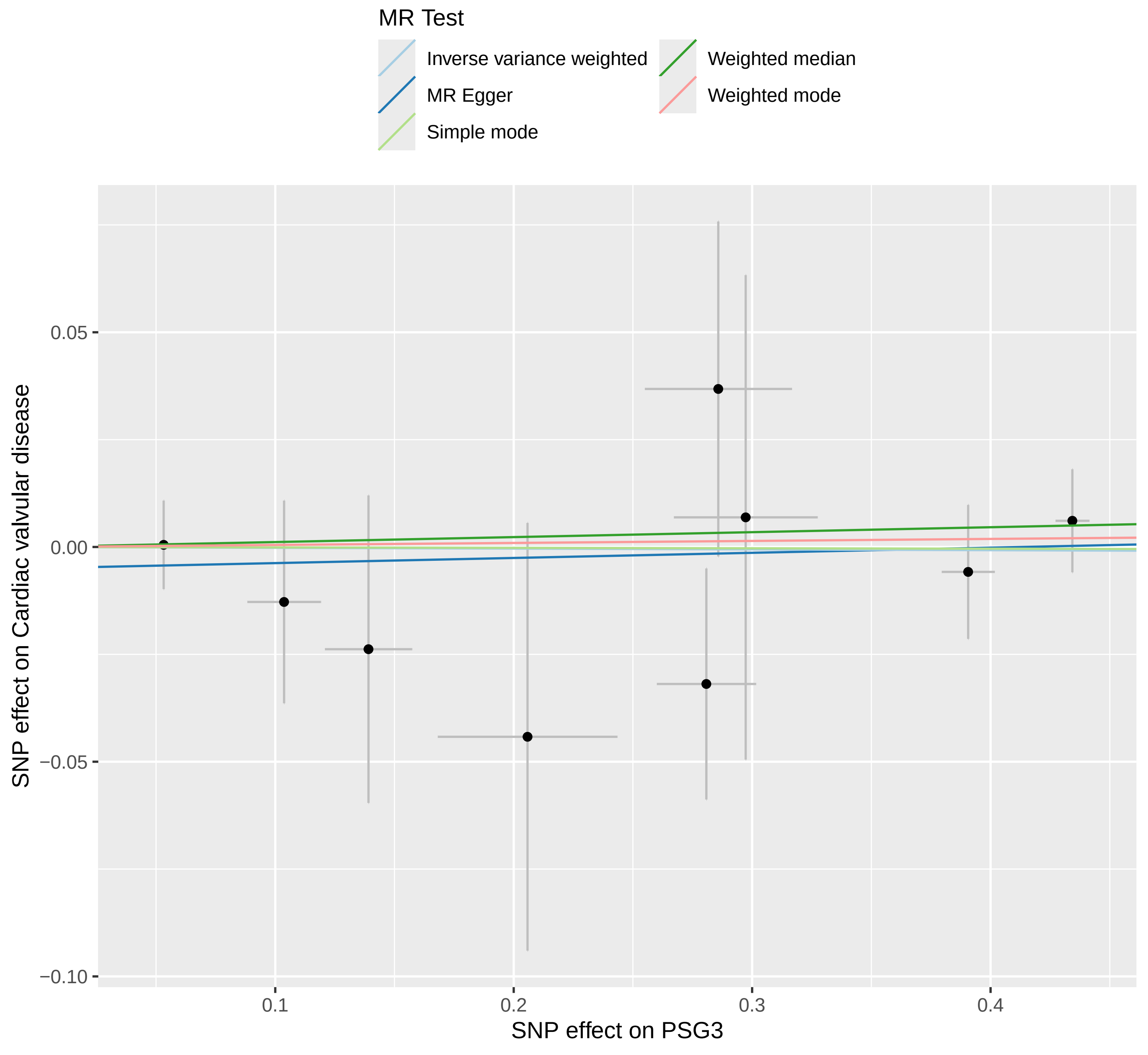

MR Test
Inverse variance weighted
MR Egger
Simple mode
Weighted median
Weighted mode
0.05
0.00
−0.05
−0.10
0.1
0.2
0.3
0.4
SNP effect on Cardiac valvular disease
SNP effect on PSG3

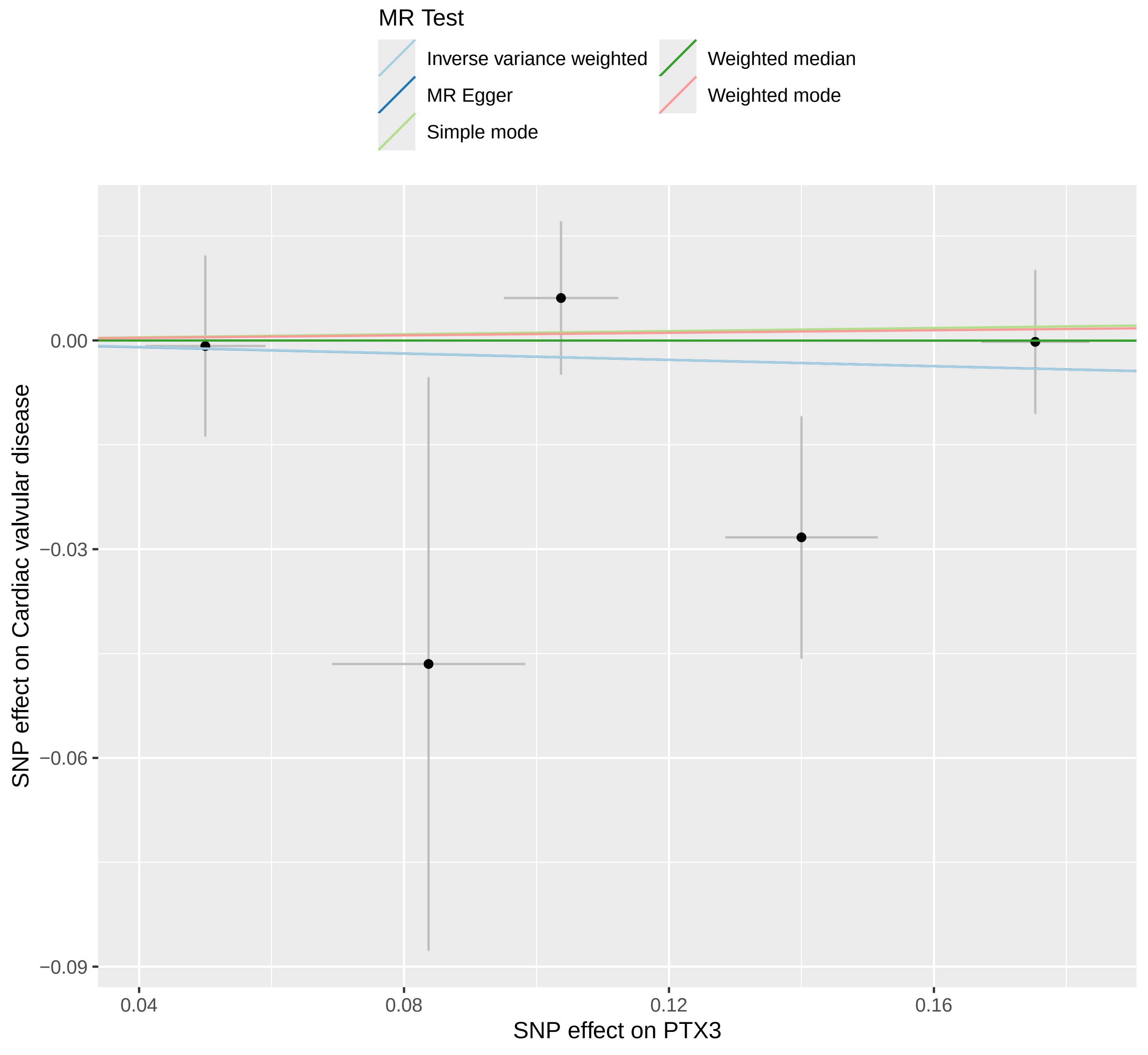
MR Test
Inverse variance weighted
MR Egger
Simple mode
Weighted median
Weighted mode
0.00
−0.03
−0.06
−0.09
SNP effect on Cardiac valvular disease
0.04
0.08
0.12
0.16
SNP effect on PTX3

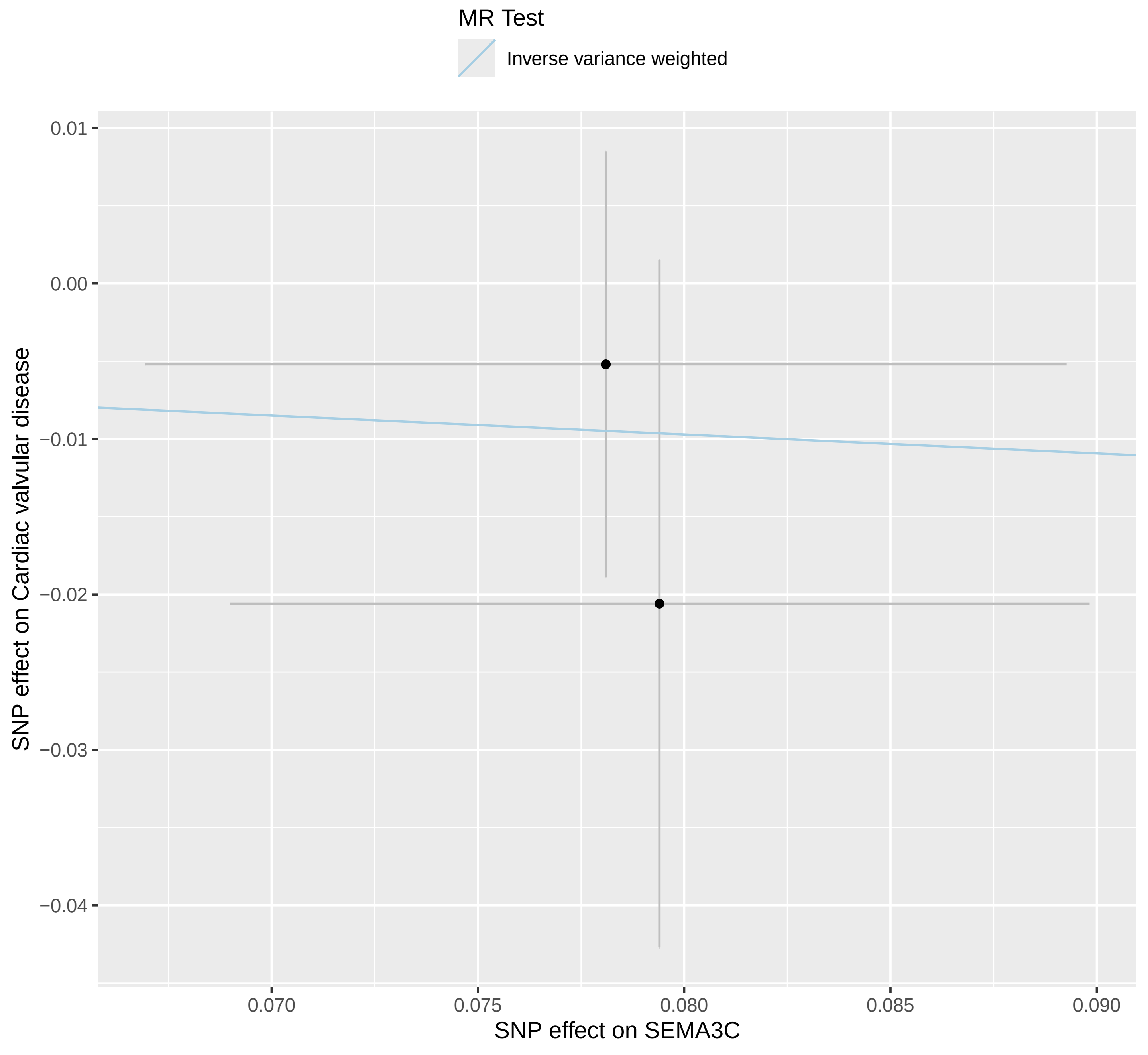

MR Test
Inverse variance weighted
0.01
0.00
−0.01
−0.02
−0.03
−0.04
SNP effect on Cardiac valvular disease
0.070
0.075
0.080
0.085
0.090
SNP effect on SEMA3C

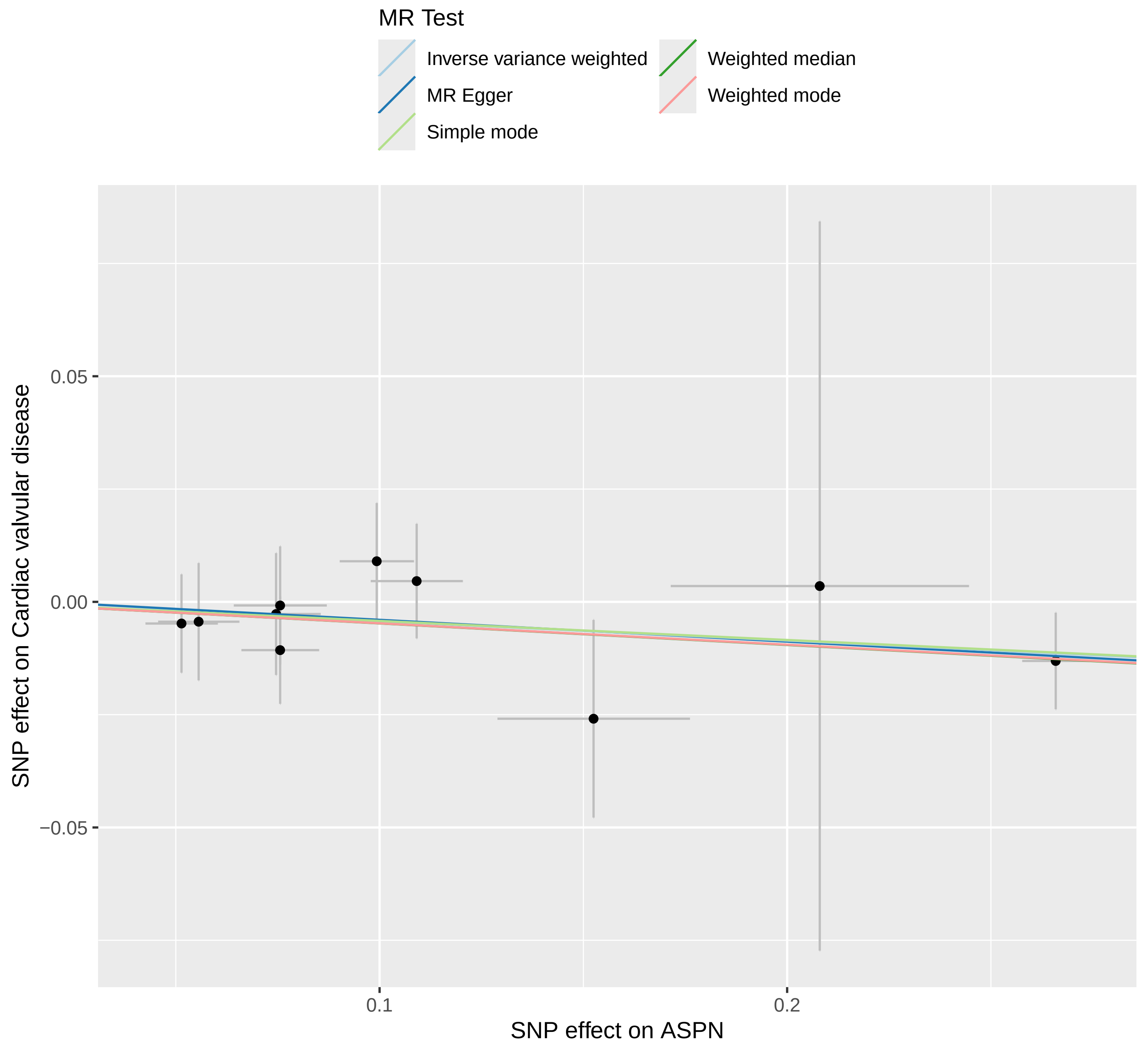

MR Test
Inverse variance weighted
MR Egger
Simple mode
Weighted median
Weighted mode
0.05
0.00
−0.05
0.1
0.2
SNP effect on Cardiac valvular disease
SNP effect on ASPN

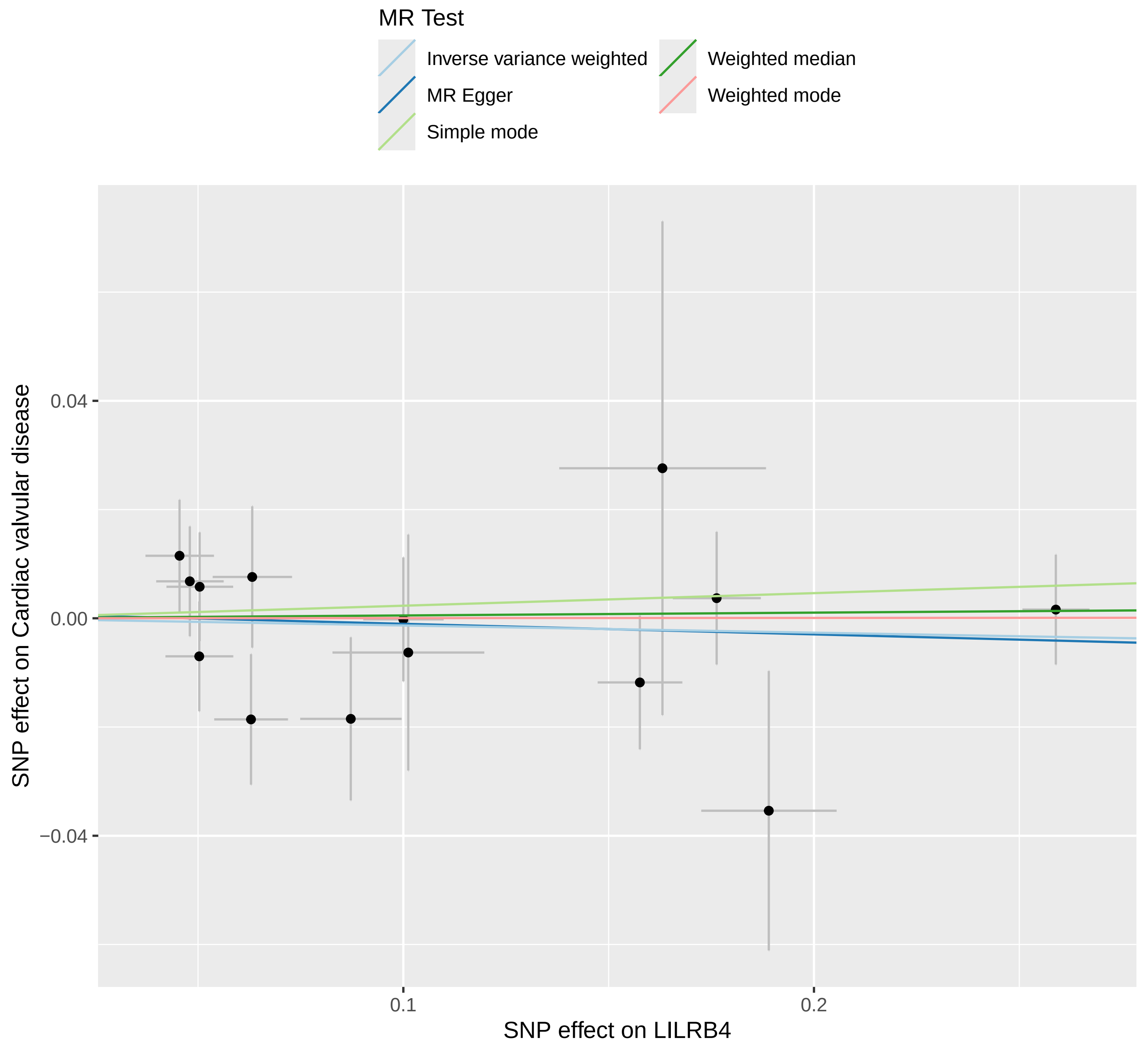

MR Test
Inverse variance weighted
MR Egger
Simple mode
Weighted median
Weighted mode
0.04
0.00
−0.04
0.1
0.2
SNP effect on Cardiac valvular disease
SNP effect on LILRB4

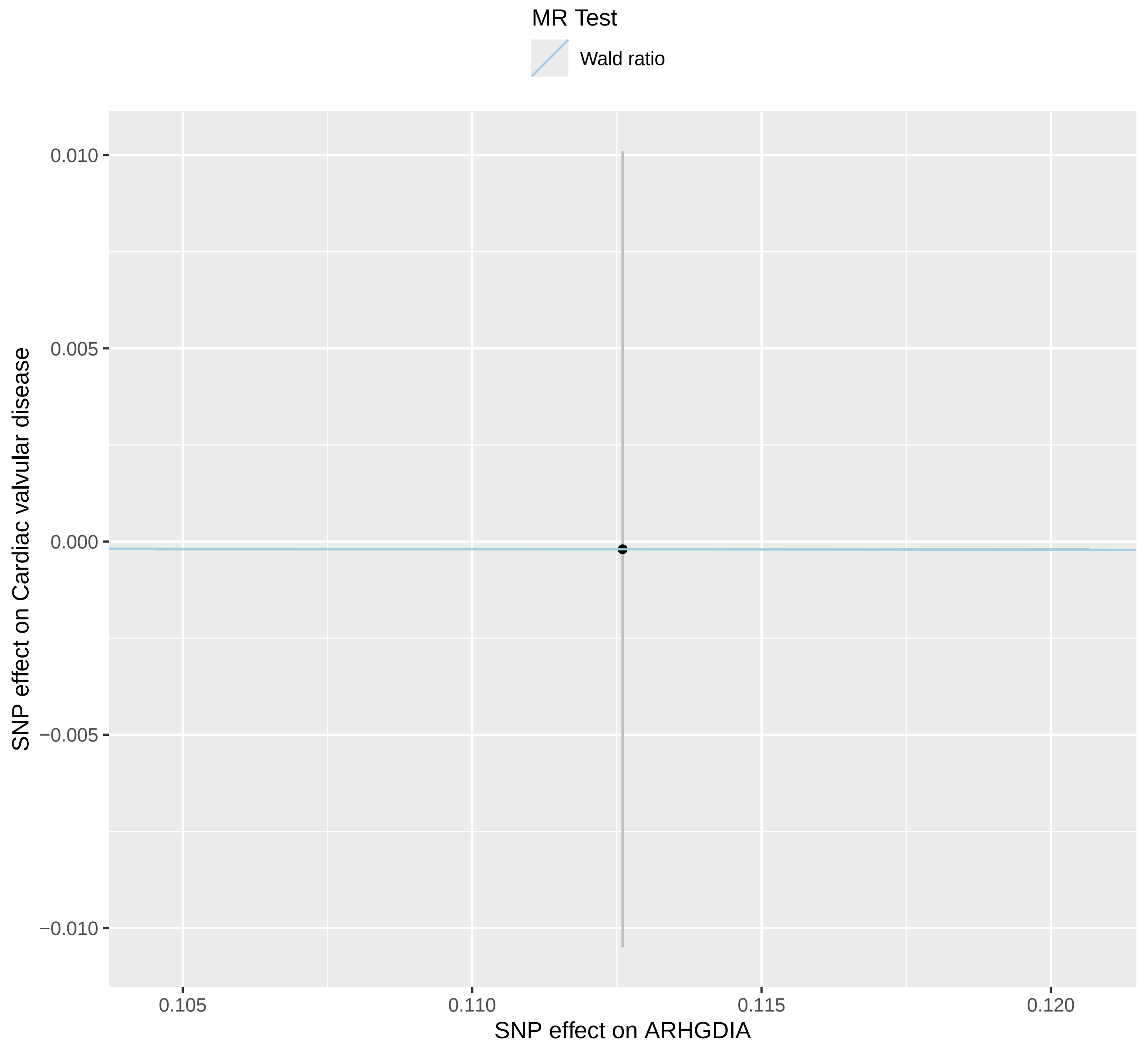
MR Test
Wald ratio
SNP effect on Cardiac valvular disease
0.010
0.005
0.000
−0.005
−0.010
0.105
0.110
0.115
0.120
SNP effect on ARHGDIA

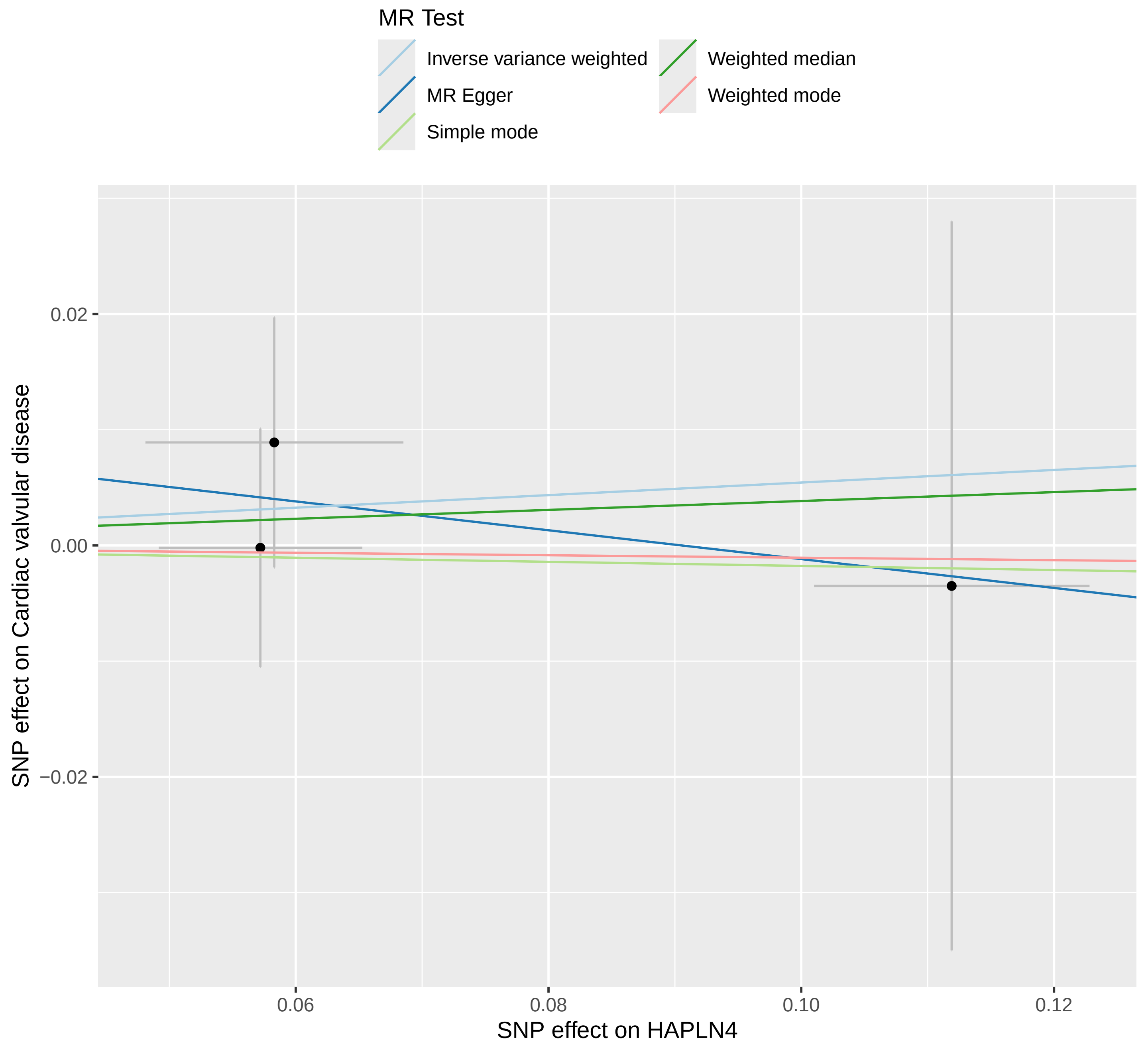
MR Test
Inverse variance weighted
MR Egger
Simple mode
Weighted median
Weighted mode
0.02
0.00
−0.02
0.06
0.08
0.10
0.12
SNP effect on Cardiac valvular disease
SNP effect on HAPLN4

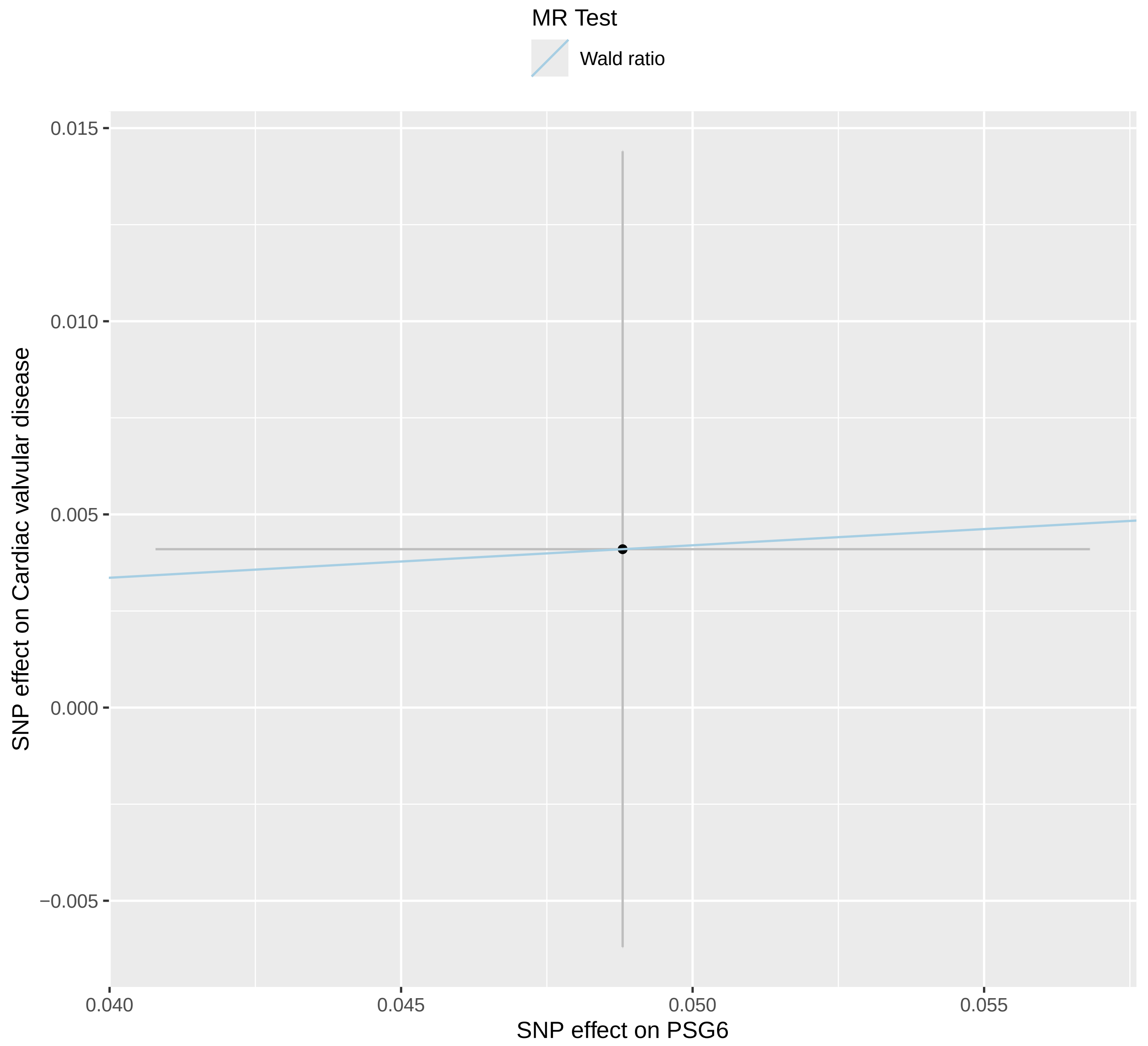
MR Test
Wald ratio
0.015
0.010
0.005
0.000
−0.005
SNP effect on Cardiac valvular disease
0.040
0.045
0.050
0.055
SNP effect on PSG6

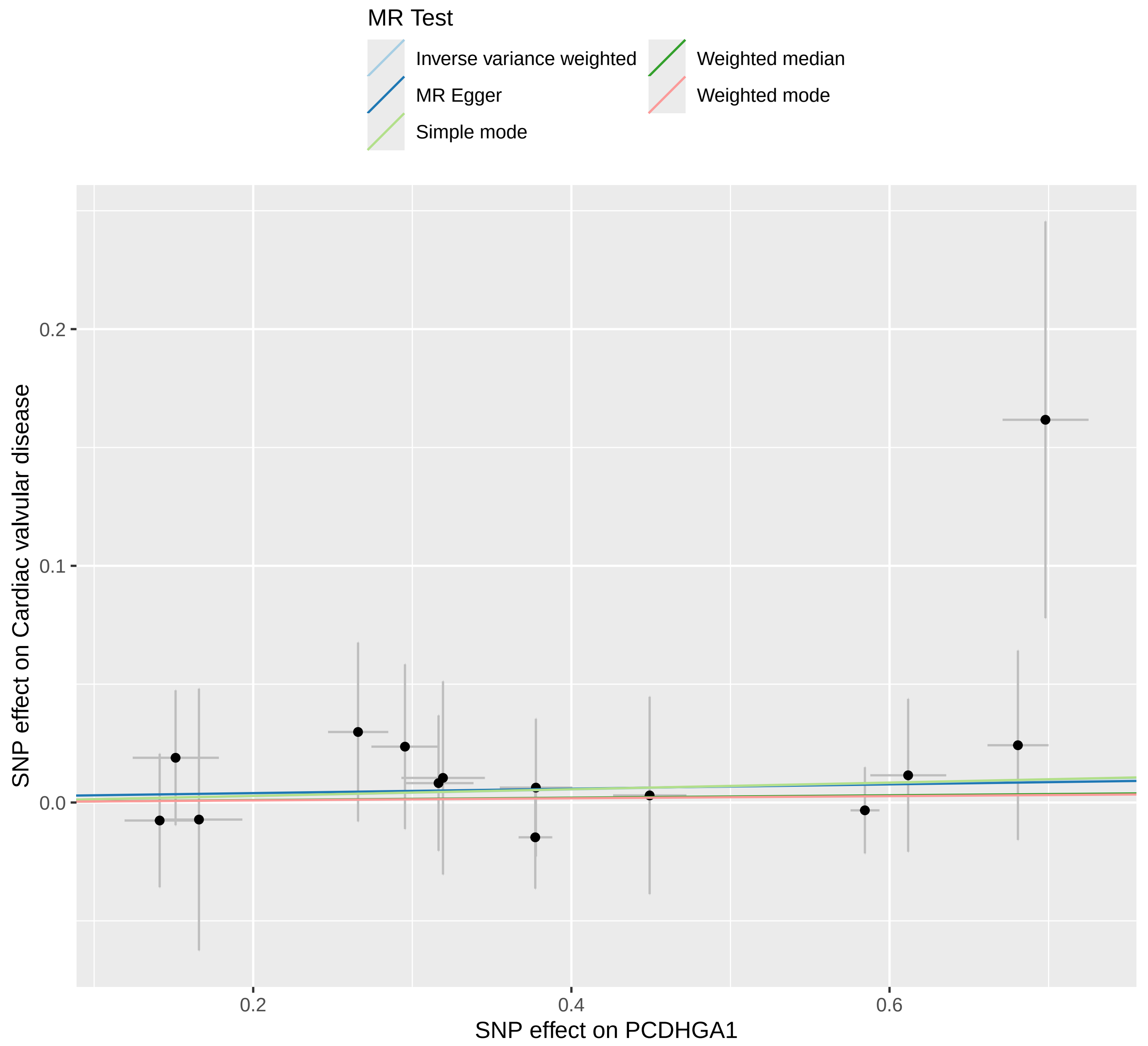
MR Test
Inverse variance weighted
MR Egger
Simple mode
Weighted median
Weighted mode
SNP effect on Cardiac valvular disease
0.2
0.1
0.0
0.2
0.4
0.6
SNP effect on PCDHGA1

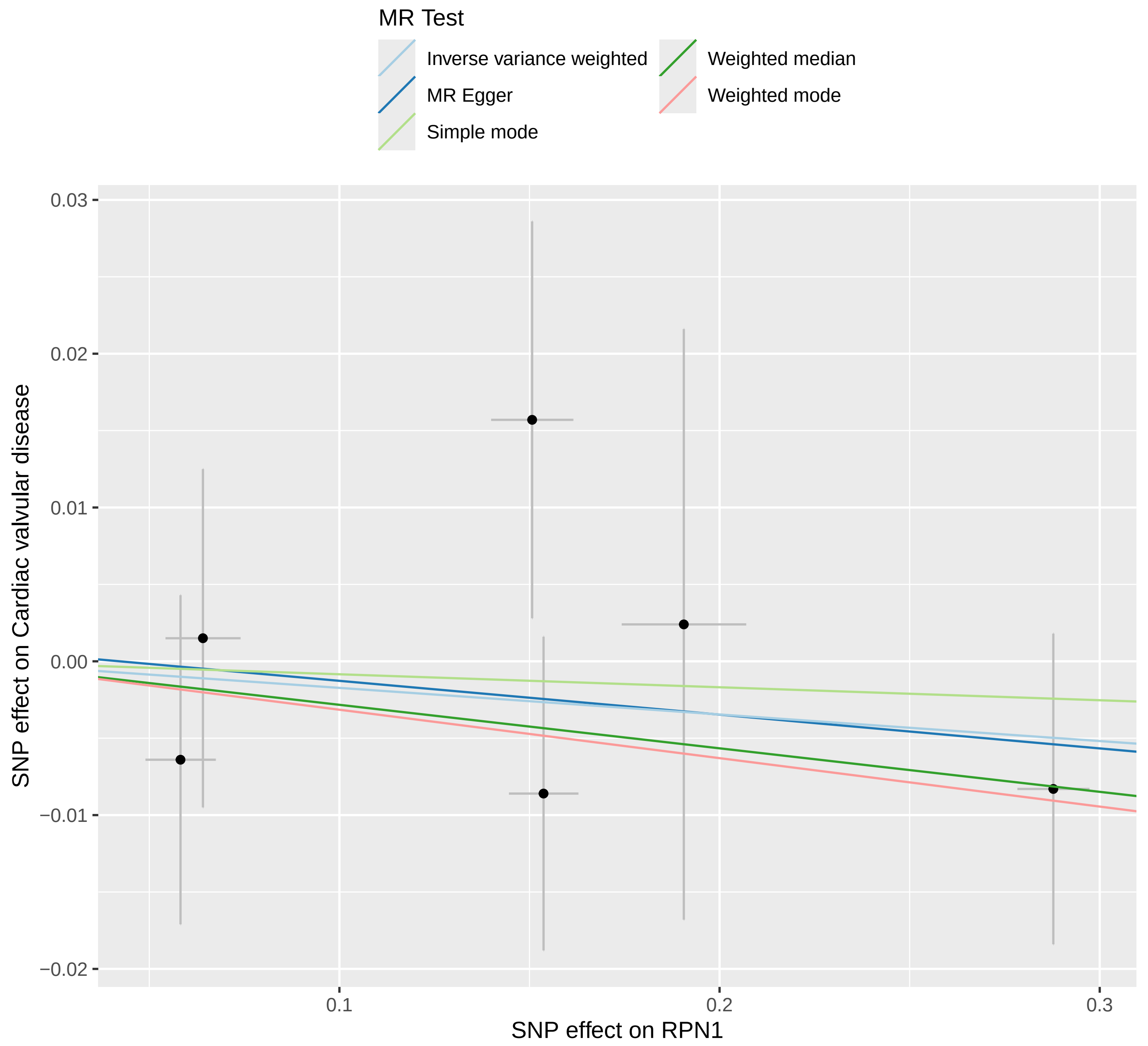
MR Test
Inverse variance weighted
MR Egger
Simple mode
Weighted median
Weighted mode
0.03
0.02
0.01
0.00
−0.01
−0.02
0.1
0.2
0.3
SNP effect on Cardiac valvular disease
SNP effect on RPN1

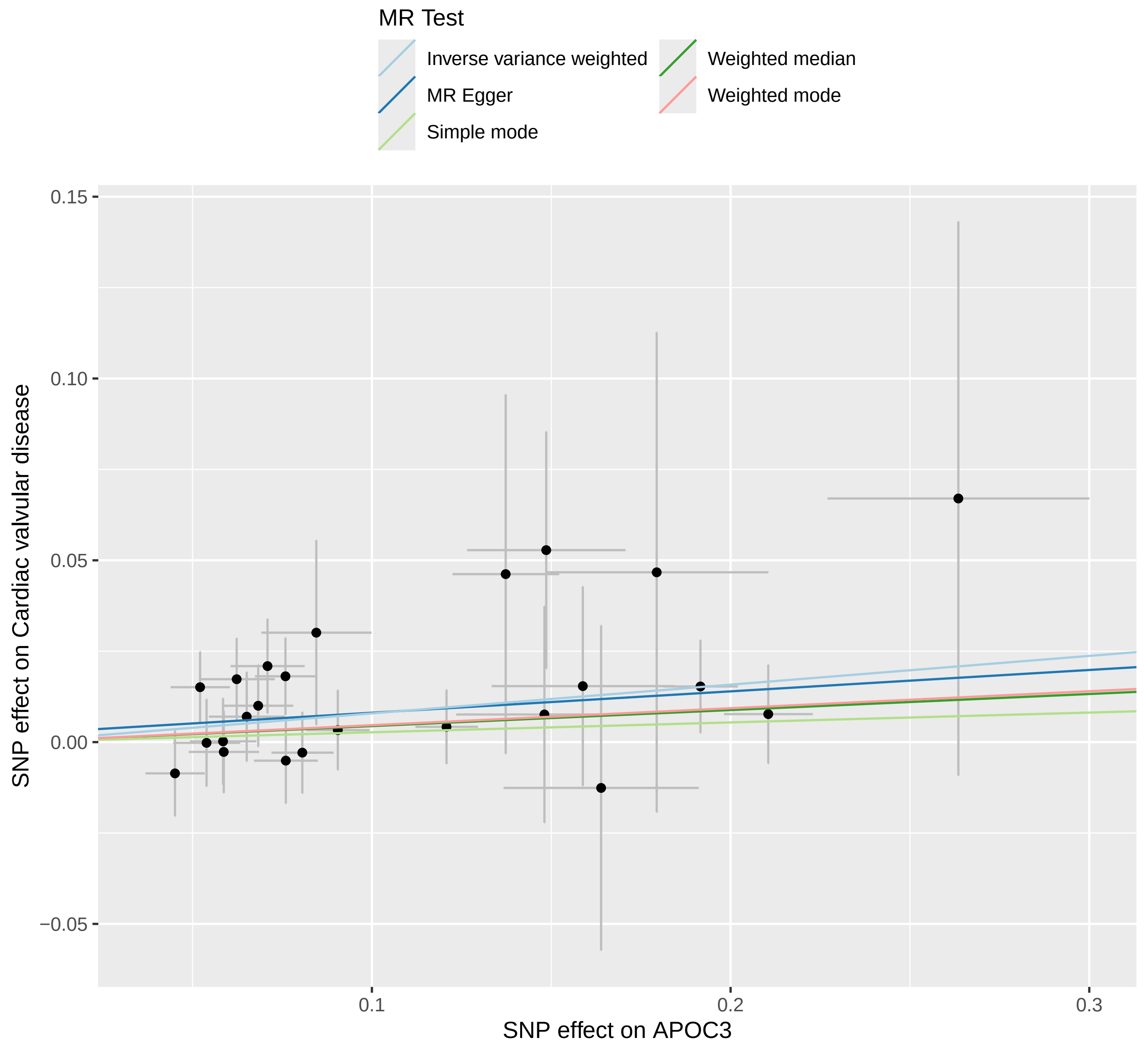

MR Test
Inverse variance weighted
MR Egger
Simple mode
Weighted median
Weighted mode
0.15
0.10
0.05
0.00
−0.05
0.1
0.2
0.3
SNP effect on APOC3
SNP effect on Cardiac valvular disease

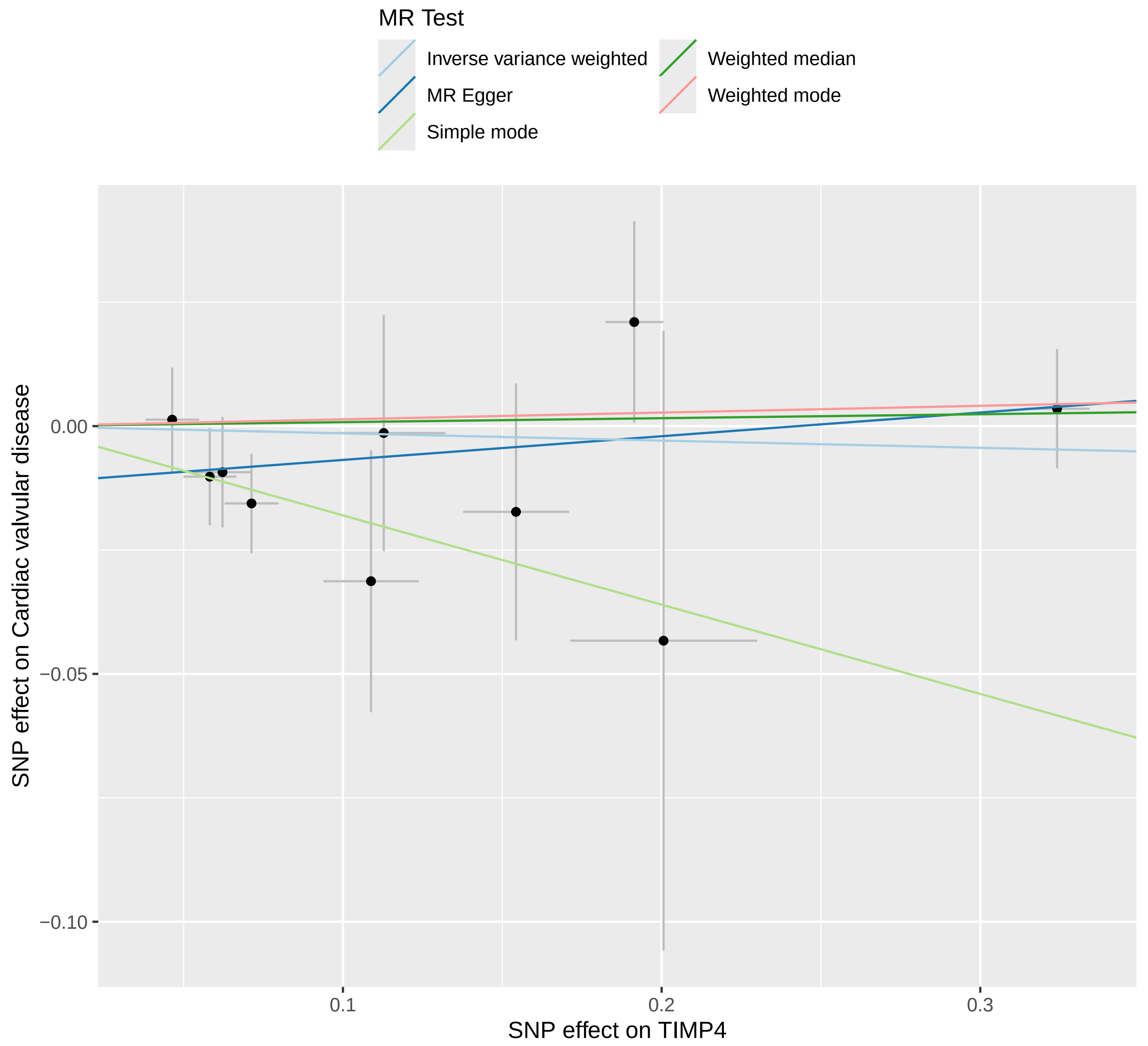
MR Test
Inverse variance weighted
MR Egger
Simple mode
Weighted median
Weighted mode
SNP effect on Cardiac valvular disease
0.00
−0.05
−0.10
0.1
0.2
0.3
SNP effect on TIMP4

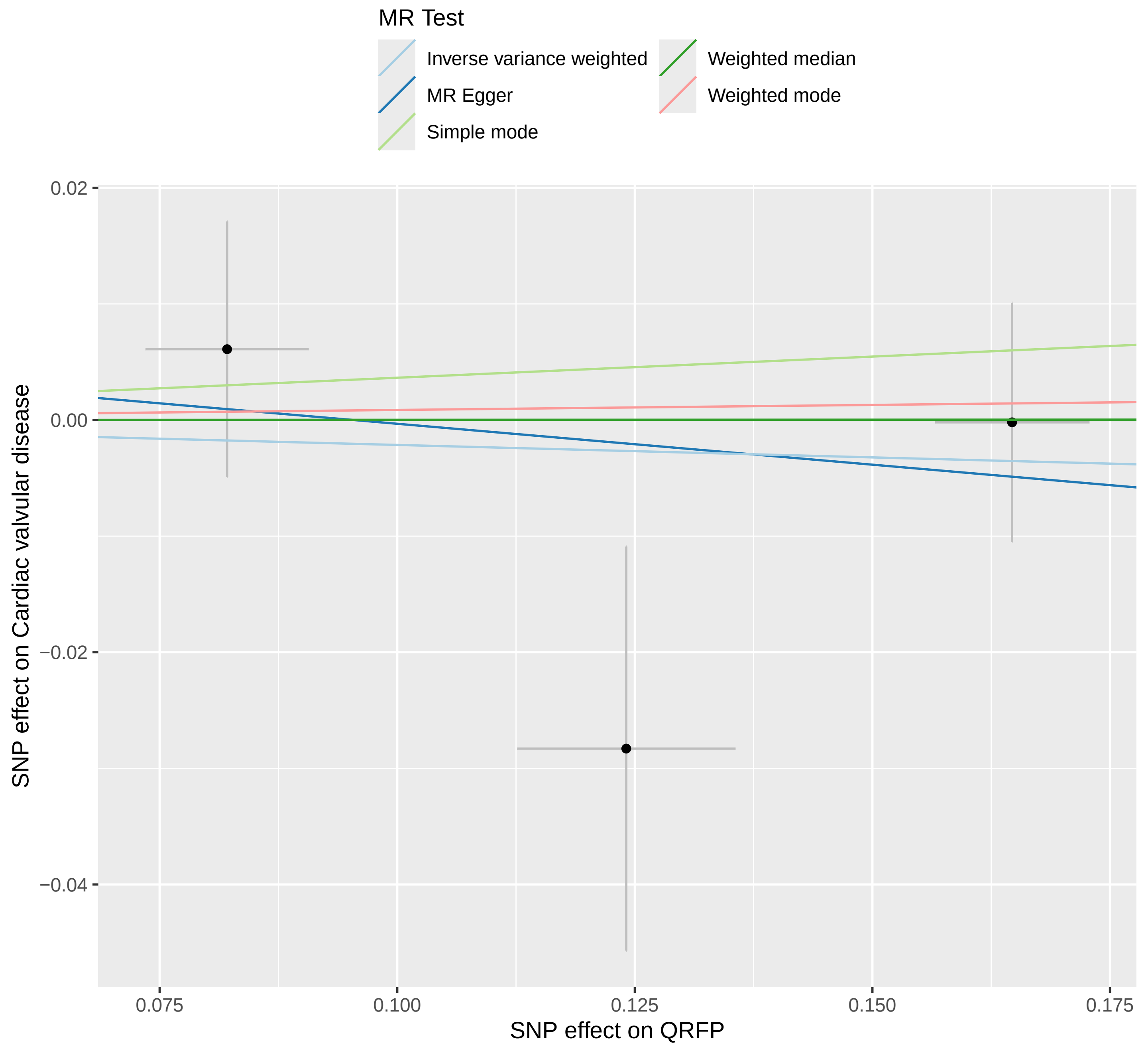
MR Test
Inverse variance weighted
MR Egger
Simple mode
Weighted median
Weighted mode
0.02
0.00
−0.02
−0.04
0.075
0.100
0.125
0.150
0.175
SNP effect on QRFP
SNP effect on Cardiac valvular disease

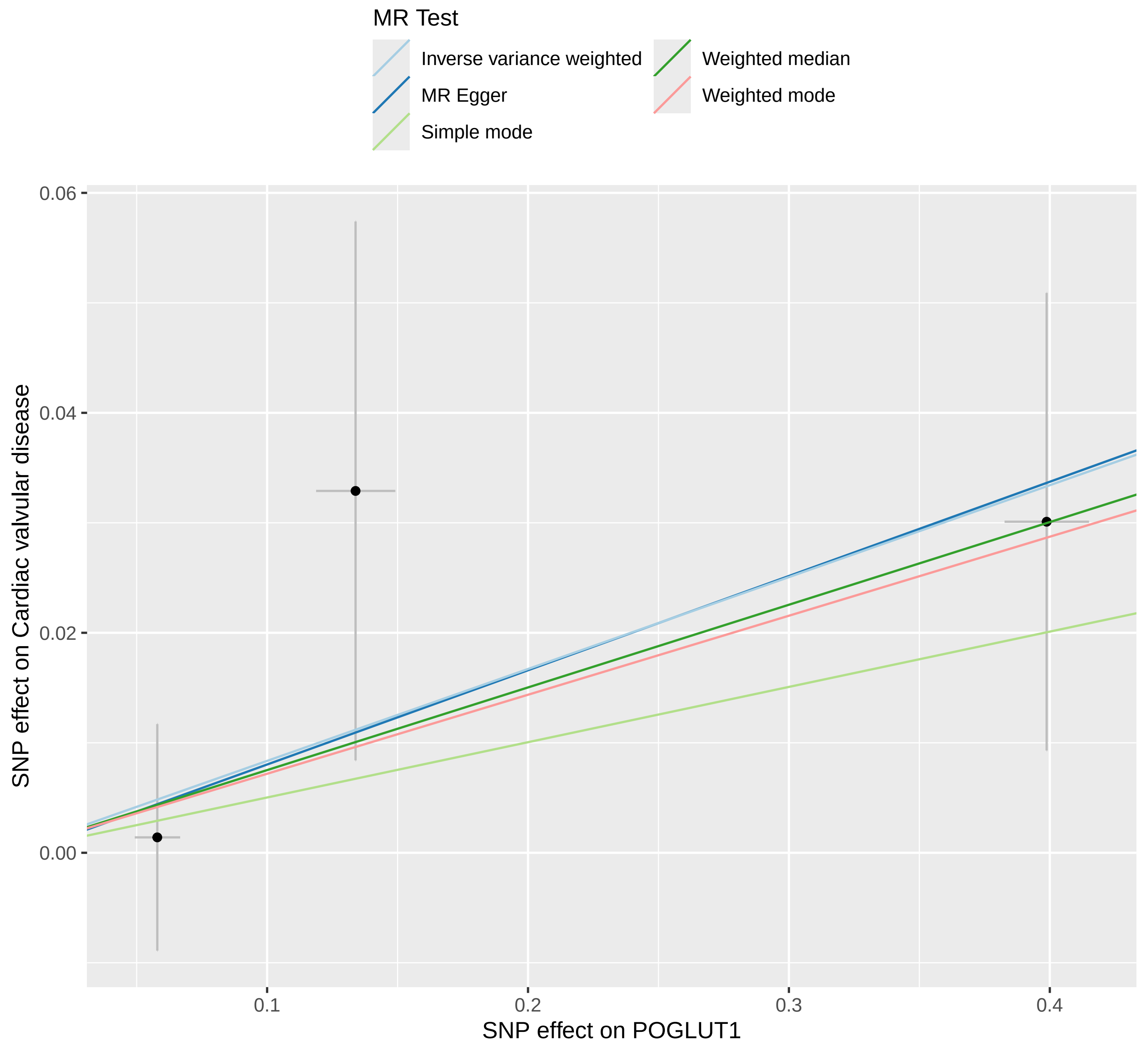
MR Test
Inverse variance weighted
MR Egger
Simple mode
Weighted median
Weighted mode
0.06
0.04
0.02
0.00
SNP effect on Cardiac valvular disease
0.1
0.2
0.3
0.4
SNP effect on POGLUT1

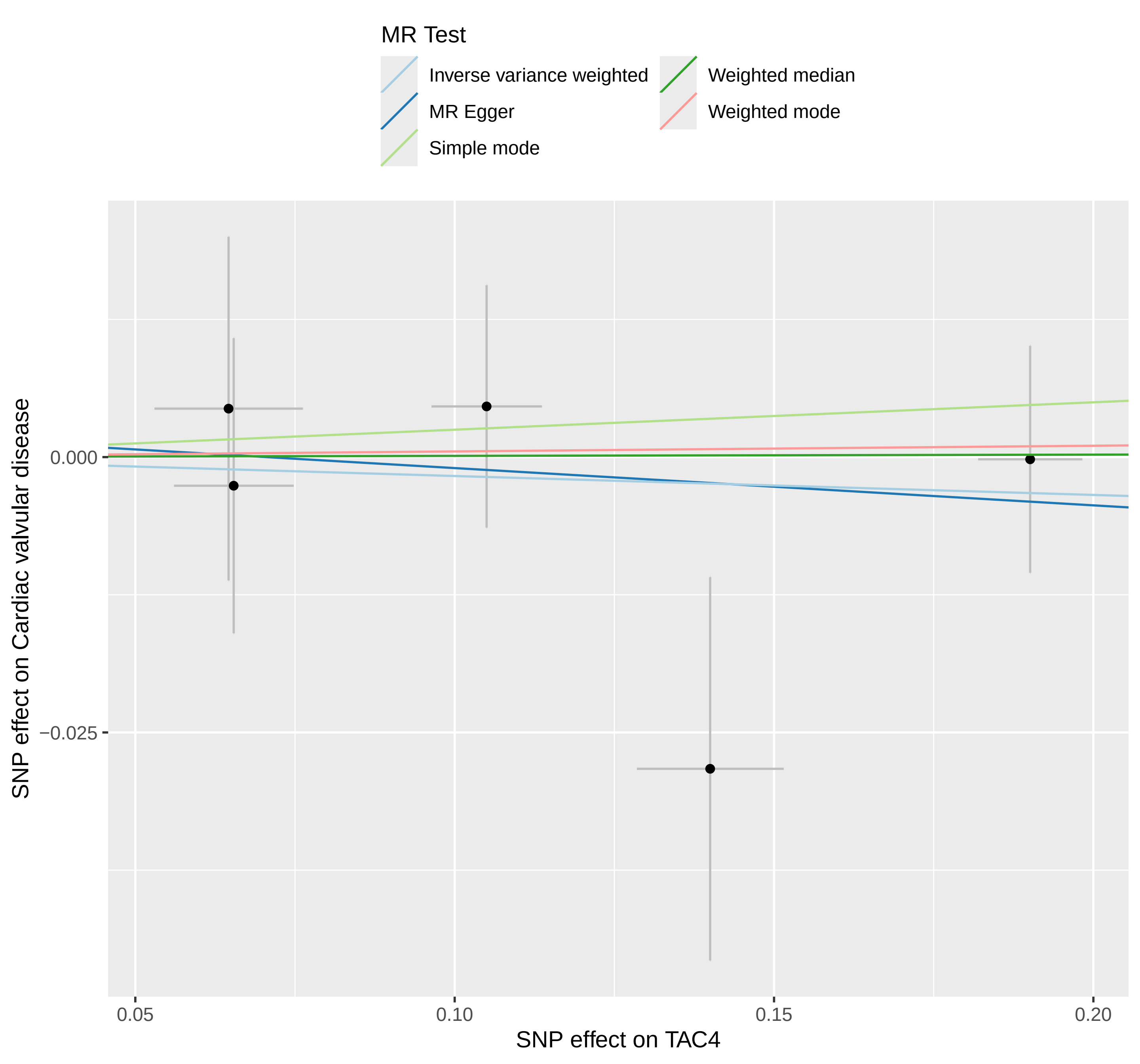
MR Test
Inverse variance weighted
MR Egger
Simple mode
Weighted median
Weighted mode
0.000
−0.025
0.05
0.10
0.15
0.20
SNP effect on TAC4
SNP effect on Cardiac valvular disease

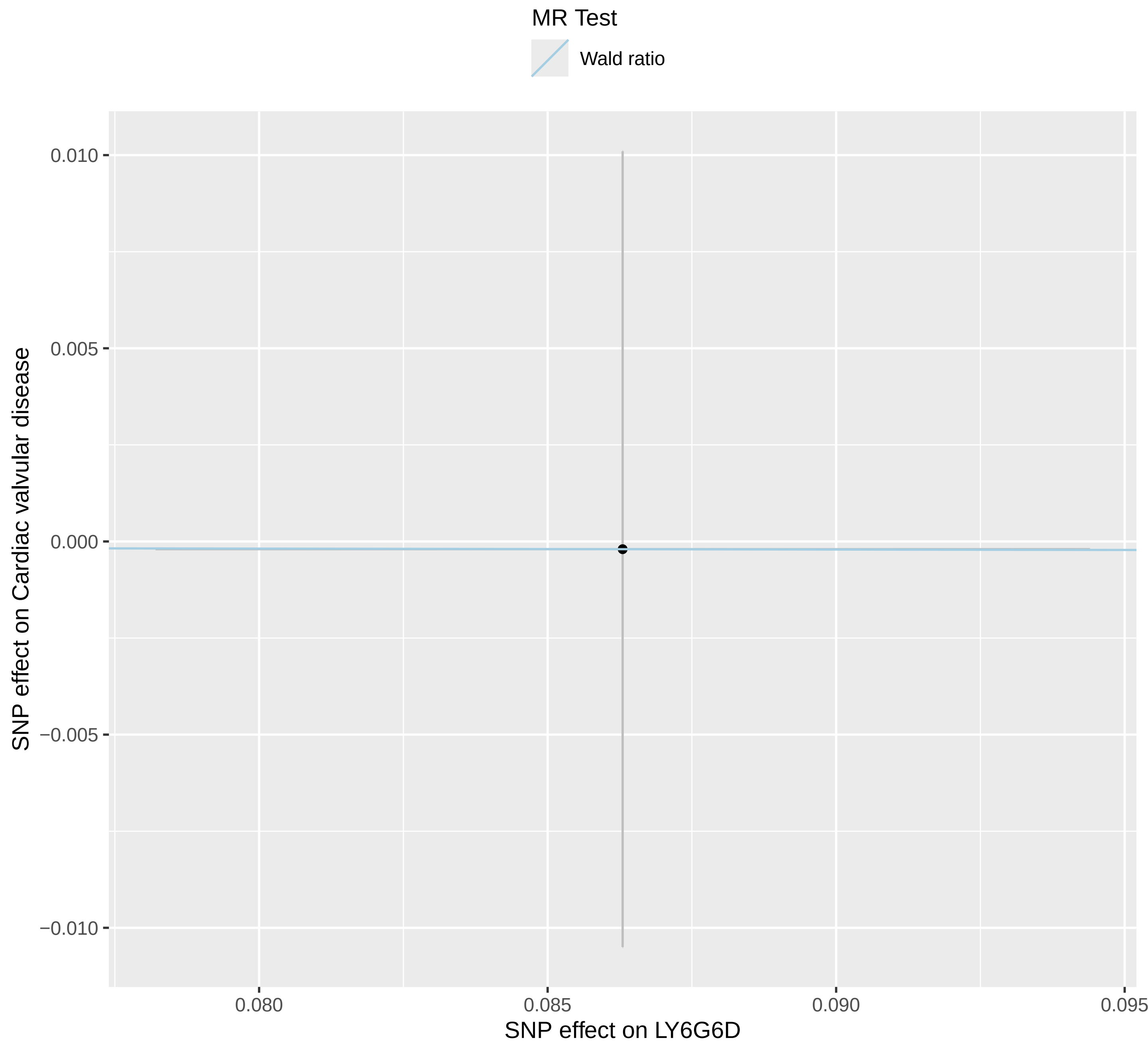
MR Test
Wald ratio
0.010
0.005
0.000
−0.005
−0.010
0.080
0.085
0.090
0.095
SNP effect on LY6G6D
SNP effect on Cardiac valvular disease

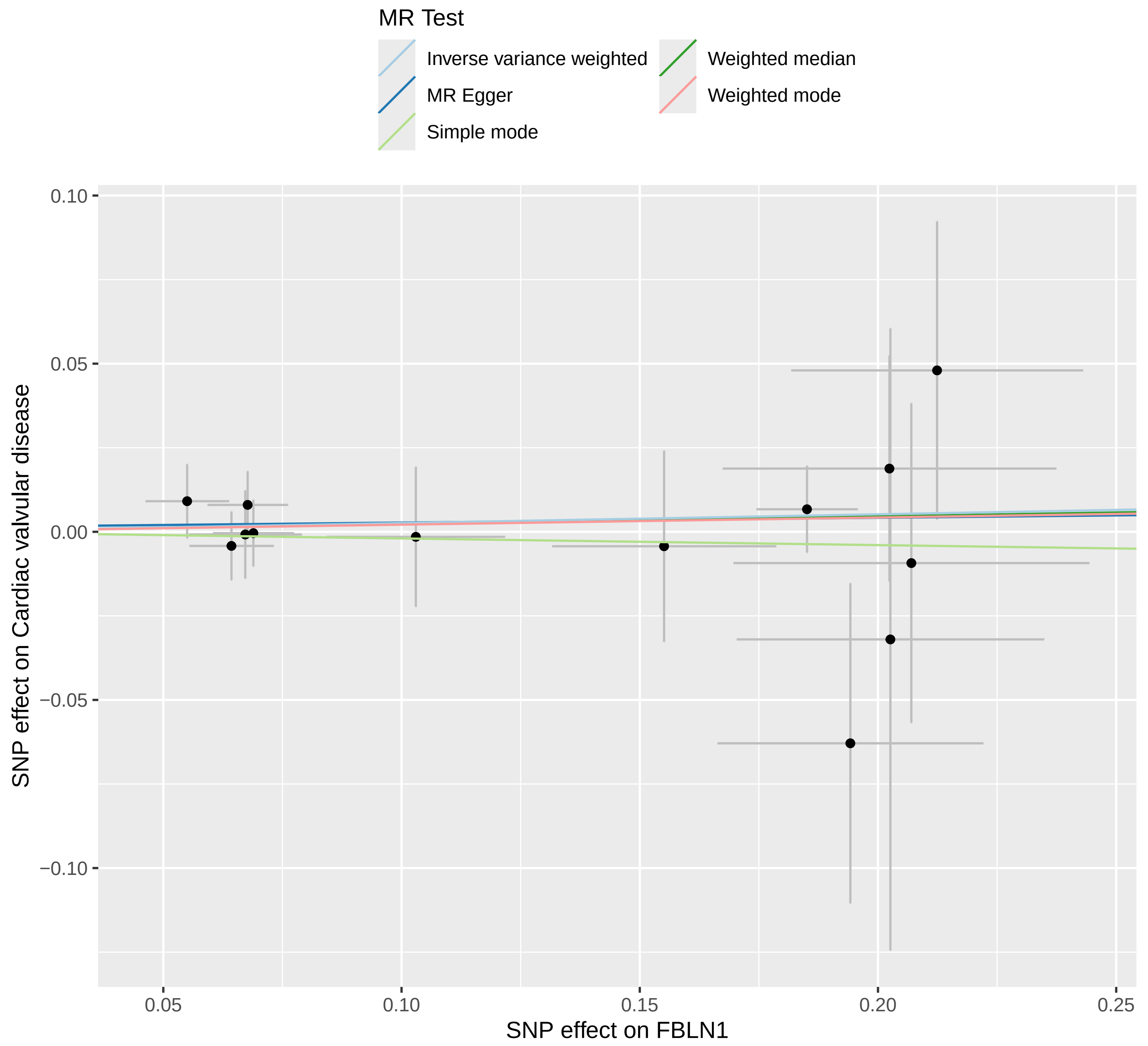

MR Test
Inverse variance weighted
MR Egger
Simple mode
Weighted median
Weighted mode
0.10
0.05
0.00
−0.05
−0.10
SNP effect on Cardiac valvular disease
0.05
0.10
0.15
0.20
0.25
SNP effect on FBLN1

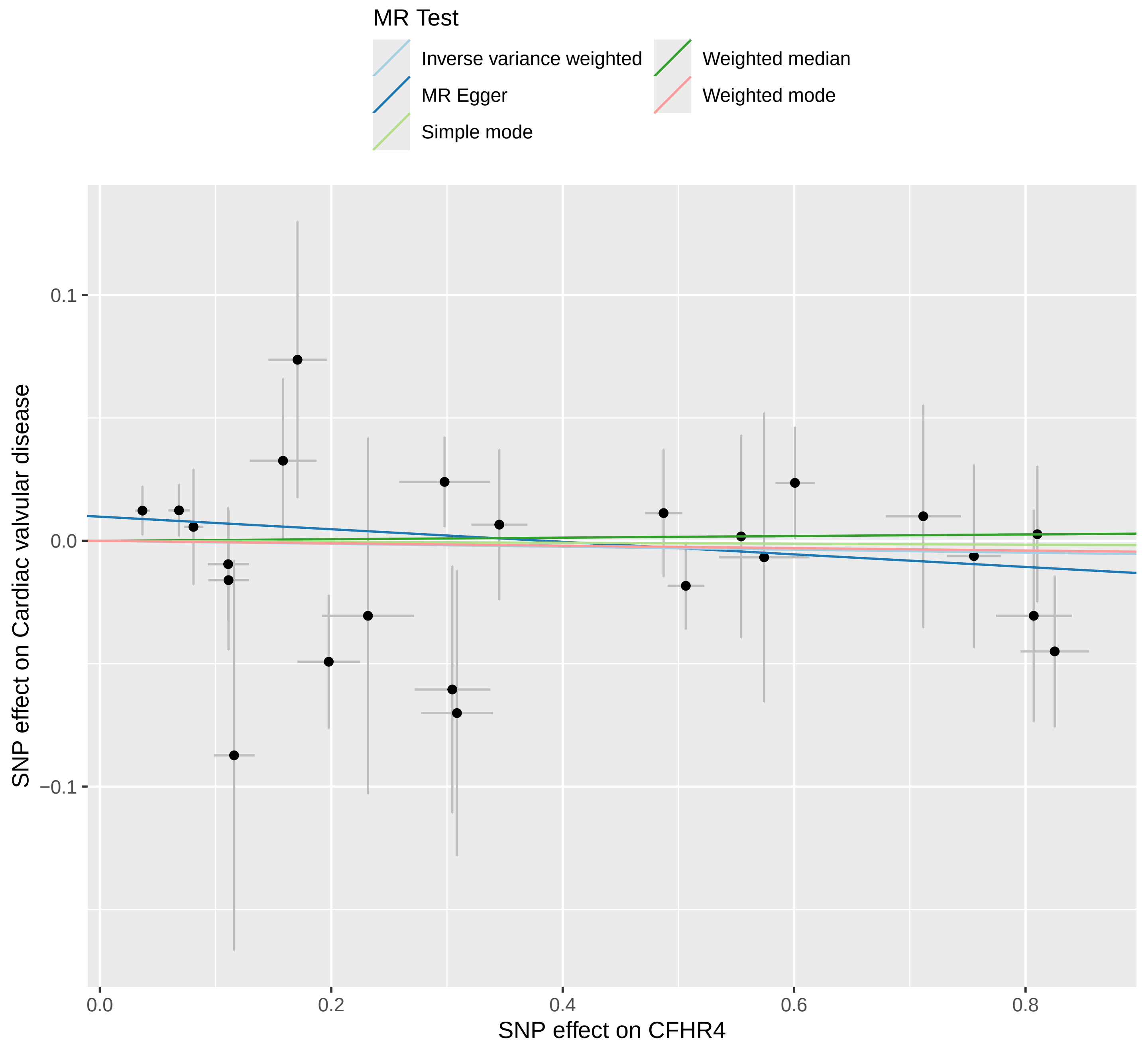
MR Test
Inverse variance weighted
MR Egger
Simple mode
Weighted median
Weighted mode
0.1
0.0
−0.1
0.0
0.2
0.4
0.6
0.8
SNP effect on Cardiac valvular disease
SNP effect on CFHR4

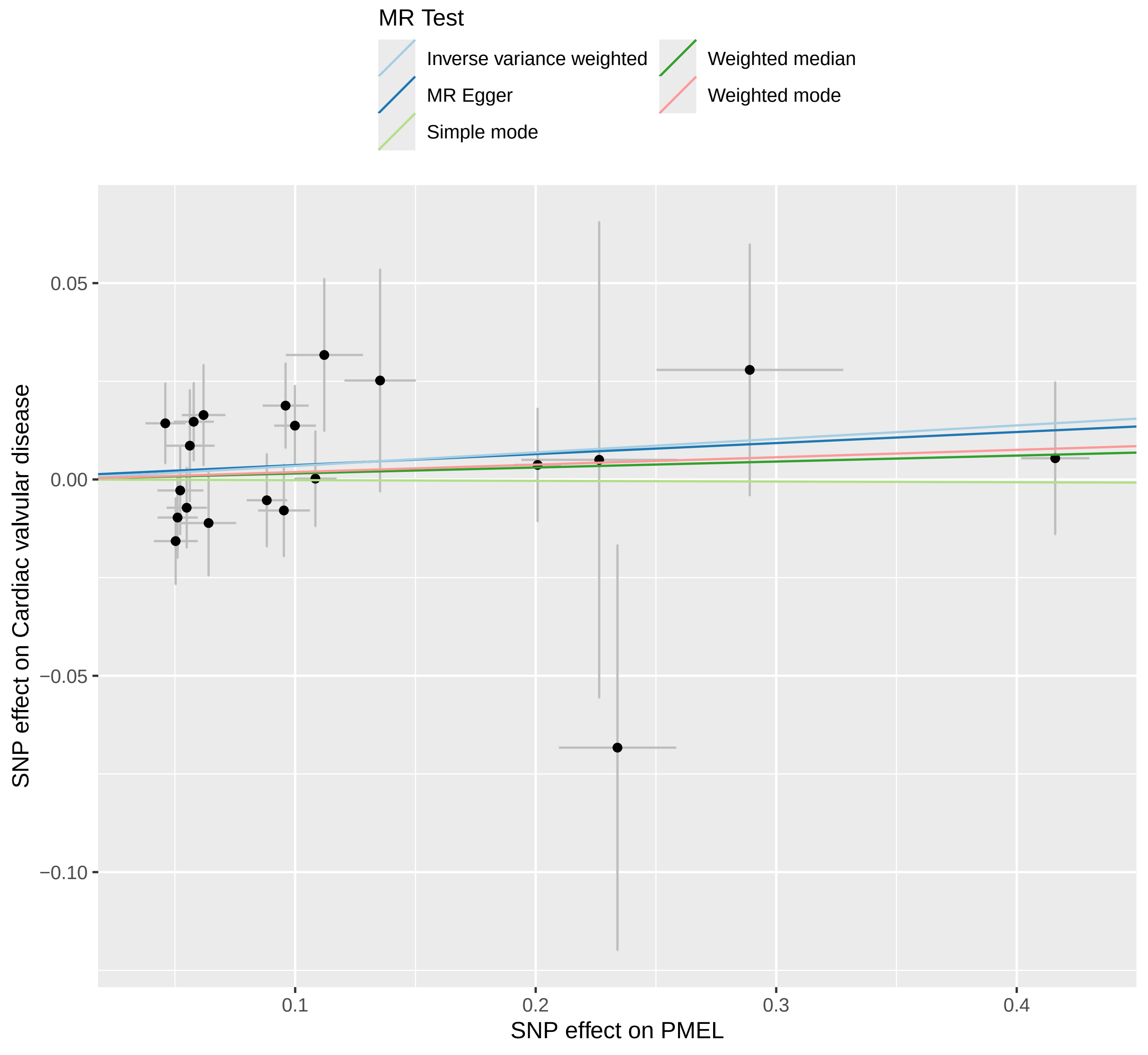
MR Test
Inverse variance weighted
MR Egger
Simple mode
Weighted median
Weighted mode
0.05
0.00
−0.05
−0.10
0.1
0.2
0.3
0.4
SNP effect on Cardiac valvular disease
SNP effect on PMEL

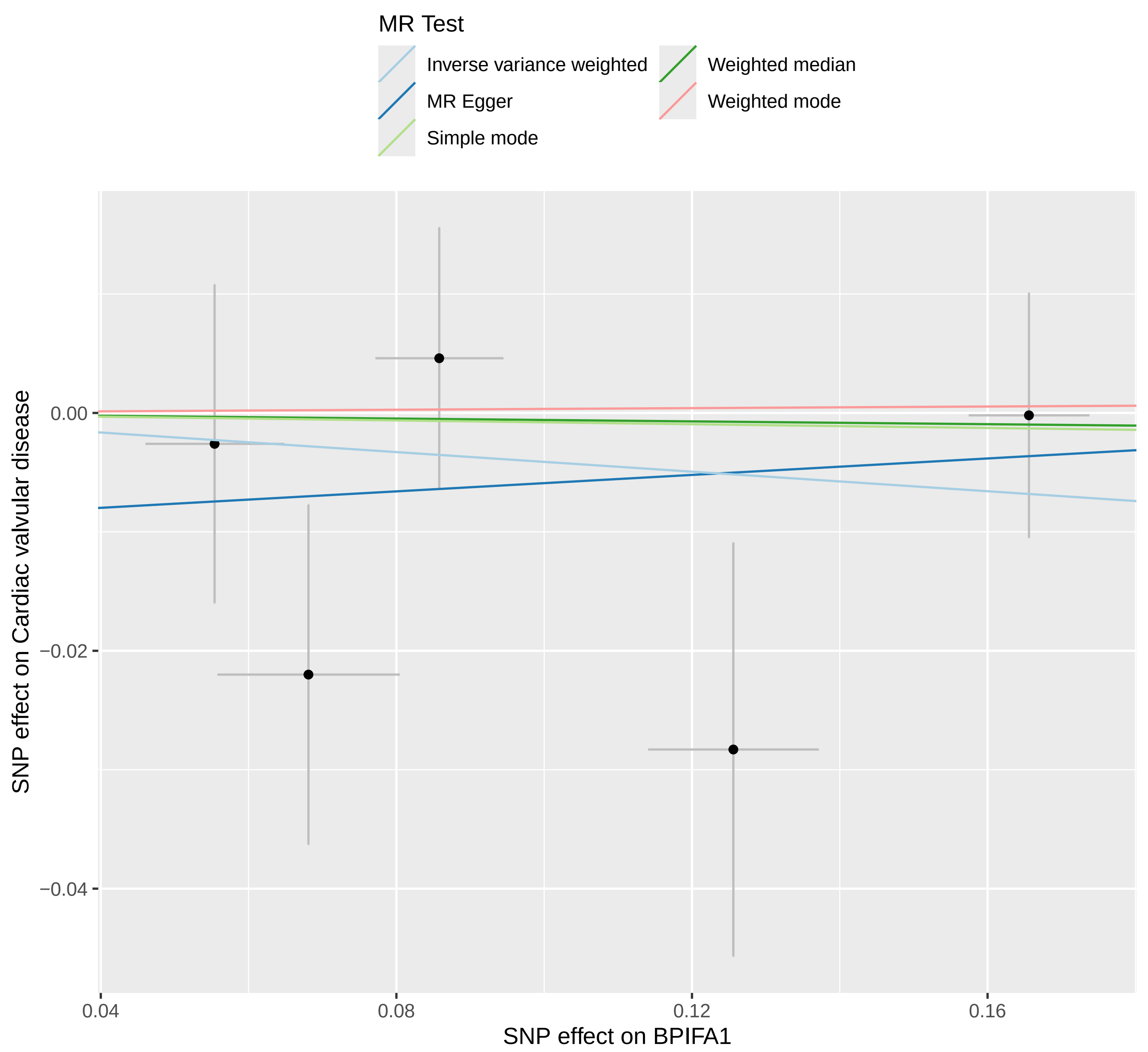

MR Test
Inverse variance weighted
MR Egger
Simple mode
Weighted median
Weighted mode
0.00
−0.02
−0.04
0.04
0.08
0.12
0.16
SNP effect on Cardiac valvular disease
SNP effect on BPIFA1

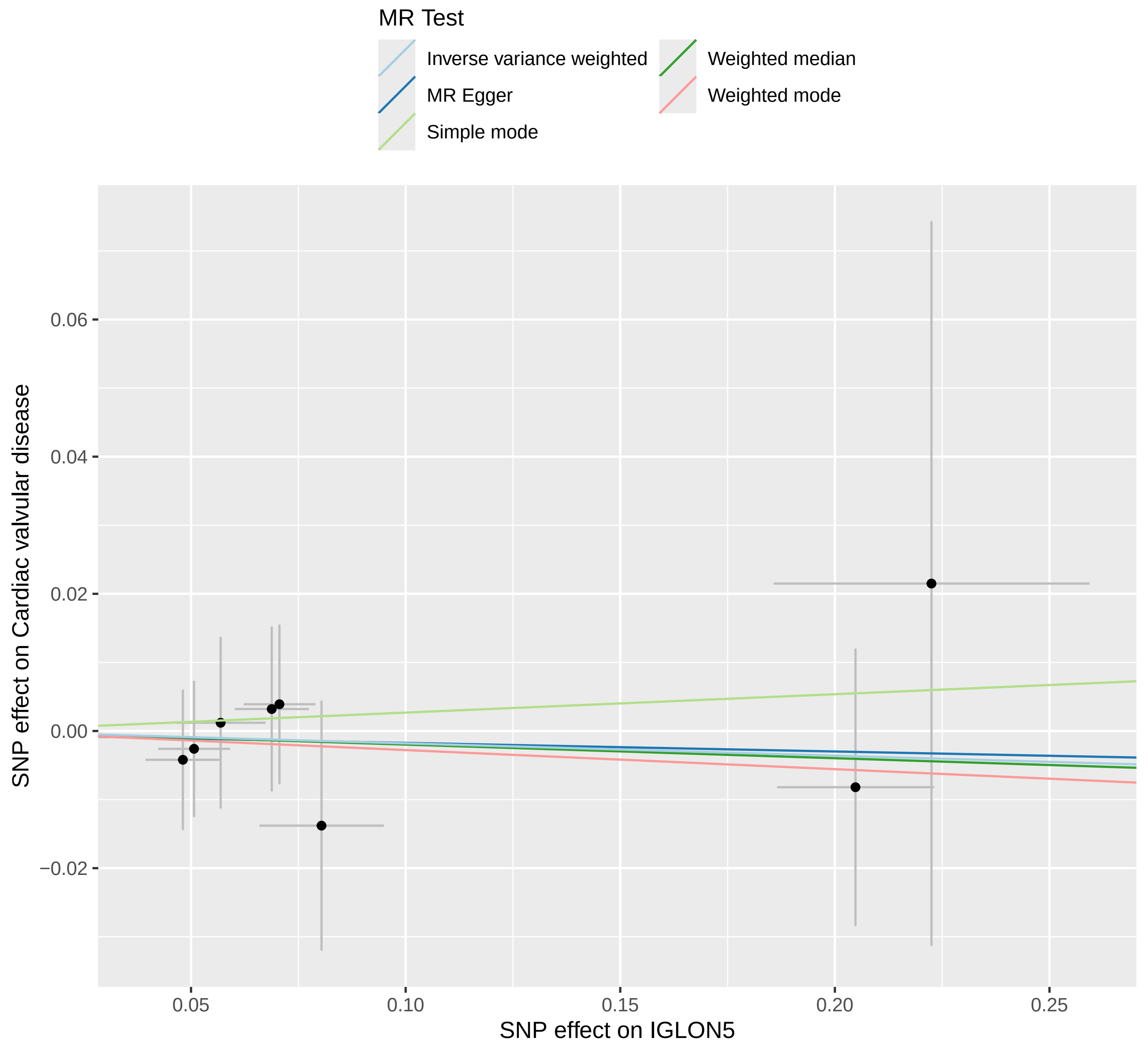

MR Test
Inverse variance weighted
MR Egger
Simple mode
Weighted median
Weighted mode
SNP effect on Cardiac valvular disease
0.06
0.04
0.02
0.00
−0.02
0.05
0.10
0.15
0.20
0.25
SNP effect on IGLON5

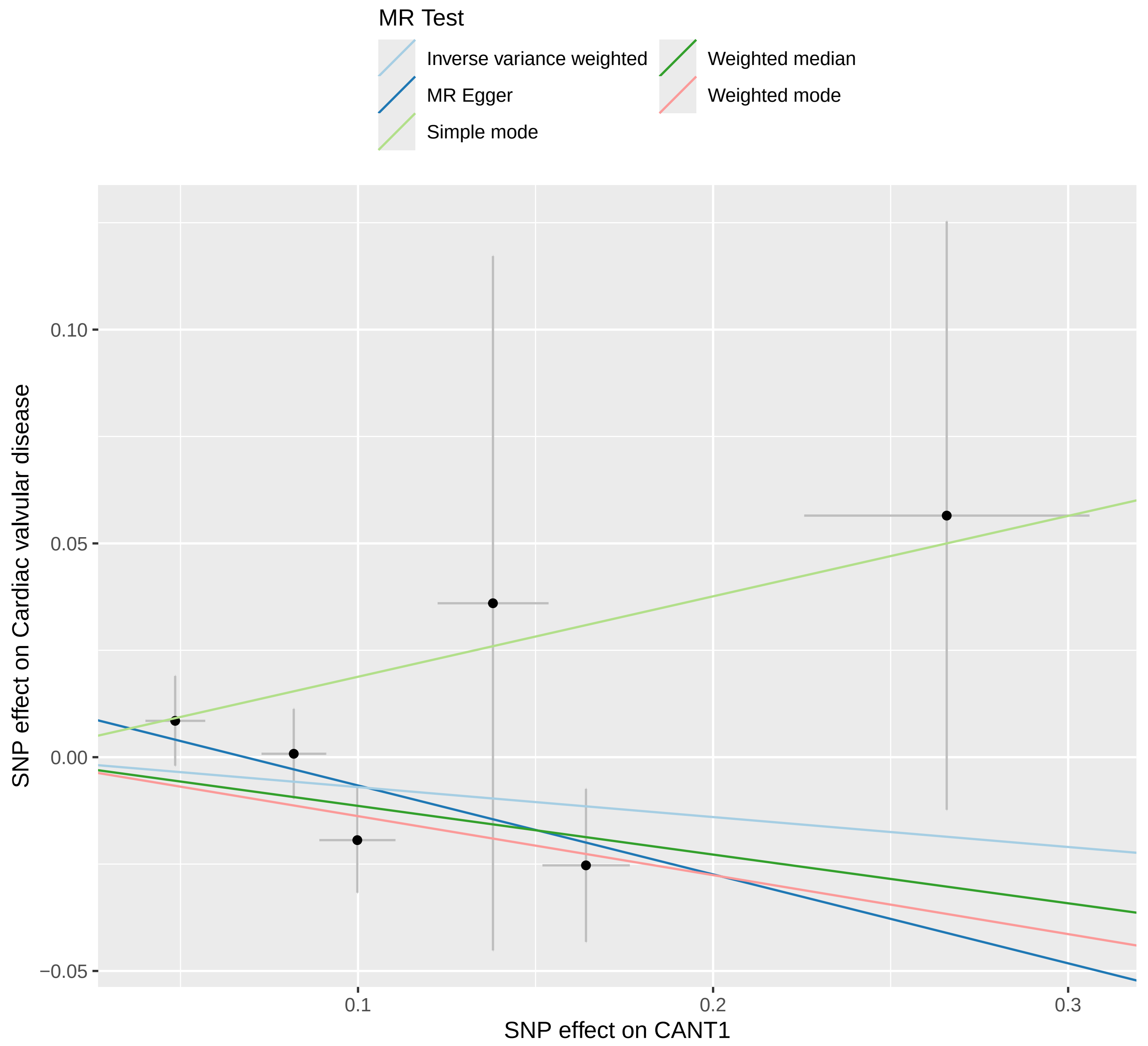

MR Test
Inverse variance weighted
MR Egger
Simple mode
Weighted median
Weighted mode
0.10
0.05
0.00
−0.05
0.1
0.2
0.3
SNP effect on Cardiac valvular disease
SNP effect on CANT1

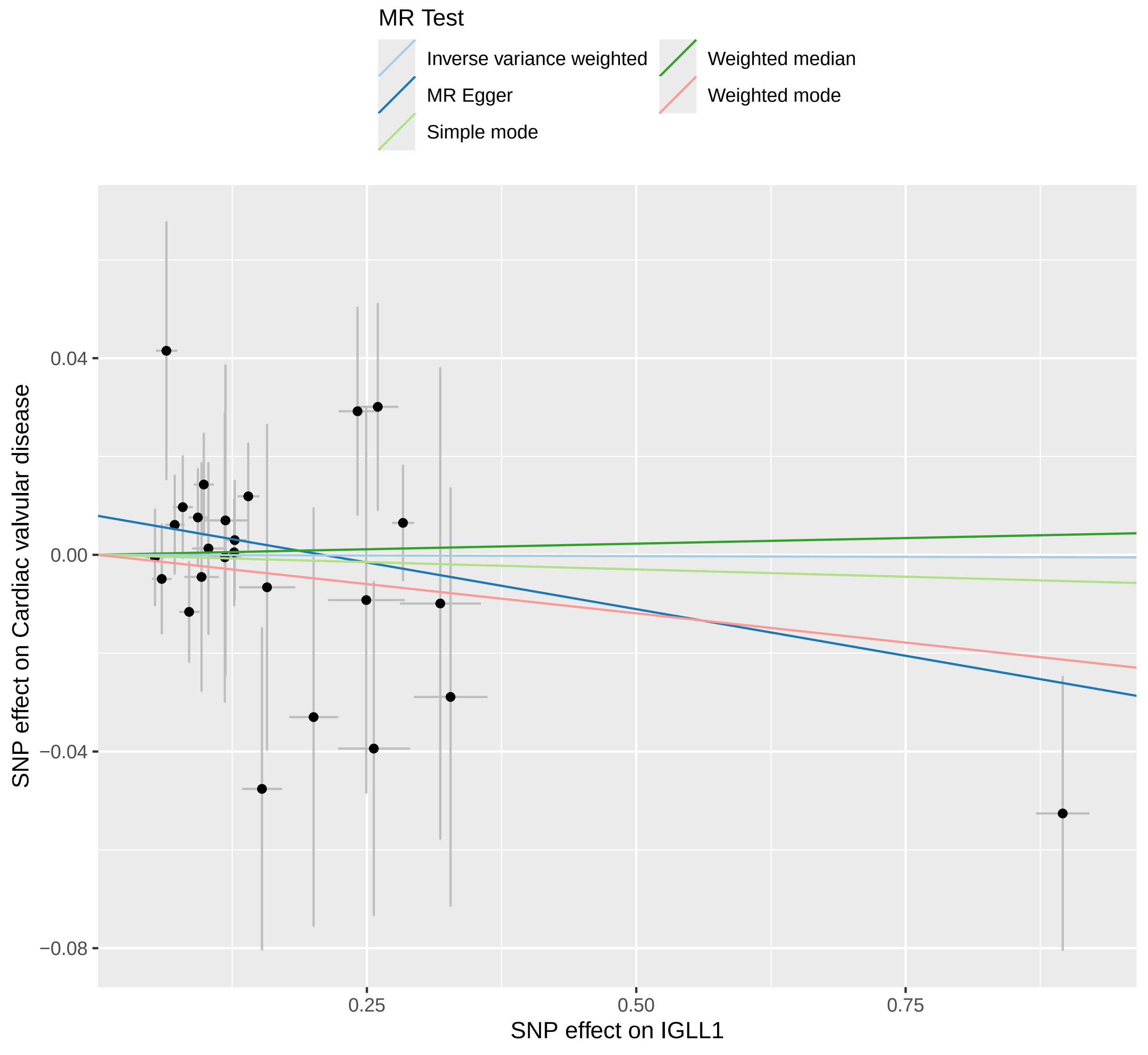

MR Test
Inverse variance weighted
MR Egger
Simple mode
Weighted median
Weighted mode
0.04
0.00
−0.04
−0.08
SNP effect on Cardiac valvular disease
0.25
0.50
0.75
SNP effect on IGLL1

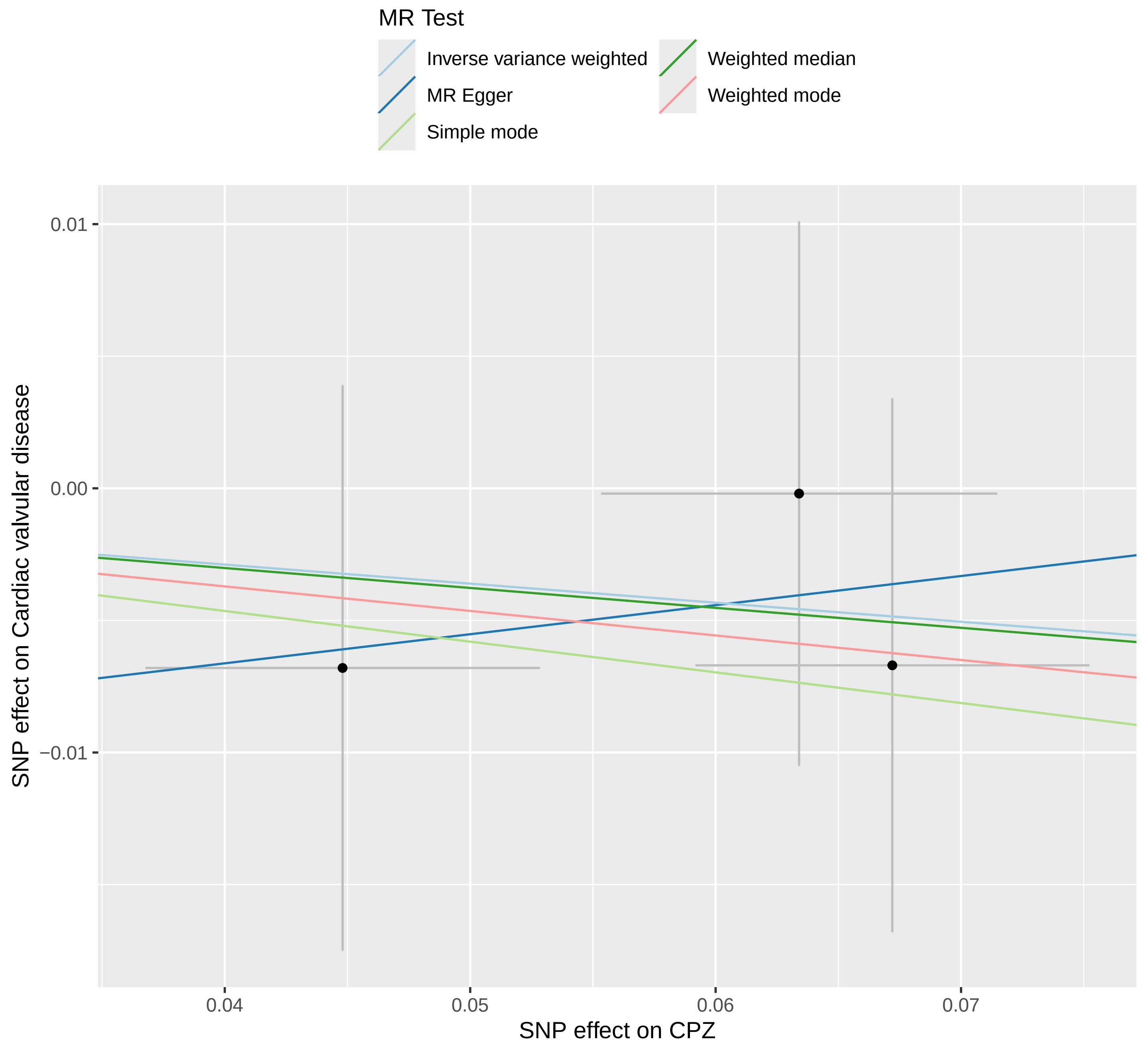
MR Test
Inverse variance weighted
MR Egger
Simple mode
Weighted median
Weighted mode
0.01
0.00
−0.01
0.04
0.05
0.06
0.07
SNP effect on CPZ
SNP effect on Cardiac valvular disease

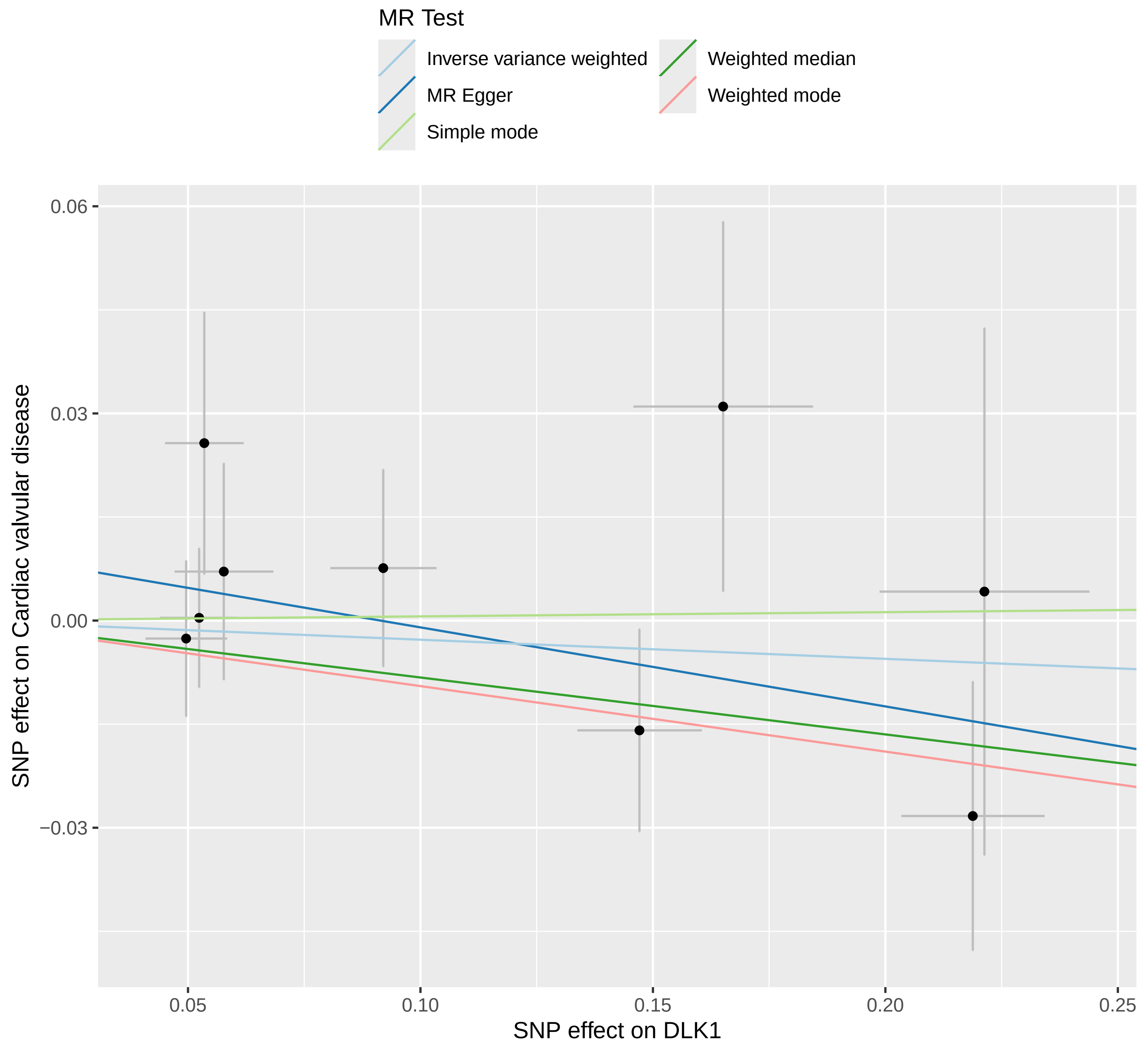

MR Test
Inverse variance weighted
MR Egger
Simple mode
Weighted median
Weighted mode
0.06
0.03
0.00
−0.03
0.05
0.10
0.15
0.20
0.25
SNP effect on DLK1
SNP effect on Cardiac valvular disease

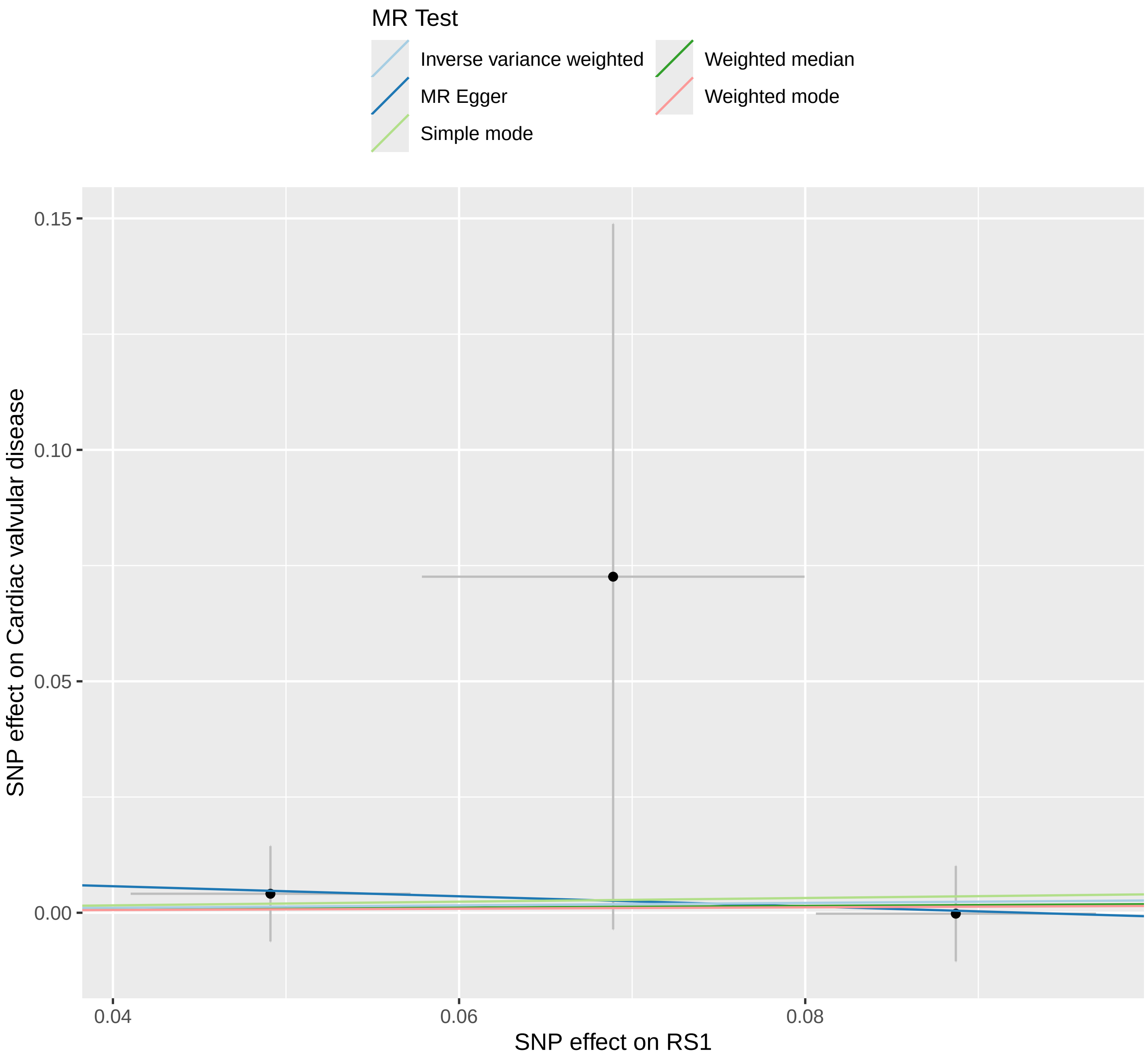
MR Test
Inverse variance weighted
MR Egger
Simple mode
Weighted median
Weighted mode
0.15
0.10
0.05
0.00
0.04
0.06
0.08
SNP effect on Cardiac valvular disease
SNP effect on RS1

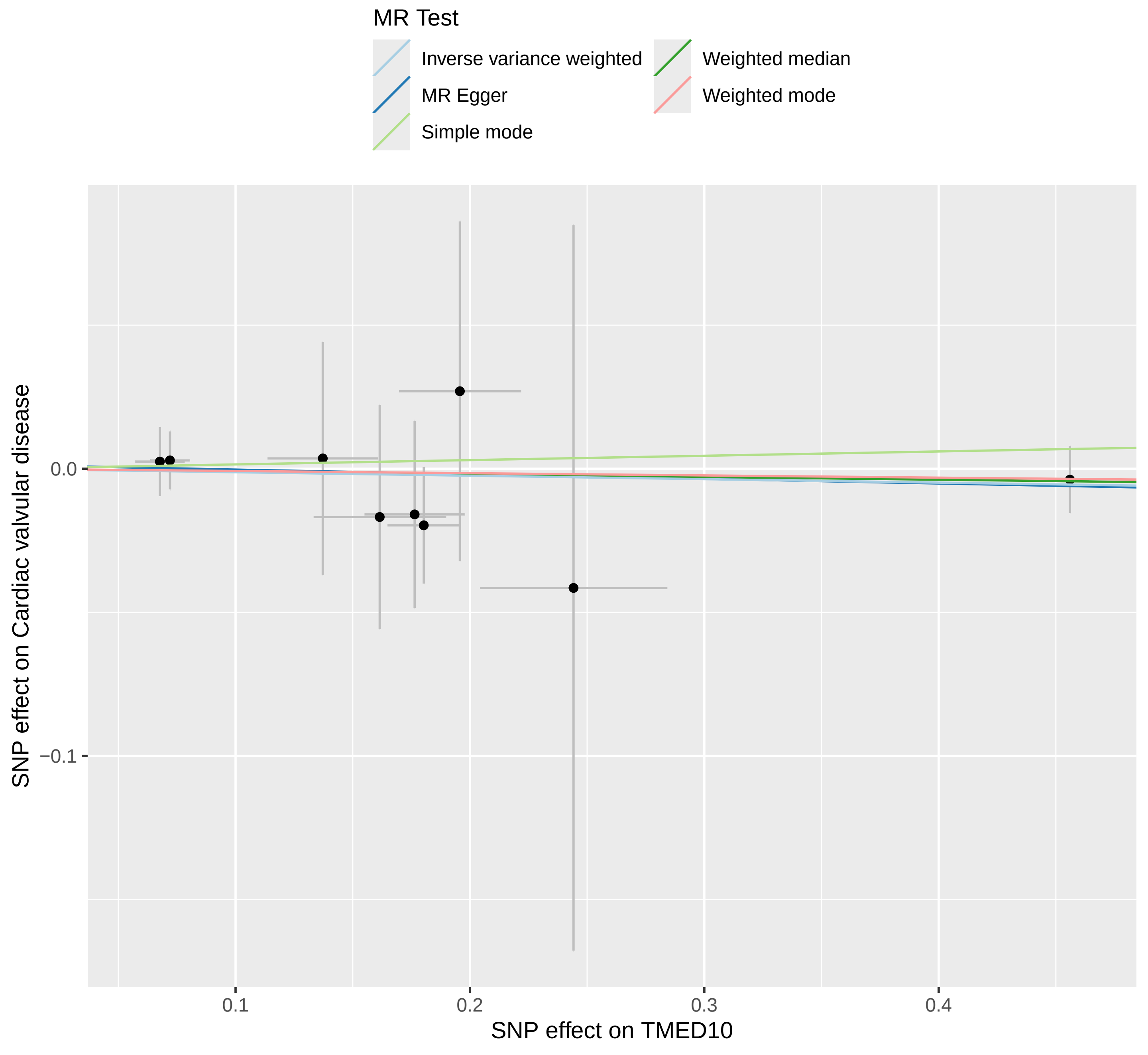
MR Test
Inverse variance weighted
MR Egger
Simple mode
Weighted median
Weighted mode
SNP effect on Cardiac valvular disease
0.0
−0.1
0.1
0.2
0.3
0.4
SNP effect on TMED10

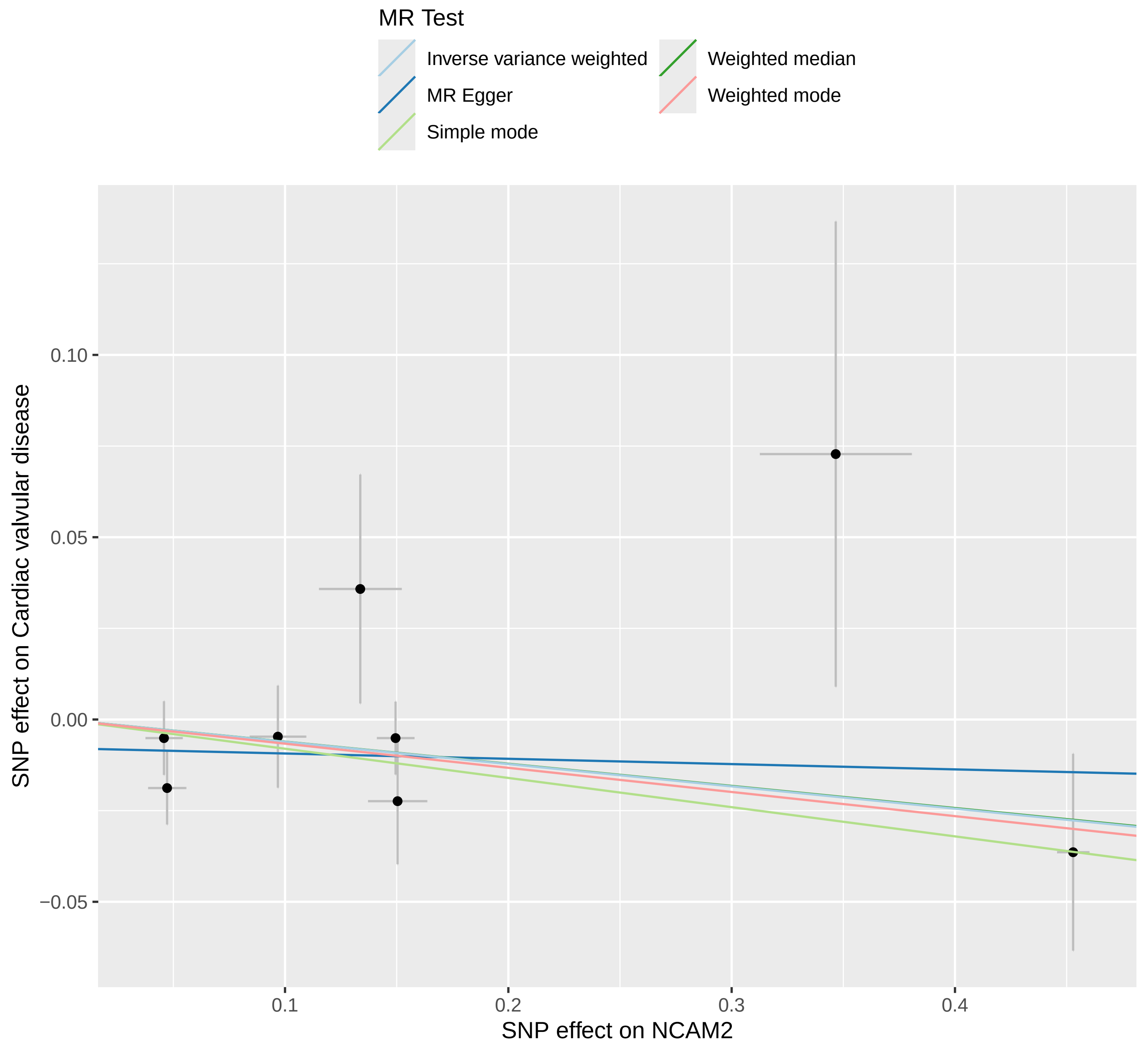

MR Test
Inverse variance weighted
MR Egger
Simple mode
Weighted median
Weighted mode
0.10
0.05
0.00
−0.05
SNP effect on Cardiac valvular disease
0.1
0.2
0.3
0.4
SNP effect on NCAM2

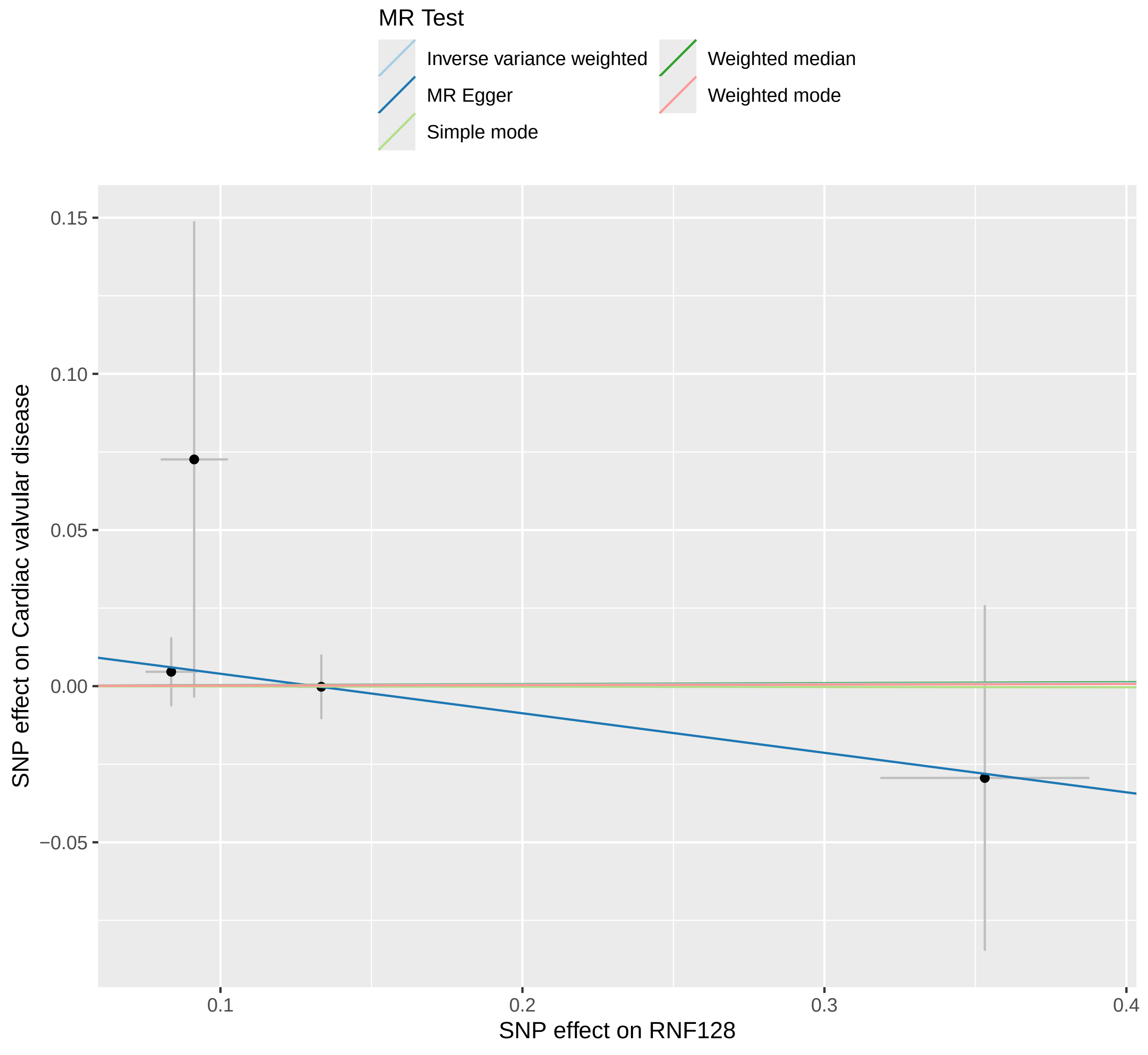
MR Test
Inverse variance weighted
MR Egger
Simple mode
Weighted median
Weighted mode
0.15
0.10
0.05
0.00
−0.05
0.1
0.2
0.3
0.4
SNP effect on Cardiac valvular disease
SNP effect on RNF128

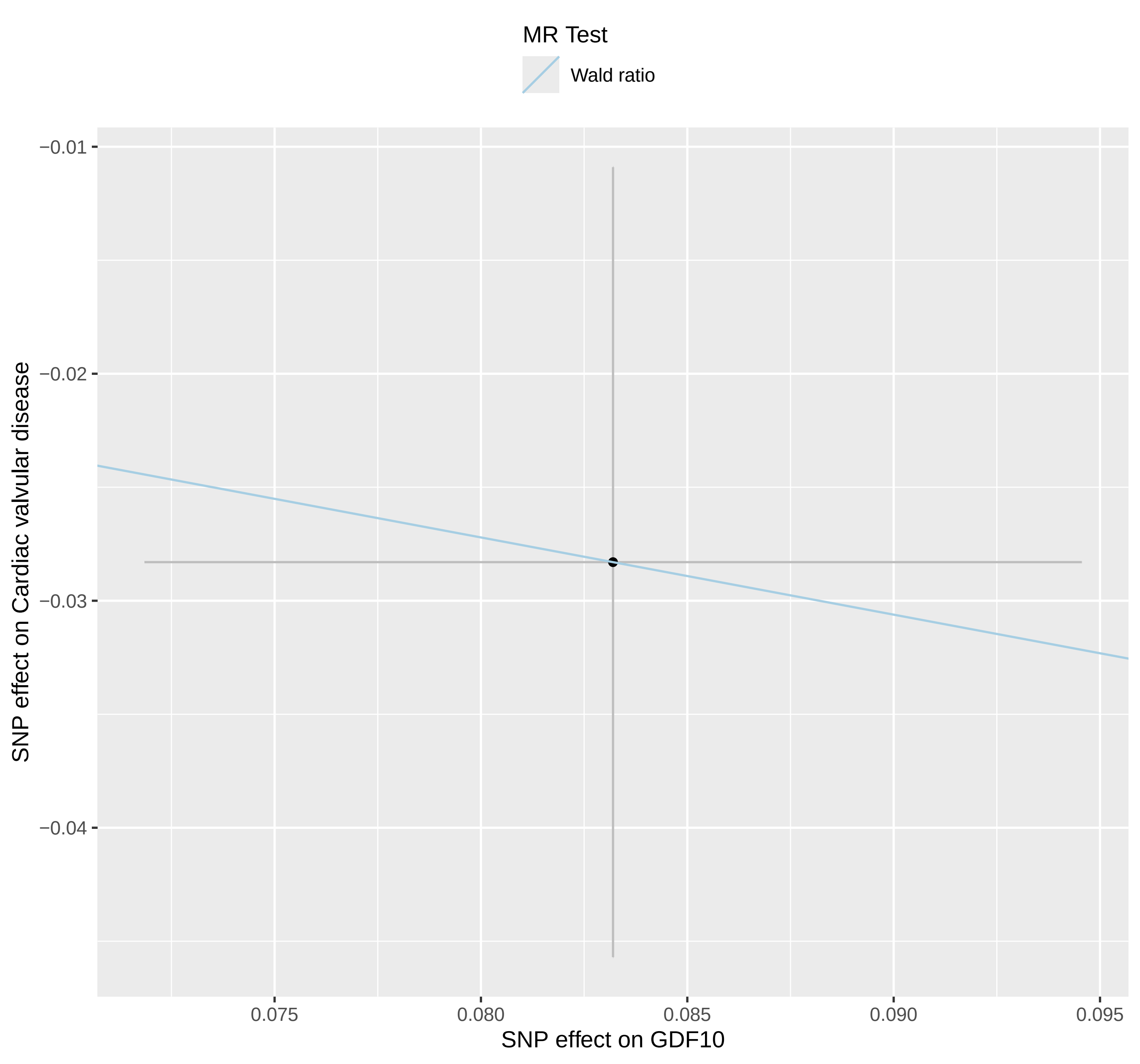
MR Test
Wald ratio
−0.01
−0.02
−0.03
−0.04
0.075
0.080
0.085
0.090
0.095
SNP effect on GDF10
SNP effect on Cardiac valvular disease

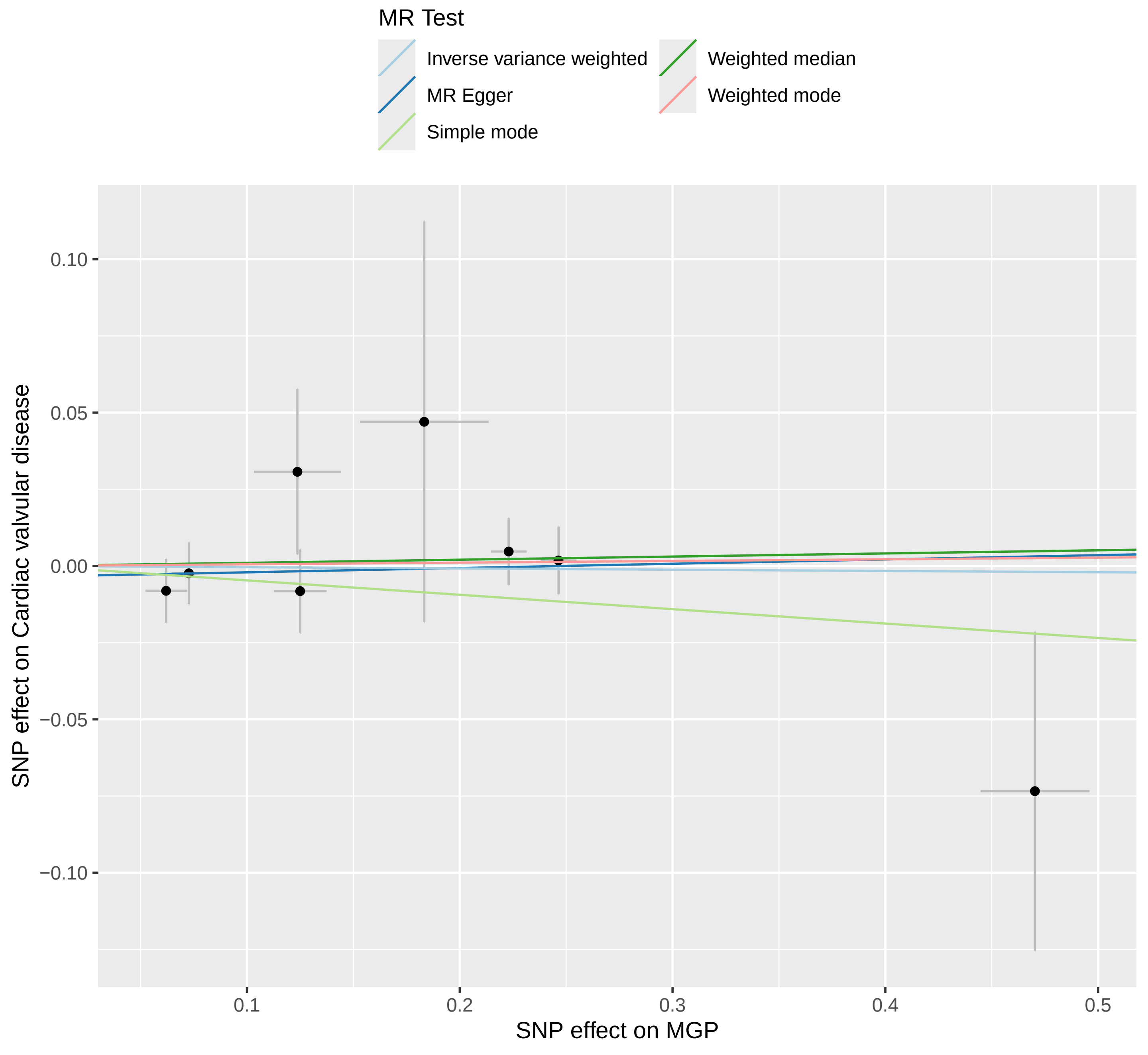

MR Test
Inverse variance weighted
MR Egger
Simple mode
Weighted median
Weighted mode
0.10
0.05
0.00
−0.05
−0.10
SNP effect on Cardiac valvular disease
0.1
0.2
0.3
0.4
0.5
SNP effect on MGP

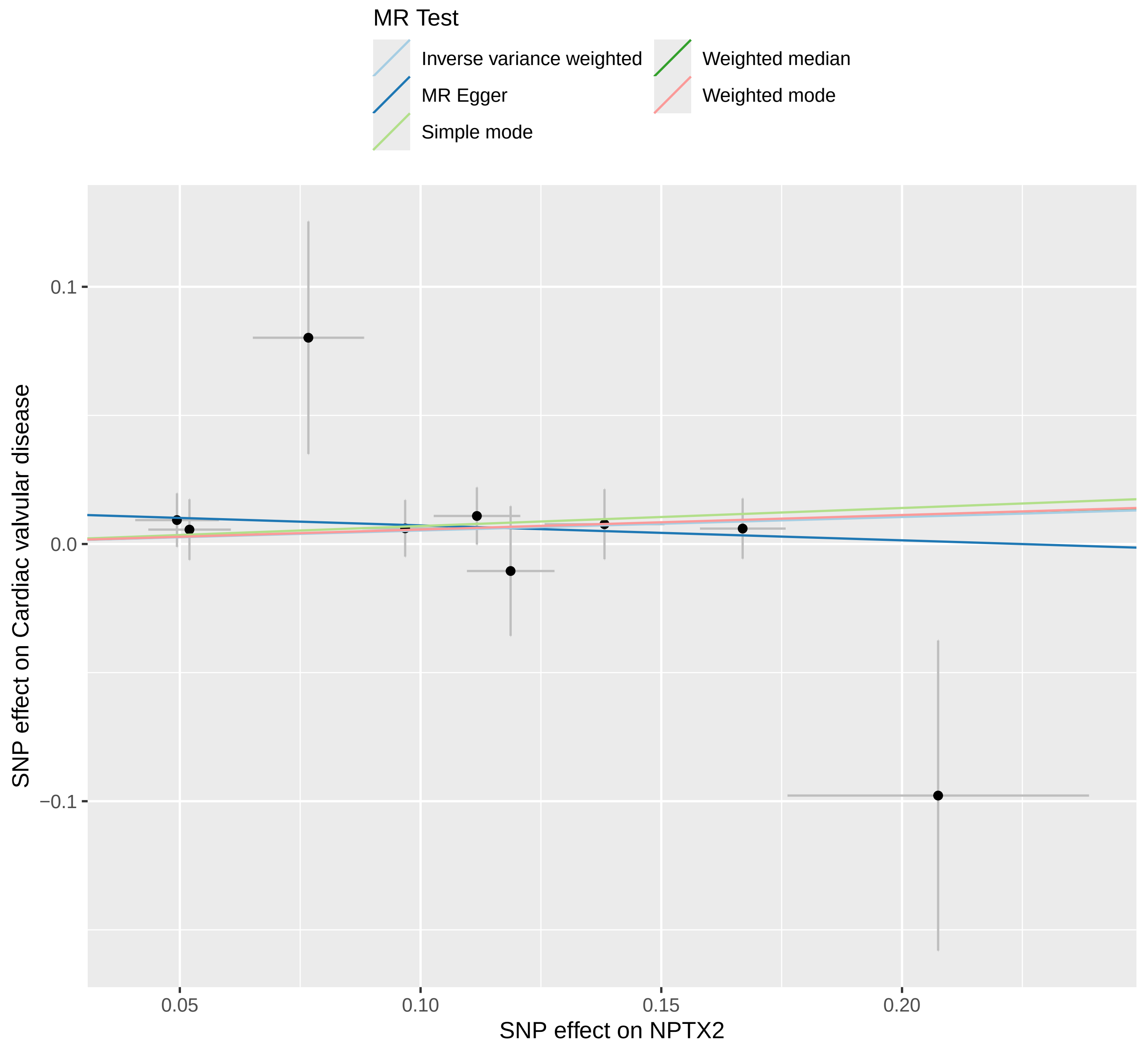
MR Test
Inverse variance weighted
MR Egger
Simple mode
Weighted median
Weighted mode
0.1
0.0
−0.1
0.05
0.10
0.15
0.20
SNP effect on NPTX2
SNP effect on Cardiac valvular disease

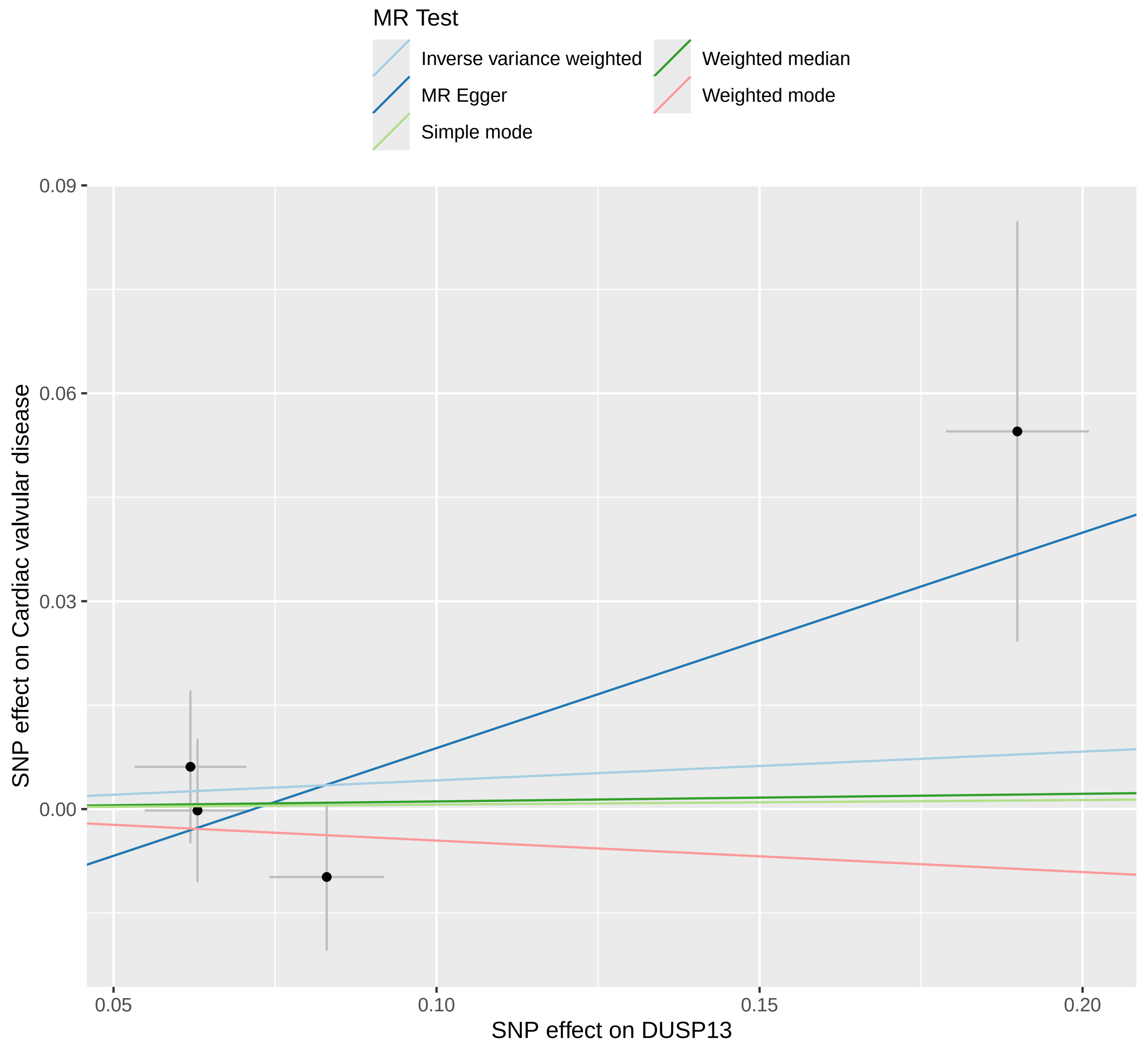
MR Test
Inverse variance weighted
MR Egger
Simple mode
Weighted median
Weighted mode
0.09
0.06
0.03
0.00
SNP effect on Cardiac valvular disease
0.05
0.10
0.15
0.20
SNP effect on DUSP13

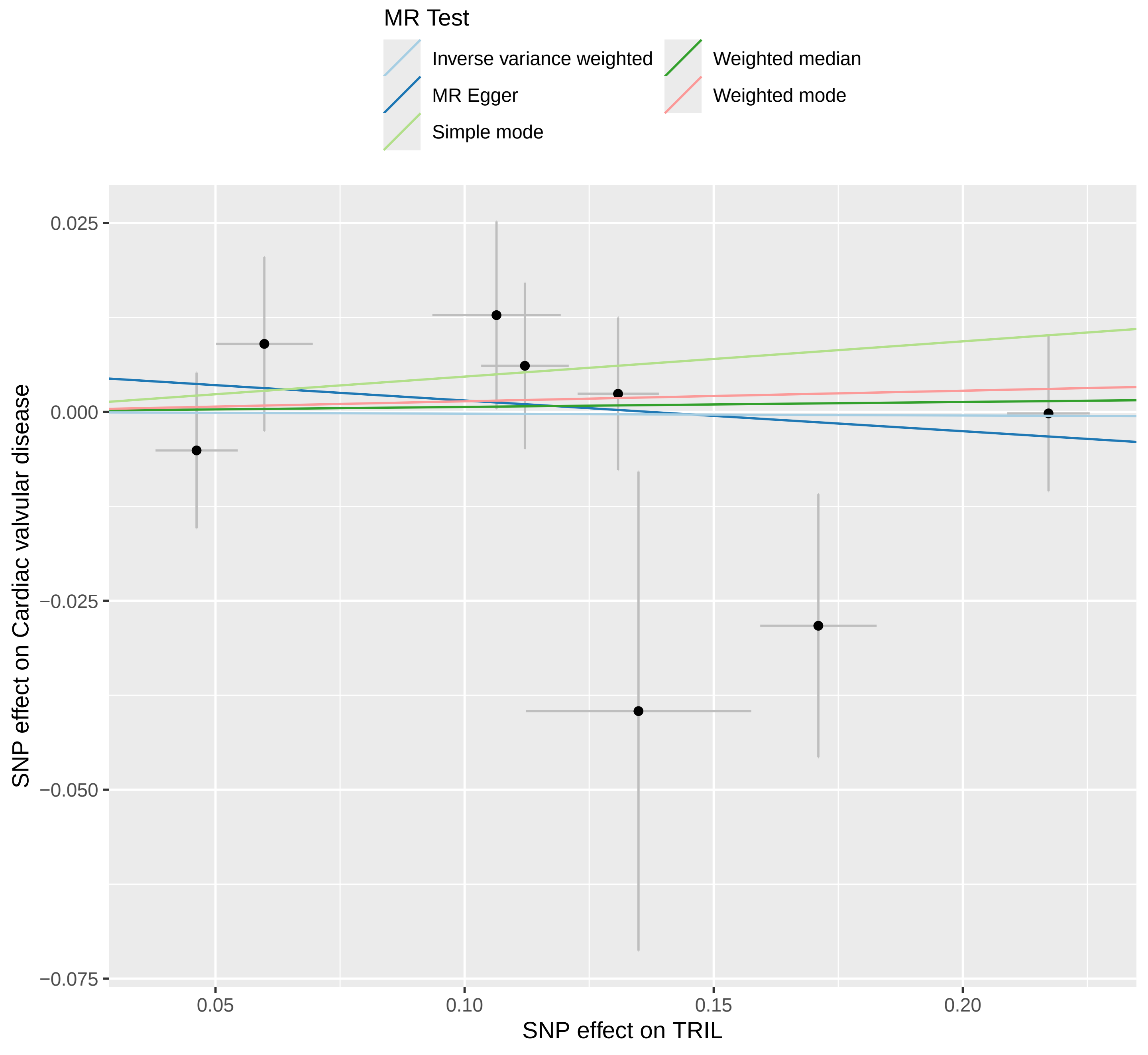

MR Test
Inverse variance weighted
MR Egger
Simple mode
Weighted median
Weighted mode
0.025
0.000
−0.025
−0.050
−0.075
0.05
0.10
0.15
0.20
SNP effect on TRIL
SNP effect on Cardiac valvular disease

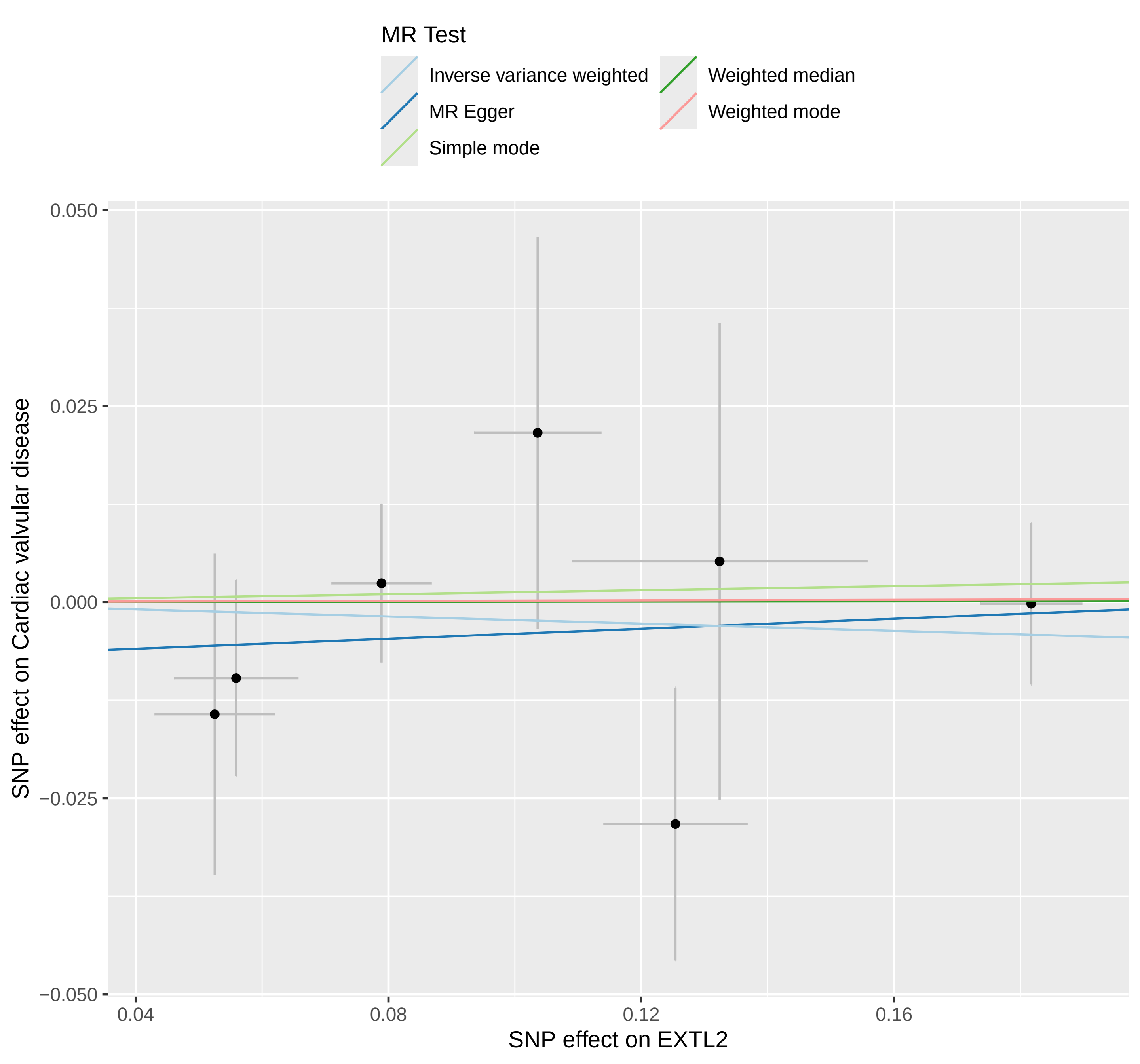
MR Test
Inverse variance weighted
MR Egger
Simple mode
Weighted median
Weighted mode
0.050
0.025
0.000
−0.025
−0.050
SNP effect on Cardiac valvular disease
0.04
0.08
0.12
0.16
SNP effect on EXTL2

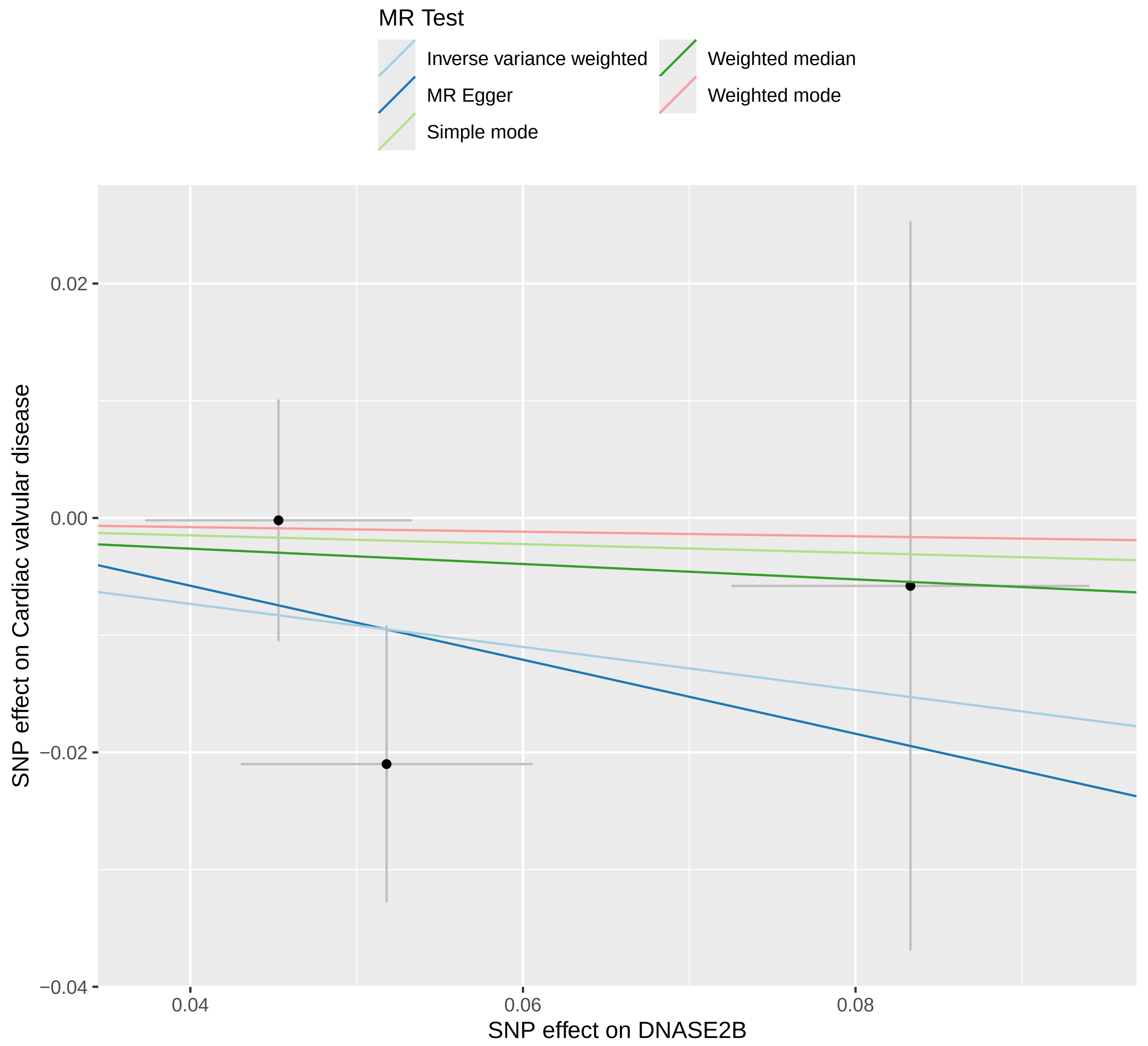

MR Test
Inverse variance weighted
MR Egger
Simple mode
Weighted median
Weighted mode
0.02
0.00
−0.02
−0.04
0.04
0.06
0.08
SNP effect on Cardiac valvular disease
SNP effect on DNASE2B

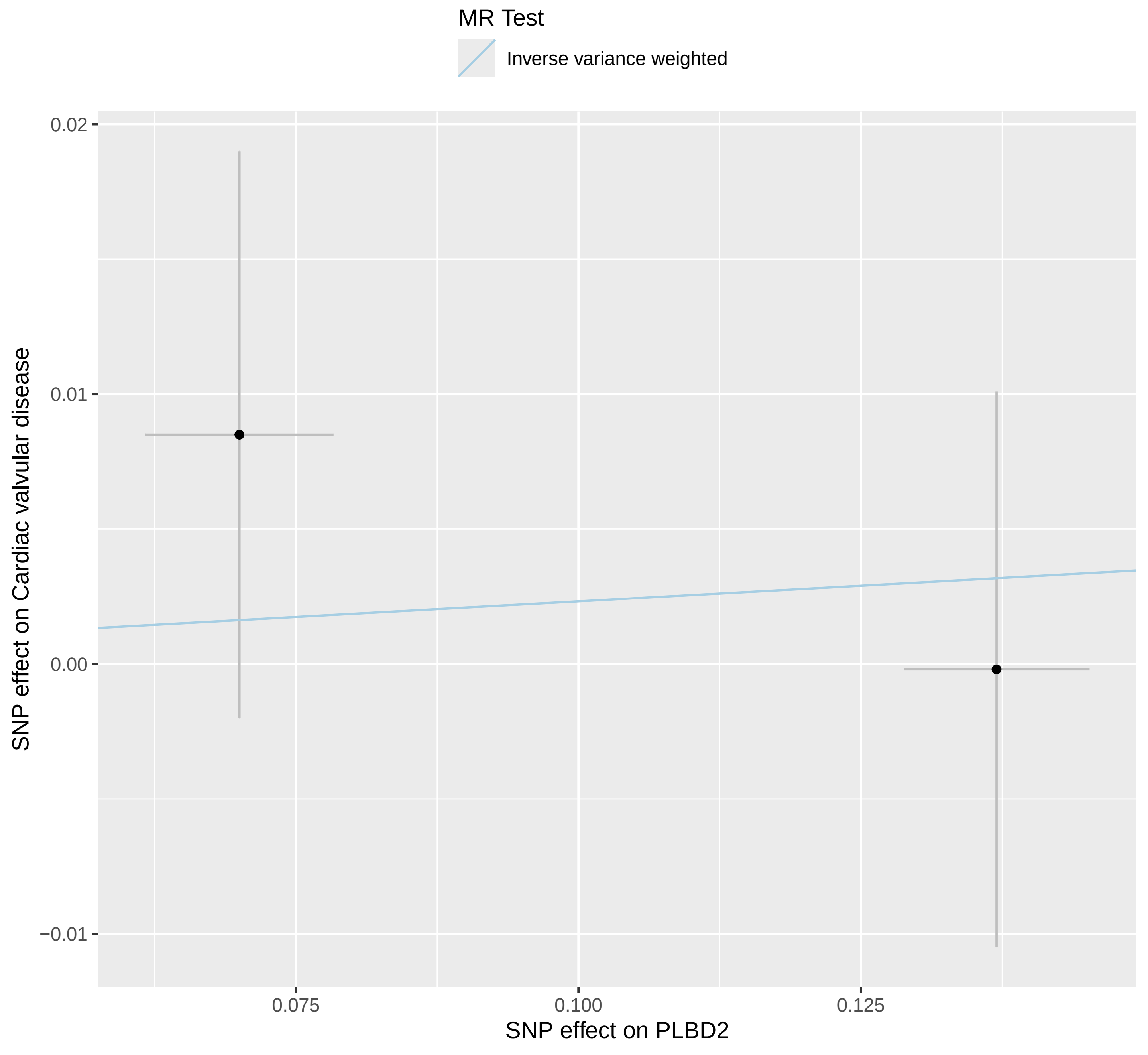

MR Test
Inverse variance weighted
0.02
0.01
0.00
−0.01
0.075
0.100
0.125
SNP effect on PLBD2
SNP effect on Cardiac valvular disease

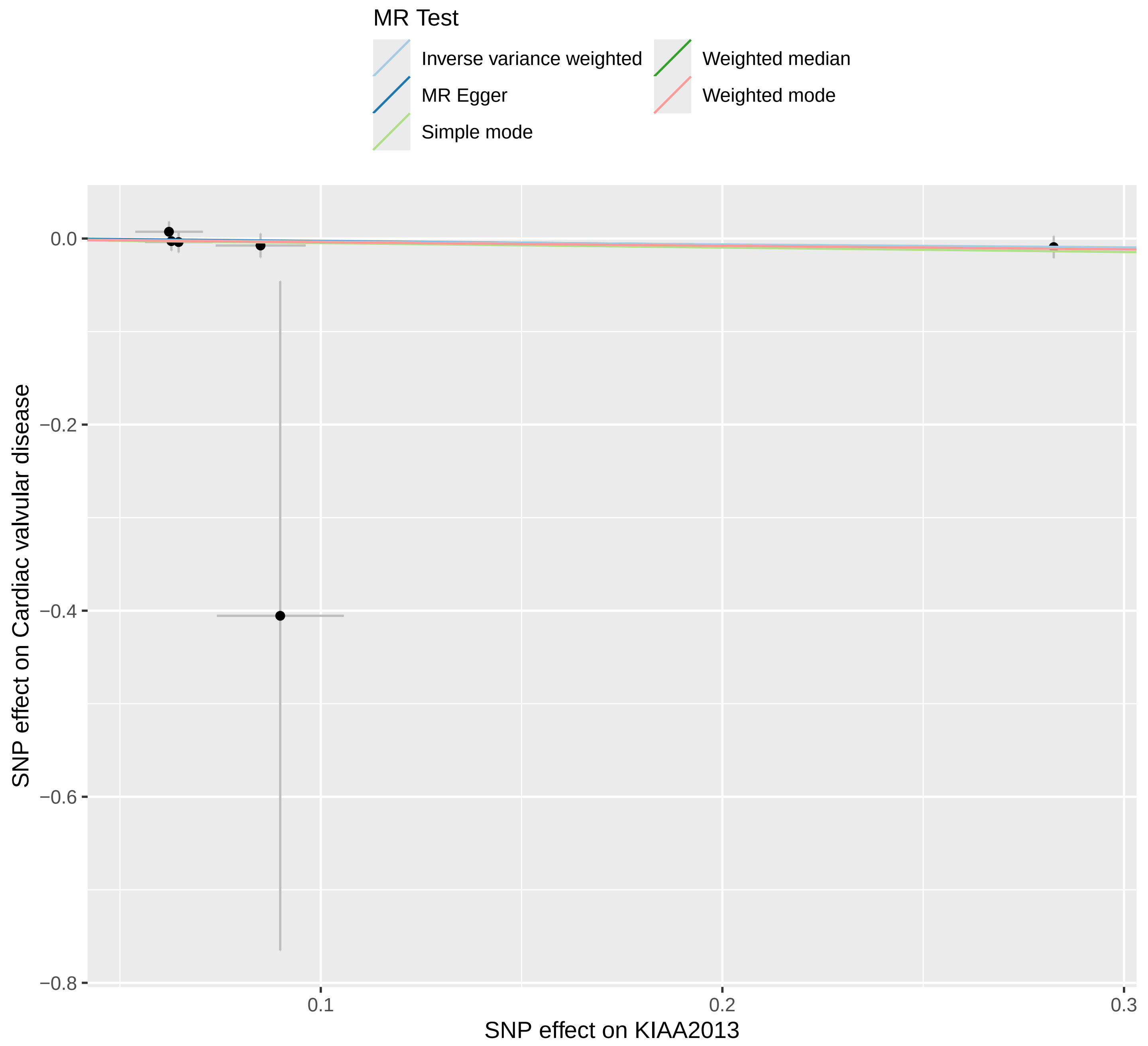
MR Test
Inverse variance weighted
MR Egger
Simple mode
Weighted median
Weighted mode
0.0
−0.2
−0.4
−0.6
−0.8
0.1
0.2
0.3
SNP effect on Cardiac valvular disease
SNP effect on KIAA2013

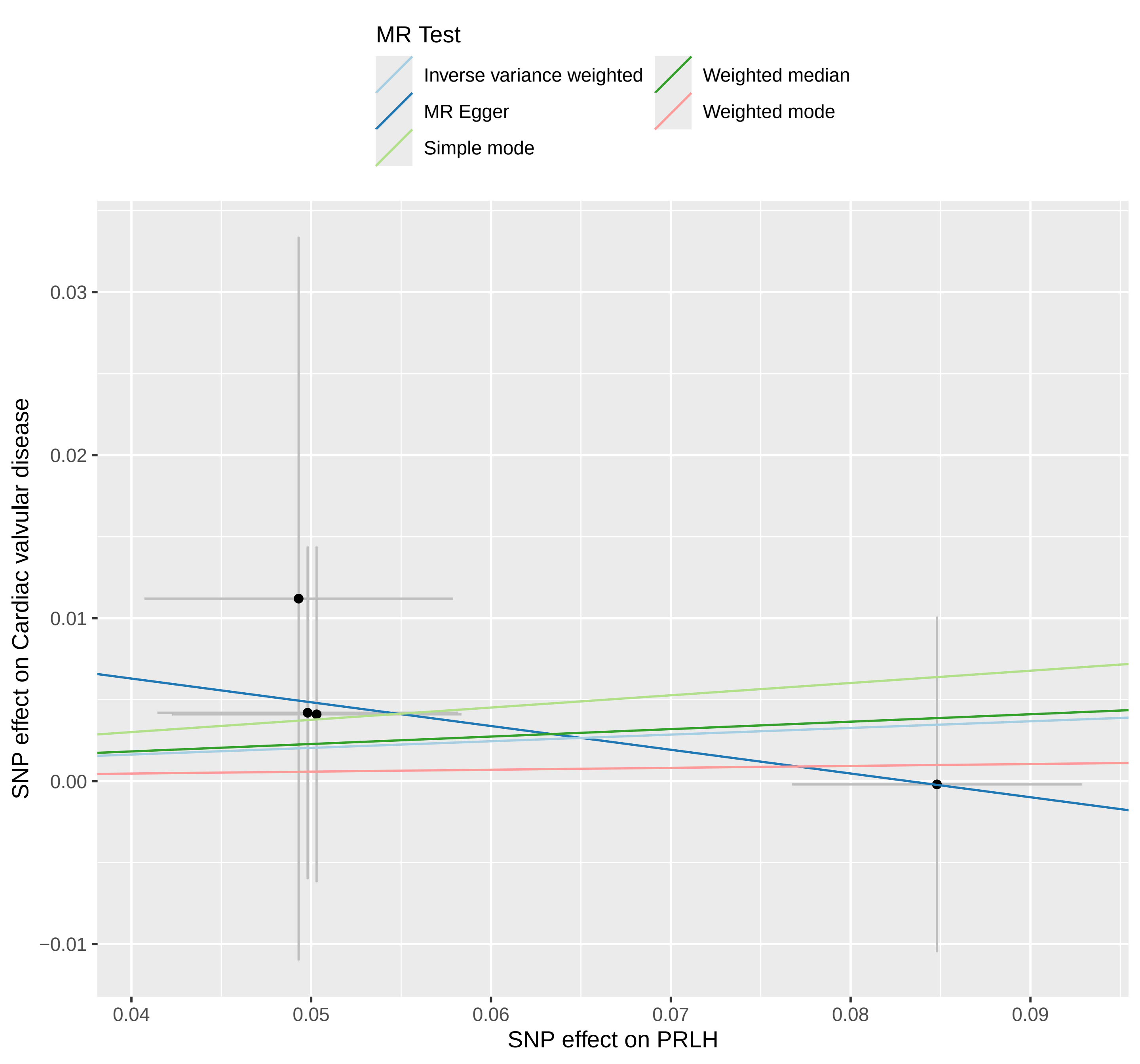
MR Test
Inverse variance weighted
MR Egger
Simple mode
Weighted median
Weighted mode
0.03
0.02
0.01
0.00
−0.01
SNP effect on Cardiac valvular disease
0.04
0.05
0.06
0.07
0.08
0.09
SNP effect on PRLH

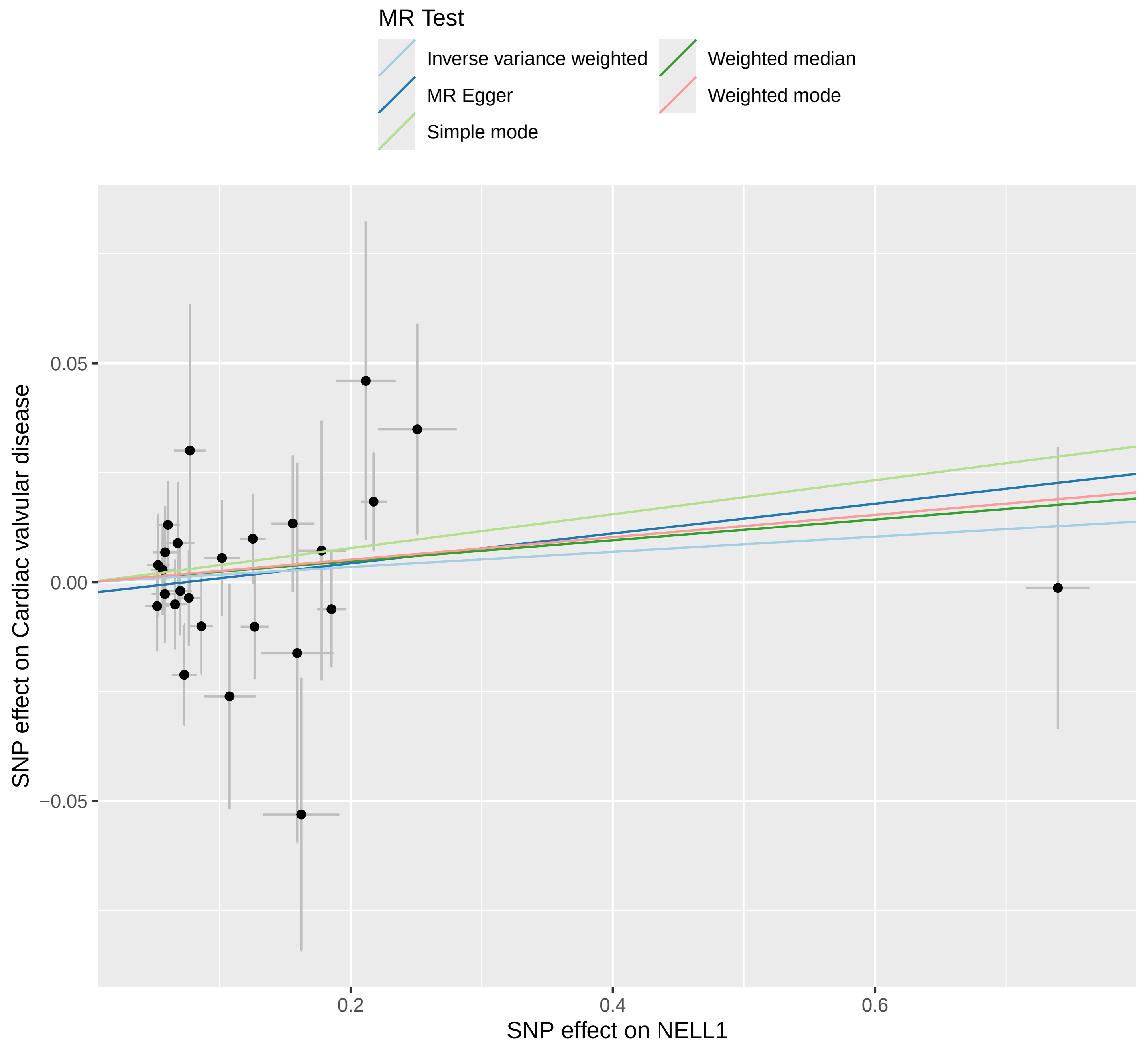
MR Test
Inverse variance weighted
MR Egger
Simple mode
Weighted median
Weighted mode
0.05
0.00
−0.05
SNP effect on Cardiac valvular disease
0.2
0.4
0.6
SNP effect on NELL1

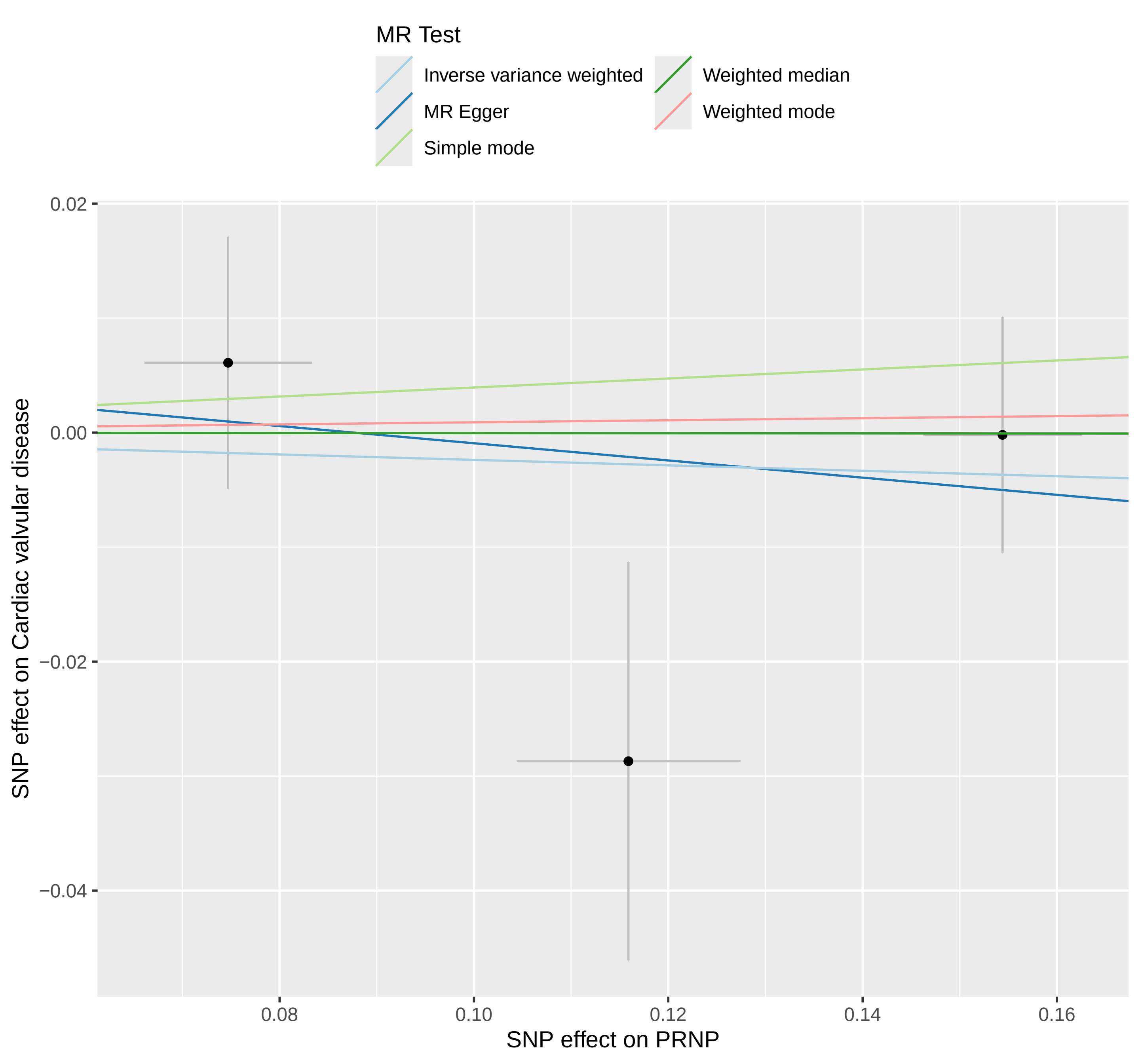

MR Test
Inverse variance weighted
MR Egger
Simple mode
Weighted median
Weighted mode
0.02
0.00
−0.02
−0.04
0.08
0.10
0.12
0.14
0.16
SNP effect on PRNP
SNP effect on Cardiac valvular disease

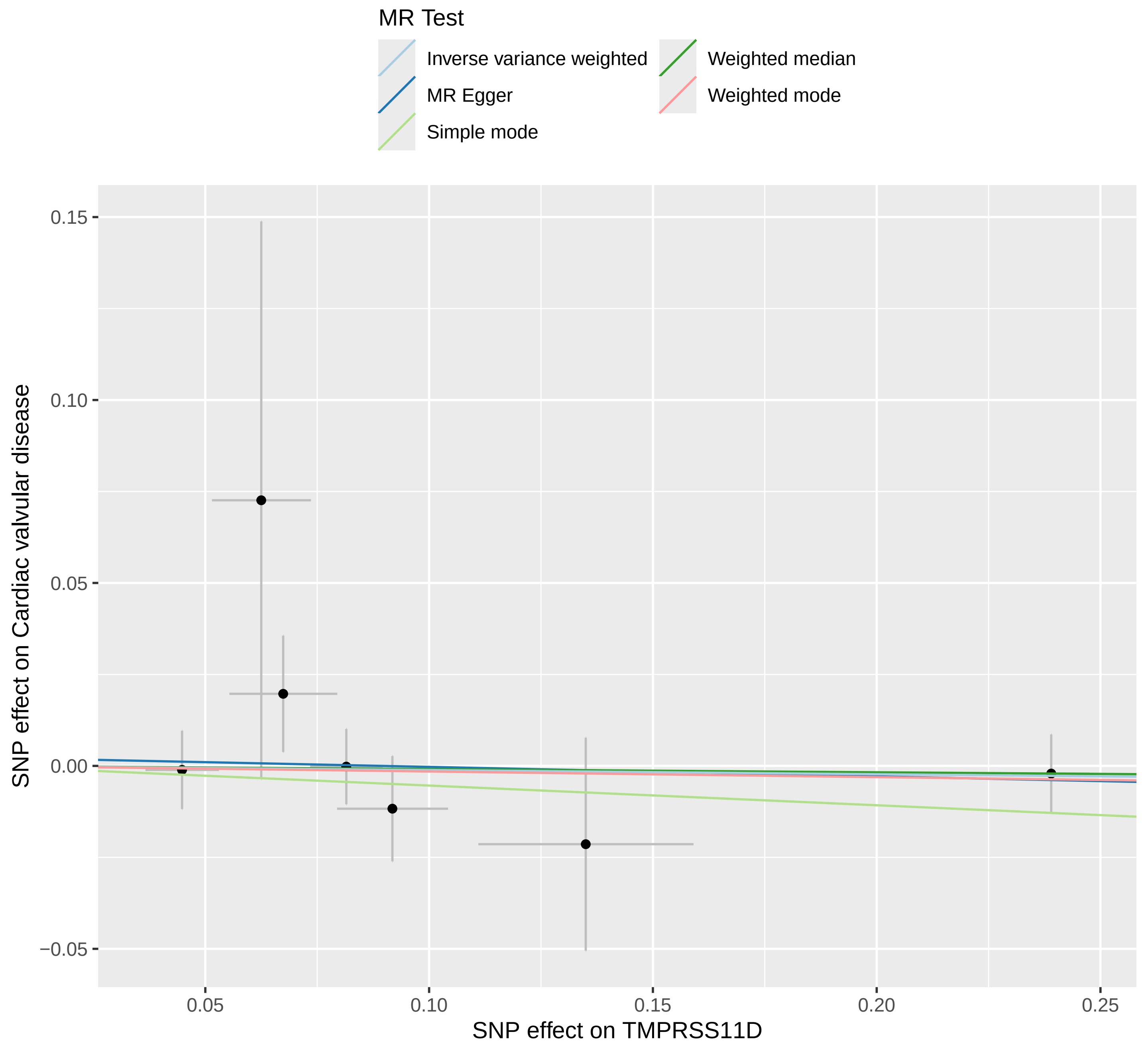

MR Test
Inverse variance weighted
MR Egger
Simple mode
Weighted median
Weighted mode
0.15
0.10
0.05
0.00
−0.05
0.05
0.10
0.15
0.20
0.25
SNP effect on Cardiac valvular disease
SNP effect on TMPRSS11D

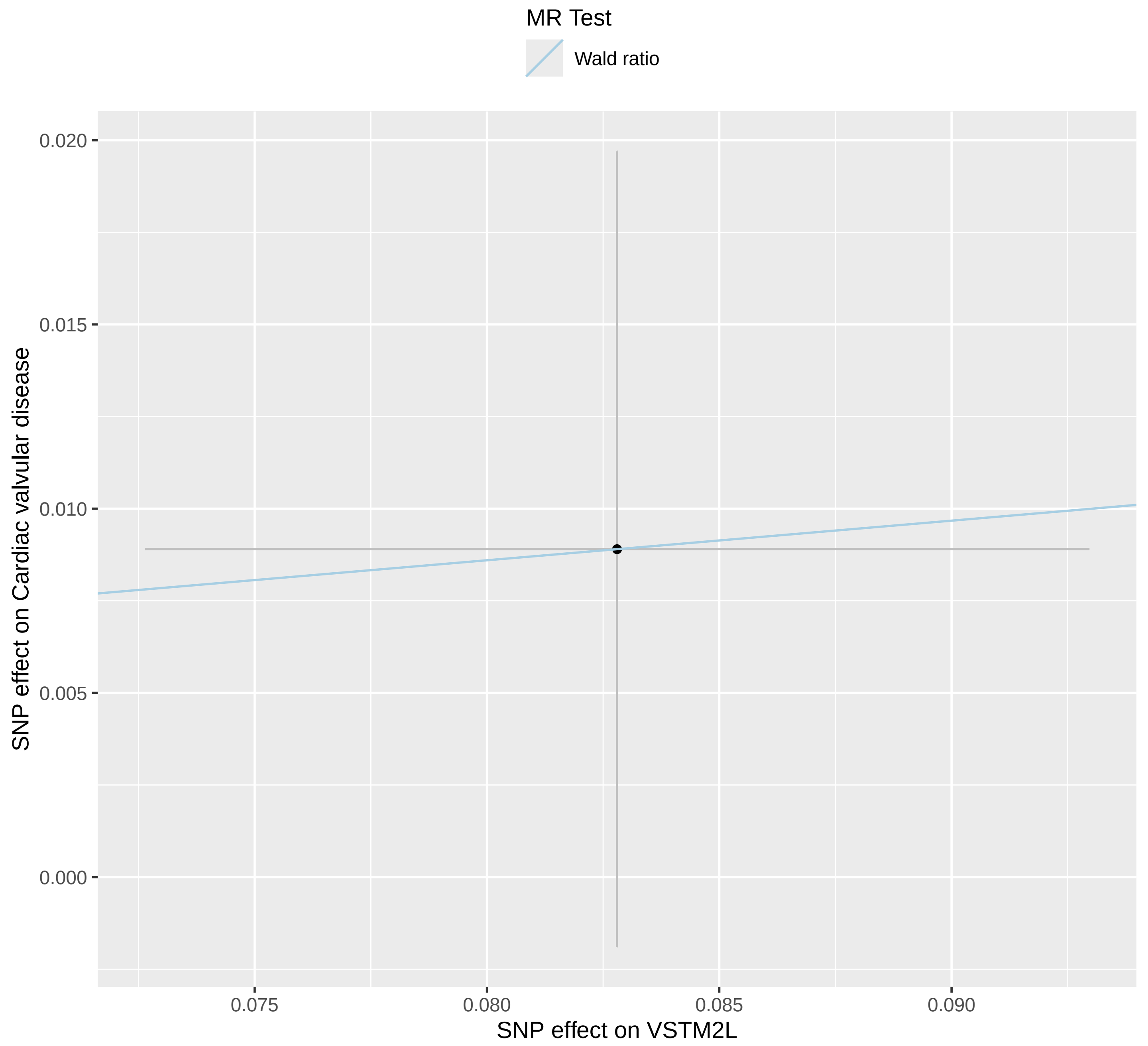
MR Test
Wald ratio
0.020
0.015
0.010
0.005
0.000
0.075
0.080
0.085
0.090
SNP effect on Cardiac valvular disease
SNP effect on VSTM2L

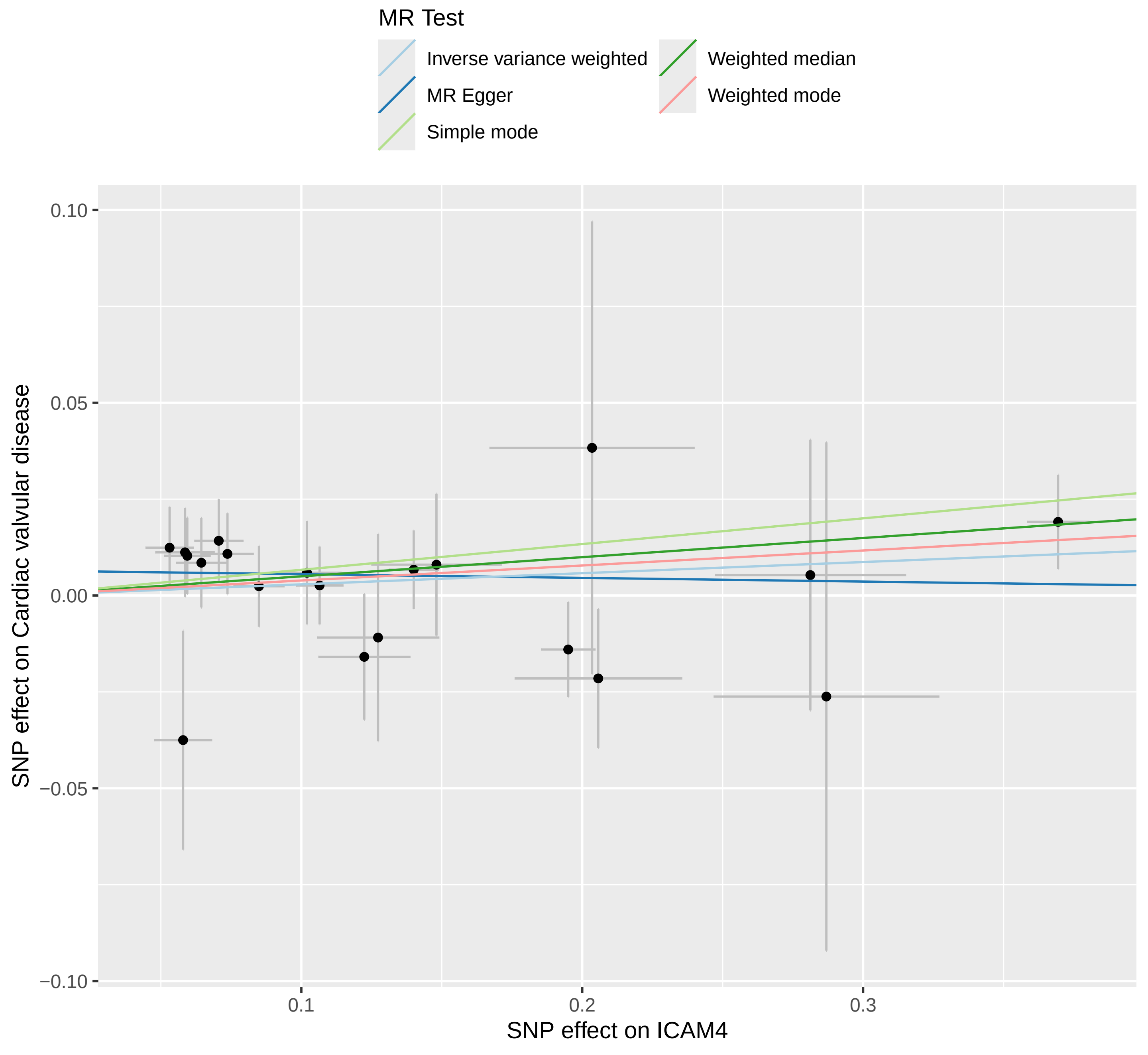
MR Test
Inverse variance weighted
MR Egger
Simple mode
Weighted median
Weighted mode
0.10
0.05
0.00
−0.05
−0.10
0.1
0.2
0.3
SNP effect on Cardiac valvular disease
SNP effect on ICAM4

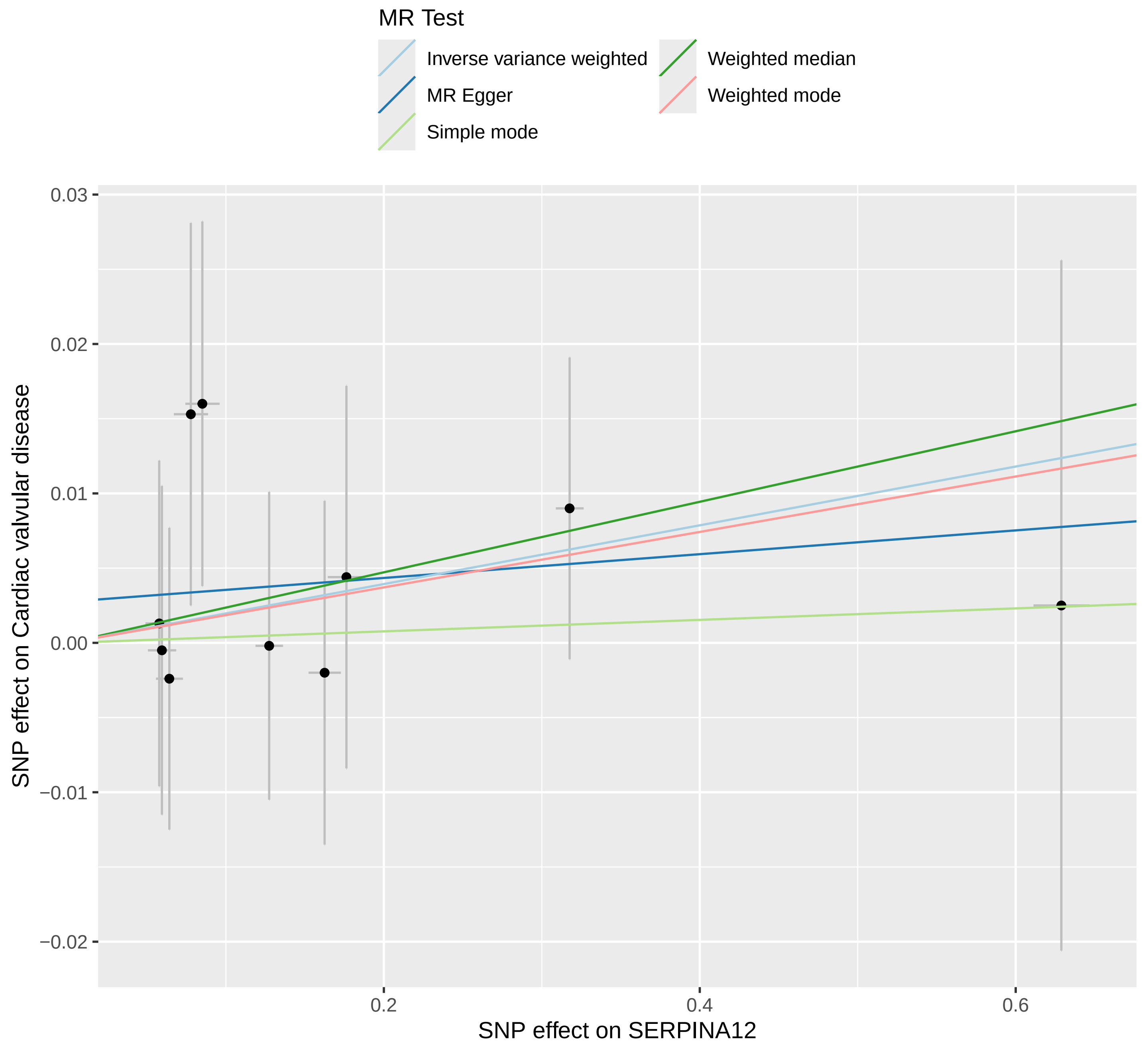
MR Test
Inverse variance weighted
MR Egger
Simple mode
Weighted median
Weighted mode
0.03
0.02
0.01
0.00
−0.01
−0.02
SNP effect on Cardiac valvular disease
0.2
0.4
0.6
SNP effect on SERPINA12

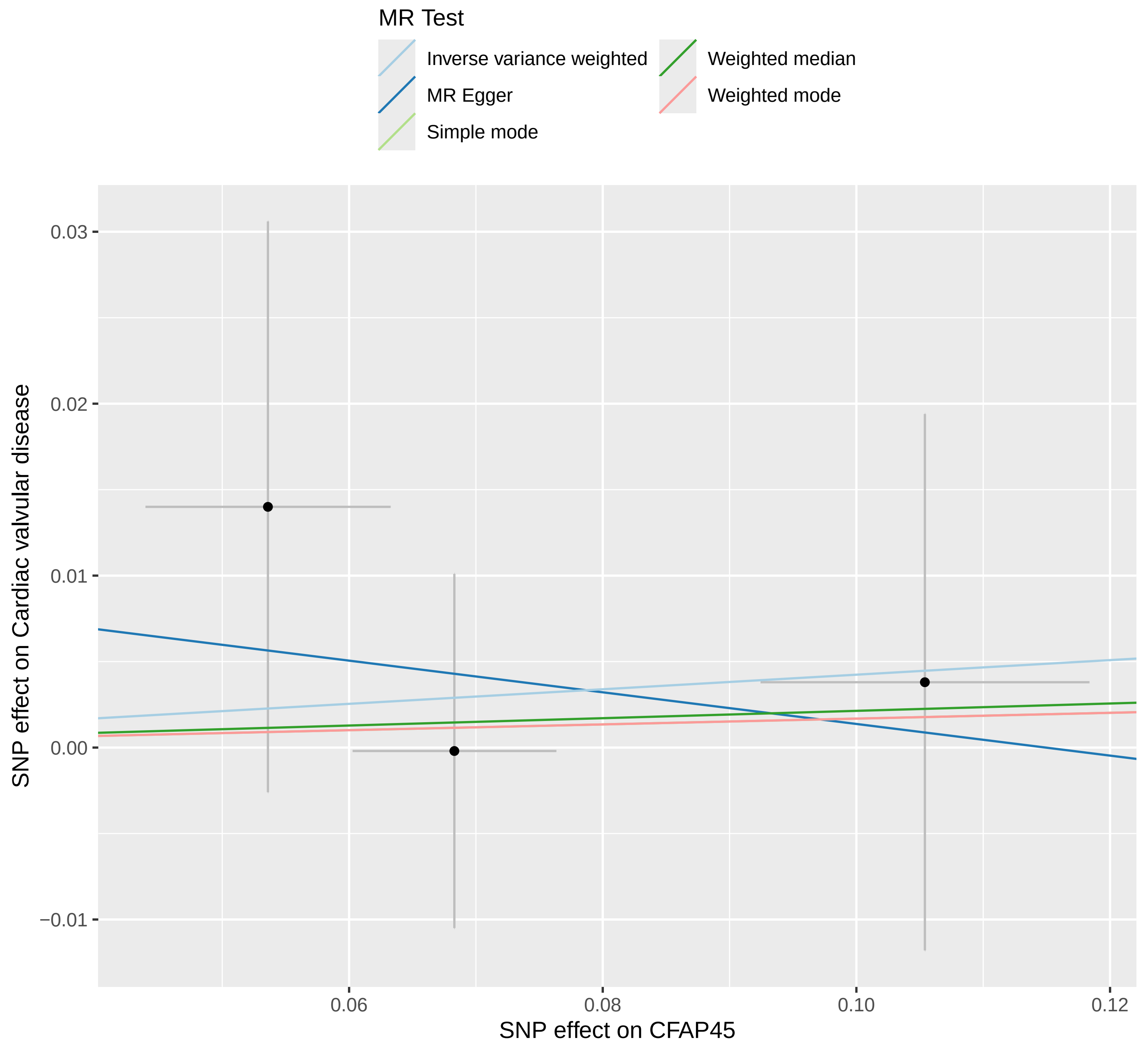

MR Test
Inverse variance weighted
MR Egger
Simple mode
Weighted median
Weighted mode
SNP effect on Cardiac valvular disease
0.03
0.02
0.01
0.00
−0.01
0.06
0.08
0.10
0.12
SNP effect on CFAP45

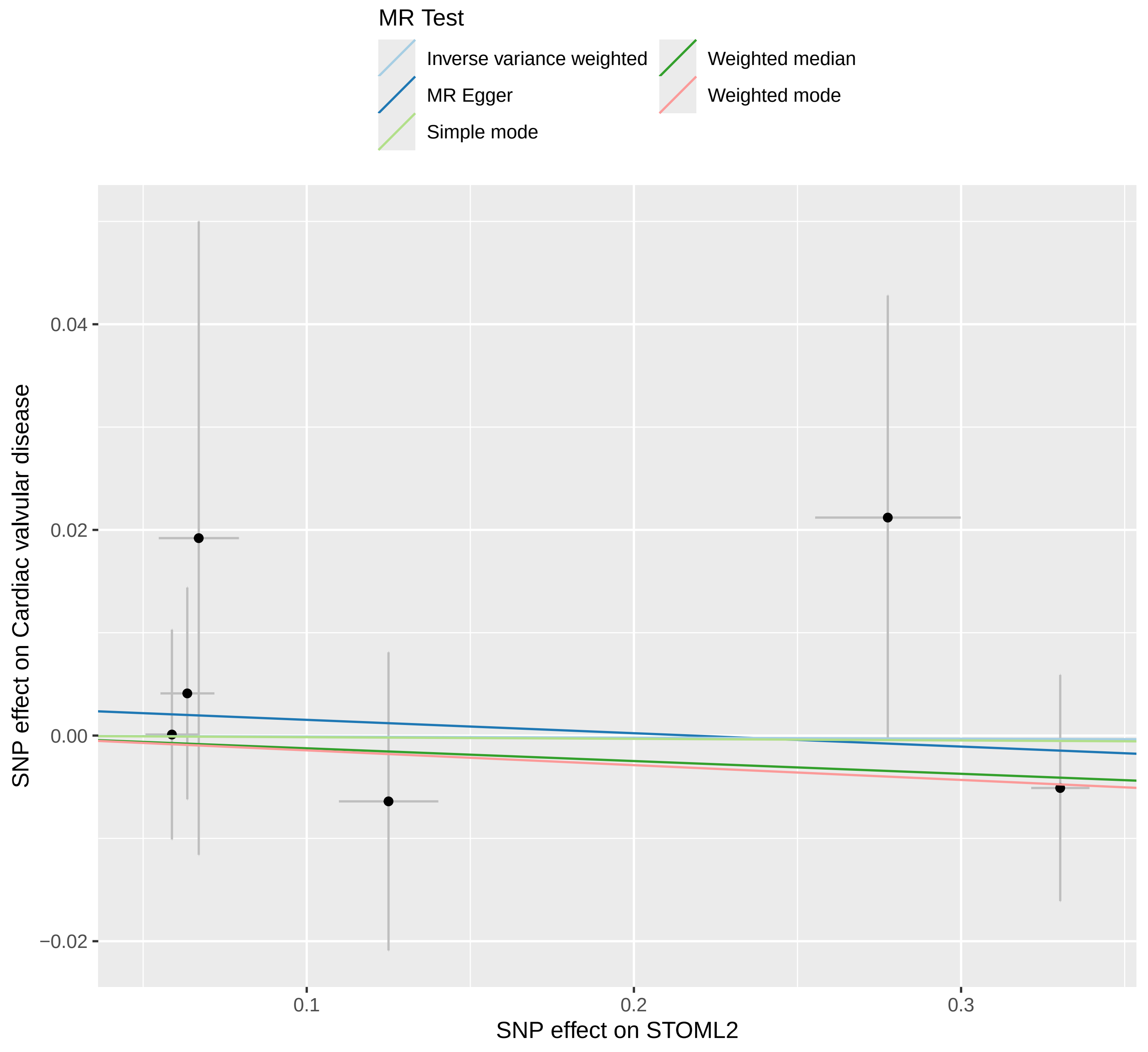
MR Test
Inverse variance weighted
MR Egger
Simple mode
Weighted median
Weighted mode
SNP effect on Cardiac valvular disease
0.04
0.02
0.00
−0.02
0.1
0.2
0.3
SNP effect on STOML2

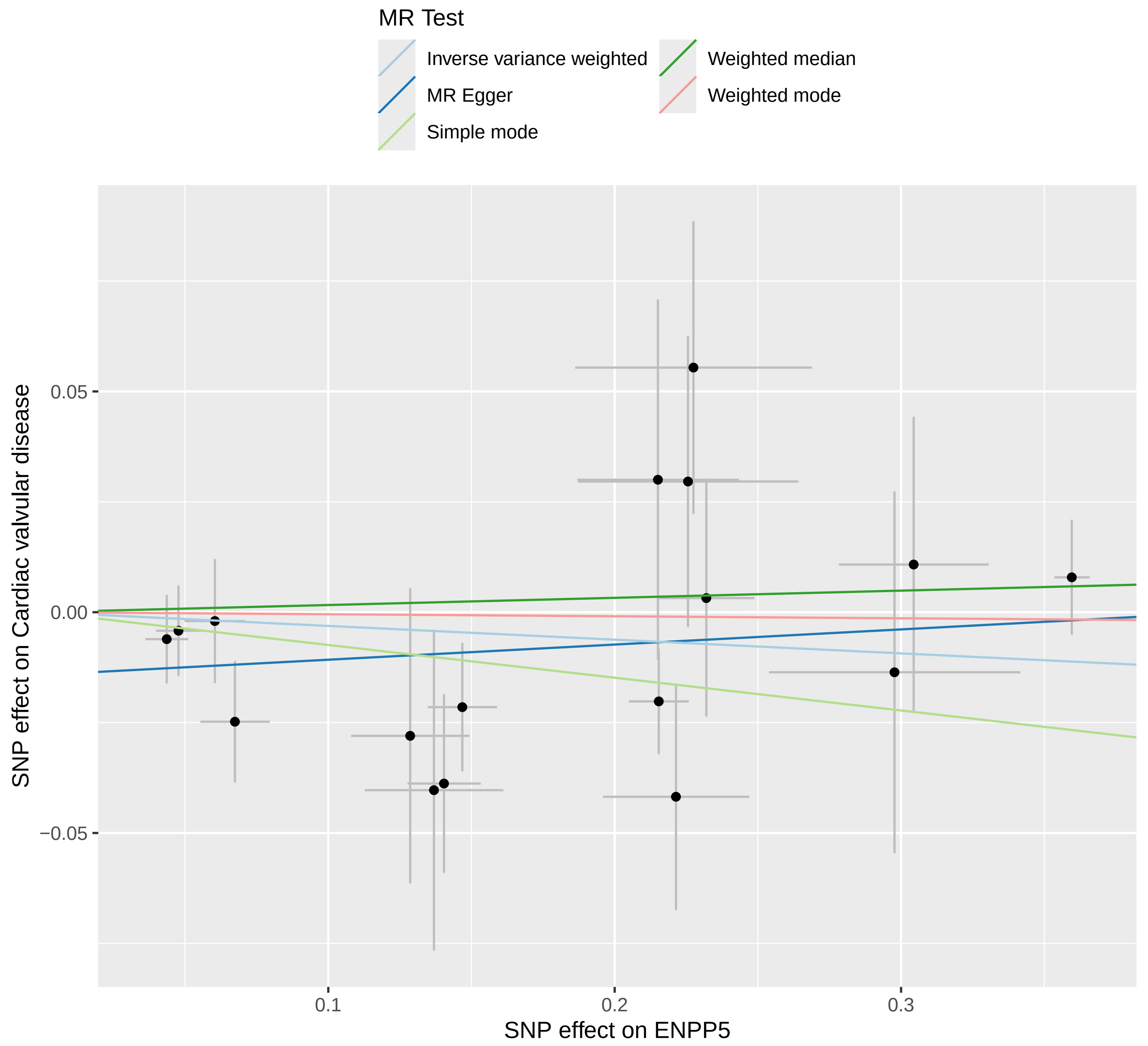

MR Test
Inverse variance weighted
MR Egger
Simple mode
Weighted median
Weighted mode
SNP effect on Cardiac valvular disease
0.05
0.00
−0.05
0.1
0.2
0.3
SNP effect on ENPP5

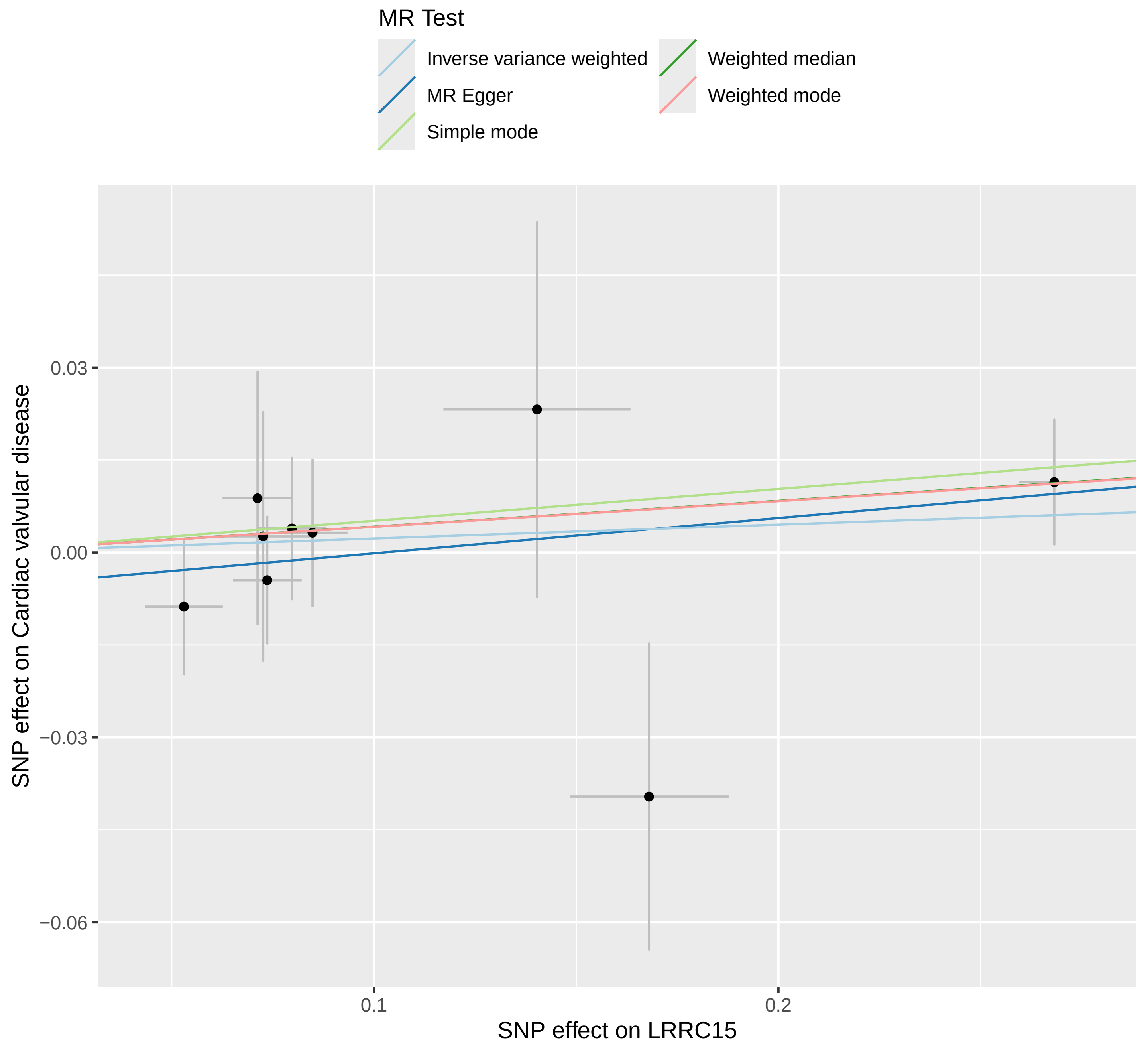

MR Test
Inverse variance weighted
MR Egger
Simple mode
Weighted median
Weighted mode
0.03
0.00
−0.03
−0.06
0.1
0.2
SNP effect on Cardiac valvular disease
SNP effect on LRRC15

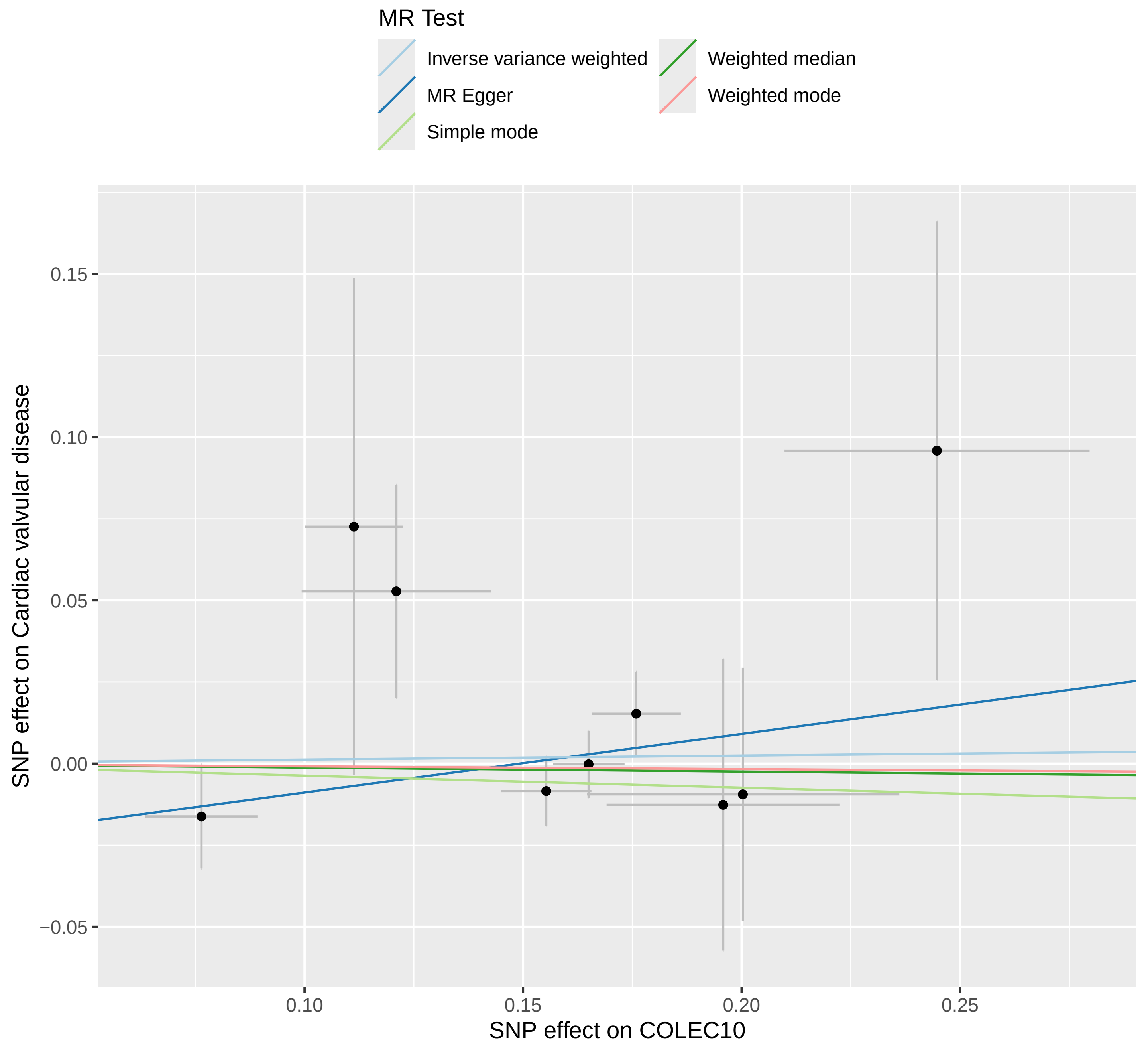
MR Test
Inverse variance weighted
MR Egger
Simple mode
Weighted median
Weighted mode
SNP effect on Cardiac valvular disease
0.15
0.10
0.05
0.00
−0.05
0.10
0.15
0.20
0.25
SNP effect on COLEC10

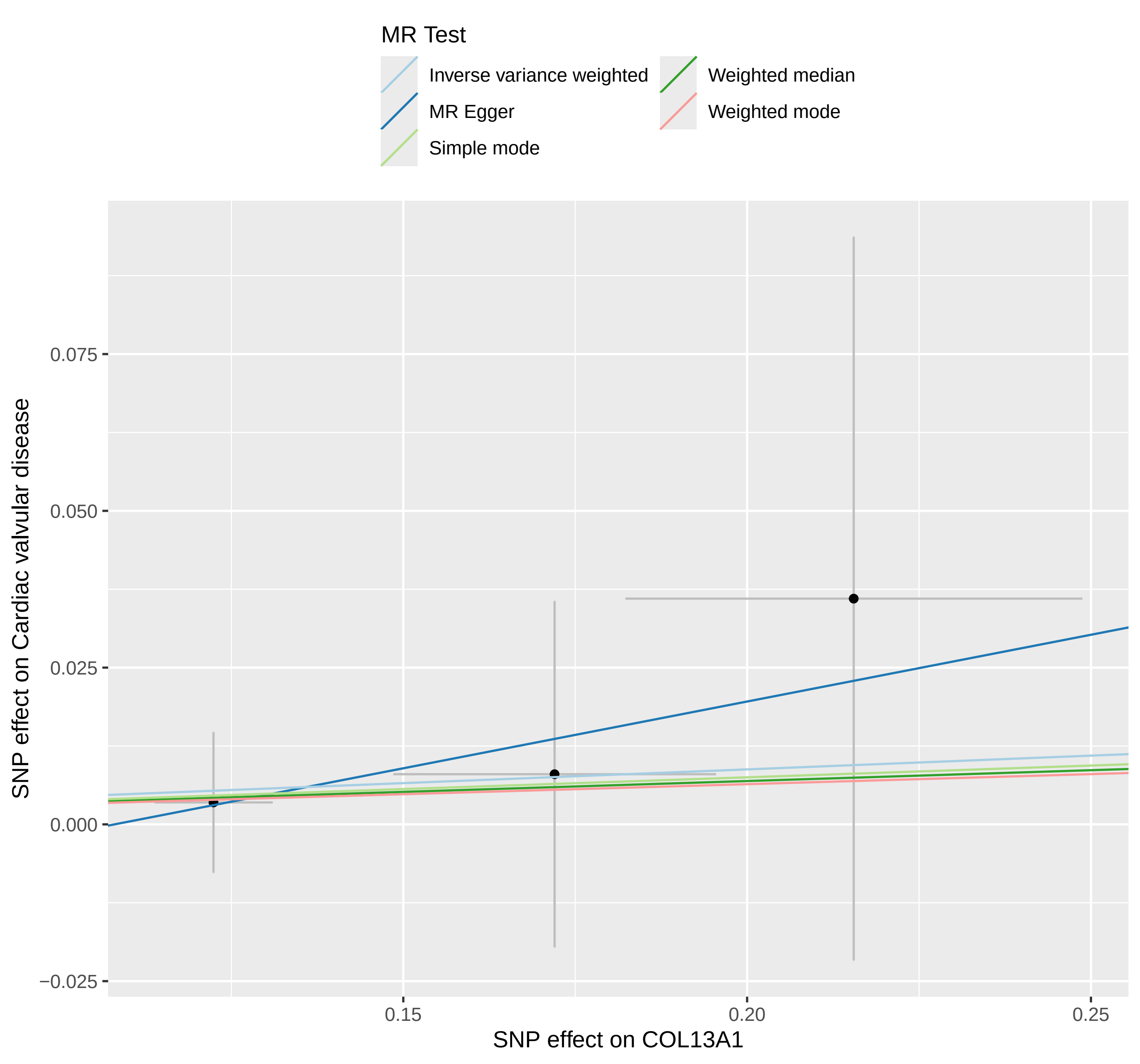
MR Test
Inverse variance weighted
MR Egger
Simple mode
Weighted median
Weighted mode
0.075
0.050
0.025
0.000
−0.025
0.15
0.20
0.25
SNP effect on Cardiac valvular disease
SNP effect on COL13A1

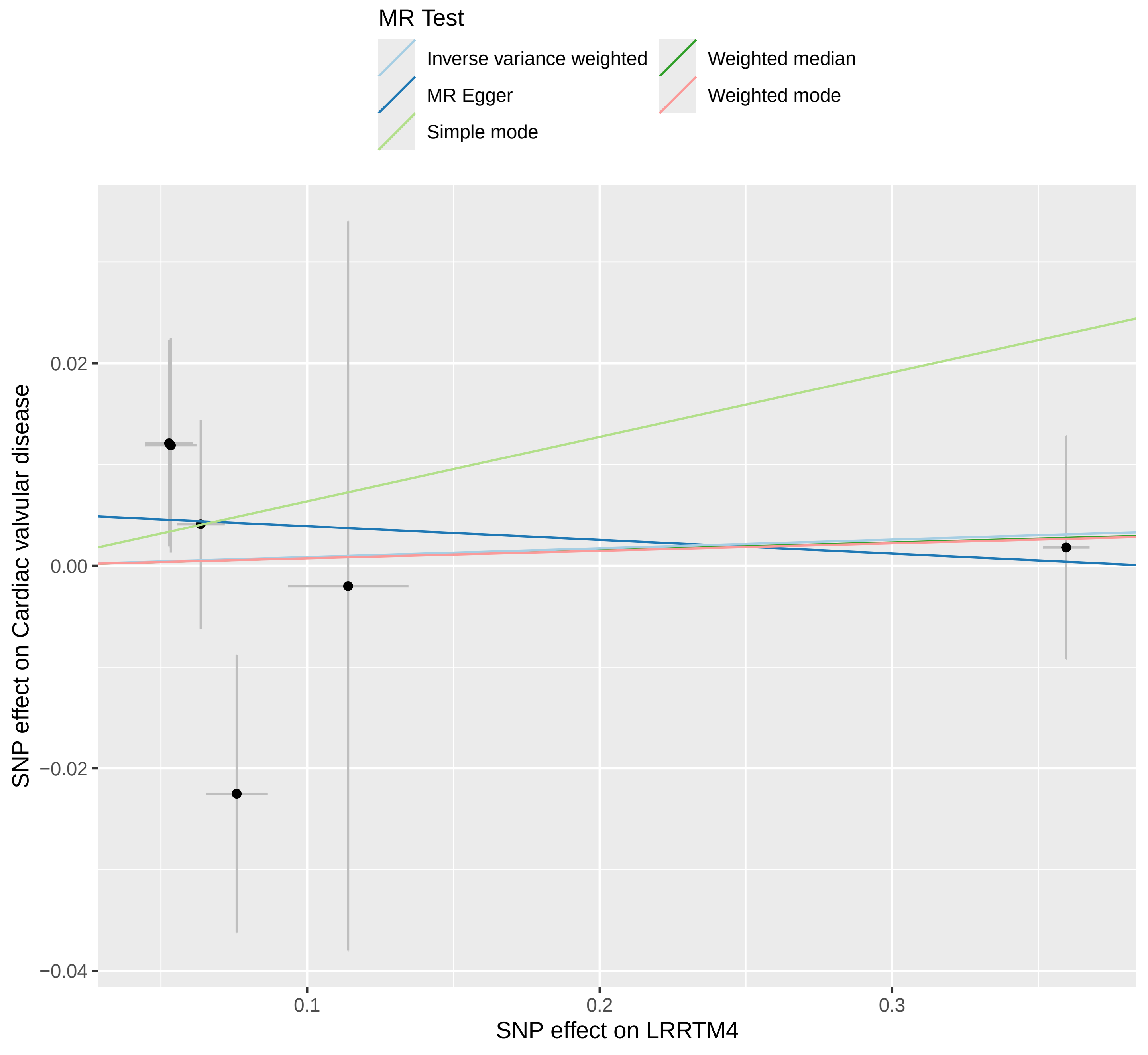
MR Test
Inverse variance weighted
MR Egger
Simple mode
Weighted median
Weighted mode
0.02
0.00
−0.02
−0.04
SNP effect on Cardiac valvular disease
0.1
0.2
0.3
SNP effect on LRRTM4

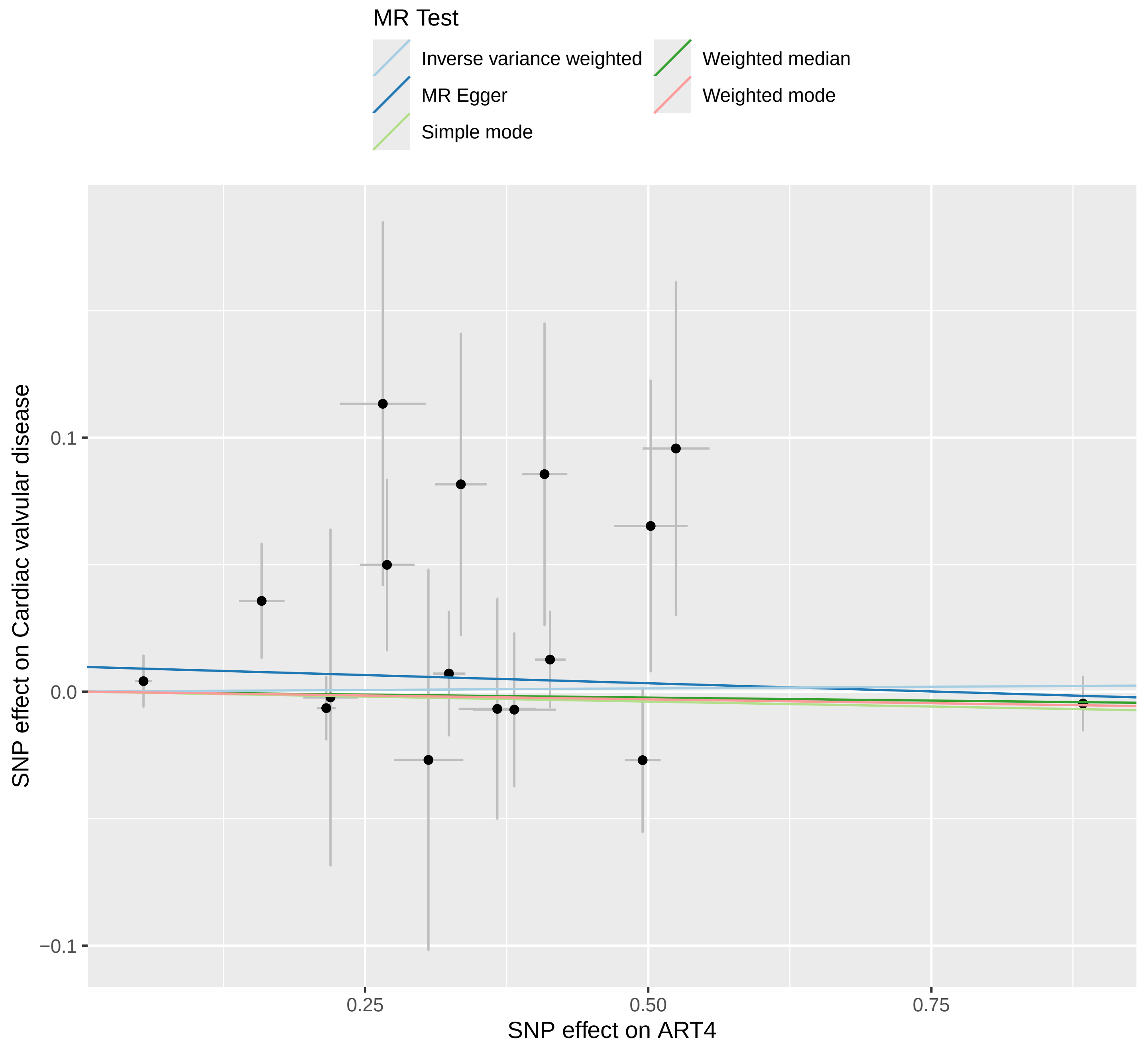
MR Test
Inverse variance weighted
MR Egger
Simple mode
Weighted median
Weighted mode
0.1
0.0
−0.1
0.25
0.50
0.75
SNP effect on ART4
SNP effect on Cardiac valvular disease

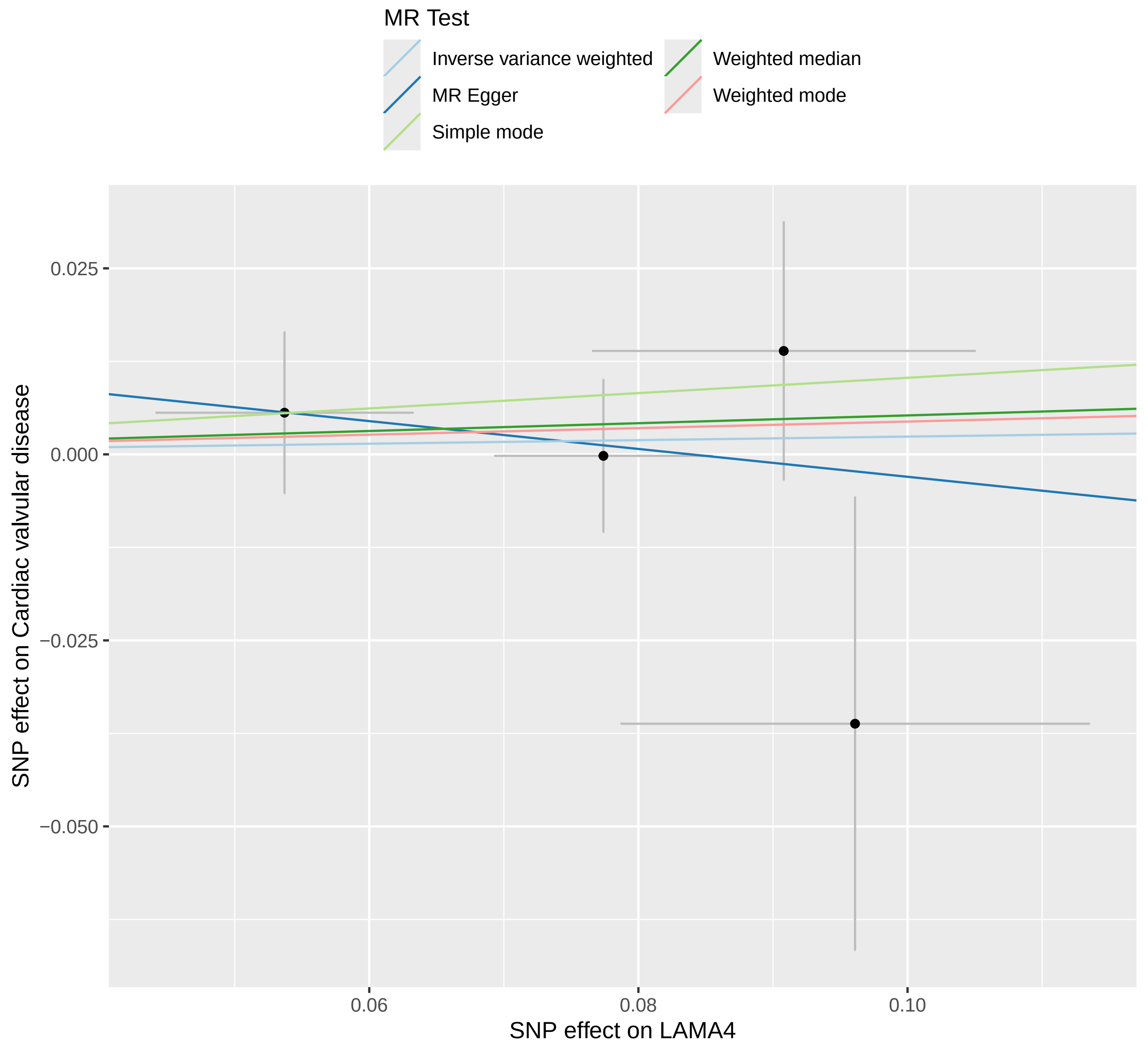
MR Test
Inverse variance weighted
MR Egger
Simple mode
Weighted median
Weighted mode
0.025
0.000
−0.025
−0.050
SNP effect on Cardiac valvular disease
0.06
0.08
0.10
SNP effect on LAMA4

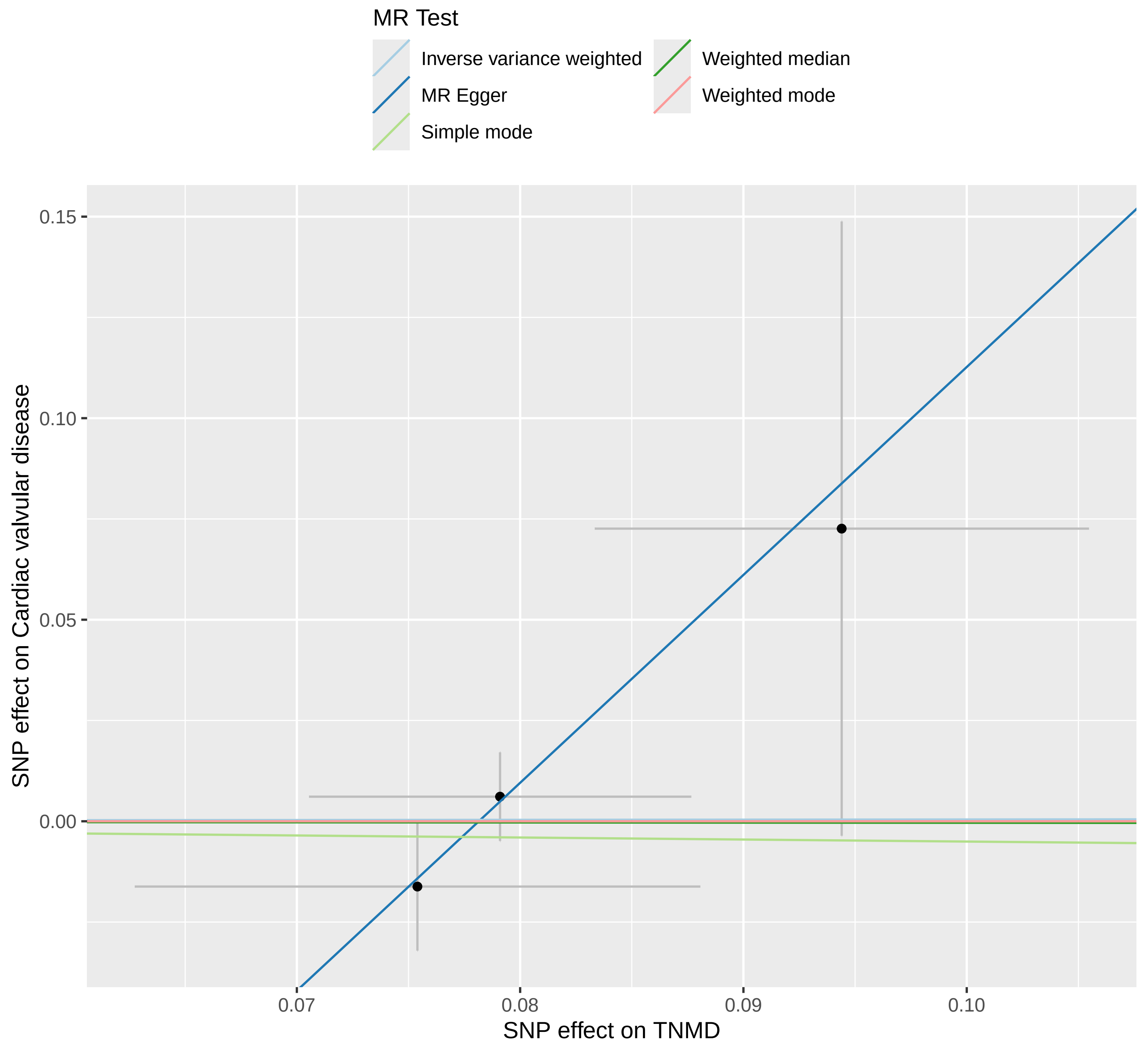
MR Test
Inverse variance weighted
MR Egger
Simple mode
Weighted median
Weighted mode
SNP effect on Cardiac valvular disease
0.15
0.10
0.05
0.00
0.07
0.08
0.09
0.10
SNP effect on TNMD

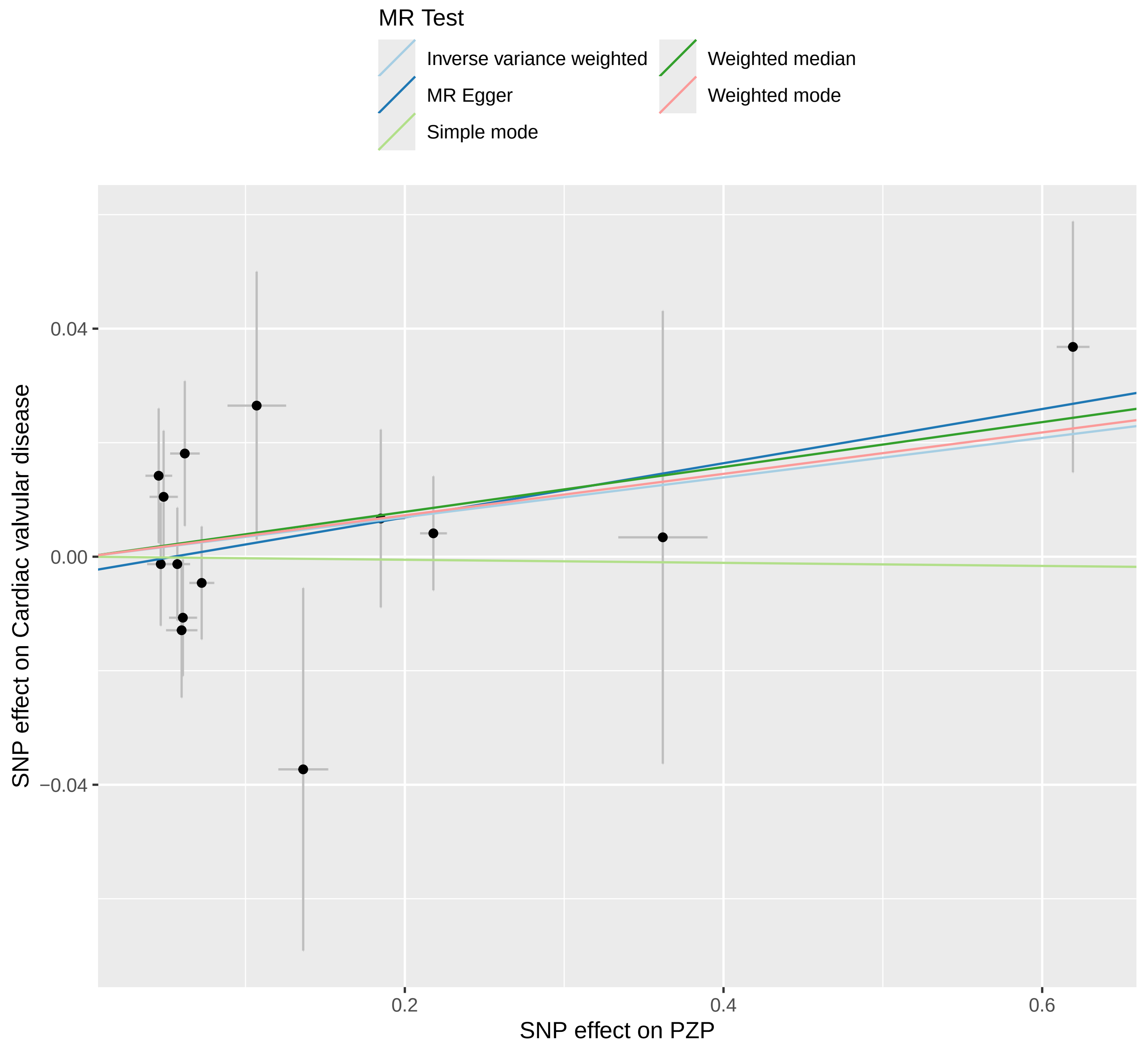

MR Test
Inverse variance weighted
MR Egger
Simple mode
Weighted median
Weighted mode
0.04
0.00
−0.04
0.2
0.4
0.6
SNP effect on Cardiac valvular disease
SNP effect on PZP

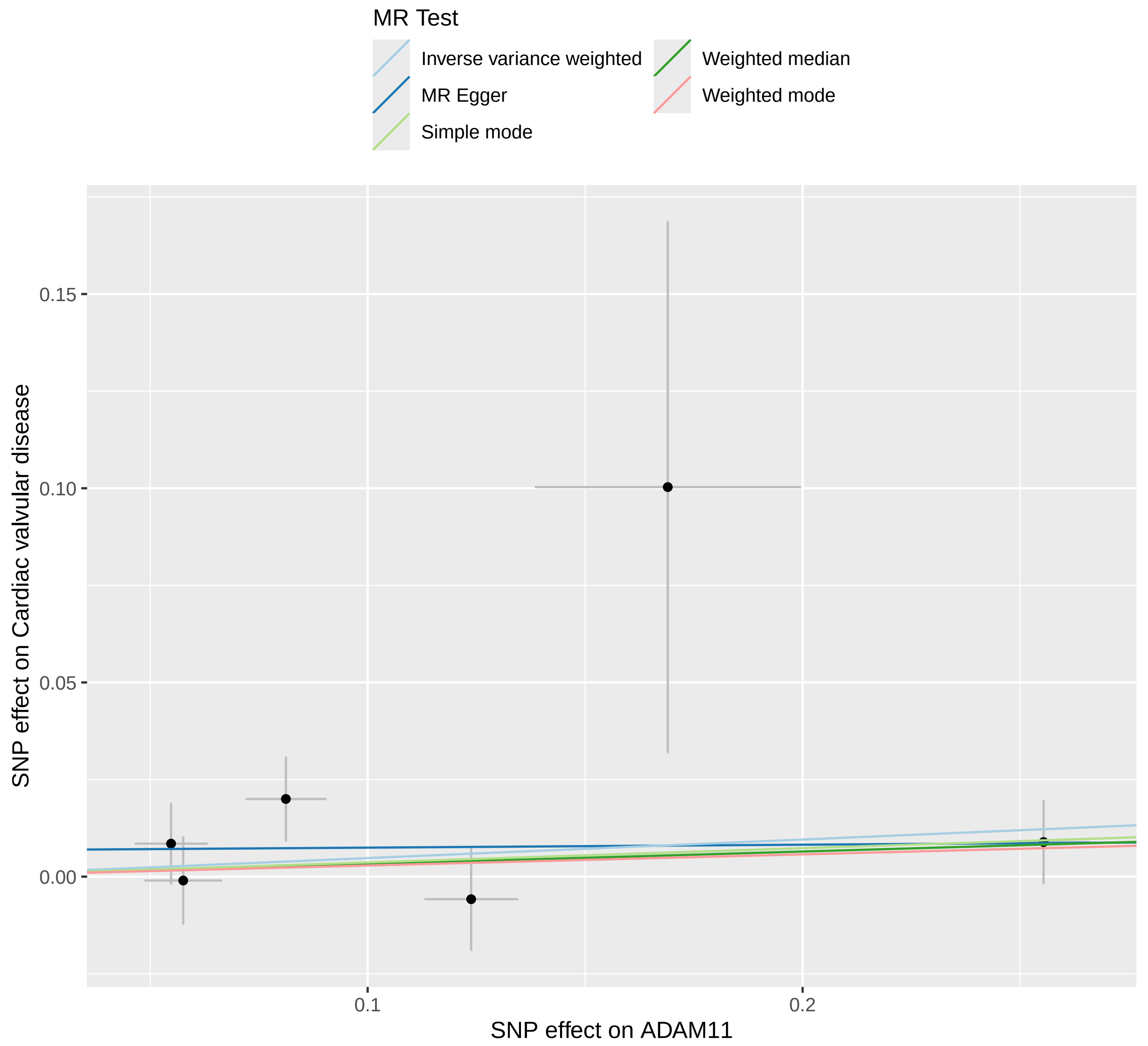
MR Test
Inverse variance weighted
MR Egger
Simple mode
Weighted median
Weighted mode
0.15
0.10
0.05
0.00
0.1
0.2
SNP effect on Cardiac valvular disease
SNP effect on ADAM11

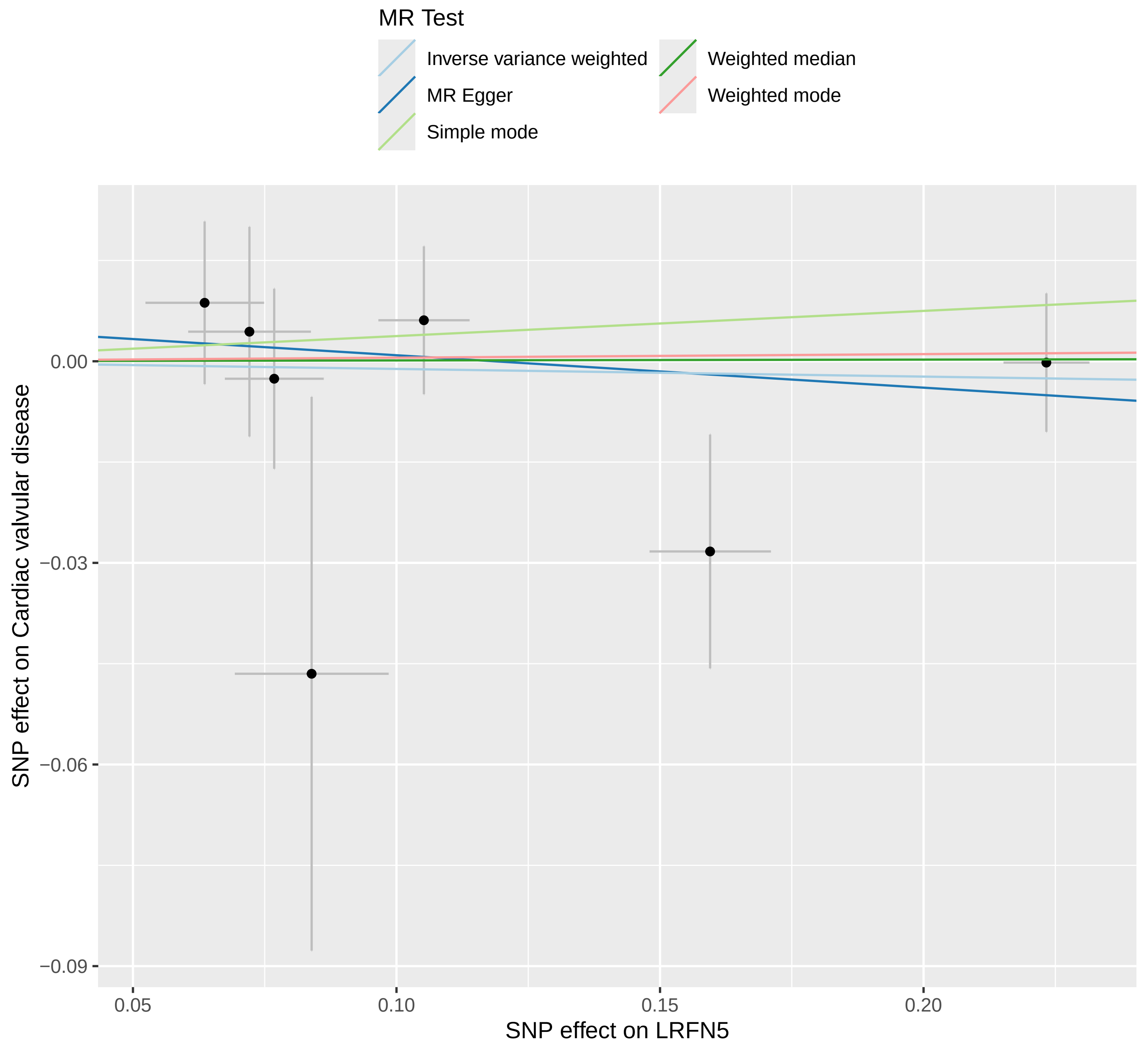

MR Test
Inverse variance weighted
MR Egger
Simple mode
Weighted median
Weighted mode
SNP effect on Cardiac valvular disease
0.00
−0.03
−0.06
−0.09
0.05
0.10
0.15
0.20
SNP effect on LRFN5

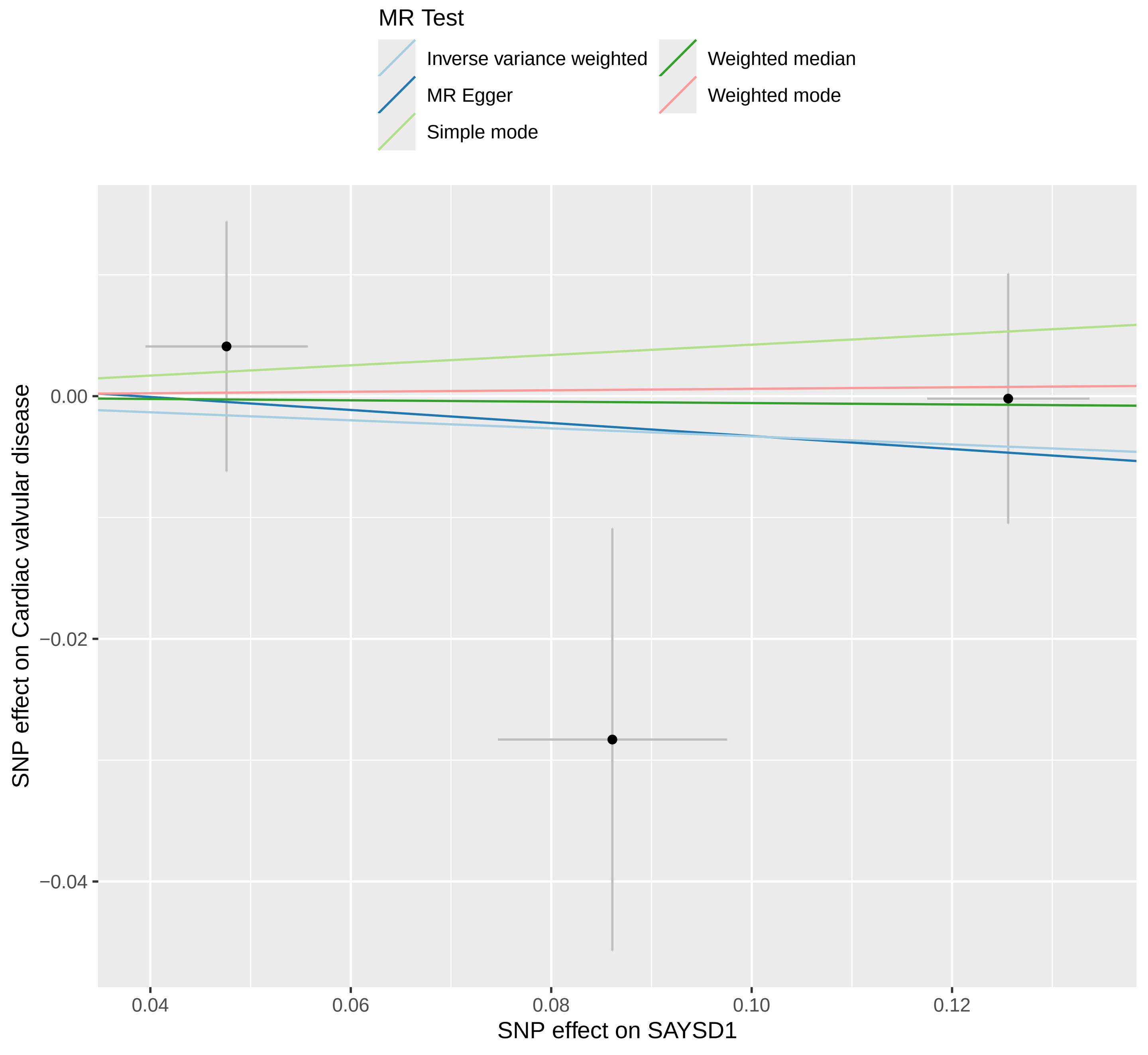
MR Test
Inverse variance weighted
MR Egger
Simple mode
Weighted median
Weighted mode
0.00
−0.02
−0.04
0.04
0.06
0.08
0.10
0.12
SNP effect on SAYSD1
SNP effect on Cardiac valvular disease

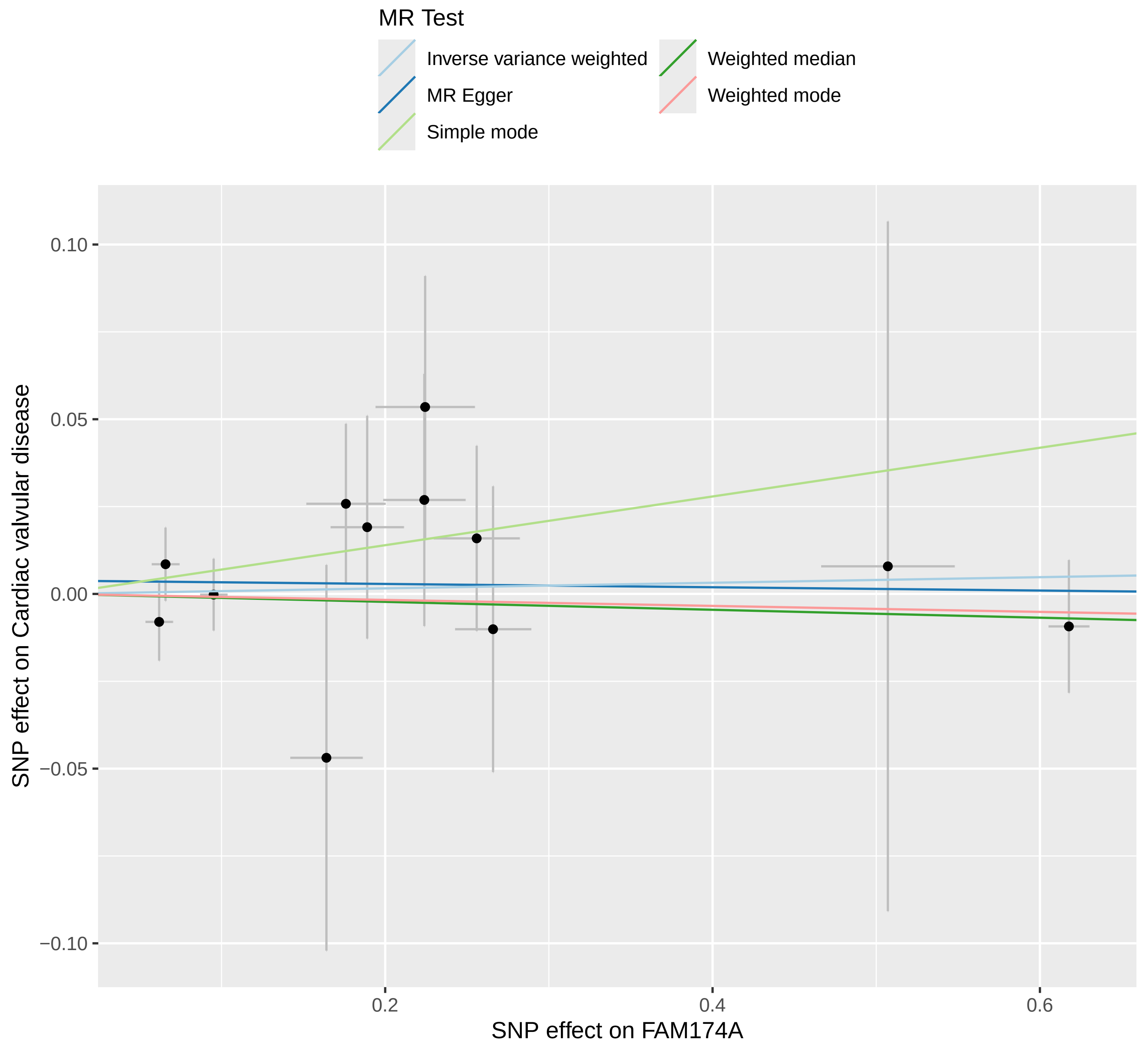
MR Test
Inverse variance weighted
MR Egger
Simple mode
Weighted median
Weighted mode
0.10
0.05
0.00
−0.05
−0.10
SNP effect on Cardiac valvular disease
0.2
0.4
0.6
SNP effect on FAM174A

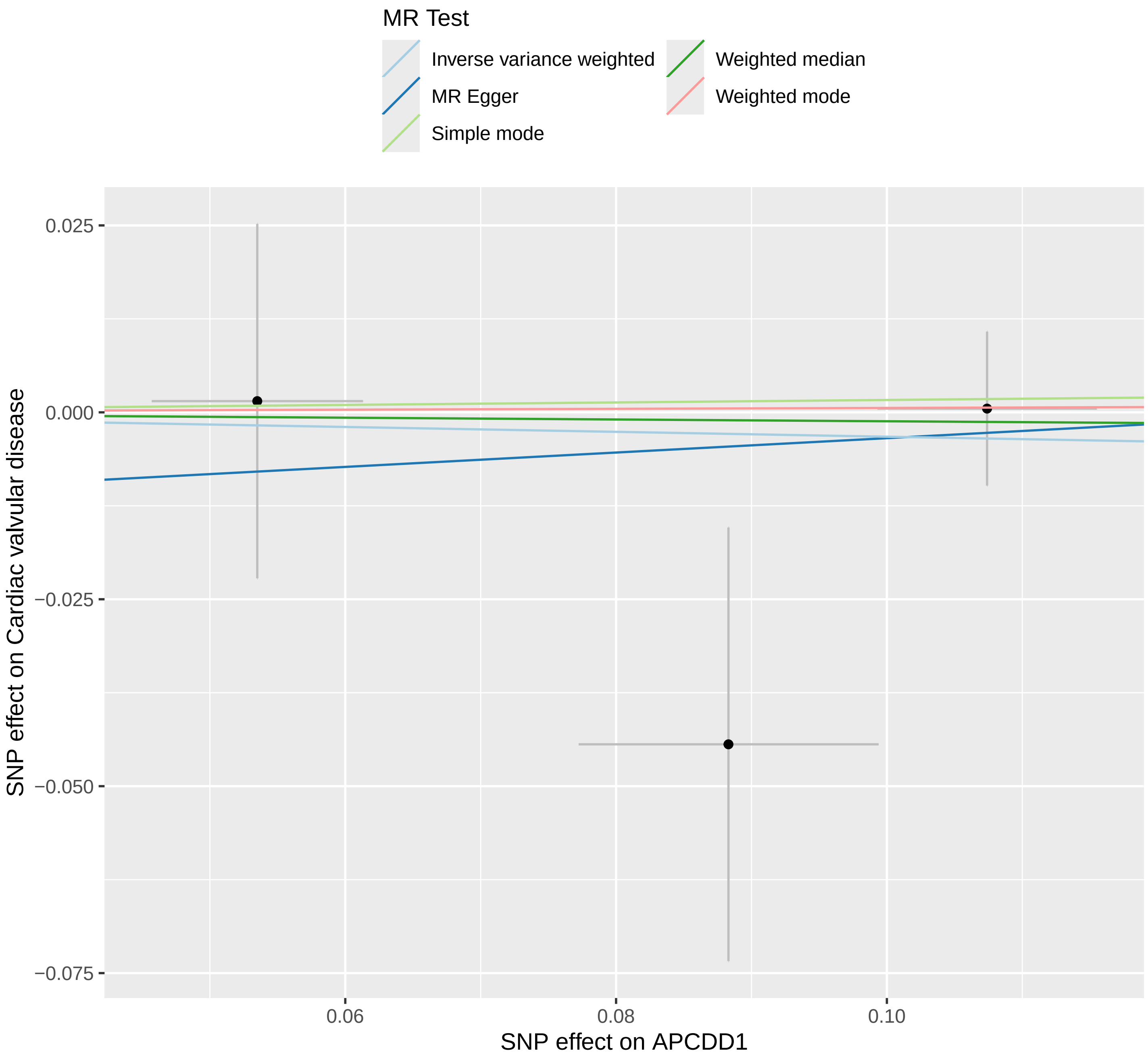
MR Test
Inverse variance weighted
MR Egger
Simple mode
Weighted median
Weighted mode
0.025
0.000
−0.025
−0.050
−0.075
0.06
0.08
0.10
SNP effect on APCDD1
SNP effect on Cardiac valvular disease

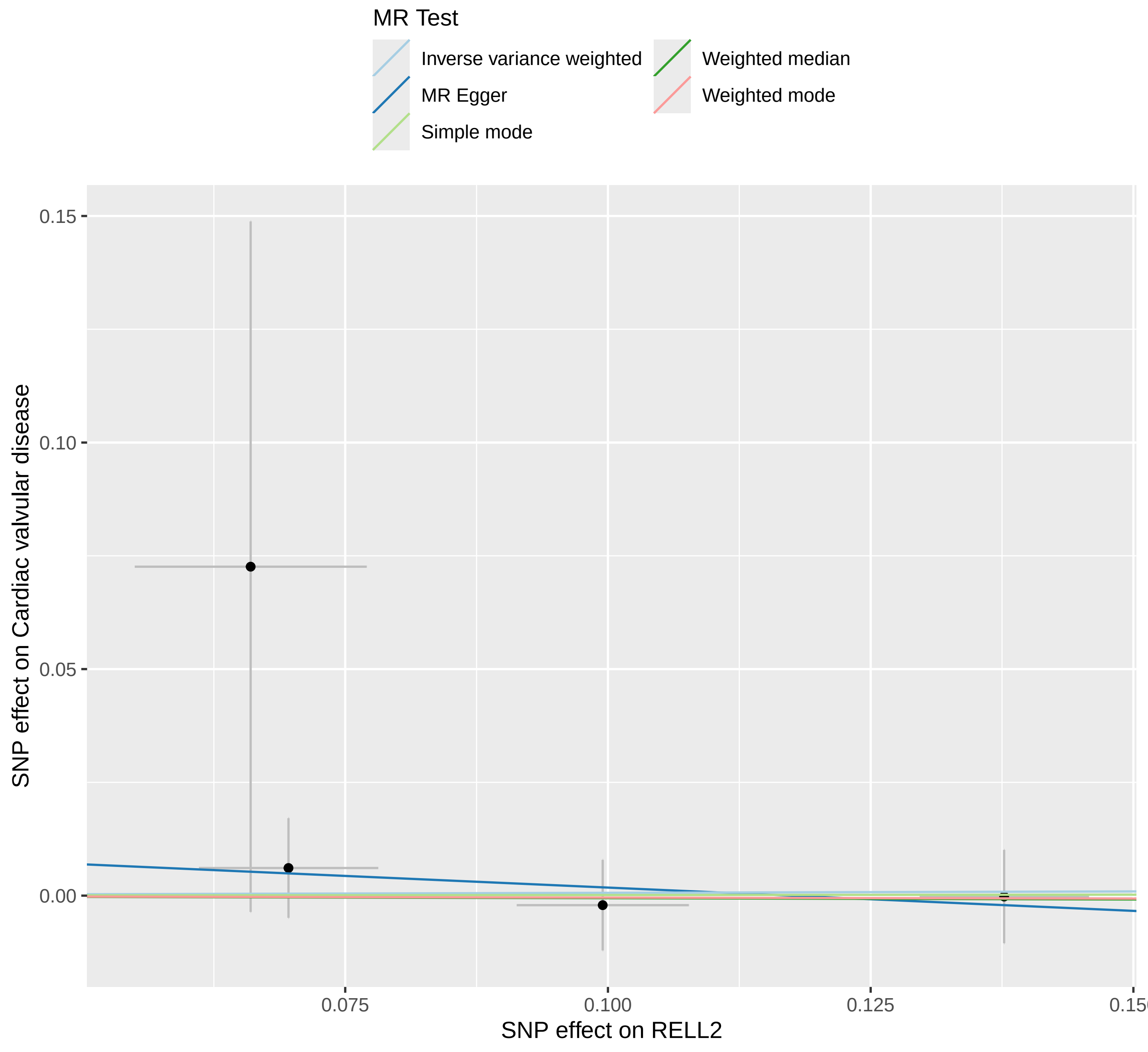

MR Test
Inverse variance weighted
MR Egger
Simple mode
Weighted median
Weighted mode
0.15
0.10
0.05
0.00
SNP effect on Cardiac valvular disease
0.075
0.100
0.125
0.15
SNP effect on RELL2

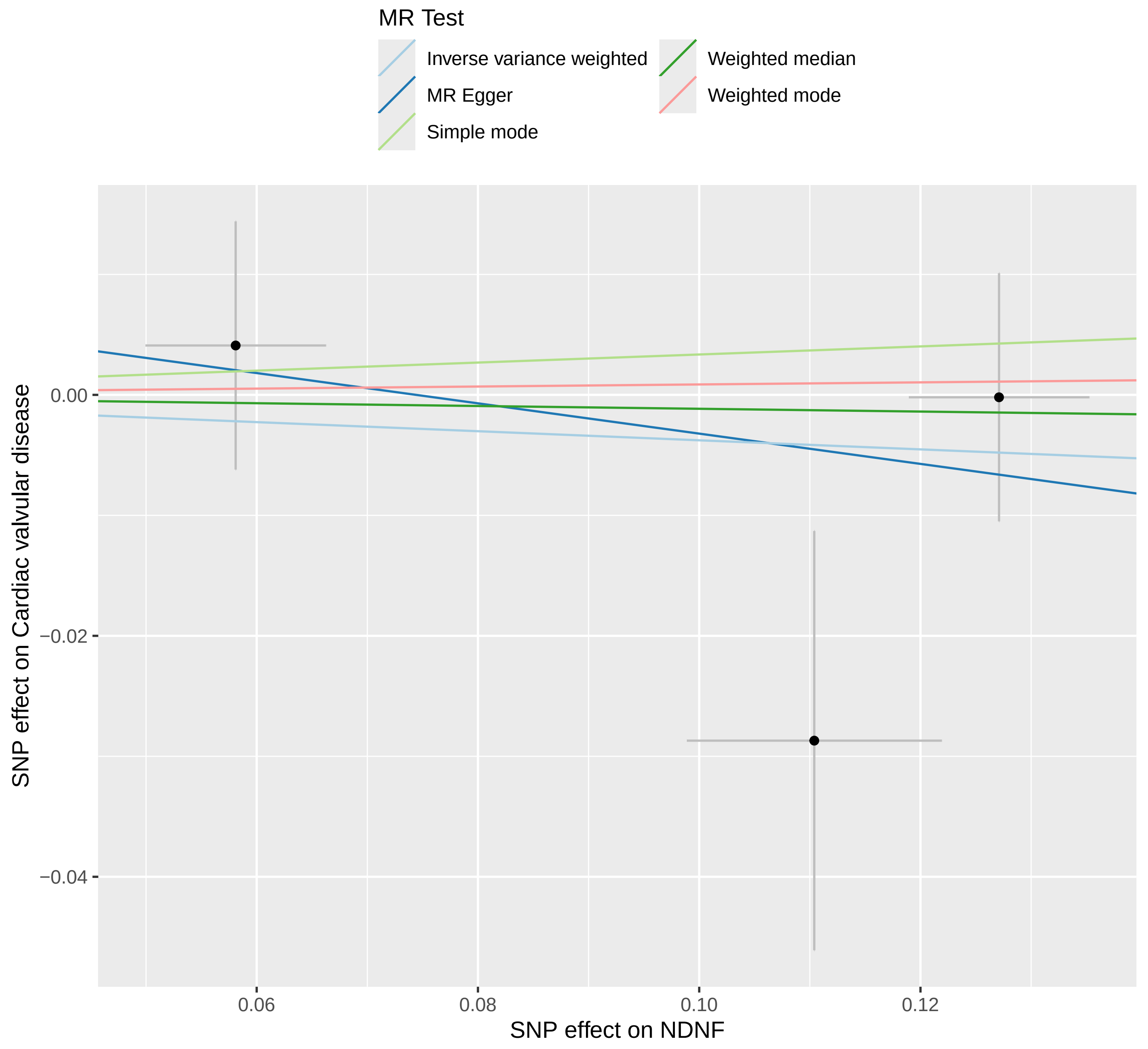

MR Test
Inverse variance weighted
MR Egger
Simple mode
Weighted median
Weighted mode
0.00
−0.02
−0.04
SNP effect on Cardiac valvular disease
0.06
0.08
0.10
0.12
SNP effect on NDNF

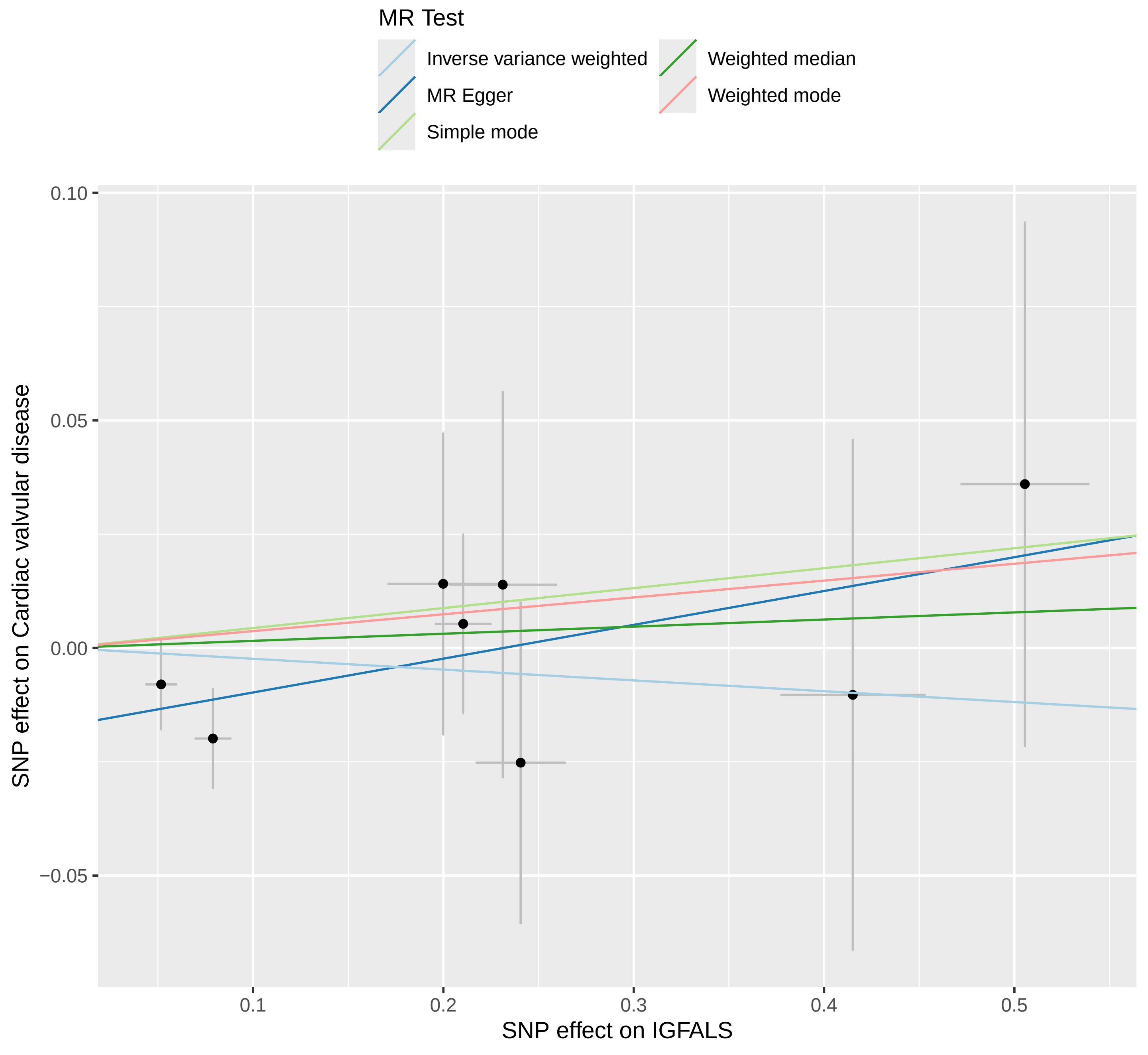
MR Test
Inverse variance weighted
MR Egger
Simple mode
Weighted median
Weighted mode
0.10
0.05
0.00
−0.05
0.1
0.2
0.3
0.4
0.5
SNP effect on IGFALS
SNP effect on Cardiac valvular disease

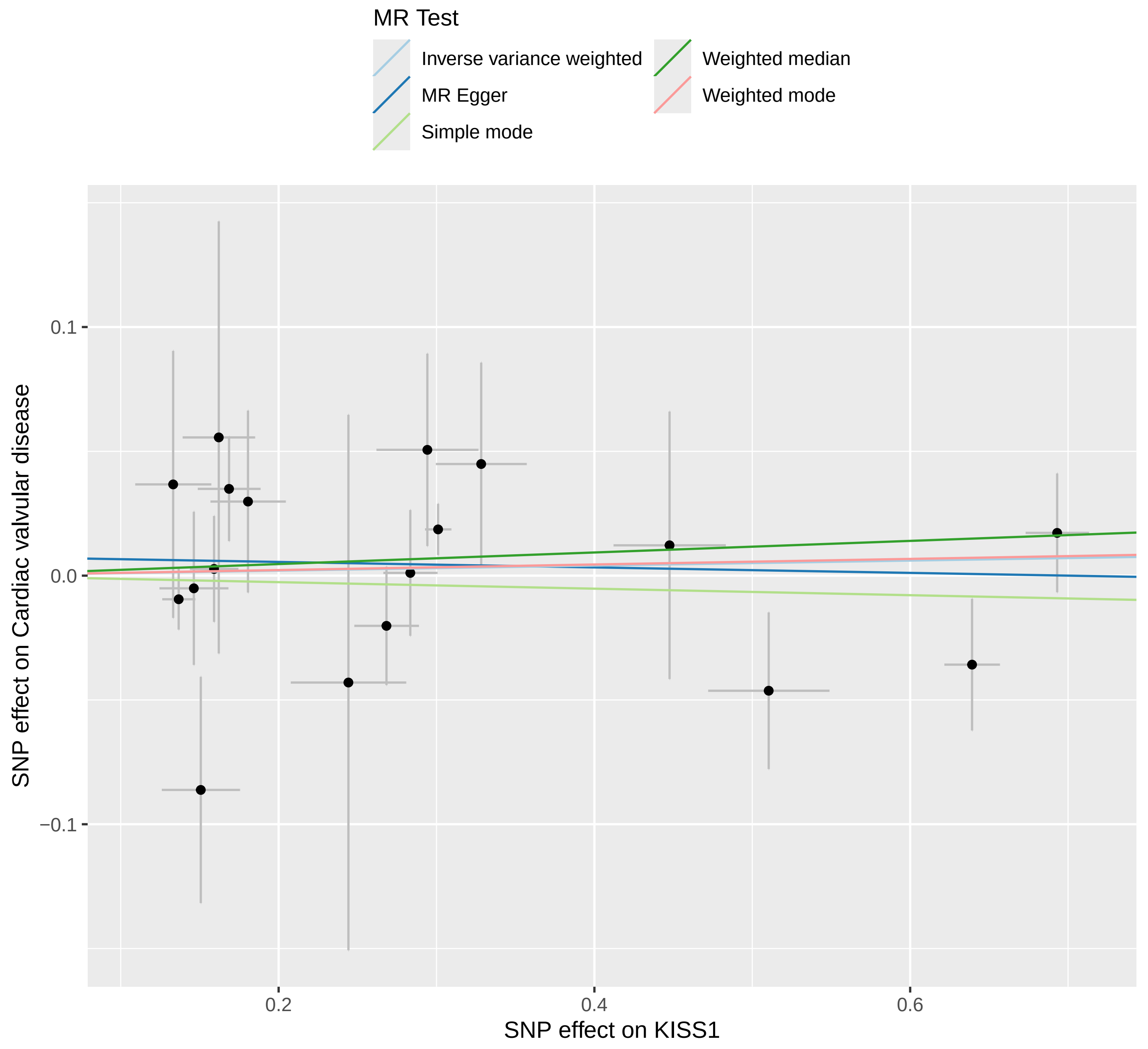
MR Test
Inverse variance weighted
MR Egger
Simple mode
Weighted median
Weighted mode
SNP effect on Cardiac valvular disease
0.1
0.0
−0.1
0.2
0.4
0.6
SNP effect on KISS1

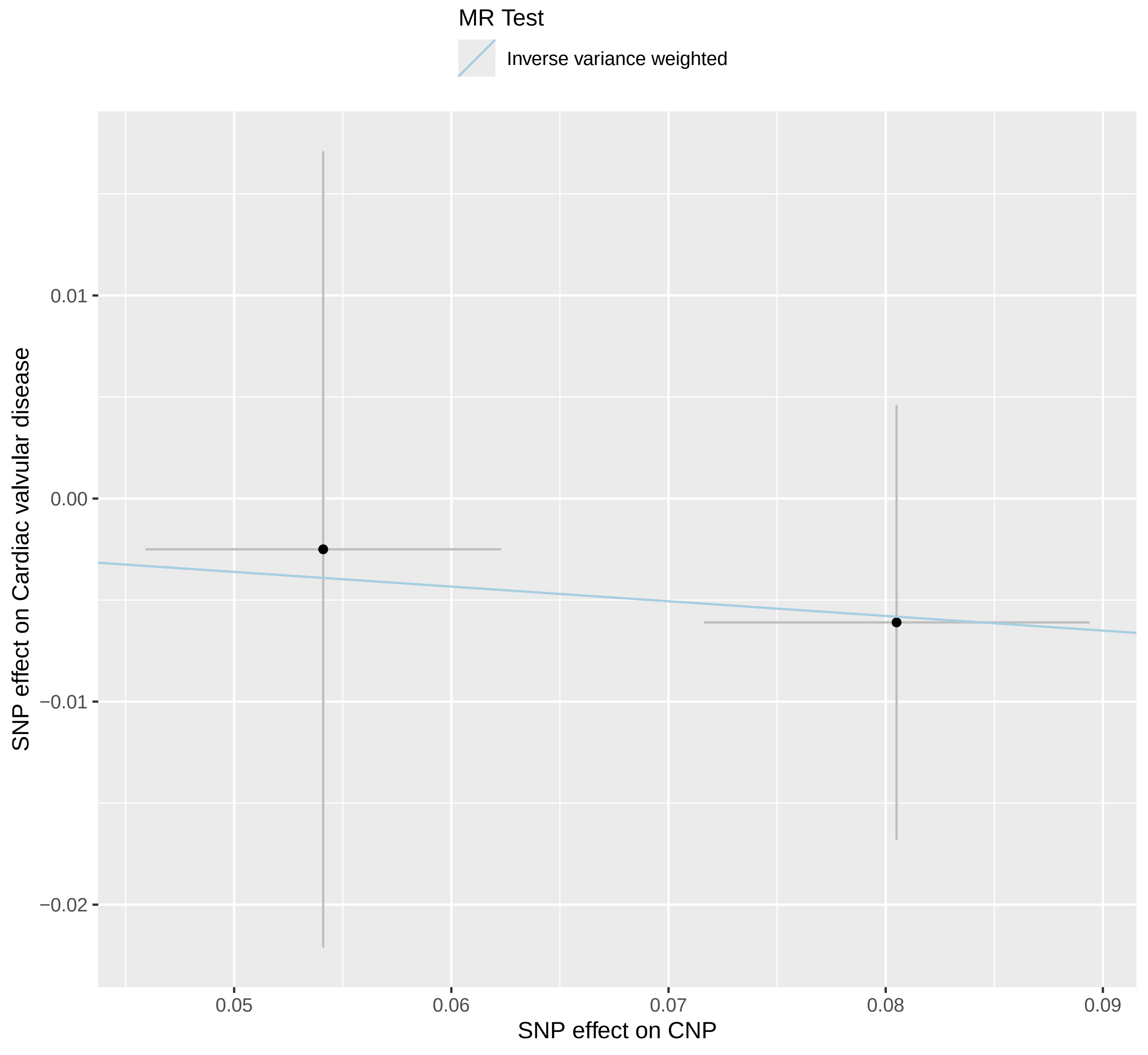

MR Test
Inverse variance weighted
0.01
0.00
−0.01
−0.02
0.05
0.06
0.07
0.08
0.09
SNP effect on CNP
SNP effect on Cardiac valvular disease

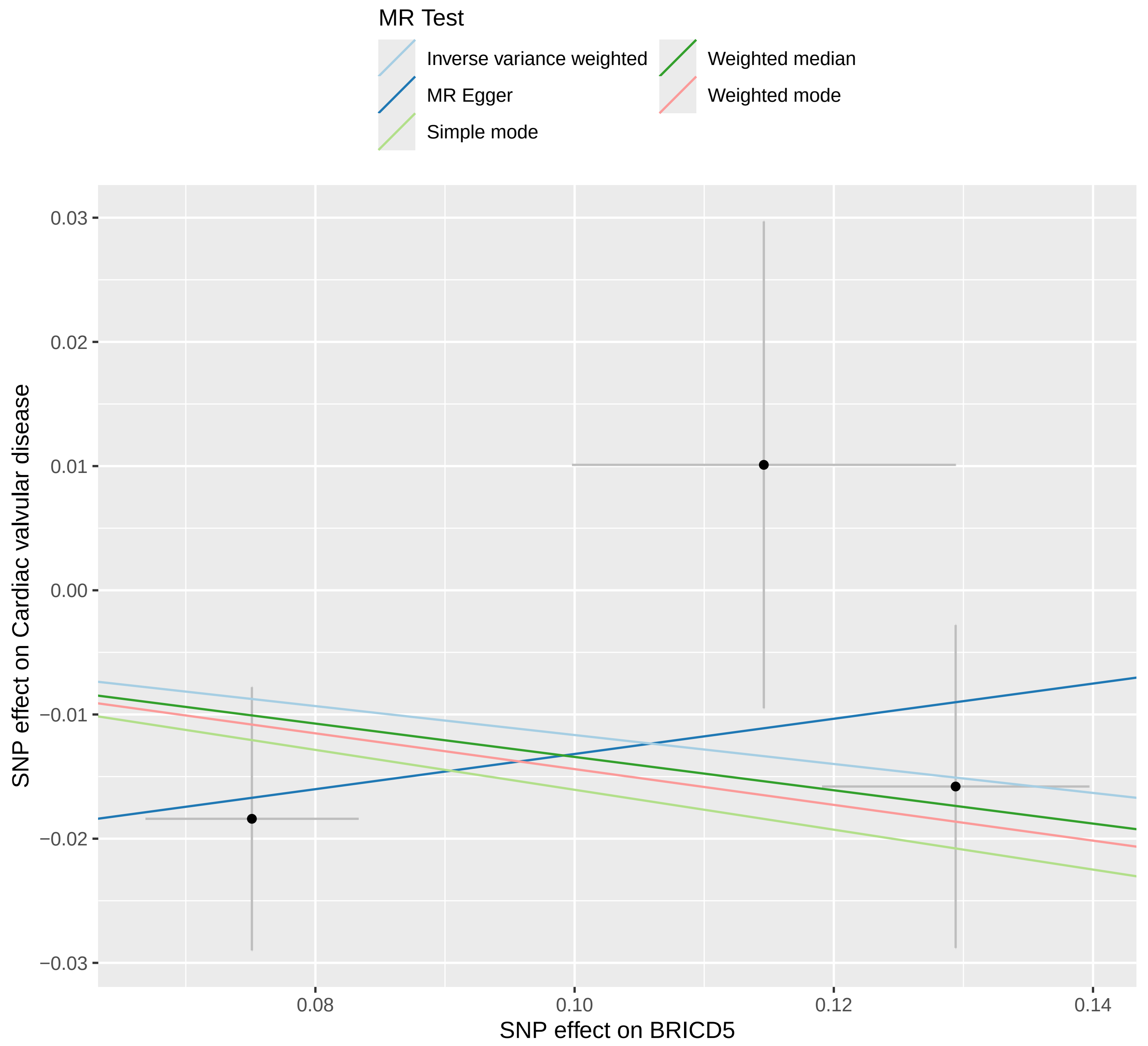
MR Test
Inverse variance weighted
MR Egger
Simple mode
Weighted median
Weighted mode
0.03
0.02
0.01
0.00
−0.01
−0.02
−0.03
0.08
0.10
0.12
0.14
SNP effect on Cardiac valvular disease
SNP effect on BRICD5

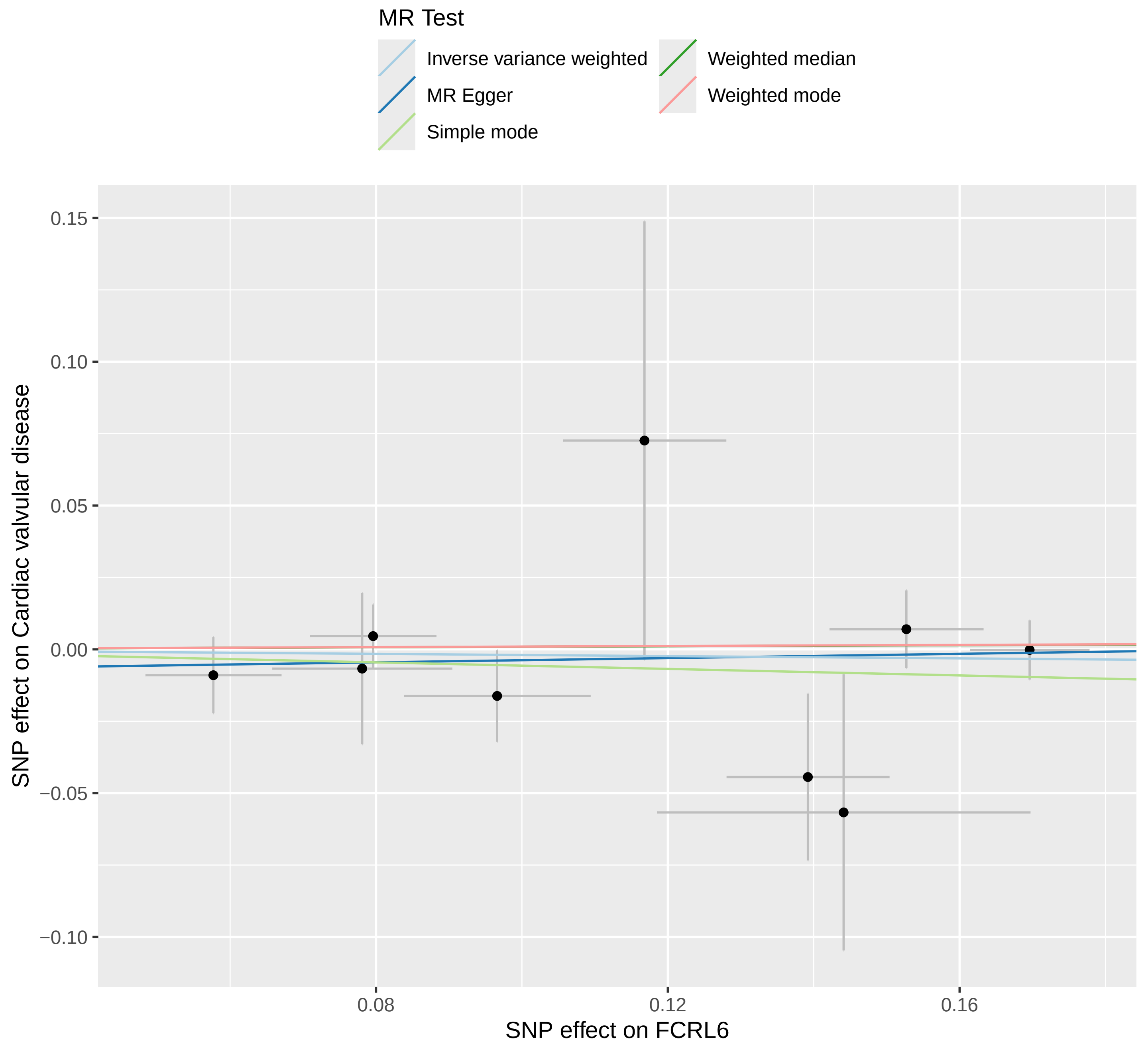

MR Test
Inverse variance weighted
MR Egger
Simple mode
Weighted median
Weighted mode
0.15
0.10
0.05
0.00
−0.05
−0.10
SNP effect on Cardiac valvular disease
0.08
0.12
0.16
SNP effect on FCRL6

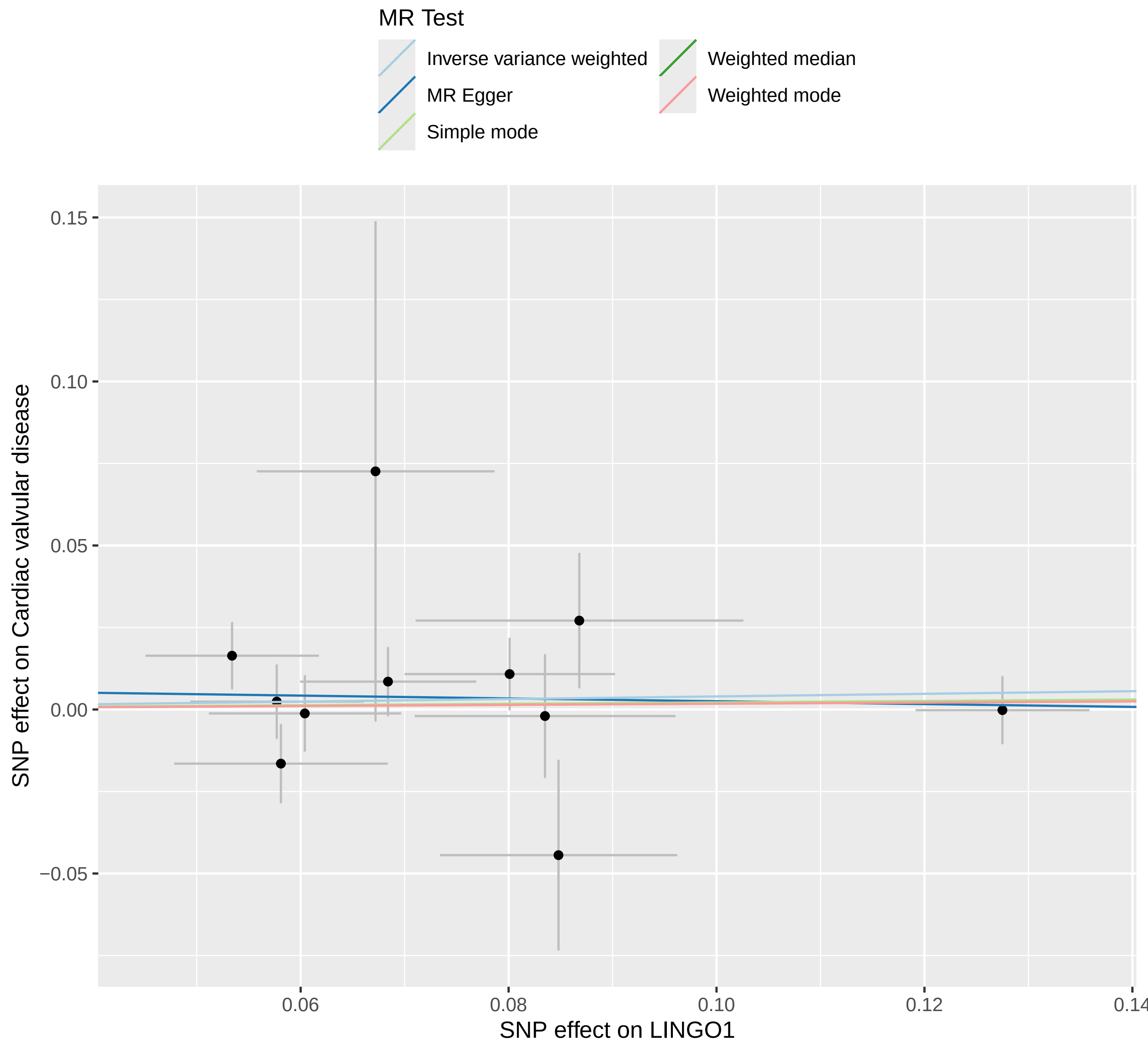
MR Test
Inverse variance weighted
MR Egger
Simple mode
Weighted median
Weighted mode
0.15
0.10
0.05
0.00
−0.05
0.06
0.08
0.10
0.12
0.14
SNP effect on Cardiac valvular disease
SNP effect on LINGO1

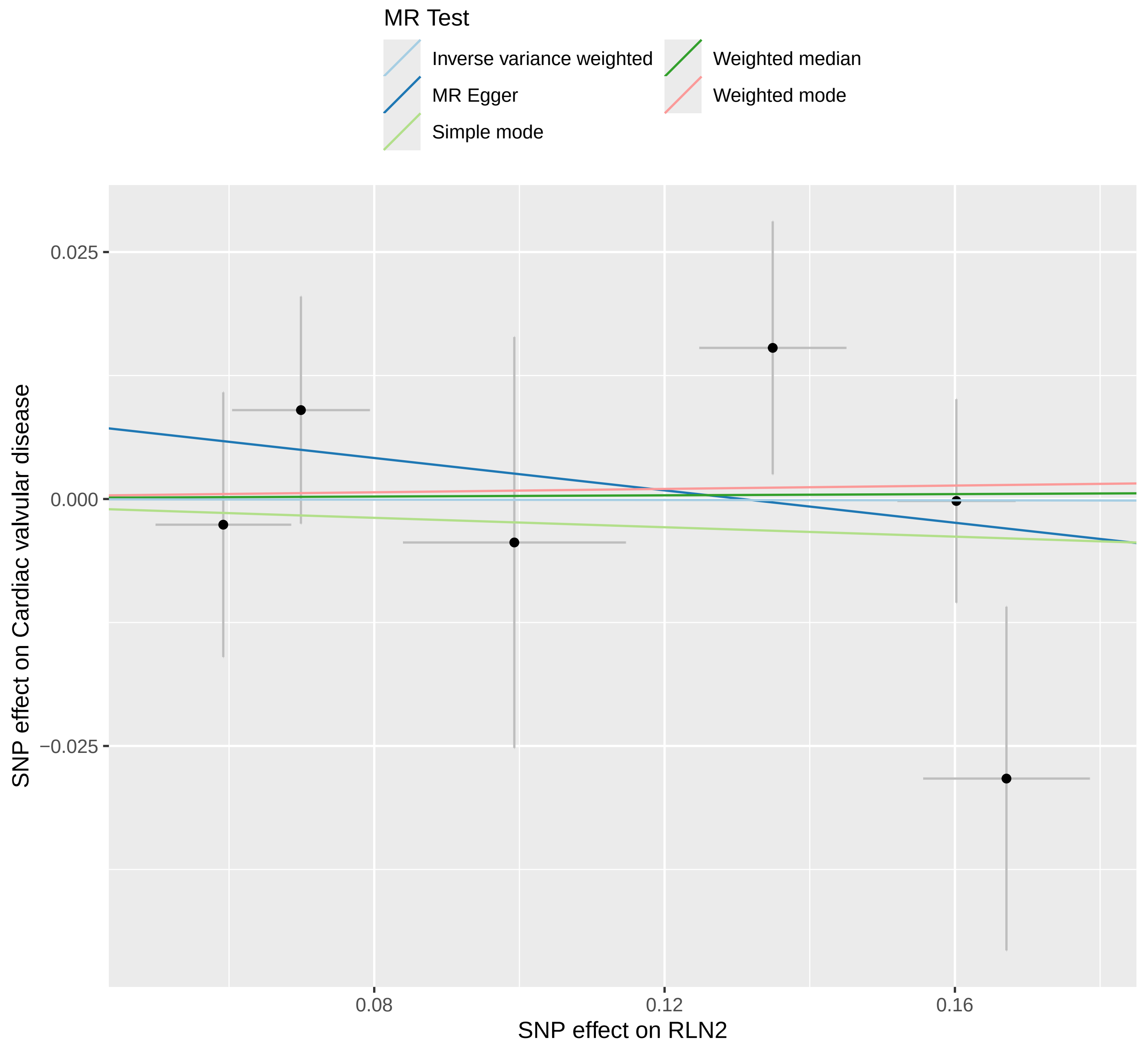

MR Test
Inverse variance weighted
MR Egger
Simple mode
Weighted median
Weighted mode
0.025
0.000
−0.025
0.08
0.12
0.16
SNP effect on RLN2
SNP effect on Cardiac valvular disease

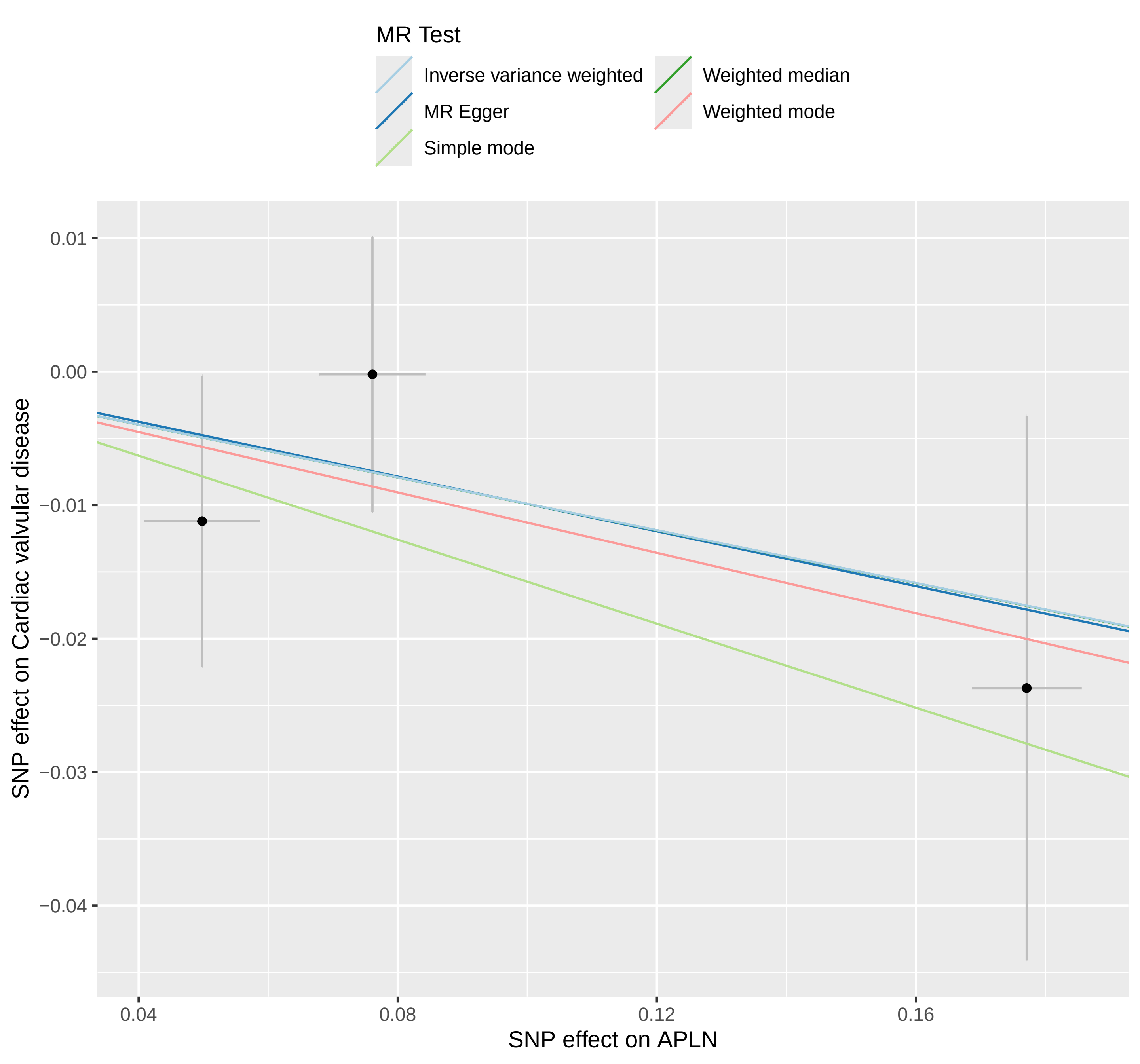

MR Test
Inverse variance weighted
MR Egger
Simple mode
Weighted median
Weighted mode
0.01
0.00
−0.01
−0.02
−0.03
−0.04
SNP effect on Cardiac valvular disease
0.04
0.08
0.12
0.16
SNP effect on APLN

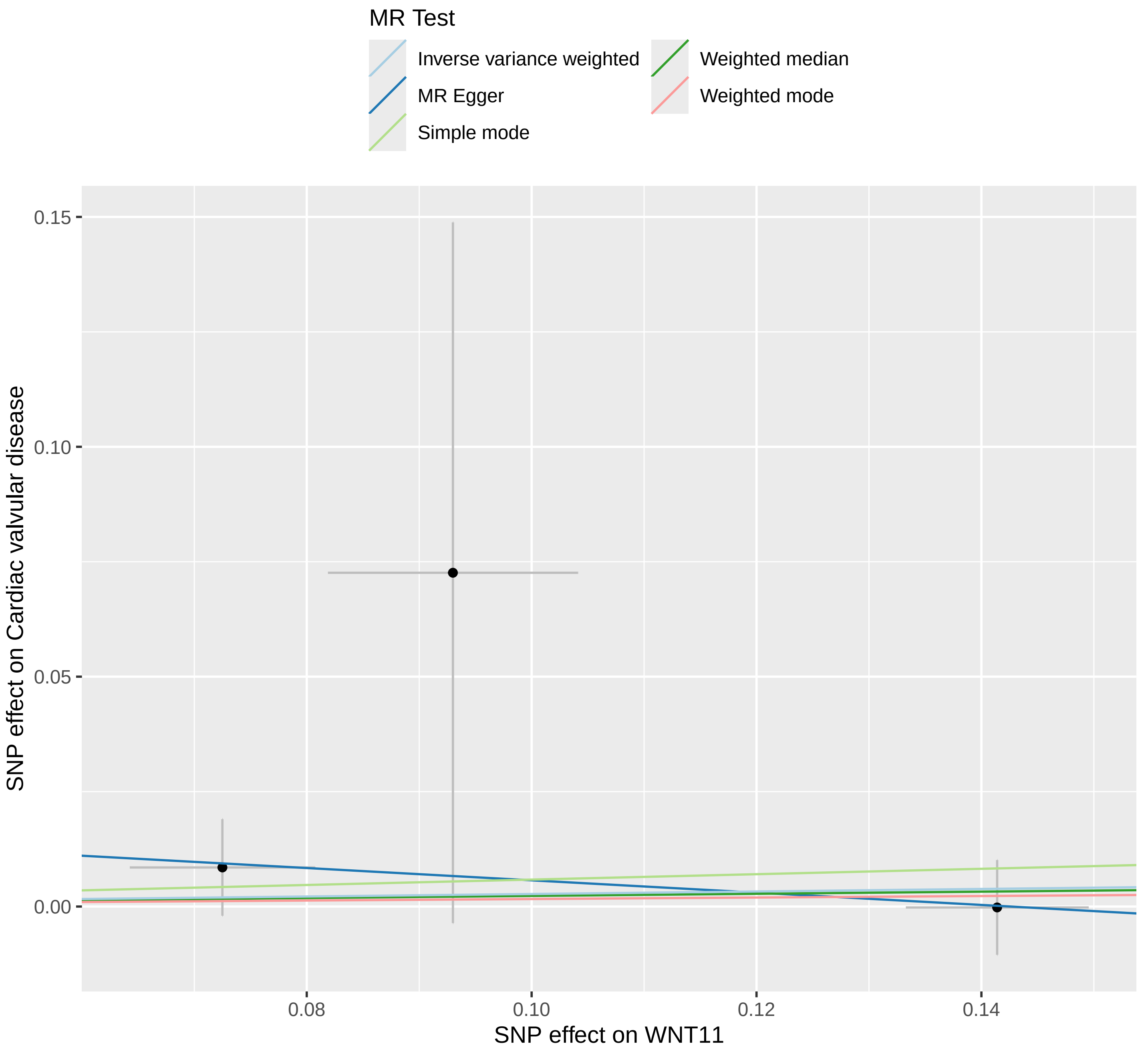

MR Test
Inverse variance weighted
MR Egger
Simple mode
Weighted median
Weighted mode
0.15
0.10
0.05
0.00
SNP effect on Cardiac valvular disease
0.08
0.10
0.12
0.14
SNP effect on WNT11

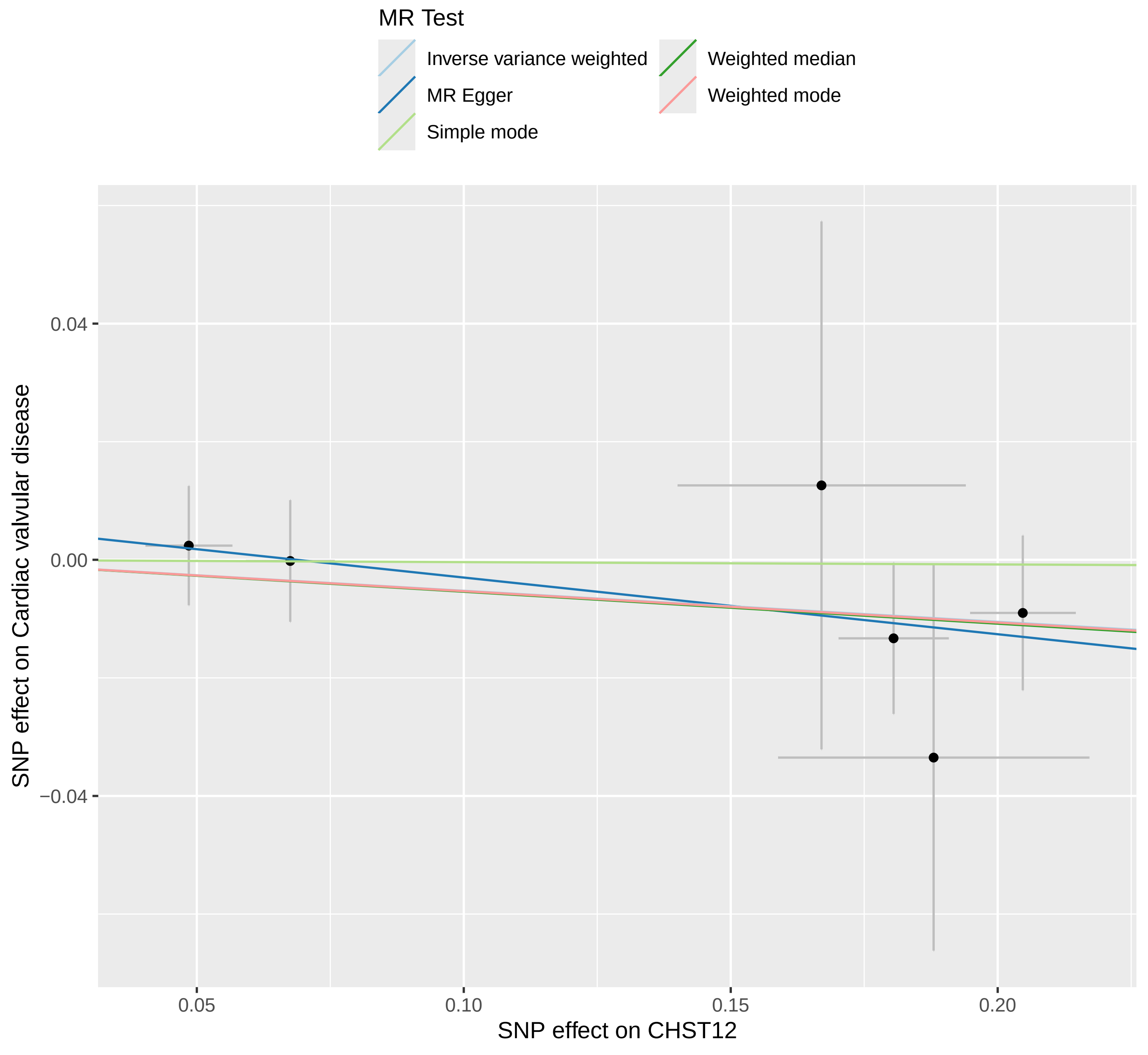
MR Test
Inverse variance weighted
MR Egger
Simple mode
Weighted median
Weighted mode
0.04
0.00
−0.04
SNP effect on Cardiac valvular disease
0.05
0.10
0.15
0.20
SNP effect on CHST12

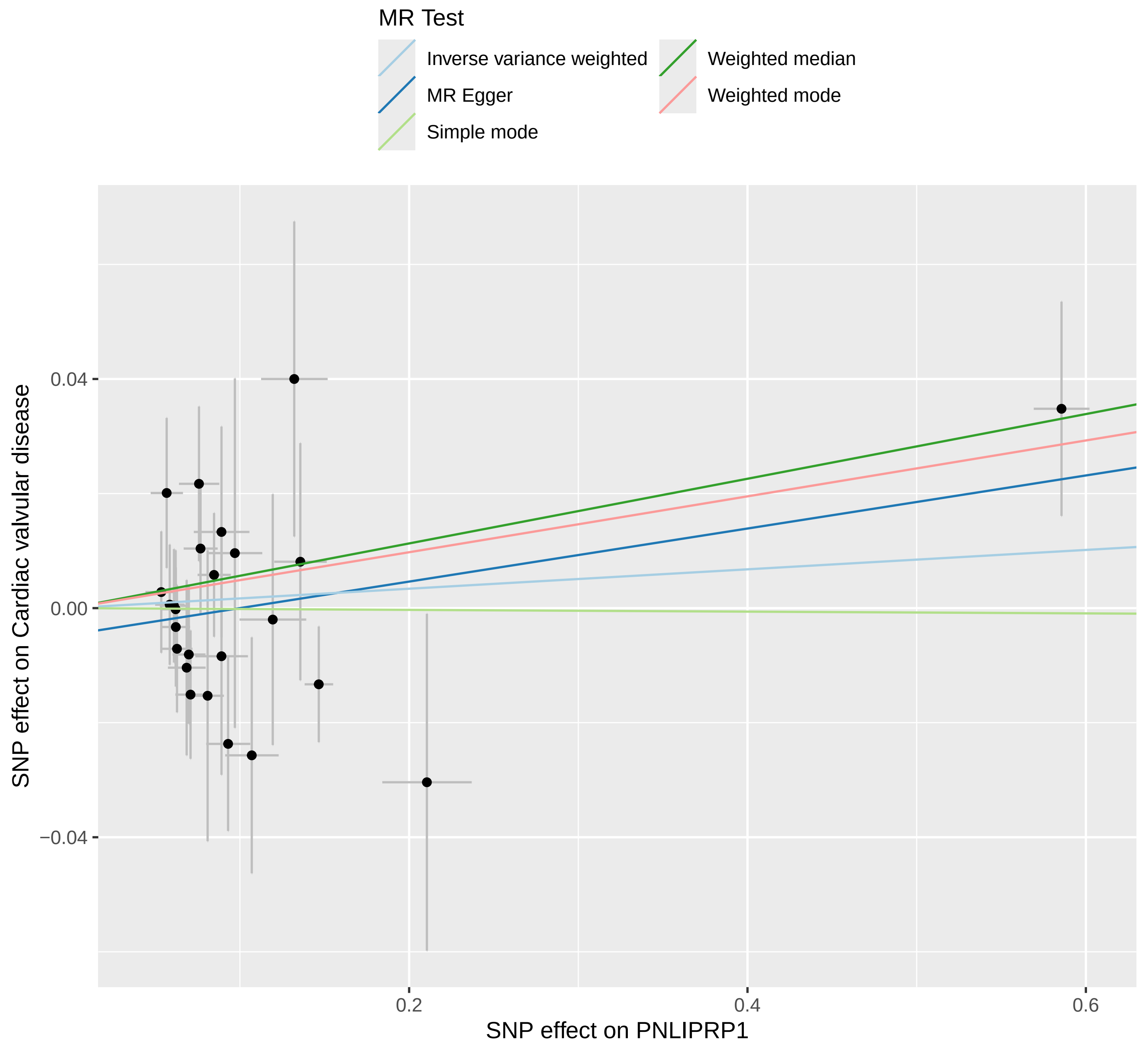
MR Test
Inverse variance weighted
MR Egger
Simple mode
Weighted median
Weighted mode
0.04
0.00
−0.04
0.2
0.4
0.6
SNP effect on Cardiac valvular disease
SNP effect on PNLIPRP1

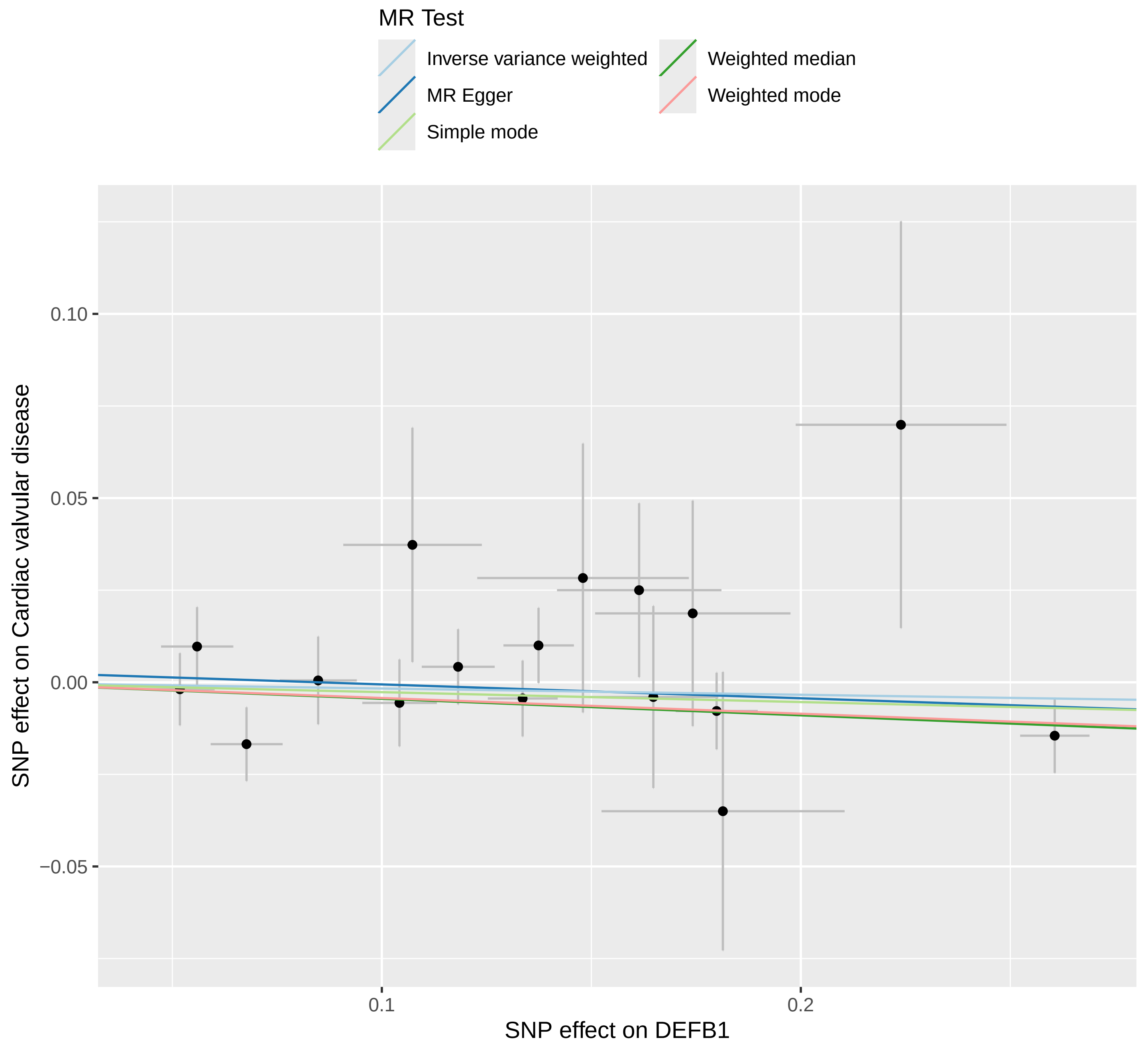

MR Test
Inverse variance weighted
MR Egger
Simple mode
Weighted median
Weighted mode
SNP effect on Cardiac valvular disease
0.10
0.05
0.00
−0.05
0.1
0.2
SNP effect on DEFB1

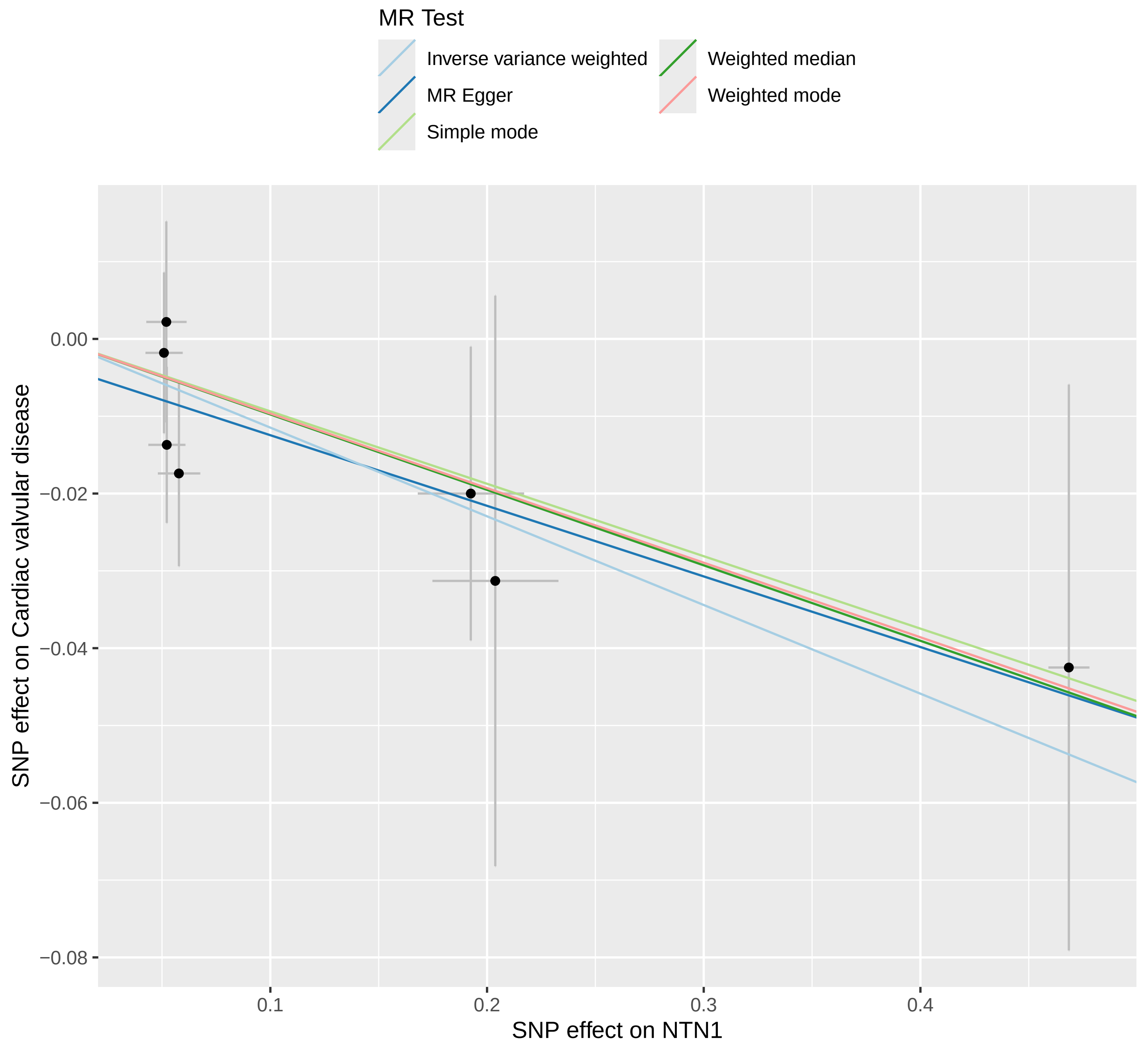
MR Test
Inverse variance weighted
MR Egger
Simple mode
Weighted median
Weighted mode
SNP effect on Cardiac valvular disease
0.00
−0.02
−0.04
−0.06
−0.08
0.1
0.2
0.3
0.4
SNP effect on NTN1

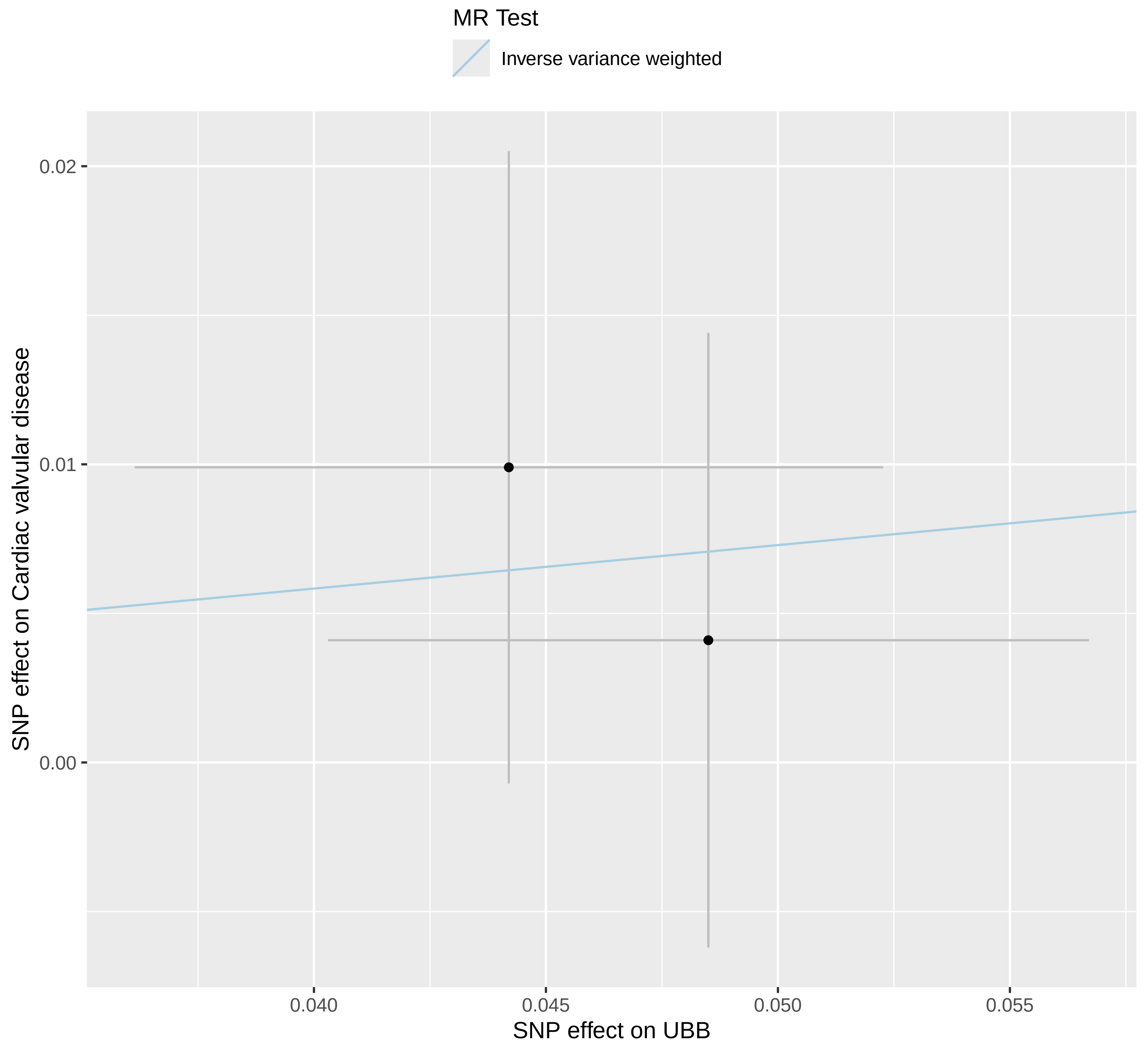
MR Test
Inverse variance weighted
SNP effect on Cardiac valvular disease
0.02
0.01
0.00
0.040
0.045
0.050
0.055
SNP effect on UBB

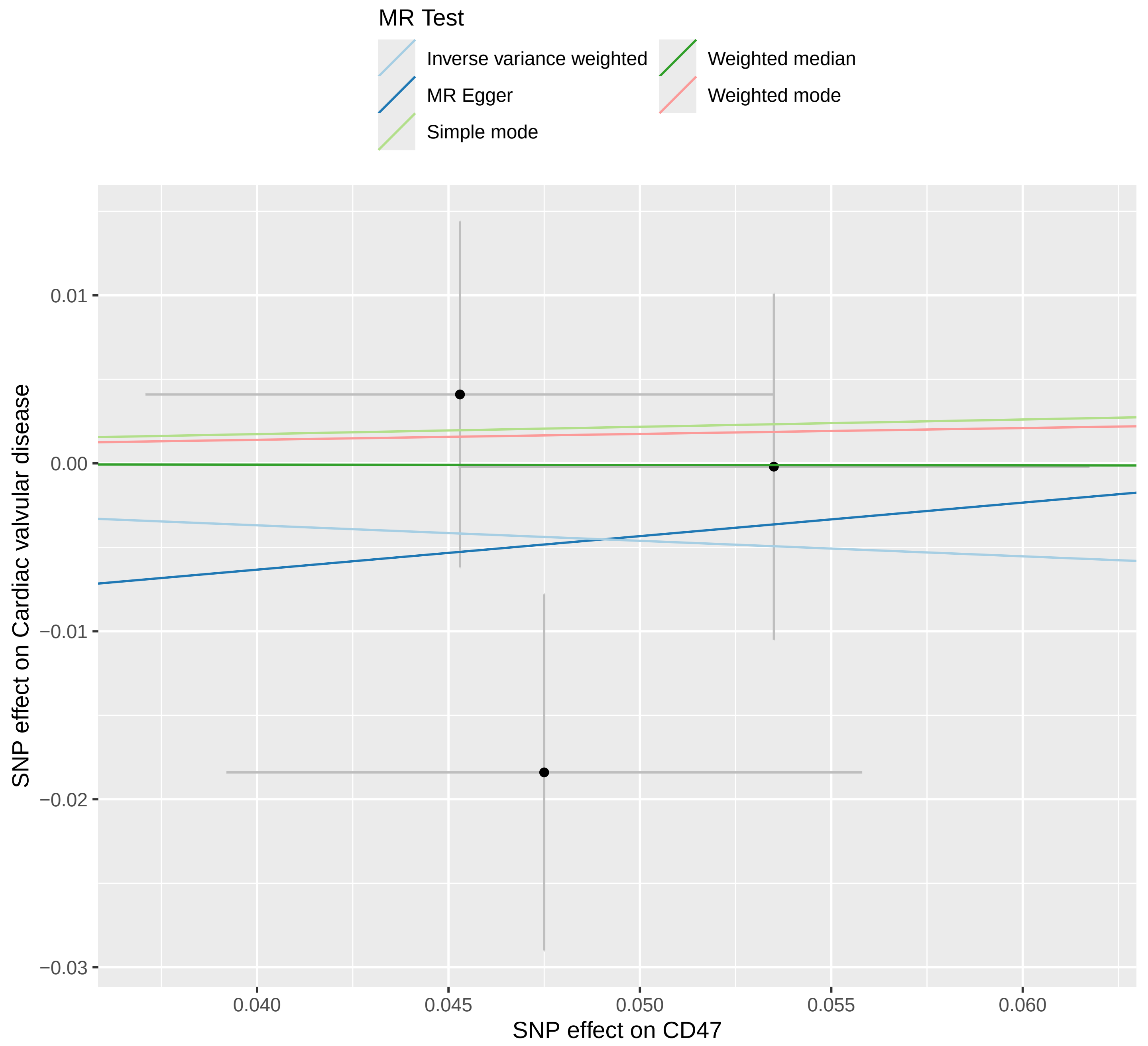
MR Test
Inverse variance weighted
MR Egger
Simple mode
Weighted median
Weighted mode
0.01
0.00
−0.01
−0.02
−0.03
SNP effect on Cardiac valvular disease
0.040
0.045
0.050
0.055
0.060
SNP effect on CD47

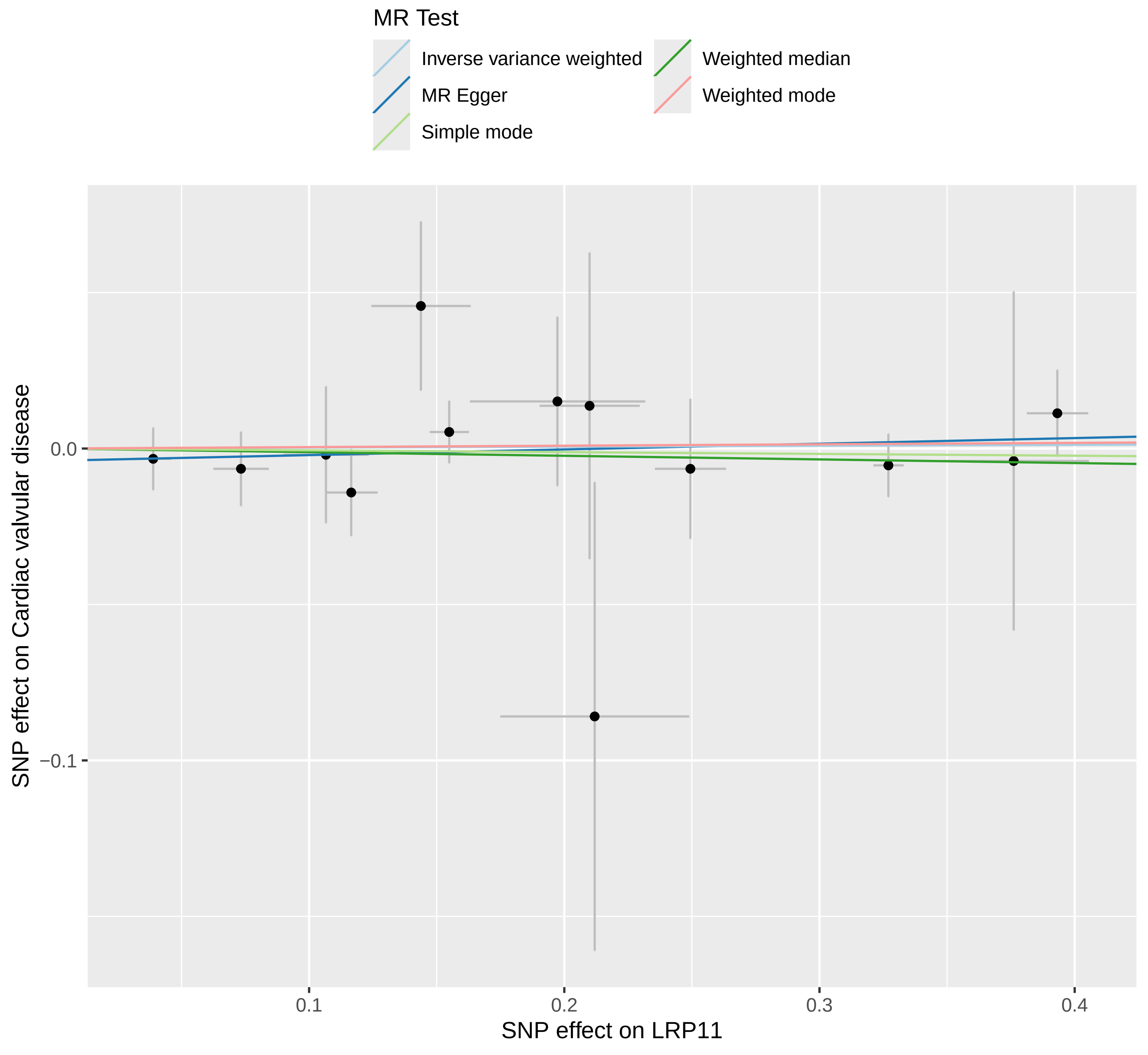
MR Test
Inverse variance weighted
MR Egger
Simple mode
Weighted median
Weighted mode
0.0
−0.1
0.1
0.2
0.3
0.4
SNP effect on LRP11
SNP effect on Cardiac valvular disease

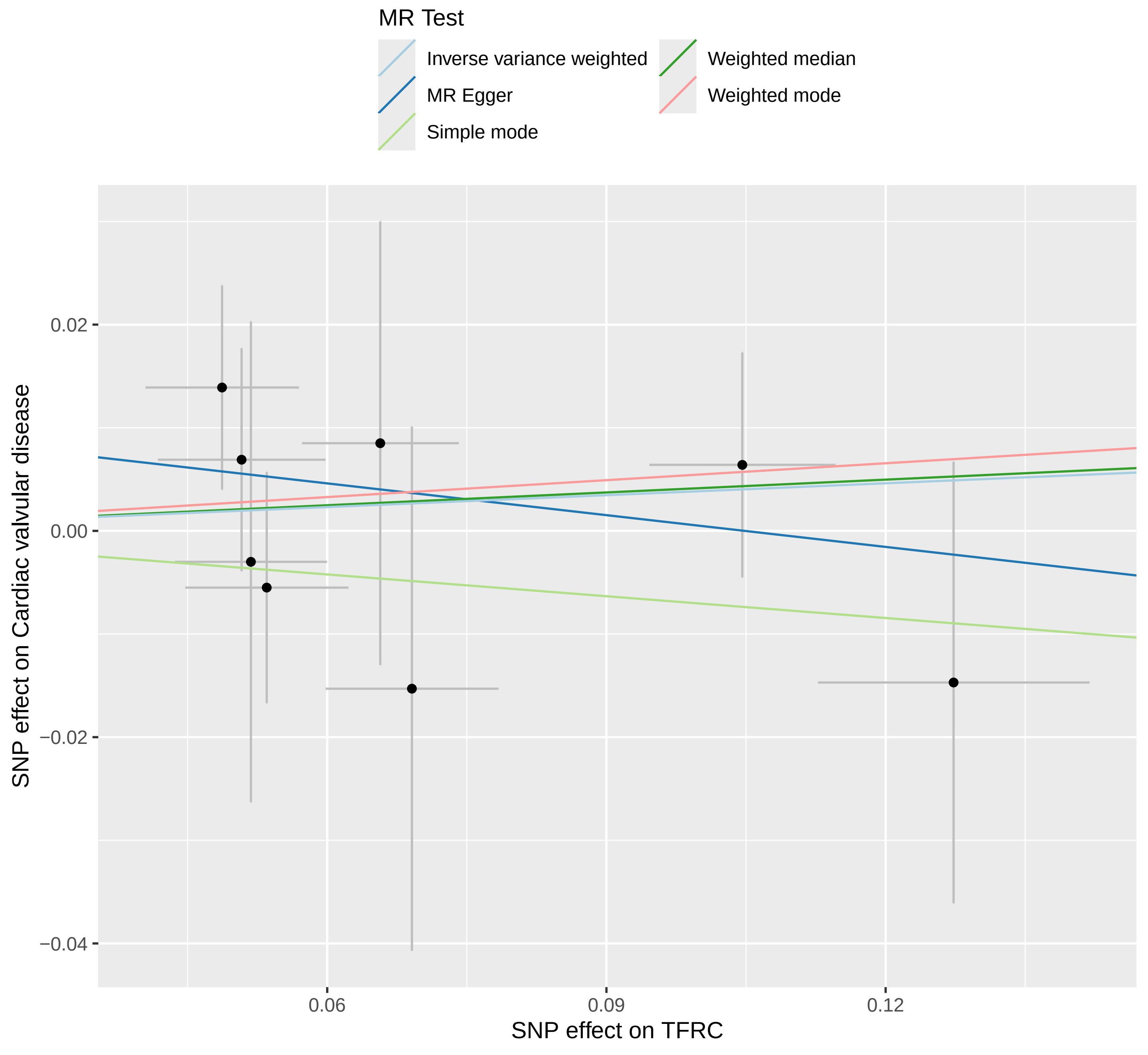
MR Test
Inverse variance weighted
MR Egger
Simple mode
Weighted median
Weighted mode
SNP effect on Cardiac valvular disease
0.02
0.00
−0.02
−0.04
0.06
0.09
0.12
SNP effect on TFRC

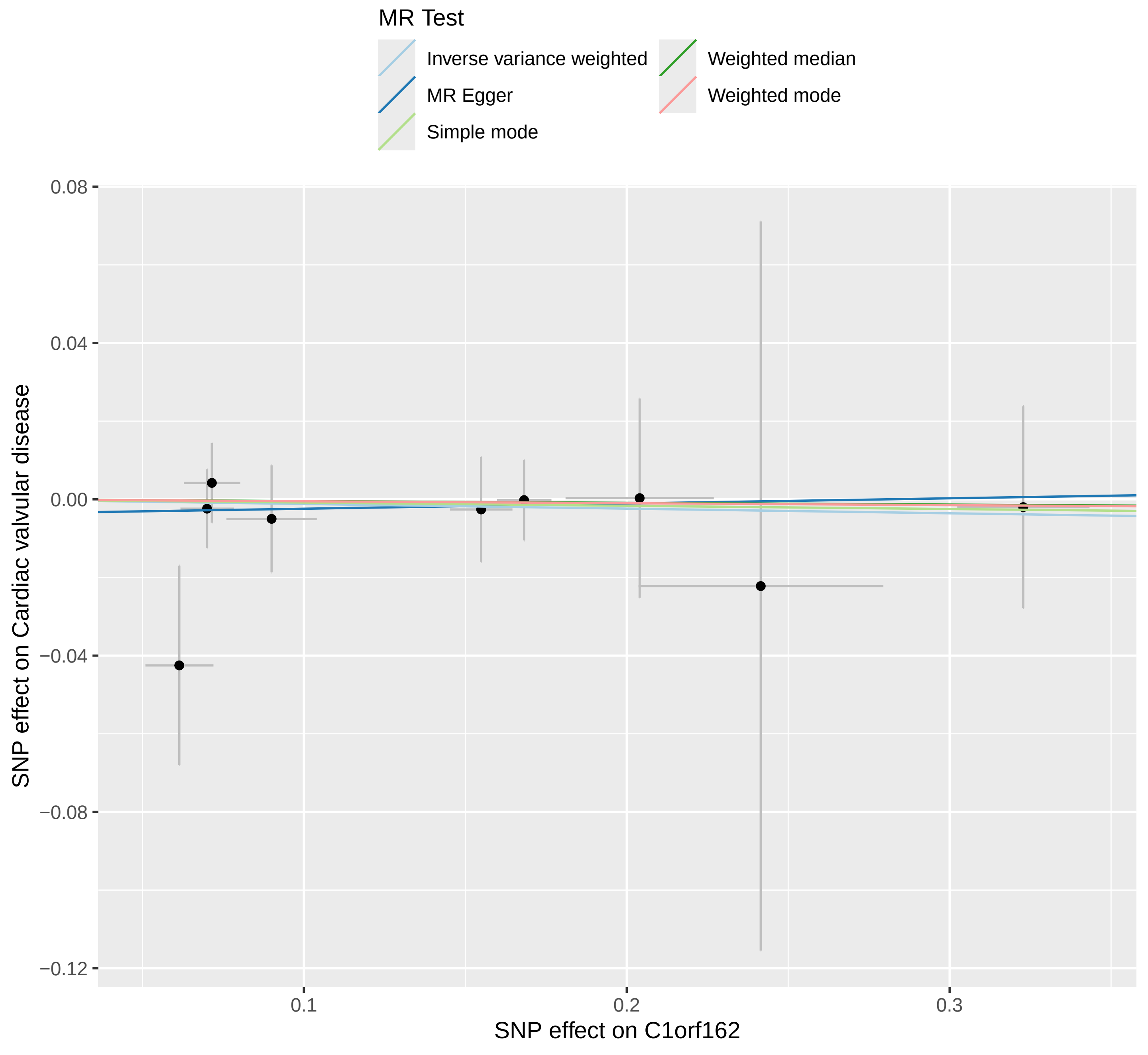
MR Test
Inverse variance weighted
MR Egger
Simple mode
Weighted median
Weighted mode
0.08
0.04
0.00
−0.04
−0.08
−0.12
SNP effect on Cardiac valvular disease
0.1
0.2
0.3
SNP effect on C1orf162

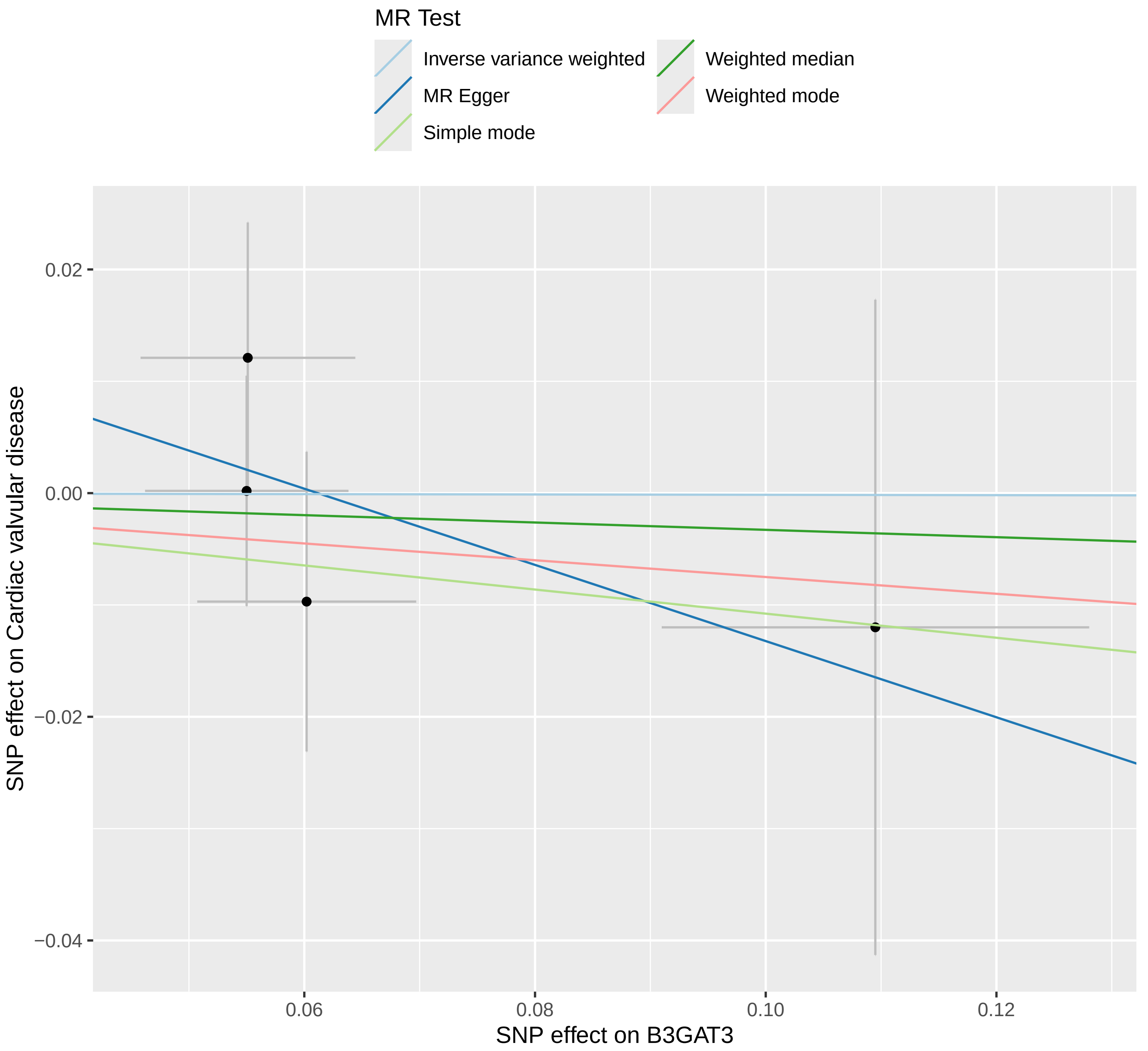
MR Test
Inverse variance weighted
MR Egger
Simple mode
Weighted median
Weighted mode
0.02
0.00
−0.02
−0.04
SNP effect on Cardiac valvular disease
0.06
0.08
0.10
0.12
SNP effect on B3GAT3

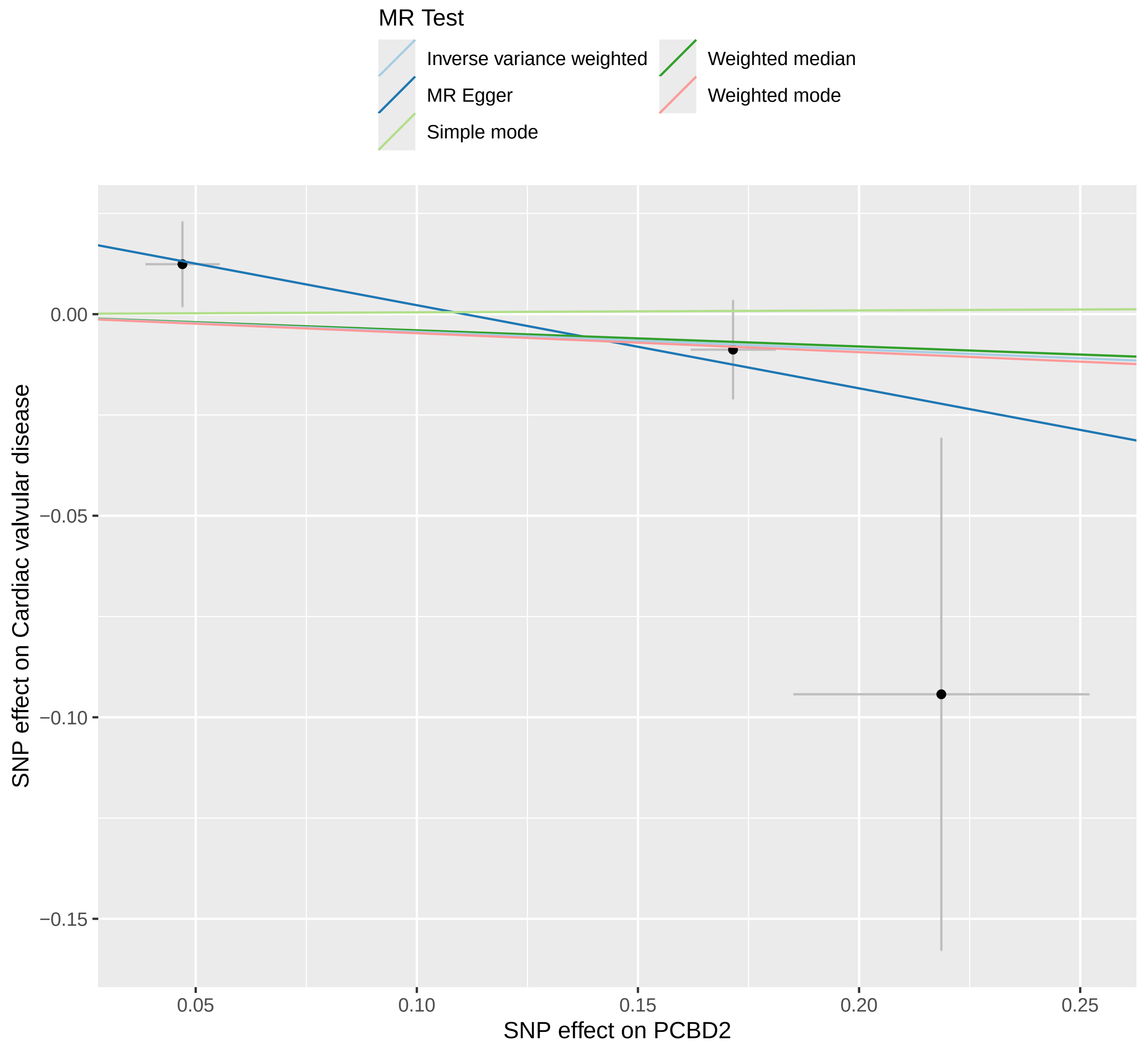

MR Test
Inverse variance weighted
MR Egger
Simple mode
Weighted median
Weighted mode
0.00
−0.05
−0.10
−0.15
0.05
0.10
0.15
0.20
0.25
SNP effect on Cardiac valvular disease
SNP effect on PCBD2

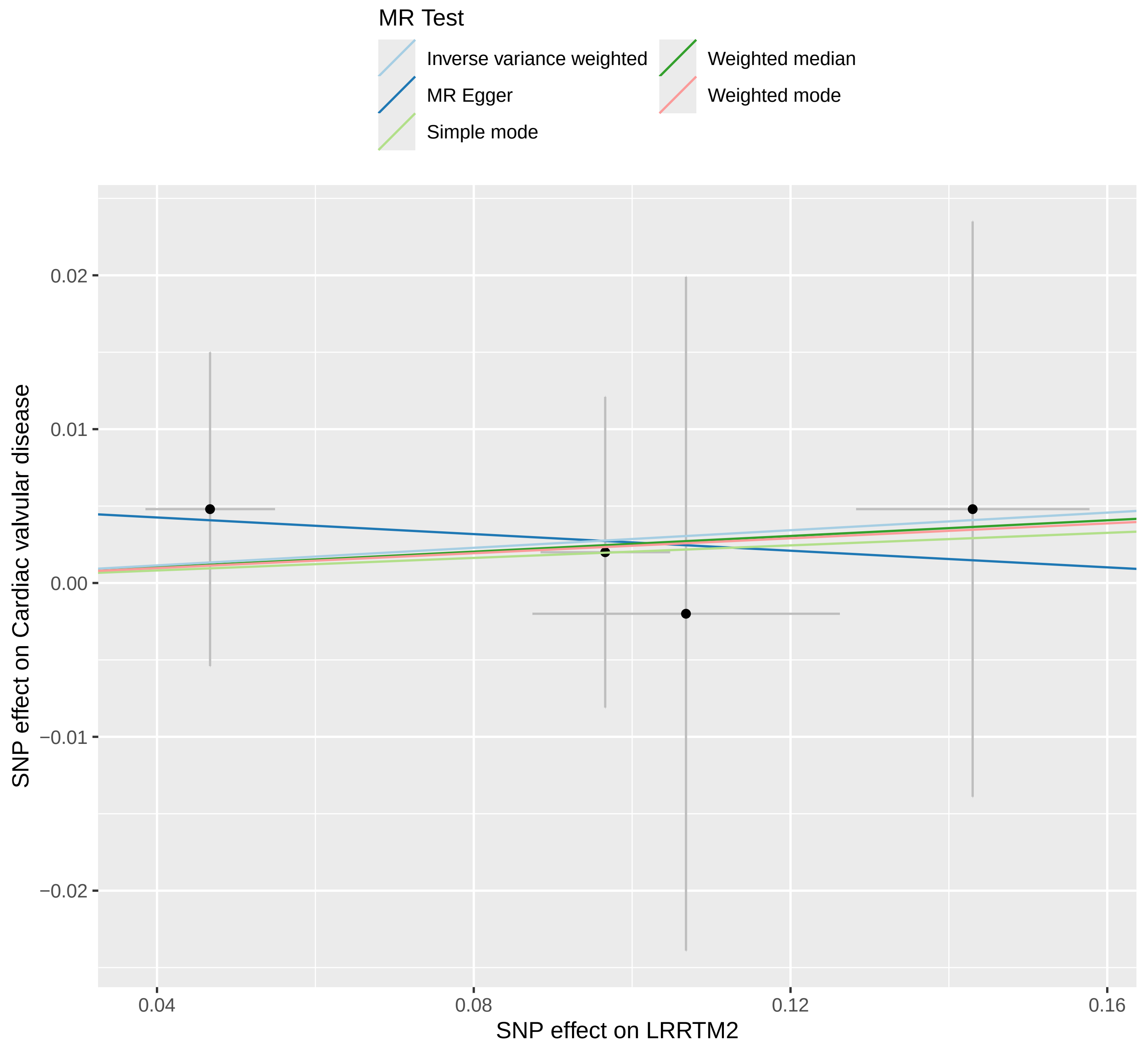

MR Test
Inverse variance weighted
MR Egger
Simple mode
Weighted median
Weighted mode
SNP effect on Cardiac valvular disease
0.02
0.01
0.00
−0.01
−0.02
0.04
0.08
0.12
0.16
SNP effect on LRRTM2

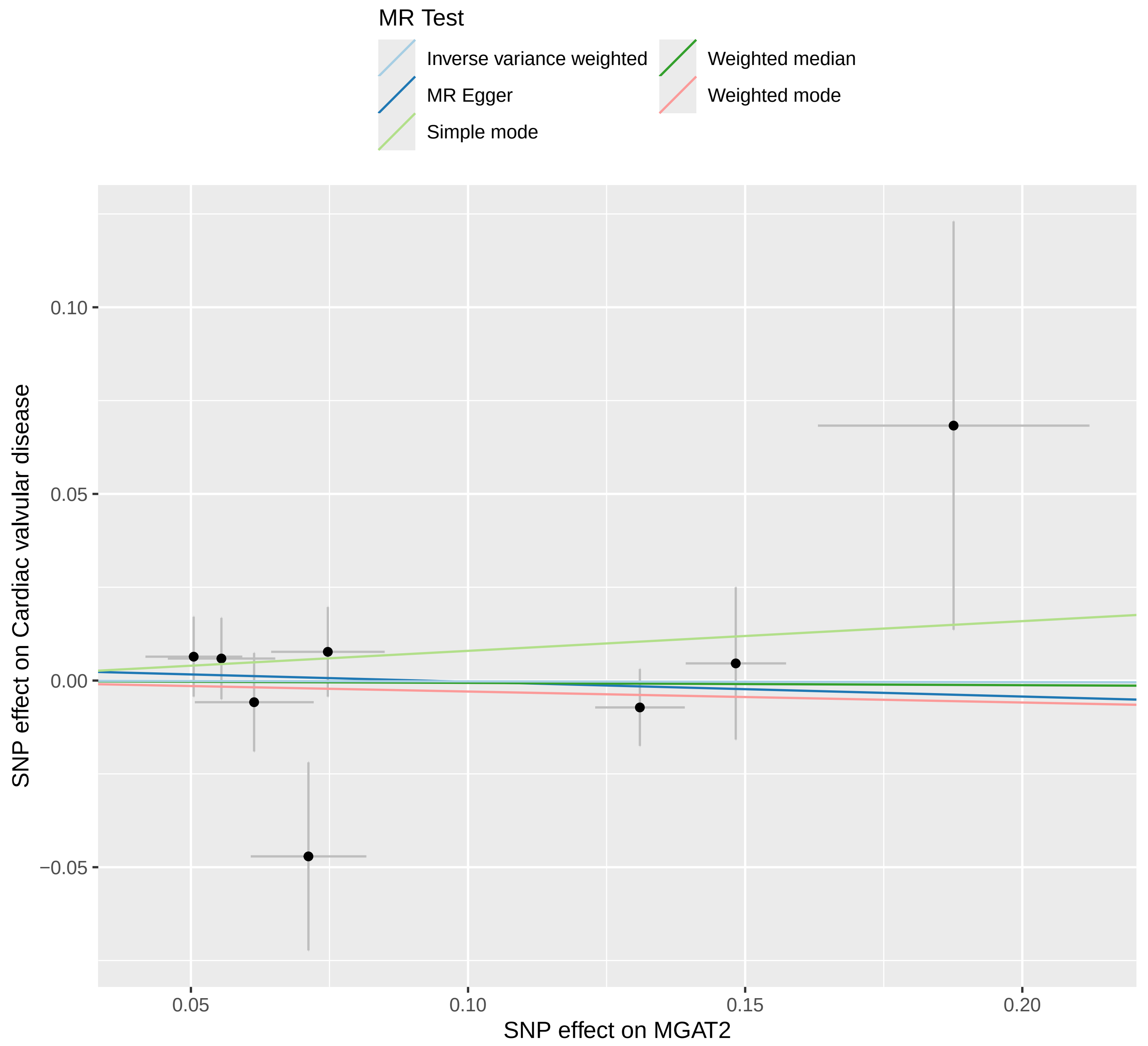
MR Test
Inverse variance weighted
MR Egger
Simple mode
Weighted median
Weighted mode
0.10
0.05
0.00
−0.05
0.05
0.10
0.15
0.20
SNP effect on MGAT2
SNP effect on Cardiac valvular disease

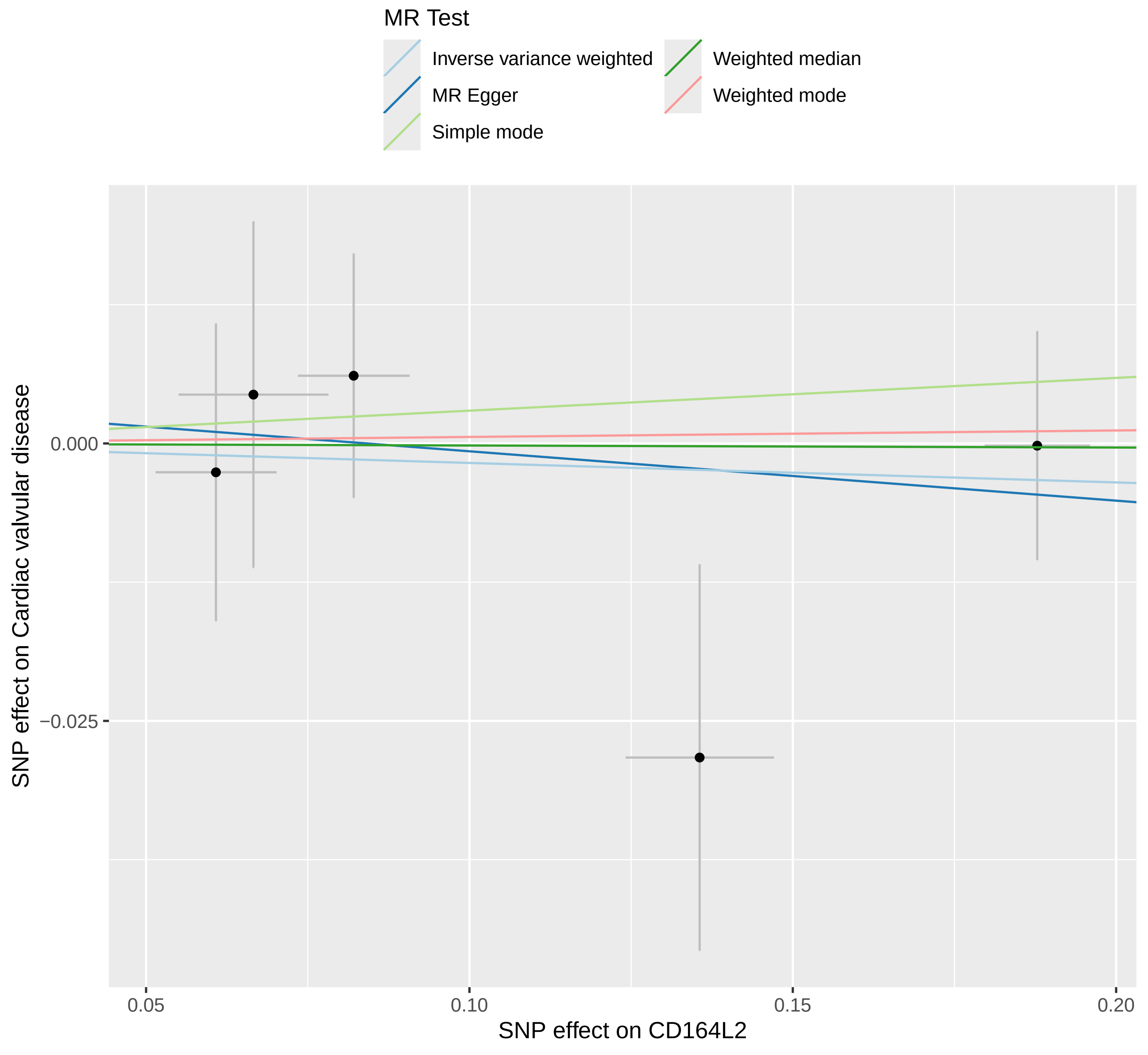
MR Test
Inverse variance weighted
MR Egger
Simple mode
Weighted median
Weighted mode
SNP effect on Cardiac valvular disease
0.000
−0.025
0.05
0.10
0.15
0.20
SNP effect on CD164L2

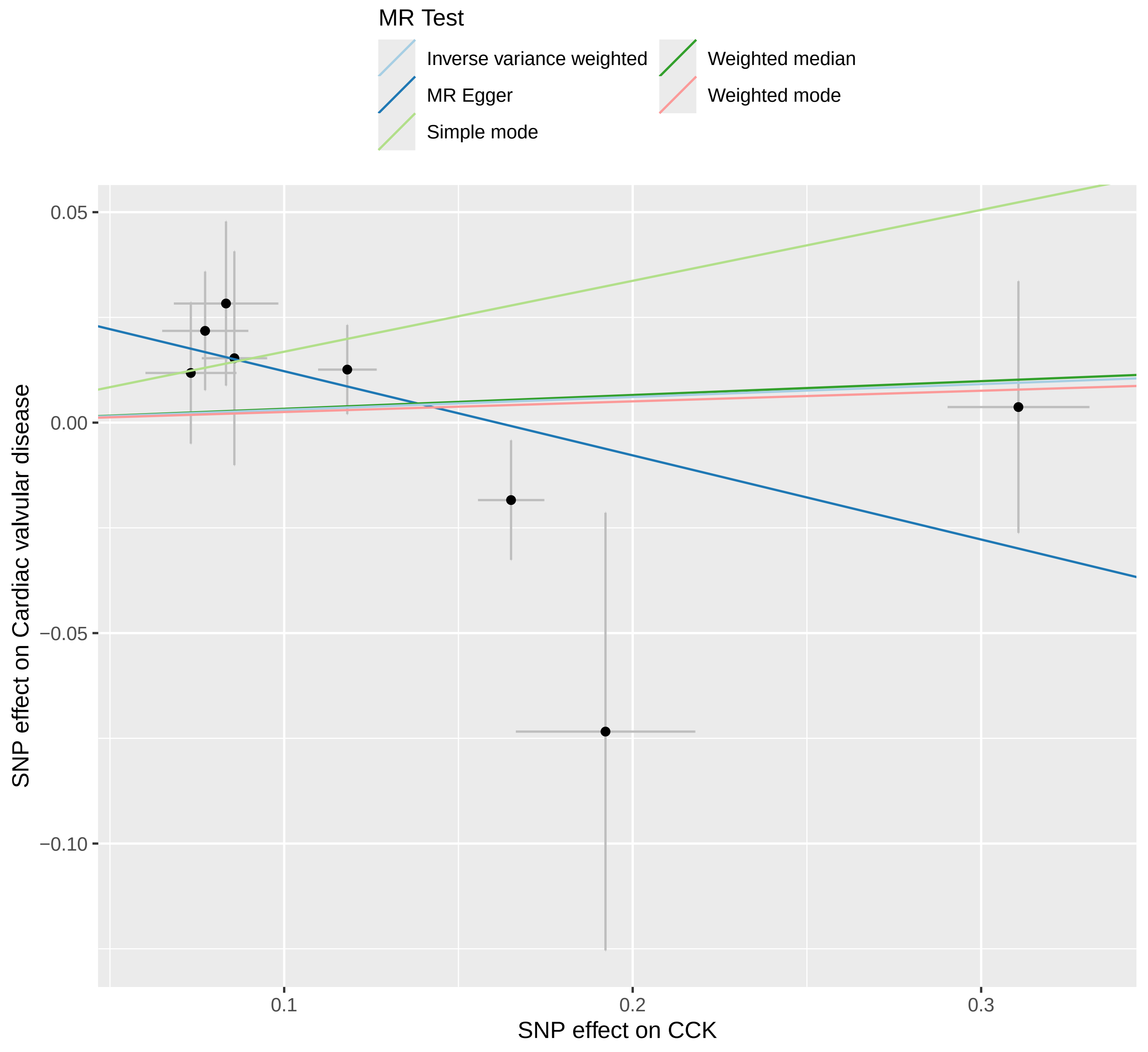

MR Test
Inverse variance weighted
MR Egger
Simple mode
Weighted median
Weighted mode
0.05
0.00
−0.05
−0.10
0.1
0.2
0.3
SNP effect on Cardiac valvular disease
SNP effect on CCK

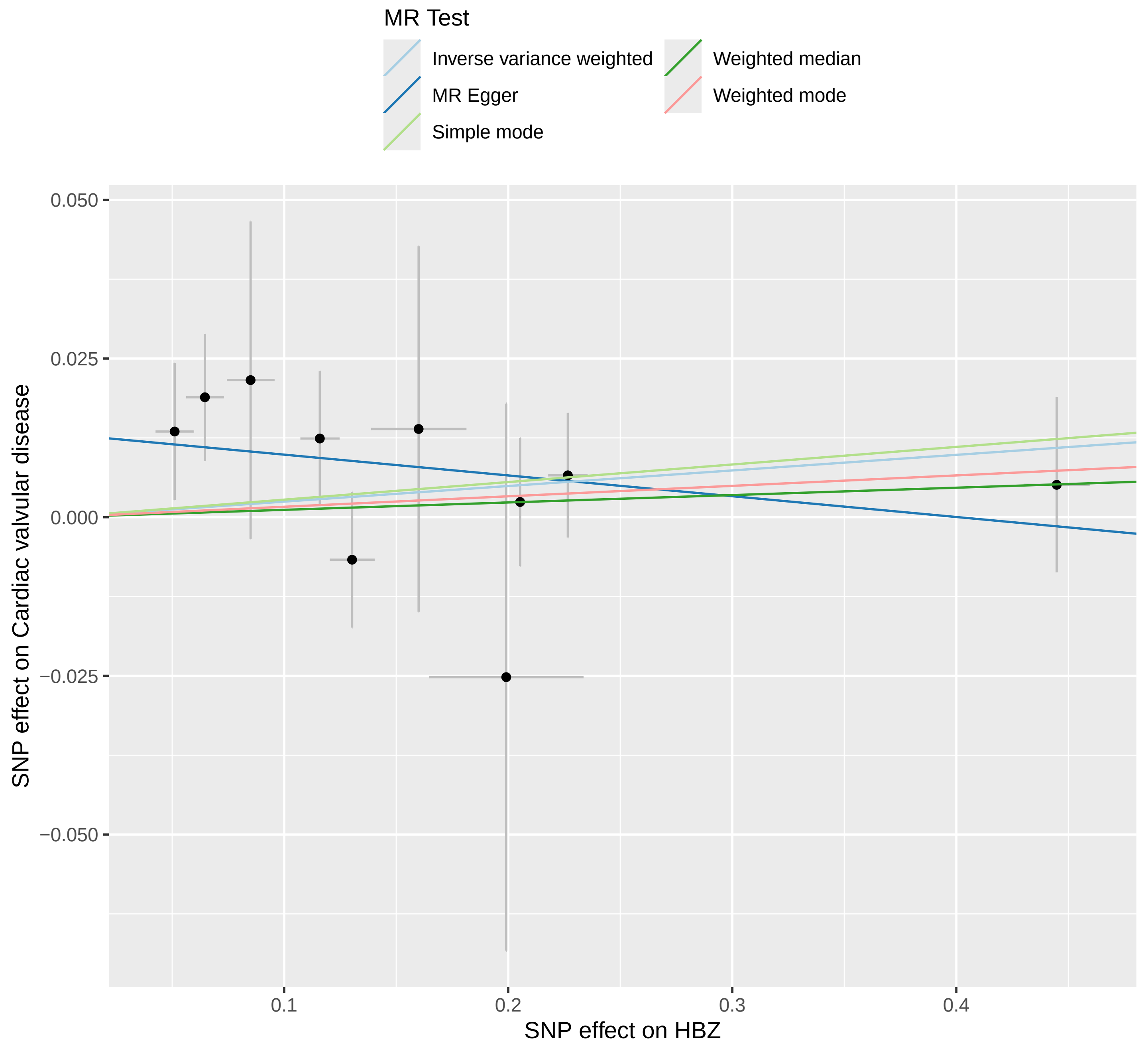

MR Test
Inverse variance weighted
MR Egger
Simple mode
Weighted median
Weighted mode
0.050
0.025
0.000
−0.025
−0.050
SNP effect on Cardiac valvular disease
0.1
0.2
0.3
0.4
SNP effect on HBZ

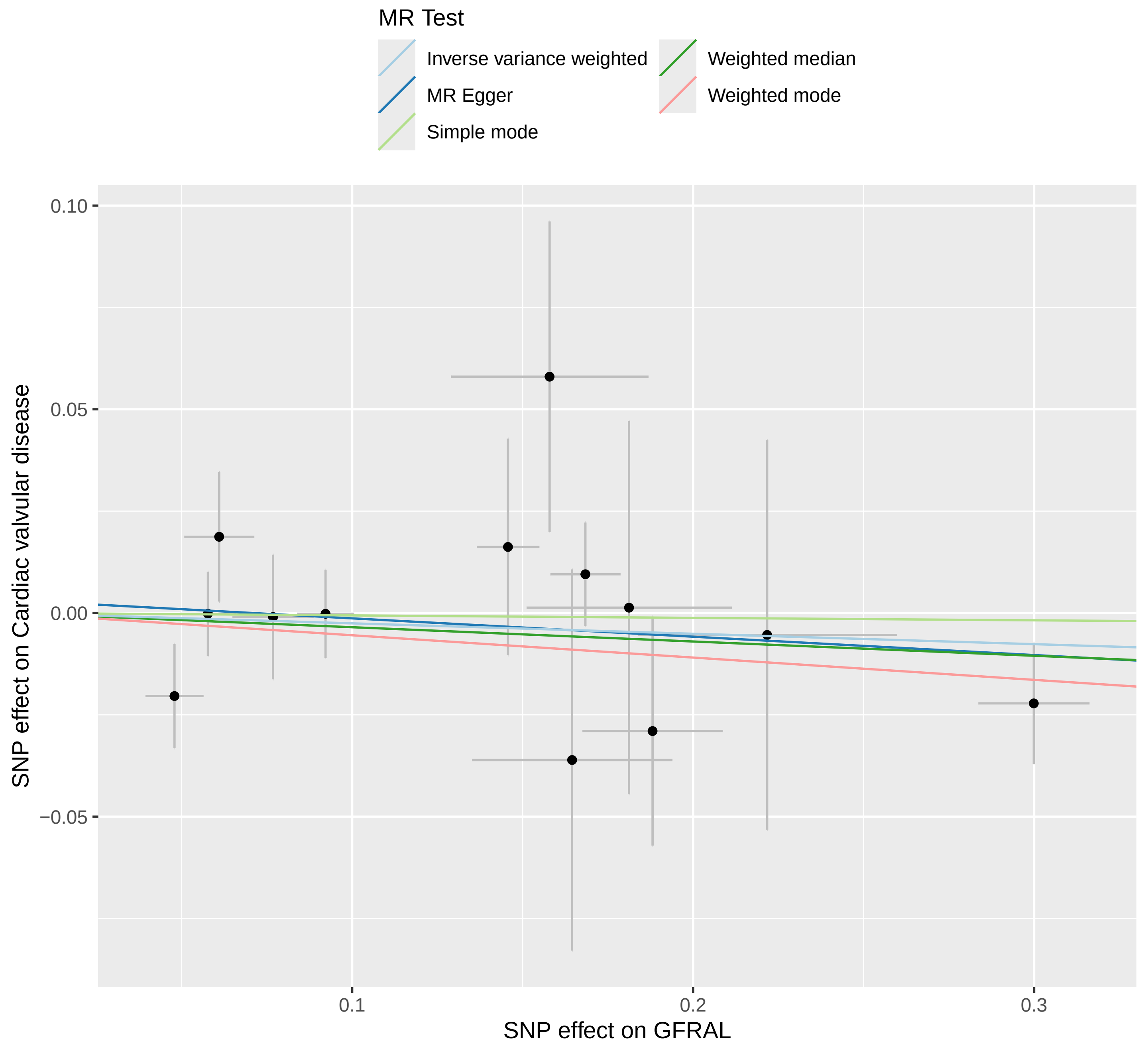
MR Test
Inverse variance weighted
MR Egger
Simple mode
Weighted median
Weighted mode
0.10
0.05
0.00
−0.05
0.1
0.2
0.3
SNP effect on Cardiac valvular disease
SNP effect on GFRAL

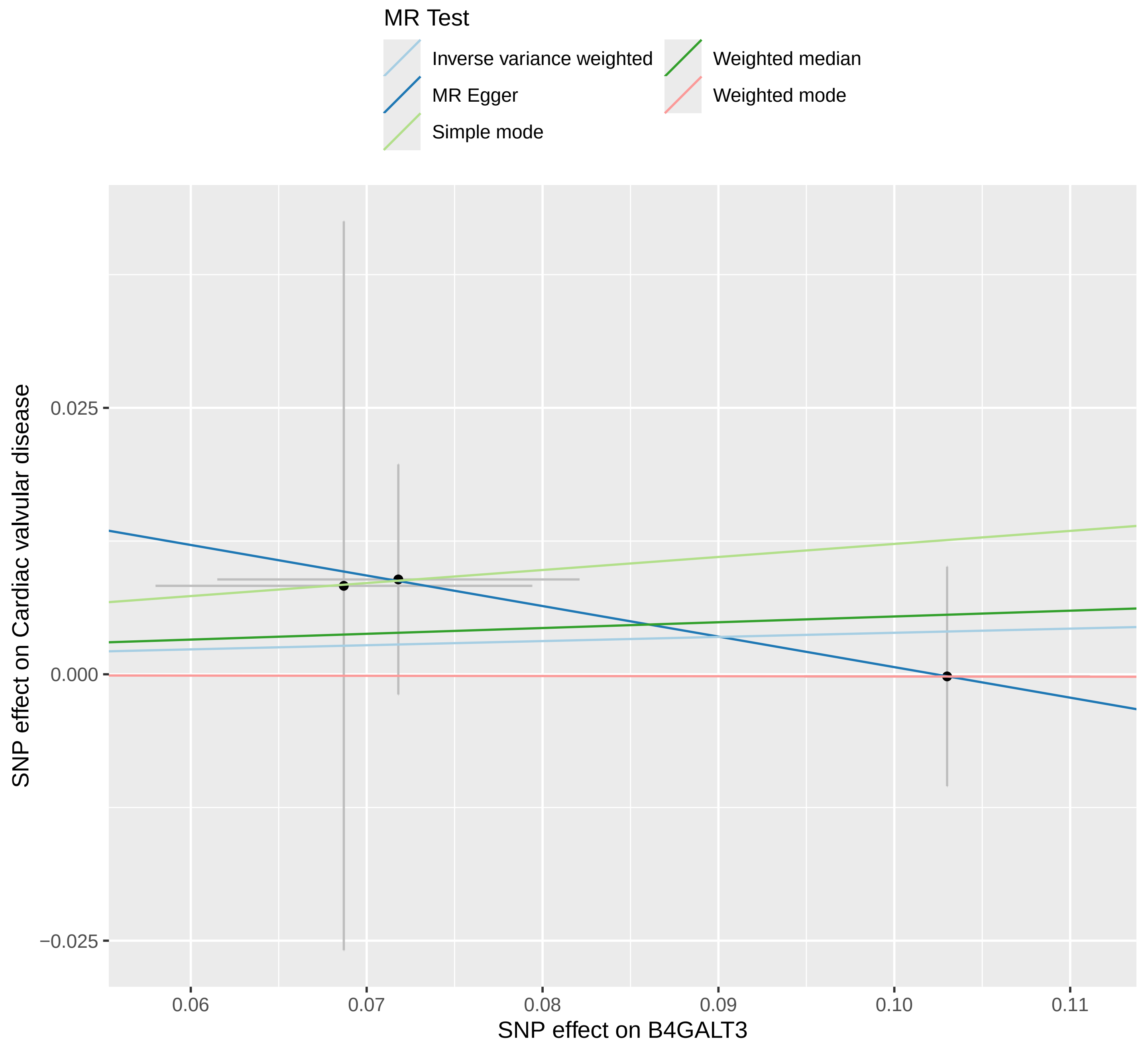
MR Test
Inverse variance weighted
MR Egger
Simple mode
Weighted median
Weighted mode
SNP effect on Cardiac valvular disease
0.025
0.000
−0.025
0.06
0.07
0.08
0.09
0.10
0.11
SNP effect on B4GALT3

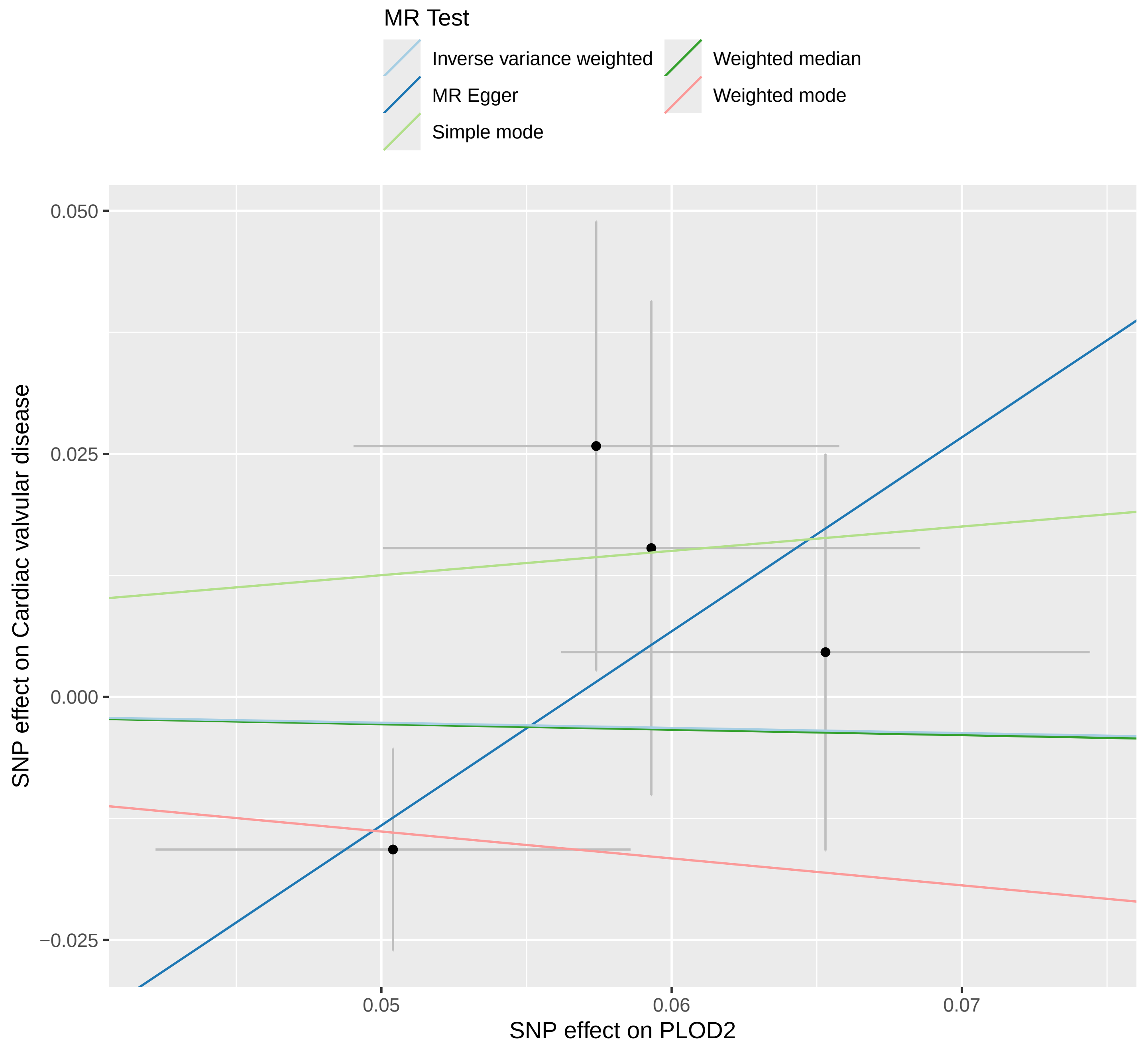
MR Test
Inverse variance weighted
MR Egger
Simple mode
Weighted median
Weighted mode
0.050
0.025
0.000
−0.025
0.05
0.06
0.07
SNP effect on Cardiac valvular disease
SNP effect on PLOD2

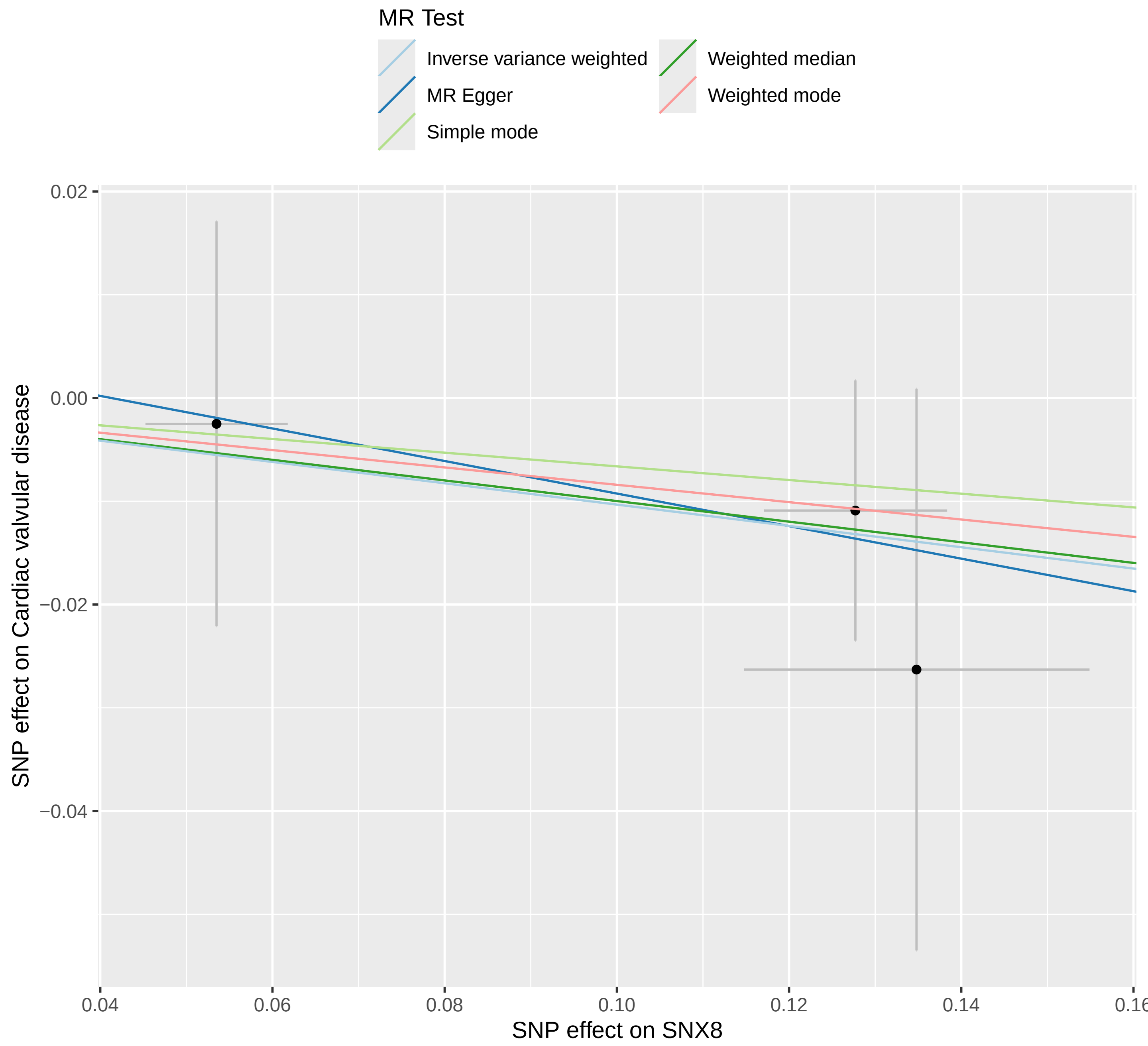

MR Test
Inverse variance weighted
MR Egger
Simple mode
Weighted median
Weighted mode
0.02
0.00
−0.02
−0.04
SNP effect on Cardiac valvular disease
0.04
0.06
0.08
0.10
0.12
0.14
0.16
SNP effect on SNX8

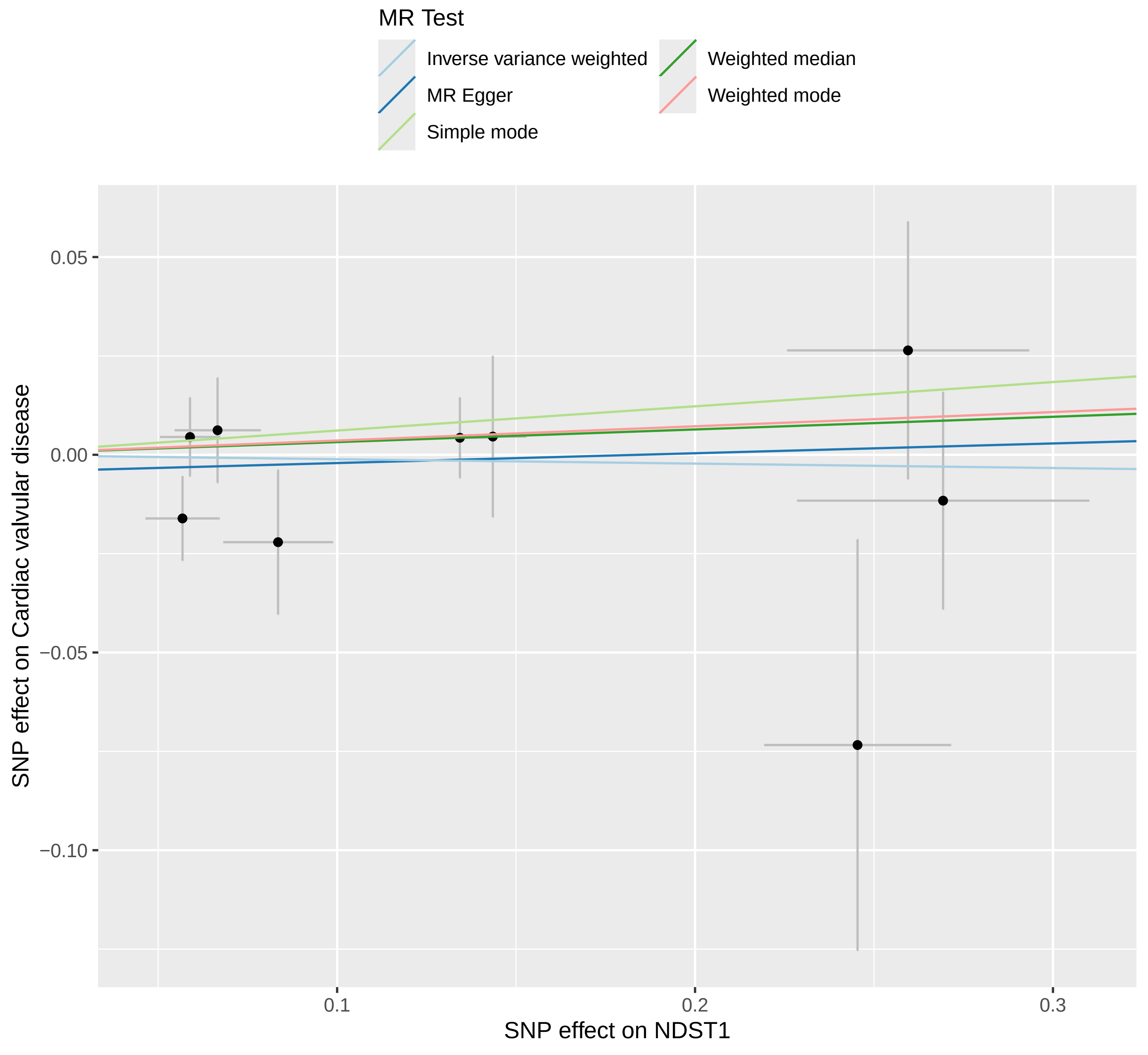
MR Test
Inverse variance weighted
MR Egger
Simple mode
Weighted median
Weighted mode
0.05
0.00
−0.05
−0.10
0.1
0.2
0.3
SNP effect on Cardiac valvular disease
SNP effect on NDST1

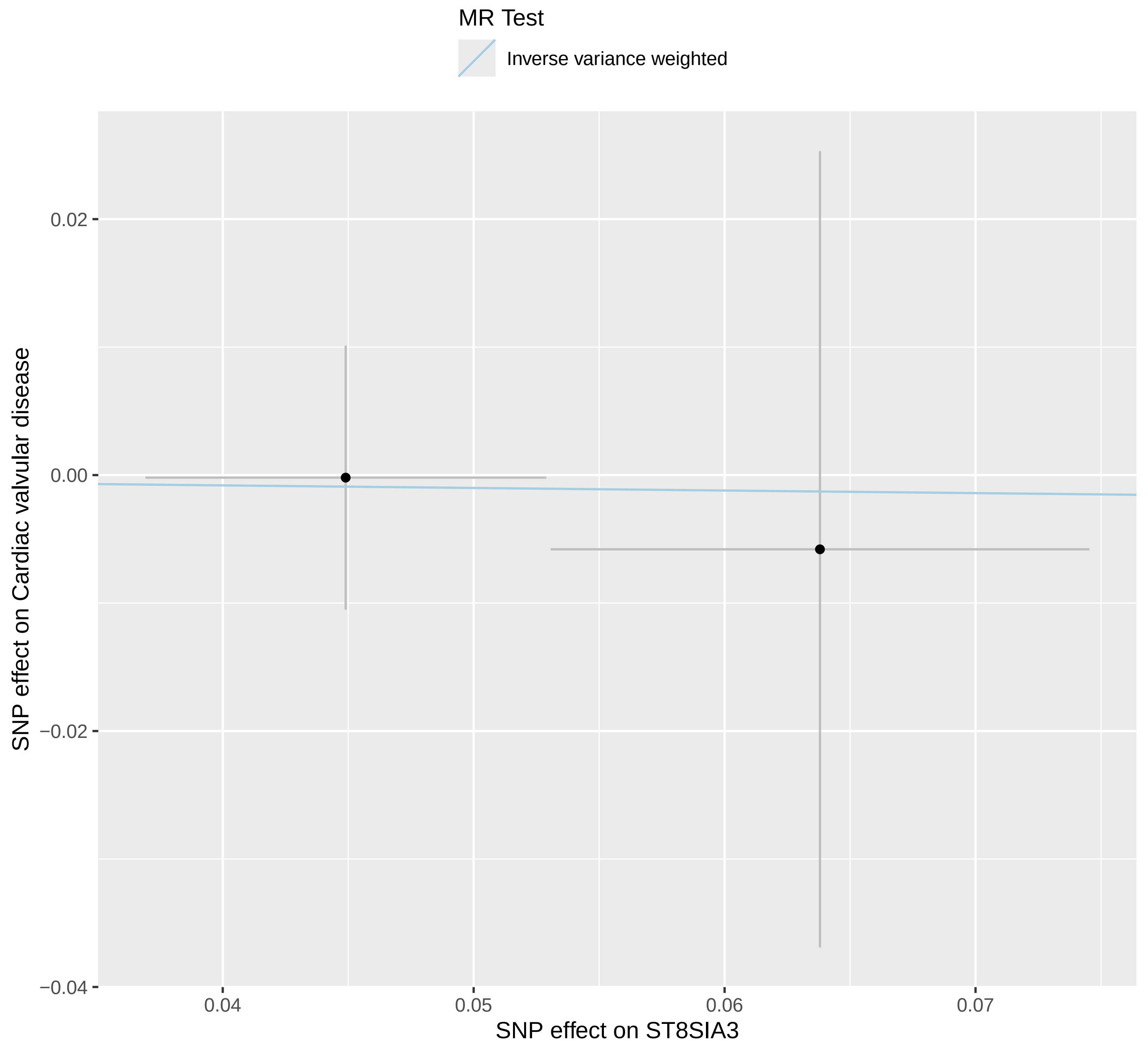
MR Test
Inverse variance weighted
0.02
0.00
−0.02
−0.04
0.04
0.05
0.06
0.07
SNP effect on Cardiac valvular disease
SNP effect on ST8SIA3

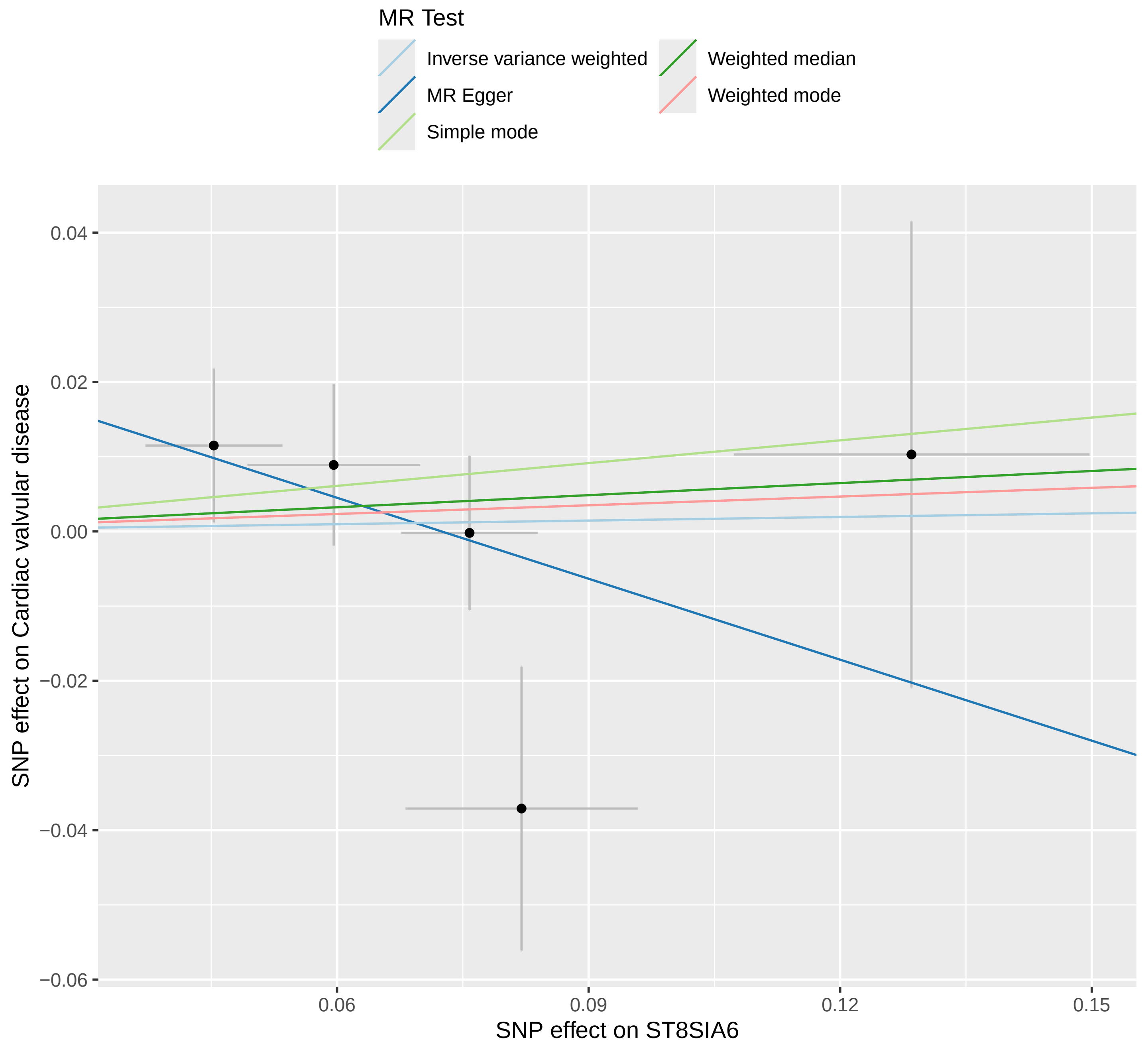
MR Test
Inverse variance weighted
MR Egger
Simple mode
Weighted median
Weighted mode
SNP effect on Cardiac valvular disease
0.04
0.02
0.00
−0.02
−0.04
−0.06
0.06
0.09
0.12
0.15
SNP effect on ST8SIA6

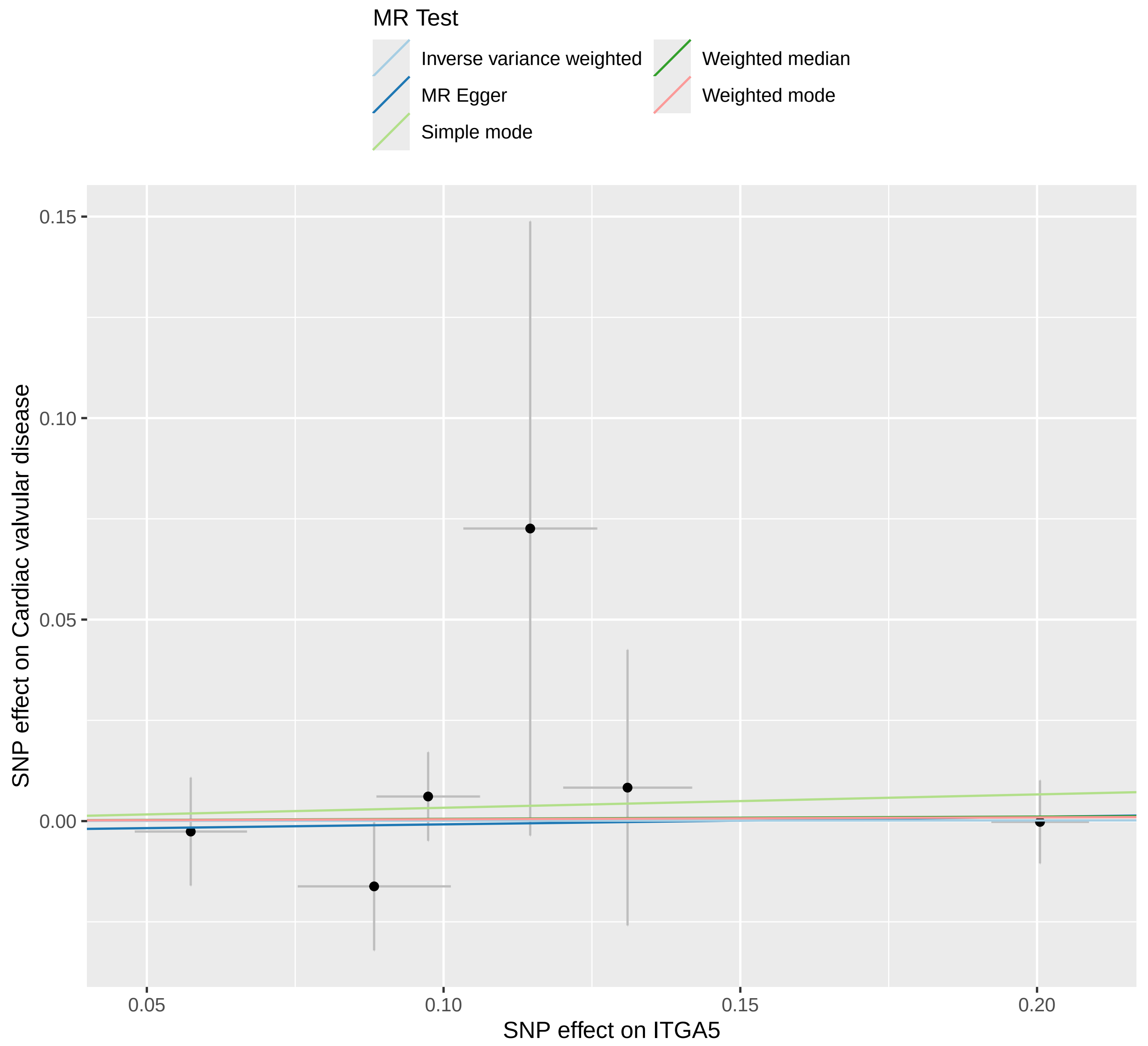

MR Test
Inverse variance weighted
MR Egger
Simple mode
Weighted median
Weighted mode
0.15
0.10
0.05
0.00
0.05
0.10
0.15
0.20
SNP effect on Cardiac valvular disease
SNP effect on ITGA5

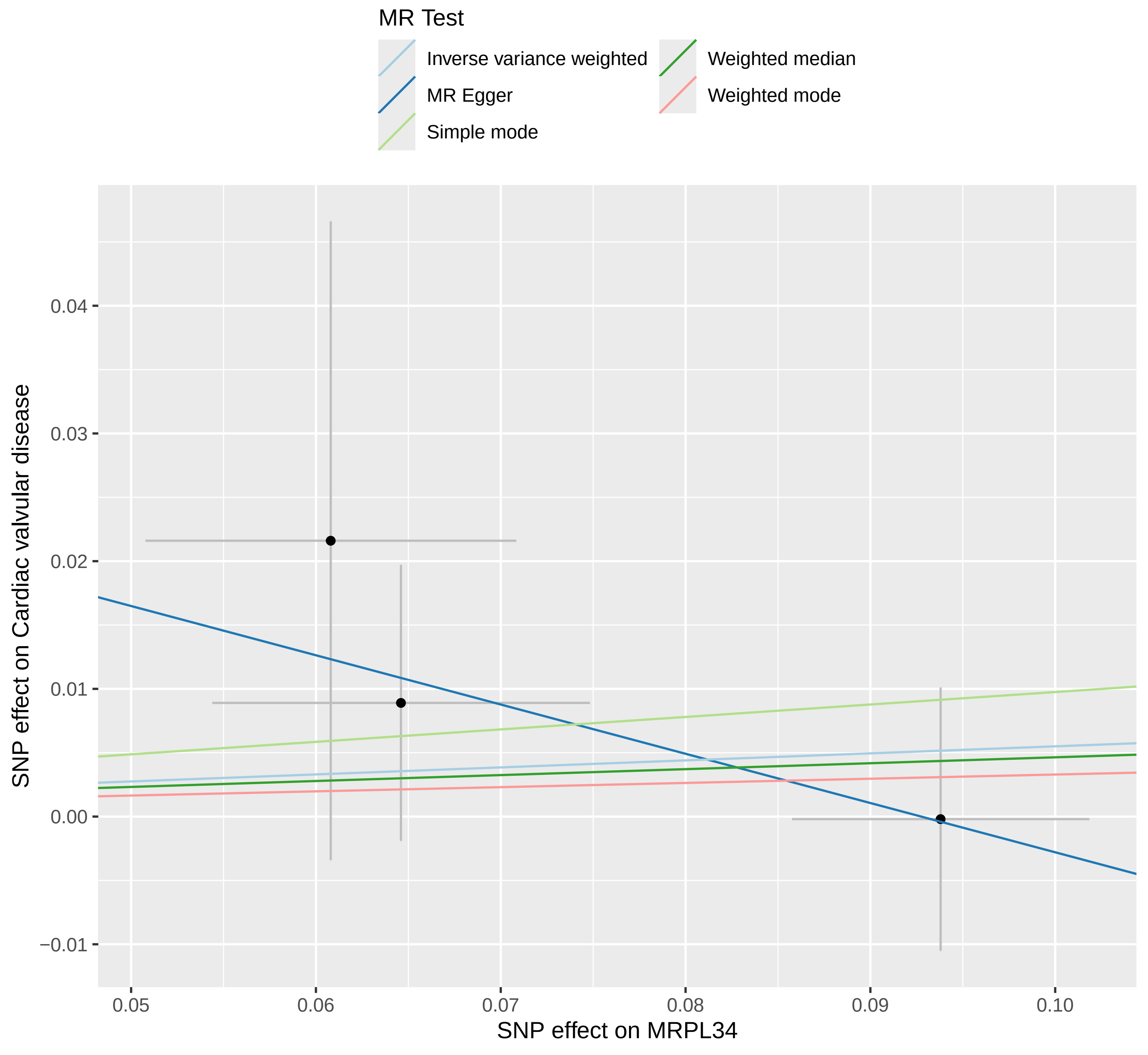
MR Test
Inverse variance weighted
MR Egger
Simple mode
Weighted median
Weighted mode
SNP effect on Cardiac valvular disease
0.04
0.03
0.02
0.01
0.00
−0.01
0.05
0.06
0.07
0.08
0.09
0.10
SNP effect on MRPL34

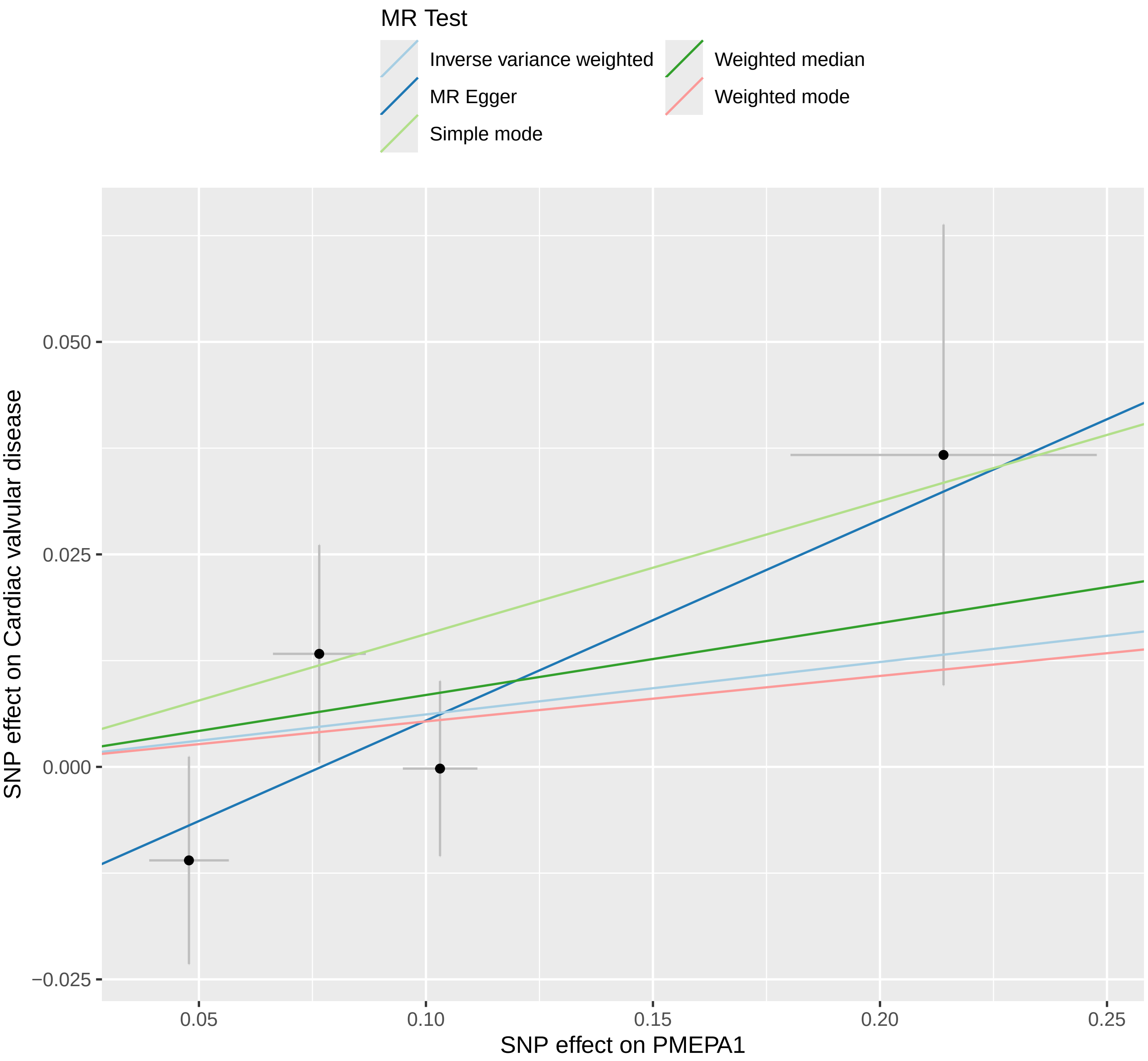
MR Test
Inverse variance weighted
MR Egger
Simple mode
Weighted median
Weighted mode
0.050
0.025
0.000
−0.025
SNP effect on Cardiac valvular disease
0.05
0.10
0.15
0.20
0.25
SNP effect on PMEPA1

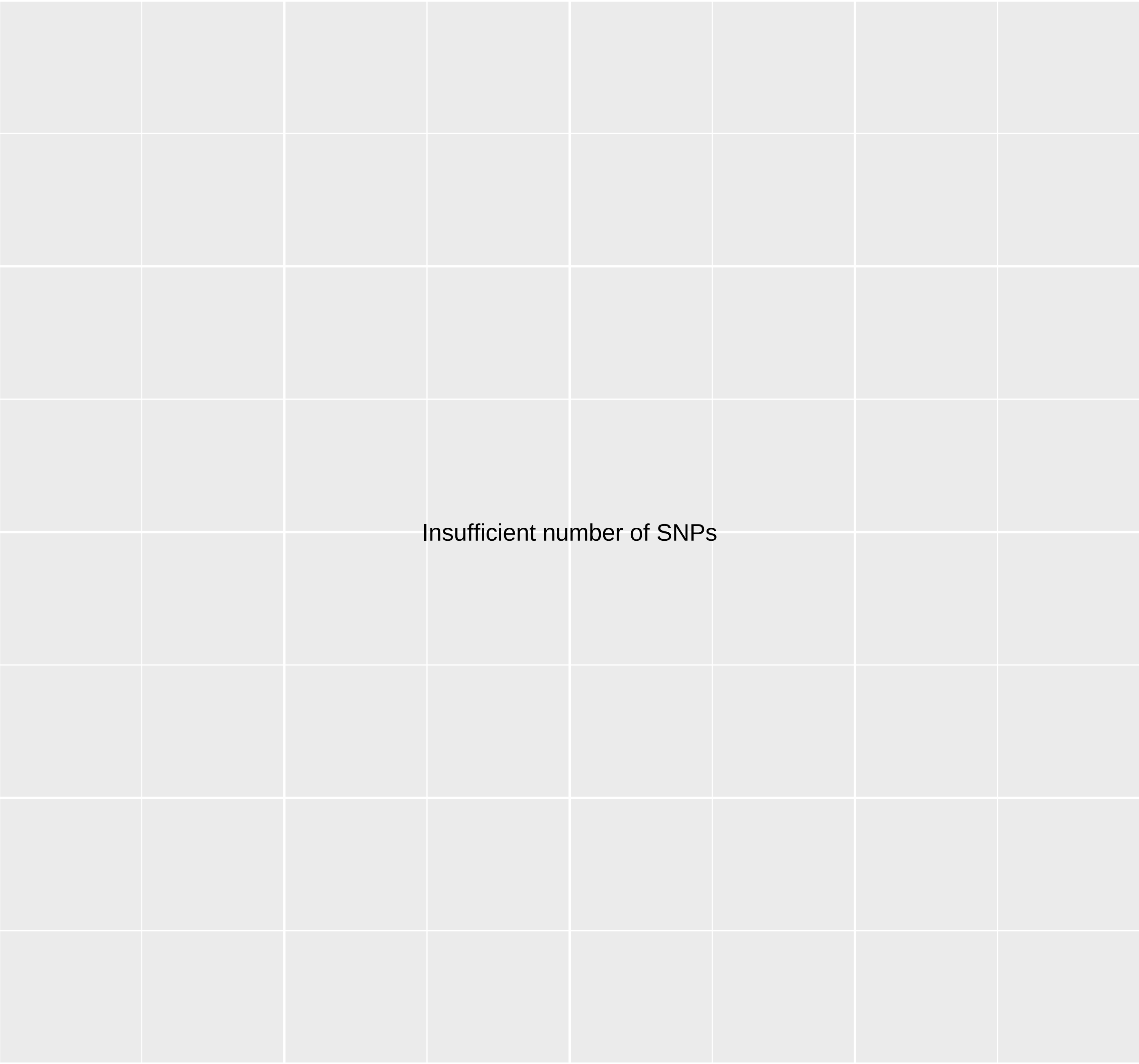
Insufficient number of SNPs

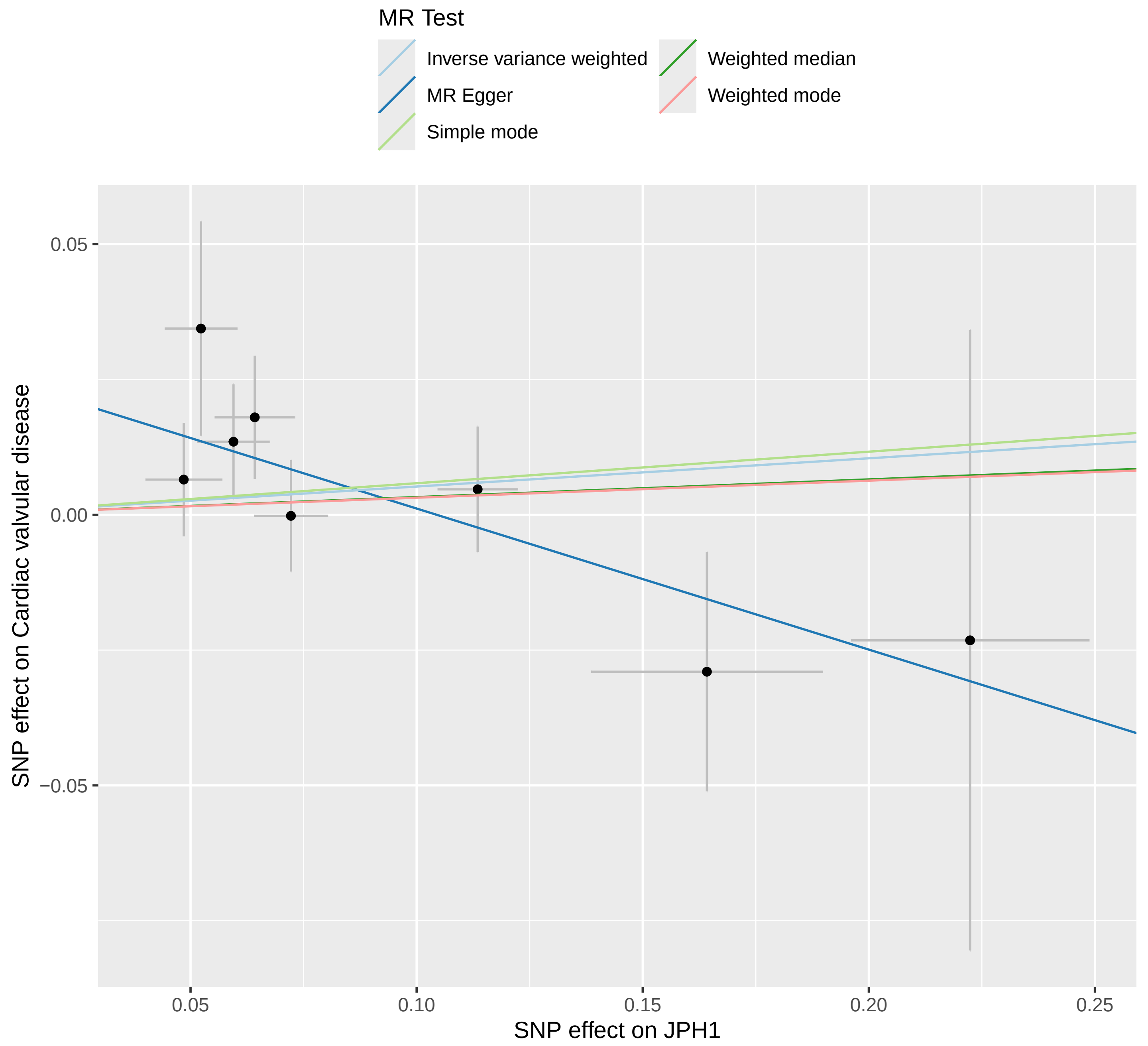
MR Test
Inverse variance weighted
MR Egger
Simple mode
Weighted median
Weighted mode
0.05
0.00
−0.05
SNP effect on Cardiac valvular disease
0.05
0.10
0.15
0.20
0.25
SNP effect on JPH1

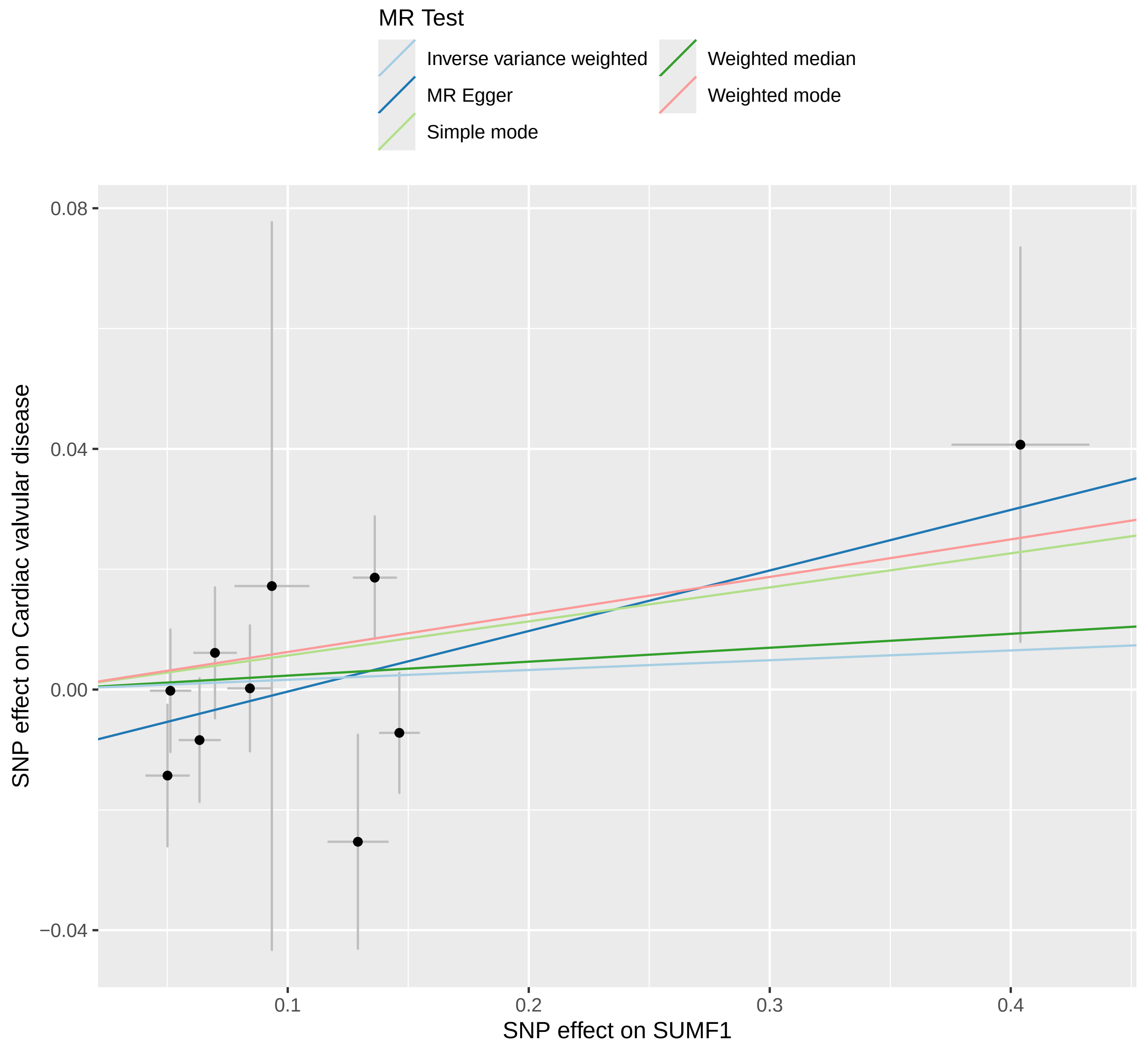
MR Test
Inverse variance weighted
MR Egger
Simple mode
Weighted median
Weighted mode
0.08
0.04
0.00
−0.04
SNP effect on Cardiac valvular disease
0.1
0.2
0.3
0.4
SNP effect on SUMF1

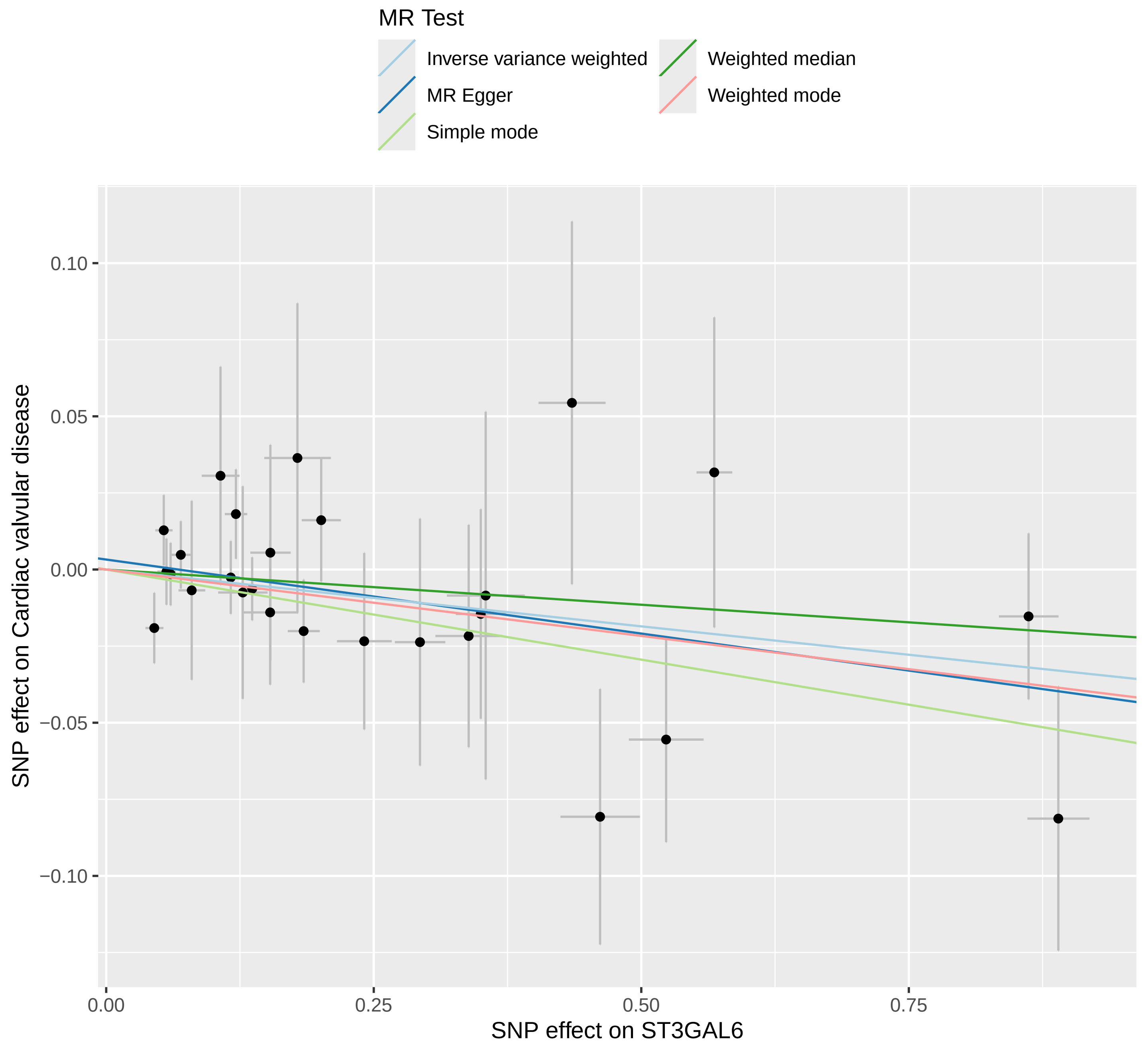

MR Test
Inverse variance weighted
MR Egger
Simple mode
Weighted median
Weighted mode
0.10
0.05
0.00
−0.05
−0.10
SNP effect on Cardiac valvular disease
0.00
0.25
0.50
0.75
SNP effect on ST3GAL6

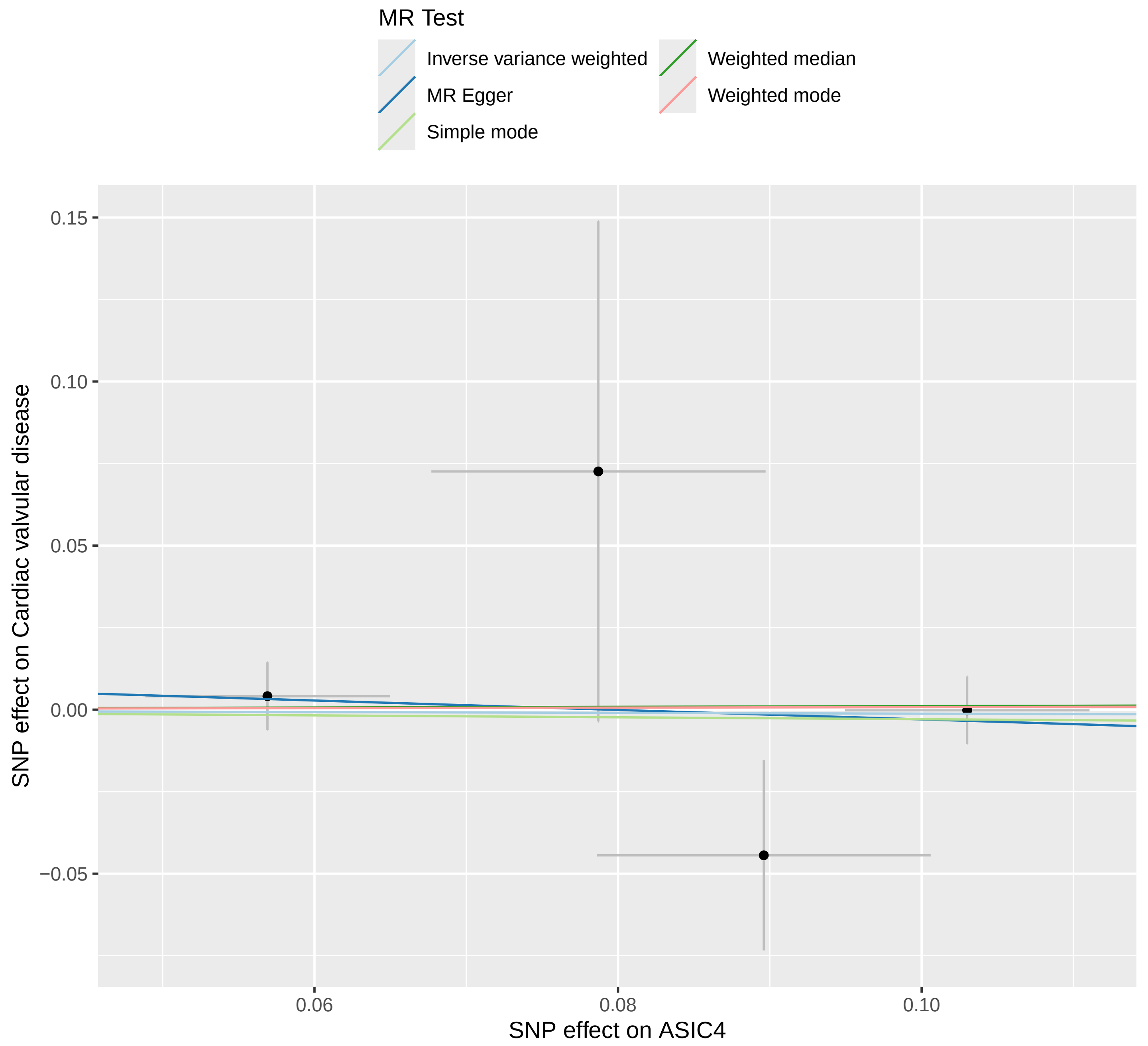
MR Test
Inverse variance weighted
MR Egger
Simple mode
Weighted median
Weighted mode
0.15
0.10
0.05
0.00
−0.05
0.06
0.08
0.10
SNP effect on Cardiac valvular disease
SNP effect on ASIC4

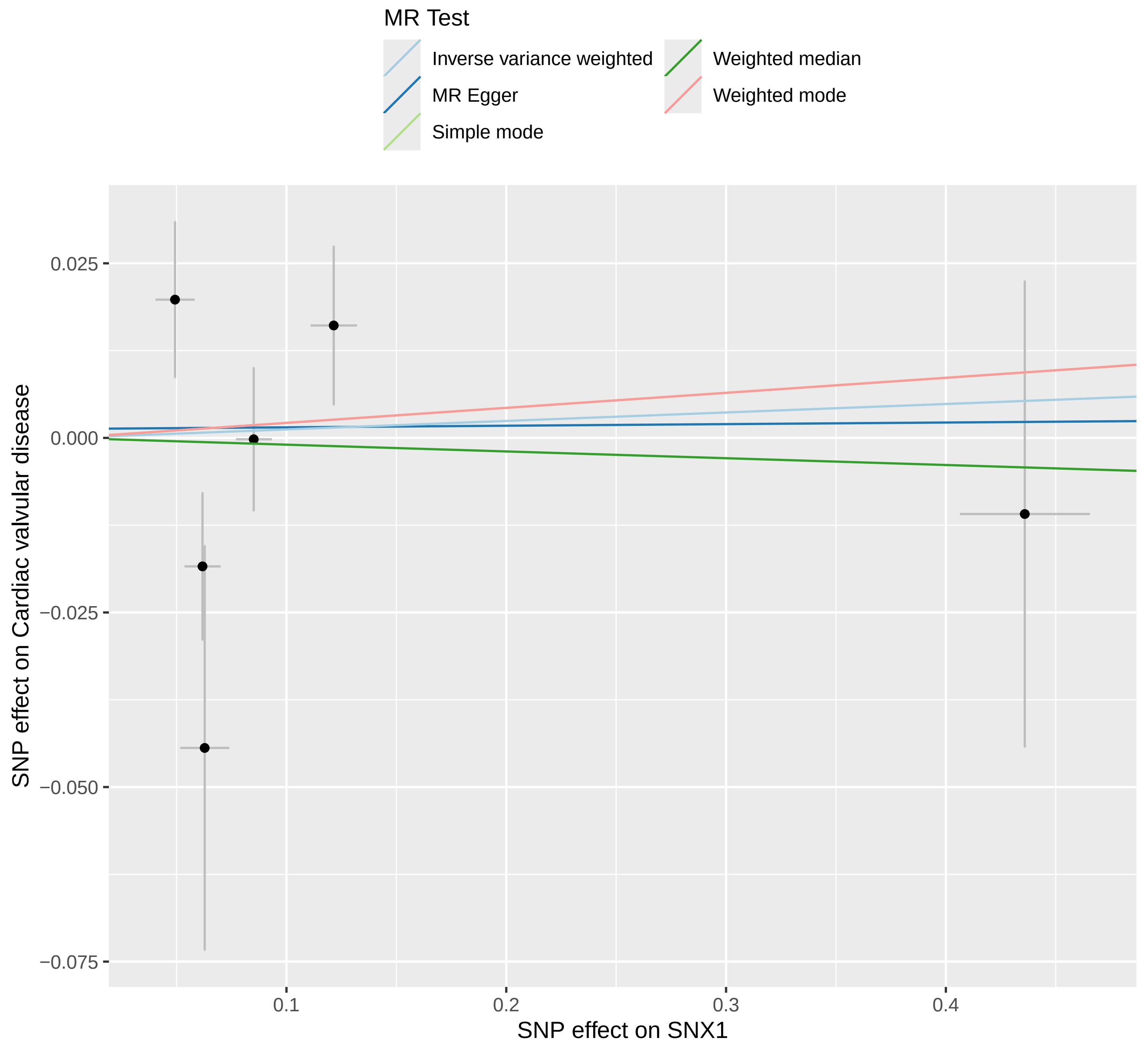
MR Test
Inverse variance weighted
MR Egger
Simple mode
Weighted median
Weighted mode
0.025
0.000
−0.025
−0.050
−0.075
SNP effect on Cardiac valvular disease
0.1
0.2
0.3
0.4
SNP effect on SNX1

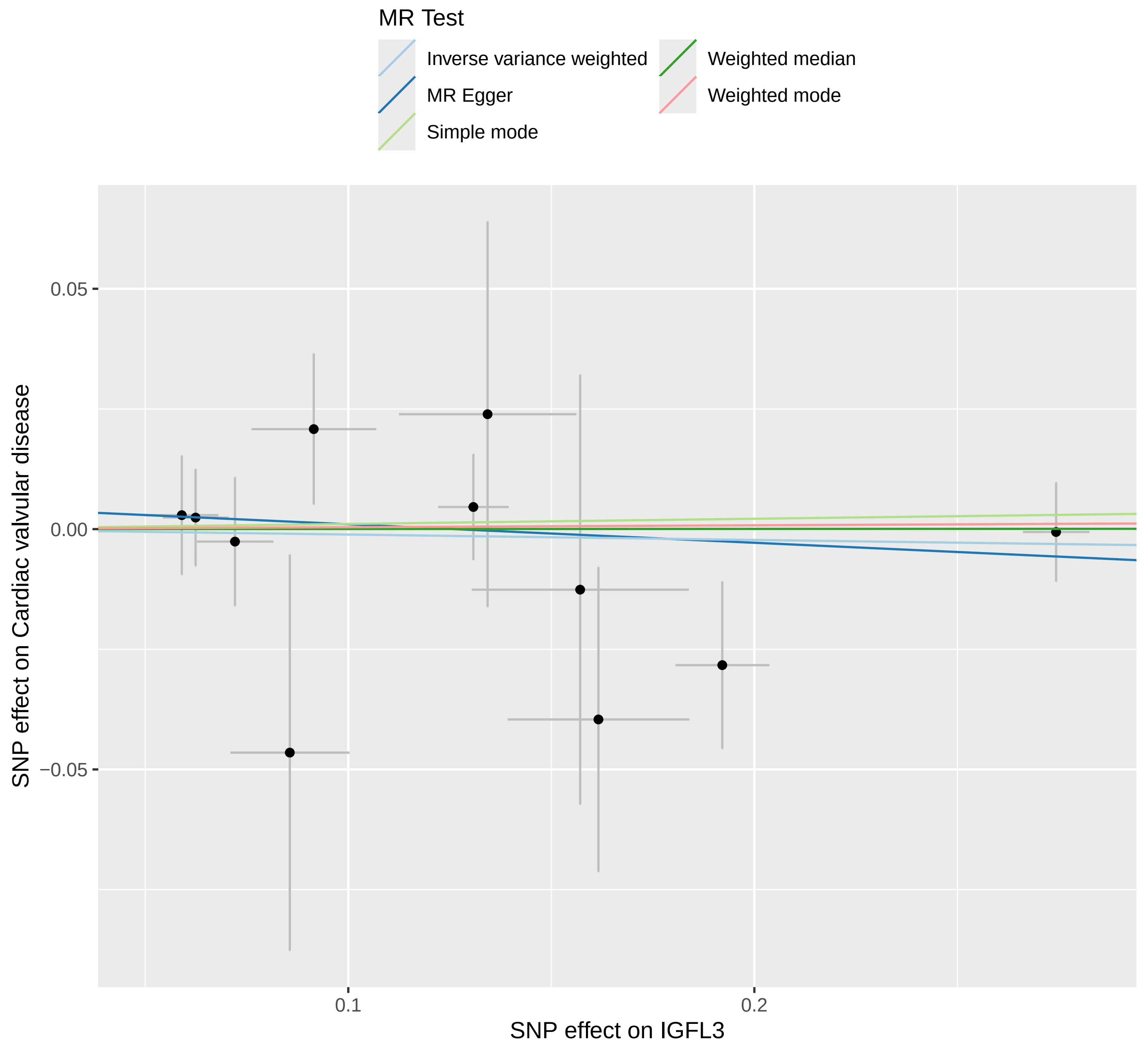
MR Test
Inverse variance weighted
MR Egger
Simple mode
Weighted median
Weighted mode
0.05
0.00
−0.05
0.1
0.2
SNP effect on Cardiac valvular disease
SNP effect on IGFL3

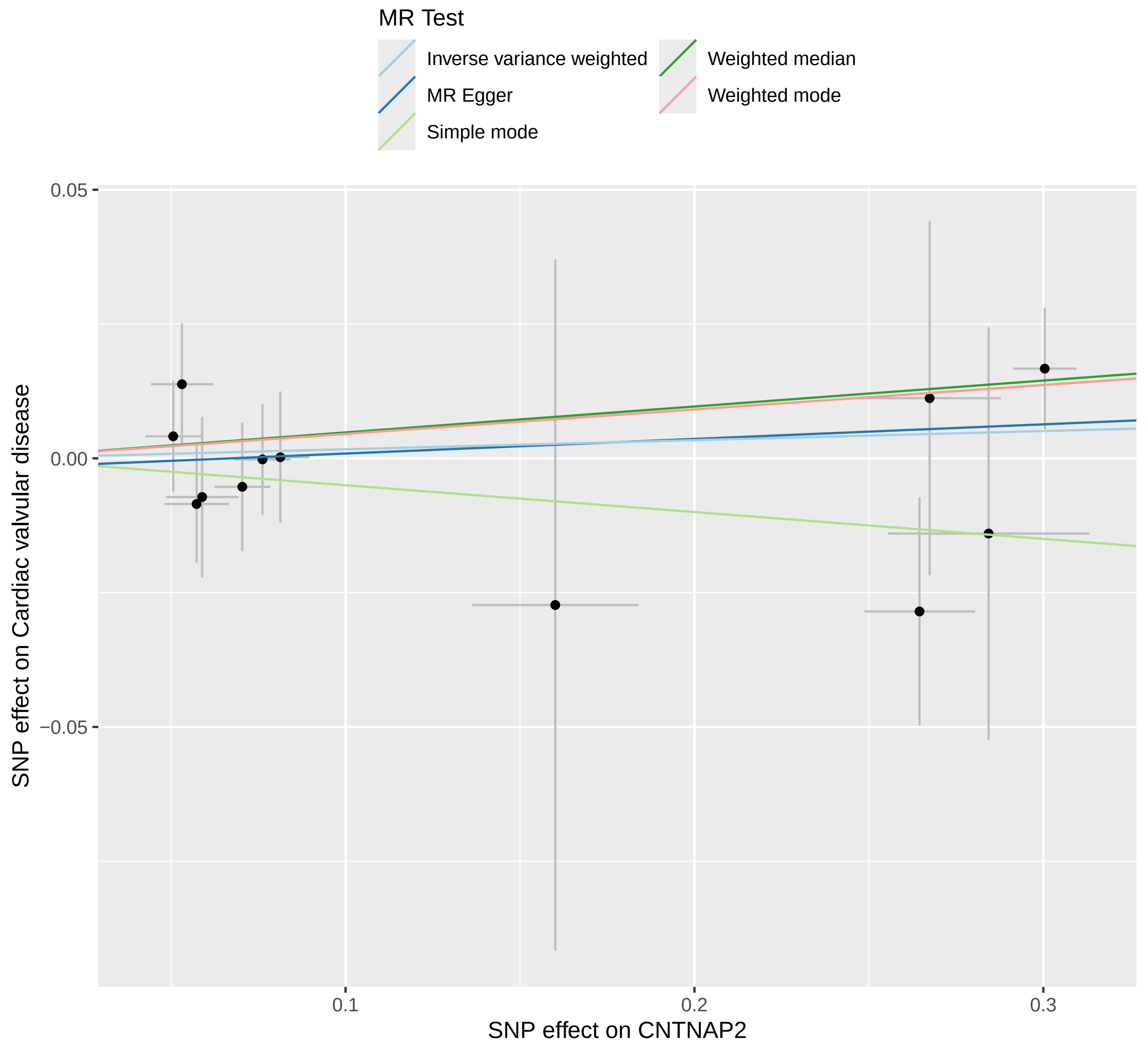

MR Test
Inverse variance weighted
MR Egger
Simple mode
Weighted median
Weighted mode
0.05
0.00
−0.05
0.1
0.2
0.3
SNP effect on Cardiac valvular disease
SNP effect on CNTNAP2

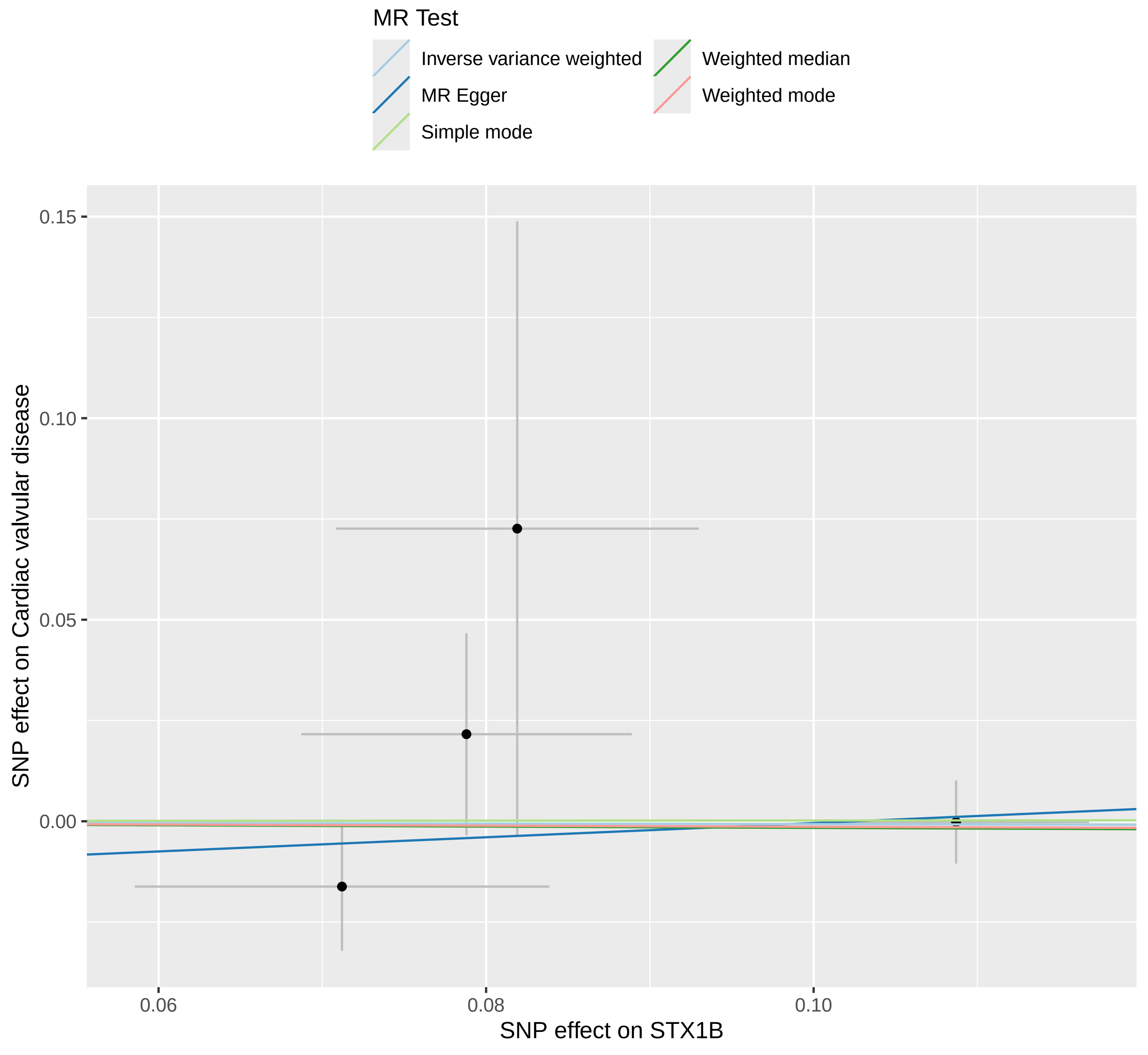

MR Test
Inverse variance weighted
MR Egger
Simple mode
Weighted median
Weighted mode
0.15
0.10
0.05
0.00
0.06
0.08
0.10
SNP effect on Cardiac valvular disease
SNP effect on STX1B

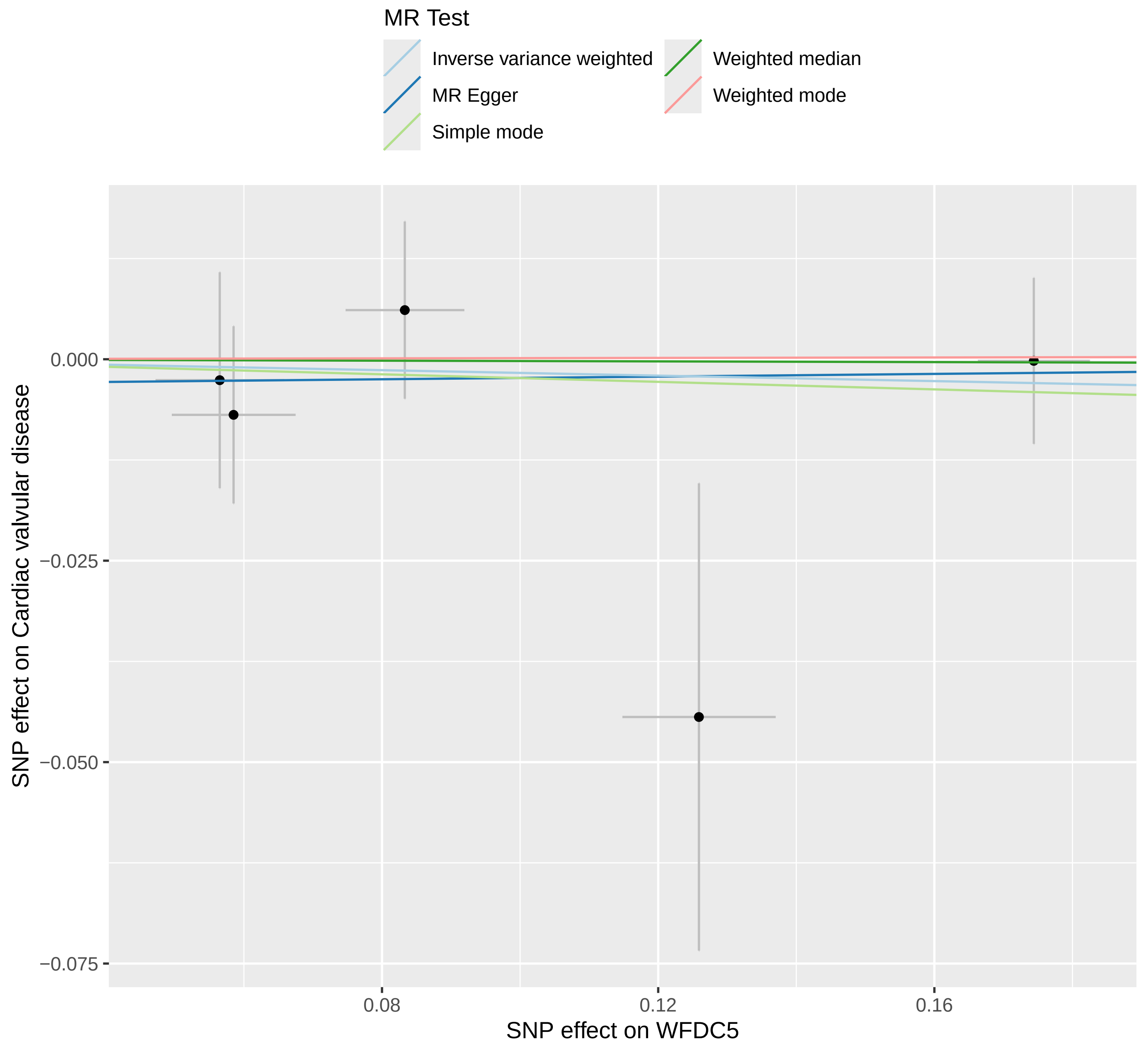

MR Test
Inverse variance weighted
MR Egger
Simple mode
Weighted median
Weighted mode
0.000
−0.025
−0.050
−0.075
0.08
0.12
0.16
SNP effect on WFDC5
SNP effect on Cardiac valvular disease

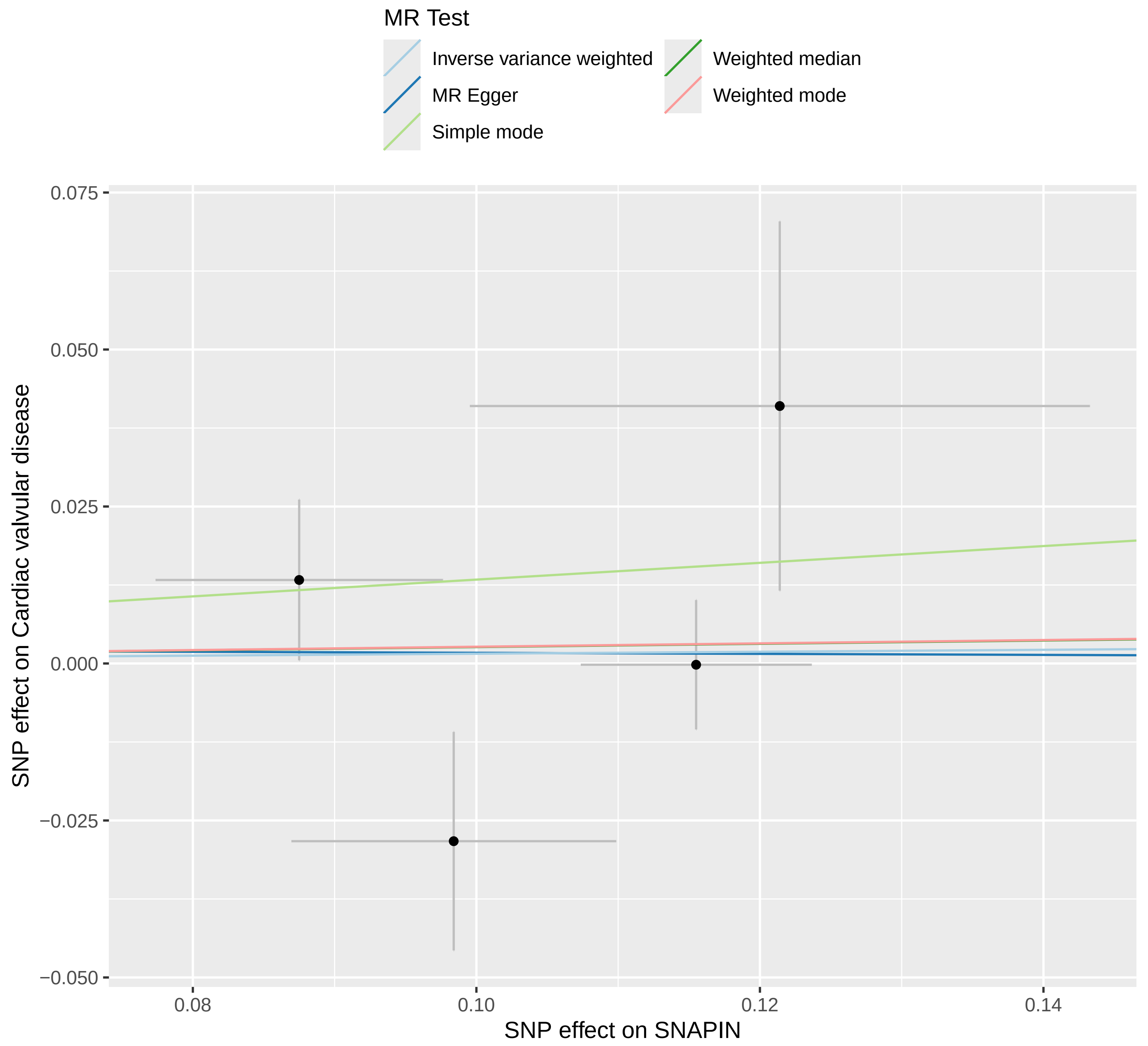

MR Test
Inverse variance weighted
MR Egger
Simple mode
Weighted median
Weighted mode
0.075
0.050
0.025
0.000
−0.025
−0.050
0.08
0.10
0.12
0.14
SNP effect on Cardiac valvular disease
SNP effect on SNAPIN

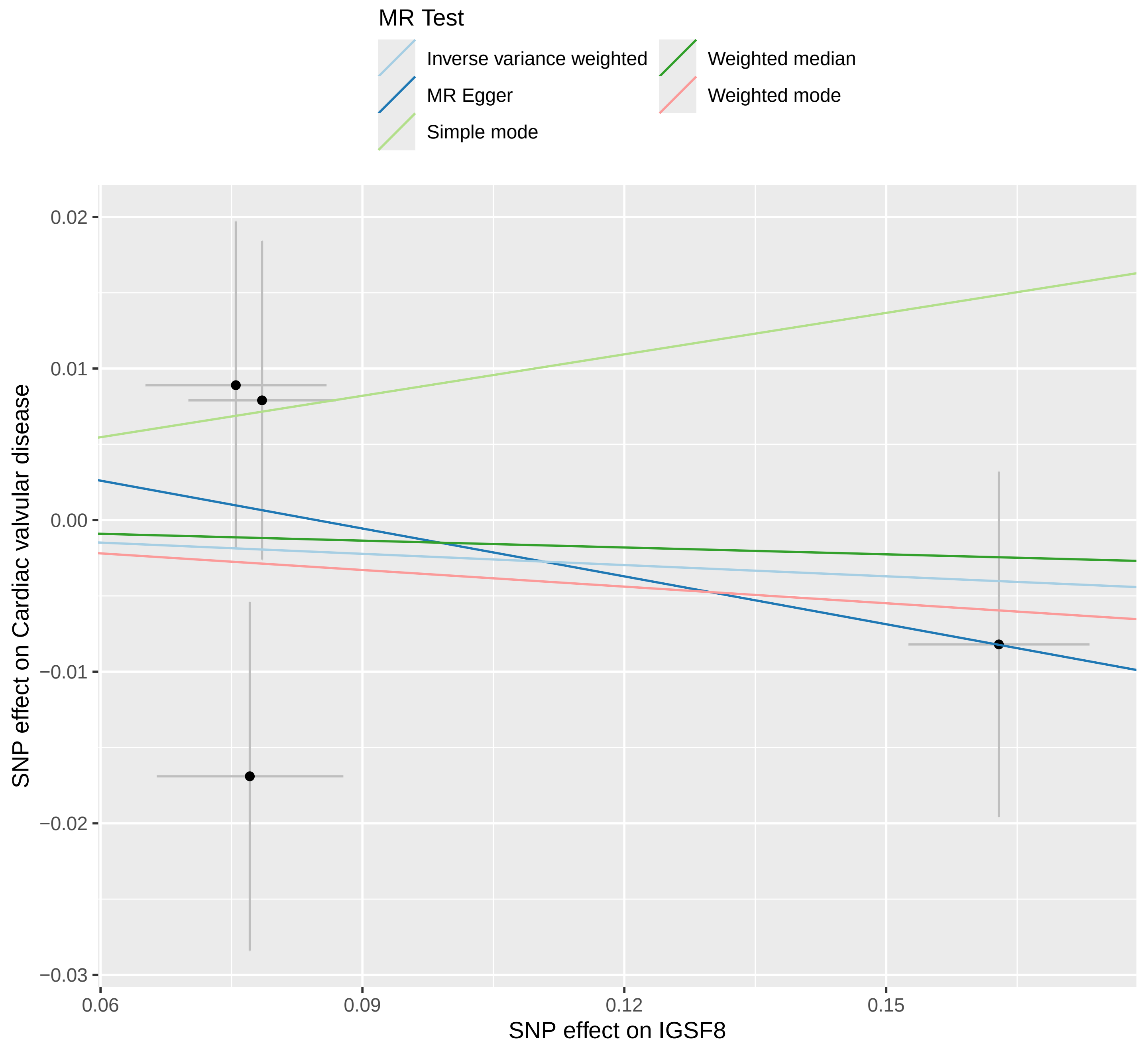
MR Test
Inverse variance weighted
MR Egger
Simple mode
Weighted median
Weighted mode
0.02
0.01
0.00
−0.01
−0.02
−0.03
SNP effect on Cardiac valvular disease
0.06
0.09
0.12
0.15
SNP effect on IGSF8

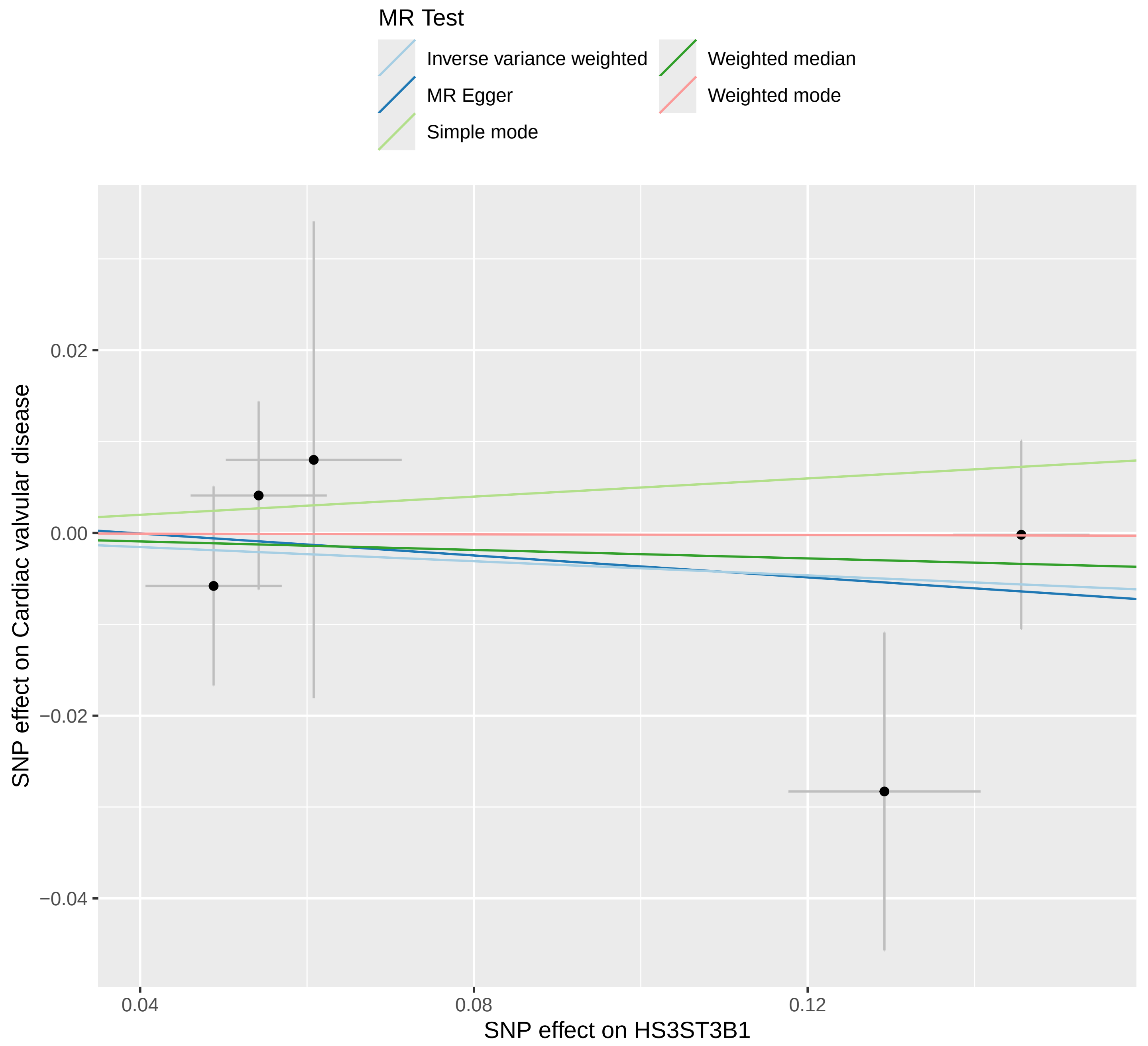
MR Test
Inverse variance weighted
MR Egger
Simple mode
Weighted median
Weighted mode
0.02
0.00
−0.02
−0.04
0.04
0.08
0.12
SNP effect on Cardiac valvular disease
SNP effect on HS3ST3B1

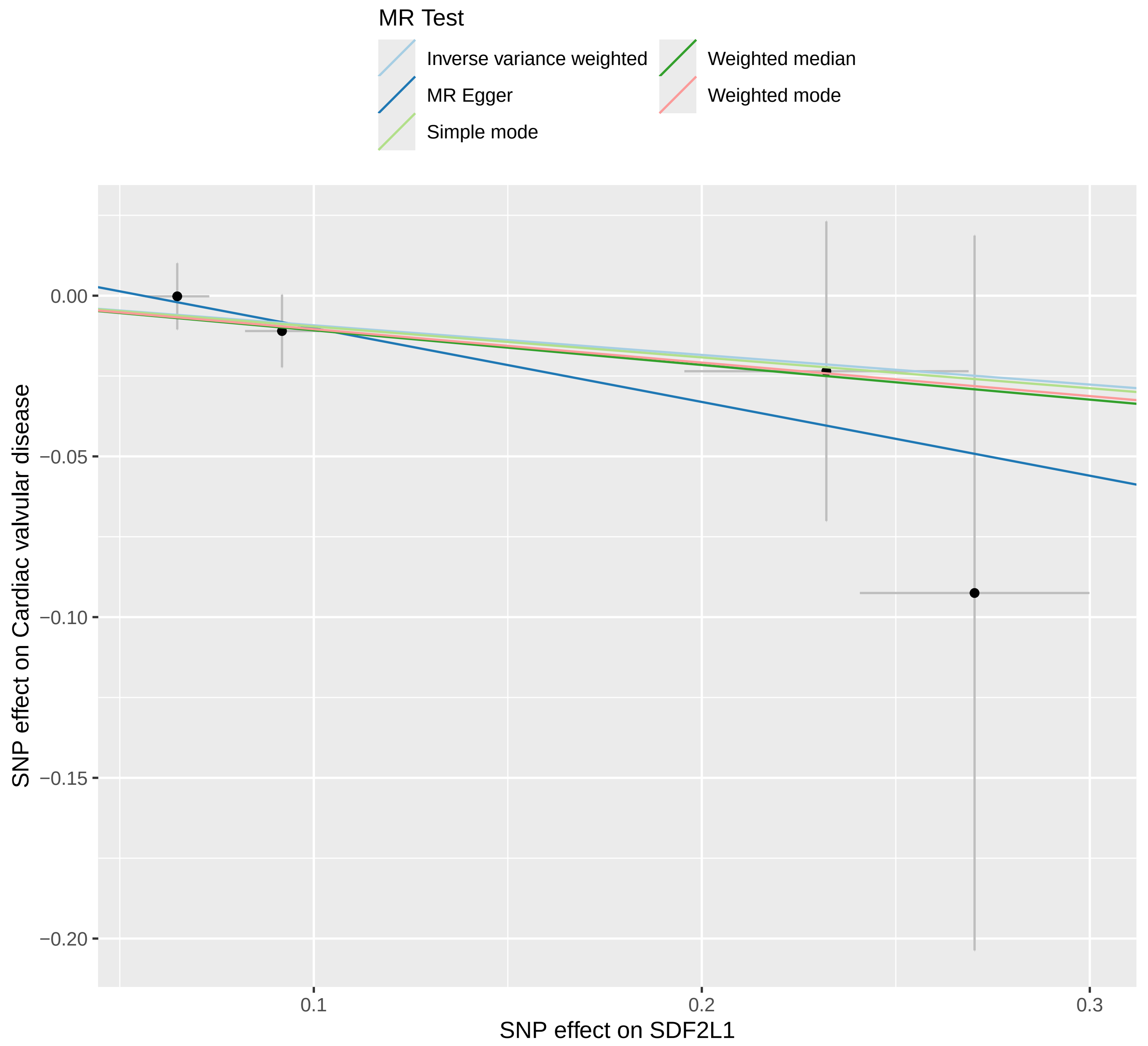
MR Test
Inverse variance weighted
MR Egger
Simple mode
Weighted median
Weighted mode
0.00
−0.05
−0.10
−0.15
−0.20
0.1
0.2
0.3
SNP effect on Cardiac valvular disease
SNP effect on SDF2L1

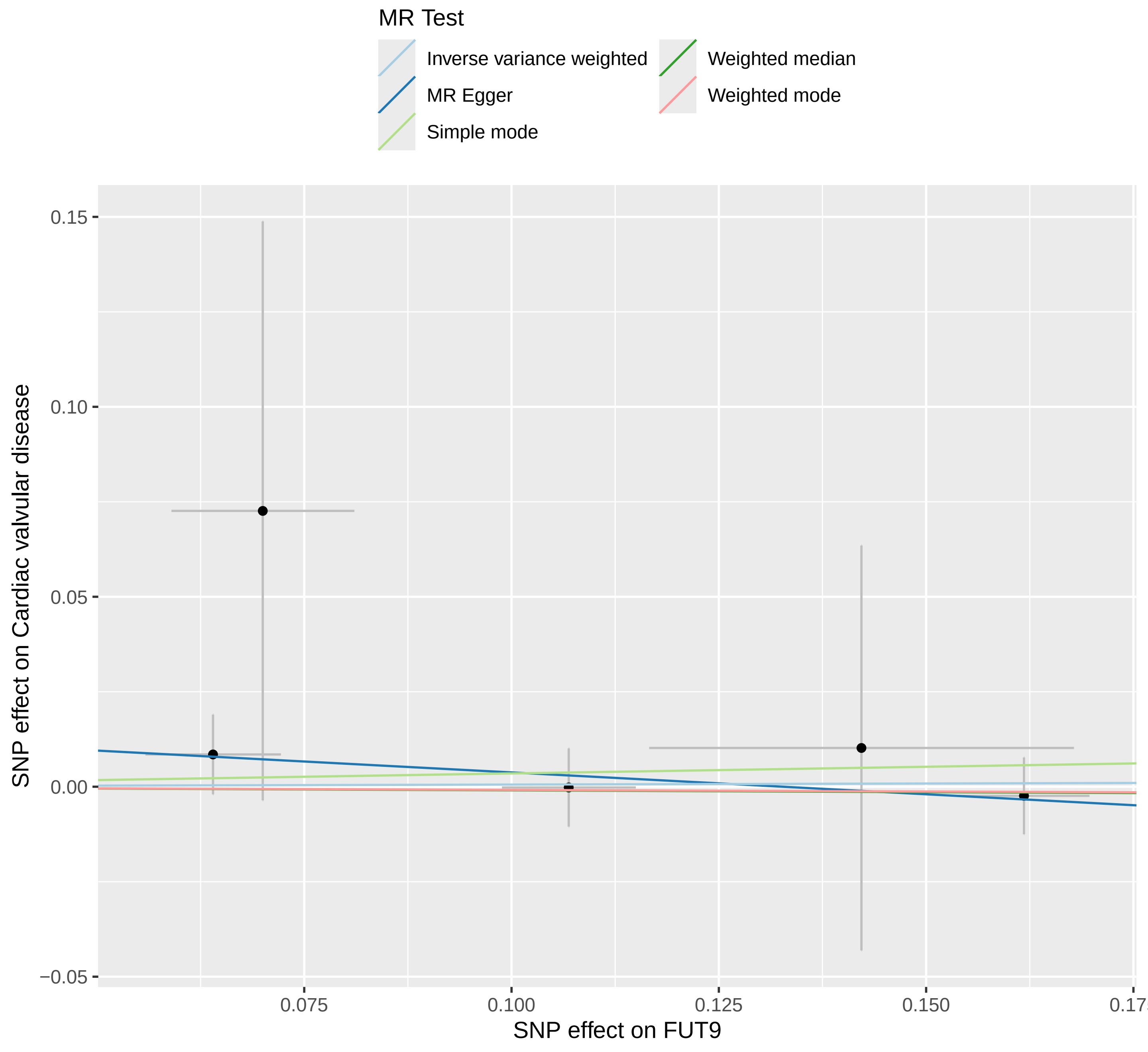
MR Test
Inverse variance weighted
MR Egger
Simple mode
Weighted median
Weighted mode
0.15
0.10
0.05
0.00
−0.05
0.075
0.100
0.125
0.150
SNP effect on Cardiac valvular disease
SNP effect on FUT9

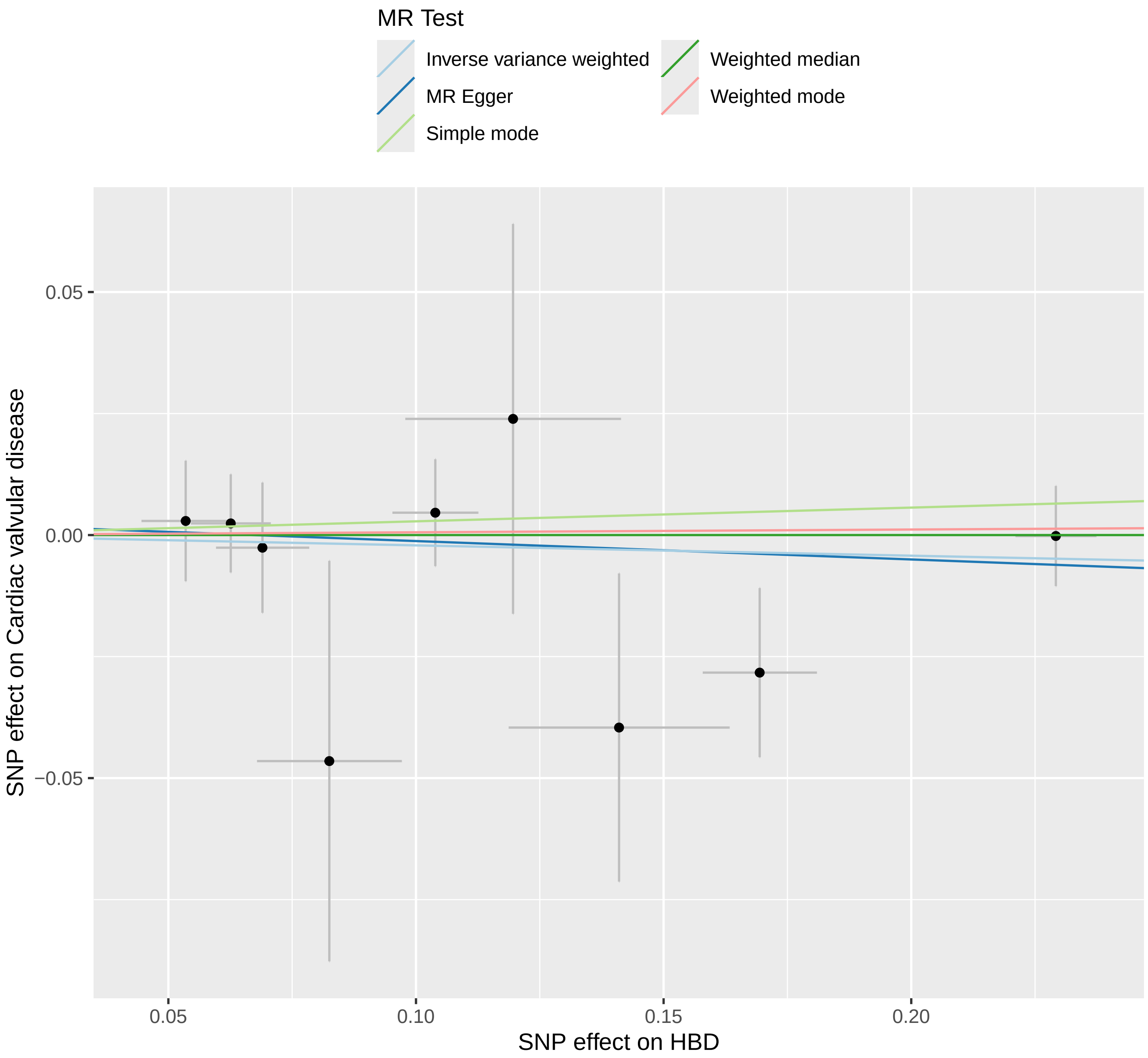
MR Test
Inverse variance weighted
MR Egger
Simple mode
Weighted median
Weighted mode
0.05
0.00
−0.05
SNP effect on Cardiac valvular disease
0.05
0.10
0.15
0.20
SNP effect on HBD

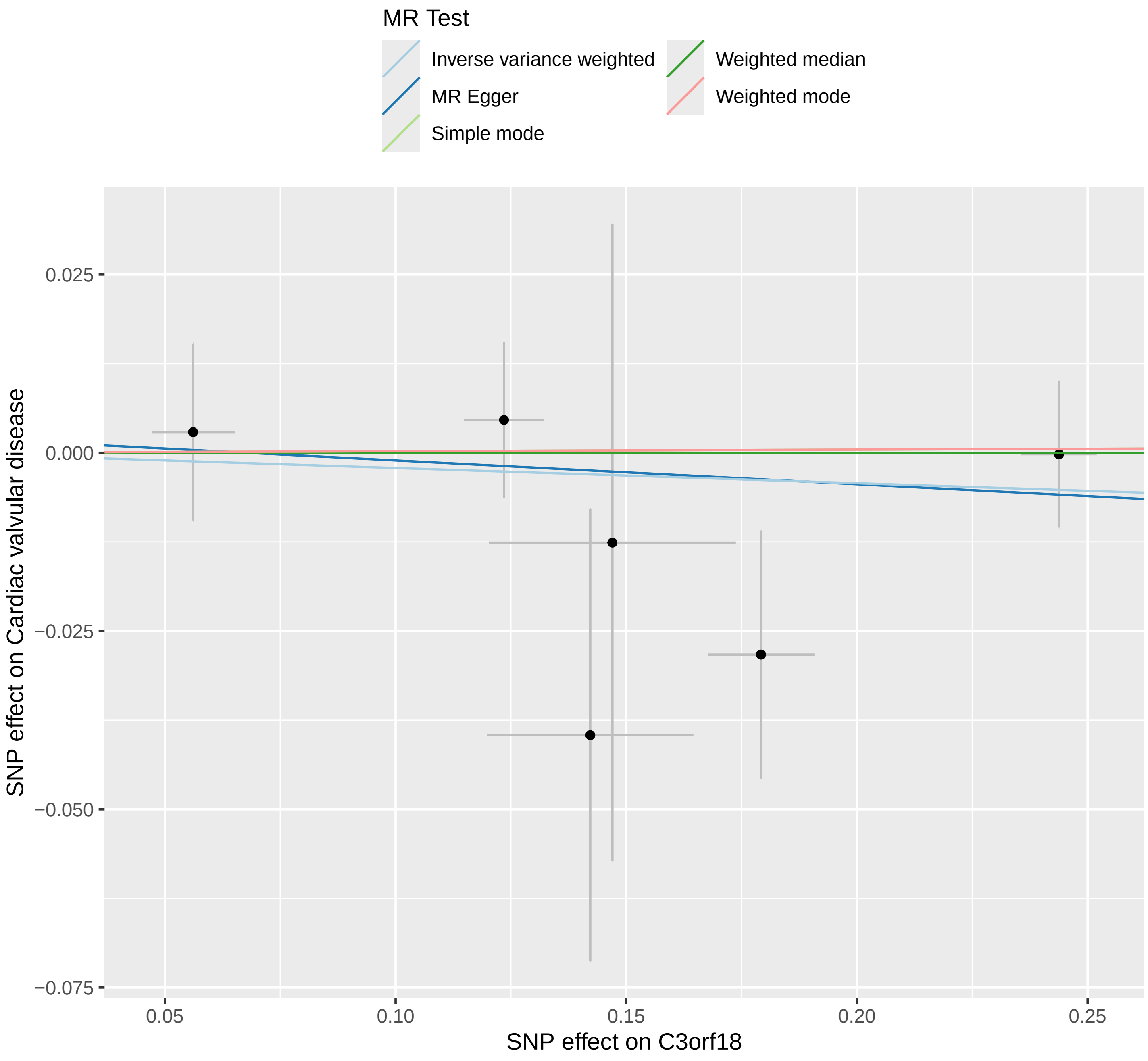
MR Test
Inverse variance weighted
MR Egger
Simple mode
Weighted median
Weighted mode
0.025
0.000
−0.025
−0.050
−0.075
0.05
0.10
0.15
0.20
0.25
SNP effect on Cardiac valvular disease
SNP effect on C3orf18

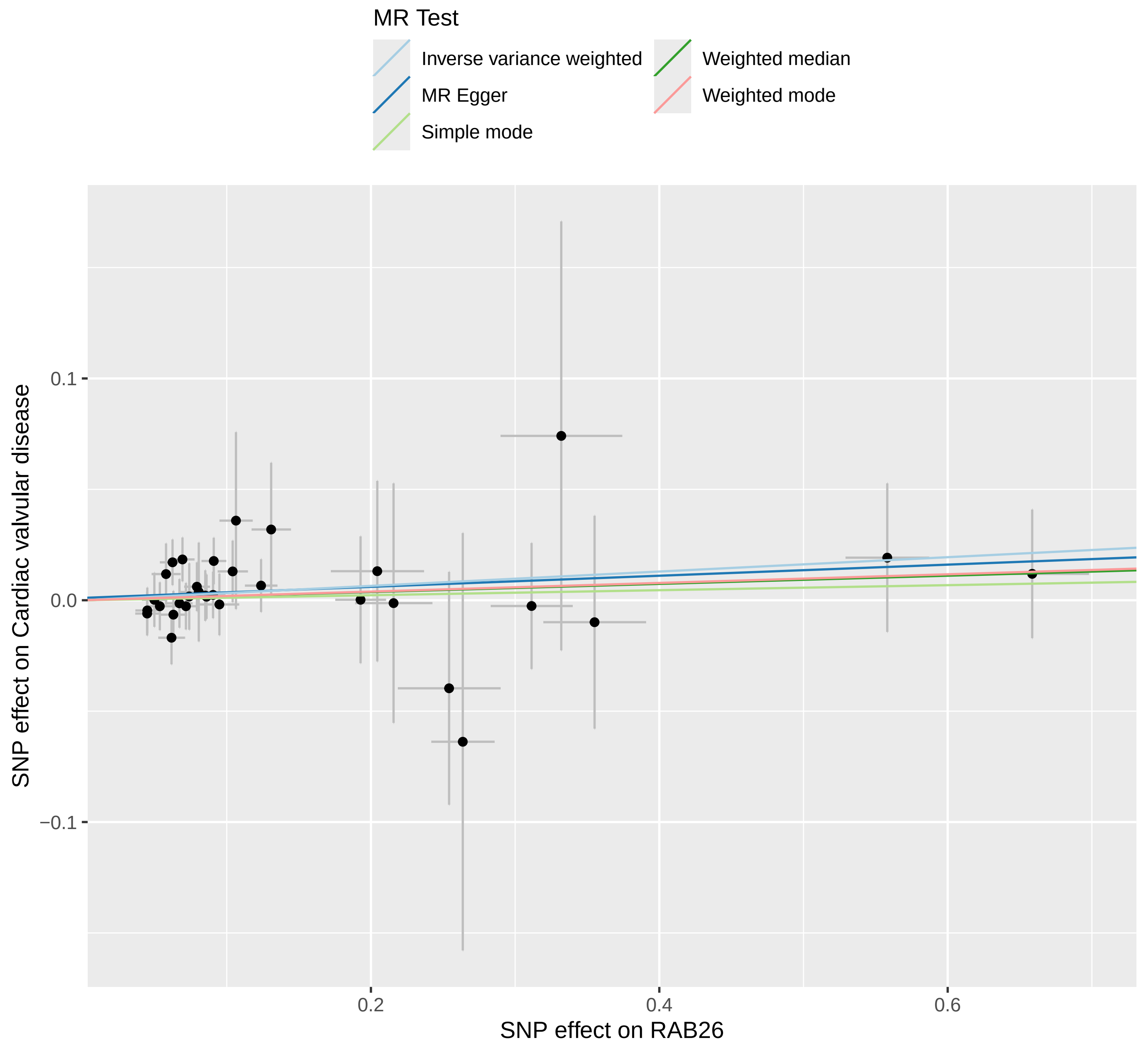

MR Test
Inverse variance weighted
MR Egger
Simple mode
Weighted median
Weighted mode
0.1
0.0
−0.1
0.2
0.4
0.6
SNP effect on Cardiac valvular disease
SNP effect on RAB26

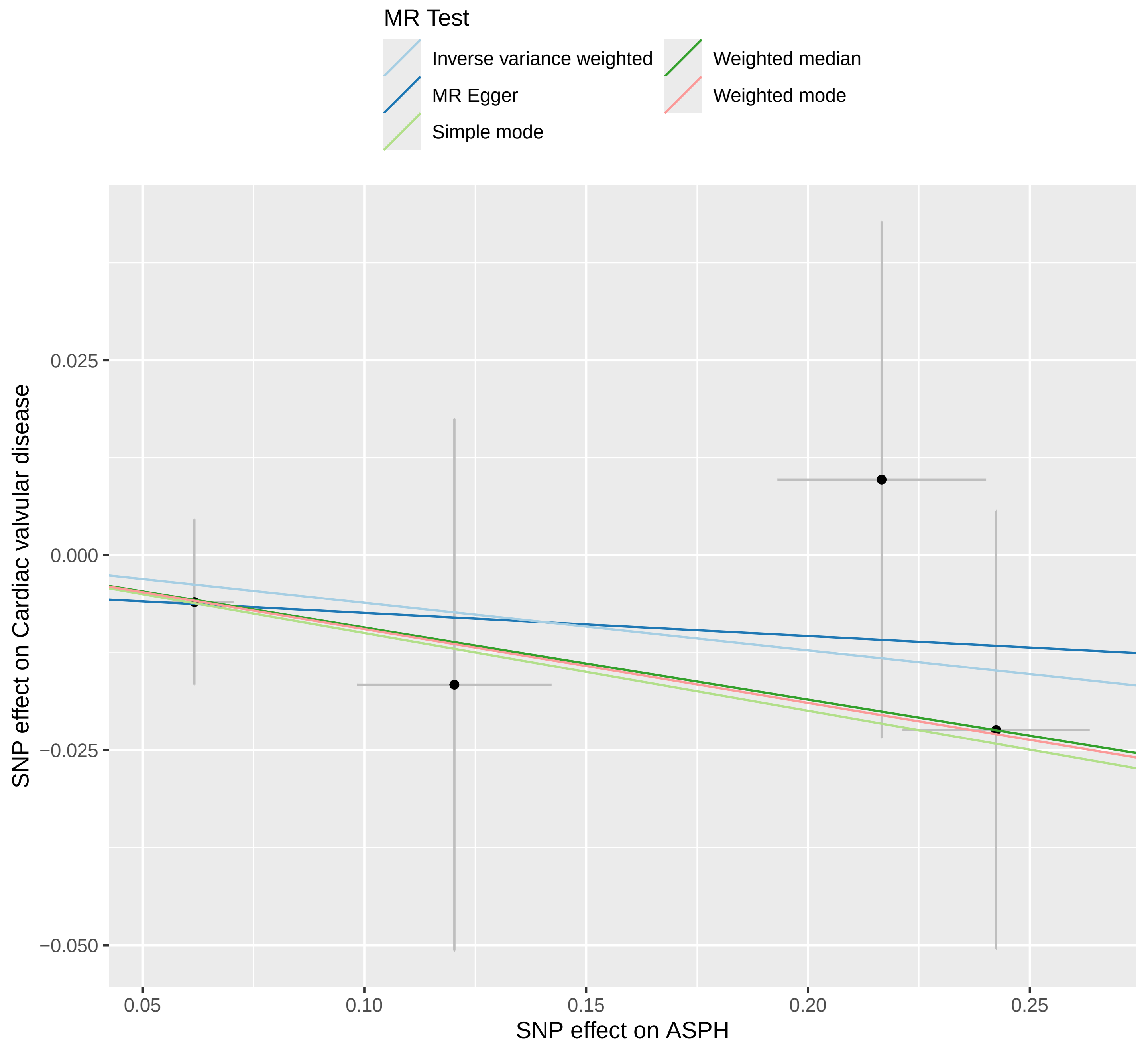
MR Test
Inverse variance weighted
MR Egger
Simple mode
Weighted median
Weighted mode
0.025
0.000
−0.025
−0.050
0.05
0.10
0.15
0.20
0.25
SNP effect on ASPH
SNP effect on Cardiac valvular disease

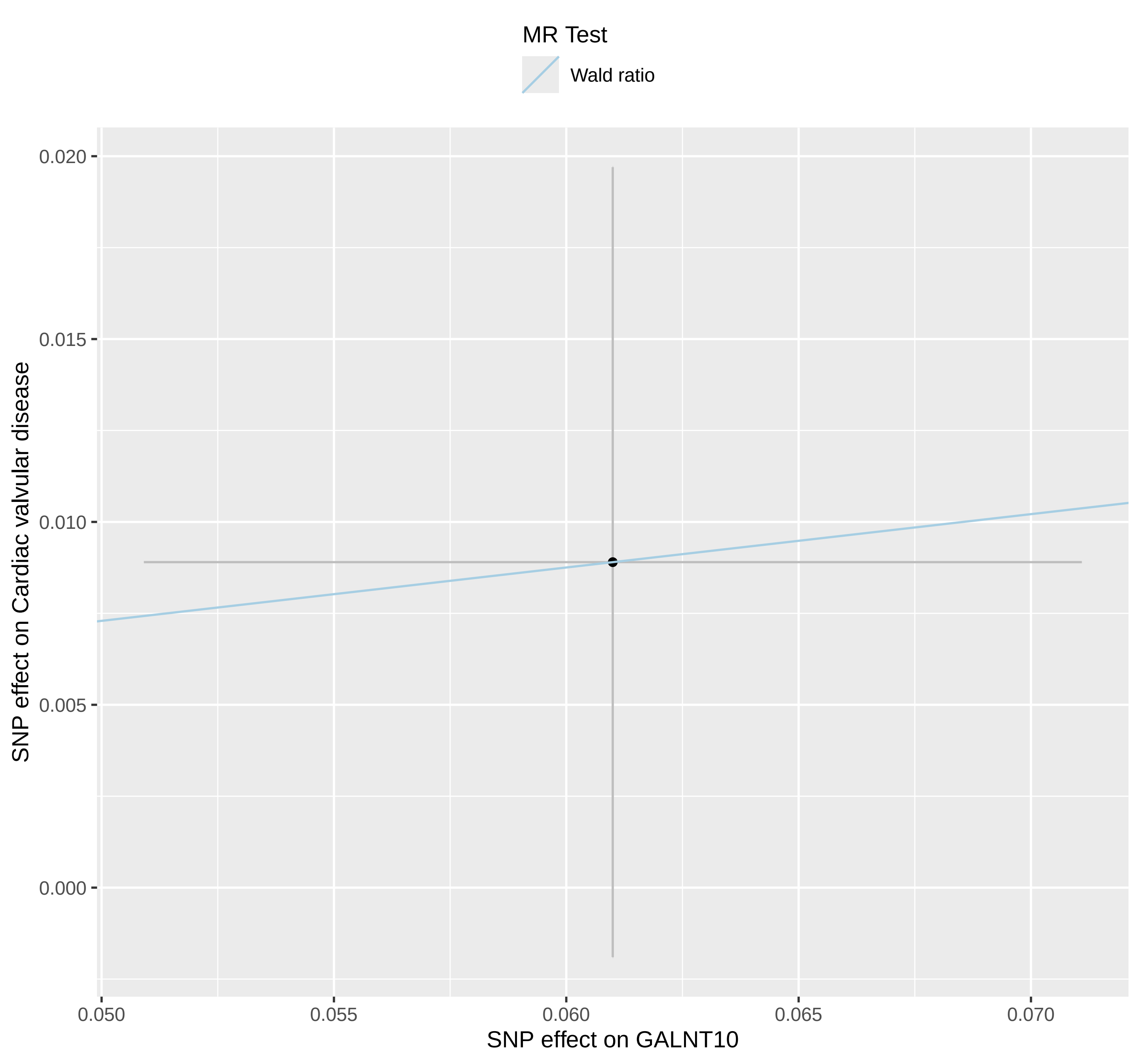
MR Test
Wald ratio
0.020
0.015
0.010
0.005
0.000
0.050
0.055
0.060
0.065
0.070
SNP effect on Cardiac valvular disease
SNP effect on GALNT10

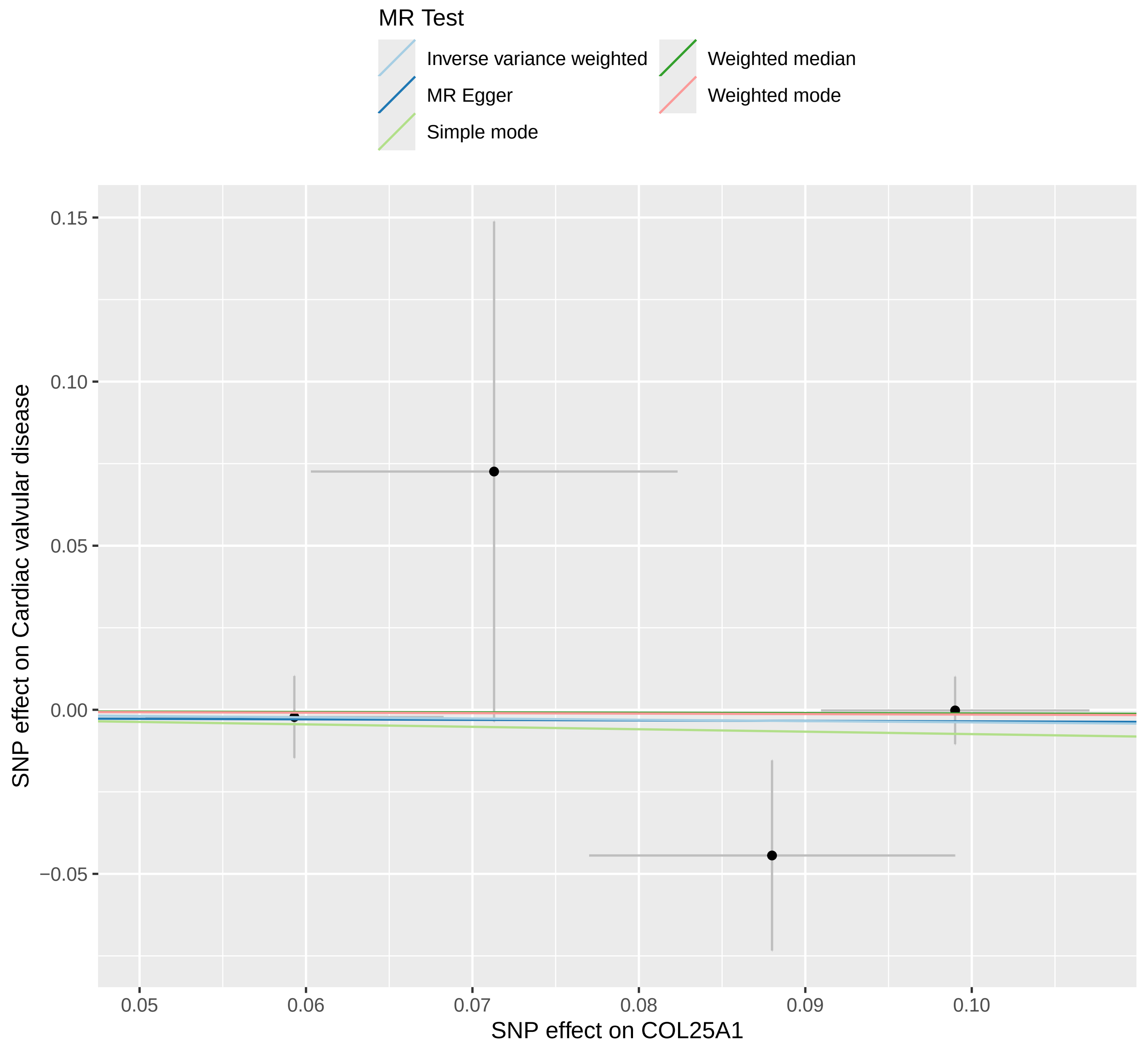
MR Test
Inverse variance weighted
MR Egger
Simple mode
Weighted median
Weighted mode
0.15
0.10
0.05
0.00
−0.05
SNP effect on Cardiac valvular disease
0.05
0.06
0.07
0.08
0.09
0.10
SNP effect on COL25A1

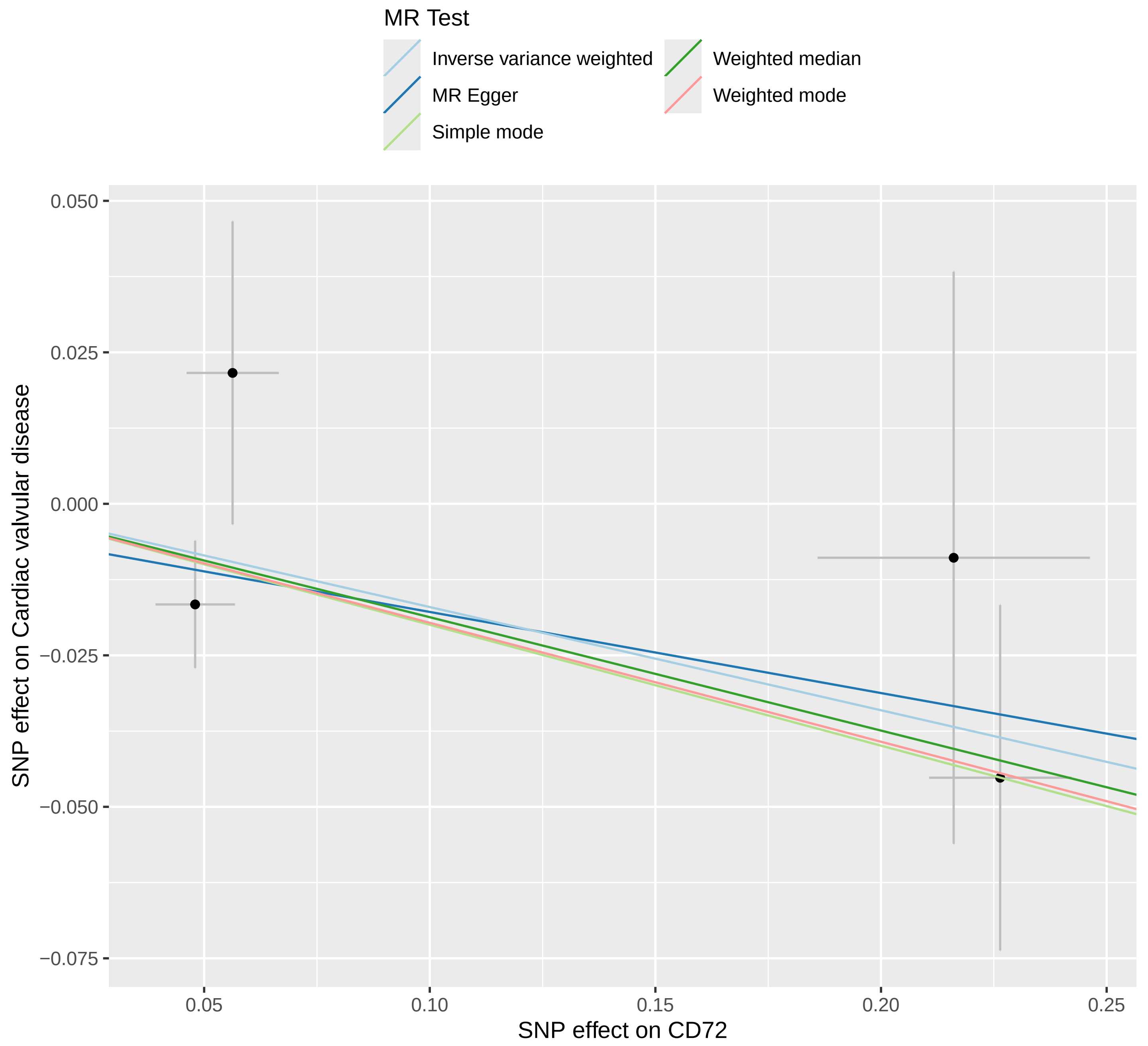
MR Test
Inverse variance weighted
MR Egger
Simple mode
Weighted median
Weighted mode
0.050
0.025
0.000
−0.025
−0.050
−0.075
SNP effect on Cardiac valvular disease
0.05
0.10
0.15
0.20
0.25
SNP effect on CD72

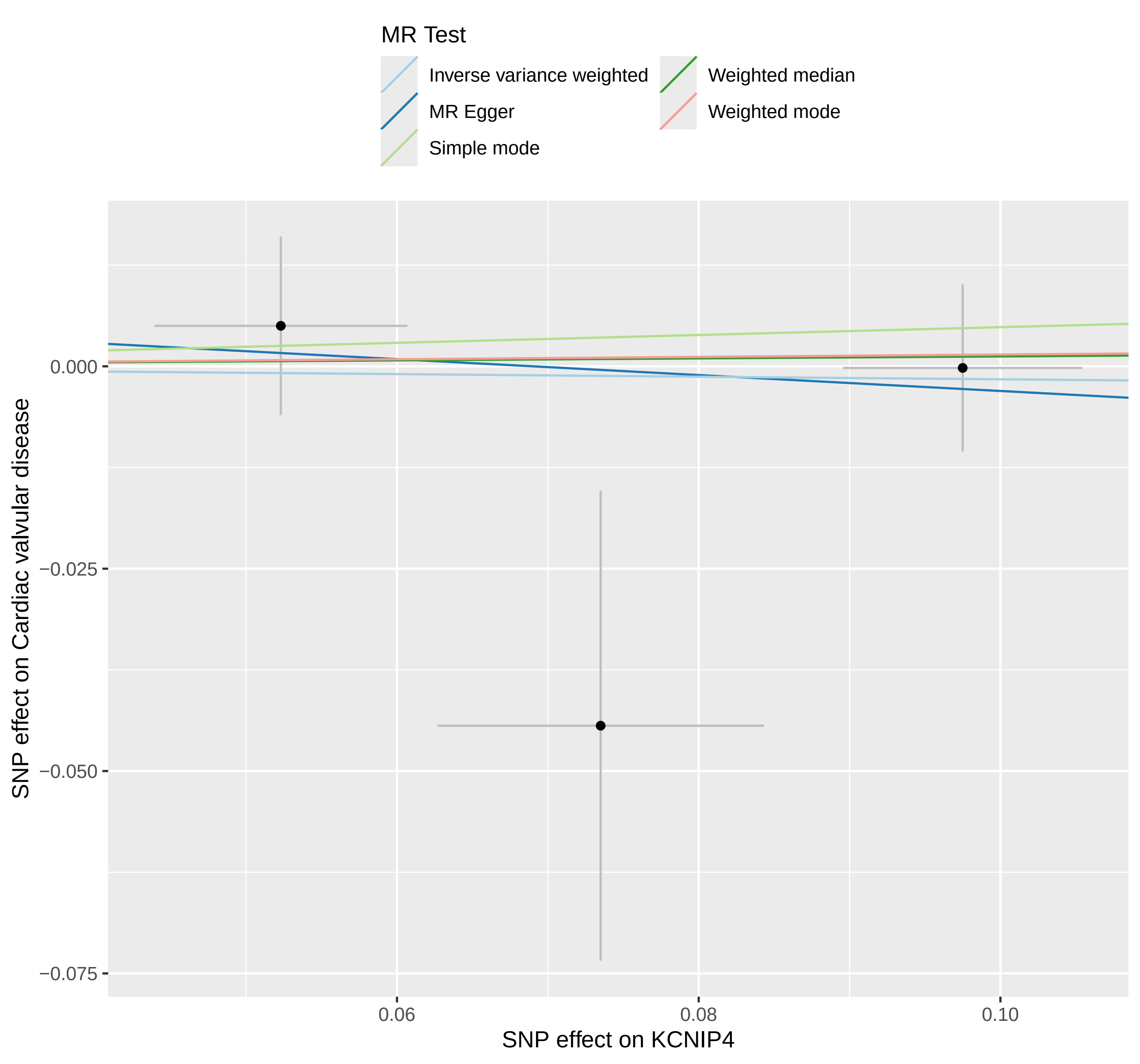
MR Test
Inverse variance weighted
MR Egger
Simple mode
Weighted median
Weighted mode
0.000
−0.025
−0.050
−0.075
0.06
0.08
0.10
SNP effect on KCNIP4
SNP effect on Cardiac valvular disease

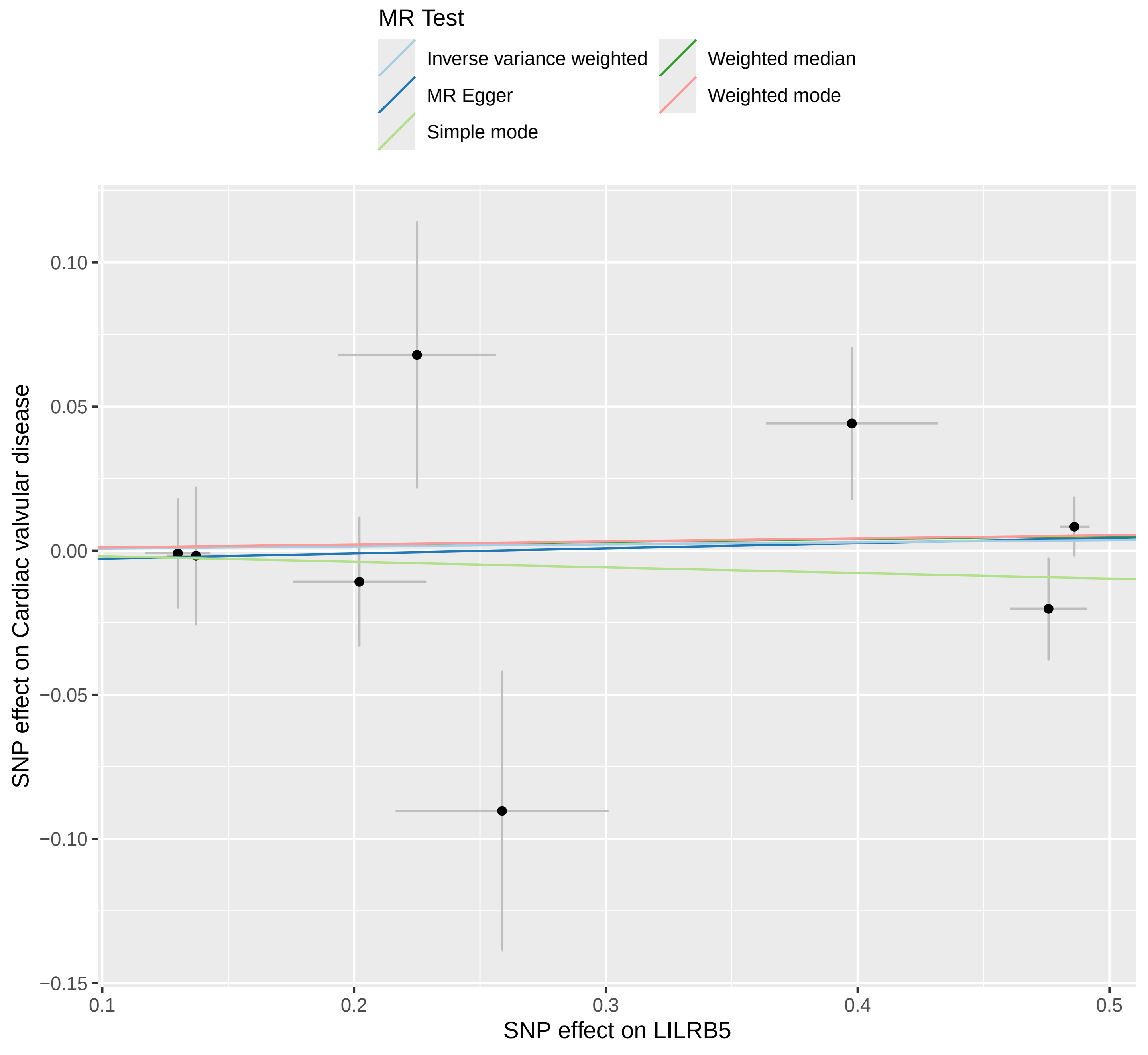

MR Test
Inverse variance weighted
MR Egger
Simple mode
Weighted median
Weighted mode
0.10
0.05
0.00
−0.05
−0.10
−0.15
0.1
0.2
0.3
0.4
0.5
SNP effect on Cardiac valvular disease
SNP effect on LILRB5

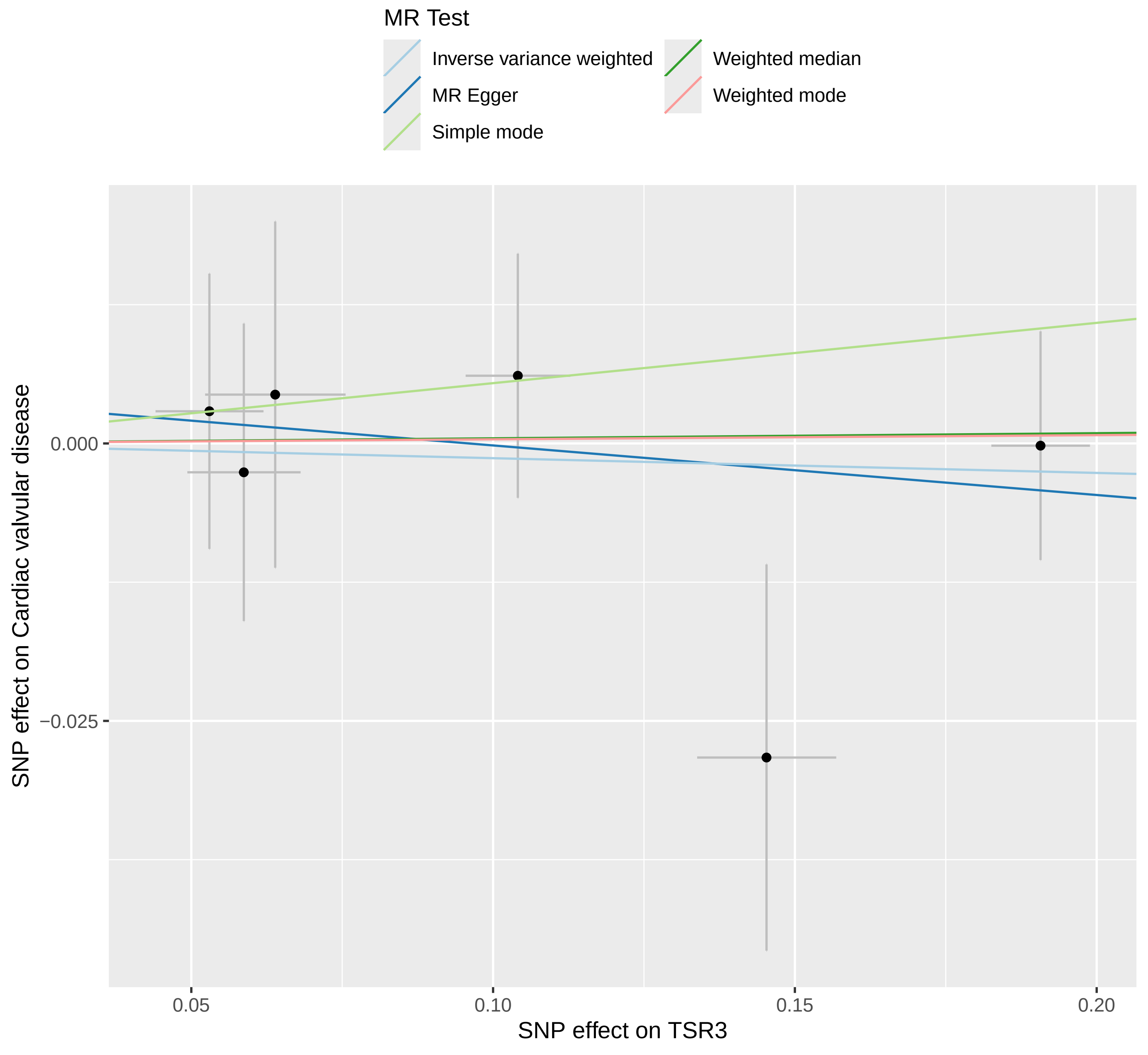
MR Test
Inverse variance weighted
MR Egger
Simple mode
Weighted median
Weighted mode
0.000
−0.025
0.05
0.10
0.15
0.20
SNP effect on TSR3
SNP effect on Cardiac valvular disease

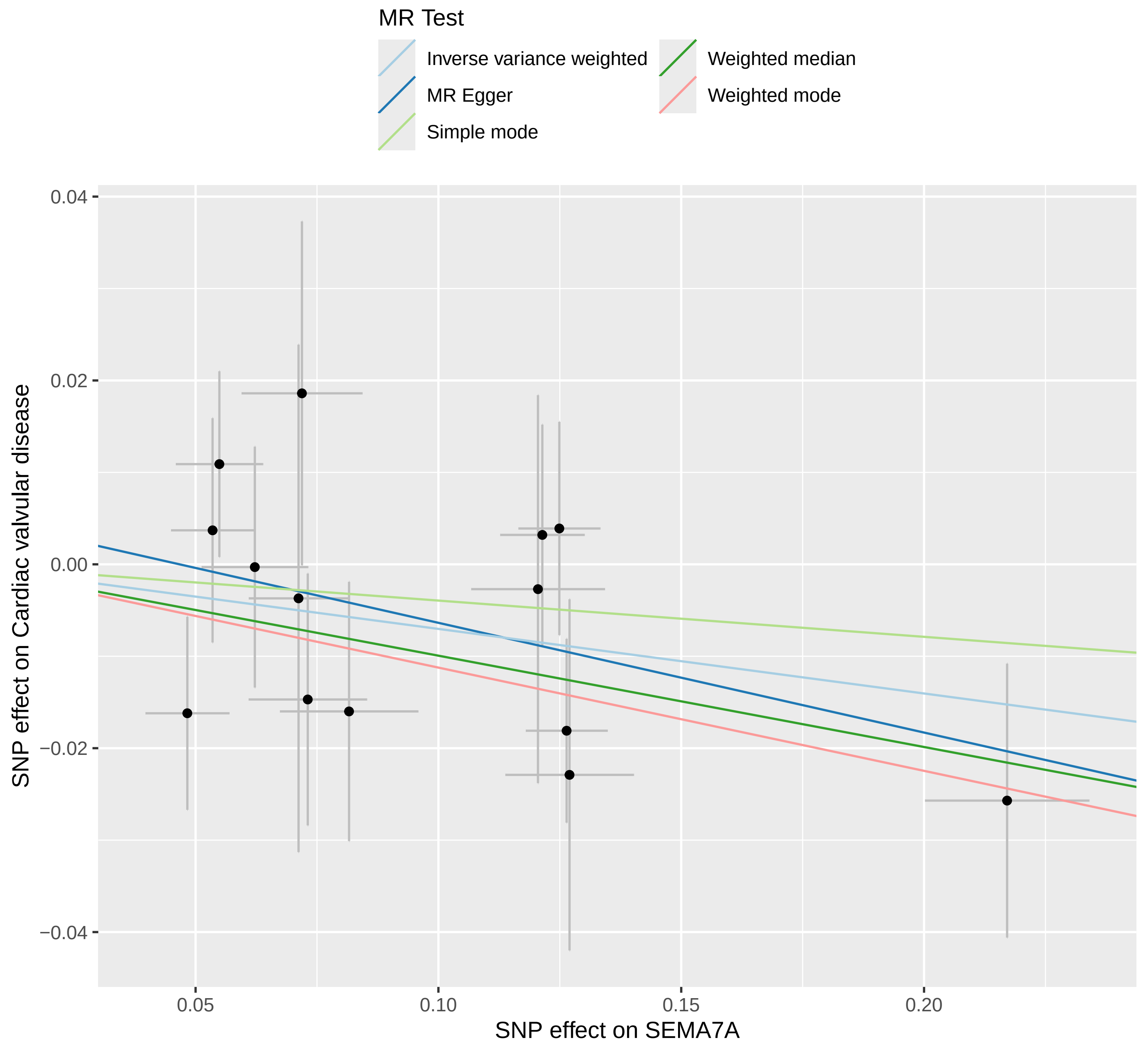

MR Test
Inverse variance weighted
MR Egger
Simple mode
Weighted median
Weighted mode
0.04
0.02
0.00
−0.02
−0.04
SNP effect on Cardiac valvular disease
0.05
0.10
0.15
0.20
SNP effect on SEMA7A

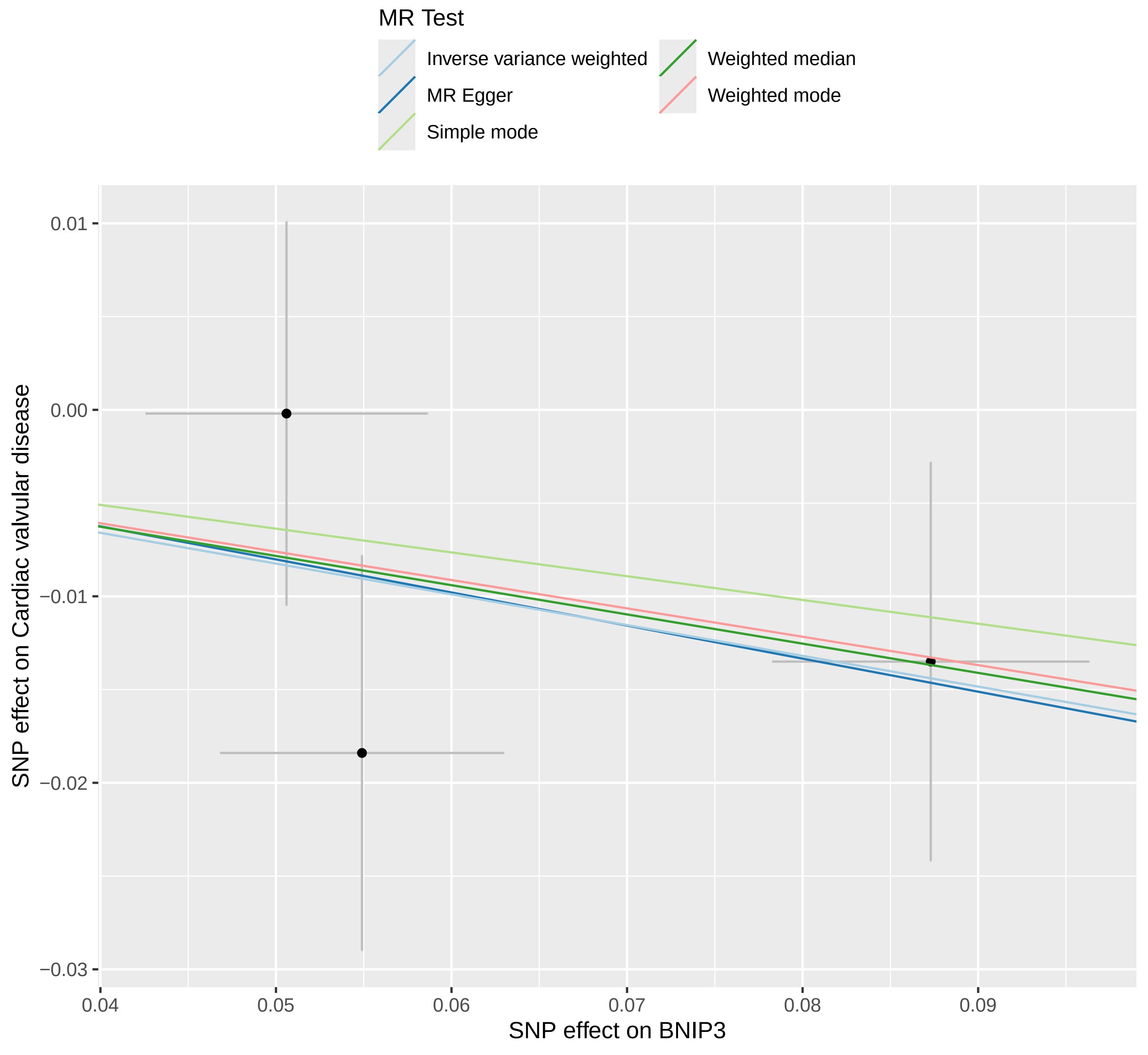

MR Test
Inverse variance weighted
MR Egger
Simple mode
Weighted median
Weighted mode
0.01
0.00
−0.01
−0.02
−0.03
SNP effect on Cardiac valvular disease
0.04
0.05
0.06
0.07
0.08
0.09
SNP effect on BNIP3

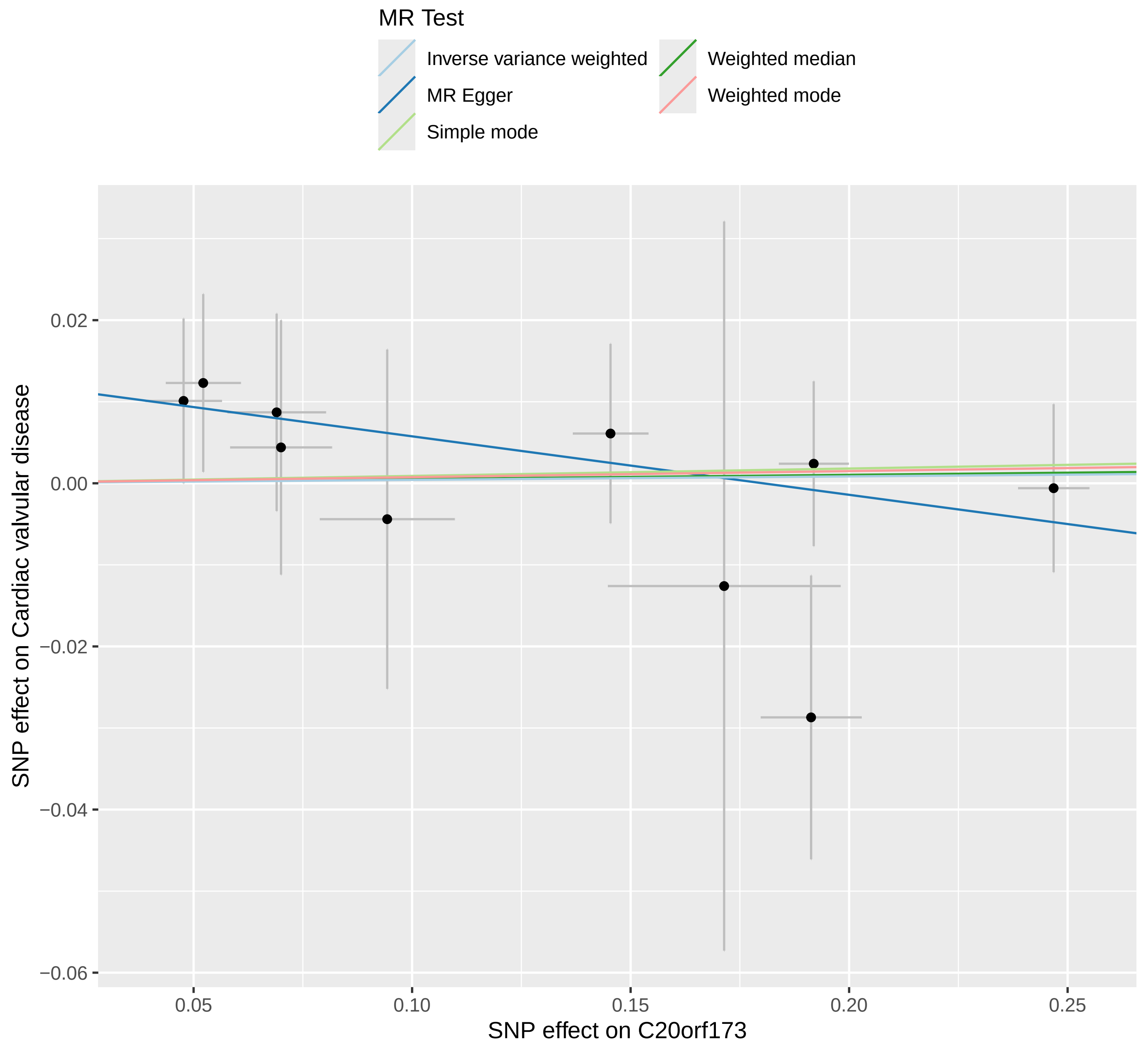

MR Test
Inverse variance weighted
MR Egger
Simple mode
Weighted median
Weighted mode
0.02
0.00
−0.02
−0.04
−0.06
SNP effect on Cardiac valvular disease
0.05
0.10
0.15
0.20
0.25
SNP effect on C20orf173

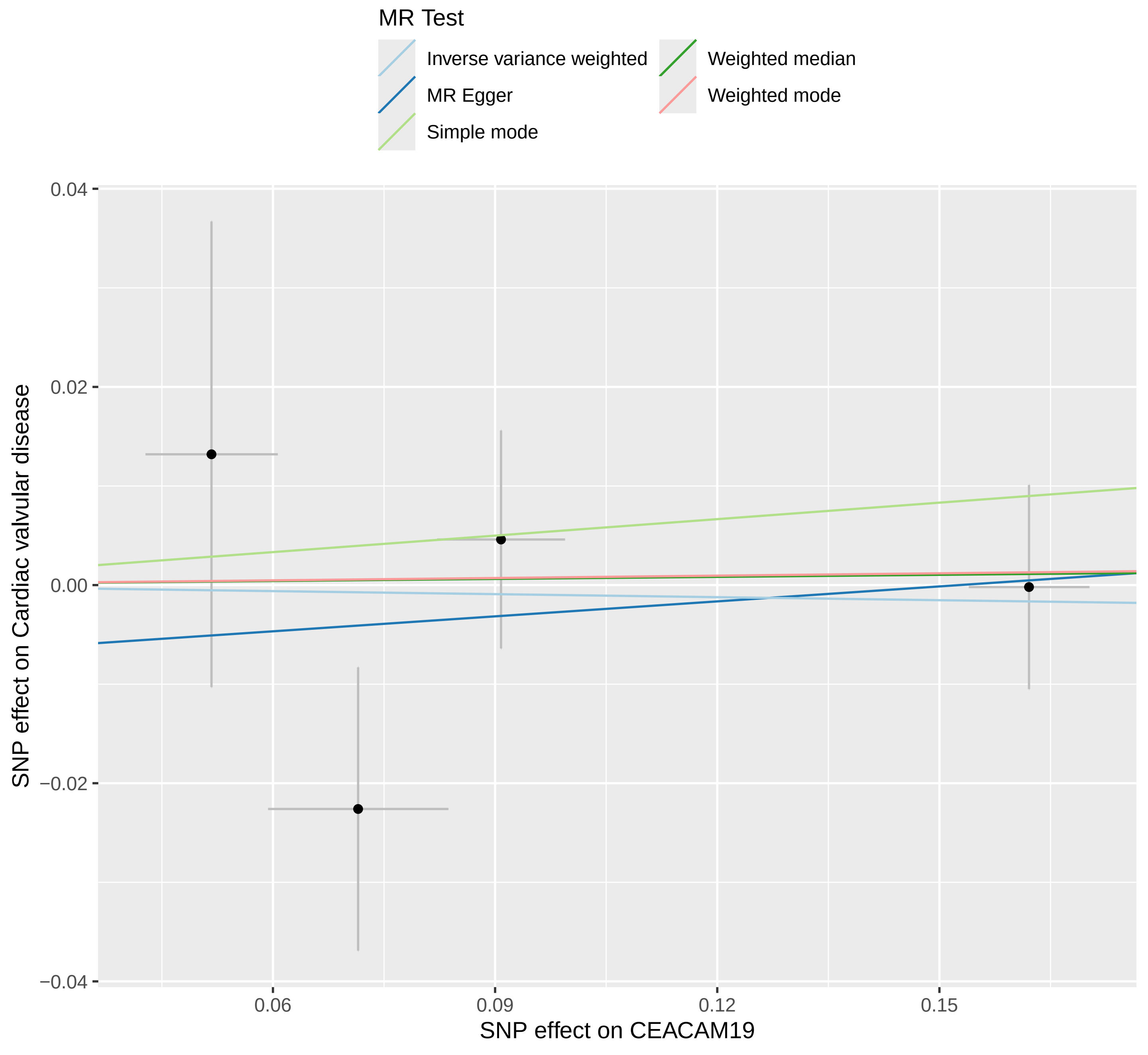
MR Test
Inverse variance weighted
MR Egger
Simple mode
Weighted median
Weighted mode
0.04
0.02
0.00
−0.02
−0.04
0.06
0.09
0.12
0.15
SNP effect on Cardiac valvular disease
SNP effect on CEACAM19

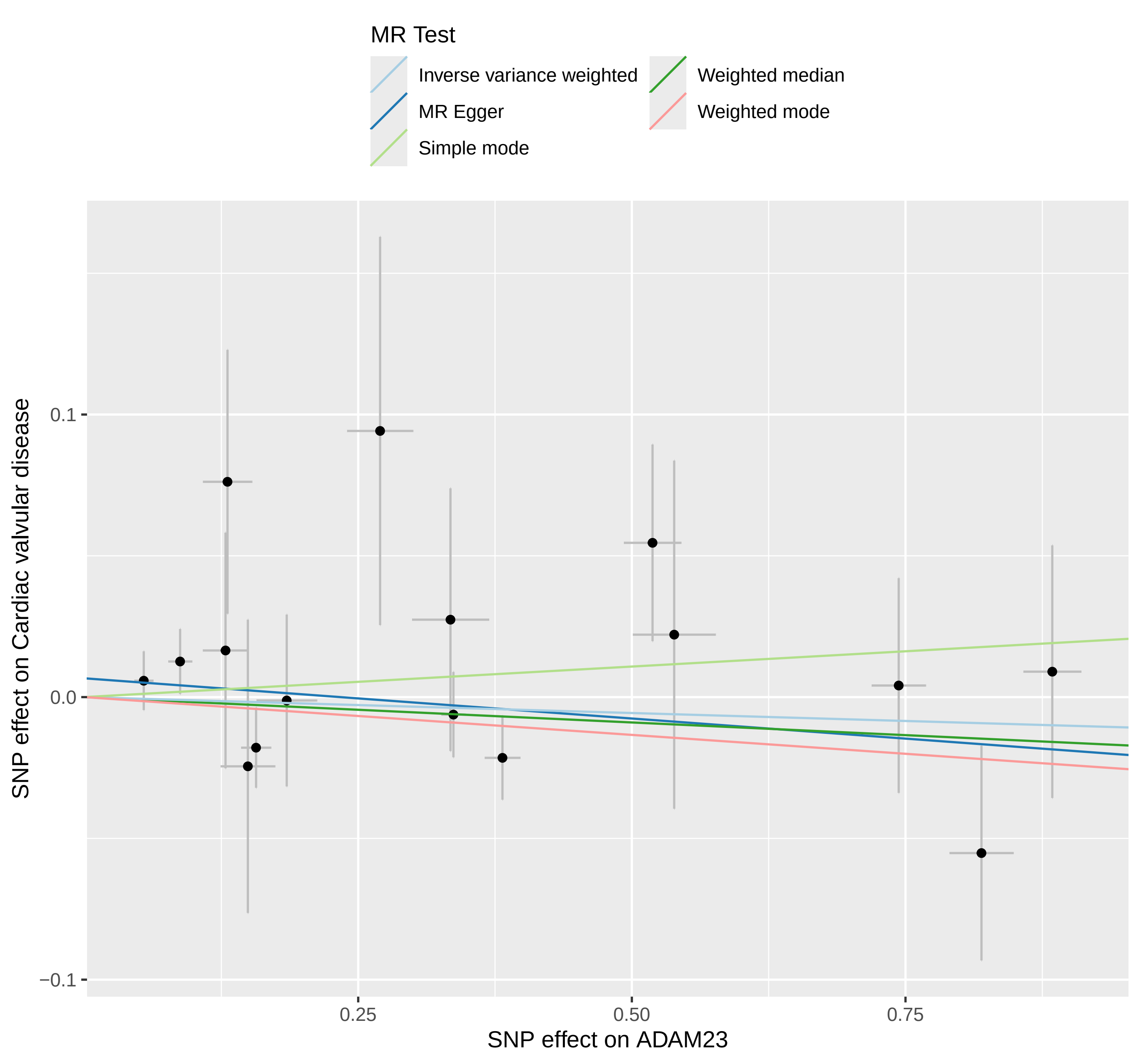
MR Test
Inverse variance weighted
MR Egger
Simple mode
Weighted median
Weighted mode
0.1
0.0
−0.1
0.25
0.50
0.75
SNP effect on ADAM23
SNP effect on Cardiac valvular disease

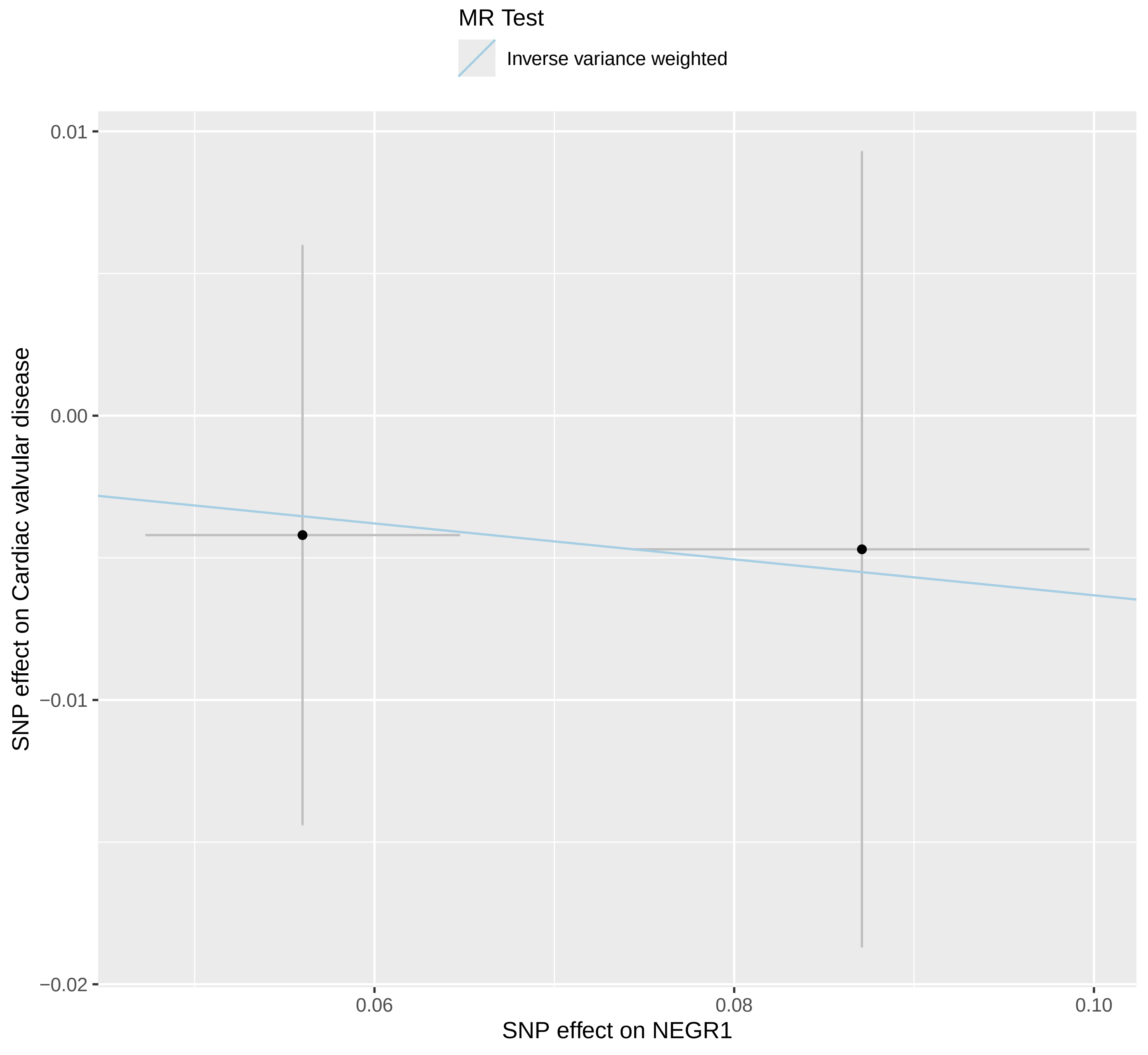
MR Test
Inverse variance weighted
0.01
0.00
−0.01
−0.02
SNP effect on Cardiac valvular disease
0.06
0.08
0.10
SNP effect on NEGR1

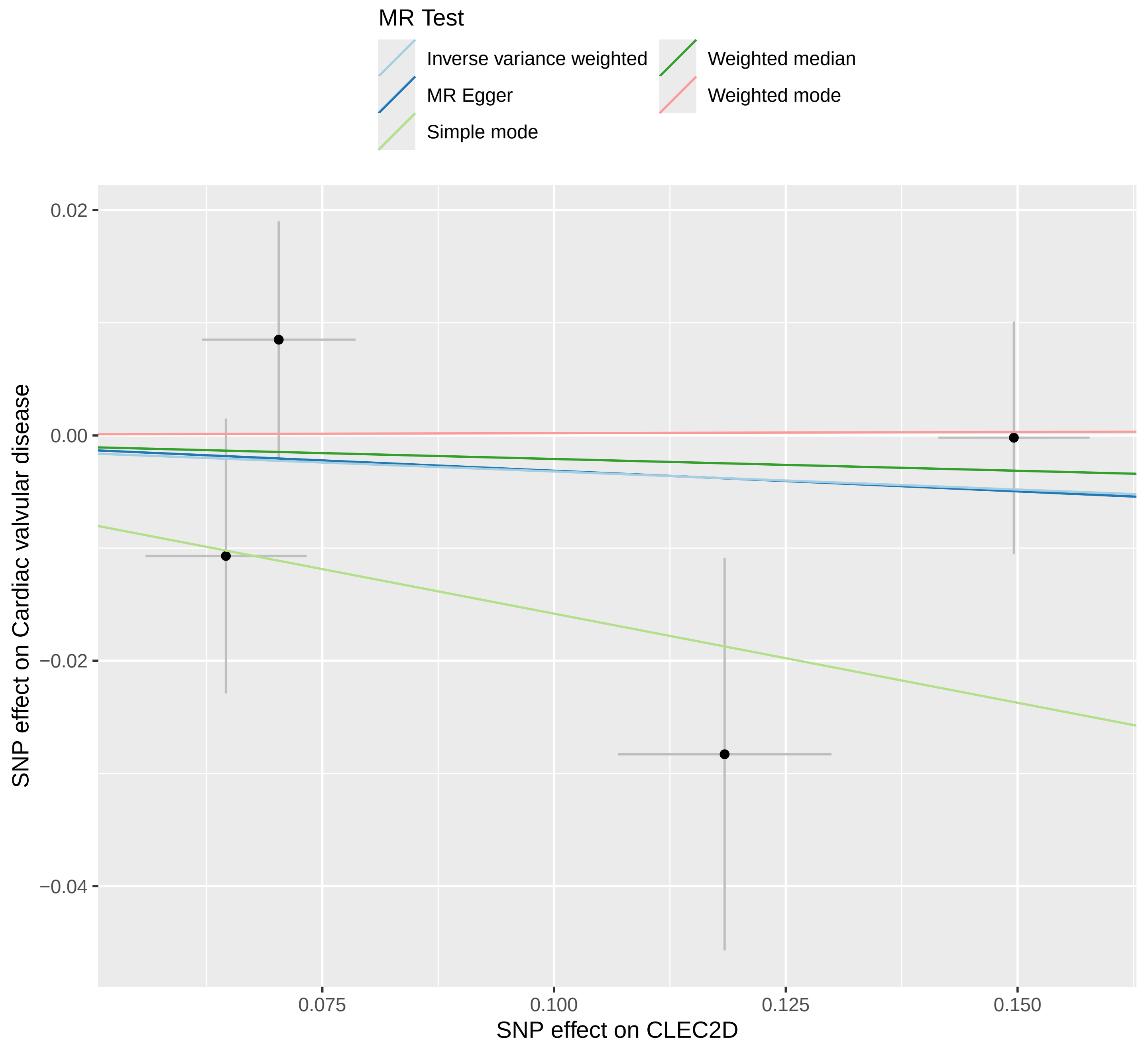

MR Test
Inverse variance weighted
MR Egger
Simple mode
Weighted median
Weighted mode
0.02
0.00
−0.02
−0.04
0.075
0.100
0.125
0.150
SNP effect on Cardiac valvular disease
SNP effect on CLEC2D

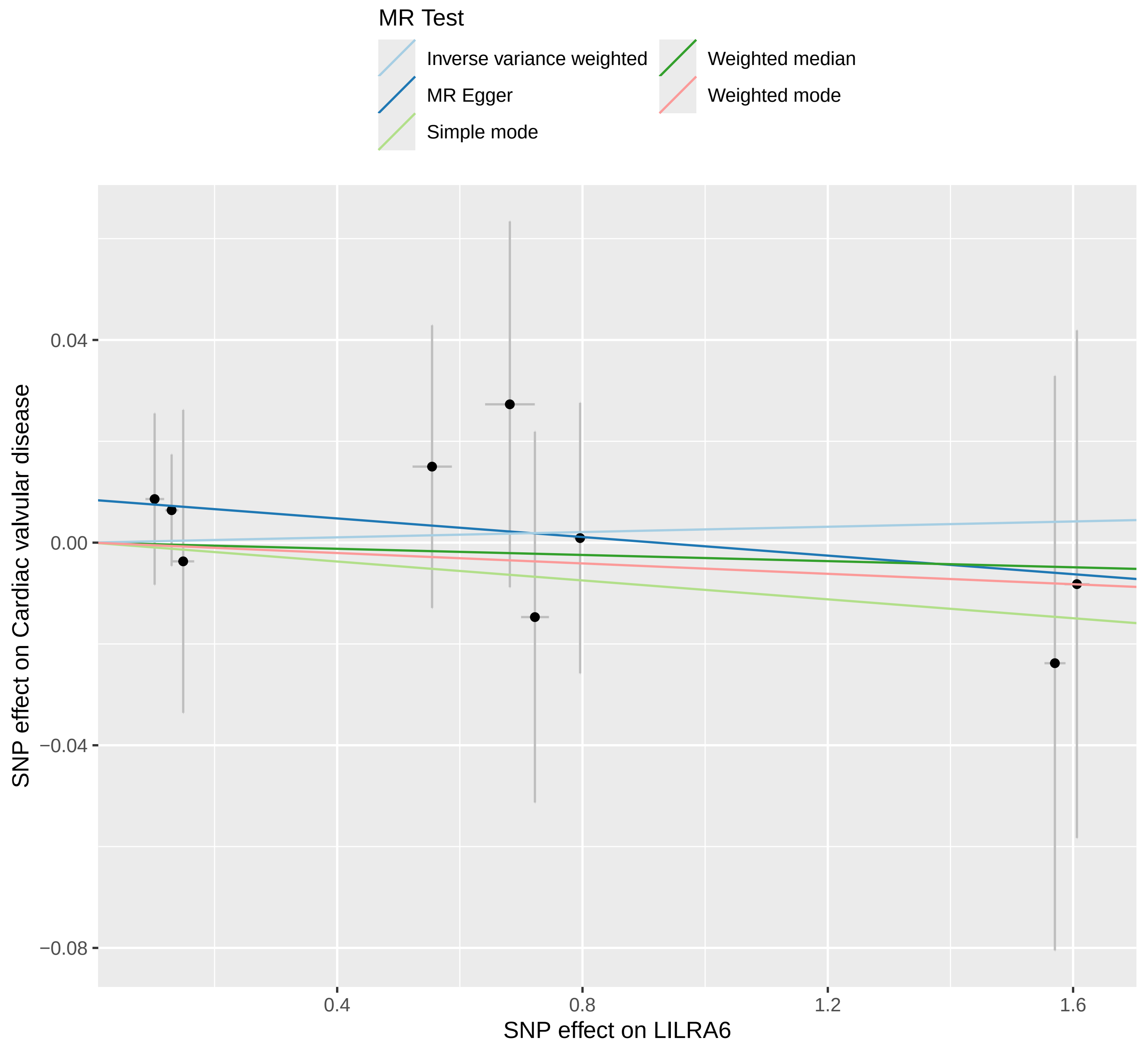
MR Test
Inverse variance weighted
MR Egger
Simple mode
Weighted median
Weighted mode
SNP effect on Cardiac valvular disease
0.04
0.00
−0.04
−0.08
0.4
0.8
1.2
1.6
SNP effect on LILRA6

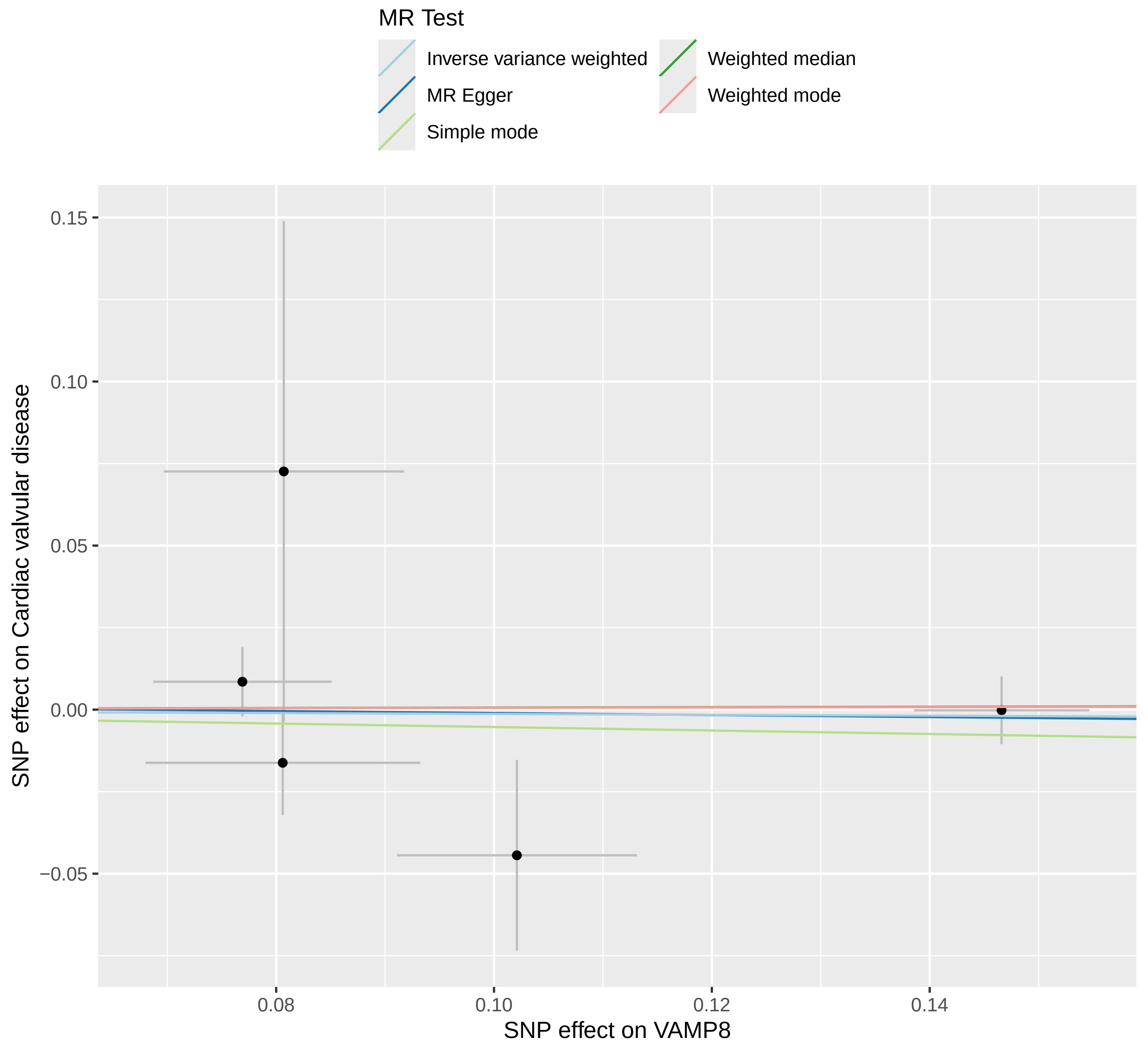

MR Test
Inverse variance weighted
MR Egger
Simple mode
Weighted median
Weighted mode
0.15
0.10
0.05
0.00
−0.05
0.08
0.10
0.12
0.14
SNP effect on VAMP8
SNP effect on Cardiac valvular disease

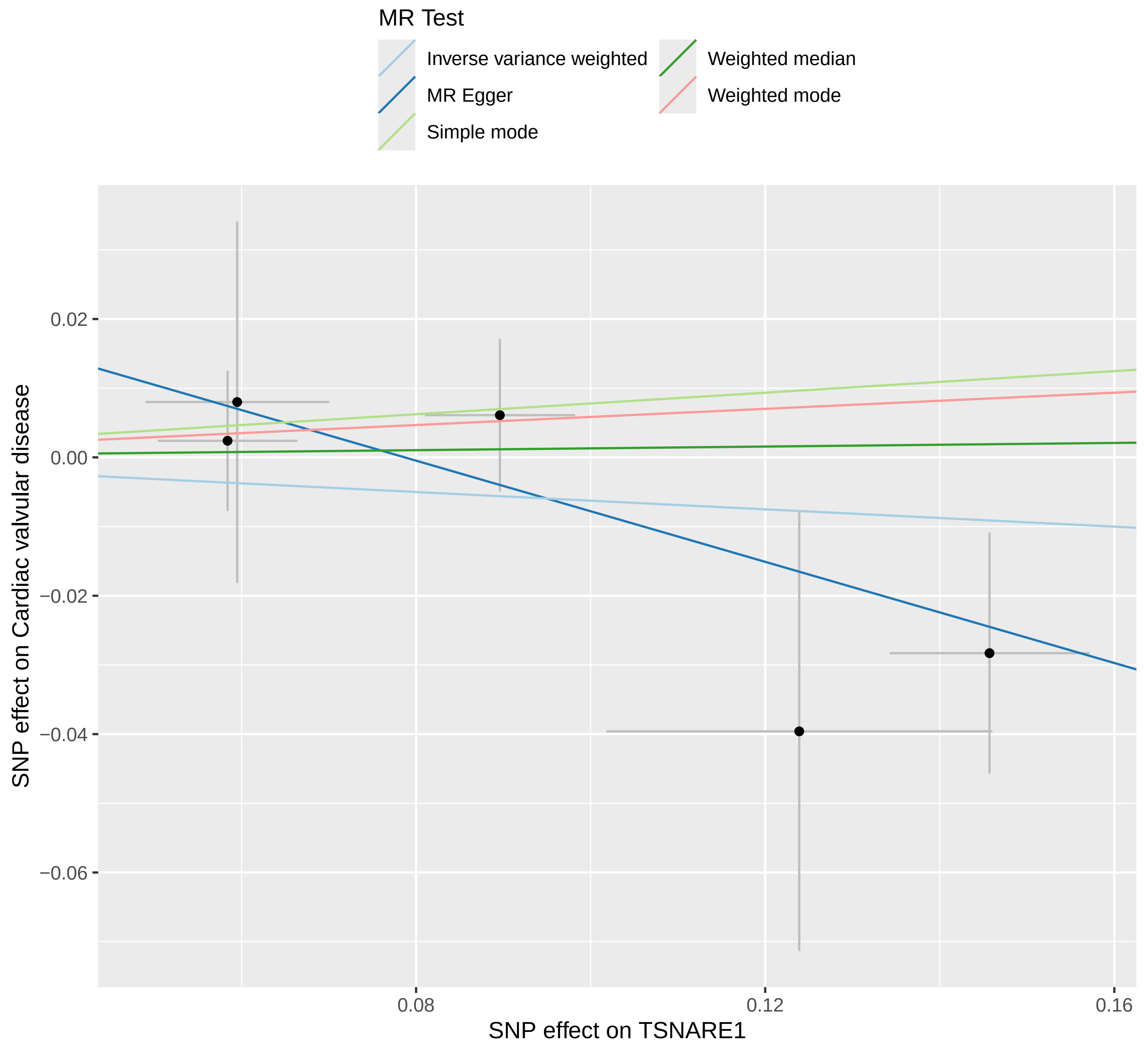
MR Test
Inverse variance weighted
MR Egger
Simple mode
Weighted median
Weighted mode
0.02
0.00
−0.02
−0.04
−0.06
SNP effect on Cardiac valvular disease
0.08
0.12
0.16
SNP effect on TSNARE1

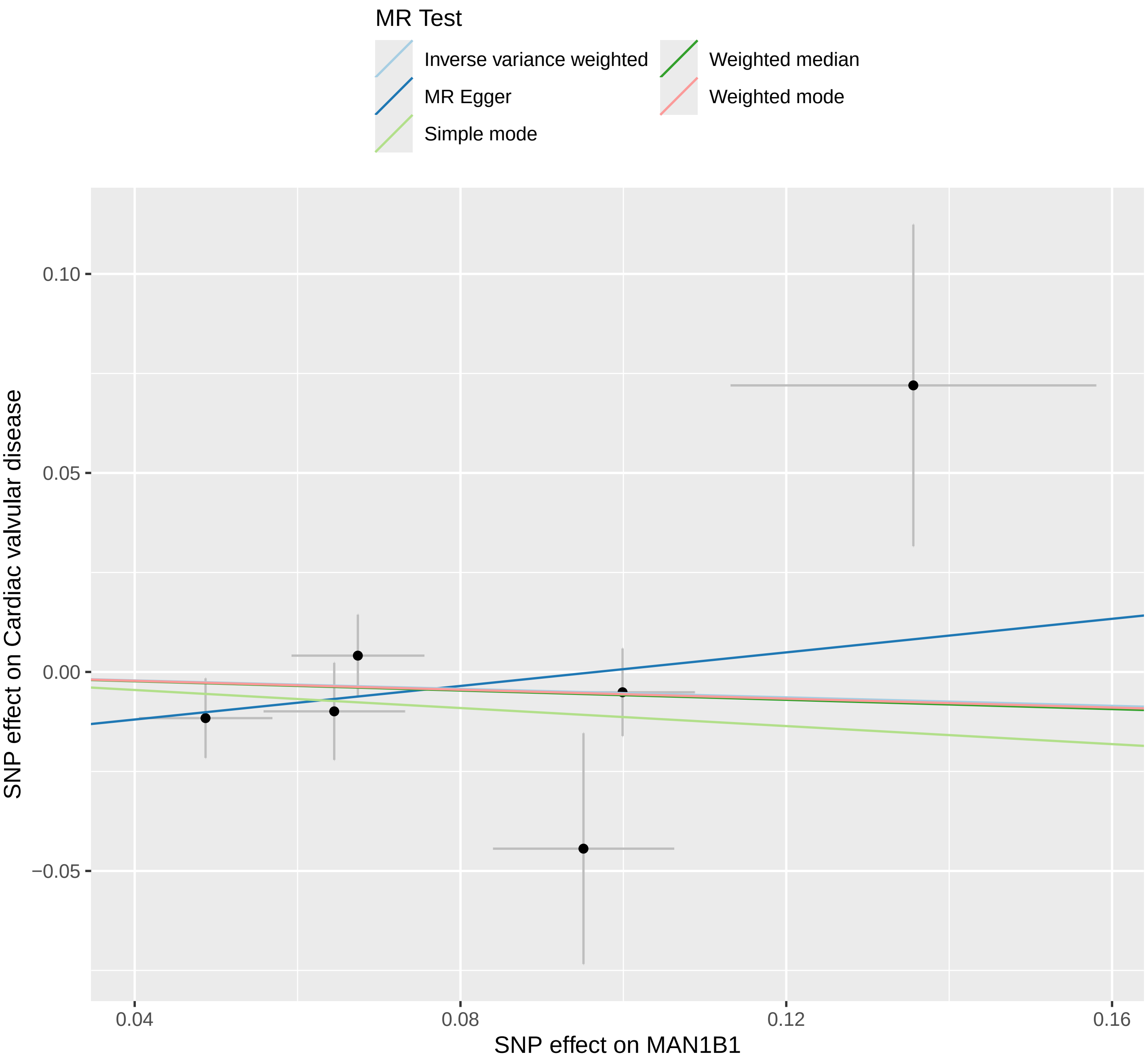

MR Test
Inverse variance weighted
MR Egger
Simple mode
Weighted median
Weighted mode
0.10
0.05
0.00
−0.05
0.04
0.08
0.12
0.16
SNP effect on Cardiac valvular disease
SNP effect on MAN1B1

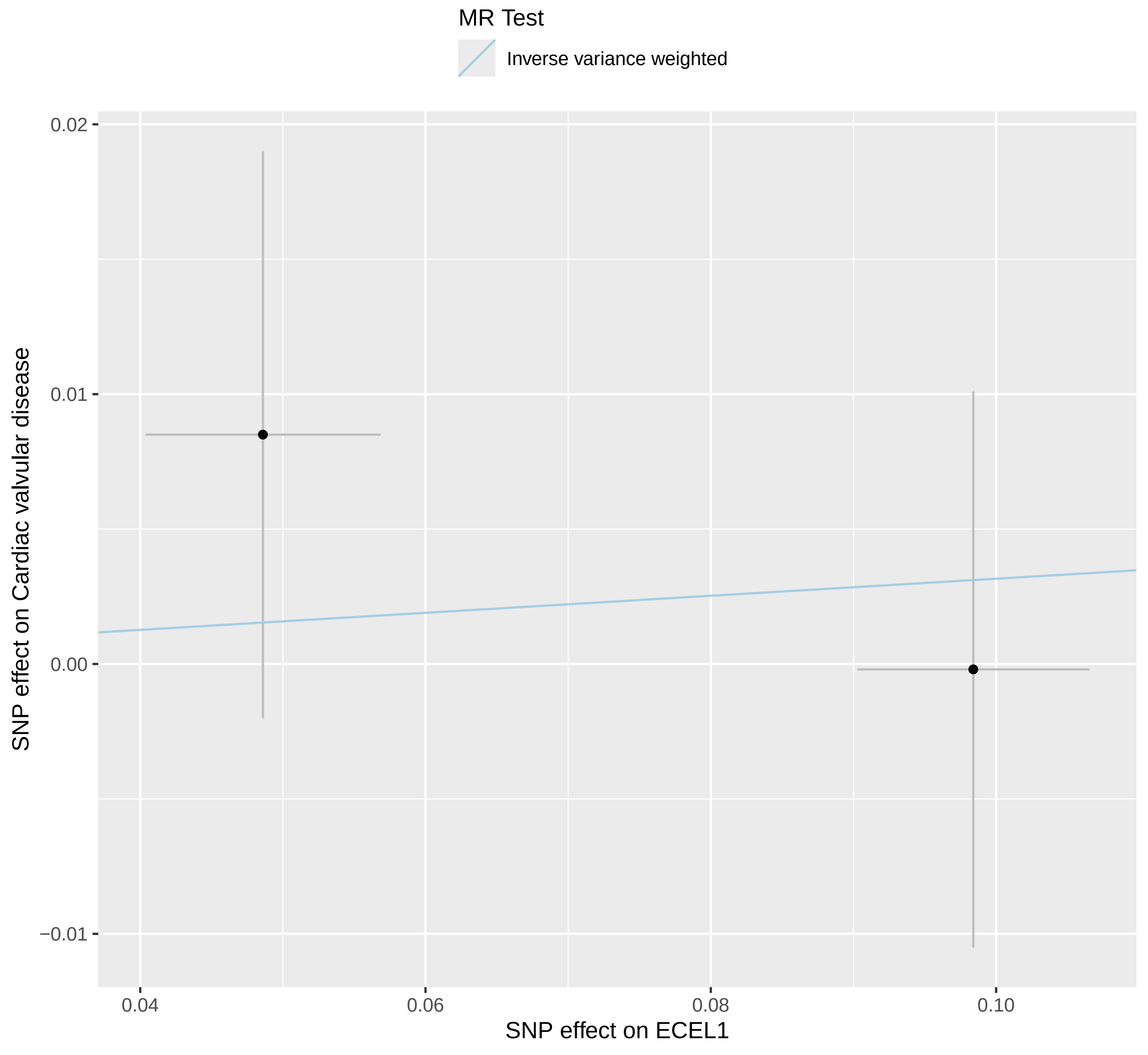
MR Test
Inverse variance weighted
0.02
0.01
0.00
−0.01
0.04
0.06
0.08
0.10
SNP effect on Cardiac valvular disease
SNP effect on ECEL1

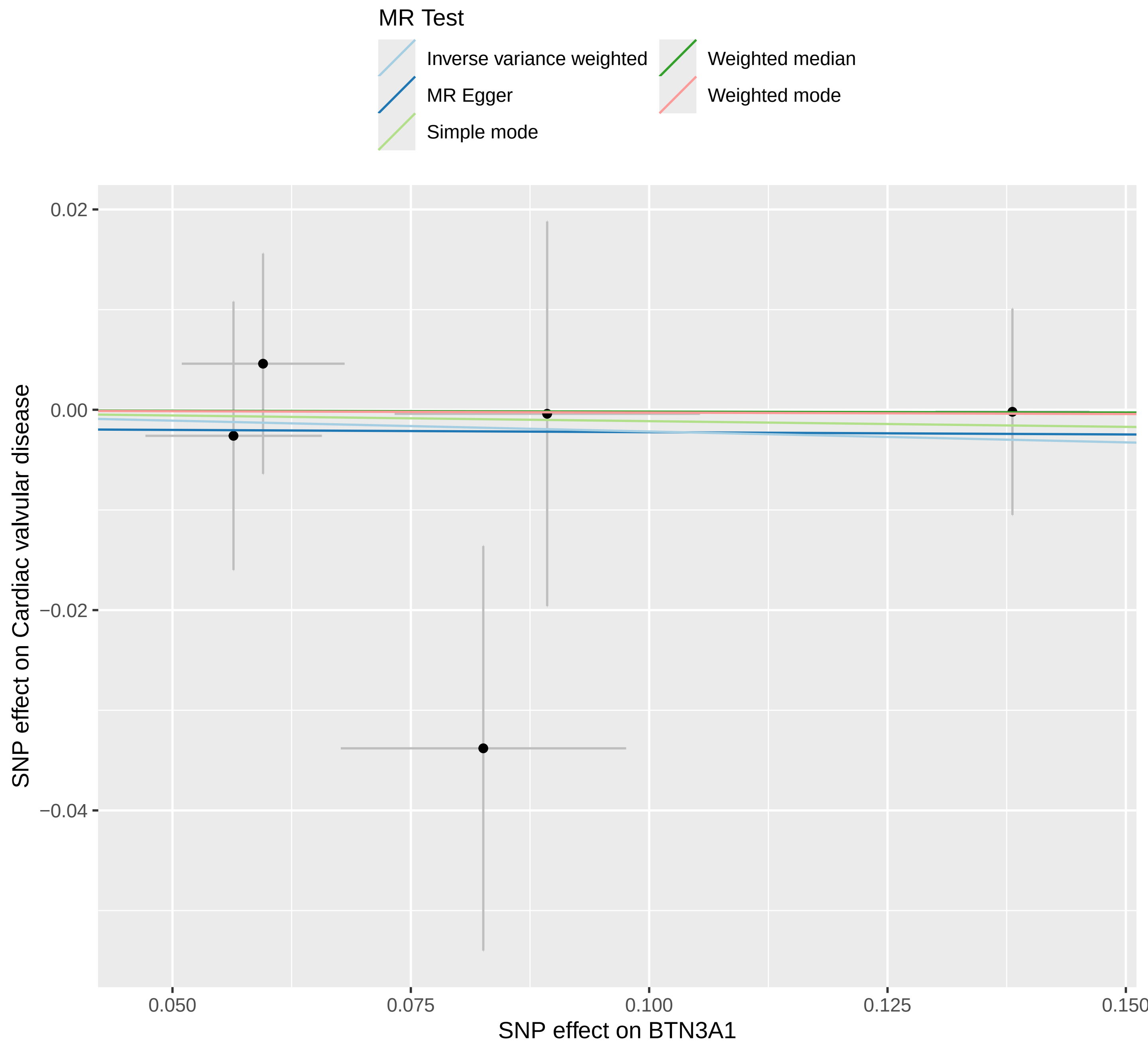

MR Test
Inverse variance weighted
MR Egger
Simple mode
Weighted median
Weighted mode
0.02
0.00
−0.02
−0.04
SNP effect on Cardiac valvular disease
0.050
0.075
0.100
0.125
0.150
SNP effect on BTN3A1

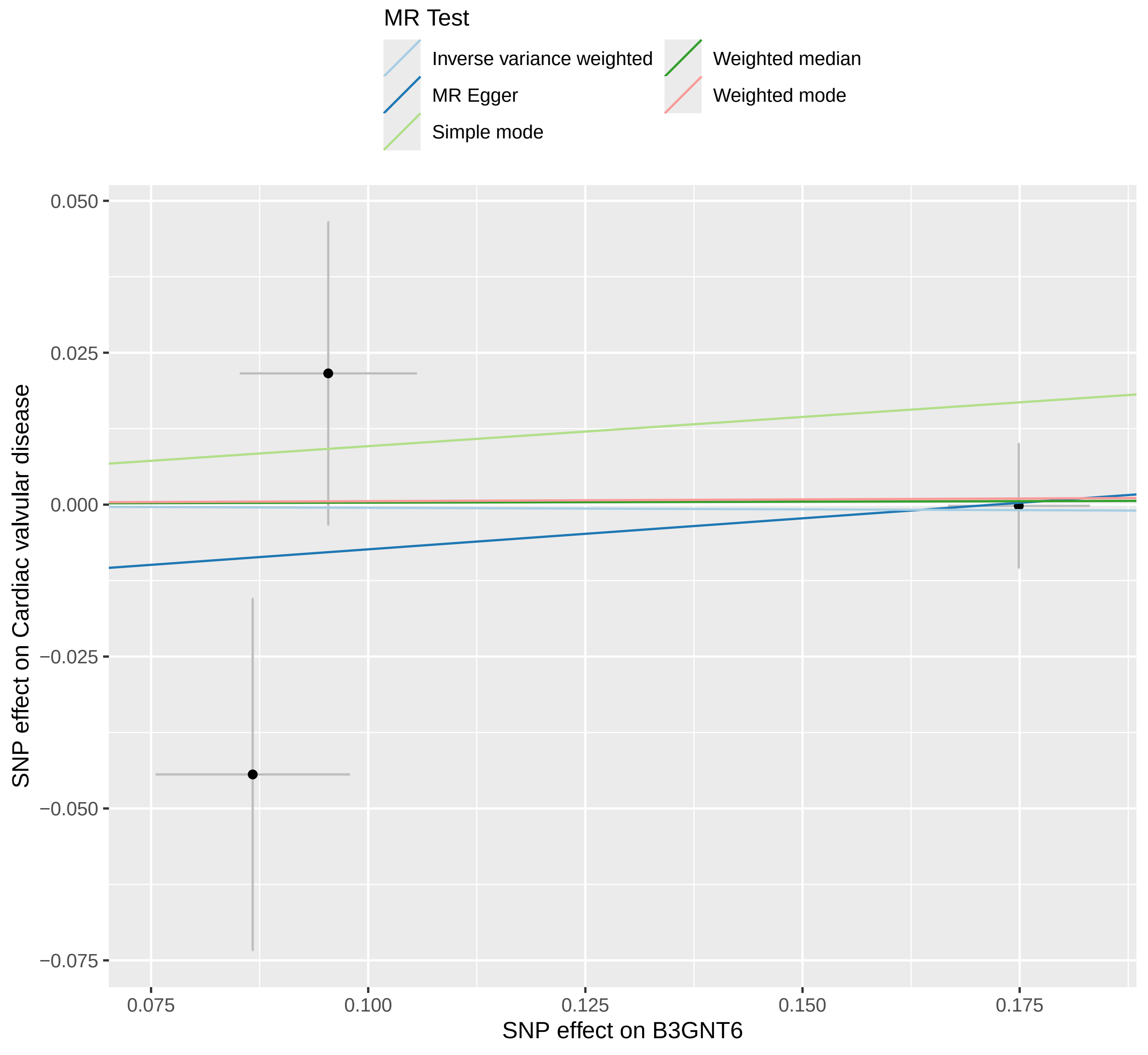
MR Test
Inverse variance weighted
MR Egger
Simple mode
Weighted median
Weighted mode
0.050
0.025
0.000
−0.025
−0.050
−0.075
SNP effect on Cardiac valvular disease
0.075
0.100
0.125
0.150
0.175
SNP effect on B3GNT6

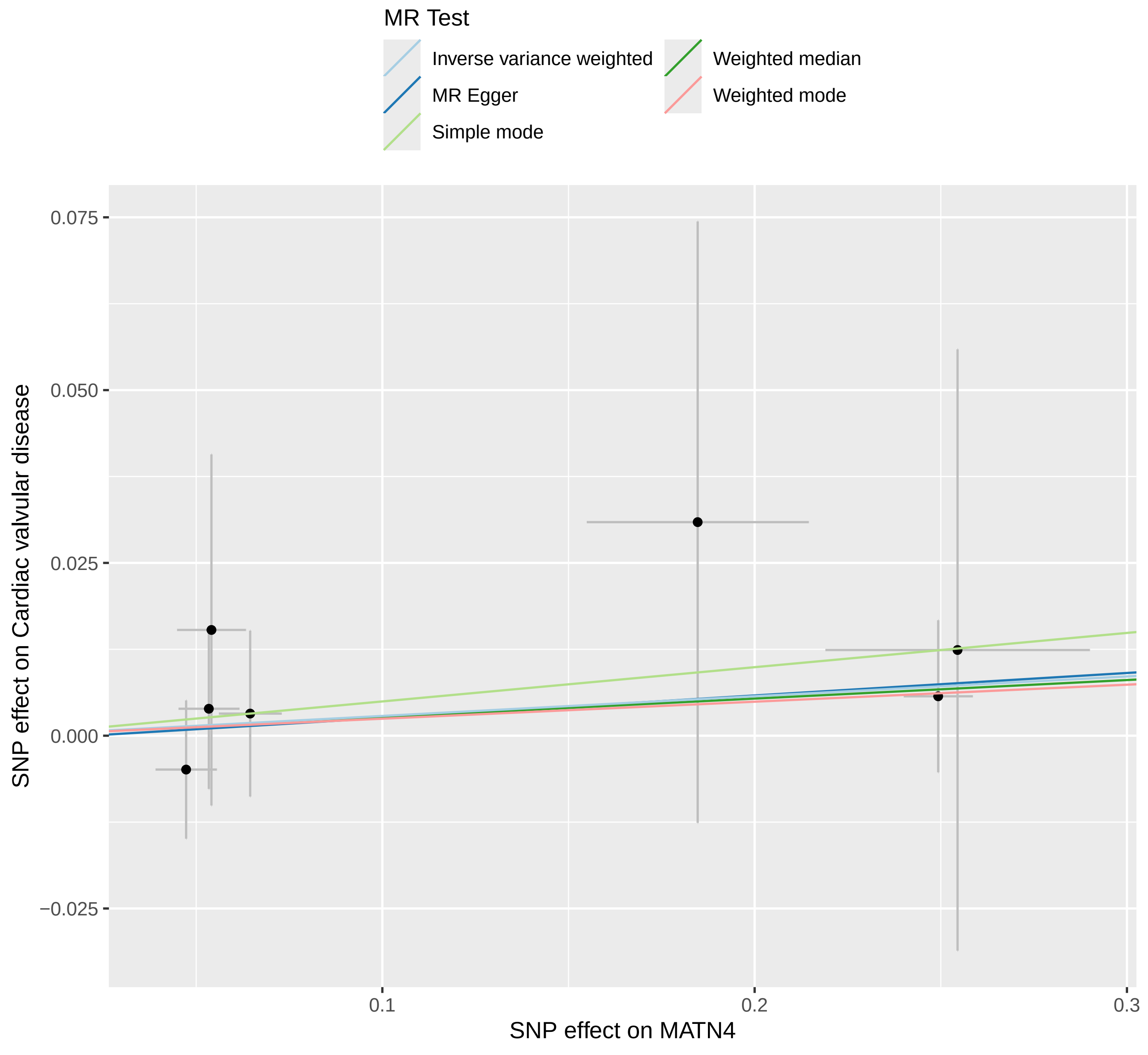

MR Test
Inverse variance weighted
MR Egger
Simple mode
Weighted median
Weighted mode
0.075
0.050
0.025
0.000
−0.025
0.1
0.2
0.3
SNP effect on MATN4
SNP effect on Cardiac valvular disease

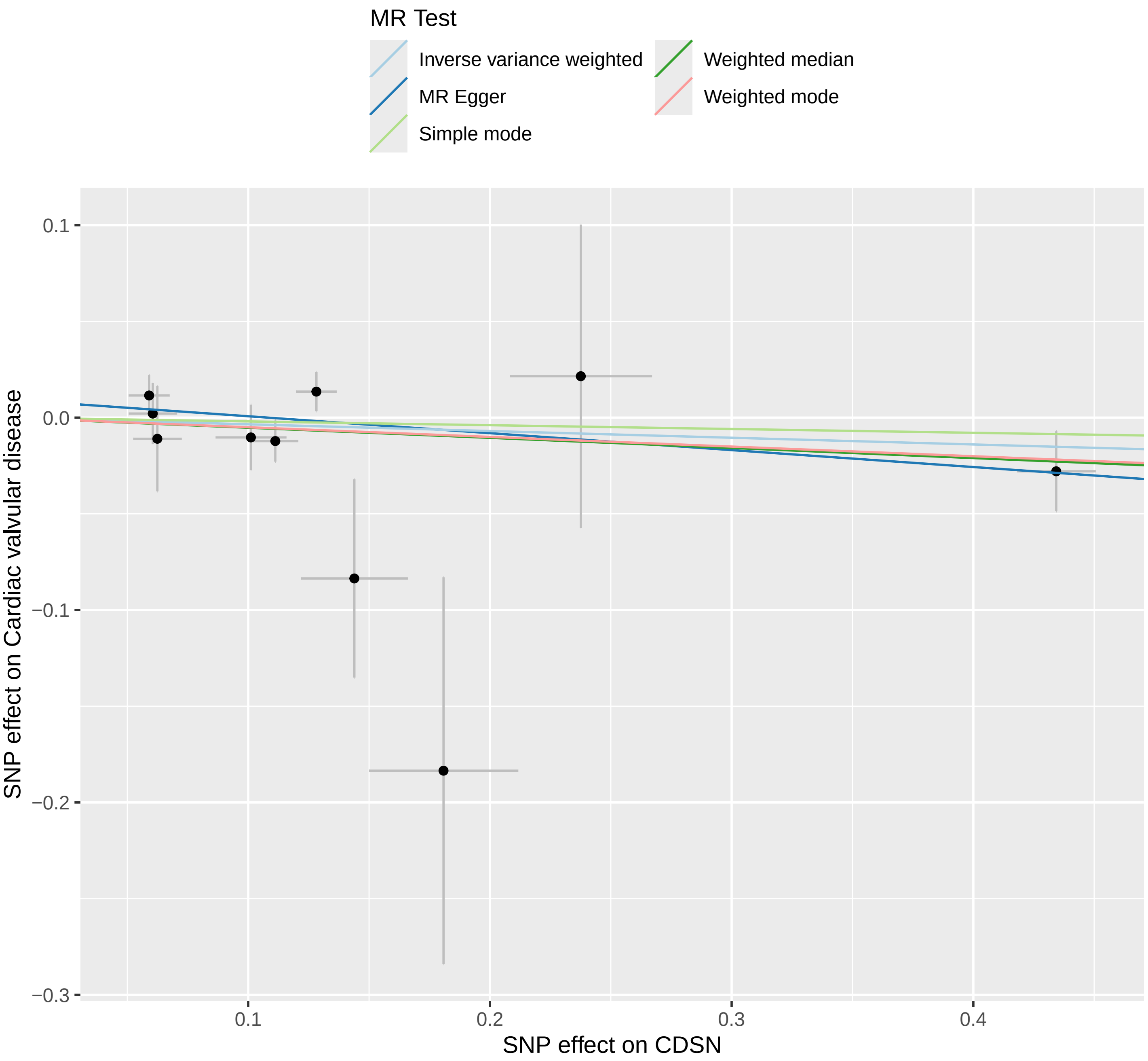
MR Test
Inverse variance weighted
MR Egger
Simple mode
Weighted median
Weighted mode
0.1
0.0
−0.1
−0.2
−0.3
SNP effect on Cardiac valvular disease
0.1
0.2
0.3
0.4
SNP effect on CDSN

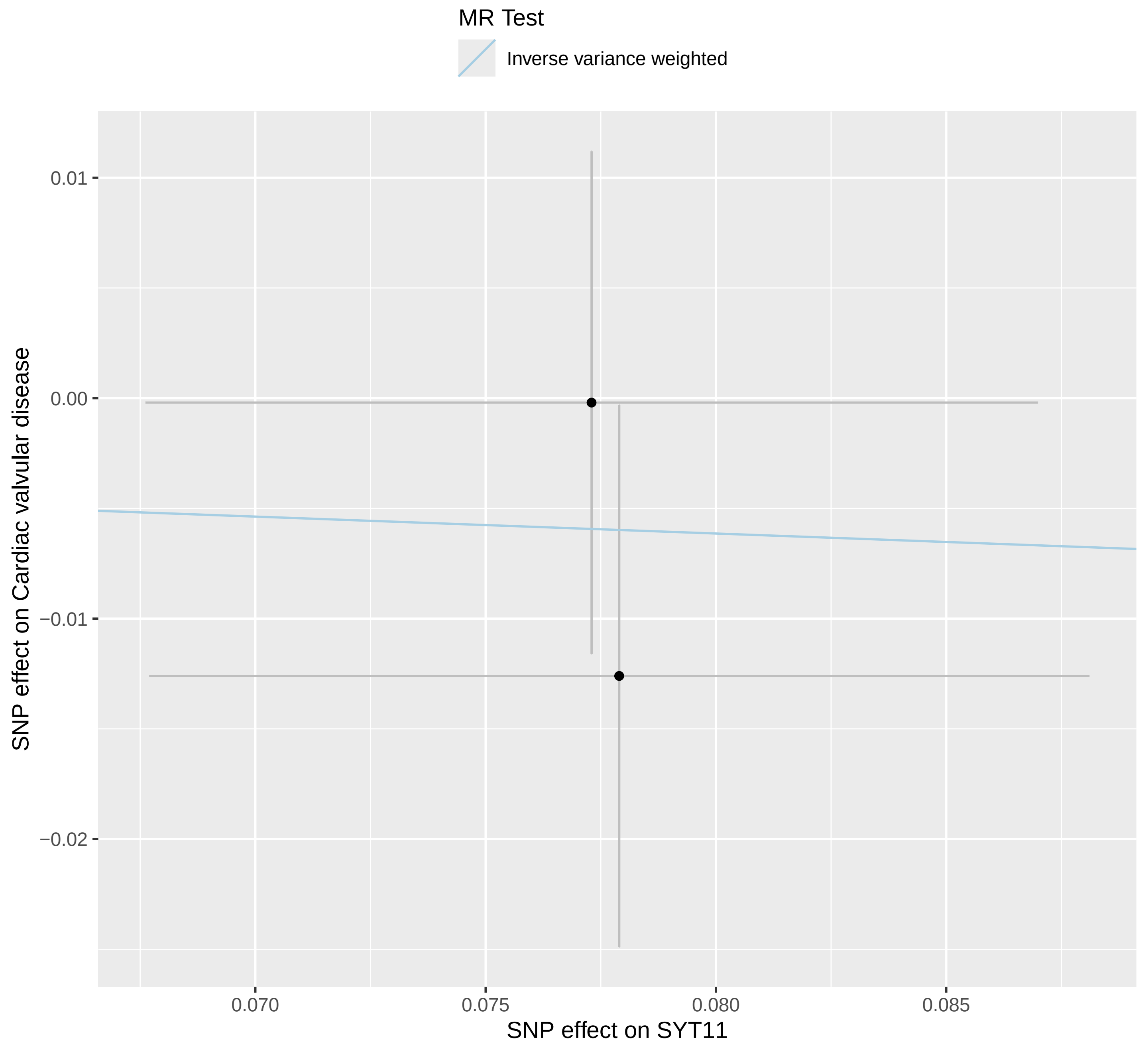

MR Test
Inverse variance weighted
0.01
0.00
−0.01
−0.02
SNP effect on Cardiac valvular disease
0.070
0.075
0.080
0.085
SNP effect on SYT11

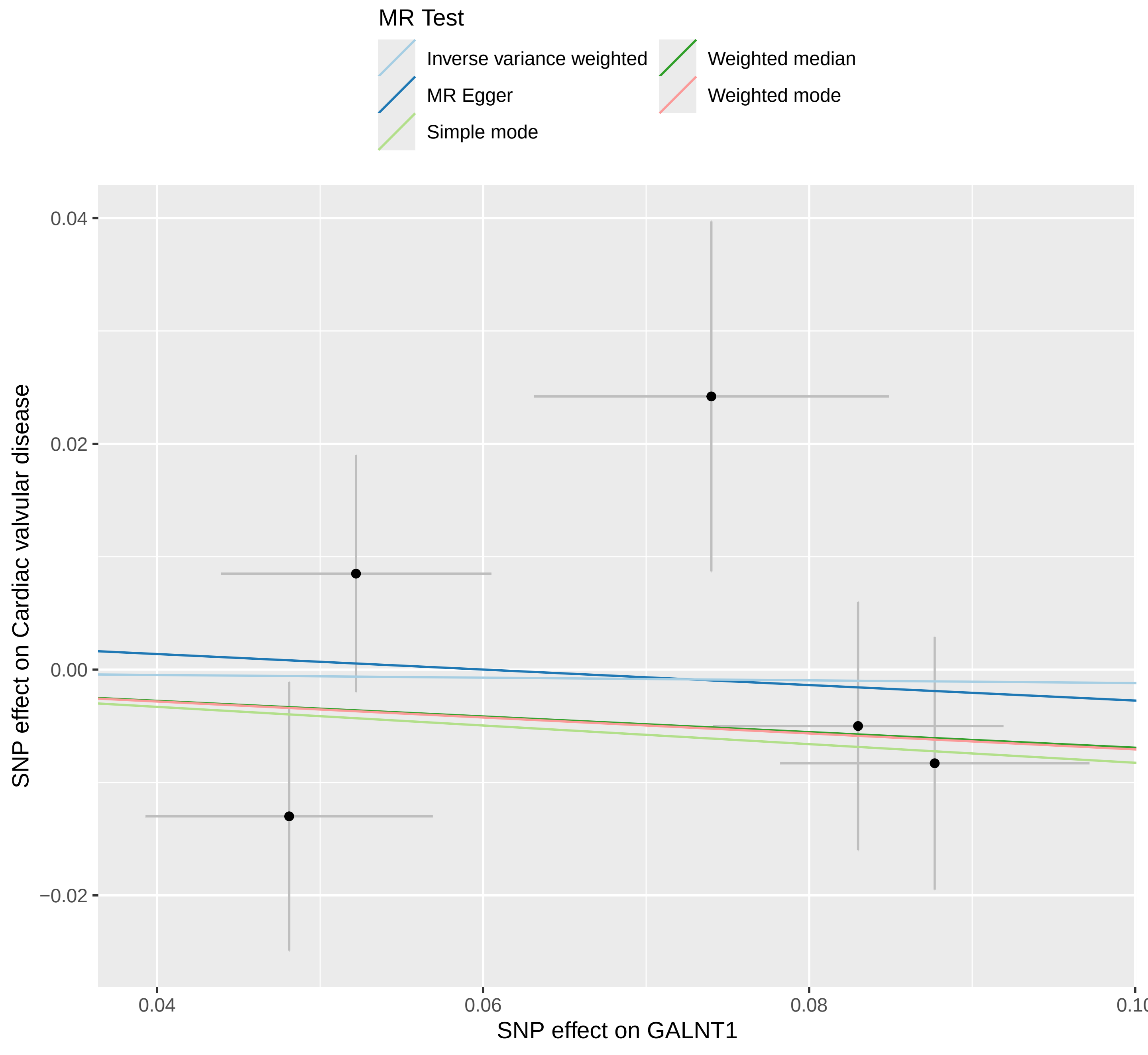
MR Test
Inverse variance weighted
MR Egger
Simple mode
Weighted median
Weighted mode
0.04
0.02
0.00
−0.02
0.04
0.06
0.08
0.10
SNP effect on GALNT1
SNP effect on Cardiac valvular disease

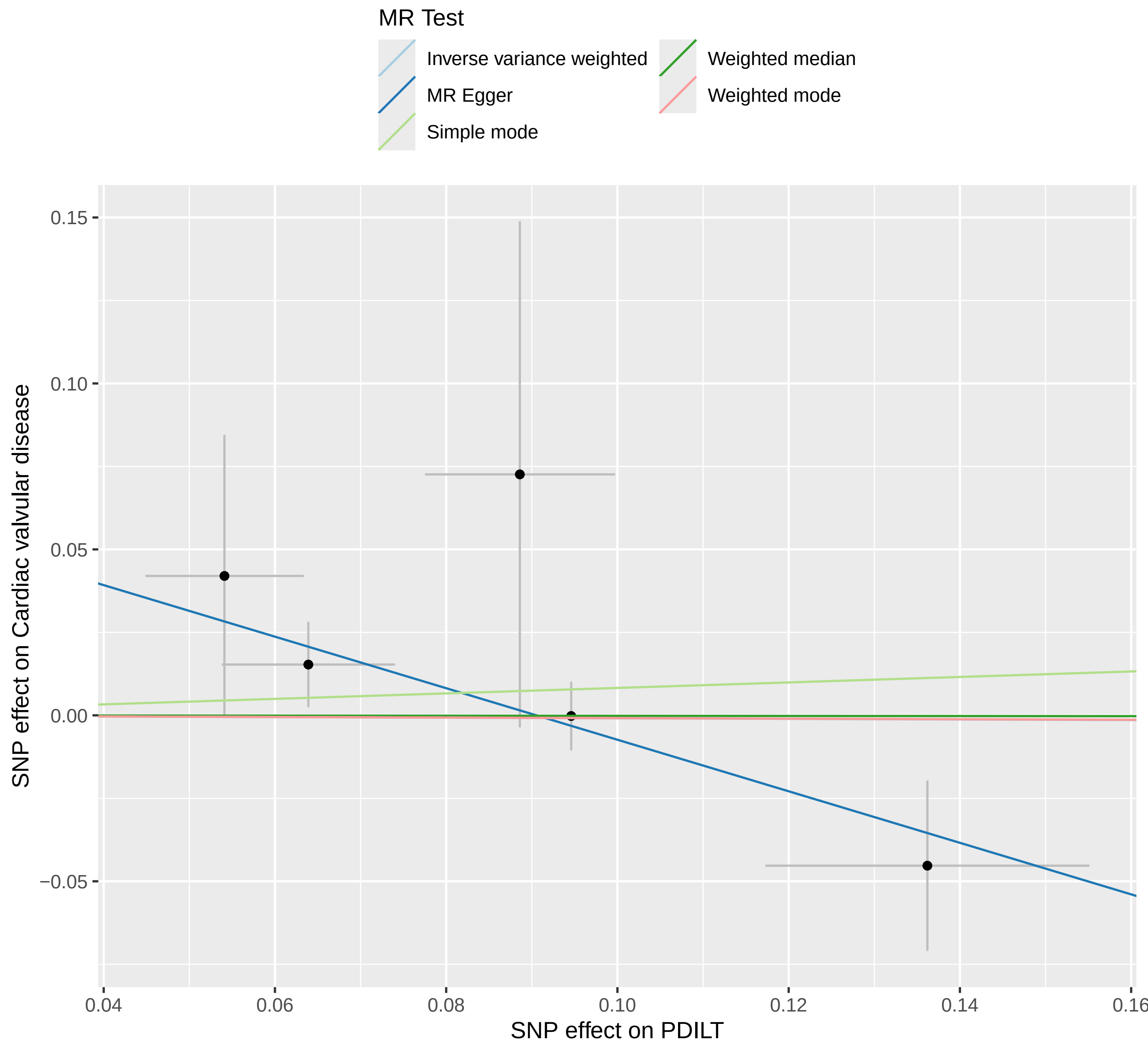
MR Test
Inverse variance weighted
MR Egger
Simple mode
Weighted median
Weighted mode
0.15
0.10
0.05
0.00
−0.05
SNP effect on Cardiac valvular disease
0.04
0.06
0.08
0.10
0.12
0.14
0.16
SNP effect on PDILT

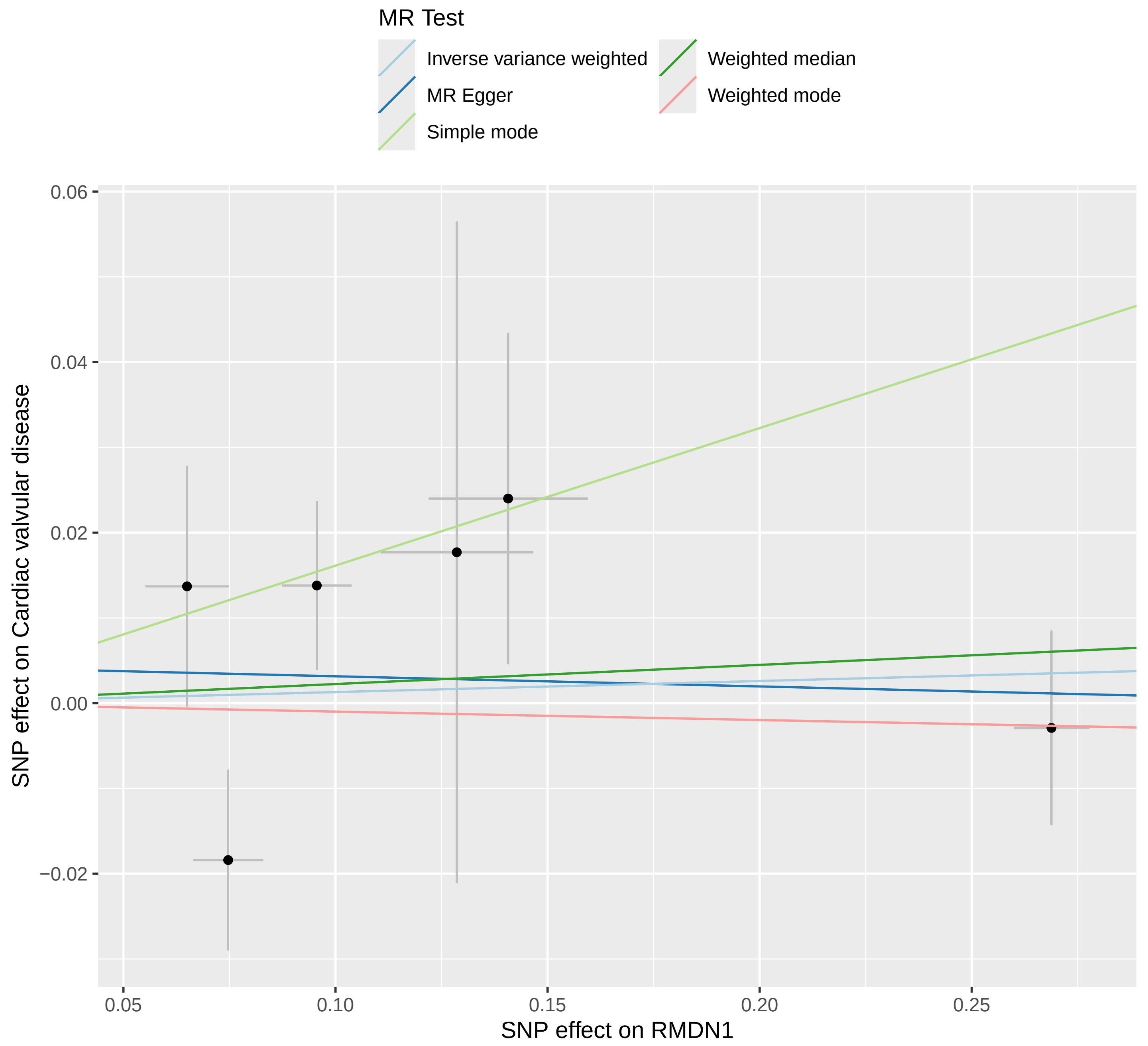

MR Test
Inverse variance weighted
MR Egger
Simple mode
Weighted median
Weighted mode
0.06
0.04
0.02
0.00
−0.02
0.05
0.10
0.15
0.20
0.25
SNP effect on Cardiac valvular disease
SNP effect on RMDN1

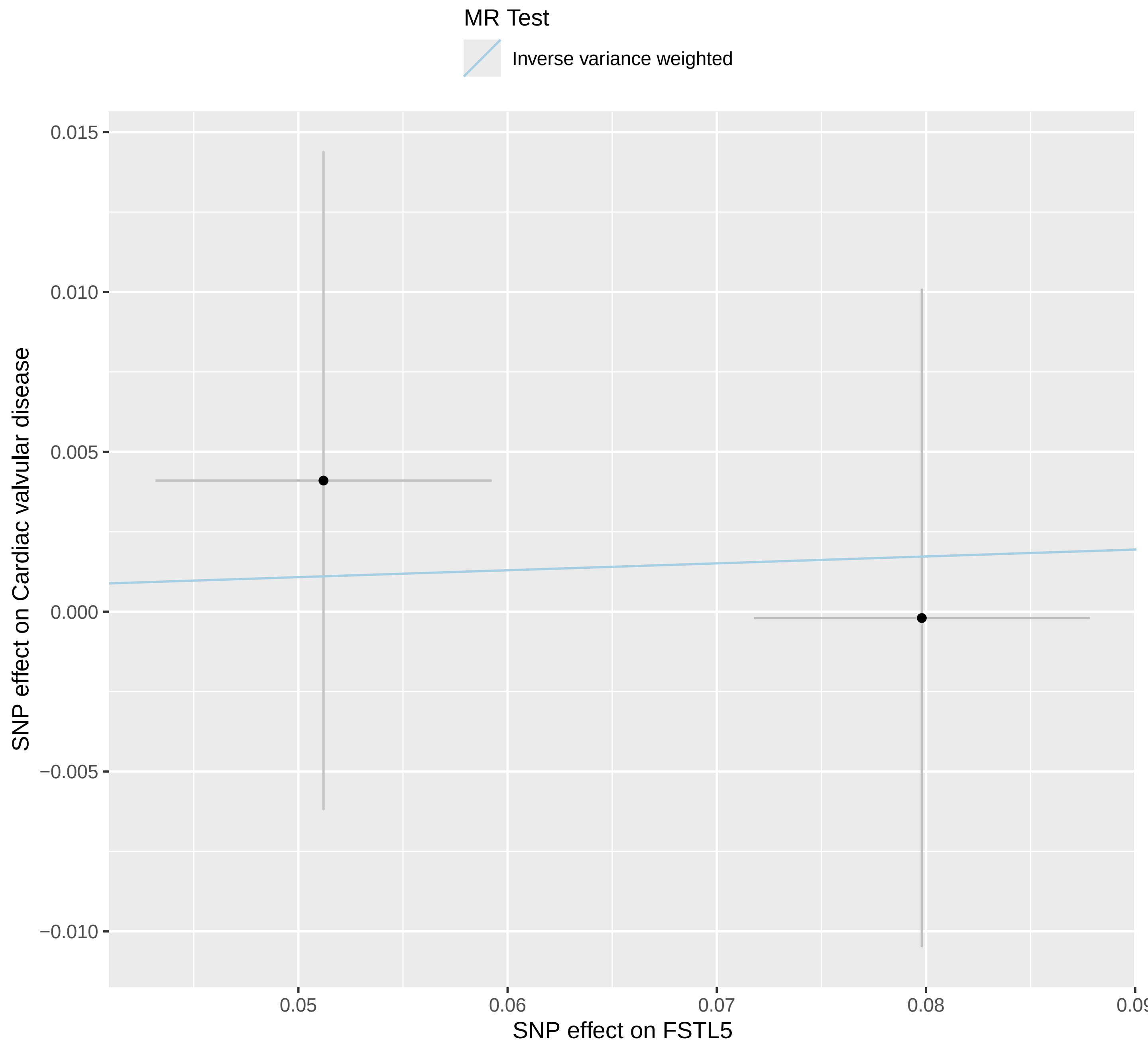

MR Test
Inverse variance weighted
0.015
0.010
0.005
0.000
−0.005
−0.010
SNP effect on Cardiac valvular disease
0.05
0.06
0.07
0.08
0.09
SNP effect on FSTL5

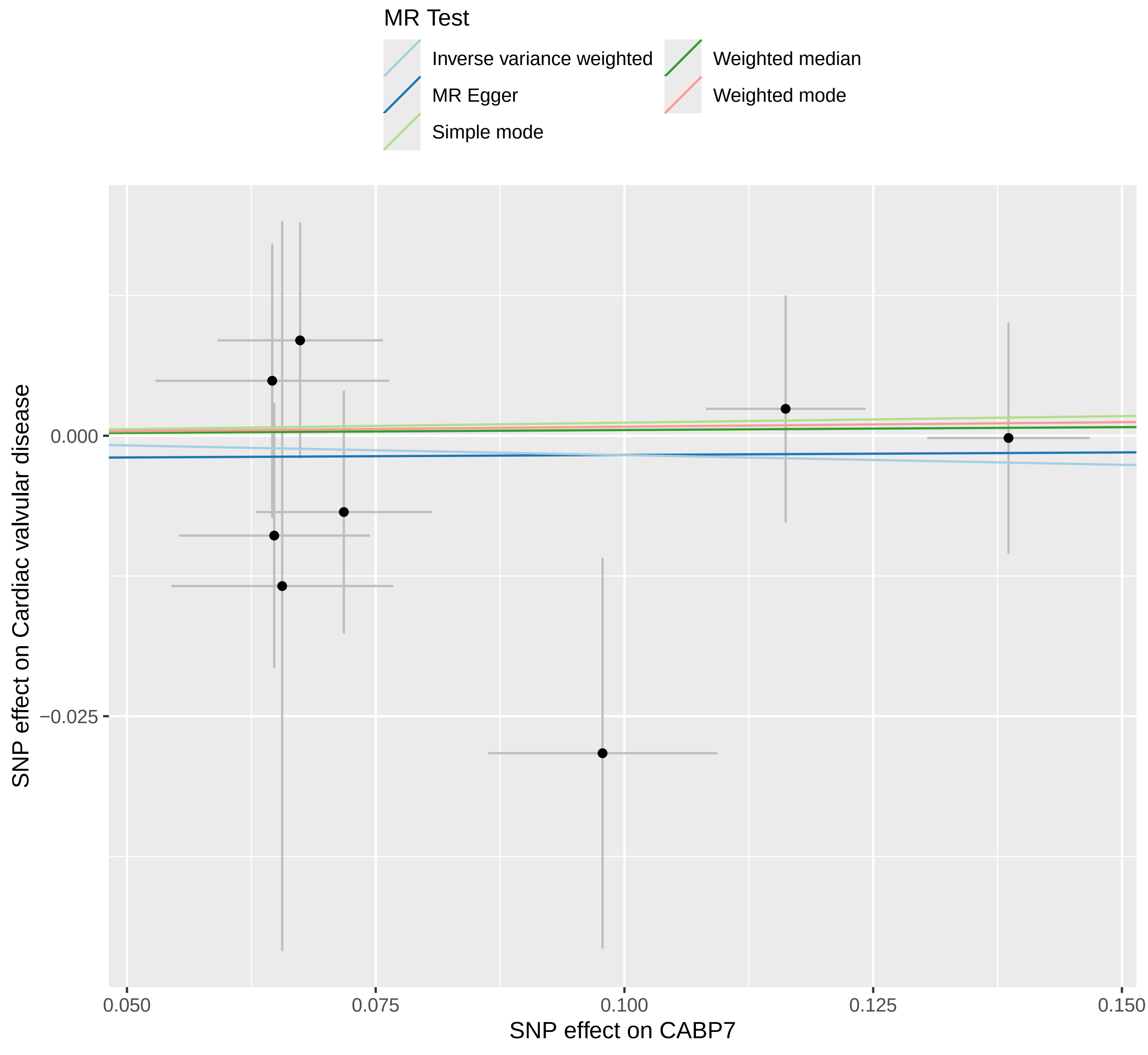
MR Test
Inverse variance weighted
MR Egger
Simple mode
Weighted median
Weighted mode
0.000
−0.025
0.050
0.075
0.100
0.125
0.150
SNP effect on CABP7
SNP effect on Cardiac valvular disease

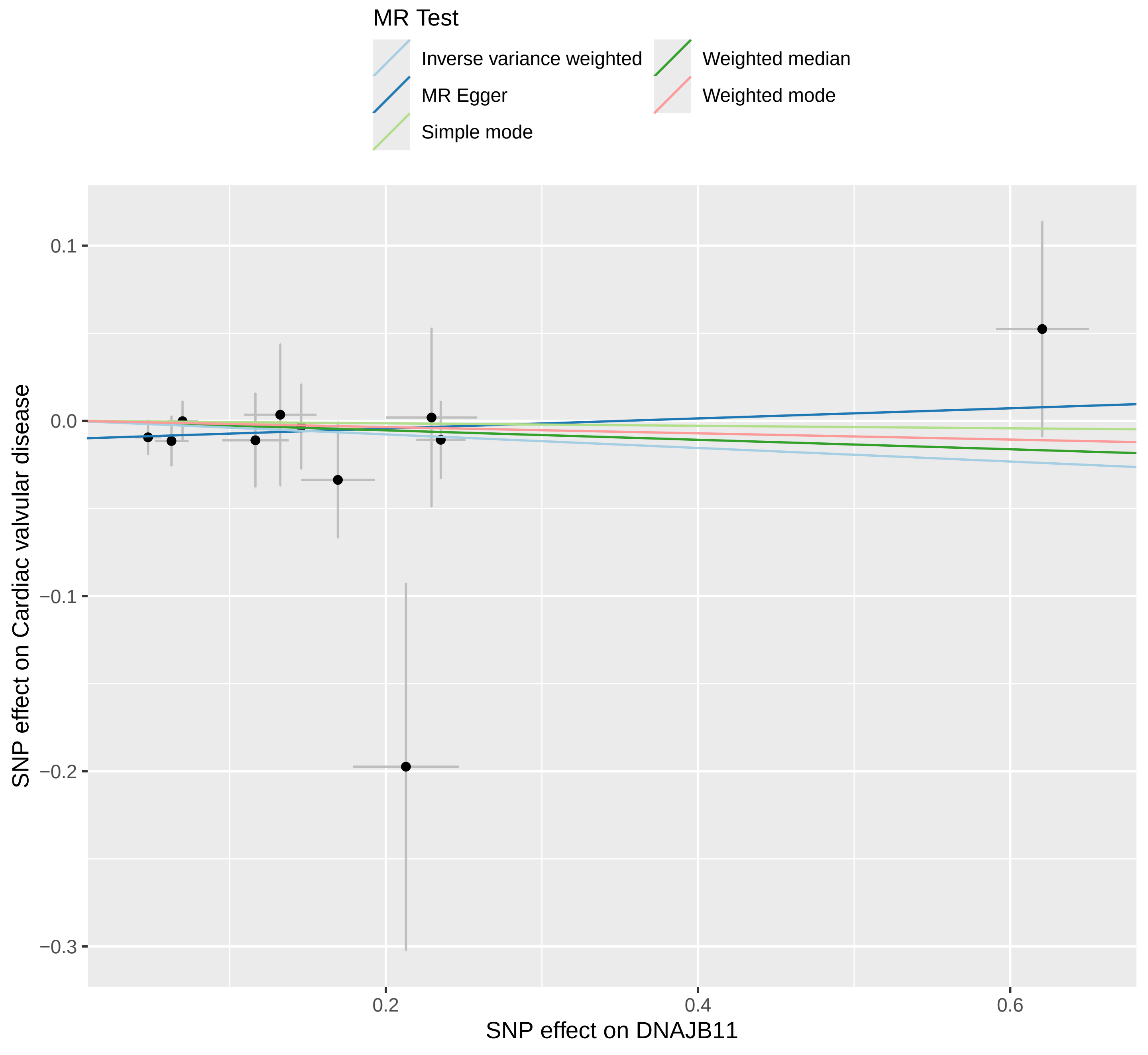
MR Test
Inverse variance weighted
MR Egger
Simple mode
Weighted median
Weighted mode
SNP effect on Cardiac valvular disease
0.1
0.0
−0.1
−0.2
−0.3
0.2
0.4
0.6
SNP effect on DNAJB11

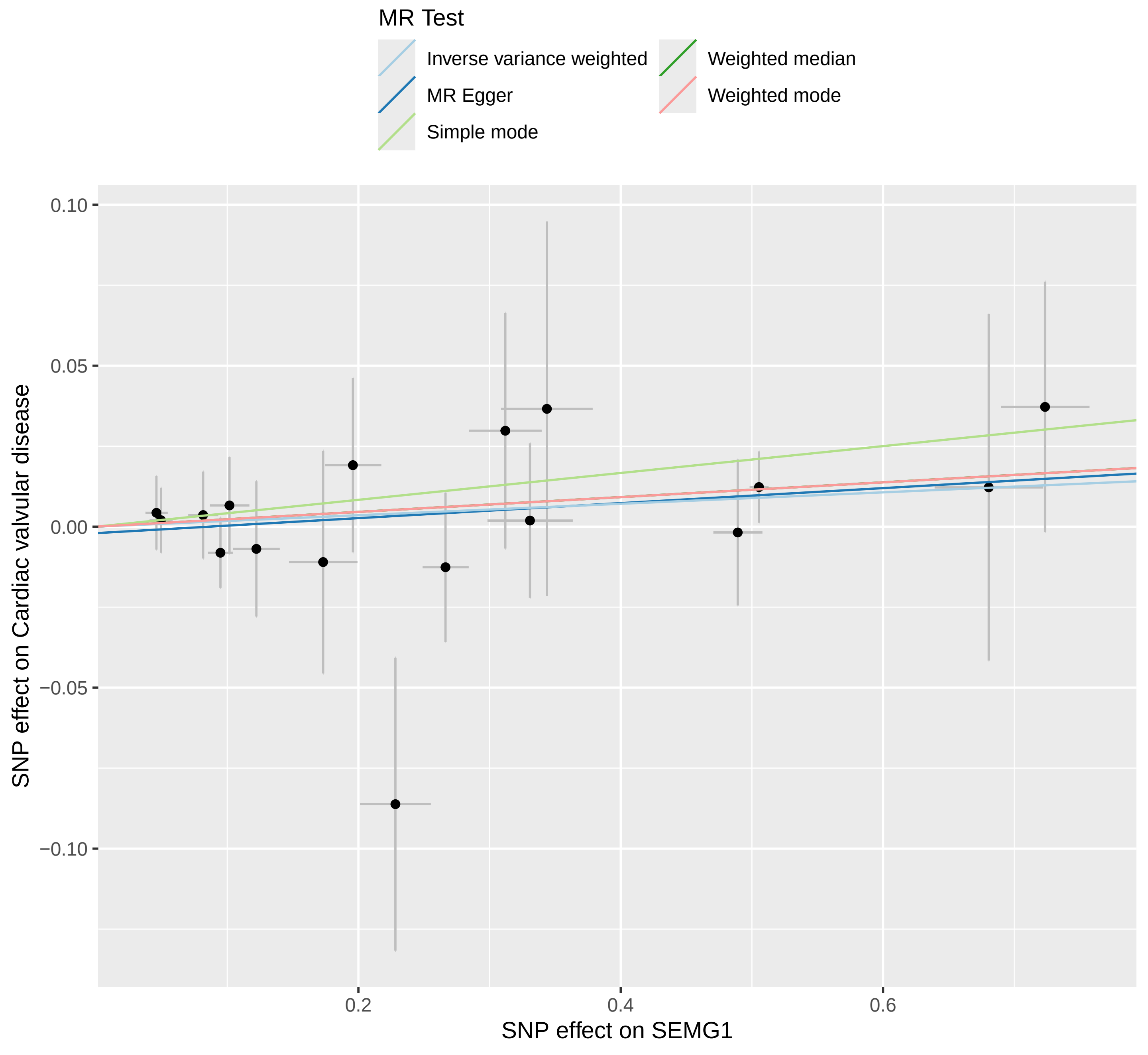

MR Test
Inverse variance weighted
MR Egger
Simple mode
Weighted median
Weighted mode
0.10
0.05
0.00
−0.05
−0.10
0.2
0.4
0.6
SNP effect on Cardiac valvular disease
SNP effect on SEMG1

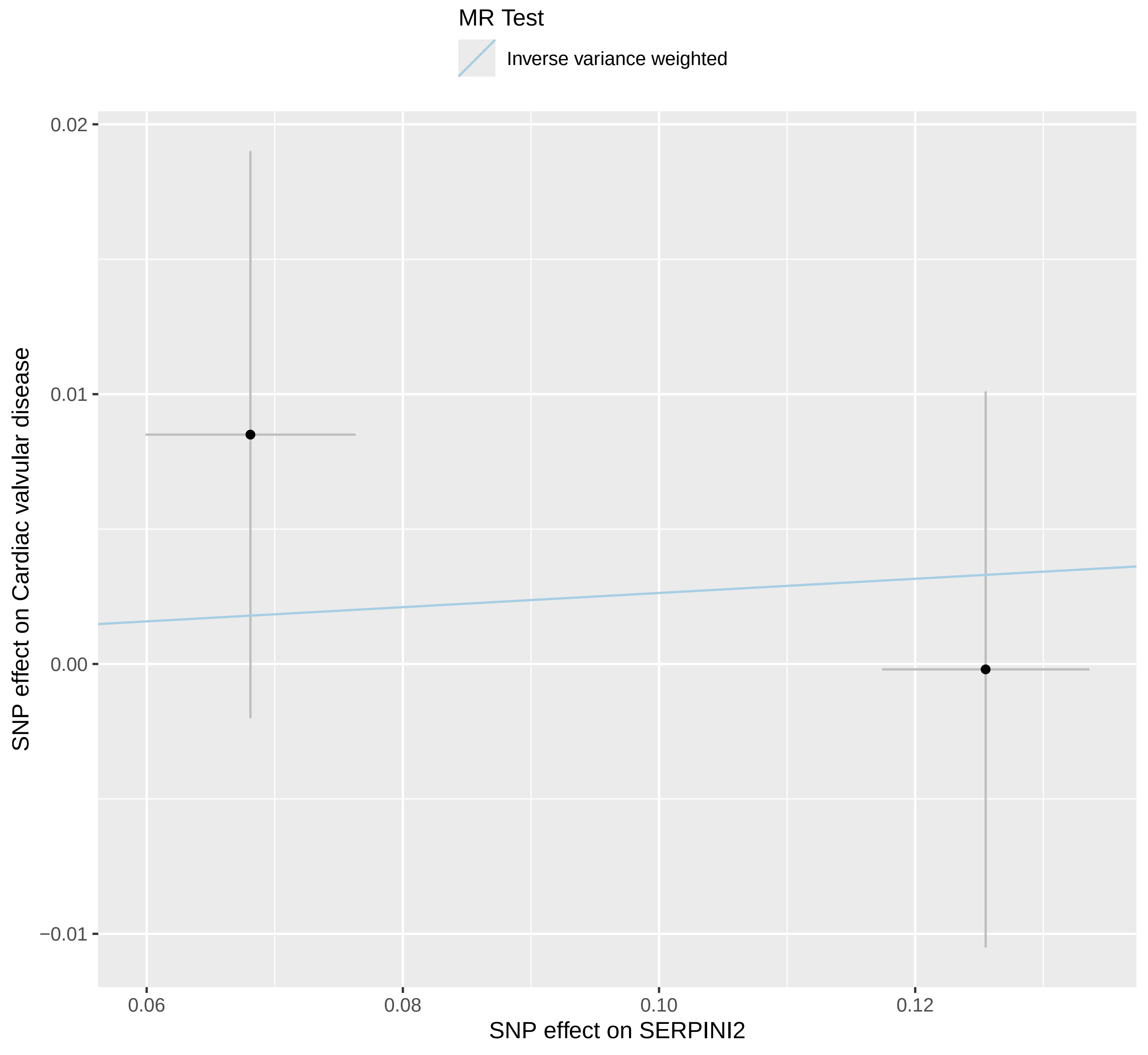
MR Test
Inverse variance weighted
0.02
0.01
0.00
−0.01
0.06
0.08
0.10
0.12
SNP effect on Cardiac valvular disease
SNP effect on SERPINI2

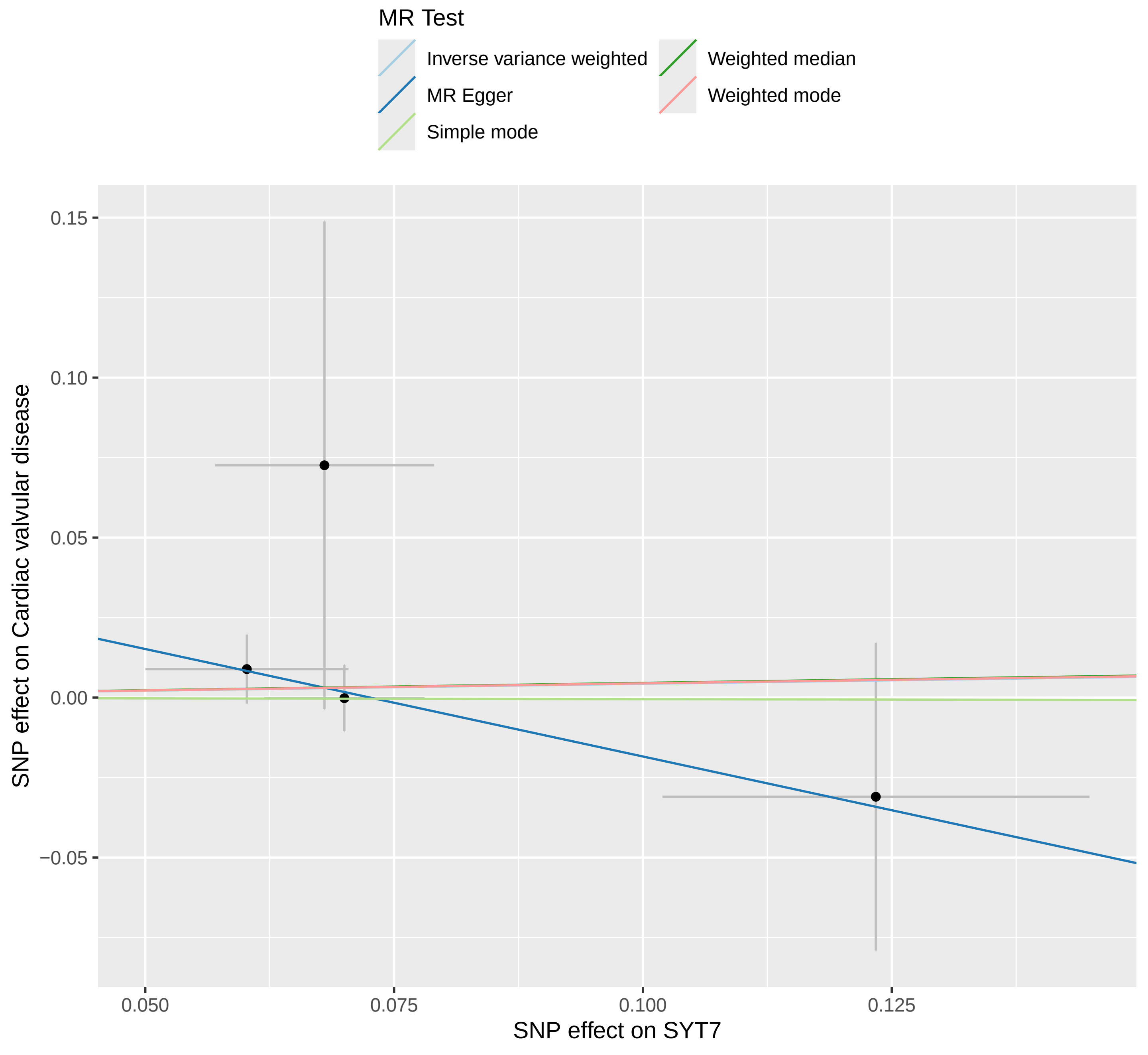

MR Test
Inverse variance weighted
MR Egger
Simple mode
Weighted median
Weighted mode
0.15
0.10
0.05
0.00
−0.05
0.050
0.075
0.100
0.125
SNP effect on Cardiac valvular disease
SNP effect on SYT7

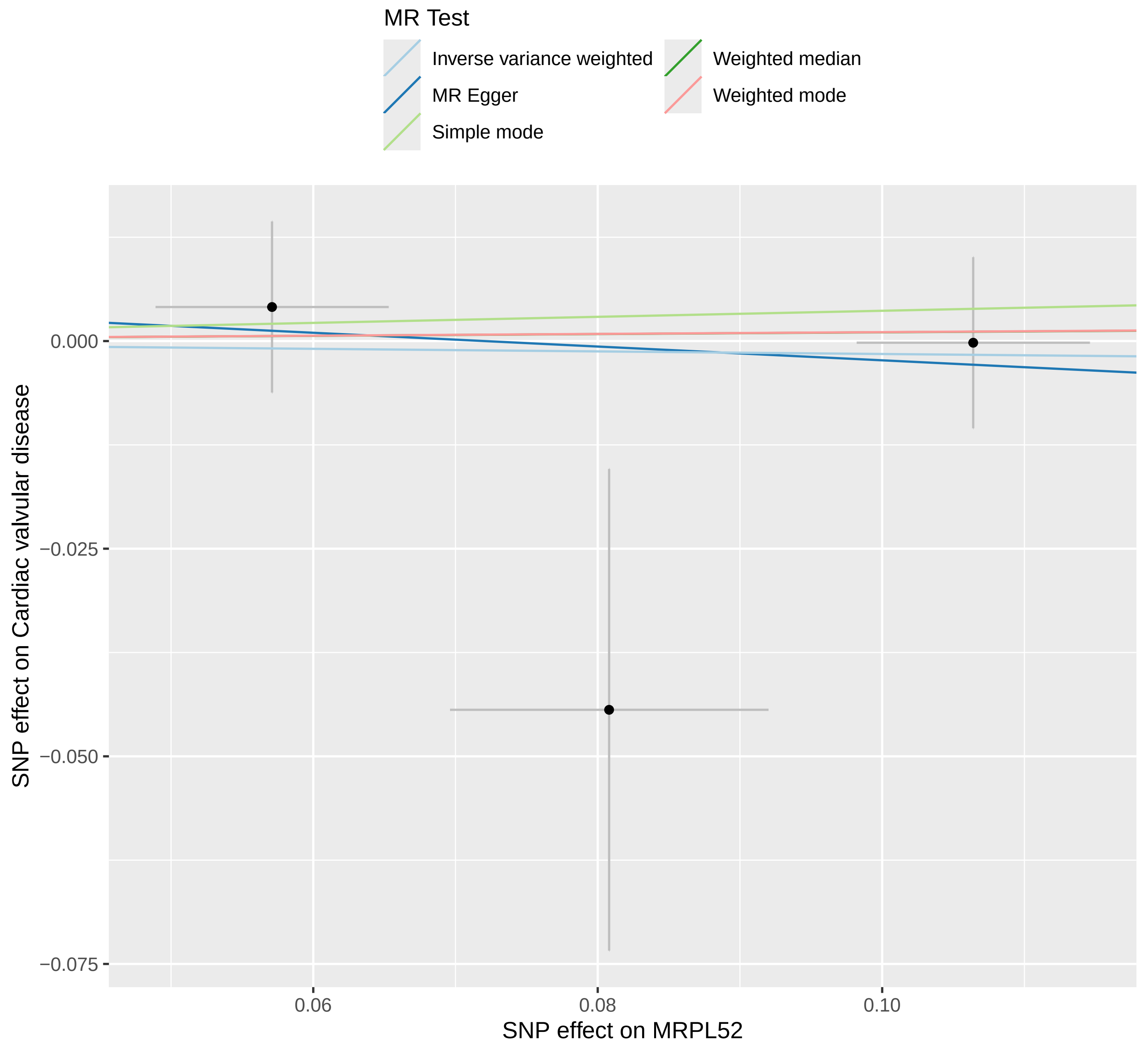

MR Test
Inverse variance weighted
MR Egger
Simple mode
Weighted median
Weighted mode
0.000
−0.025
−0.050
−0.075
0.06
0.08
0.10
SNP effect on Cardiac valvular disease
SNP effect on MRPL52

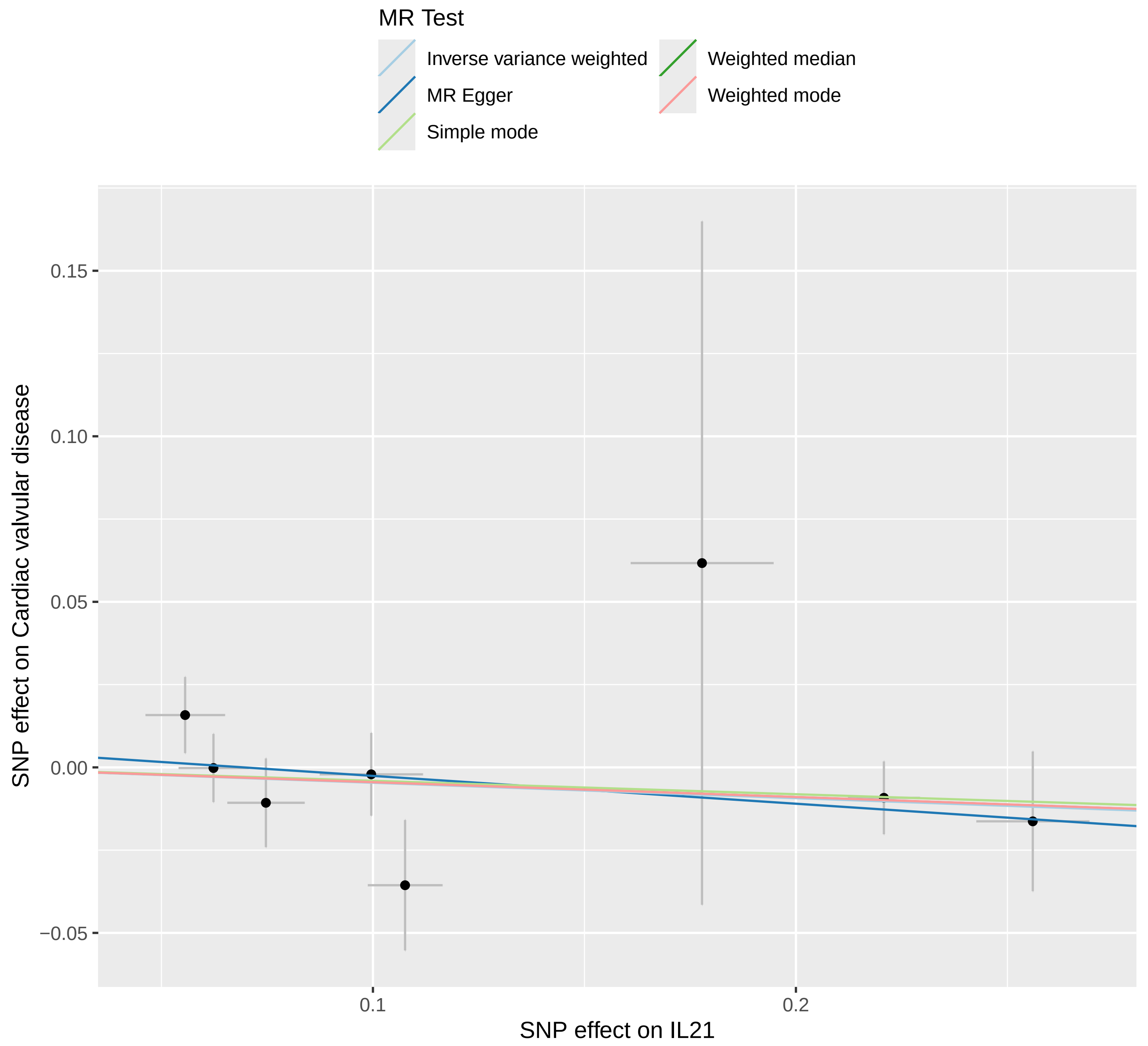

MR Test
Inverse variance weighted
MR Egger
Simple mode
Weighted median
Weighted mode
0.15
0.10
0.05
0.00
−0.05
0.1
0.2
SNP effect on Cardiac valvular disease
SNP effect on IL21

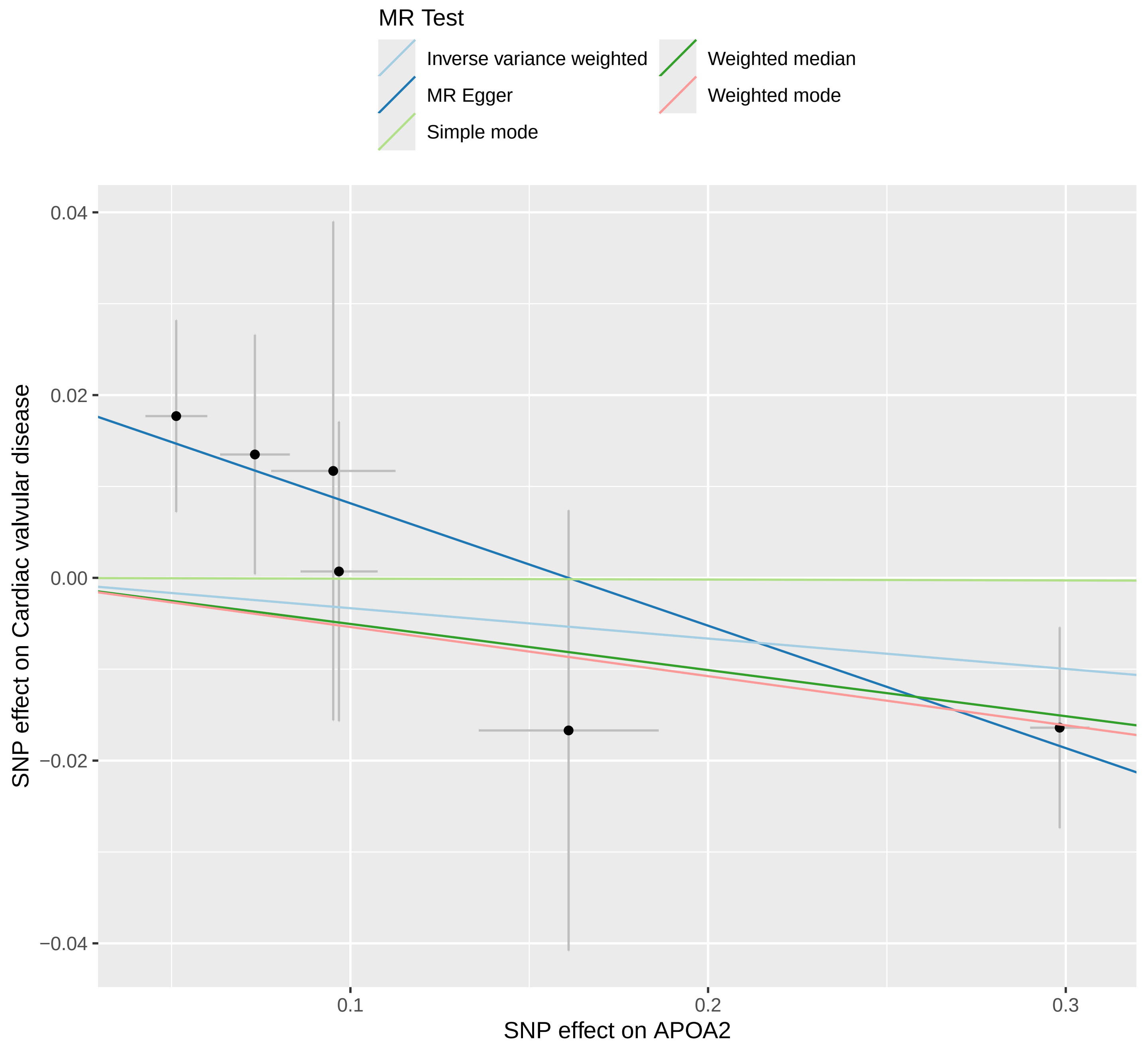

MR Test
Inverse variance weighted
MR Egger
Simple mode
Weighted median
Weighted mode
0.04
0.02
0.00
−0.02
−0.04
SNP effect on Cardiac valvular disease
0.1
0.2
0.3
SNP effect on APOA2

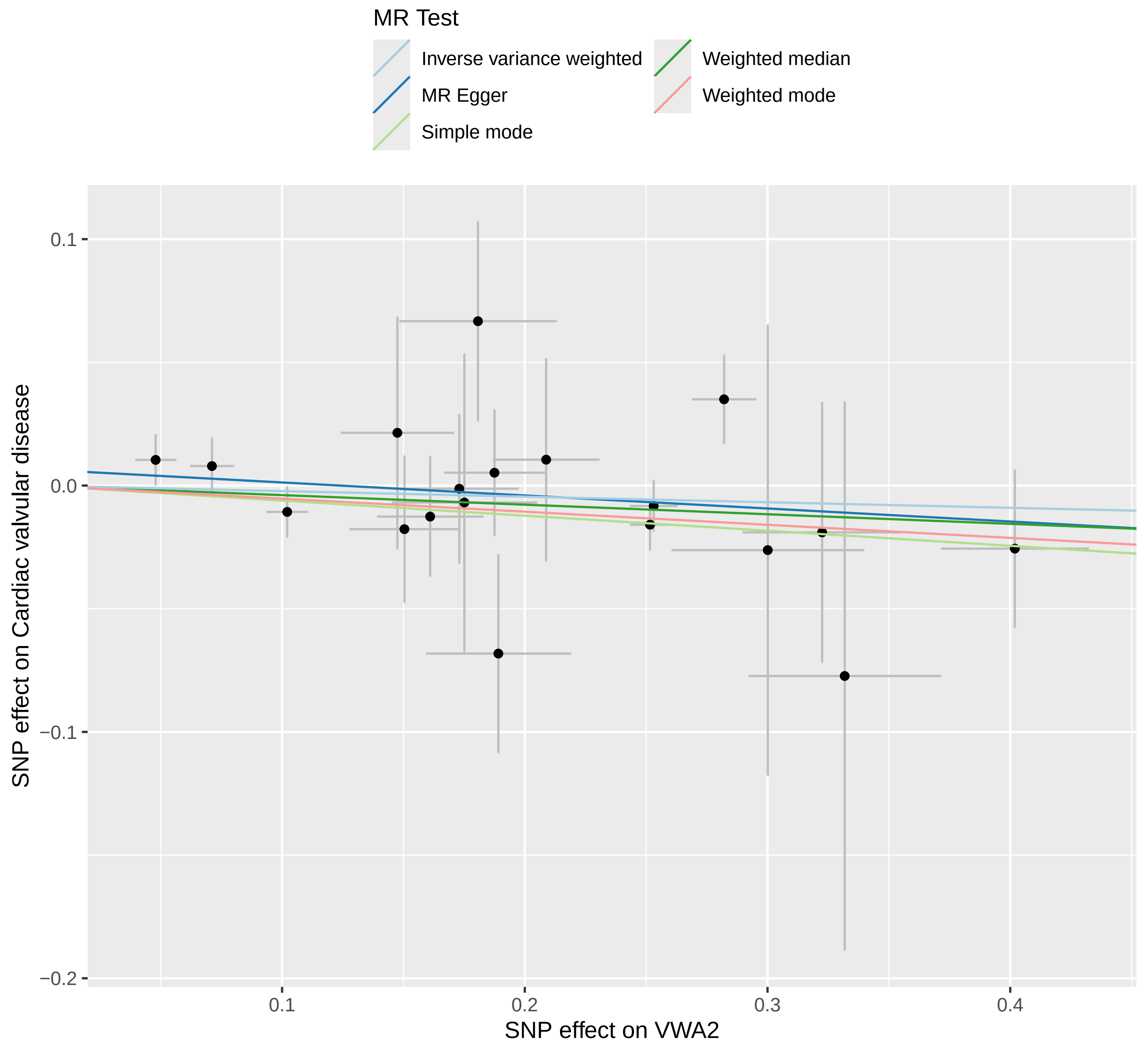
MR Test
Inverse variance weighted
MR Egger
Simple mode
Weighted median
Weighted mode
SNP effect on Cardiac valvular disease
0.1
0.0
−0.1
−0.2
0.1
0.2
0.3
0.4
SNP effect on VWA2

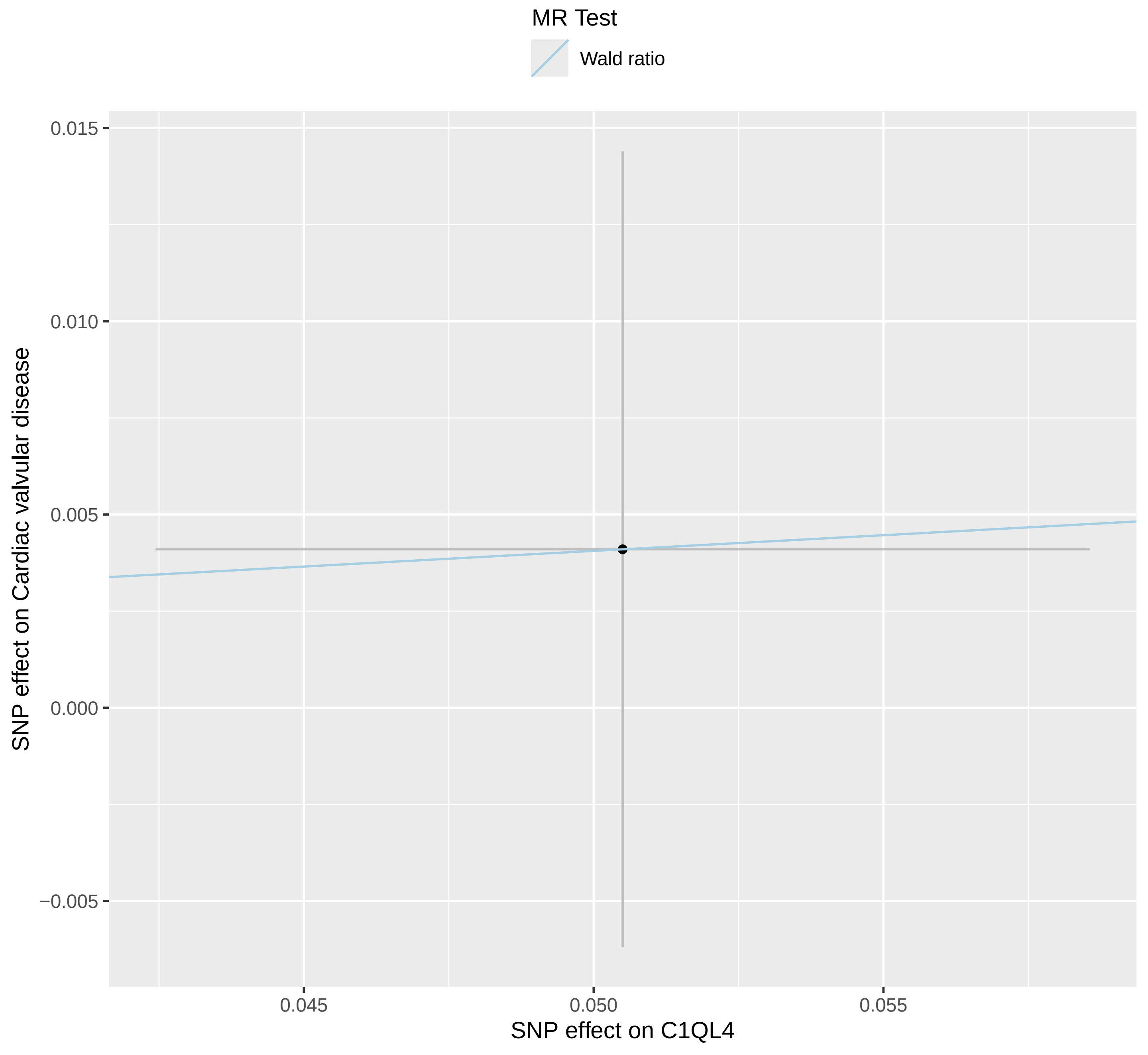

MR Test
Wald ratio
0.015
0.010
0.005
0.000
−0.005
SNP effect on Cardiac valvular disease
0.045
0.050
0.055
SNP effect on C1QL4

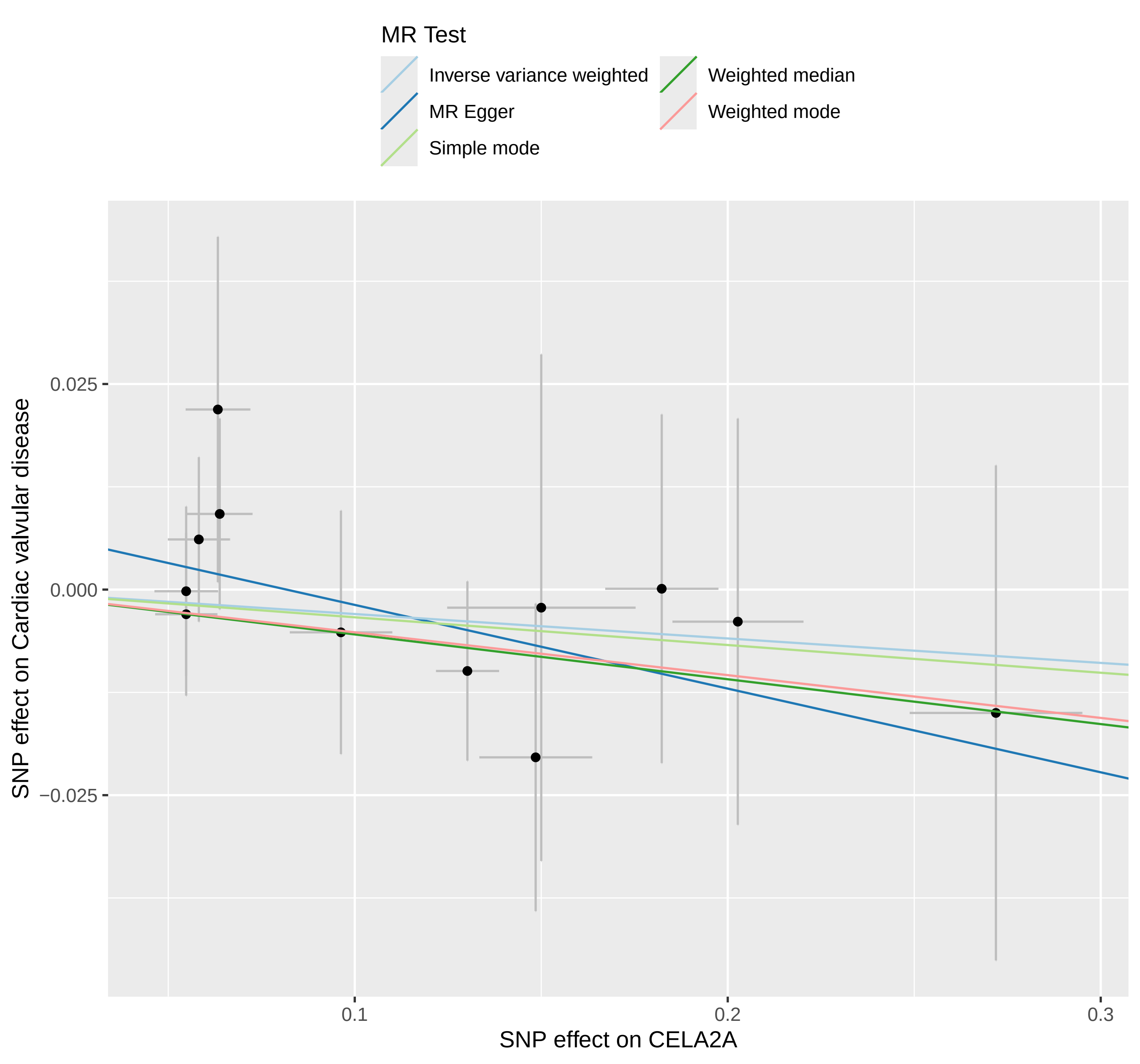
MR Test
Inverse variance weighted
MR Egger
Simple mode
Weighted median
Weighted mode
0.025
0.000
−0.025
SNP effect on Cardiac valvular disease
0.1
0.2
0.3
SNP effect on CELA2A

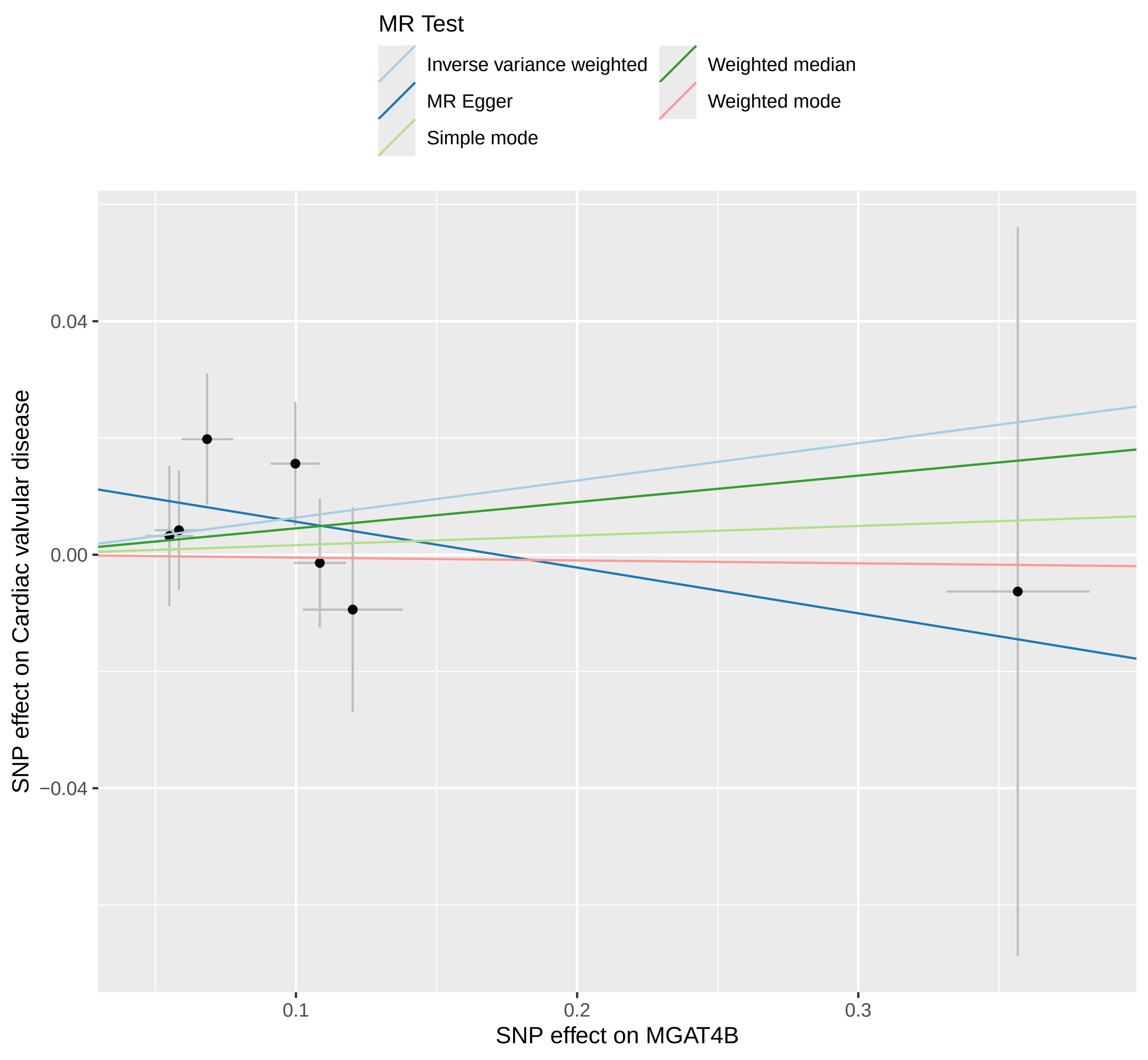
MR Test
Inverse variance weighted
MR Egger
Simple mode
Weighted median
Weighted mode
0.04
0.00
−0.04
0.1
0.2
0.3
SNP effect on Cardiac valvular disease
SNP effect on MGAT4B

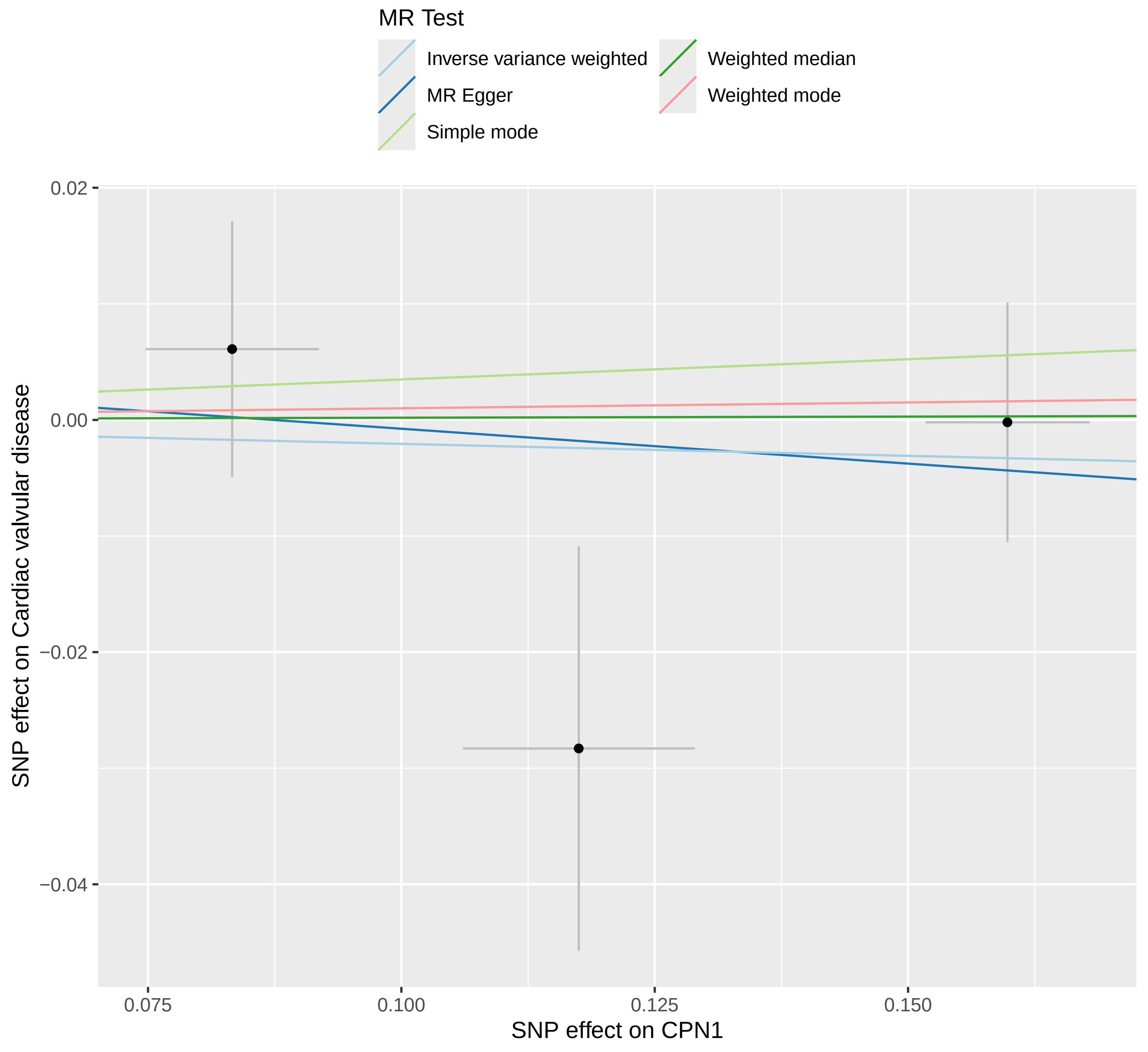
MR Test
Inverse variance weighted
MR Egger
Simple mode
Weighted median
Weighted mode
0.02
0.00
−0.02
−0.04
SNP effect on Cardiac valvular disease
0.075
0.100
0.125
0.150
SNP effect on CPN1

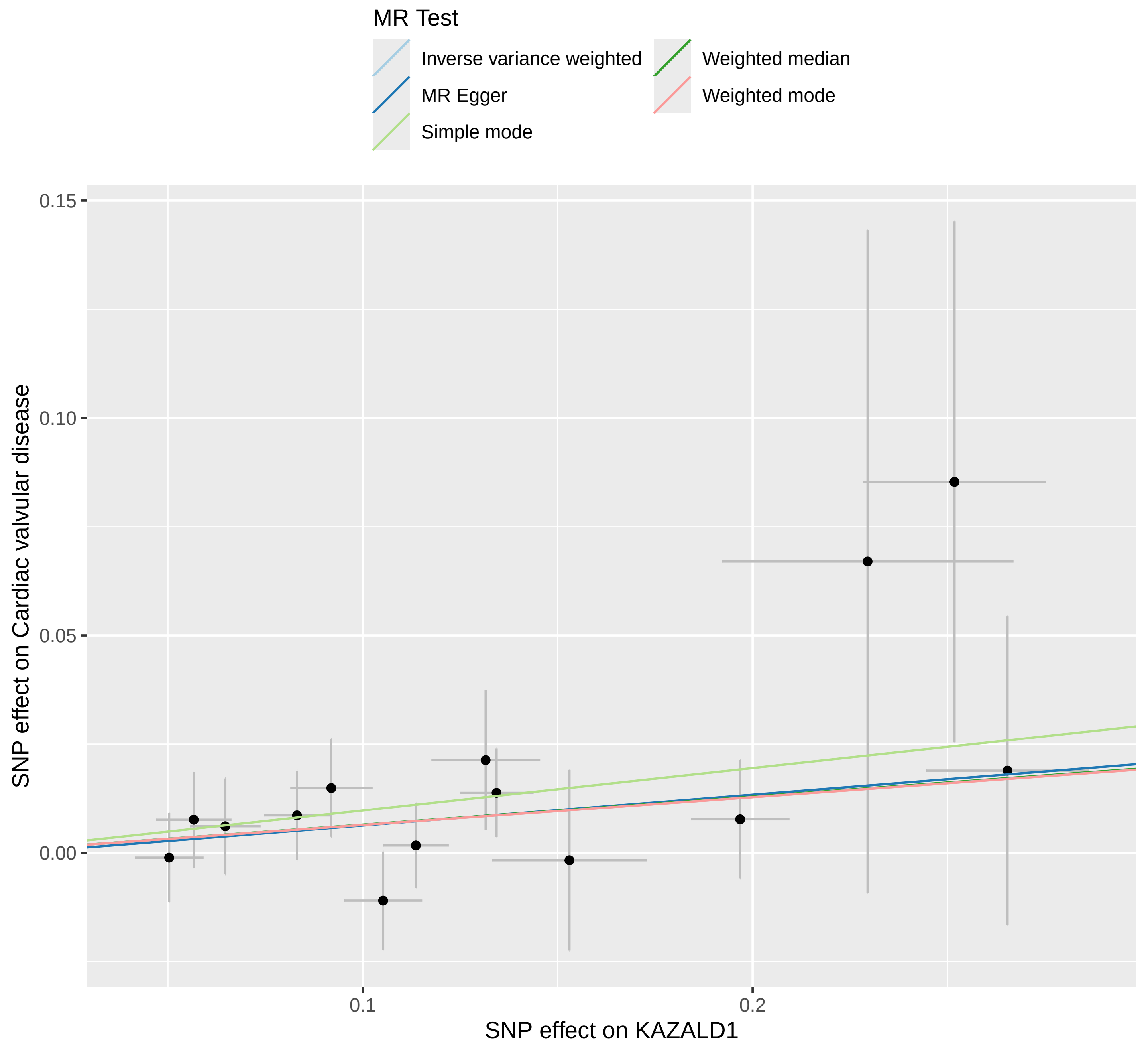
MR Test
Inverse variance weighted
MR Egger
Simple mode
Weighted median
Weighted mode
0.15
0.10
0.05
0.00
0.1
0.2
SNP effect on Cardiac valvular disease
SNP effect on KAZALD1

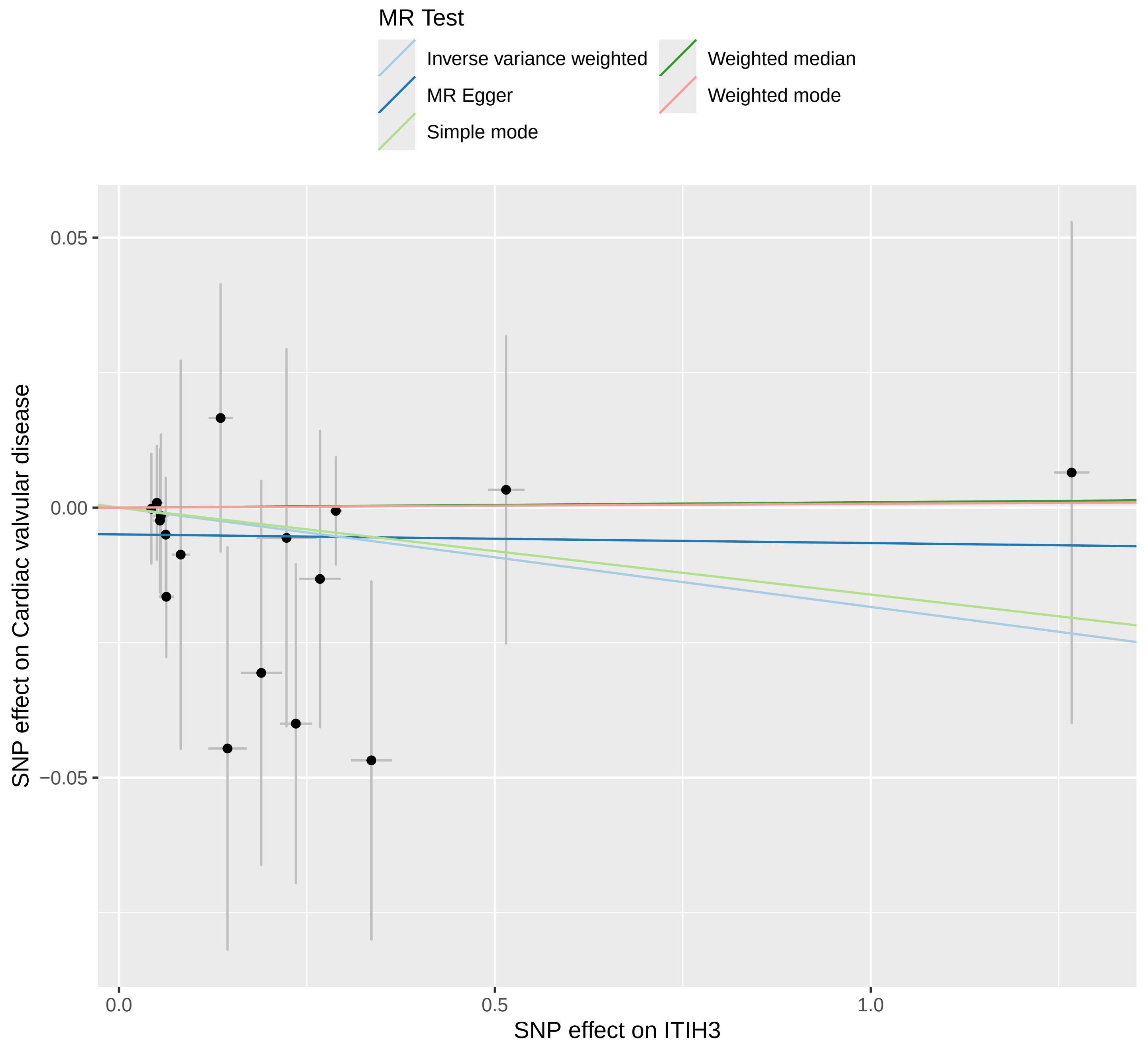
MR Test
Inverse variance weighted
MR Egger
Simple mode
Weighted median
Weighted mode
0.05
0.00
−0.05
0.0
0.5
1.0
SNP effect on Cardiac valvular disease
SNP effect on ITIH3

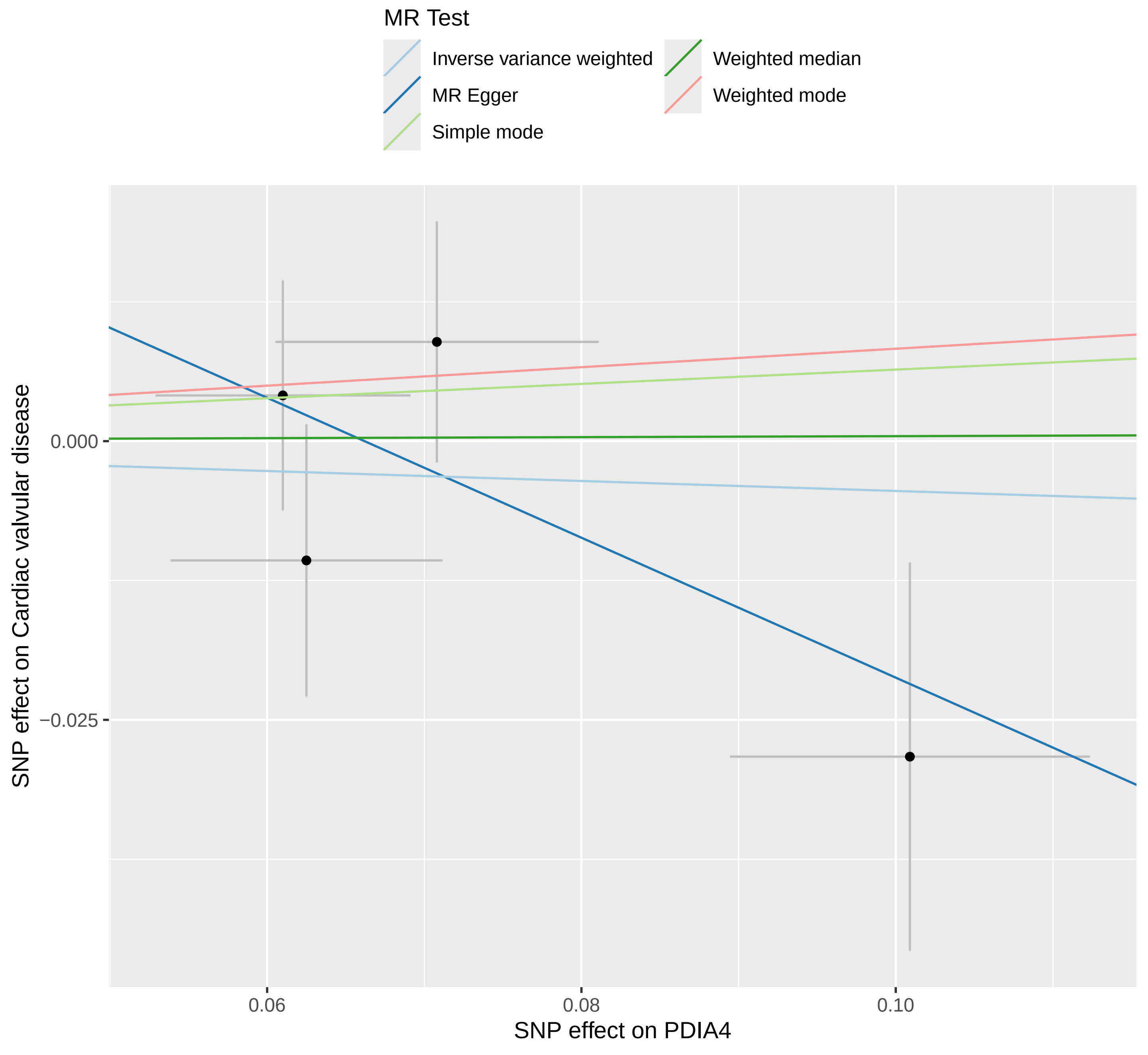
MR Test
Inverse variance weighted
MR Egger
Simple mode
Weighted median
Weighted mode
SNP effect on Cardiac valvular disease
0.000
−0.025
0.06
0.08
0.10
SNP effect on PDIA4

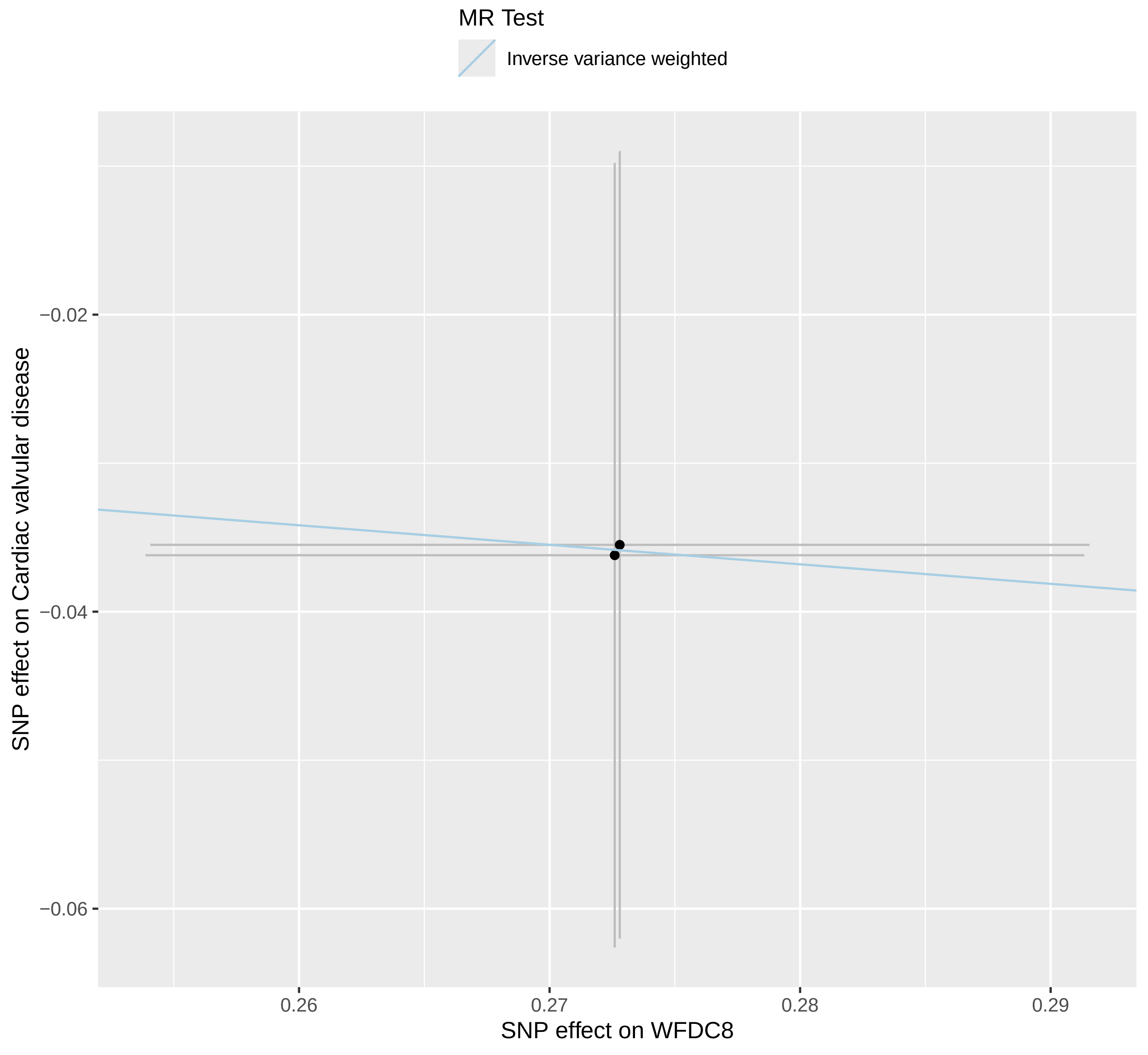
MR Test
Inverse variance weighted
SNP effect on Cardiac valvular disease
−0.02
−0.04
−0.06
0.26
0.27
0.28
0.29
SNP effect on WFDC8

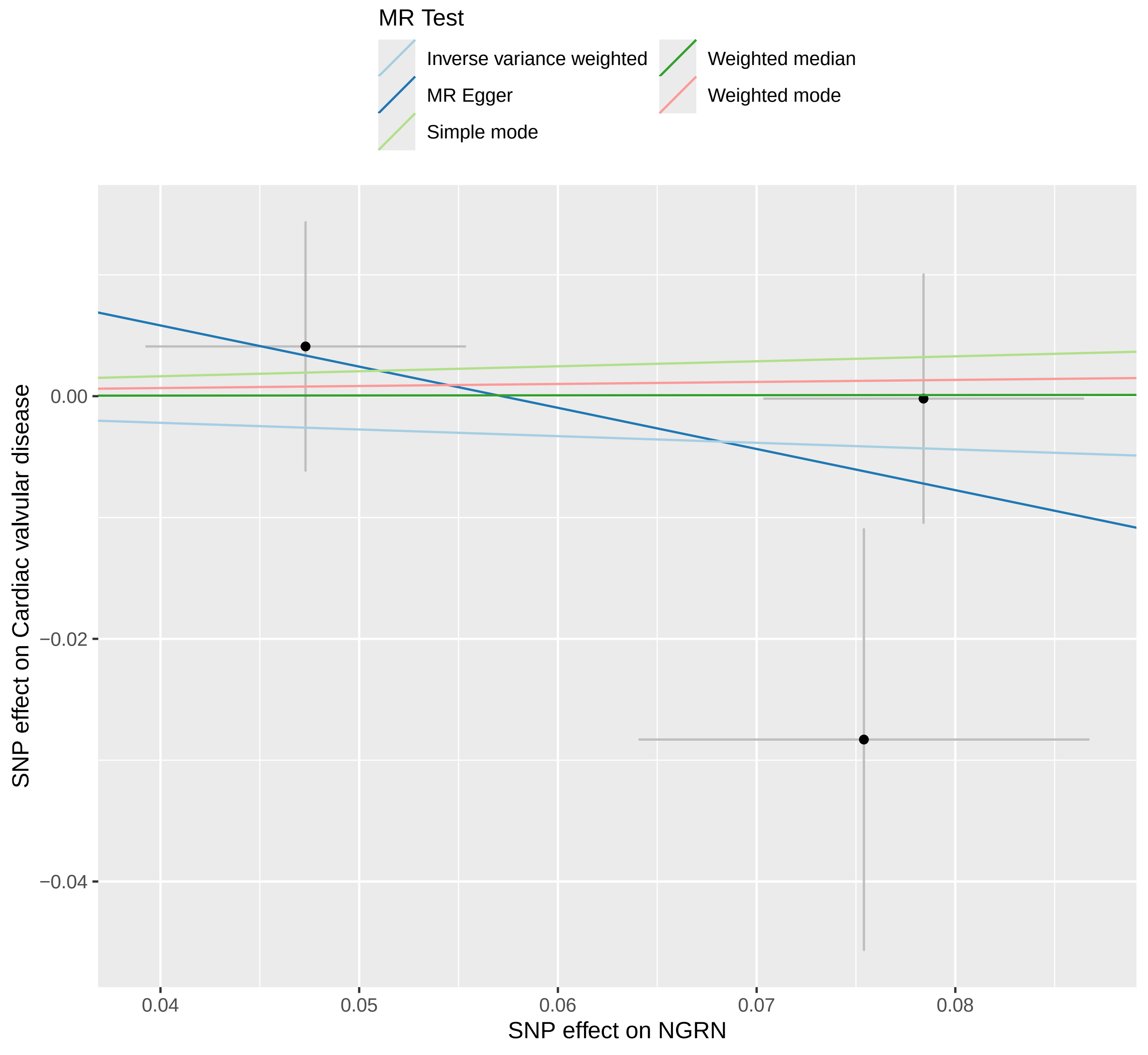
MR Test
Inverse variance weighted
MR Egger
Simple mode
Weighted median
Weighted mode
0.00
−0.02
−0.04
0.04
0.05
0.06
0.07
0.08
SNP effect on NGRN
SNP effect on Cardiac valvular disease

Insufficient number of SNPs

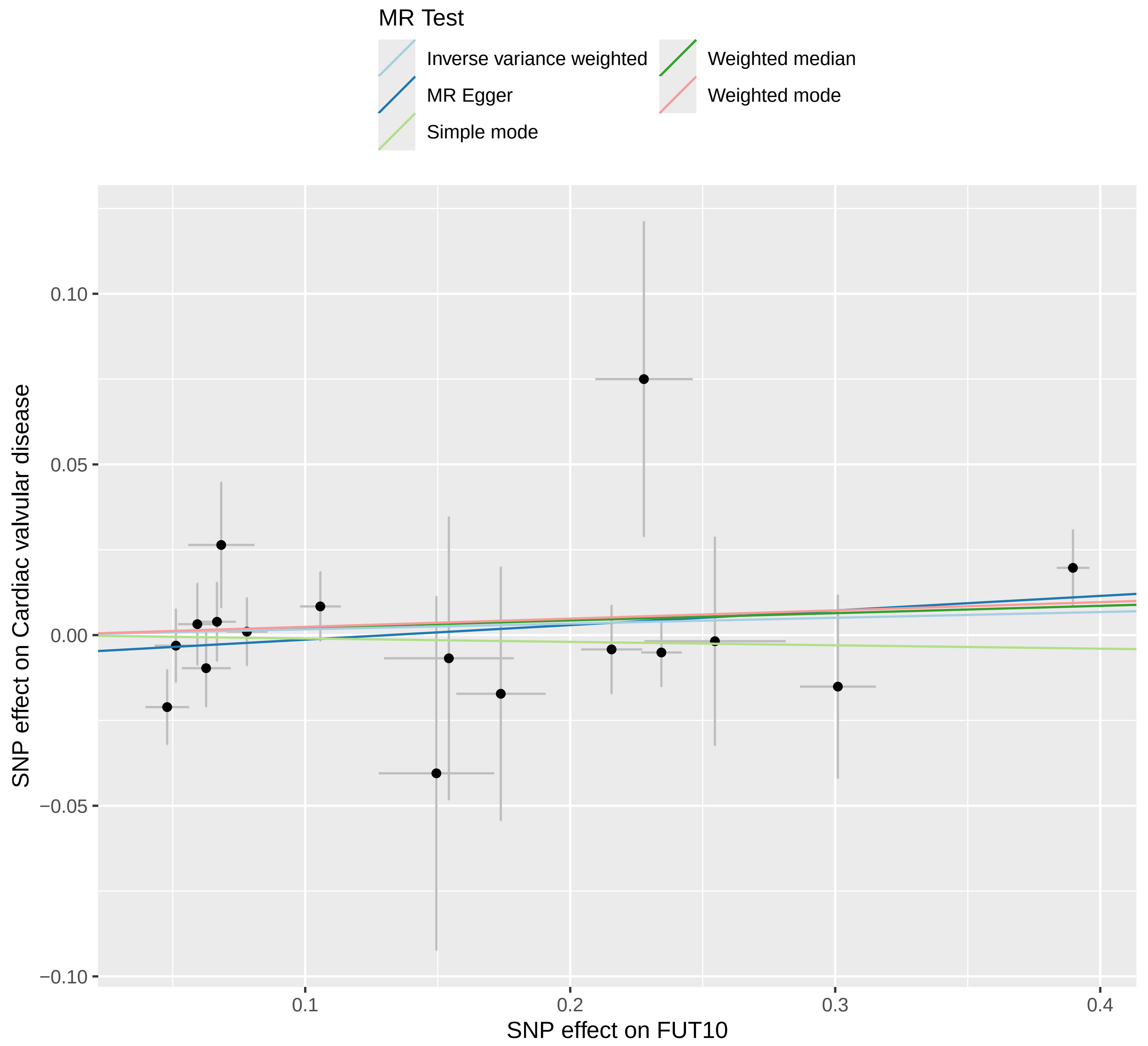
MR Test
Inverse variance weighted
MR Egger
Simple mode
Weighted median
Weighted mode
0.10
0.05
0.00
−0.05
−0.10
SNP effect on Cardiac valvular disease
0.1
0.2
0.3
0.4
SNP effect on FUT10

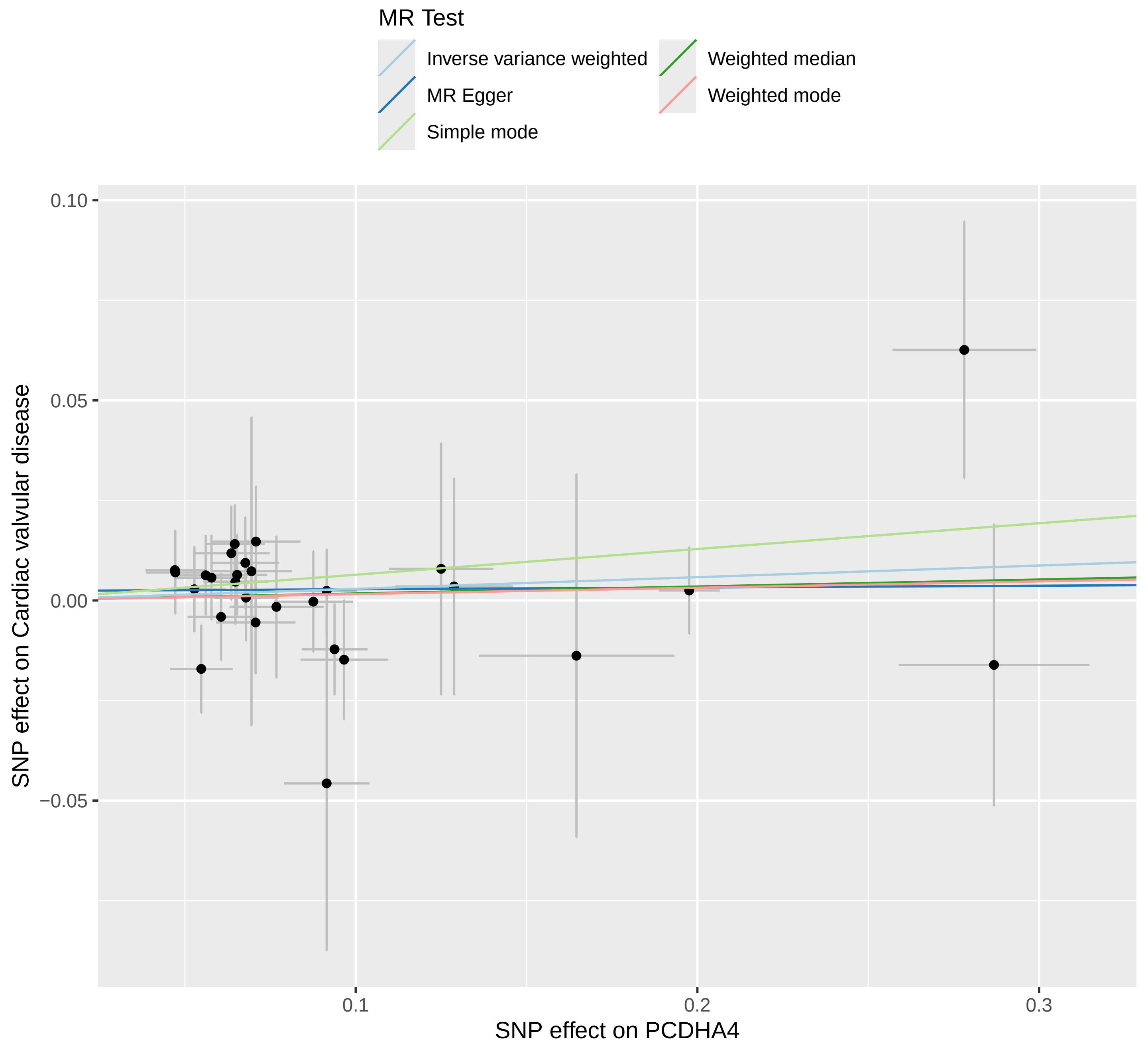
MR Test
Inverse variance weighted
MR Egger
Simple mode
Weighted median
Weighted mode
0.10
0.05
0.00
−0.05
SNP effect on Cardiac valvular disease
0.1
0.2
0.3
SNP effect on PCDHA4

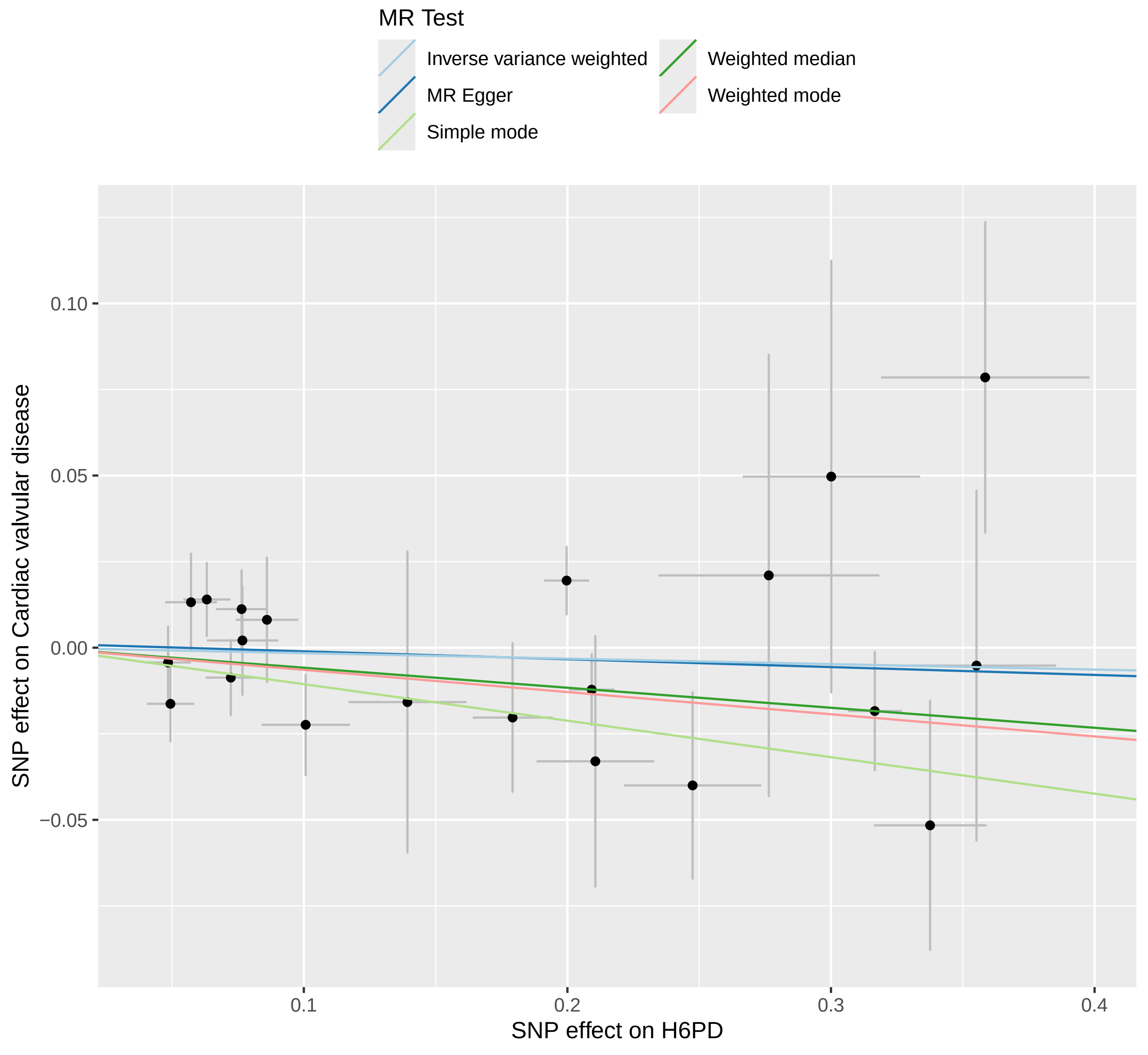

MR Test
Inverse variance weighted
MR Egger
Simple mode
Weighted median
Weighted mode
0.10
0.05
0.00
−0.05
SNP effect on Cardiac valvular disease
0.1
0.2
0.3
0.4
SNP effect on H6PD

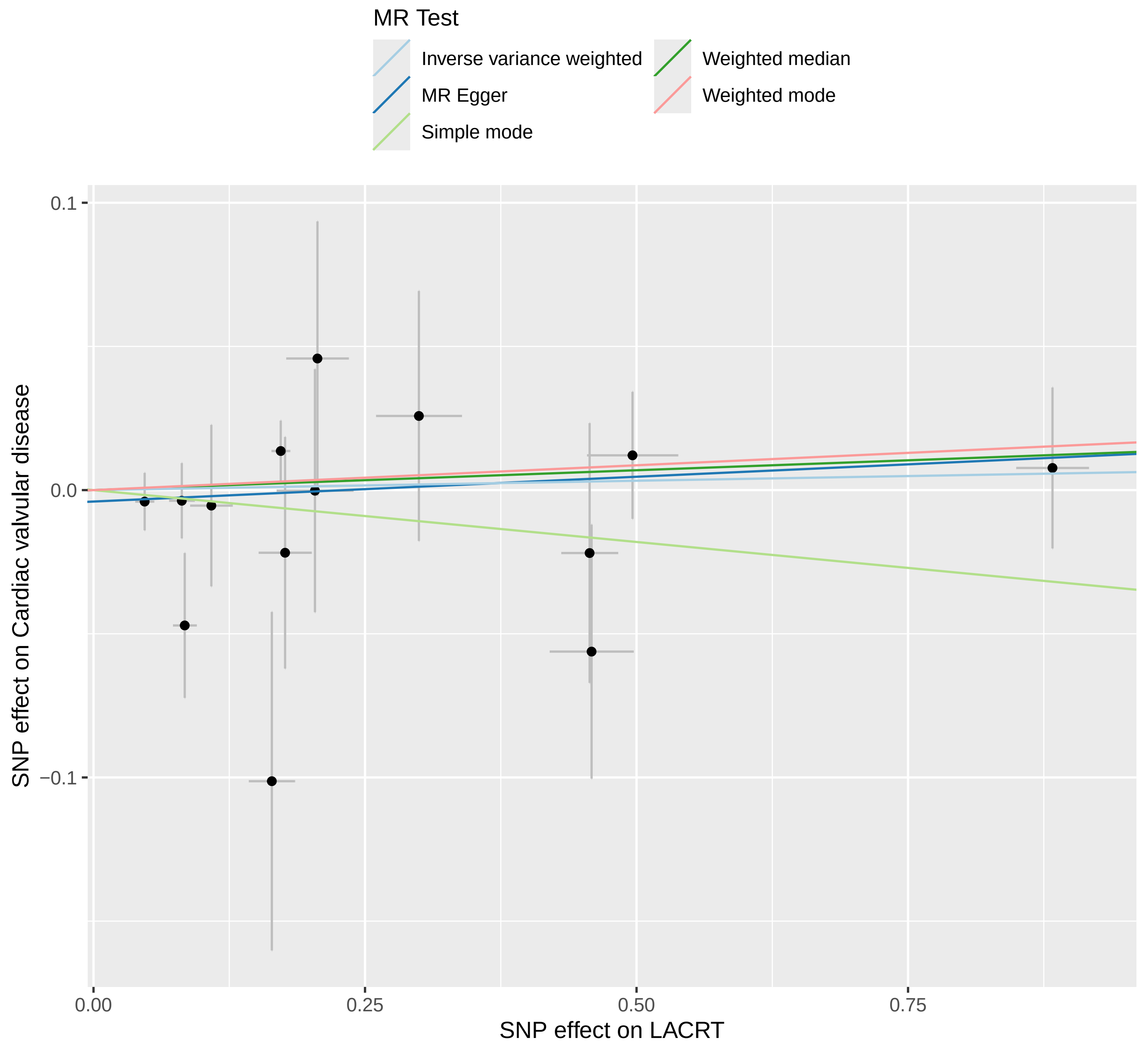

MR Test
Inverse variance weighted
MR Egger
Simple mode
Weighted median
Weighted mode
0.1
0.0
−0.1
SNP effect on Cardiac valvular disease
0.00
0.25
0.50
0.75
SNP effect on LACRT

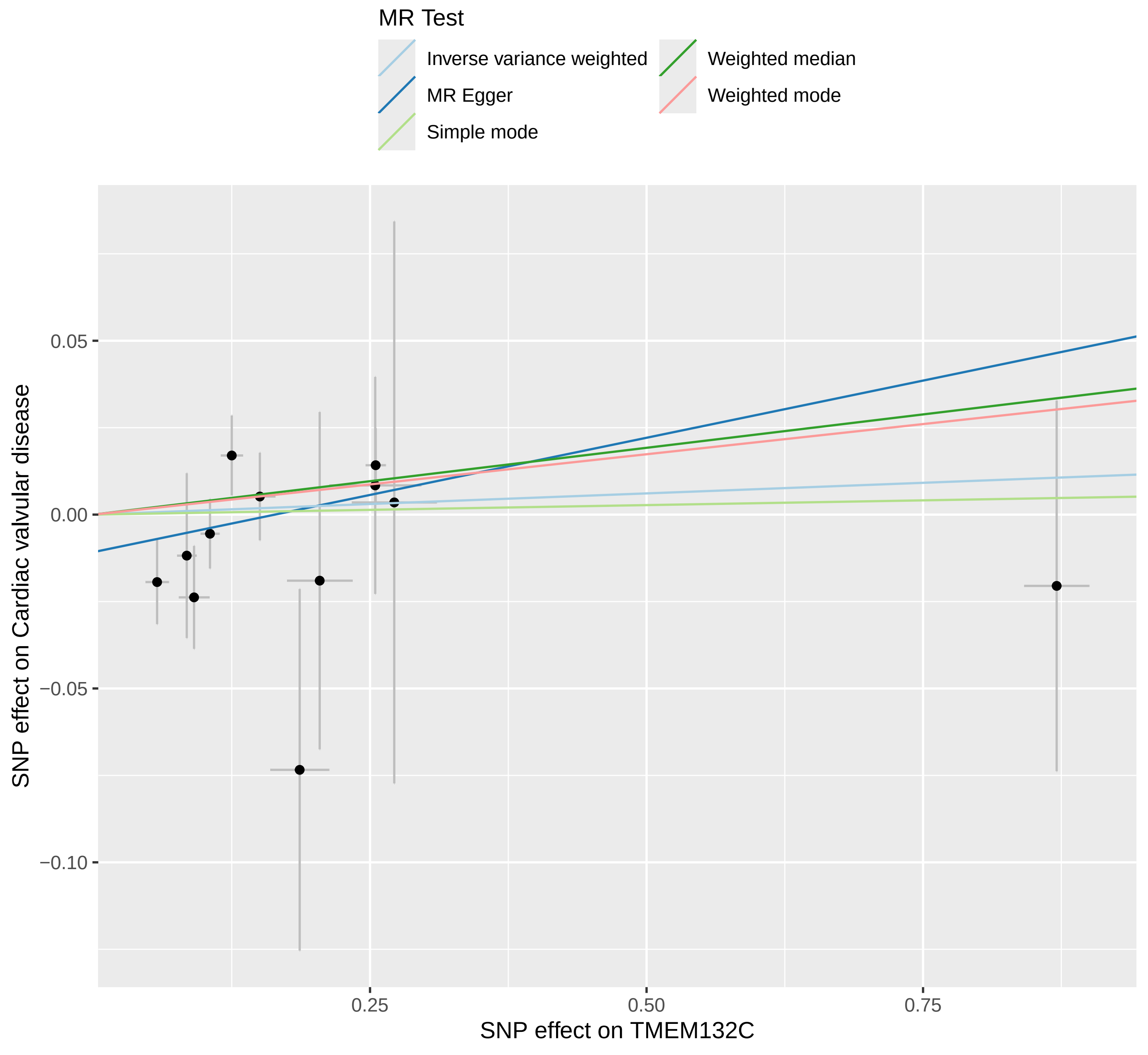
MR Test
Inverse variance weighted
MR Egger
Simple mode
Weighted median
Weighted mode
SNP effect on Cardiac valvular disease
0.05
0.00
−0.05
−0.10
0.25
0.50
0.75
SNP effect on TMEM132C

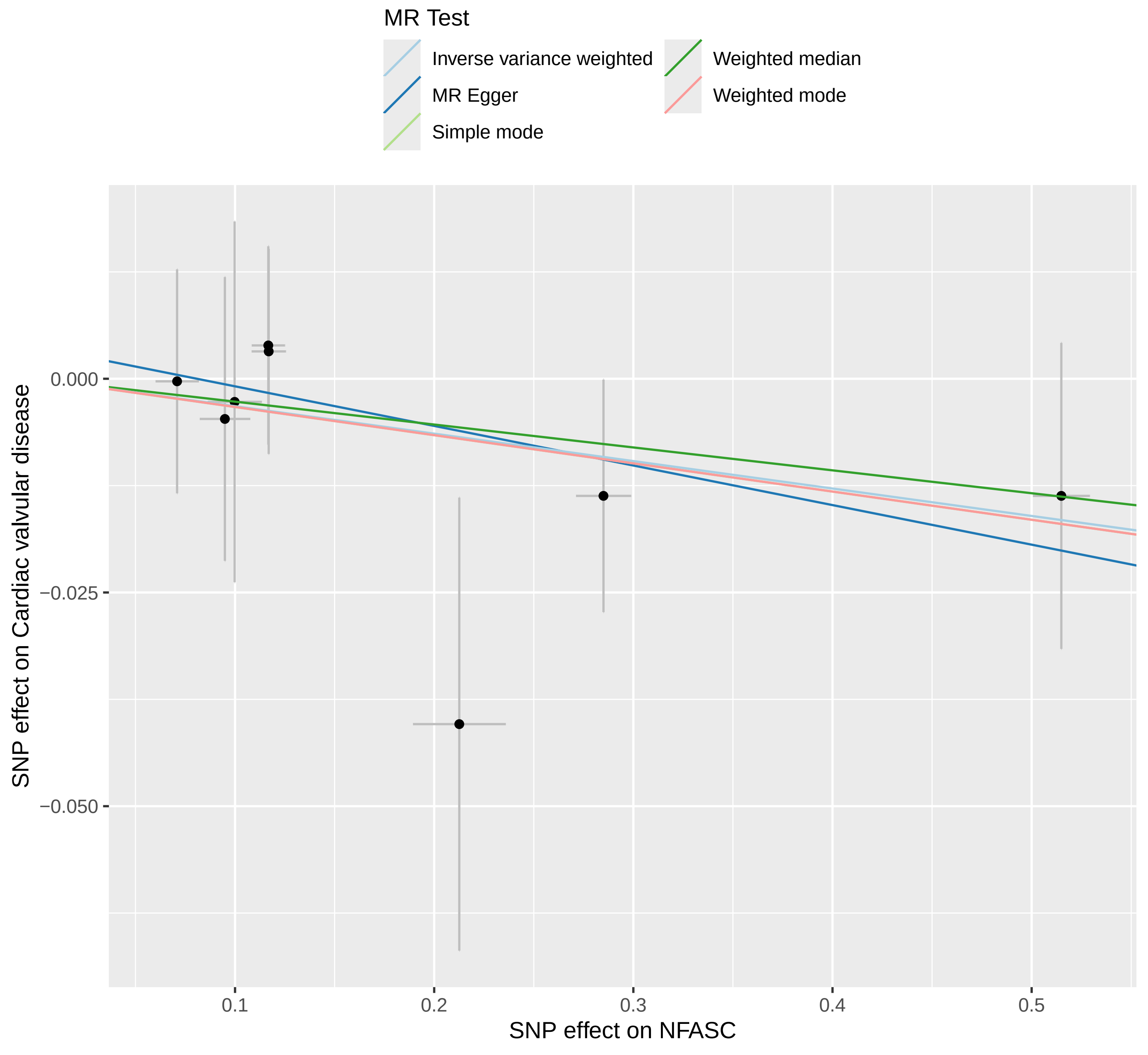
MR Test
Inverse variance weighted
MR Egger
Simple mode
Weighted median
Weighted mode
0.000
−0.025
−0.050
0.1
0.2
0.3
0.4
0.5
SNP effect on Cardiac valvular disease
SNP effect on NFASC

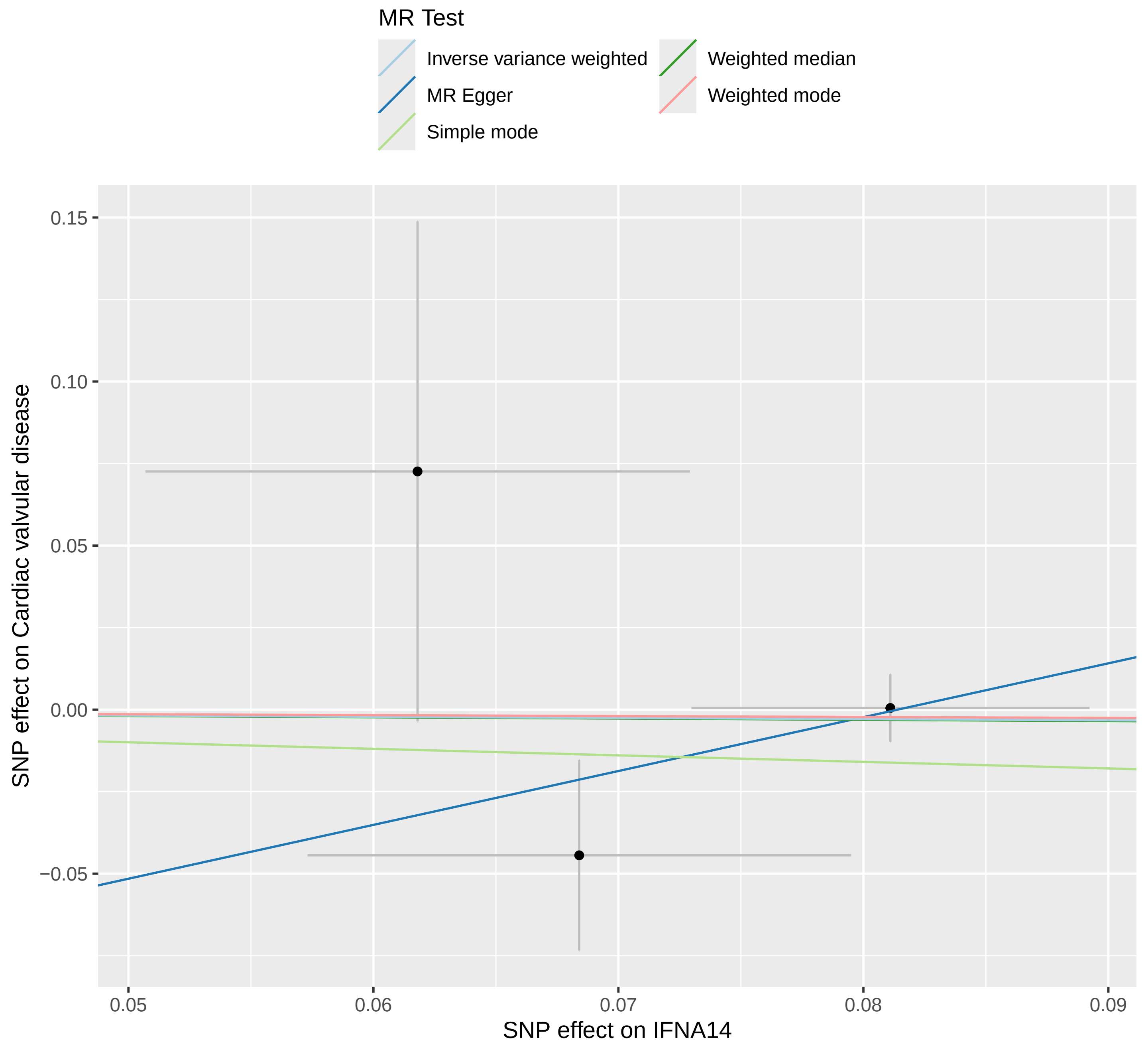
MR Test
Inverse variance weighted
MR Egger
Simple mode
Weighted median
Weighted mode
0.15
0.10
0.05
0.00
−0.05
0.05
0.06
0.07
0.08
0.09
SNP effect on Cardiac valvular disease
SNP effect on IFNA14

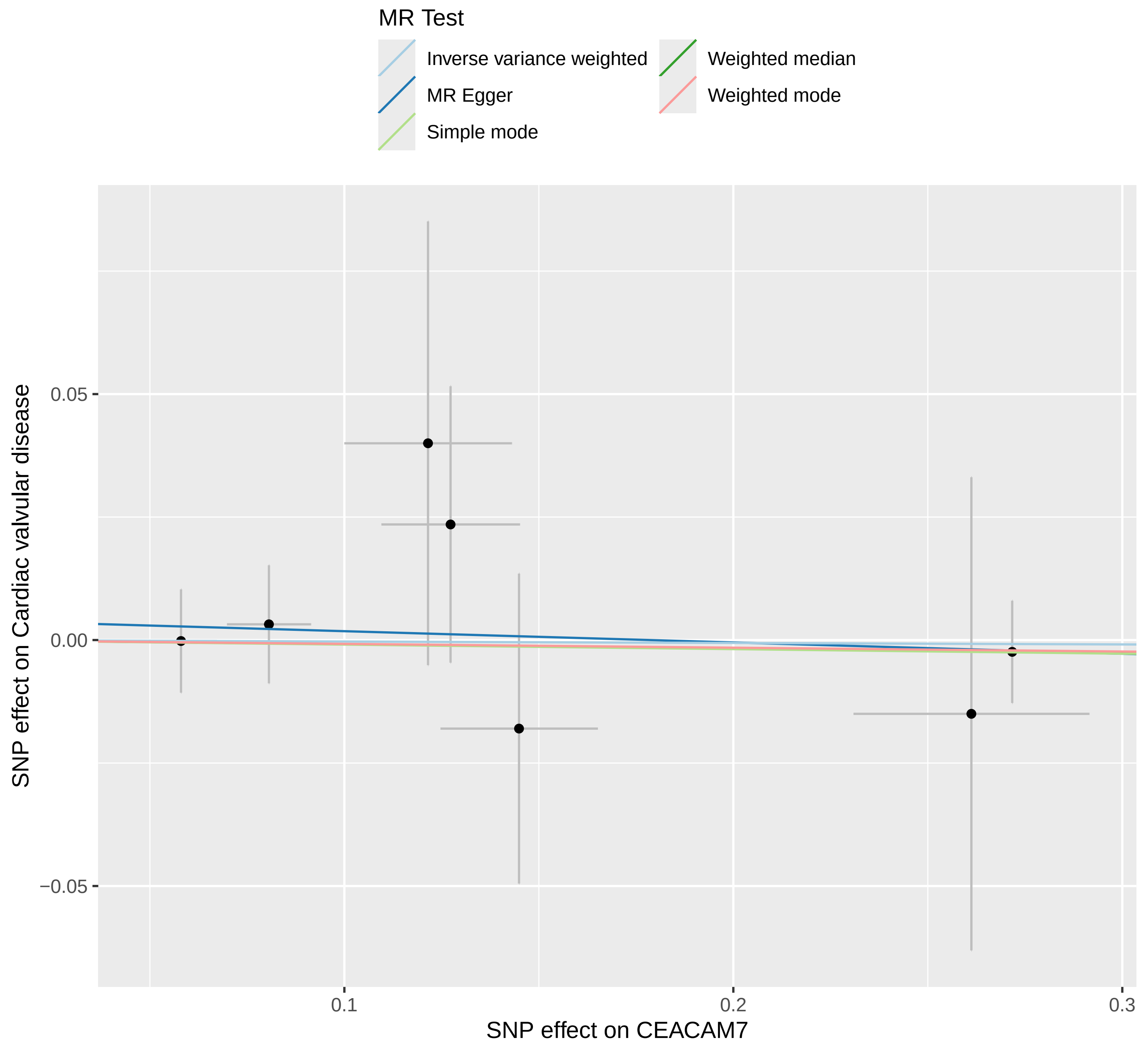

MR Test
Inverse variance weighted
MR Egger
Simple mode
Weighted median
Weighted mode
0.05
0.00
−0.05
SNP effect on Cardiac valvular disease
0.1
0.2
0.3
SNP effect on CEACAM7

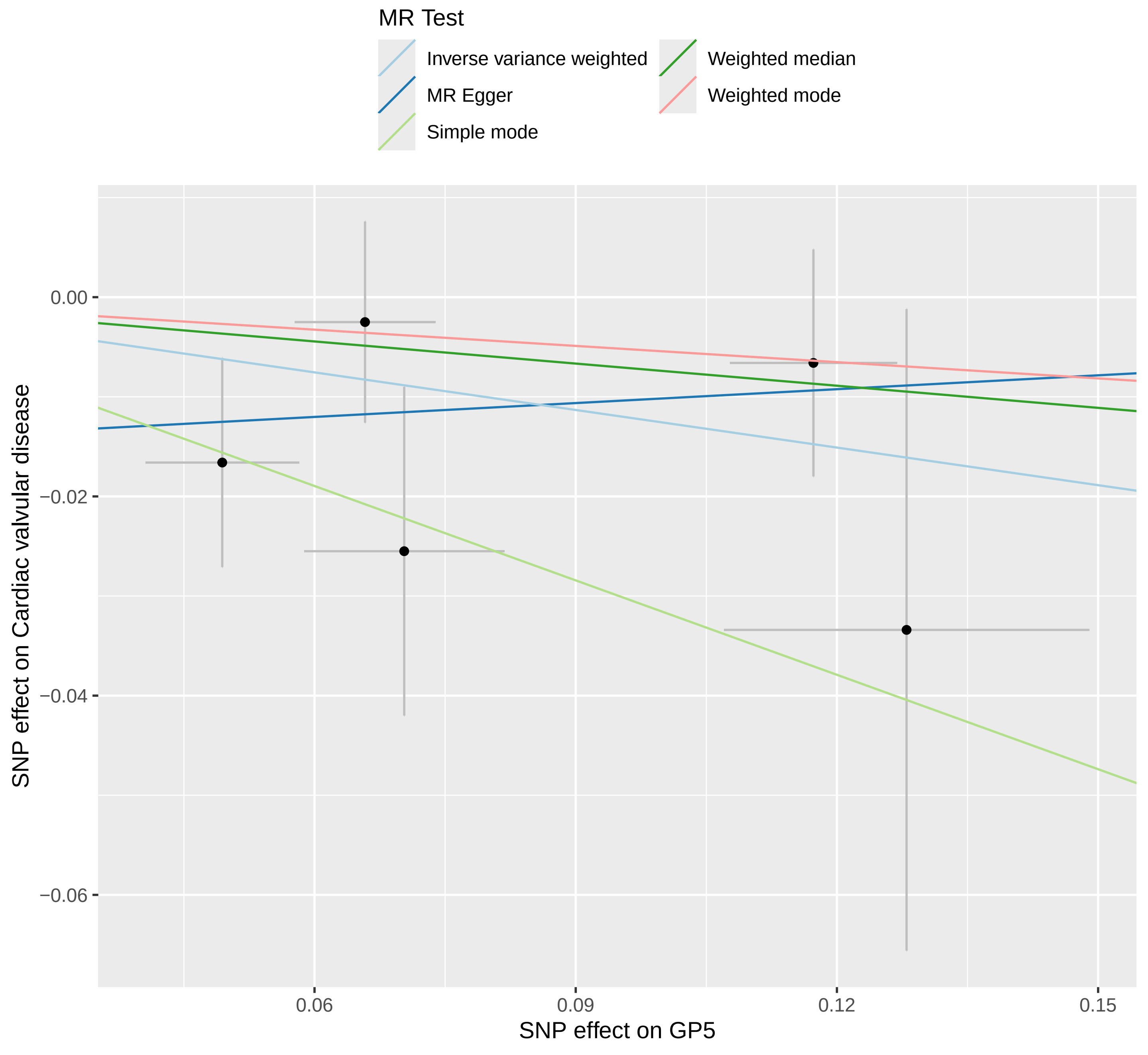

MR Test
Inverse variance weighted
MR Egger
Simple mode
Weighted median
Weighted mode
0.00
−0.02
−0.04
−0.06
0.06
0.09
0.12
0.15
SNP effect on GP5
SNP effect on Cardiac valvular disease

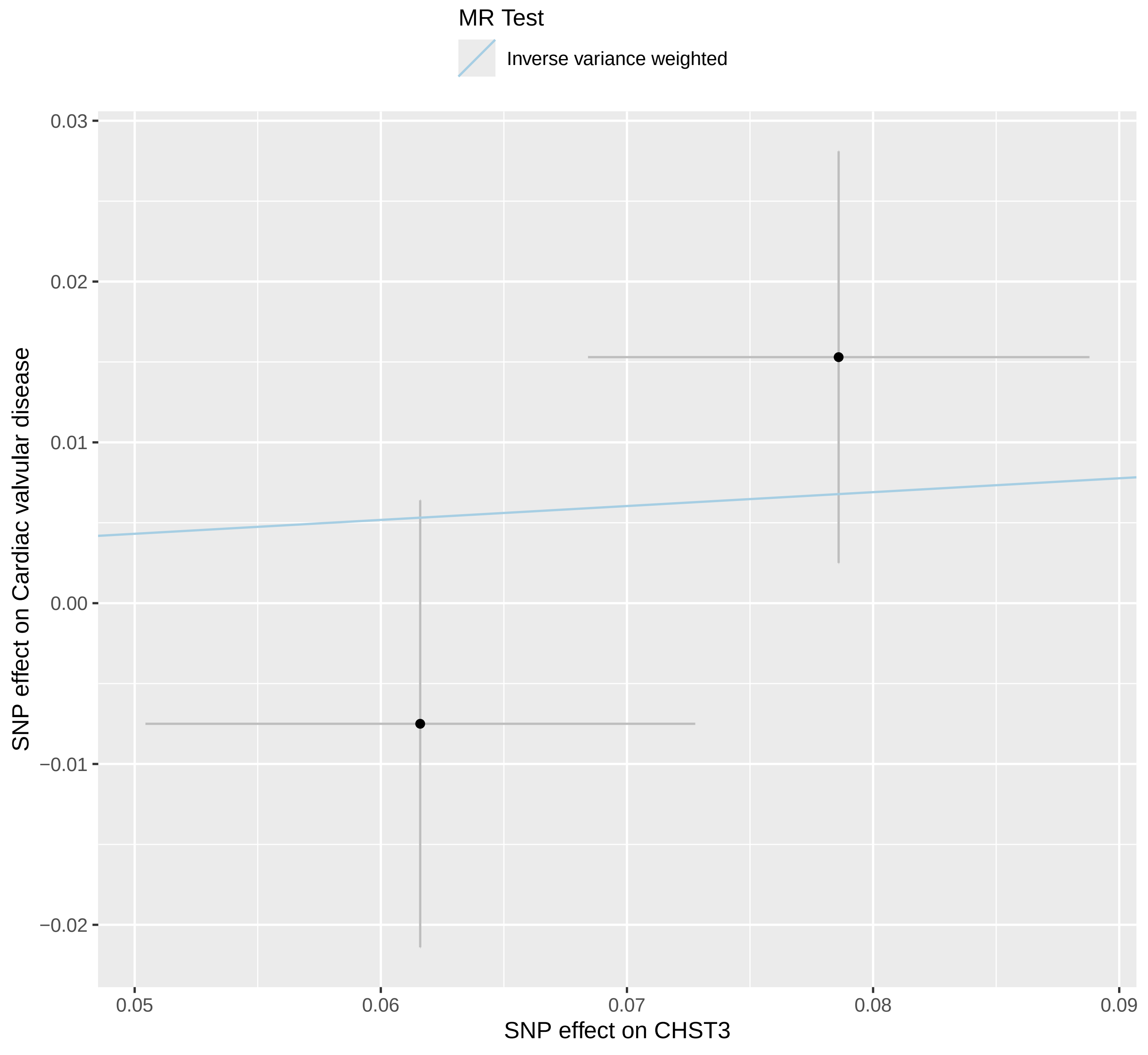

MR Test
Inverse variance weighted
0.03
0.02
0.01
0.00
−0.01
−0.02
SNP effect on Cardiac valvular disease
0.05
0.06
0.07
0.08
0.09
SNP effect on CHST3

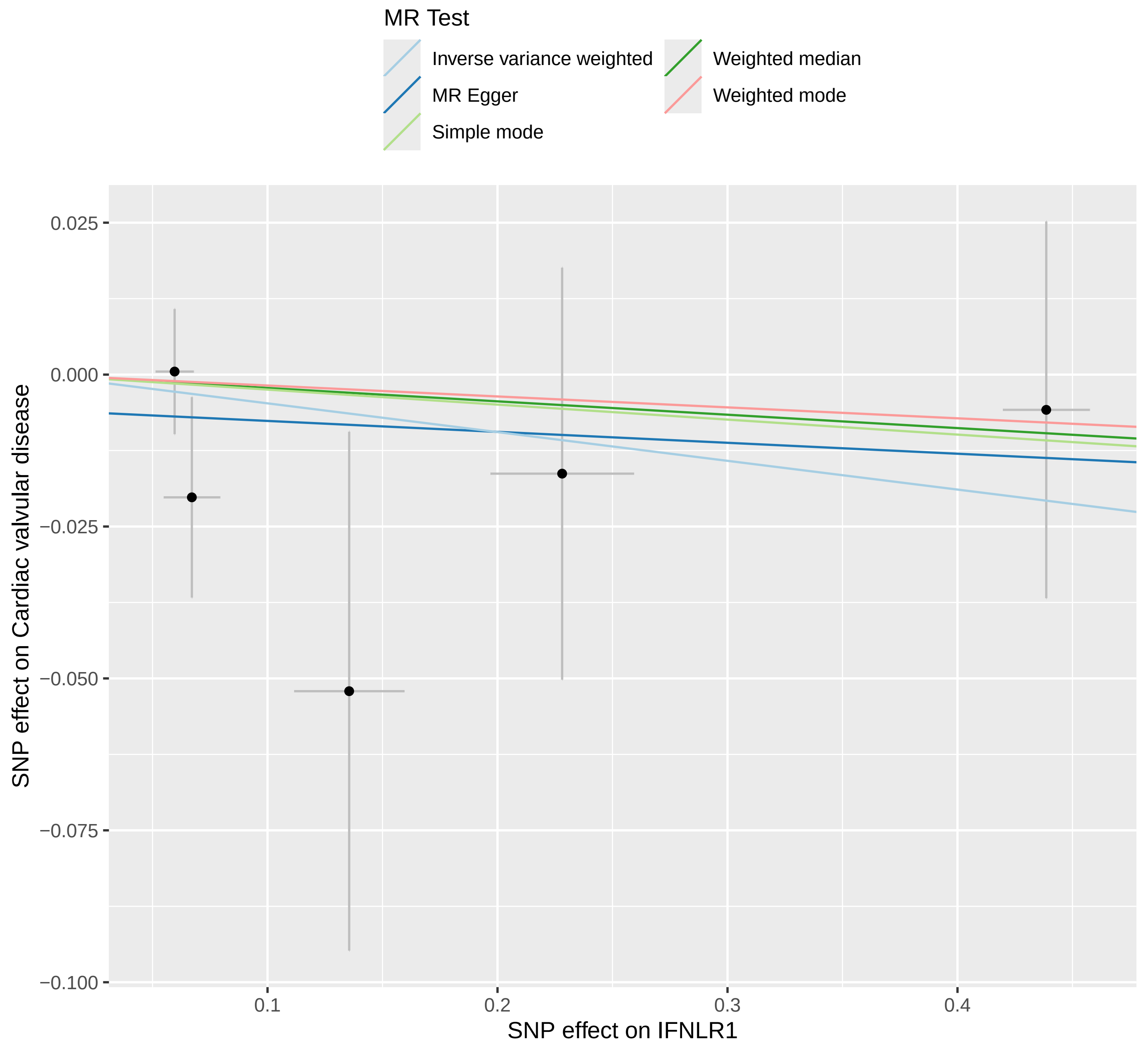

MR Test
Inverse variance weighted
MR Egger
Simple mode
Weighted median
Weighted mode
0.025
0.000
−0.025
−0.050
−0.075
−0.100
0.1
0.2
0.3
0.4
SNP effect on Cardiac valvular disease
SNP effect on IFNLR1

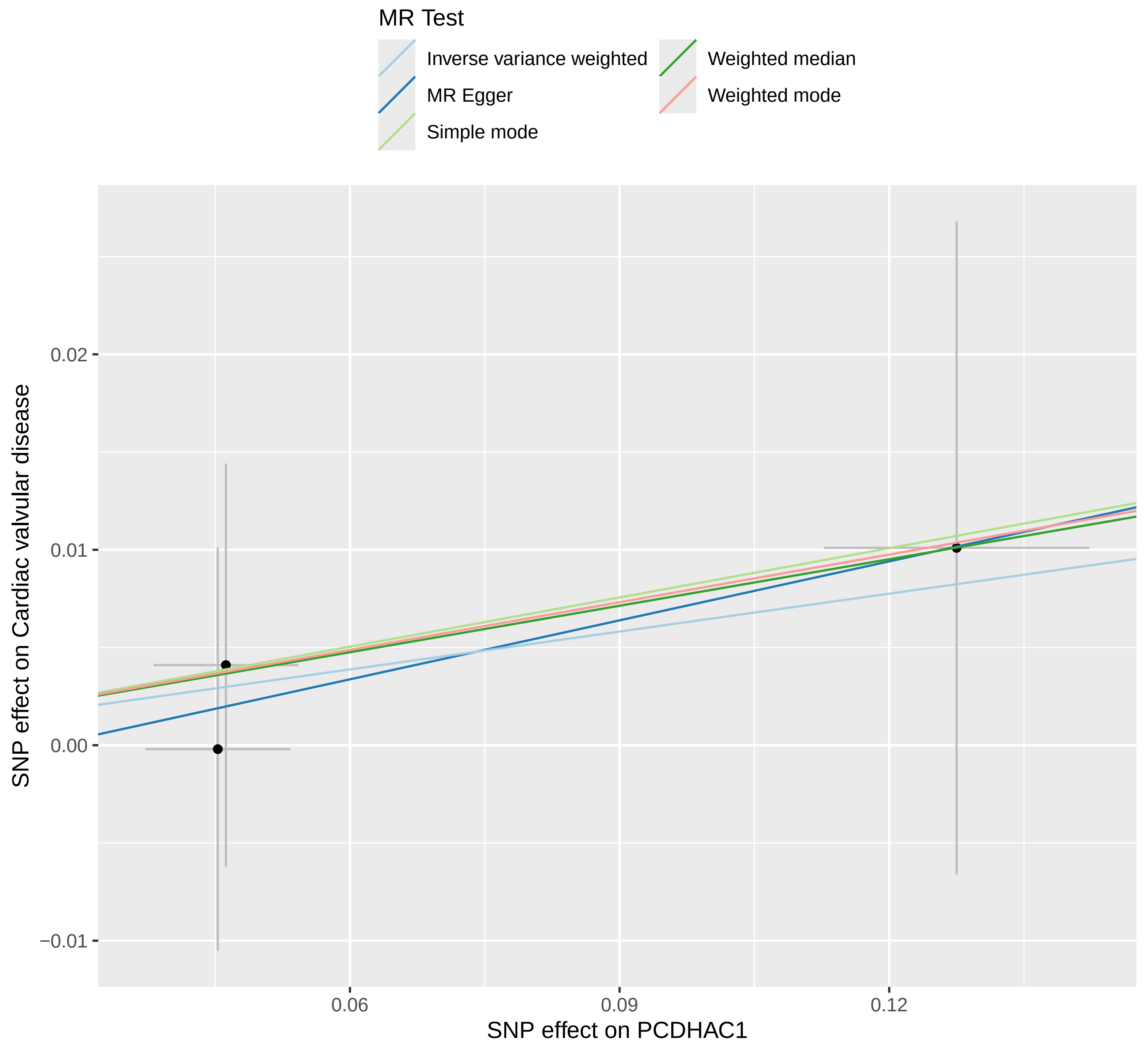
MR Test
Inverse variance weighted
MR Egger
Simple mode
Weighted median
Weighted mode
0.02
0.01
0.00
−0.01
0.06
0.09
0.12
SNP effect on Cardiac valvular disease
SNP effect on PCDHAC1

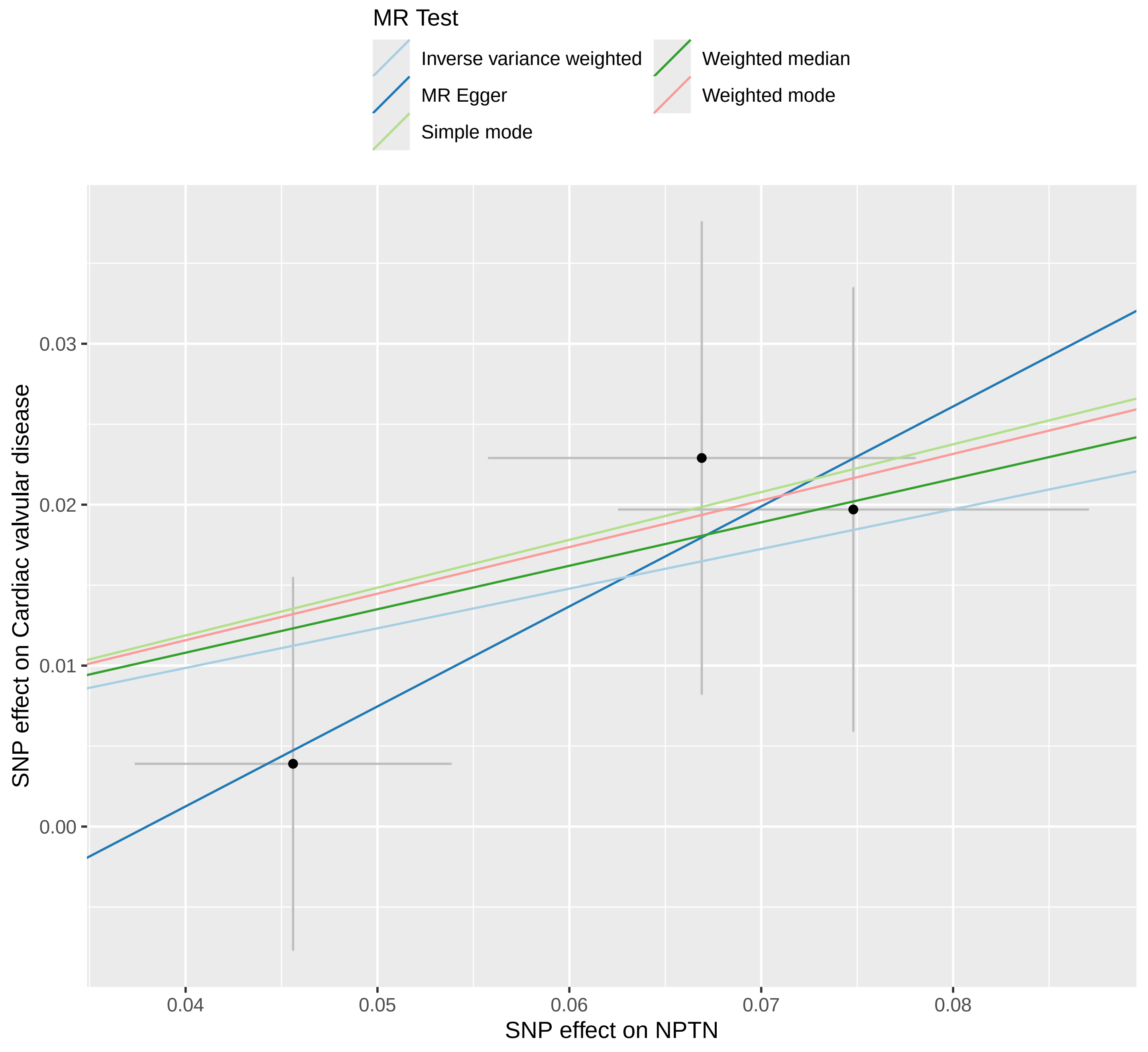

MR Test
Inverse variance weighted
MR Egger
Simple mode
Weighted median
Weighted mode
SNP effect on Cardiac valvular disease
0.03
0.02
0.01
0.00
0.04
0.05
0.06
0.07
0.08
SNP effect on NPTN

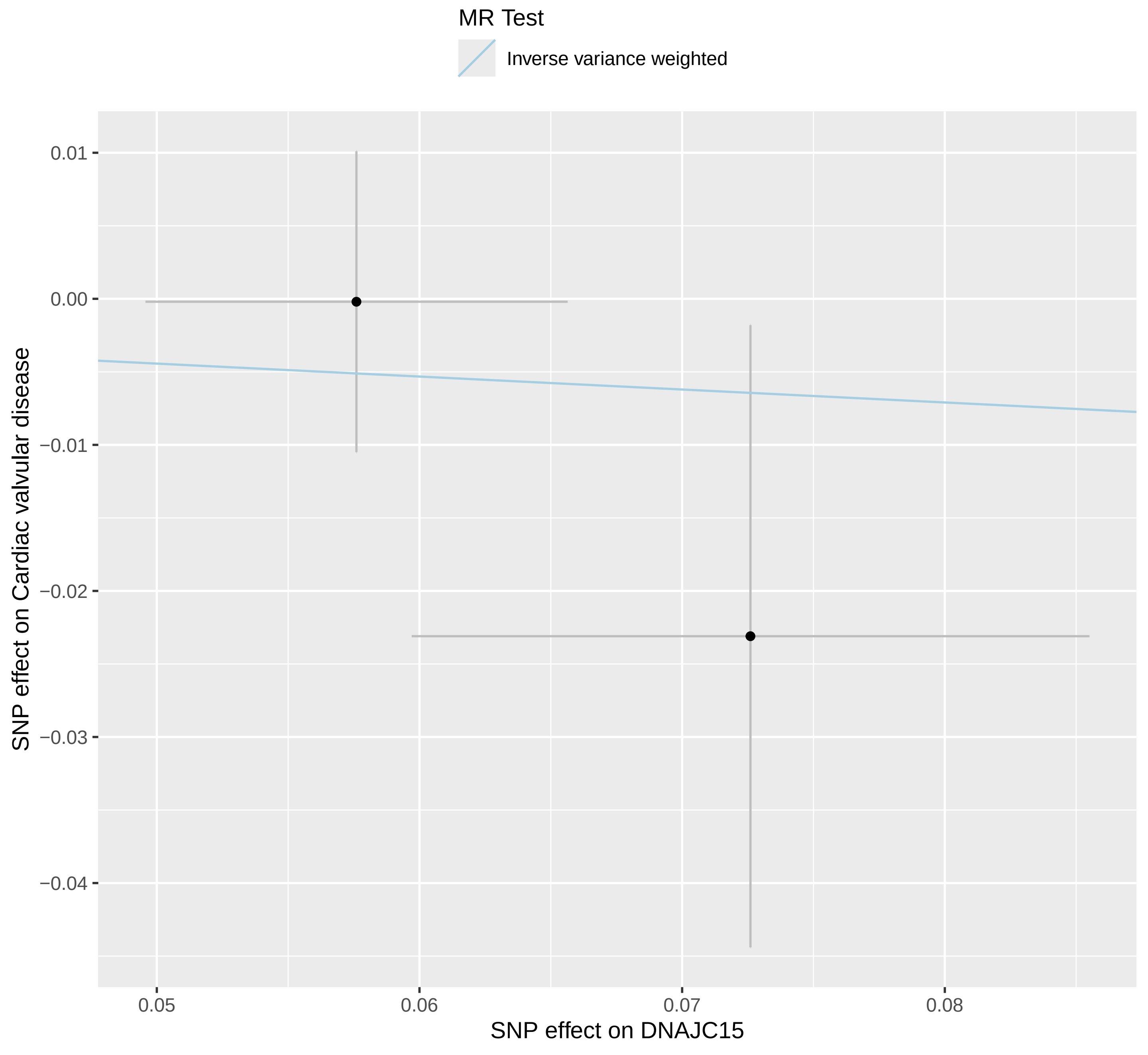

MR Test
Inverse variance weighted
0.01
0.00
−0.01
−0.02
−0.03
−0.04
SNP effect on Cardiac valvular disease
0.05
0.06
0.07
0.08
SNP effect on DNAJC15

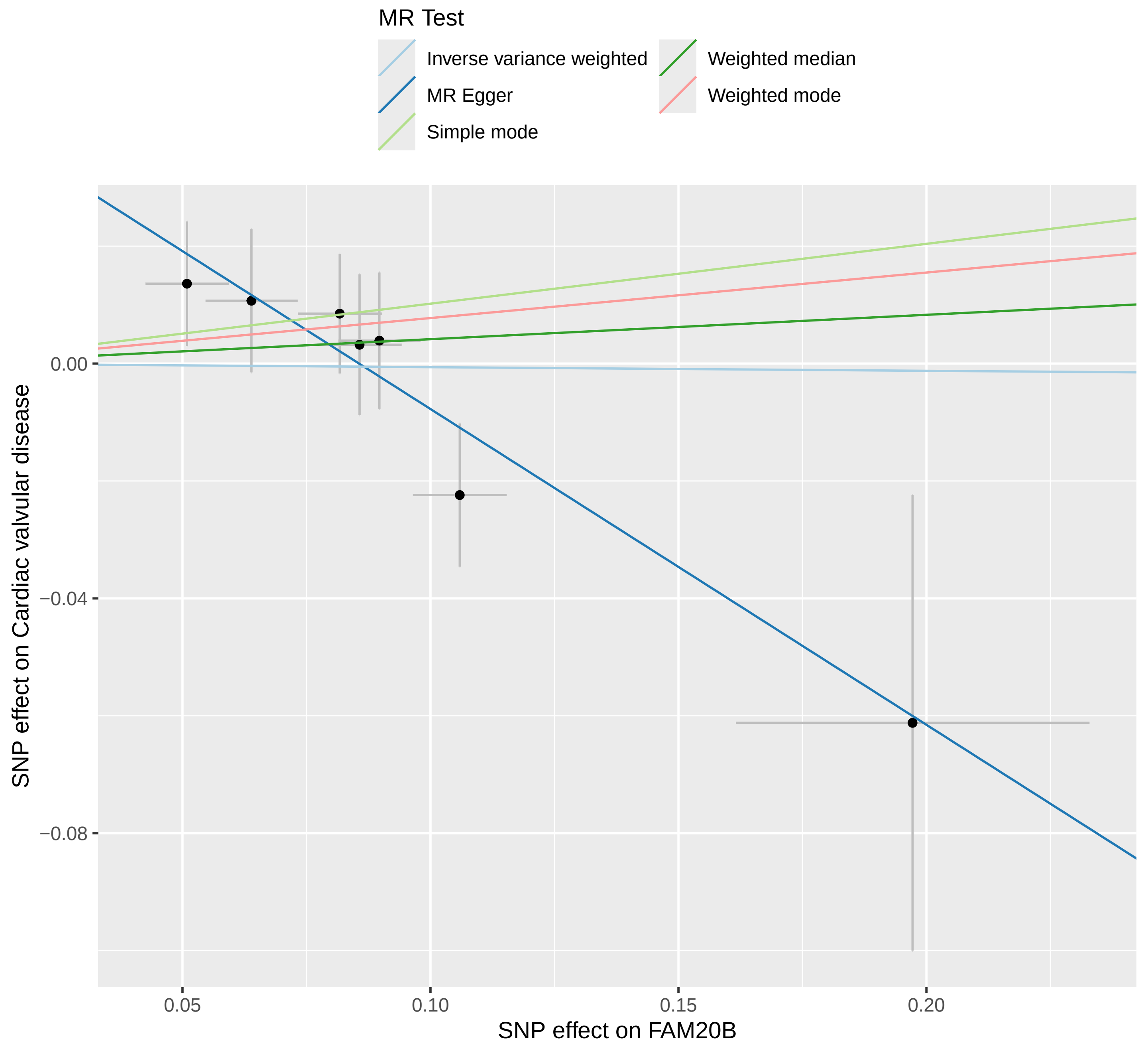

MR Test
Inverse variance weighted
MR Egger
Simple mode
Weighted median
Weighted mode
0.00
−0.04
−0.08
0.05
0.10
0.15
0.20
SNP effect on FAM20B
SNP effect on Cardiac valvular disease

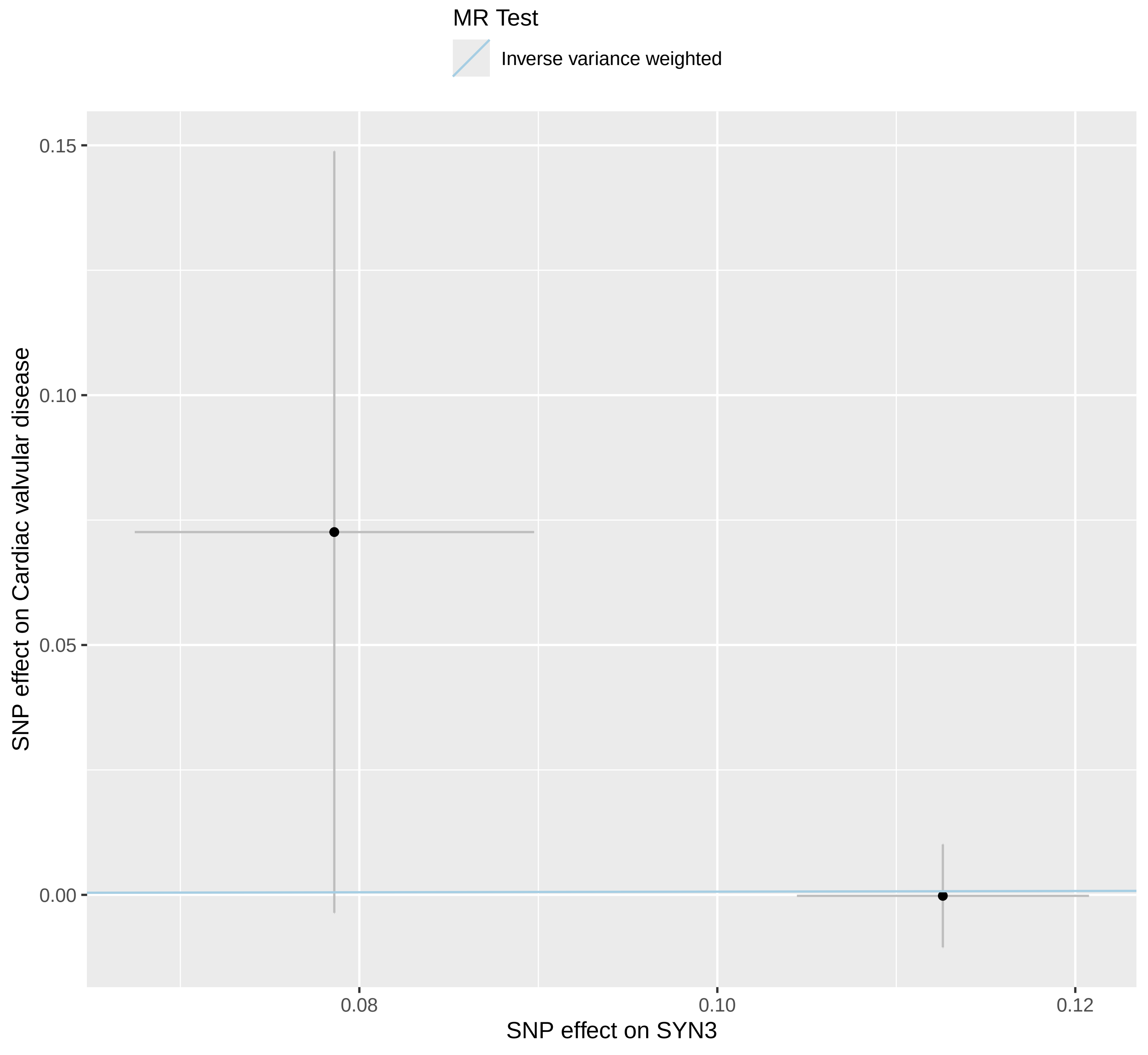

MR Test
Inverse variance weighted
0.15
0.10
0.05
0.00
SNP effect on Cardiac valvular disease
0.08
0.10
0.12
SNP effect on SYN3

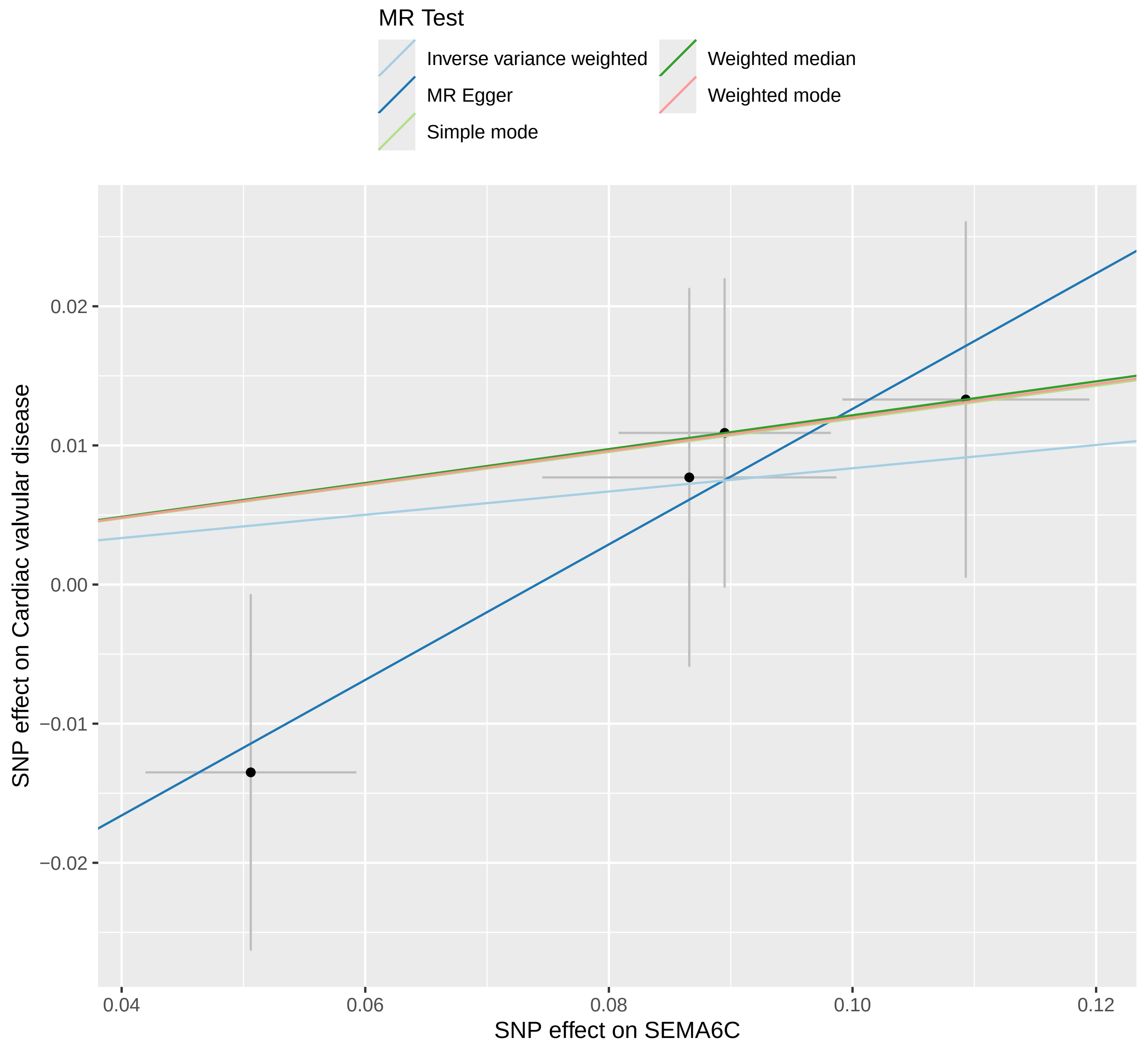
MR Test
Inverse variance weighted
MR Egger
Simple mode
Weighted median
Weighted mode
0.02
0.01
0.00
−0.01
−0.02
SNP effect on Cardiac valvular disease
0.04
0.06
0.08
0.10
0.12
SNP effect on SEMA6C

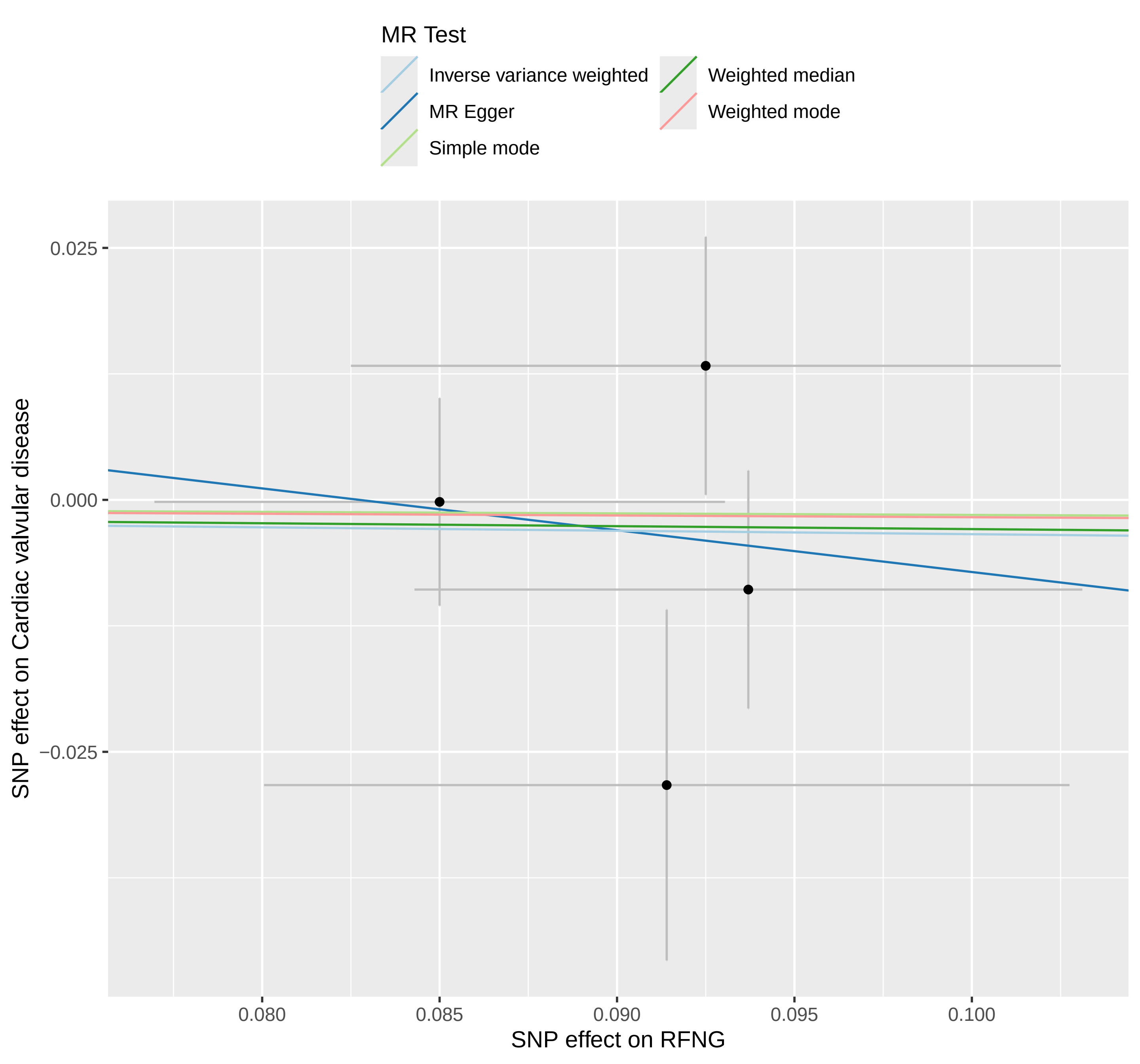

MR Test
Inverse variance weighted
MR Egger
Simple mode
Weighted median
Weighted mode
0.025
0.000
−0.025
0.080
0.085
0.090
0.095
0.100
SNP effect on Cardiac valvular disease
SNP effect on RFNG

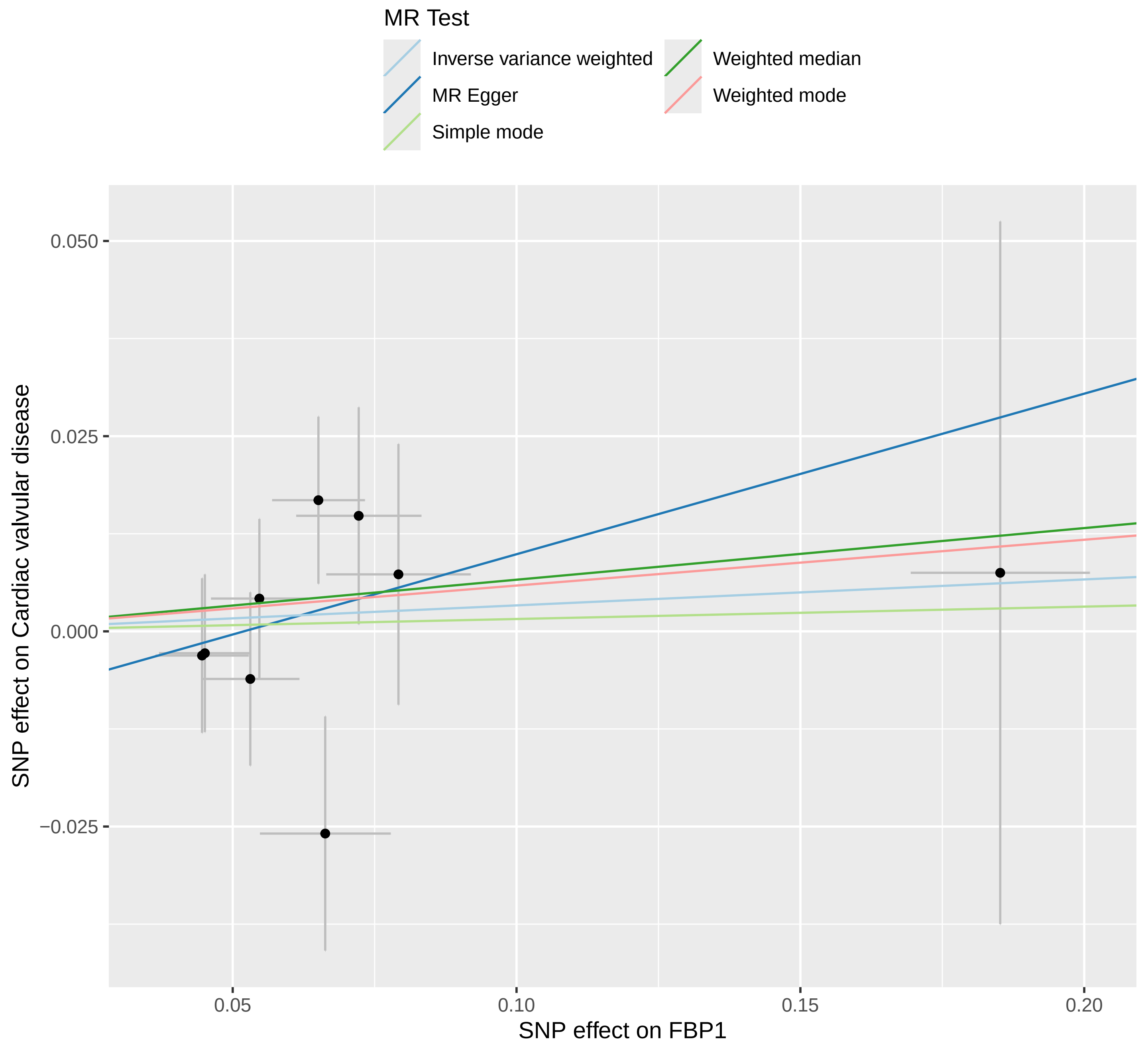

MR Test
Inverse variance weighted
MR Egger
Simple mode
Weighted median
Weighted mode
0.050
0.025
0.000
−0.025
0.05
0.10
0.15
0.20
SNP effect on FBP1
SNP effect on Cardiac valvular disease

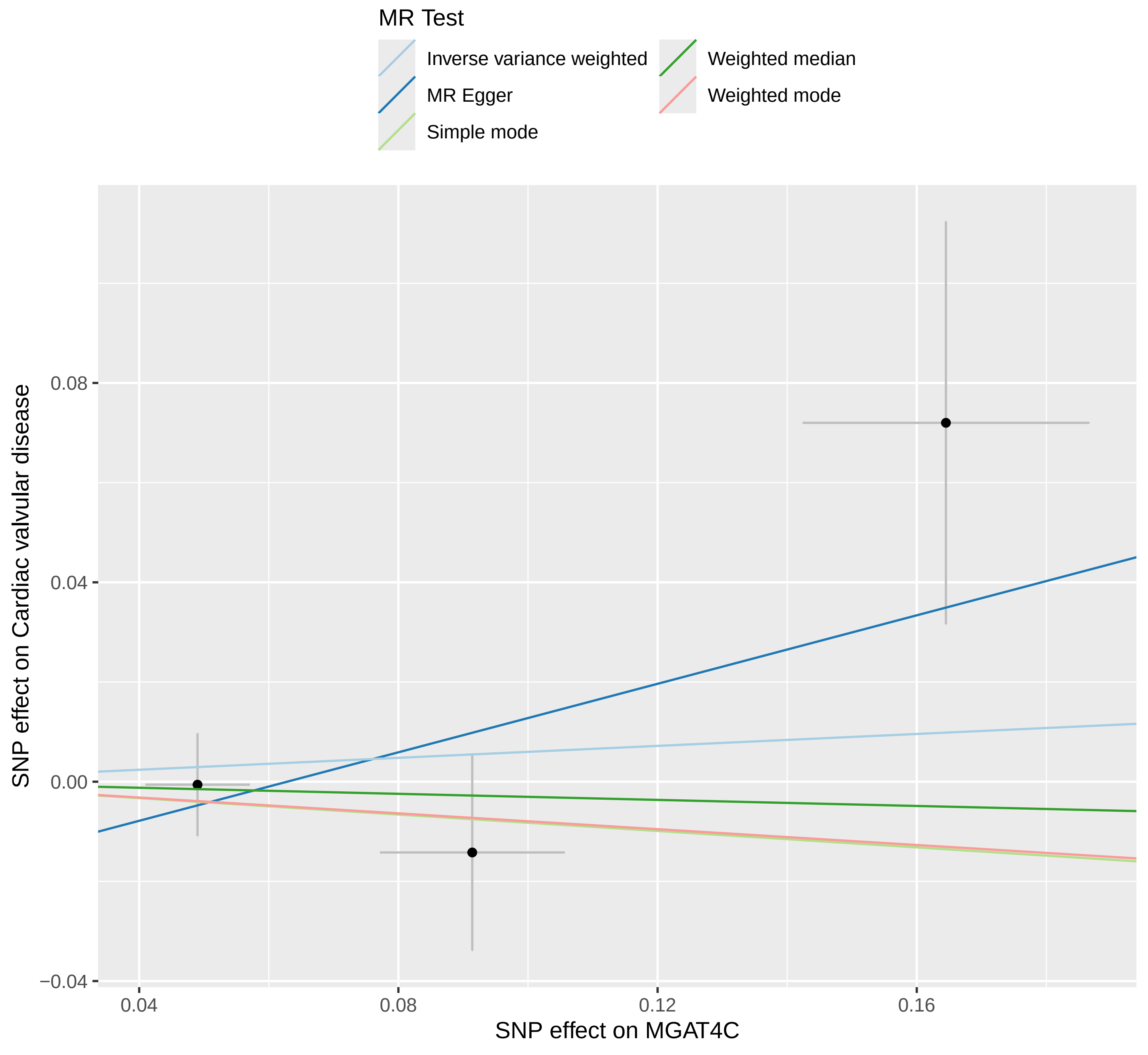
MR Test
Inverse variance weighted
MR Egger
Simple mode
Weighted median
Weighted mode
0.08
0.04
0.00
−0.04
0.04
0.08
0.12
0.16
SNP effect on Cardiac valvular disease
SNP effect on MGAT4C

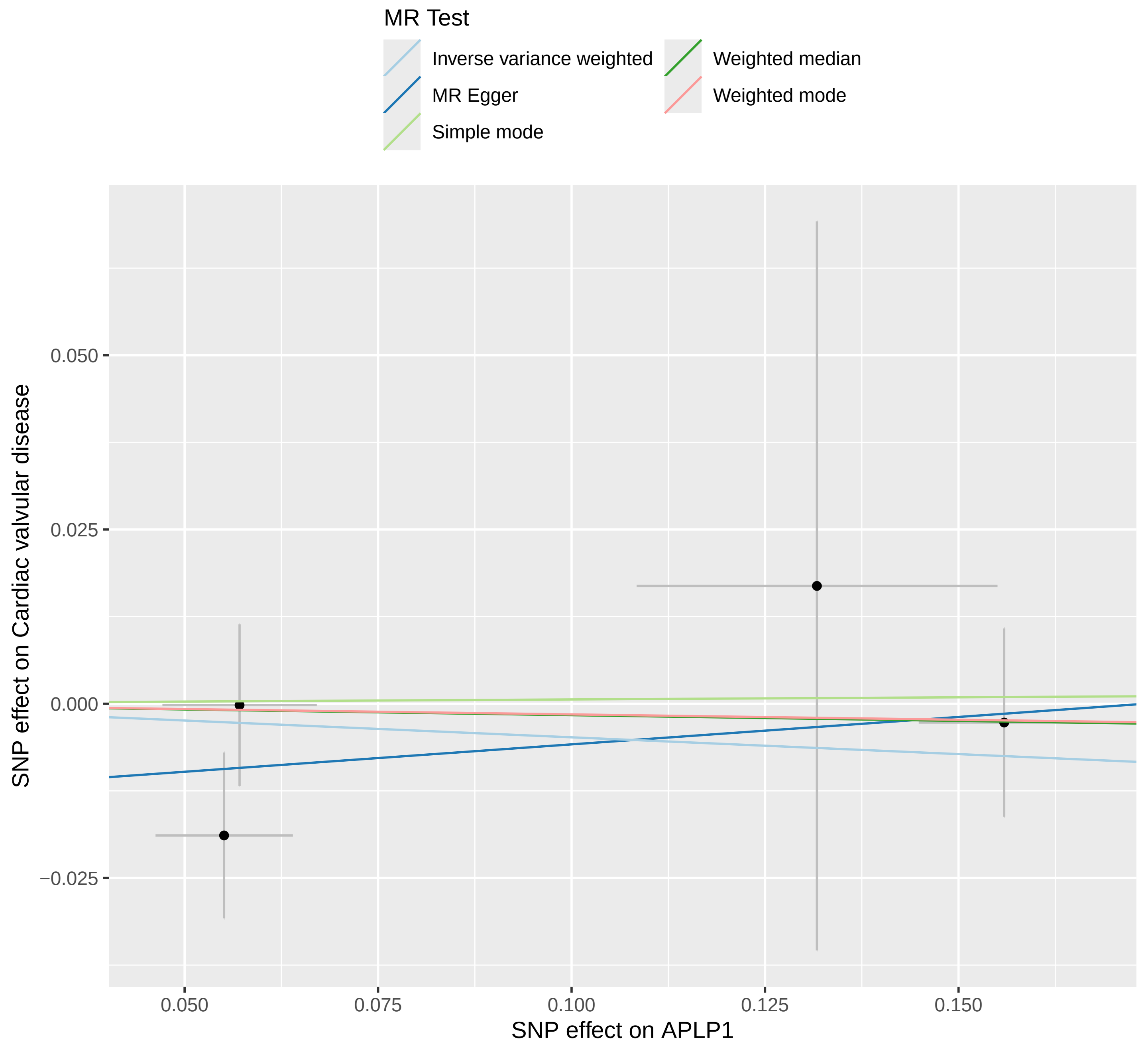
MR Test
Inverse variance weighted
MR Egger
Simple mode
Weighted median
Weighted mode
SNP effect on Cardiac valvular disease
0.050
0.025
0.000
−0.025
0.050
0.075
0.100
0.125
0.150
SNP effect on APLP1

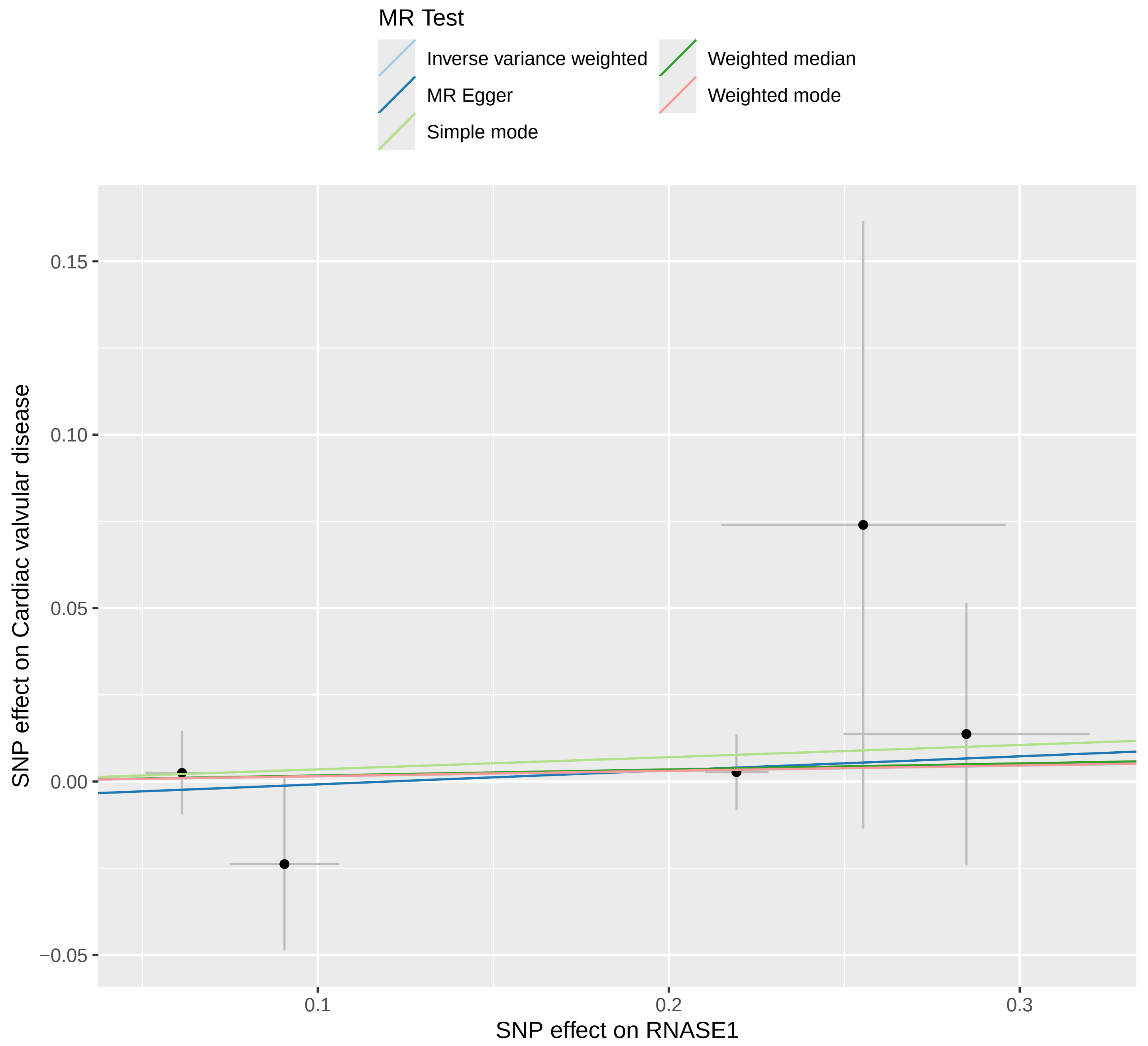

MR Test
Inverse variance weighted
MR Egger
Simple mode
Weighted median
Weighted mode
0.15
0.10
0.05
0.00
−0.05
0.1
0.2
0.3
SNP effect on Cardiac valvular disease
SNP effect on RNASE1

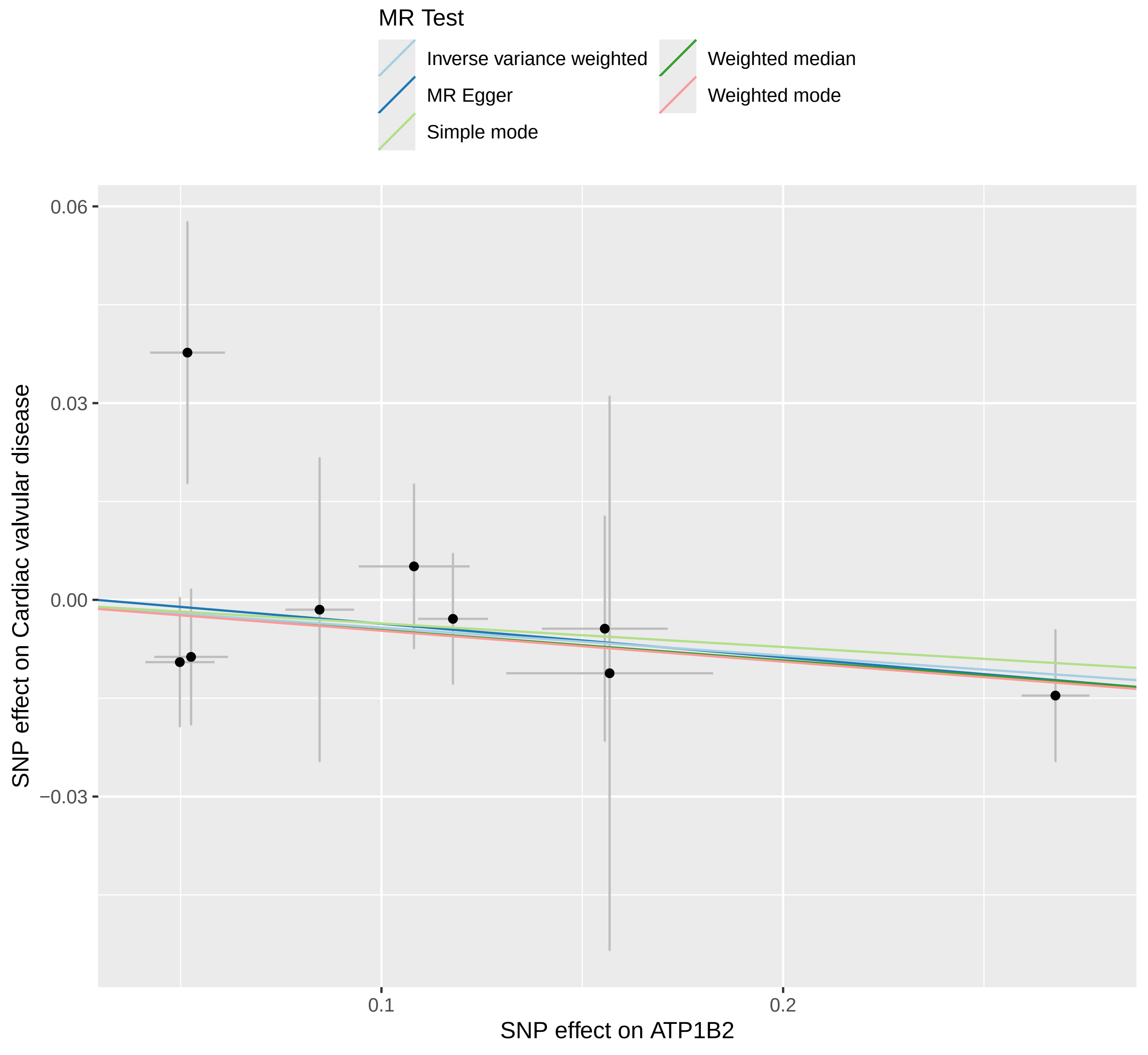
MR Test
Inverse variance weighted
MR Egger
Simple mode
Weighted median
Weighted mode
0.06
0.03
0.00
−0.03
0.1
0.2
SNP effect on Cardiac valvular disease
SNP effect on ATP1B2

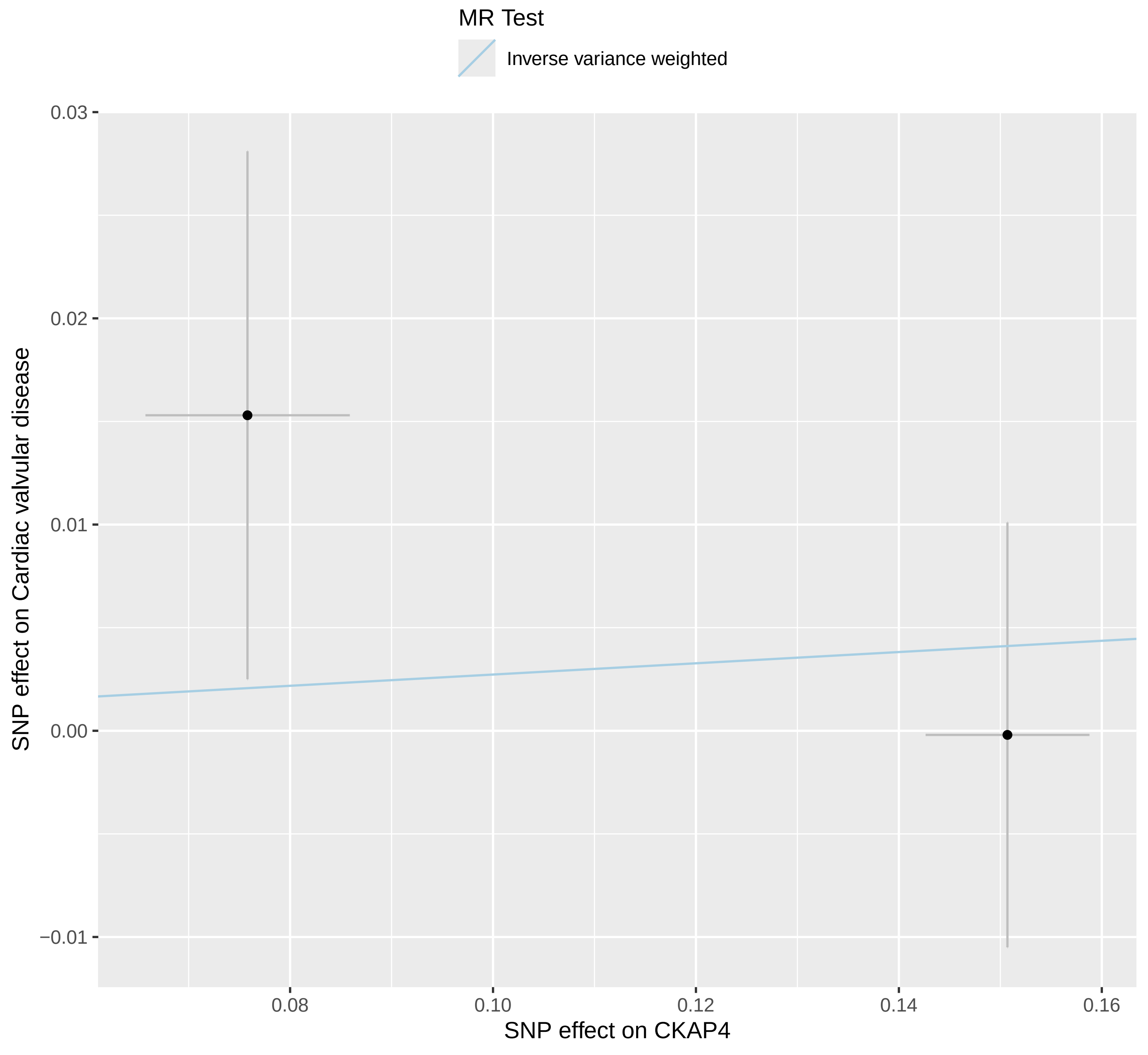

MR Test
Inverse variance weighted
0.03
0.02
0.01
0.00
−0.01
SNP effect on Cardiac valvular disease
0.08
0.10
0.12
0.14
0.16
SNP effect on CKAP4

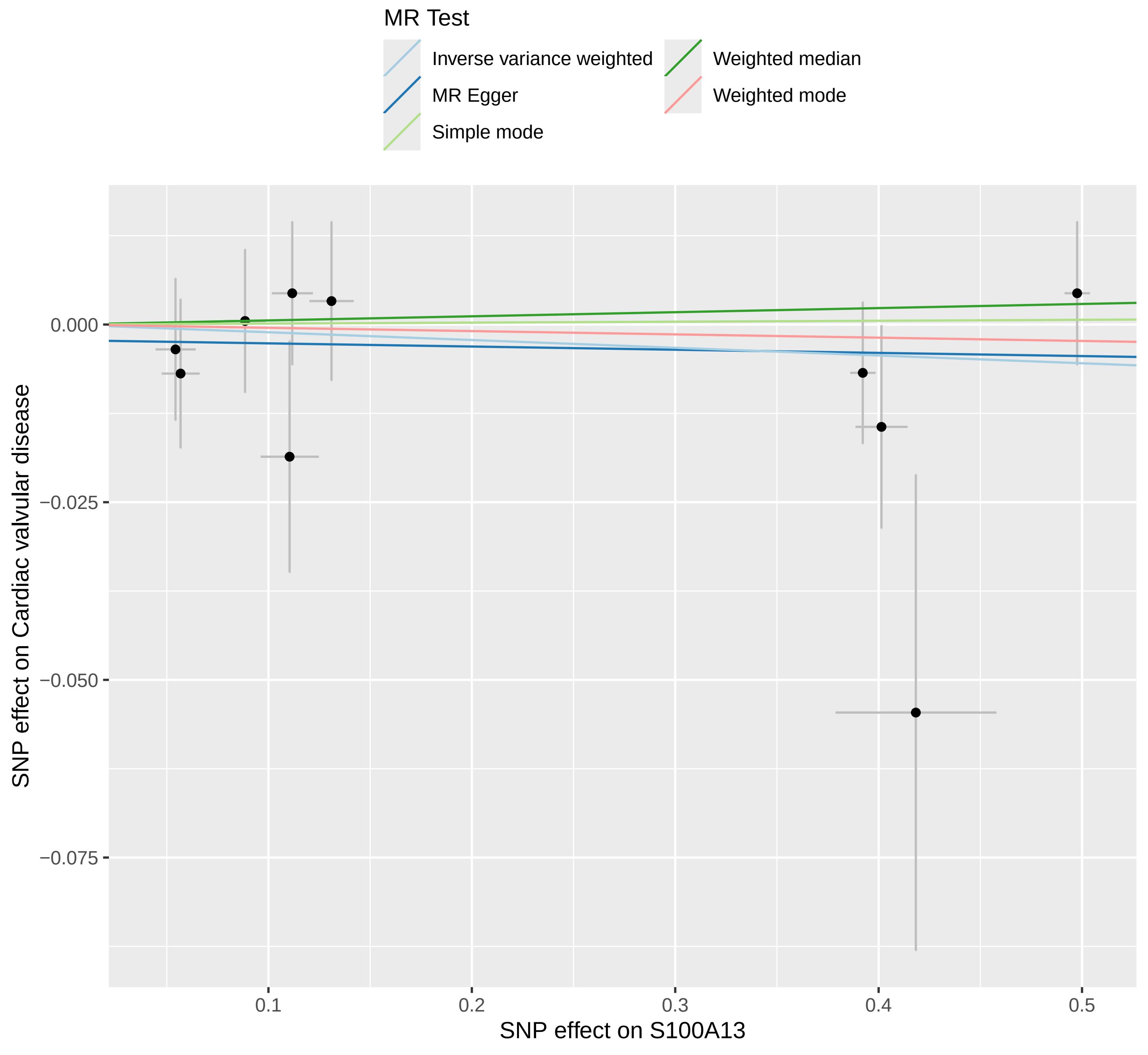
MR Test
Inverse variance weighted
MR Egger
Simple mode
Weighted median
Weighted mode
0.000
−0.025
−0.050
−0.075
0.1
0.2
0.3
0.4
0.5
SNP effect on S100A13
SNP effect on Cardiac valvular disease

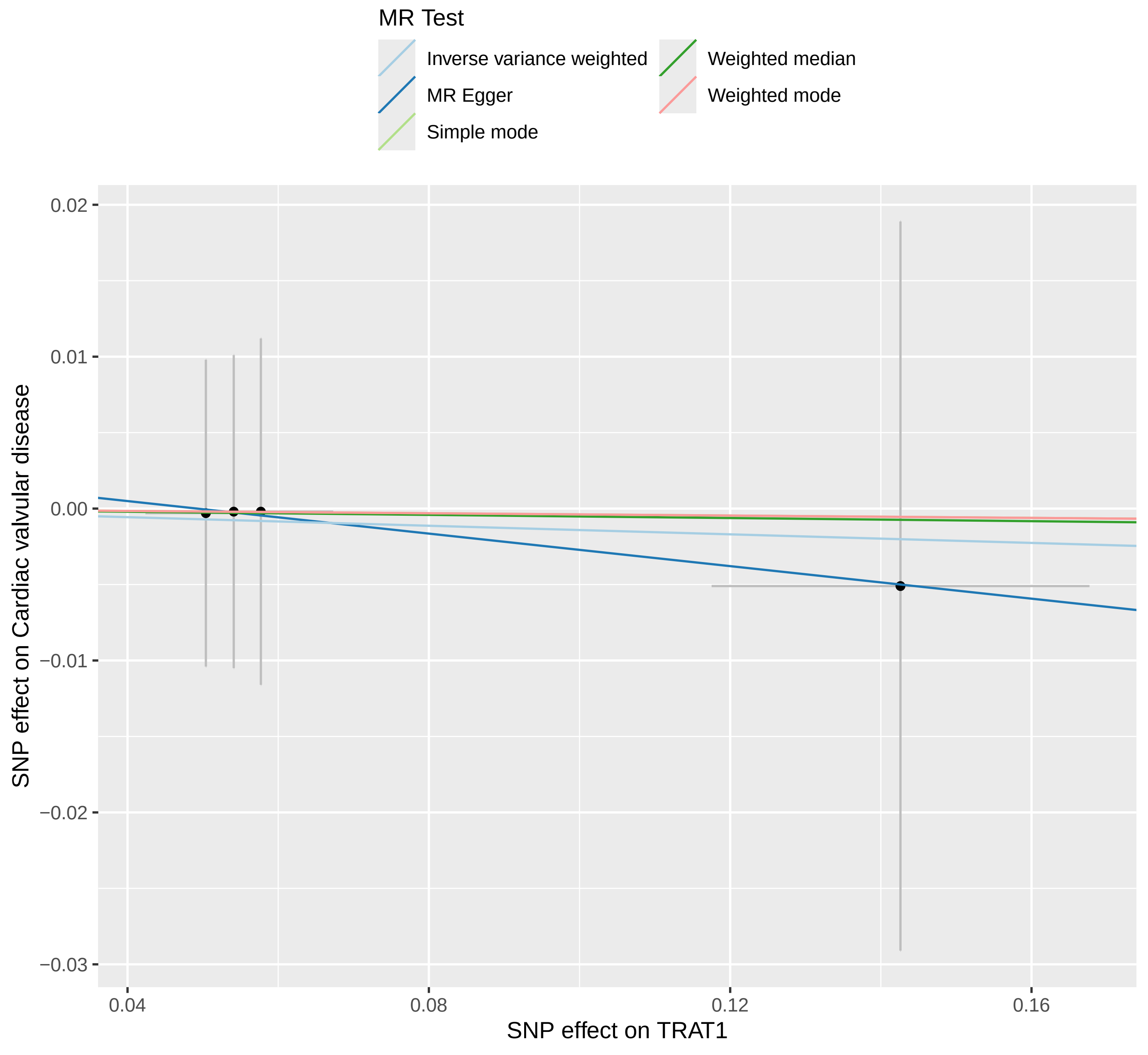
MR Test
Inverse variance weighted
MR Egger
Simple mode
Weighted median
Weighted mode
0.02
0.01
0.00
−0.01
−0.02
−0.03
SNP effect on Cardiac valvular disease
0.04
0.08
0.12
0.16
SNP effect on TRAT1

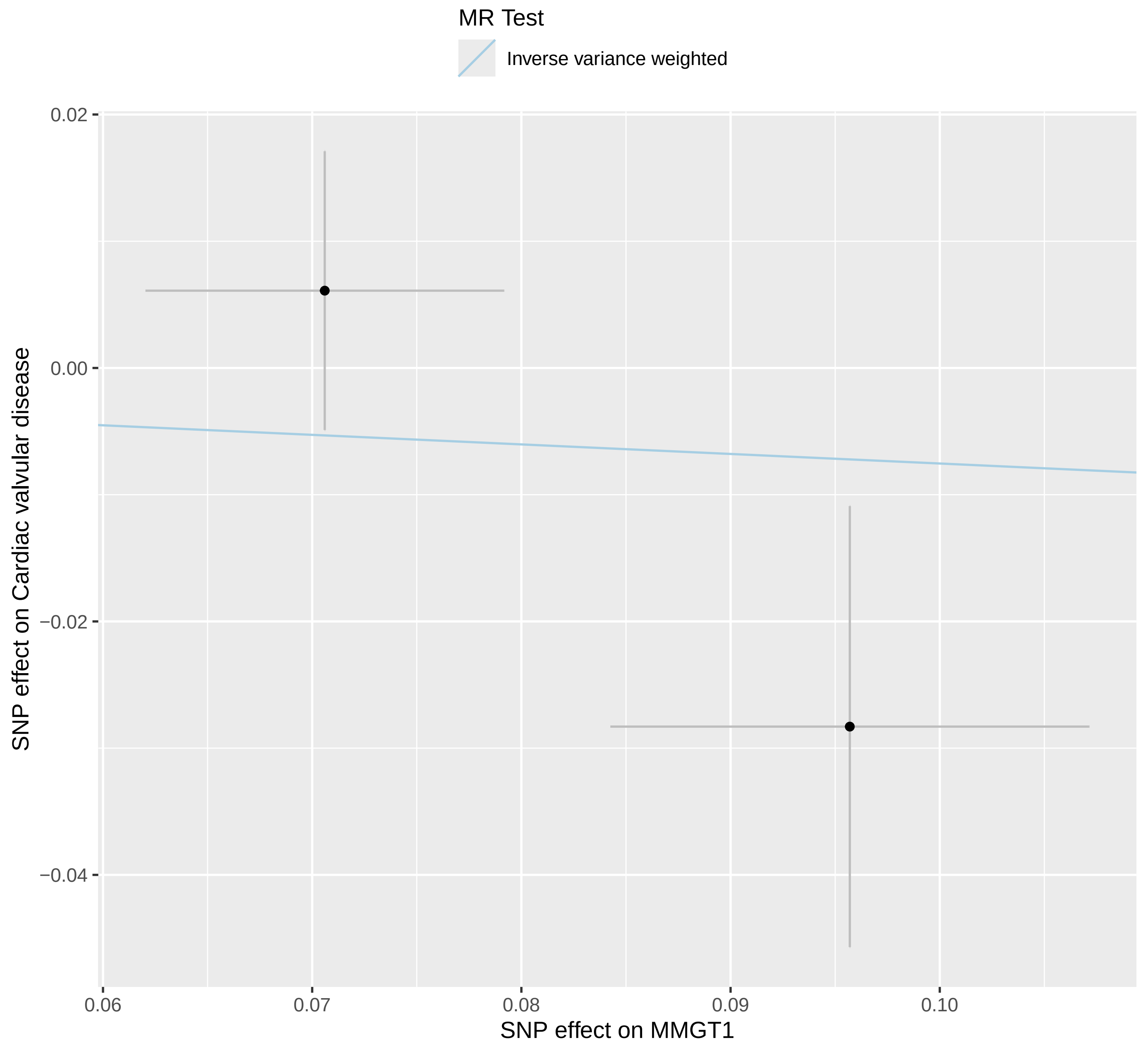
MR Test
Inverse variance weighted
0.02
0.00
−0.02
−0.04
0.06
0.07
0.08
0.09
0.10
SNP effect on MMGT1
SNP effect on Cardiac valvular disease

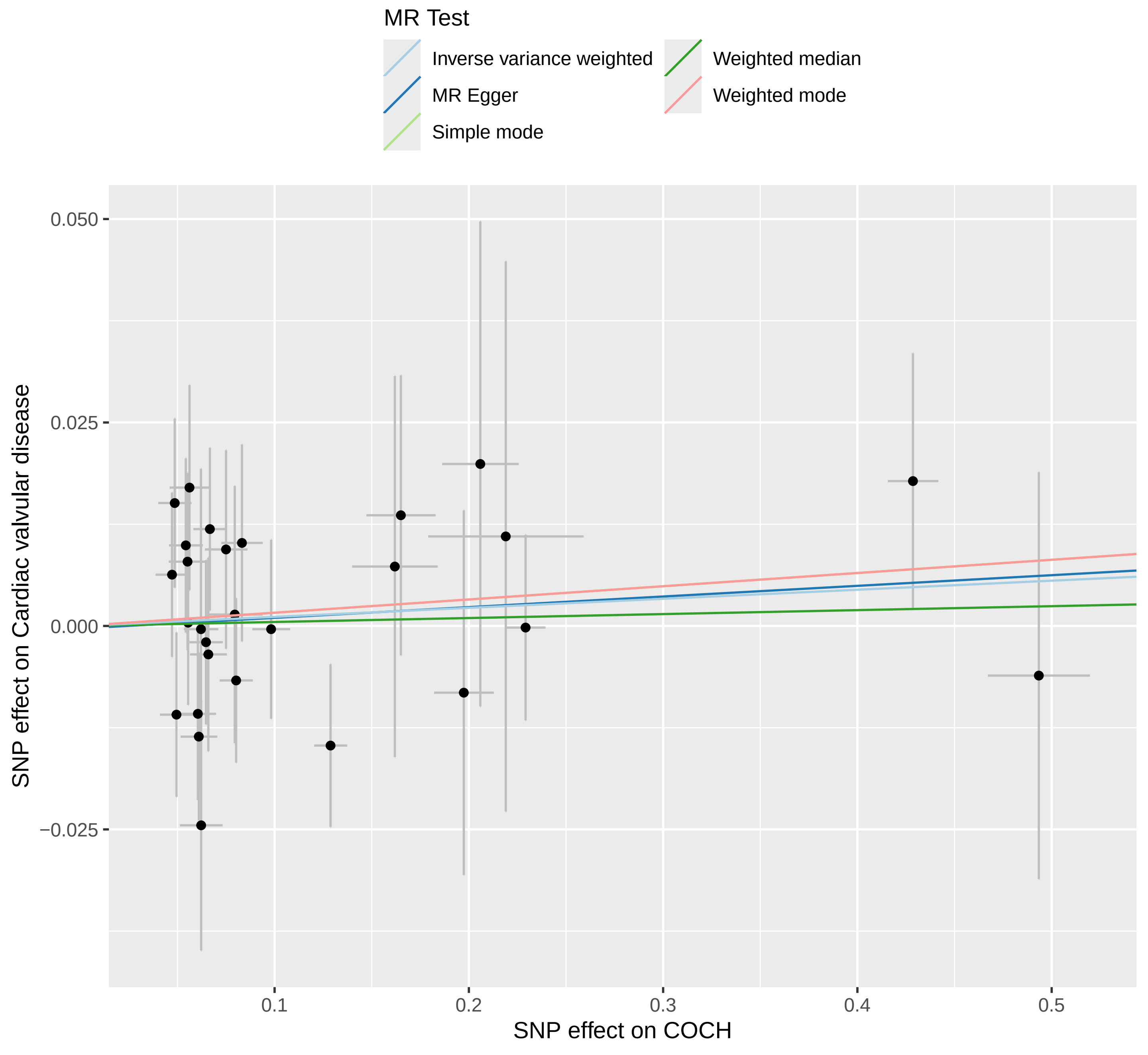

MR Test
Inverse variance weighted
MR Egger
Simple mode
Weighted median
Weighted mode
0.050
0.025
0.000
−0.025
SNP effect on Cardiac valvular disease
0.1
0.2
0.3
0.4
0.5
SNP effect on COCH

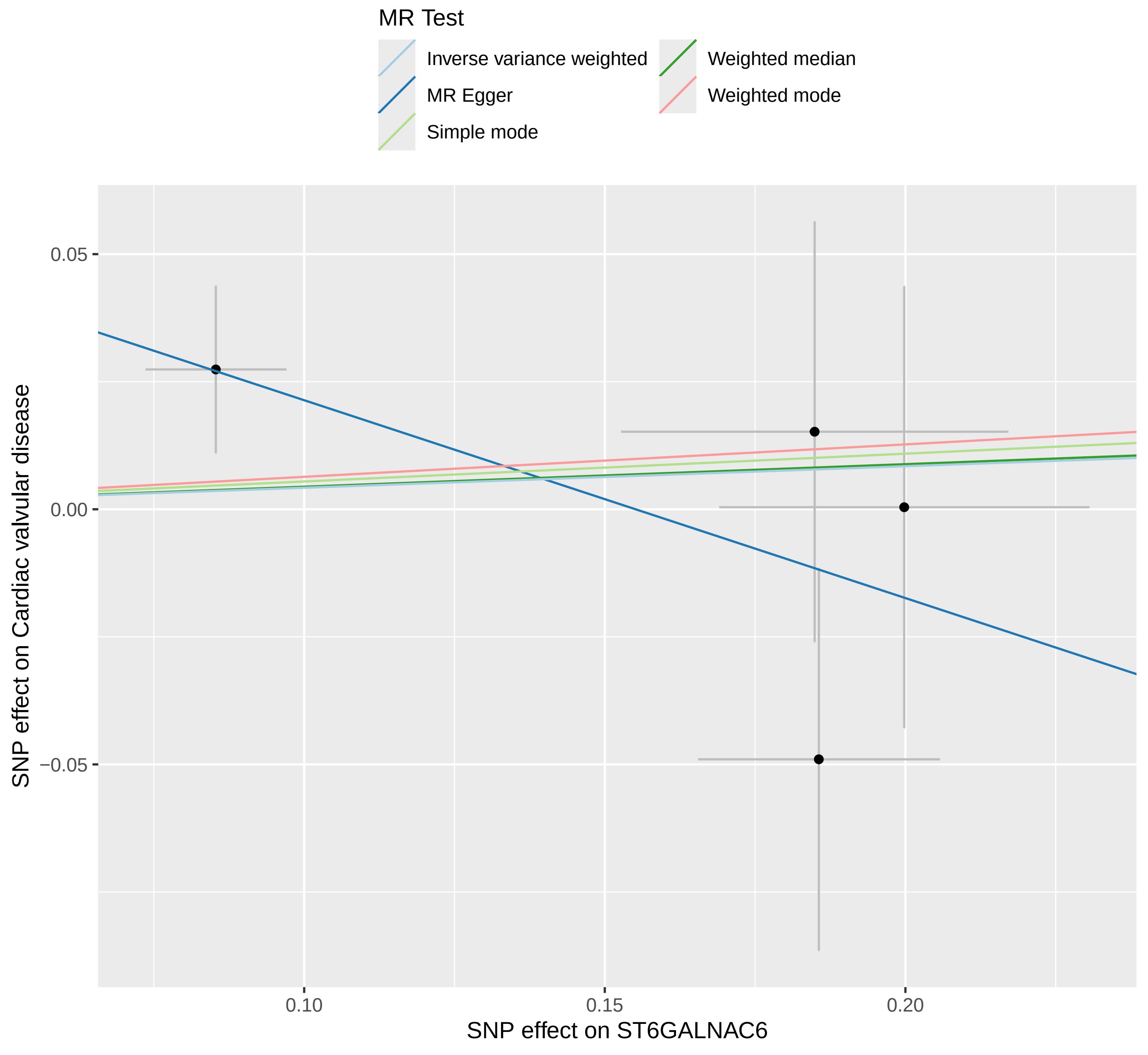
MR Test
Inverse variance weighted
MR Egger
Simple mode
Weighted median
Weighted mode
0.05
0.00
−0.05
0.10
0.15
0.20
SNP effect on Cardiac valvular disease
SNP effect on ST6GALNAC6

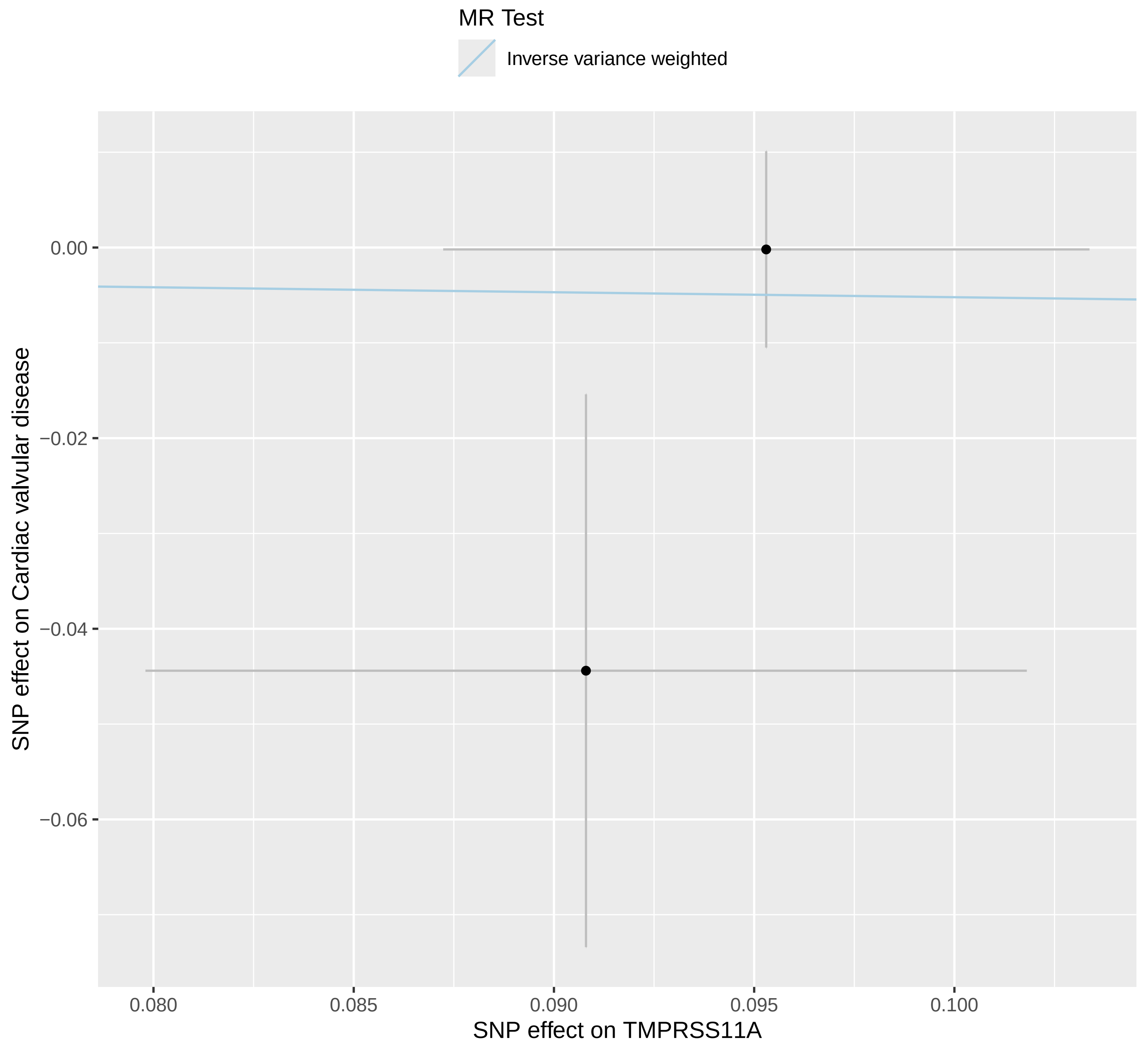
MR Test
Inverse variance weighted
0.00
−0.02
−0.04
−0.06
0.080
0.085
0.090
0.095
0.100
SNP effect on TMPRSS11A
SNP effect on Cardiac valvular disease

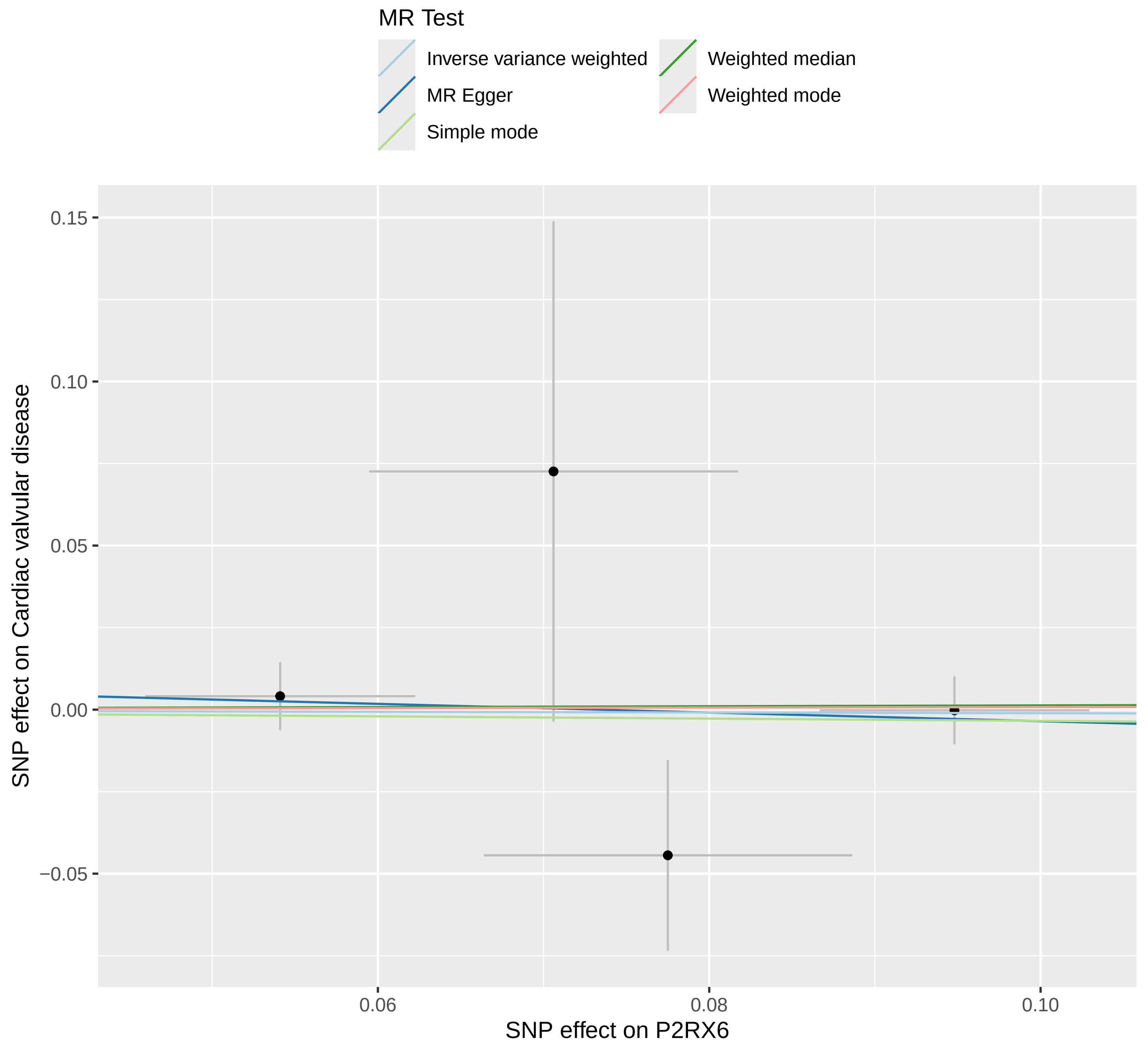
MR Test
Inverse variance weighted
MR Egger
Simple mode
Weighted median
Weighted mode
0.15
0.10
0.05
0.00
−0.05
0.06
0.08
0.10
SNP effect on Cardiac valvular disease
SNP effect on P2RX6

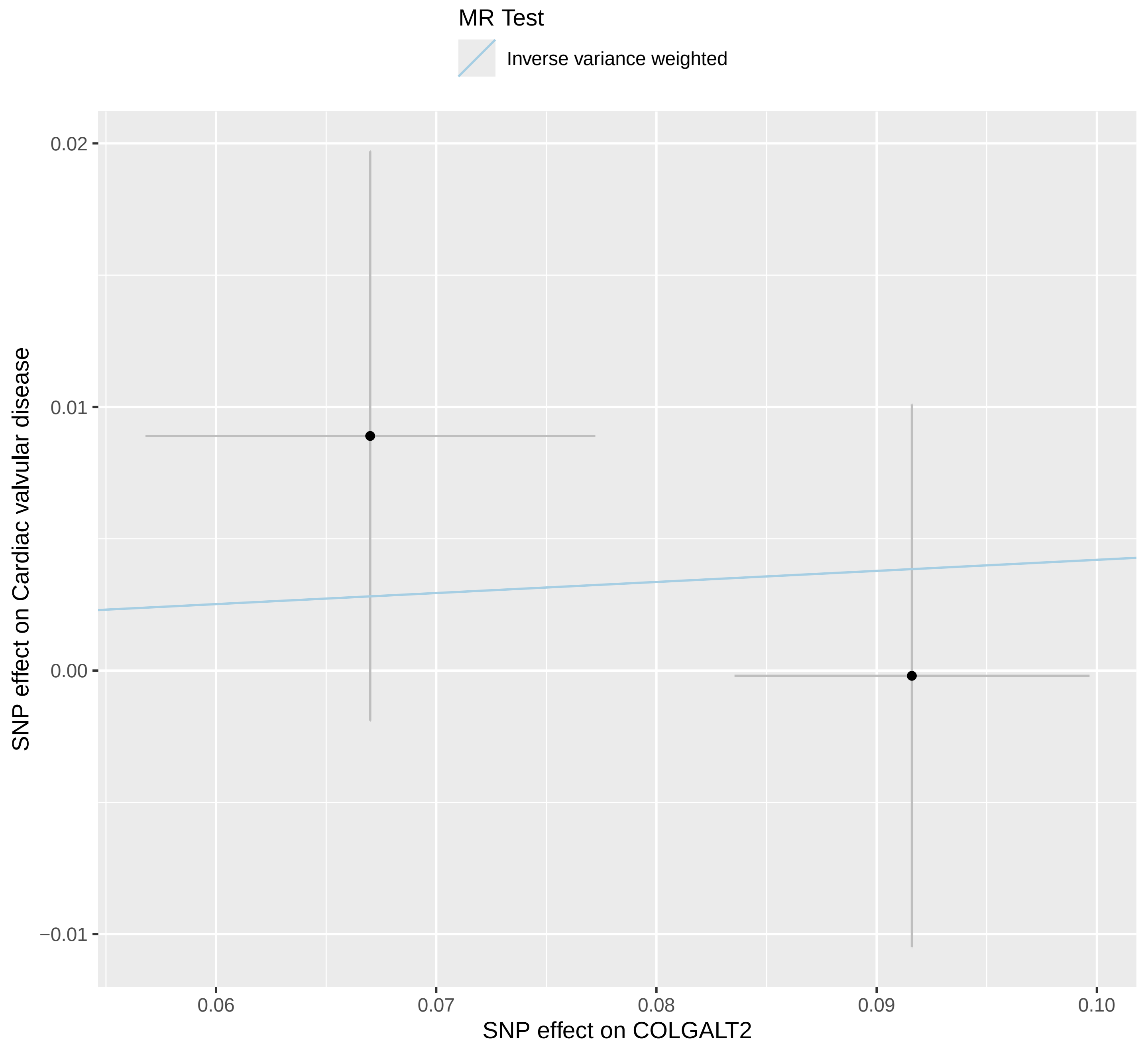
MR Test
Inverse variance weighted
0.02
0.01
0.00
−0.01
0.06
0.07
0.08
0.09
0.10
SNP effect on Cardiac valvular disease
SNP effect on COLGALT2

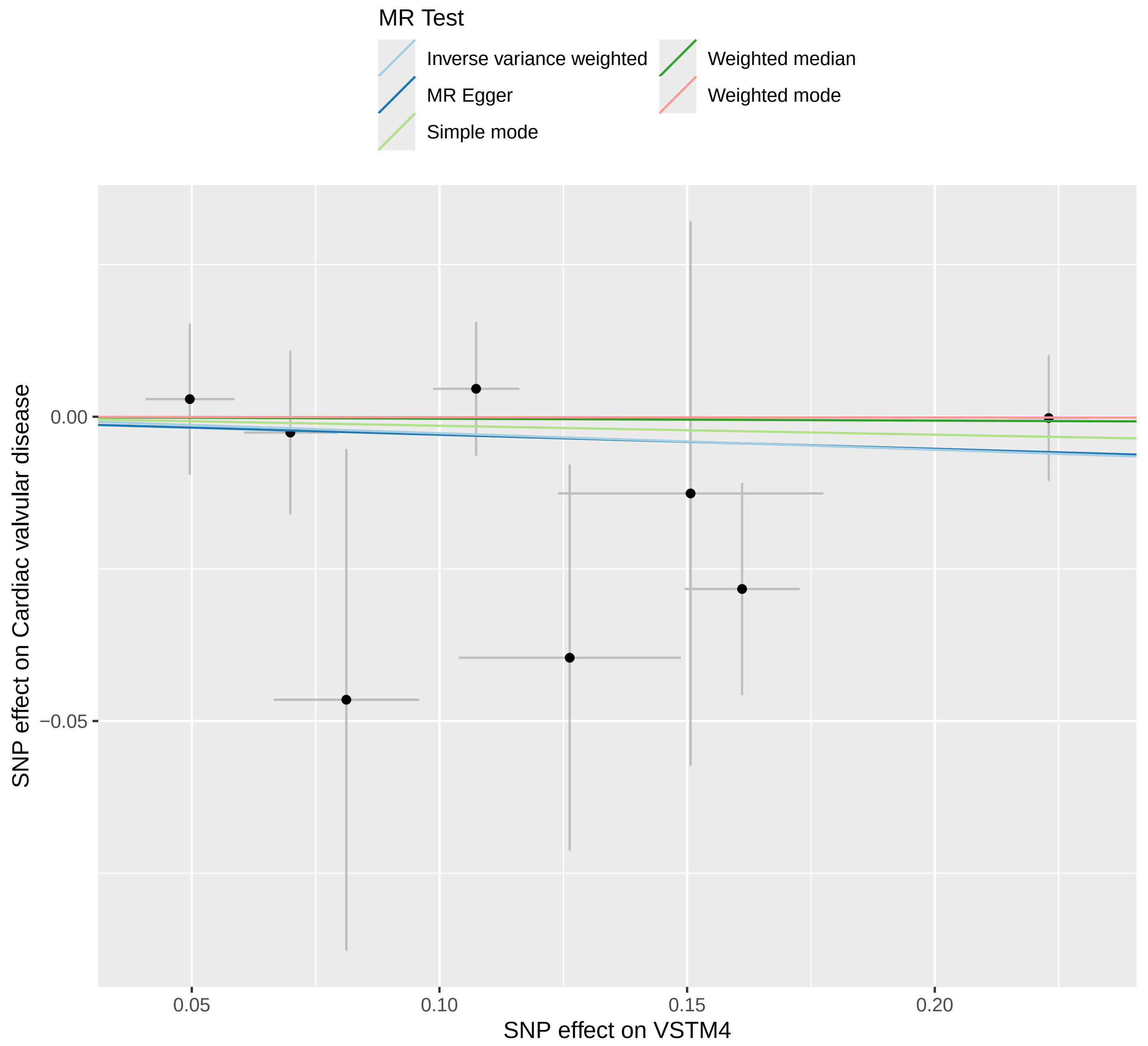
MR Test
Inverse variance weighted
MR Egger
Simple mode
Weighted median
Weighted mode
0.00
−0.05
SNP effect on Cardiac valvular disease
0.05
0.10
0.15
0.20
SNP effect on VSTM4

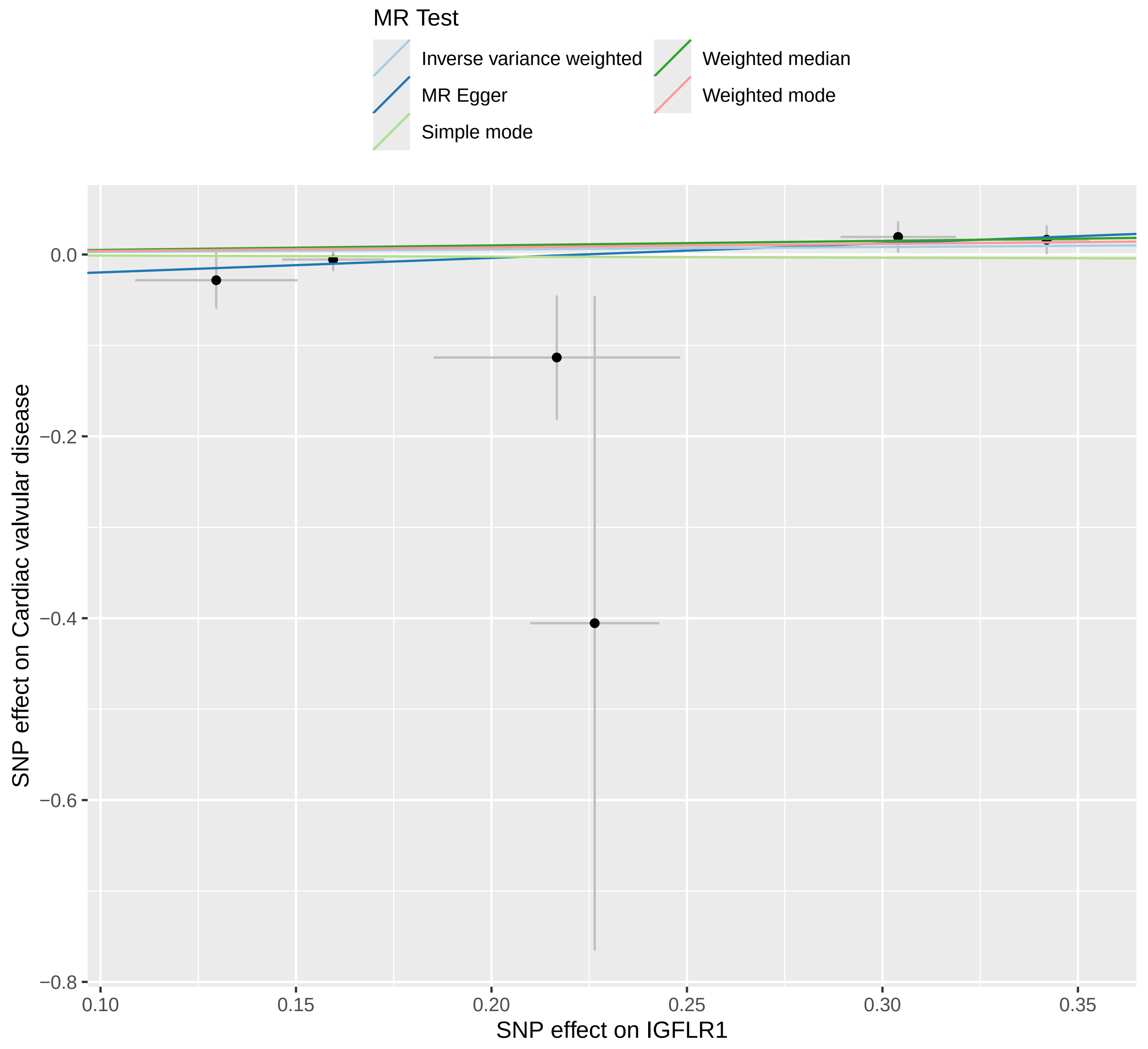

MR Test
Inverse variance weighted
MR Egger
Simple mode
Weighted median
Weighted mode
0.0
−0.2
−0.4
−0.6
−0.8
0.10
0.15
0.20
0.25
0.30
0.35
SNP effect on Cardiac valvular disease
SNP effect on IGFLR1

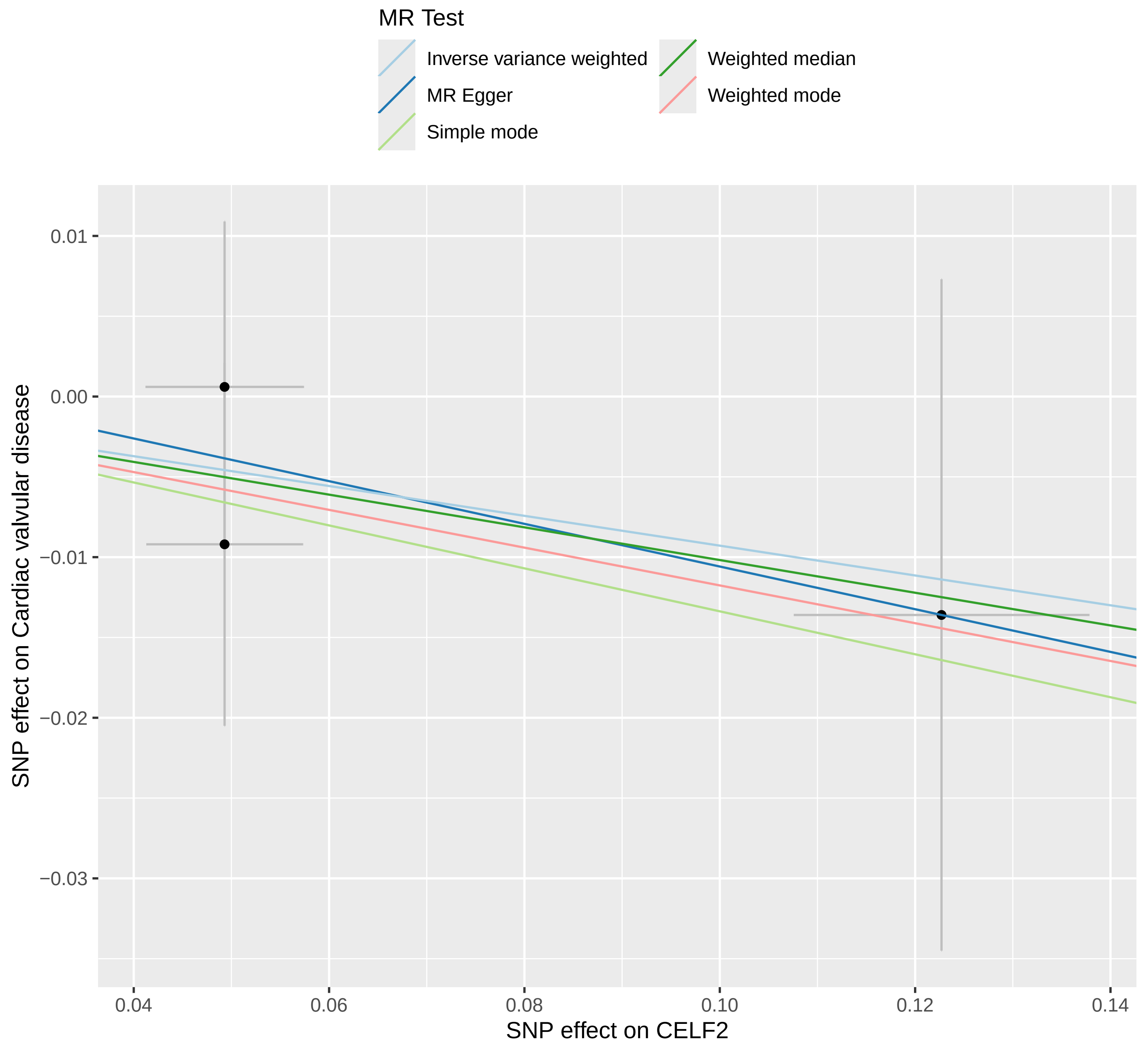
MR Test
Inverse variance weighted
MR Egger
Simple mode
Weighted median
Weighted mode
SNP effect on Cardiac valvular disease
0.01
0.00
−0.01
−0.02
−0.03
0.04
0.06
0.08
0.10
0.12
0.14
SNP effect on CELF2

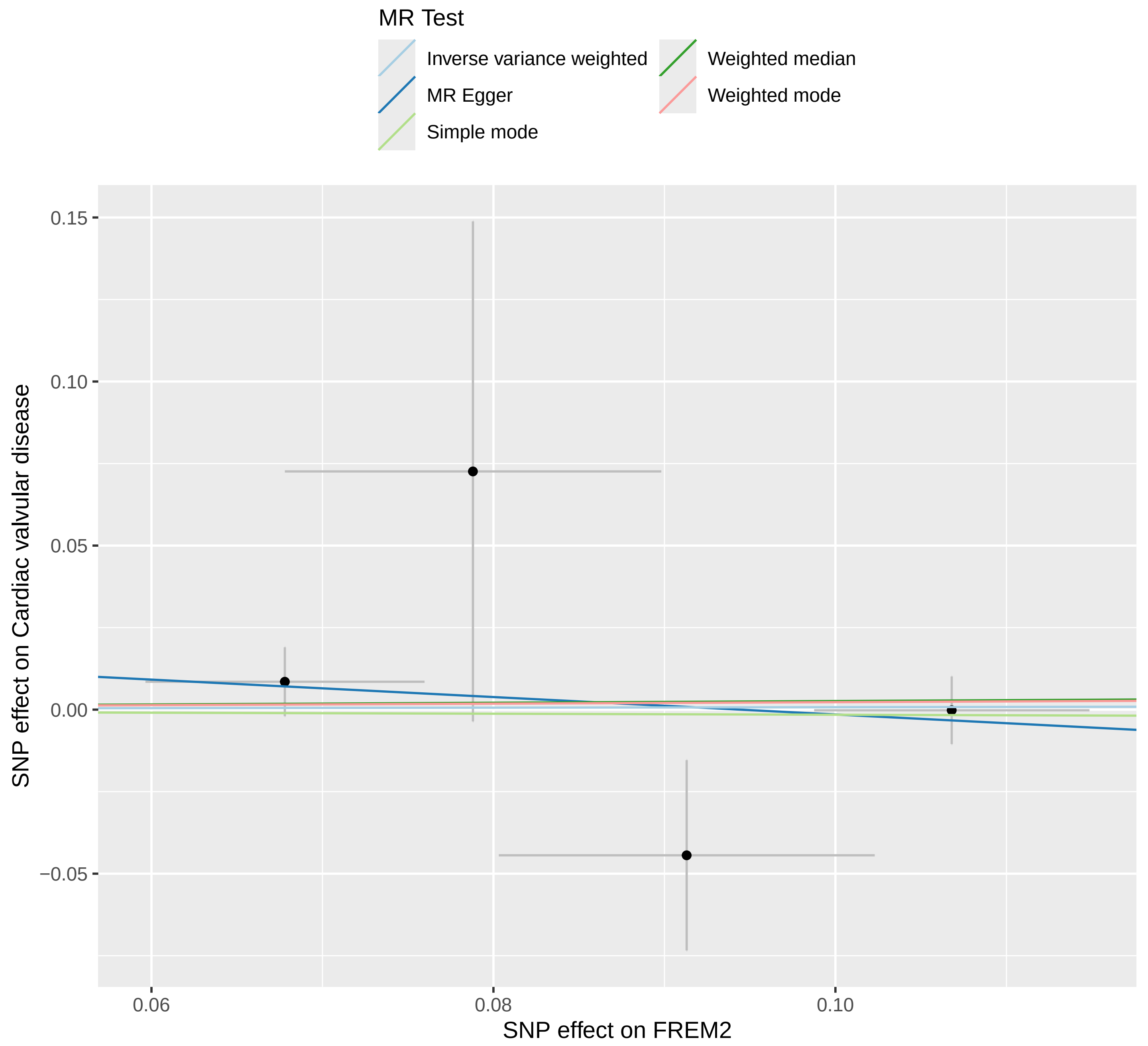
MR Test
Inverse variance weighted
MR Egger
Simple mode
Weighted median
Weighted mode
0.15
0.10
0.05
0.00
−0.05
0.06
0.08
0.10
SNP effect on FREM2
SNP effect on Cardiac valvular disease

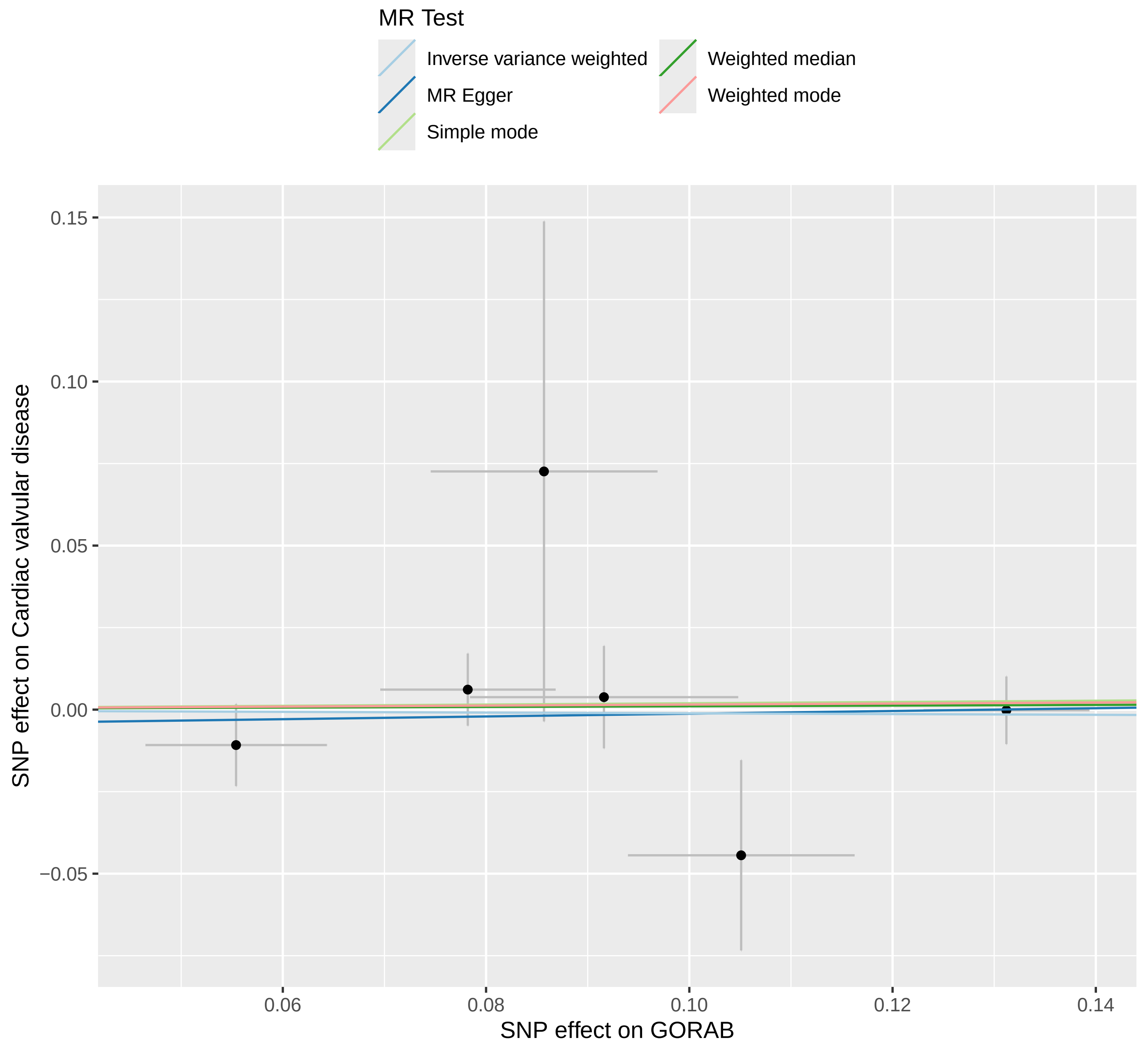
MR Test
Inverse variance weighted
MR Egger
Simple mode
Weighted median
Weighted mode
0.15
0.10
0.05
0.00
−0.05
0.06
0.08
0.10
0.12
0.14
SNP effect on Cardiac valvular disease
SNP effect on GORAB

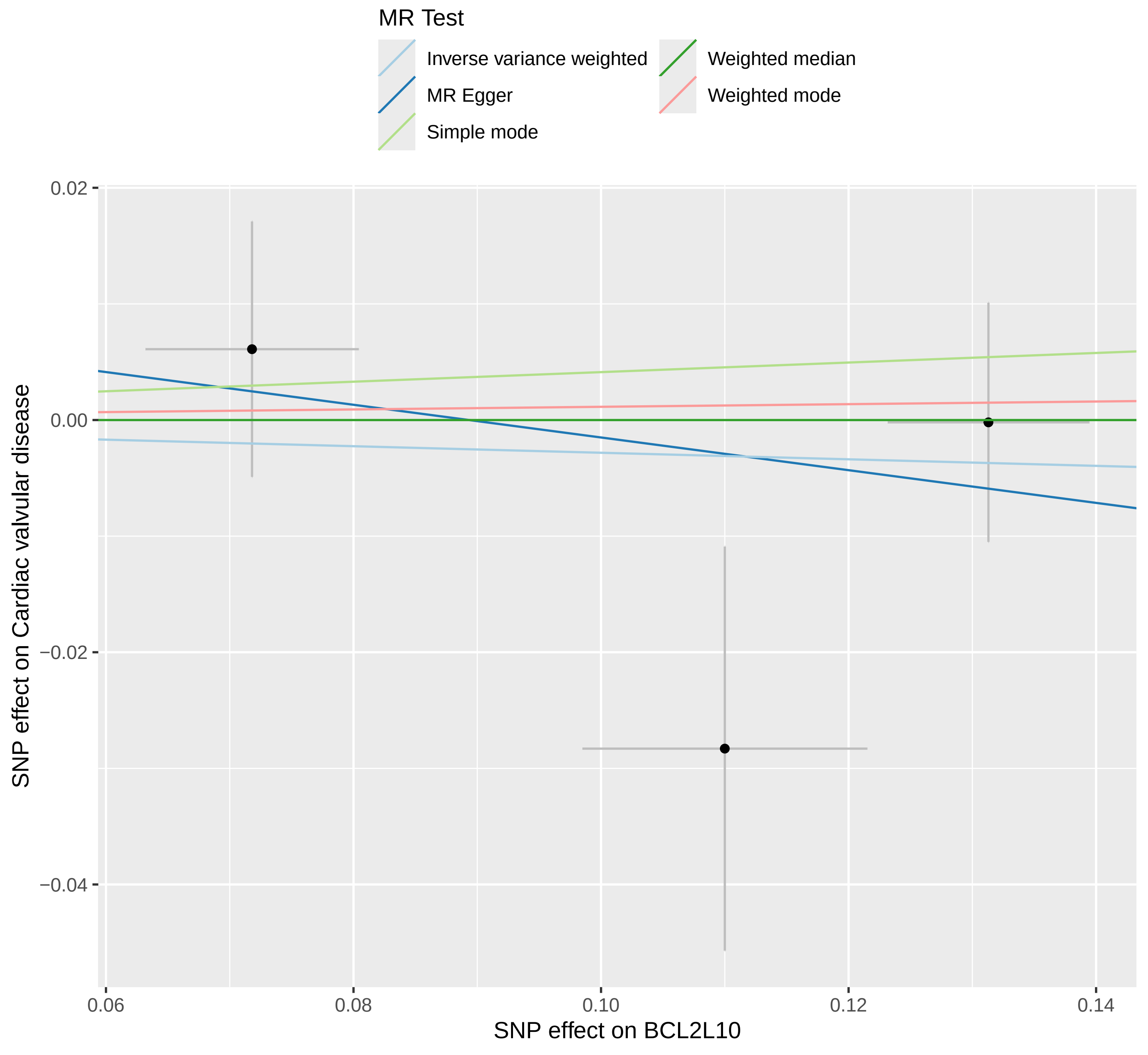

MR Test
Inverse variance weighted
MR Egger
Simple mode
Weighted median
Weighted mode
0.02
0.00
−0.02
−0.04
SNP effect on Cardiac valvular disease
0.06
0.08
0.10
0.12
0.14
SNP effect on BCL2L10

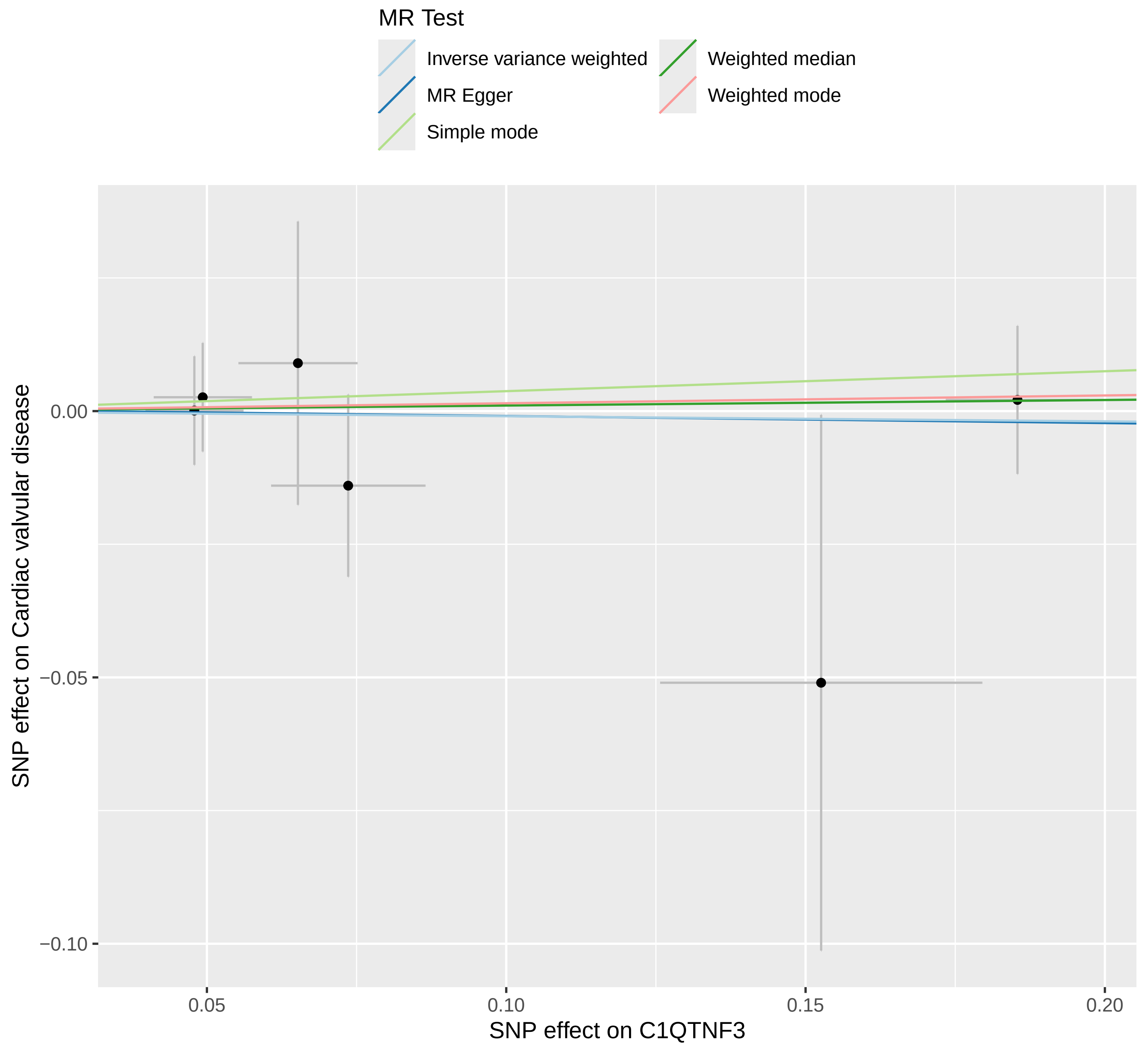
MR Test
Inverse variance weighted
MR Egger
Simple mode
Weighted median
Weighted mode
0.00
−0.05
−0.10
0.05
0.10
0.15
0.20
SNP effect on C1QTNF3
SNP effect on Cardiac valvular disease

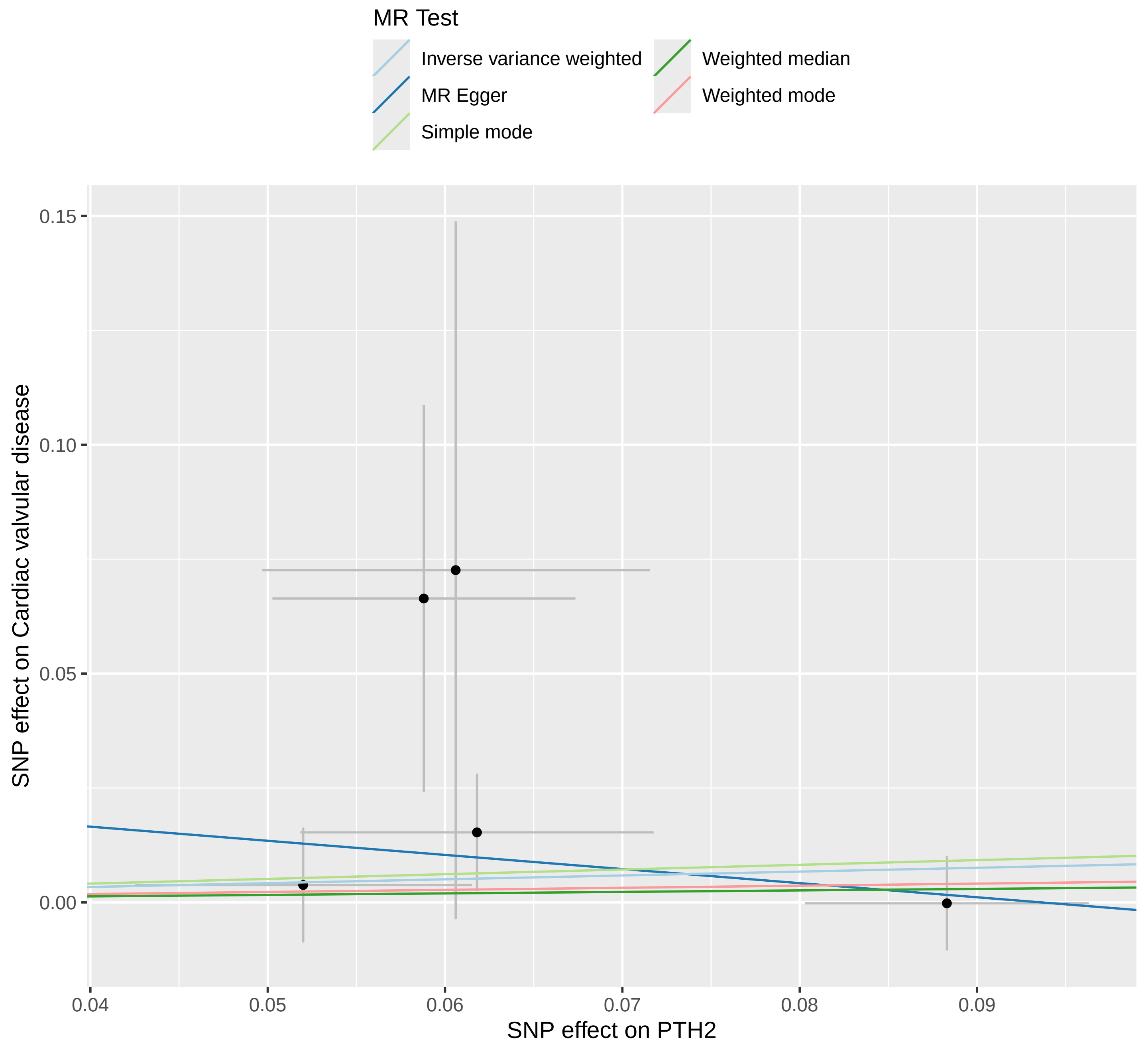

MR Test
Inverse variance weighted
MR Egger
Simple mode
Weighted median
Weighted mode
0.15
0.10
0.05
0.00
SNP effect on Cardiac valvular disease
0.04
0.05
0.06
0.07
0.08
0.09
SNP effect on PTH2

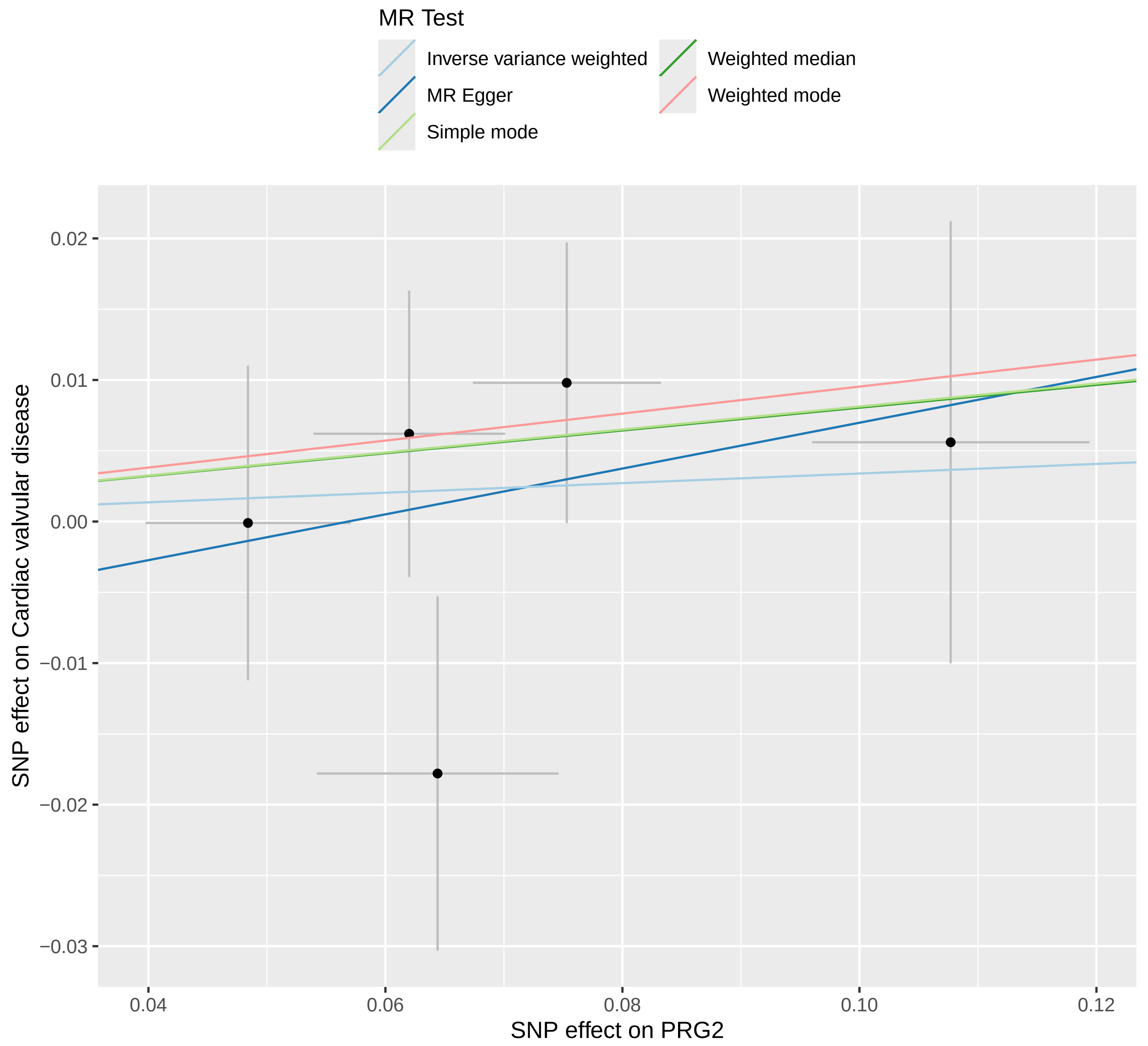
MR Test
Inverse variance weighted
MR Egger
Simple mode
Weighted median
Weighted mode
0.02
0.01
0.00
−0.01
−0.02
−0.03
SNP effect on Cardiac valvular disease
0.04
0.06
0.08
0.10
0.12
SNP effect on PRG2

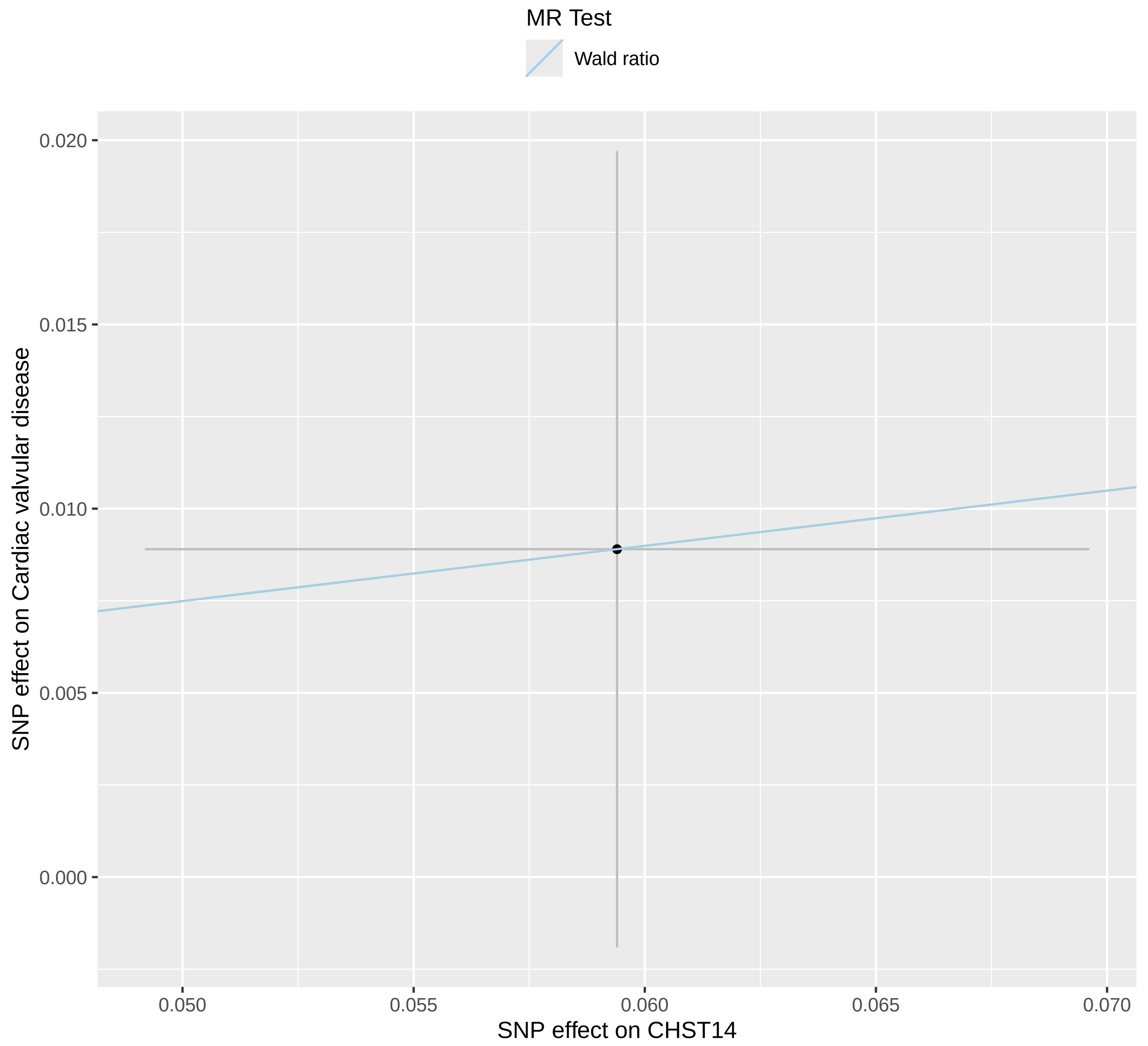
MR Test
Wald ratio
SNP effect on Cardiac valvular disease
0.020
0.015
0.010
0.005
0.000
0.050
0.055
0.060
0.065
0.070
SNP effect on CHST14

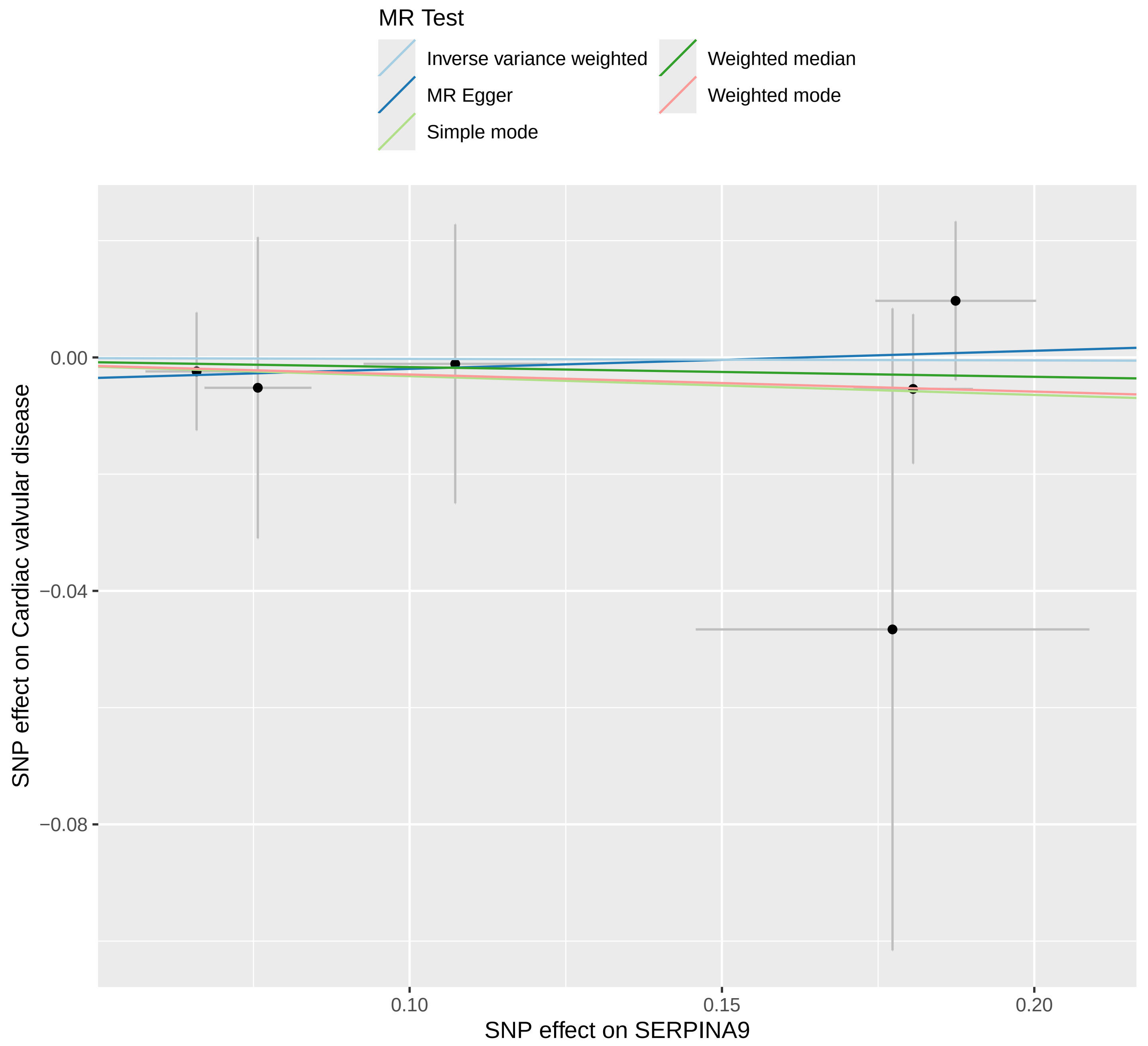
MR Test
Inverse variance weighted
MR Egger
Simple mode
Weighted median
Weighted mode
0.00
−0.04
−0.08
0.10
0.15
0.20
SNP effect on Cardiac valvular disease
SNP effect on SERPINA9

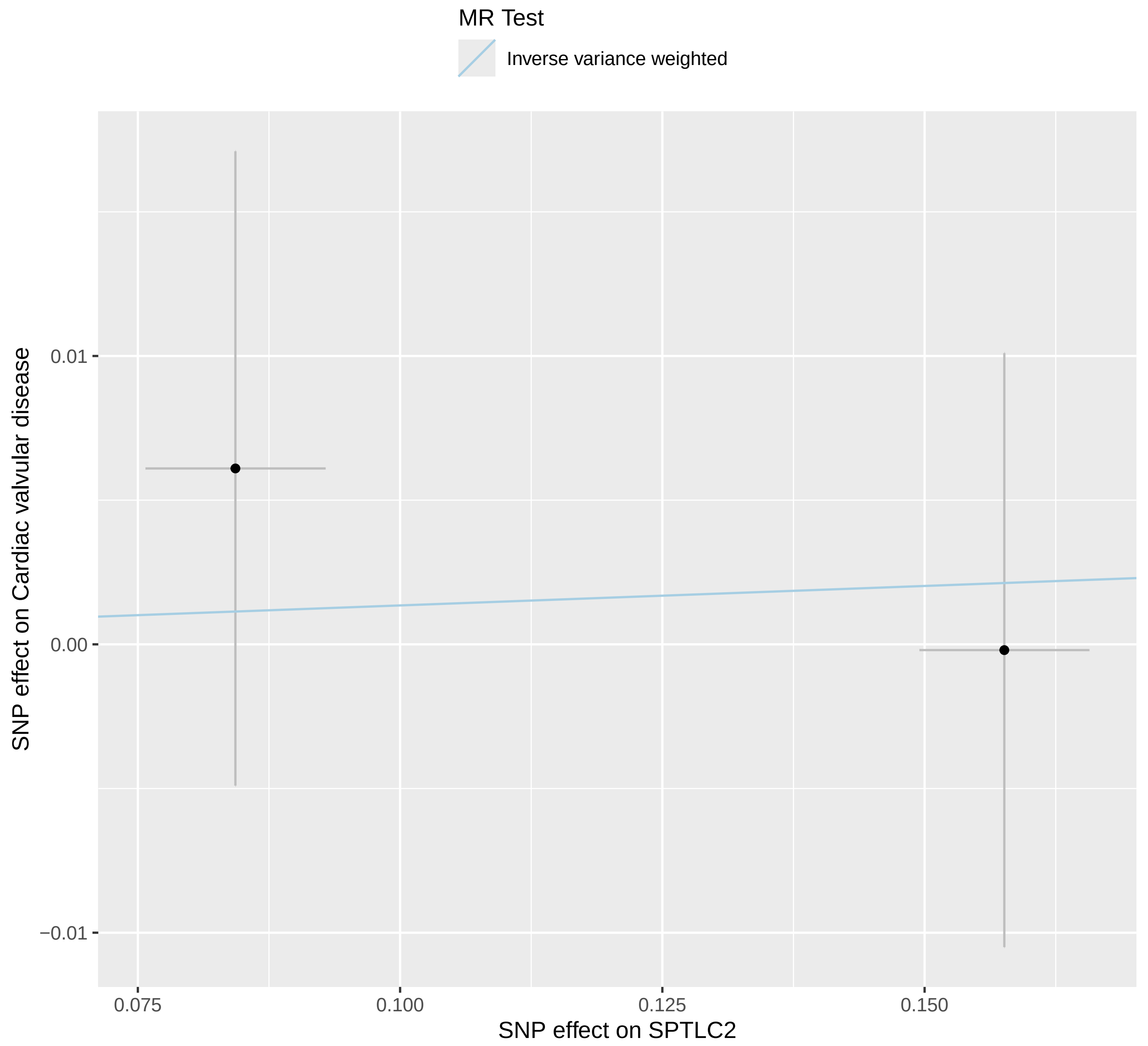
MR Test
Inverse variance weighted
SNP effect on Cardiac valvular disease
0.01
0.00
−0.01
0.075
0.100
0.125
0.150
SNP effect on SPTLC2

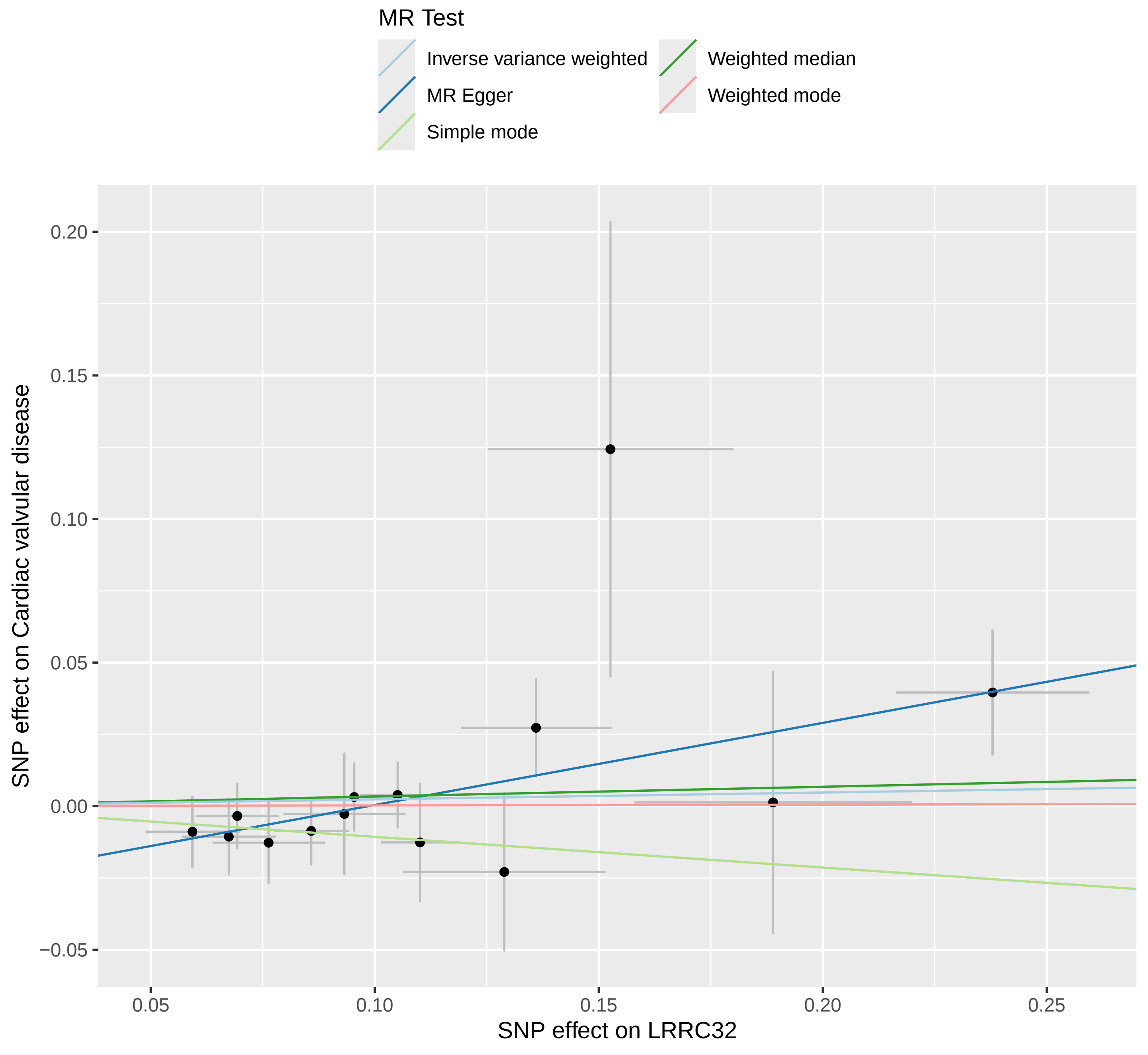
MR Test
Inverse variance weighted
MR Egger
Simple mode
Weighted median
Weighted mode
SNP effect on Cardiac valvular disease
0.20
0.15
0.10
0.05
0.00
−0.05
0.05
0.10
0.15
0.20
0.25
SNP effect on LRRC32

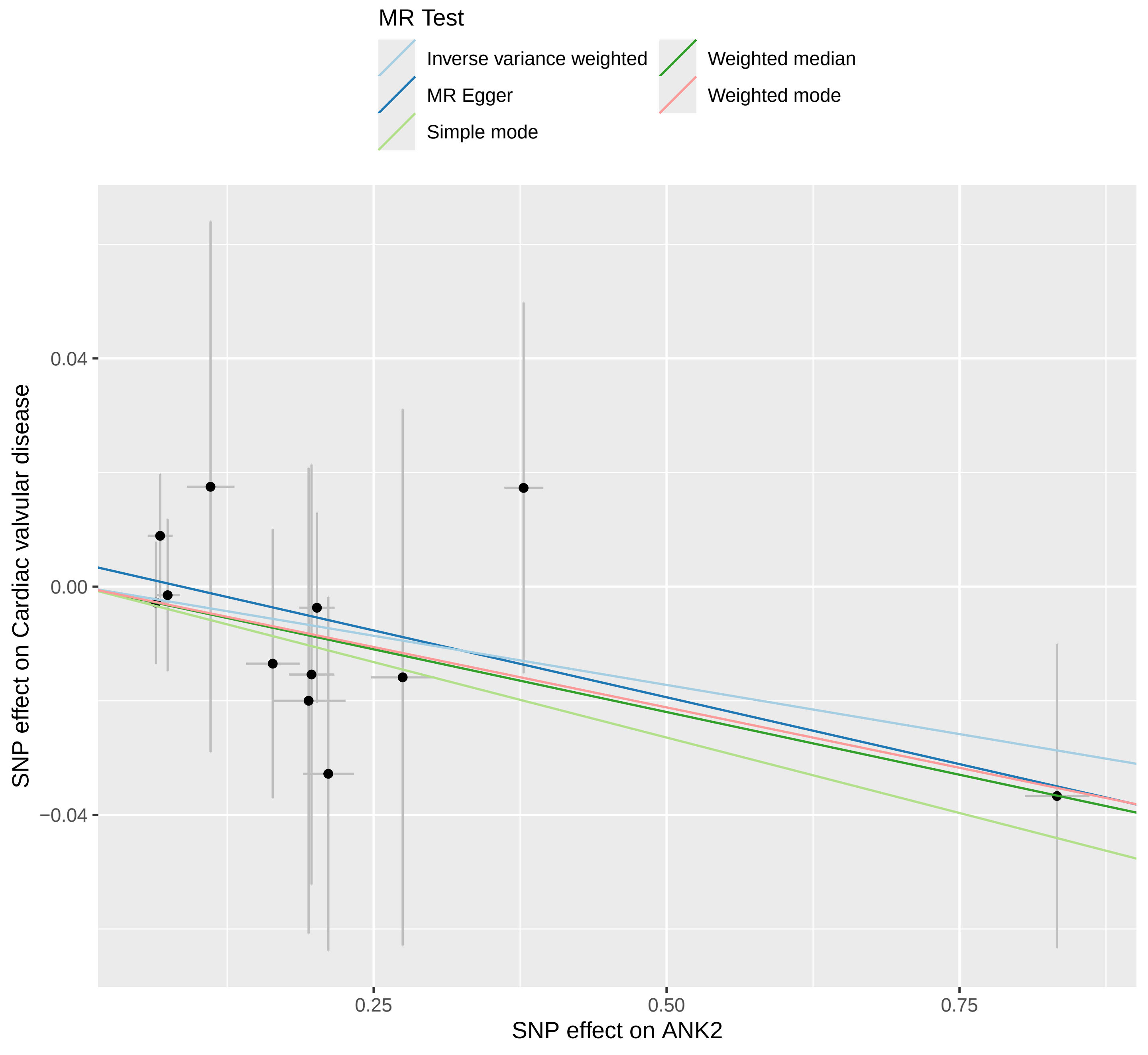
MR Test
Inverse variance weighted
MR Egger
Simple mode
Weighted median
Weighted mode
0.04
0.00
−0.04
SNP effect on Cardiac valvular disease
0.25
0.50
0.75
SNP effect on ANK2

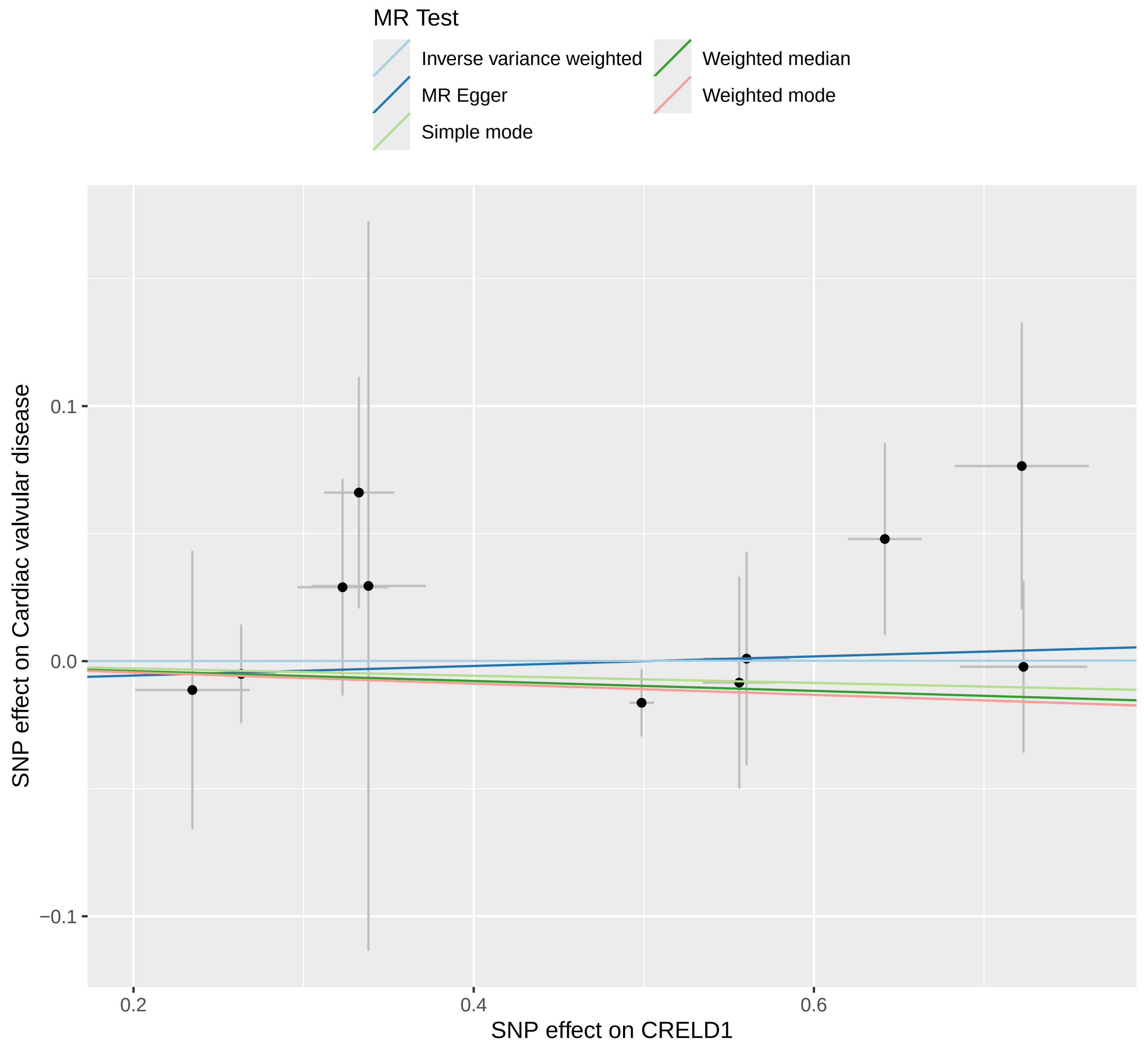
MR Test
Inverse variance weighted
MR Egger
Simple mode
Weighted median
Weighted mode
SNP effect on Cardiac valvular disease
0.1
0.0
−0.1
0.2
0.4
0.6
SNP effect on CRELD1

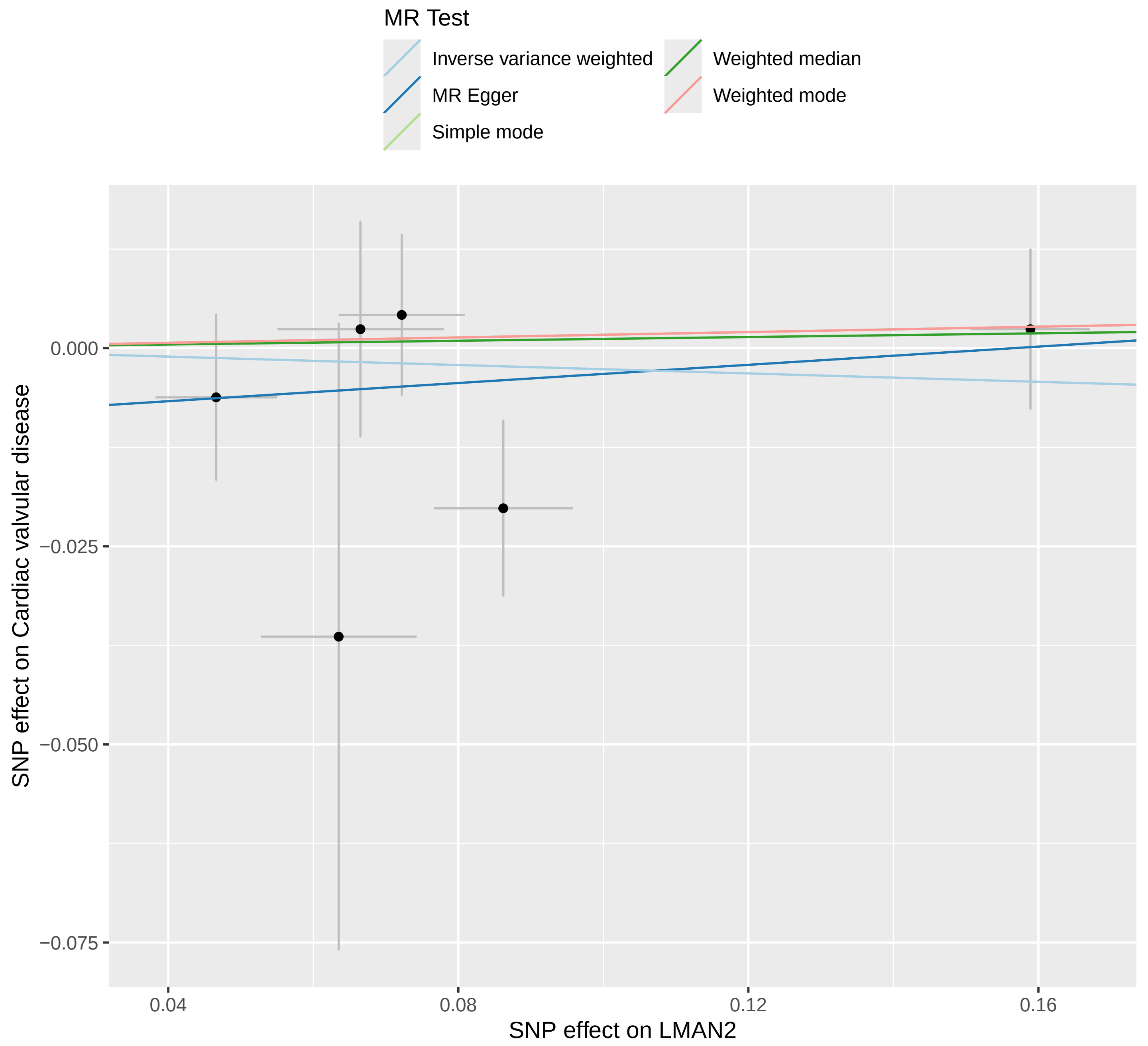
MR Test
Inverse variance weighted
MR Egger
Simple mode
Weighted median
Weighted mode
0.000
−0.025
−0.050
−0.075
0.04
0.08
0.12
0.16
SNP effect on LMAN2
SNP effect on Cardiac valvular disease

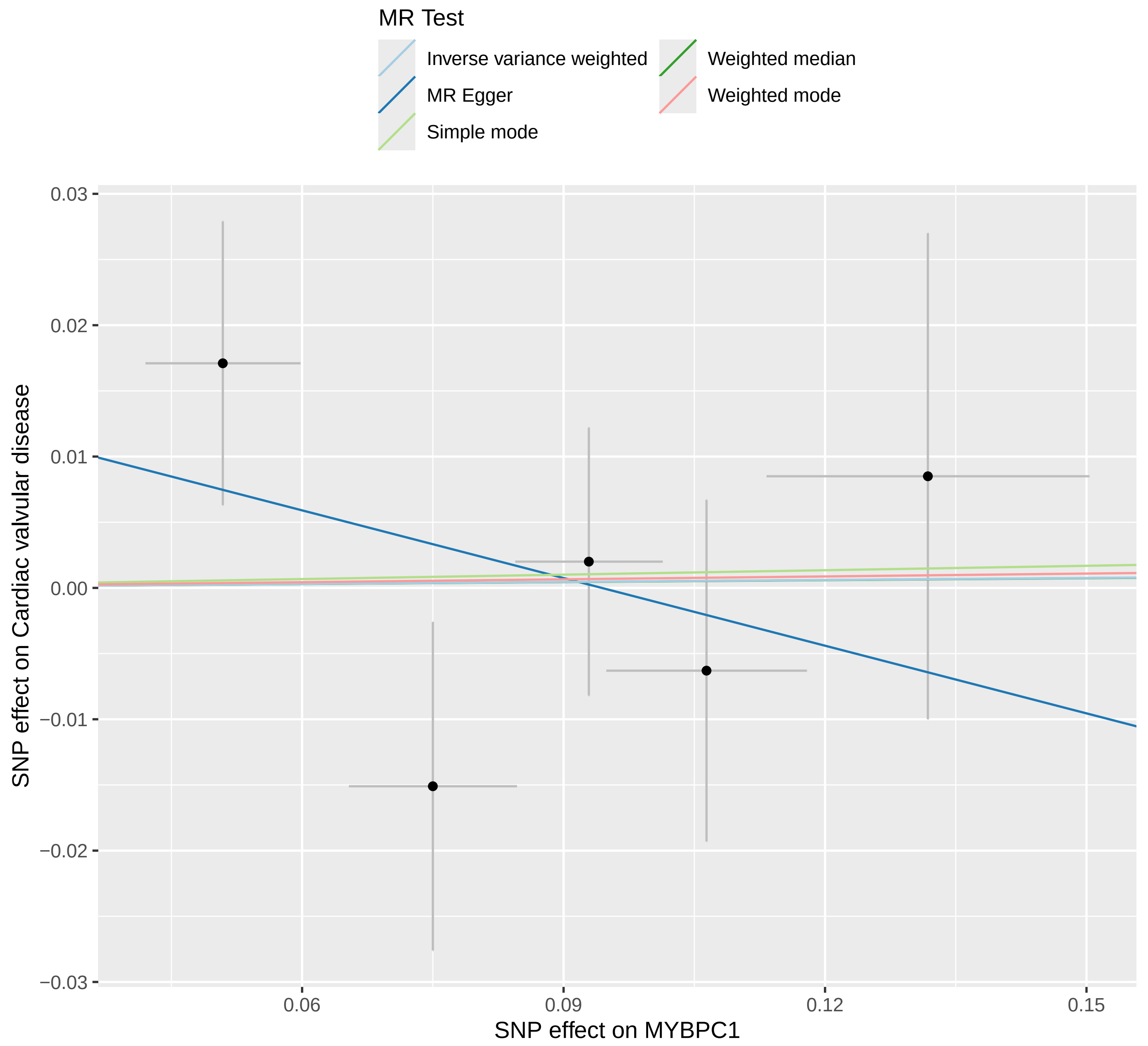
MR Test
Inverse variance weighted
MR Egger
Simple mode
Weighted median
Weighted mode
0.03
0.02
0.01
0.00
−0.01
−0.02
−0.03
0.06
0.09
0.12
0.15
SNP effect on Cardiac valvular disease
SNP effect on MYBPC1

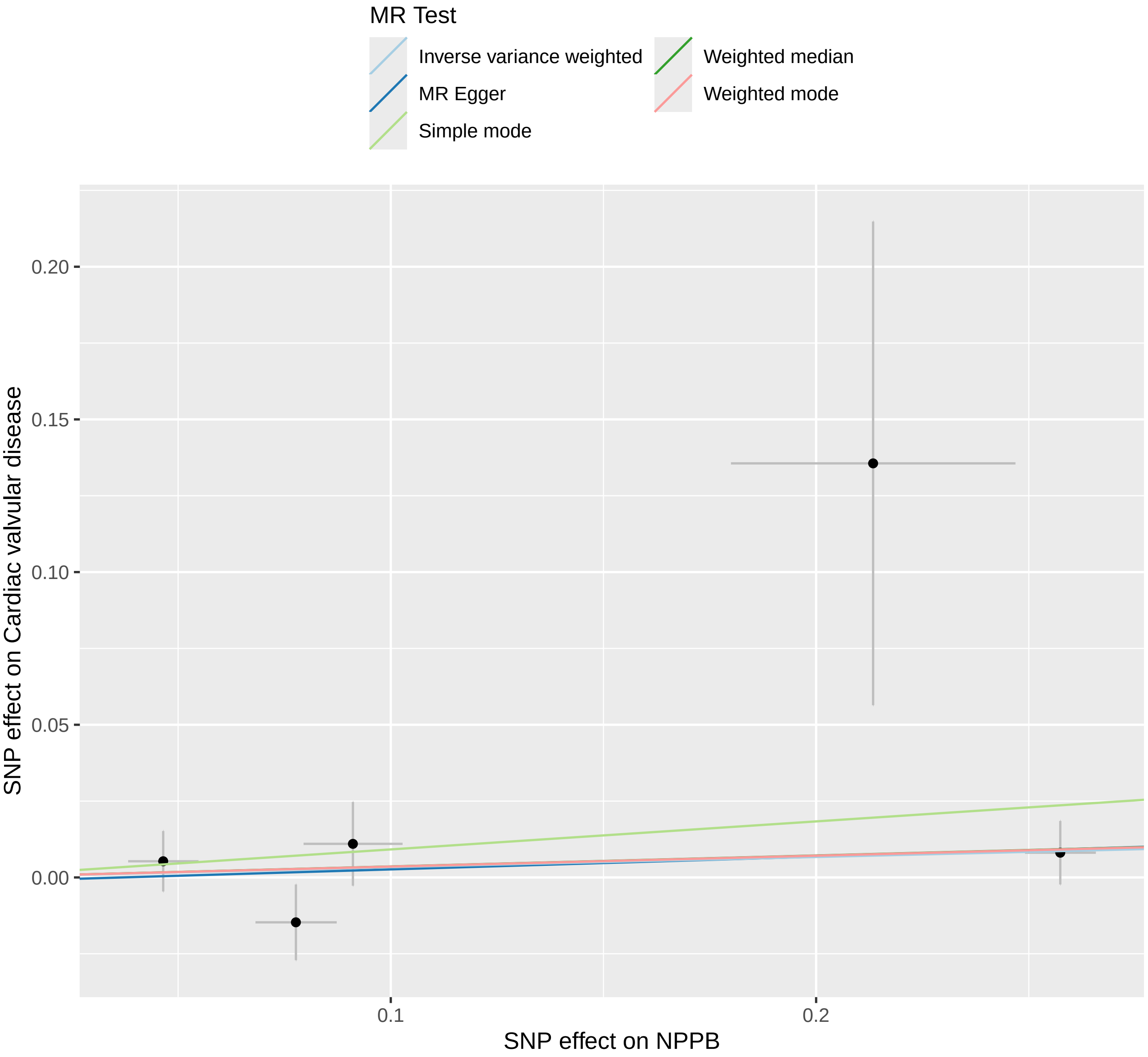

MR Test
Inverse variance weighted
MR Egger
Simple mode
Weighted median
Weighted mode
SNP effect on Cardiac valvular disease
0.20
0.15
0.10
0.05
0.00
0.1
0.2
SNP effect on NPPB

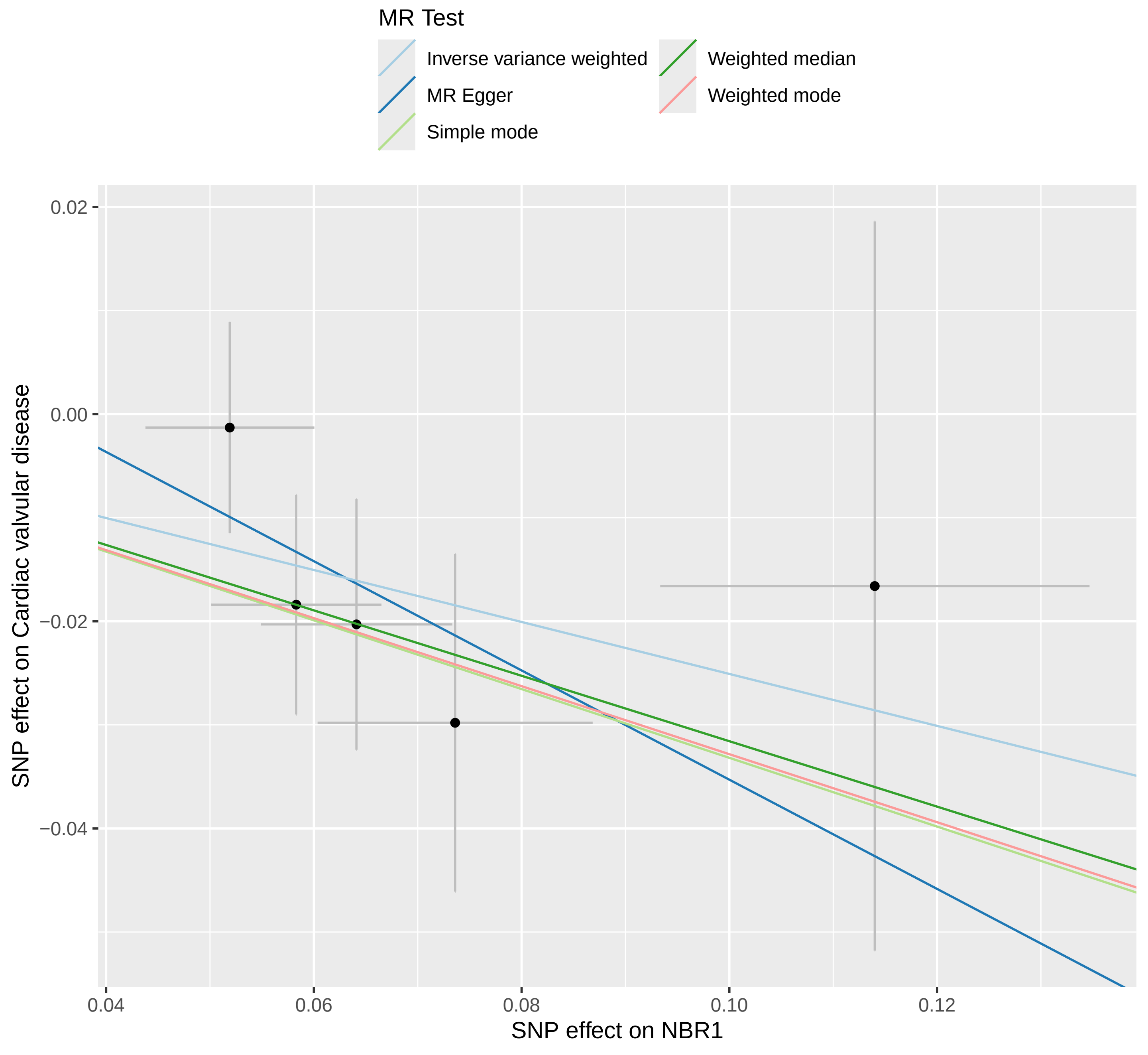

MR Test
Inverse variance weighted
MR Egger
Simple mode
Weighted median
Weighted mode
0.02
0.00
−0.02
−0.04
0.04
0.06
0.08
0.10
0.12
SNP effect on Cardiac valvular disease
SNP effect on NBR1

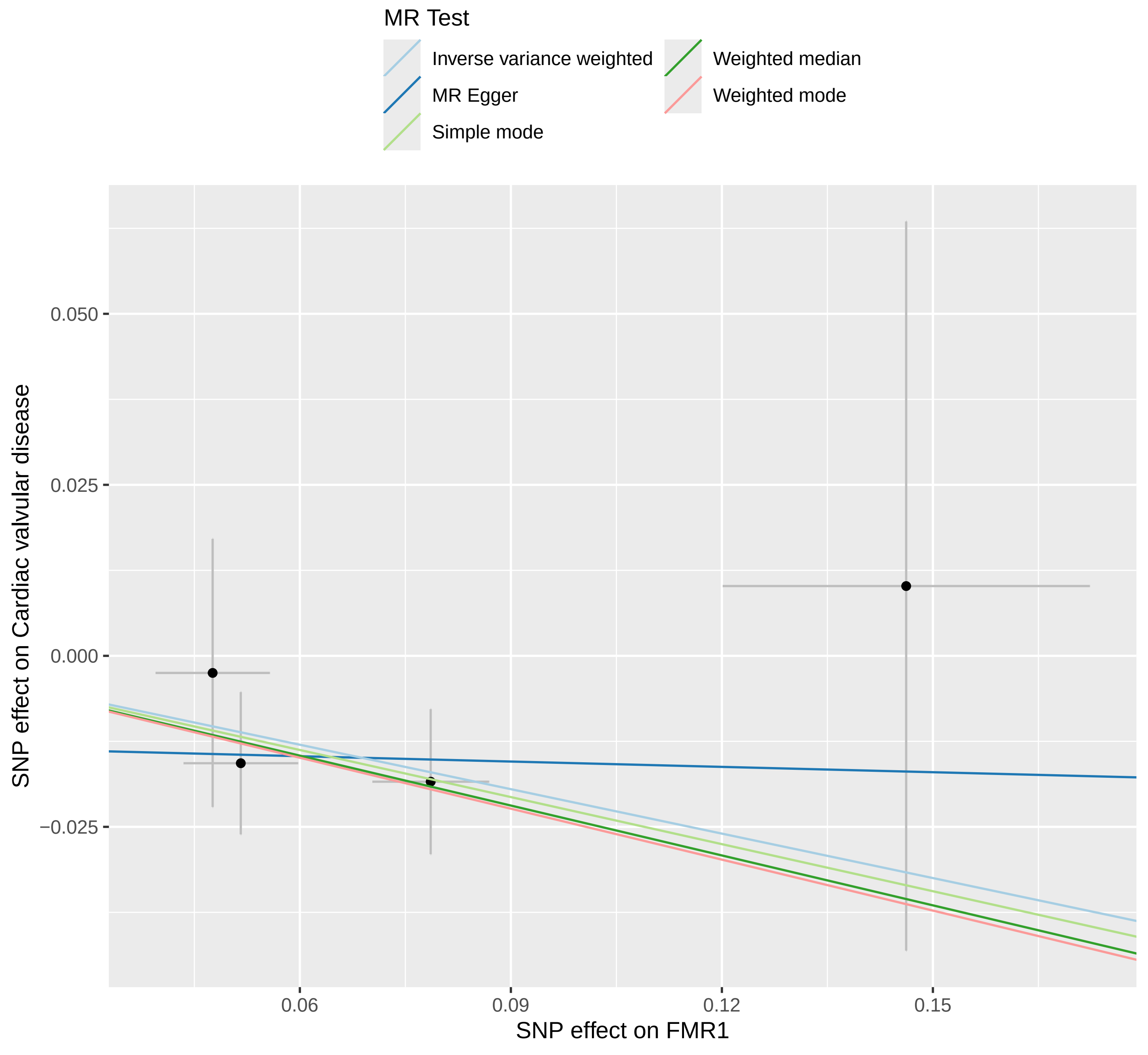
MR Test
Inverse variance weighted
MR Egger
Simple mode
Weighted median
Weighted mode
0.050
0.025
0.000
−0.025
SNP effect on Cardiac valvular disease
0.06
0.09
0.12
0.15
SNP effect on FMR1

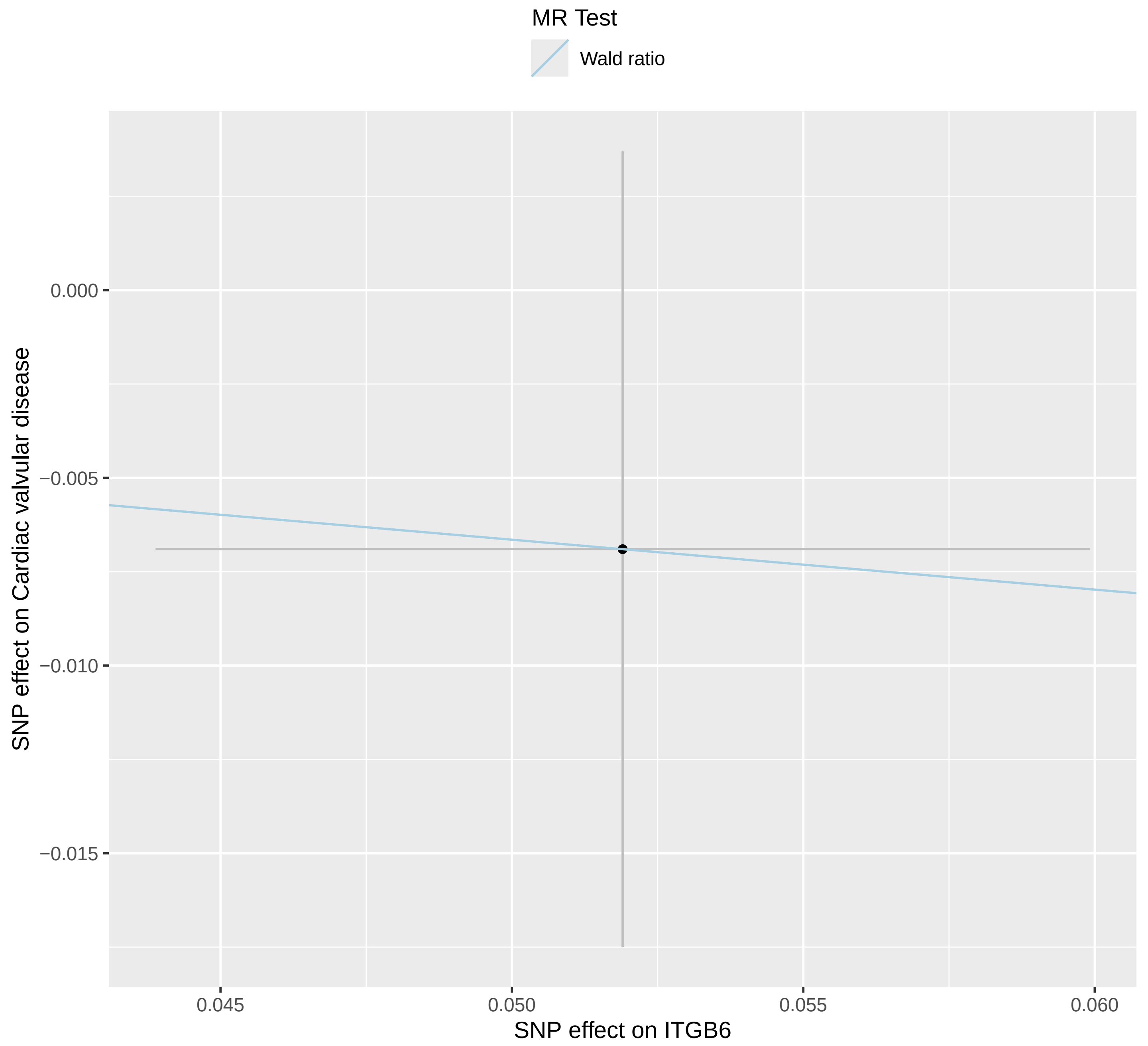
MR Test
Wald ratio
0.000
−0.005
−0.010
−0.015
SNP effect on Cardiac valvular disease
0.045
0.050
0.055
0.060
SNP effect on ITGB6

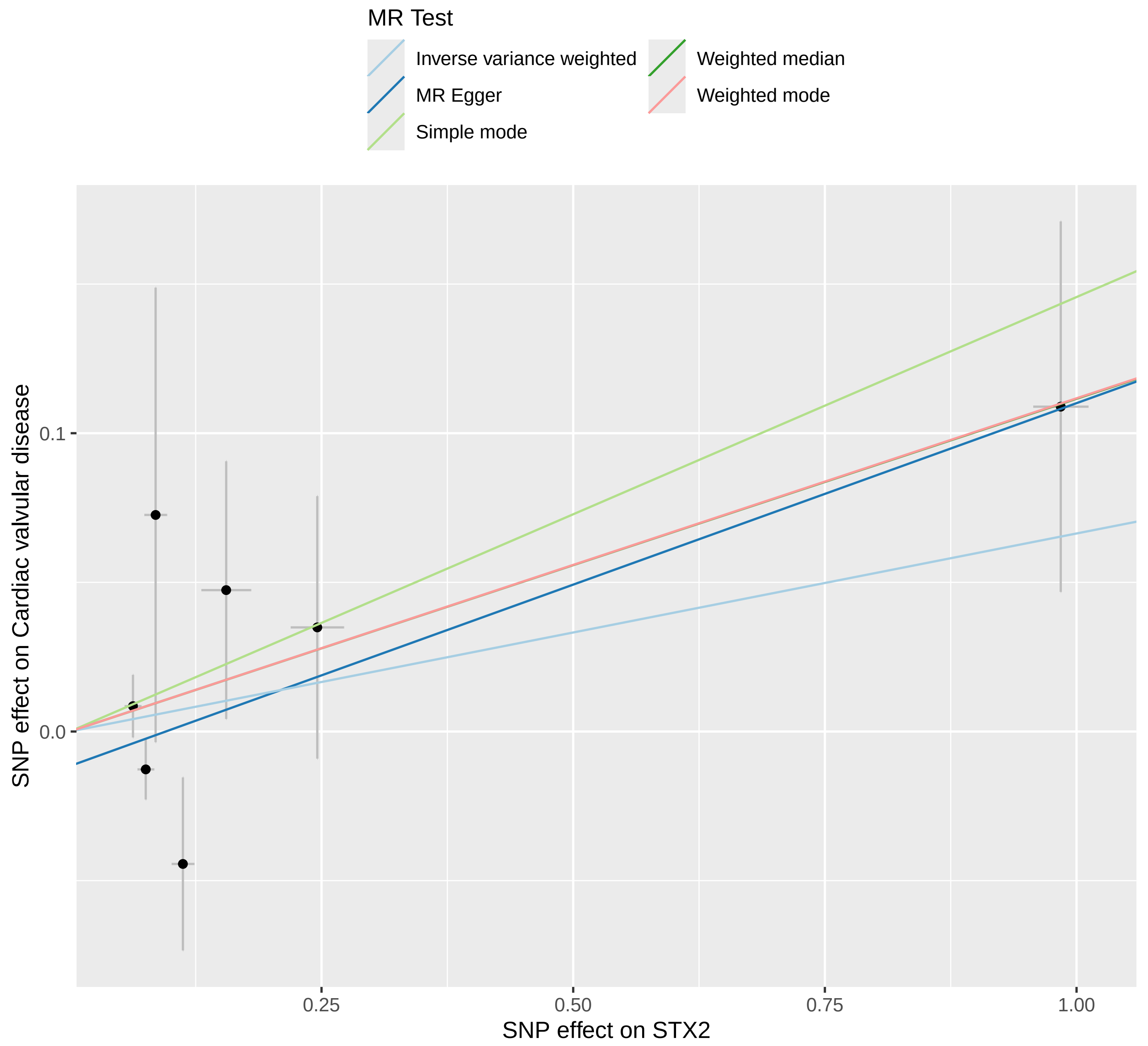
MR Test
Inverse variance weighted
MR Egger
Simple mode
Weighted median
Weighted mode
SNP effect on Cardiac valvular disease
0.1
0.0
0.25
0.50
0.75
1.00
SNP effect on STX2

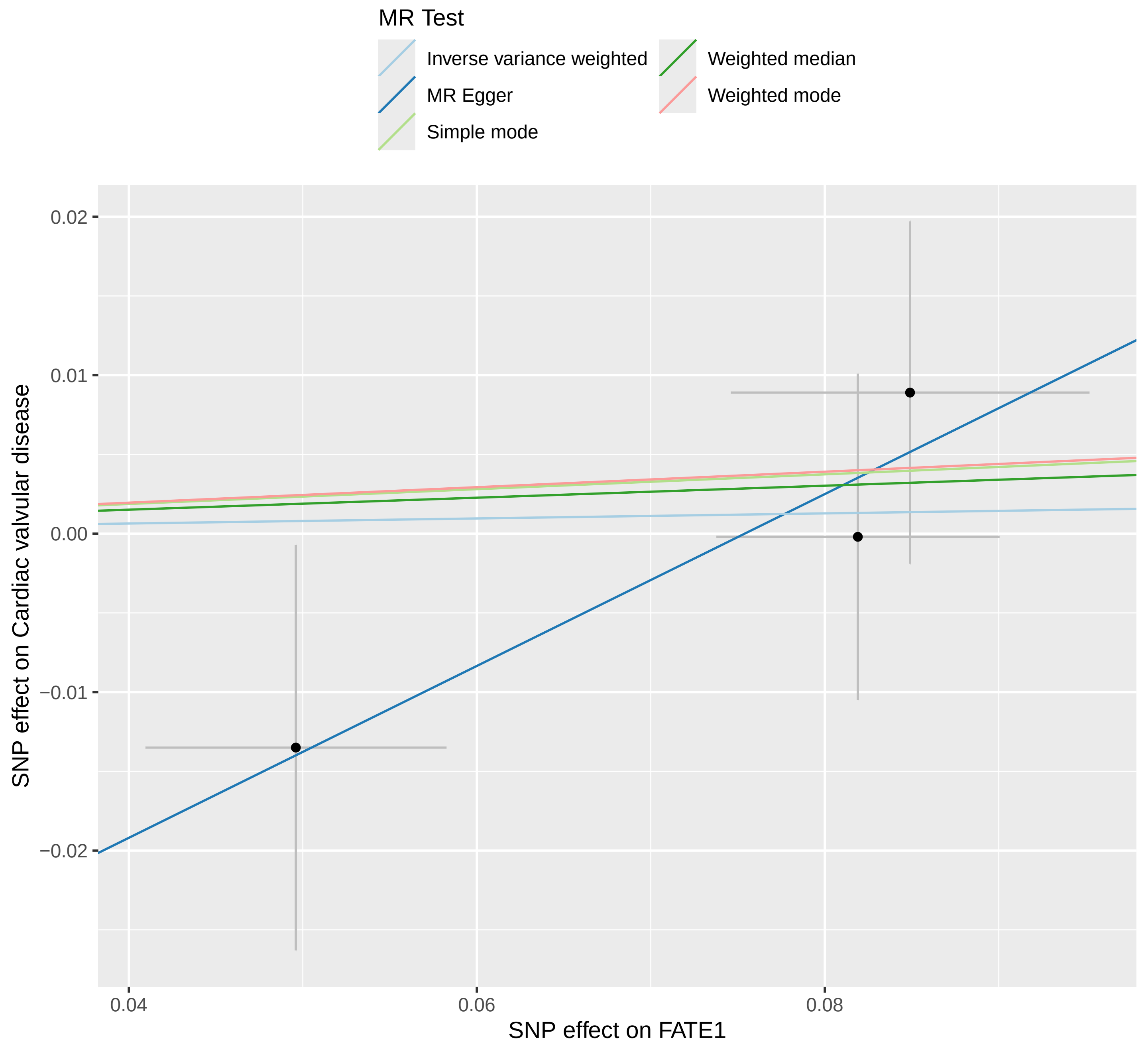

MR Test
Inverse variance weighted
MR Egger
Simple mode
Weighted median
Weighted mode
0.02
0.01
0.00
−0.01
−0.02
SNP effect on Cardiac valvular disease
0.04
0.06
0.08
SNP effect on FATE1

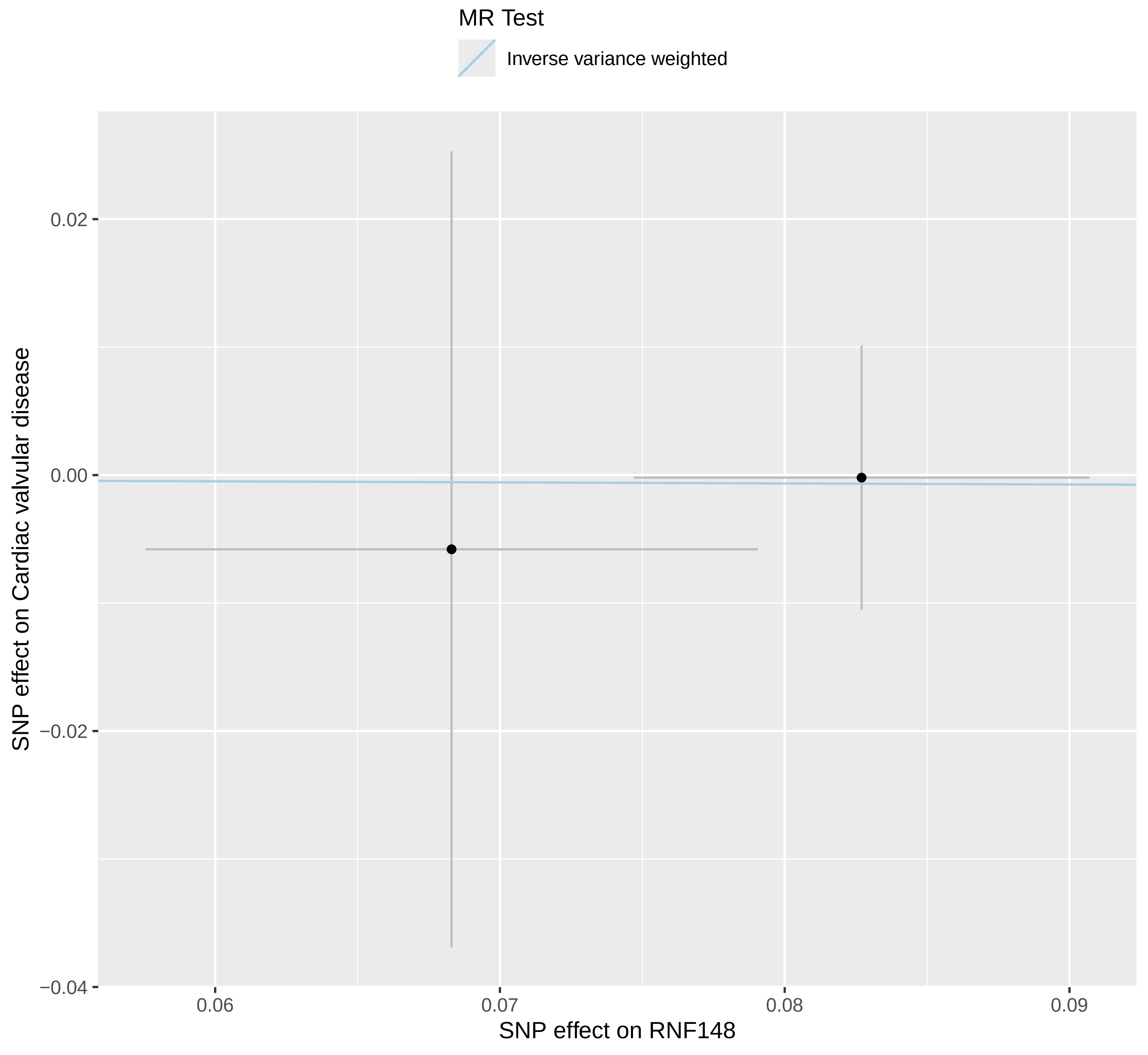

MR Test
Inverse variance weighted
0.02
0.00
−0.02
−0.04
SNP effect on Cardiac valvular disease
0.06
0.07
0.08
0.09
SNP effect on RNF148

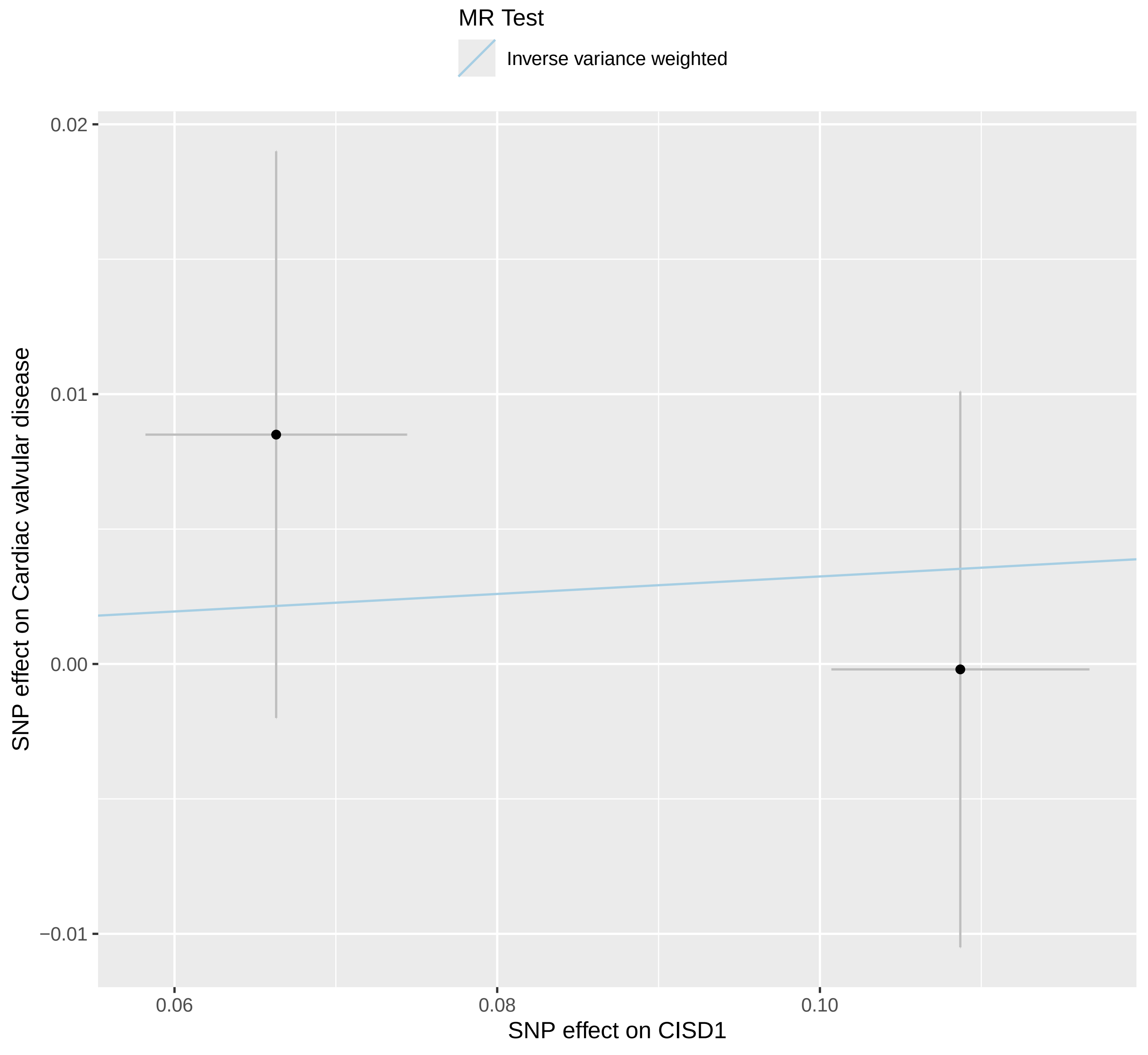

MR Test
Inverse variance weighted
0.02
0.01
0.00
−0.01
0.06
0.08
0.10
SNP effect on Cardiac valvular disease
SNP effect on CISD1

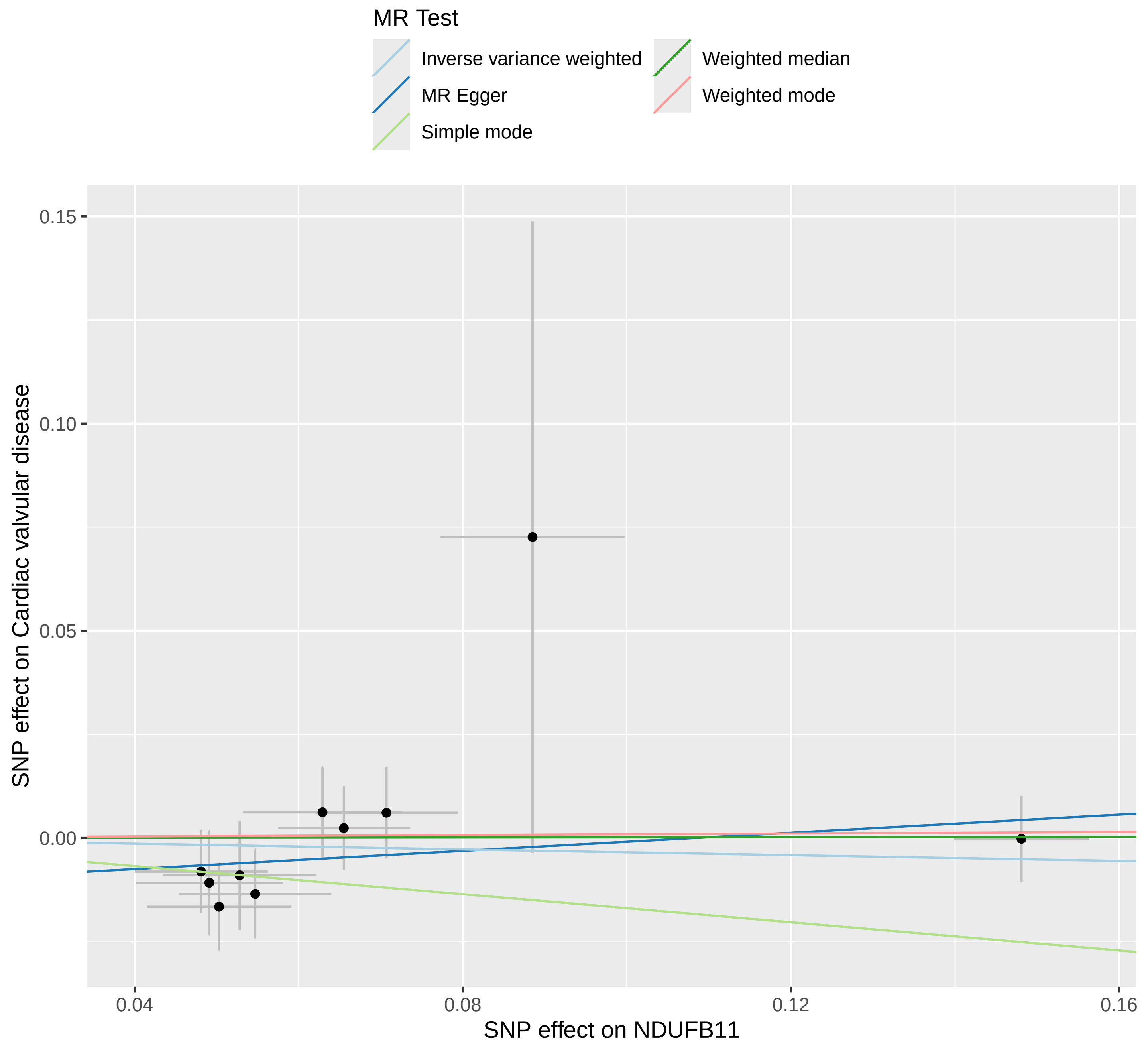
MR Test
Inverse variance weighted
MR Egger
Simple mode
Weighted median
Weighted mode
SNP effect on Cardiac valvular disease
0.15
0.10
0.05
0.00
0.04
0.08
0.12
0.16
SNP effect on NDUFB11

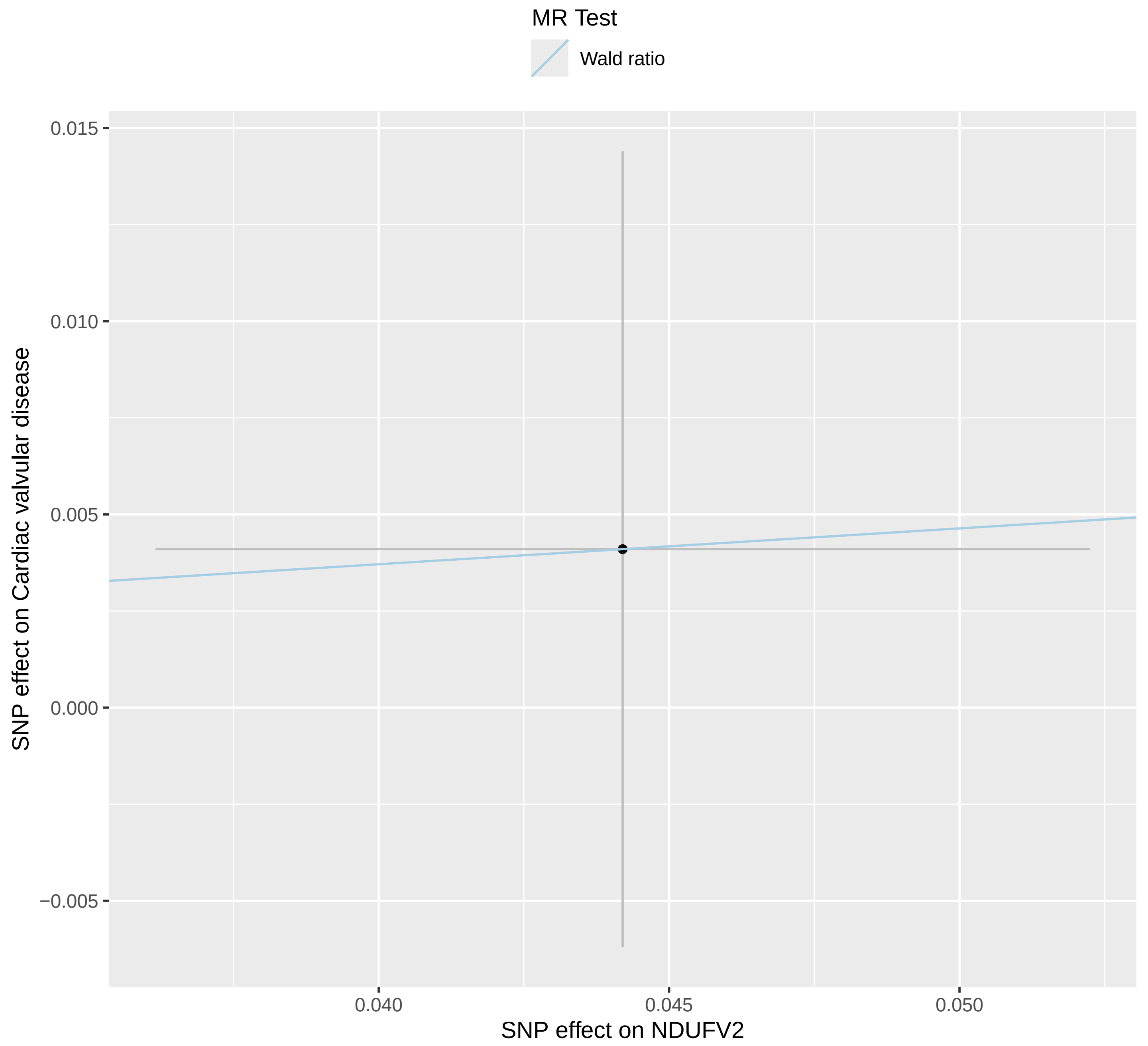

MR Test
Wald ratio
0.015
0.010
0.005
0.000
−0.005
0.040
0.045
0.050
SNP effect on NDUFV2
SNP effect on Cardiac valvular disease

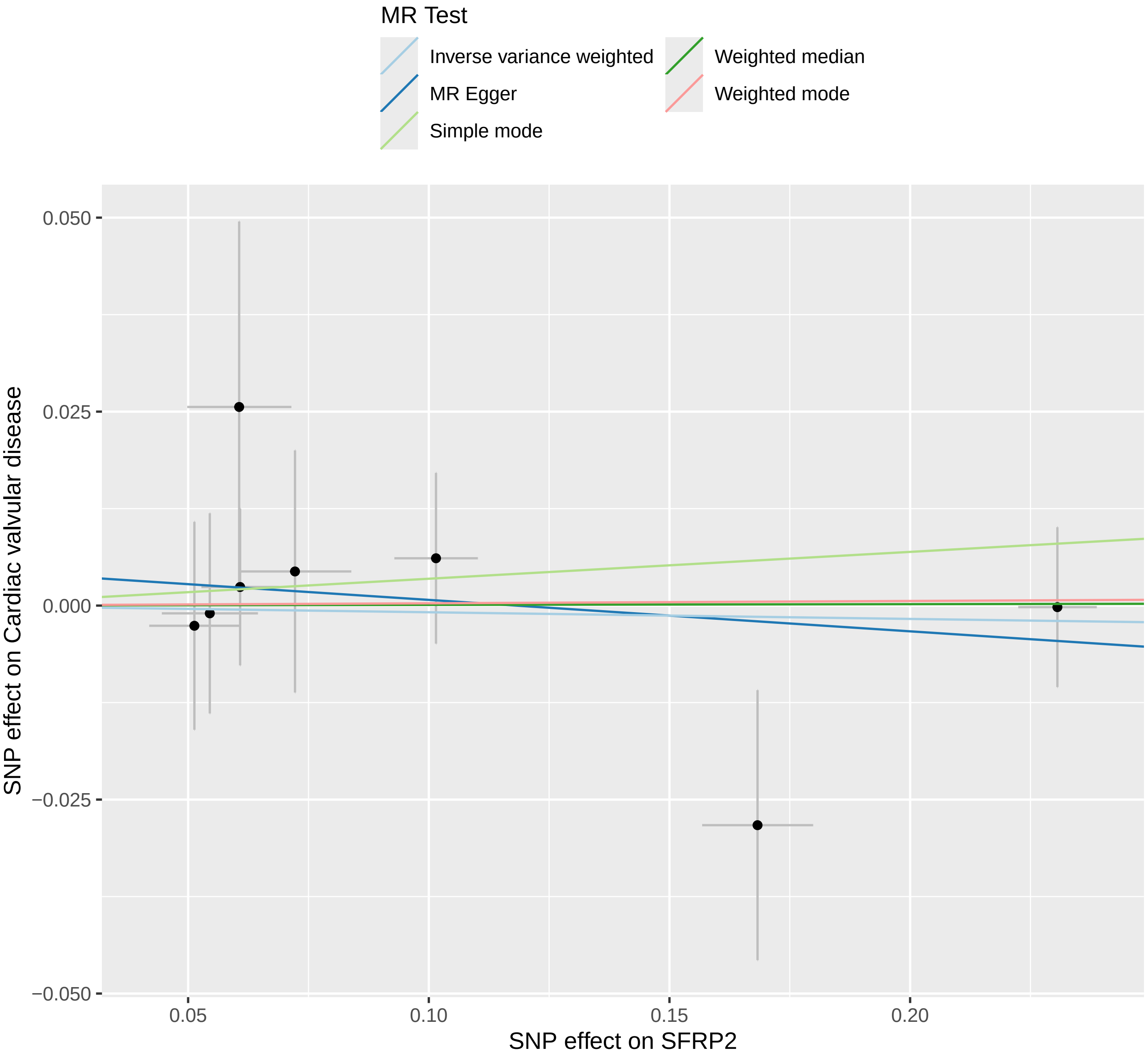
MR Test
Inverse variance weighted
MR Egger
Simple mode
Weighted median
Weighted mode
0.050
0.025
0.000
−0.025
−0.050
SNP effect on Cardiac valvular disease
0.05
0.10
0.15
0.20
SNP effect on SFRP2

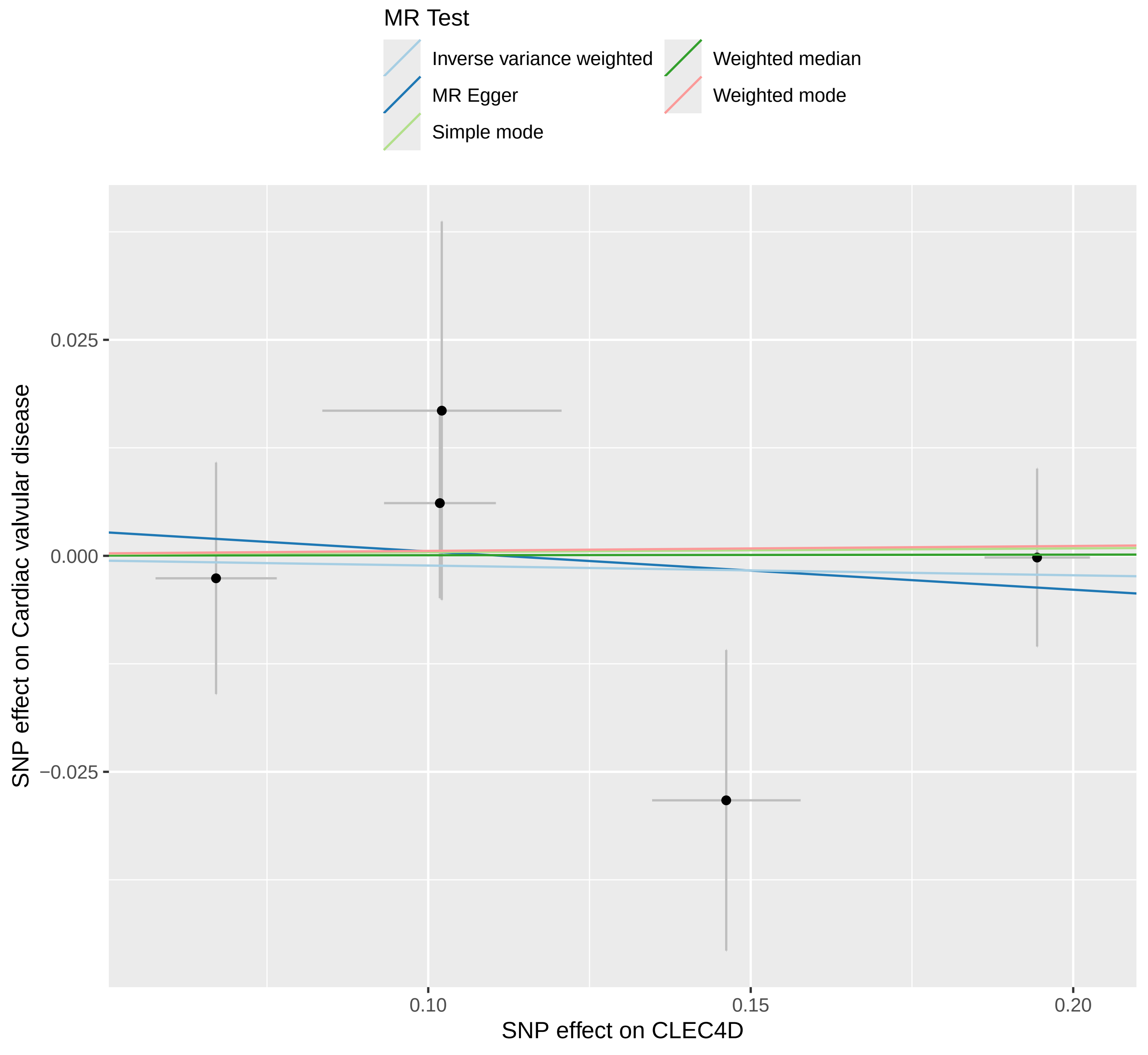
MR Test
Inverse variance weighted
MR Egger
Simple mode
Weighted median
Weighted mode
0.025
0.000
−0.025
0.10
0.15
0.20
SNP effect on CLEC4D
SNP effect on Cardiac valvular disease

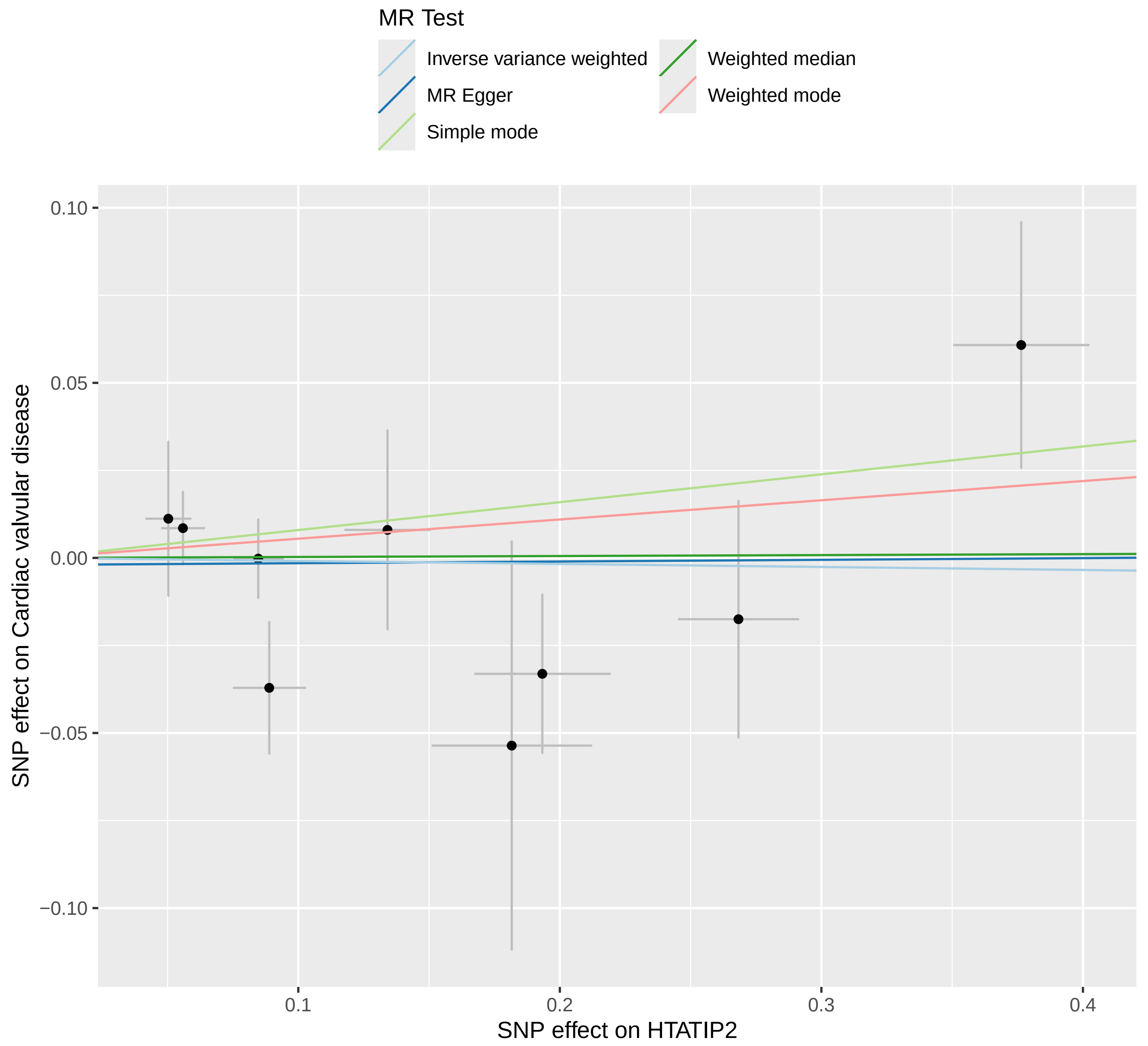
MR Test
Inverse variance weighted
MR Egger
Simple mode
Weighted median
Weighted mode
0.10
0.05
0.00
−0.05
−0.10
SNP effect on Cardiac valvular disease
0.1
0.2
0.3
0.4
SNP effect on HTATIP2

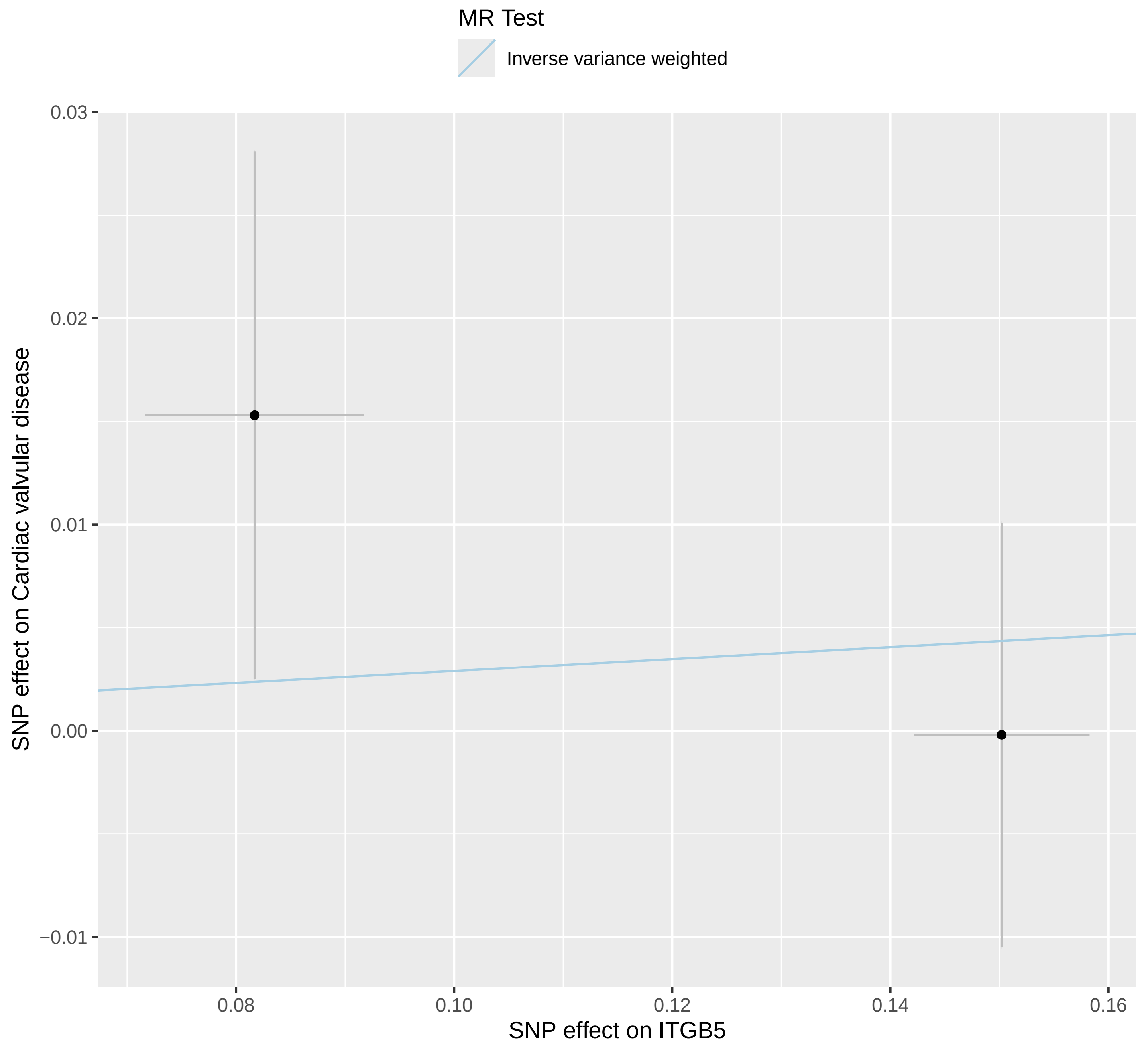

MR Test
Inverse variance weighted
0.03
0.02
0.01
0.00
−0.01
0.08
0.10
0.12
0.14
0.16
SNP effect on Cardiac valvular disease
SNP effect on ITGB5

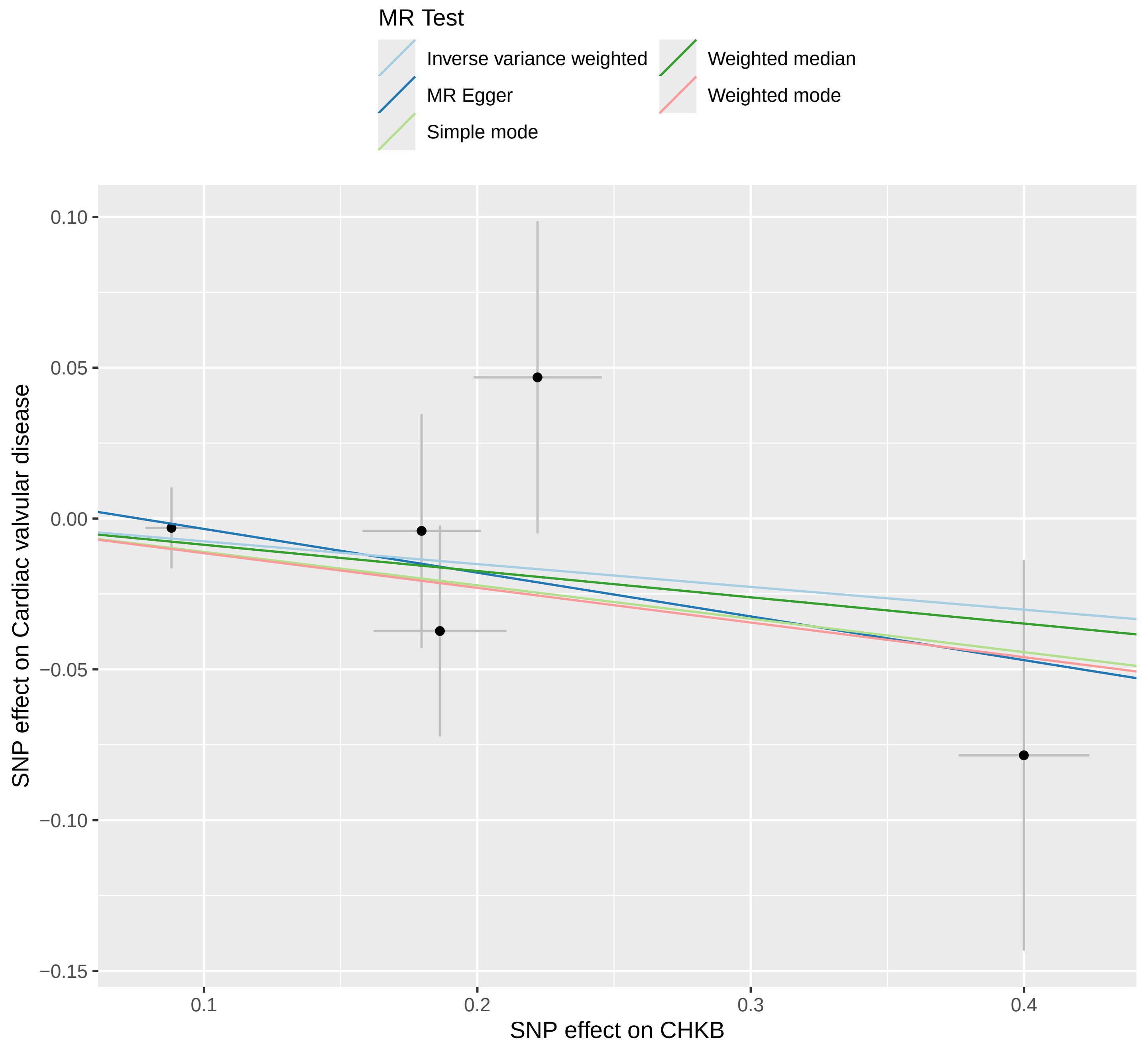
MR Test
Inverse variance weighted
MR Egger
Simple mode
Weighted median
Weighted mode
0.10
0.05
0.00
−0.05
−0.10
−0.15
SNP effect on Cardiac valvular disease
0.1
0.2
0.3
0.4
SNP effect on CHKB

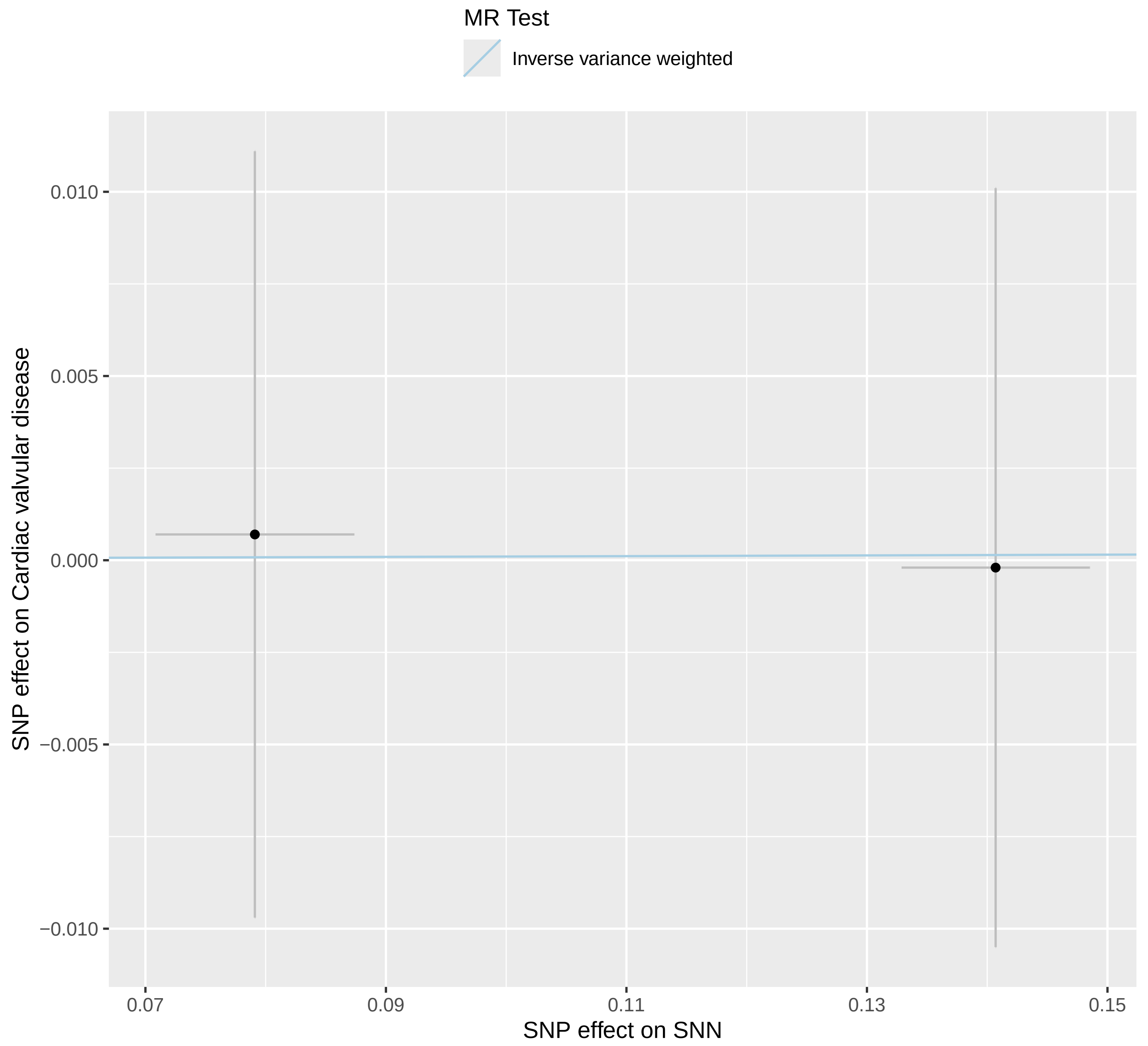
MR Test
Inverse variance weighted
0.010
0.005
0.000
−0.005
−0.010
0.07
0.09
0.11
0.13
0.15
SNP effect on SNN
SNP effect on Cardiac valvular disease

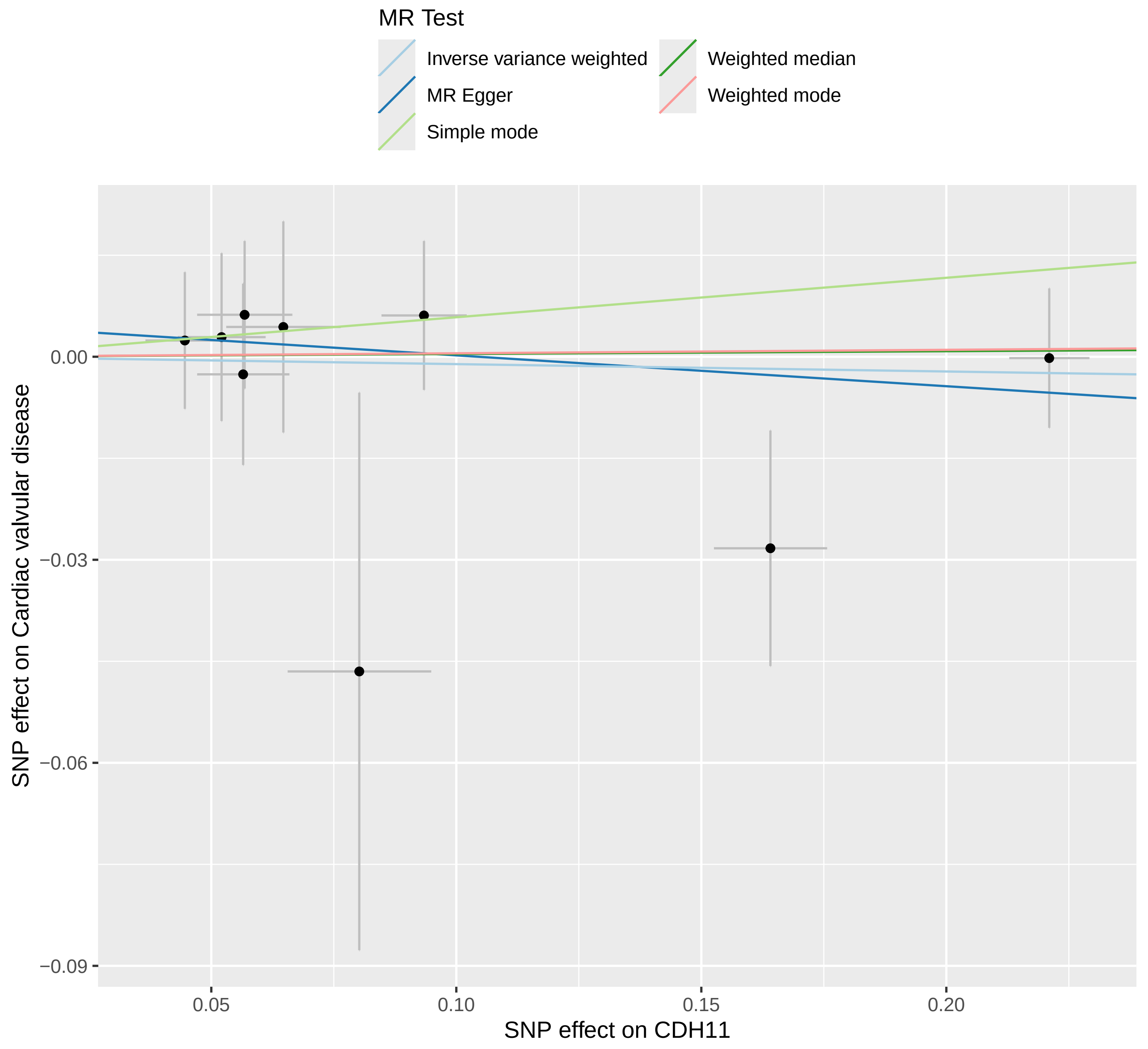

MR Test
Inverse variance weighted
MR Egger
Simple mode
Weighted median
Weighted mode
SNP effect on Cardiac valvular disease
0.00
−0.03
−0.06
−0.09
0.05
0.10
0.15
0.20
SNP effect on CDH11

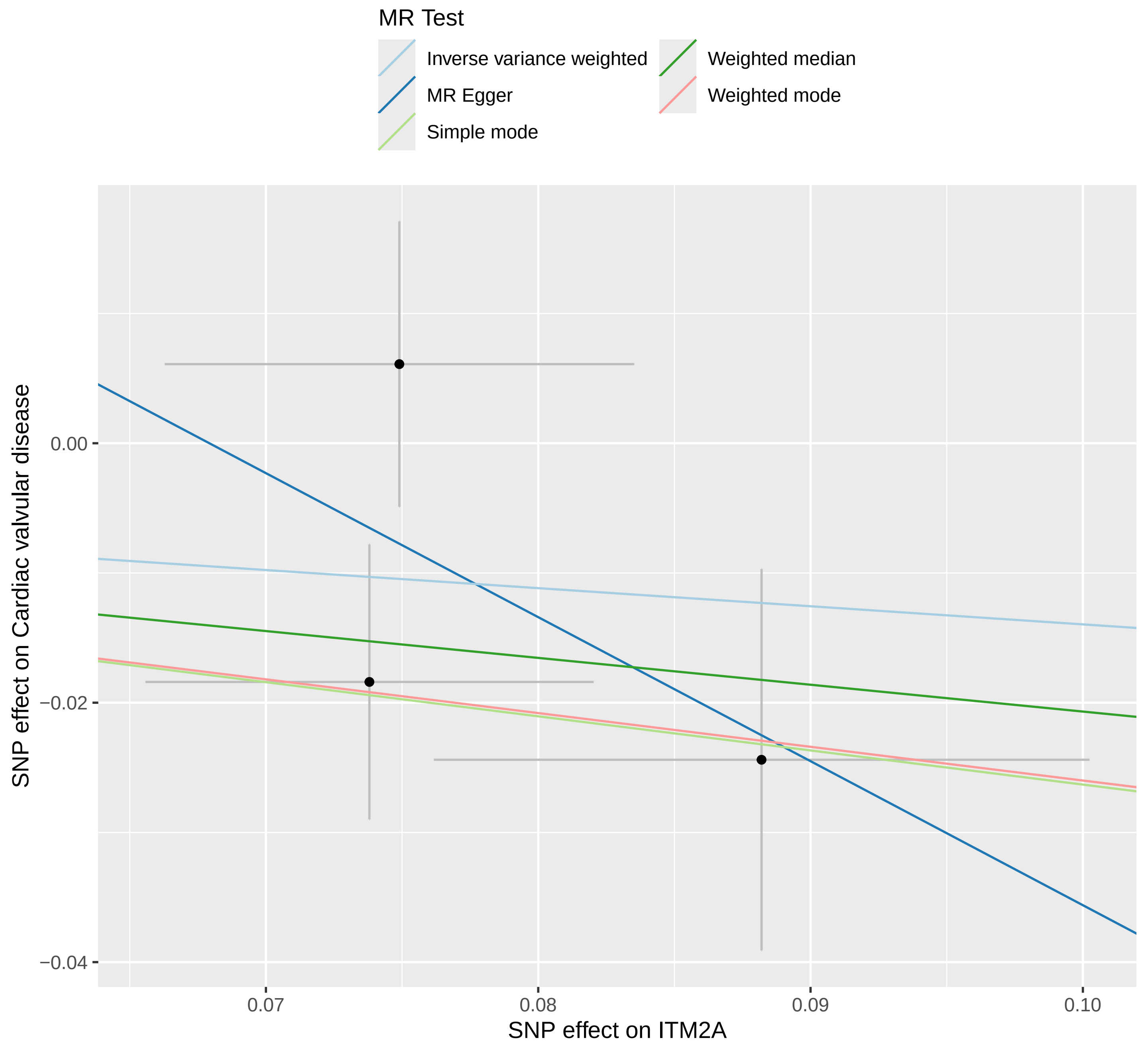

MR Test
Inverse variance weighted
MR Egger
Simple mode
Weighted median
Weighted mode
SNP effect on Cardiac valvular disease
0.00
−0.02
−0.04
0.07
0.08
0.09
0.10
SNP effect on ITM2A

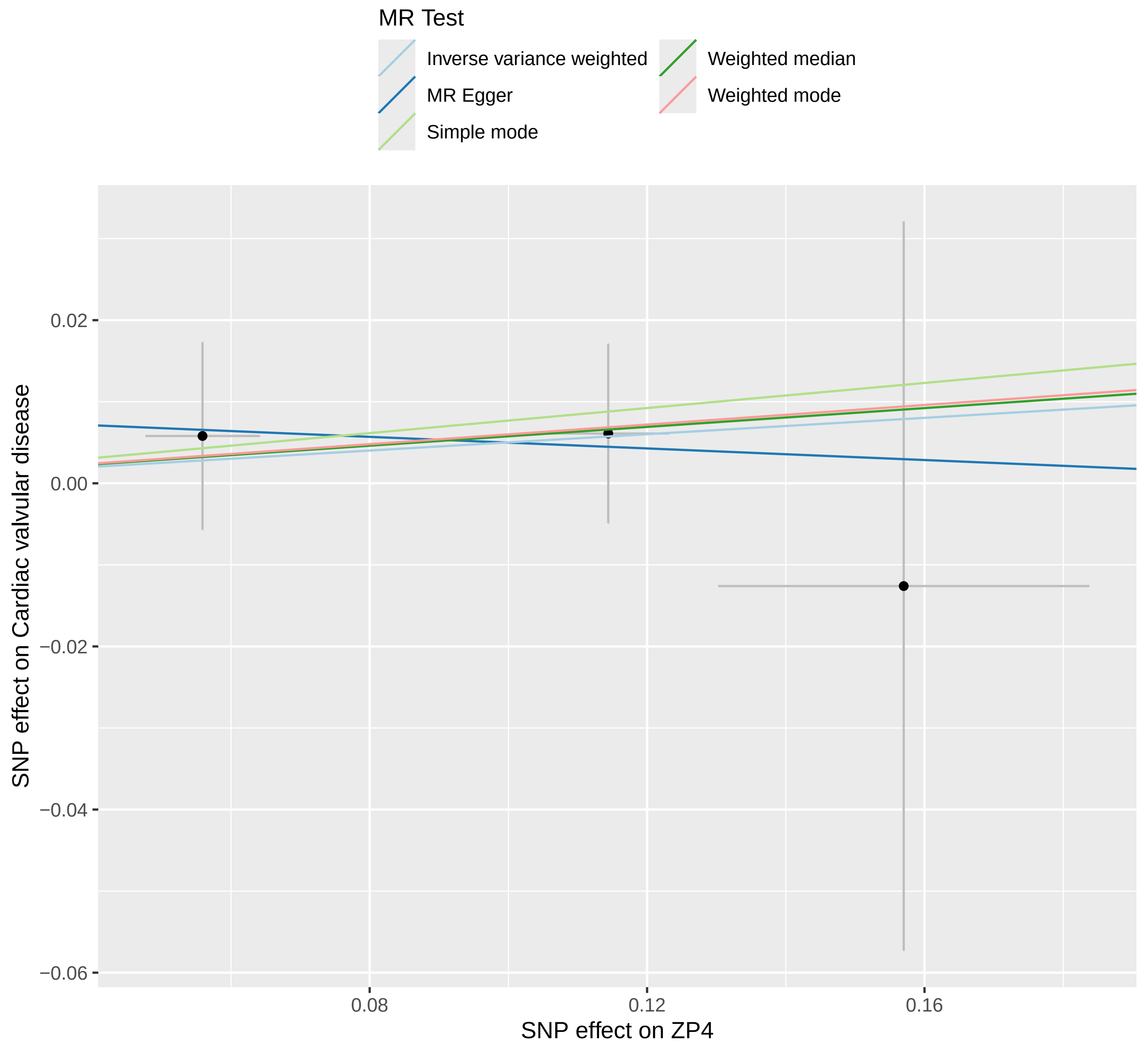
MR Test
Inverse variance weighted
MR Egger
Simple mode
Weighted median
Weighted mode
SNP effect on Cardiac valvular disease
0.02
0.00
−0.02
−0.04
−0.06
0.08
0.12
0.16
SNP effect on ZP4

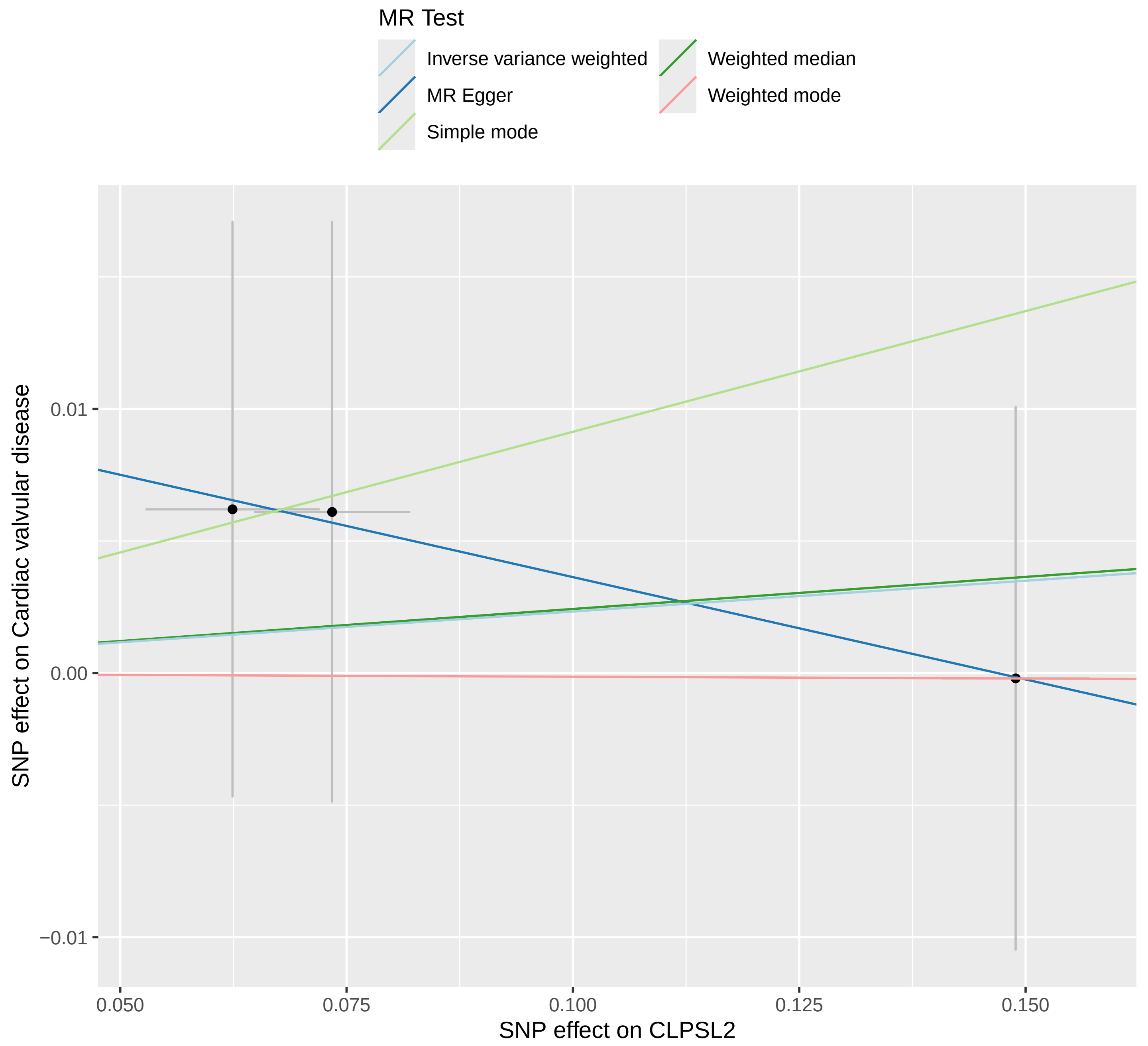
MR Test
Inverse variance weighted
MR Egger
Simple mode
Weighted median
Weighted mode
SNP effect on Cardiac valvular disease
0.01
0.00
−0.01
0.050
0.075
0.100
0.125
0.150
SNP effect on CLPSL2

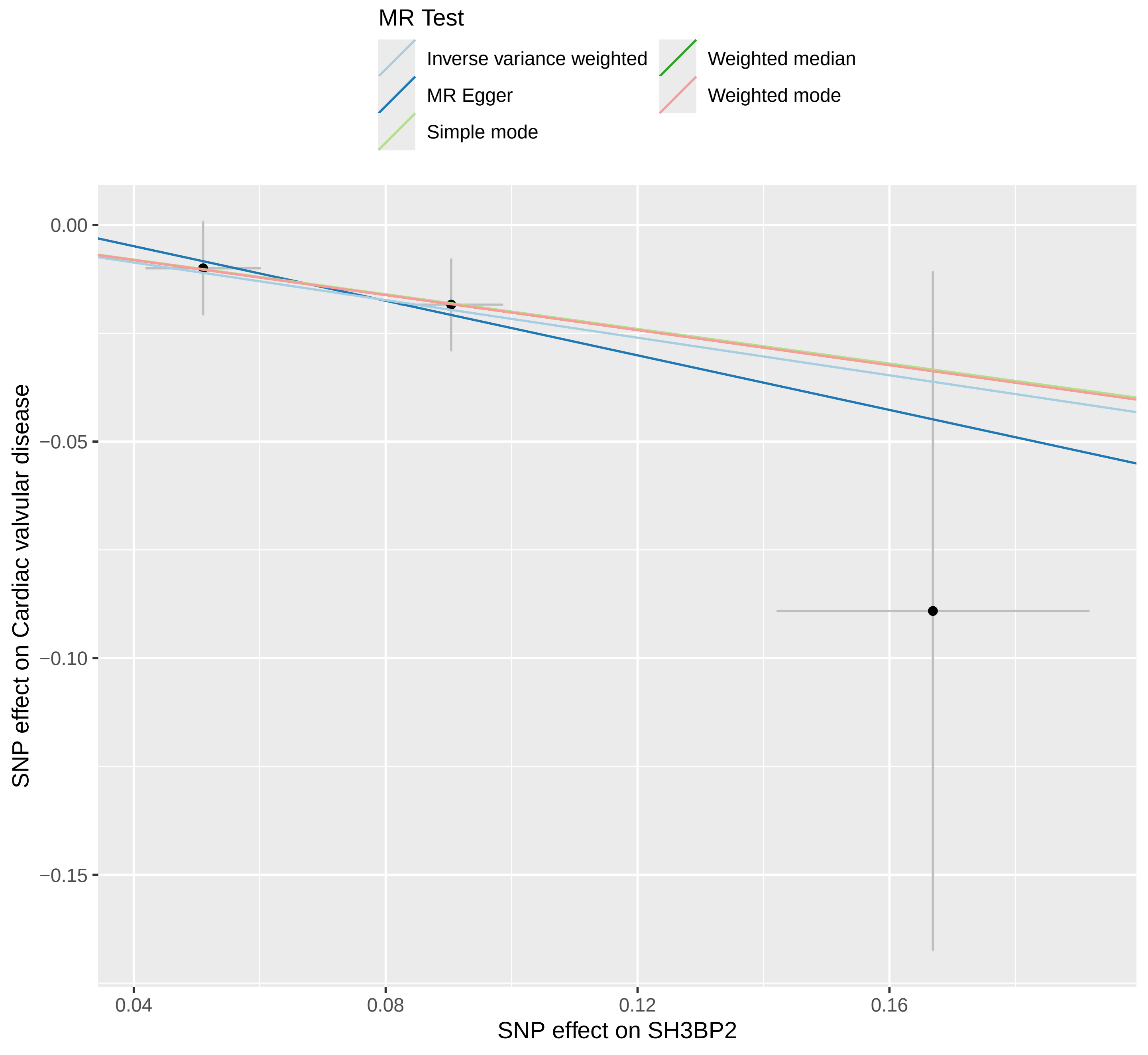

MR Test
Inverse variance weighted
MR Egger
Simple mode
Weighted median
Weighted mode
0.00
−0.05
−0.10
−0.15
0.04
0.08
0.12
0.16
SNP effect on SH3BP2
SNP effect on Cardiac valvular disease

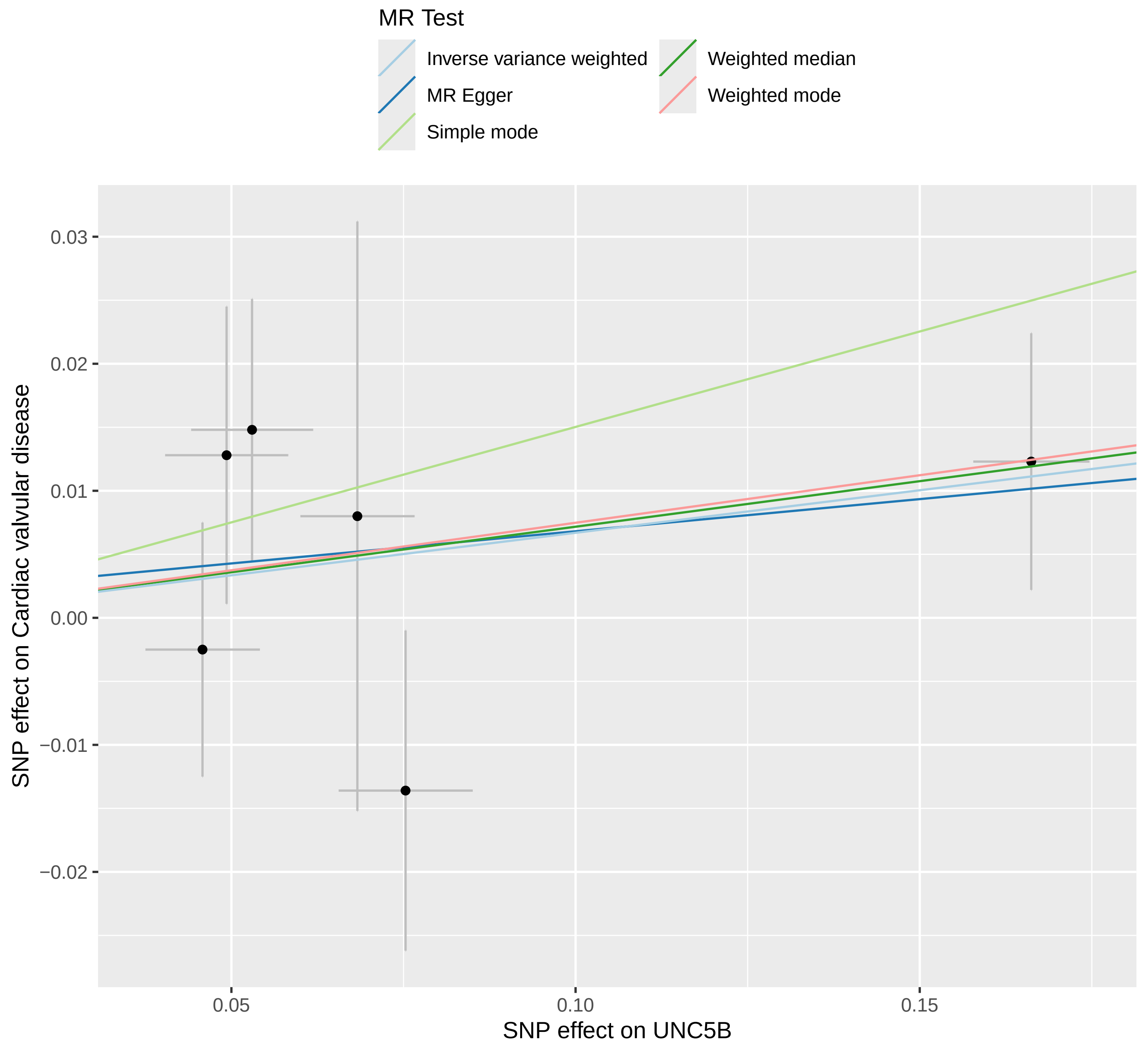
MR Test
Inverse variance weighted
MR Egger
Simple mode
Weighted median
Weighted mode
0.03
0.02
0.01
0.00
−0.01
−0.02
0.05
0.10
0.15
SNP effect on Cardiac valvular disease
SNP effect on UNC5B

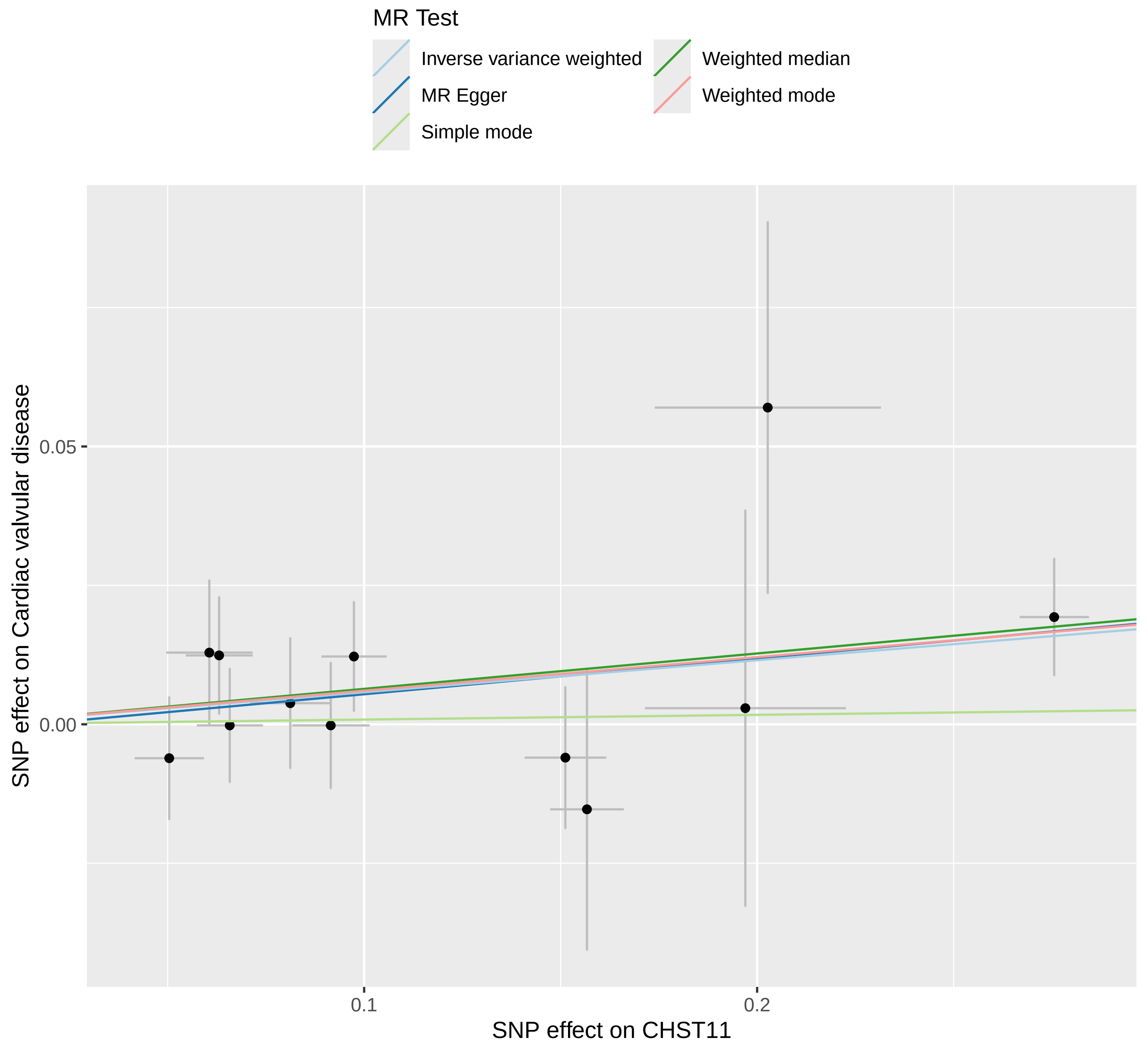
MR Test
Inverse variance weighted
MR Egger
Simple mode
Weighted median
Weighted mode
0.05
0.00
0.1
0.2
SNP effect on Cardiac valvular disease
SNP effect on CHST11

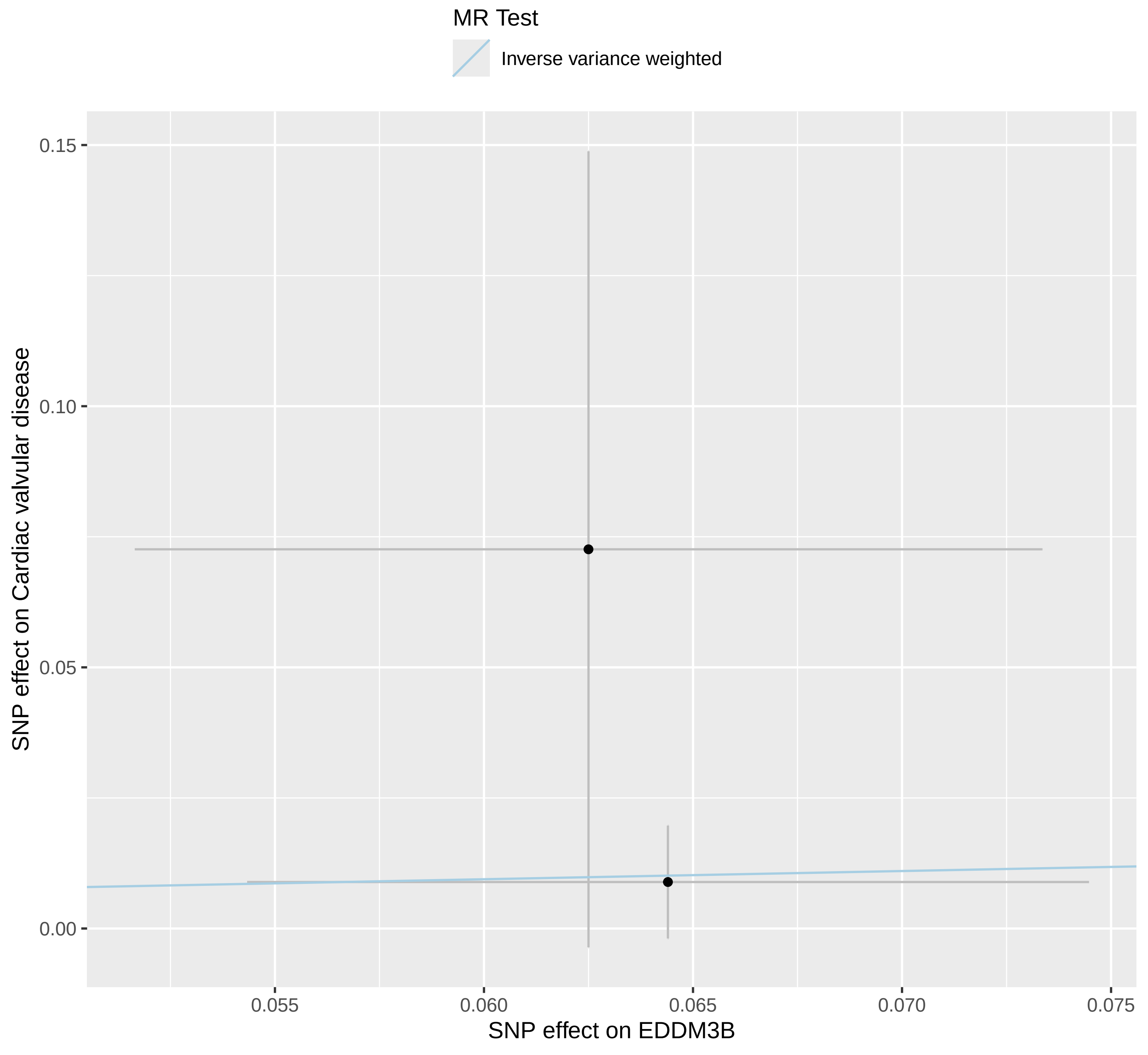

MR Test
Inverse variance weighted
0.15
0.10
0.05
0.00
0.055
0.060
0.065
0.070
0.075
SNP effect on EDDM3B
SNP effect on Cardiac valvular disease

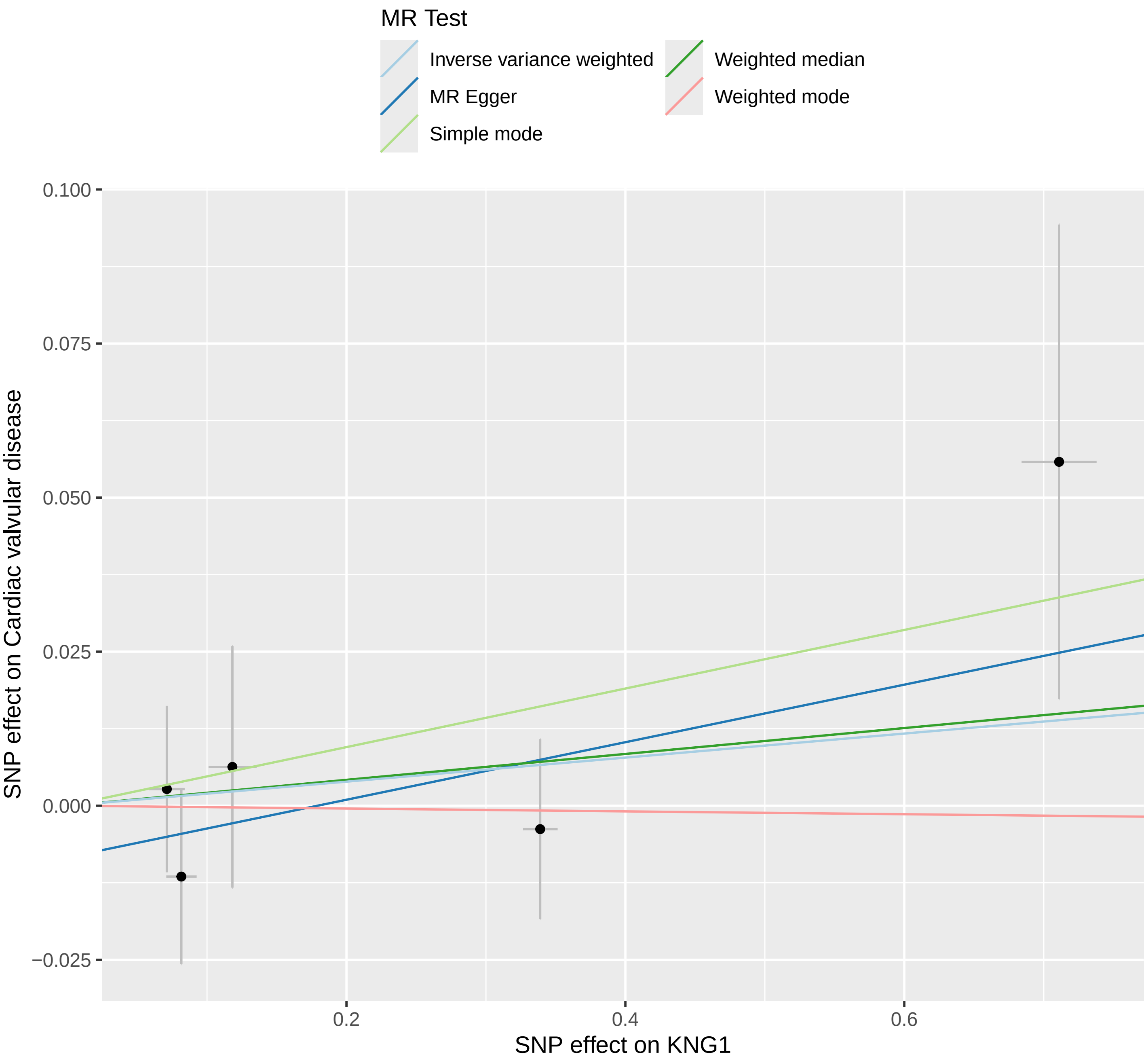
MR Test
Inverse variance weighted
MR Egger
Simple mode
Weighted median
Weighted mode
0.100
0.075
0.050
0.025
0.000
−0.025
SNP effect on Cardiac valvular disease
0.2
0.4
0.6
SNP effect on KNG1

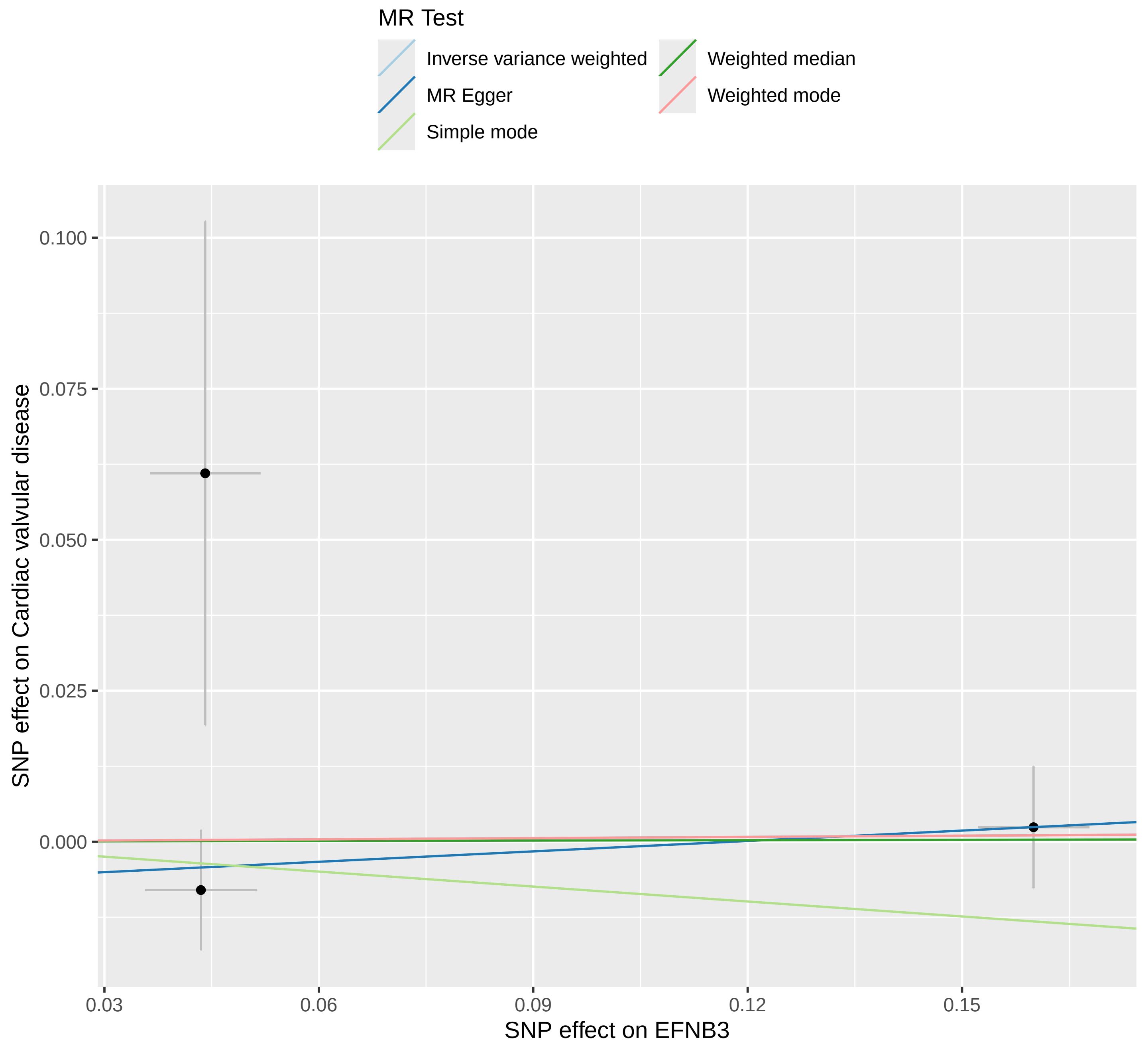
MR Test
Inverse variance weighted
MR Egger
Simple mode
Weighted median
Weighted mode
SNP effect on Cardiac valvular disease
0.100
0.075
0.050
0.025
0.000
0.03
0.06
0.09
0.12
0.15
SNP effect on EFNB3

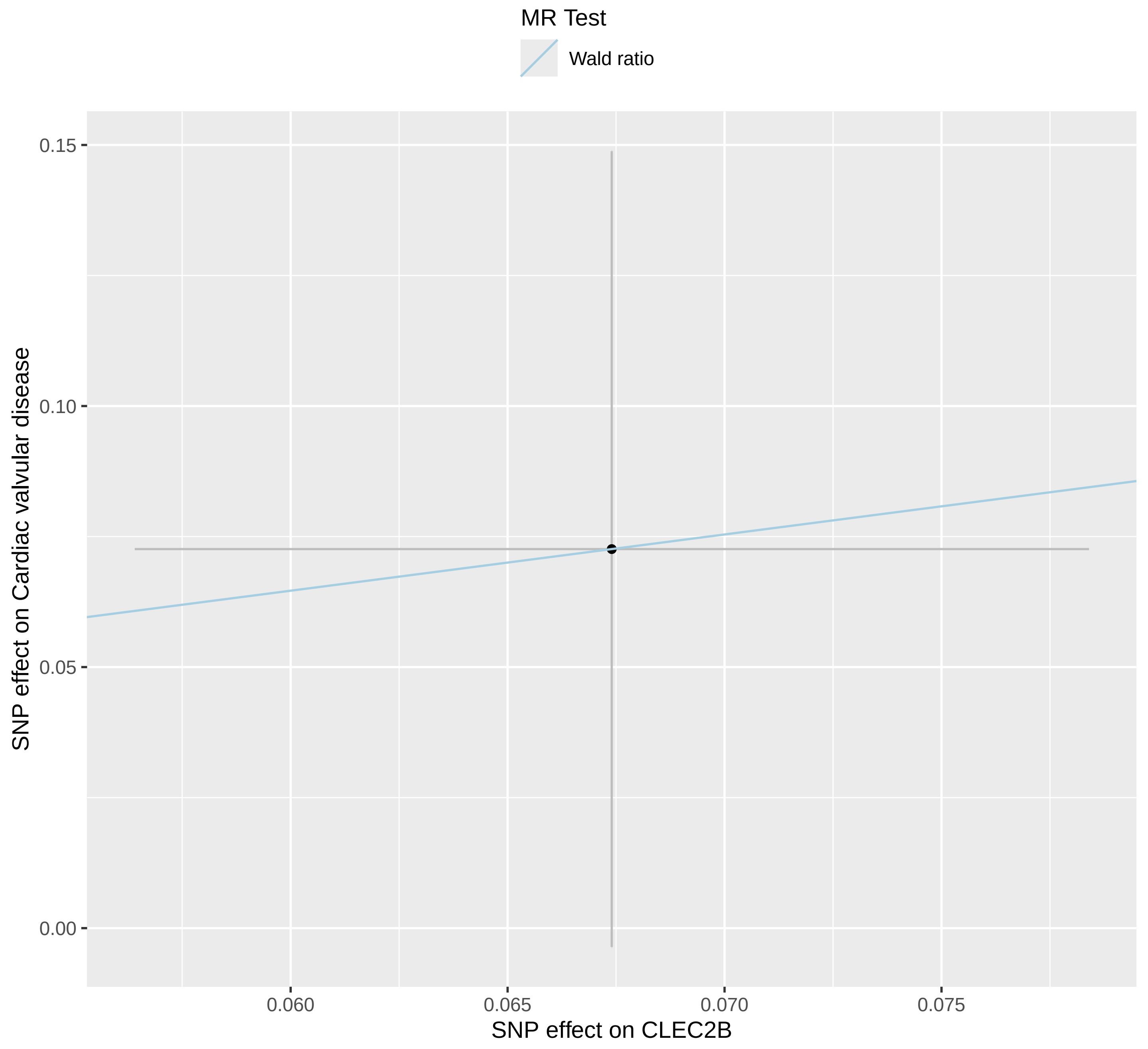

MR Test
Wald ratio
SNP effect on Cardiac valvular disease
0.15
0.10
0.05
0.00
0.060
0.065
0.070
0.075
SNP effect on CLEC2B

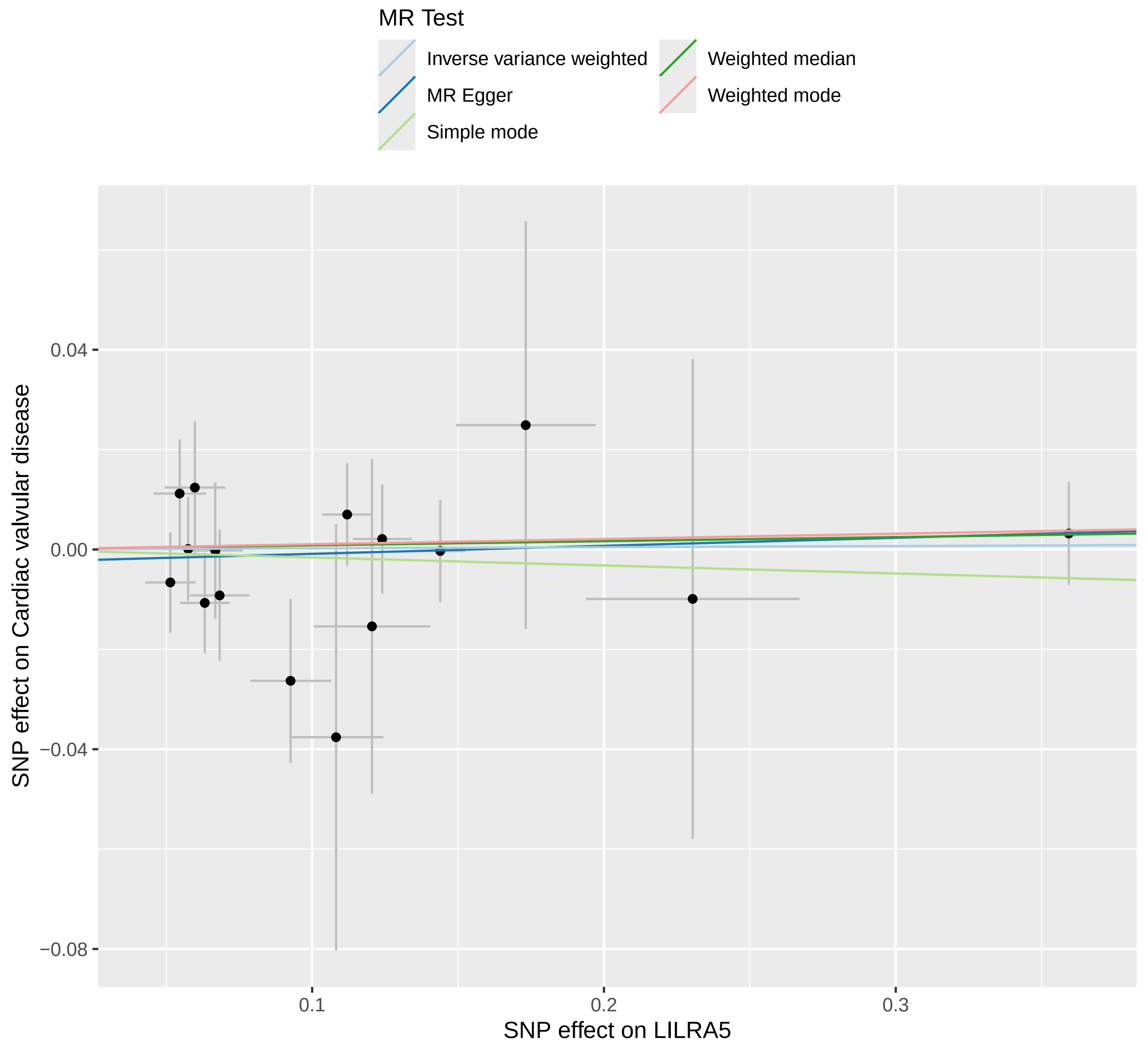
MR Test
Inverse variance weighted
MR Egger
Simple mode
Weighted median
Weighted mode
0.04
0.00
−0.04
−0.08
0.1
0.2
0.3
SNP effect on Cardiac valvular disease
SNP effect on LILRA5

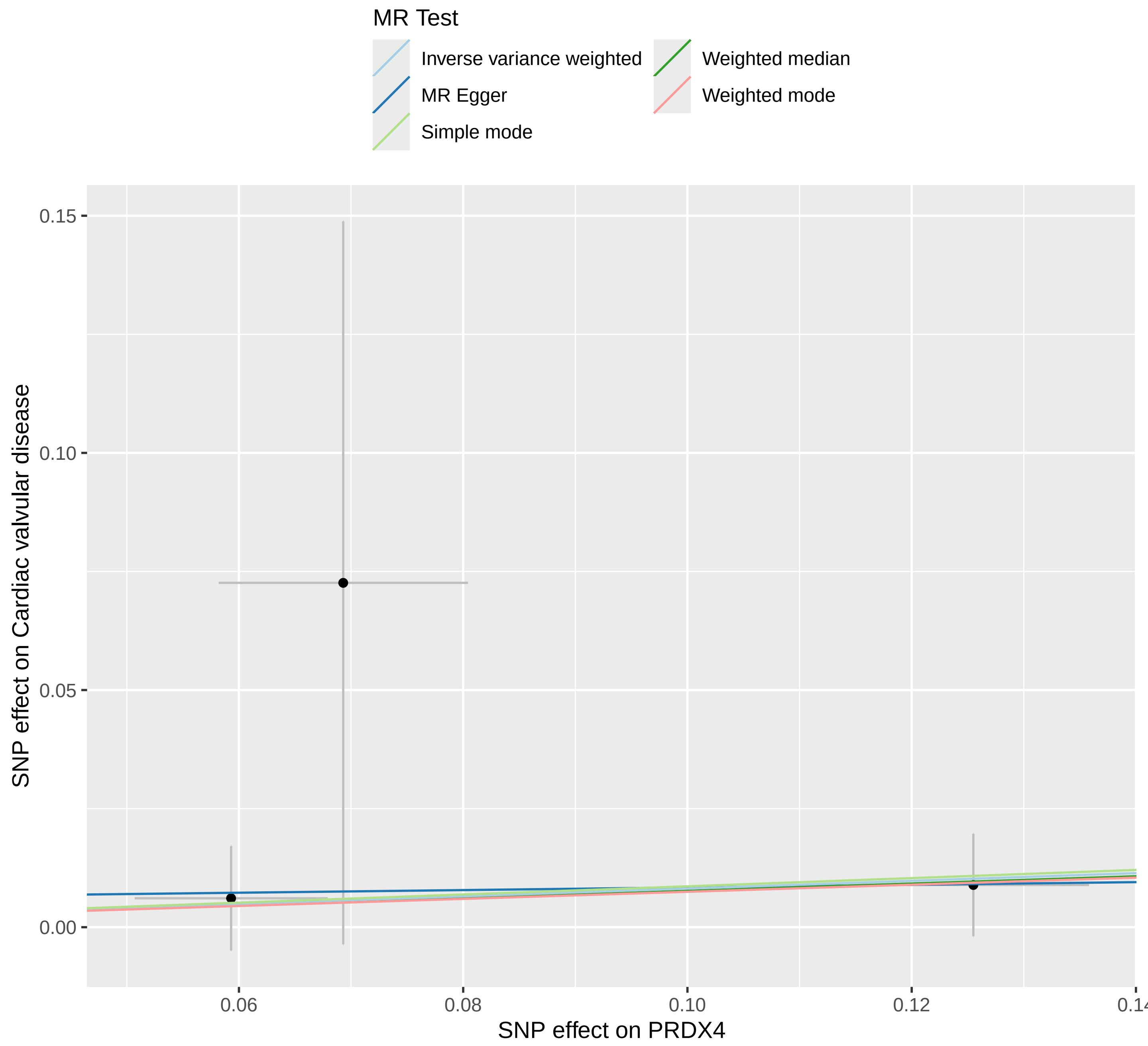
MR Test
Inverse variance weighted
MR Egger
Simple mode
Weighted median
Weighted mode
SNP effect on Cardiac valvular disease
0.15
0.10
0.05
0.00
0.06
0.08
0.10
0.12
SNP effect on PRDX4

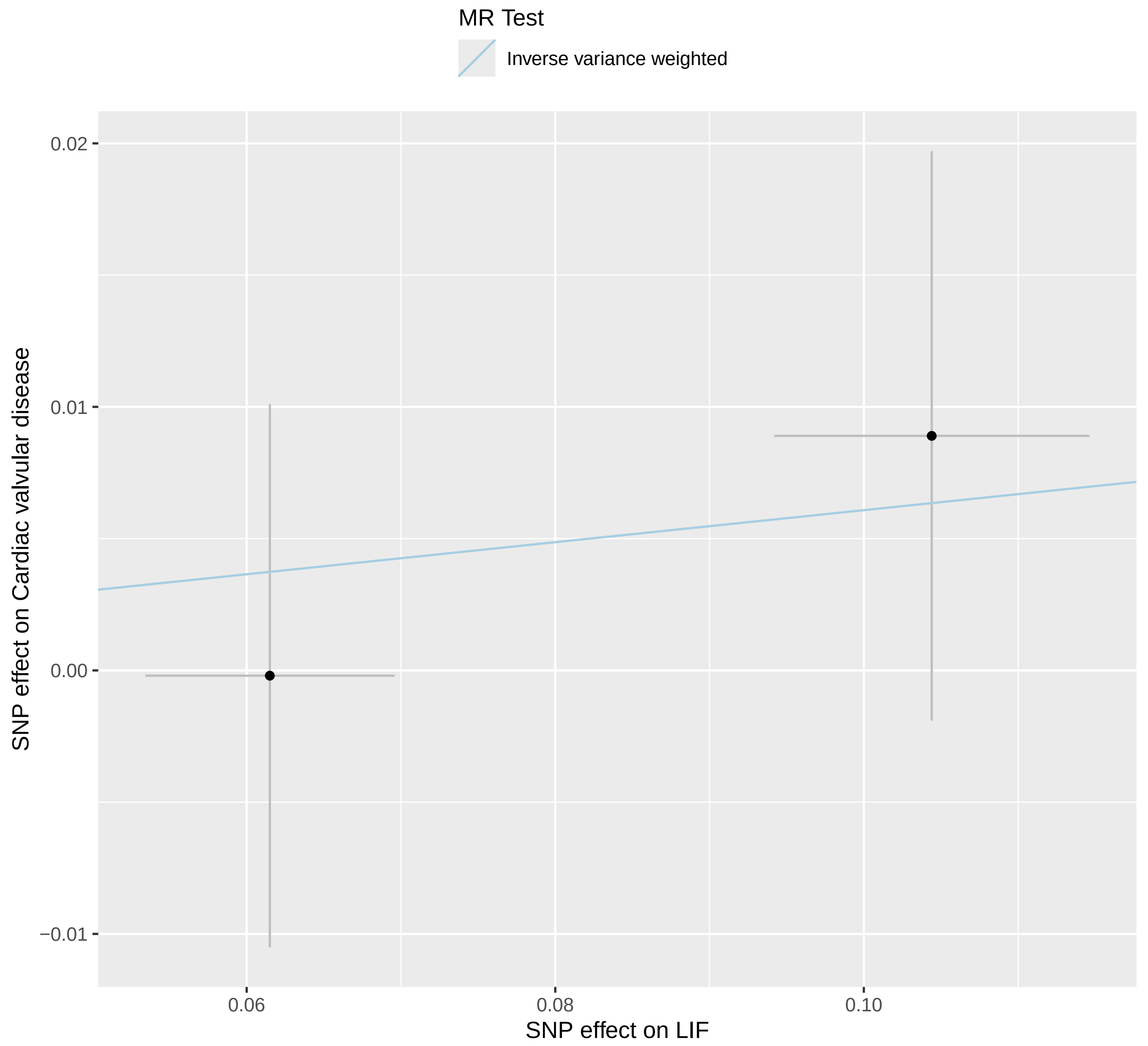

MR Test
Inverse variance weighted
0.02
0.01
0.00
−0.01
SNP effect on Cardiac valvular disease
0.06
0.08
0.10
SNP effect on LIF

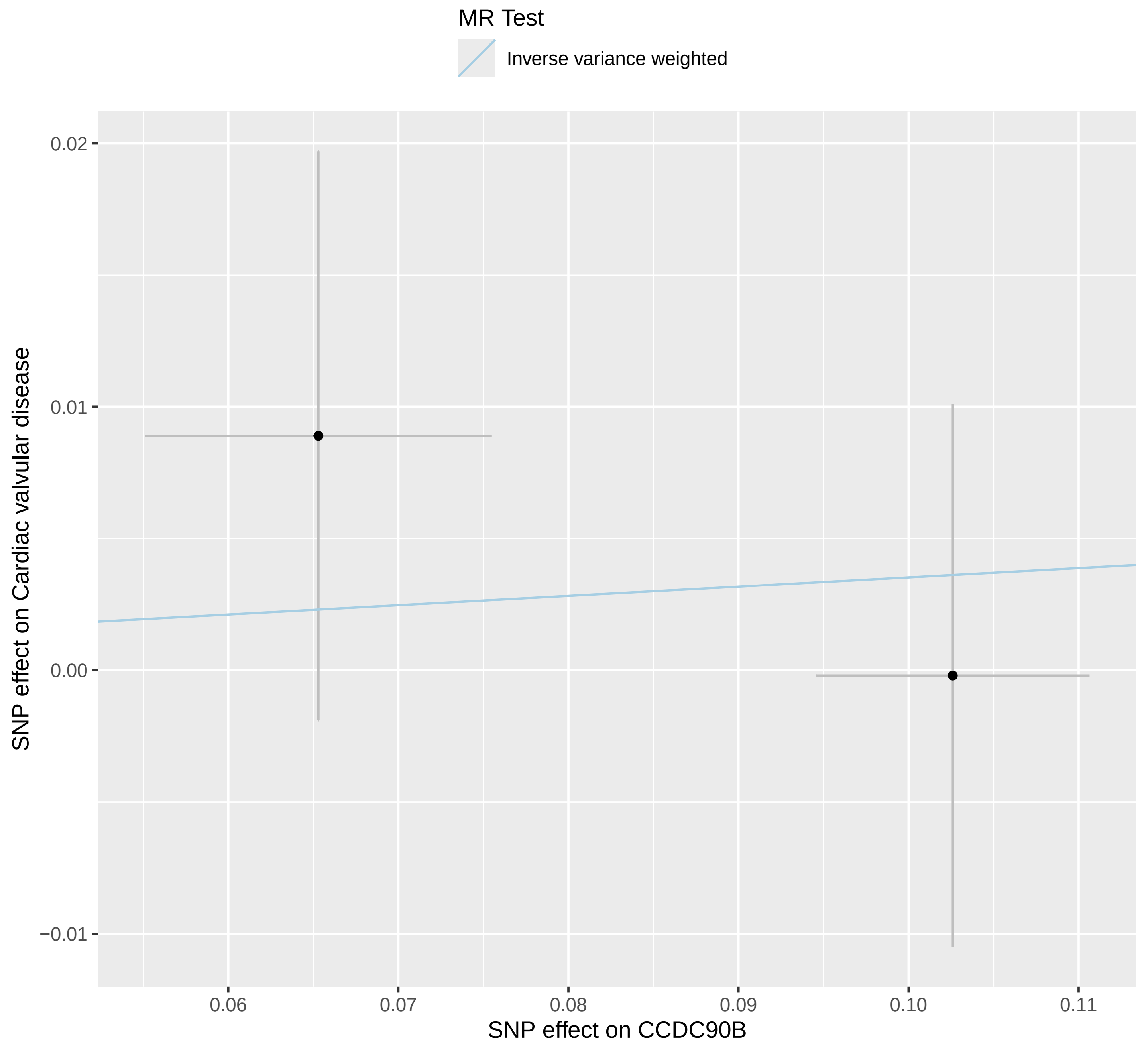
MR Test
Inverse variance weighted
0.02
0.01
0.00
−0.01
0.06
0.07
0.08
0.09
0.10
0.11
SNP effect on CCDC90B
SNP effect on Cardiac valvular disease

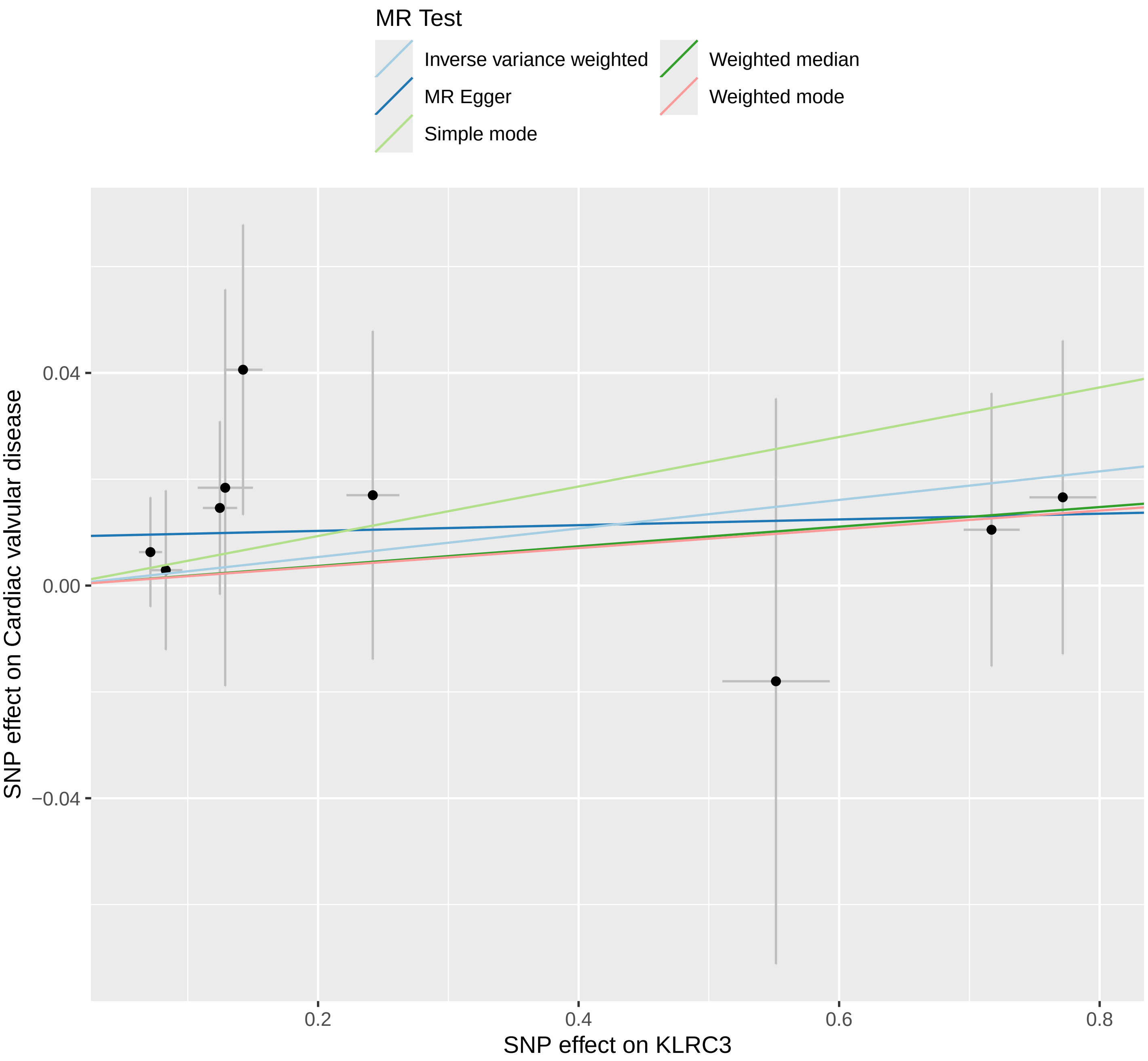
MR Test
Inverse variance weighted
MR Egger
Simple mode
Weighted median
Weighted mode
0.04
0.00
−0.04
SNP effect on Cardiac valvular disease
0.2
0.4
0.6
0.8
SNP effect on KLRC3

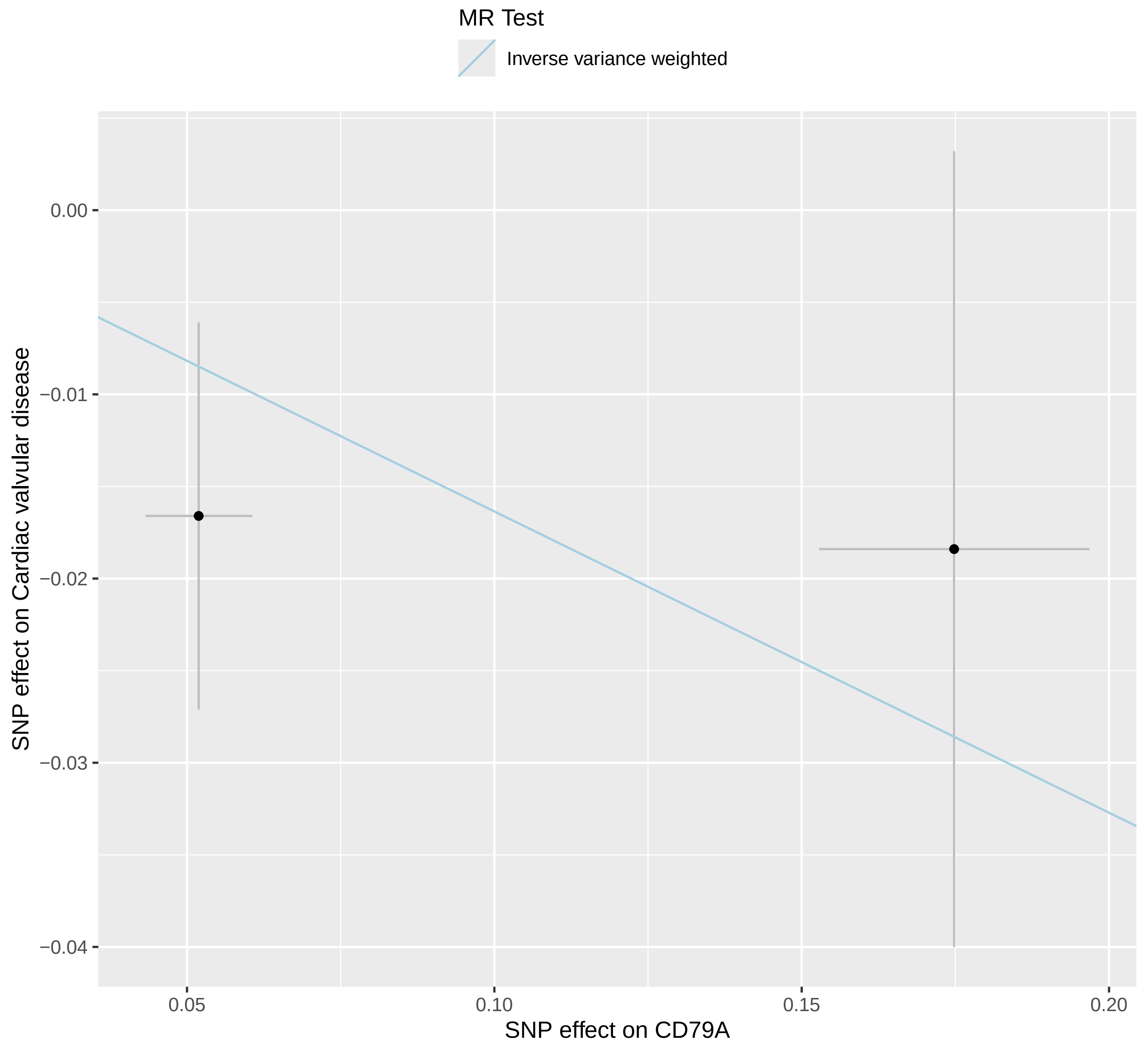
MR Test
Inverse variance weighted
0.00
−0.01
−0.02
−0.03
−0.04
SNP effect on Cardiac valvular disease
0.05
0.10
0.15
0.20
SNP effect on CD79A

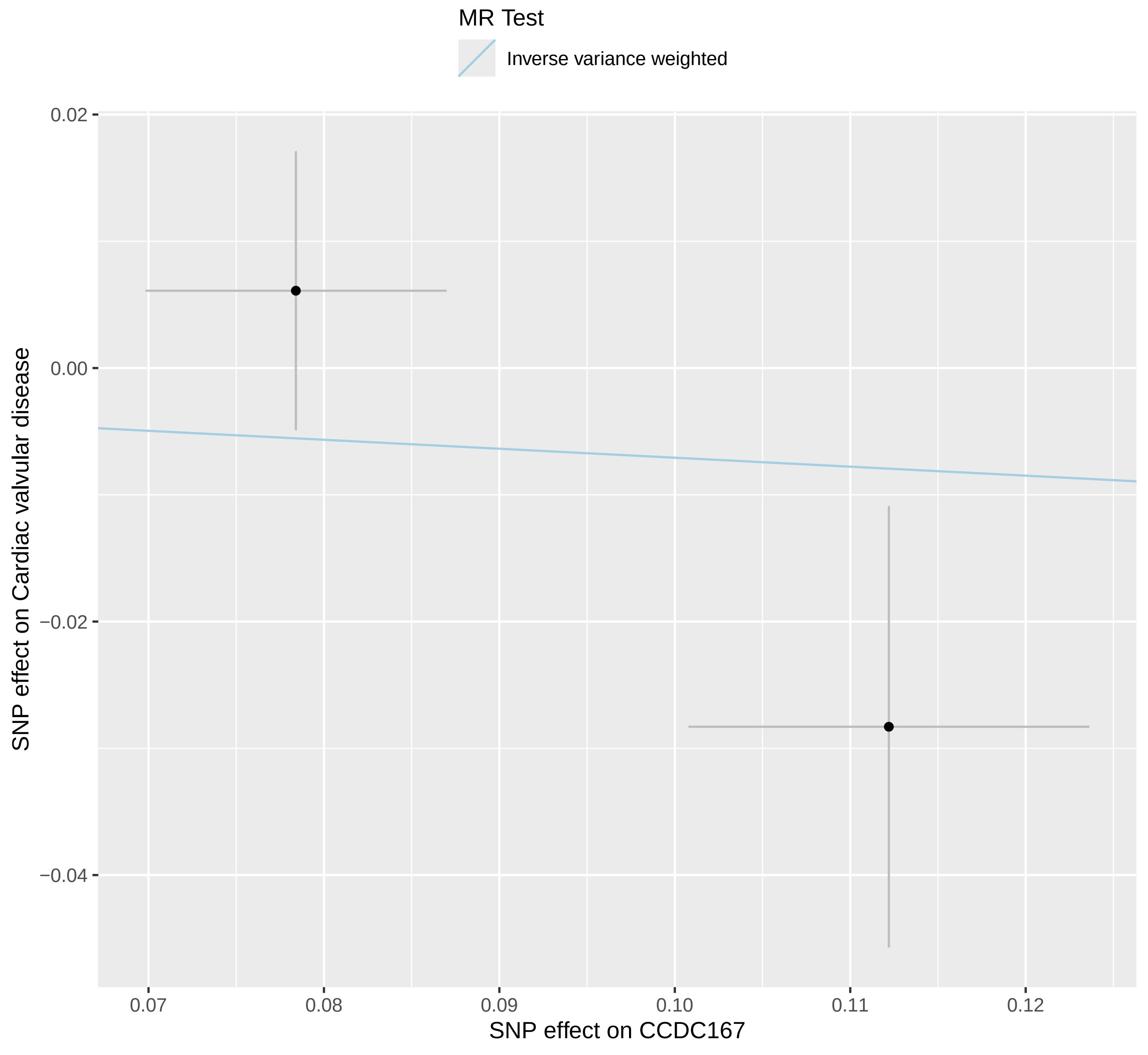
MR Test
Inverse variance weighted
0.02
0.00
−0.02
−0.04
0.07
0.08
0.09
0.10
0.11
0.12
SNP effect on CCDC167
SNP effect on Cardiac valvular disease

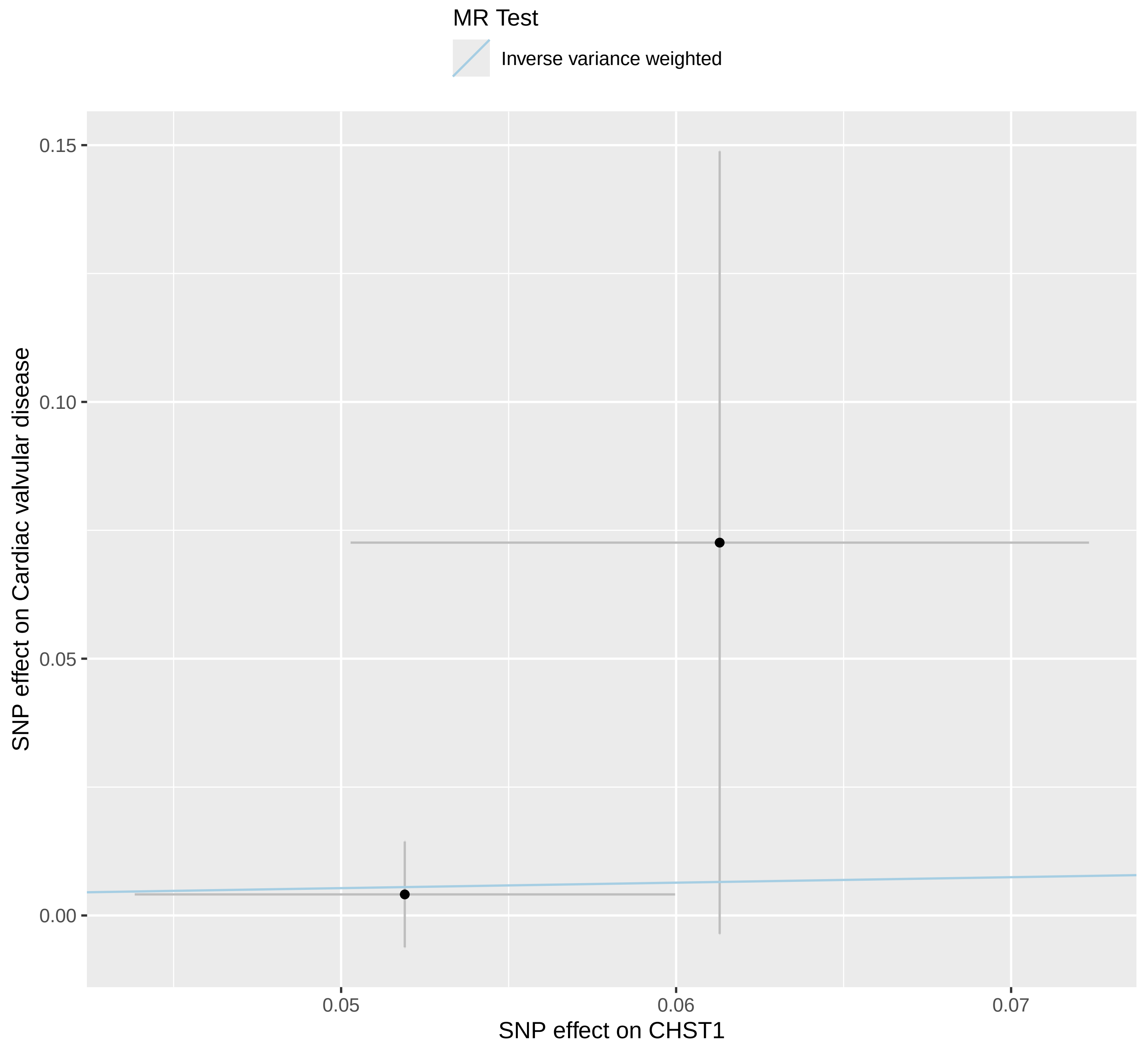
MR Test
Inverse variance weighted
0.15
0.10
0.05
0.00
0.05
0.06
0.07
SNP effect on Cardiac valvular disease
SNP effect on CHST1

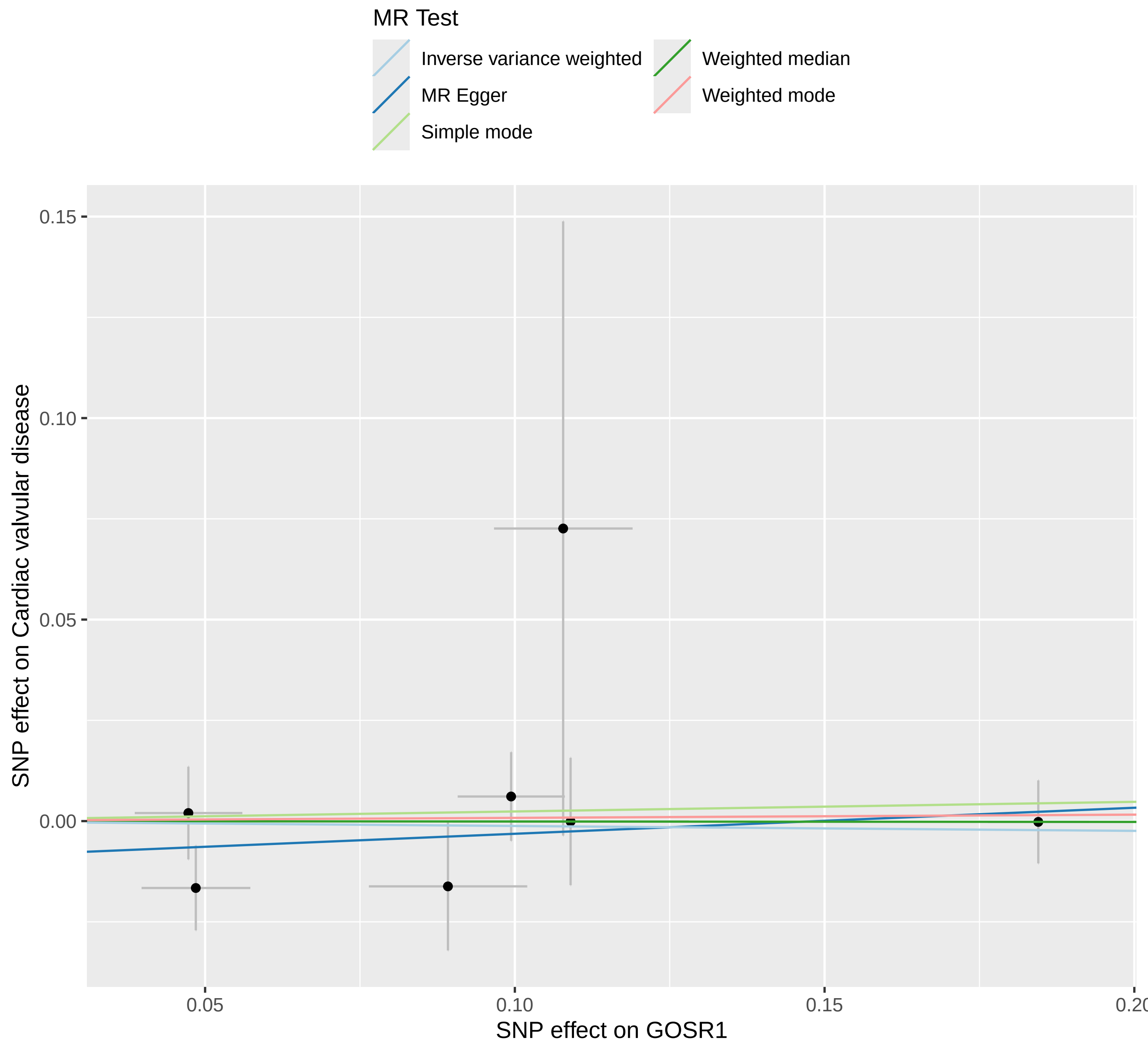
MR Test
Inverse variance weighted
MR Egger
Simple mode
Weighted median
Weighted mode
0.15
0.10
0.05
0.00
SNP effect on Cardiac valvular disease
0.05
0.10
0.15
0.20
SNP effect on GOSR1

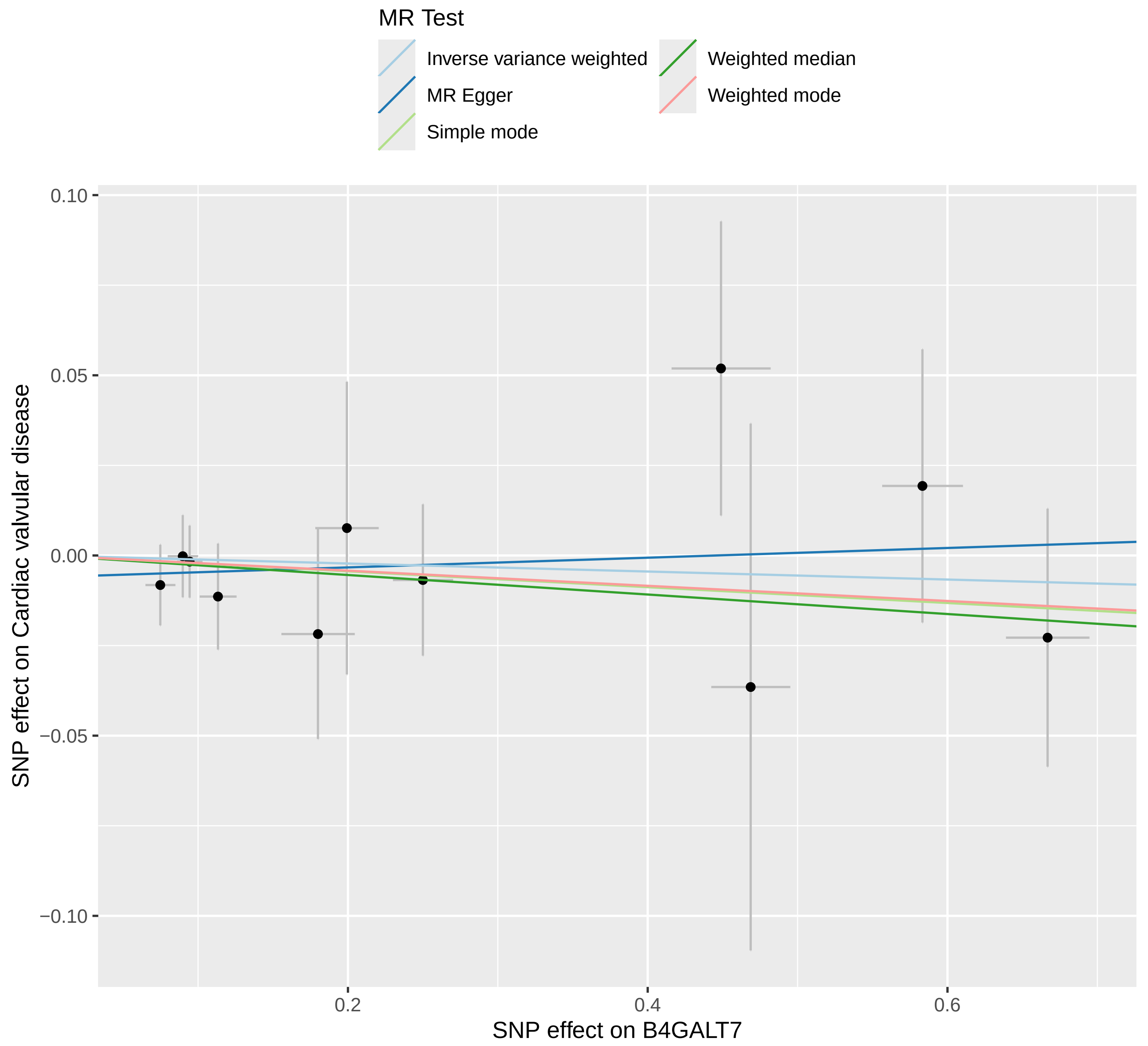
MR Test
Inverse variance weighted
MR Egger
Simple mode
Weighted median
Weighted mode
0.10
0.05
0.00
−0.05
−0.10
0.2
0.4
0.6
SNP effect on Cardiac valvular disease
SNP effect on B4GALT7

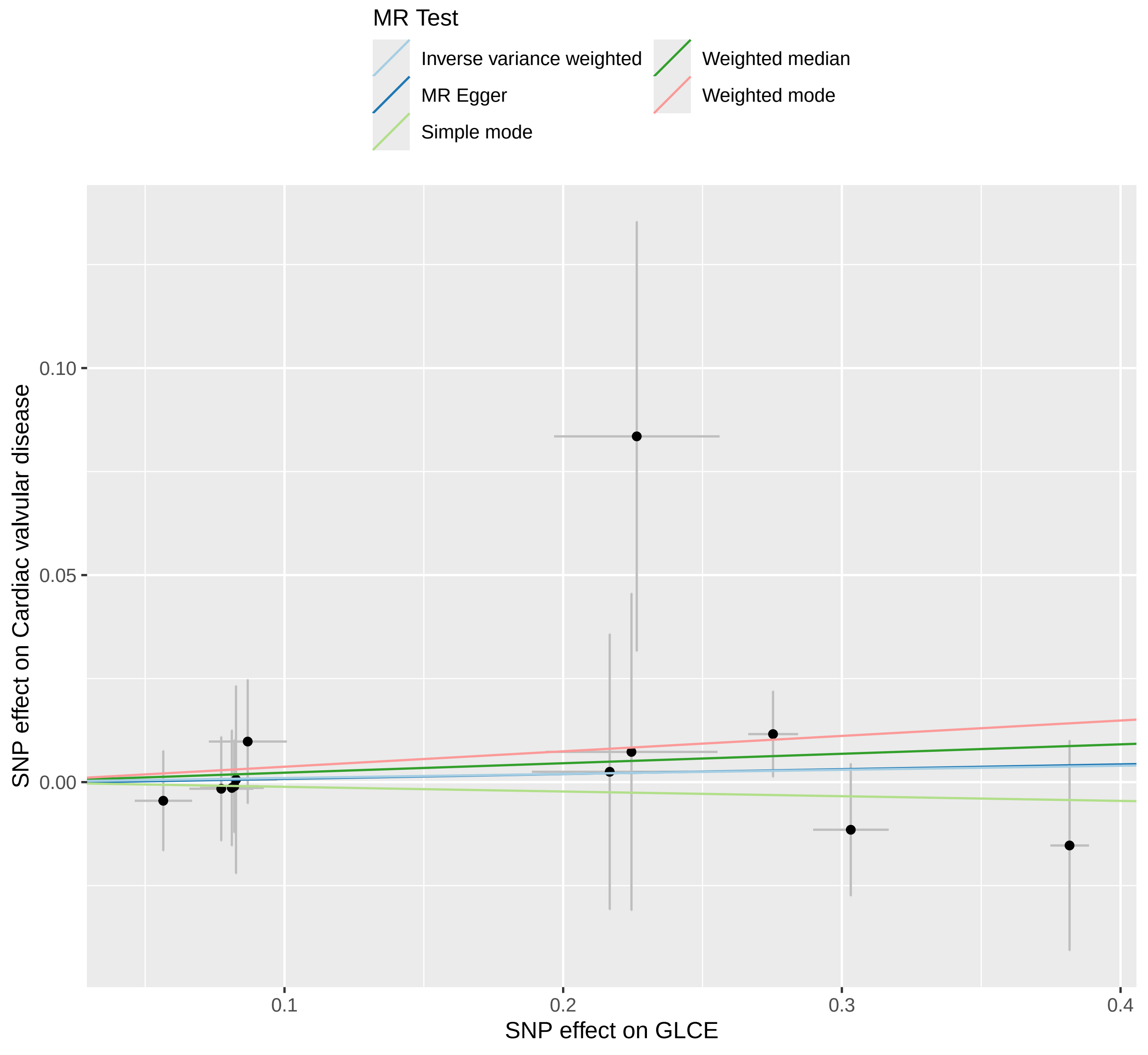
MR Test
Inverse variance weighted
MR Egger
Simple mode
Weighted median
Weighted mode
0.10
0.05
0.00
SNP effect on Cardiac valvular disease
0.1
0.2
0.3
0.4
SNP effect on GLCE

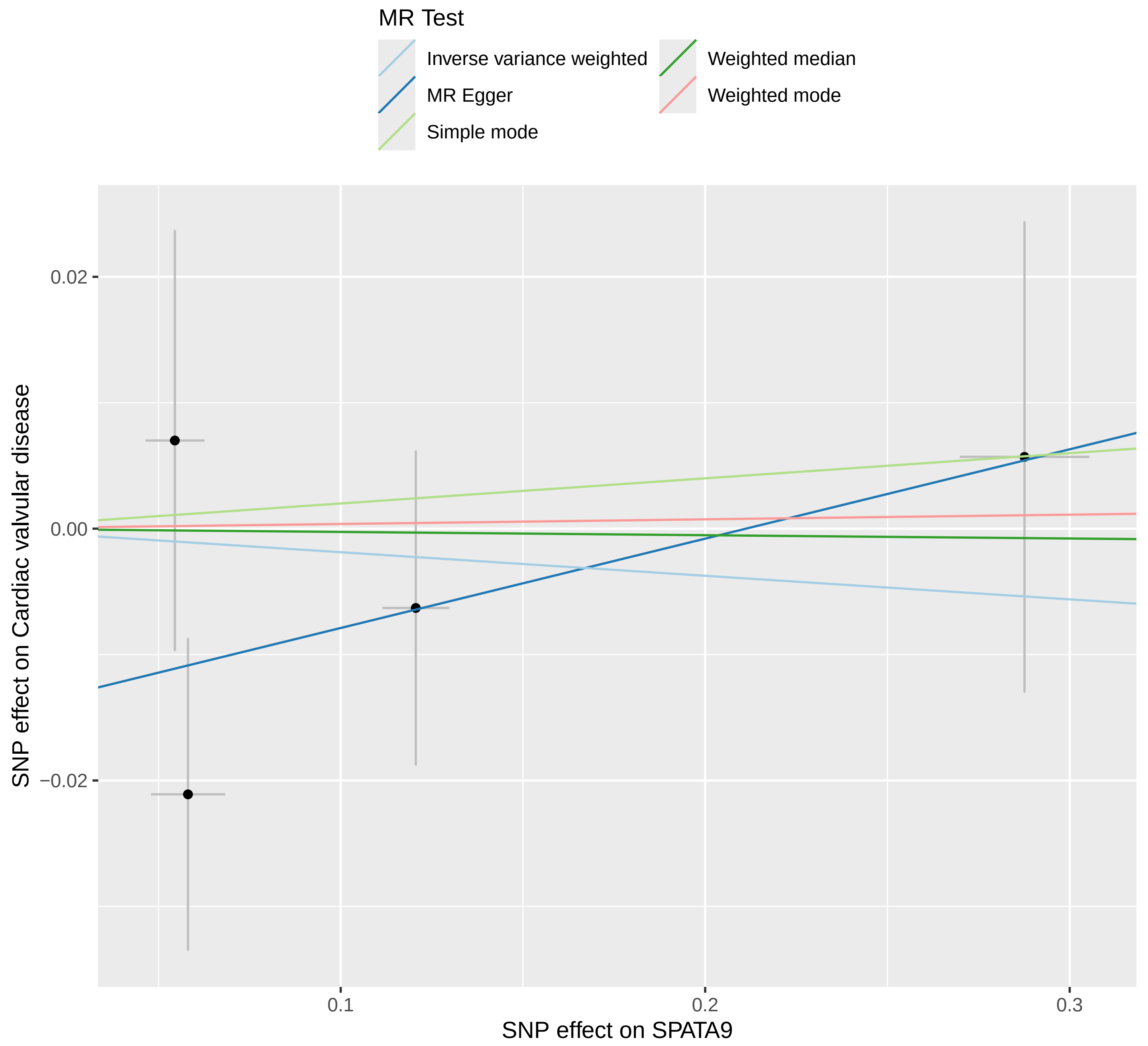
MR Test
Inverse variance weighted
MR Egger
Simple mode
Weighted median
Weighted mode
0.02
0.00
−0.02
0.1
0.2
0.3
SNP effect on Cardiac valvular disease
SNP effect on SPATA9

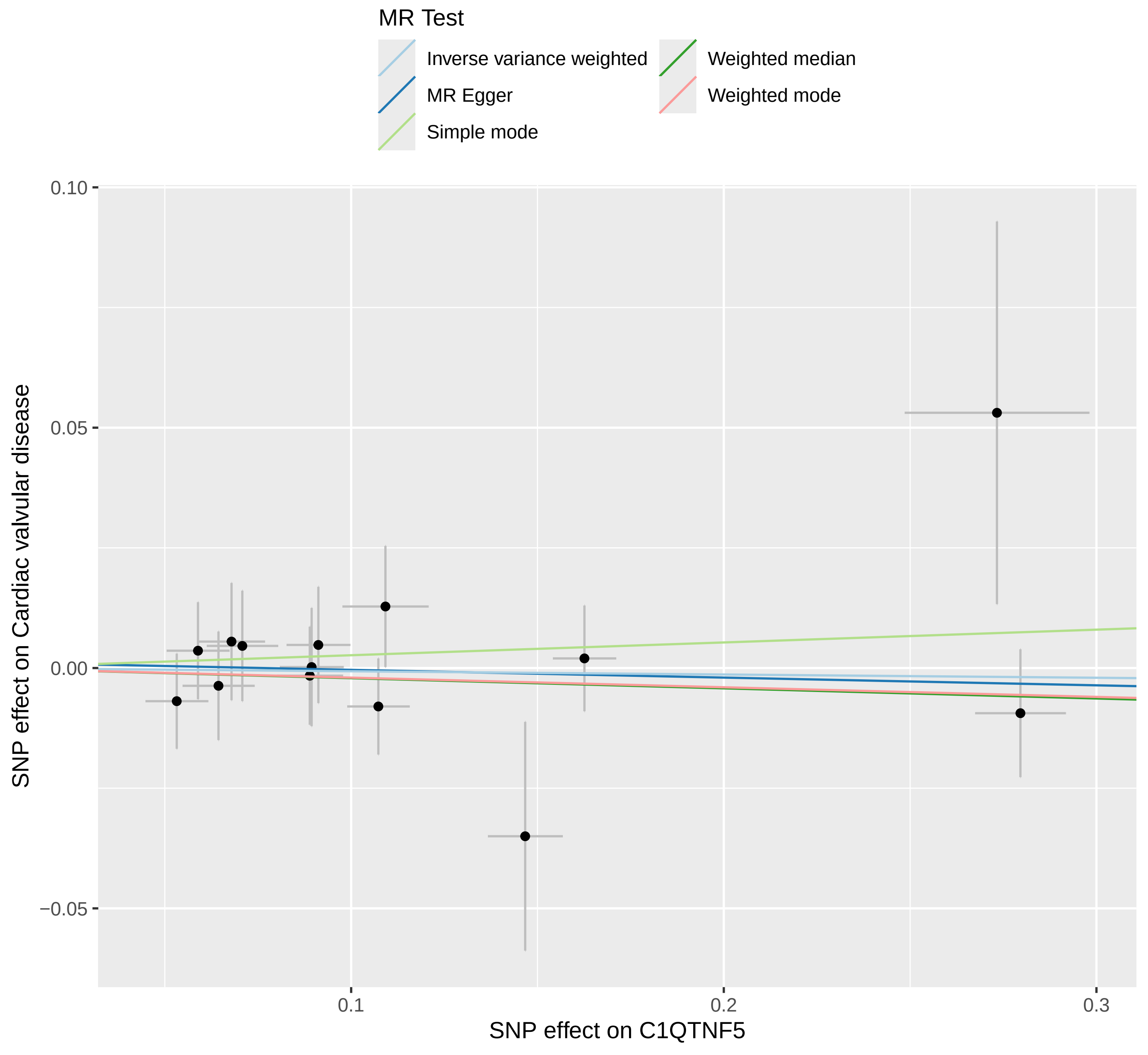
MR Test
Inverse variance weighted
MR Egger
Simple mode
Weighted median
Weighted mode
0.10
0.05
0.00
−0.05
0.1
0.2
0.3
SNP effect on Cardiac valvular disease
SNP effect on C1QTNF5

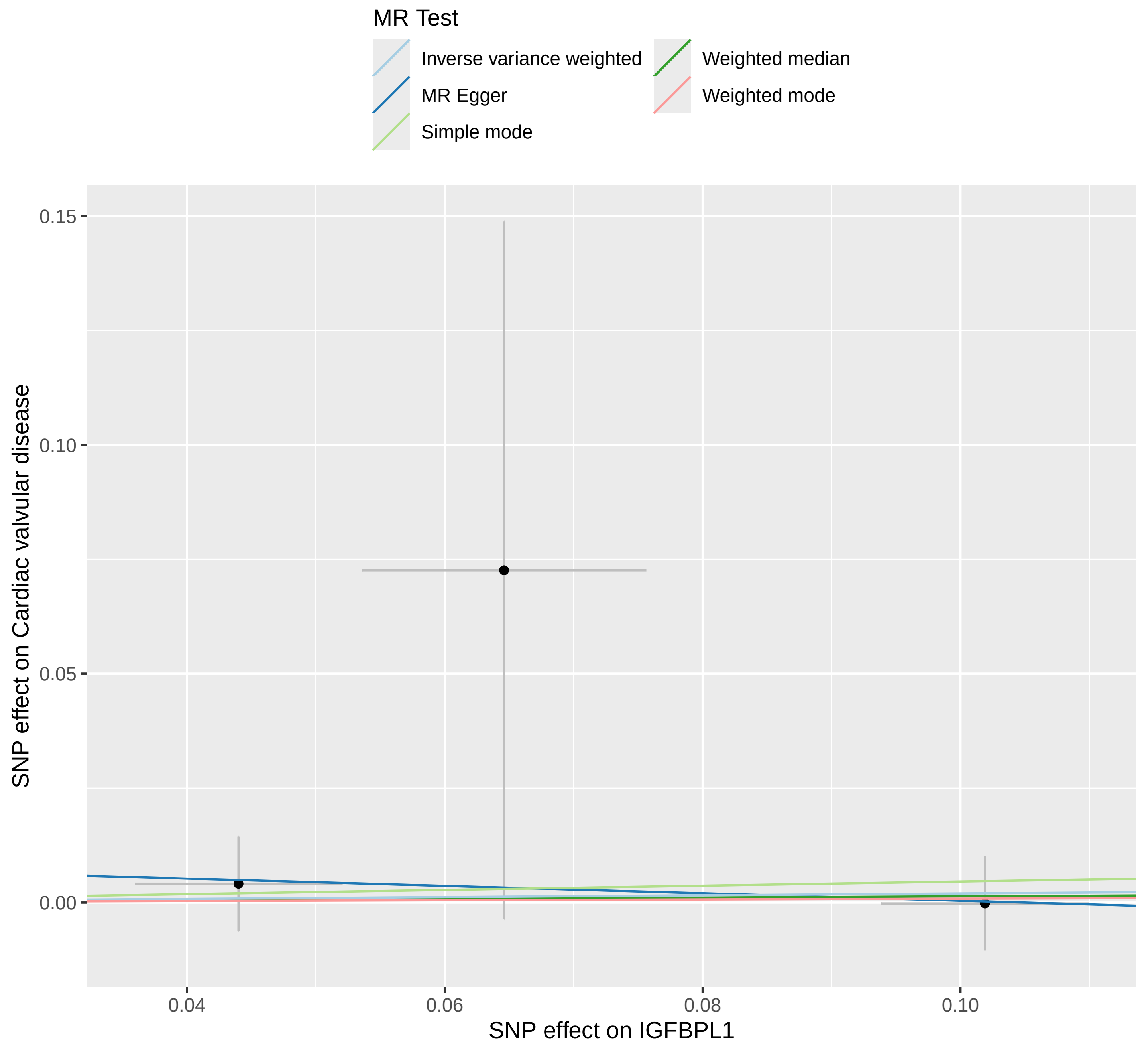
MR Test
Inverse variance weighted
MR Egger
Simple mode
Weighted median
Weighted mode
0.15
0.10
0.05
0.00
SNP effect on Cardiac valvular disease
0.04
0.06
0.08
0.10
SNP effect on IGFBPL1

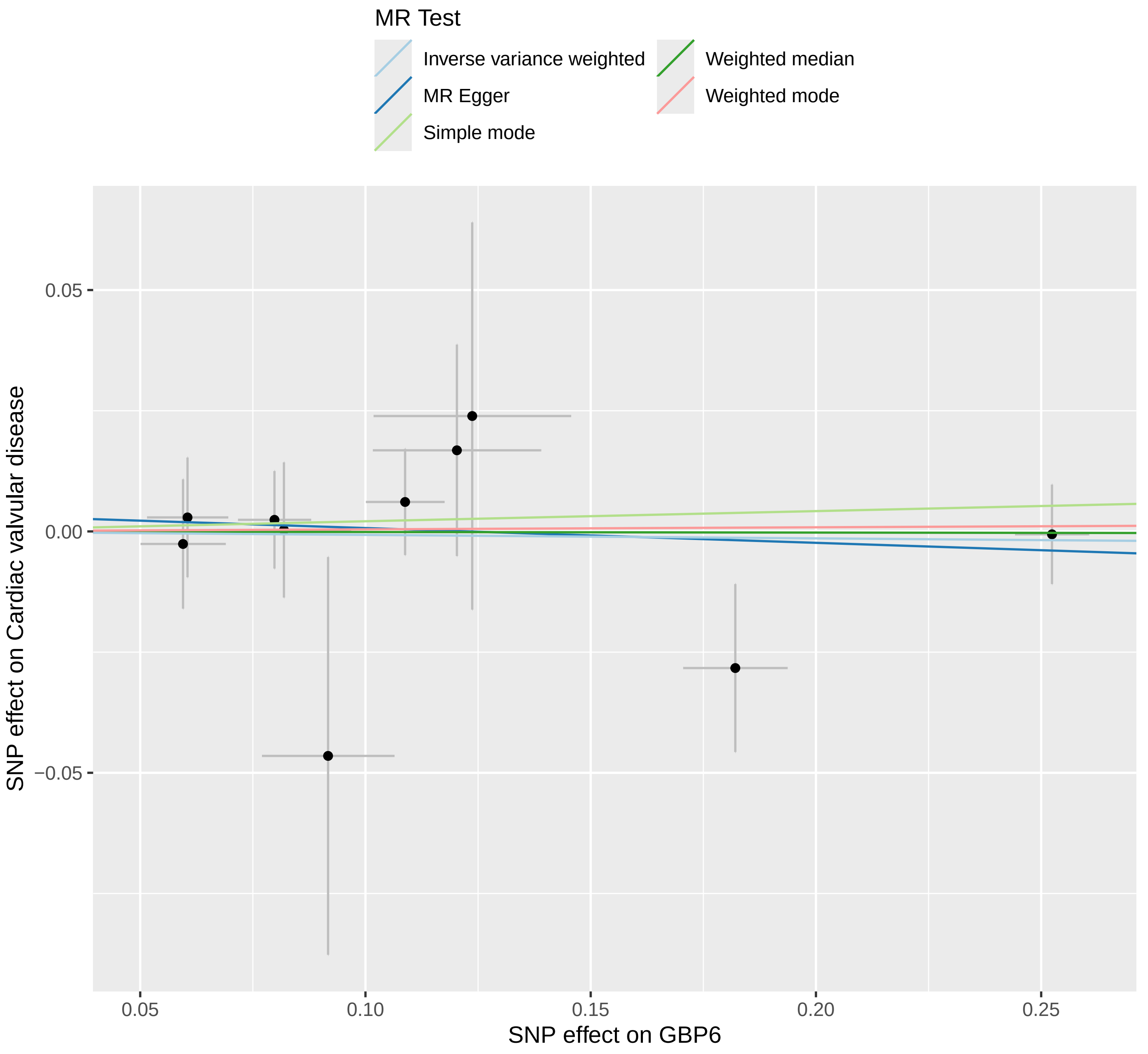
MR Test
Inverse variance weighted
MR Egger
Simple mode
Weighted median
Weighted mode
0.05
0.00
−0.05
0.05
0.10
0.15
0.20
0.25
SNP effect on Cardiac valvular disease
SNP effect on GBP6

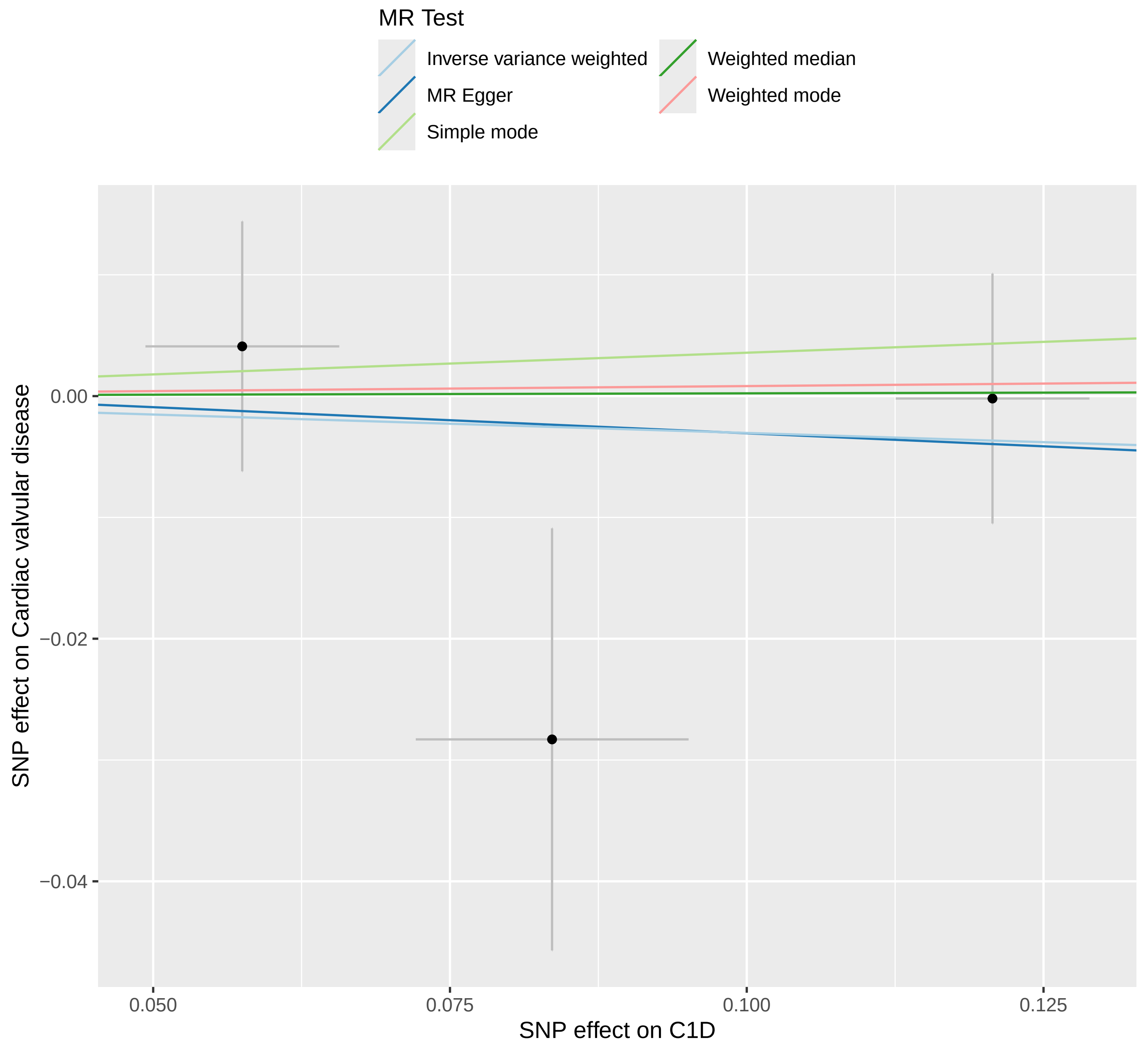
MR Test
Inverse variance weighted
MR Egger
Simple mode
Weighted median
Weighted mode
0.00
−0.02
−0.04
0.050
0.075
0.100
0.125
SNP effect on Cardiac valvular disease
SNP effect on C1D

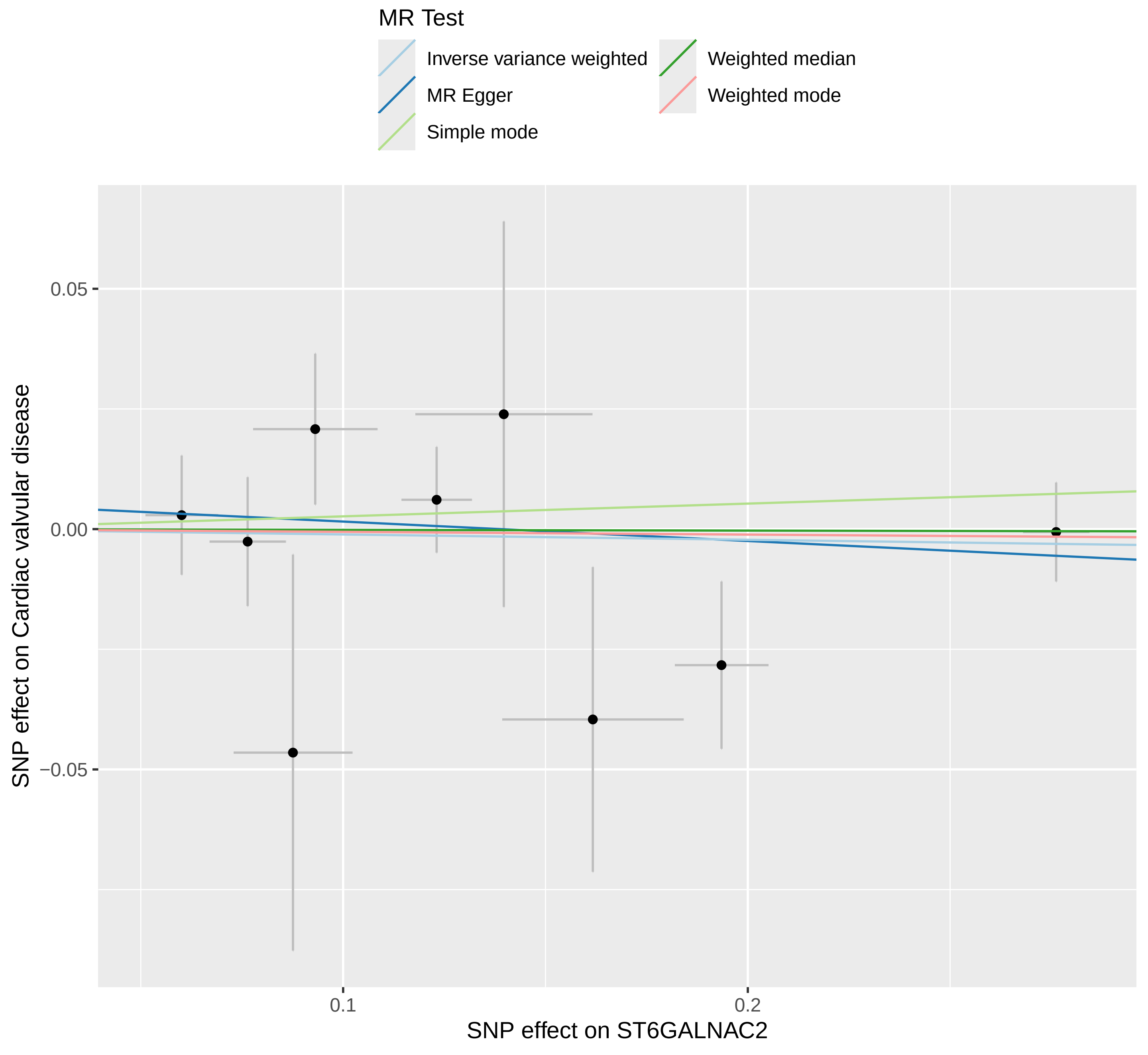

MR Test
Inverse variance weighted
MR Egger
Simple mode
Weighted median
Weighted mode
0.05
0.00
−0.05
0.1
0.2
SNP effect on Cardiac valvular disease
SNP effect on ST6GALNAC2

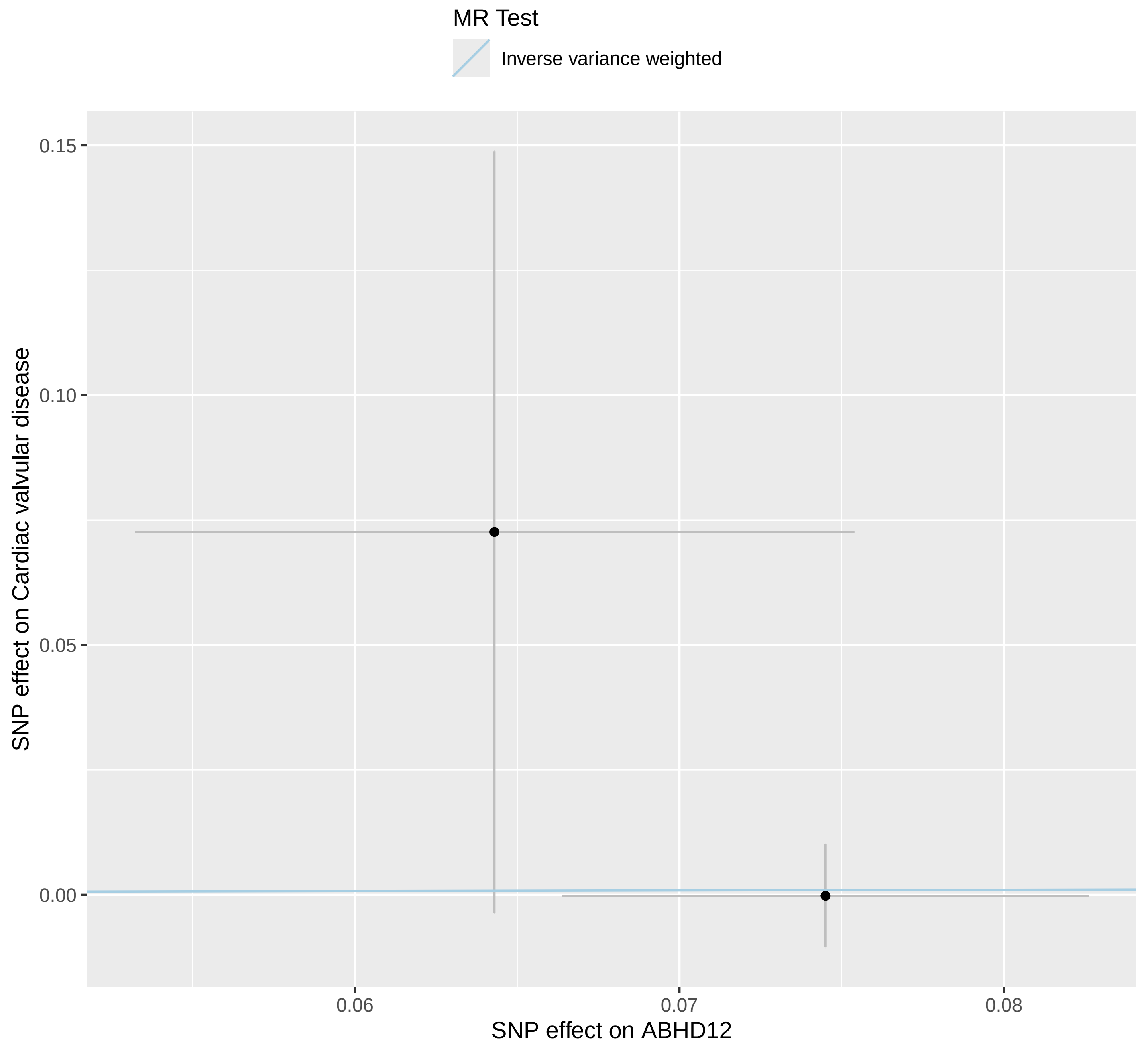
MR Test
Inverse variance weighted
0.15
0.10
0.05
0.00
0.06
0.07
0.08
SNP effect on Cardiac valvular disease
SNP effect on ABHD12

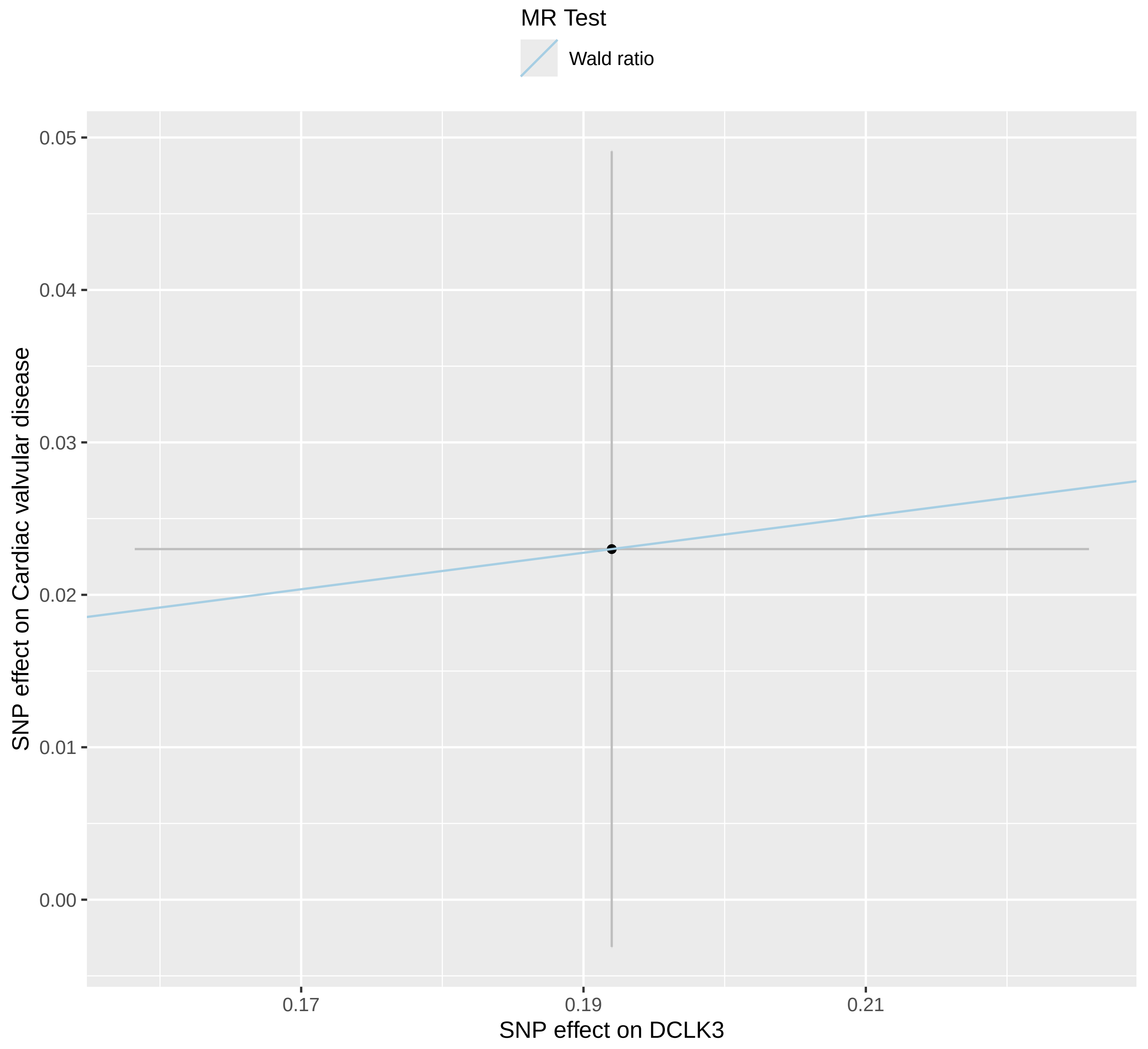
MR Test
Wald ratio
0.05
0.04
0.03
0.02
0.01
0.00
SNP effect on Cardiac valvular disease
0.17
0.19
0.21
SNP effect on DCLK3

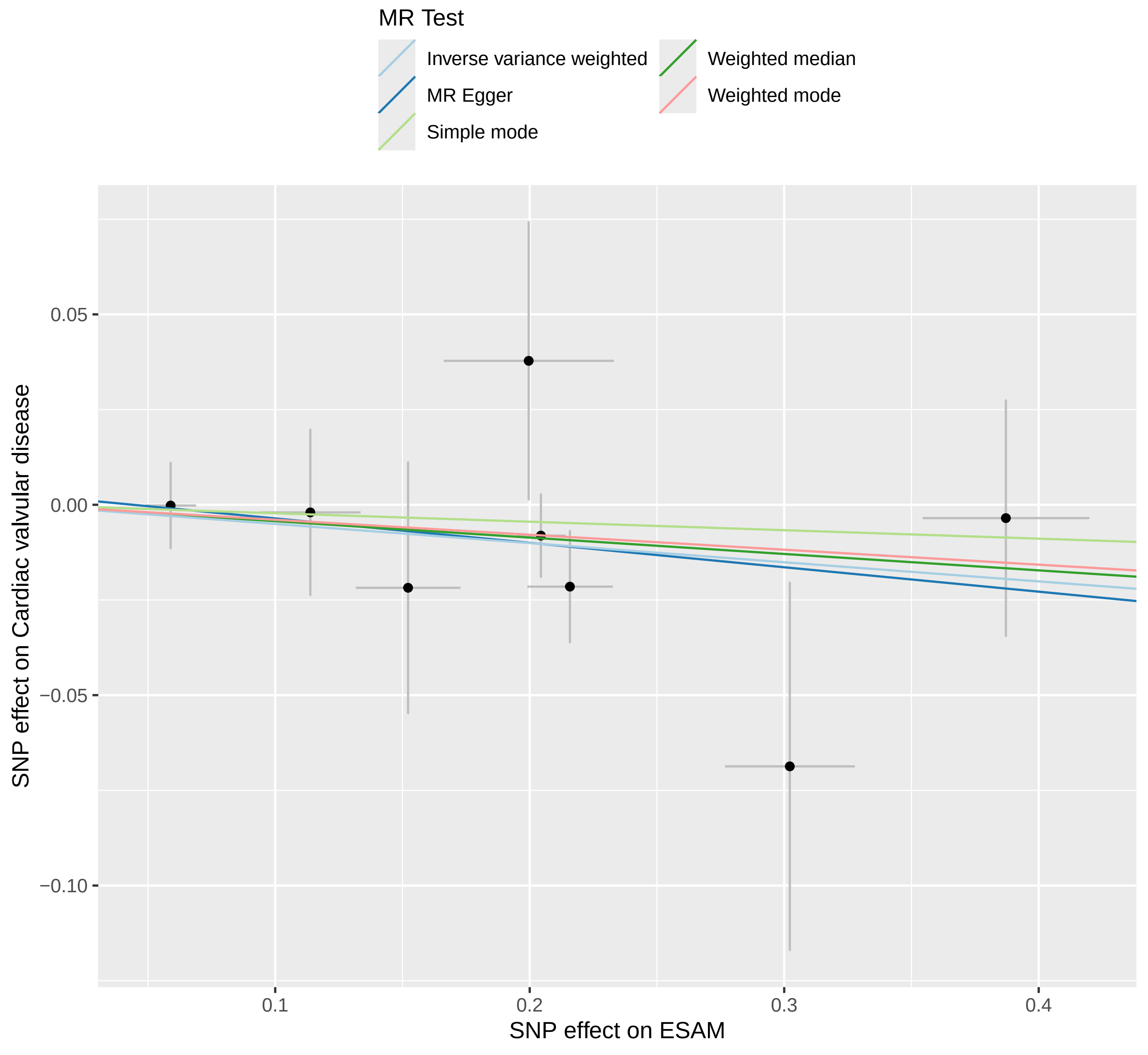
MR Test
Inverse variance weighted
MR Egger
Simple mode
Weighted median
Weighted mode
0.05
0.00
−0.05
−0.10
0.1
0.2
0.3
0.4
SNP effect on Cardiac valvular disease
SNP effect on ESAM

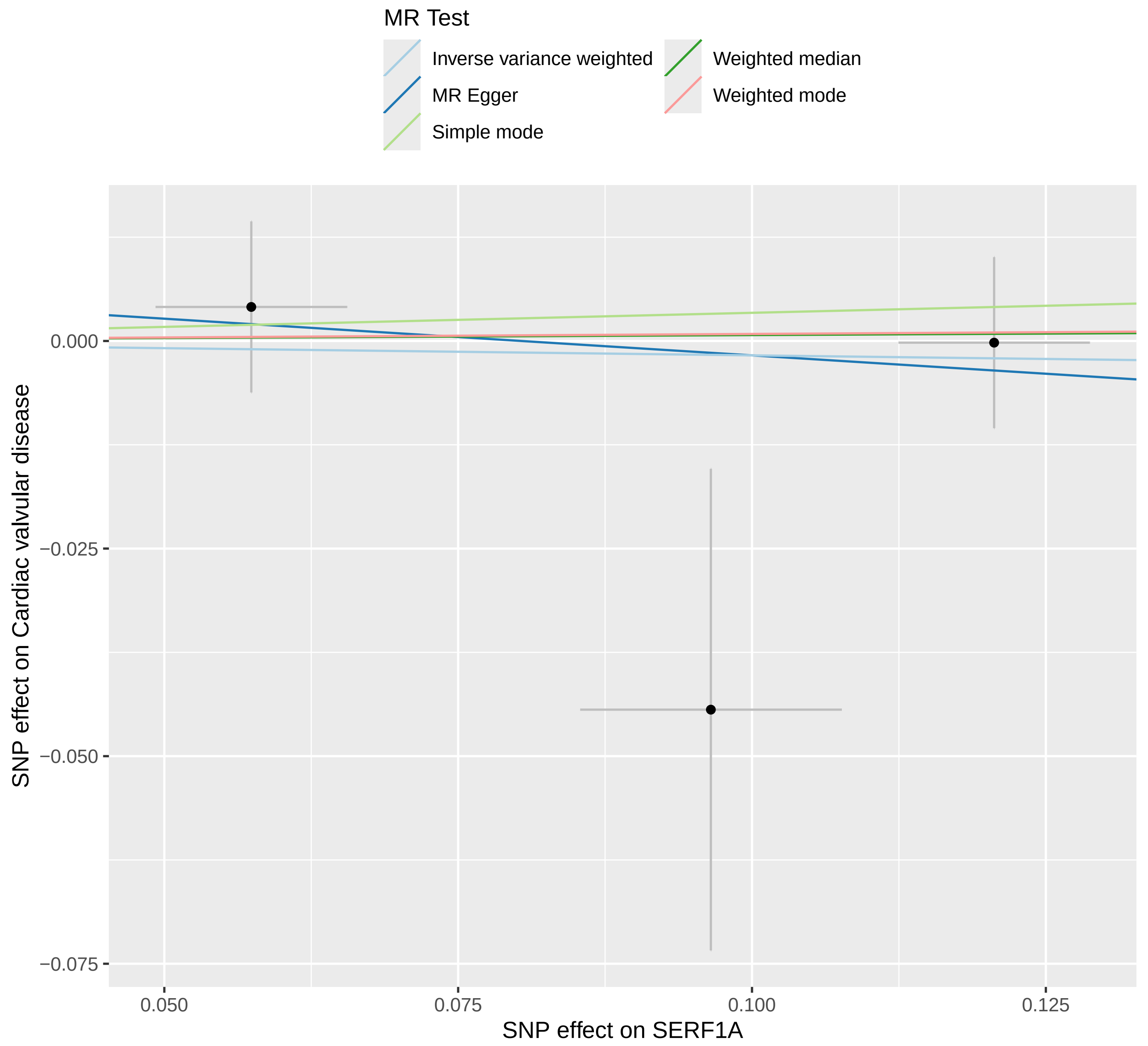
MR Test
Inverse variance weighted
MR Egger
Simple mode
Weighted median
Weighted mode
SNP effect on Cardiac valvular disease
0.000
−0.025
−0.050
−0.075
0.050
0.075
0.100
0.125
SNP effect on SERF1A

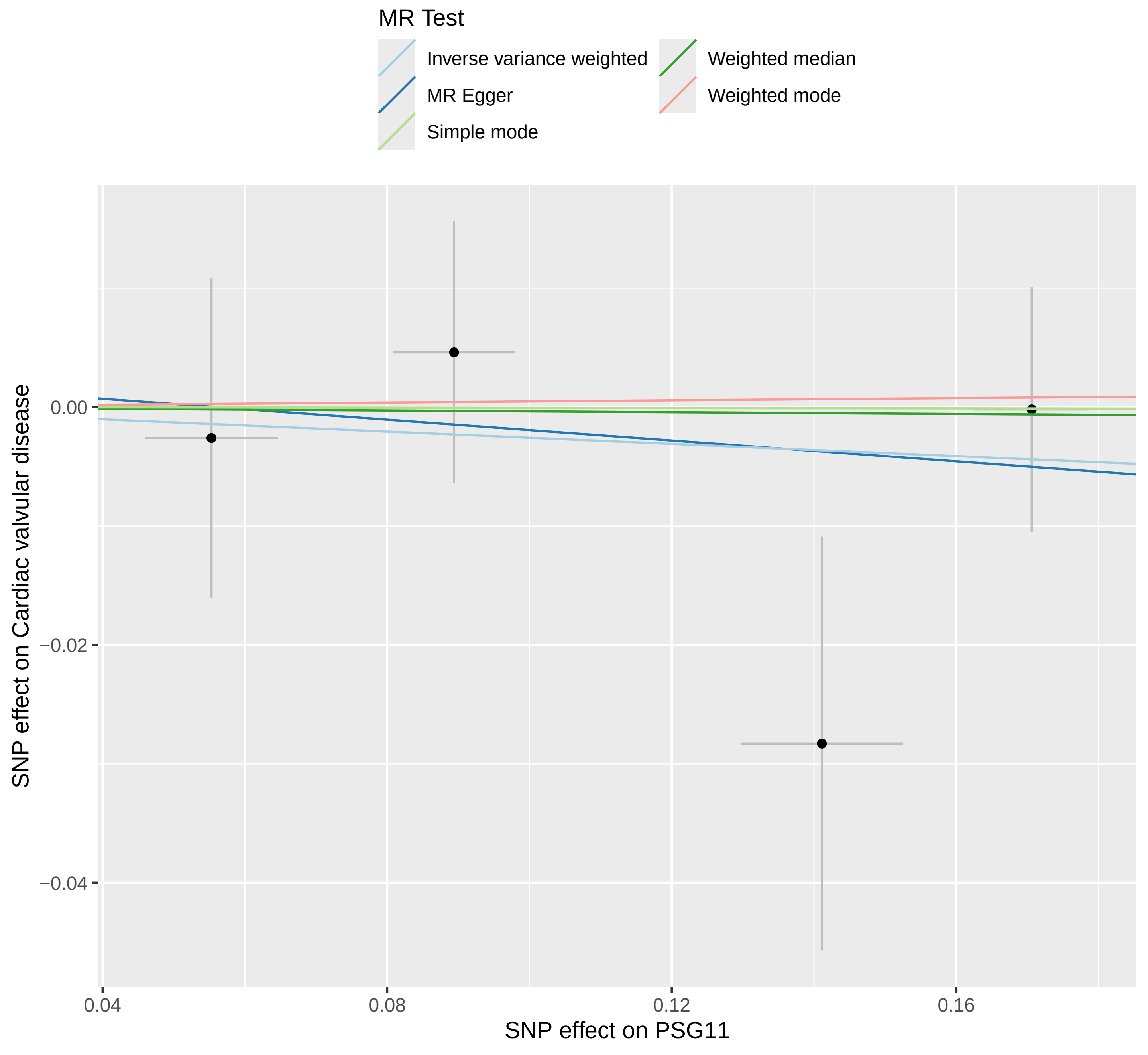
MR Test
Inverse variance weighted
MR Egger
Simple mode
Weighted median
Weighted mode
SNP effect on Cardiac valvular disease
0.00
−0.02
−0.04
0.04
0.08
0.12
0.16
SNP effect on PSG11

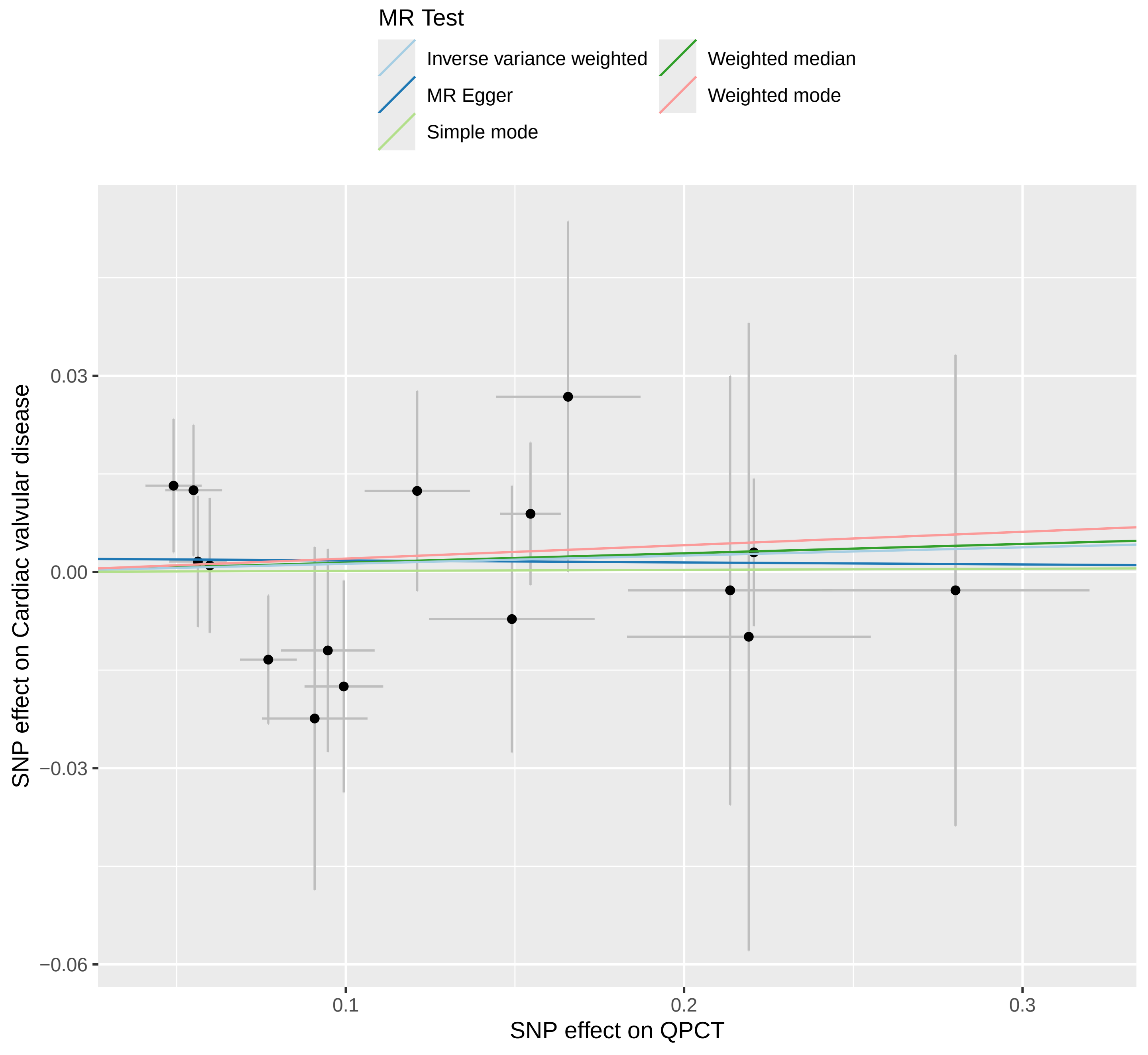
MR Test
Inverse variance weighted
MR Egger
Simple mode
Weighted median
Weighted mode
0.03
0.00
−0.03
−0.06
0.1
0.2
0.3
SNP effect on Cardiac valvular disease
SNP effect on QPCT

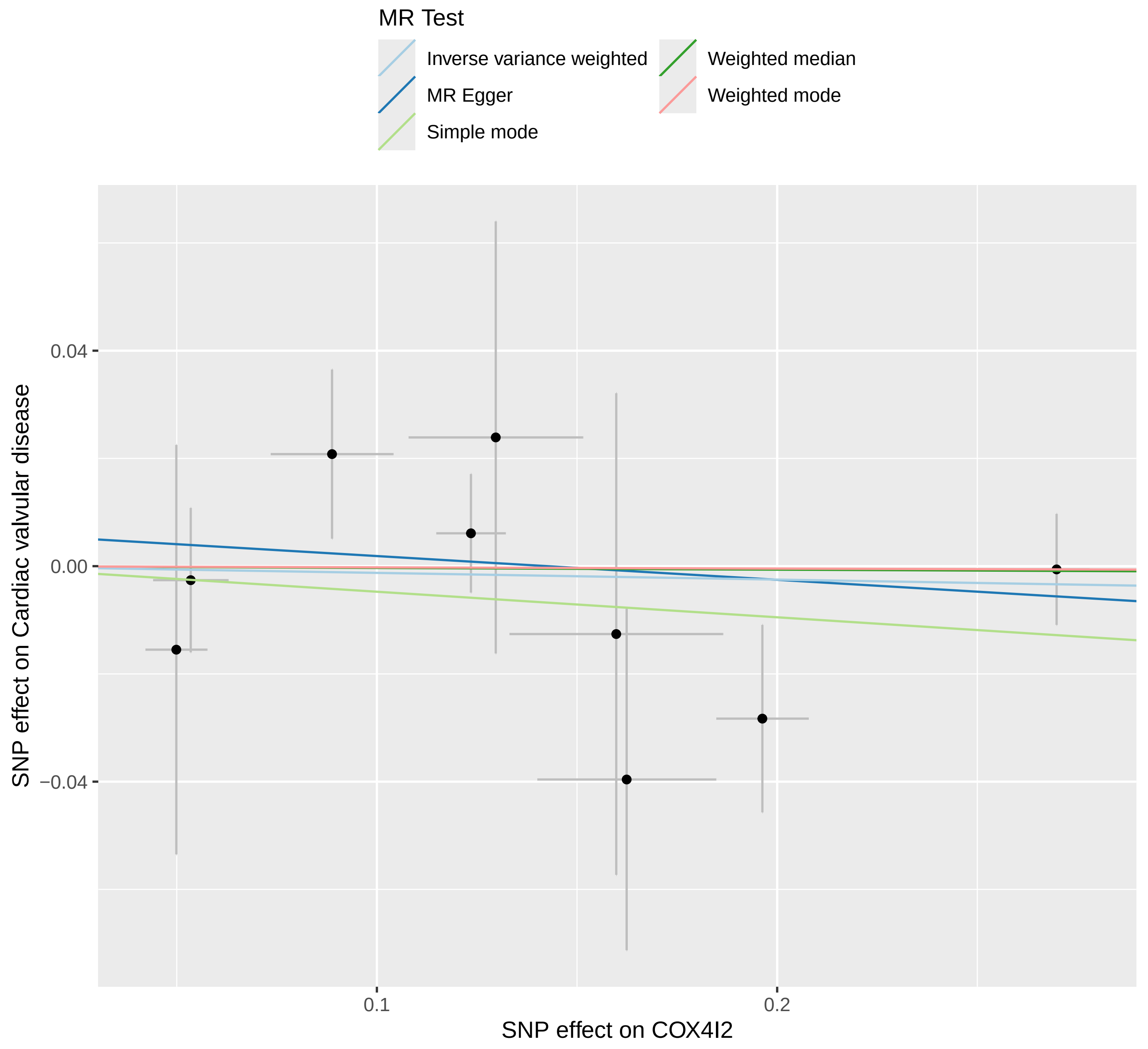
MR Test
Inverse variance weighted
MR Egger
Simple mode
Weighted median
Weighted mode
0.04
0.00
−0.04
0.1
0.2
SNP effect on Cardiac valvular disease
SNP effect on COX4I2

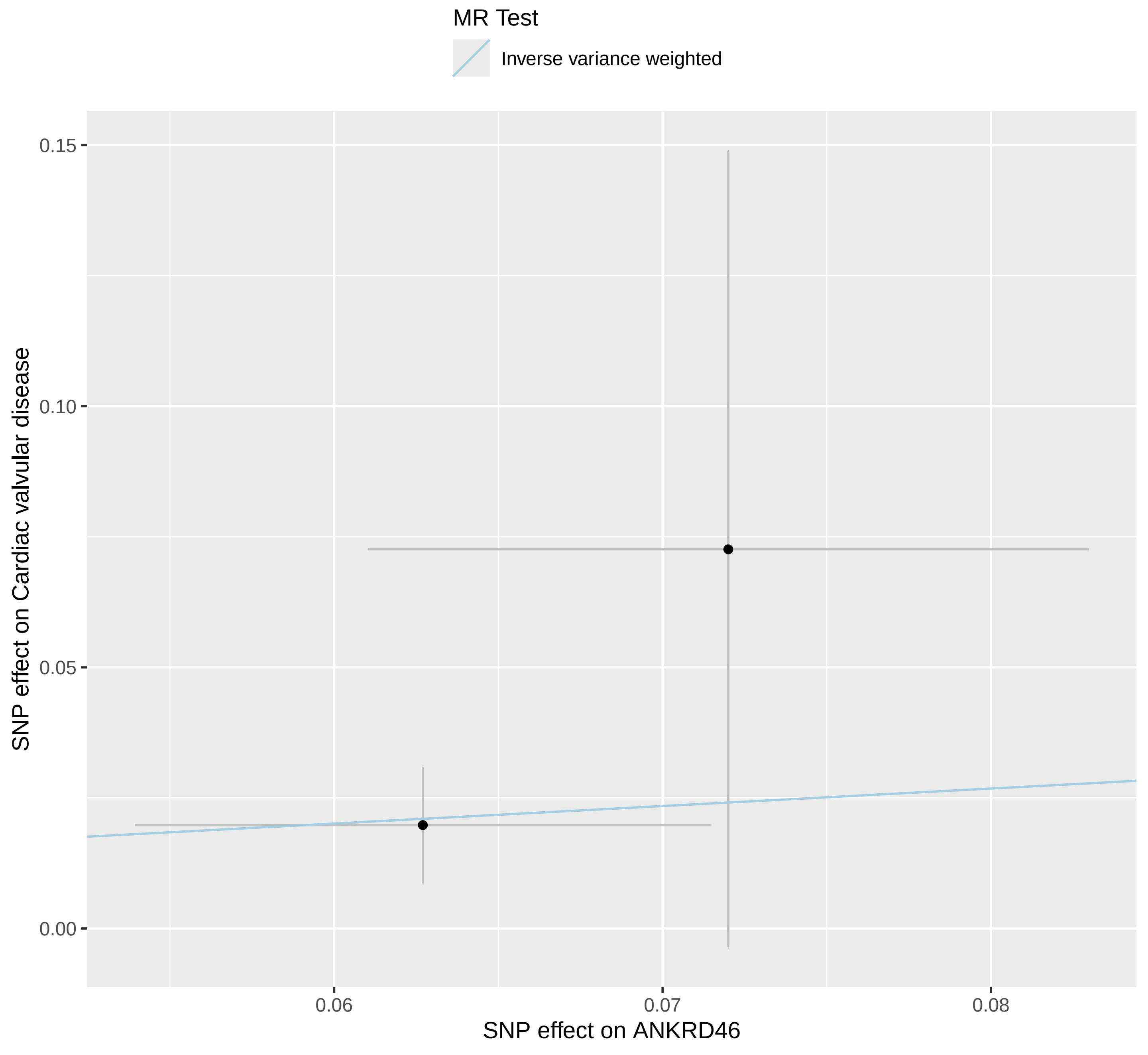

MR Test
Inverse variance weighted
0.15
0.10
0.05
0.00
SNP effect on Cardiac valvular disease
0.06
0.07
0.08
SNP effect on ANKRD46

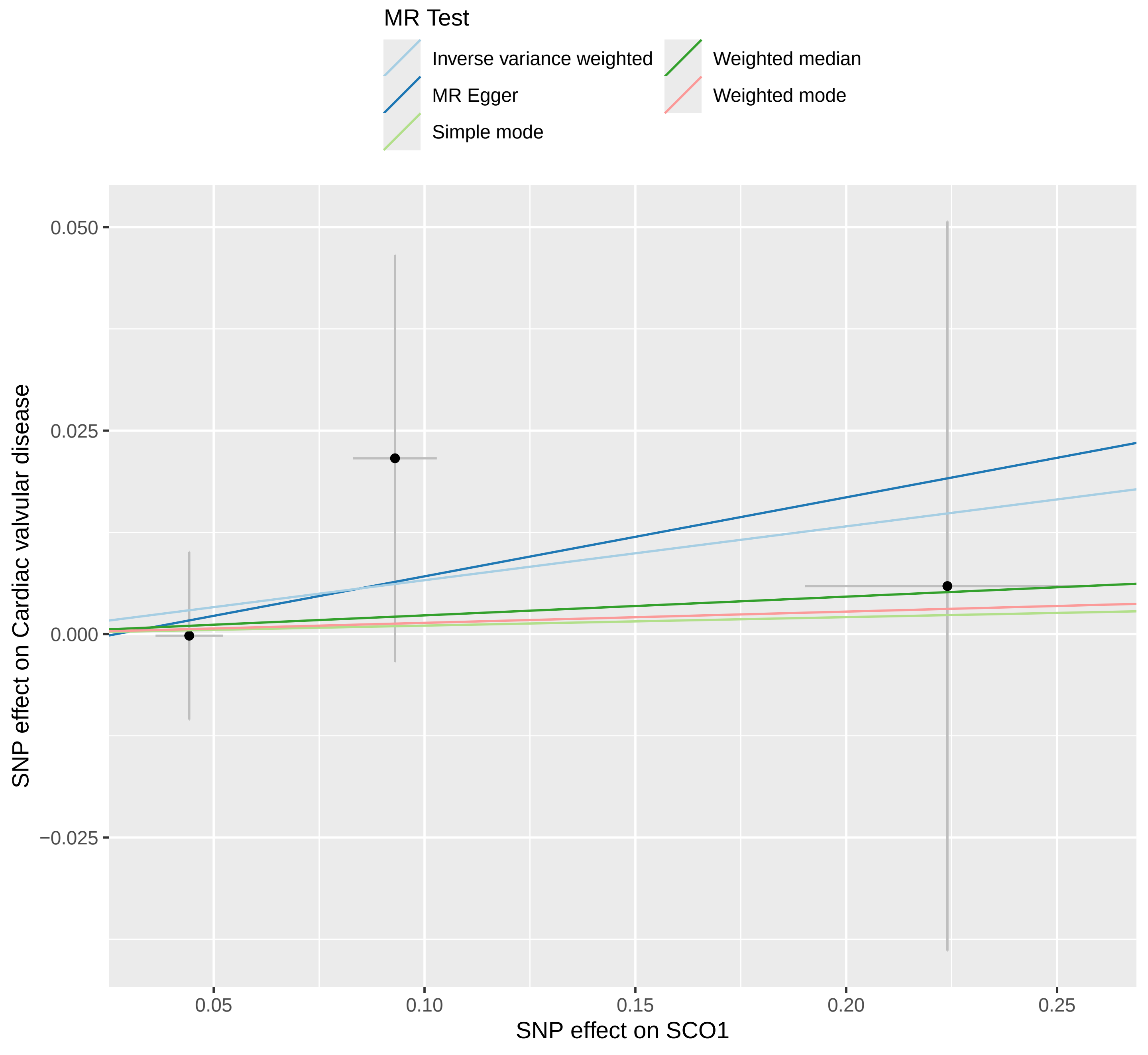

MR Test
Inverse variance weighted
MR Egger
Simple mode
Weighted median
Weighted mode
0.050
0.025
0.000
−0.025
0.05
0.10
0.15
0.20
0.25
SNP effect on Cardiac valvular disease
SNP effect on SCO1

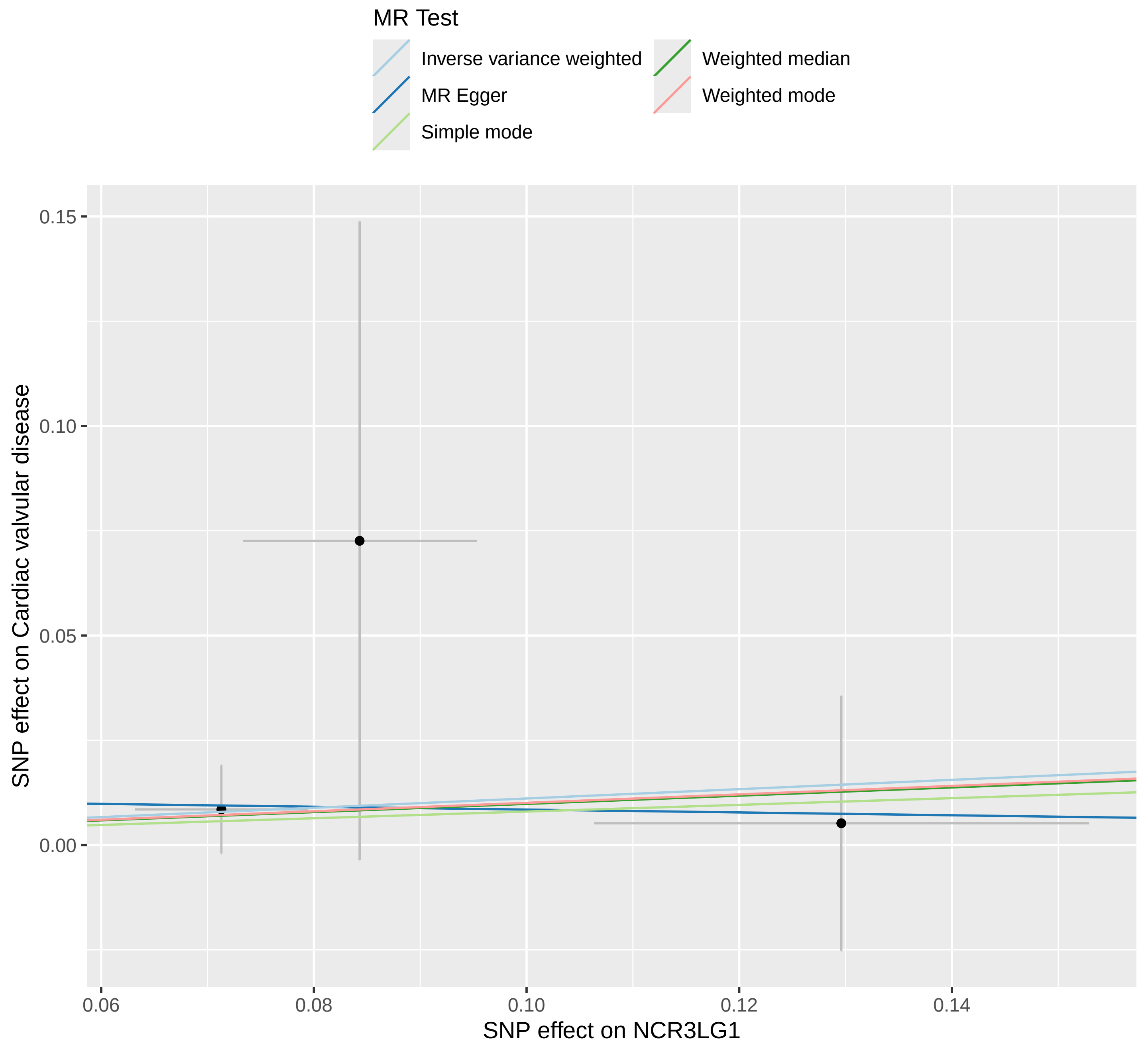
MR Test
Inverse variance weighted
MR Egger
Simple mode
Weighted median
Weighted mode
SNP effect on Cardiac valvular disease
0.15
0.10
0.05
0.00
0.06
0.08
0.10
0.12
0.14
SNP effect on NCR3LG1

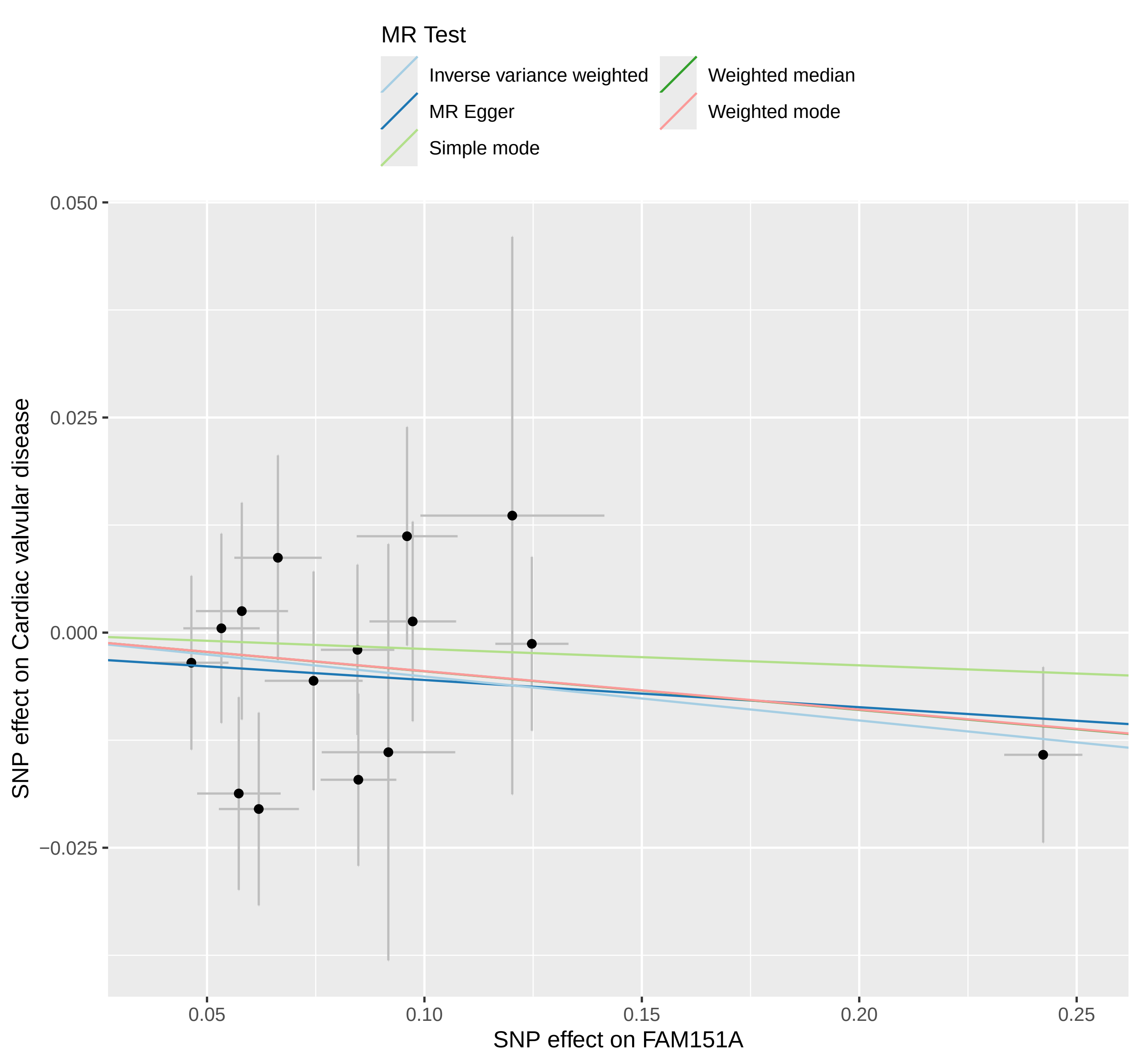

MR Test
Inverse variance weighted
MR Egger
Simple mode
Weighted median
Weighted mode
0.050
0.025
0.000
−0.025
SNP effect on Cardiac valvular disease
0.05
0.10
0.15
0.20
0.25
SNP effect on FAM151A

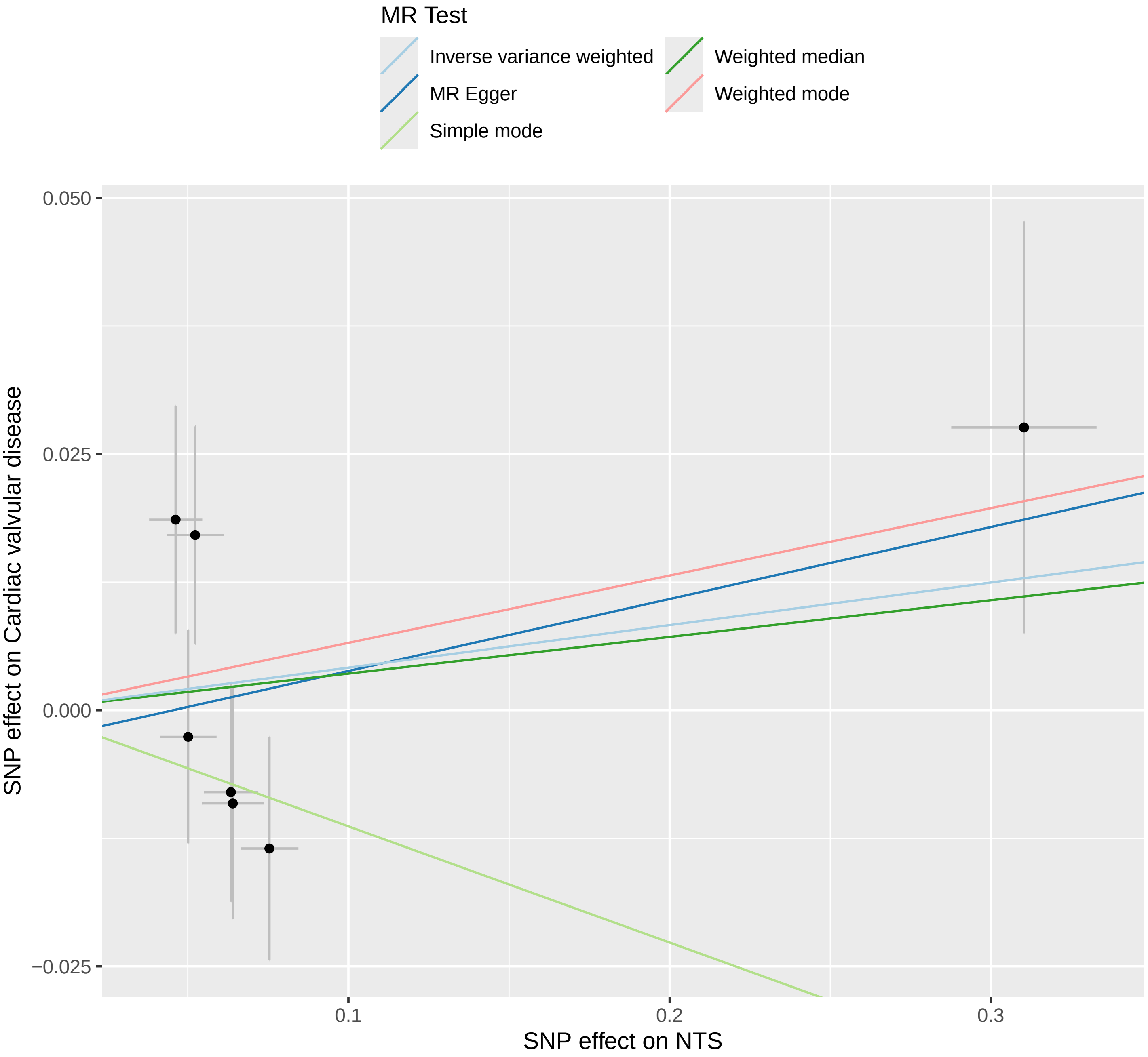

MR Test
Inverse variance weighted
MR Egger
Simple mode
Weighted median
Weighted mode
0.050
0.025
0.000
−0.025
0.1
0.2
0.3
SNP effect on Cardiac valvular disease
SNP effect on NTS

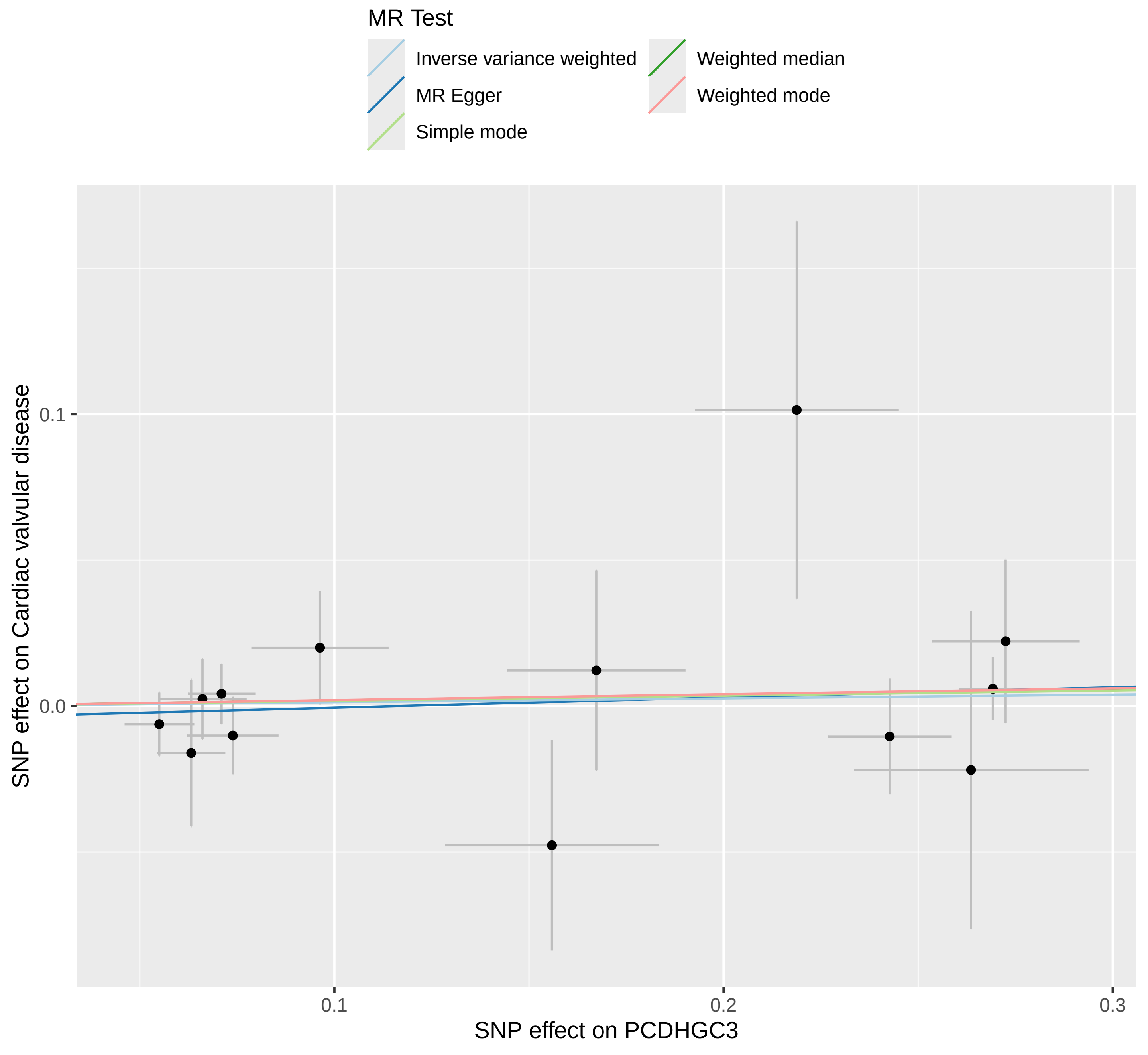

MR Test
Inverse variance weighted
MR Egger
Simple mode
Weighted median
Weighted mode
SNP effect on Cardiac valvular disease
0.1
0.0
0.1
0.2
0.3
SNP effect on PCDHGC3

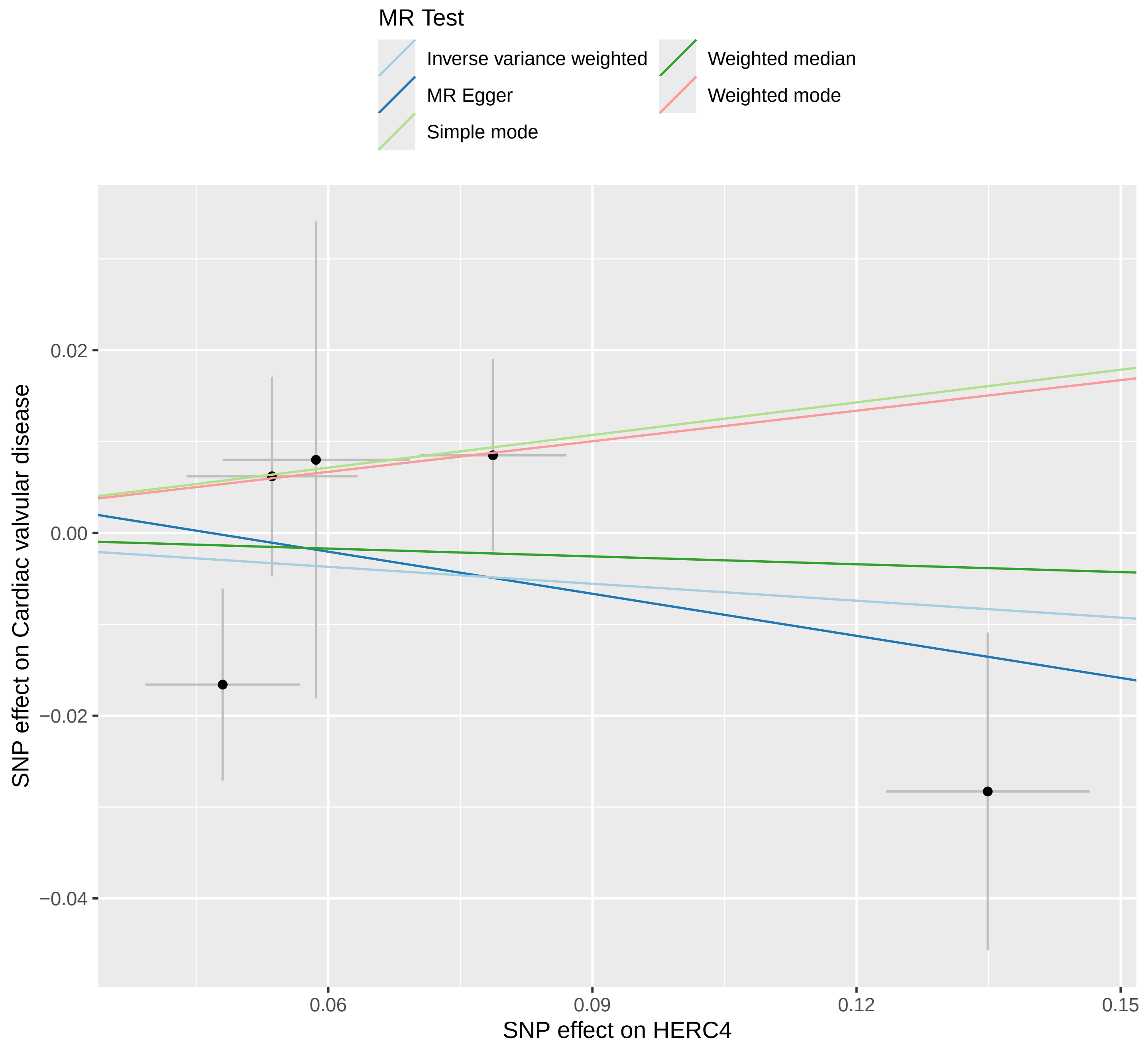
MR Test
Inverse variance weighted
MR Egger
Simple mode
Weighted median
Weighted mode
0.02
0.00
−0.02
−0.04
0.06
0.09
0.12
0.15
SNP effect on Cardiac valvular disease
SNP effect on HERC4

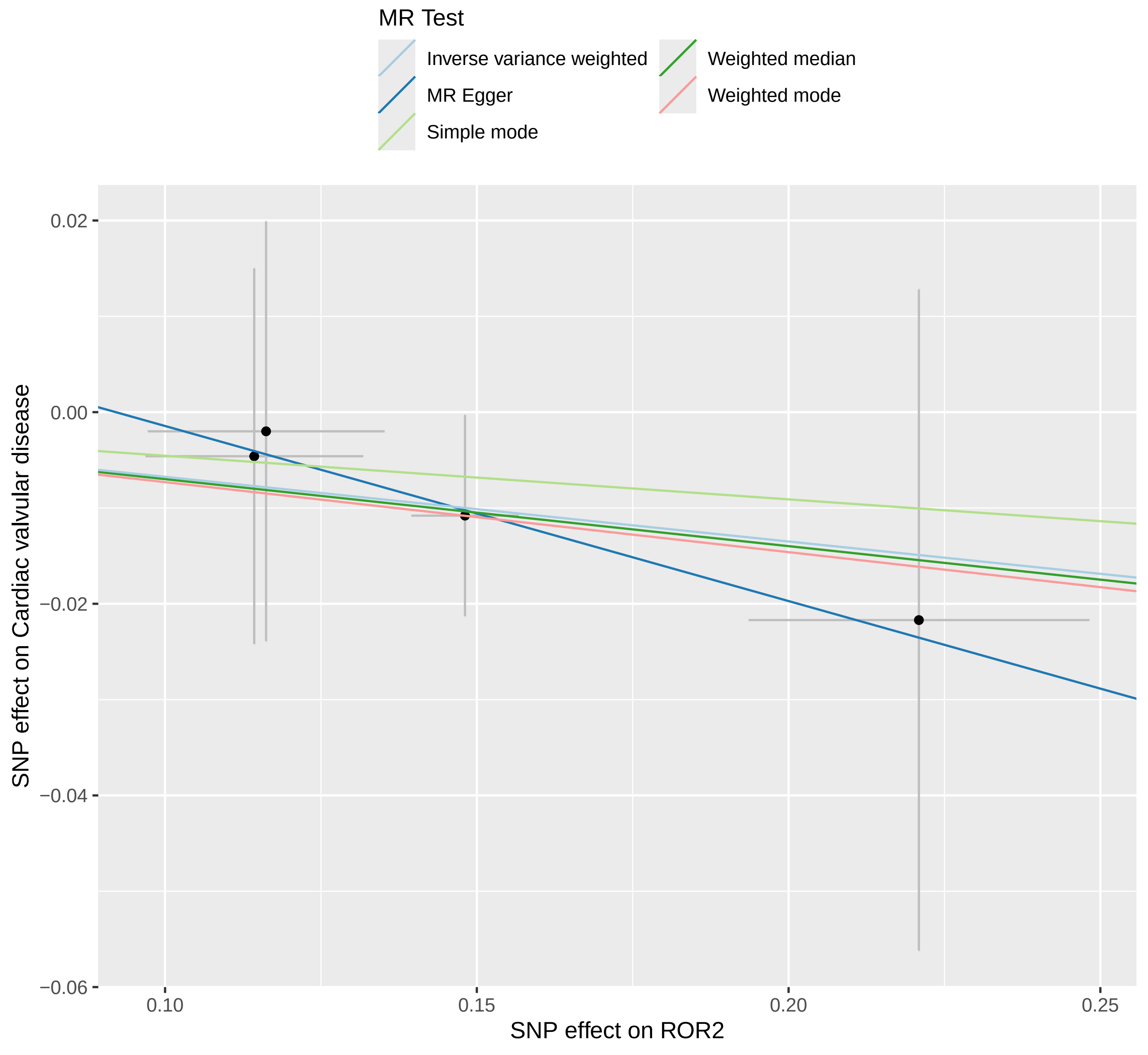

MR Test
Inverse variance weighted
MR Egger
Simple mode
Weighted median
Weighted mode
0.02
0.00
−0.02
−0.04
−0.06
0.10
0.15
0.20
0.25
SNP effect on Cardiac valvular disease
SNP effect on ROR2

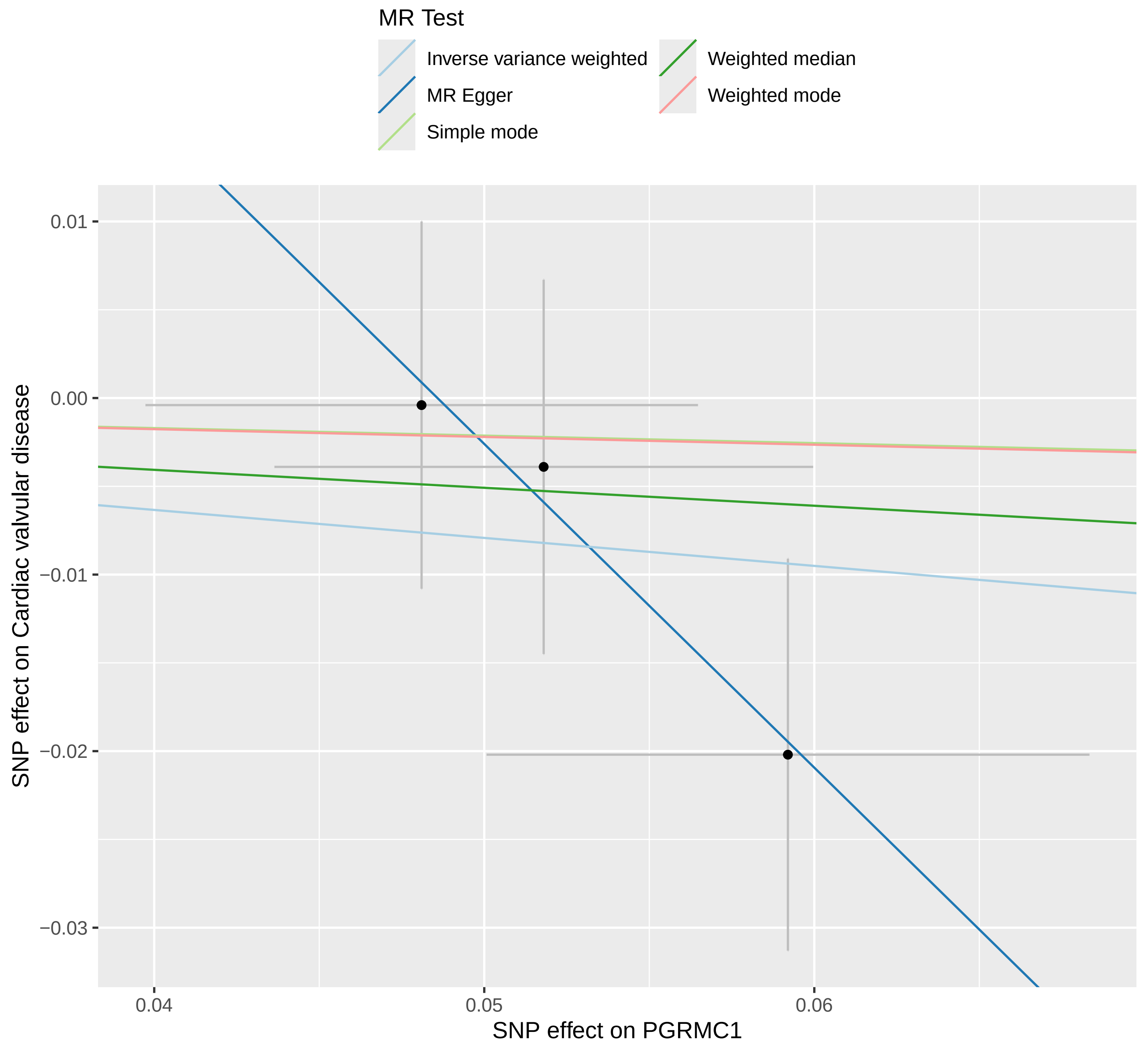
MR Test
Inverse variance weighted
MR Egger
Simple mode
Weighted median
Weighted mode
0.01
0.00
−0.01
−0.02
−0.03
0.04
0.05
0.06
SNP effect on Cardiac valvular disease
SNP effect on PGRMC1

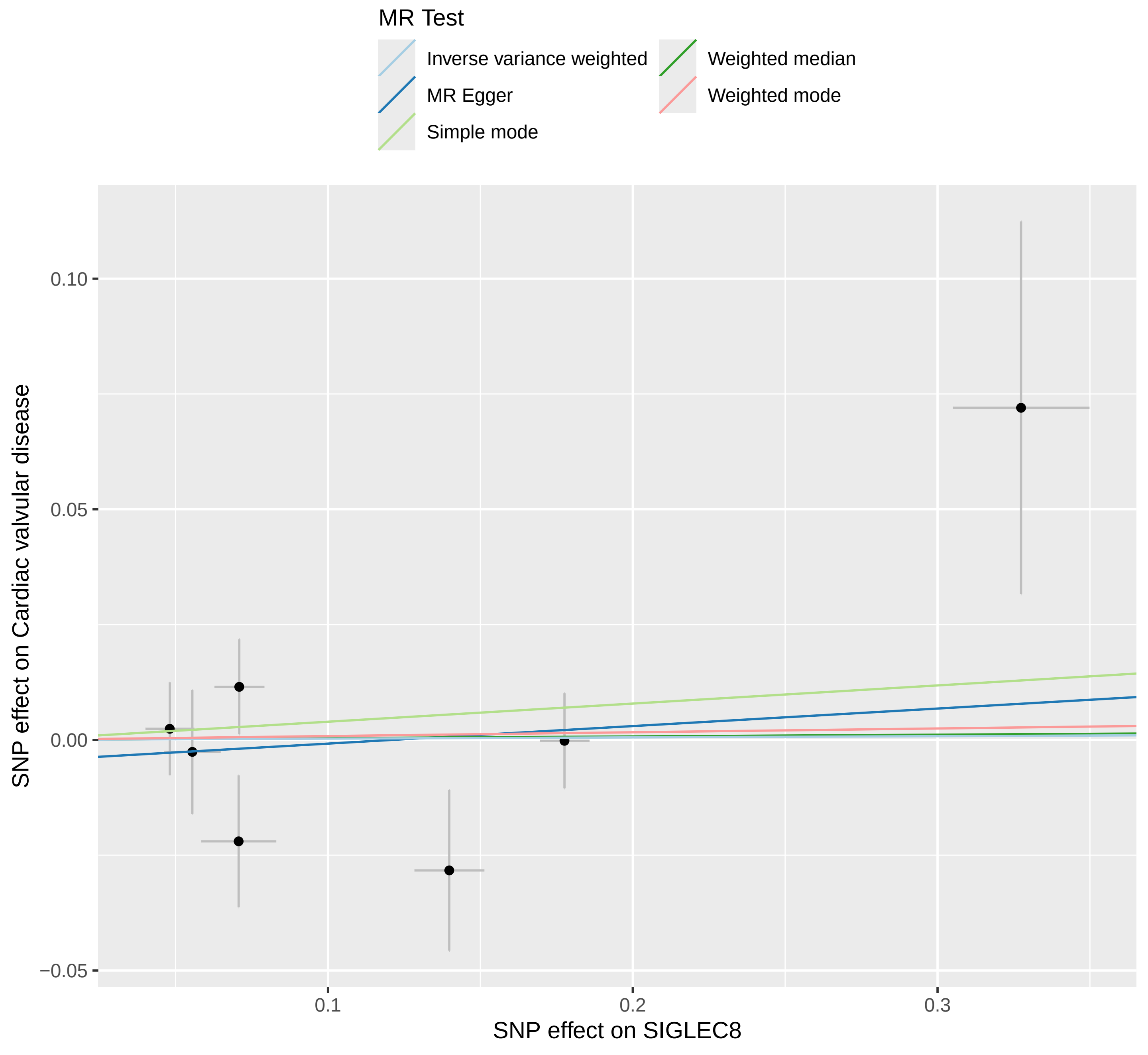

MR Test
Inverse variance weighted
MR Egger
Simple mode
Weighted median
Weighted mode
0.10
0.05
0.00
−0.05
0.1
0.2
0.3
SNP effect on Cardiac valvular disease
SNP effect on SIGLEC8

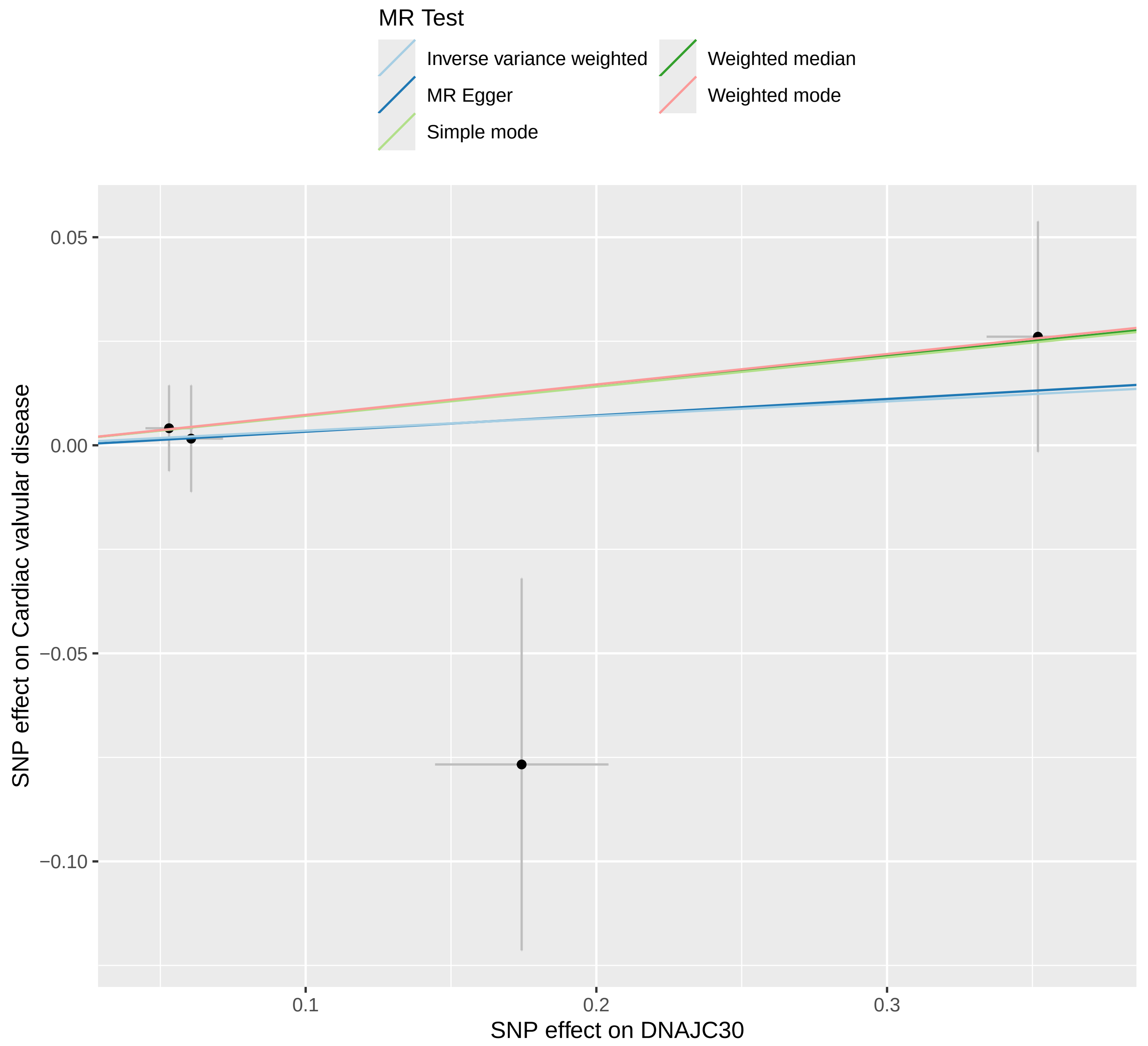
MR Test
Inverse variance weighted
MR Egger
Simple mode
Weighted median
Weighted mode
0.05
0.00
−0.05
−0.10
0.1
0.2
0.3
SNP effect on DNAJC30
SNP effect on Cardiac valvular disease

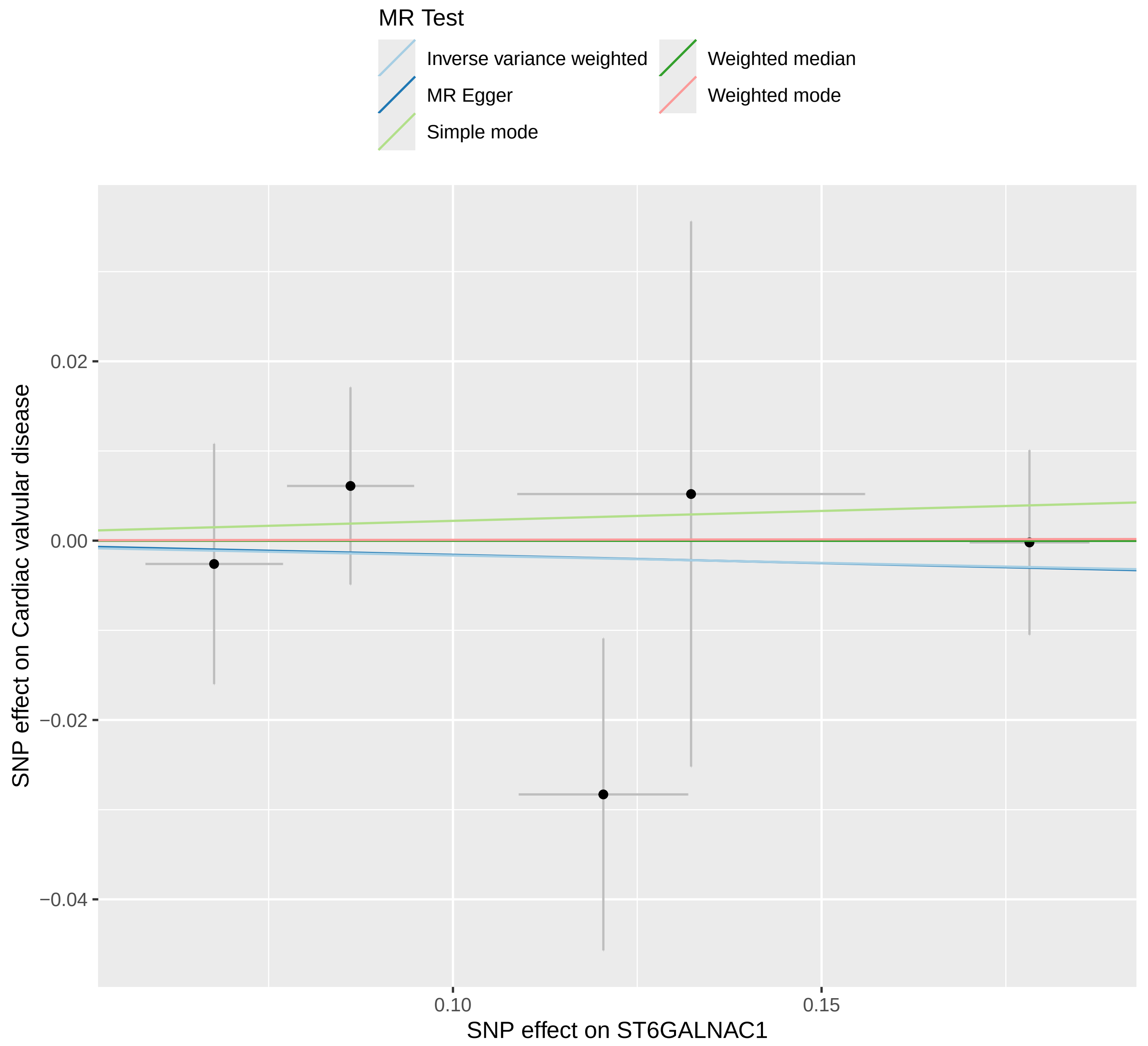
MR Test
Inverse variance weighted
MR Egger
Simple mode
Weighted median
Weighted mode
0.02
0.00
−0.02
−0.04
0.10
0.15
SNP effect on Cardiac valvular disease
SNP effect on ST6GALNAC1

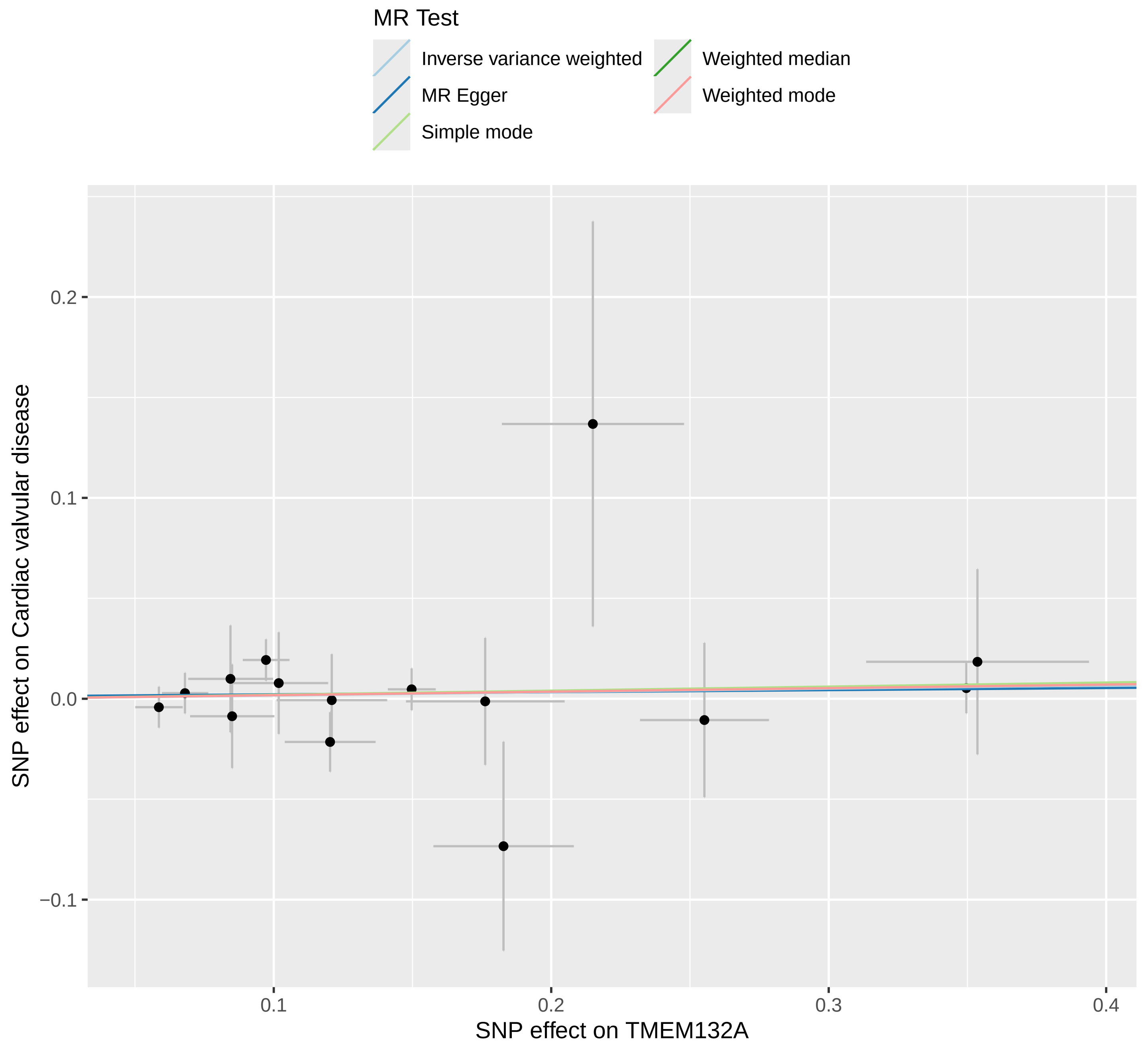

MR Test
Inverse variance weighted
MR Egger
Simple mode
Weighted median
Weighted mode
SNP effect on Cardiac valvular disease
0.2
0.1
0.0
−0.1
0.1
0.2
0.3
0.4
SNP effect on TMEM132A

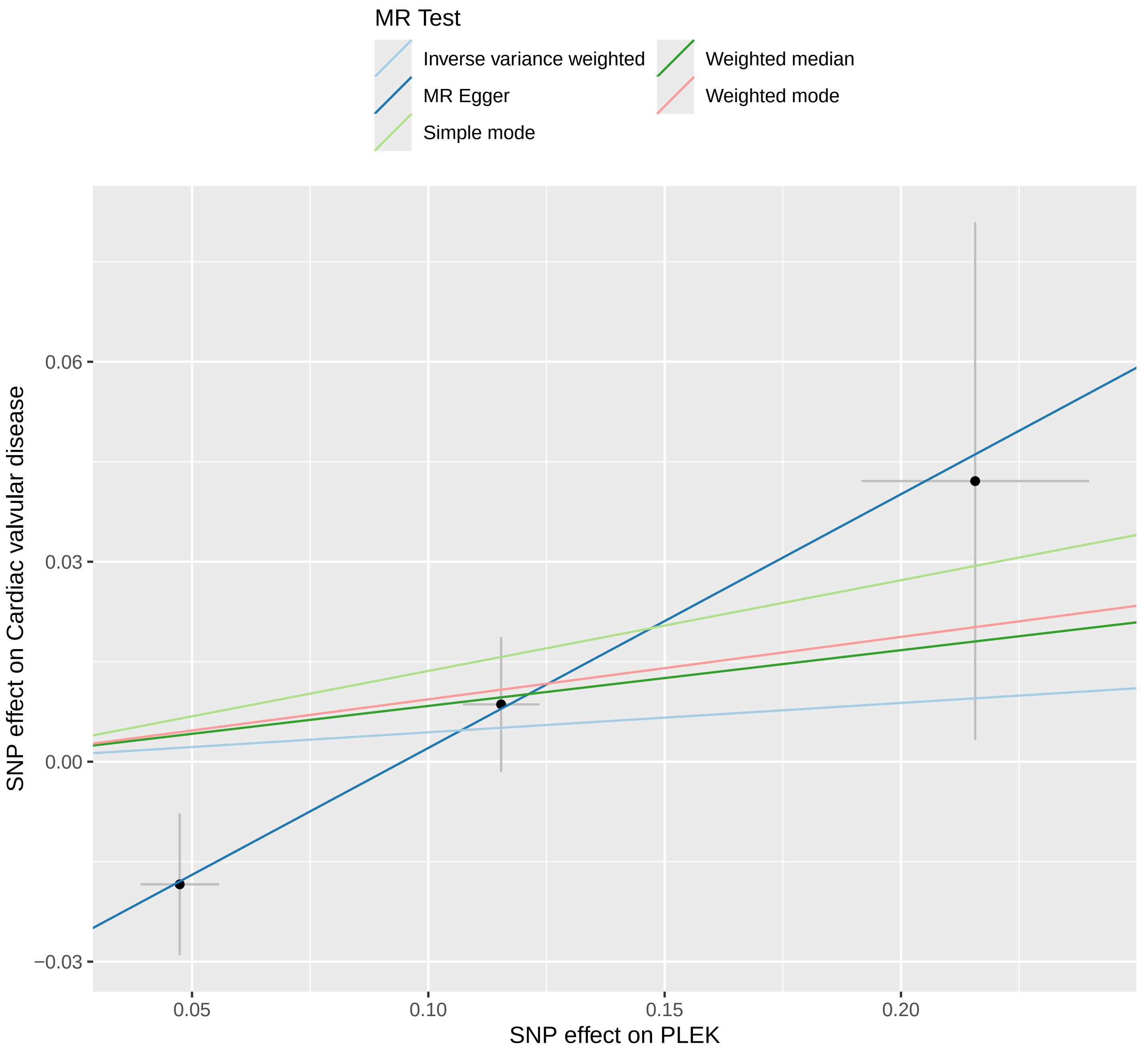
MR Test
Inverse variance weighted
MR Egger
Simple mode
Weighted median
Weighted mode
0.06
0.03
0.00
−0.03
0.05
0.10
0.15
0.20
SNP effect on Cardiac valvular disease
SNP effect on PLEK

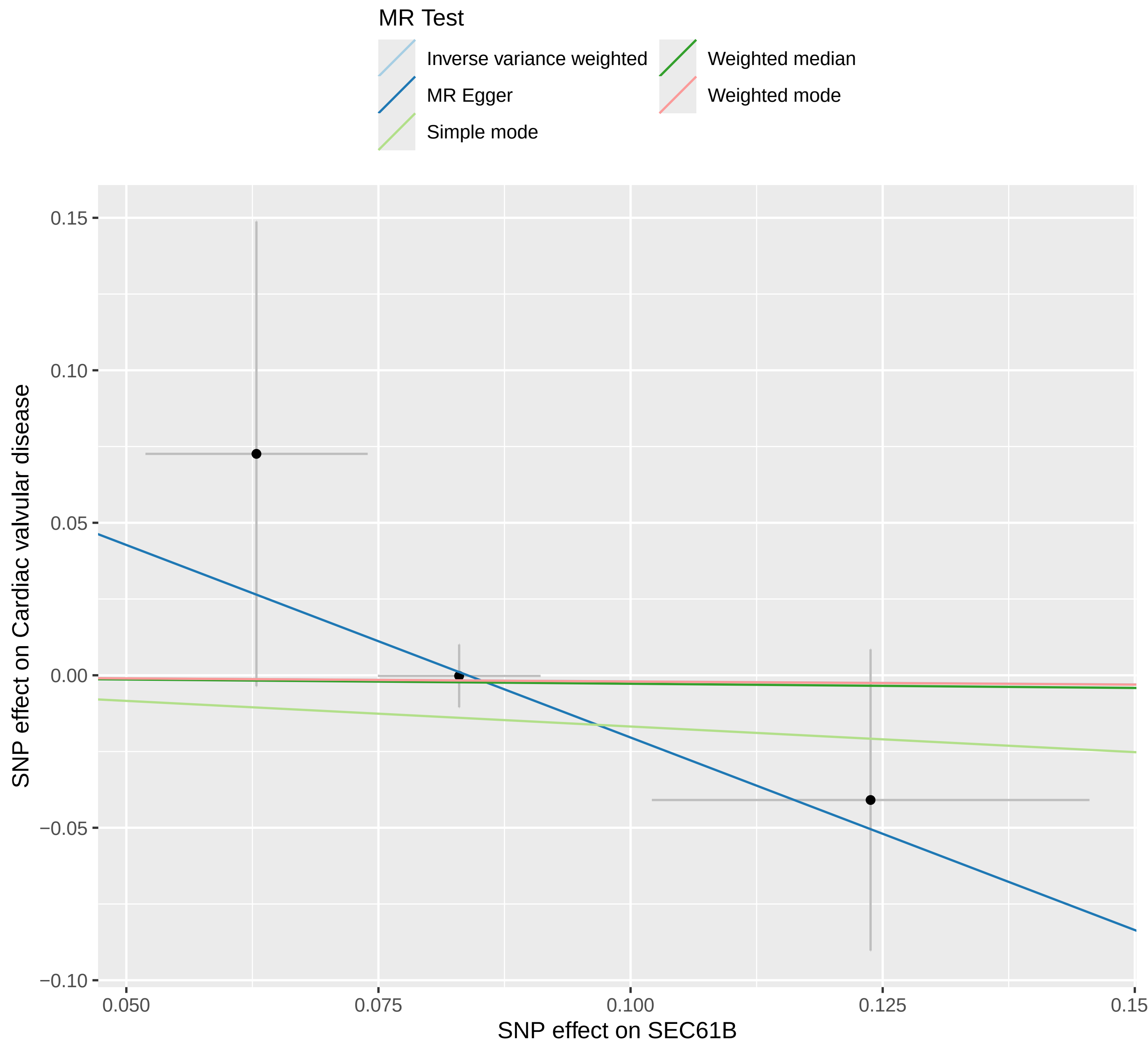
MR Test
Inverse variance weighted
MR Egger
Simple mode
Weighted median
Weighted mode
0.15
0.10
0.05
0.00
−0.05
−0.10
0.050
0.075
0.100
0.125
0.15
SNP effect on Cardiac valvular disease
SNP effect on SEC61B

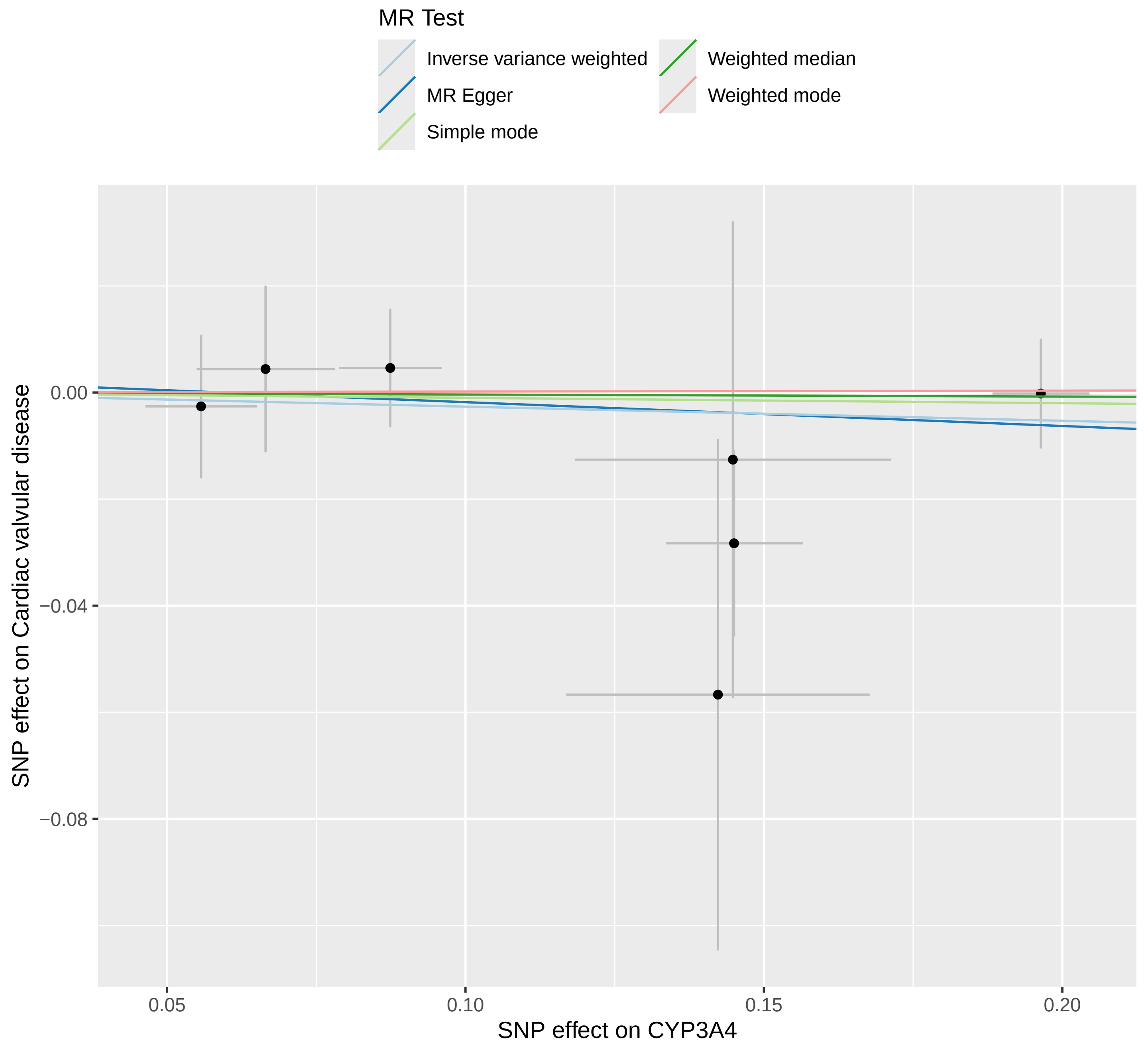

MR Test
Inverse variance weighted
MR Egger
Simple mode
Weighted median
Weighted mode
SNP effect on Cardiac valvular disease
0.00
−0.04
−0.08
0.05
0.10
0.15
0.20
SNP effect on CYP3A4

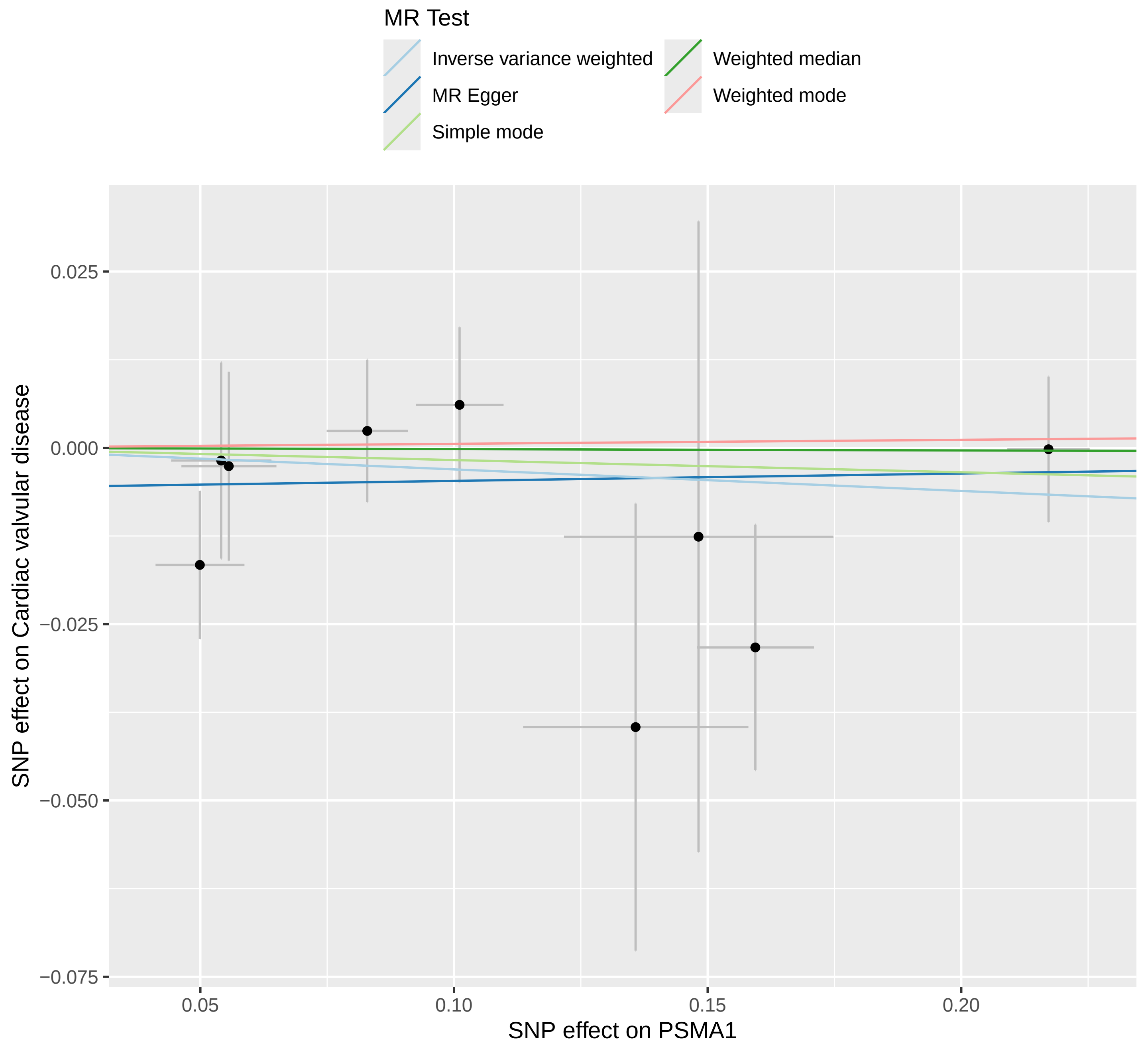
MR Test
Inverse variance weighted
MR Egger
Simple mode
Weighted median
Weighted mode
0.025
0.000
−0.025
−0.050
−0.075
0.05
0.10
0.15
0.20
SNP effect on Cardiac valvular disease
SNP effect on PSMA1

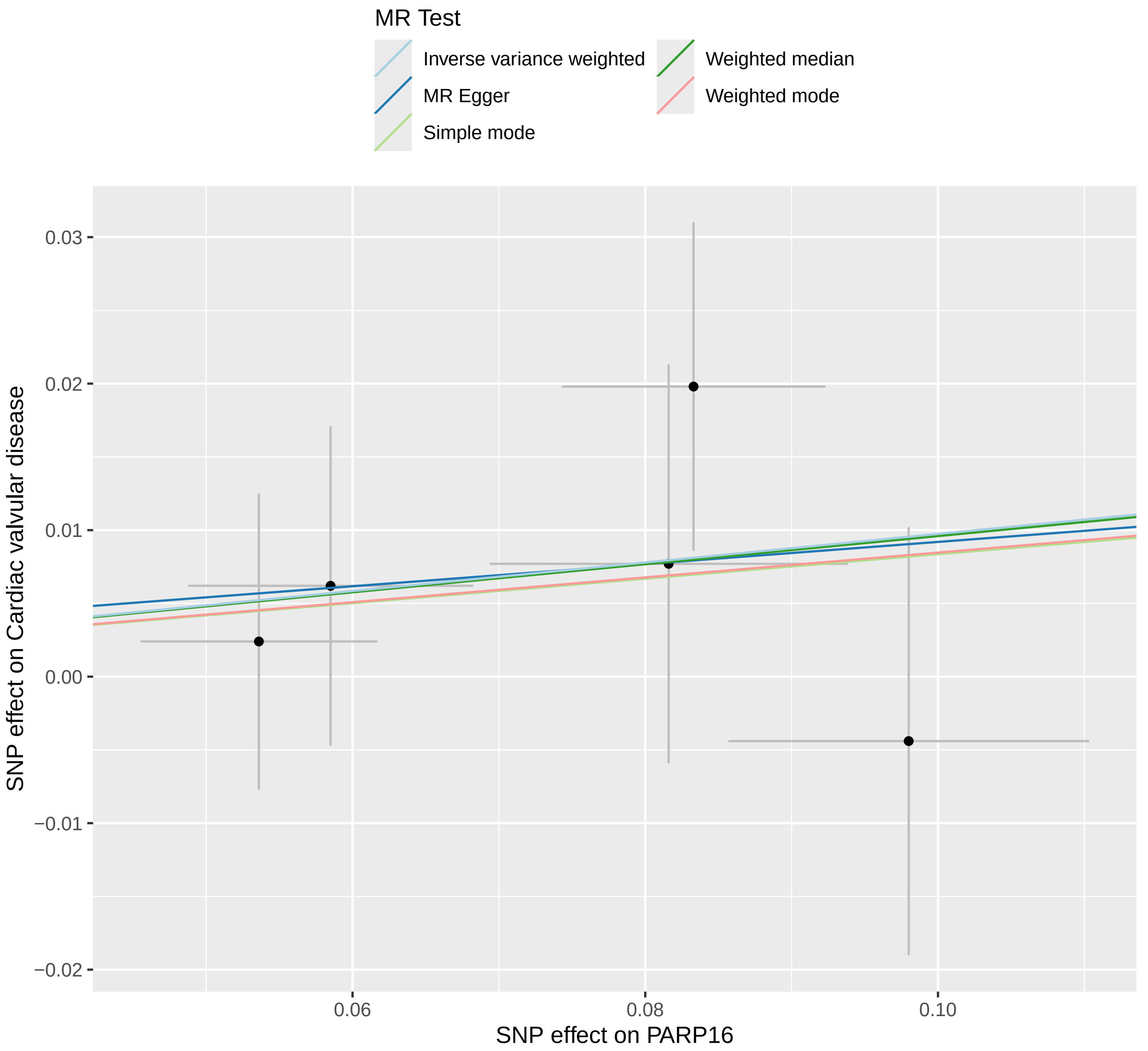
MR Test
Inverse variance weighted
MR Egger
Simple mode
Weighted median
Weighted mode
0.03
0.02
0.01
0.00
−0.01
−0.02
SNP effect on Cardiac valvular disease
0.06
0.08
0.10
SNP effect on PARP16

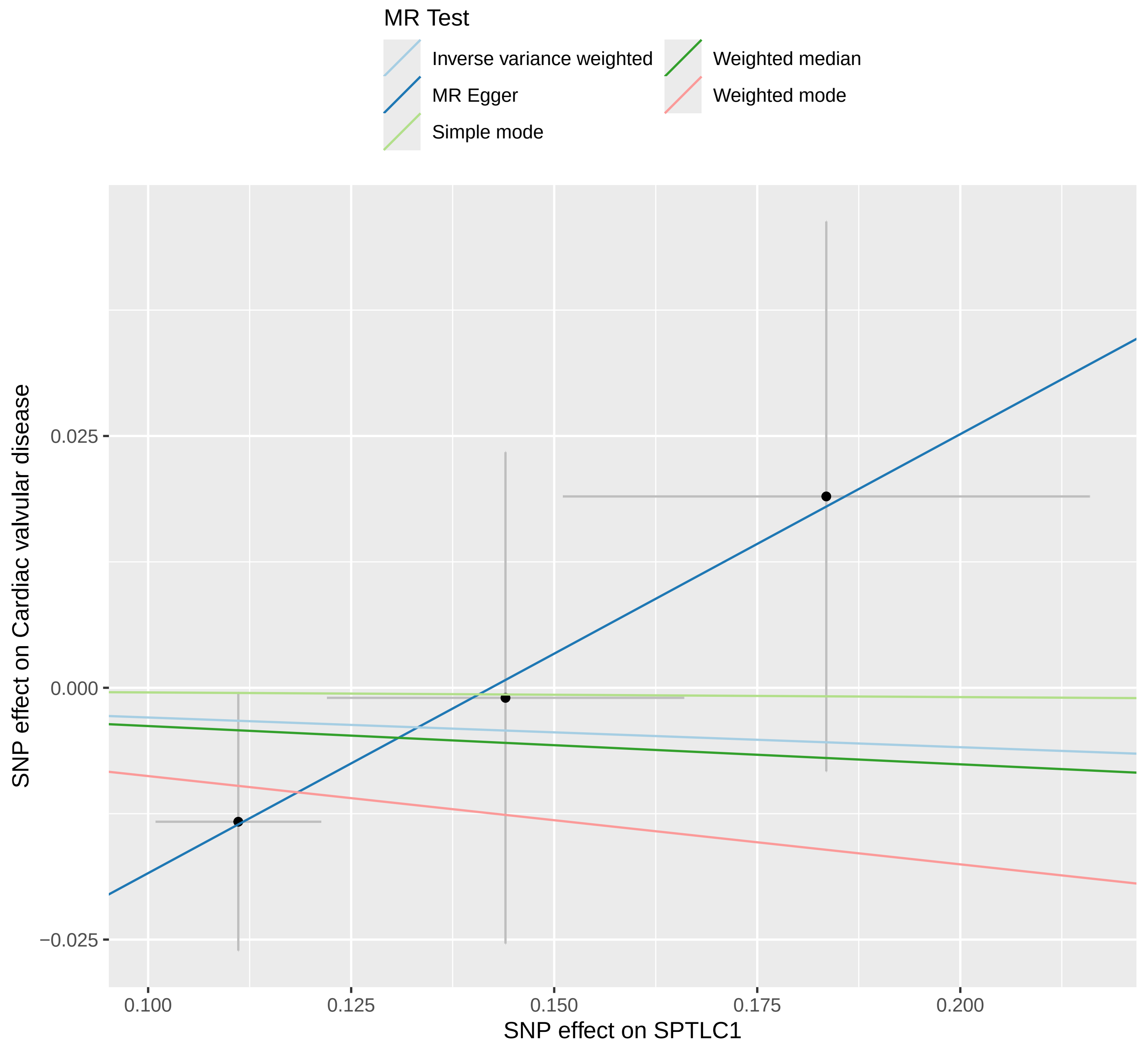
MR Test
Inverse variance weighted
MR Egger
Simple mode
Weighted median
Weighted mode
SNP effect on Cardiac valvular disease
0.025
0.000
−0.025
0.100
0.125
0.150
0.175
0.200
SNP effect on SPTLC1

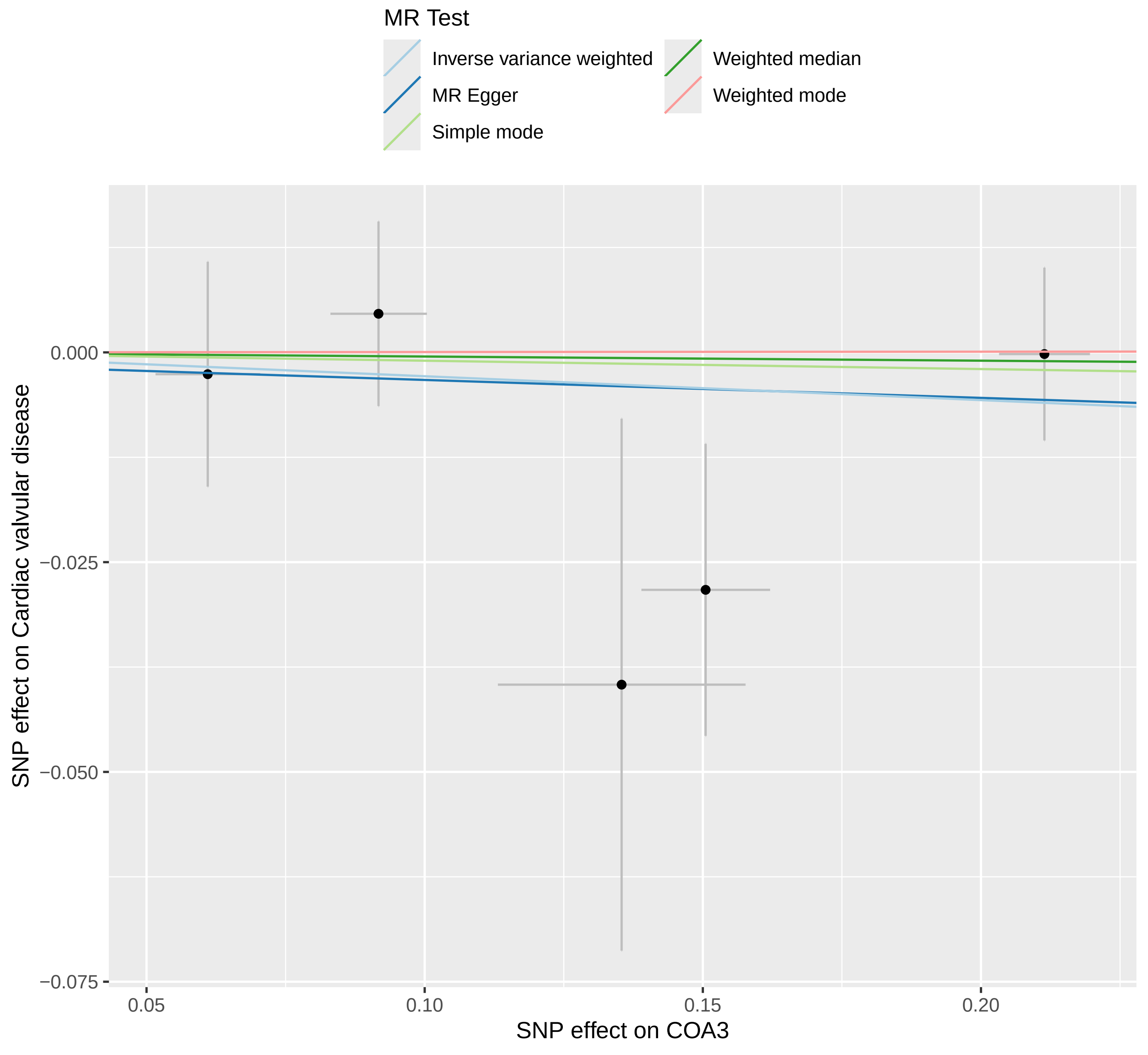

MR Test
Inverse variance weighted
MR Egger
Simple mode
Weighted median
Weighted mode
0.000
−0.025
−0.050
−0.075
SNP effect on Cardiac valvular disease
0.05
0.10
0.15
0.20
SNP effect on COA3

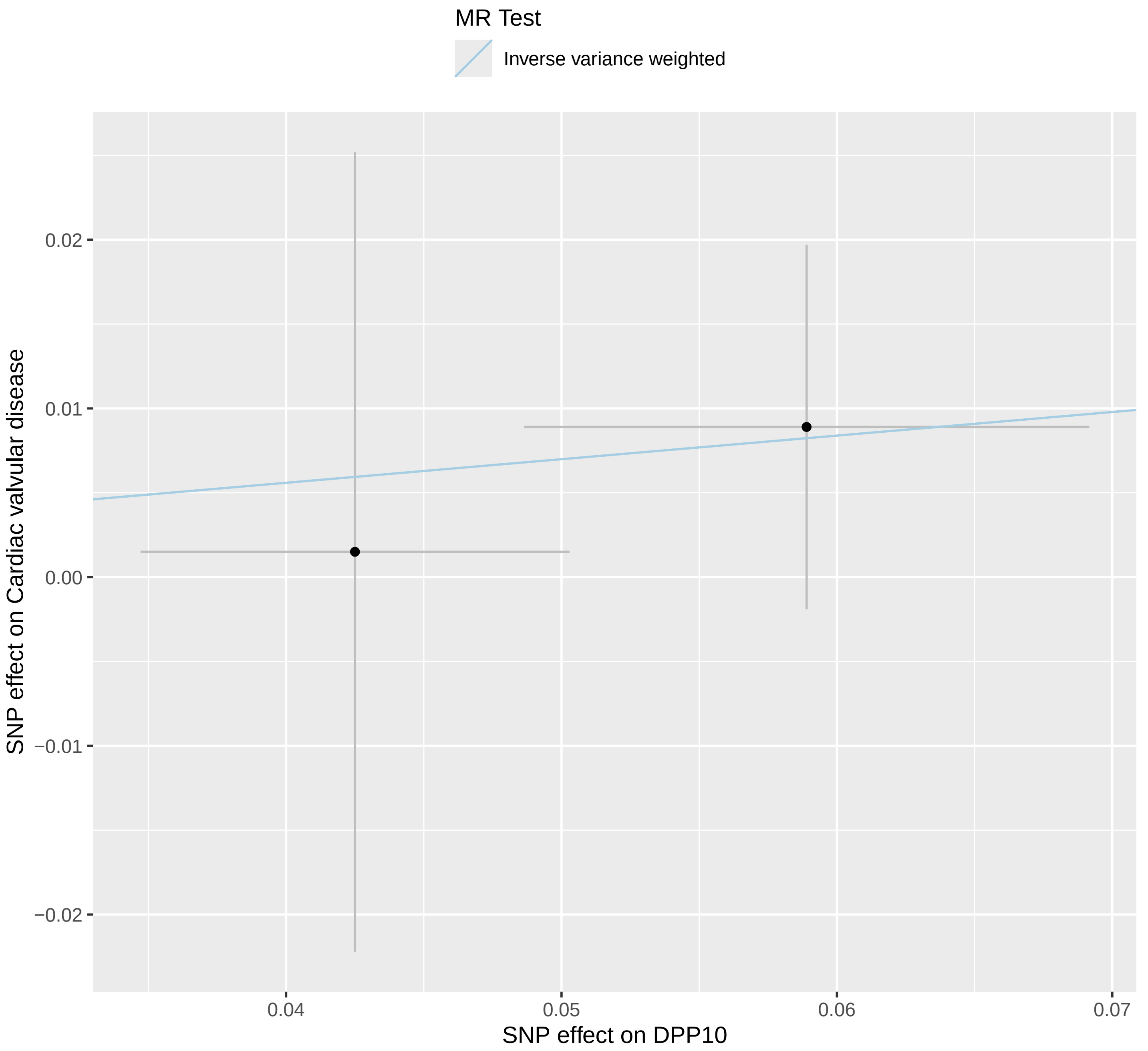
MR Test
Inverse variance weighted
0.02
0.01
0.00
−0.01
−0.02
0.04
0.05
0.06
0.07
SNP effect on DPP10
SNP effect on Cardiac valvular disease

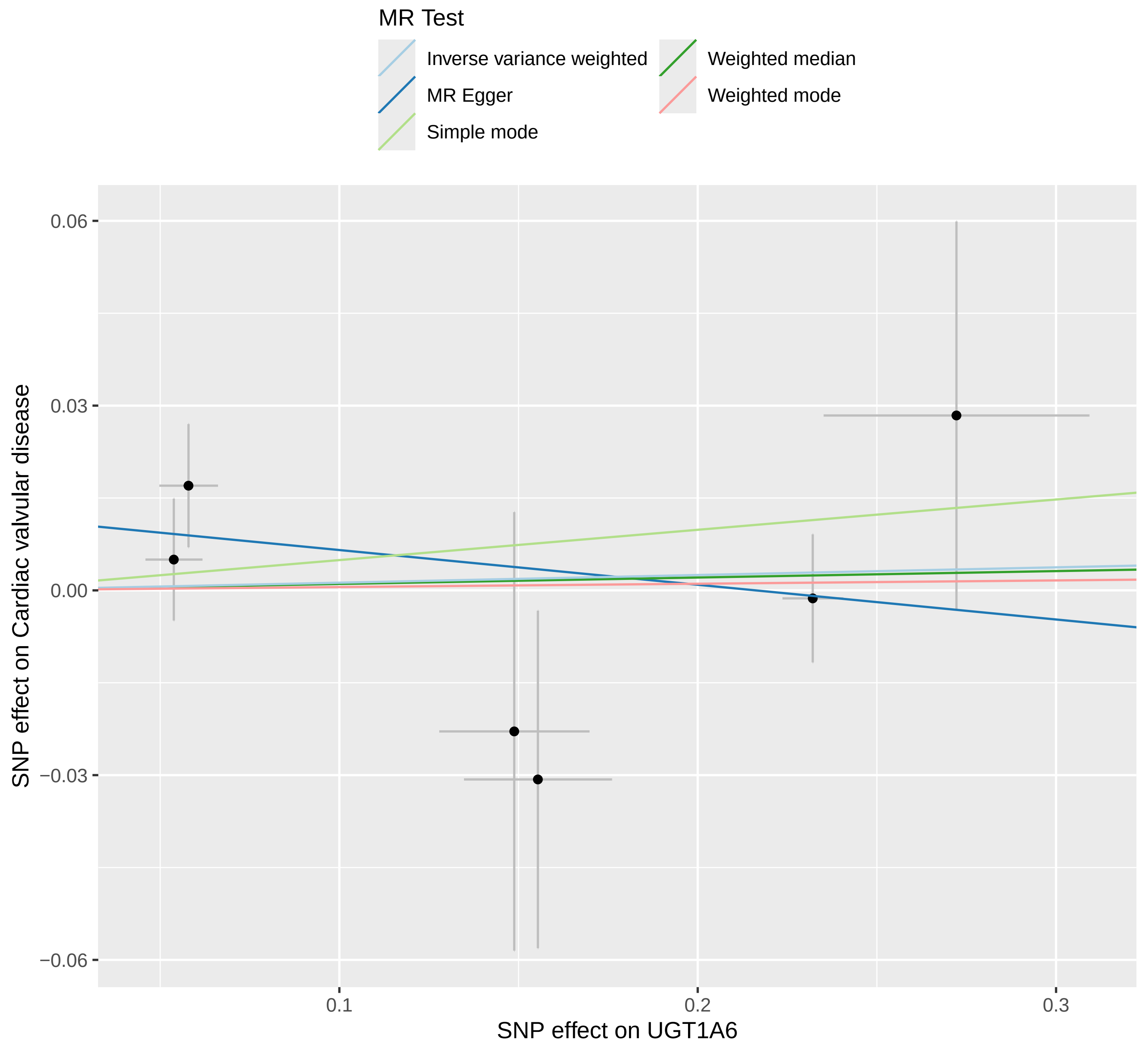
MR Test
Inverse variance weighted
MR Egger
Simple mode
Weighted median
Weighted mode
SNP effect on Cardiac valvular disease
0.06
0.03
0.00
−0.03
−0.06
0.1
0.2
0.3
SNP effect on UGT1A6

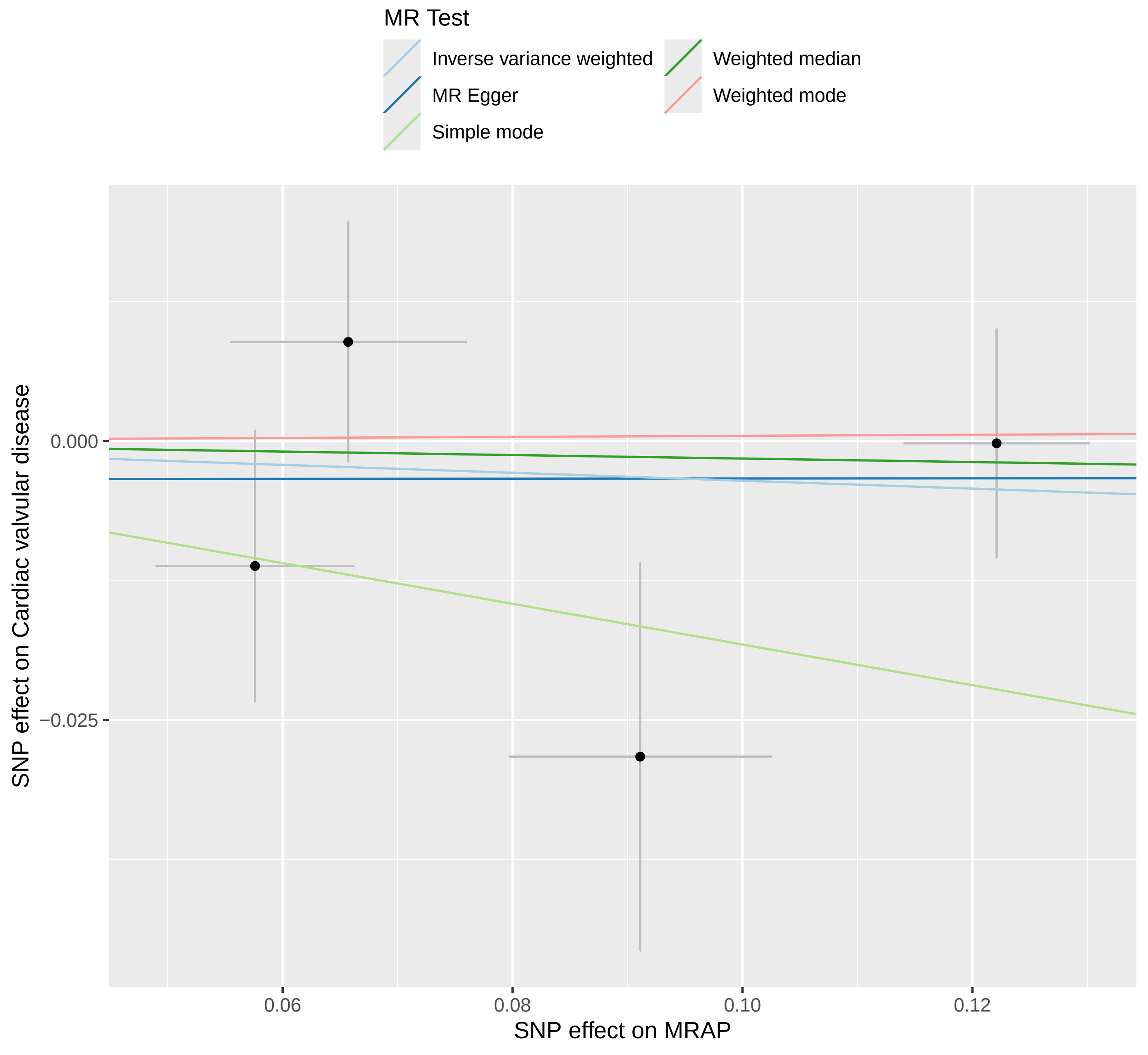
MR Test
Inverse variance weighted
MR Egger
Simple mode
Weighted median
Weighted mode
0.000
−0.025
0.06
0.08
0.10
0.12
SNP effect on MRAP
SNP effect on Cardiac valvular disease

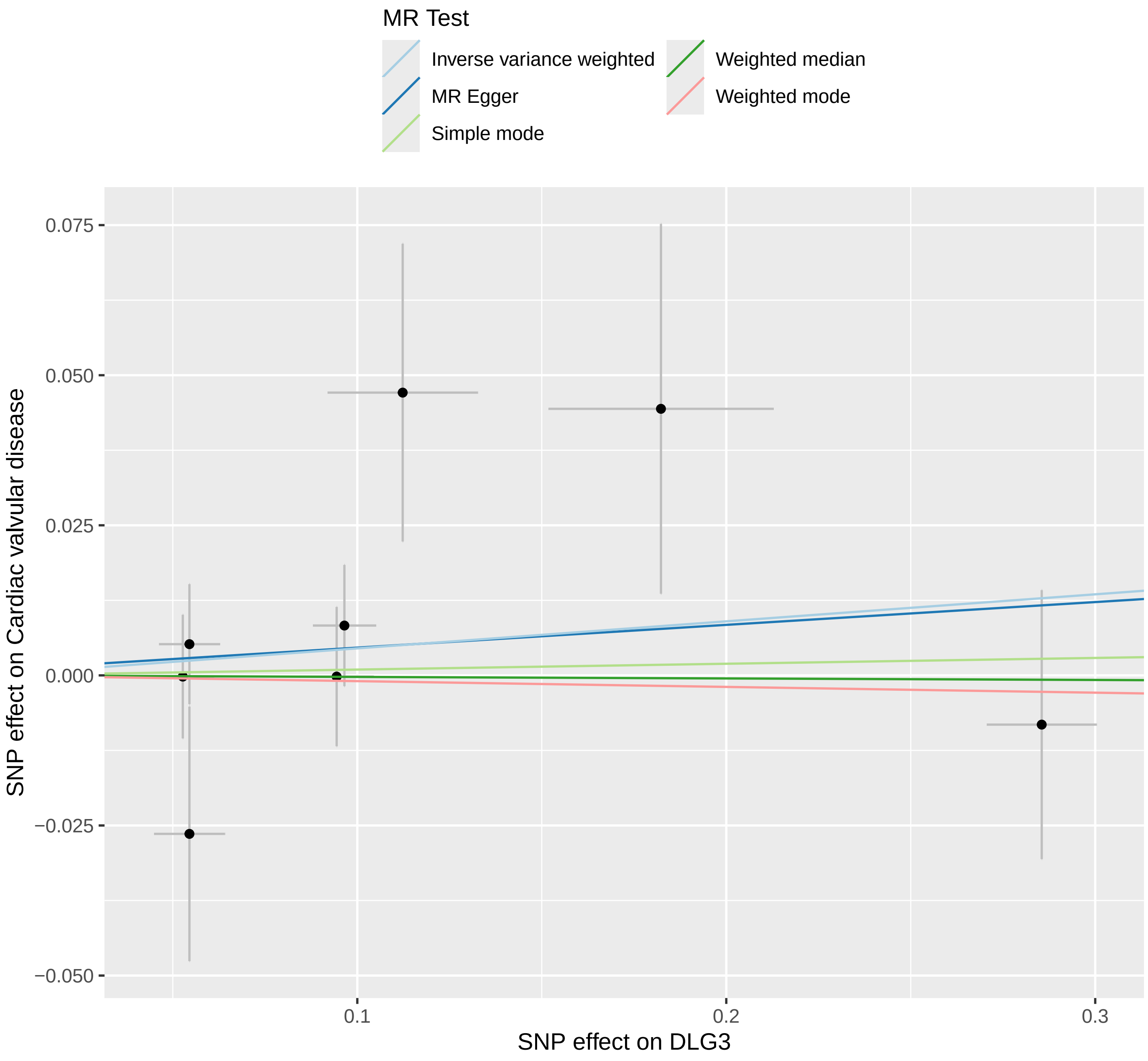
MR Test
Inverse variance weighted
MR Egger
Simple mode
Weighted median
Weighted mode
0.075
0.050
0.025
0.000
−0.025
−0.050
SNP effect on Cardiac valvular disease
0.1
0.2
0.3
SNP effect on DLG3

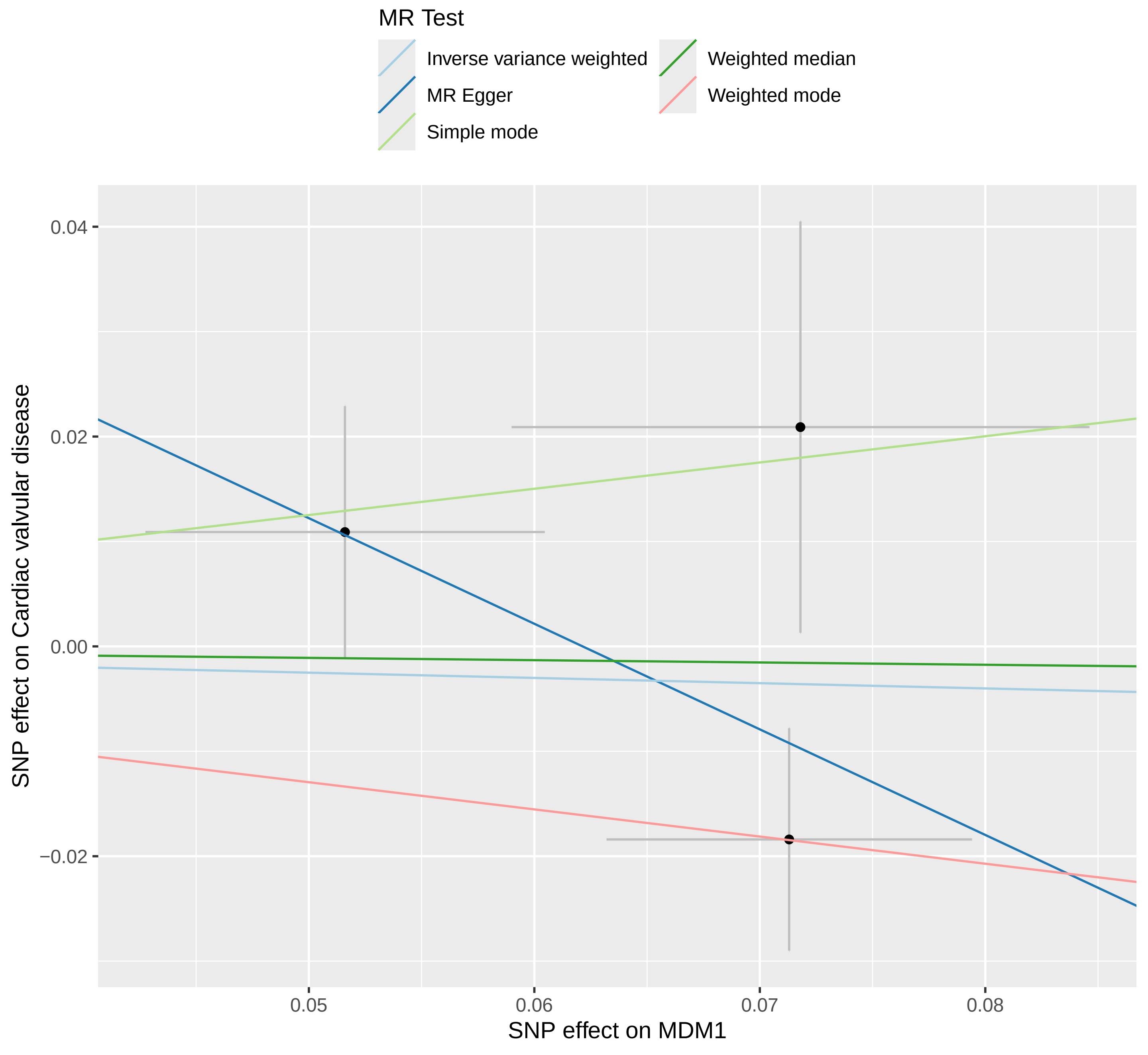
MR Test
Inverse variance weighted
MR Egger
Simple mode
Weighted median
Weighted mode
0.04
0.02
0.00
−0.02
0.05
0.06
0.07
0.08
SNP effect on Cardiac valvular disease
SNP effect on MDM1

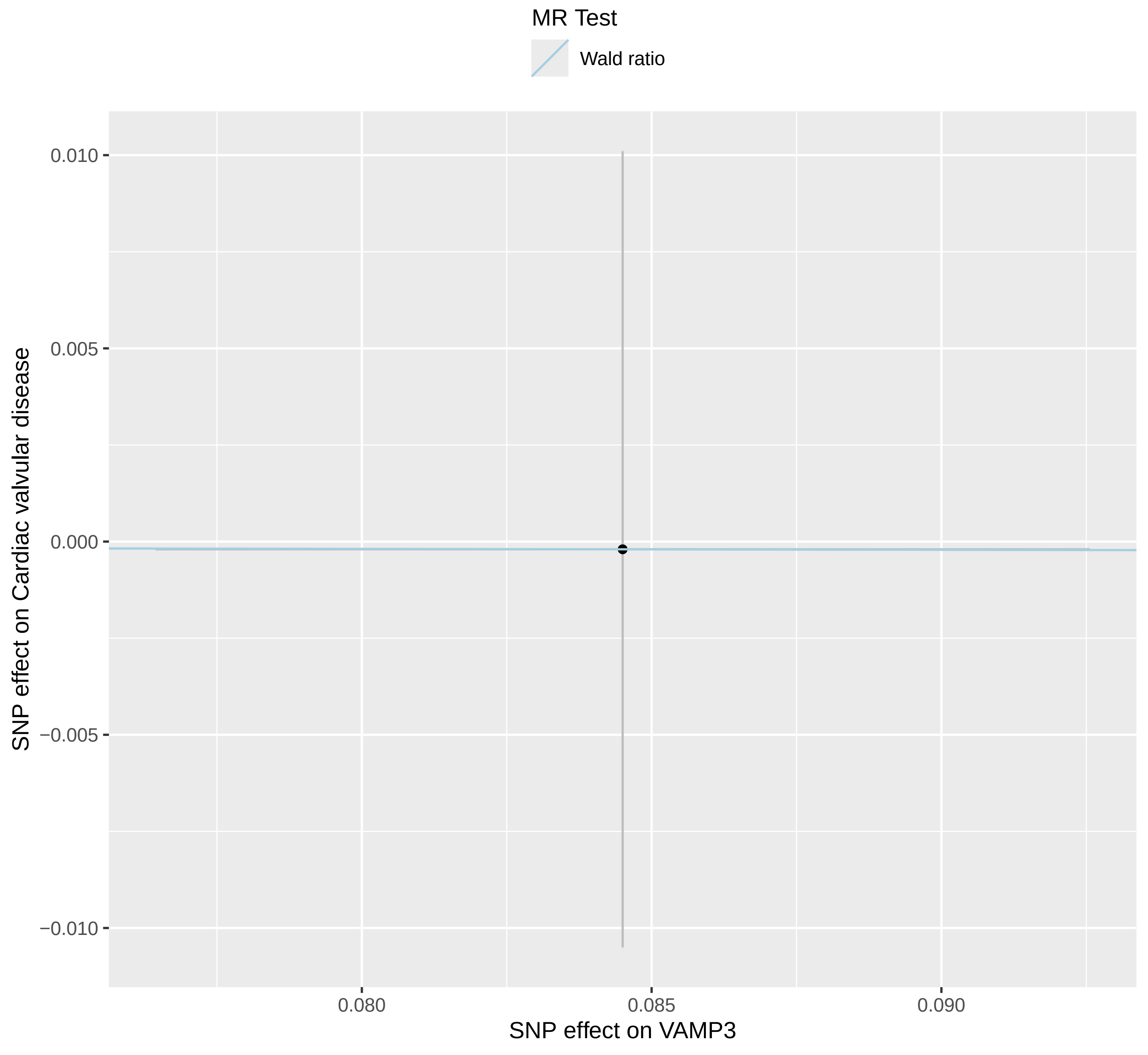
MR Test
Wald ratio
0.010
0.005
0.000
−0.005
−0.010
0.080
0.085
0.090
SNP effect on VAMP3
SNP effect on Cardiac valvular disease

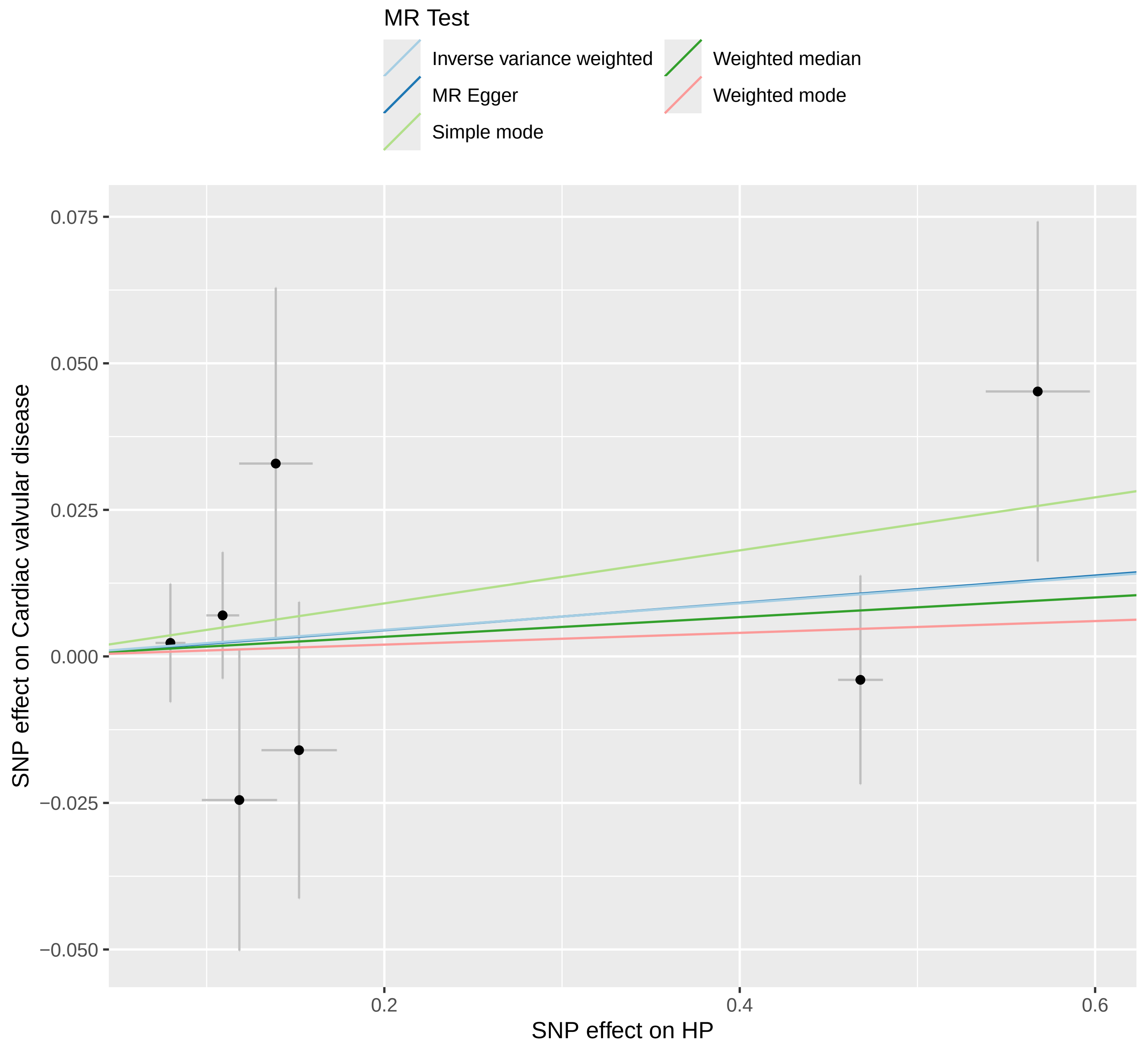

MR Test
Inverse variance weighted
MR Egger
Simple mode
Weighted median
Weighted mode
0.075
0.050
0.025
0.000
−0.025
−0.050
0.2
0.4
0.6
SNP effect on Cardiac valvular disease
SNP effect on HP

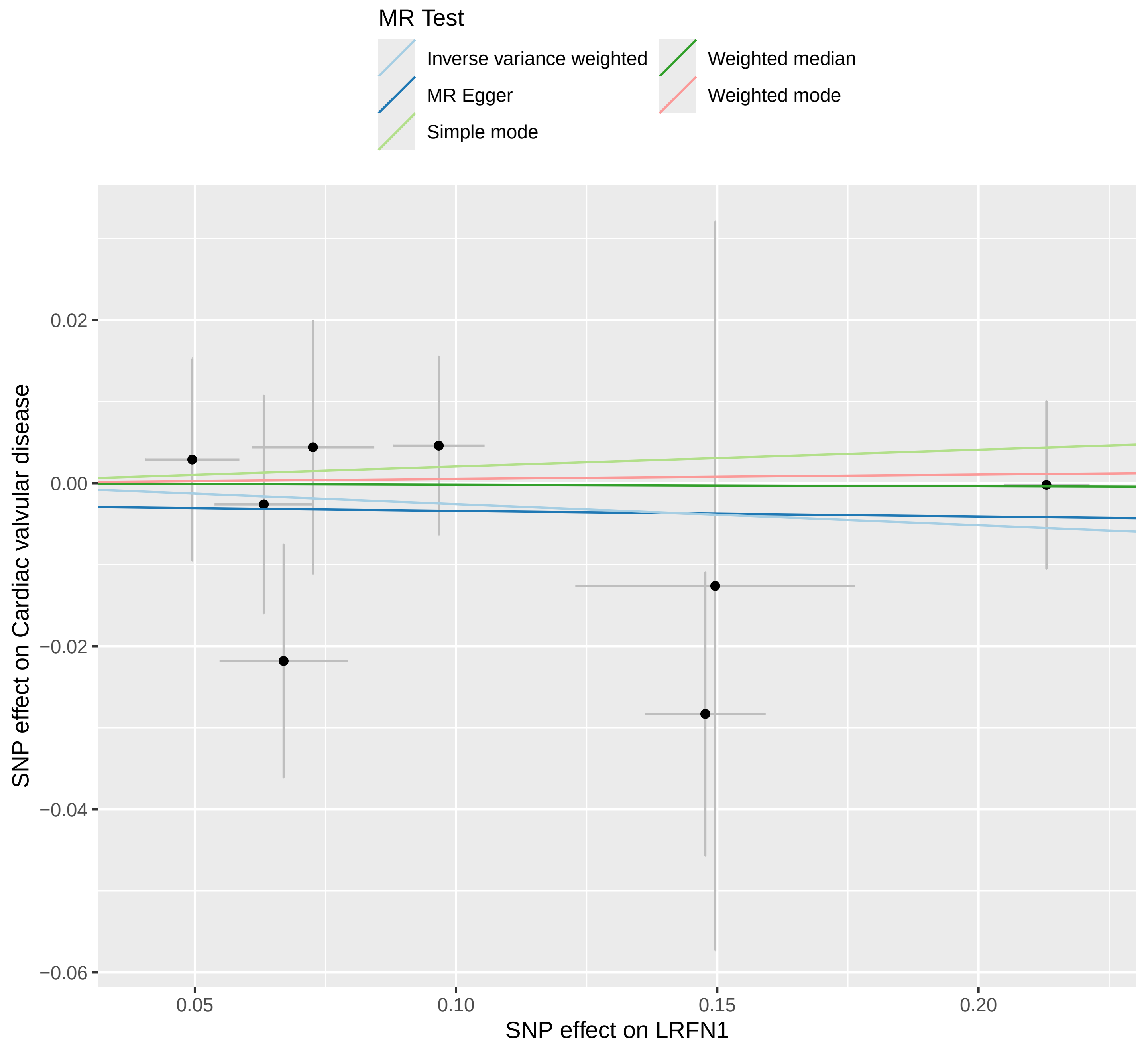
MR Test
Inverse variance weighted
MR Egger
Simple mode
Weighted median
Weighted mode
0.02
0.00
−0.02
−0.04
−0.06
SNP effect on Cardiac valvular disease
0.05
0.10
0.15
0.20
SNP effect on LRFN1

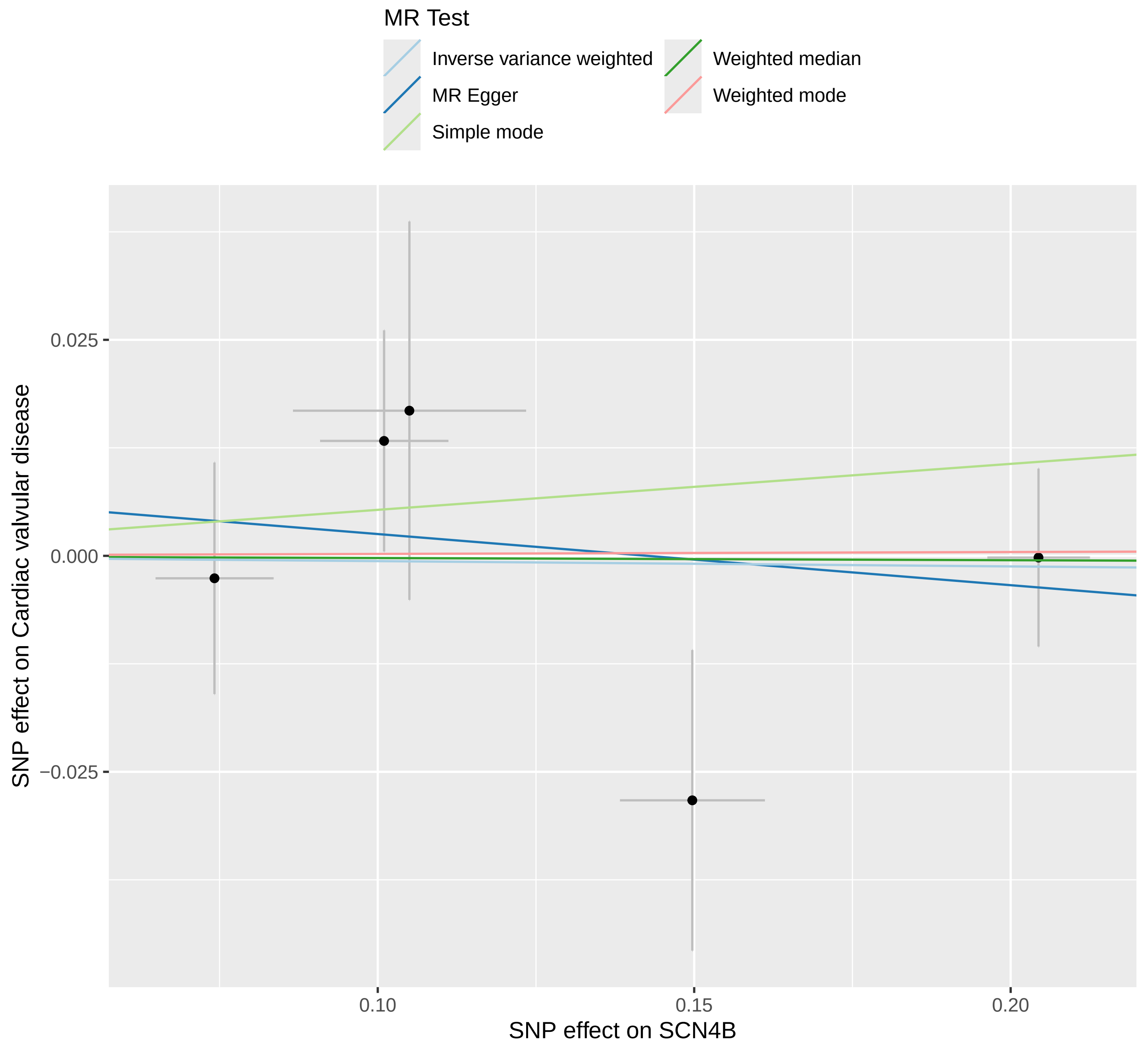
MR Test
Inverse variance weighted
MR Egger
Simple mode
Weighted median
Weighted mode
0.025
0.000
−0.025
0.10
0.15
0.20
SNP effect on Cardiac valvular disease
SNP effect on SCN4B

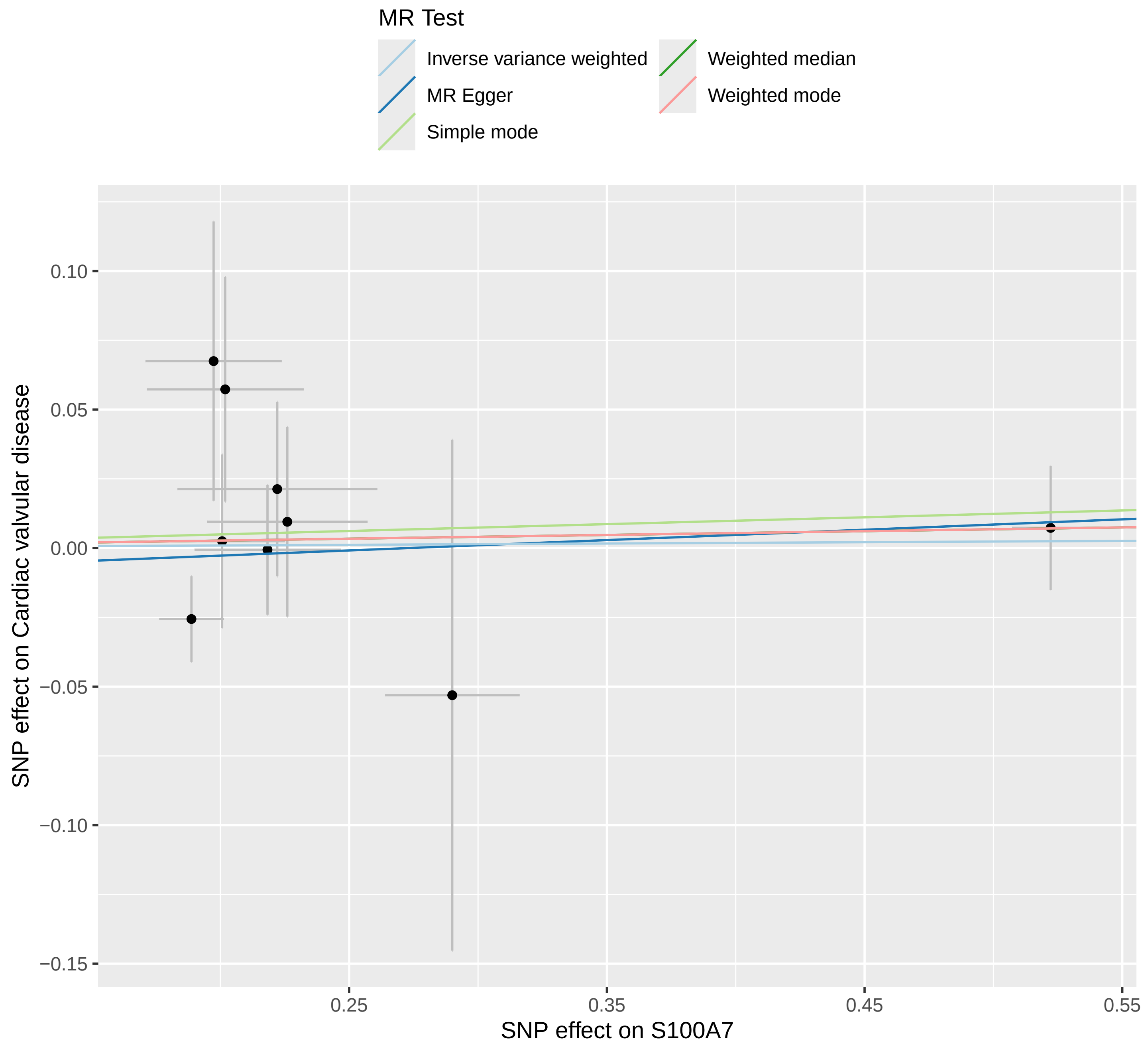
MR Test
Inverse variance weighted
MR Egger
Simple mode
Weighted median
Weighted mode
SNP effect on Cardiac valvular disease
0.10
0.05
0.00
−0.05
−0.10
−0.15
0.25
0.35
0.45
0.55
SNP effect on S100A7

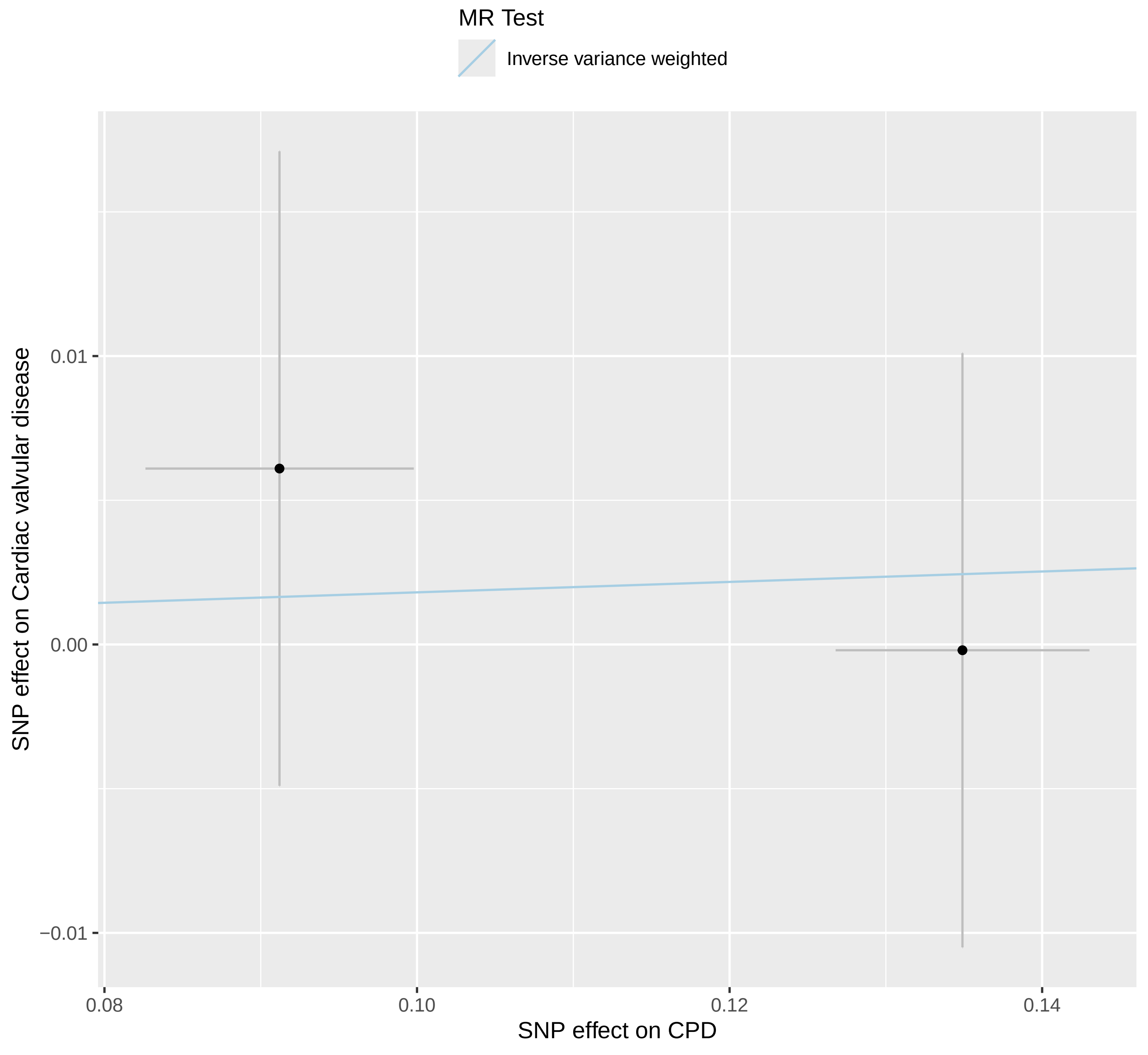

MR Test
Inverse variance weighted
SNP effect on Cardiac valvular disease
0.01
0.00
−0.01
0.08
0.10
0.12
0.14
SNP effect on CPD

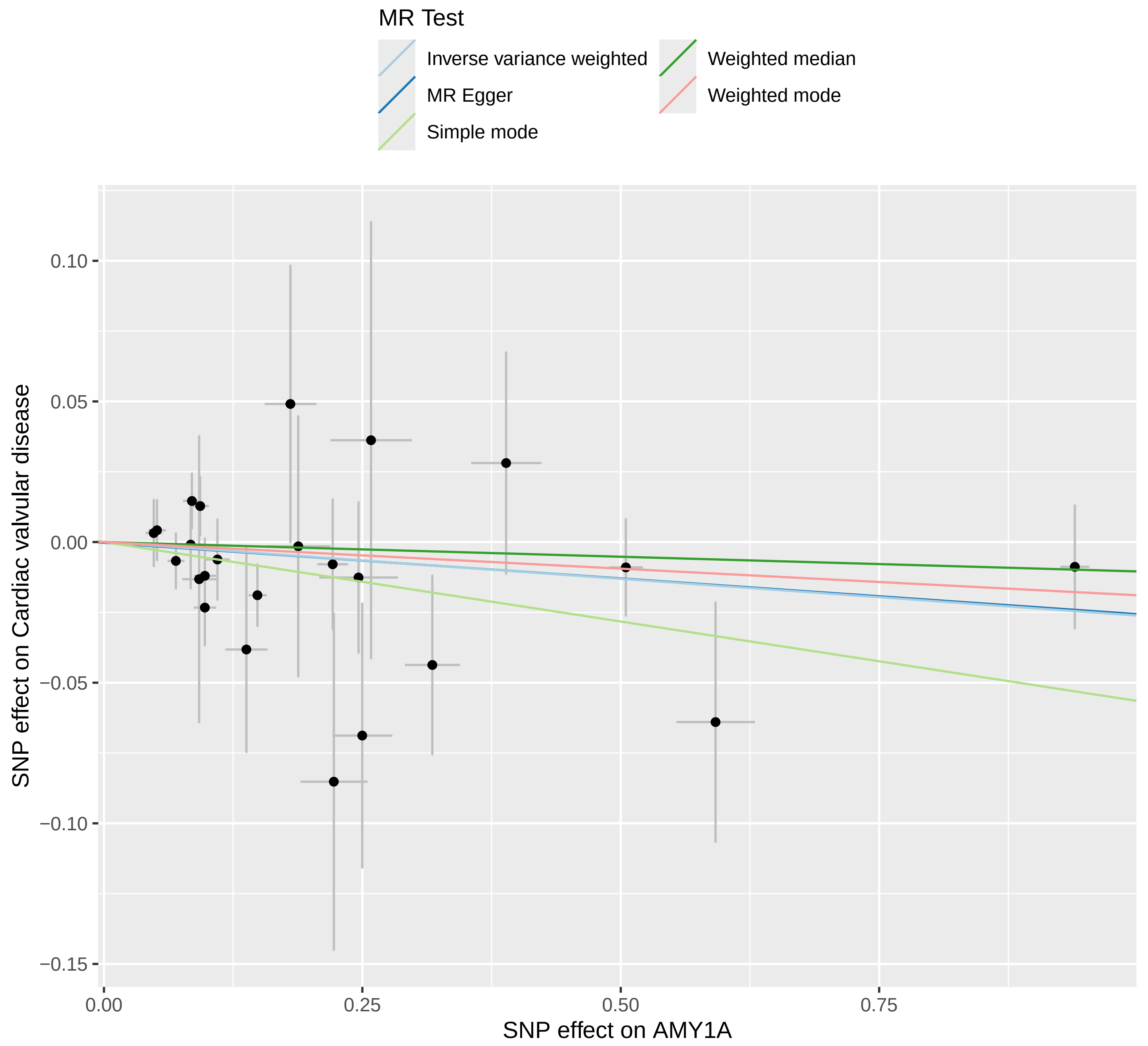
MR Test
Inverse variance weighted
MR Egger
Simple mode
Weighted median
Weighted mode
SNP effect on Cardiac valvular disease
SNP effect on AMY1A
0.10
0.05
0.00
−0.05
−0.10
−0.15
0.00
0.25
0.50
0.75

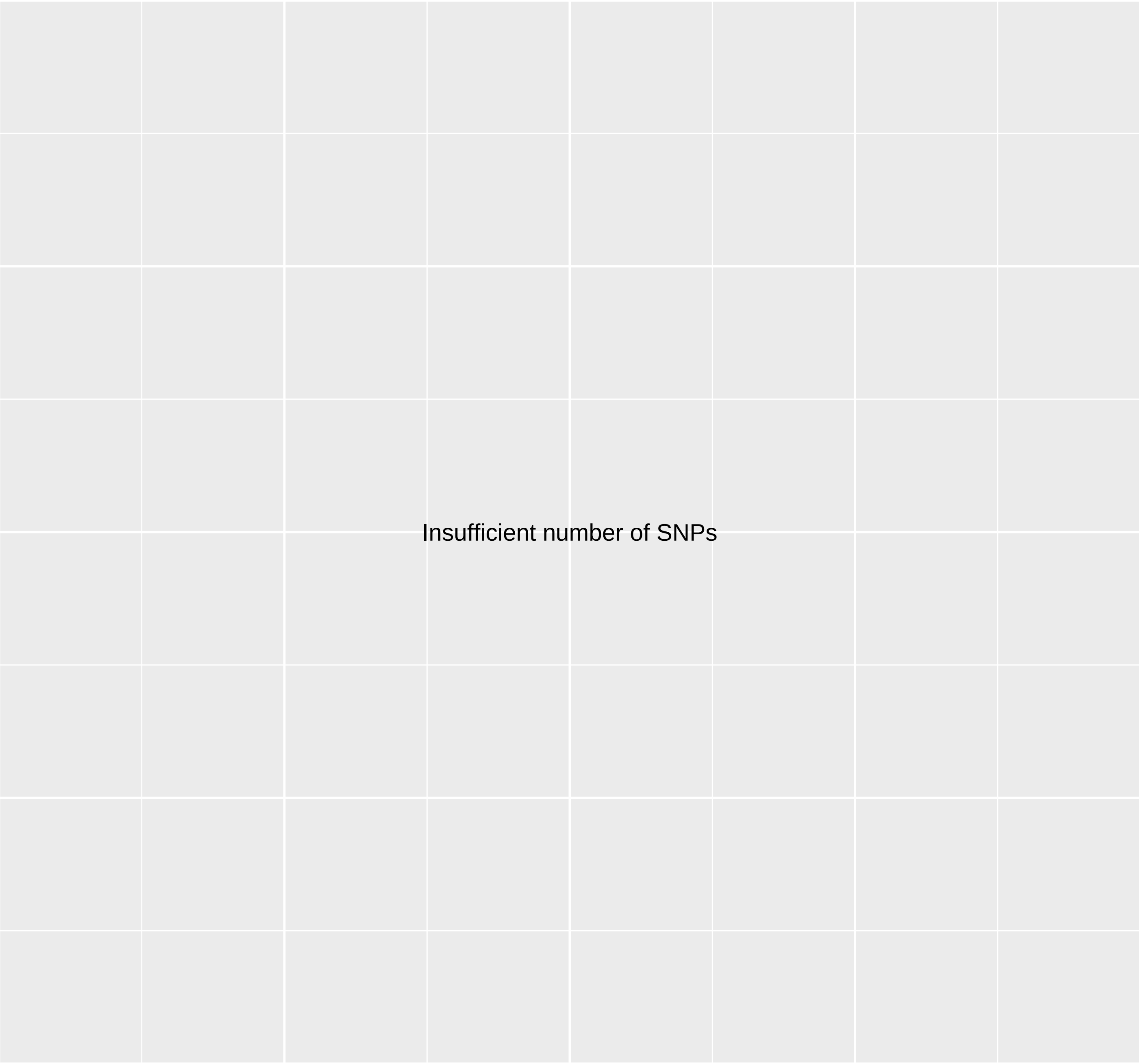
Insufficient number of SNPs

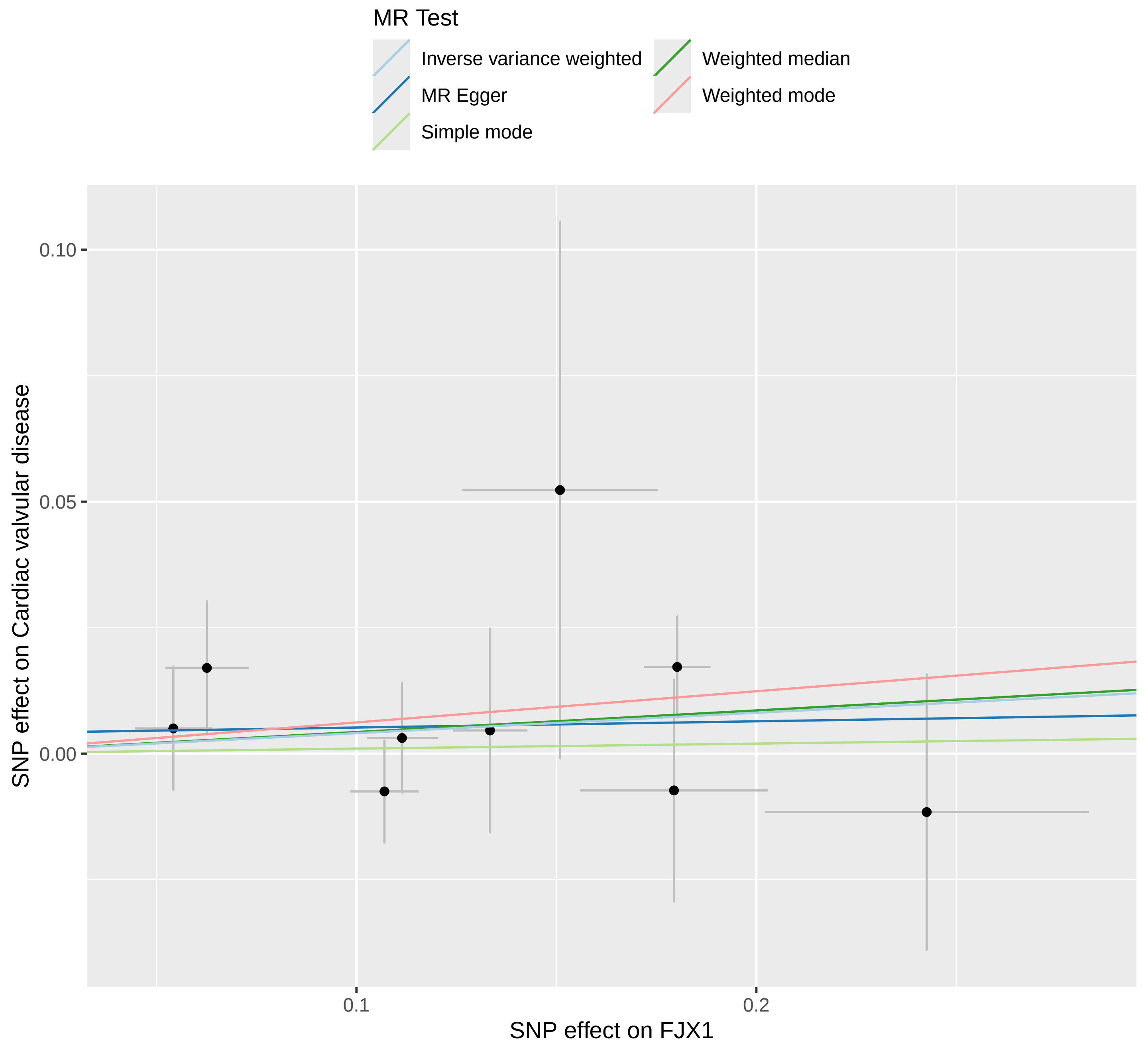

MR Test
Inverse variance weighted
MR Egger
Simple mode
Weighted median
Weighted mode
0.10
0.05
0.00
SNP effect on Cardiac valvular disease
0.1
0.2
SNP effect on FJX1

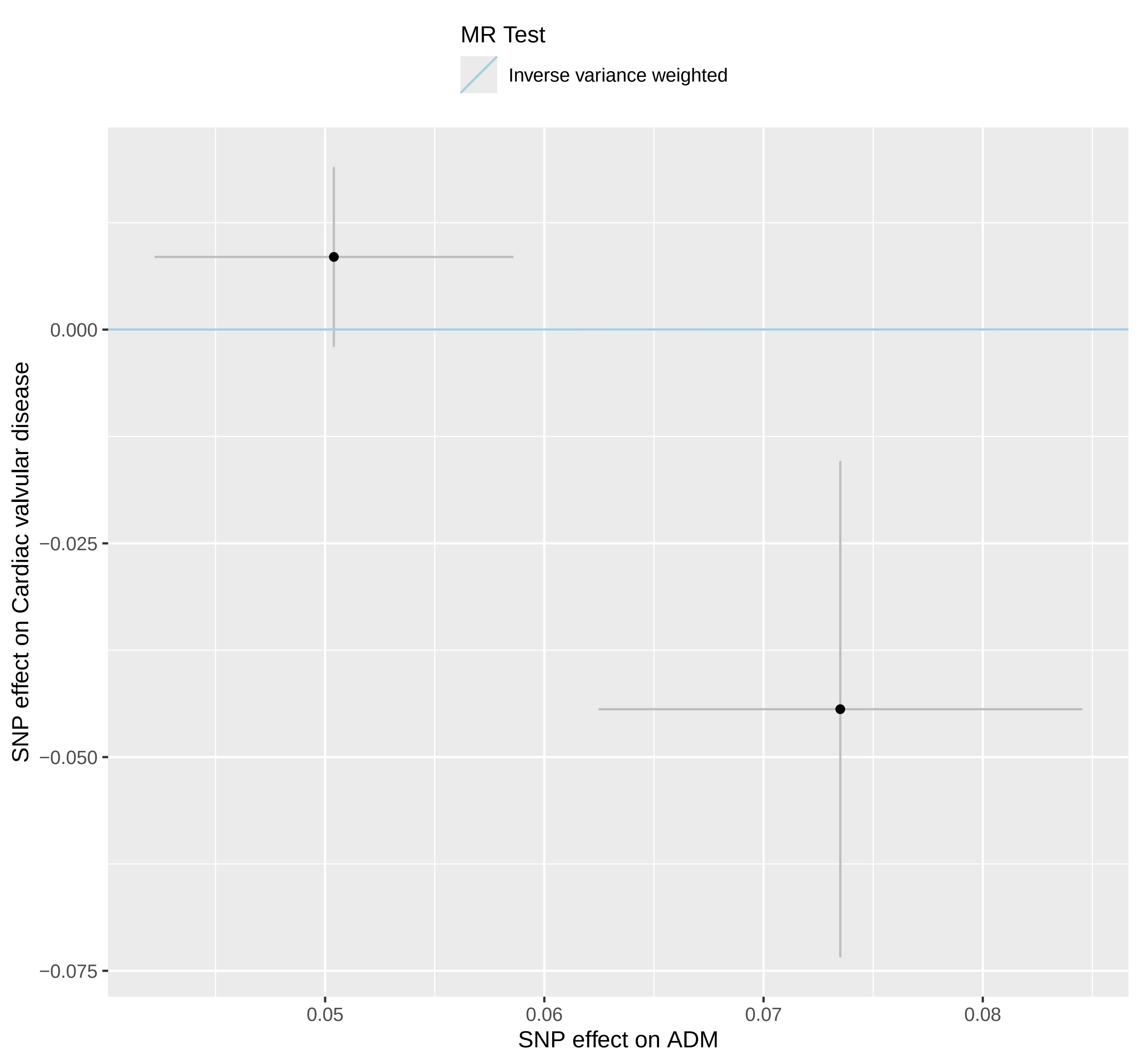
MR Test
Inverse variance weighted
0.000
−0.025
−0.050
−0.075
0.05
0.06
0.07
0.08
SNP effect on ADM
SNP effect on Cardiac valvular disease

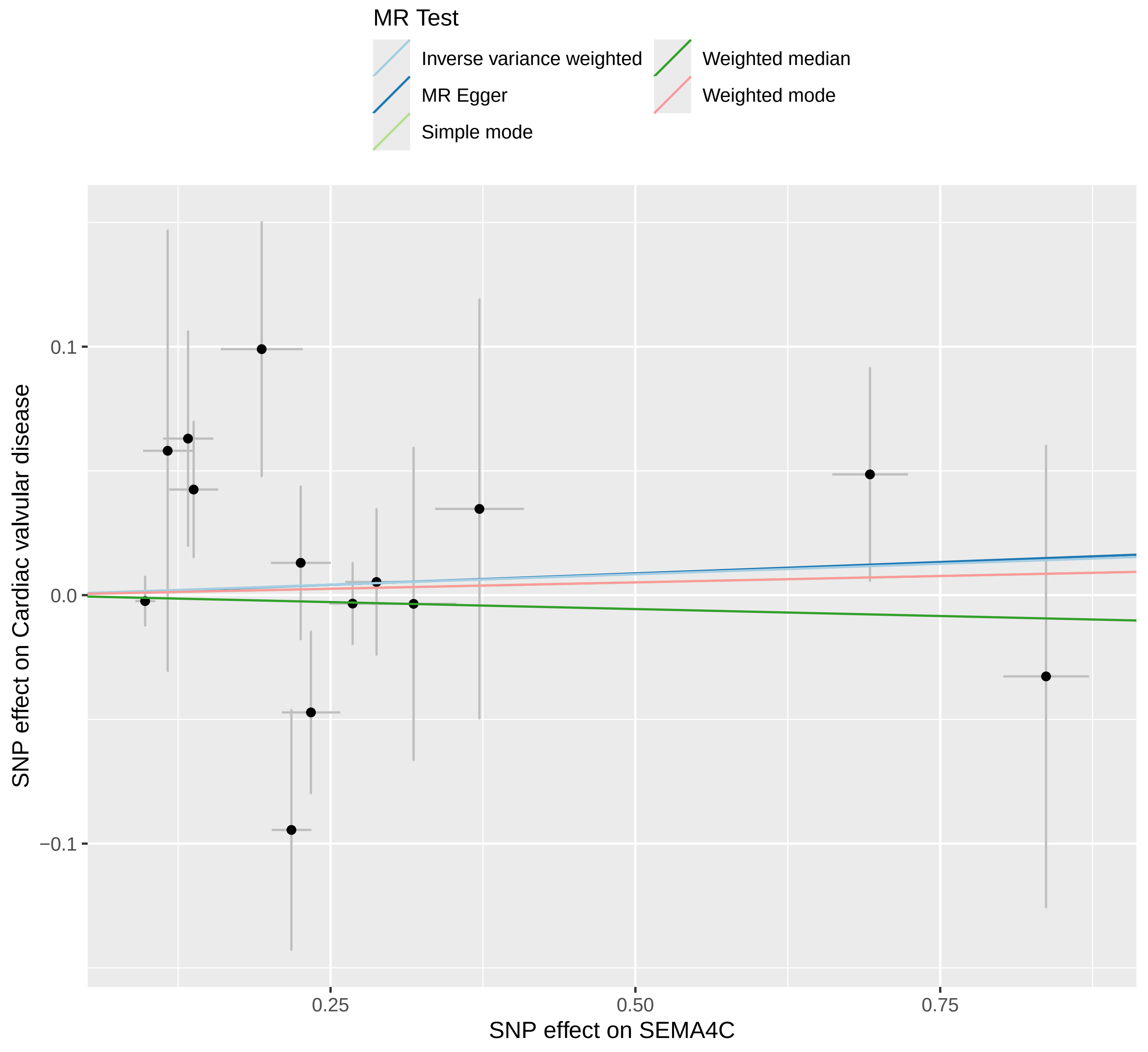
MR Test
Inverse variance weighted
MR Egger
Simple mode
Weighted median
Weighted mode
SNP effect on Cardiac valvular disease
0.1
0.0
−0.1
0.25
0.50
0.75
SNP effect on SEMA4C

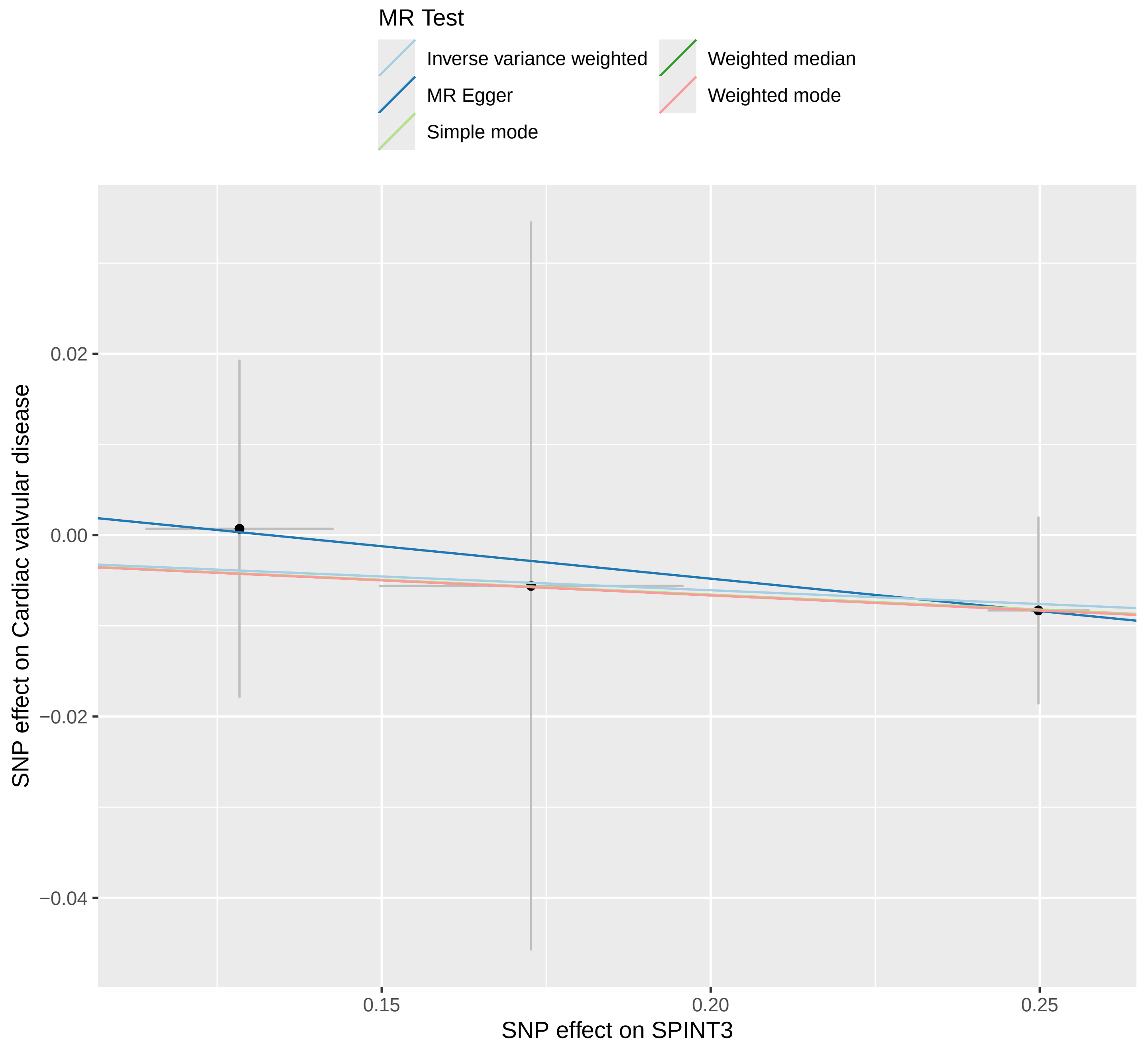
MR Test
Inverse variance weighted
MR Egger
Simple mode
Weighted median
Weighted mode
0.02
0.00
−0.02
−0.04
0.15
0.20
0.25
SNP effect on Cardiac valvular disease
SNP effect on SPINT3

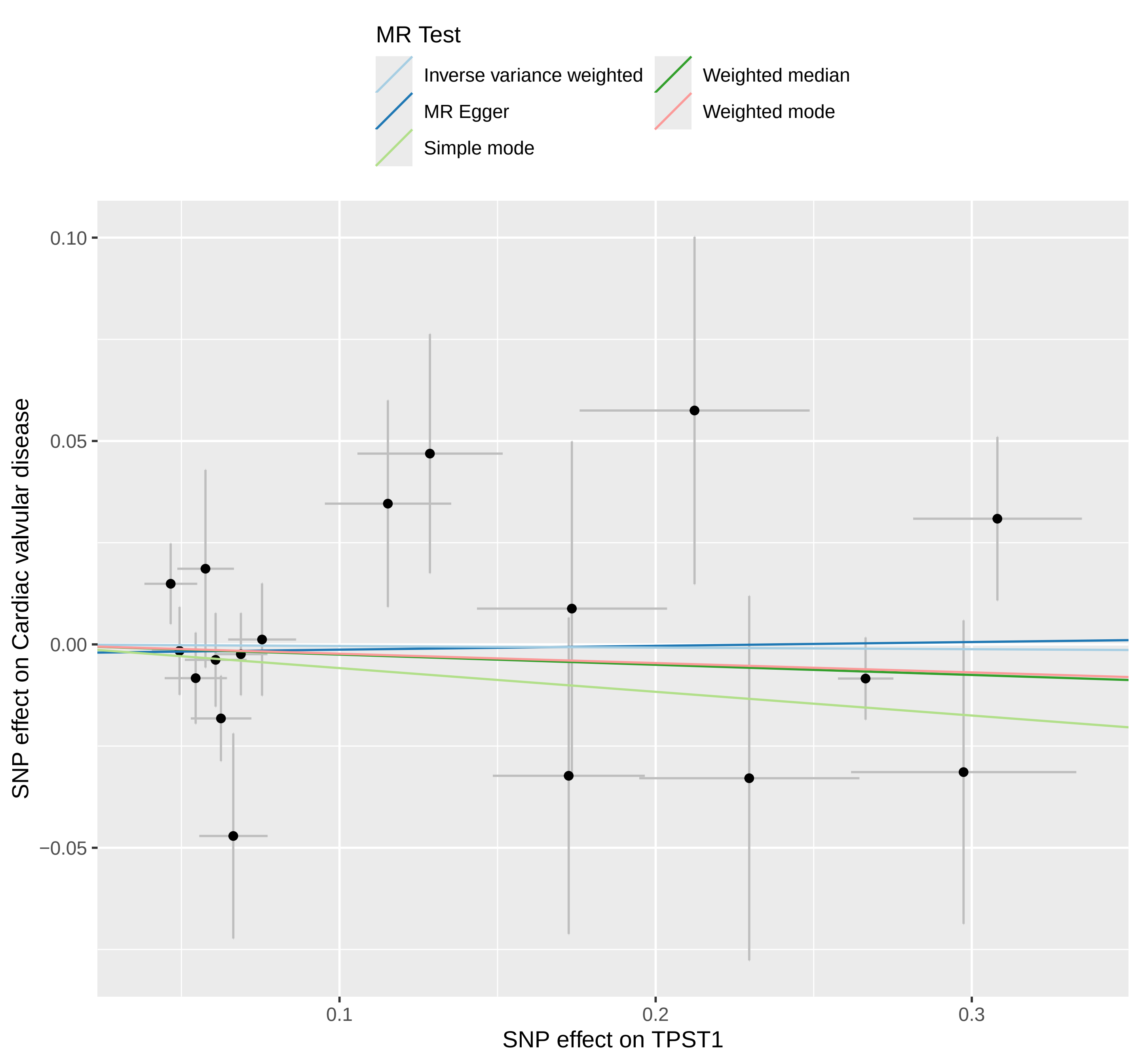
MR Test
Inverse variance weighted
MR Egger
Simple mode
Weighted median
Weighted mode
0.10
0.05
0.00
−0.05
SNP effect on Cardiac valvular disease
0.1
0.2
0.3
SNP effect on TPST1

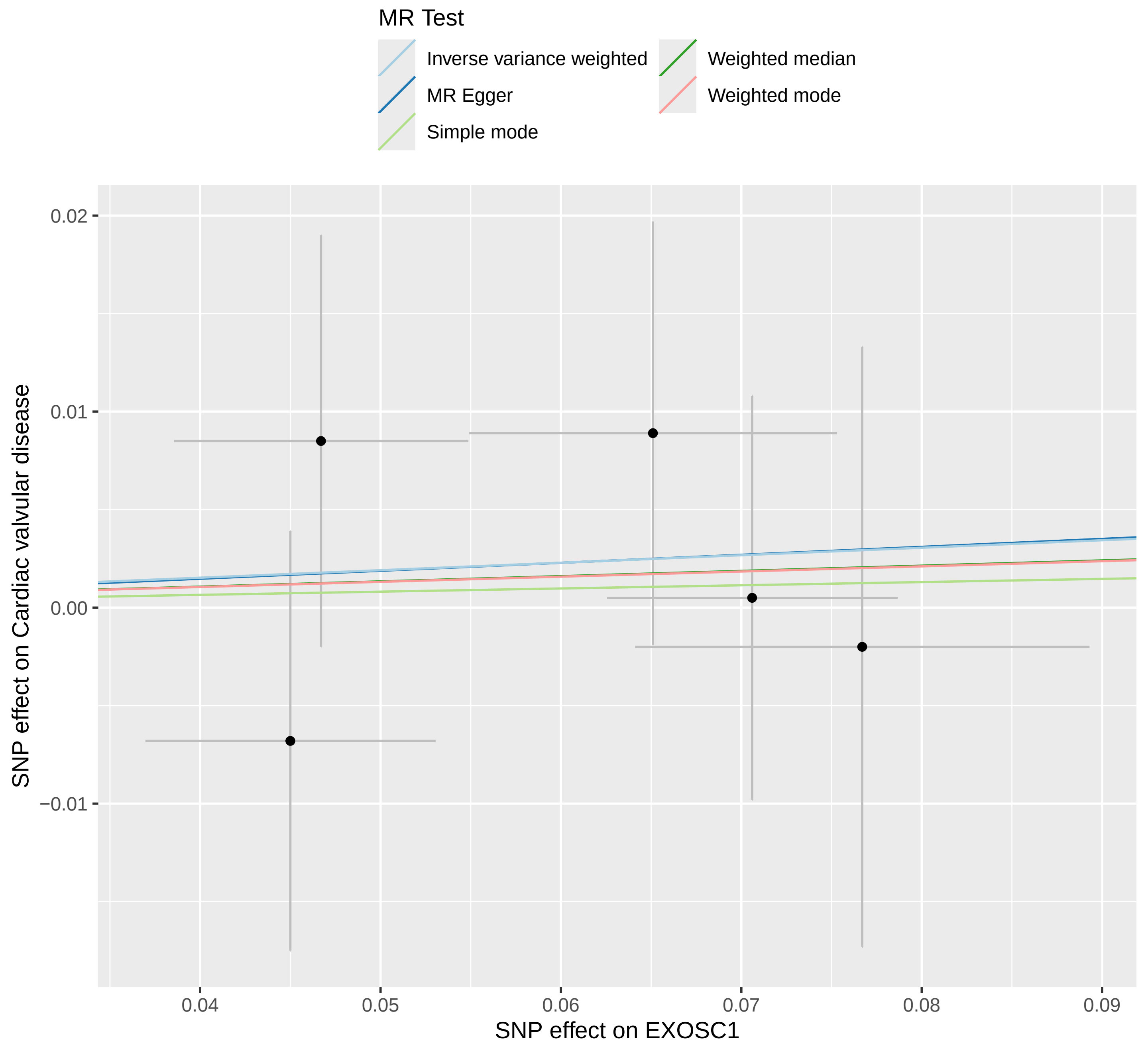
MR Test
Inverse variance weighted
MR Egger
Simple mode
Weighted median
Weighted mode
0.02
0.01
0.00
−0.01
0.04
0.05
0.06
0.07
0.08
0.09
SNP effect on Cardiac valvular disease
SNP effect on EXOSC1

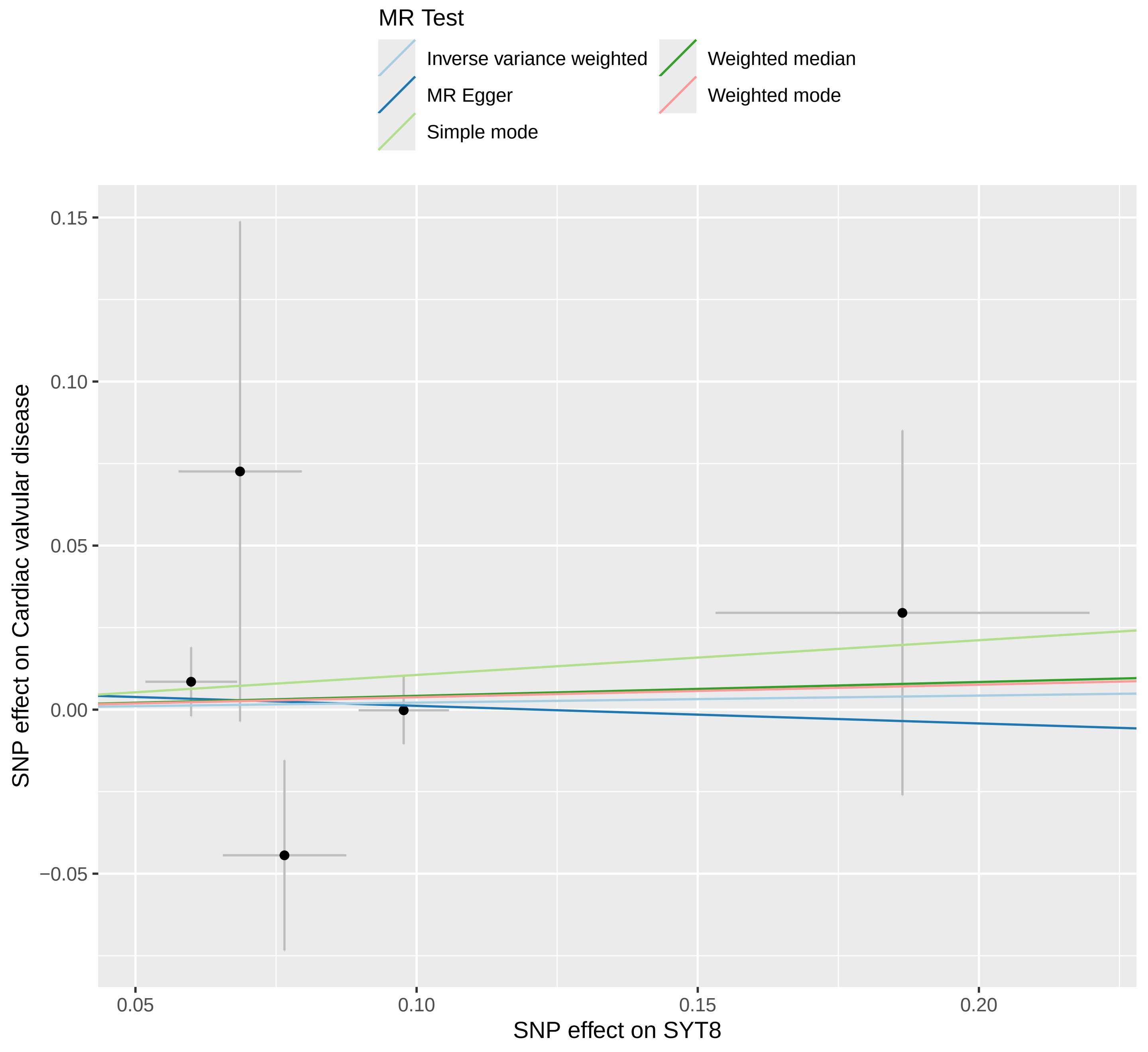
MR Test
Inverse variance weighted
MR Egger
Simple mode
Weighted median
Weighted mode
SNP effect on Cardiac valvular disease
0.15
0.10
0.05
0.00
−0.05
SNP effect on SYT8
0.05
0.10
0.15
0.20

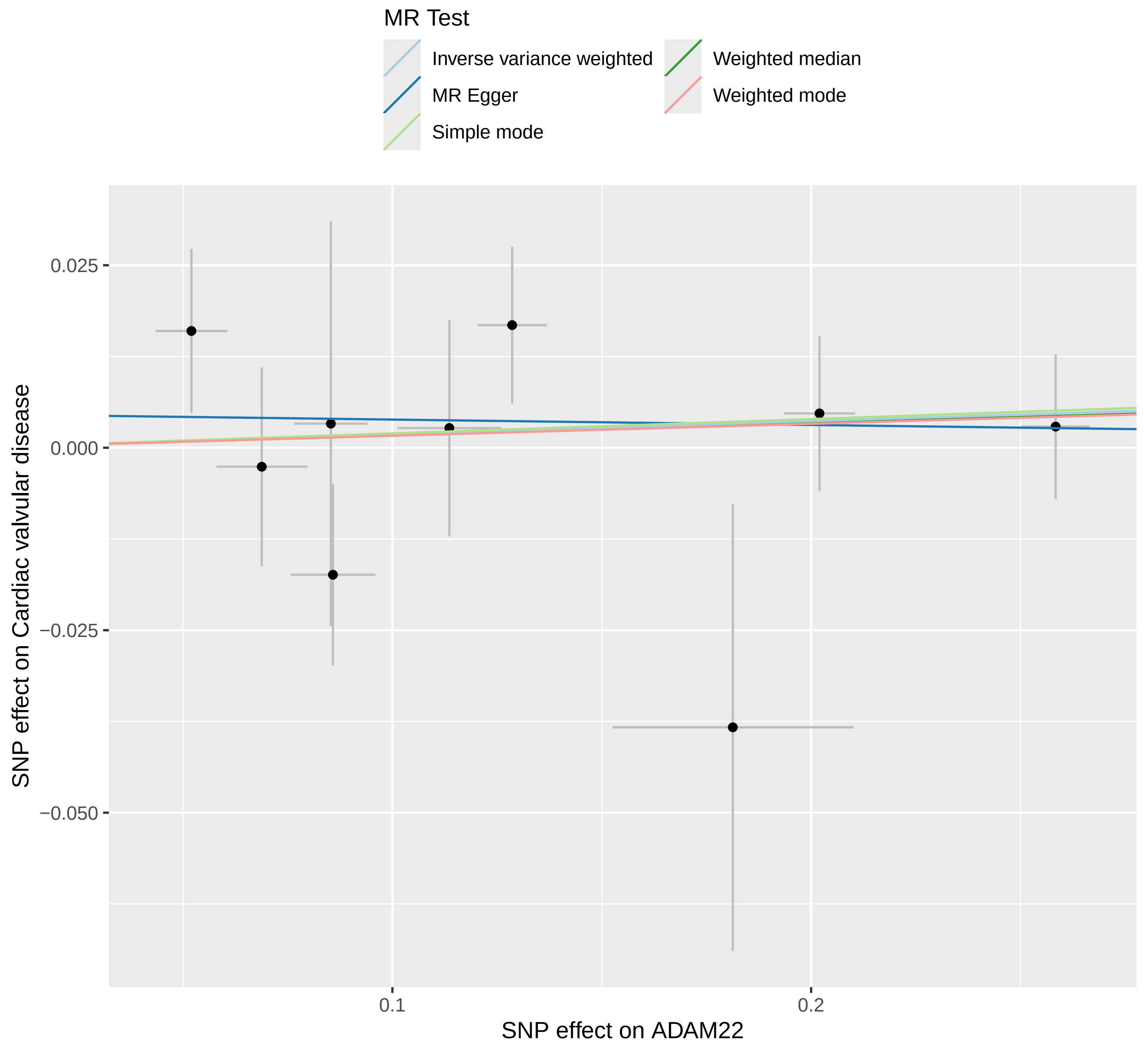
MR Test
Inverse variance weighted
MR Egger
Simple mode
Weighted median
Weighted mode
0.025
0.000
−0.025
−0.050
0.1
0.2
SNP effect on Cardiac valvular disease
SNP effect on ADAM22

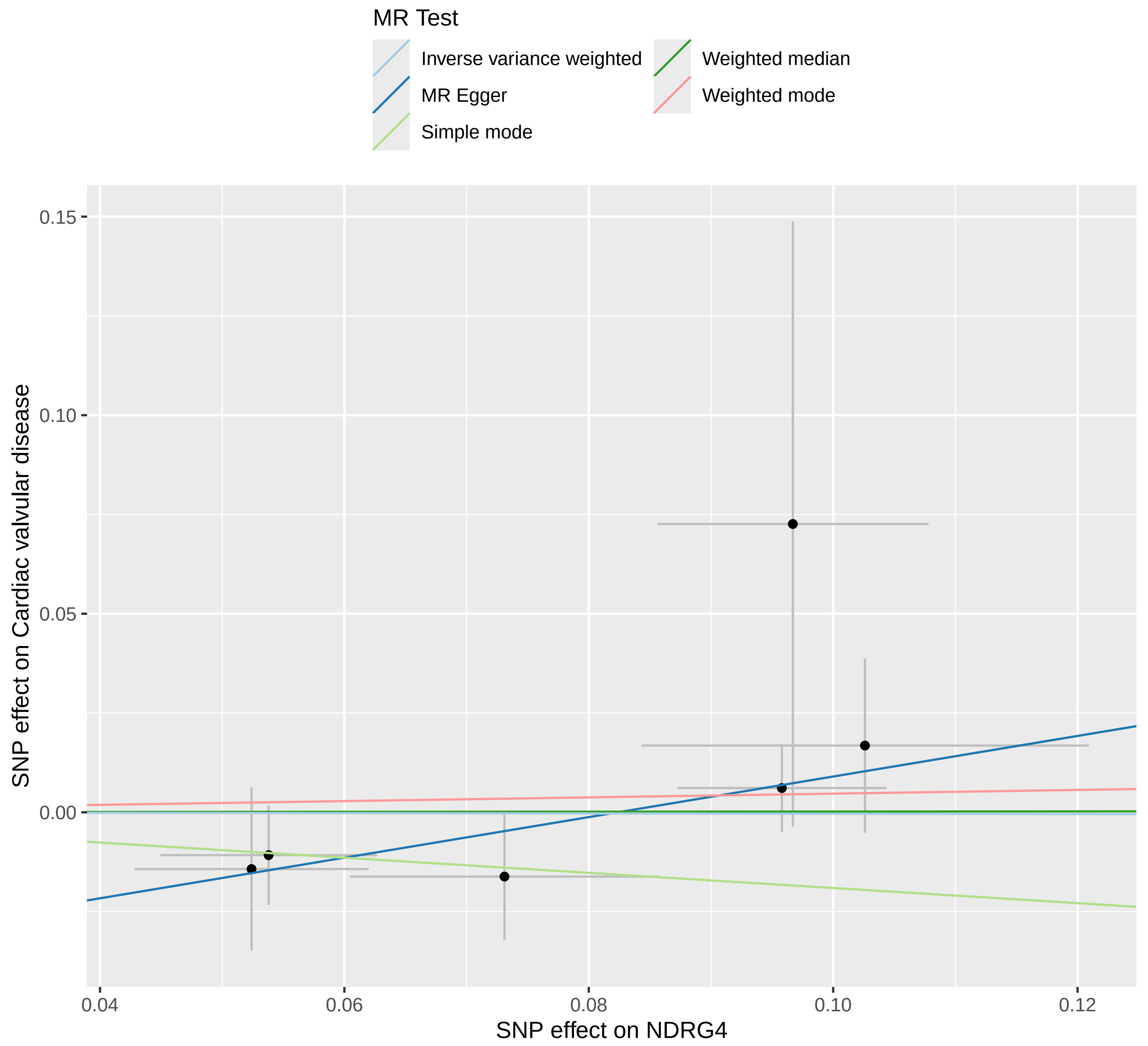

MR Test
Inverse variance weighted
MR Egger
Simple mode
Weighted median
Weighted mode
0.15
0.10
0.05
0.00
0.04
0.06
0.08
0.10
0.12
SNP effect on Cardiac valvular disease
SNP effect on NDRG4

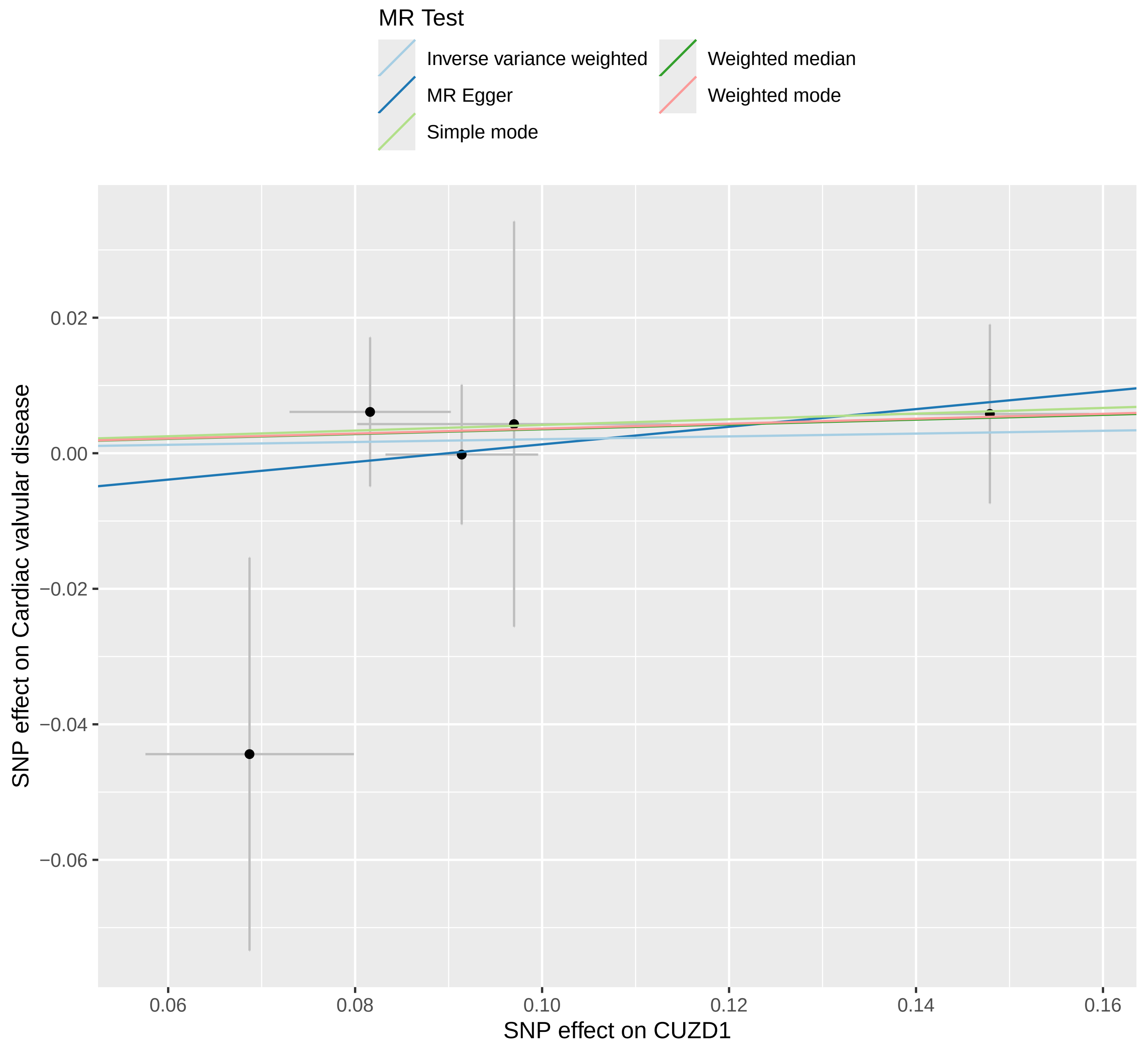
MR Test
Inverse variance weighted
MR Egger
Simple mode
Weighted median
Weighted mode
SNP effect on Cardiac valvular disease
0.02
0.00
−0.02
−0.04
−0.06
0.06
0.08
0.10
0.12
0.14
0.16
SNP effect on CUZD1

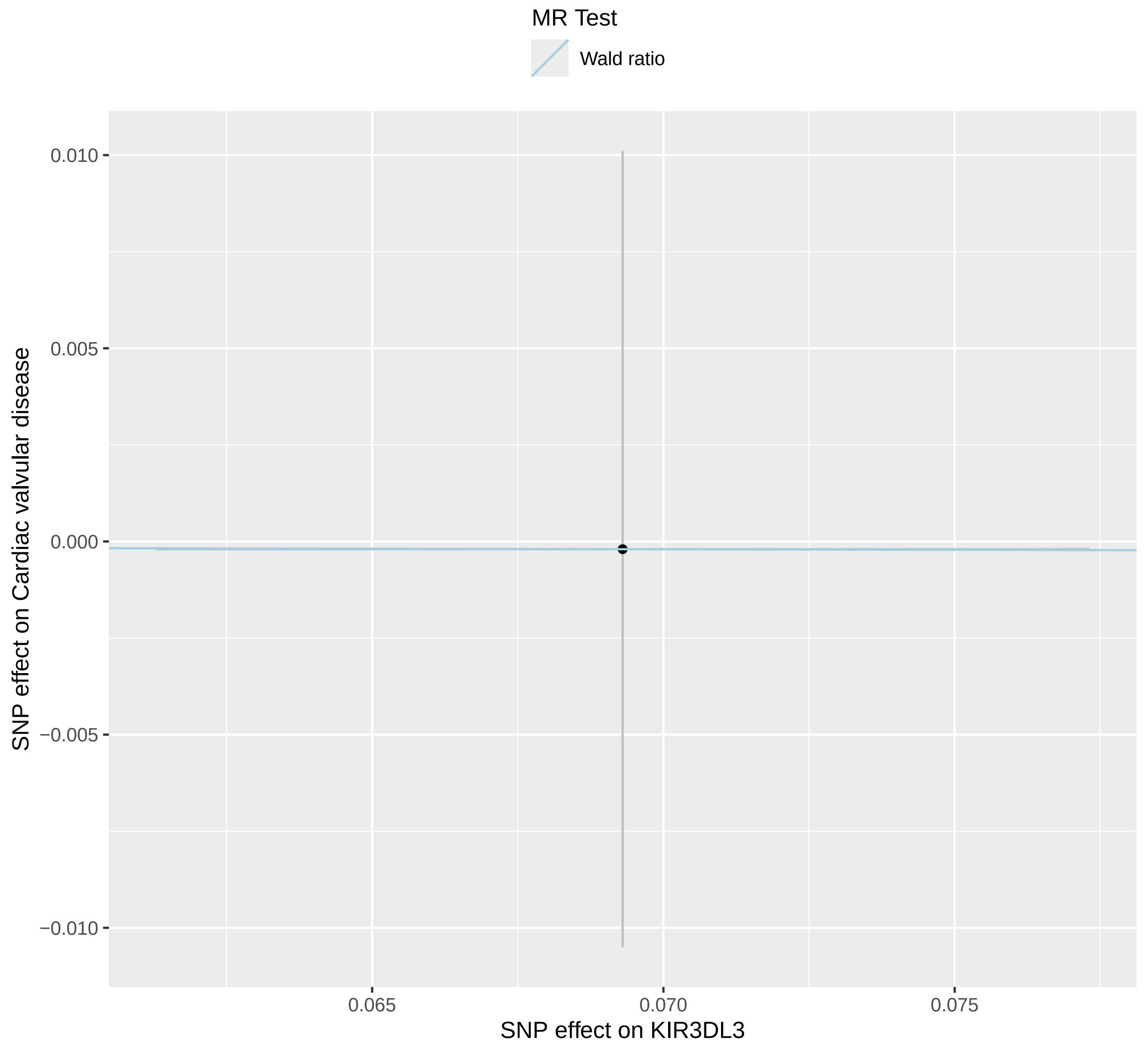

MR Test
Wald ratio
0.010
0.005
0.000
−0.005
−0.010
0.065
0.070
0.075
SNP effect on KIR3DL3
SNP effect on Cardiac valvular disease

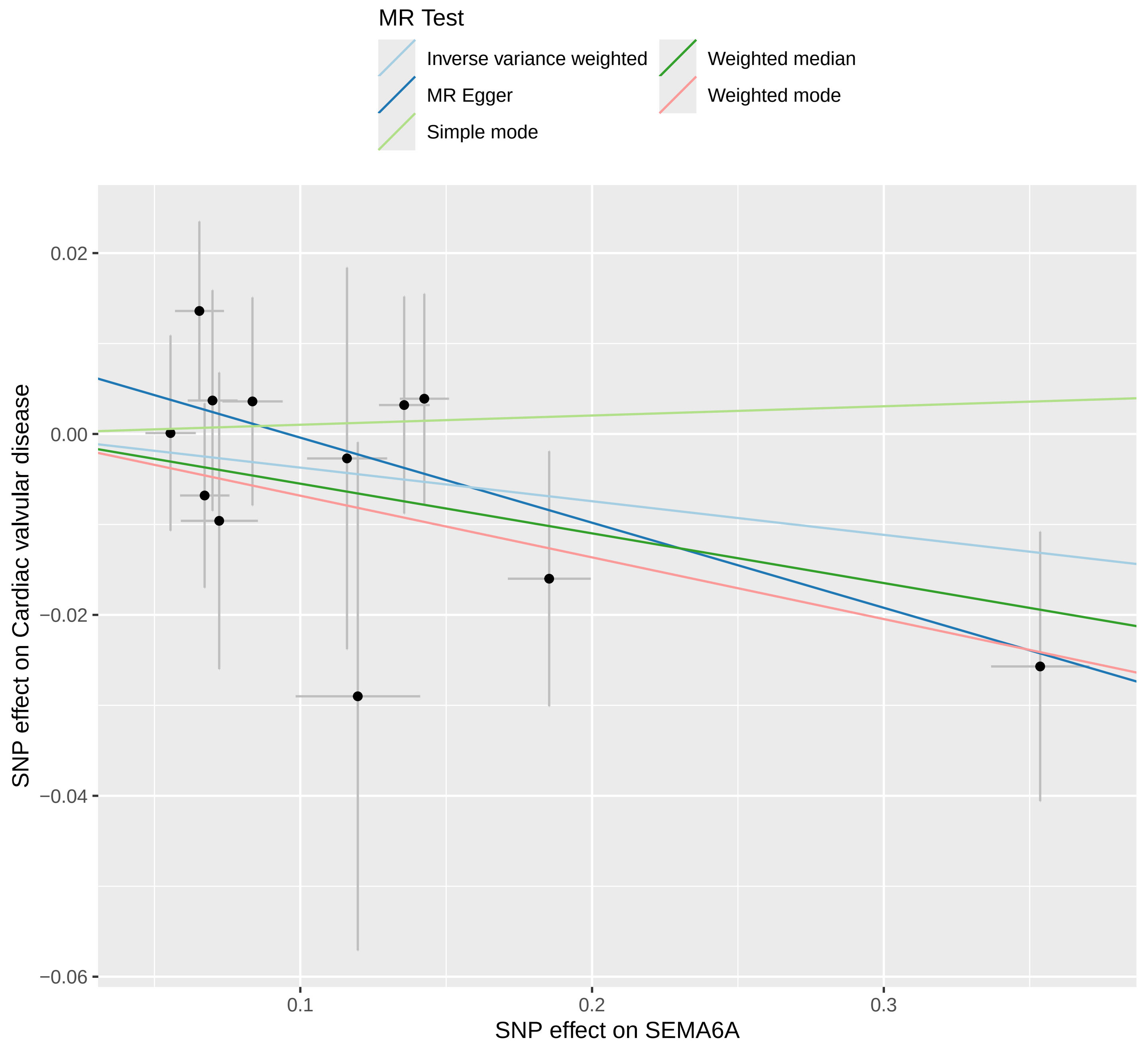
MR Test
Inverse variance weighted
MR Egger
Simple mode
Weighted median
Weighted mode
0.02
0.00
−0.02
−0.04
−0.06
SNP effect on Cardiac valvular disease
0.1
0.2
0.3
SNP effect on SEMA6A

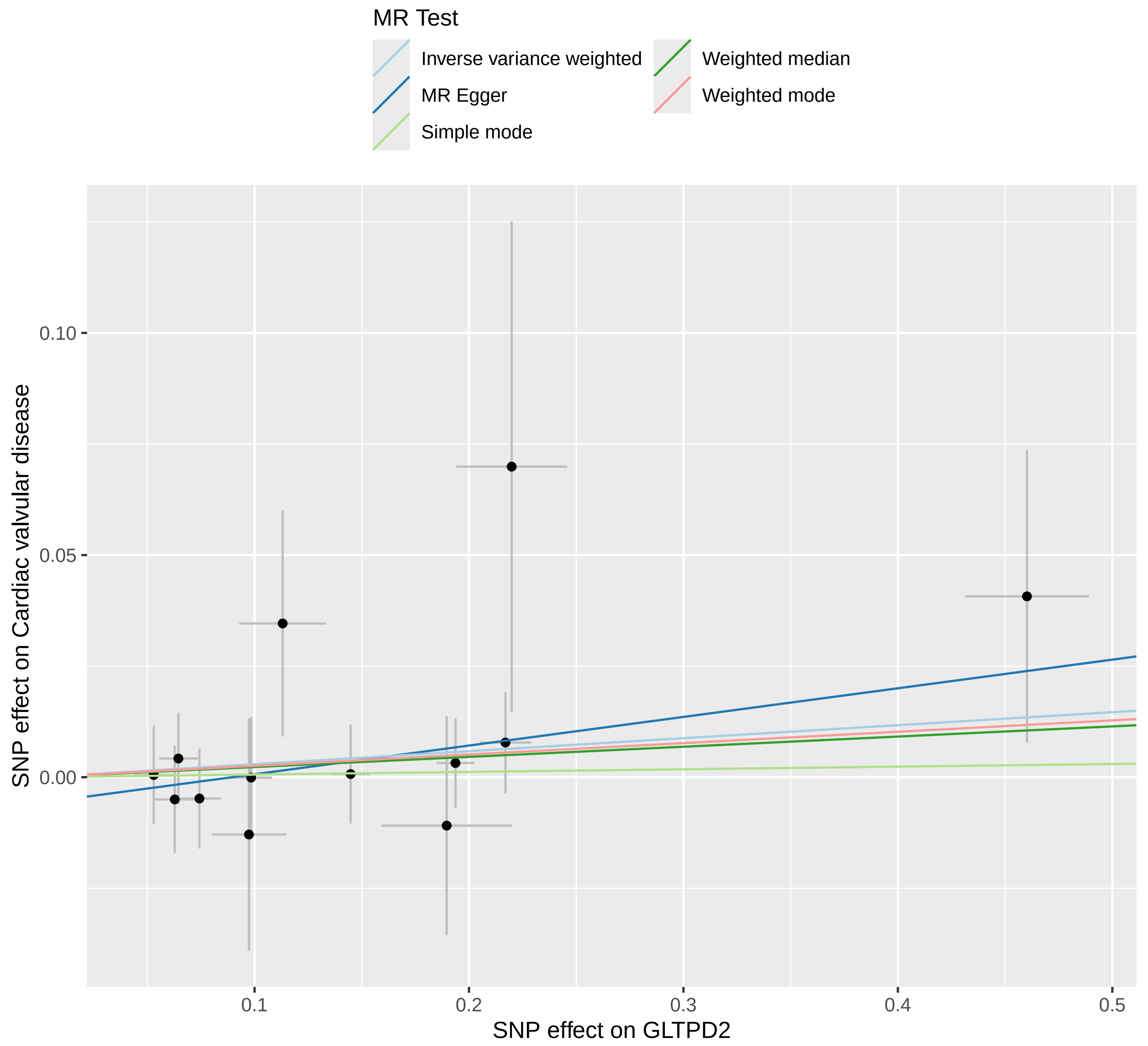
MR Test
Inverse variance weighted
MR Egger
Simple mode
Weighted median
Weighted mode
SNP effect on Cardiac valvular disease
0.10
0.05
0.00
0.1
0.2
0.3
0.4
0.5
SNP effect on GLTPD2

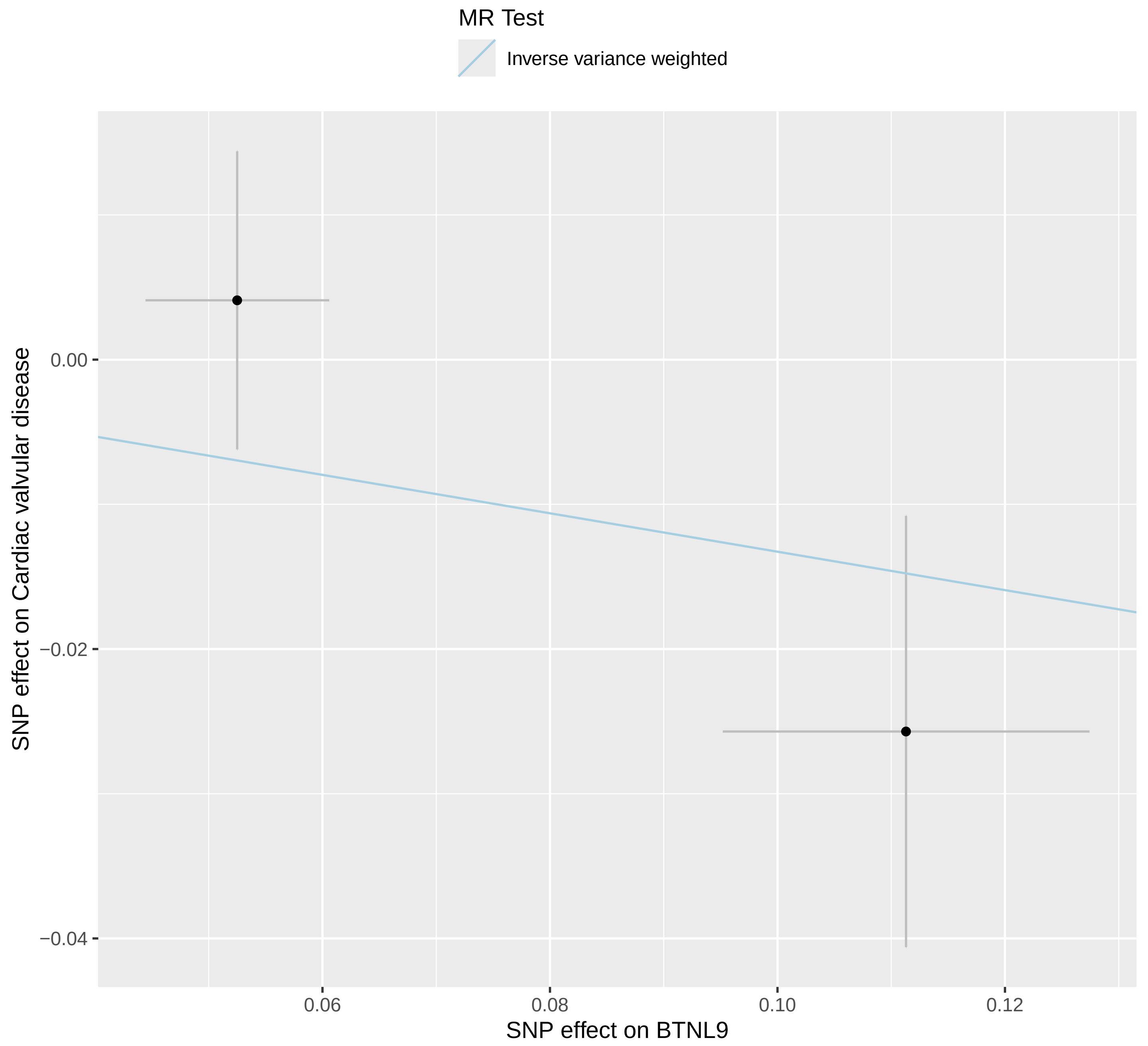

MR Test
Inverse variance weighted
SNP effect on Cardiac valvular disease
0.00
−0.02
−0.04
0.06
0.08
0.10
0.12
SNP effect on BTNL9

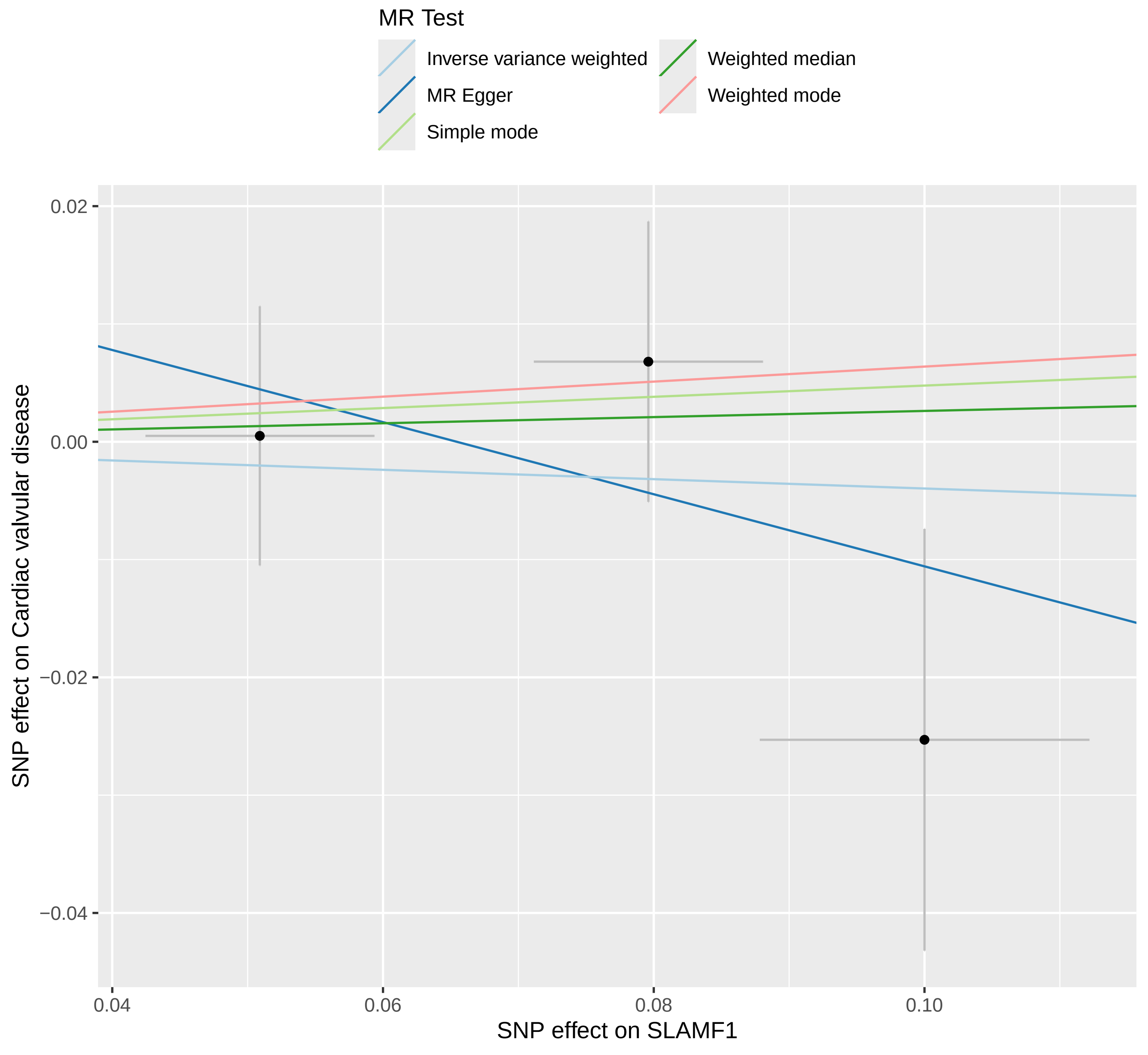

MR Test
Inverse variance weighted
MR Egger
Simple mode
Weighted median
Weighted mode
0.02
0.00
−0.02
−0.04
0.04
0.06
0.08
0.10
SNP effect on Cardiac valvular disease
SNP effect on SLAMF1

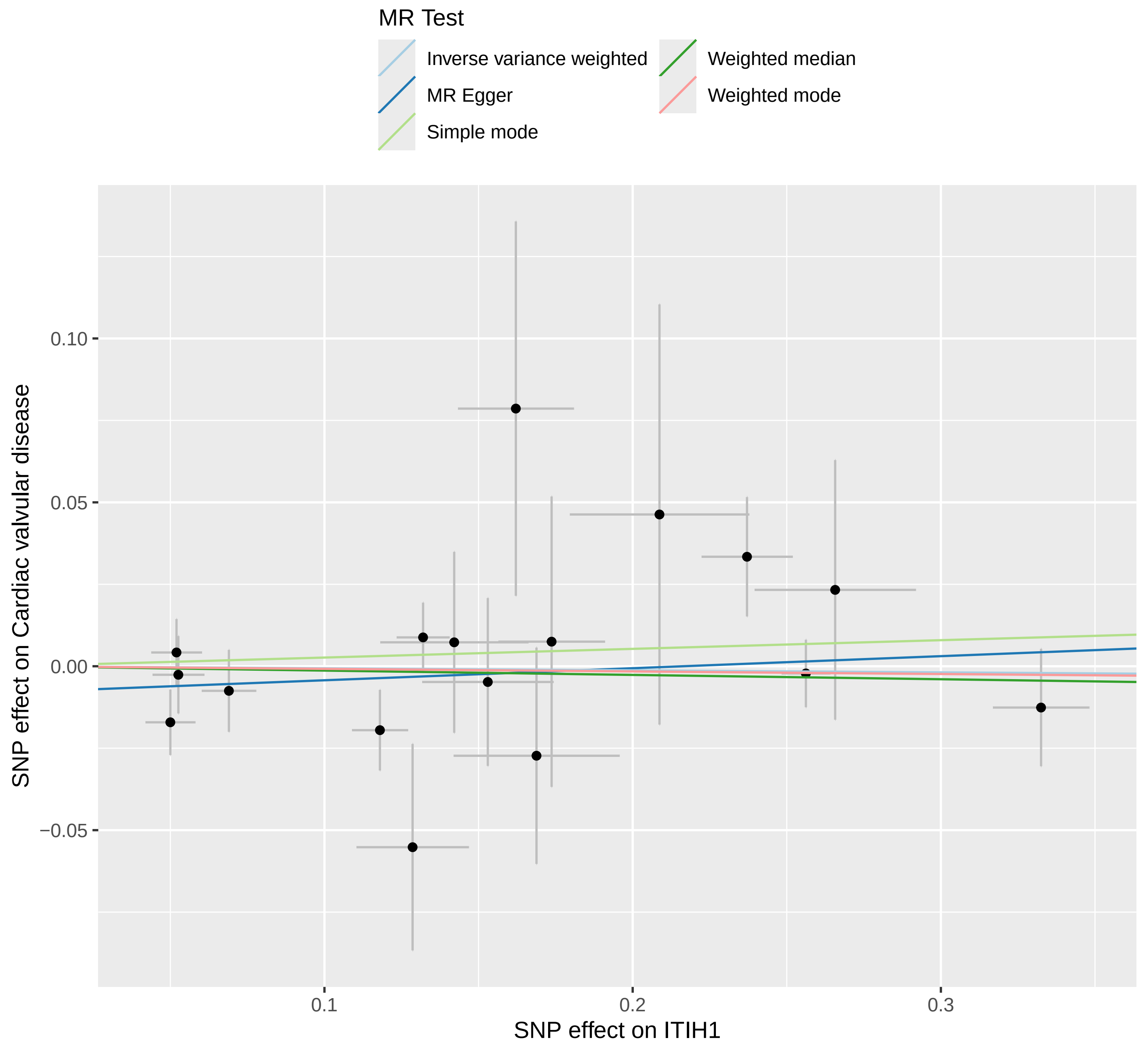
MR Test
Inverse variance weighted
MR Egger
Simple mode
Weighted median
Weighted mode
SNP effect on Cardiac valvular disease
0.10
0.05
0.00
−0.05
0.1
0.2
0.3
SNP effect on ITIH1

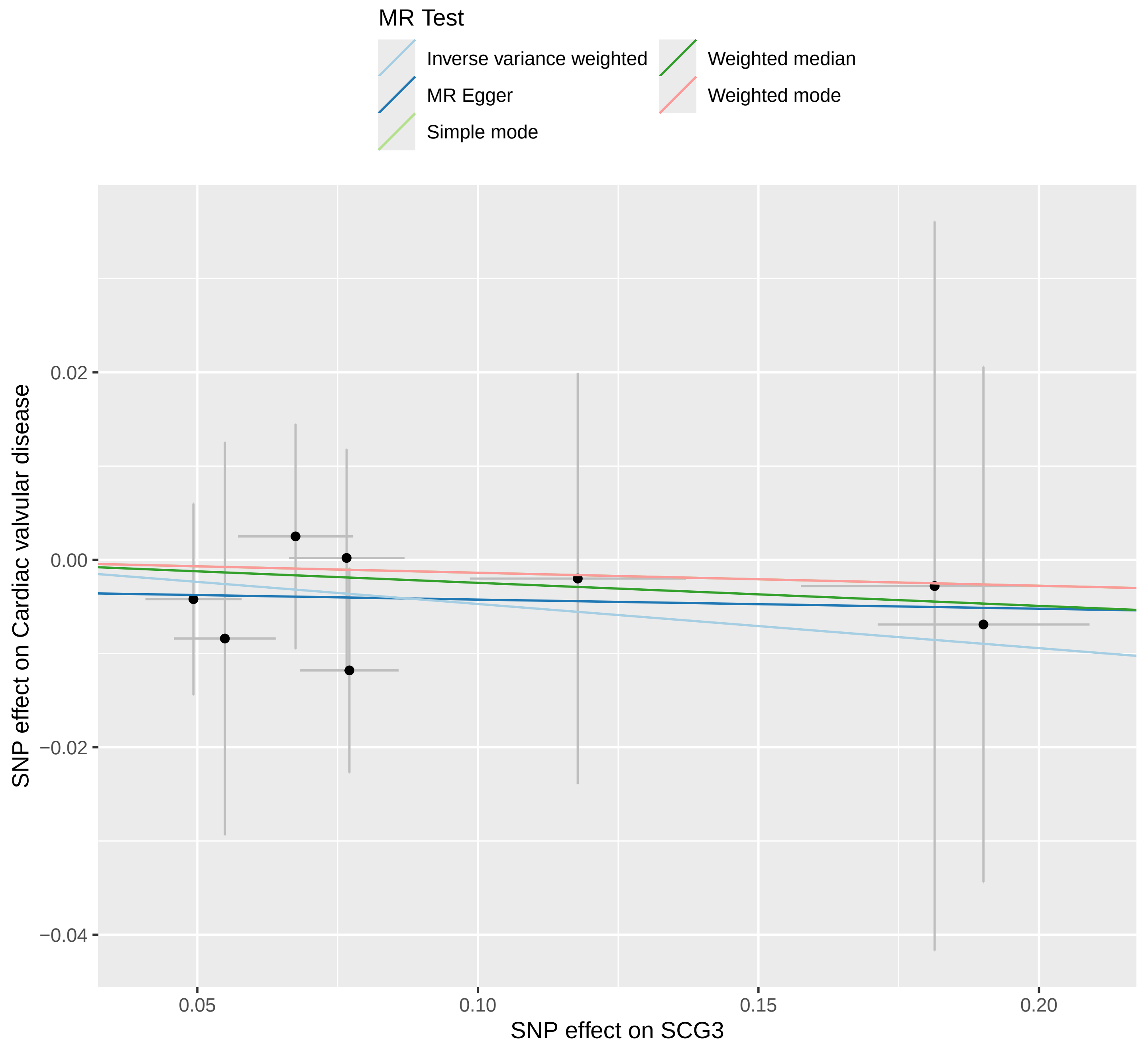
MR Test
Inverse variance weighted
MR Egger
Simple mode
Weighted median
Weighted mode
0.02
0.00
−0.02
−0.04
SNP effect on Cardiac valvular disease
0.05
0.10
0.15
0.20
SNP effect on SCG3

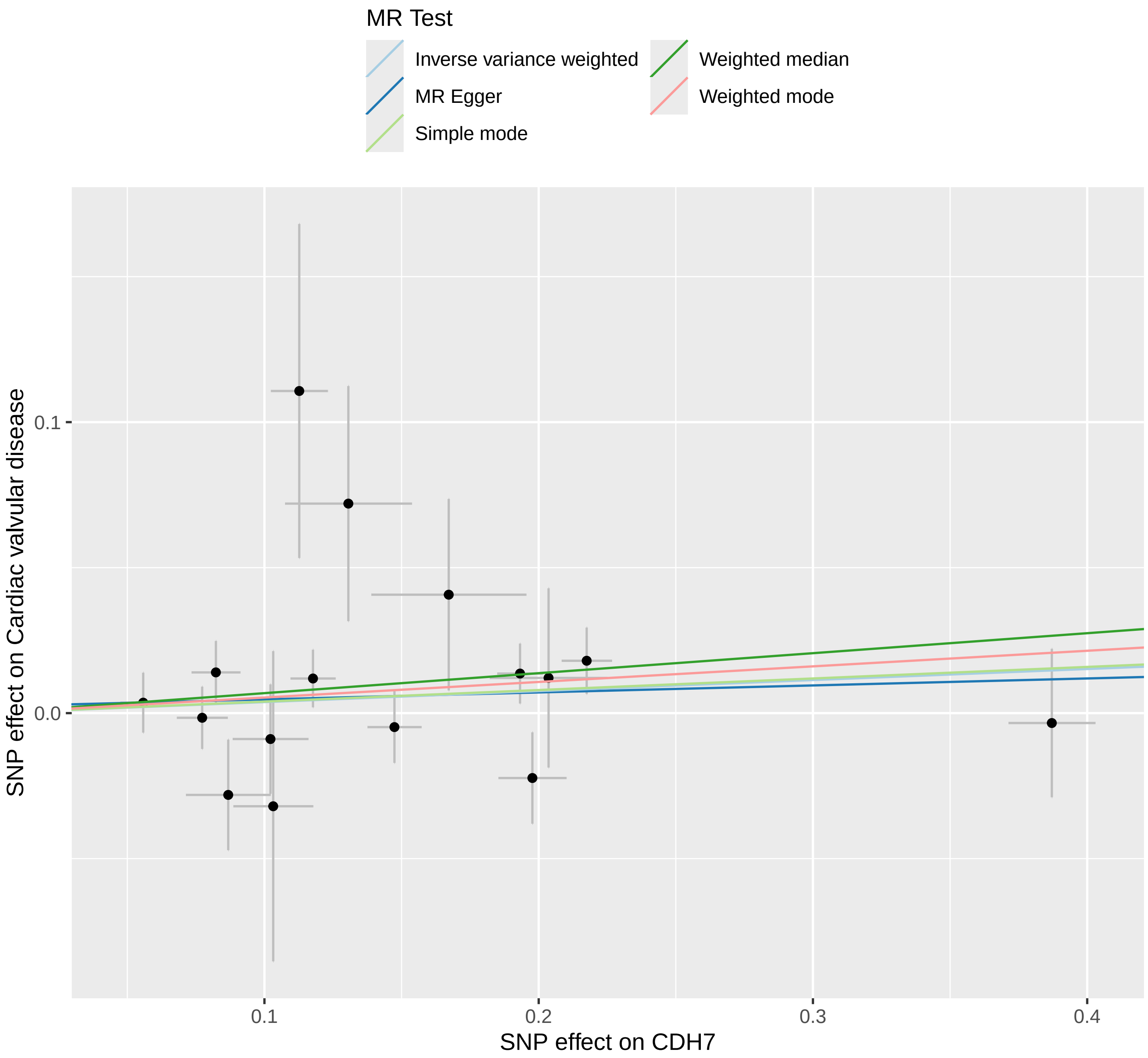
MR Test
Inverse variance weighted
MR Egger
Simple mode
Weighted median
Weighted mode
SNP effect on Cardiac valvular disease
0.1
0.0
0.1
0.2
0.3
0.4
SNP effect on CDH7

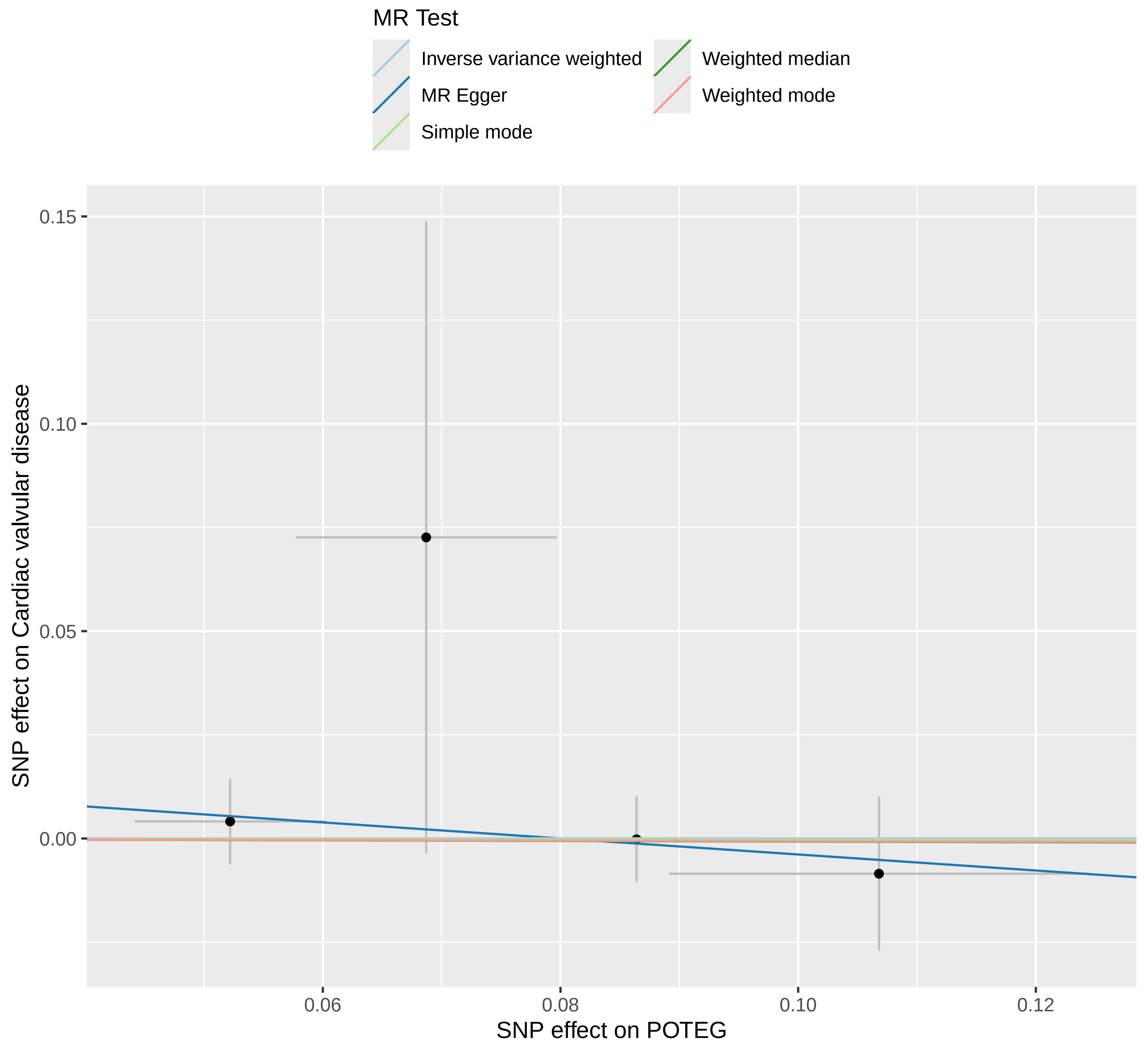
MR Test
Inverse variance weighted
MR Egger
Simple mode
Weighted median
Weighted mode
0.15
0.10
0.05
0.00
0.06
0.08
0.10
0.12
SNP effect on Cardiac valvular disease
SNP effect on POTEG

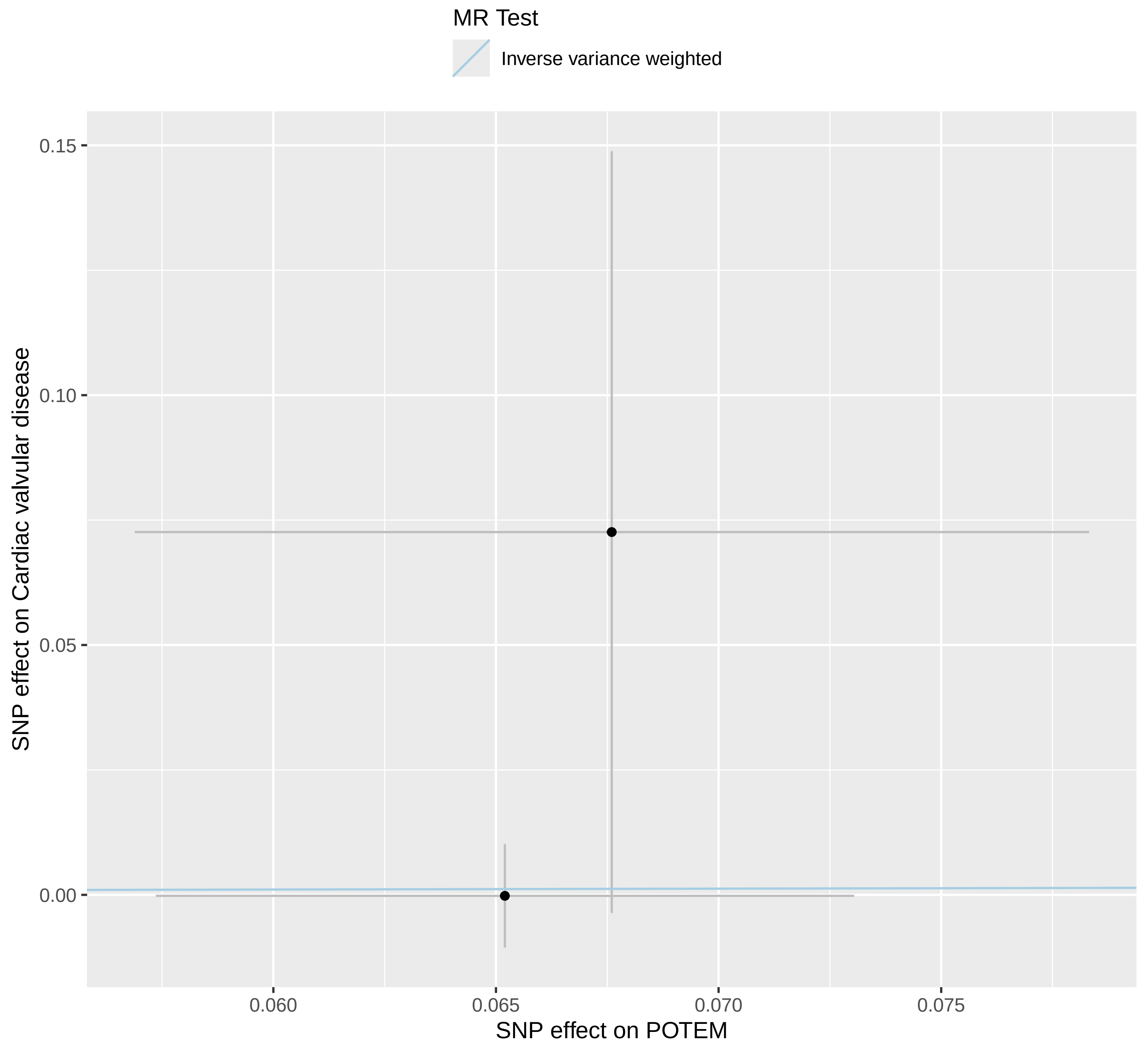

MR Test
Inverse variance weighted
0.15
0.10
0.05
0.00
0.060
0.065
0.070
0.075
SNP effect on POTEM
SNP effect on Cardiac valvular disease

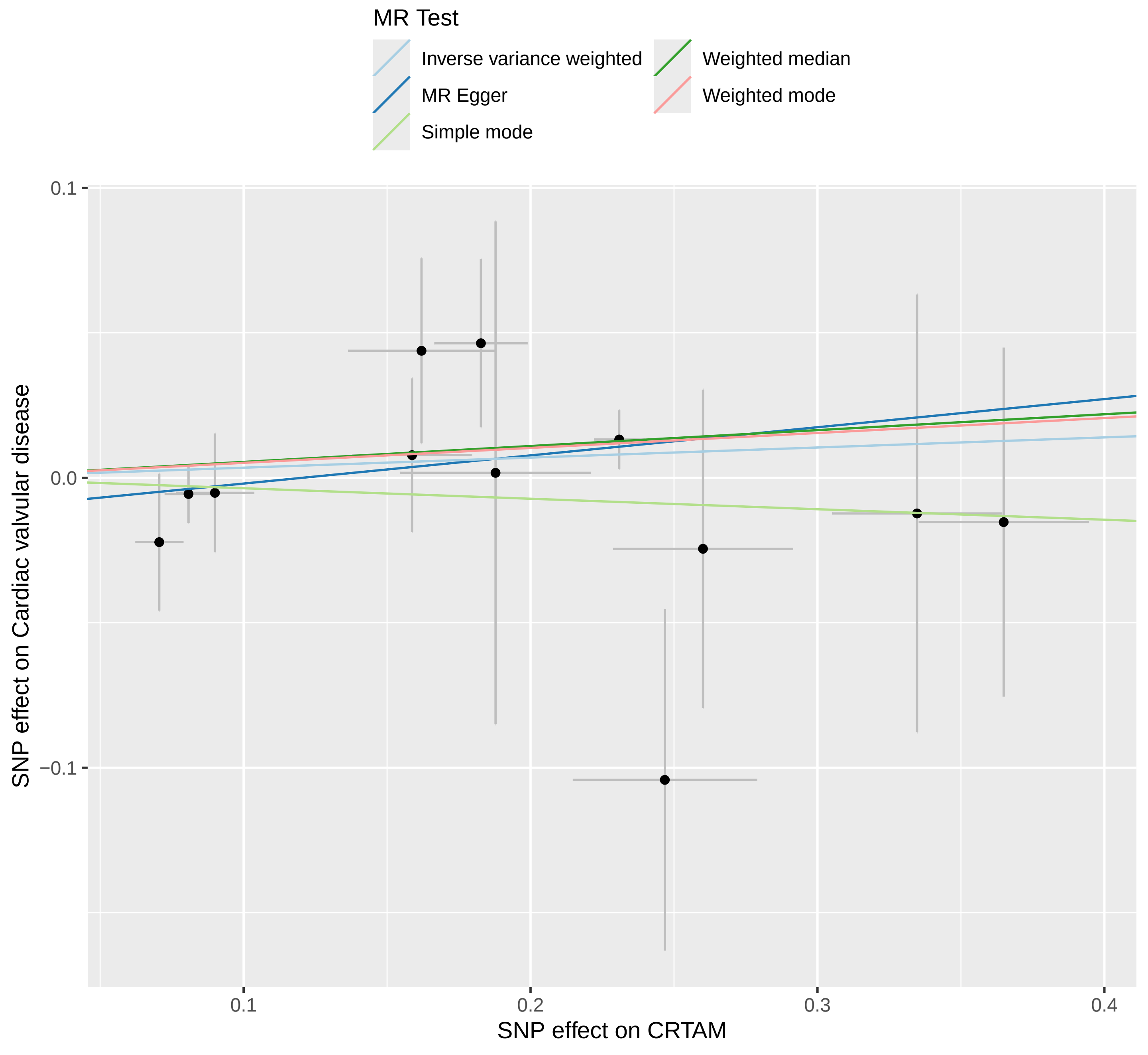
MR Test
Inverse variance weighted
MR Egger
Simple mode
Weighted median
Weighted mode
0.1
0.0
−0.1
0.1
0.2
0.3
0.4
SNP effect on Cardiac valvular disease
SNP effect on CRTAM

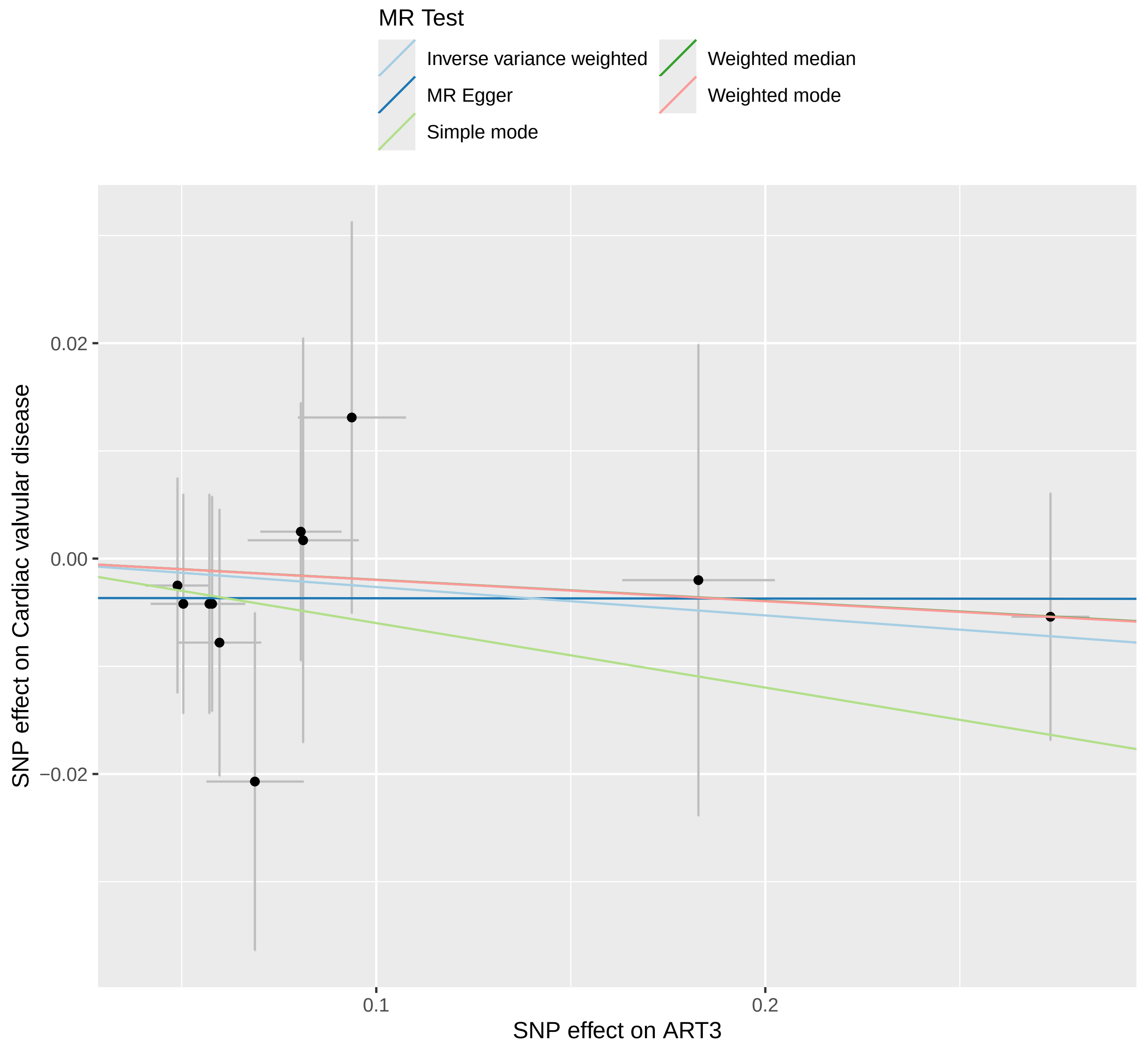

MR Test
Inverse variance weighted
MR Egger
Simple mode
Weighted median
Weighted mode
SNP effect on Cardiac valvular disease
0.02
0.00
−0.02
0.1
0.2
SNP effect on ART3

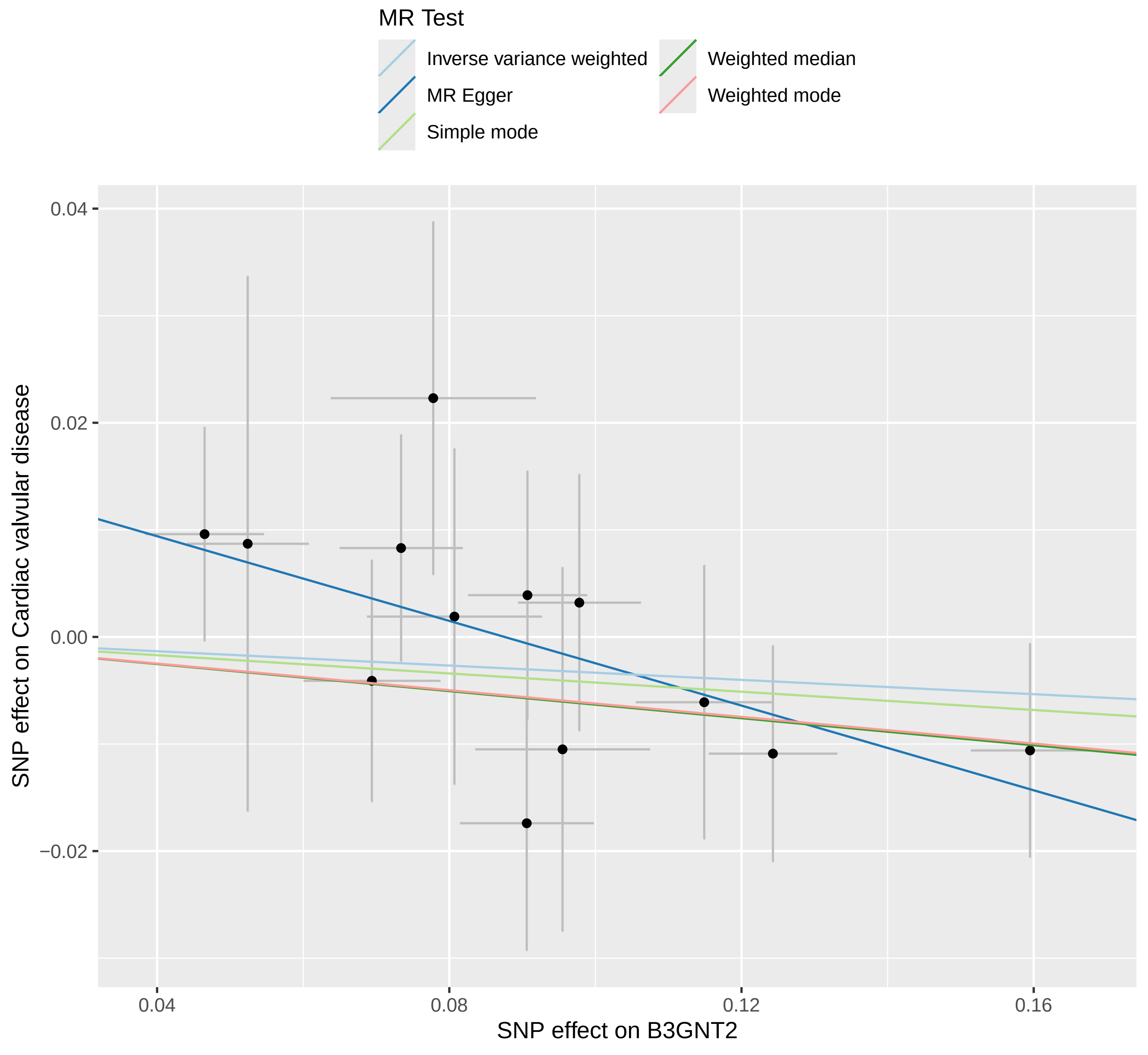
MR Test
Inverse variance weighted
MR Egger
Simple mode
Weighted median
Weighted mode
0.04
0.02
0.00
−0.02
0.04
0.08
0.12
0.16
SNP effect on Cardiac valvular disease
SNP effect on B3GNT2

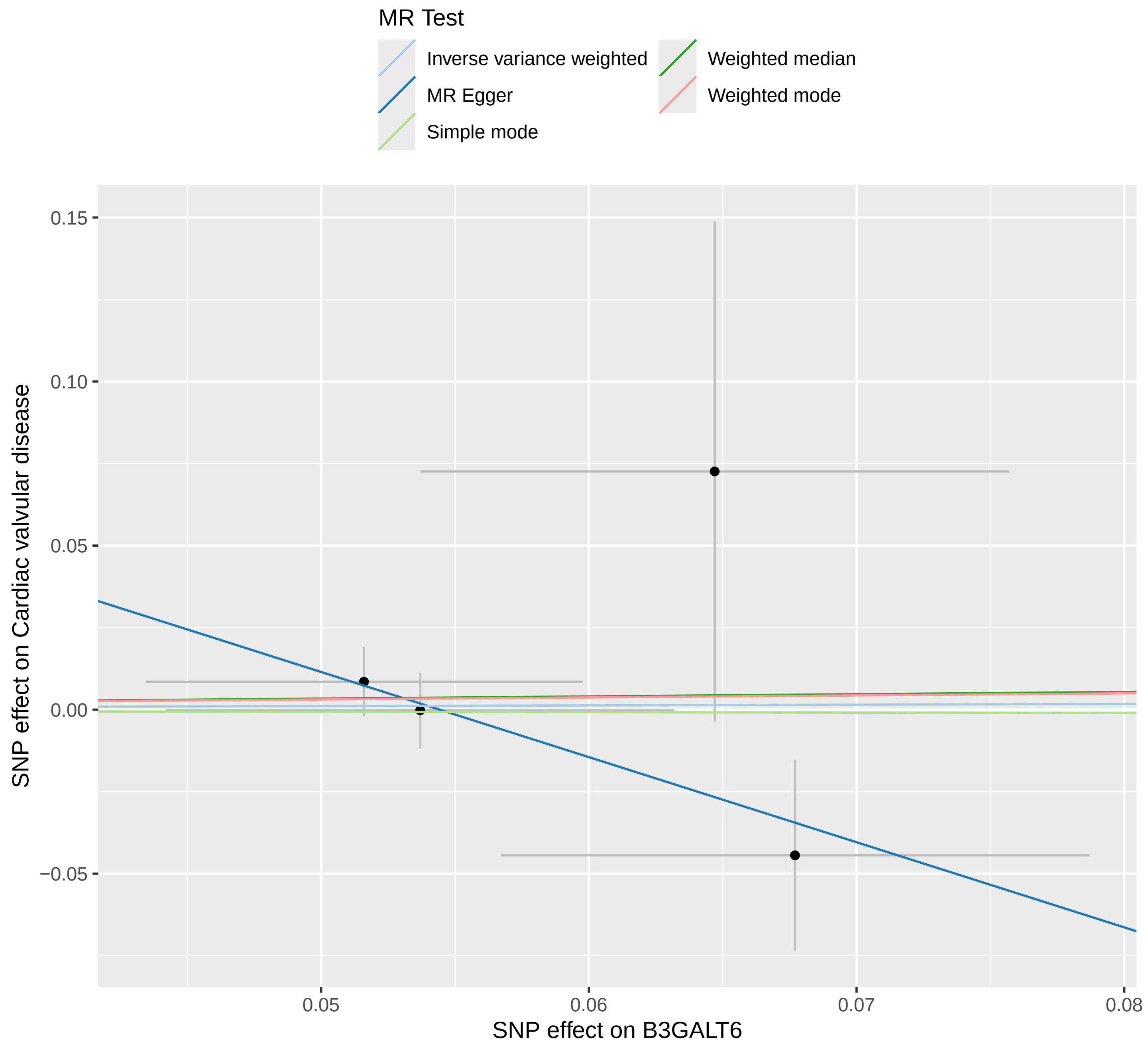

MR Test
Inverse variance weighted
MR Egger
Simple mode
Weighted median
Weighted mode
SNP effect on Cardiac valvular disease
0.15
0.10
0.05
0.00
−0.05
0.05
0.06
0.07
0.08
SNP effect on B3GALT6

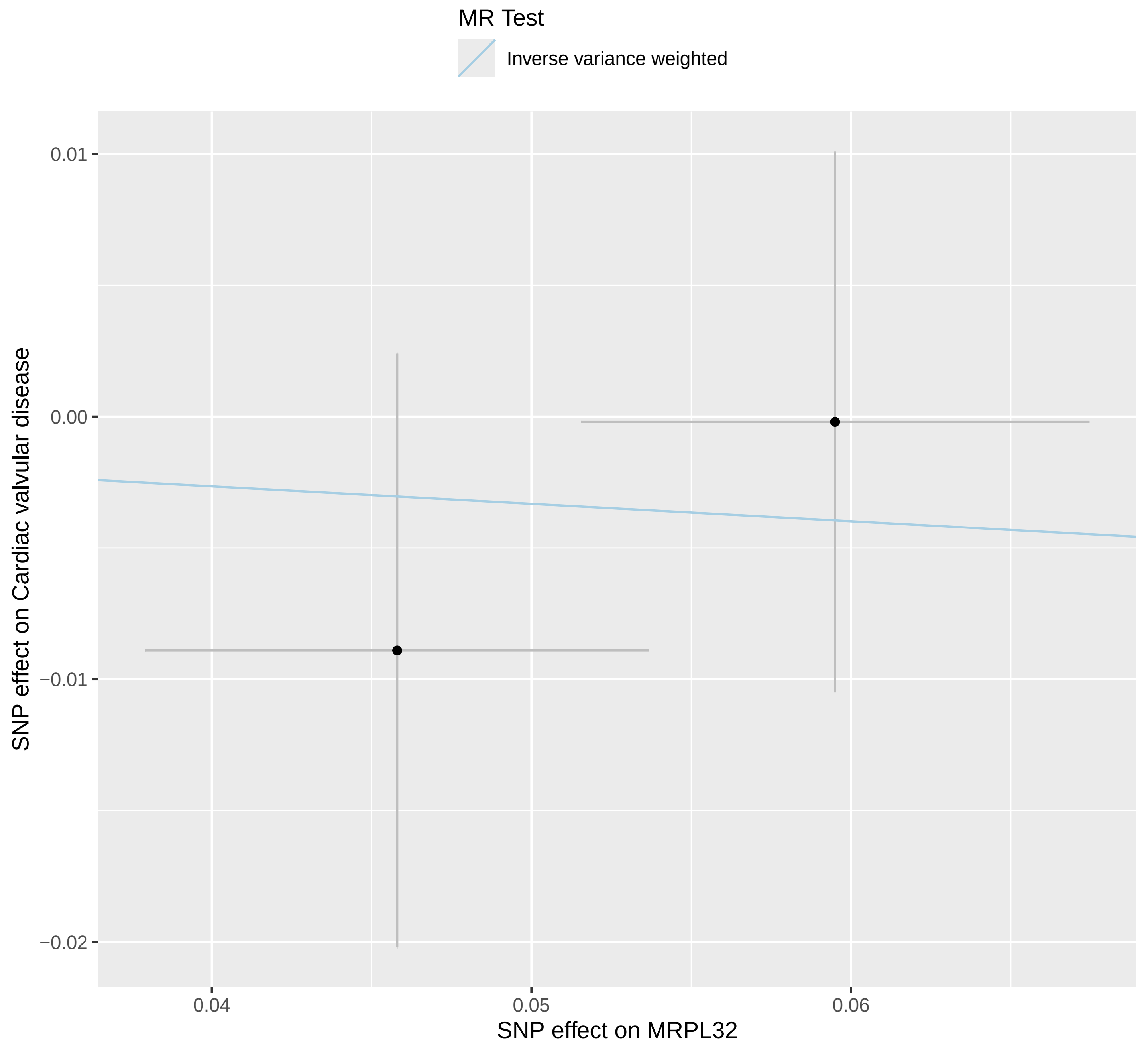
MR Test
Inverse variance weighted
0.01
0.00
−0.01
−0.02
0.04
0.05
0.06
SNP effect on MRPL32
SNP effect on Cardiac valvular disease

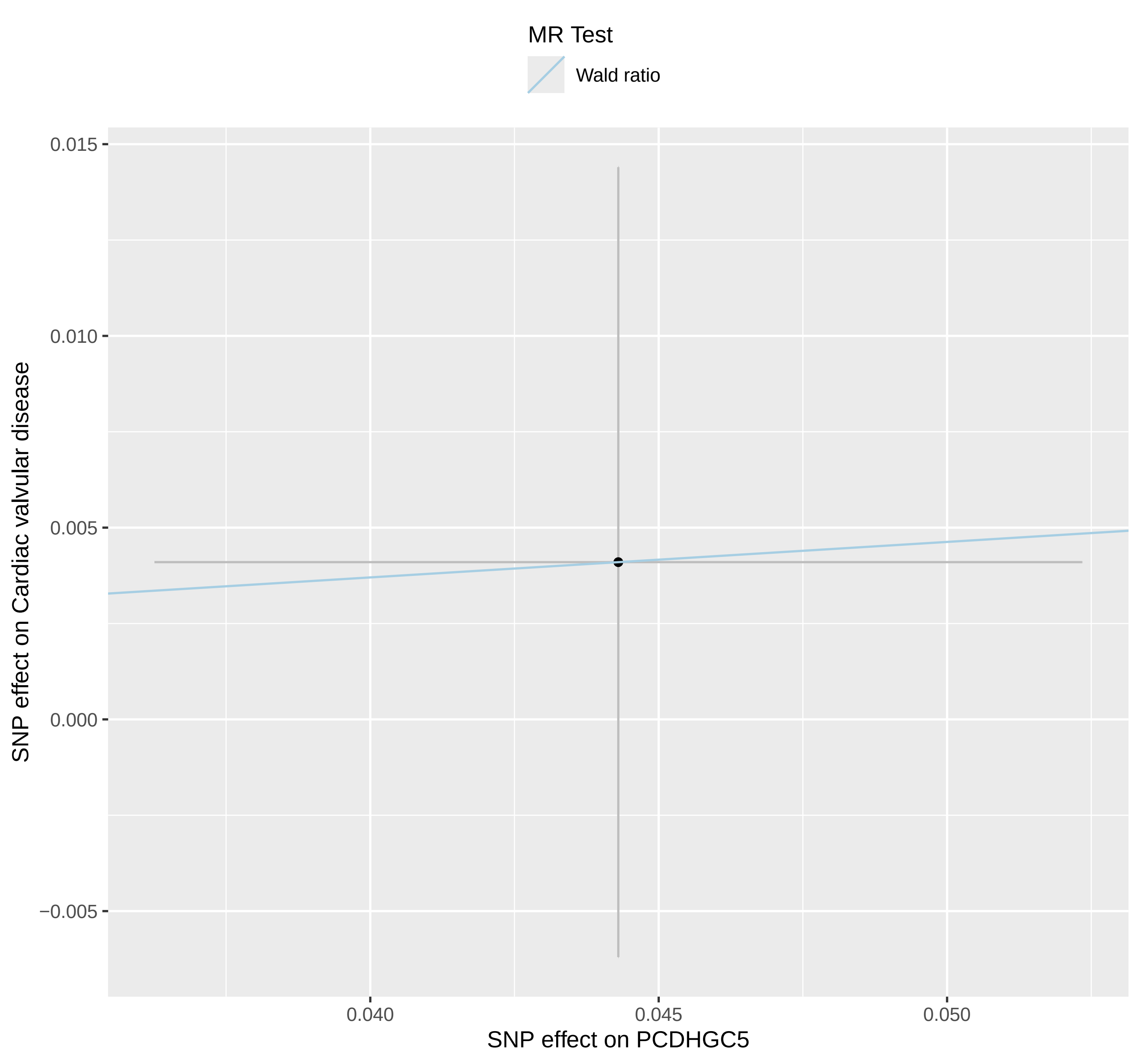
MR Test
Wald ratio
0.015
0.010
0.005
0.000
−0.005
SNP effect on Cardiac valvular disease
0.040
0.045
0.050
SNP effect on PCDHGC5

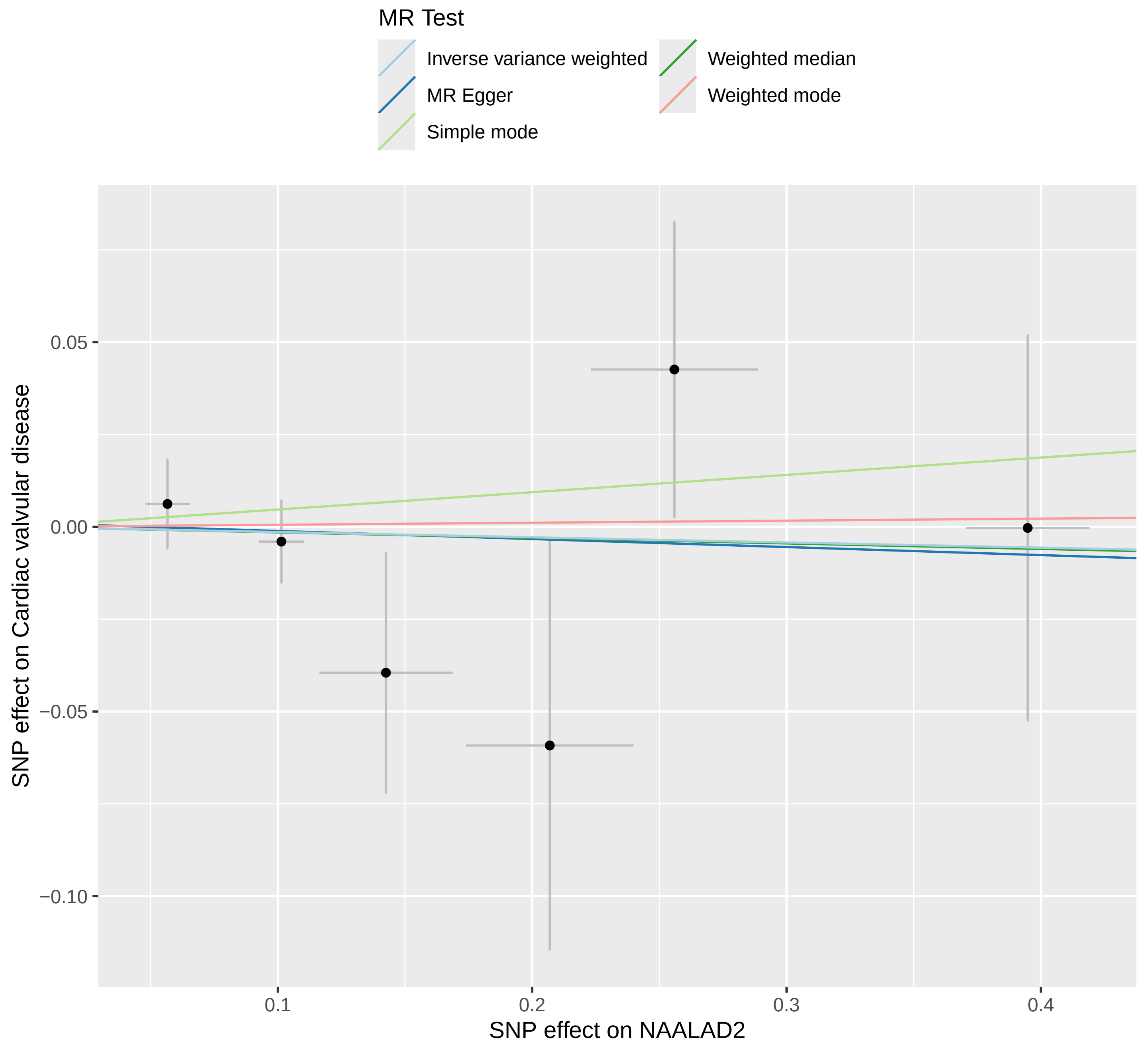
MR Test
Inverse variance weighted
MR Egger
Simple mode
Weighted median
Weighted mode
0.05
0.00
−0.05
−0.10
0.1
0.2
0.3
0.4
SNP effect on Cardiac valvular disease
SNP effect on NAALAD2

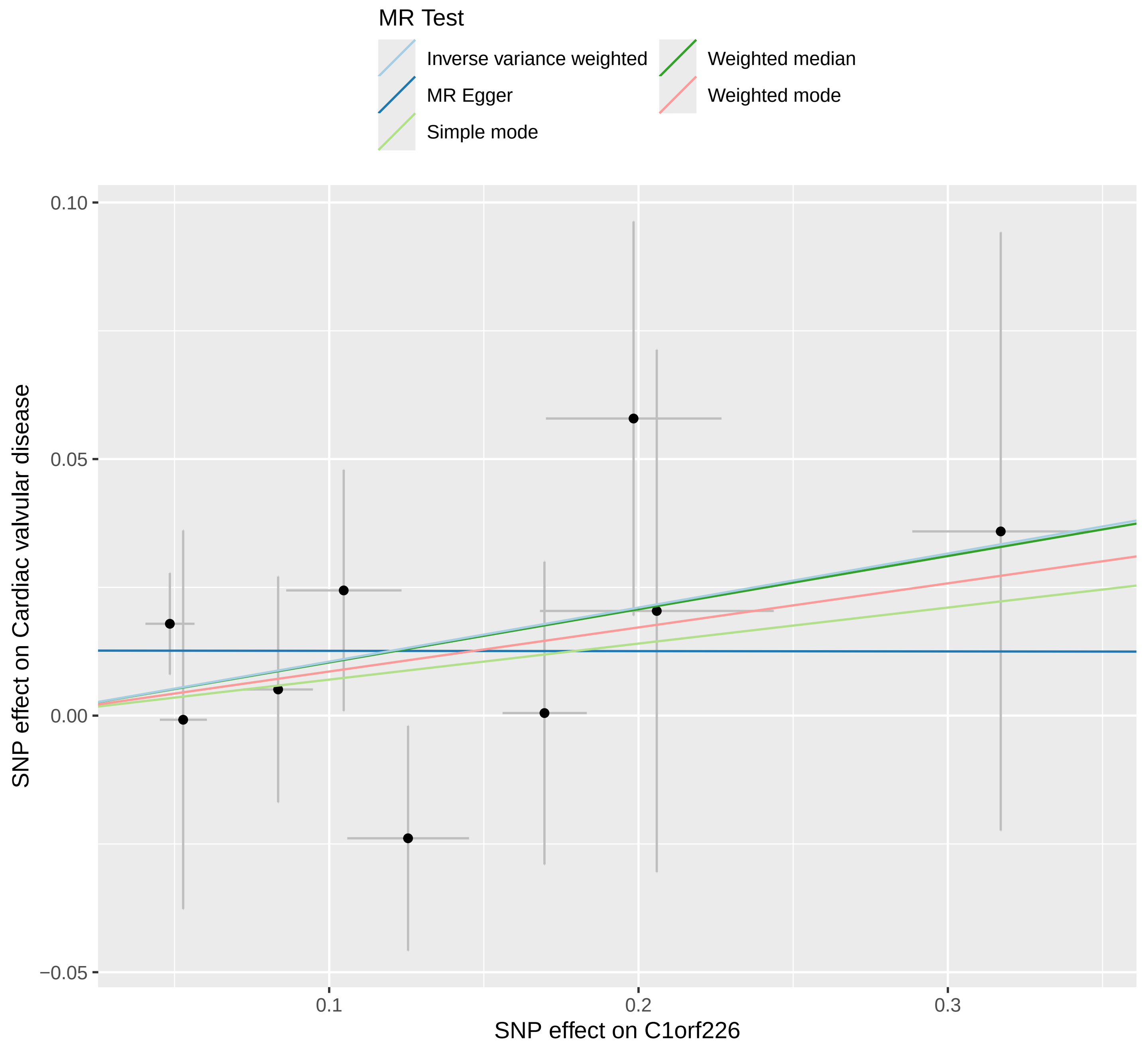
MR Test
Inverse variance weighted
MR Egger
Simple mode
Weighted median
Weighted mode
0.10
0.05
0.00
−0.05
0.1
0.2
0.3
SNP effect on Cardiac valvular disease
SNP effect on C1orf226

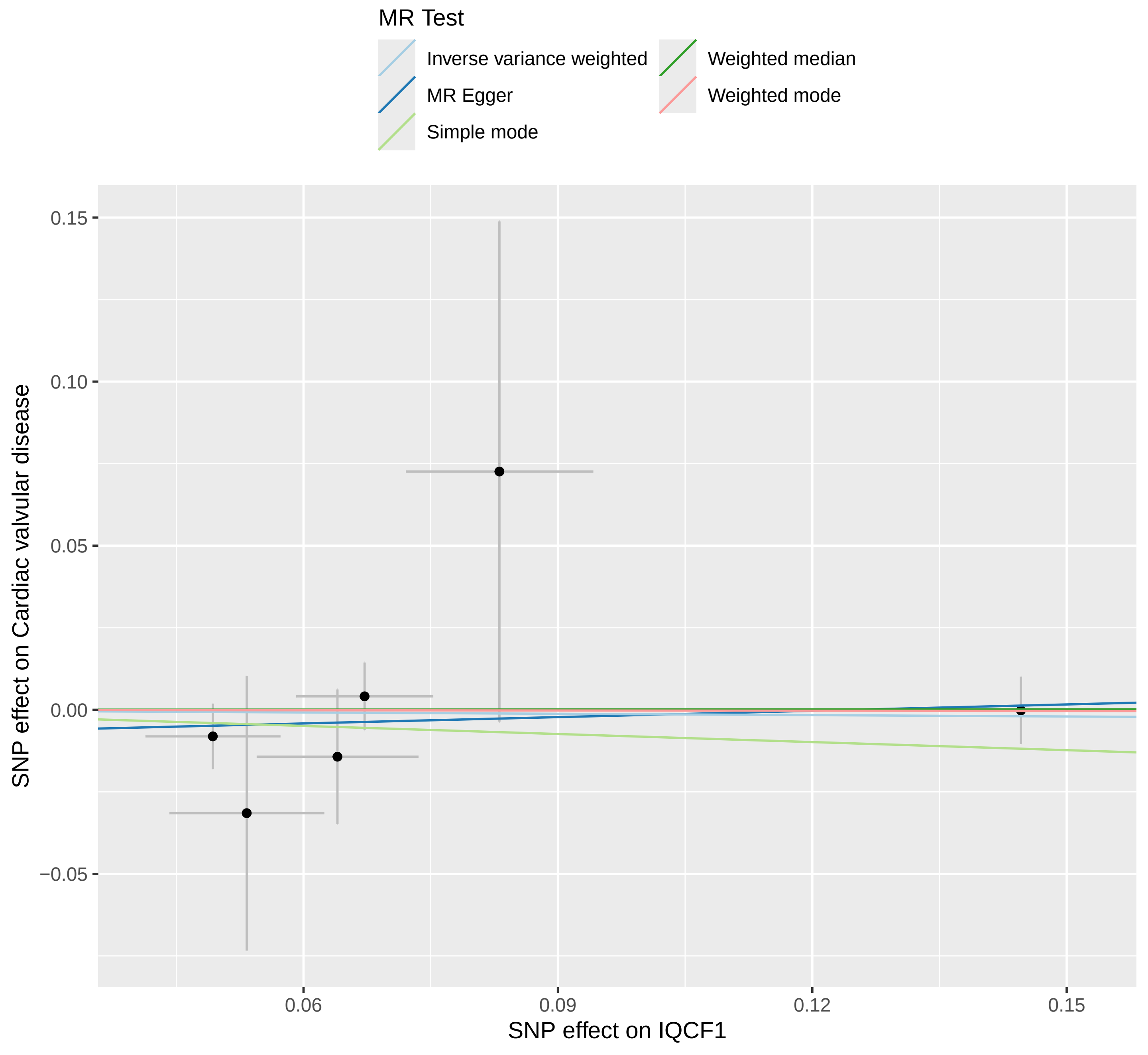
MR Test
Inverse variance weighted
MR Egger
Simple mode
Weighted median
Weighted mode
0.15
0.10
0.05
0.00
−0.05
SNP effect on Cardiac valvular disease
0.06
0.09
0.12
0.15
SNP effect on IQCF1

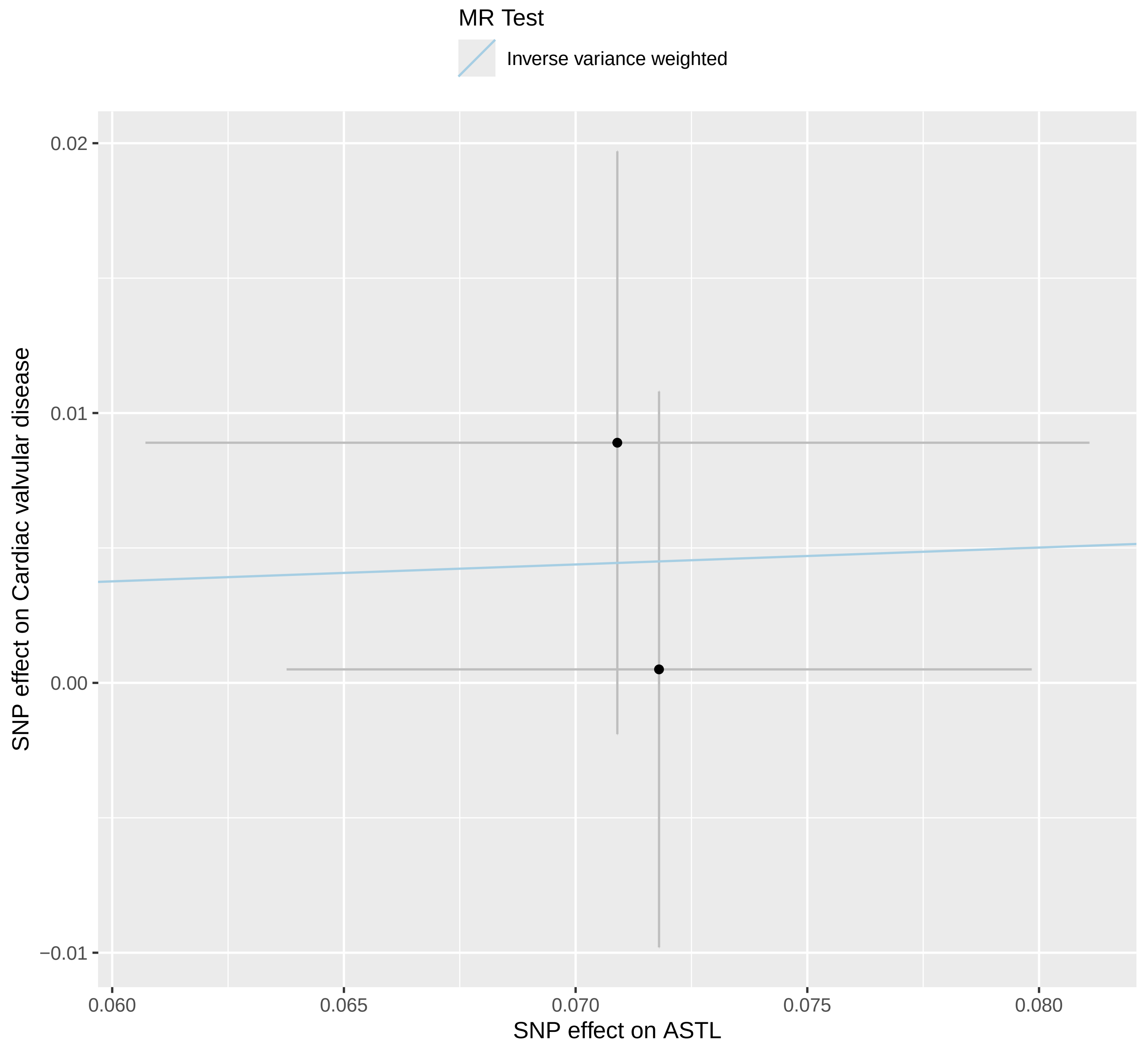

MR Test
Inverse variance weighted
0.02
0.01
0.00
−0.01
0.060
0.065
0.070
0.075
0.080
SNP effect on ASTL
SNP effect on Cardiac valvular disease

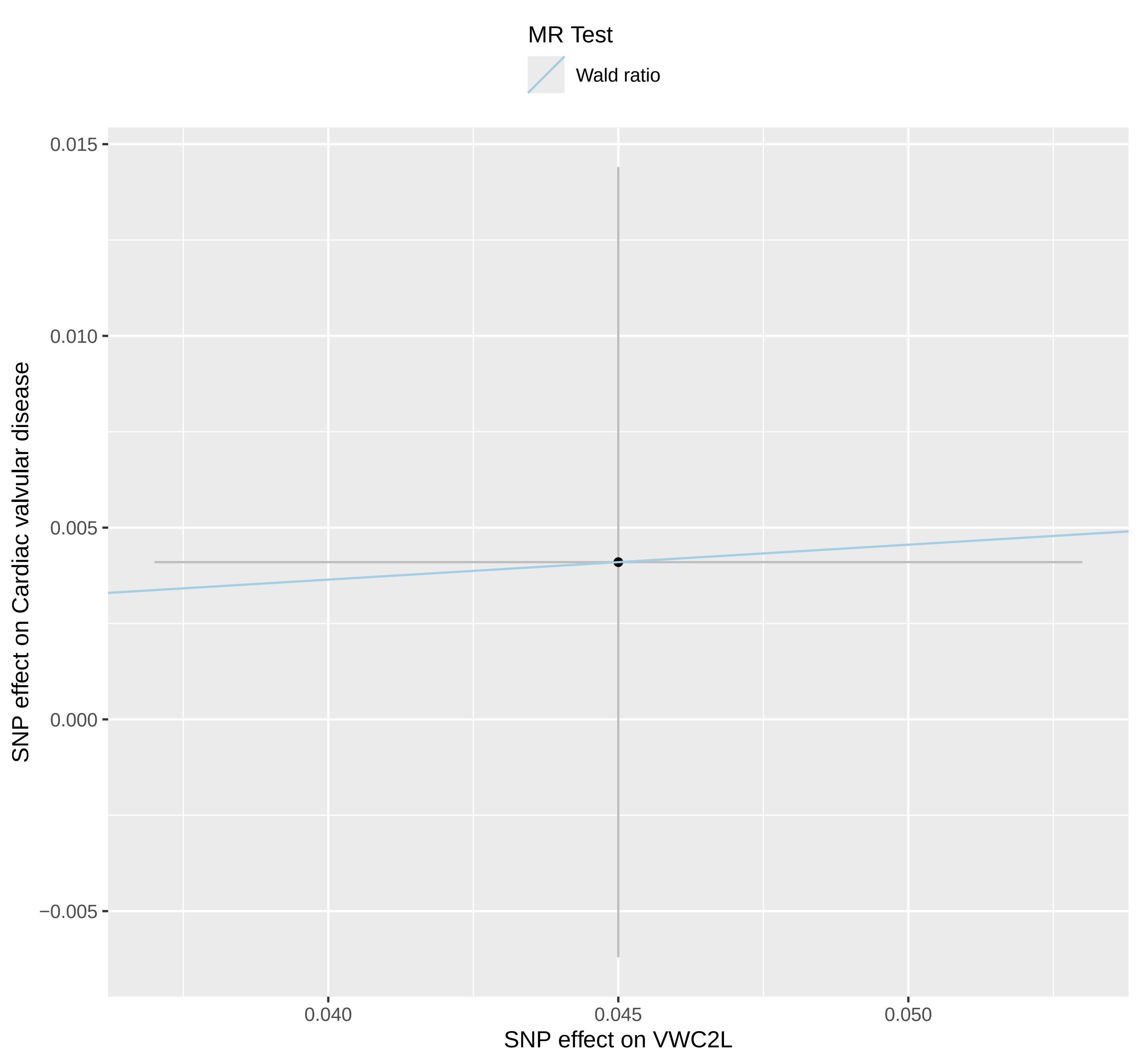
MR Test
Wald ratio
0.015
0.010
0.005
0.000
−0.005
0.040
0.045
0.050
SNP effect on Cardiac valvular disease
SNP effect on VWC2L

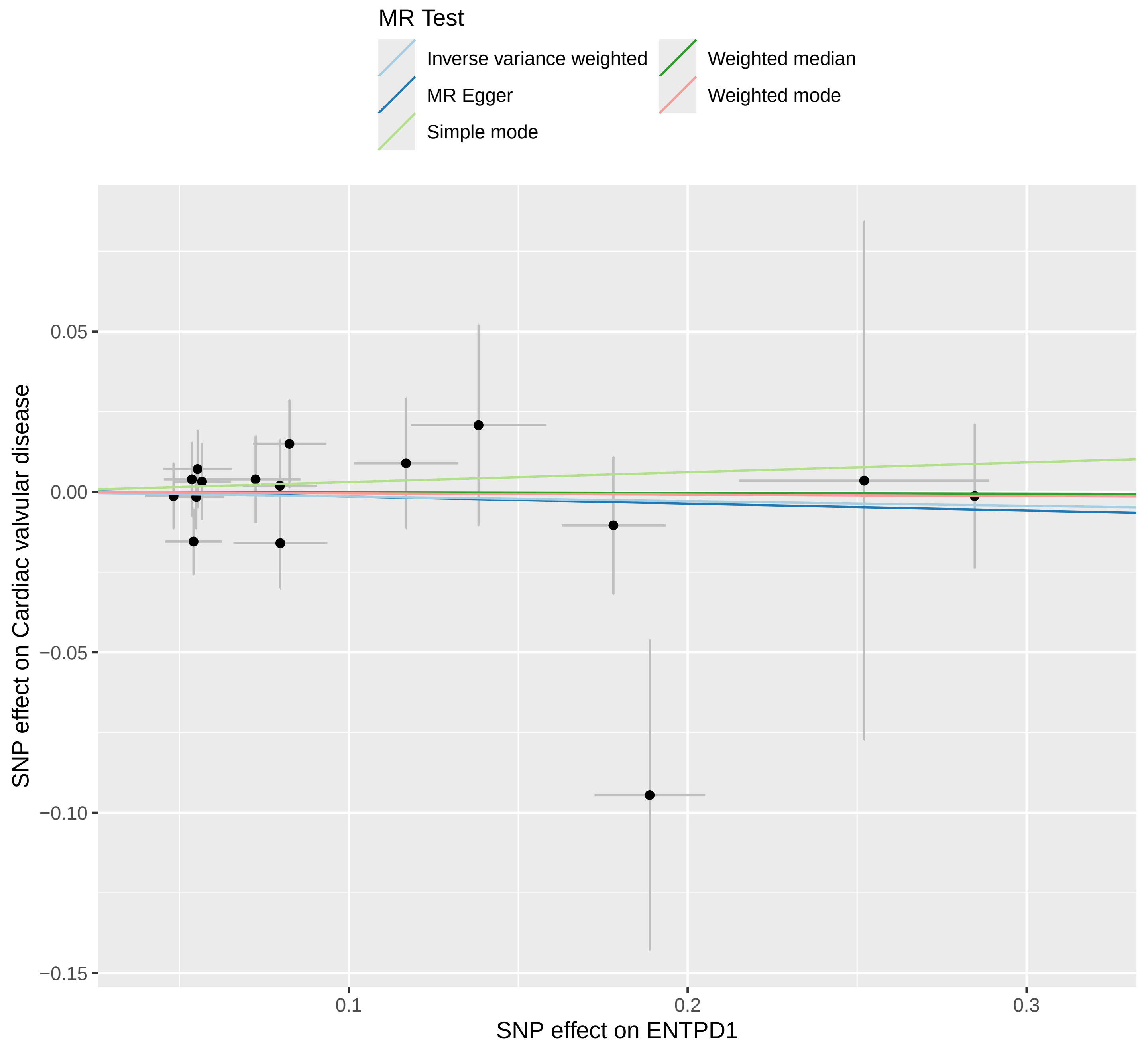
MR Test
Inverse variance weighted
MR Egger
Simple mode
Weighted median
Weighted mode
0.05
0.00
−0.05
−0.10
−0.15
0.1
0.2
0.3
SNP effect on Cardiac valvular disease
SNP effect on ENTPD1

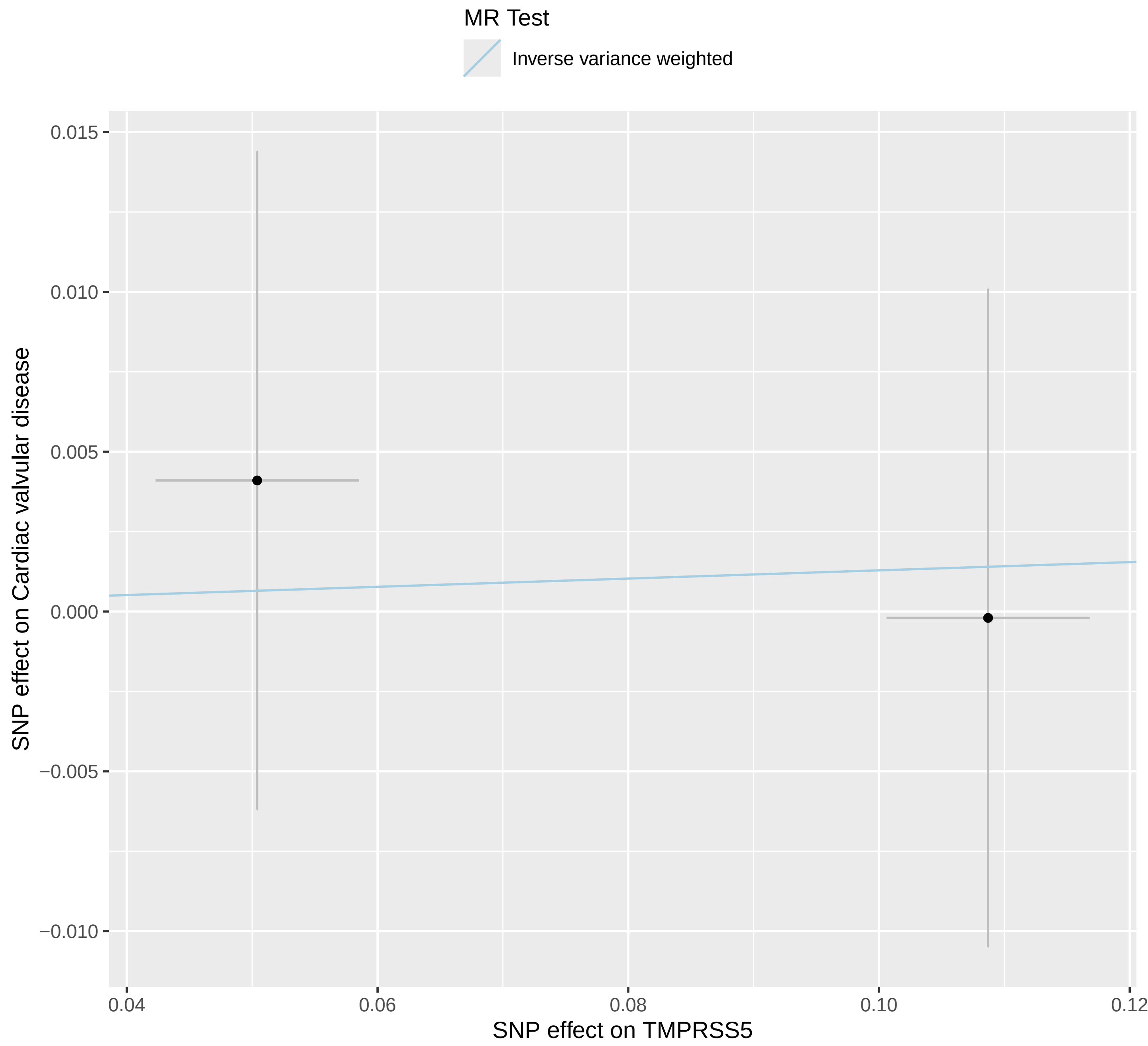
MR Test
Inverse variance weighted
0.015
0.010
0.005
0.000
−0.005
−0.010
0.04
0.06
0.08
0.10
0.12
SNP effect on TMPRSS5
SNP effect on Cardiac valvular disease

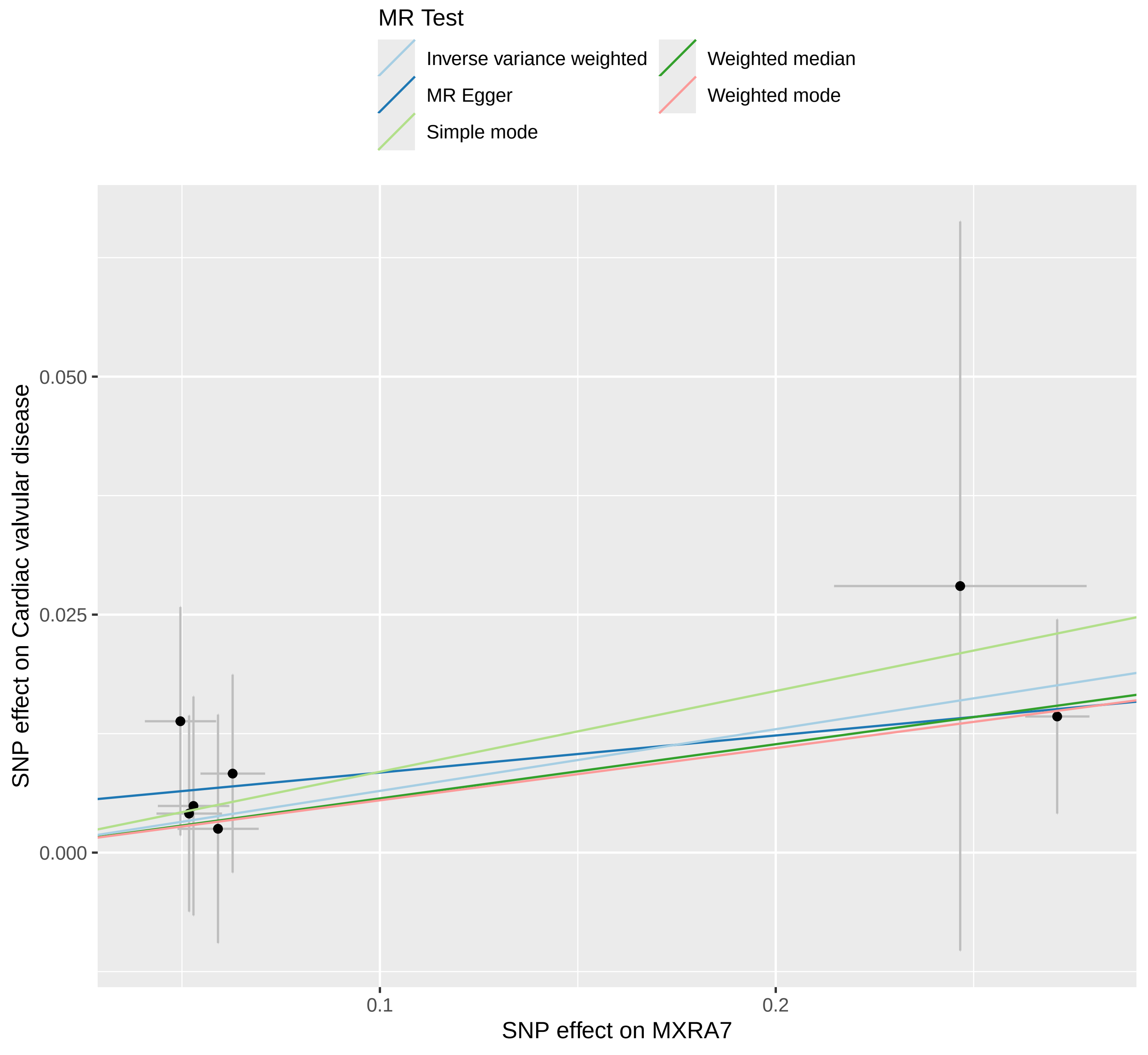
MR Test
Inverse variance weighted
MR Egger
Simple mode
Weighted median
Weighted mode
0.050
0.025
0.000
0.1
0.2
SNP effect on Cardiac valvular disease
SNP effect on MXRA7

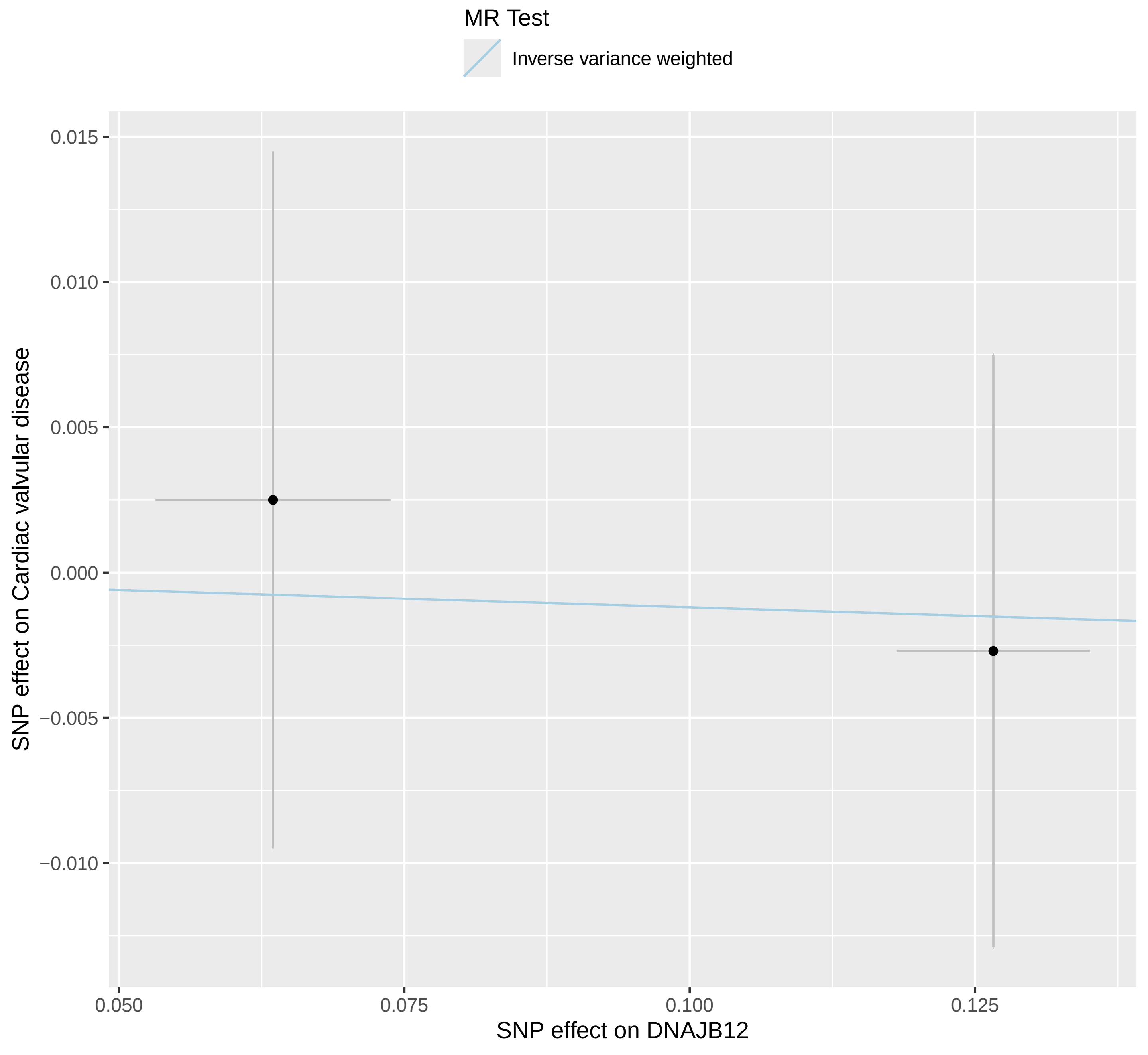

MR Test
Inverse variance weighted
0.015
0.010
0.005
0.000
−0.005
−0.010
SNP effect on Cardiac valvular disease
0.050
0.075
0.100
0.125
SNP effect on DNAJB12

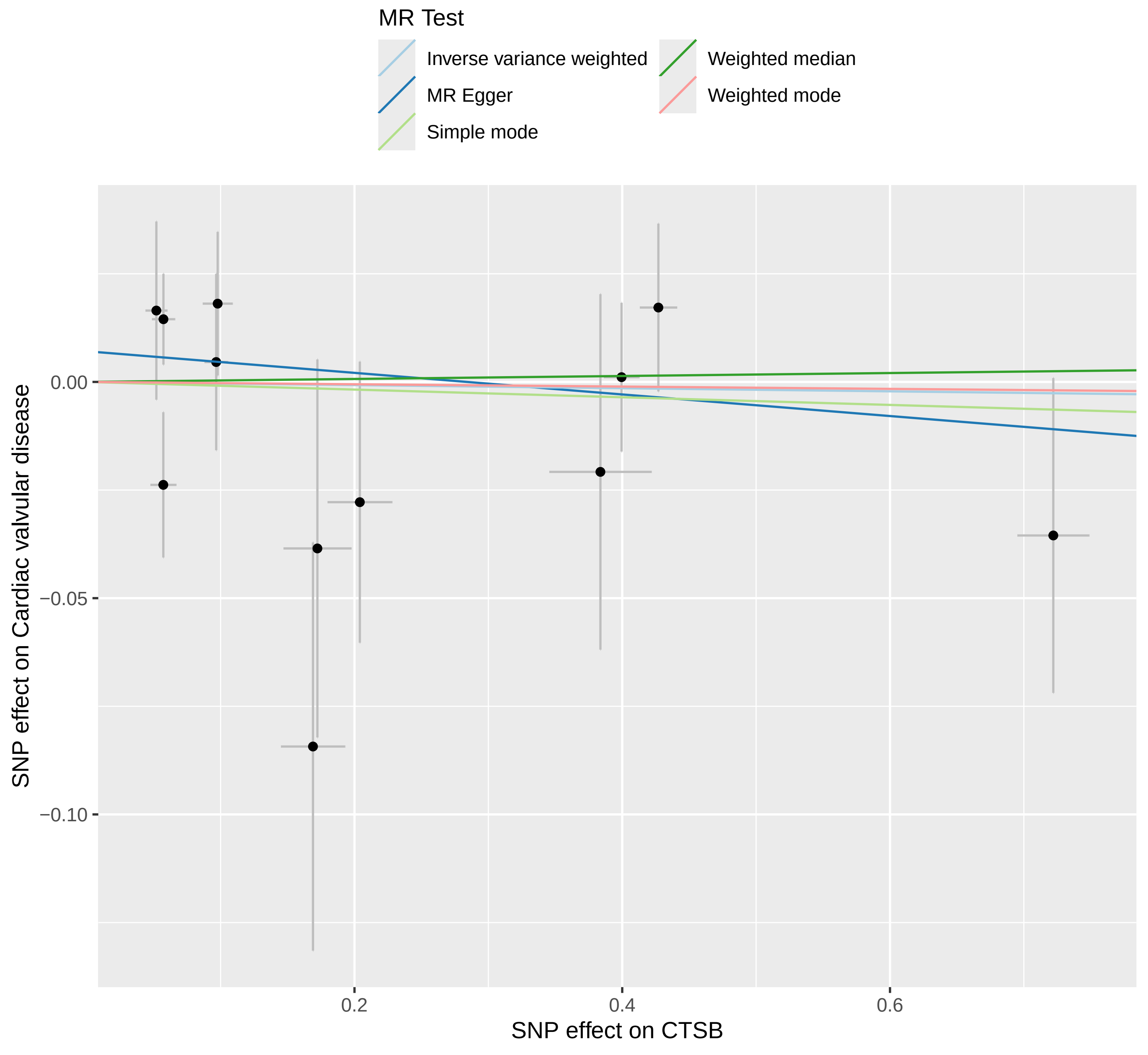

MR Test
Inverse variance weighted
MR Egger
Simple mode
Weighted median
Weighted mode
0.00
−0.05
−0.10
0.2
0.4
0.6
SNP effect on Cardiac valvular disease
SNP effect on CTSB

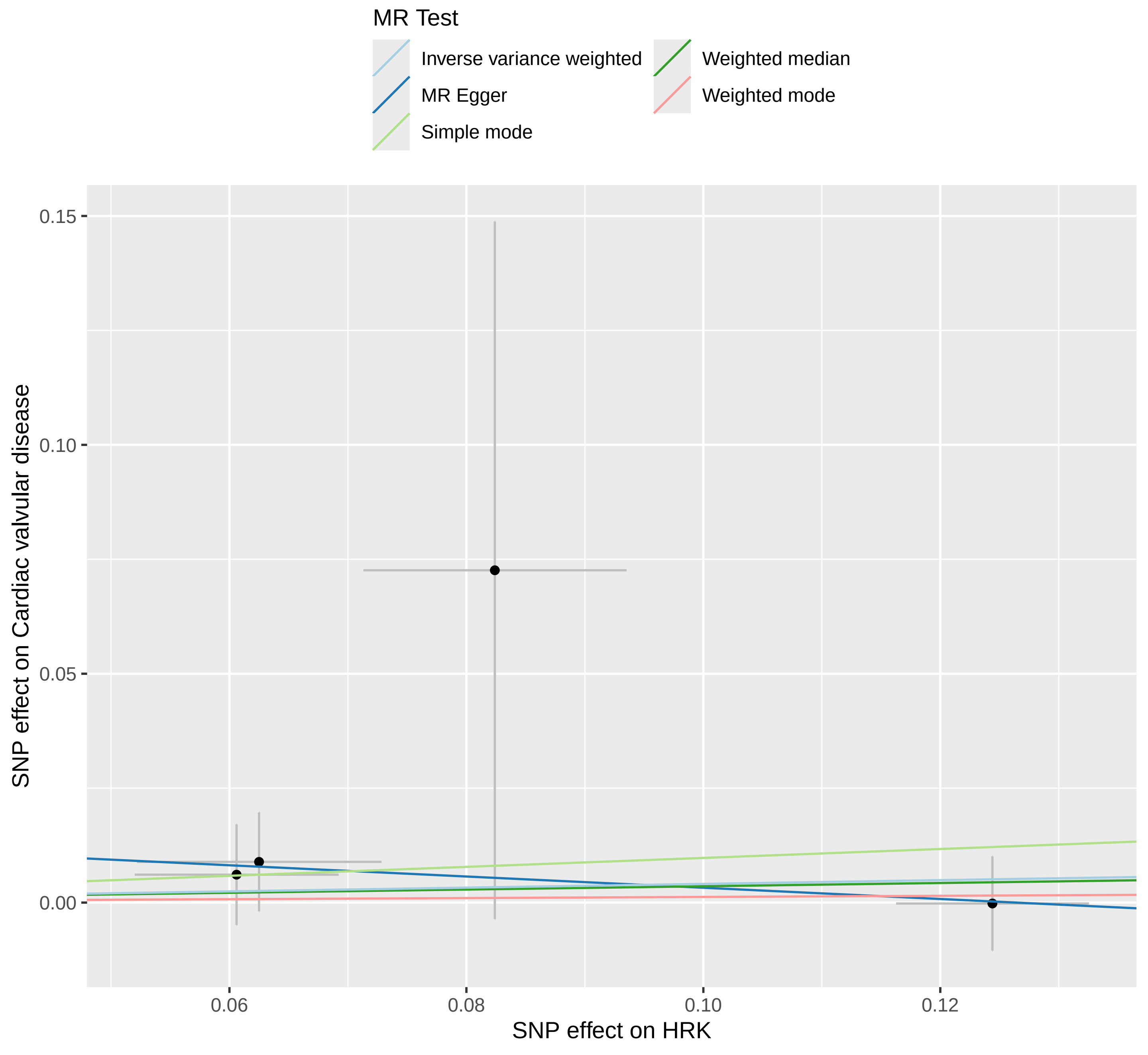
MR Test
Inverse variance weighted
MR Egger
Simple mode
Weighted median
Weighted mode
0.15
0.10
0.05
0.00
0.06
0.08
0.10
0.12
SNP effect on Cardiac valvular disease
SNP effect on HRK

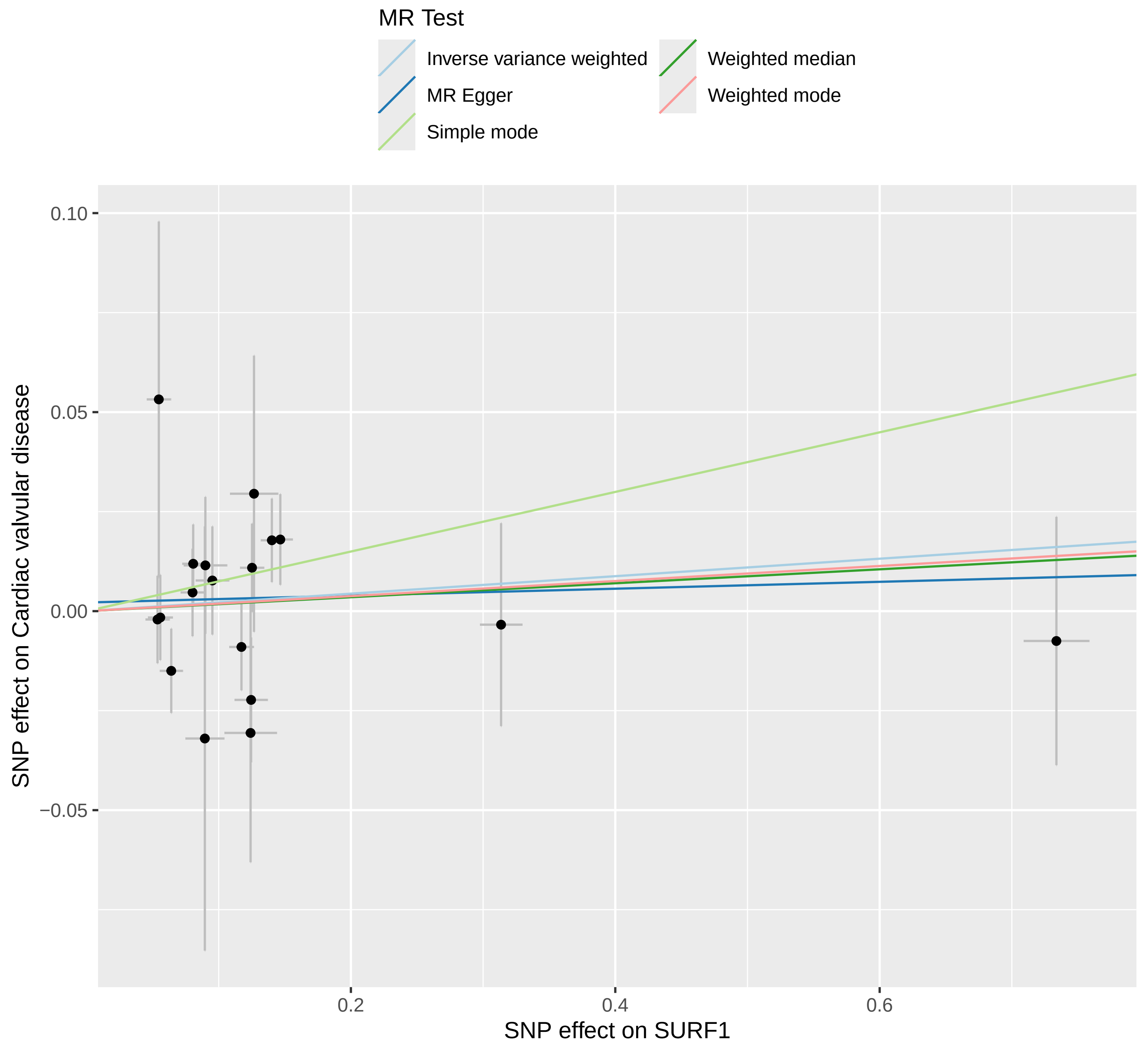
MR Test
Inverse variance weighted
MR Egger
Simple mode
Weighted median
Weighted mode
0.10
0.05
0.00
−0.05
SNP effect on Cardiac valvular disease
0.2
0.4
0.6
SNP effect on SURF1

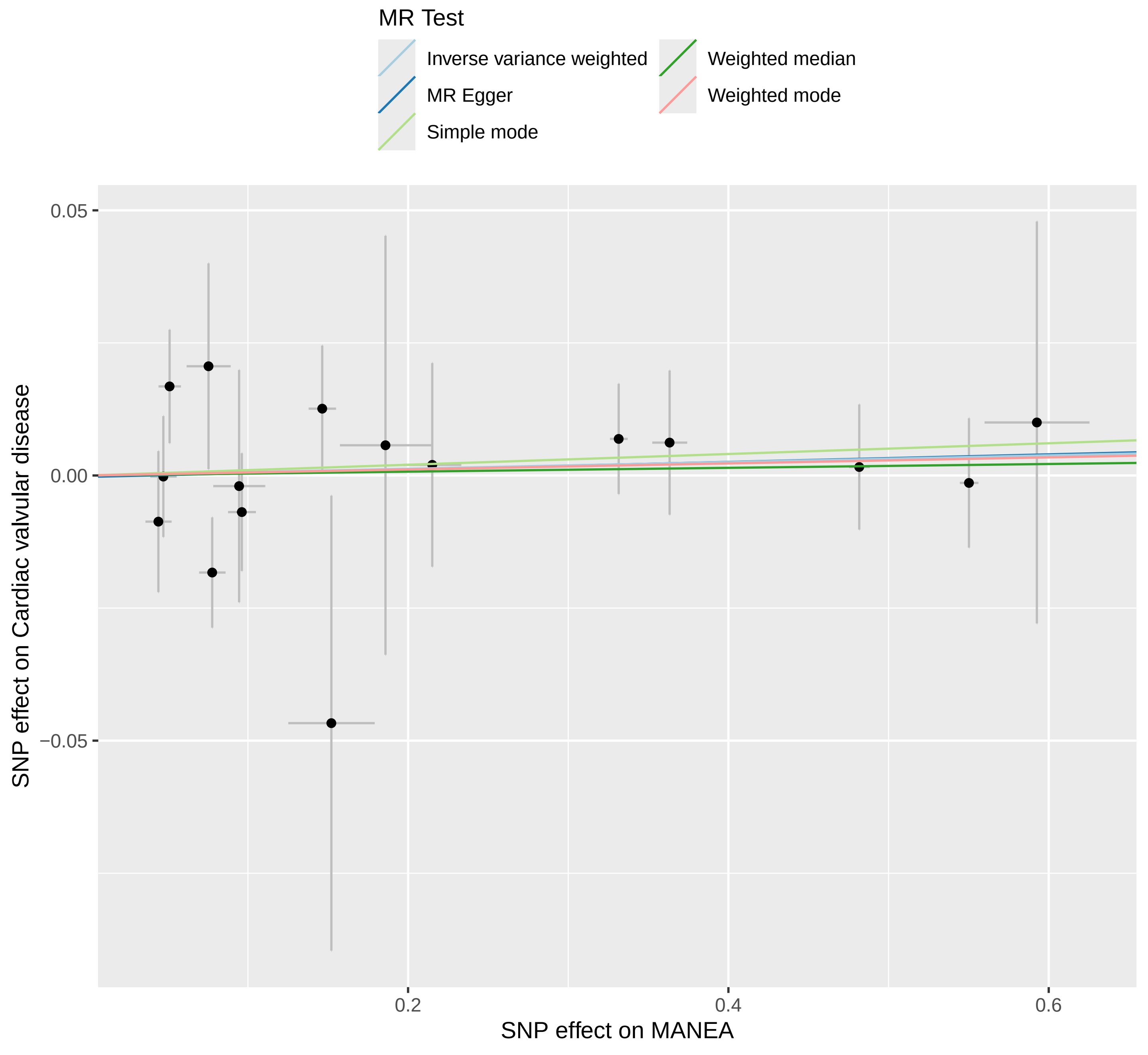
MR Test
Inverse variance weighted
MR Egger
Simple mode
Weighted median
Weighted mode
0.05
0.00
−0.05
SNP effect on Cardiac valvular disease
0.2
0.4
0.6
SNP effect on MANEA

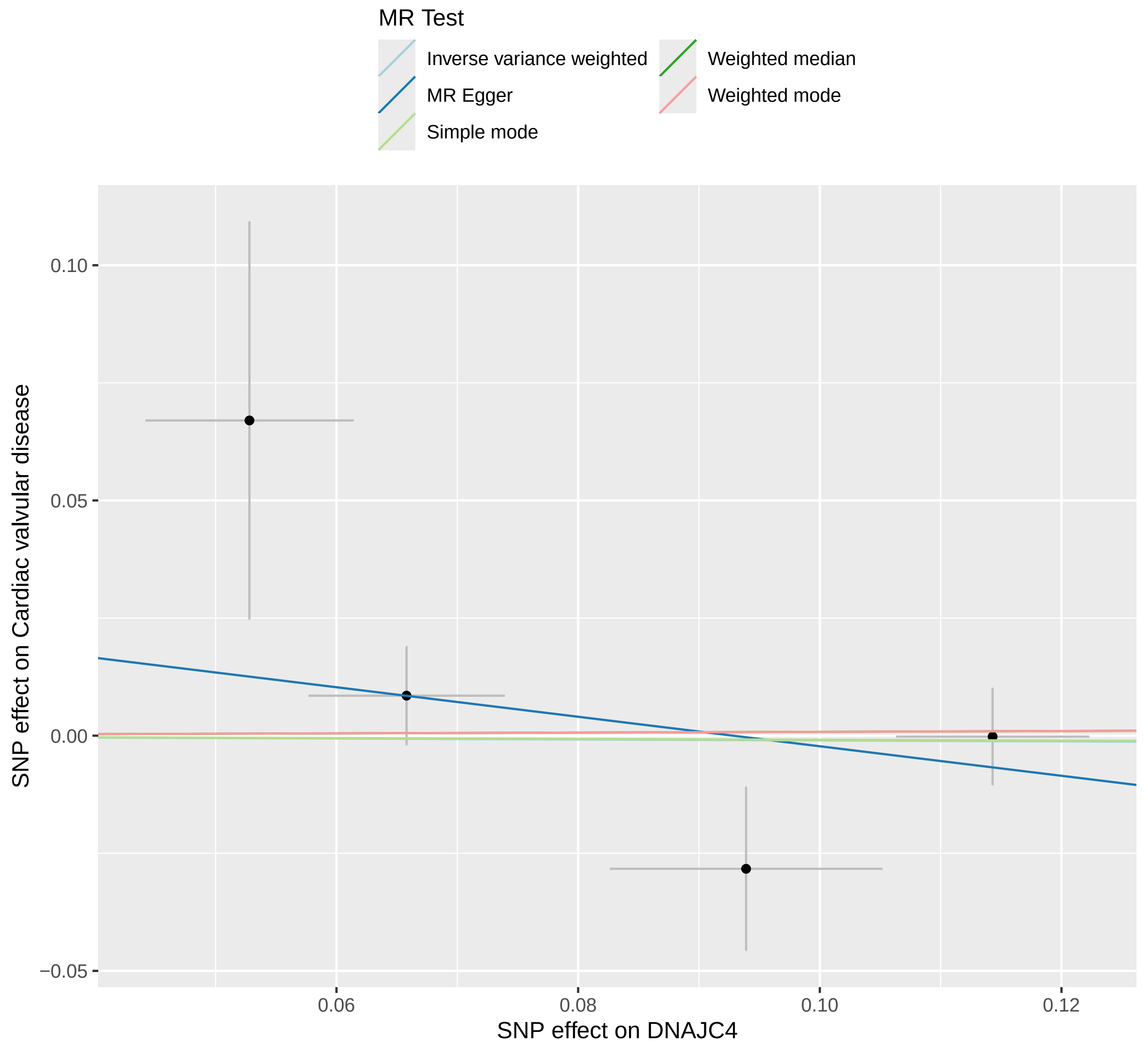
MR Test
Inverse variance weighted
MR Egger
Simple mode
Weighted median
Weighted mode
SNP effect on Cardiac valvular disease
0.10
0.05
0.00
−0.05
0.06
0.08
0.10
0.12
SNP effect on DNAJC4

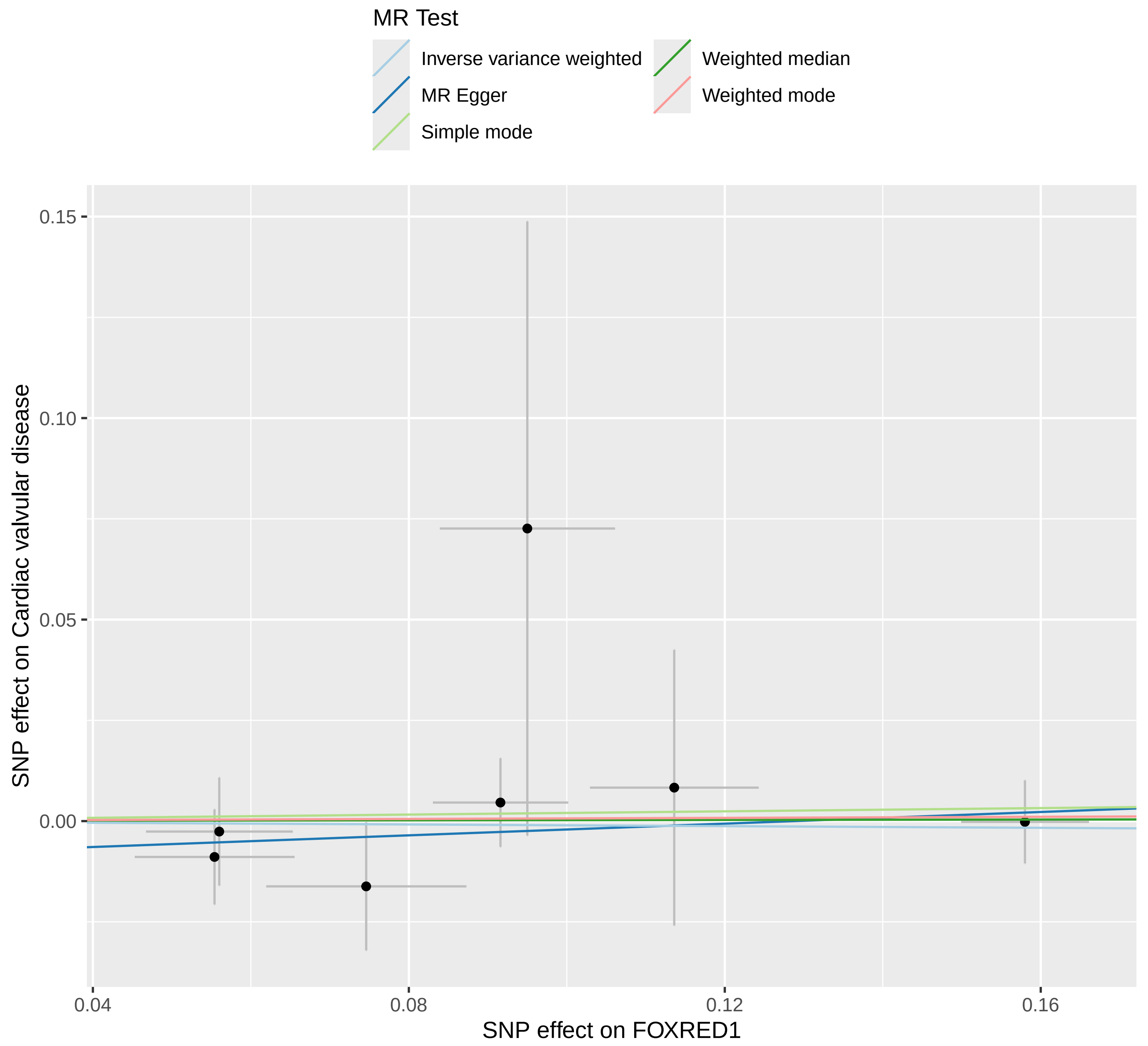

MR Test
Inverse variance weighted
MR Egger
Simple mode
Weighted median
Weighted mode
0.15
0.10
0.05
0.00
0.04
0.08
0.12
0.16
SNP effect on Cardiac valvular disease
SNP effect on FOXRED1

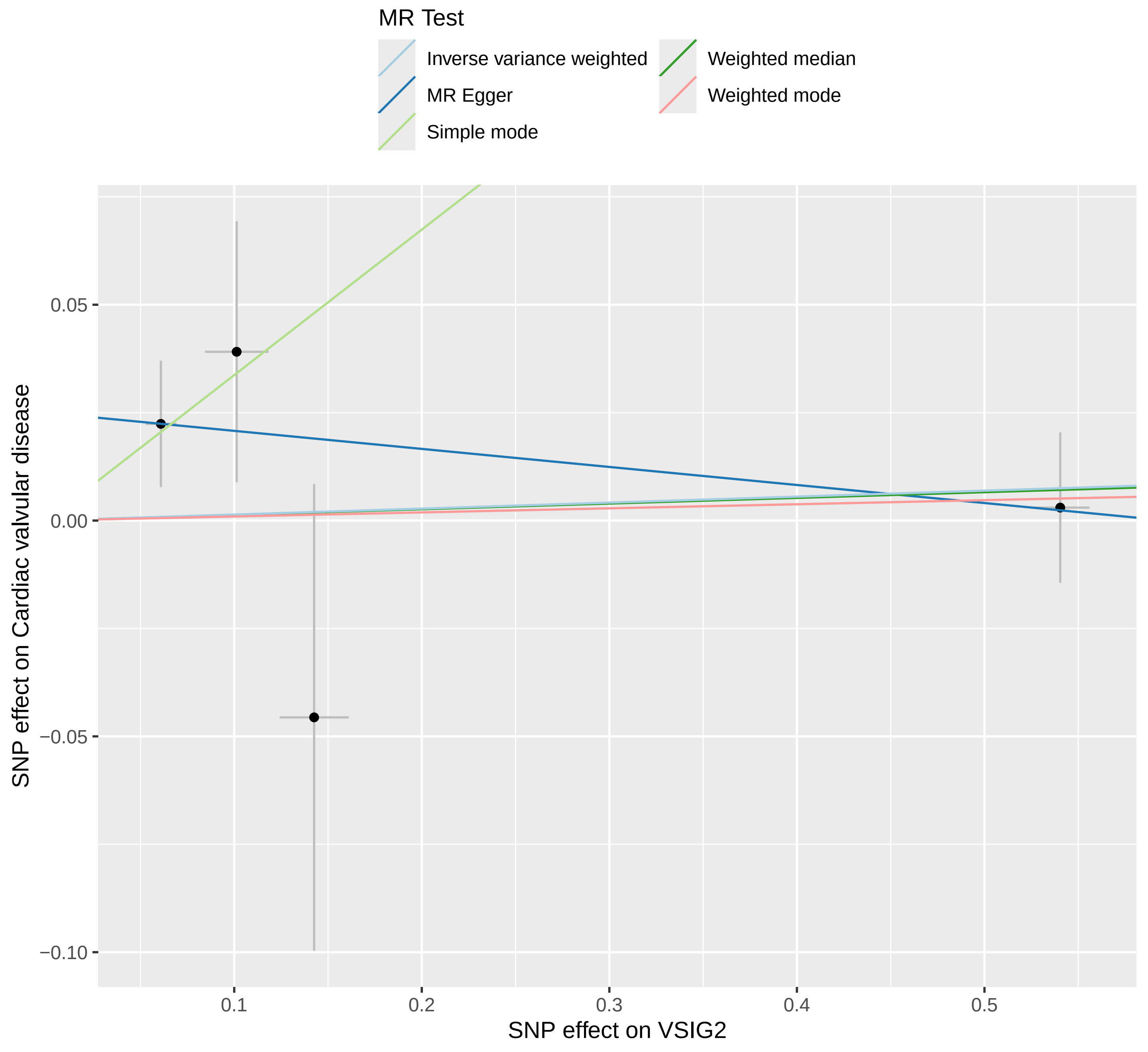

MR Test
Inverse variance weighted
MR Egger
Simple mode
Weighted median
Weighted mode
0.05
0.00
−0.05
−0.10
0.1
0.2
0.3
0.4
0.5
SNP effect on Cardiac valvular disease
SNP effect on VSIG2

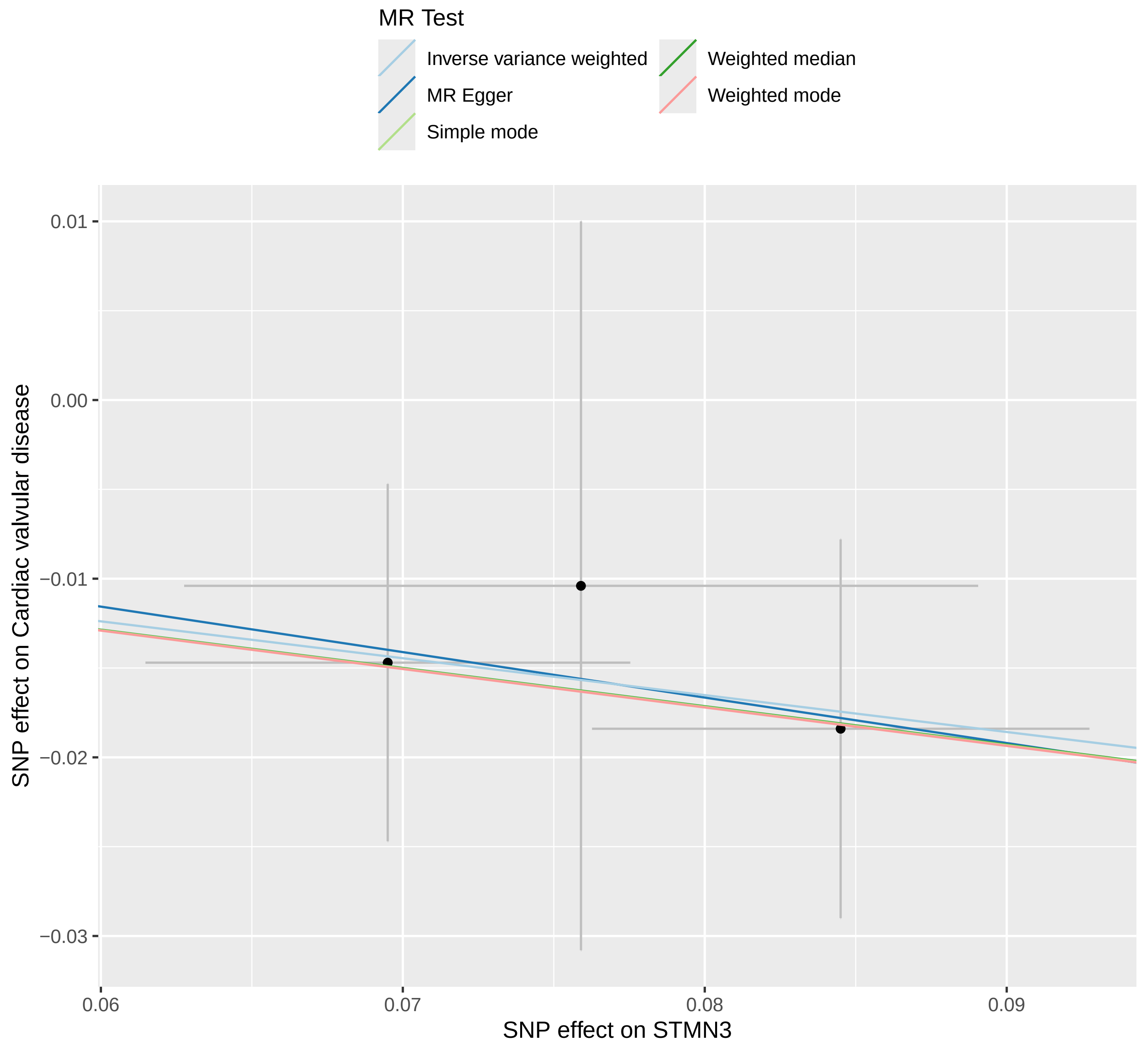

MR Test
Inverse variance weighted
MR Egger
Simple mode
Weighted median
Weighted mode
0.01
0.00
−0.01
−0.02
−0.03
SNP effect on Cardiac valvular disease
0.06
0.07
0.08
0.09
SNP effect on STMN3

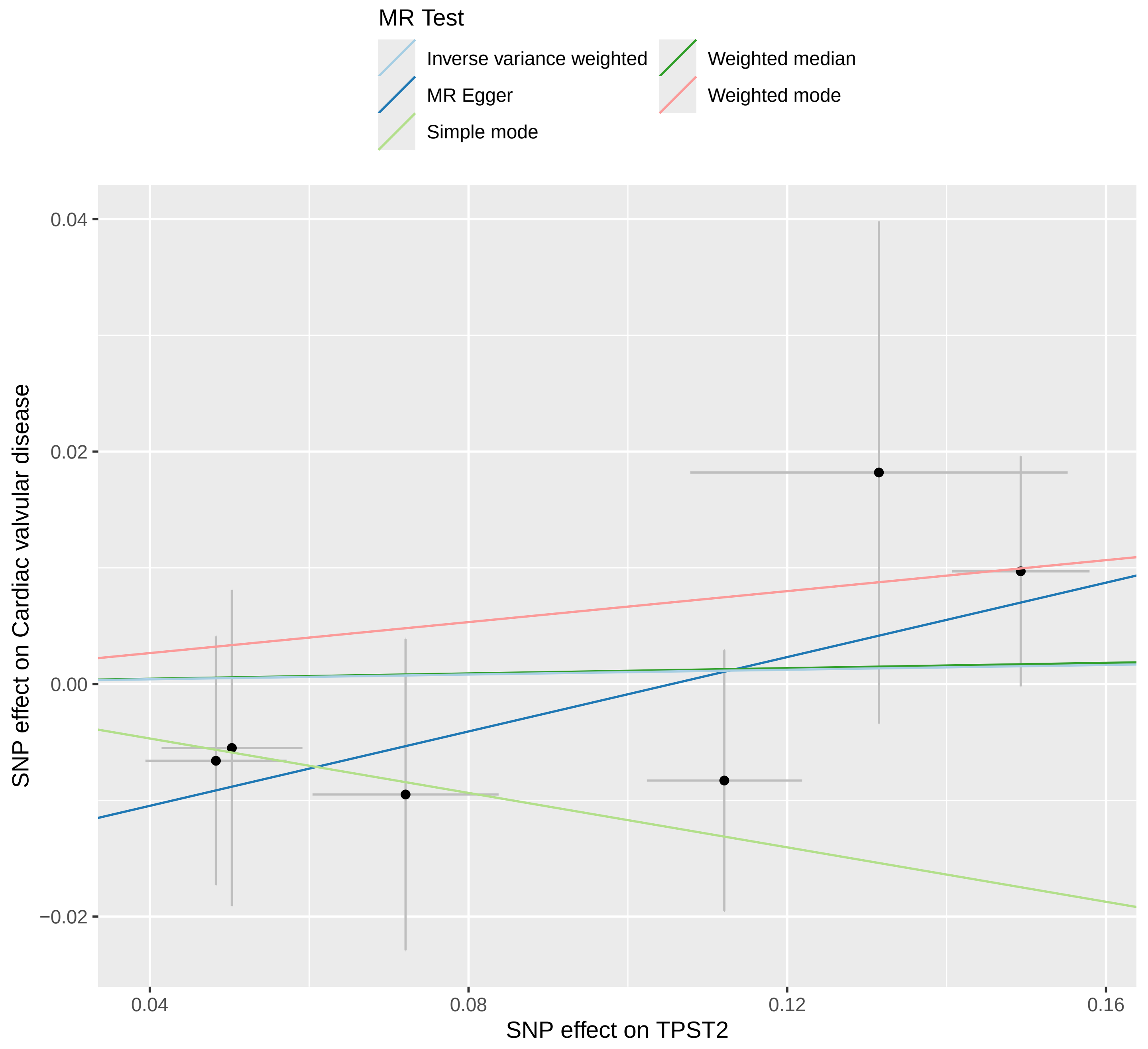
MR Test
Inverse variance weighted
MR Egger
Simple mode
Weighted median
Weighted mode
0.04
0.02
0.00
−0.02
0.04
0.08
0.12
0.16
SNP effect on Cardiac valvular disease
SNP effect on TPST2

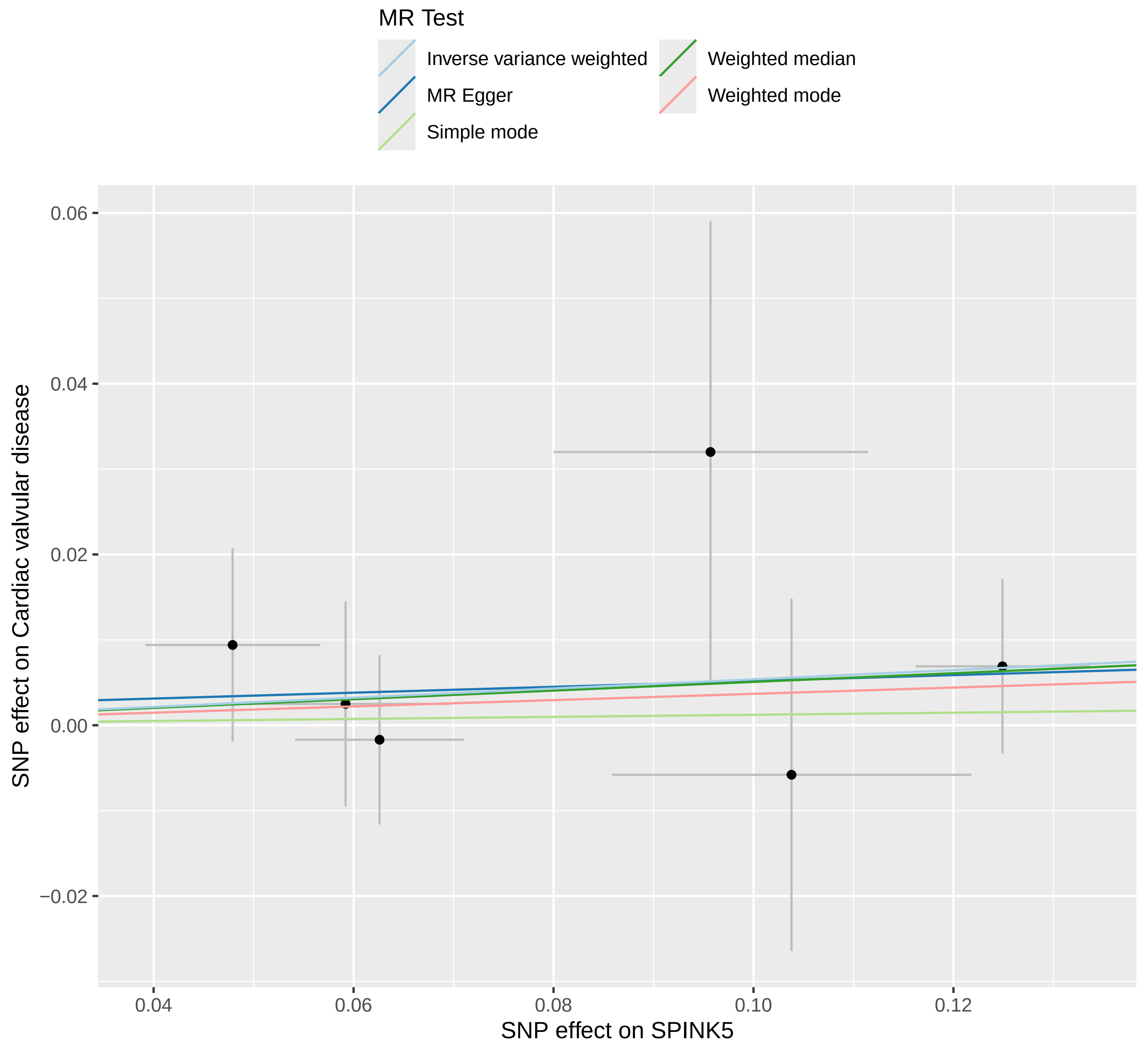
MR Test
Inverse variance weighted
MR Egger
Simple mode
Weighted median
Weighted mode
0.06
0.04
0.02
0.00
−0.02
SNP effect on Cardiac valvular disease
0.04
0.06
0.08
0.10
0.12
SNP effect on SPINK5

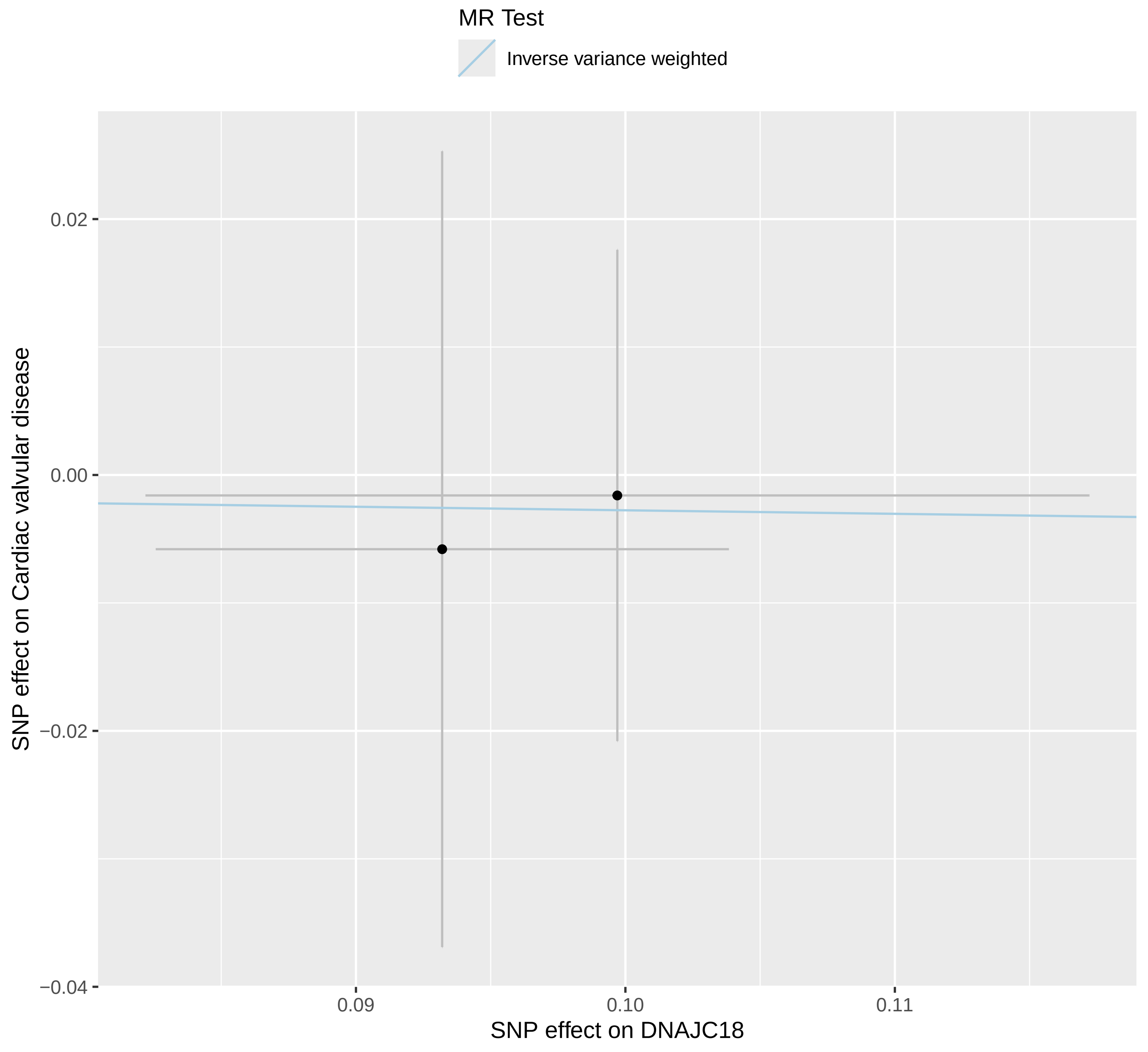

MR Test
Inverse variance weighted
0.02
0.00
−0.02
−0.04
0.09
0.10
0.11
SNP effect on DNAJC18
SNP effect on Cardiac valvular disease

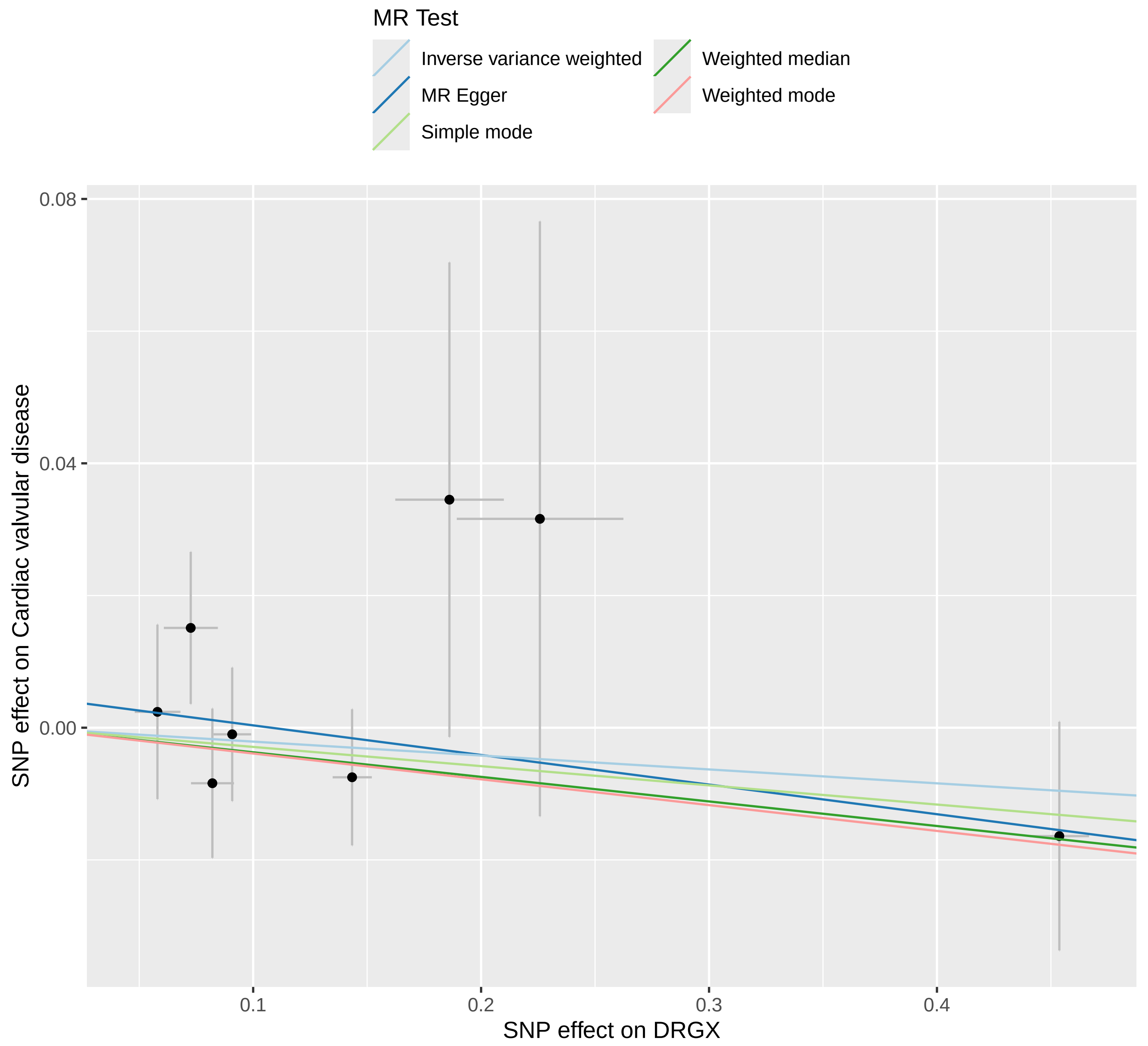

MR Test
Inverse variance weighted
MR Egger
Simple mode
Weighted median
Weighted mode
0.08
0.04
0.00
SNP effect on Cardiac valvular disease
0.1
0.2
0.3
0.4
SNP effect on DRGX

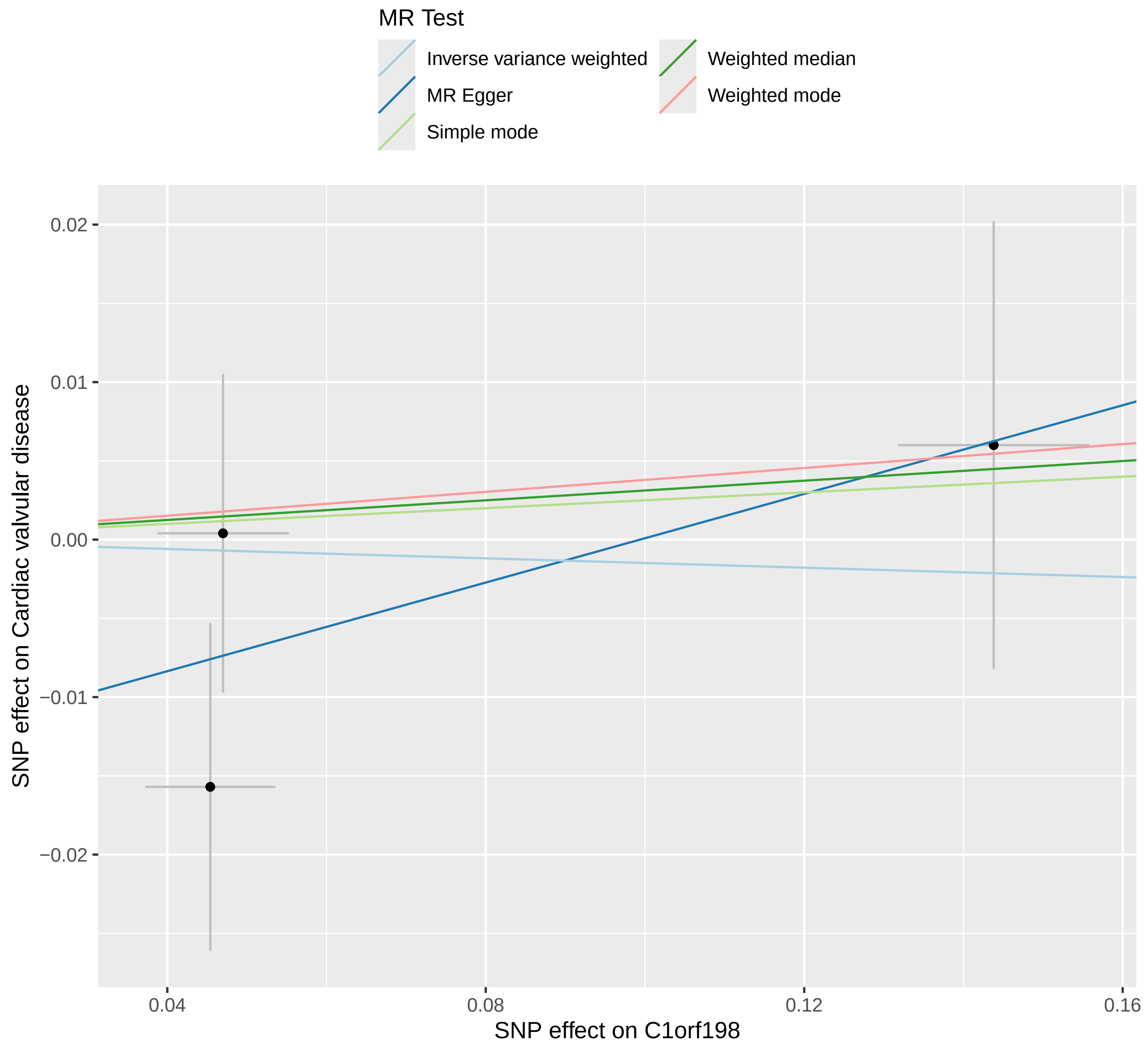
MR Test
Inverse variance weighted
MR Egger
Simple mode
Weighted median
Weighted mode
0.02
0.01
0.00
−0.01
−0.02
SNP effect on Cardiac valvular disease
0.04
0.08
0.12
0.16
SNP effect on C1orf198

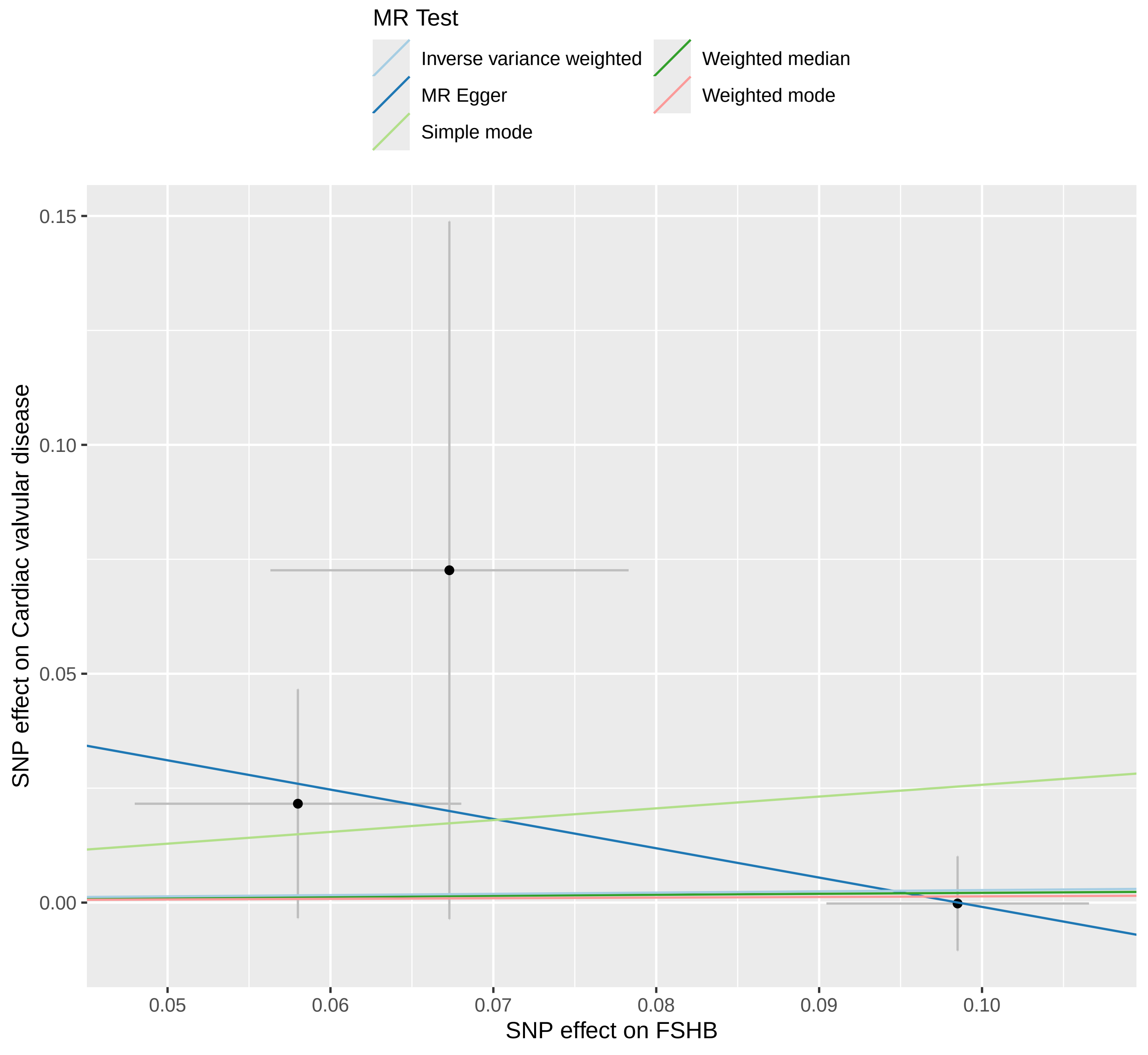

MR Test
Inverse variance weighted
MR Egger
Simple mode
Weighted median
Weighted mode
0.15
0.10
0.05
0.00
0.05
0.06
0.07
0.08
0.09
0.10
SNP effect on Cardiac valvular disease
SNP effect on FSHB

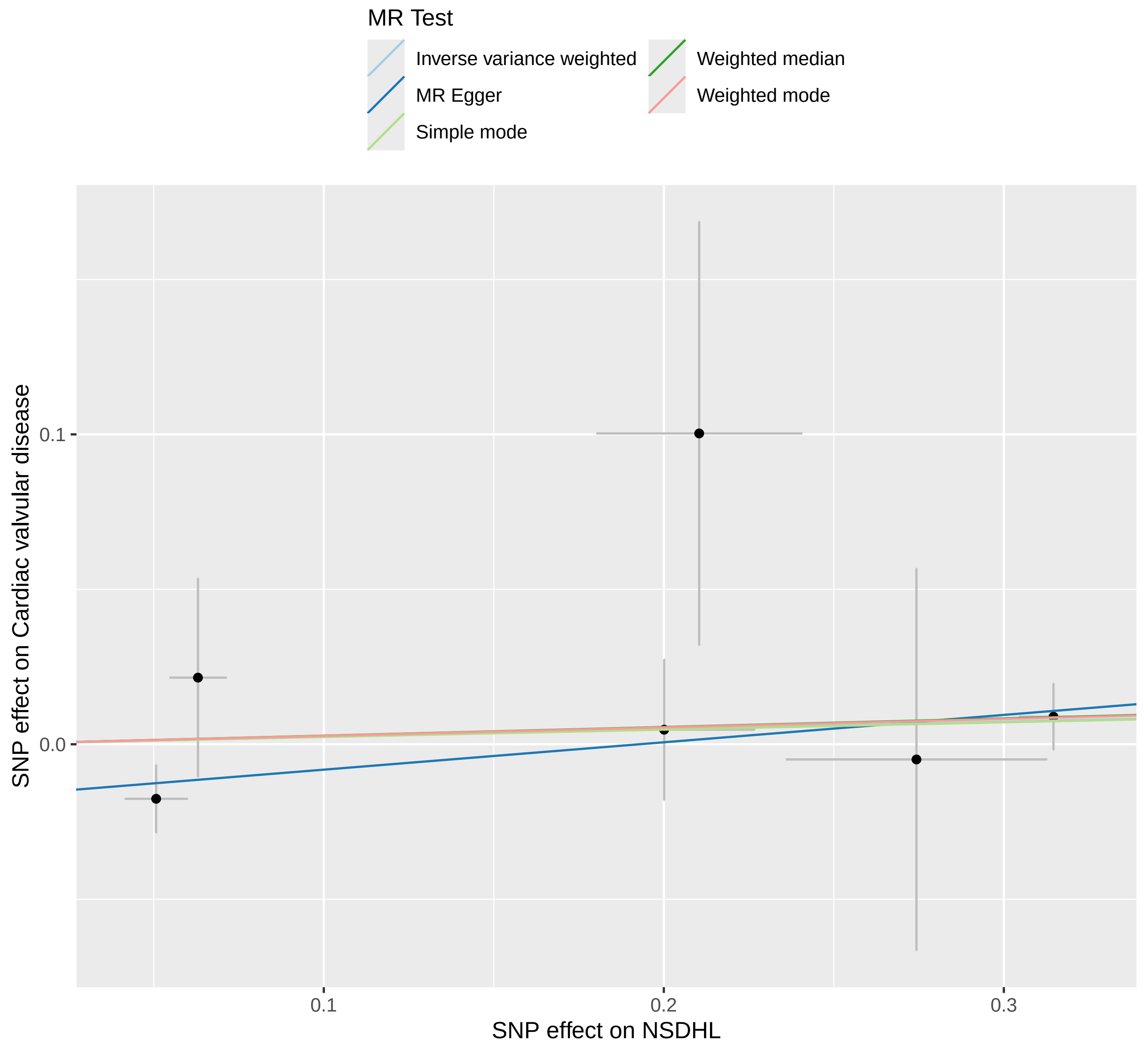
MR Test
Inverse variance weighted
MR Egger
Simple mode
Weighted median
Weighted mode
SNP effect on Cardiac valvular disease
0.1
0.0
0.1
0.2
0.3
SNP effect on NSDHL

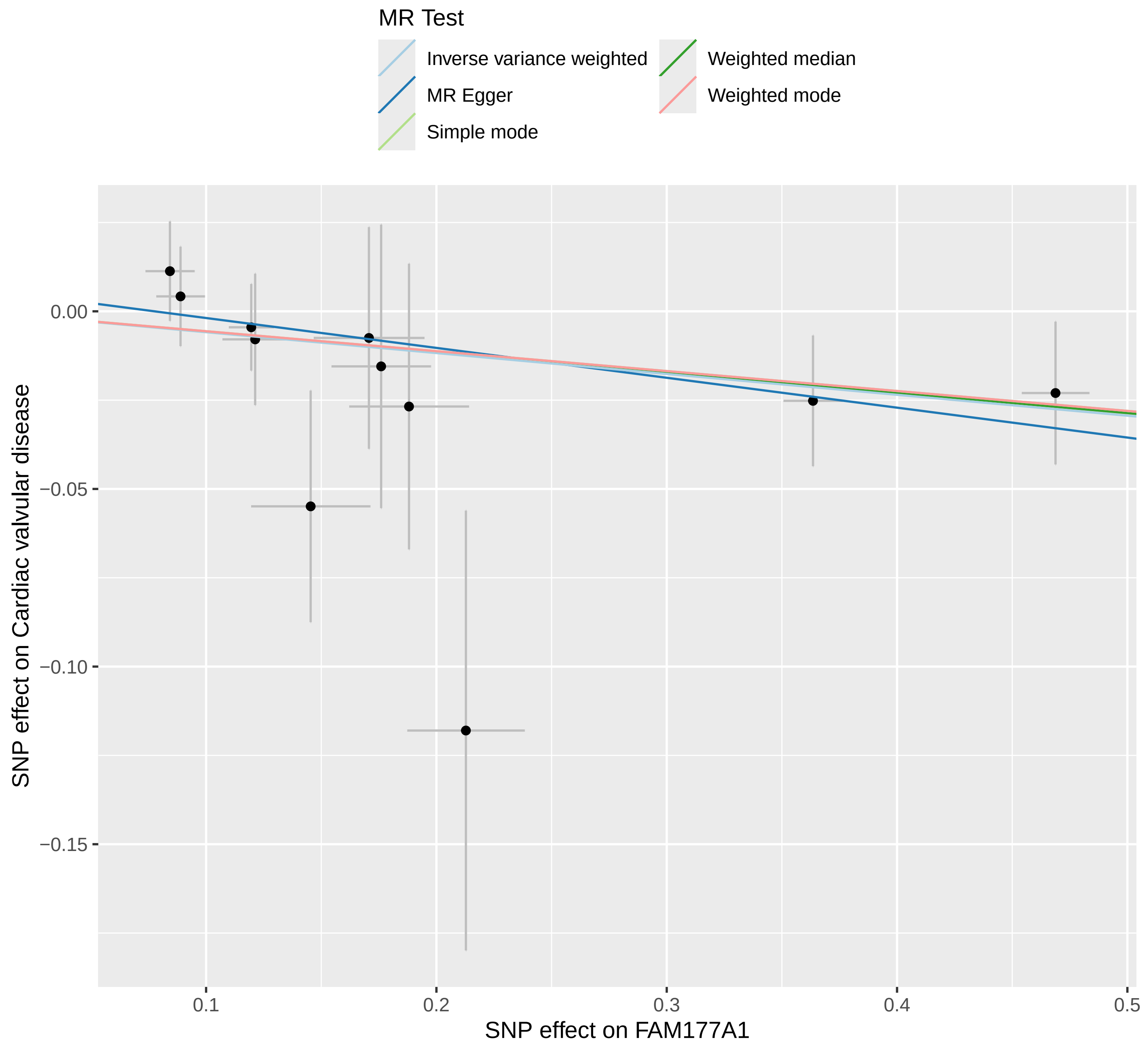
MR Test
Inverse variance weighted
MR Egger
Simple mode
Weighted median
Weighted mode
SNP effect on Cardiac valvular disease
0.00
−0.05
−0.10
−0.15
0.1
0.2
0.3
0.4
0.5
SNP effect on FAM177A1

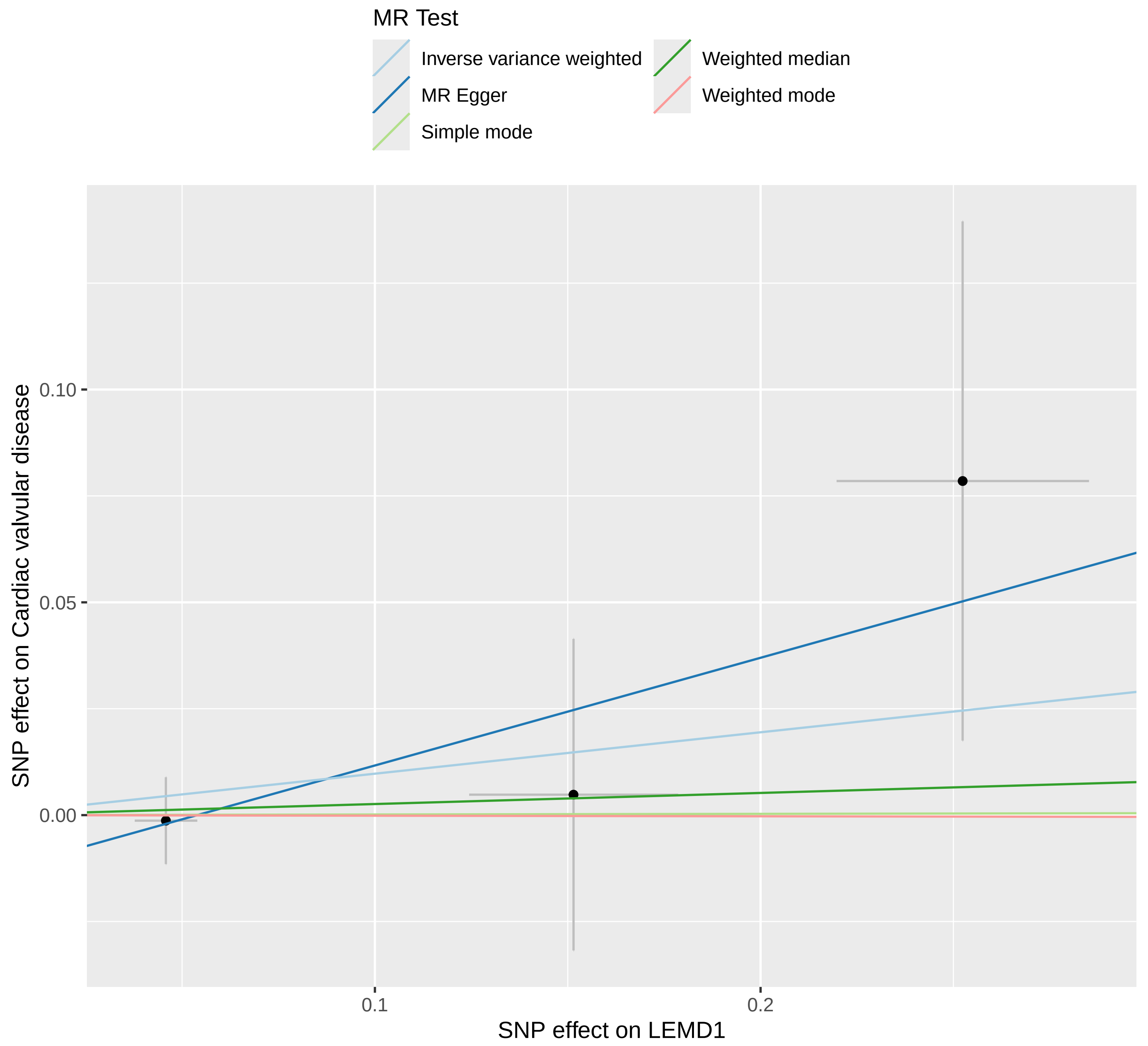
MR Test
Inverse variance weighted
MR Egger
Simple mode
Weighted median
Weighted mode
0.10
0.05
0.00
0.1
0.2
SNP effect on Cardiac valvular disease
SNP effect on LEMD1

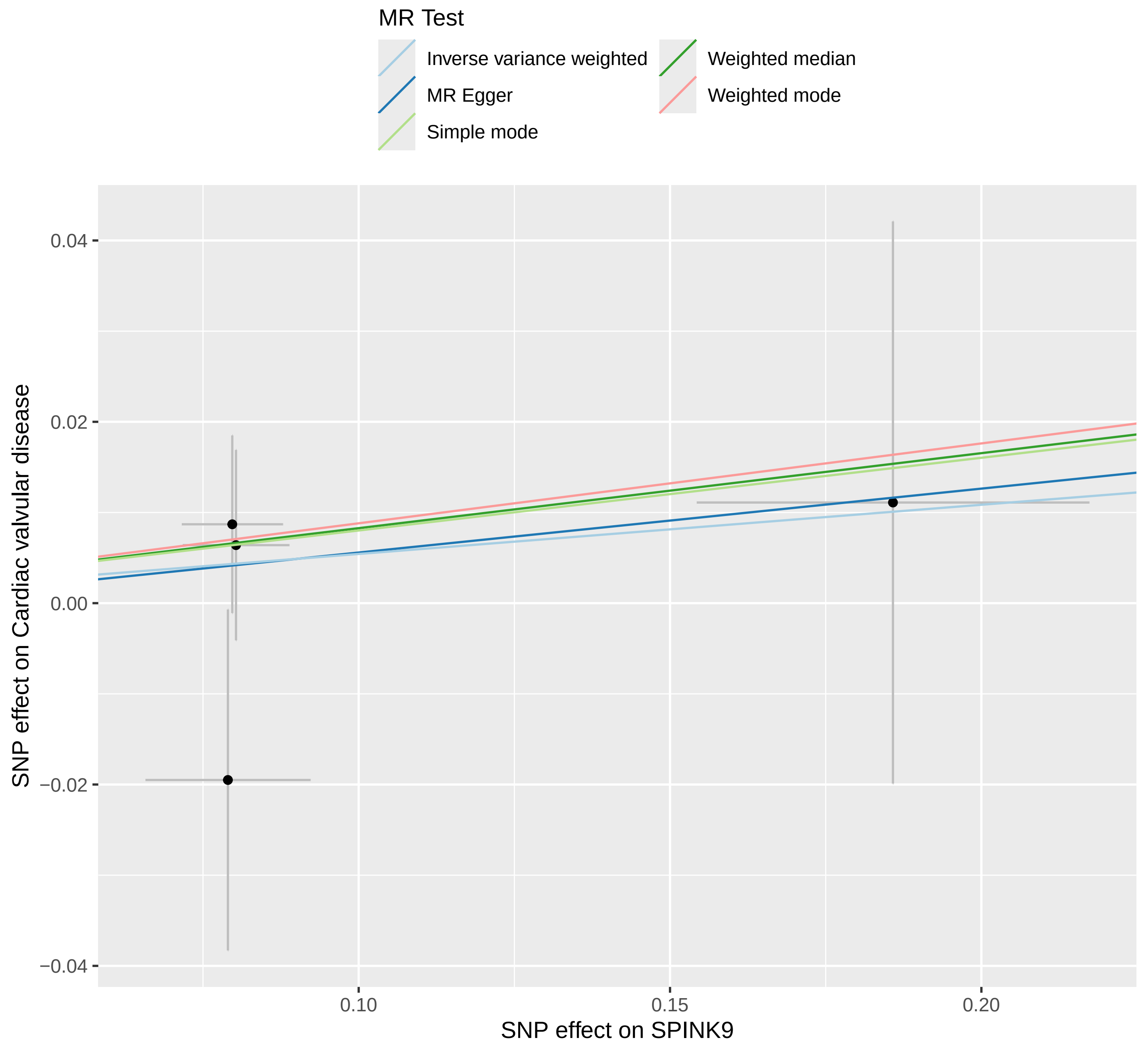
MR Test
Inverse variance weighted
MR Egger
Simple mode
Weighted median
Weighted mode
0.04
0.02
0.00
−0.02
−0.04
SNP effect on Cardiac valvular disease
0.10
0.15
0.20
SNP effect on SPINK9

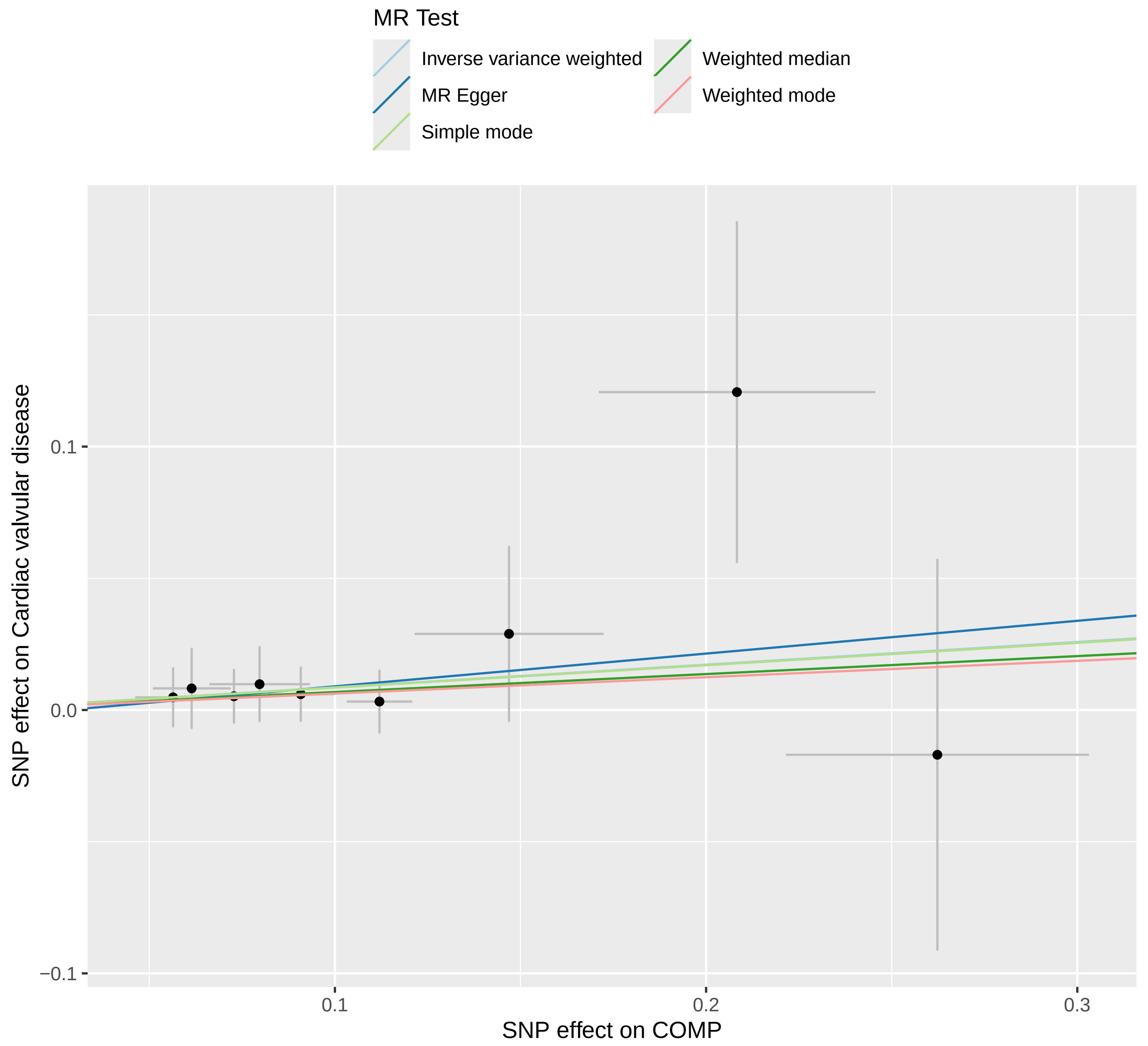
MR Test
Inverse variance weighted
MR Egger
Simple mode
Weighted median
Weighted mode
0.1
0.0
−0.1
0.1
0.2
0.3
SNP effect on COMP
SNP effect on Cardiac valvular disease

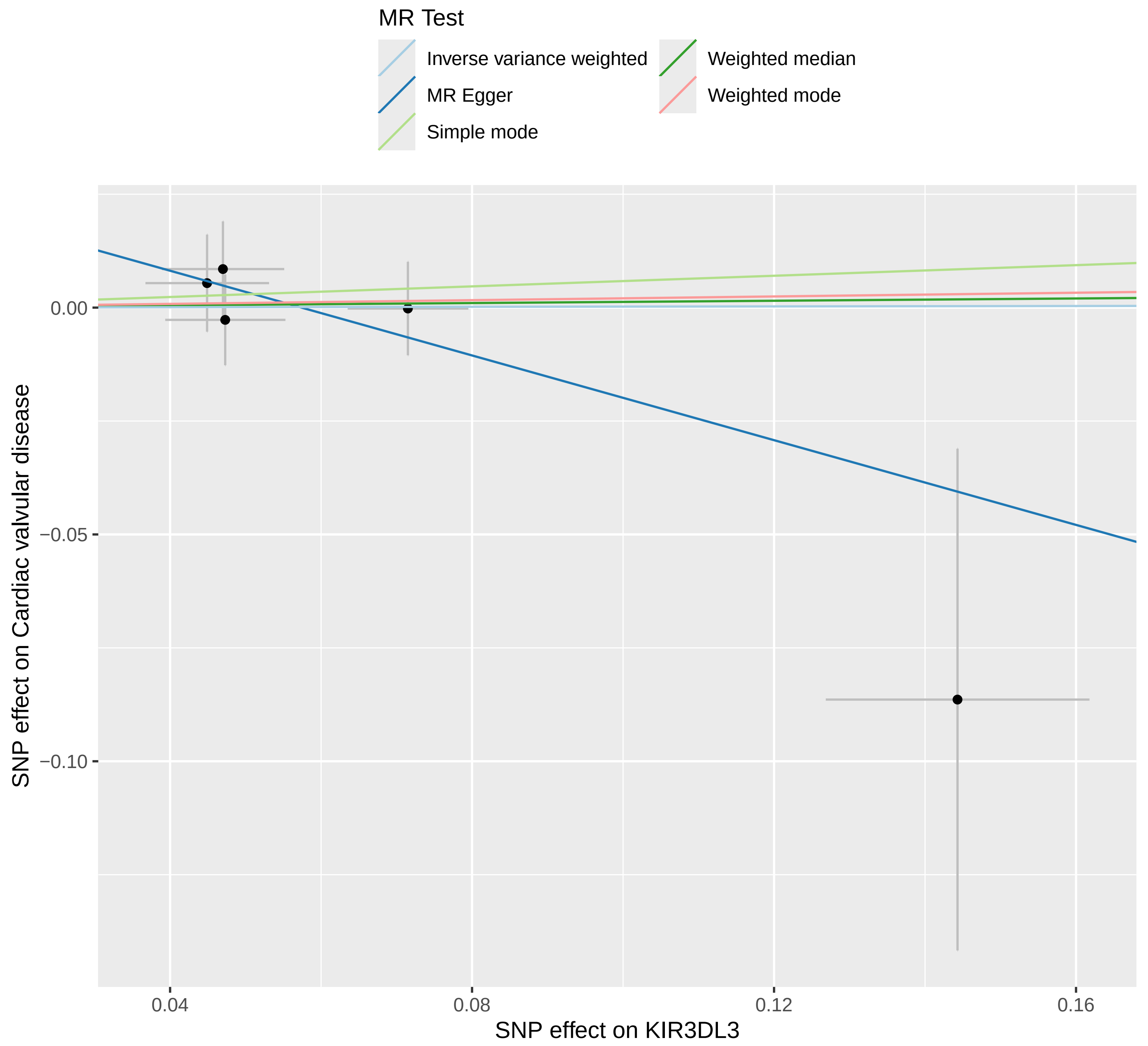
MR Test
Inverse variance weighted
MR Egger
Simple mode
Weighted median
Weighted mode
0.00
−0.05
−0.10
0.04
0.08
0.12
0.16
SNP effect on KIR3DL3
SNP effect on Cardiac valvular disease

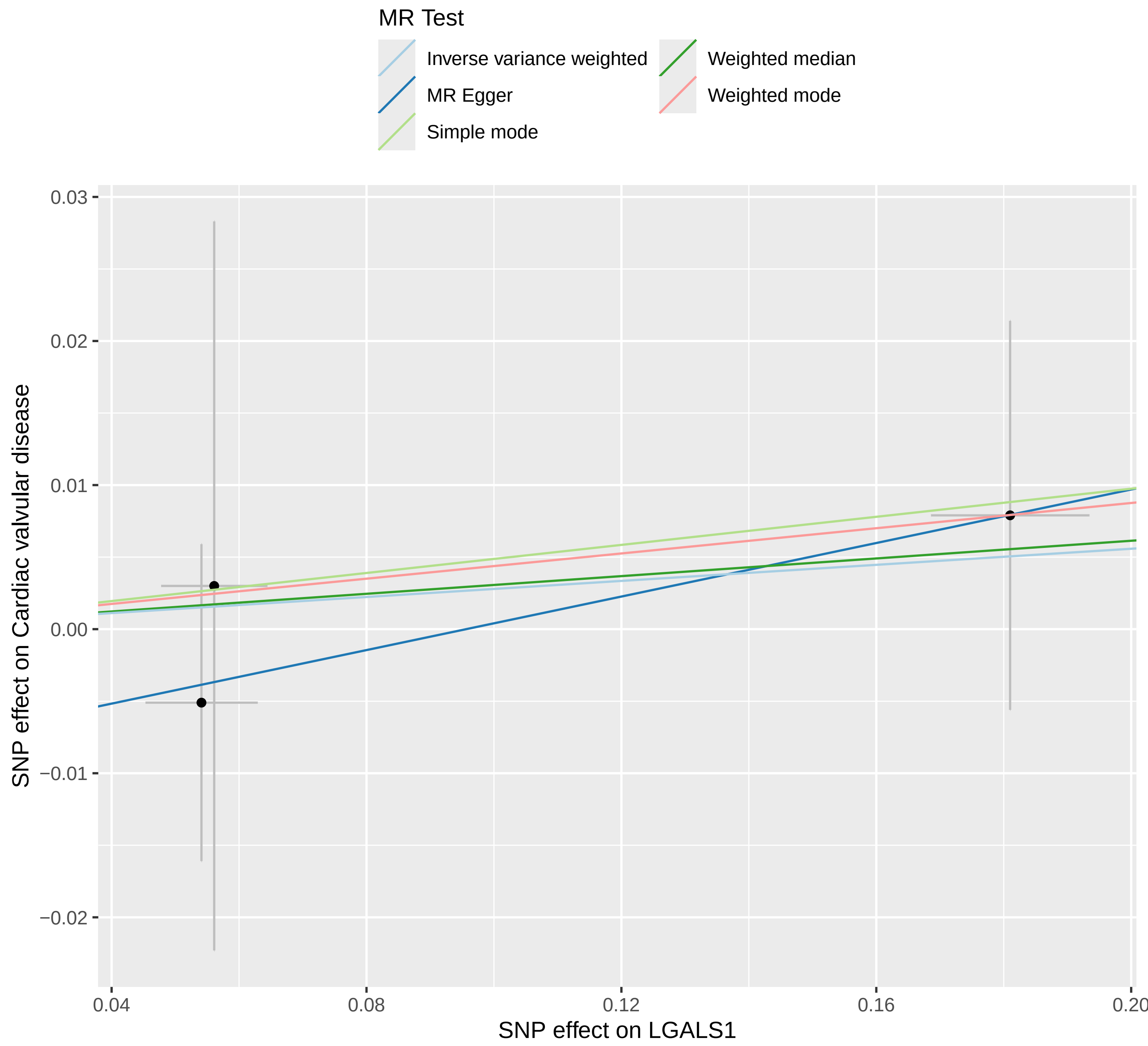

MR Test
Inverse variance weighted
MR Egger
Simple mode
Weighted median
Weighted mode
0.03
0.02
0.01
0.00
−0.01
−0.02
SNP effect on Cardiac valvular disease
0.04
0.08
0.12
0.16
0.20
SNP effect on LGALS1

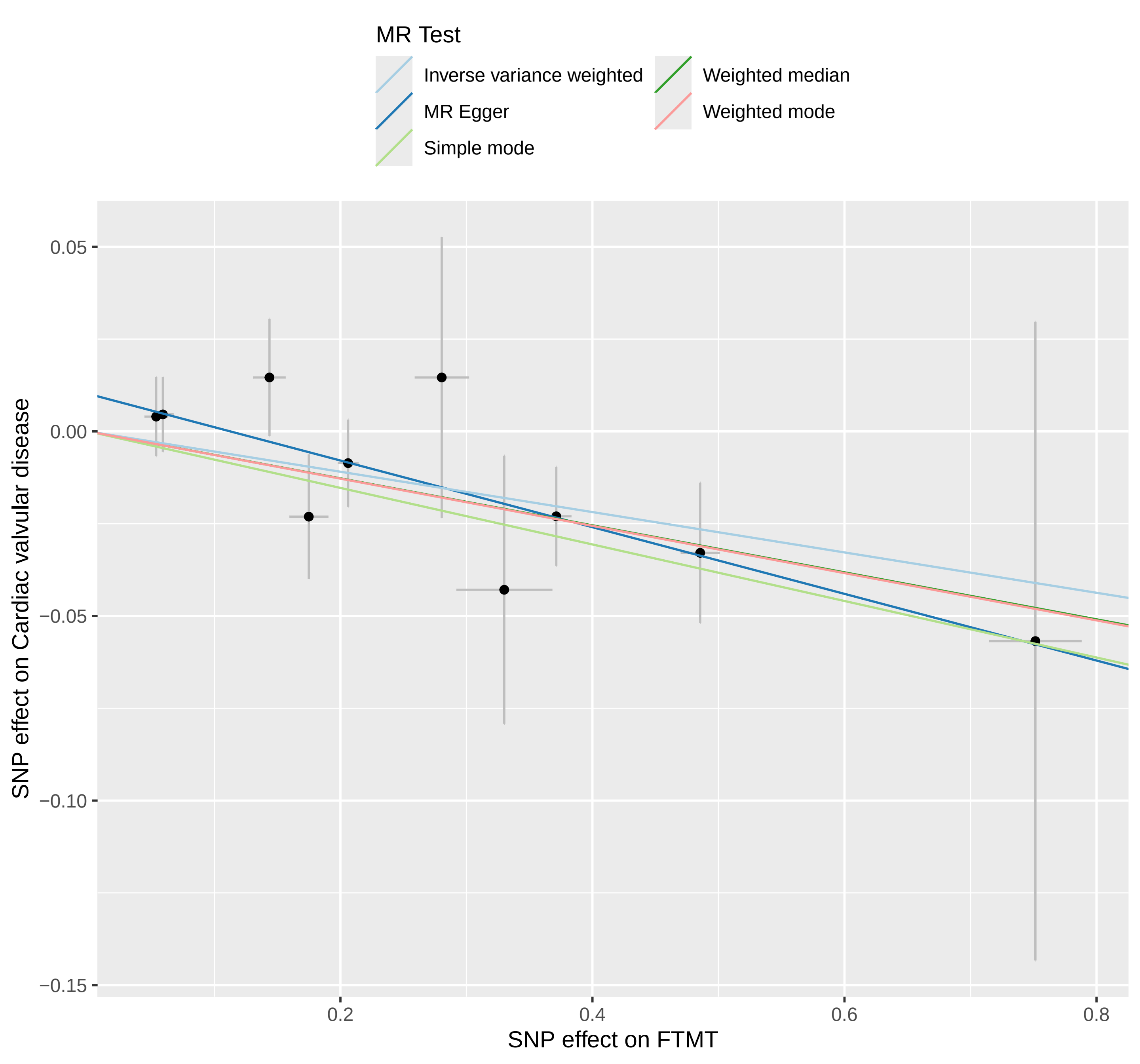
MR Test
Inverse variance weighted
MR Egger
Simple mode
Weighted median
Weighted mode
0.05
0.00
−0.05
−0.10
−0.15
0.2
0.4
0.6
0.8
SNP effect on Cardiac valvular disease
SNP effect on FTMT

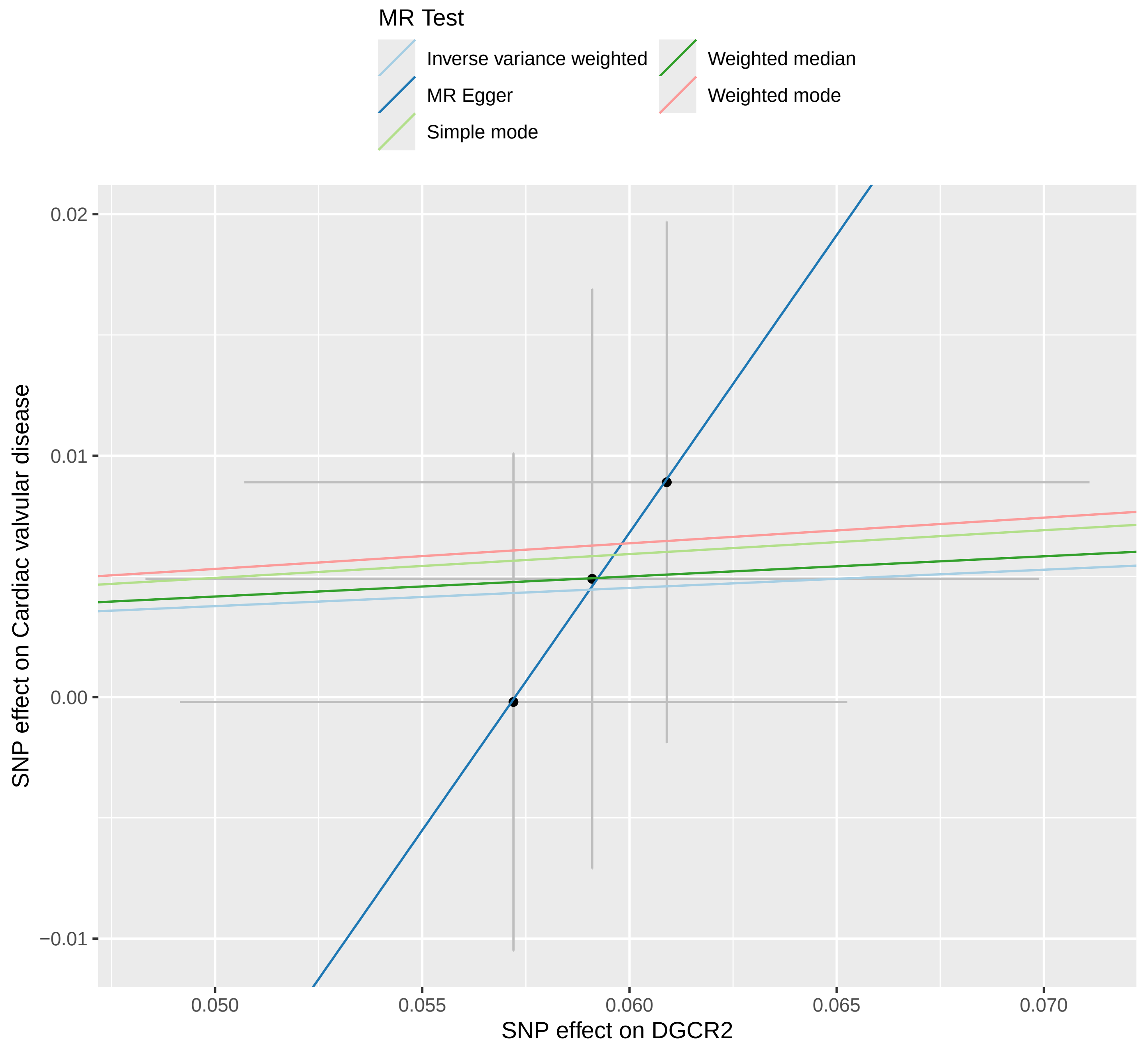
MR Test
Inverse variance weighted
MR Egger
Simple mode
Weighted median
Weighted mode
0.02
0.01
0.00
−0.01
0.050
0.055
0.060
0.065
0.070
SNP effect on DGCR2
SNP effect on Cardiac valvular disease

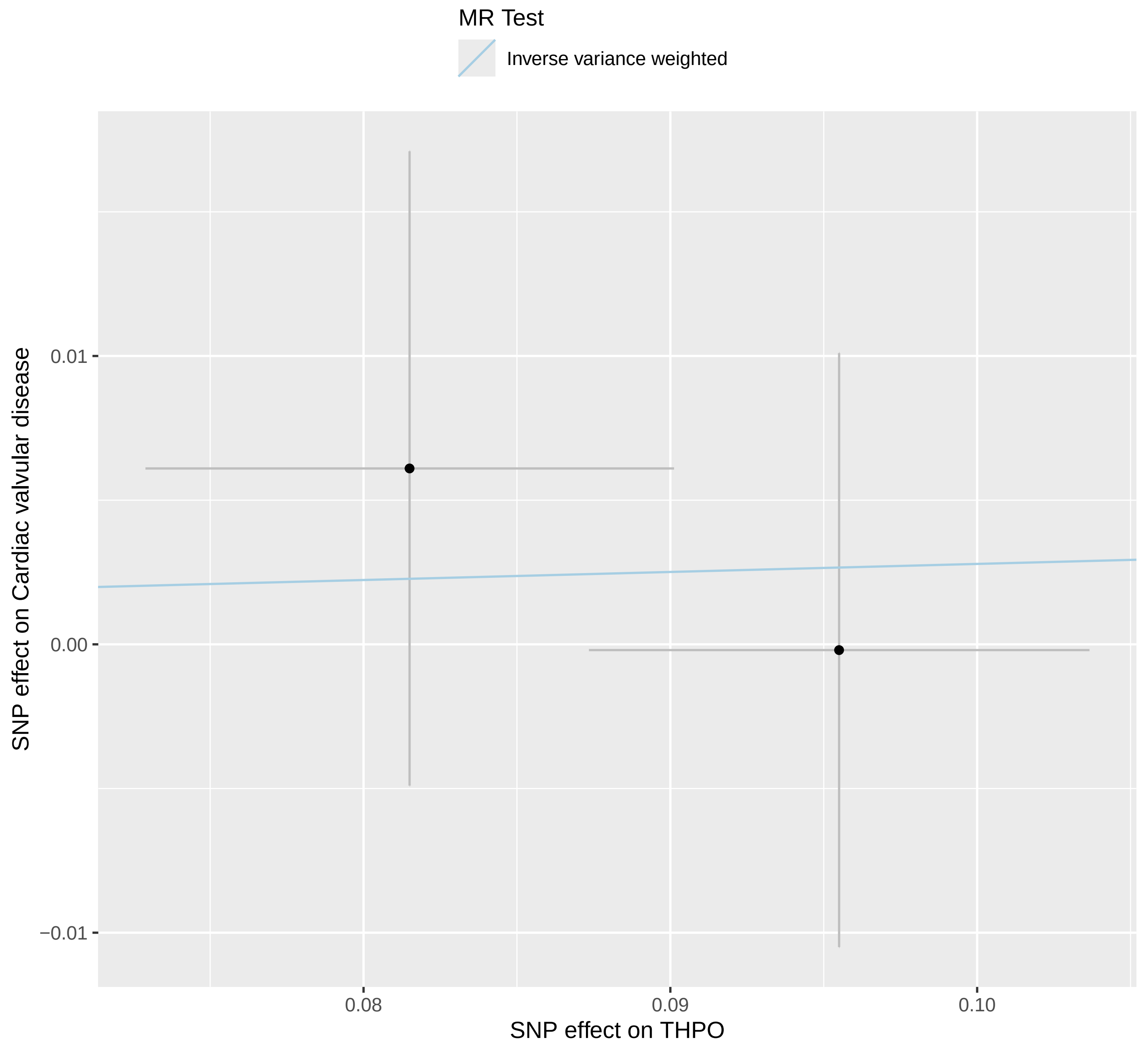

MR Test
Inverse variance weighted
0.01
0.00
−0.01
SNP effect on Cardiac valvular disease
0.08
0.09
0.10
SNP effect on THPO

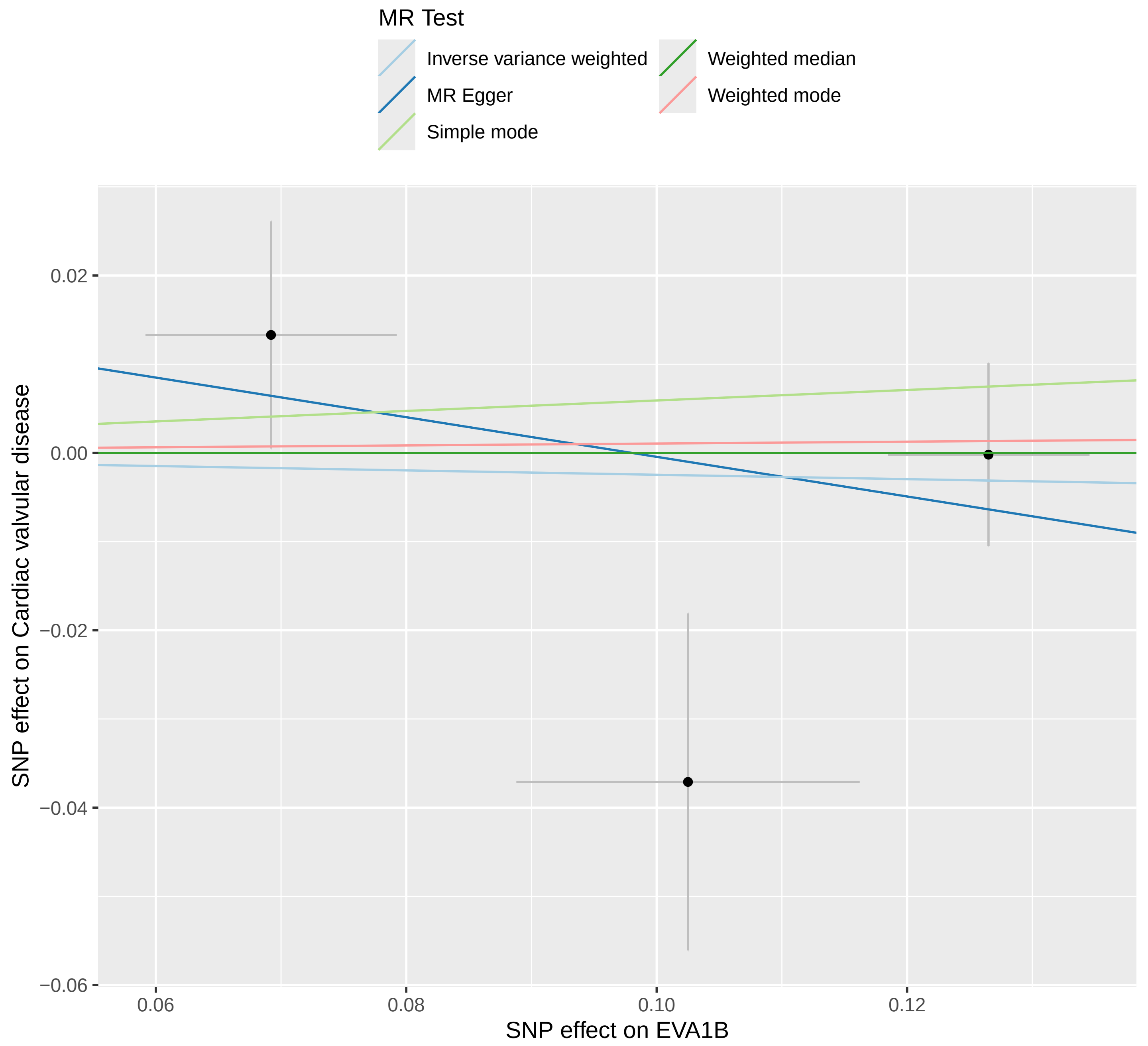
MR Test
Inverse variance weighted
MR Egger
Simple mode
Weighted median
Weighted mode
0.02
0.00
−0.02
−0.04
−0.06
SNP effect on Cardiac valvular disease
0.06
0.08
0.10
0.12
SNP effect on EVA1B

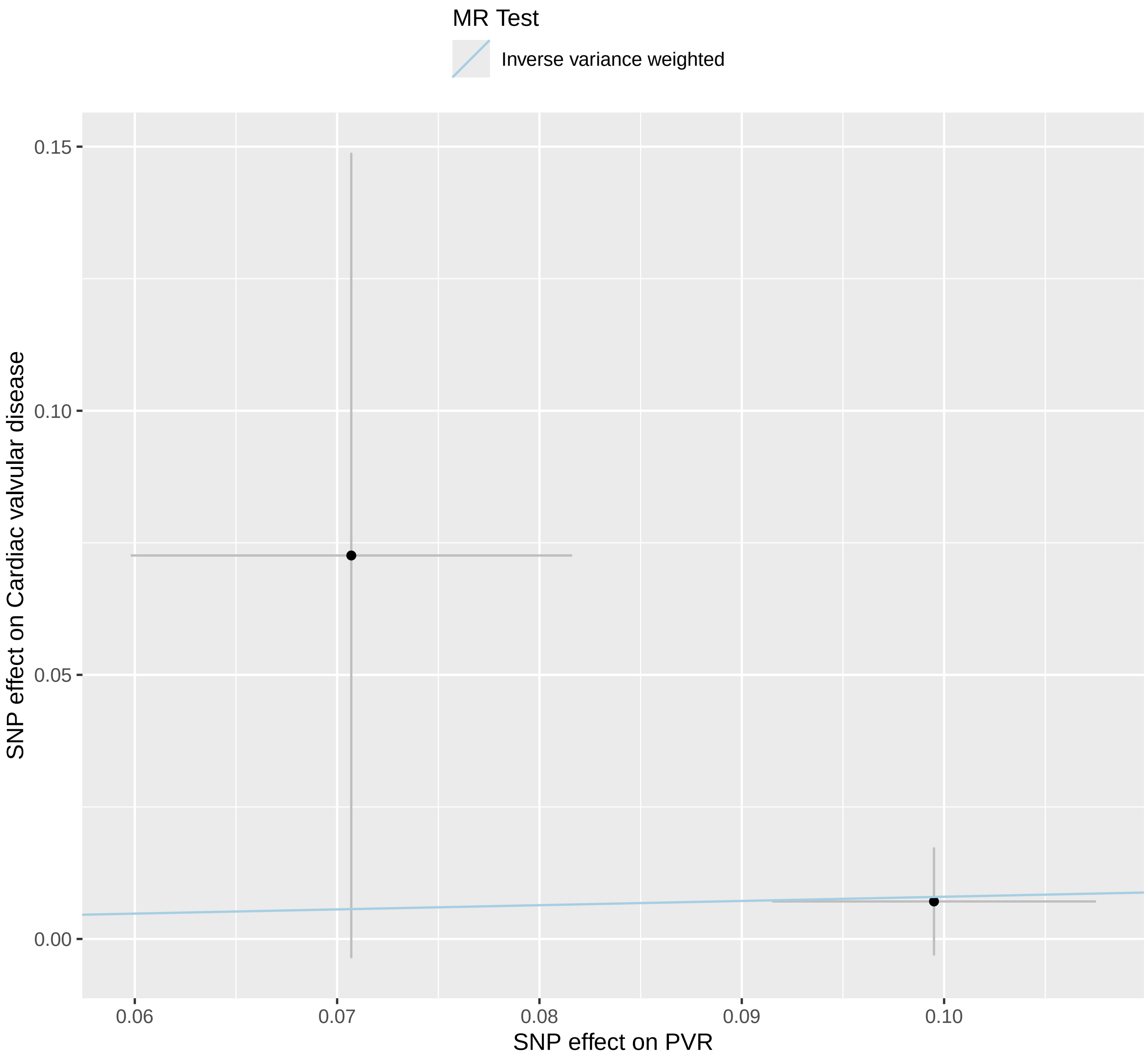
MR Test
Inverse variance weighted
0.15
0.10
0.05
0.00
0.06
0.07
0.08
0.09
0.10
SNP effect on Cardiac valvular disease
SNP effect on PVR

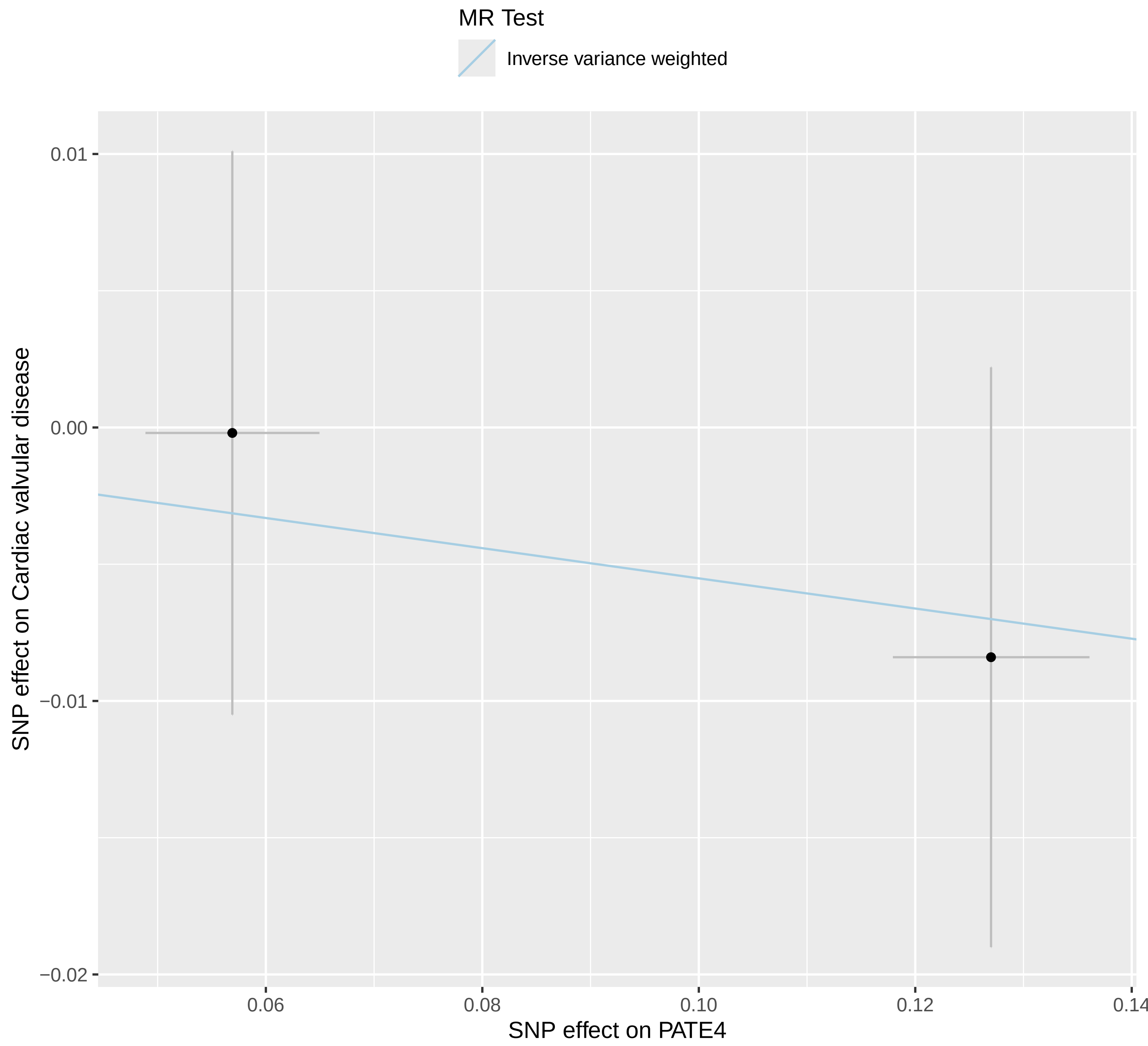
MR Test
Inverse variance weighted
0.01
0.00
−0.01
−0.02
SNP effect on Cardiac valvular disease
0.06
0.08
0.10
0.12
0.14
SNP effect on PATE4

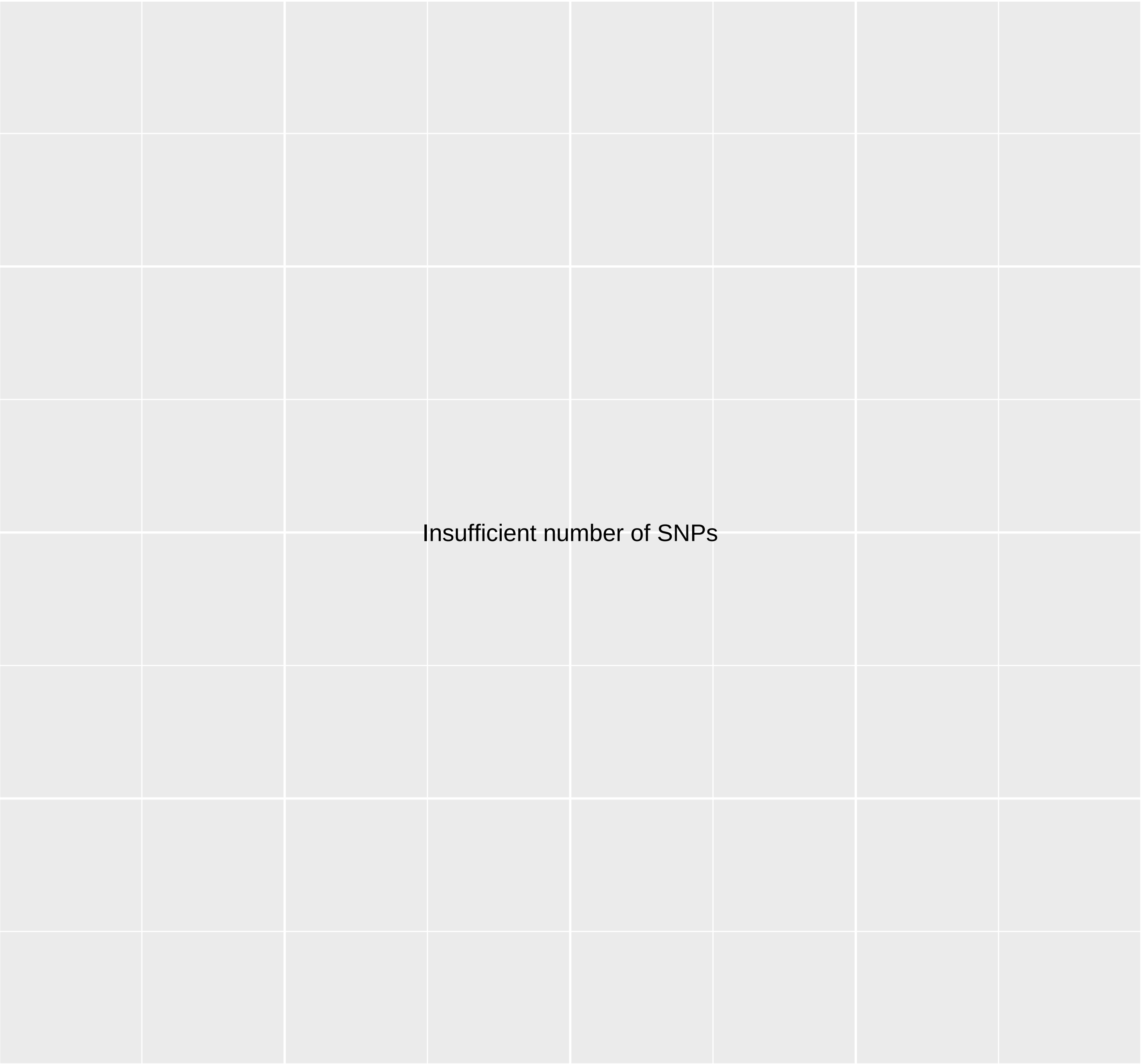
Insufficient number of SNPs

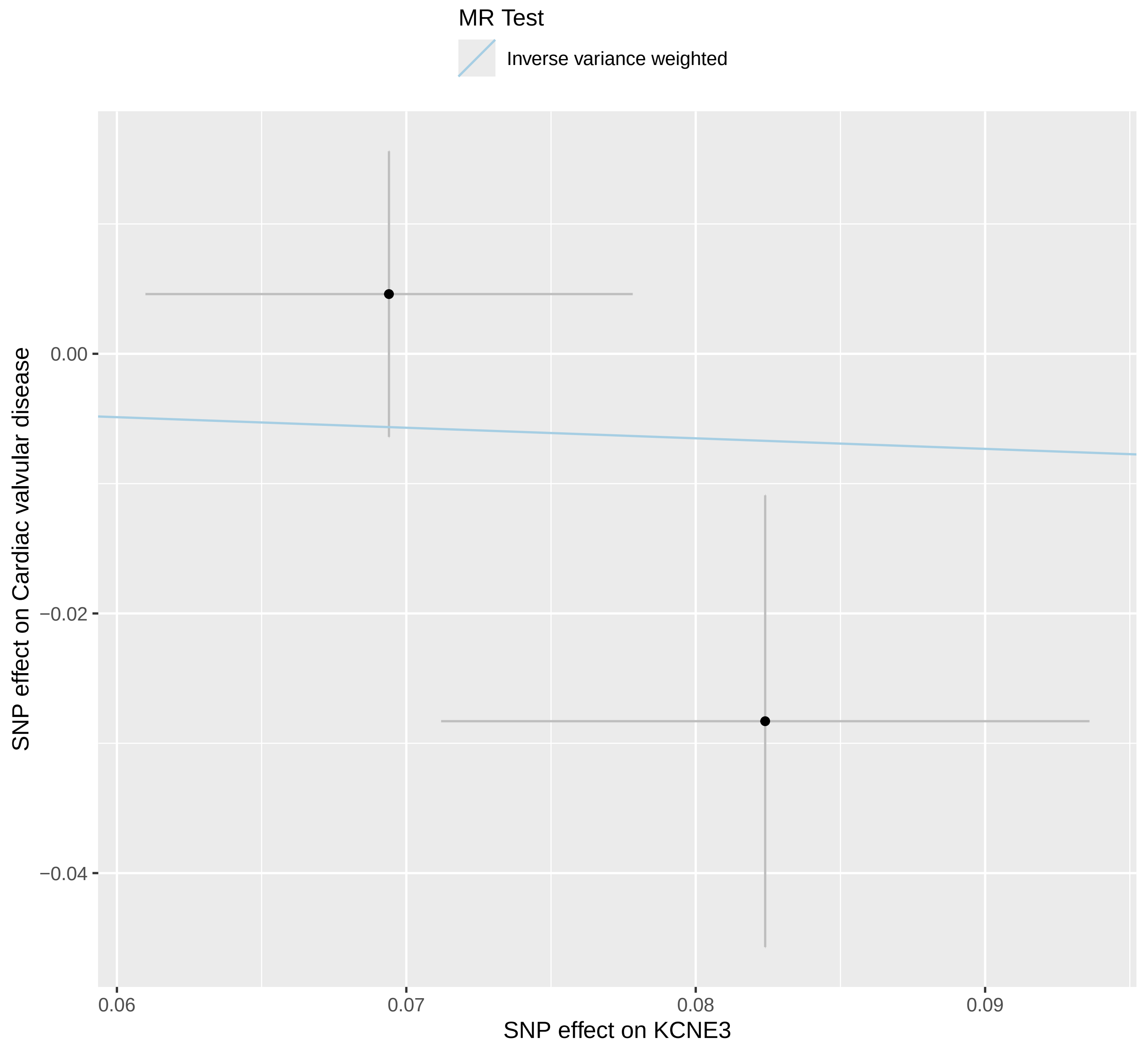

MR Test
Inverse variance weighted
0.00
−0.02
−0.04
0.06
0.07
0.08
0.09
SNP effect on Cardiac valvular disease
SNP effect on KCNE3

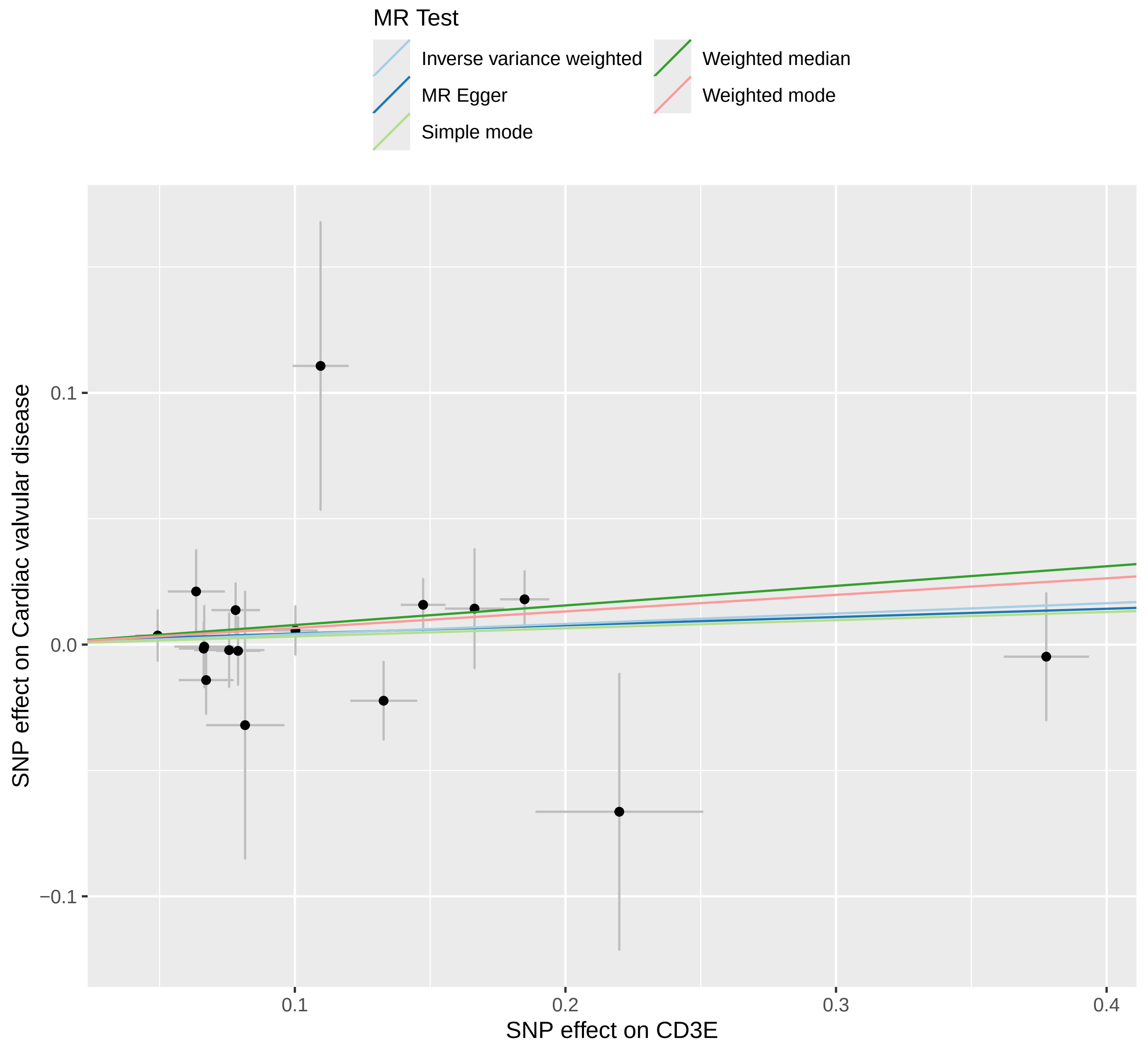
MR Test
Inverse variance weighted
MR Egger
Simple mode
Weighted median
Weighted mode
SNP effect on Cardiac valvular disease
0.1
0.0
−0.1
0.1
0.2
0.3
0.4
SNP effect on CD3E

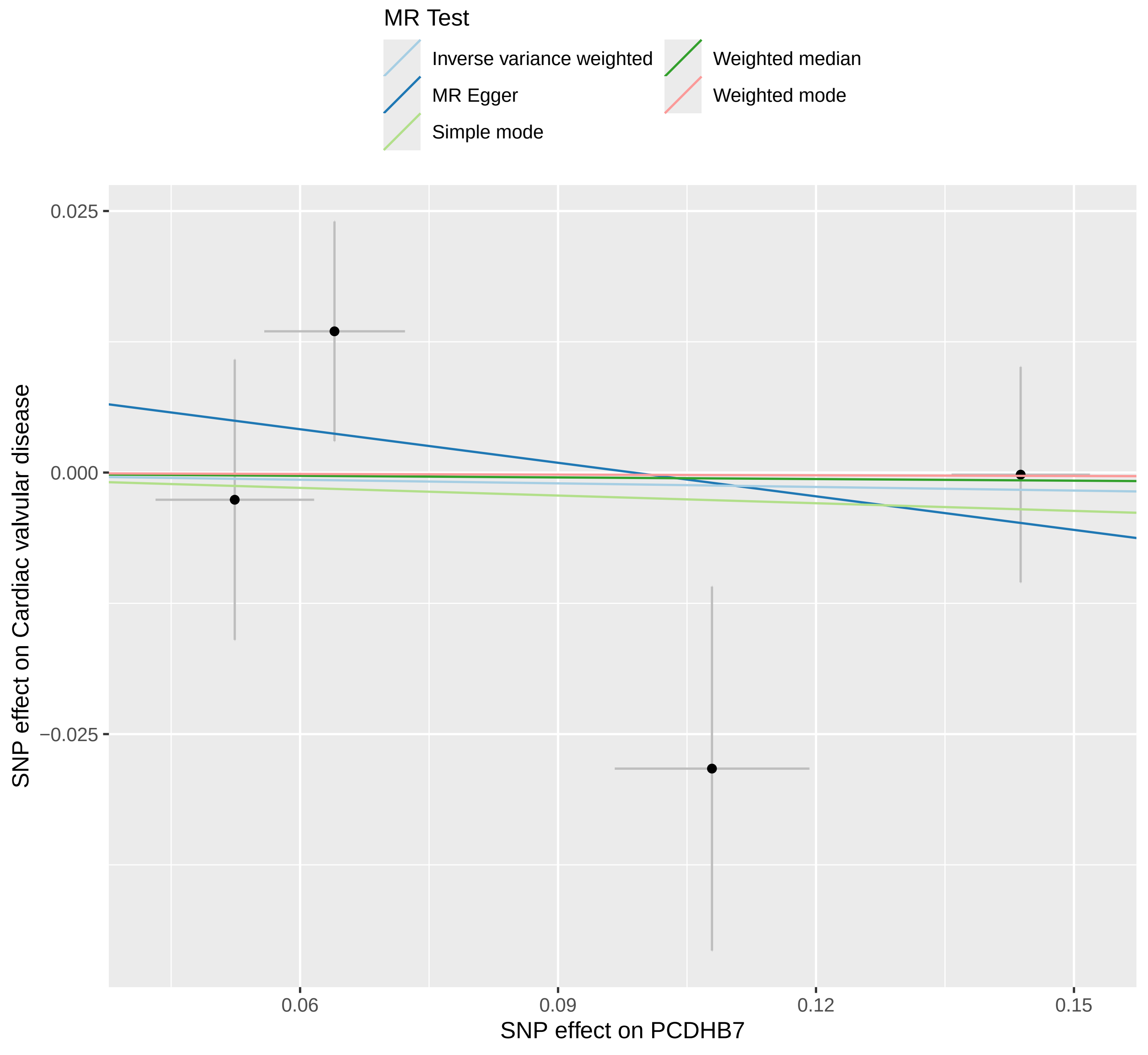
MR Test
Inverse variance weighted
MR Egger
Simple mode
Weighted median
Weighted mode
0.025
0.000
−0.025
0.06
0.09
0.12
0.15
SNP effect on PCDHB7
SNP effect on Cardiac valvular disease

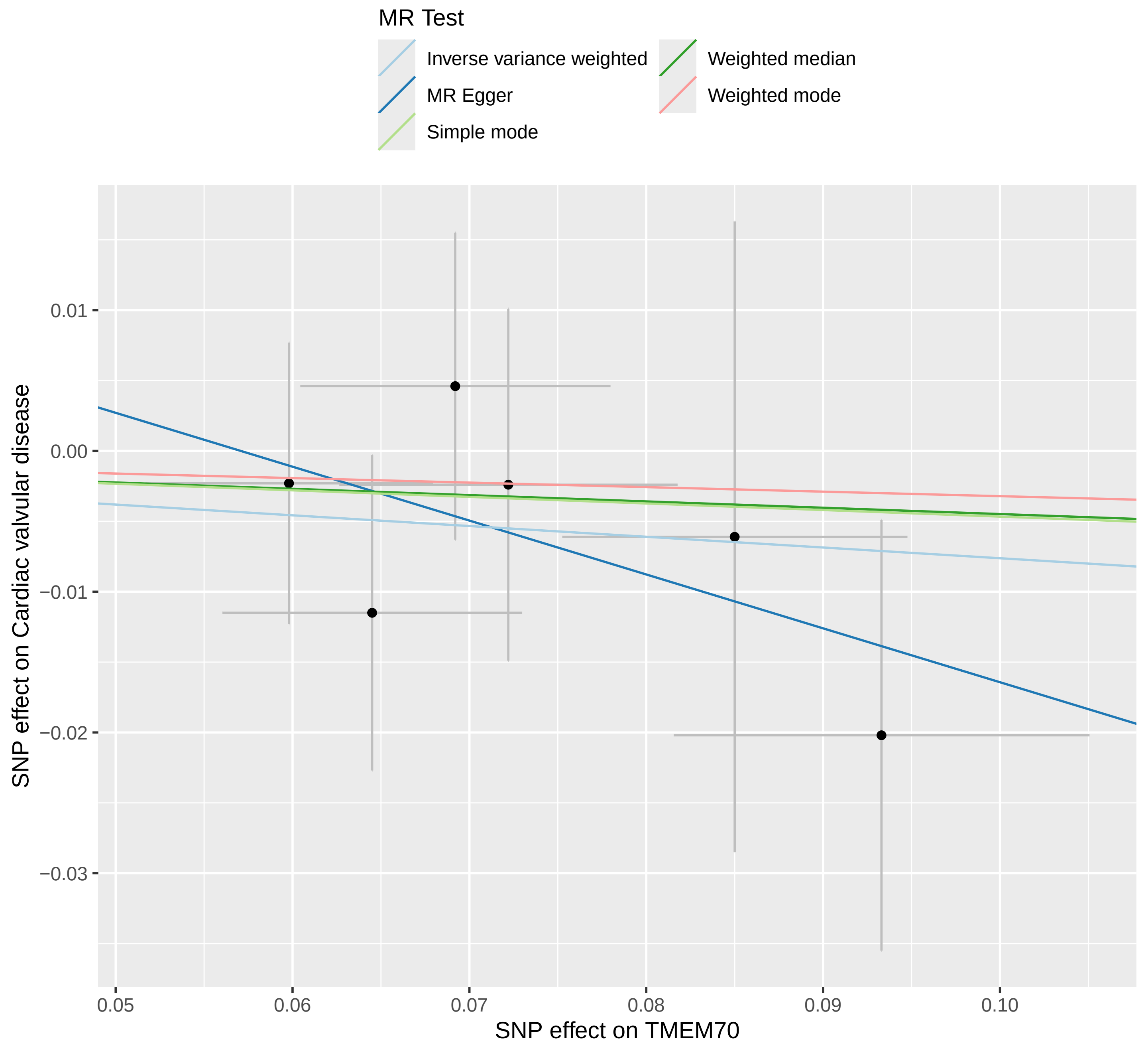
MR Test
Inverse variance weighted
MR Egger
Simple mode
Weighted median
Weighted mode
SNP effect on Cardiac valvular disease
0.01
0.00
−0.01
−0.02
−0.03
0.05
0.06
0.07
0.08
0.09
0.10
SNP effect on TMEM70

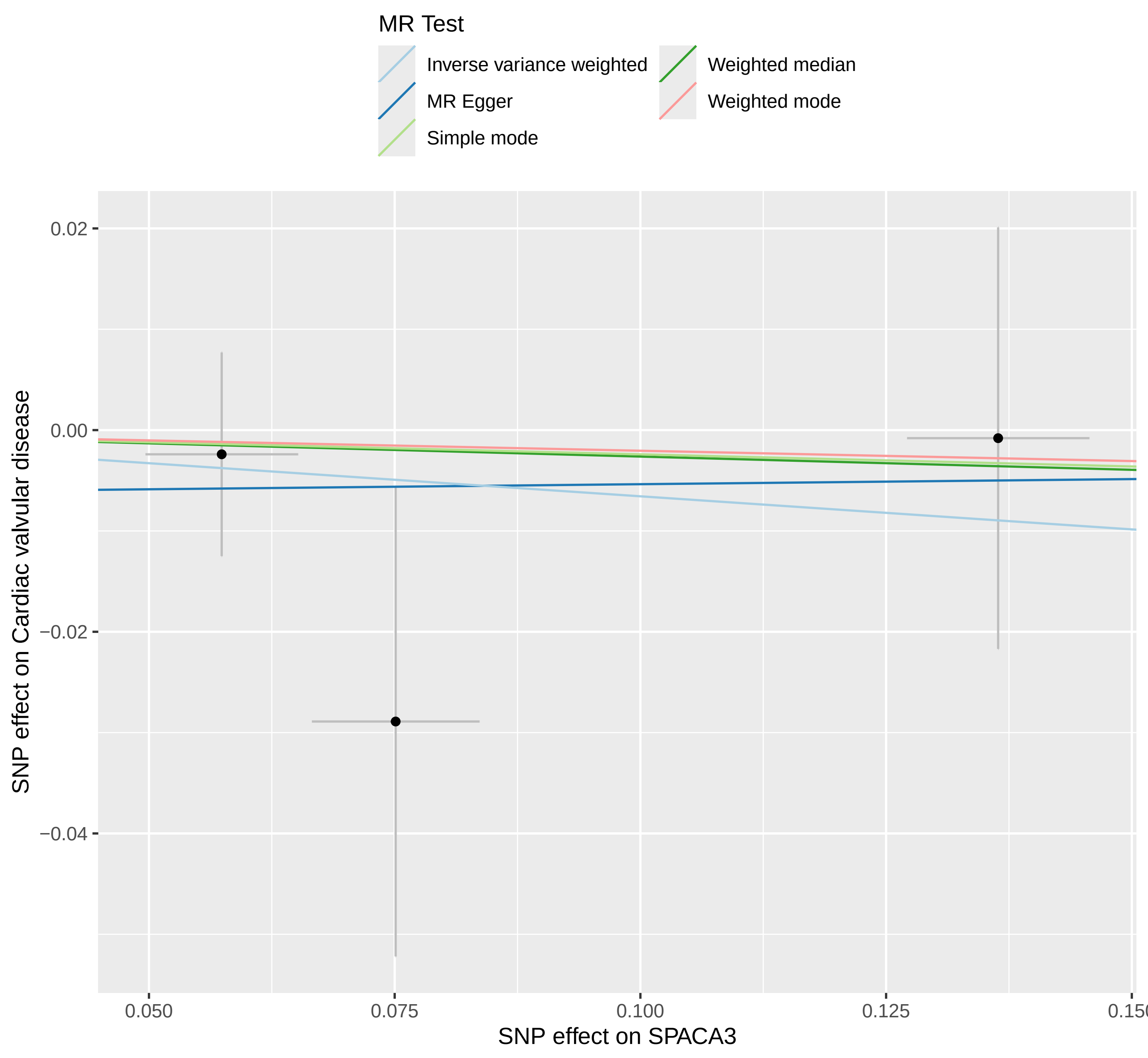

MR Test
Inverse variance weighted
MR Egger
Simple mode
Weighted median
Weighted mode
0.02
0.00
−0.02
−0.04
SNP effect on Cardiac valvular disease
0.050
0.075
0.100
0.125
SNP effect on SPACA3

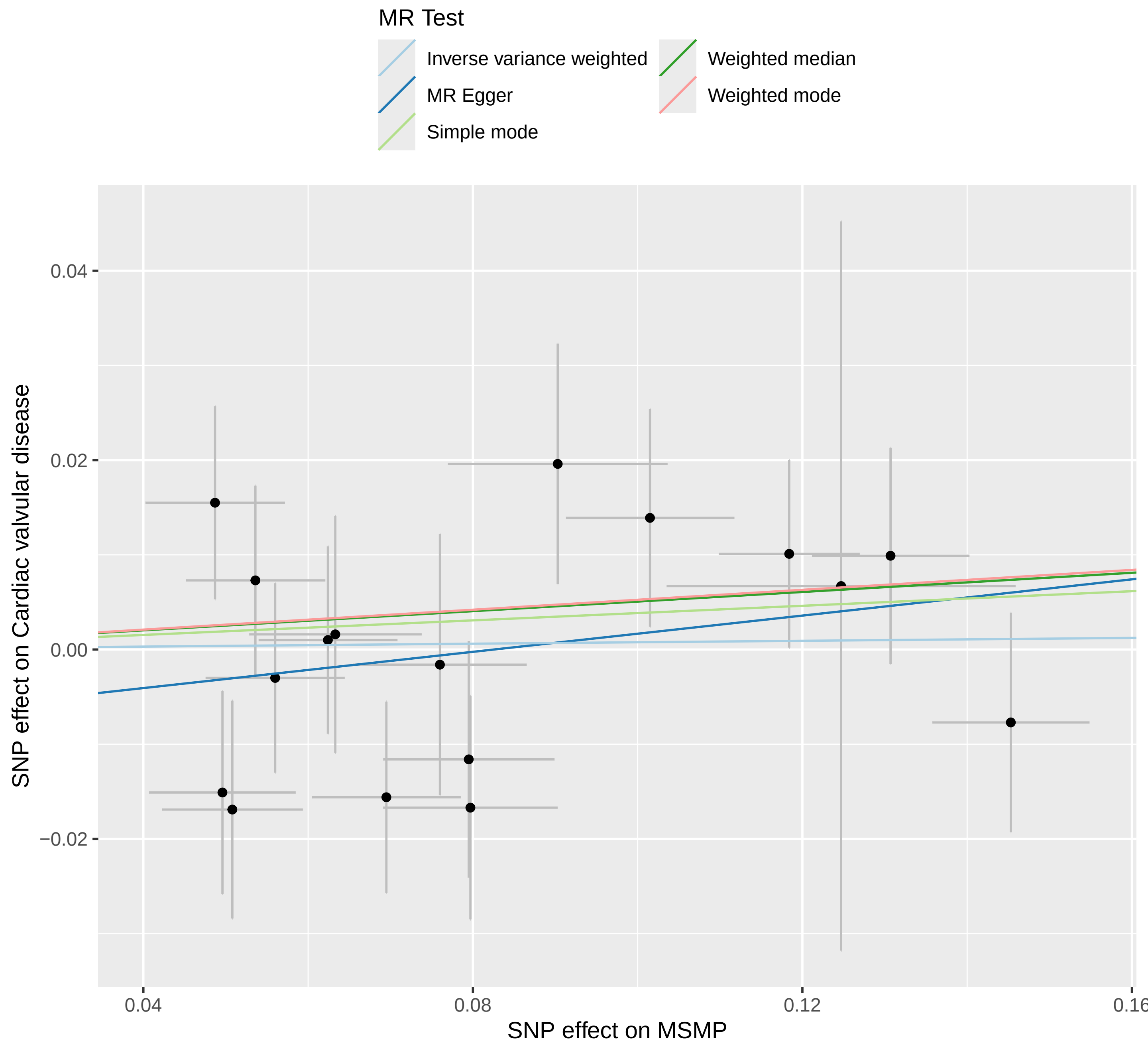

MR Test
Inverse variance weighted
MR Egger
Simple mode
Weighted median
Weighted mode
0.04
0.02
0.00
−0.02
SNP effect on Cardiac valvular disease
0.04
0.08
0.12
0.16
SNP effect on MSMP

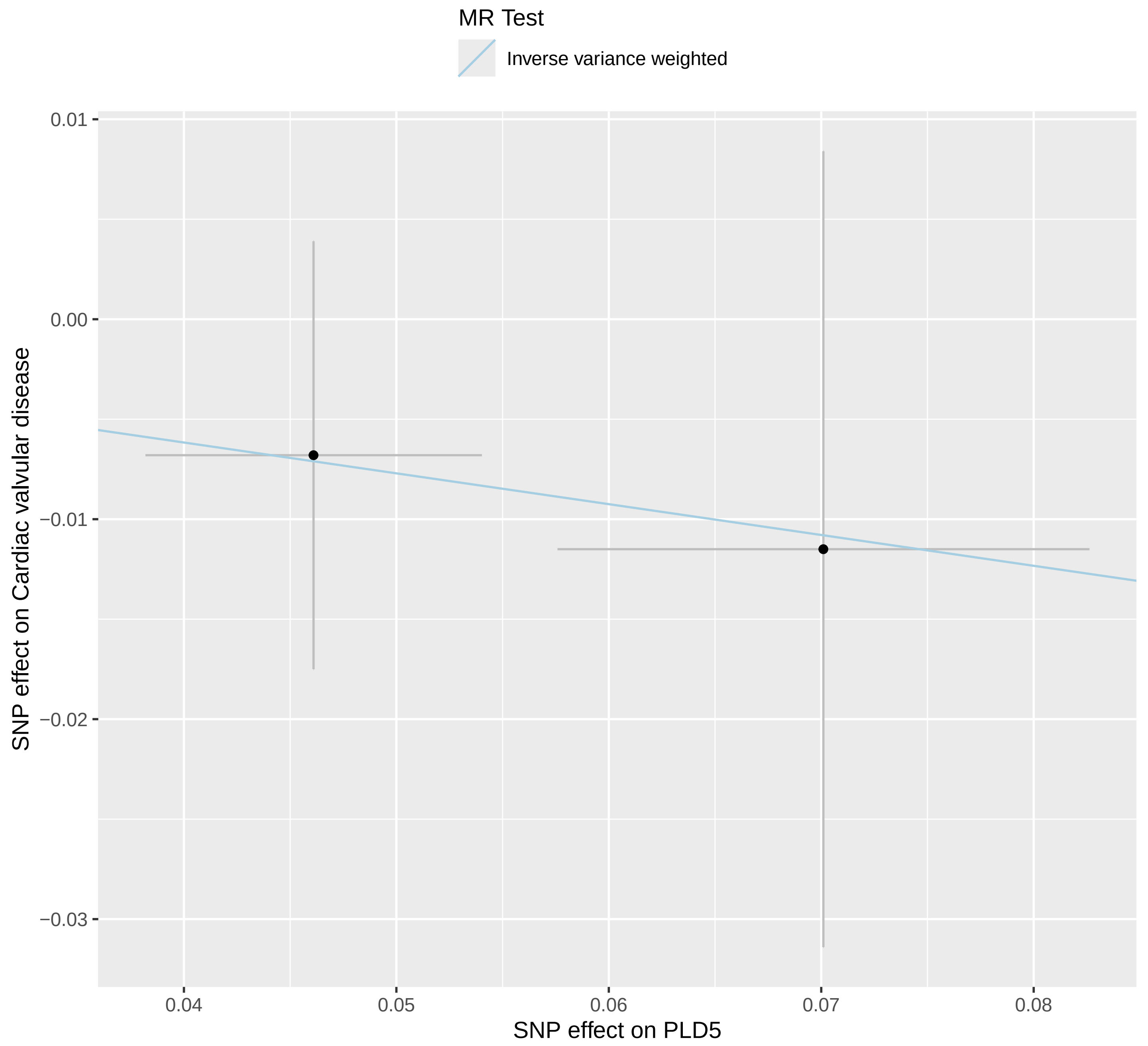
MR Test
Inverse variance weighted
0.01
0.00
−0.01
−0.02
−0.03
0.04
0.05
0.06
0.07
0.08
SNP effect on Cardiac valvular disease
SNP effect on PLD5

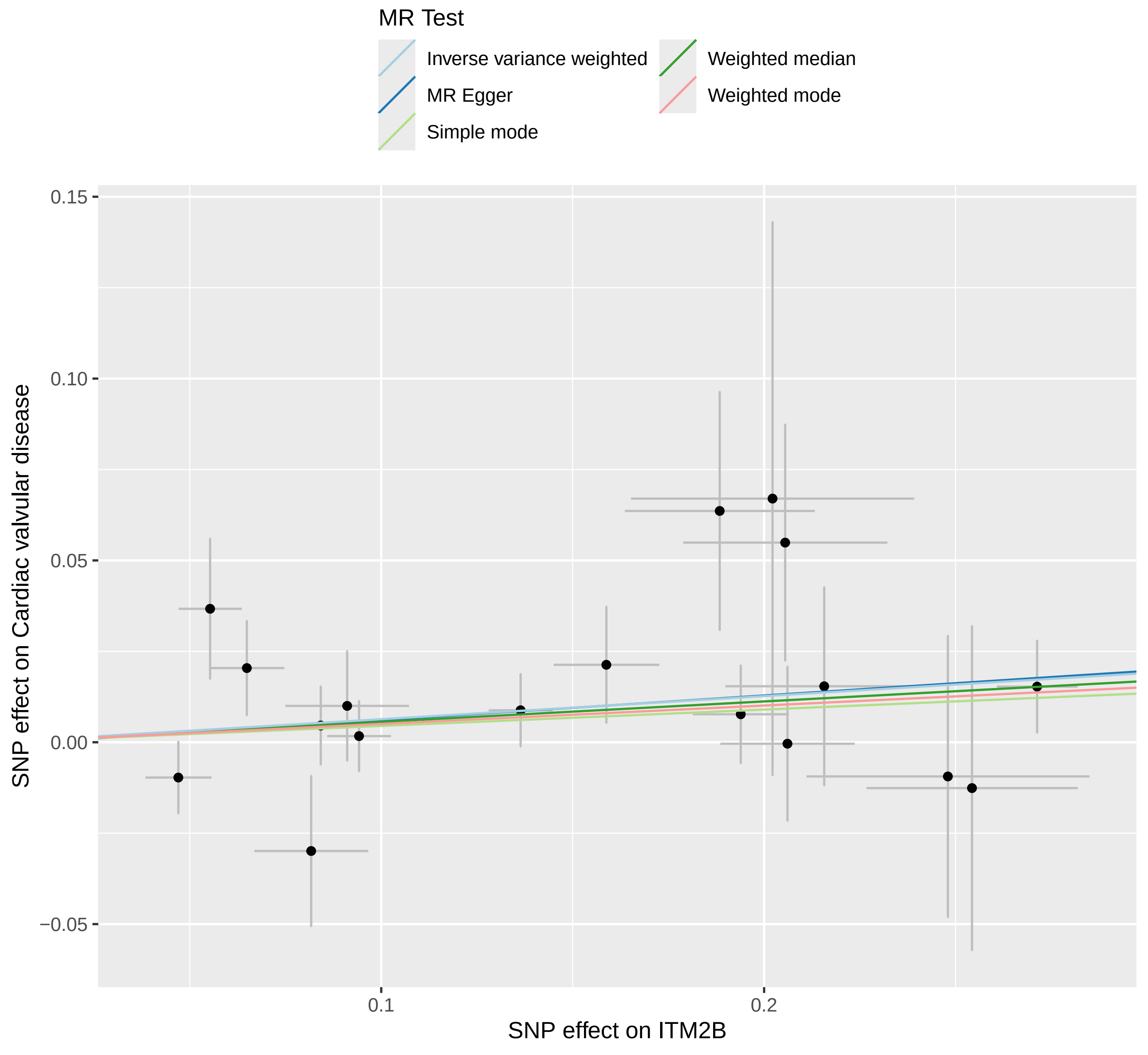
MR Test
Inverse variance weighted
MR Egger
Simple mode
Weighted median
Weighted mode
0.15
0.10
0.05
0.00
−0.05
SNP effect on Cardiac valvular disease
0.1
0.2
SNP effect on ITM2B

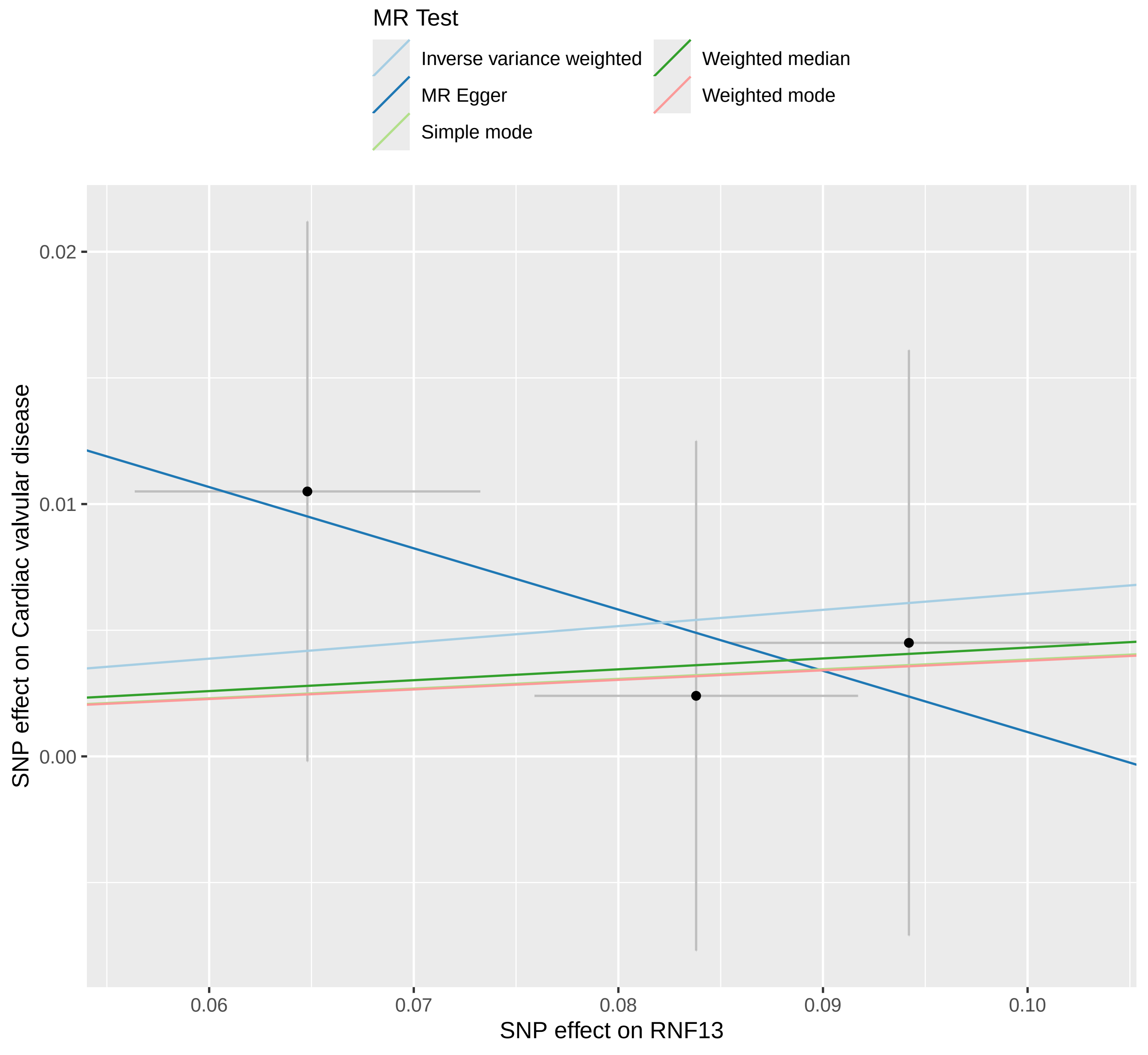
MR Test
Inverse variance weighted
MR Egger
Simple mode
Weighted median
Weighted mode
0.02
0.01
0.00
SNP effect on Cardiac valvular disease
0.06
0.07
0.08
0.09
0.10
SNP effect on RNF13

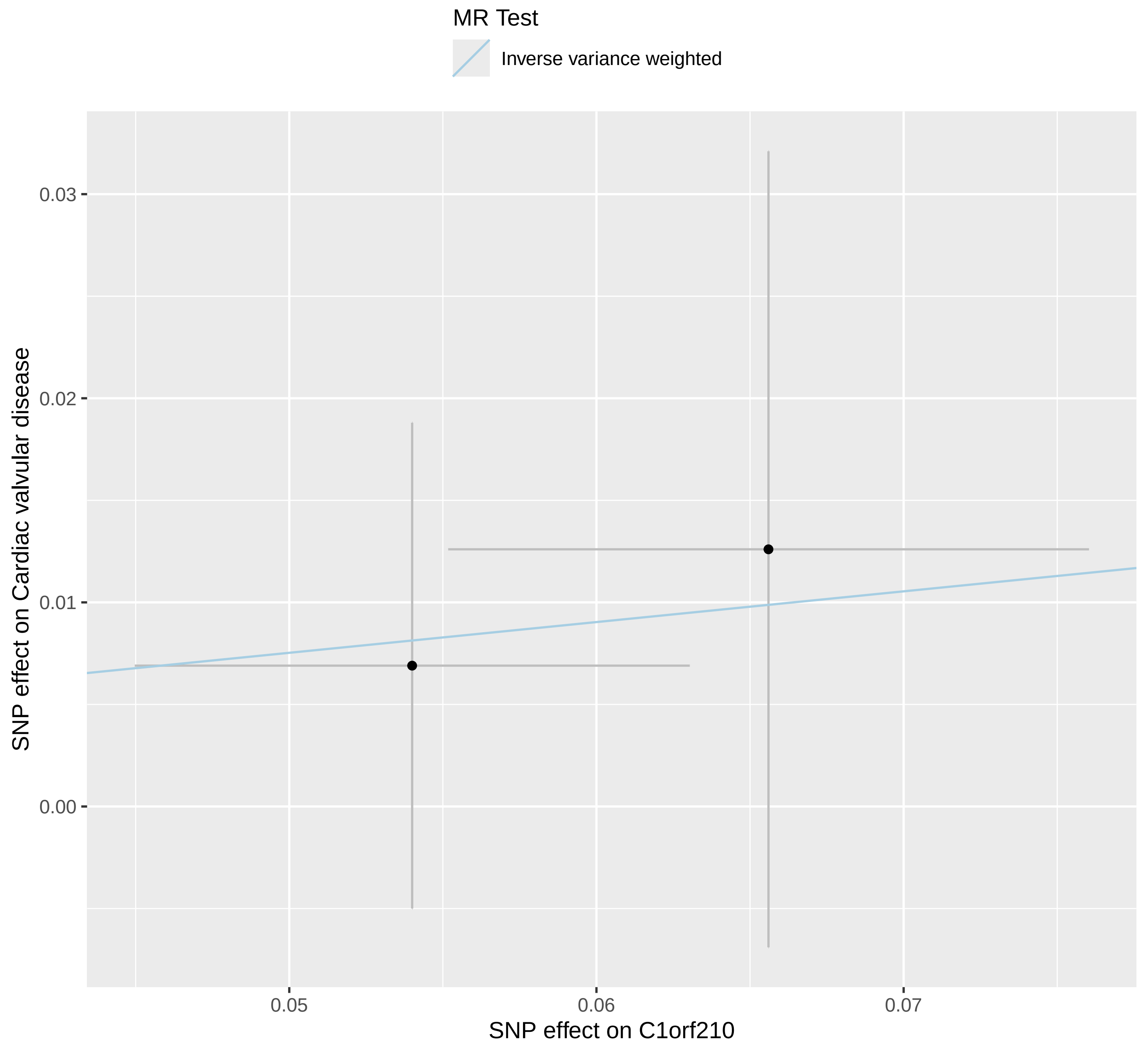
MR Test
Inverse variance weighted
0.03
0.02
0.01
0.00
0.05
0.06
0.07
SNP effect on Cardiac valvular disease
SNP effect on C1orf210

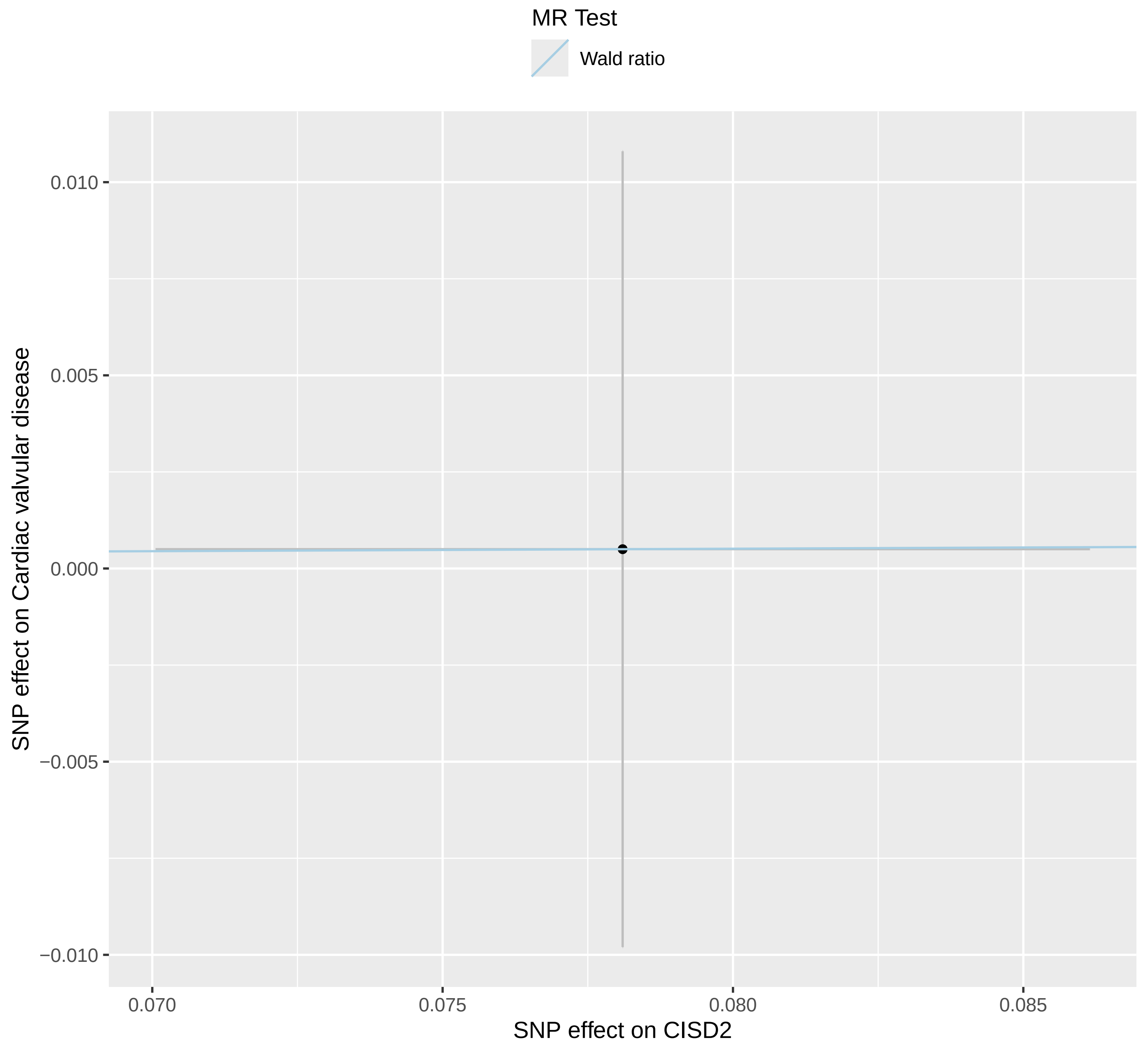
MR Test
Wald ratio
0.010
0.005
0.000
−0.005
−0.010
0.070
0.075
0.080
0.085
SNP effect on CISD2
SNP effect on Cardiac valvular disease

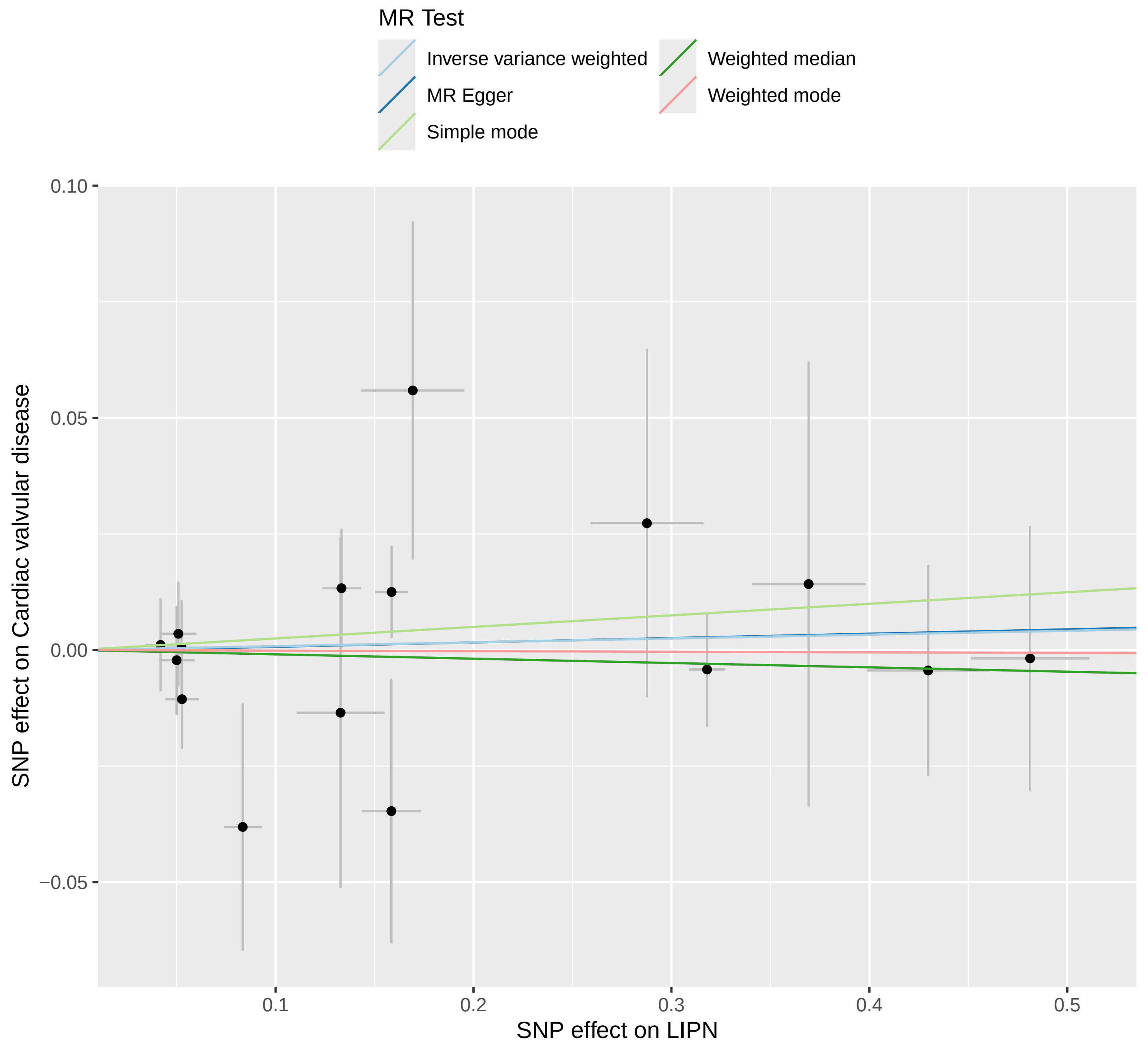
MR Test
Inverse variance weighted
MR Egger
Simple mode
Weighted median
Weighted mode
0.10
0.05
0.00
−0.05
0.1
0.2
0.3
0.4
0.5
SNP effect on Cardiac valvular disease
SNP effect on LIPN

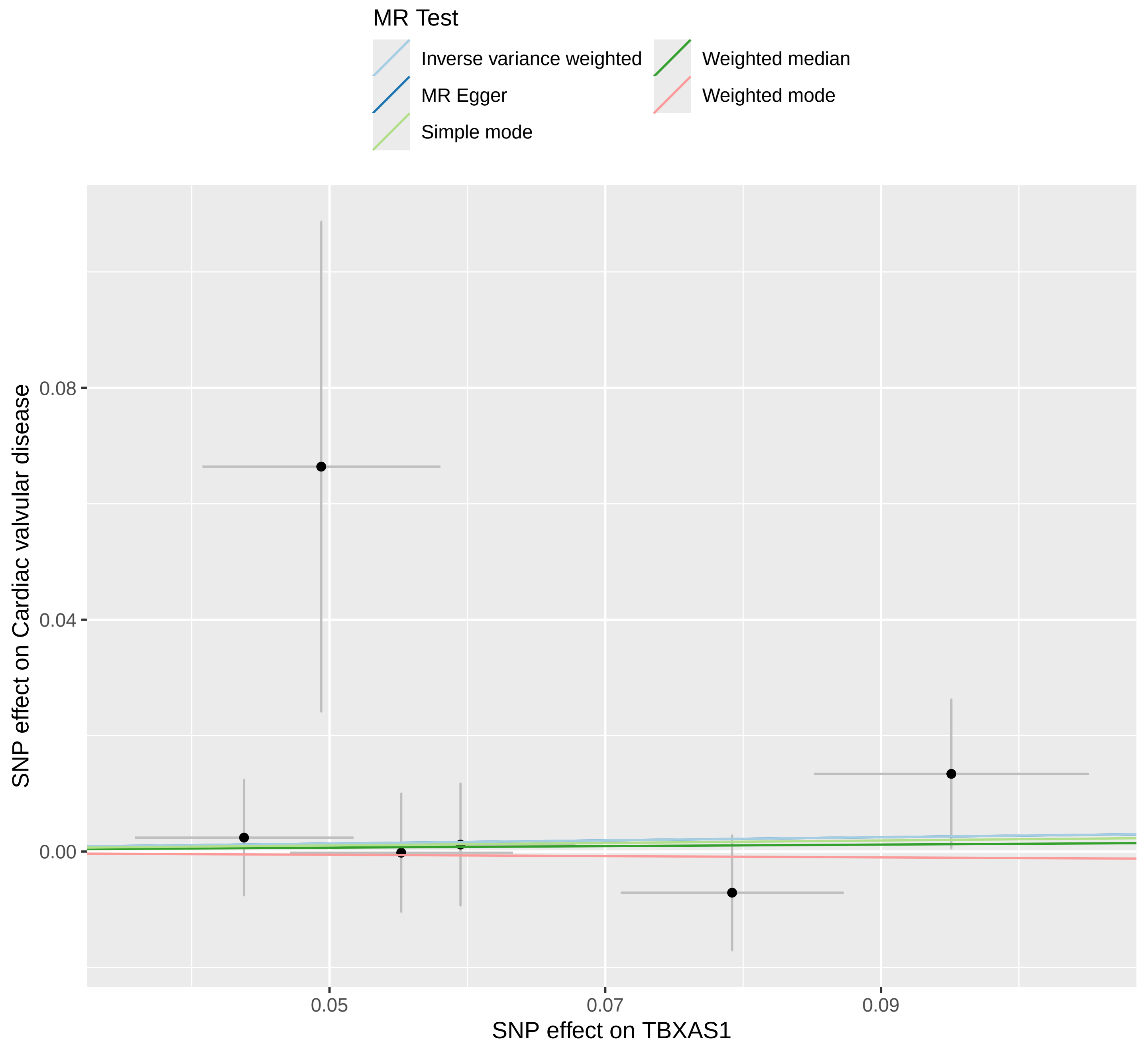
MR Test
Inverse variance weighted
MR Egger
Simple mode
Weighted median
Weighted mode
0.08
0.04
0.00
0.05
0.07
0.09
SNP effect on Cardiac valvular disease
SNP effect on TBXAS1

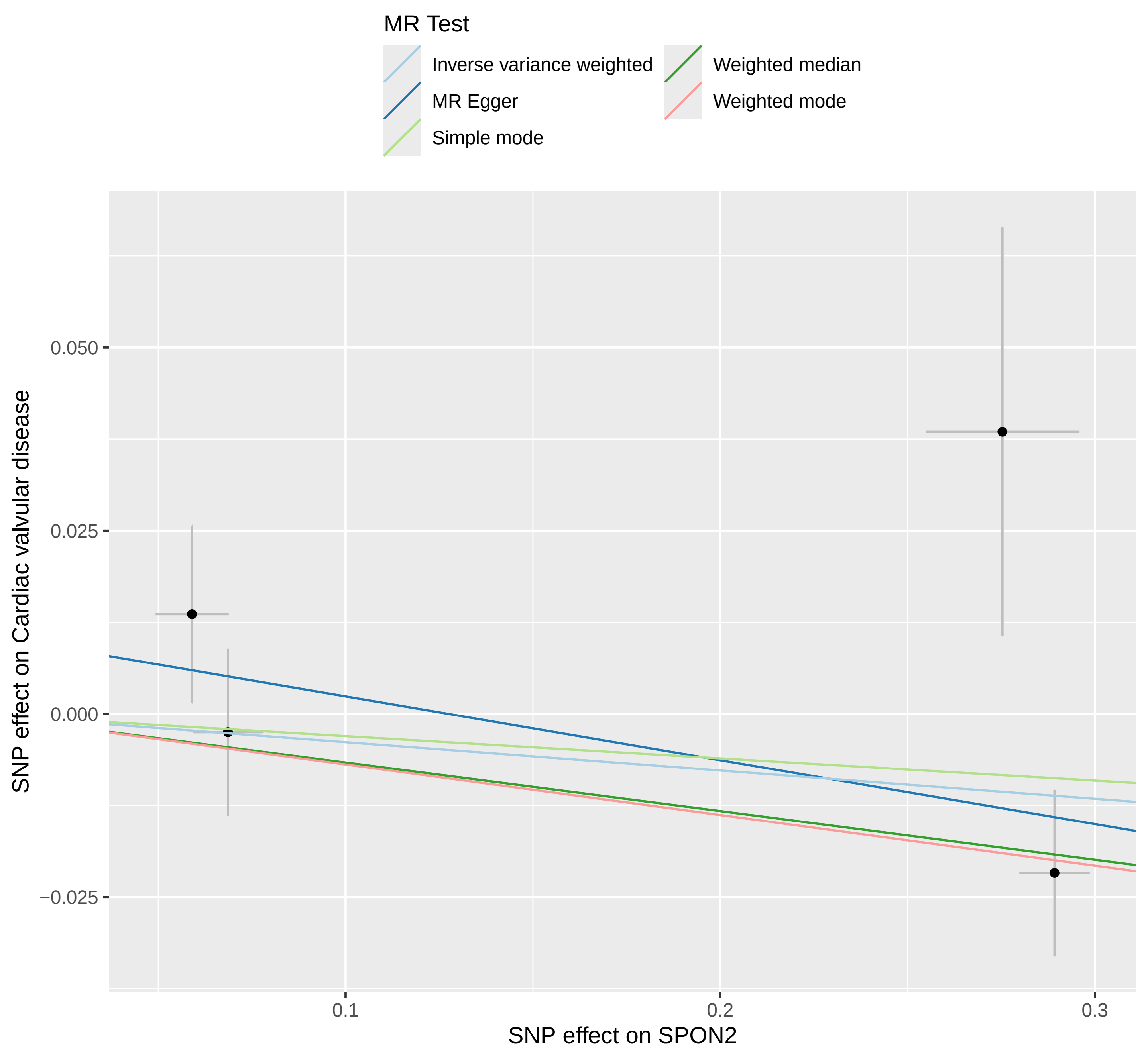
MR Test
Inverse variance weighted
MR Egger
Simple mode
Weighted median
Weighted mode
0.050
0.025
0.000
−0.025
0.1
0.2
0.3
SNP effect on Cardiac valvular disease
SNP effect on SPON2

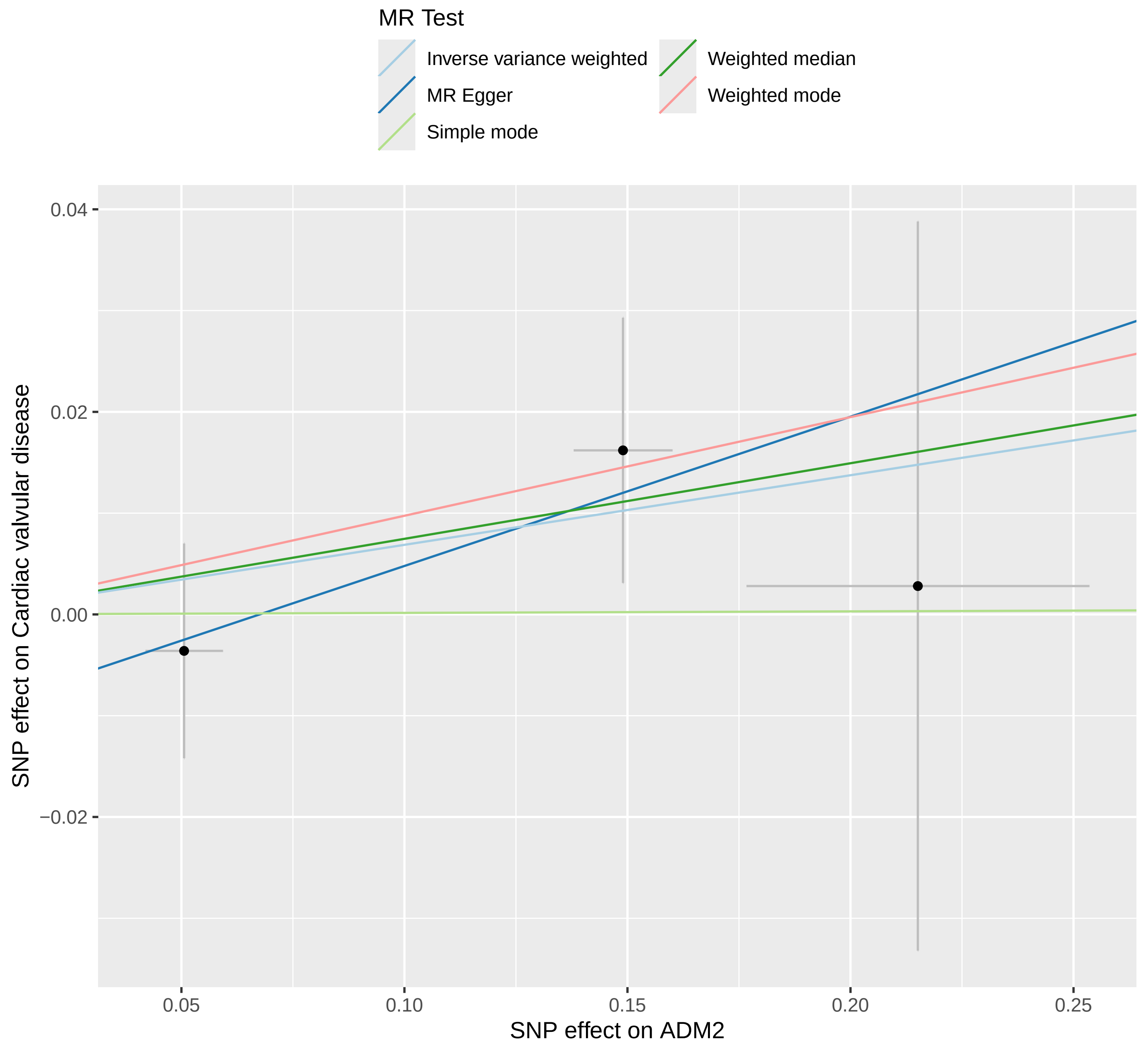
MR Test
Inverse variance weighted
MR Egger
Simple mode
Weighted median
Weighted mode
0.04
0.02
0.00
−0.02
SNP effect on Cardiac valvular disease
0.05
0.10
0.15
0.20
0.25
SNP effect on ADM2

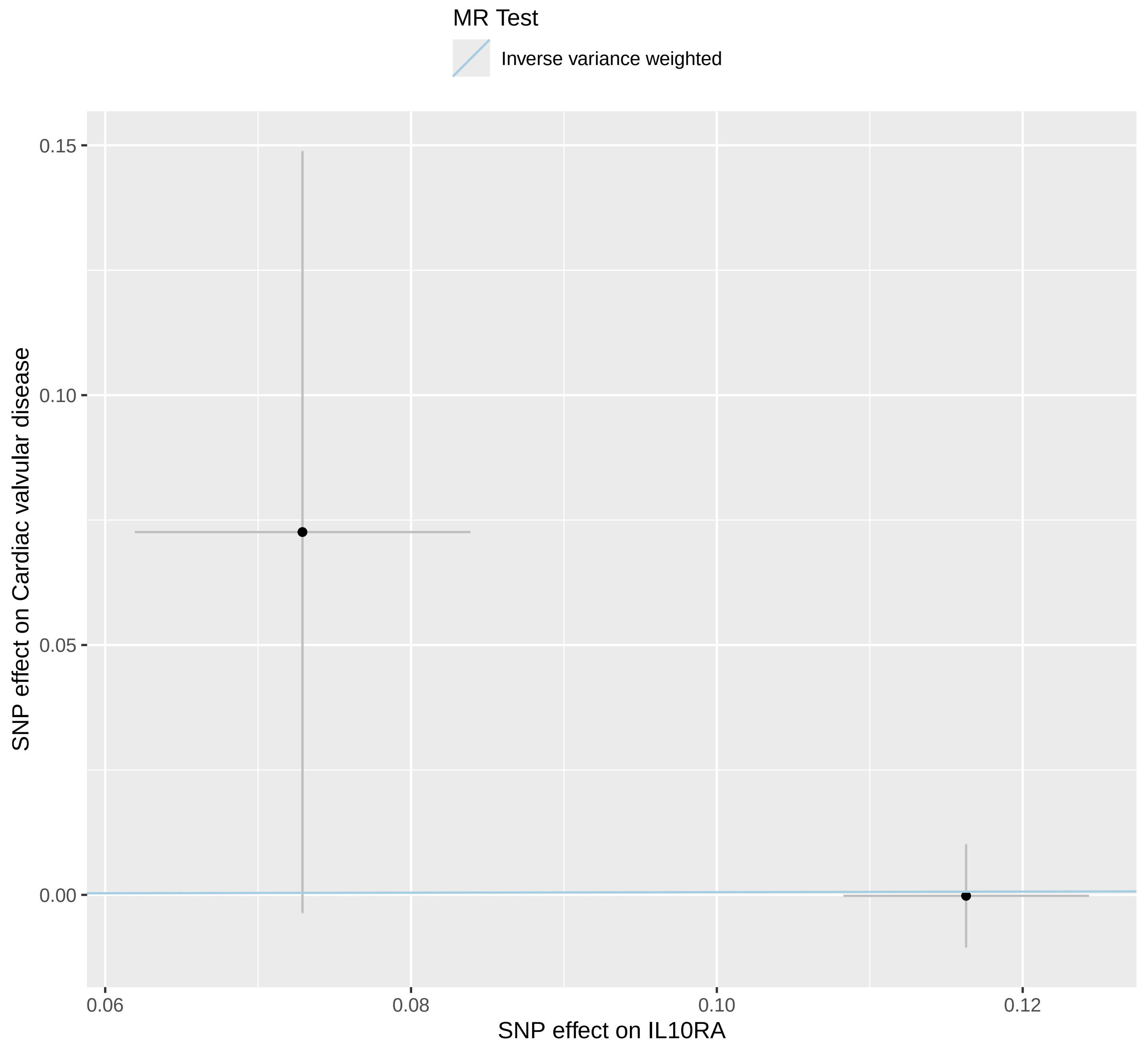

MR Test
Inverse variance weighted
0.15
0.10
0.05
0.00
0.06
0.08
0.10
0.12
SNP effect on Cardiac valvular disease
SNP effect on IL10RA

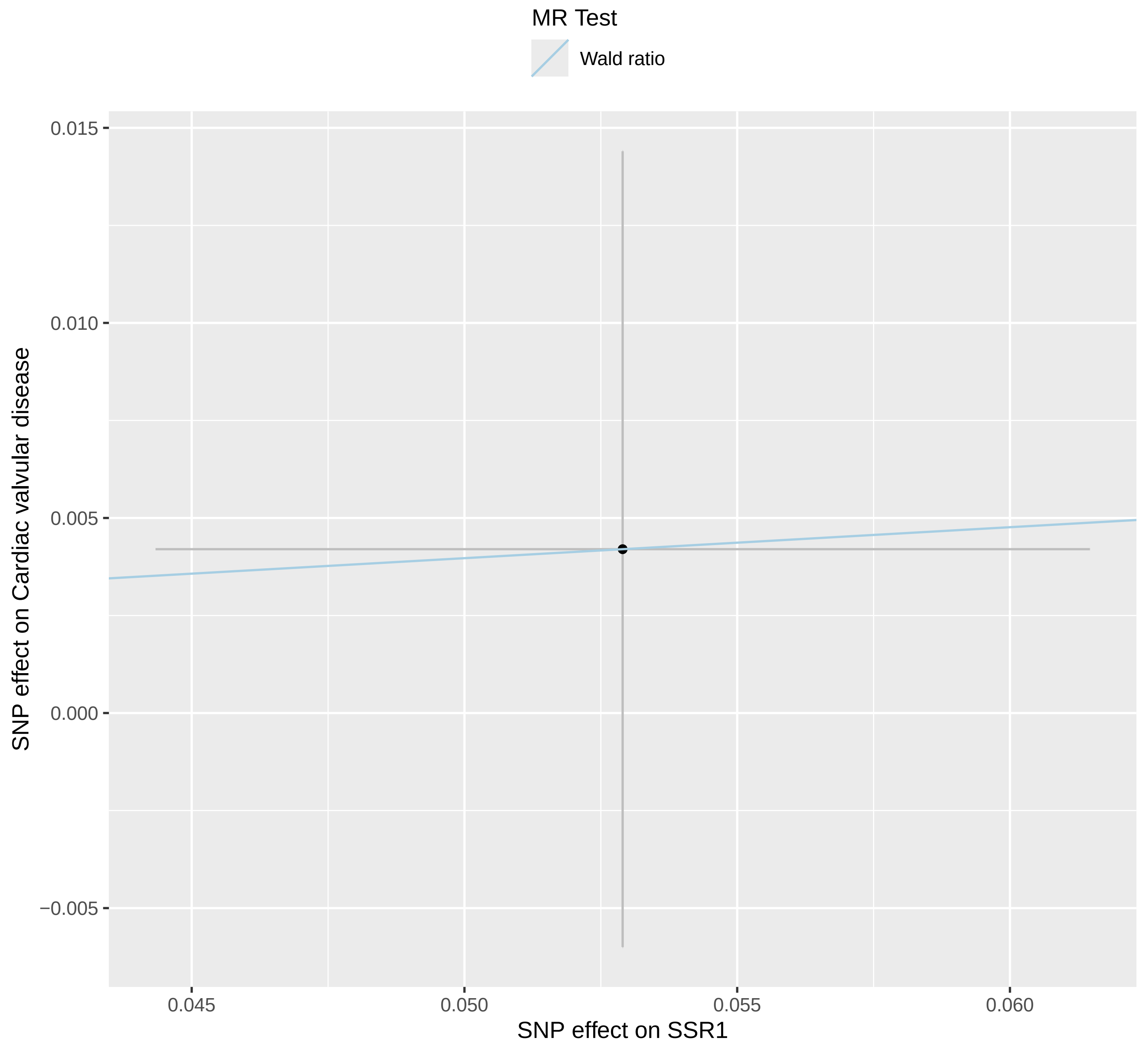

MR Test
Wald ratio
0.015
0.010
0.005
0.000
−0.005
0.045
0.050
0.055
0.060
SNP effect on Cardiac valvular disease
SNP effect on SSR1

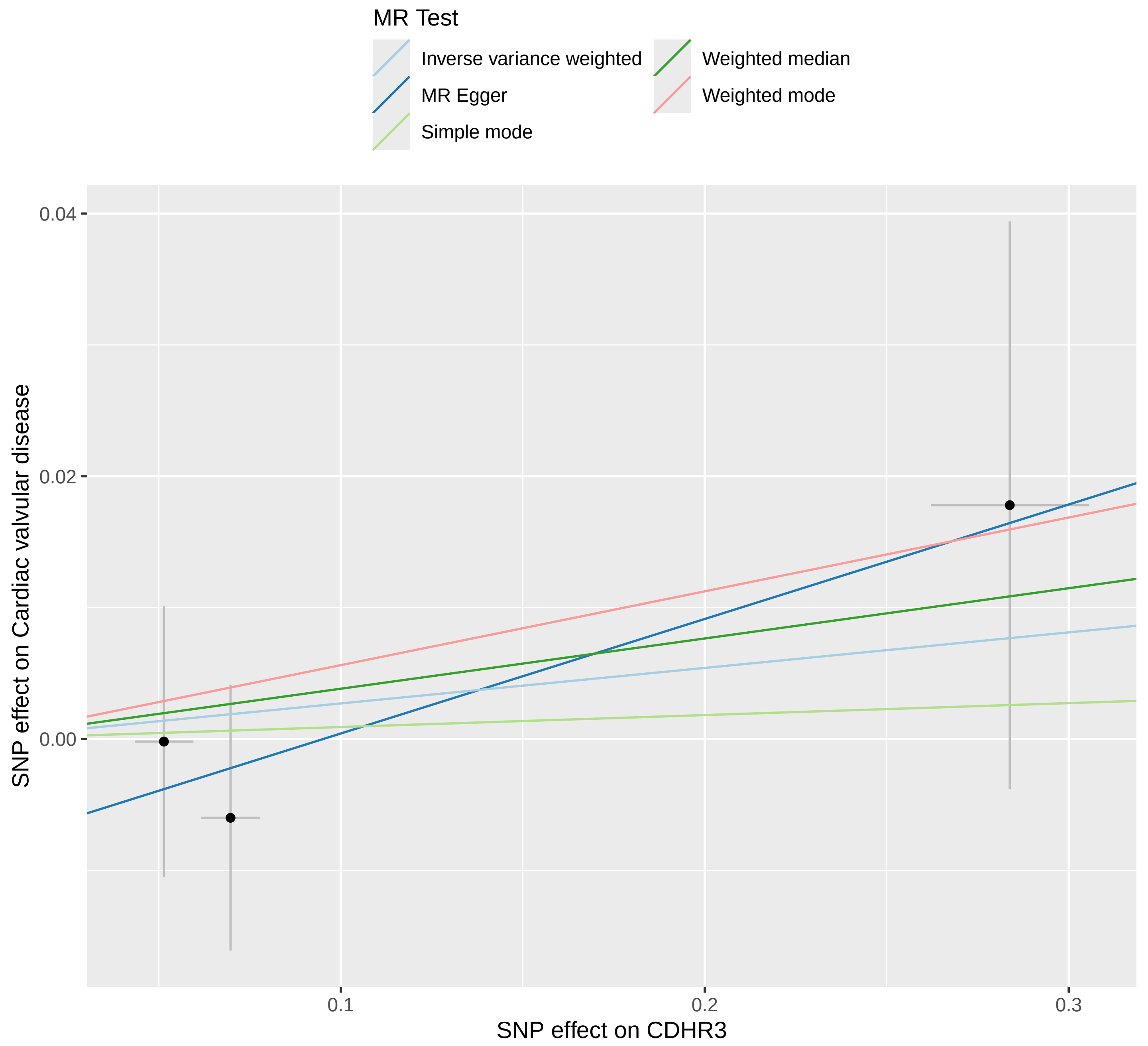
MR Test
Inverse variance weighted
MR Egger
Simple mode
Weighted median
Weighted mode
0.04
0.02
0.00
SNP effect on Cardiac valvular disease
0.1
0.2
0.3
SNP effect on CDHR3

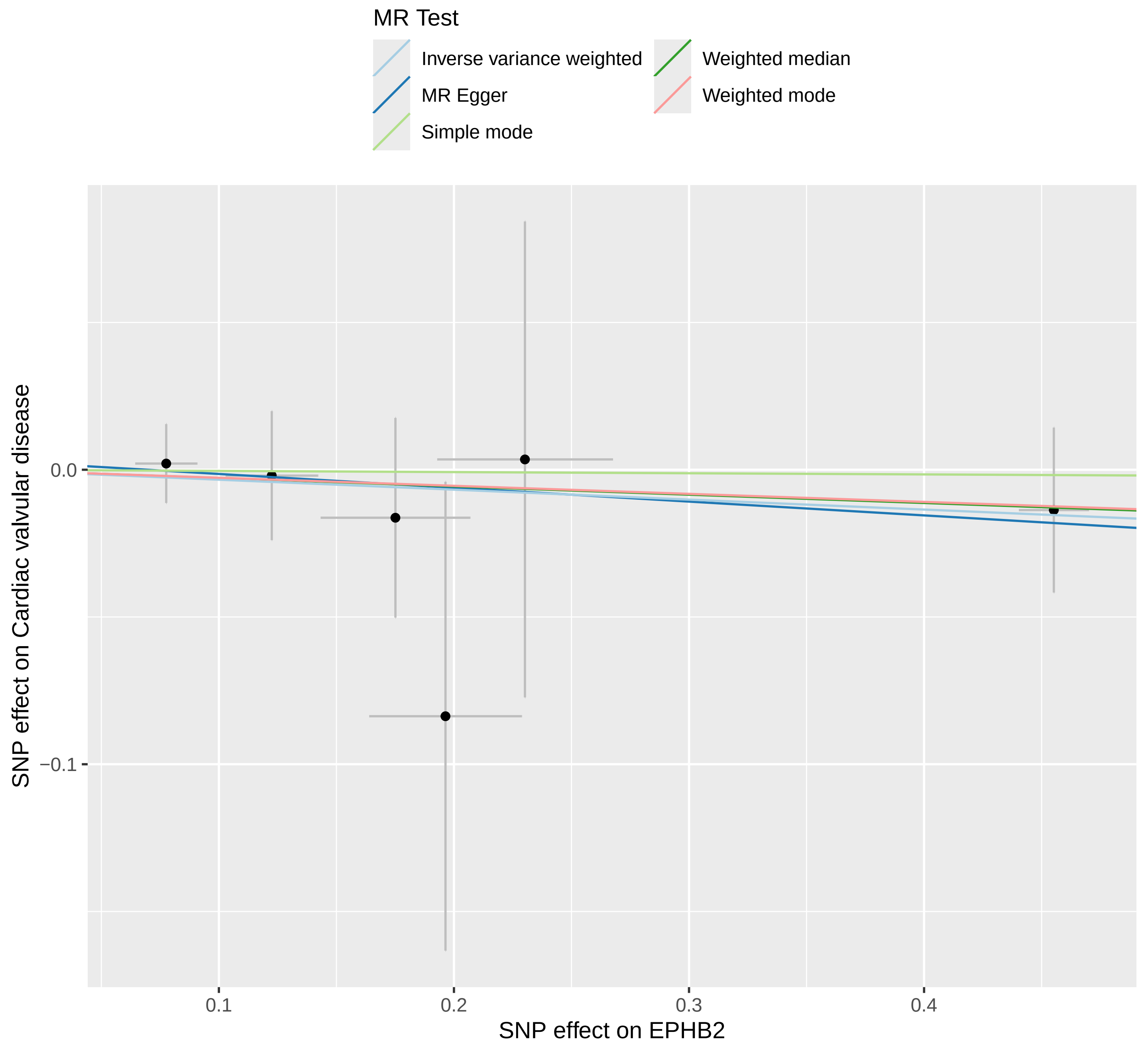
MR Test
Inverse variance weighted
MR Egger
Simple mode
Weighted median
Weighted mode
SNP effect on Cardiac valvular disease
0.0
−0.1
0.1
0.2
0.3
0.4
SNP effect on EPHB2

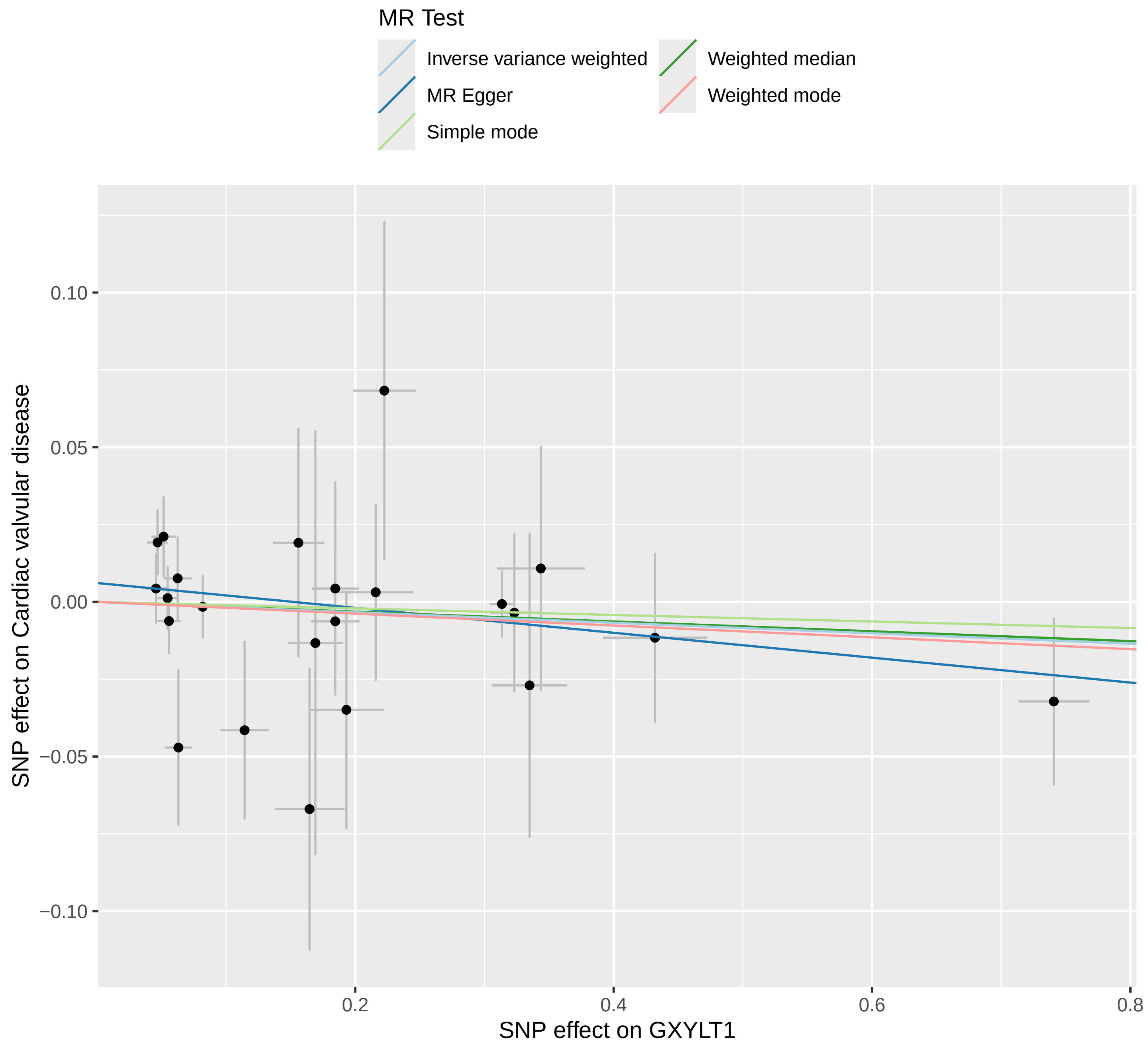

MR Test
Inverse variance weighted
MR Egger
Simple mode
Weighted median
Weighted mode
0.10
0.05
0.00
−0.05
−0.10
SNP effect on Cardiac valvular disease
0.2
0.4
0.6
0.8
SNP effect on GXYLT1

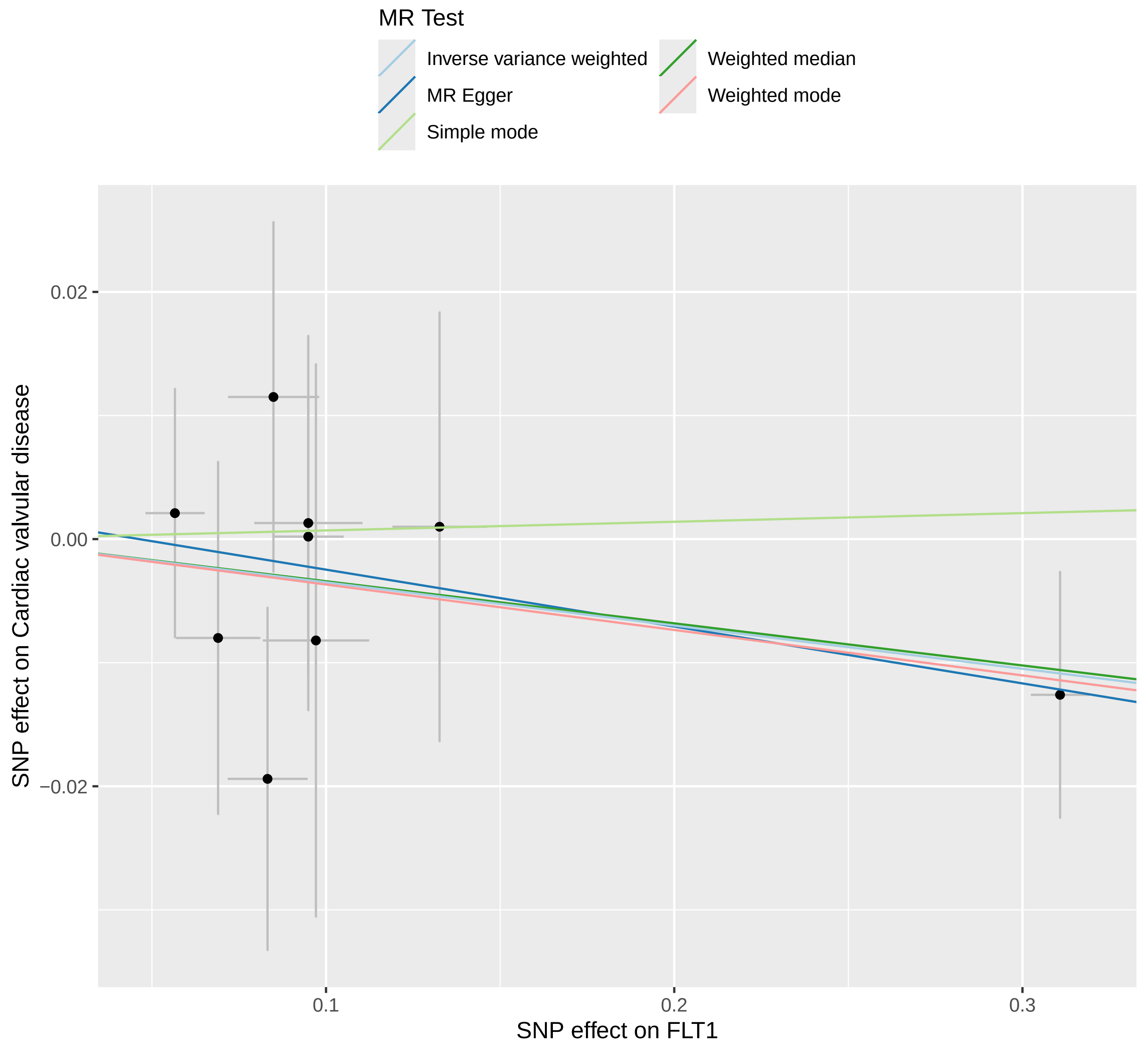
MR Test
Inverse variance weighted
MR Egger
Simple mode
Weighted median
Weighted mode
0.02
0.00
−0.02
0.1
0.2
0.3
SNP effect on Cardiac valvular disease
SNP effect on FLT1

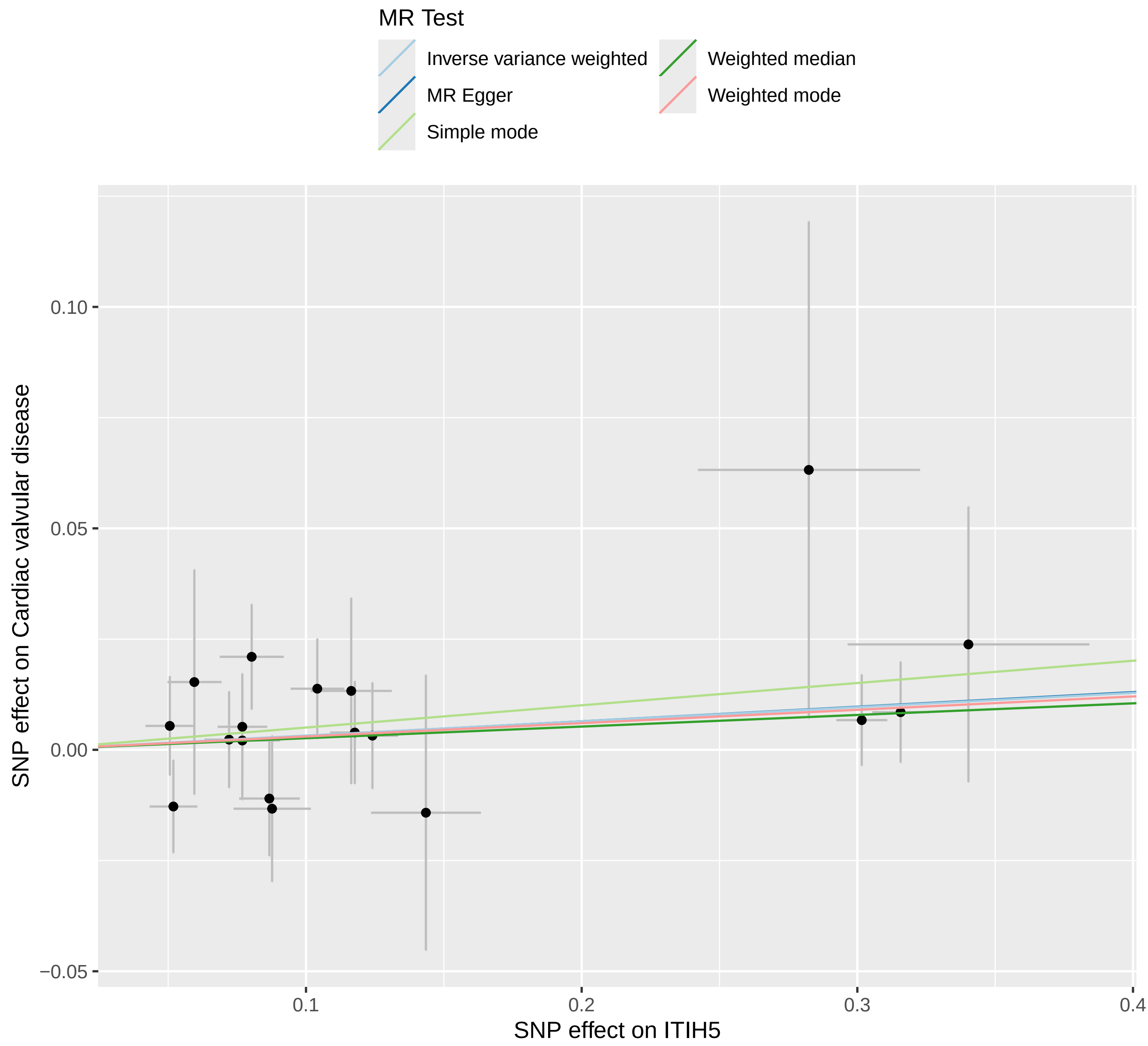

MR Test
Inverse variance weighted
MR Egger
Simple mode
Weighted median
Weighted mode
SNP effect on Cardiac valvular disease
0.10
0.05
0.00
−0.05
0.1
0.2
0.3
0.4
SNP effect on ITIH5

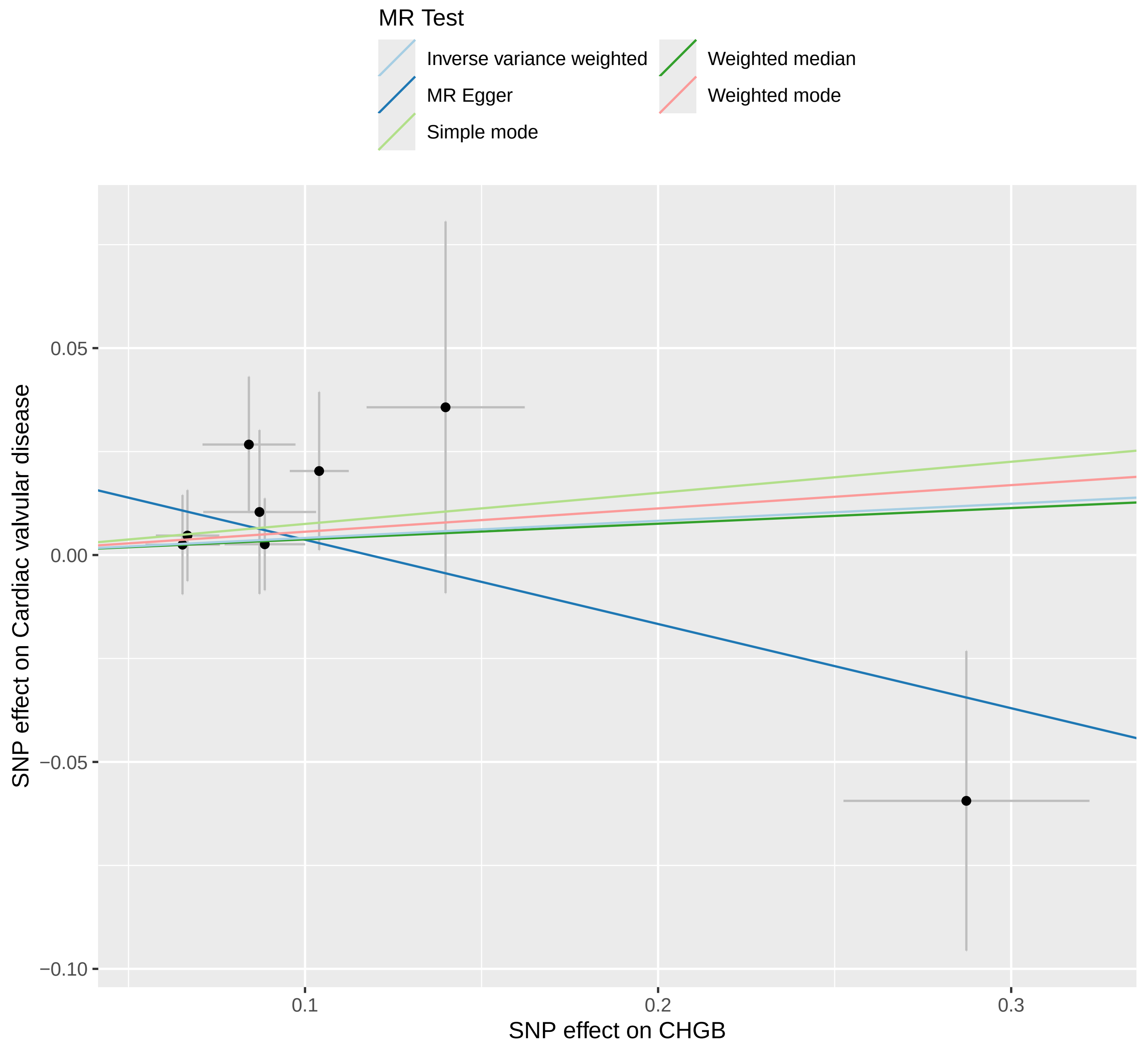
MR Test
Inverse variance weighted
MR Egger
Simple mode
Weighted median
Weighted mode
SNP effect on Cardiac valvular disease
0.05
0.00
−0.05
−0.10
0.1
0.2
0.3
SNP effect on CHGB

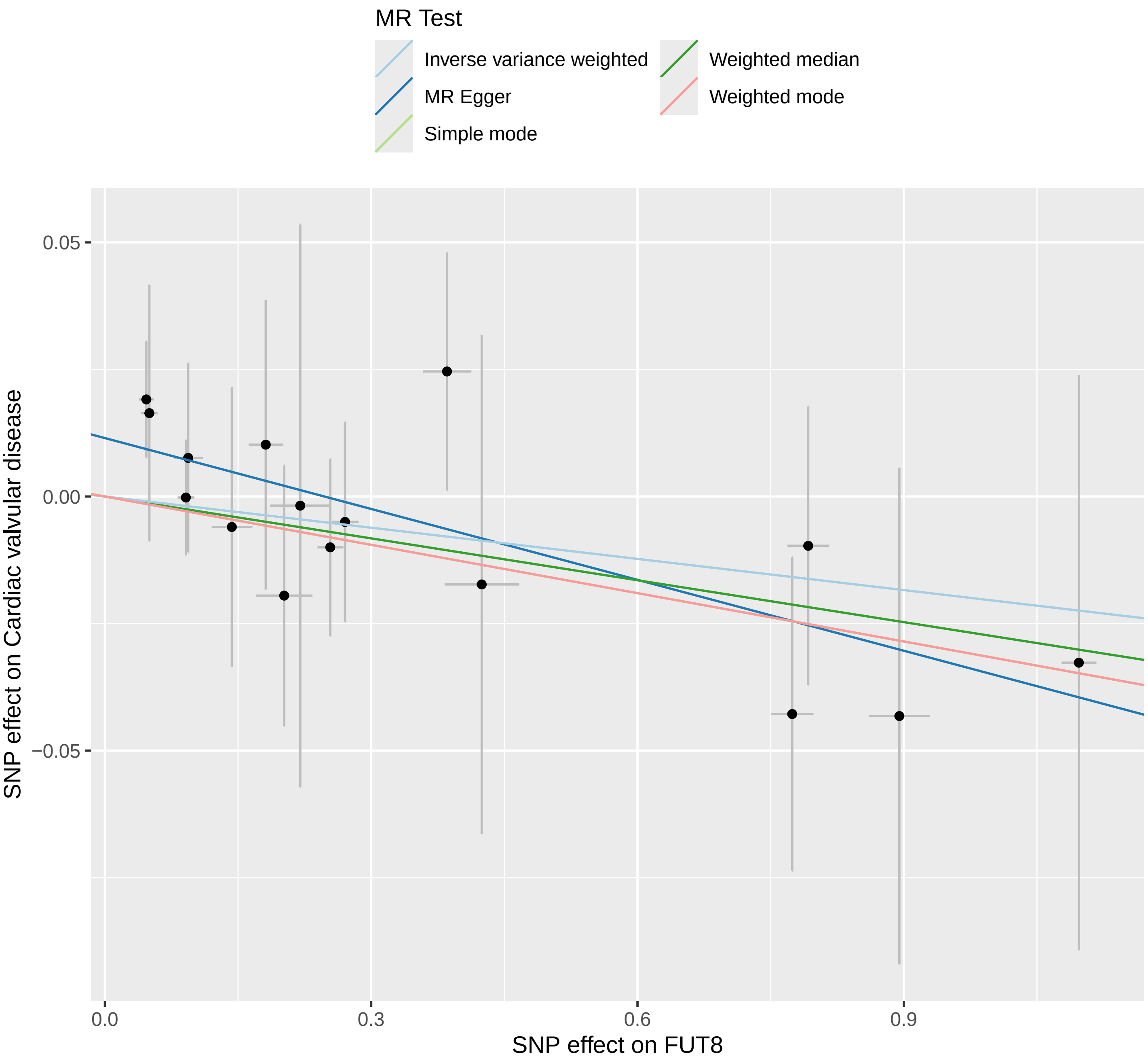

MR Test
Inverse variance weighted
MR Egger
Simple mode
Weighted median
Weighted mode
0.05
0.00
−0.05
0.0
0.3
0.6
0.9
SNP effect on Cardiac valvular disease
SNP effect on FUT8

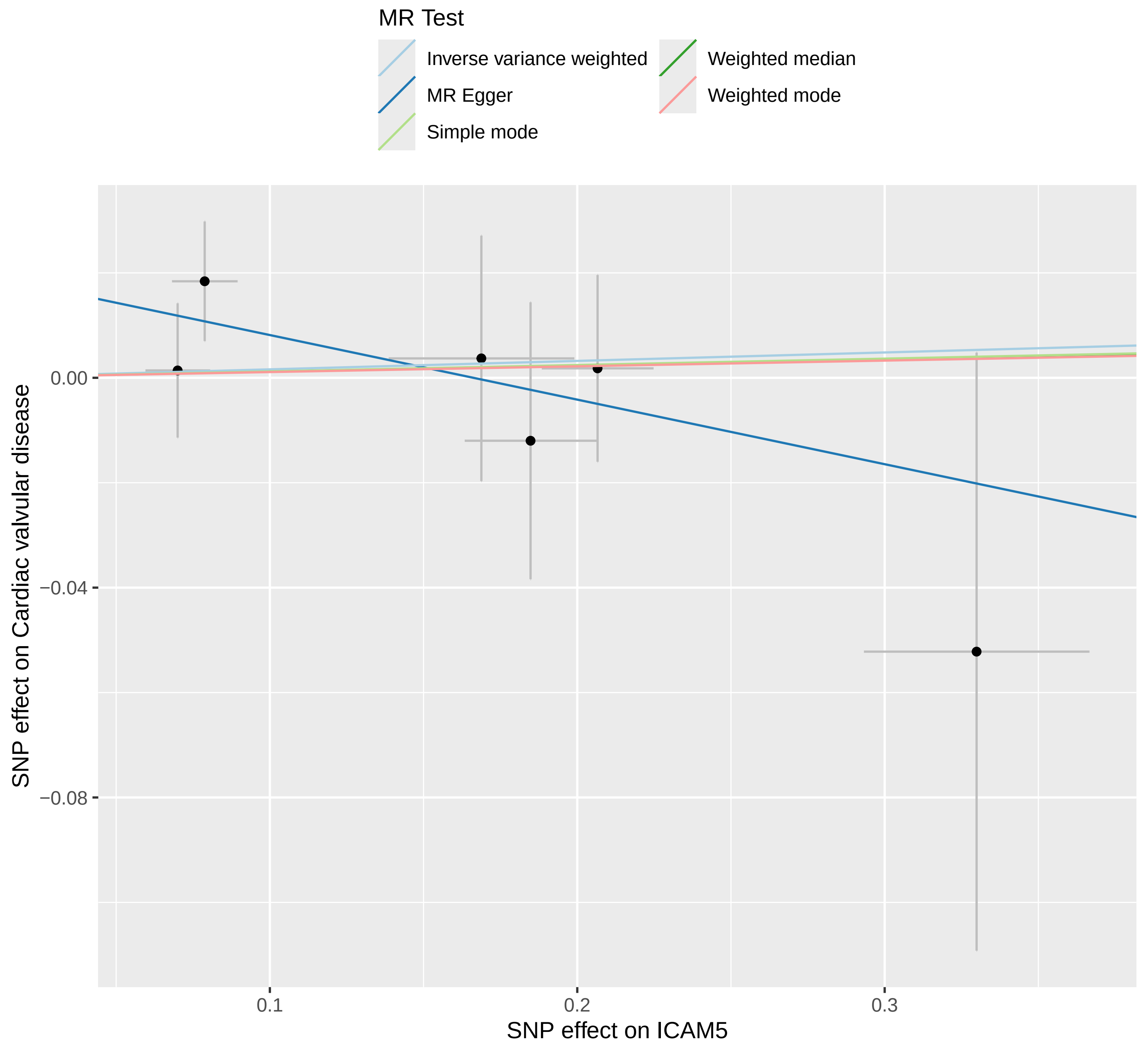
MR Test
Inverse variance weighted
MR Egger
Simple mode
Weighted median
Weighted mode
0.00
−0.04
−0.08
0.1
0.2
0.3
SNP effect on ICAM5
SNP effect on Cardiac valvular disease

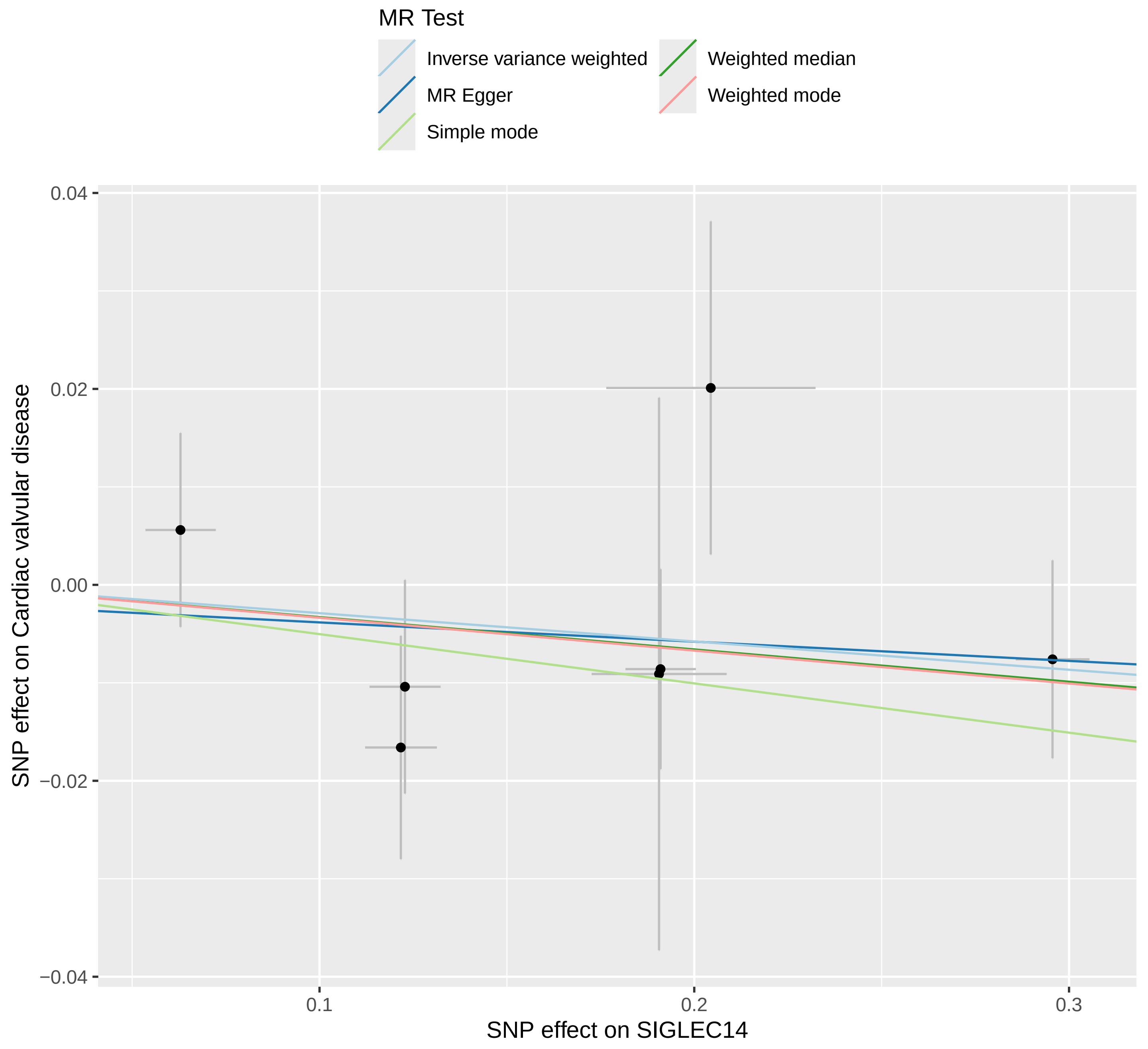
MR Test
Inverse variance weighted
MR Egger
Simple mode
Weighted median
Weighted mode
0.04
0.02
0.00
−0.02
−0.04
0.1
0.2
0.3
SNP effect on Cardiac valvular disease
SNP effect on SIGLEC14

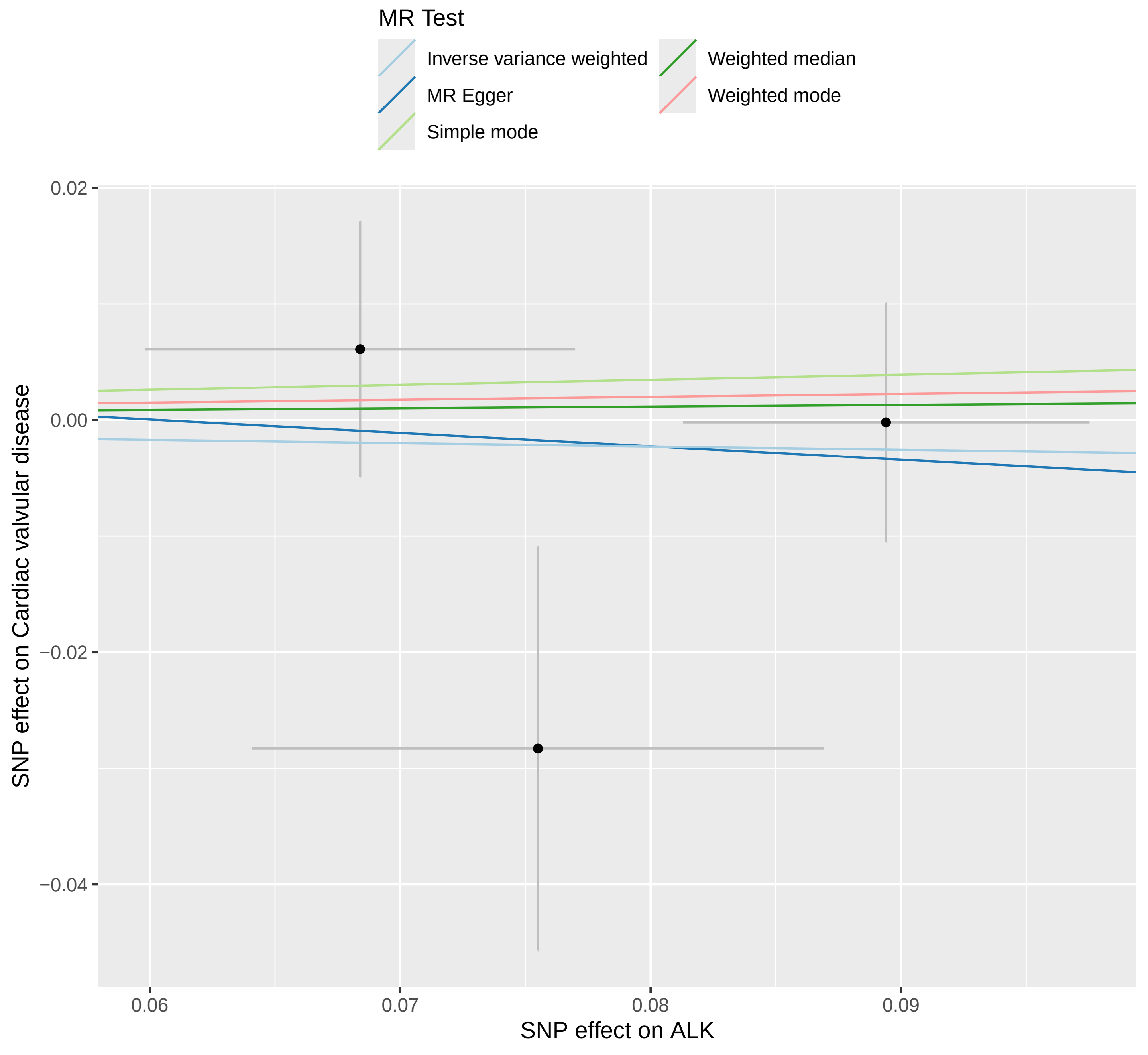
MR Test
Inverse variance weighted
MR Egger
Simple mode
Weighted median
Weighted mode
0.02
0.00
−0.02
−0.04
SNP effect on Cardiac valvular disease
0.06
0.07
0.08
0.09
SNP effect on ALK

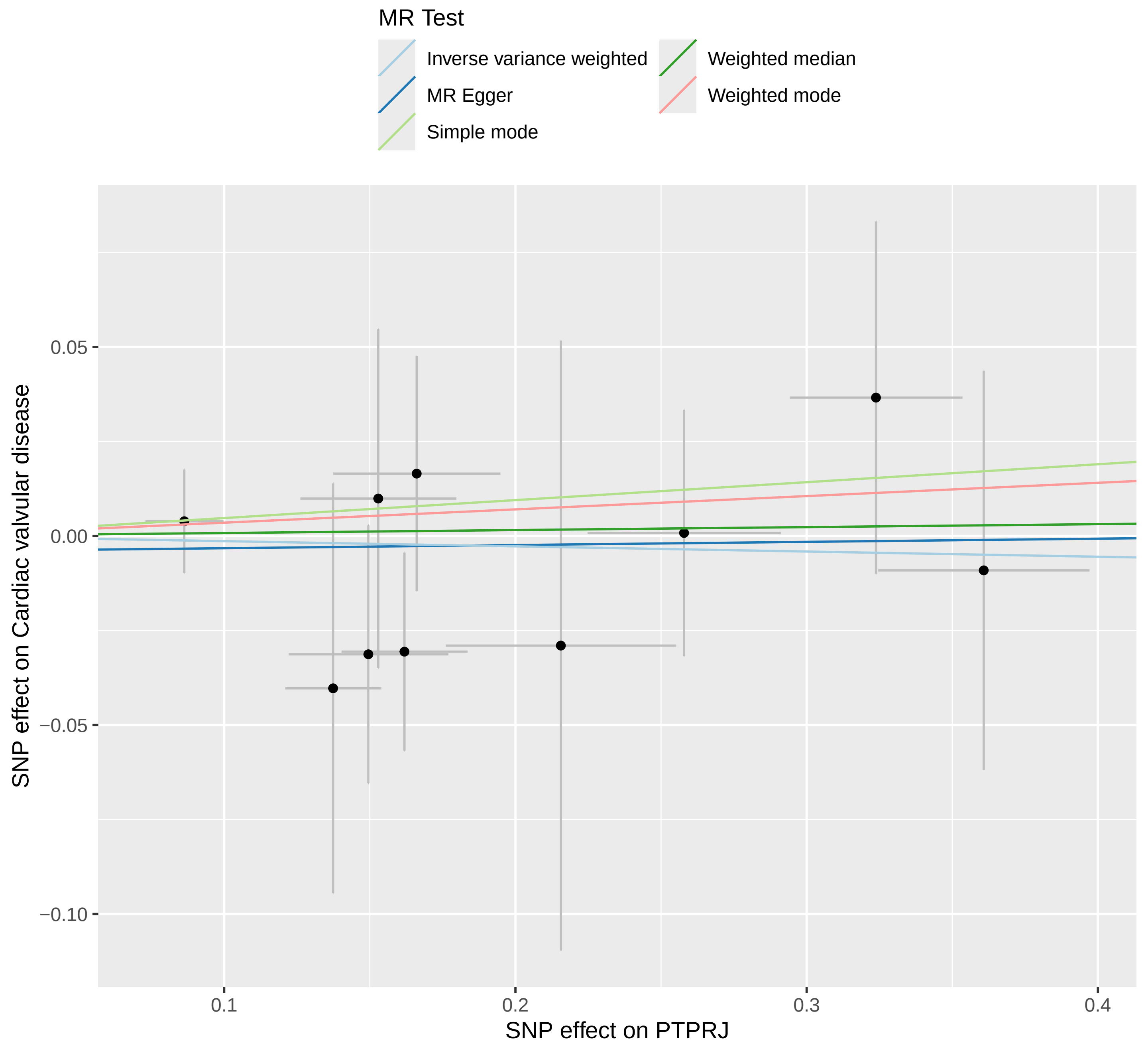
MR Test
Inverse variance weighted
MR Egger
Simple mode
Weighted median
Weighted mode
0.05
0.00
−0.05
−0.10
0.1
0.2
0.3
0.4
SNP effect on Cardiac valvular disease
SNP effect on PTPRJ

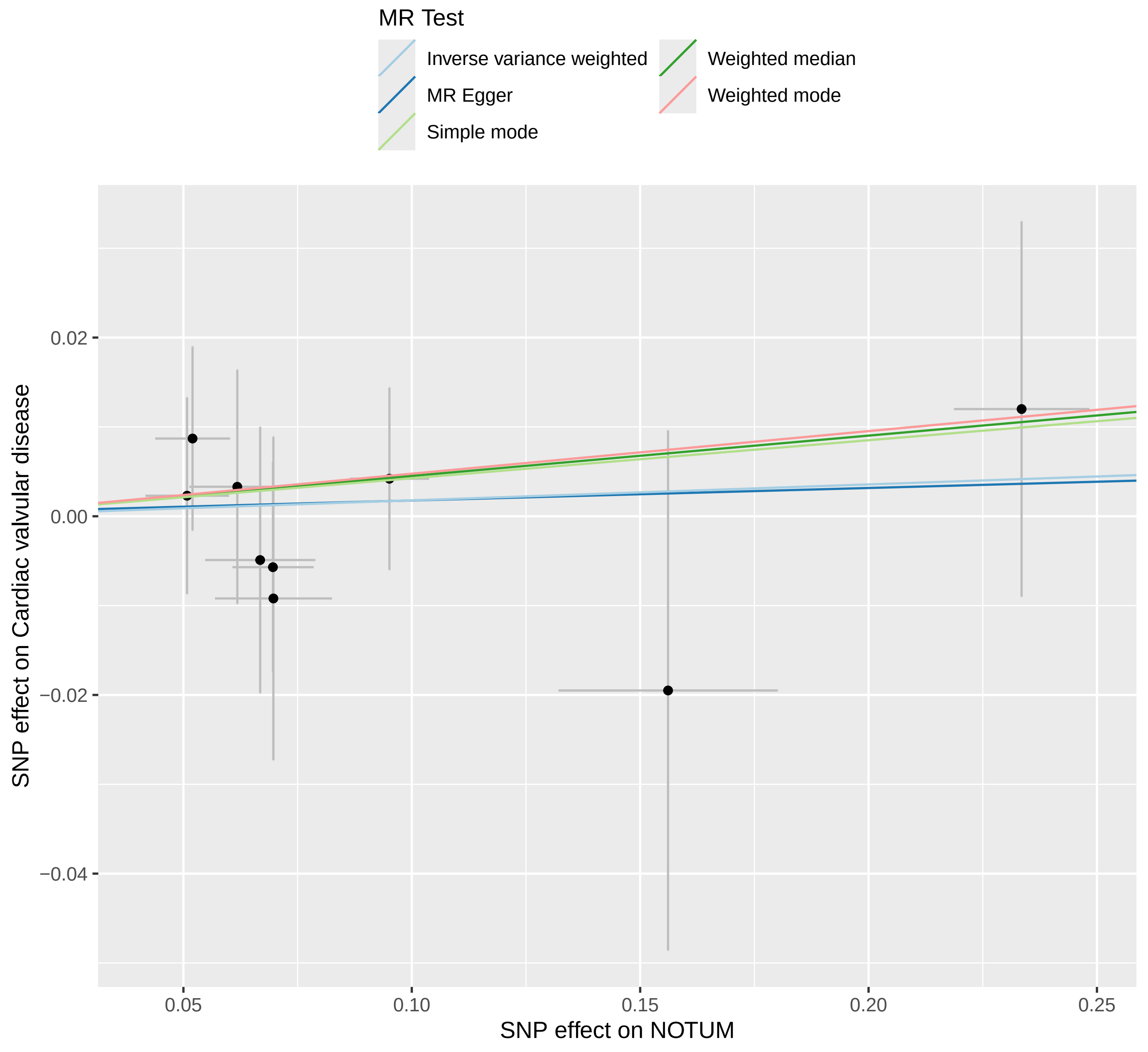
MR Test
Inverse variance weighted
MR Egger
Simple mode
Weighted median
Weighted mode
0.02
0.00
−0.02
−0.04
SNP effect on Cardiac valvular disease
0.05
0.10
0.15
0.20
0.25
SNP effect on NOTUM

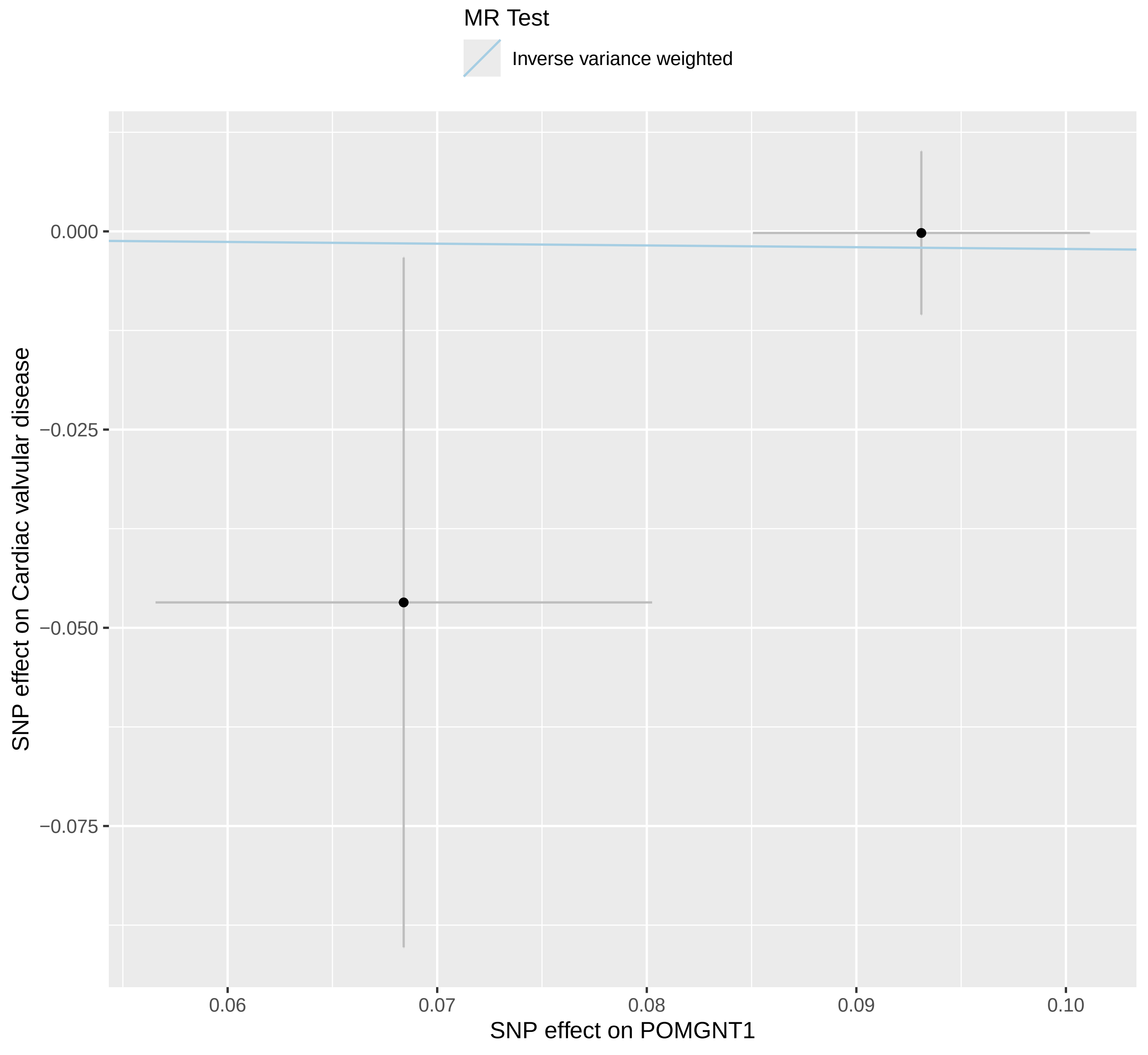
MR Test
Inverse variance weighted
0.000
−0.025
−0.050
−0.075
0.06
0.07
0.08
0.09
0.10
SNP effect on POMGNT1
SNP effect on Cardiac valvular disease

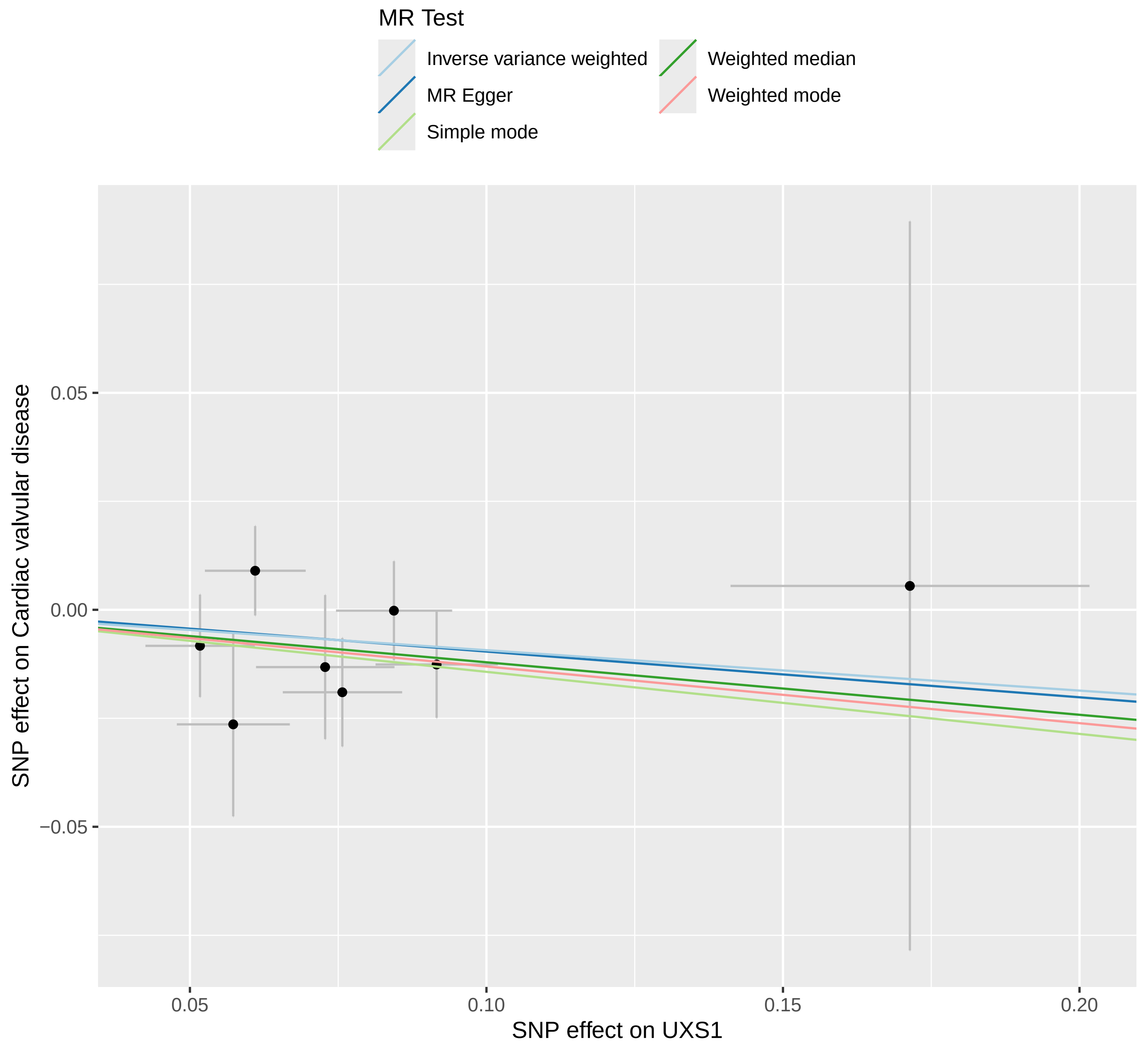

MR Test
Inverse variance weighted
MR Egger
Simple mode
Weighted median
Weighted mode
0.05
0.00
−0.05
SNP effect on Cardiac valvular disease
0.05
0.10
0.15
0.20
SNP effect on UXS1

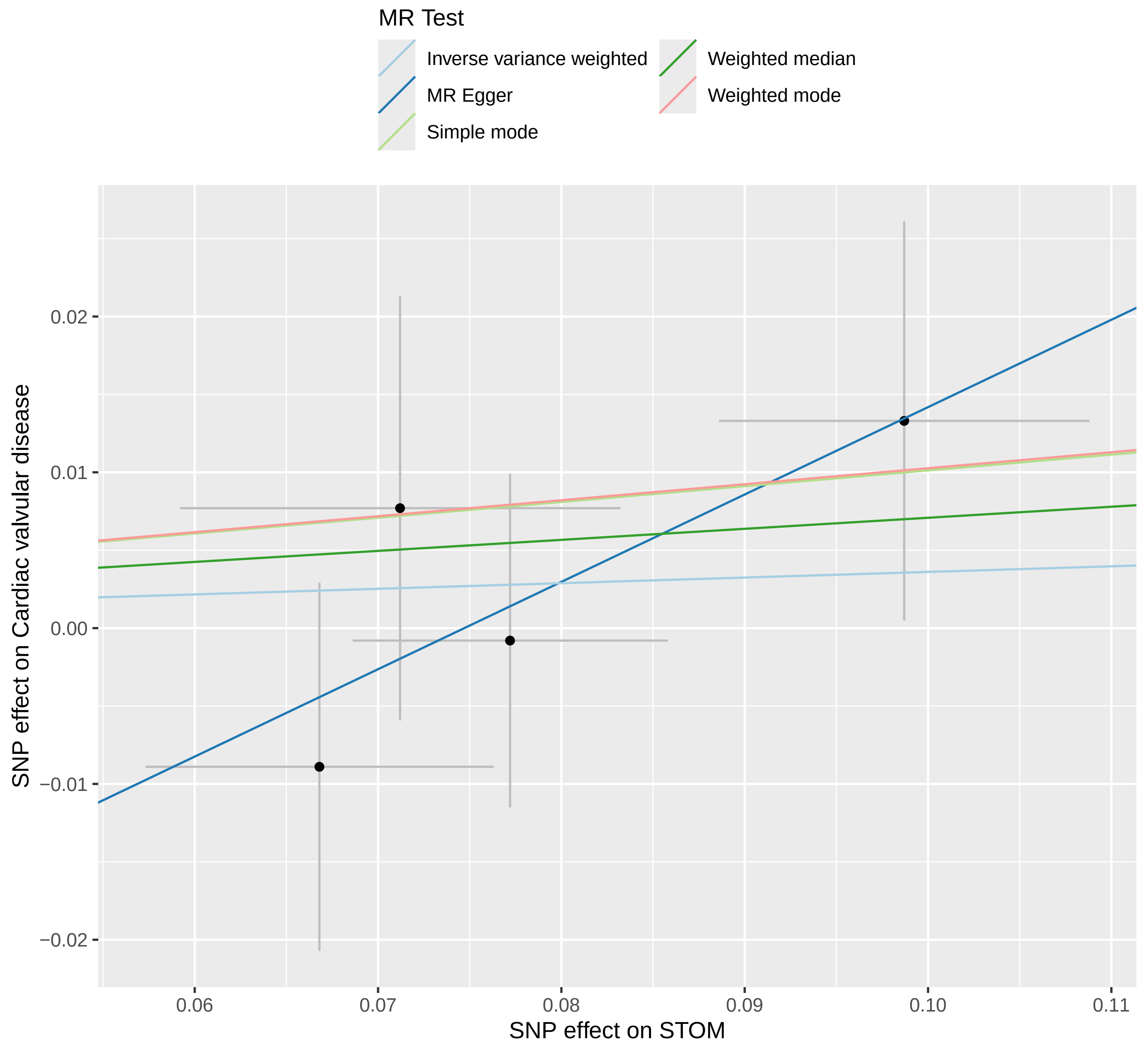
MR Test
Inverse variance weighted
MR Egger
Simple mode
Weighted median
Weighted mode
0.02
0.01
0.00
−0.01
−0.02
SNP effect on Cardiac valvular disease
0.06
0.07
0.08
0.09
0.10
0.11
SNP effect on STOM

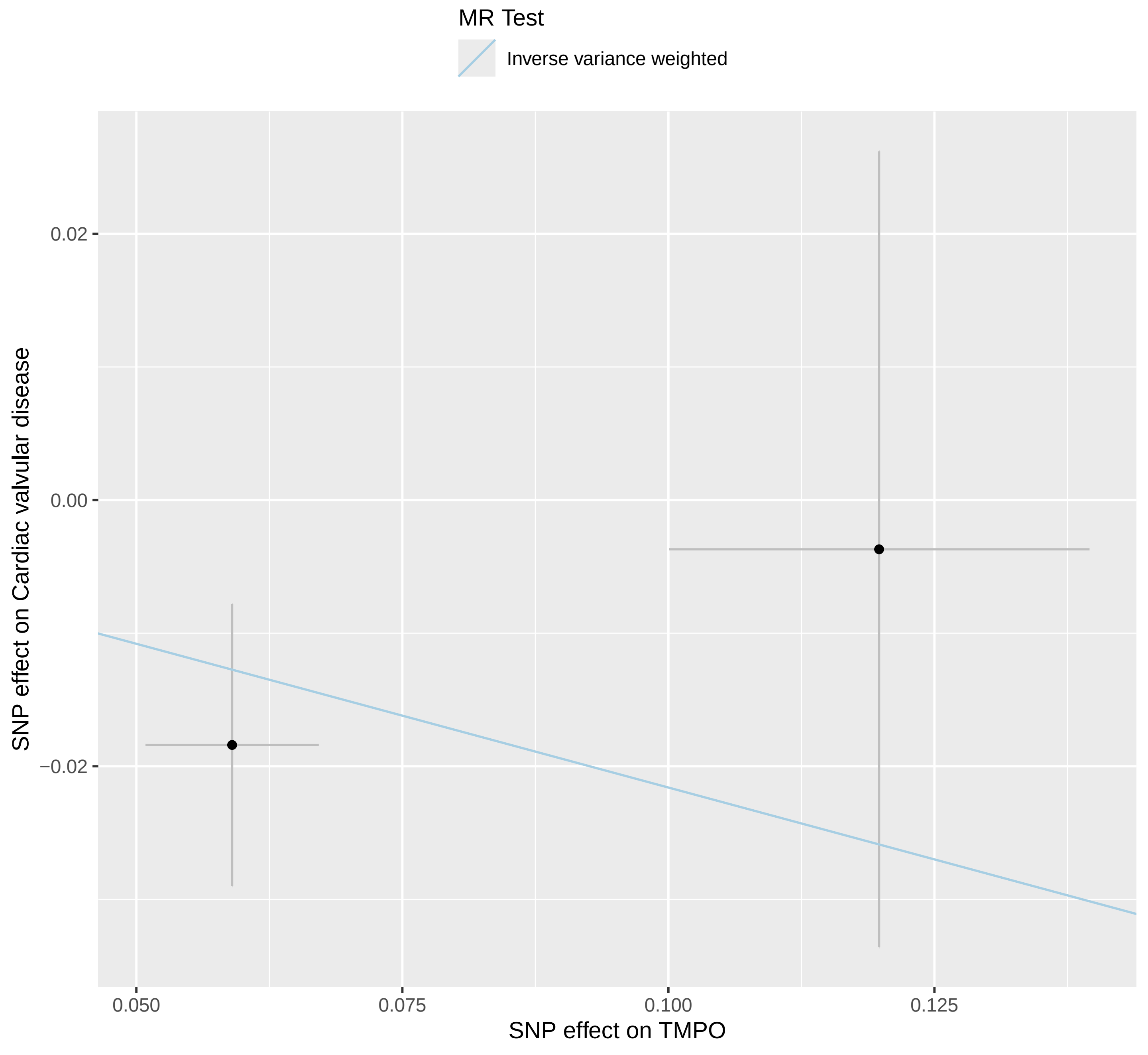
MR Test
Inverse variance weighted
0.02
0.00
−0.02
0.050
0.075
0.100
0.125
SNP effect on TMPO
SNP effect on Cardiac valvular disease

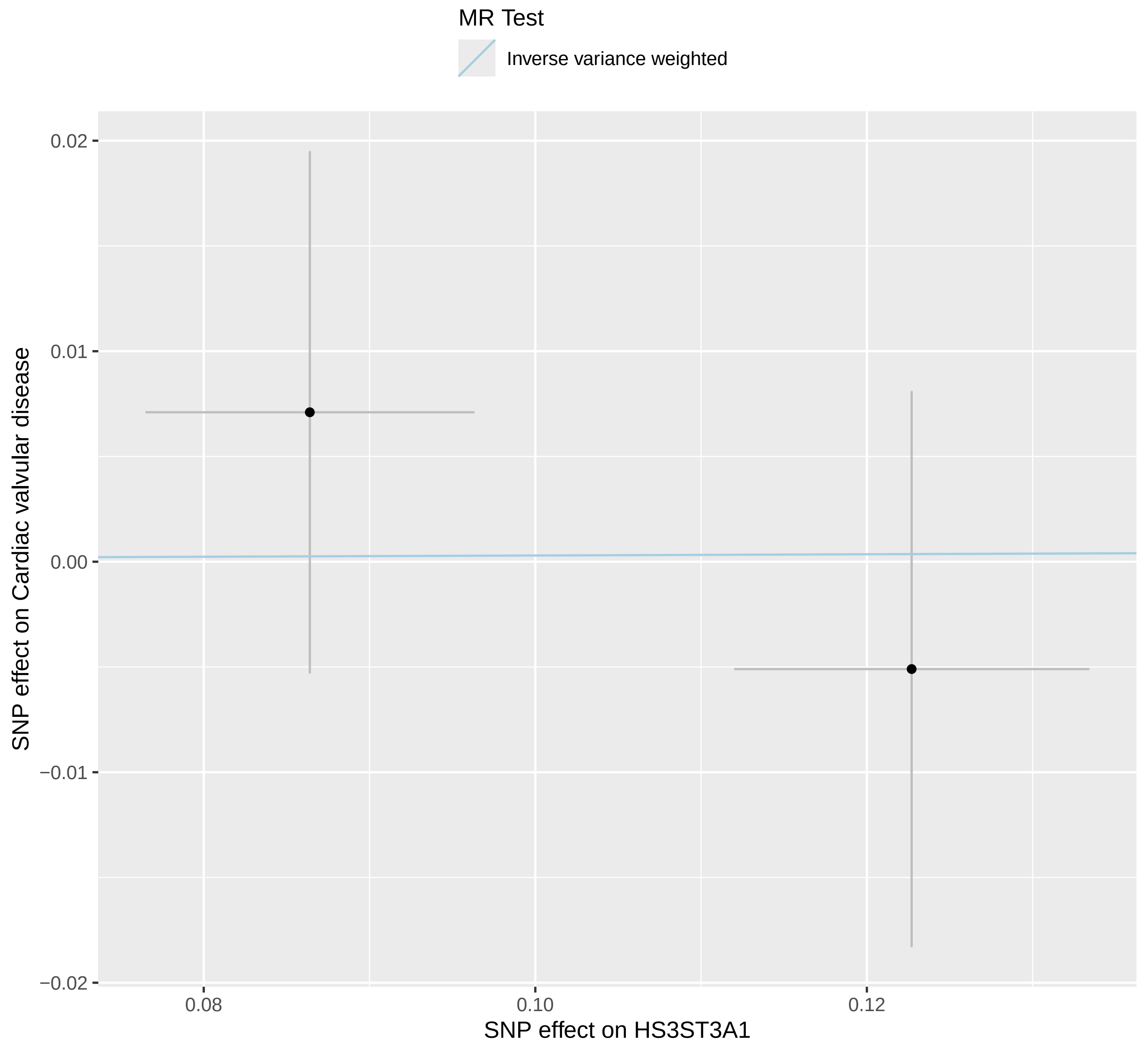

MR Test
Inverse variance weighted
0.02
0.01
0.00
−0.01
−0.02
0.08
0.10
0.12
SNP effect on HS3ST3A1
SNP effect on Cardiac valvular disease

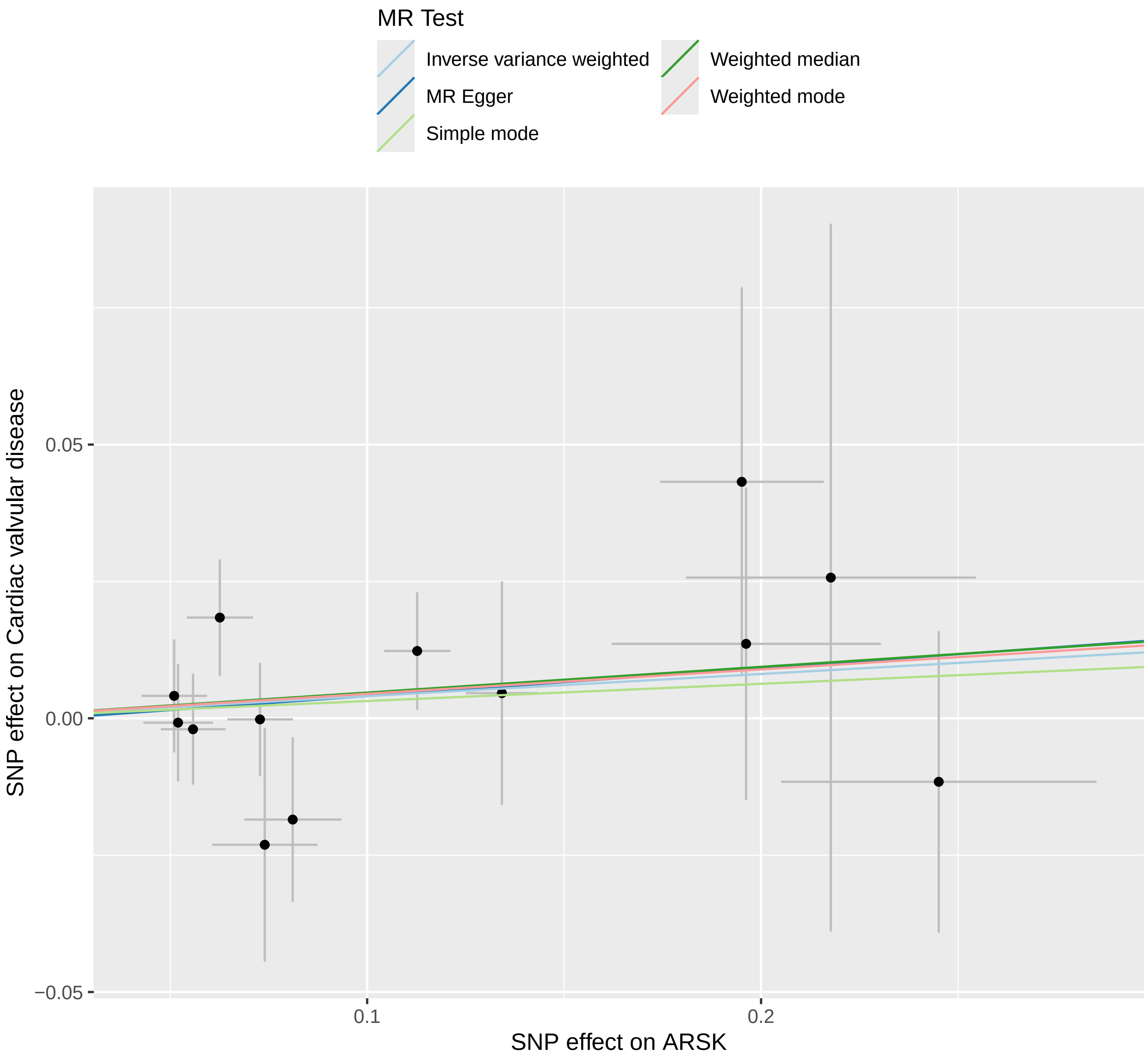
MR Test
Inverse variance weighted
MR Egger
Simple mode
Weighted median
Weighted mode
0.05
0.00
−0.05
0.1
0.2
SNP effect on Cardiac valvular disease
SNP effect on ARSK

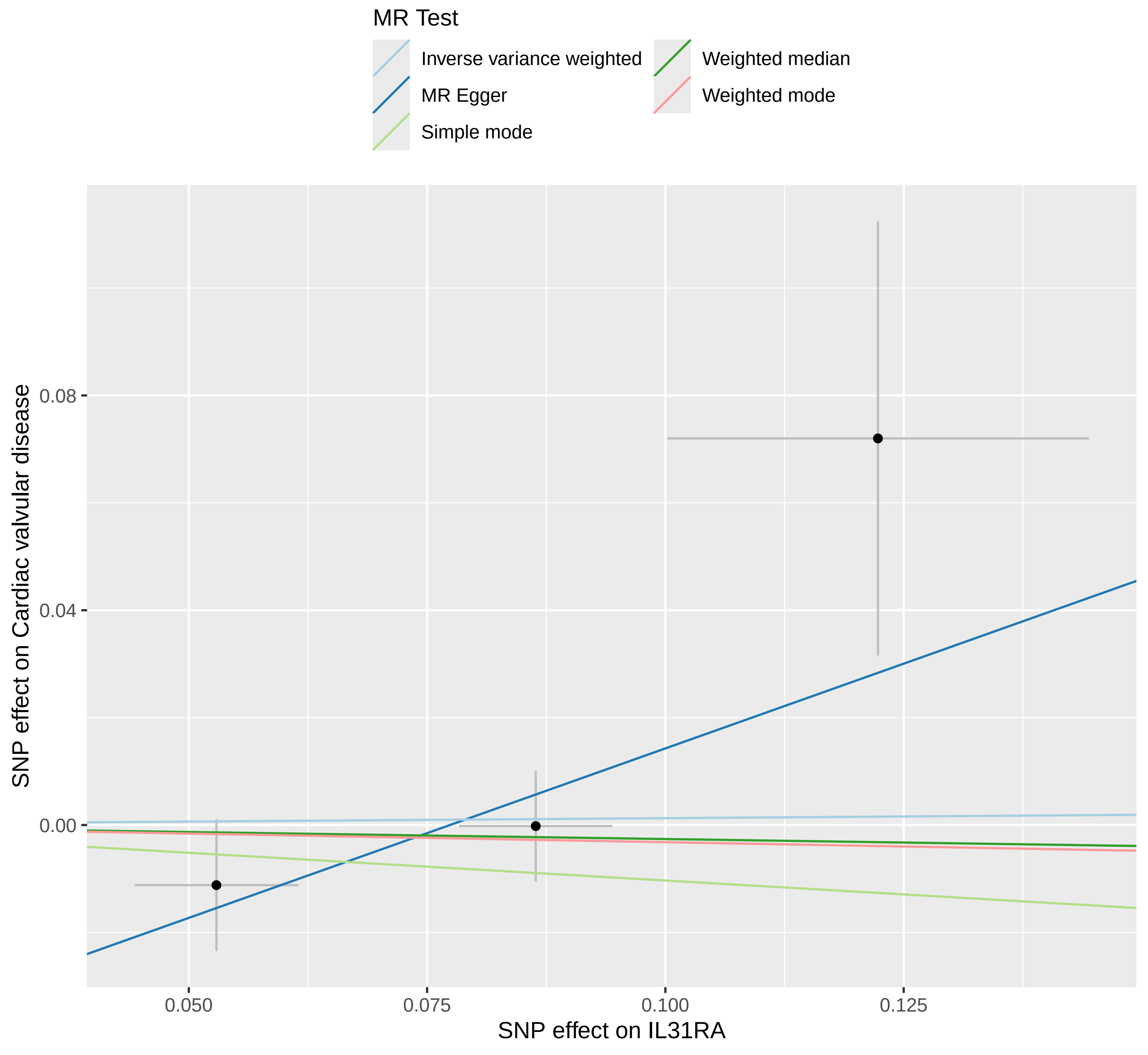
MR Test
Inverse variance weighted
MR Egger
Simple mode
Weighted median
Weighted mode
SNP effect on Cardiac valvular disease
0.08
0.04
0.00
0.050
0.075
0.100
0.125
SNP effect on IL31RA

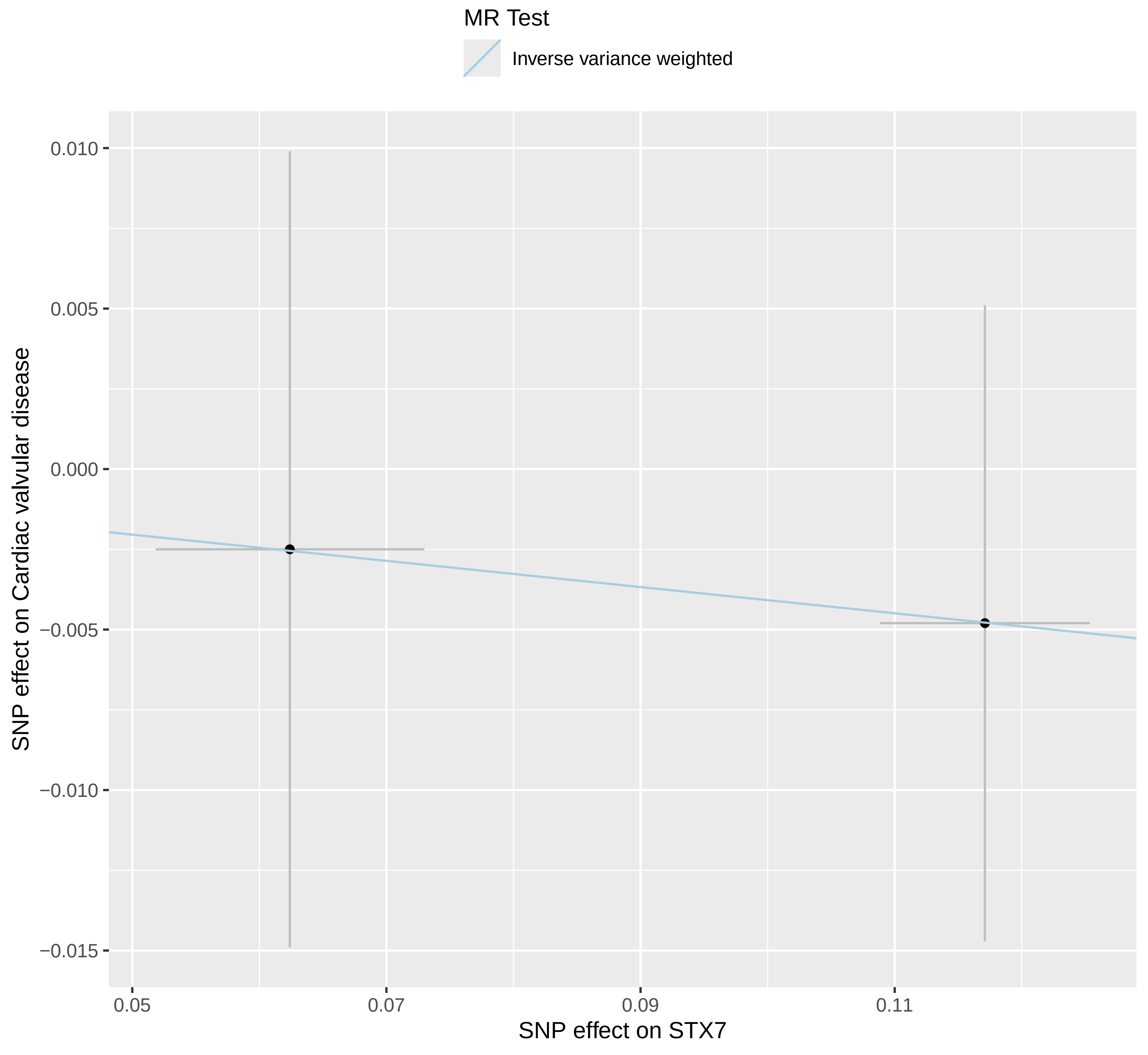

MR Test
Inverse variance weighted
0.010
0.005
0.000
−0.005
−0.010
−0.015
0.05
0.07
0.09
0.11
SNP effect on Cardiac valvular disease
SNP effect on STX7

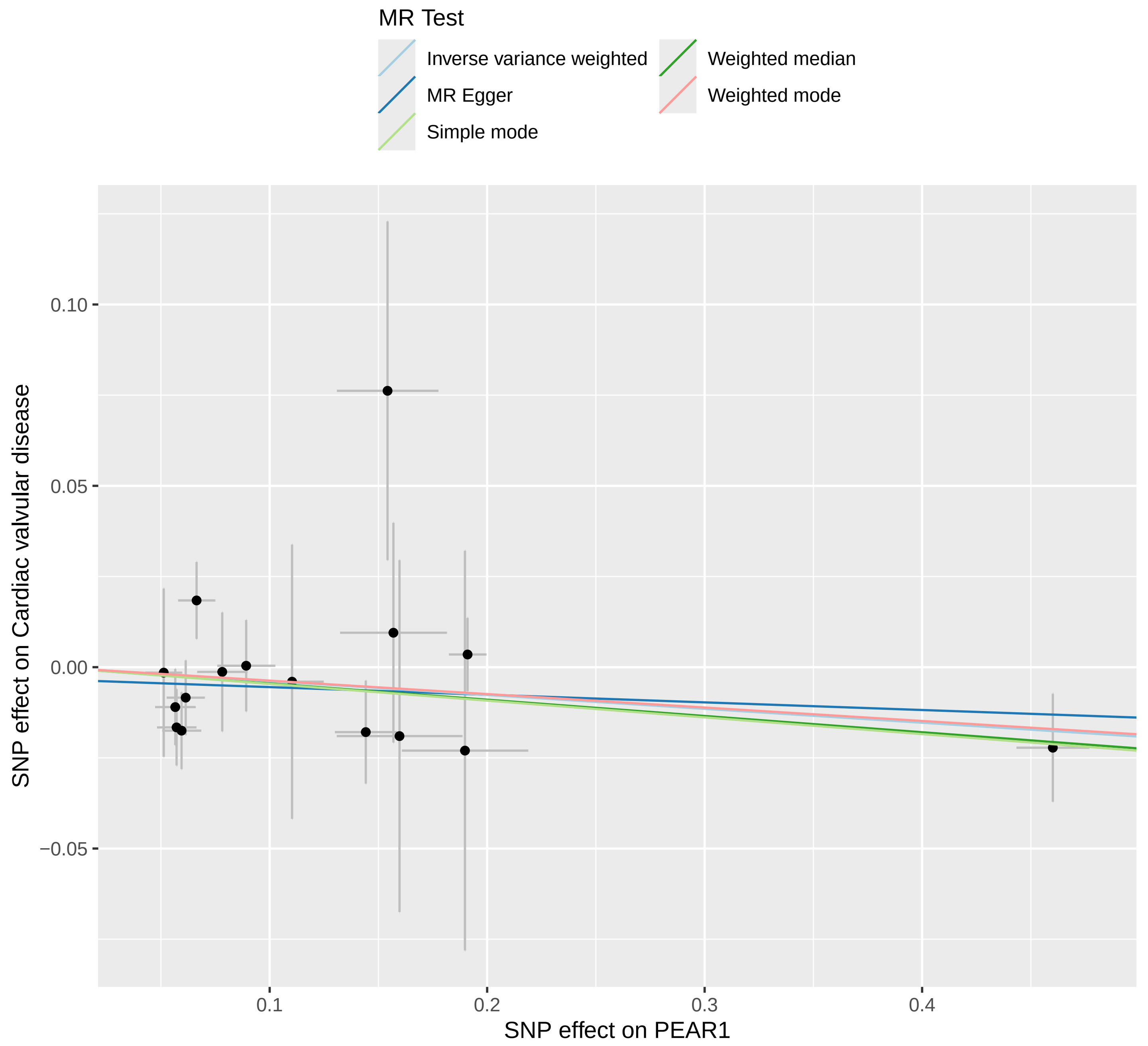
MR Test
Inverse variance weighted
MR Egger
Simple mode
Weighted median
Weighted mode
SNP effect on Cardiac valvular disease
0.10
0.05
0.00
−0.05
0.1
0.2
0.3
0.4
SNP effect on PEAR1

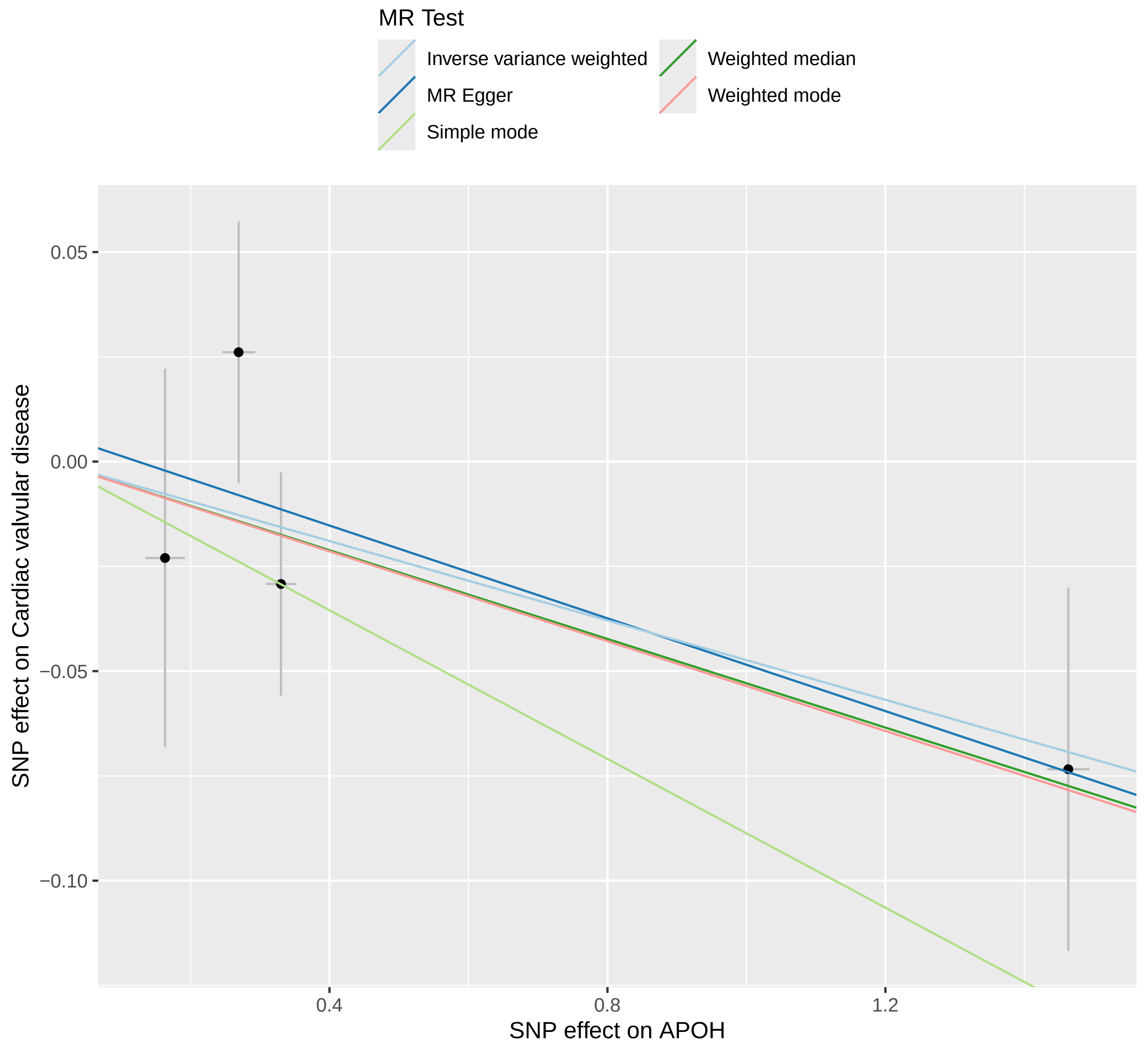
MR Test
Inverse variance weighted
MR Egger
Simple mode
Weighted median
Weighted mode
0.05
0.00
−0.05
−0.10
0.4
0.8
1.2
SNP effect on Cardiac valvular disease
SNP effect on APOH

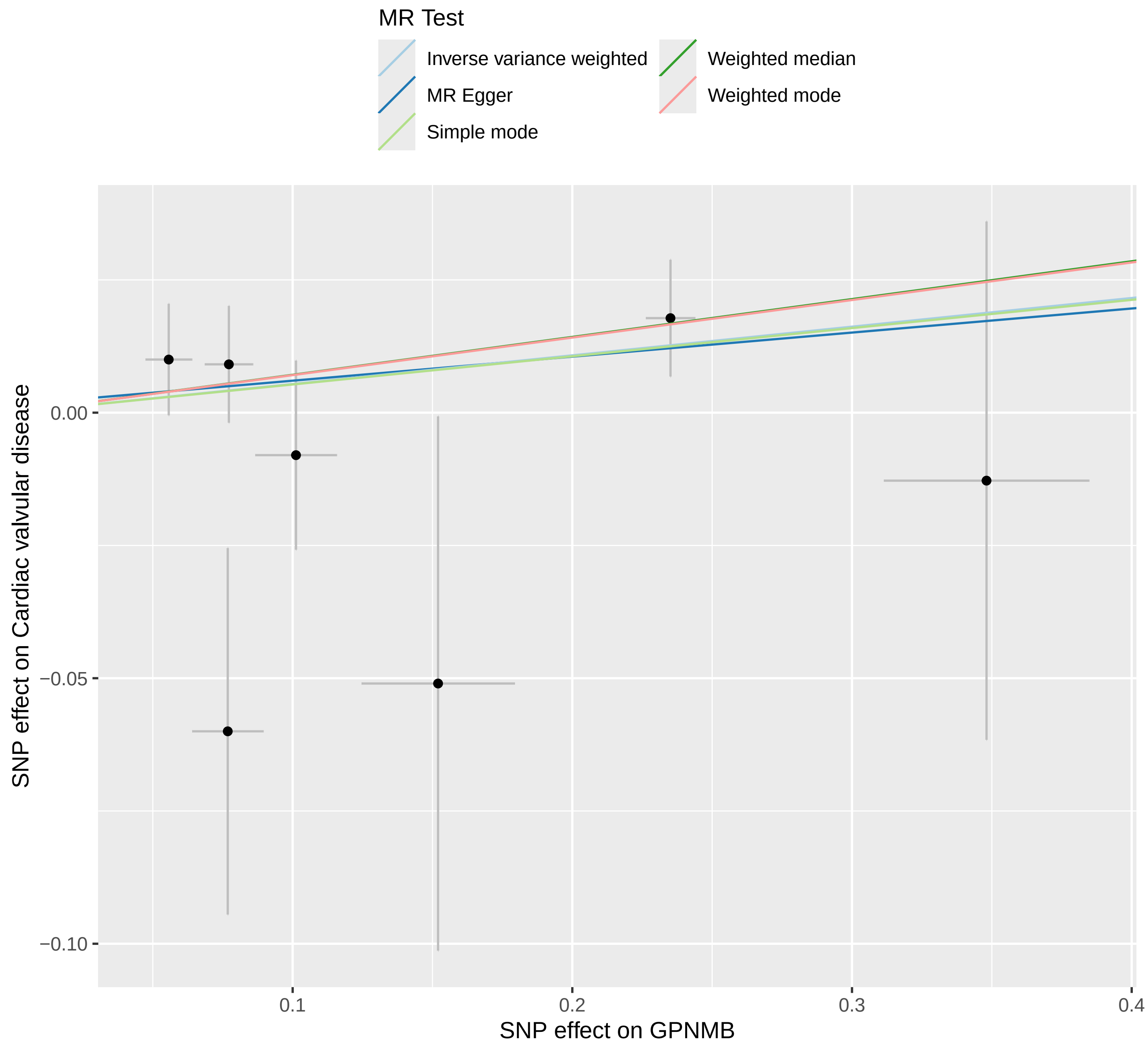
MR Test
Inverse variance weighted
MR Egger
Simple mode
Weighted median
Weighted mode
SNP effect on Cardiac valvular disease
0.00
−0.05
−0.10
0.1
0.2
0.3
0.4
SNP effect on GPNMB

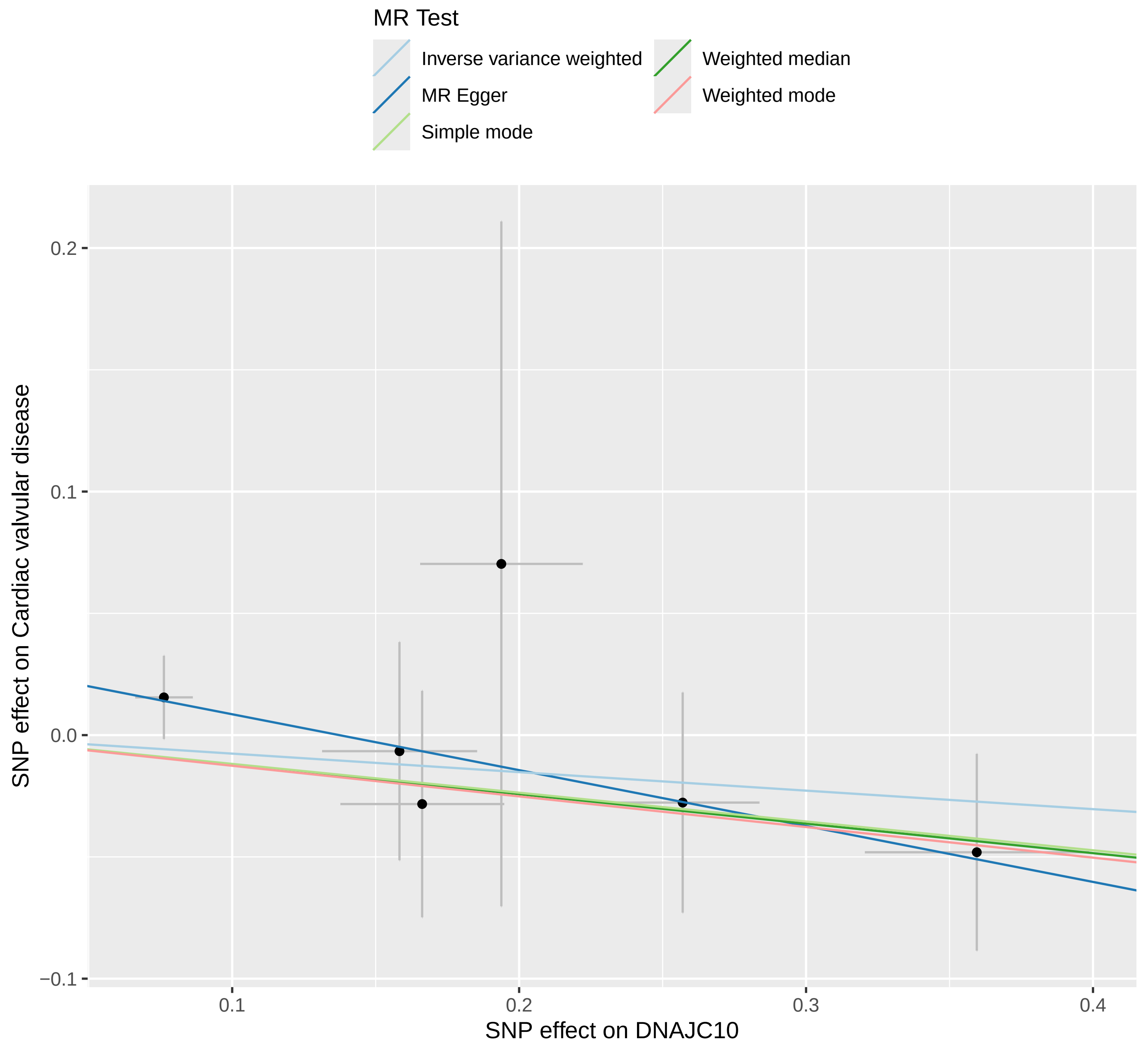
MR Test
Inverse variance weighted
MR Egger
Simple mode
Weighted median
Weighted mode
0.2
0.1
0.0
−0.1
0.1
0.2
0.3
0.4
SNP effect on Cardiac valvular disease
SNP effect on DNAJC10

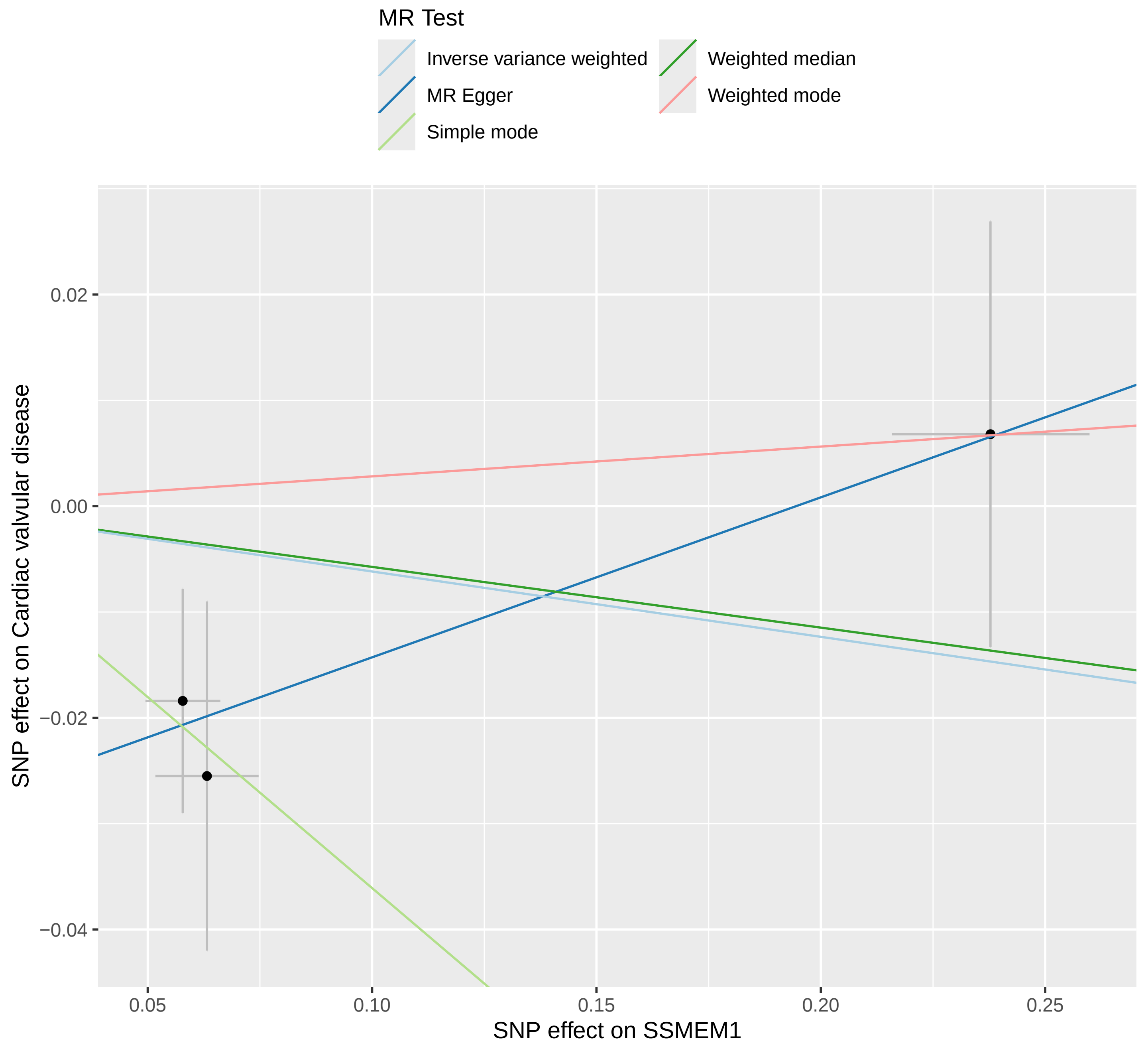
MR Test
Inverse variance weighted
MR Egger
Simple mode
Weighted median
Weighted mode
0.02
0.00
−0.02
−0.04
0.05
0.10
0.15
0.20
0.25
SNP effect on SSMEM1
SNP effect on Cardiac valvular disease

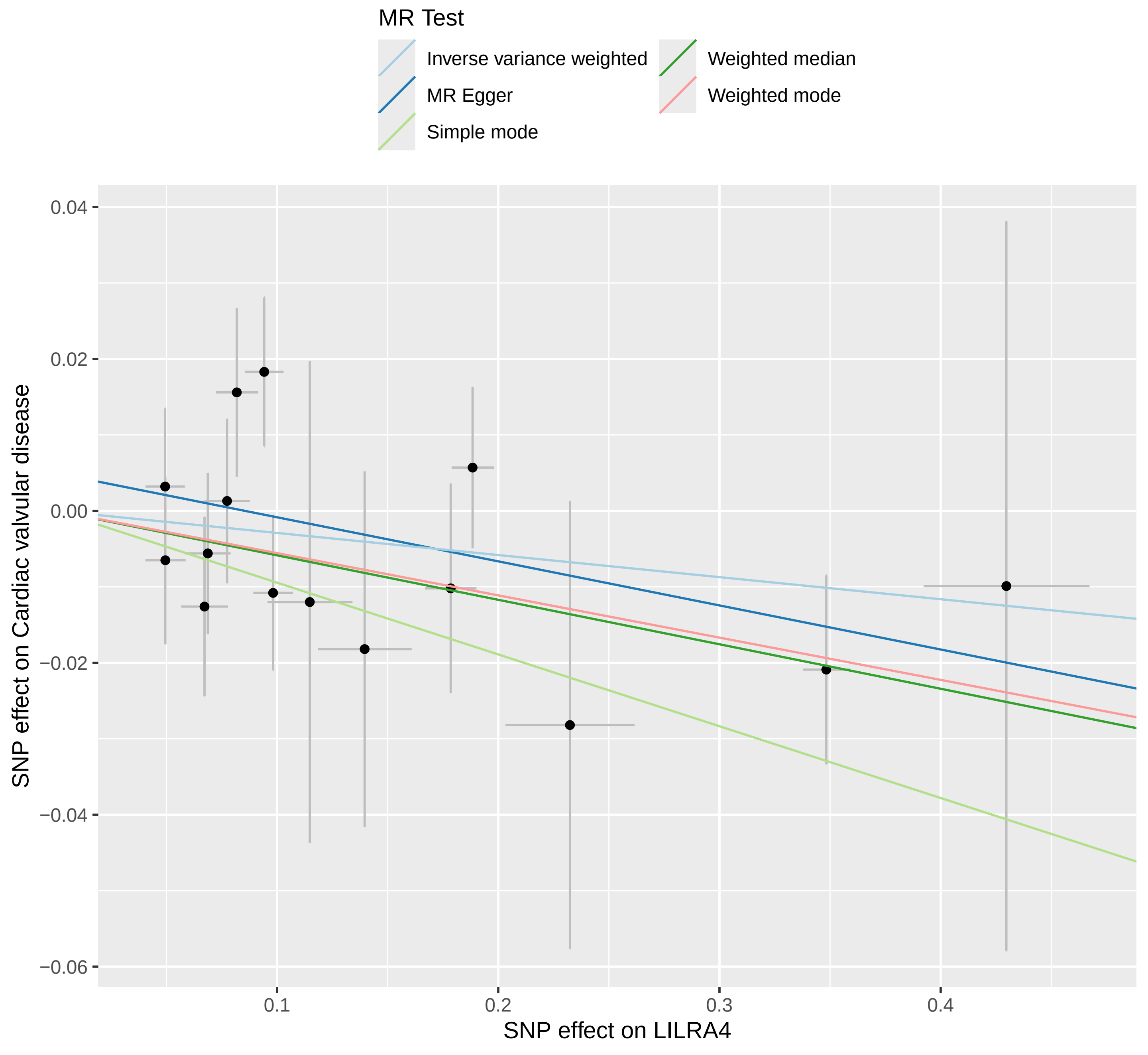
MR Test
Inverse variance weighted
MR Egger
Simple mode
Weighted median
Weighted mode
0.04
0.02
0.00
−0.02
−0.04
−0.06
SNP effect on Cardiac valvular disease
0.1
0.2
0.3
0.4
SNP effect on LILRA4

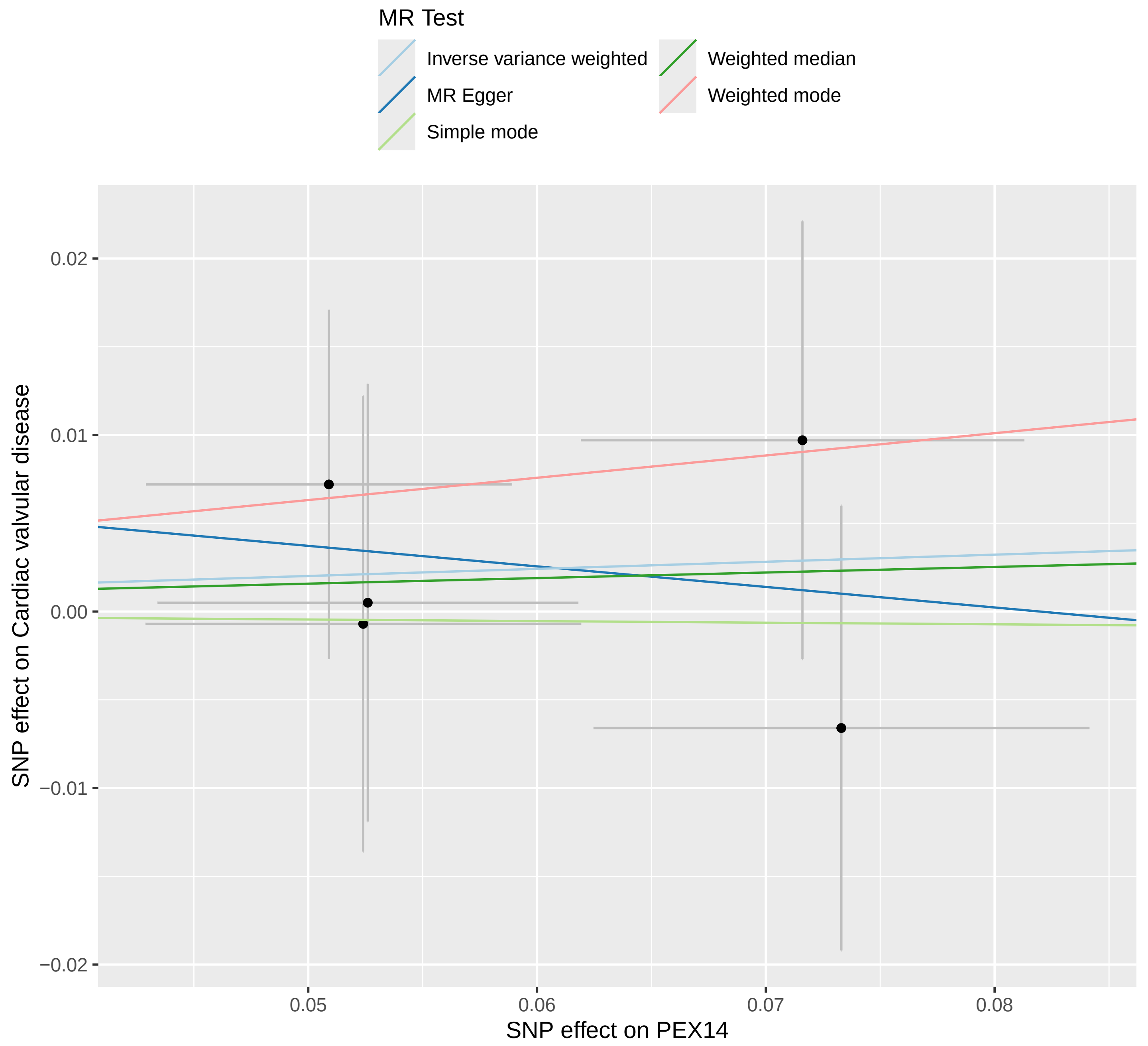
MR Test
Inverse variance weighted
MR Egger
Simple mode
Weighted median
Weighted mode
SNP effect on Cardiac valvular disease
0.02
0.01
0.00
−0.01
−0.02
0.05
0.06
0.07
0.08
SNP effect on PEX14

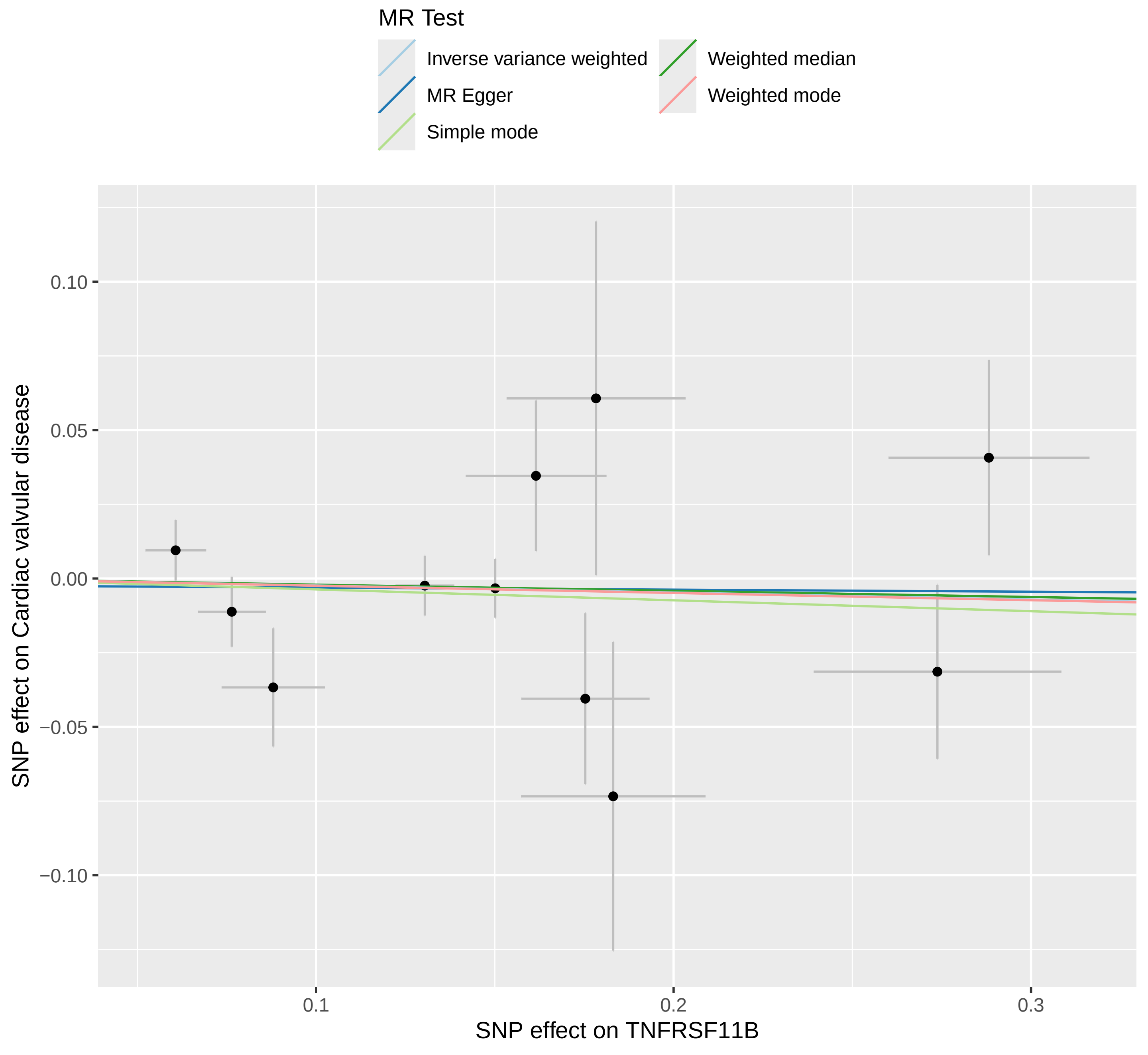
MR Test
Inverse variance weighted
MR Egger
Simple mode
Weighted median
Weighted mode
0.10
0.05
0.00
−0.05
−0.10
SNP effect on Cardiac valvular disease
0.1
0.2
0.3
SNP effect on TNFRSF11B

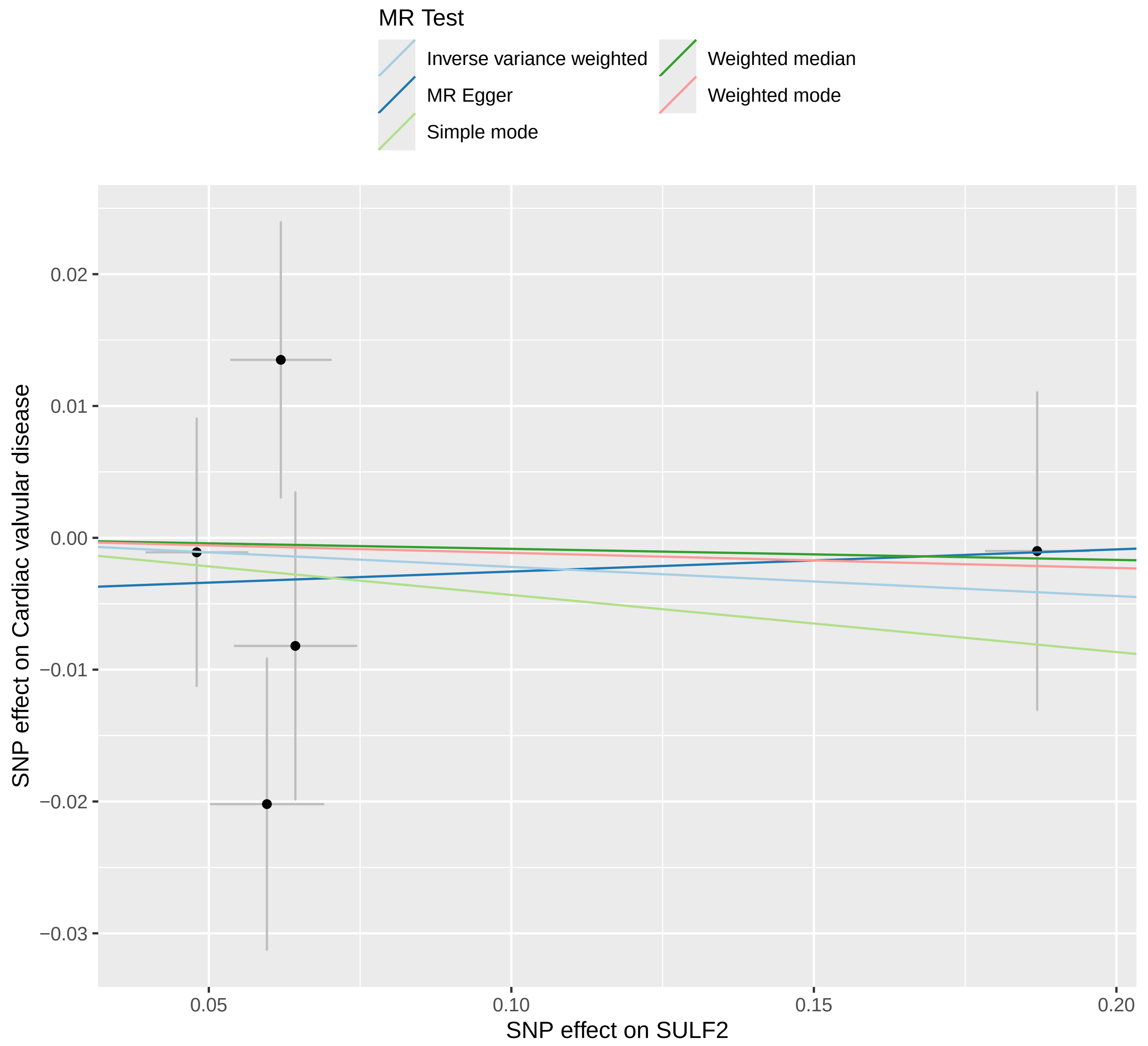
MR Test
Inverse variance weighted
MR Egger
Simple mode
Weighted median
Weighted mode
0.02
0.01
0.00
−0.01
−0.02
−0.03
SNP effect on Cardiac valvular disease
0.05
0.10
0.15
0.20
SNP effect on SULF2

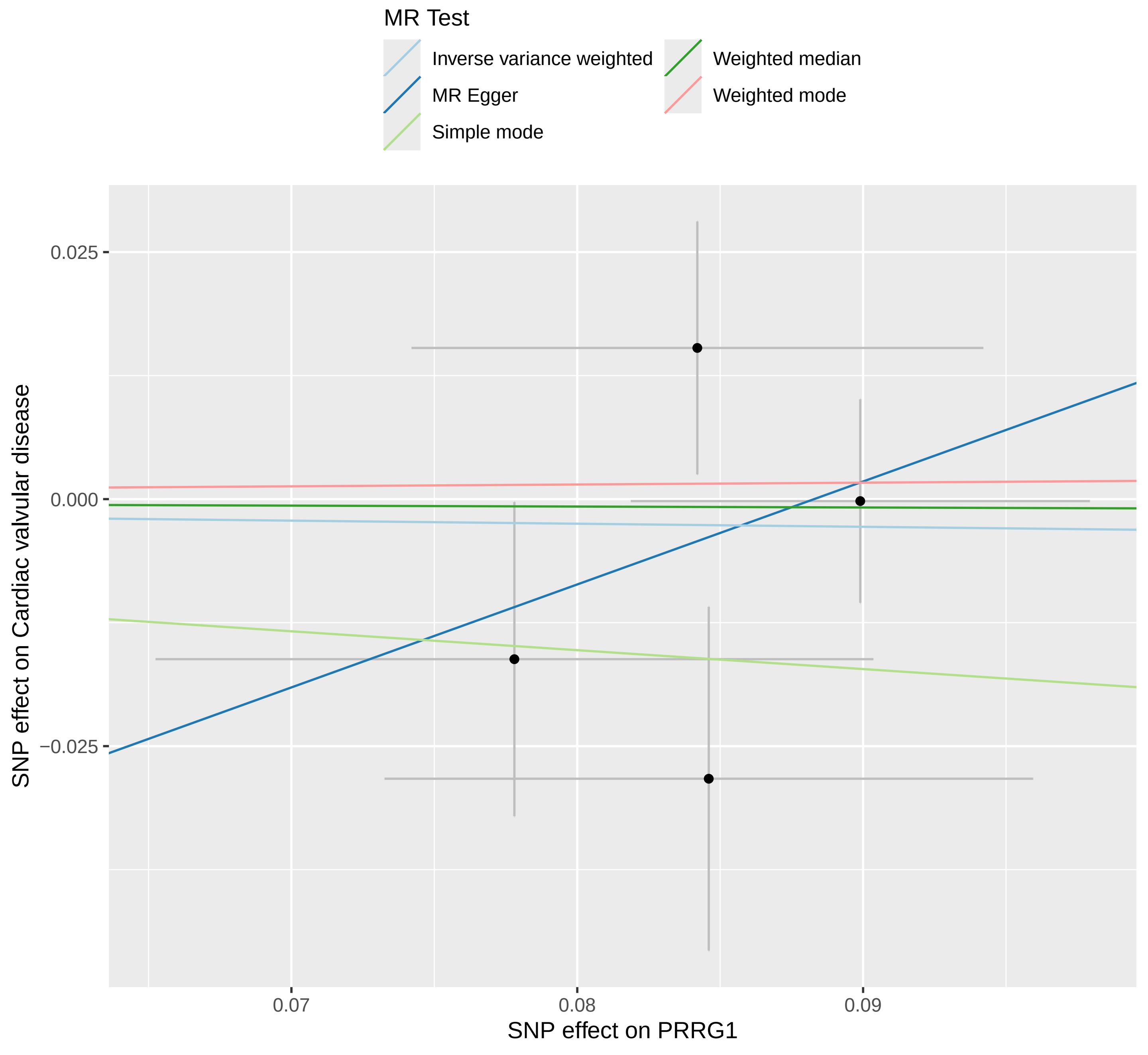

MR Test
Inverse variance weighted
MR Egger
Simple mode
Weighted median
Weighted mode
0.025
0.000
−0.025
0.07
0.08
0.09
SNP effect on PRRG1
SNP effect on Cardiac valvular disease

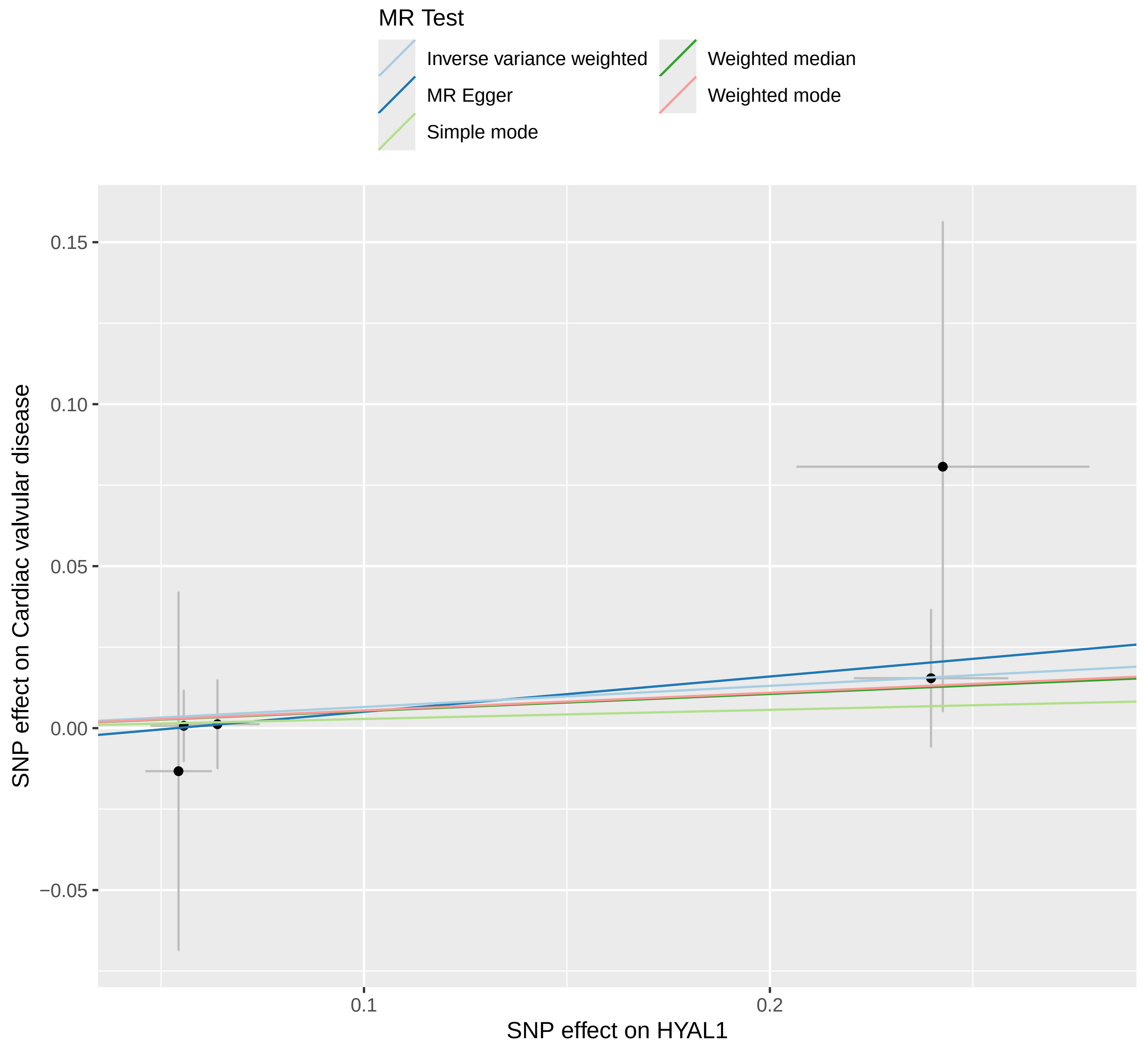

MR Test
Inverse variance weighted
MR Egger
Simple mode
Weighted median
Weighted mode
0.15
0.10
0.05
0.00
−0.05
0.1
0.2
SNP effect on Cardiac valvular disease
SNP effect on HYAL1

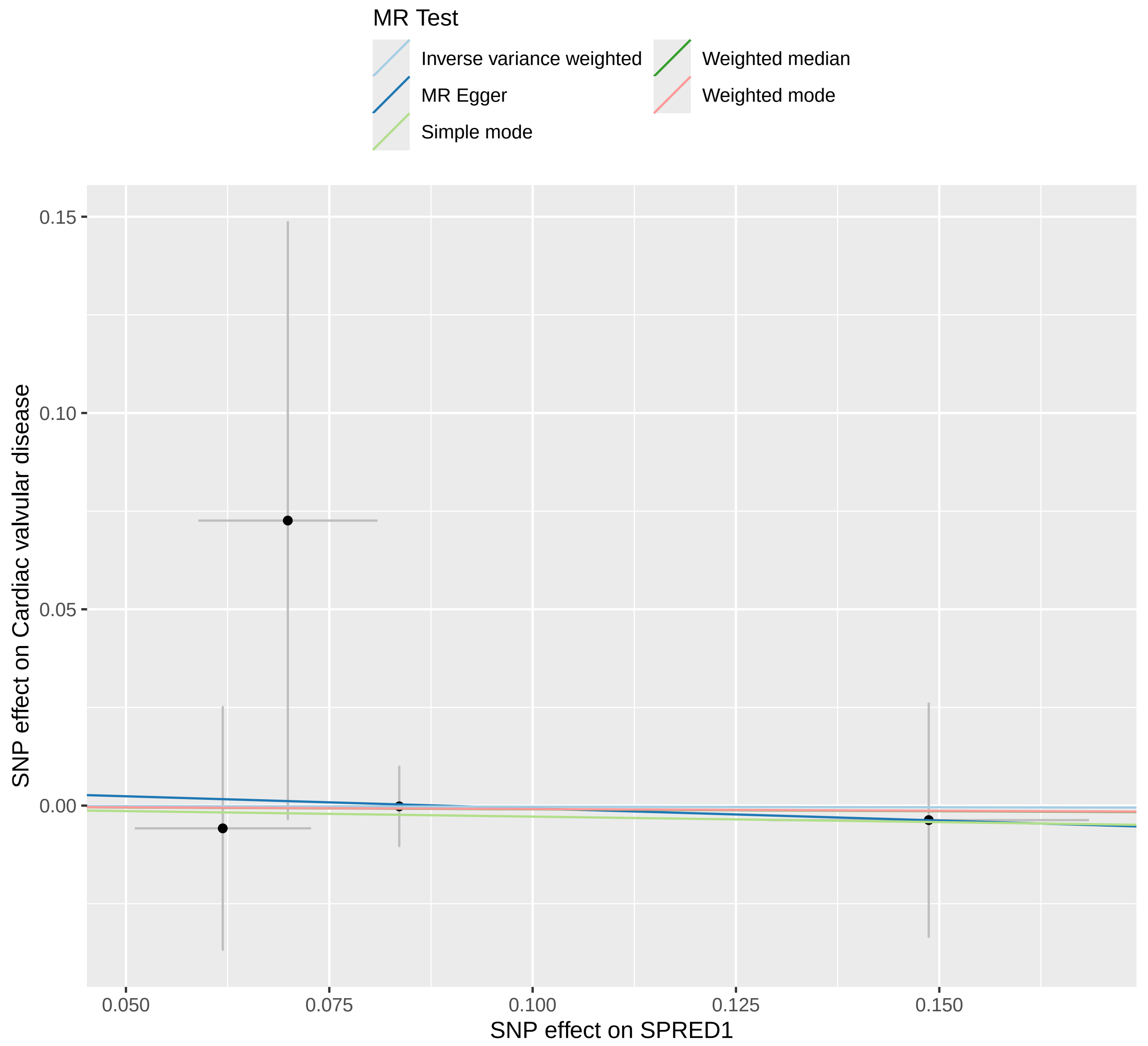
MR Test
Inverse variance weighted
MR Egger
Simple mode
Weighted median
Weighted mode
0.15
0.10
0.05
0.00
SNP effect on Cardiac valvular disease
0.050
0.075
0.100
0.125
0.150
SNP effect on SPRED1

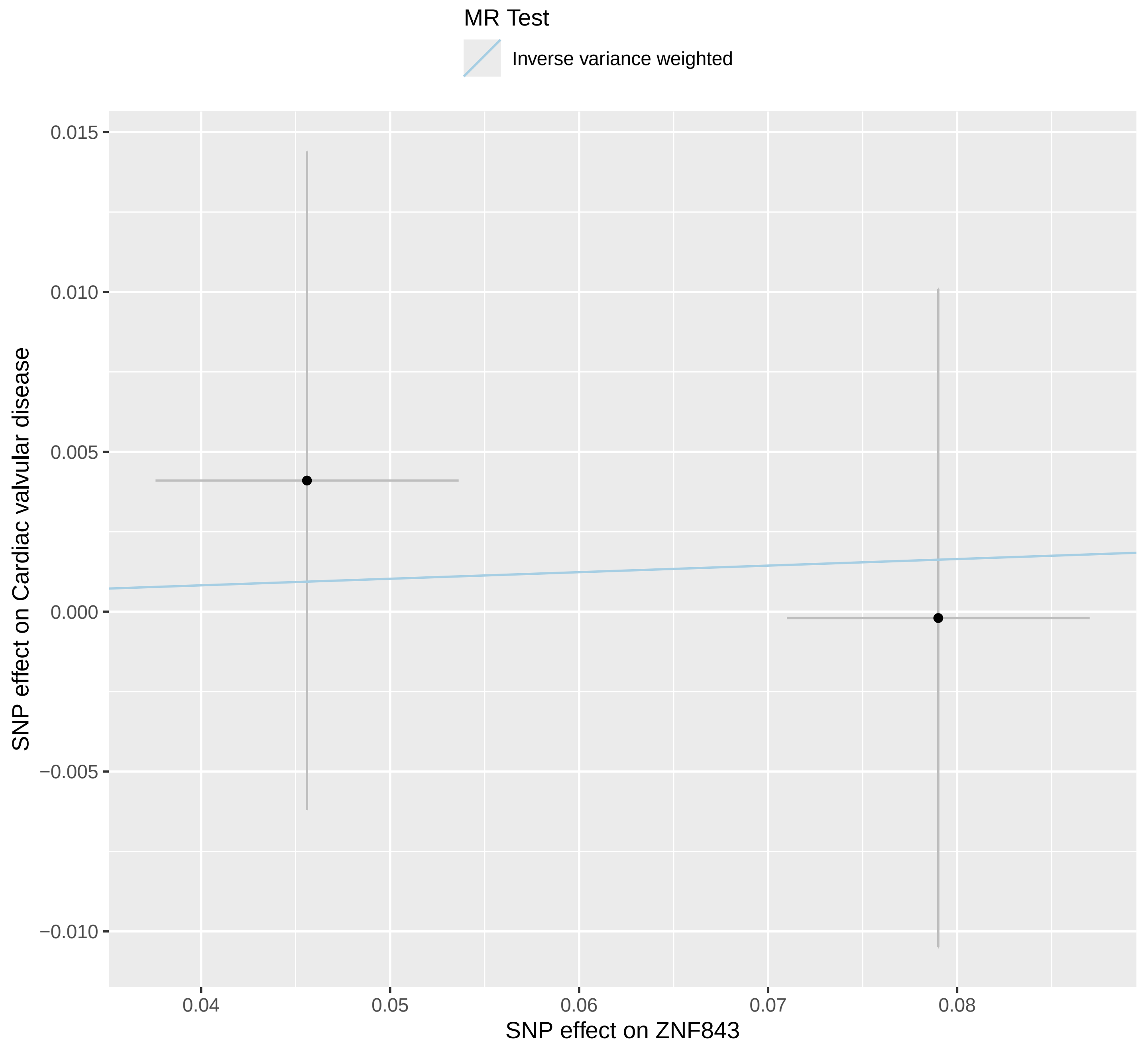

MR Test
Inverse variance weighted
0.015
0.010
0.005
0.000
−0.005
−0.010
0.04
0.05
0.06
0.07
0.08
SNP effect on Cardiac valvular disease
SNP effect on ZNF843

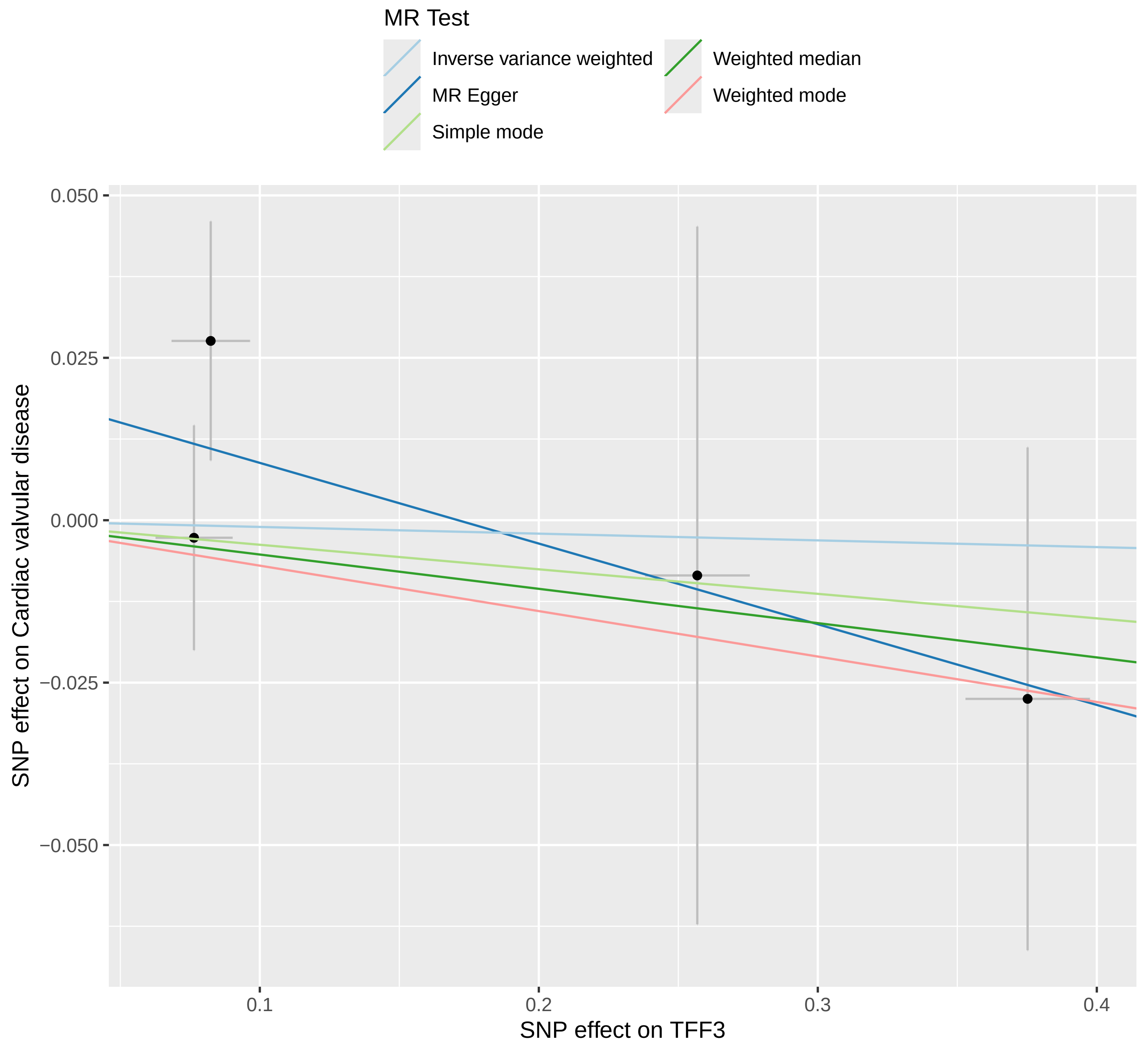

MR Test
Inverse variance weighted
MR Egger
Simple mode
Weighted median
Weighted mode
0.050
0.025
0.000
−0.025
−0.050
0.1
0.2
0.3
0.4
SNP effect on Cardiac valvular disease
SNP effect on TFF3

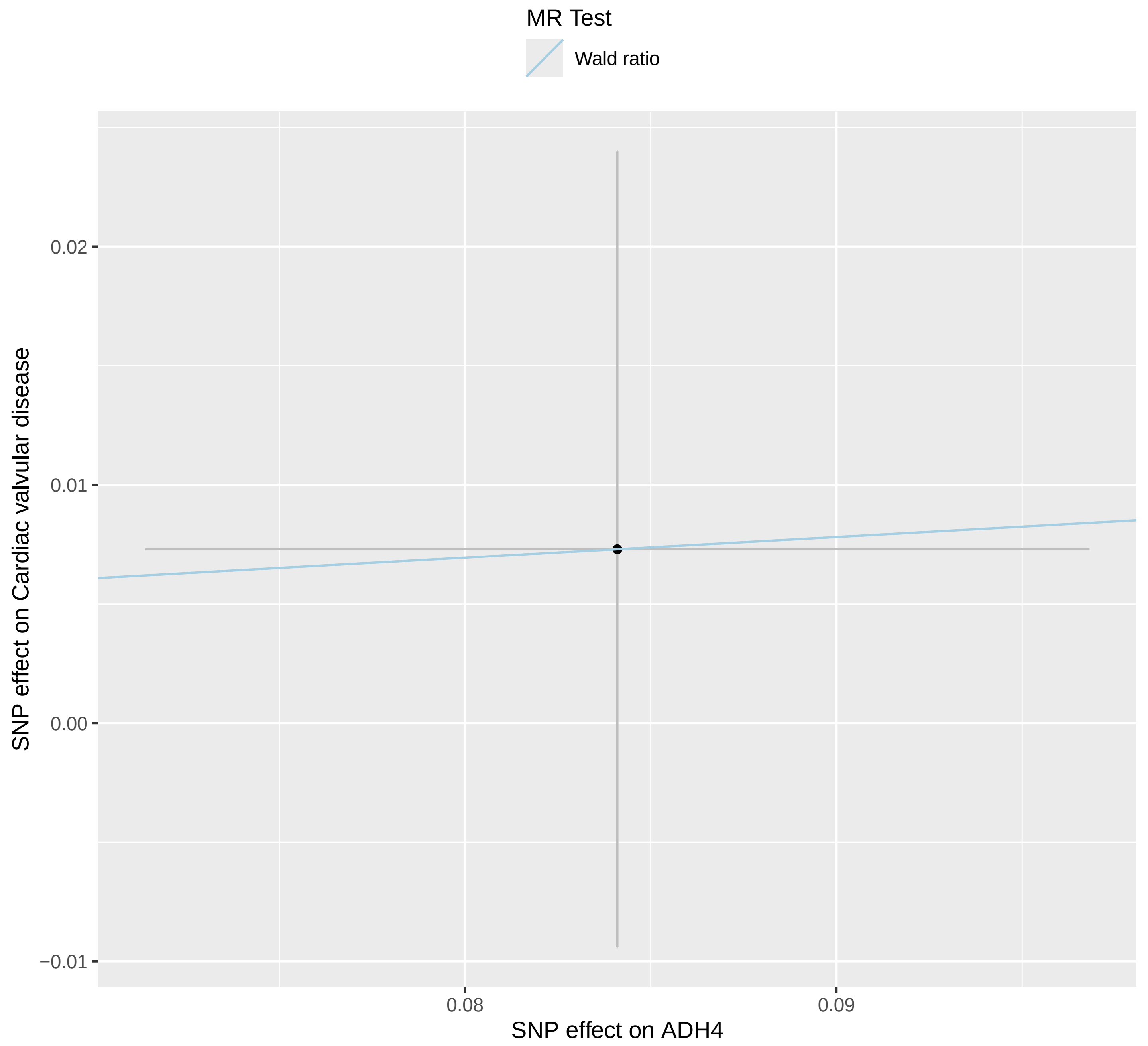
MR Test
Wald ratio
0.02
0.01
0.00
−0.01
SNP effect on Cardiac valvular disease
0.08
0.09
SNP effect on ADH4

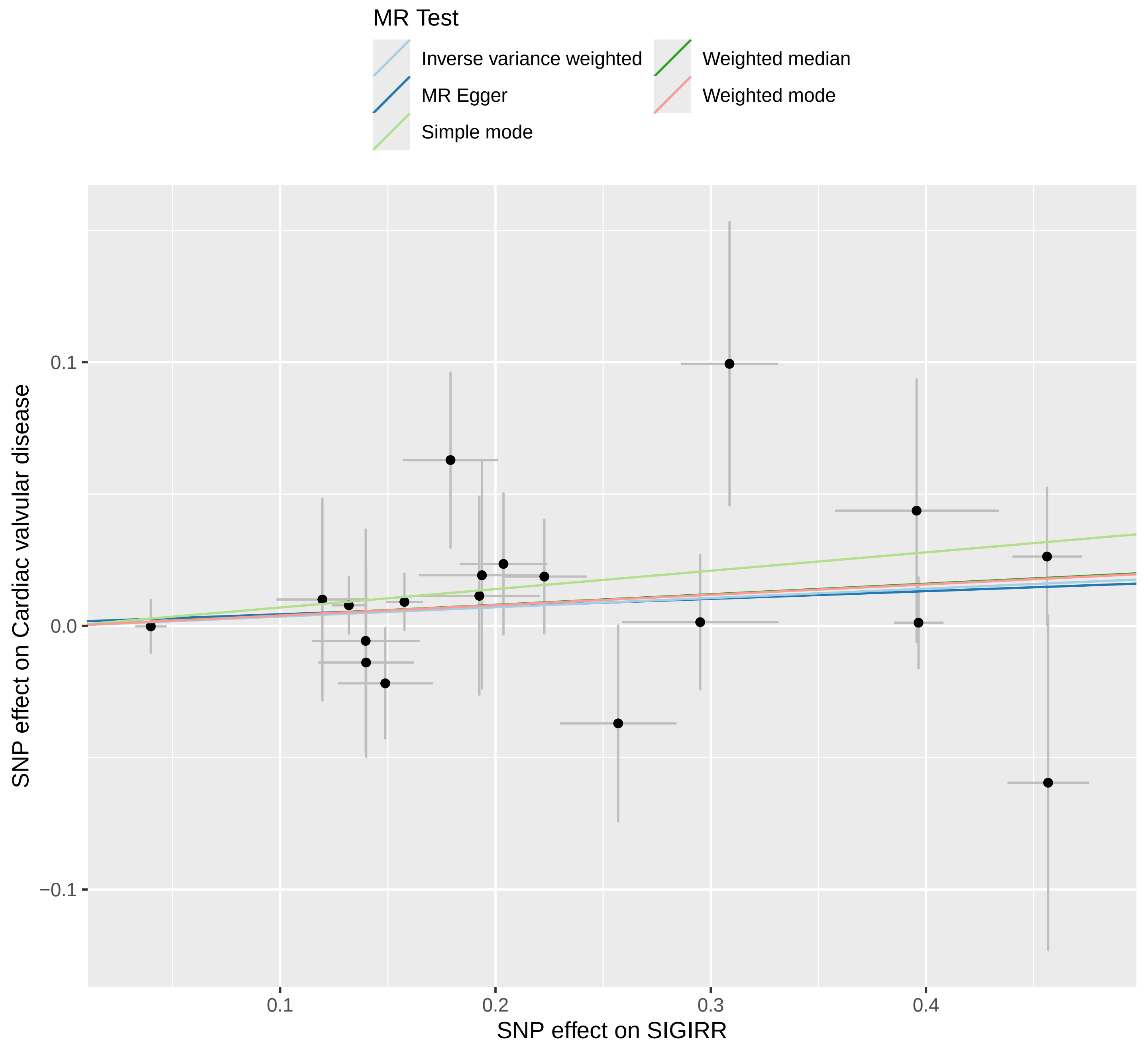
MR Test
Inverse variance weighted
MR Egger
Simple mode
Weighted median
Weighted mode
0.1
0.0
−0.1
SNP effect on Cardiac valvular disease
0.1
0.2
0.3
0.4
SNP effect on SIGIRR

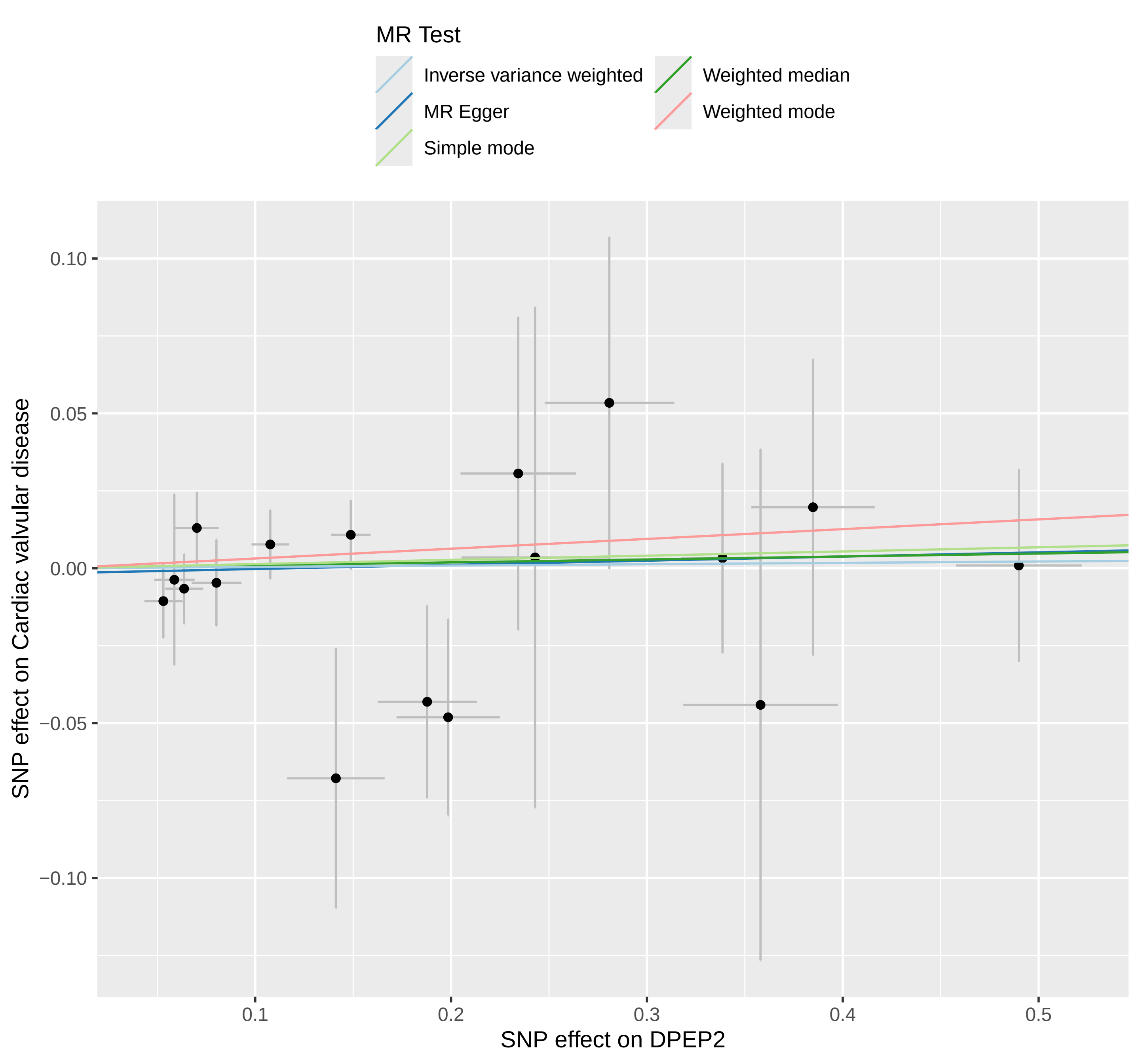
MR Test
Inverse variance weighted
MR Egger
Simple mode
Weighted median
Weighted mode
SNP effect on Cardiac valvular disease
0.10
0.05
0.00
−0.05
−0.10
0.1
0.2
0.3
0.4
0.5
SNP effect on DPEP2

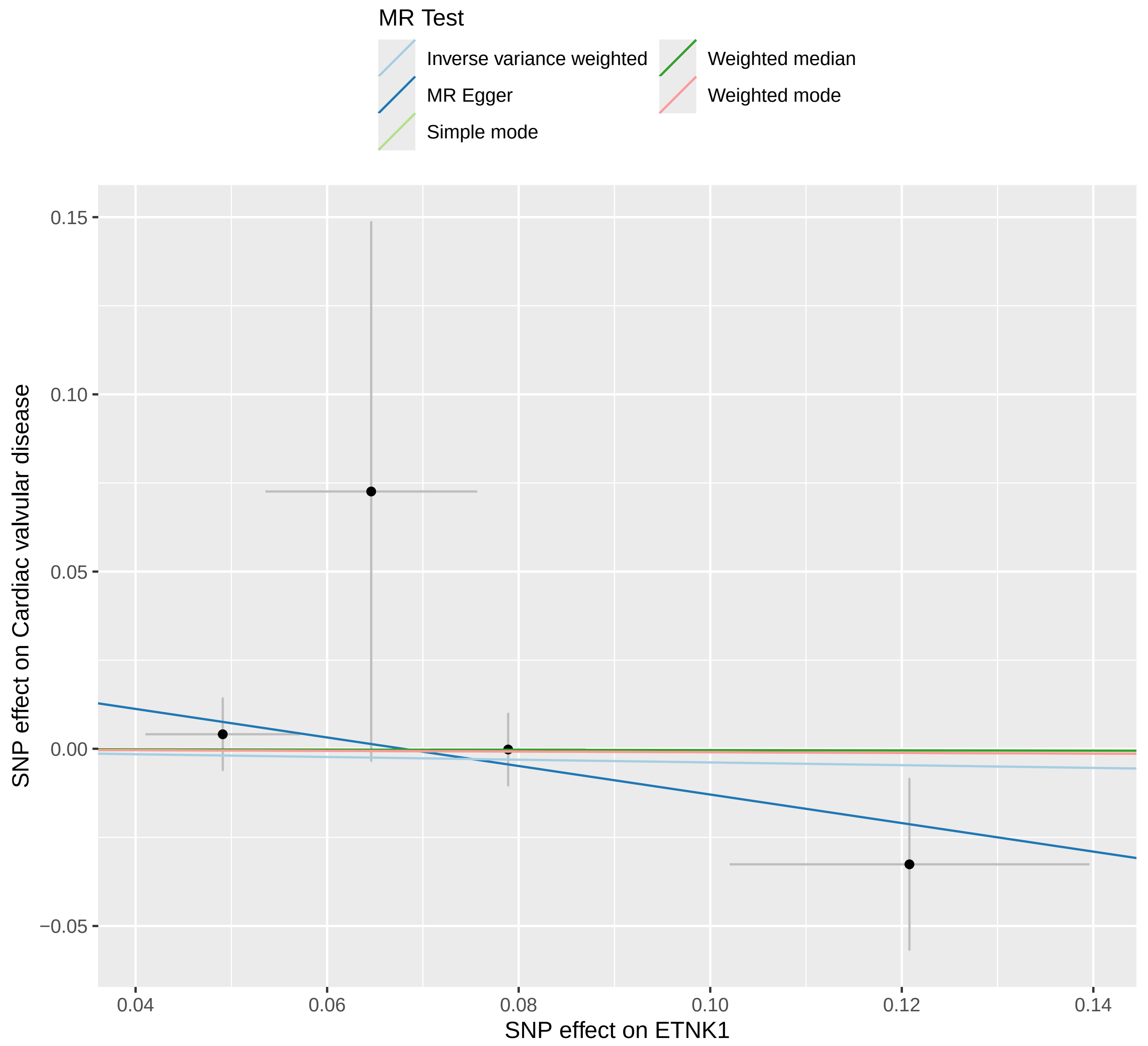
MR Test
Inverse variance weighted
MR Egger
Simple mode
Weighted median
Weighted mode
0.15
0.10
0.05
0.00
−0.05
SNP effect on Cardiac valvular disease
0.04
0.06
0.08
0.10
0.12
0.14
SNP effect on ETNK1

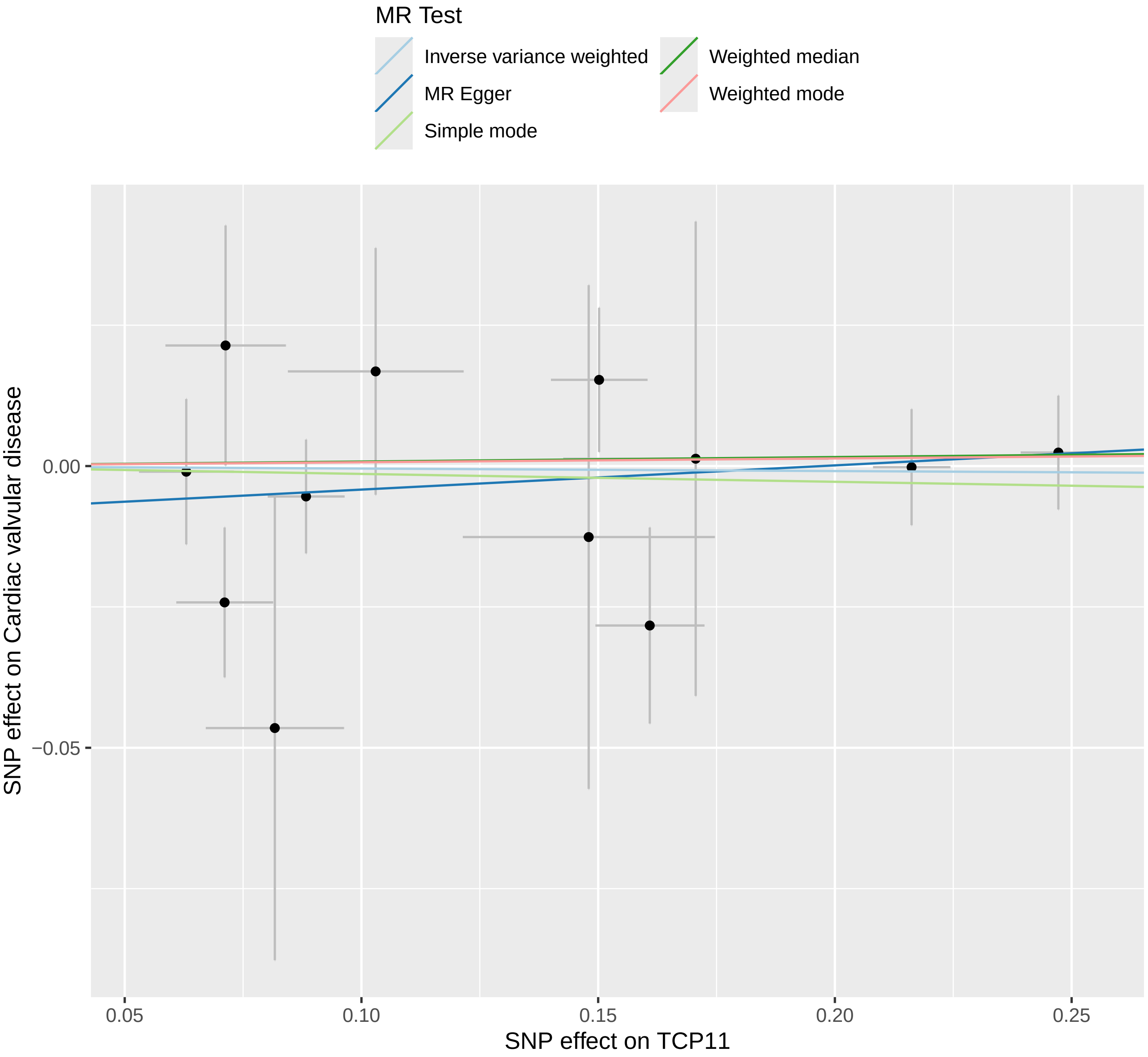
MR Test
Inverse variance weighted
MR Egger
Simple mode
Weighted median
Weighted mode
0.00
−0.05
0.05
0.10
0.15
0.20
0.25
SNP effect on Cardiac valvular disease
SNP effect on TCP11

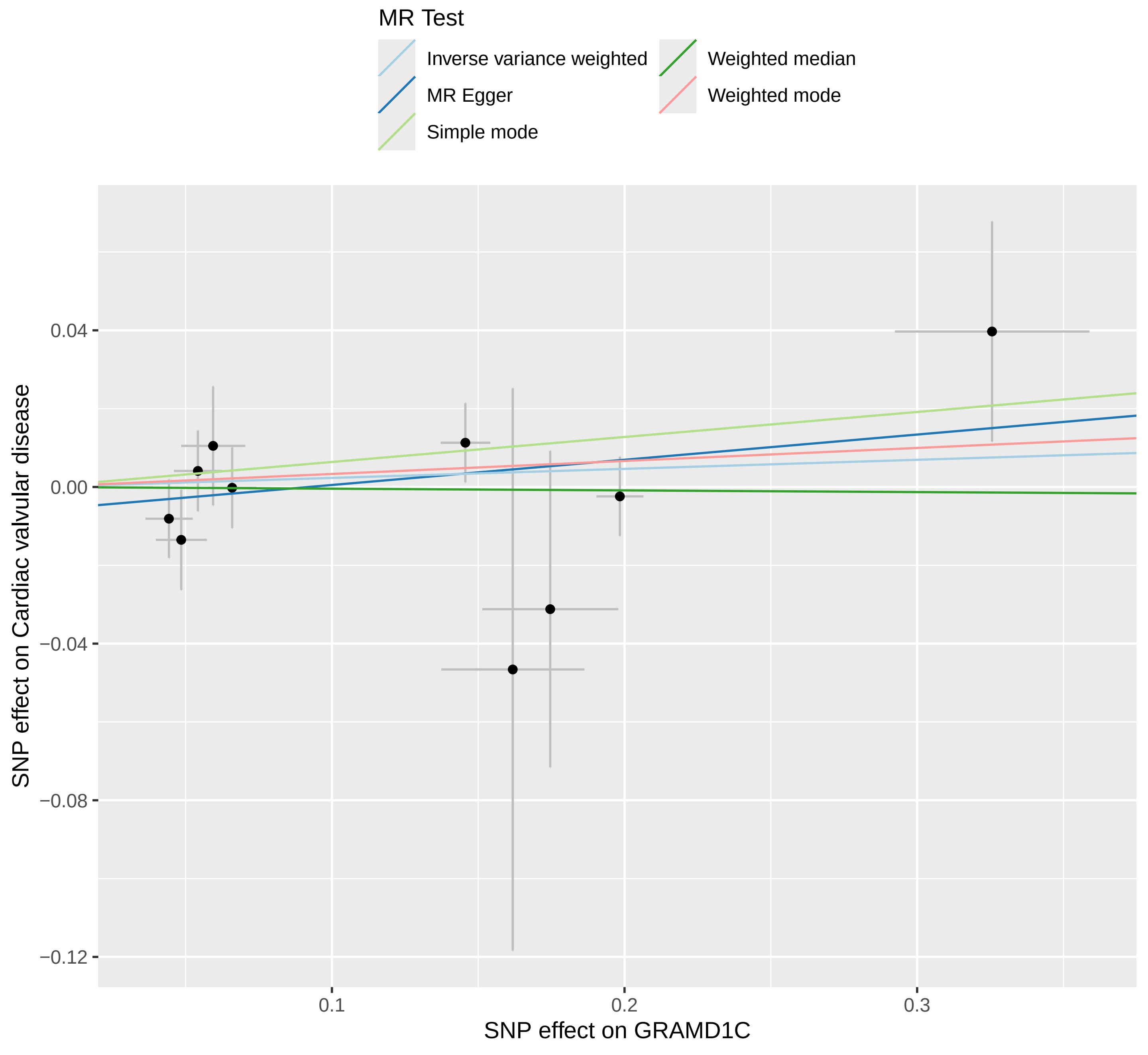

MR Test
Inverse variance weighted
MR Egger
Simple mode
Weighted median
Weighted mode
0.04
0.00
−0.04
−0.08
−0.12
0.1
0.2
0.3
SNP effect on Cardiac valvular disease
SNP effect on GRAMD1C

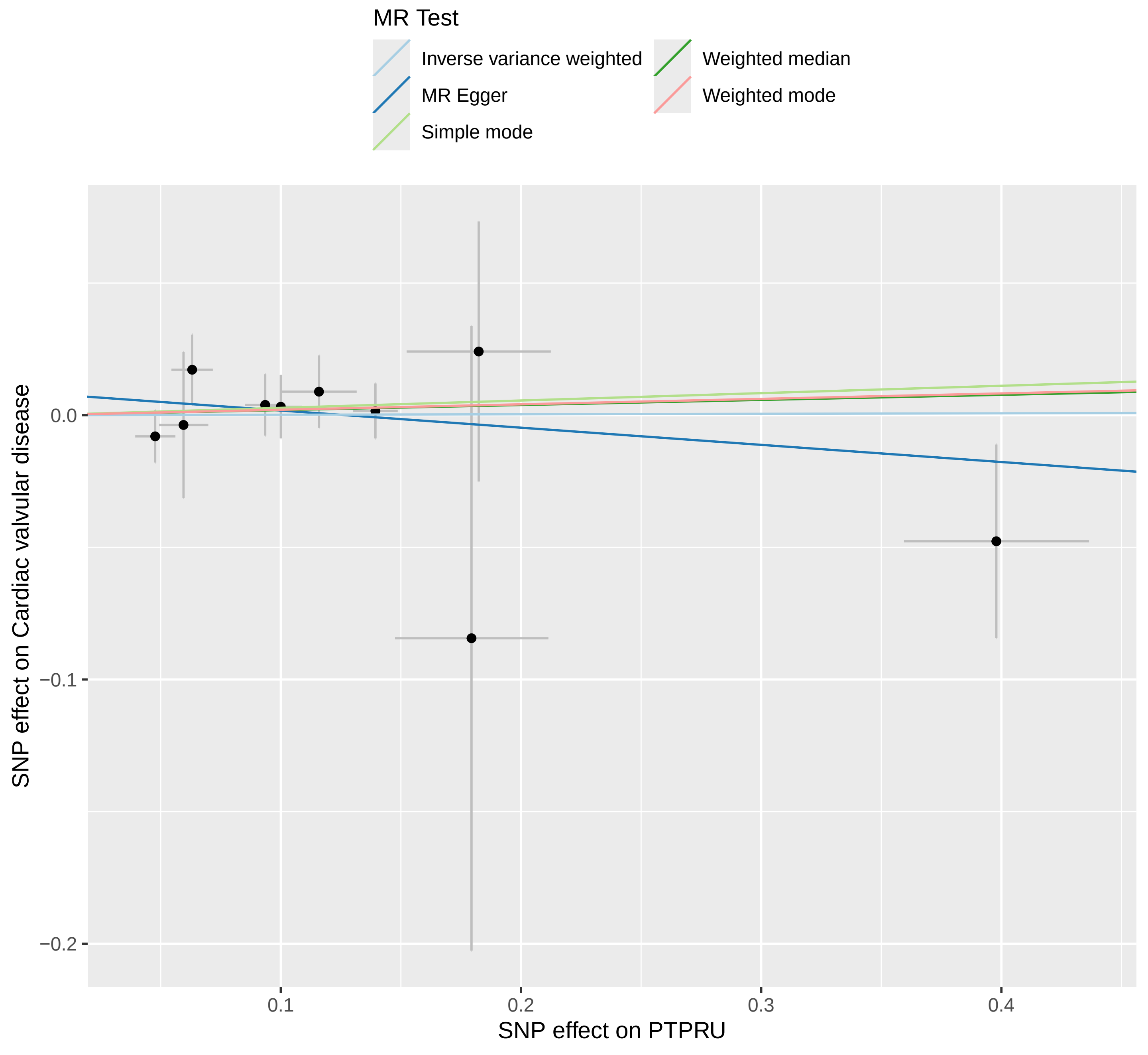
MR Test
Inverse variance weighted
MR Egger
Simple mode
Weighted median
Weighted mode
0.0
−0.1
−0.2
SNP effect on Cardiac valvular disease
0.1
0.2
0.3
0.4
SNP effect on PTPRU

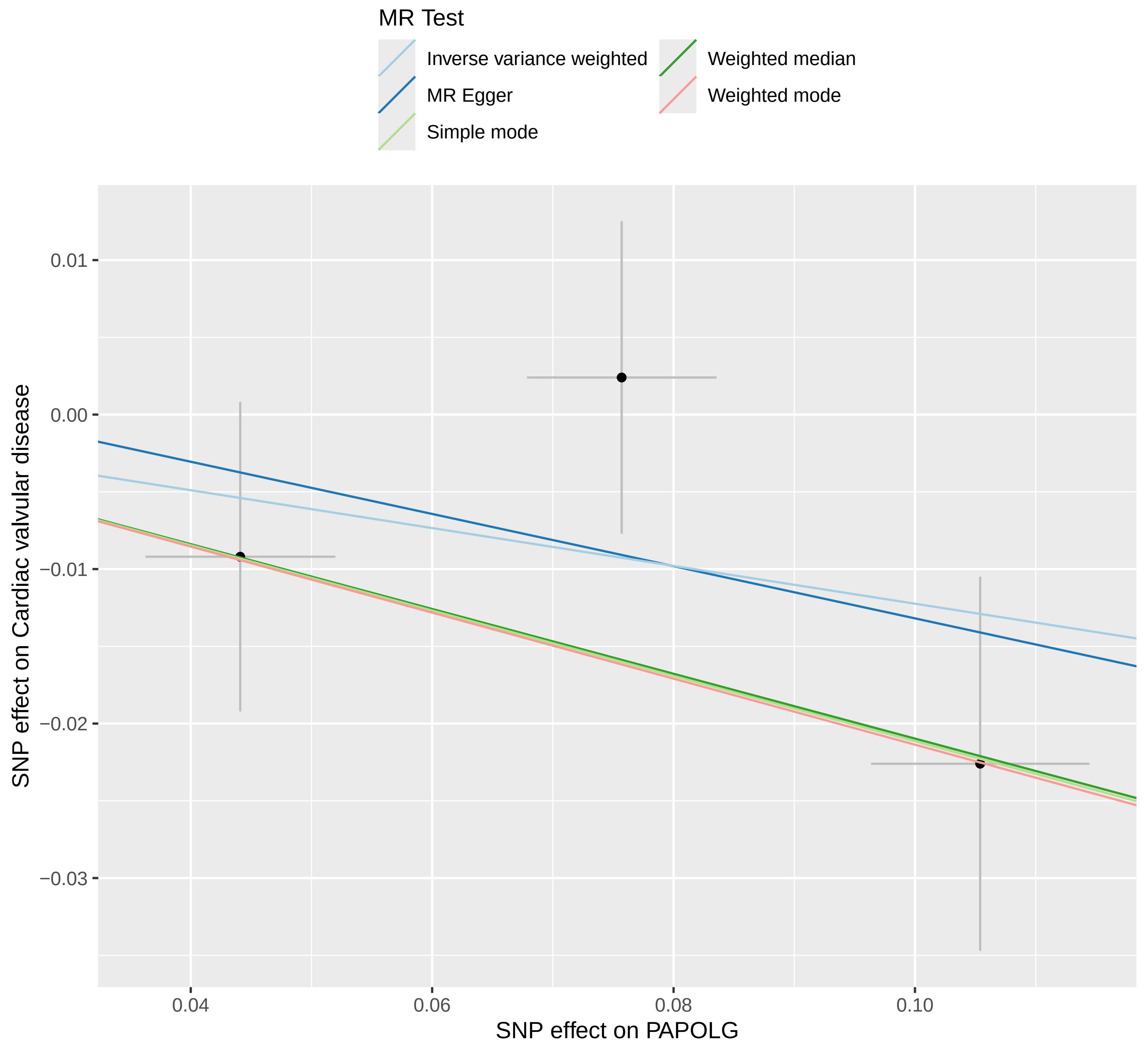

MR Test
Inverse variance weighted
MR Egger
Simple mode
Weighted median
Weighted mode
0.01
0.00
−0.01
−0.02
−0.03
SNP effect on Cardiac valvular disease
0.04
0.06
0.08
0.10
SNP effect on PAPOLG

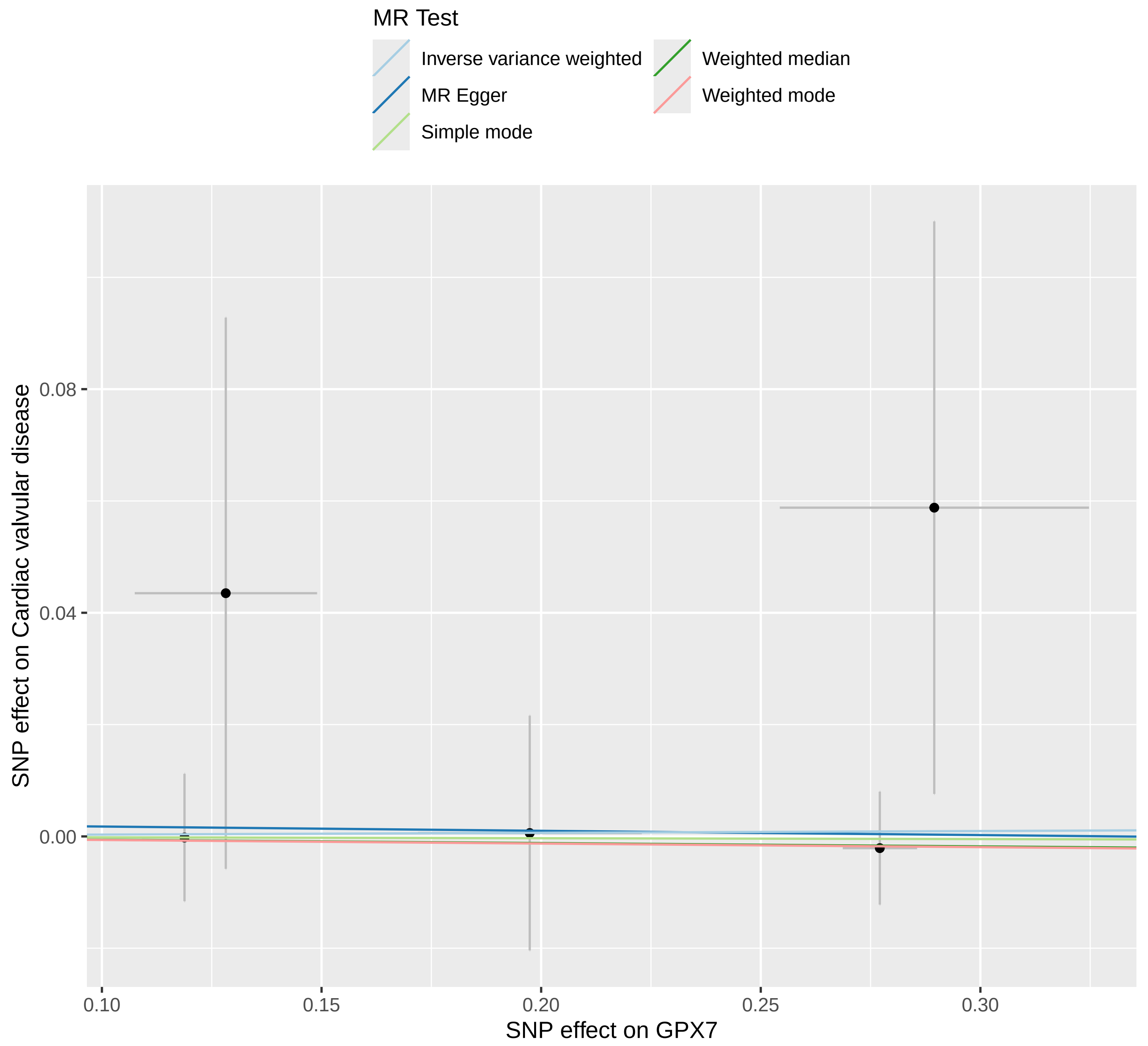
MR Test
Inverse variance weighted
MR Egger
Simple mode
Weighted median
Weighted mode
0.08
0.04
0.00
0.10
0.15
0.20
0.25
0.30
SNP effect on GPX7
SNP effect on Cardiac valvular disease

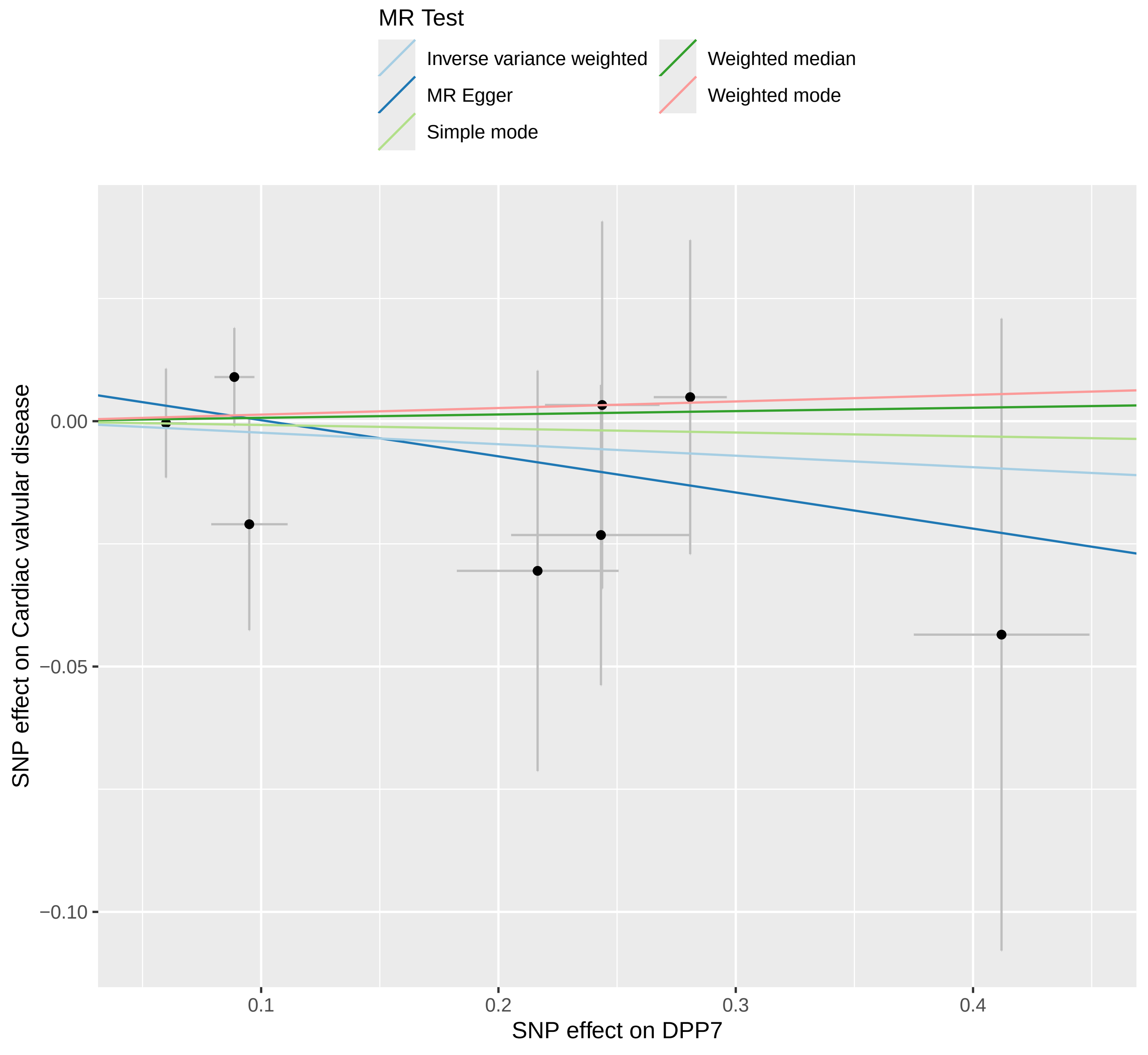
MR Test
Inverse variance weighted
MR Egger
Simple mode
Weighted median
Weighted mode
SNP effect on Cardiac valvular disease
0.00
−0.05
−0.10
0.1
0.2
0.3
0.4
SNP effect on DPP7

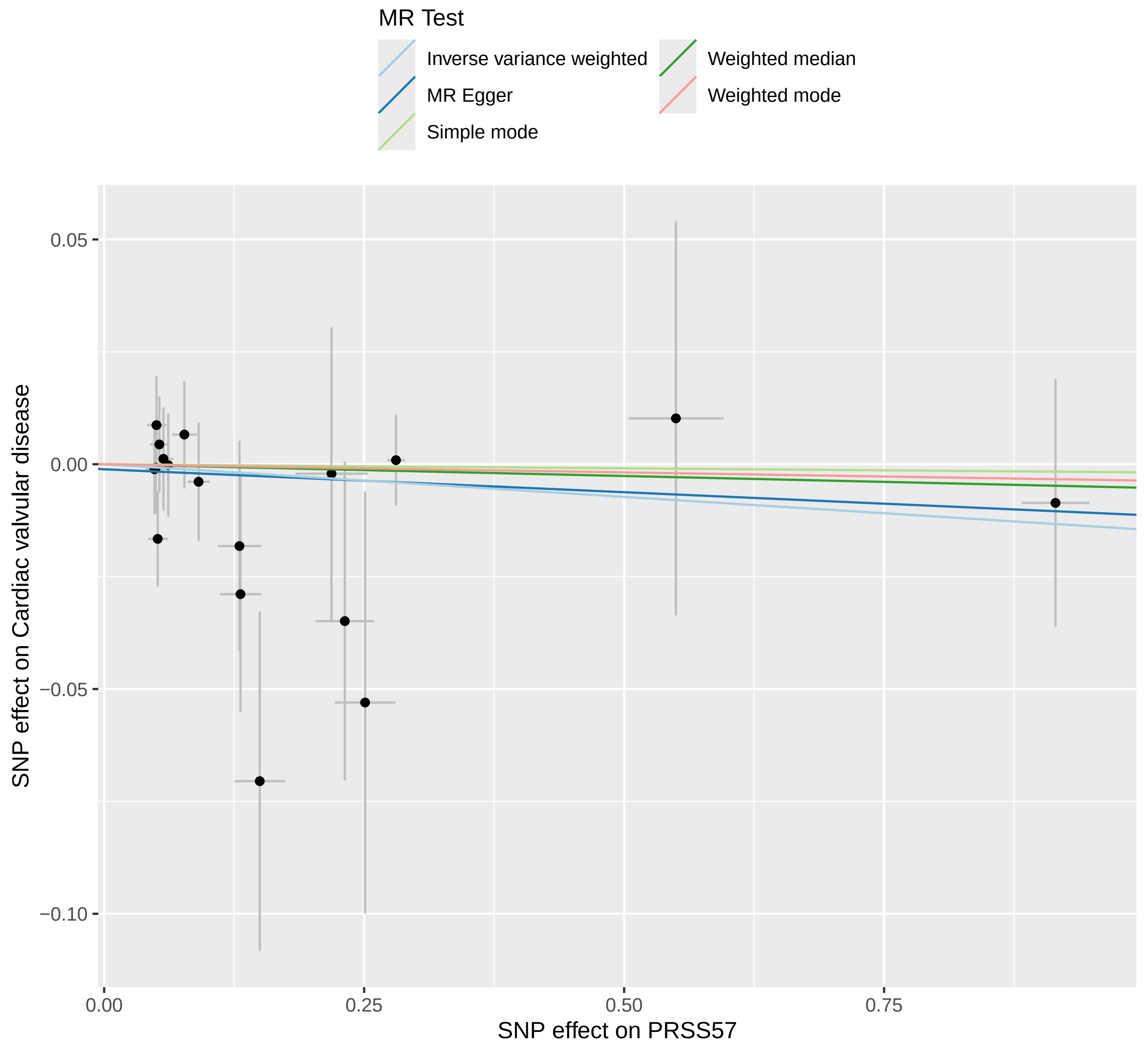

MR Test
Inverse variance weighted
MR Egger
Simple mode
Weighted median
Weighted mode
0.05
0.00
−0.05
−0.10
SNP effect on Cardiac valvular disease
0.00
0.25
0.50
0.75
SNP effect on PRSS57

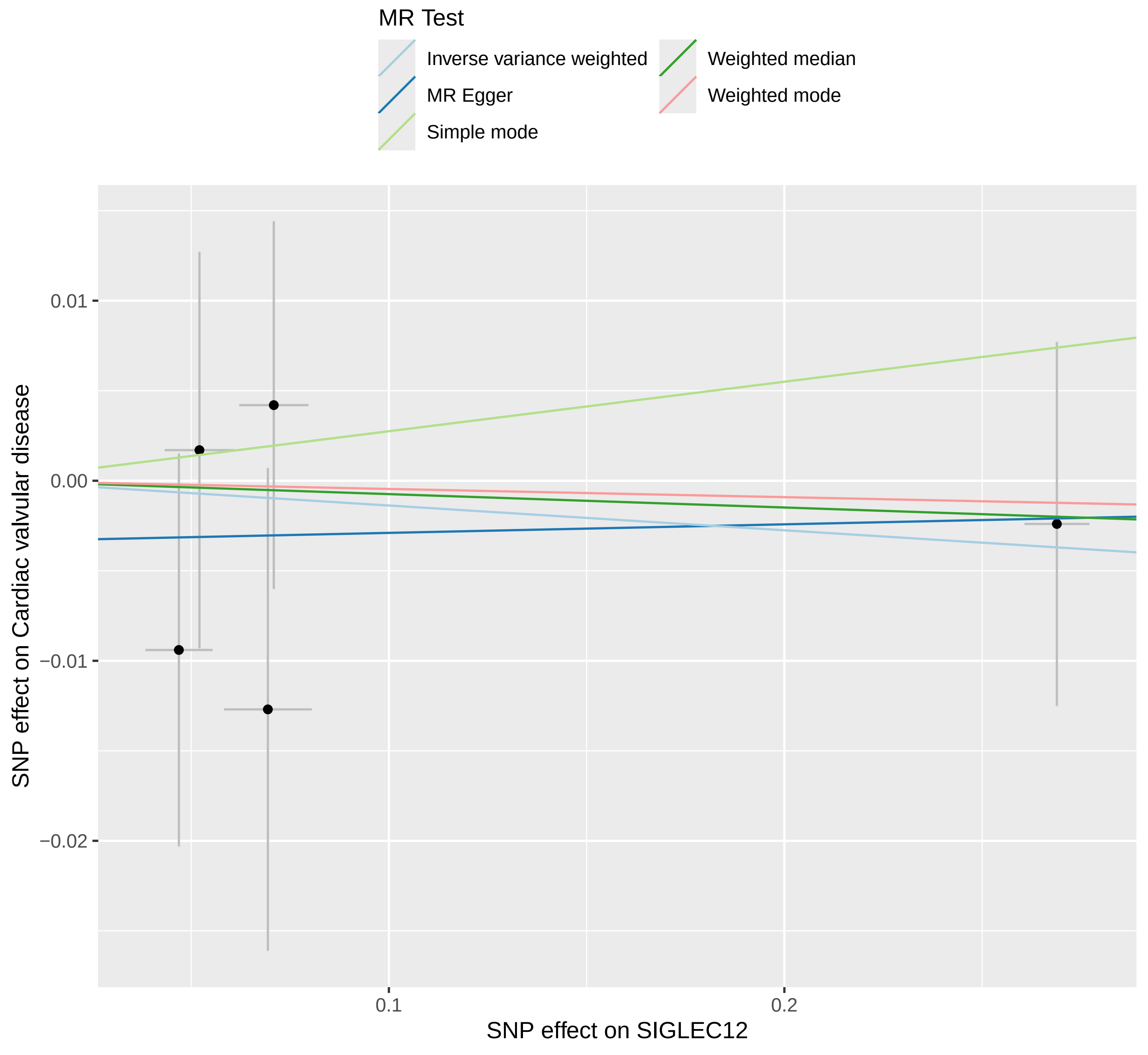
MR Test
Inverse variance weighted
MR Egger
Simple mode
Weighted median
Weighted mode
0.01
0.00
−0.01
−0.02
0.1
0.2
SNP effect on Cardiac valvular disease
SNP effect on SIGLEC12

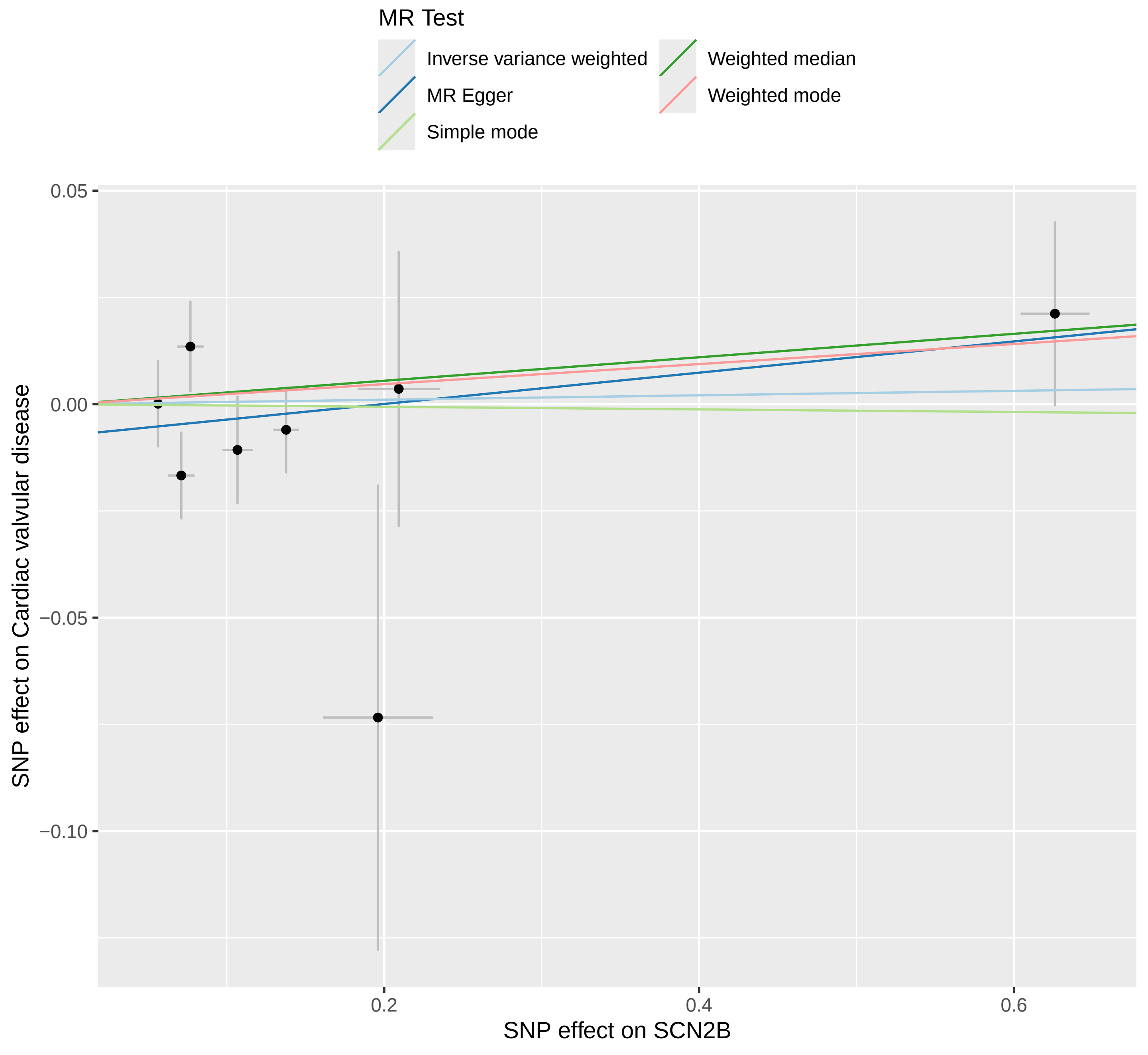
MR Test
Inverse variance weighted
MR Egger
Simple mode
Weighted median
Weighted mode
0.05
0.00
−0.05
−0.10
0.2
0.4
0.6
SNP effect on Cardiac valvular disease
SNP effect on SCN2B

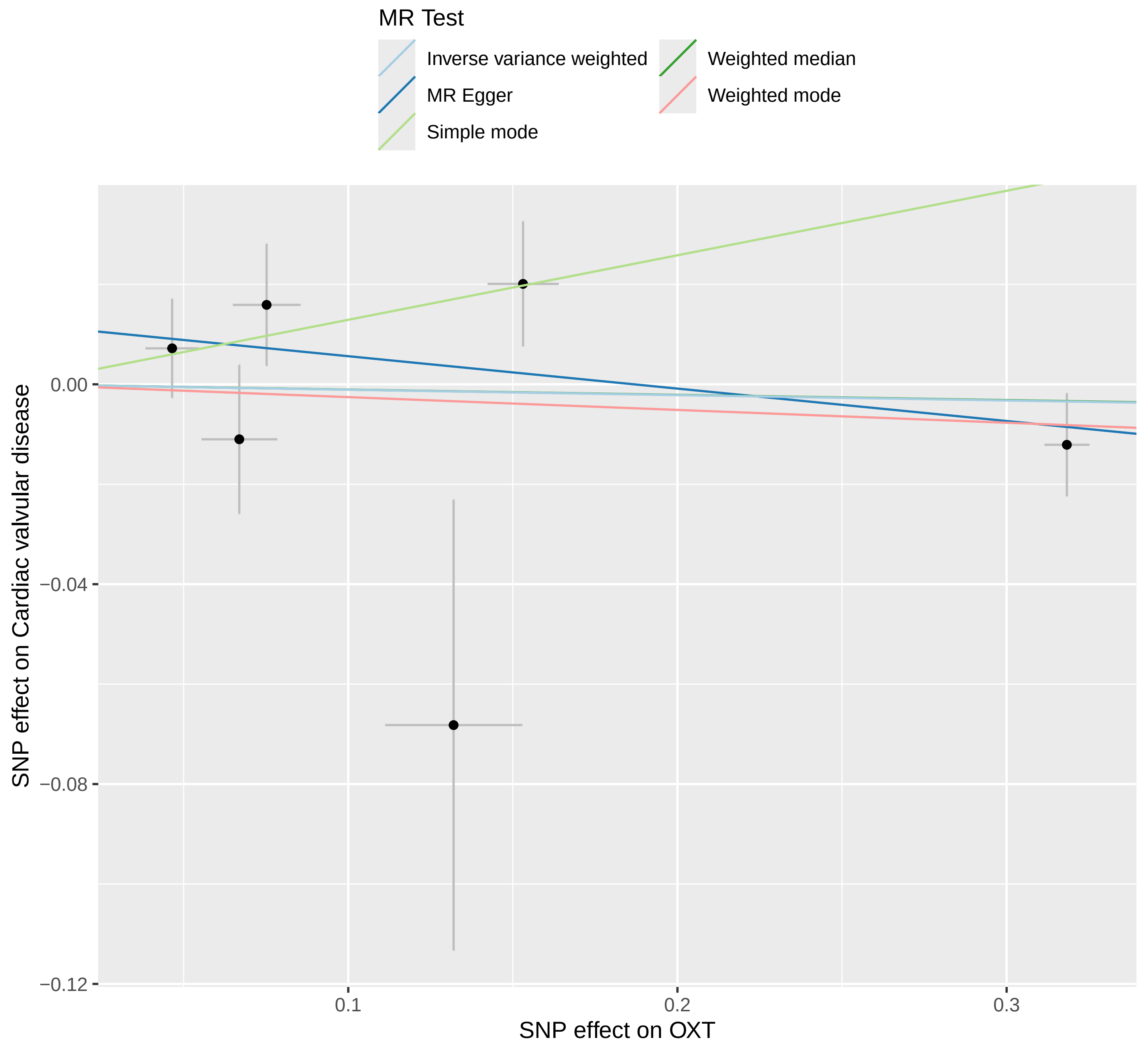
MR Test
Inverse variance weighted
MR Egger
Simple mode
Weighted median
Weighted mode
SNP effect on Cardiac valvular disease
0.00
−0.04
−0.08
−0.12
0.1
0.2
0.3
SNP effect on OXT

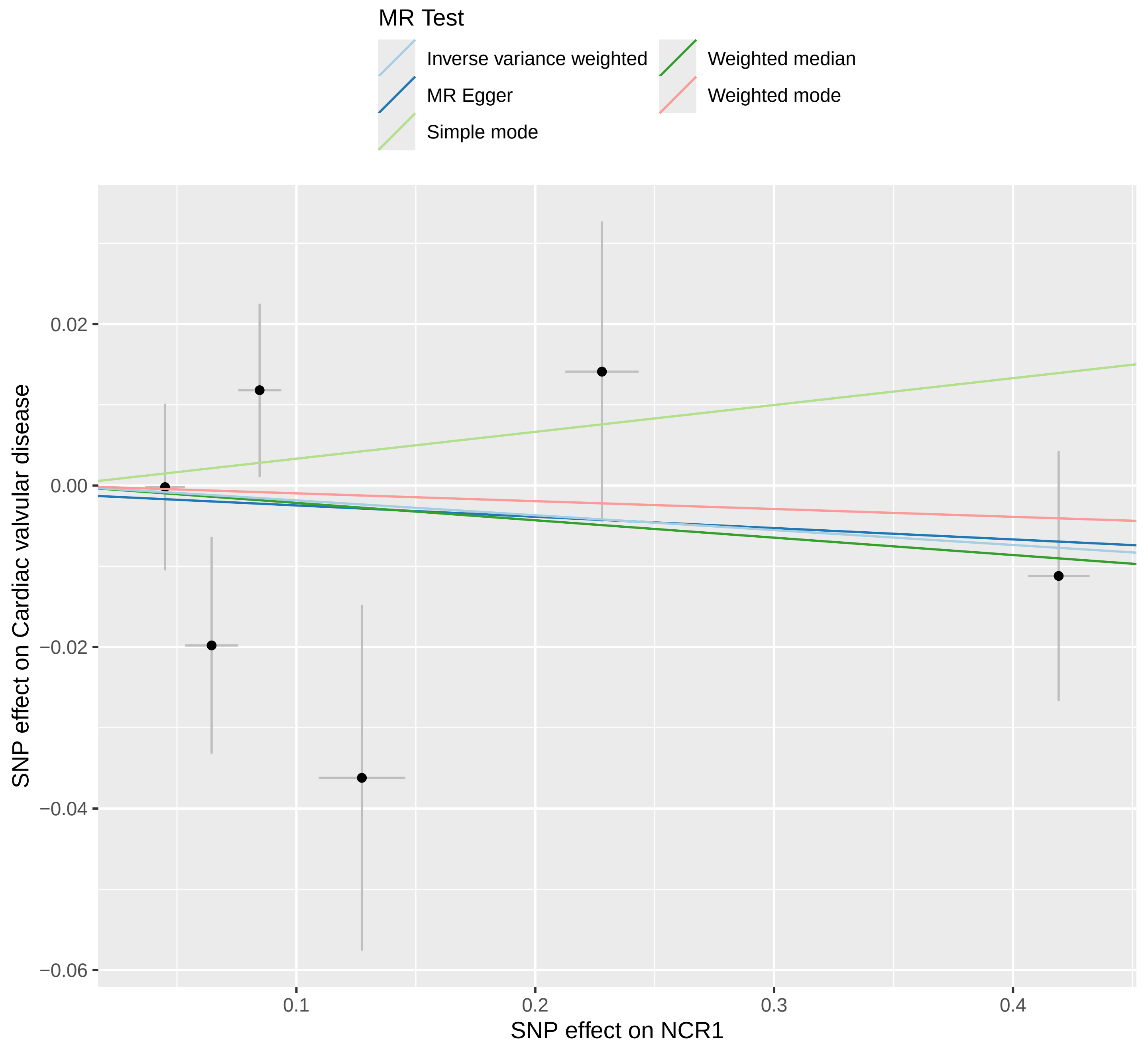

MR Test
Inverse variance weighted
MR Egger
Simple mode
Weighted median
Weighted mode
0.02
0.00
−0.02
−0.04
−0.06
0.1
0.2
0.3
0.4
SNP effect on Cardiac valvular disease
SNP effect on NCR1

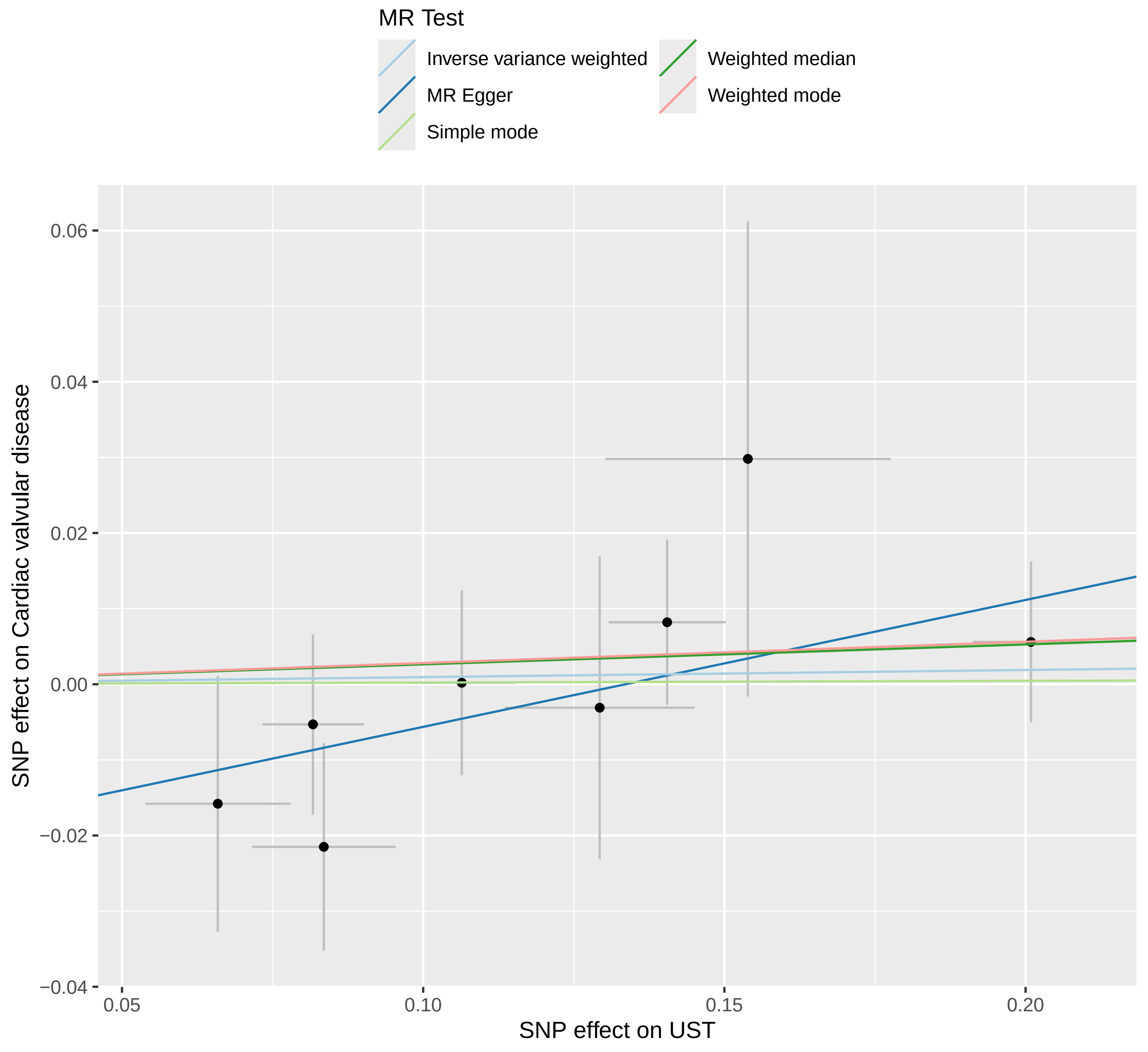
MR Test
Inverse variance weighted
MR Egger
Simple mode
Weighted median
Weighted mode
0.06
0.04
0.02
0.00
−0.02
−0.04
SNP effect on Cardiac valvular disease
0.05
0.10
0.15
0.20
SNP effect on UST

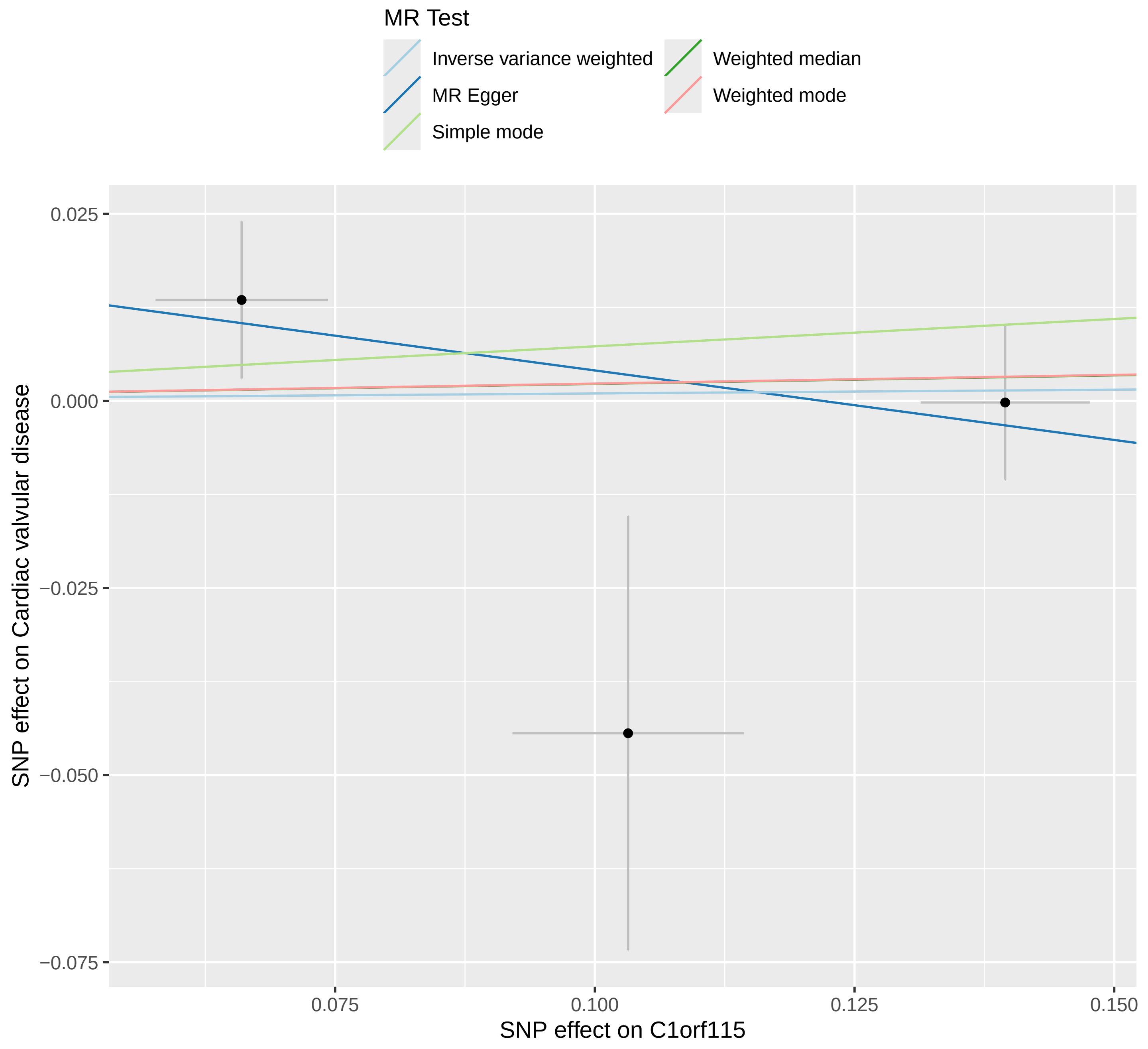
MR Test
Inverse variance weighted
MR Egger
Simple mode
Weighted median
Weighted mode
0.025
0.000
−0.025
−0.050
−0.075
0.075
0.100
0.125
0.150
SNP effect on Cardiac valvular disease
SNP effect on C1orf115

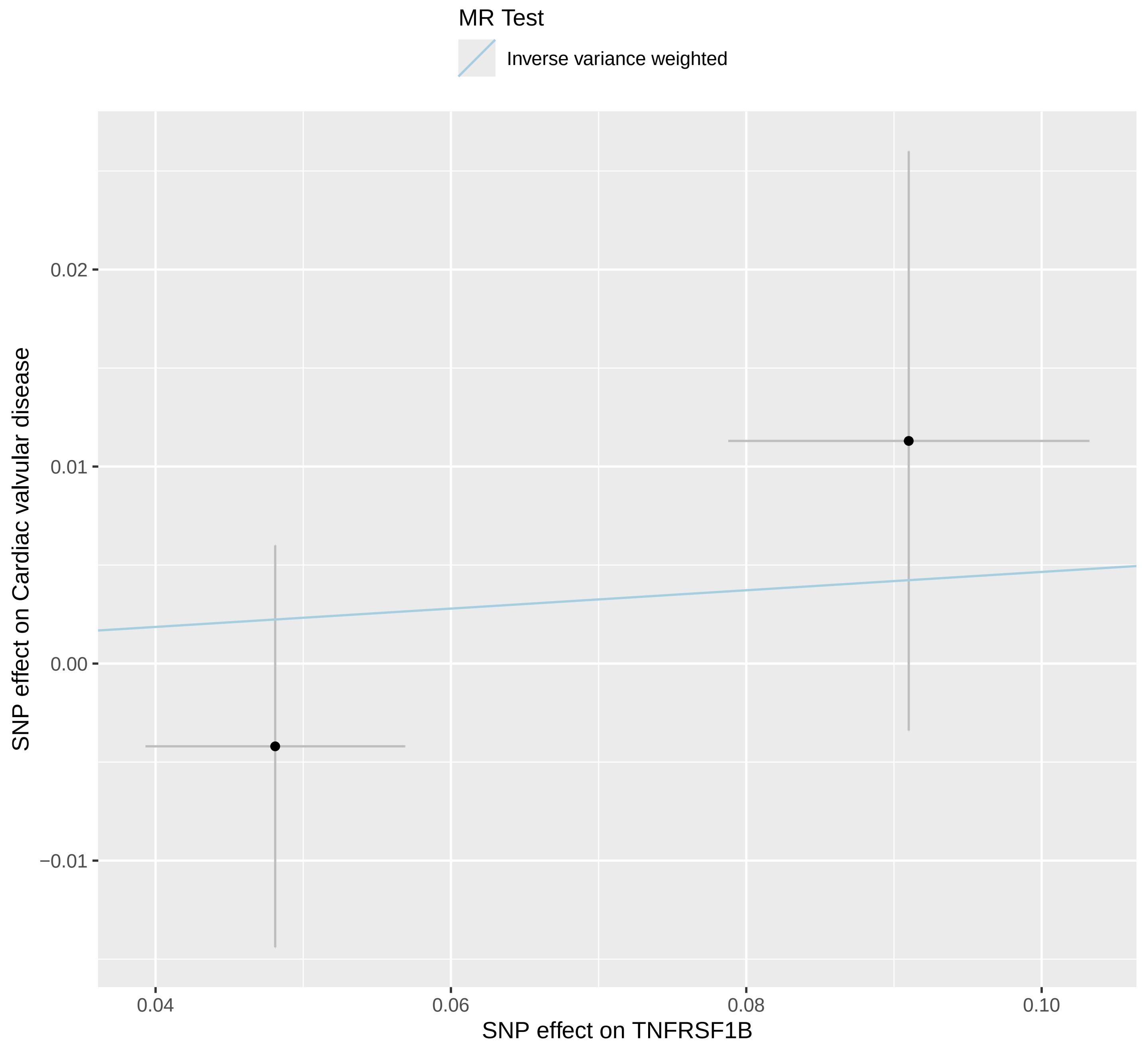
MR Test
Inverse variance weighted
0.02
0.01
0.00
−0.01
0.04
0.06
0.08
0.10
SNP effect on Cardiac valvular disease
SNP effect on TNFRSF1B

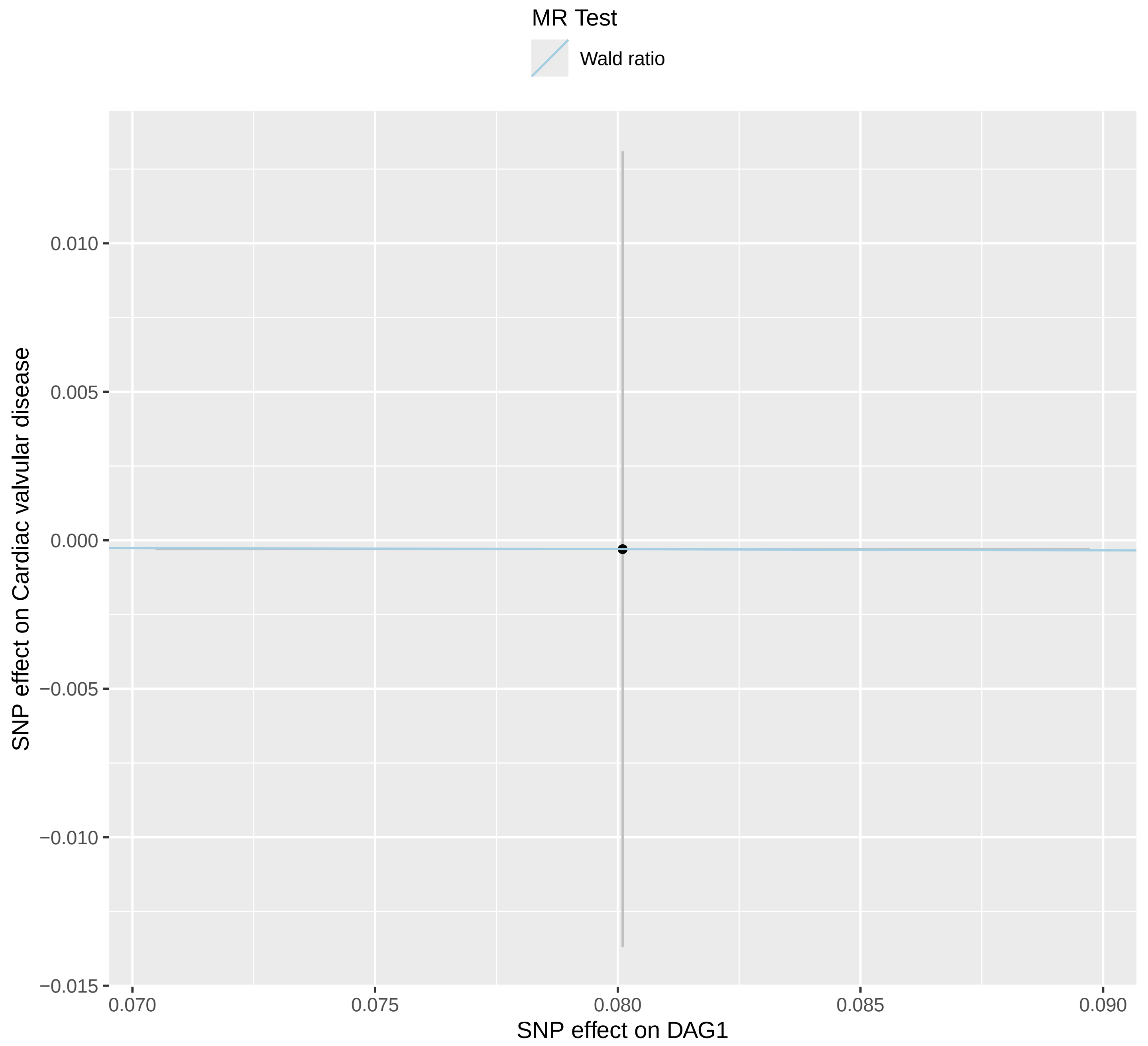
MR Test
Wald ratio
SNP effect on Cardiac valvular disease
0.010
0.005
0.000
−0.005
−0.010
−0.015
0.070
0.075
0.080
0.085
0.090
SNP effect on DAG1

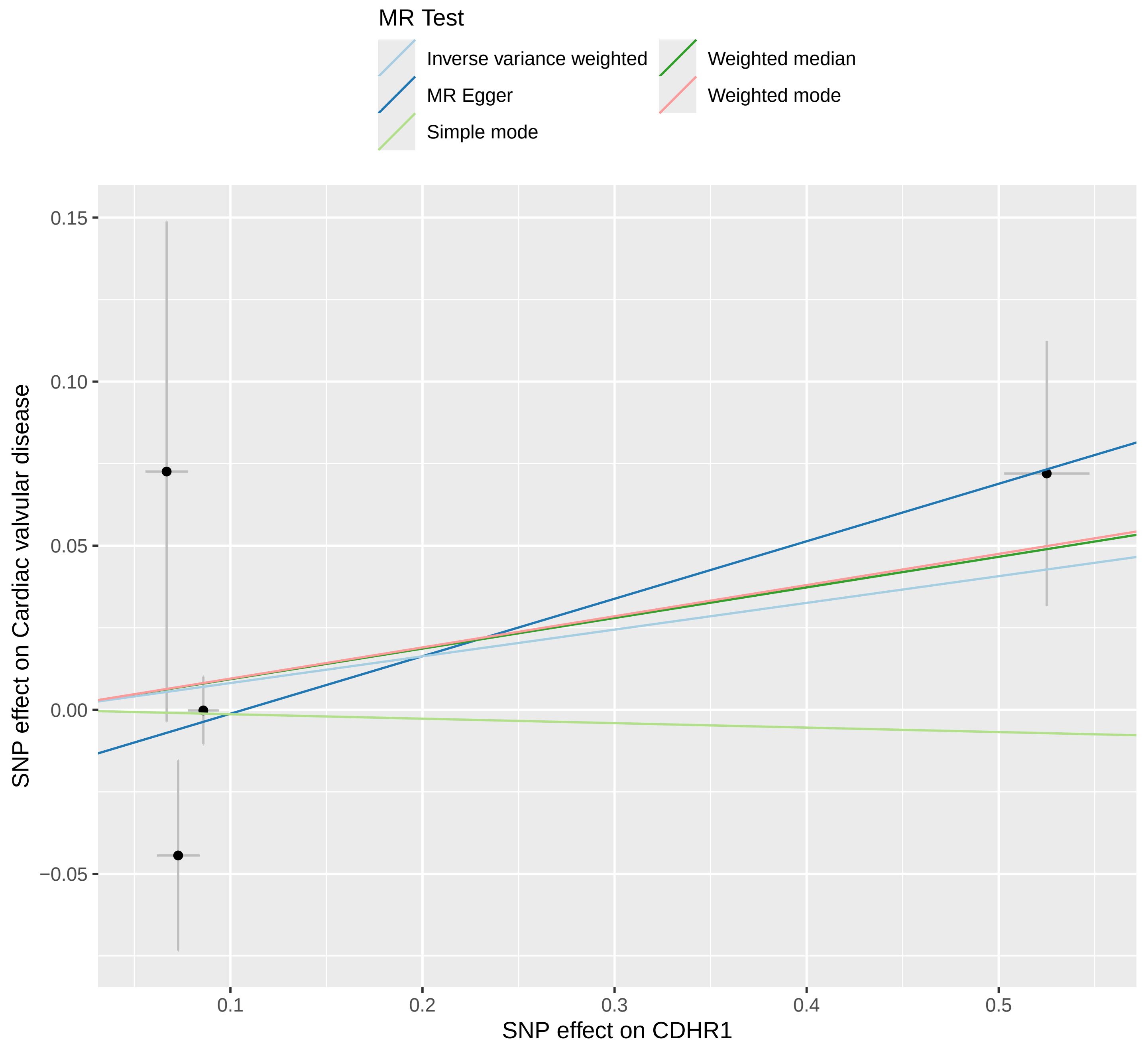
MR Test
Inverse variance weighted
MR Egger
Simple mode
Weighted median
Weighted mode
SNP effect on Cardiac valvular disease
0.15
0.10
0.05
0.00
−0.05
0.1
0.2
0.3
0.4
0.5
SNP effect on CDHR1

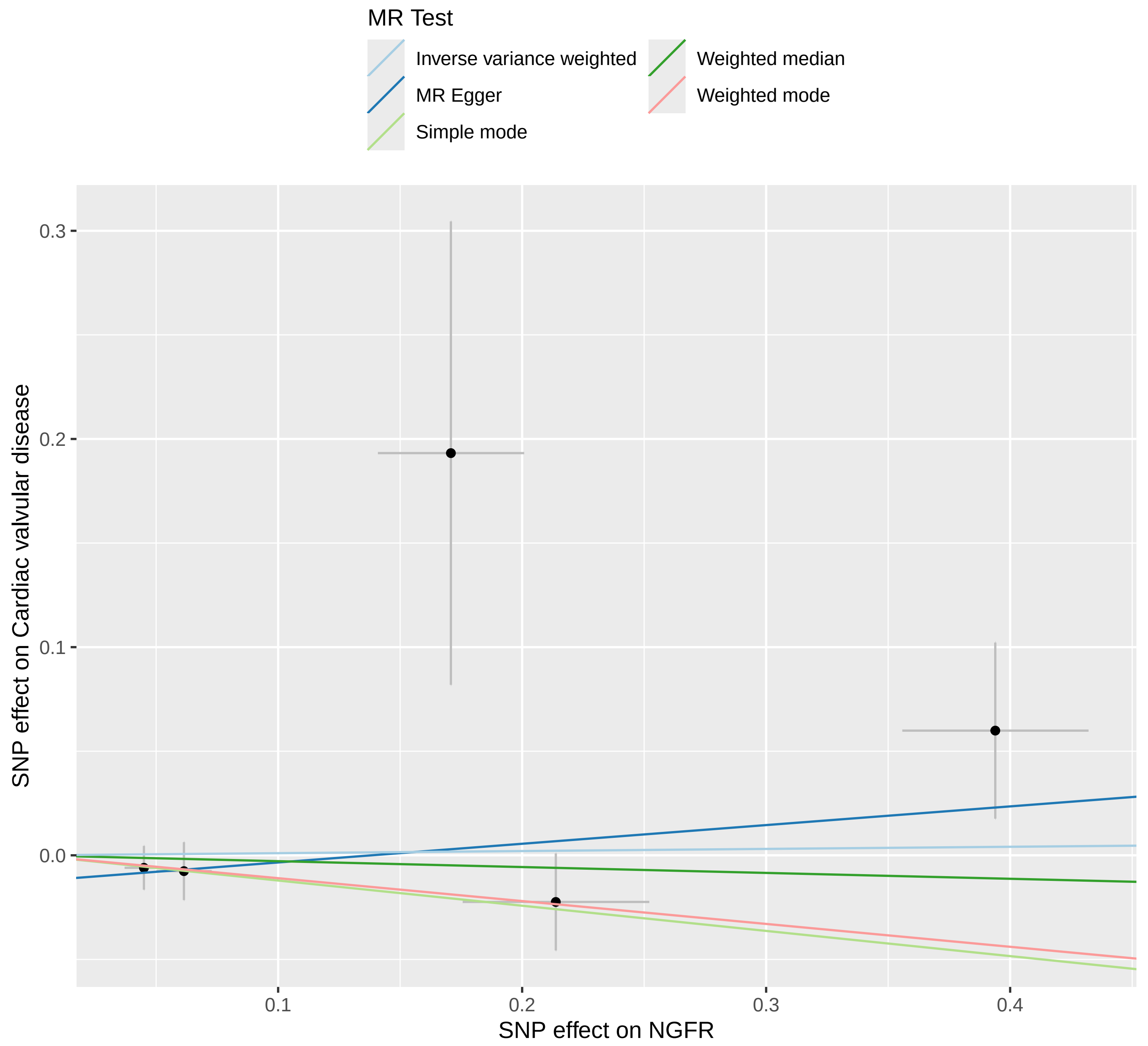
MR Test
Inverse variance weighted
MR Egger
Simple mode
Weighted median
Weighted mode
0.3
0.2
0.1
0.0
0.1
0.2
0.3
0.4
SNP effect on Cardiac valvular disease
SNP effect on NGFR

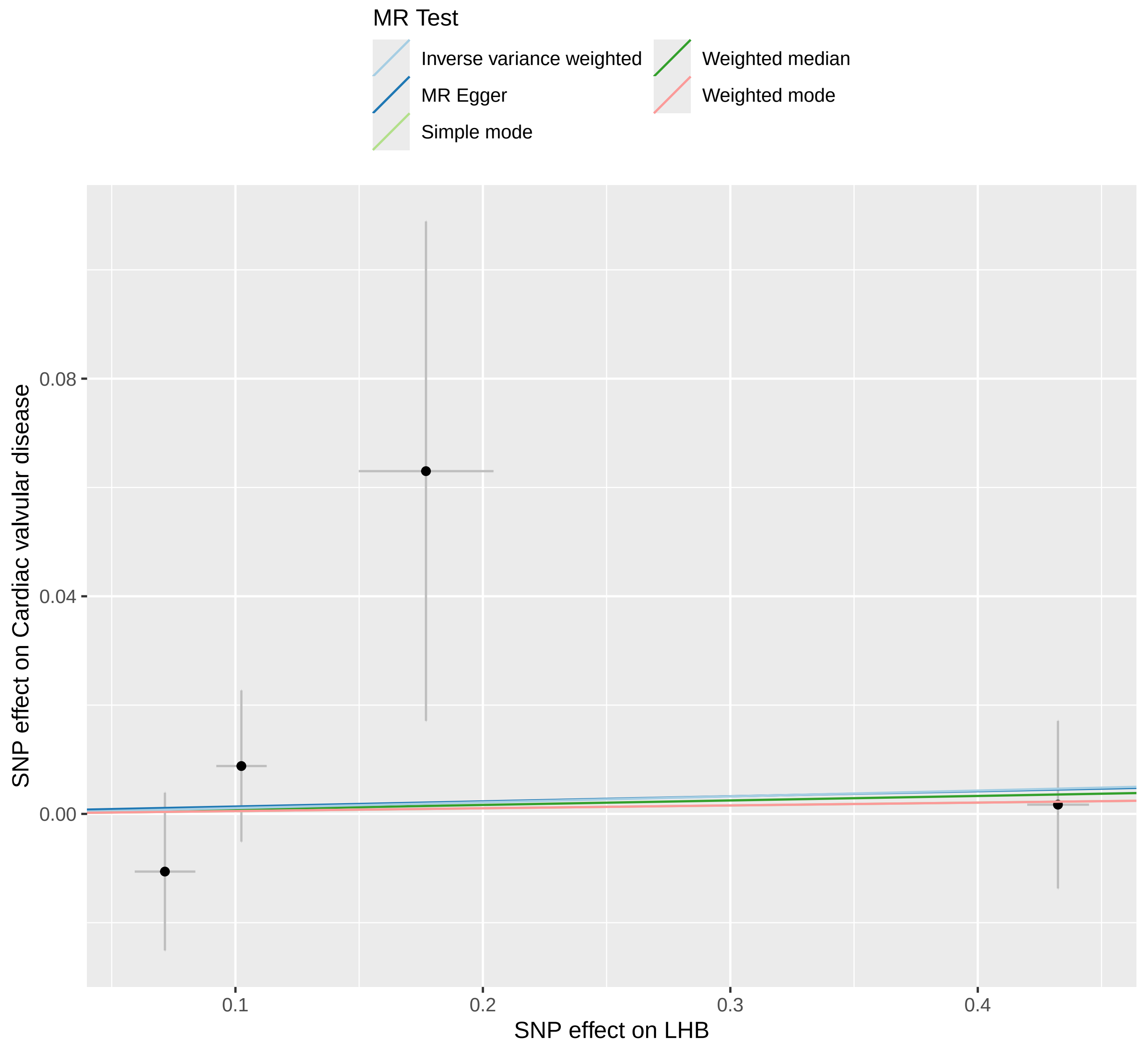

MR Test
Inverse variance weighted
MR Egger
Simple mode
Weighted median
Weighted mode
0.08
0.04
0.00
0.1
0.2
0.3
0.4
SNP effect on Cardiac valvular disease
SNP effect on LHB

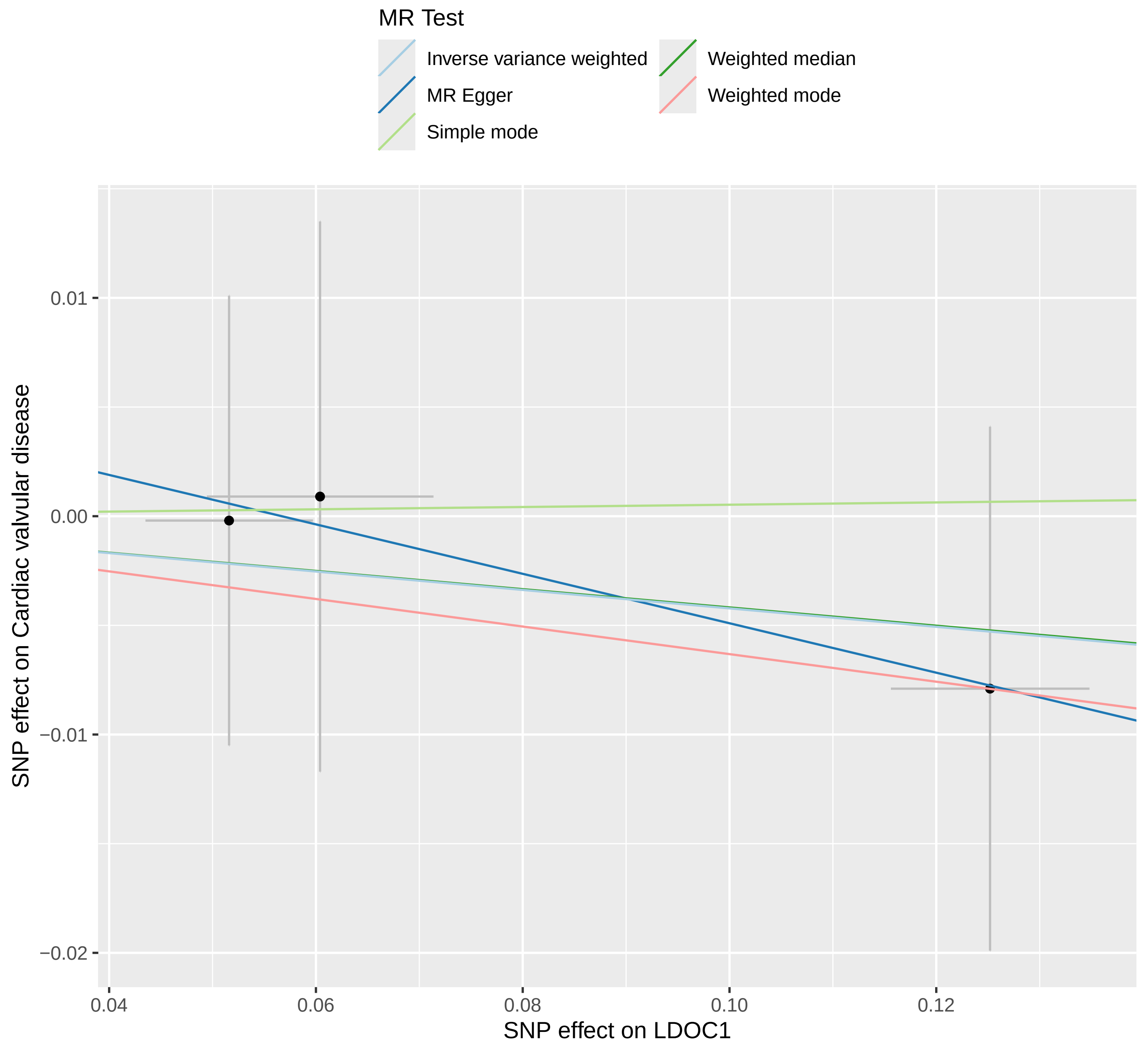

MR Test
Inverse variance weighted
MR Egger
Simple mode
Weighted median
Weighted mode
0.01
0.00
−0.01
−0.02
SNP effect on Cardiac valvular disease
0.04
0.06
0.08
0.10
0.12
SNP effect on LDOC1

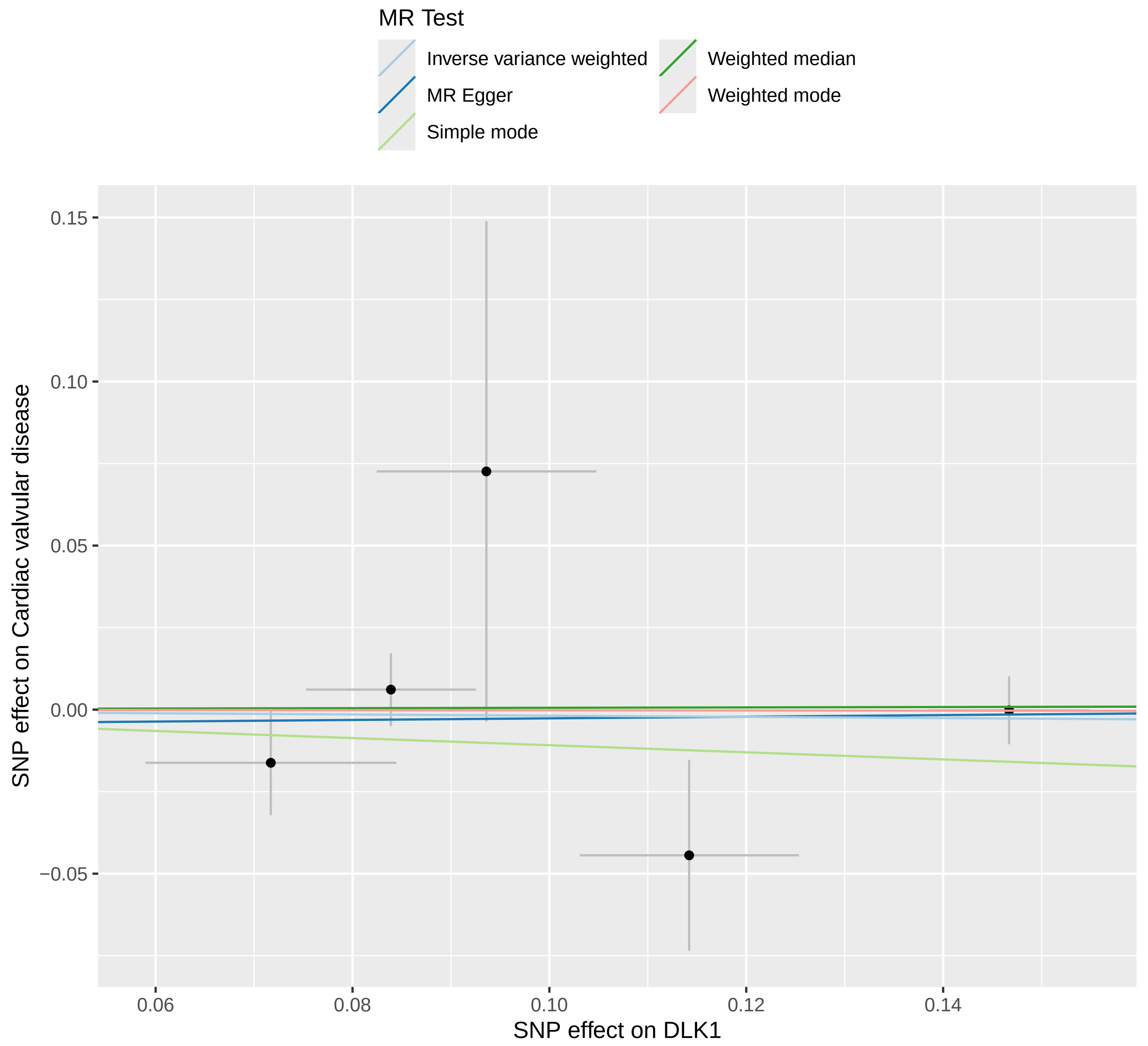
MR Test
Inverse variance weighted
MR Egger
Simple mode
Weighted median
Weighted mode
SNP effect on Cardiac valvular disease
0.15
0.10
0.05
0.00
−0.05
0.06
0.08
0.10
0.12
0.14
SNP effect on DLK1

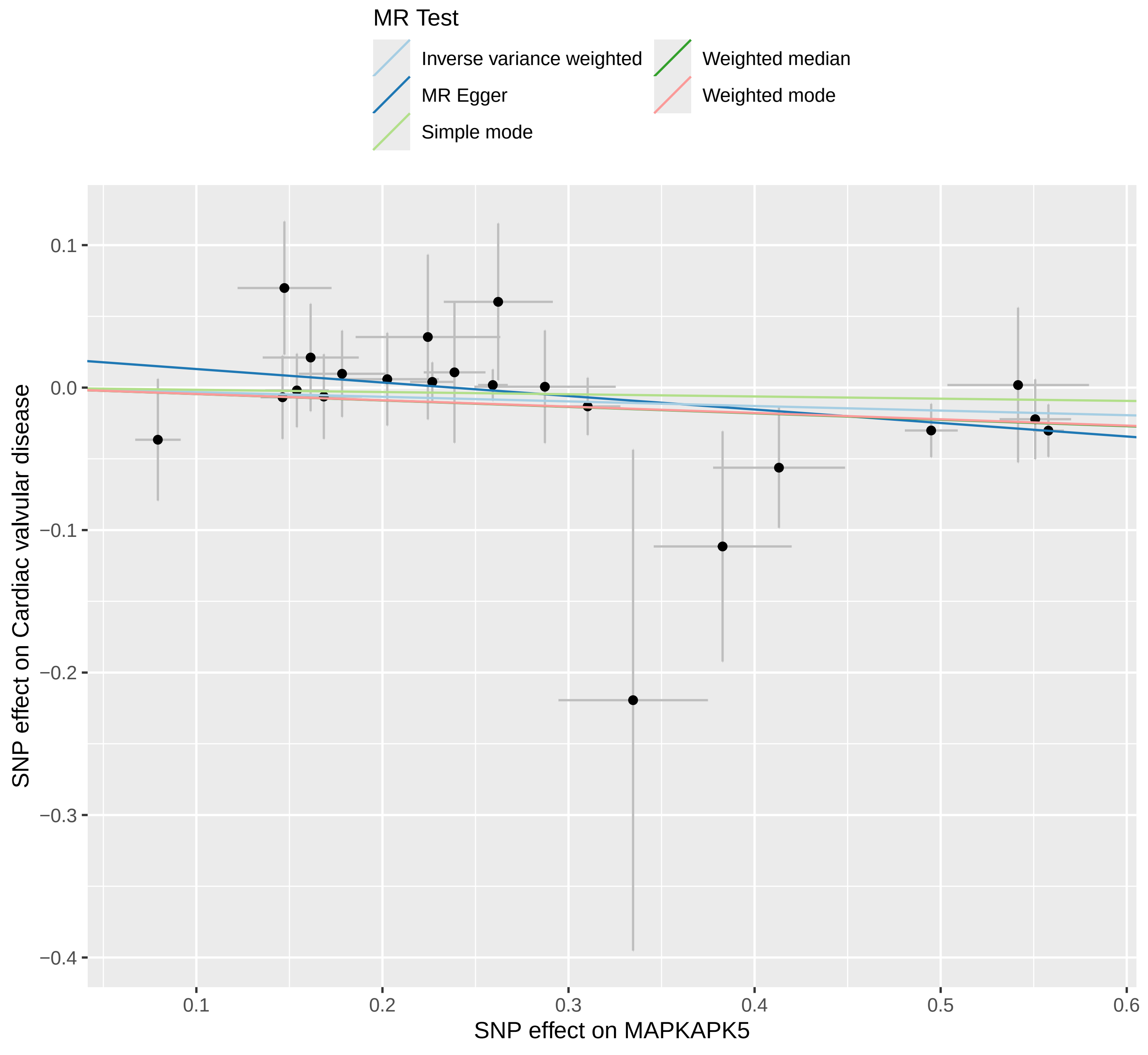

MR Test
Inverse variance weighted
MR Egger
Simple mode
Weighted median
Weighted mode
0.1
0.0
−0.1
−0.2
−0.3
−0.4
SNP effect on Cardiac valvular disease
0.1
0.2
0.3
0.4
0.5
0.6
SNP effect on MAPKAPK5

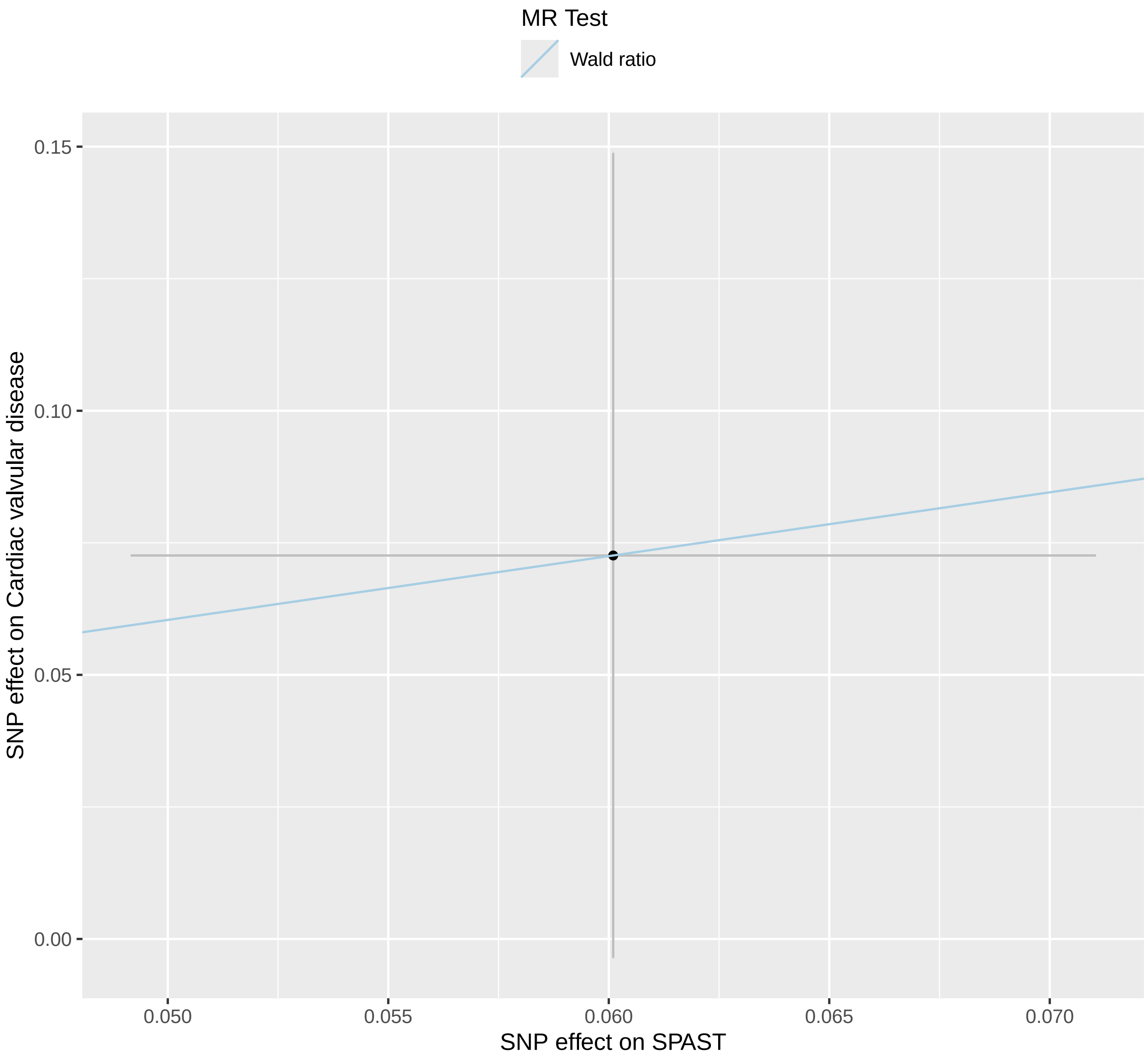
MR Test
Wald ratio
0.15
0.10
0.05
0.00
SNP effect on Cardiac valvular disease
0.050
0.055
0.060
0.065
0.070
SNP effect on SPAST

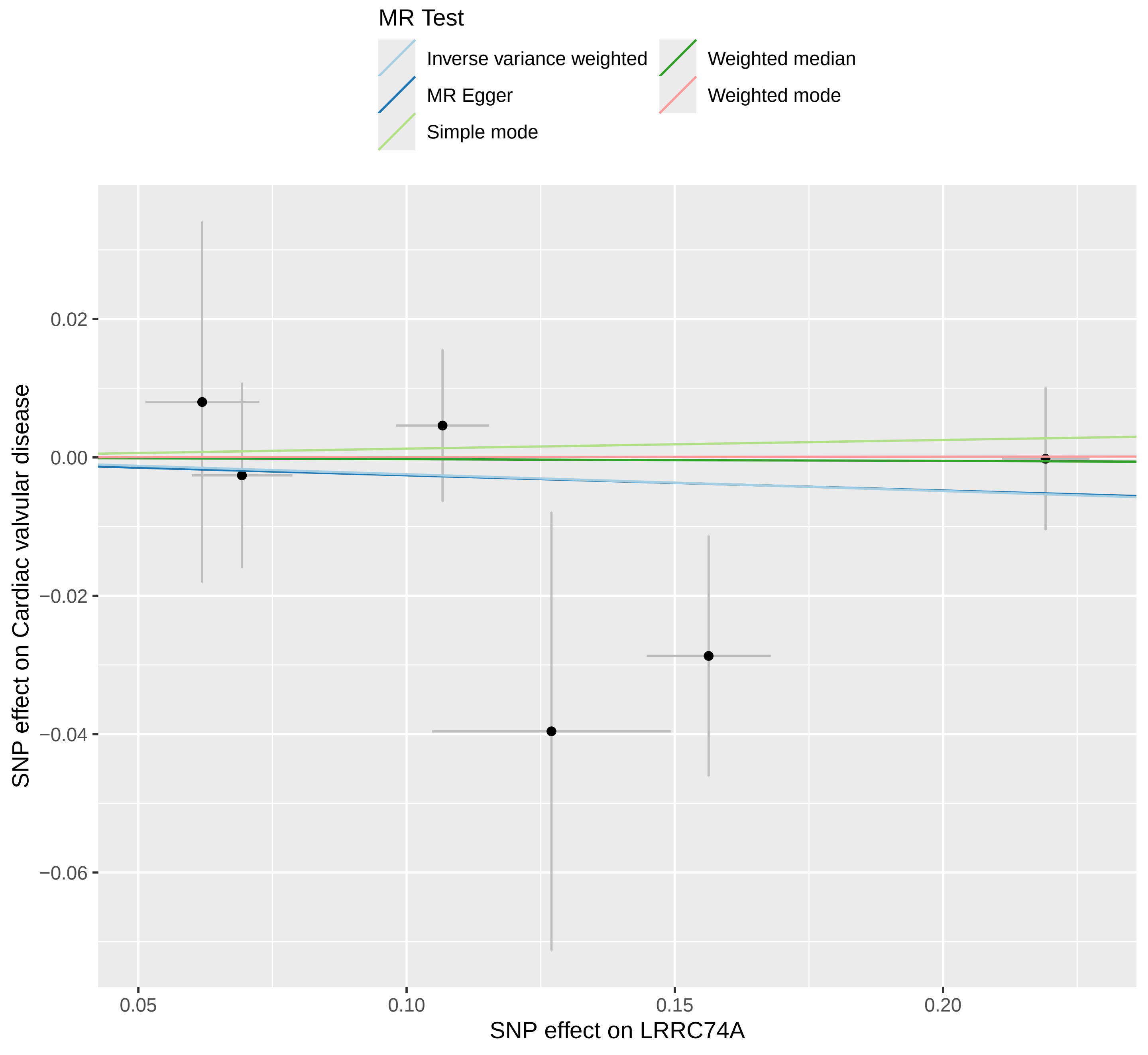

MR Test
Inverse variance weighted
MR Egger
Simple mode
Weighted median
Weighted mode
0.02
0.00
−0.02
−0.04
−0.06
0.05
0.10
0.15
0.20
SNP effect on Cardiac valvular disease
SNP effect on LRRC74A

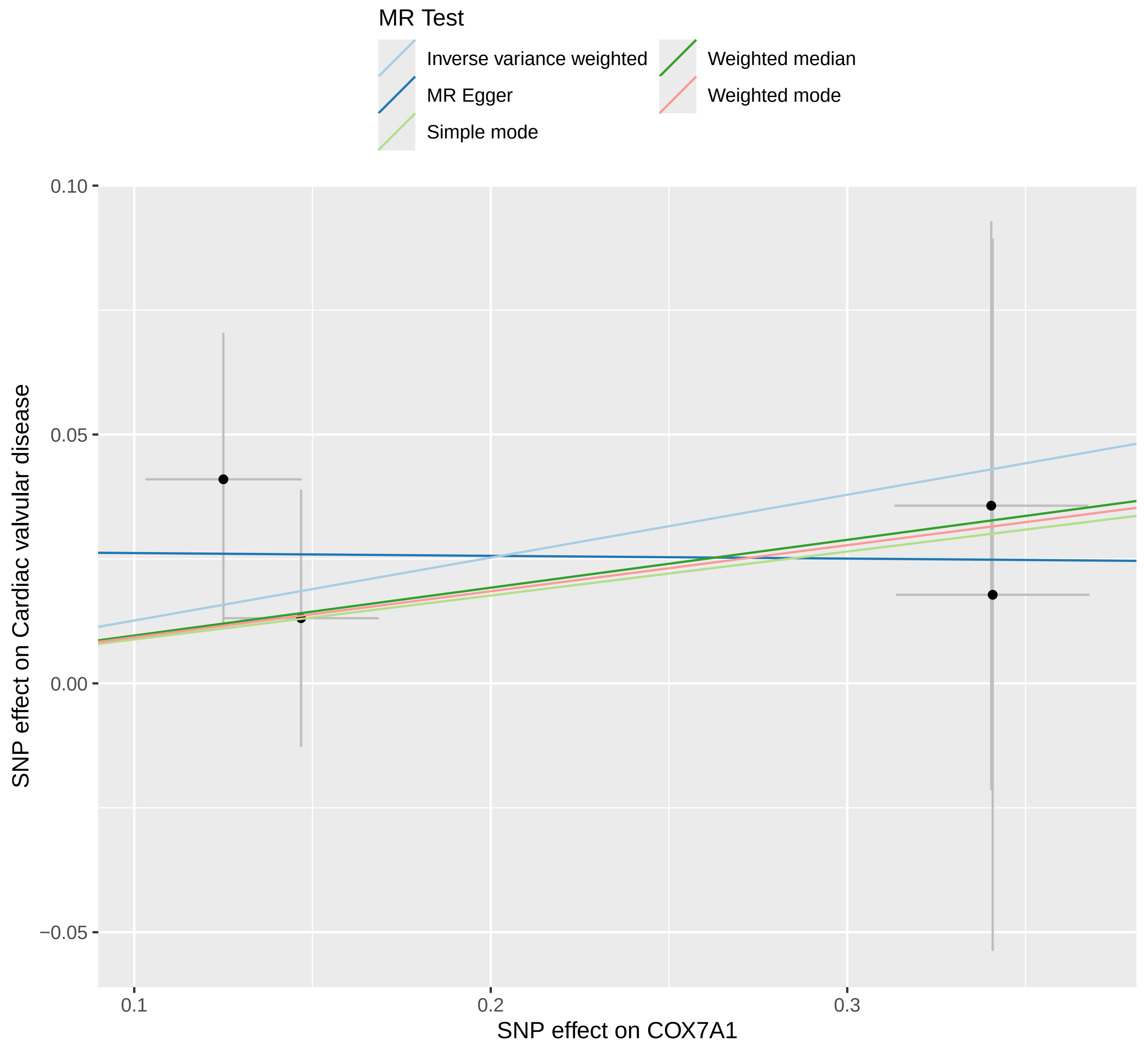
MR Test
Inverse variance weighted
MR Egger
Simple mode
Weighted median
Weighted mode
0.10
0.05
0.00
−0.05
0.1
0.2
0.3
SNP effect on Cardiac valvular disease
SNP effect on COX7A1

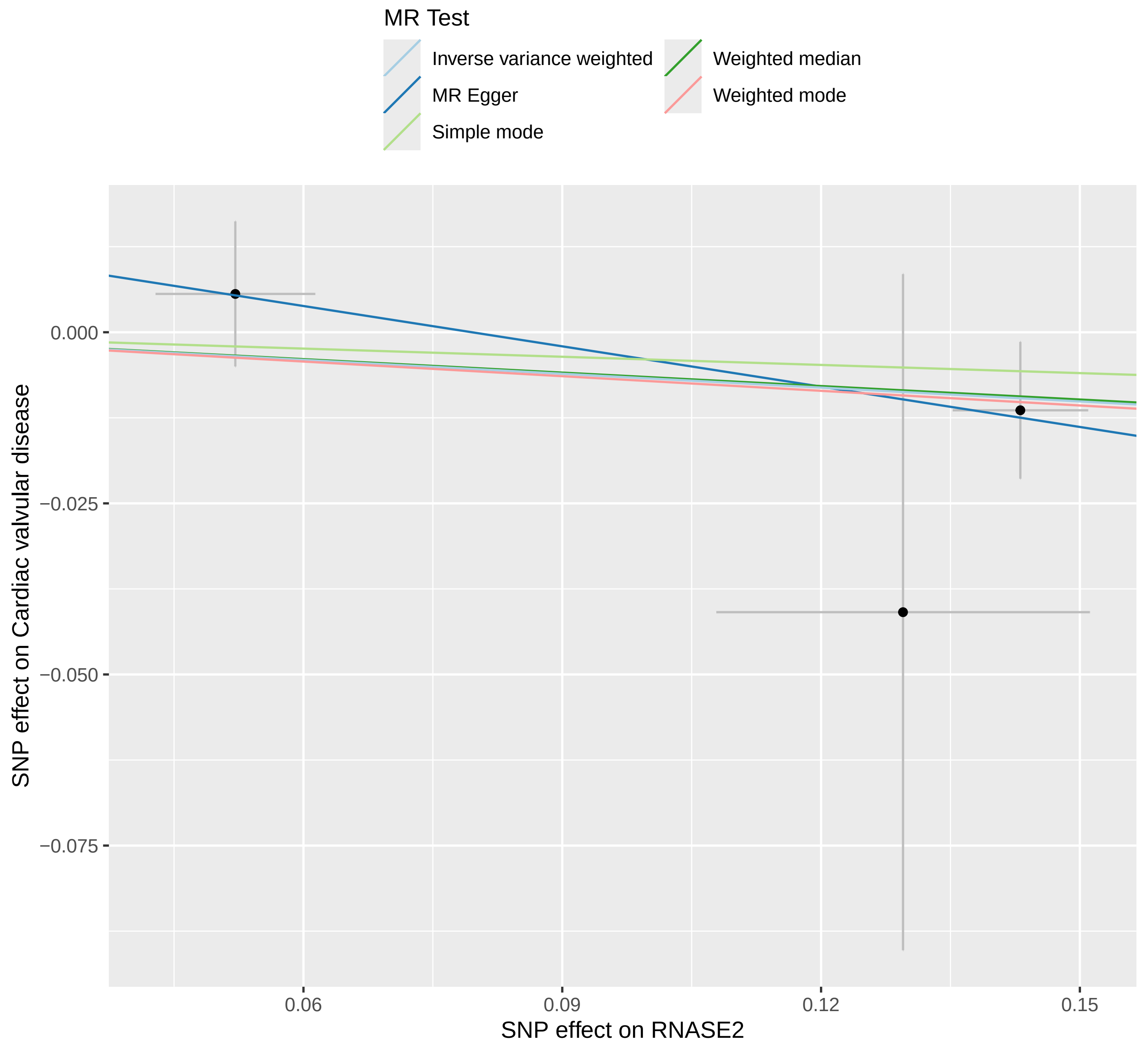

MR Test
Inverse variance weighted
MR Egger
Simple mode
Weighted median
Weighted mode
0.000
−0.025
−0.050
−0.075
SNP effect on Cardiac valvular disease
0.06
0.09
0.12
0.15
SNP effect on RNASE2

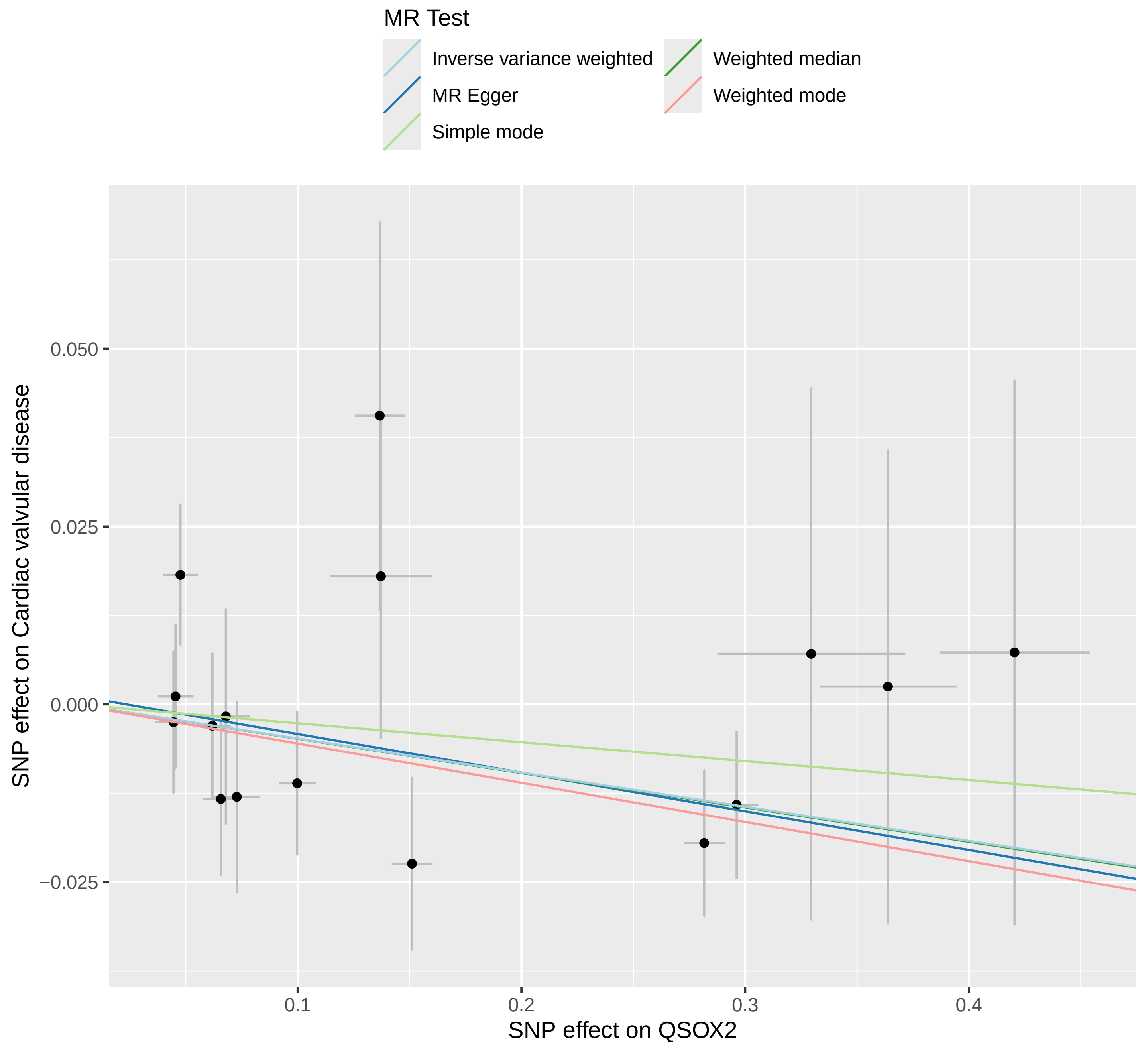
MR Test
Inverse variance weighted
MR Egger
Simple mode
Weighted median
Weighted mode
0.050
0.025
0.000
−0.025
SNP effect on Cardiac valvular disease
0.1
0.2
0.3
0.4
SNP effect on QSOX2

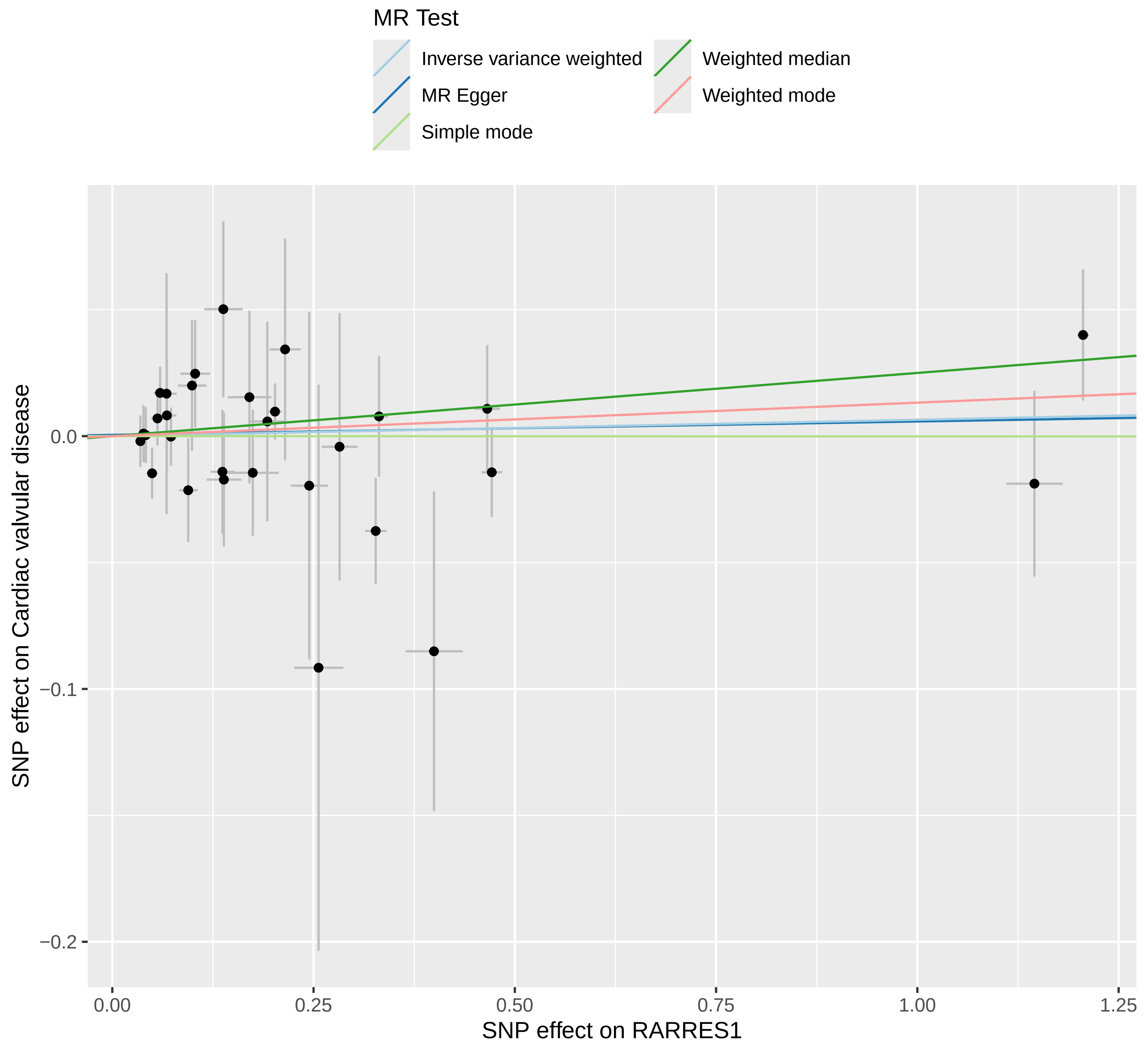
MR Test
Inverse variance weighted
MR Egger
Simple mode
Weighted median
Weighted mode
SNP effect on Cardiac valvular disease
0.0
−0.1
−0.2
0.00
0.25
0.50
0.75
1.00
1.25
SNP effect on RARRES1

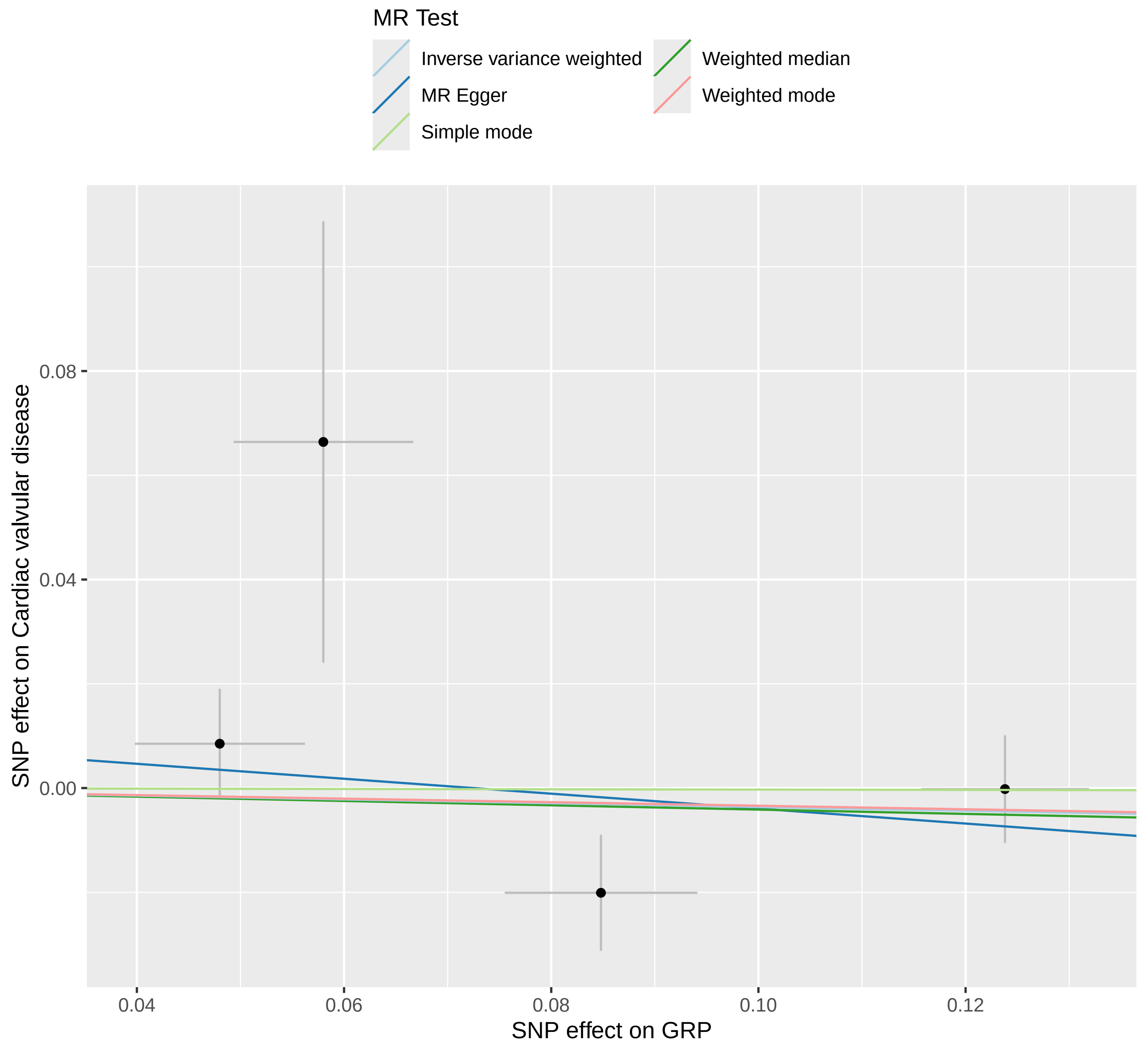
MR Test
Inverse variance weighted
MR Egger
Simple mode
Weighted median
Weighted mode
SNP effect on Cardiac valvular disease
0.08
0.04
0.00
0.04
0.06
0.08
0.10
0.12
SNP effect on GRP

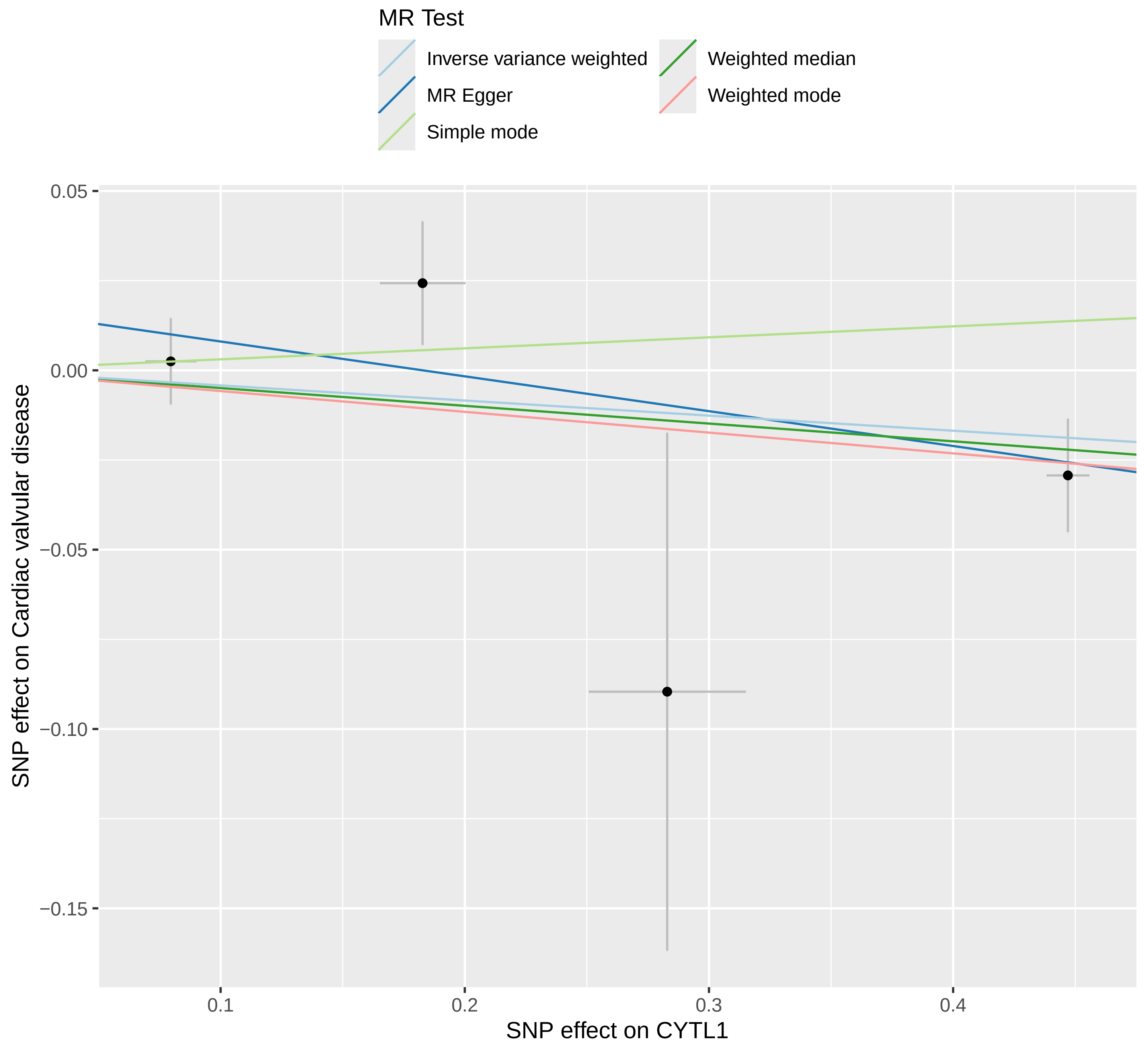
MR Test
Inverse variance weighted
MR Egger
Simple mode
Weighted median
Weighted mode
0.05
0.00
−0.05
−0.10
−0.15
0.1
0.2
0.3
0.4
SNP effect on Cardiac valvular disease
SNP effect on CYTL1

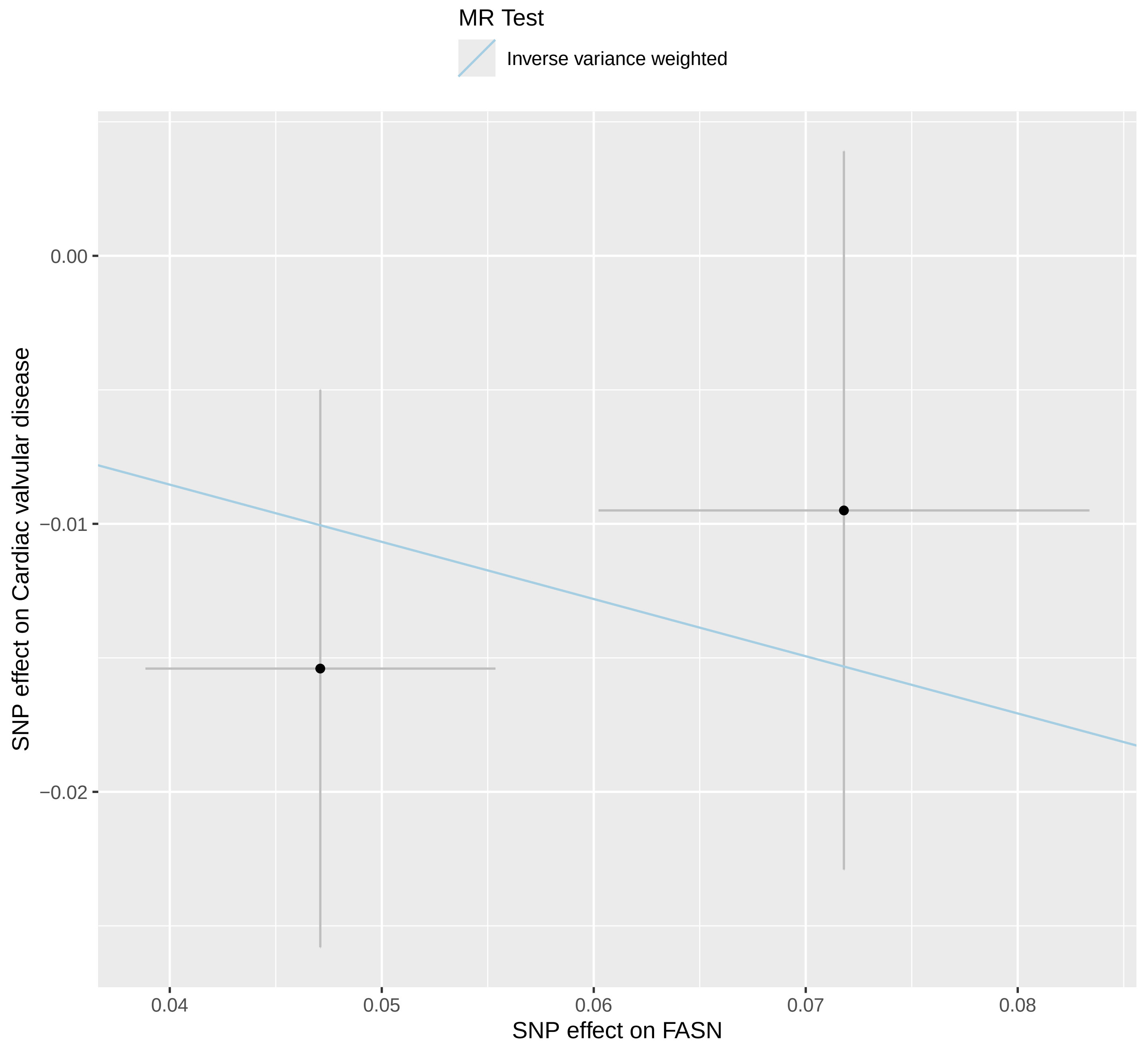
MR Test
Inverse variance weighted
0.00
−0.01
−0.02
SNP effect on Cardiac valvular disease
0.04
0.05
0.06
0.07
0.08
SNP effect on FASN

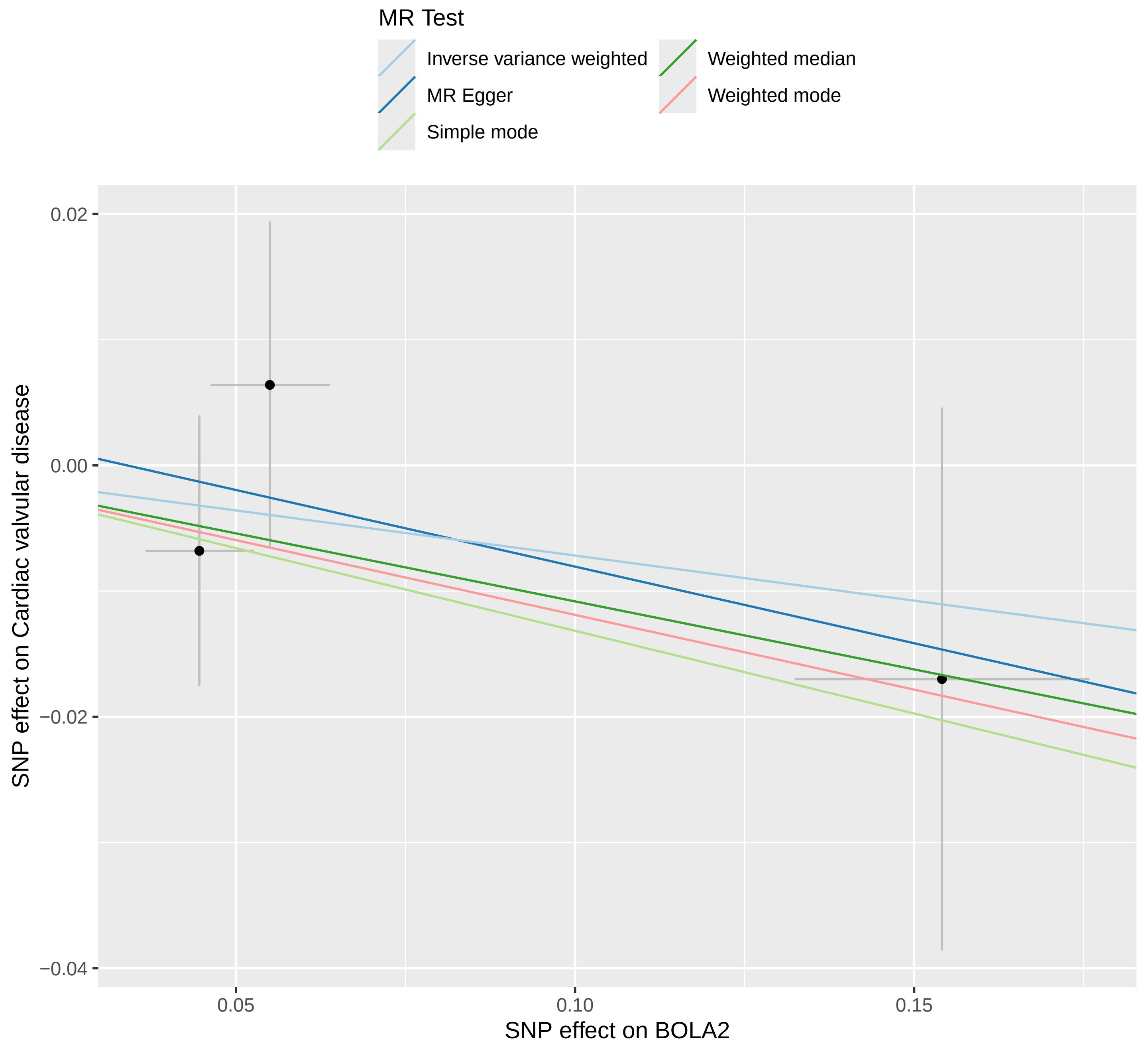
MR Test
Inverse variance weighted
MR Egger
Simple mode
Weighted median
Weighted mode
0.02
0.00
−0.02
−0.04
0.05
0.10
0.15
SNP effect on Cardiac valvular disease
SNP effect on BOLA2

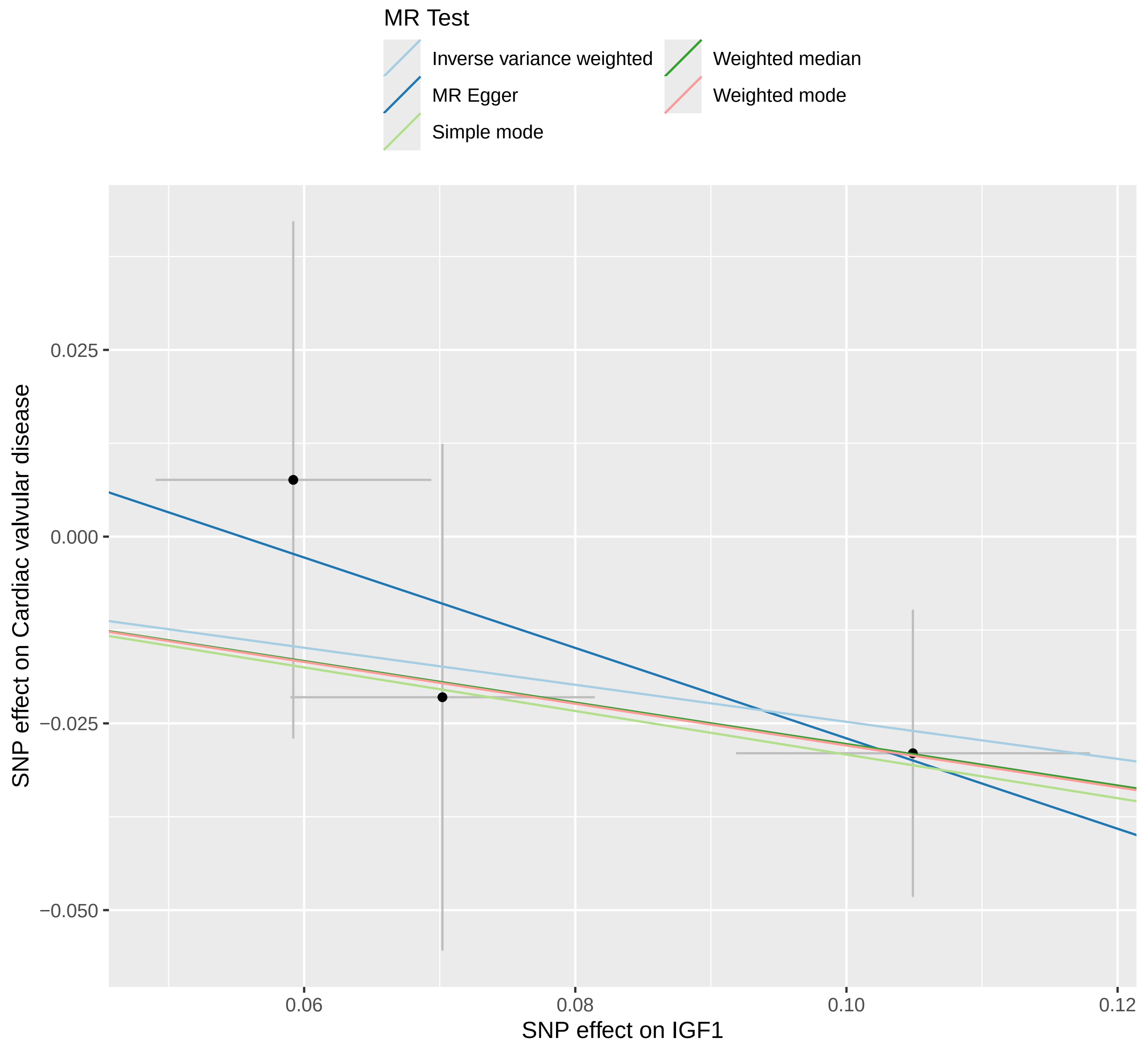
MR Test
Inverse variance weighted
MR Egger
Simple mode
Weighted median
Weighted mode
SNP effect on Cardiac valvular disease
0.025
0.000
−0.025
−0.050
0.06
0.08
0.10
0.12
SNP effect on IGF1

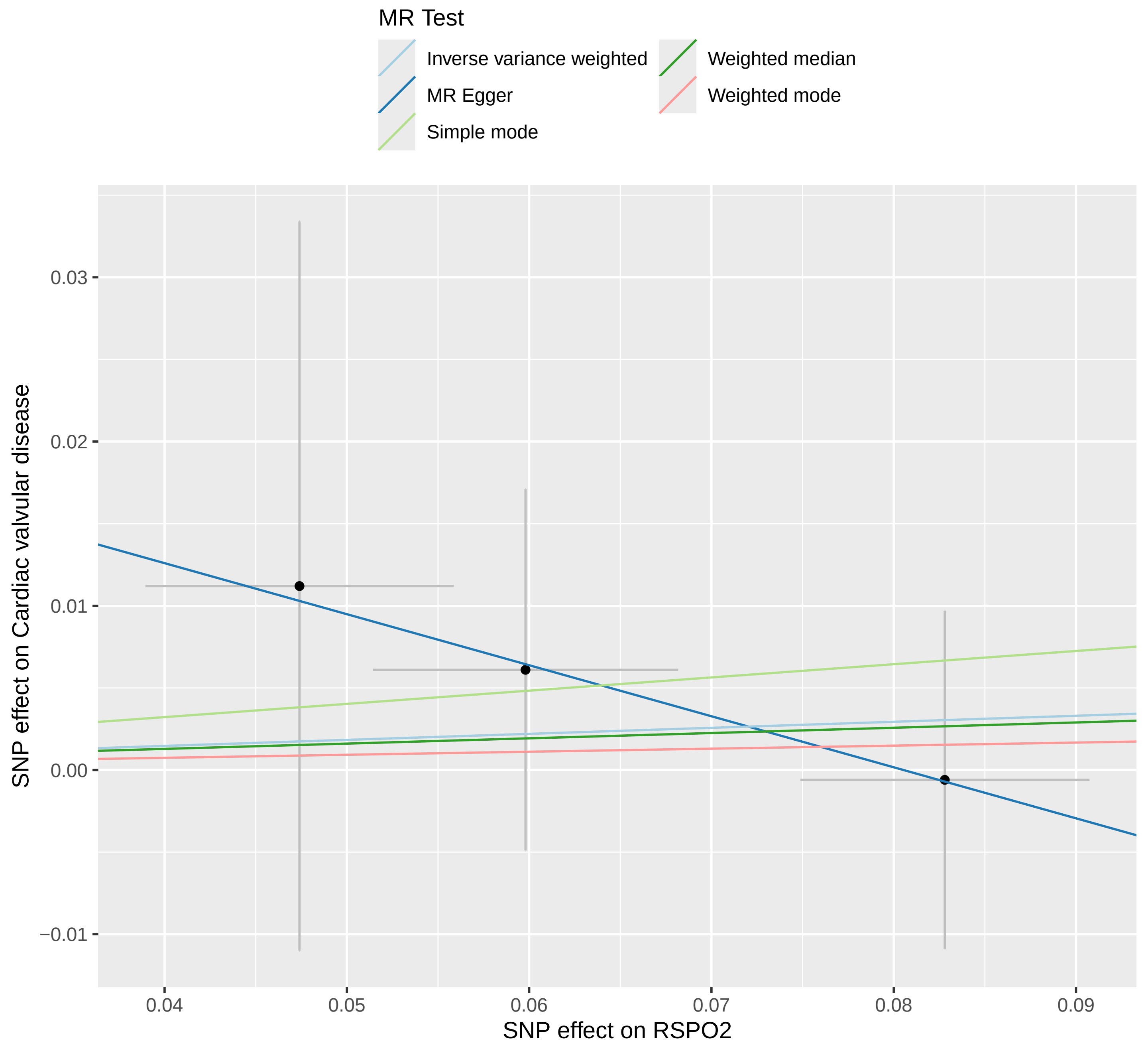

MR Test
Inverse variance weighted
MR Egger
Simple mode
Weighted median
Weighted mode
0.03
0.02
0.01
0.00
−0.01
SNP effect on Cardiac valvular disease
0.04
0.05
0.06
0.07
0.08
0.09
SNP effect on RSPO2

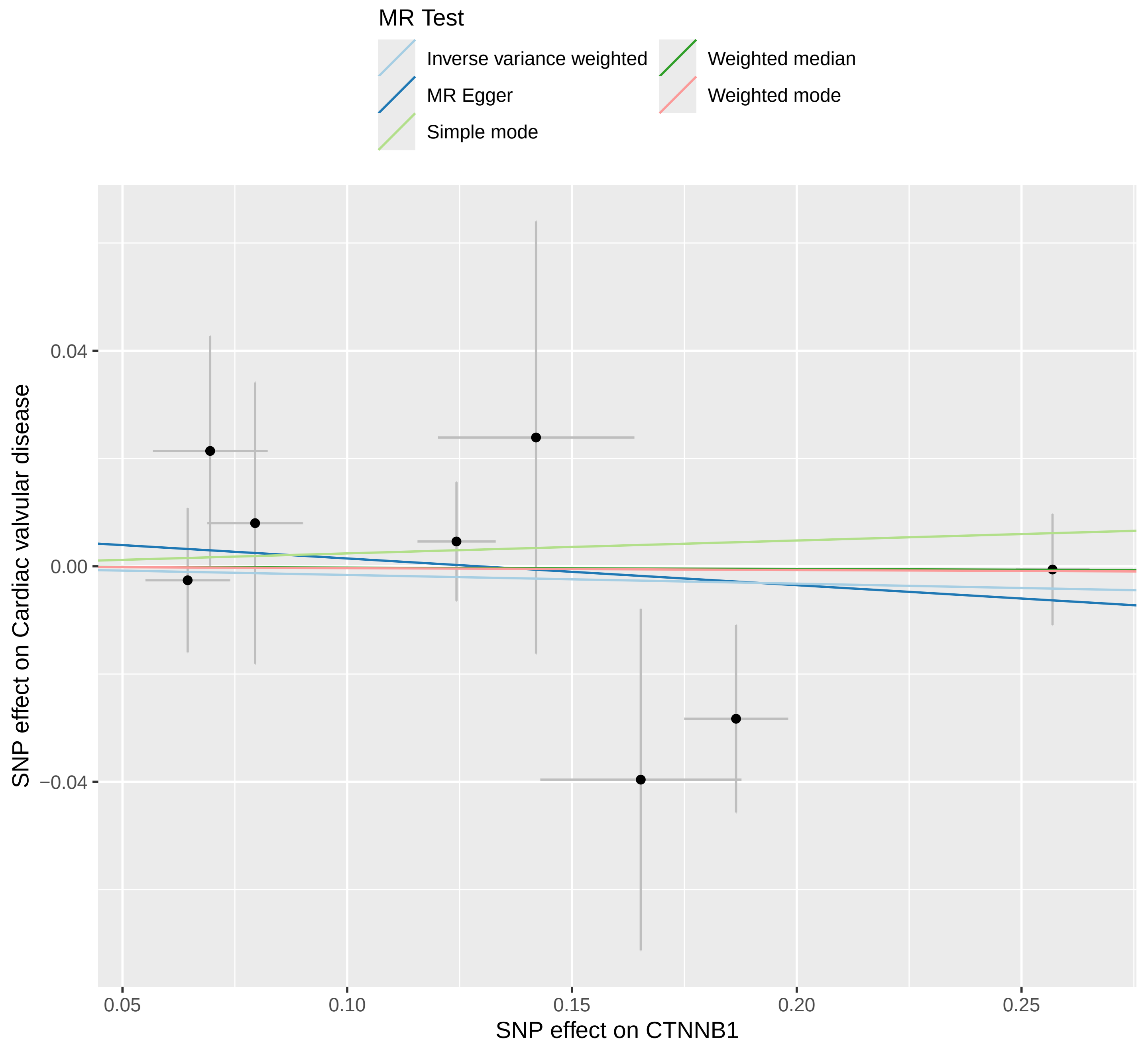
MR Test
Inverse variance weighted
MR Egger
Simple mode
Weighted median
Weighted mode
0.04
0.00
−0.04
0.05
0.10
0.15
0.20
0.25
SNP effect on Cardiac valvular disease
SNP effect on CTNNB1

Insufficient number of SNPs

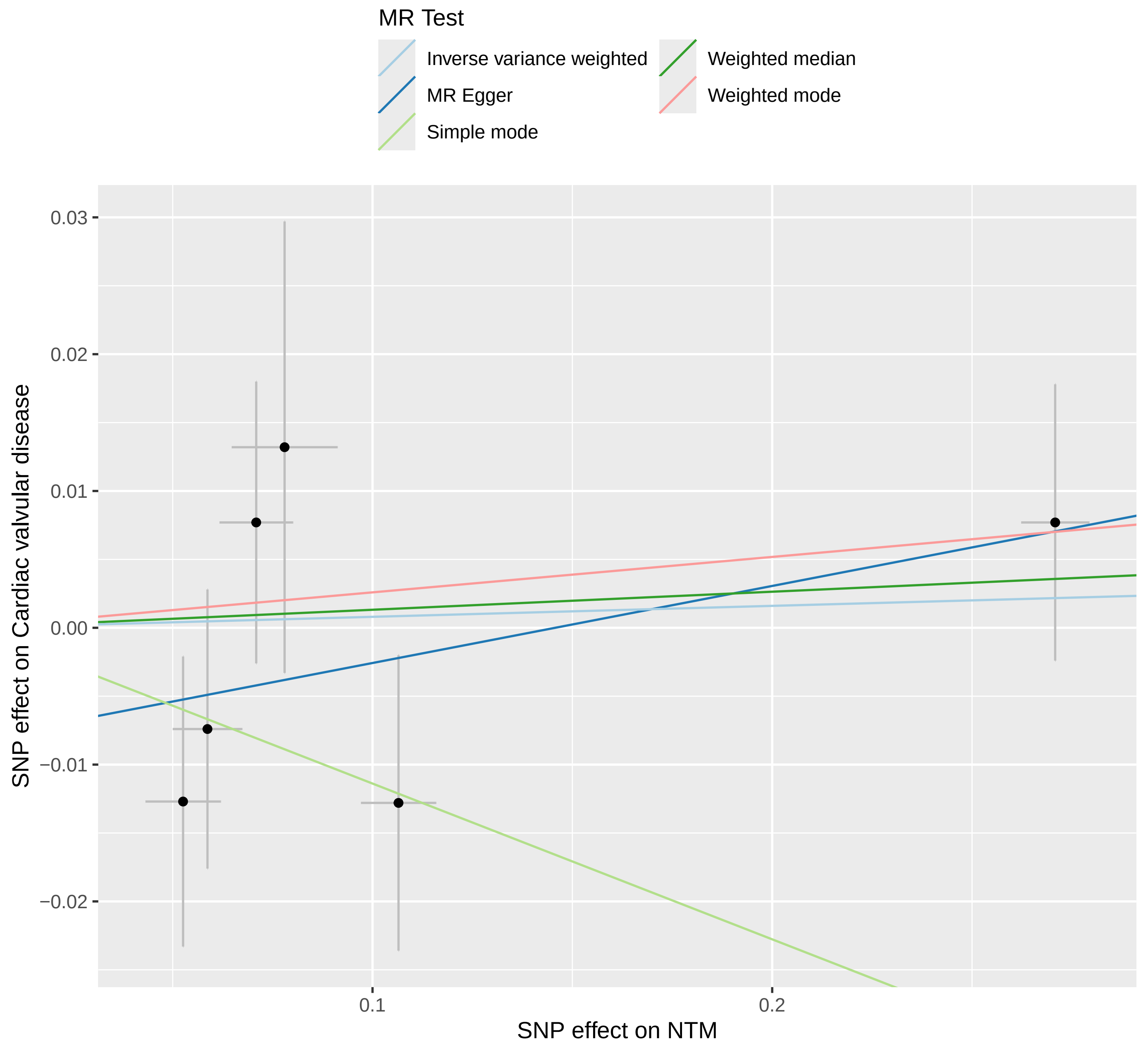
MR Test
Inverse variance weighted
MR Egger
Simple mode
Weighted median
Weighted mode
0.03
0.02
0.01
0.00
−0.01
−0.02
0.1
0.2
SNP effect on Cardiac valvular disease
SNP effect on NTM

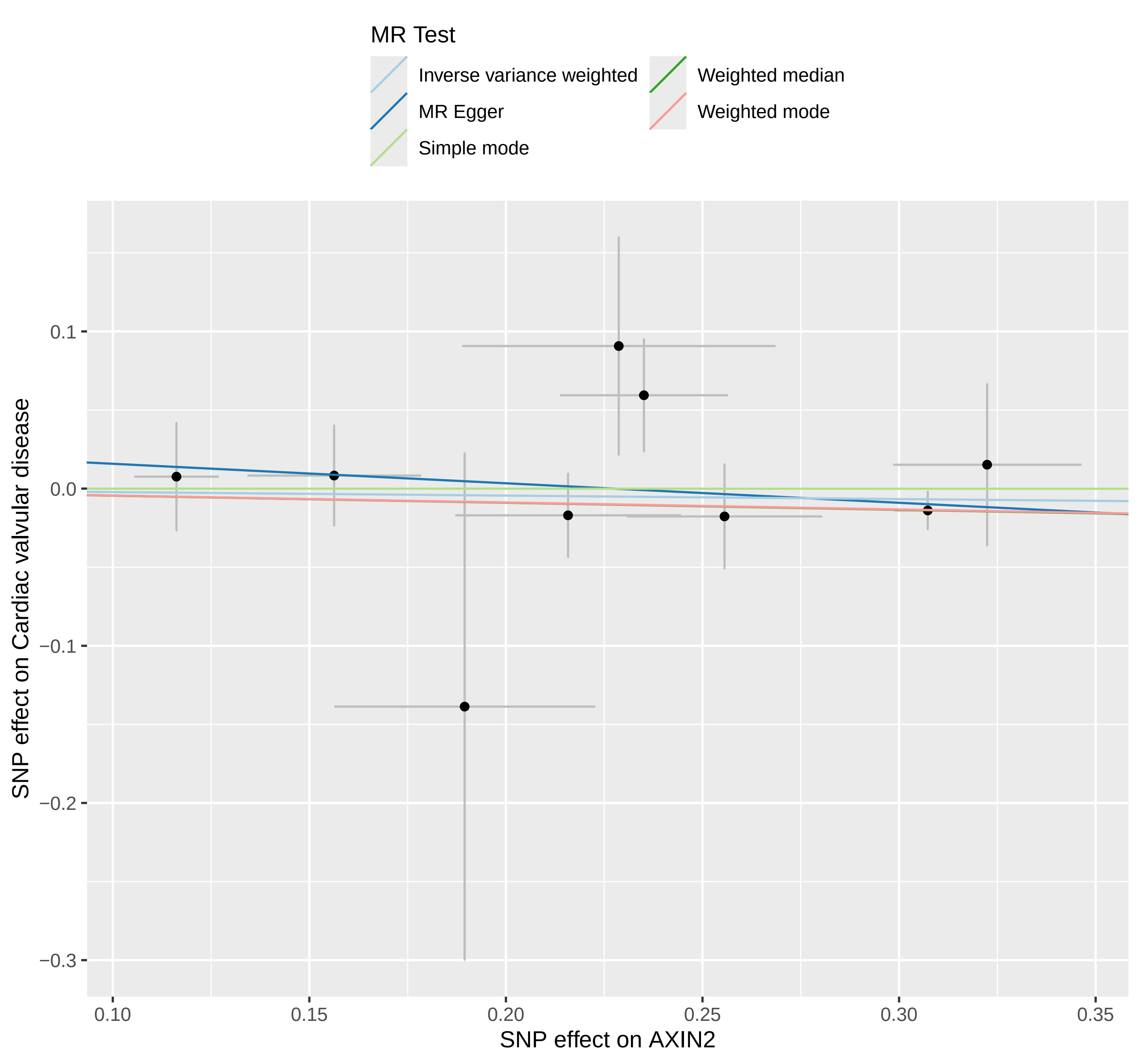
MR Test
Inverse variance weighted
MR Egger
Simple mode
Weighted median
Weighted mode
SNP effect on Cardiac valvular disease
0.1
0.0
−0.1
−0.2
−0.3
0.10
0.15
0.20
0.25
0.30
0.35
SNP effect on AXIN2

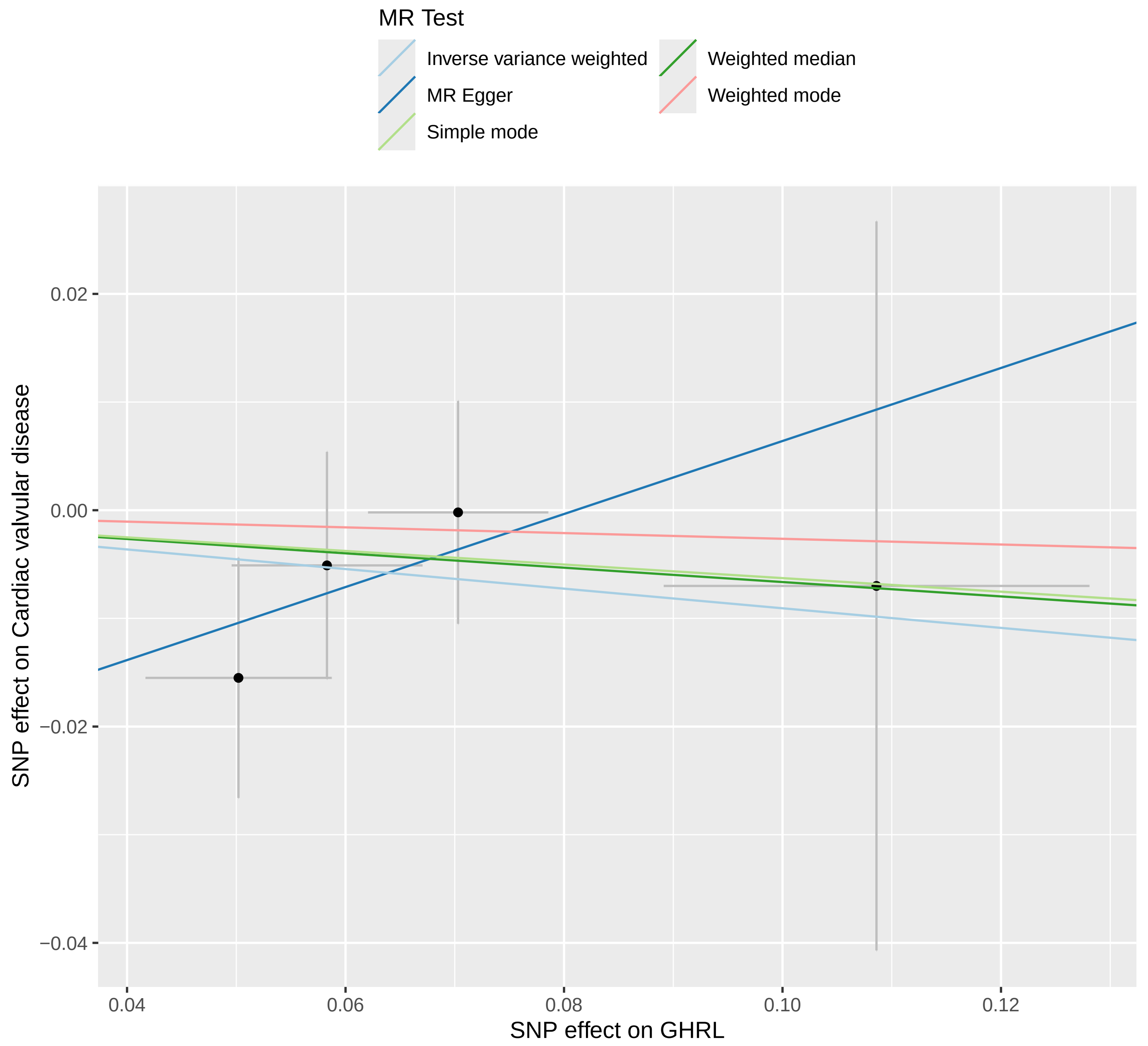
MR Test
Inverse variance weighted
MR Egger
Simple mode
Weighted median
Weighted mode
0.02
0.00
−0.02
−0.04
SNP effect on Cardiac valvular disease
0.04
0.06
0.08
0.10
0.12
SNP effect on GHRL

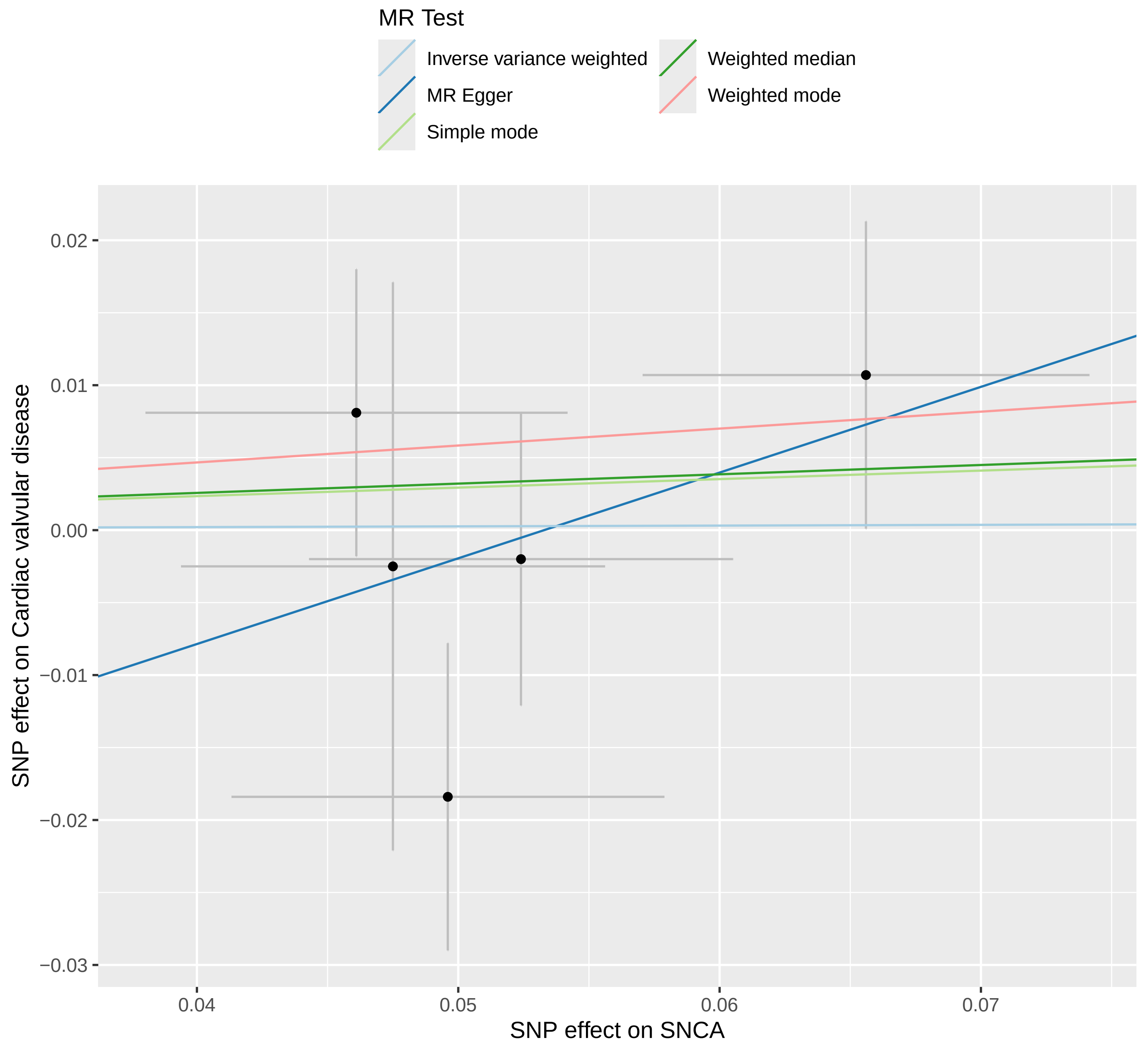
MR Test
Inverse variance weighted
MR Egger
Simple mode
Weighted median
Weighted mode
0.02
0.01
0.00
−0.01
−0.02
−0.03
SNP effect on Cardiac valvular disease
0.04
0.05
0.06
0.07
SNP effect on SNCA

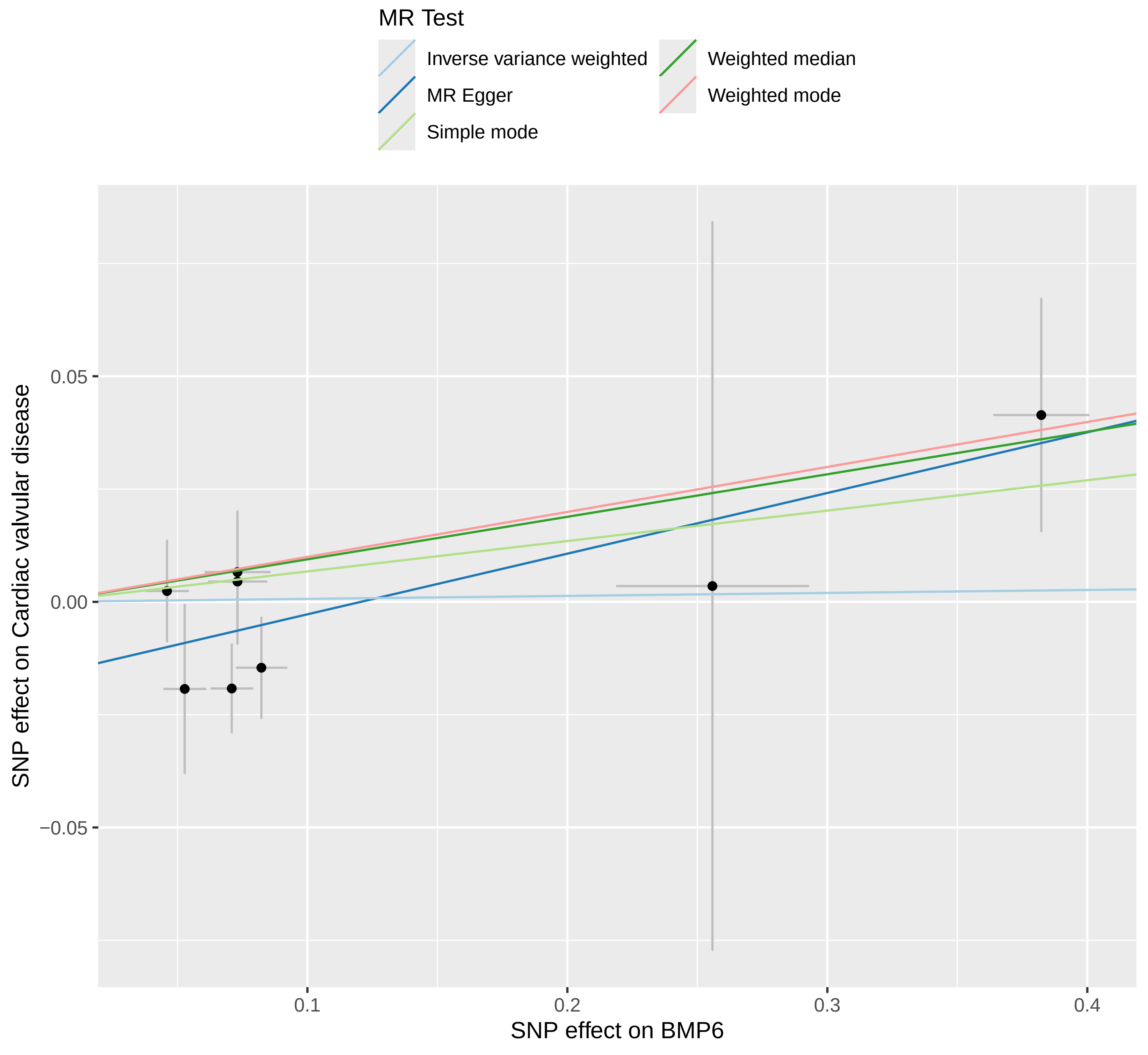
MR Test
Inverse variance weighted
MR Egger
Simple mode
Weighted median
Weighted mode
0.05
0.00
−0.05
0.1
0.2
0.3
0.4
SNP effect on Cardiac valvular disease
SNP effect on BMP6

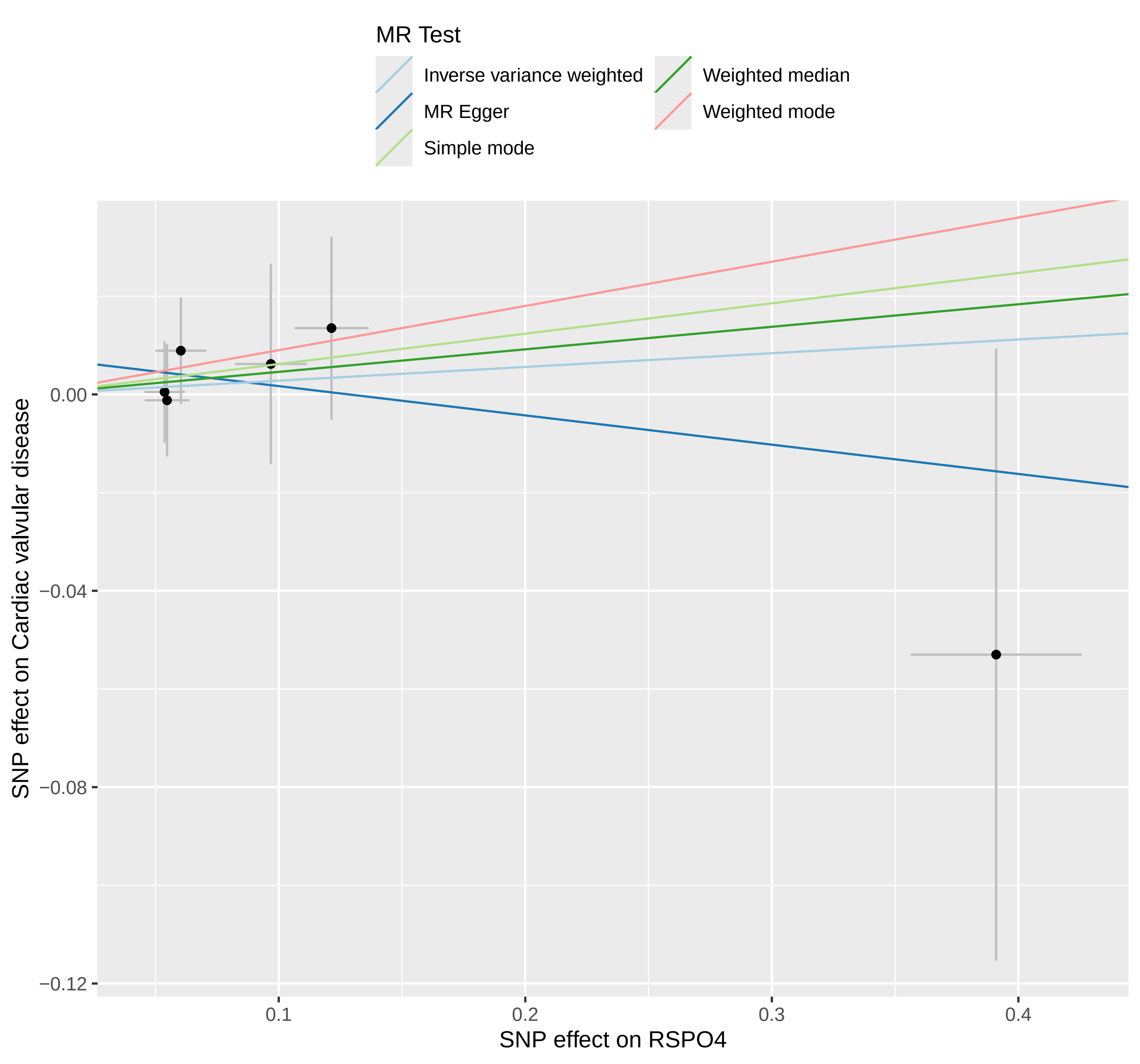
MR Test
Inverse variance weighted
MR Egger
Simple mode
Weighted median
Weighted mode
0.00
−0.04
−0.08
−0.12
0.1
0.2
0.3
0.4
SNP effect on RSPO4
SNP effect on Cardiac valvular disease

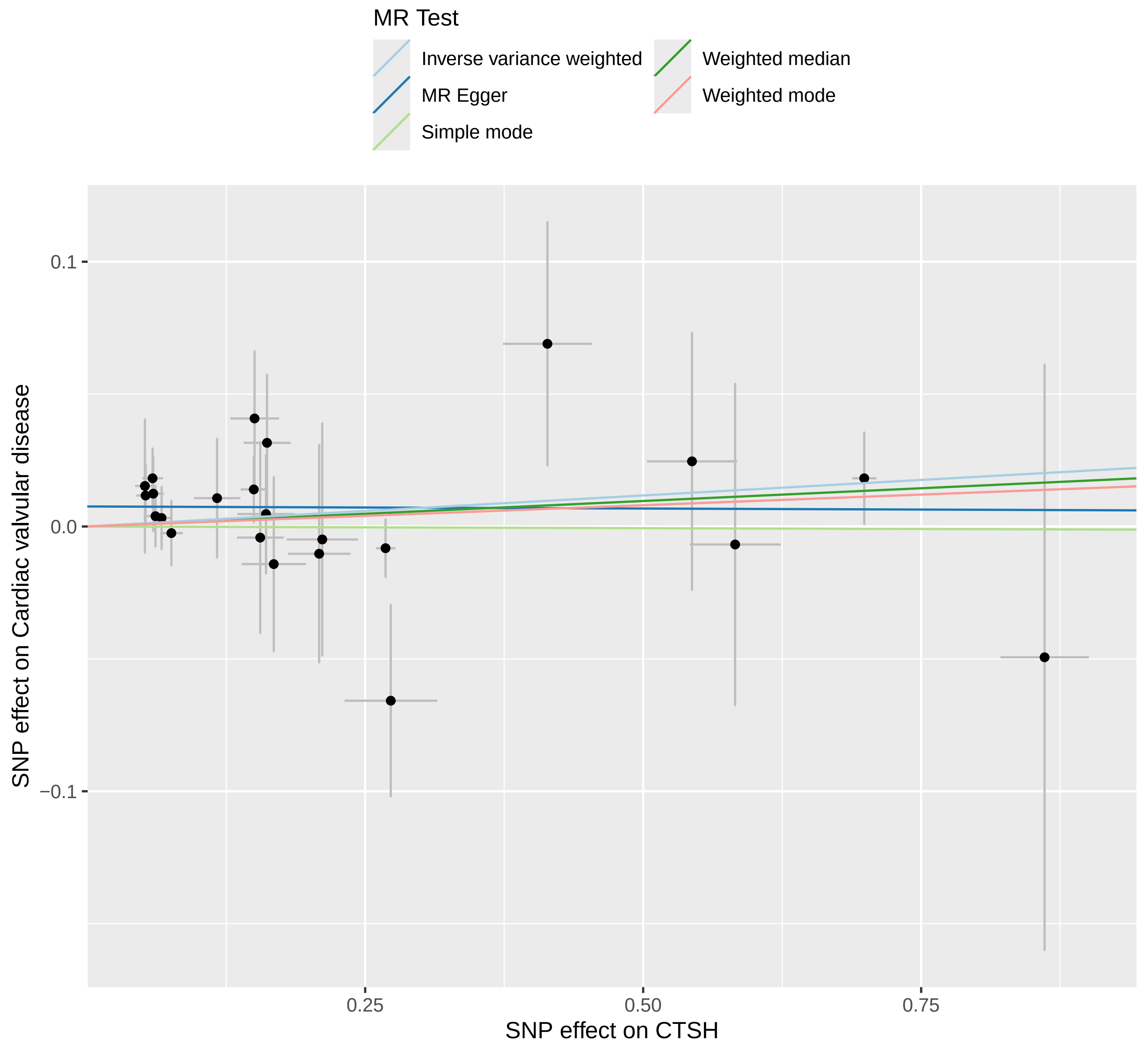

MR Test
Inverse variance weighted
MR Egger
Simple mode
Weighted median
Weighted mode
0.1
0.0
−0.1
0.25
0.50
0.75
SNP effect on Cardiac valvular disease
SNP effect on CTSH

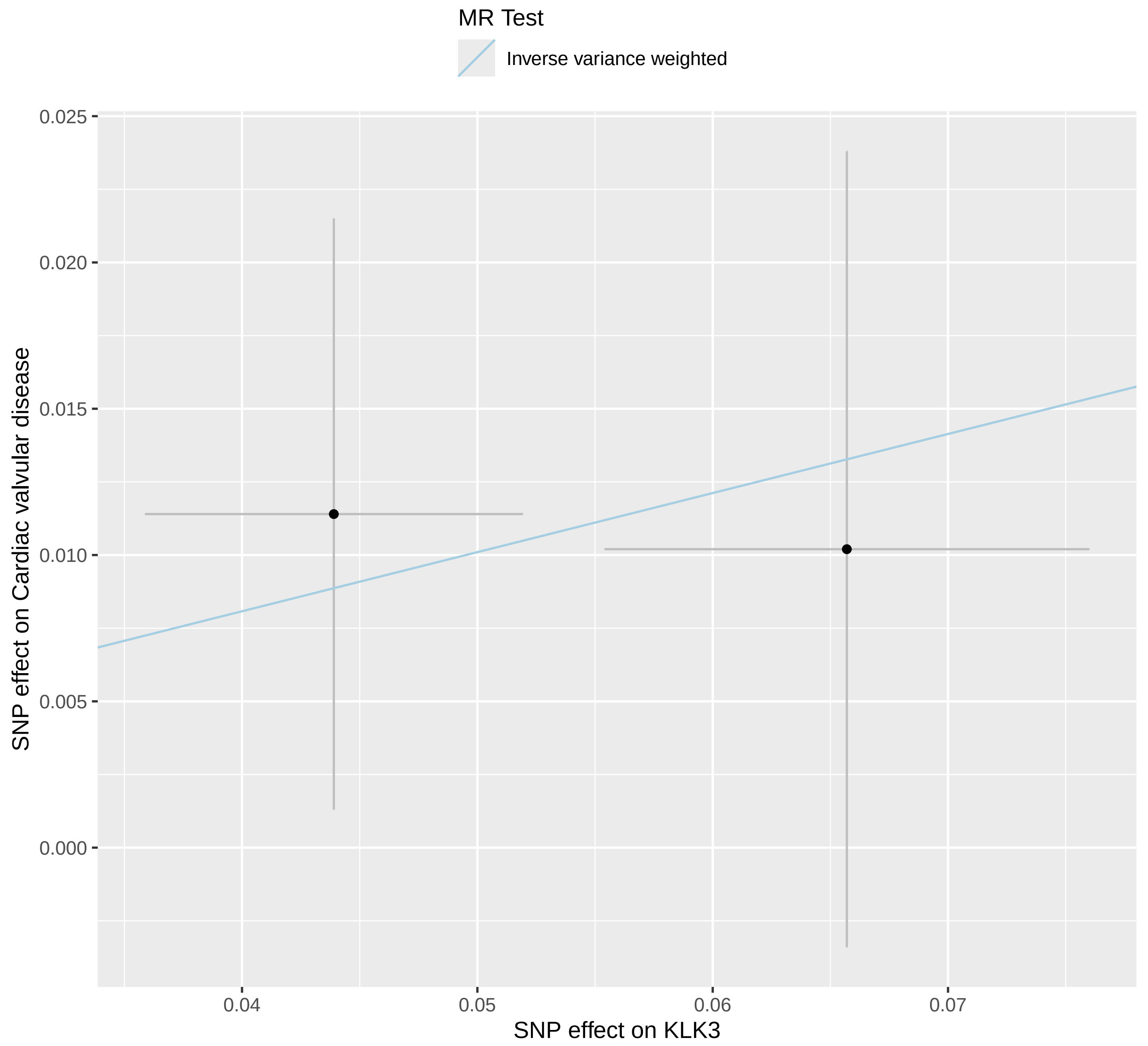

MR Test
Inverse variance weighted
0.025
0.020
0.015
0.010
0.005
0.000
SNP effect on Cardiac valvular disease
0.04
0.05
0.06
0.07
SNP effect on KLK3

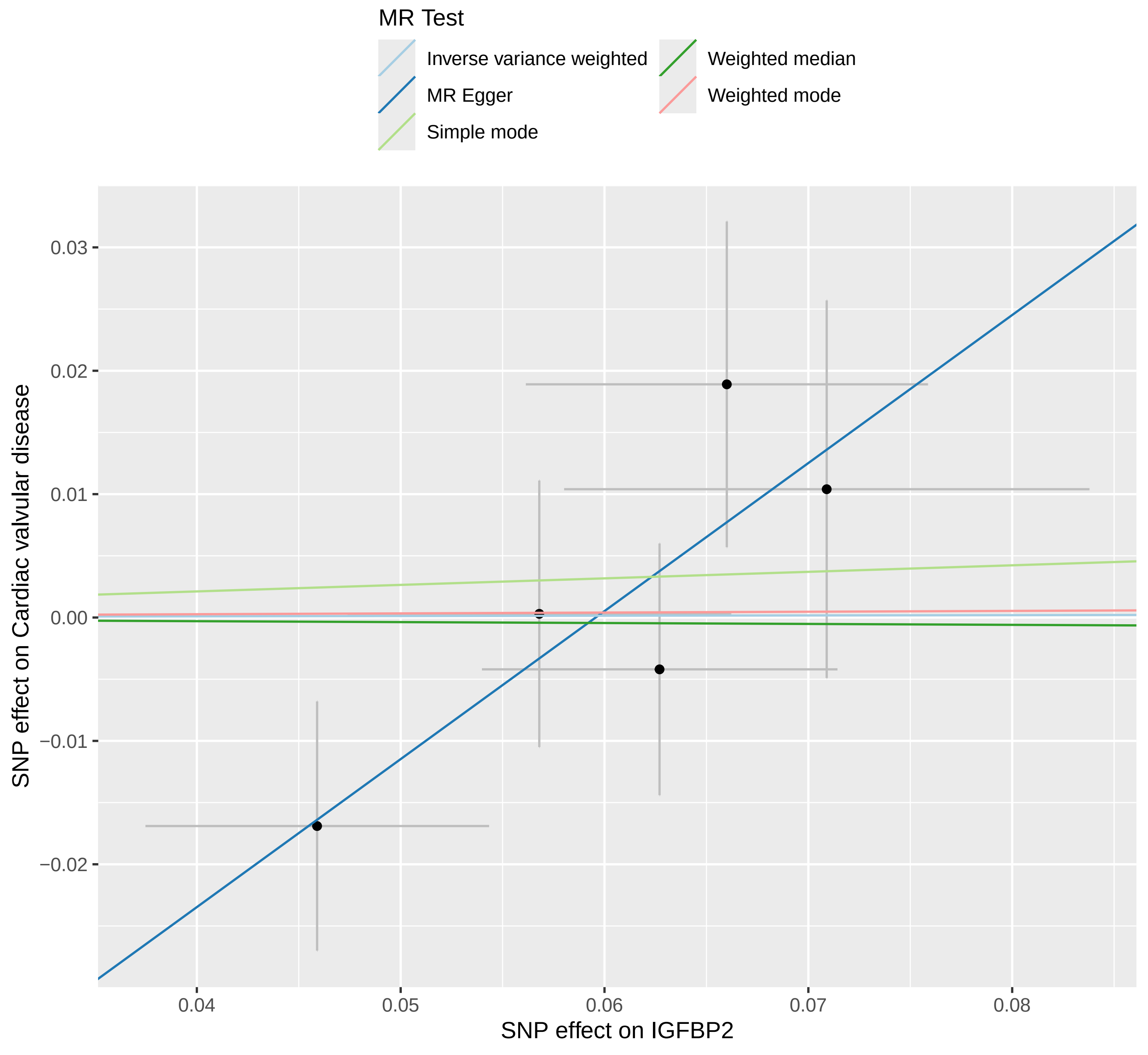

MR Test
Inverse variance weighted
MR Egger
Simple mode
Weighted median
Weighted mode
0.03
0.02
0.01
0.00
−0.01
−0.02
SNP effect on Cardiac valvular disease
0.04
0.05
0.06
0.07
0.08
SNP effect on IGFBP2

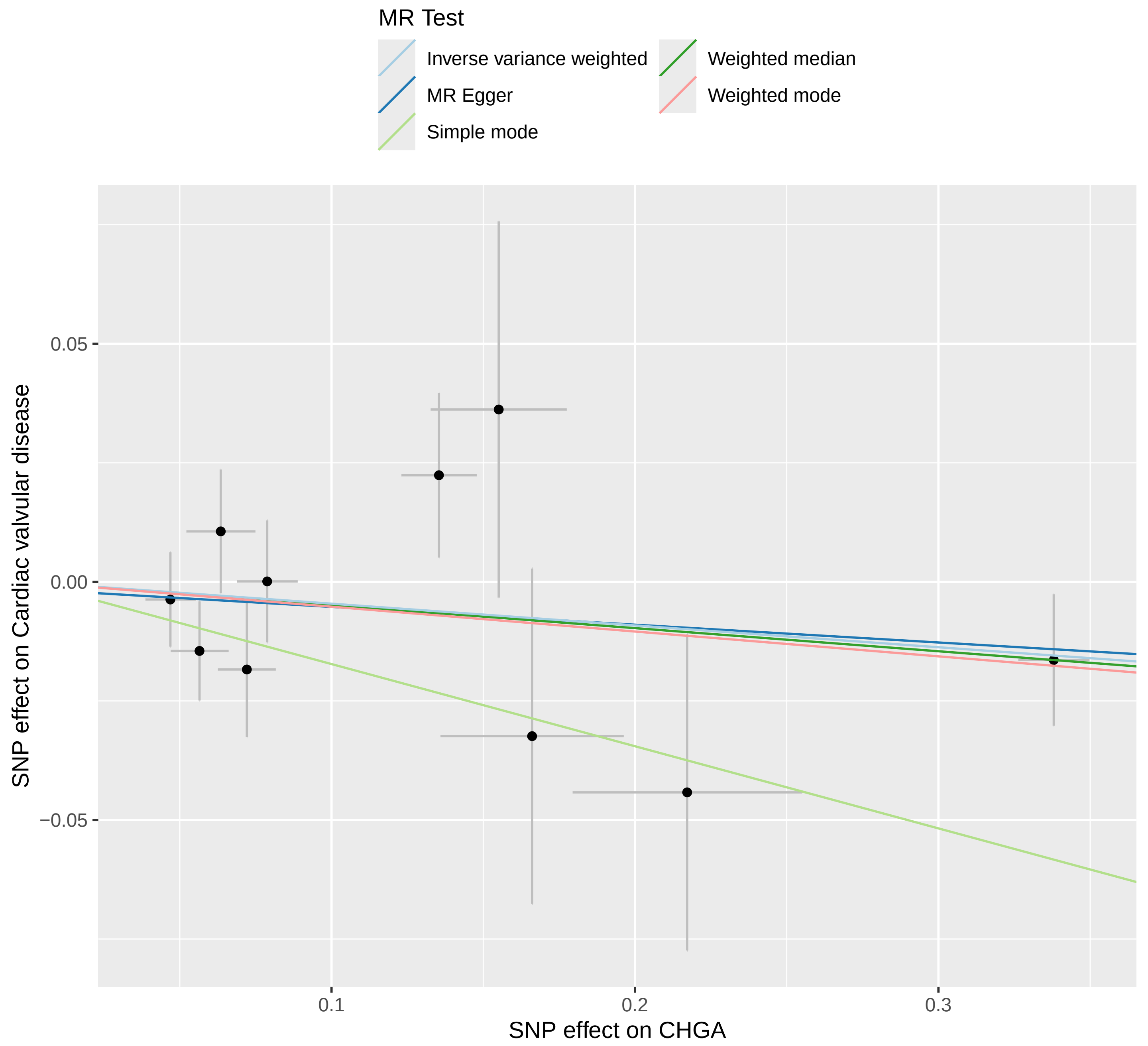
MR Test
Inverse variance weighted
MR Egger
Simple mode
Weighted median
Weighted mode
0.05
0.00
−0.05
0.1
0.2
0.3
SNP effect on Cardiac valvular disease
SNP effect on CHGA

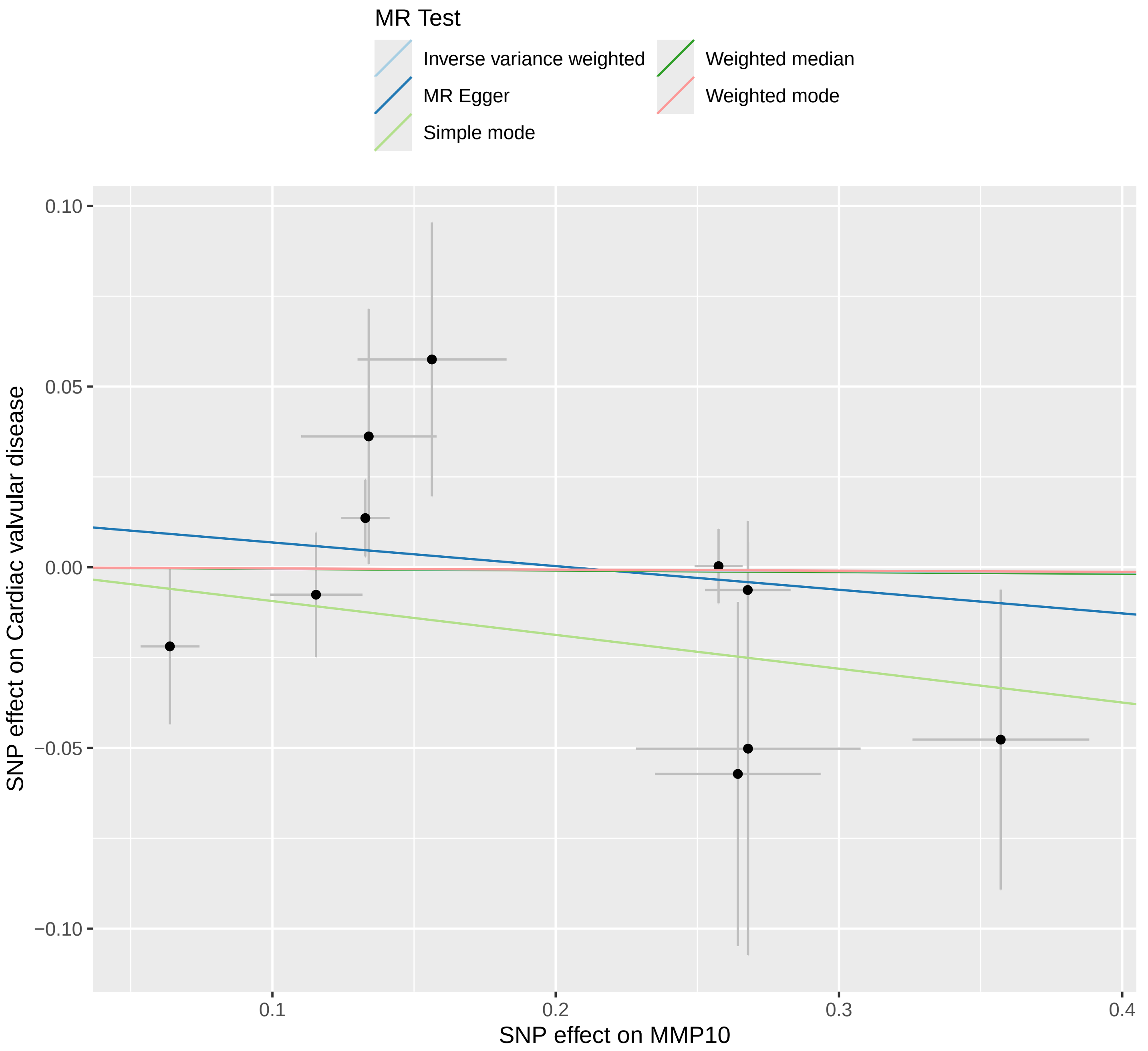
MR Test
Inverse variance weighted
MR Egger
Simple mode
Weighted median
Weighted mode
0.10
0.05
0.00
−0.05
−0.10
SNP effect on Cardiac valvular disease
0.1
0.2
0.3
0.4
SNP effect on MMP10

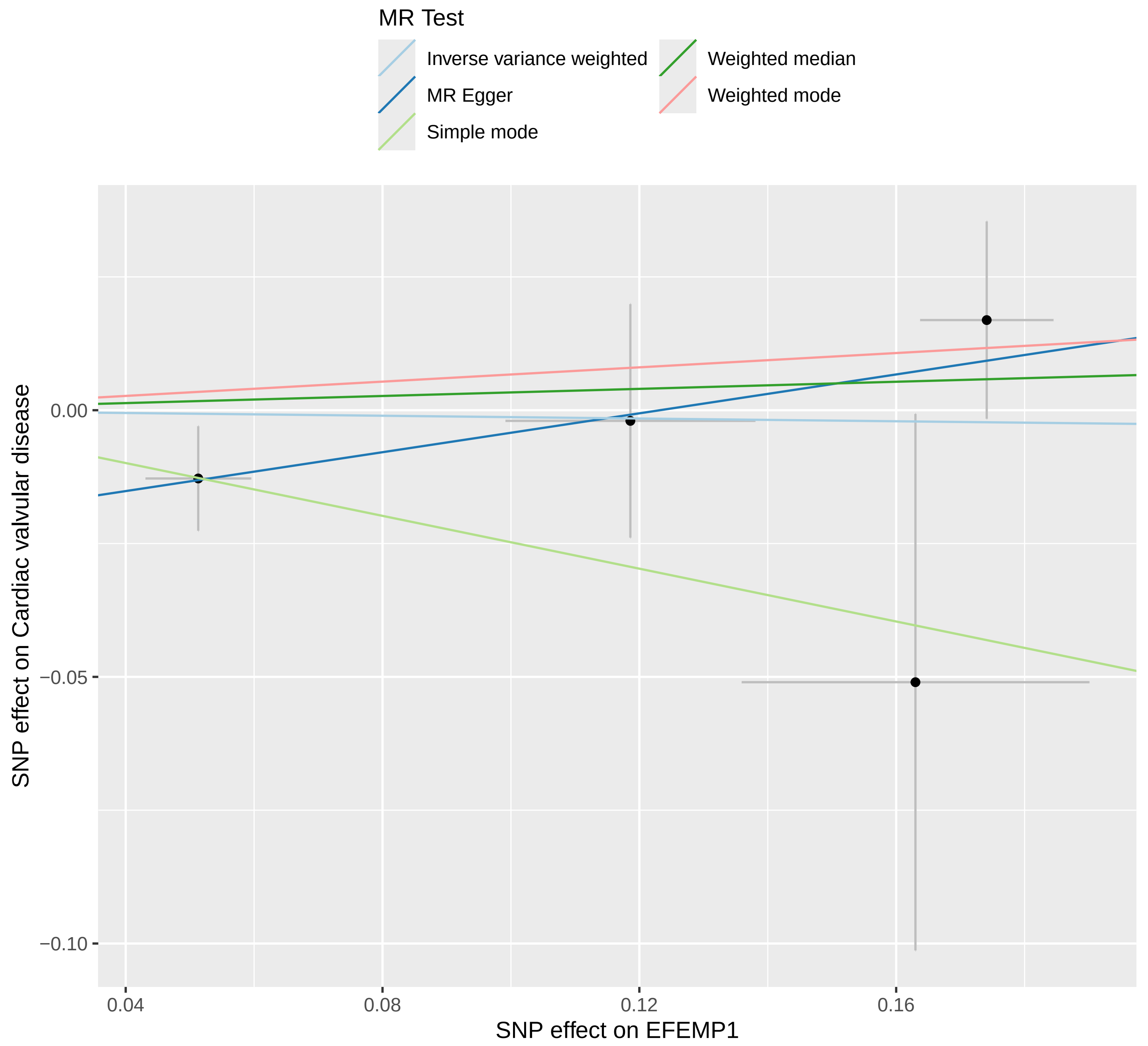

MR Test
Inverse variance weighted
MR Egger
Simple mode
Weighted median
Weighted mode
0.00
−0.05
−0.10
SNP effect on Cardiac valvular disease
0.04
0.08
0.12
0.16
SNP effect on EFEMP1

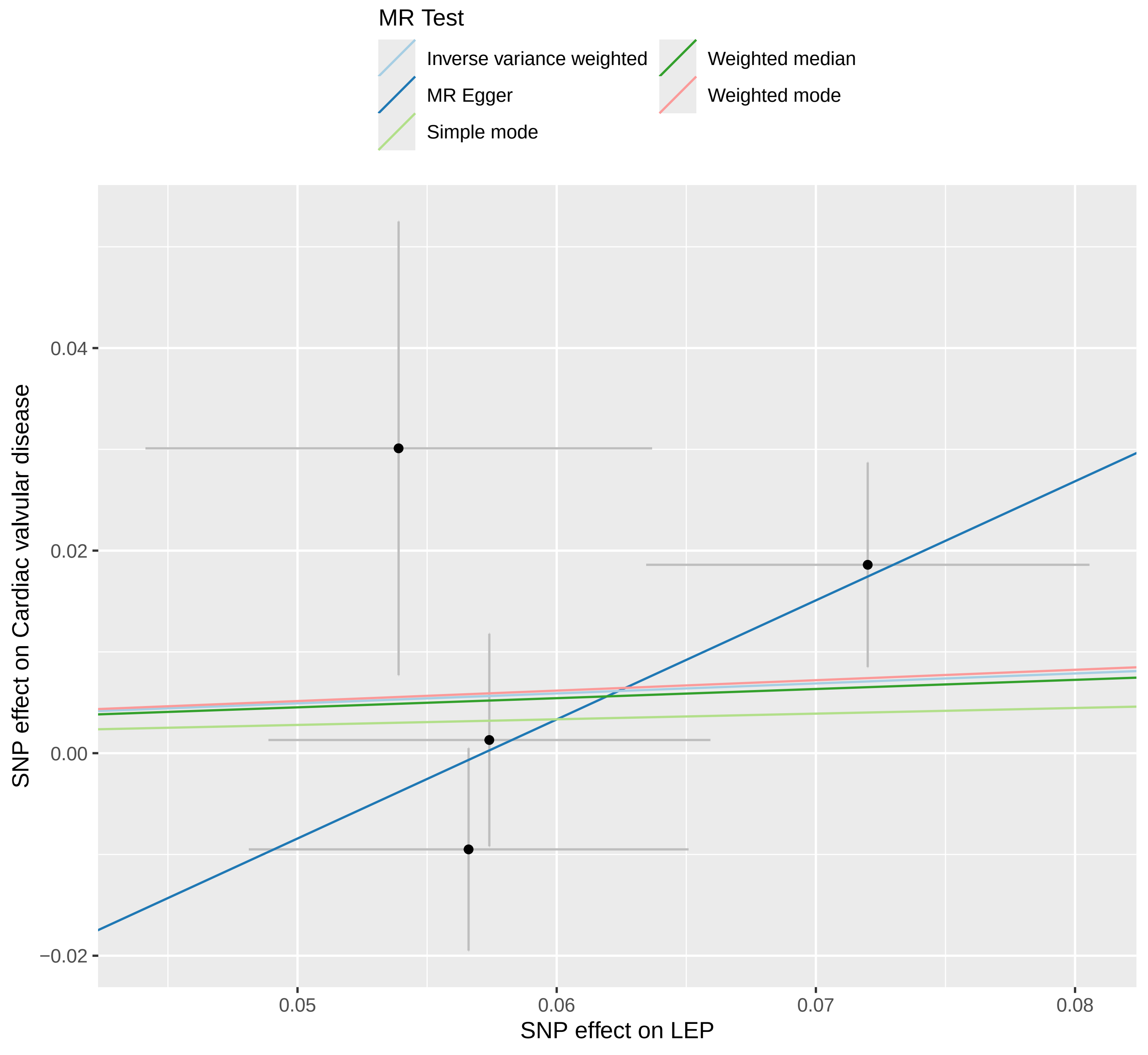

MR Test
Inverse variance weighted
MR Egger
Simple mode
Weighted median
Weighted mode
0.04
0.02
0.00
−0.02
SNP effect on Cardiac valvular disease
0.05
0.06
0.07
0.08
SNP effect on LEP

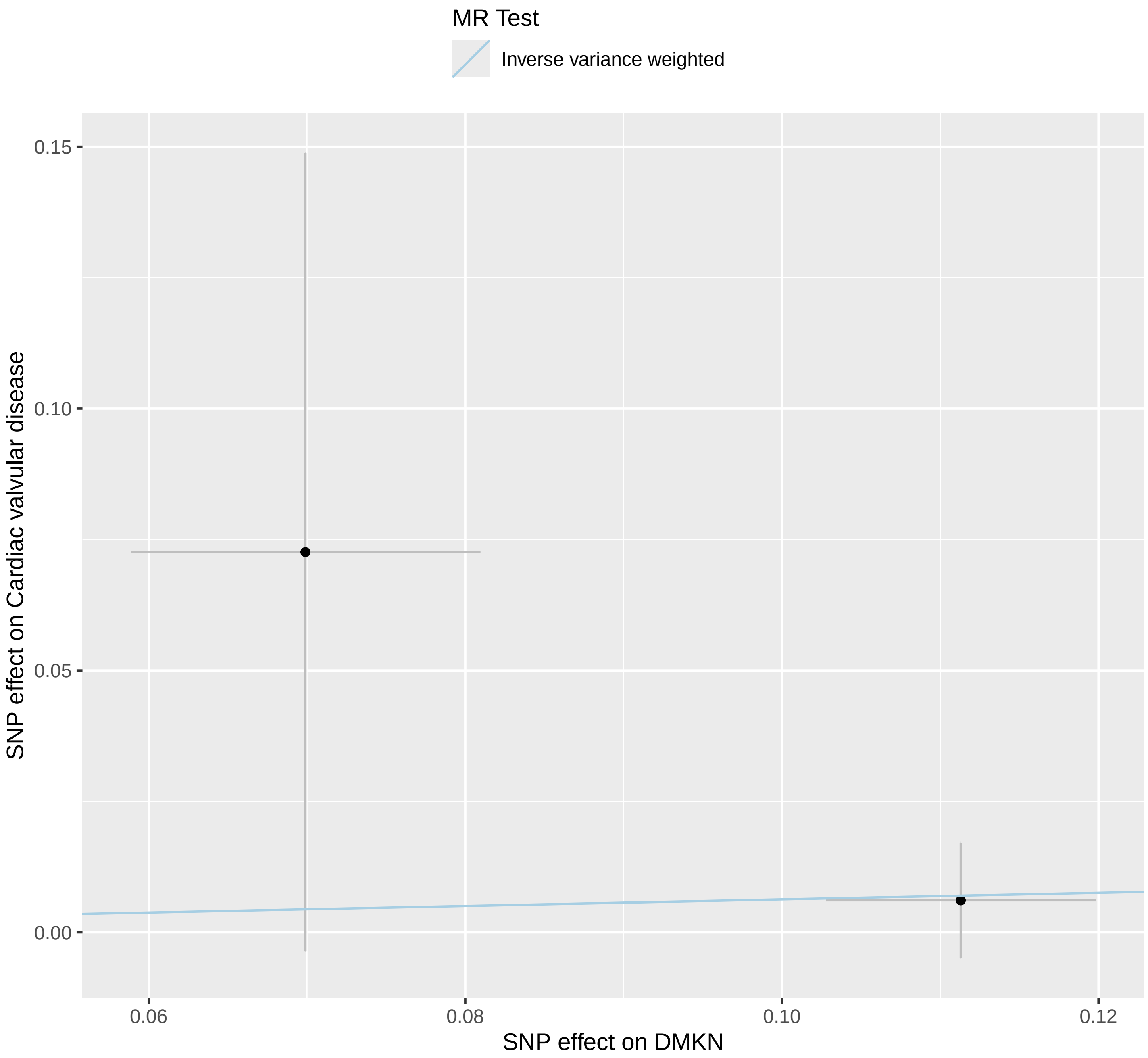

MR Test
Inverse variance weighted
SNP effect on Cardiac valvular disease
SNP effect on DMKN
0.15
0.10
0.05
0.00
0.06
0.08
0.10
0.12

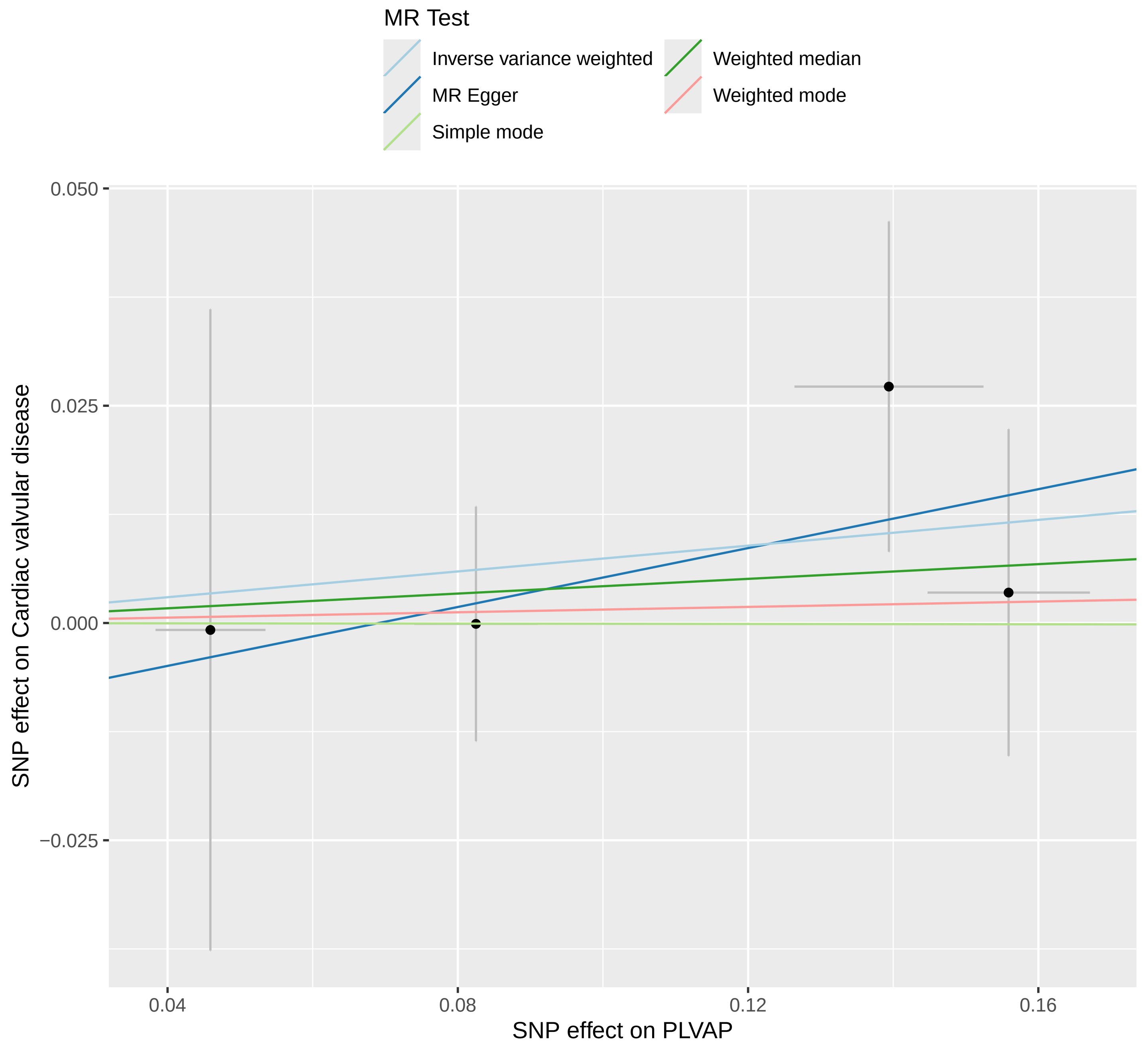

MR Test
Inverse variance weighted
MR Egger
Simple mode
Weighted median
Weighted mode
0.050
0.025
0.000
−0.025
SNP effect on Cardiac valvular disease
0.04
0.08
0.12
0.16
SNP effect on PLVAP

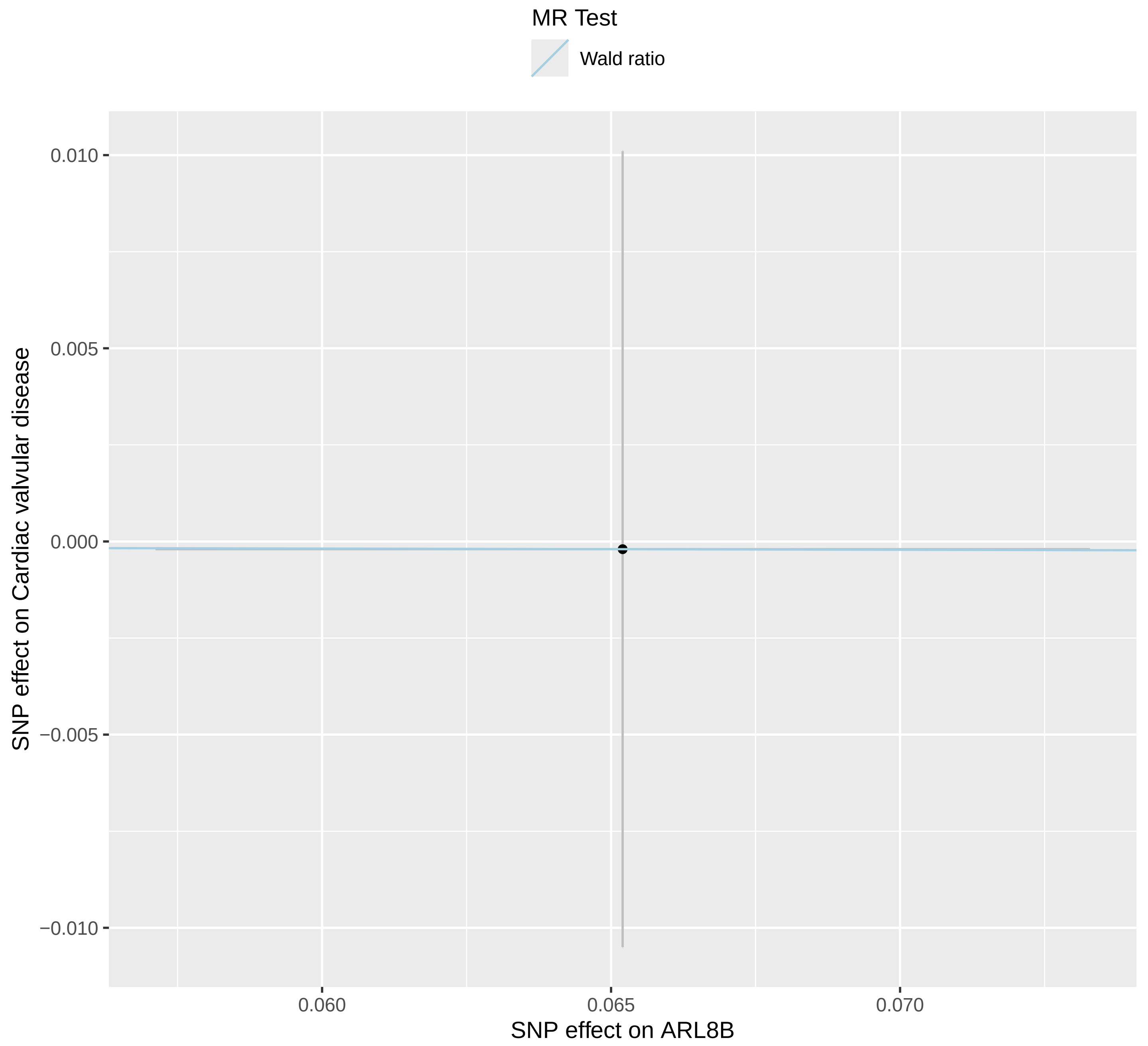
MR Test
Wald ratio
0.010
0.005
0.000
−0.005
−0.010
0.060
0.065
0.070
SNP effect on ARL8B
SNP effect on Cardiac valvular disease

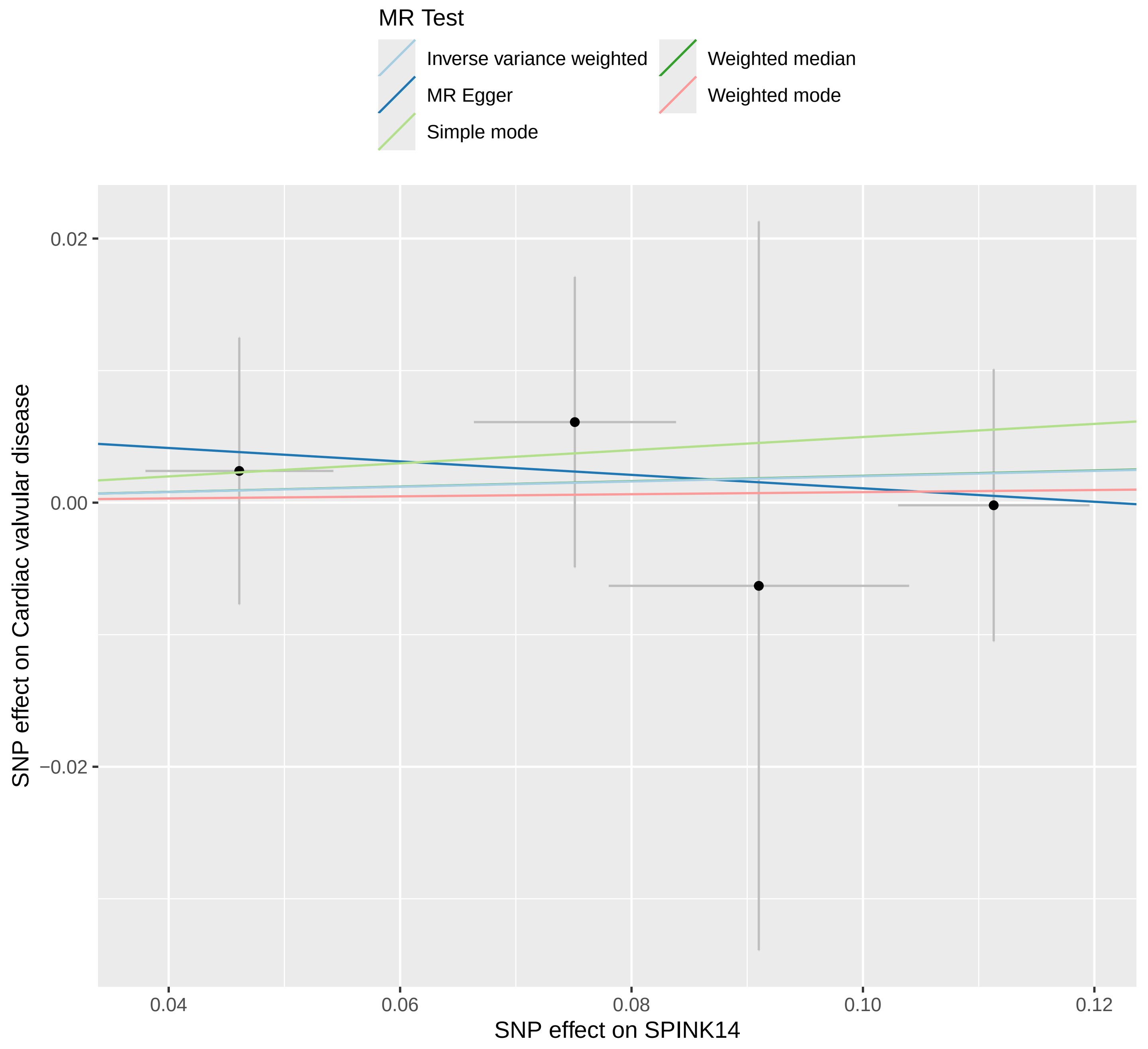
MR Test
Inverse variance weighted
MR Egger
Simple mode
Weighted median
Weighted mode
0.02
0.00
−0.02
0.04
0.06
0.08
0.10
0.12
SNP effect on Cardiac valvular disease
SNP effect on SPINK14

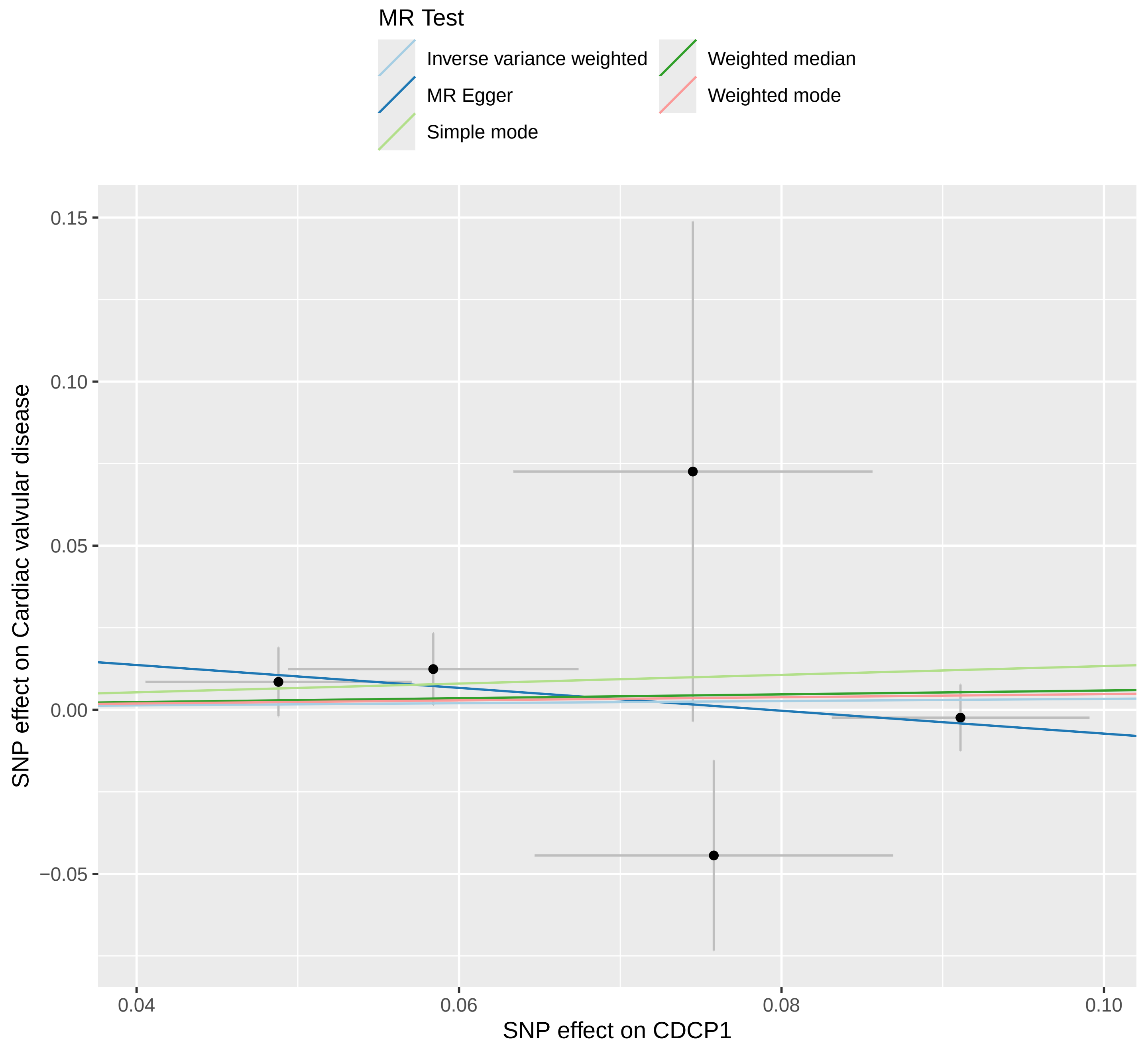
MR Test
Inverse variance weighted
MR Egger
Simple mode
Weighted median
Weighted mode
0.15
0.10
0.05
0.00
−0.05
0.04
0.06
0.08
0.10
SNP effect on Cardiac valvular disease
SNP effect on CDCP1

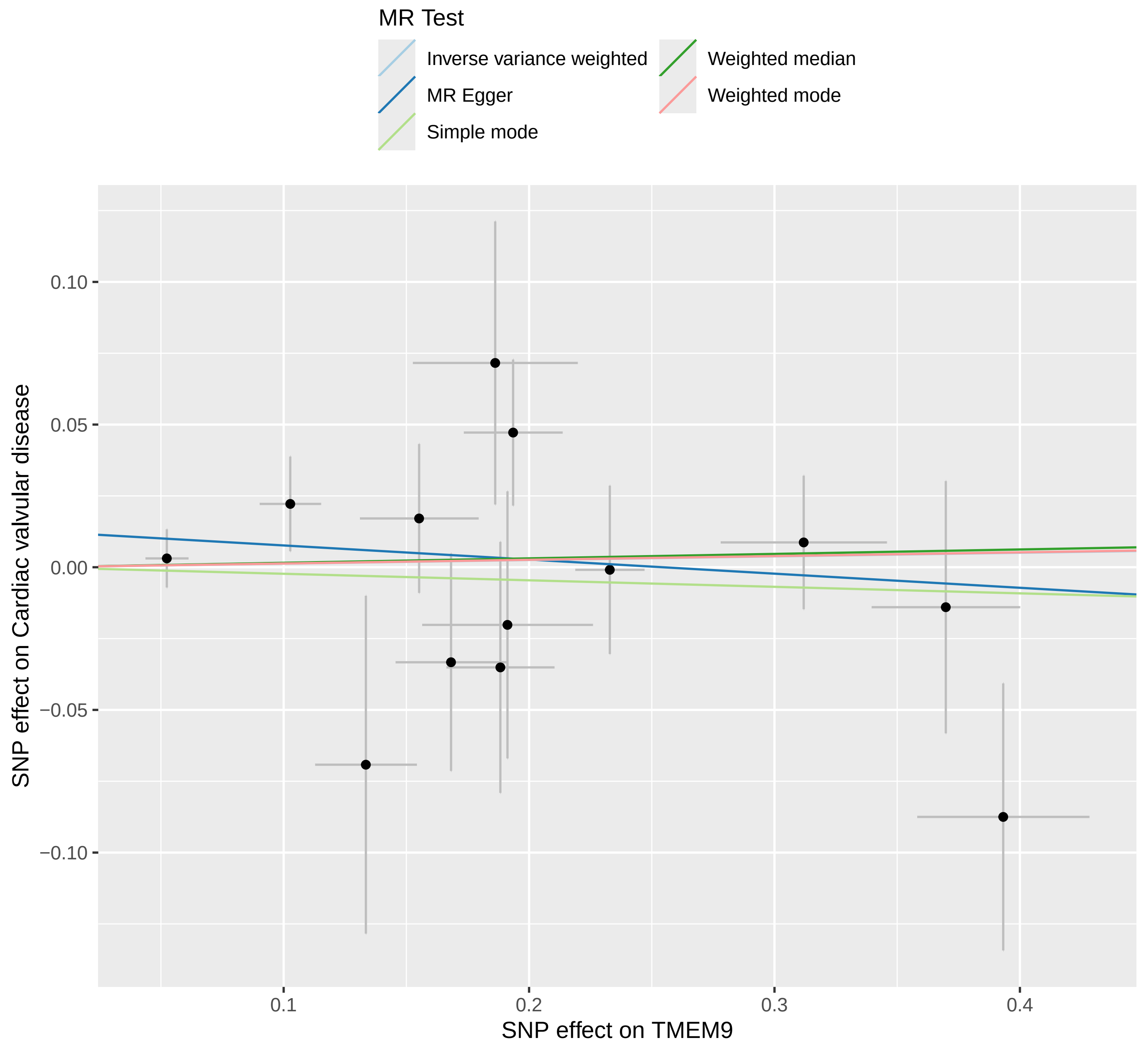
MR Test
Inverse variance weighted
MR Egger
Simple mode
Weighted median
Weighted mode
0.10
0.05
0.00
−0.05
−0.10
0.1
0.2
0.3
0.4
SNP effect on Cardiac valvular disease
SNP effect on TMEM9

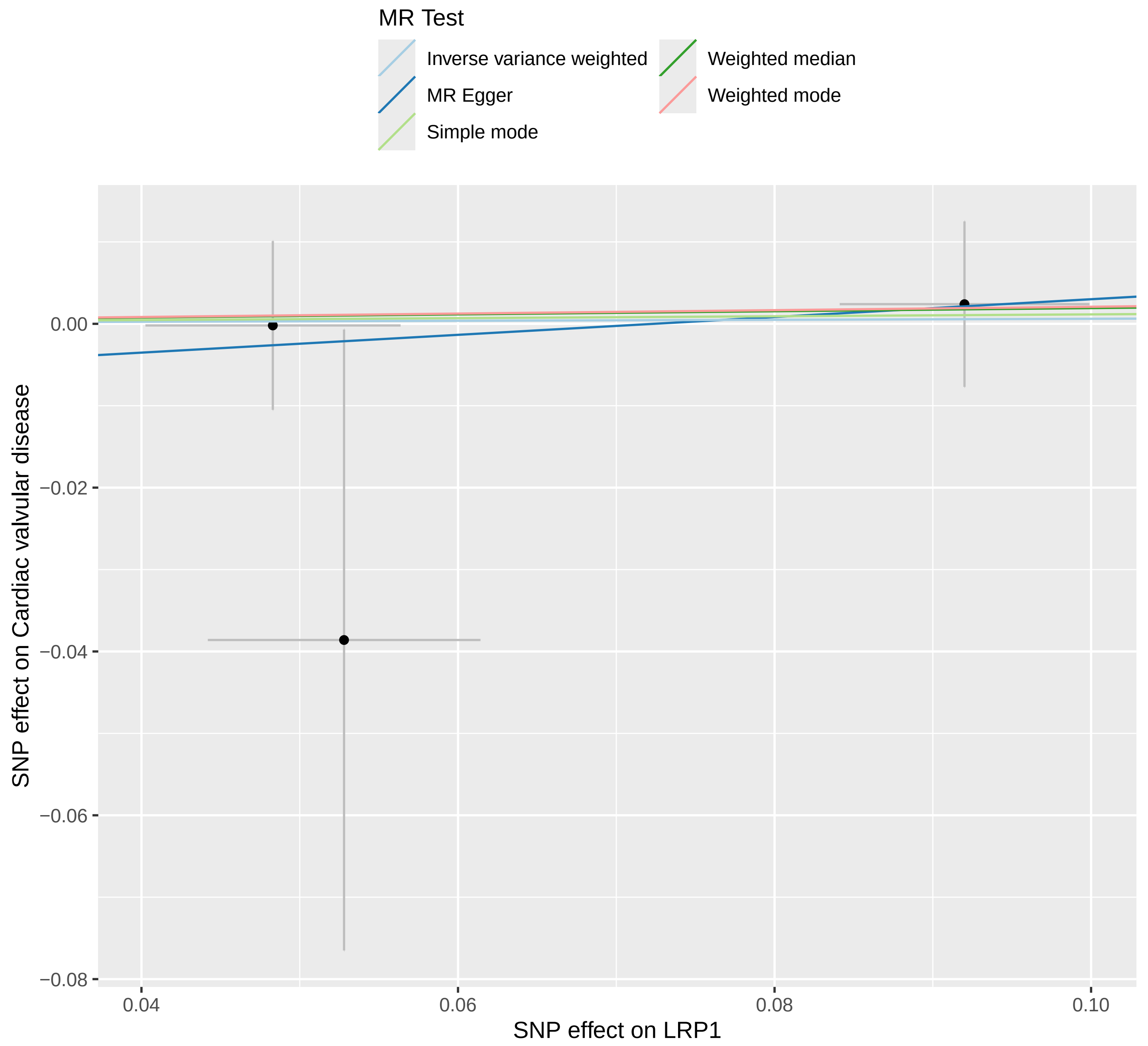
MR Test
Inverse variance weighted
MR Egger
Simple mode
Weighted median
Weighted mode
SNP effect on Cardiac valvular disease
0.00
−0.02
−0.04
−0.06
−0.08
0.04
0.06
0.08
0.10
SNP effect on LRP1

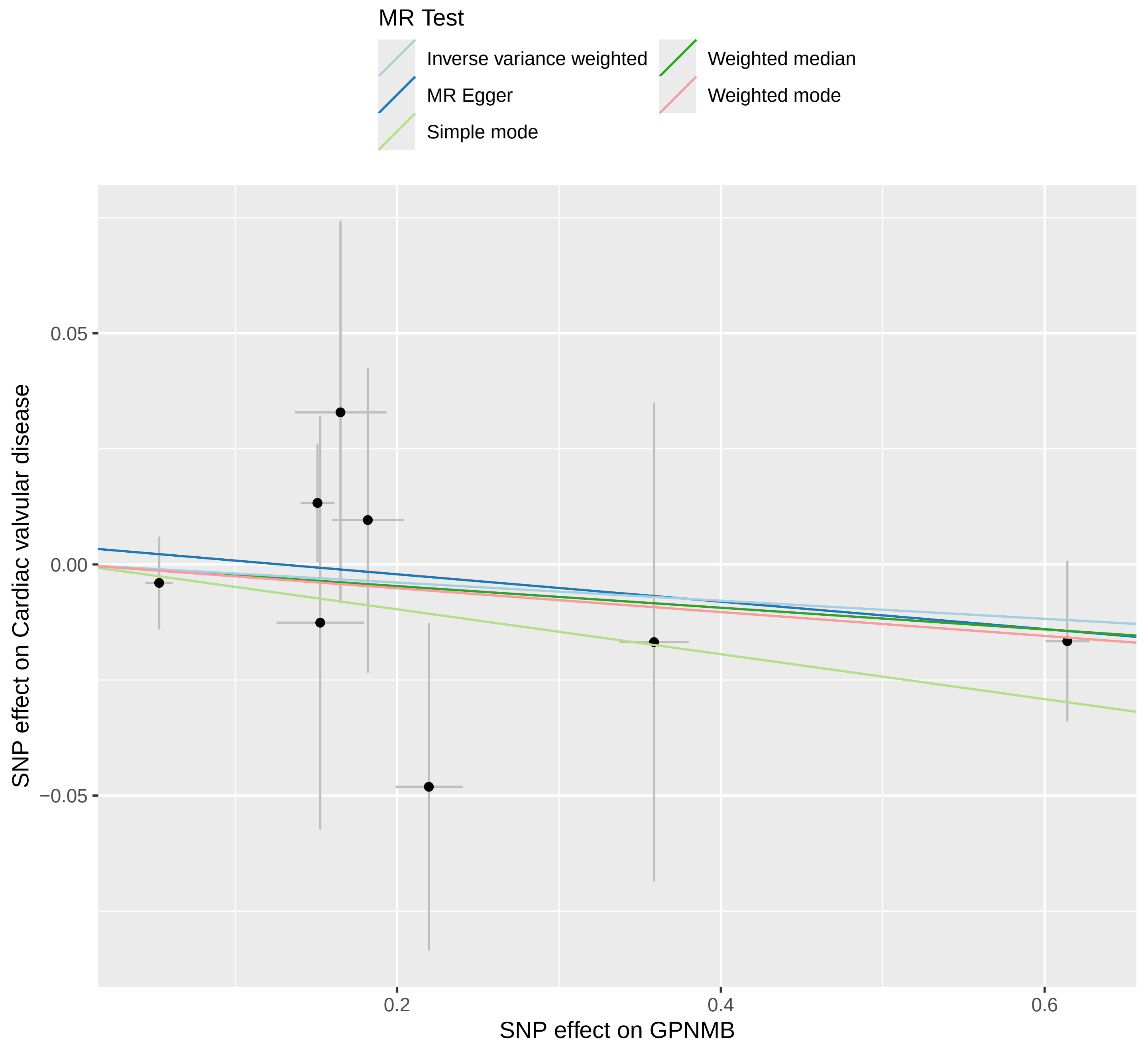
MR Test
Inverse variance weighted
MR Egger
Simple mode
Weighted median
Weighted mode
0.05
0.00
−0.05
0.2
0.4
0.6
SNP effect on Cardiac valvular disease
SNP effect on GPNMB

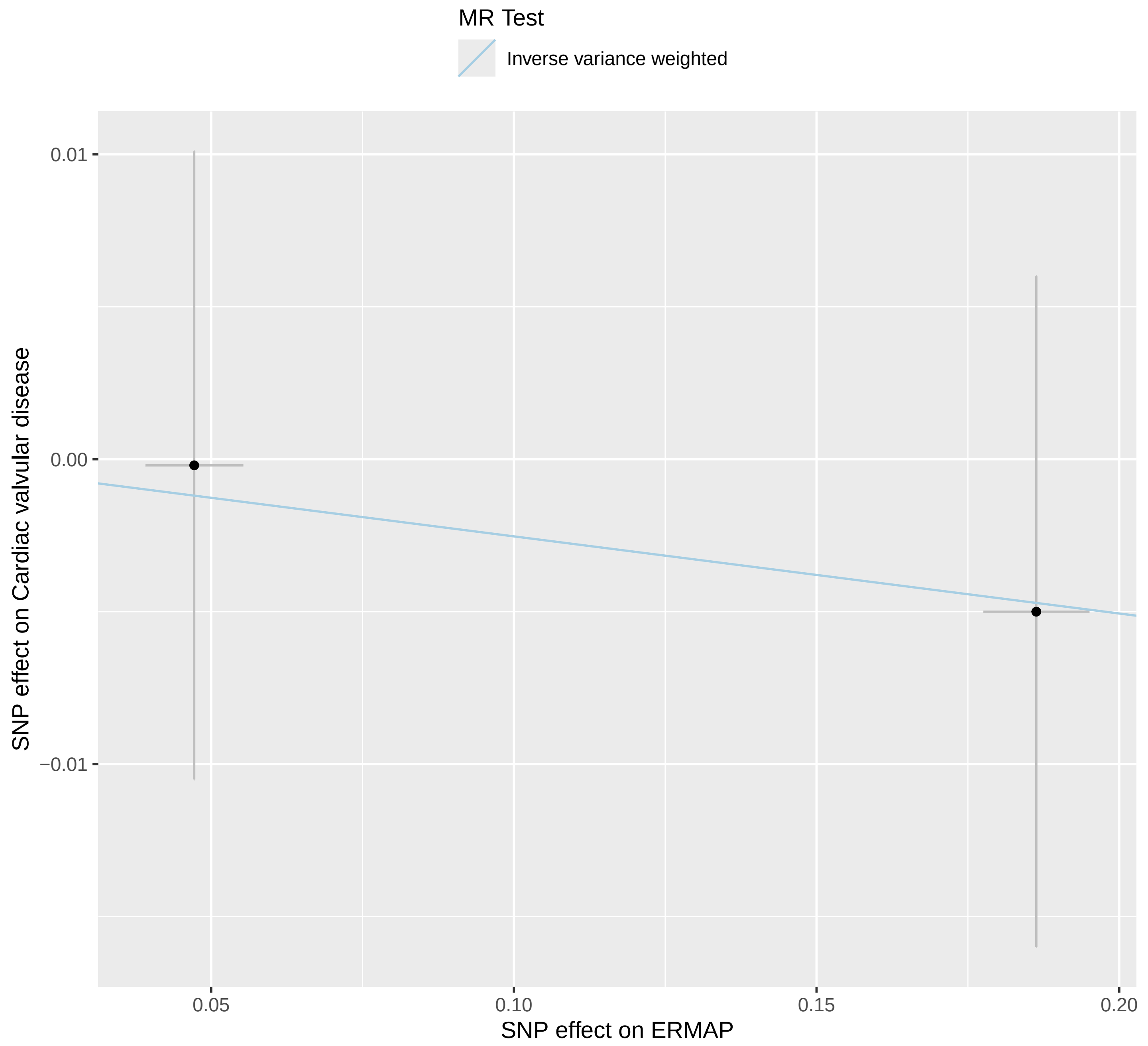

MR Test
Inverse variance weighted
0.01
0.00
−0.01
0.05
0.10
0.15
0.20
SNP effect on ERMAP
SNP effect on Cardiac valvular disease

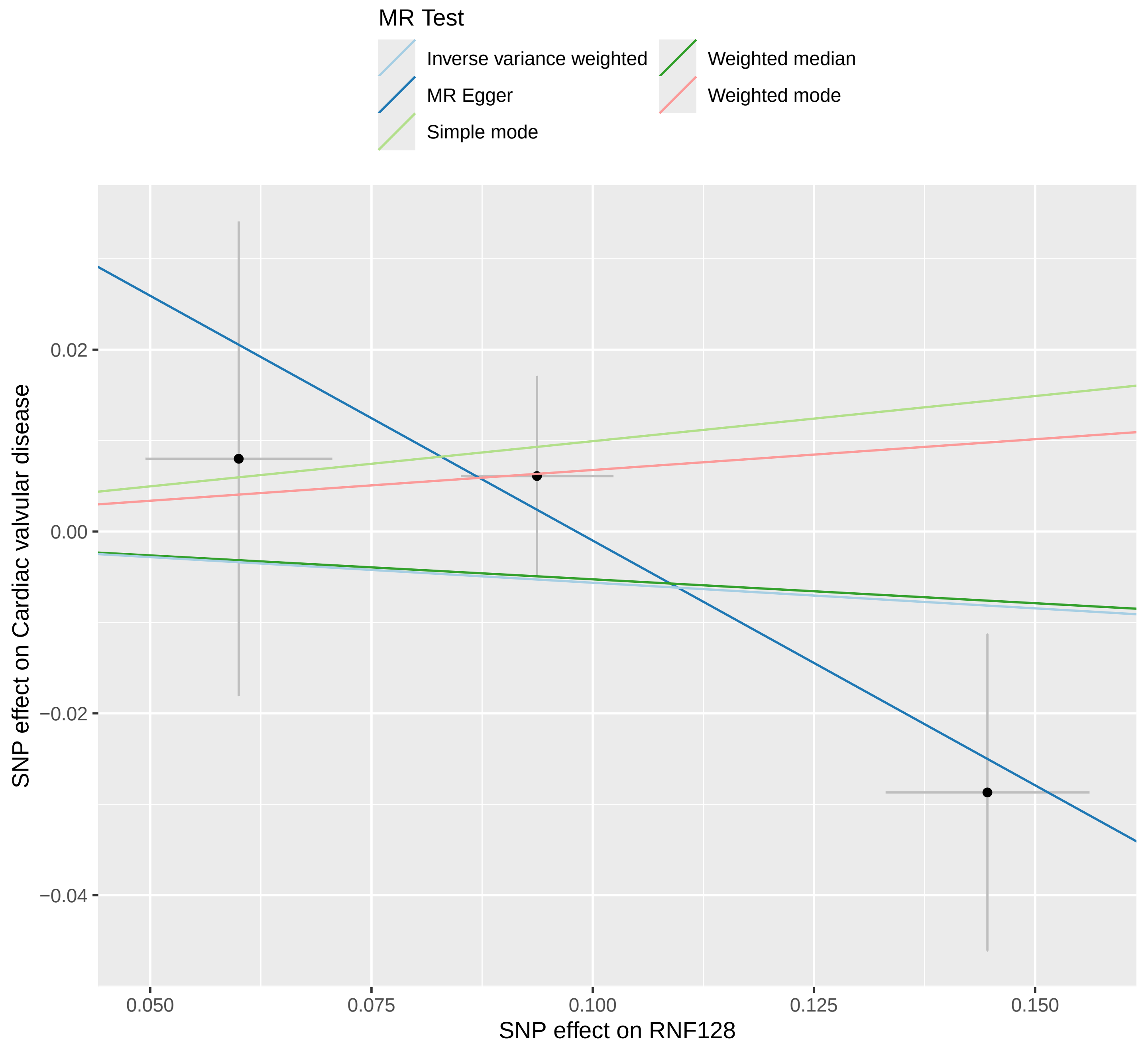
MR Test
Inverse variance weighted
MR Egger
Simple mode
Weighted median
Weighted mode
0.02
0.00
−0.02
−0.04
0.050
0.075
0.100
0.125
0.150
SNP effect on Cardiac valvular disease
SNP effect on RNF128

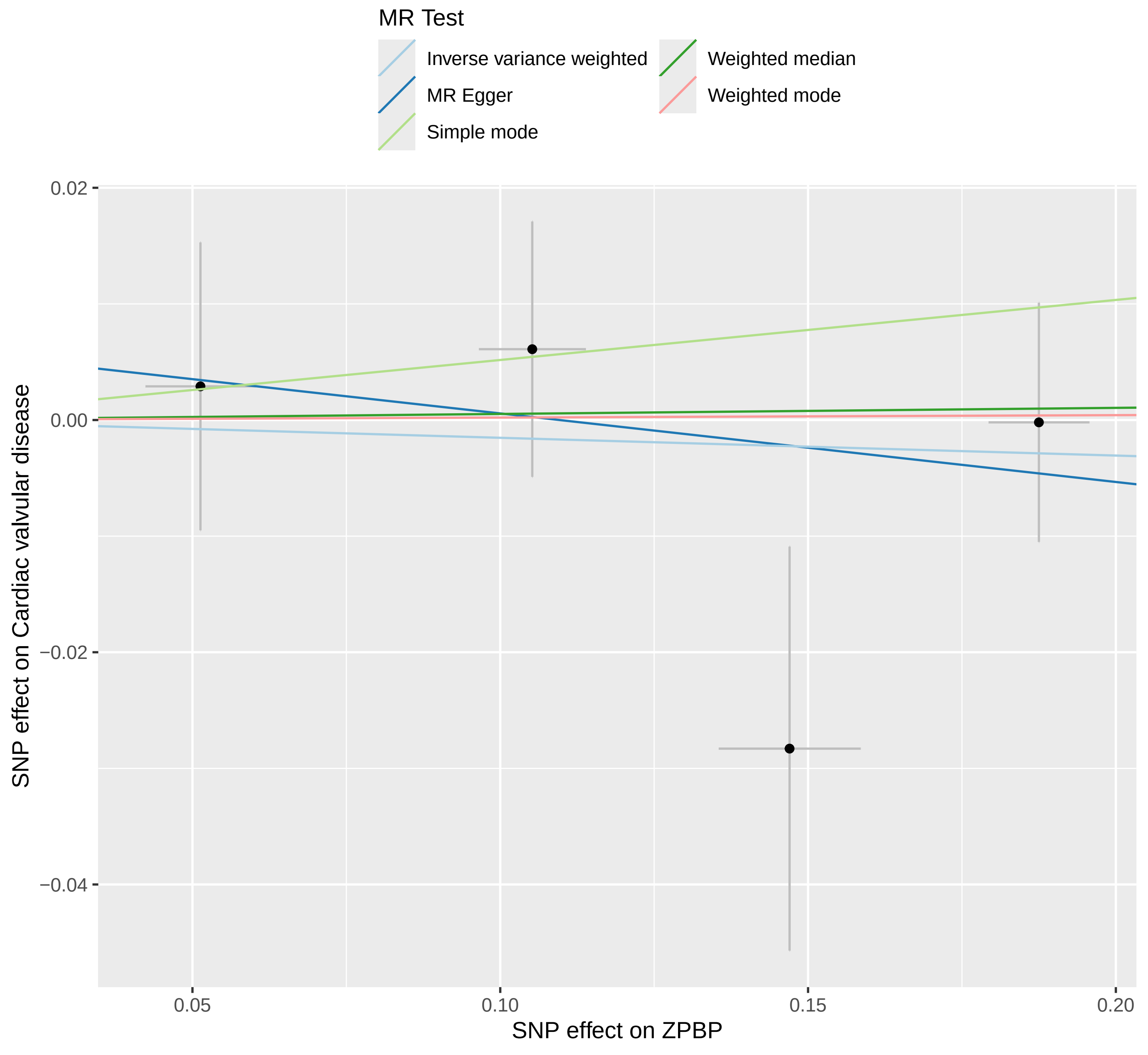
MR Test
Inverse variance weighted
MR Egger
Simple mode
Weighted median
Weighted mode
0.02
0.00
−0.02
−0.04
0.05
0.10
0.15
0.20
SNP effect on Cardiac valvular disease
SNP effect on ZPBP

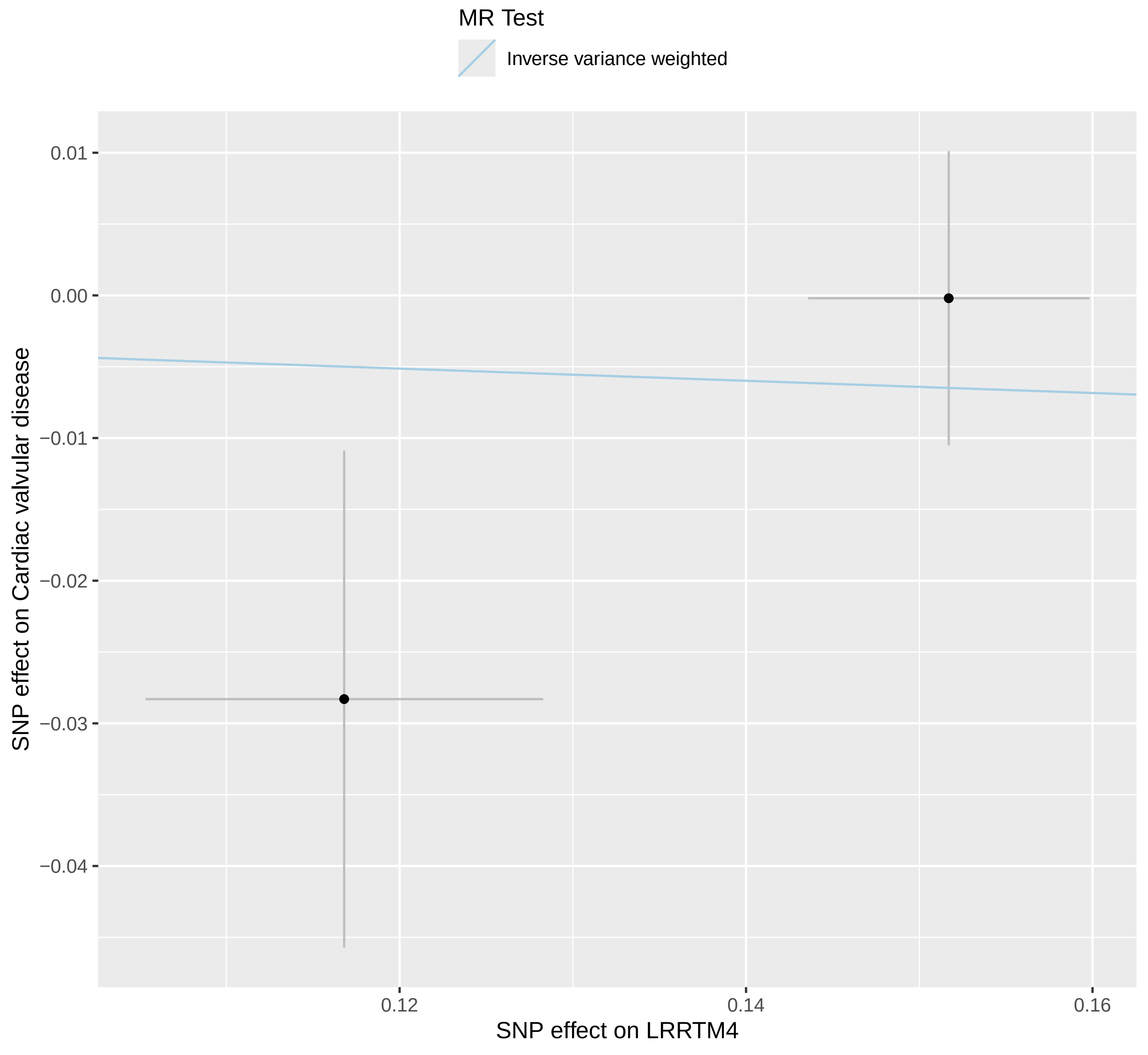

MR Test
Inverse variance weighted
0.01
0.00
−0.01
−0.02
−0.03
−0.04
SNP effect on Cardiac valvular disease
0.12
0.14
0.16
SNP effect on LRRTM4

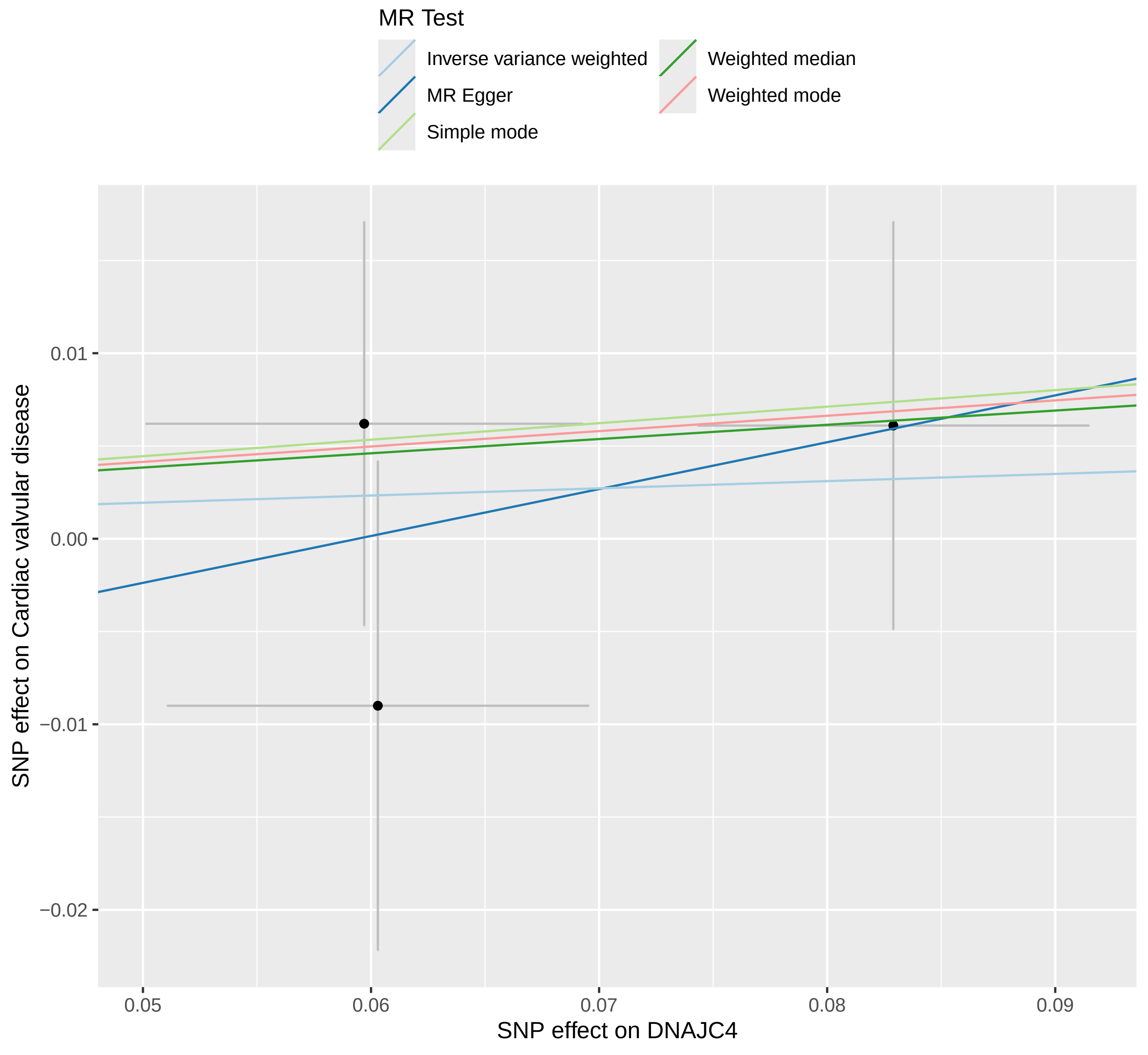

MR Test
Inverse variance weighted
MR Egger
Simple mode
Weighted median
Weighted mode
0.01
0.00
−0.01
−0.02
SNP effect on Cardiac valvular disease
0.05
0.06
0.07
0.08
0.09
SNP effect on DNAJC4

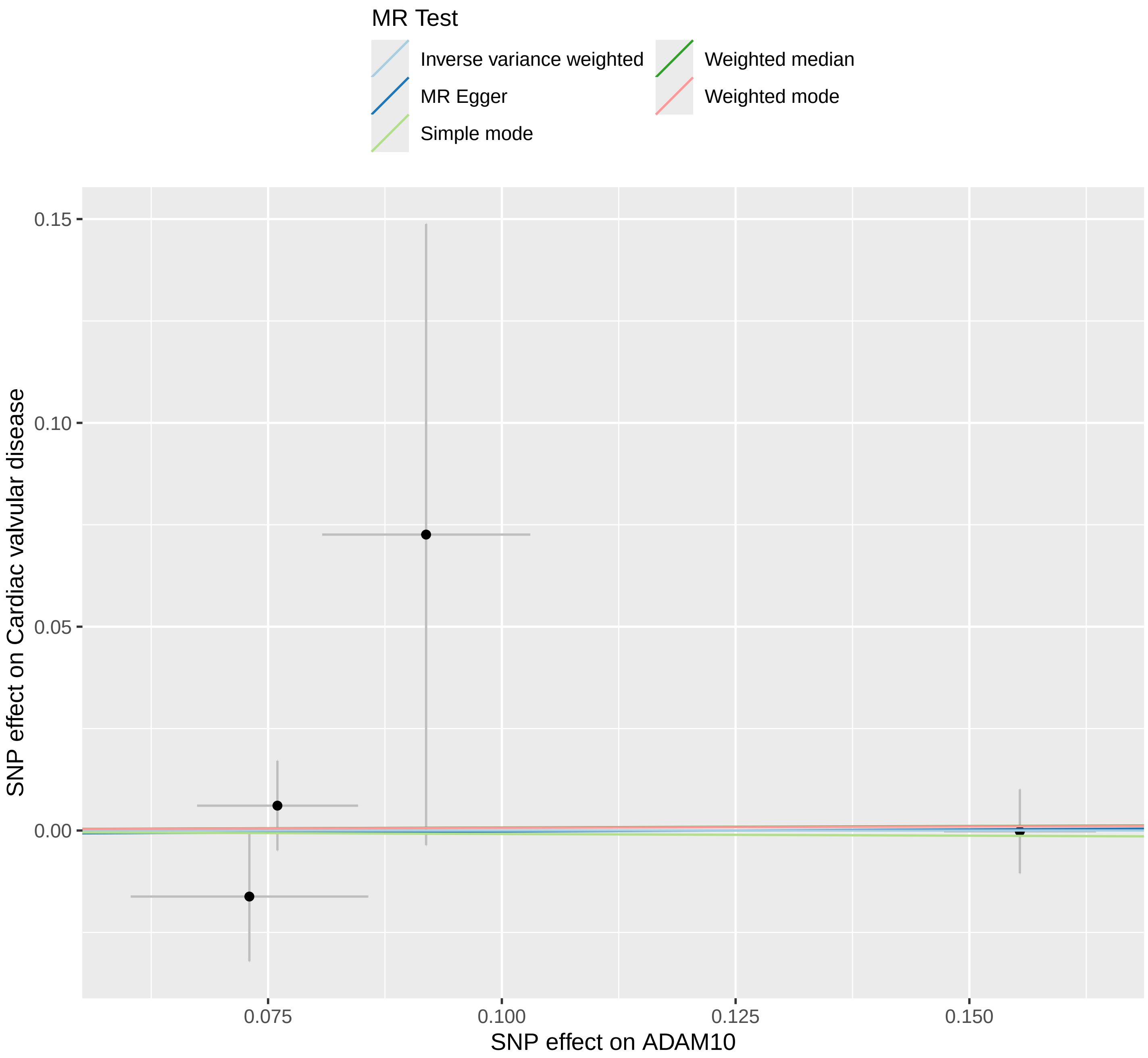
MR Test
Inverse variance weighted
MR Egger
Simple mode
Weighted median
Weighted mode
0.15
0.10
0.05
0.00
SNP effect on Cardiac valvular disease
0.075
0.100
0.125
0.150
SNP effect on ADAM10

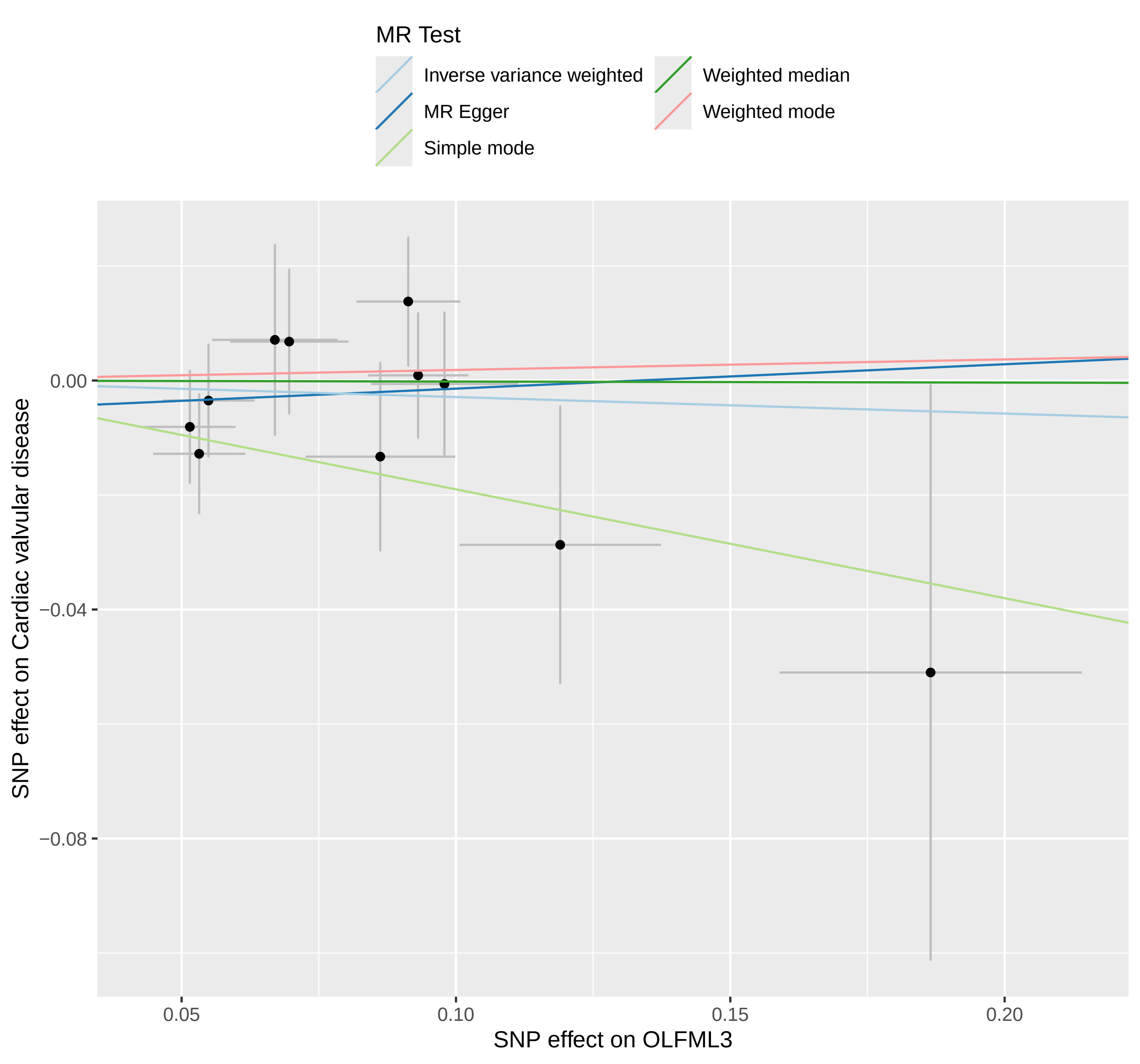
MR Test
Inverse variance weighted
MR Egger
Simple mode
Weighted median
Weighted mode
0.00
−0.04
−0.08
0.05
0.10
0.15
0.20
SNP effect on OLFML3
SNP effect on Cardiac valvular disease

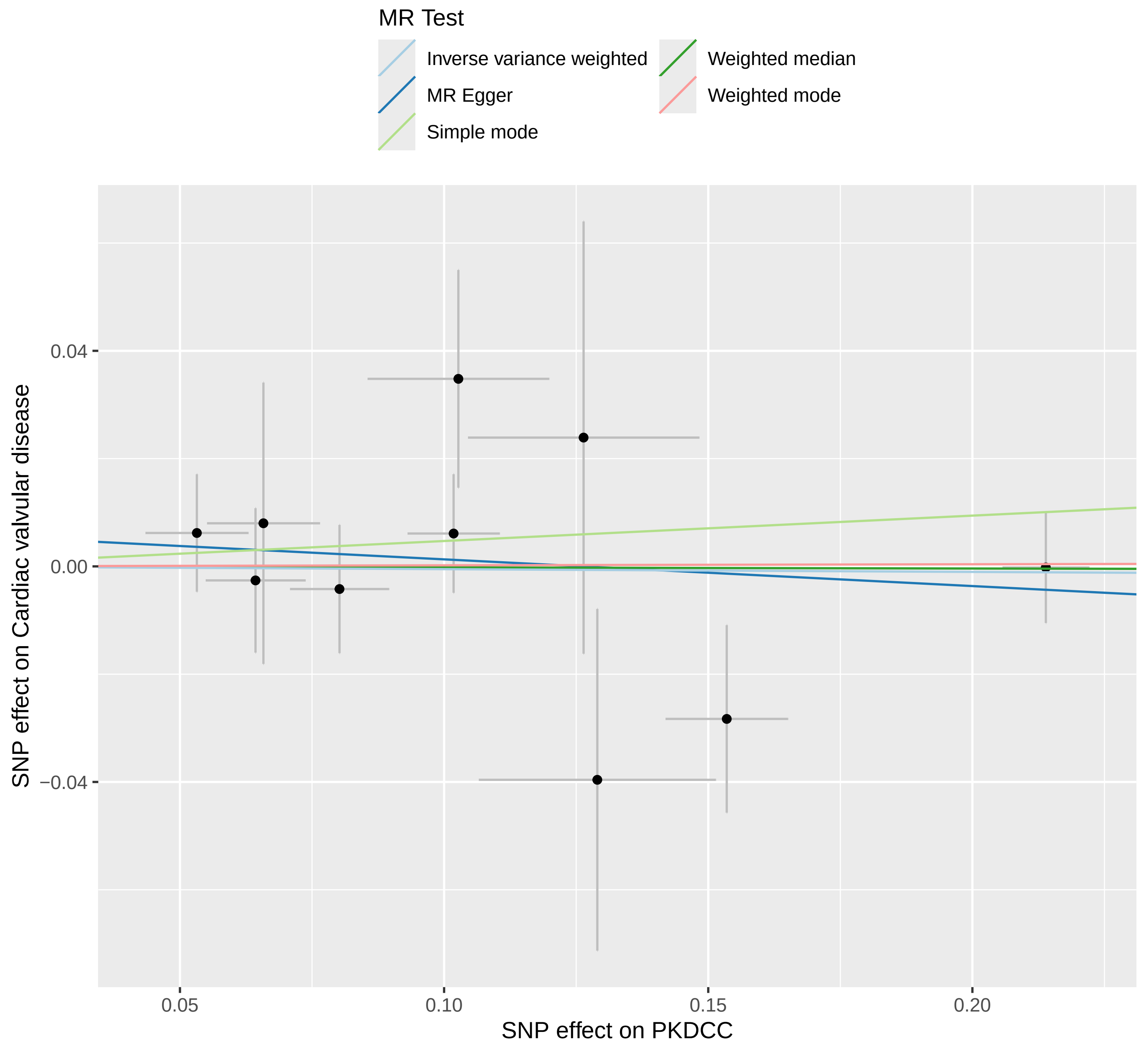
MR Test
Inverse variance weighted
MR Egger
Simple mode
Weighted median
Weighted mode
0.04
0.00
−0.04
0.05
0.10
0.15
0.20
SNP effect on Cardiac valvular disease
SNP effect on PKDCC

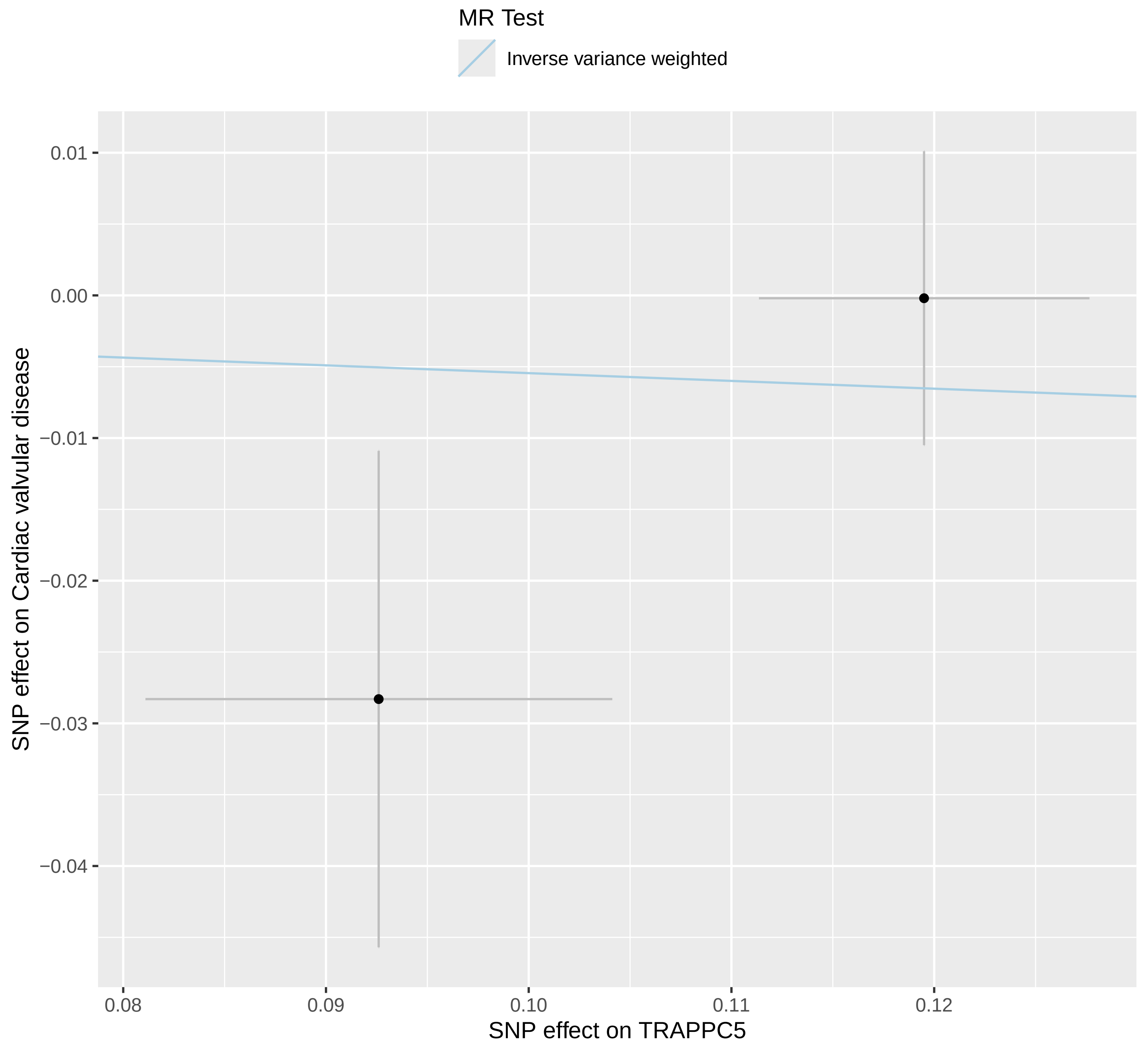
MR Test
Inverse variance weighted
0.01
0.00
−0.01
−0.02
−0.03
−0.04
SNP effect on Cardiac valvular disease
0.08
0.09
0.10
0.11
0.12
SNP effect on TRAPPC5

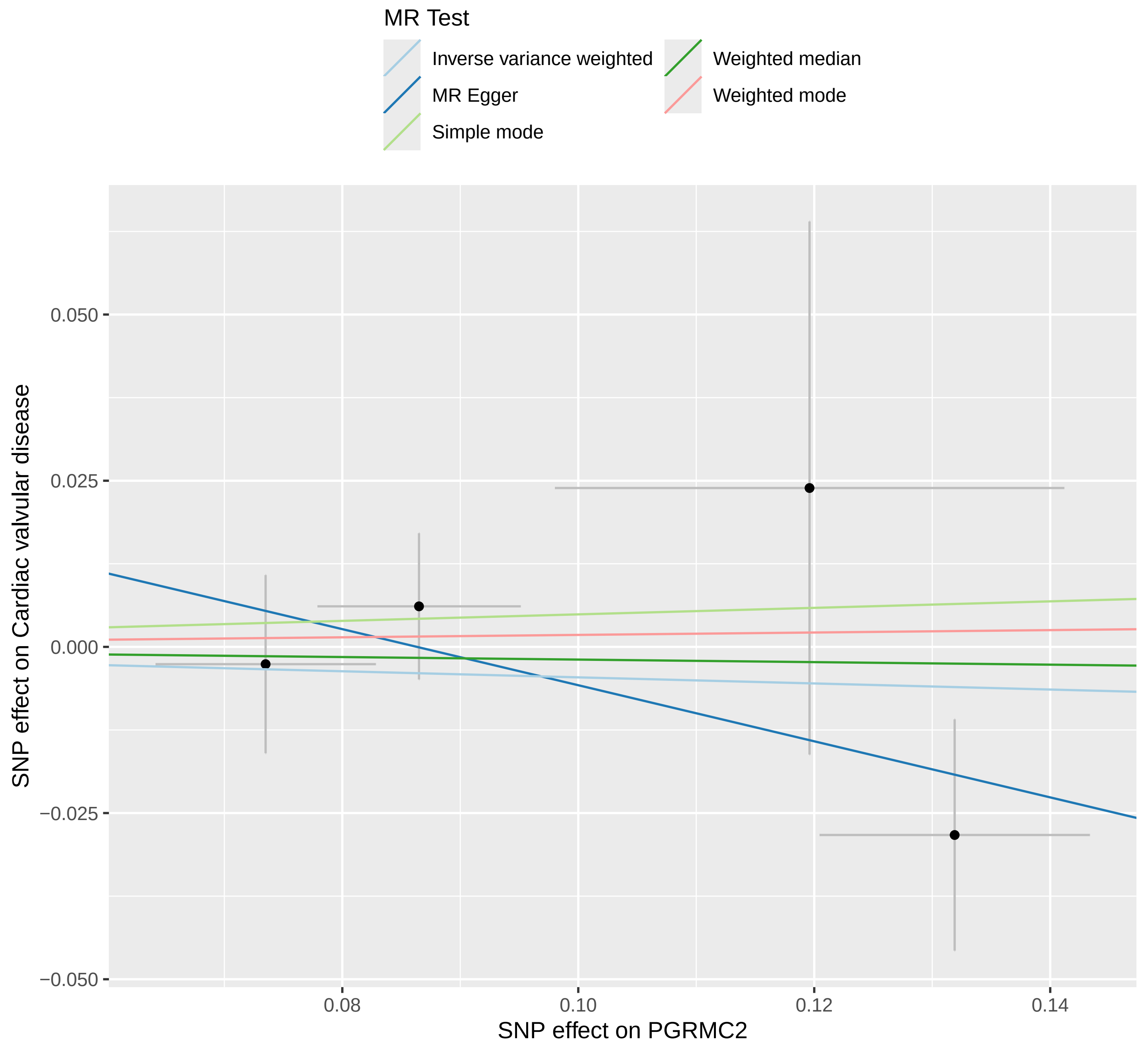
MR Test
Inverse variance weighted
MR Egger
Simple mode
Weighted median
Weighted mode
0.050
0.025
0.000
−0.025
−0.050
SNP effect on Cardiac valvular disease
0.08
0.10
0.12
0.14
SNP effect on PGRMC2

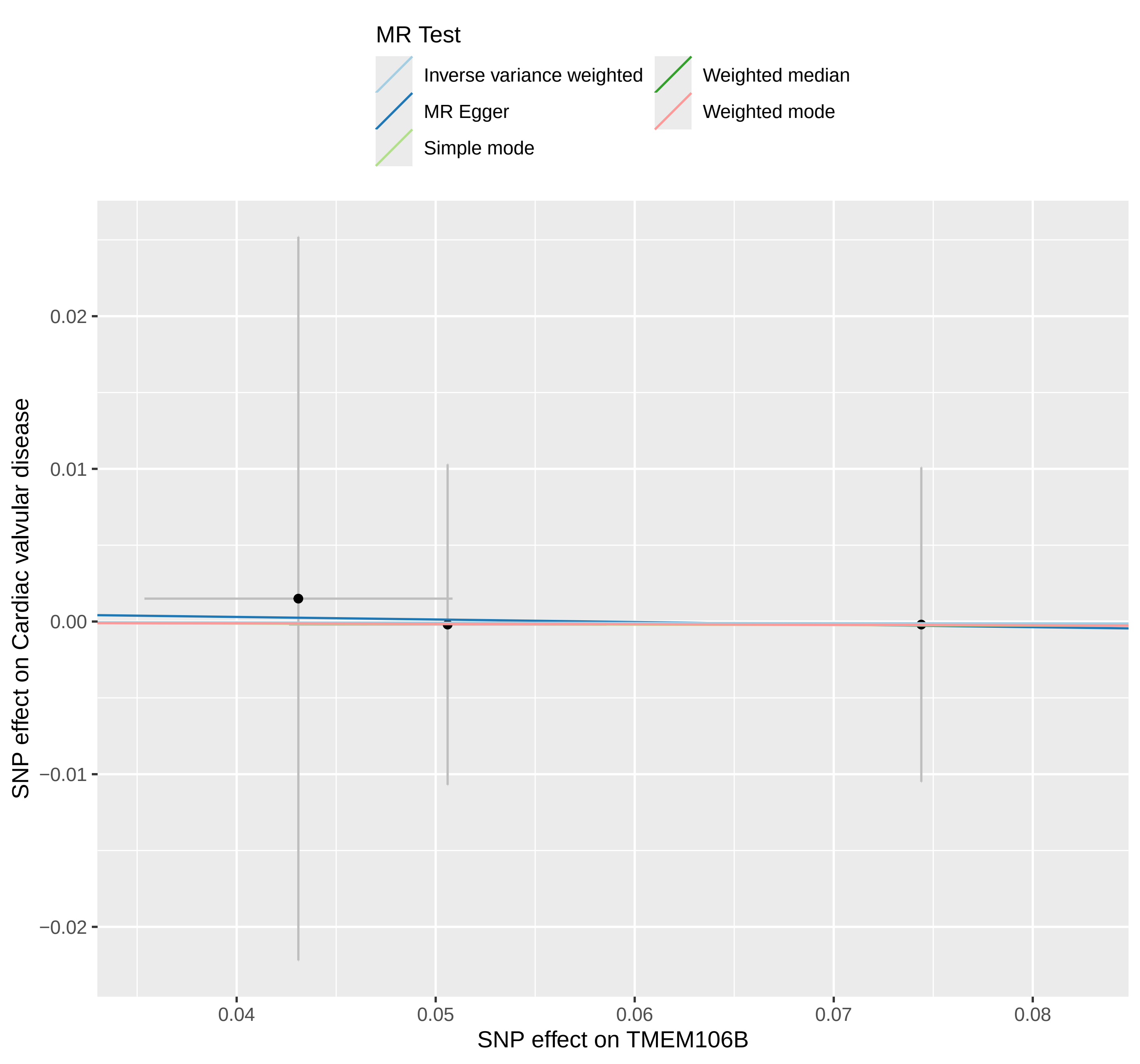
MR Test
Inverse variance weighted
MR Egger
Simple mode
Weighted median
Weighted mode
0.02
0.01
0.00
−0.01
−0.02
SNP effect on Cardiac valvular disease
0.04
0.05
0.06
0.07
0.08
SNP effect on TMEM106B

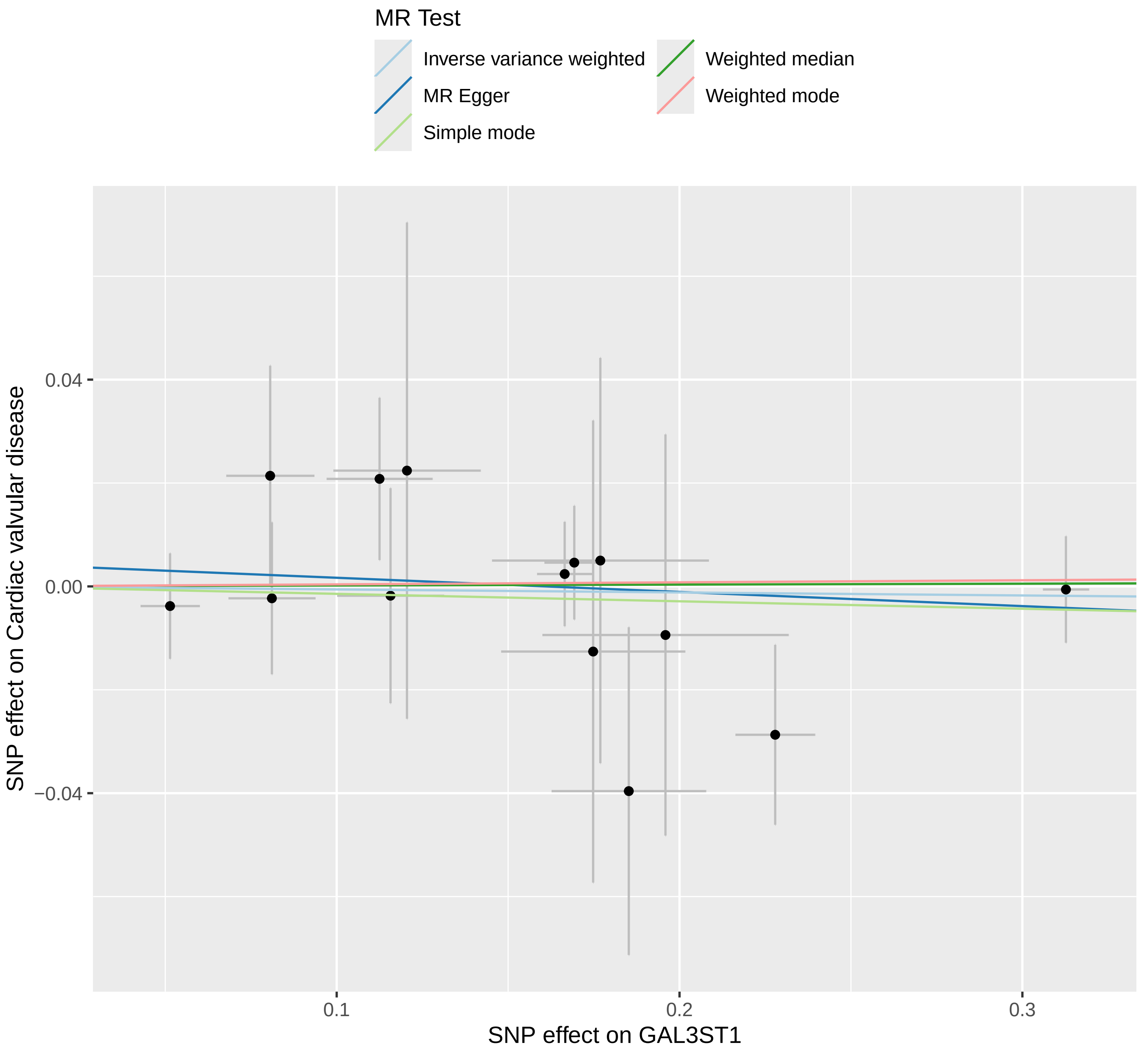
MR Test
Inverse variance weighted
MR Egger
Simple mode
Weighted median
Weighted mode
0.04
0.00
−0.04
0.1
0.2
0.3
SNP effect on Cardiac valvular disease
SNP effect on GAL3ST1

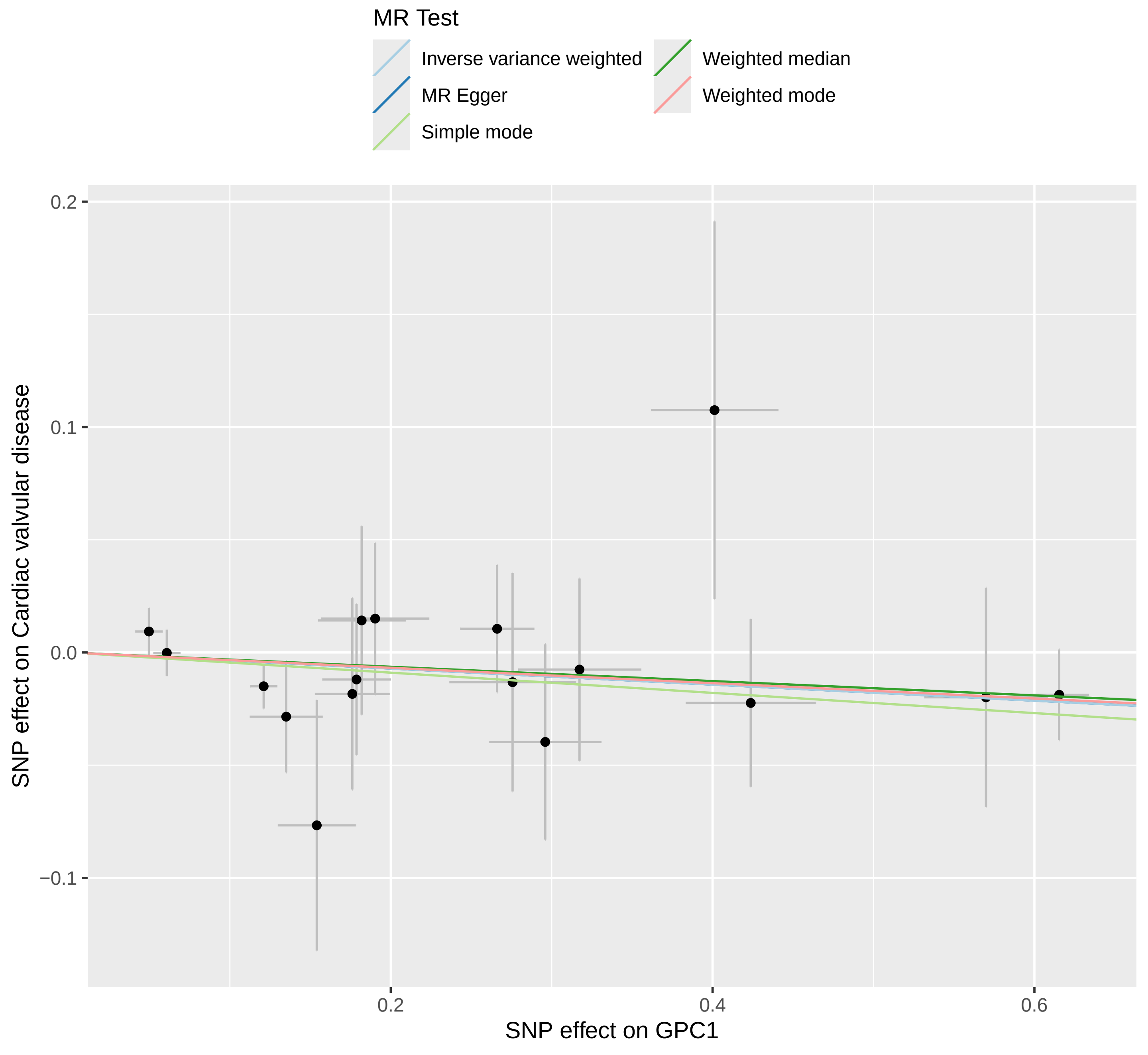
MR Test
Inverse variance weighted
MR Egger
Simple mode
Weighted median
Weighted mode
0.2
0.1
0.0
−0.1
SNP effect on Cardiac valvular disease
0.2
0.4
0.6
SNP effect on GPC1

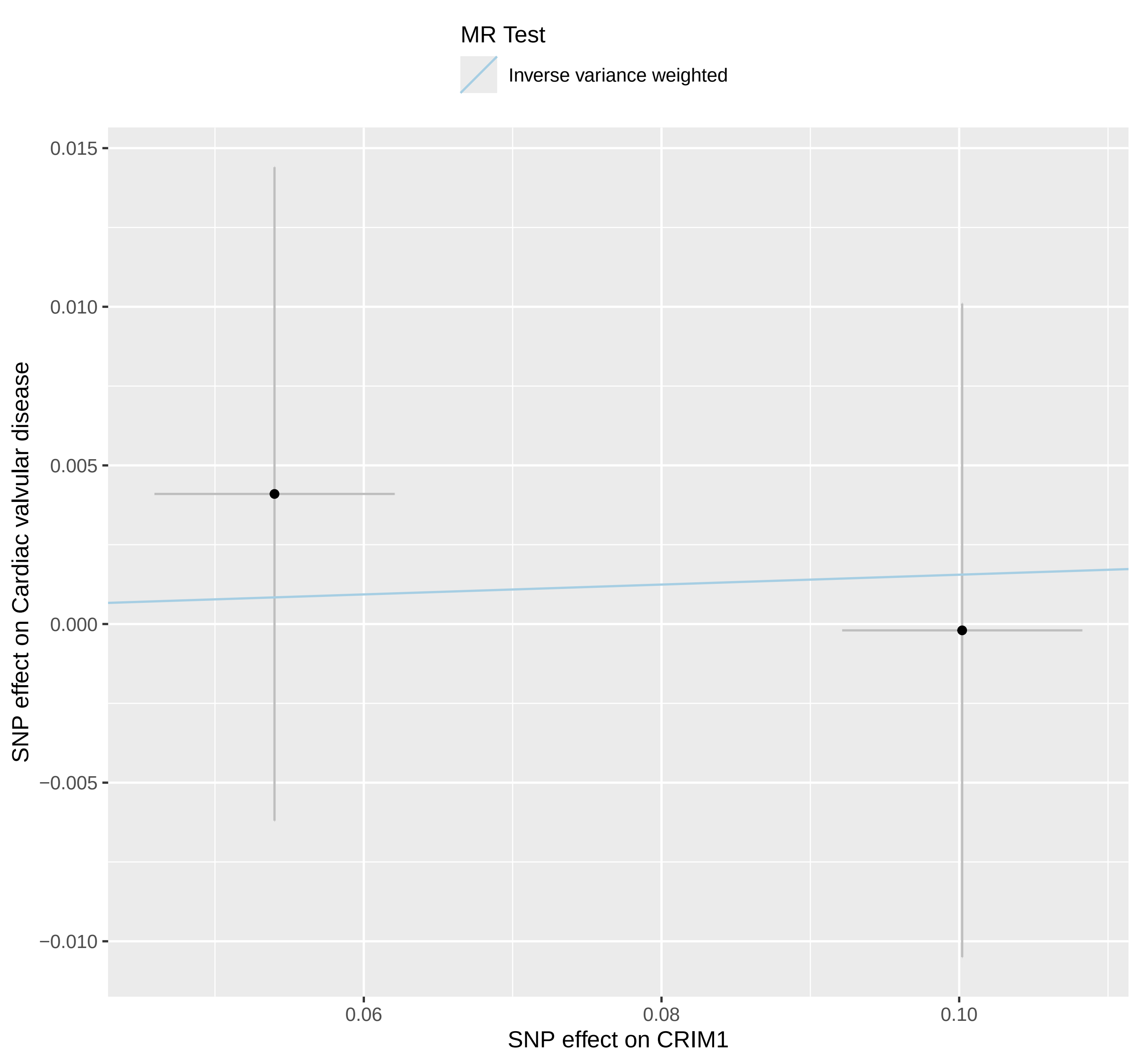

MR Test
Inverse variance weighted
0.015
0.010
0.005
0.000
−0.005
−0.010
SNP effect on Cardiac valvular disease
0.06
0.08
0.10
SNP effect on CRIM1

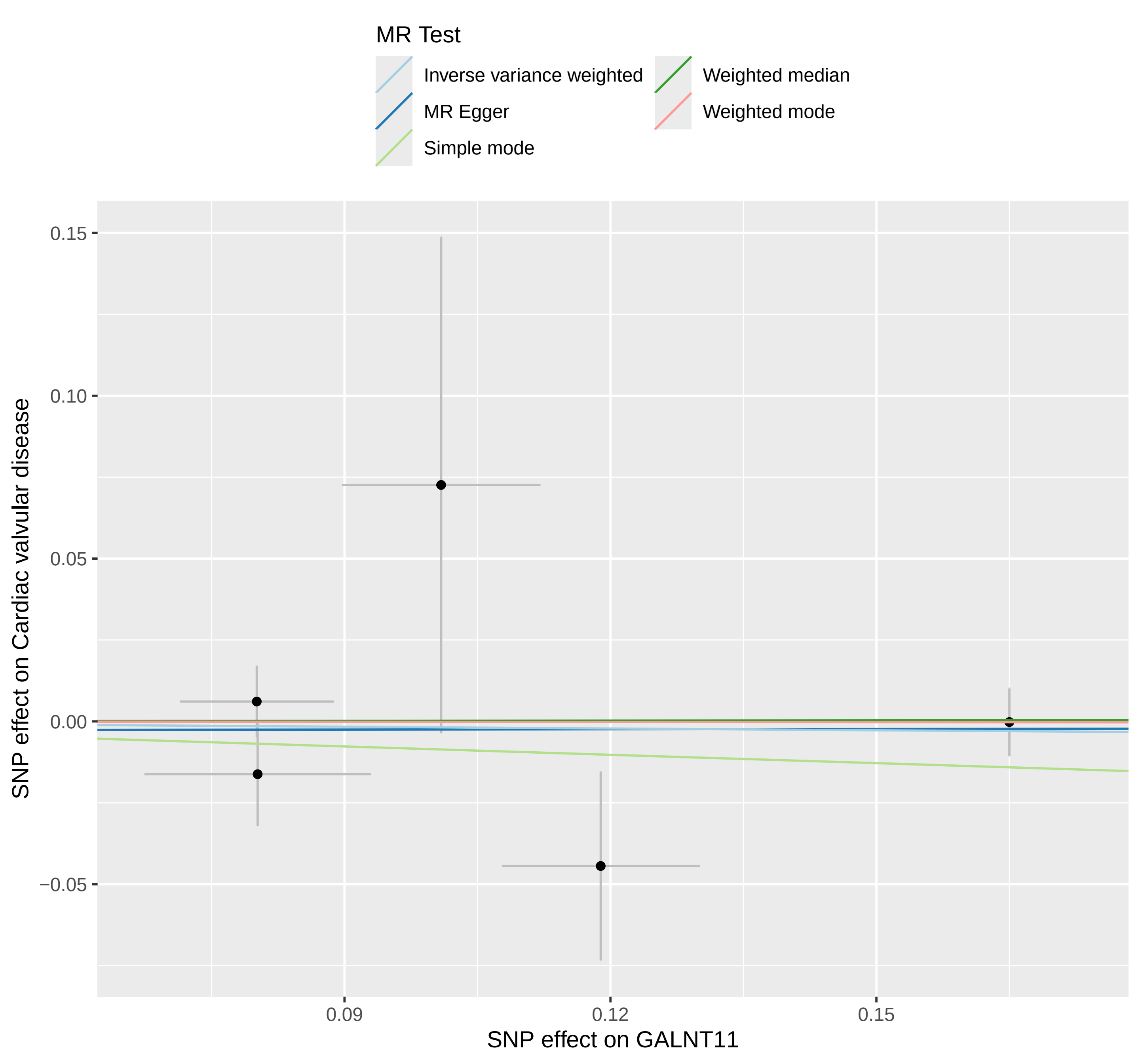

MR Test
Inverse variance weighted
MR Egger
Simple mode
Weighted median
Weighted mode
0.15
0.10
0.05
0.00
−0.05
0.09
0.12
0.15
SNP effect on Cardiac valvular disease
SNP effect on GALNT11

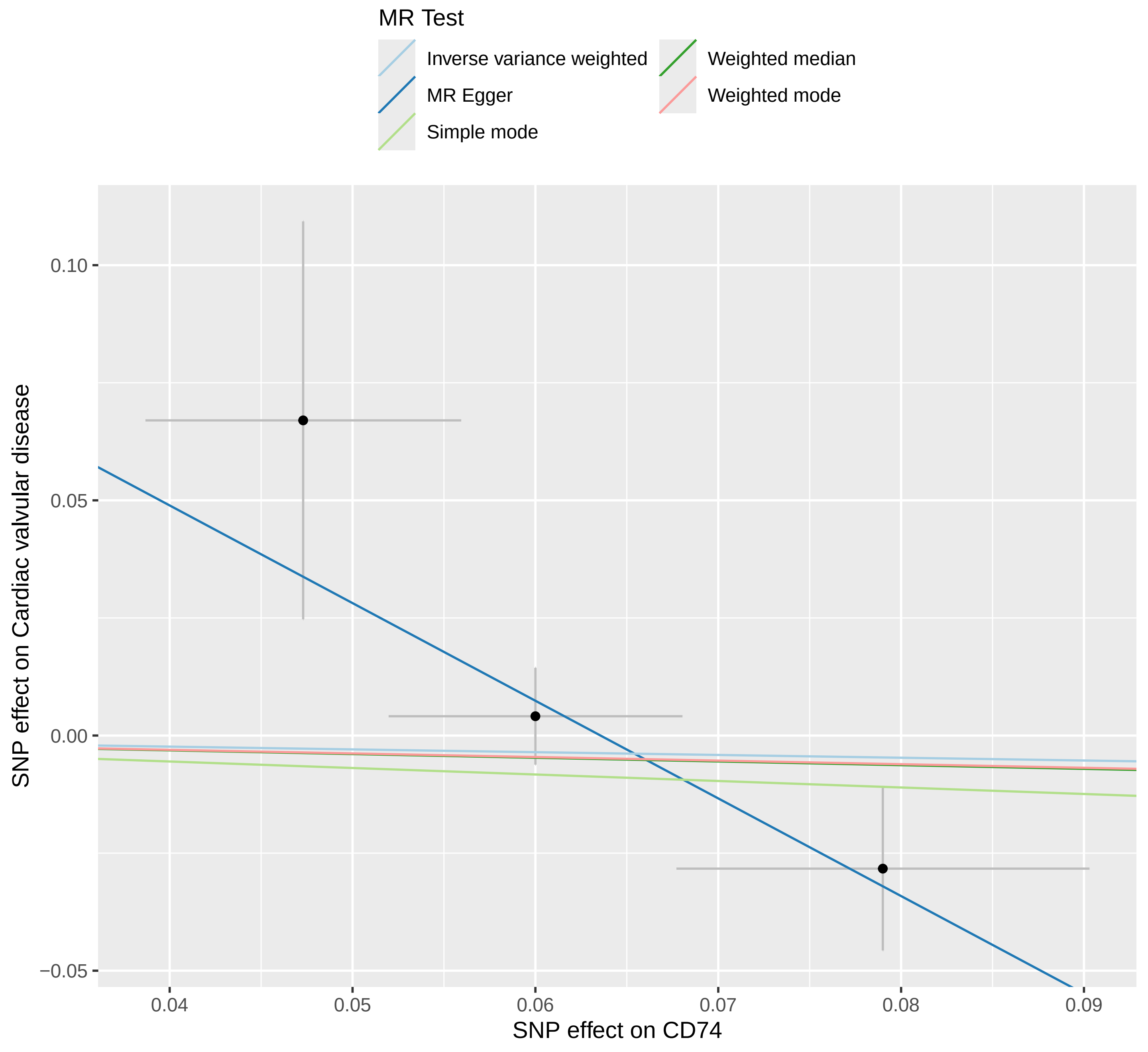
MR Test
Inverse variance weighted
MR Egger
Simple mode
Weighted median
Weighted mode
0.10
0.05
0.00
−0.05
SNP effect on Cardiac valvular disease
0.04
0.05
0.06
0.07
0.08
0.09
SNP effect on CD74

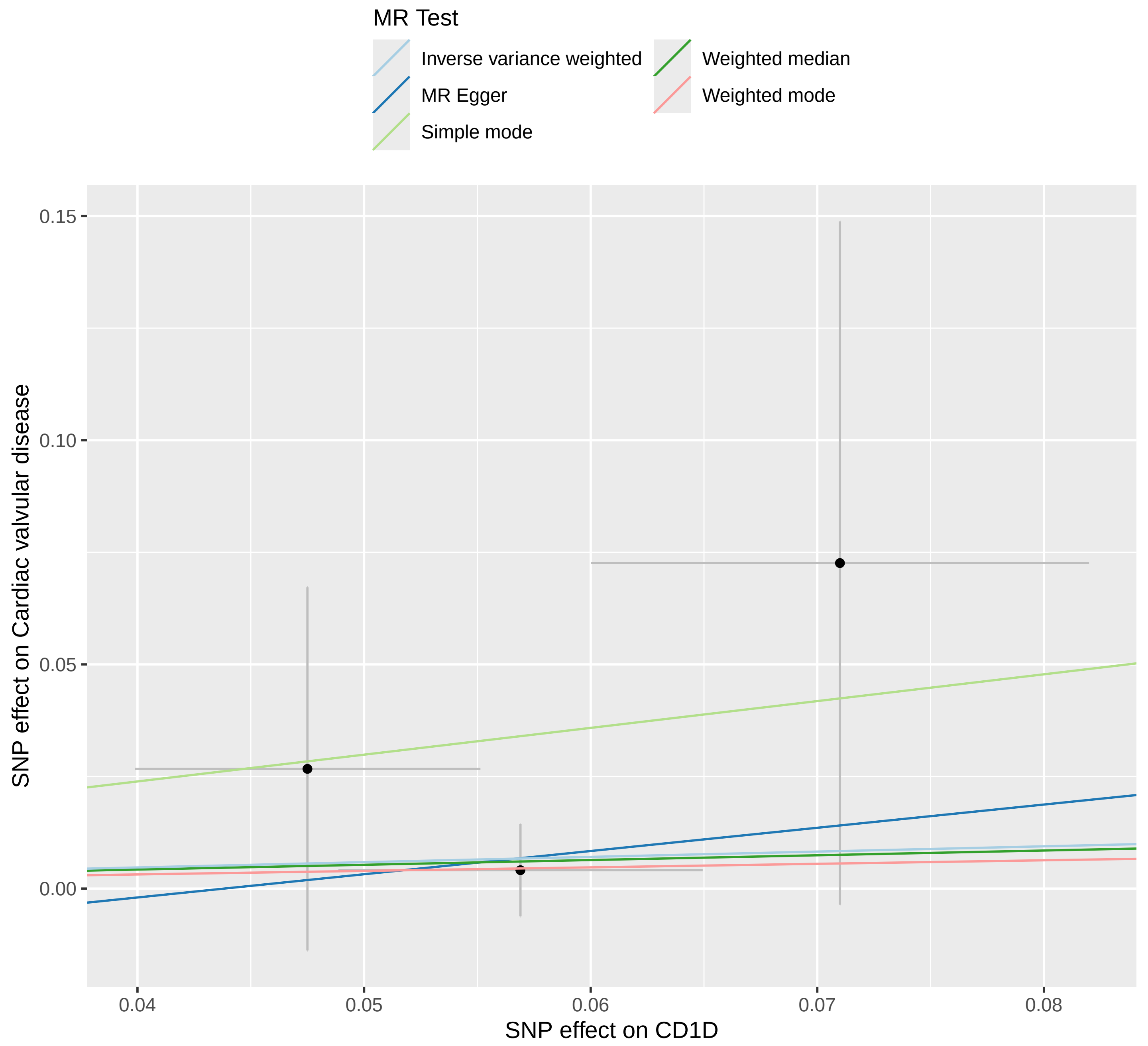
MR Test
Inverse variance weighted
MR Egger
Simple mode
Weighted median
Weighted mode
0.15
0.10
0.05
0.00
SNP effect on Cardiac valvular disease
0.04
0.05
0.06
0.07
0.08
SNP effect on CD1D

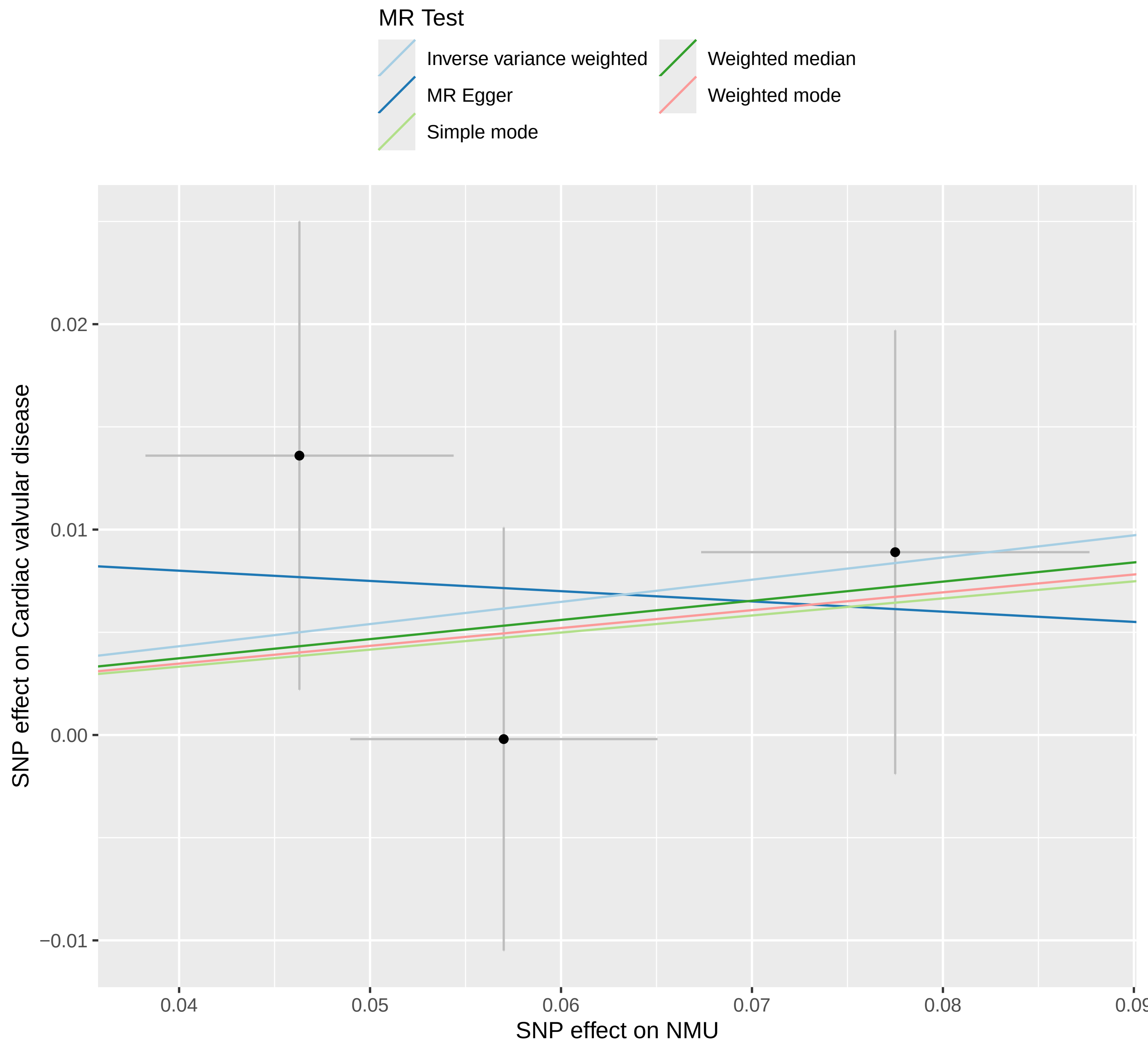
MR Test
Inverse variance weighted
MR Egger
Simple mode
Weighted median
Weighted mode
0.02
0.01
0.00
−0.01
0.04
0.05
0.06
0.07
0.08
0.09
SNP effect on Cardiac valvular disease
SNP effect on NMU

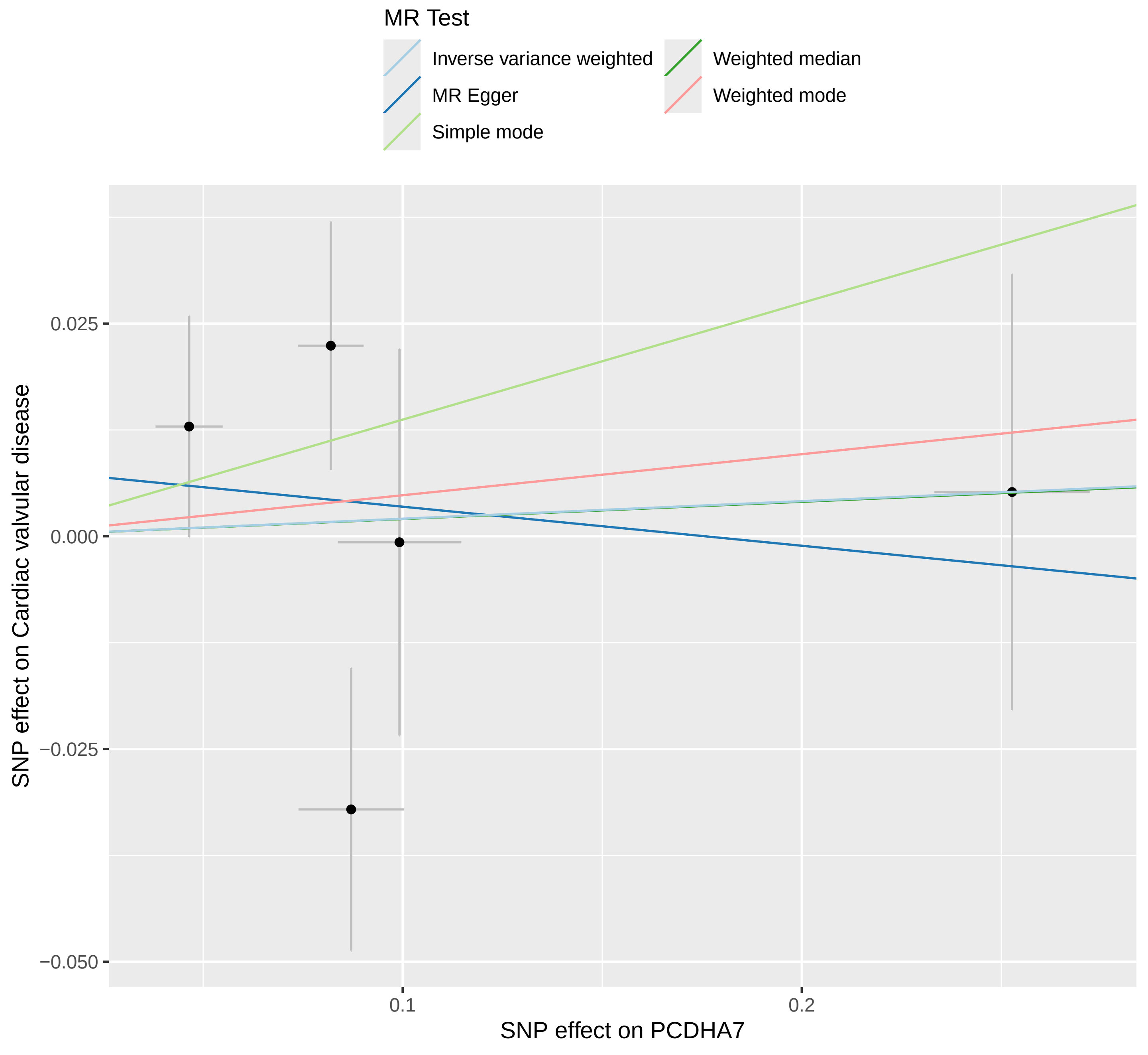
MR Test
Inverse variance weighted
MR Egger
Simple mode
Weighted median
Weighted mode
0.025
0.000
−0.025
−0.050
SNP effect on Cardiac valvular disease
0.1
0.2
SNP effect on PCDHA7

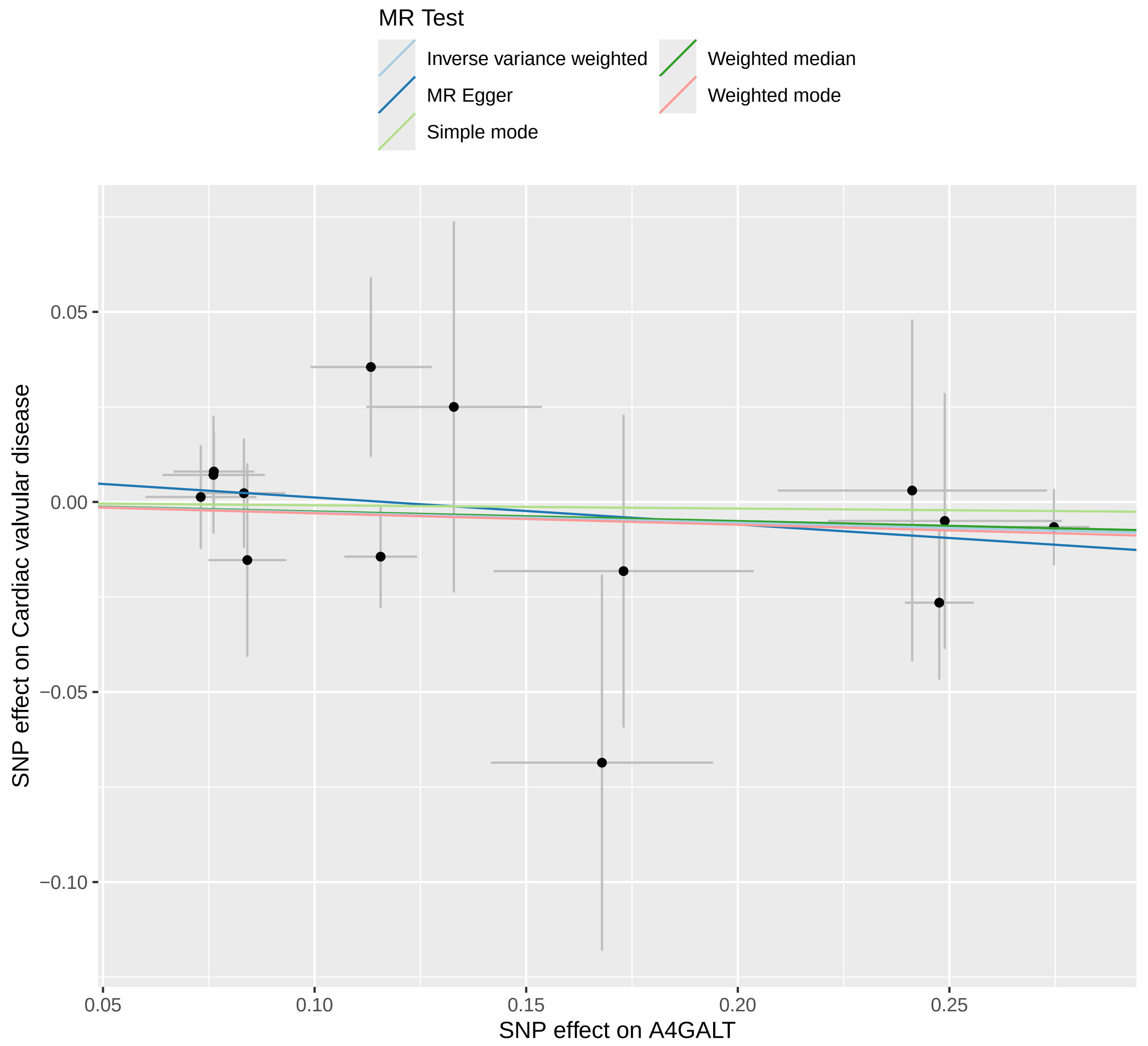

MR Test
Inverse variance weighted
MR Egger
Simple mode
Weighted median
Weighted mode
0.05
0.00
−0.05
−0.10
SNP effect on Cardiac valvular disease
0.05
0.10
0.15
0.20
0.25
SNP effect on A4GALT

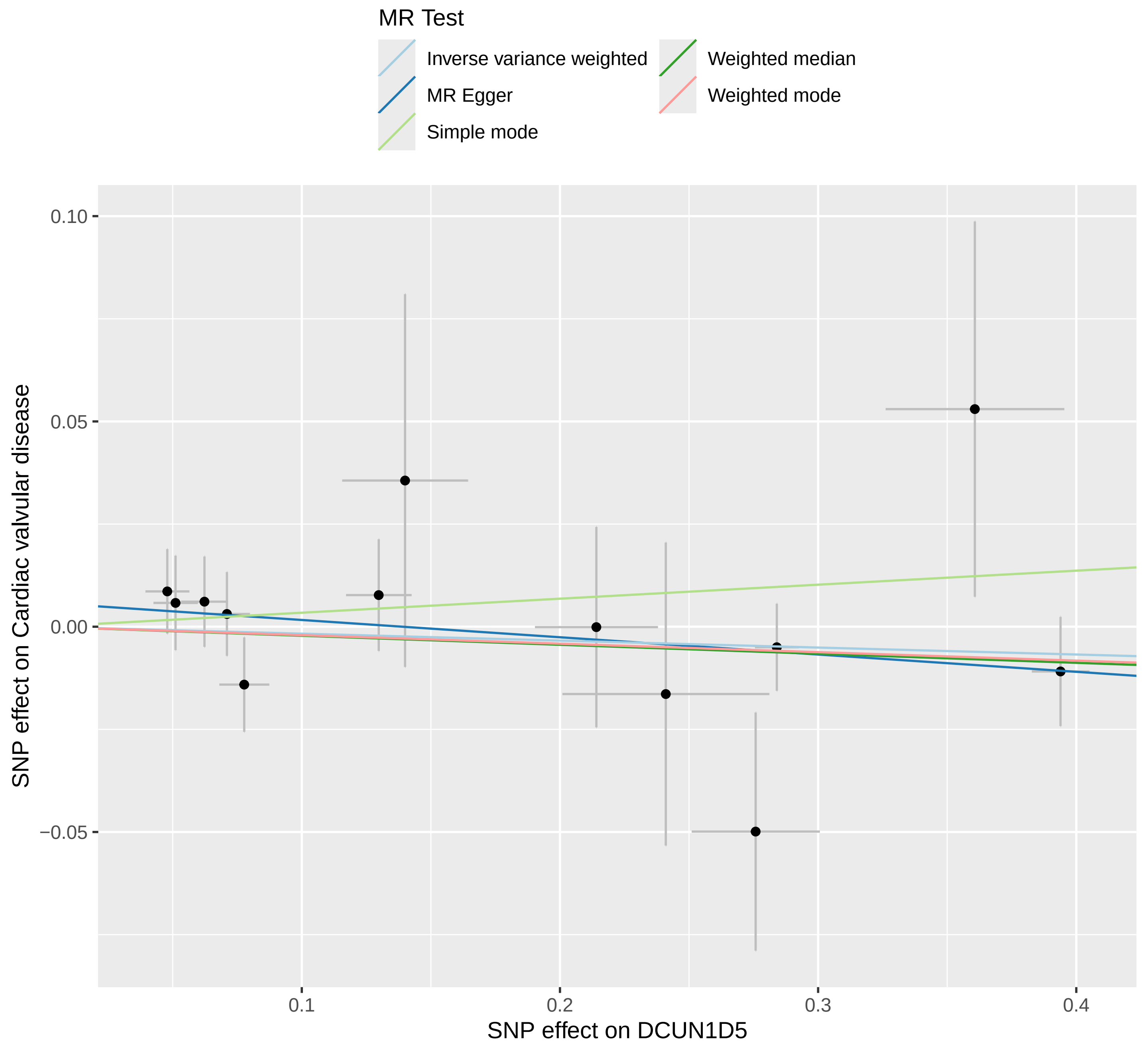
MR Test
Inverse variance weighted
MR Egger
Simple mode
Weighted median
Weighted mode
0.10
0.05
0.00
−0.05
SNP effect on Cardiac valvular disease
0.1
0.2
0.3
0.4
SNP effect on DCUN1D5

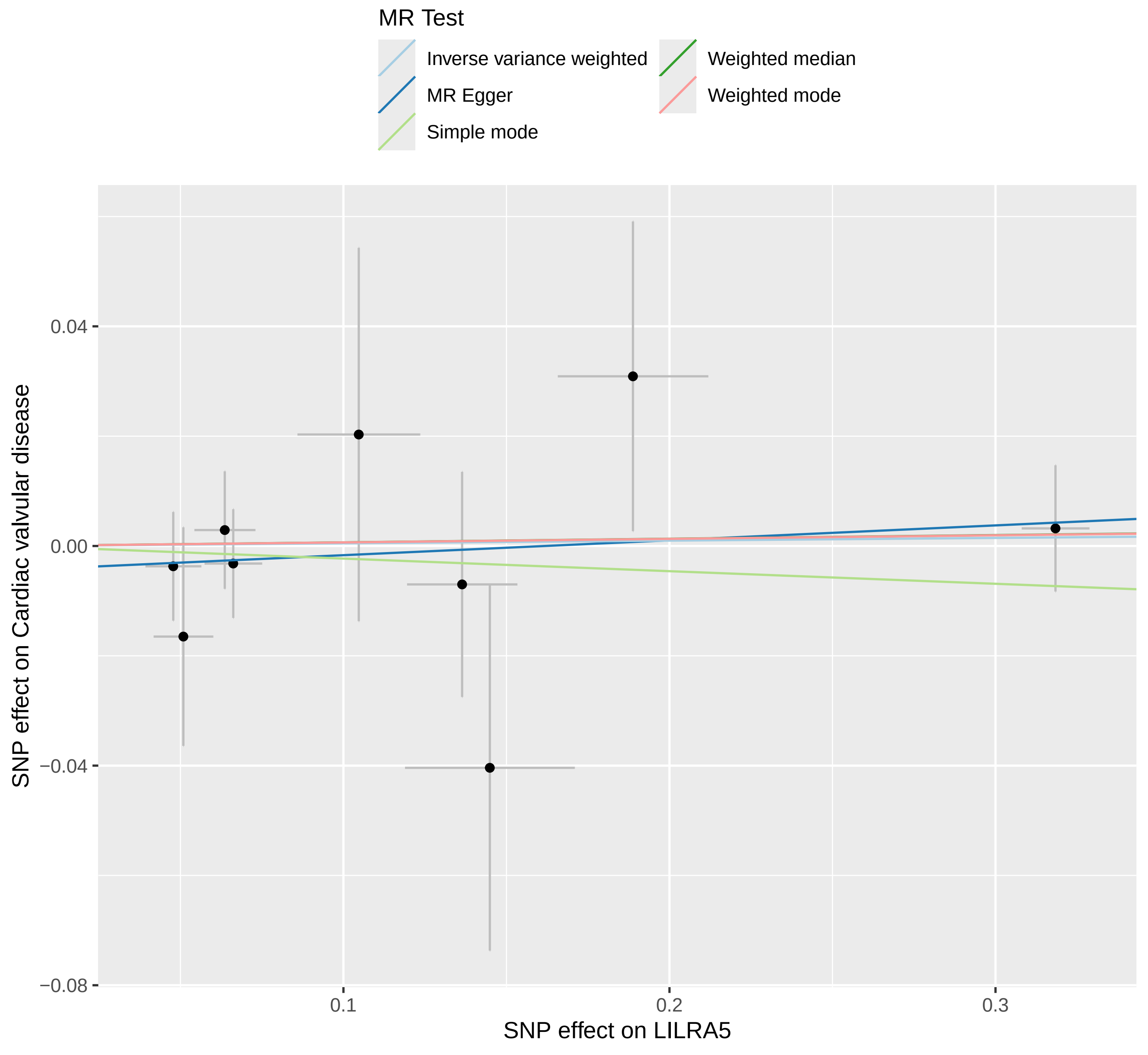
MR Test
Inverse variance weighted
MR Egger
Simple mode
Weighted median
Weighted mode
SNP effect on Cardiac valvular disease
0.04
0.00
−0.04
−0.08
0.1
0.2
0.3
SNP effect on LILRA5

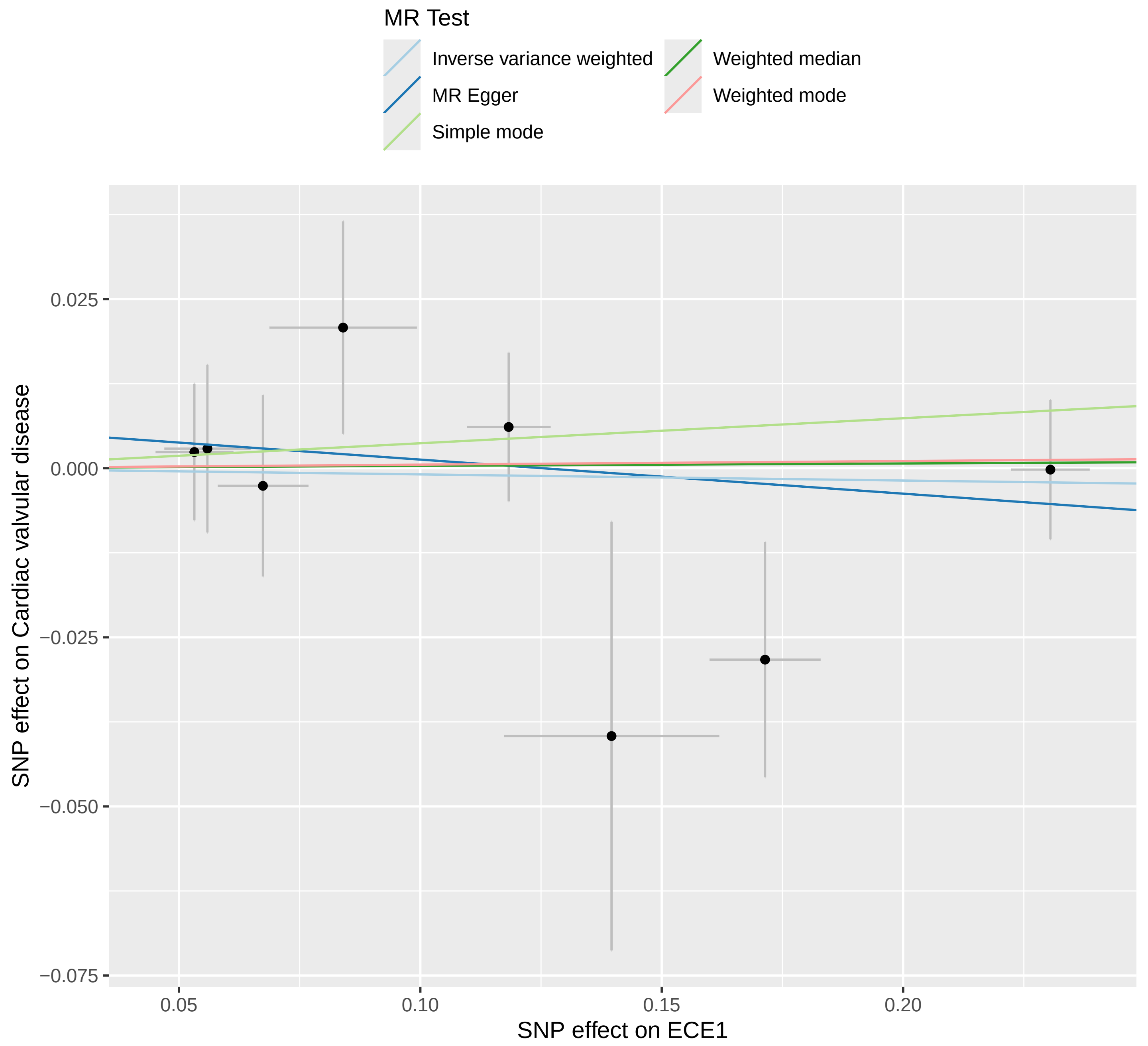
MR Test
Inverse variance weighted
MR Egger
Simple mode
Weighted median
Weighted mode
0.025
0.000
−0.025
−0.050
−0.075
SNP effect on Cardiac valvular disease
0.05
0.10
0.15
0.20
SNP effect on ECE1

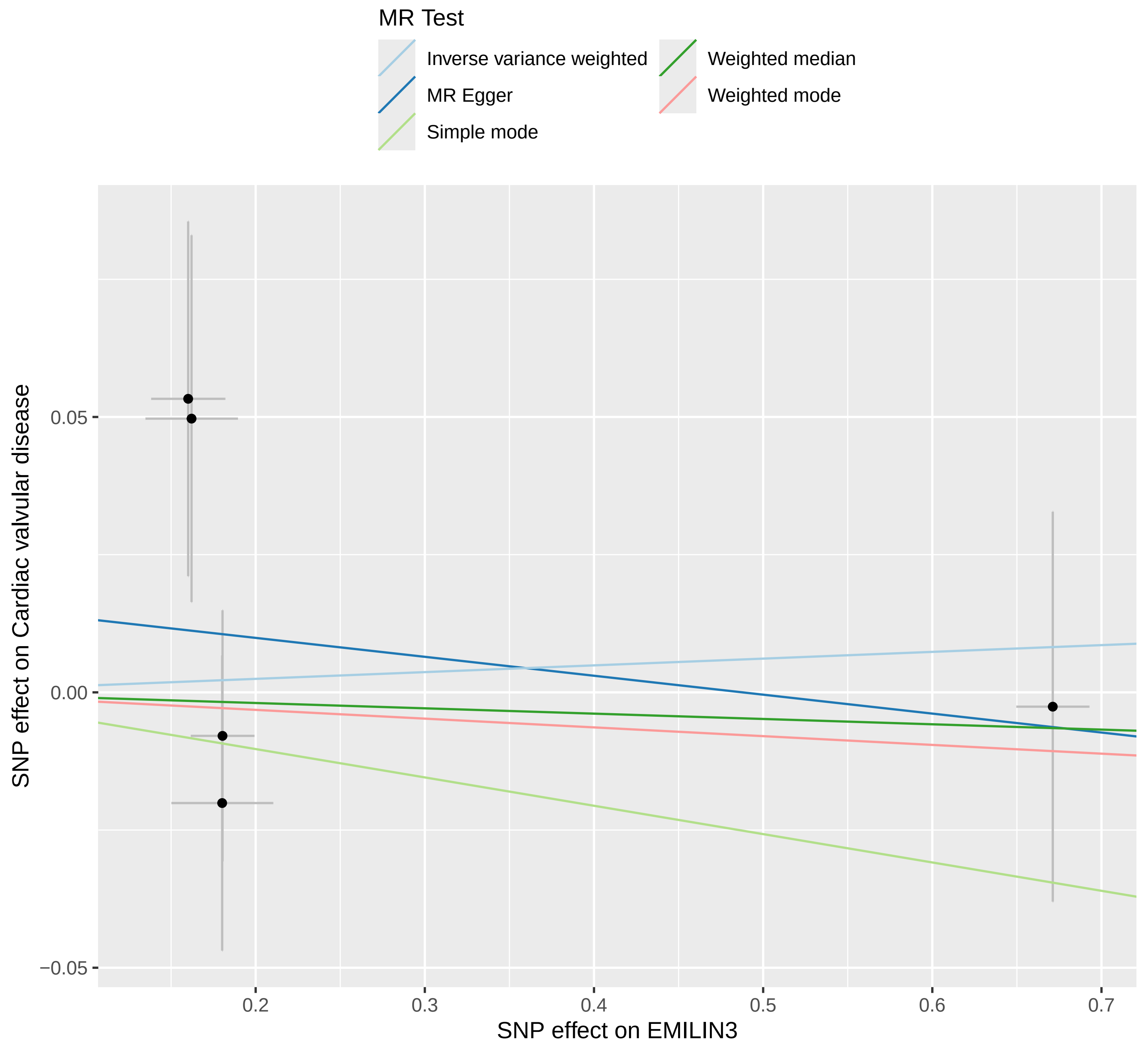
MR Test
Inverse variance weighted
MR Egger
Simple mode
Weighted median
Weighted mode
SNP effect on Cardiac valvular disease
0.05
0.00
−0.05
0.2
0.3
0.4
0.5
0.6
0.7
SNP effect on EMILIN3

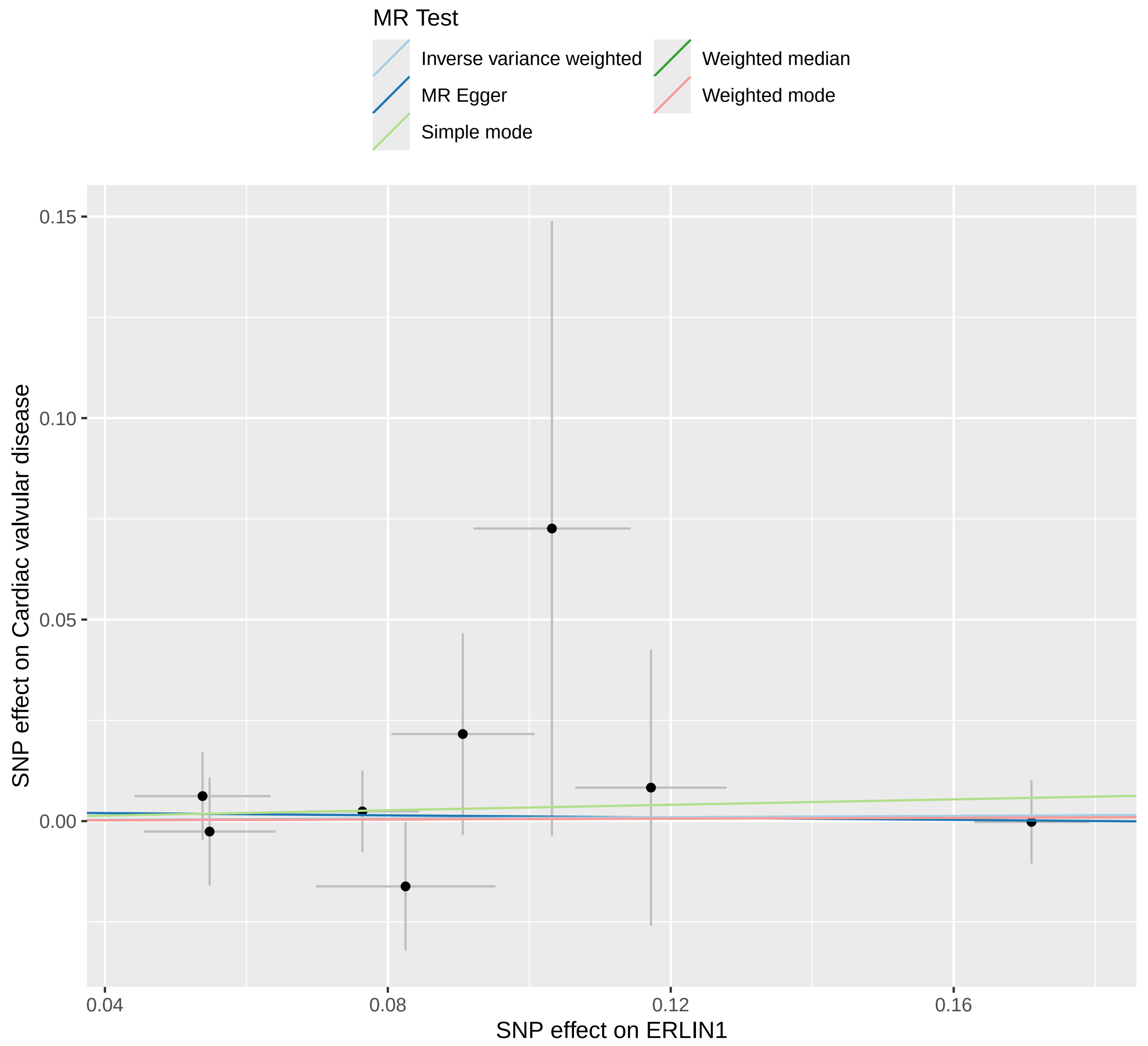
MR Test
Inverse variance weighted
Weighted median
MR Egger
Weighted mode
Simple mode
0.15
0.10
0.05
0.00
SNP effect on Cardiac valvular disease
0.04
0.08
0.12
0.16
SNP effect on ERLIN1

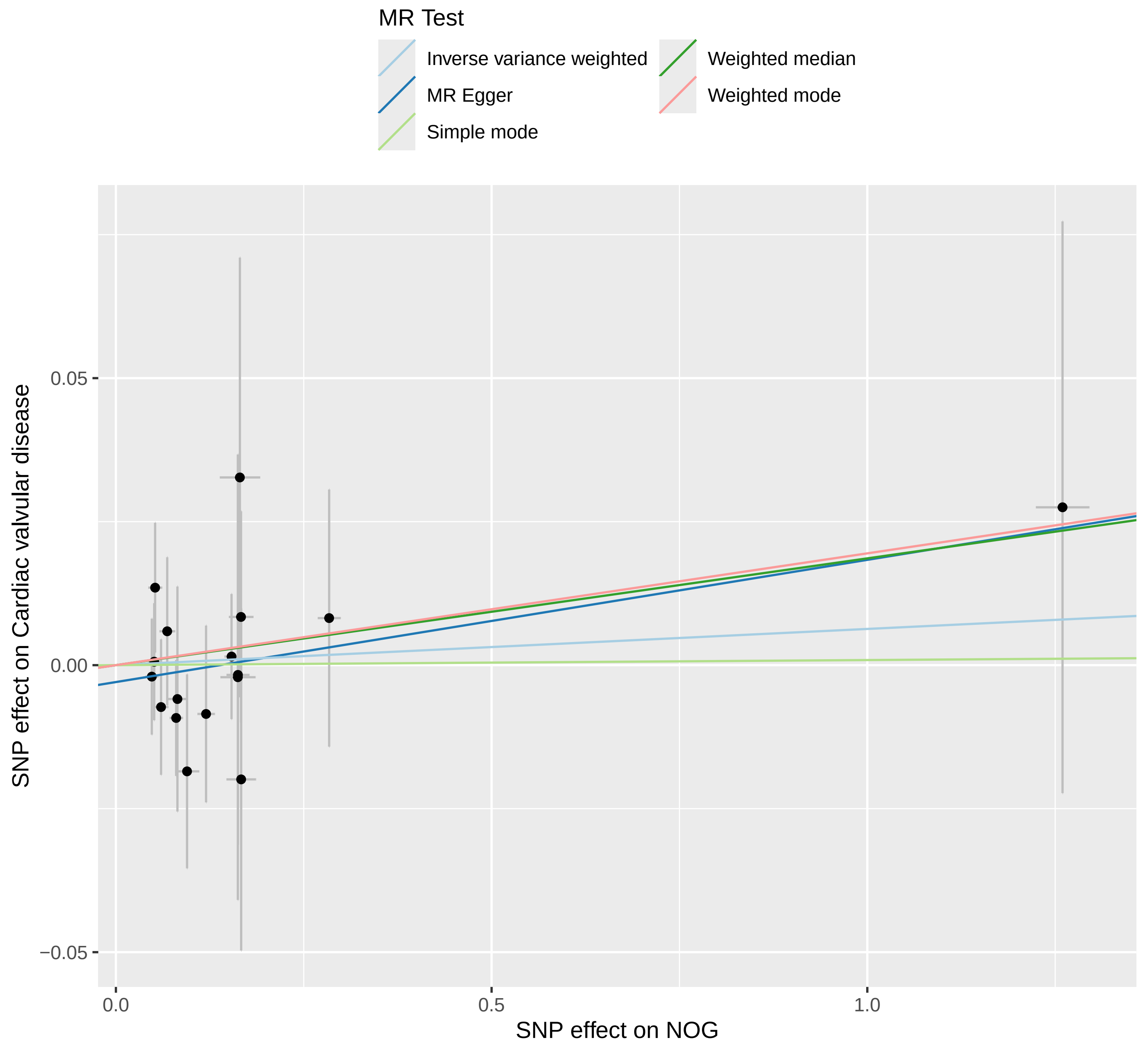
MR Test
Inverse variance weighted
MR Egger
Simple mode
Weighted median
Weighted mode
0.05
0.00
−0.05
0.0
0.5
1.0
SNP effect on Cardiac valvular disease
SNP effect on NOG

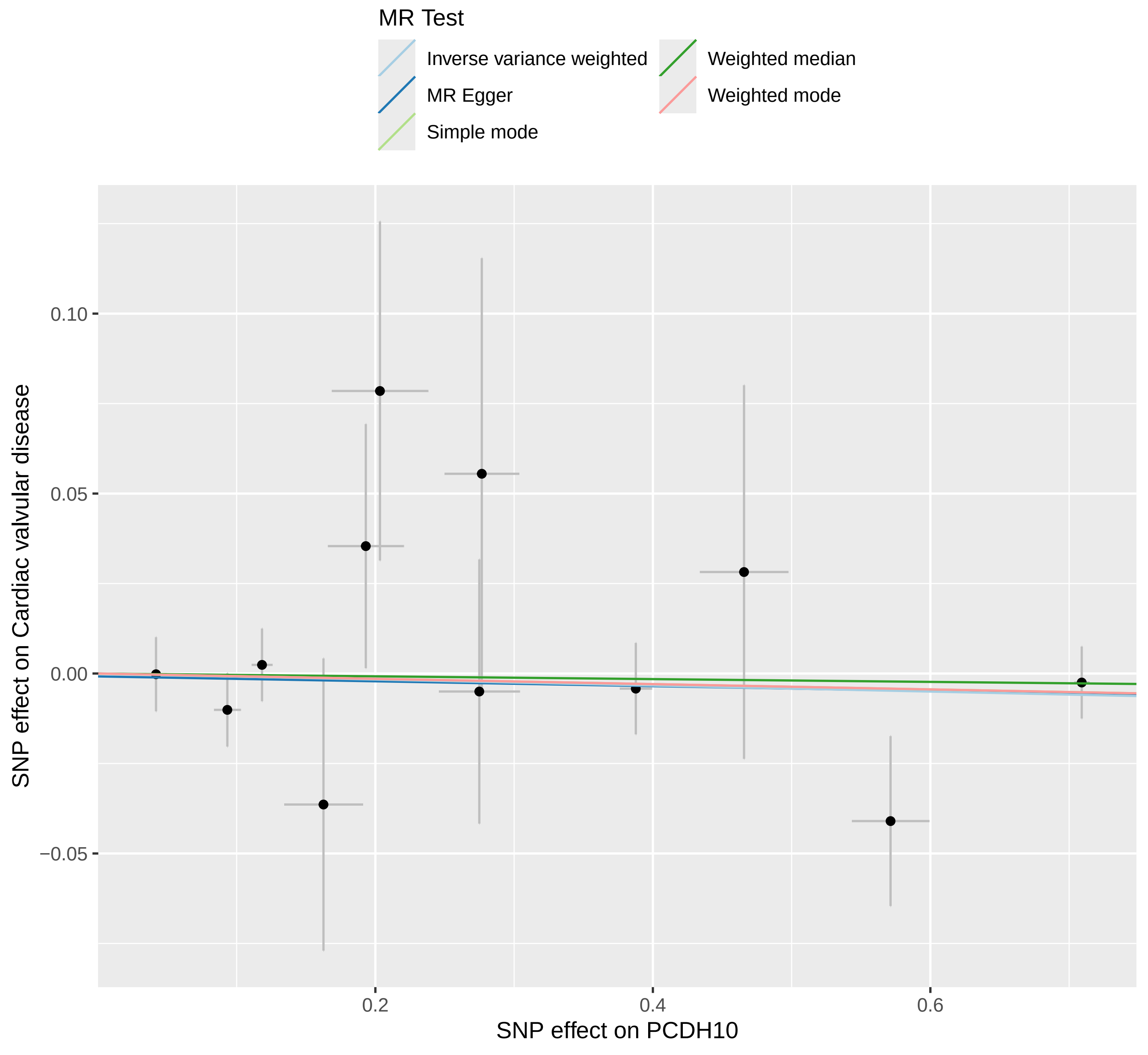
MR Test
Inverse variance weighted
MR Egger
Simple mode
Weighted median
Weighted mode
0.10
0.05
0.00
−0.05
0.2
0.4
0.6
SNP effect on Cardiac valvular disease
SNP effect on PCDH10

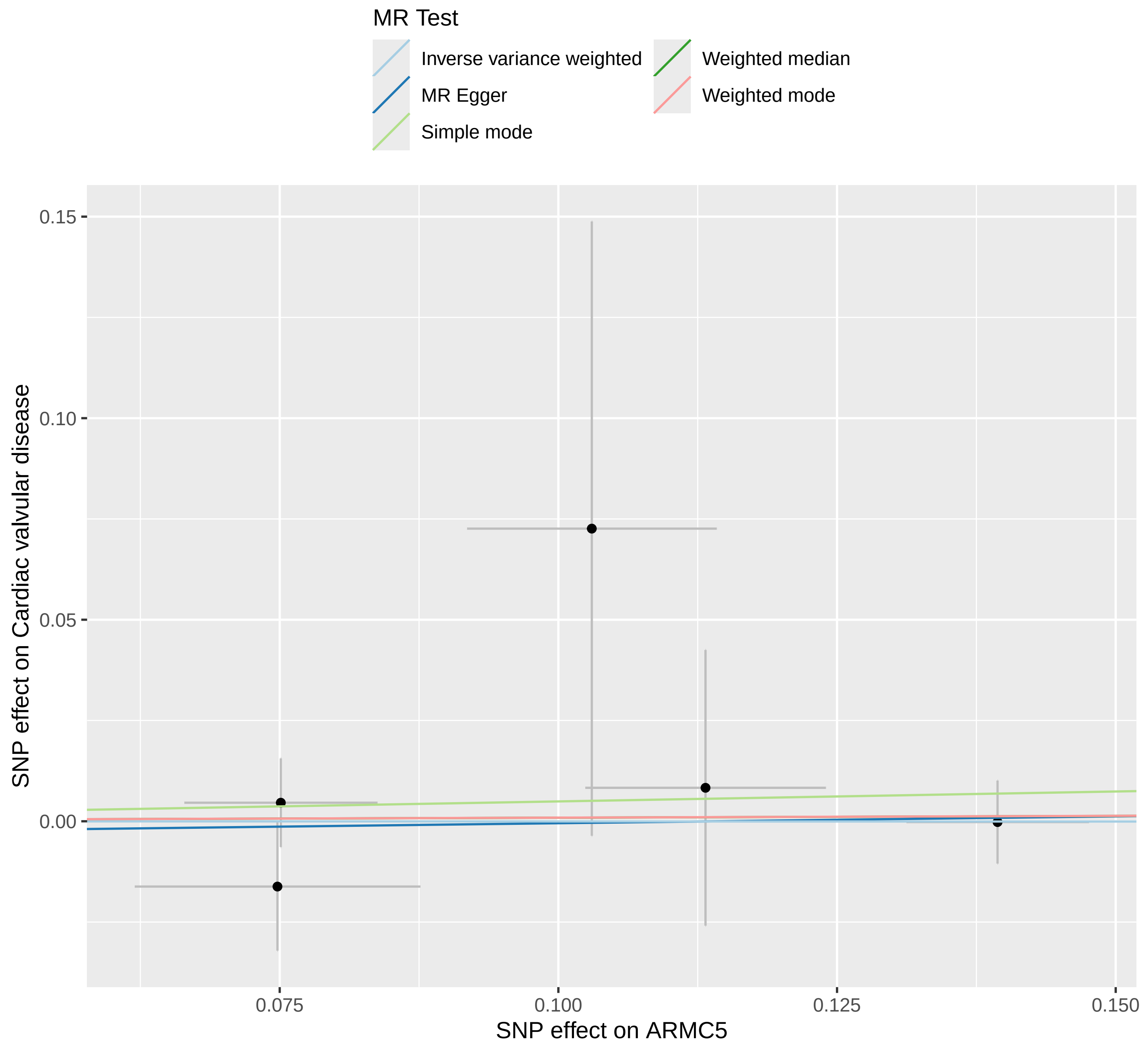
MR Test
Inverse variance weighted
MR Egger
Simple mode
Weighted median
Weighted mode
0.15
0.10
0.05
0.00
SNP effect on Cardiac valvular disease
0.075
0.100
0.125
0.150
SNP effect on ARMC5

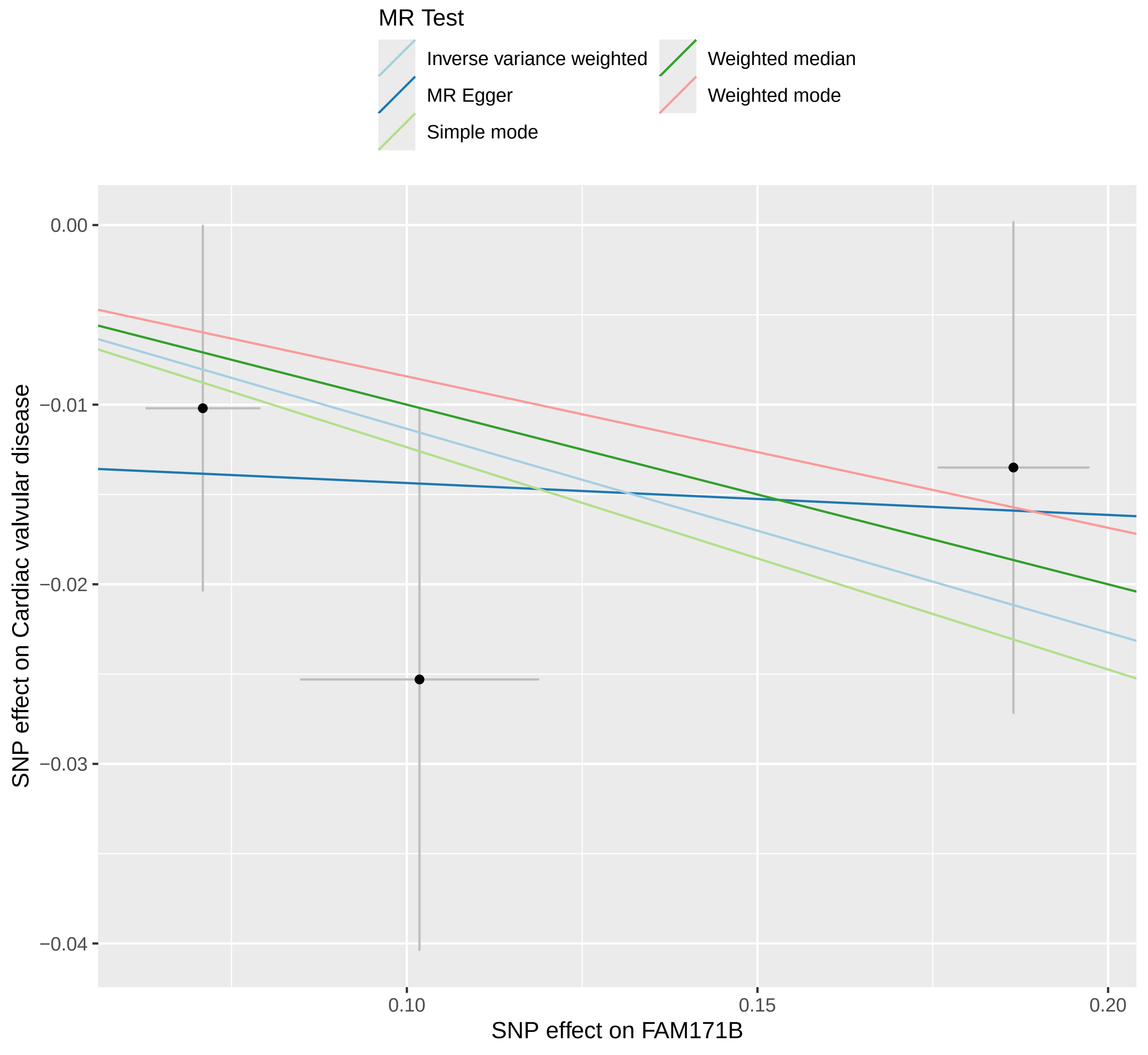

MR Test
Inverse variance weighted
MR Egger
Simple mode
Weighted median
Weighted mode
0.00
−0.01
−0.02
−0.03
−0.04
0.10
0.15
0.20
SNP effect on Cardiac valvular disease
SNP effect on FAM171B

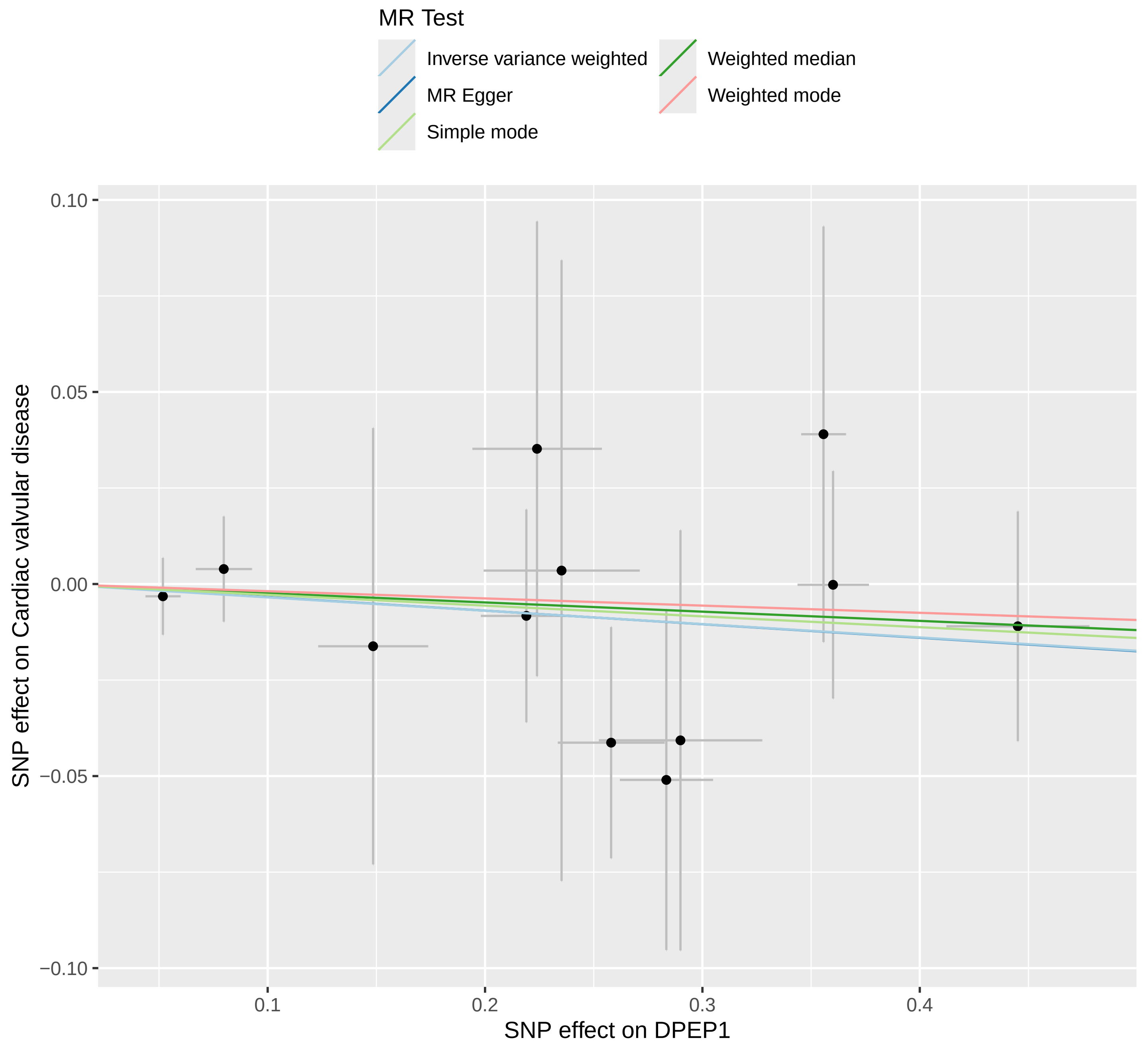
MR Test
Inverse variance weighted
MR Egger
Simple mode
Weighted median
Weighted mode
0.10
0.05
0.00
−0.05
−0.10
SNP effect on Cardiac valvular disease
0.1
0.2
0.3
0.4
SNP effect on DPEP1

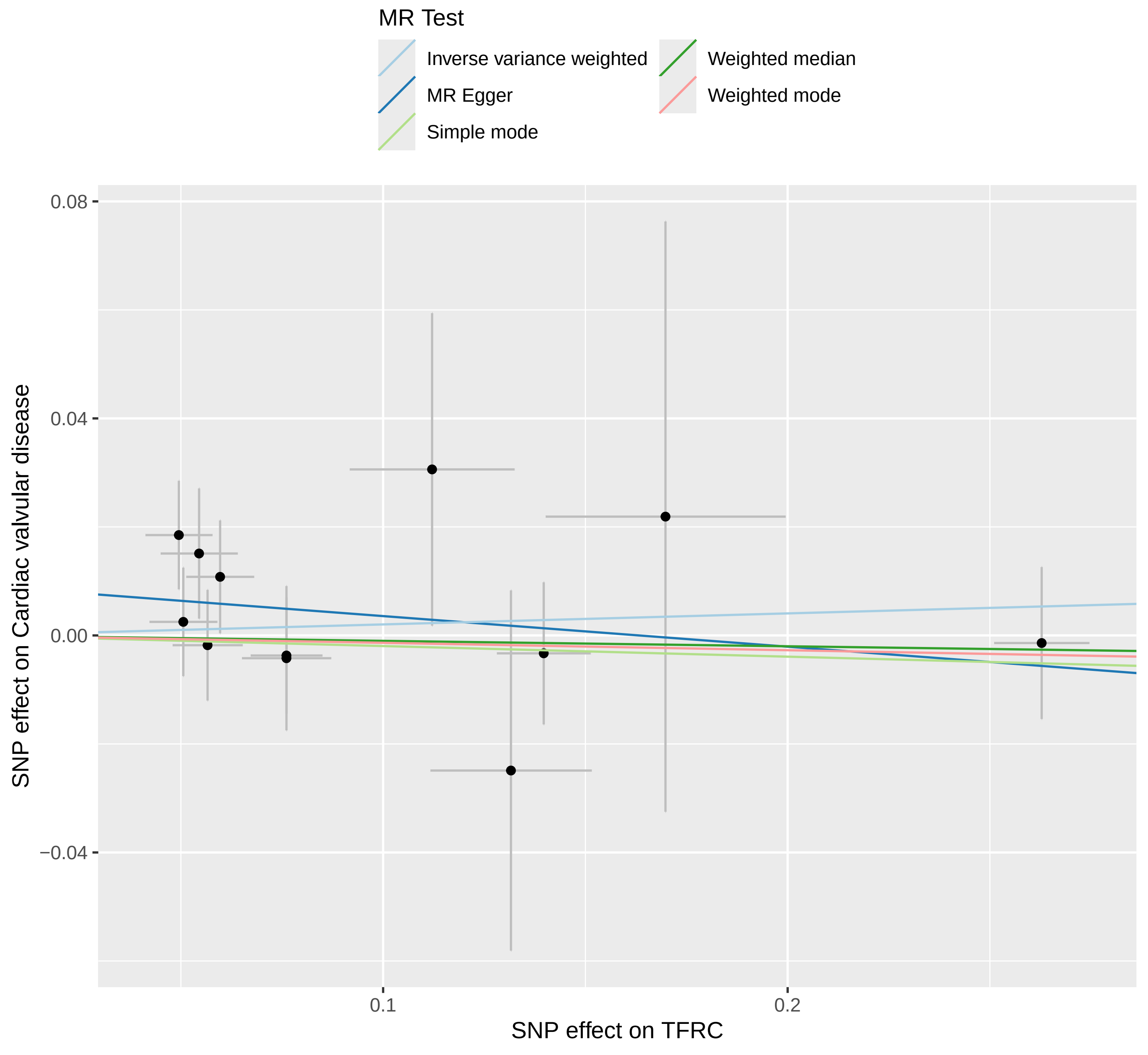
MR Test
Inverse variance weighted
MR Egger
Simple mode
Weighted median
Weighted mode
0.08
0.04
0.00
−0.04
SNP effect on Cardiac valvular disease
0.1
0.2
SNP effect on TFRC

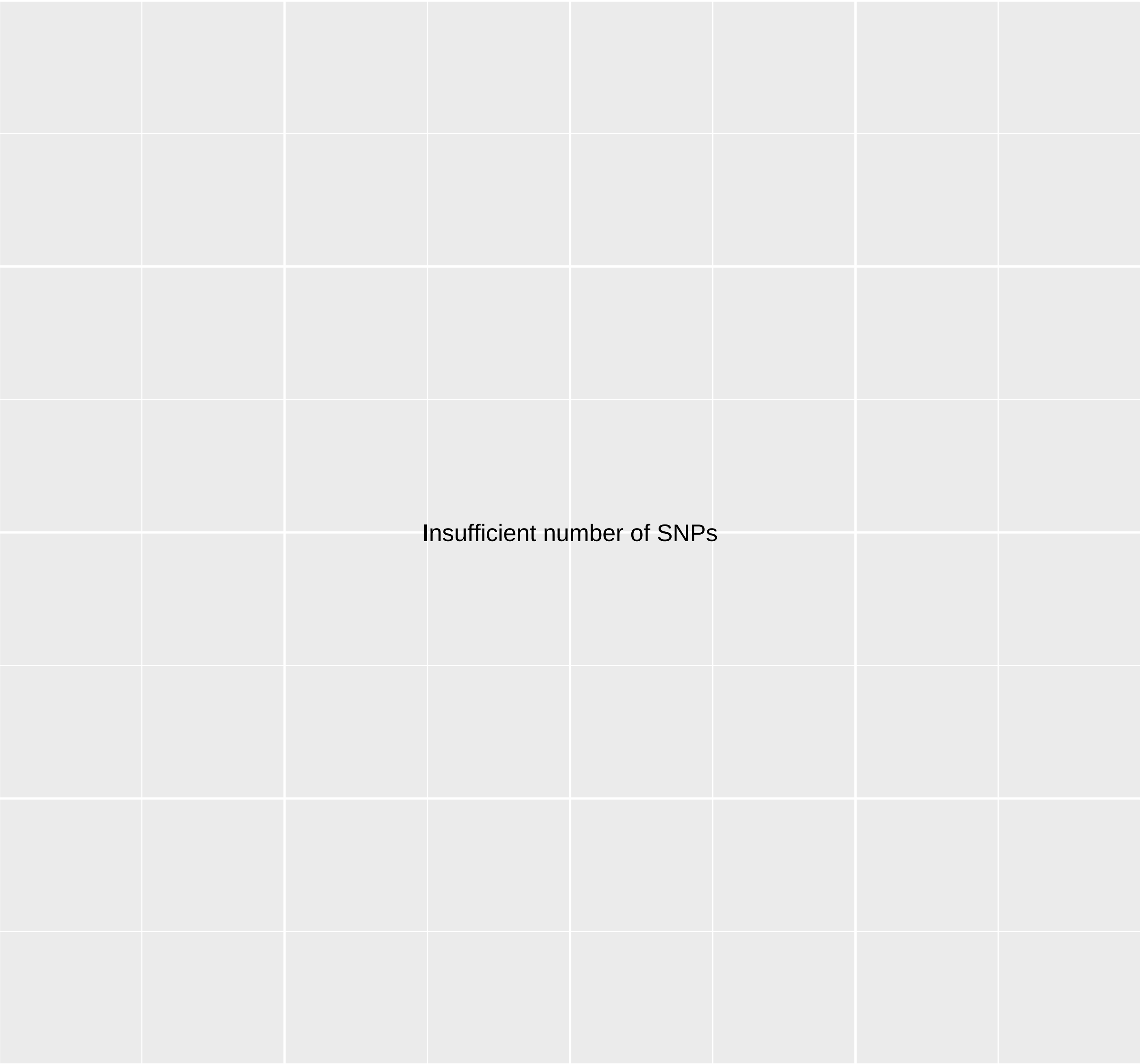
Insufficient number of SNPs

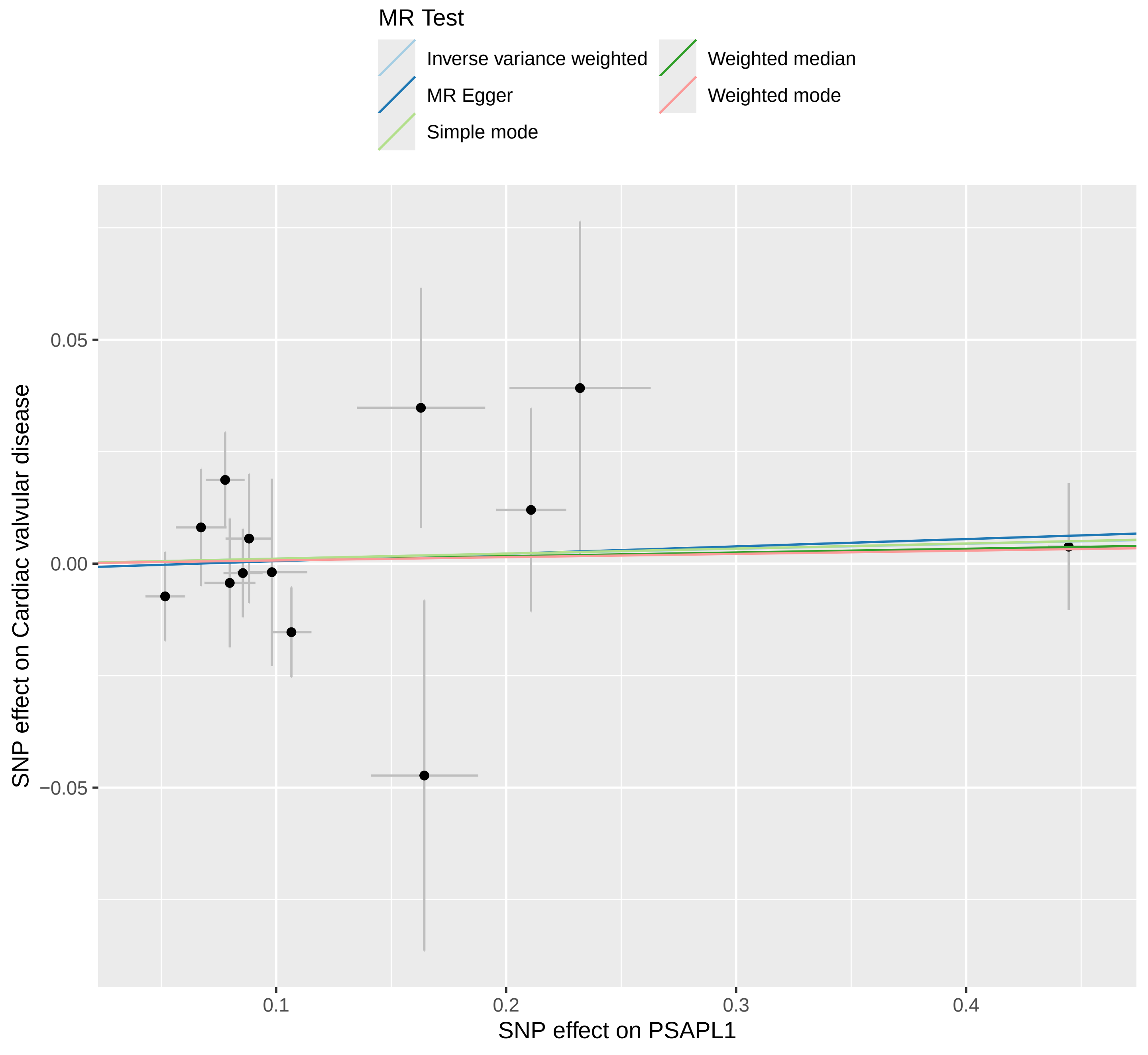
MR Test
Inverse variance weighted
MR Egger
Simple mode
Weighted median
Weighted mode
SNP effect on Cardiac valvular disease
0.05
0.00
−0.05
0.1
0.2
0.3
0.4
SNP effect on PSAPL1

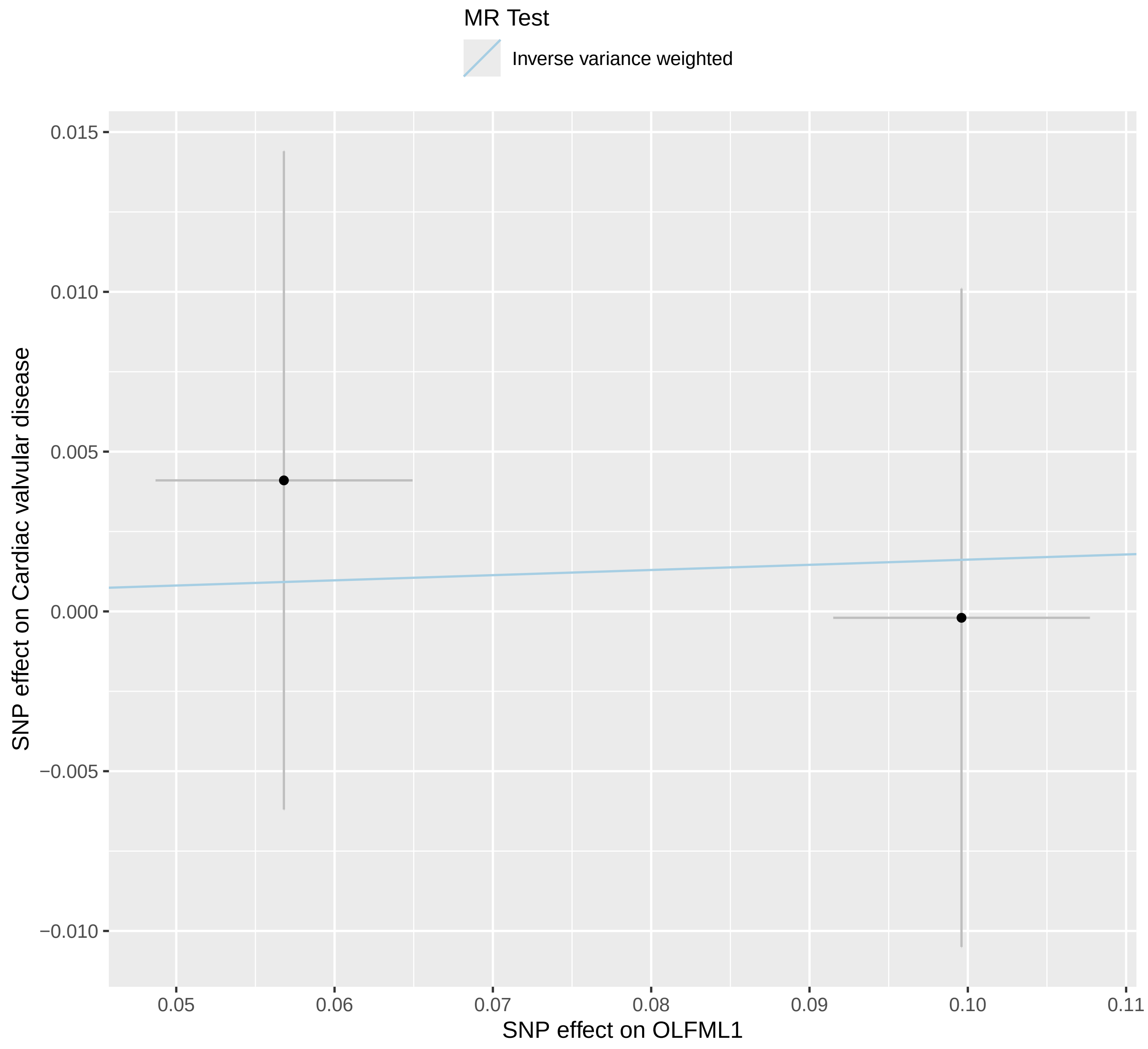

MR Test
Inverse variance weighted
0.015
0.010
0.005
0.000
−0.005
−0.010
0.05
0.06
0.07
0.08
0.09
0.10
0.11
SNP effect on OLFML1
SNP effect on Cardiac valvular disease

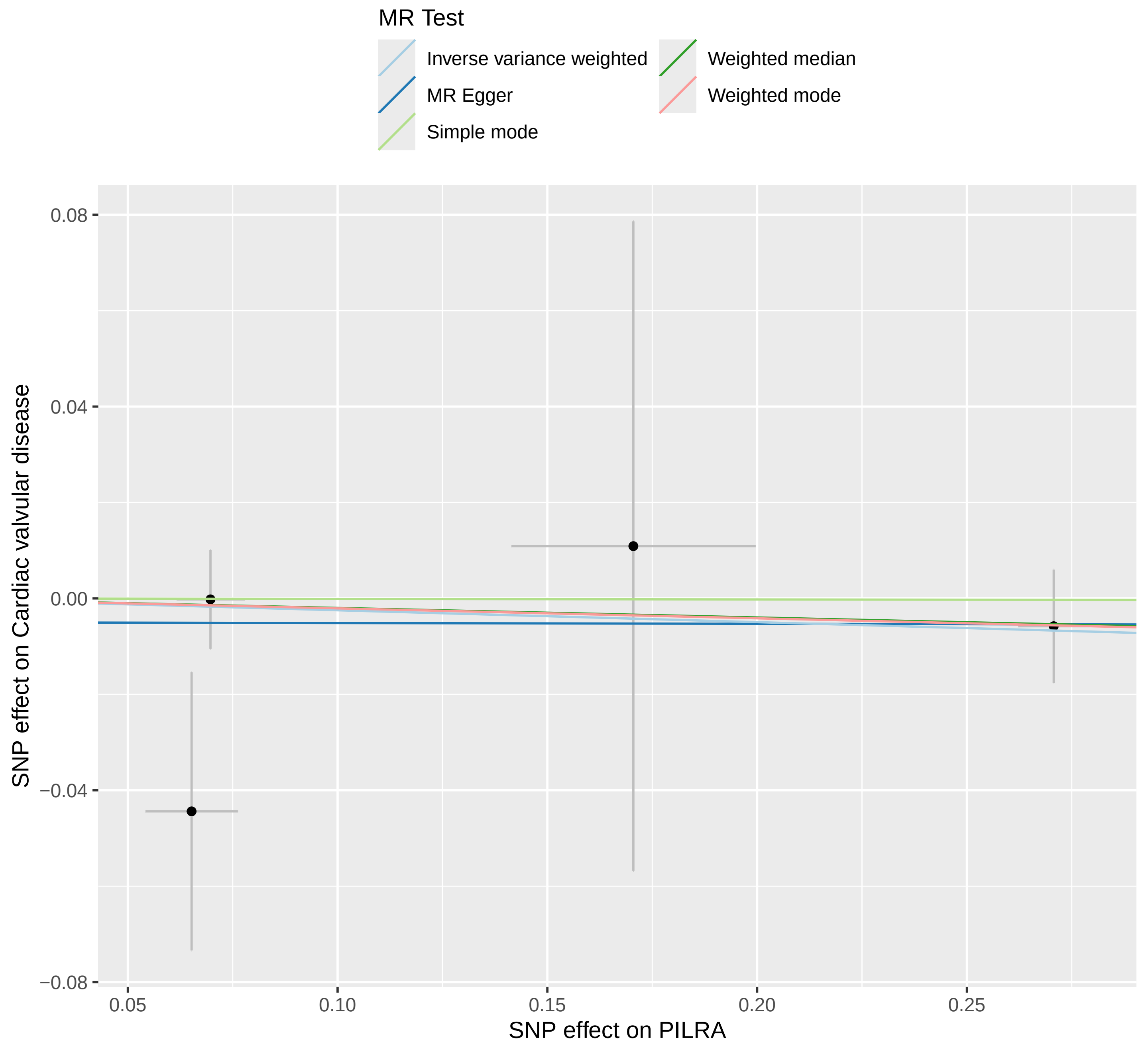
MR Test
Inverse variance weighted
MR Egger
Simple mode
Weighted median
Weighted mode
SNP effect on Cardiac valvular disease
0.08
0.04
0.00
−0.04
−0.08
0.05
0.10
0.15
0.20
0.25
SNP effect on PILRA

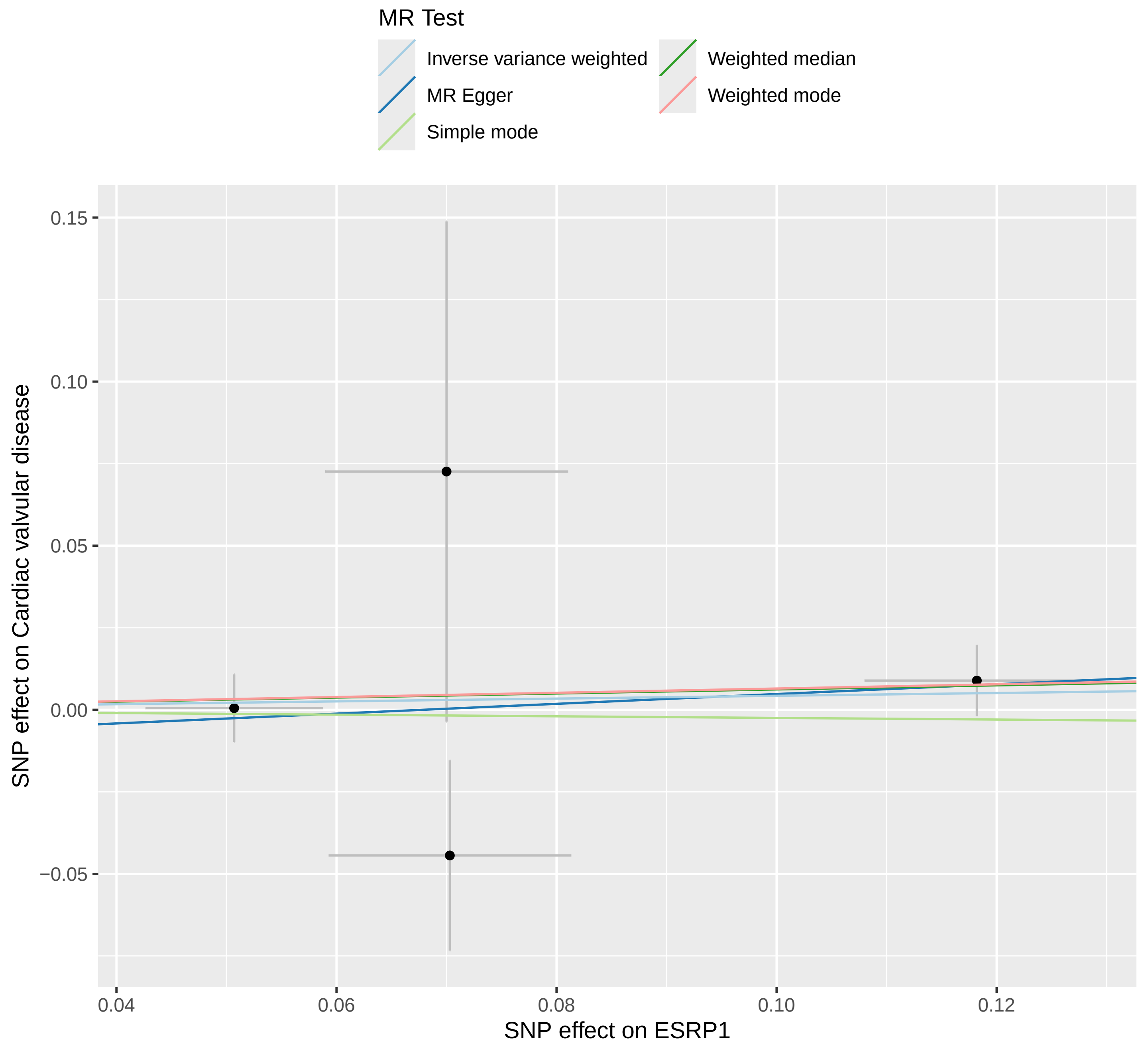
MR Test
Inverse variance weighted
MR Egger
Simple mode
Weighted median
Weighted mode
0.15
0.10
0.05
0.00
−0.05
0.04
0.06
0.08
0.10
0.12
SNP effect on Cardiac valvular disease
SNP effect on ESRP1

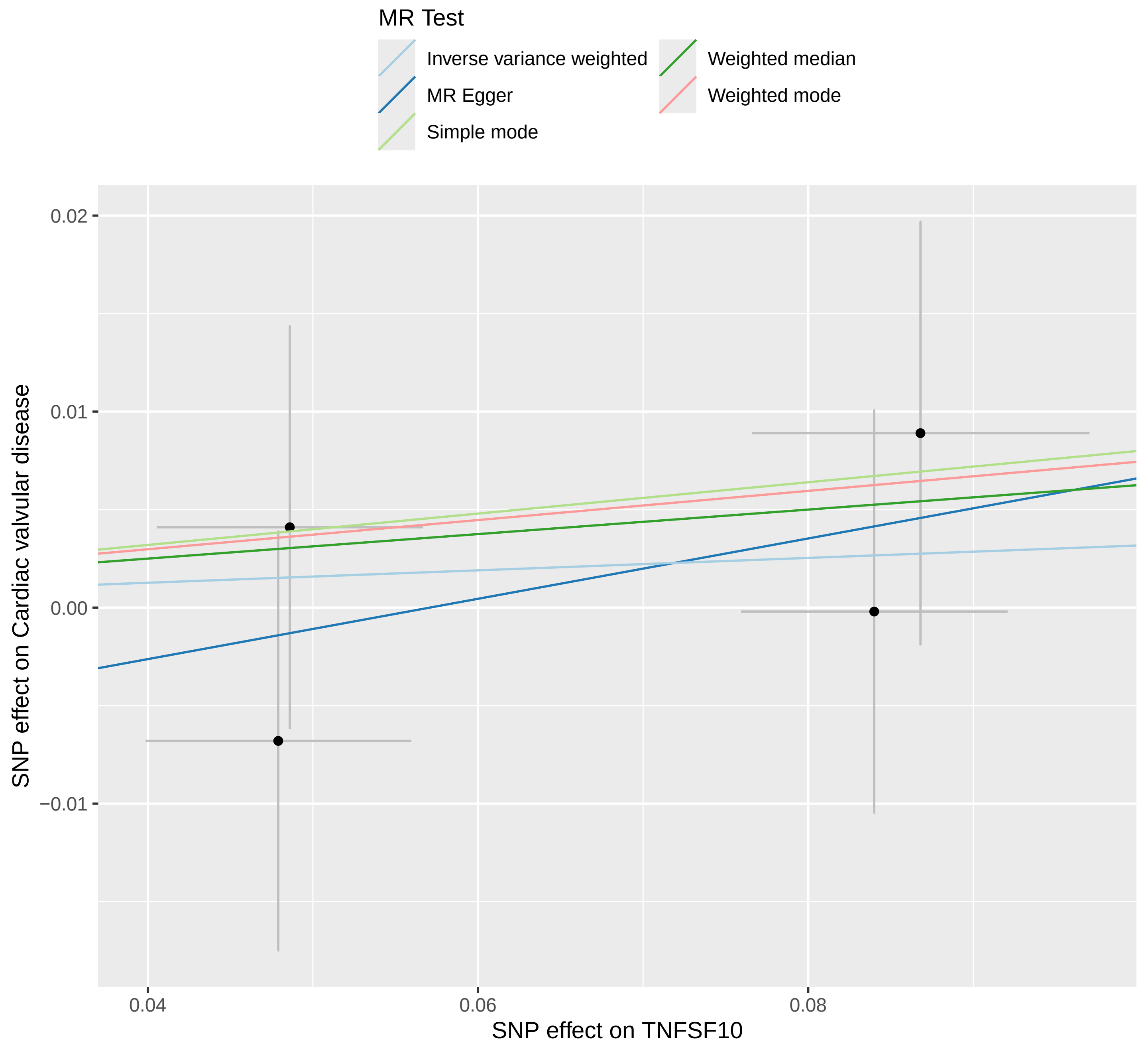
MR Test
Inverse variance weighted
MR Egger
Simple mode
Weighted median
Weighted mode
0.02
0.01
0.00
−0.01
0.04
0.06
0.08
SNP effect on Cardiac valvular disease
SNP effect on TNFSF10

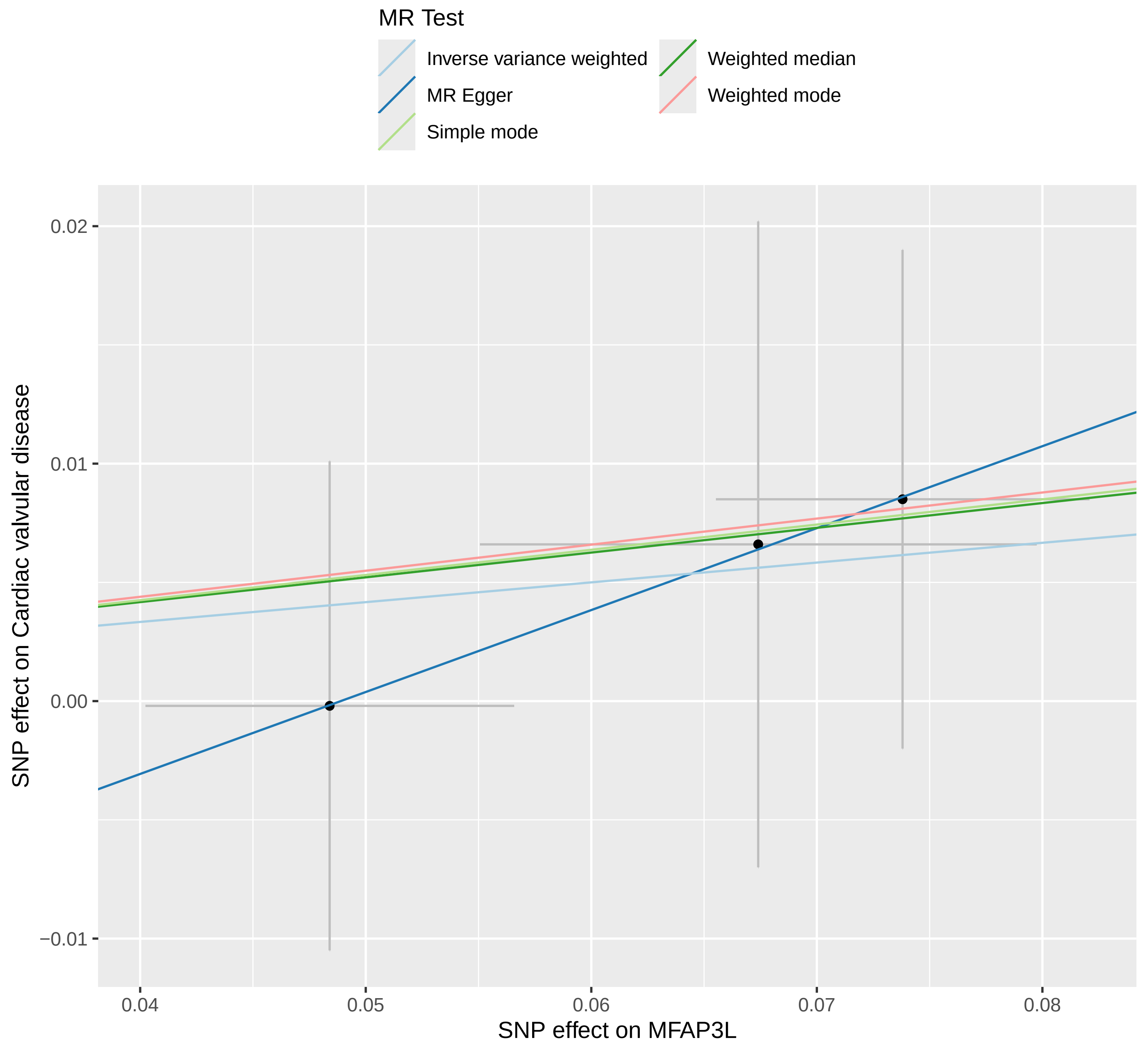
MR Test
Inverse variance weighted
MR Egger
Simple mode
Weighted median
Weighted mode
0.02
0.01
0.00
−0.01
0.04
0.05
0.06
0.07
0.08
SNP effect on Cardiac valvular disease
SNP effect on MFAP3L

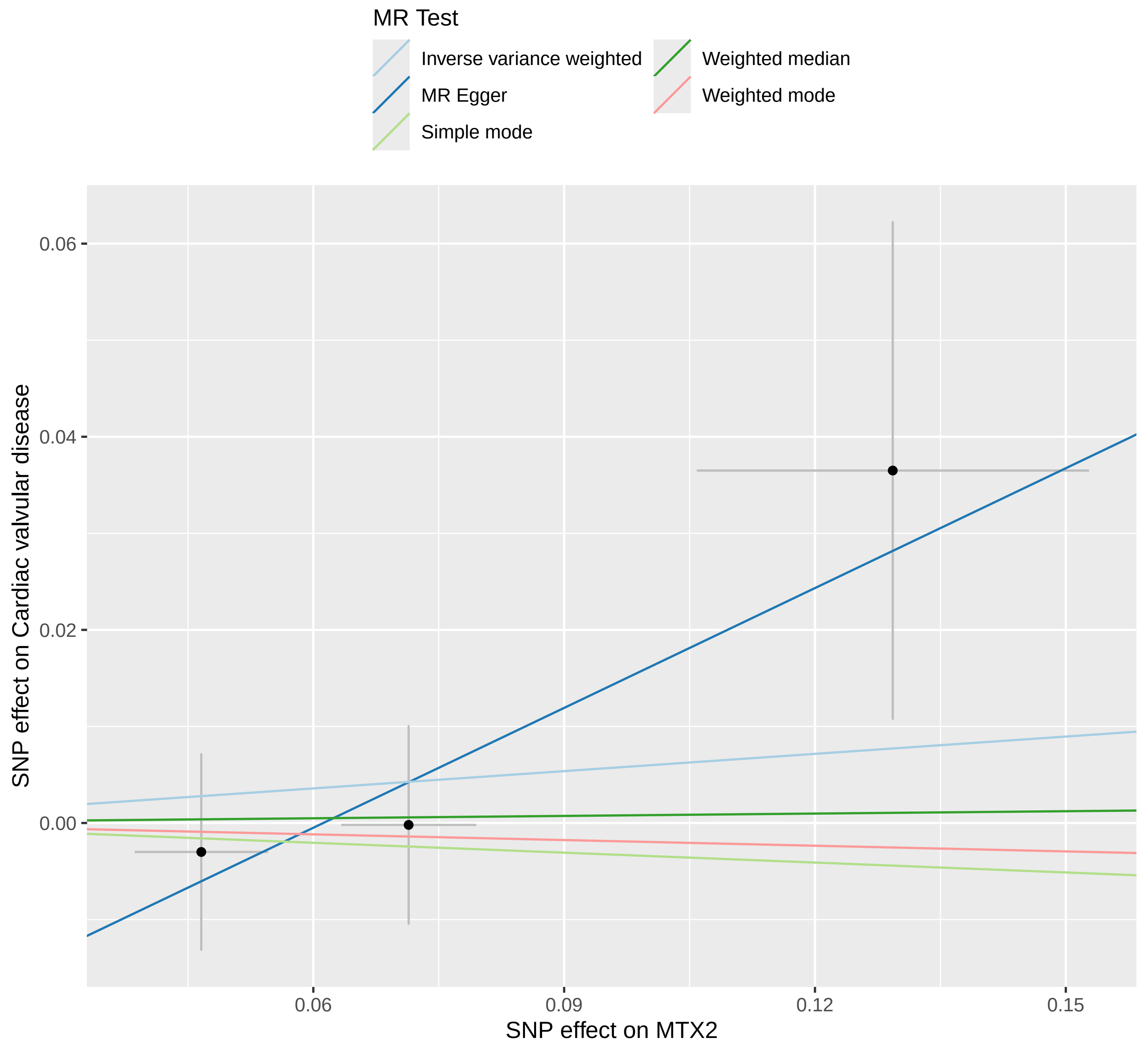

MR Test
Inverse variance weighted
MR Egger
Simple mode
Weighted median
Weighted mode
0.06
0.04
0.02
0.00
SNP effect on Cardiac valvular disease
0.06
0.09
0.12
0.15
SNP effect on MTX2

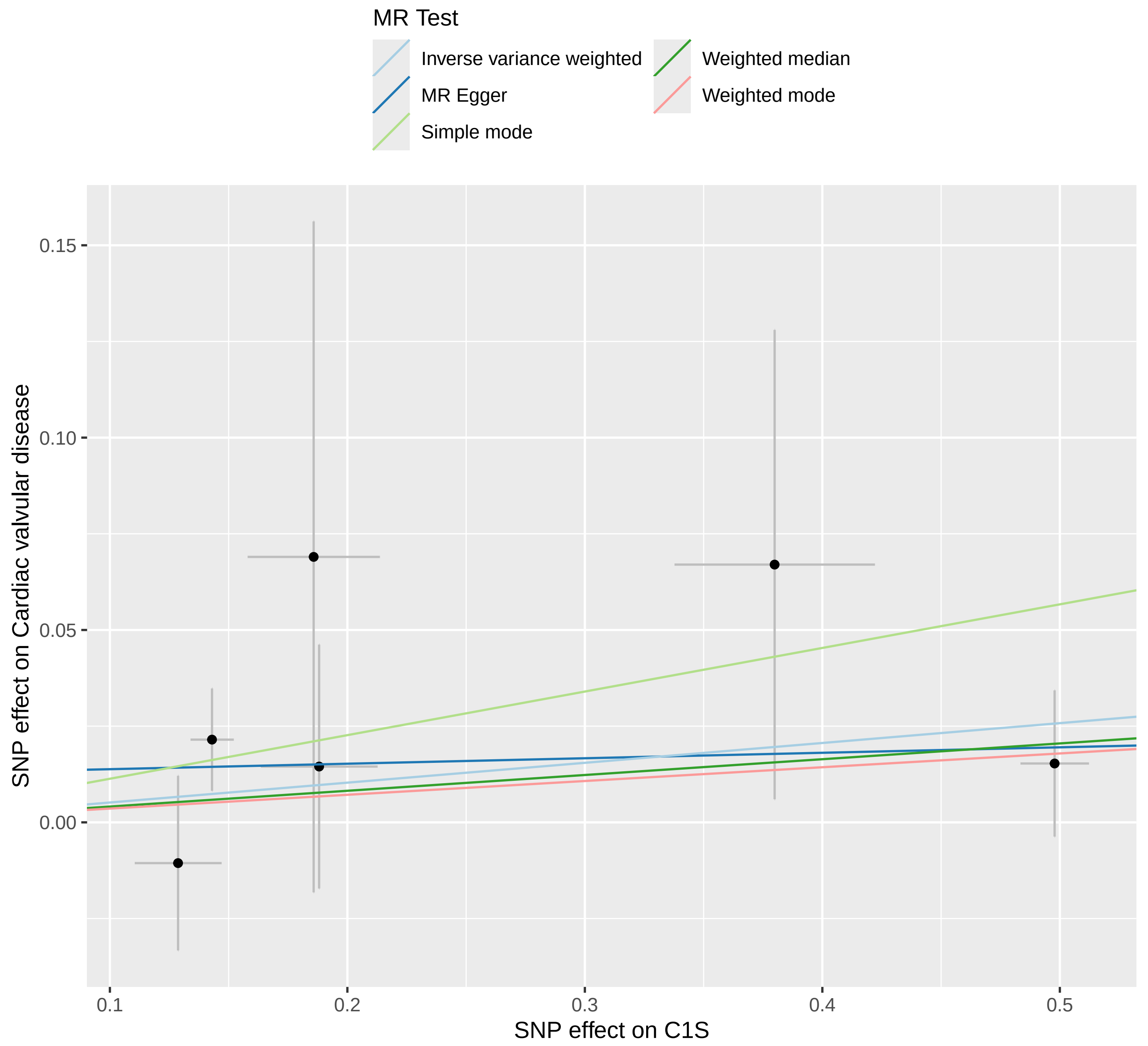
MR Test
Inverse variance weighted
MR Egger
Simple mode
Weighted median
Weighted mode
SNP effect on Cardiac valvular disease
0.15
0.10
0.05
0.00
0.1
0.2
0.3
0.4
0.5
SNP effect on C1S

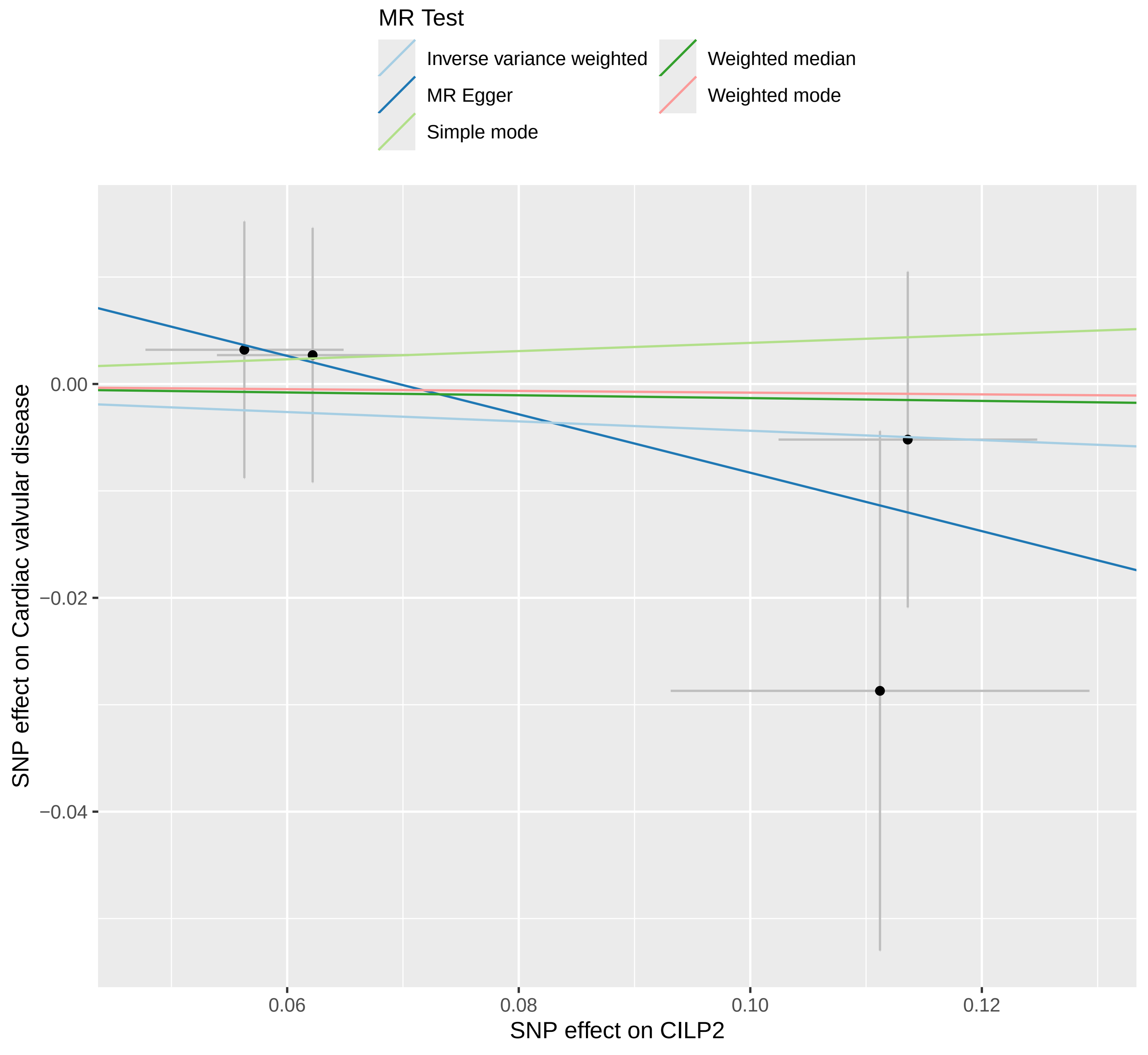
MR Test
Inverse variance weighted
MR Egger
Simple mode
Weighted median
Weighted mode
0.00
−0.02
−0.04
0.06
0.08
0.10
0.12
SNP effect on Cardiac valvular disease
SNP effect on CILP2

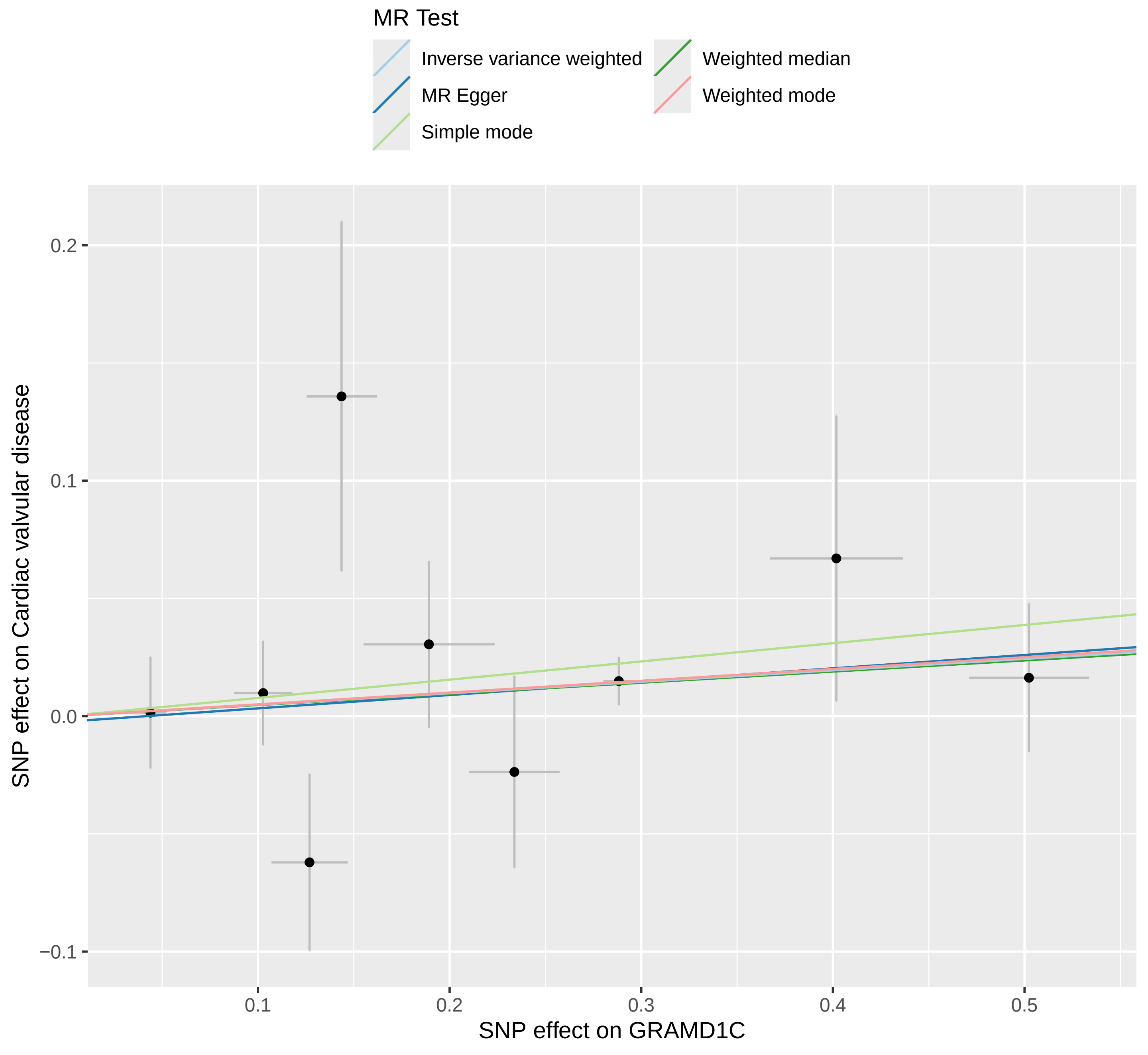
MR Test
Inverse variance weighted
MR Egger
Simple mode
Weighted median
Weighted mode
0.2
0.1
0.0
−0.1
0.1
0.2
0.3
0.4
0.5
SNP effect on Cardiac valvular disease
SNP effect on GRAMD1C

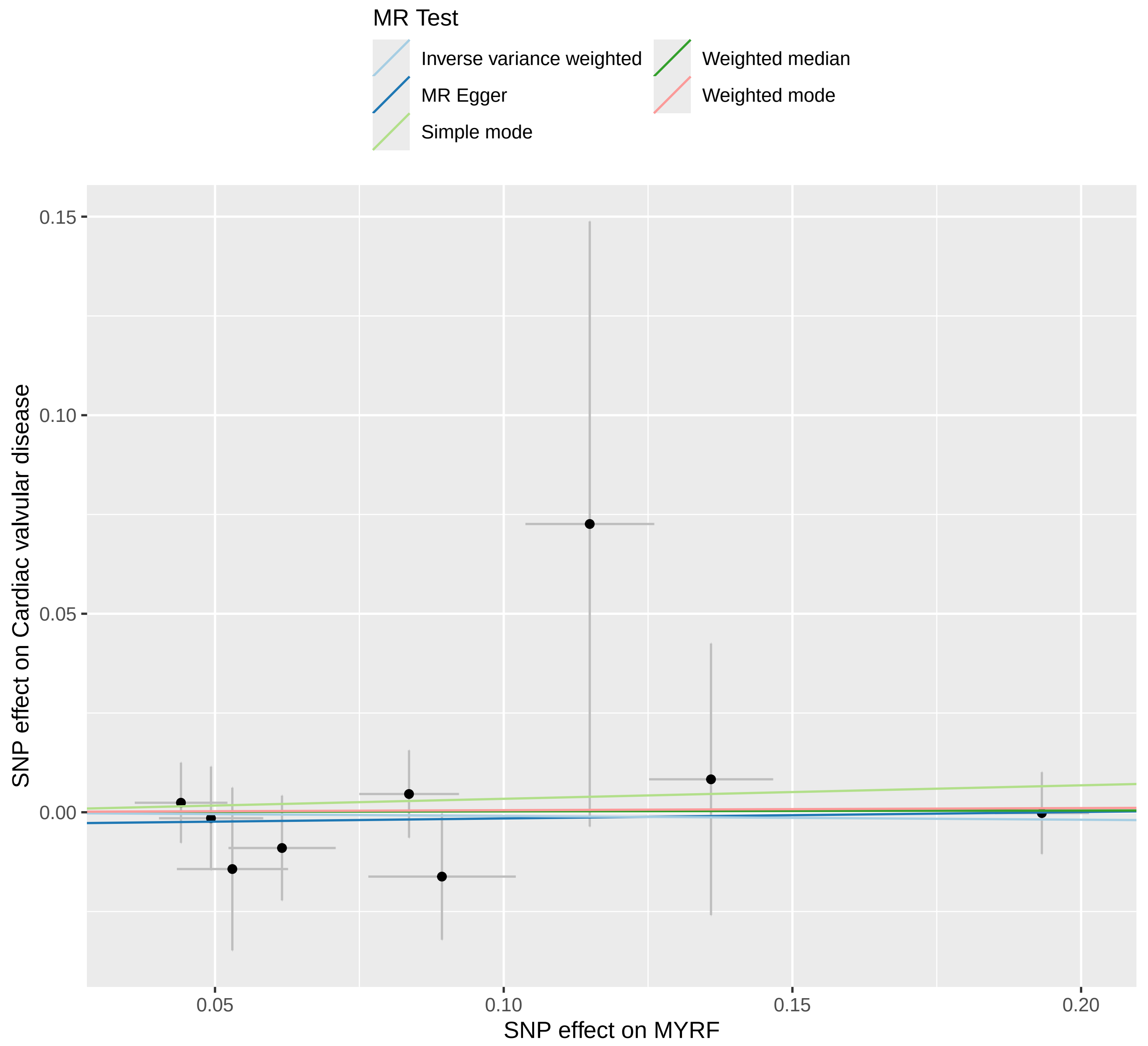

MR Test
Inverse variance weighted
MR Egger
Simple mode
Weighted median
Weighted mode
SNP effect on Cardiac valvular disease
0.15
0.10
0.05
0.00
0.05
0.10
0.15
0.20
SNP effect on MYRF

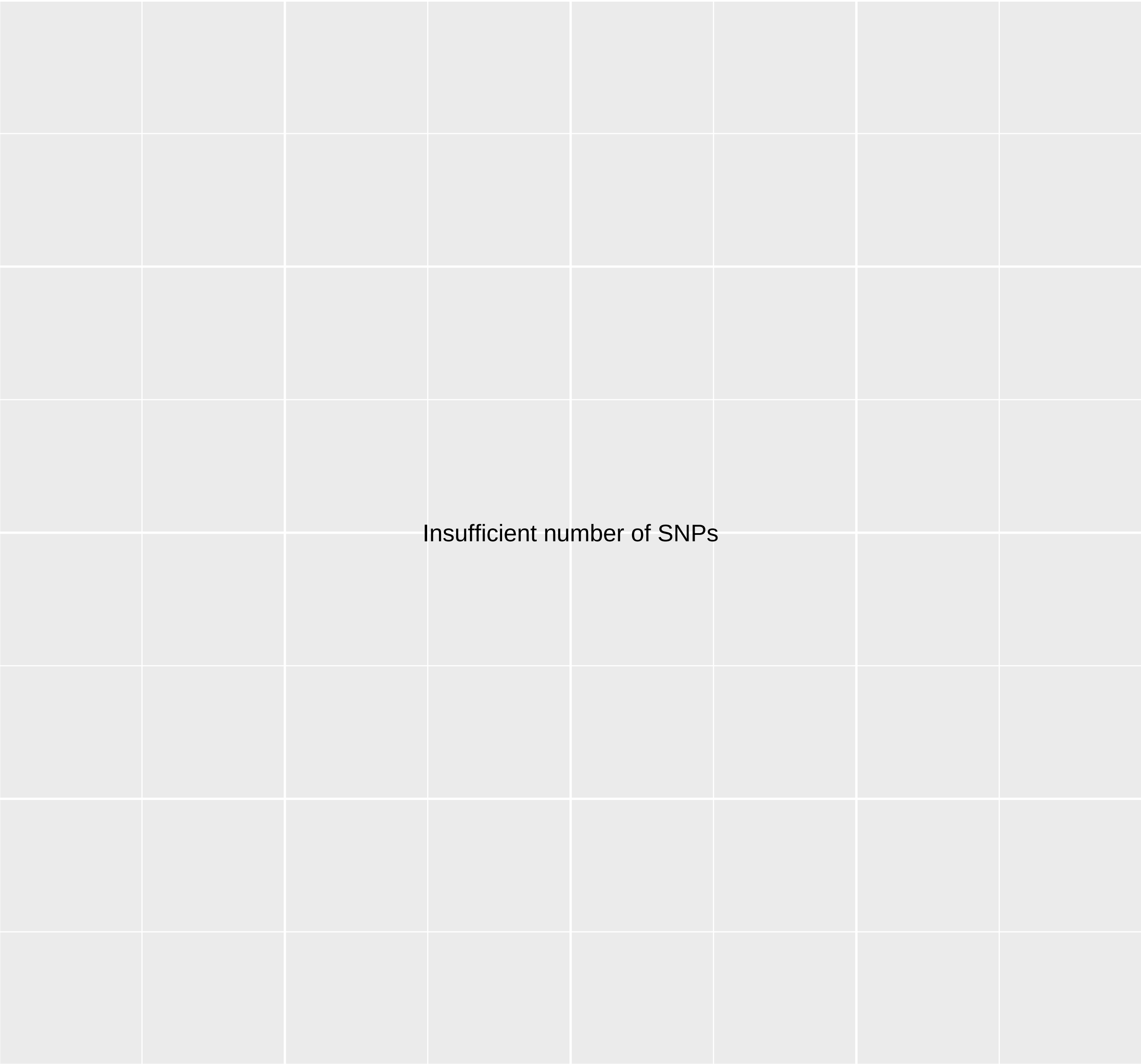
Insufficient number of SNPs

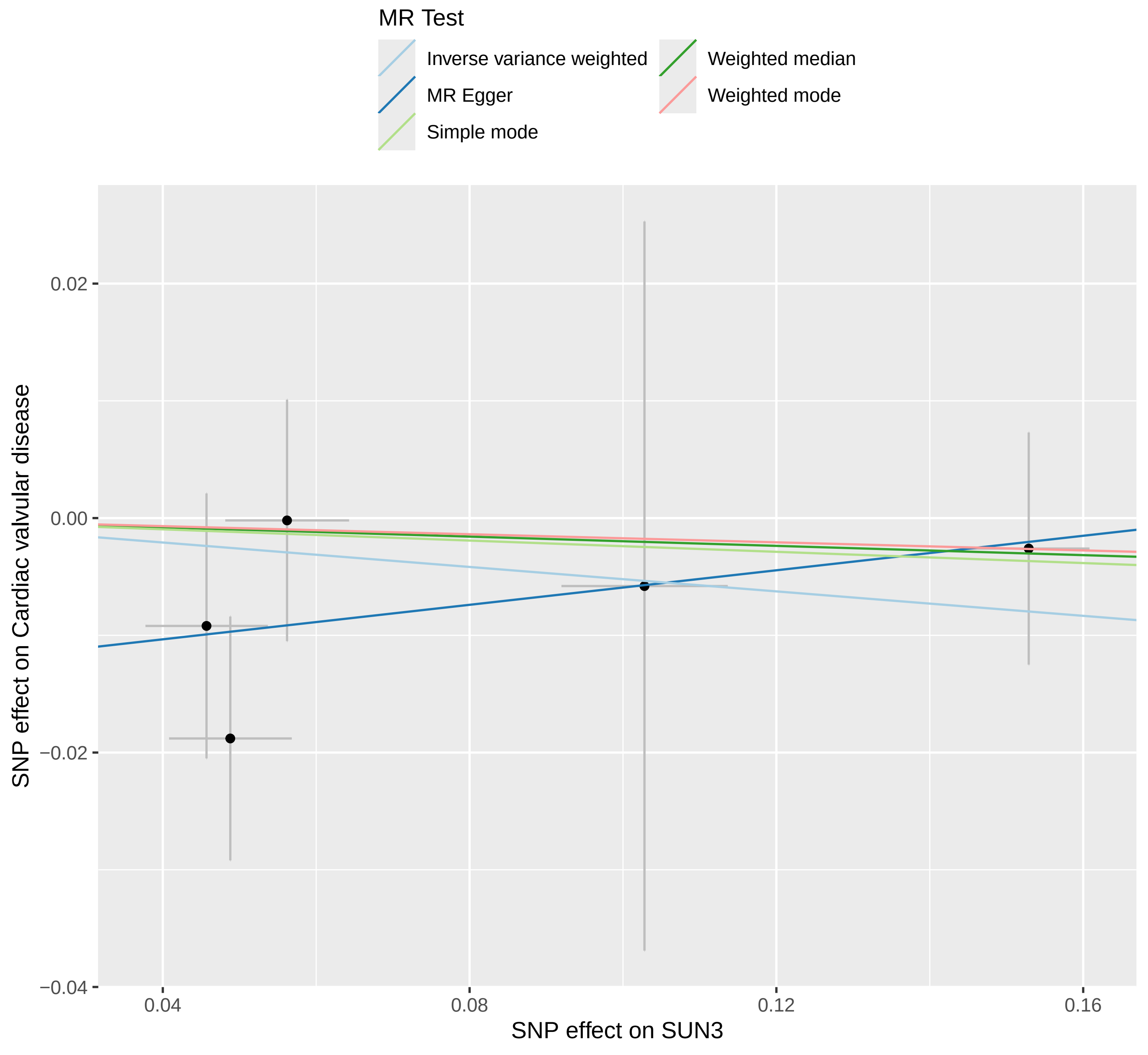

MR Test
Inverse variance weighted
MR Egger
Simple mode
Weighted median
Weighted mode
0.02
0.00
−0.02
−0.04
0.04
0.08
0.12
0.16
SNP effect on Cardiac valvular disease
SNP effect on SUN3

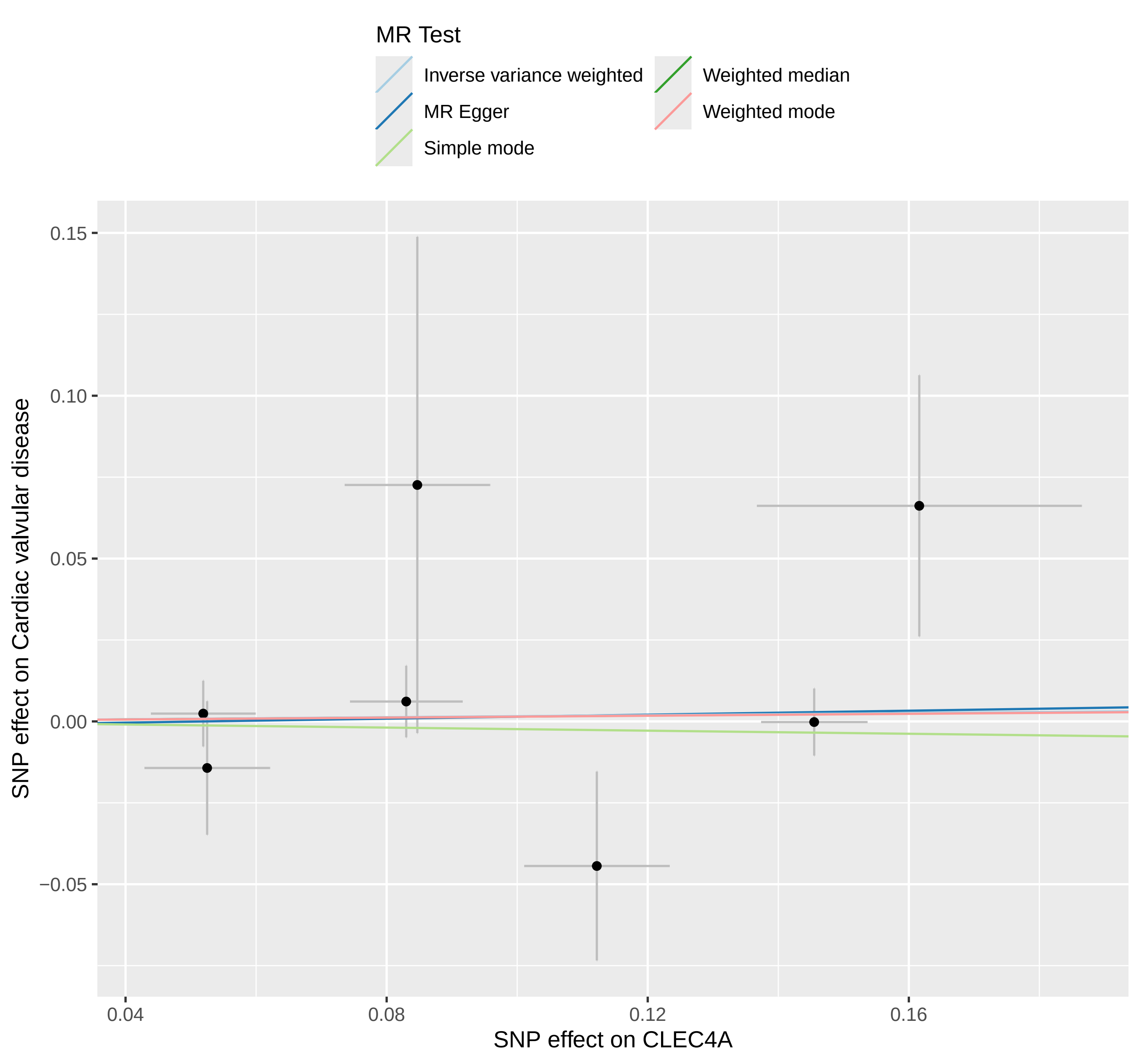

MR Test
Inverse variance weighted
MR Egger
Simple mode
Weighted median
Weighted mode
0.15
0.10
0.05
0.00
−0.05
0.04
0.08
0.12
0.16
SNP effect on CLEC4A
SNP effect on Cardiac valvular disease

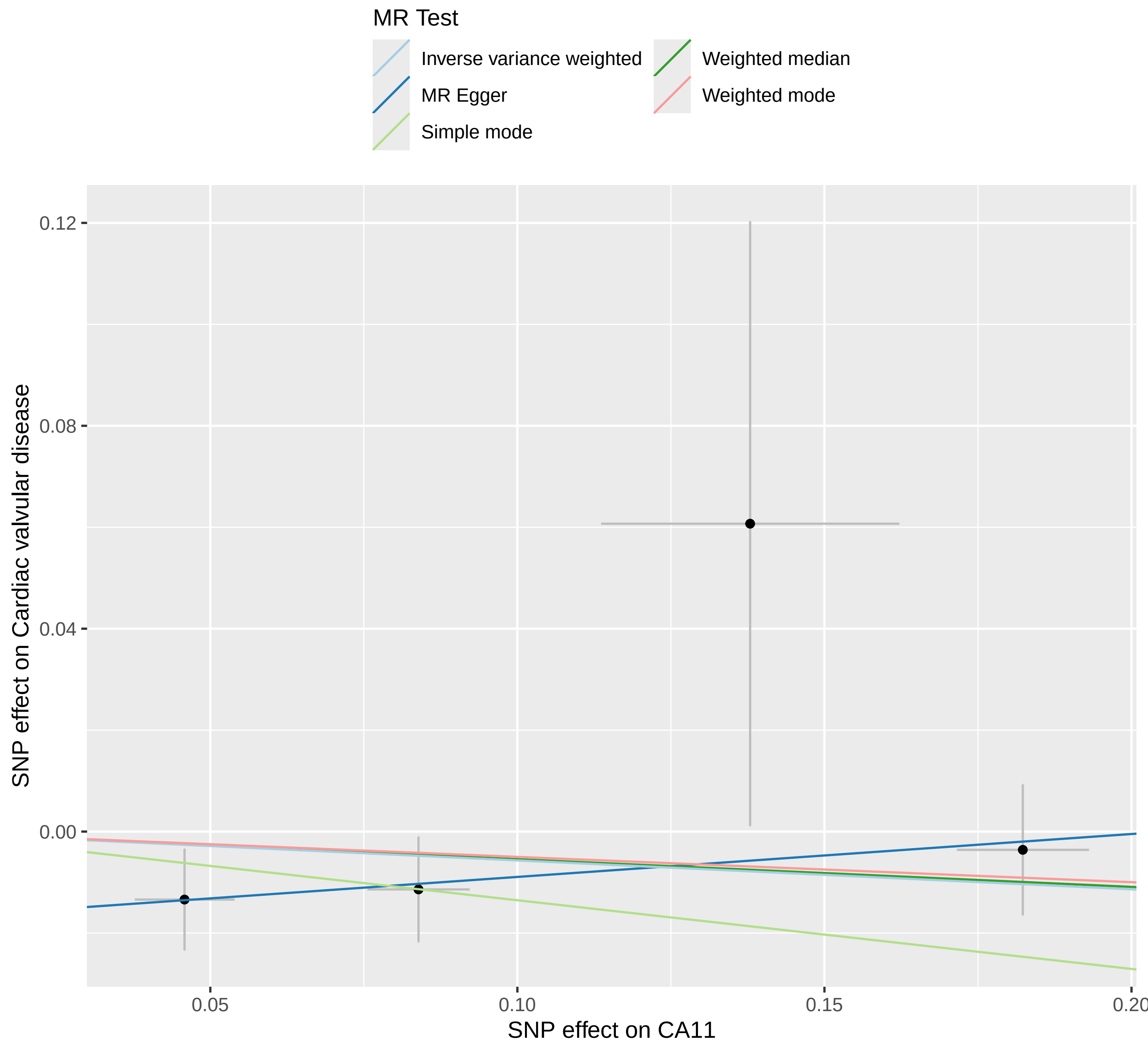
MR Test
Inverse variance weighted
MR Egger
Simple mode
Weighted median
Weighted mode
SNP effect on Cardiac valvular disease
0.12
0.08
0.04
0.00
0.05
0.10
0.15
0.20
SNP effect on CA11

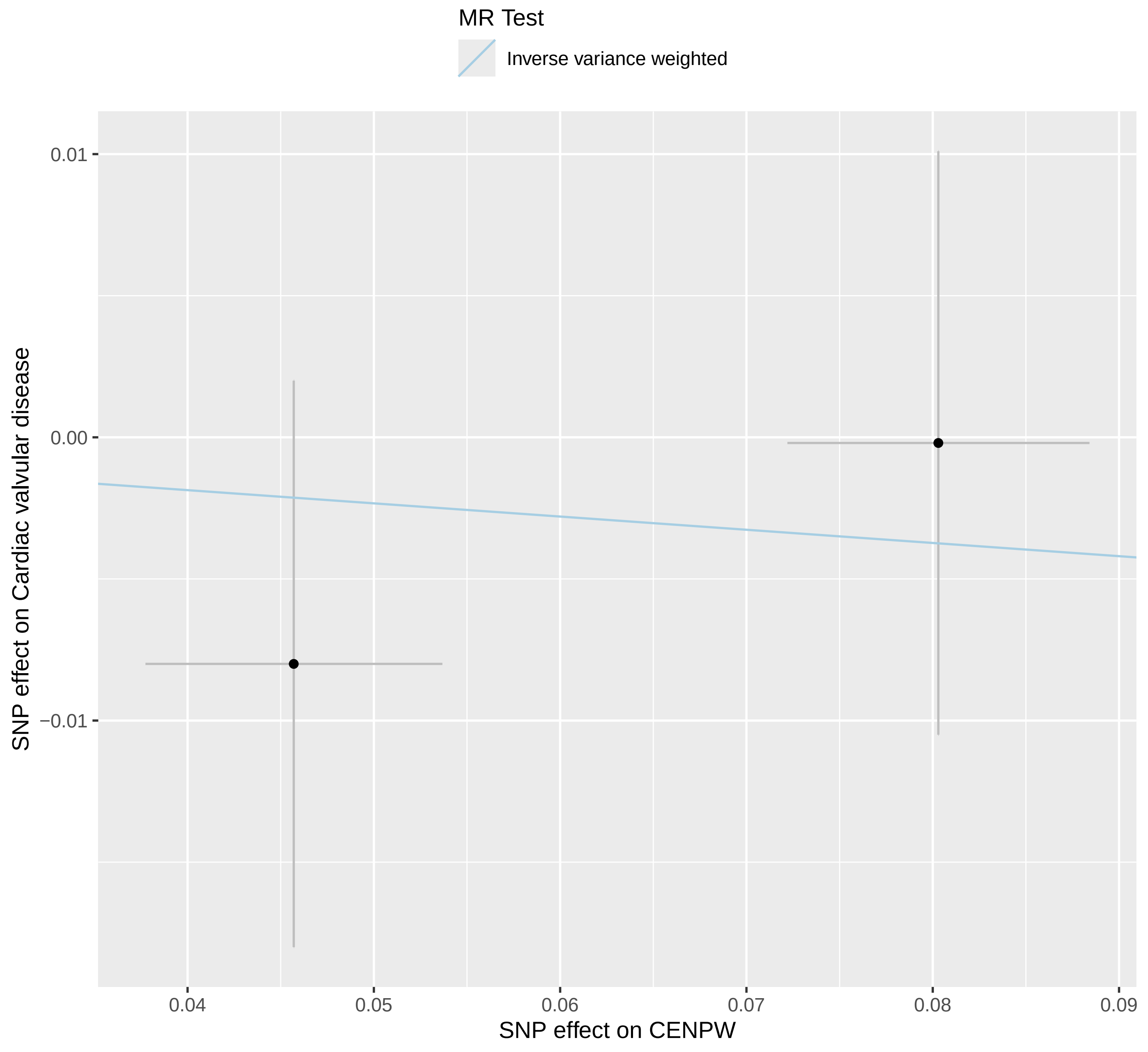

MR Test
Inverse variance weighted
0.01
0.00
−0.01
0.04
0.05
0.06
0.07
0.08
0.09
SNP effect on CENPW
SNP effect on Cardiac valvular disease

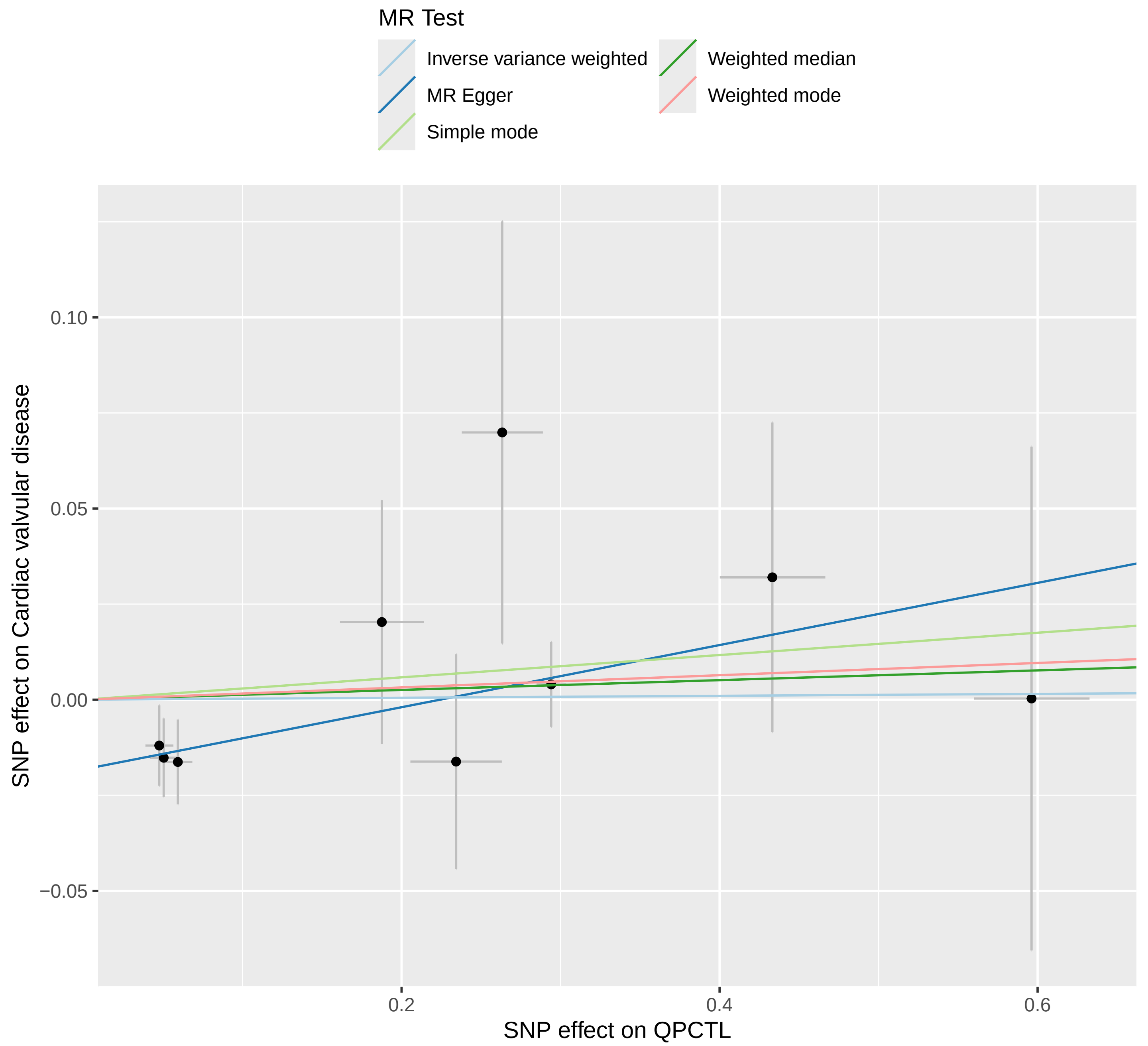
MR Test
Inverse variance weighted
MR Egger
Simple mode
Weighted median
Weighted mode
0.10
0.05
0.00
−0.05
0.2
0.4
0.6
SNP effect on QPCTL
SNP effect on Cardiac valvular disease

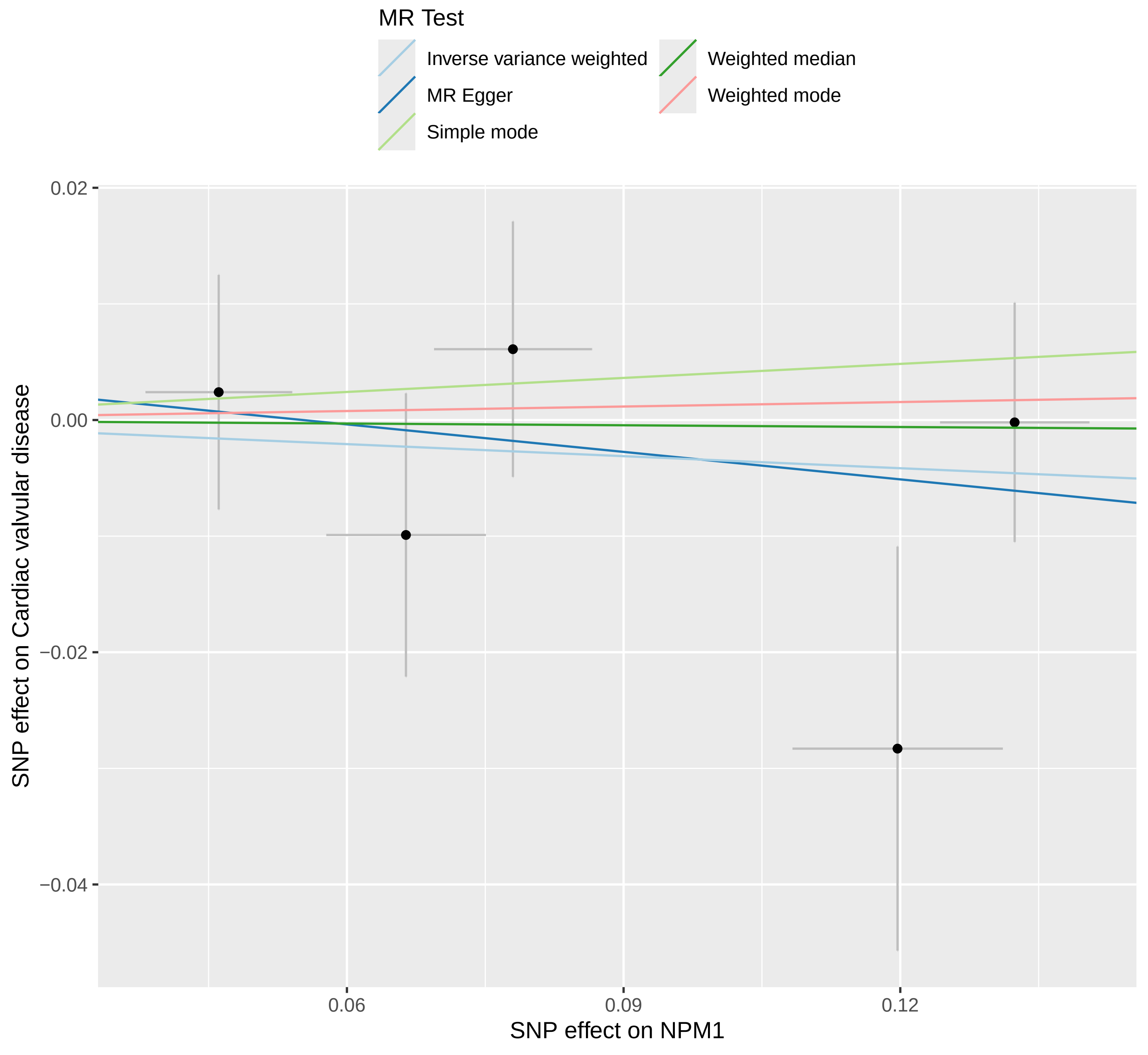
MR Test
Inverse variance weighted
MR Egger
Simple mode
Weighted median
Weighted mode
0.02
0.00
−0.02
−0.04
0.06
0.09
0.12
SNP effect on NPM1
SNP effect on Cardiac valvular disease

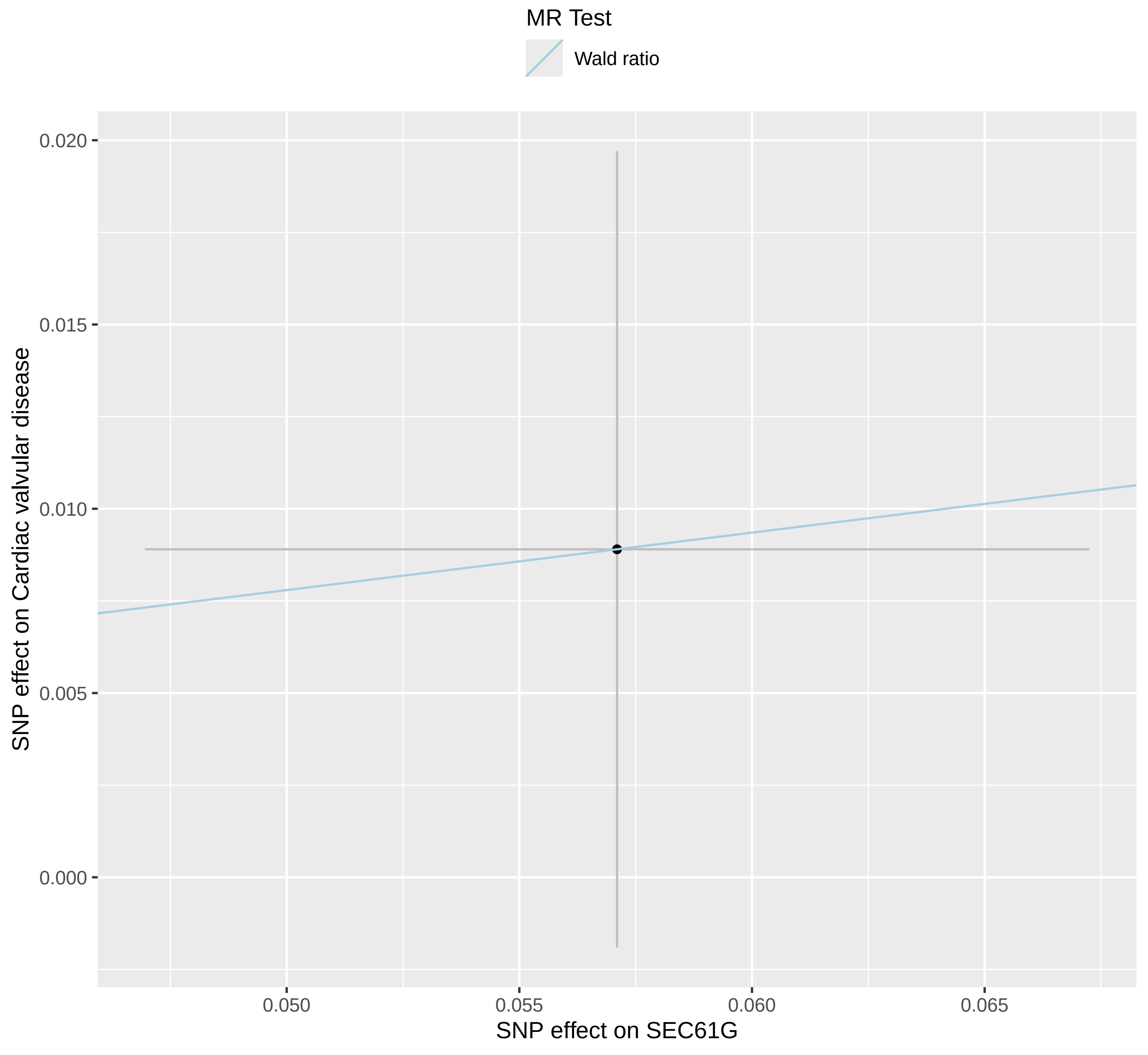
MR Test
Wald ratio
0.020
0.015
0.010
0.005
0.000
SNP effect on Cardiac valvular disease
0.050
0.055
0.060
0.065
SNP effect on SEC61G

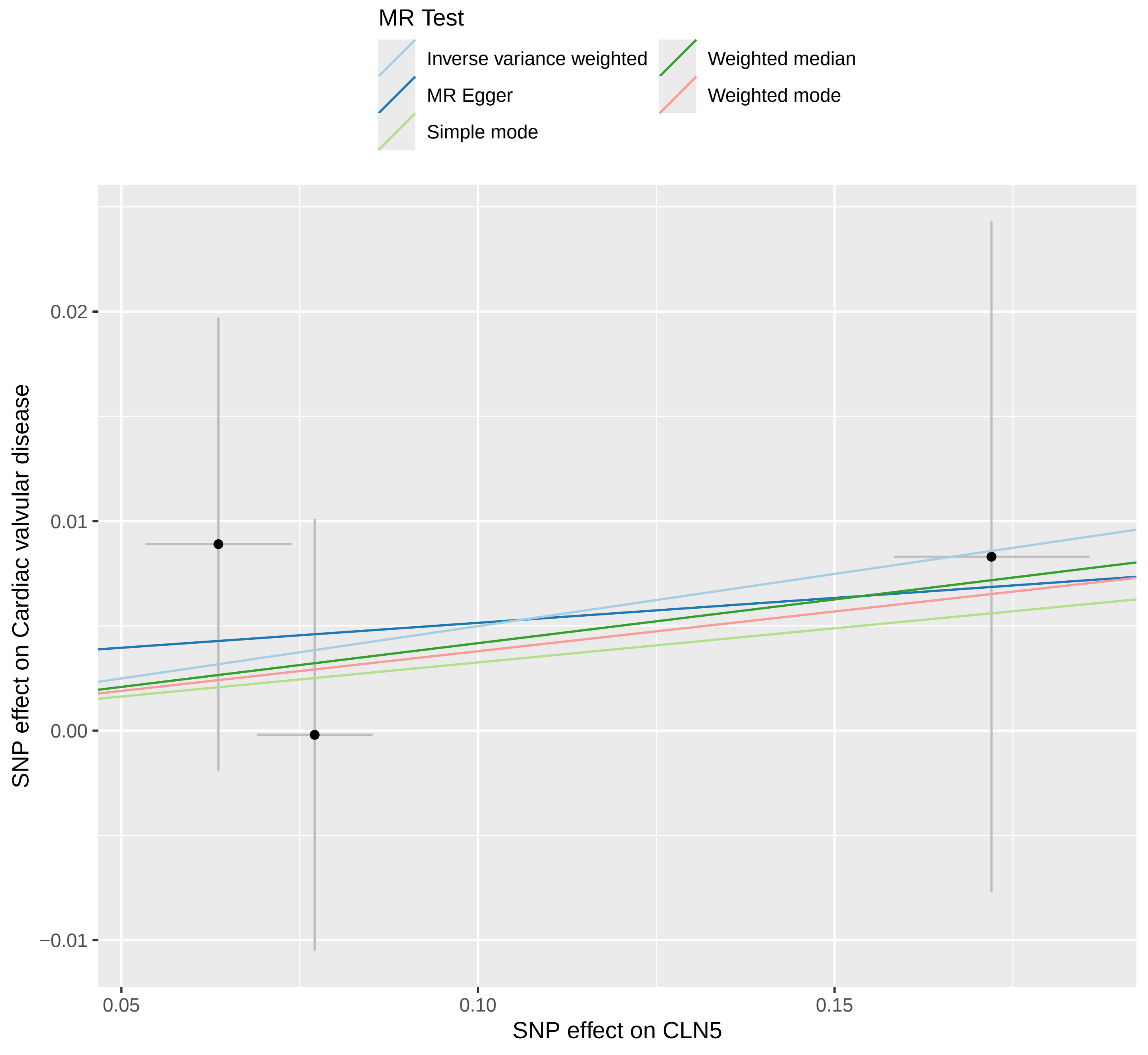

MR Test
Inverse variance weighted
MR Egger
Simple mode
Weighted median
Weighted mode
SNP effect on Cardiac valvular disease
0.02
0.01
0.00
−0.01
0.05
0.10
0.15
SNP effect on CLN5

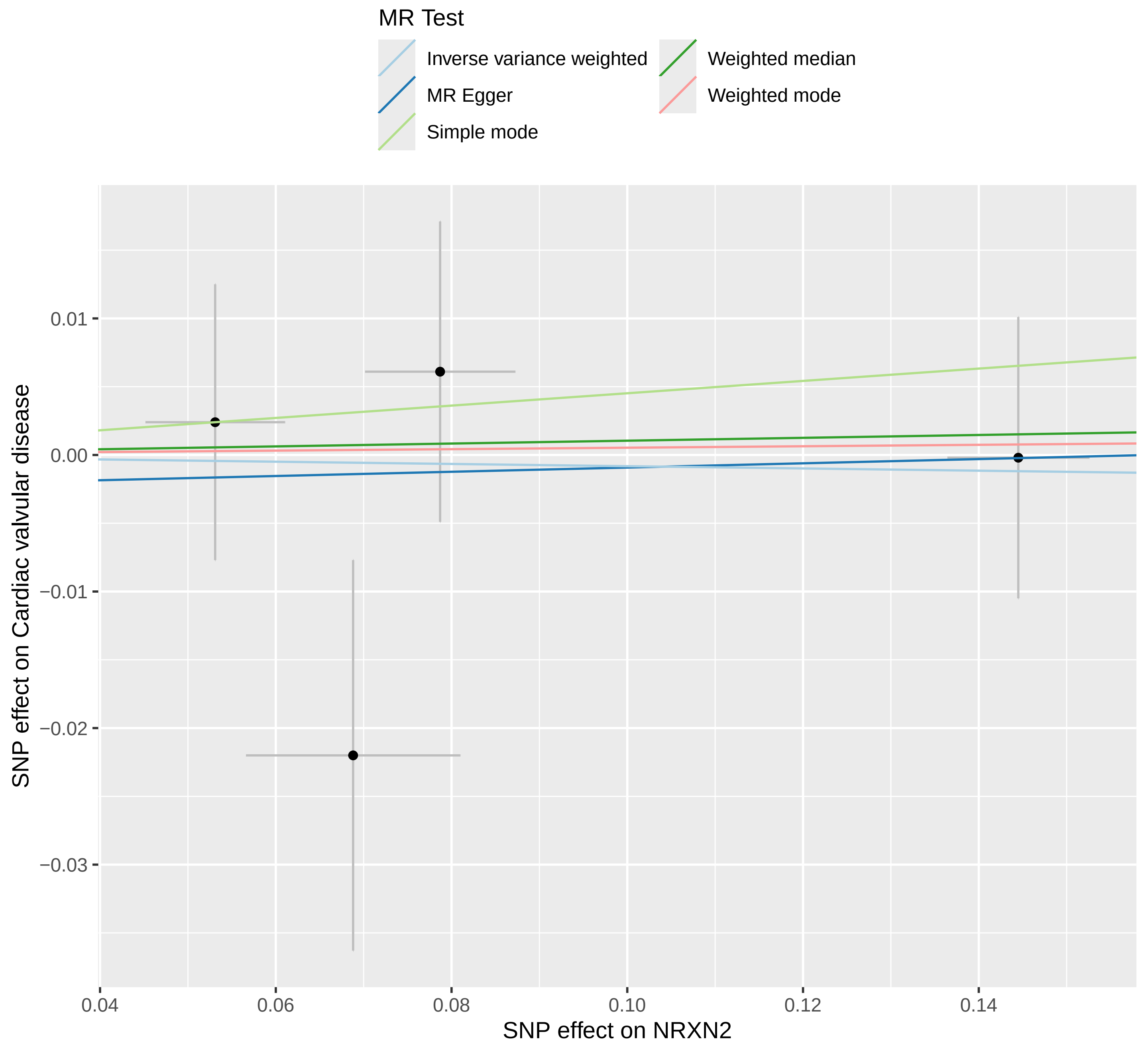
MR Test
Inverse variance weighted
MR Egger
Simple mode
Weighted median
Weighted mode
0.01
0.00
−0.01
−0.02
−0.03
SNP effect on Cardiac valvular disease
0.04
0.06
0.08
0.10
0.12
0.14
SNP effect on NRXN2

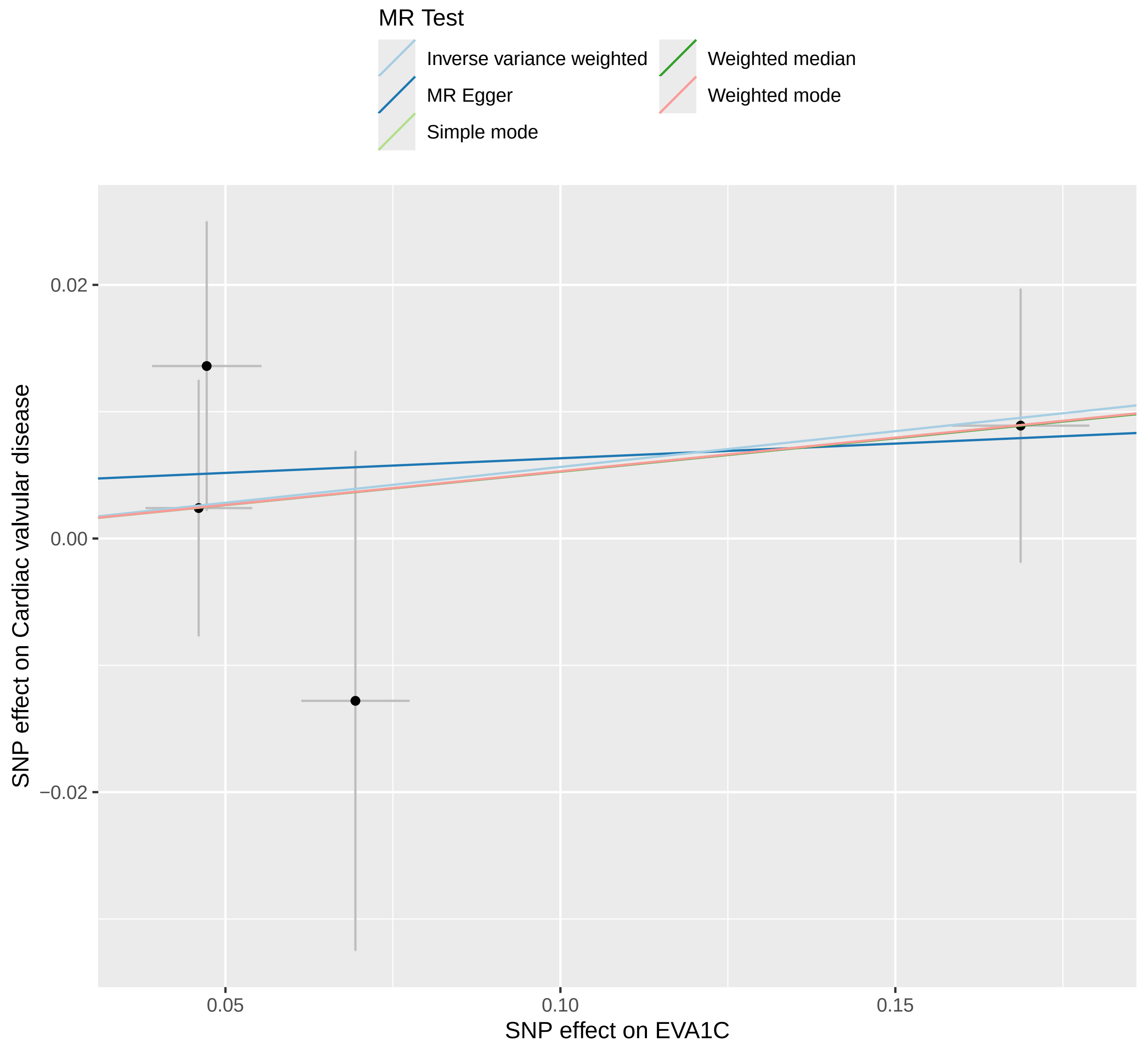
MR Test
Inverse variance weighted
MR Egger
Simple mode
Weighted median
Weighted mode
0.02
0.00
−0.02
0.05
0.10
0.15
SNP effect on Cardiac valvular disease
SNP effect on EVA1C

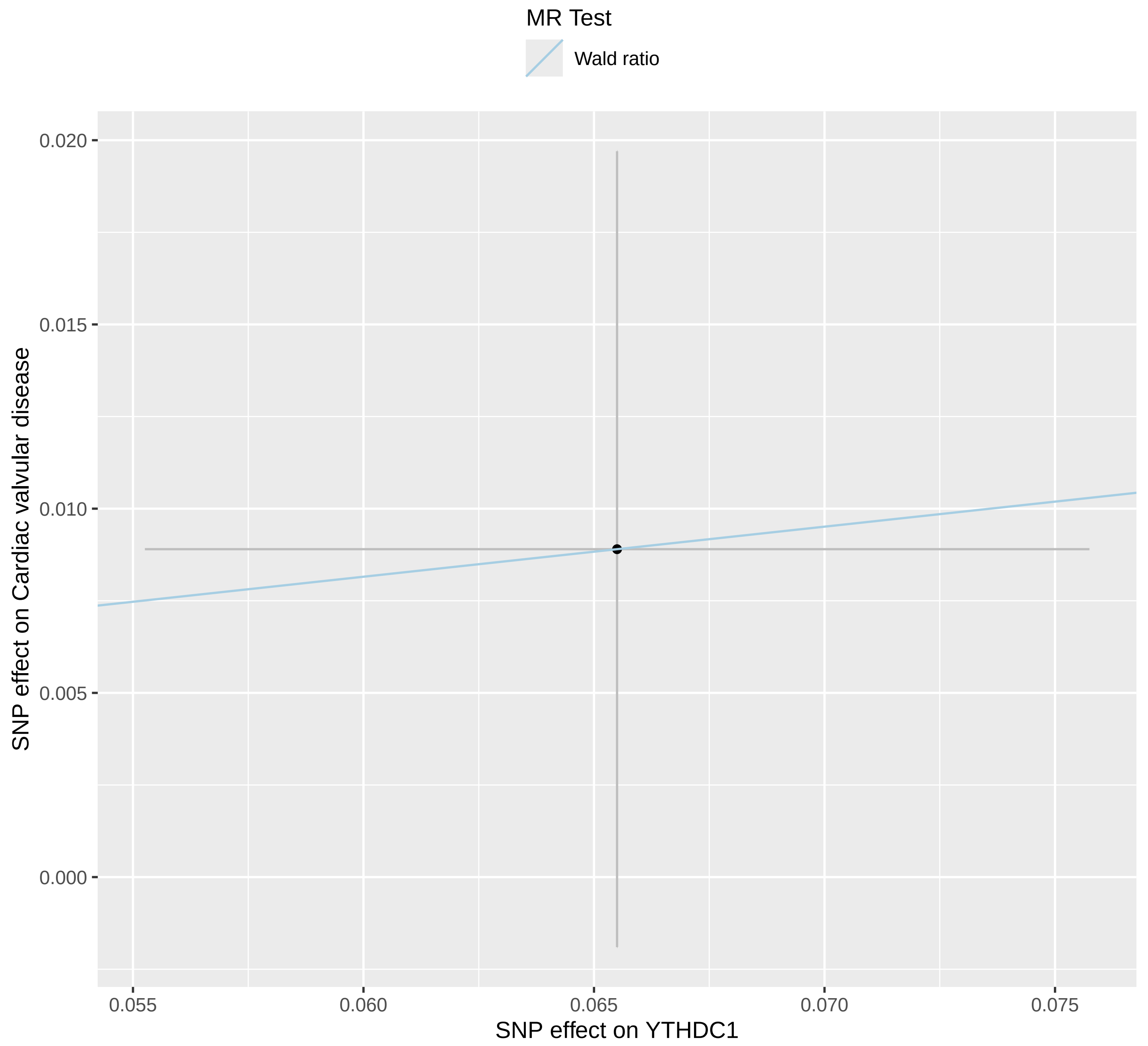
MR Test
Wald ratio
0.020
0.015
0.010
0.005
0.000
SNP effect on Cardiac valvular disease
0.055
0.060
0.065
0.070
0.075
SNP effect on YTHDC1

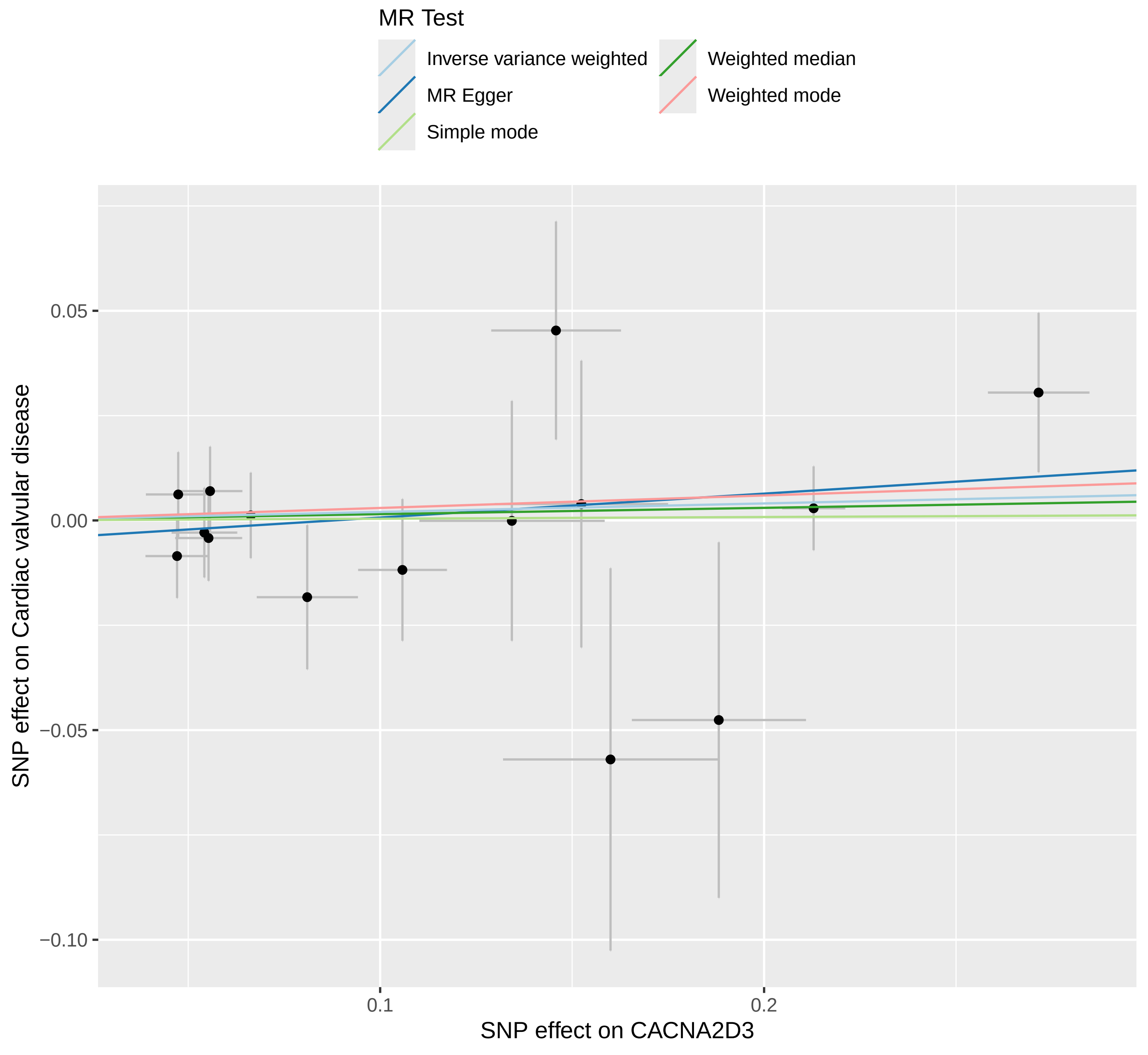
MR Test
Inverse variance weighted
MR Egger
Simple mode
Weighted median
Weighted mode
0.05
0.00
−0.05
−0.10
0.1
0.2
SNP effect on Cardiac valvular disease
SNP effect on CACNA2D3

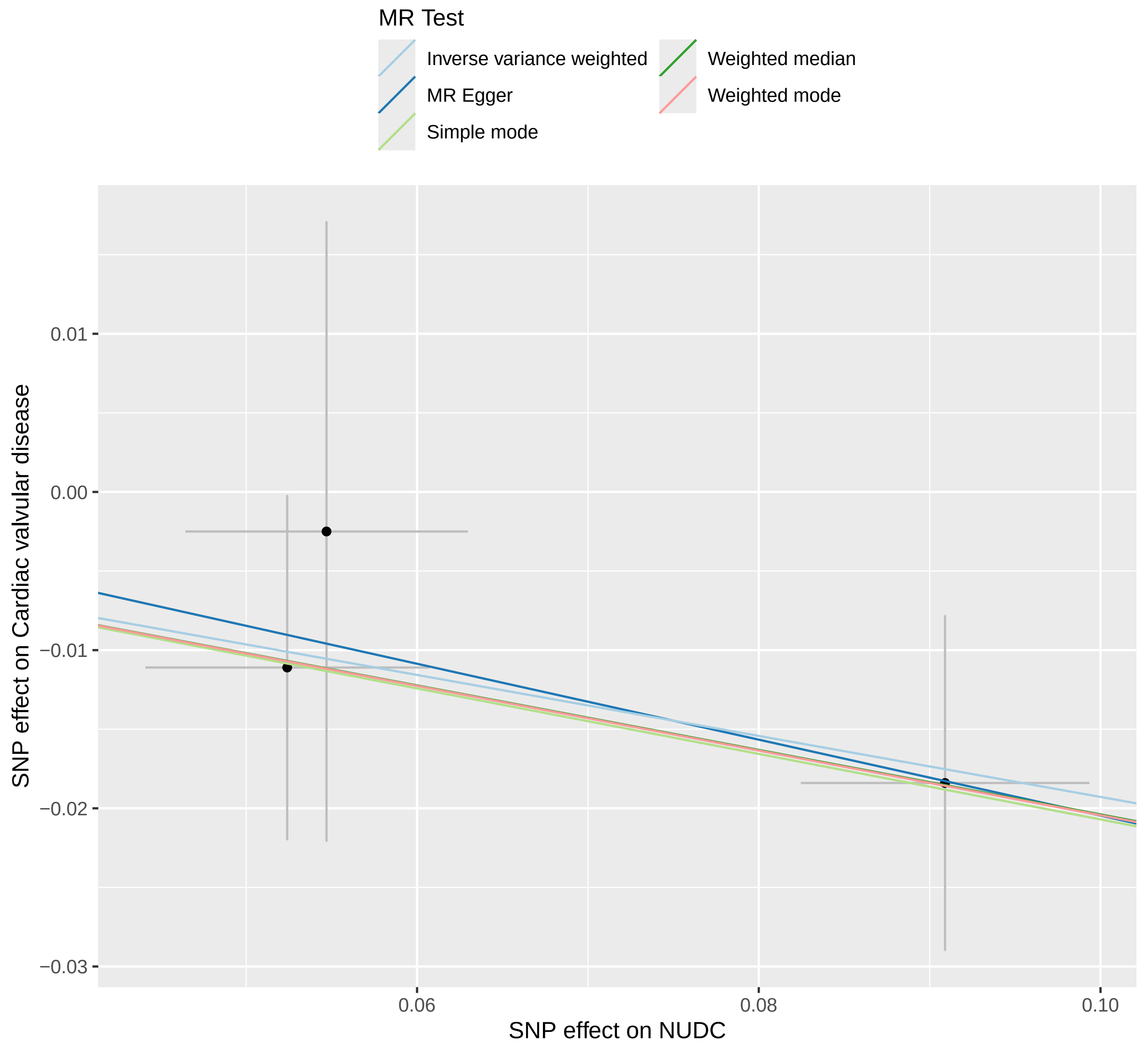
MR Test
Inverse variance weighted
MR Egger
Simple mode
Weighted median
Weighted mode
0.01
0.00
−0.01
−0.02
−0.03
0.06
0.08
0.10
SNP effect on Cardiac valvular disease
SNP effect on NUDC

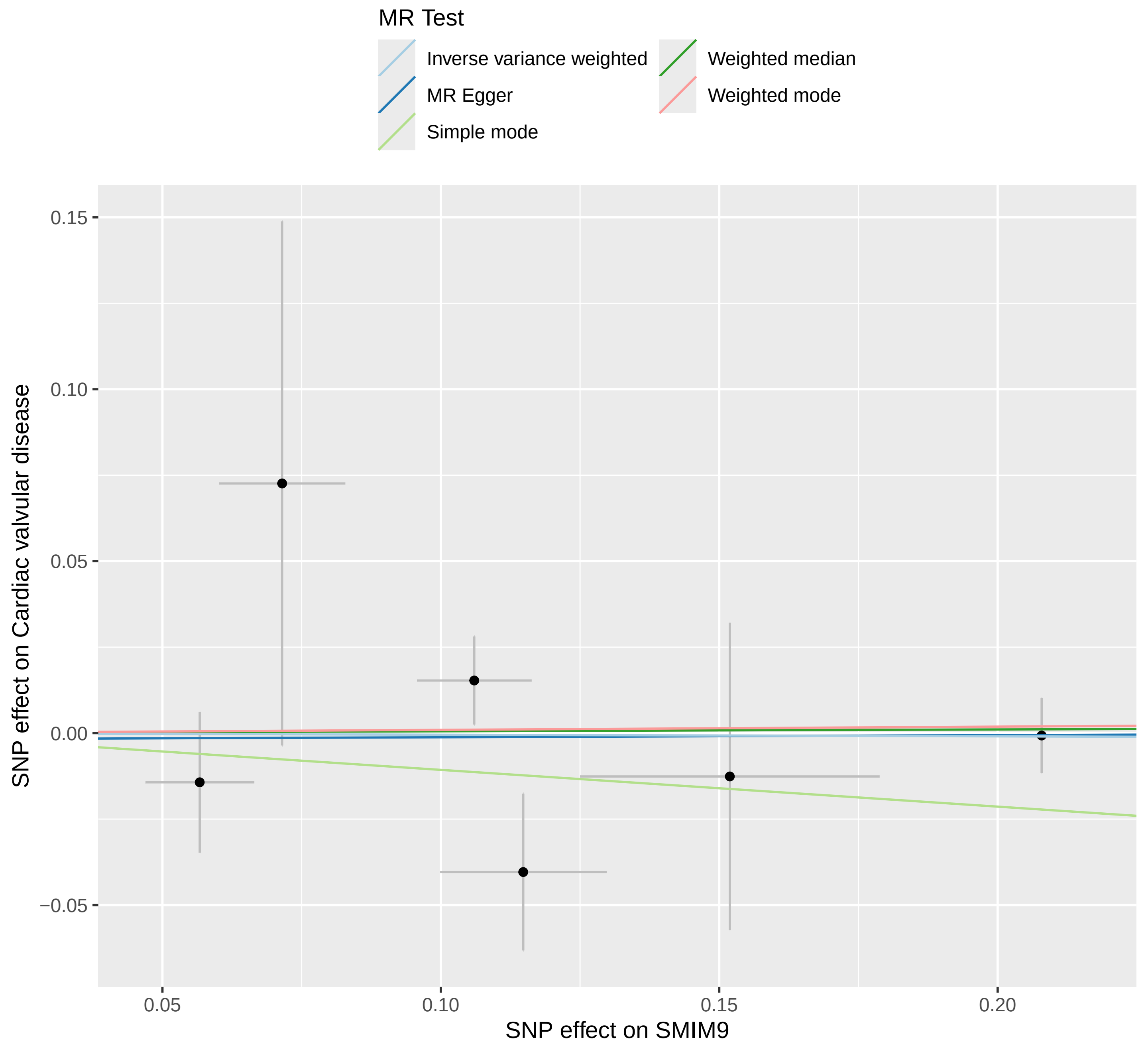

MR Test
Inverse variance weighted
MR Egger
Simple mode
Weighted median
Weighted mode
0.15
0.10
0.05
0.00
−0.05
SNP effect on Cardiac valvular disease
0.05
0.10
0.15
0.20
SNP effect on SMIM9

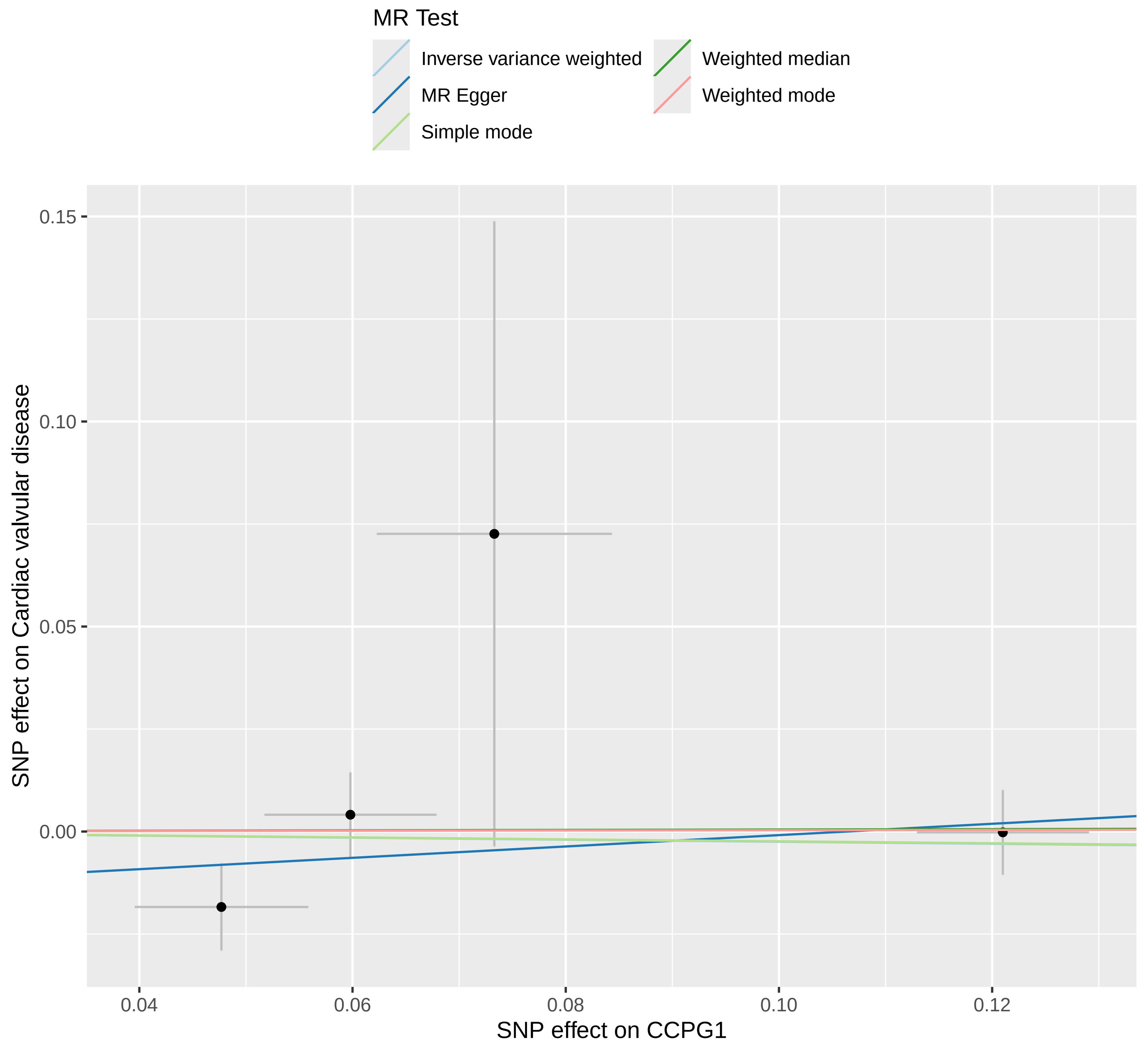
MR Test
Inverse variance weighted
MR Egger
Simple mode
Weighted median
Weighted mode
0.15
0.10
0.05
0.00
SNP effect on Cardiac valvular disease
0.04
0.06
0.08
0.10
0.12
SNP effect on CCPG1

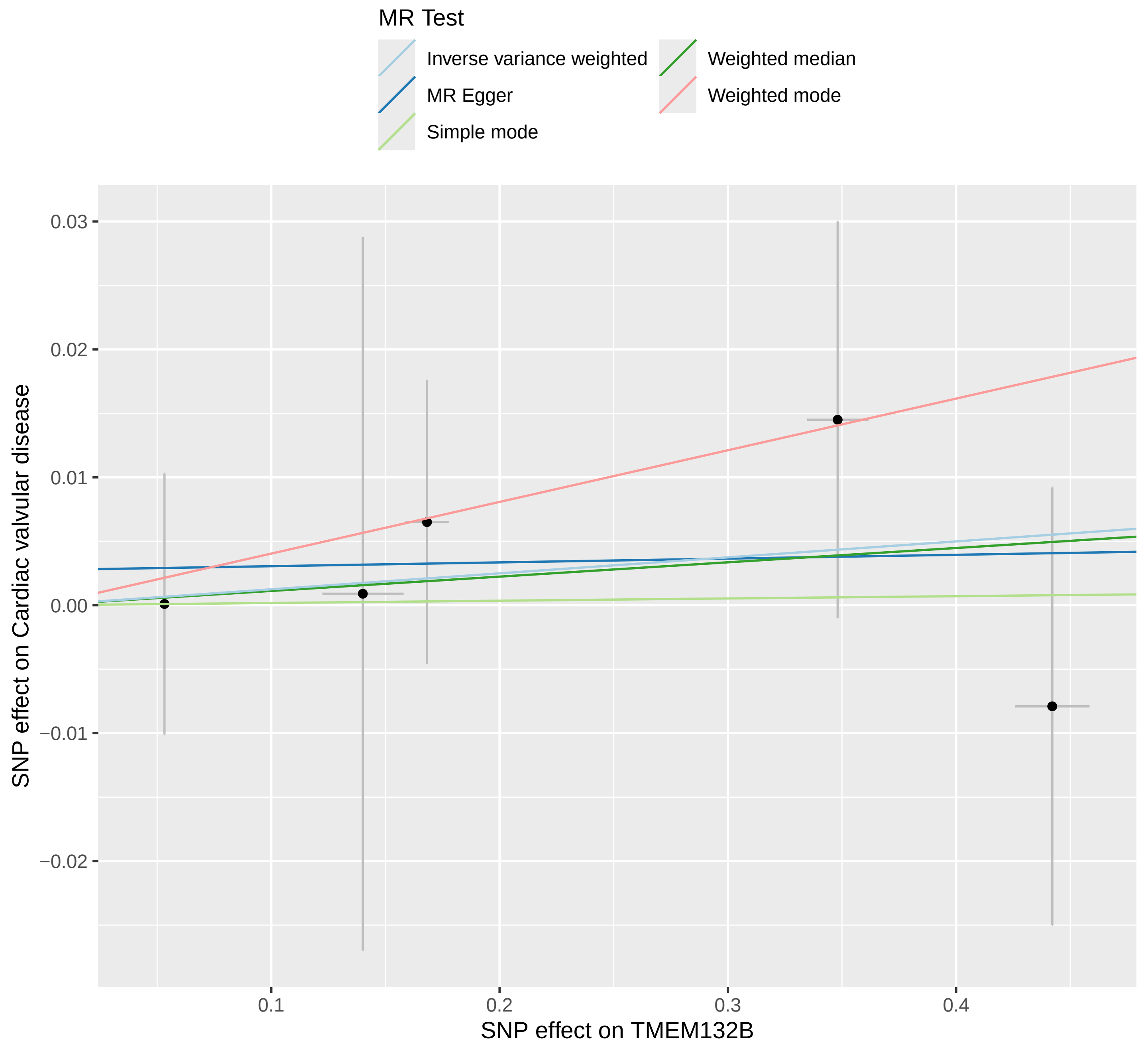

MR Test
Inverse variance weighted
MR Egger
Simple mode
Weighted median
Weighted mode
0.03
0.02
0.01
0.00
−0.01
−0.02
0.1
0.2
0.3
0.4
SNP effect on Cardiac valvular disease
SNP effect on TMEM132B

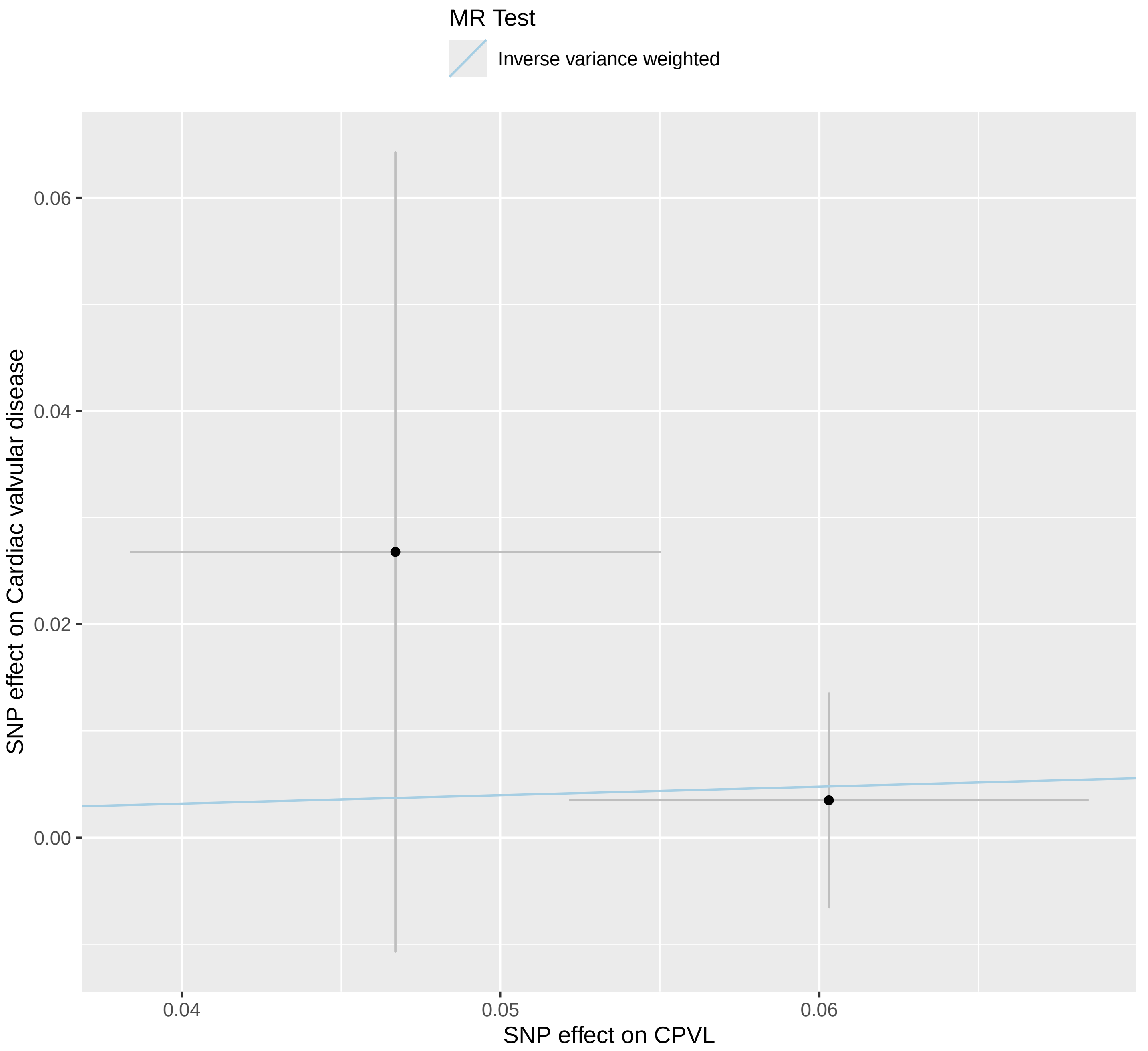
MR Test
Inverse variance weighted
SNP effect on Cardiac valvular disease
0.06
0.04
0.02
0.00
0.04
0.05
0.06
SNP effect on CPVL

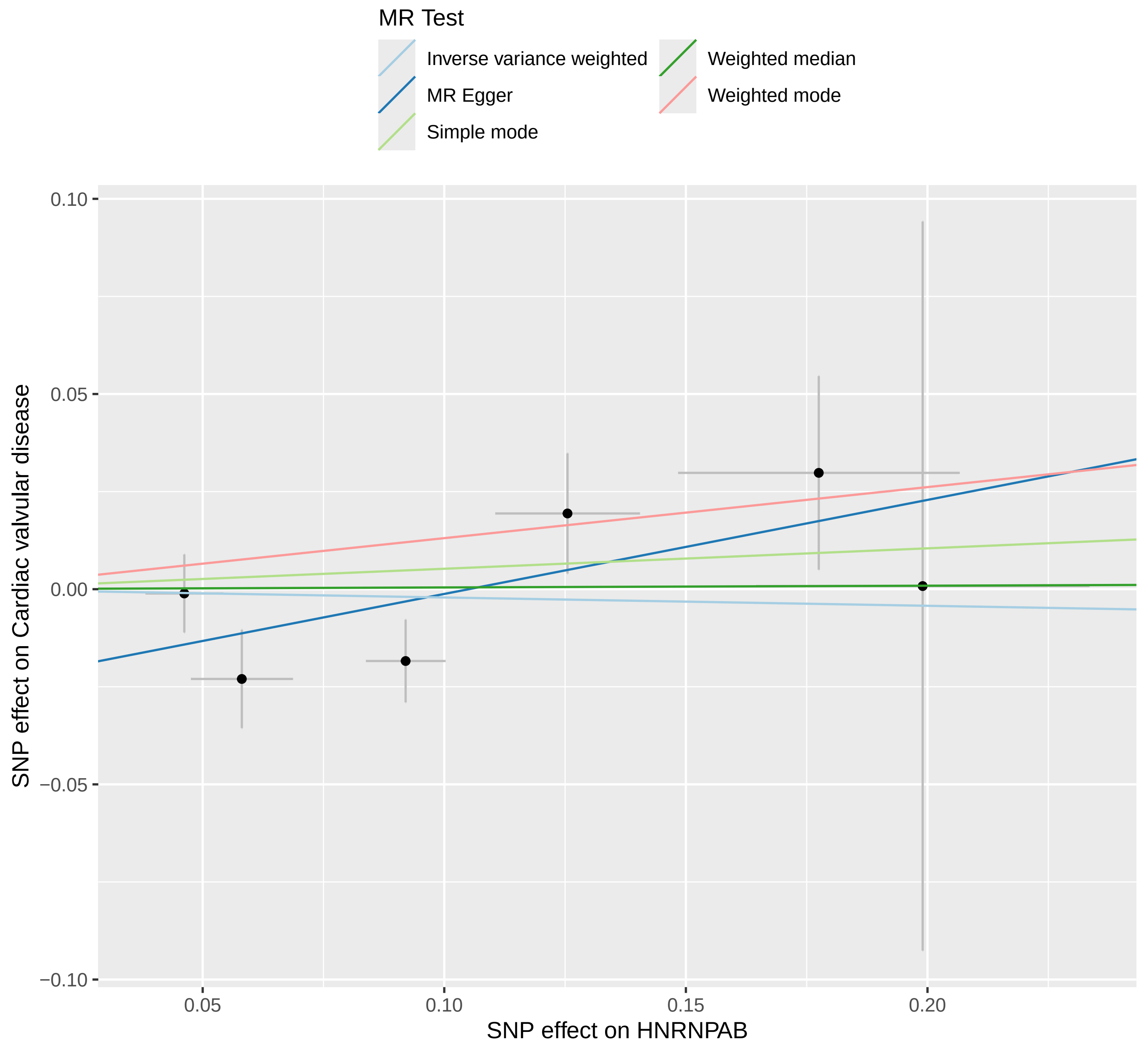
MR Test
Inverse variance weighted
MR Egger
Simple mode
Weighted median
Weighted mode
0.10
0.05
0.00
−0.05
−0.10
SNP effect on Cardiac valvular disease
0.05
0.10
0.15
0.20
SNP effect on HNRNPAB

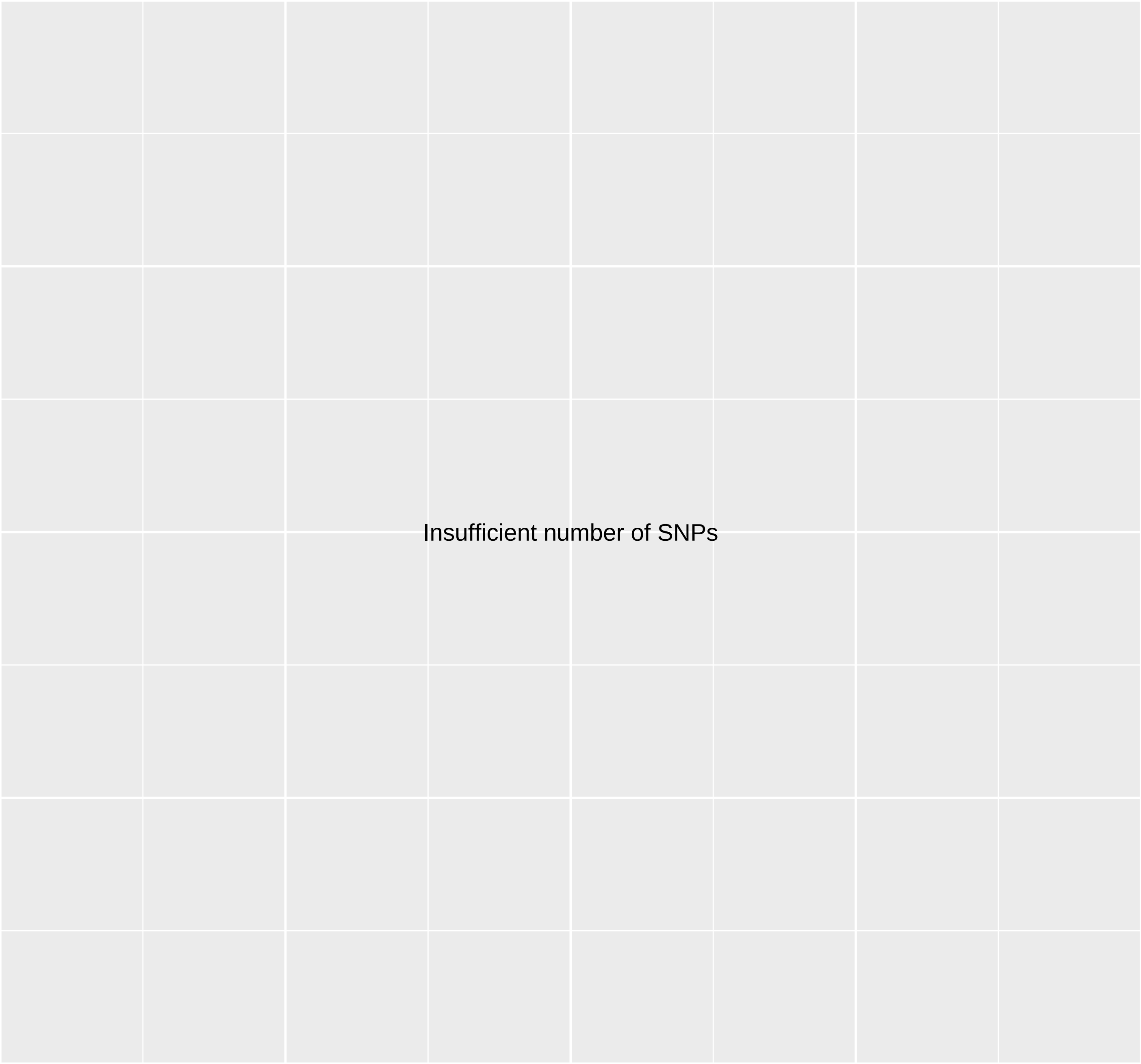
Insufficient number of SNPs

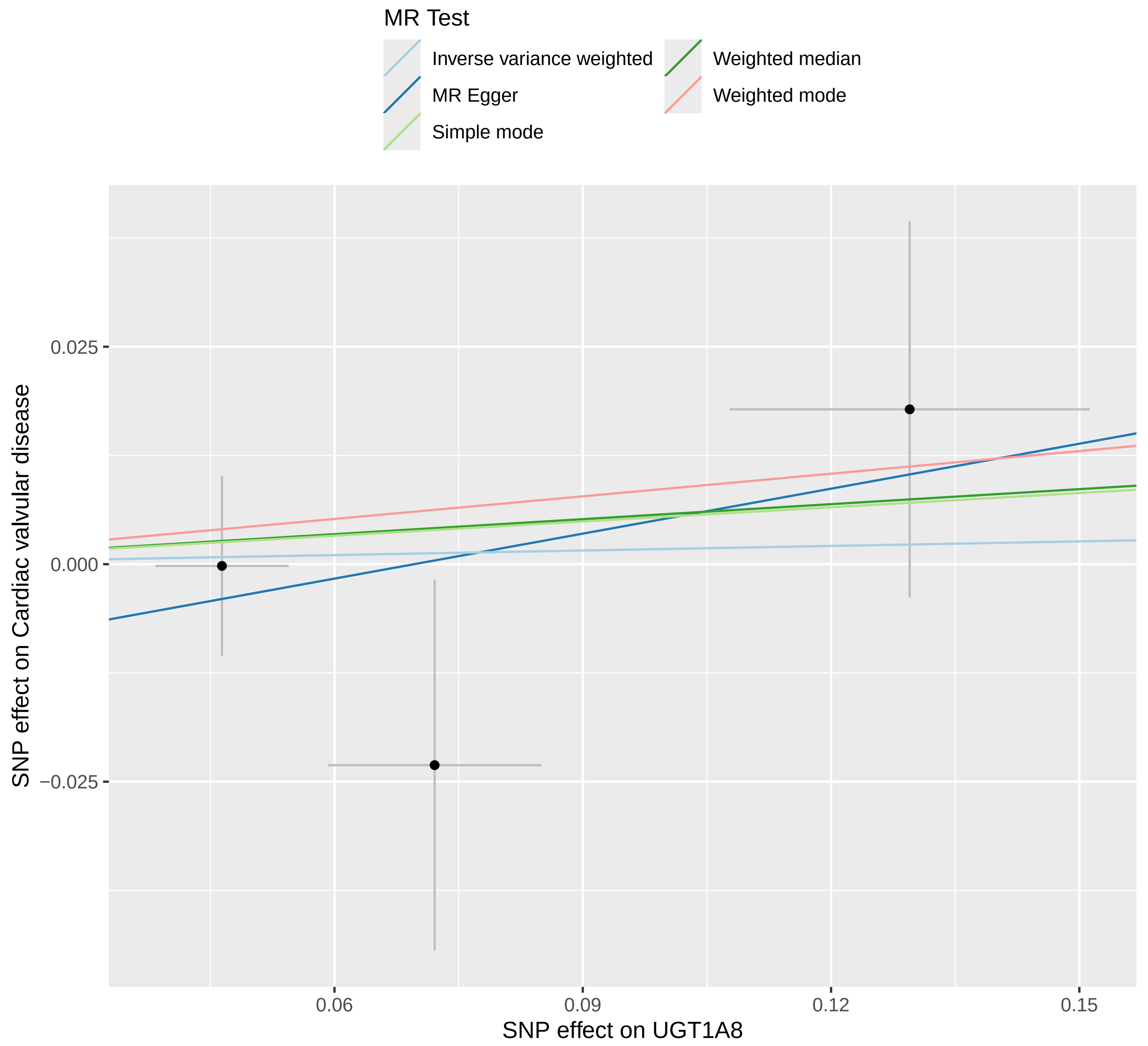
MR Test
Inverse variance weighted
MR Egger
Simple mode
Weighted median
Weighted mode
0.025
0.000
−0.025
0.06
0.09
0.12
0.15
SNP effect on Cardiac valvular disease
SNP effect on UGT1A8

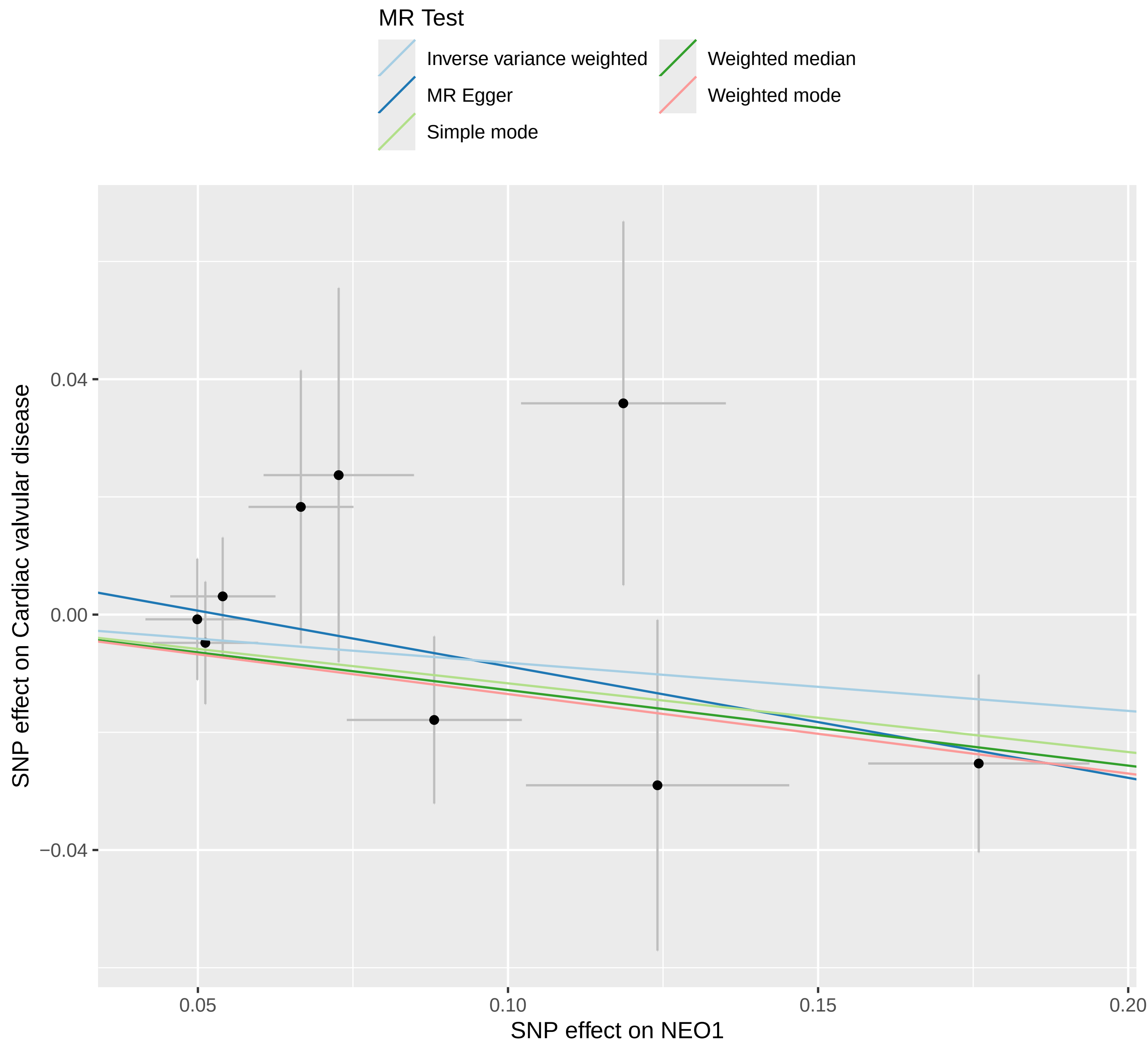

MR Test
Inverse variance weighted
MR Egger
Simple mode
Weighted median
Weighted mode
SNP effect on Cardiac valvular disease
0.04
0.00
−0.04
0.05
0.10
0.15
0.20
SNP effect on NEO1

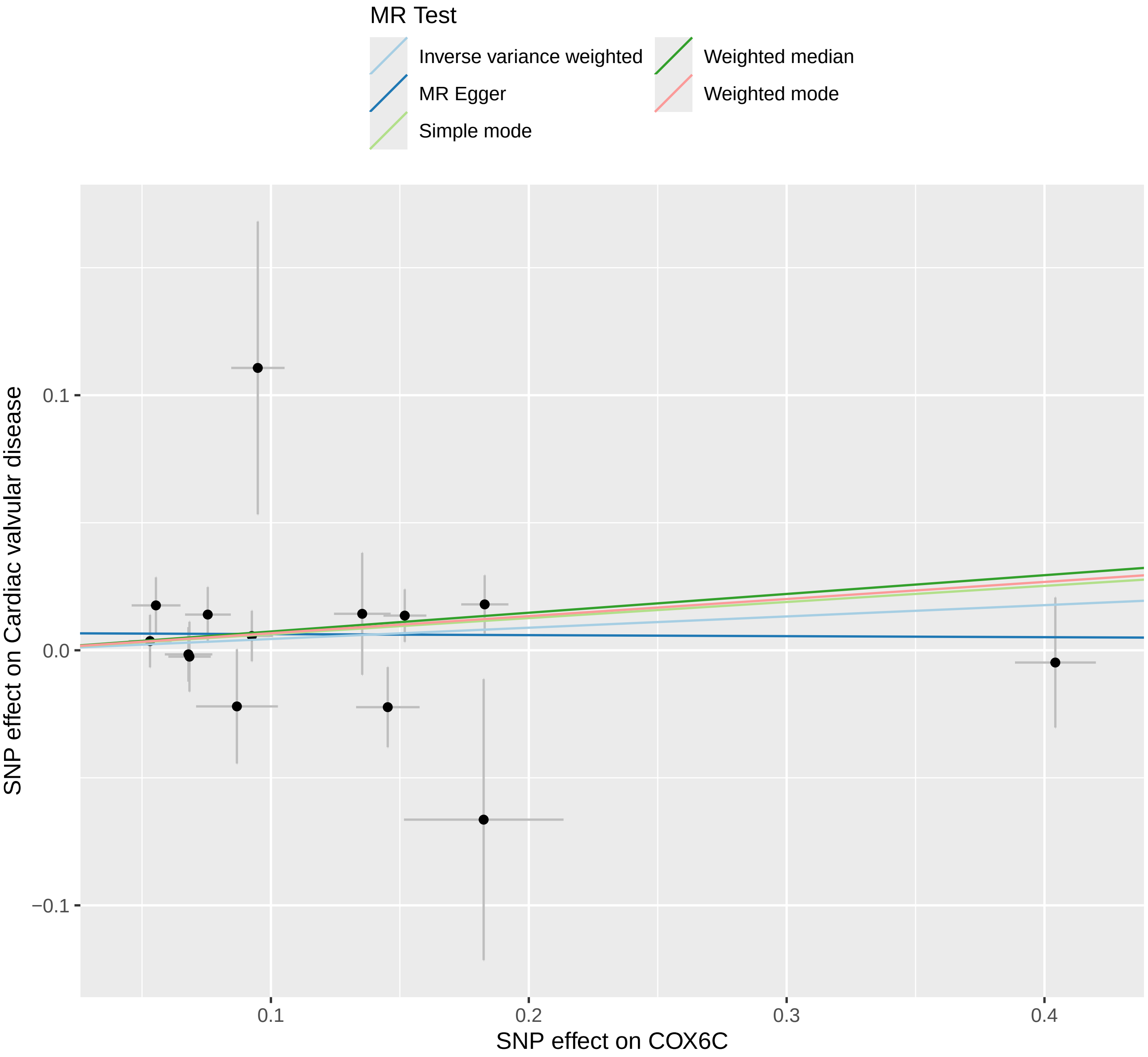

MR Test
Inverse variance weighted
MR Egger
Simple mode
Weighted median
Weighted mode
0.1
0.0
−0.1
SNP effect on Cardiac valvular disease
0.1
0.2
0.3
0.4
SNP effect on COX6C

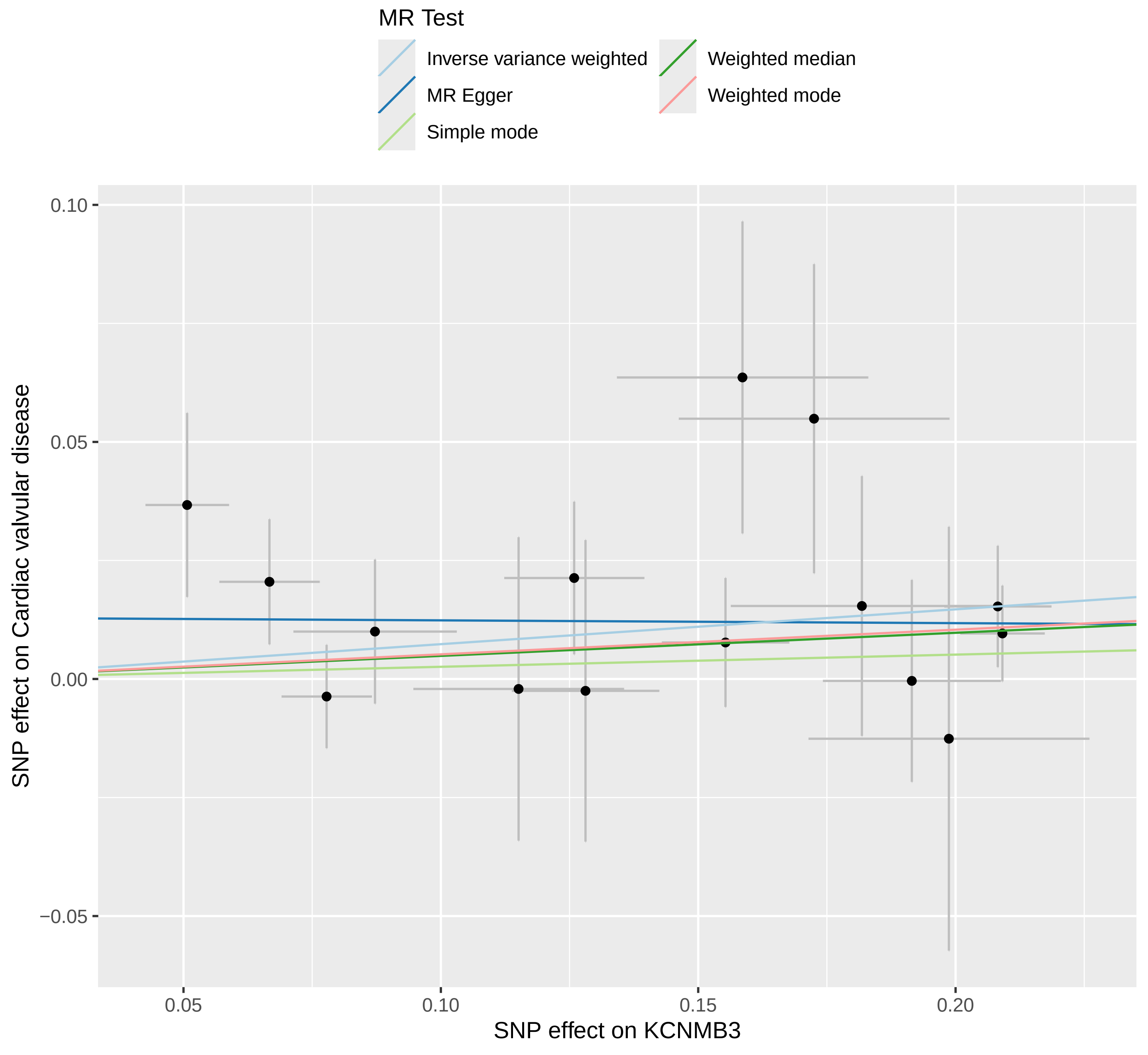
MR Test
Inverse variance weighted
MR Egger
Simple mode
Weighted median
Weighted mode
0.10
0.05
0.00
−0.05
SNP effect on Cardiac valvular disease
0.05
0.10
0.15
0.20
SNP effect on KCNMB3

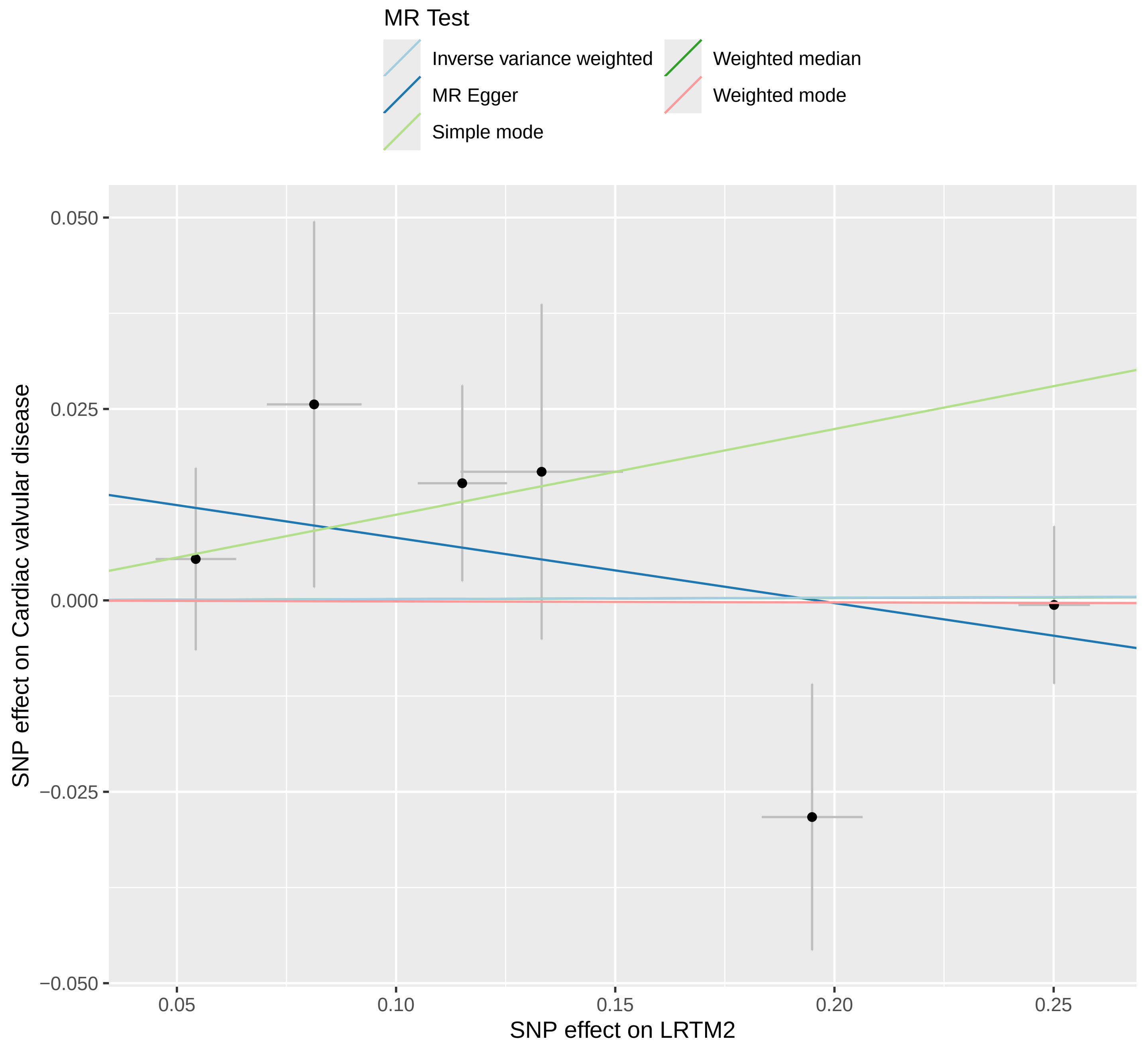

MR Test
Inverse variance weighted
MR Egger
Simple mode
Weighted median
Weighted mode
0.050
0.025
0.000
−0.025
−0.050
SNP effect on Cardiac valvular disease
0.05
0.10
0.15
0.20
0.25
SNP effect on LRTM2

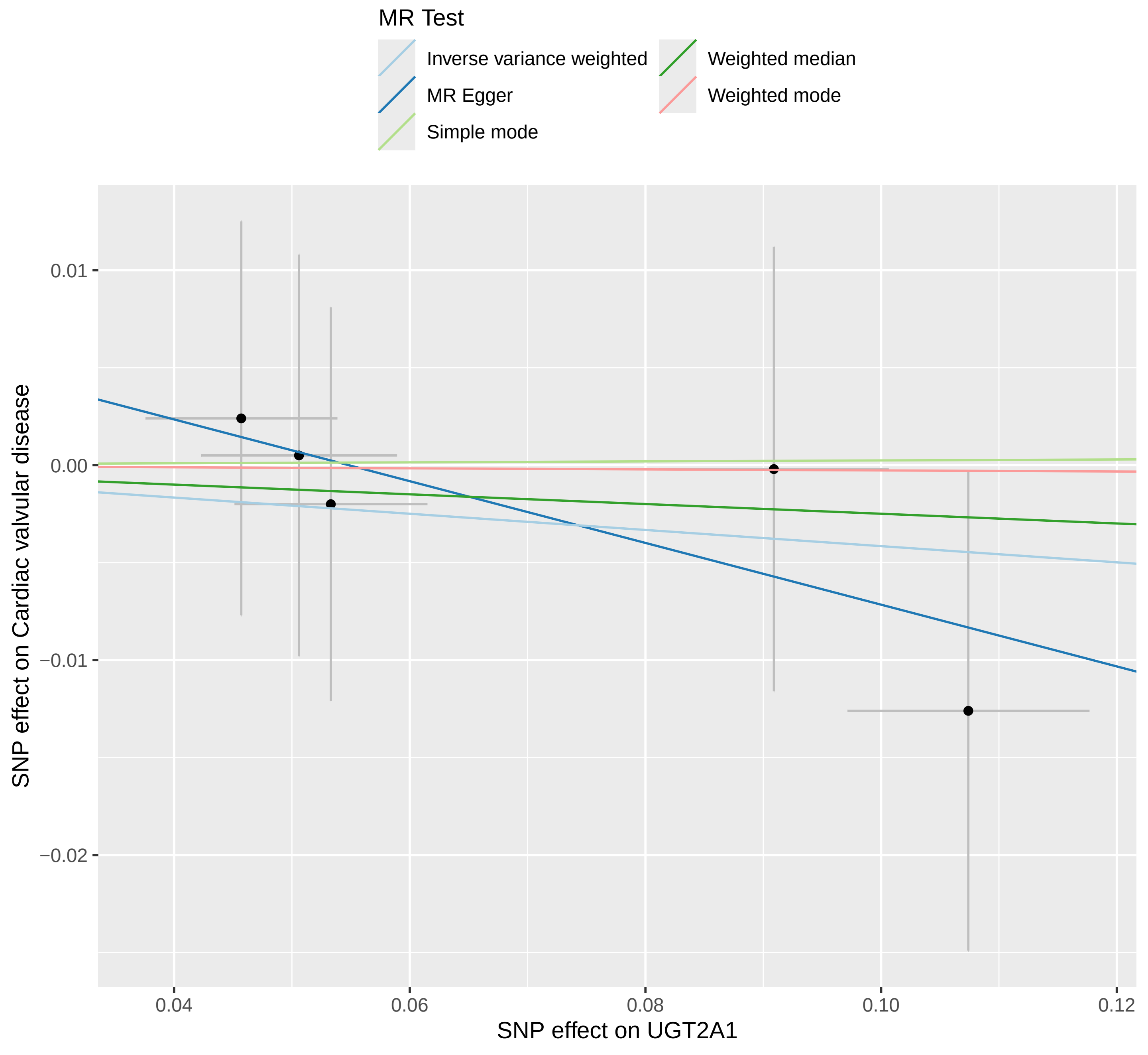
MR Test
Inverse variance weighted
MR Egger
Simple mode
Weighted median
Weighted mode
0.01
0.00
−0.01
−0.02
SNP effect on Cardiac valvular disease
0.04
0.06
0.08
0.10
0.12
SNP effect on UGT2A1

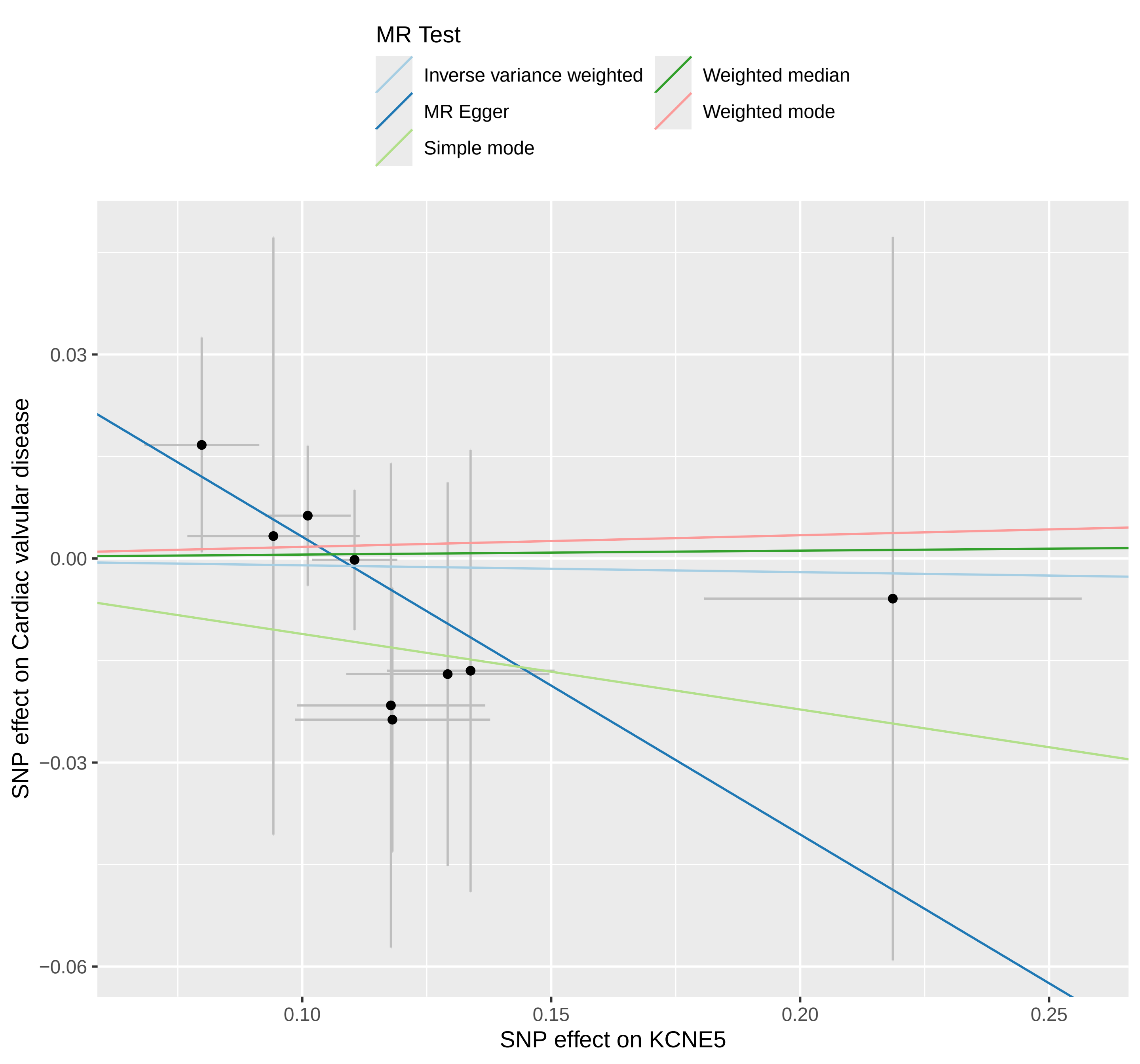

MR Test
Inverse variance weighted
MR Egger
Simple mode
Weighted median
Weighted mode
0.03
0.00
−0.03
−0.06
0.10
0.15
0.20
0.25
SNP effect on KCNE5
SNP effect on Cardiac valvular disease

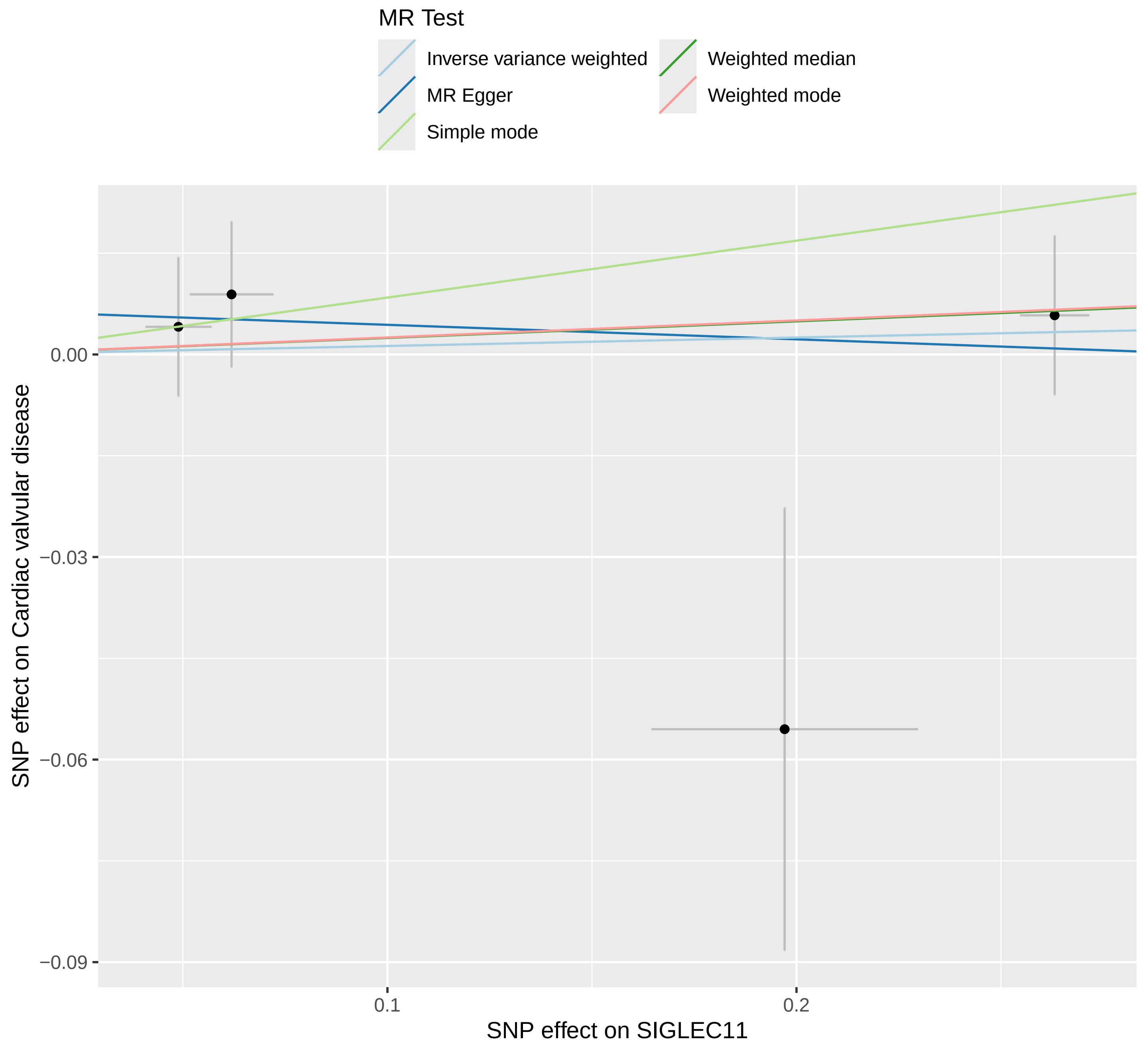
MR Test
Inverse variance weighted
MR Egger
Simple mode
Weighted median
Weighted mode
0.00
−0.03
−0.06
−0.09
0.1
0.2
SNP effect on Cardiac valvular disease
SNP effect on SIGLEC11

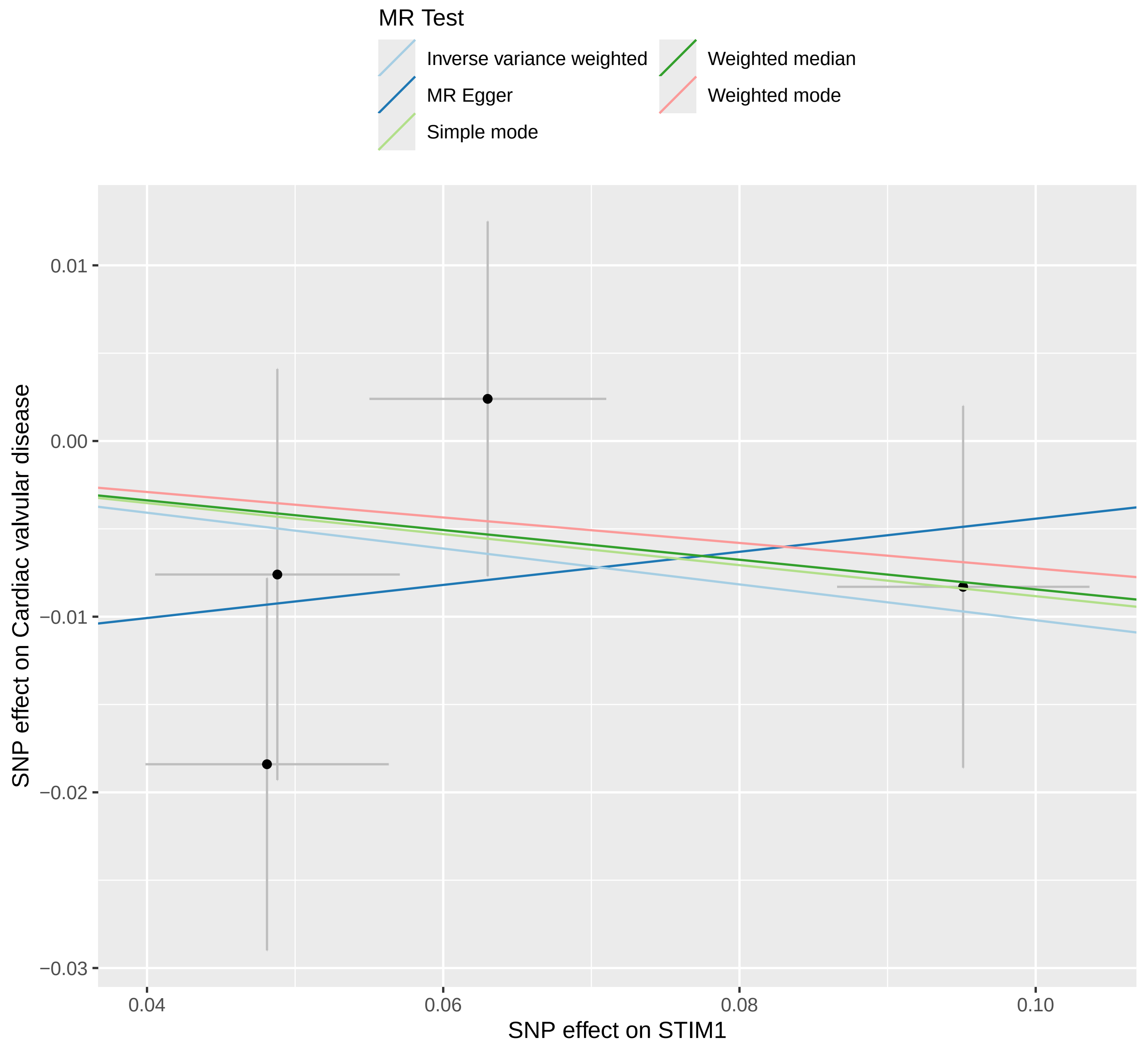
MR Test
Inverse variance weighted
MR Egger
Simple mode
Weighted median
Weighted mode
0.01
0.00
−0.01
−0.02
−0.03
0.04
0.06
0.08
0.10
SNP effect on Cardiac valvular disease
SNP effect on STIM1

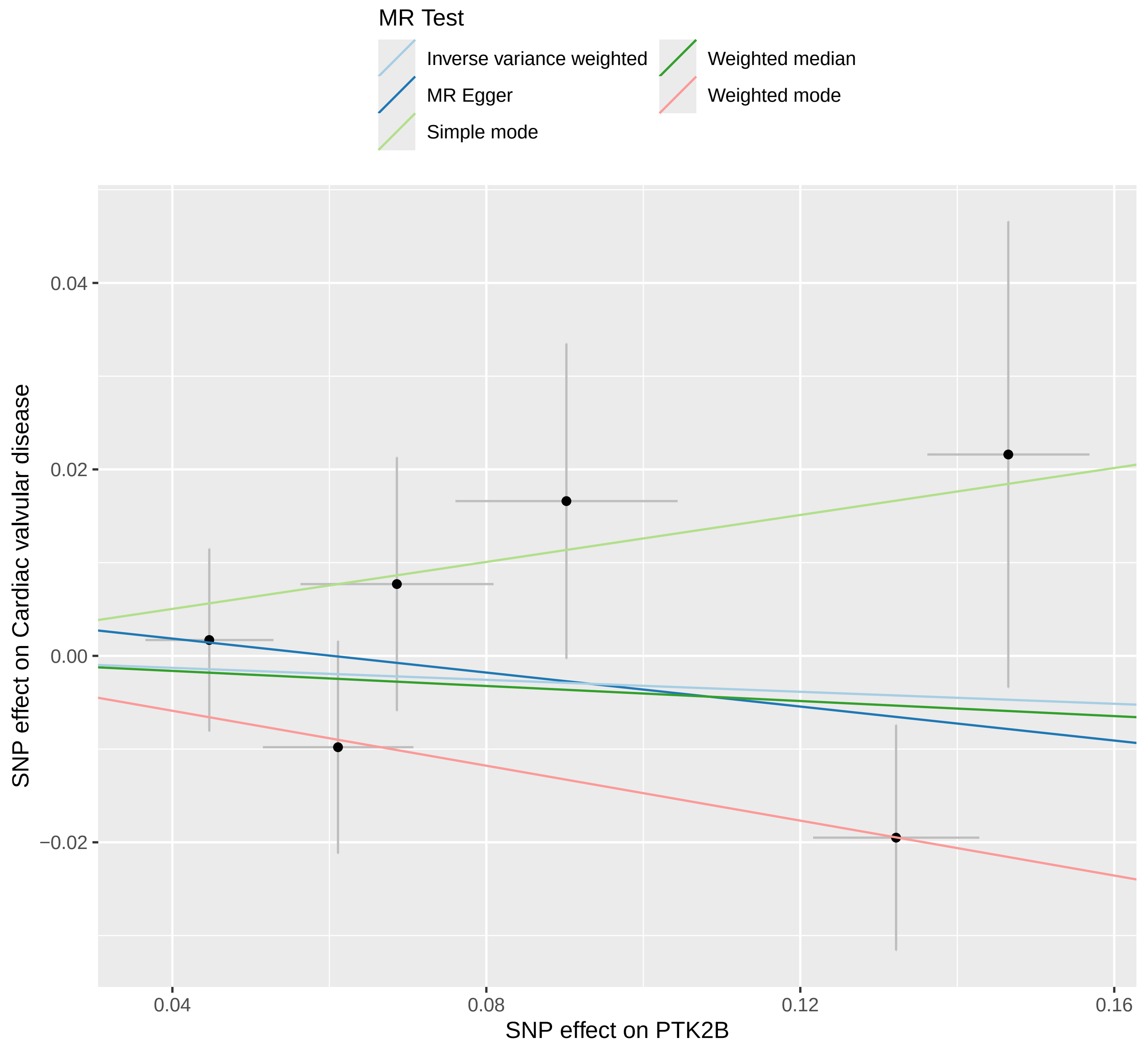
MR Test
Inverse variance weighted
MR Egger
Simple mode
Weighted median
Weighted mode
0.04
0.02
0.00
−0.02
SNP effect on Cardiac valvular disease
0.04
0.08
0.12
0.16
SNP effect on PTK2B

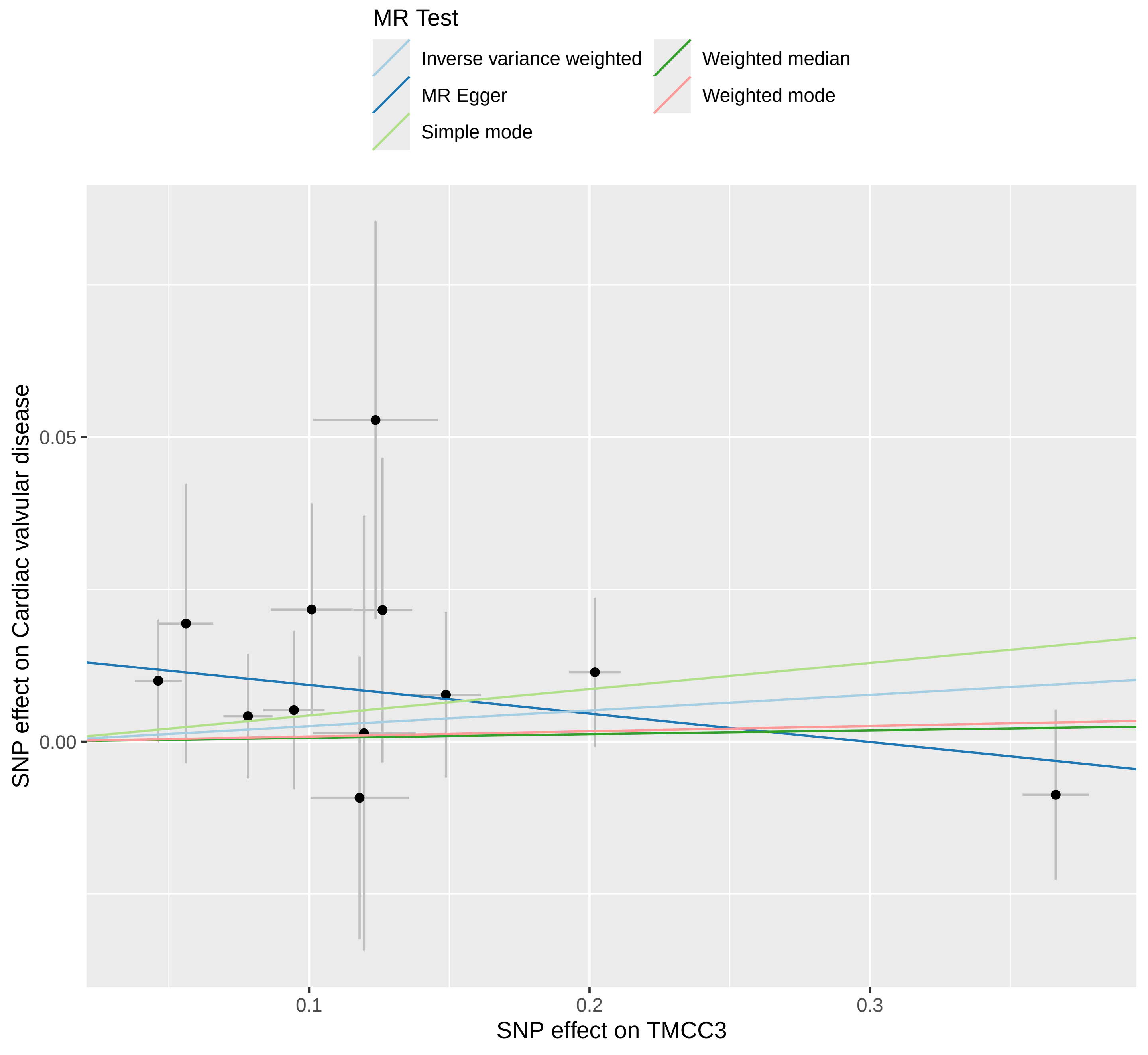
MR Test
Inverse variance weighted
MR Egger
Simple mode
Weighted median
Weighted mode
SNP effect on Cardiac valvular disease
0.05
0.00
0.1
0.2
0.3
SNP effect on TMCC3

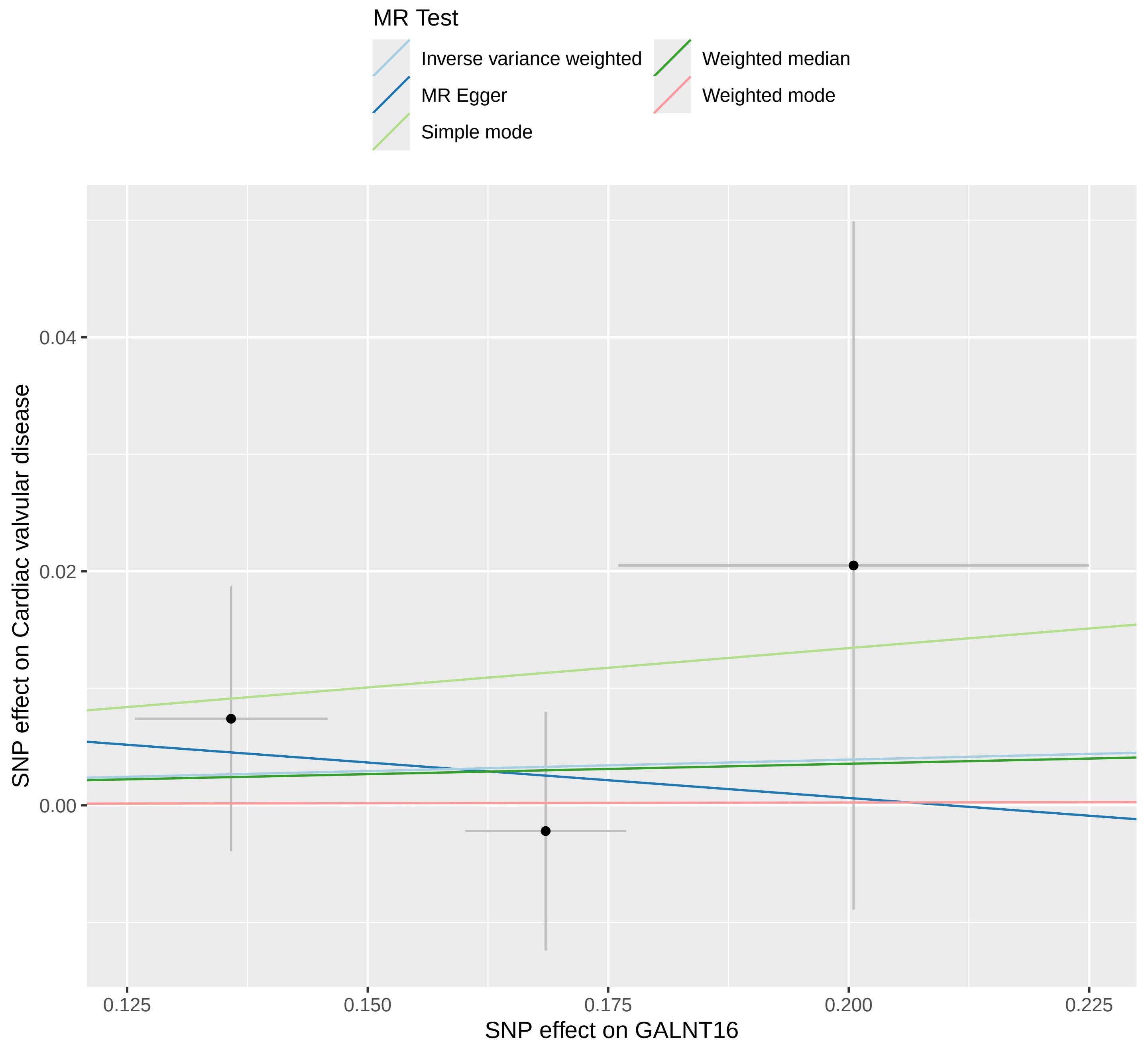

MR Test
Inverse variance weighted
MR Egger
Simple mode
Weighted median
Weighted mode
0.04
0.02
0.00
SNP effect on Cardiac valvular disease
0.125
0.150
0.175
0.200
0.225
SNP effect on GALNT16

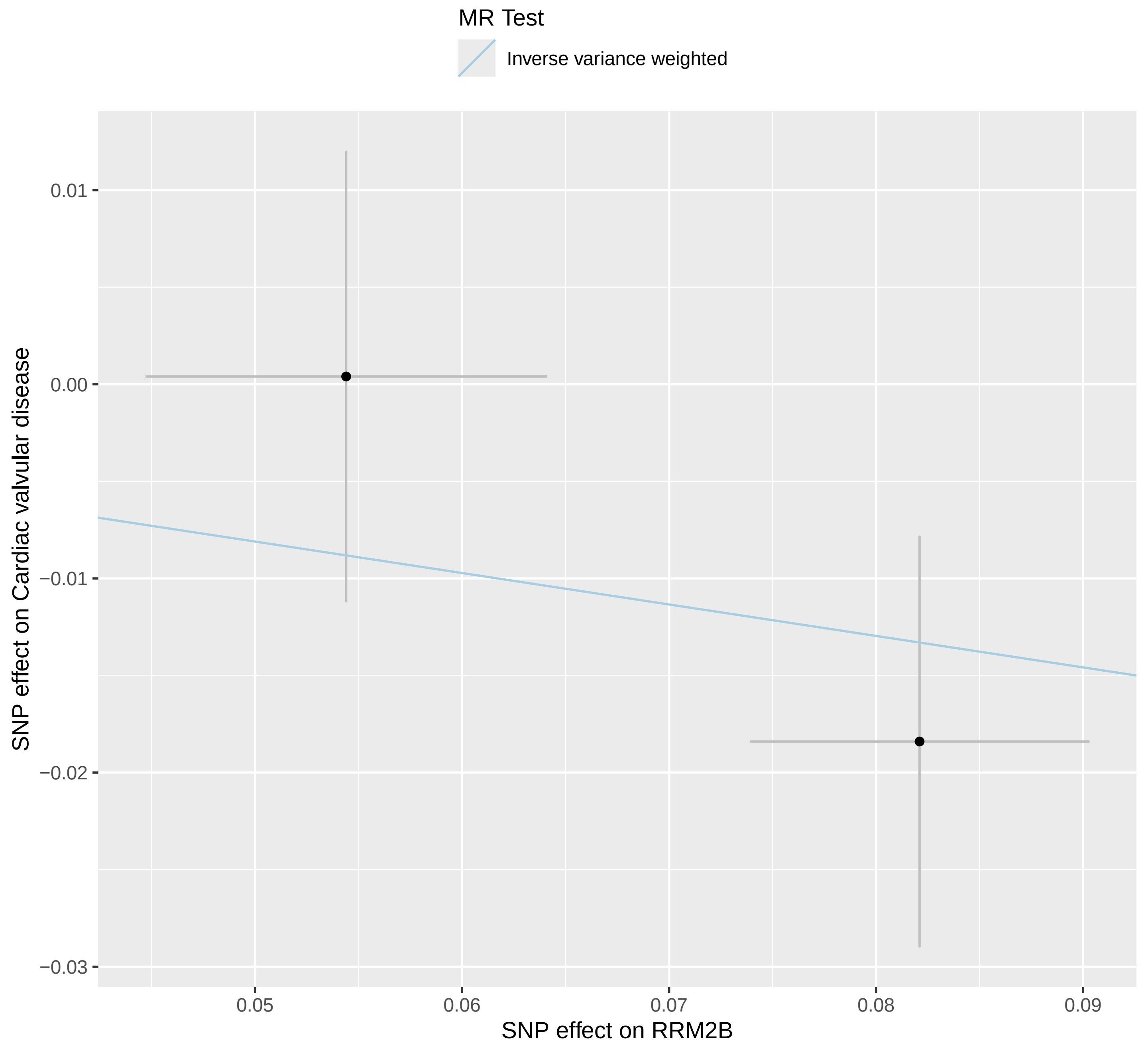
MR Test
Inverse variance weighted
SNP effect on Cardiac valvular disease
0.01
0.00
−0.01
−0.02
−0.03
0.05
0.06
0.07
0.08
0.09
SNP effect on RRM2B

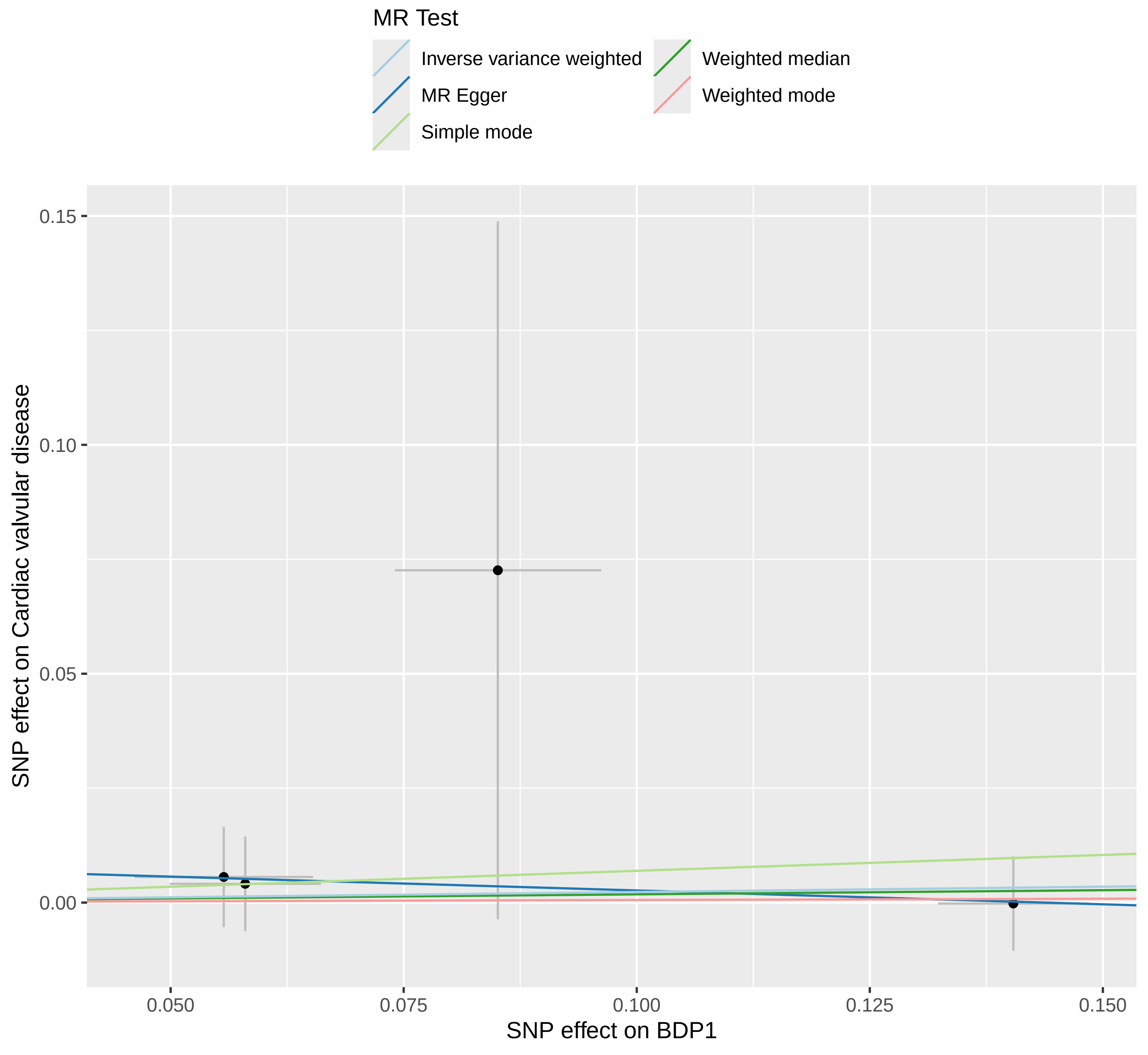
MR Test
Inverse variance weighted
MR Egger
Simple mode
Weighted median
Weighted mode
SNP effect on Cardiac valvular disease
0.15
0.10
0.05
0.00
0.050
0.075
0.100
0.125
0.150
SNP effect on BDP1

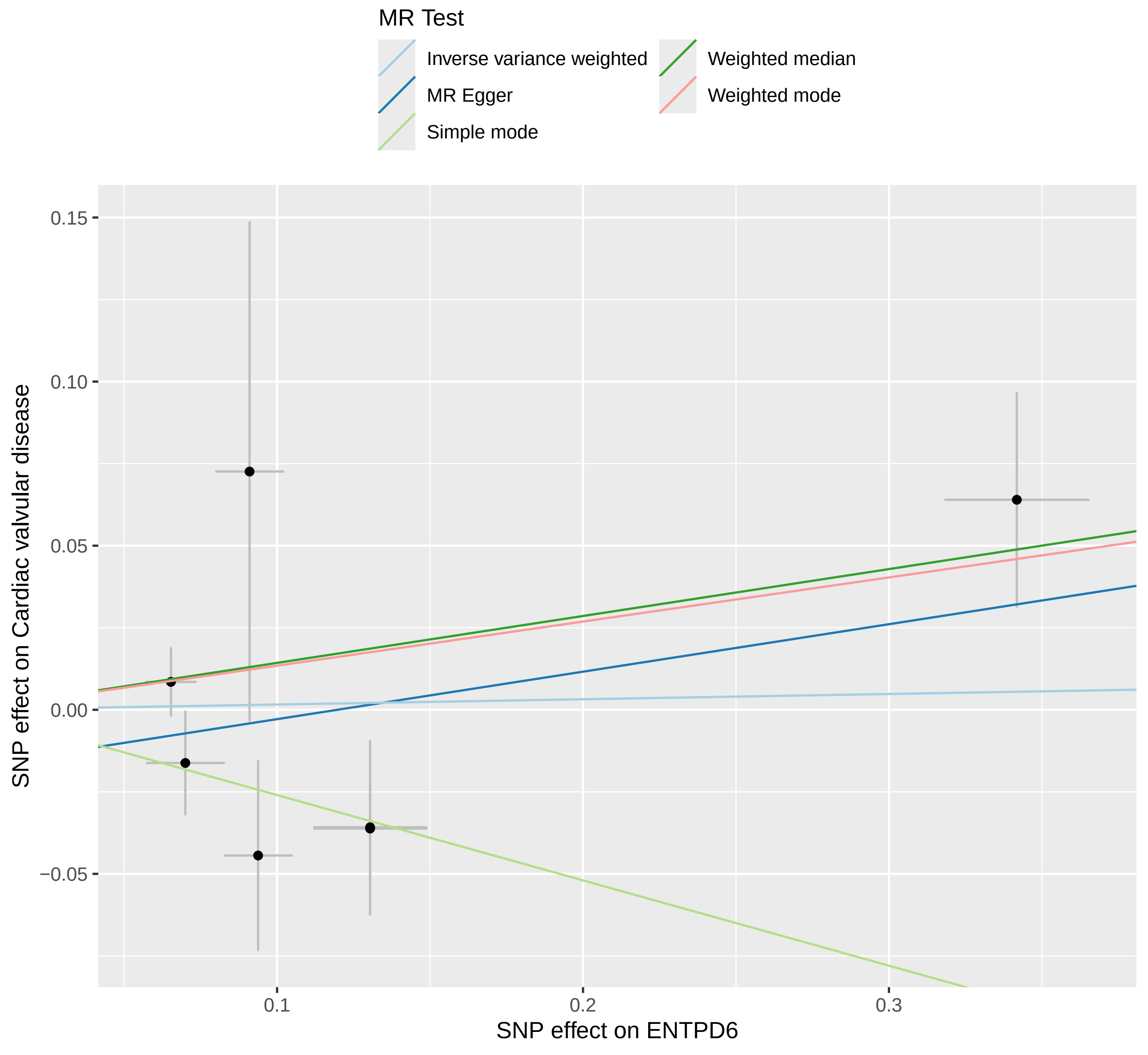
MR Test
Inverse variance weighted
MR Egger
Simple mode
Weighted median
Weighted mode
SNP effect on Cardiac valvular disease
0.15
0.10
0.05
0.00
−0.05
0.1
0.2
0.3
SNP effect on ENTPD6

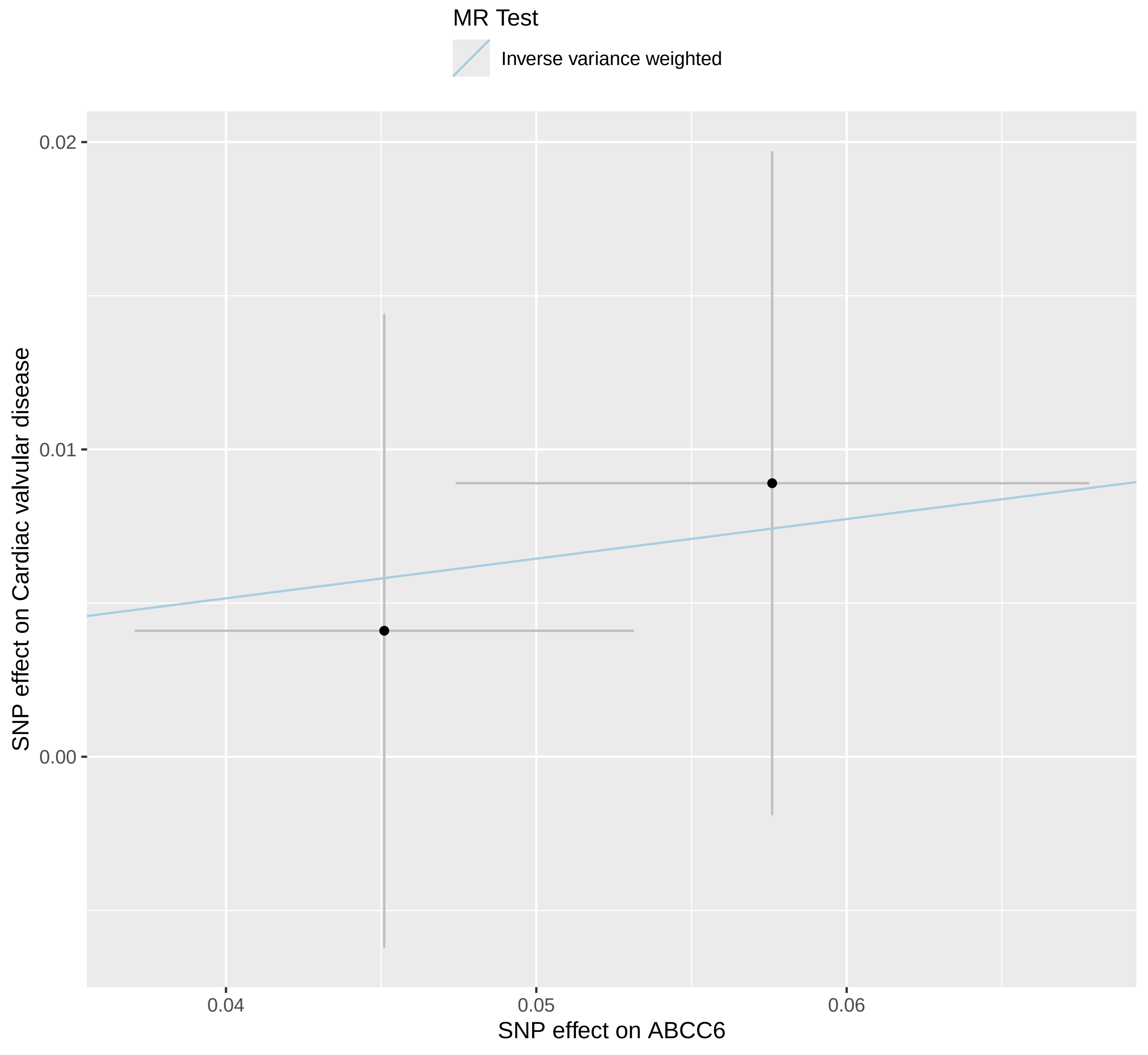
MR Test
Inverse variance weighted
0.02
0.01
0.00
SNP effect on Cardiac valvular disease
0.04
0.05
0.06
SNP effect on ABCC6

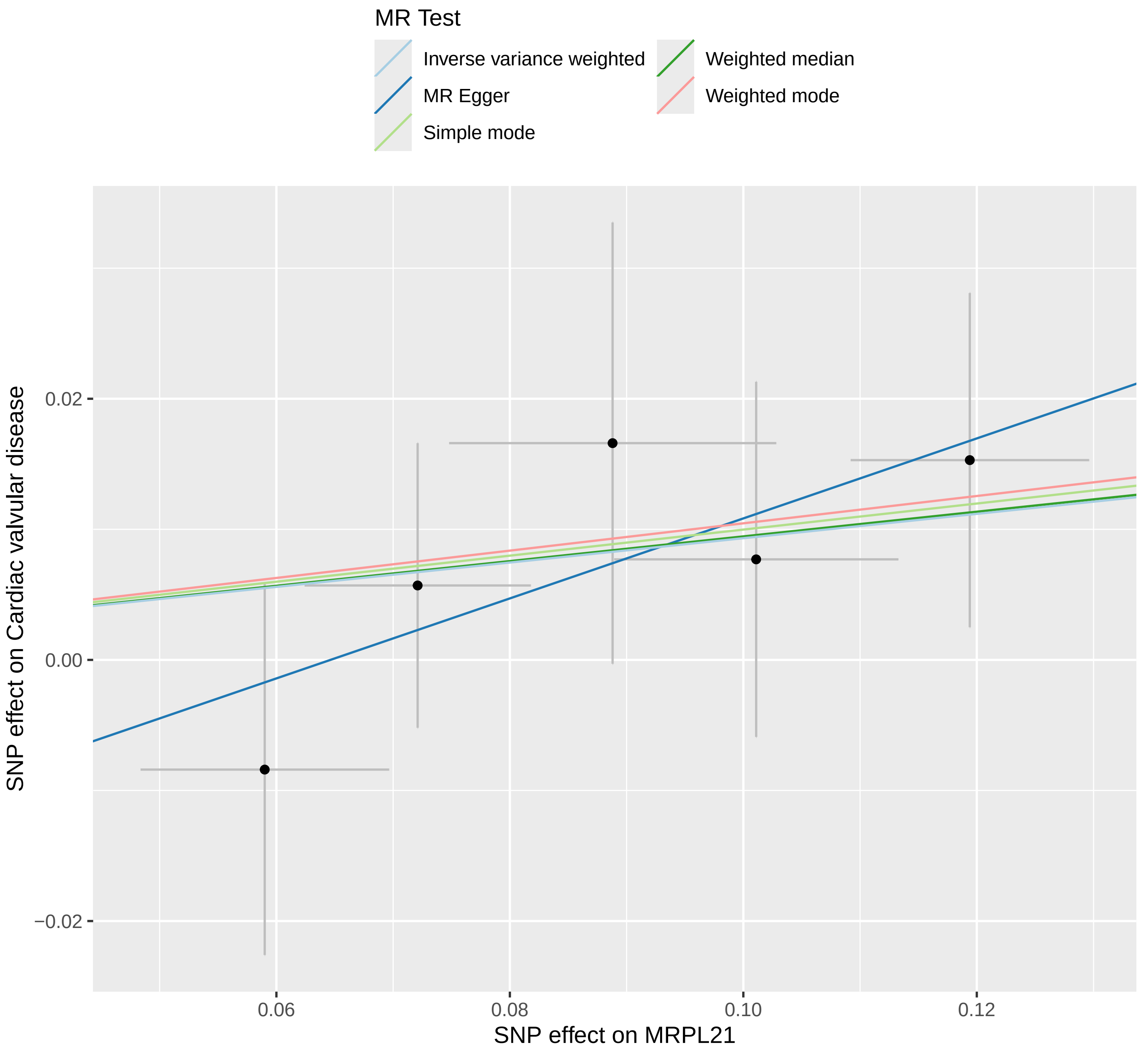

MR Test
Inverse variance weighted
MR Egger
Simple mode
Weighted median
Weighted mode
0.02
0.00
−0.02
SNP effect on Cardiac valvular disease
0.06
0.08
0.10
0.12
SNP effect on MRPL21

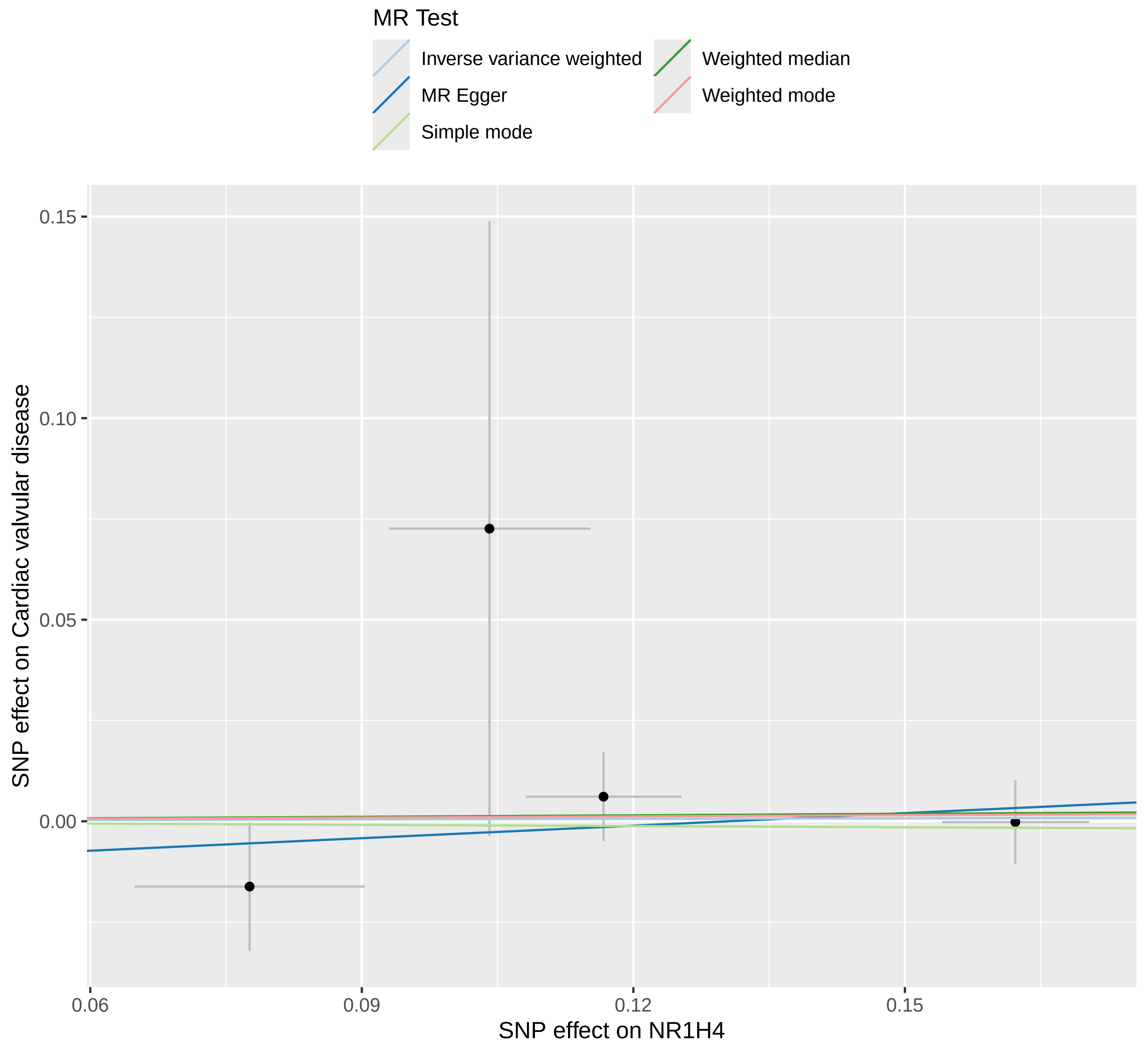
MR Test
Inverse variance weighted
MR Egger
Simple mode
Weighted median
Weighted mode
0.15
0.10
0.05
0.00
SNP effect on Cardiac valvular disease
0.06
0.09
0.12
0.15
SNP effect on NR1H4

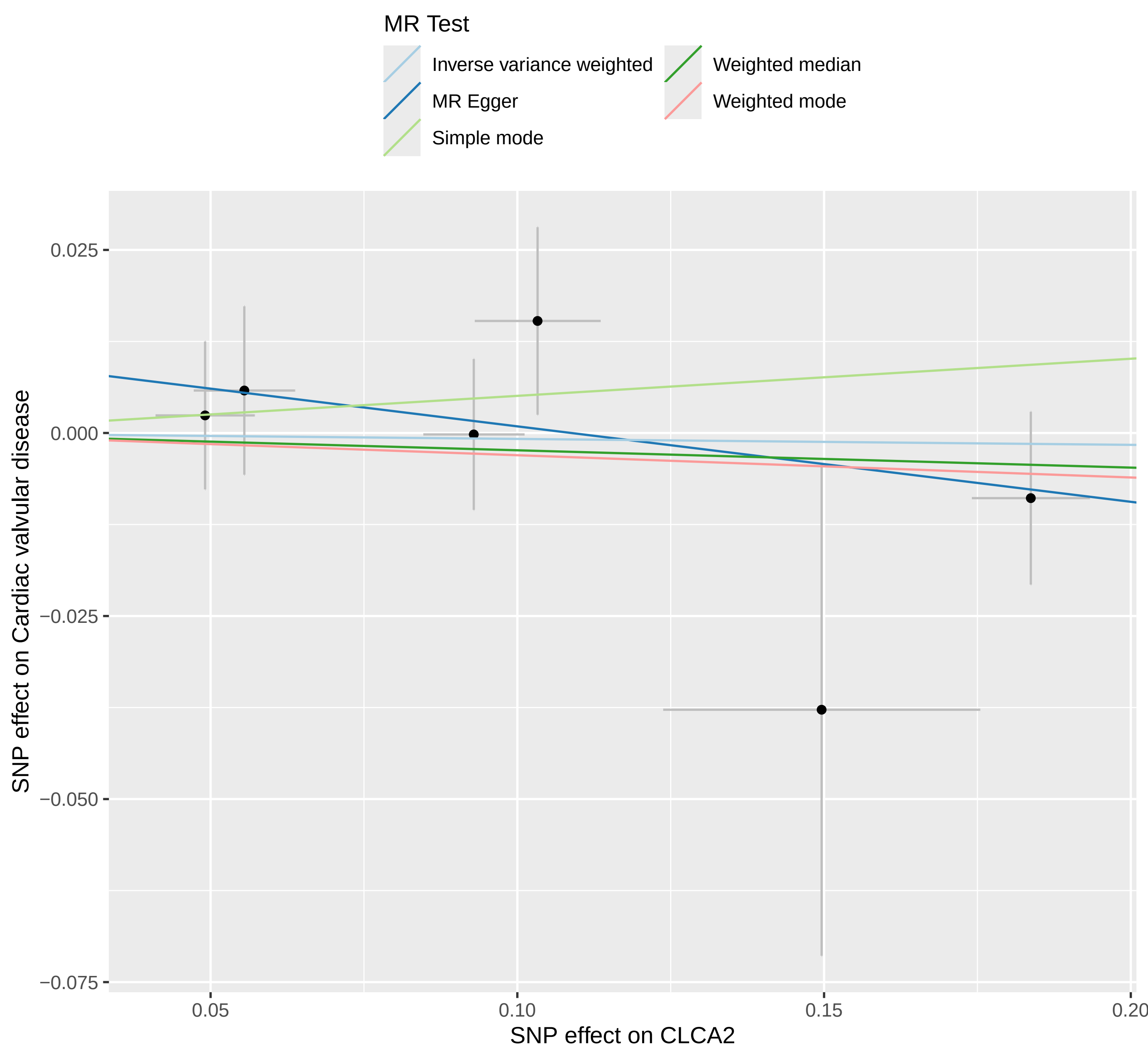
MR Test
Inverse variance weighted
MR Egger
Simple mode
Weighted median
Weighted mode
0.025
0.000
−0.025
−0.050
−0.075
0.05
0.10
0.15
0.20
SNP effect on CLCA2
SNP effect on Cardiac valvular disease

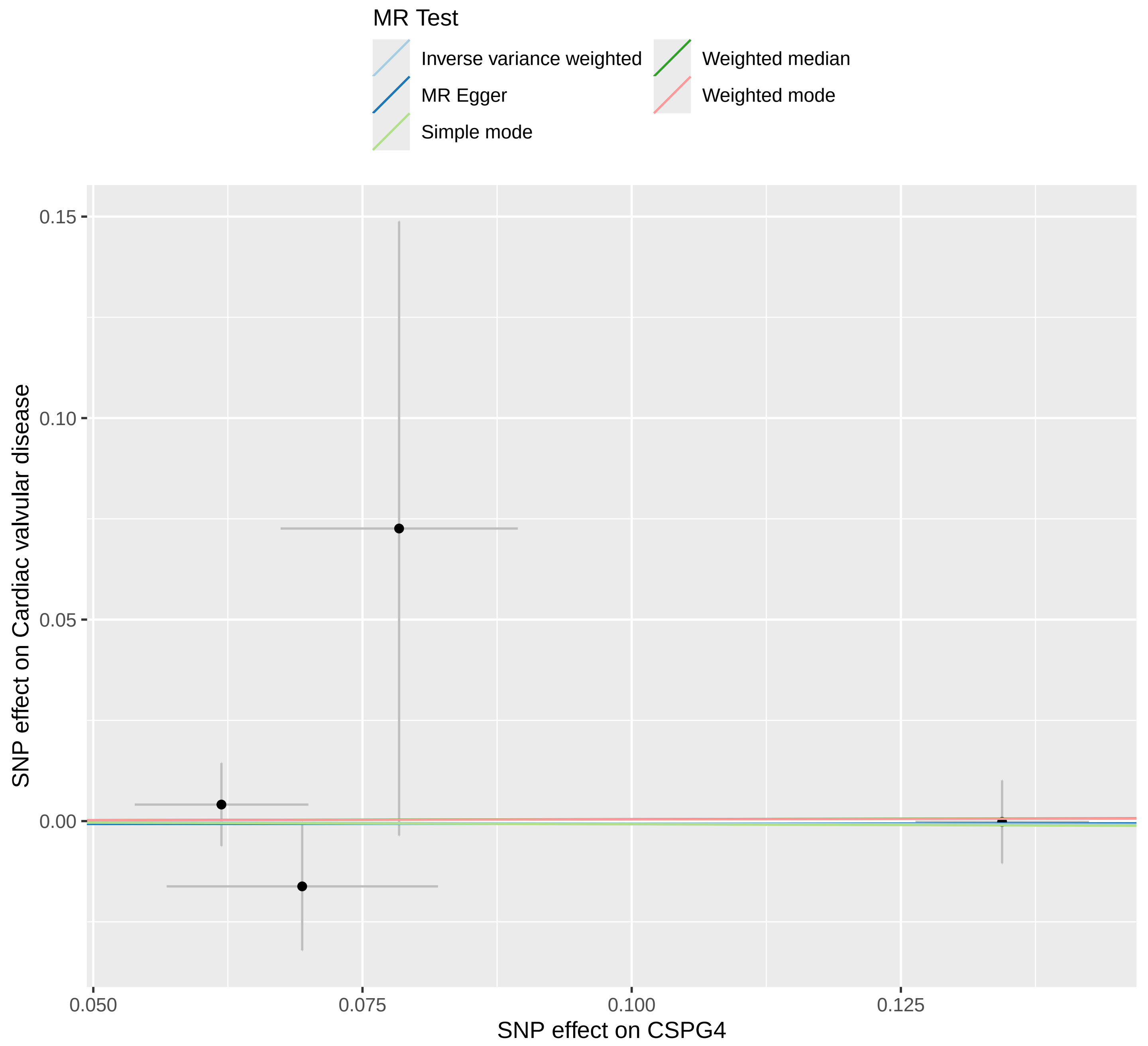

MR Test
Inverse variance weighted
MR Egger
Simple mode
Weighted median
Weighted mode
0.15
0.10
0.05
0.00
SNP effect on Cardiac valvular disease
0.050
0.075
0.100
0.125
SNP effect on CSPG4

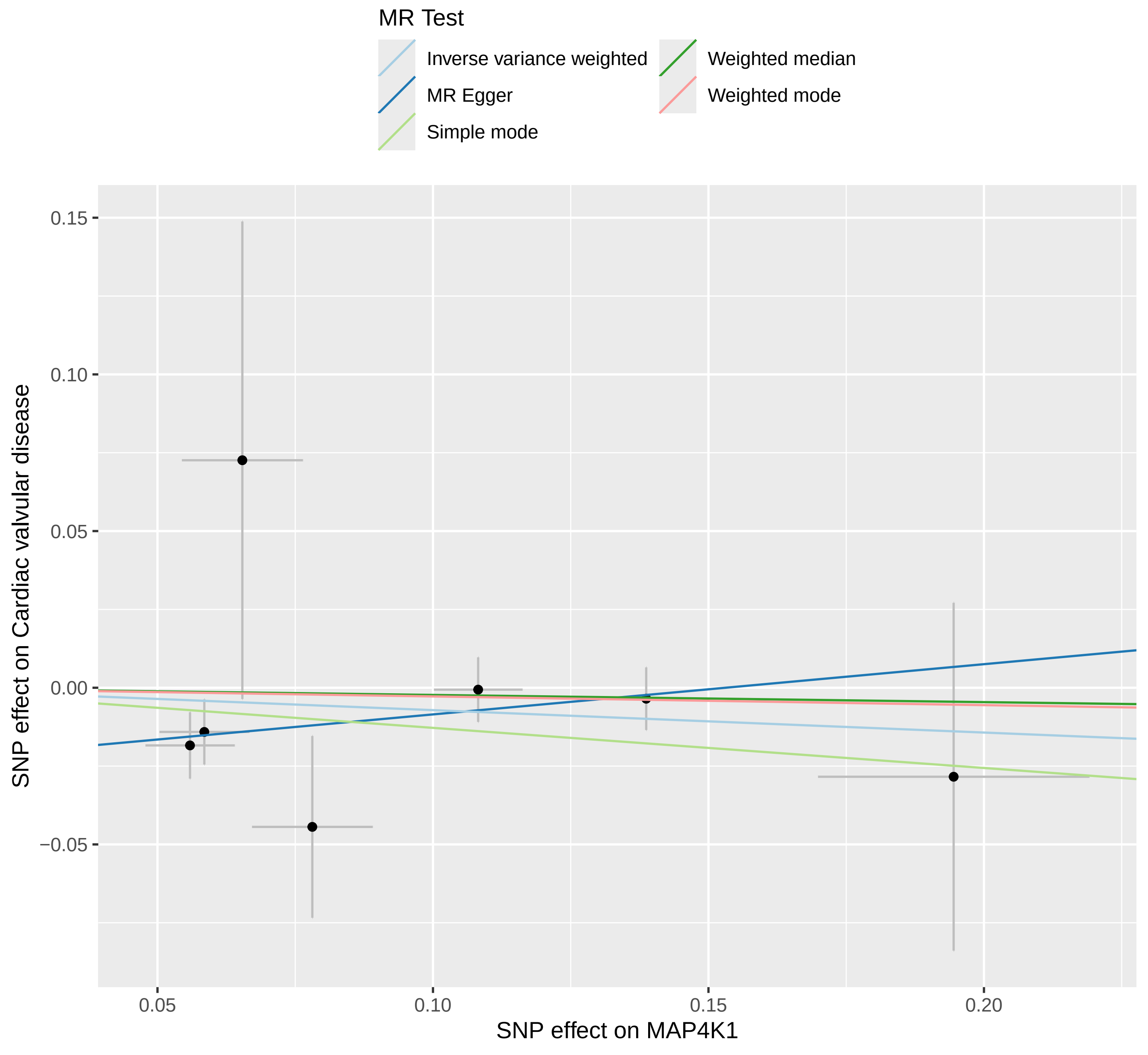
MR Test
Inverse variance weighted
MR Egger
Simple mode
Weighted median
Weighted mode
0.15
0.10
0.05
0.00
−0.05
SNP effect on Cardiac valvular disease
0.05
0.10
0.15
0.20
SNP effect on MAP4K1

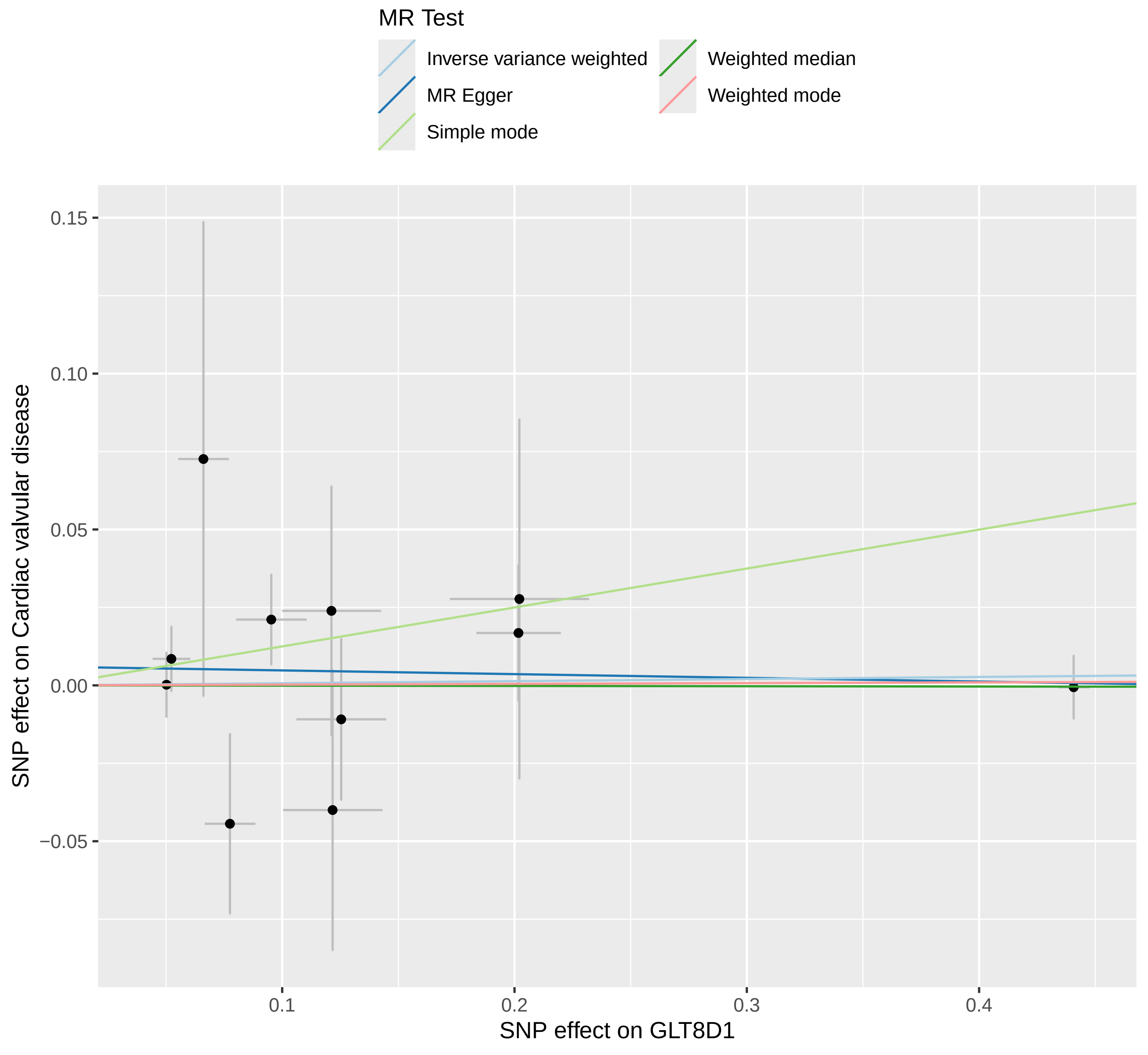
MR Test
Inverse variance weighted
MR Egger
Simple mode
Weighted median
Weighted mode
0.15
0.10
0.05
0.00
−0.05
0.1
0.2
0.3
0.4
SNP effect on Cardiac valvular disease
SNP effect on GLT8D1

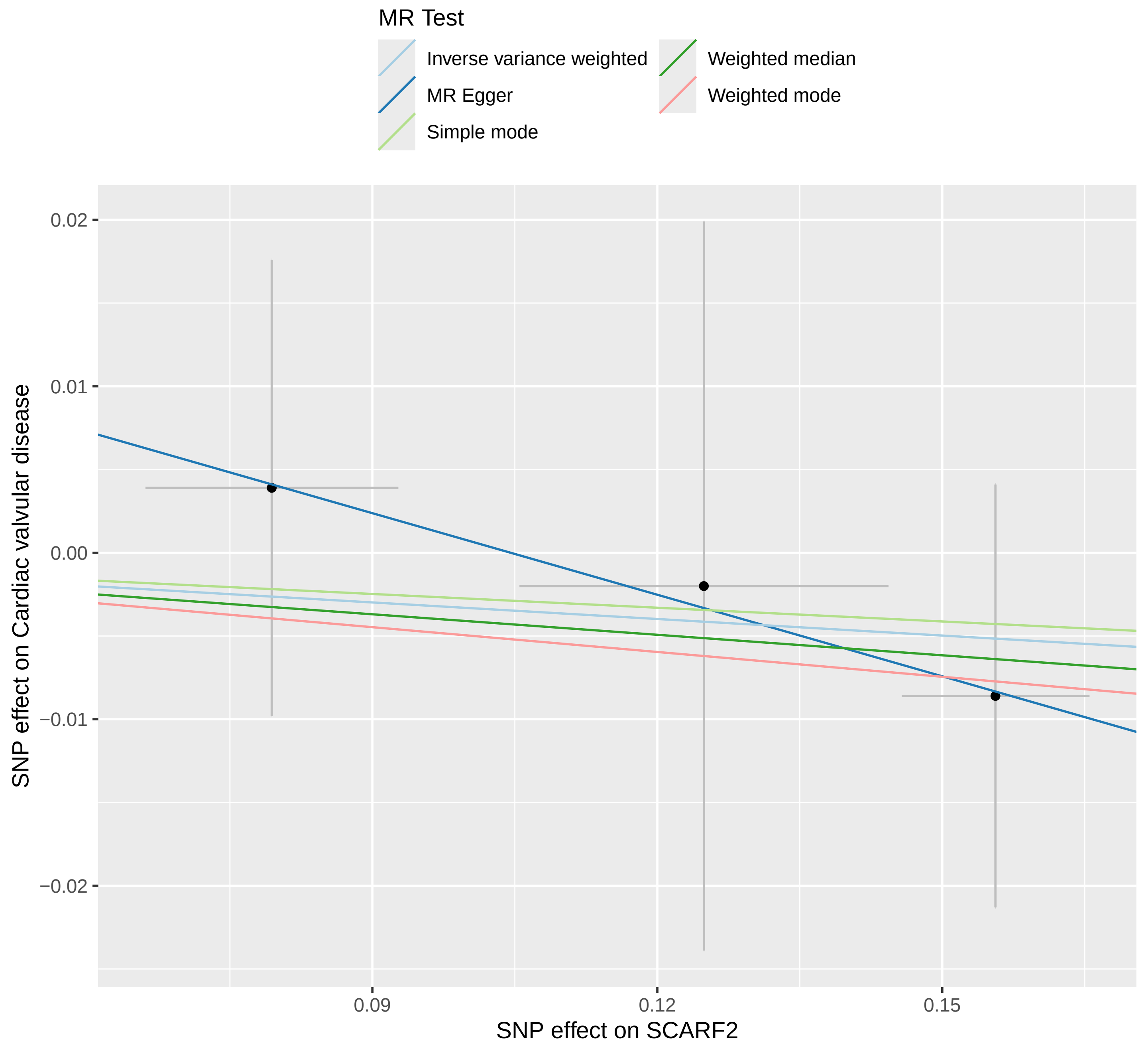

MR Test
Inverse variance weighted
MR Egger
Simple mode
Weighted median
Weighted mode
0.02
0.01
0.00
−0.01
−0.02
SNP effect on Cardiac valvular disease
0.09
0.12
0.15
SNP effect on SCARF2

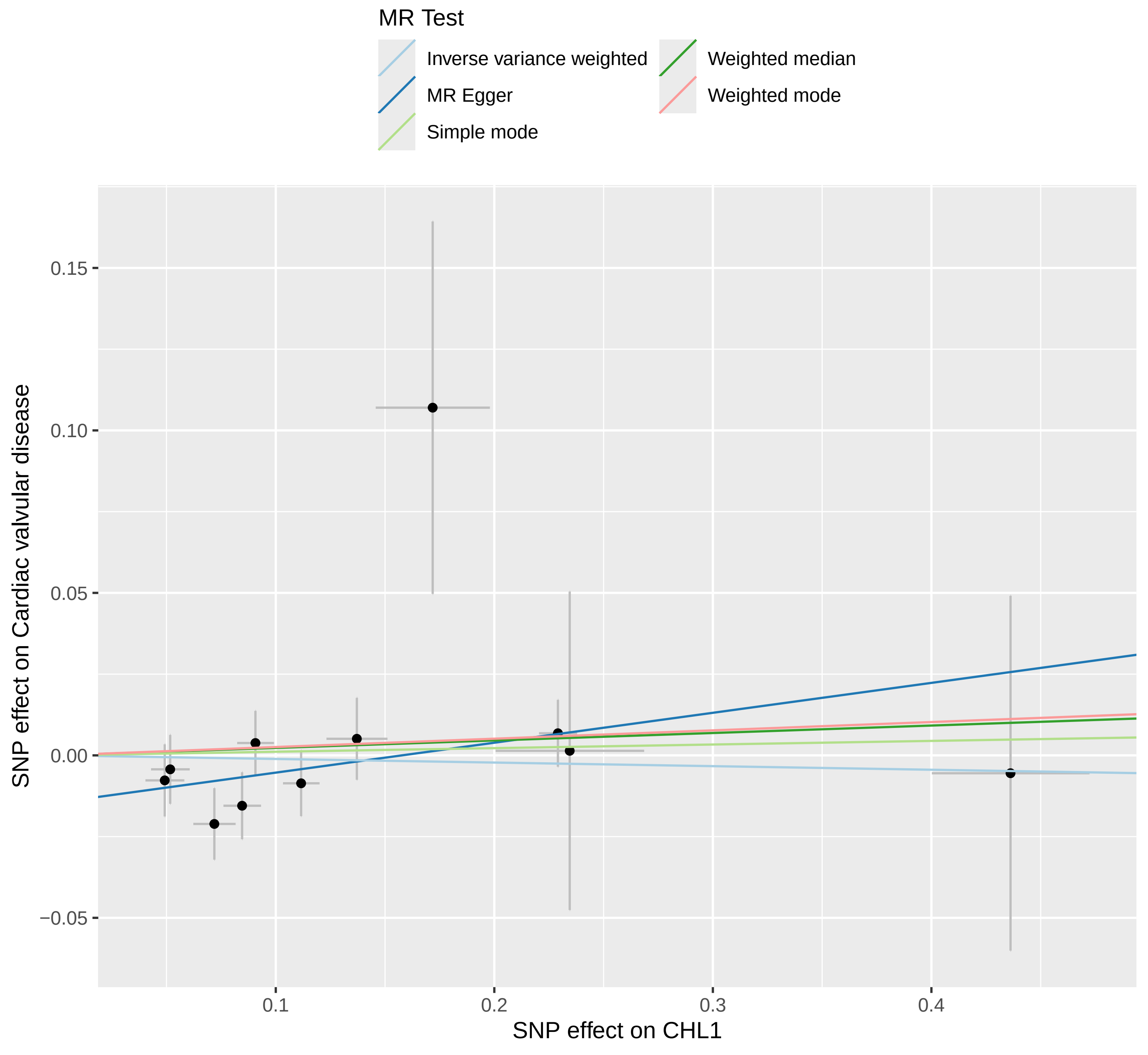
MR Test
Inverse variance weighted
MR Egger
Simple mode
Weighted median
Weighted mode
0.15
0.10
0.05
0.00
−0.05
SNP effect on Cardiac valvular disease
0.1
0.2
0.3
0.4
SNP effect on CHL1

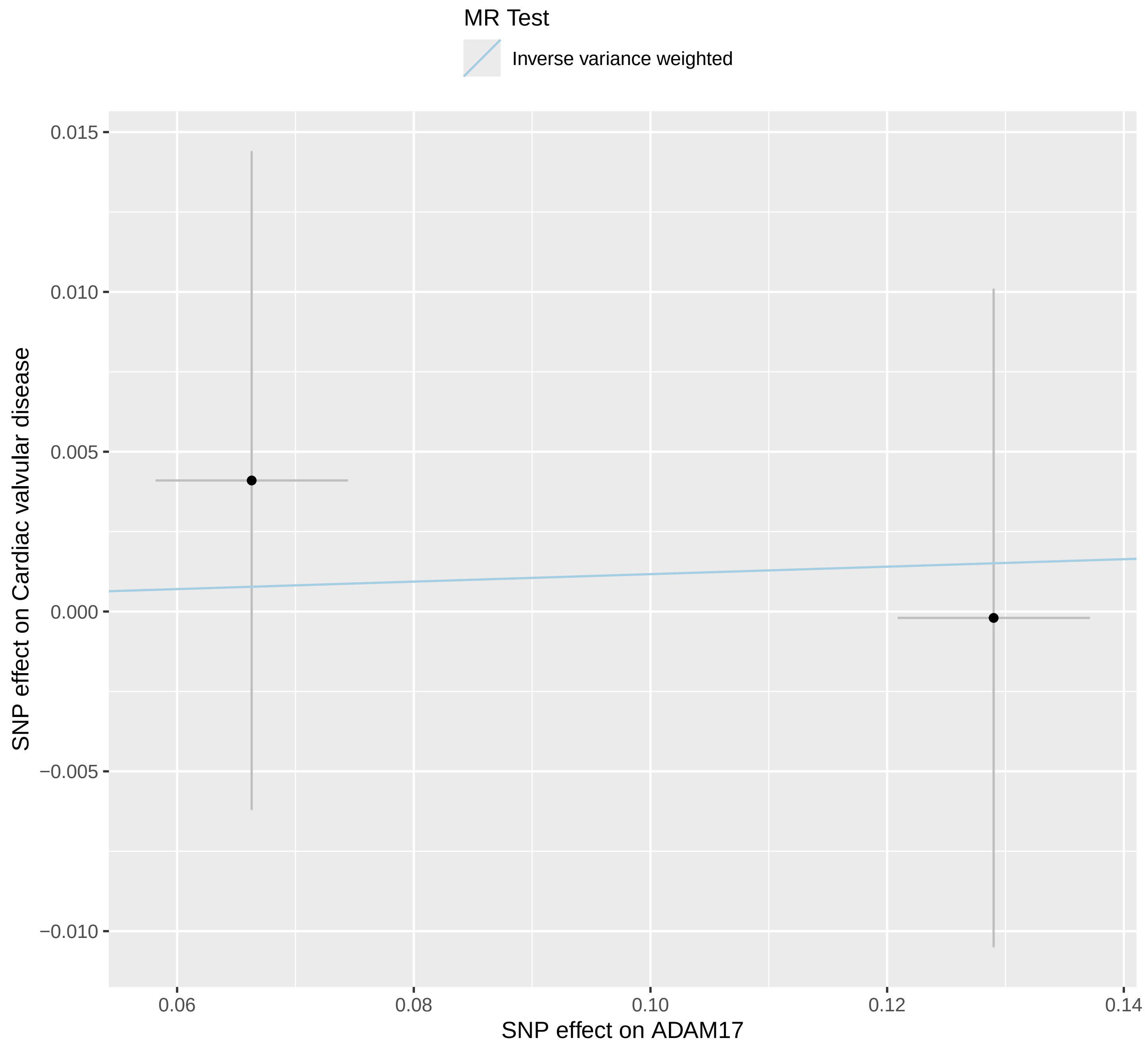
MR Test
Inverse variance weighted
0.015
0.010
0.005
0.000
−0.005
−0.010
SNP effect on Cardiac valvular disease
0.06
0.08
0.10
0.12
0.14
SNP effect on ADAM17

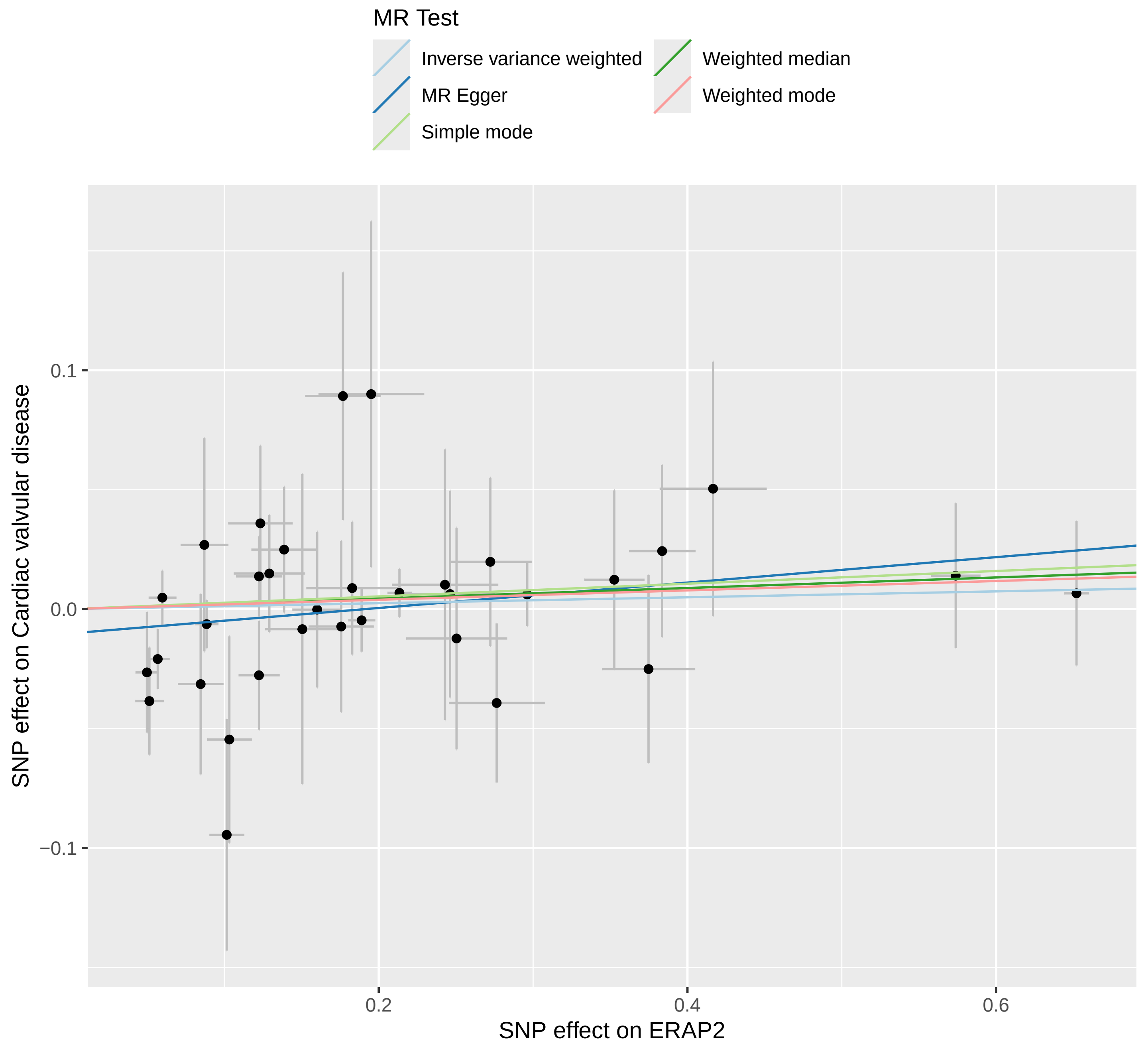

MR Test
Inverse variance weighted
MR Egger
Simple mode
Weighted median
Weighted mode
SNP effect on Cardiac valvular disease
0.1
0.0
−0.1
0.2
0.4
0.6
SNP effect on ERAP2

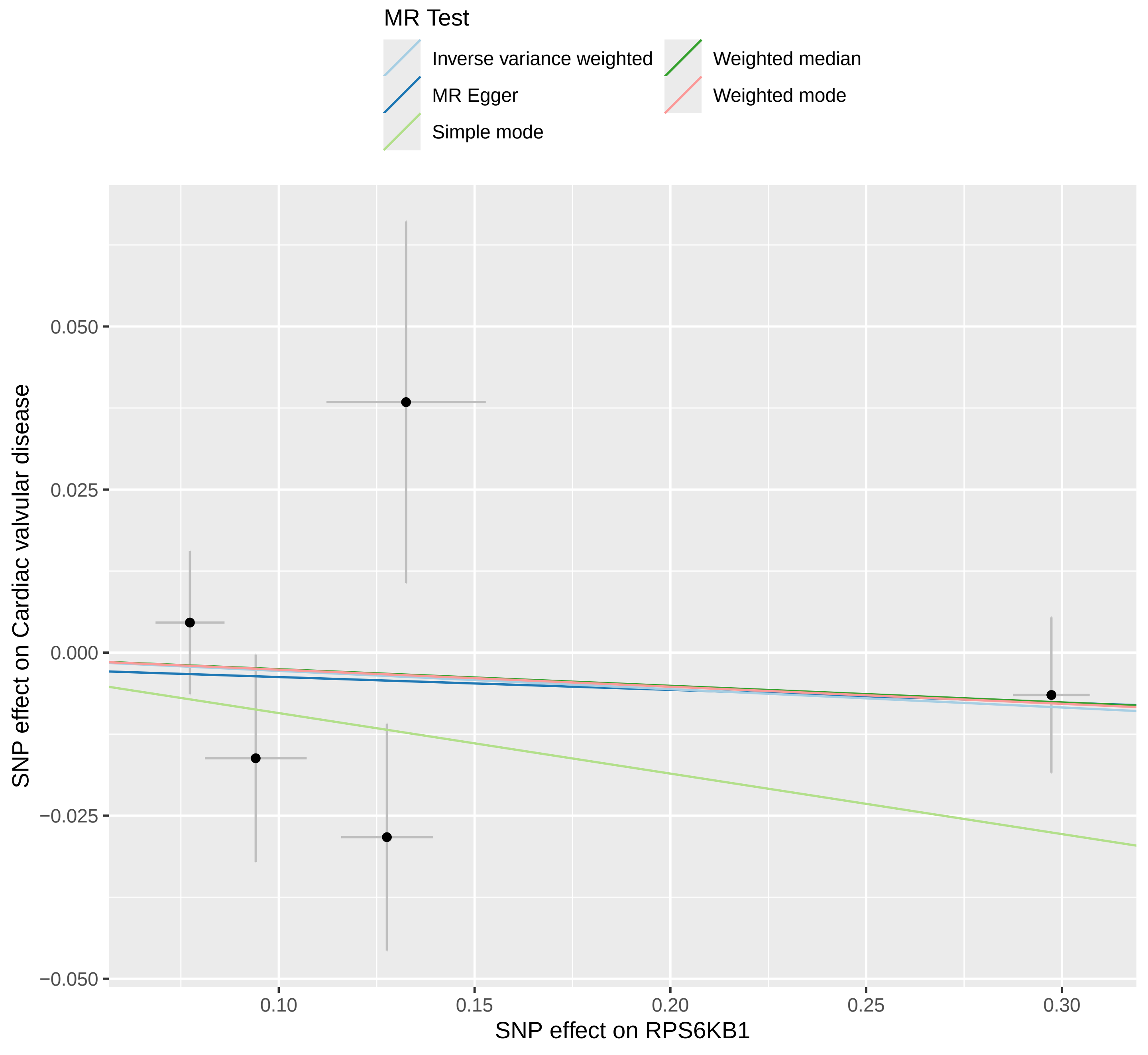
MR Test
Inverse variance weighted
MR Egger
Simple mode
Weighted median
Weighted mode
0.050
0.025
0.000
−0.025
−0.050
SNP effect on Cardiac valvular disease
0.10
0.15
0.20
0.25
0.30
SNP effect on RPS6KB1

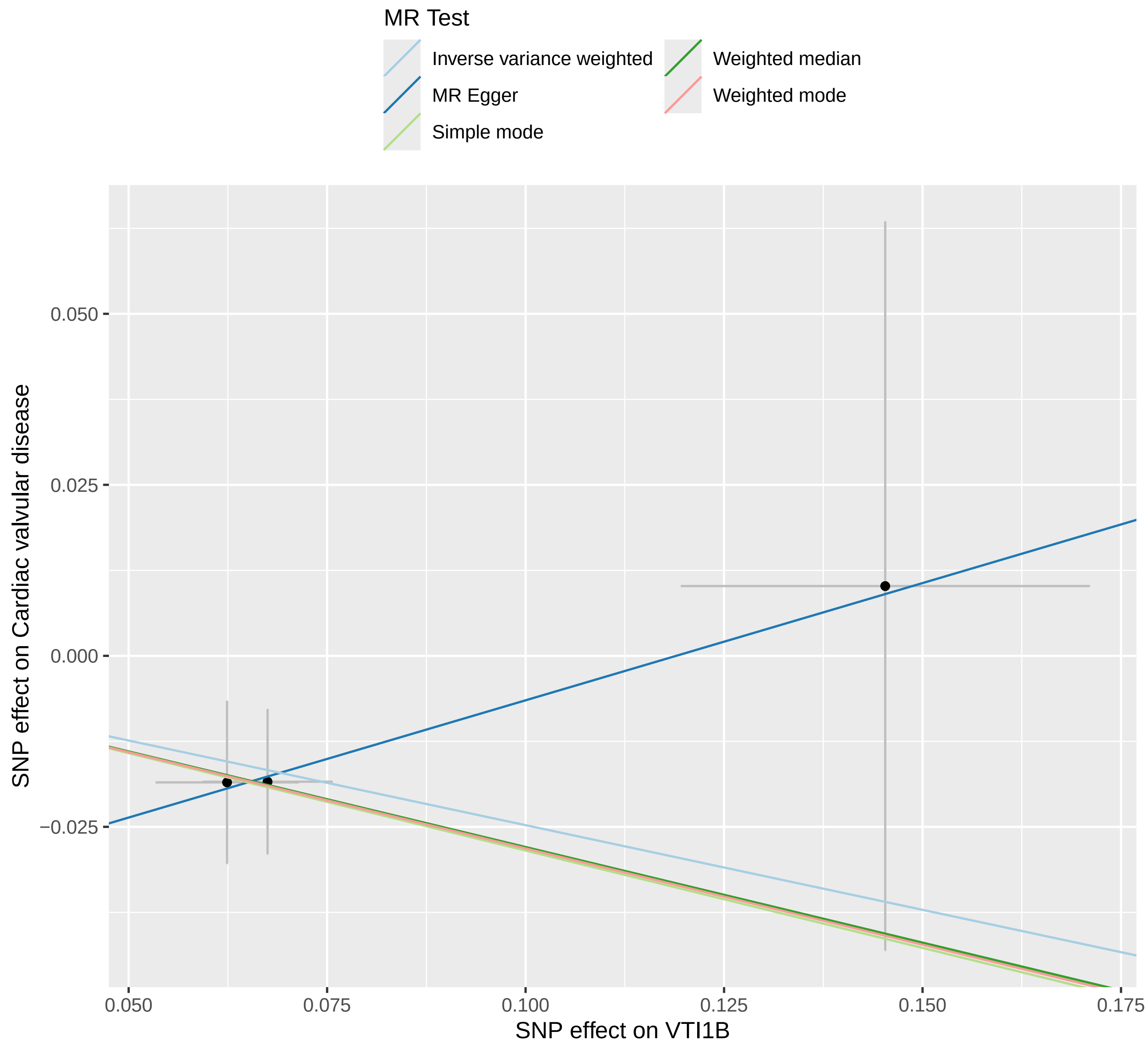
MR Test
Inverse variance weighted
MR Egger
Simple mode
Weighted median
Weighted mode
SNP effect on Cardiac valvular disease
0.050
0.025
0.000
−0.025
0.050
0.075
0.100
0.125
0.150
0.175
SNP effect on VTI1B

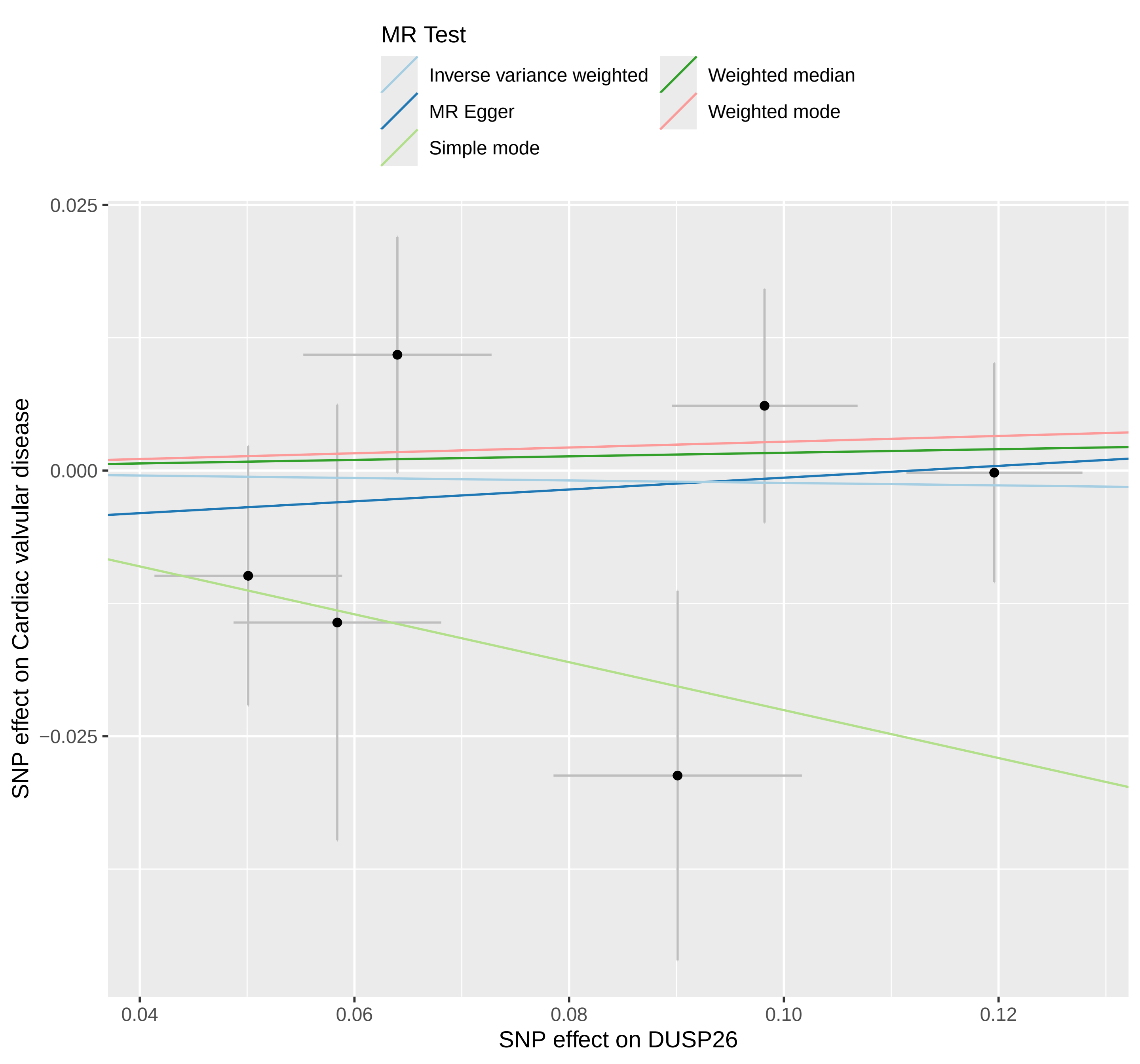
MR Test
Inverse variance weighted
MR Egger
Simple mode
Weighted median
Weighted mode
0.025
0.000
−0.025
0.04
0.06
0.08
0.10
0.12
SNP effect on DUSP26
SNP effect on Cardiac valvular disease

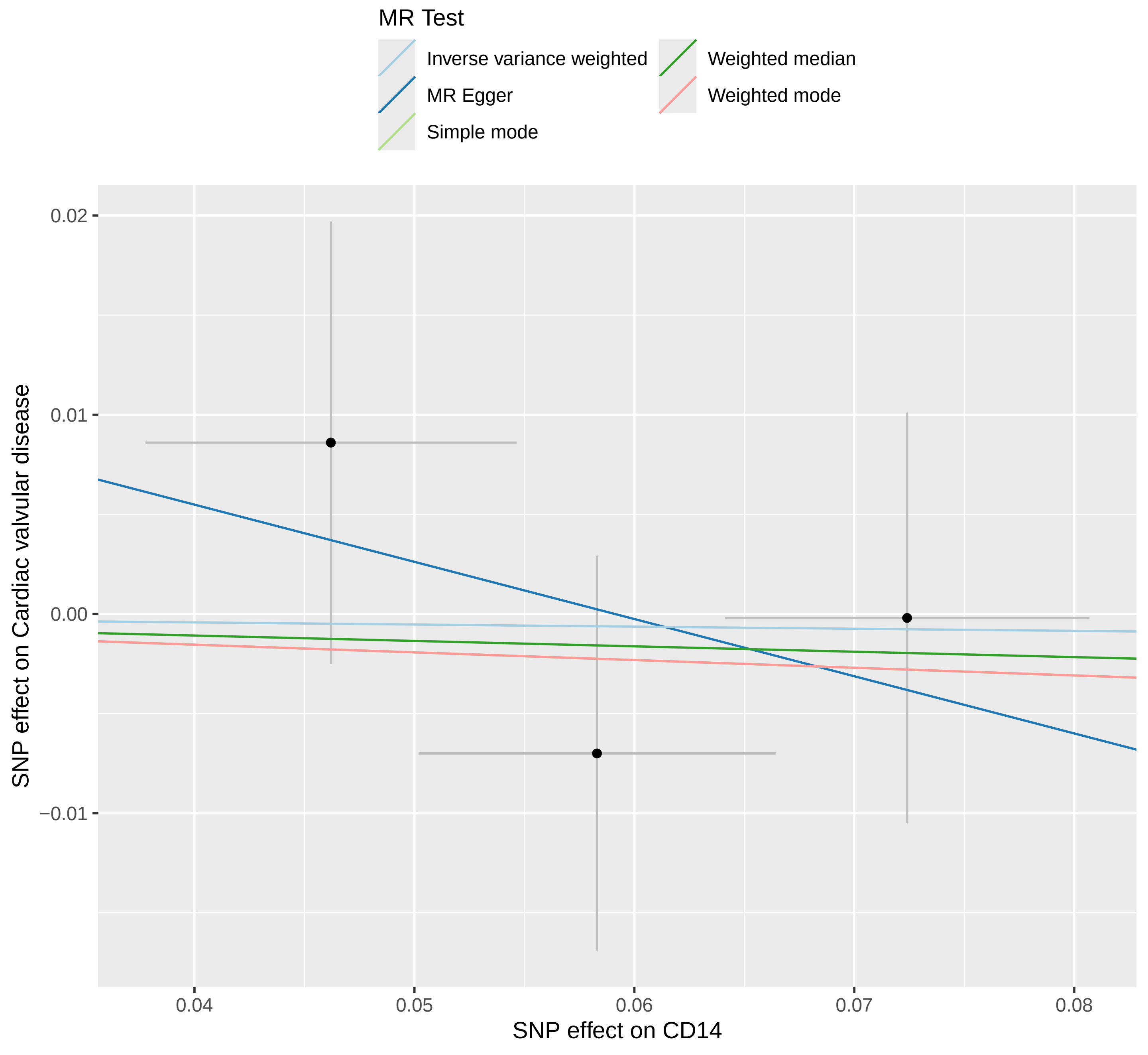

MR Test
Inverse variance weighted
MR Egger
Simple mode
Weighted median
Weighted mode
0.02
0.01
0.00
−0.01
0.04
0.05
0.06
0.07
0.08
SNP effect on Cardiac valvular disease
SNP effect on CD14

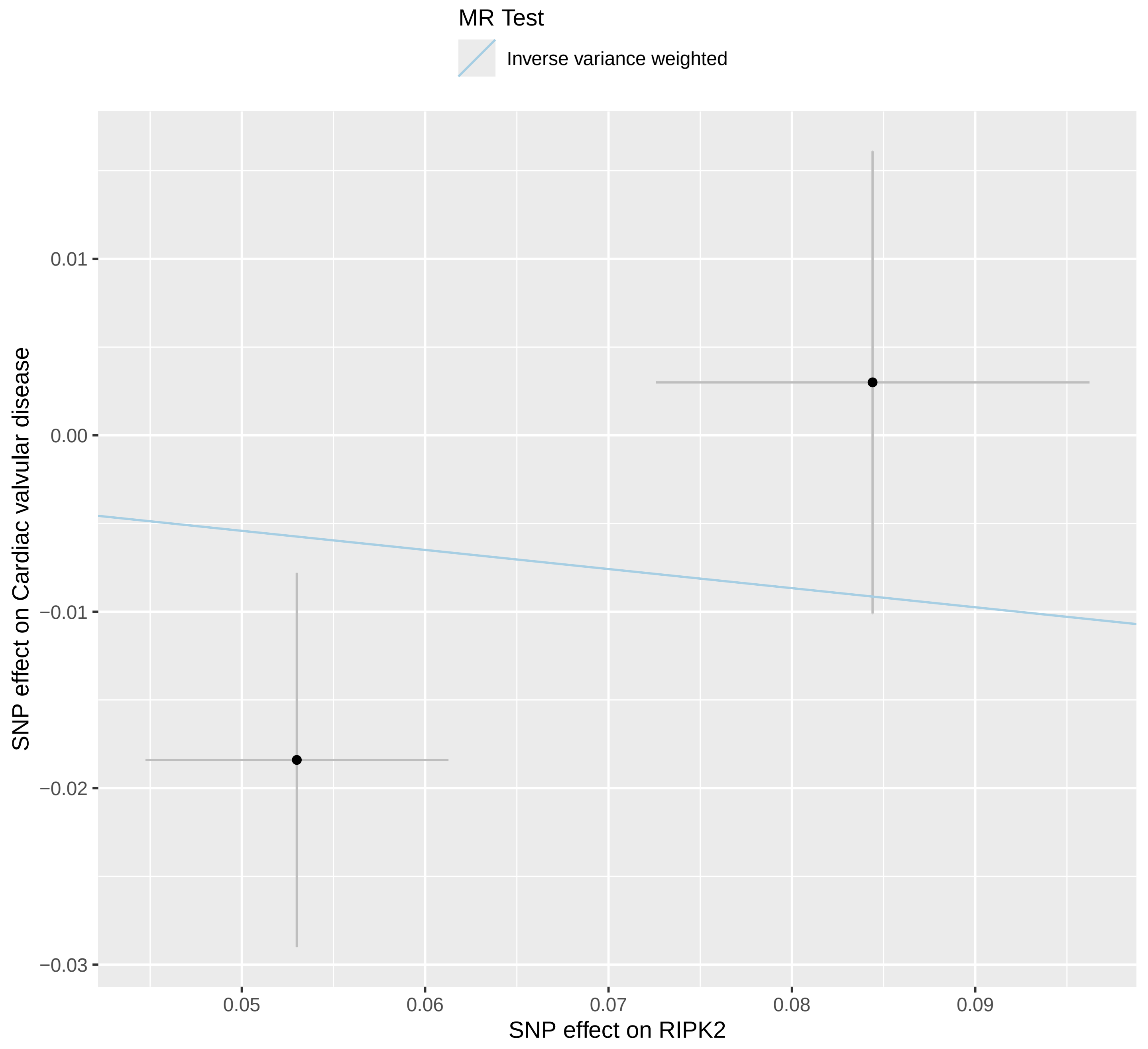
MR Test
Inverse variance weighted
0.01
0.00
−0.01
−0.02
−0.03
0.05
0.06
0.07
0.08
0.09
SNP effect on RIPK2
SNP effect on Cardiac valvular disease

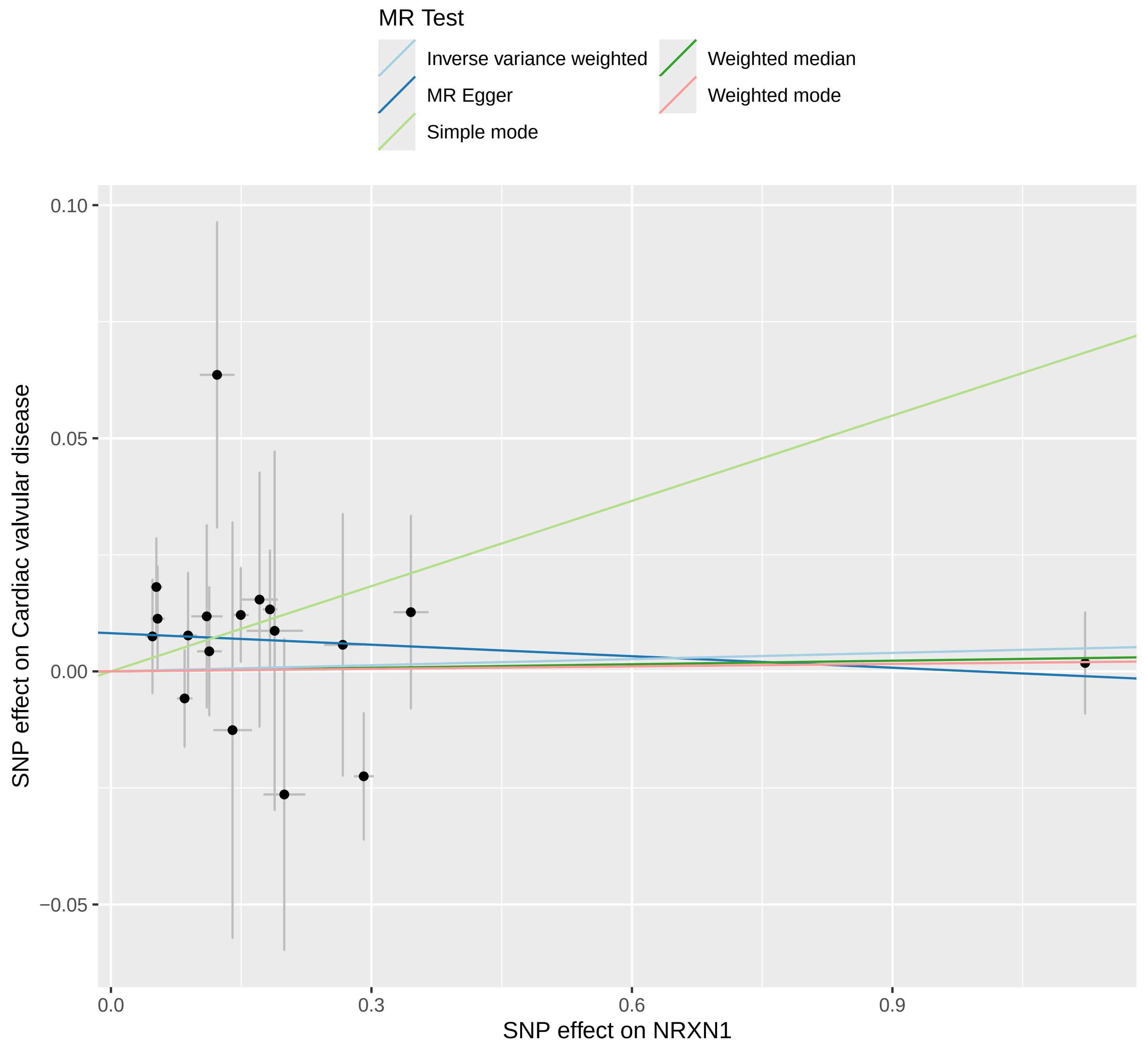

MR Test
Inverse variance weighted
MR Egger
Simple mode
Weighted median
Weighted mode
0.10
0.05
0.00
−0.05
0.0
0.3
0.6
0.9
SNP effect on NRXN1
SNP effect on Cardiac valvular disease

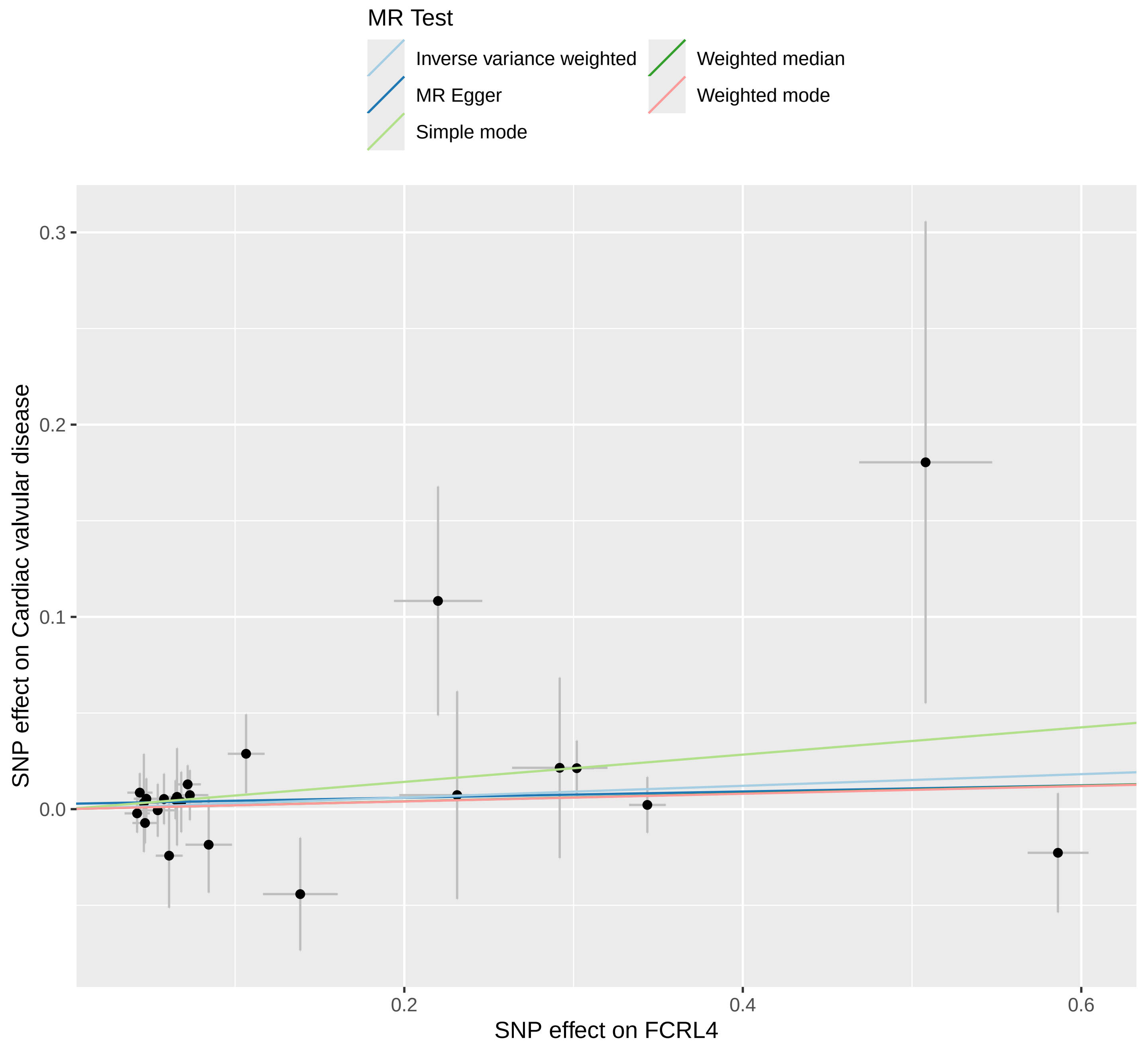
MR Test
Inverse variance weighted
MR Egger
Simple mode
Weighted median
Weighted mode
SNP effect on Cardiac valvular disease
0.3
0.2
0.1
0.0
0.2
0.4
0.6
SNP effect on FCRL4

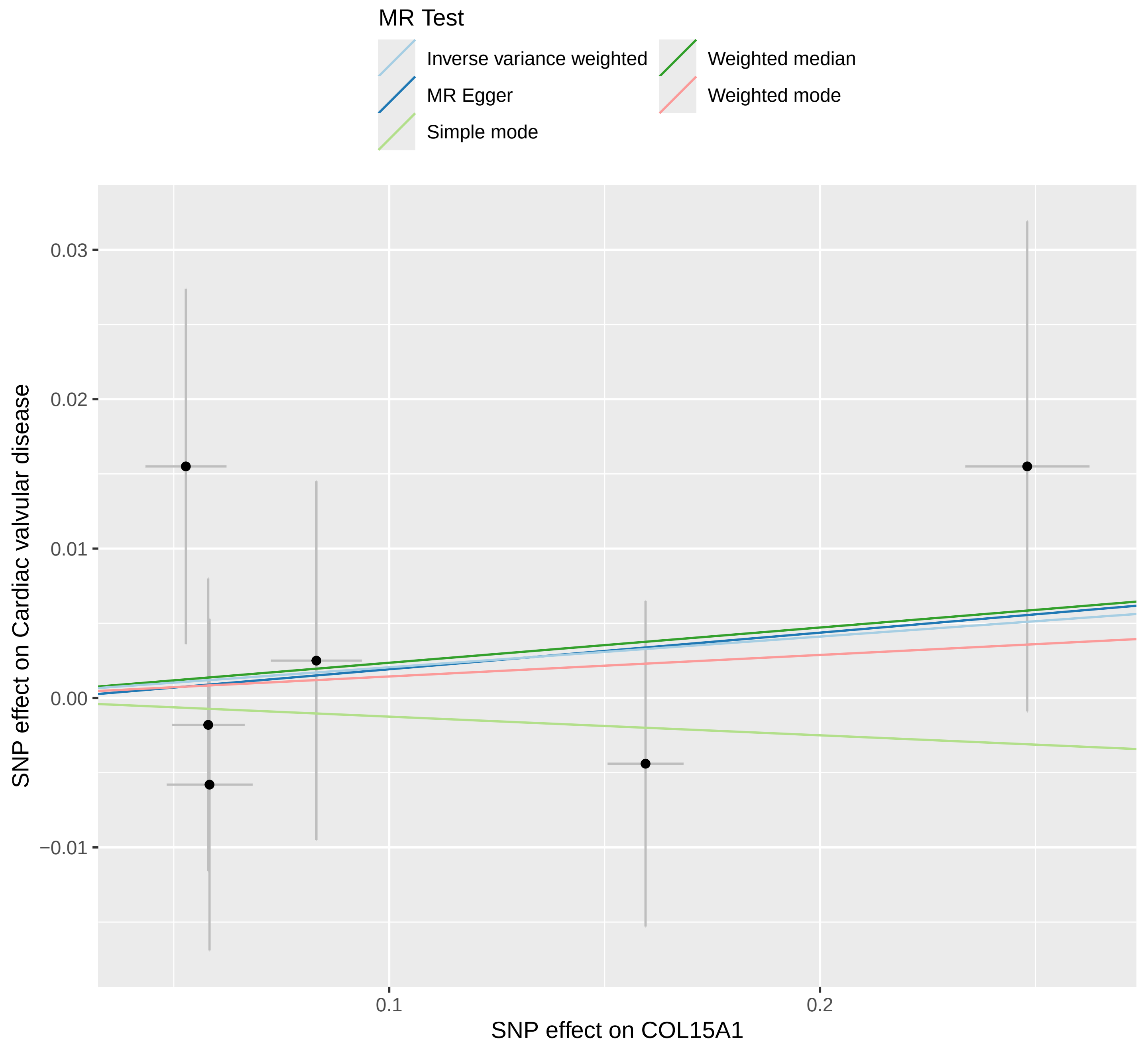

MR Test
Inverse variance weighted
MR Egger
Simple mode
Weighted median
Weighted mode
0.03
0.02
0.01
0.00
−0.01
0.1
0.2
SNP effect on Cardiac valvular disease
SNP effect on COL15A1

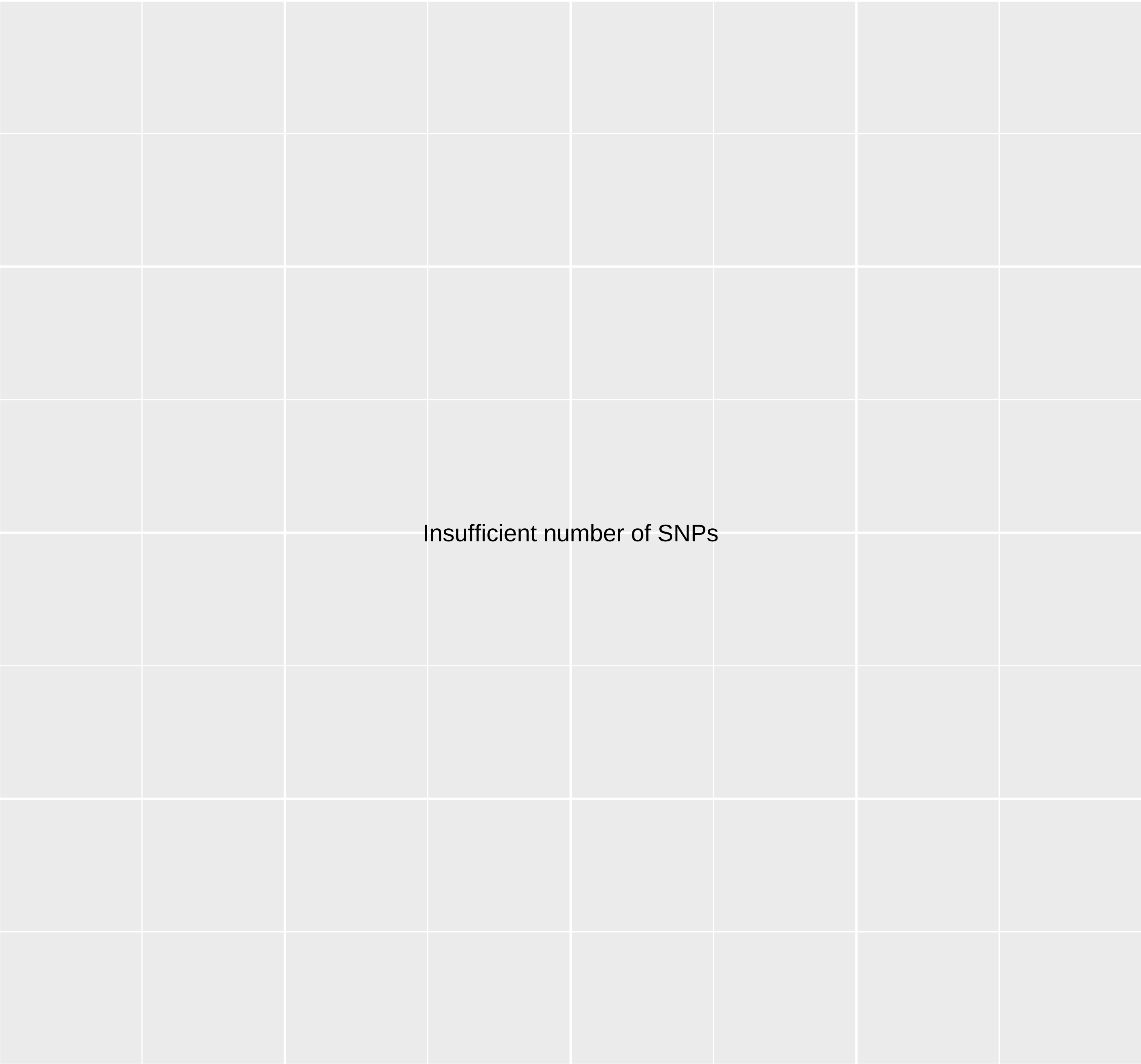
Insufficient number of SNPs

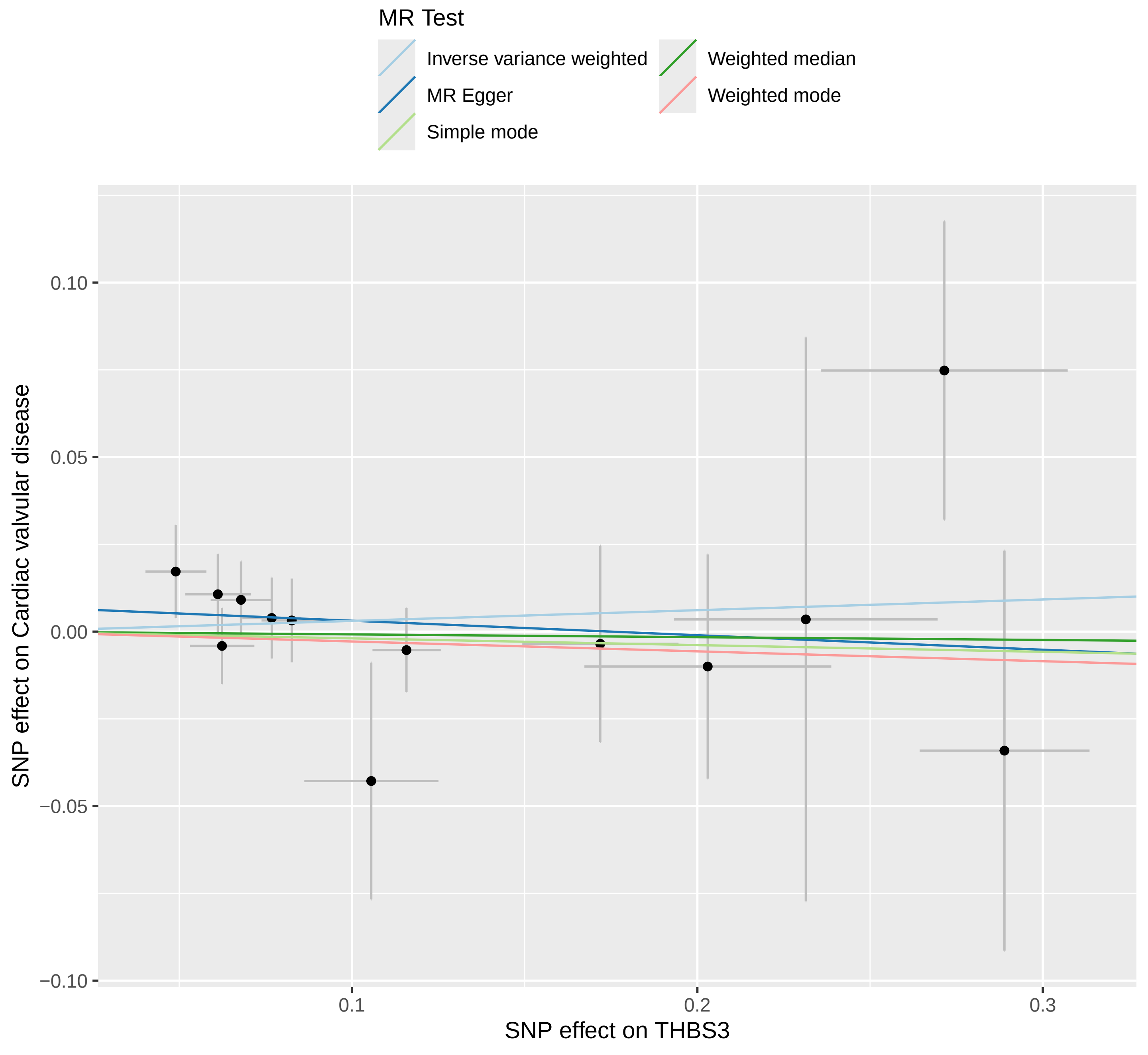
MR Test
Inverse variance weighted
MR Egger
Simple mode
Weighted median
Weighted mode
0.10
0.05
0.00
−0.05
−0.10
0.1
0.2
0.3
SNP effect on Cardiac valvular disease
SNP effect on THBS3

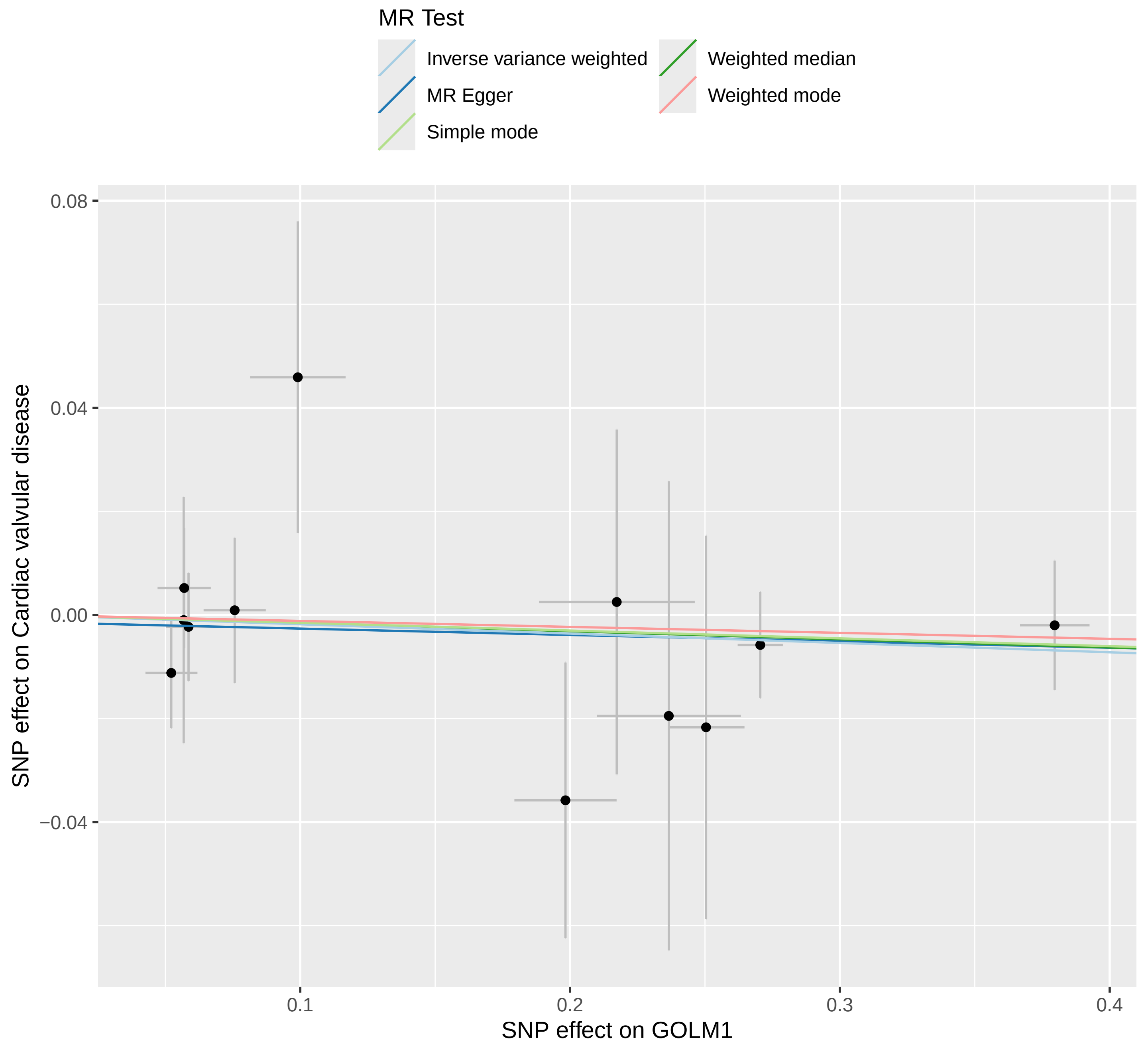
MR Test
Inverse variance weighted
MR Egger
Simple mode
Weighted median
Weighted mode
SNP effect on Cardiac valvular disease
0.08
0.04
0.00
−0.04
0.1
0.2
0.3
0.4
SNP effect on GOLM1

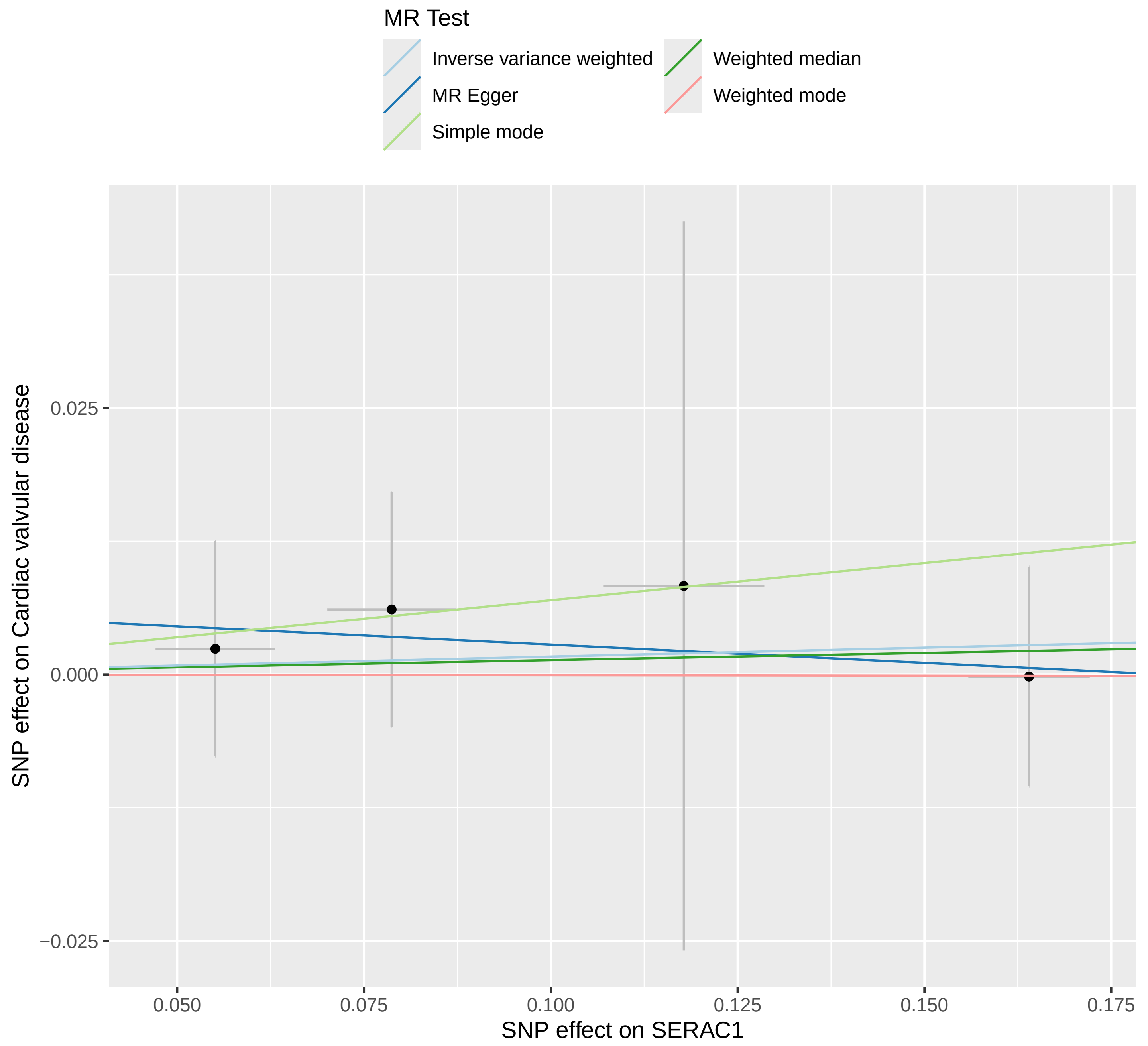

MR Test
Inverse variance weighted
MR Egger
Simple mode
Weighted median
Weighted mode
0.025
0.000
−0.025
SNP effect on Cardiac valvular disease
0.050
0.075
0.100
0.125
0.150
0.175
SNP effect on SERAC1

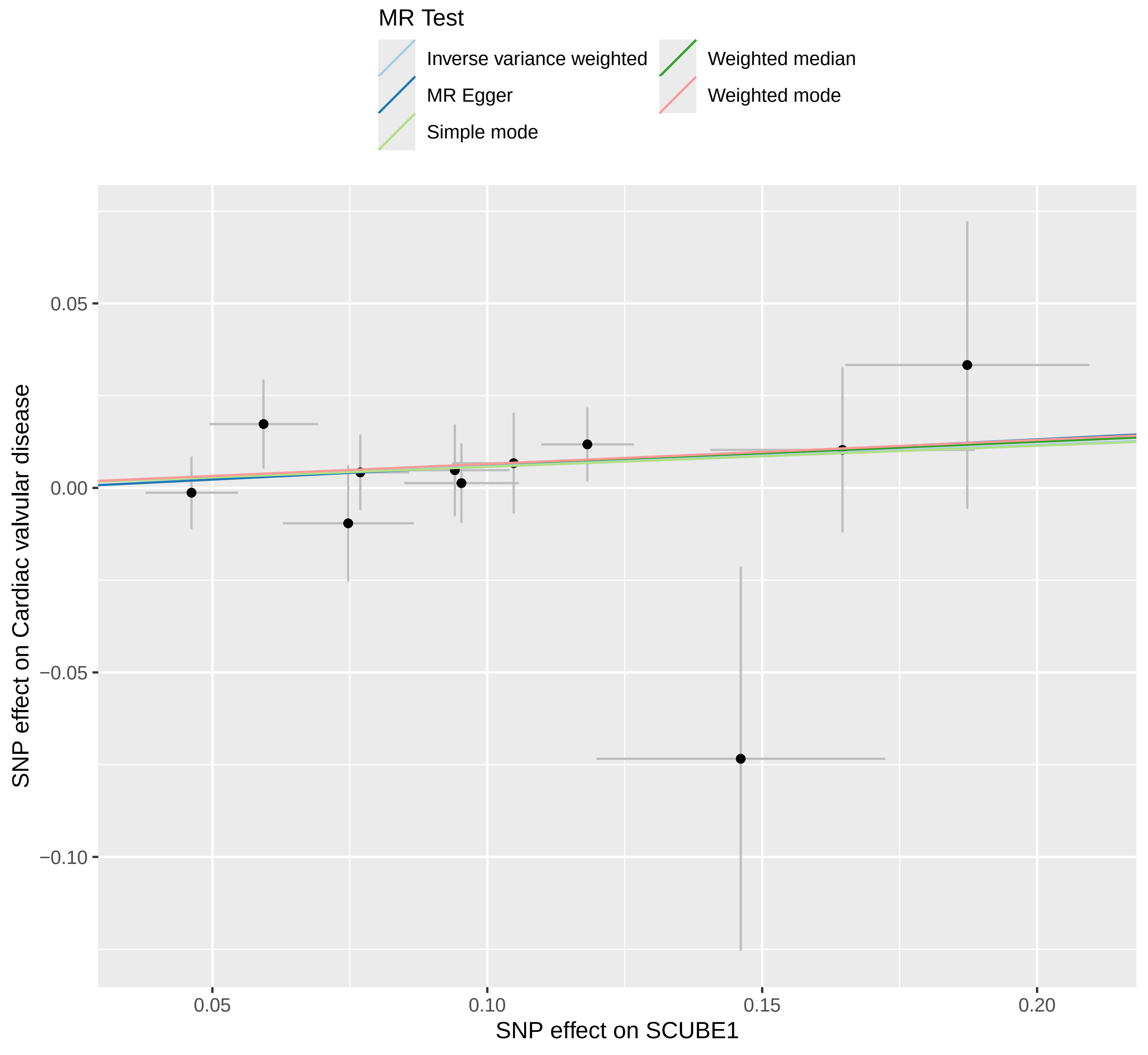
MR Test
Inverse variance weighted
MR Egger
Simple mode
Weighted median
Weighted mode
0.05
0.00
−0.05
−0.10
SNP effect on Cardiac valvular disease
0.05
0.10
0.15
0.20
SNP effect on SCUBE1

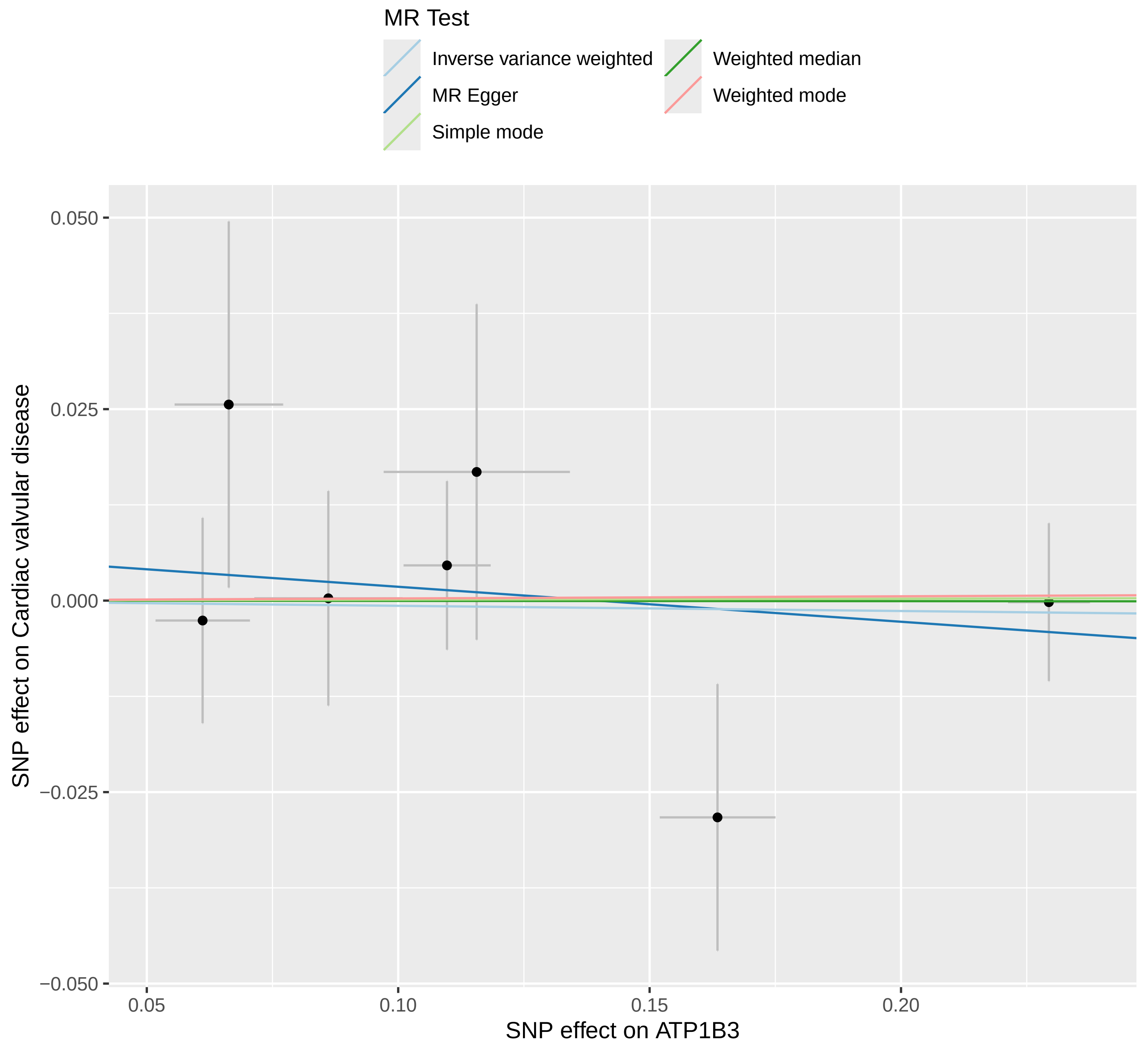

MR Test
Inverse variance weighted
MR Egger
Simple mode
Weighted median
Weighted mode
0.050
0.025
0.000
−0.025
−0.050
SNP effect on Cardiac valvular disease
0.05
0.10
0.15
0.20
SNP effect on ATP1B3

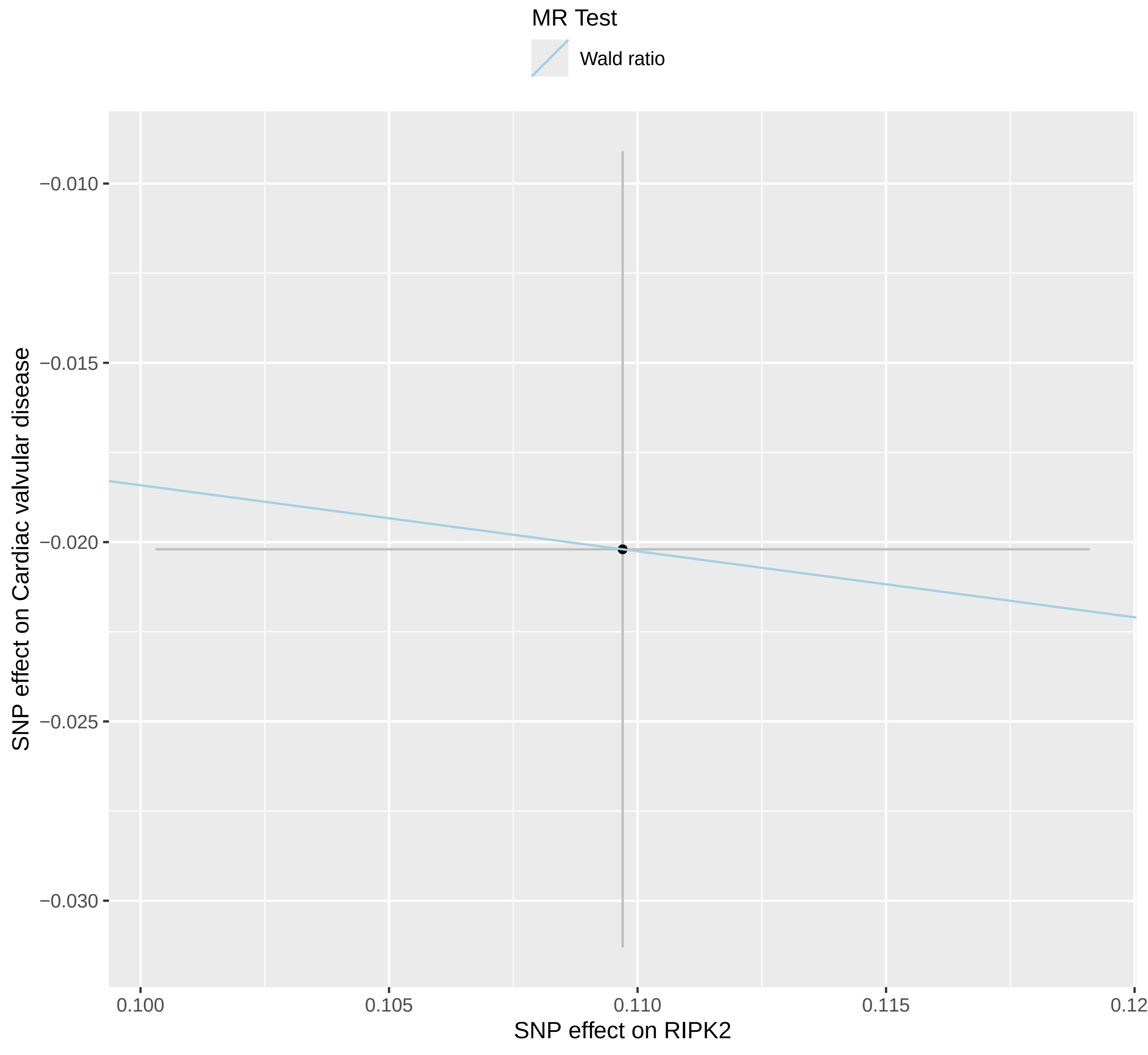
MR Test
Wald ratio
−0.010
−0.015
−0.020
−0.025
−0.030
SNP effect on Cardiac valvular disease
0.100
0.105
0.110
0.115
0.12
SNP effect on RIPK2

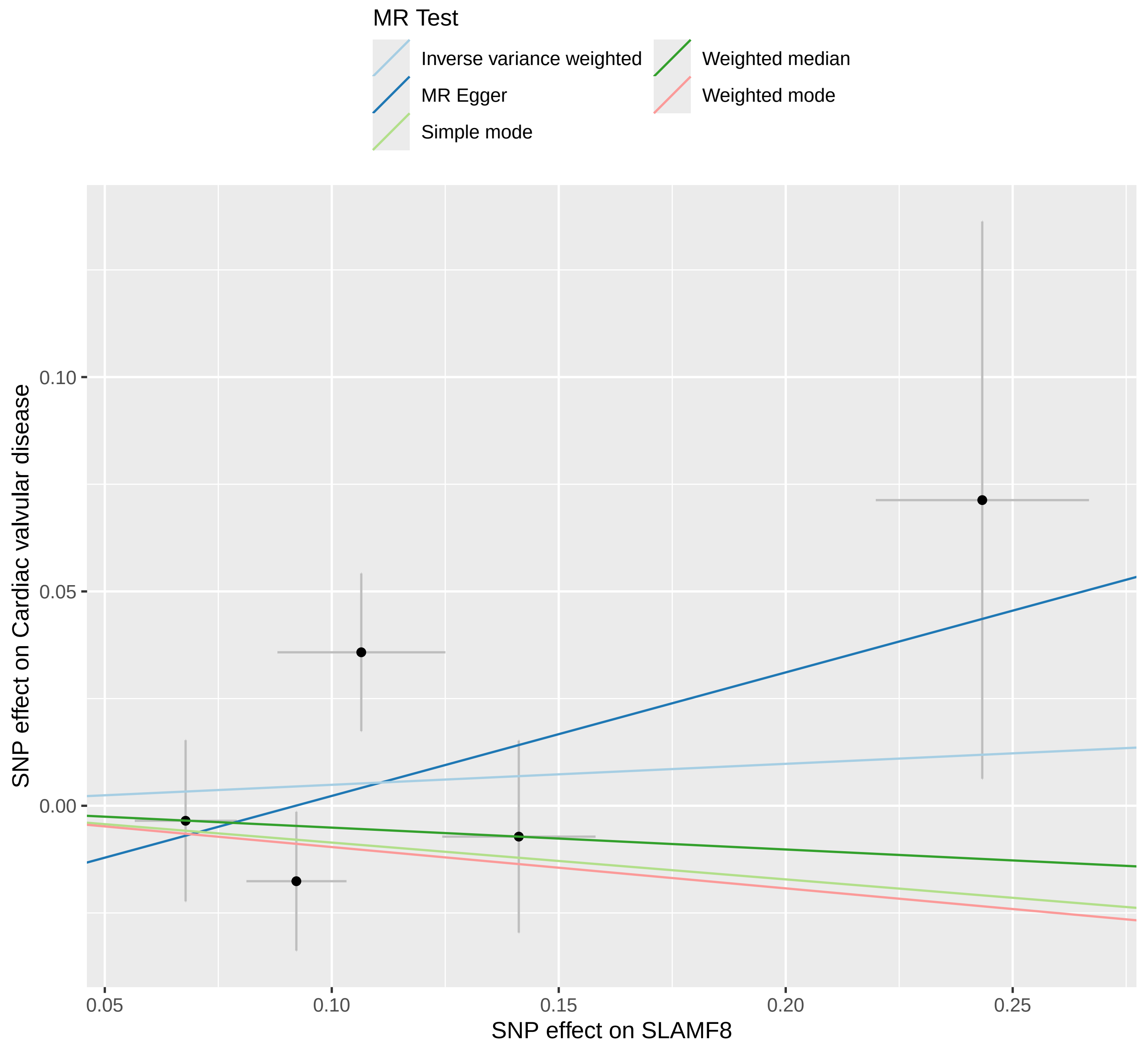
MR Test
Inverse variance weighted
MR Egger
Simple mode
Weighted median
Weighted mode
SNP effect on Cardiac valvular disease
0.10
0.05
0.00
0.05
0.10
0.15
0.20
0.25
SNP effect on SLAMF8

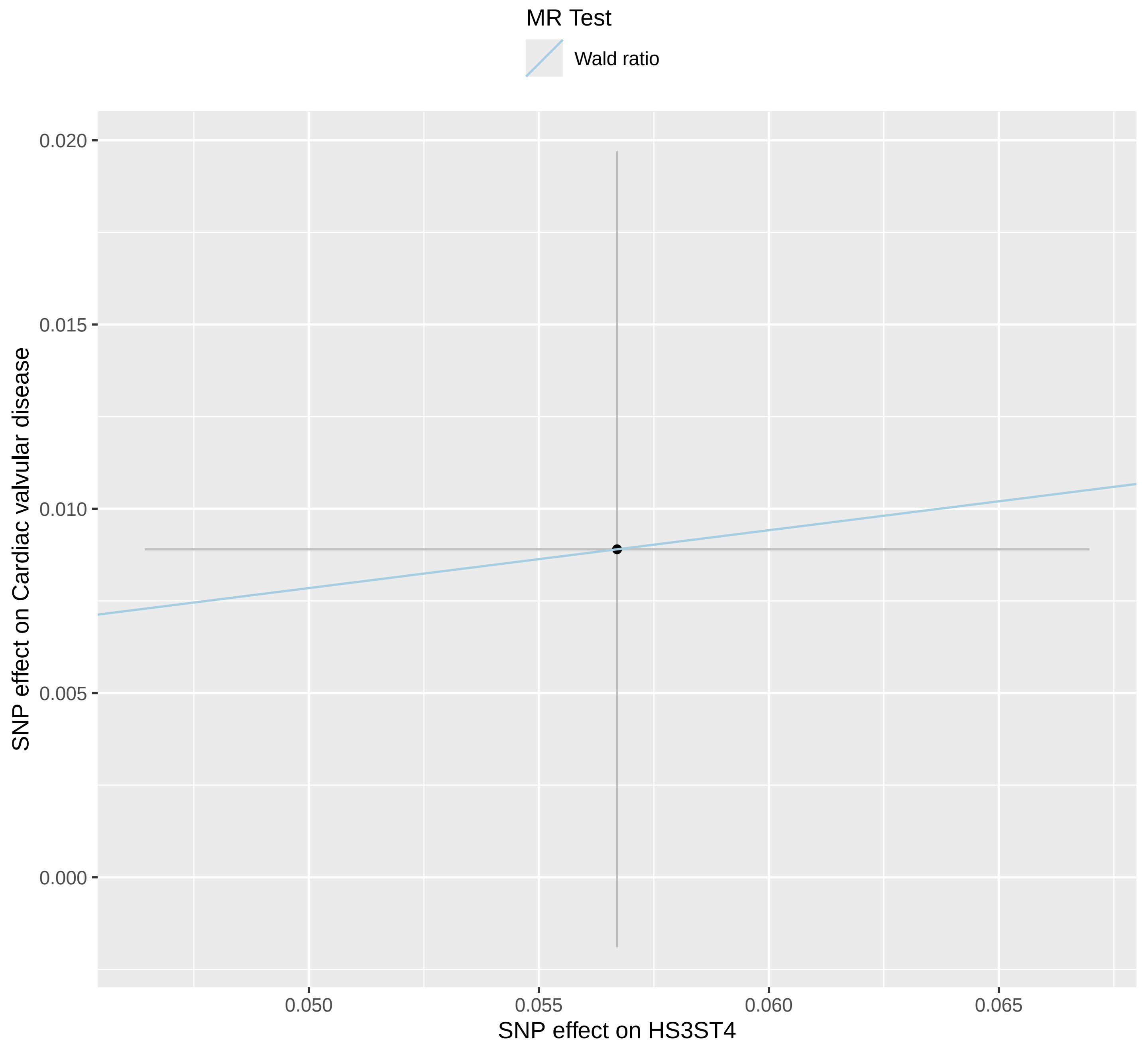
MR Test
Wald ratio
SNP effect on Cardiac valvular disease
0.020
0.015
0.010
0.005
0.000
0.050
0.055
0.060
0.065
SNP effect on HS3ST4

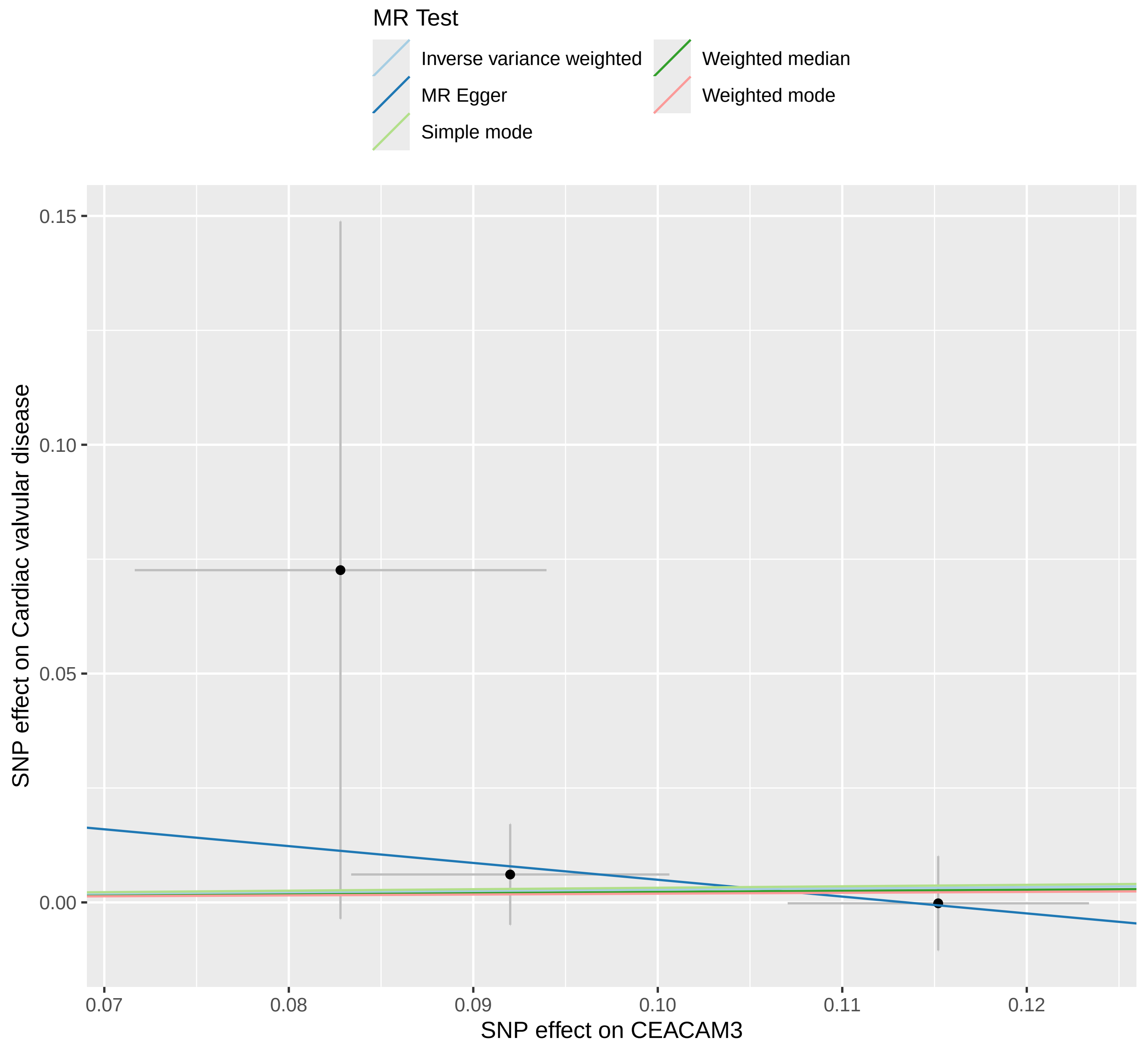
MR Test
Inverse variance weighted
MR Egger
Simple mode
Weighted median
Weighted mode
SNP effect on Cardiac valvular disease
0.15
0.10
0.05
0.00
0.07
0.08
0.09
0.10
0.11
0.12
SNP effect on CEACAM3

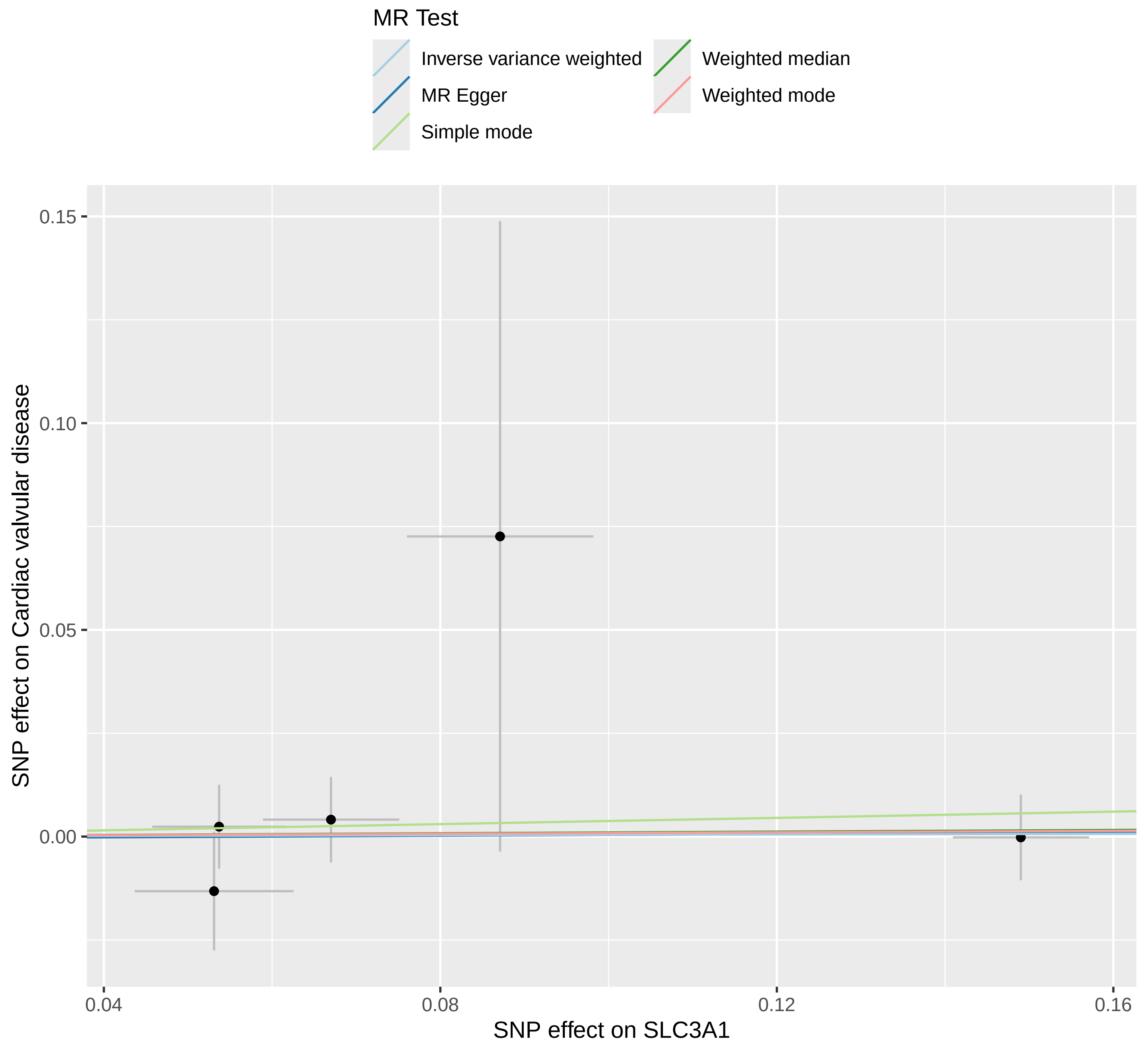
MR Test
Inverse variance weighted
MR Egger
Simple mode
Weighted median
Weighted mode
0.15
0.10
0.05
0.00
0.04
0.08
0.12
0.16
SNP effect on Cardiac valvular disease
SNP effect on SLC3A1

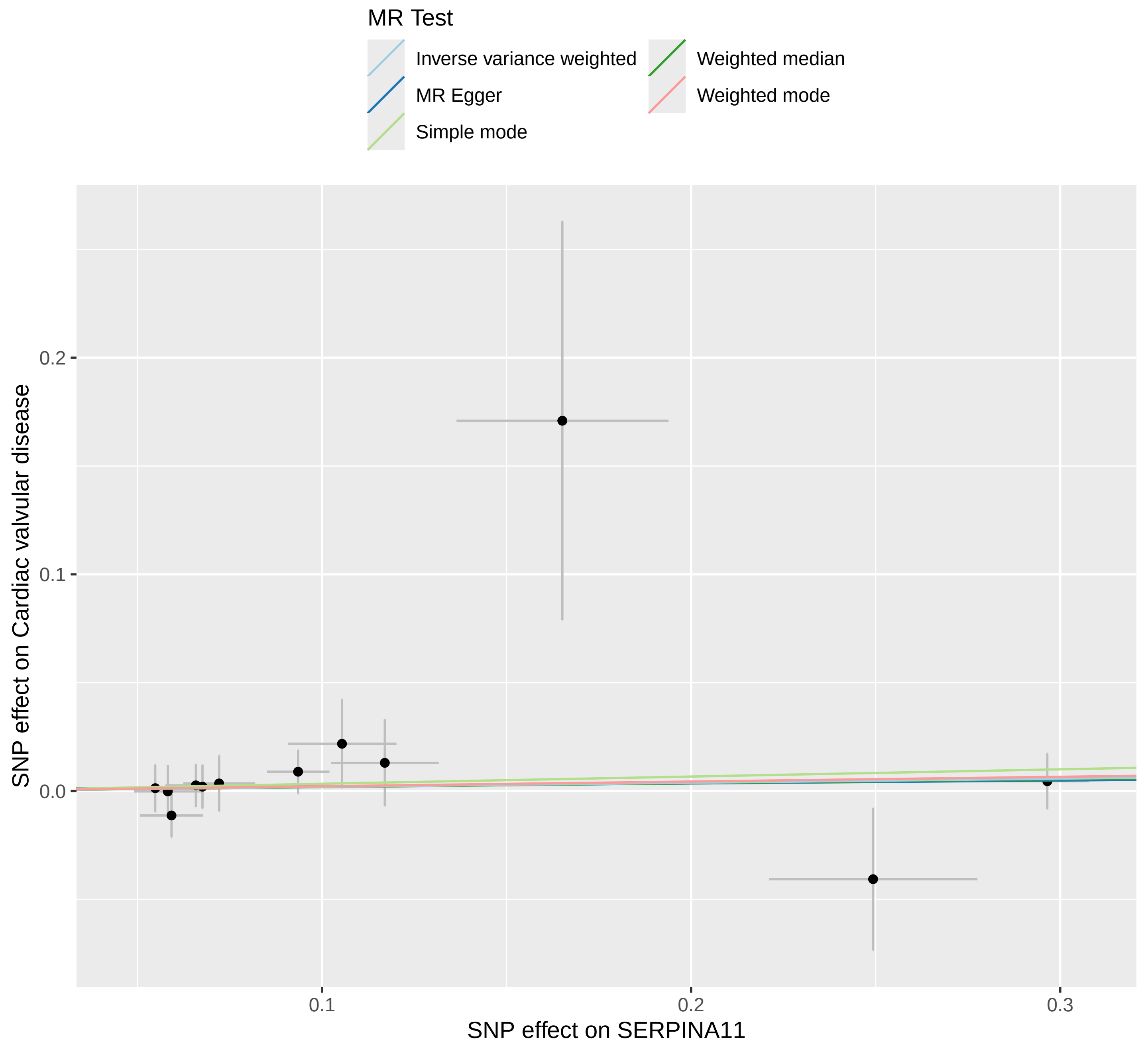
MR Test
Inverse variance weighted
MR Egger
Simple mode
Weighted median
Weighted mode
SNP effect on Cardiac valvular disease
0.2
0.1
0.0
0.1
0.2
0.3
SNP effect on SERPINA11

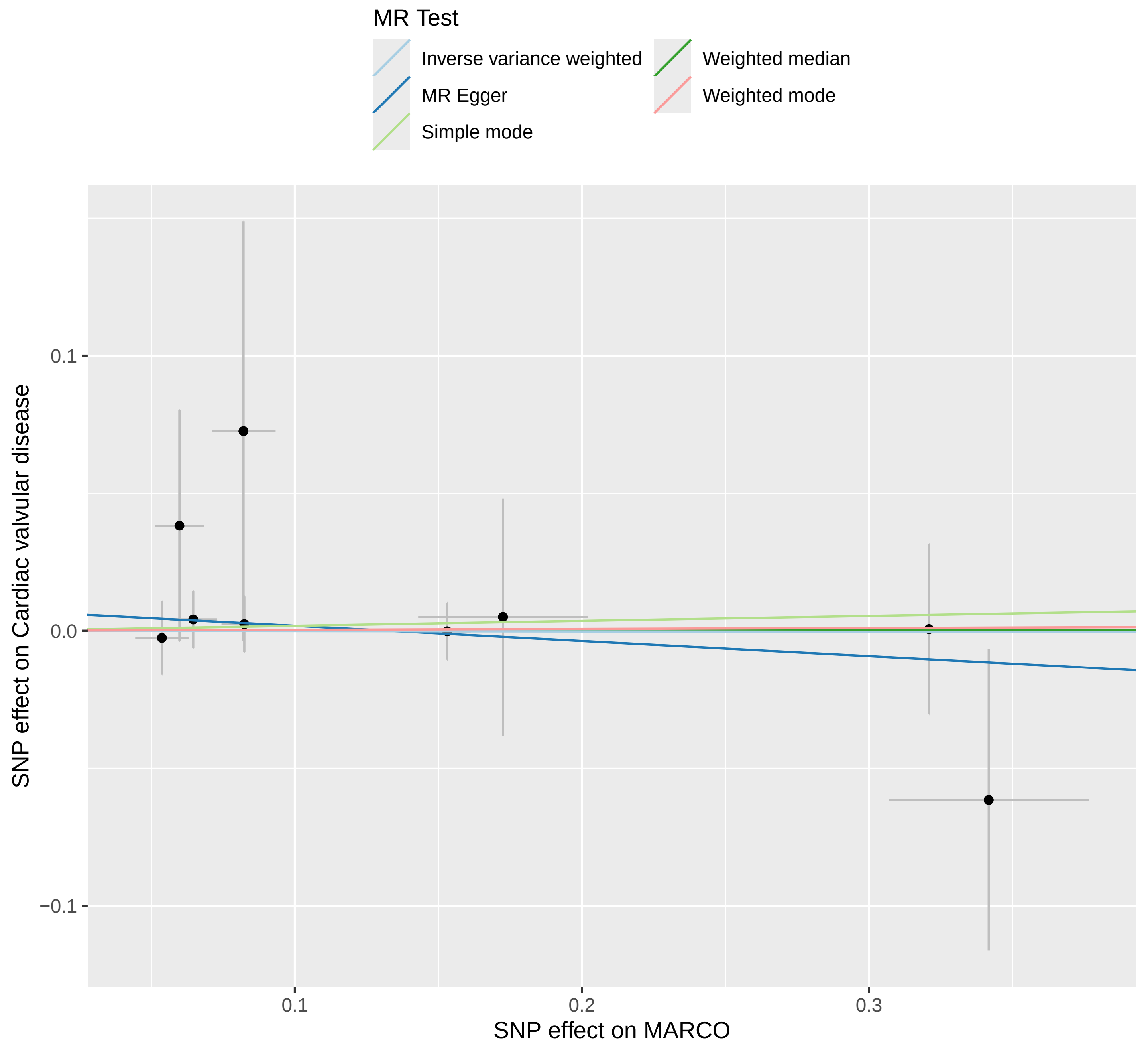

MR Test
Inverse variance weighted
MR Egger
Simple mode
Weighted median
Weighted mode
SNP effect on Cardiac valvular disease
0.1
0.0
−0.1
0.1
0.2
0.3
SNP effect on MARCO

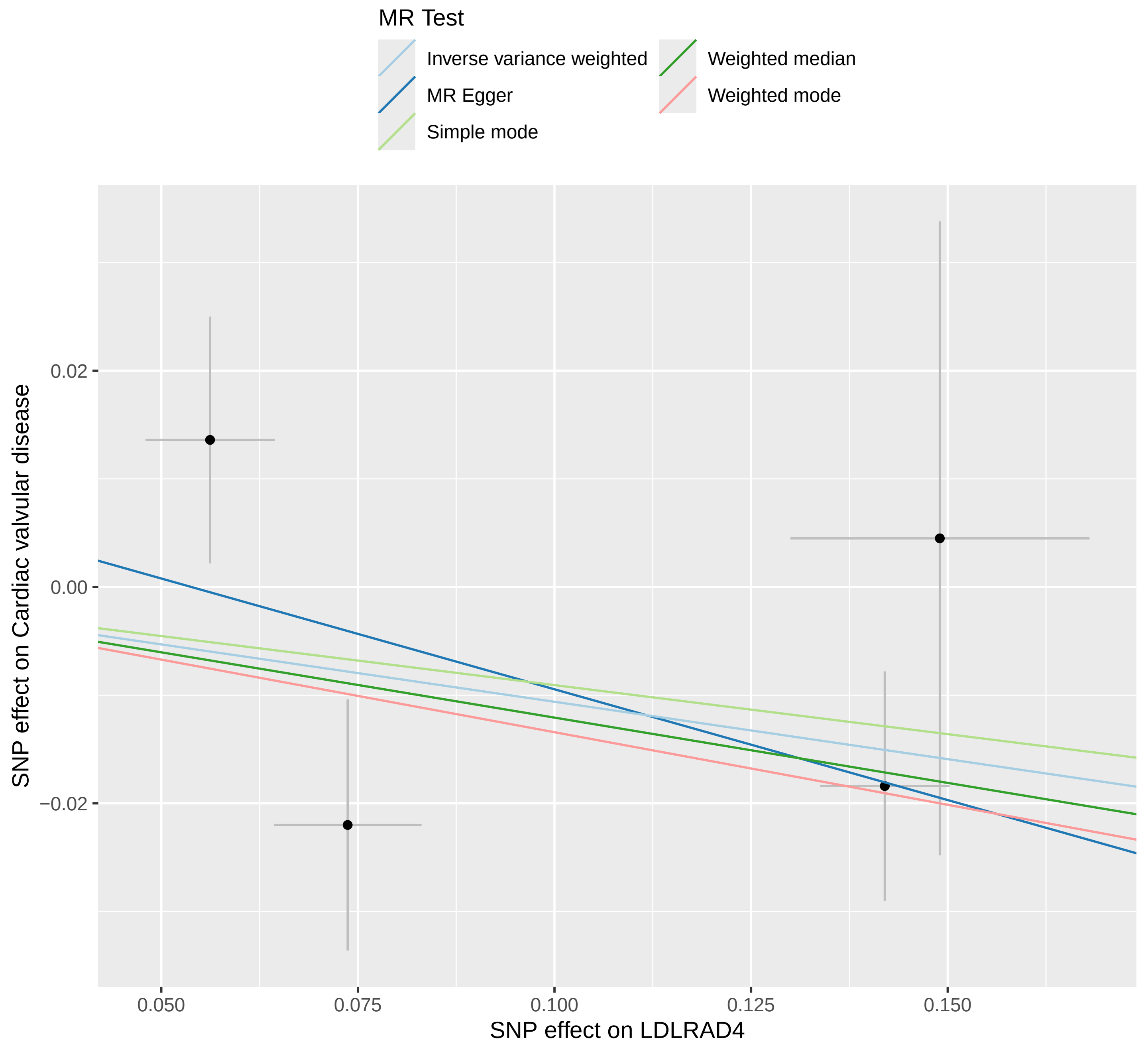
MR Test
Inverse variance weighted
MR Egger
Simple mode
Weighted median
Weighted mode
0.02
0.00
−0.02
SNP effect on Cardiac valvular disease
0.050
0.075
0.100
0.125
0.150
SNP effect on LDLRAD4

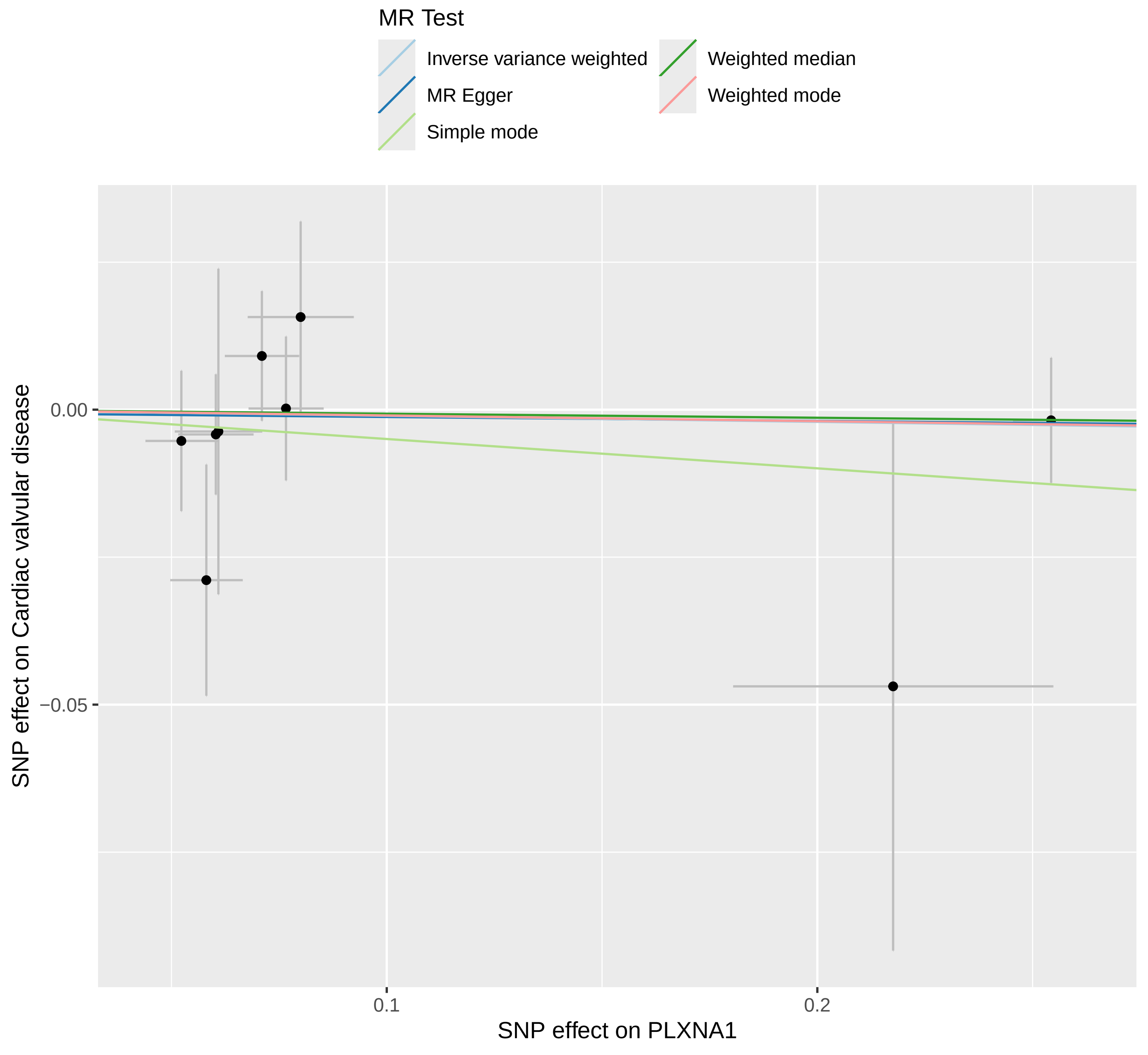
MR Test
Inverse variance weighted
MR Egger
Simple mode
Weighted median
Weighted mode
0.00
−0.05
0.1
0.2
SNP effect on PLXNA1
SNP effect on Cardiac valvular disease

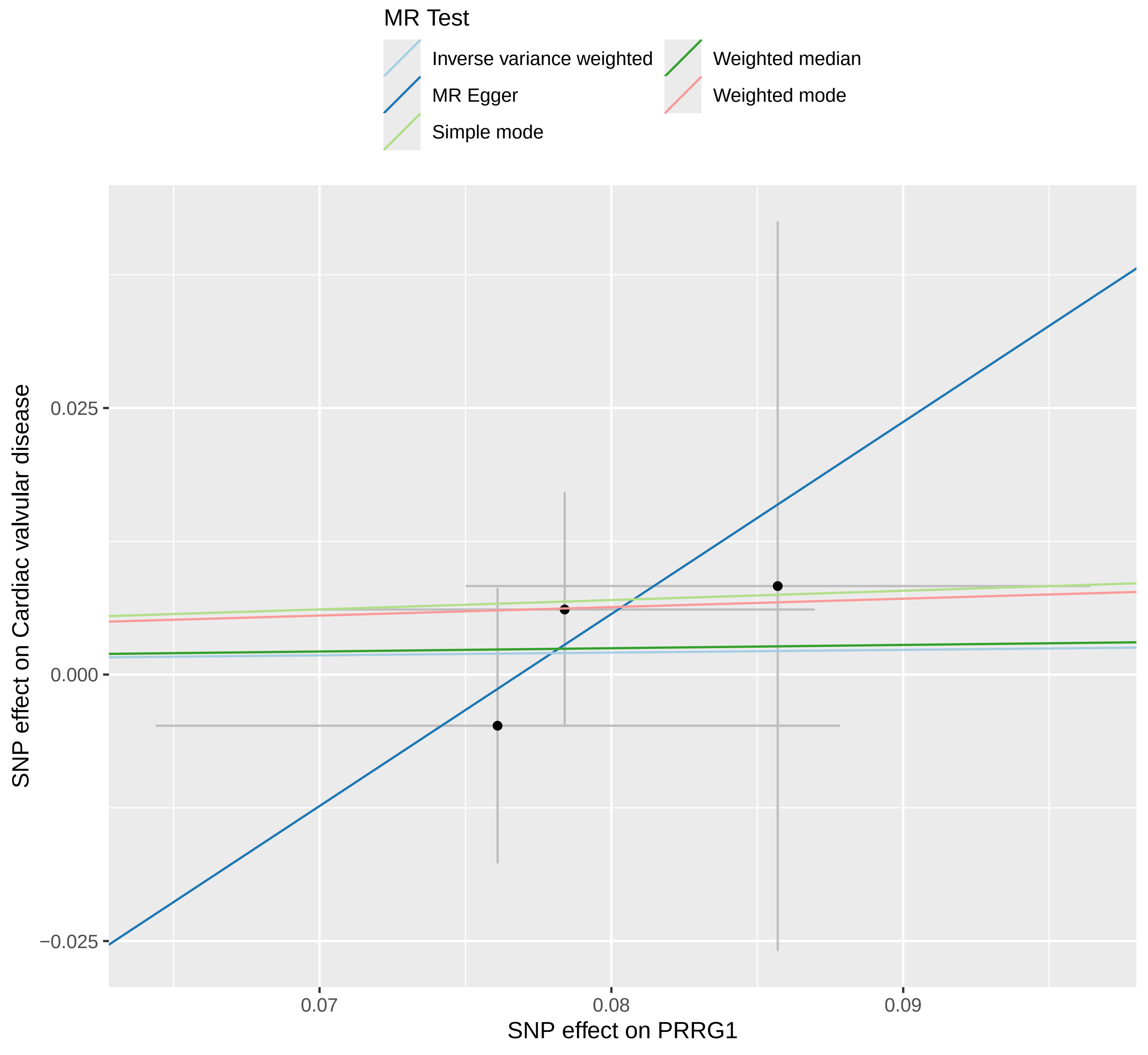

MR Test
Inverse variance weighted
MR Egger
Simple mode
Weighted median
Weighted mode
SNP effect on Cardiac valvular disease
0.025
0.000
−0.025
0.07
0.08
0.09
SNP effect on PRRG1

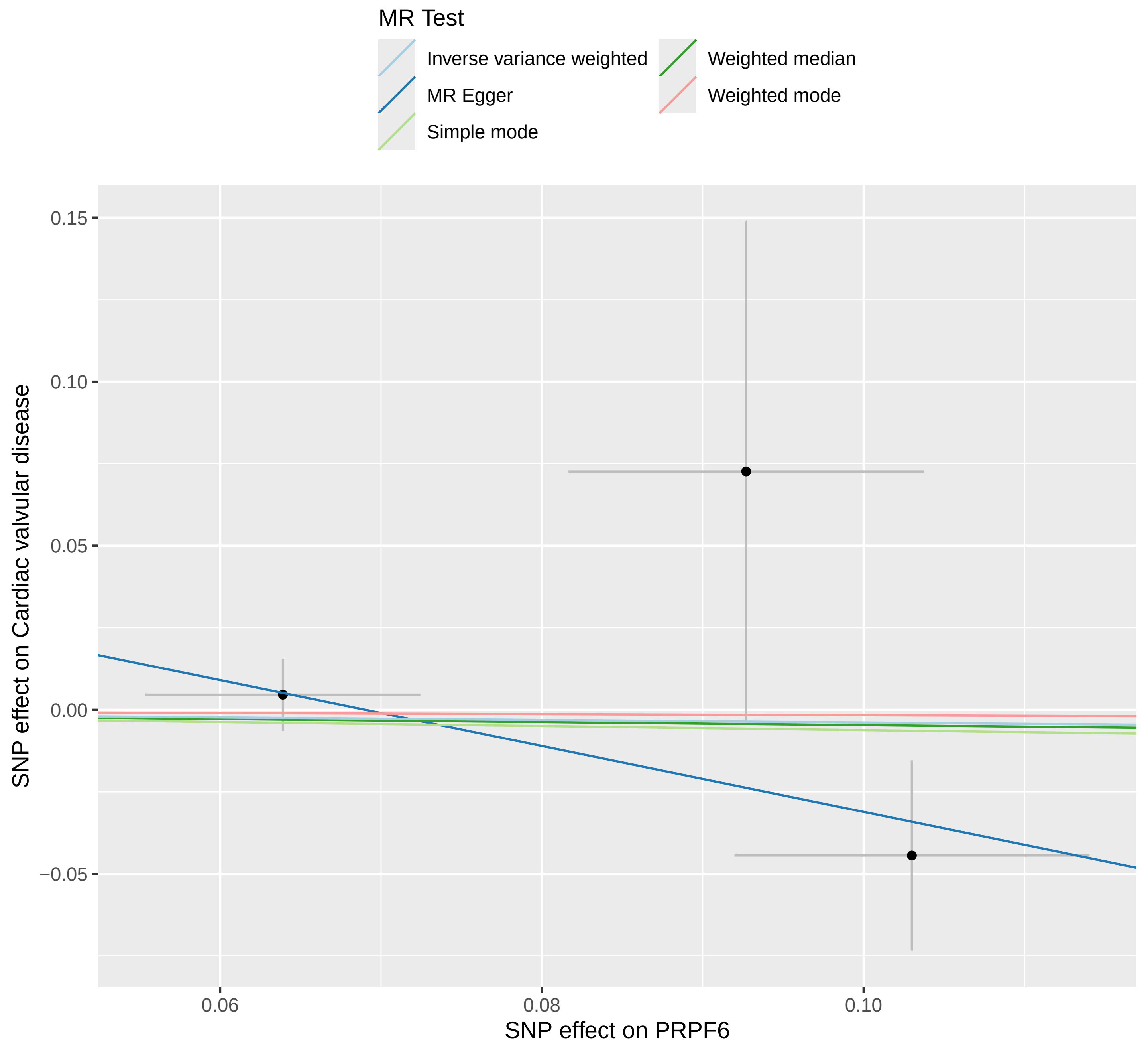
MR Test
Inverse variance weighted
MR Egger
Simple mode
Weighted median
Weighted mode
0.15
0.10
0.05
0.00
−0.05
SNP effect on Cardiac valvular disease
0.06
0.08
0.10
SNP effect on PRPF6

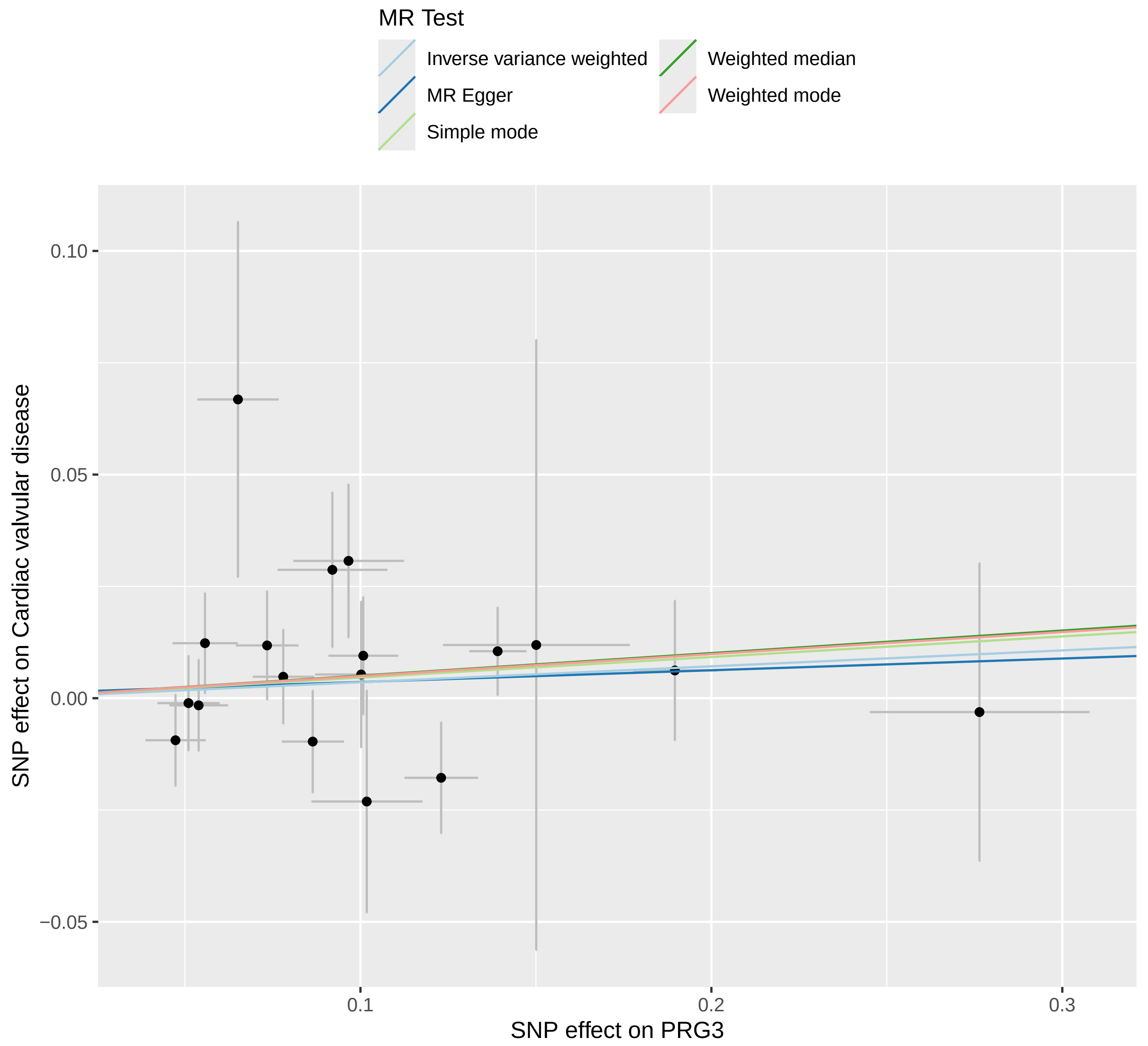

MR Test
Inverse variance weighted
MR Egger
Simple mode
Weighted median
Weighted mode
0.10
0.05
0.00
−0.05
0.1
0.2
0.3
SNP effect on Cardiac valvular disease
SNP effect on PRG3

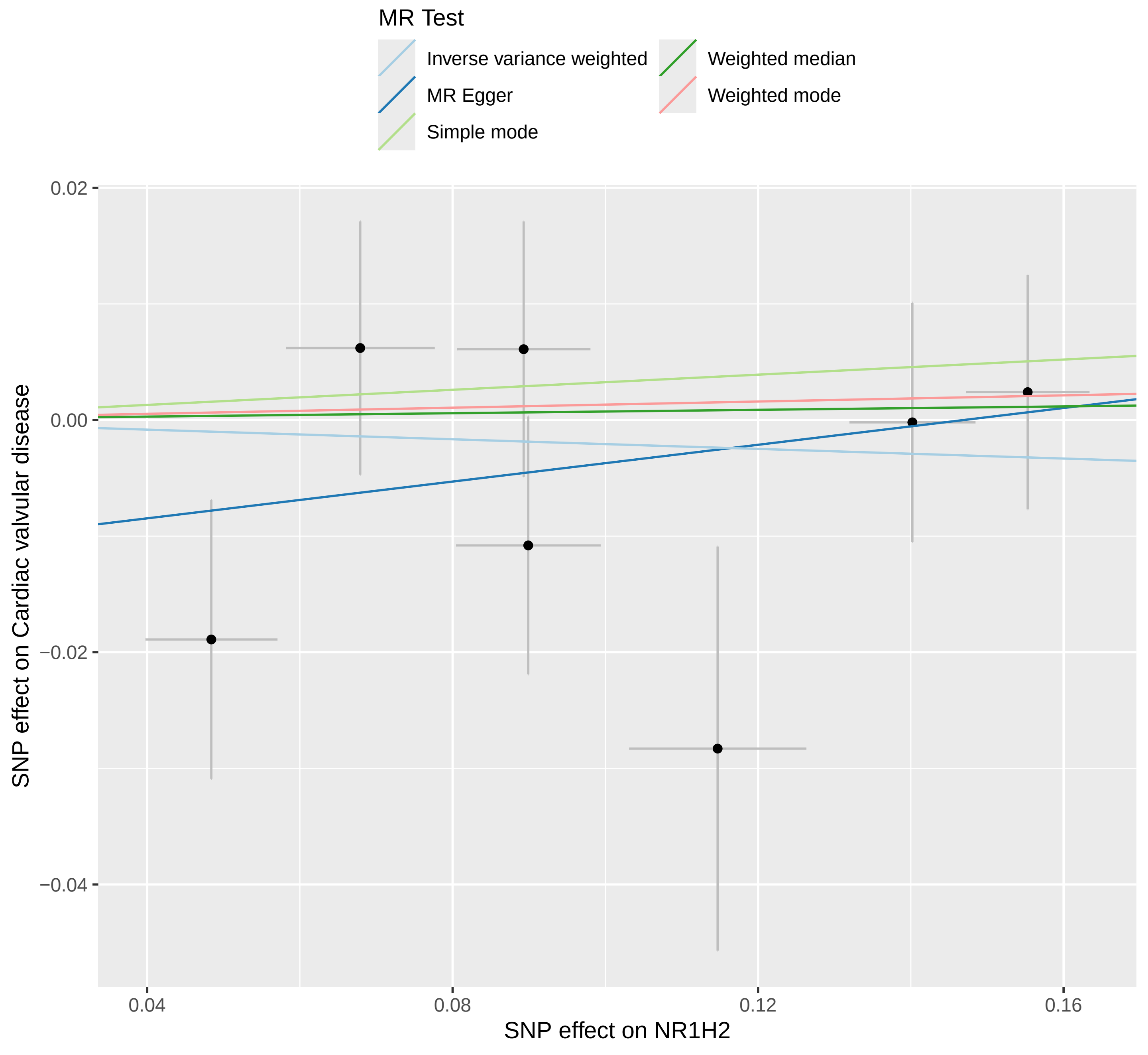
MR Test
Inverse variance weighted
MR Egger
Simple mode
Weighted median
Weighted mode
0.02
0.00
−0.02
−0.04
SNP effect on Cardiac valvular disease
0.04
0.08
0.12
0.16
SNP effect on NR1H2

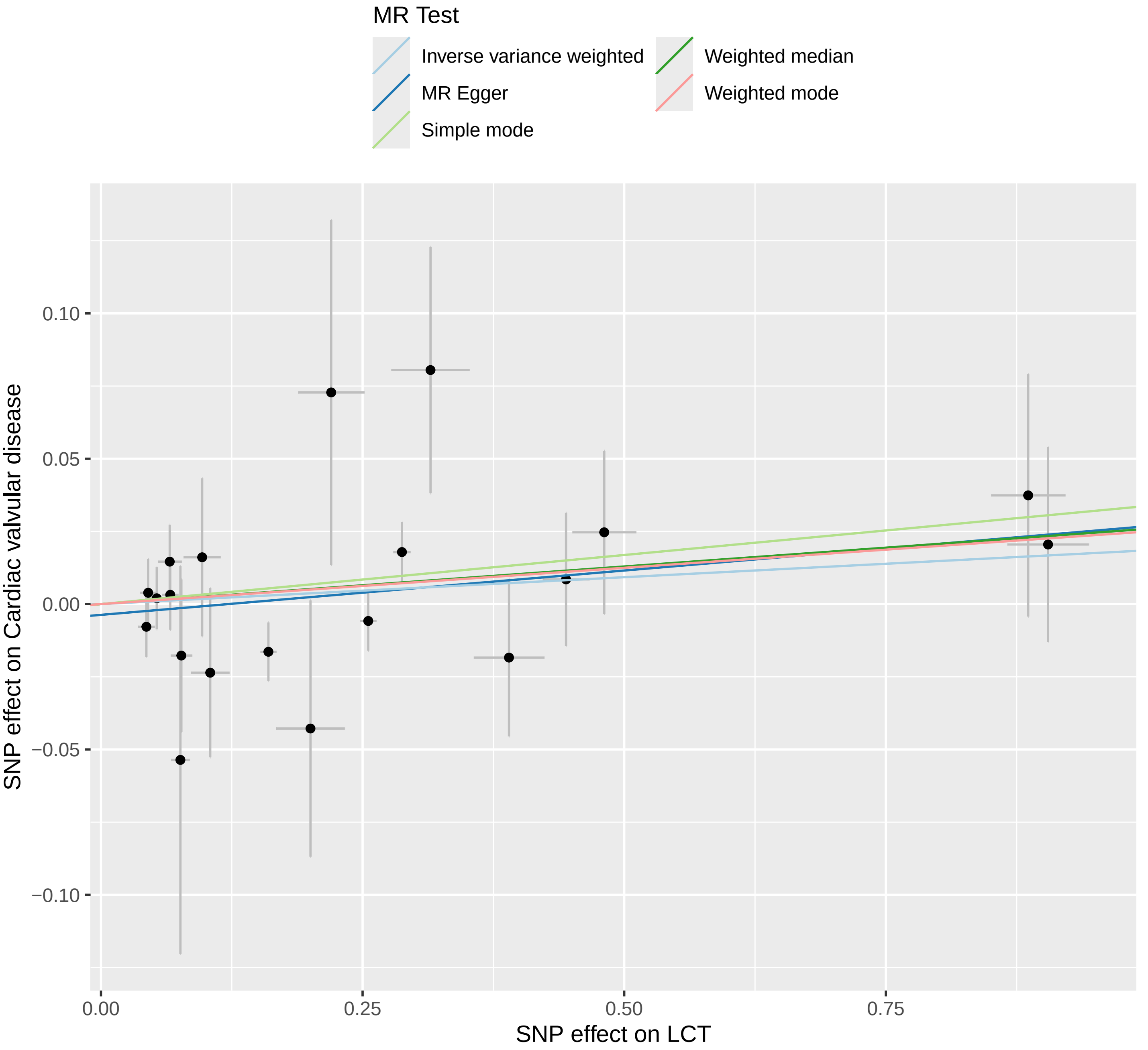
MR Test
Inverse variance weighted
MR Egger
Simple mode
Weighted median
Weighted mode
0.10
0.05
0.00
−0.05
−0.10
0.00
0.25
0.50
0.75
SNP effect on Cardiac valvular disease
SNP effect on LCT

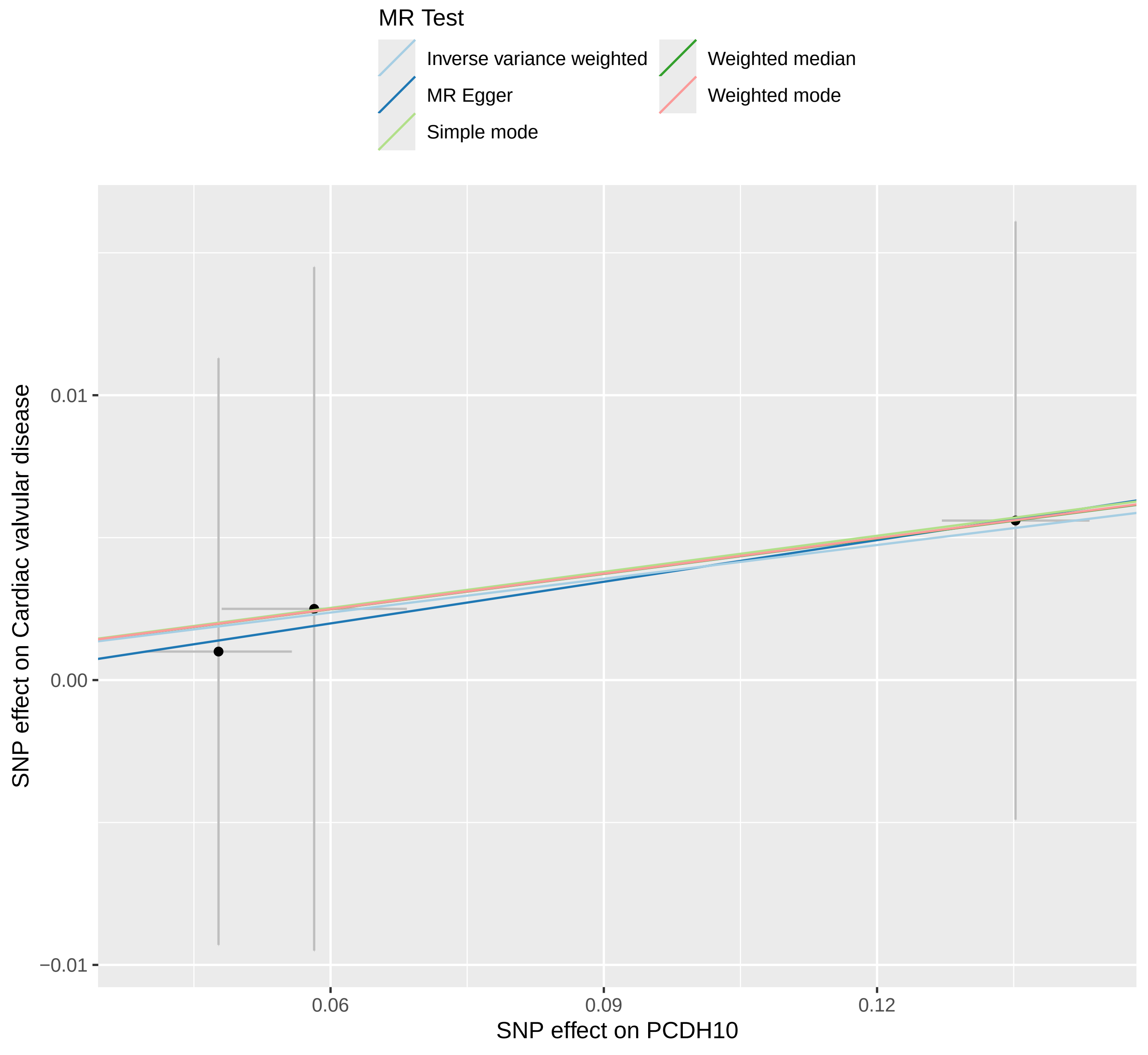

MR Test
Inverse variance weighted
MR Egger
Simple mode
Weighted median
Weighted mode
0.01
0.00
−0.01
0.06
0.09
0.12
SNP effect on Cardiac valvular disease
SNP effect on PCDH10

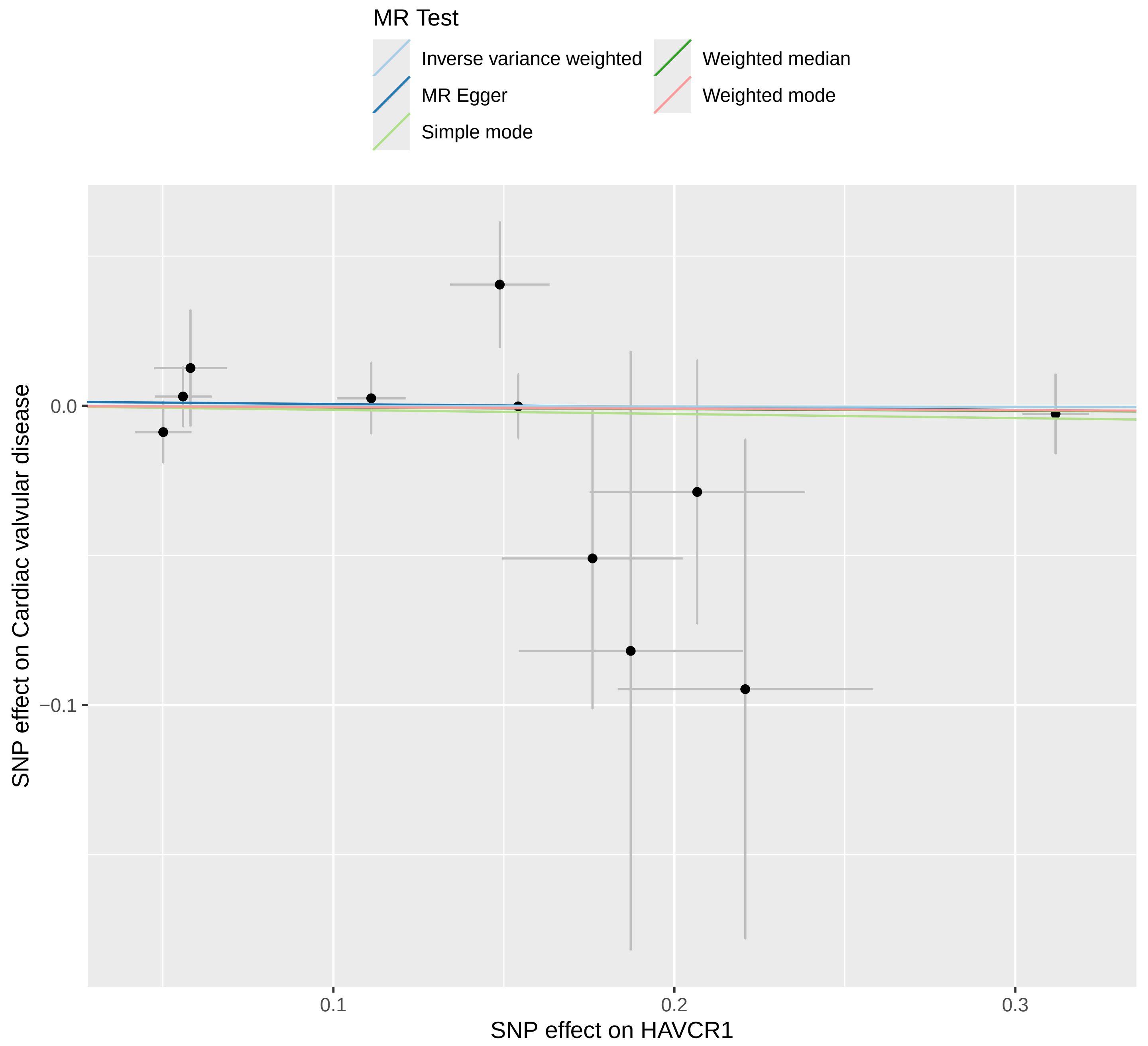
MR Test
Inverse variance weighted
MR Egger
Simple mode
Weighted median
Weighted mode
0.0
−0.1
0.1
0.2
0.3
SNP effect on HAVCR1
SNP effect on Cardiac valvular disease

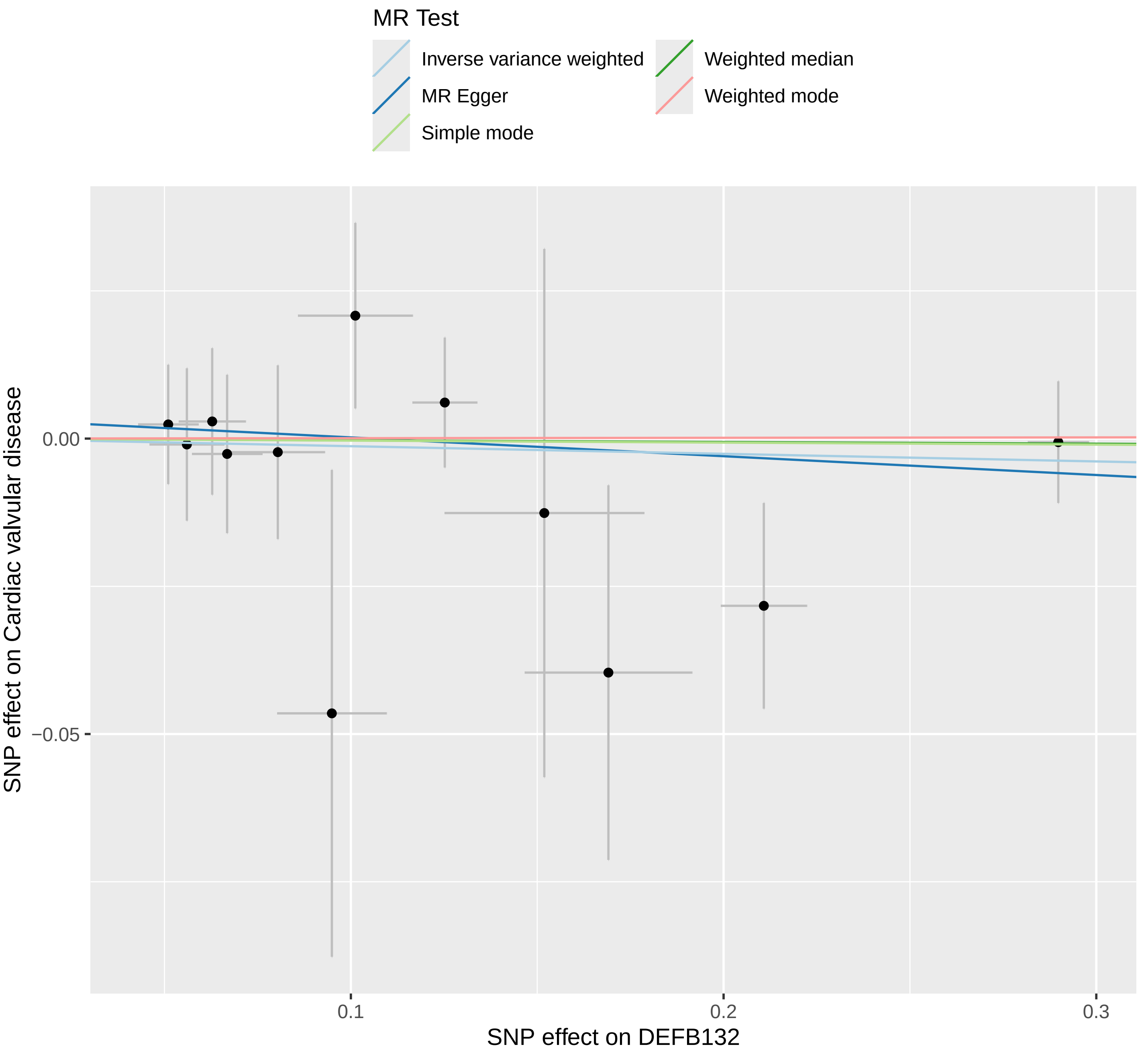
MR Test
Inverse variance weighted
MR Egger
Simple mode
Weighted median
Weighted mode
SNP effect on Cardiac valvular disease
0.00
−0.05
0.1
0.2
0.3
SNP effect on DEFB132

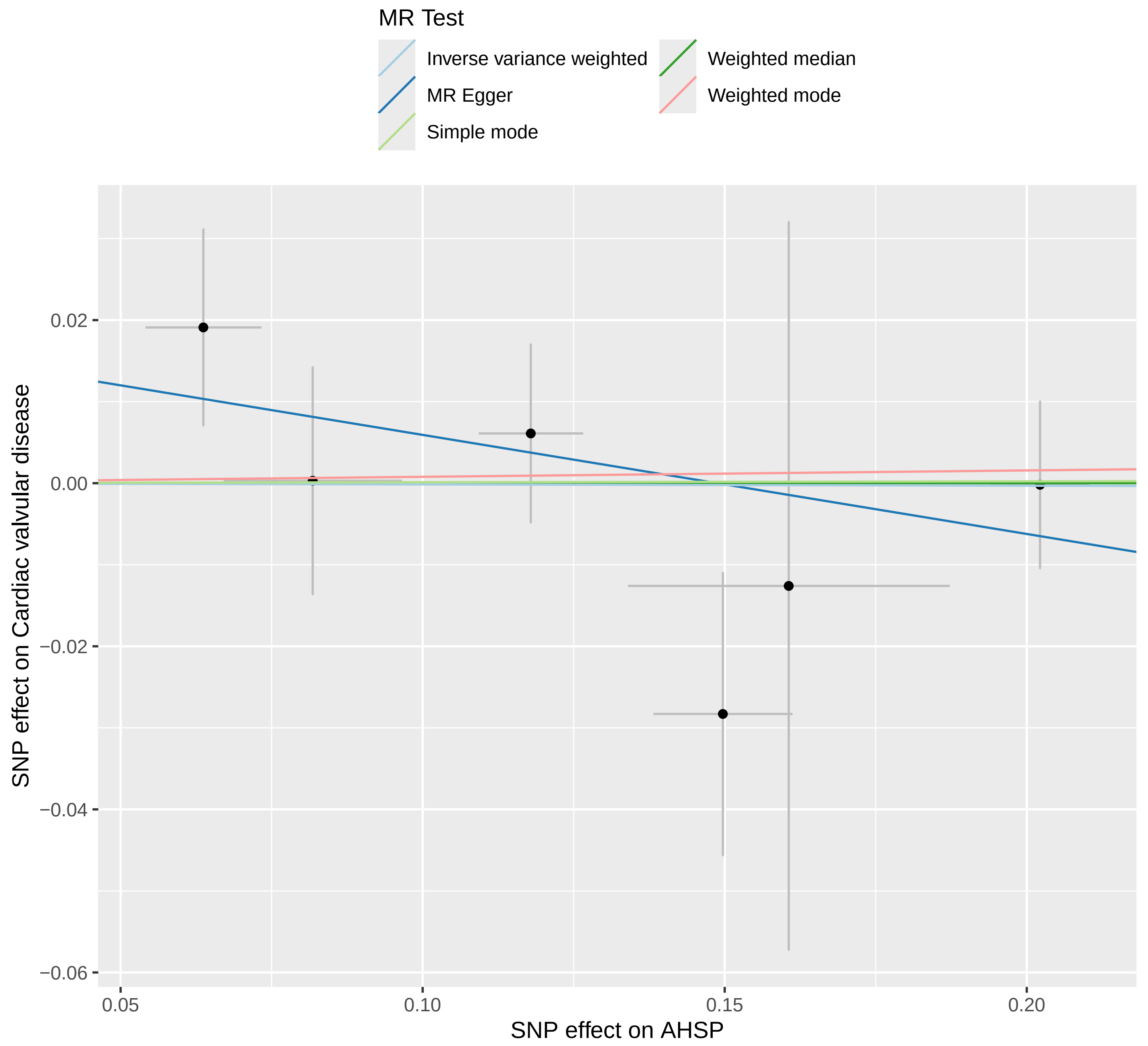
MR Test
Inverse variance weighted
MR Egger
Simple mode
Weighted median
Weighted mode
SNP effect on Cardiac valvular disease
0.02
0.00
−0.02
−0.04
−0.06
0.05
0.10
0.15
0.20
SNP effect on AHSP

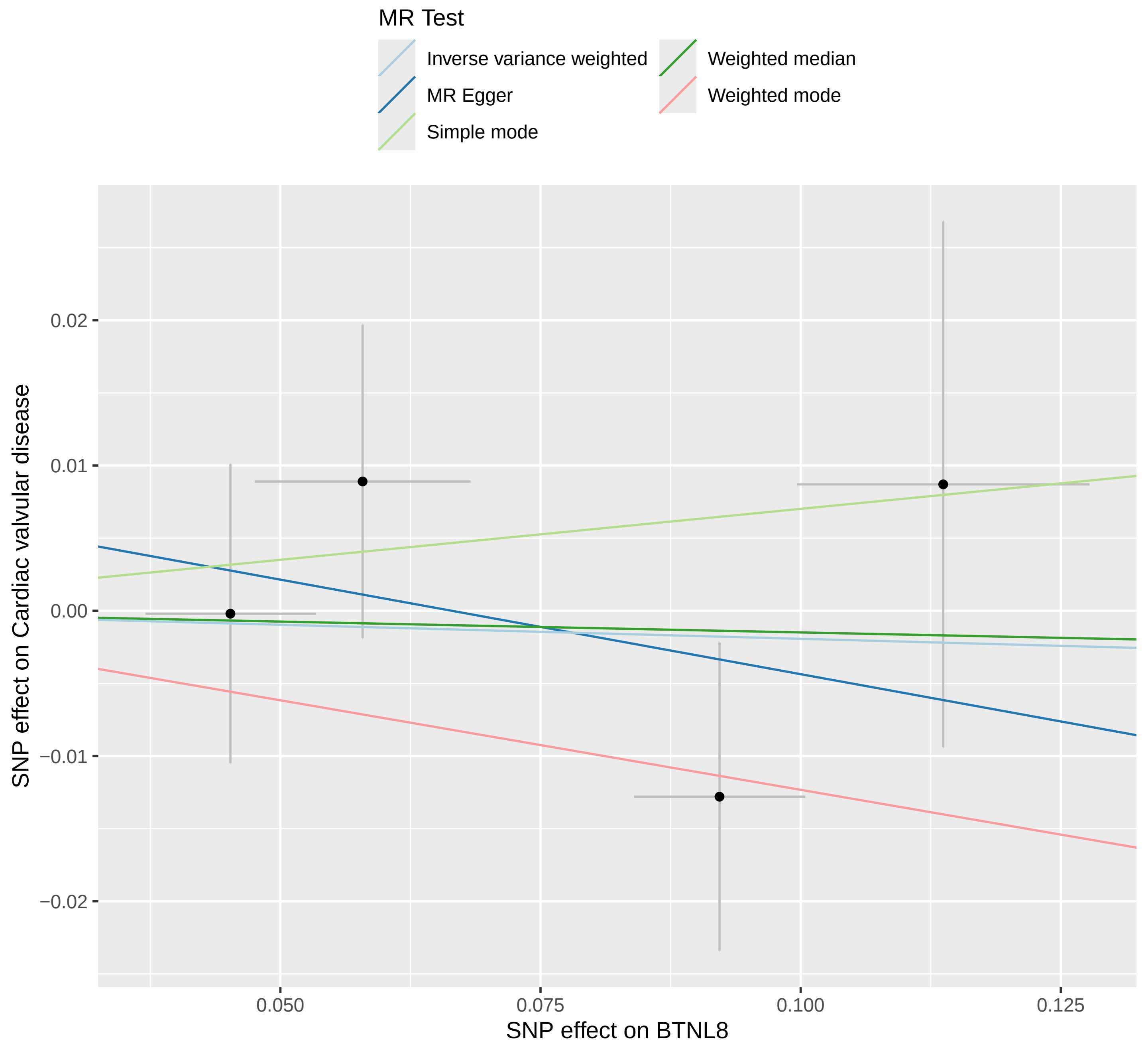

MR Test
Inverse variance weighted
MR Egger
Simple mode
Weighted median
Weighted mode
0.02
0.01
0.00
−0.01
−0.02
SNP effect on Cardiac valvular disease
0.050
0.075
0.100
0.125
SNP effect on BTNL8

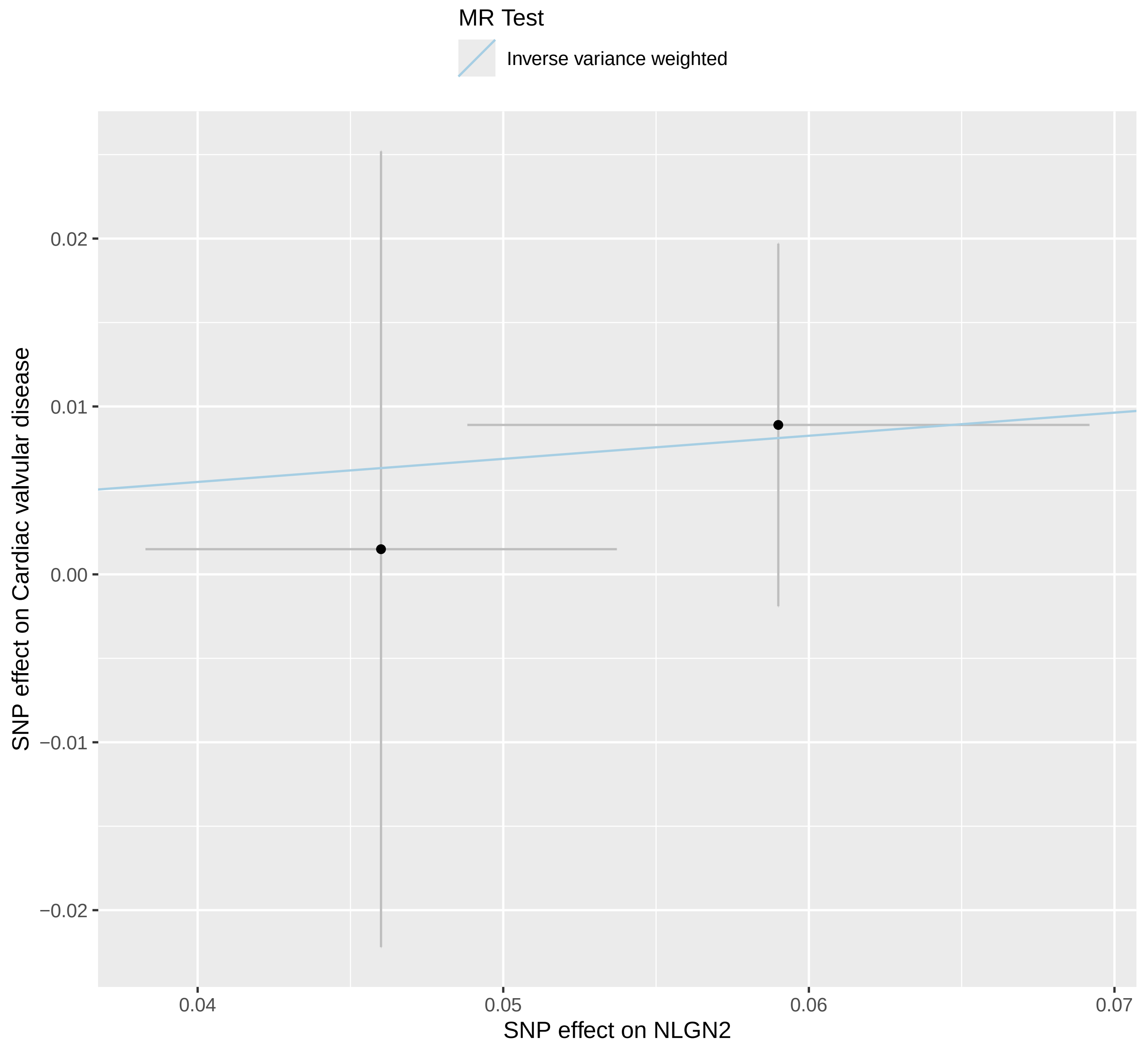
MR Test
Inverse variance weighted
0.02
0.01
0.00
−0.01
−0.02
SNP effect on Cardiac valvular disease
0.04
0.05
0.06
0.07
SNP effect on NLGN2

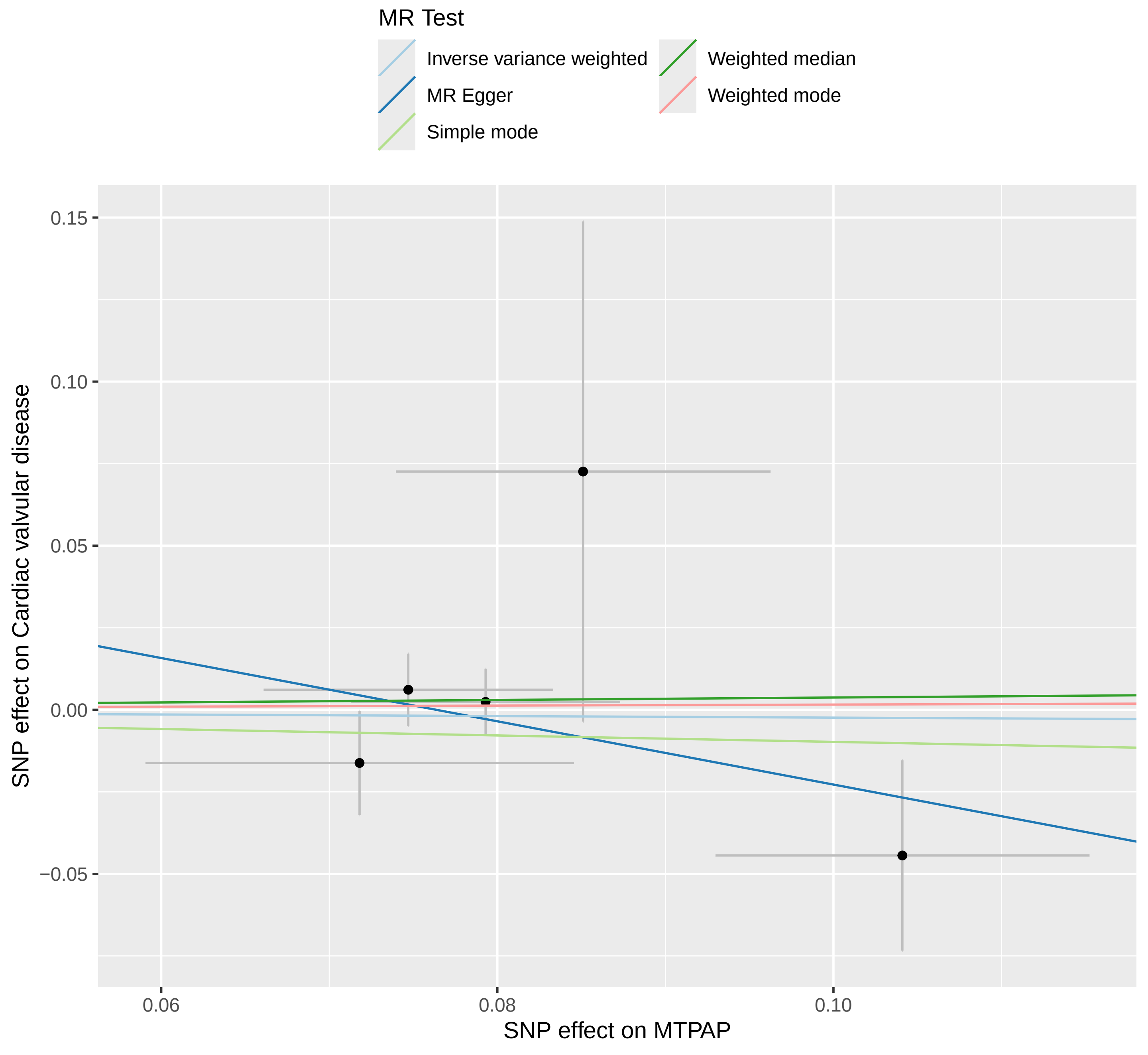
MR Test
Inverse variance weighted
MR Egger
Simple mode
Weighted median
Weighted mode
0.15
0.10
0.05
0.00
−0.05
0.06
0.08
0.10
SNP effect on Cardiac valvular disease
SNP effect on MTPAP

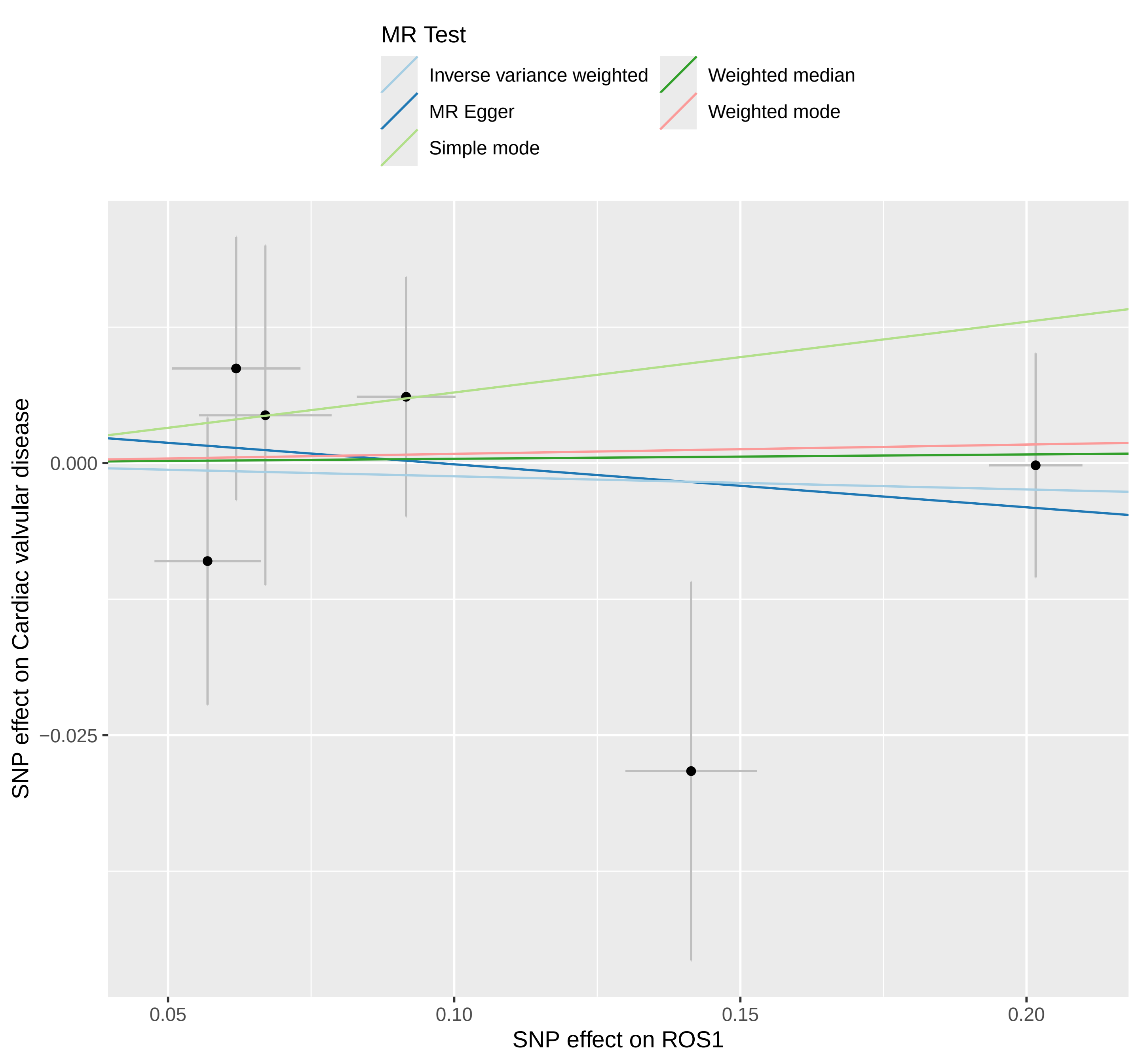
MR Test
Inverse variance weighted
MR Egger
Simple mode
Weighted median
Weighted mode
SNP effect on Cardiac valvular disease
0.000
−0.025
0.05
0.10
0.15
0.20
SNP effect on ROS1

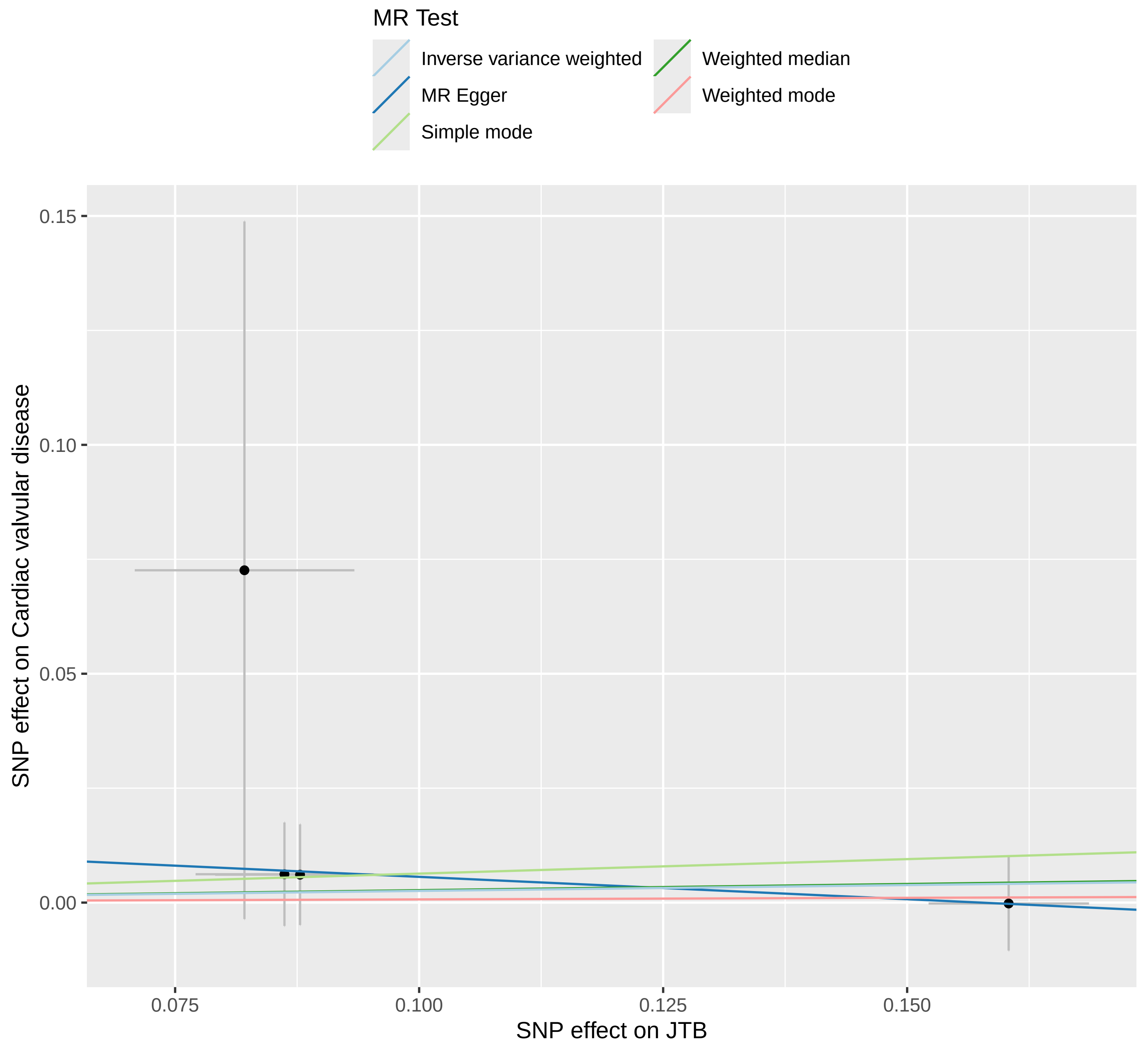

MR Test
Inverse variance weighted
MR Egger
Simple mode
Weighted median
Weighted mode
SNP effect on Cardiac valvular disease
0.15
0.10
0.05
0.00
0.075
0.100
0.125
0.150
SNP effect on JTB

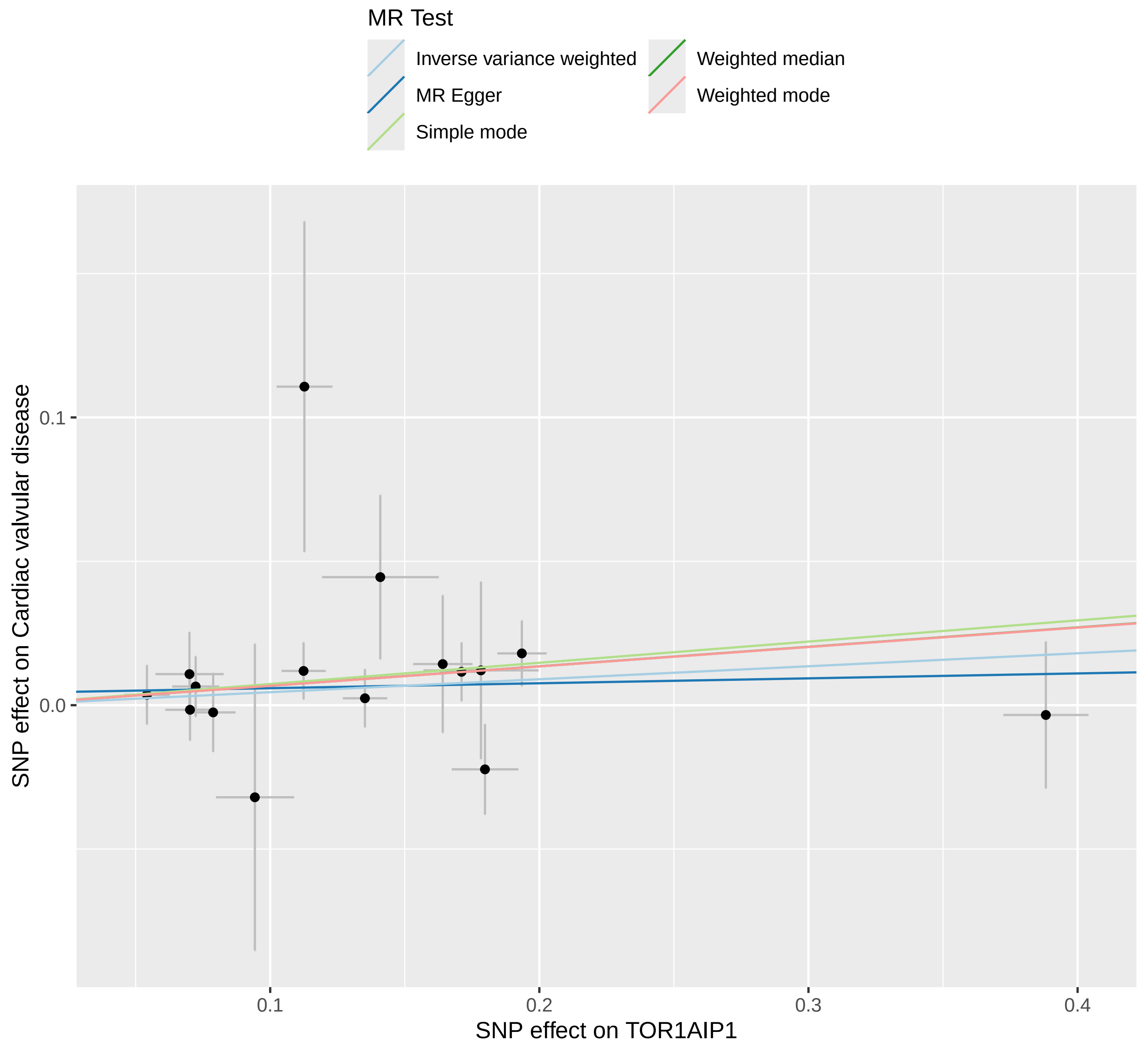
MR Test
Inverse variance weighted
MR Egger
Simple mode
Weighted median
Weighted mode
0.1
0.0
0.1
0.2
0.3
0.4
SNP effect on Cardiac valvular disease
SNP effect on TOR1AIP1

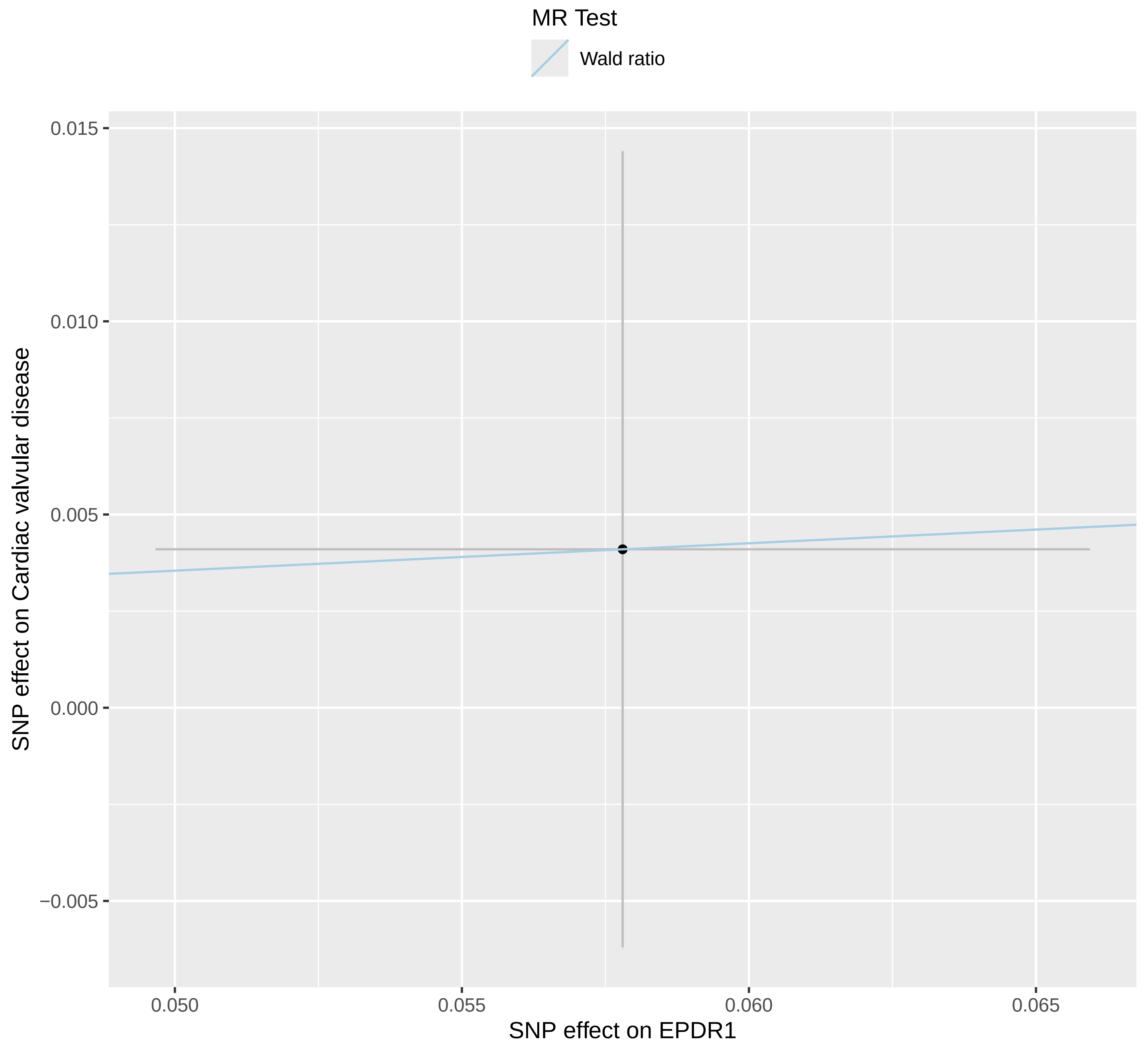
MR Test
Wald ratio
0.015
0.010
0.005
0.000
−0.005
0.050
0.055
0.060
0.065
SNP effect on Cardiac valvular disease
SNP effect on EPDR1

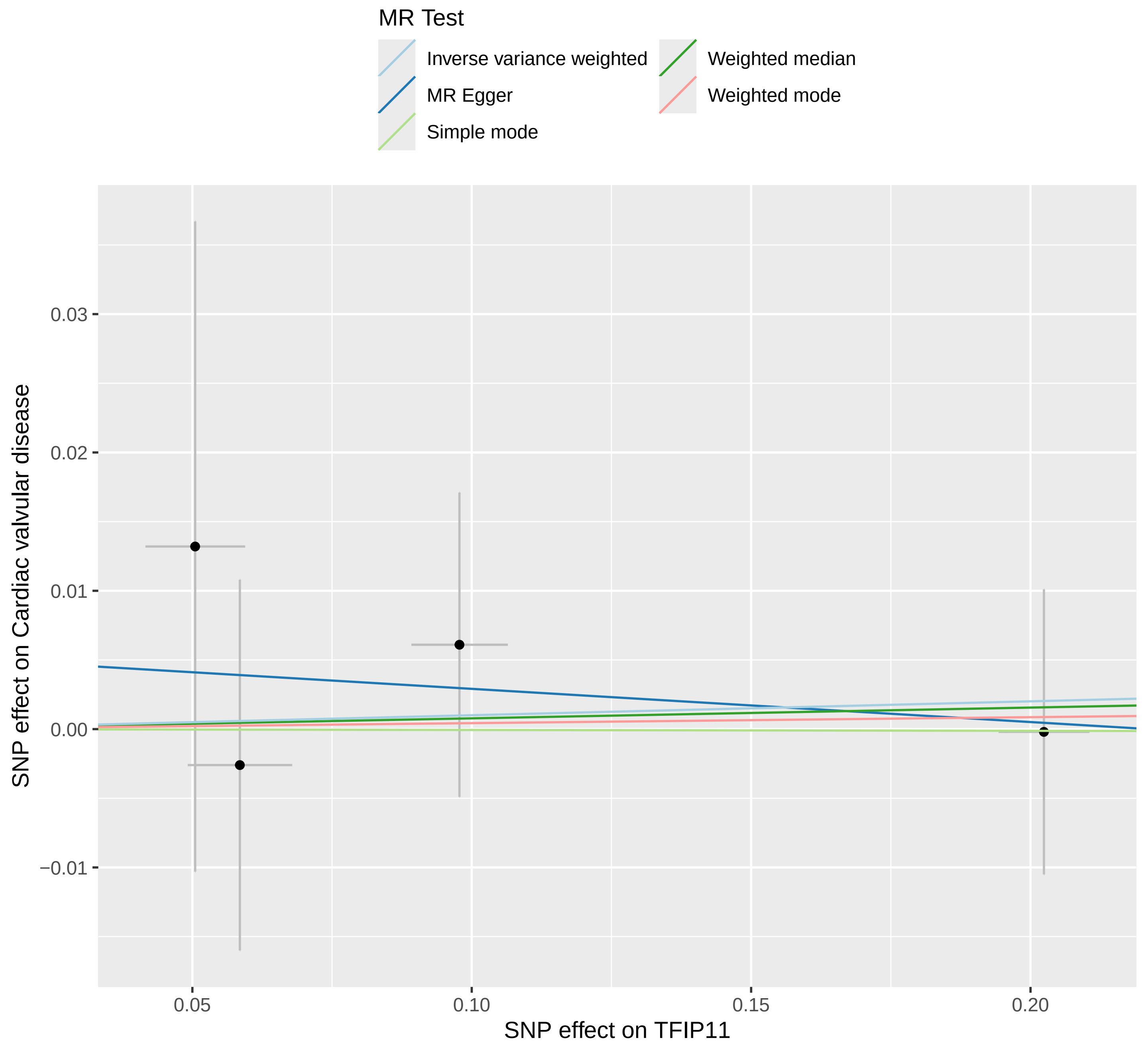
MR Test
Inverse variance weighted
MR Egger
Simple mode
Weighted median
Weighted mode
0.03
0.02
0.01
0.00
−0.01
0.05
0.10
0.15
0.20
SNP effect on Cardiac valvular disease
SNP effect on TFIP11

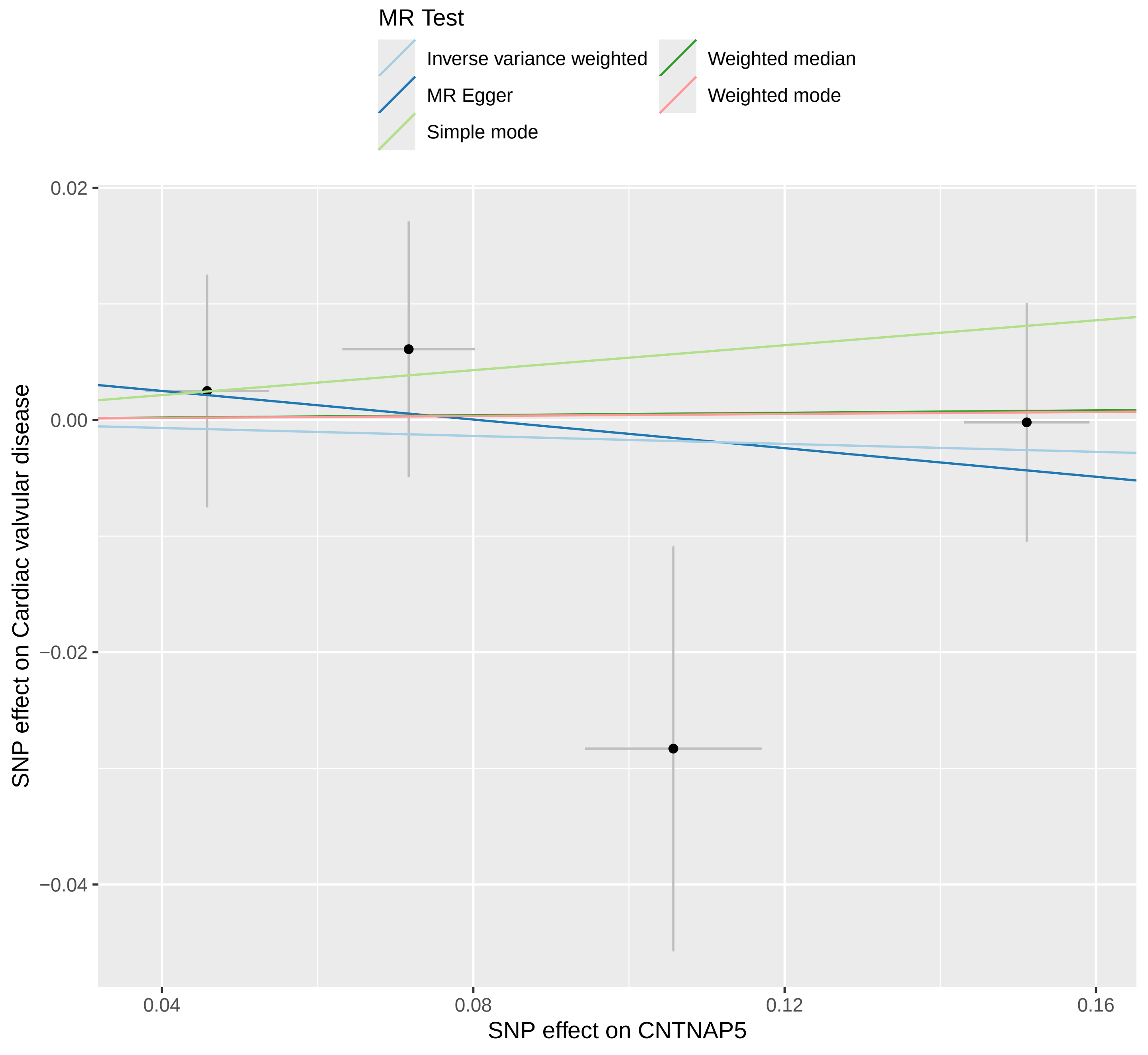
MR Test
Inverse variance weighted
MR Egger
Simple mode
Weighted median
Weighted mode
0.02
0.00
−0.02
−0.04
0.04
0.08
0.12
0.16
SNP effect on Cardiac valvular disease
SNP effect on CNTNAP5

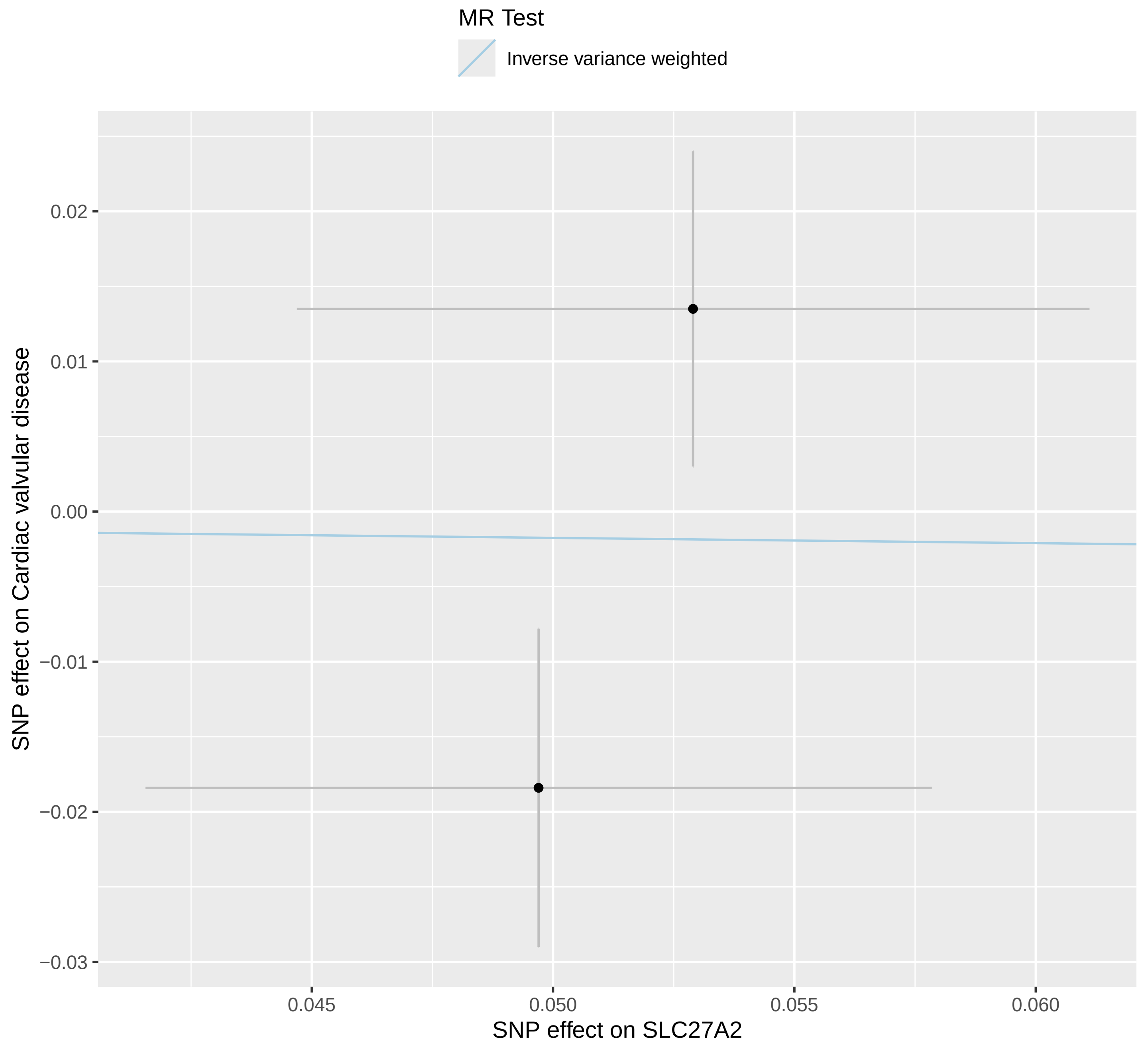

MR Test
Inverse variance weighted
0.02
0.01
0.00
−0.01
−0.02
−0.03
0.045
0.050
0.055
0.060
SNP effect on SLC27A2
SNP effect on Cardiac valvular disease

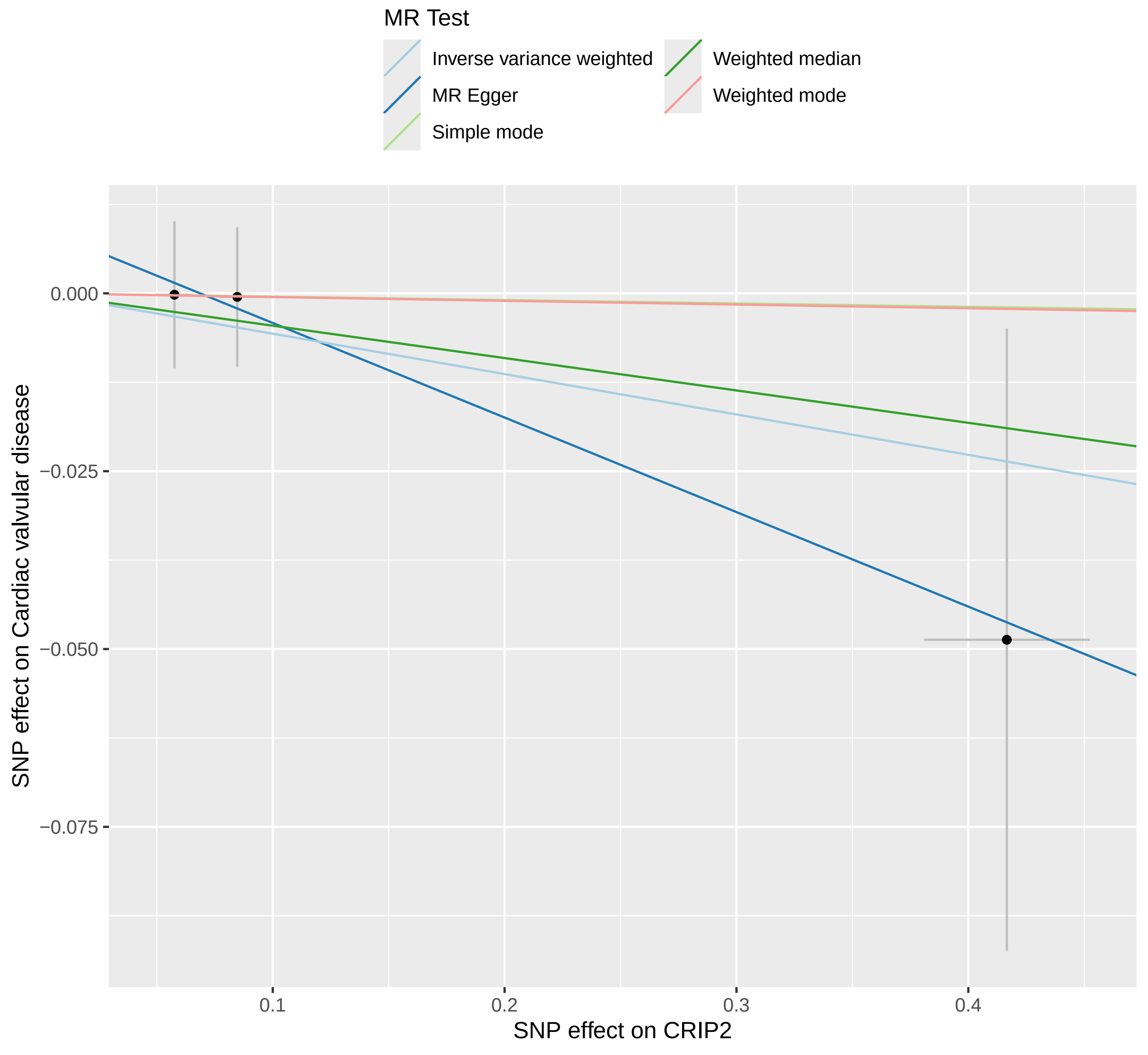
MR Test
Inverse variance weighted
MR Egger
Simple mode
Weighted median
Weighted mode
0.000
−0.025
−0.050
−0.075
0.1
0.2
0.3
0.4
SNP effect on CRIP2
SNP effect on Cardiac valvular disease

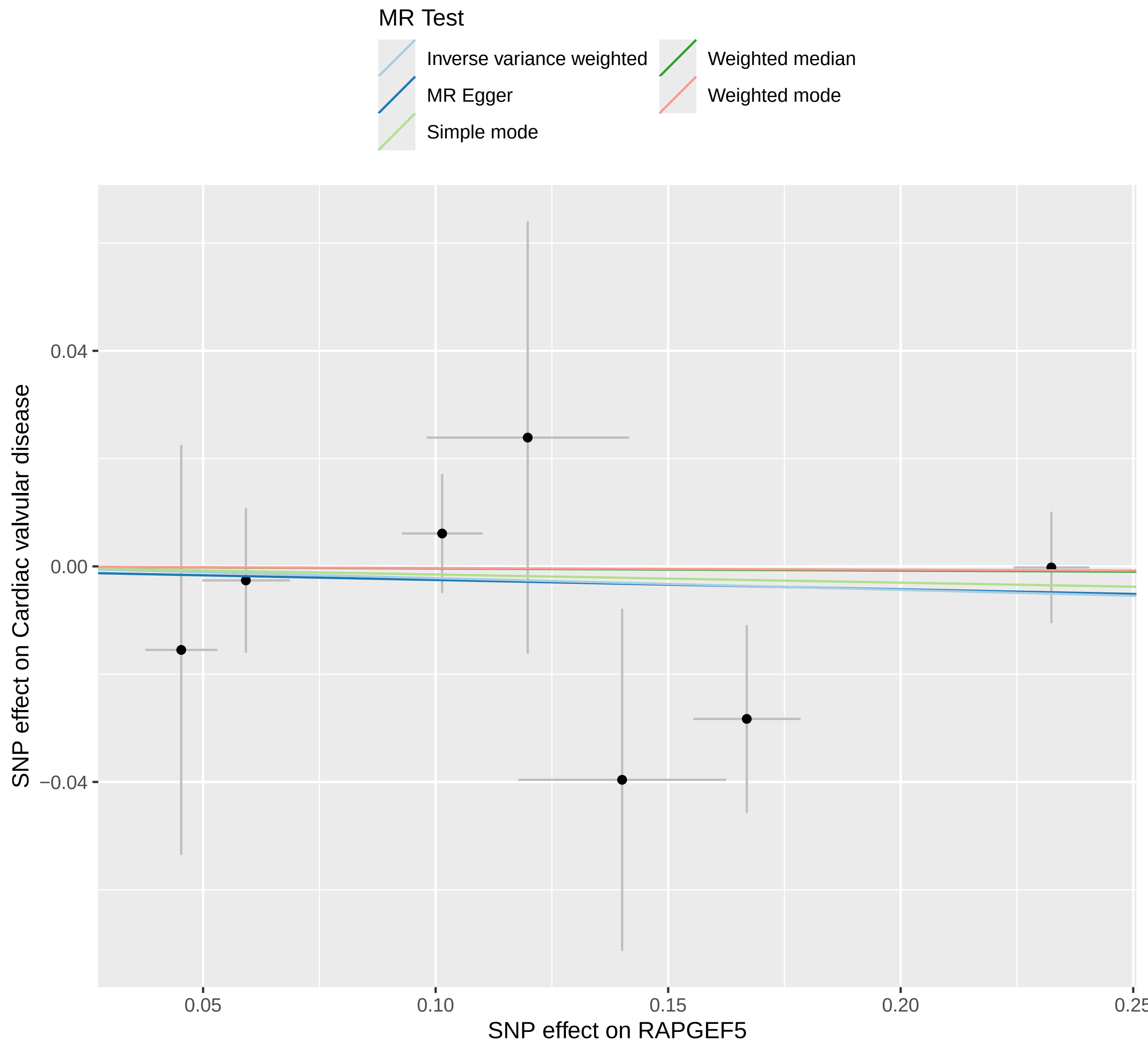
MR Test
Inverse variance weighted
MR Egger
Simple mode
Weighted median
Weighted mode
0.04
0.00
−0.04
SNP effect on Cardiac valvular disease
0.05
0.10
0.15
0.20
0.25
SNP effect on RAPGEF5

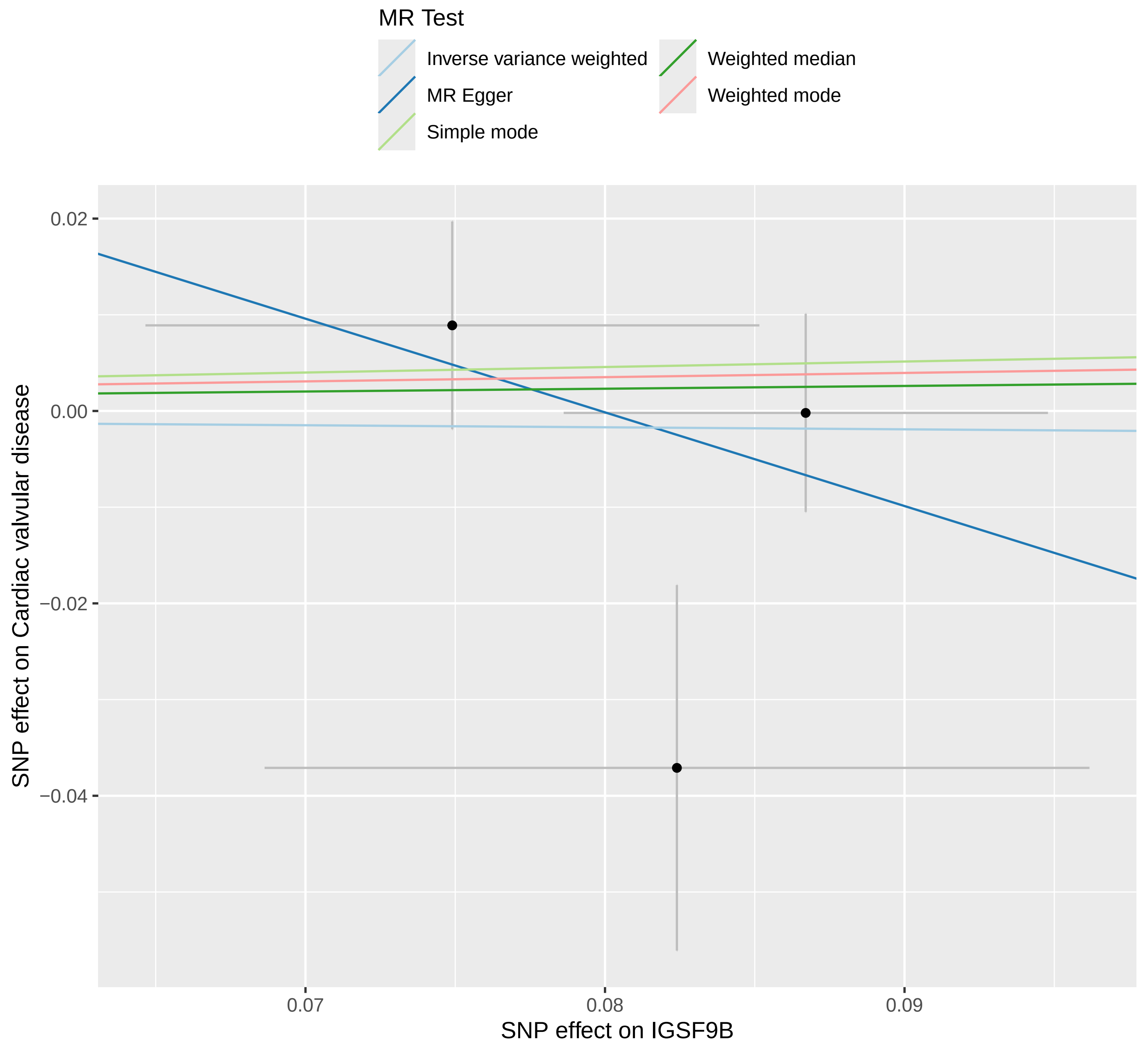

MR Test
Inverse variance weighted
MR Egger
Simple mode
Weighted median
Weighted mode
0.02
0.00
−0.02
−0.04
0.07
0.08
0.09
SNP effect on Cardiac valvular disease
SNP effect on IGSF9B

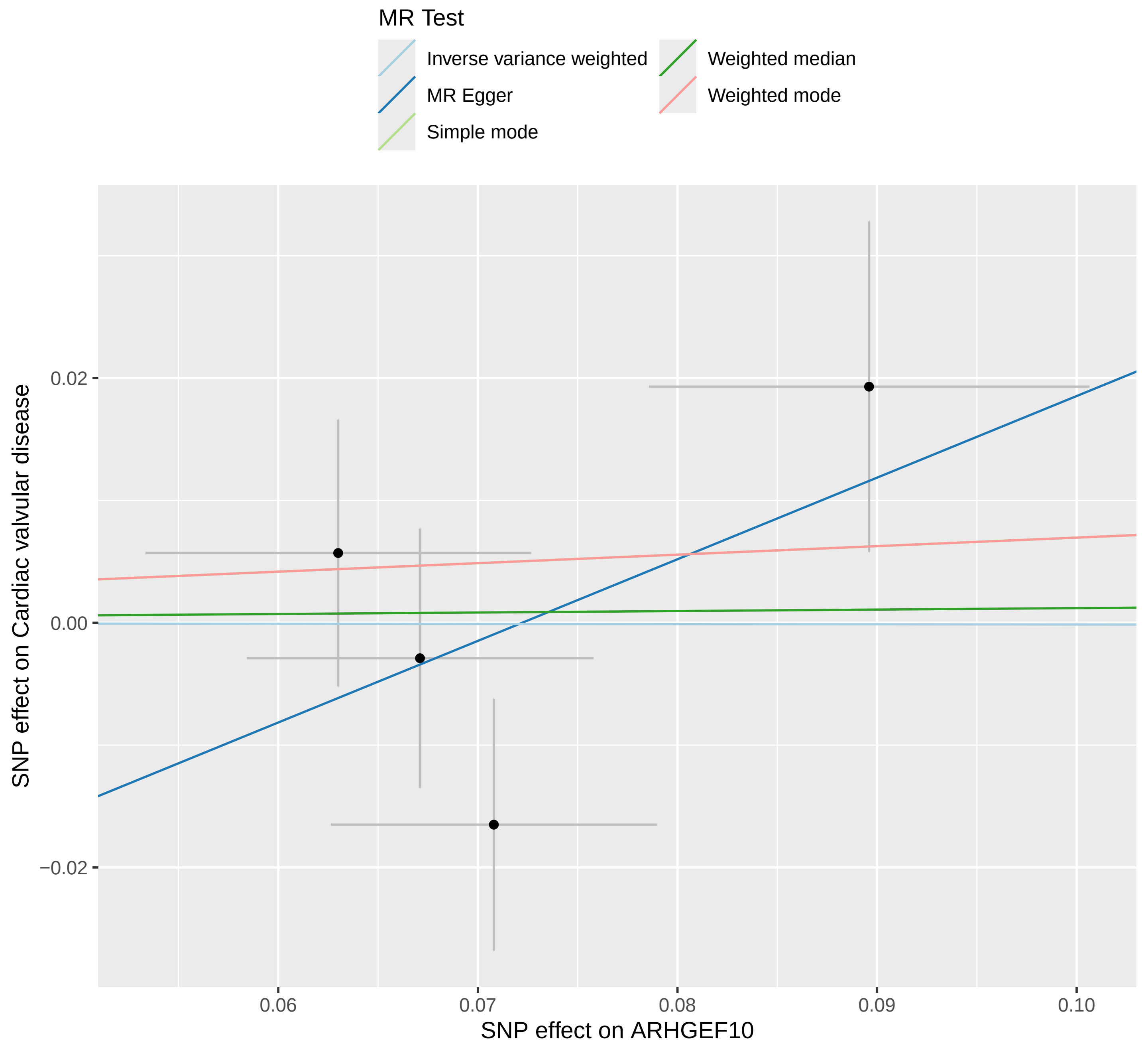

MR Test
Inverse variance weighted
MR Egger
Simple mode
Weighted median
Weighted mode
0.02
0.00
−0.02
0.06
0.07
0.08
0.09
0.10
SNP effect on Cardiac valvular disease
SNP effect on ARHGEF10

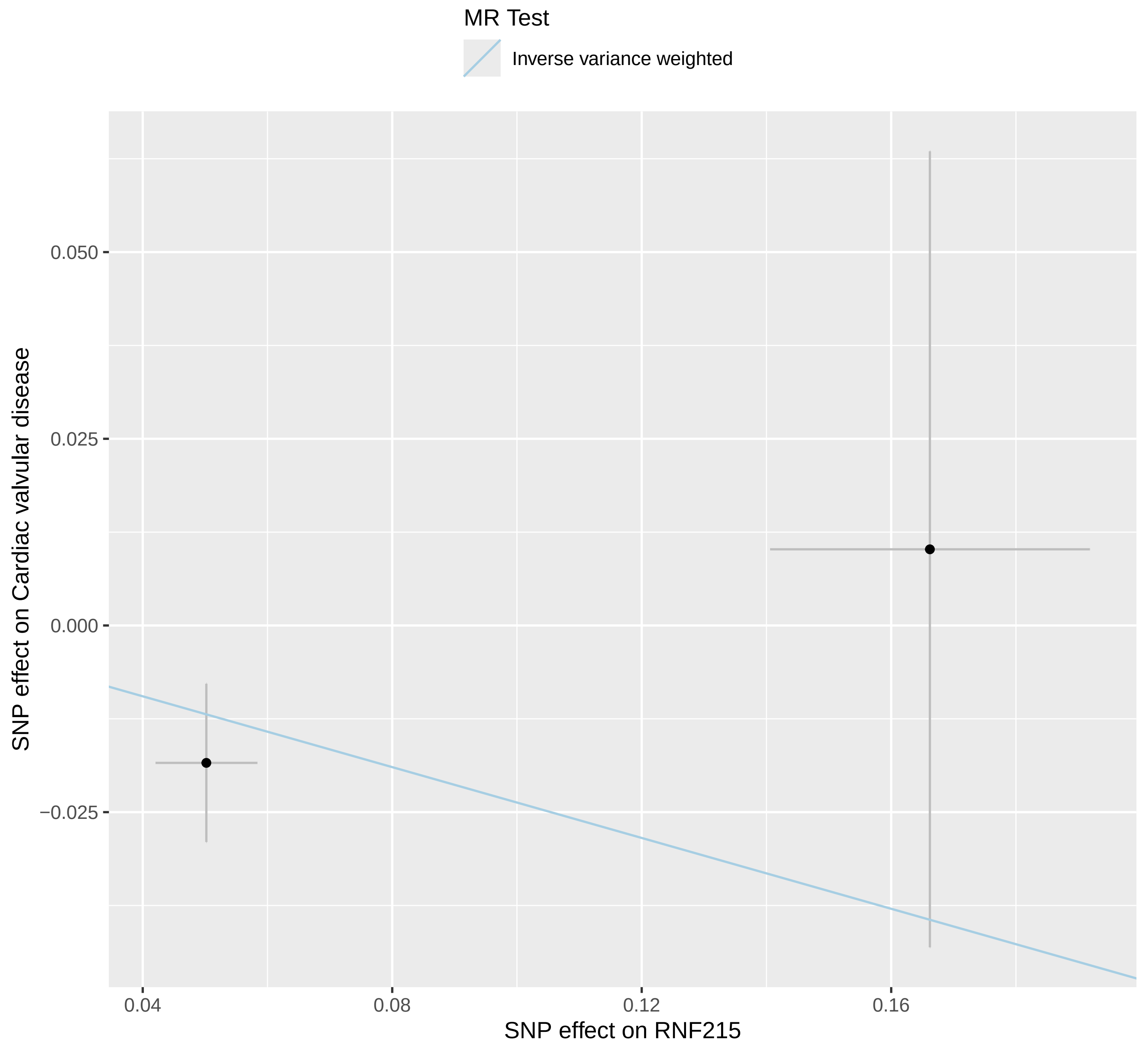

MR Test
Inverse variance weighted
0.050
0.025
0.000
−0.025
SNP effect on Cardiac valvular disease
0.04
0.08
0.12
0.16
SNP effect on RNF215

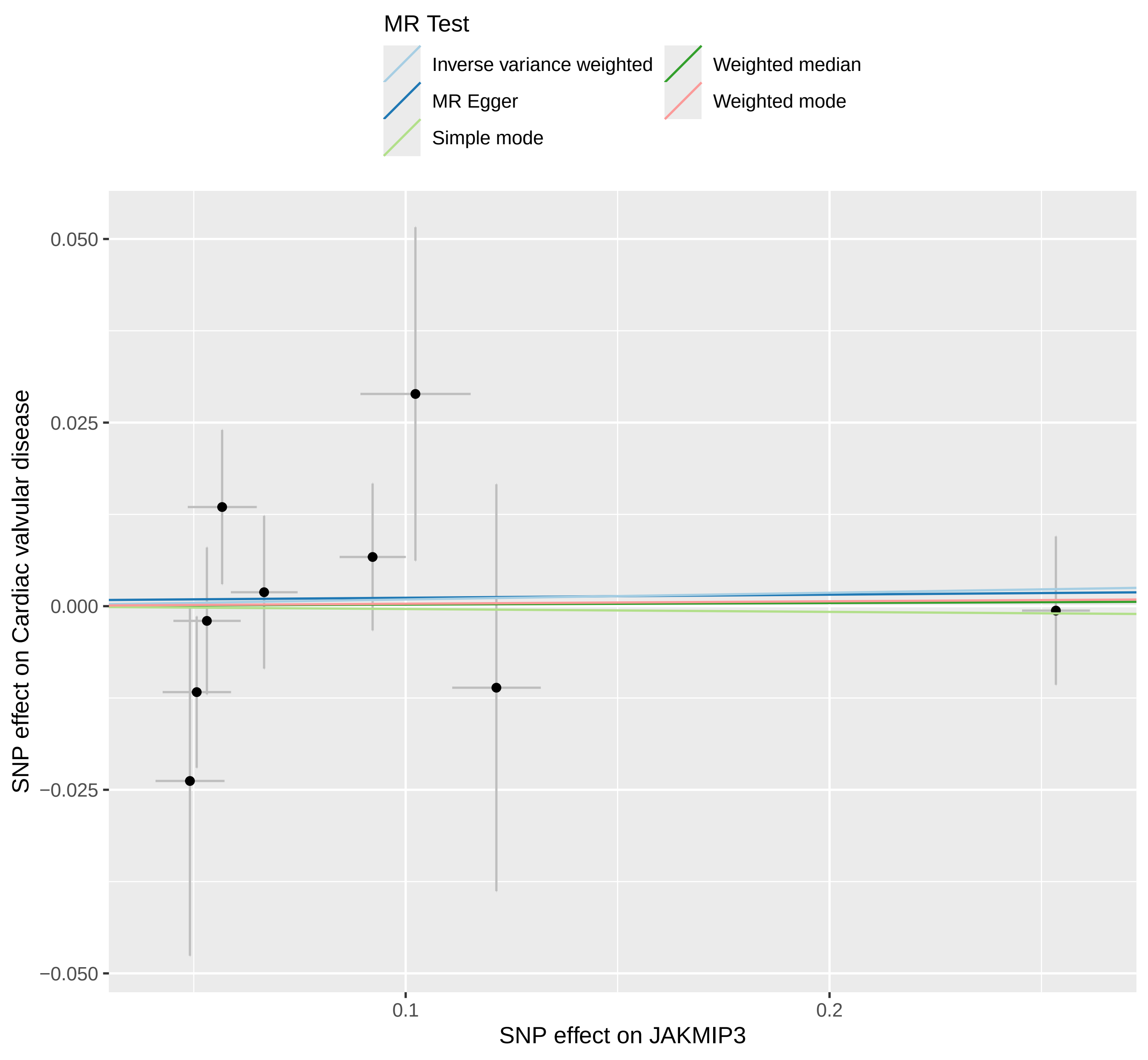
MR Test
Inverse variance weighted
MR Egger
Simple mode
Weighted median
Weighted mode
0.050
0.025
0.000
−0.025
−0.050
SNP effect on Cardiac valvular disease
0.1
0.2
SNP effect on JAKMIP3

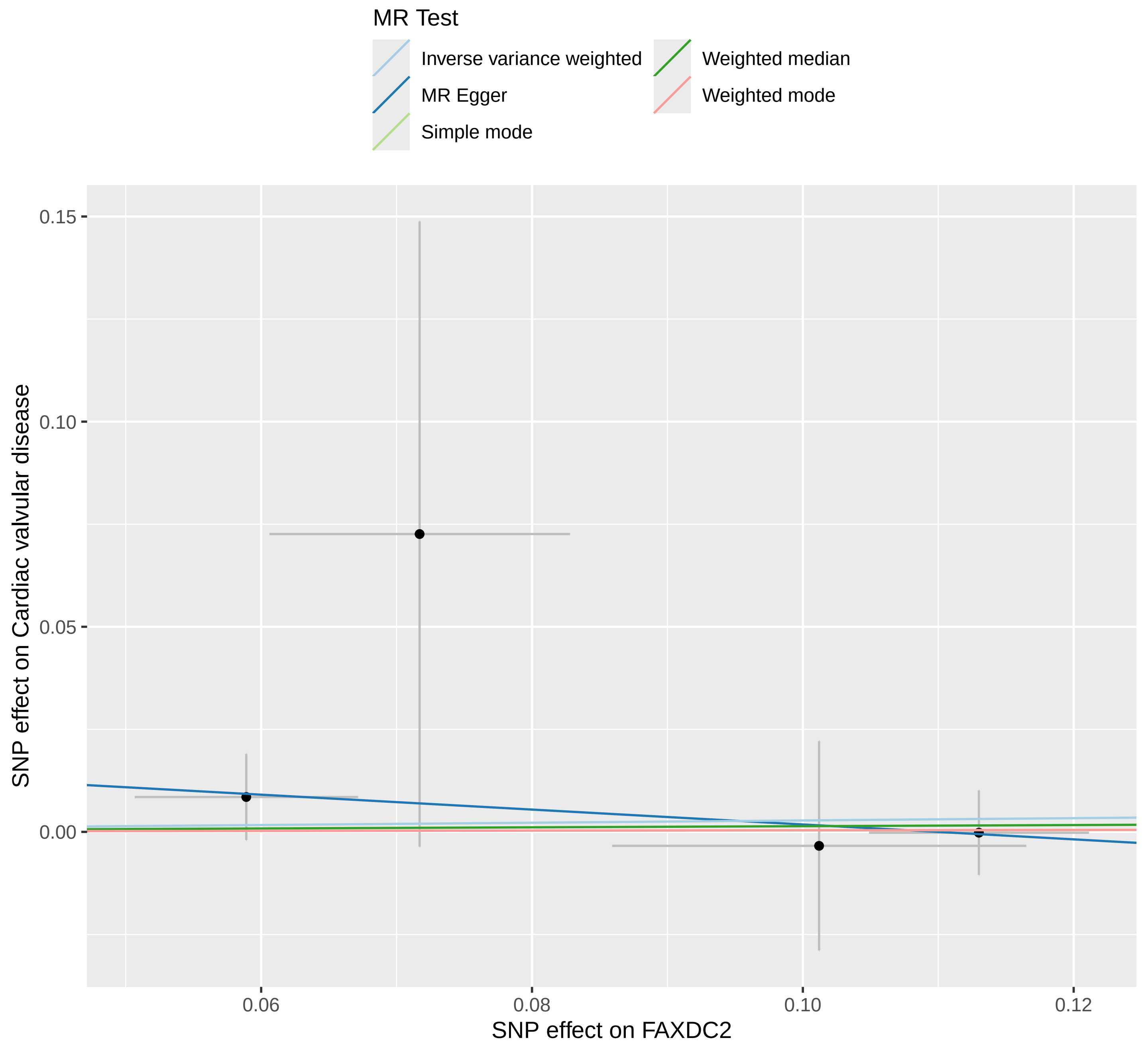
MR Test
Inverse variance weighted
MR Egger
Simple mode
Weighted median
Weighted mode
0.15
0.10
0.05
0.00
SNP effect on Cardiac valvular disease
0.06
0.08
0.10
0.12
SNP effect on FAXDC2

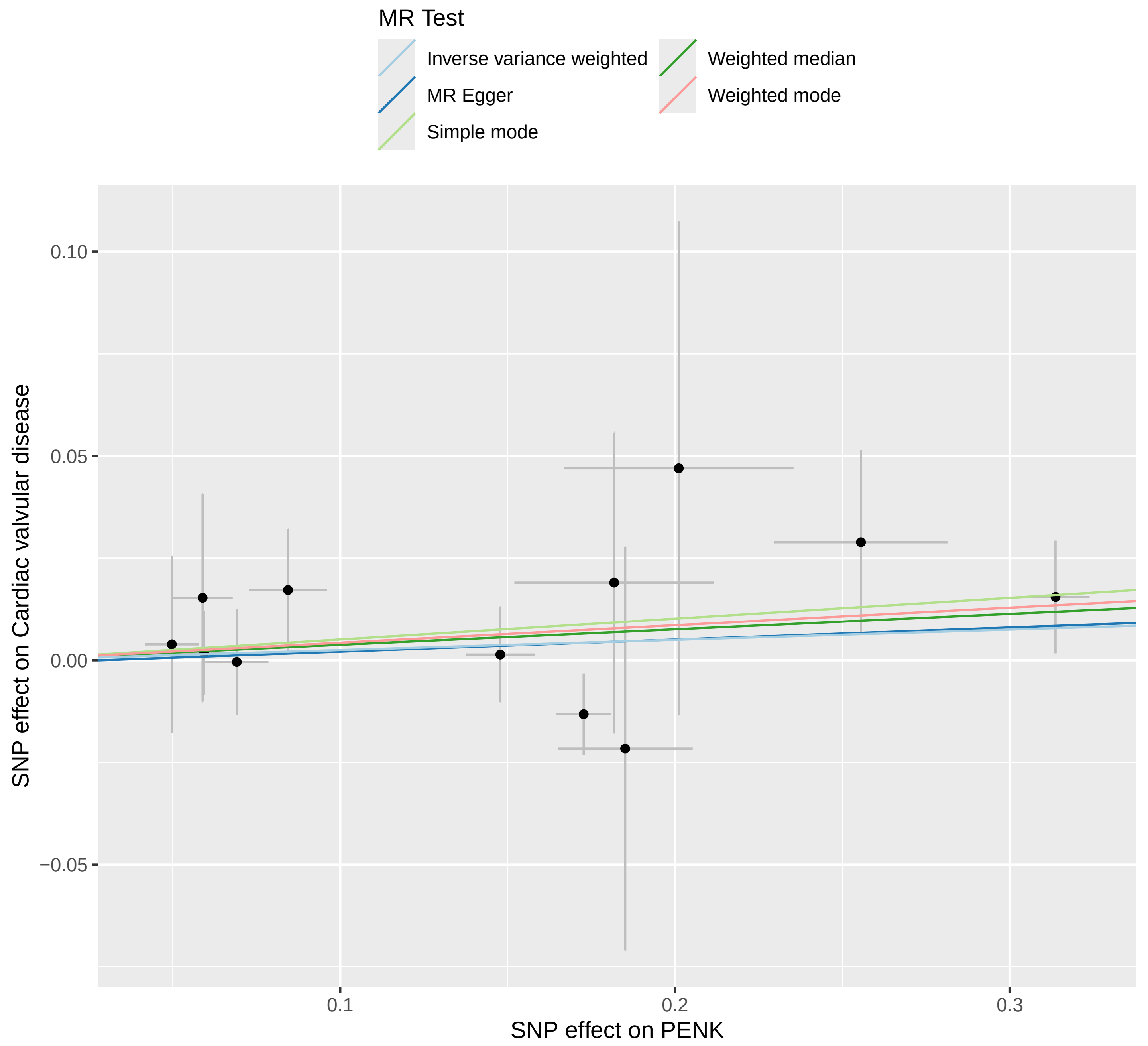
MR Test
Inverse variance weighted
MR Egger
Simple mode
Weighted median
Weighted mode
0.10
0.05
0.00
−0.05
0.1
0.2
0.3
SNP effect on Cardiac valvular disease
SNP effect on PENK

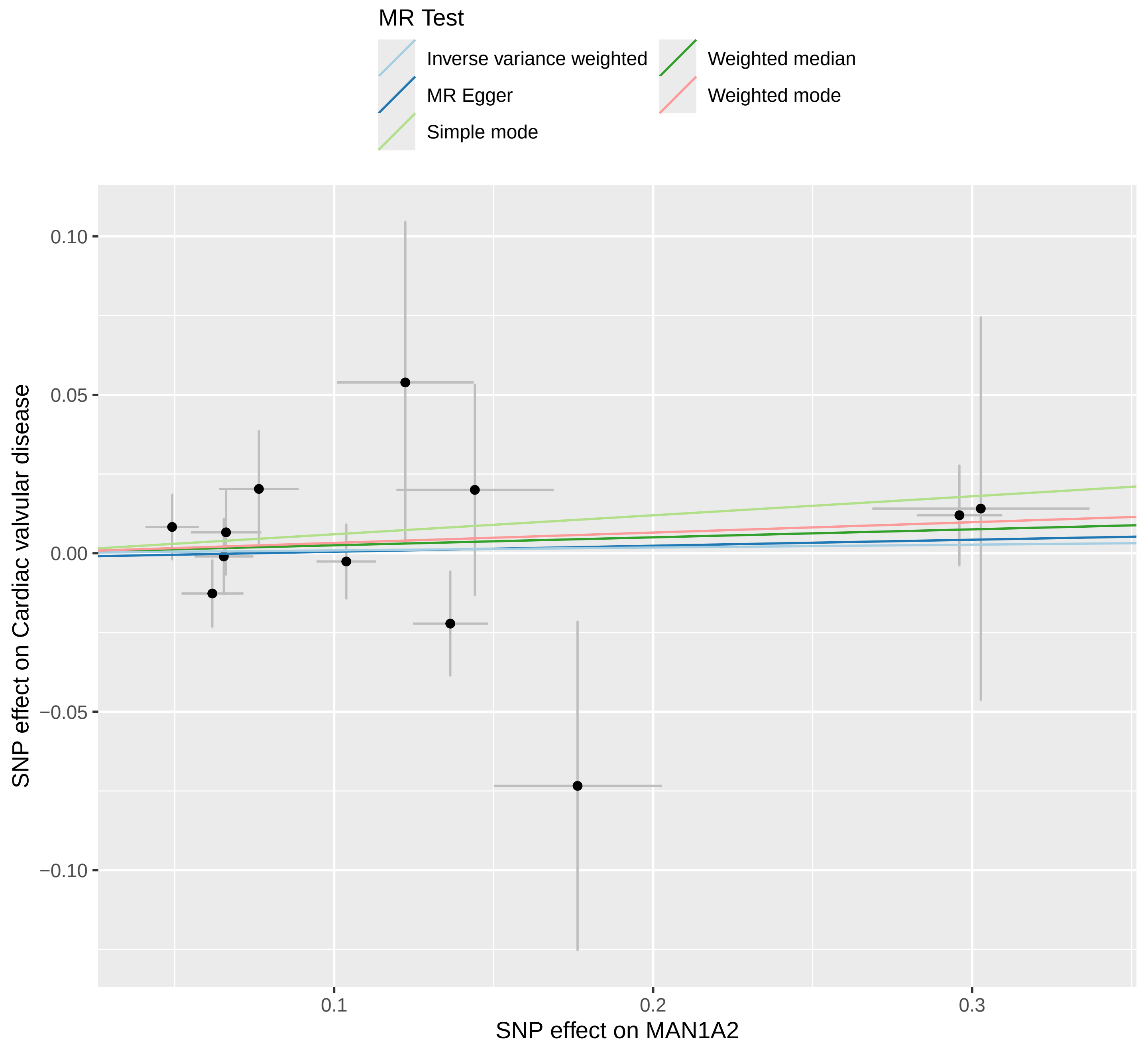

MR Test
Inverse variance weighted
MR Egger
Simple mode
Weighted median
Weighted mode
0.10
0.05
0.00
−0.05
−0.10
SNP effect on Cardiac valvular disease
0.1
0.2
0.3
SNP effect on MAN1A2

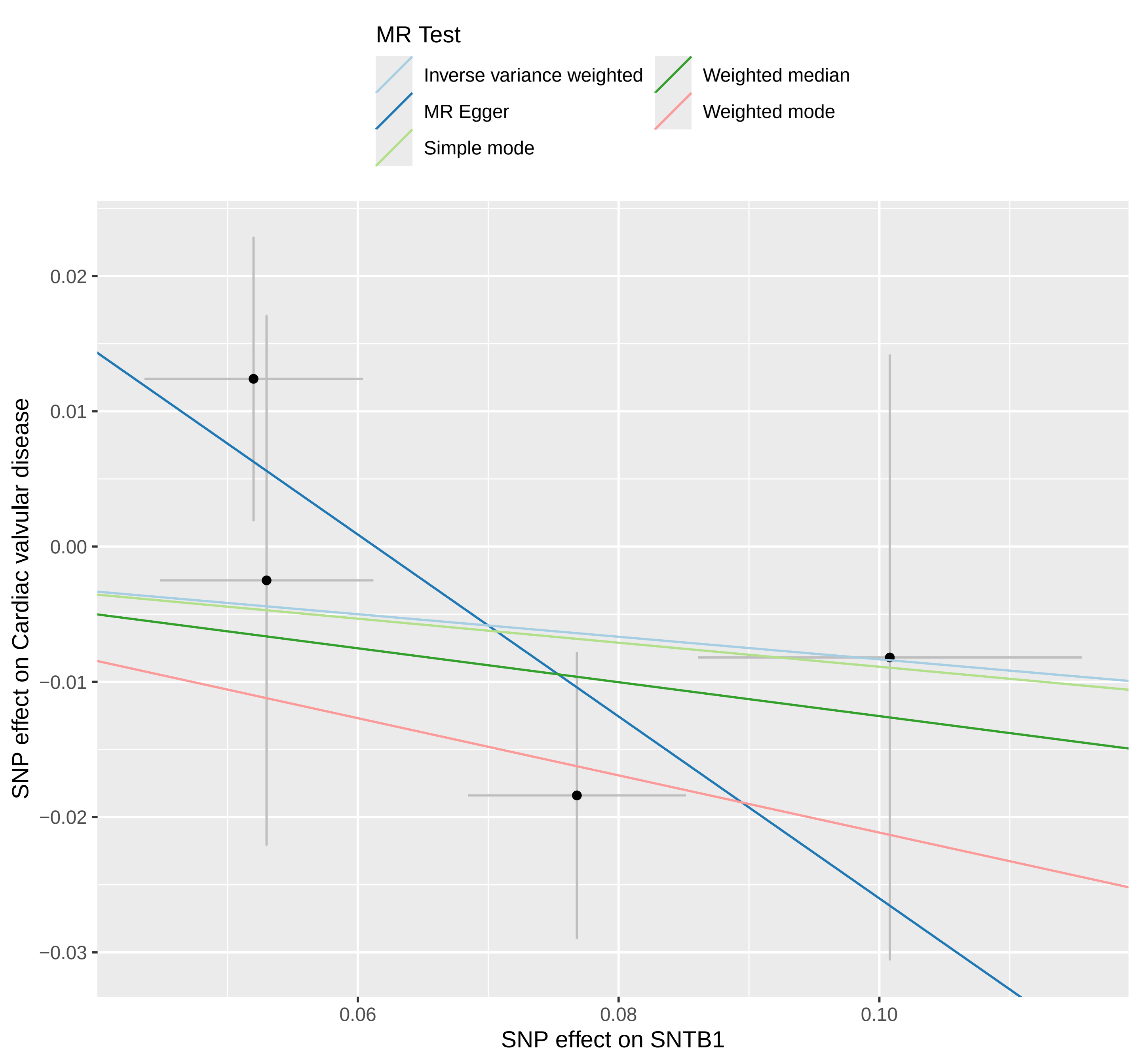
MR Test
Inverse variance weighted
MR Egger
Simple mode
Weighted median
Weighted mode
0.02
0.01
0.00
−0.01
−0.02
−0.03
SNP effect on Cardiac valvular disease
0.06
0.08
0.10
SNP effect on SNTB1

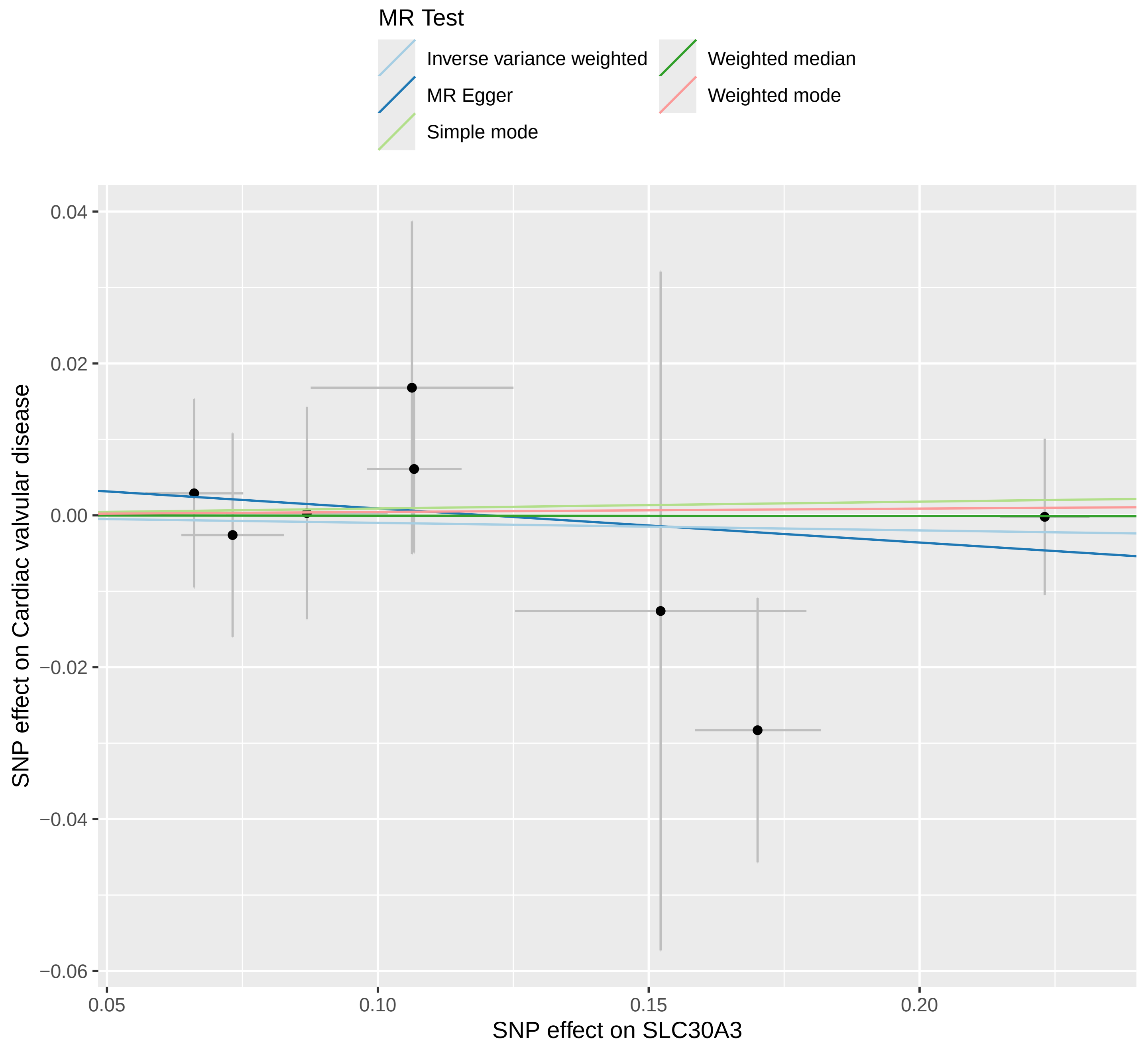

MR Test
Inverse variance weighted
MR Egger
Simple mode
Weighted median
Weighted mode
0.04
0.02
0.00
−0.02
−0.04
−0.06
SNP effect on Cardiac valvular disease
0.05
0.10
0.15
0.20
SNP effect on SLC30A3

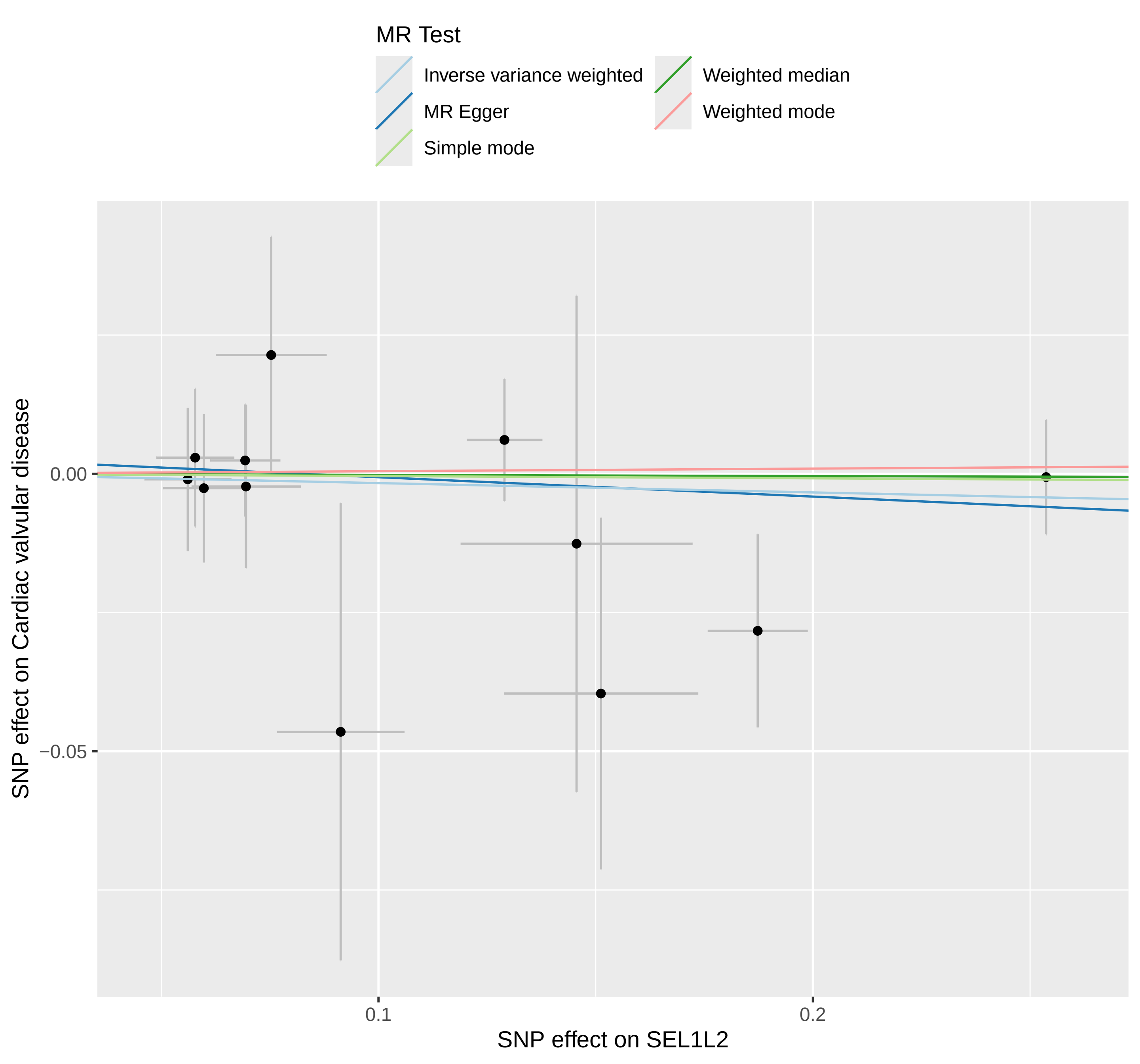
MR Test
Inverse variance weighted
MR Egger
Simple mode
Weighted median
Weighted mode
0.00
−0.05
0.1
0.2
SNP effect on Cardiac valvular disease
SNP effect on SEL1L2

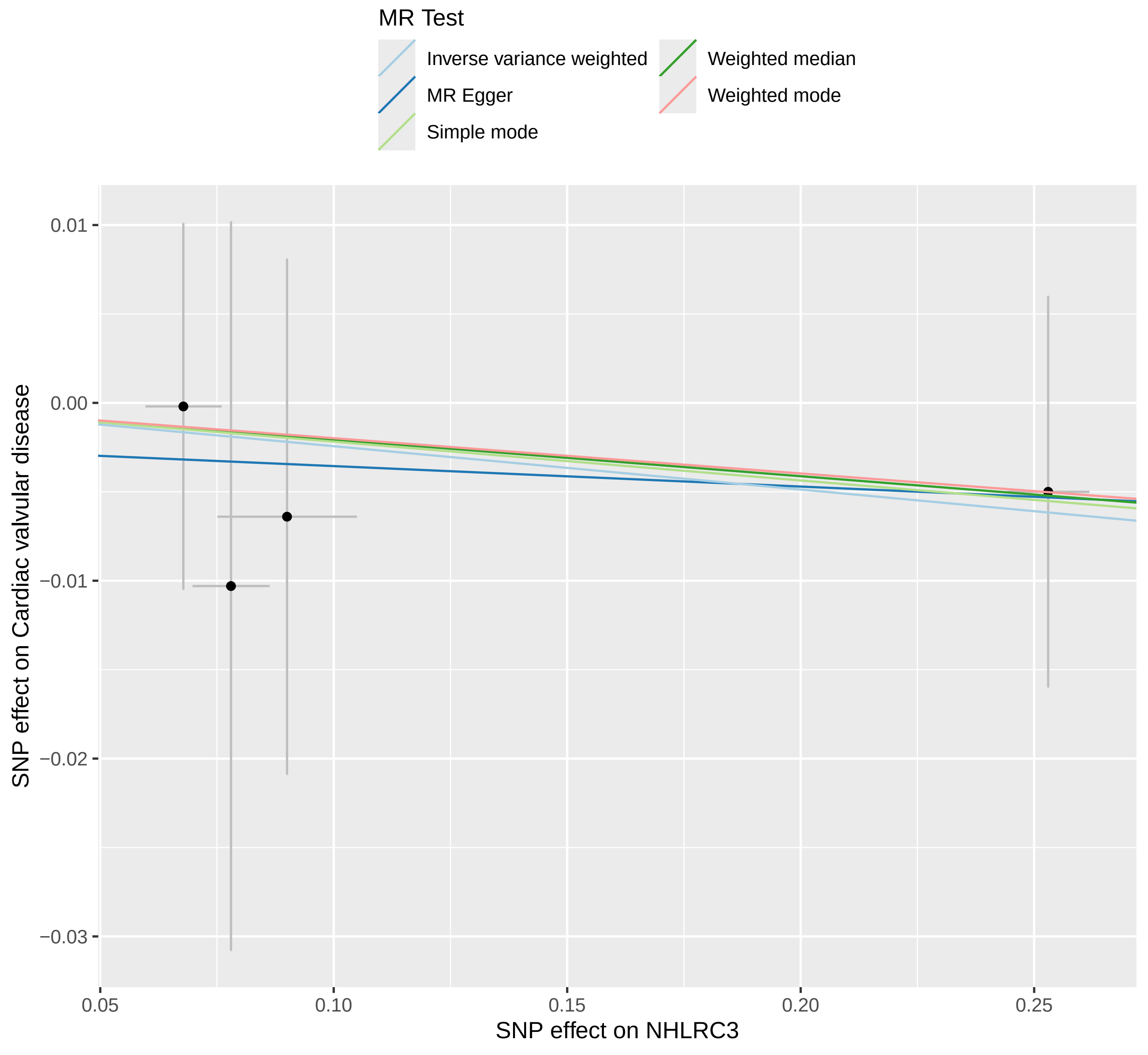
MR Test
Inverse variance weighted
MR Egger
Simple mode
Weighted median
Weighted mode
0.01
0.00
−0.01
−0.02
−0.03
0.05
0.10
0.15
0.20
0.25
SNP effect on Cardiac valvular disease
SNP effect on NHLRC3

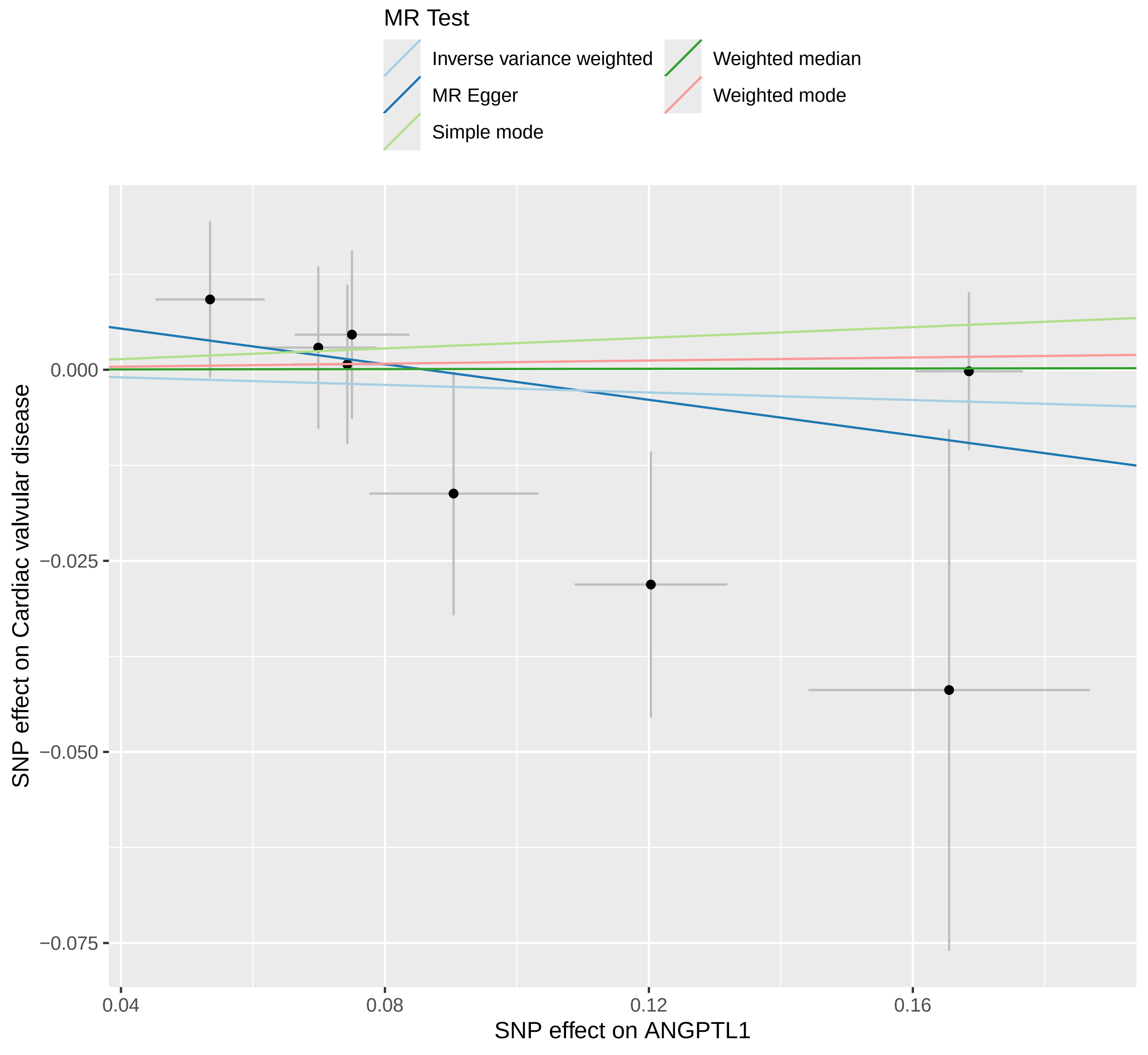
MR Test
Inverse variance weighted
MR Egger
Simple mode
Weighted median
Weighted mode
0.000
−0.025
−0.050
−0.075
SNP effect on Cardiac valvular disease
0.04
0.08
0.12
0.16
SNP effect on ANGPTL1

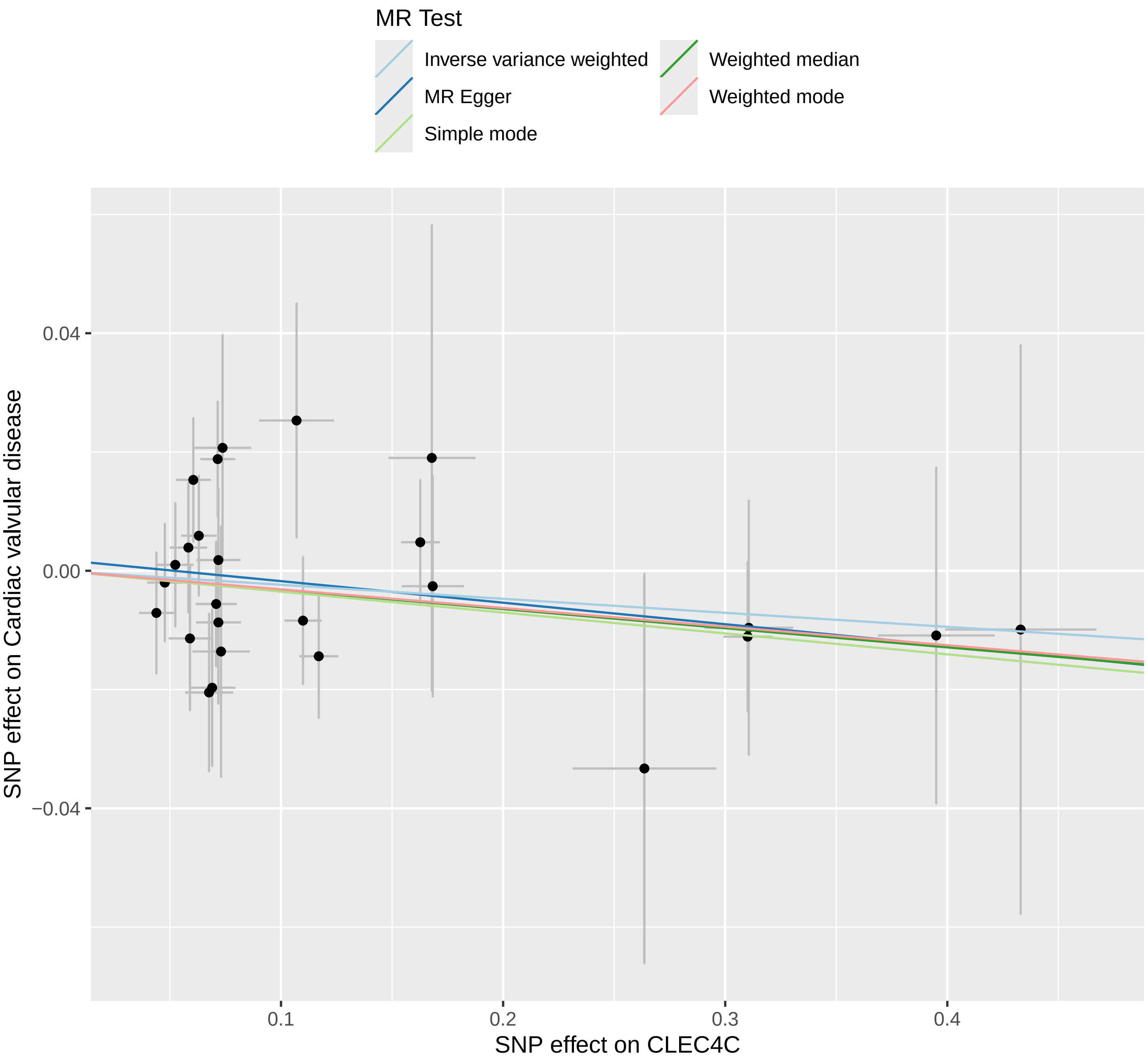

MR Test
Inverse variance weighted
MR Egger
Simple mode
Weighted median
Weighted mode
0.04
0.00
−0.04
0.1
0.2
0.3
0.4
SNP effect on Cardiac valvular disease
SNP effect on CLEC4C

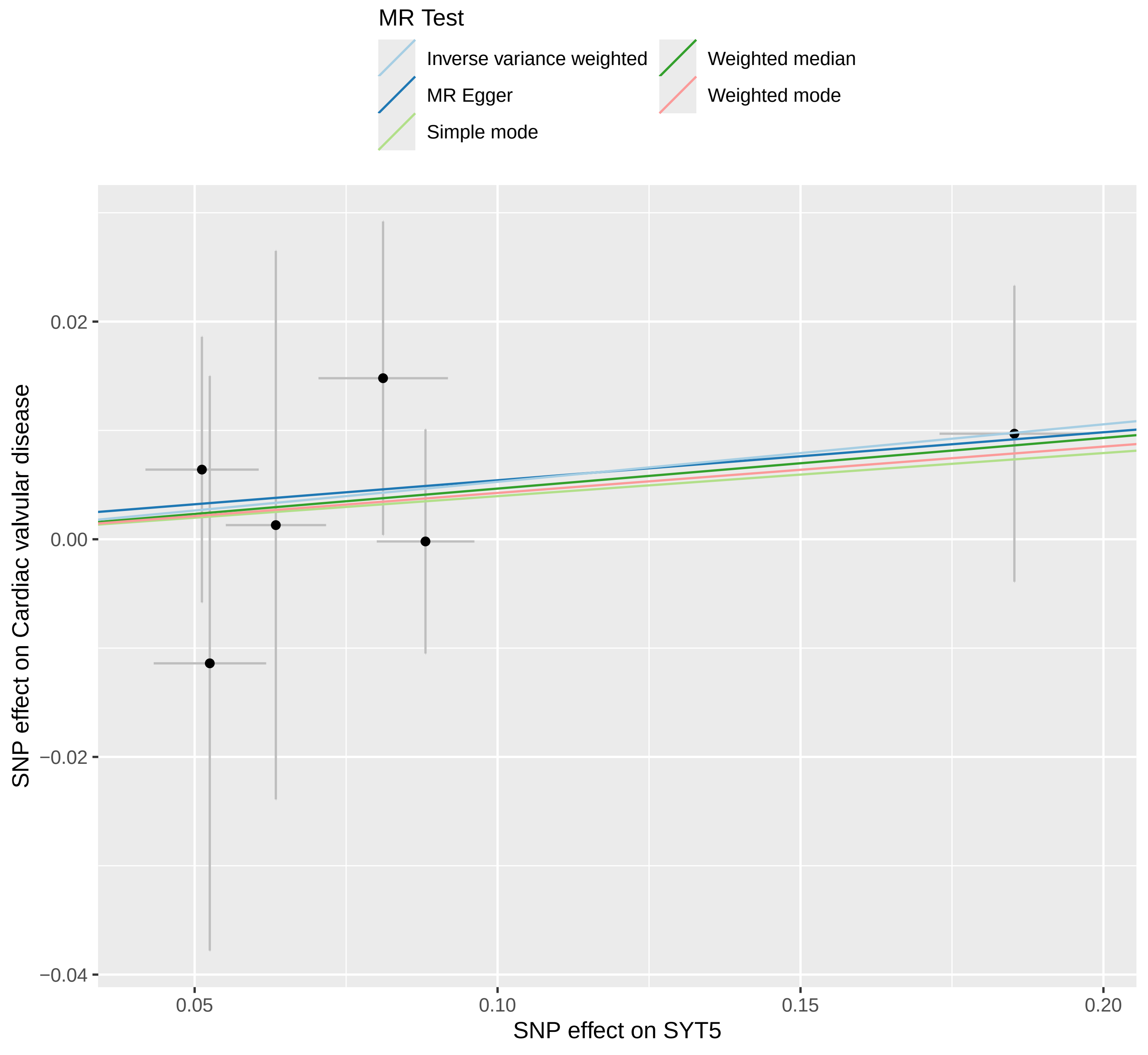

MR Test
Inverse variance weighted
MR Egger
Simple mode
Weighted median
Weighted mode
0.02
0.00
−0.02
−0.04
0.05
0.10
0.15
0.20
SNP effect on Cardiac valvular disease
SNP effect on SYT5

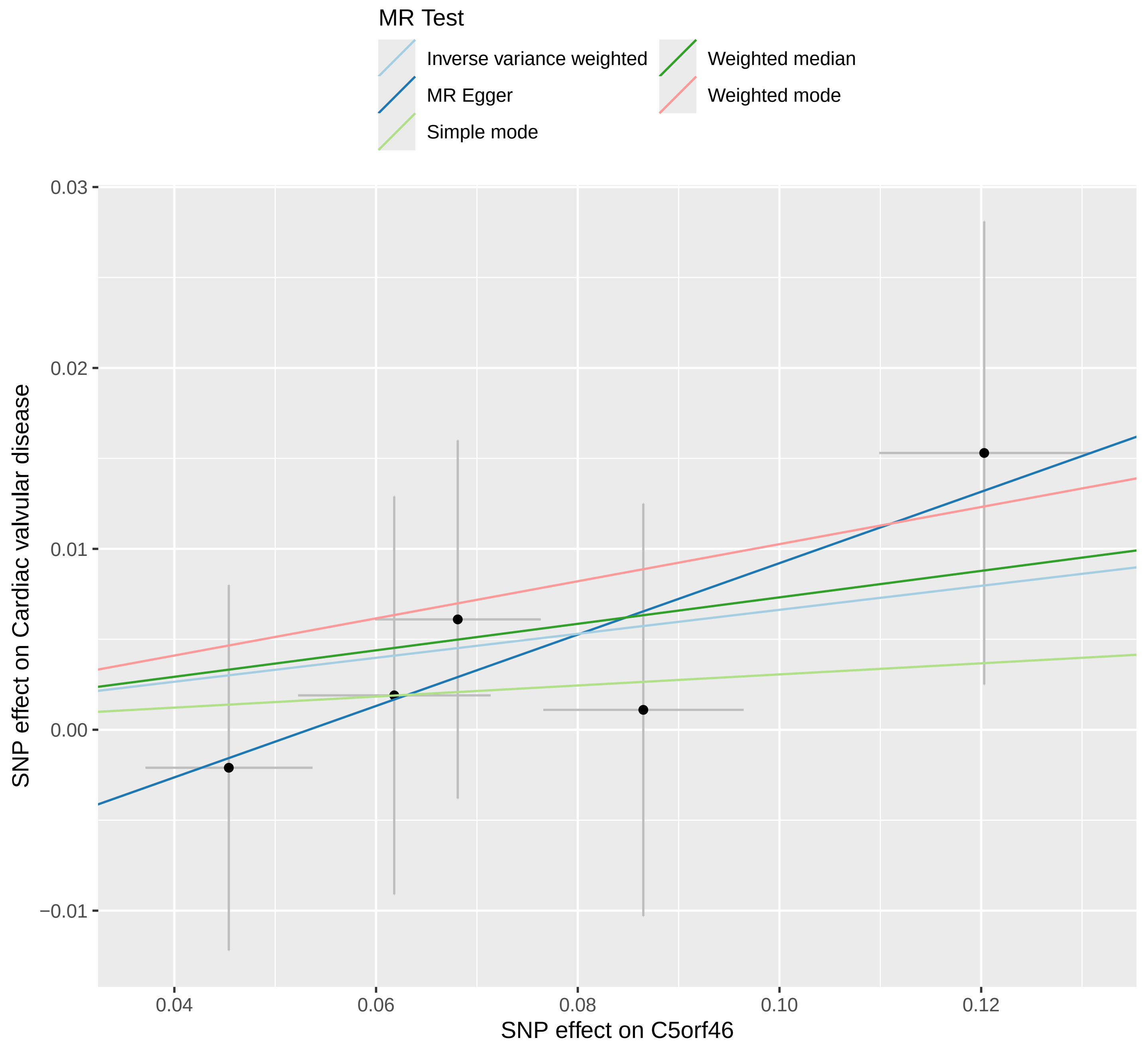

MR Test
Inverse variance weighted
MR Egger
Simple mode
Weighted median
Weighted mode
0.03
0.02
0.01
0.00
−0.01
0.04
0.06
0.08
0.10
0.12
SNP effect on C5orf46
SNP effect on Cardiac valvular disease

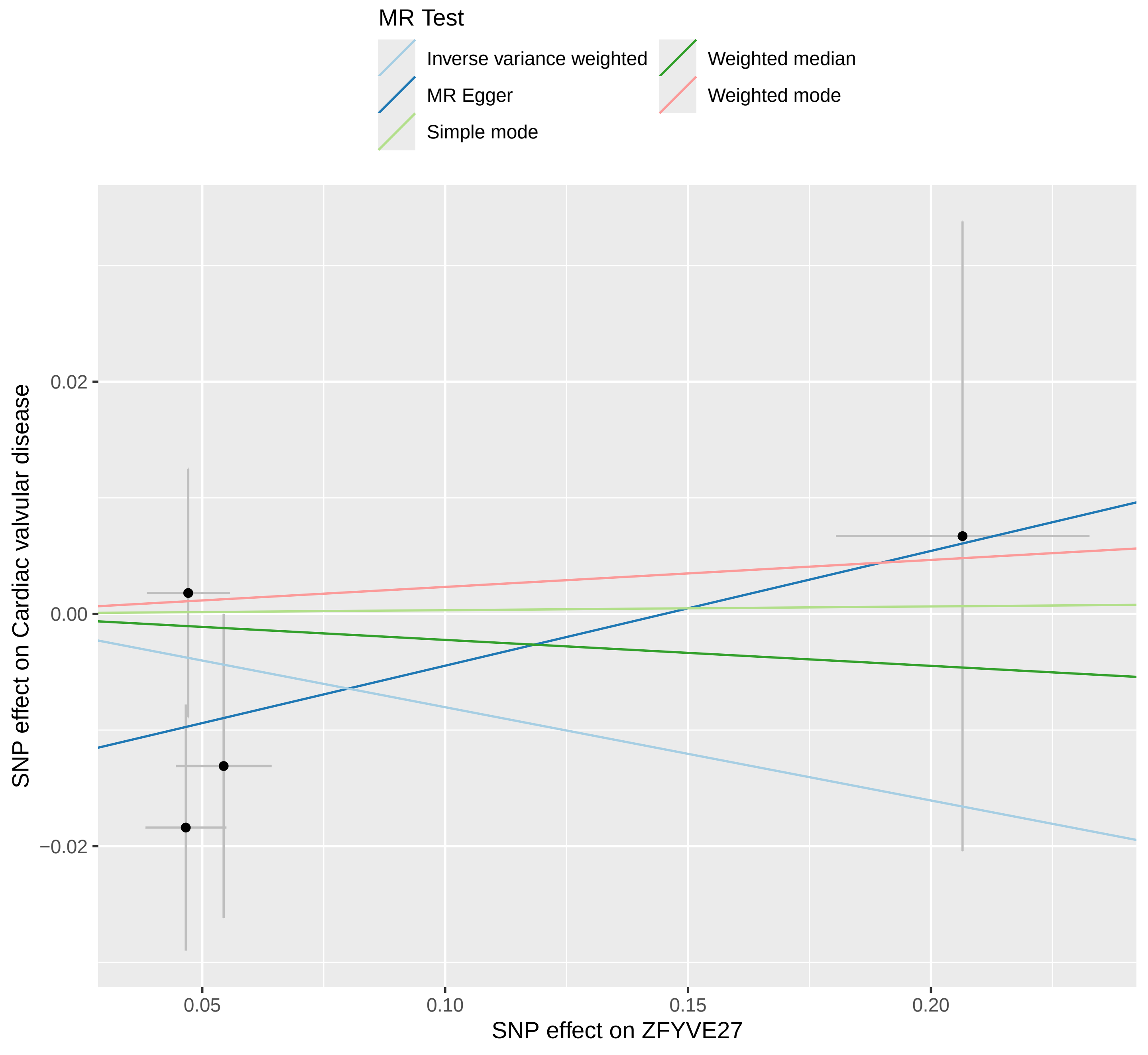
MR Test
Inverse variance weighted
MR Egger
Simple mode
Weighted median
Weighted mode
0.02
0.00
−0.02
0.05
0.10
0.15
0.20
SNP effect on Cardiac valvular disease
SNP effect on ZFYVE27

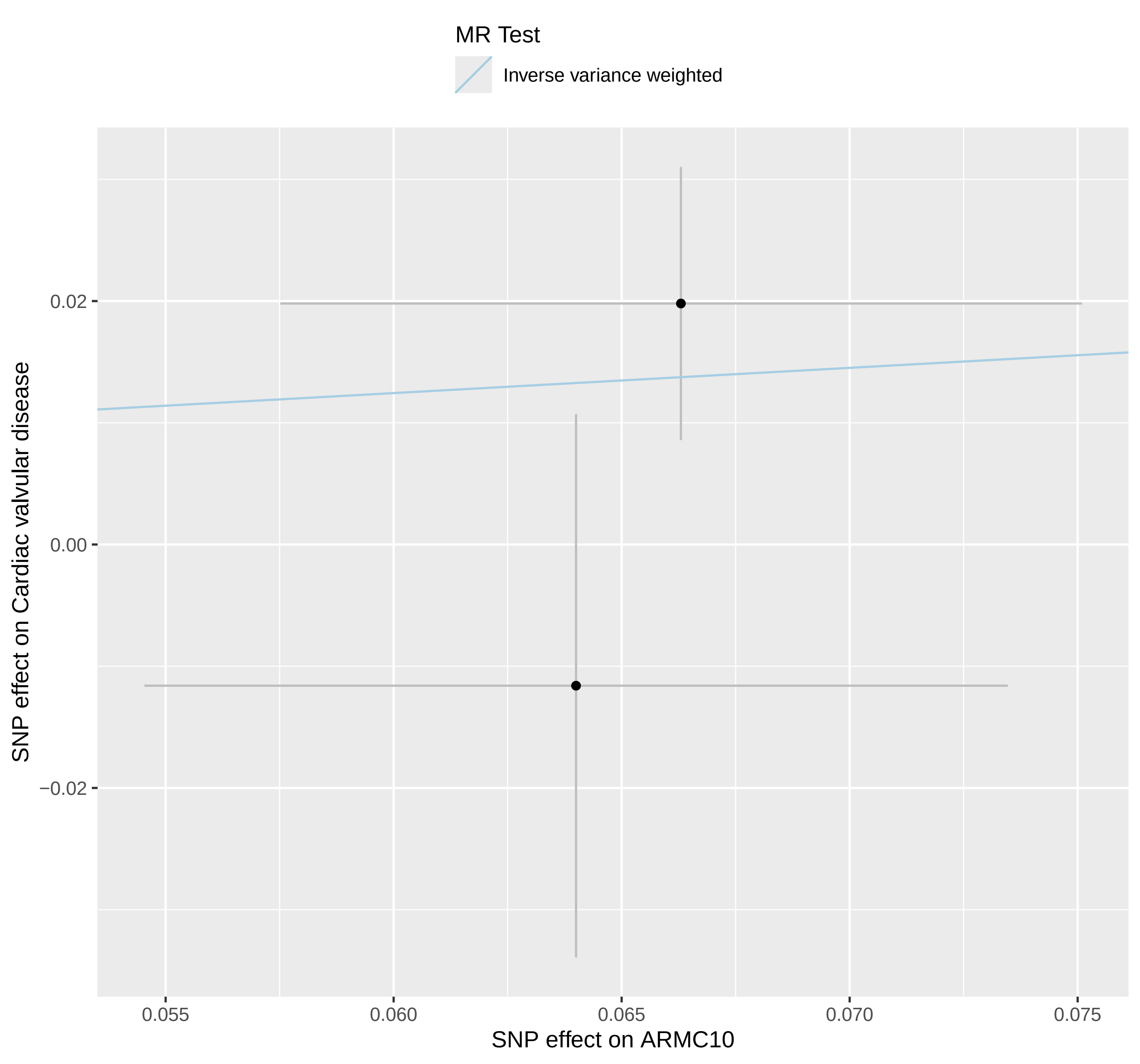
MR Test
Inverse variance weighted
0.02
0.00
−0.02
0.055
0.060
0.065
0.070
0.075
SNP effect on Cardiac valvular disease
SNP effect on ARMC10

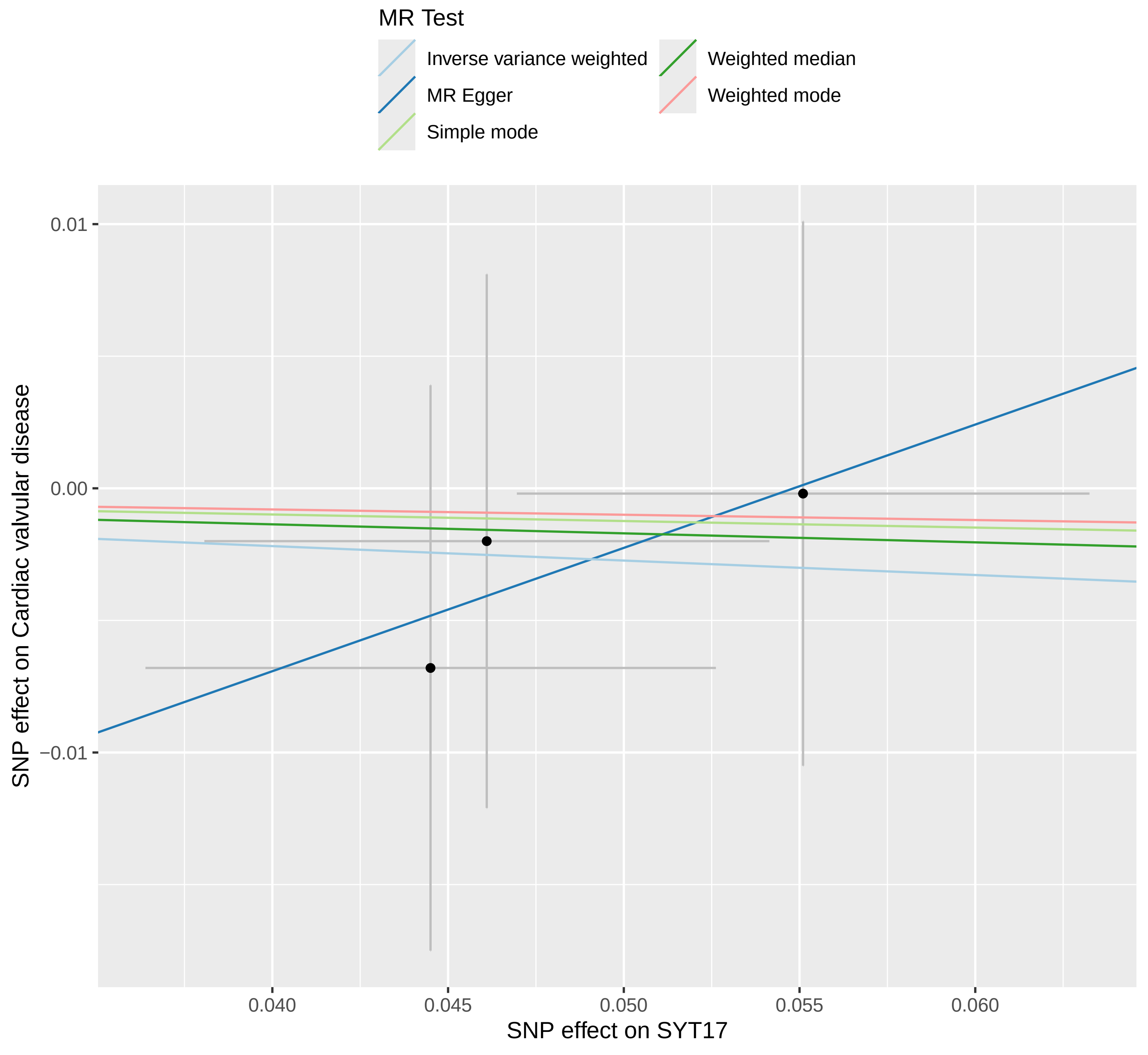
MR Test
Inverse variance weighted
MR Egger
Simple mode
Weighted median
Weighted mode
0.01
0.00
−0.01
0.040
0.045
0.050
0.055
0.060
SNP effect on Cardiac valvular disease
SNP effect on SYT17

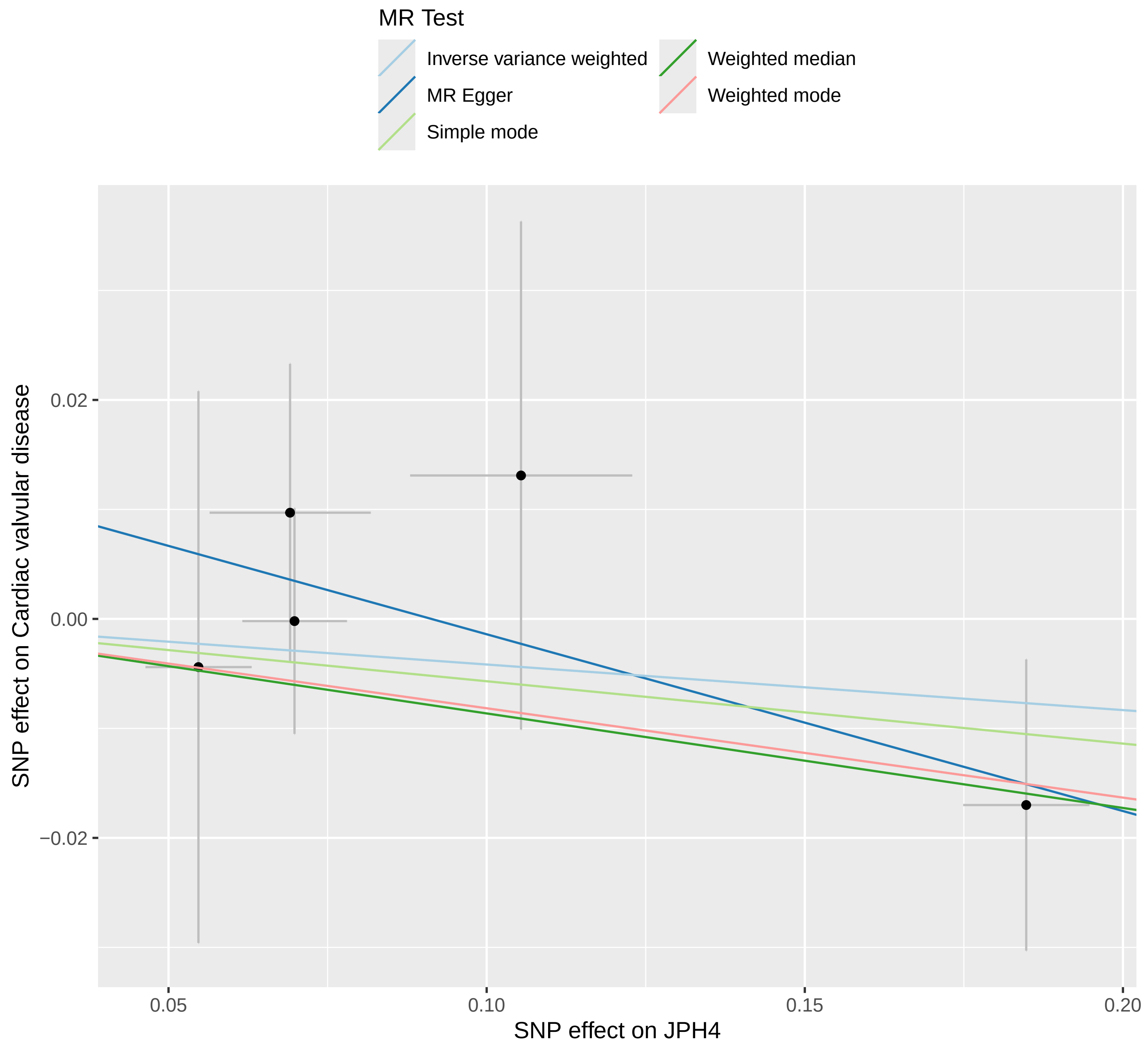
MR Test
Inverse variance weighted
MR Egger
Simple mode
Weighted median
Weighted mode
0.02
0.00
−0.02
0.05
0.10
0.15
0.20
SNP effect on Cardiac valvular disease
SNP effect on JPH4

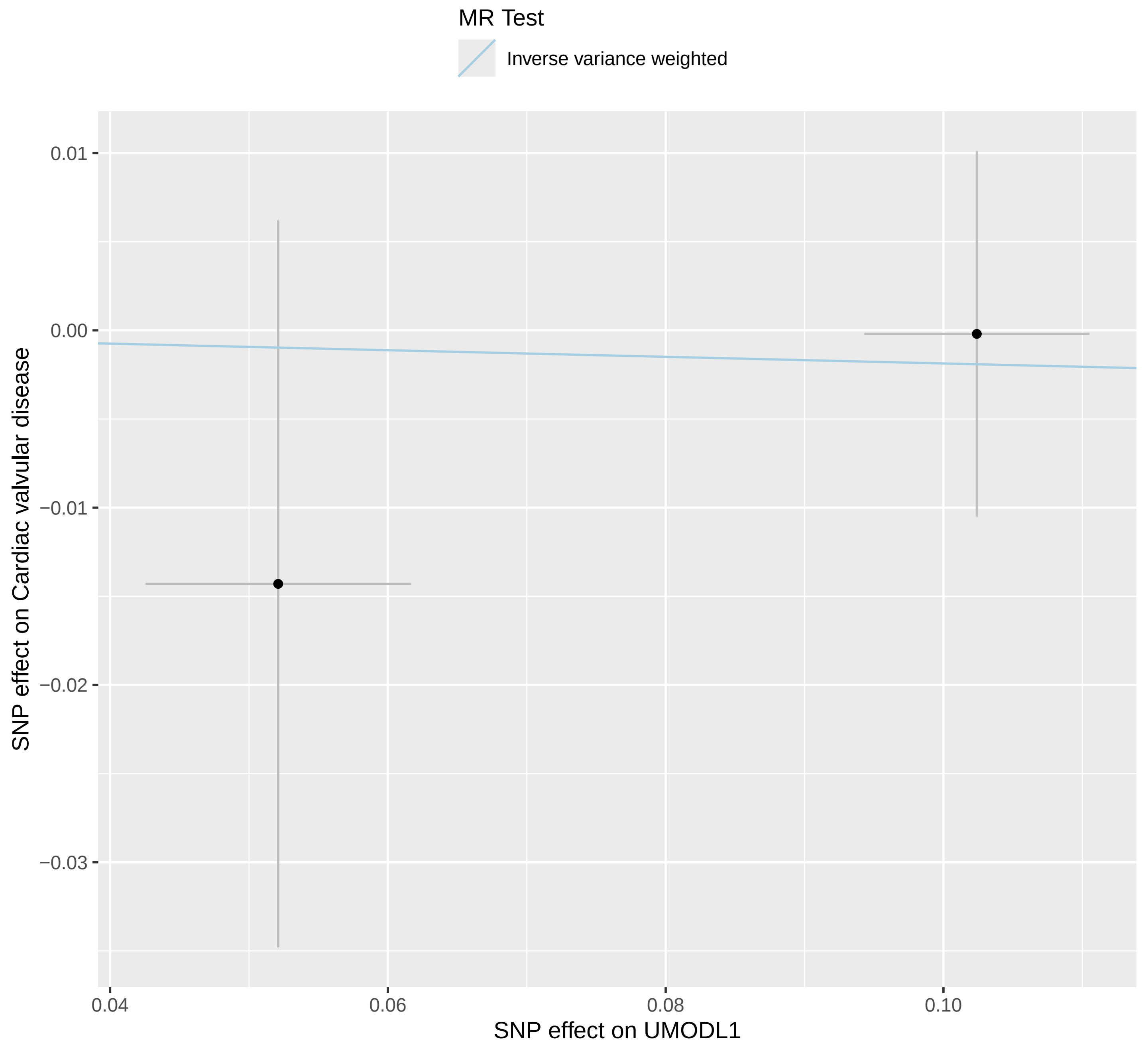

MR Test
Inverse variance weighted
0.01
0.00
−0.01
−0.02
−0.03
SNP effect on Cardiac valvular disease
0.04
0.06
0.08
0.10
SNP effect on UMODL1

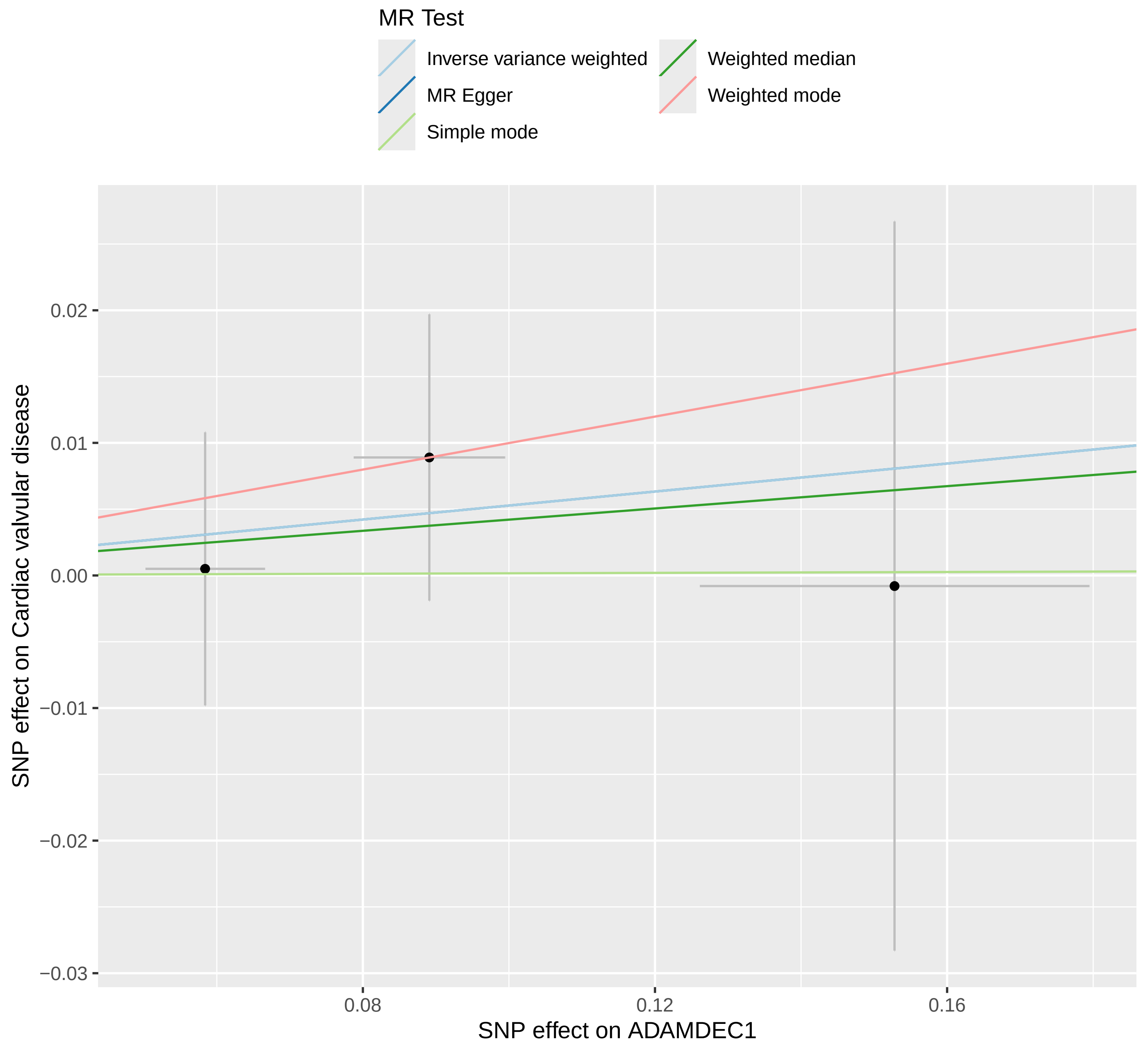
MR Test
Inverse variance weighted
MR Egger
Simple mode
Weighted median
Weighted mode
0.02
0.01
0.00
−0.01
−0.02
−0.03
SNP effect on Cardiac valvular disease
0.08
0.12
0.16
SNP effect on ADAMDEC1

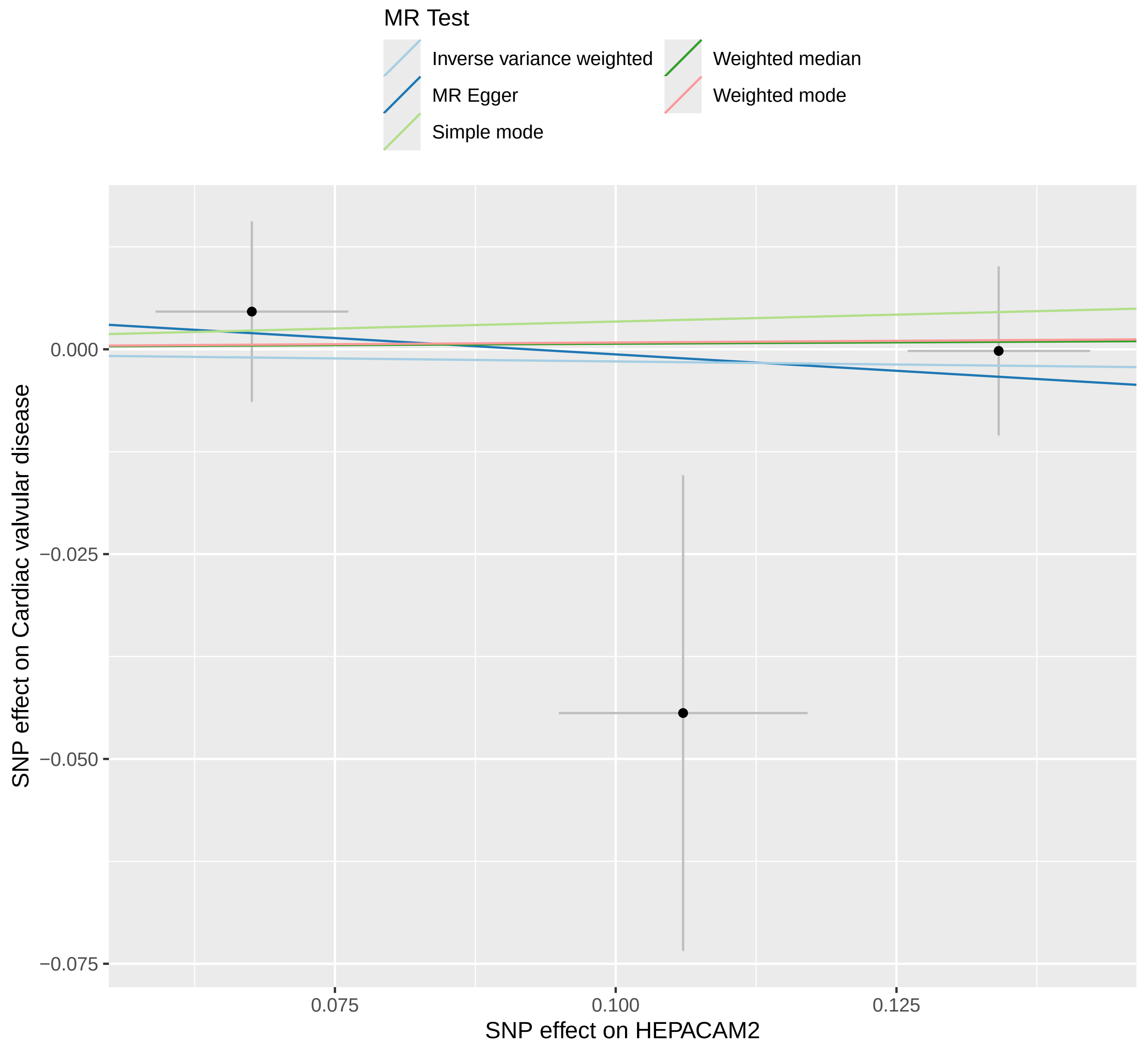
MR Test
Inverse variance weighted
MR Egger
Simple mode
Weighted median
Weighted mode
0.000
−0.025
−0.050
−0.075
0.075
0.100
0.125
SNP effect on HEPACAM2
SNP effect on Cardiac valvular disease

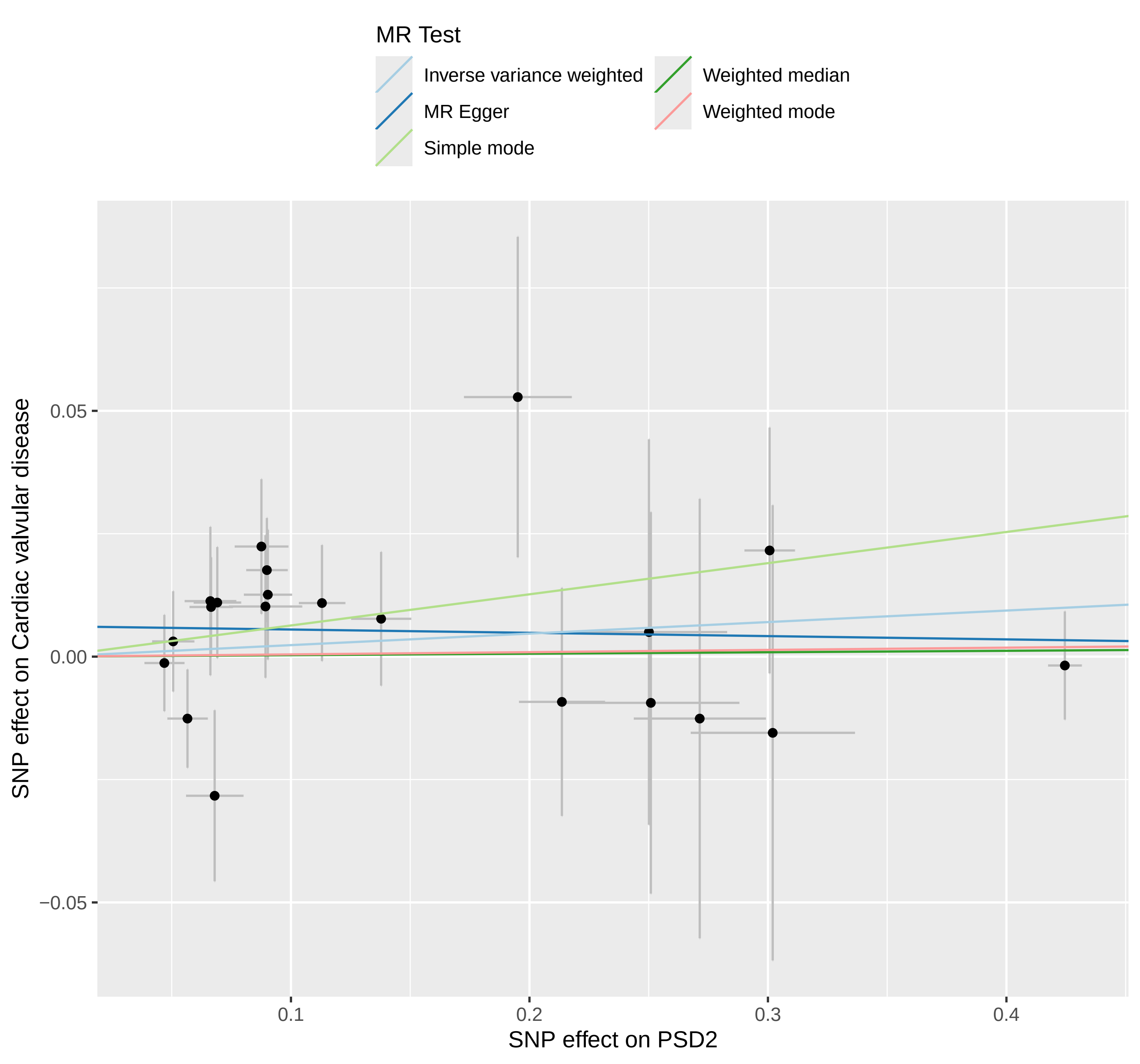
MR Test
Inverse variance weighted
MR Egger
Simple mode
Weighted median
Weighted mode
SNP effect on Cardiac valvular disease
0.05
0.00
−0.05
0.1
0.2
0.3
0.4
SNP effect on PSD2

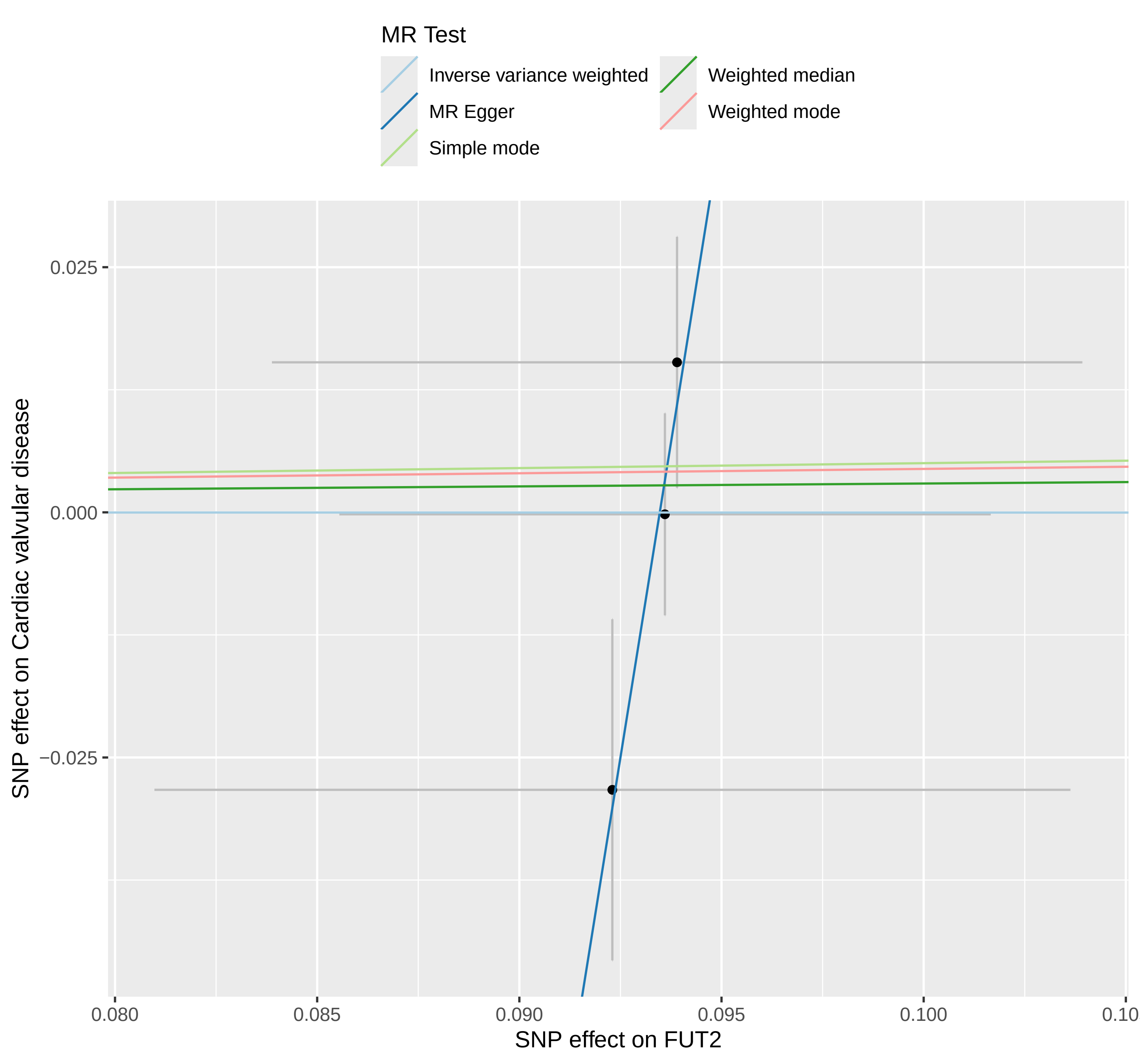
MR Test
Inverse variance weighted
MR Egger
Simple mode
Weighted median
Weighted mode
SNP effect on Cardiac valvular disease
0.025
0.000
−0.025
0.080
0.085
0.090
0.095
0.100
0.10
SNP effect on FUT2

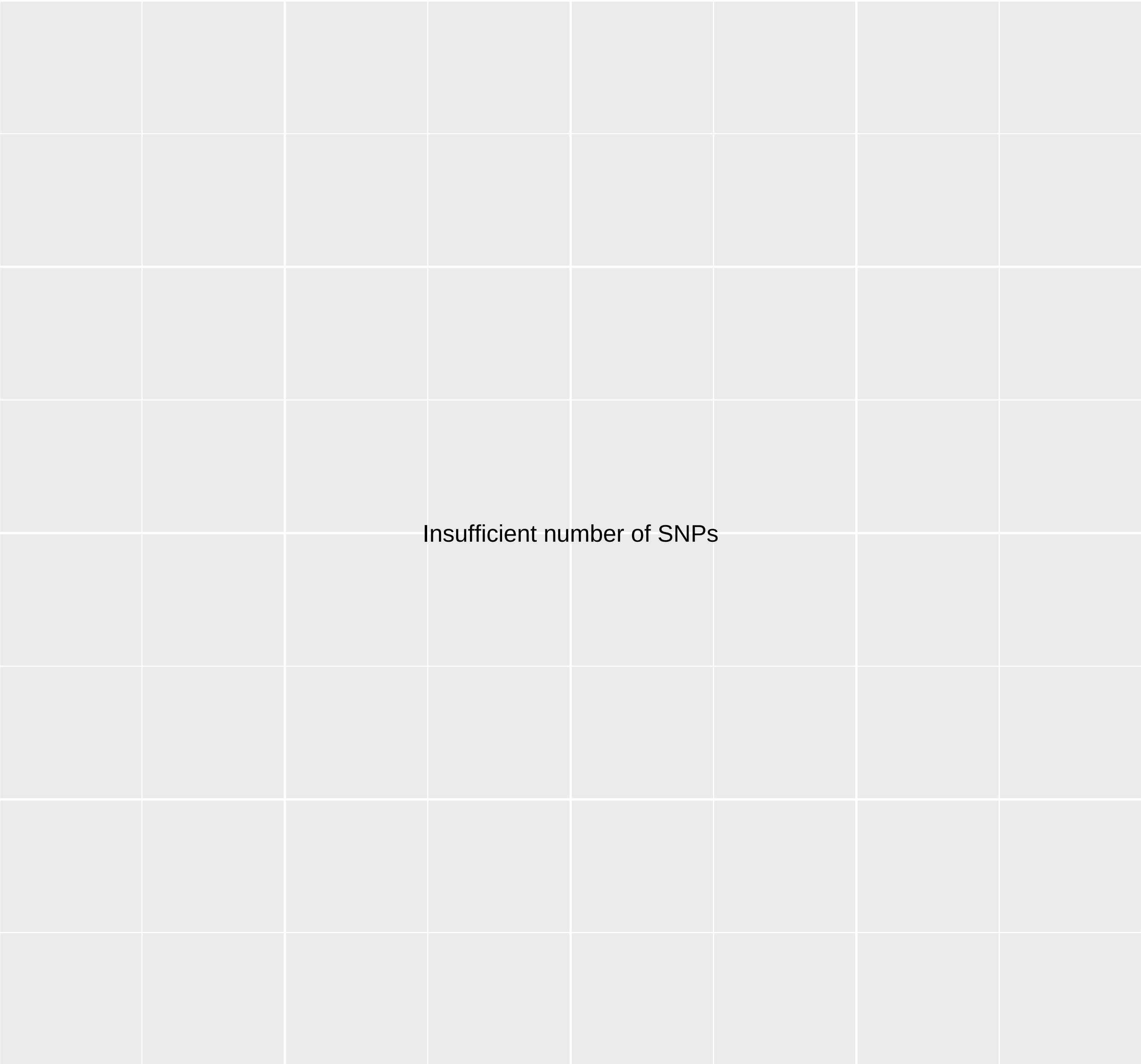
Insufficient number of SNPs

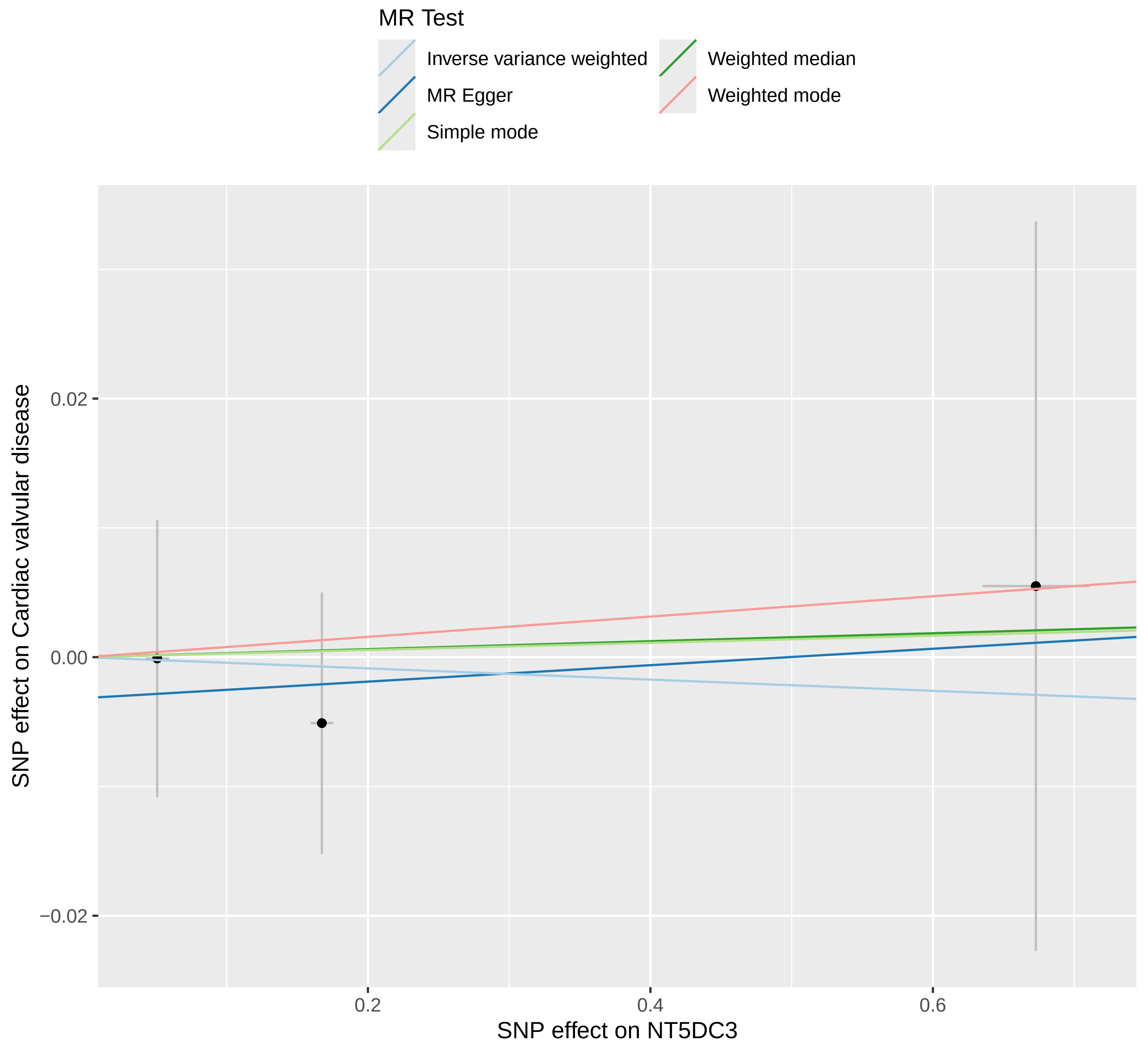
MR Test
Inverse variance weighted
MR Egger
Simple mode
Weighted median
Weighted mode
0.02
0.00
−0.02
0.2
0.4
0.6
SNP effect on NT5DC3
SNP effect on Cardiac valvular disease

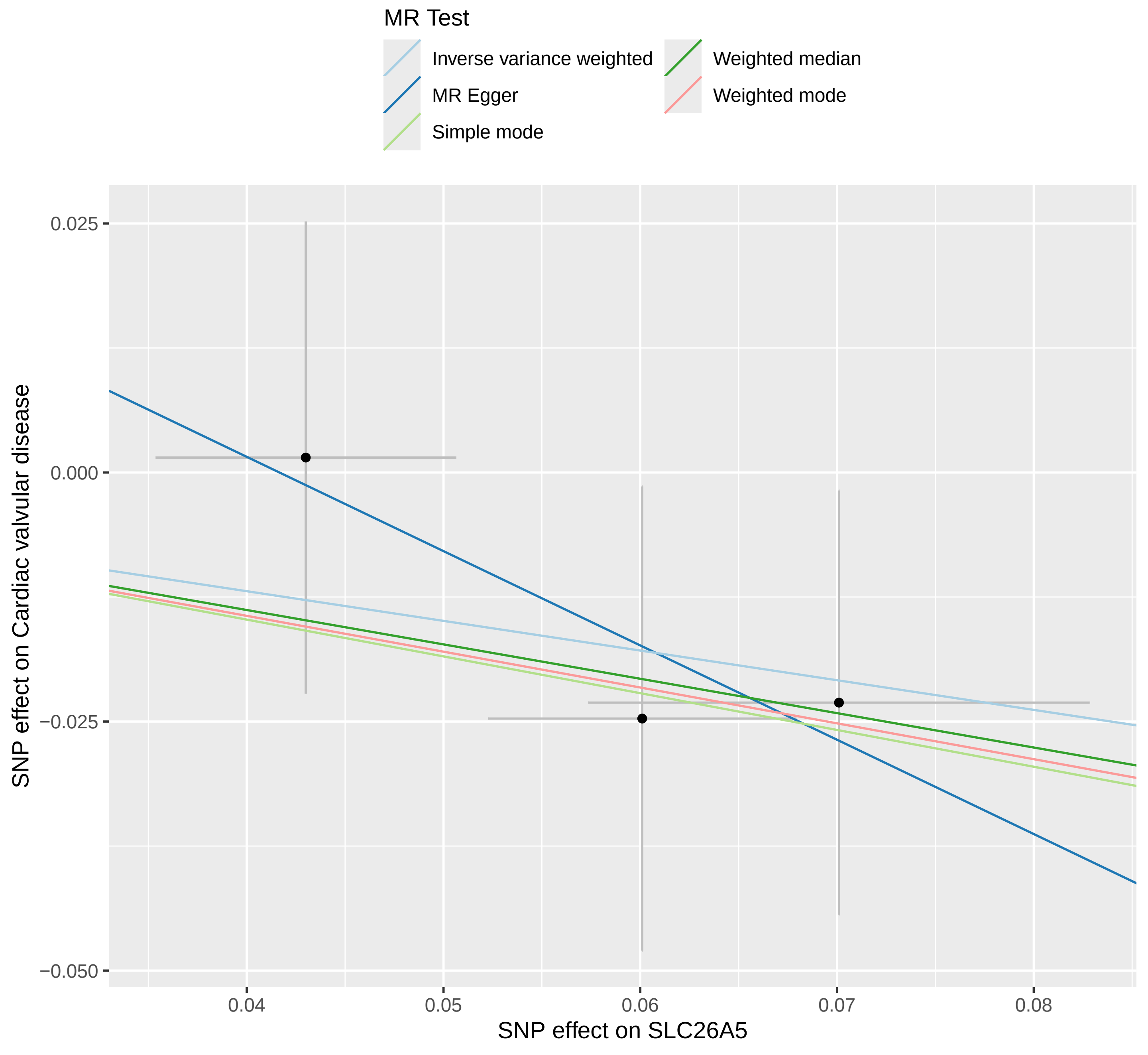
MR Test
Inverse variance weighted
MR Egger
Simple mode
Weighted median
Weighted mode
0.025
0.000
−0.025
−0.050
0.04
0.05
0.06
0.07
0.08
SNP effect on SLC26A5
SNP effect on Cardiac valvular disease

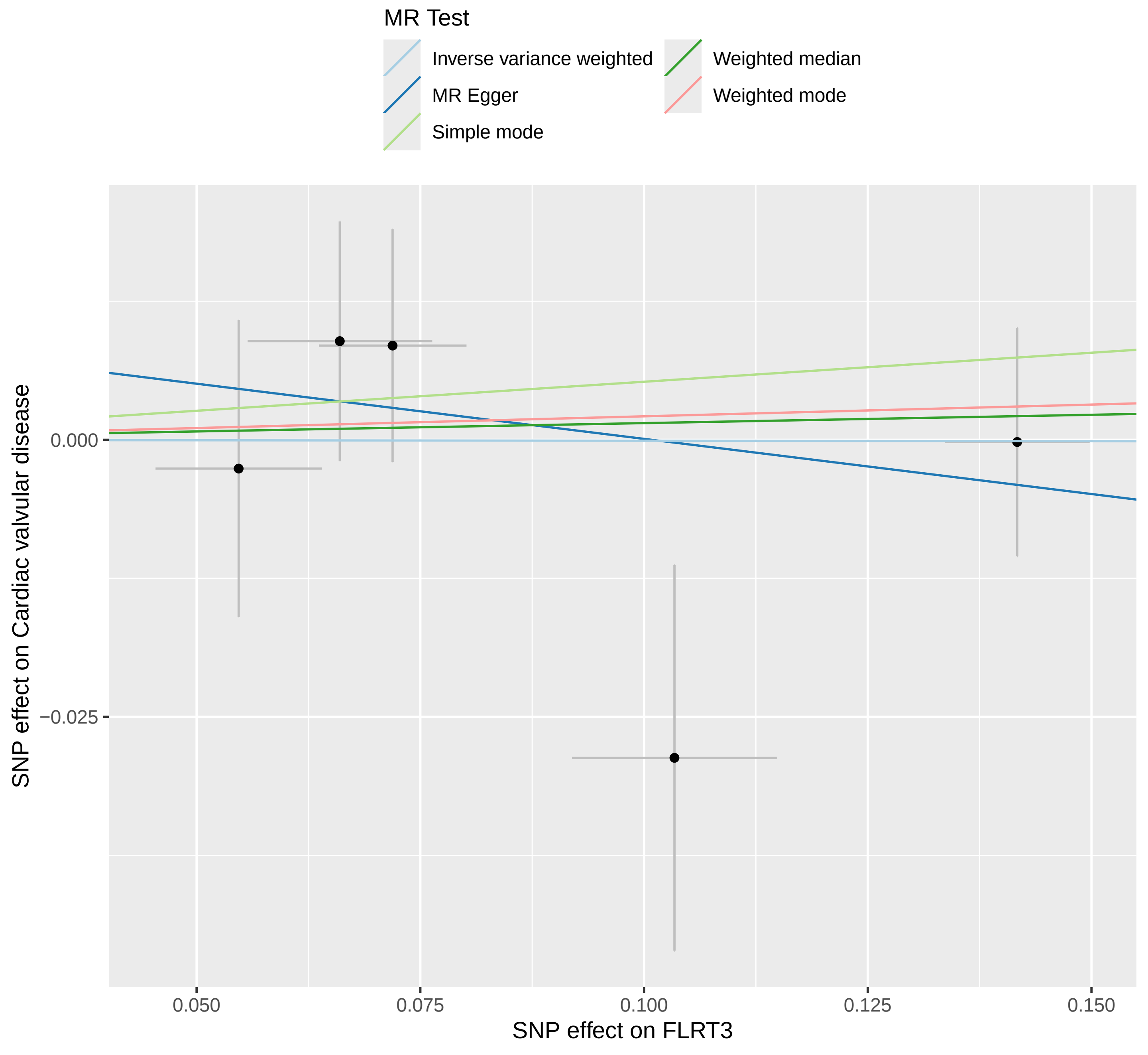

MR Test
Inverse variance weighted
MR Egger
Simple mode
Weighted median
Weighted mode
SNP effect on Cardiac valvular disease
0.000
−0.025
0.050
0.075
0.100
0.125
0.150
SNP effect on FLRT3

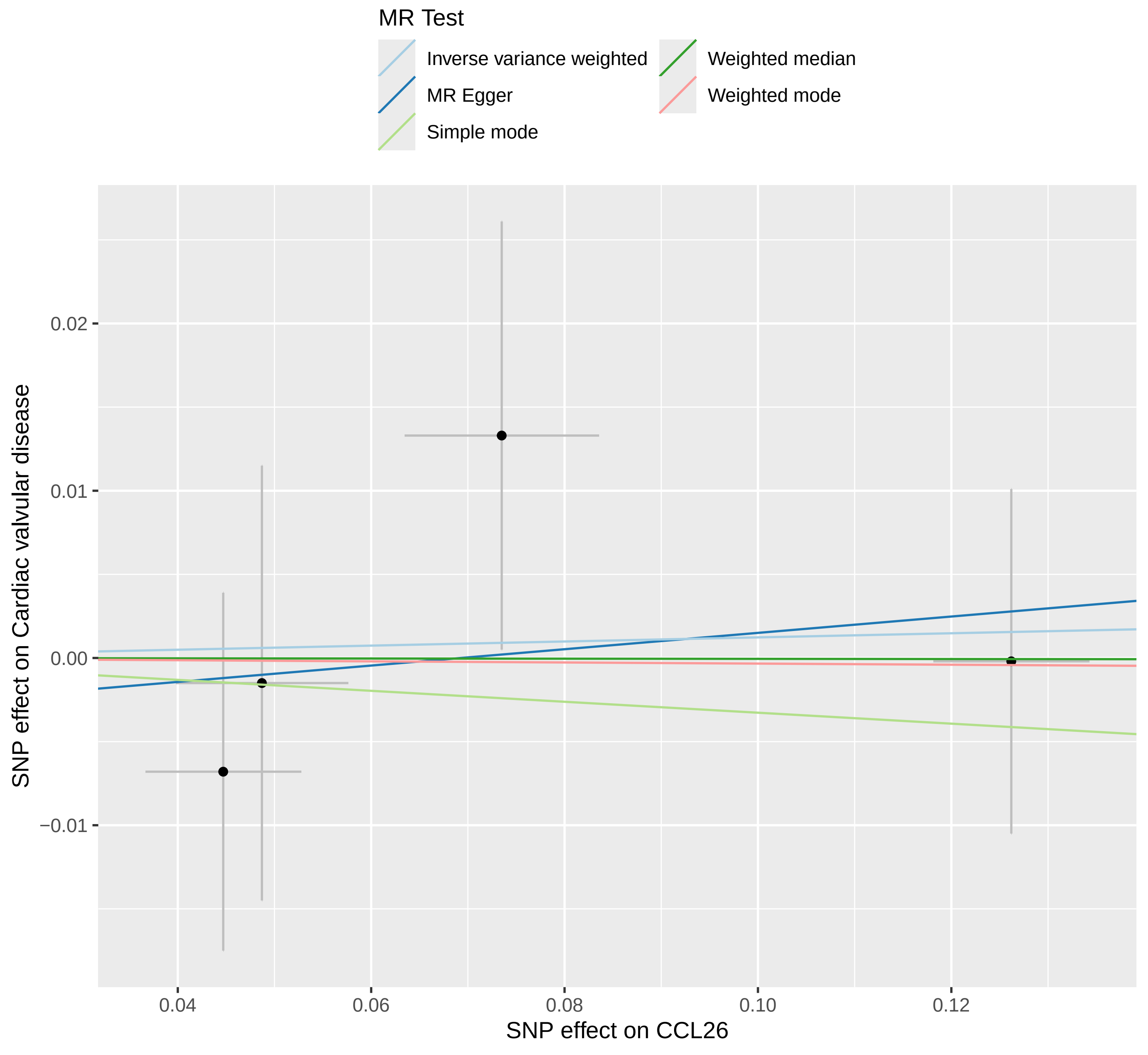
MR Test
Inverse variance weighted
MR Egger
Simple mode
Weighted median
Weighted mode
0.02
0.01
0.00
−0.01
0.04
0.06
0.08
0.10
0.12
SNP effect on Cardiac valvular disease
SNP effect on CCL26

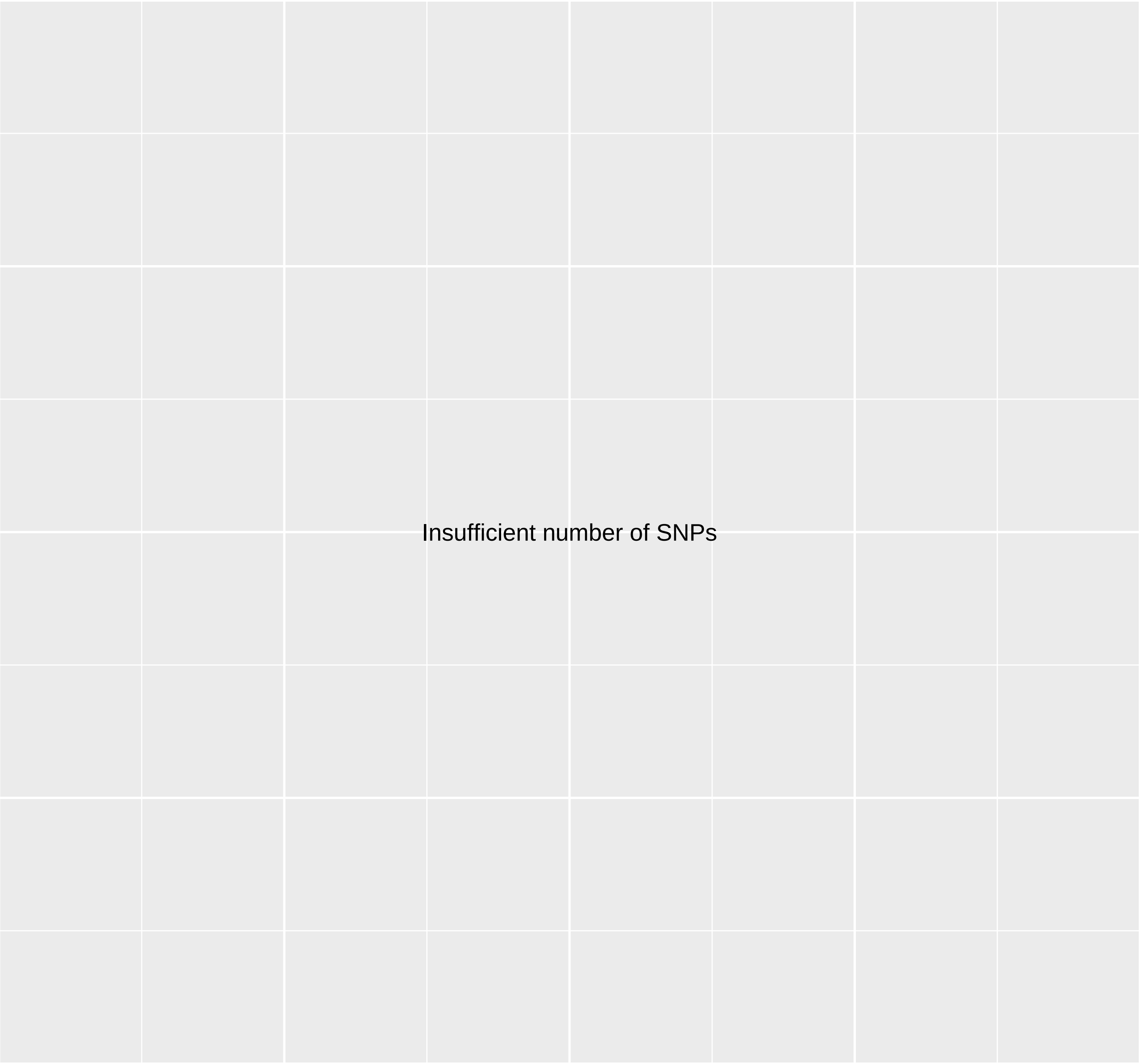
Insufficient number of SNPs

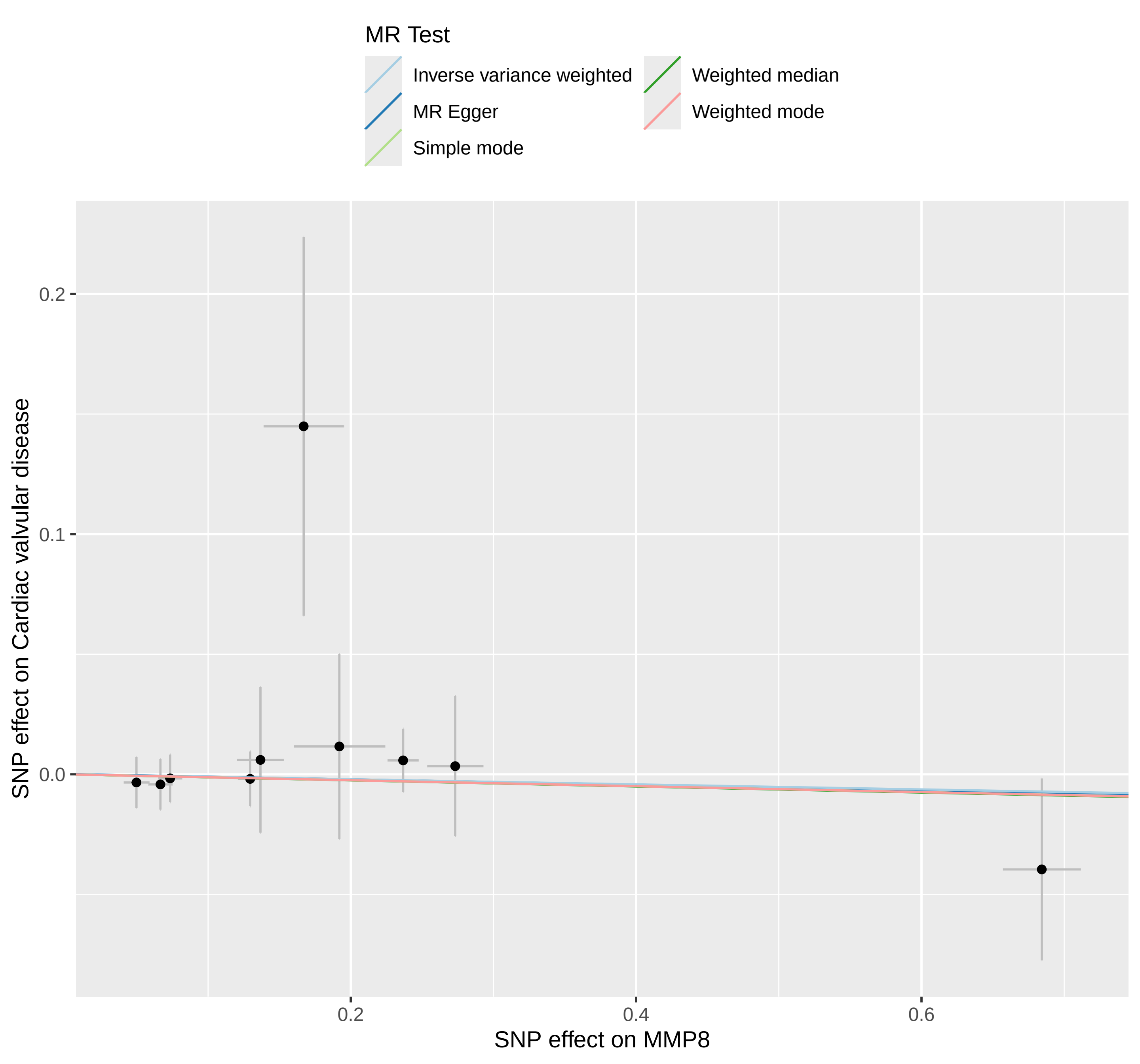
MR Test
Inverse variance weighted
MR Egger
Simple mode
Weighted median
Weighted mode
SNP effect on Cardiac valvular disease
0.2
0.1
0.0
0.2
0.4
0.6
SNP effect on MMP8

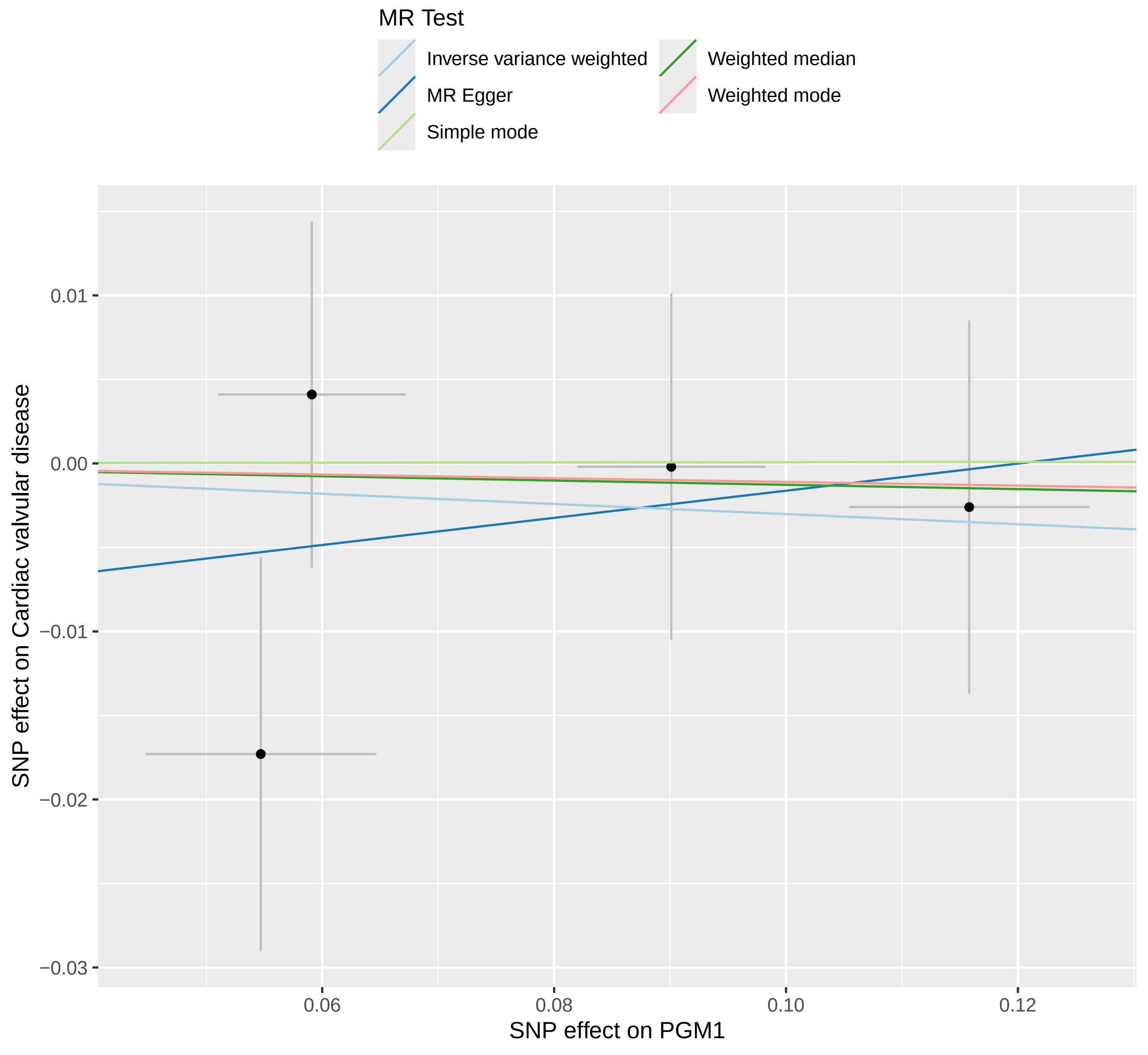
MR Test
Inverse variance weighted
MR Egger
Simple mode
Weighted median
Weighted mode
0.01
0.00
−0.01
−0.02
−0.03
SNP effect on Cardiac valvular disease
0.06
0.08
0.10
0.12
SNP effect on PGM1

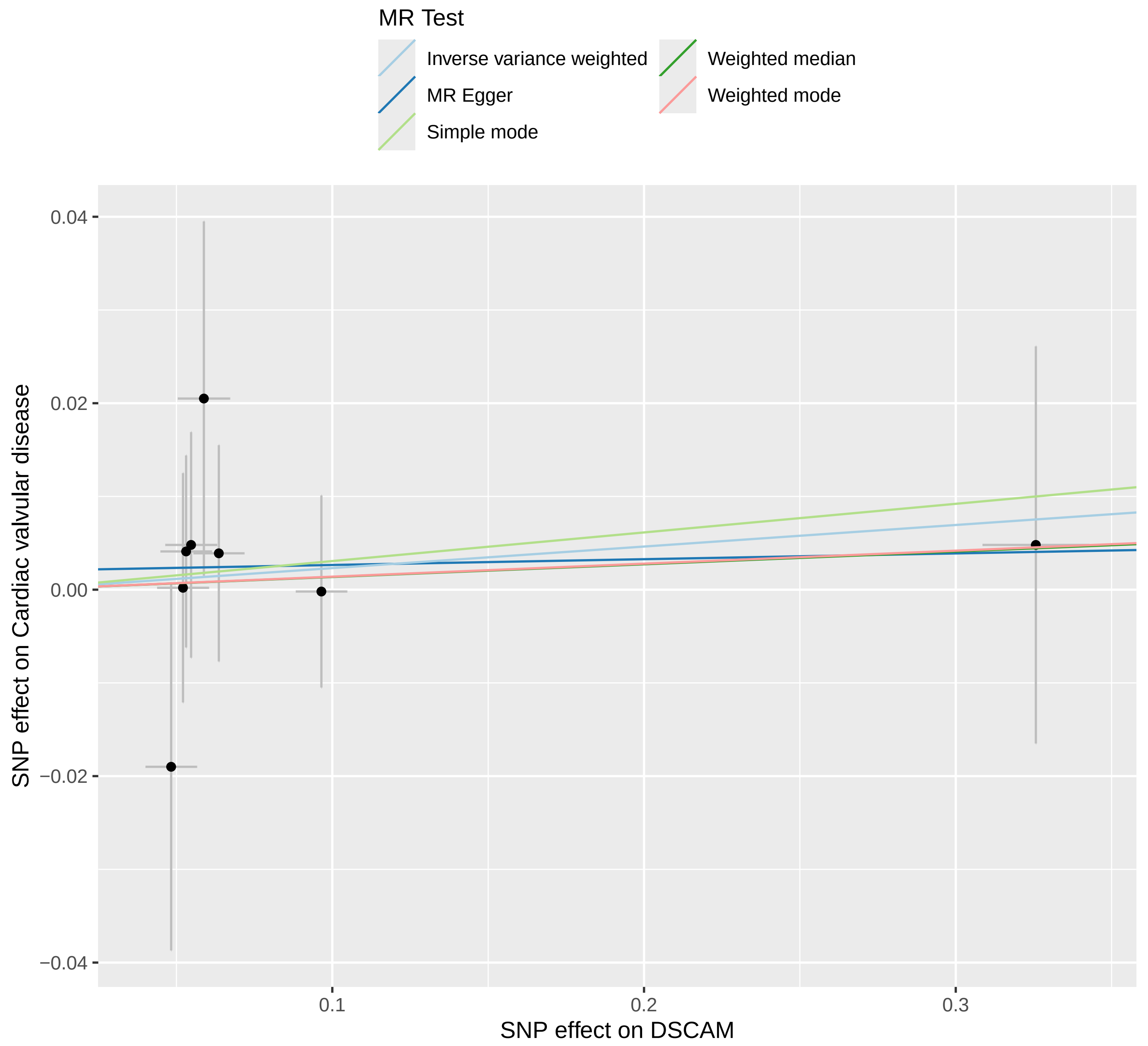
MR Test
Inverse variance weighted
MR Egger
Simple mode
Weighted median
Weighted mode
0.04
0.02
0.00
−0.02
−0.04
0.1
0.2
0.3
SNP effect on Cardiac valvular disease
SNP effect on DSCAM

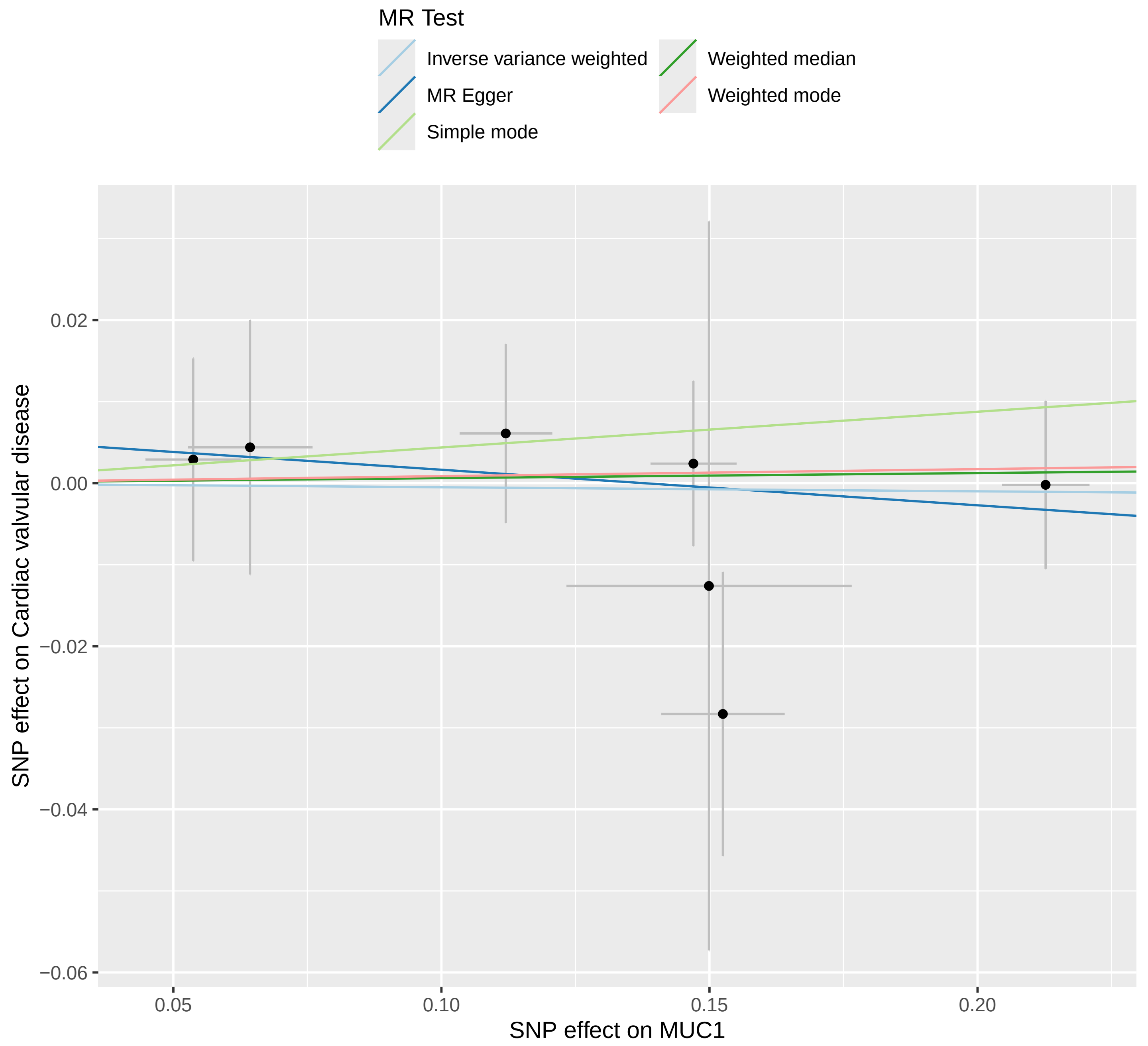
MR Test
Inverse variance weighted
MR Egger
Simple mode
Weighted median
Weighted mode
SNP effect on Cardiac valvular disease
0.02
0.00
−0.02
−0.04
−0.06
0.05
0.10
0.15
0.20
SNP effect on MUC1

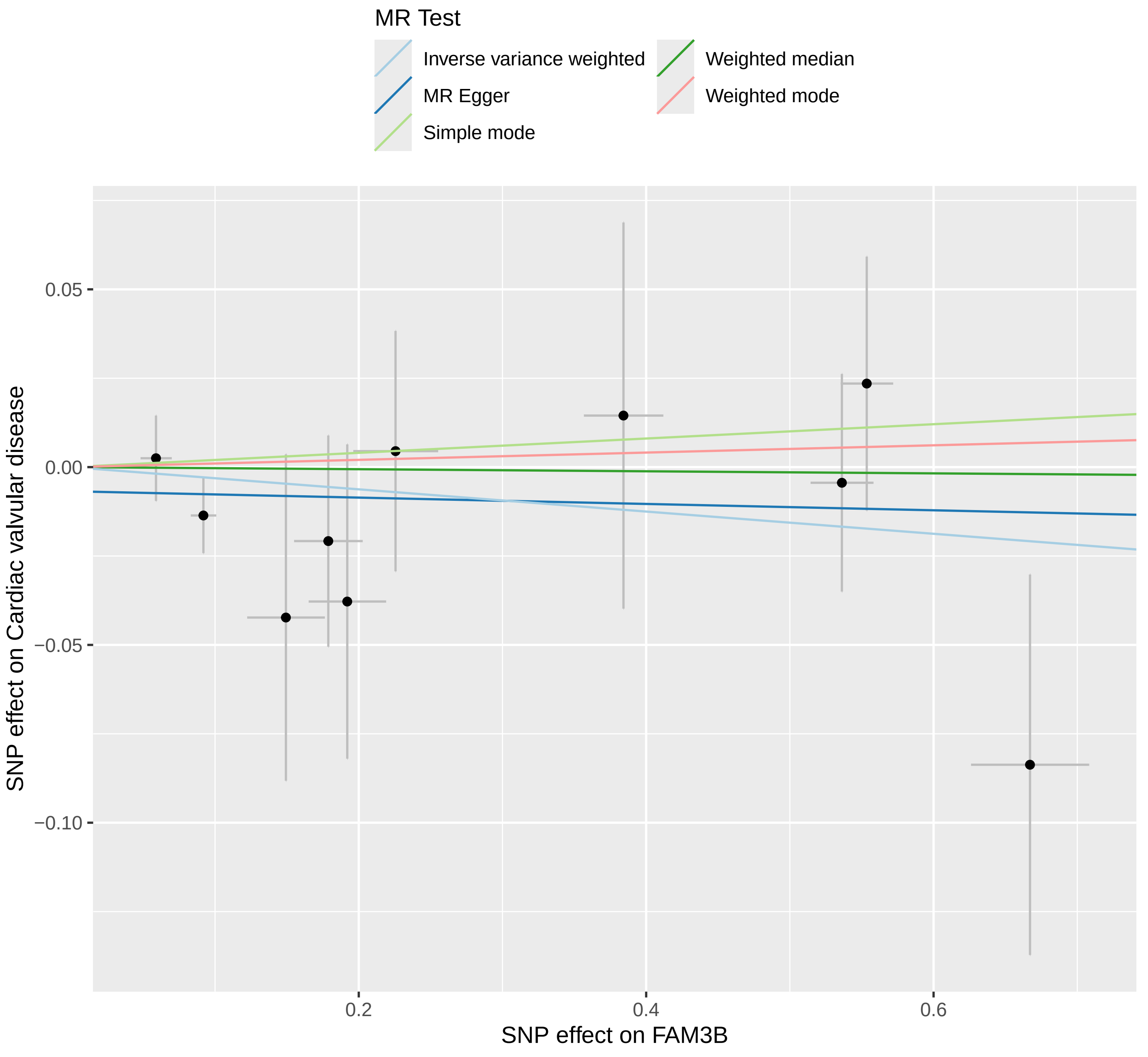

MR Test
Inverse variance weighted
MR Egger
Simple mode
Weighted median
Weighted mode
0.05
0.00
−0.05
−0.10
0.2
0.4
0.6
SNP effect on Cardiac valvular disease
SNP effect on FAM3B

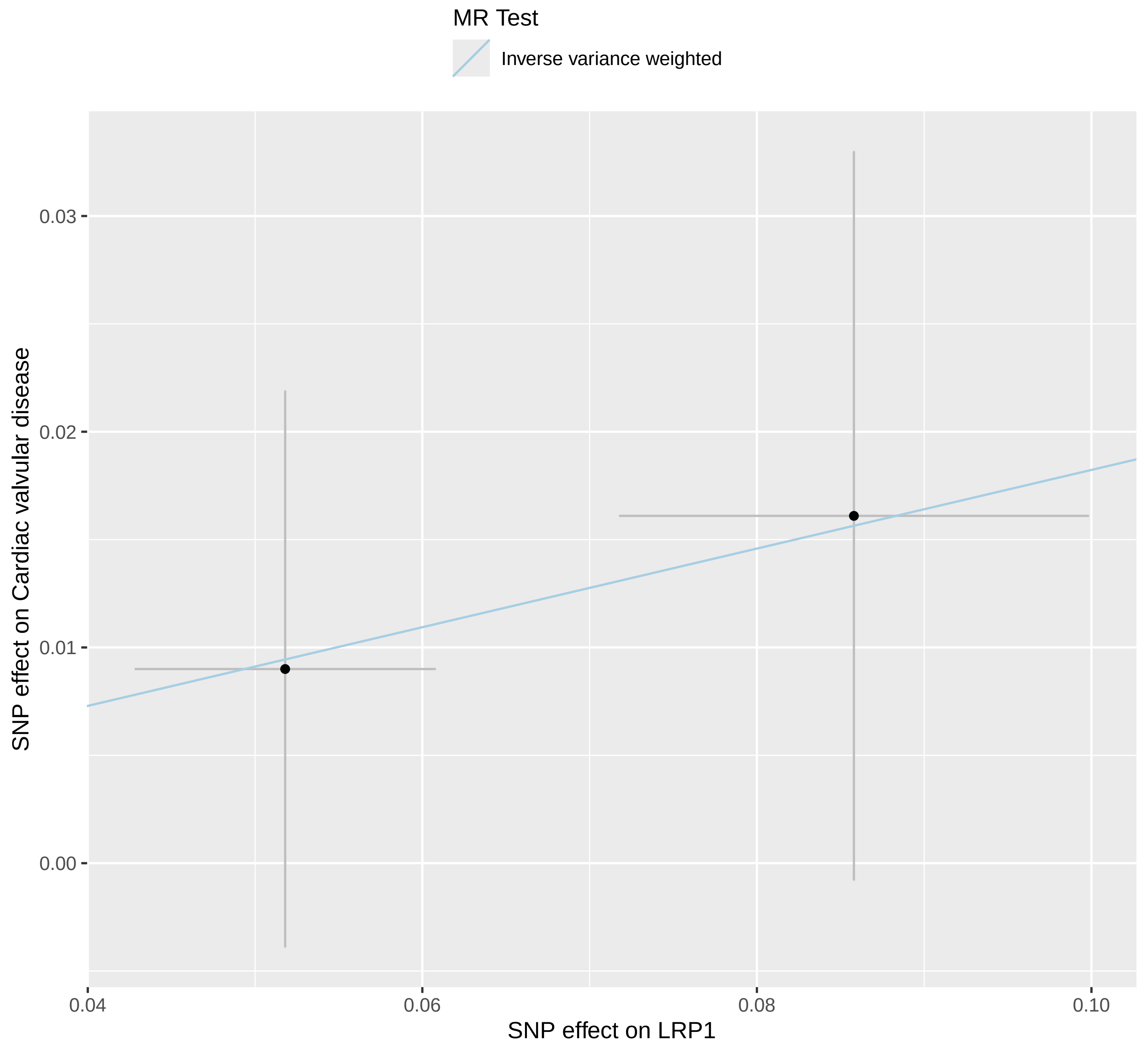

MR Test
Inverse variance weighted
SNP effect on Cardiac valvular disease
0.03
0.02
0.01
0.00
0.04
0.06
0.08
0.10
SNP effect on LRP1

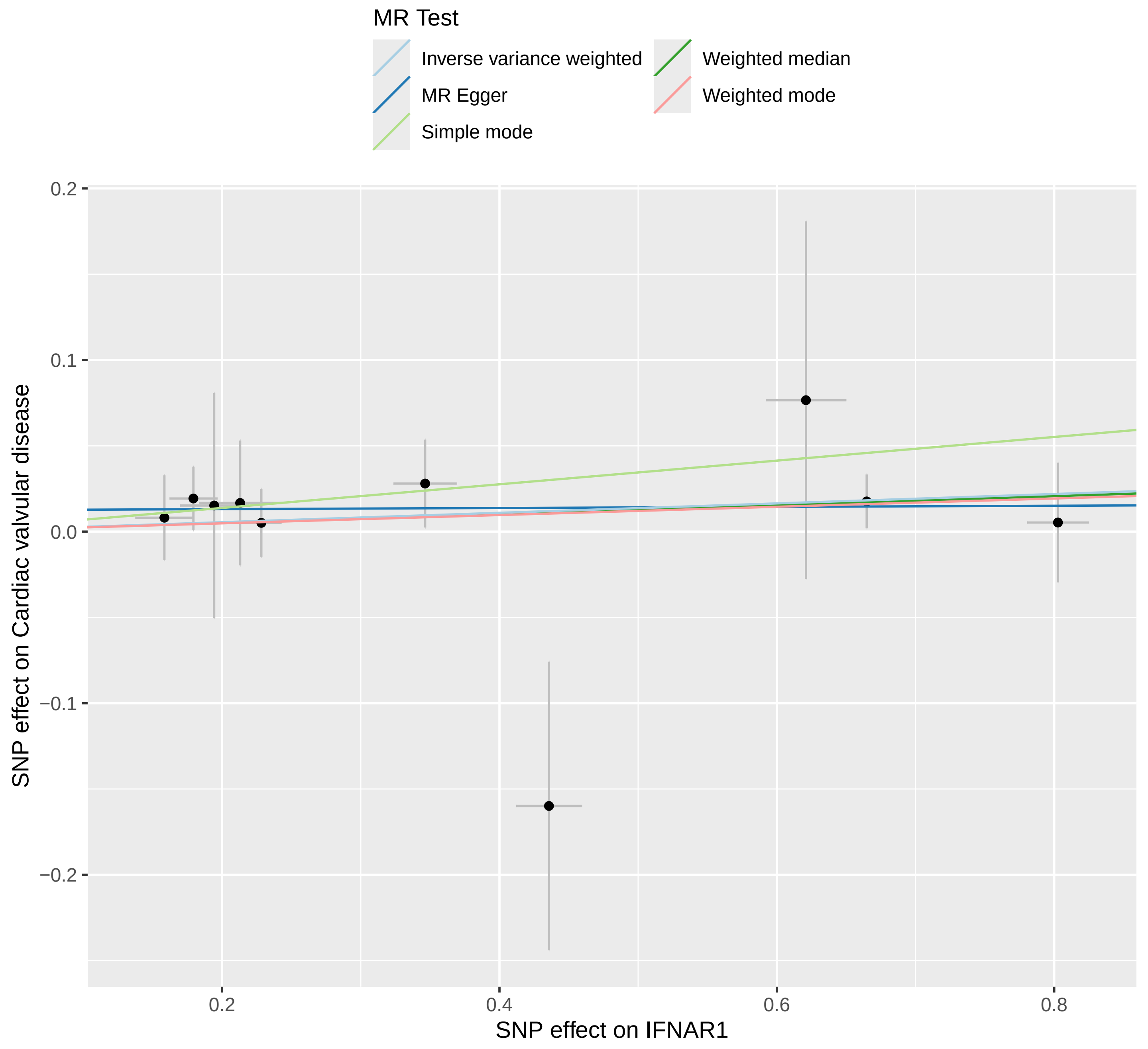

MR Test
Inverse variance weighted
MR Egger
Simple mode
Weighted median
Weighted mode
0.2
0.1
0.0
−0.1
−0.2
SNP effect on Cardiac valvular disease
0.2
0.4
0.6
0.8
SNP effect on IFNAR1

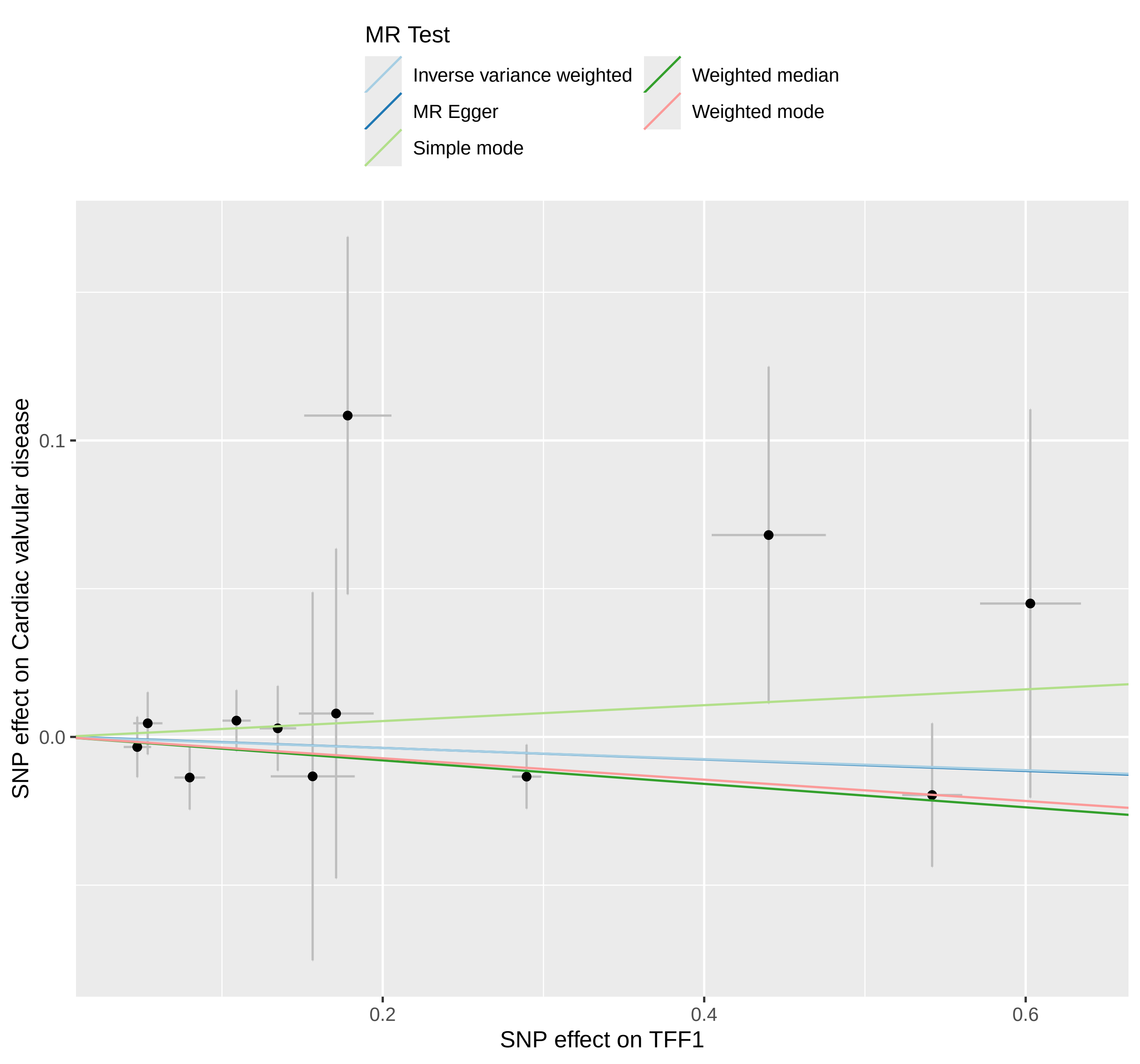
MR Test
Inverse variance weighted
MR Egger
Simple mode
Weighted median
Weighted mode
0.1
0.0
0.2
0.4
0.6
SNP effect on TFF1
SNP effect on Cardiac valvular disease

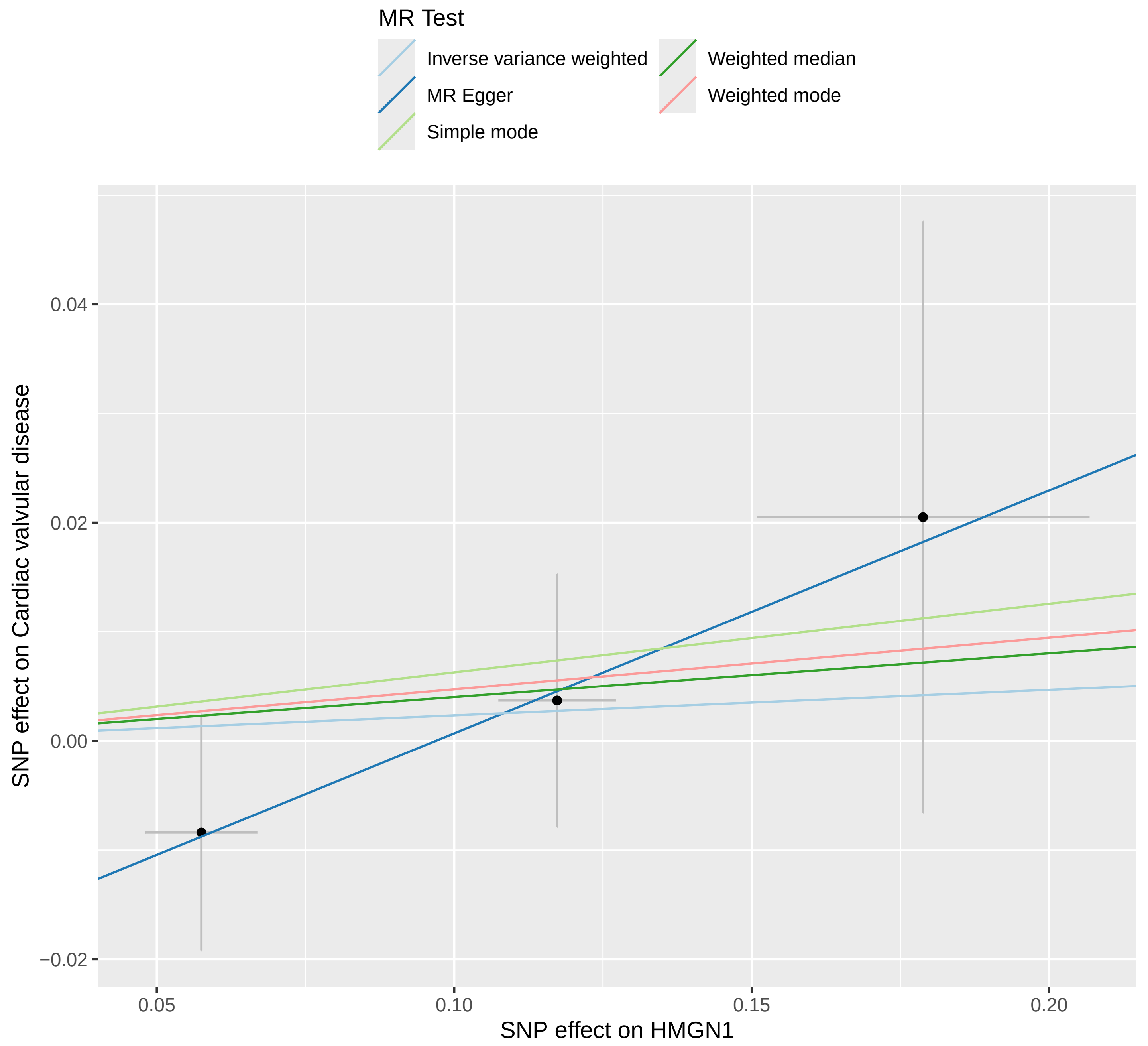

MR Test
Inverse variance weighted
MR Egger
Simple mode
Weighted median
Weighted mode
SNP effect on Cardiac valvular disease
0.04
0.02
0.00
−0.02
0.05
0.10
0.15
0.20
SNP effect on HMGN1

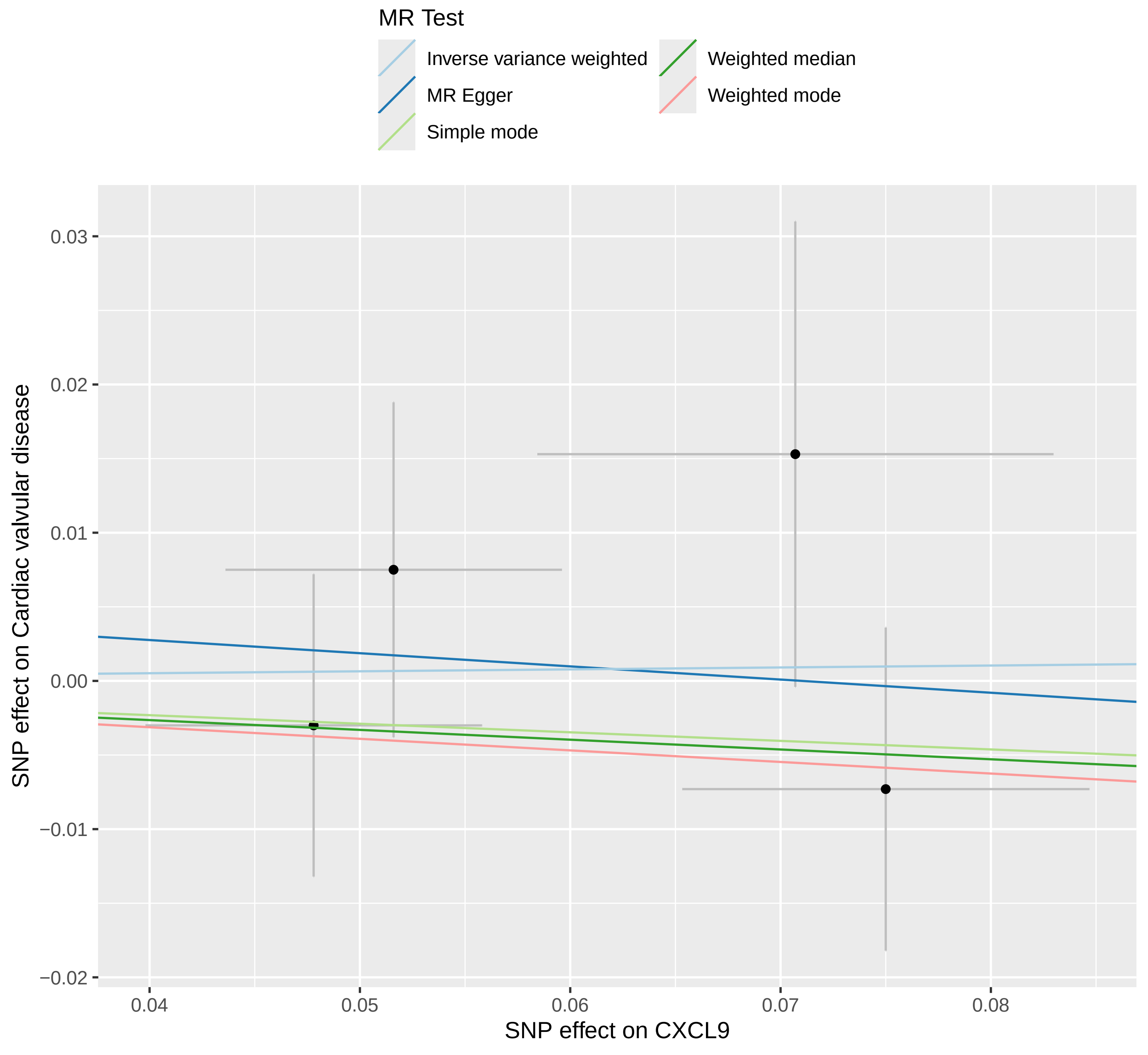

MR Test
Inverse variance weighted
MR Egger
Simple mode
Weighted median
Weighted mode
0.03
0.02
0.01
0.00
−0.01
−0.02
SNP effect on Cardiac valvular disease
0.04
0.05
0.06
0.07
0.08
SNP effect on CXCL9

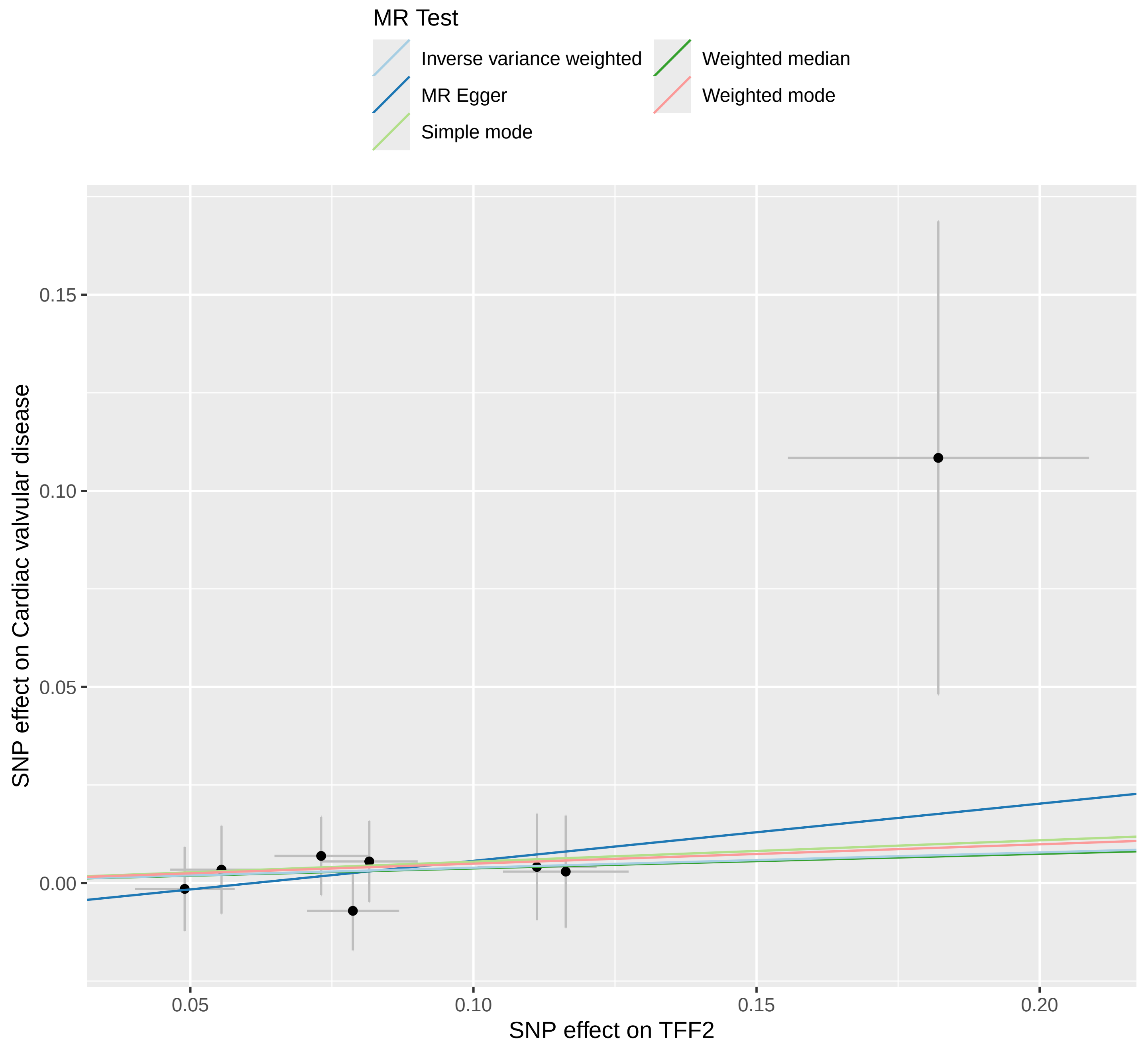
MR Test
Inverse variance weighted
MR Egger
Simple mode
Weighted median
Weighted mode
SNP effect on Cardiac valvular disease
0.15
0.10
0.05
0.00
0.05
0.10
0.15
0.20
SNP effect on TFF2

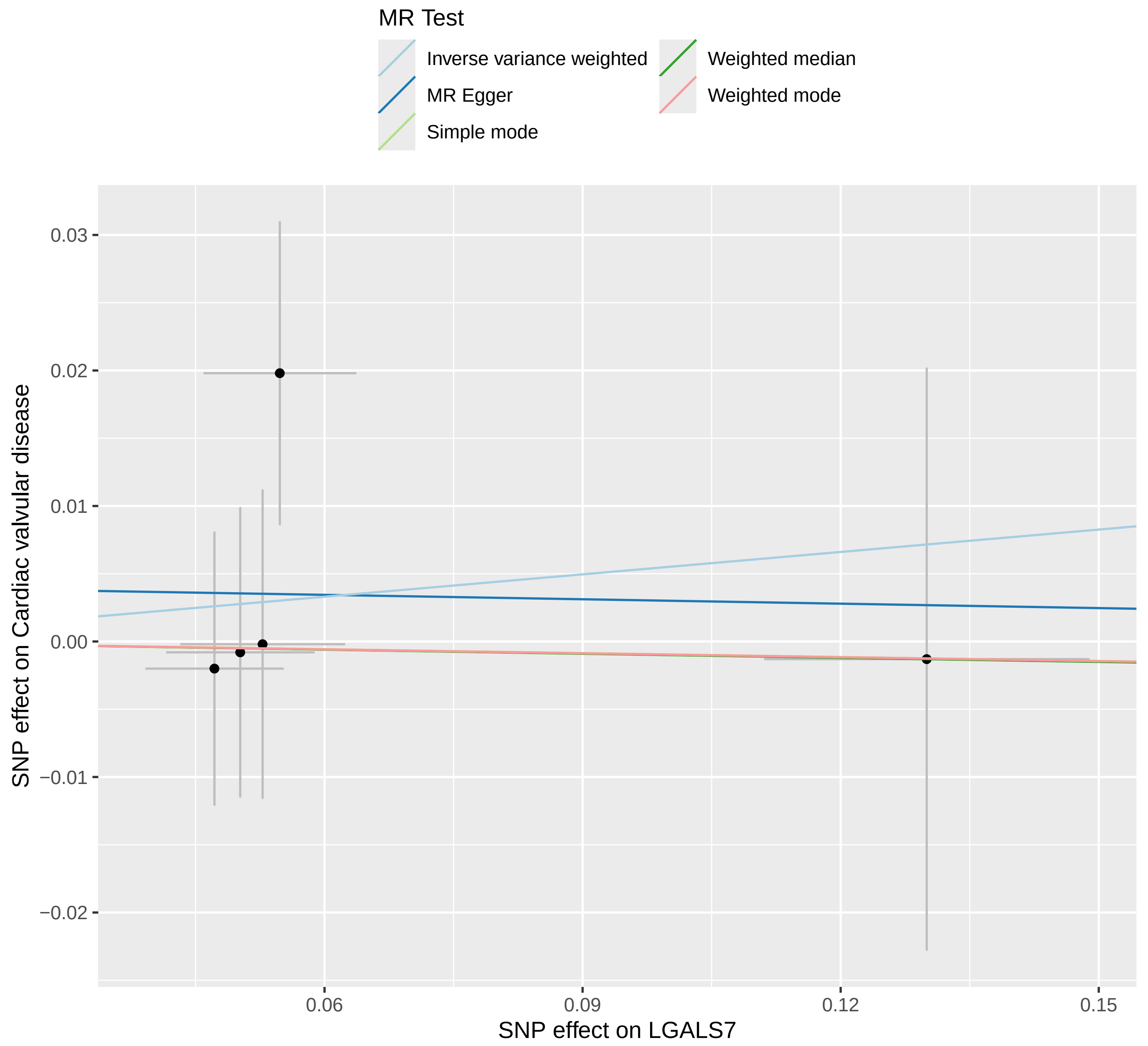
MR Test
Inverse variance weighted
MR Egger
Simple mode
Weighted median
Weighted mode
SNP effect on Cardiac valvular disease
0.03
0.02
0.01
0.00
−0.01
−0.02
SNP effect on LGALS7
0.06
0.09
0.12
0.15

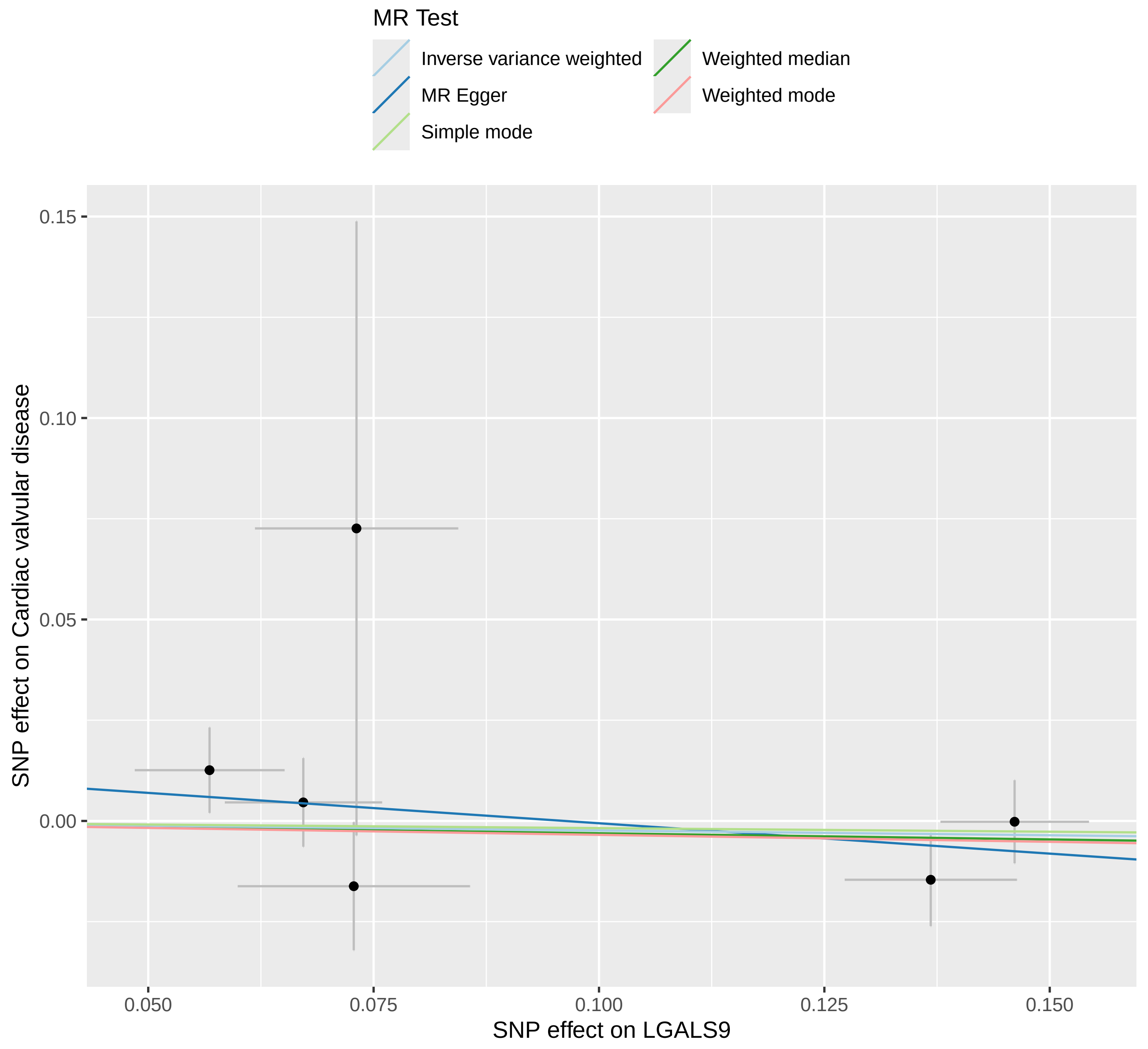
MR Test
Inverse variance weighted
MR Egger
Simple mode
Weighted median
Weighted mode
0.15
0.10
0.05
0.00
SNP effect on Cardiac valvular disease
0.050
0.075
0.100
0.125
0.150
SNP effect on LGALS9

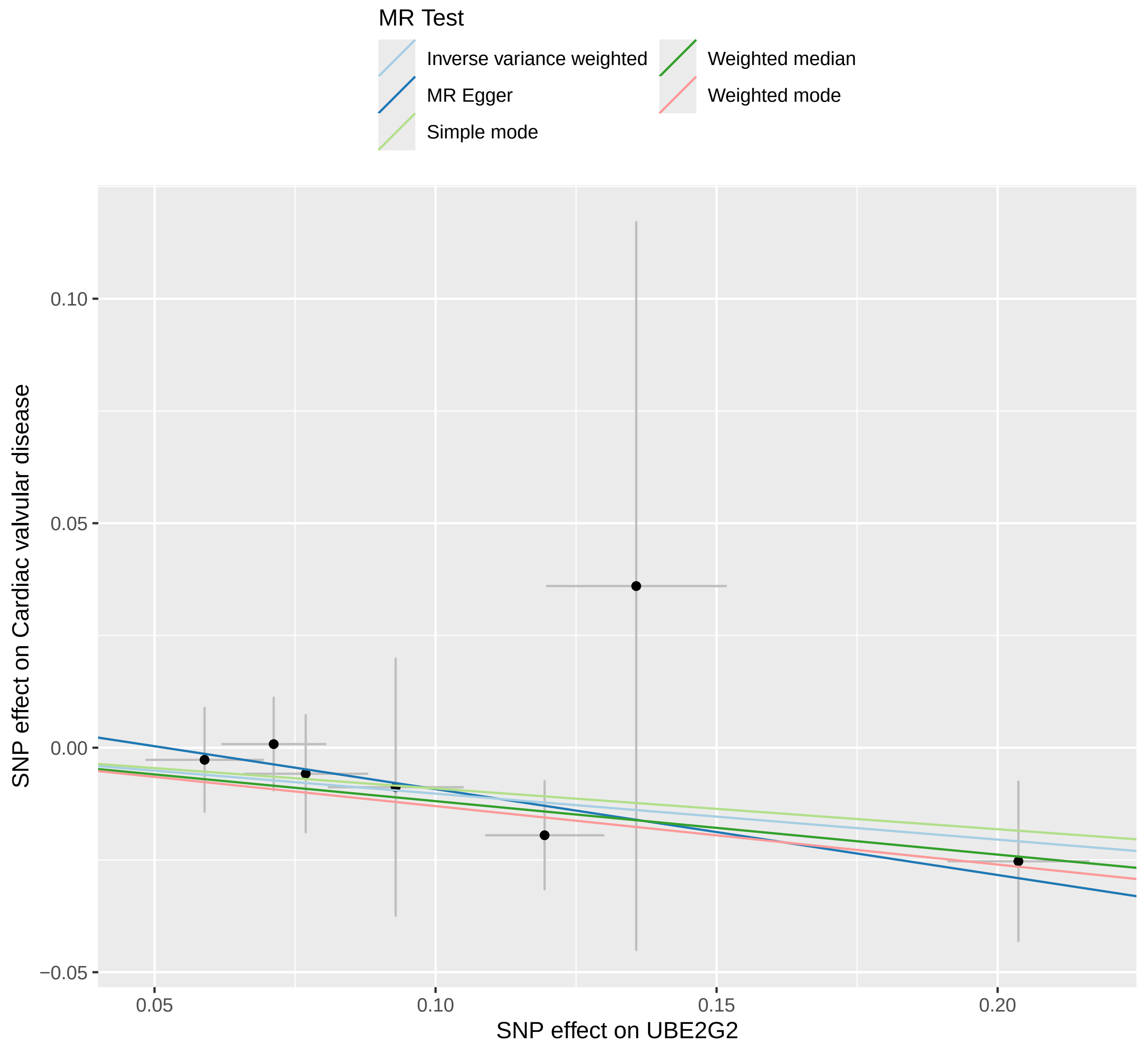

MR Test
Inverse variance weighted
MR Egger
Simple mode
Weighted median
Weighted mode
SNP effect on Cardiac valvular disease
0.10
0.05
0.00
−0.05
0.05
0.10
0.15
0.20
SNP effect on UBE2G2

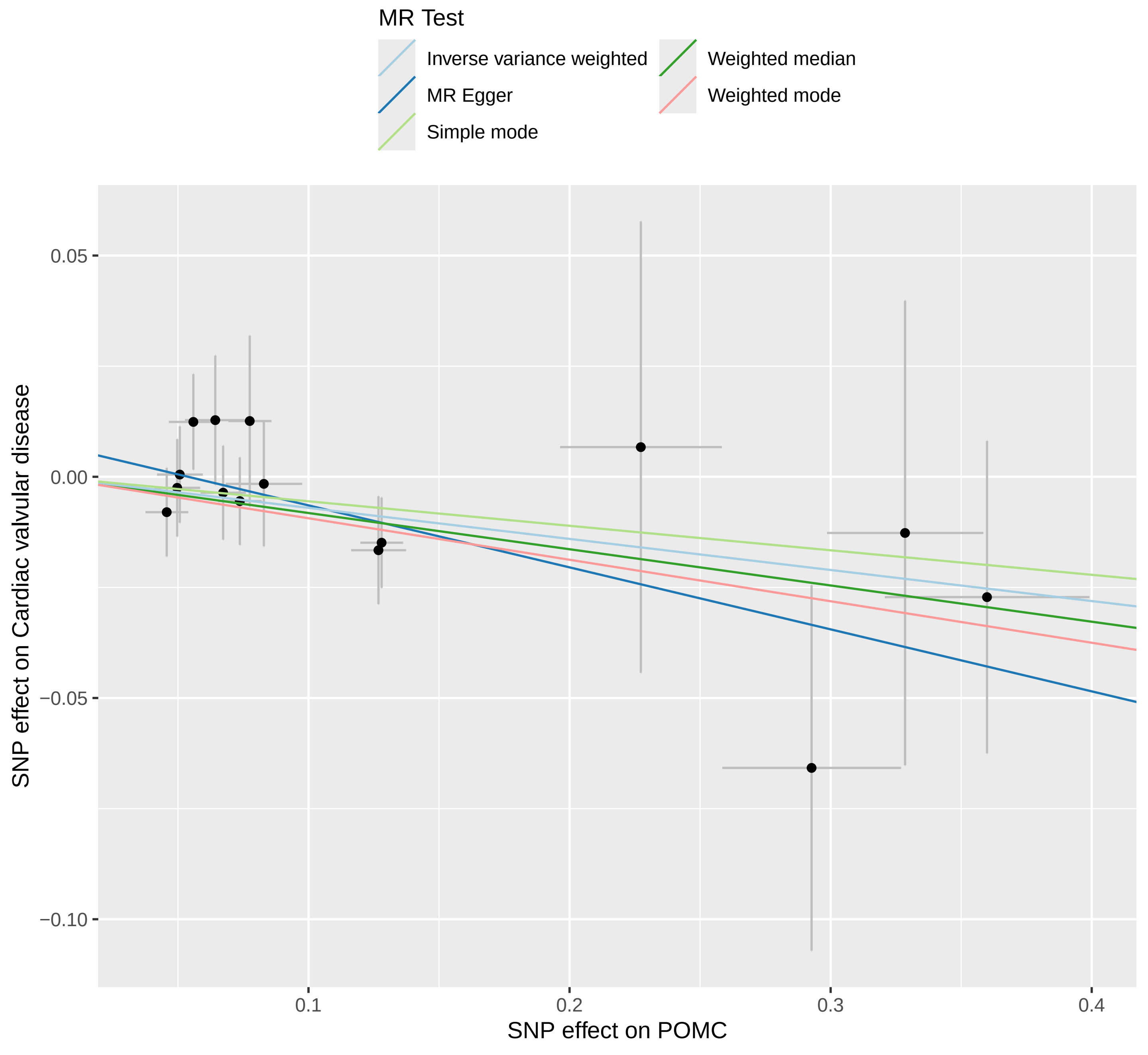
MR Test
Inverse variance weighted
MR Egger
Simple mode
Weighted median
Weighted mode
0.05
0.00
−0.05
−0.10
0.1
0.2
0.3
0.4
SNP effect on Cardiac valvular disease
SNP effect on POMC

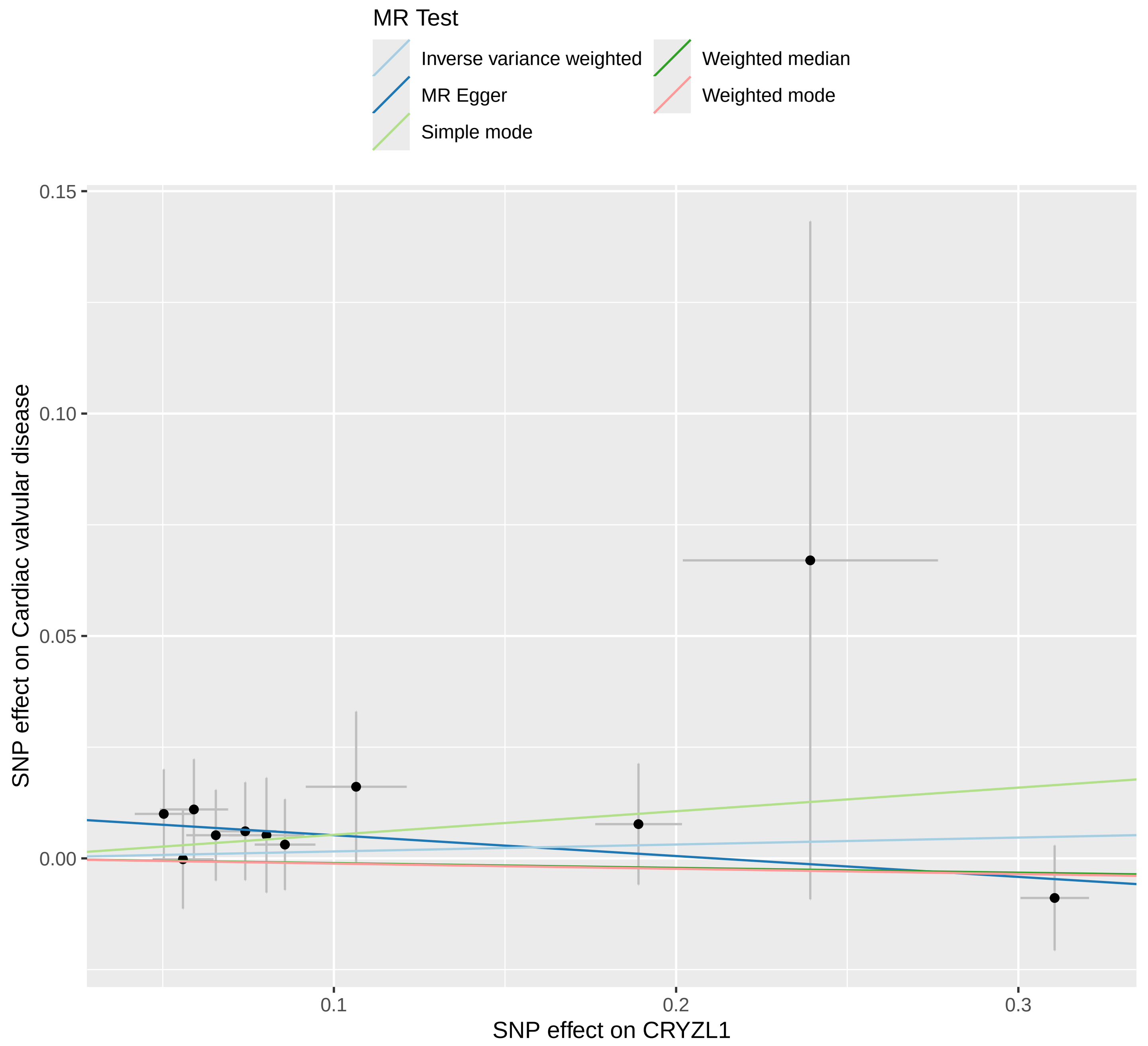

MR Test
Inverse variance weighted
MR Egger
Simple mode
Weighted median
Weighted mode
0.15
0.10
0.05
0.00
0.1
0.2
0.3
SNP effect on Cardiac valvular disease
SNP effect on CRYZL1

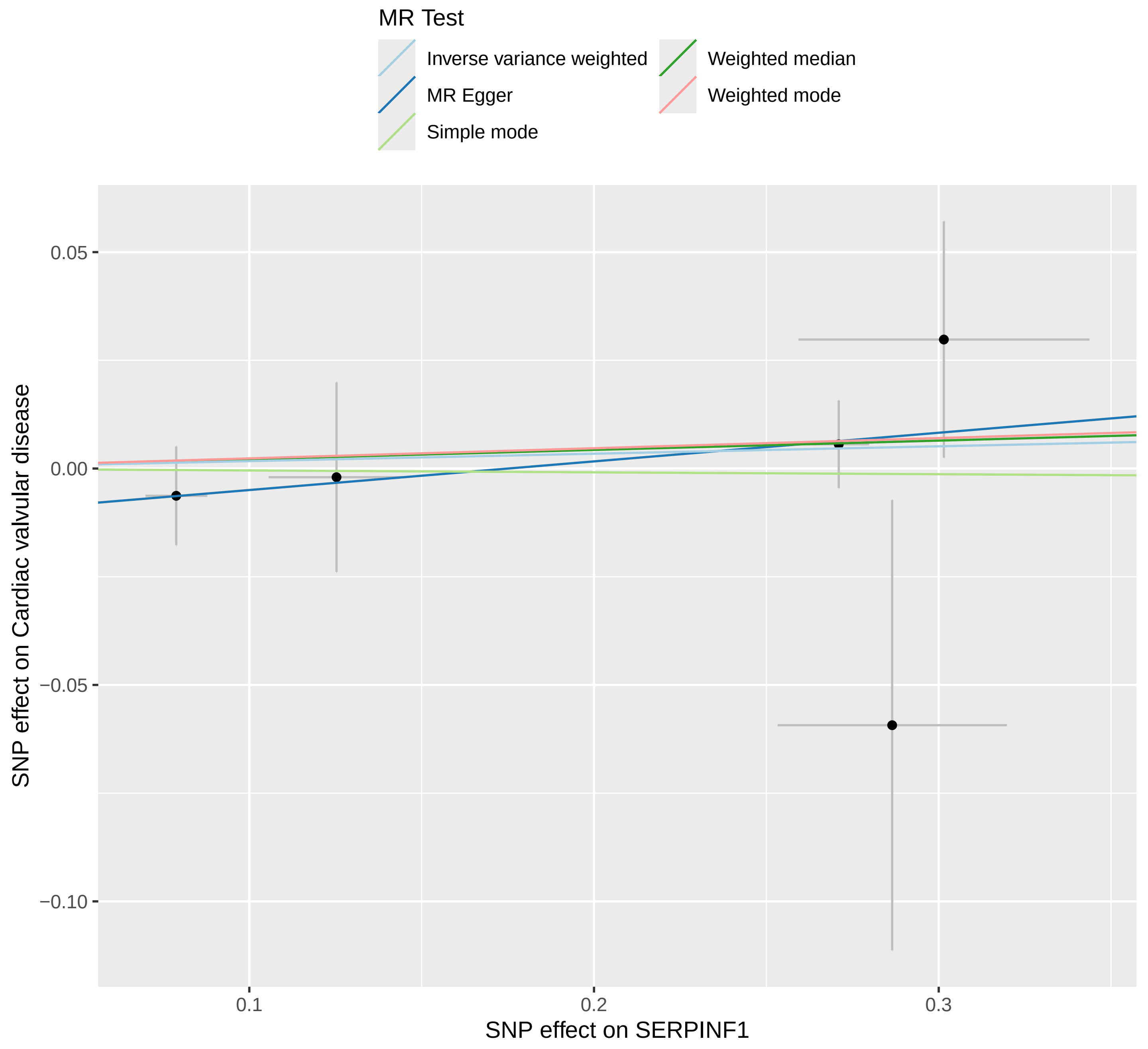

MR Test
Inverse variance weighted
MR Egger
Simple mode
Weighted median
Weighted mode
0.05
0.00
−0.05
−0.10
0.1
0.2
0.3
SNP effect on Cardiac valvular disease
SNP effect on SERPINF1

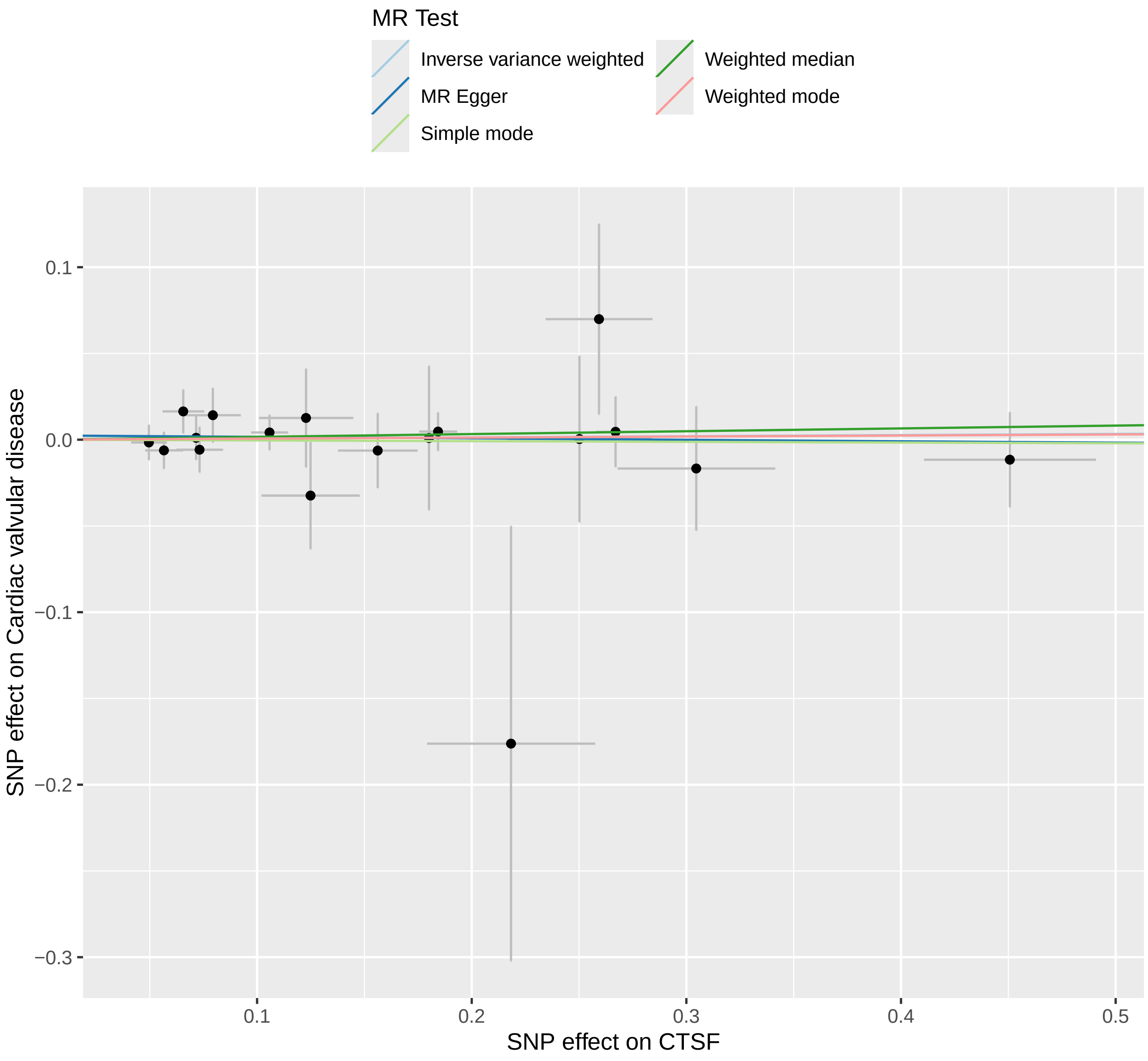
MR Test
Inverse variance weighted
MR Egger
Simple mode
Weighted median
Weighted mode
SNP effect on Cardiac valvular disease
0.1
0.0
−0.1
−0.2
−0.3
0.1
0.2
0.3
0.4
0.5
SNP effect on CTSF

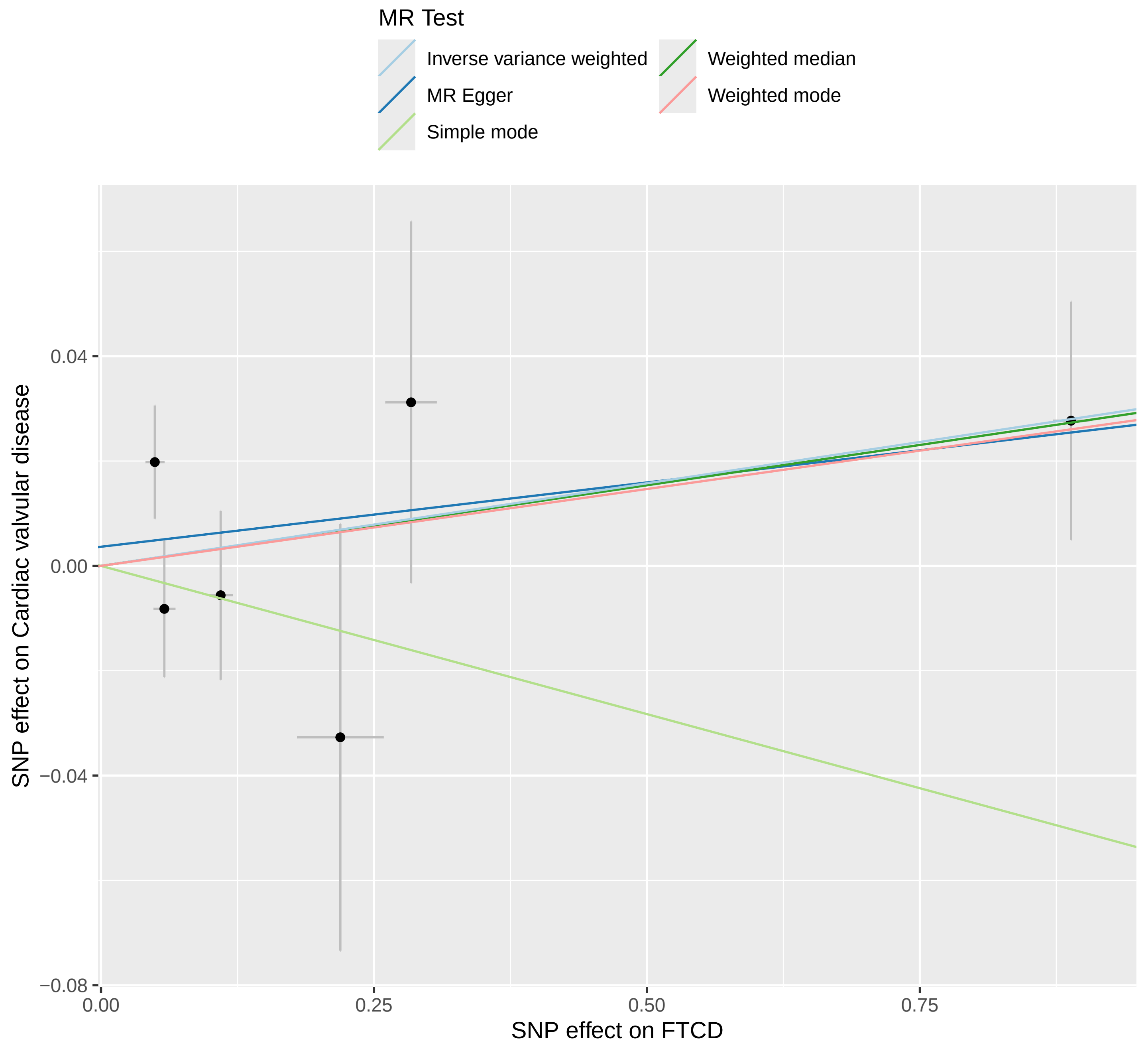
MR Test
Inverse variance weighted
MR Egger
Simple mode
Weighted median
Weighted mode
0.04
0.00
−0.04
−0.08
SNP effect on Cardiac valvular disease
0.00
0.25
0.50
0.75
SNP effect on FTCD

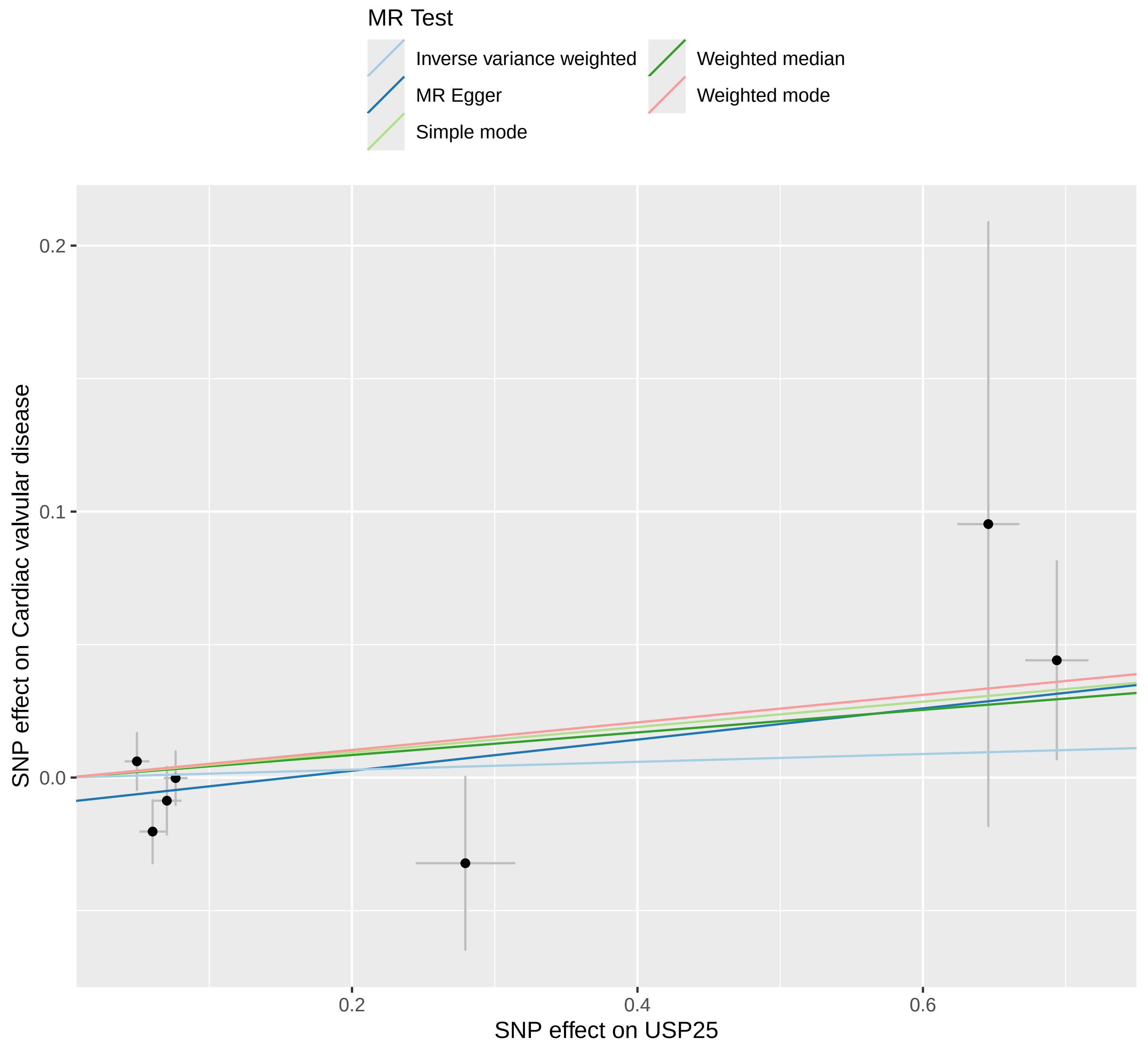
MR Test
Inverse variance weighted
MR Egger
Simple mode
Weighted median
Weighted mode
0.2
0.1
0.0
SNP effect on Cardiac valvular disease
0.2
0.4
0.6
SNP effect on USP25

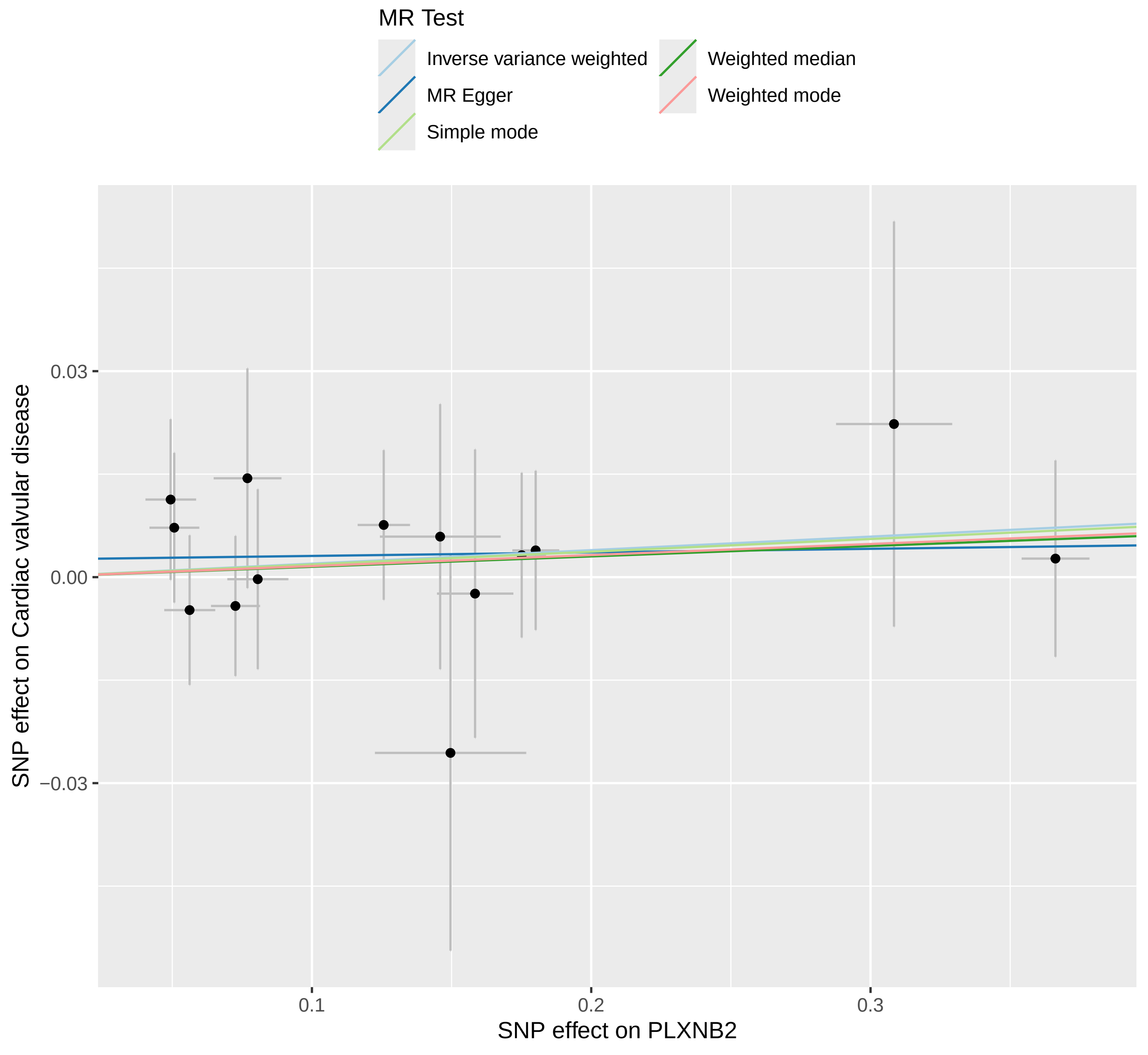

MR Test
Inverse variance weighted
MR Egger
Simple mode
Weighted median
Weighted mode
0.03
0.00
−0.03
0.1
0.2
0.3
SNP effect on Cardiac valvular disease
SNP effect on PLXNB2

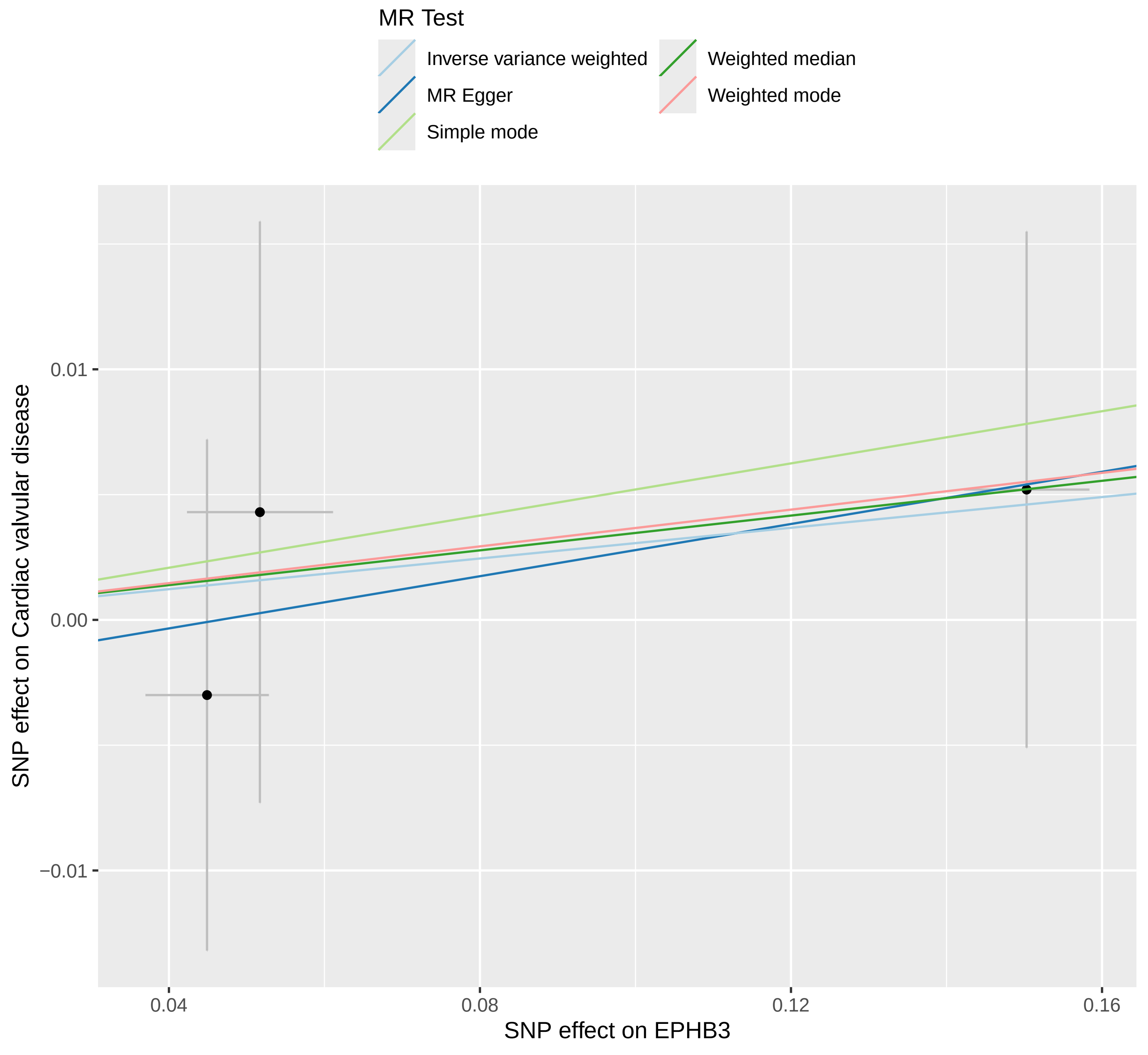
MR Test
Inverse variance weighted
MR Egger
Simple mode
Weighted median
Weighted mode
SNP effect on Cardiac valvular disease
0.01
0.00
−0.01
0.04
0.08
0.12
0.16
SNP effect on EPHB3

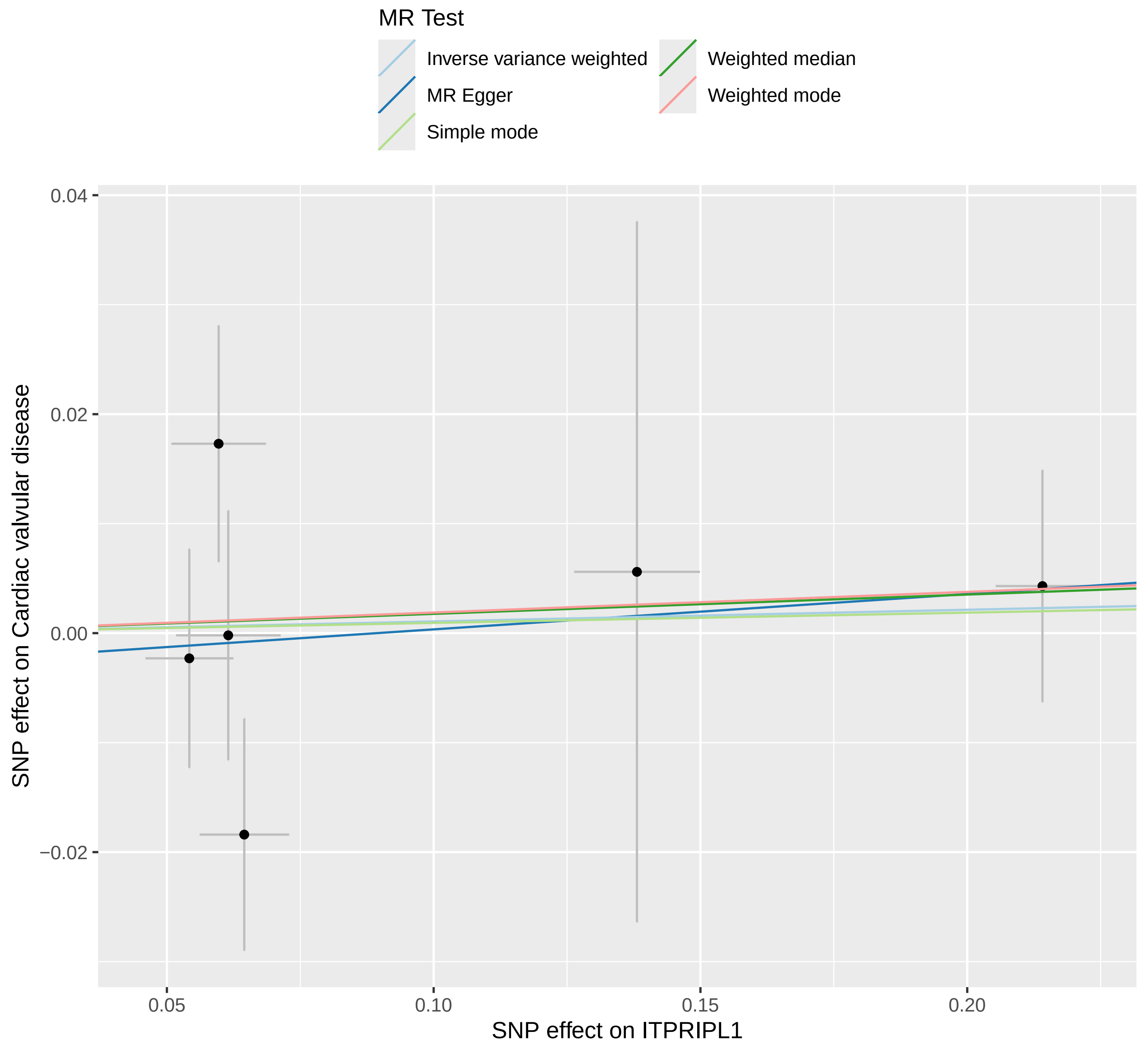
MR Test
Inverse variance weighted
MR Egger
Simple mode
Weighted median
Weighted mode
0.04
0.02
0.00
−0.02
0.05
0.10
0.15
0.20
SNP effect on Cardiac valvular disease
SNP effect on ITPRIPL1

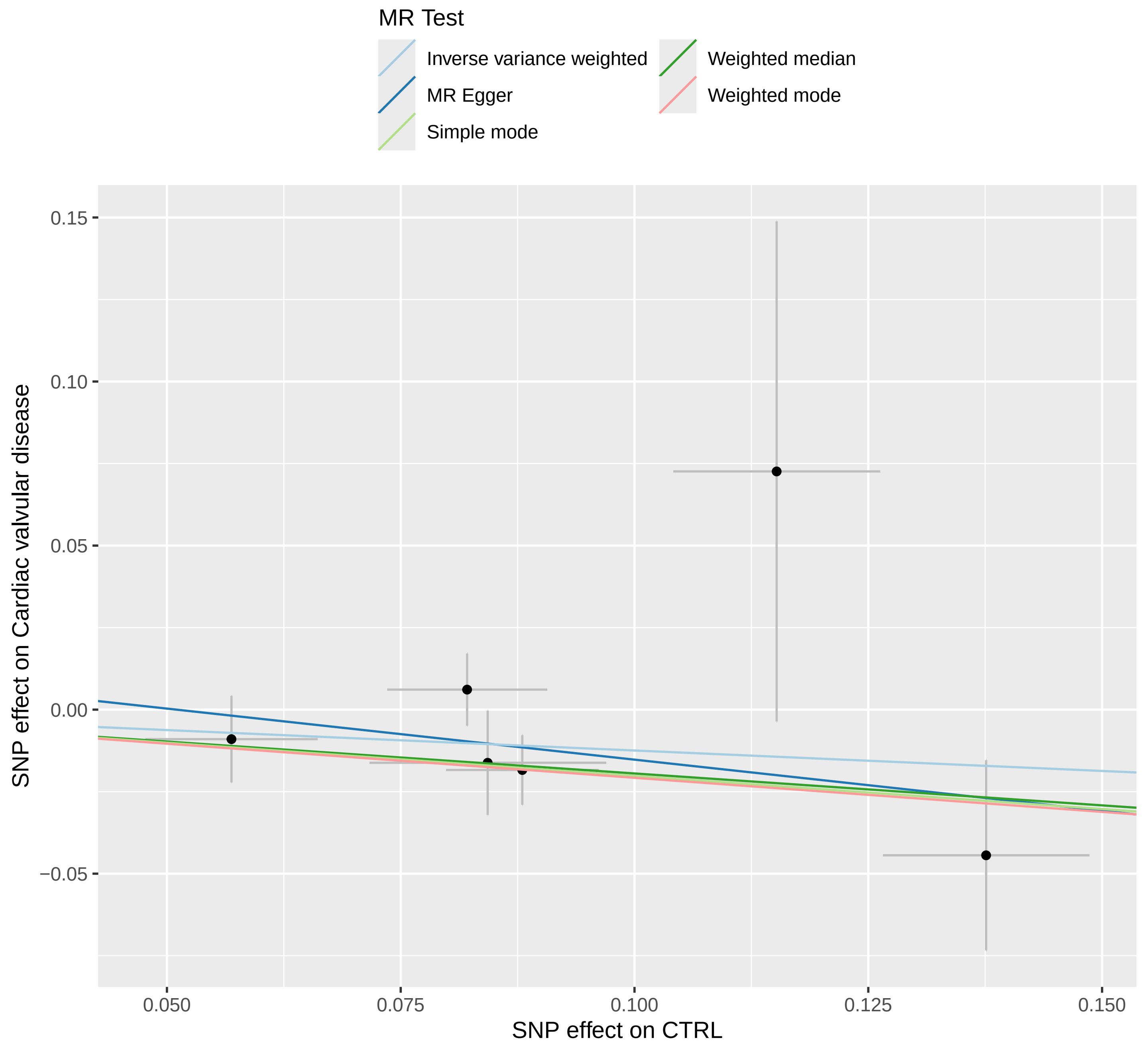

MR Test
Inverse variance weighted
MR Egger
Simple mode
Weighted median
Weighted mode
SNP effect on Cardiac valvular disease
0.15
0.10
0.05
0.00
−0.05
0.050
0.075
0.100
0.125
0.150
SNP effect on CTRL

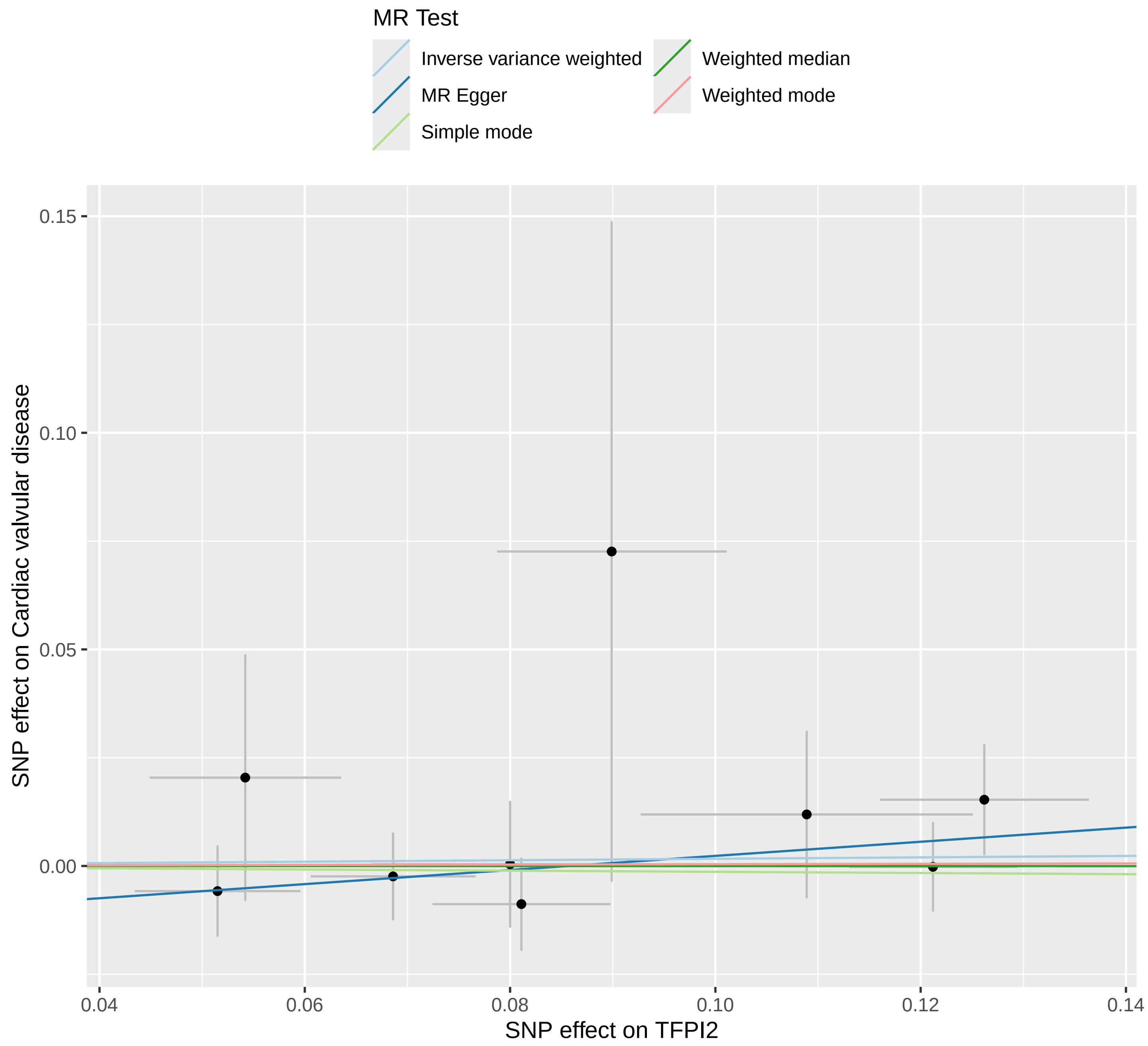
MR Test
Inverse variance weighted
MR Egger
Simple mode
Weighted median
Weighted mode
0.15
0.10
0.05
0.00
SNP effect on Cardiac valvular disease
0.04
0.06
0.08
0.10
0.12
0.14
SNP effect on TFPI2

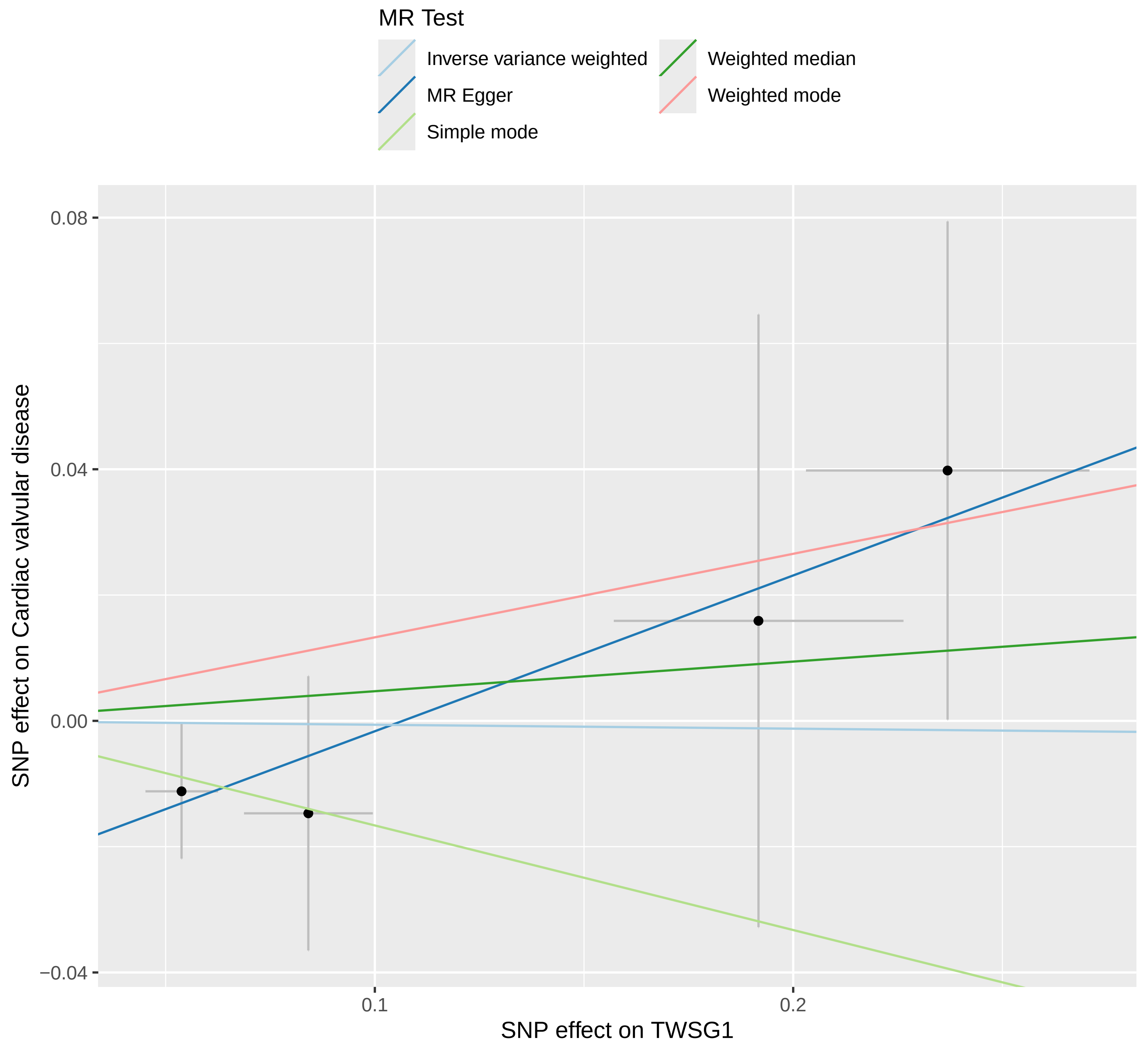
MR Test
Inverse variance weighted
MR Egger
Simple mode
Weighted median
Weighted mode
0.08
0.04
0.00
−0.04
SNP effect on Cardiac valvular disease
0.1
0.2
SNP effect on TWSG1

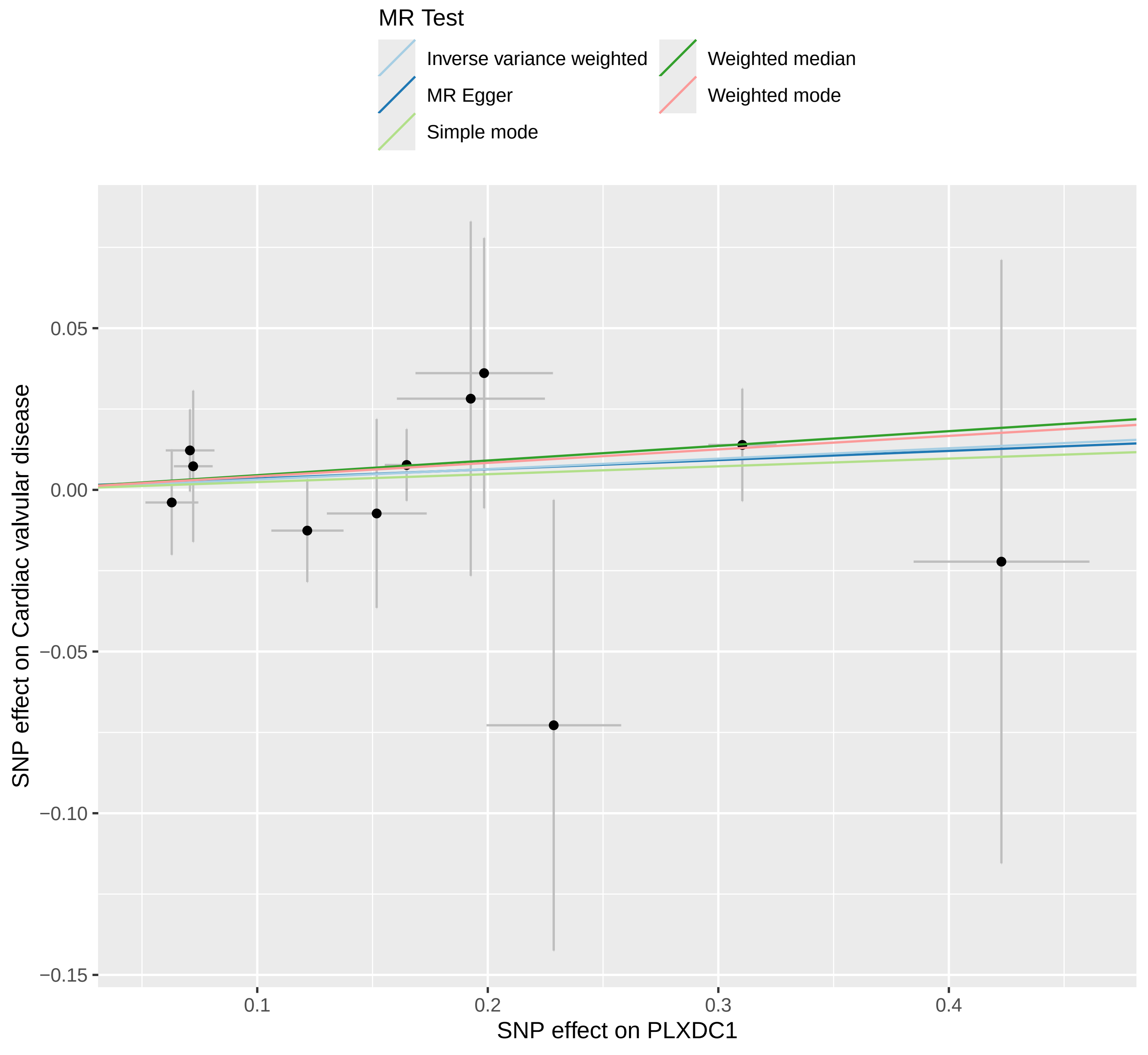
MR Test
Inverse variance weighted
MR Egger
Simple mode
Weighted median
Weighted mode
SNP effect on Cardiac valvular disease
0.05
0.00
−0.05
−0.10
−0.15
0.1
0.2
0.3
0.4
SNP effect on PLXDC1

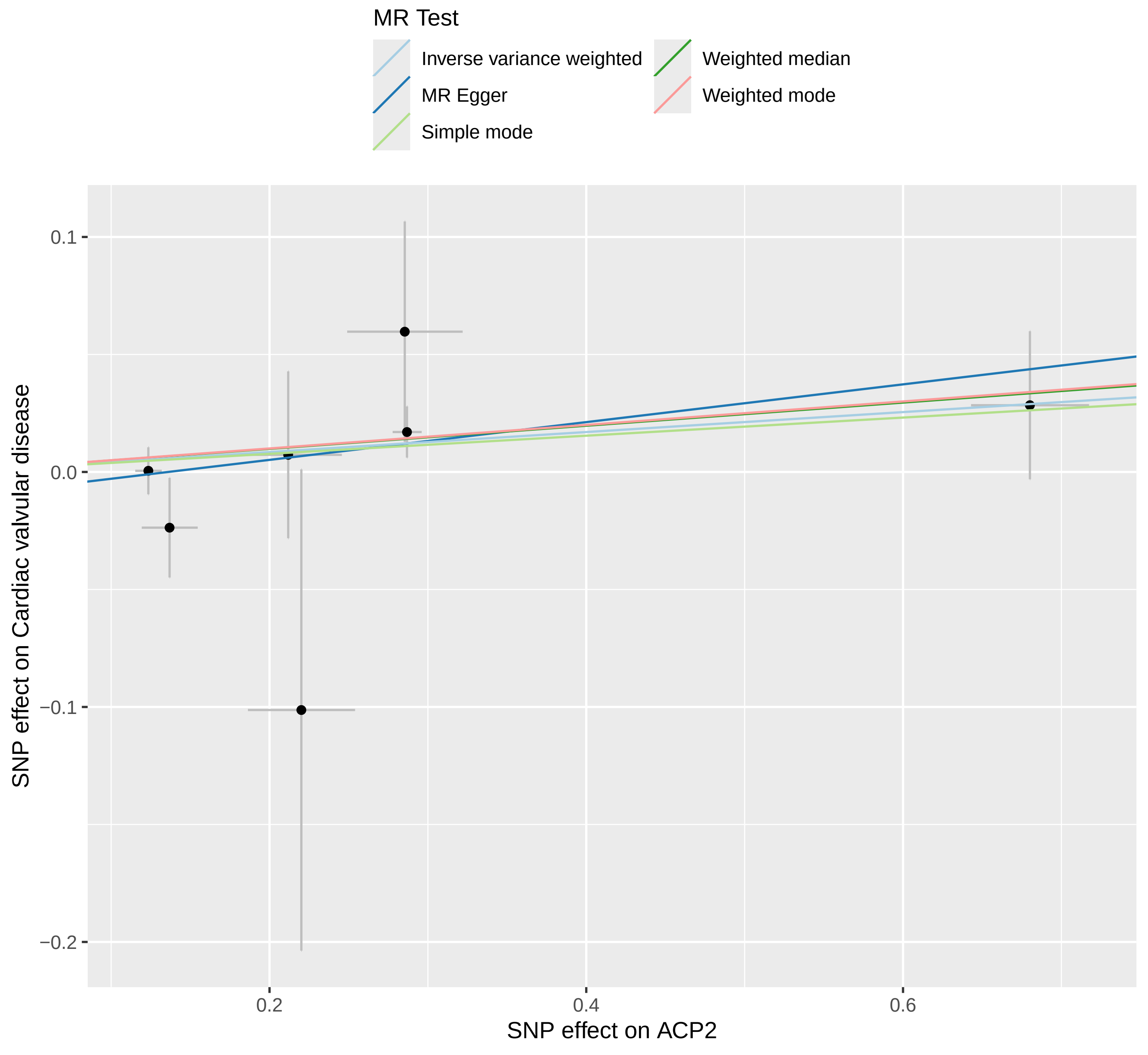
MR Test
Inverse variance weighted
MR Egger
Simple mode
Weighted median
Weighted mode
0.1
0.0
−0.1
−0.2
0.2
0.4
0.6
SNP effect on Cardiac valvular disease
SNP effect on ACP2

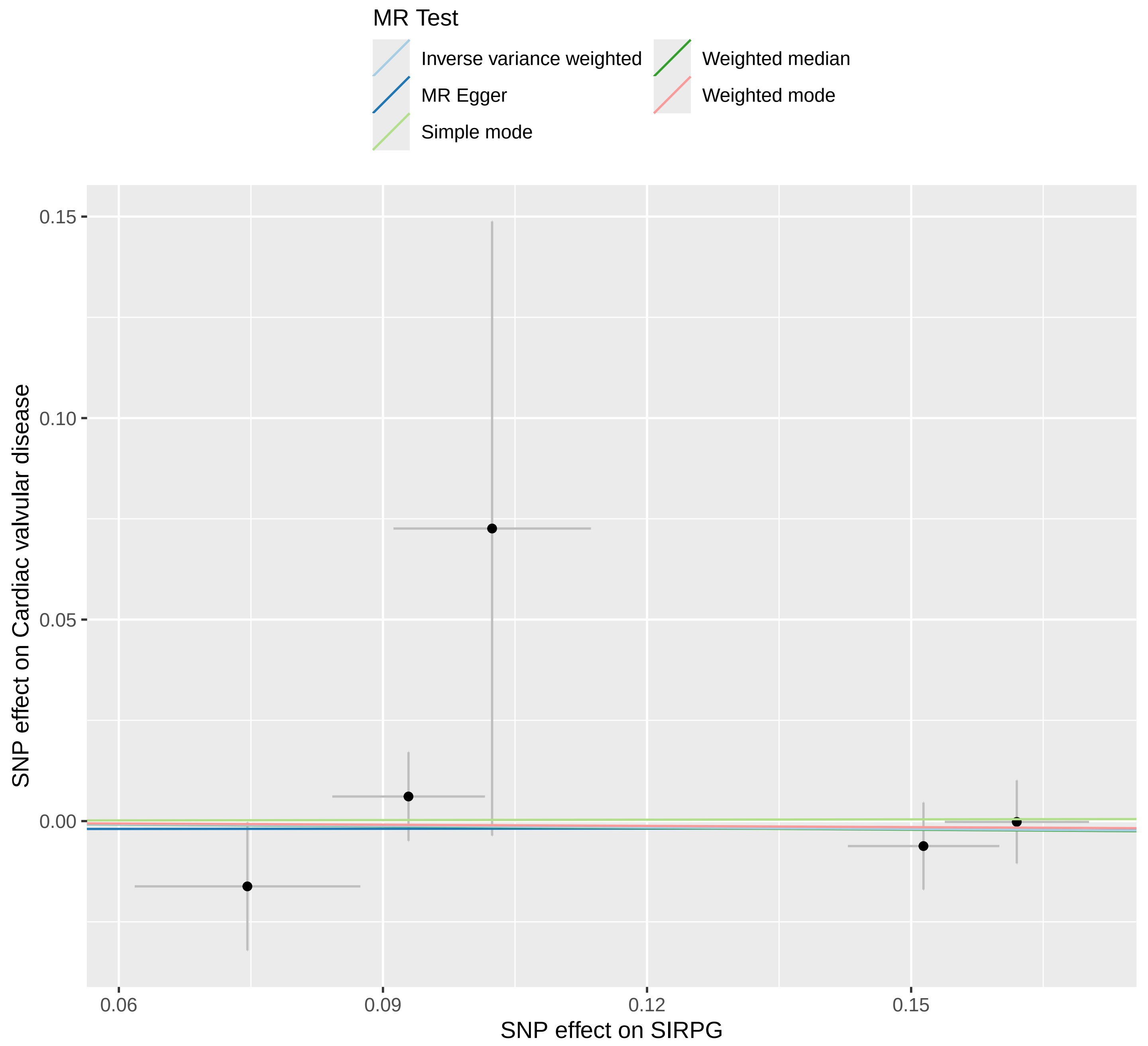
MR Test
Inverse variance weighted
MR Egger
Simple mode
Weighted median
Weighted mode
0.15
0.10
0.05
0.00
SNP effect on Cardiac valvular disease
0.06
0.09
0.12
0.15
SNP effect on SIRPG

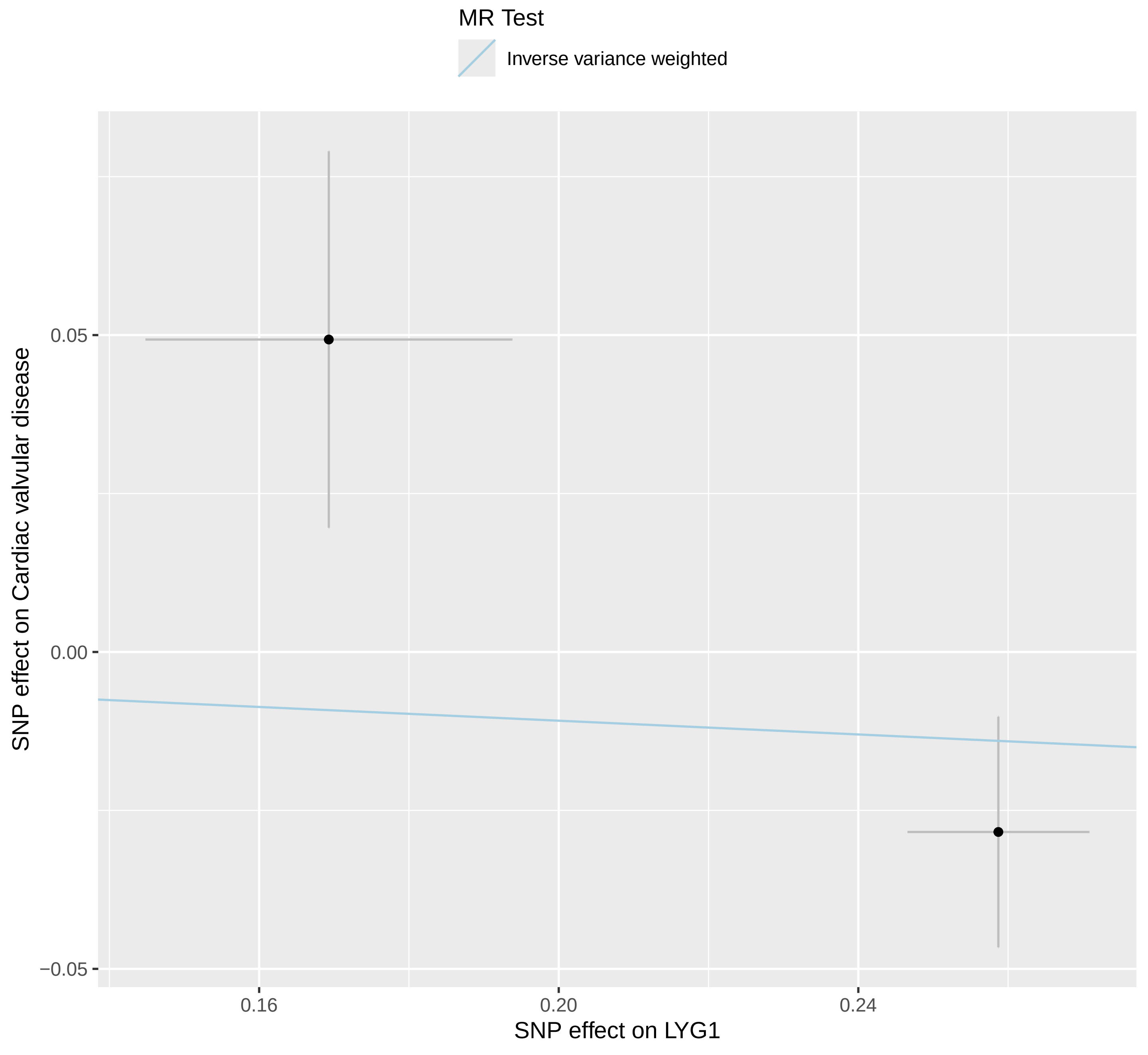

MR Test
Inverse variance weighted
0.05
0.00
−0.05
0.16
0.20
0.24
SNP effect on Cardiac valvular disease
SNP effect on LYG1

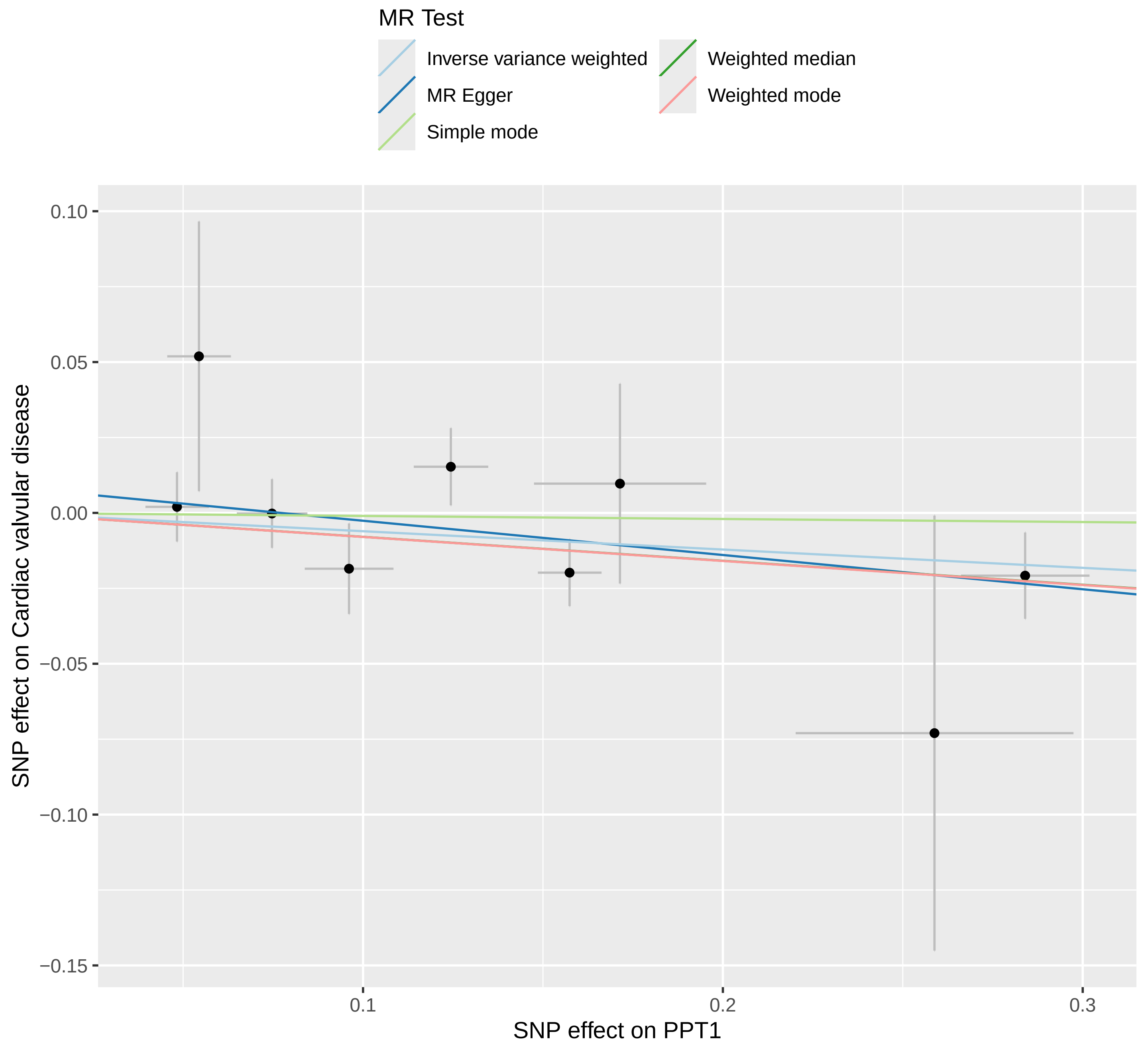
MR Test
Inverse variance weighted
MR Egger
Simple mode
Weighted median
Weighted mode
SNP effect on Cardiac valvular disease
0.10
0.05
0.00
−0.05
−0.10
−0.15
0.1
0.2
0.3
SNP effect on PPT1

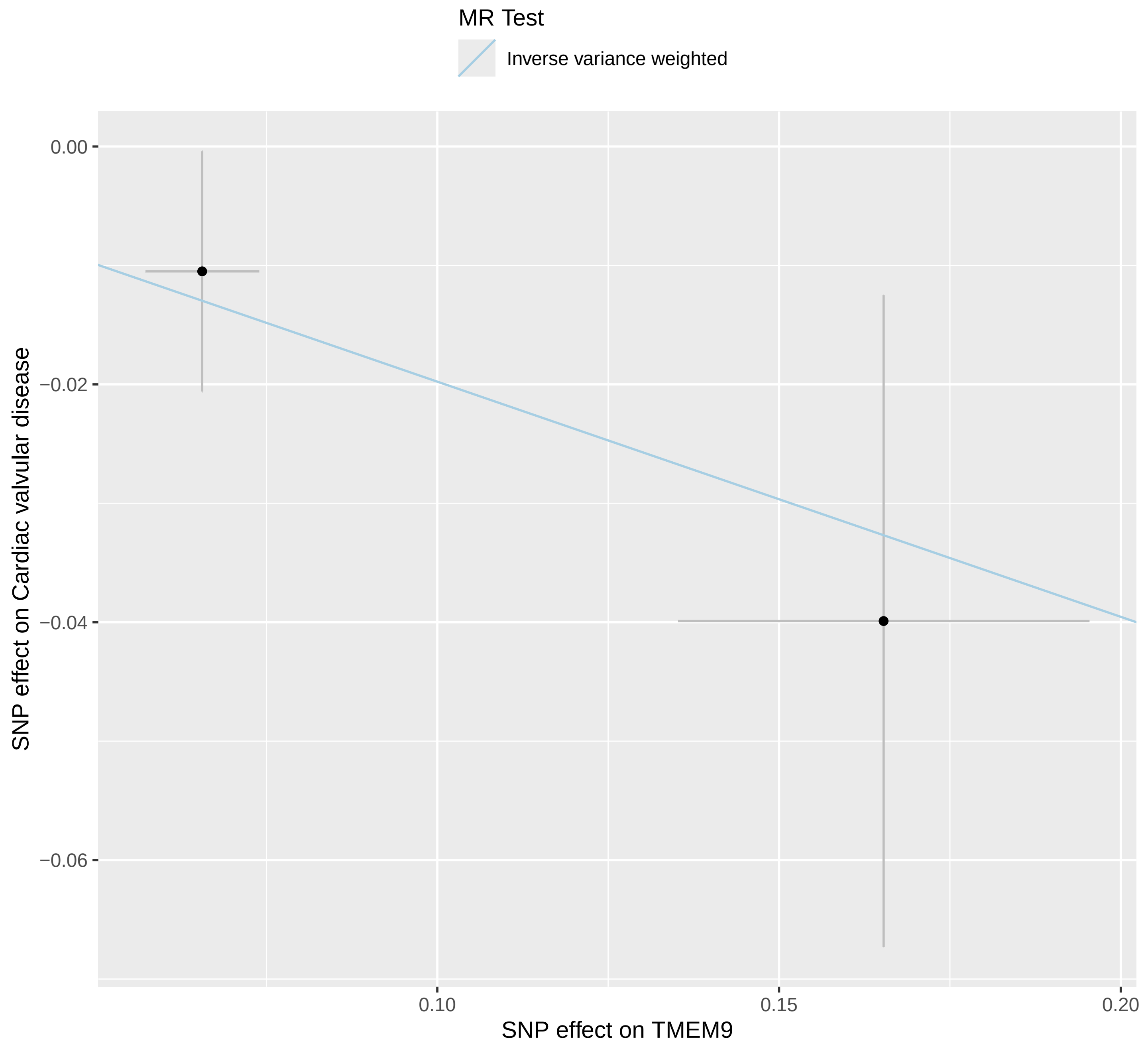

MR Test
Inverse variance weighted
0.00
−0.02
−0.04
−0.06
0.10
0.15
0.20
SNP effect on TMEM9
SNP effect on Cardiac valvular disease

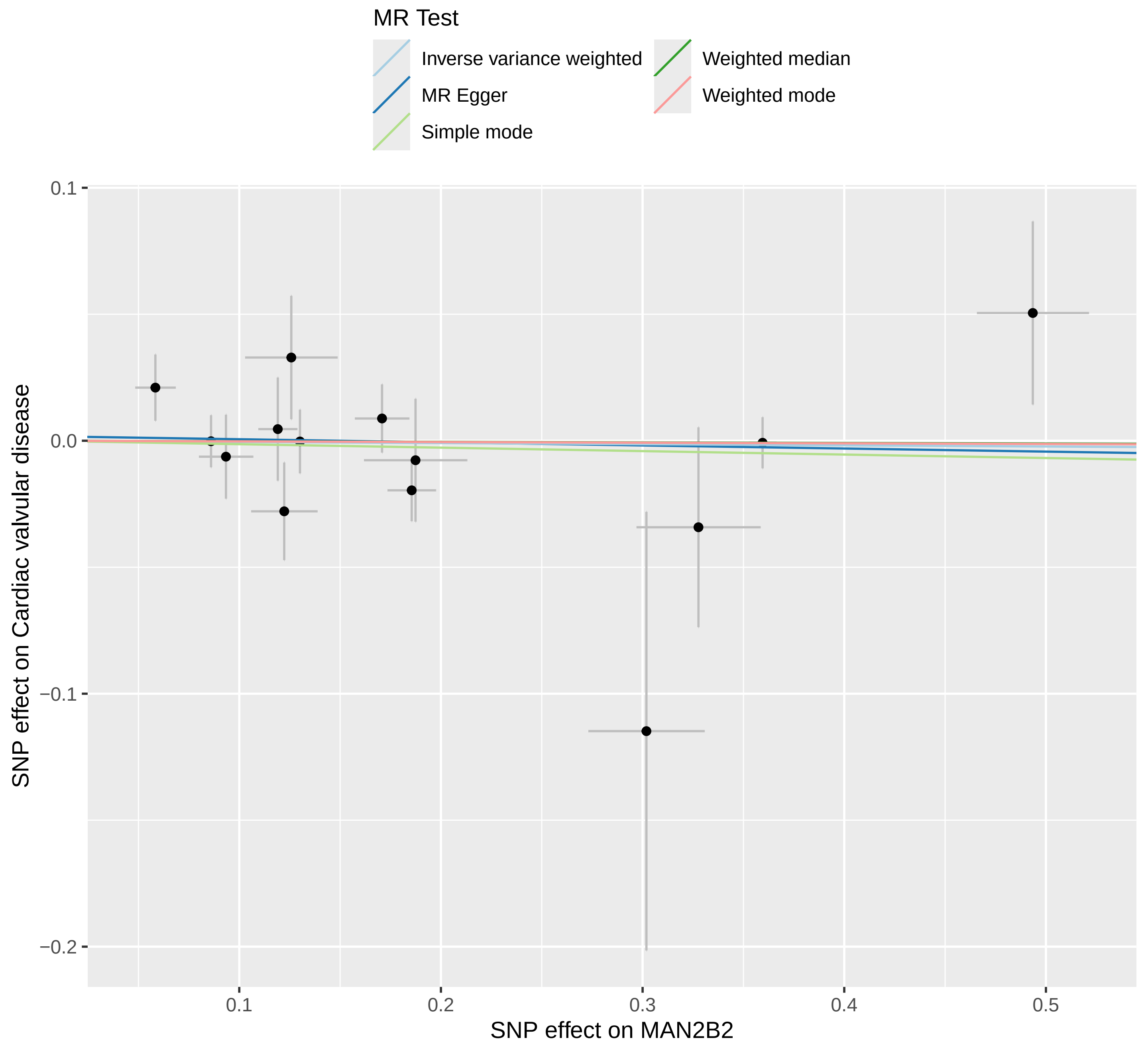

MR Test
Inverse variance weighted
MR Egger
Simple mode
Weighted median
Weighted mode
0.1
0.0
−0.1
−0.2
0.1
0.2
0.3
0.4
0.5
SNP effect on Cardiac valvular disease
SNP effect on MAN2B2

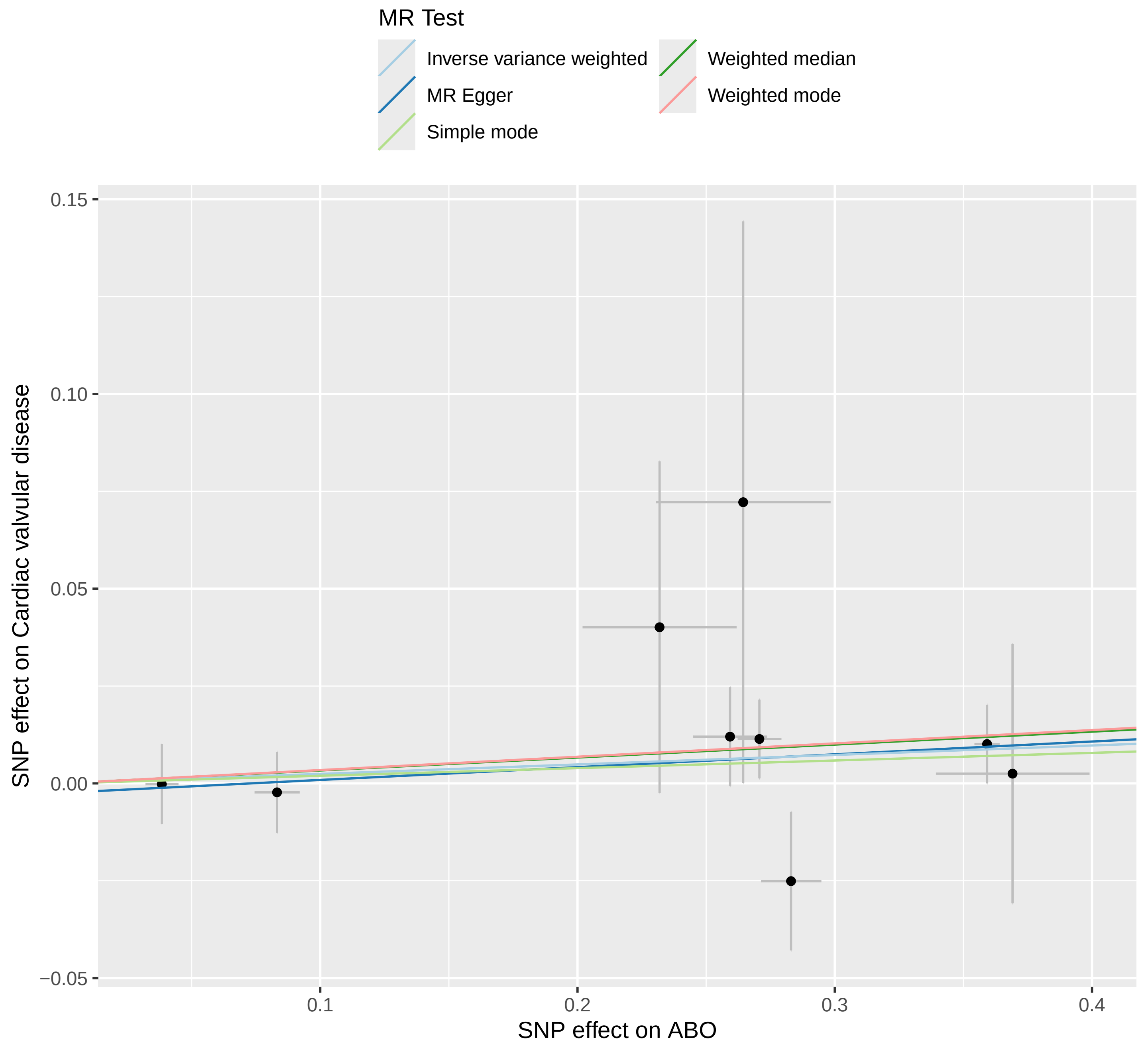
MR Test
Inverse variance weighted
MR Egger
Simple mode
Weighted median
Weighted mode
SNP effect on Cardiac valvular disease
0.15
0.10
0.05
0.00
−0.05
0.1
0.2
0.3
0.4
SNP effect on ABO

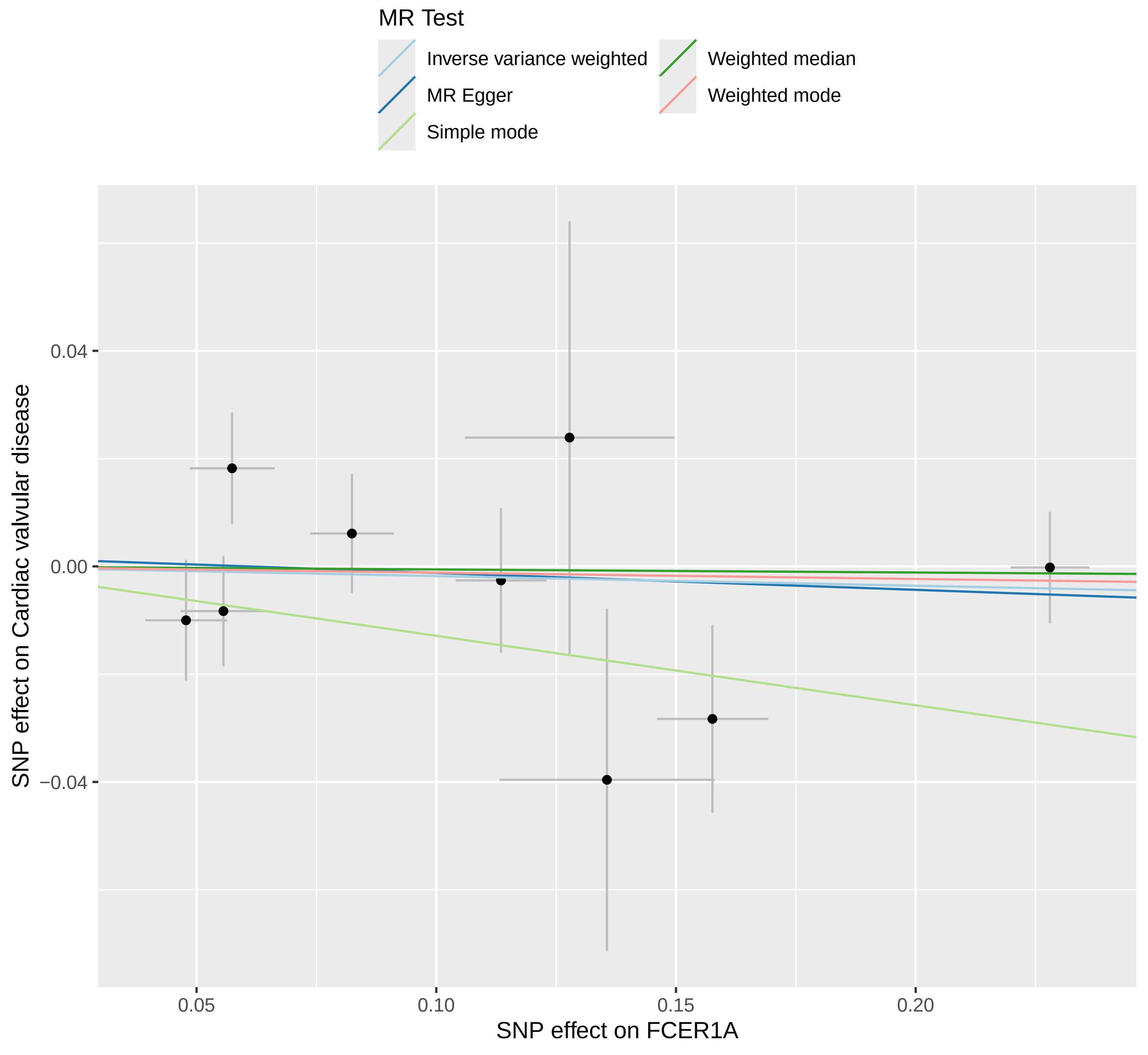

MR Test
Inverse variance weighted
MR Egger
Simple mode
Weighted median
Weighted mode
0.04
0.00
−0.04
SNP effect on Cardiac valvular disease
0.05
0.10
0.15
0.20
SNP effect on FCER1A

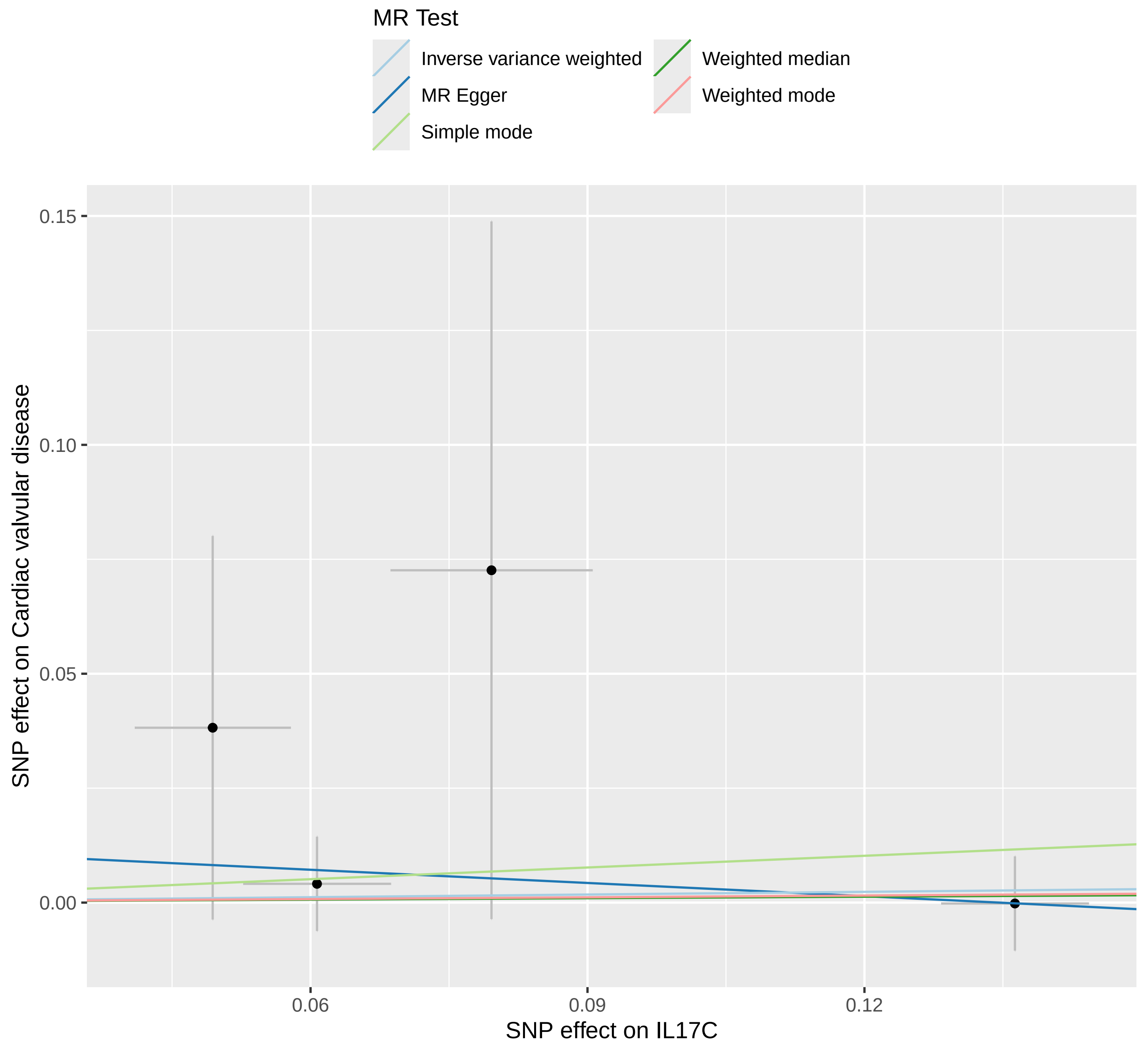
MR Test
Inverse variance weighted
MR Egger
Simple mode
Weighted median
Weighted mode
0.15
0.10
0.05
0.00
0.06
0.09
0.12
SNP effect on Cardiac valvular disease
SNP effect on IL17C

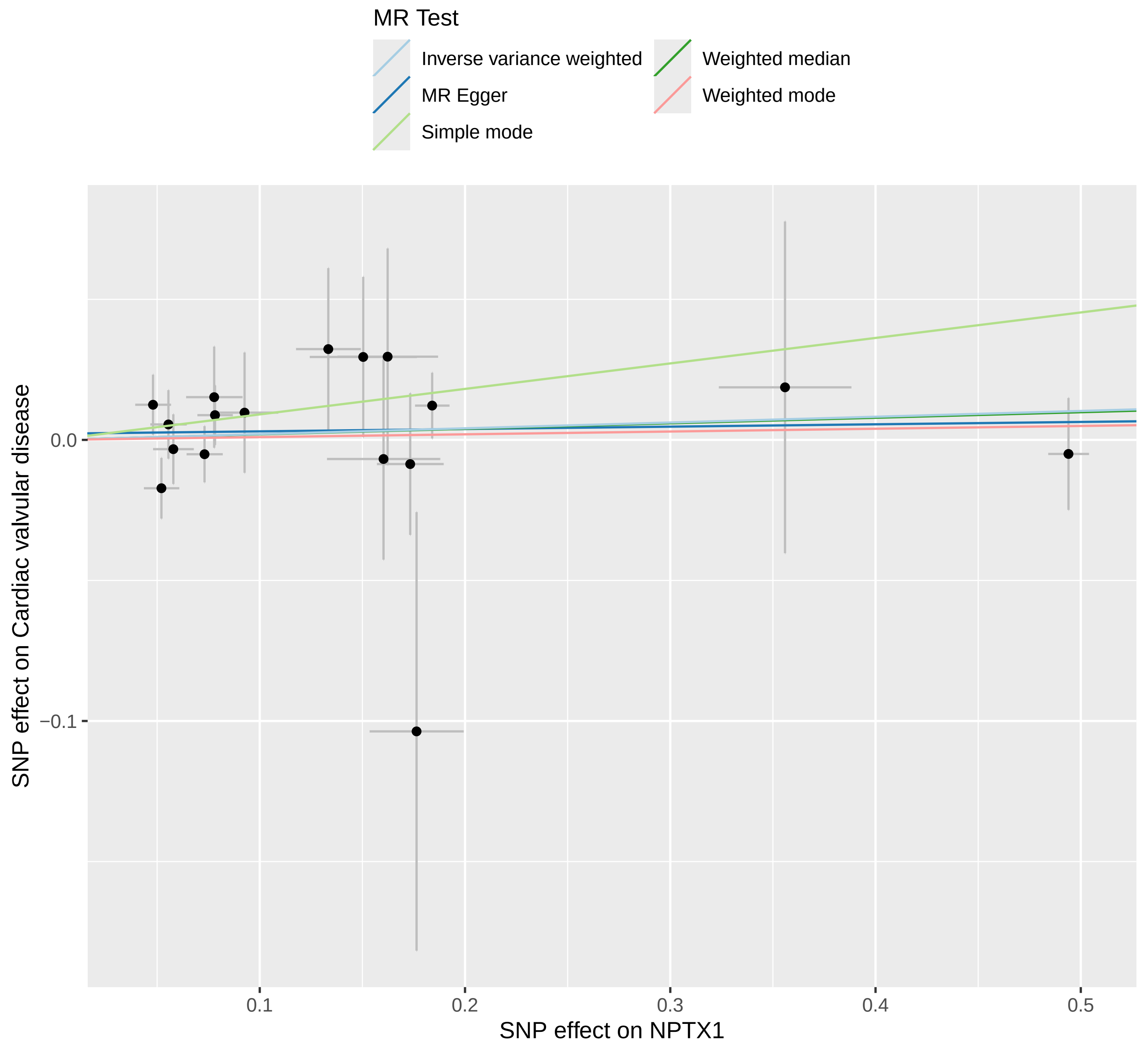
MR Test
Inverse variance weighted
MR Egger
Simple mode
Weighted median
Weighted mode
SNP effect on Cardiac valvular disease
0.0
−0.1
0.1
0.2
0.3
0.4
0.5
SNP effect on NPTX1

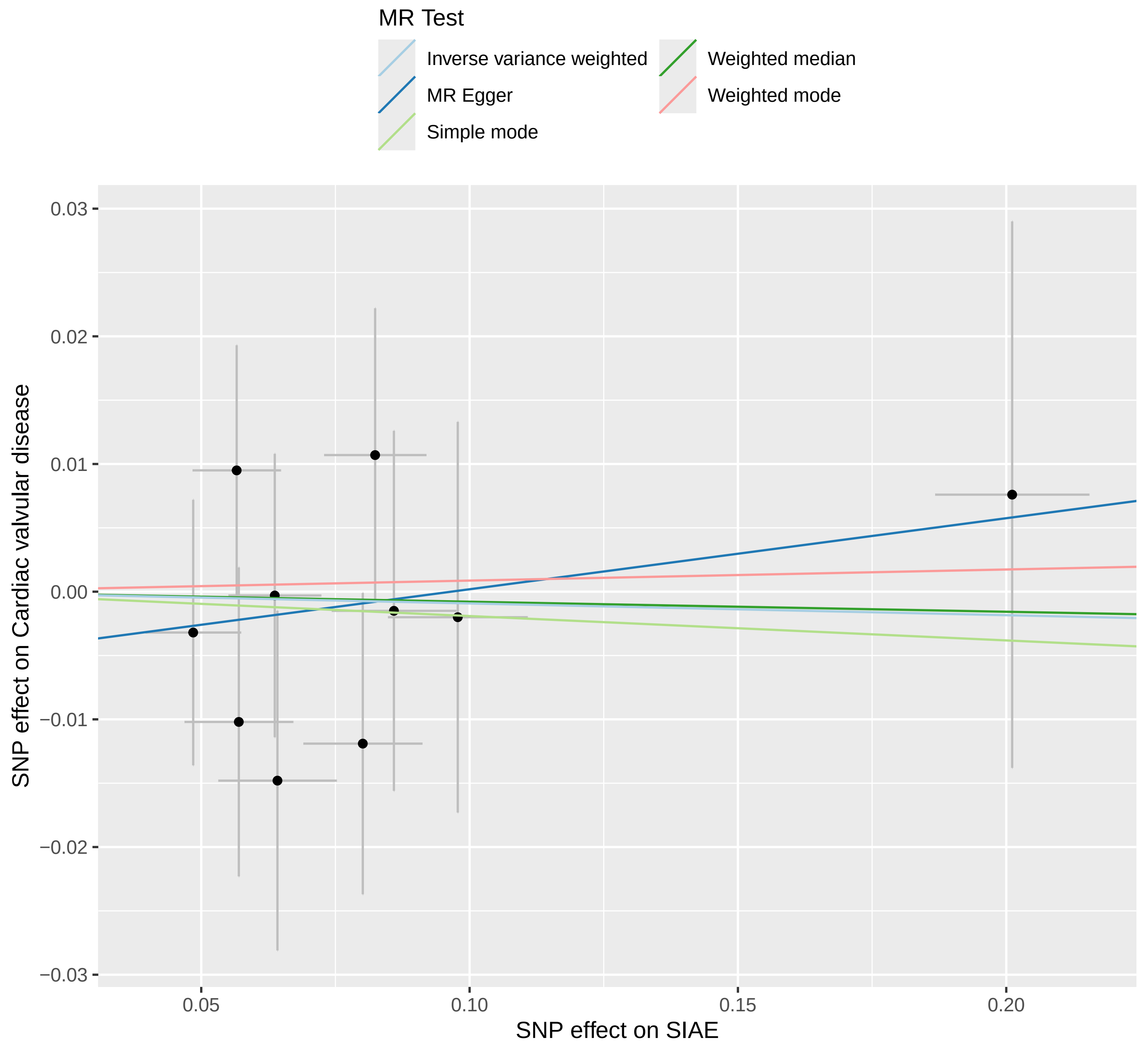

MR Test
Inverse variance weighted
MR Egger
Simple mode
Weighted median
Weighted mode
0.03
0.02
0.01
0.00
−0.01
−0.02
−0.03
SNP effect on Cardiac valvular disease
0.05
0.10
0.15
0.20
SNP effect on SIAE

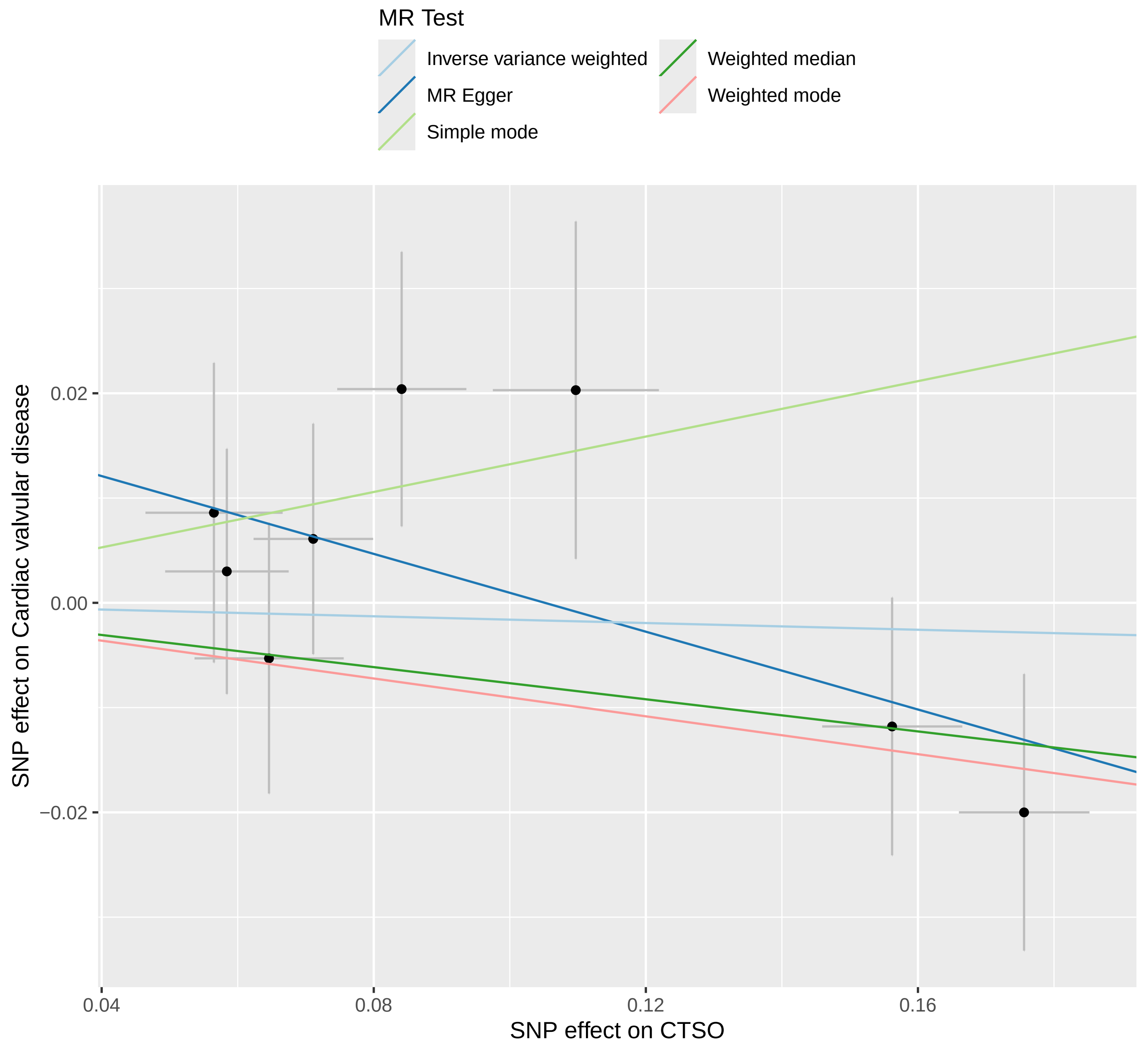
MR Test
Inverse variance weighted
MR Egger
Simple mode
Weighted median
Weighted mode
SNP effect on Cardiac valvular disease
0.02
0.00
−0.02
0.04
0.08
0.12
0.16
SNP effect on CTSO

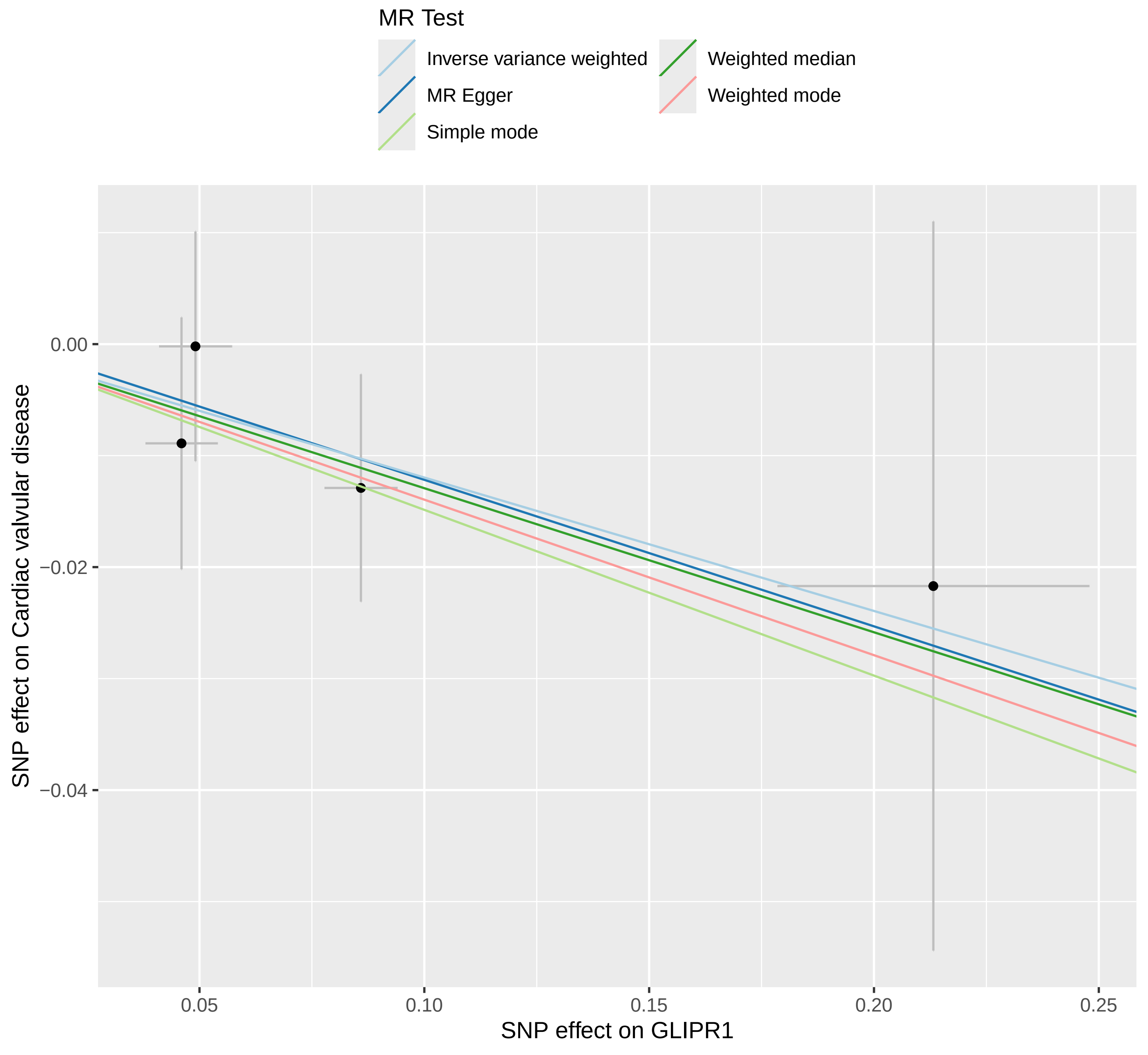
MR Test
Inverse variance weighted
MR Egger
Simple mode
Weighted median
Weighted mode
0.00
−0.02
−0.04
0.05
0.10
0.15
0.20
0.25
SNP effect on GLIPR1
SNP effect on Cardiac valvular disease

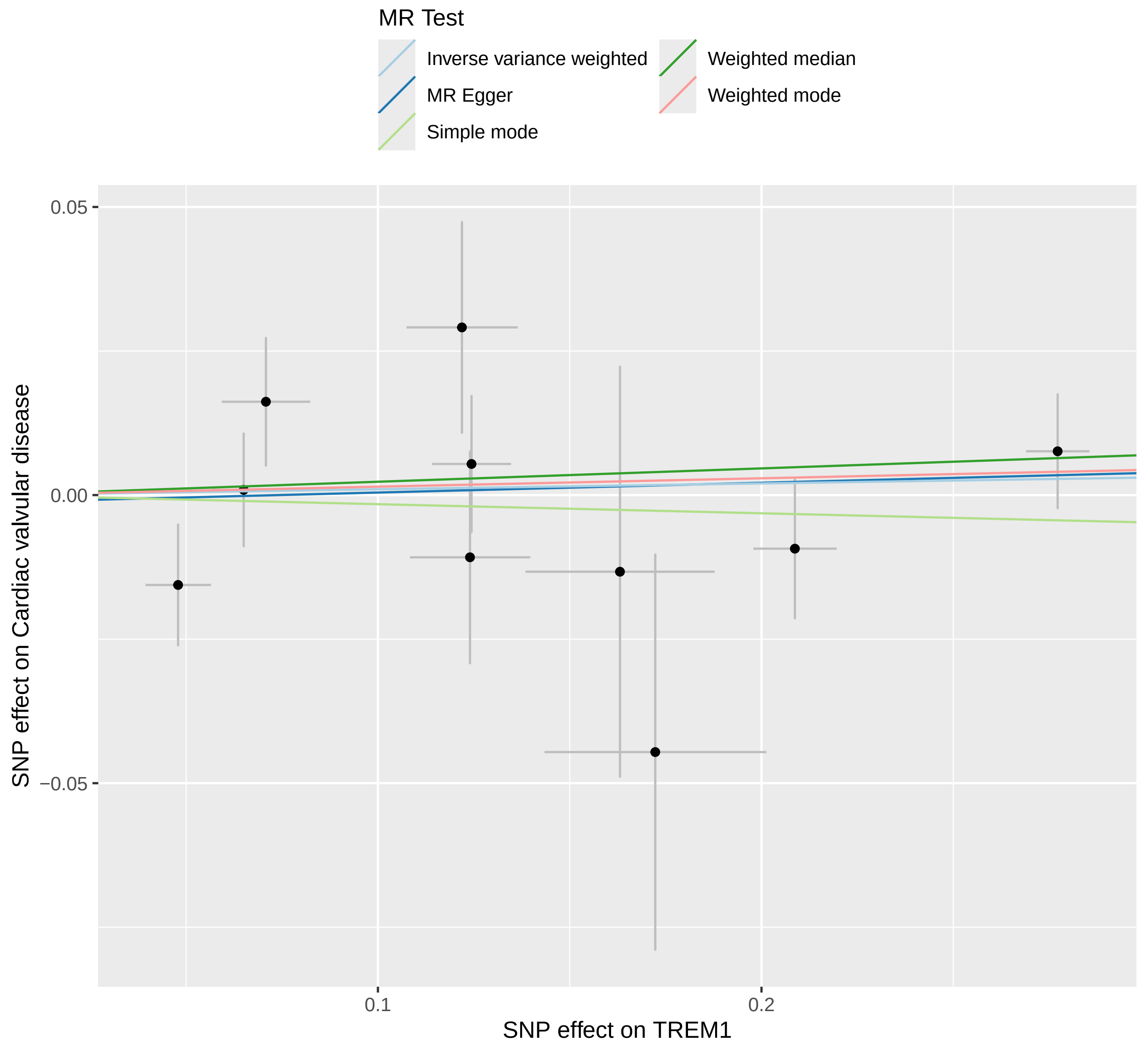
MR Test
Inverse variance weighted
MR Egger
Simple mode
Weighted median
Weighted mode
0.05
0.00
−0.05
0.1
0.2
SNP effect on Cardiac valvular disease
SNP effect on TREM1

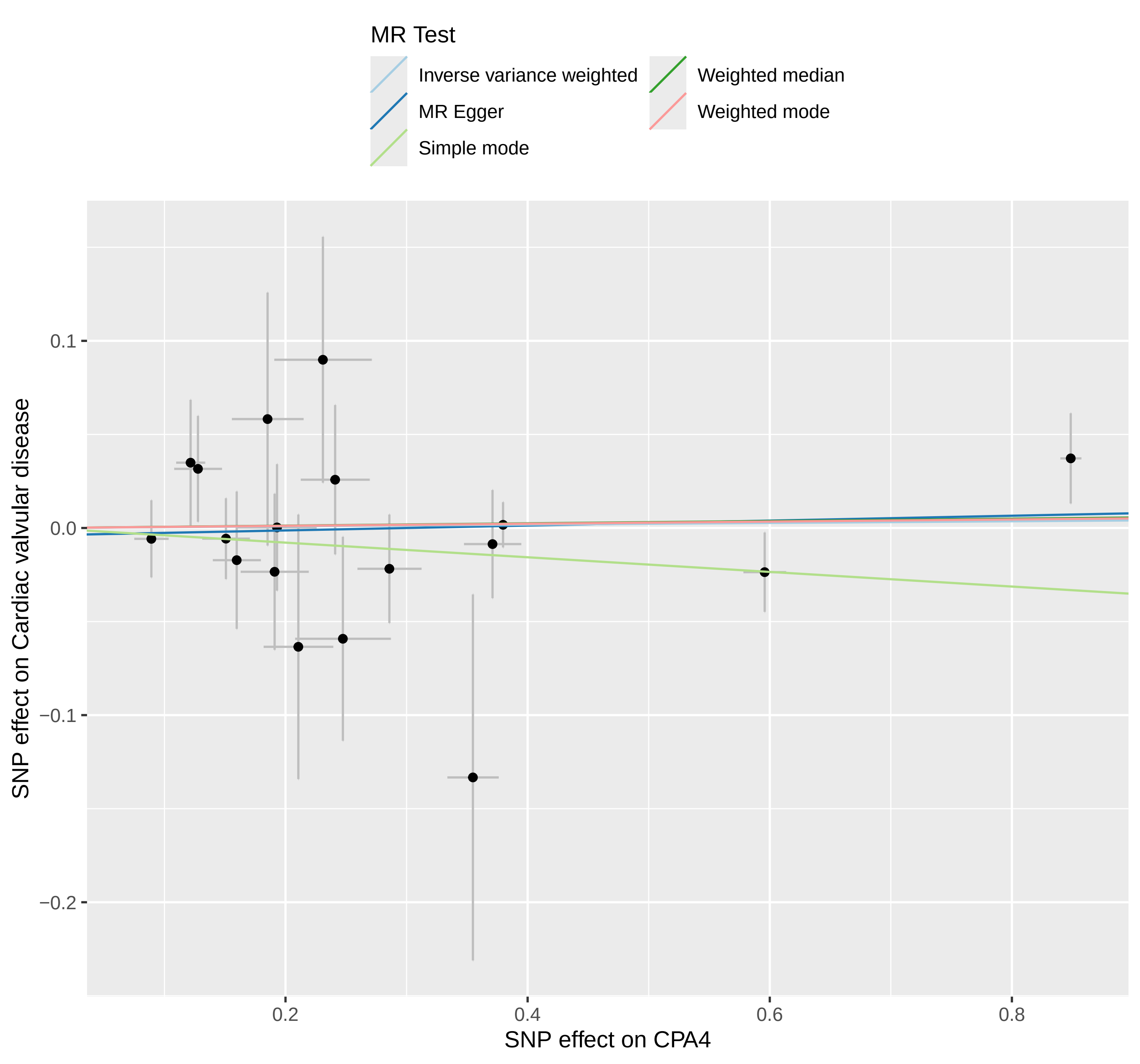
MR Test
Inverse variance weighted
MR Egger
Simple mode
Weighted median
Weighted mode
0.1
0.0
−0.1
−0.2
SNP effect on Cardiac valvular disease
0.2
0.4
0.6
0.8
SNP effect on CPA4

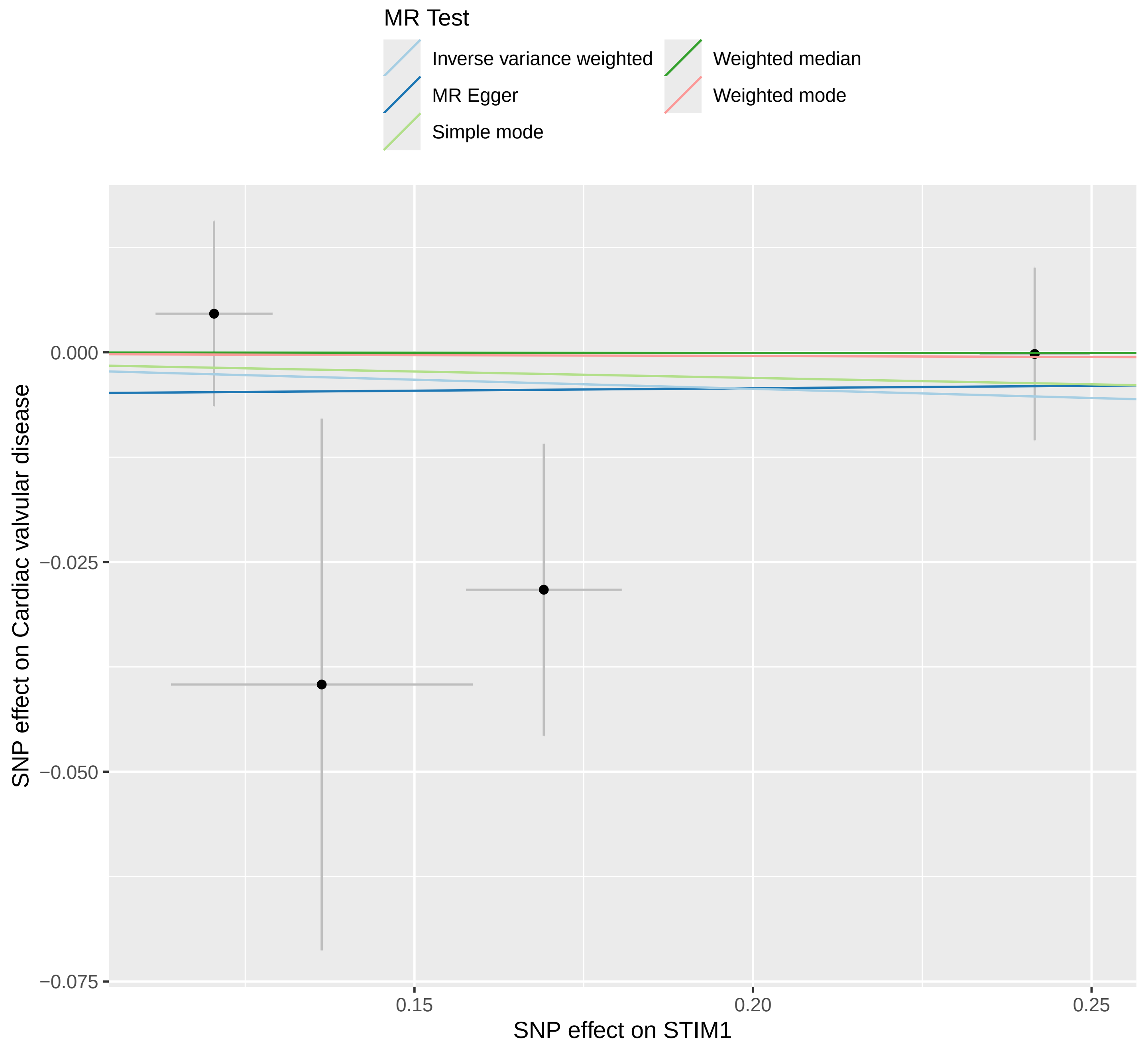

MR Test
Inverse variance weighted
MR Egger
Simple mode
Weighted median
Weighted mode
0.000
−0.025
−0.050
−0.075
0.15
0.20
0.25
SNP effect on Cardiac valvular disease
SNP effect on STIM1

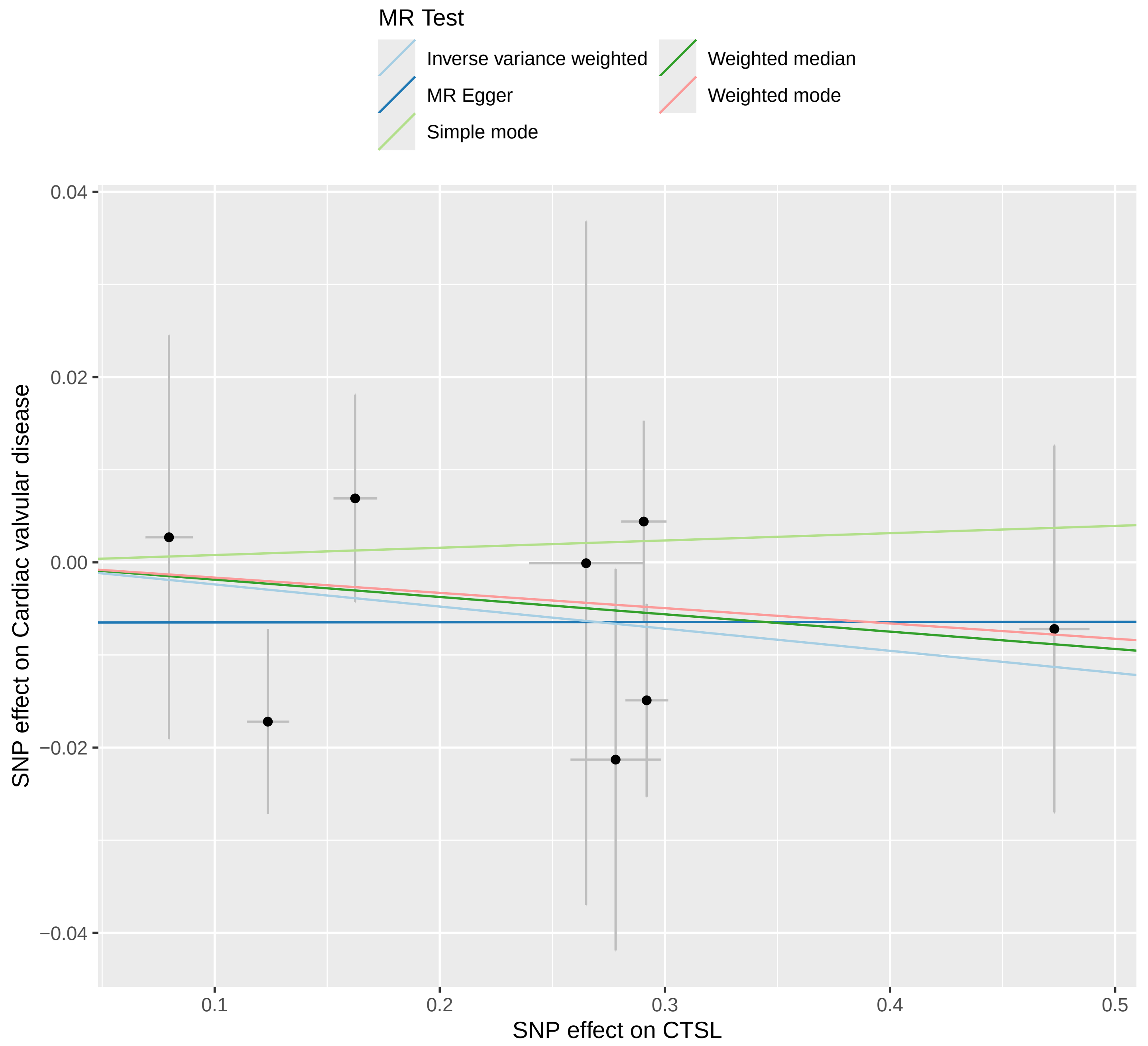
MR Test
Inverse variance weighted
MR Egger
Simple mode
Weighted median
Weighted mode
0.04
0.02
0.00
−0.02
−0.04
SNP effect on Cardiac valvular disease
0.1
0.2
0.3
0.4
0.5
SNP effect on CTSL

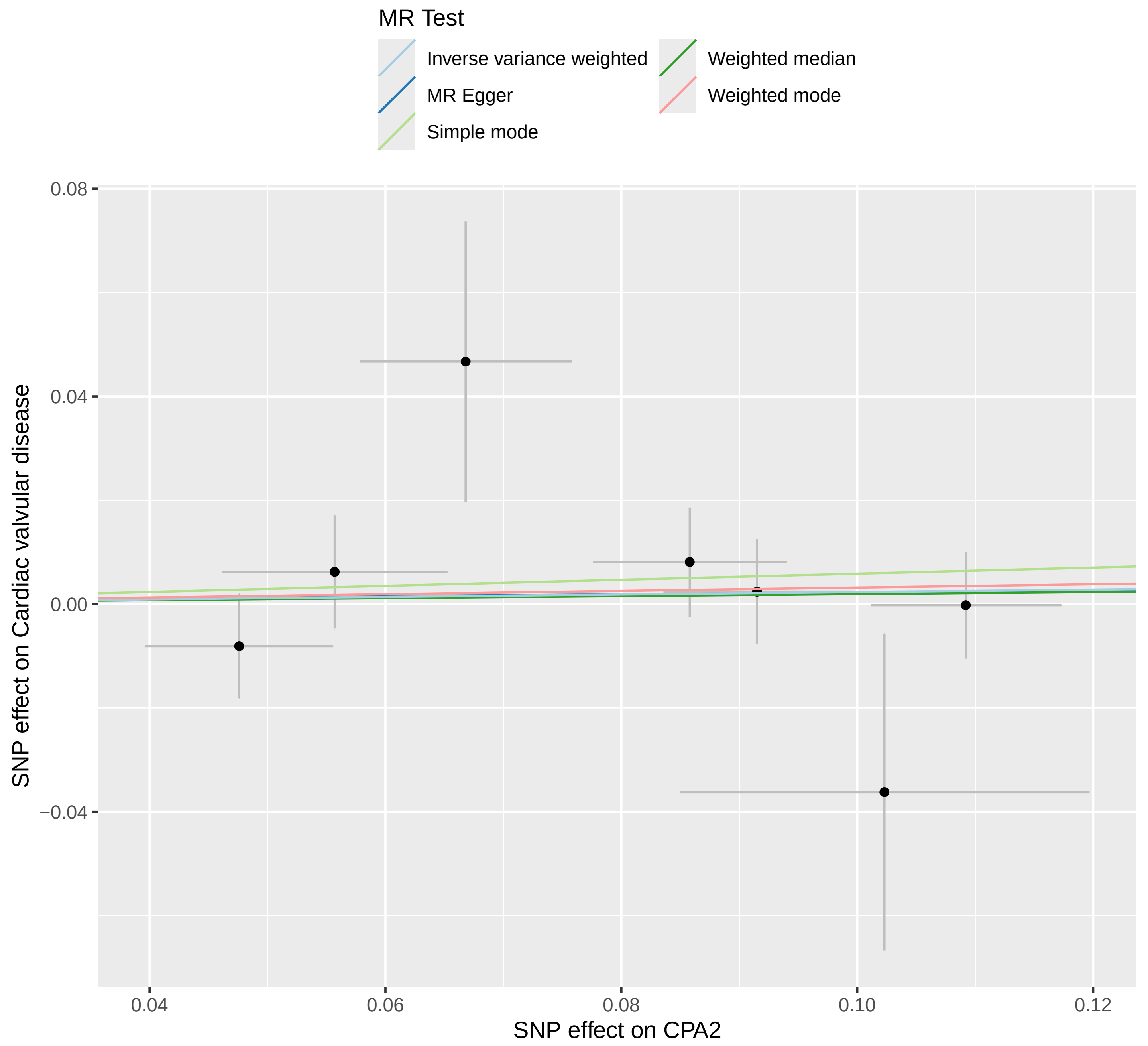
MR Test
Inverse variance weighted
MR Egger
Simple mode
Weighted median
Weighted mode
0.08
0.04
0.00
−0.04
0.04
0.06
0.08
0.10
0.12
SNP effect on Cardiac valvular disease
SNP effect on CPA2

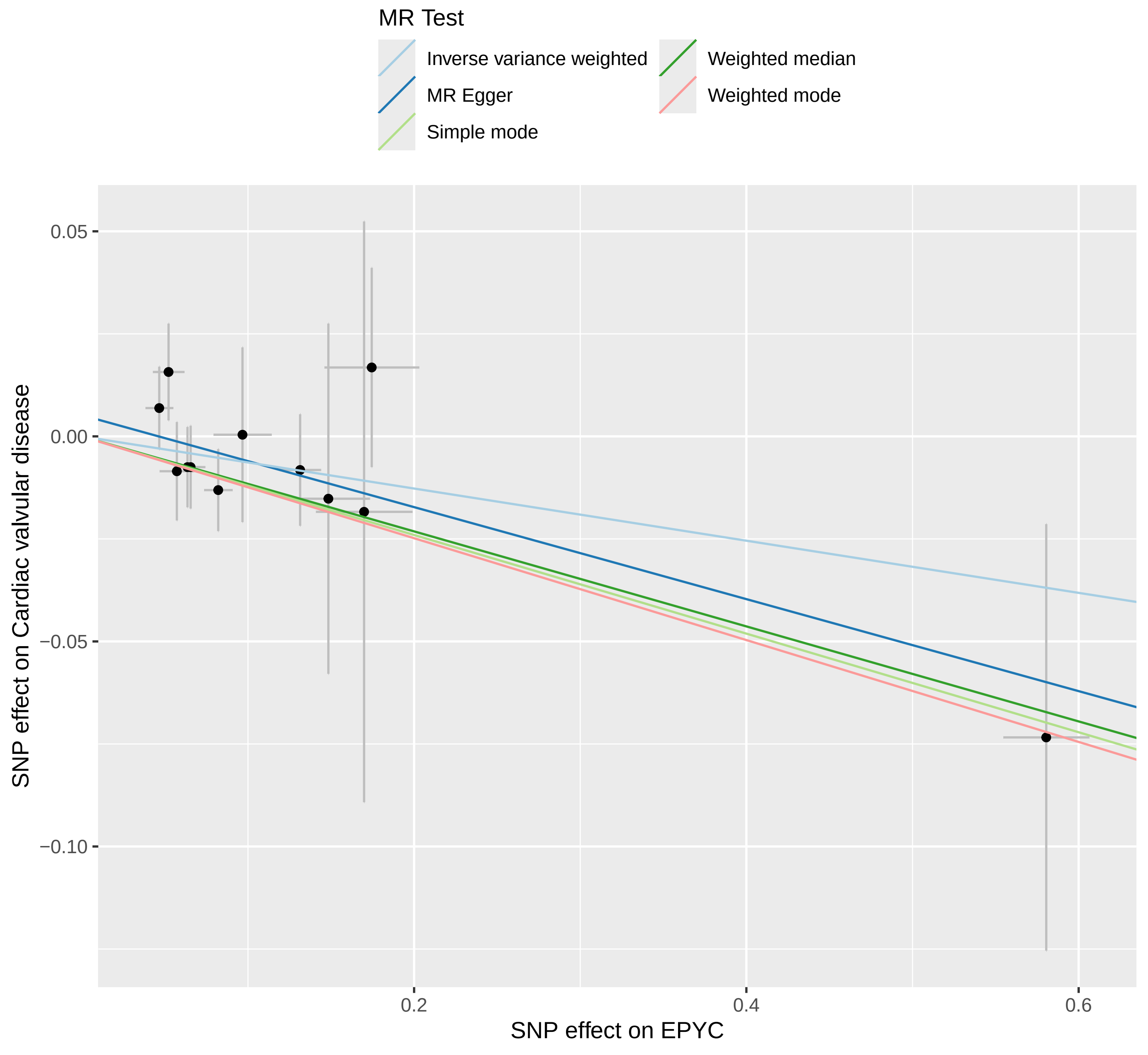
MR Test
Inverse variance weighted
MR Egger
Simple mode
Weighted median
Weighted mode
0.05
0.00
−0.05
−0.10
SNP effect on Cardiac valvular disease
0.2
0.4
0.6
SNP effect on EPYC

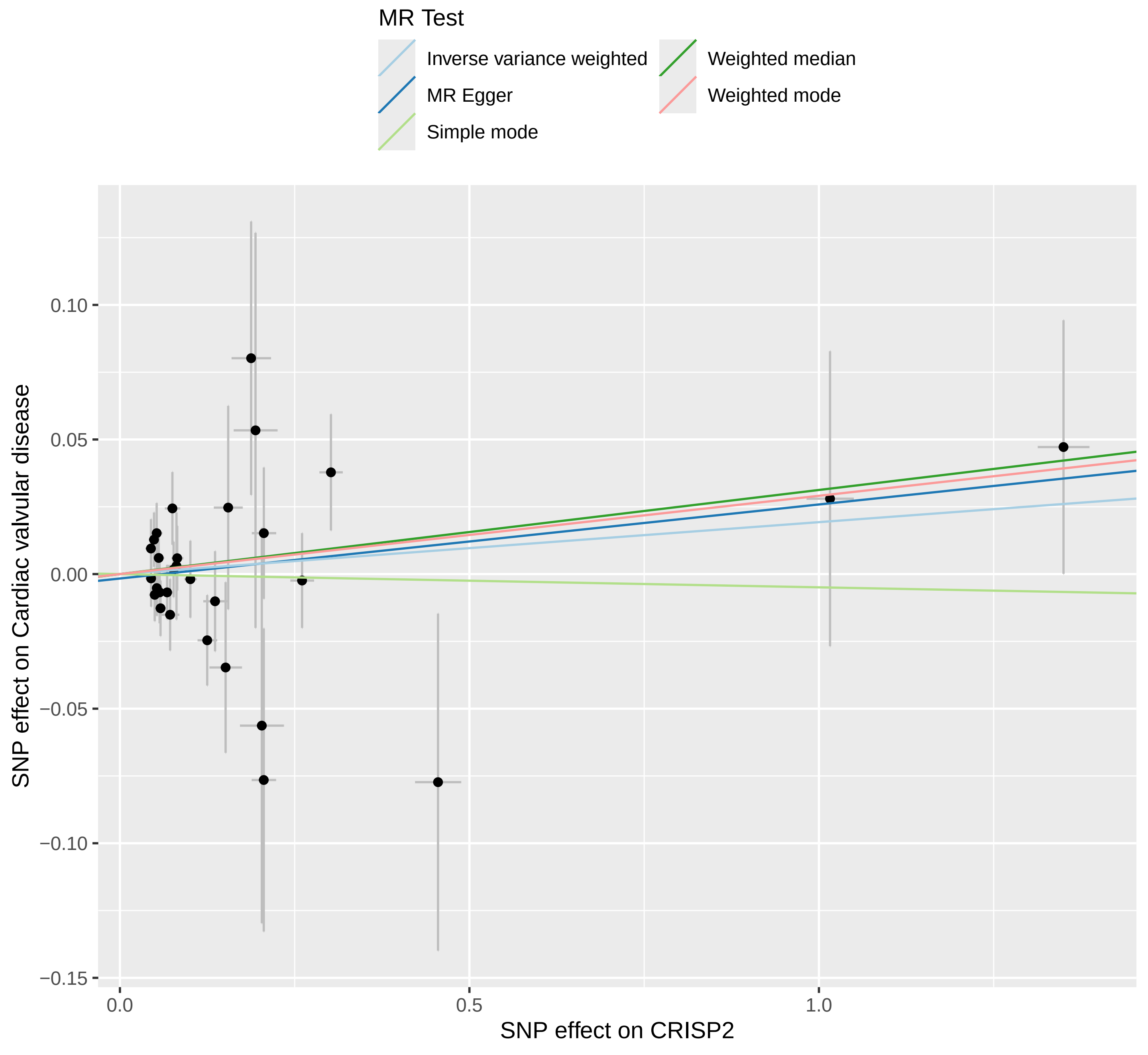
MR Test
Inverse variance weighted
MR Egger
Simple mode
Weighted median
Weighted mode
SNP effect on Cardiac valvular disease
0.10
0.05
0.00
−0.05
−0.10
−0.15
0.0
0.5
1.0
SNP effect on CRISP2

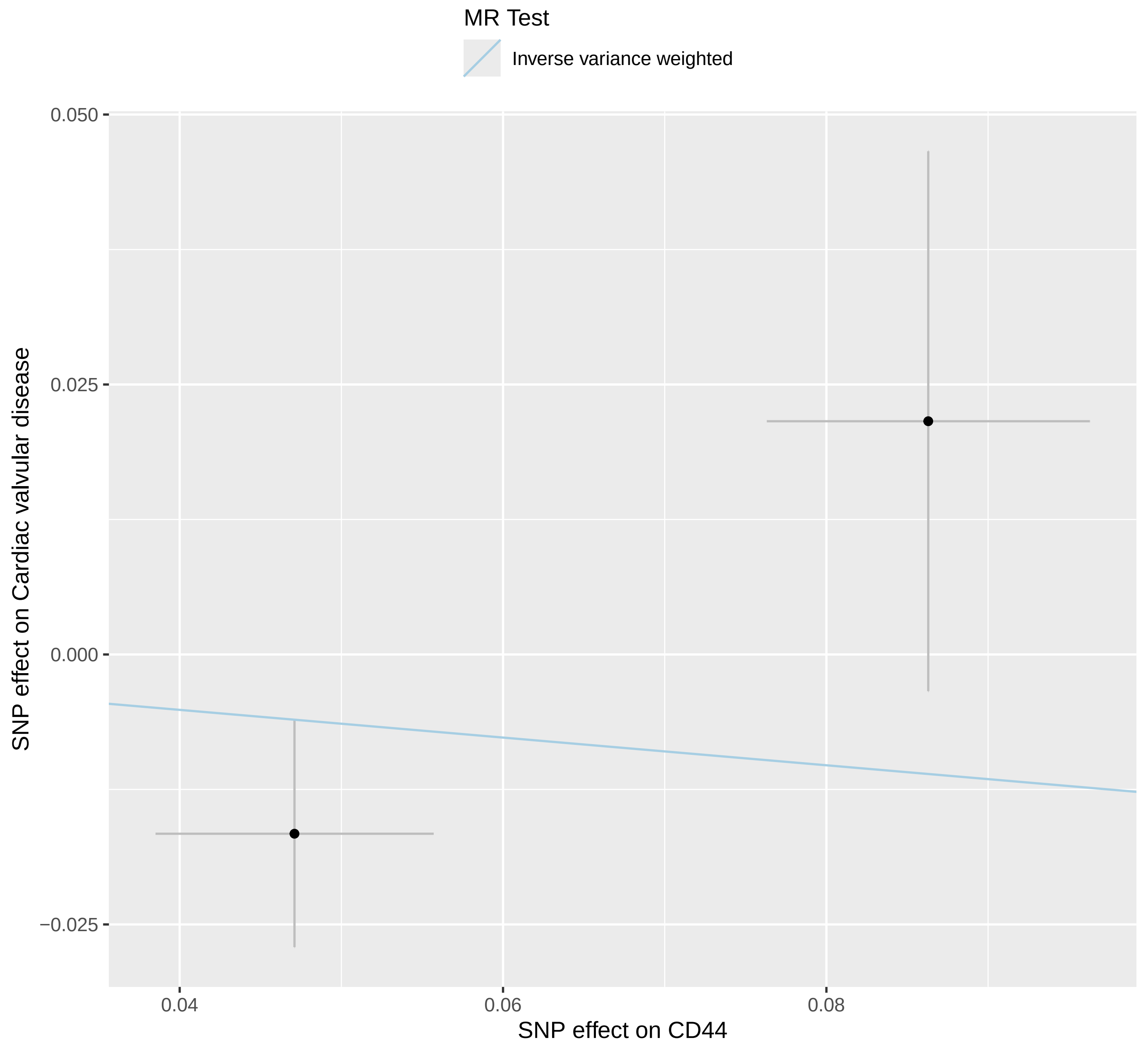
MR Test
Inverse variance weighted
0.050
0.025
0.000
−0.025
SNP effect on Cardiac valvular disease
0.04
0.06
0.08
SNP effect on CD44

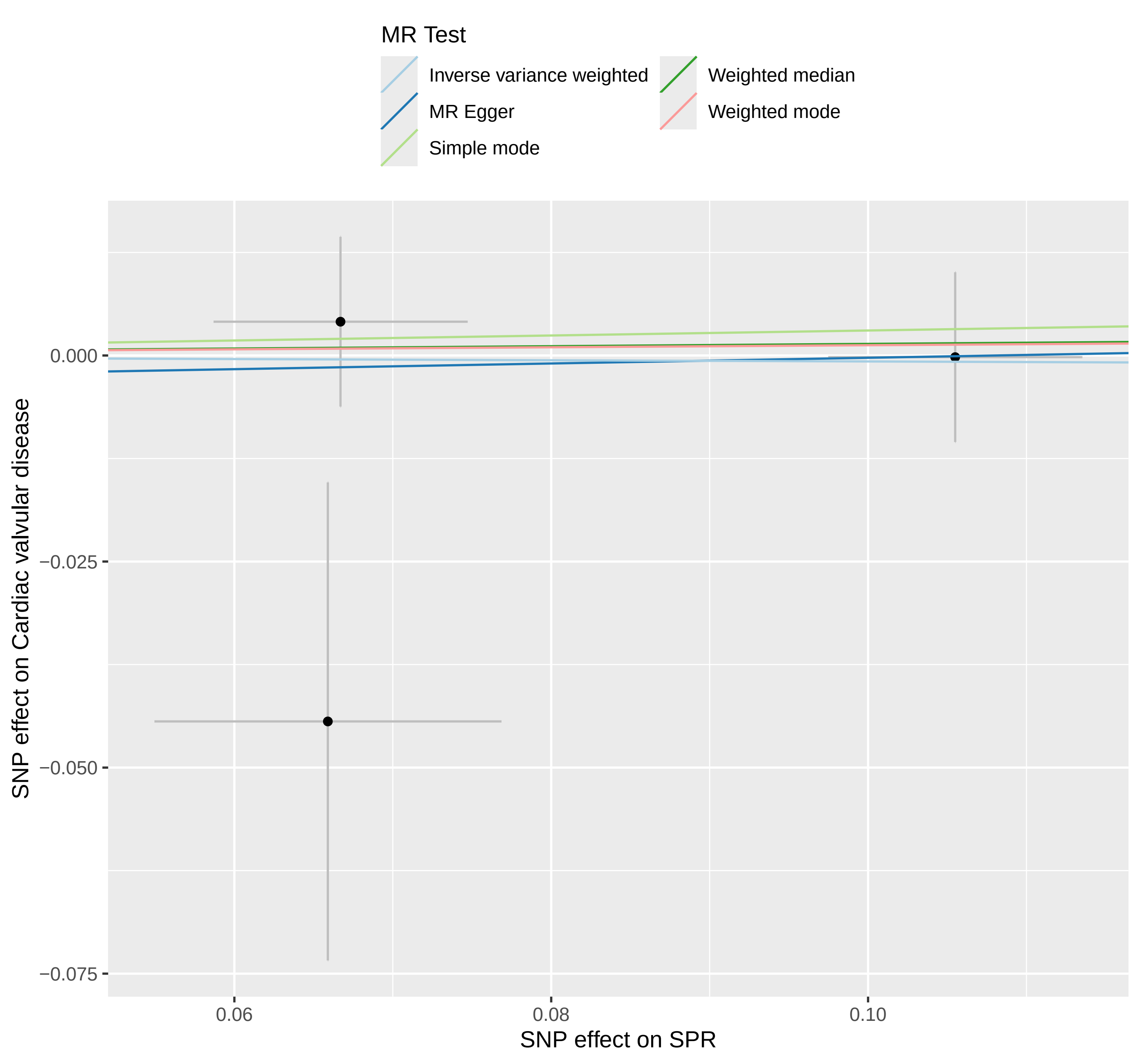

MR Test
Inverse variance weighted
MR Egger
Simple mode
Weighted median
Weighted mode
SNP effect on Cardiac valvular disease
0.000
−0.025
−0.050
−0.075
0.06
0.08
0.10
SNP effect on SPR

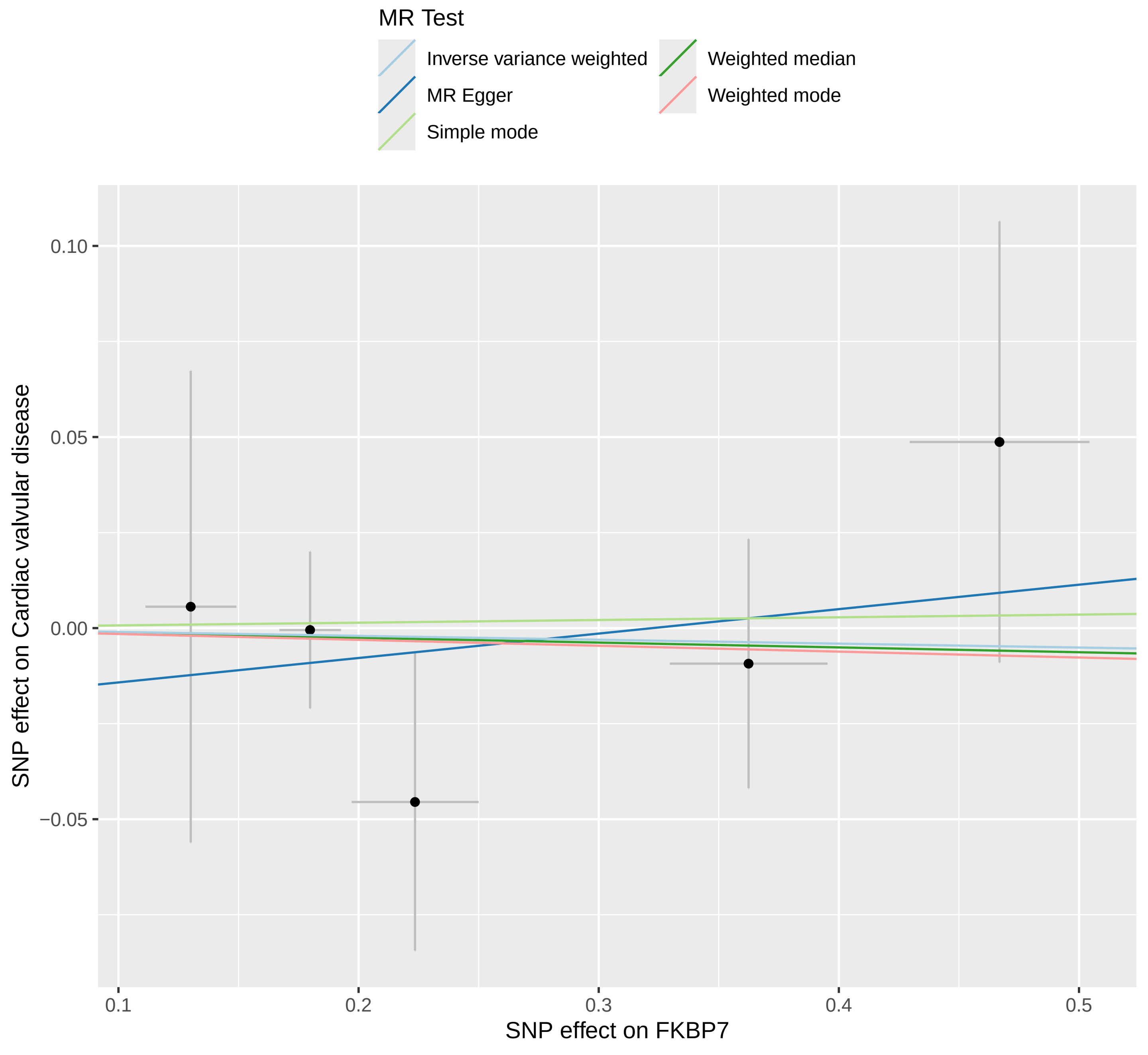

MR Test
Inverse variance weighted
MR Egger
Simple mode
Weighted median
Weighted mode
0.10
0.05
0.00
−0.05
SNP effect on Cardiac valvular disease
0.1
0.2
0.3
0.4
0.5
SNP effect on FKBP7

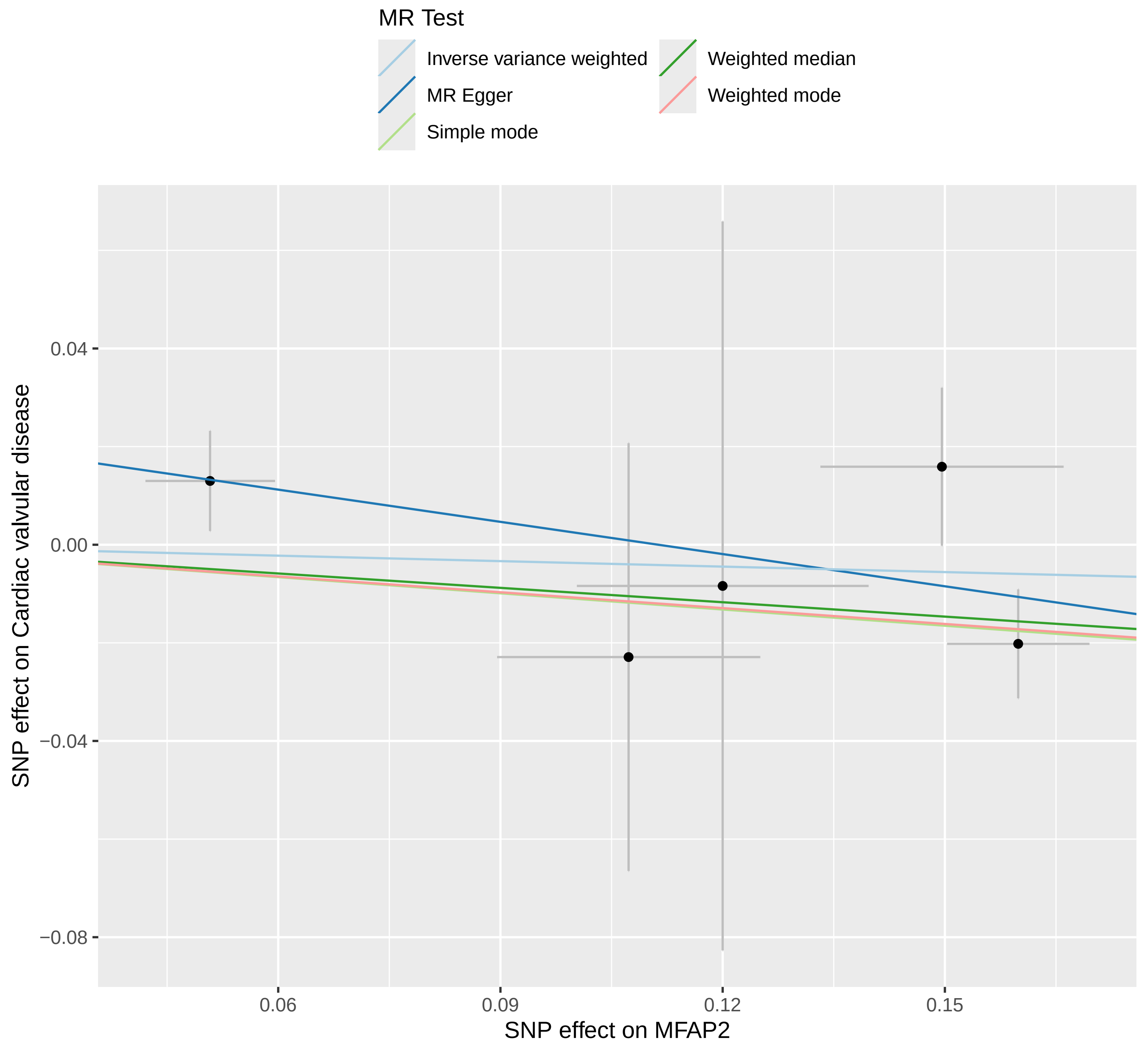

MR Test
Inverse variance weighted
MR Egger
Simple mode
Weighted median
Weighted mode
0.04
0.00
−0.04
−0.08
0.06
0.09
0.12
0.15
SNP effect on MFAP2
SNP effect on Cardiac valvular disease

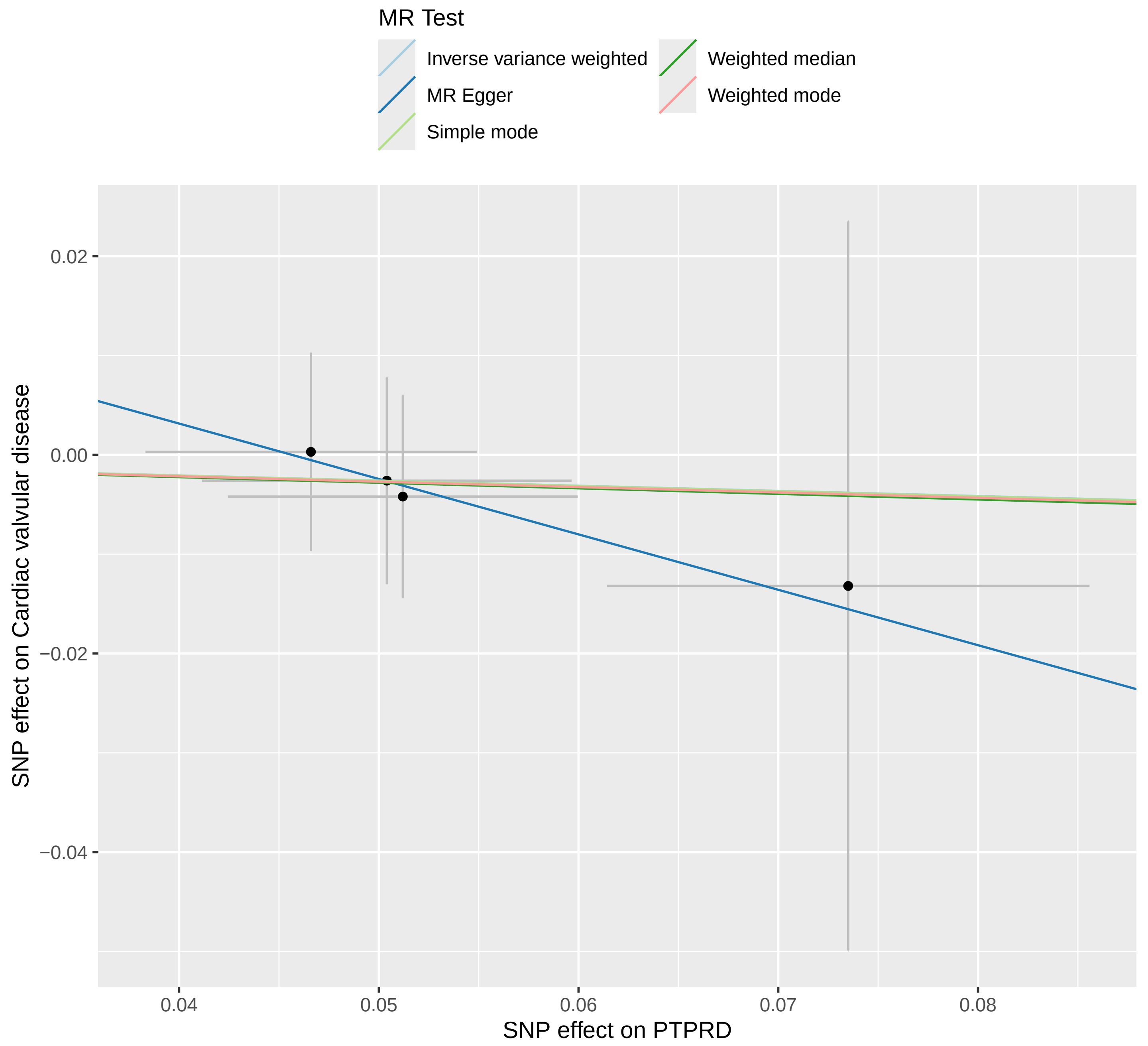

MR Test
Inverse variance weighted
MR Egger
Simple mode
Weighted median
Weighted mode
0.02
0.00
−0.02
−0.04
0.04
0.05
0.06
0.07
0.08
SNP effect on PTPRD
SNP effect on Cardiac valvular disease

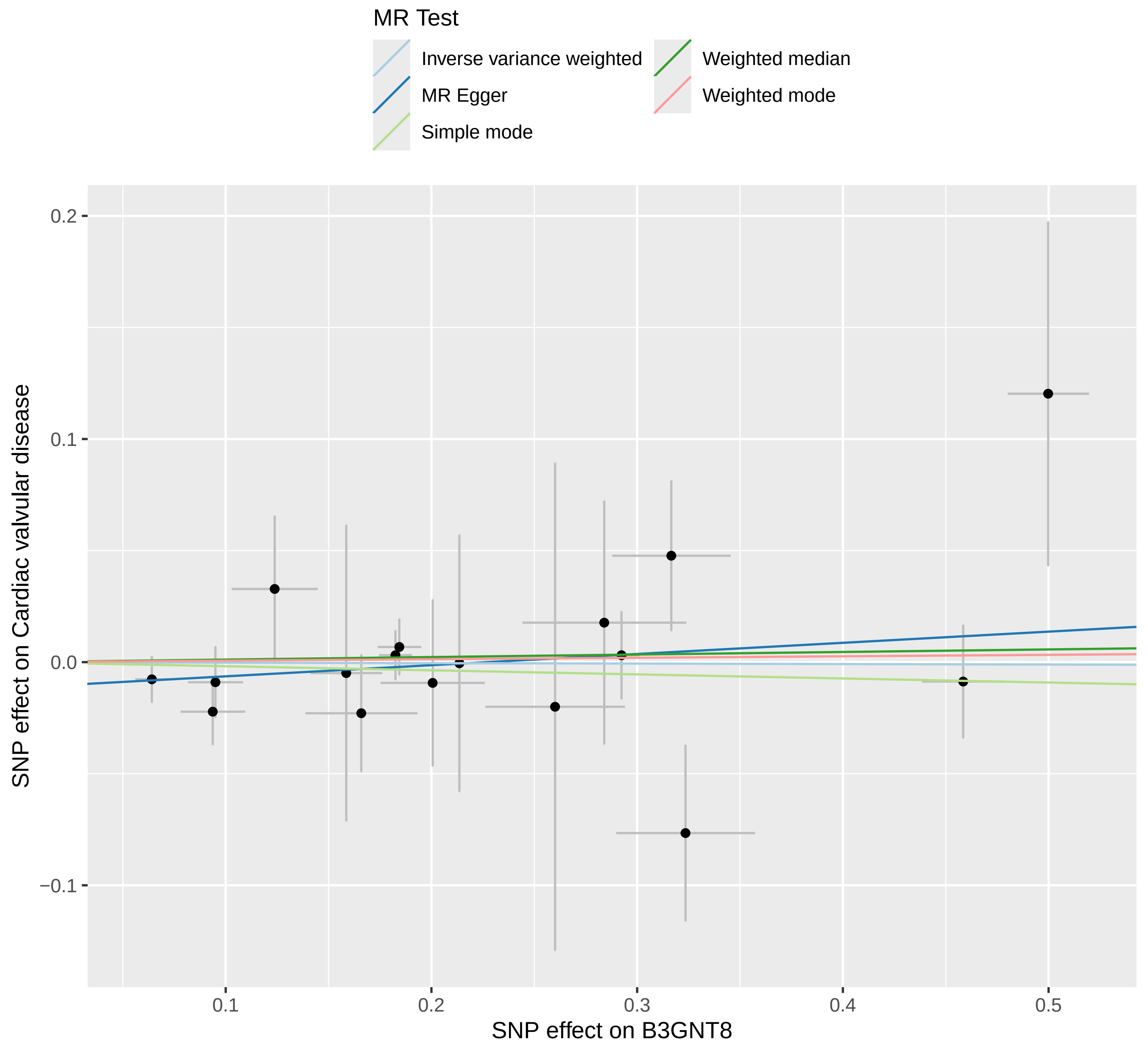
MR Test
Inverse variance weighted
MR Egger
Simple mode
Weighted median
Weighted mode
0.2
0.1
0.0
−0.1
0.1
0.2
0.3
0.4
0.5
SNP effect on Cardiac valvular disease
SNP effect on B3GNT8

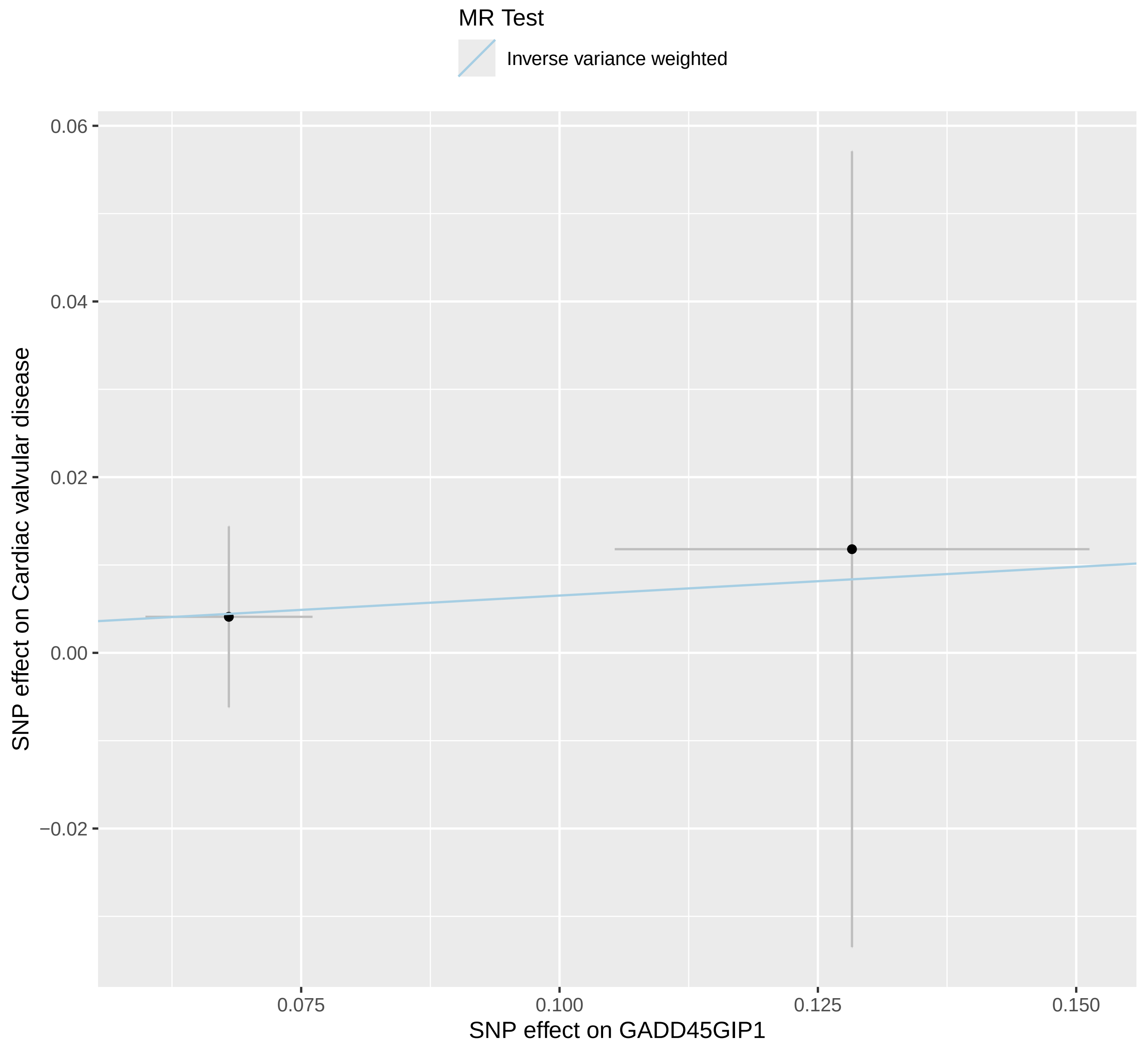
MR Test
Inverse variance weighted
0.06
0.04
0.02
0.00
−0.02
0.075
0.100
0.125
0.150
SNP effect on Cardiac valvular disease
SNP effect on GADD45GIP1

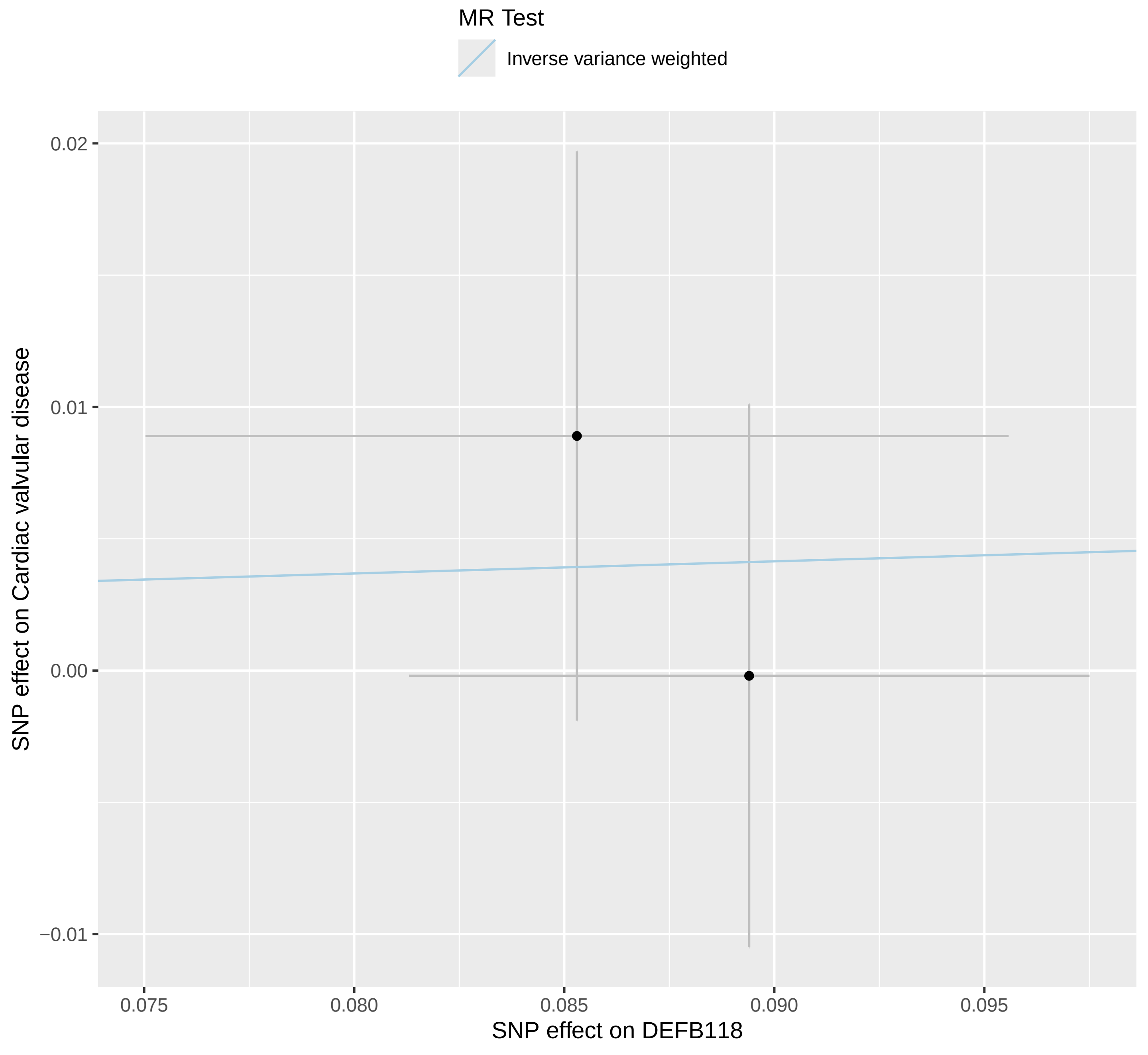

MR Test
Inverse variance weighted
0.02
0.01
0.00
−0.01
SNP effect on Cardiac valvular disease
0.075
0.080
0.085
0.090
0.095
SNP effect on DEFB118

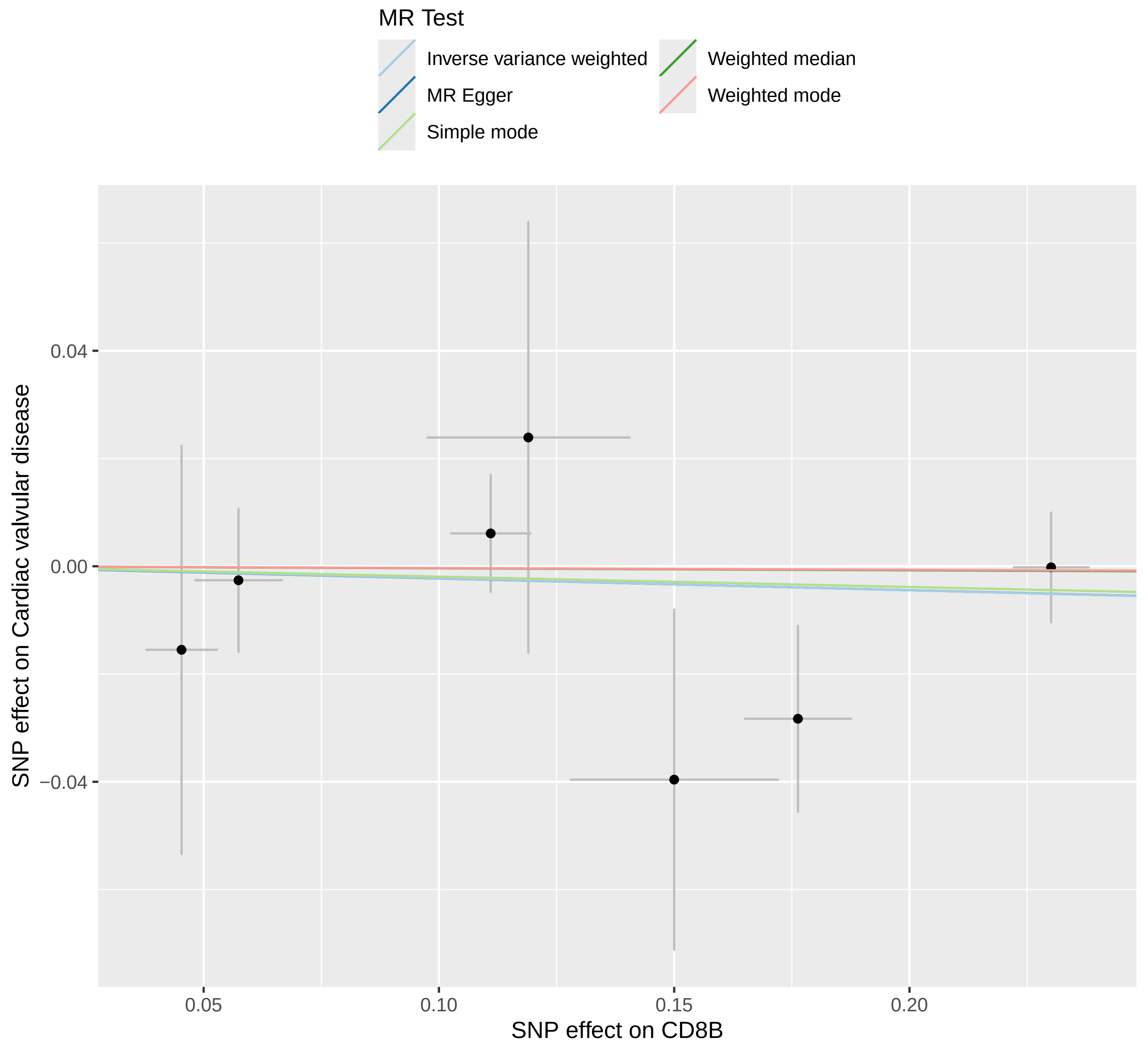

MR Test
Inverse variance weighted
MR Egger
Simple mode
Weighted median
Weighted mode
0.04
0.00
−0.04
0.05
0.10
0.15
0.20
SNP effect on Cardiac valvular disease
SNP effect on CD8B

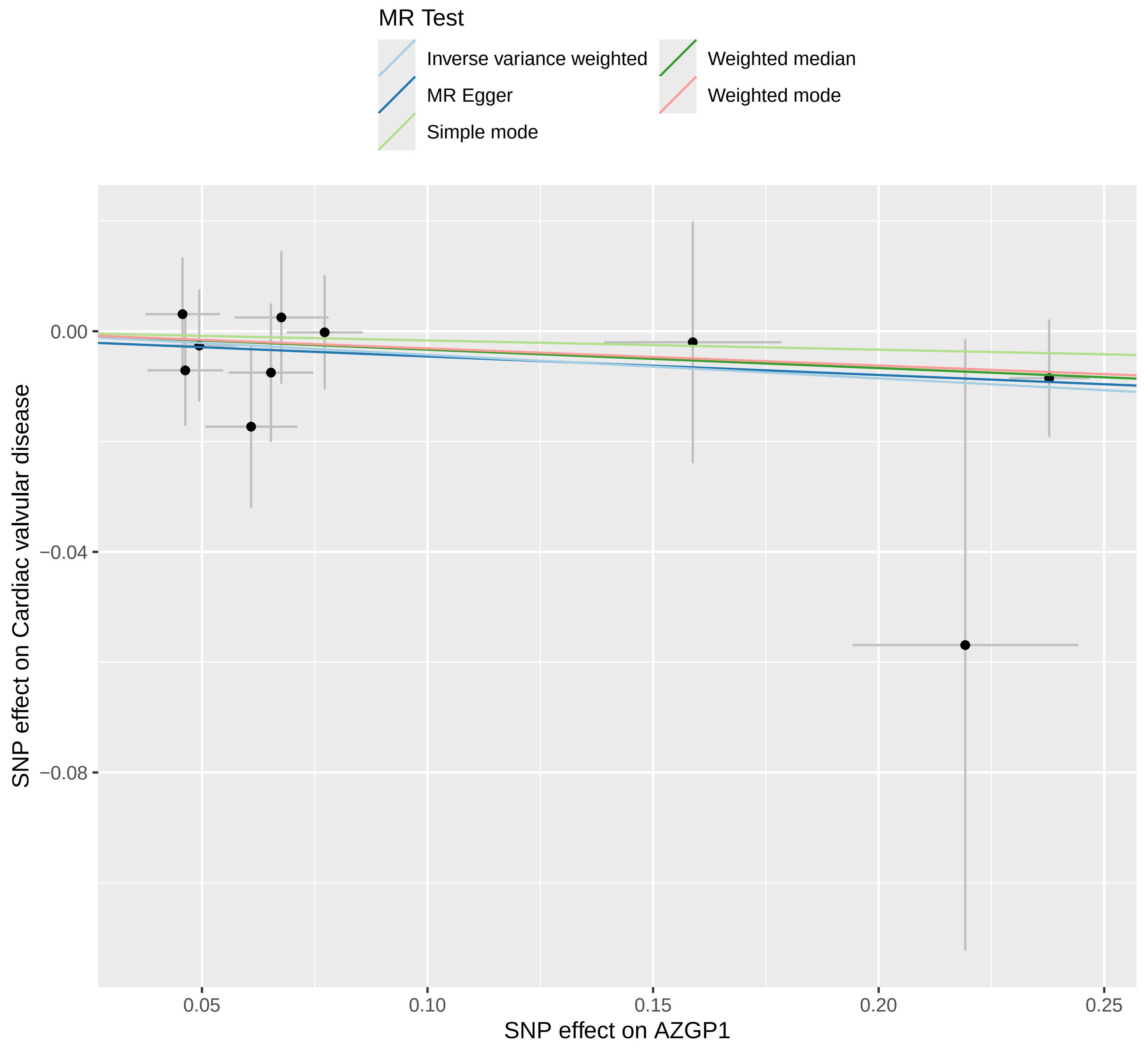

MR Test
Inverse variance weighted
MR Egger
Simple mode
Weighted median
Weighted mode
0.00
−0.04
−0.08
SNP effect on Cardiac valvular disease
0.05
0.10
0.15
0.20
0.25
SNP effect on AZGP1

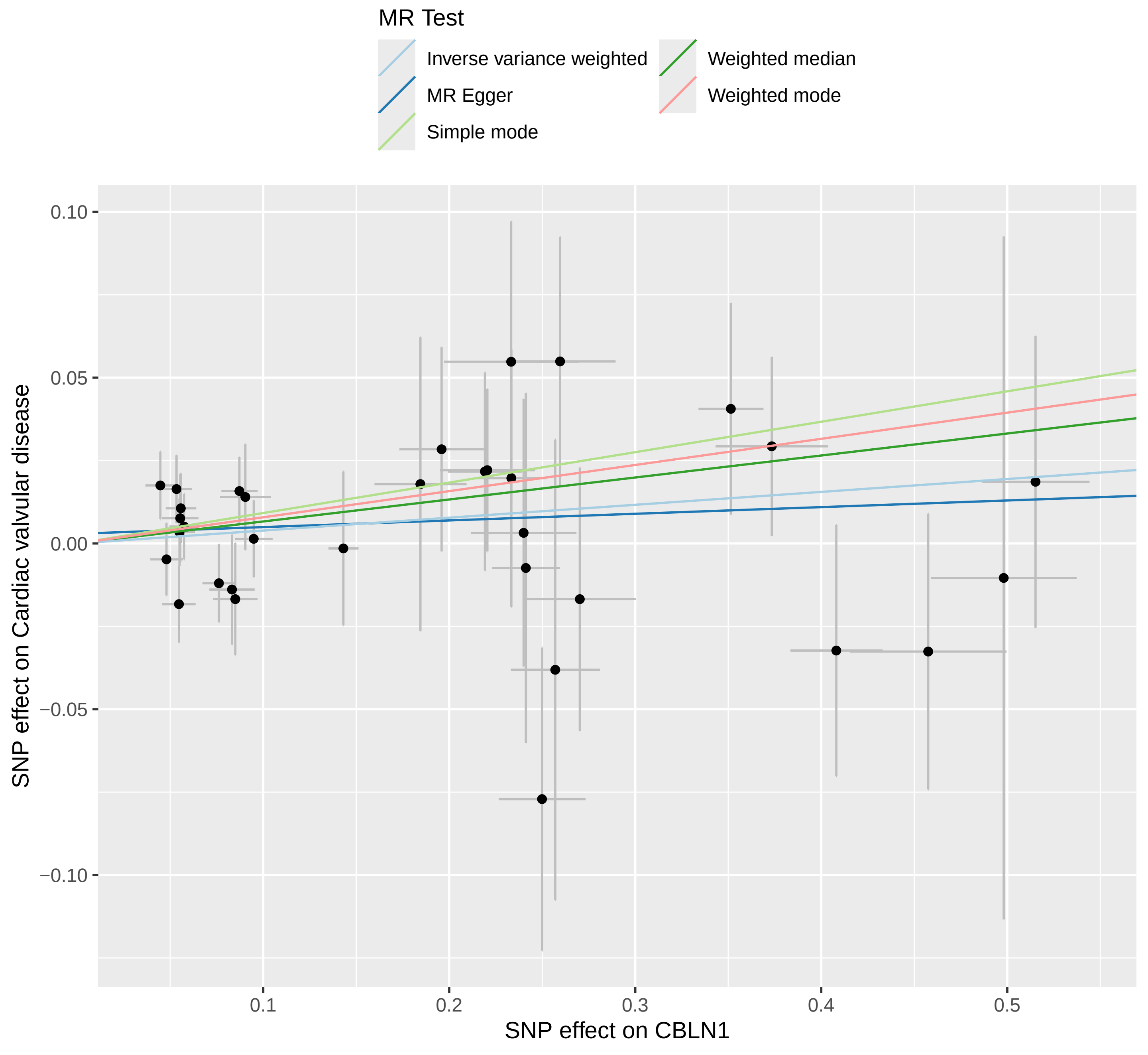
MR Test
Inverse variance weighted
MR Egger
Simple mode
Weighted median
Weighted mode
0.10
0.05
0.00
−0.05
−0.10
SNP effect on Cardiac valvular disease
0.1
0.2
0.3
0.4
0.5
SNP effect on CBLN1

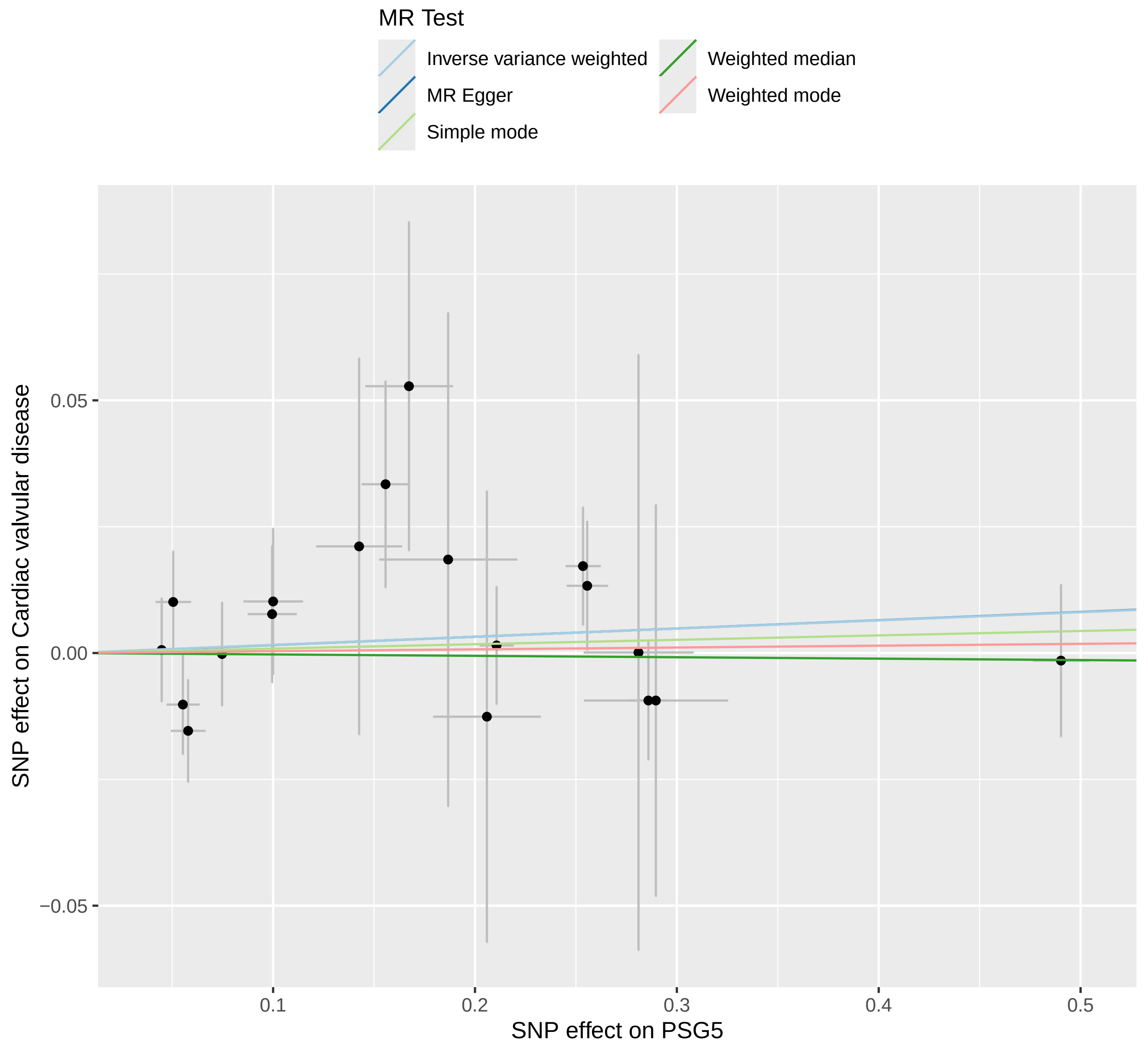
MR Test
Inverse variance weighted
MR Egger
Simple mode
Weighted median
Weighted mode
0.05
0.00
−0.05
SNP effect on Cardiac valvular disease
0.1
0.2
0.3
0.4
0.5
SNP effect on PSG5

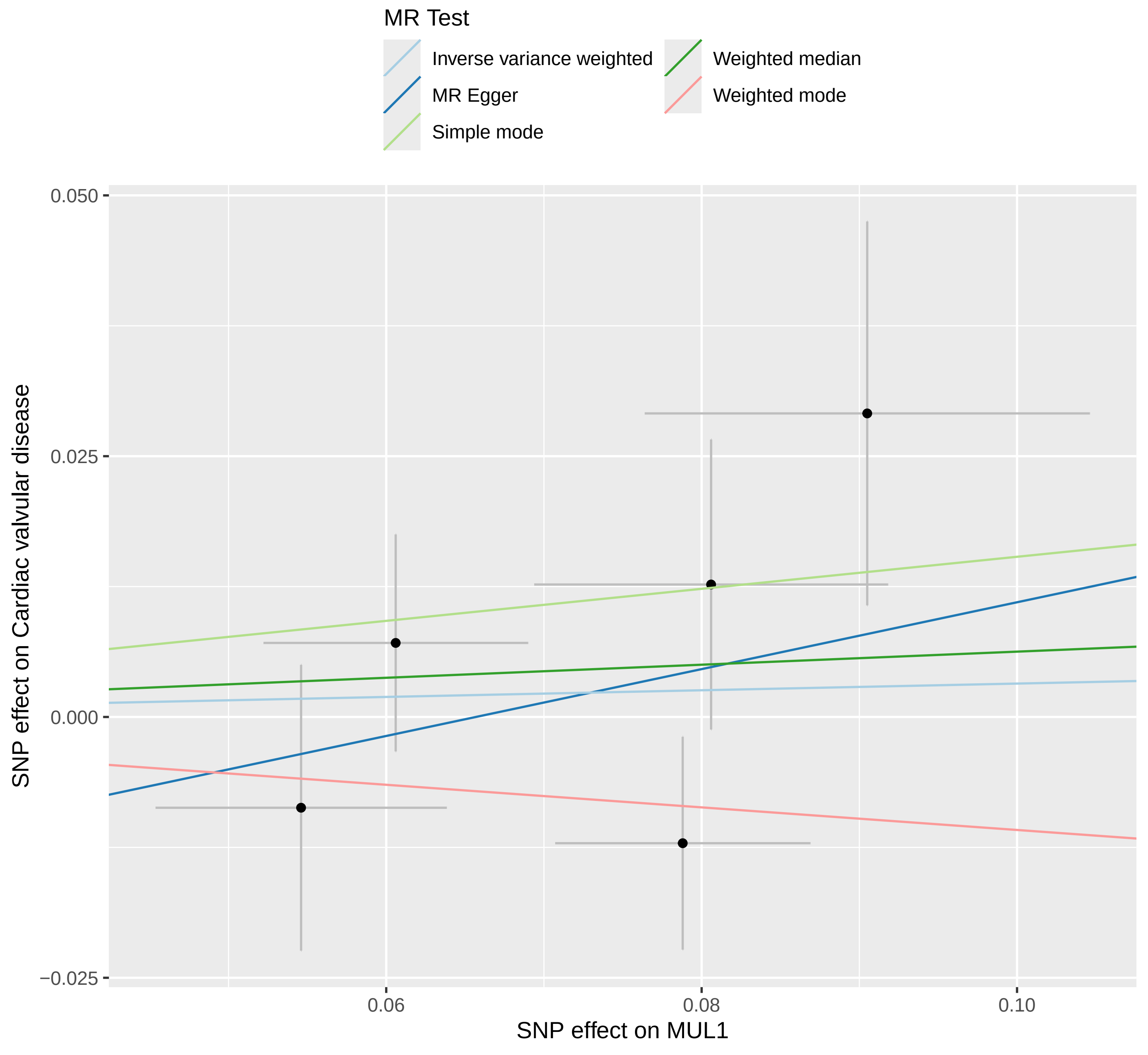
MR Test
Inverse variance weighted
MR Egger
Simple mode
Weighted median
Weighted mode
0.050
0.025
0.000
−0.025
0.06
0.08
0.10
SNP effect on Cardiac valvular disease
SNP effect on MUL1

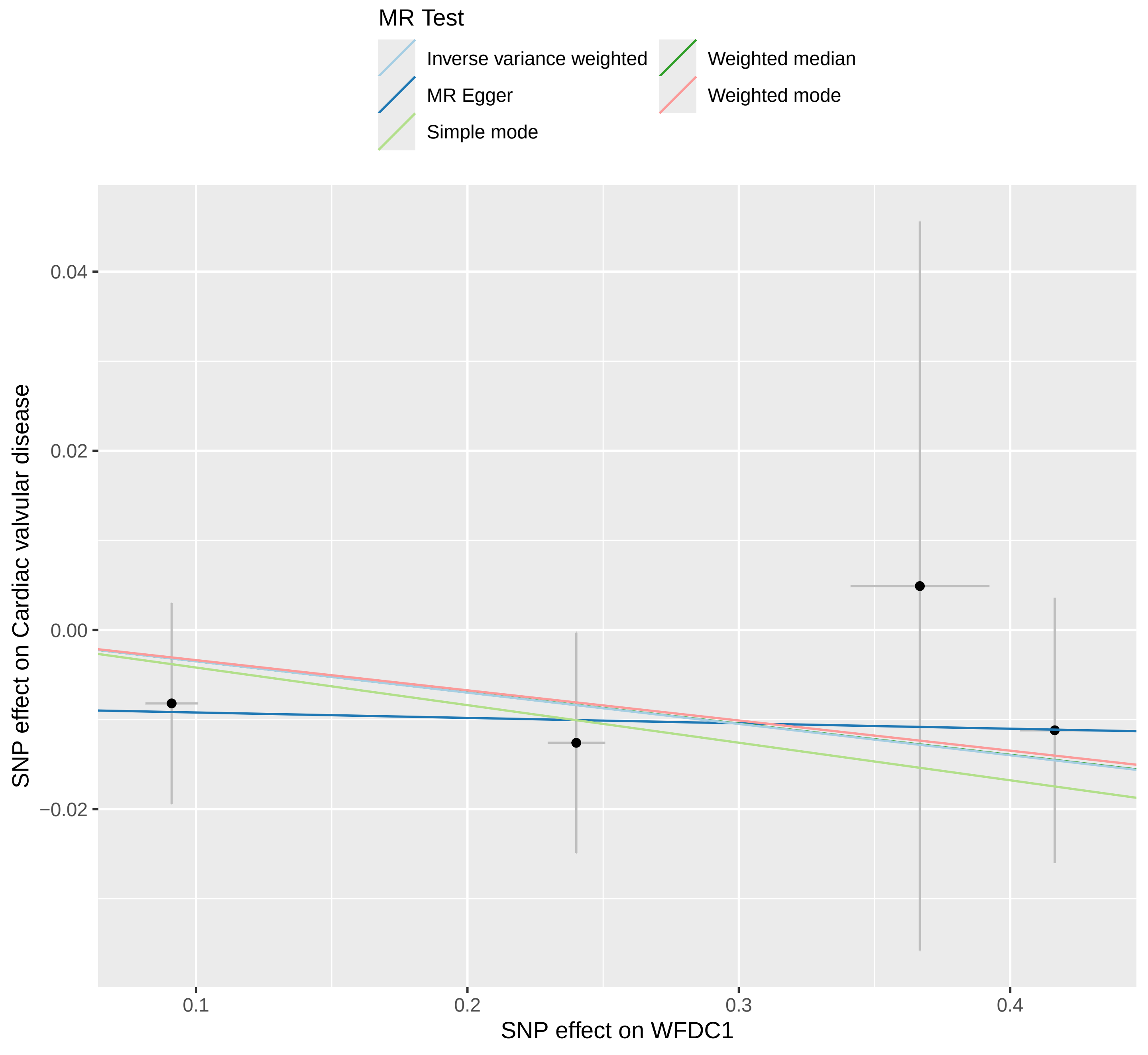

MR Test
Inverse variance weighted
MR Egger
Simple mode
Weighted median
Weighted mode
0.04
0.02
0.00
−0.02
0.1
0.2
0.3
0.4
SNP effect on Cardiac valvular disease
SNP effect on WFDC1

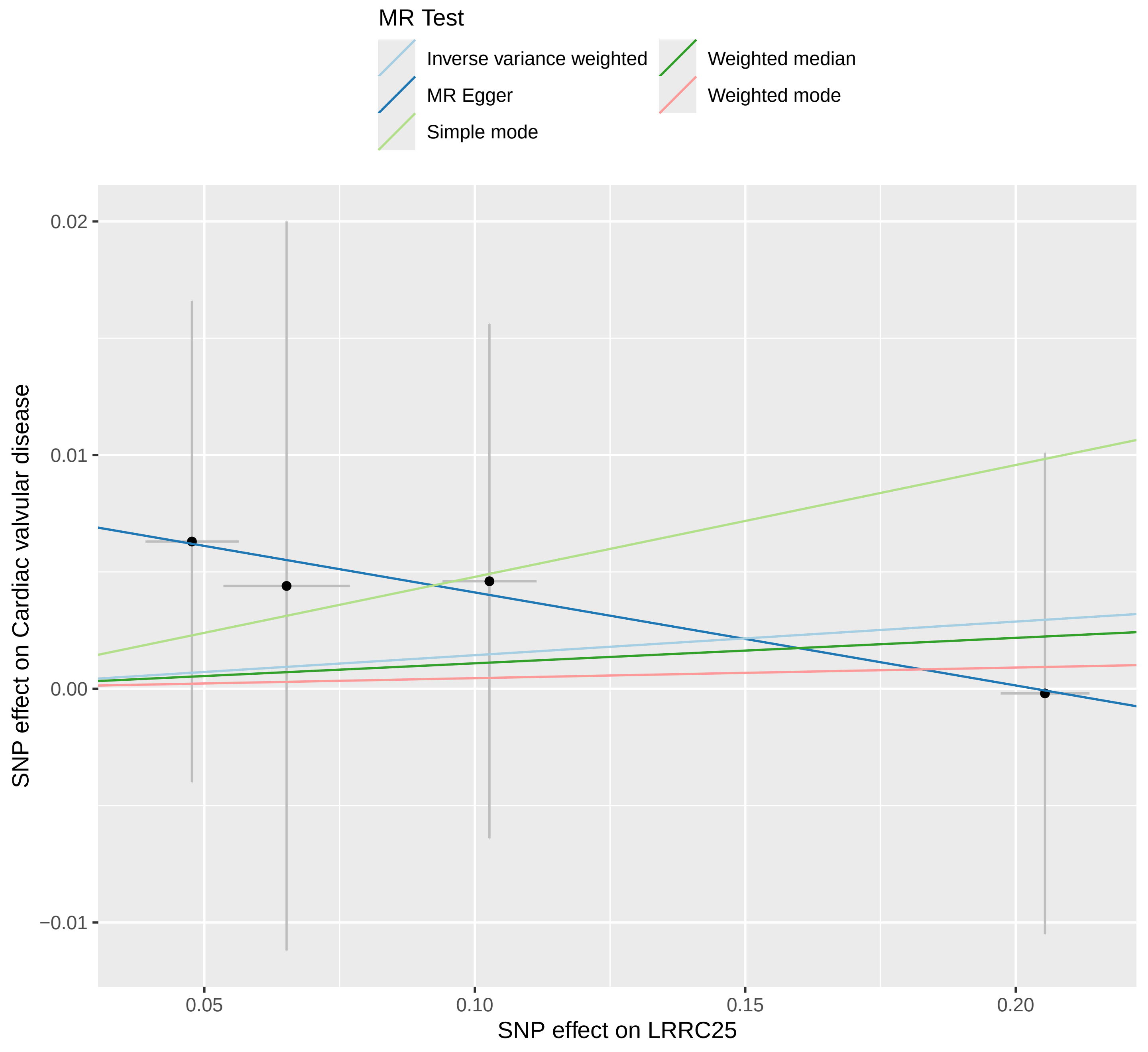

MR Test
Inverse variance weighted
MR Egger
Simple mode
Weighted median
Weighted mode
0.02
0.01
0.00
−0.01
0.05
0.10
0.15
0.20
SNP effect on Cardiac valvular disease
SNP effect on LRRC25

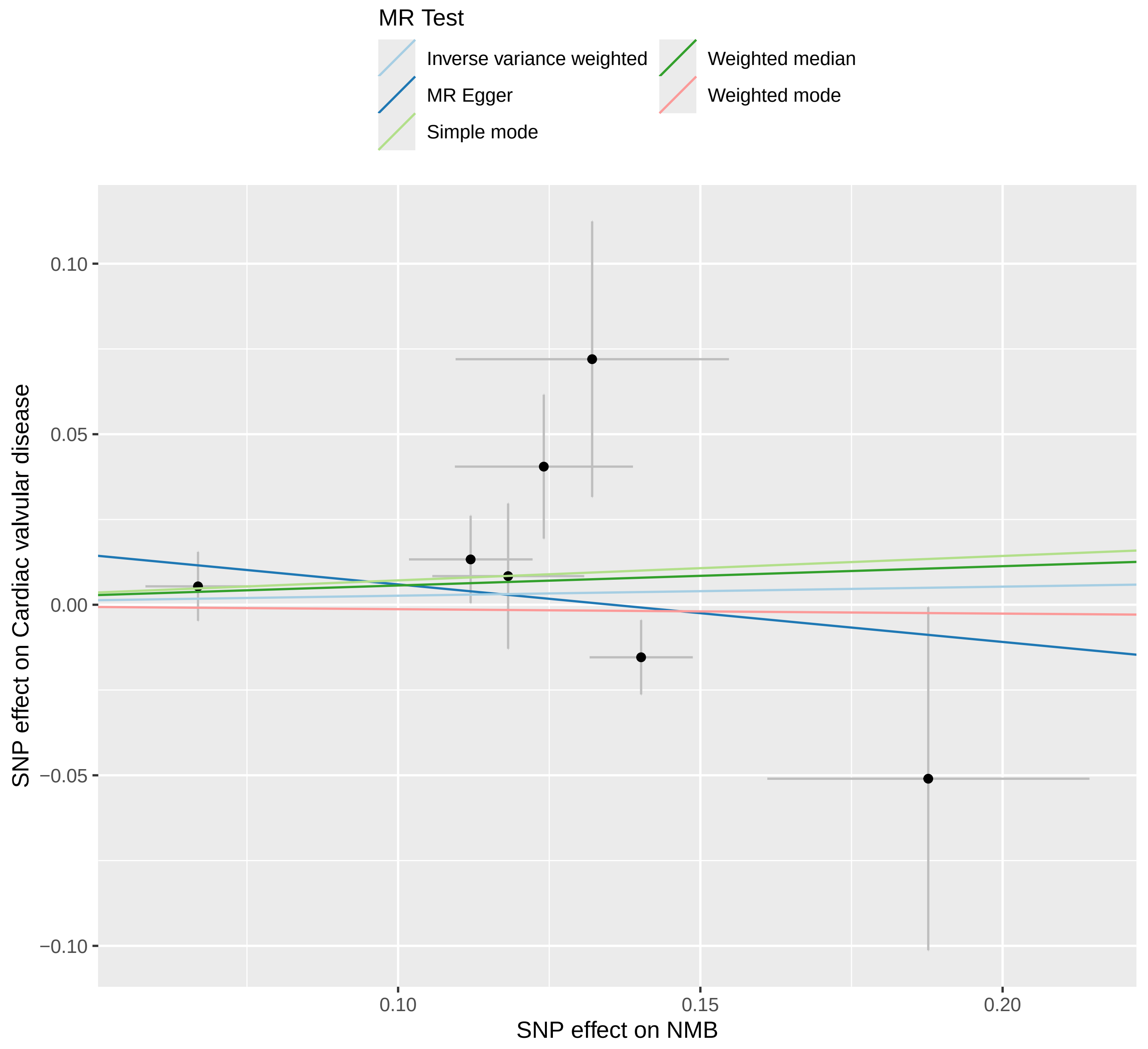
MR Test
Inverse variance weighted
MR Egger
Simple mode
Weighted median
Weighted mode
0.10
0.05
0.00
−0.05
−0.10
0.10
0.15
0.20
SNP effect on Cardiac valvular disease
SNP effect on NMB

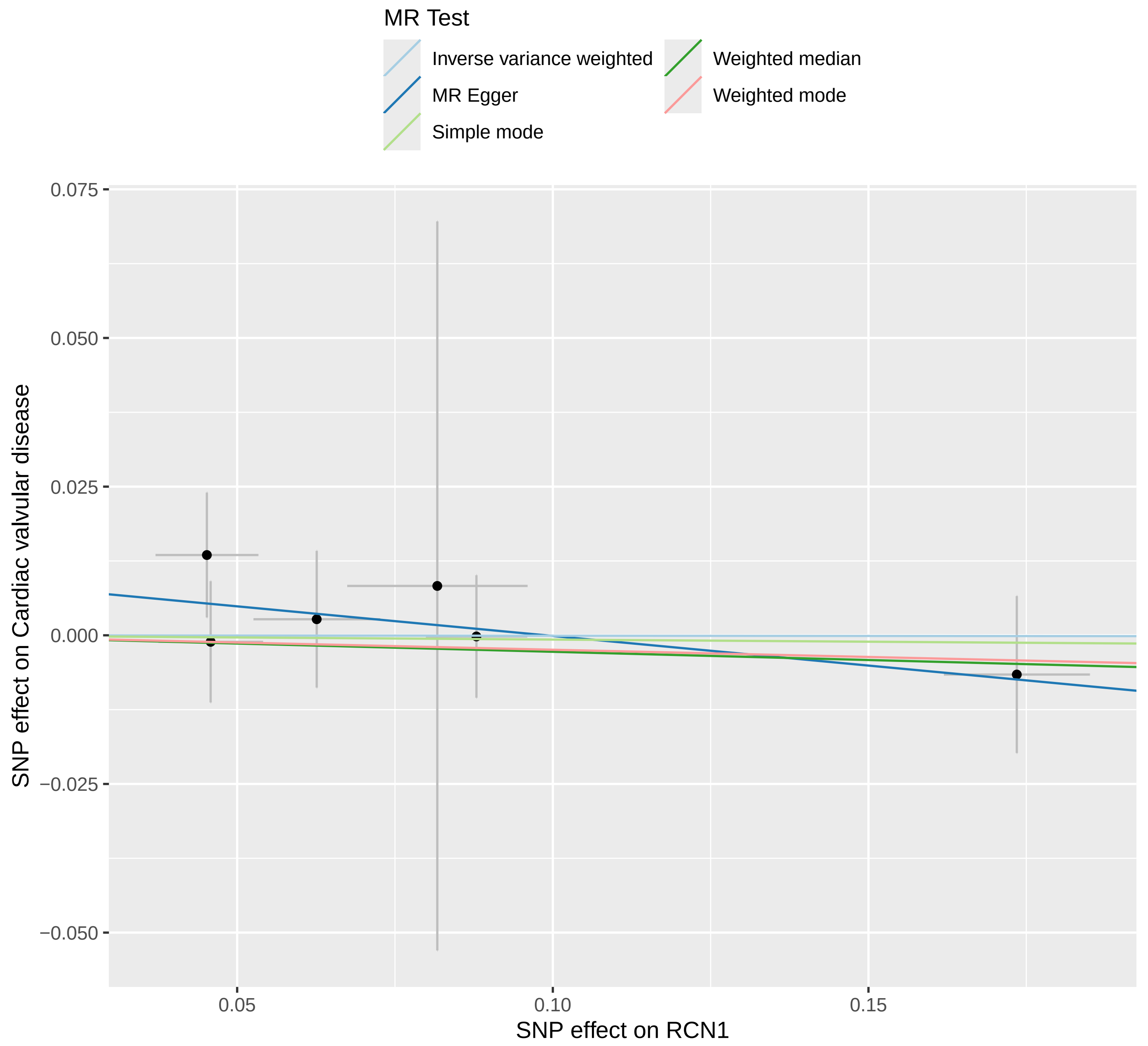
MR Test
Inverse variance weighted
MR Egger
Simple mode
Weighted median
Weighted mode
0.075
0.050
0.025
0.000
−0.025
−0.050
SNP effect on Cardiac valvular disease
0.05
0.10
0.15
SNP effect on RCN1

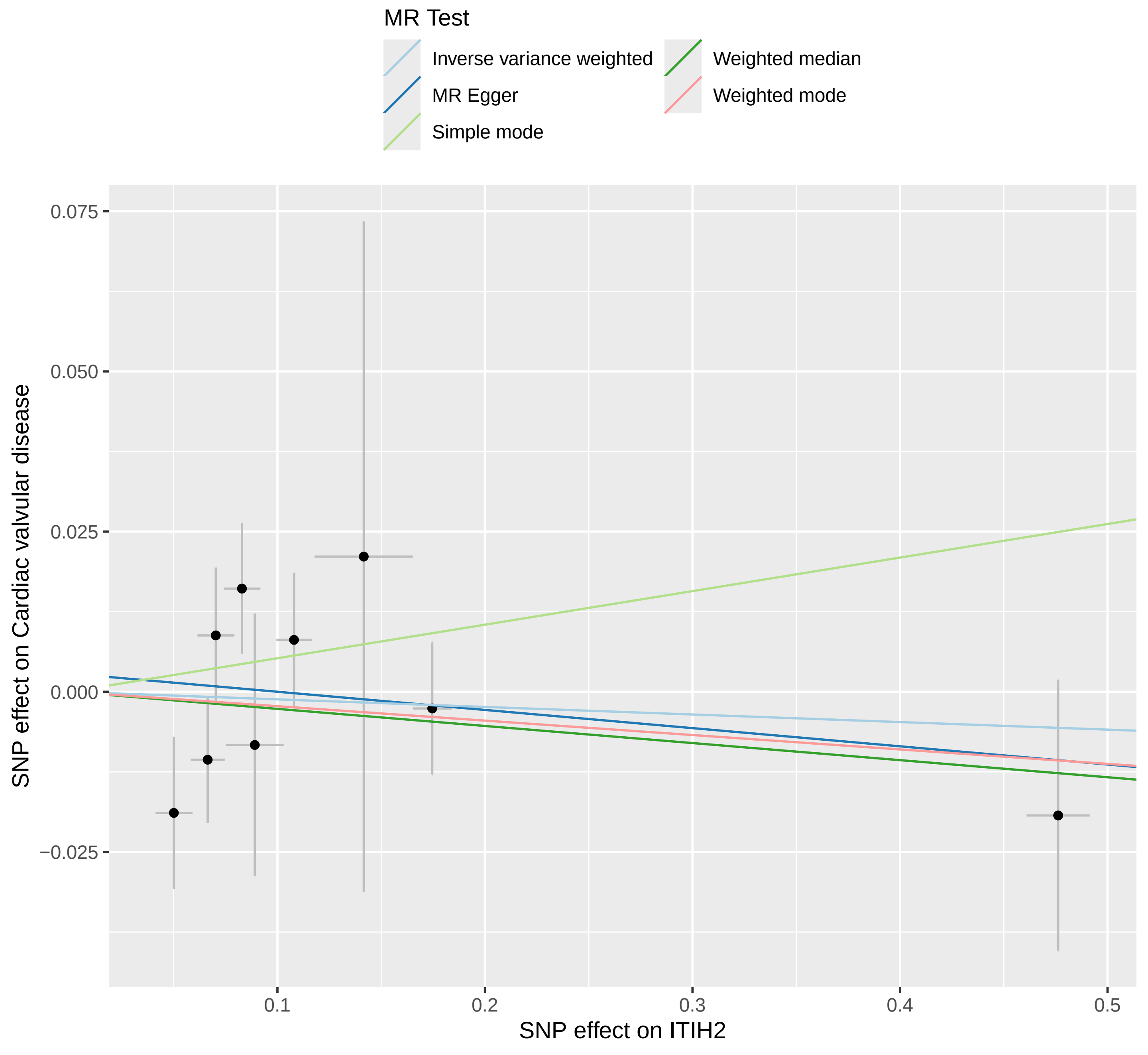
MR Test
Inverse variance weighted
MR Egger
Simple mode
Weighted median
Weighted mode
0.075
0.050
0.025
0.000
−0.025
0.1
0.2
0.3
0.4
0.5
SNP effect on Cardiac valvular disease
SNP effect on ITIH2

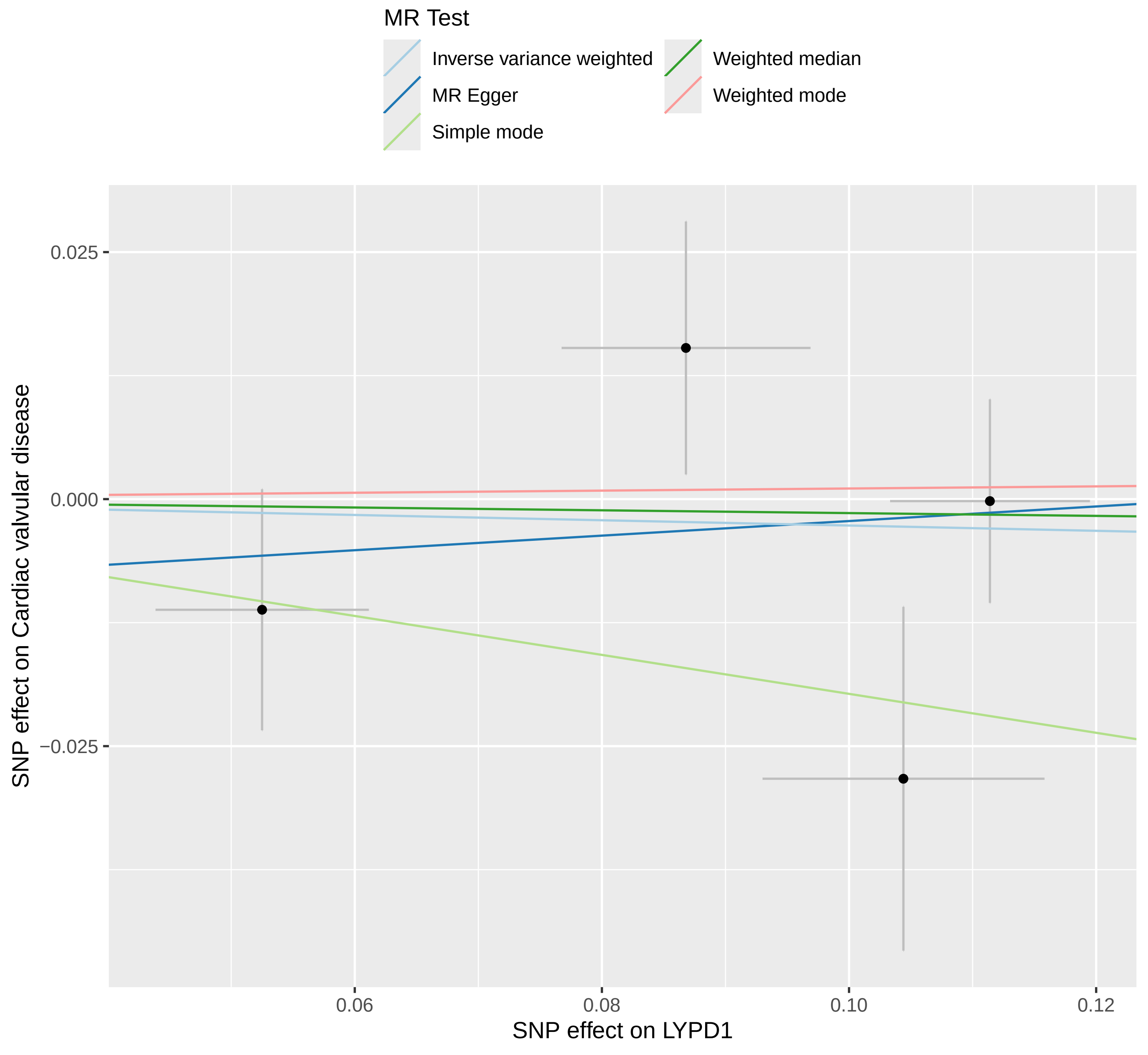
MR Test
Inverse variance weighted
MR Egger
Simple mode
Weighted median
Weighted mode
0.025
0.000
−0.025
0.06
0.08
0.10
0.12
SNP effect on Cardiac valvular disease
SNP effect on LYPD1

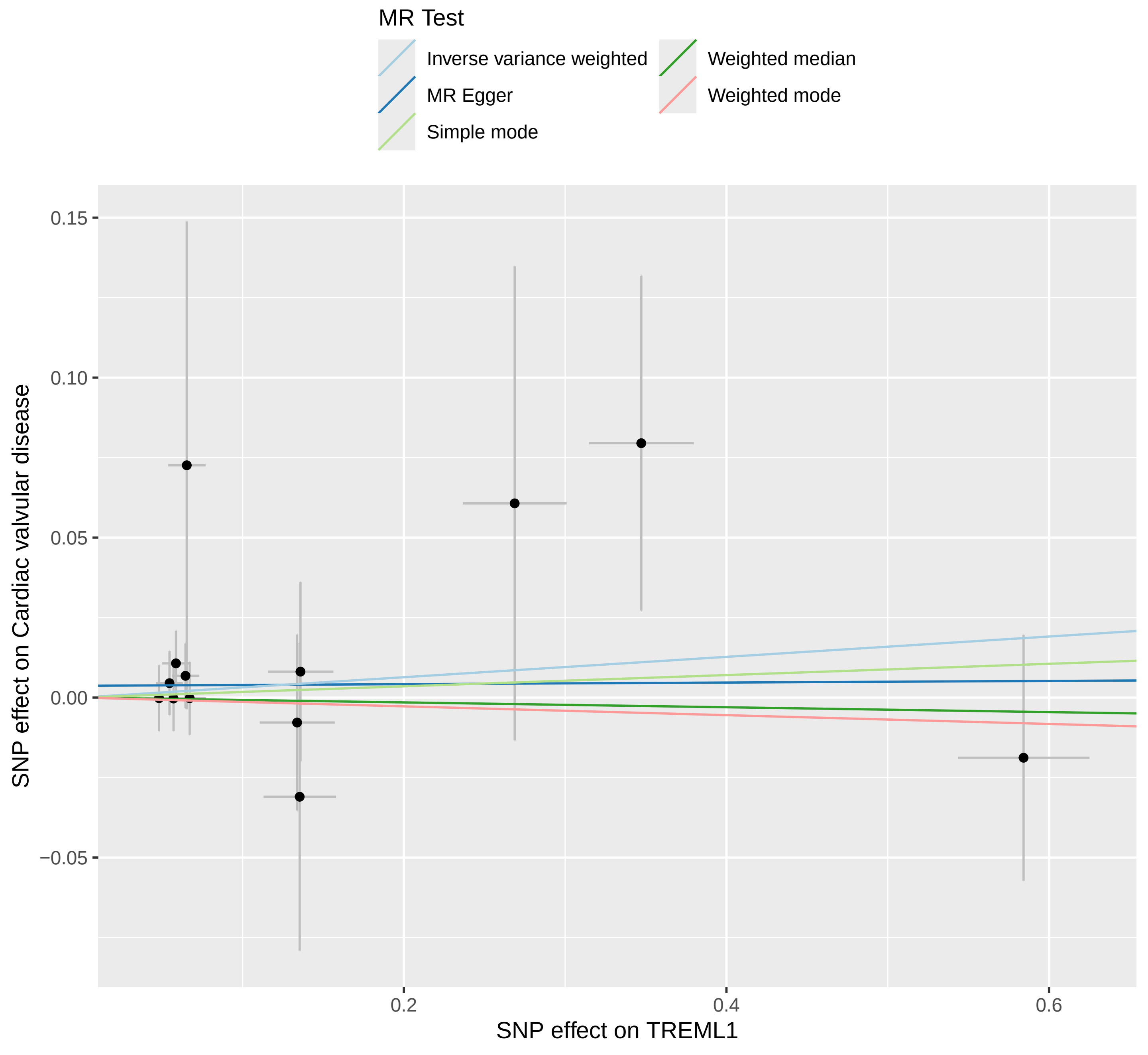
MR Test
Inverse variance weighted
MR Egger
Simple mode
Weighted median
Weighted mode
0.15
0.10
0.05
0.00
−0.05
SNP effect on Cardiac valvular disease
0.2
0.4
0.6
SNP effect on TREML1

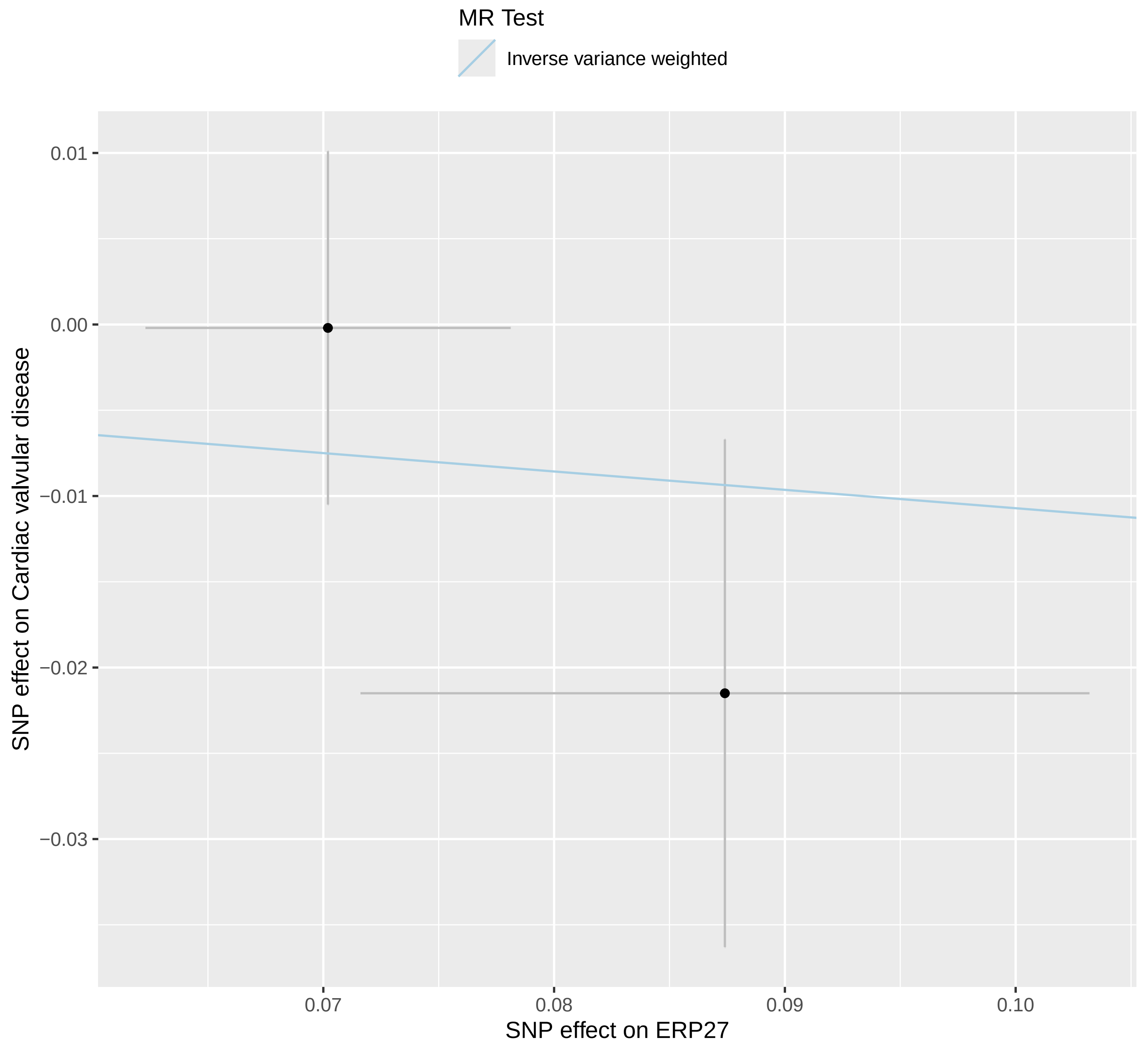

MR Test
Inverse variance weighted
0.01
0.00
−0.01
−0.02
−0.03
SNP effect on Cardiac valvular disease
0.07
0.08
0.09
0.10
SNP effect on ERP27

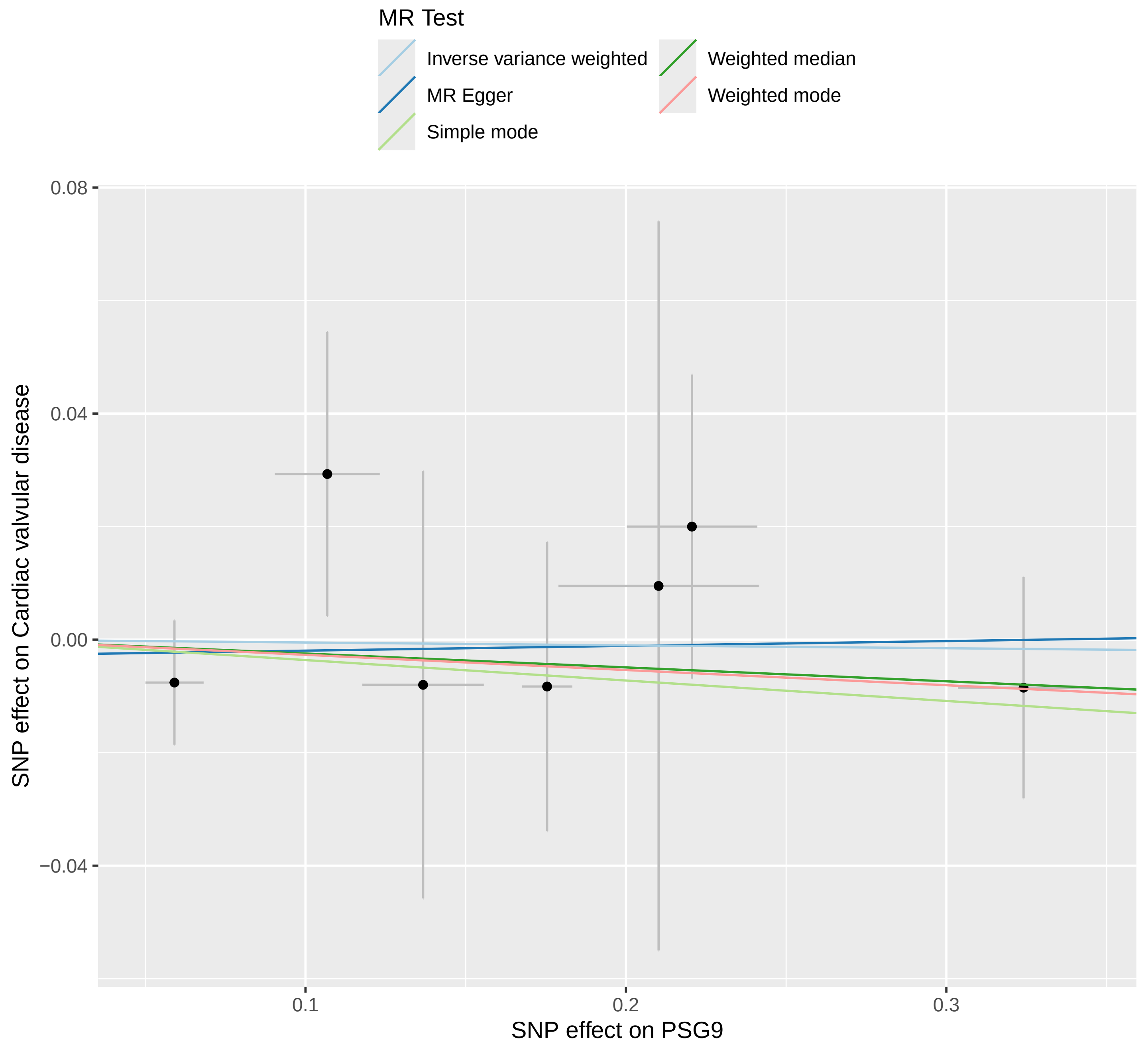
MR Test
Inverse variance weighted
MR Egger
Simple mode
Weighted median
Weighted mode
0.08
0.04
0.00
−0.04
SNP effect on Cardiac valvular disease
0.1
0.2
0.3
SNP effect on PSG9

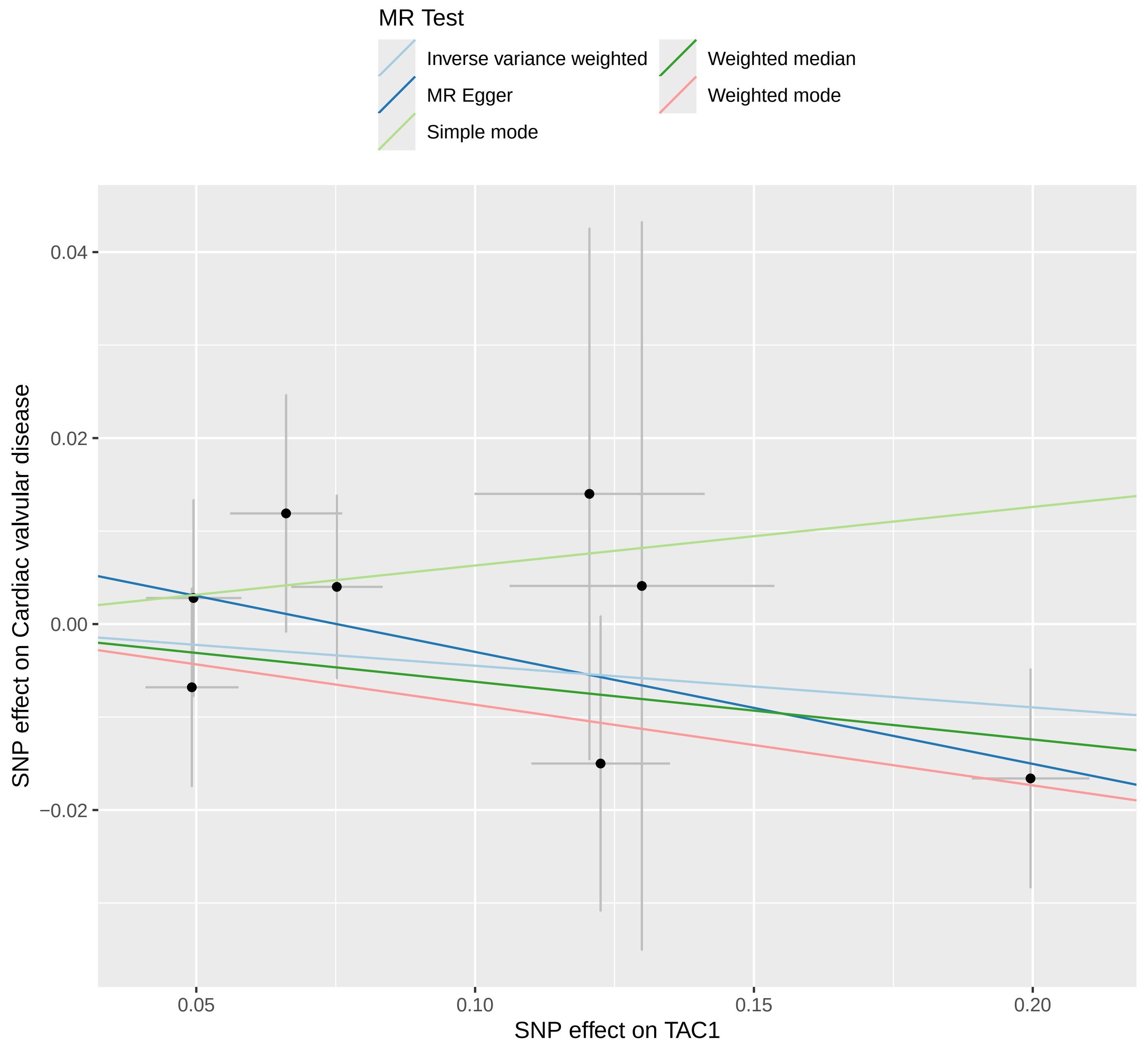

MR Test
Inverse variance weighted
MR Egger
Simple mode
Weighted median
Weighted mode
0.04
0.02
0.00
−0.02
SNP effect on Cardiac valvular disease
0.05
0.10
0.15
0.20
SNP effect on TAC1

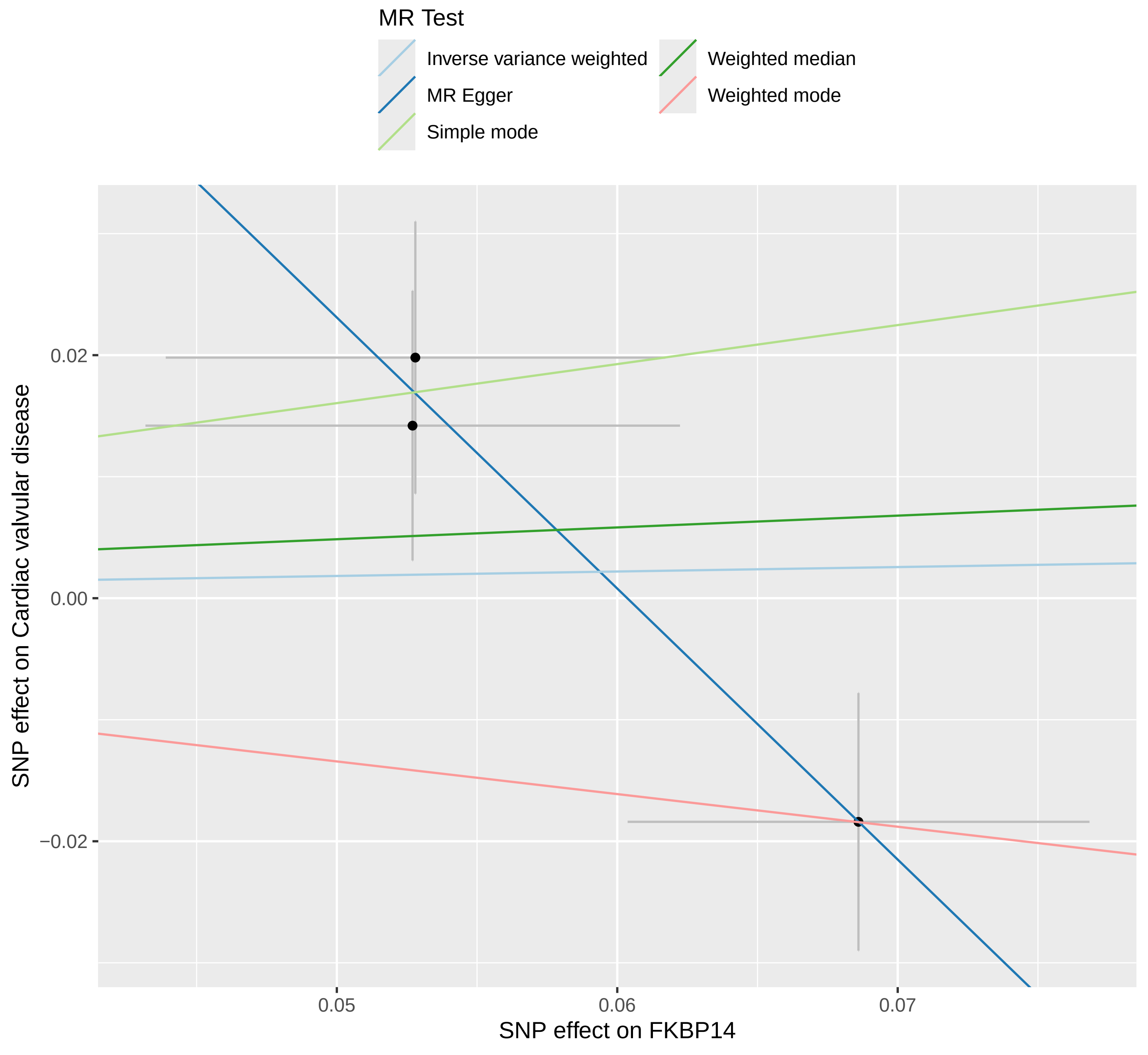
MR Test
Inverse variance weighted
MR Egger
Simple mode
Weighted median
Weighted mode
0.02
0.00
−0.02
0.05
0.06
0.07
SNP effect on Cardiac valvular disease
SNP effect on FKBP14

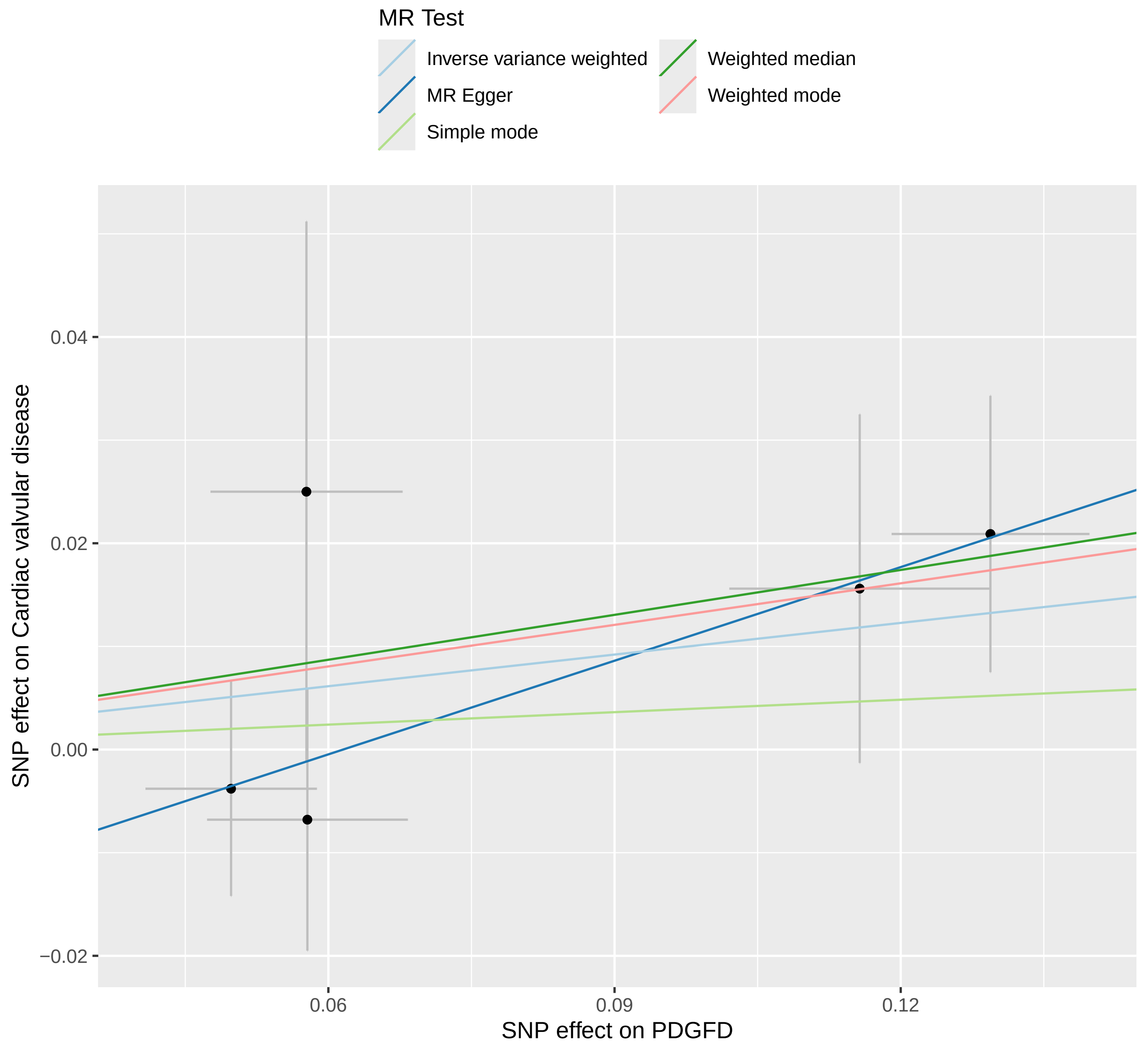
MR Test
Inverse variance weighted
MR Egger
Simple mode
Weighted median
Weighted mode
0.04
0.02
0.00
−0.02
0.06
0.09
0.12
SNP effect on Cardiac valvular disease
SNP effect on PDGFD

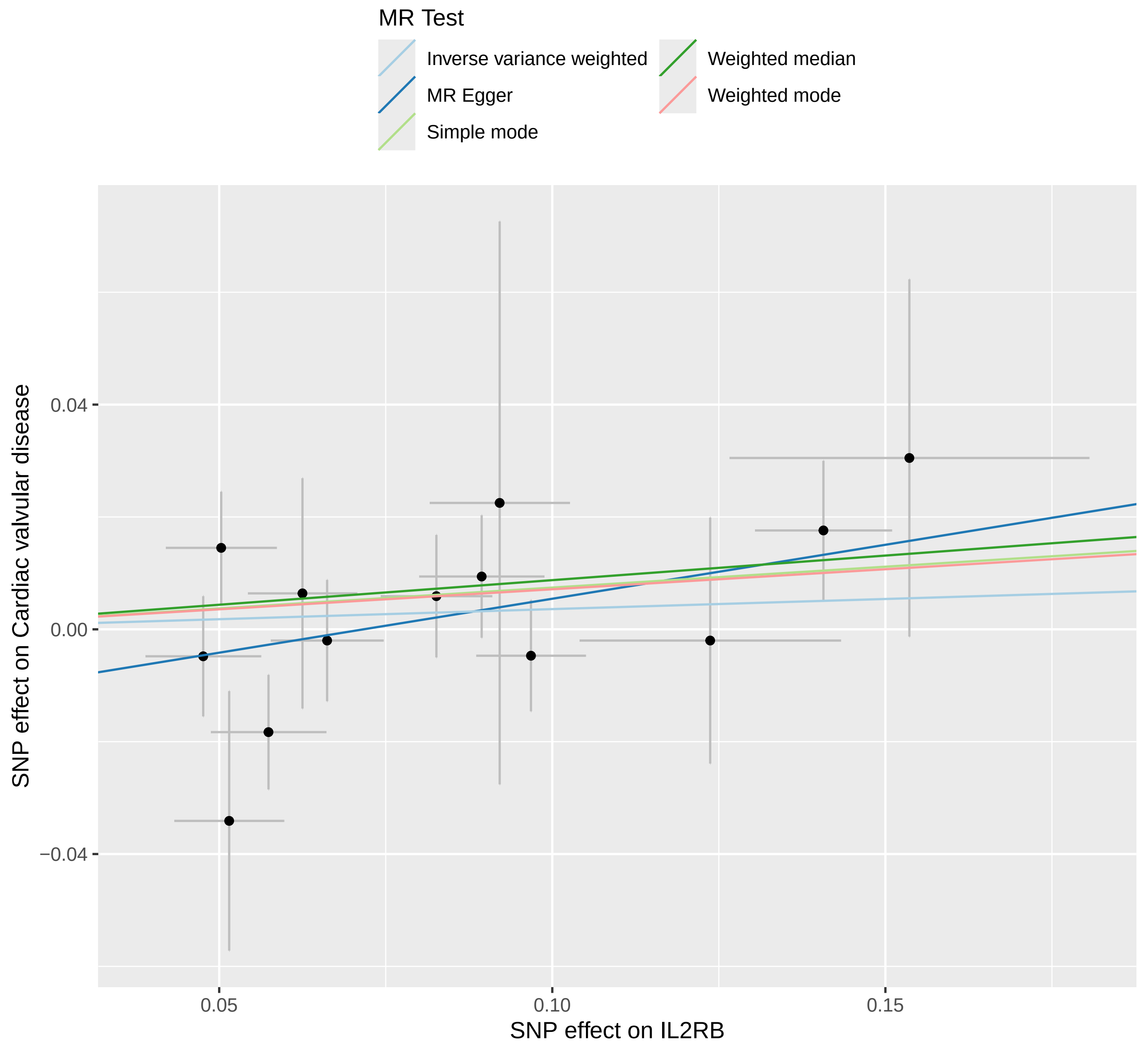

MR Test
Inverse variance weighted
MR Egger
Simple mode
Weighted median
Weighted mode
0.04
0.00
−0.04
SNP effect on Cardiac valvular disease
0.05
0.10
0.15
SNP effect on IL2RB

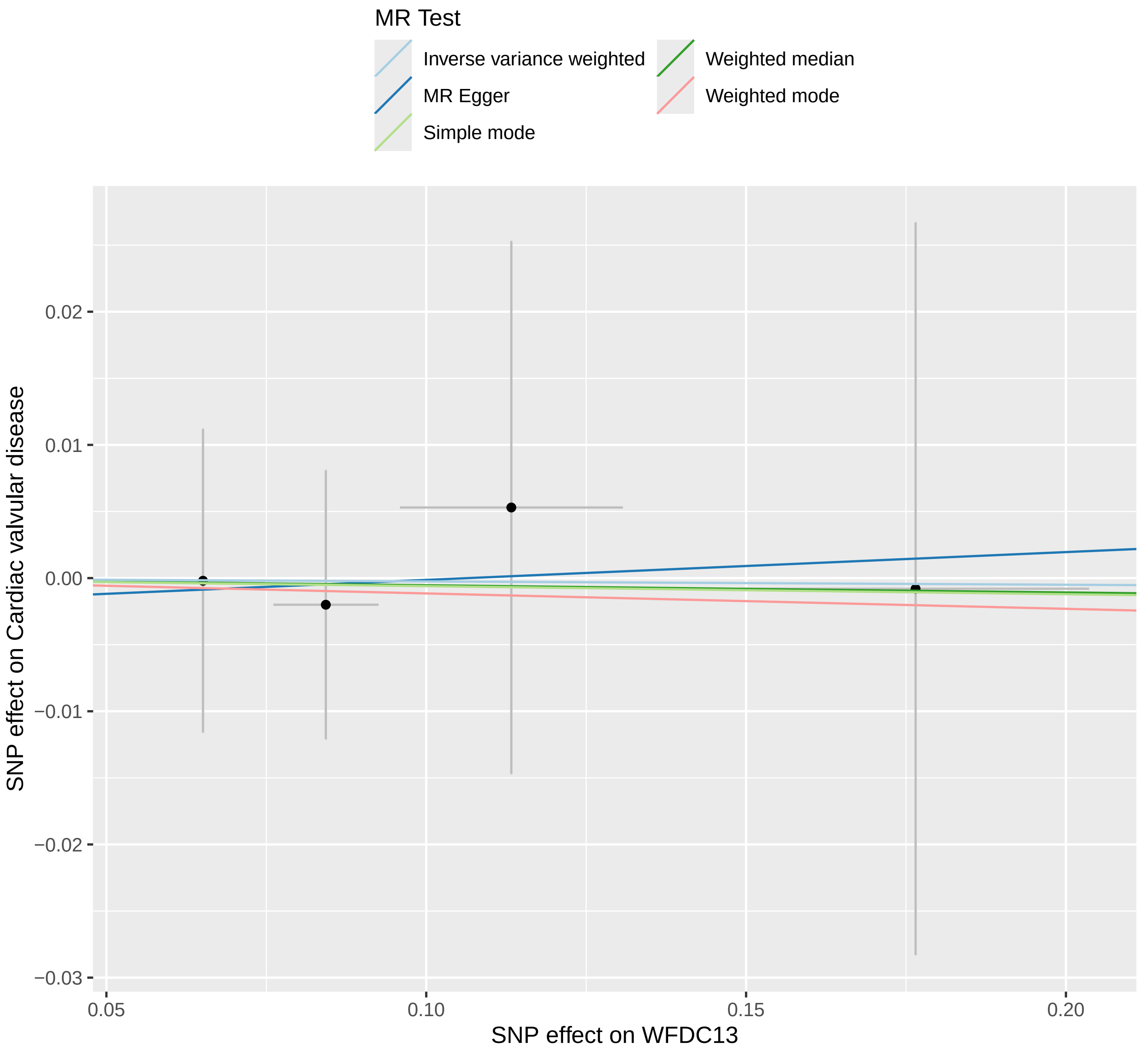
MR Test
Inverse variance weighted
MR Egger
Simple mode
Weighted median
Weighted mode
SNP effect on Cardiac valvular disease
0.02
0.01
0.00
−0.01
−0.02
−0.03
0.05
0.10
0.15
0.20
SNP effect on WFDC13

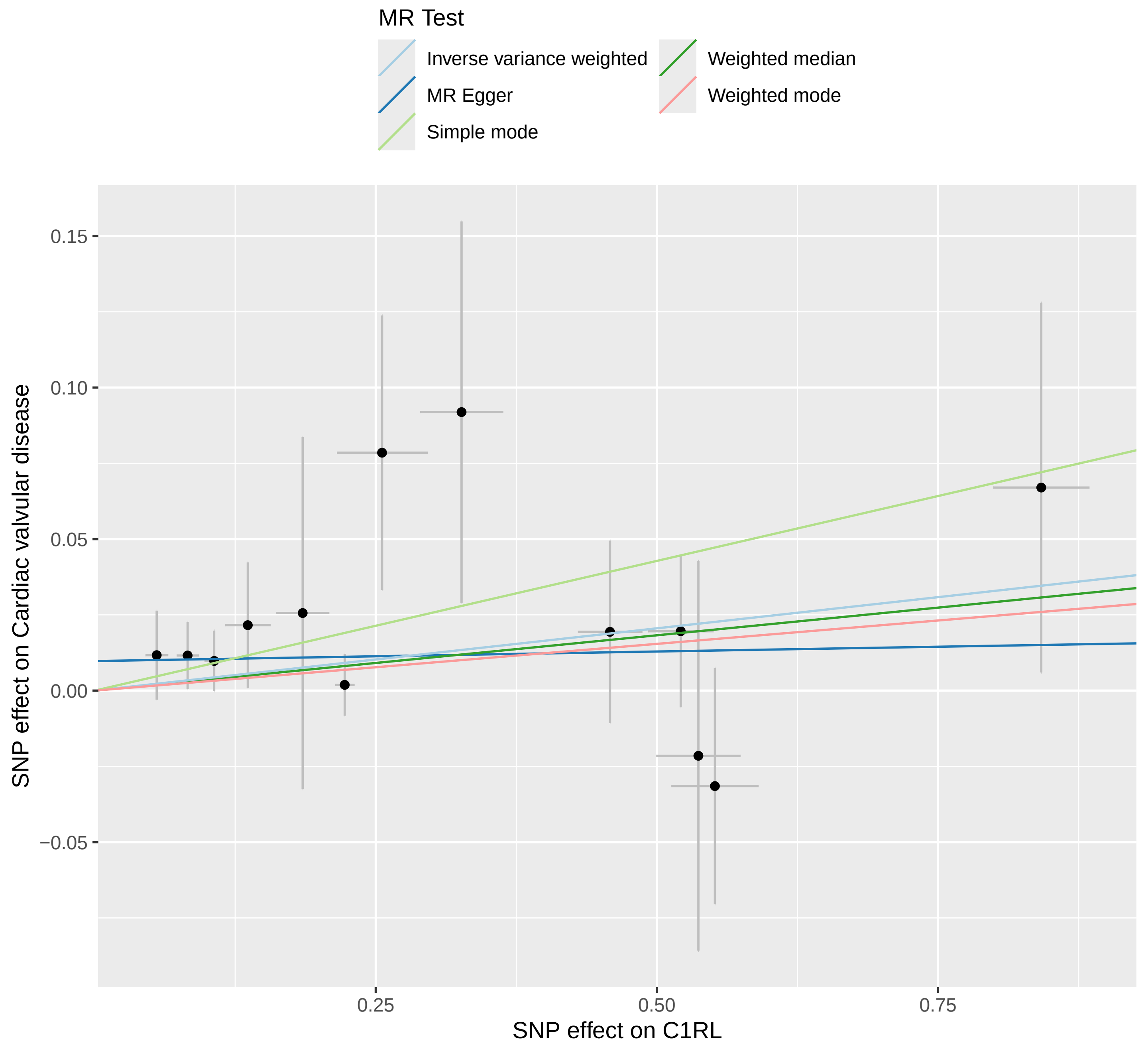

MR Test
Inverse variance weighted
MR Egger
Simple mode
Weighted median
Weighted mode
0.15
0.10
0.05
0.00
−0.05
0.25
0.50
0.75
SNP effect on Cardiac valvular disease
SNP effect on C1RL

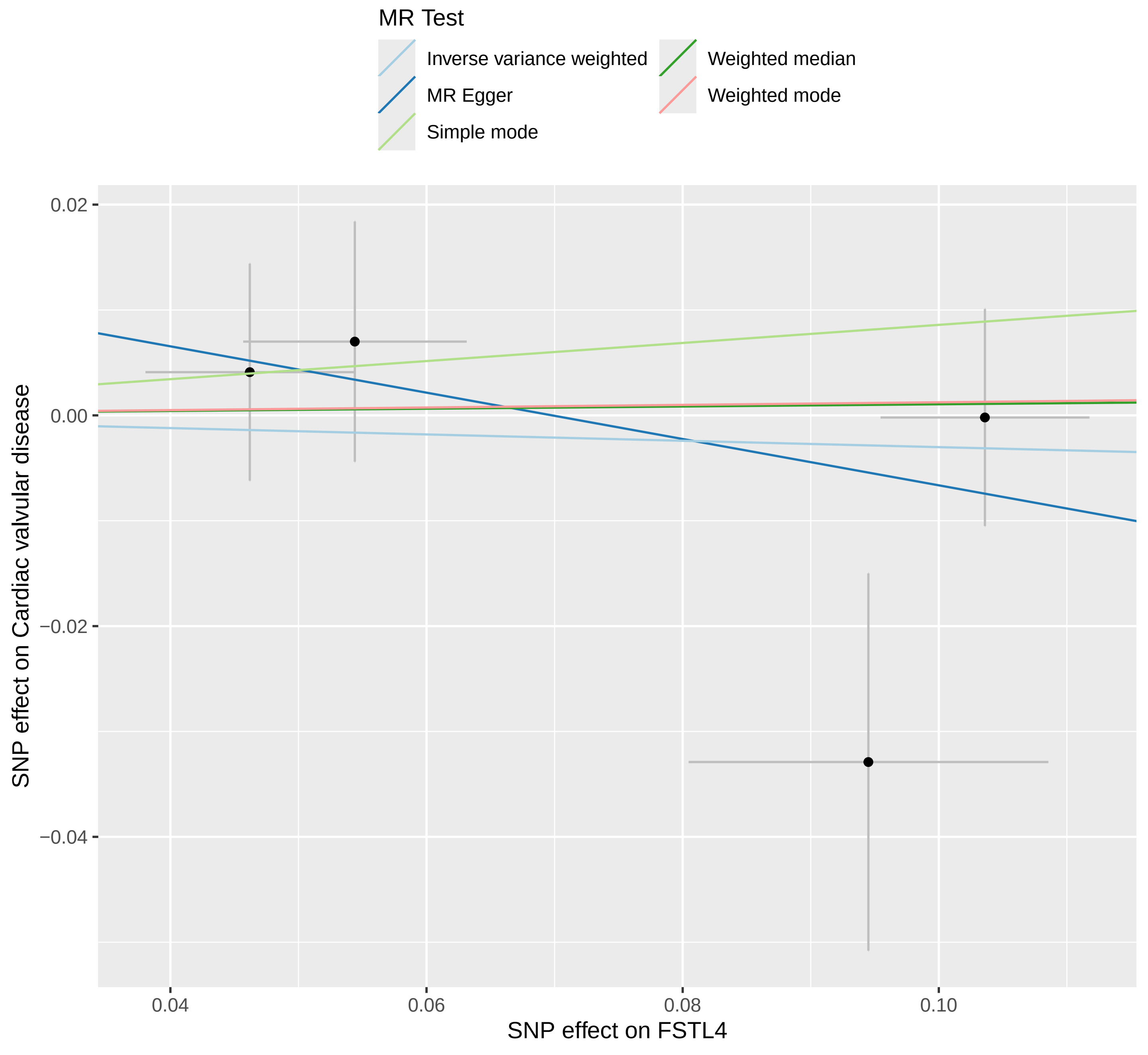

MR Test
Inverse variance weighted
MR Egger
Simple mode
Weighted median
Weighted mode
0.02
0.00
−0.02
−0.04
0.04
0.06
0.08
0.10
SNP effect on Cardiac valvular disease
SNP effect on FSTL4

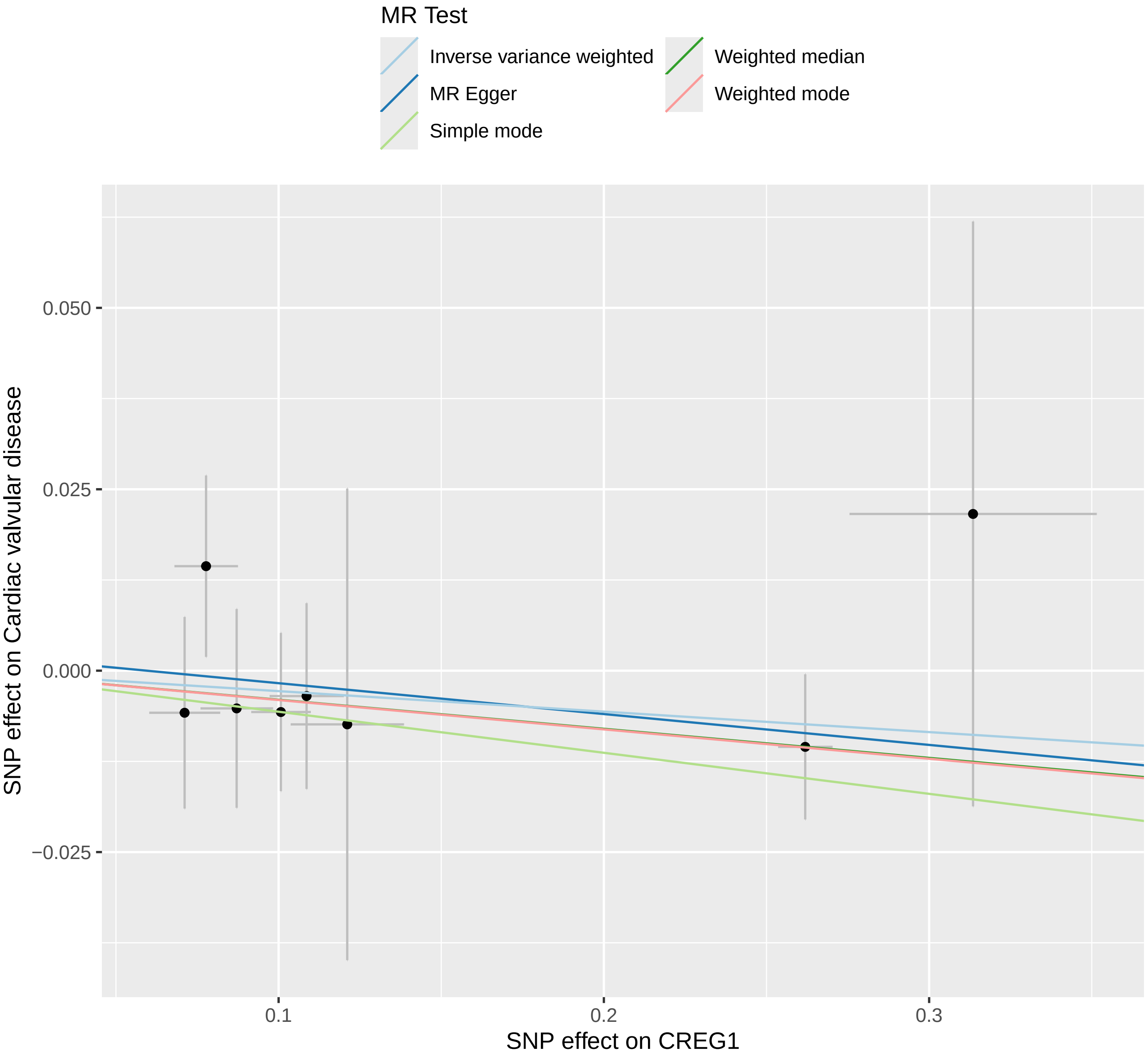
MR Test
Inverse variance weighted
MR Egger
Simple mode
Weighted median
Weighted mode
0.050
0.025
0.000
−0.025
0.1
0.2
0.3
SNP effect on Cardiac valvular disease
SNP effect on CREG1

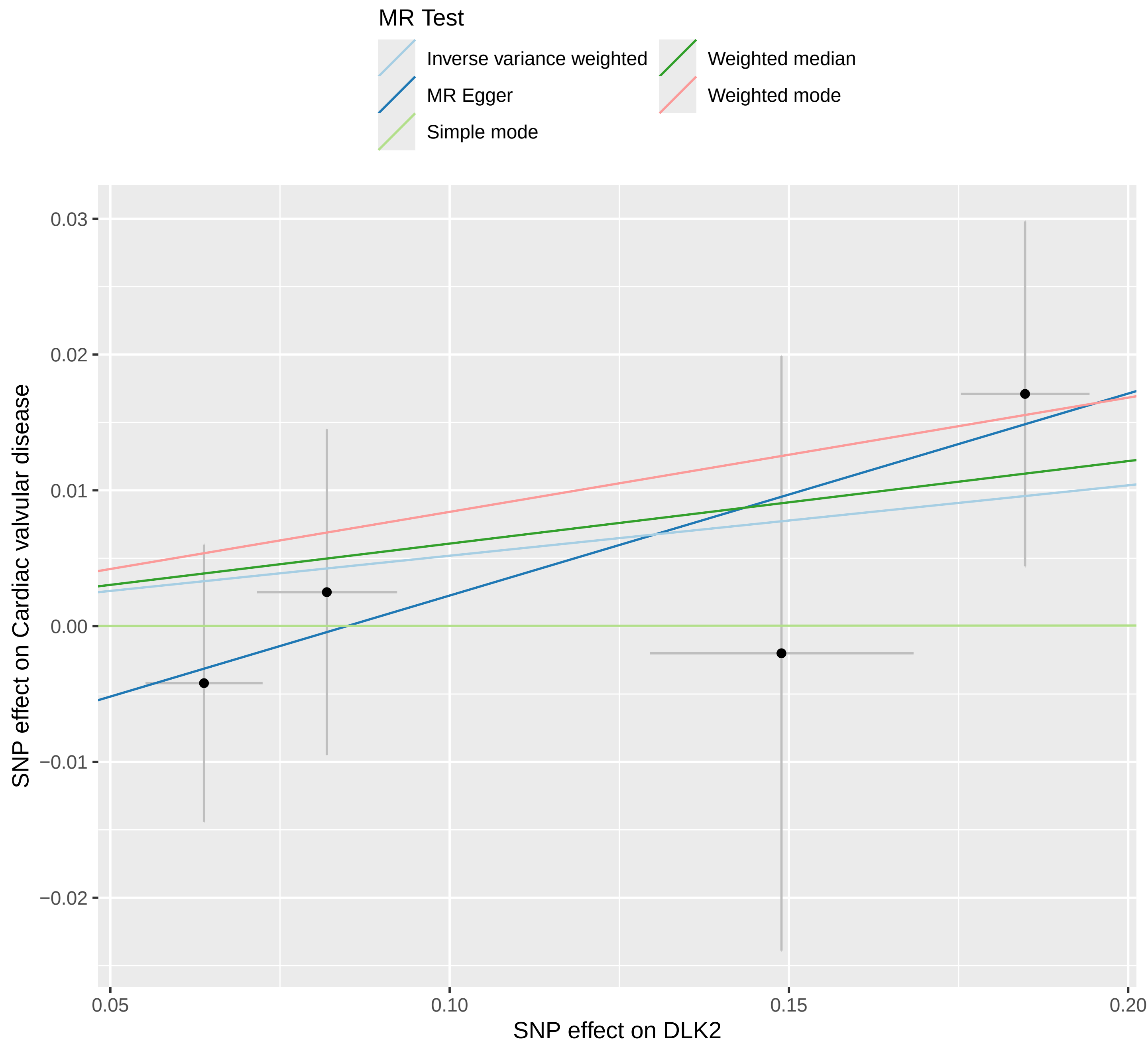

MR Test
Inverse variance weighted
MR Egger
Simple mode
Weighted median
Weighted mode
0.03
0.02
0.01
0.00
−0.01
−0.02
SNP effect on Cardiac valvular disease
0.05
0.10
0.15
0.20
SNP effect on DLK2

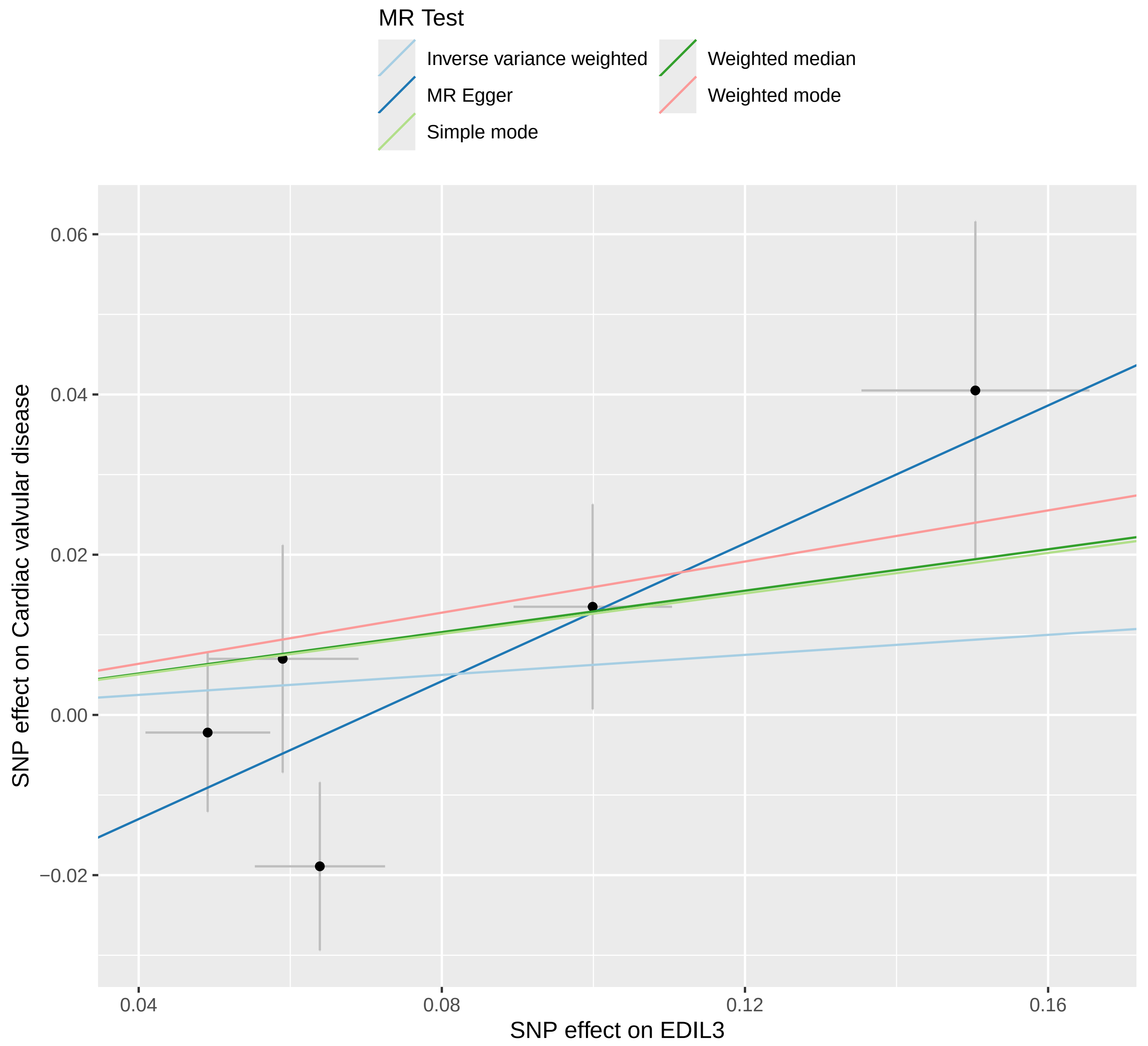
MR Test
Inverse variance weighted
MR Egger
Simple mode
Weighted median
Weighted mode
0.06
0.04
0.02
0.00
−0.02
SNP effect on Cardiac valvular disease
0.04
0.08
0.12
0.16
SNP effect on EDIL3

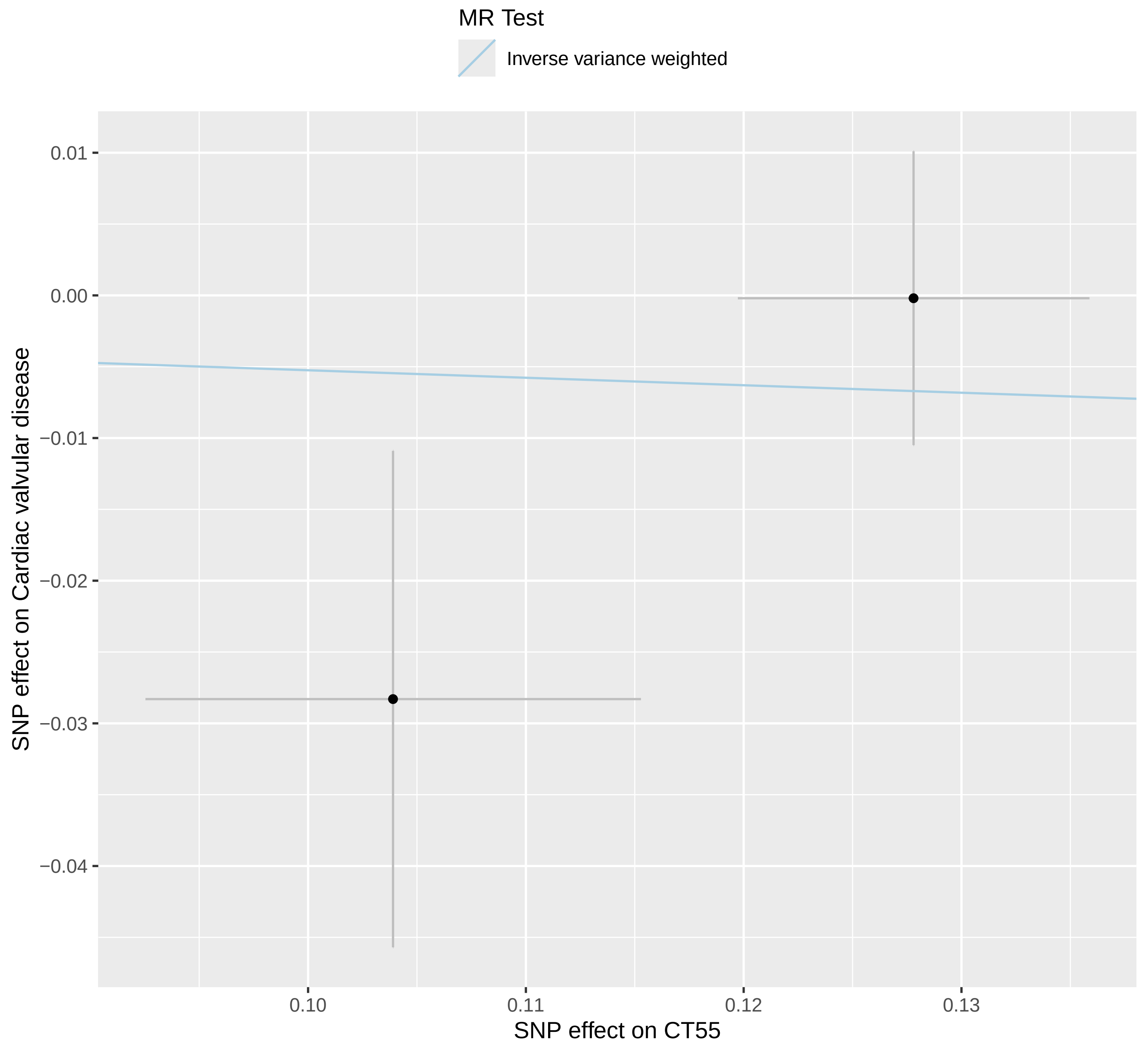
MR Test
Inverse variance weighted
0.01
0.00
−0.01
−0.02
−0.03
−0.04
0.10
0.11
0.12
0.13
SNP effect on CT55
SNP effect on Cardiac valvular disease

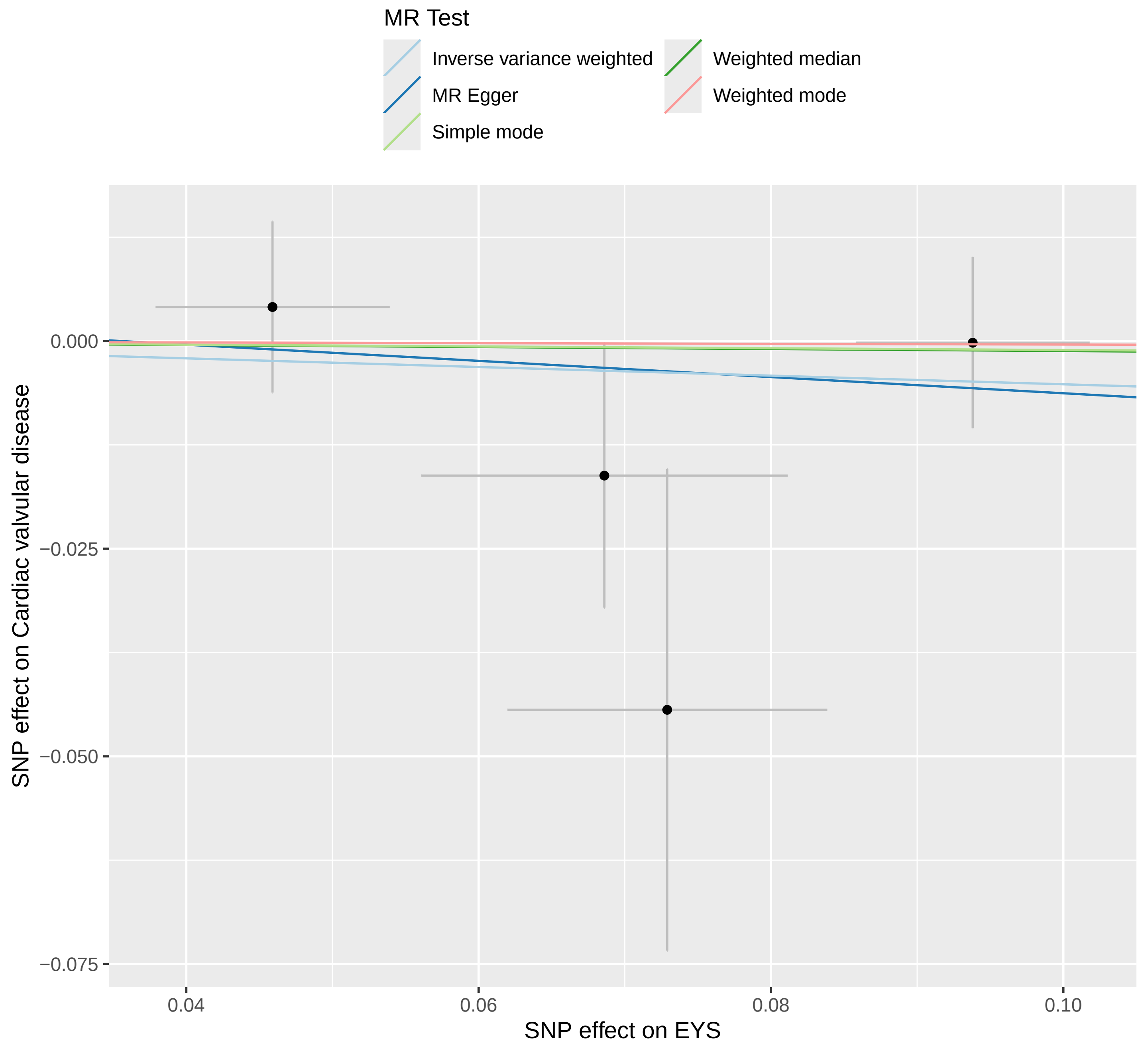
MR Test
Inverse variance weighted
MR Egger
Simple mode
Weighted median
Weighted mode
0.000
−0.025
−0.050
−0.075
0.04
0.06
0.08
0.10
SNP effect on Cardiac valvular disease
SNP effect on EYS

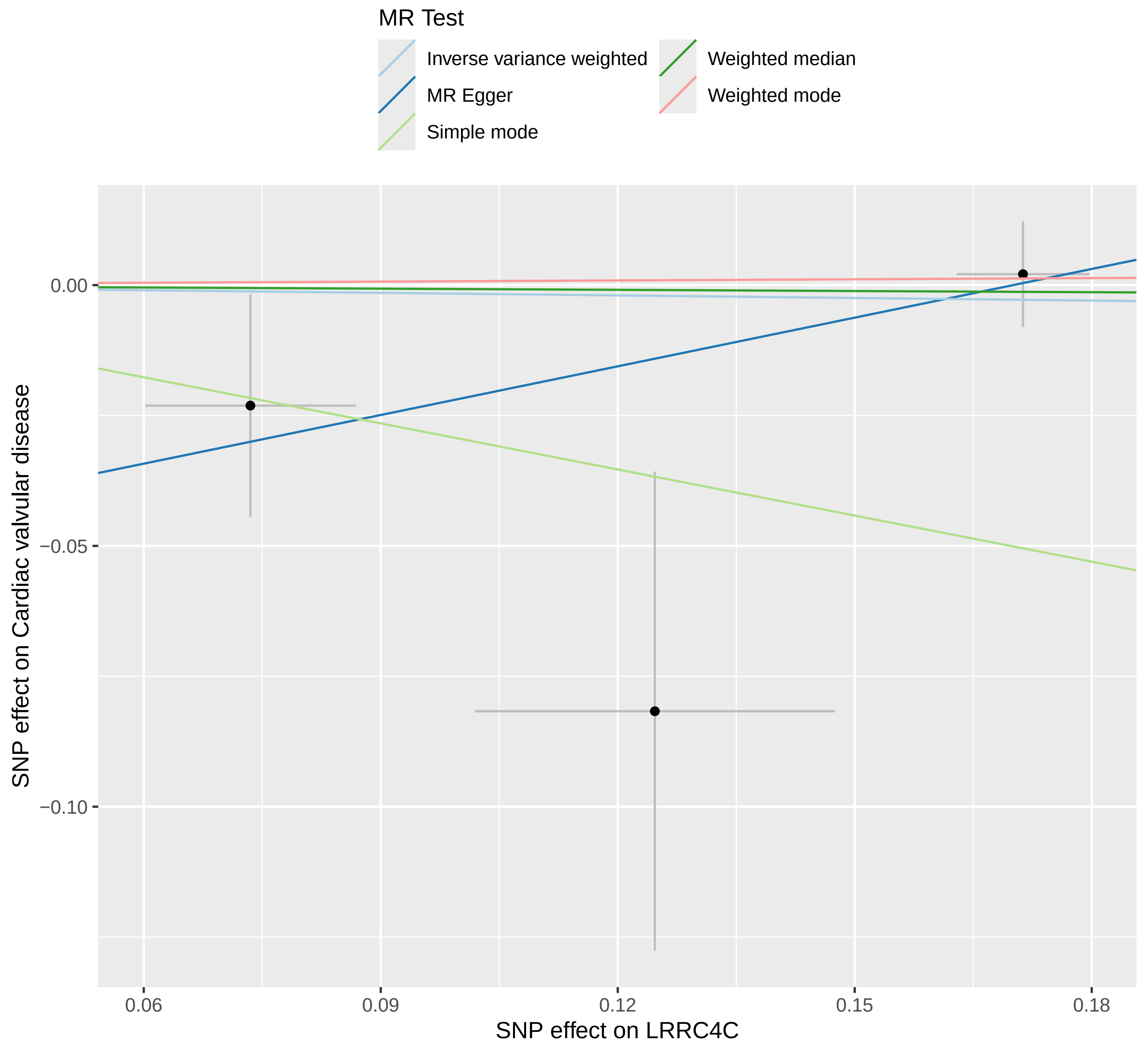
MR Test
Inverse variance weighted
MR Egger
Simple mode
Weighted median
Weighted mode
0.00
−0.05
−0.10
0.06
0.09
0.12
0.15
0.18
SNP effect on Cardiac valvular disease
SNP effect on LRRC4C

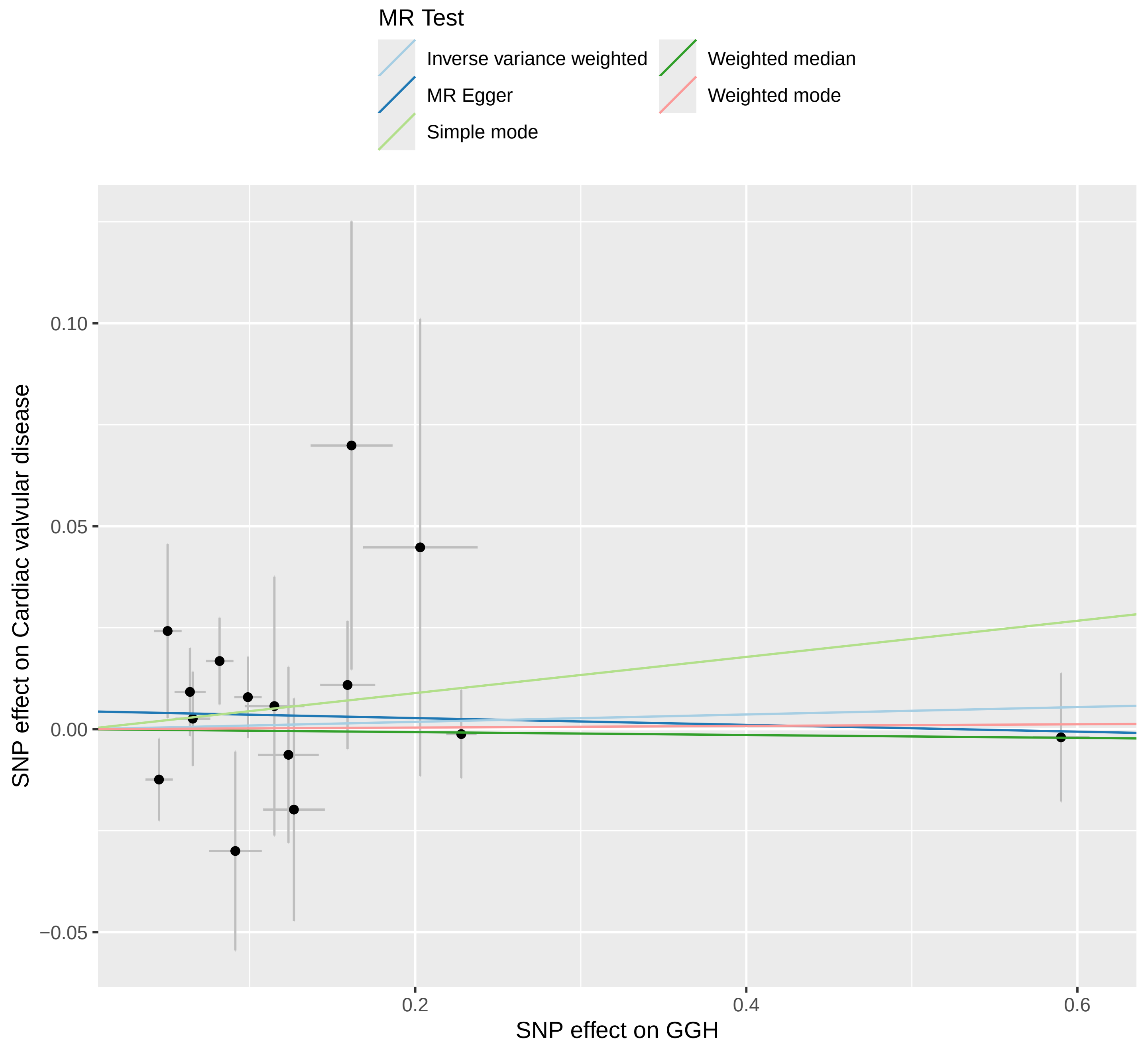

MR Test
Inverse variance weighted
MR Egger
Simple mode
Weighted median
Weighted mode
SNP effect on Cardiac valvular disease
0.10
0.05
0.00
−0.05
SNP effect on GGH
0.2
0.4
0.6

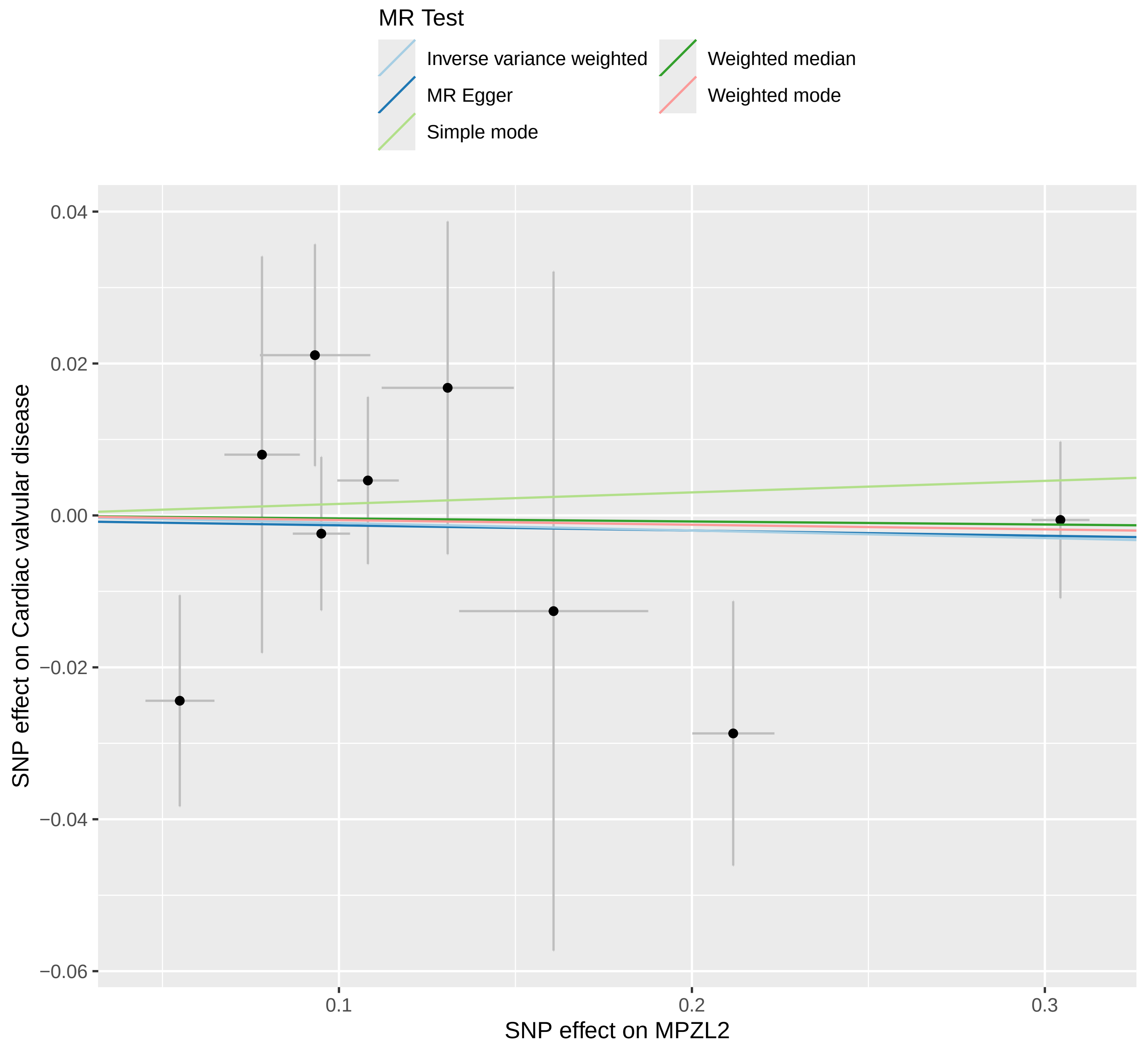
MR Test
Inverse variance weighted
MR Egger
Simple mode
Weighted median
Weighted mode
0.04
0.02
0.00
−0.02
−0.04
−0.06
0.1
0.2
0.3
SNP effect on Cardiac valvular disease
SNP effect on MPZL2

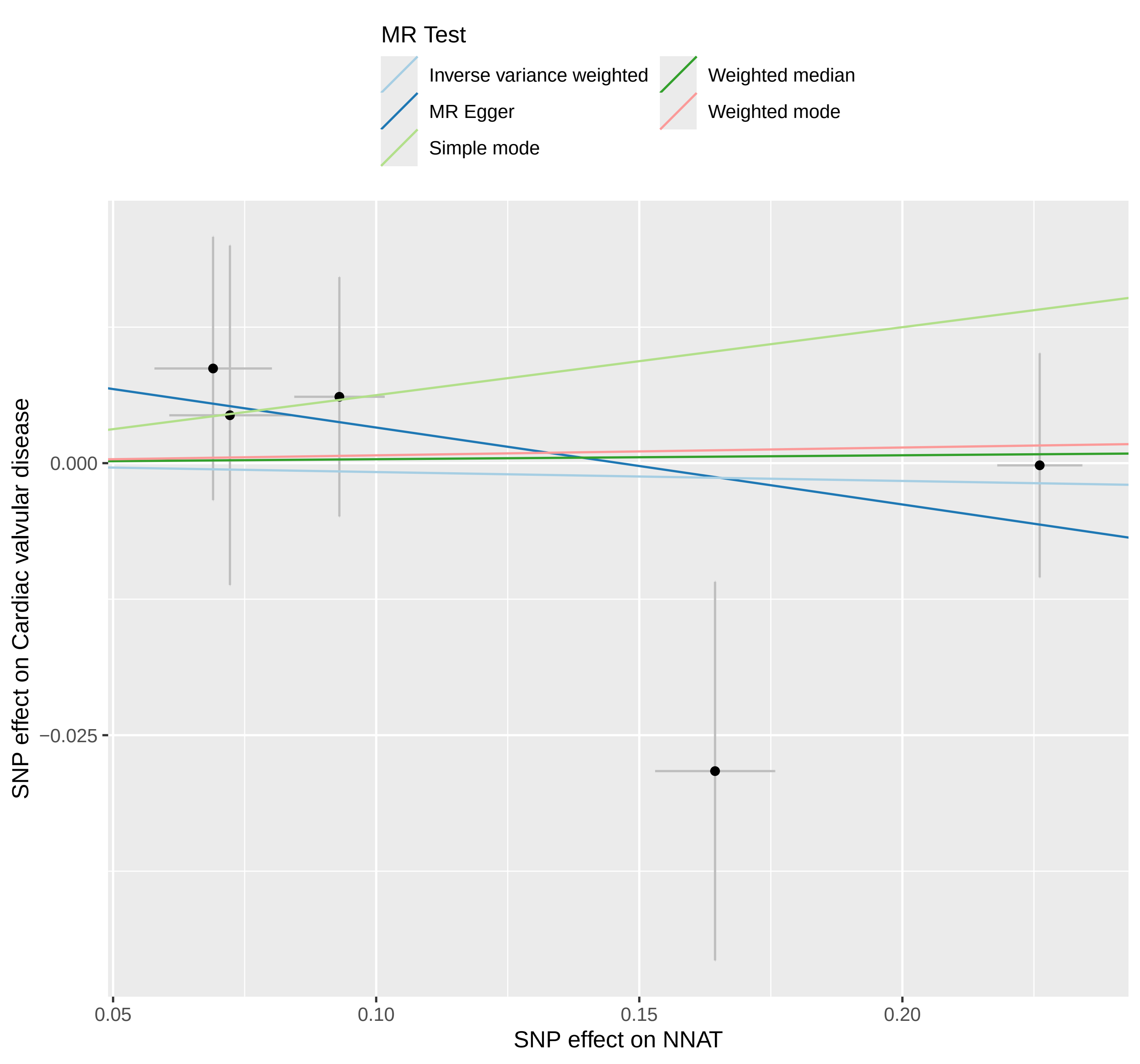

MR Test
Inverse variance weighted
MR Egger
Simple mode
Weighted median
Weighted mode
SNP effect on Cardiac valvular disease
0.000
−0.025
0.05
0.10
0.15
0.20
SNP effect on NNAT

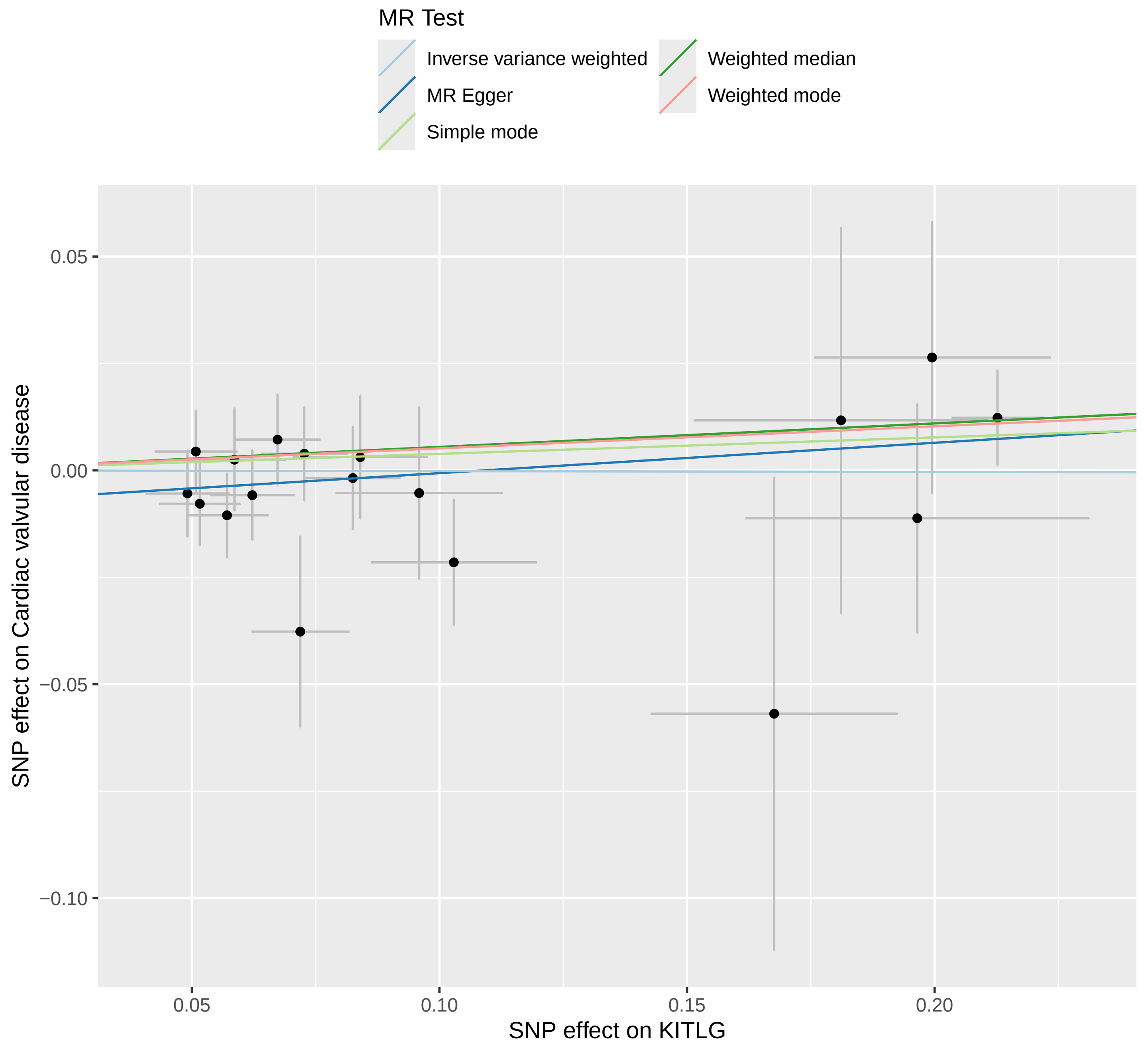
MR Test
Inverse variance weighted
MR Egger
Simple mode
Weighted median
Weighted mode
0.05
0.00
−0.05
−0.10
0.05
0.10
0.15
0.20
SNP effect on Cardiac valvular disease
SNP effect on KITLG

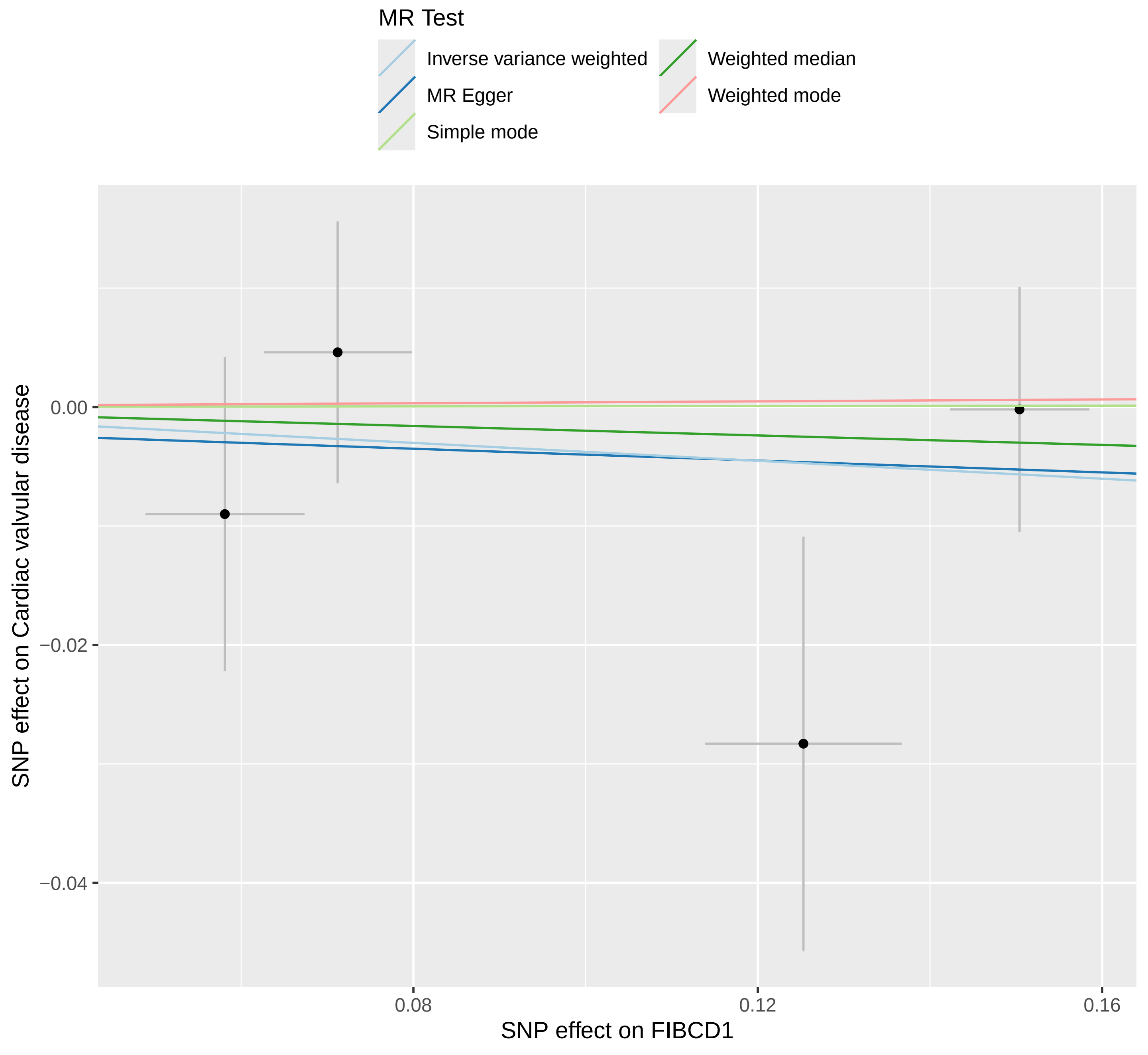

MR Test
Inverse variance weighted
MR Egger
Simple mode
Weighted median
Weighted mode
0.00
−0.02
−0.04
0.08
0.12
0.16
SNP effect on FIBCD1
SNP effect on Cardiac valvular disease

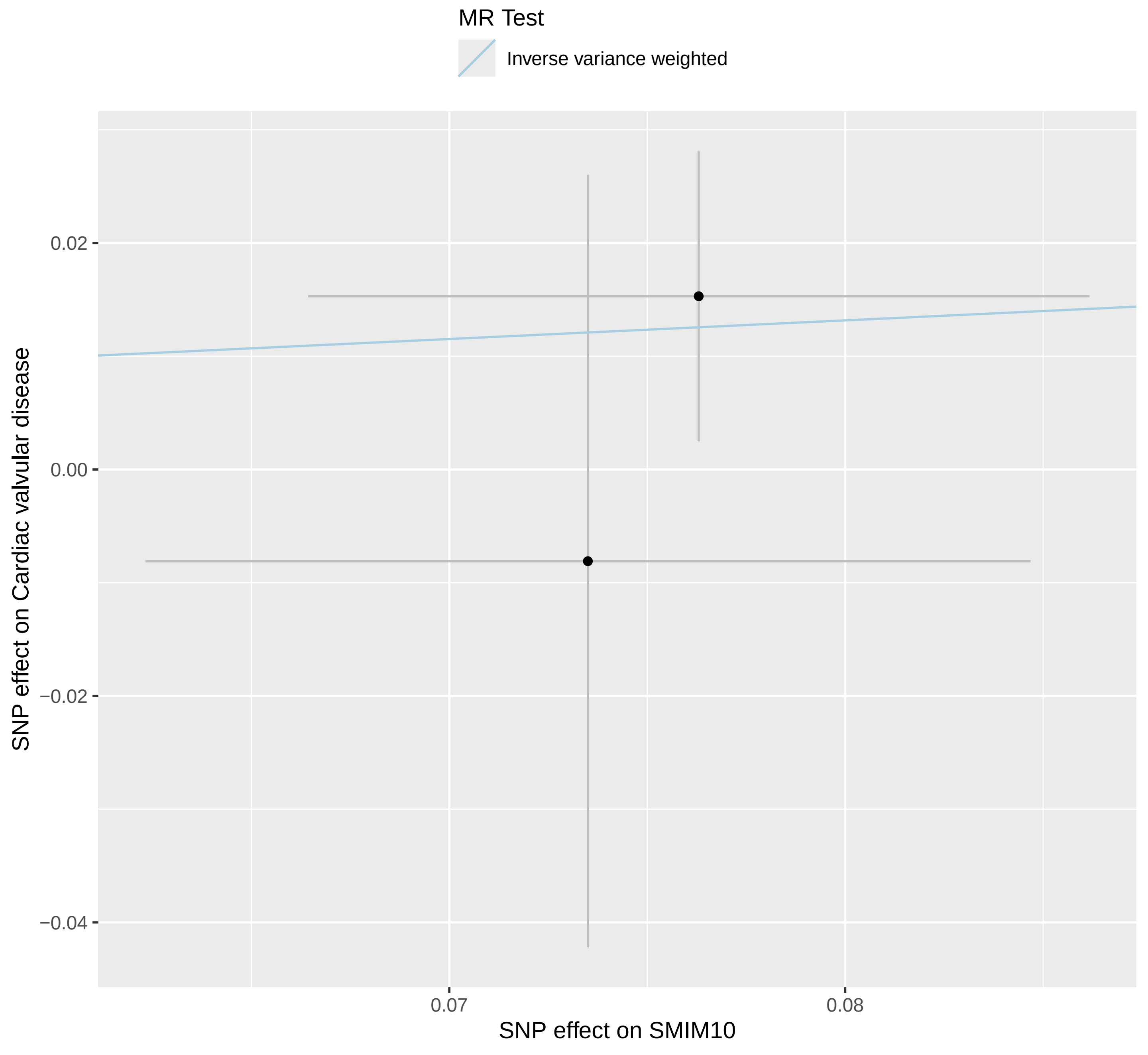

MR Test
Inverse variance weighted
SNP effect on Cardiac valvular disease
0.02
0.00
−0.02
−0.04
0.07
0.08
SNP effect on SMIM10

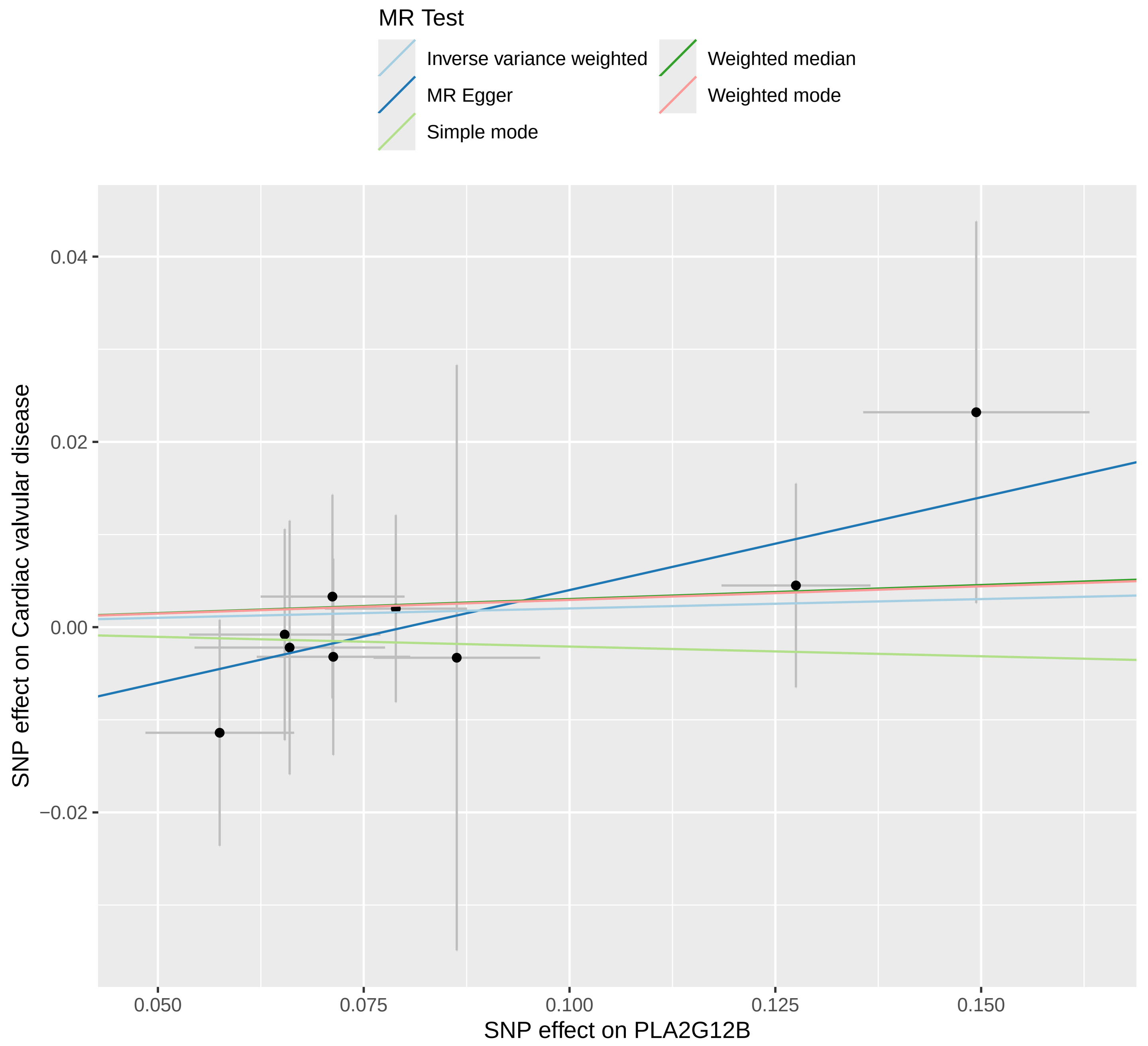

MR Test
Inverse variance weighted
MR Egger
Simple mode
Weighted median
Weighted mode
0.04
0.02
0.00
−0.02
SNP effect on Cardiac valvular disease
0.050
0.075
0.100
0.125
0.150
SNP effect on PLA2G12B

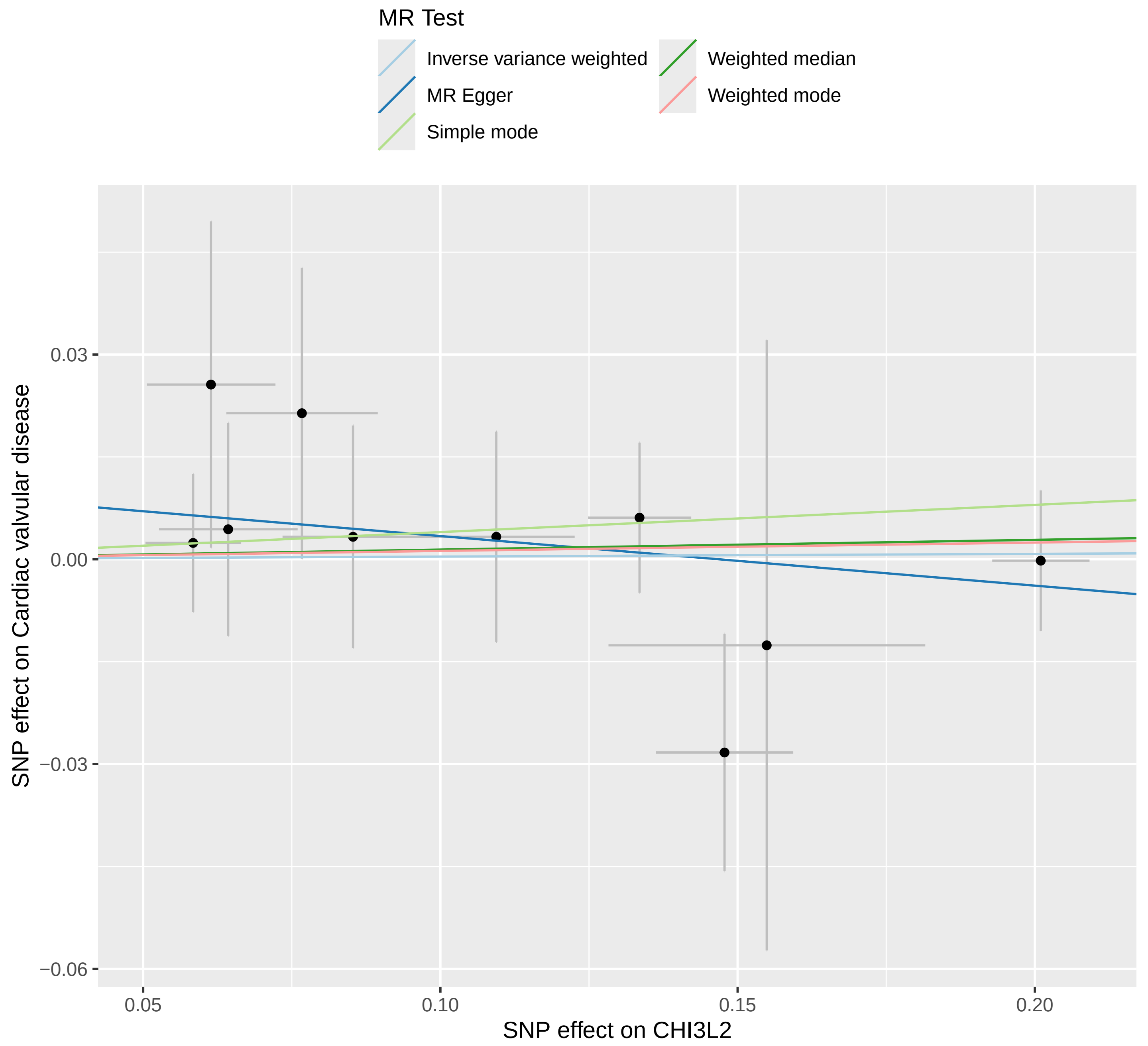
MR Test
Inverse variance weighted
MR Egger
Simple mode
Weighted median
Weighted mode
SNP effect on Cardiac valvular disease
0.03
0.00
−0.03
−0.06
0.05
0.10
0.15
0.20
SNP effect on CHI3L2

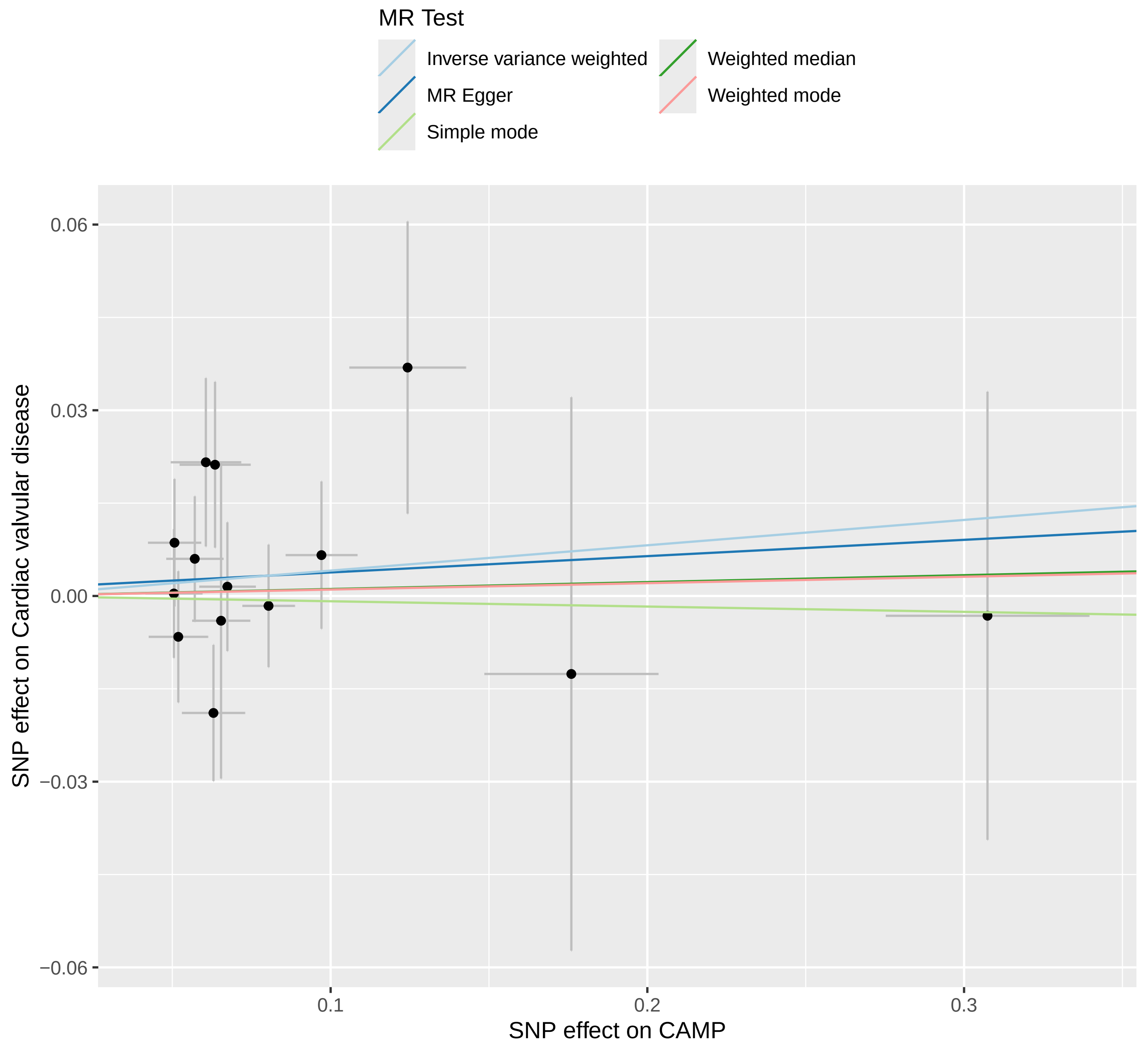
MR Test
Inverse variance weighted
MR Egger
Simple mode
Weighted median
Weighted mode
SNP effect on Cardiac valvular disease
0.06
0.03
0.00
−0.03
−0.06
0.1
0.2
0.3
SNP effect on CAMP

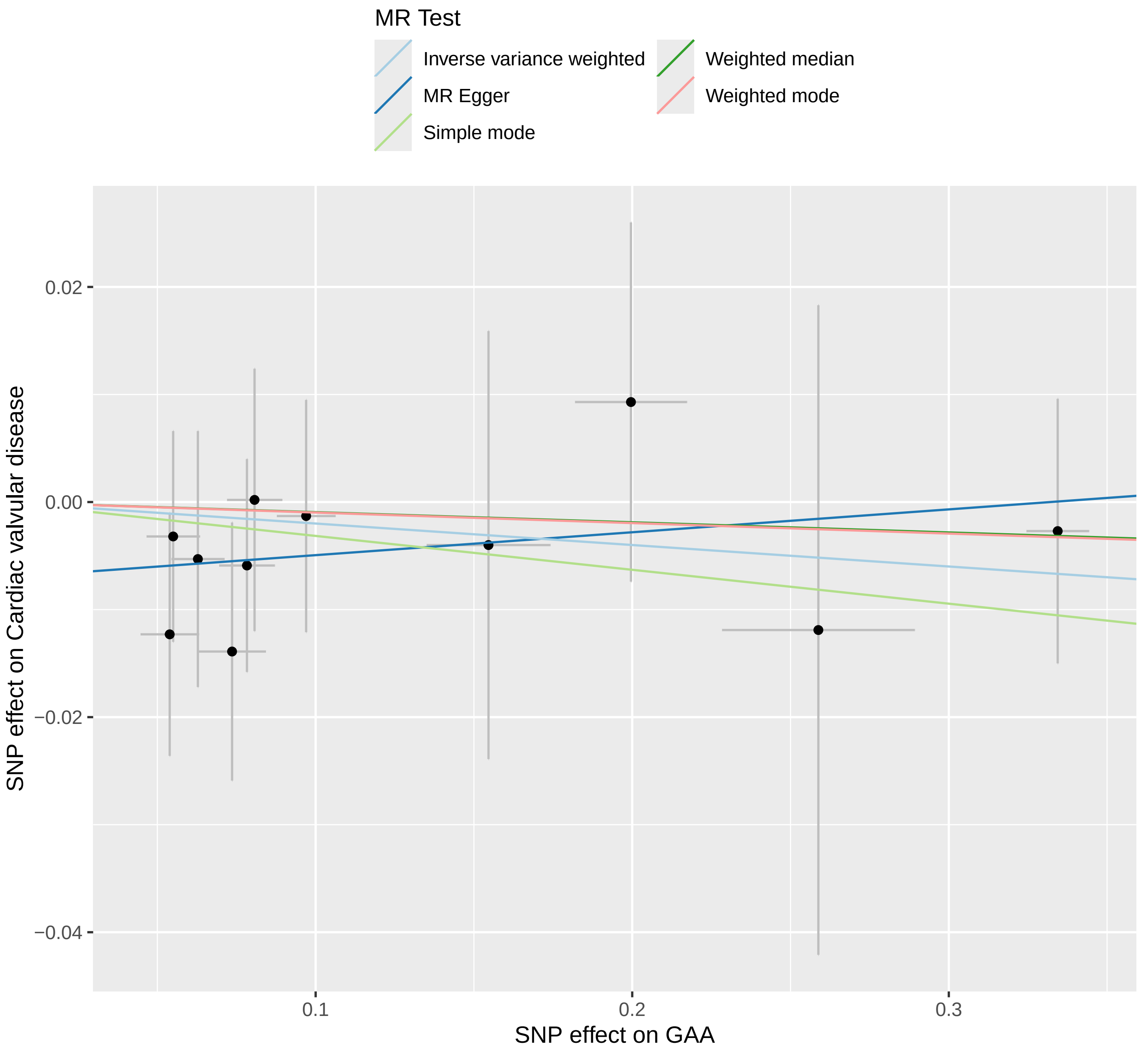

MR Test
Inverse variance weighted
MR Egger
Simple mode
Weighted median
Weighted mode
0.02
0.00
−0.02
−0.04
SNP effect on Cardiac valvular disease
0.1
0.2
0.3
SNP effect on GAA

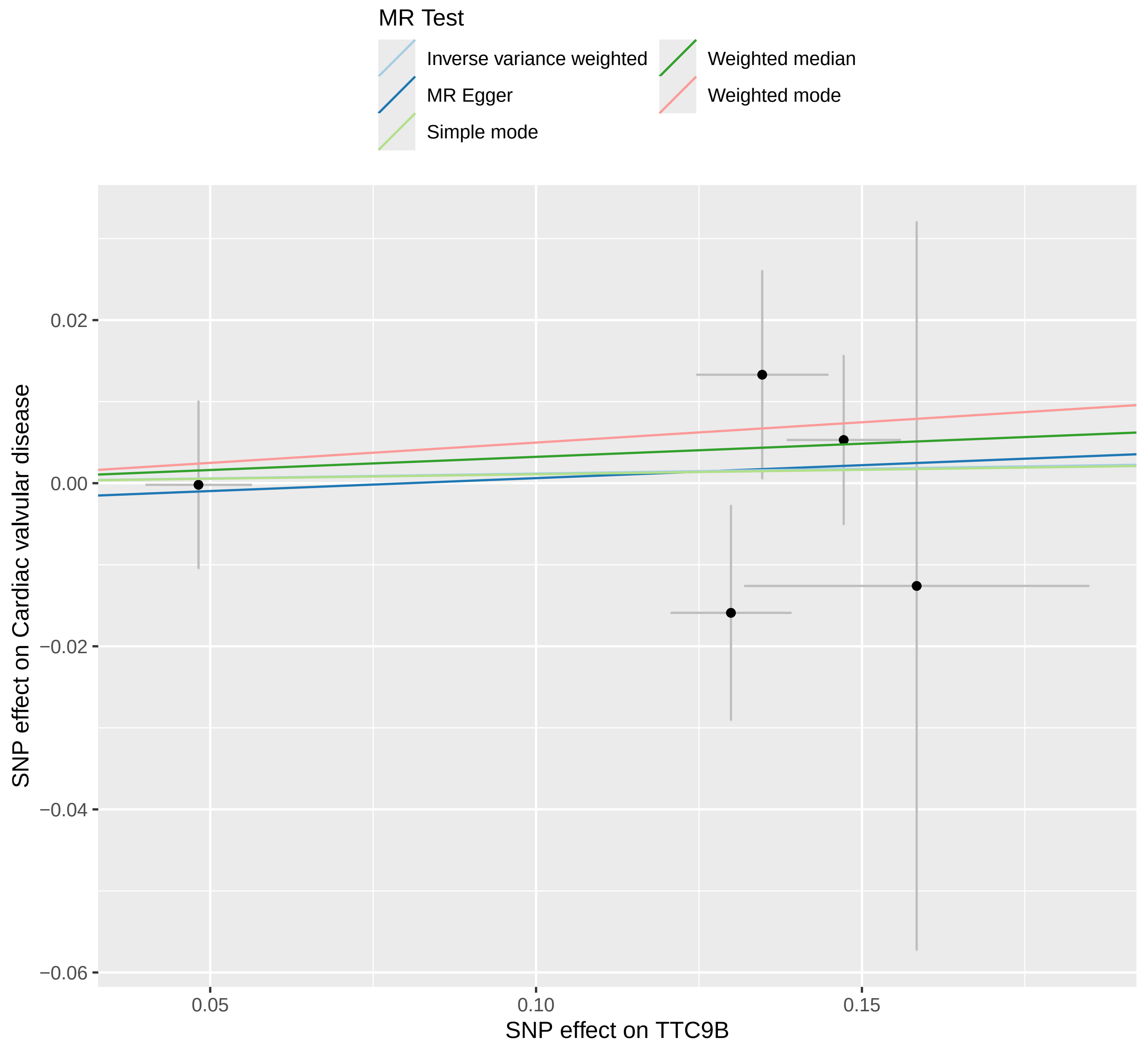
MR Test
Inverse variance weighted
MR Egger
Simple mode
Weighted median
Weighted mode
SNP effect on Cardiac valvular disease
0.02
0.00
−0.02
−0.04
−0.06
0.05
0.10
0.15
SNP effect on TTC9B

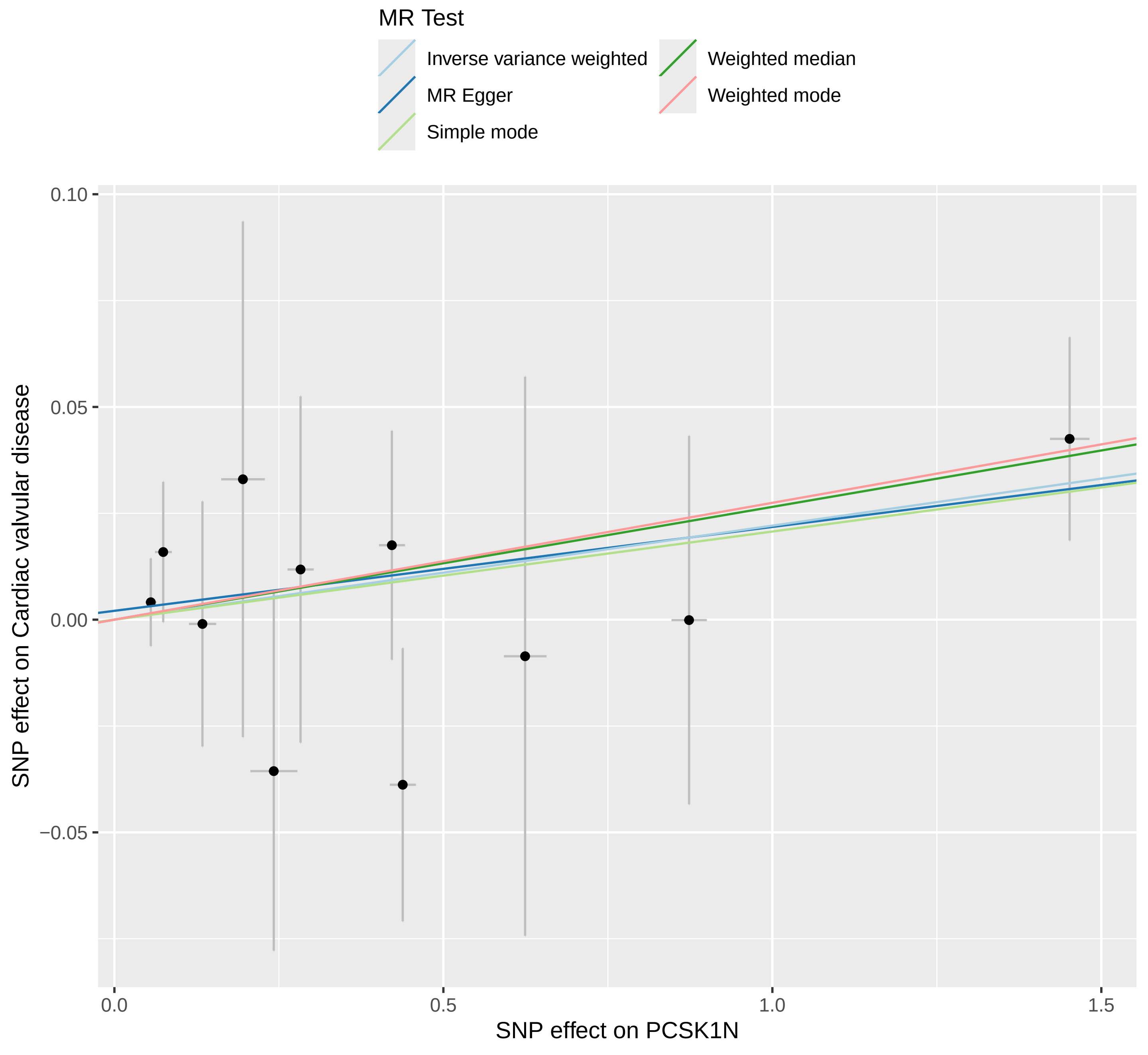
MR Test
Inverse variance weighted
MR Egger
Simple mode
Weighted median
Weighted mode
0.10
0.05
0.00
−0.05
0.0
0.5
1.0
1.5
SNP effect on Cardiac valvular disease
SNP effect on PCSK1N

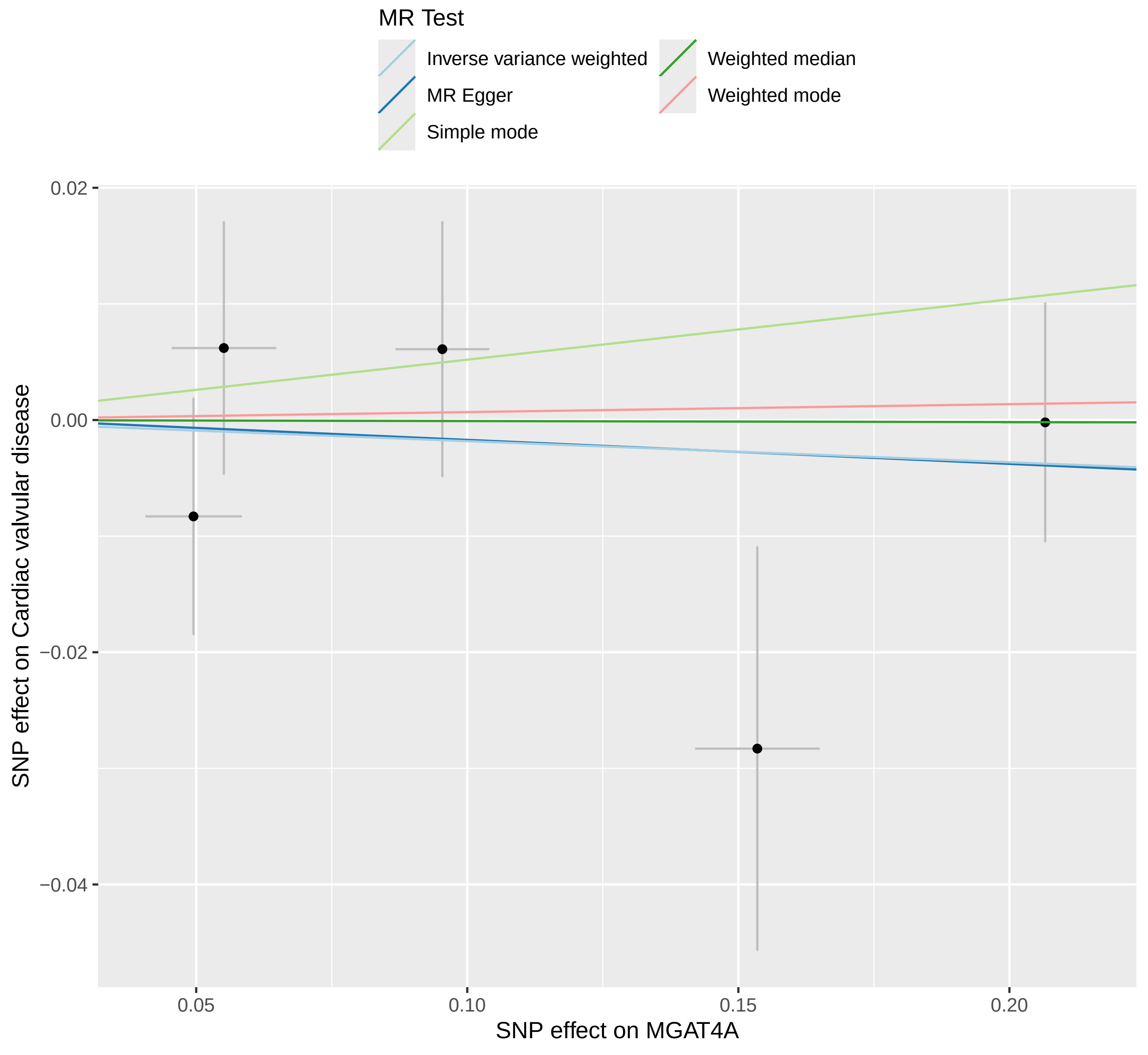

MR Test
Inverse variance weighted
MR Egger
Simple mode
Weighted median
Weighted mode
0.02
0.00
−0.02
−0.04
0.05
0.10
0.15
0.20
SNP effect on Cardiac valvular disease
SNP effect on MGAT4A

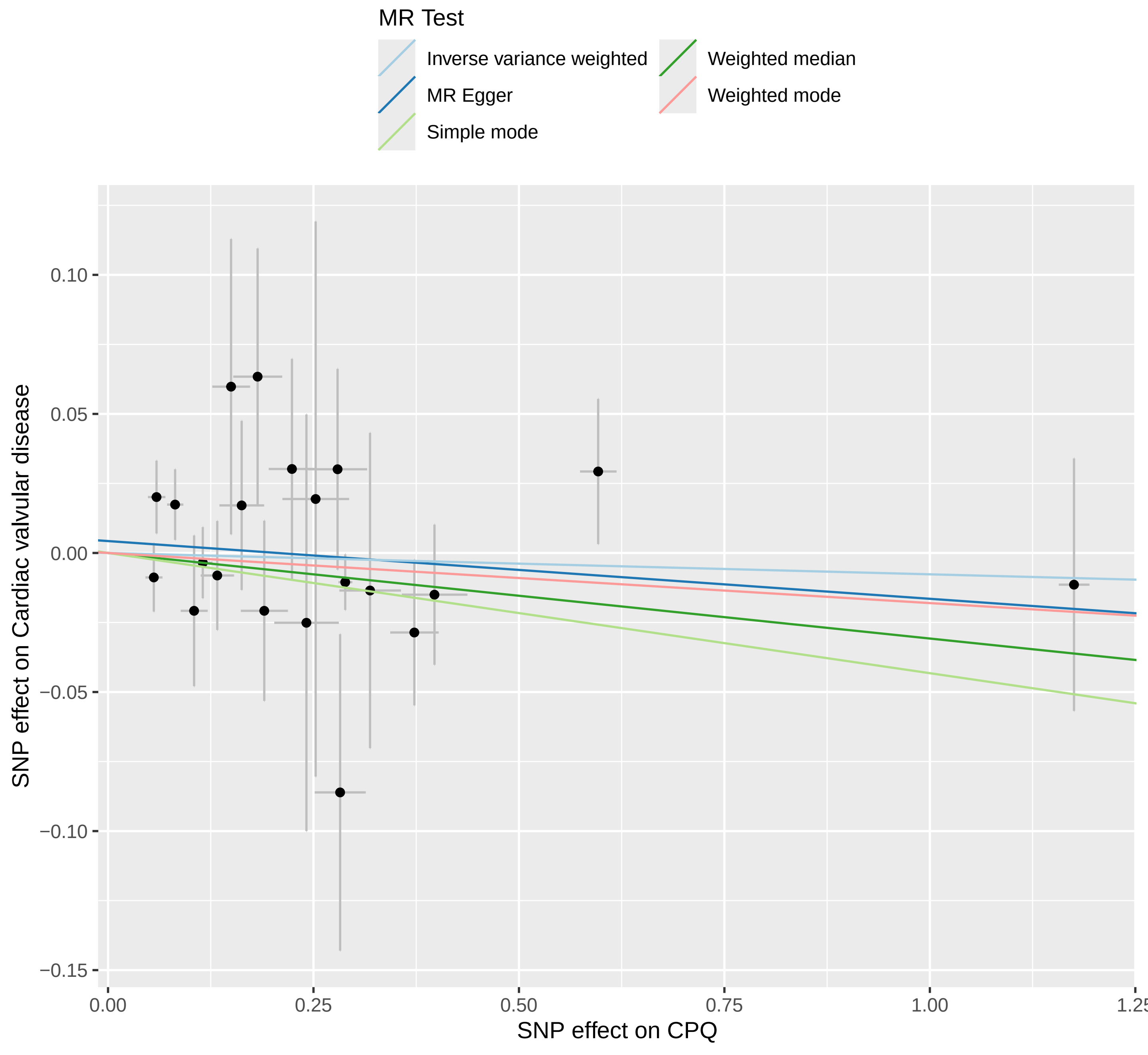
MR Test
Inverse variance weighted
MR Egger
Simple mode
Weighted median
Weighted mode
0.10
0.05
0.00
−0.05
−0.10
−0.15
SNP effect on Cardiac valvular disease
0.00
0.25
0.50
0.75
1.00
1.25
SNP effect on CPQ

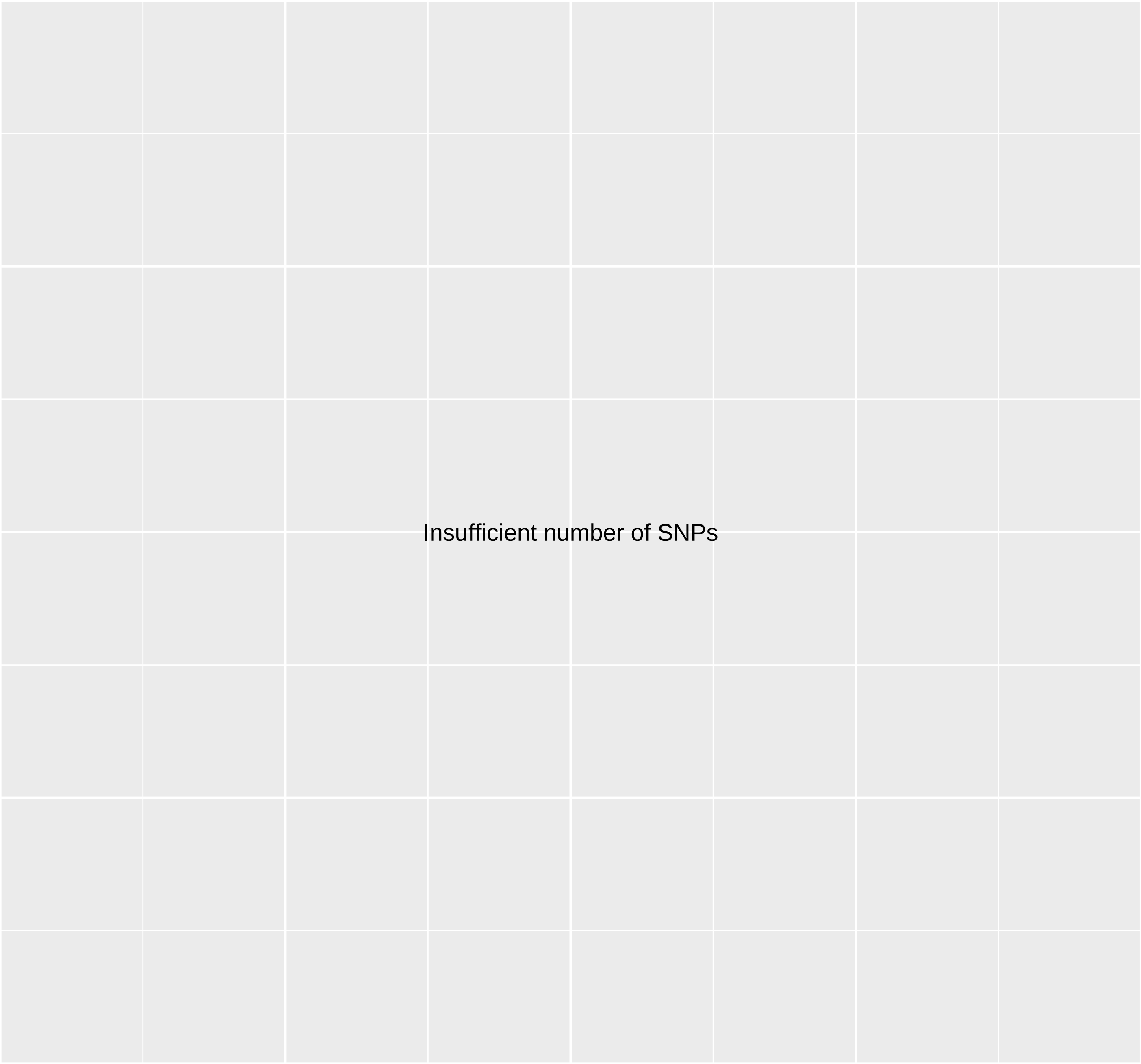
Insufficient number of SNPs

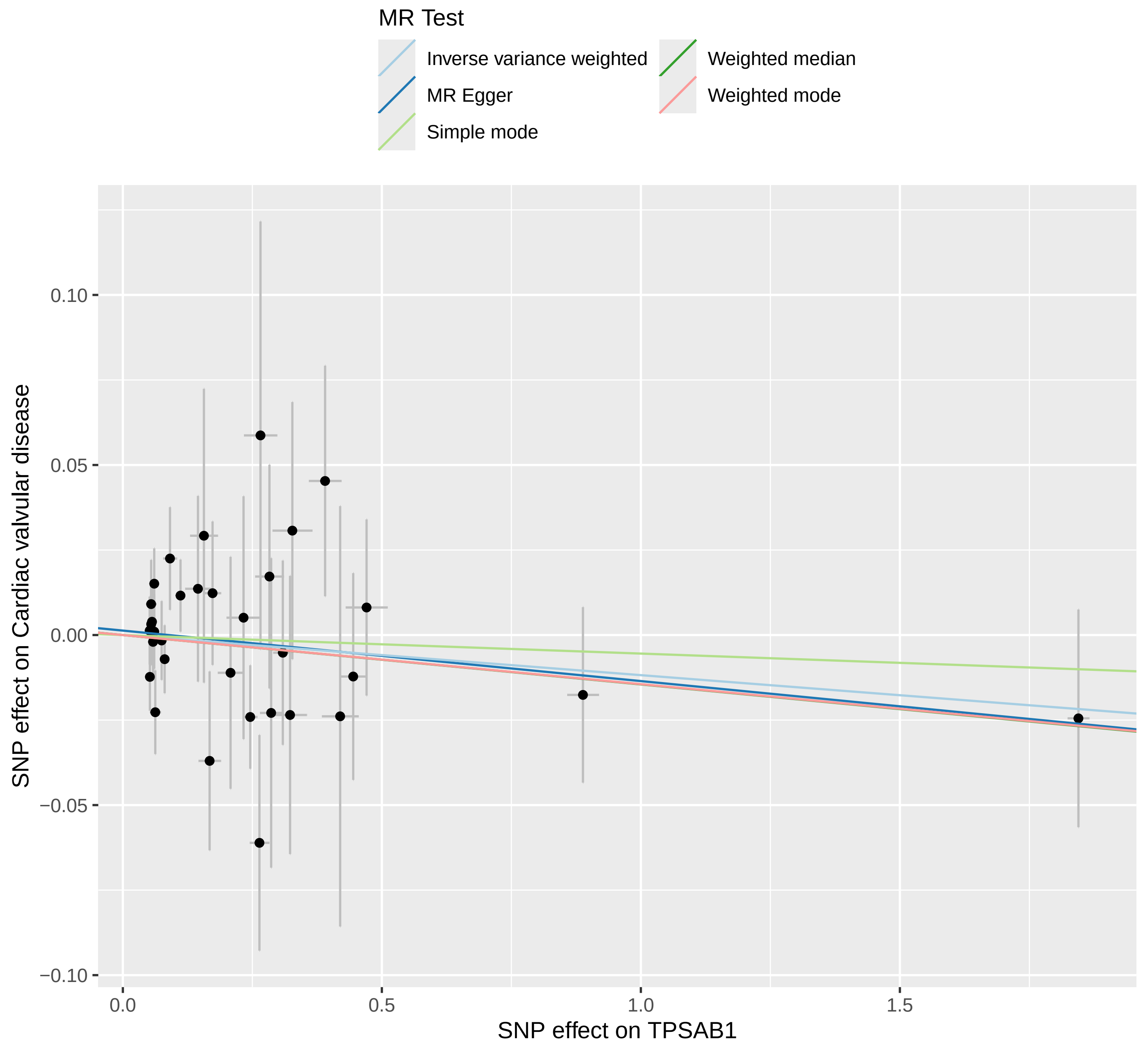

MR Test
Inverse variance weighted
MR Egger
Simple mode
Weighted median
Weighted mode
0.10
0.05
0.00
−0.05
−0.10
SNP effect on Cardiac valvular disease
0.0
0.5
1.0
1.5
SNP effect on TPSAB1

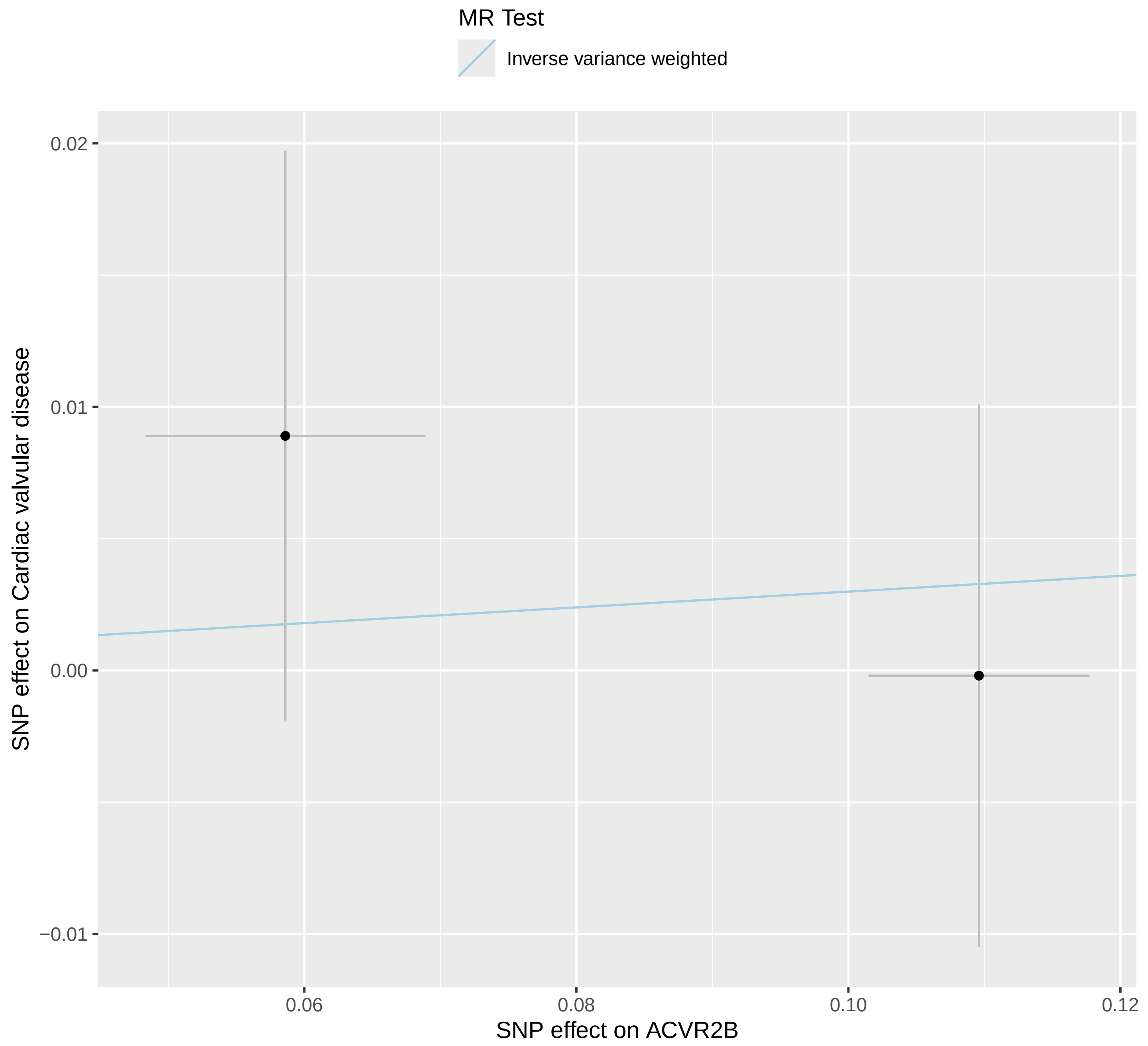
MR Test
Inverse variance weighted
0.02
0.01
0.00
−0.01
0.06
0.08
0.10
0.12
SNP effect on ACVR2B
SNP effect on Cardiac valvular disease

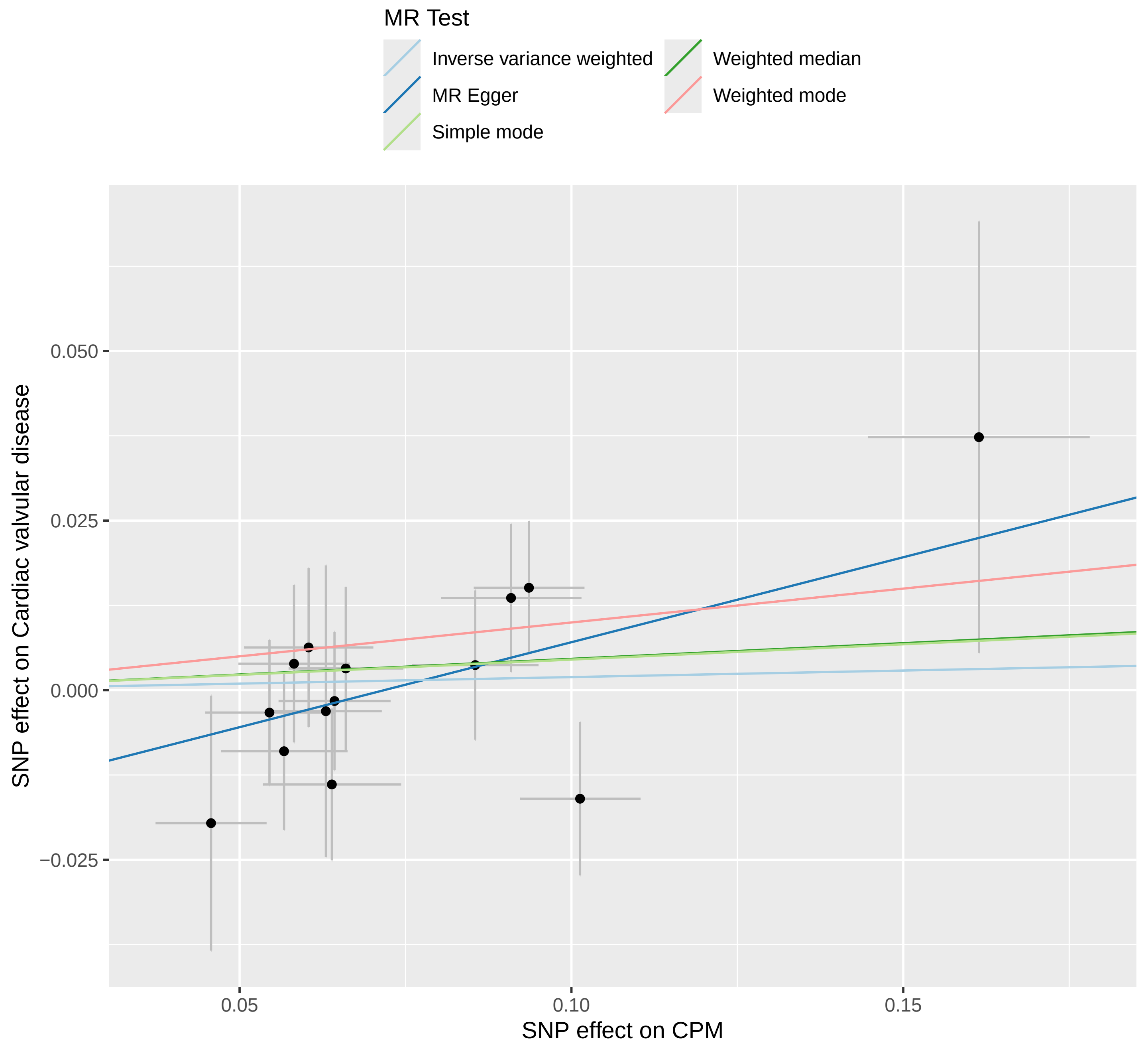
MR Test
Inverse variance weighted
MR Egger
Simple mode
Weighted median
Weighted mode
0.050
0.025
0.000
−0.025
SNP effect on Cardiac valvular disease
0.05
0.10
0.15
SNP effect on CPM

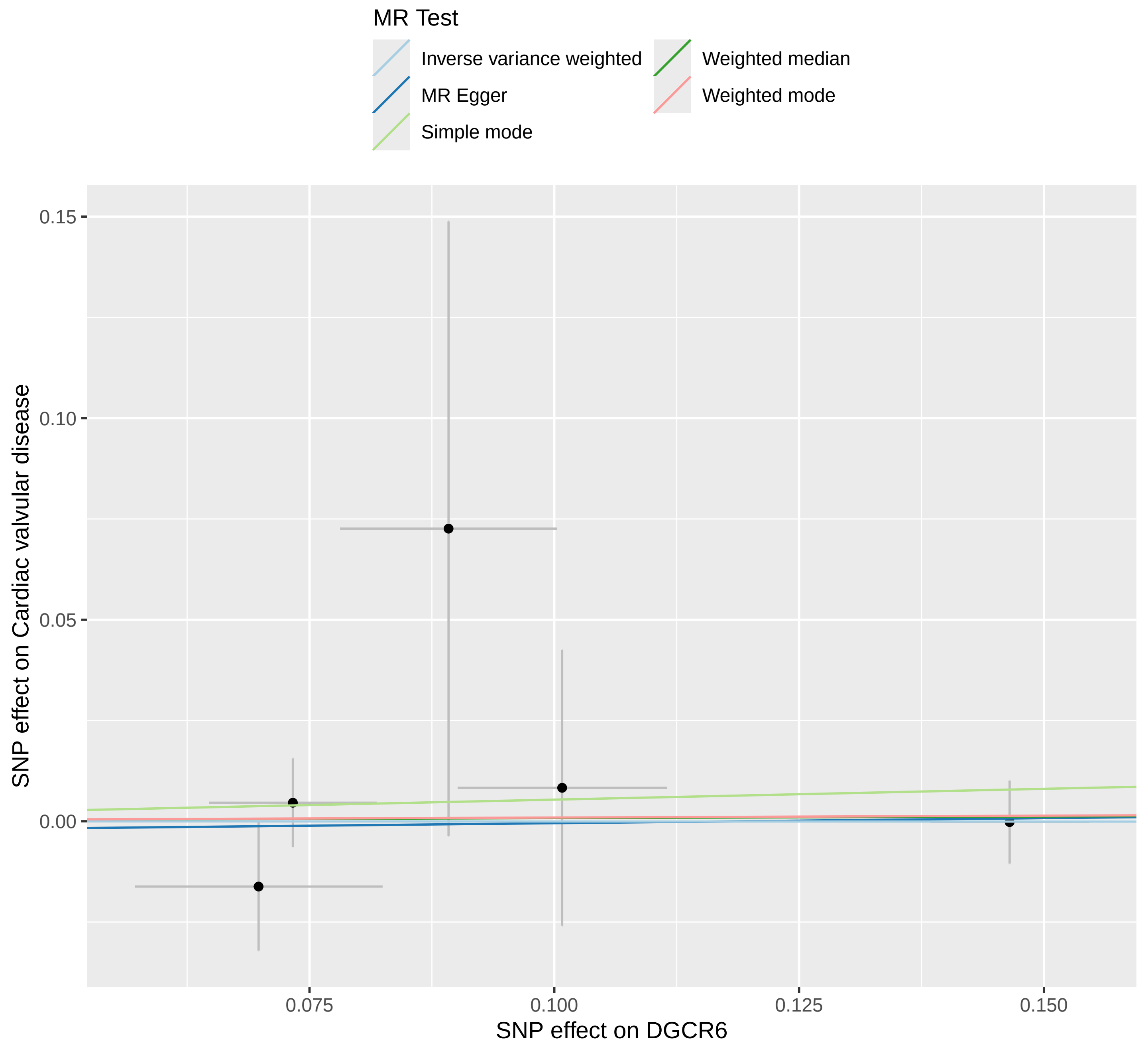

MR Test
Inverse variance weighted
MR Egger
Simple mode
Weighted median
Weighted mode
0.15
0.10
0.05
0.00
0.075
0.100
0.125
0.150
SNP effect on Cardiac valvular disease
SNP effect on DGCR6

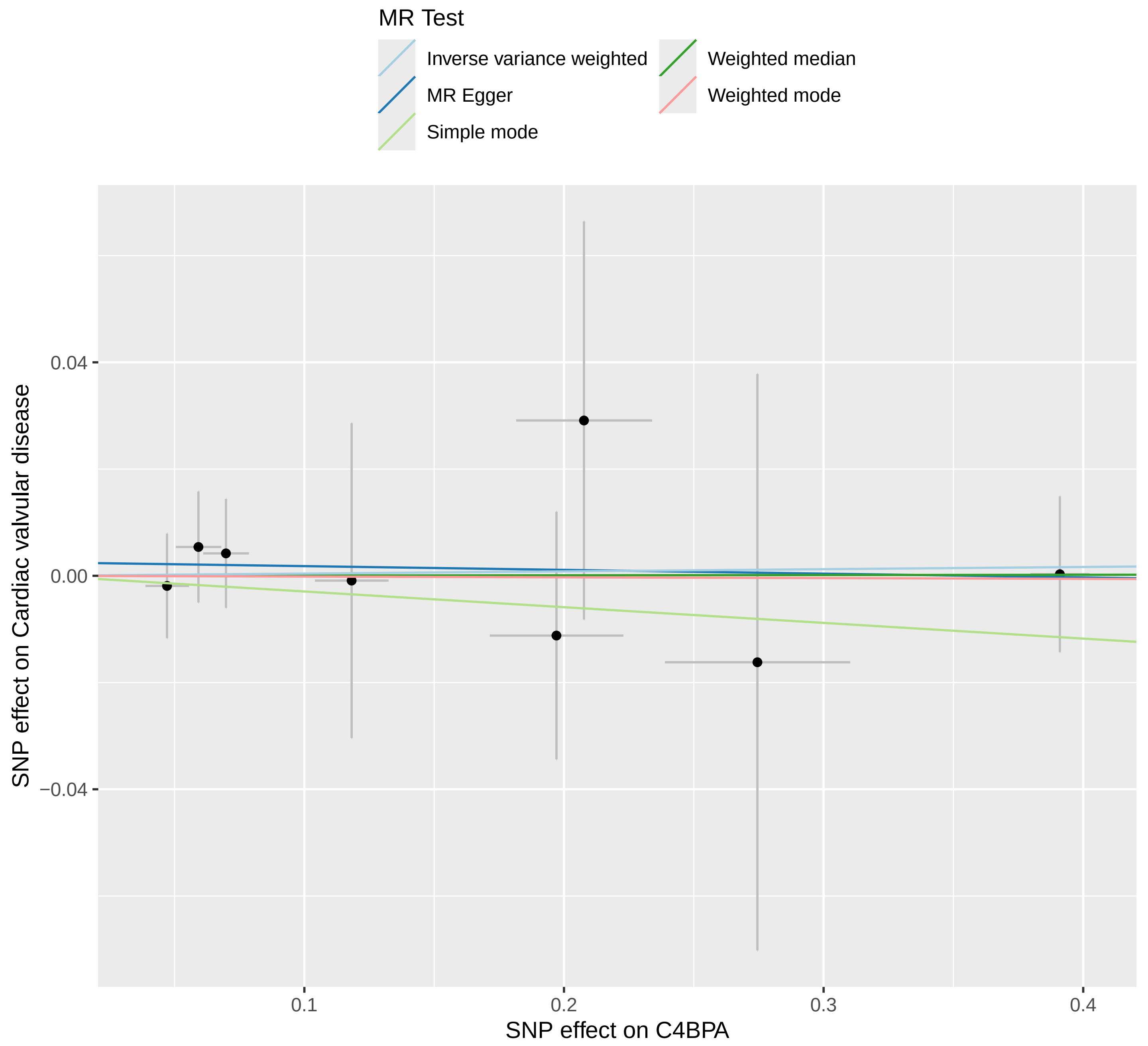

MR Test
Inverse variance weighted
MR Egger
Simple mode
Weighted median
Weighted mode
0.04
0.00
−0.04
0.1
0.2
0.3
0.4
SNP effect on Cardiac valvular disease
SNP effect on C4BPA

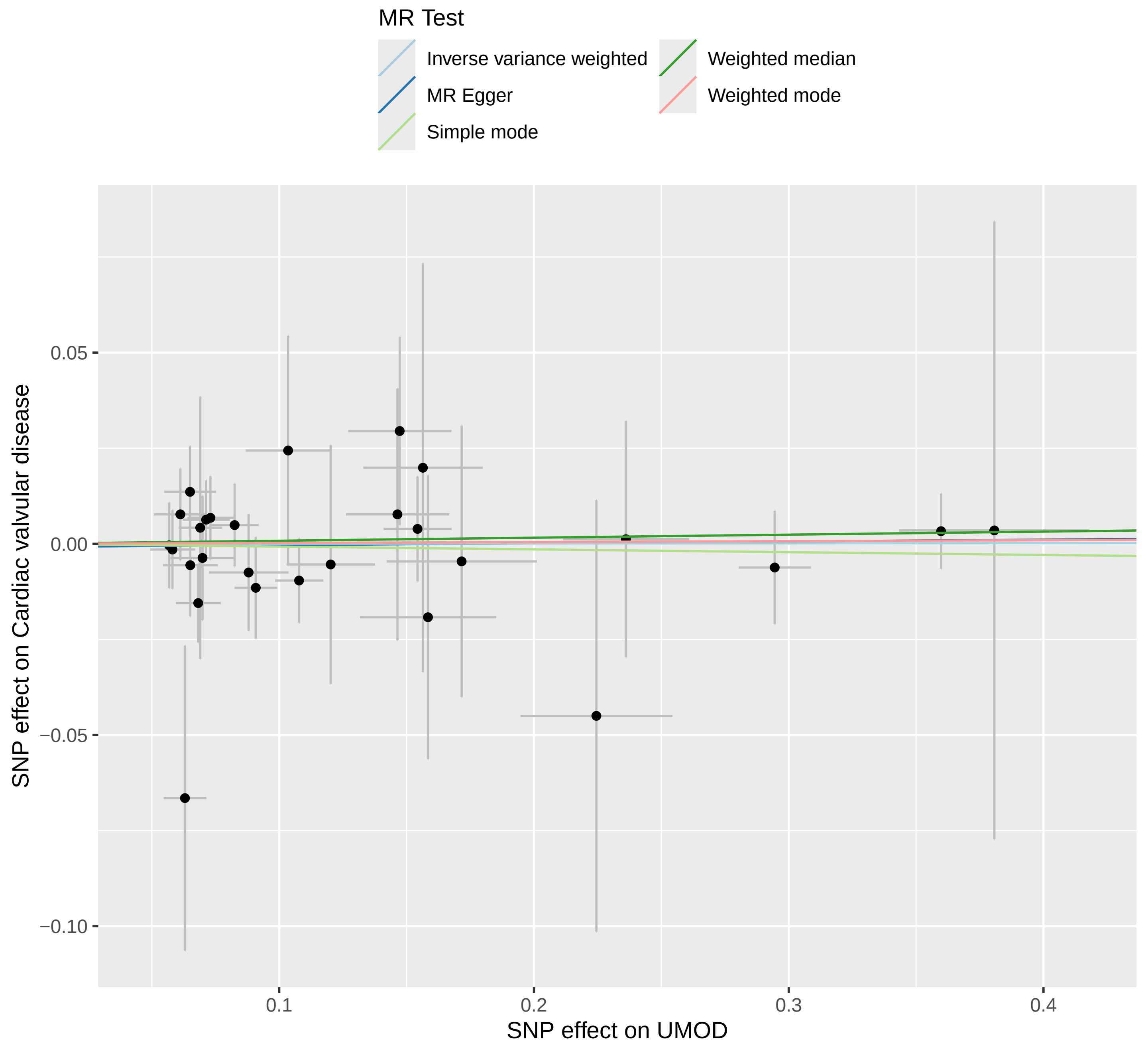
MR Test
Inverse variance weighted
MR Egger
Simple mode
Weighted median
Weighted mode
SNP effect on Cardiac valvular disease
0.05
0.00
−0.05
−0.10
0.1
0.2
0.3
0.4
SNP effect on UMOD

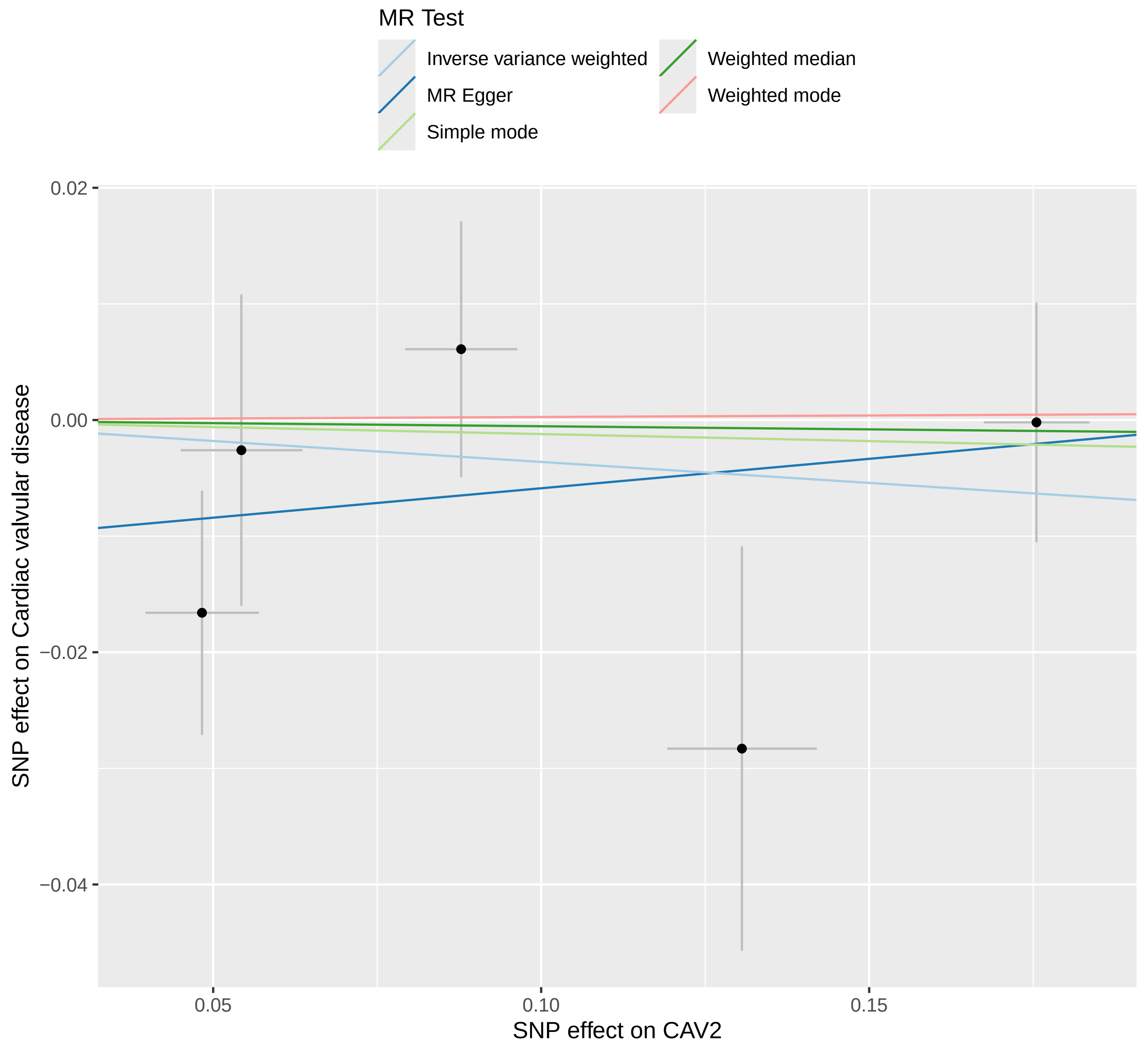
MR Test
Inverse variance weighted
MR Egger
Simple mode
Weighted median
Weighted mode
0.02
0.00
−0.02
−0.04
SNP effect on Cardiac valvular disease
0.05
0.10
0.15
SNP effect on CAV2

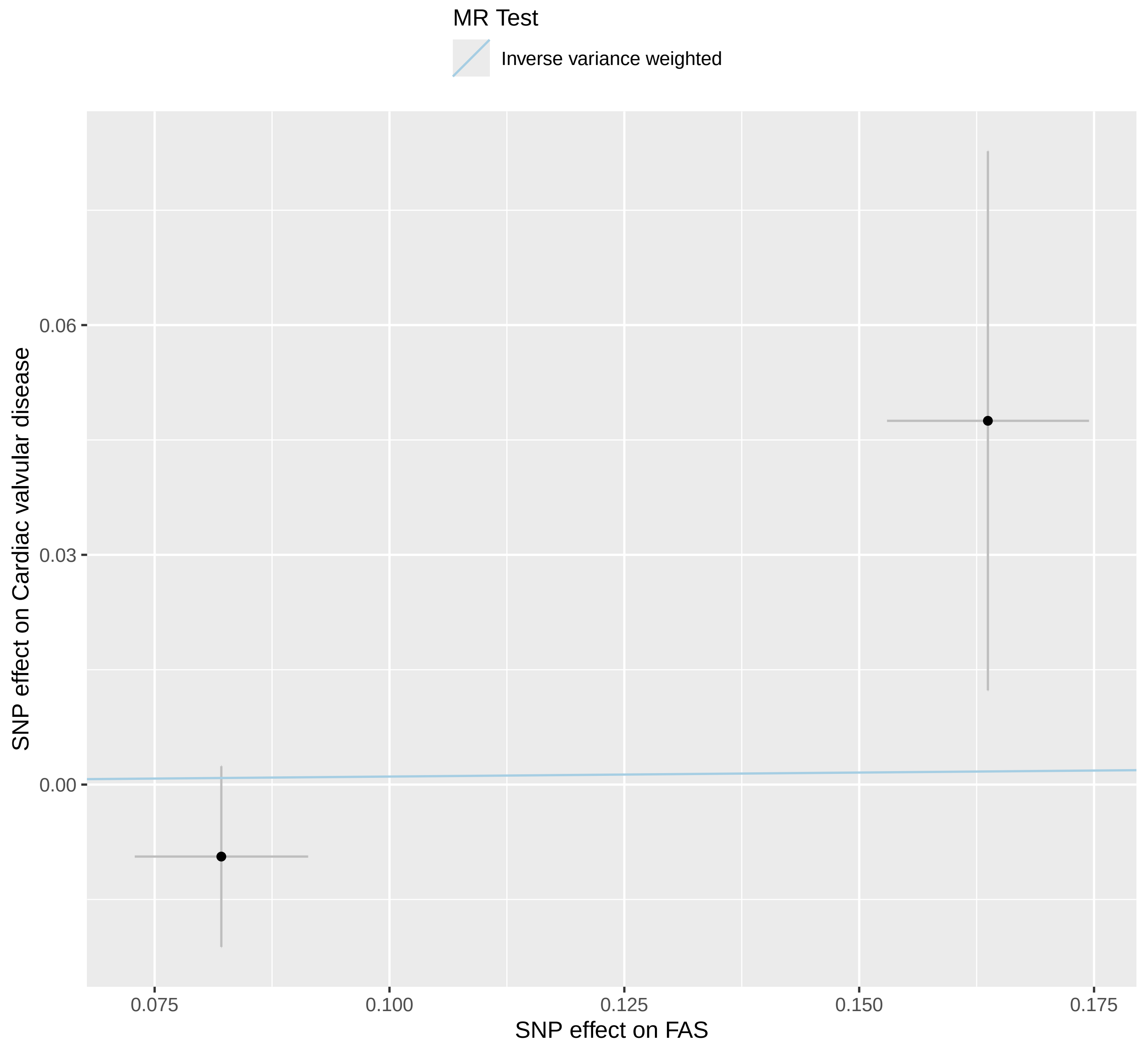
MR Test
Inverse variance weighted
SNP effect on Cardiac valvular disease
0.06
0.03
0.00
0.075
0.100
0.125
0.150
0.175
SNP effect on FAS

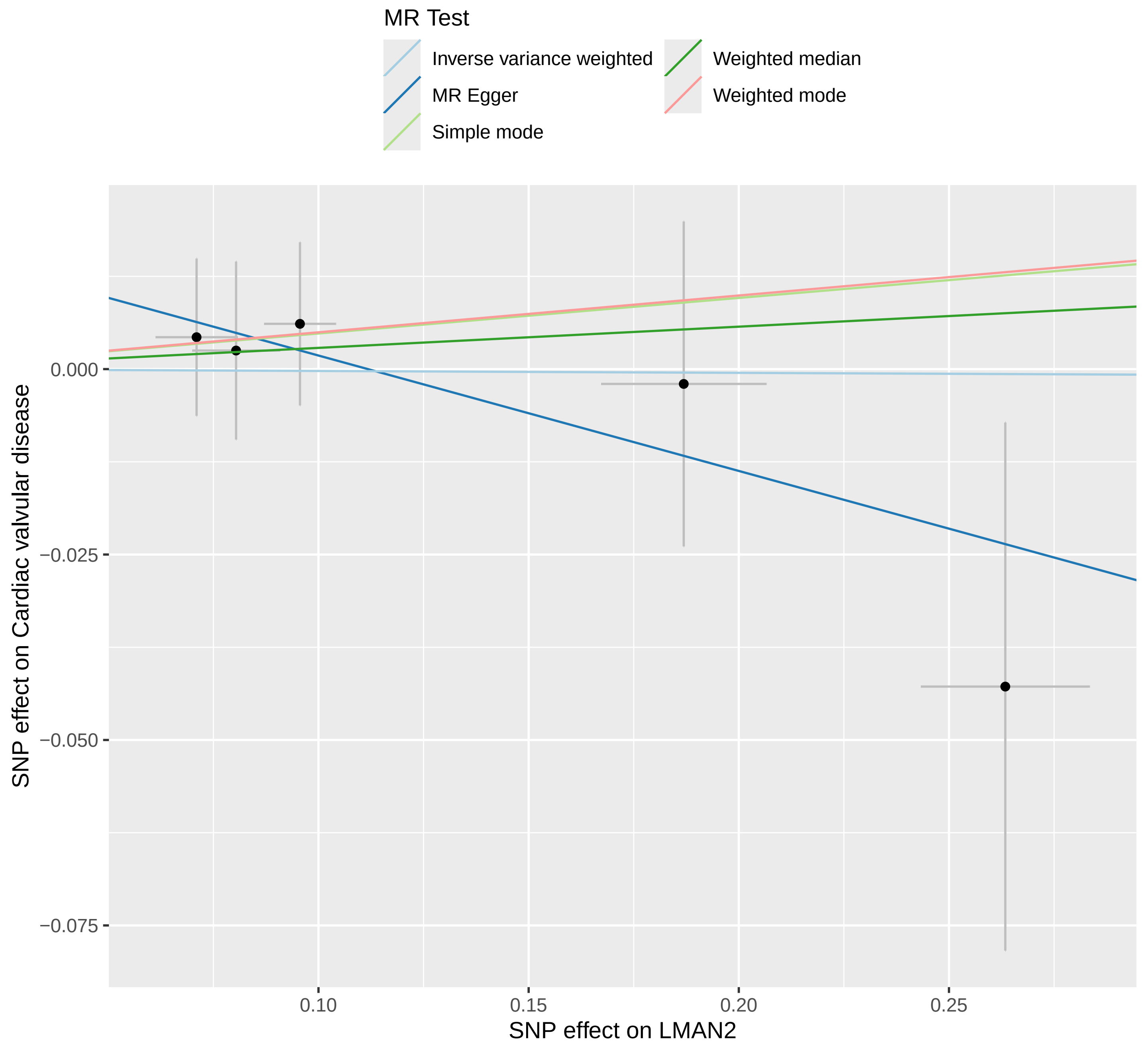
MR Test
Inverse variance weighted
MR Egger
Simple mode
Weighted median
Weighted mode
0.000
−0.025
−0.050
−0.075
0.10
0.15
0.20
0.25
SNP effect on Cardiac valvular disease
SNP effect on LMAN2

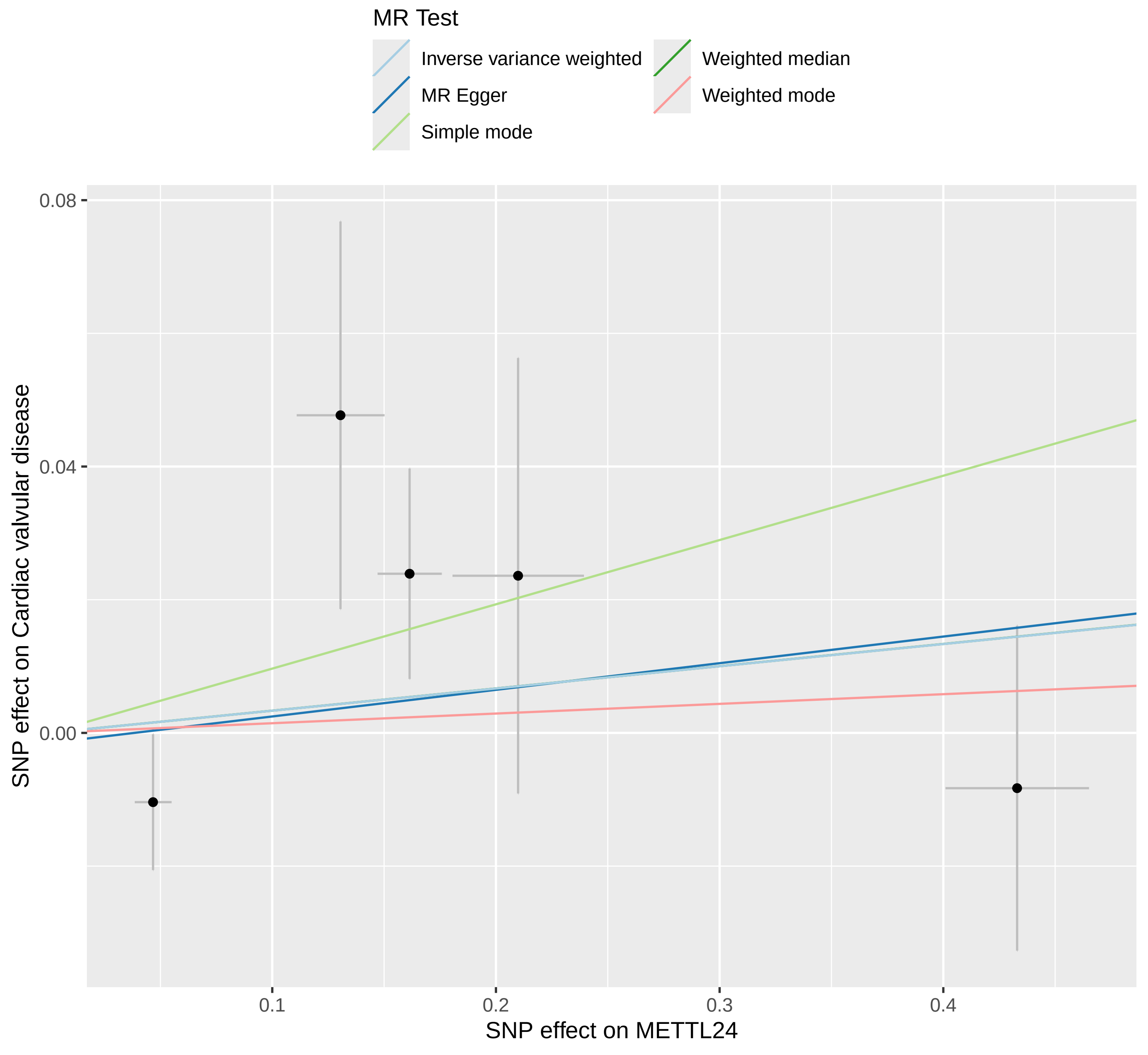
MR Test
Inverse variance weighted
MR Egger
Simple mode
Weighted median
Weighted mode
0.08
0.04
0.00
0.1
0.2
0.3
0.4
SNP effect on METTL24
SNP effect on Cardiac valvular disease

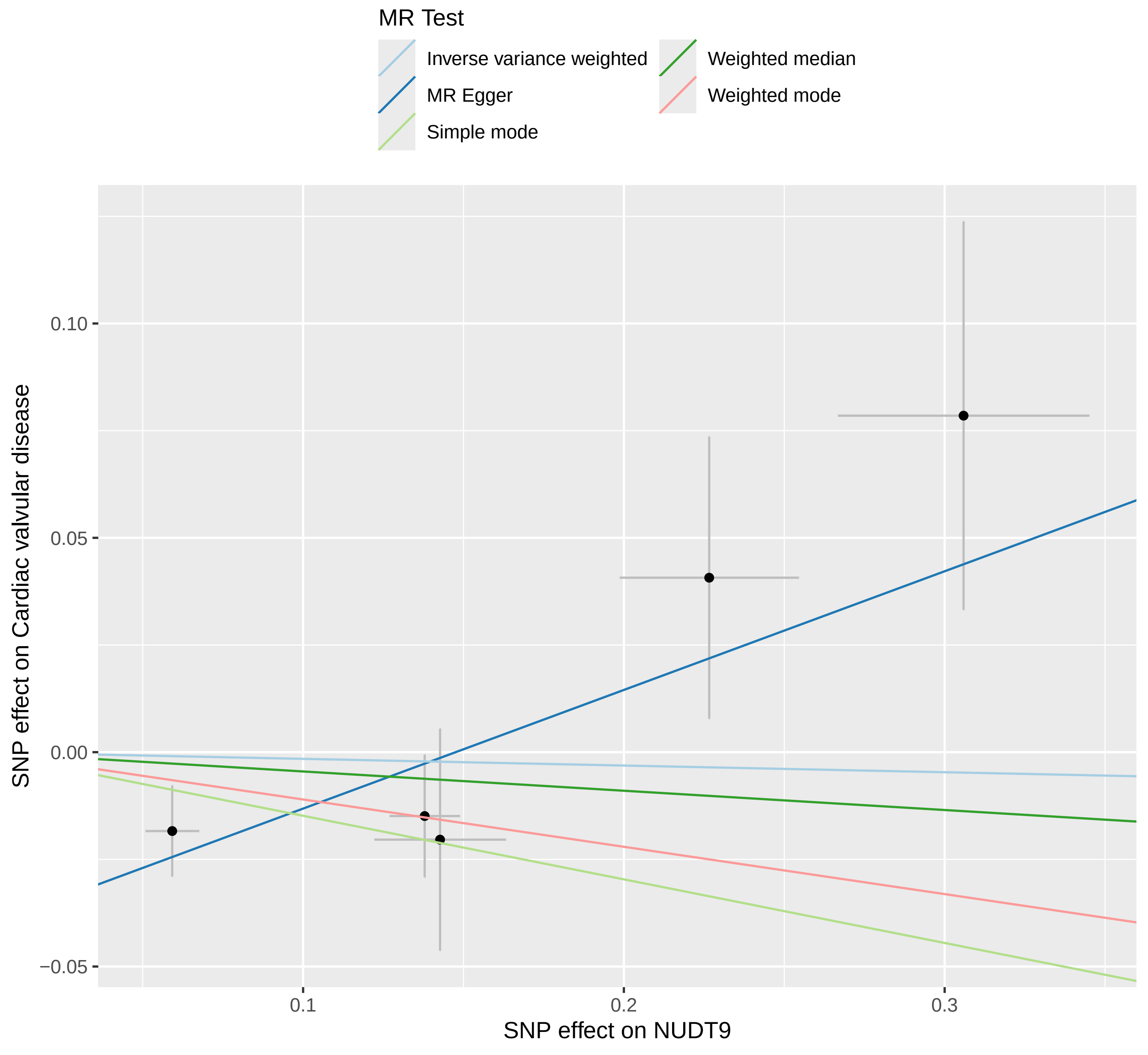

MR Test
Inverse variance weighted
MR Egger
Simple mode
Weighted median
Weighted mode
0.10
0.05
0.00
−0.05
SNP effect on Cardiac valvular disease
0.1
0.2
0.3
SNP effect on NUDT9

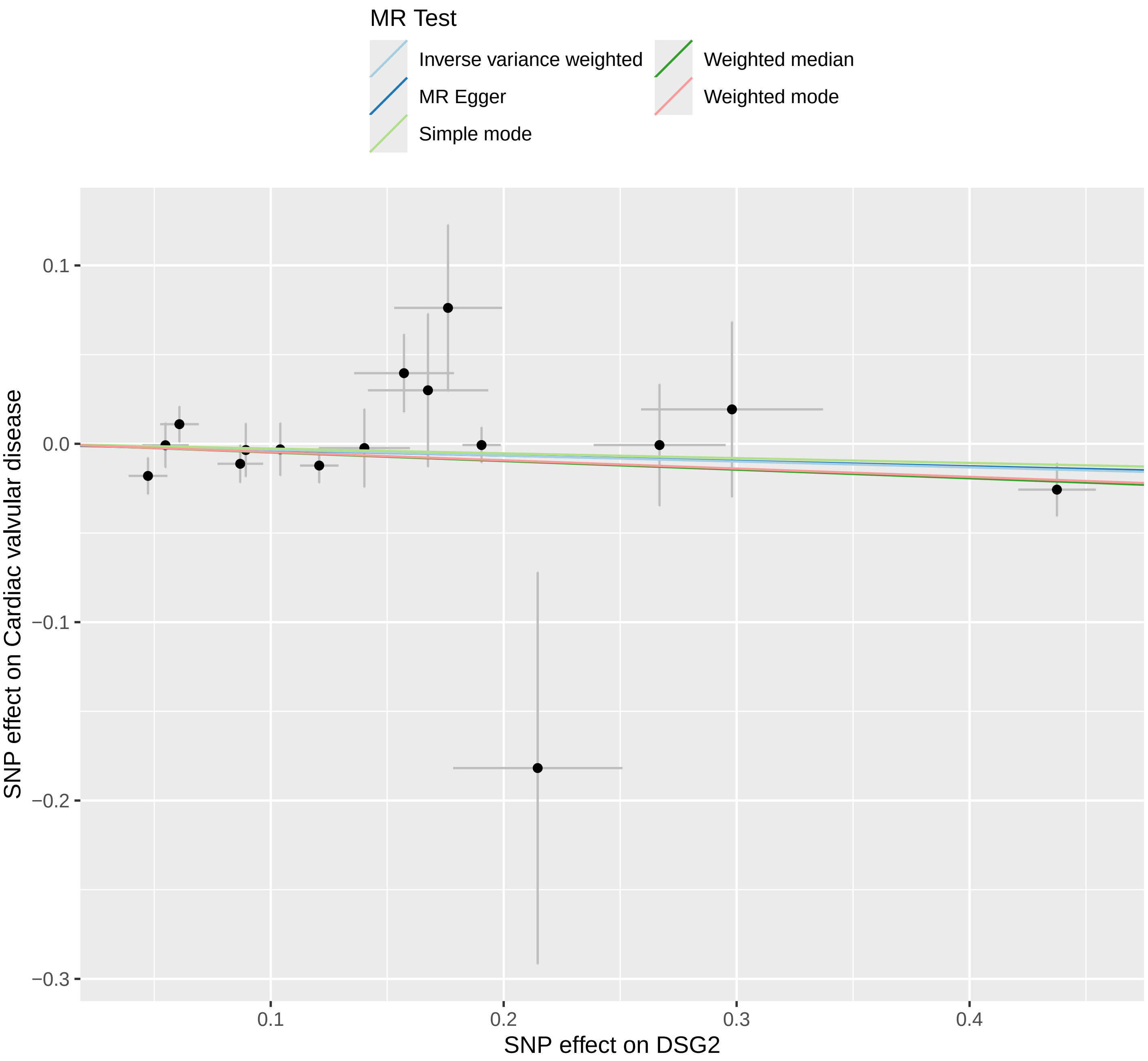
MR Test
Inverse variance weighted
MR Egger
Simple mode
Weighted median
Weighted mode
0.1
0.0
−0.1
−0.2
−0.3
SNP effect on Cardiac valvular disease
0.1
0.2
0.3
0.4
SNP effect on DSG2

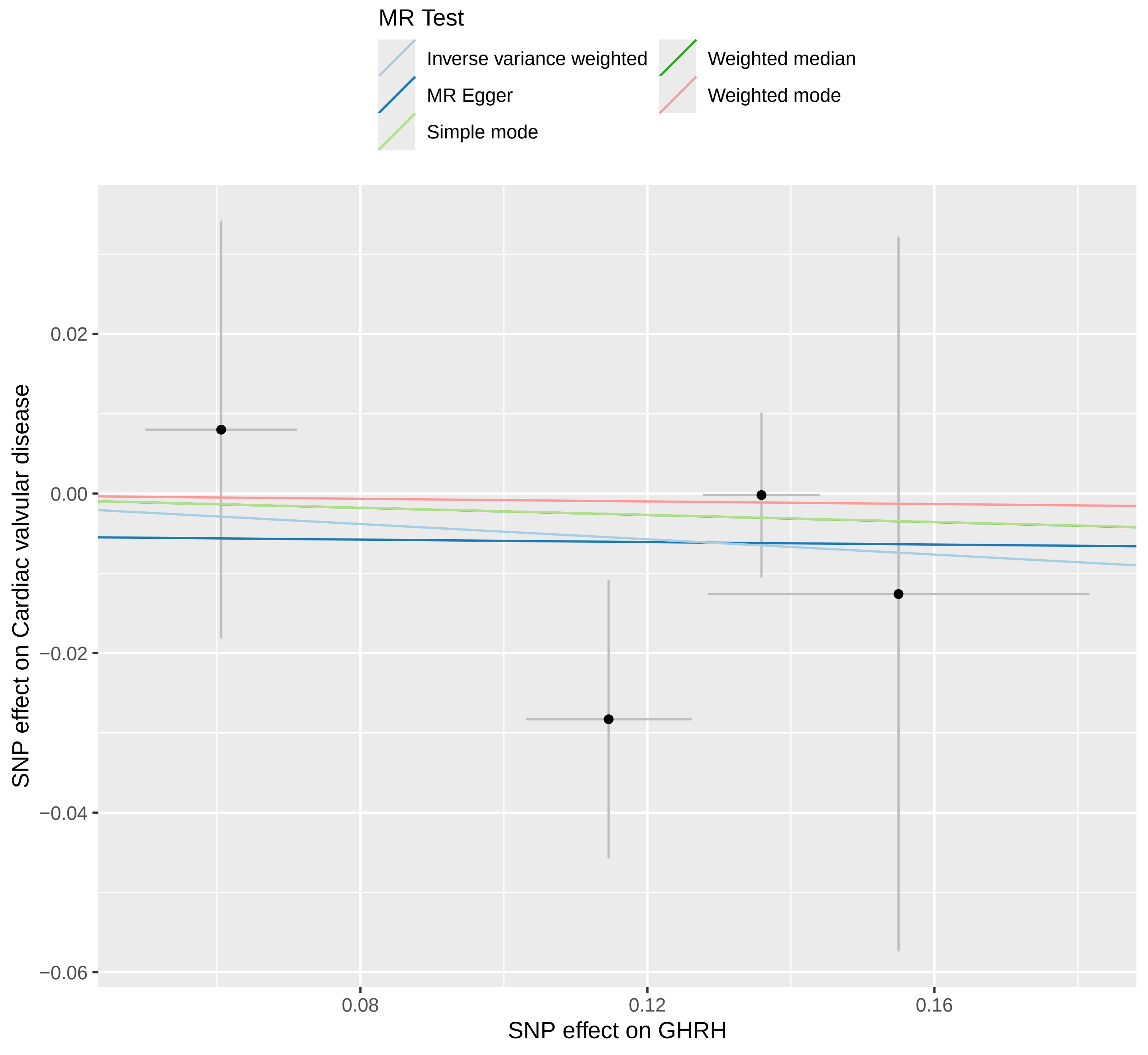
MR Test
Inverse variance weighted
MR Egger
Simple mode
Weighted median
Weighted mode
0.02
0.00
−0.02
−0.04
−0.06
SNP effect on Cardiac valvular disease
0.08
0.12
0.16
SNP effect on GHRH

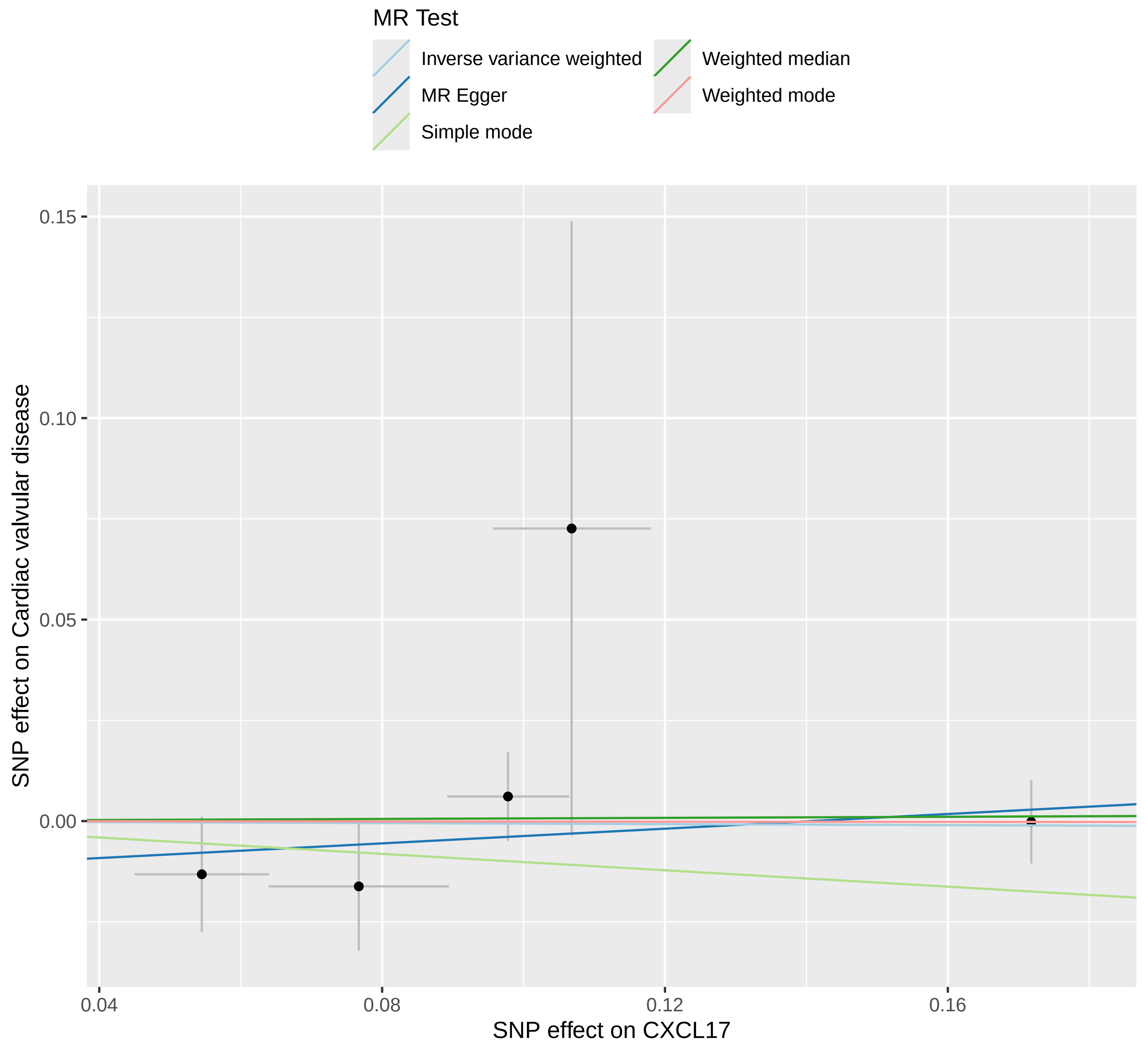
MR Test
Inverse variance weighted
MR Egger
Simple mode
Weighted median
Weighted mode
0.15
0.10
0.05
0.00
0.04
0.08
0.12
0.16
SNP effect on CXCL17
SNP effect on Cardiac valvular disease

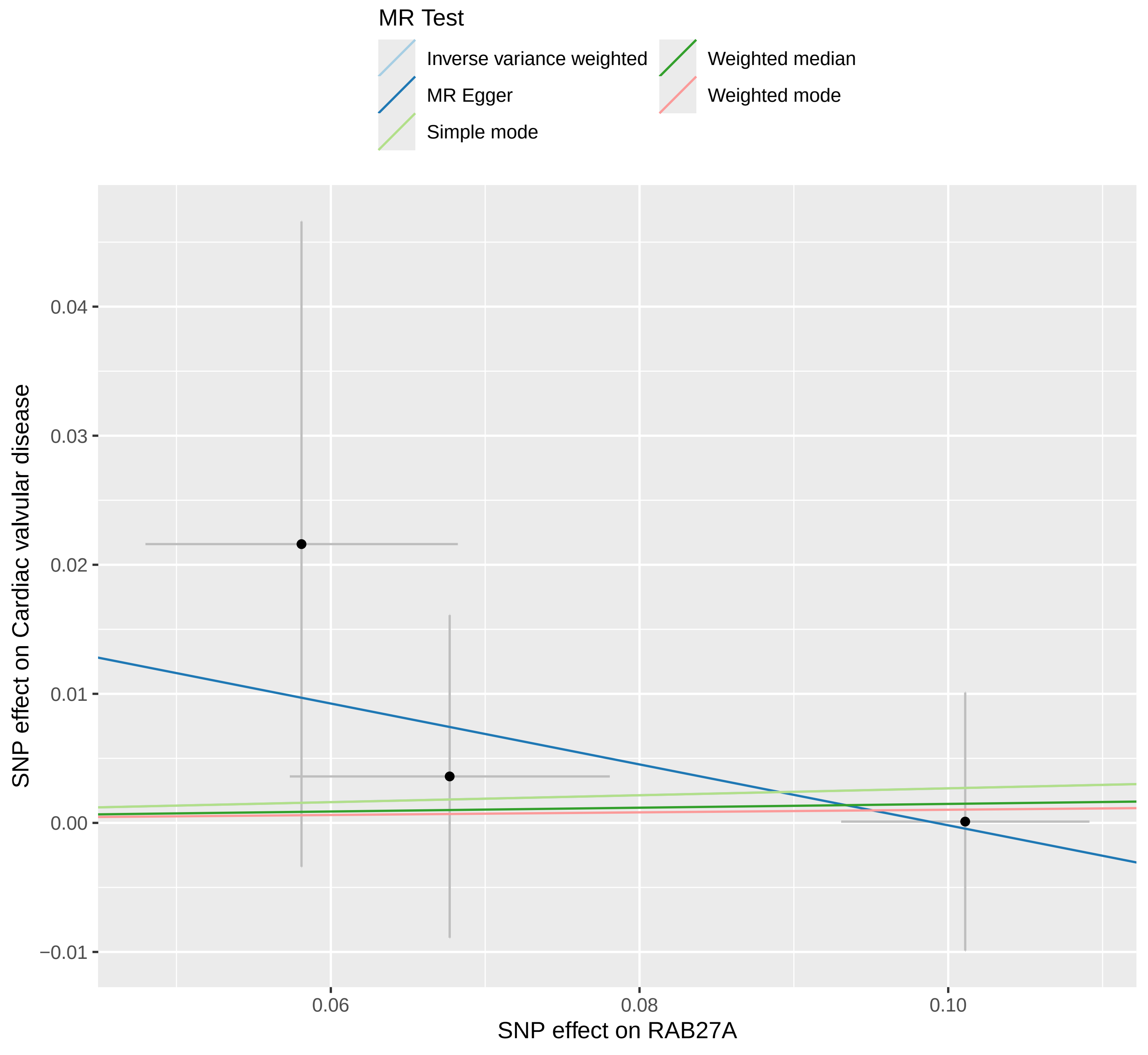

MR Test
Inverse variance weighted
MR Egger
Simple mode
Weighted median
Weighted mode
0.04
0.03
0.02
0.01
0.00
−0.01
0.06
0.08
0.10
SNP effect on Cardiac valvular disease
SNP effect on RAB27A

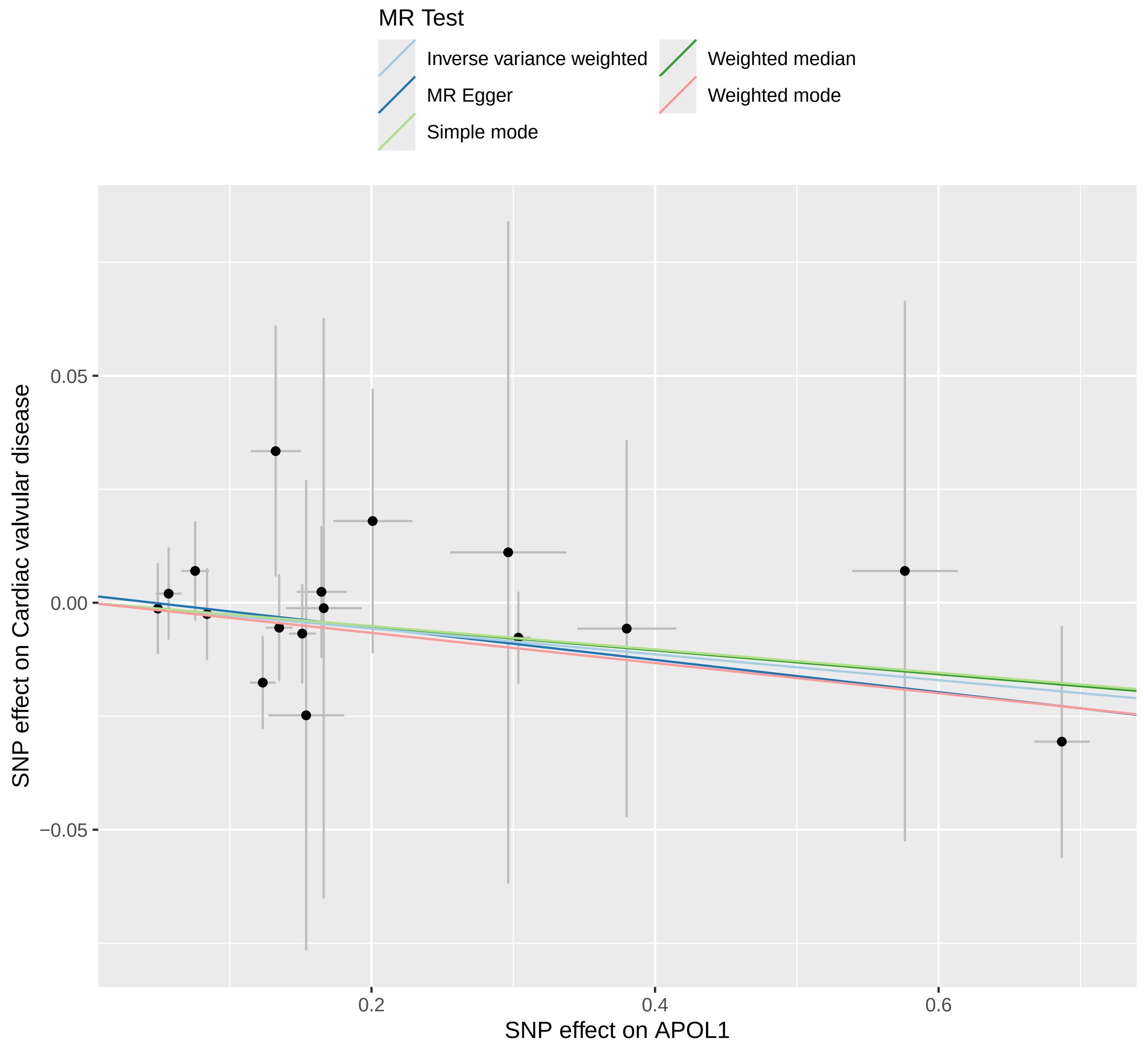
MR Test
Inverse variance weighted
MR Egger
Simple mode
Weighted median
Weighted mode
0.05
0.00
−0.05
0.2
0.4
0.6
SNP effect on Cardiac valvular disease
SNP effect on APOL1

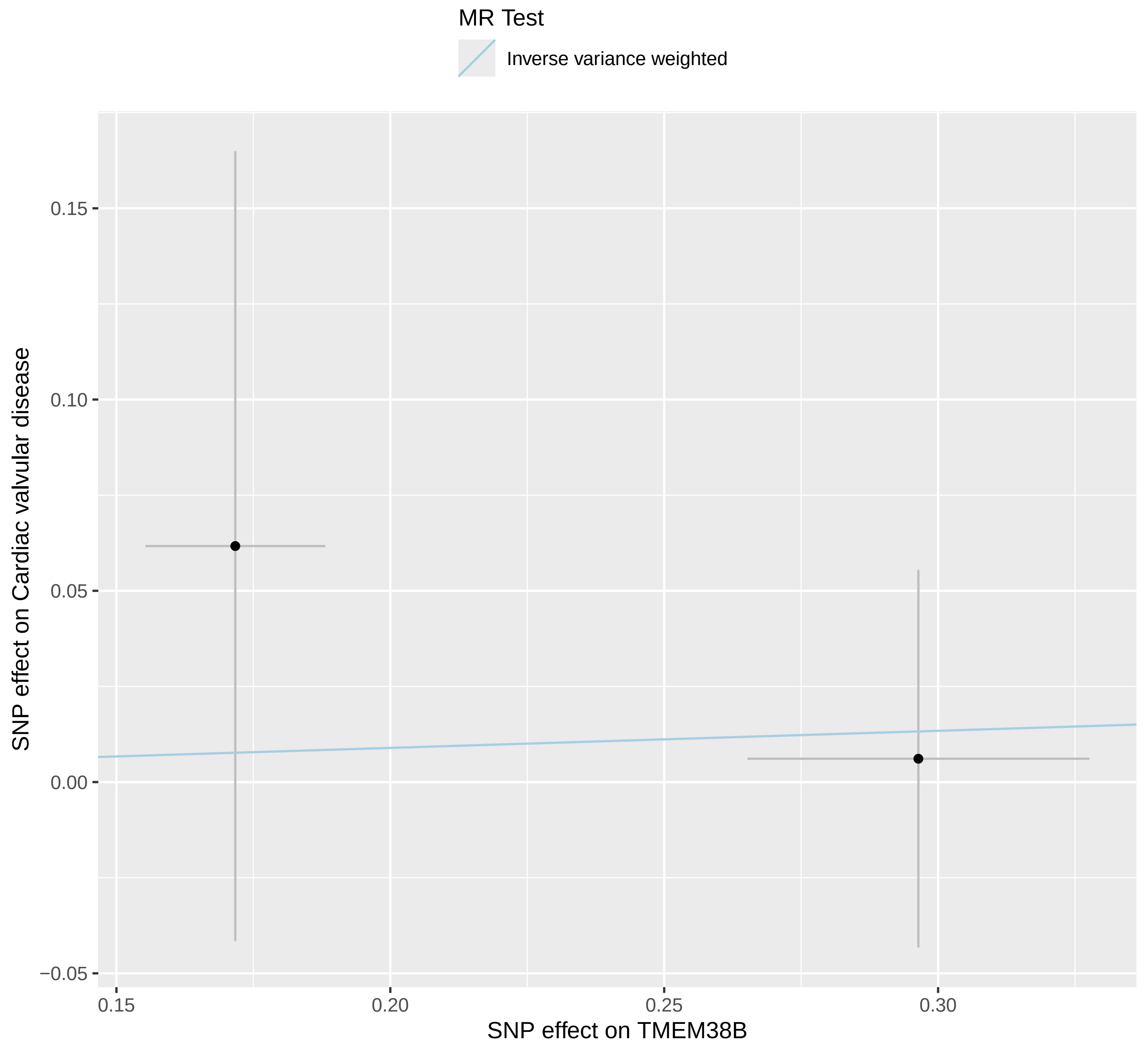

MR Test
Inverse variance weighted
0.15
0.10
0.05
0.00
−0.05
0.15
0.20
0.25
0.30
SNP effect on TMEM38B
SNP effect on Cardiac valvular disease

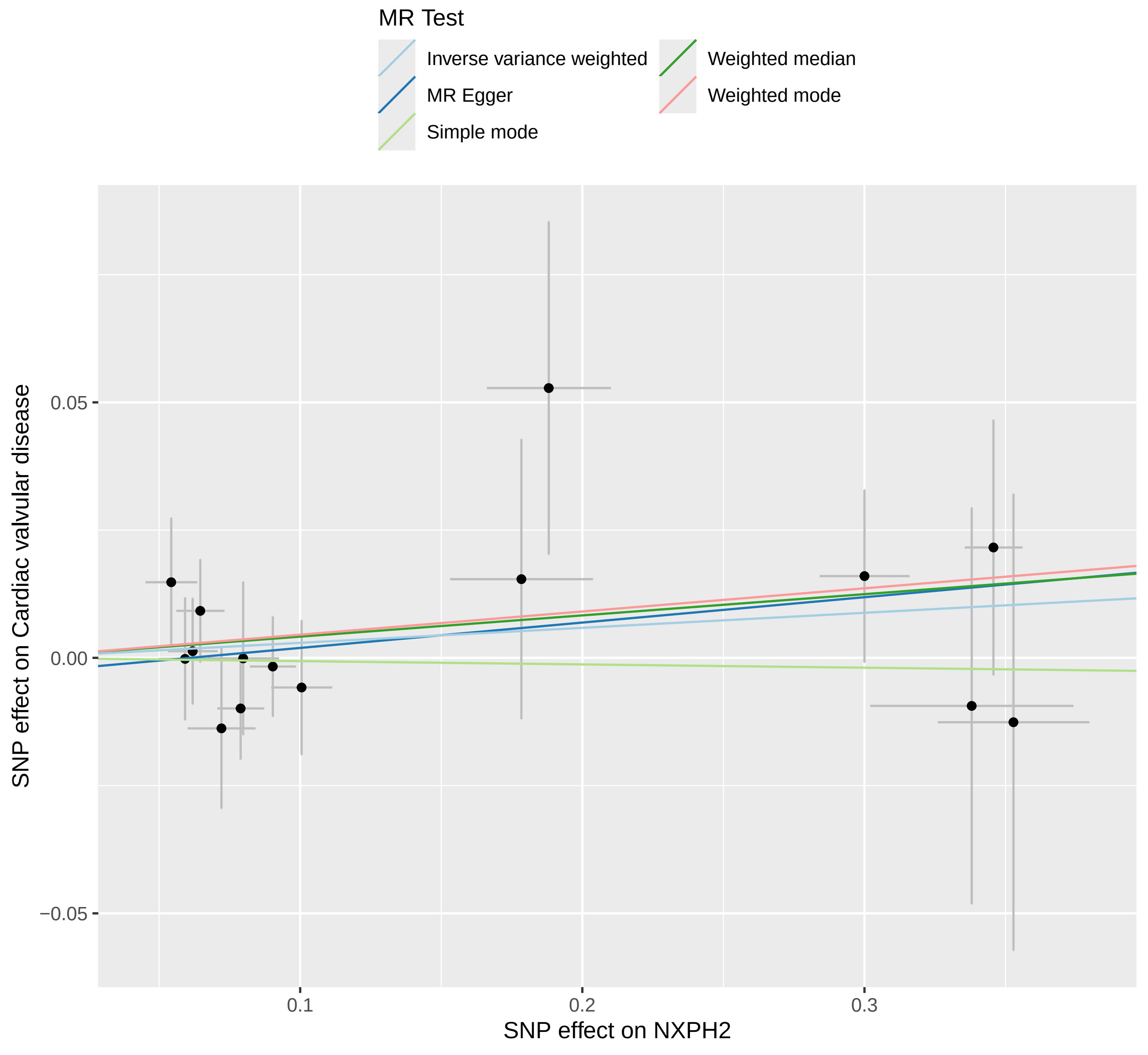
MR Test
Inverse variance weighted
MR Egger
Simple mode
Weighted median
Weighted mode
0.05
0.00
−0.05
0.1
0.2
0.3
SNP effect on Cardiac valvular disease
SNP effect on NXPH2

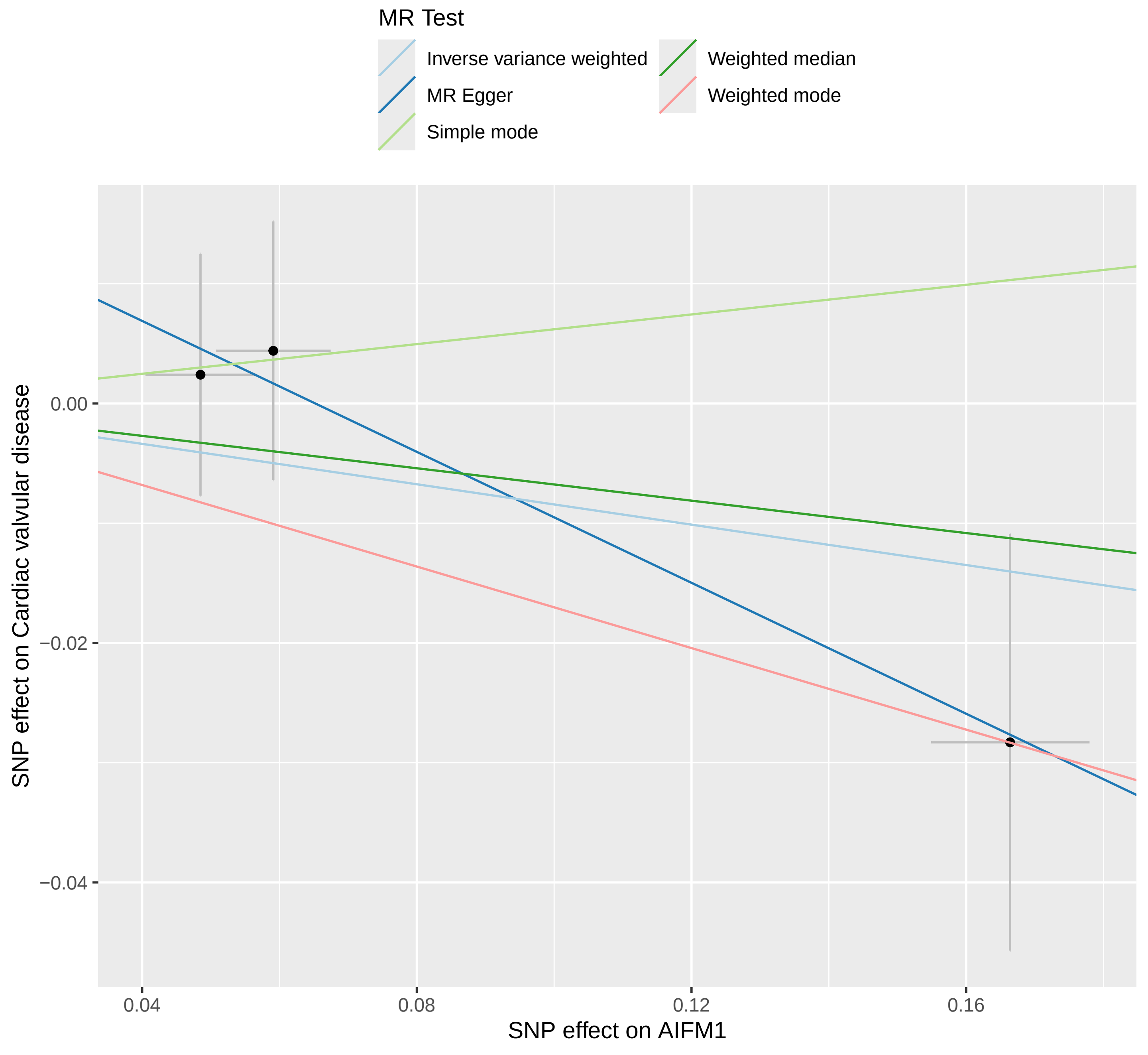

MR Test
Inverse variance weighted
MR Egger
Simple mode
Weighted median
Weighted mode
0.00
−0.02
−0.04
0.04
0.08
0.12
0.16
SNP effect on Cardiac valvular disease
SNP effect on AIFM1

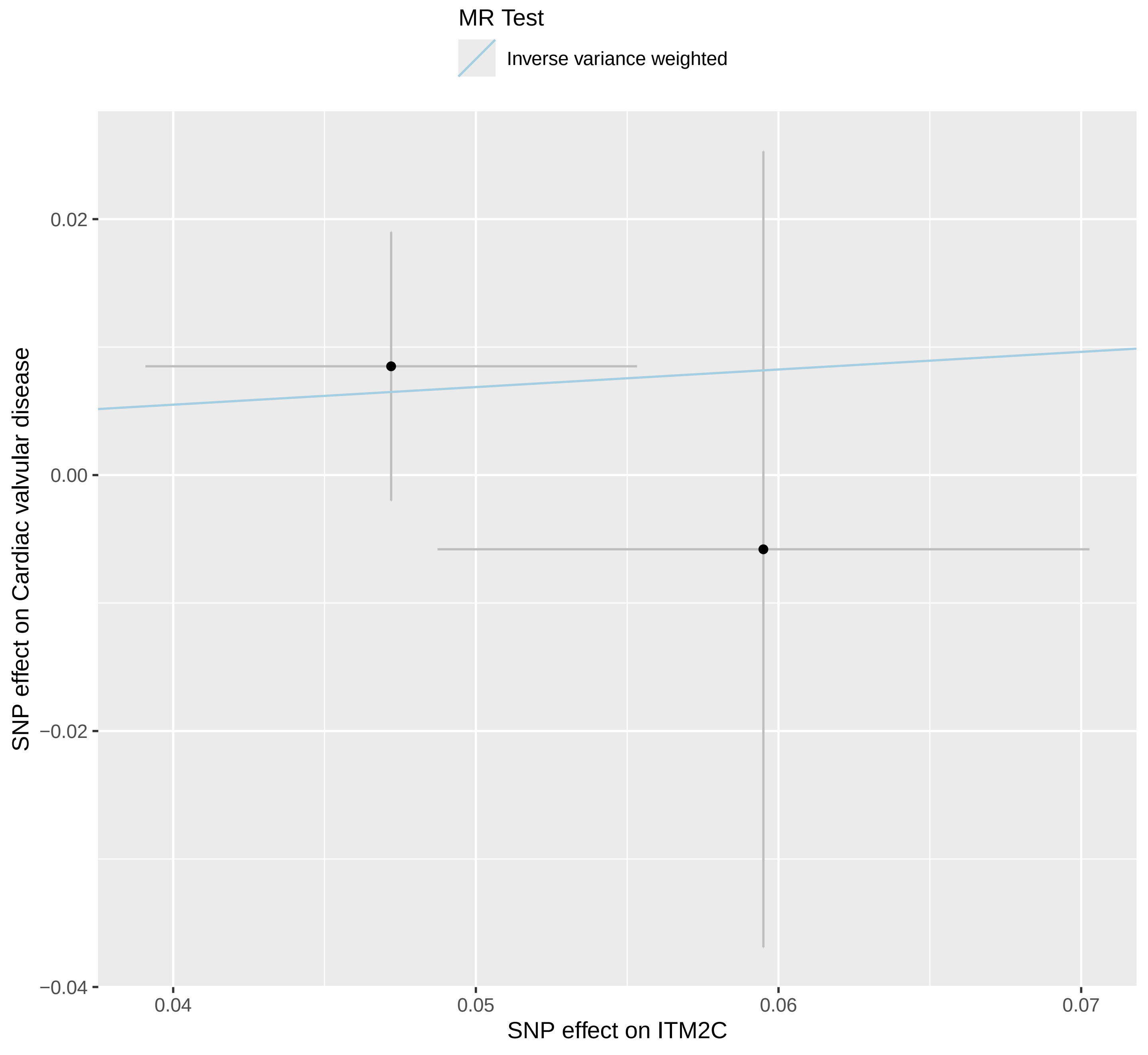
MR Test
Inverse variance weighted
SNP effect on Cardiac valvular disease
0.02
0.00
−0.02
−0.04
0.04
0.05
0.06
0.07
SNP effect on ITM2C

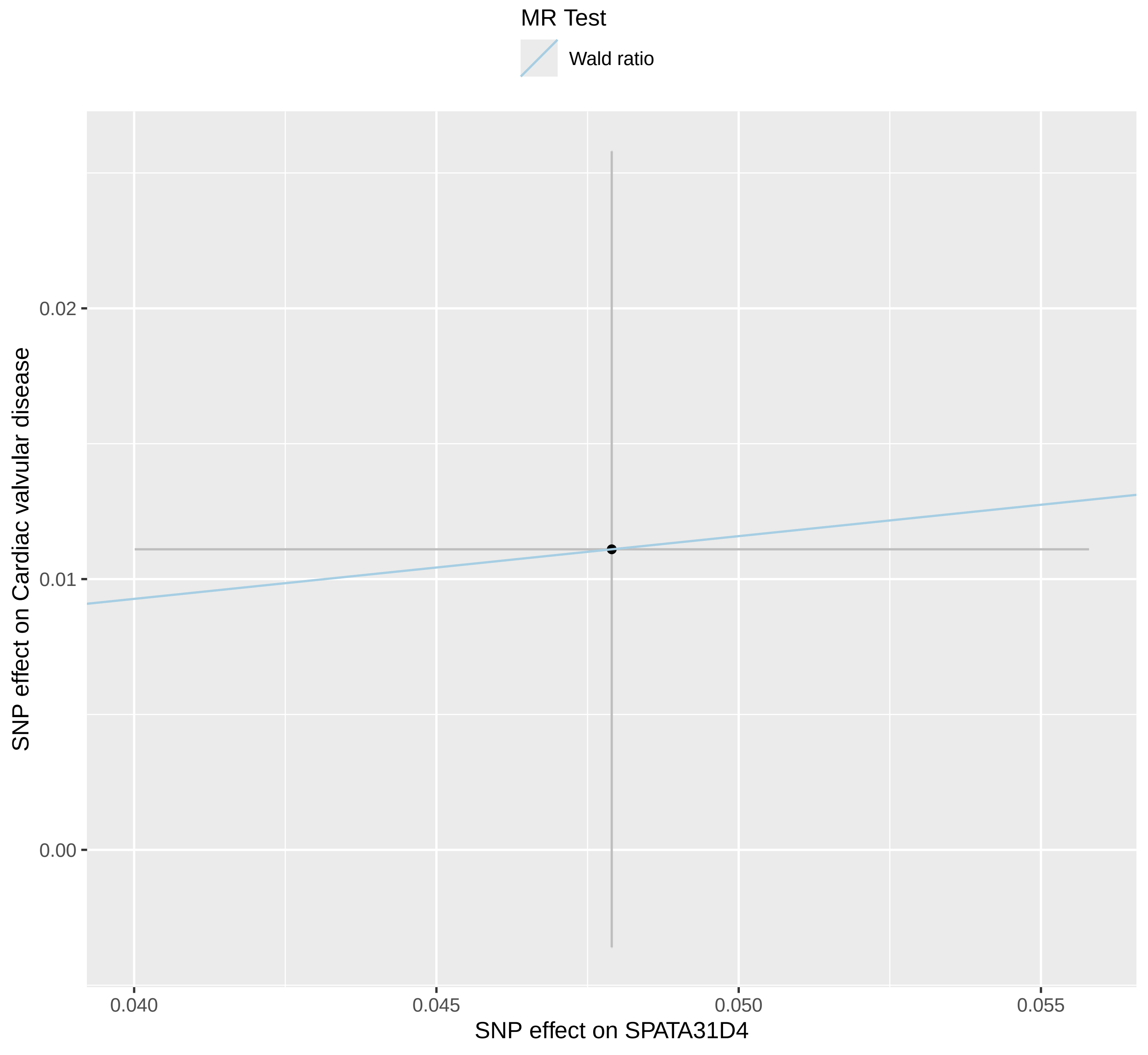

MR Test
Wald ratio
0.02
0.01
0.00
SNP effect on Cardiac valvular disease
0.040
0.045
0.050
0.055
SNP effect on SPATA31D4

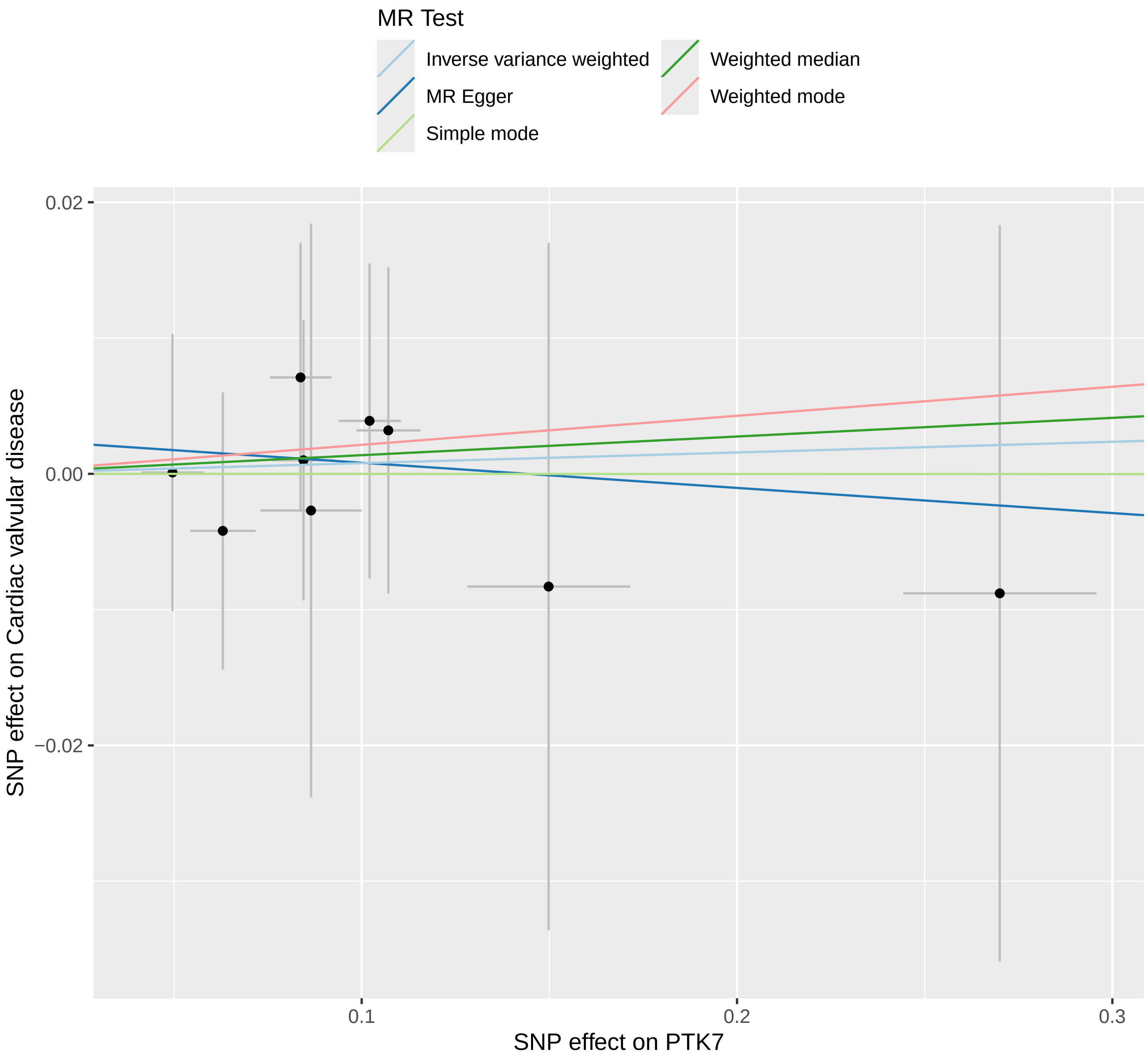
MR Test
Inverse variance weighted
MR Egger
Simple mode
Weighted median
Weighted mode
0.02
0.00
−0.02
0.1
0.2
0.3
SNP effect on Cardiac valvular disease
SNP effect on PTK7

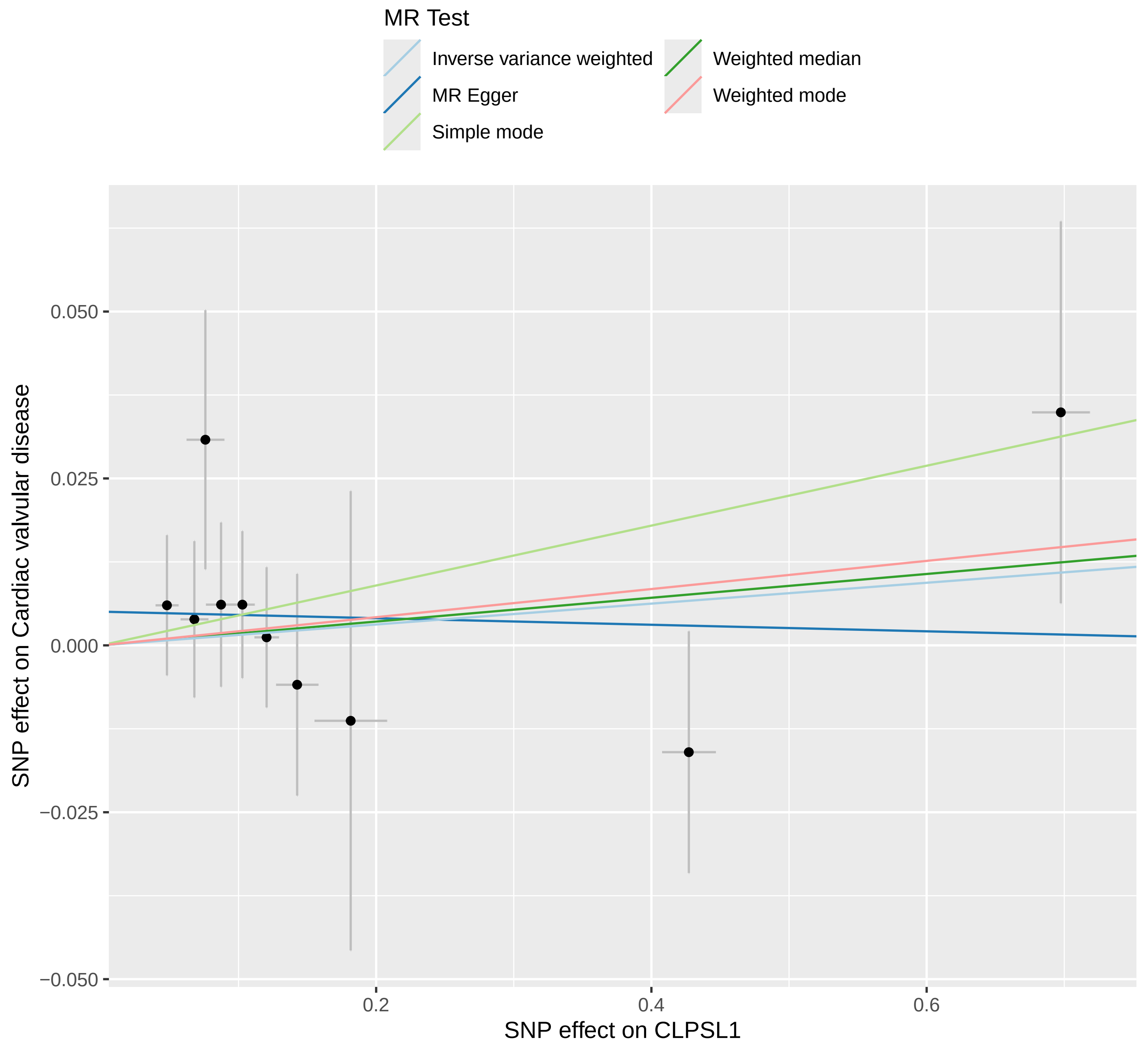

MR Test
Inverse variance weighted
MR Egger
Simple mode
Weighted median
Weighted mode
0.050
0.025
0.000
−0.025
−0.050
SNP effect on Cardiac valvular disease
0.2
0.4
0.6
SNP effect on CLPSL1

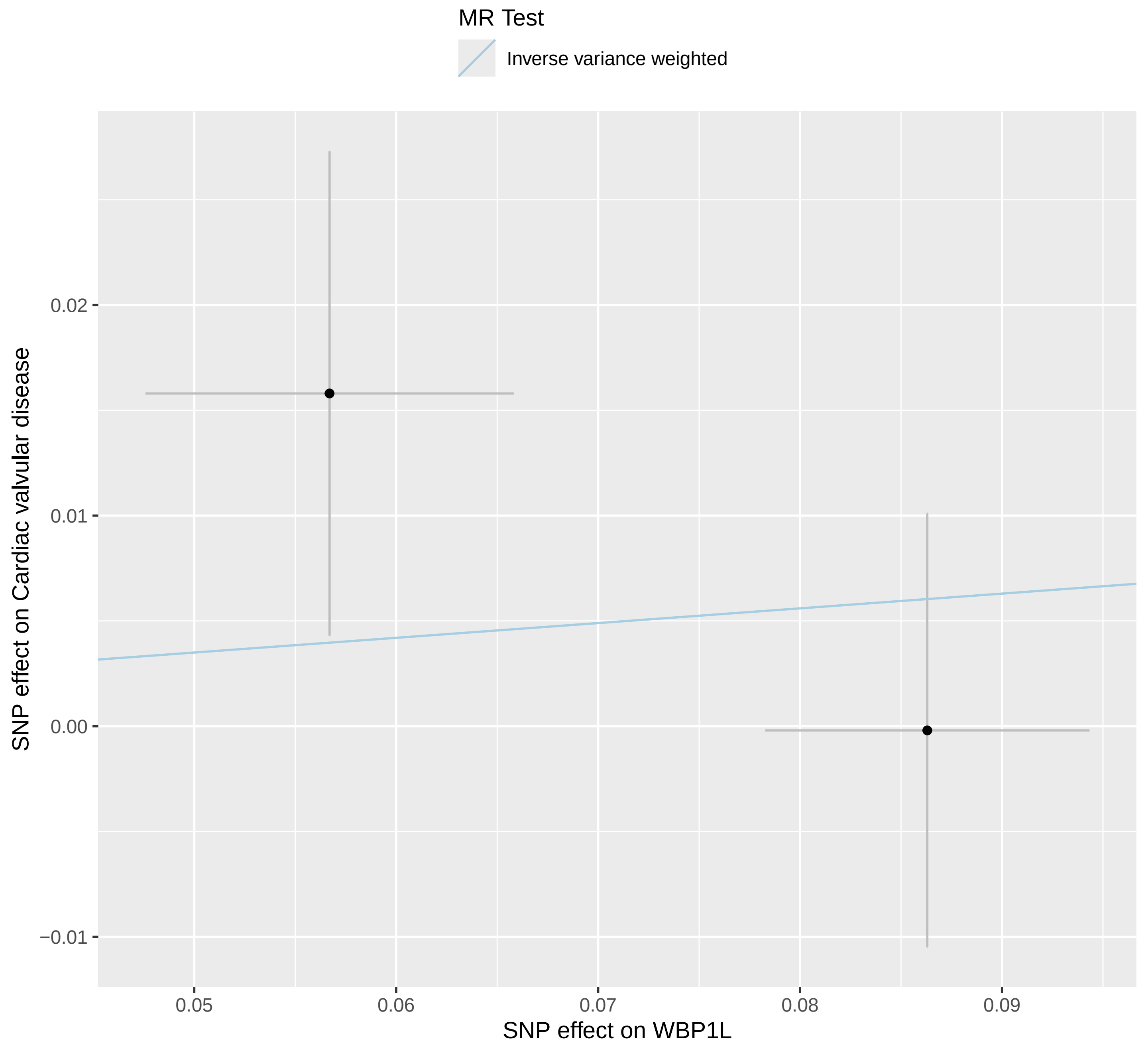
MR Test
Inverse variance weighted
SNP effect on Cardiac valvular disease
0.02
0.01
0.00
−0.01
0.05
0.06
0.07
0.08
0.09
SNP effect on WBP1L

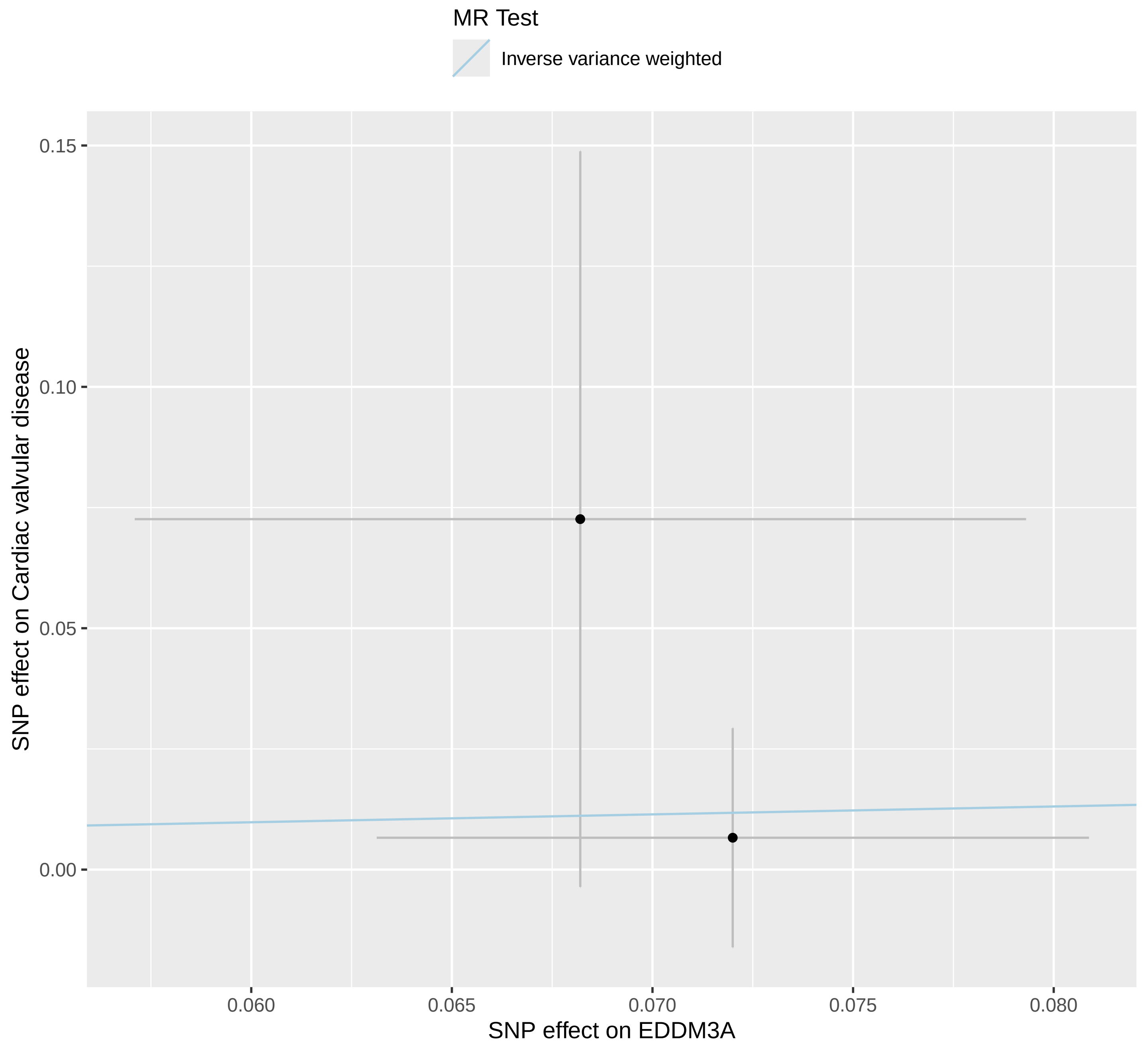
MR Test
Inverse variance weighted
0.15
0.10
0.05
0.00
0.060
0.065
0.070
0.075
0.080
SNP effect on EDDM3A
SNP effect on Cardiac valvular disease

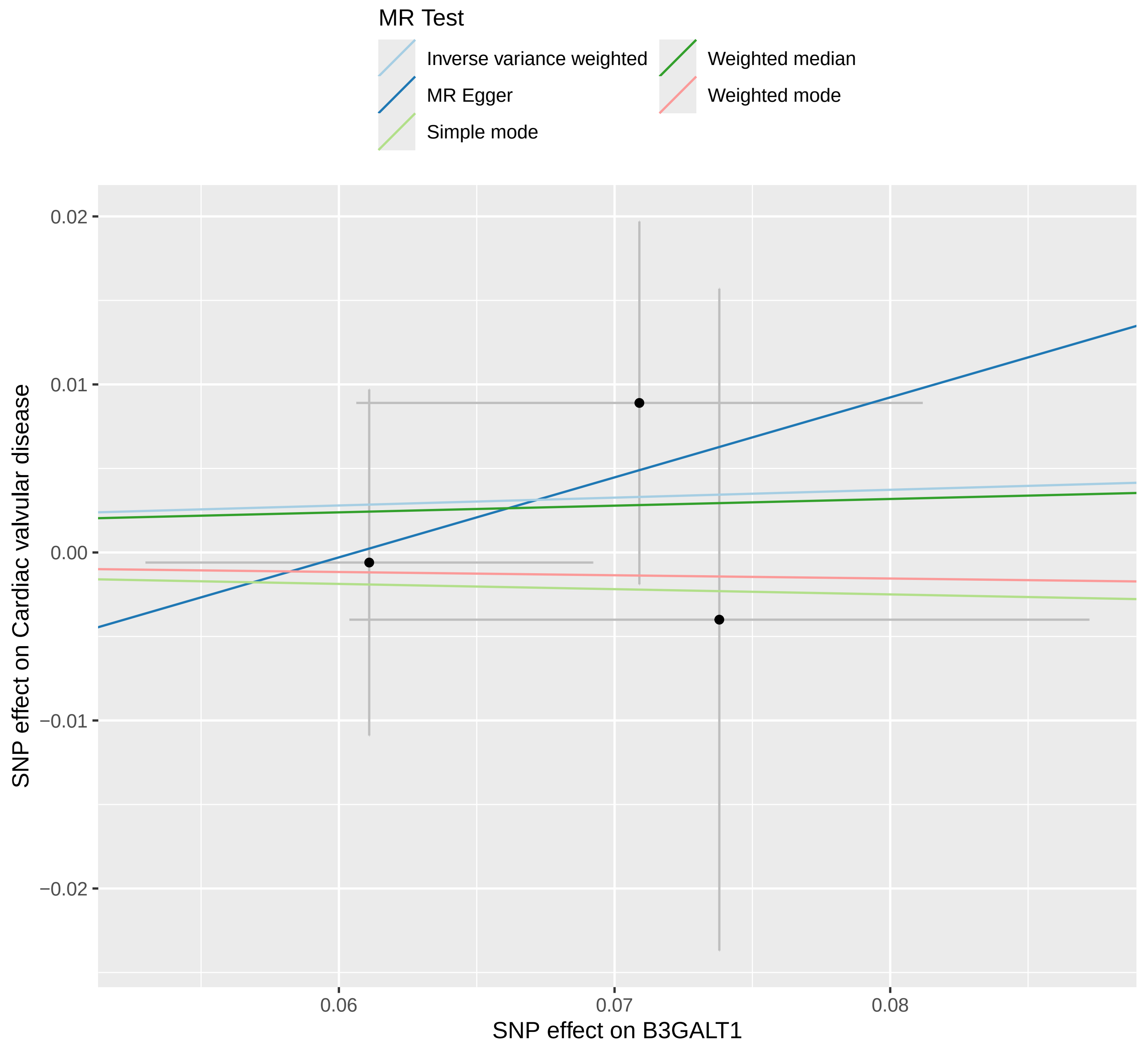
MR Test
Inverse variance weighted
MR Egger
Simple mode
Weighted median
Weighted mode
0.02
0.01
0.00
−0.01
−0.02
0.06
0.07
0.08
SNP effect on Cardiac valvular disease
SNP effect on B3GALT1

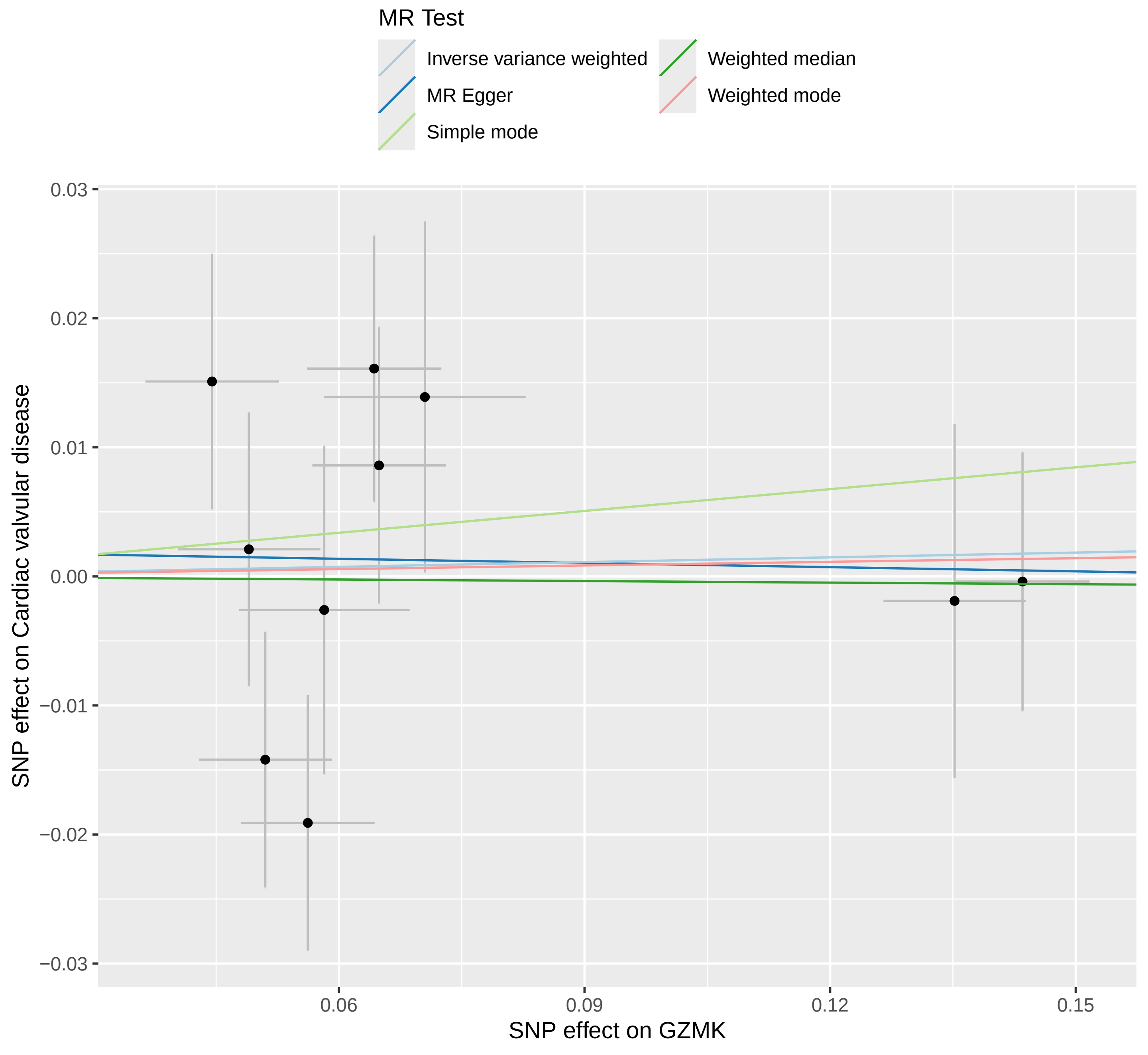

MR Test
Inverse variance weighted
MR Egger
Simple mode
Weighted median
Weighted mode
0.03
0.02
0.01
0.00
−0.01
−0.02
−0.03
0.06
0.09
0.12
0.15
SNP effect on Cardiac valvular disease
SNP effect on GZMK

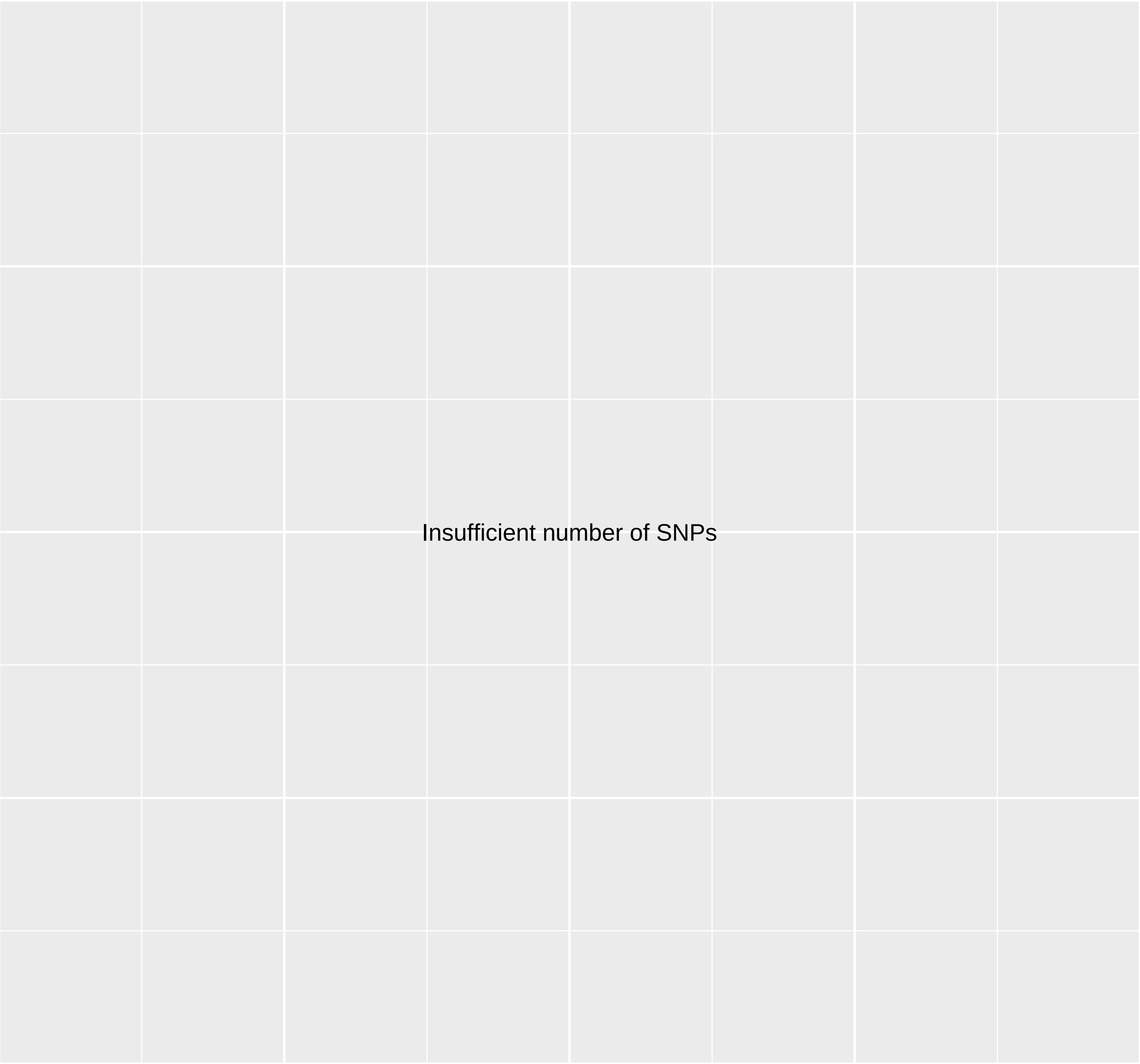
Insufficient number of SNPs

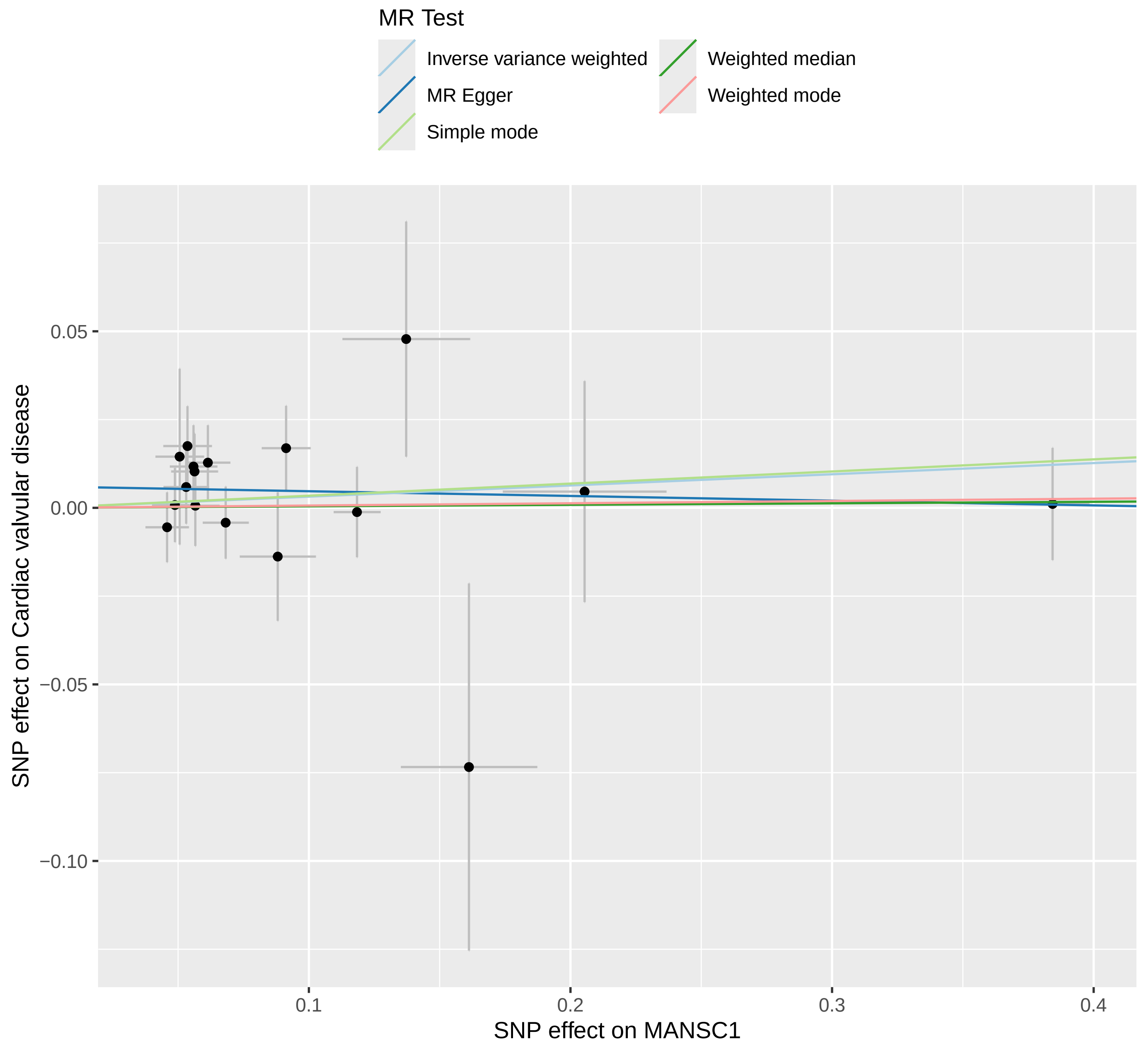

MR Test
Inverse variance weighted
MR Egger
Simple mode
Weighted median
Weighted mode
SNP effect on Cardiac valvular disease
0.05
0.00
−0.05
−0.10
0.1
0.2
0.3
0.4
SNP effect on MANSC1

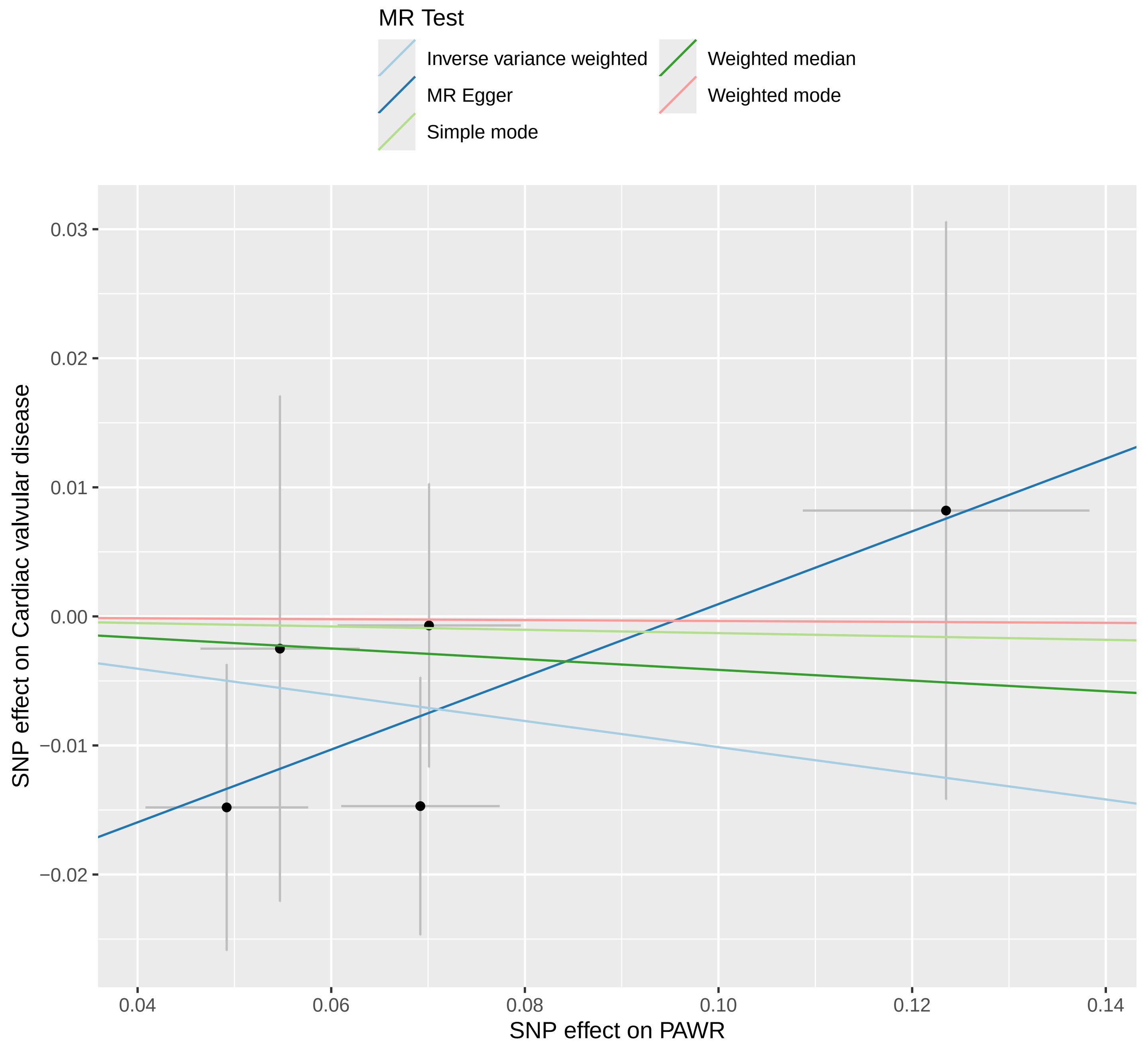
MR Test
Inverse variance weighted
MR Egger
Simple mode
Weighted median
Weighted mode
SNP effect on Cardiac valvular disease
0.03
0.02
0.01
0.00
−0.01
−0.02
0.04
0.06
0.08
0.10
0.12
0.14
SNP effect on PAWR

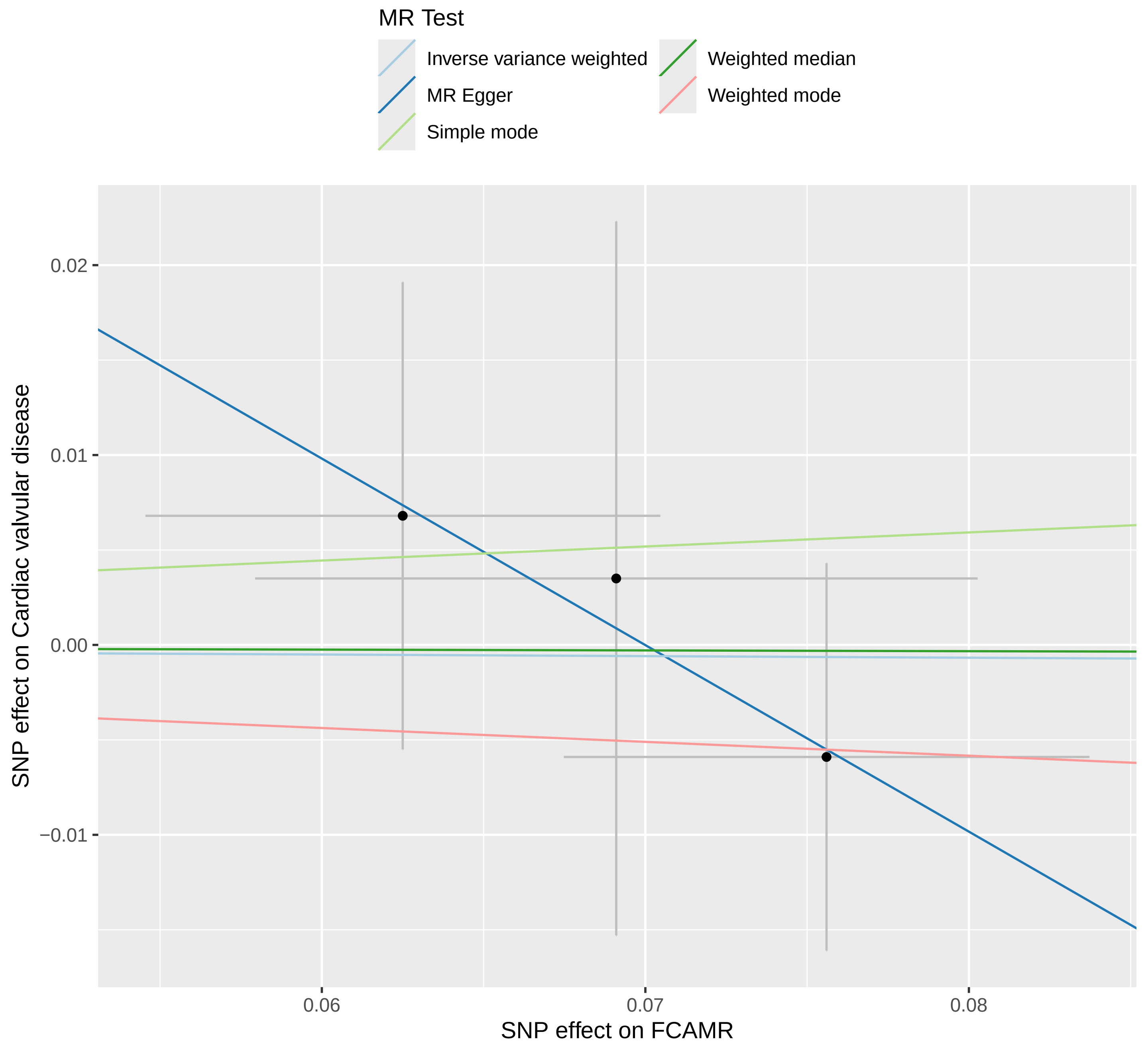
MR Test
Inverse variance weighted
MR Egger
Simple mode
Weighted median
Weighted mode
0.02
0.01
0.00
−0.01
SNP effect on Cardiac valvular disease
0.06
0.07
0.08
SNP effect on FCAMR

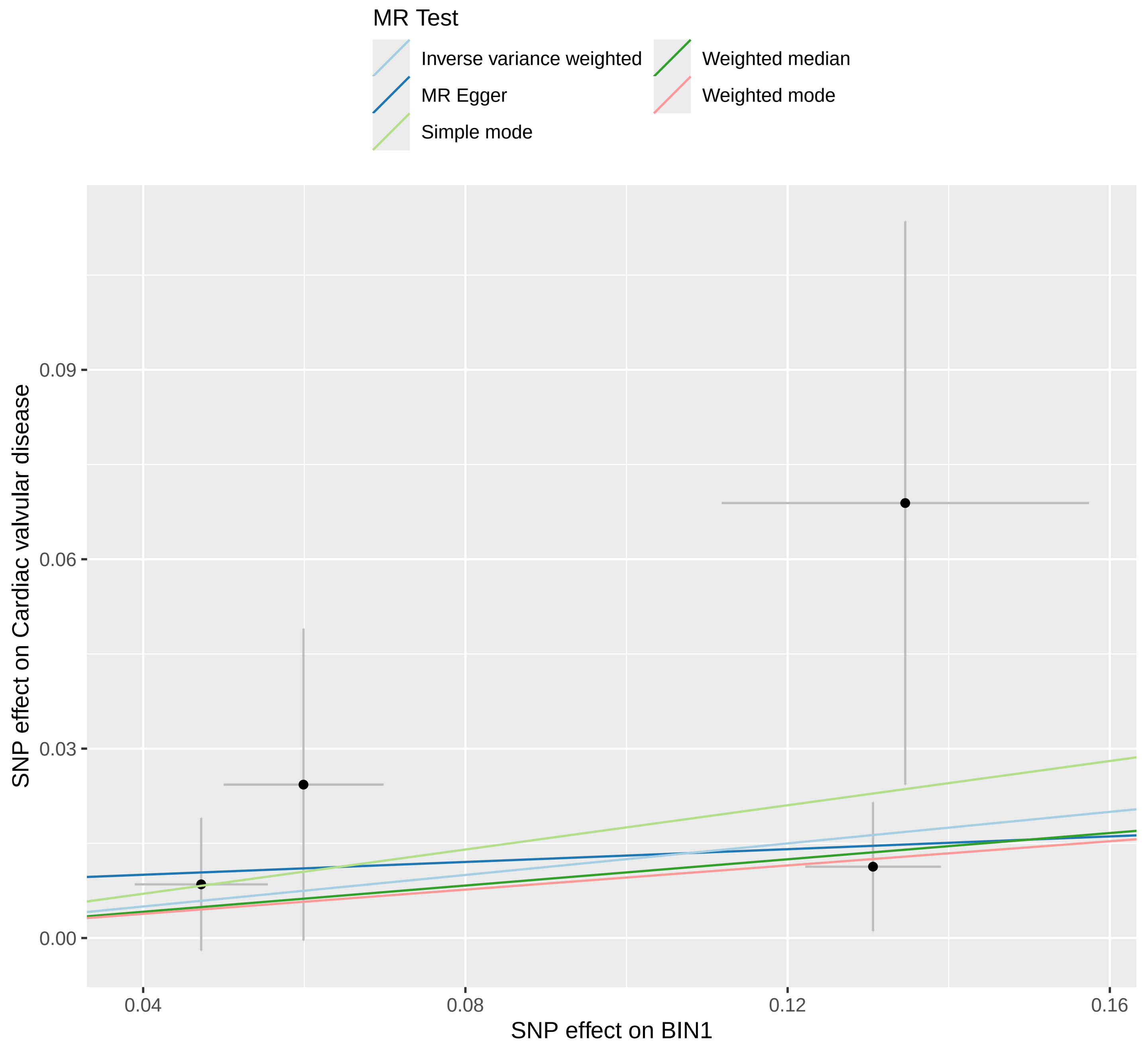
MR Test
Inverse variance weighted
MR Egger
Simple mode
Weighted median
Weighted mode
0.09
0.06
0.03
0.00
SNP effect on Cardiac valvular disease
0.04
0.08
0.12
0.16
SNP effect on BIN1

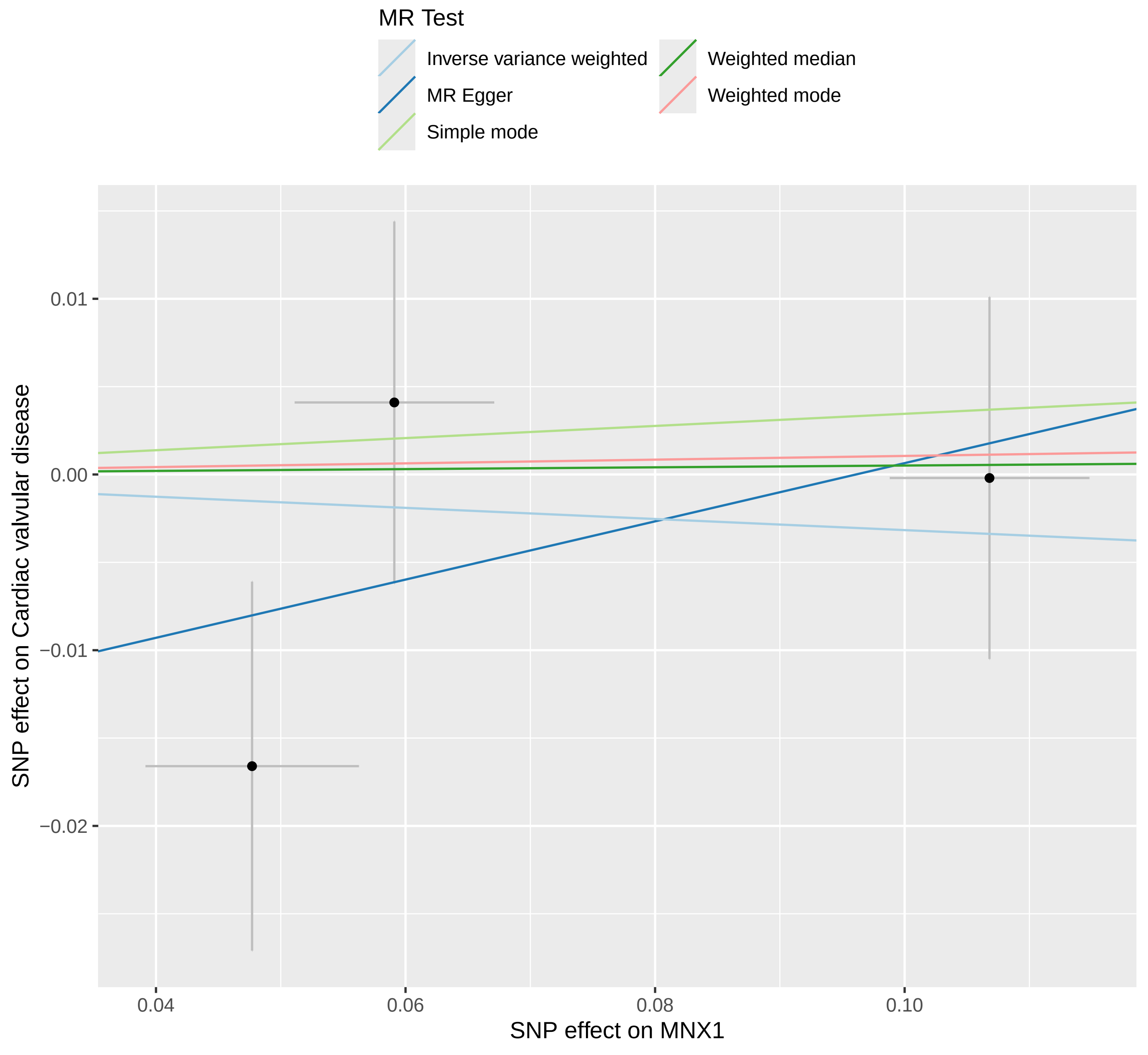
MR Test
Inverse variance weighted
MR Egger
Simple mode
Weighted median
Weighted mode
0.01
0.00
−0.01
−0.02
SNP effect on Cardiac valvular disease
0.04
0.06
0.08
0.10
SNP effect on MNX1

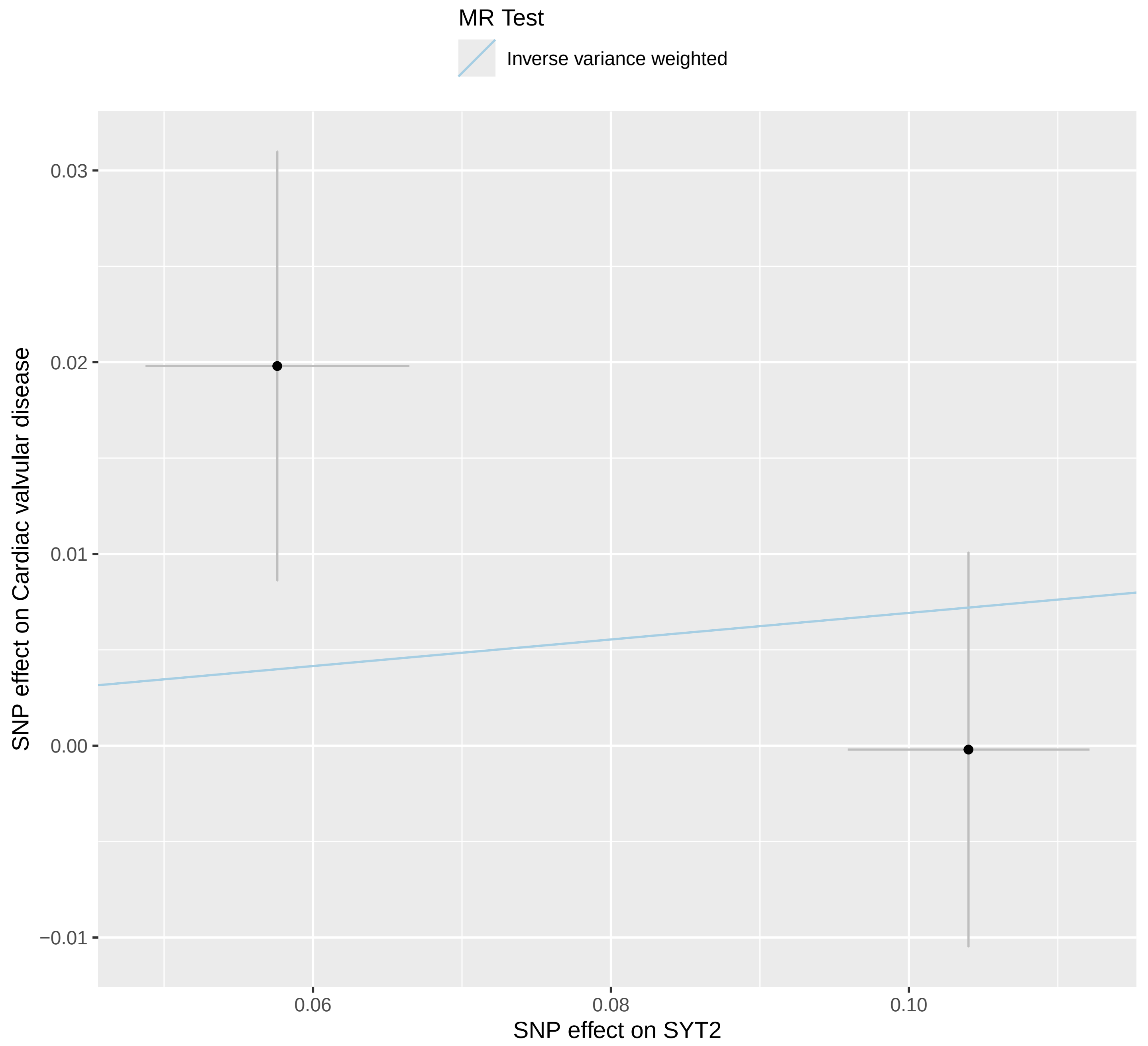

MR Test
Inverse variance weighted
SNP effect on Cardiac valvular disease
0.03
0.02
0.01
0.00
−0.01
0.06
0.08
0.10
SNP effect on SYT2

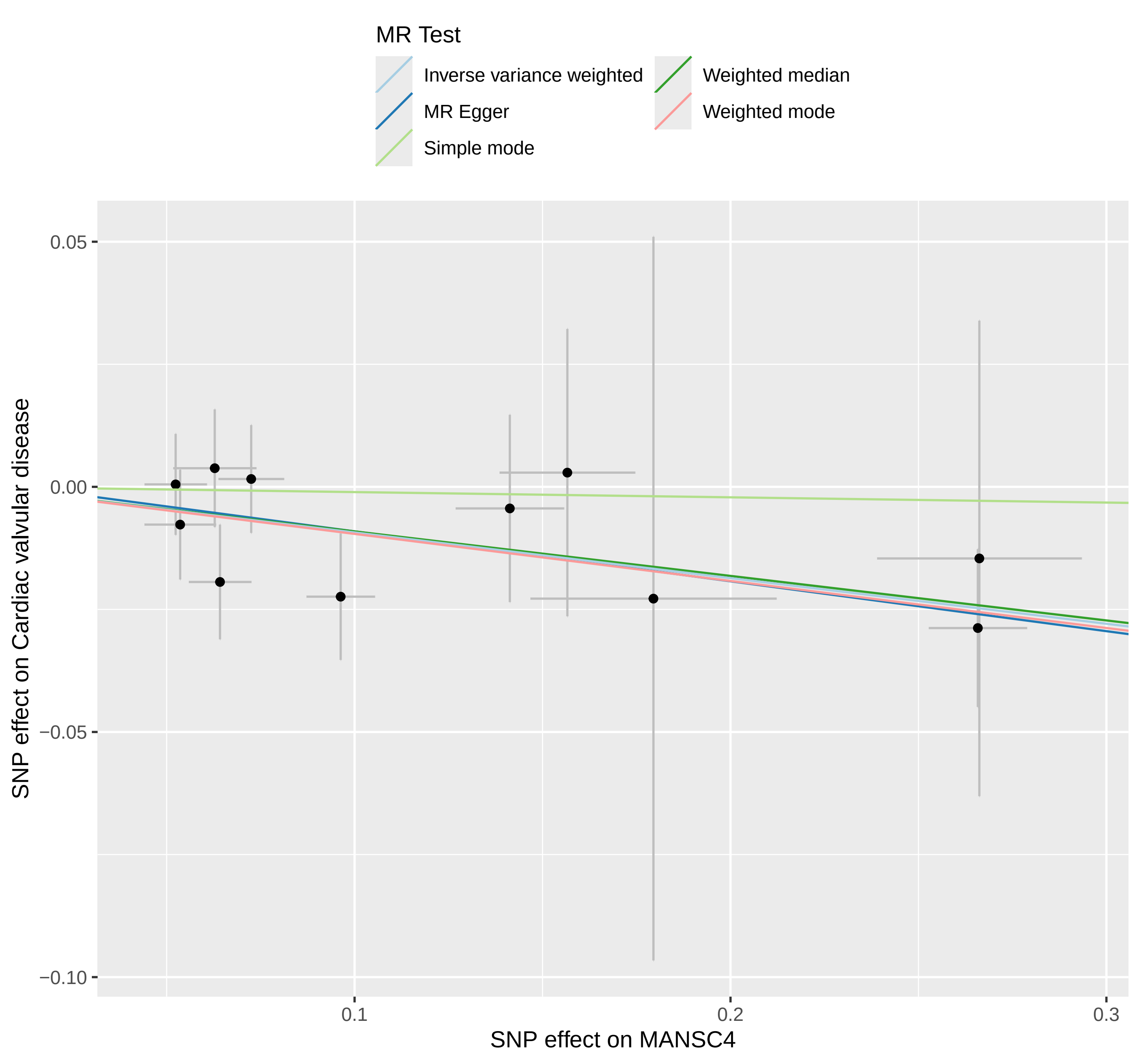
MR Test
Inverse variance weighted
MR Egger
Simple mode
Weighted median
Weighted mode
0.05
0.00
−0.05
−0.10
0.1
0.2
0.3
SNP effect on Cardiac valvular disease
SNP effect on MANSC4

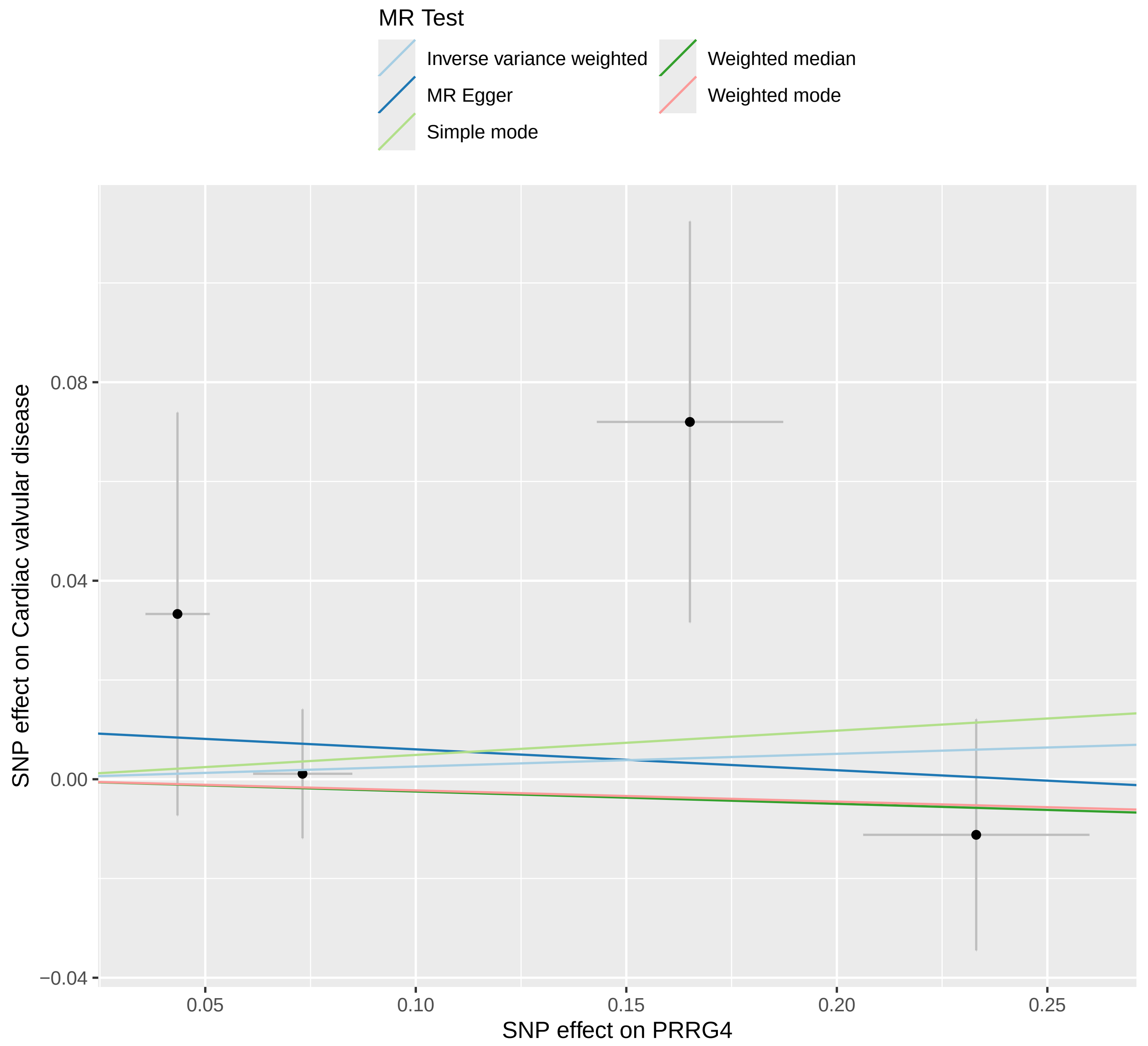
MR Test
Inverse variance weighted
MR Egger
Simple mode
Weighted median
Weighted mode
SNP effect on Cardiac valvular disease
0.08
0.04
0.00
−0.04
0.05
0.10
0.15
0.20
0.25
SNP effect on PRRG4

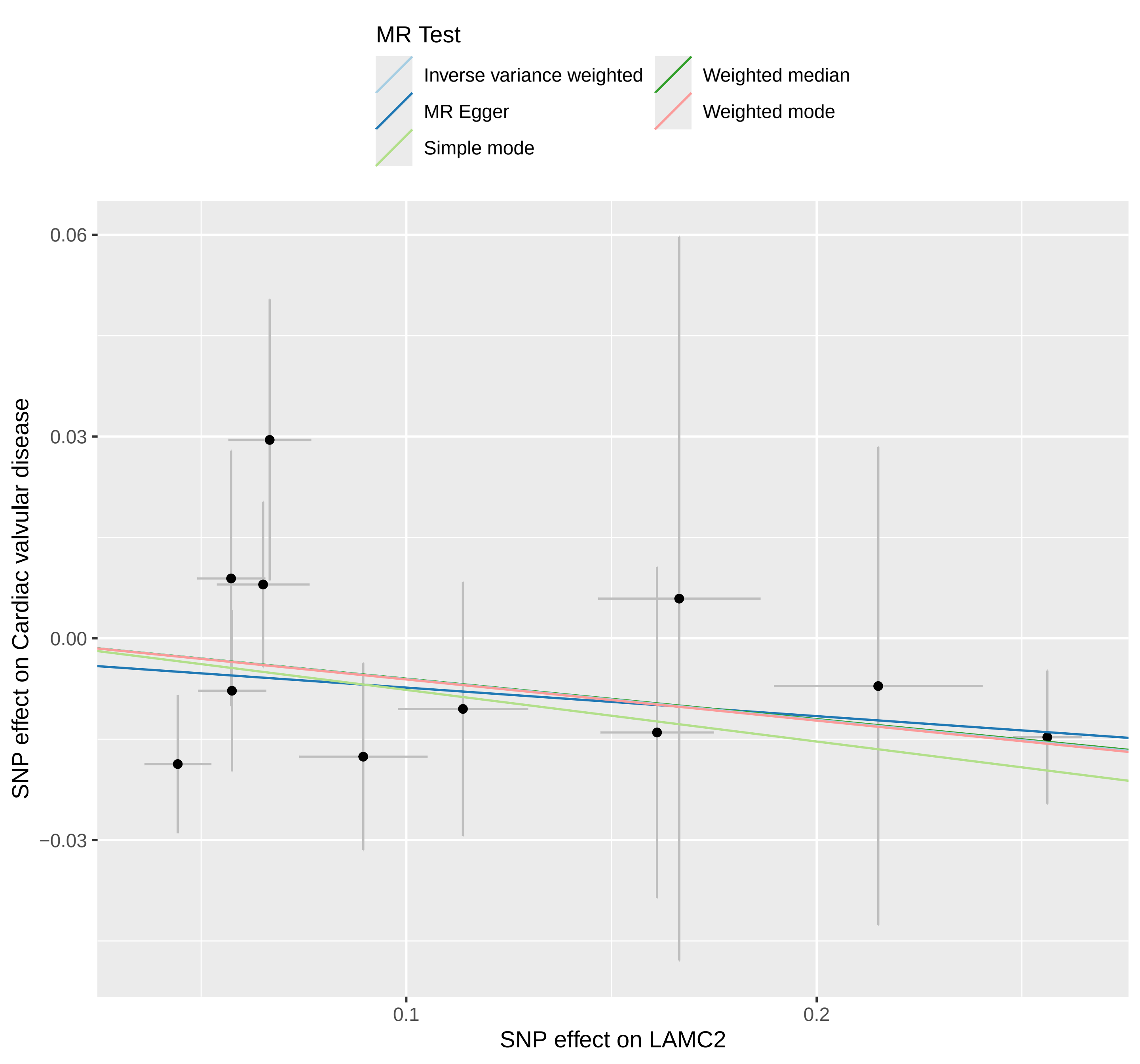
MR Test
Inverse variance weighted
MR Egger
Simple mode
Weighted median
Weighted mode
0.06
0.03
0.00
−0.03
SNP effect on Cardiac valvular disease
0.1
0.2
SNP effect on LAMC2

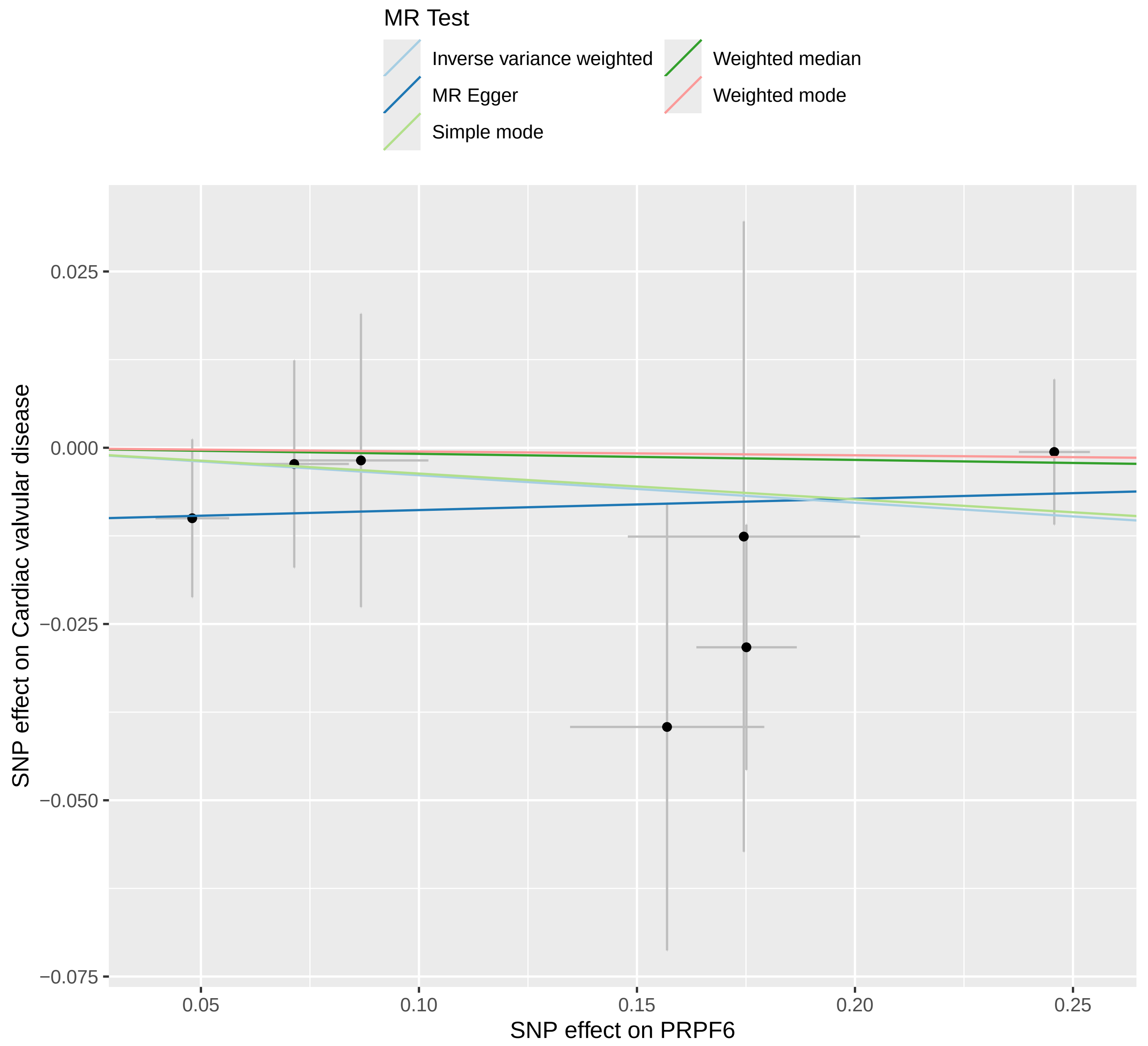
MR Test
Inverse variance weighted
MR Egger
Simple mode
Weighted median
Weighted mode
SNP effect on Cardiac valvular disease
0.025
0.000
−0.025
−0.050
−0.075
0.05
0.10
0.15
0.20
0.25
SNP effect on PRPF6

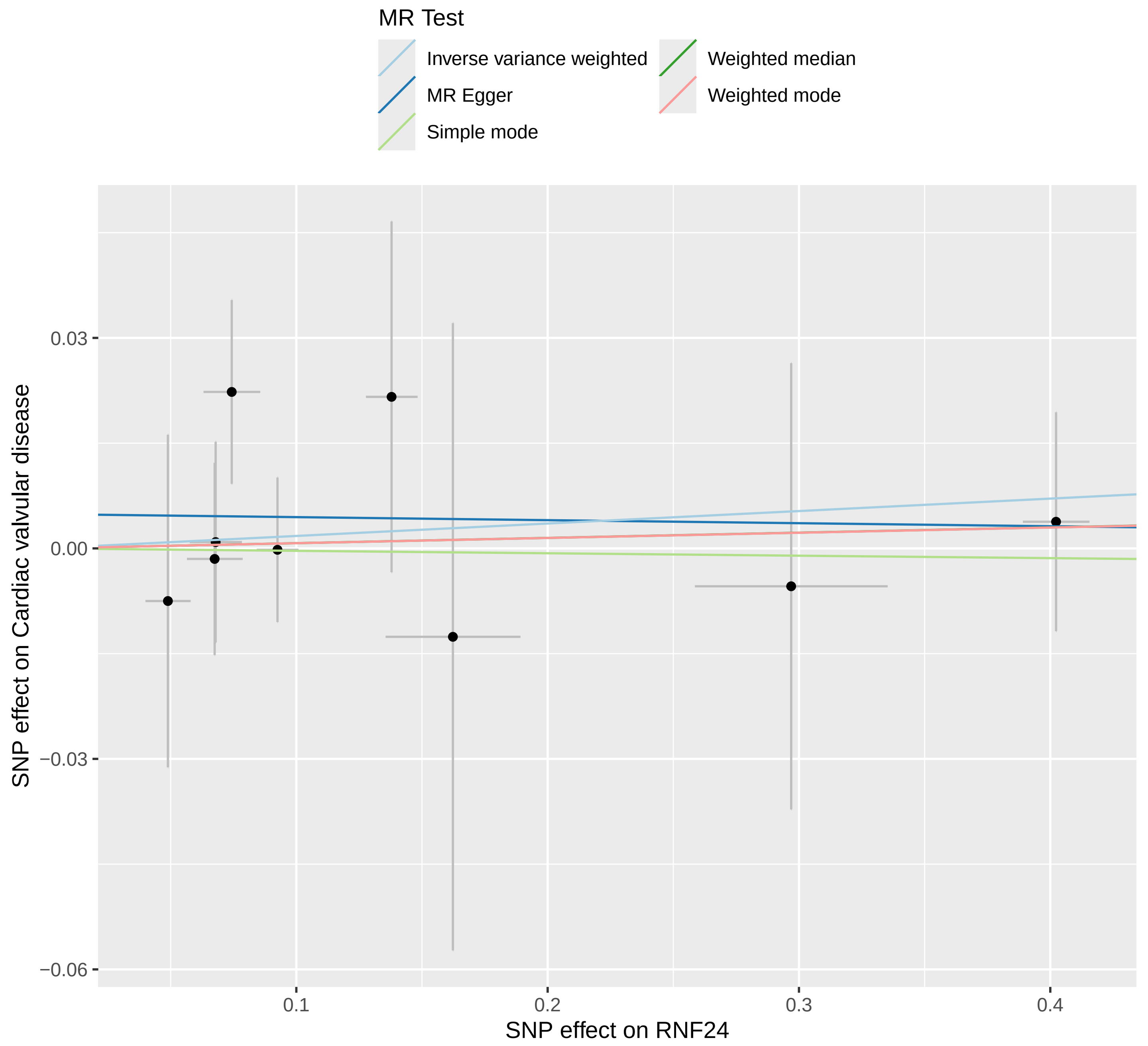
MR Test
Inverse variance weighted
MR Egger
Simple mode
Weighted median
Weighted mode
0.03
0.00
−0.03
−0.06
0.1
0.2
0.3
0.4
SNP effect on Cardiac valvular disease
SNP effect on RNF24

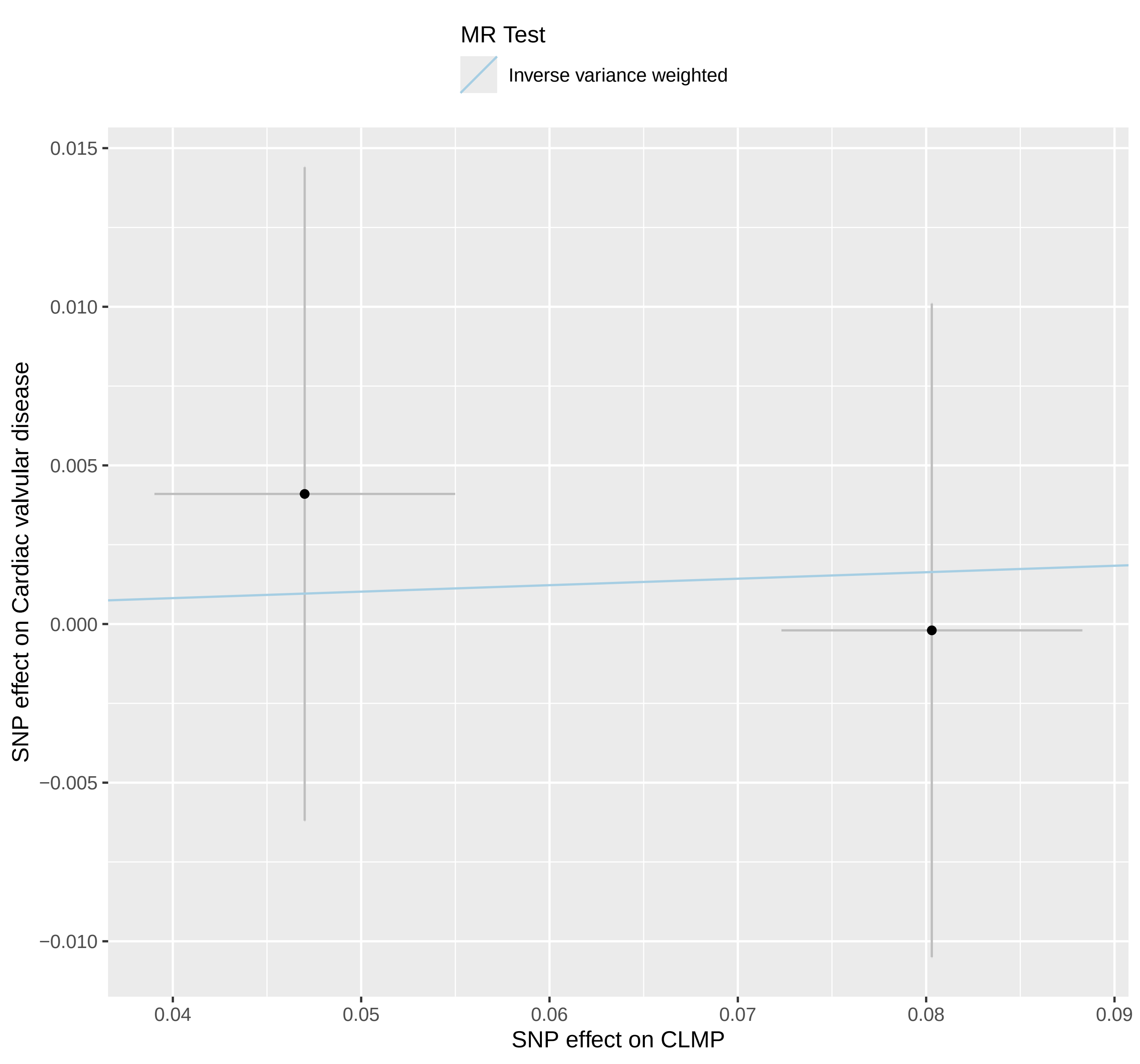
MR Test
Inverse variance weighted
0.015
0.010
0.005
0.000
−0.005
−0.010
SNP effect on Cardiac valvular disease
0.04
0.05
0.06
0.07
0.08
0.09
SNP effect on CLMP

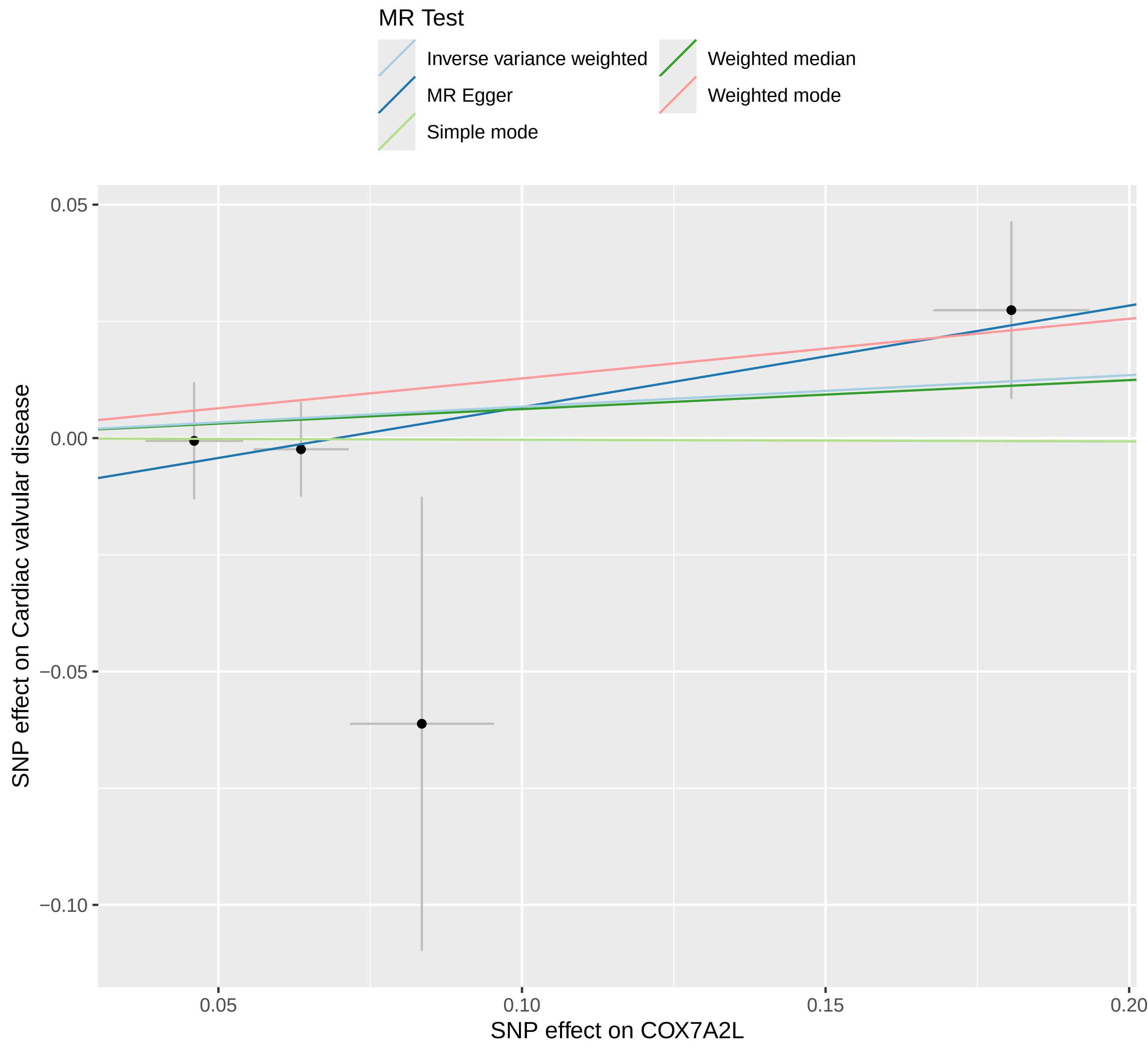

MR Test
Inverse variance weighted
MR Egger
Simple mode
Weighted median
Weighted mode
0.05
0.00
−0.05
−0.10
SNP effect on Cardiac valvular disease
0.05
0.10
0.15
0.20
SNP effect on COX7A2L

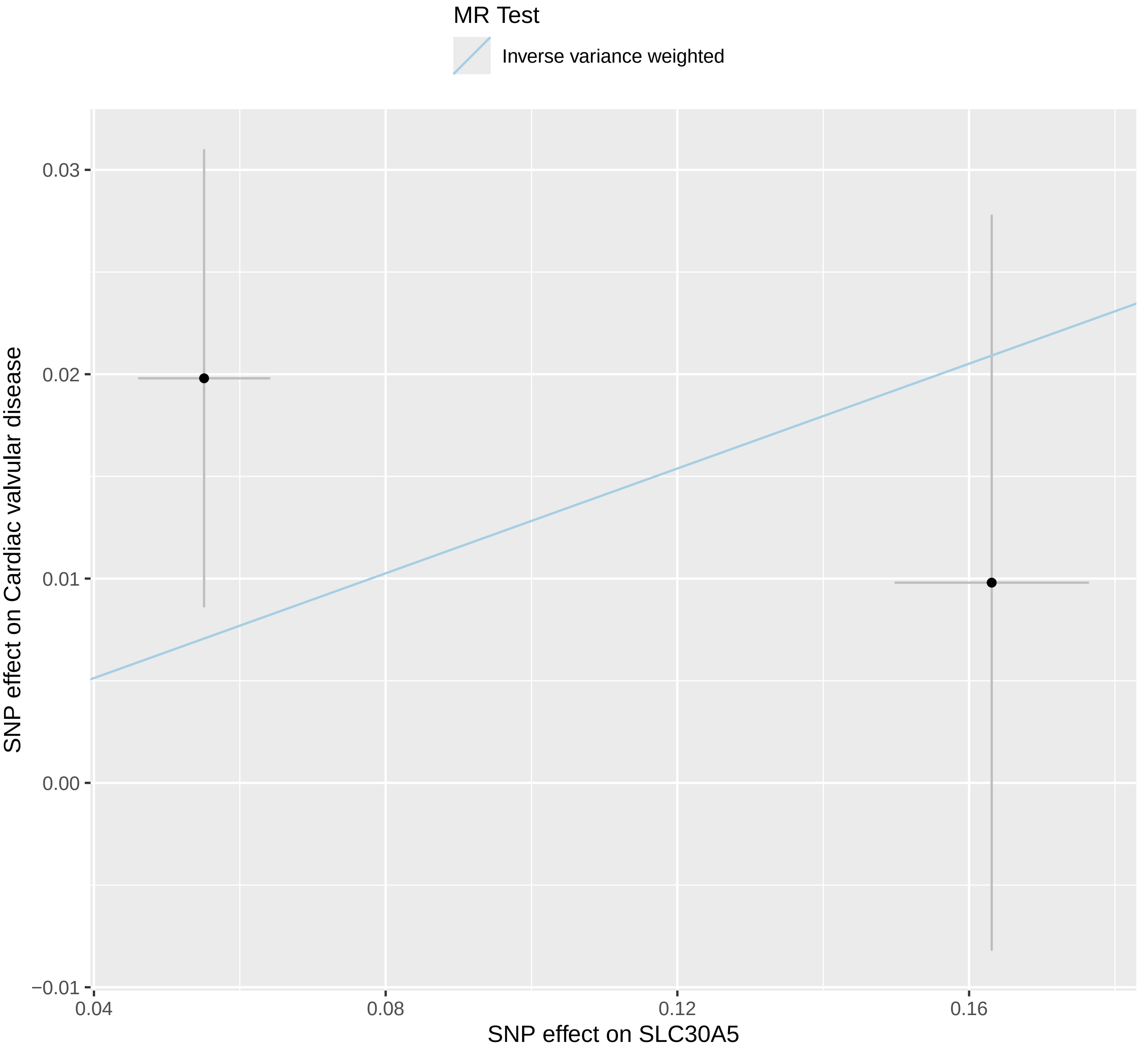
MR Test
Inverse variance weighted
0.03
0.02
0.01
0.00
−0.01
0.04
0.08
0.12
0.16
SNP effect on Cardiac valvular disease
SNP effect on SLC30A5

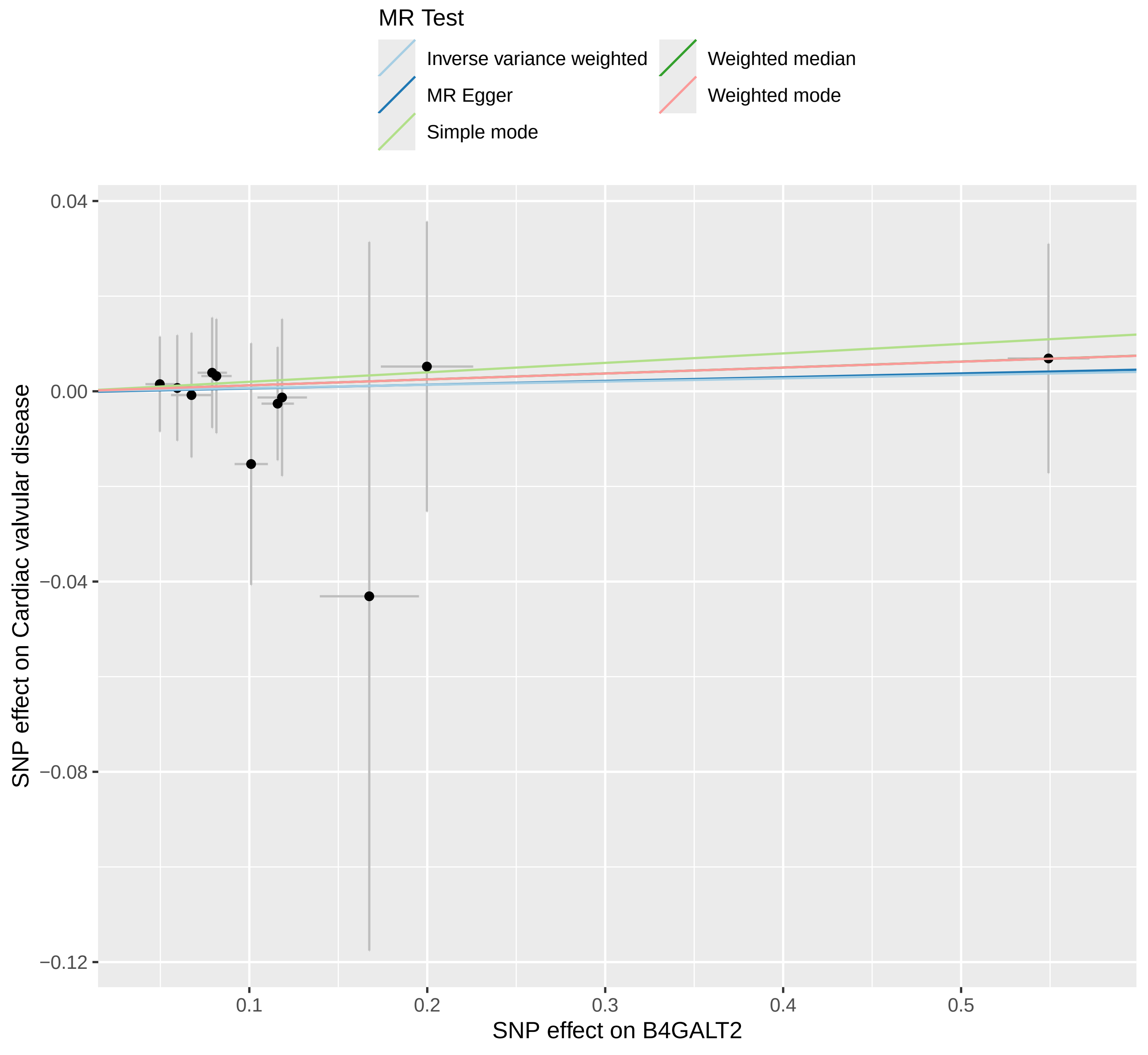
MR Test
Inverse variance weighted
MR Egger
Simple mode
Weighted median
Weighted mode
0.04
0.00
−0.04
−0.08
−0.12
0.1
0.2
0.3
0.4
0.5
SNP effect on Cardiac valvular disease
SNP effect on B4GALT2

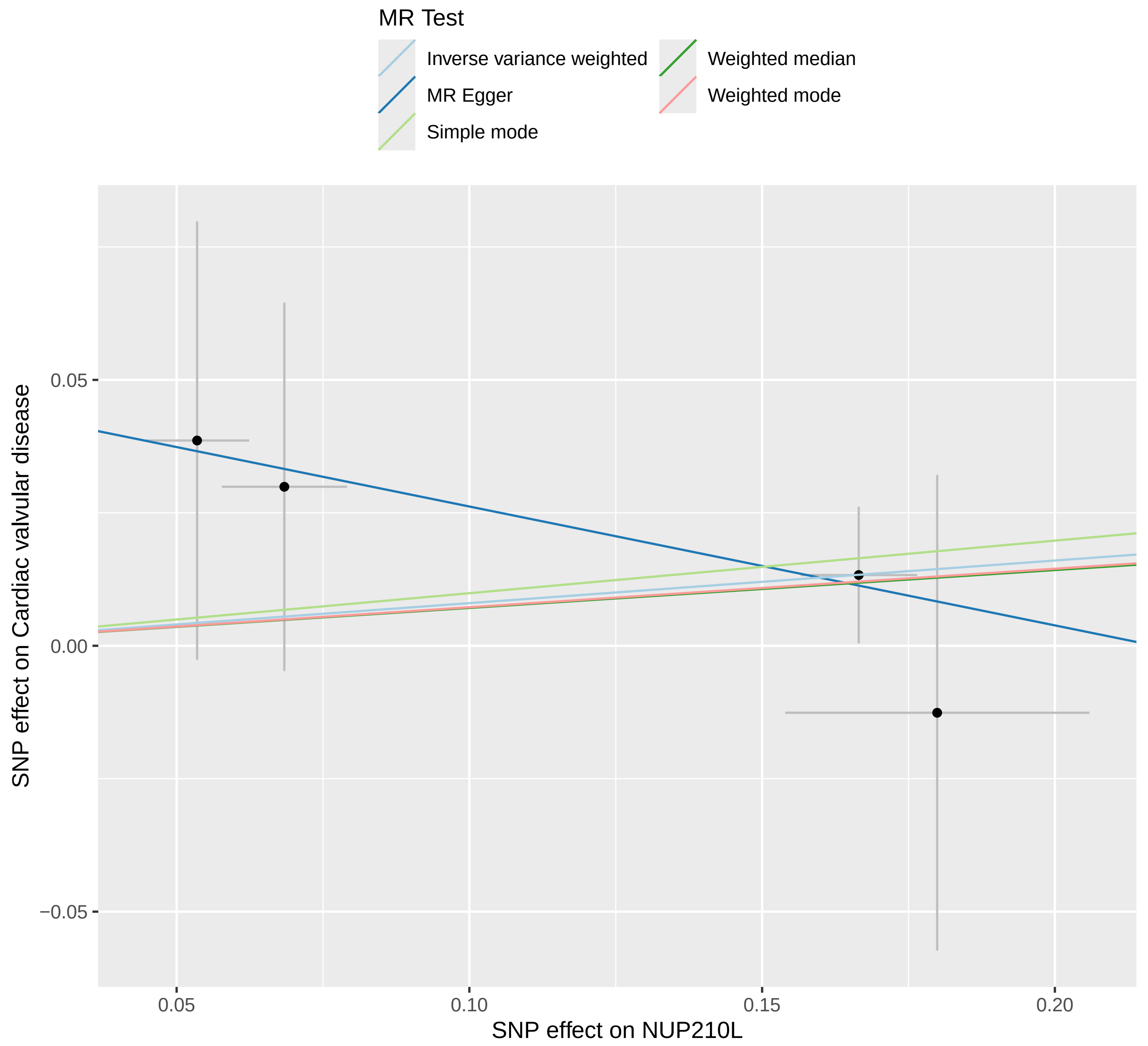
MR Test
Inverse variance weighted
MR Egger
Simple mode
Weighted median
Weighted mode
SNP effect on Cardiac valvular disease
0.05
0.00
−0.05
0.05
0.10
0.15
0.20
SNP effect on NUP210L

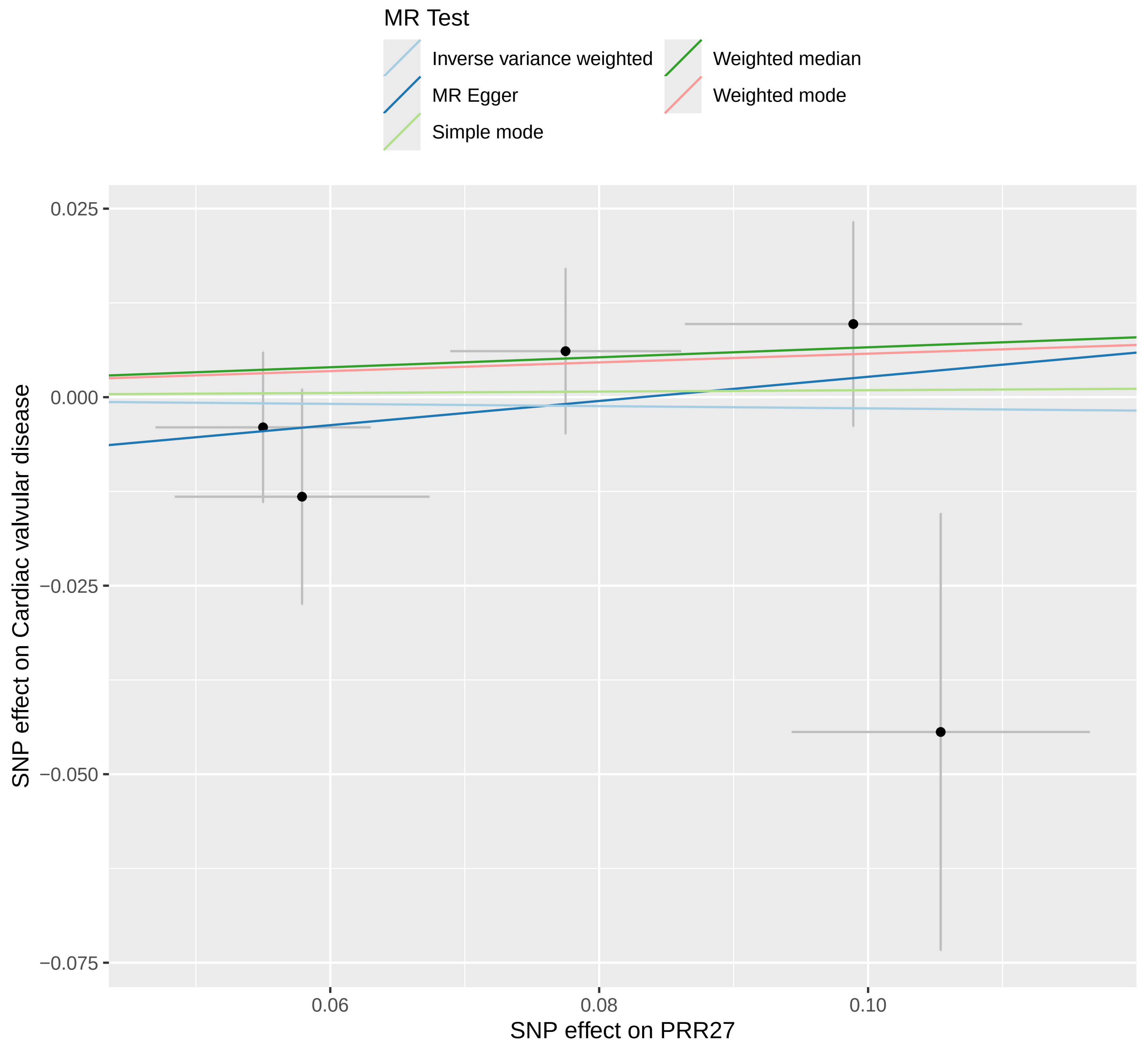
MR Test
Inverse variance weighted
MR Egger
Simple mode
Weighted median
Weighted mode
0.025
0.000
−0.025
−0.050
−0.075
0.06
0.08
0.10
SNP effect on Cardiac valvular disease
SNP effect on PRR27

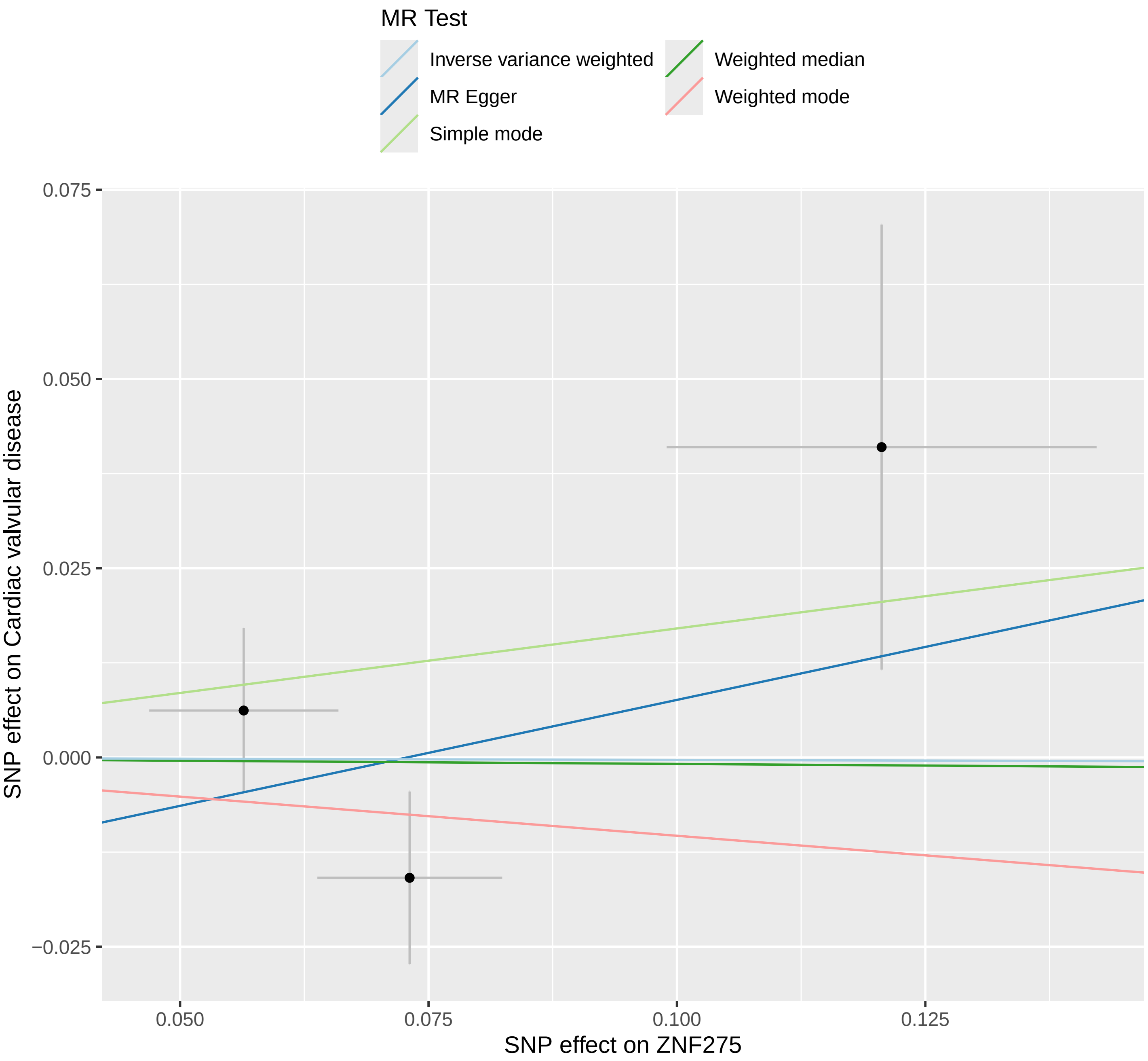
MR Test
Inverse variance weighted
MR Egger
Simple mode
Weighted median
Weighted mode
0.075
0.050
0.025
0.000
−0.025
0.050
0.075
0.100
0.125
SNP effect on Cardiac valvular disease
SNP effect on ZNF275

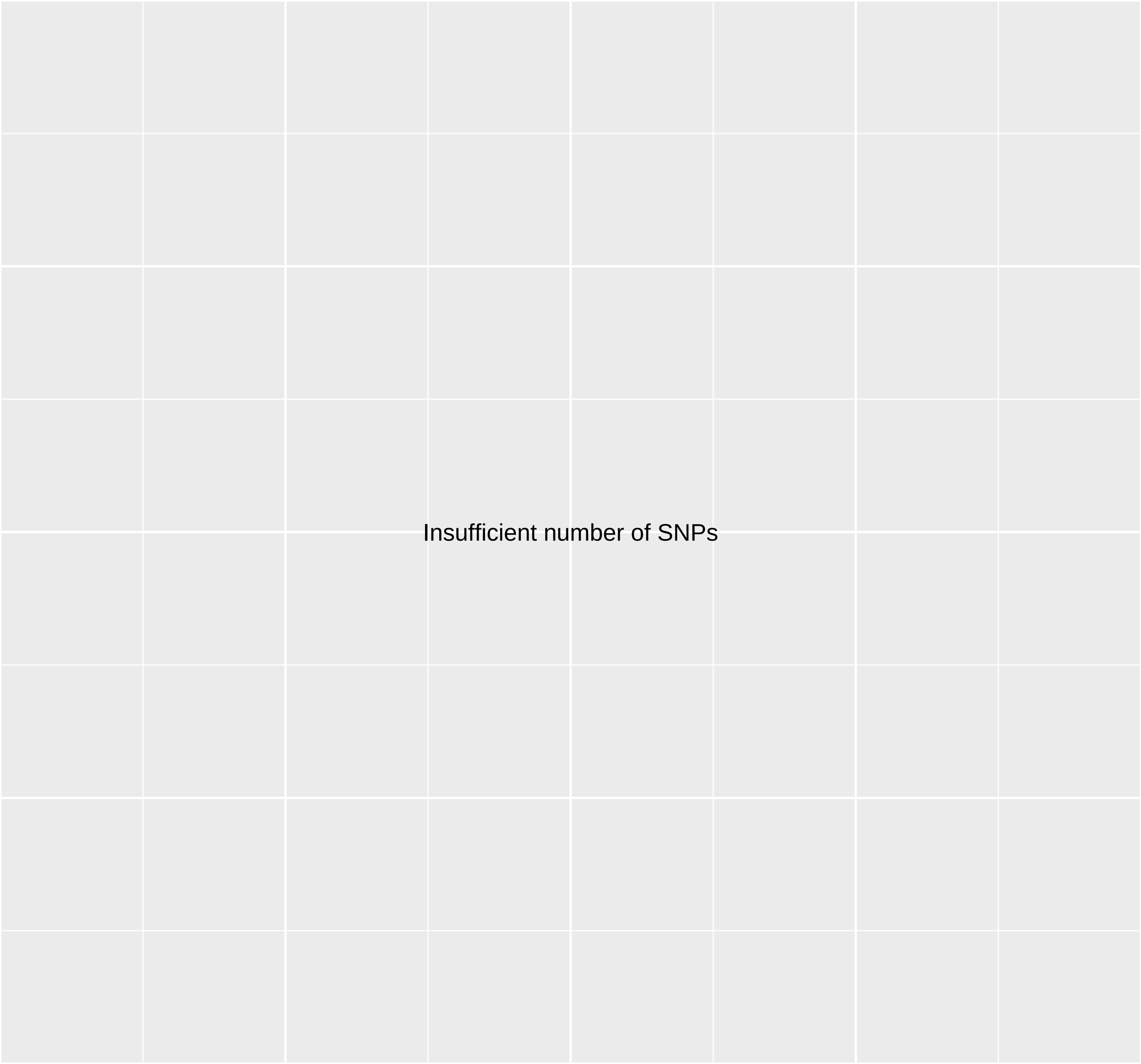
Insufficient number of SNPs

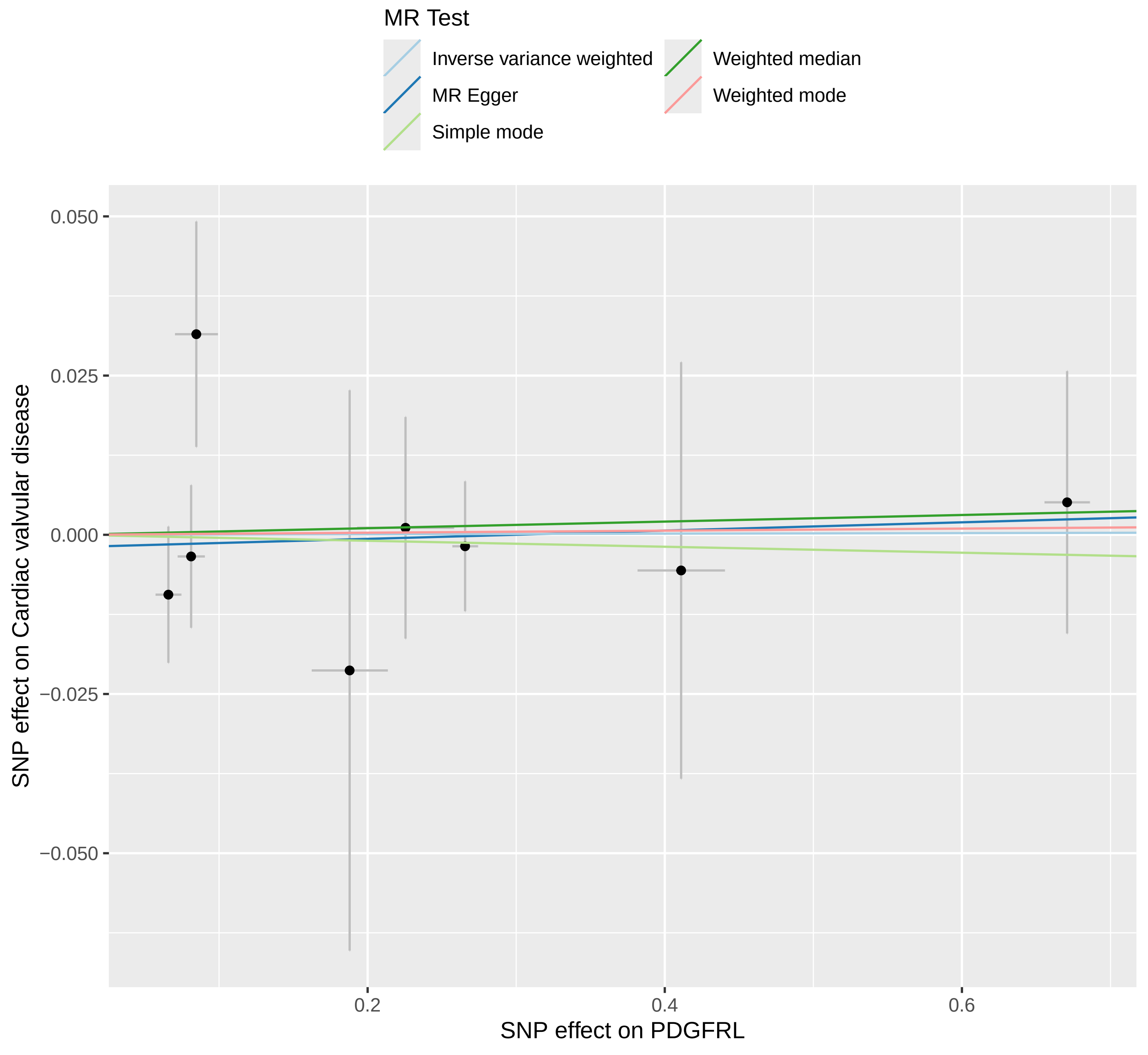

MR Test
Inverse variance weighted
MR Egger
Simple mode
Weighted median
Weighted mode
0.050
0.025
0.000
−0.025
−0.050
SNP effect on Cardiac valvular disease
0.2
0.4
0.6
SNP effect on PDGFRL

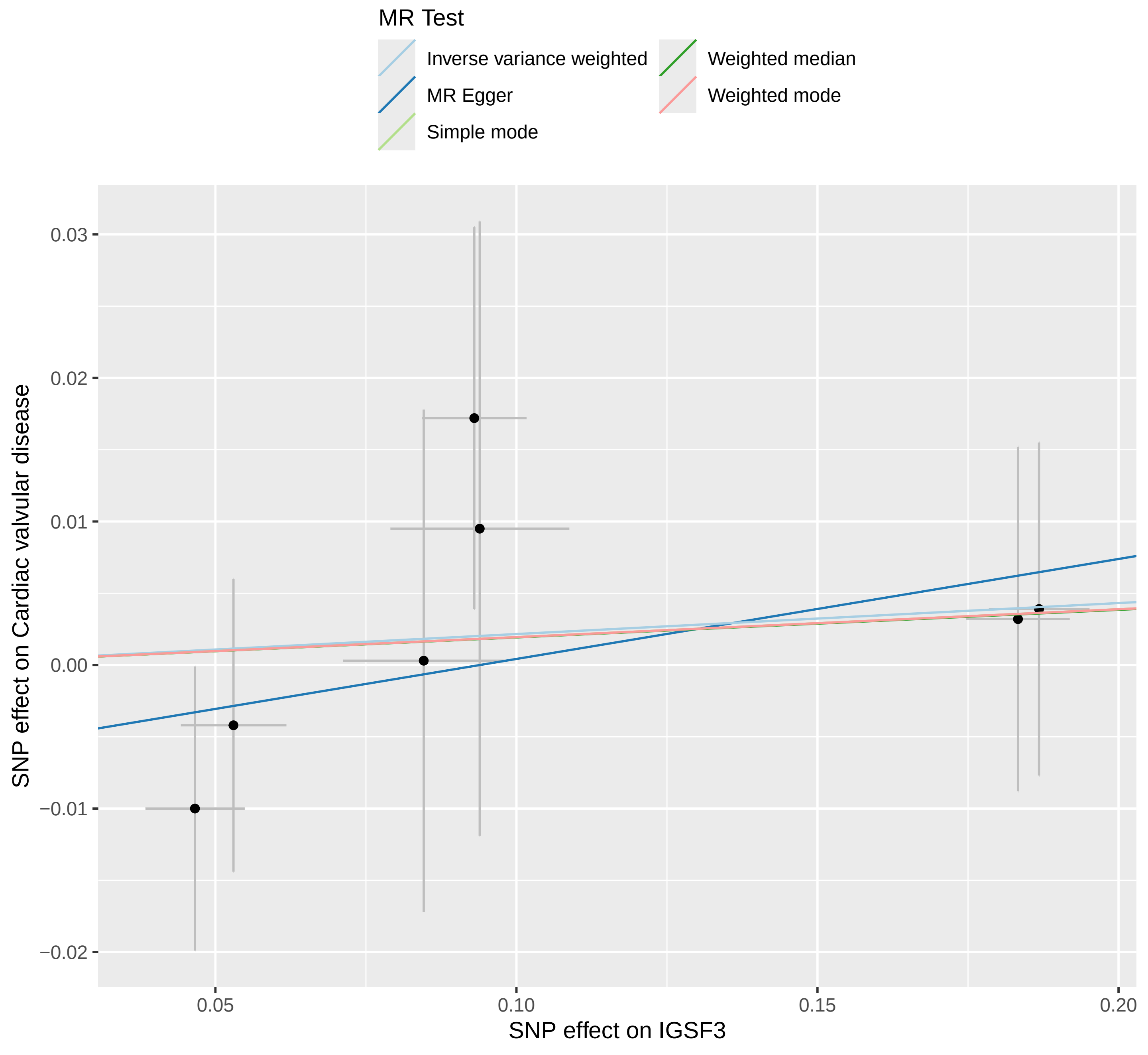

MR Test
Inverse variance weighted
MR Egger
Simple mode
Weighted median
Weighted mode
0.03
0.02
0.01
0.00
−0.01
−0.02
0.05
0.10
0.15
0.20
SNP effect on IGSF3
SNP effect on Cardiac valvular disease

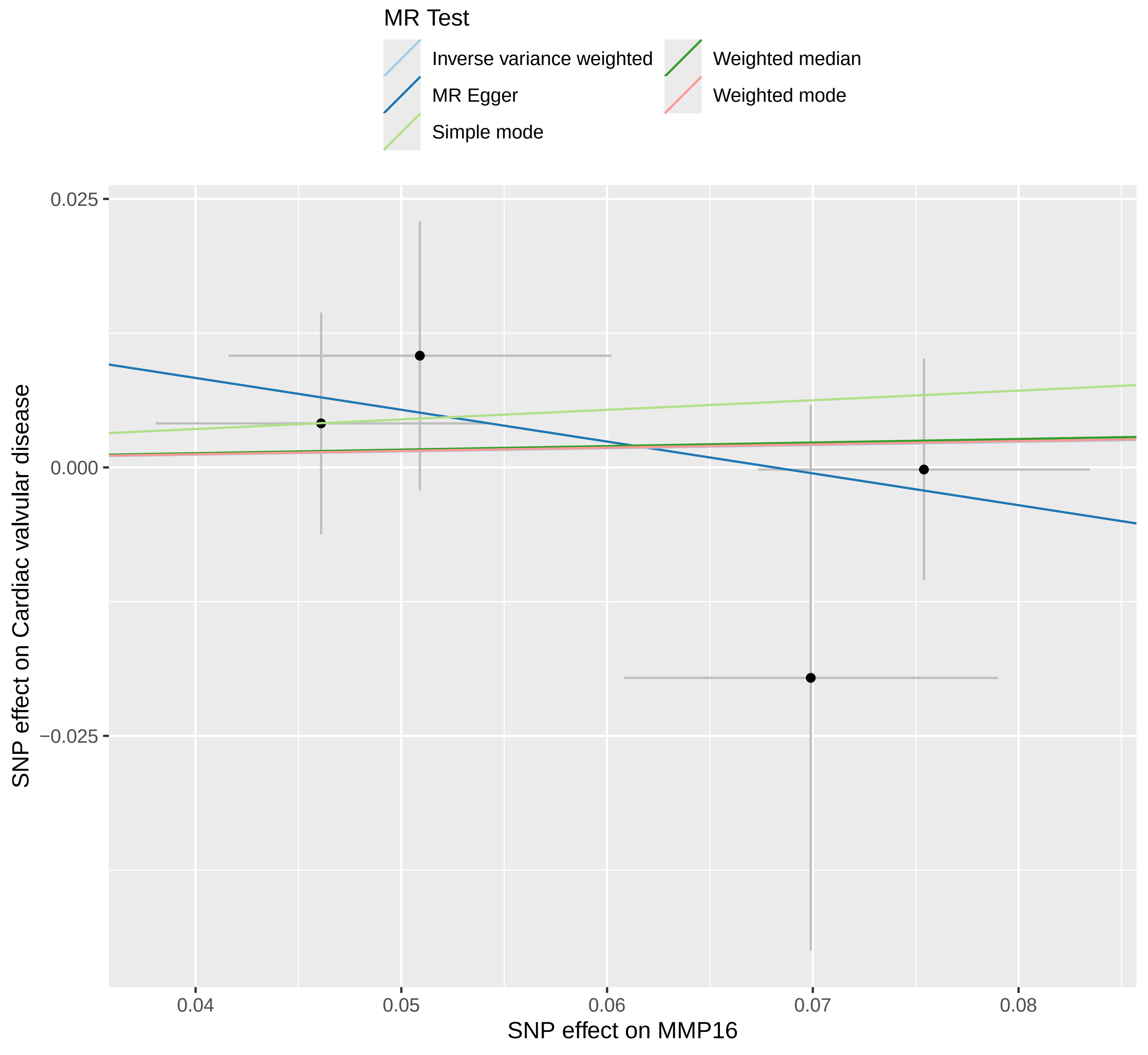
MR Test
Inverse variance weighted
MR Egger
Simple mode
Weighted median
Weighted mode
0.025
0.000
−0.025
0.04
0.05
0.06
0.07
0.08
SNP effect on MMP16
SNP effect on Cardiac valvular disease

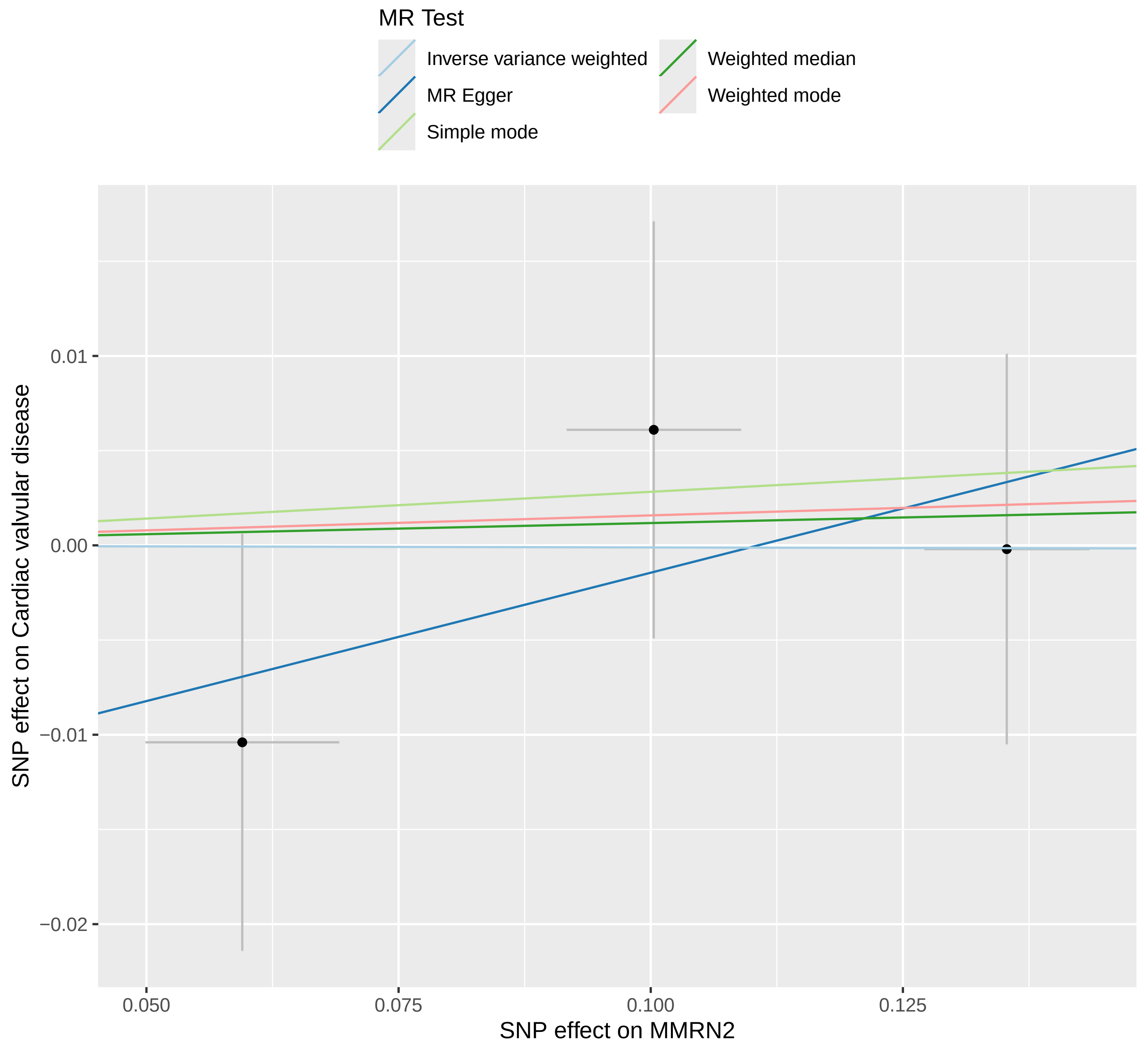
MR Test
Inverse variance weighted
MR Egger
Simple mode
Weighted median
Weighted mode
SNP effect on Cardiac valvular disease
0.01
0.00
−0.01
−0.02
0.050
0.075
0.100
0.125
SNP effect on MMRN2

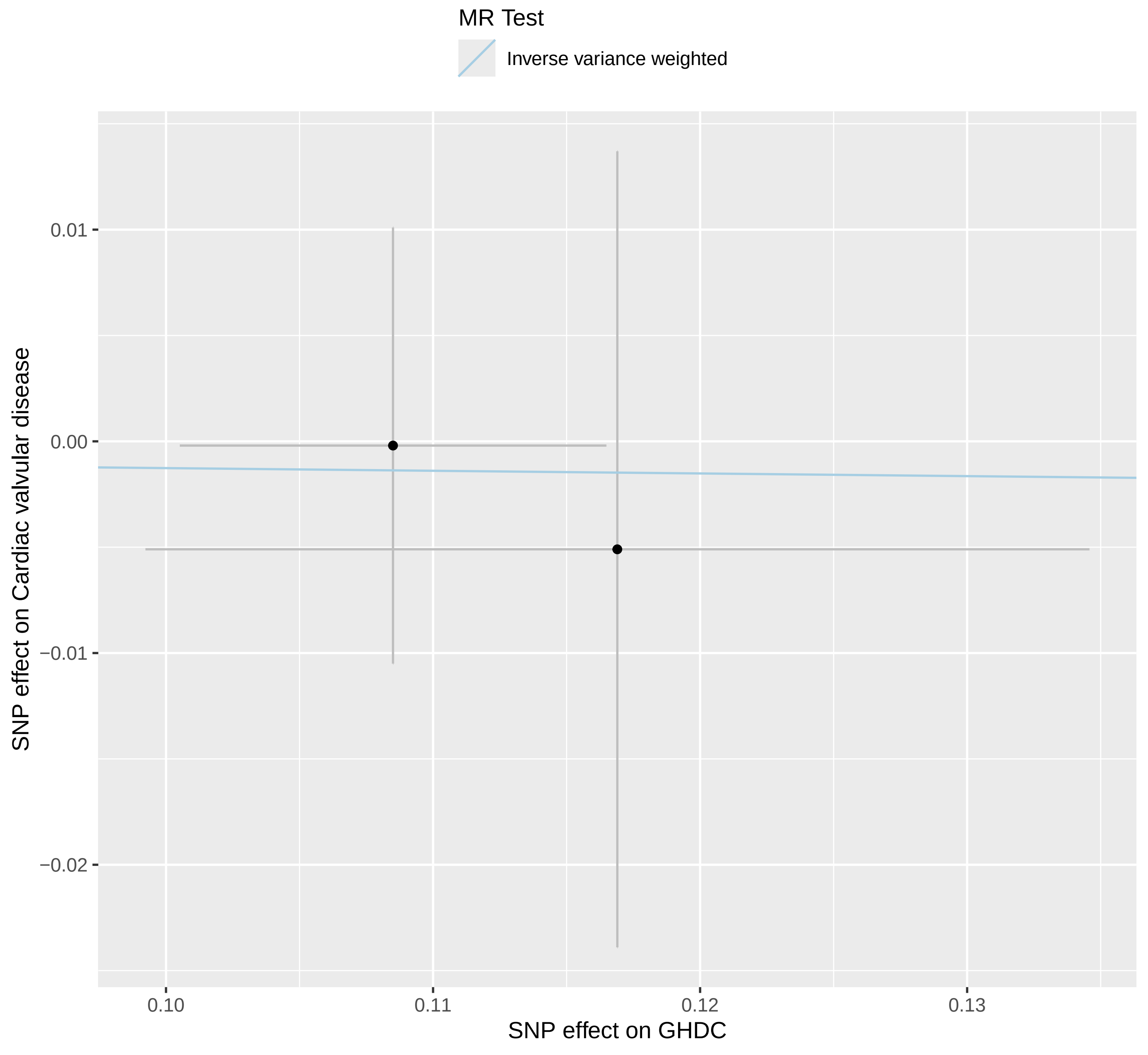

MR Test
Inverse variance weighted
0.01
0.00
−0.01
−0.02
0.10
0.11
0.12
0.13
SNP effect on GHDC
SNP effect on Cardiac valvular disease

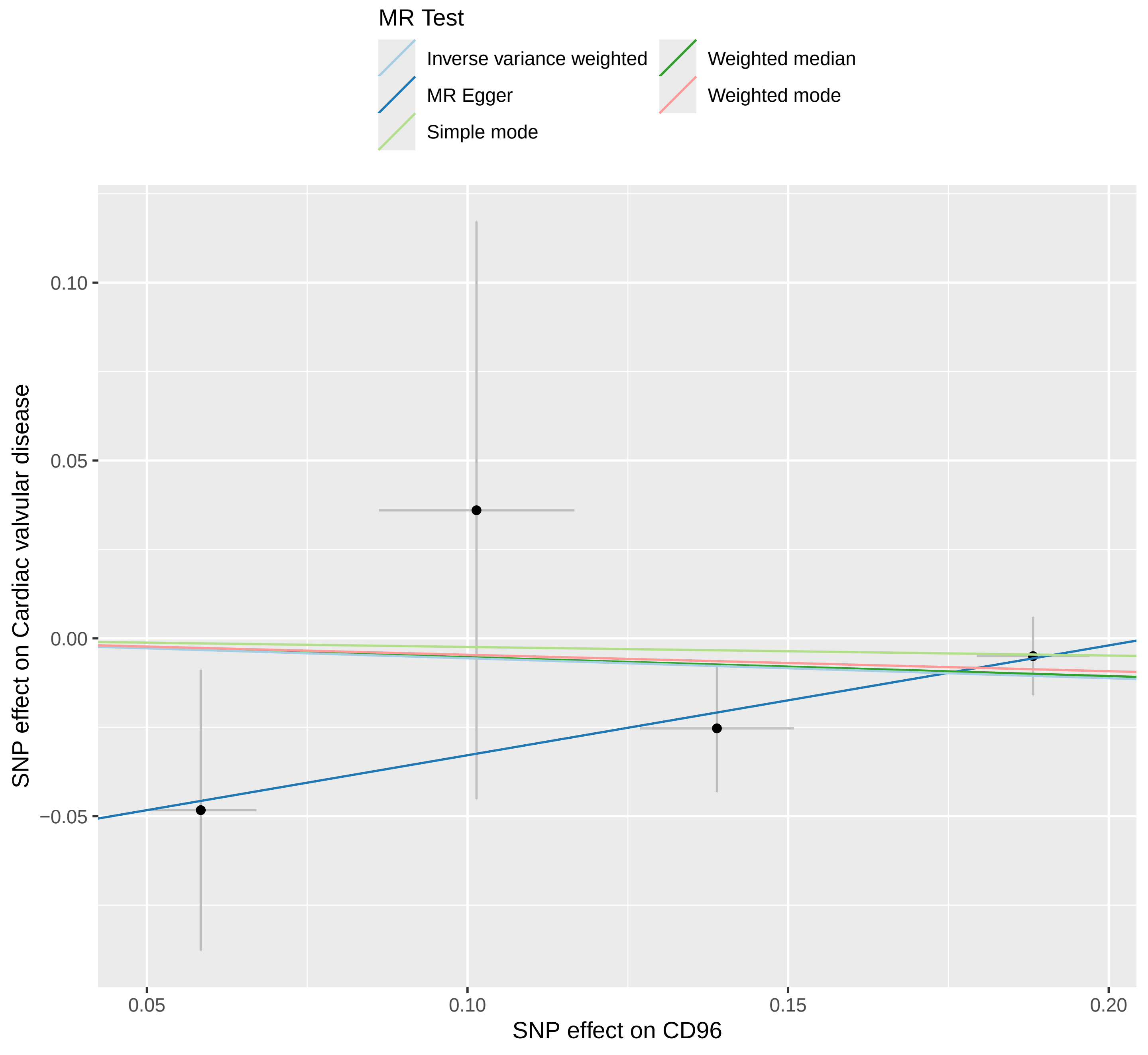
MR Test
Inverse variance weighted
MR Egger
Simple mode
Weighted median
Weighted mode
0.10
0.05
0.00
−0.05
0.05
0.10
0.15
0.20
SNP effect on Cardiac valvular disease
SNP effect on CD96

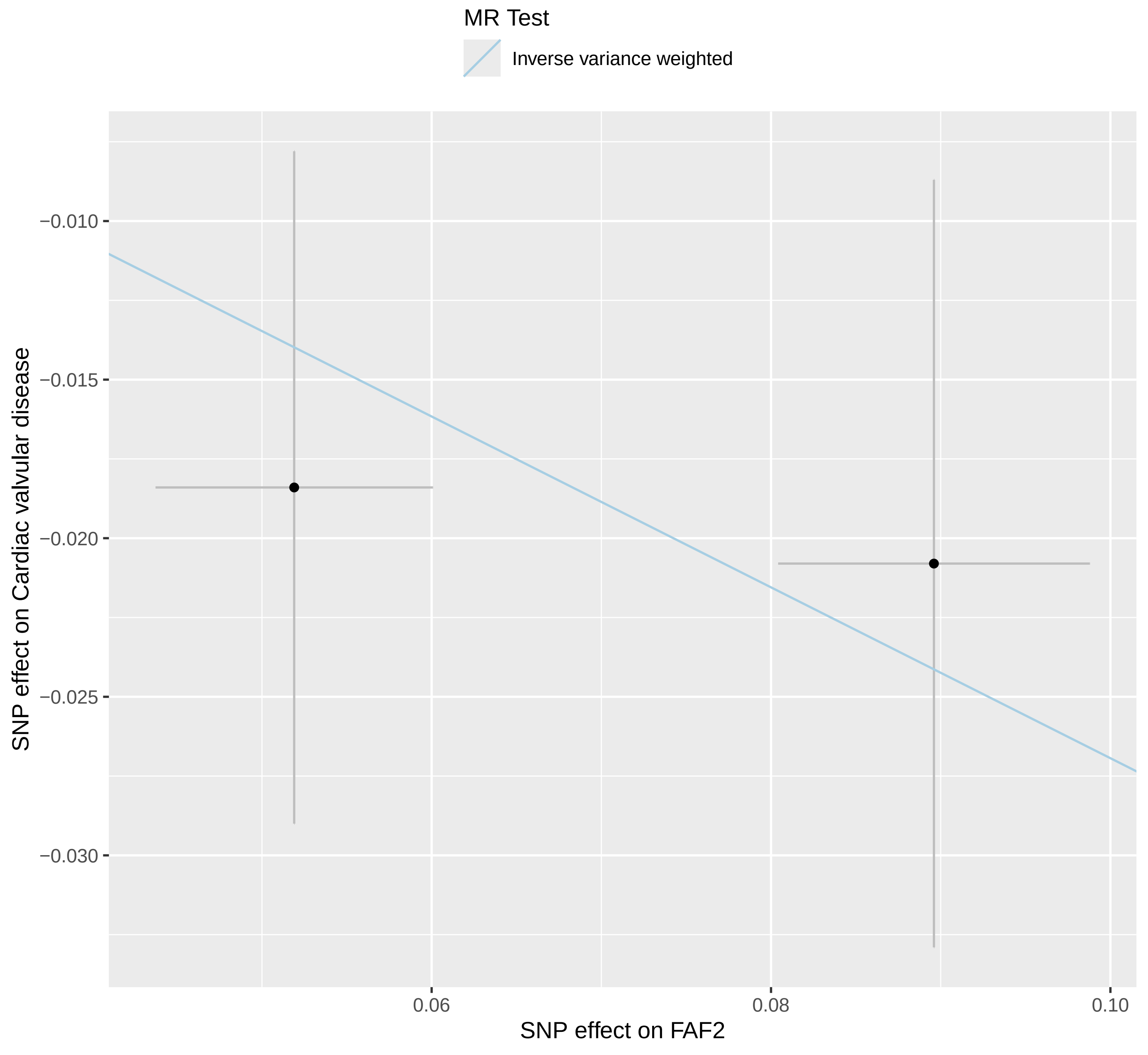
MR Test
Inverse variance weighted
−0.010
−0.015
−0.020
−0.025
−0.030
SNP effect on Cardiac valvular disease
0.06
0.08
0.10
SNP effect on FAF2

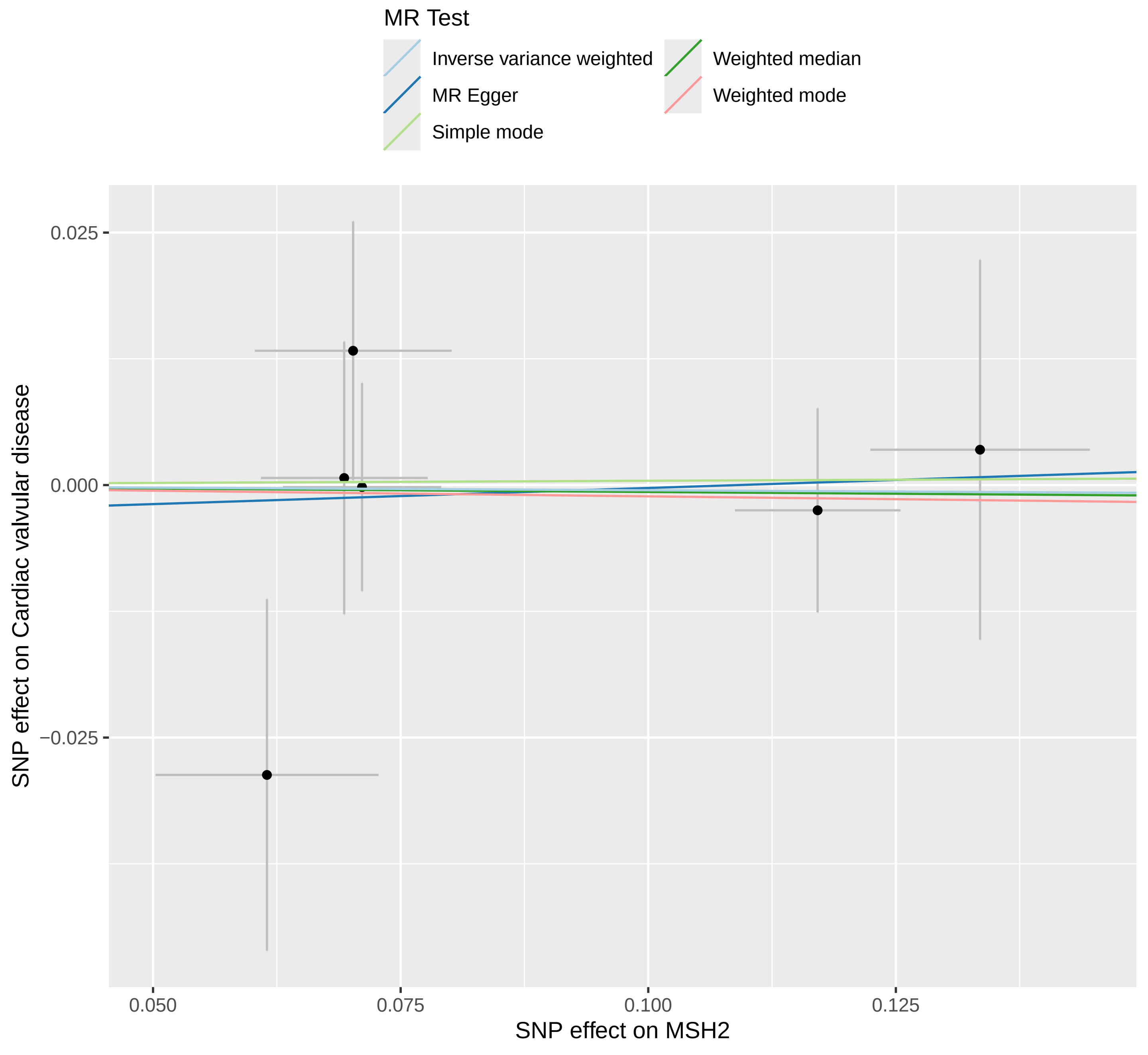

MR Test
Inverse variance weighted
MR Egger
Simple mode
Weighted median
Weighted mode
0.025
0.000
−0.025
SNP effect on Cardiac valvular disease
0.050
0.075
0.100
0.125
SNP effect on MSH2

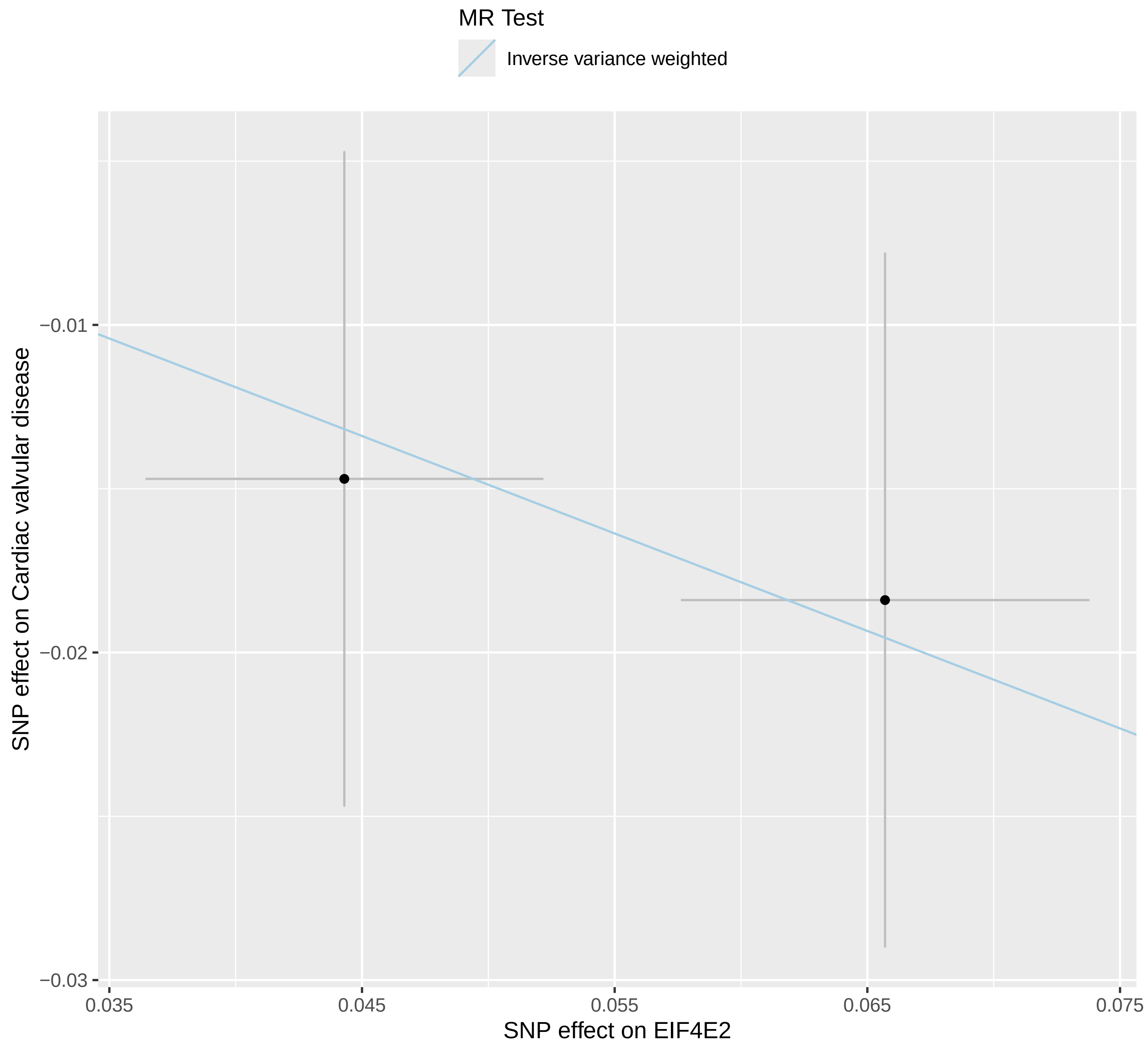
MR Test
Inverse variance weighted
SNP effect on Cardiac valvular disease
−0.01
−0.02
−0.03
0.035
0.045
0.055
0.065
0.075
SNP effect on EIF4E2

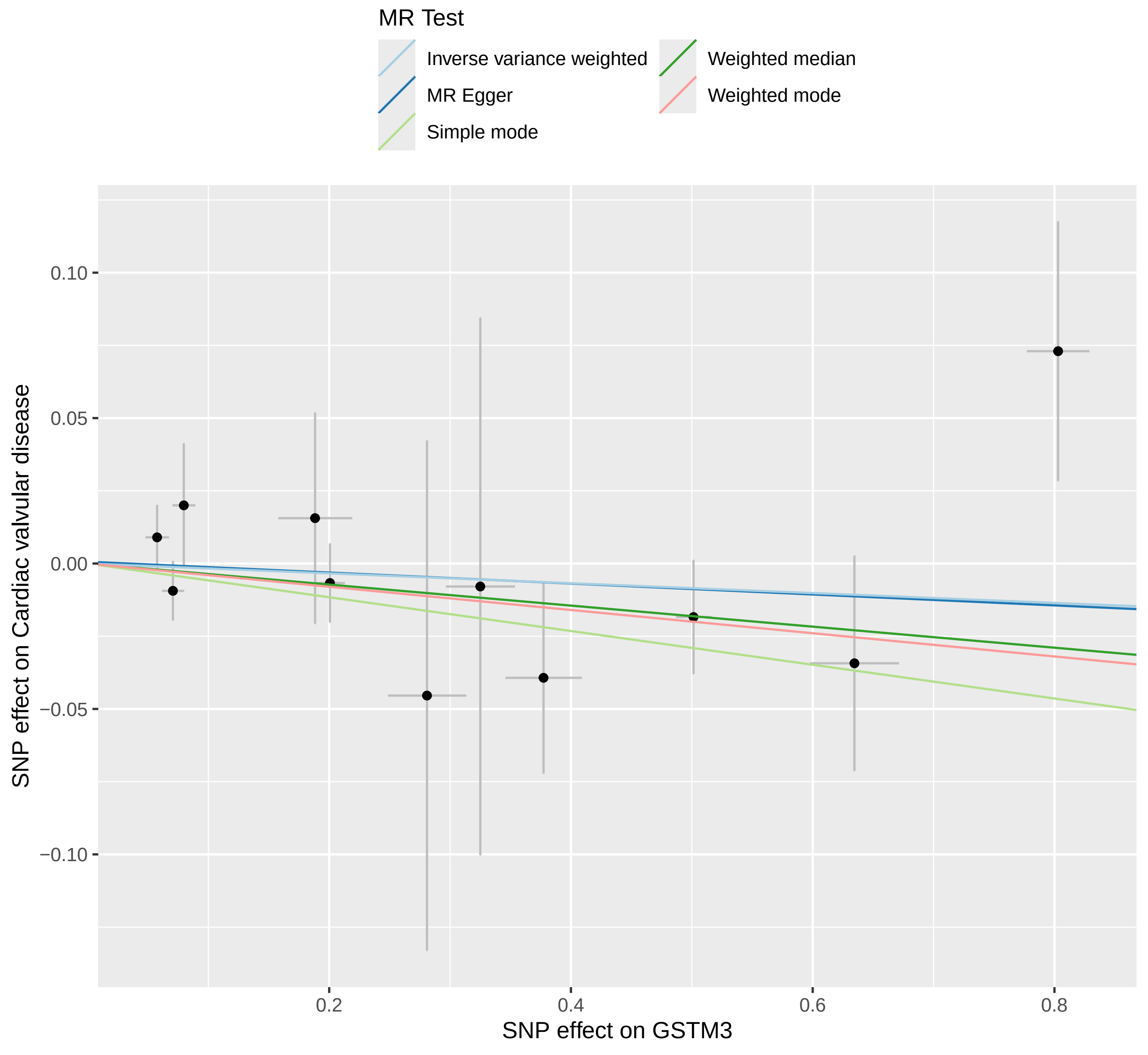
MR Test
Inverse variance weighted
MR Egger
Simple mode
Weighted median
Weighted mode
0.10
0.05
0.00
−0.05
−0.10
SNP effect on Cardiac valvular disease
0.2
0.4
0.6
0.8
SNP effect on GSTM3

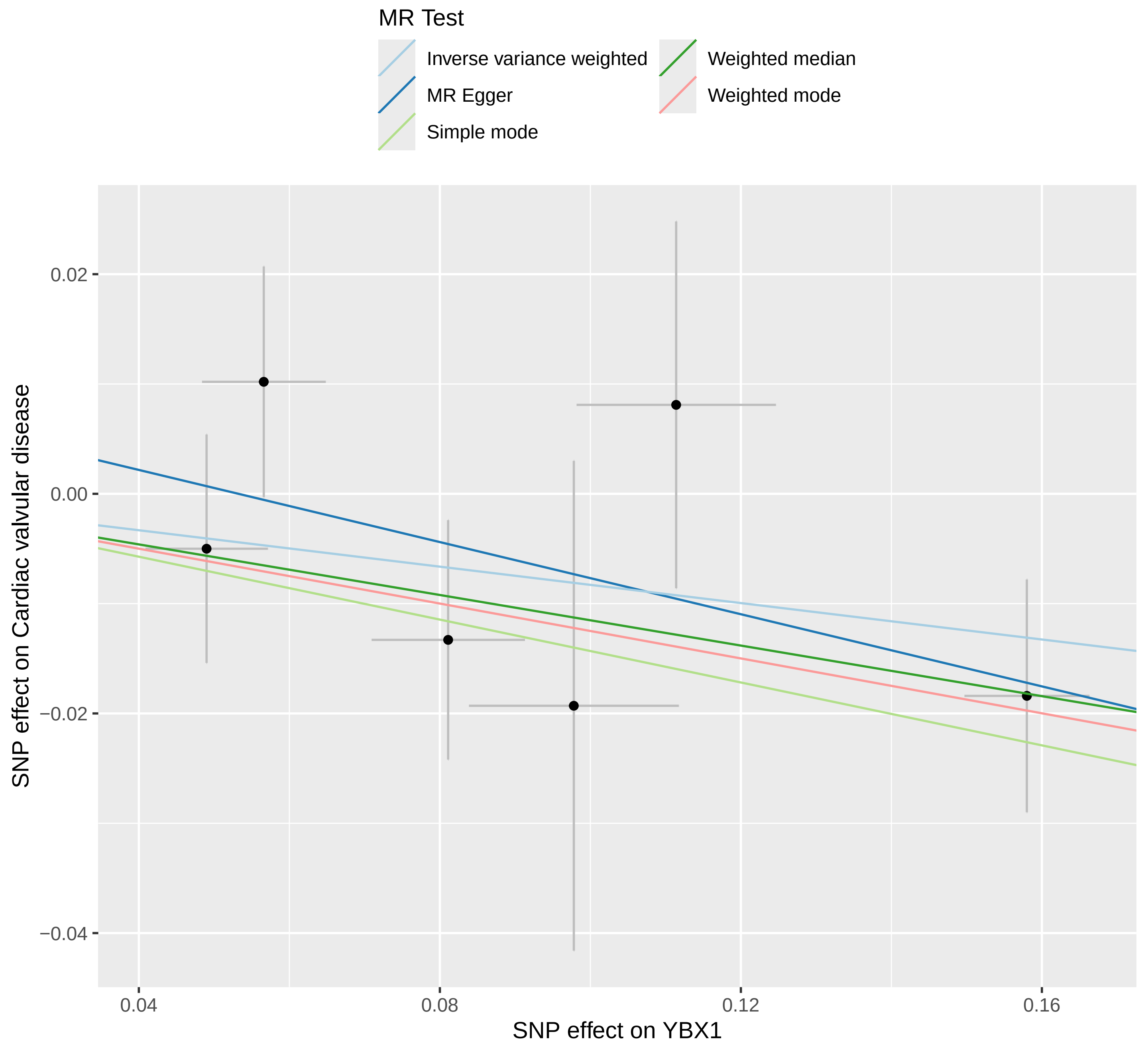
MR Test
Inverse variance weighted
MR Egger
Simple mode
Weighted median
Weighted mode
0.02
0.00
−0.02
−0.04
0.04
0.08
0.12
0.16
SNP effect on Cardiac valvular disease
SNP effect on YBX1

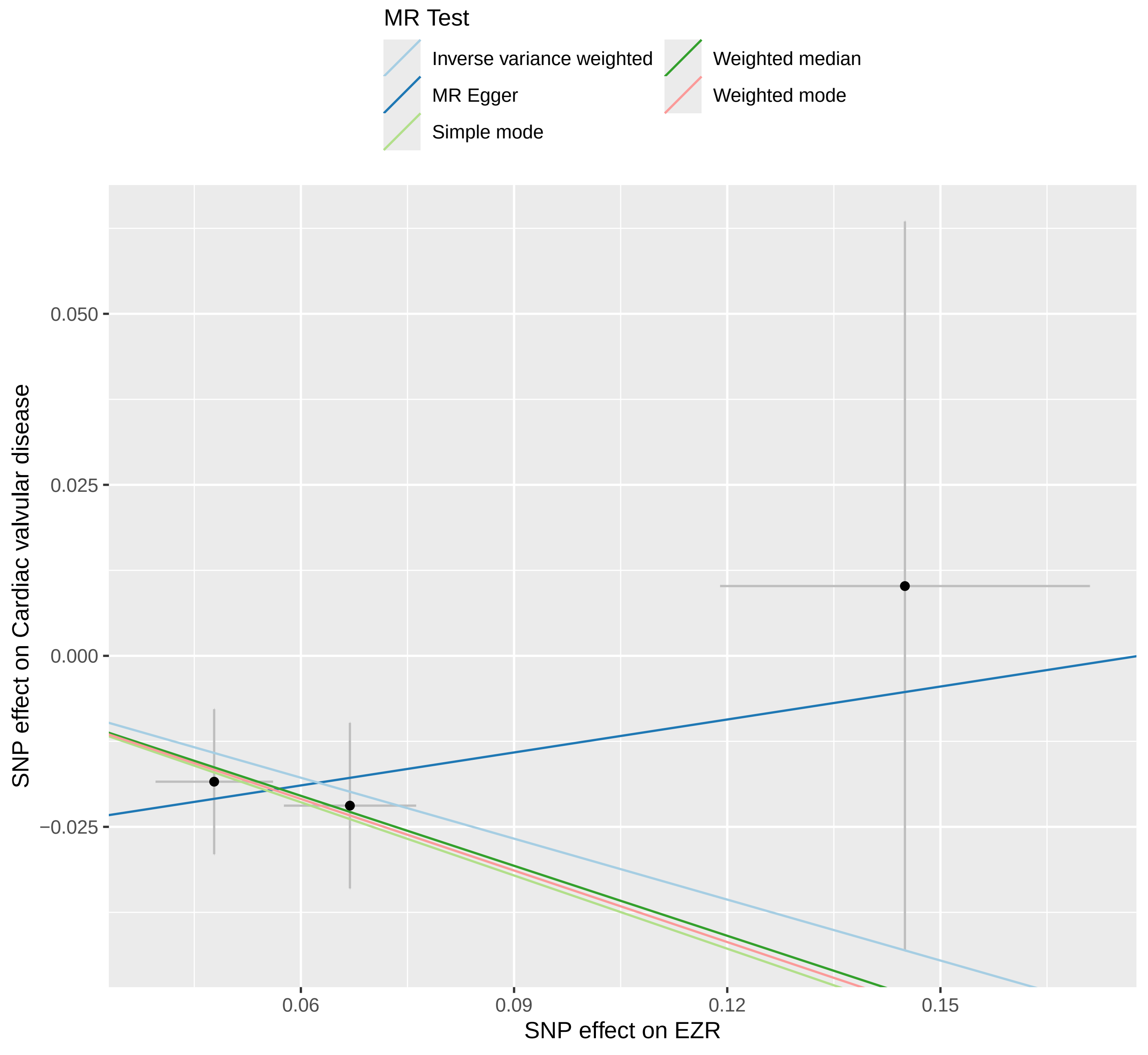
MR Test
Inverse variance weighted
MR Egger
Simple mode
Weighted median
Weighted mode
SNP effect on Cardiac valvular disease
0.050
0.025
0.000
−0.025
0.06
0.09
0.12
0.15
SNP effect on EZR

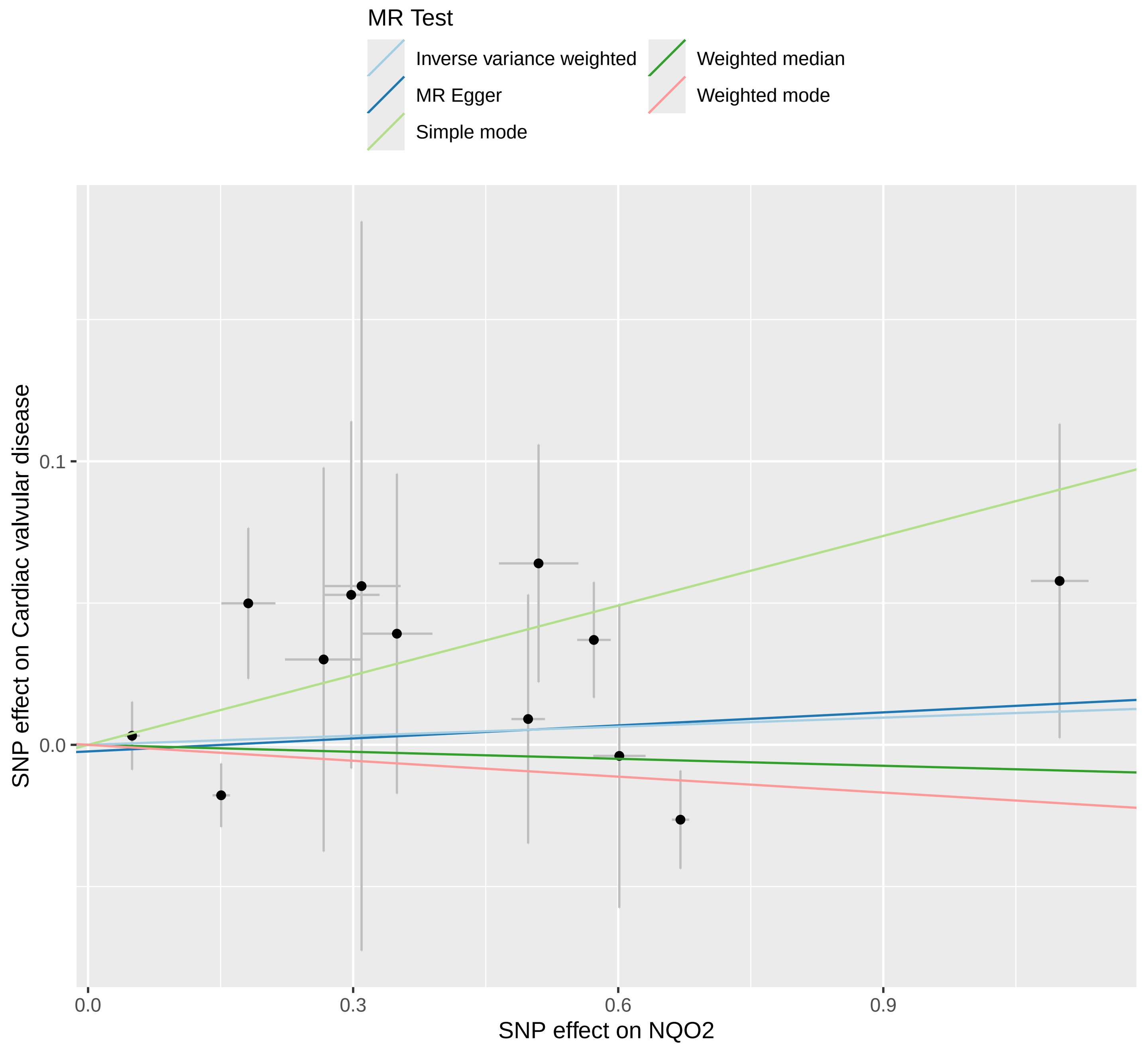
MR Test
Inverse variance weighted
MR Egger
Simple mode
Weighted median
Weighted mode
SNP effect on Cardiac valvular disease
0.1
0.0
0.0
0.3
0.6
0.9
SNP effect on NQO2

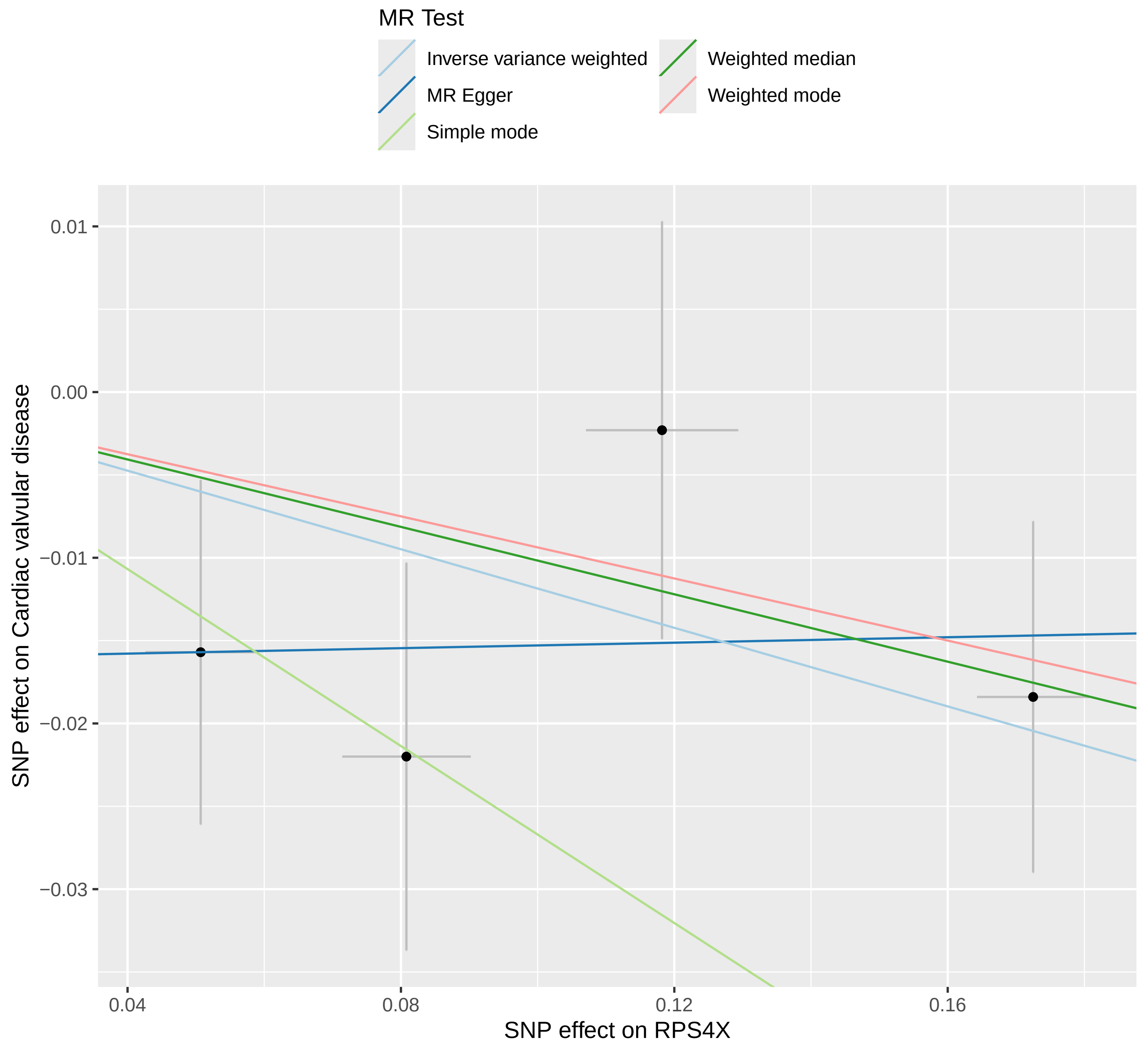

MR Test
Inverse variance weighted
MR Egger
Simple mode
Weighted median
Weighted mode
0.01
0.00
−0.01
−0.02
−0.03
SNP effect on Cardiac valvular disease
0.04
0.08
0.12
0.16
SNP effect on RPS4X

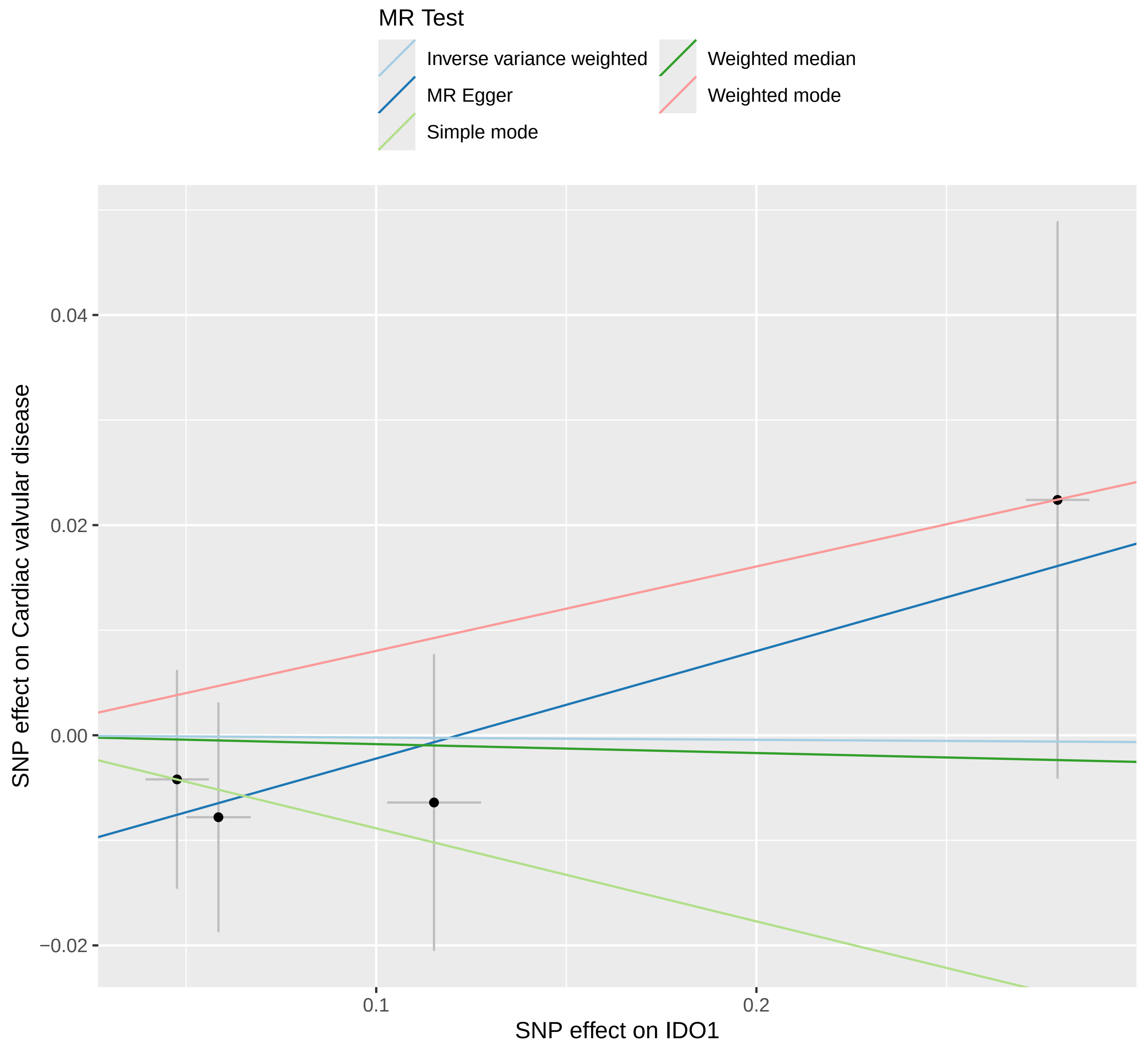
MR Test
Inverse variance weighted
MR Egger
Simple mode
Weighted median
Weighted mode
0.04
0.02
0.00
−0.02
SNP effect on Cardiac valvular disease
0.1
0.2
SNP effect on IDO1

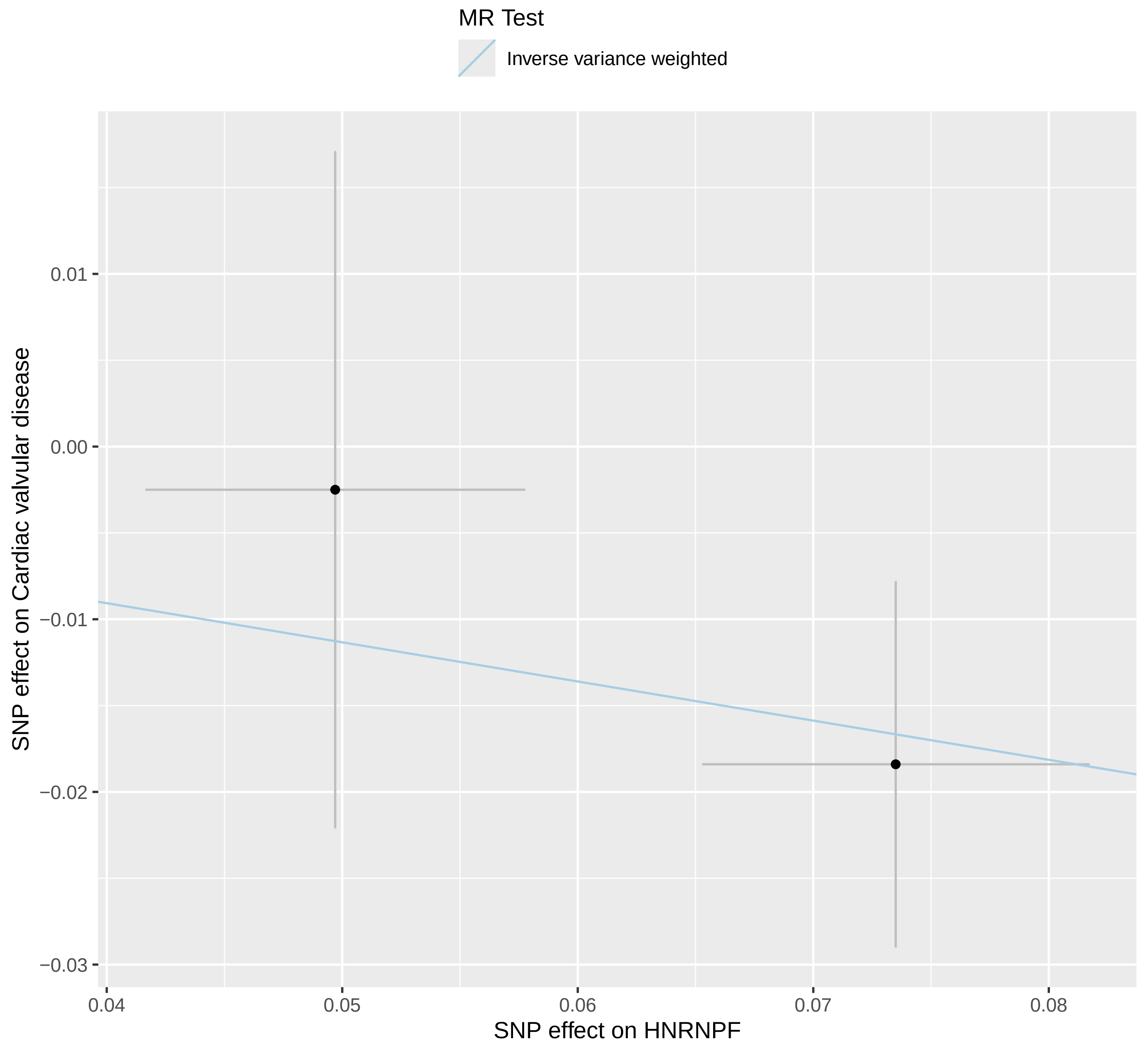
MR Test
Inverse variance weighted
SNP effect on Cardiac valvular disease
0.01
0.00
−0.01
−0.02
−0.03
0.04
0.05
0.06
0.07
0.08
SNP effect on HNRNPF

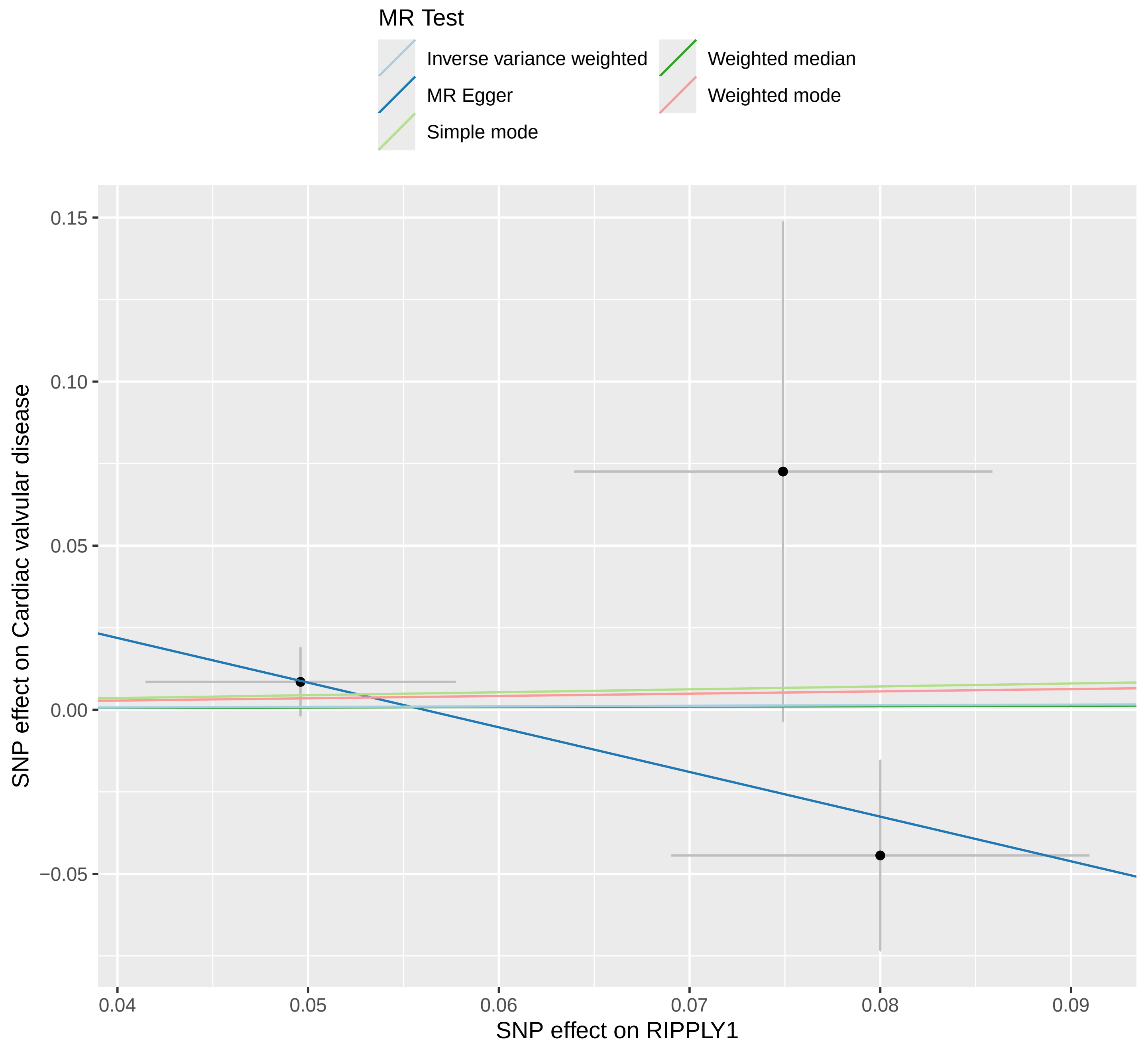
MR Test
Inverse variance weighted
MR Egger
Simple mode
Weighted median
Weighted mode
0.15
0.10
0.05
0.00
−0.05
0.04
0.05
0.06
0.07
0.08
0.09
SNP effect on Cardiac valvular disease
SNP effect on RIPPLY1

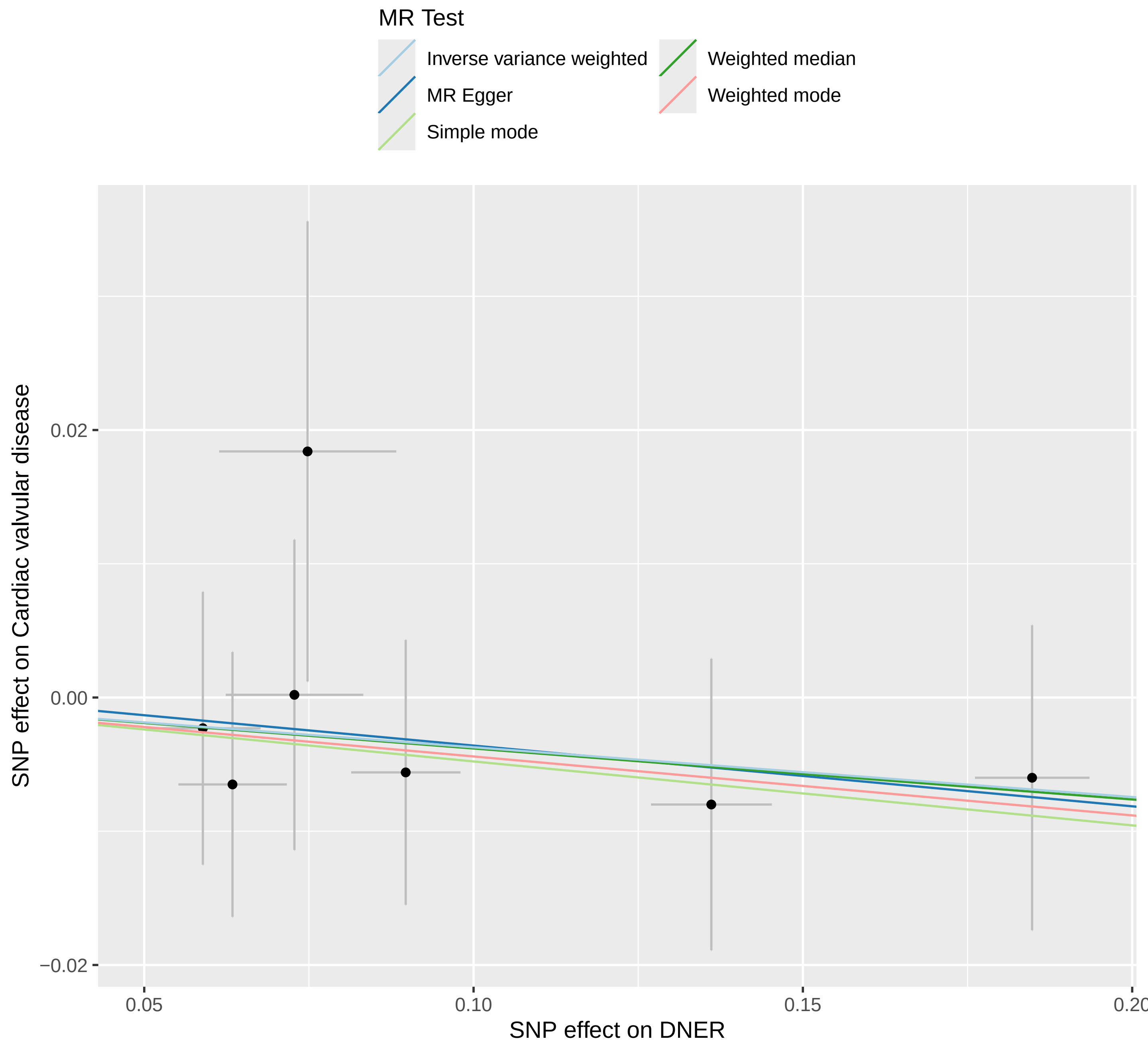

MR Test
Inverse variance weighted
MR Egger
Simple mode
Weighted median
Weighted mode
0.02
0.00
−0.02
0.05
0.10
0.15
0.20
SNP effect on DNER
SNP effect on Cardiac valvular disease

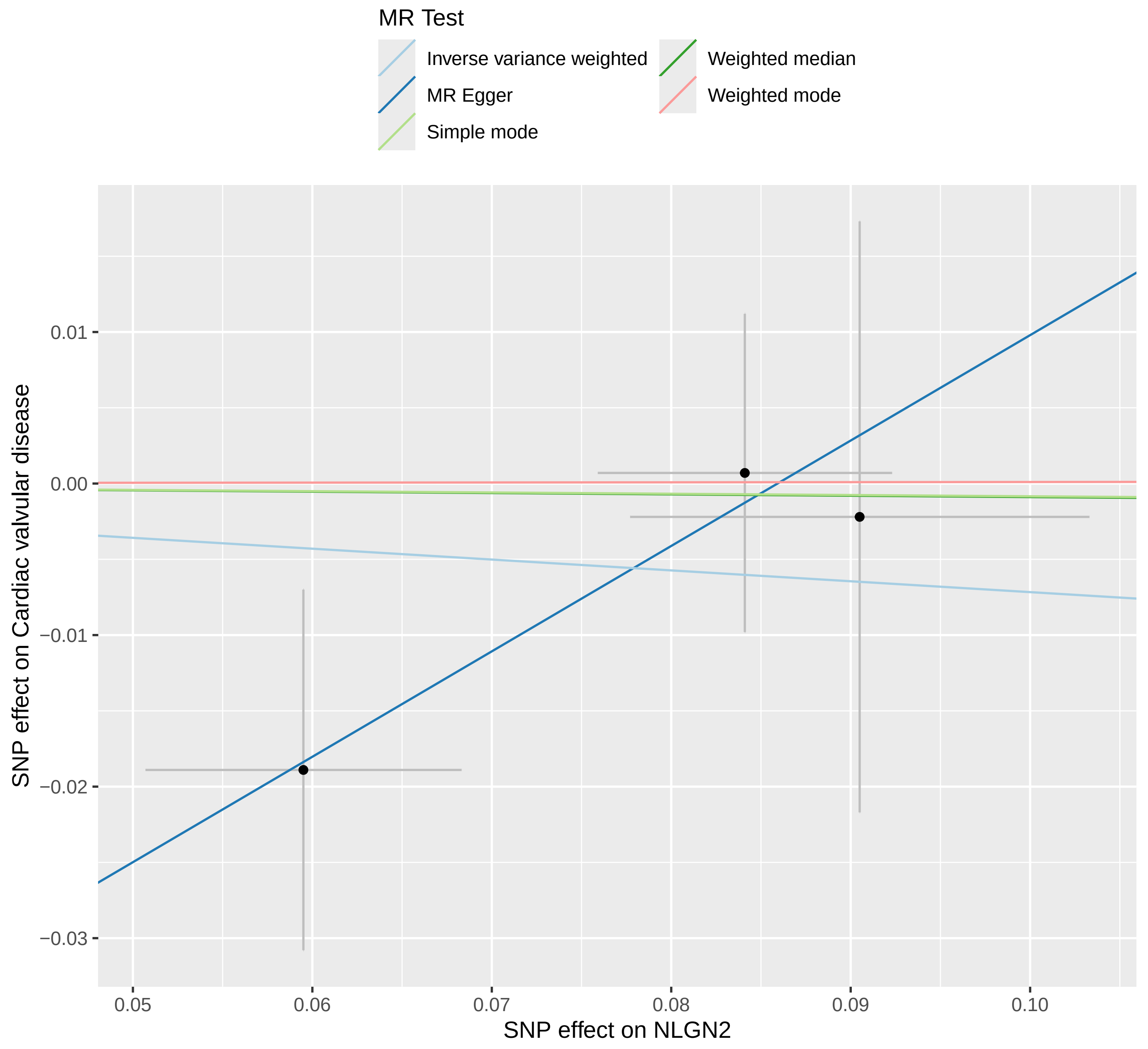
MR Test
Inverse variance weighted
MR Egger
Simple mode
Weighted median
Weighted mode
SNP effect on Cardiac valvular disease
0.01
0.00
−0.01
−0.02
−0.03
0.05
0.06
0.07
0.08
0.09
0.10
SNP effect on NLGN2

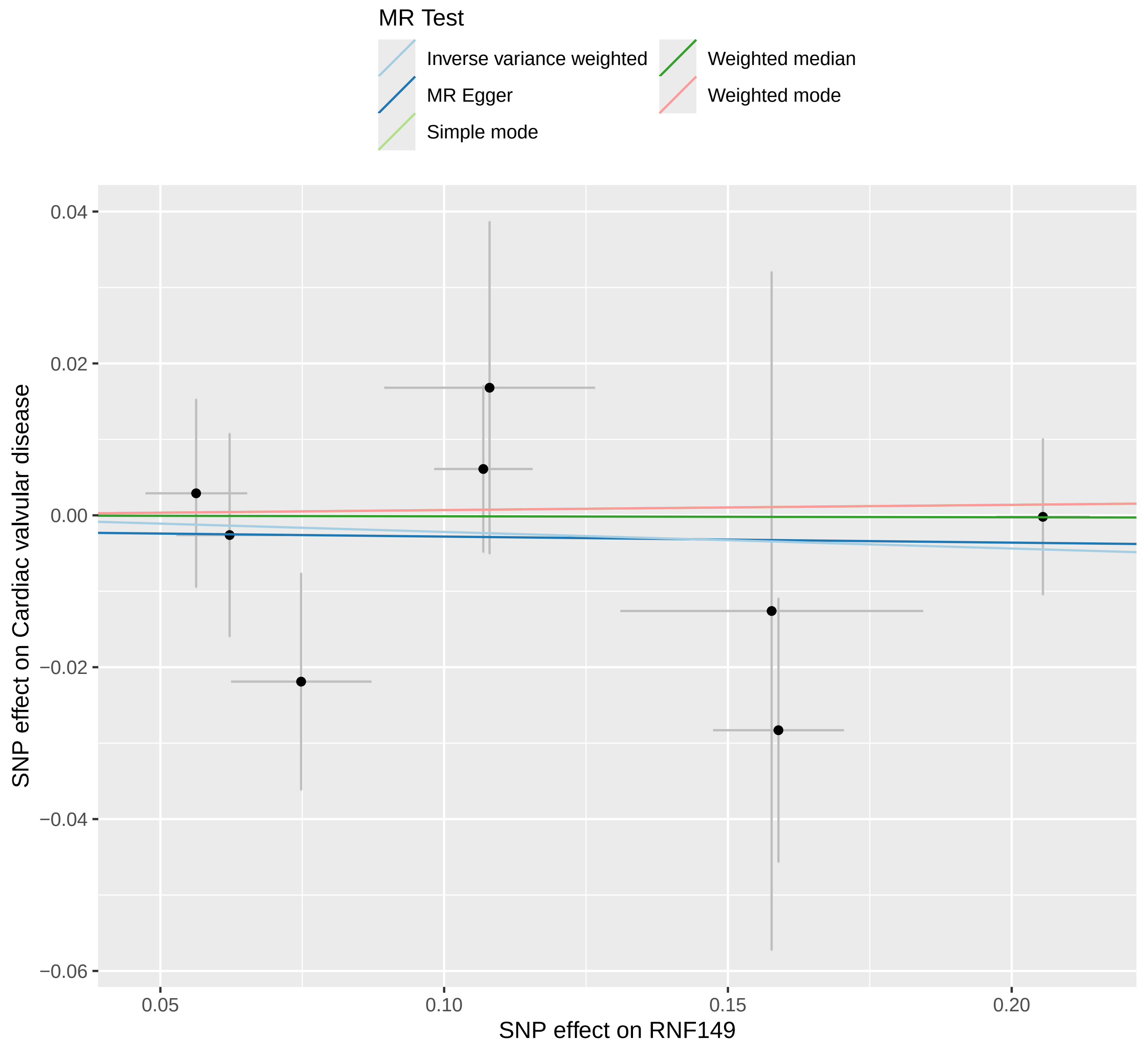
MR Test
Inverse variance weighted
MR Egger
Simple mode
Weighted median
Weighted mode
0.04
0.02
0.00
−0.02
−0.04
−0.06
SNP effect on Cardiac valvular disease
0.05
0.10
0.15
0.20
SNP effect on RNF149

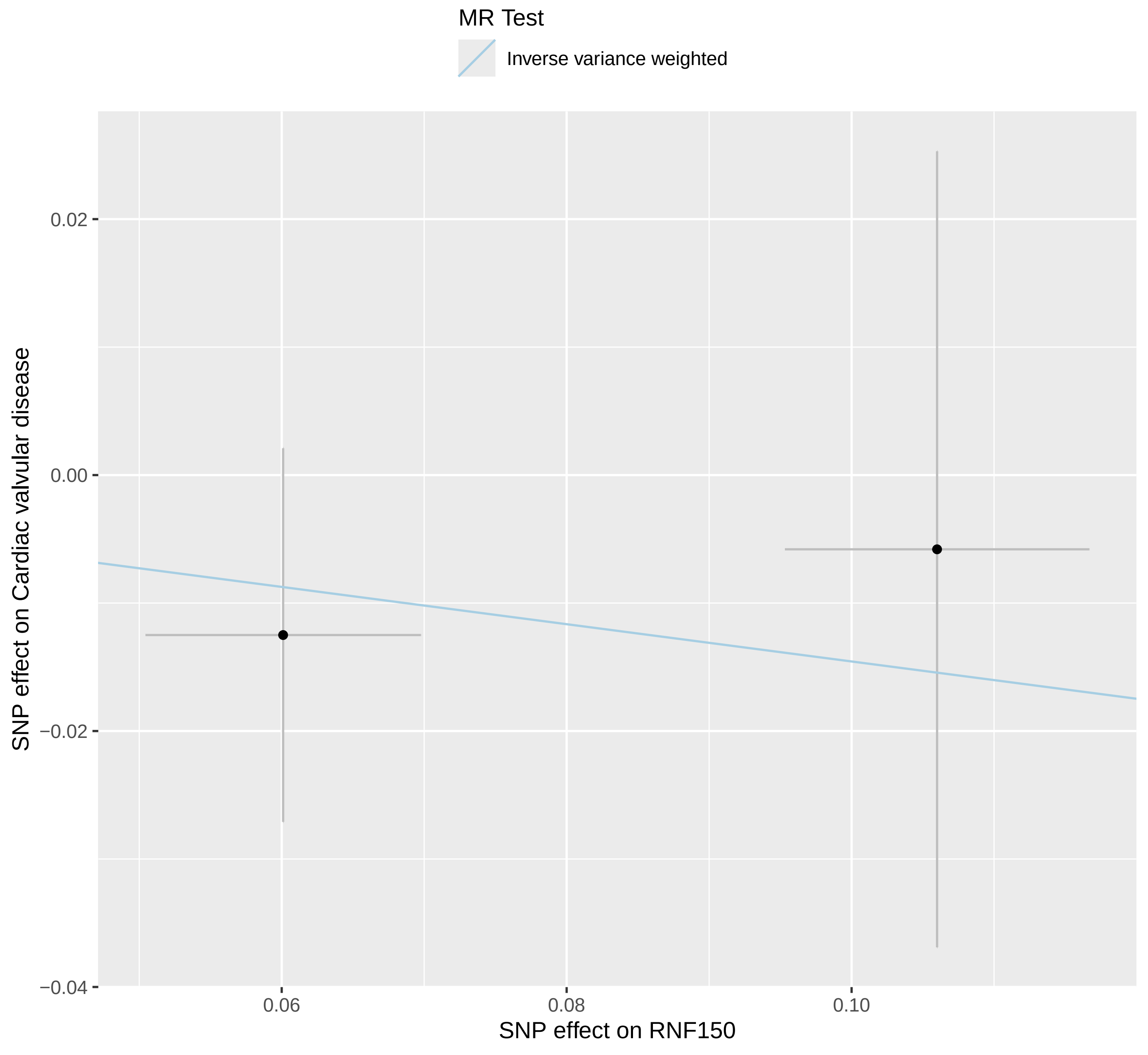
MR Test
Inverse variance weighted
0.02
0.00
−0.02
−0.04
0.06
0.08
0.10
SNP effect on Cardiac valvular disease
SNP effect on RNF150

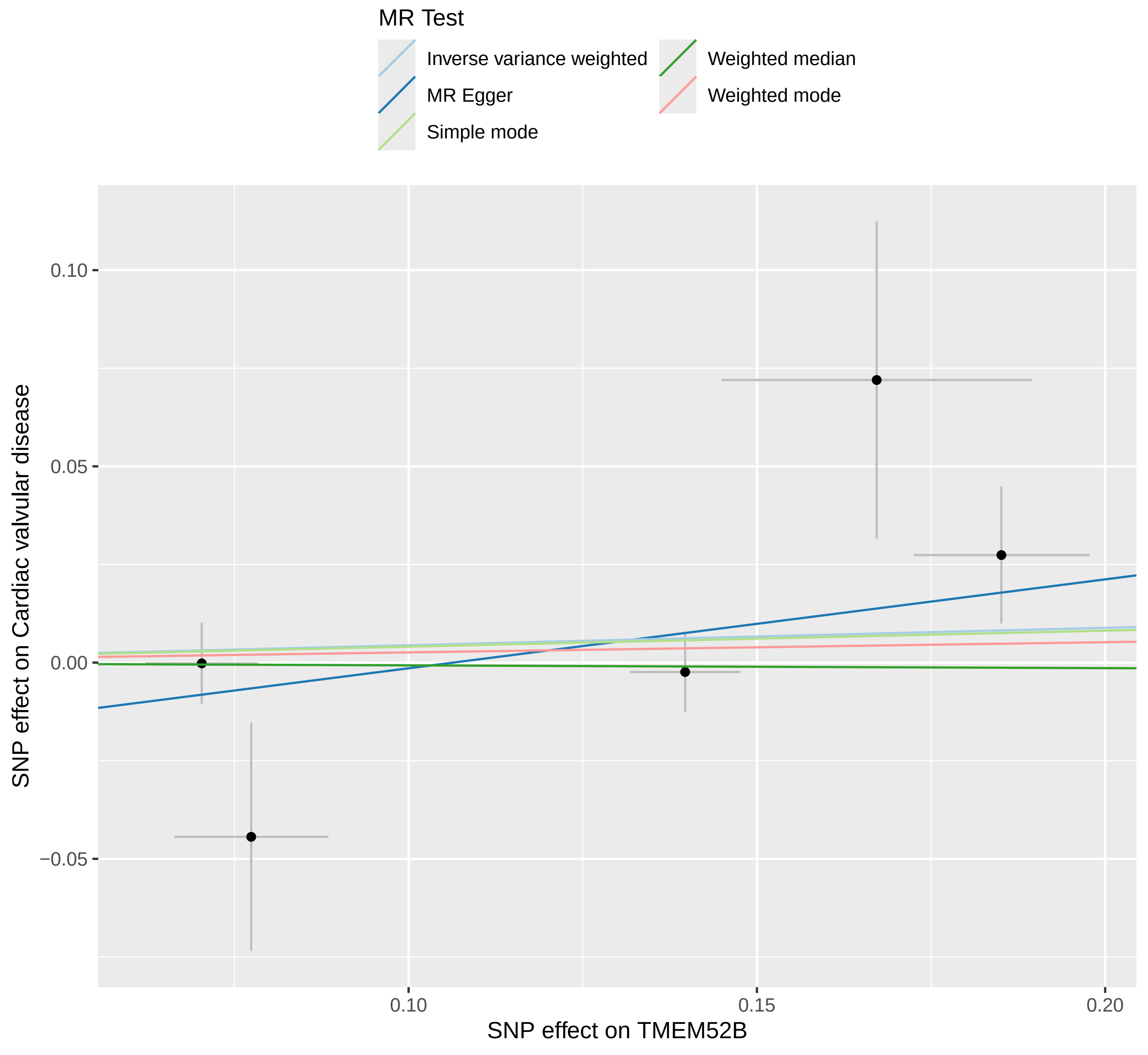
MR Test
Inverse variance weighted
MR Egger
Simple mode
Weighted median
Weighted mode
0.10
0.05
0.00
−0.05
SNP effect on Cardiac valvular disease
0.10
0.15
0.20
SNP effect on TMEM52B

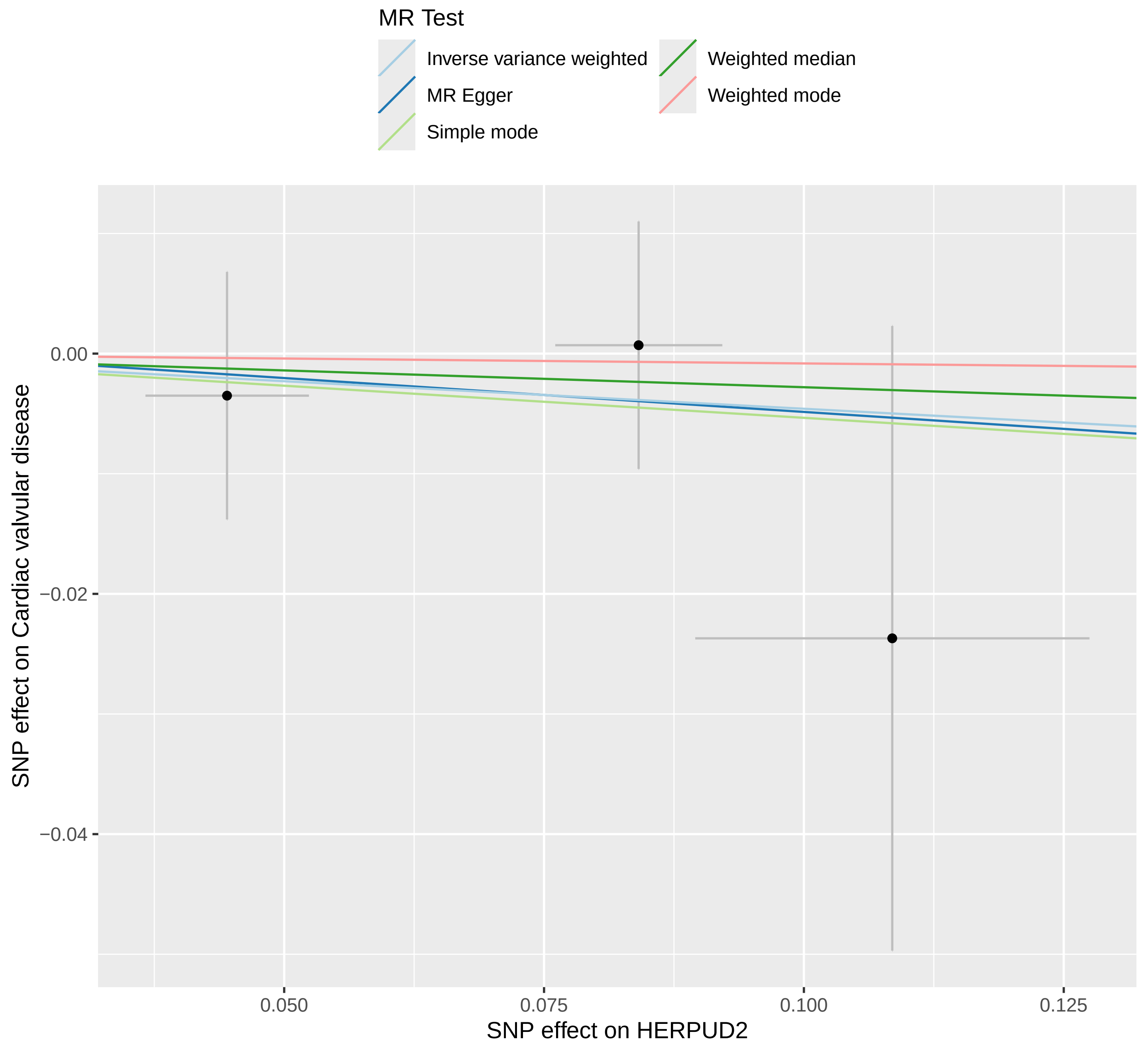
MR Test
Inverse variance weighted
MR Egger
Simple mode
Weighted median
Weighted mode
0.00
−0.02
−0.04
0.050
0.075
0.100
0.125
SNP effect on Cardiac valvular disease
SNP effect on HERPUD2

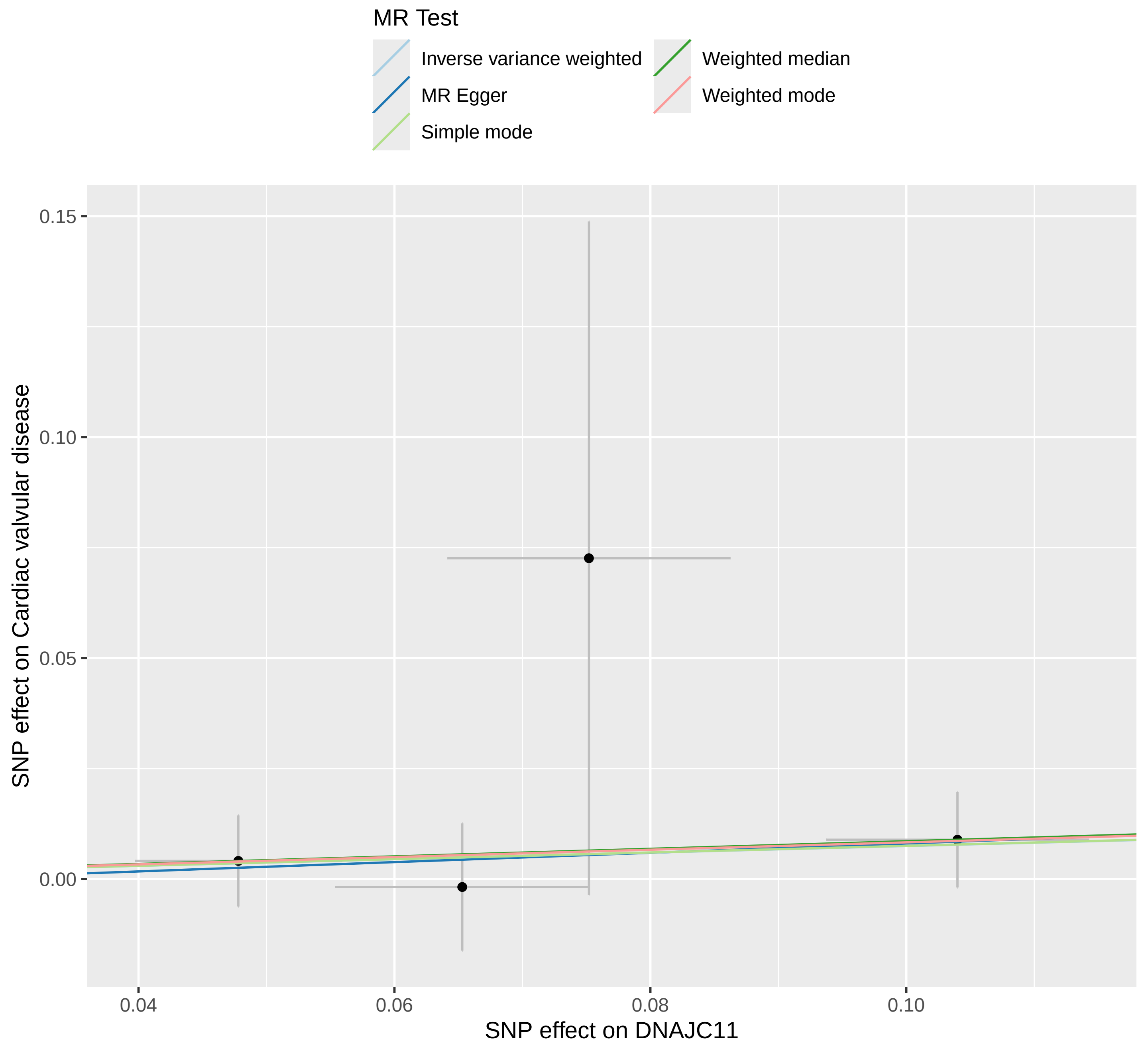
MR Test
Inverse variance weighted
MR Egger
Simple mode
Weighted median
Weighted mode
0.15
0.10
0.05
0.00
0.04
0.06
0.08
0.10
SNP effect on Cardiac valvular disease
SNP effect on DNAJC11

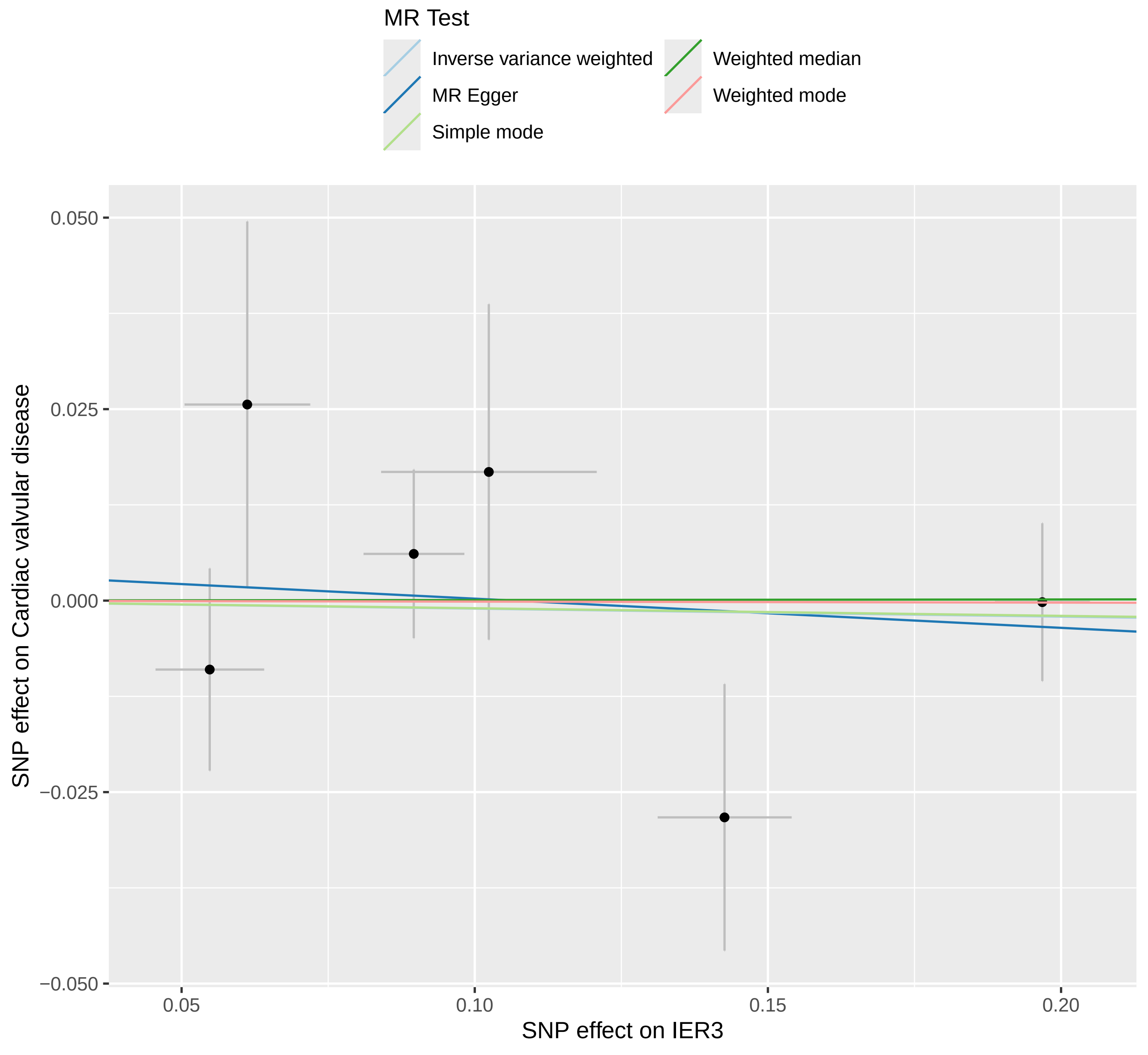
MR Test
Inverse variance weighted
MR Egger
Simple mode
Weighted median
Weighted mode
0.050
0.025
0.000
−0.025
−0.050
0.05
0.10
0.15
0.20
SNP effect on Cardiac valvular disease
SNP effect on IER3

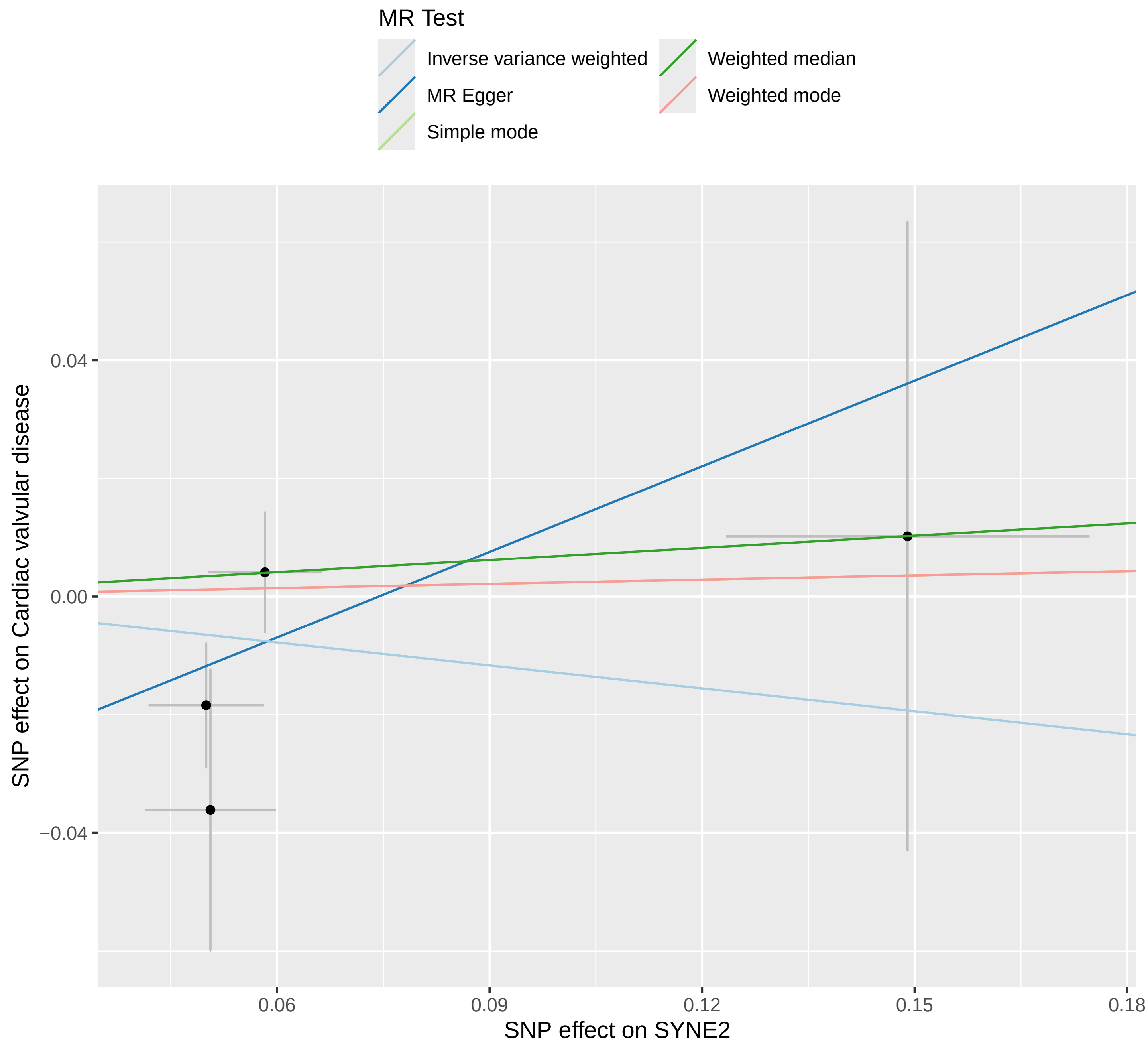

MR Test
Inverse variance weighted
MR Egger
Simple mode
Weighted median
Weighted mode
0.04
0.00
−0.04
0.06
0.09
0.12
0.15
0.18
SNP effect on SYNE2
SNP effect on Cardiac valvular disease

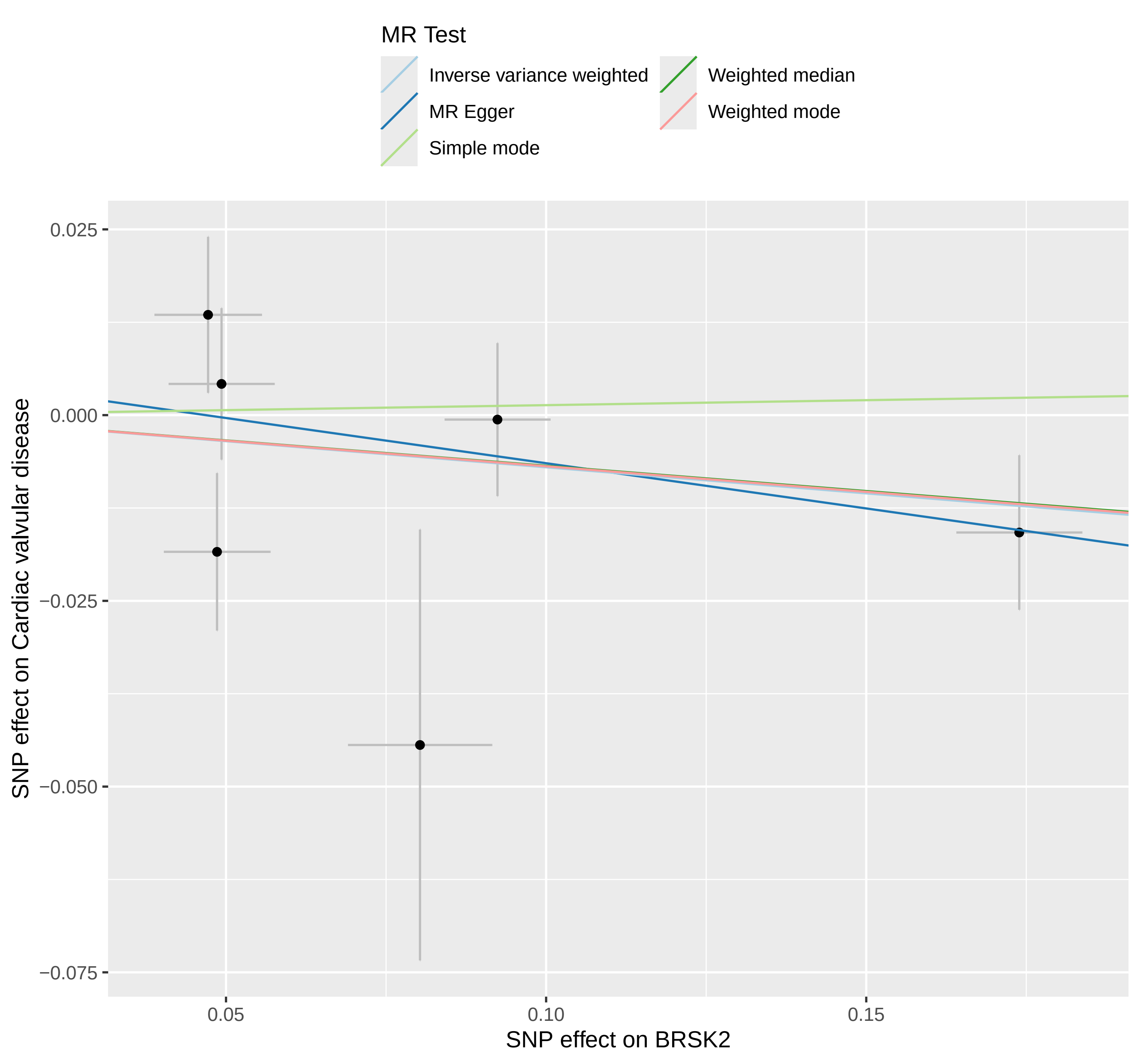
MR Test
Inverse variance weighted
MR Egger
Simple mode
Weighted median
Weighted mode
0.025
0.000
−0.025
−0.050
−0.075
SNP effect on Cardiac valvular disease
0.05
0.10
0.15
SNP effect on BRSK2

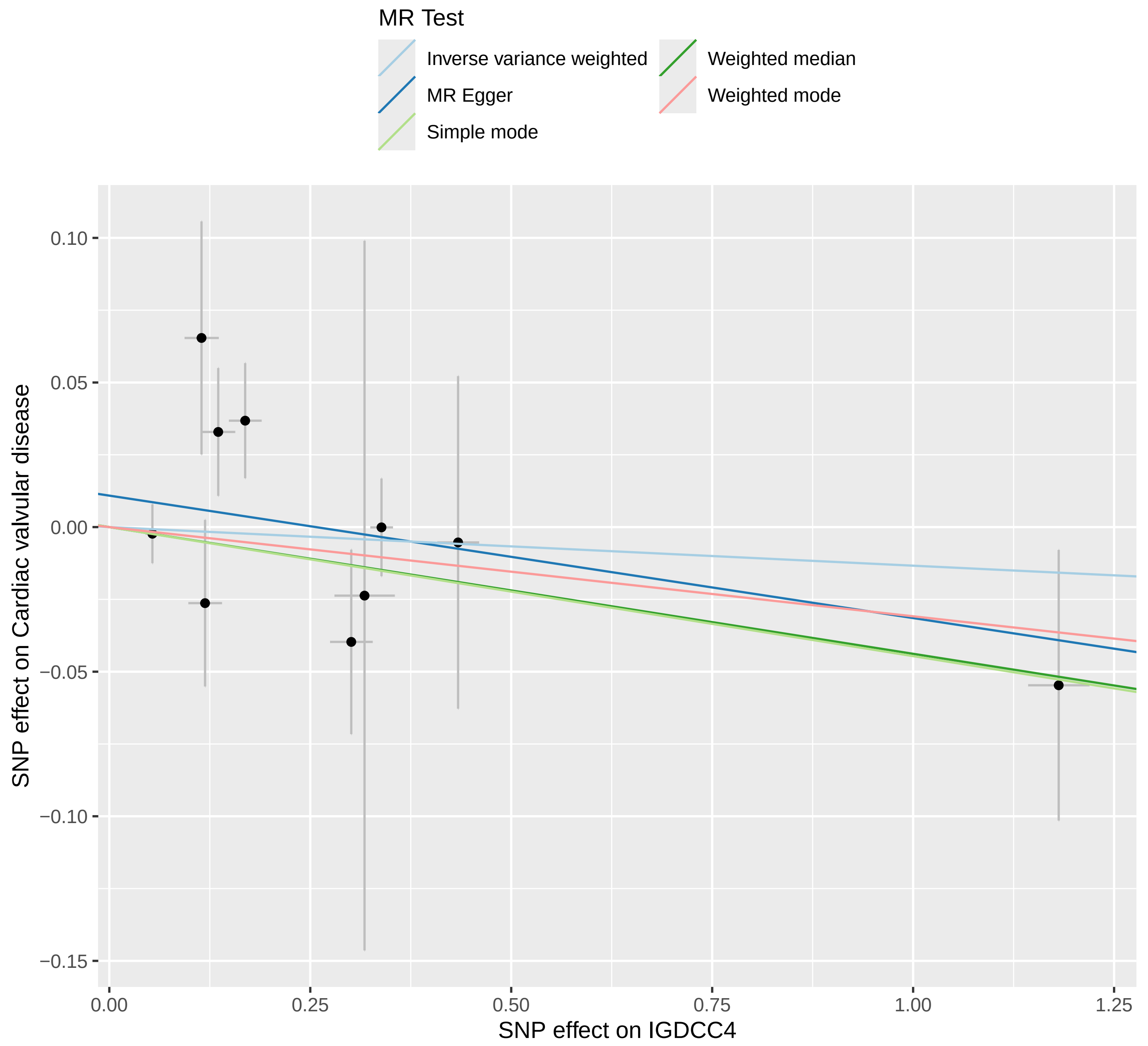

MR Test
Inverse variance weighted
MR Egger
Simple mode
Weighted median
Weighted mode
SNP effect on Cardiac valvular disease
0.10
0.05
0.00
−0.05
−0.10
−0.15
0.00
0.25
0.50
0.75
1.00
1.25
SNP effect on IGDCC4

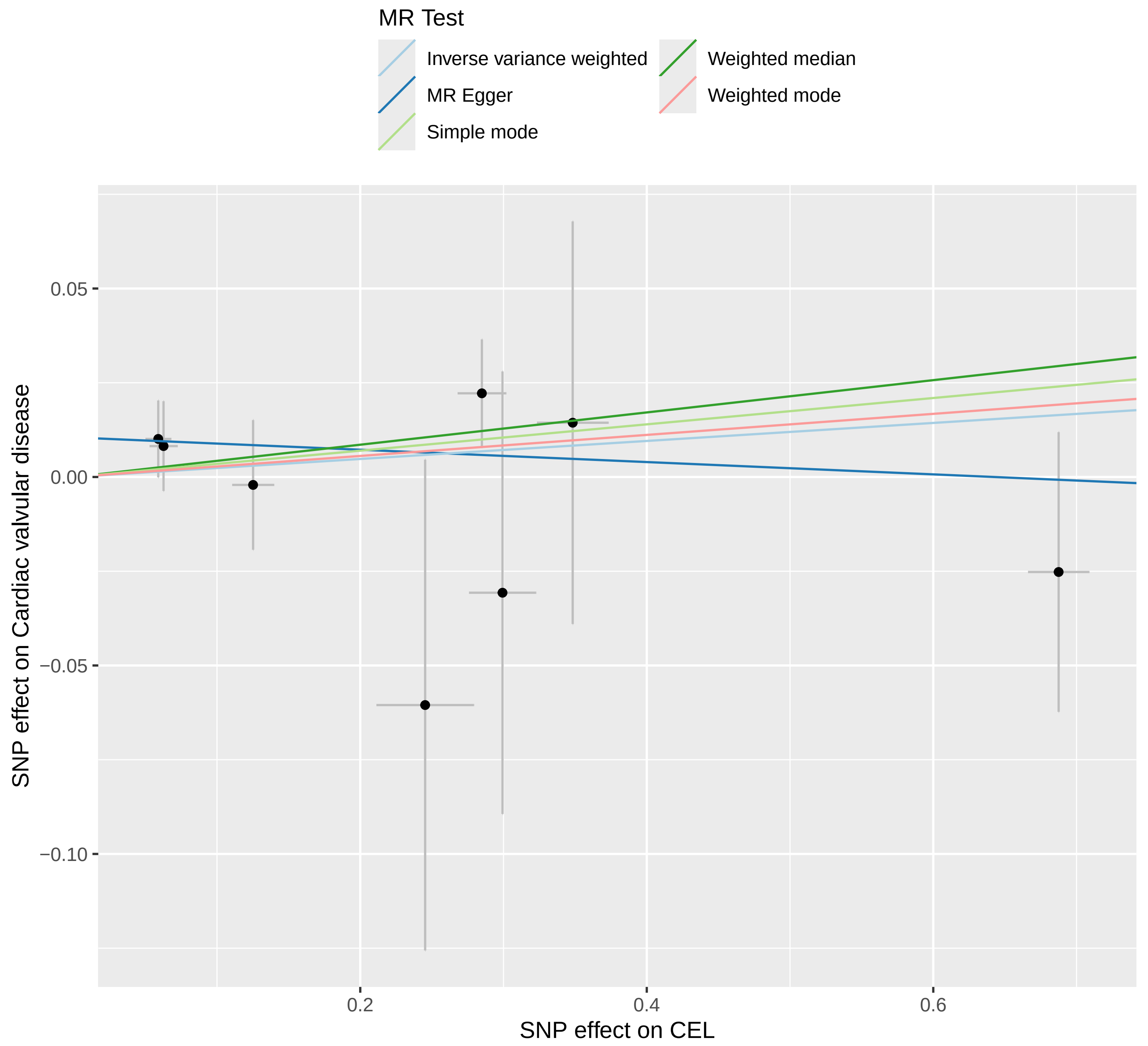
MR Test
Inverse variance weighted
MR Egger
Simple mode
Weighted median
Weighted mode
SNP effect on Cardiac valvular disease
0.05
0.00
−0.05
−0.10
0.2
0.4
0.6
SNP effect on CEL

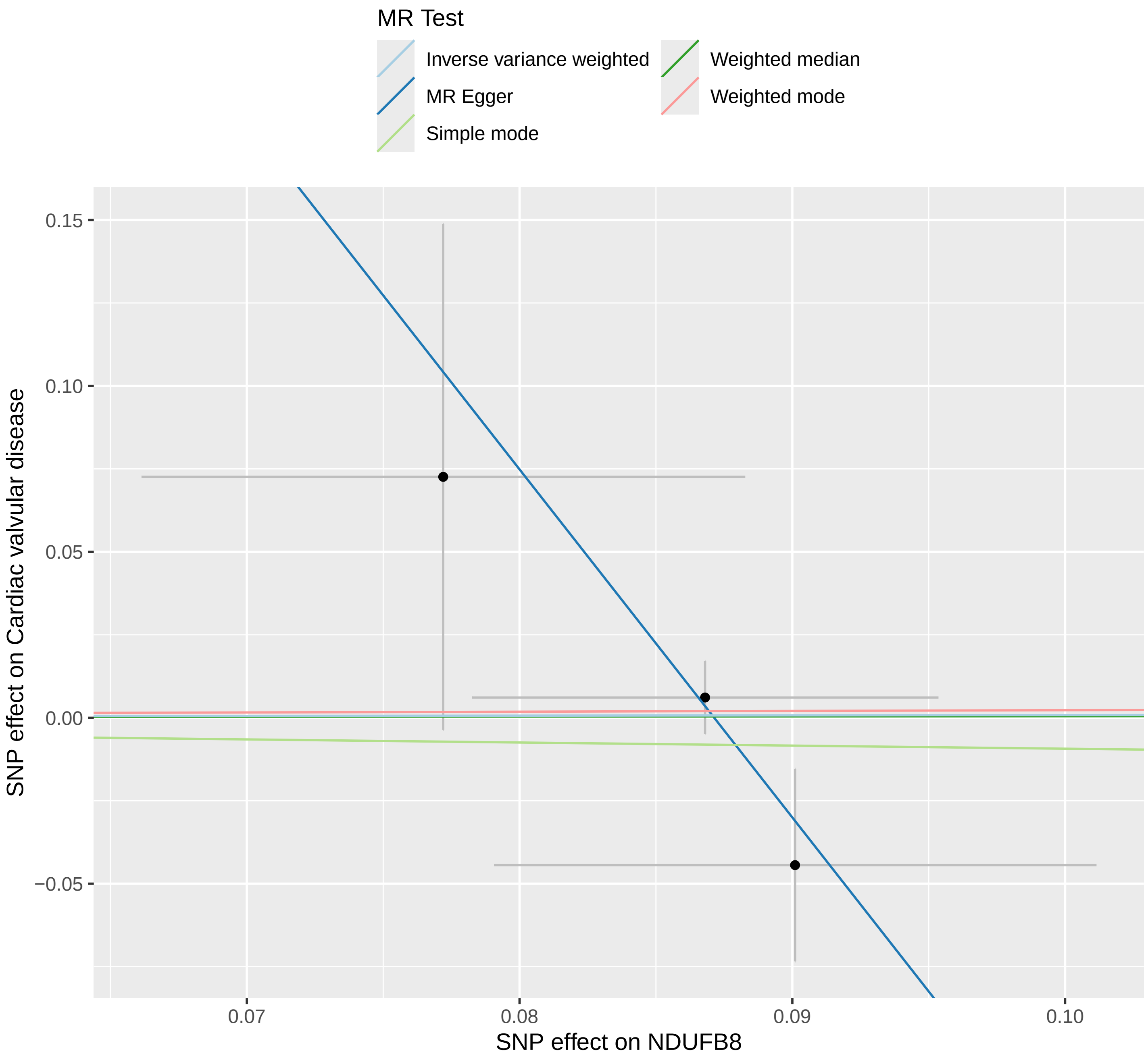

MR Test
Inverse variance weighted
MR Egger
Simple mode
Weighted median
Weighted mode
0.15
0.10
0.05
0.00
−0.05
SNP effect on Cardiac valvular disease
0.07
0.08
0.09
0.10
SNP effect on NDUFB8

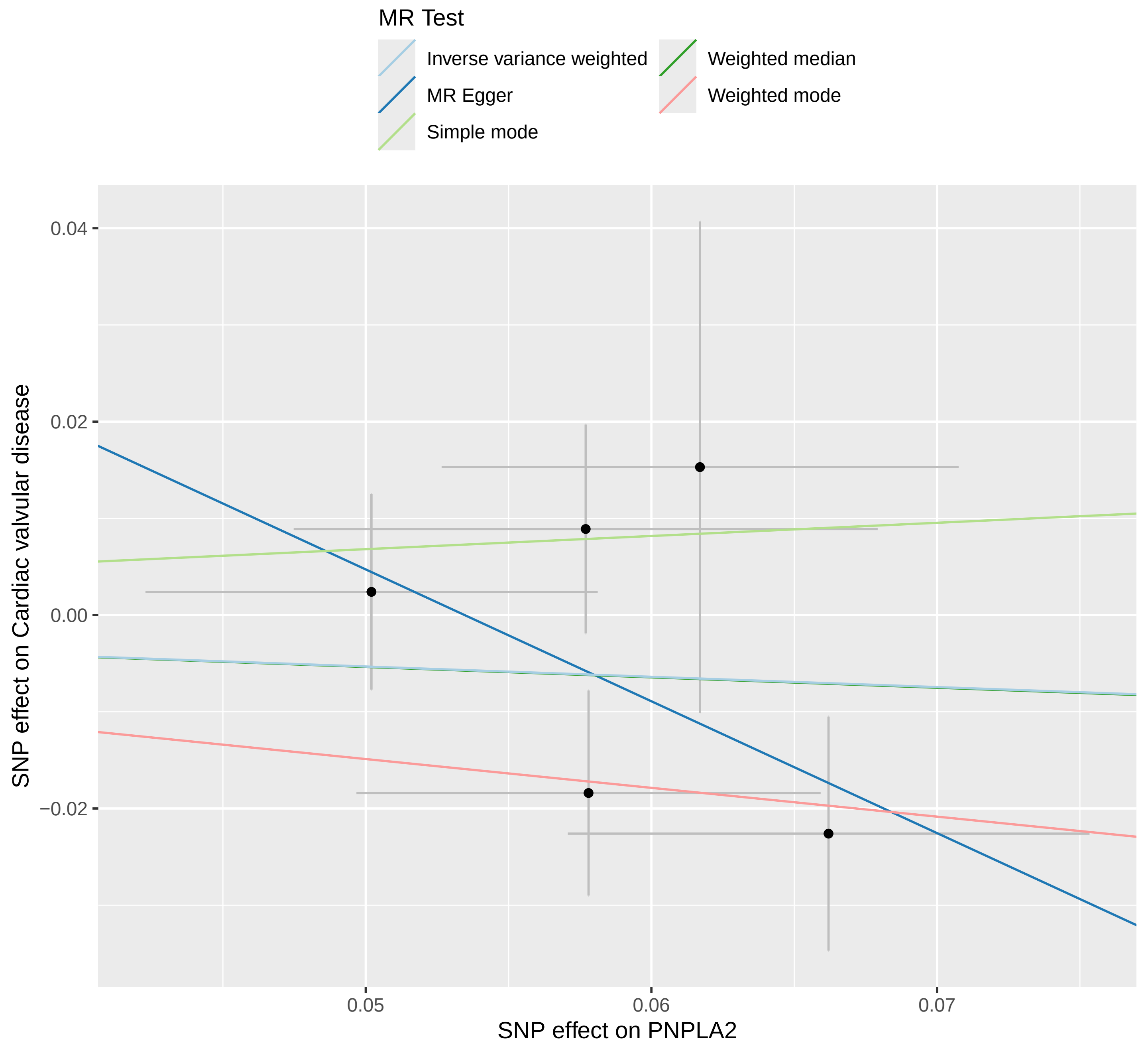
MR Test
Inverse variance weighted
MR Egger
Simple mode
Weighted median
Weighted mode
0.04
0.02
0.00
−0.02
SNP effect on Cardiac valvular disease
0.05
0.06
0.07
SNP effect on PNPLA2

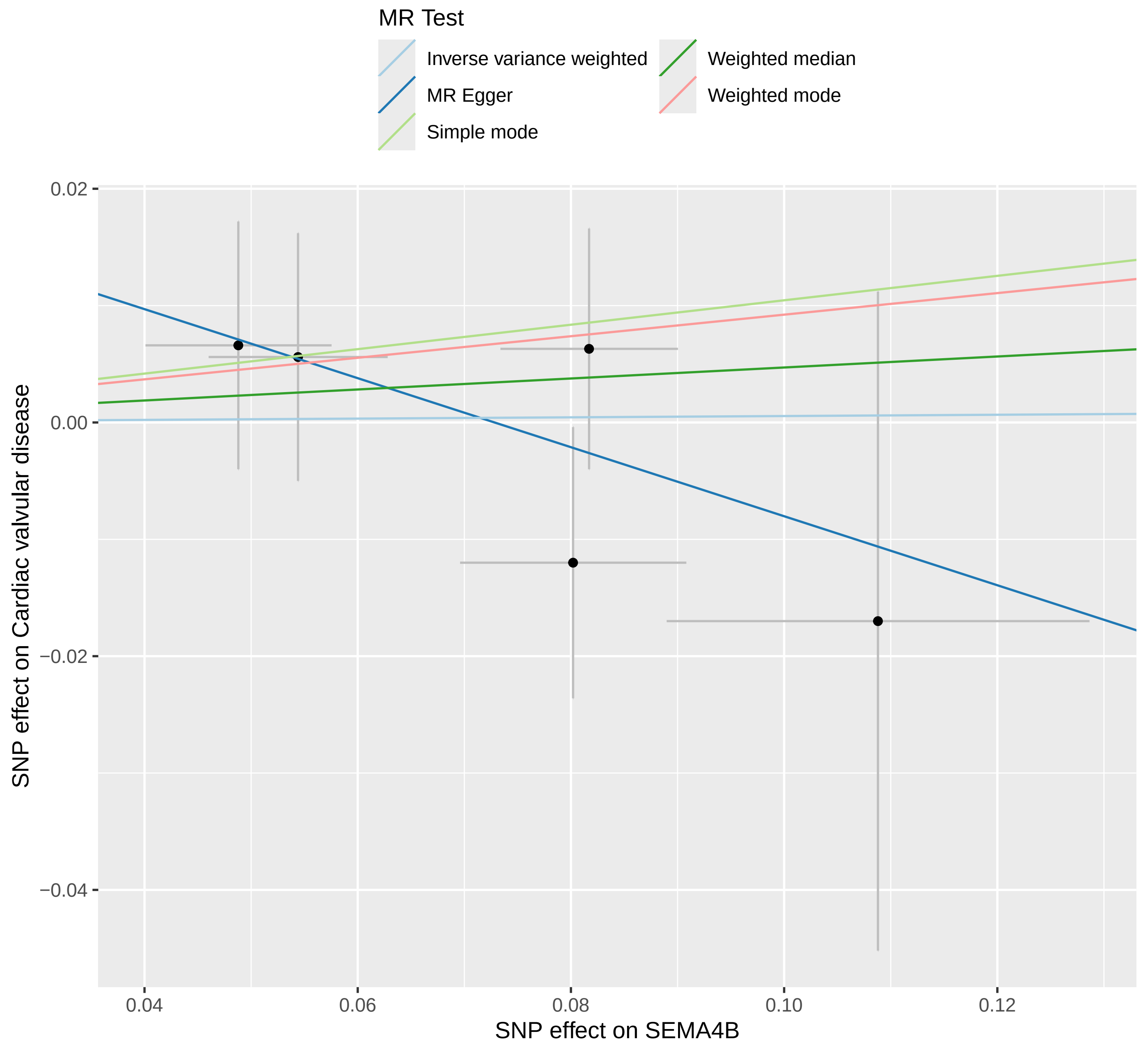
MR Test
Inverse variance weighted
MR Egger
Simple mode
Weighted median
Weighted mode
0.02
0.00
−0.02
−0.04
0.04
0.06
0.08
0.10
0.12
SNP effect on Cardiac valvular disease
SNP effect on SEMA4B

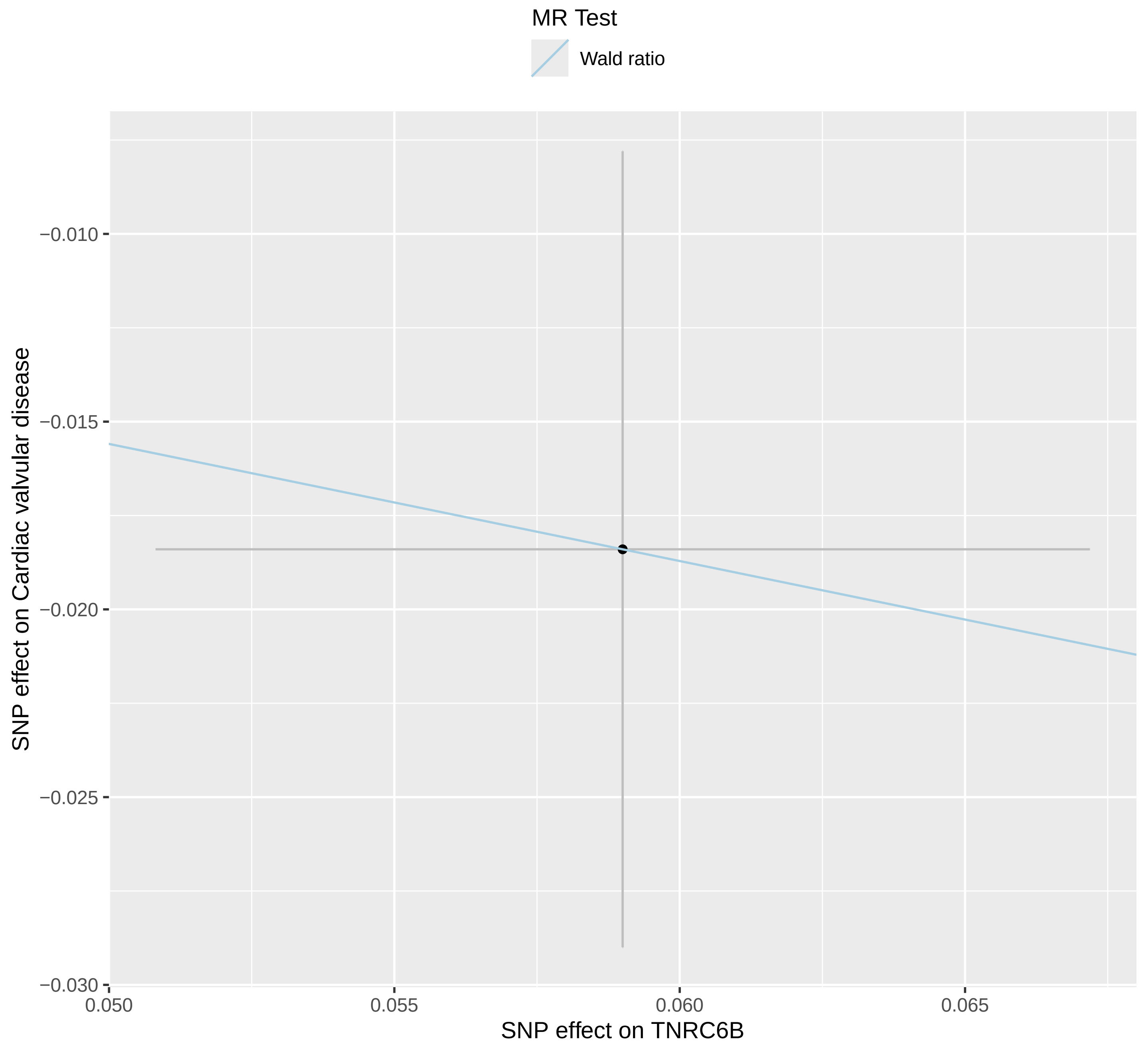
MR Test
Wald ratio
SNP effect on Cardiac valvular disease
−0.010
−0.015
−0.020
−0.025
−0.030
0.050
0.055
0.060
0.065
SNP effect on TNRC6B

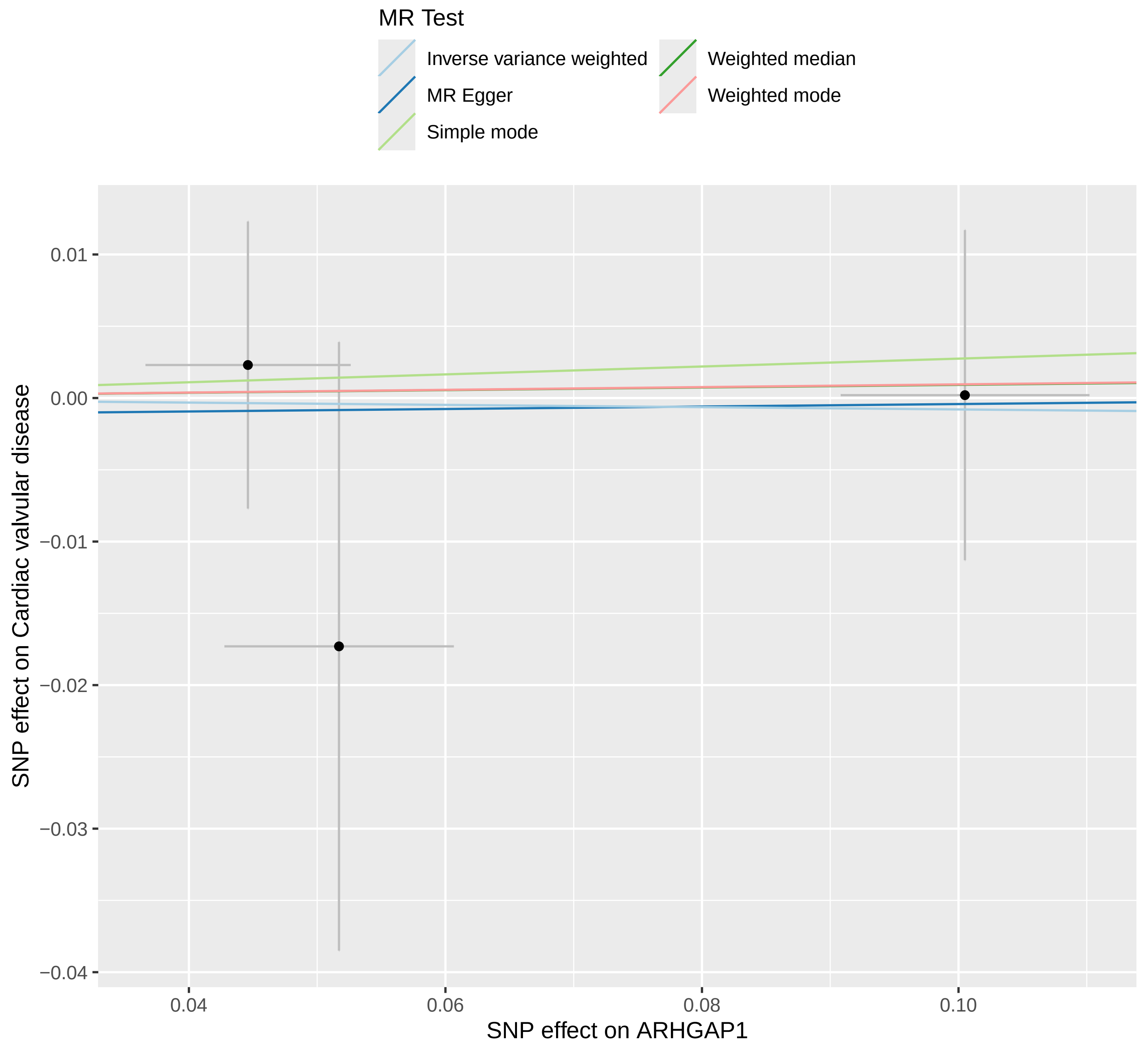
MR Test
Inverse variance weighted
MR Egger
Simple mode
Weighted median
Weighted mode
0.01
0.00
−0.01
−0.02
−0.03
−0.04
0.04
0.06
0.08
0.10
SNP effect on Cardiac valvular disease
SNP effect on ARHGAP1

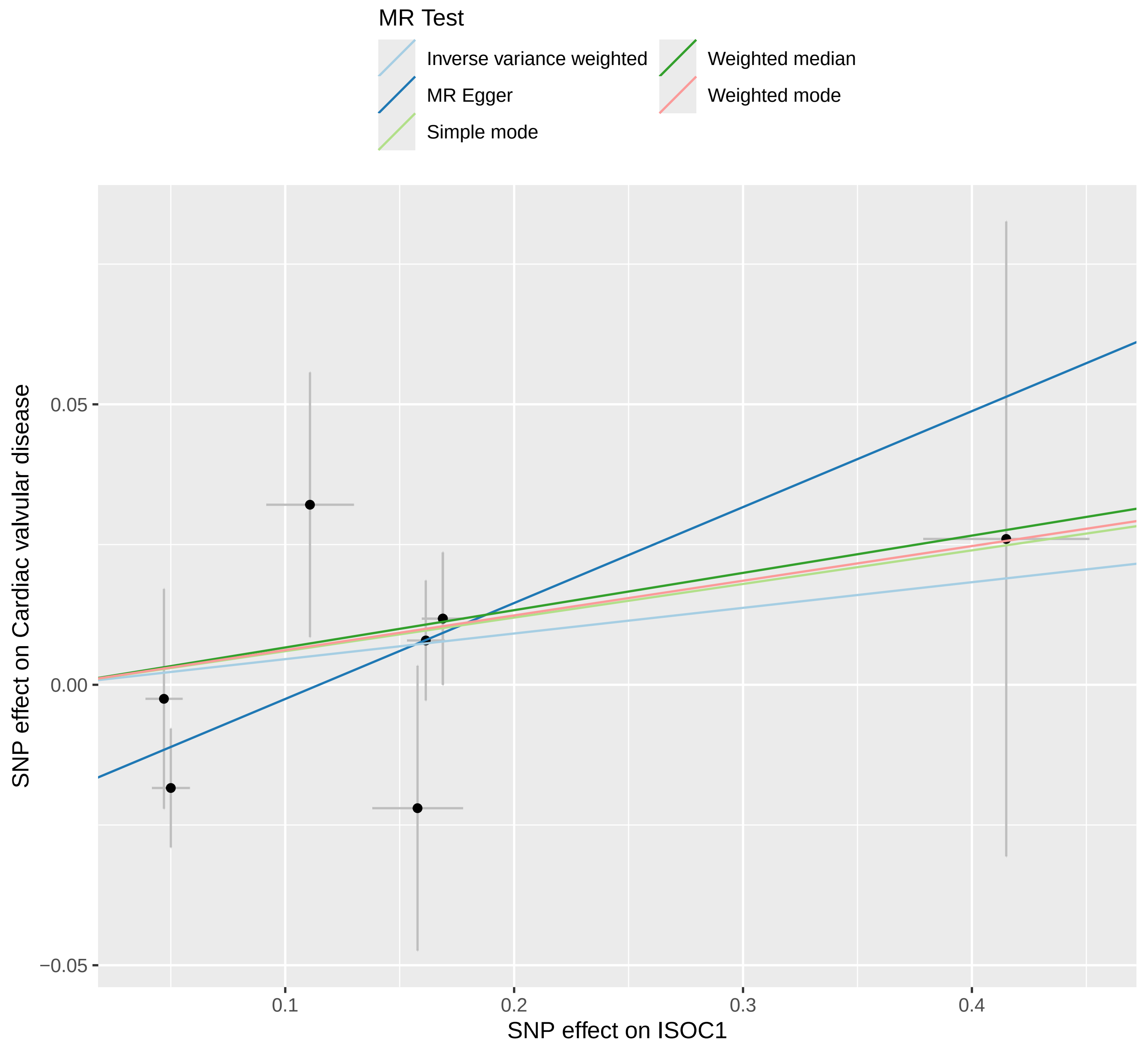

MR Test
Inverse variance weighted
MR Egger
Simple mode
Weighted median
Weighted mode
SNP effect on Cardiac valvular disease
0.05
0.00
−0.05
0.1
0.2
0.3
0.4
SNP effect on ISOC1

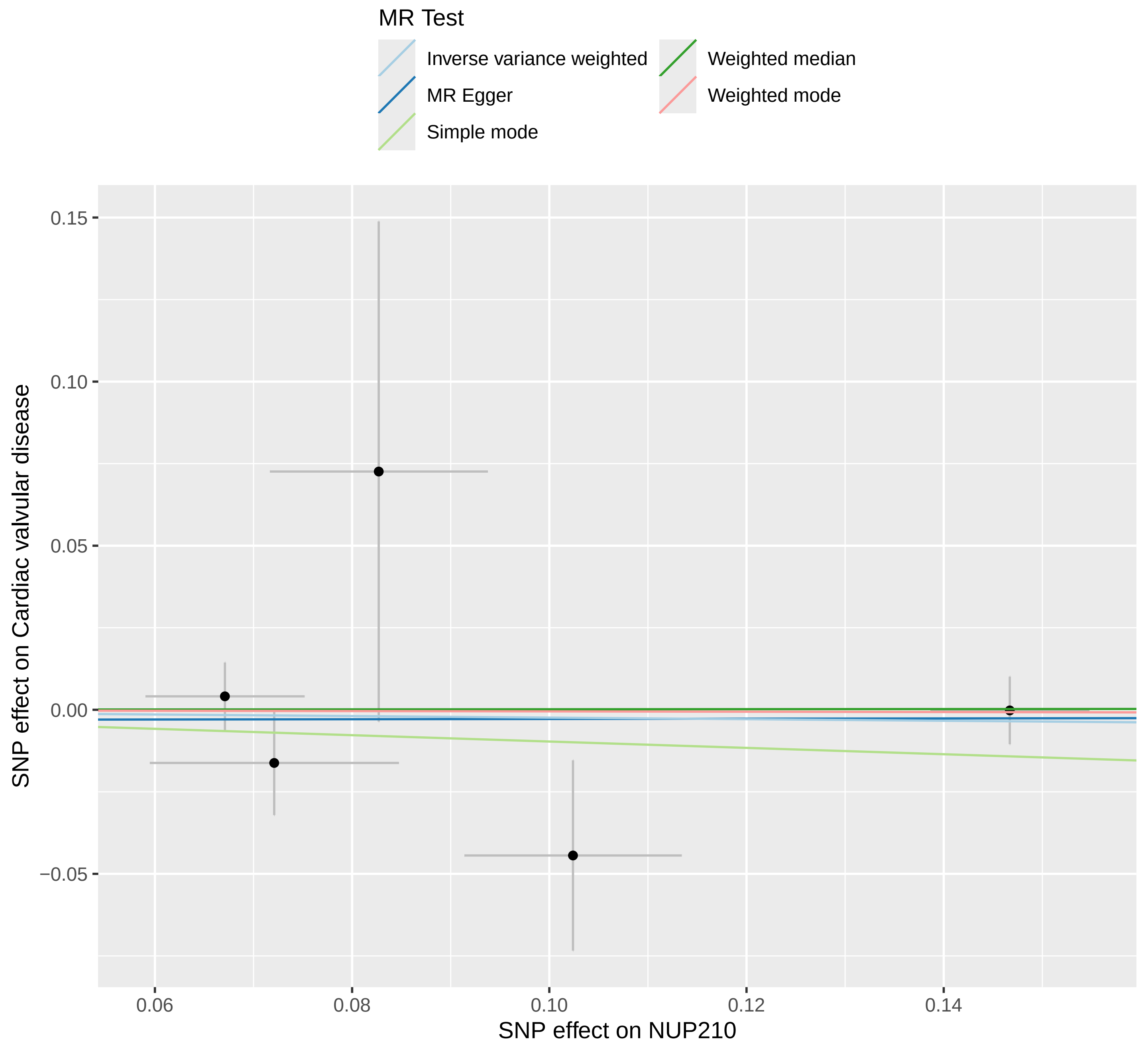

MR Test
Inverse variance weighted
MR Egger
Simple mode
Weighted median
Weighted mode
0.15
0.10
0.05
0.00
−0.05
0.06
0.08
0.10
0.12
0.14
SNP effect on Cardiac valvular disease
SNP effect on NUP210

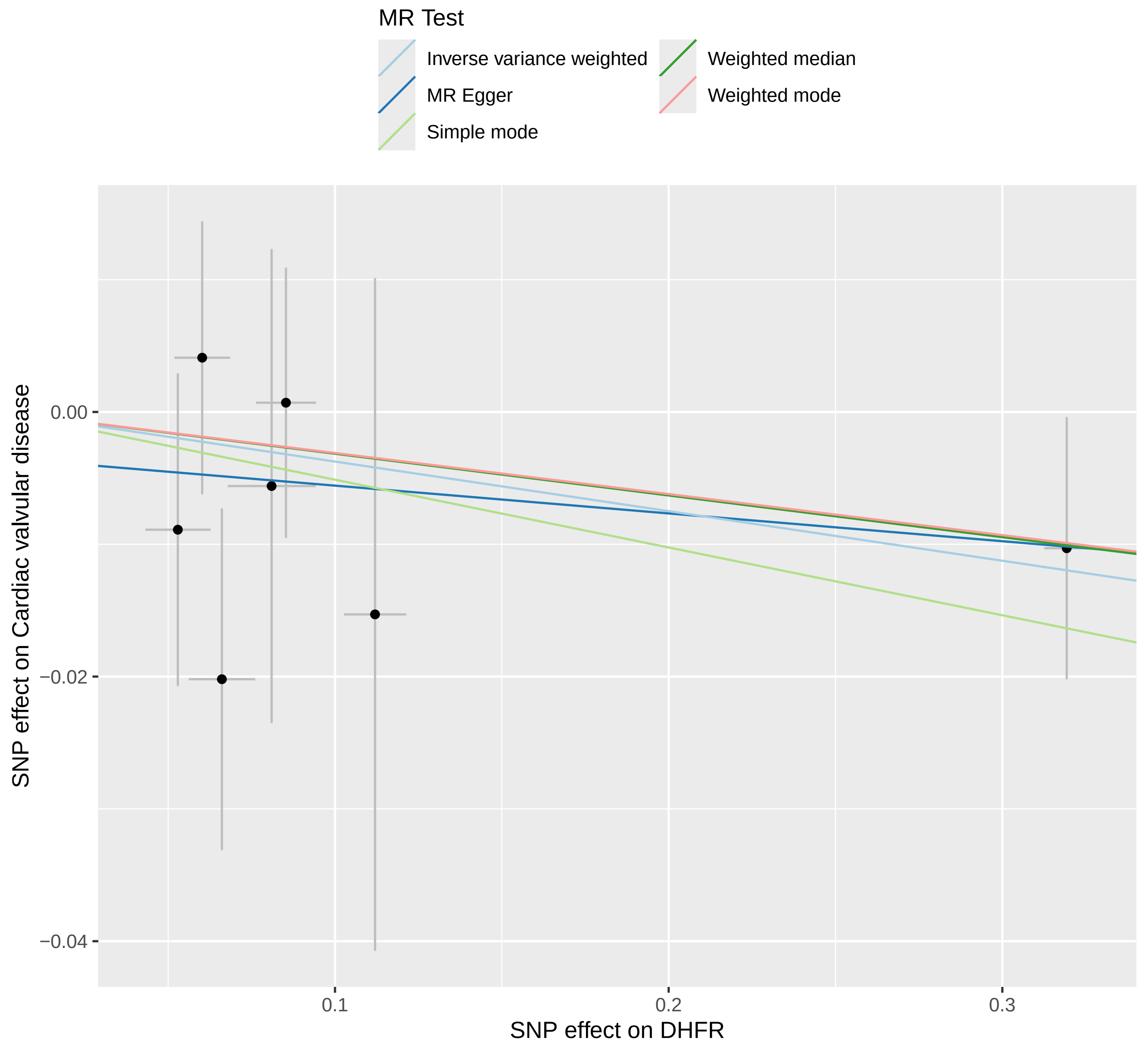
MR Test
Inverse variance weighted
MR Egger
Simple mode
Weighted median
Weighted mode
0.00
−0.02
−0.04
0.1
0.2
0.3
SNP effect on Cardiac valvular disease
SNP effect on DHFR

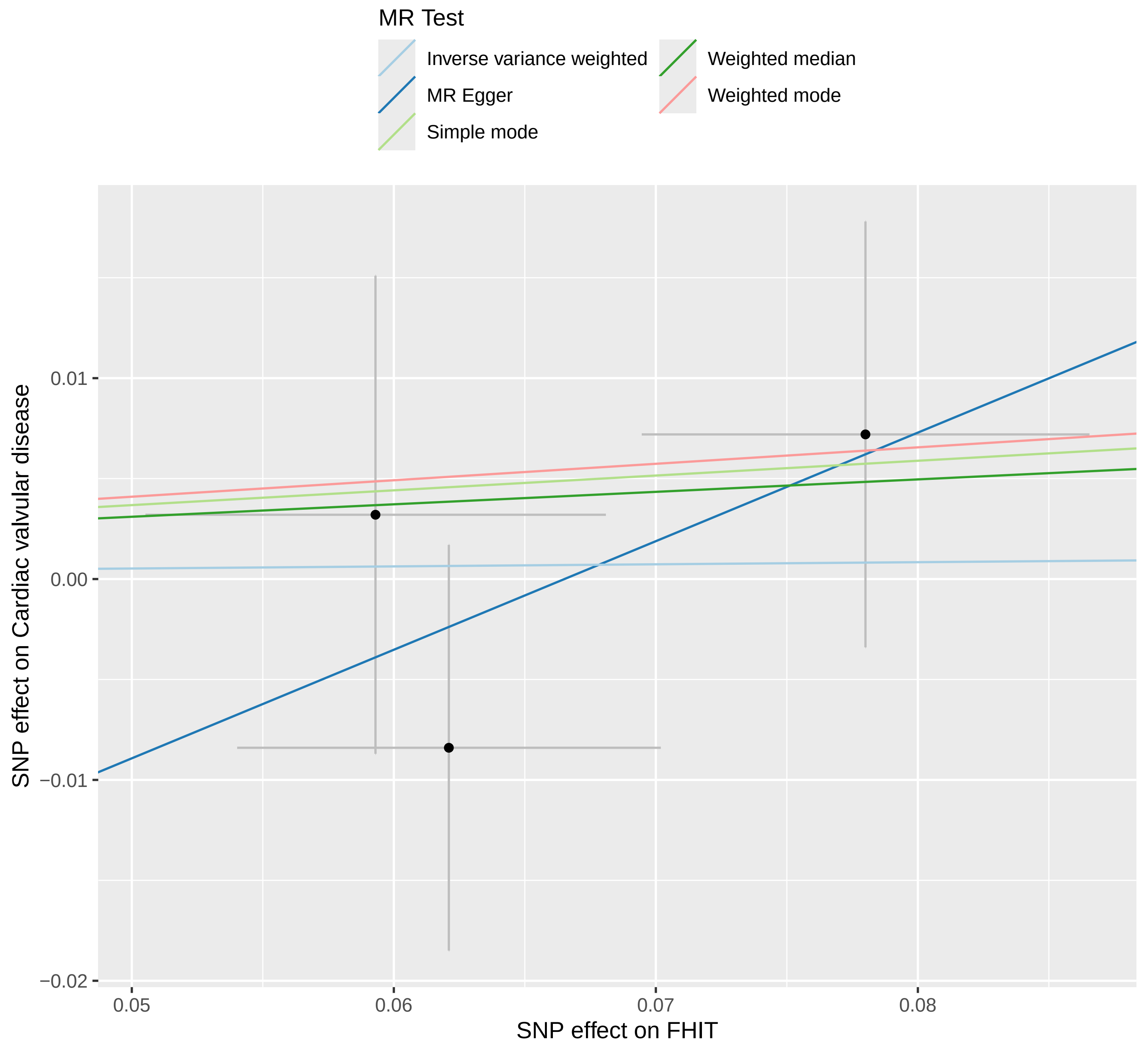
MR Test
Inverse variance weighted
MR Egger
Simple mode
Weighted median
Weighted mode
0.01
0.00
−0.01
−0.02
0.05
0.06
0.07
0.08
SNP effect on Cardiac valvular disease
SNP effect on FHIT

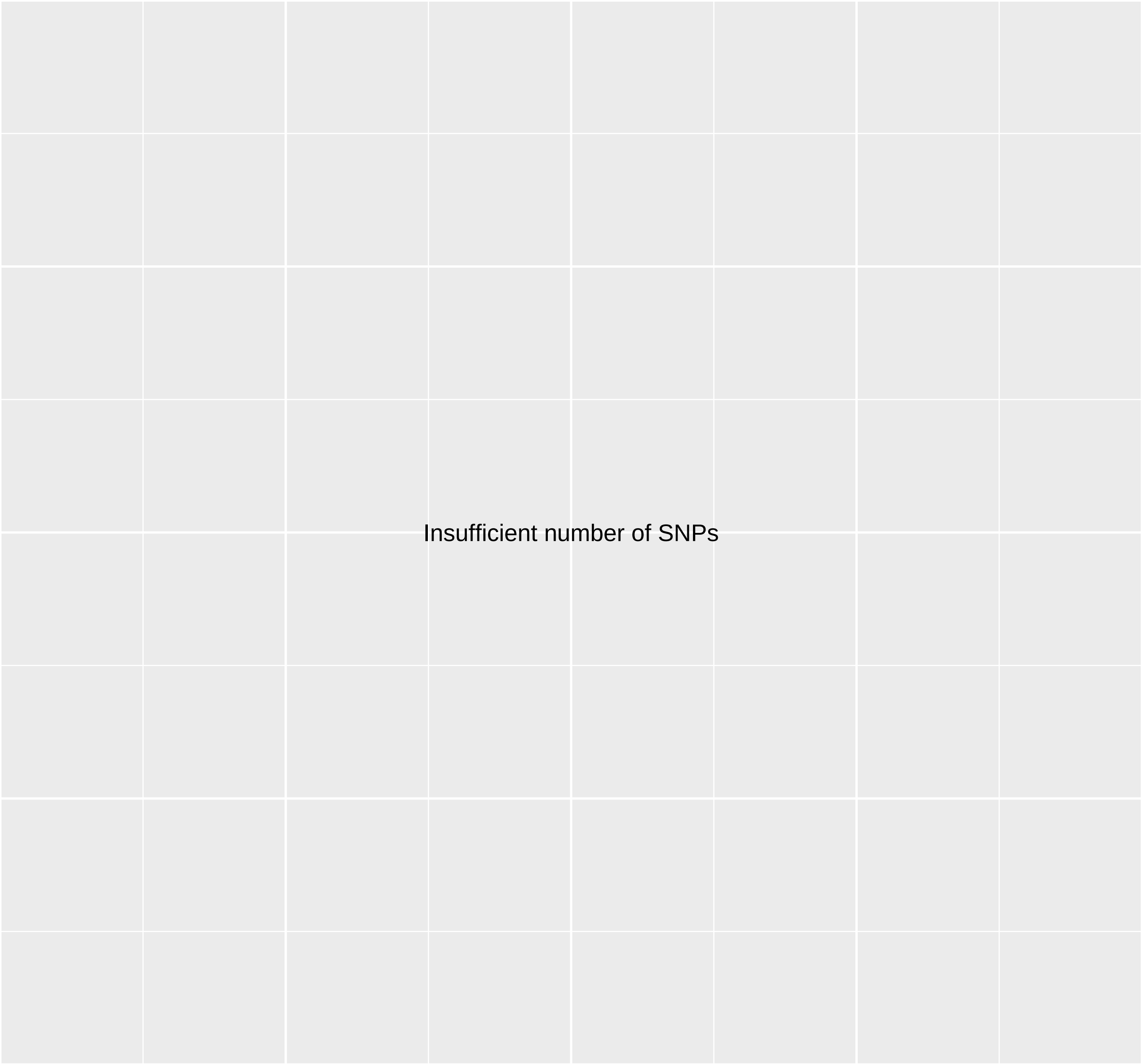
Insufficient number of SNPs

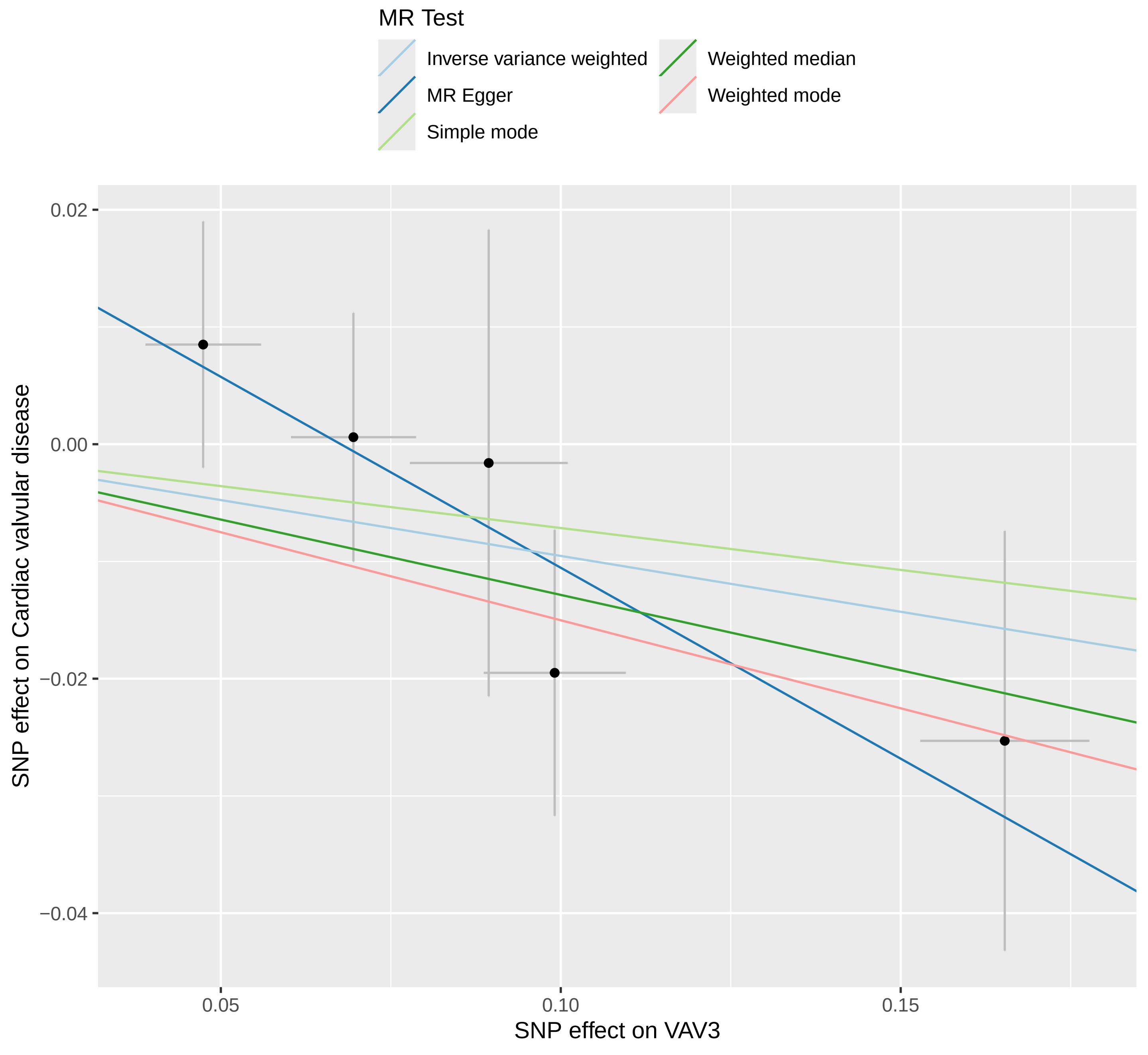

MR Test
Inverse variance weighted
MR Egger
Simple mode
Weighted median
Weighted mode
0.02
0.00
−0.02
−0.04
0.05
0.10
0.15
SNP effect on Cardiac valvular disease
SNP effect on VAV3

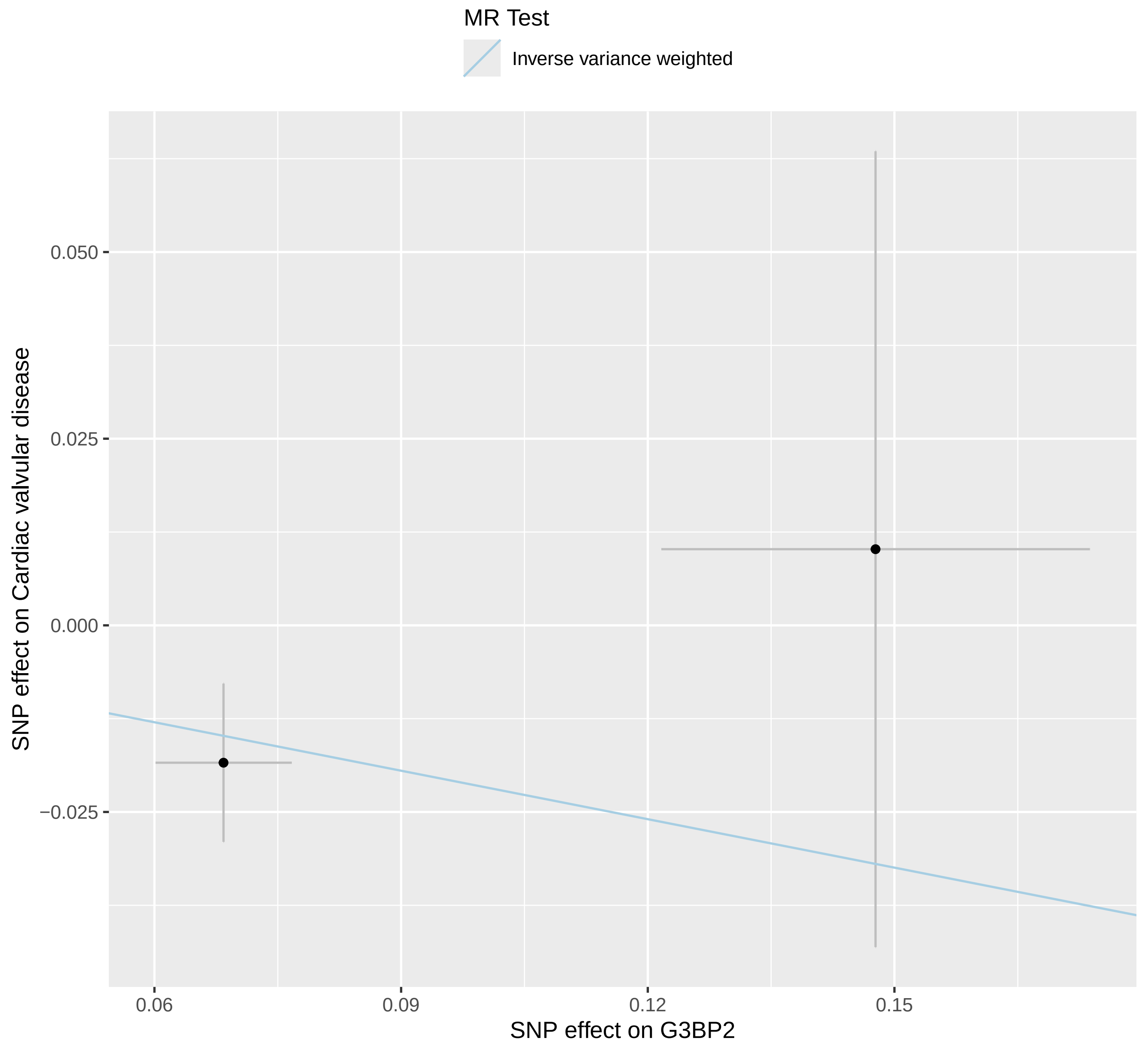
MR Test
Inverse variance weighted
0.050
0.025
0.000
−0.025
SNP effect on Cardiac valvular disease
0.06
0.09
0.12
0.15
SNP effect on G3BP2

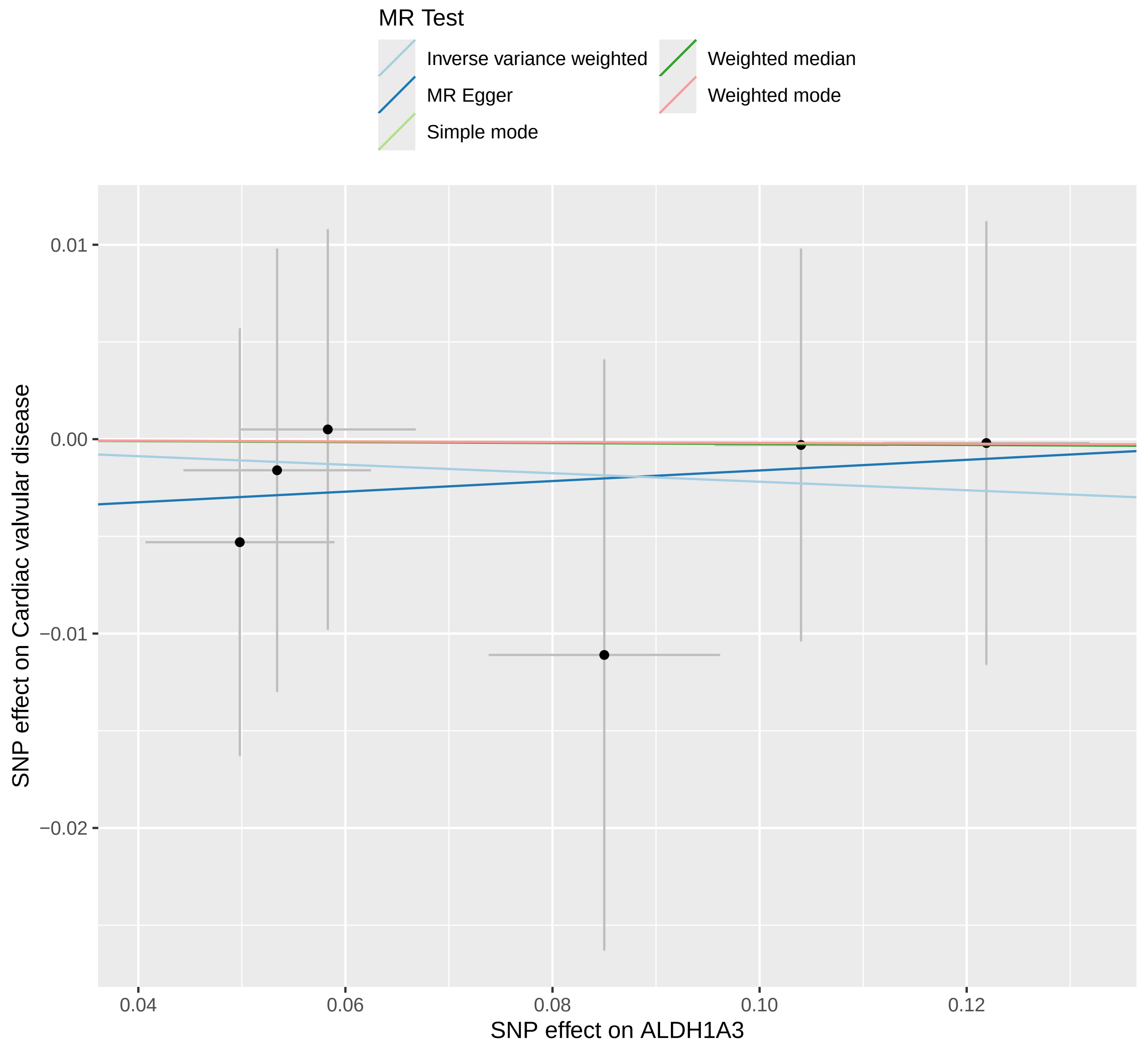

MR Test
Inverse variance weighted
MR Egger
Simple mode
Weighted median
Weighted mode
0.01
0.00
−0.01
−0.02
SNP effect on Cardiac valvular disease
0.04
0.06
0.08
0.10
0.12
SNP effect on ALDH1A3

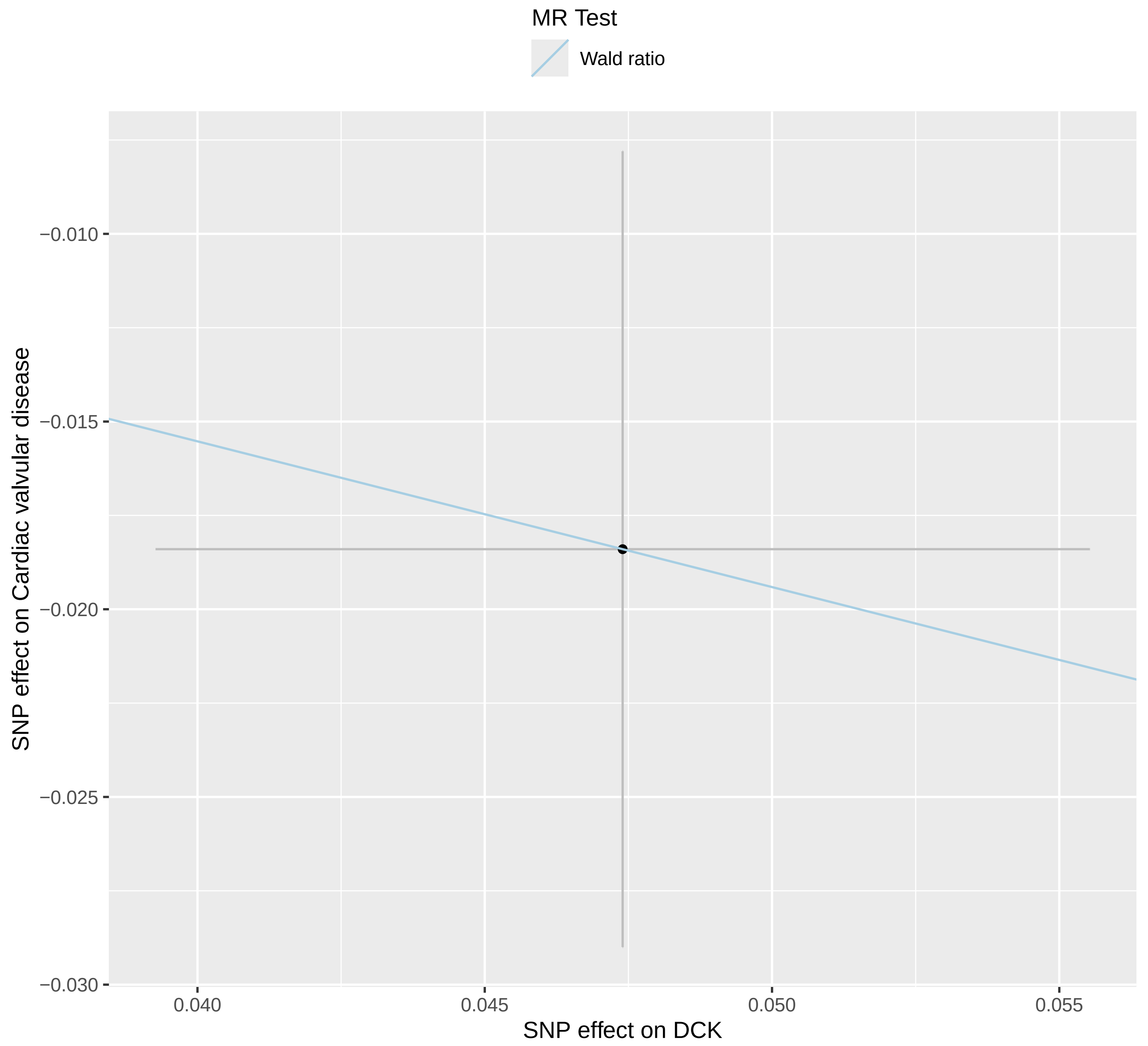

MR Test
Wald ratio
−0.010
−0.015
−0.020
−0.025
−0.030
SNP effect on Cardiac valvular disease
0.040
0.045
0.050
0.055
SNP effect on DCK

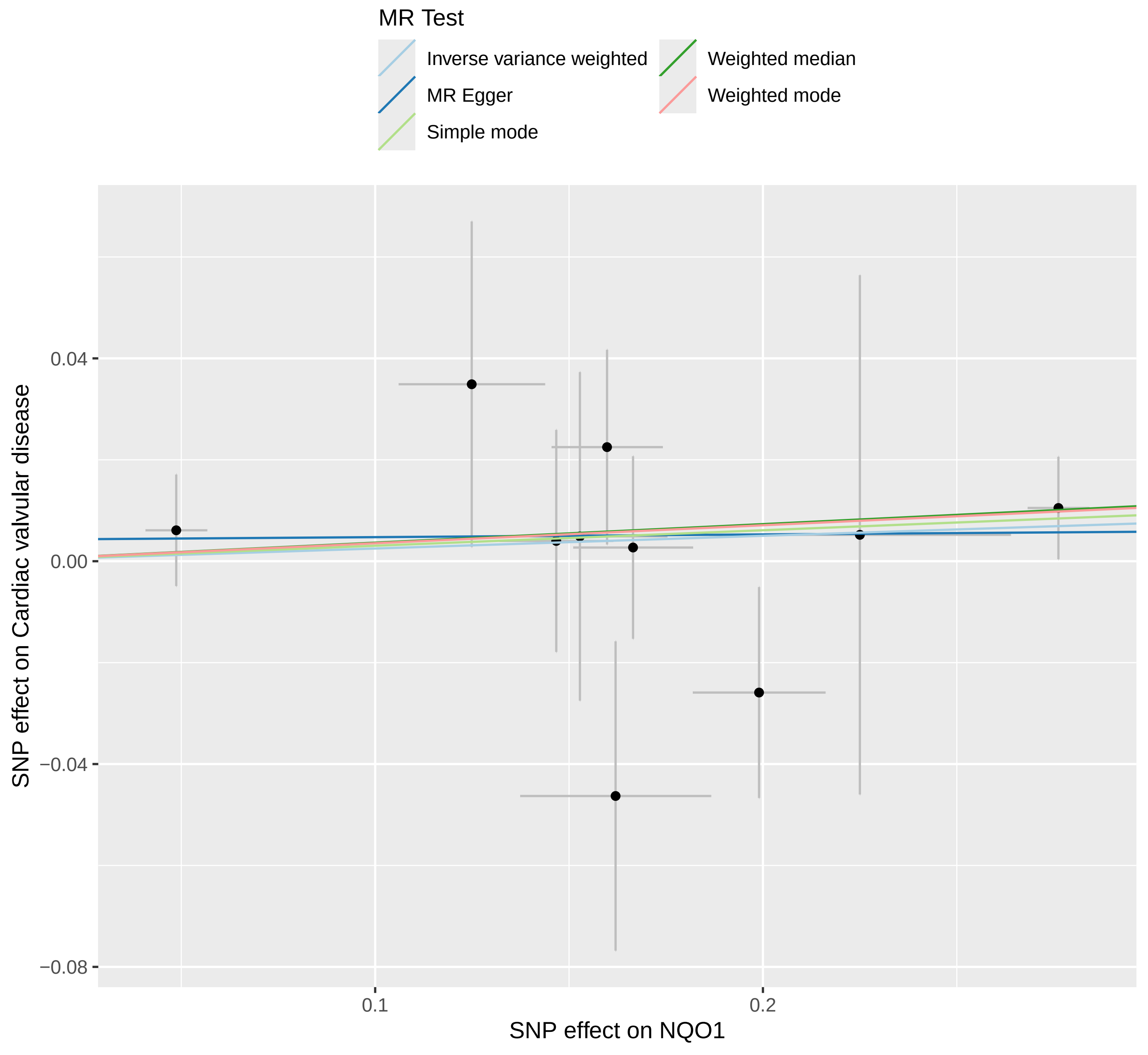

MR Test
Inverse variance weighted
MR Egger
Simple mode
Weighted median
Weighted mode
0.04
0.00
−0.04
−0.08
0.1
0.2
SNP effect on Cardiac valvular disease
SNP effect on NQO1

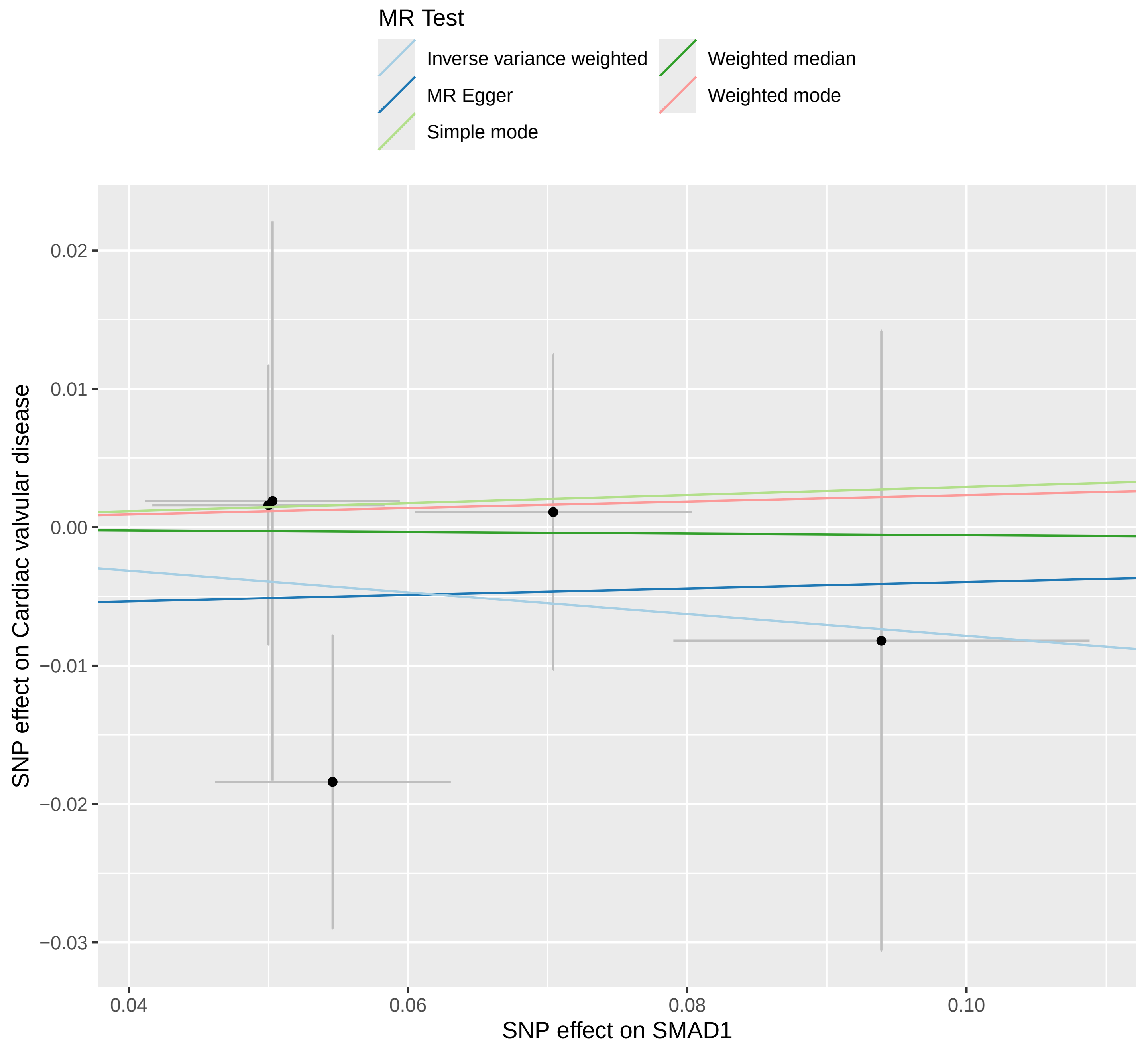
MR Test
Inverse variance weighted
MR Egger
Simple mode
Weighted median
Weighted mode
SNP effect on Cardiac valvular disease
0.02
0.01
0.00
−0.01
−0.02
−0.03
0.04
0.06
0.08
0.10
SNP effect on SMAD1

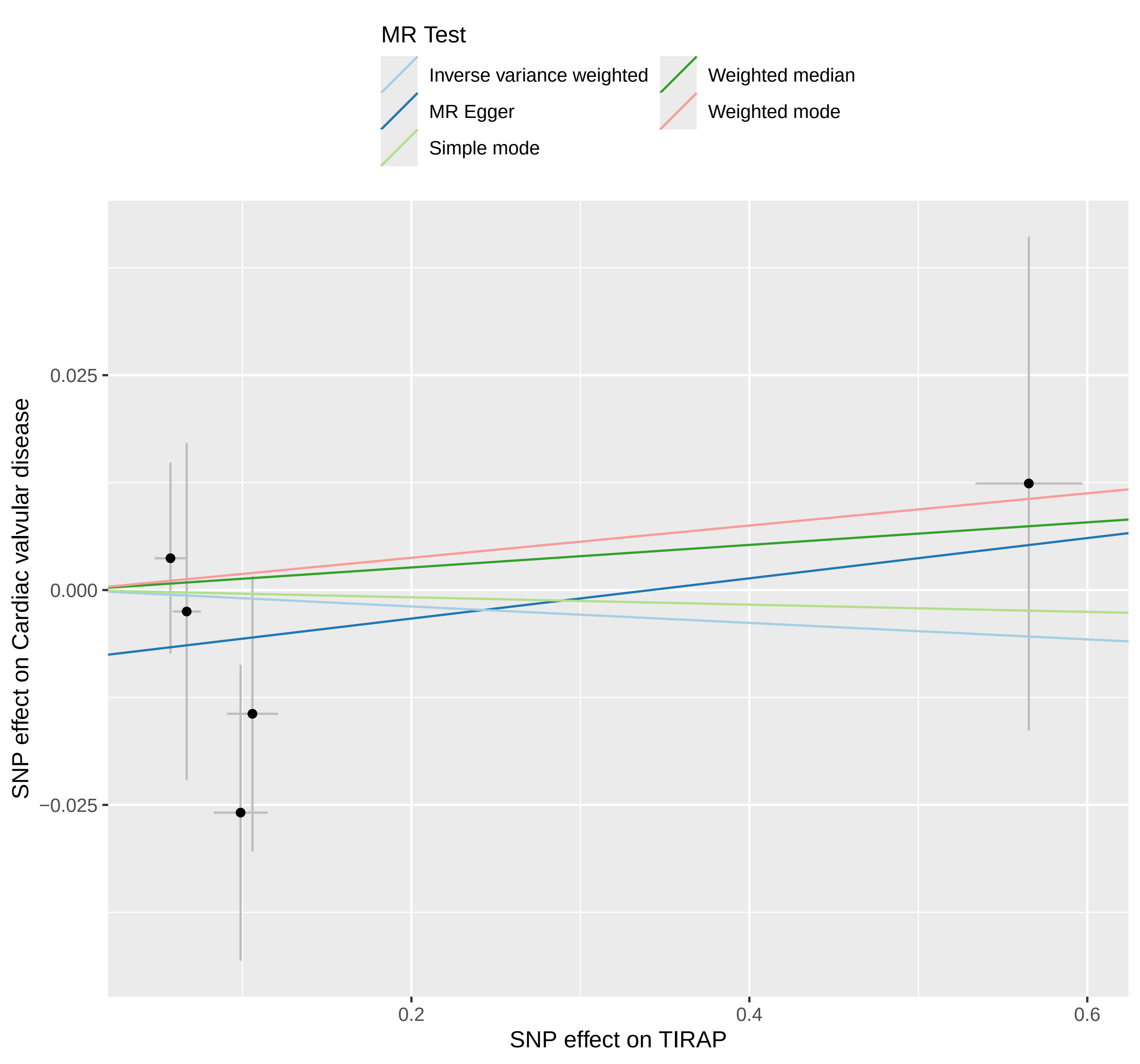

MR Test
Inverse variance weighted
MR Egger
Simple mode
Weighted median
Weighted mode
0.025
0.000
−0.025
SNP effect on Cardiac valvular disease
0.2
0.4
0.6
SNP effect on TIRAP

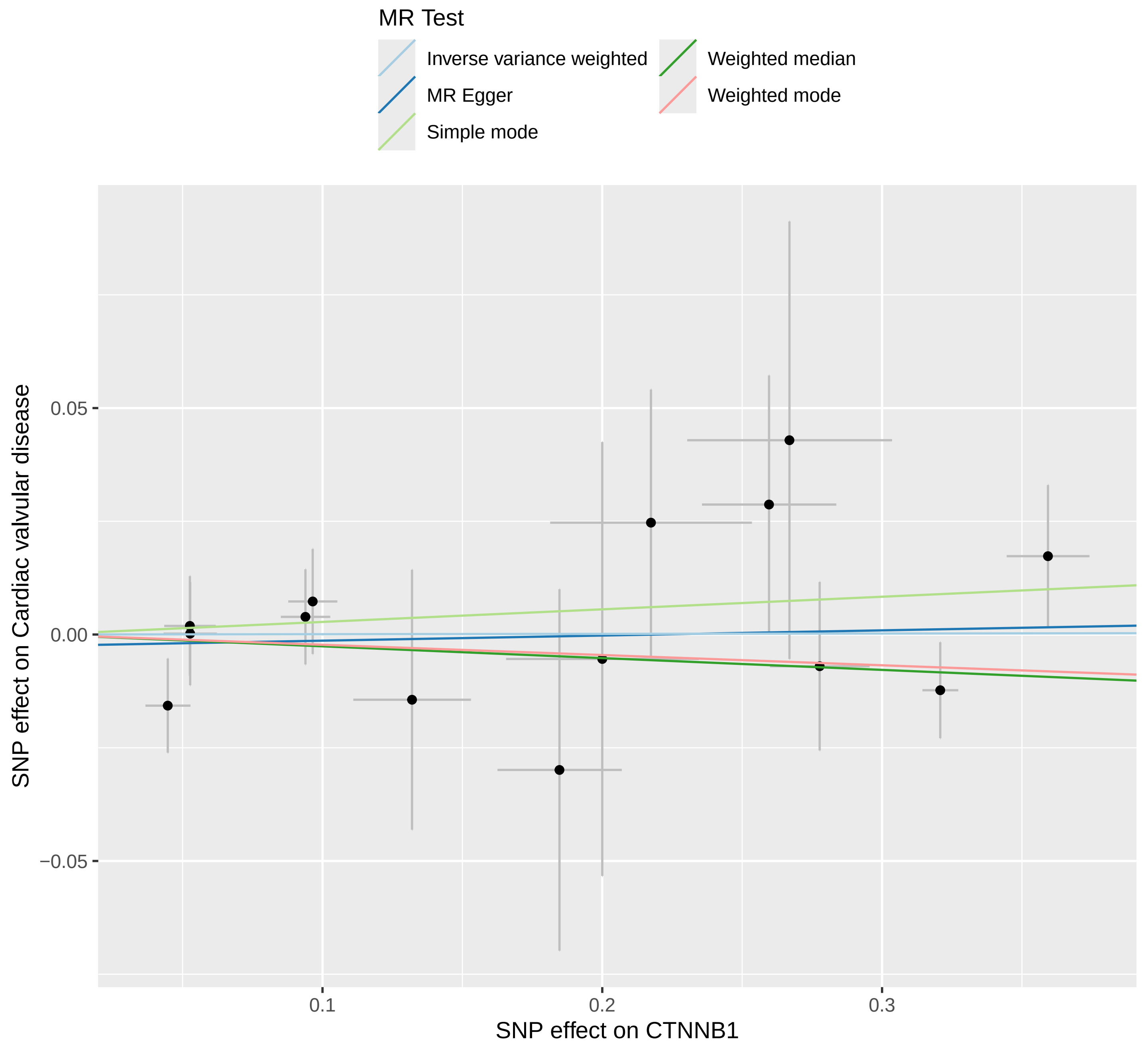
MR Test
Inverse variance weighted
MR Egger
Simple mode
Weighted median
Weighted mode
SNP effect on Cardiac valvular disease
0.05
0.00
−0.05
0.1
0.2
0.3
SNP effect on CTNNB1

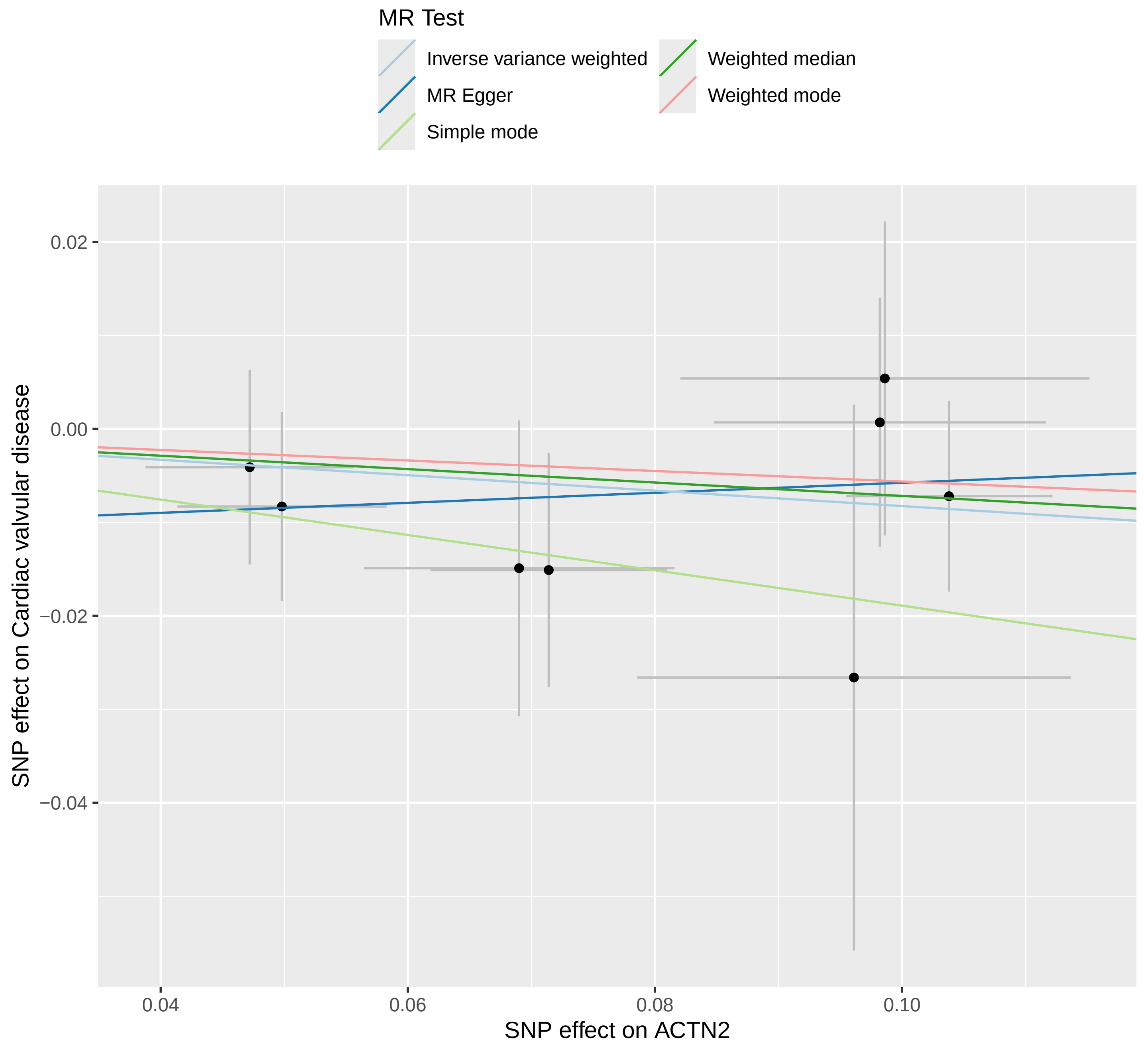

MR Test
Inverse variance weighted
MR Egger
Simple mode
Weighted median
Weighted mode
0.02
0.00
−0.02
−0.04
SNP effect on Cardiac valvular disease
0.04
0.06
0.08
0.10
SNP effect on ACTN2

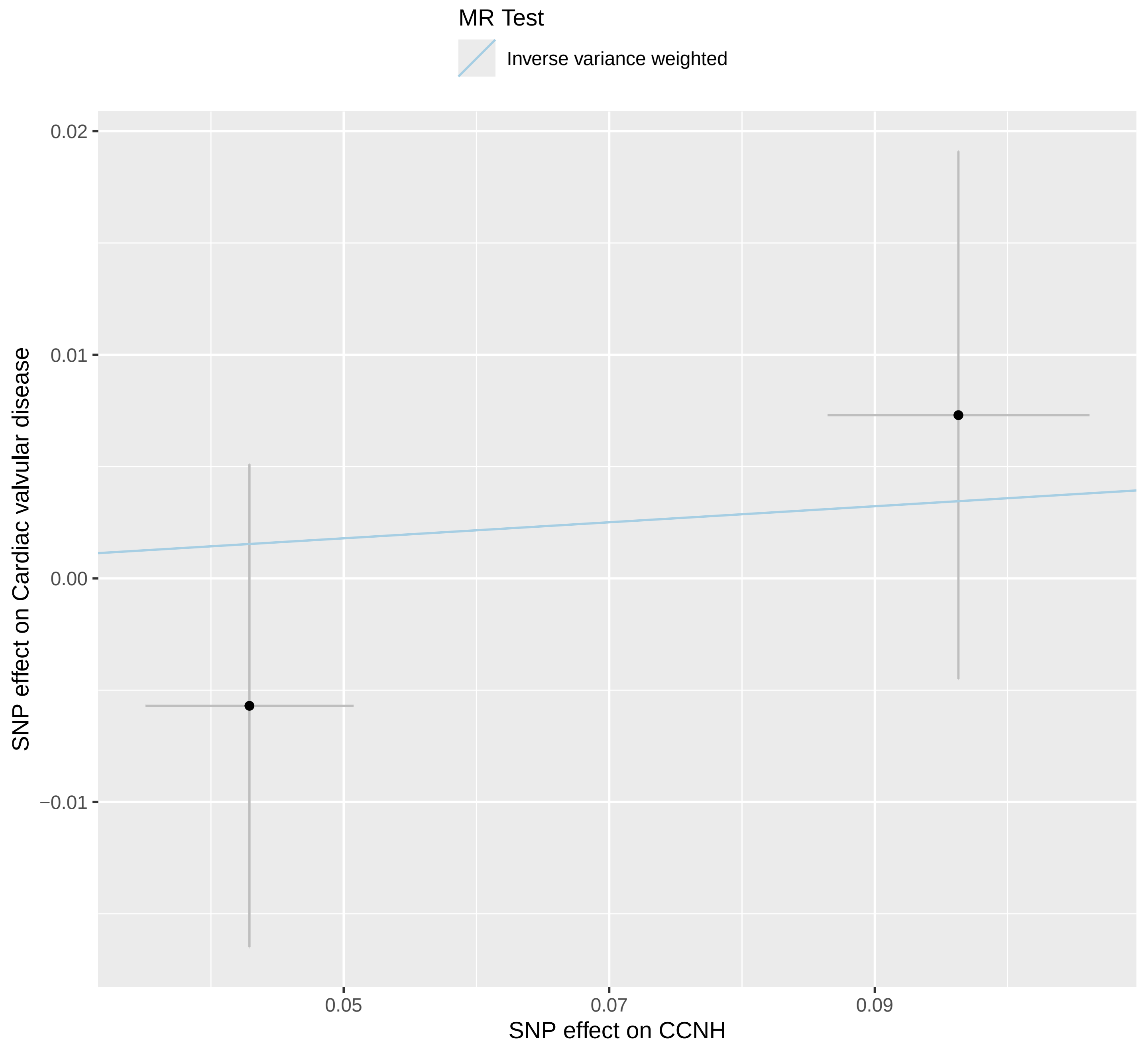
MR Test
Inverse variance weighted
0.02
0.01
0.00
−0.01
0.05
0.07
0.09
SNP effect on Cardiac valvular disease
SNP effect on CCNH

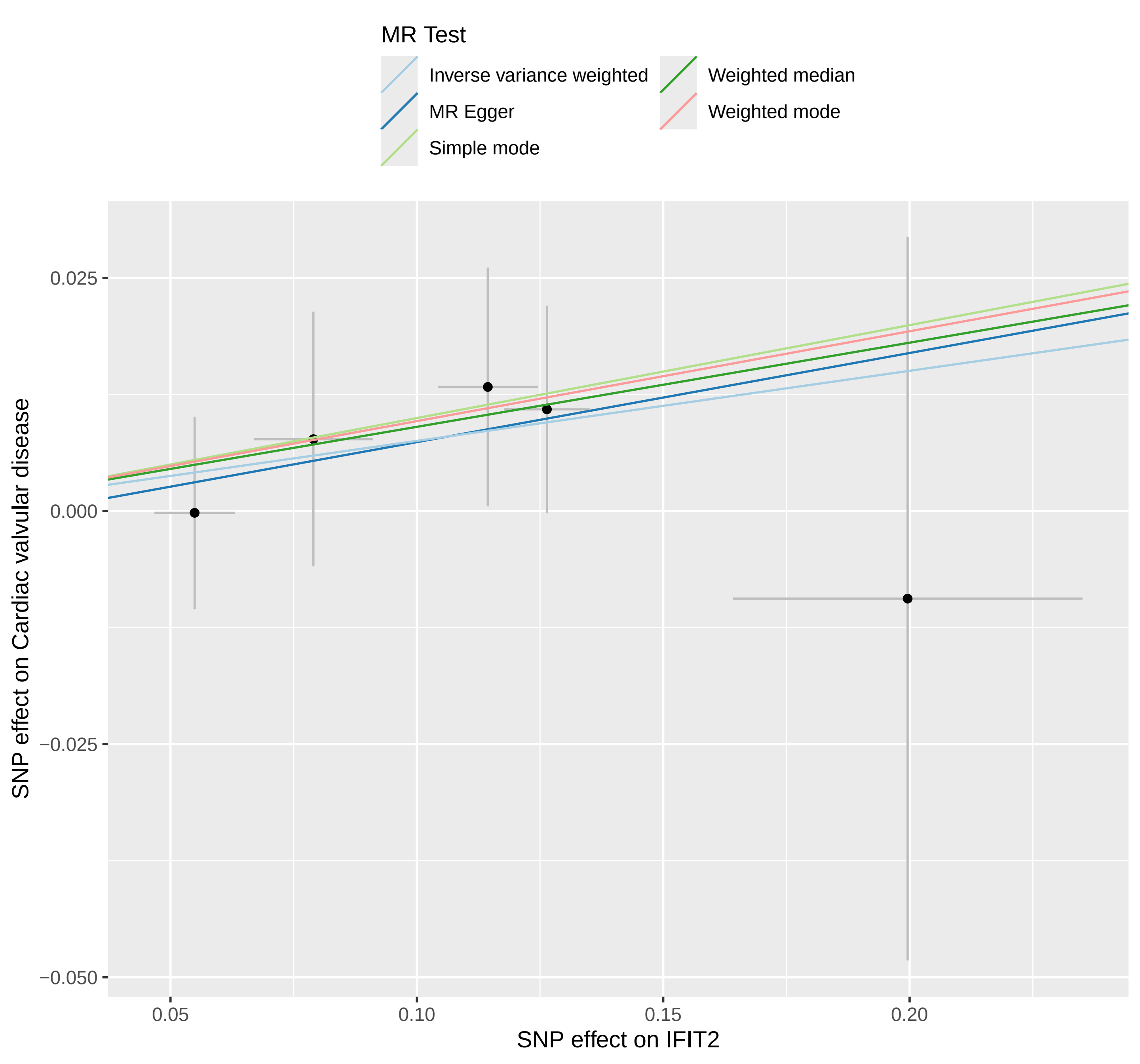

MR Test
Inverse variance weighted
MR Egger
Simple mode
Weighted median
Weighted mode
0.025
0.000
−0.025
−0.050
0.05
0.10
0.15
0.20
SNP effect on Cardiac valvular disease
SNP effect on IFIT2

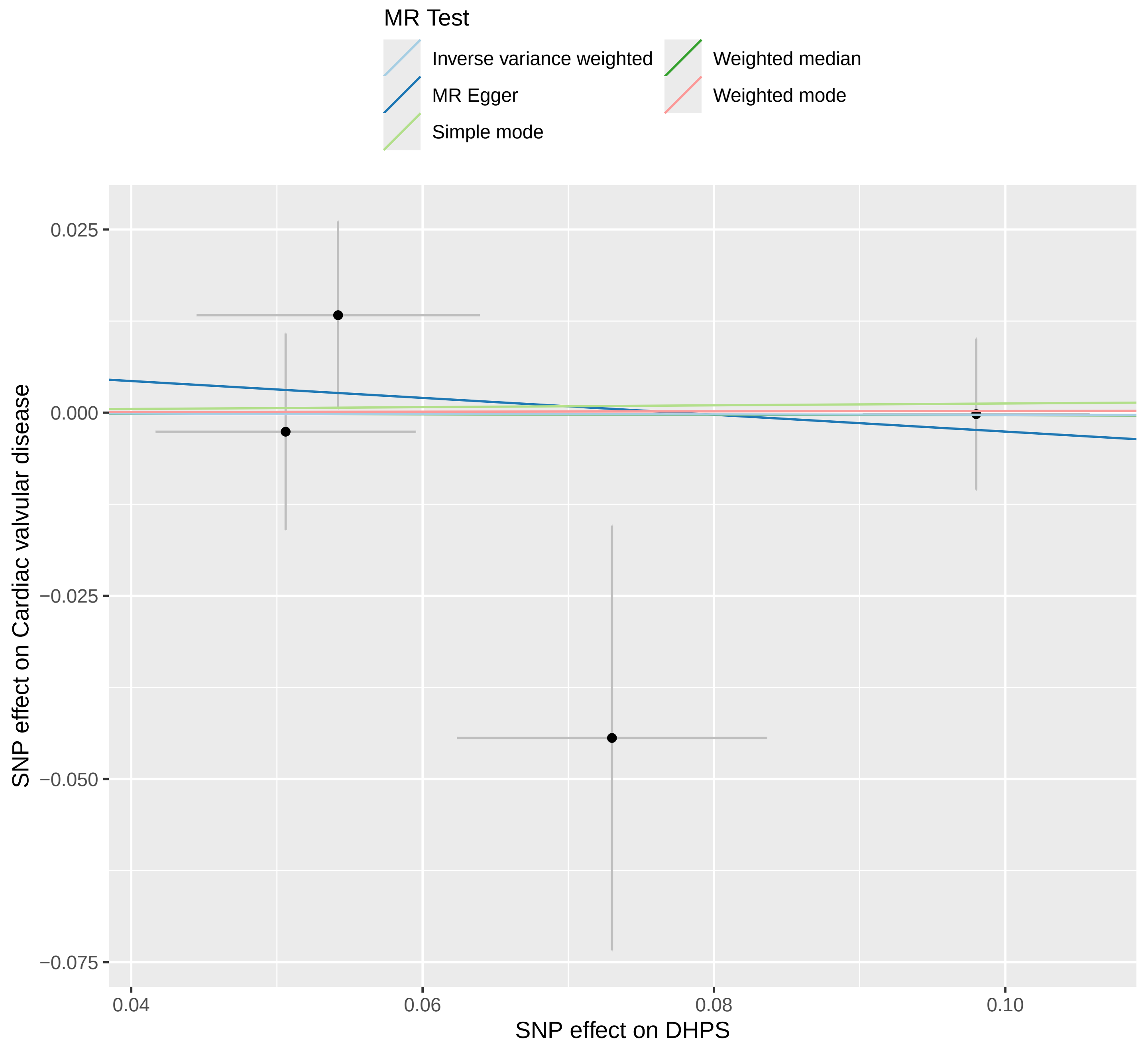
MR Test
Inverse variance weighted
MR Egger
Simple mode
Weighted median
Weighted mode
0.025
0.000
−0.025
−0.050
−0.075
SNP effect on Cardiac valvular disease
0.04
0.06
0.08
0.10
SNP effect on DHPS

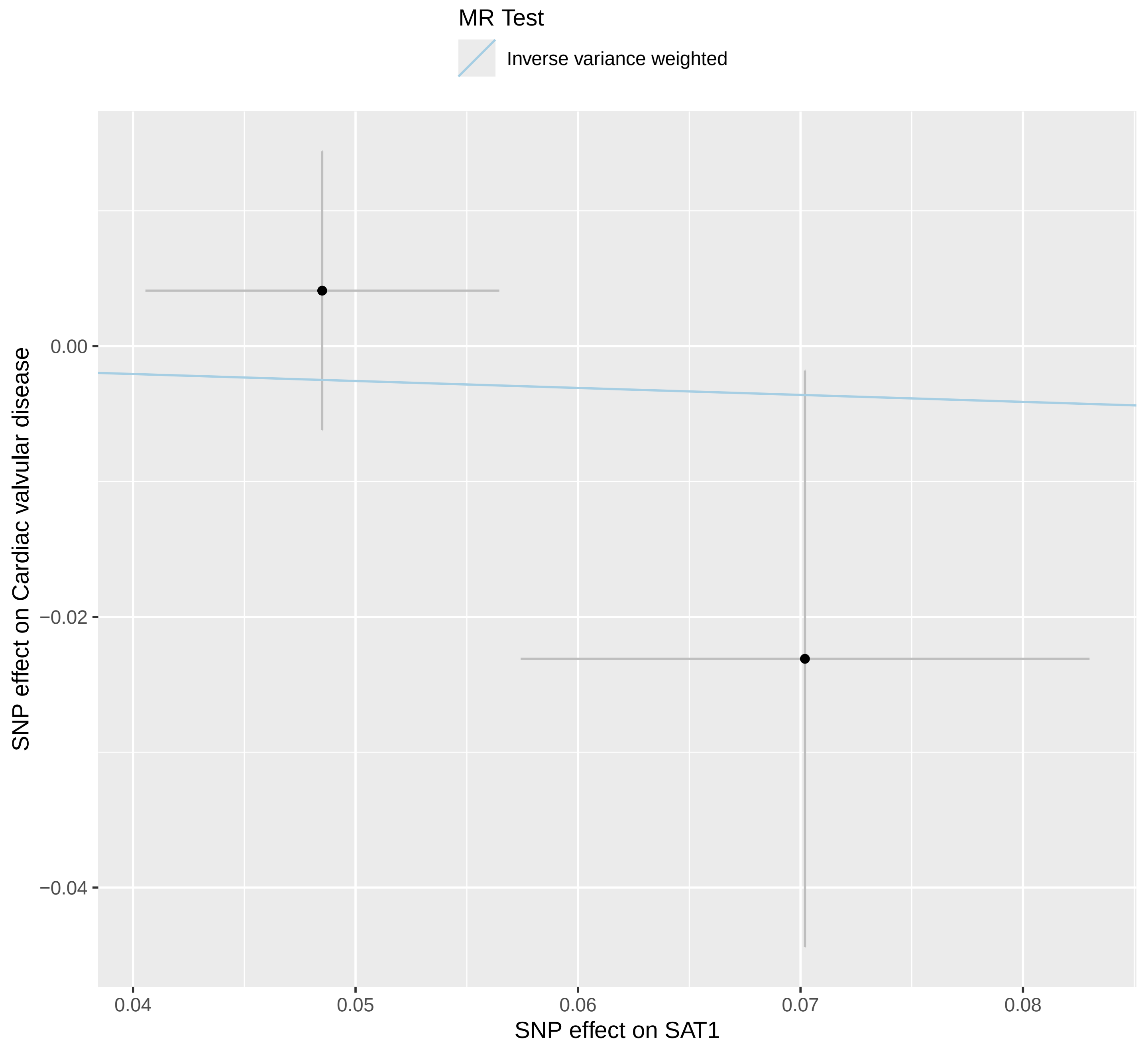
MR Test
Inverse variance weighted
SNP effect on Cardiac valvular disease
0.00
−0.02
−0.04
0.04
0.05
0.06
0.07
0.08
SNP effect on SAT1

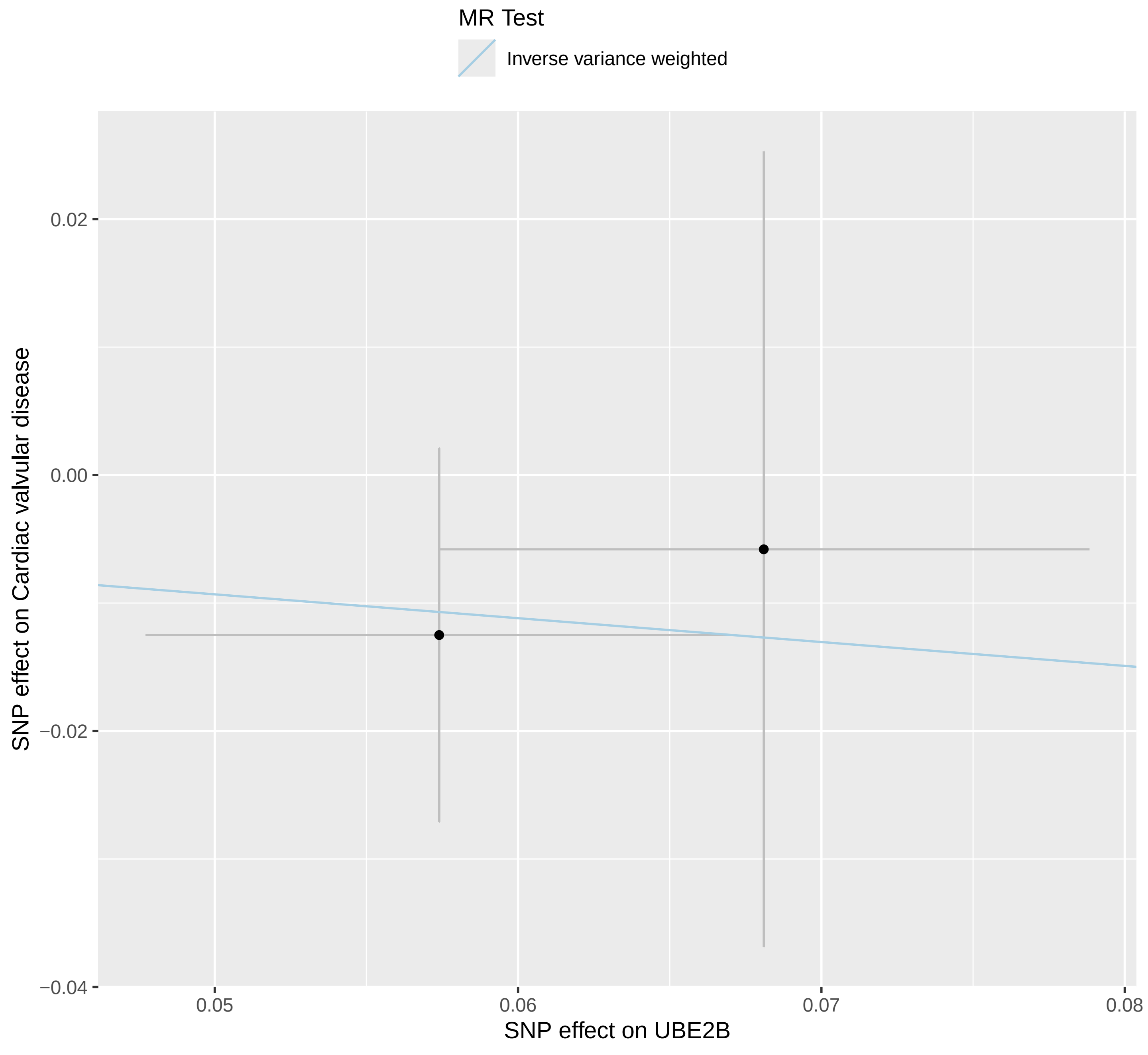

MR Test
Inverse variance weighted
0.02
0.00
−0.02
−0.04
0.05
0.06
0.07
0.08
SNP effect on UBE2B
SNP effect on Cardiac valvular disease

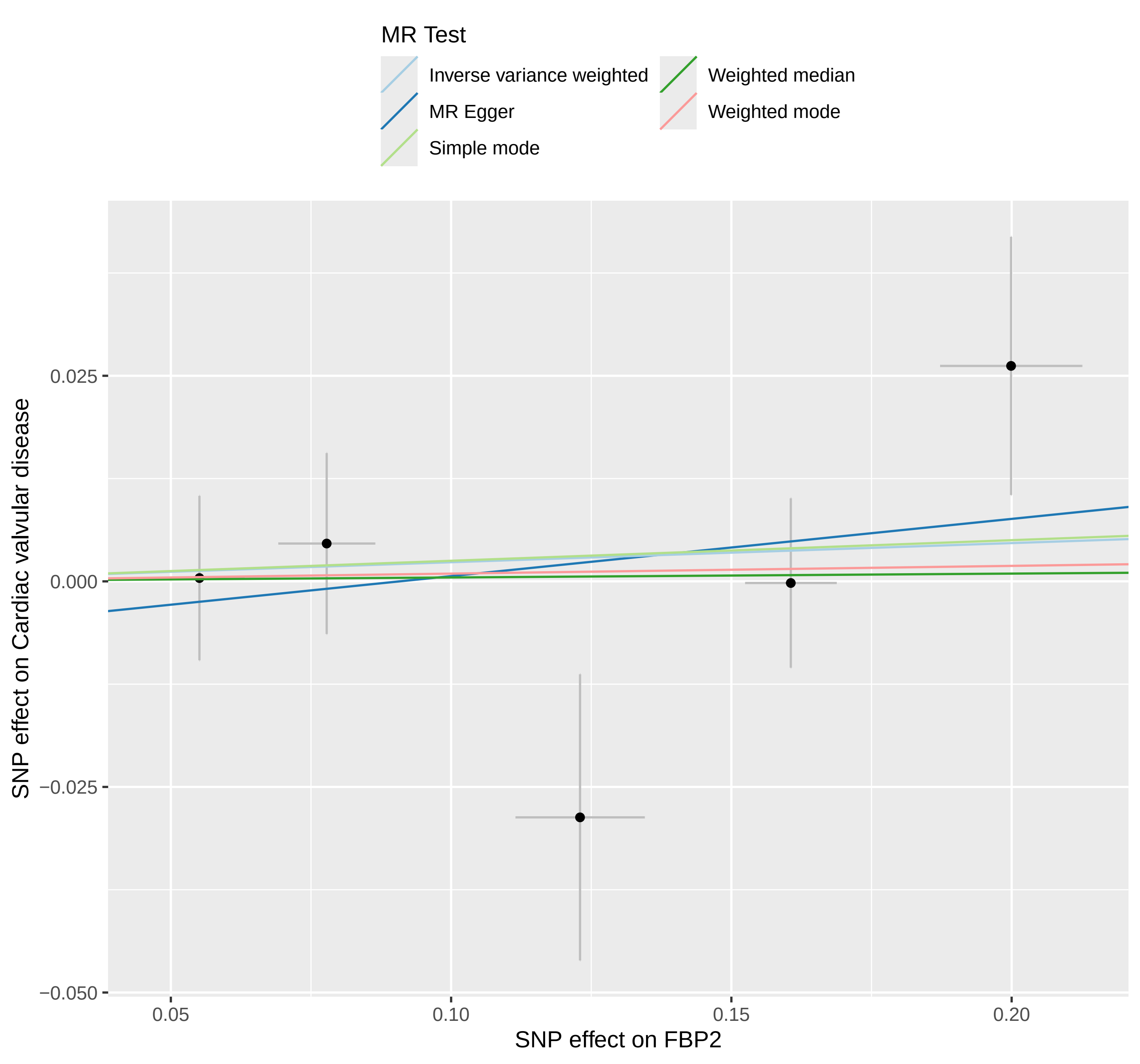
MR Test
Inverse variance weighted
MR Egger
Simple mode
Weighted median
Weighted mode
0.025
0.000
−0.025
−0.050
0.05
0.10
0.15
0.20
SNP effect on Cardiac valvular disease
SNP effect on FBP2

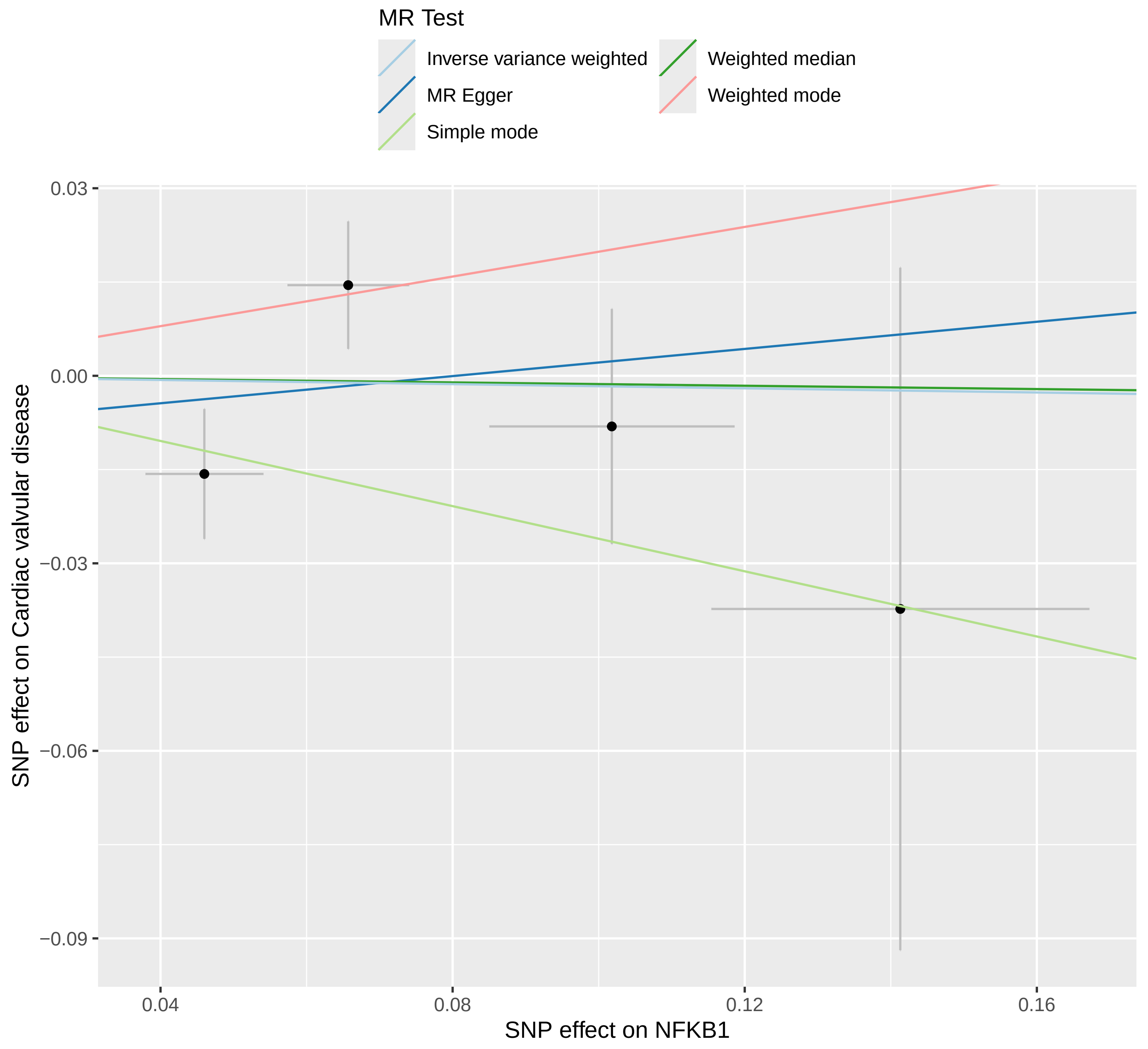
MR Test
Inverse variance weighted
MR Egger
Simple mode
Weighted median
Weighted mode
0.03
0.00
−0.03
−0.06
−0.09
SNP effect on Cardiac valvular disease
0.04
0.08
0.12
0.16
SNP effect on NFKB1

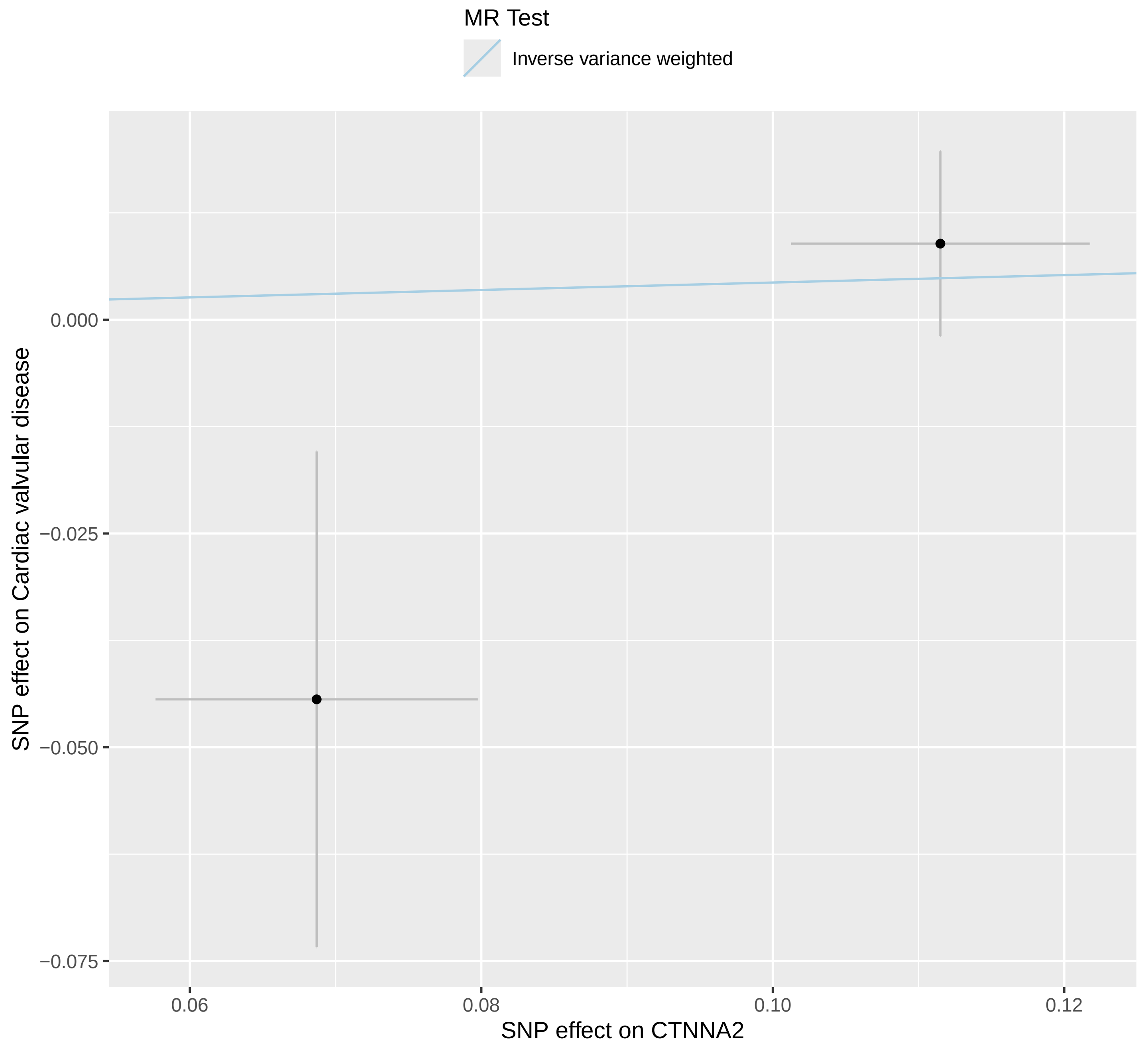
MR Test
Inverse variance weighted
0.000
−0.025
−0.050
−0.075
0.06
0.08
0.10
0.12
SNP effect on Cardiac valvular disease
SNP effect on CTNNA2

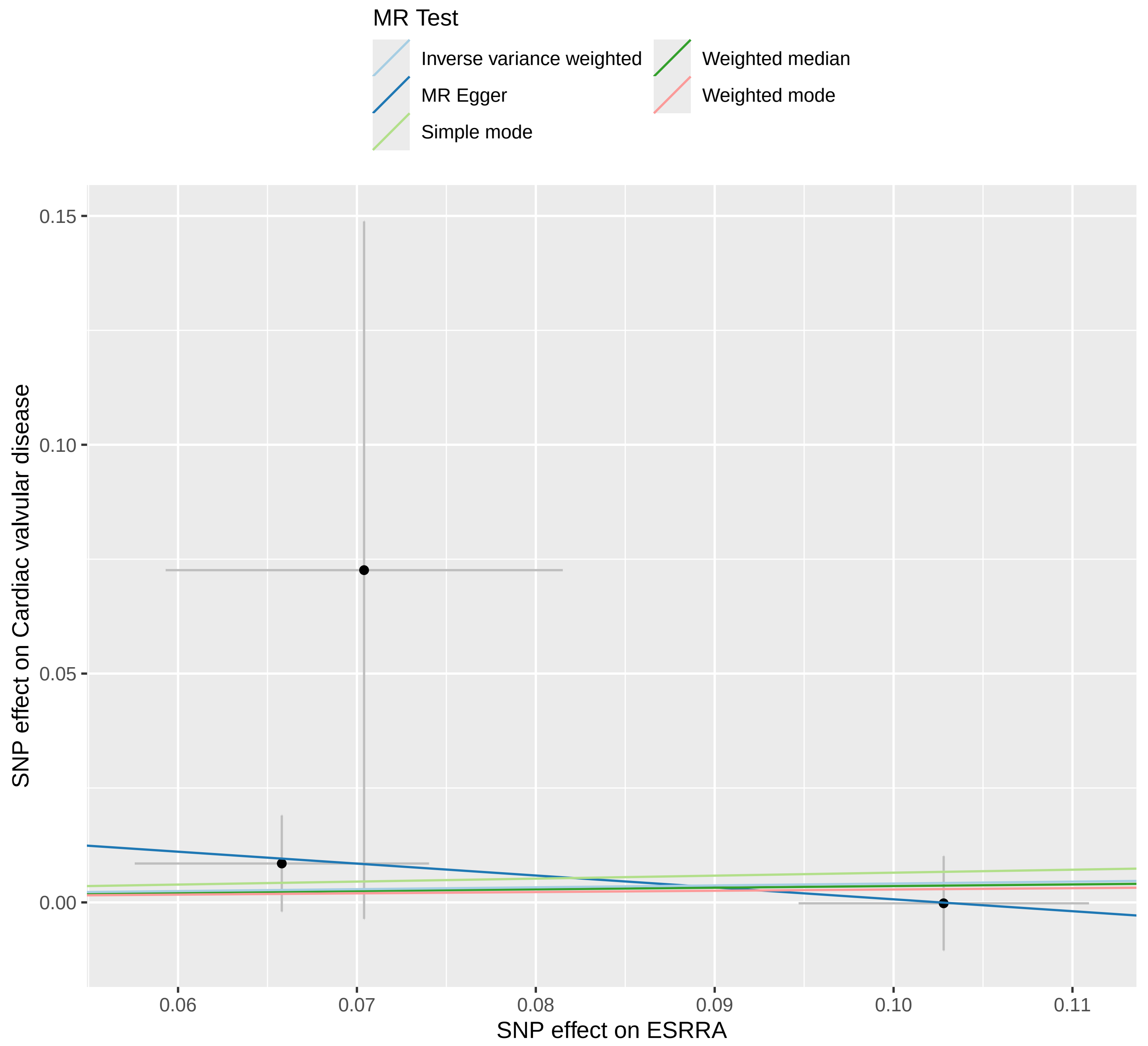

MR Test
Inverse variance weighted
MR Egger
Simple mode
Weighted median
Weighted mode
0.15
0.10
0.05
0.00
SNP effect on Cardiac valvular disease
0.06
0.07
0.08
0.09
0.10
0.11
SNP effect on ESRRA

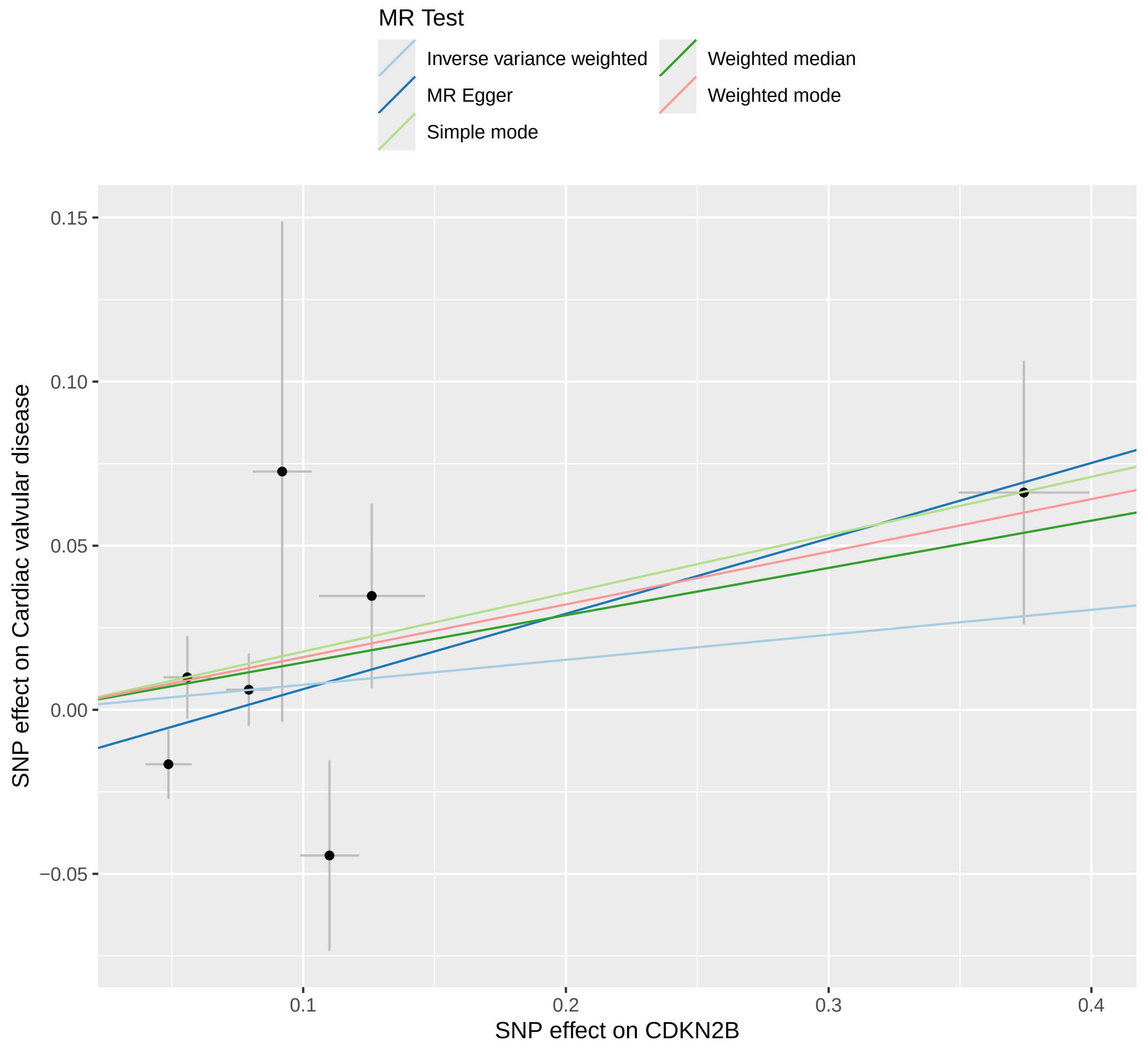
MR Test
Inverse variance weighted
MR Egger
Simple mode
Weighted median
Weighted mode
0.15
0.10
0.05
0.00
−0.05
SNP effect on Cardiac valvular disease
0.1
0.2
0.3
0.4
SNP effect on CDKN2B

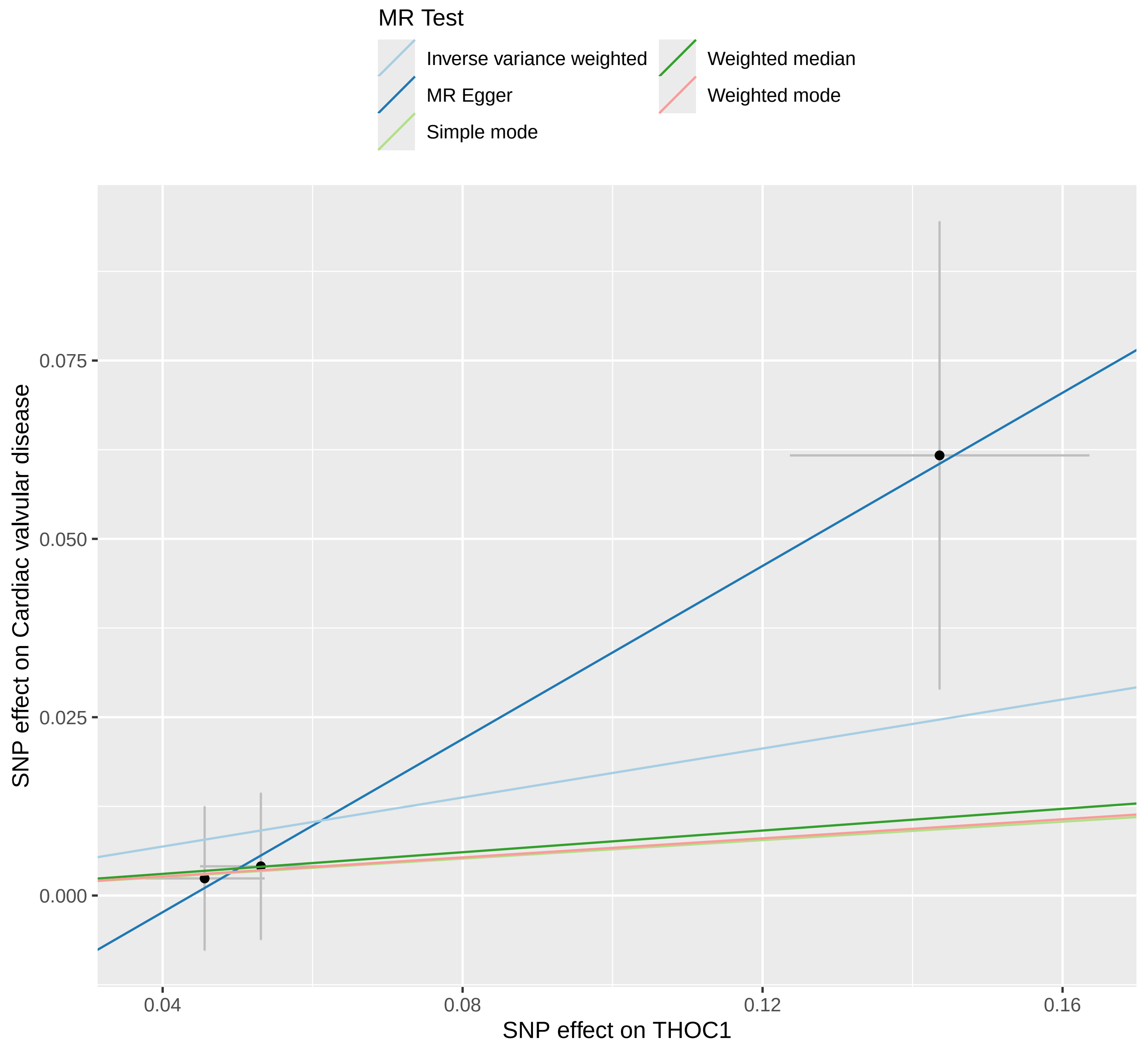
MR Test
Inverse variance weighted
MR Egger
Simple mode
Weighted median
Weighted mode
0.075
0.050
0.025
0.000
SNP effect on Cardiac valvular disease
0.04
0.08
0.12
0.16
SNP effect on THOC1

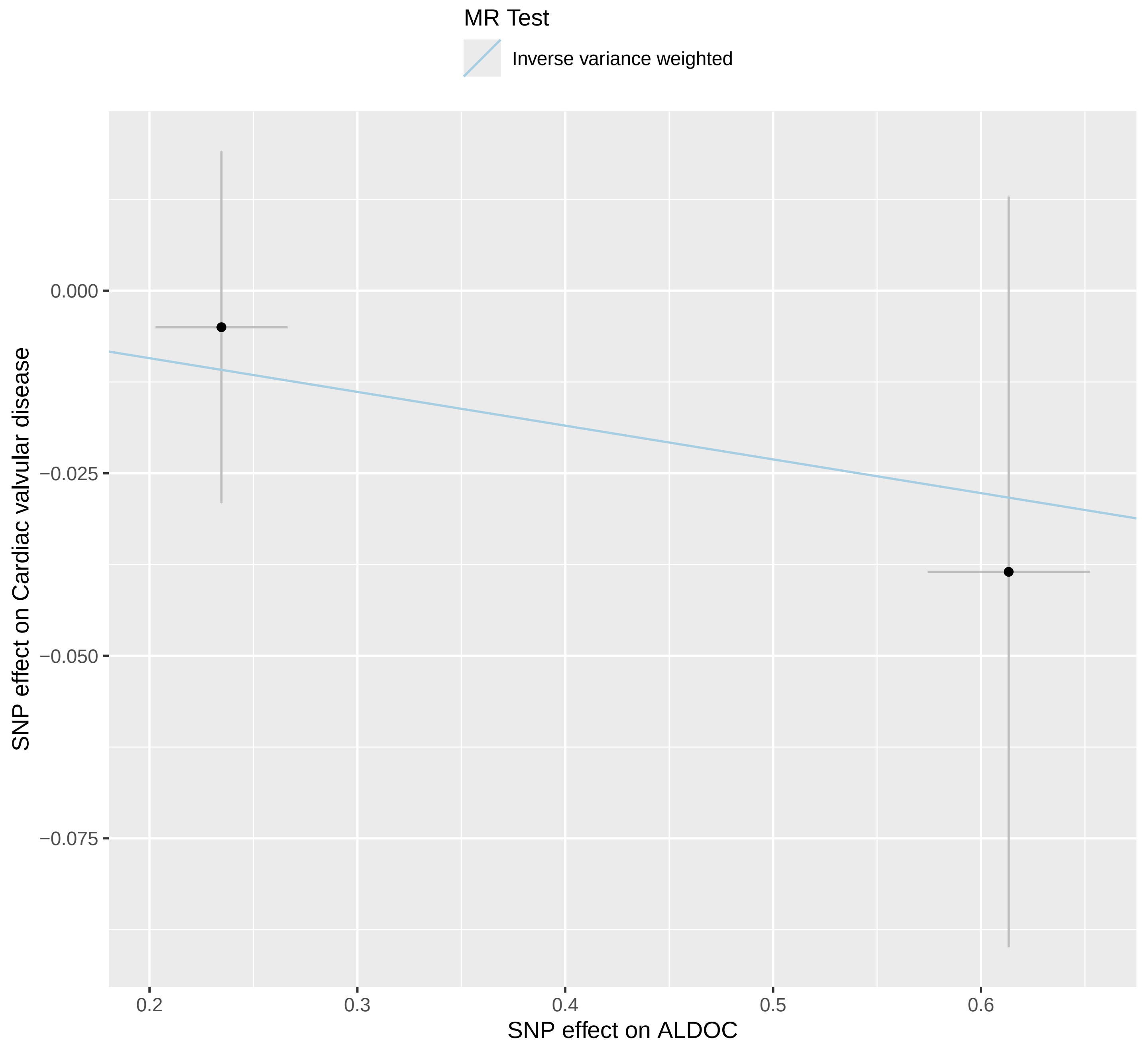

MR Test
Inverse variance weighted
0.000
−0.025
−0.050
−0.075
SNP effect on Cardiac valvular disease
0.2
0.3
0.4
0.5
0.6
SNP effect on ALDOC

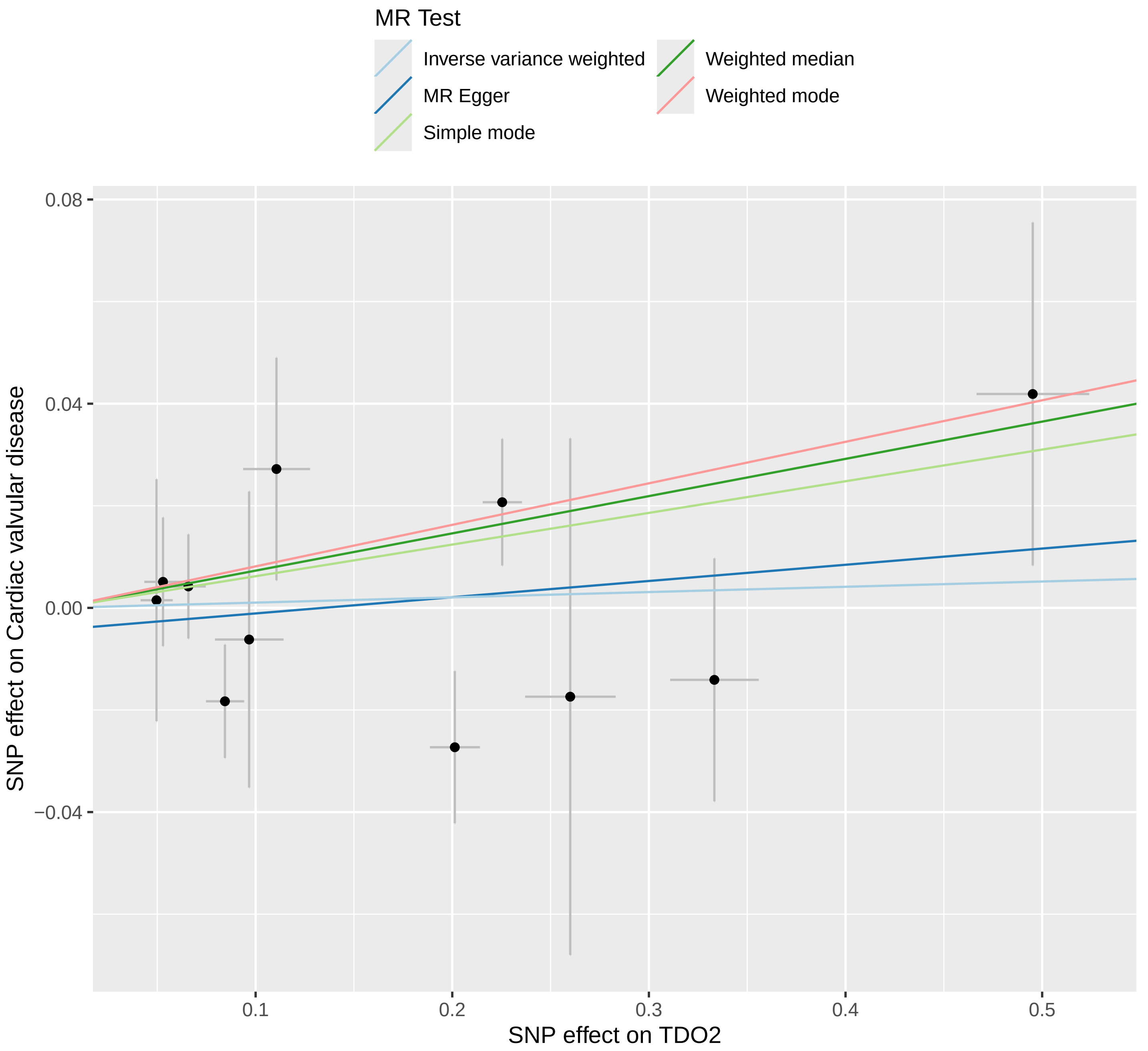
MR Test
Inverse variance weighted
MR Egger
Simple mode
Weighted median
Weighted mode
SNP effect on Cardiac valvular disease
0.08
0.04
0.00
−0.04
0.1
0.2
0.3
0.4
0.5
SNP effect on TDO2

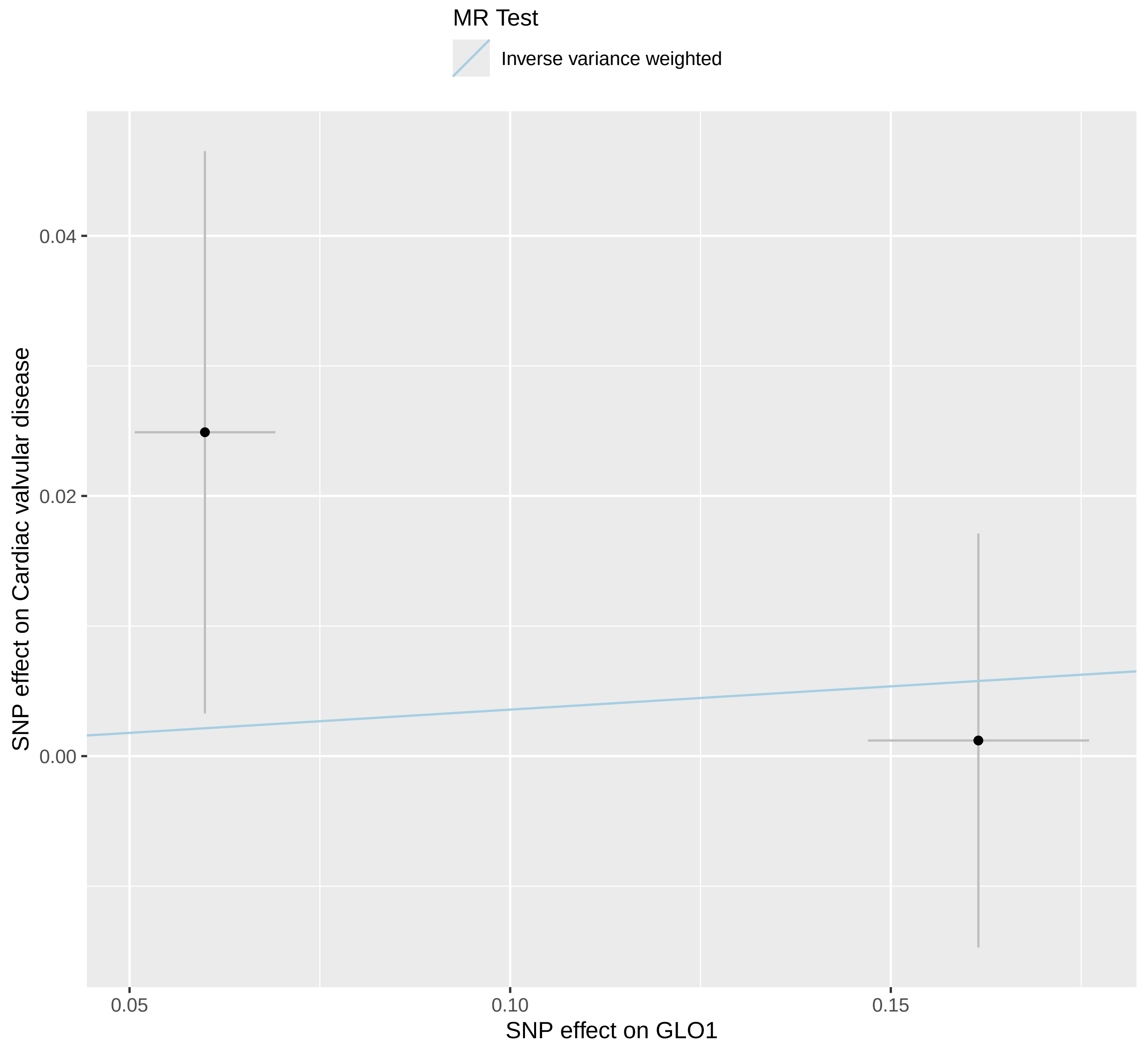
MR Test
Inverse variance weighted
0.04
0.02
0.00
SNP effect on Cardiac valvular disease
0.05
0.10
0.15
SNP effect on GLO1

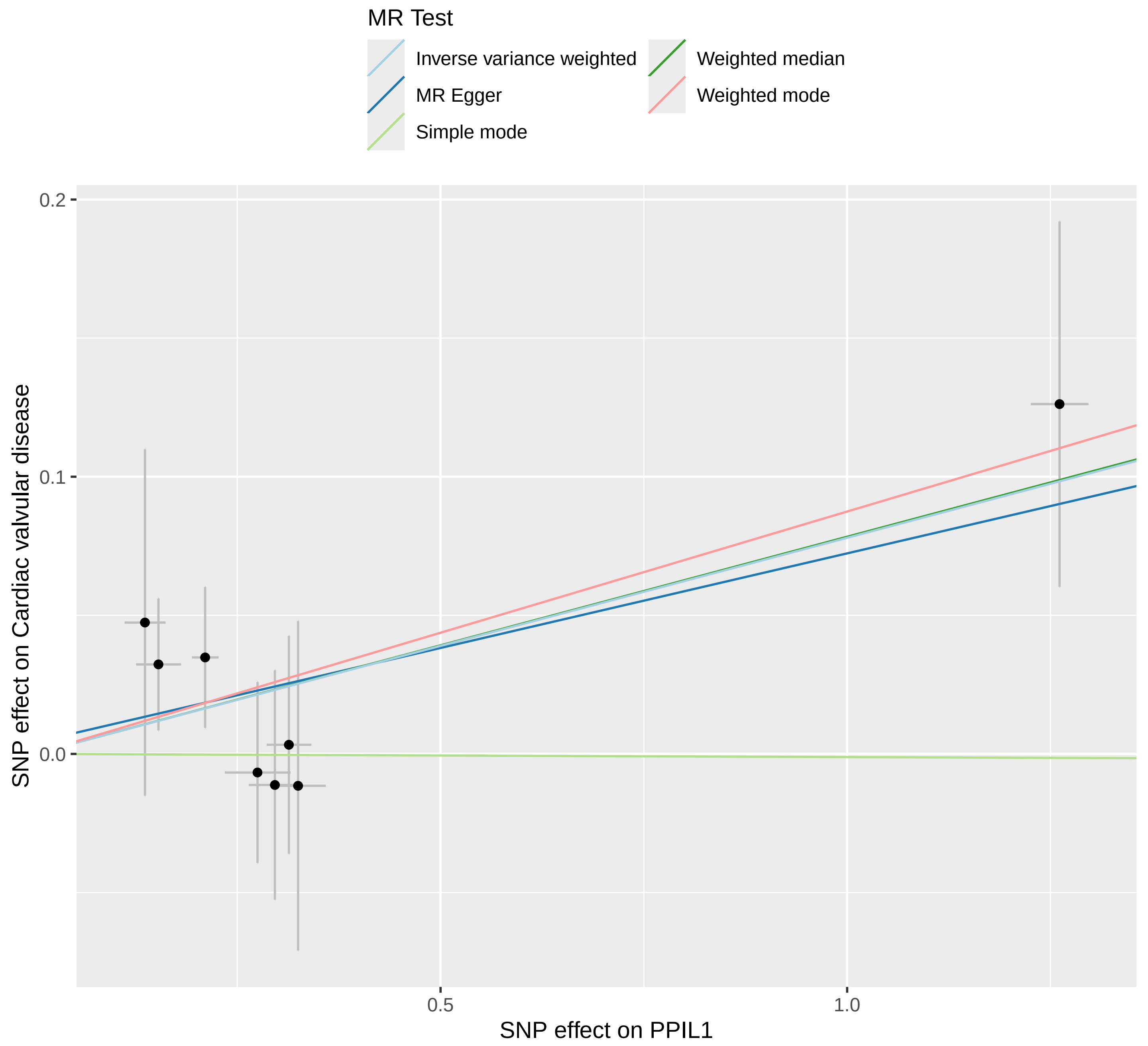

MR Test
Inverse variance weighted
MR Egger
Simple mode
Weighted median
Weighted mode
0.2
0.1
0.0
0.5
1.0
SNP effect on Cardiac valvular disease
SNP effect on PPIL1

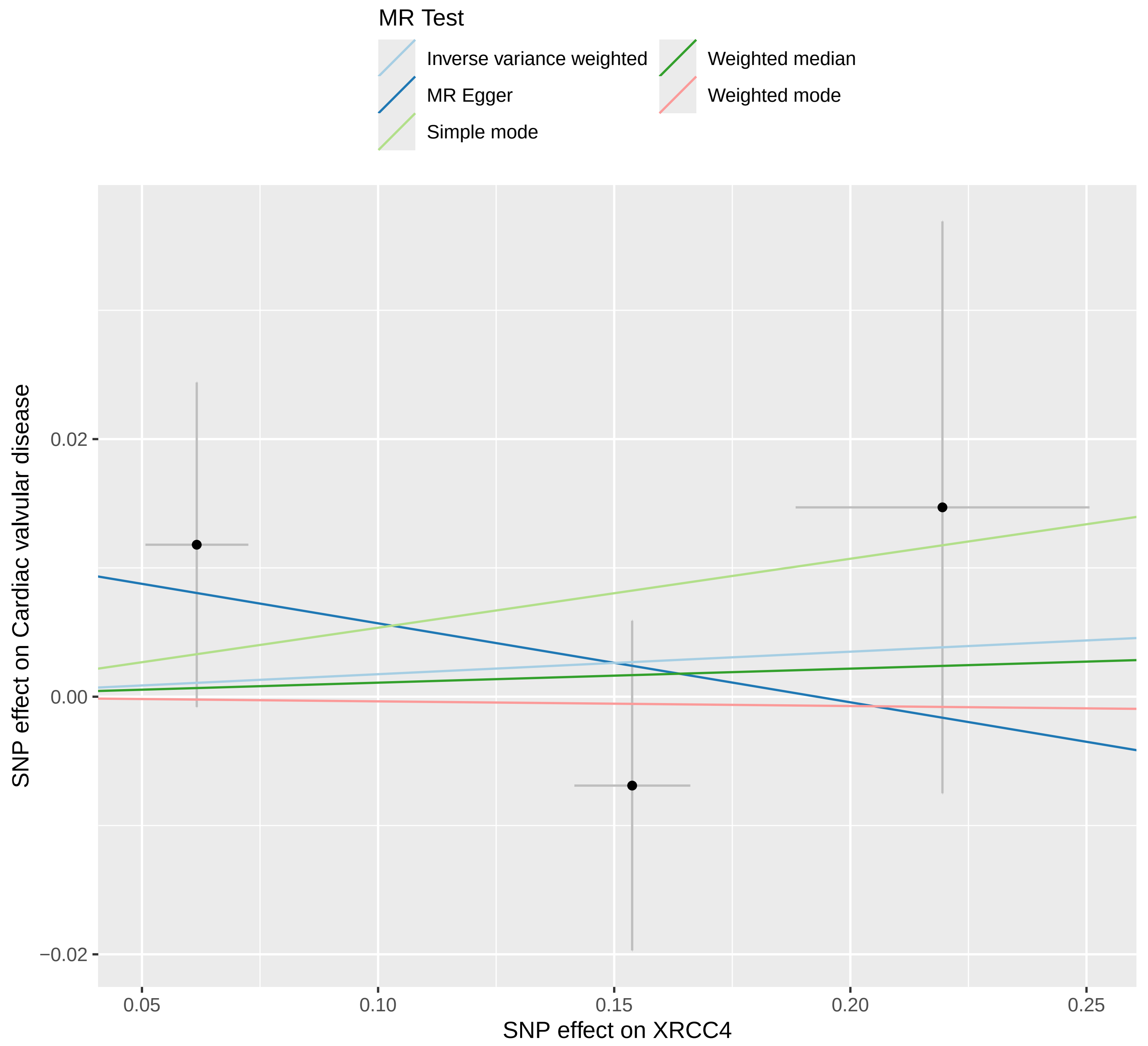
MR Test
Inverse variance weighted
MR Egger
Simple mode
Weighted median
Weighted mode
SNP effect on Cardiac valvular disease
0.02
0.00
−0.02
0.05
0.10
0.15
0.20
0.25
SNP effect on XRCC4

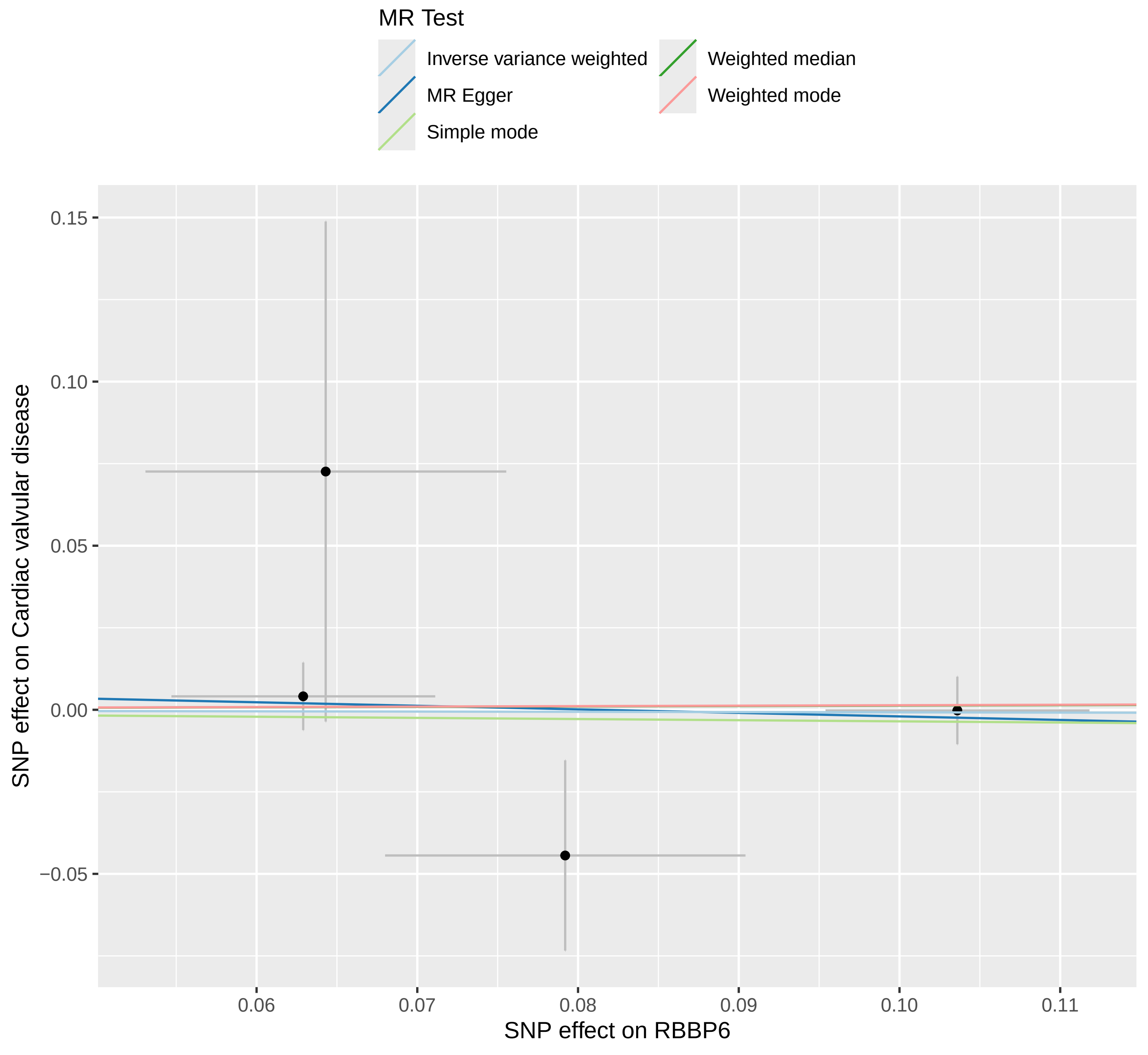
MR Test
Inverse variance weighted
MR Egger
Simple mode
Weighted median
Weighted mode
0.15
0.10
0.05
0.00
−0.05
0.06
0.07
0.08
0.09
0.10
0.11
SNP effect on Cardiac valvular disease
SNP effect on RBBP6

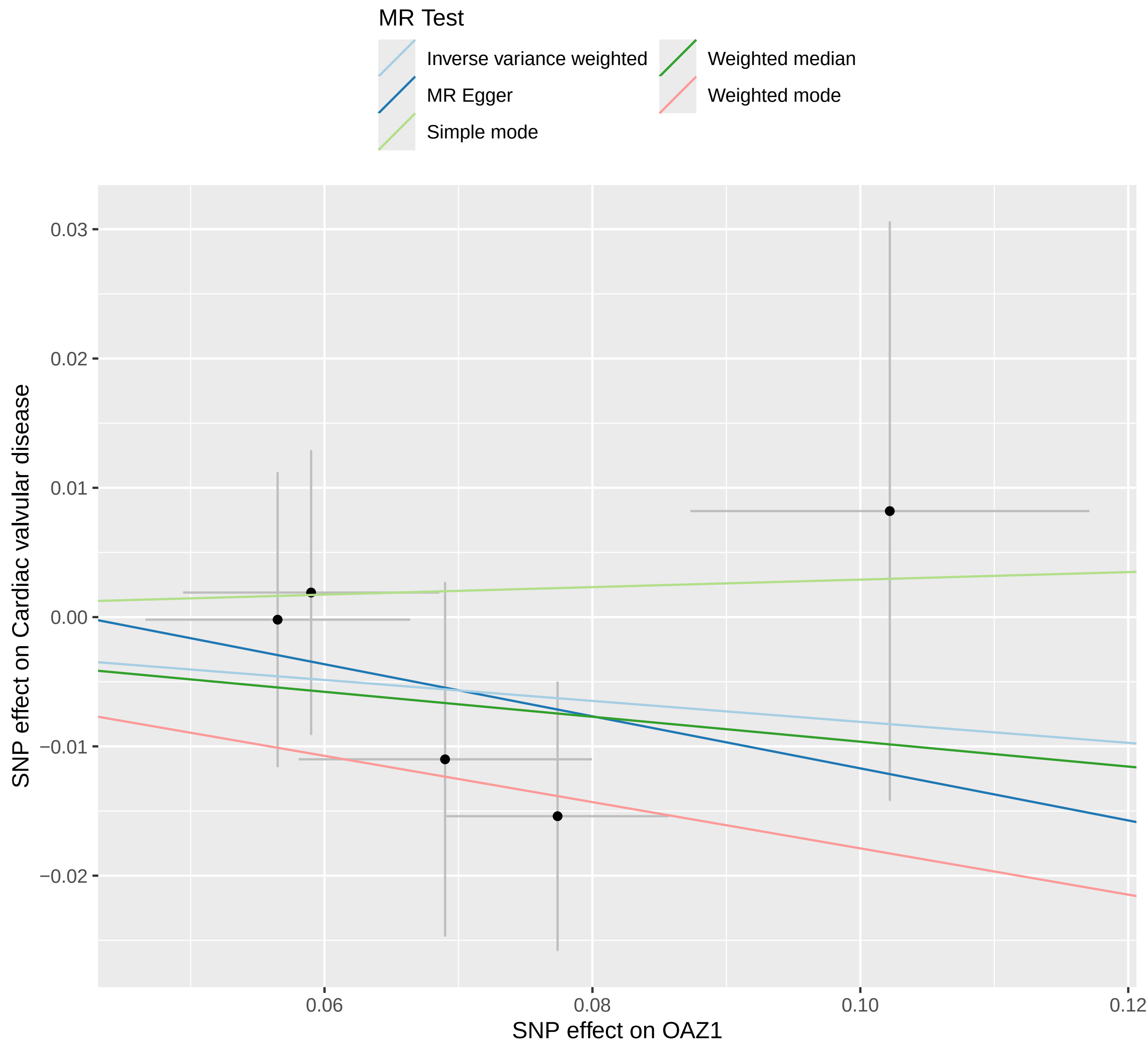
MR Test
Inverse variance weighted
MR Egger
Simple mode
Weighted median
Weighted mode
0.03
0.02
0.01
0.00
−0.01
−0.02
SNP effect on Cardiac valvular disease
0.06
0.08
0.10
0.12
SNP effect on OAZ1

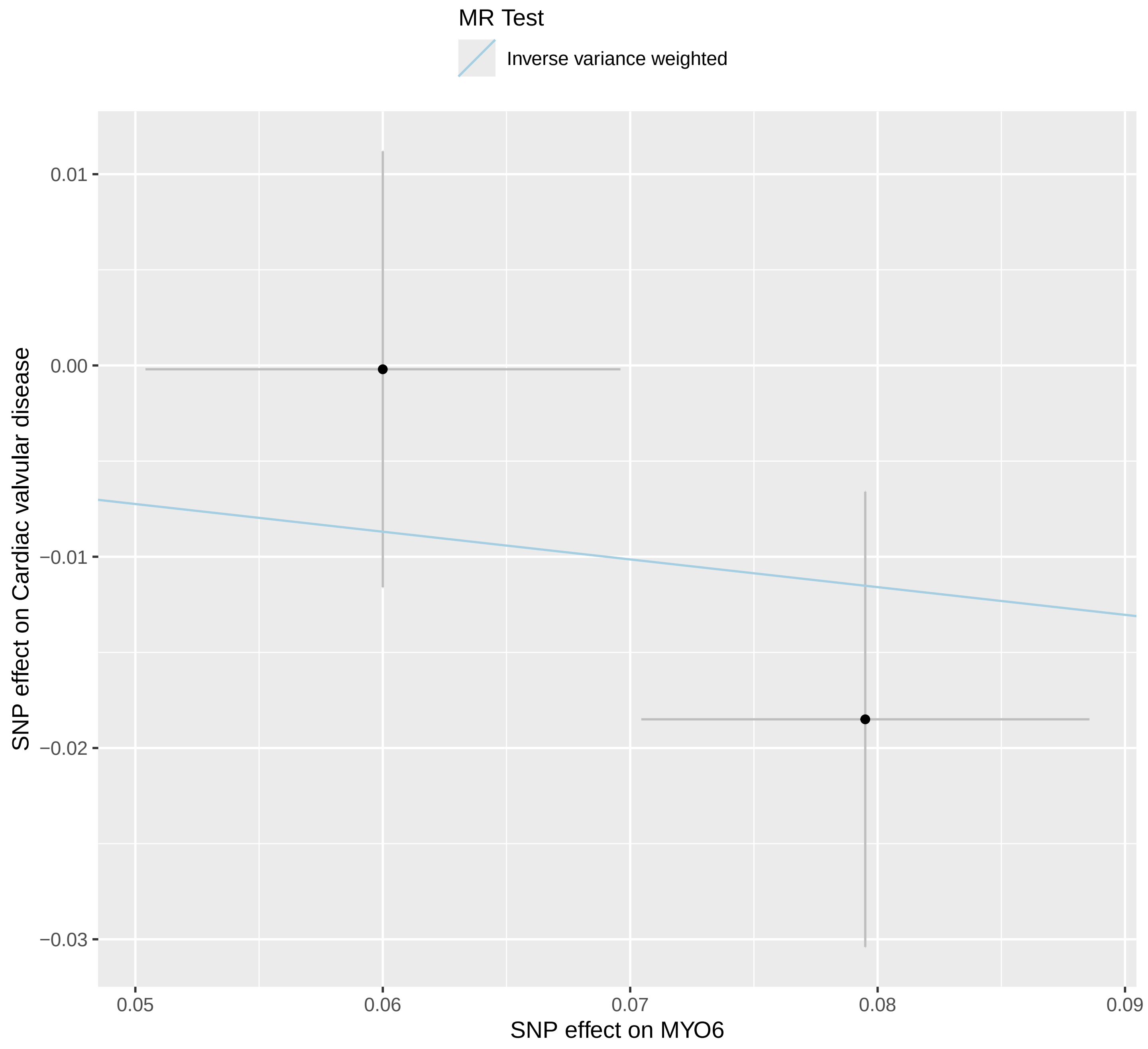
MR Test
Inverse variance weighted
0.01
0.00
−0.01
−0.02
−0.03
SNP effect on Cardiac valvular disease
0.05
0.06
0.07
0.08
0.09
SNP effect on MYO6

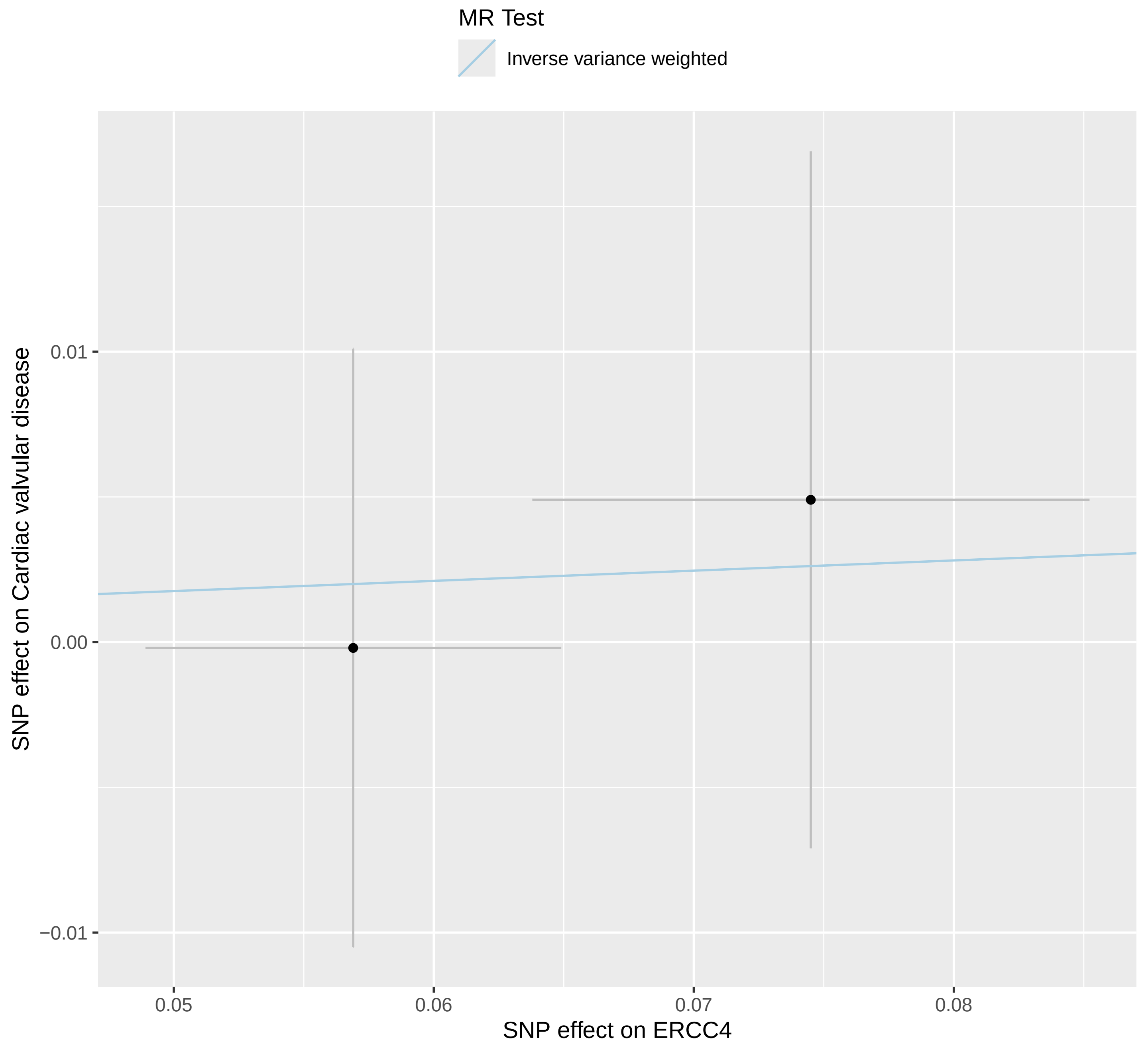
MR Test
Inverse variance weighted
SNP effect on Cardiac valvular disease
0.01
0.00
−0.01
0.05
0.06
0.07
0.08
SNP effect on ERCC4

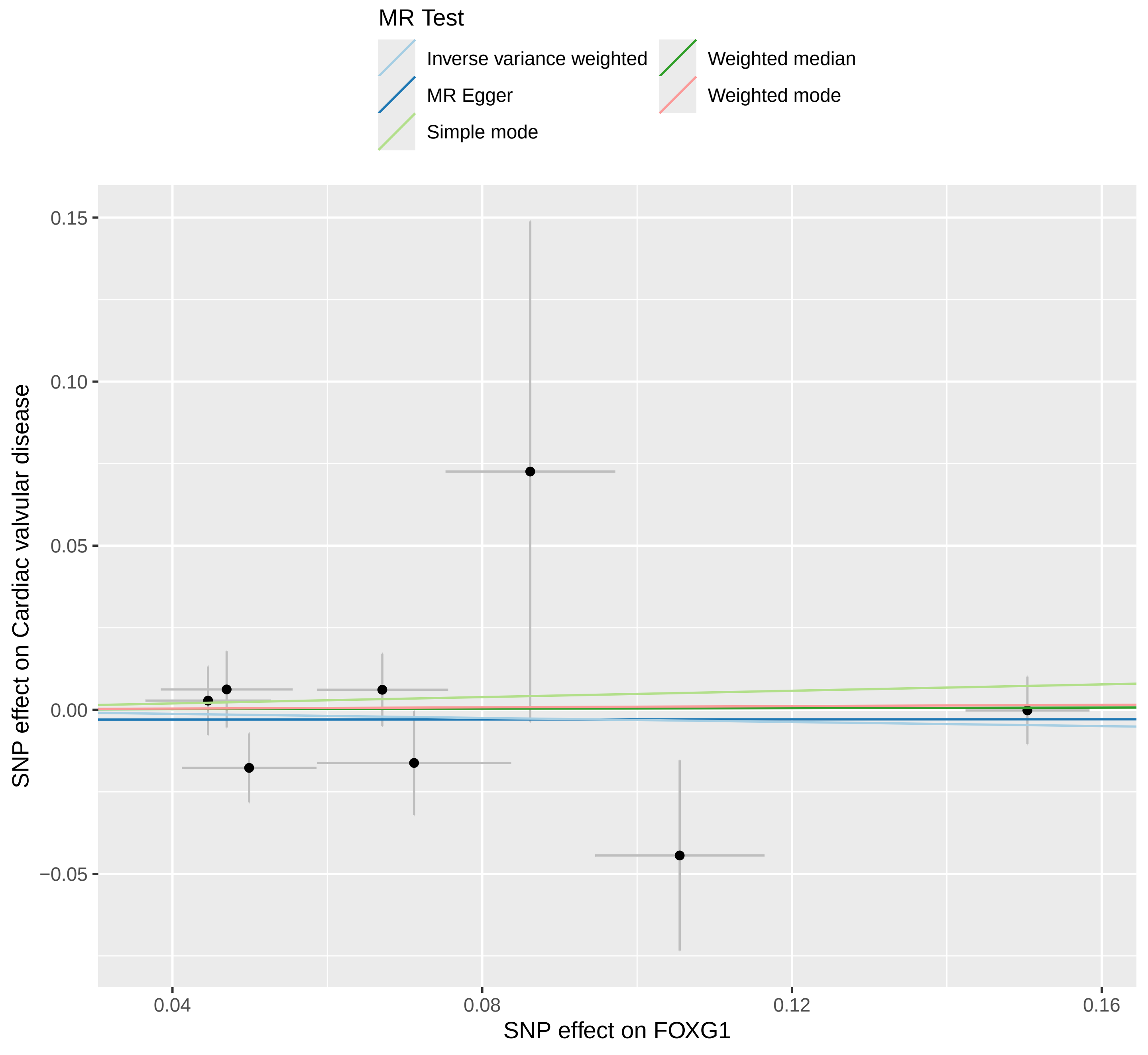
MR Test
Inverse variance weighted
MR Egger
Simple mode
Weighted median
Weighted mode
0.15
0.10
0.05
0.00
−0.05
0.04
0.08
0.12
0.16
SNP effect on Cardiac valvular disease
SNP effect on FOXG1

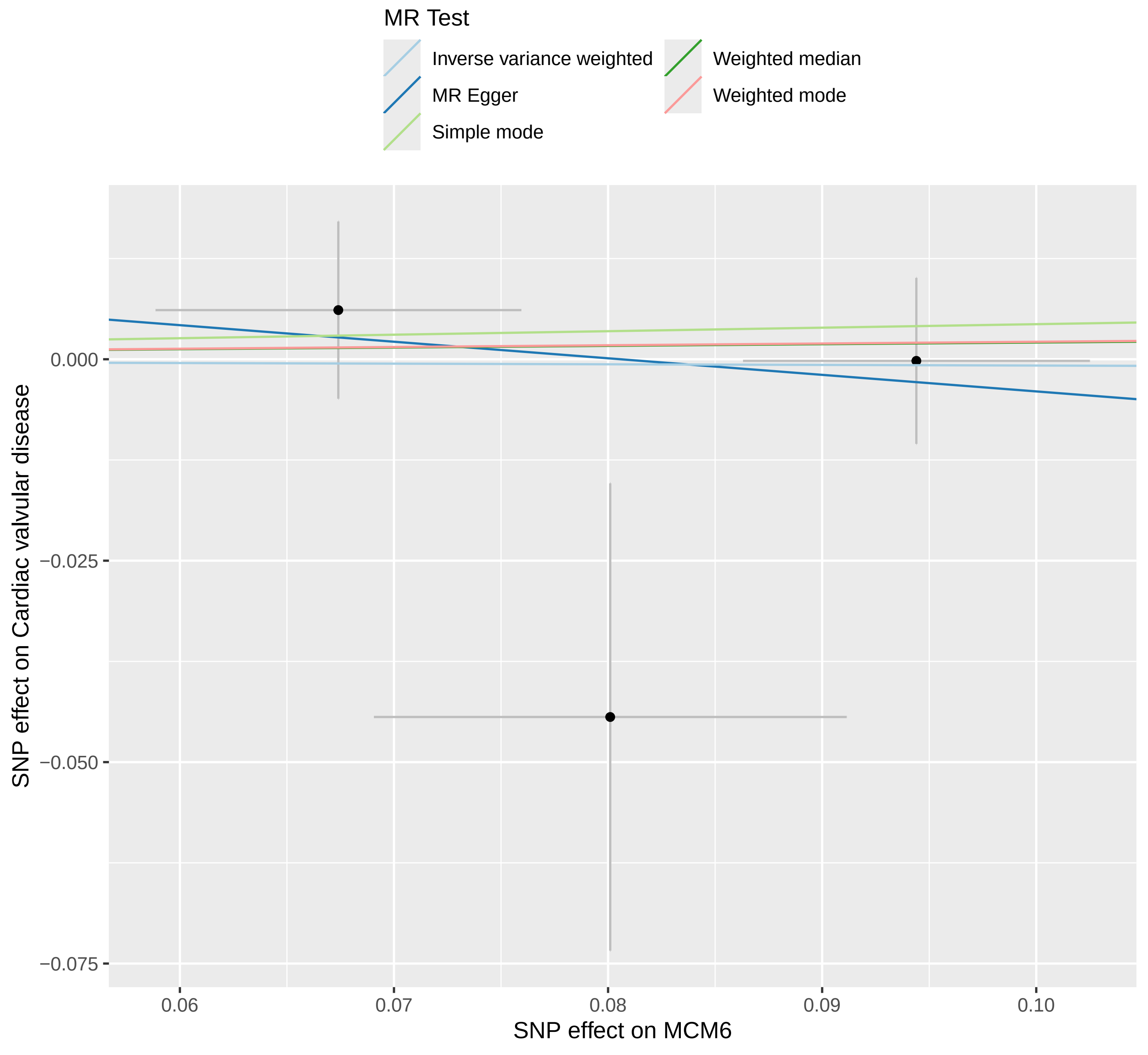

MR Test
Inverse variance weighted
MR Egger
Simple mode
Weighted median
Weighted mode
0.000
−0.025
−0.050
−0.075
SNP effect on Cardiac valvular disease
0.06
0.07
0.08
0.09
0.10
SNP effect on MCM6

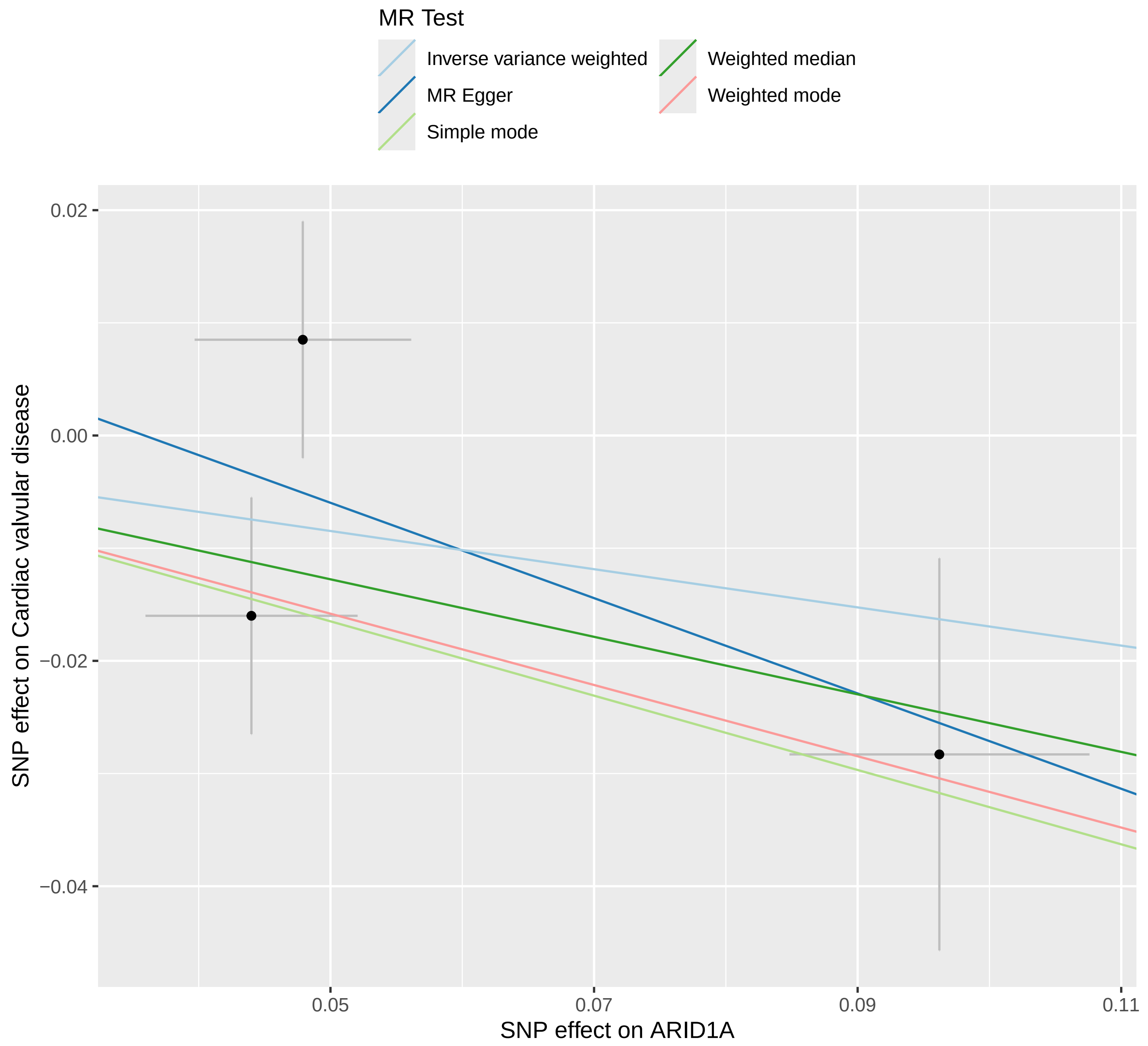

MR Test
Inverse variance weighted
MR Egger
Simple mode
Weighted median
Weighted mode
0.02
0.00
−0.02
−0.04
SNP effect on Cardiac valvular disease
0.05
0.07
0.09
0.11
SNP effect on ARID1A

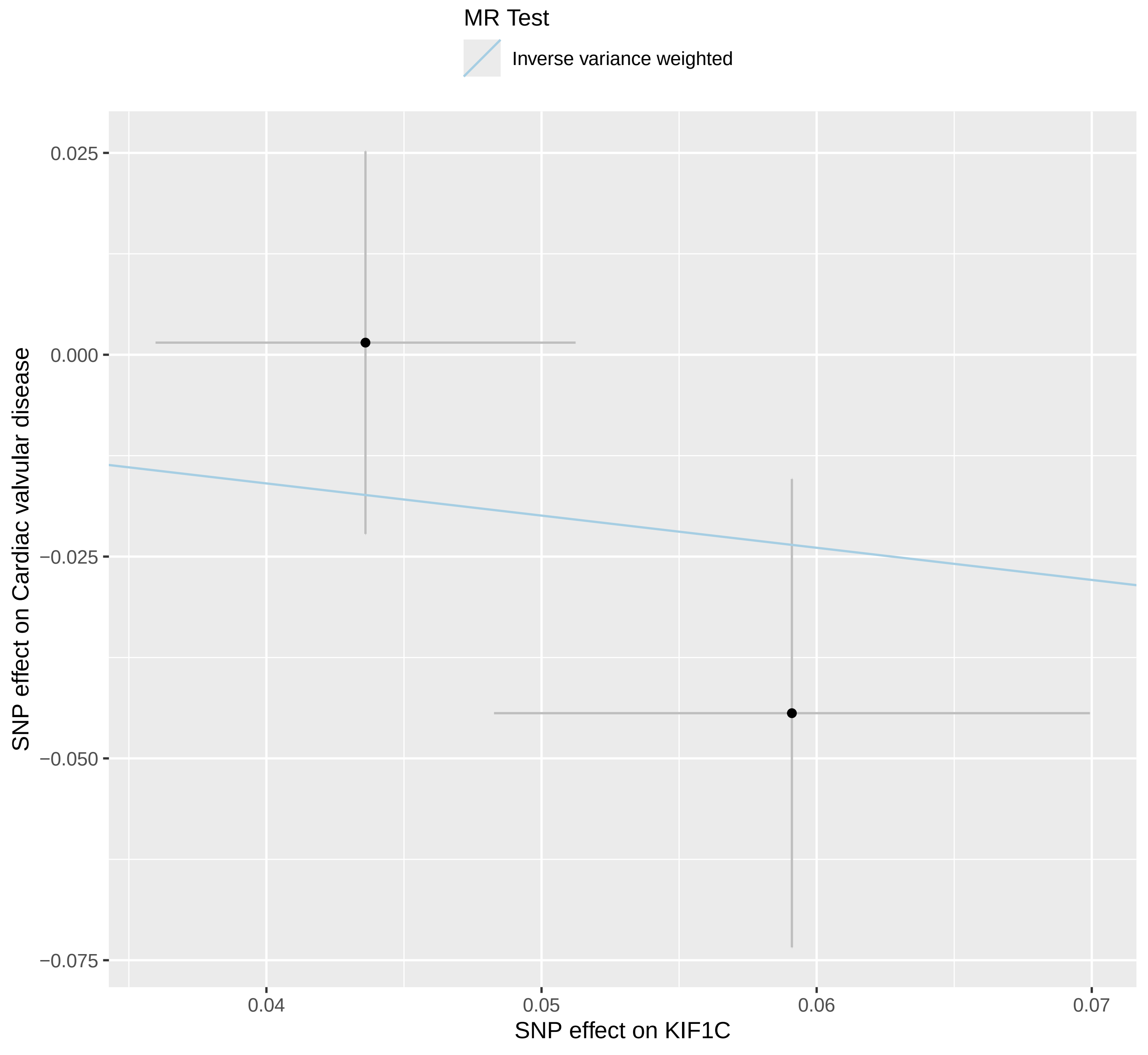
MR Test
Inverse variance weighted
0.025
0.000
−0.025
−0.050
−0.075
0.04
0.05
0.06
0.07
SNP effect on KIF1C
SNP effect on Cardiac valvular disease

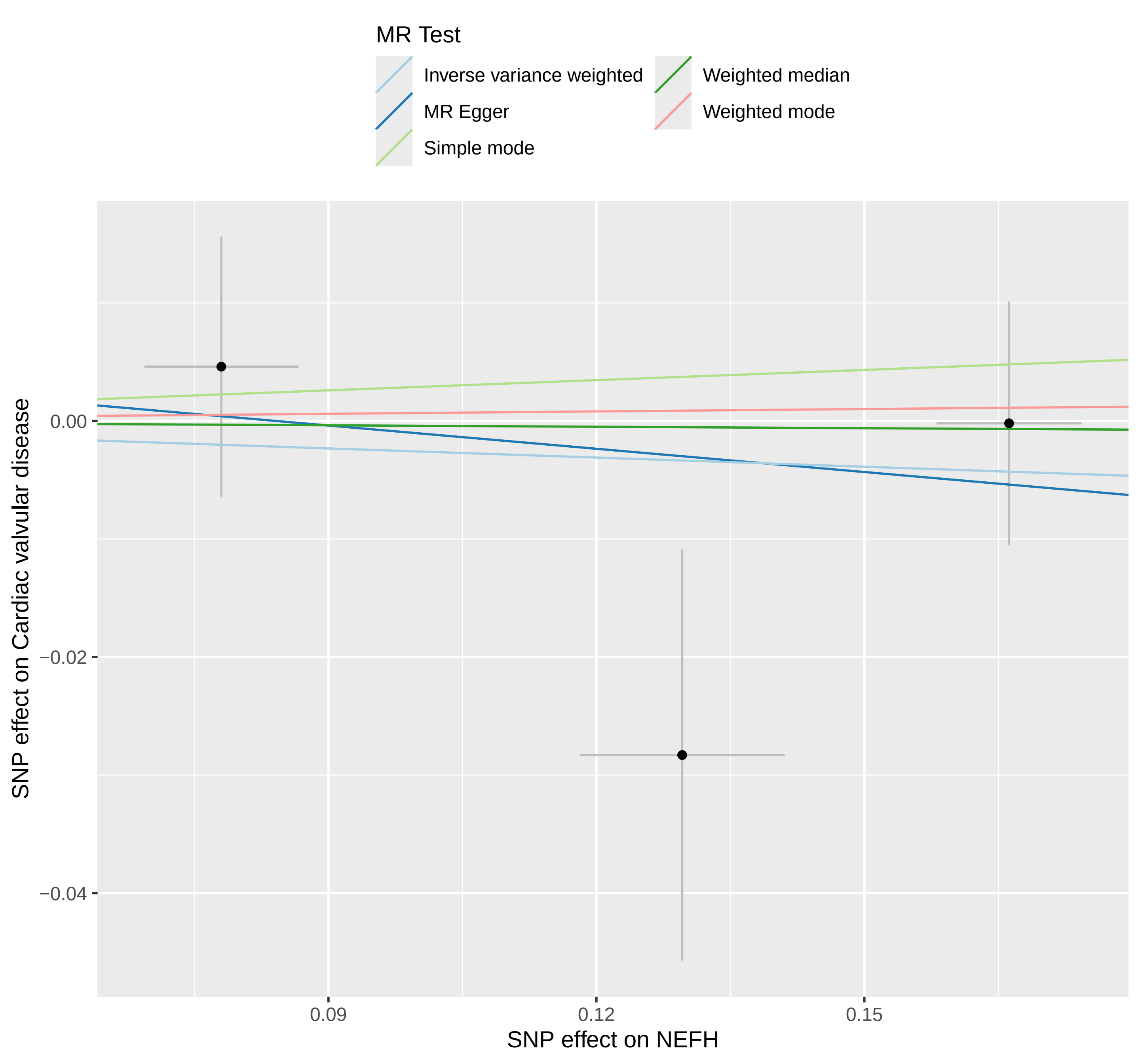
MR Test
Inverse variance weighted
MR Egger
Simple mode
Weighted median
Weighted mode
0.00
−0.02
−0.04
0.09
0.12
0.15
SNP effect on NEFH
SNP effect on Cardiac valvular disease

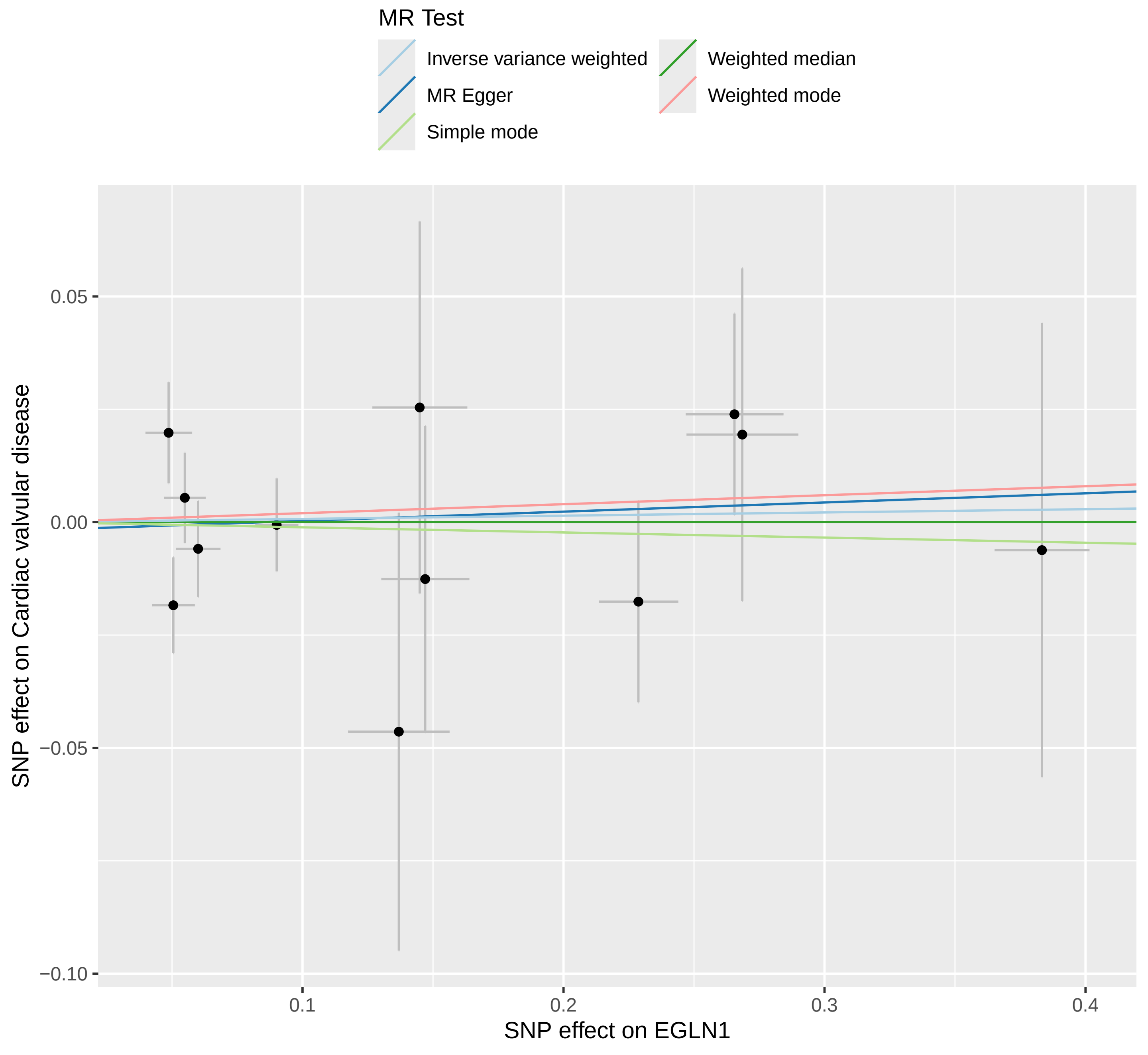

MR Test
Inverse variance weighted
MR Egger
Simple mode
Weighted median
Weighted mode
0.05
0.00
−0.05
−0.10
0.1
0.2
0.3
0.4
SNP effect on Cardiac valvular disease
SNP effect on EGLN1

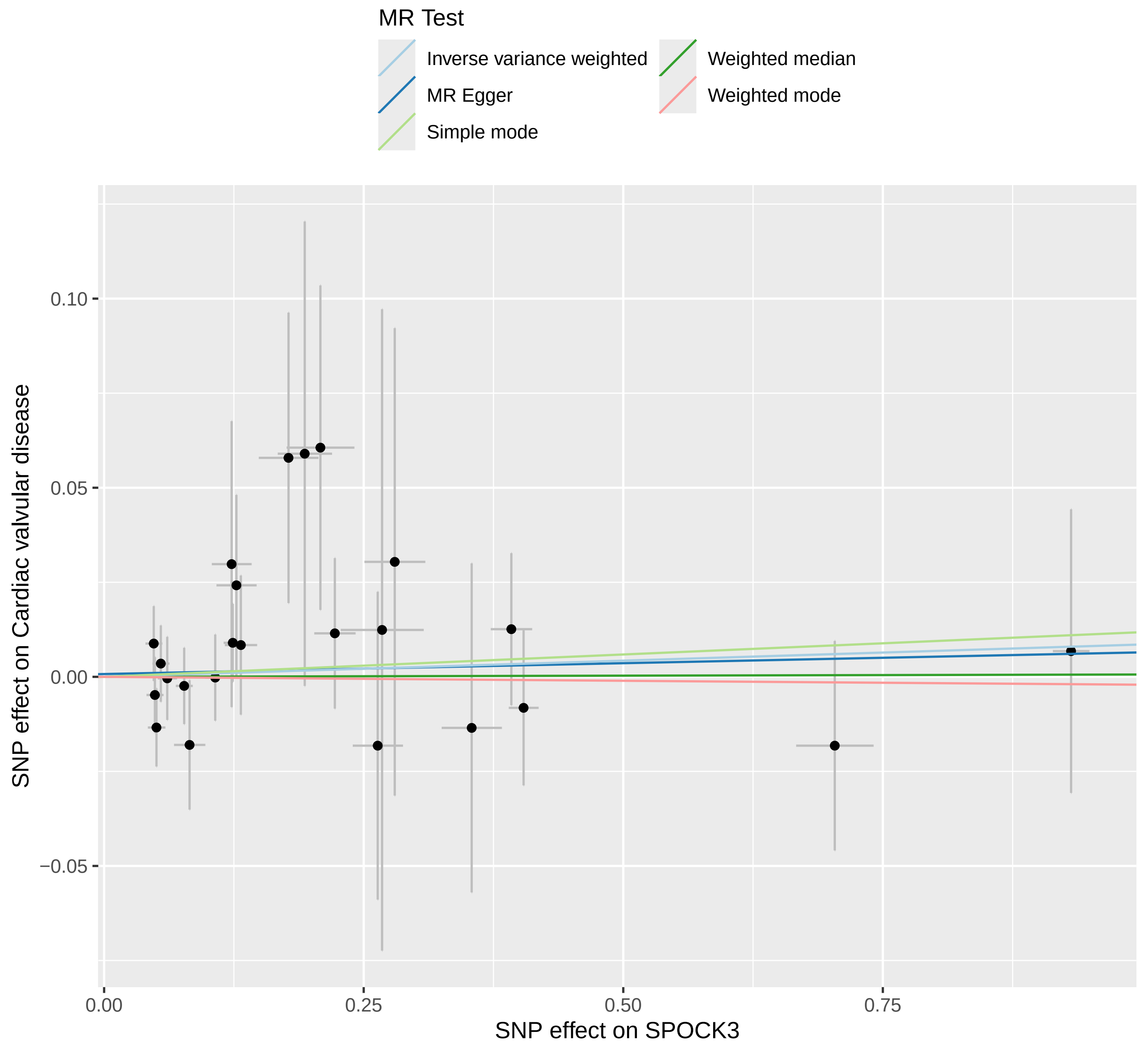
MR Test
Inverse variance weighted
MR Egger
Simple mode
Weighted median
Weighted mode
0.10
0.05
0.00
−0.05
0.00
0.25
0.50
0.75
SNP effect on Cardiac valvular disease
SNP effect on SPOCK3

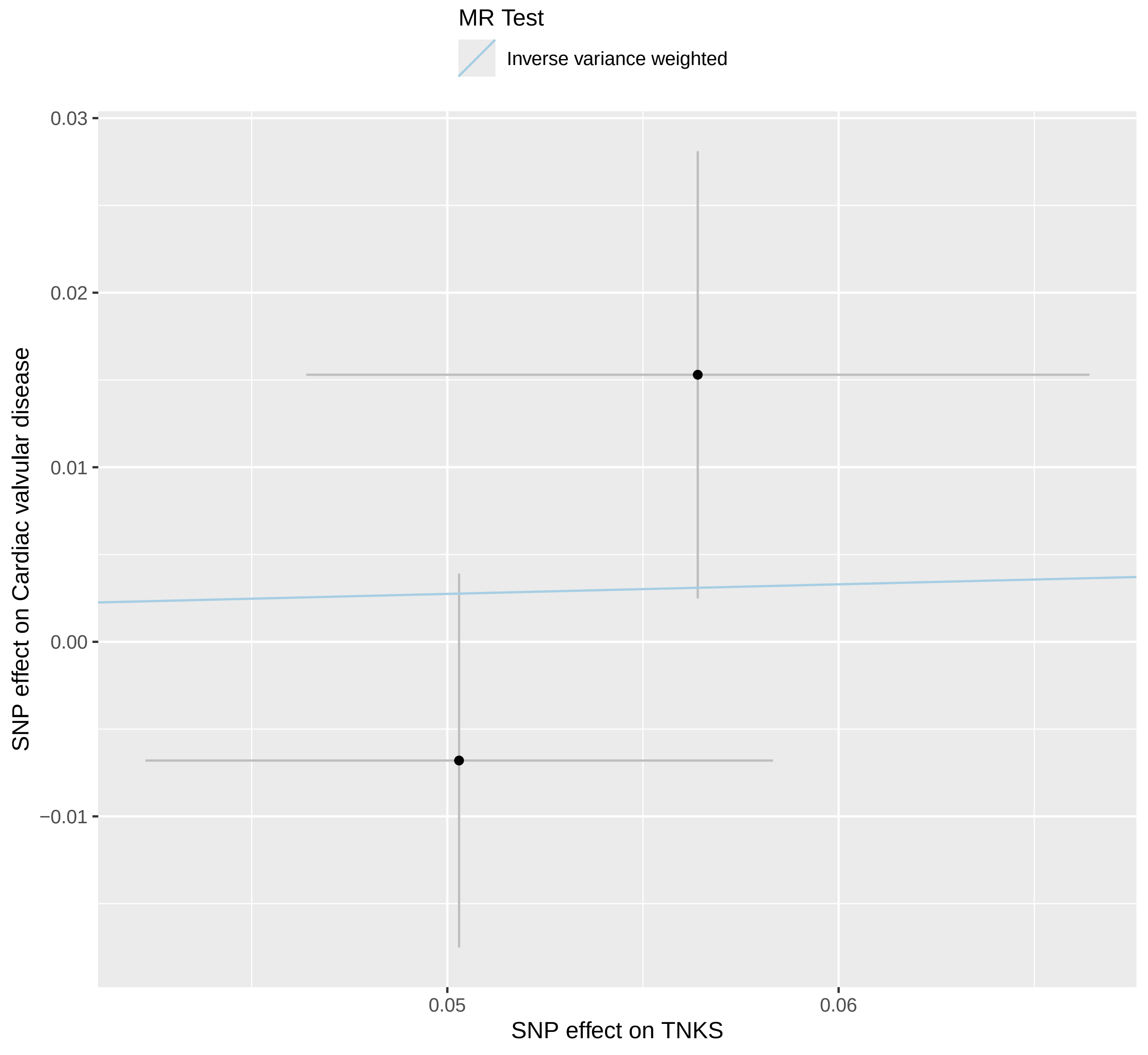

MR Test
Inverse variance weighted
0.03
0.02
0.01
0.00
−0.01
0.05
0.06
SNP effect on Cardiac valvular disease
SNP effect on TNKS

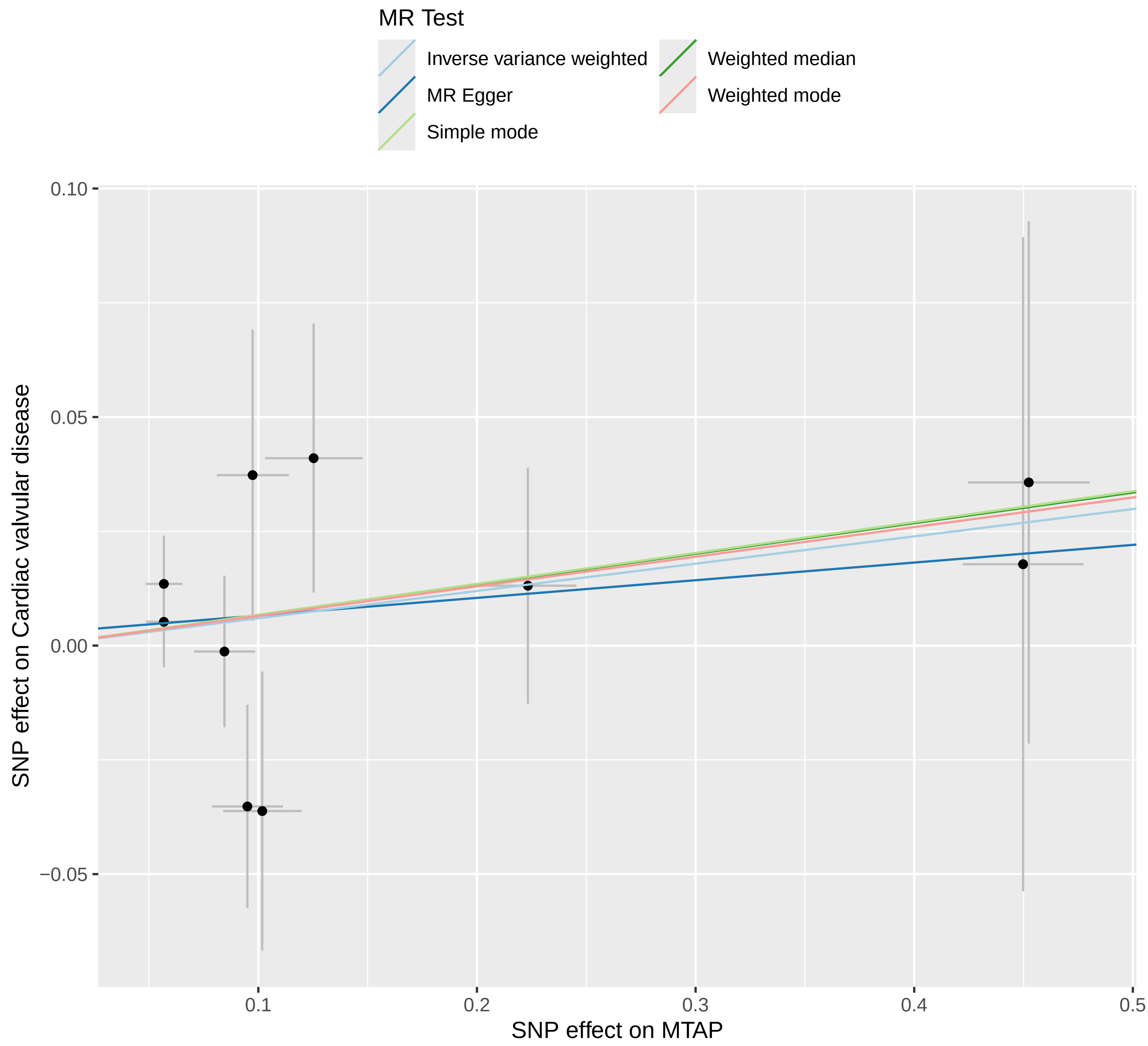
MR Test
Inverse variance weighted
MR Egger
Simple mode
Weighted median
Weighted mode
0.10
0.05
0.00
−0.05
0.1
0.2
0.3
0.4
0.5
SNP effect on MTAP
SNP effect on Cardiac valvular disease

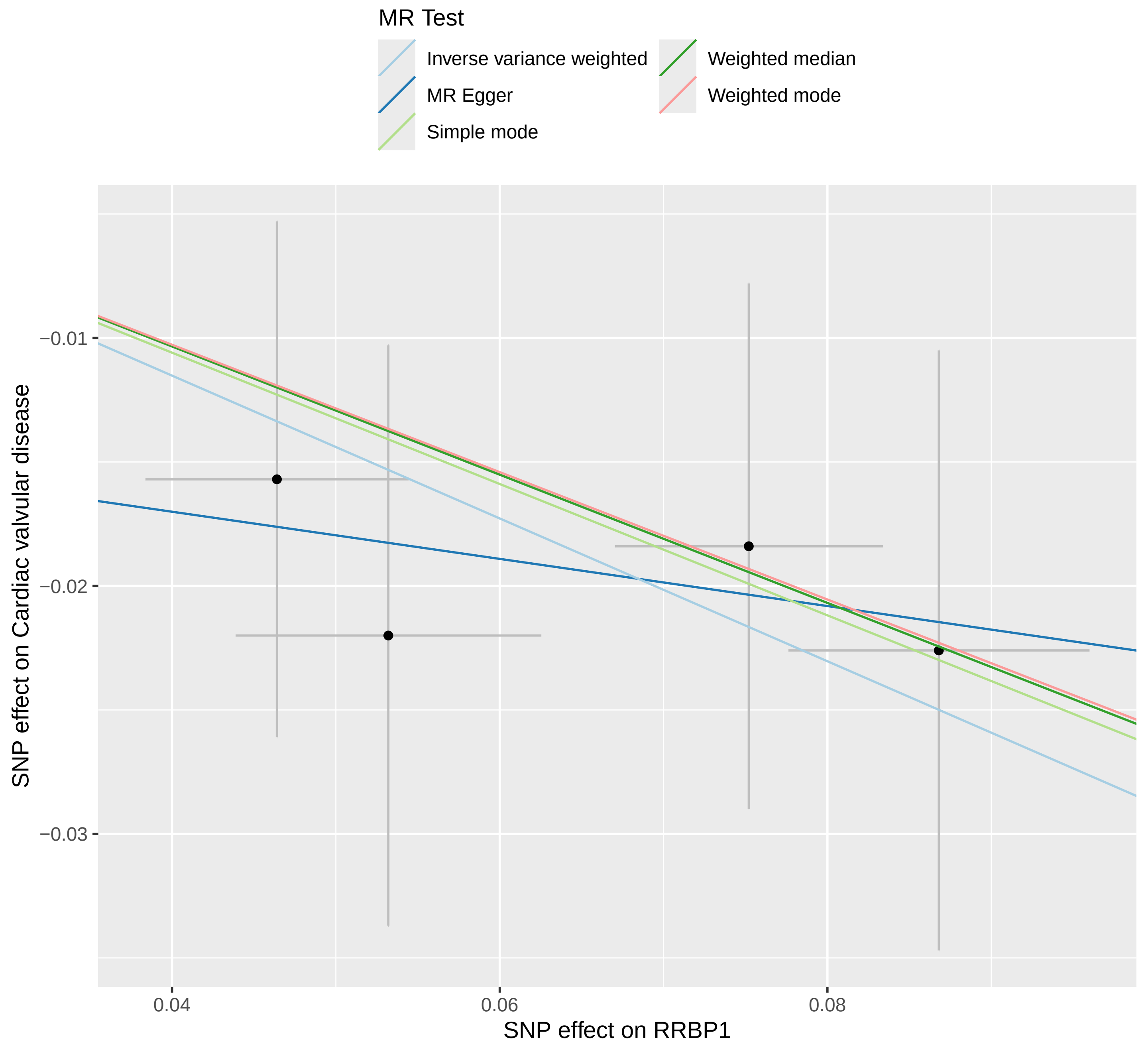
MR Test
Inverse variance weighted
MR Egger
Simple mode
Weighted median
Weighted mode
−0.01
−0.02
−0.03
0.04
0.06
0.08
SNP effect on RRBP1
SNP effect on Cardiac valvular disease

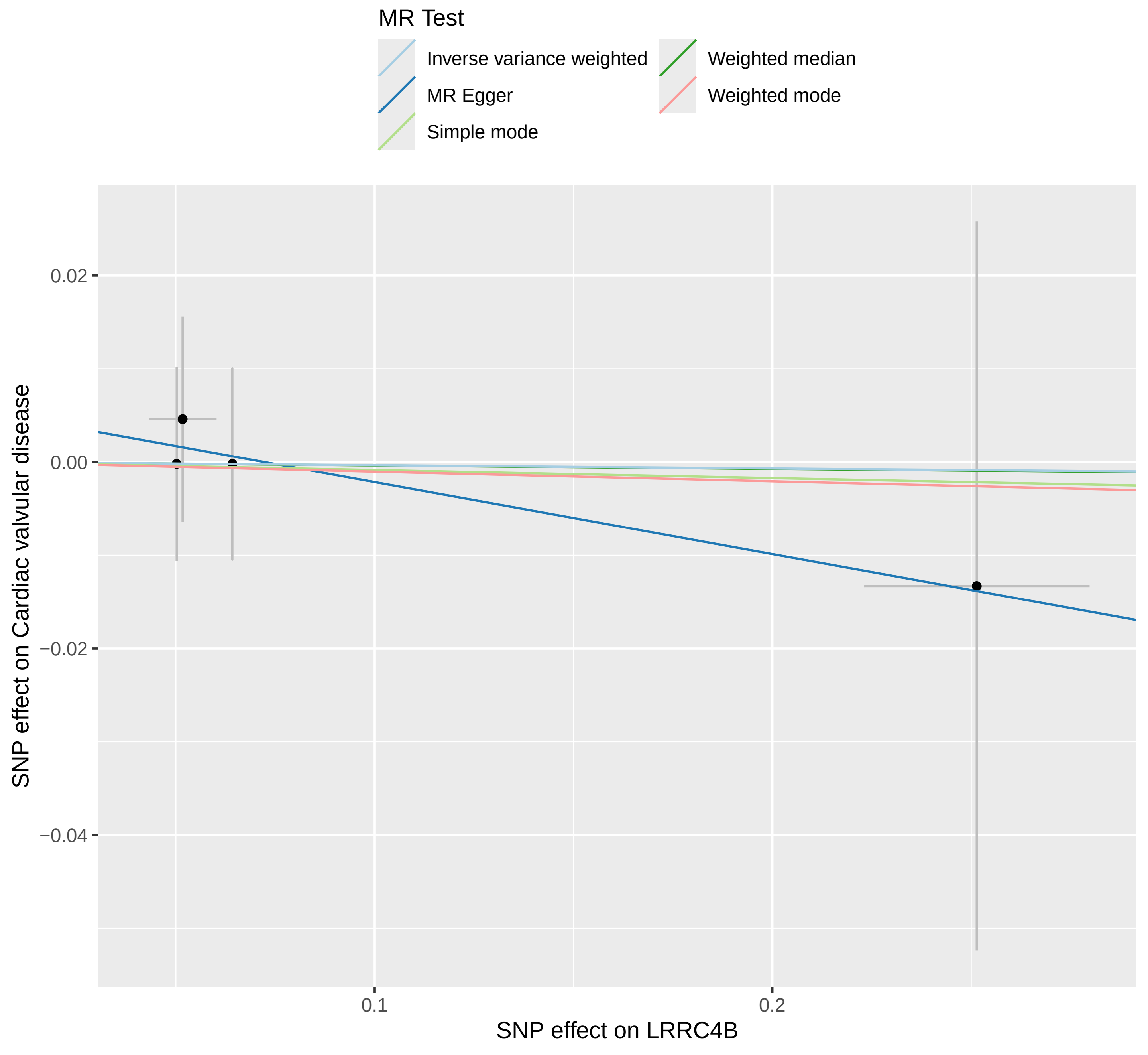
MR Test
Inverse variance weighted
MR Egger
Simple mode
Weighted median
Weighted mode
0.02
0.00
−0.02
−0.04
0.1
0.2
SNP effect on Cardiac valvular disease
SNP effect on LRRC4B

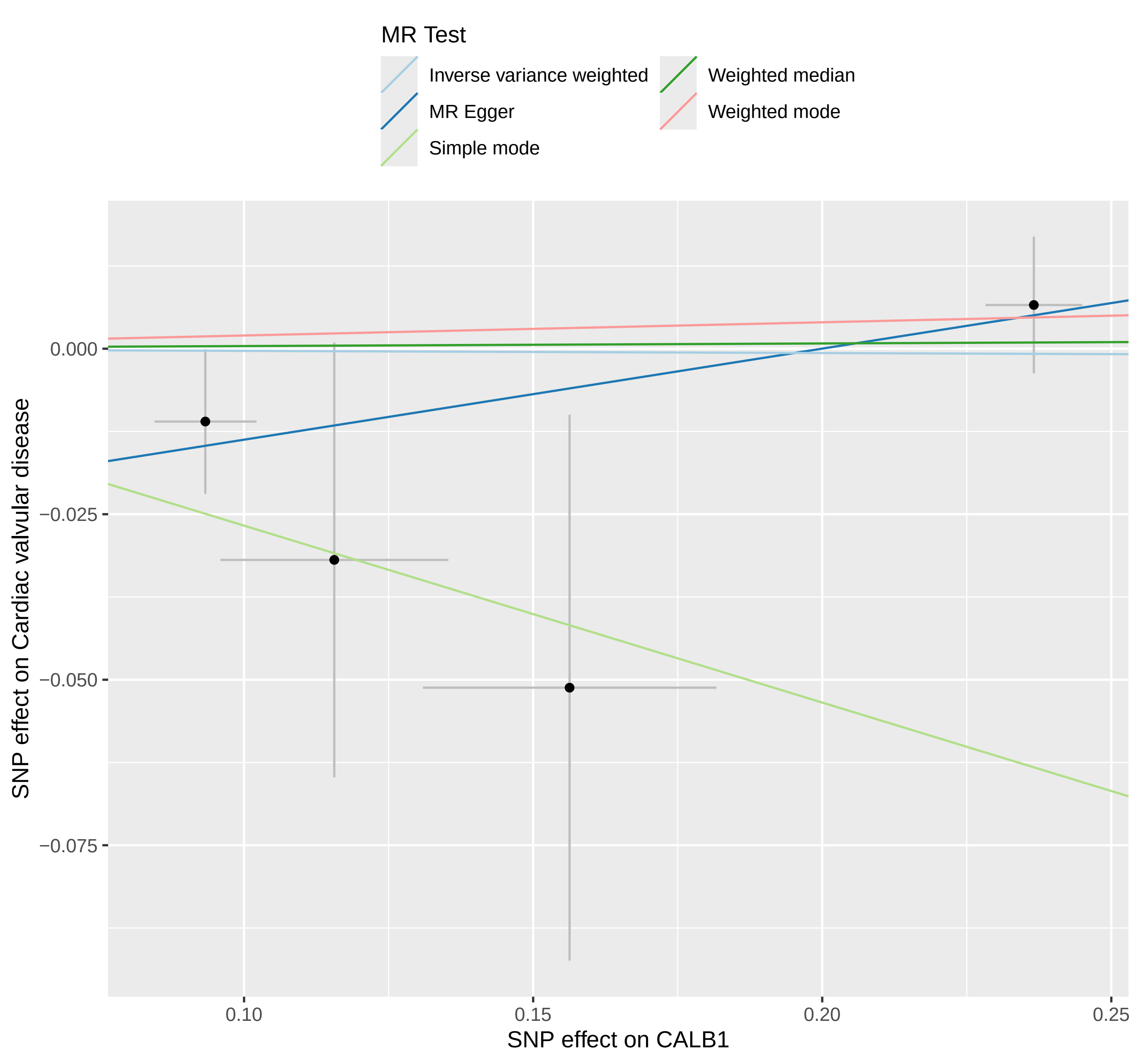

MR Test
Inverse variance weighted
MR Egger
Simple mode
Weighted median
Weighted mode
0.000
−0.025
−0.050
−0.075
0.10
0.15
0.20
0.25
SNP effect on Cardiac valvular disease
SNP effect on CALB1

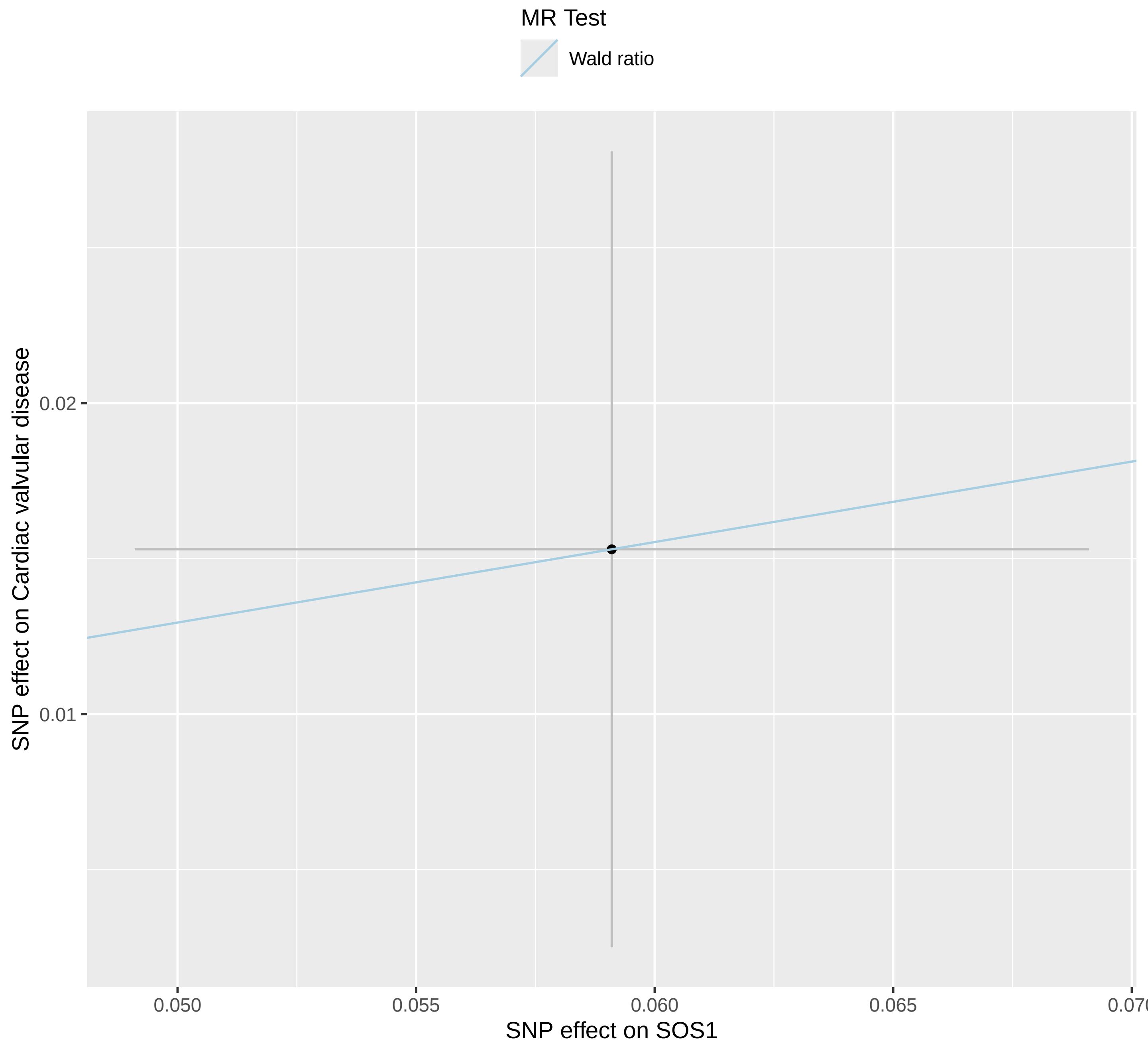
MR Test
Wald ratio
SNP effect on Cardiac valvular disease
0.02
0.01
0.050
0.055
0.060
0.065
SNP effect on SOS1

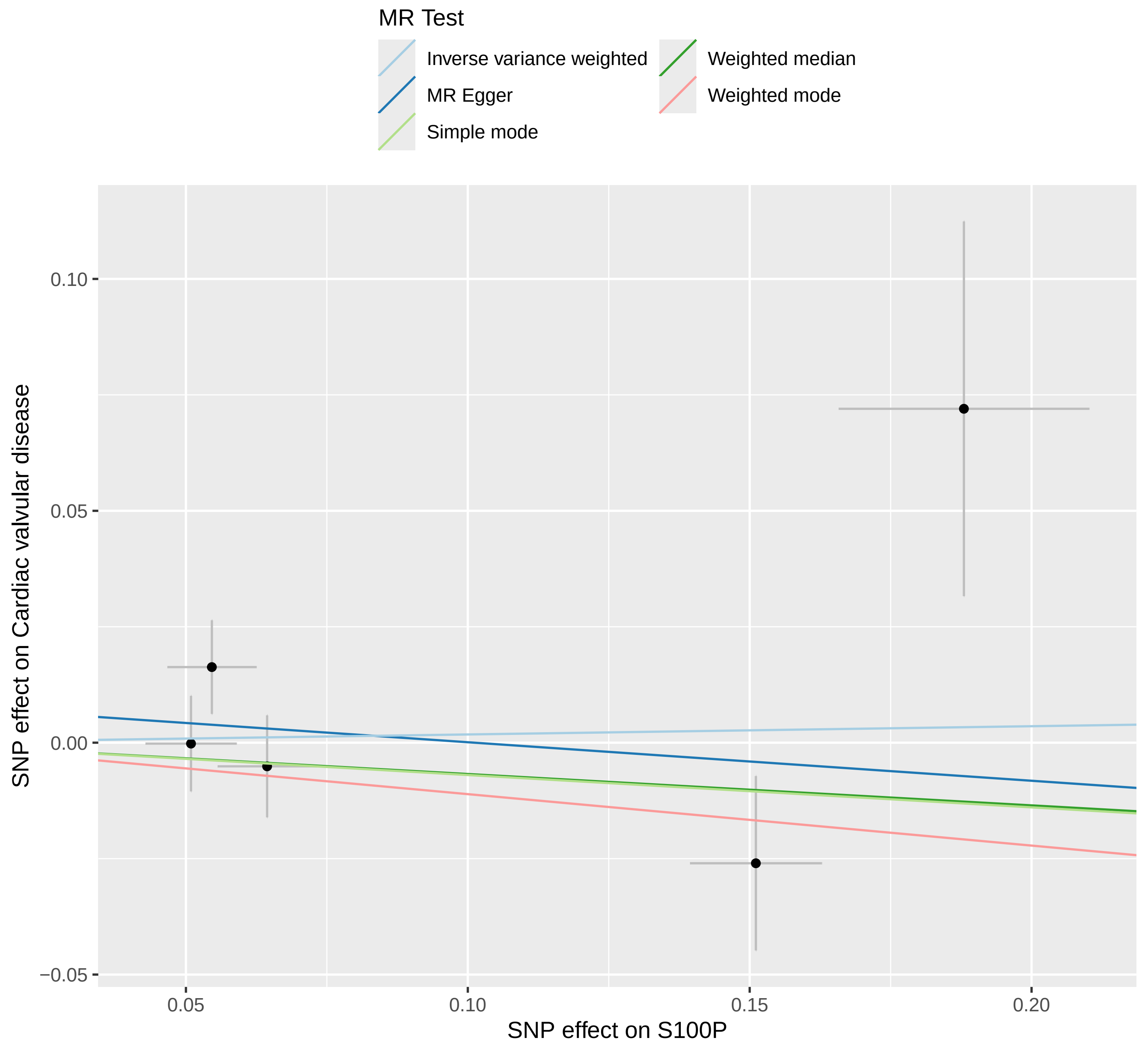
MR Test
Inverse variance weighted
MR Egger
Simple mode
Weighted median
Weighted mode
0.10
0.05
0.00
−0.05
0.05
0.10
0.15
0.20
SNP effect on Cardiac valvular disease
SNP effect on S100P

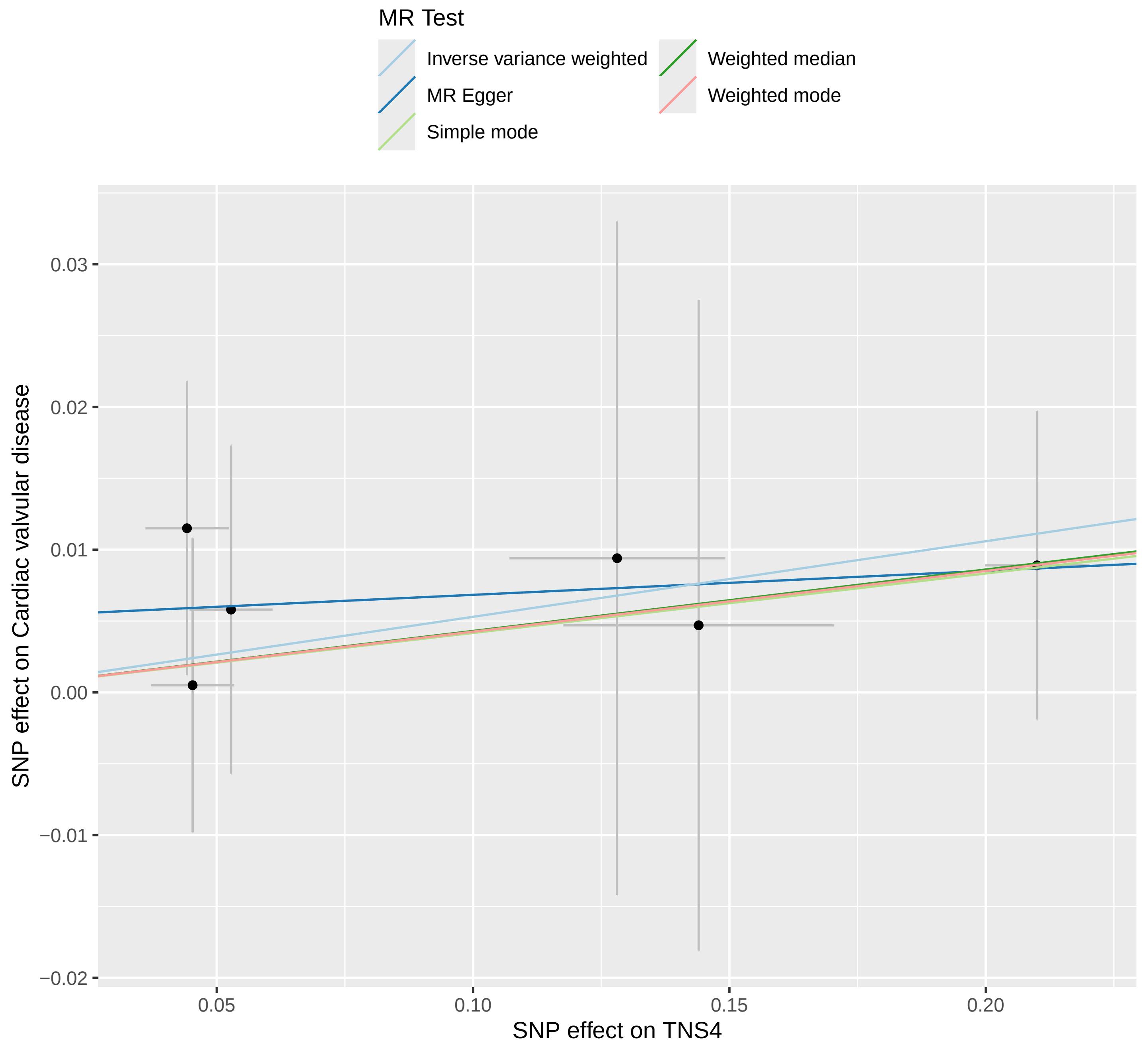

MR Test
Inverse variance weighted
MR Egger
Simple mode
Weighted median
Weighted mode
0.03
0.02
0.01
0.00
−0.01
−0.02
0.05
0.10
0.15
0.20
SNP effect on Cardiac valvular disease
SNP effect on TNS4

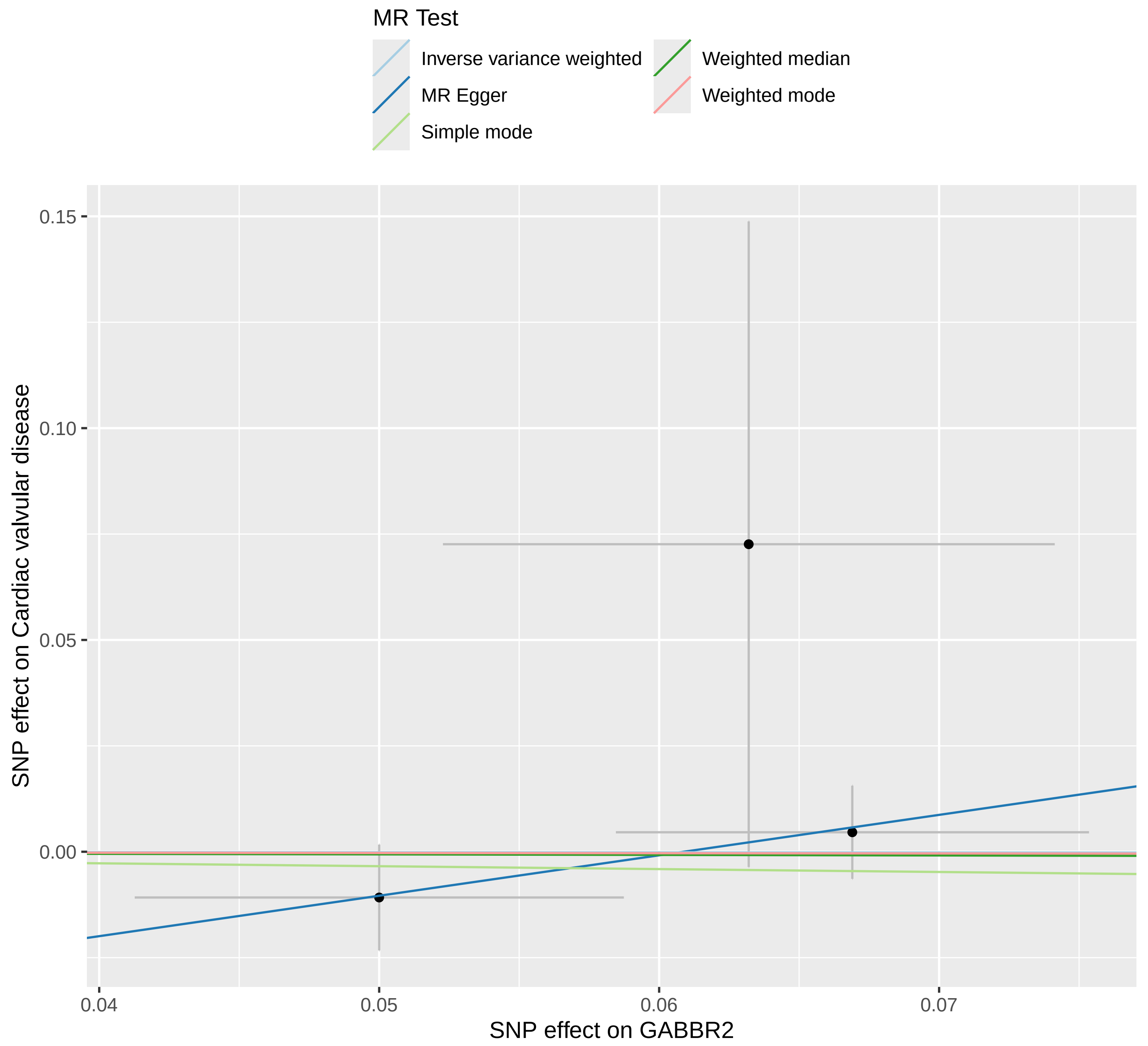

MR Test
Inverse variance weighted
MR Egger
Simple mode
Weighted median
Weighted mode
0.15
0.10
0.05
0.00
SNP effect on Cardiac valvular disease
0.04
0.05
0.06
0.07
SNP effect on GABBR2

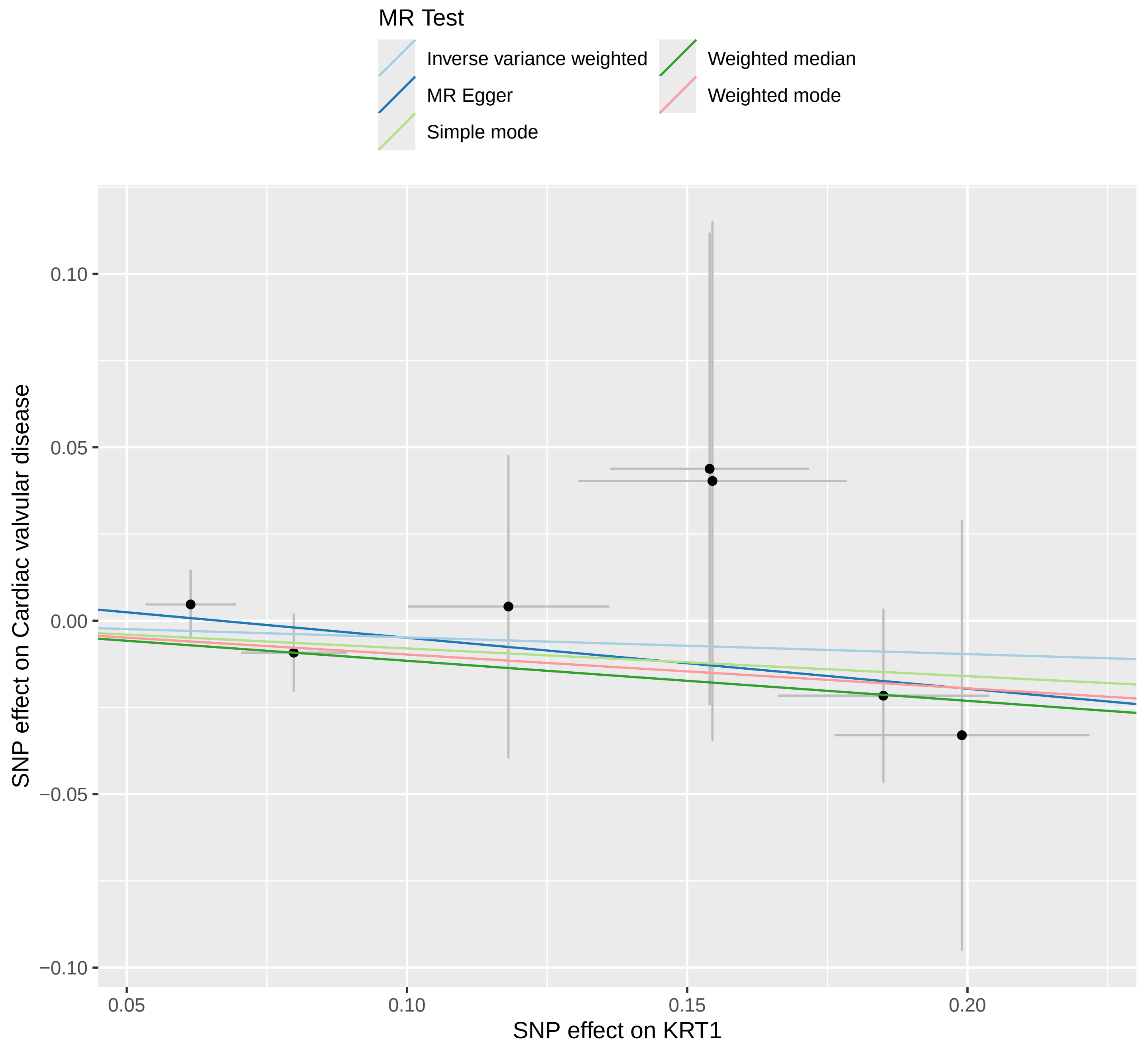
MR Test
Inverse variance weighted
MR Egger
Simple mode
Weighted median
Weighted mode
SNP effect on Cardiac valvular disease
0.10
0.05
0.00
−0.05
−0.10
0.05
0.10
0.15
0.20
SNP effect on KRT1

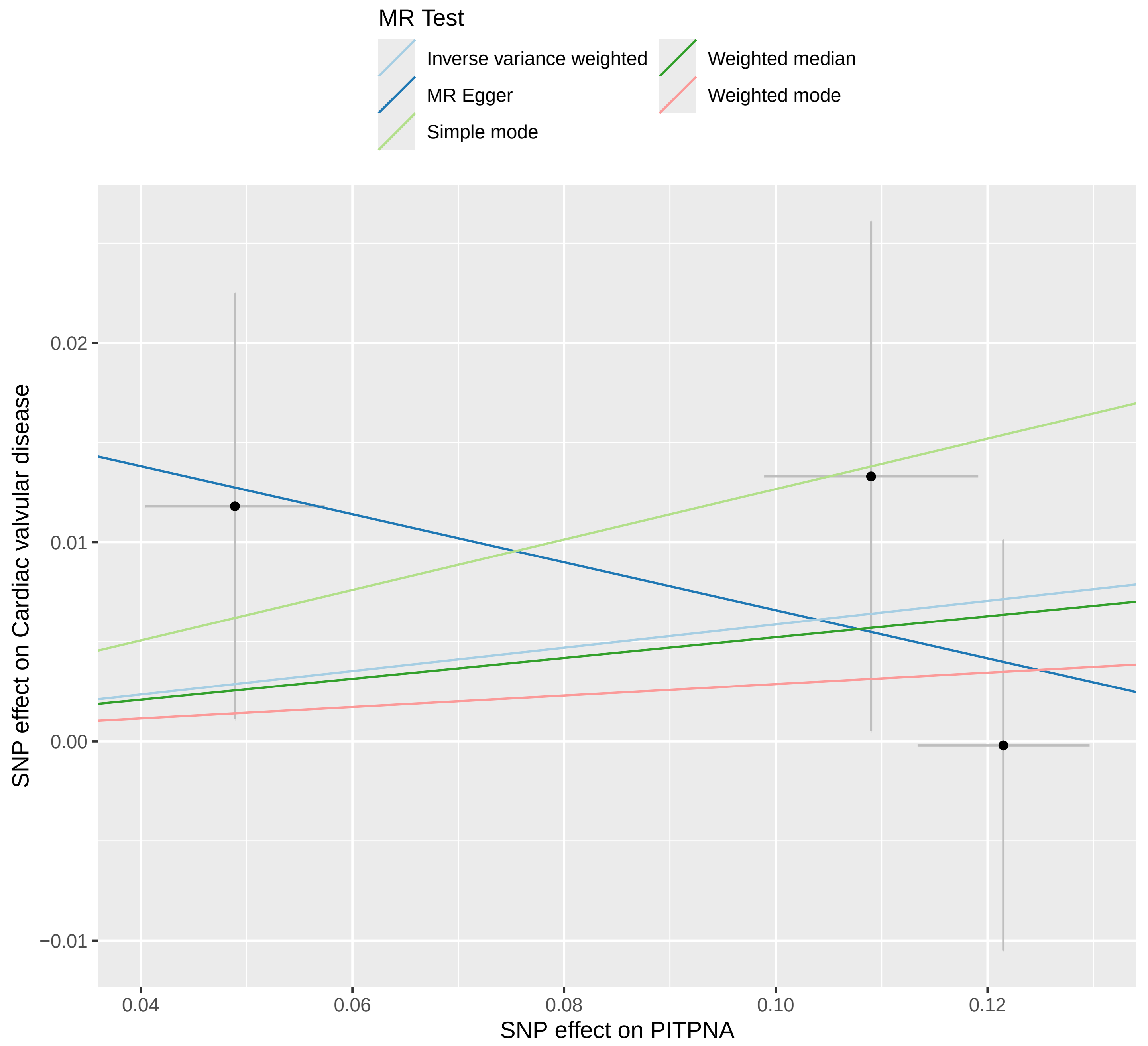
MR Test
Inverse variance weighted
MR Egger
Simple mode
Weighted median
Weighted mode
SNP effect on Cardiac valvular disease
0.02
0.01
0.00
−0.01
0.04
0.06
0.08
0.10
0.12
SNP effect on PITPNA

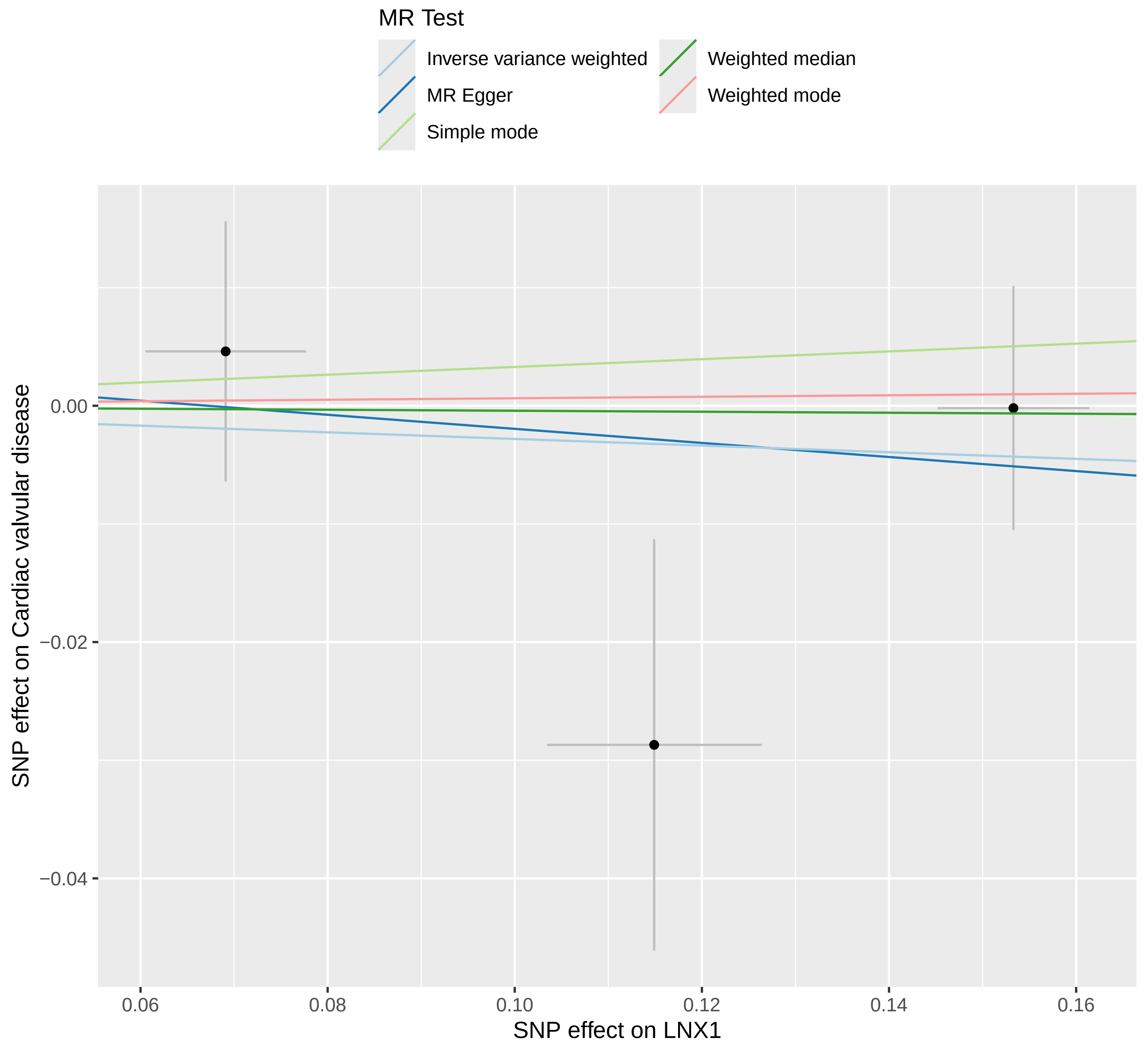
MR Test
Inverse variance weighted
MR Egger
Simple mode
Weighted median
Weighted mode
SNP effect on Cardiac valvular disease
0.00
−0.02
−0.04
0.06
0.08
0.10
0.12
0.14
0.16
SNP effect on LNX1

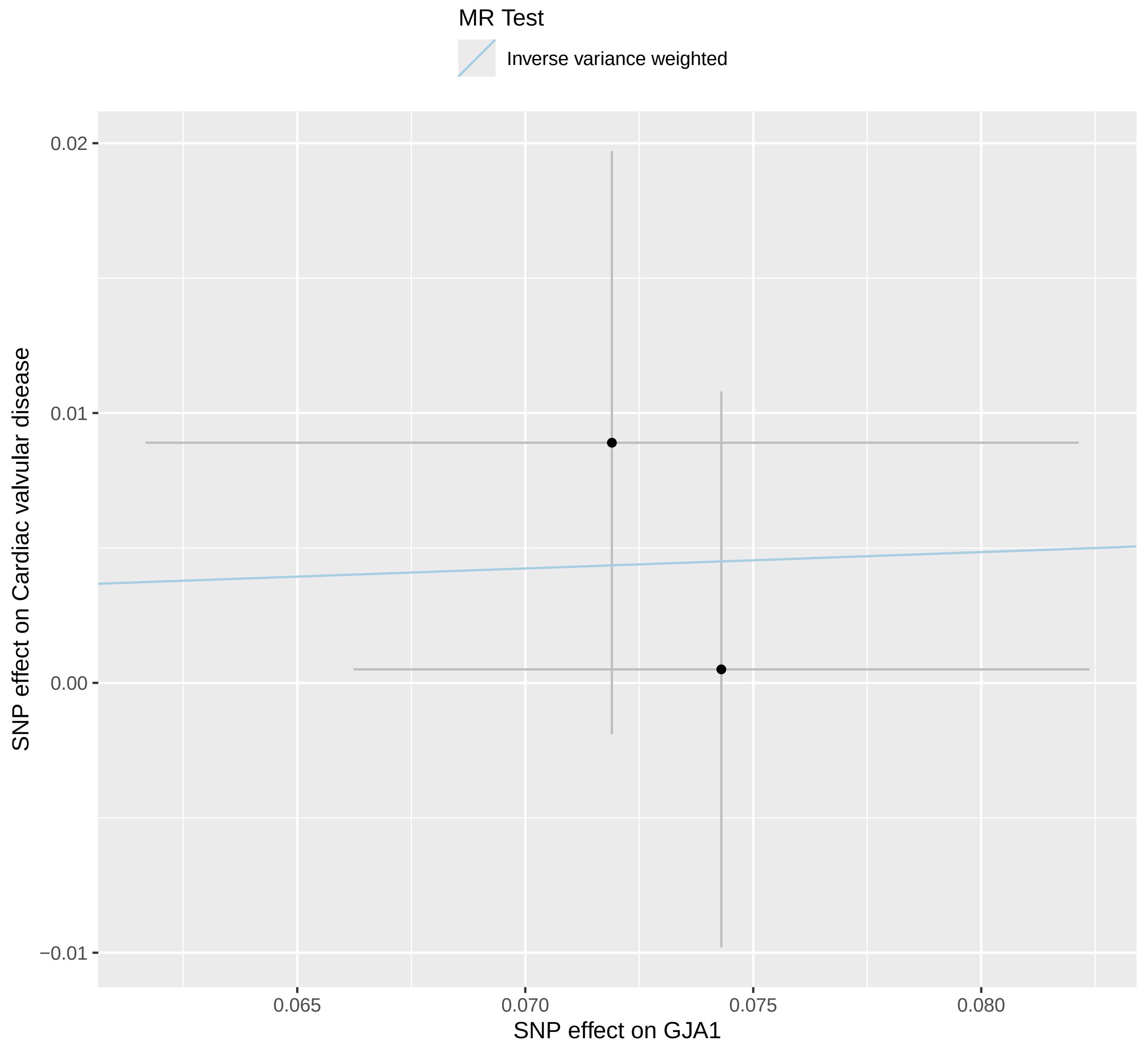
MR Test
Inverse variance weighted
0.02
0.01
0.00
−0.01
0.065
0.070
0.075
0.080
SNP effect on GJA1
SNP effect on Cardiac valvular disease

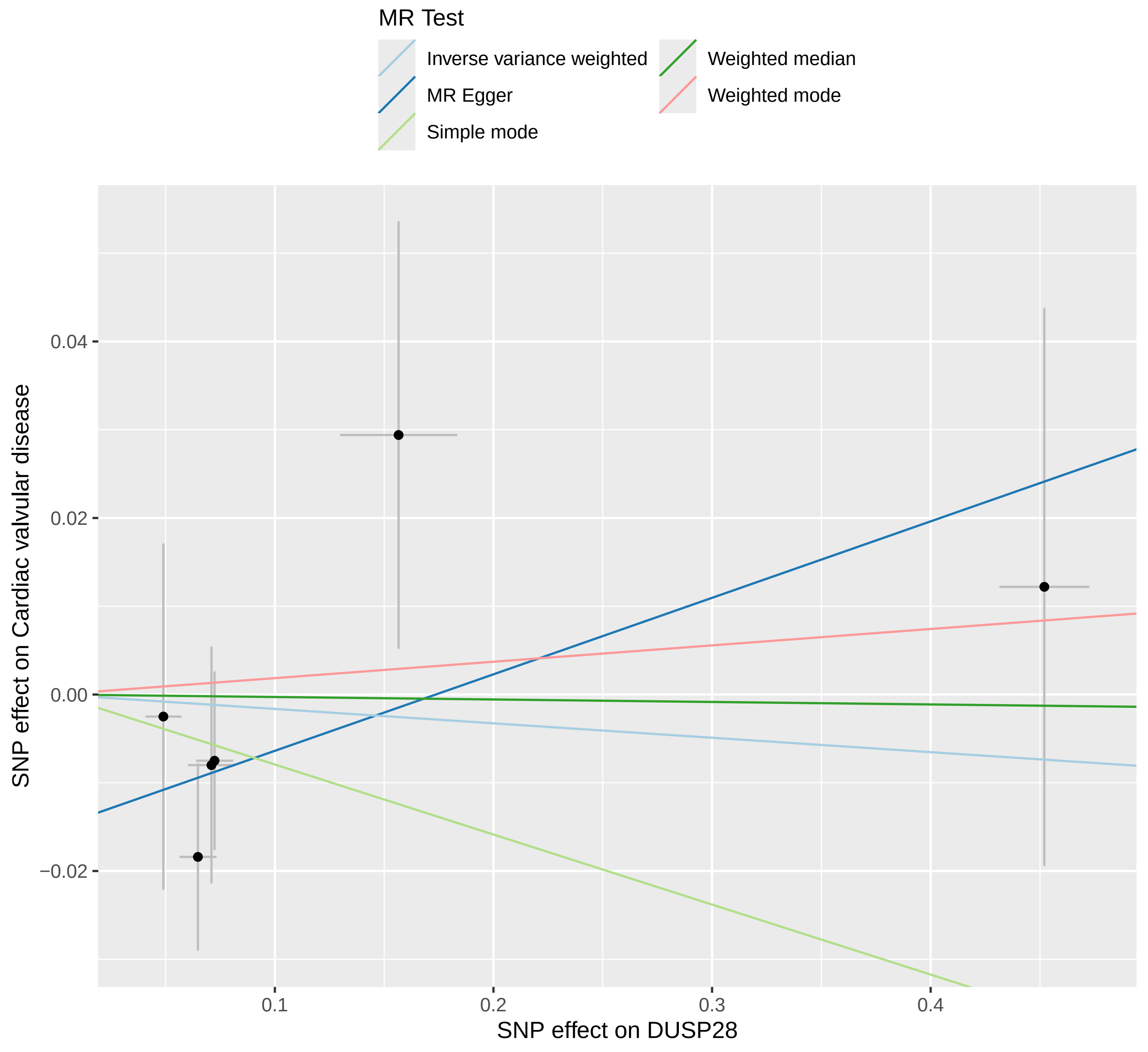
MR Test
Inverse variance weighted
MR Egger
Simple mode
Weighted median
Weighted mode
0.04
0.02
0.00
−0.02
0.1
0.2
0.3
0.4
SNP effect on DUSP28
SNP effect on Cardiac valvular disease

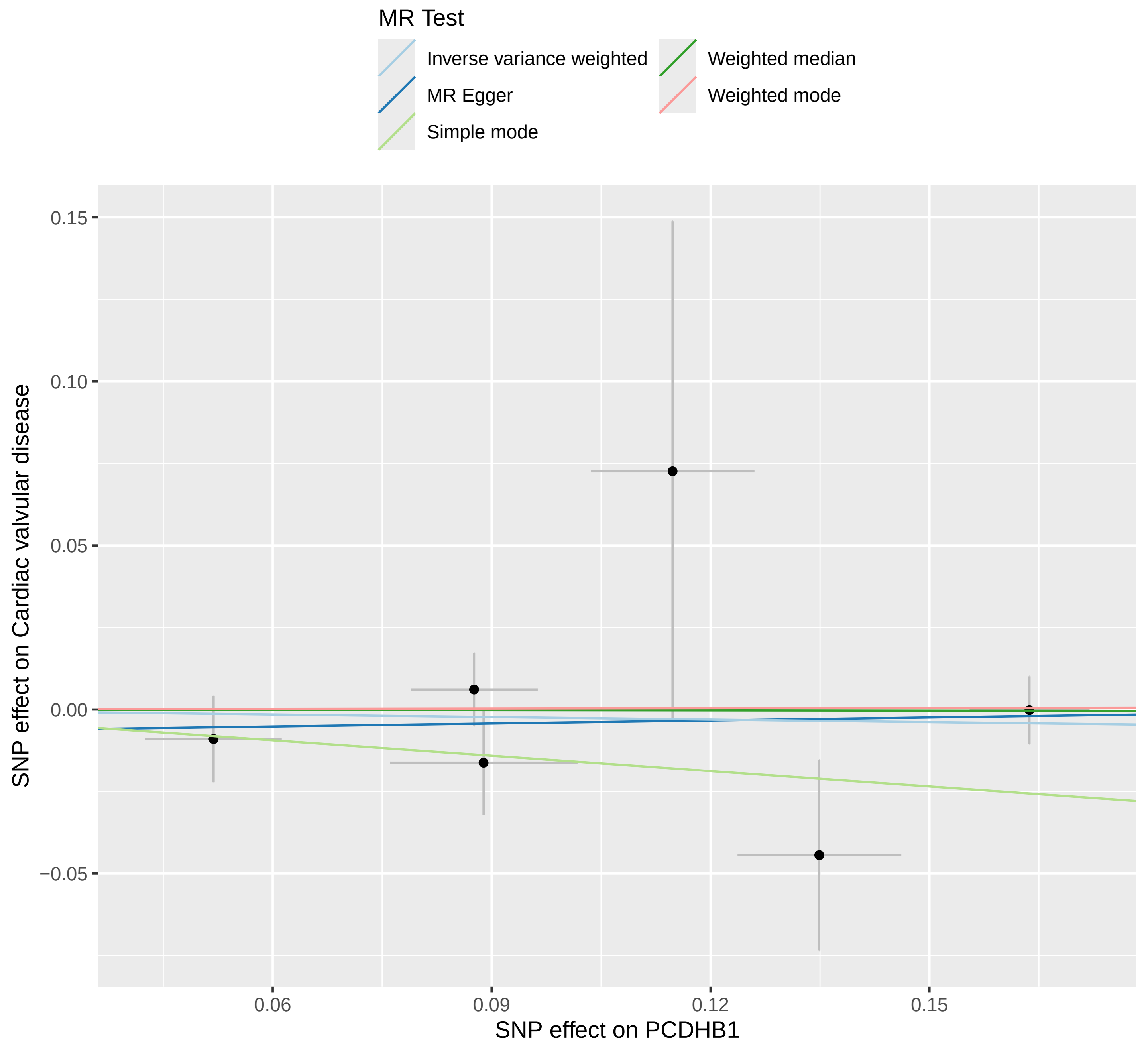
MR Test
Inverse variance weighted
MR Egger
Simple mode
Weighted median
Weighted mode
0.15
0.10
0.05
0.00
−0.05
0.06
0.09
0.12
0.15
SNP effect on Cardiac valvular disease
SNP effect on PCDHB1

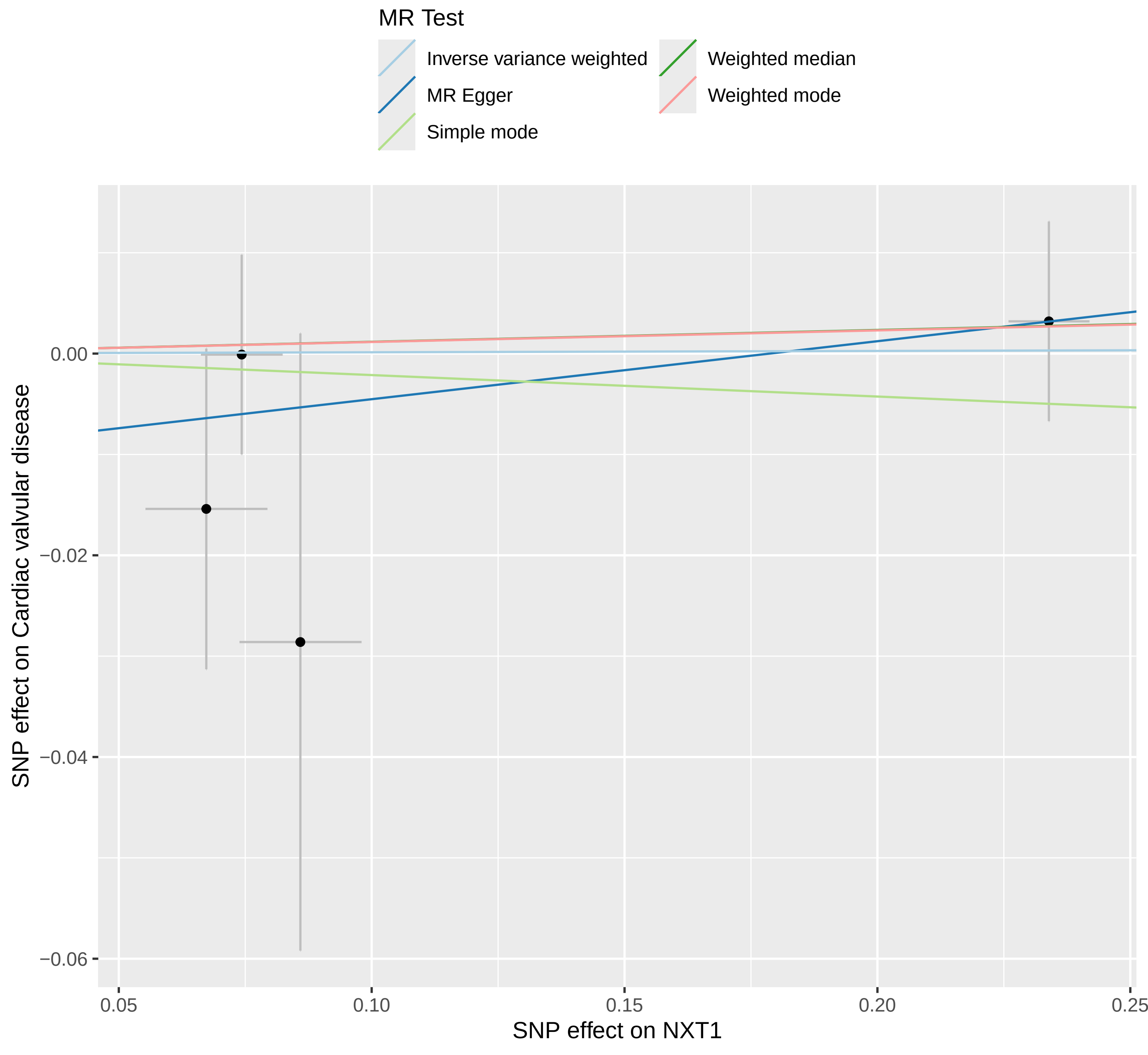
MR Test
Inverse variance weighted
MR Egger
Simple mode
Weighted median
Weighted mode
SNP effect on Cardiac valvular disease
0.00
−0.02
−0.04
−0.06
0.05
0.10
0.15
0.20
0.25
SNP effect on NXT1

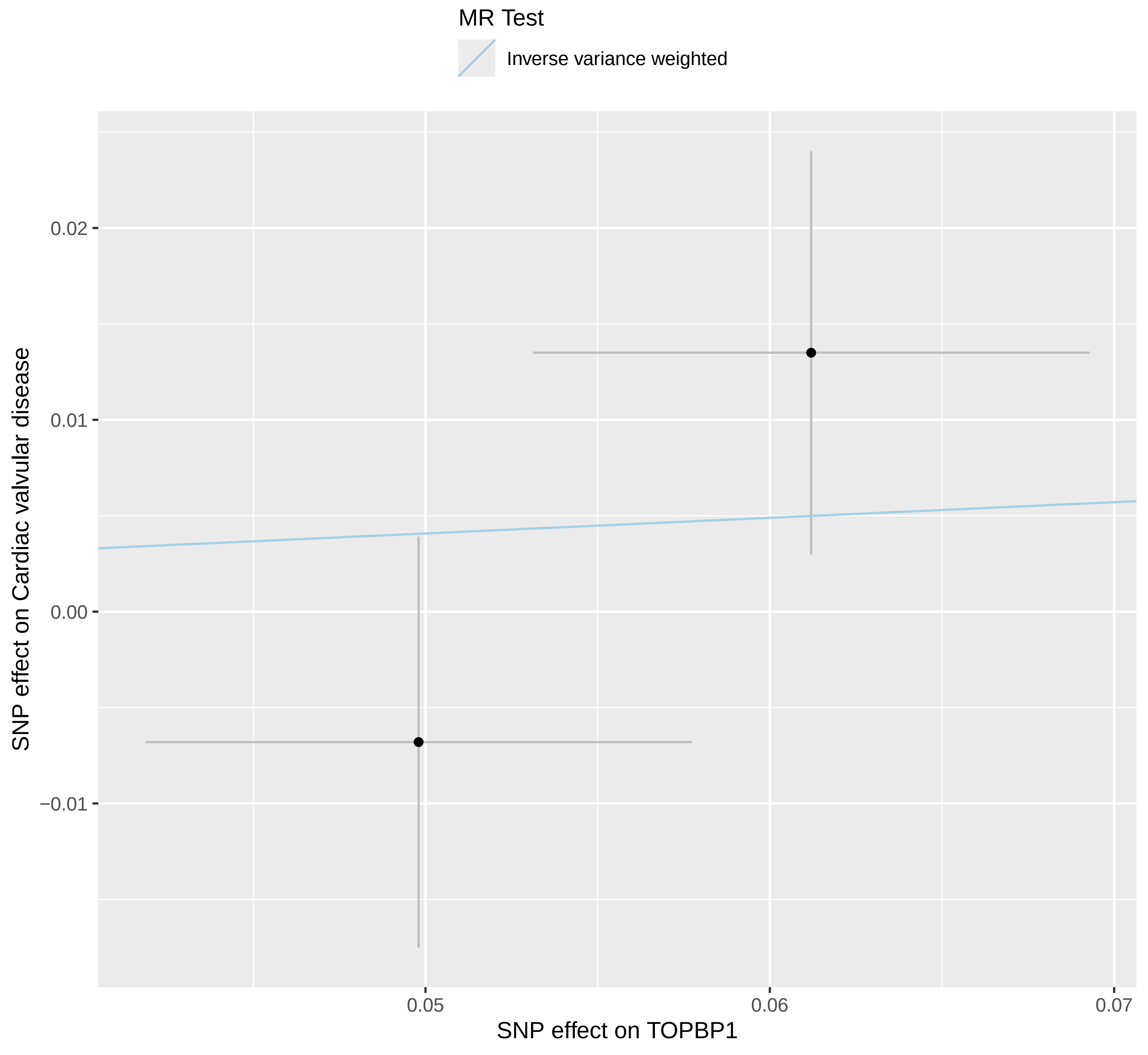
MR Test
Inverse variance weighted
0.02
0.01
0.00
−0.01
0.05
0.06
0.07
SNP effect on Cardiac valvular disease
SNP effect on TOPBP1

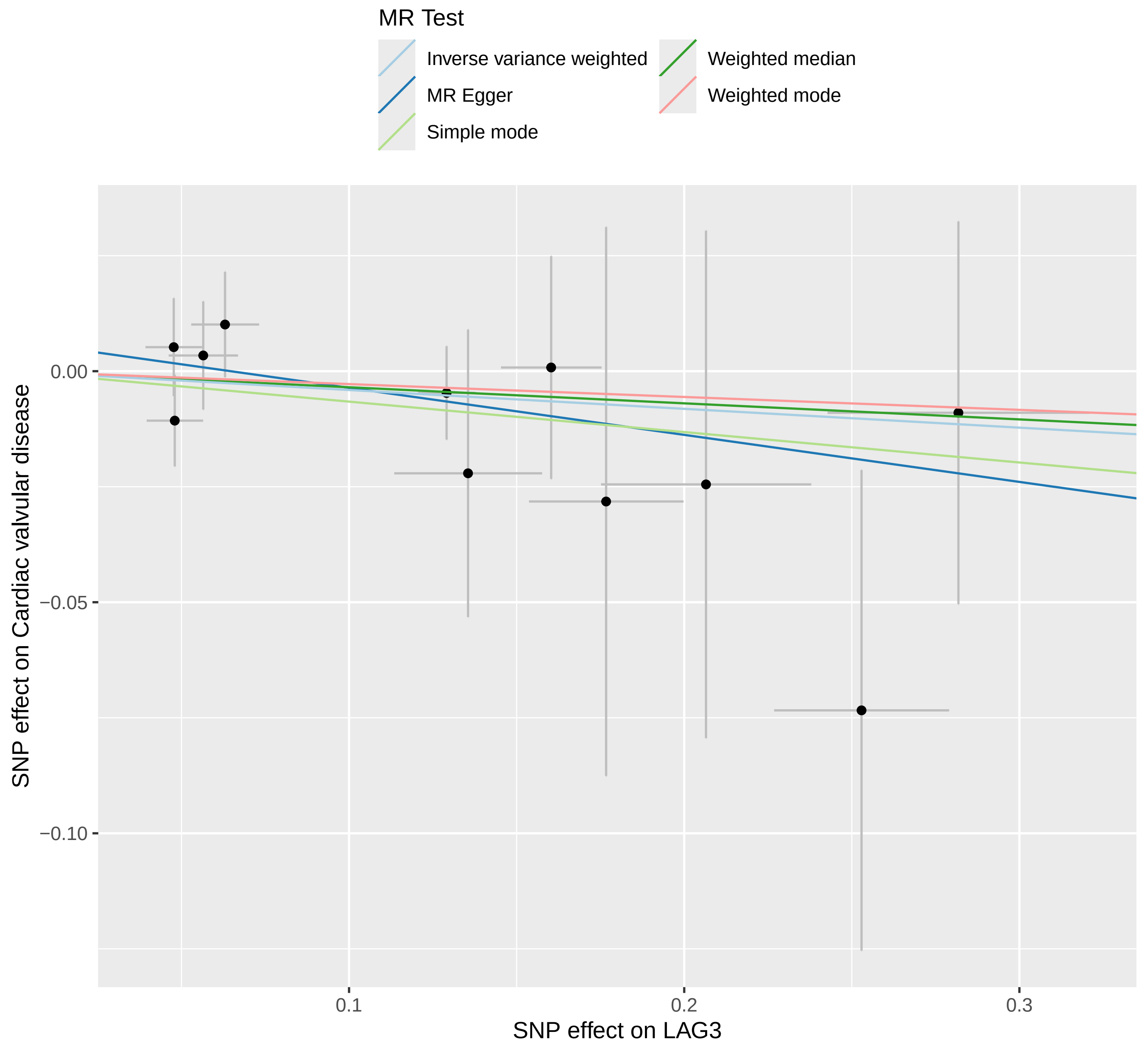
MR Test
Inverse variance weighted
MR Egger
Simple mode
Weighted median
Weighted mode
SNP effect on Cardiac valvular disease
0.00
−0.05
−0.10
0.1
0.2
0.3
SNP effect on LAG3

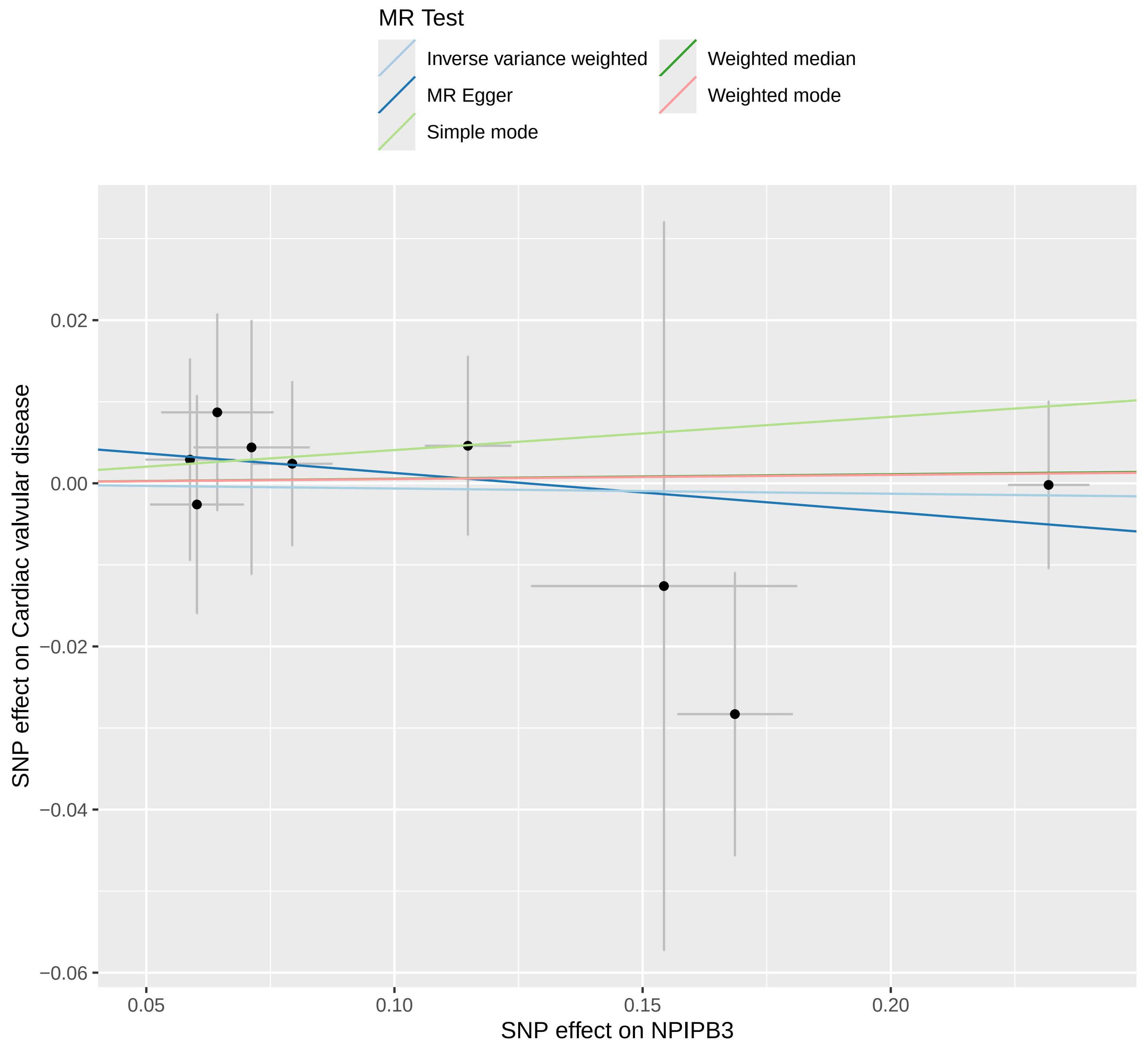

MR Test
Inverse variance weighted
MR Egger
Simple mode
Weighted median
Weighted mode
0.02
0.00
−0.02
−0.04
−0.06
0.05
0.10
0.15
0.20
SNP effect on Cardiac valvular disease
SNP effect on NPIPB3

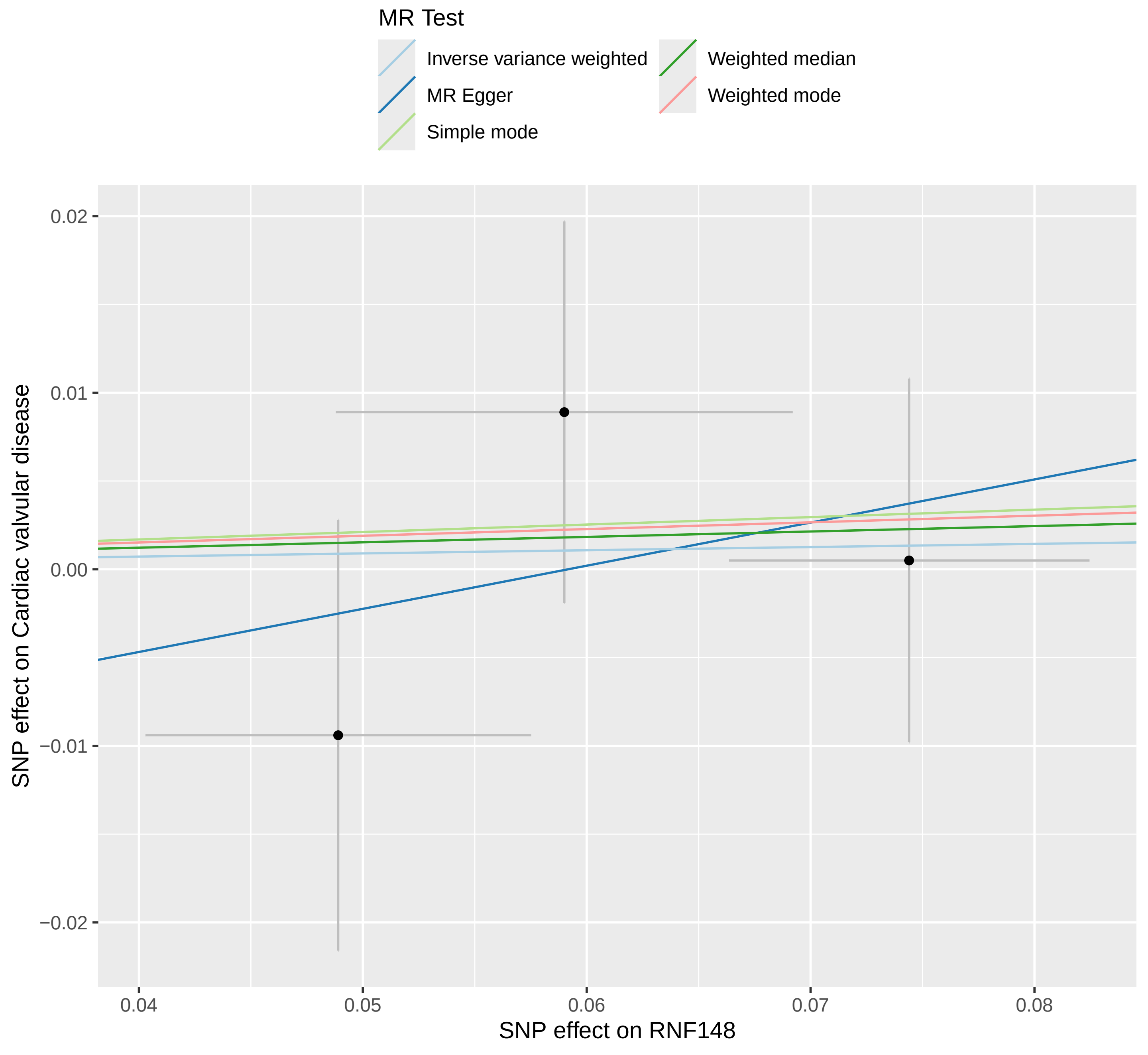
MR Test
Inverse variance weighted
MR Egger
Simple mode
Weighted median
Weighted mode
0.02
0.01
0.00
−0.01
−0.02
SNP effect on Cardiac valvular disease
0.04
0.05
0.06
0.07
0.08
SNP effect on RNF148

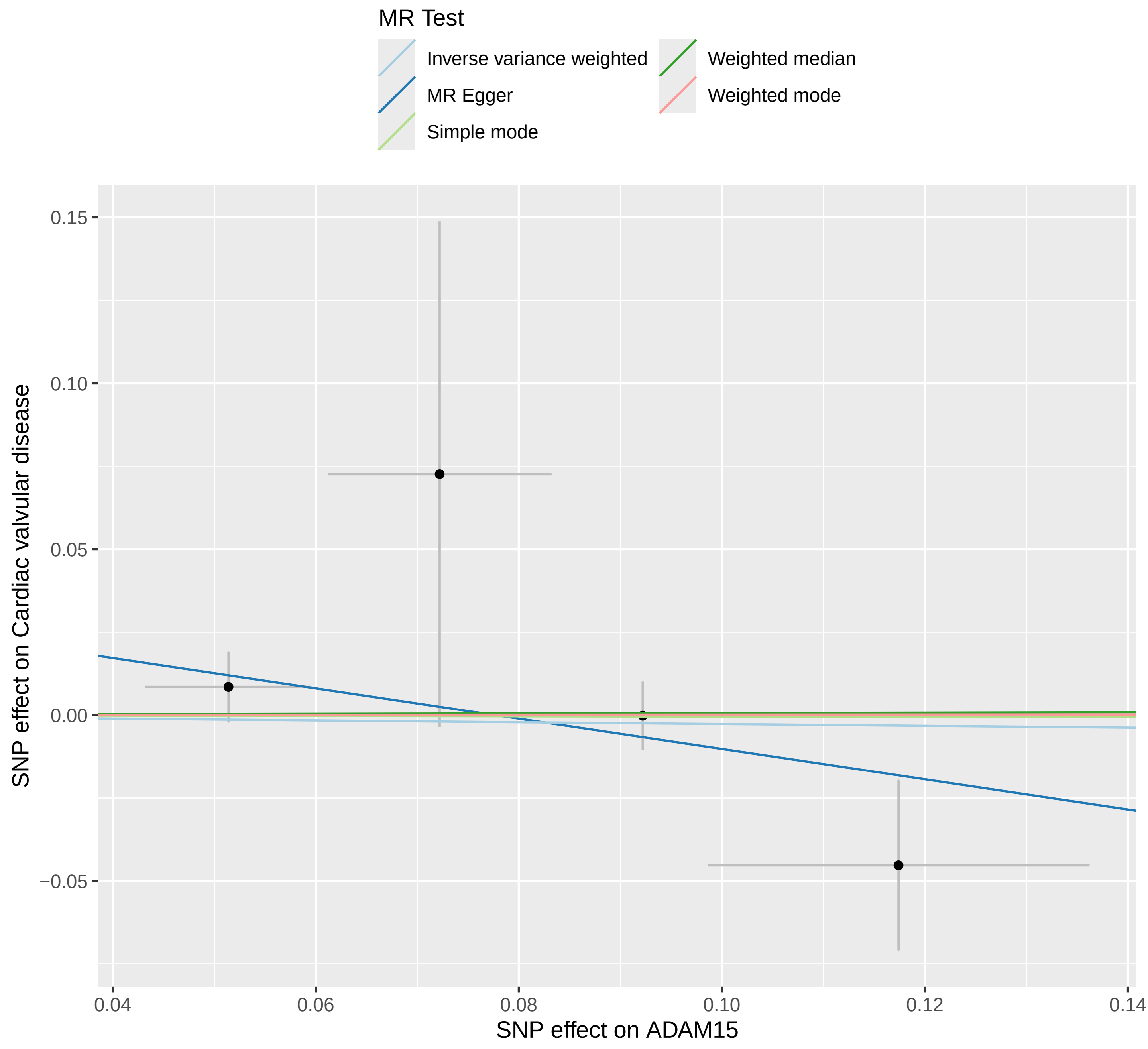
MR Test
Inverse variance weighted
MR Egger
Simple mode
Weighted median
Weighted mode
0.15
0.10
0.05
0.00
−0.05
0.04
0.06
0.08
0.10
0.12
0.14
SNP effect on Cardiac valvular disease
SNP effect on ADAM15

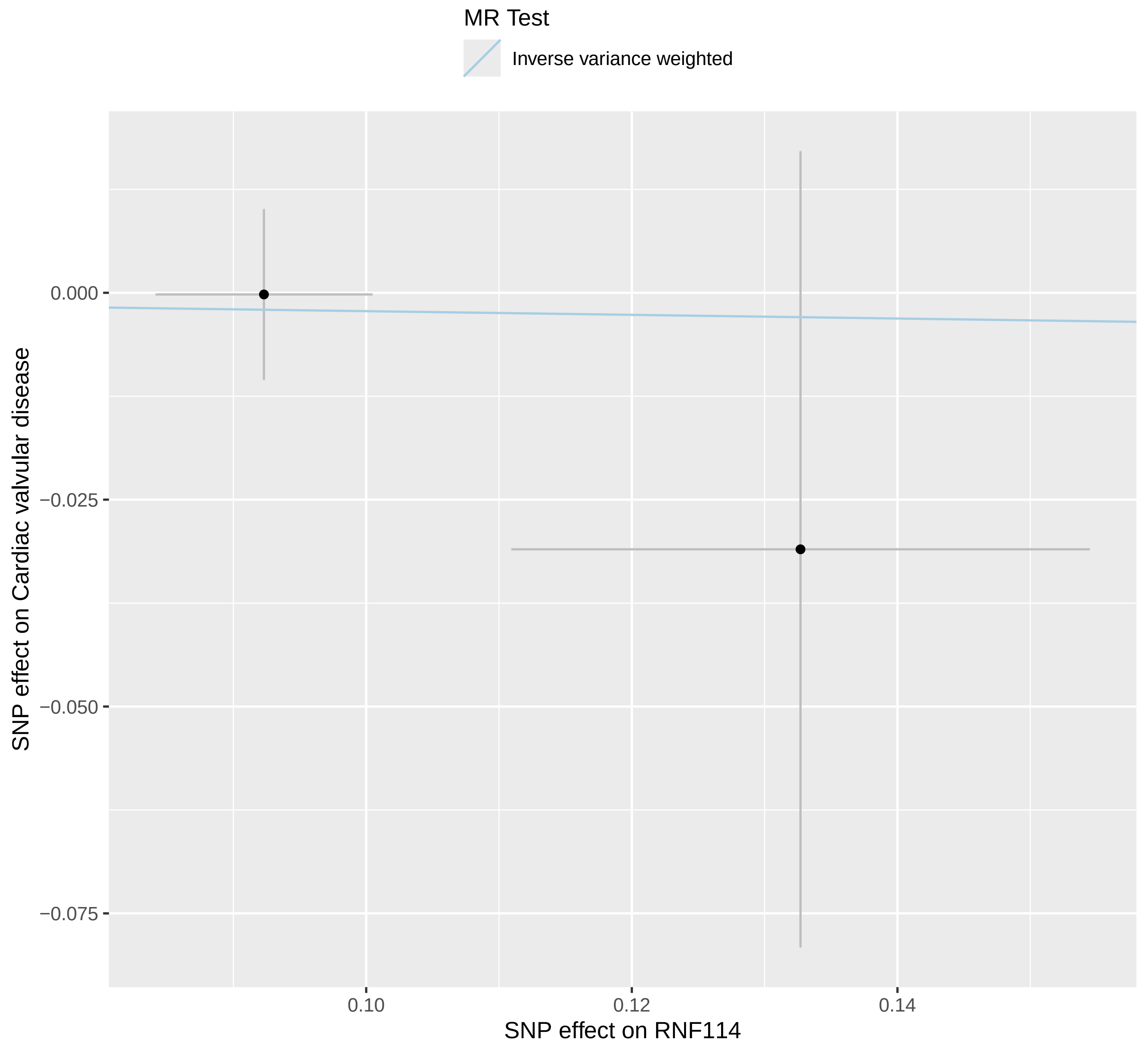
MR Test
Inverse variance weighted
0.000
−0.025
−0.050
−0.075
SNP effect on Cardiac valvular disease
0.10
0.12
0.14
SNP effect on RNF114

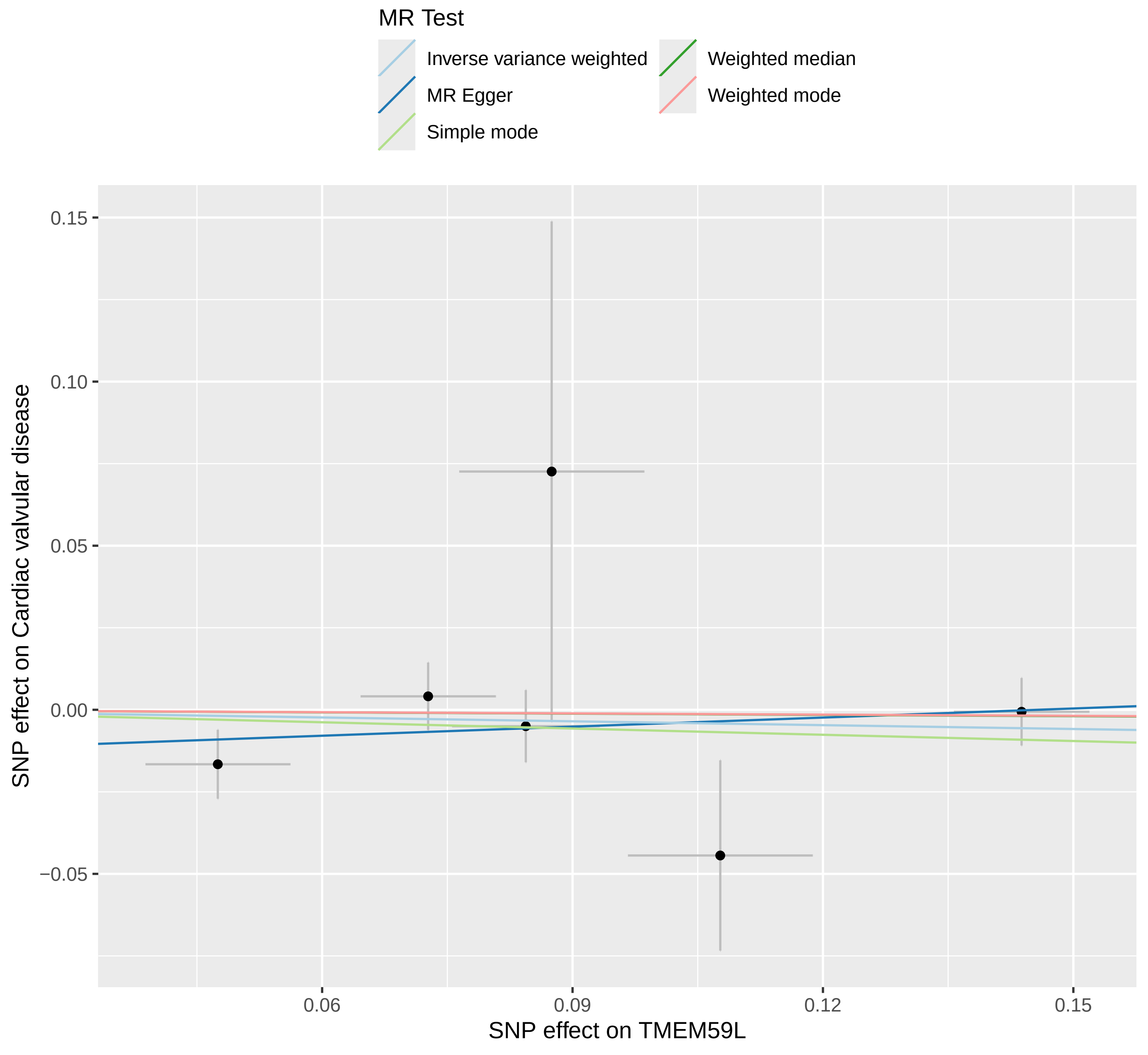
MR Test
Inverse variance weighted
MR Egger
Simple mode
Weighted median
Weighted mode
0.15
0.10
0.05
0.00
−0.05
0.06
0.09
0.12
0.15
SNP effect on Cardiac valvular disease
SNP effect on TMEM59L

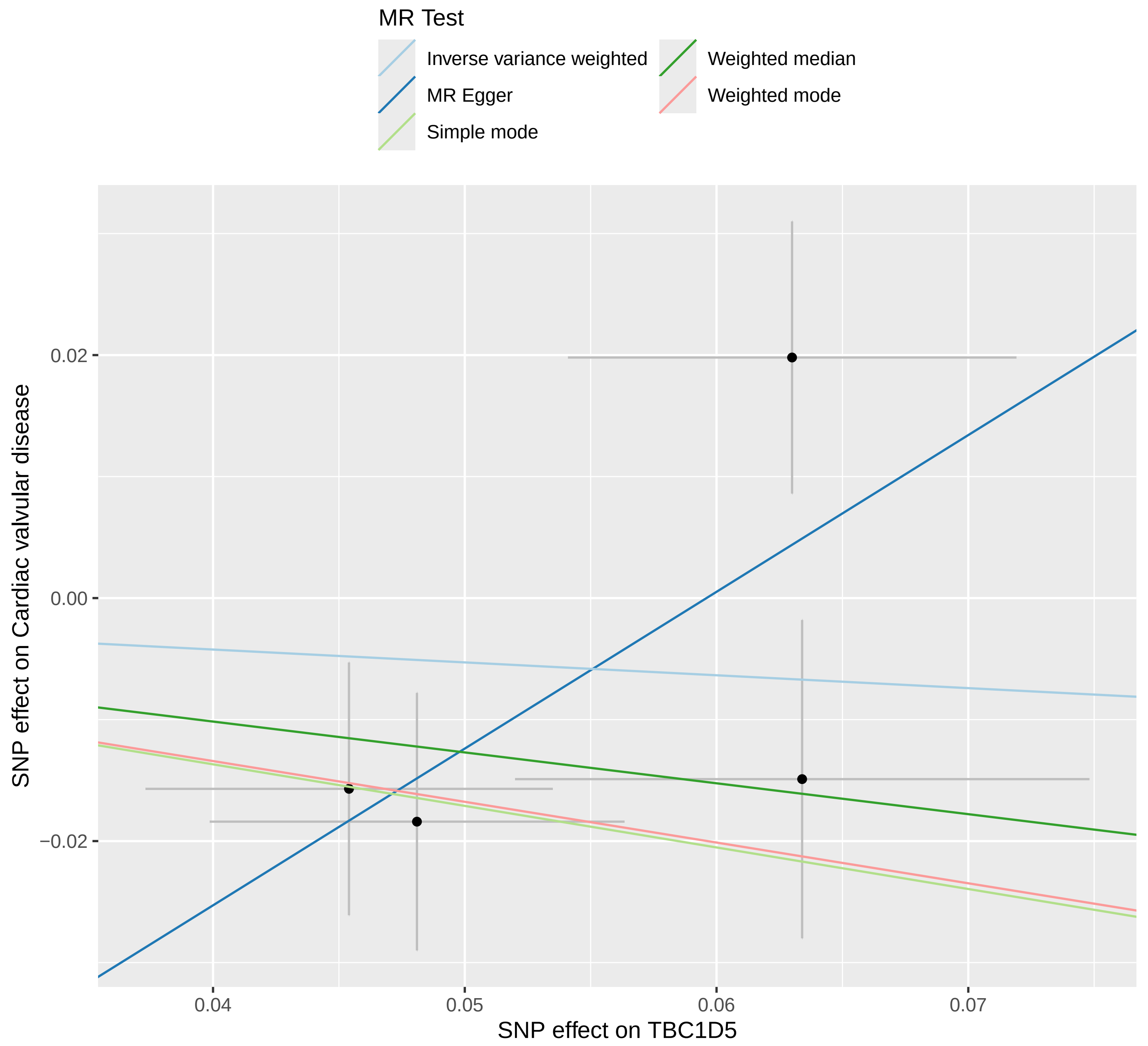

MR Test
Inverse variance weighted
MR Egger
Simple mode
Weighted median
Weighted mode
0.02
0.00
−0.02
SNP effect on Cardiac valvular disease
0.04
0.05
0.06
0.07
SNP effect on TBC1D5

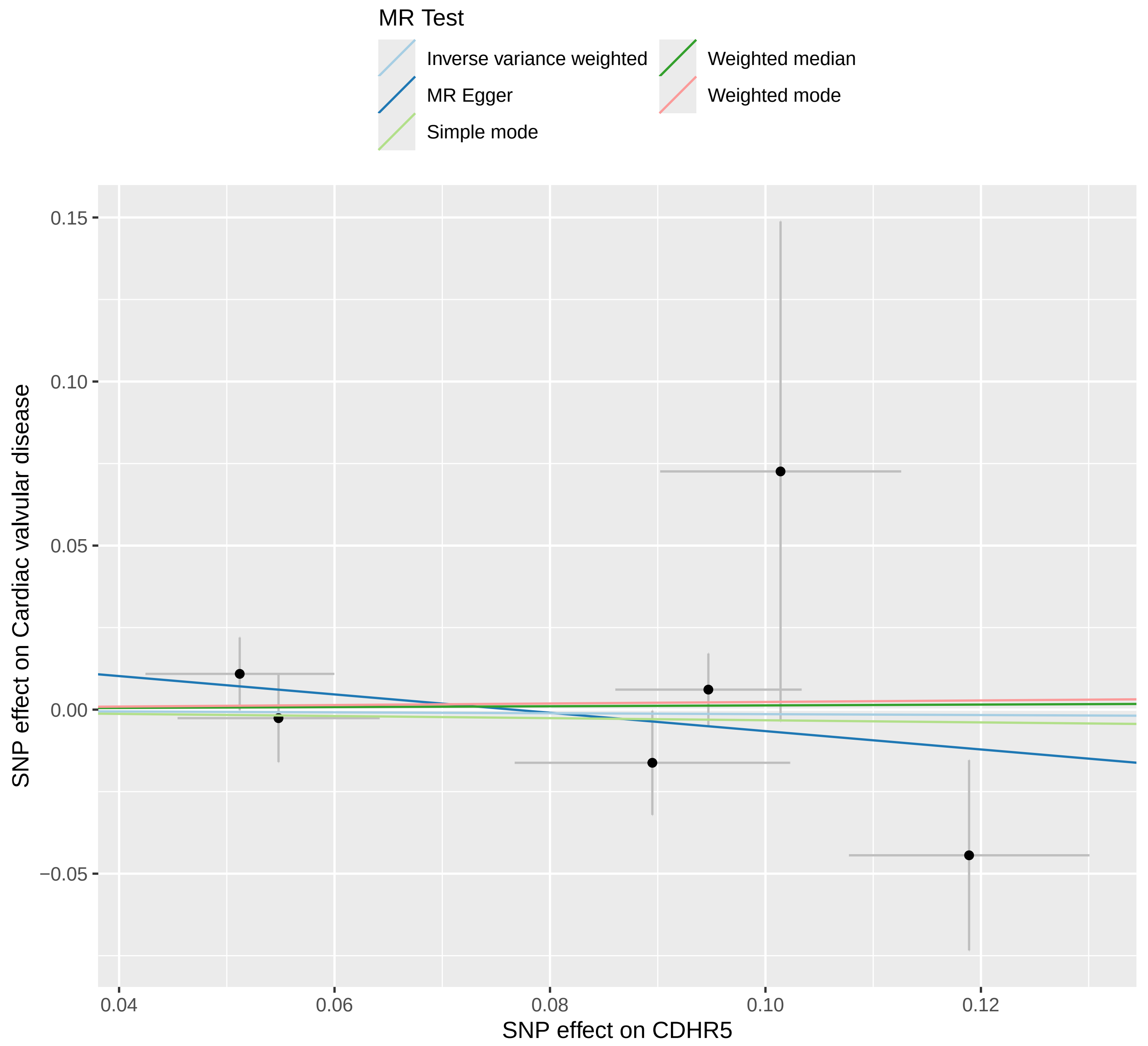

MR Test
Inverse variance weighted
MR Egger
Simple mode
Weighted median
Weighted mode
0.15
0.10
0.05
0.00
−0.05
0.04
0.06
0.08
0.10
0.12
SNP effect on CDHR5
SNP effect on Cardiac valvular disease

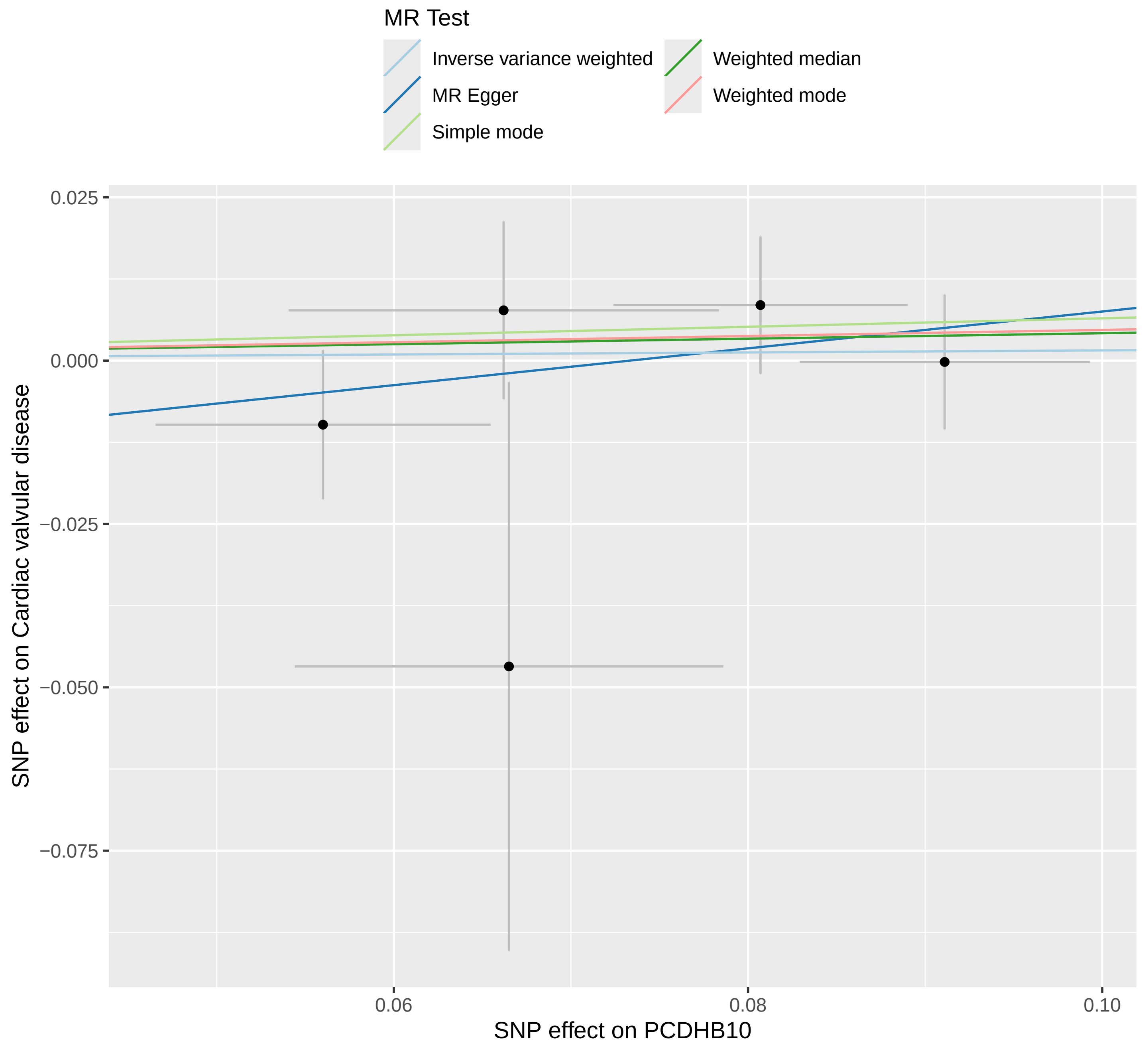

MR Test
Inverse variance weighted
MR Egger
Simple mode
Weighted median
Weighted mode
0.025
0.000
−0.025
−0.050
−0.075
0.06
0.08
0.10
SNP effect on PCDHB10
SNP effect on Cardiac valvular disease

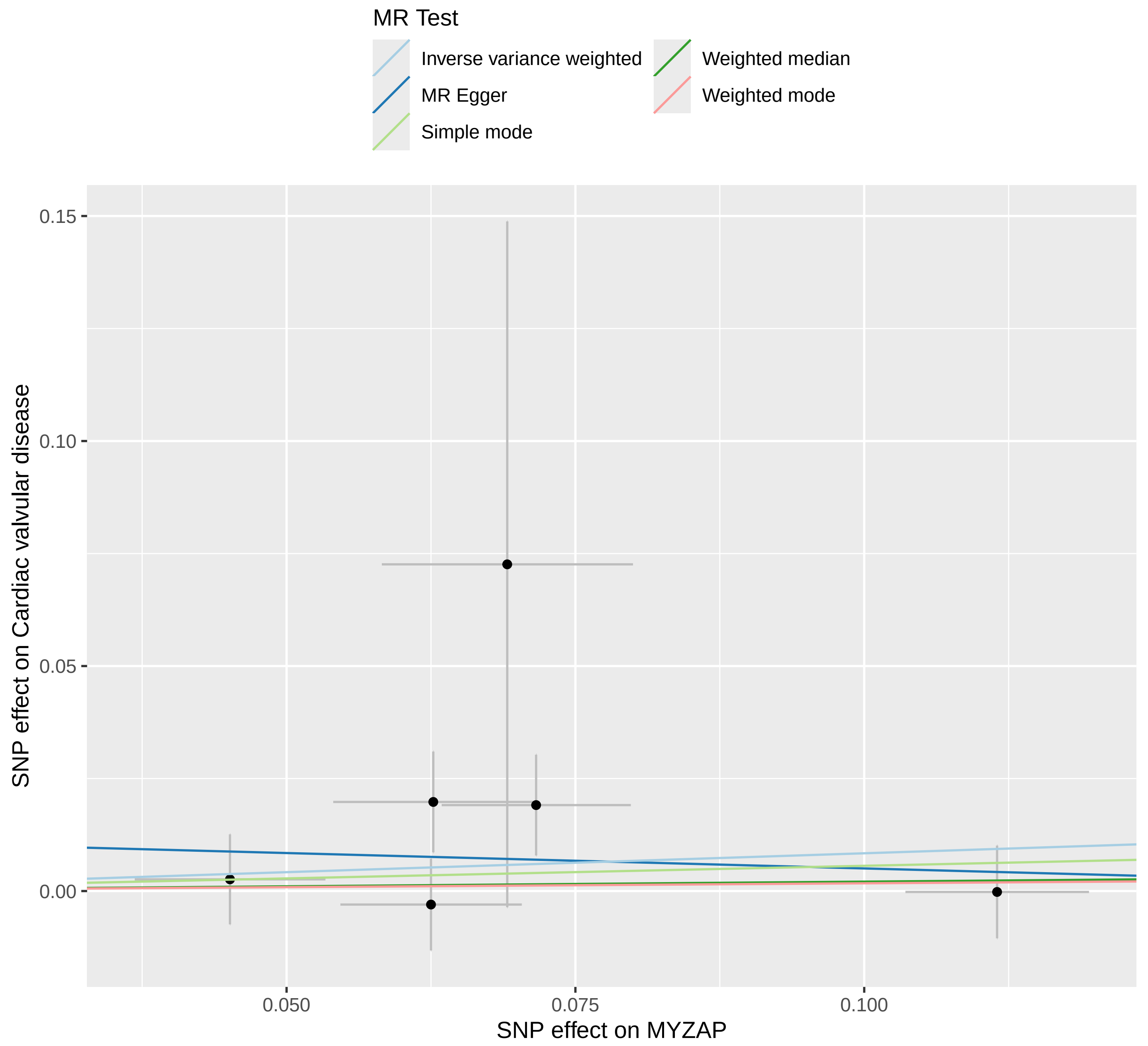
MR Test
Inverse variance weighted
MR Egger
Simple mode
Weighted median
Weighted mode
0.15
0.10
0.05
0.00
0.050
0.075
0.100
SNP effect on Cardiac valvular disease
SNP effect on MYZAP

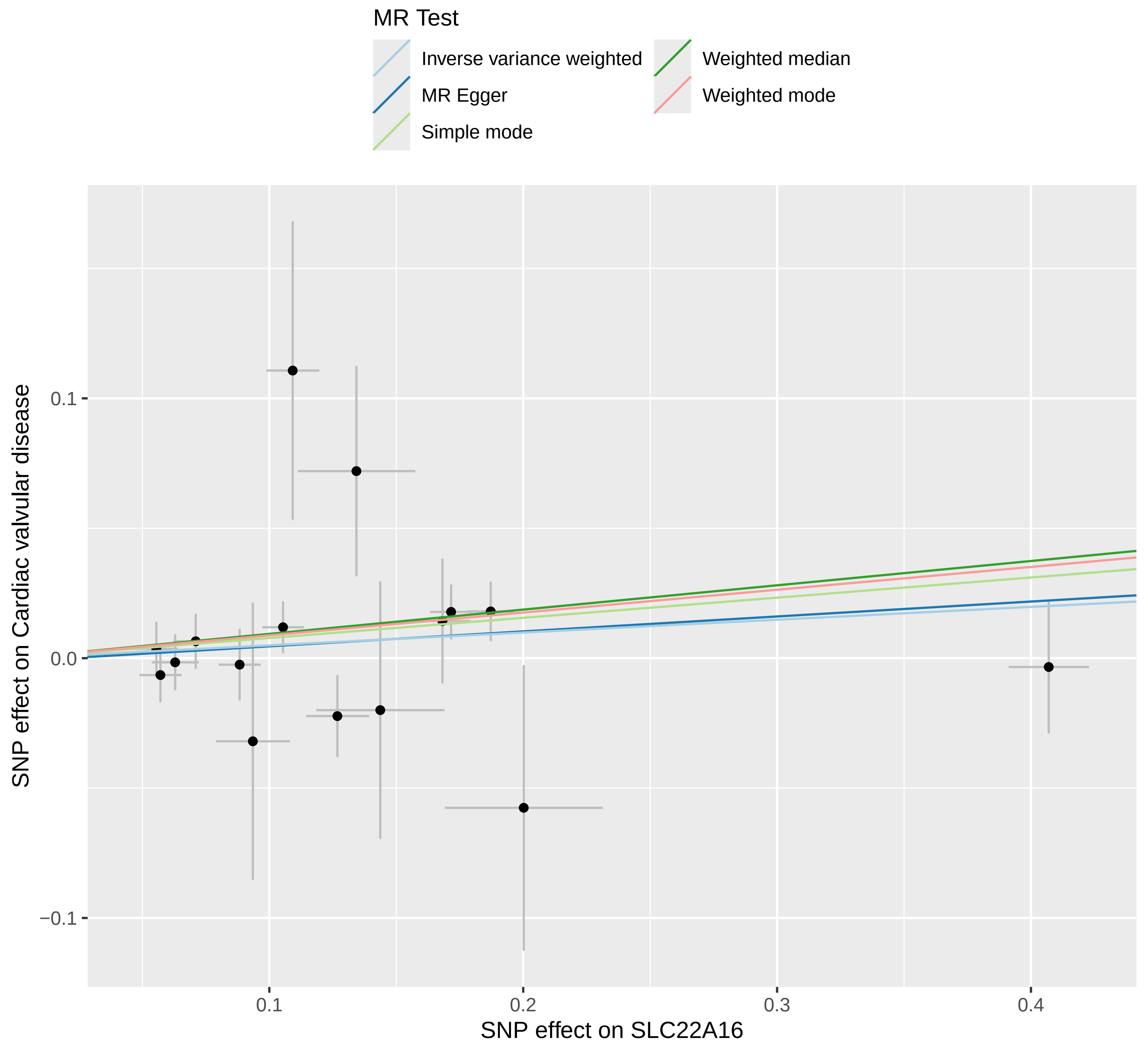
MR Test
Inverse variance weighted
MR Egger
Simple mode
Weighted median
Weighted mode
0.1
0.0
−0.1
0.1
0.2
0.3
0.4
SNP effect on Cardiac valvular disease
SNP effect on SLC22A16

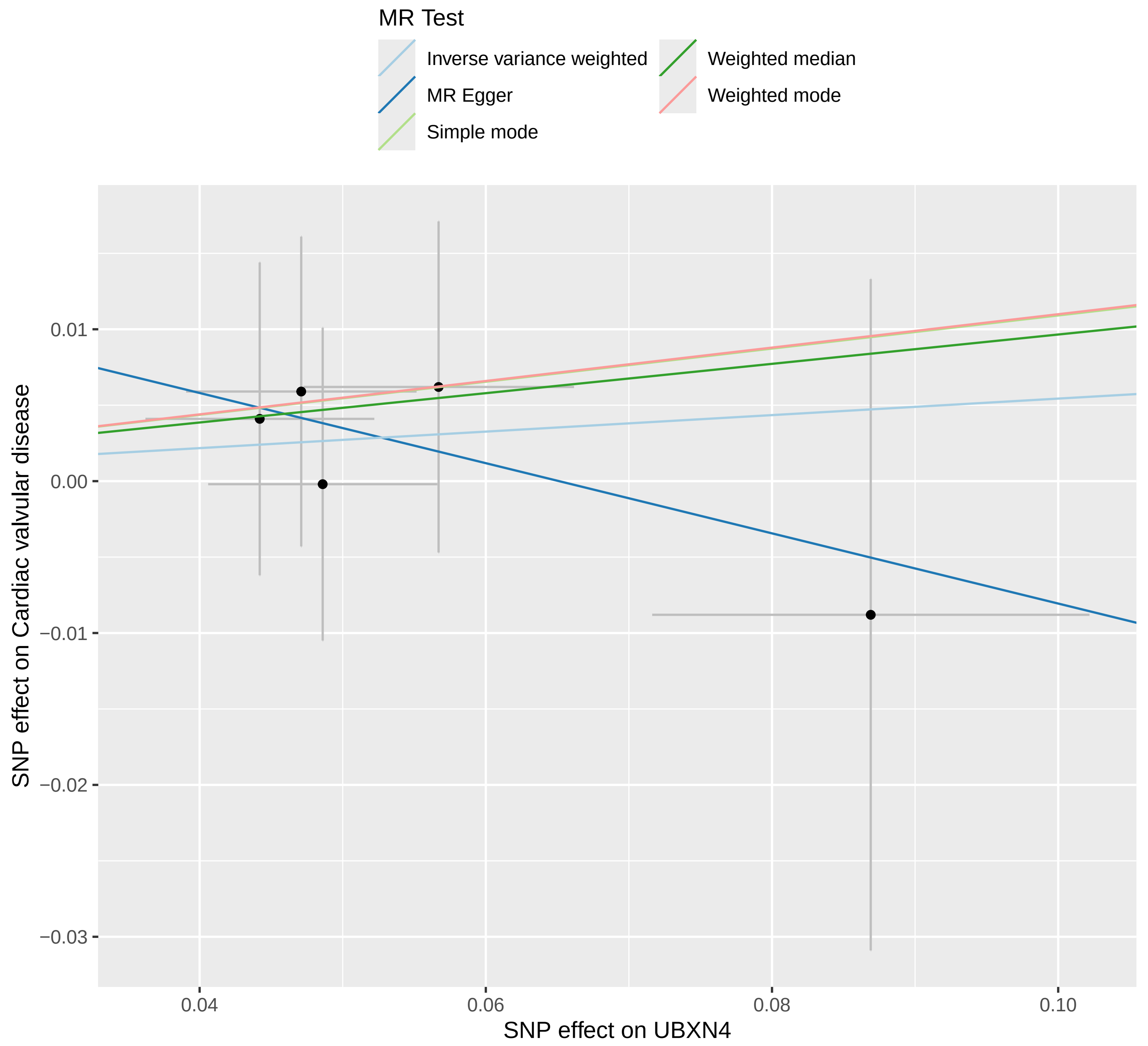
MR Test
Inverse variance weighted
MR Egger
Simple mode
Weighted median
Weighted mode
0.01
0.00
−0.01
−0.02
−0.03
0.04
0.06
0.08
0.10
SNP effect on Cardiac valvular disease
SNP effect on UBXN4

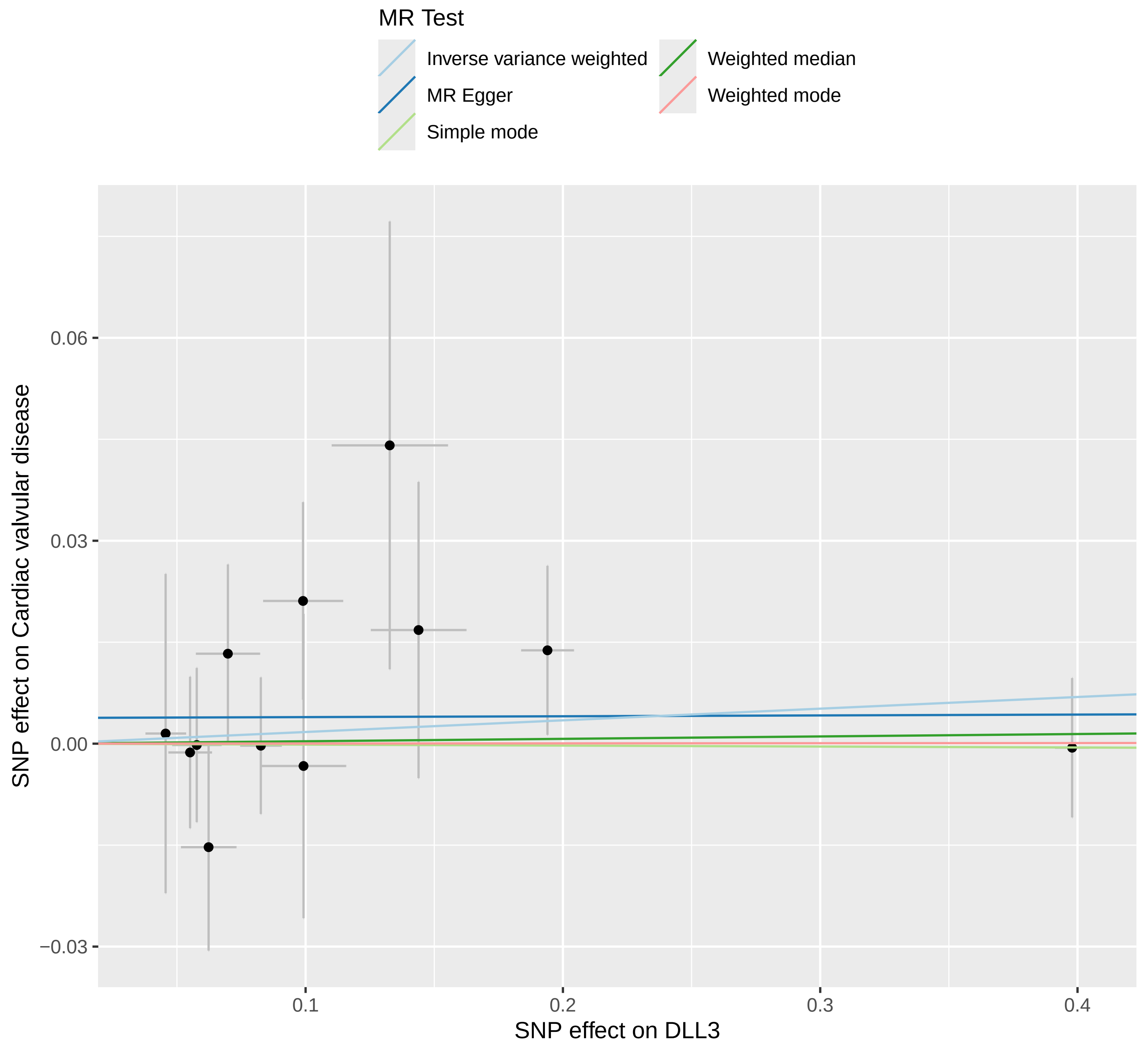
MR Test
Inverse variance weighted
MR Egger
Simple mode
Weighted median
Weighted mode
SNP effect on Cardiac valvular disease
0.06
0.03
0.00
−0.03
0.1
0.2
0.3
0.4
SNP effect on DLL3

Insufficient number of SNPs

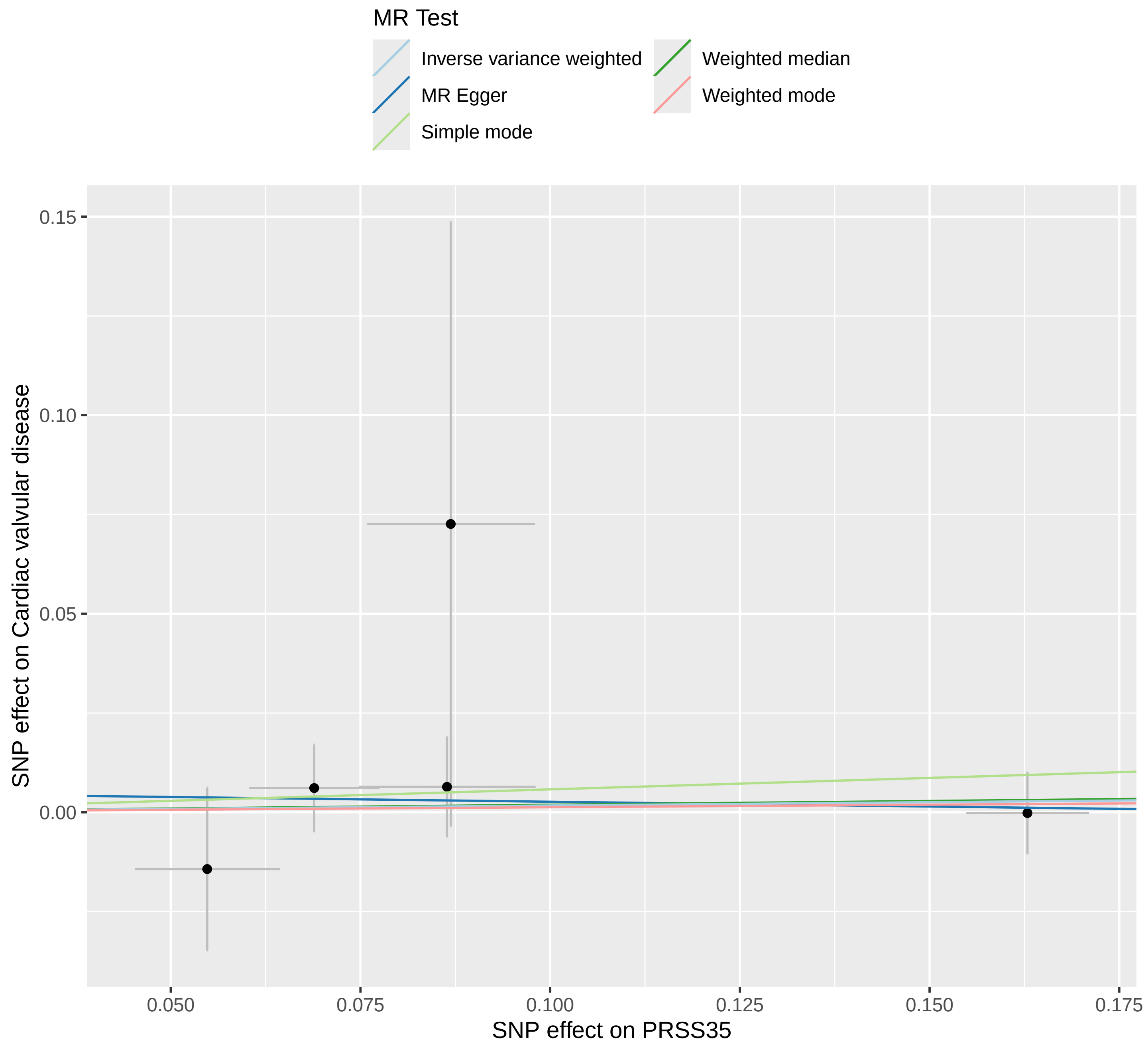
MR Test
Inverse variance weighted
MR Egger
Simple mode
Weighted median
Weighted mode
0.15
0.10
0.05
0.00
SNP effect on Cardiac valvular disease
0.050
0.075
0.100
0.125
0.150
0.175
SNP effect on PRSS35

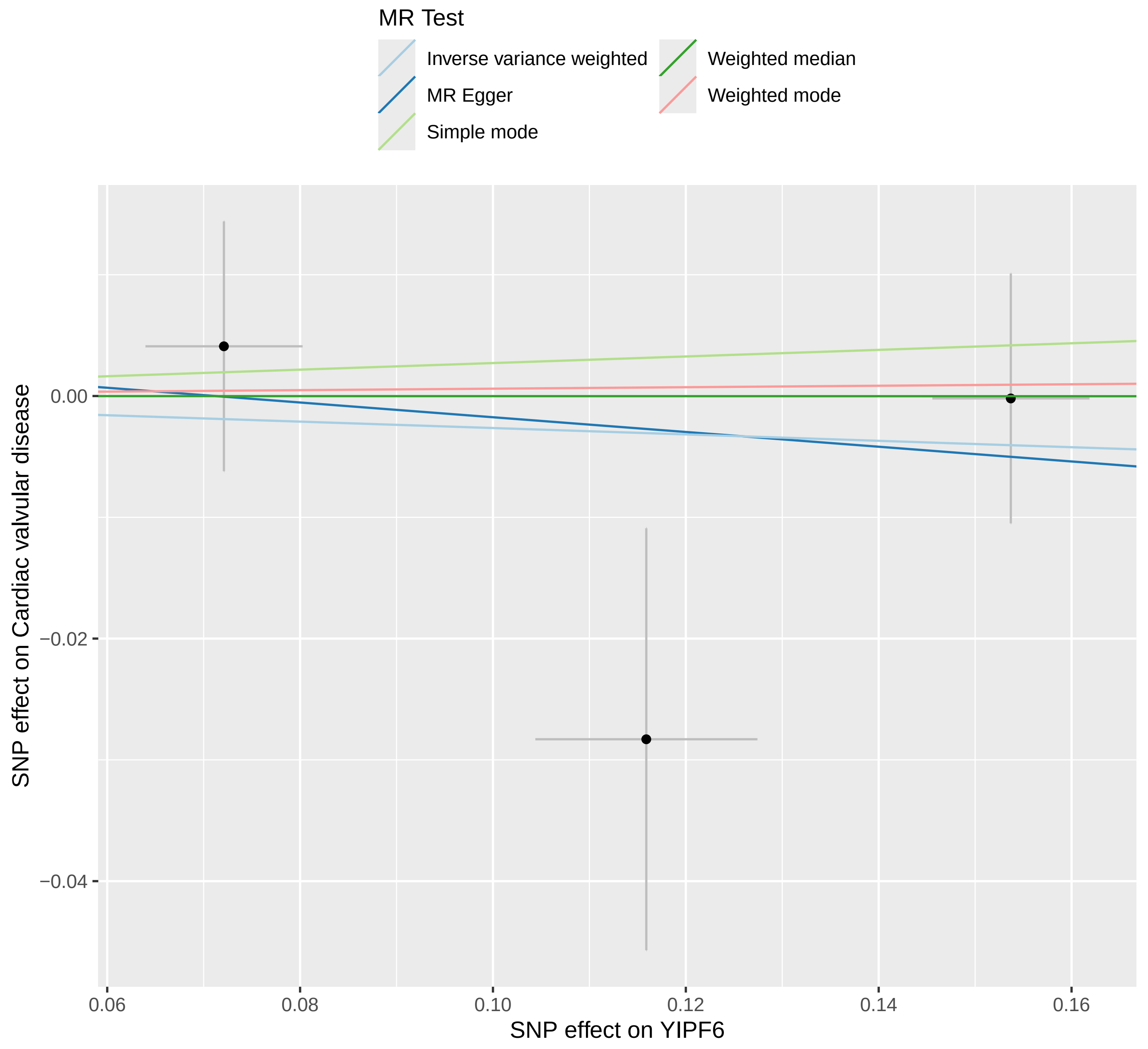
MR Test
Inverse variance weighted
MR Egger
Simple mode
Weighted median
Weighted mode
0.00
−0.02
−0.04
SNP effect on Cardiac valvular disease
0.06
0.08
0.10
0.12
0.14
0.16
SNP effect on YIPF6

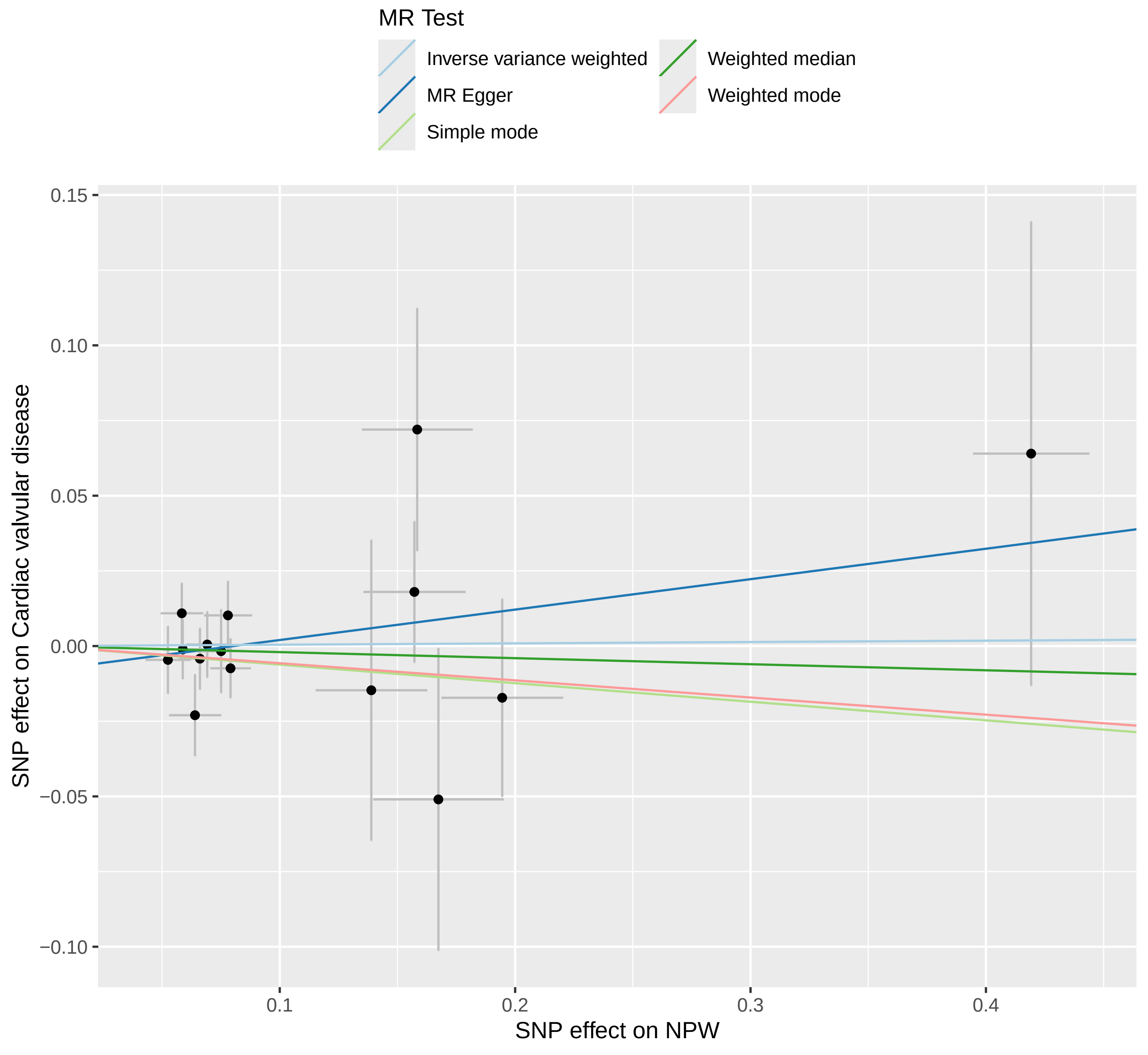
MR Test
Inverse variance weighted
MR Egger
Simple mode
Weighted median
Weighted mode
0.15
0.10
0.05
0.00
−0.05
−0.10
0.1
0.2
0.3
0.4
SNP effect on Cardiac valvular disease
SNP effect on NPW

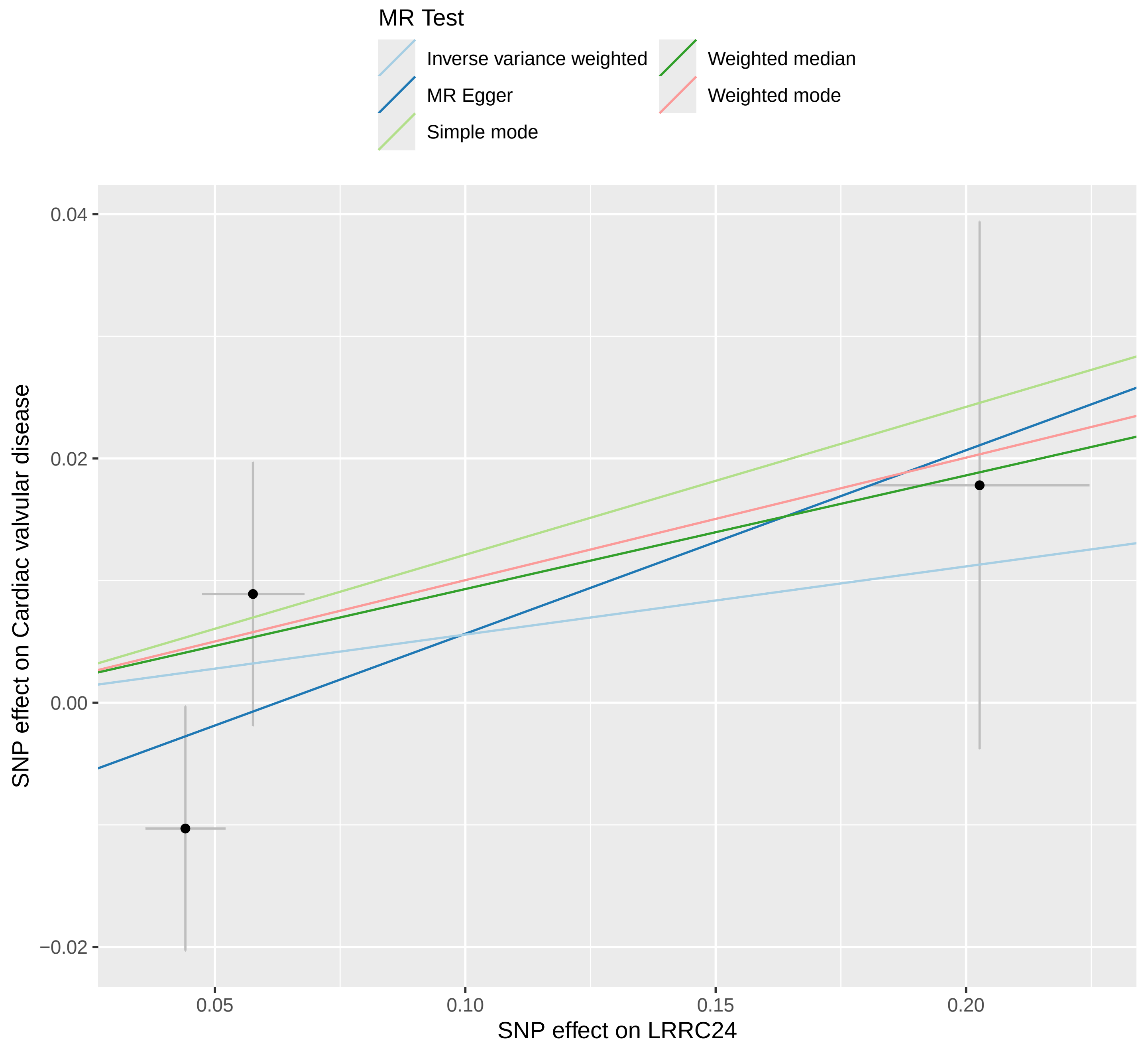

MR Test
Inverse variance weighted
MR Egger
Simple mode
Weighted median
Weighted mode
SNP effect on Cardiac valvular disease
0.04
0.02
0.00
−0.02
0.05
0.10
0.15
0.20
SNP effect on LRRC24

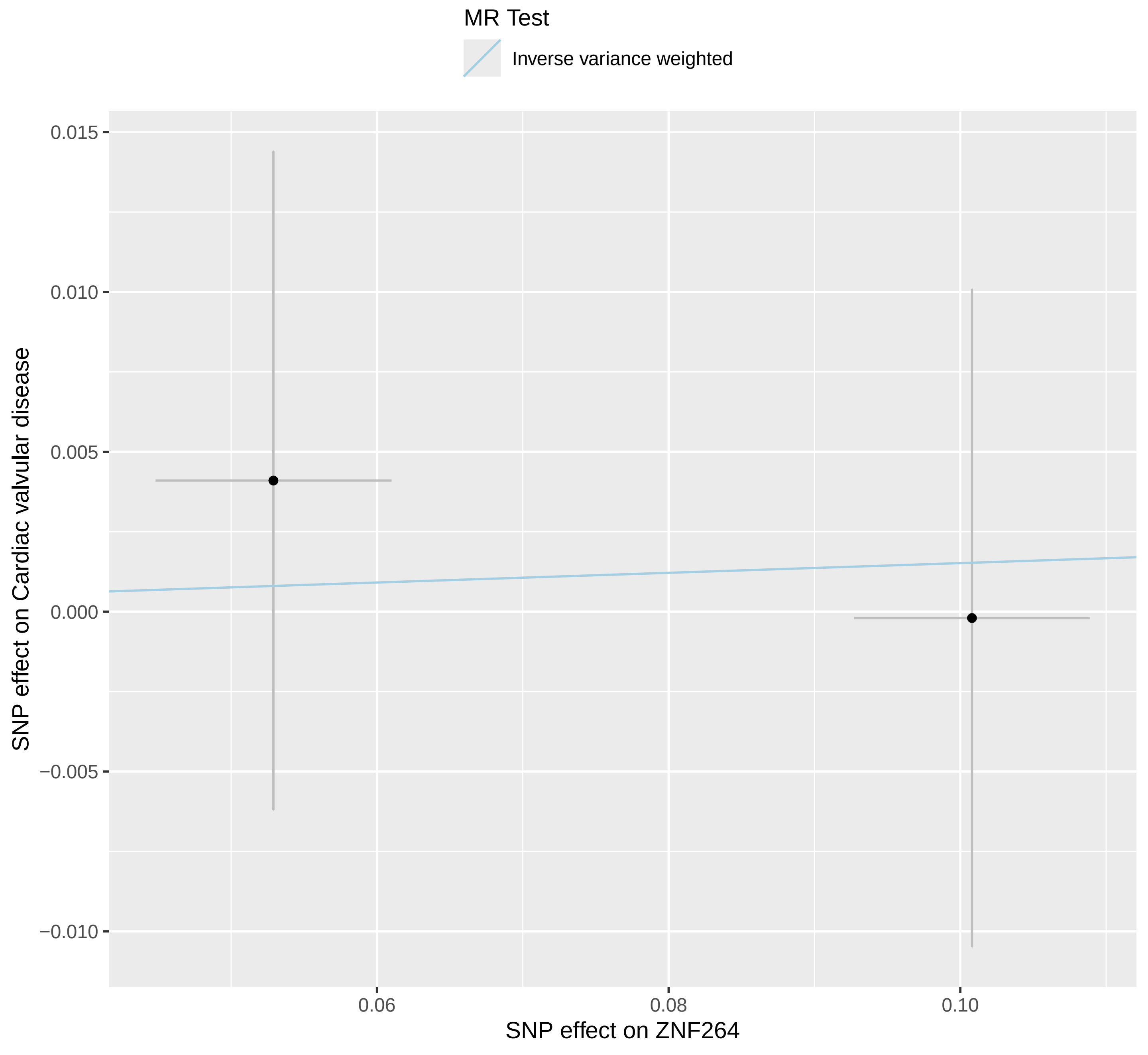
MR Test
Inverse variance weighted
0.015
0.010
0.005
0.000
−0.005
−0.010
SNP effect on Cardiac valvular disease
0.06
0.08
0.10
SNP effect on ZNF264

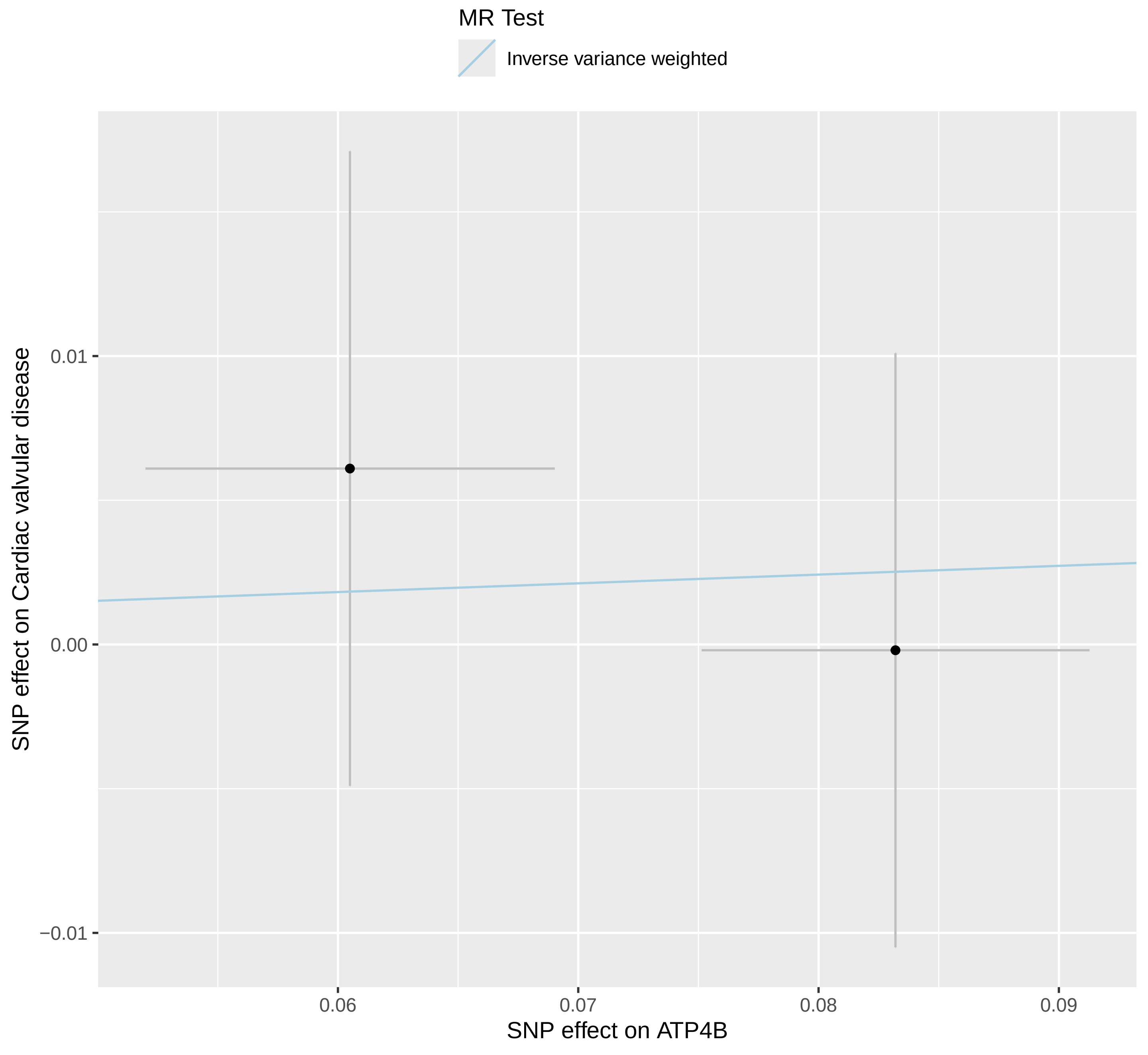

MR Test
Inverse variance weighted
SNP effect on Cardiac valvular disease
0.01
0.00
−0.01
0.06
0.07
0.08
0.09
SNP effect on ATP4B

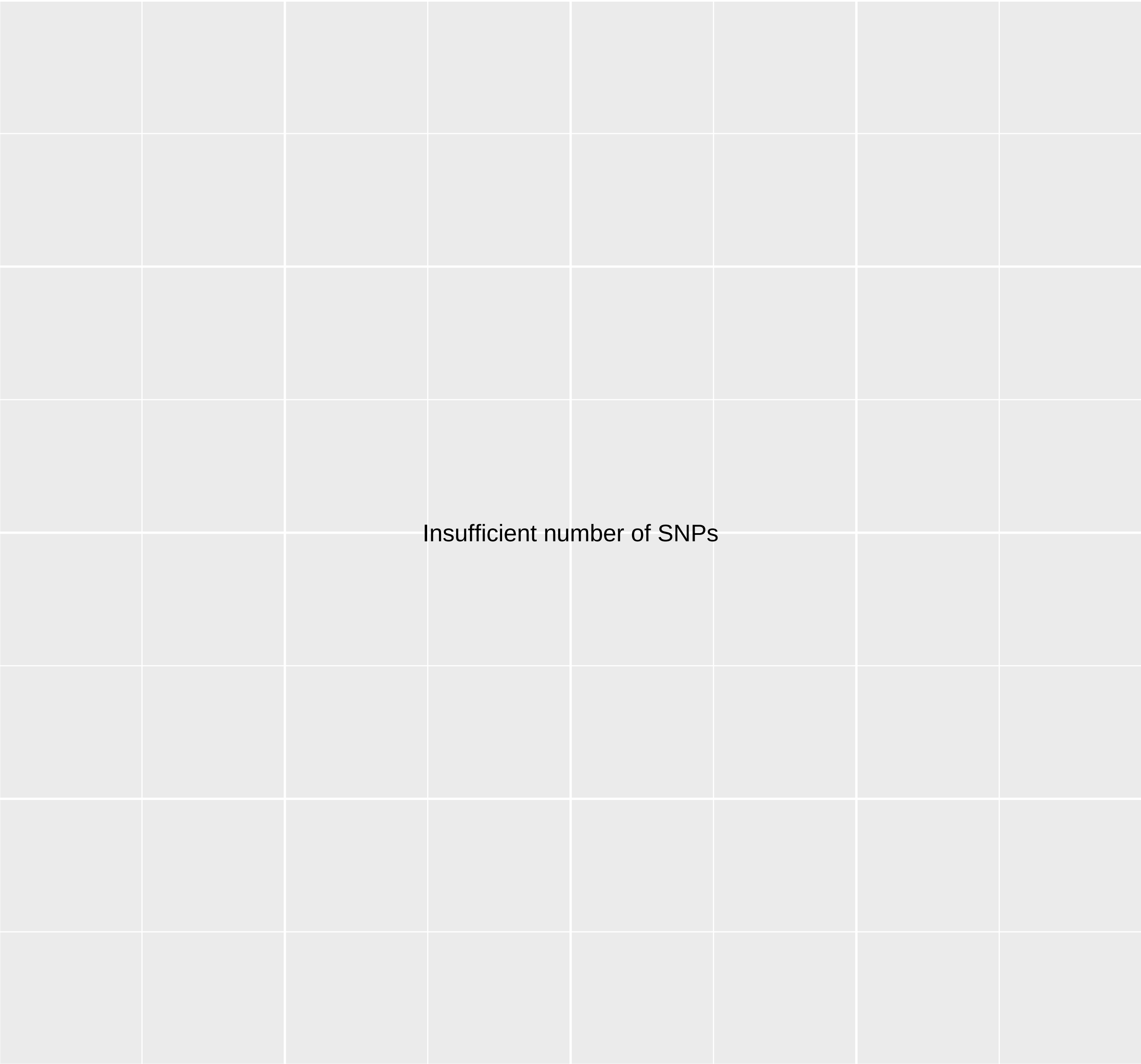
Insufficient number of SNPs

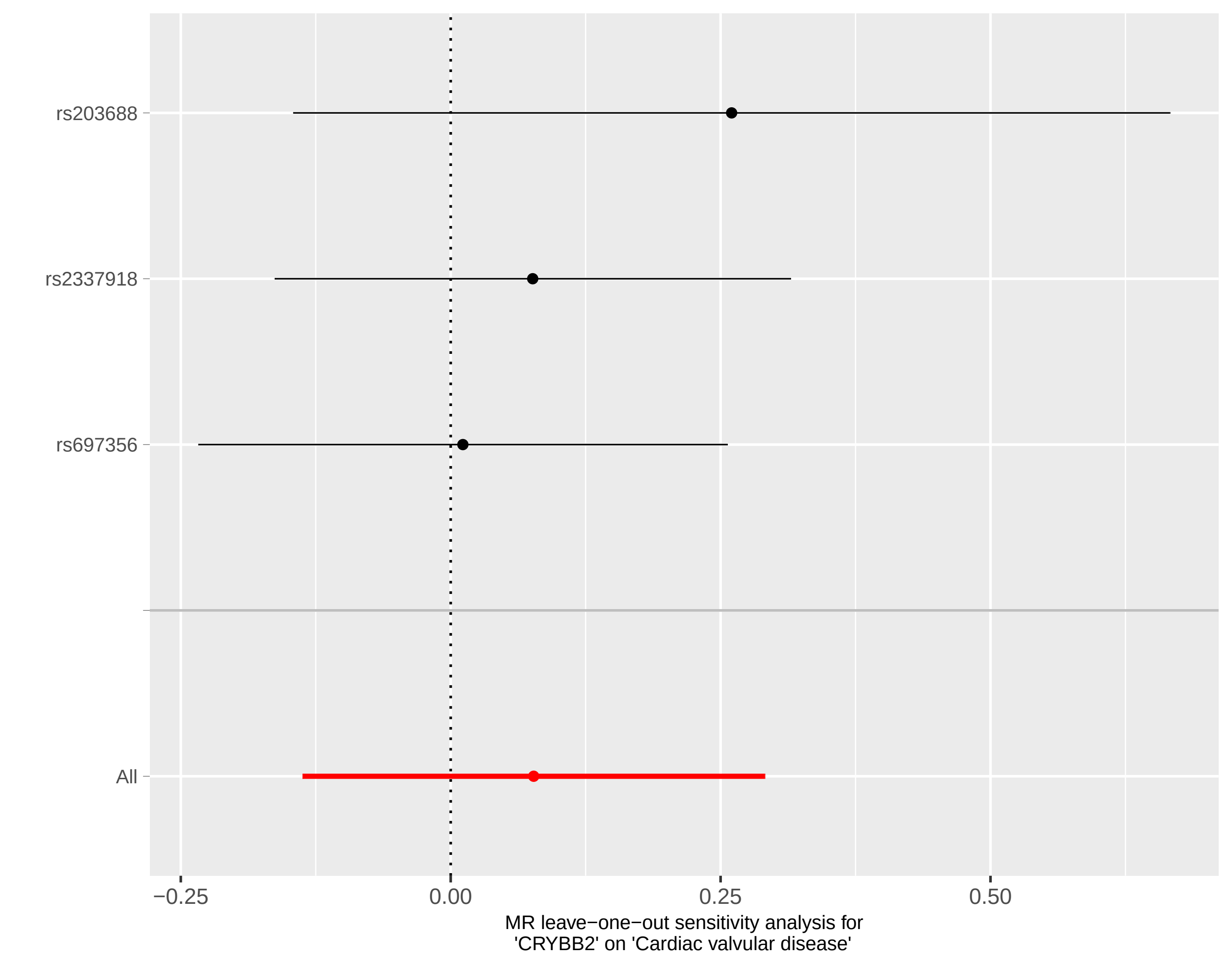

rs203688
rs2337918
rs697356
All
−0.25
0.00
0.25
0.50
MR leave−one−out sensitivity analysis for
'CRYBB2' on 'Cardiac valvular disease'

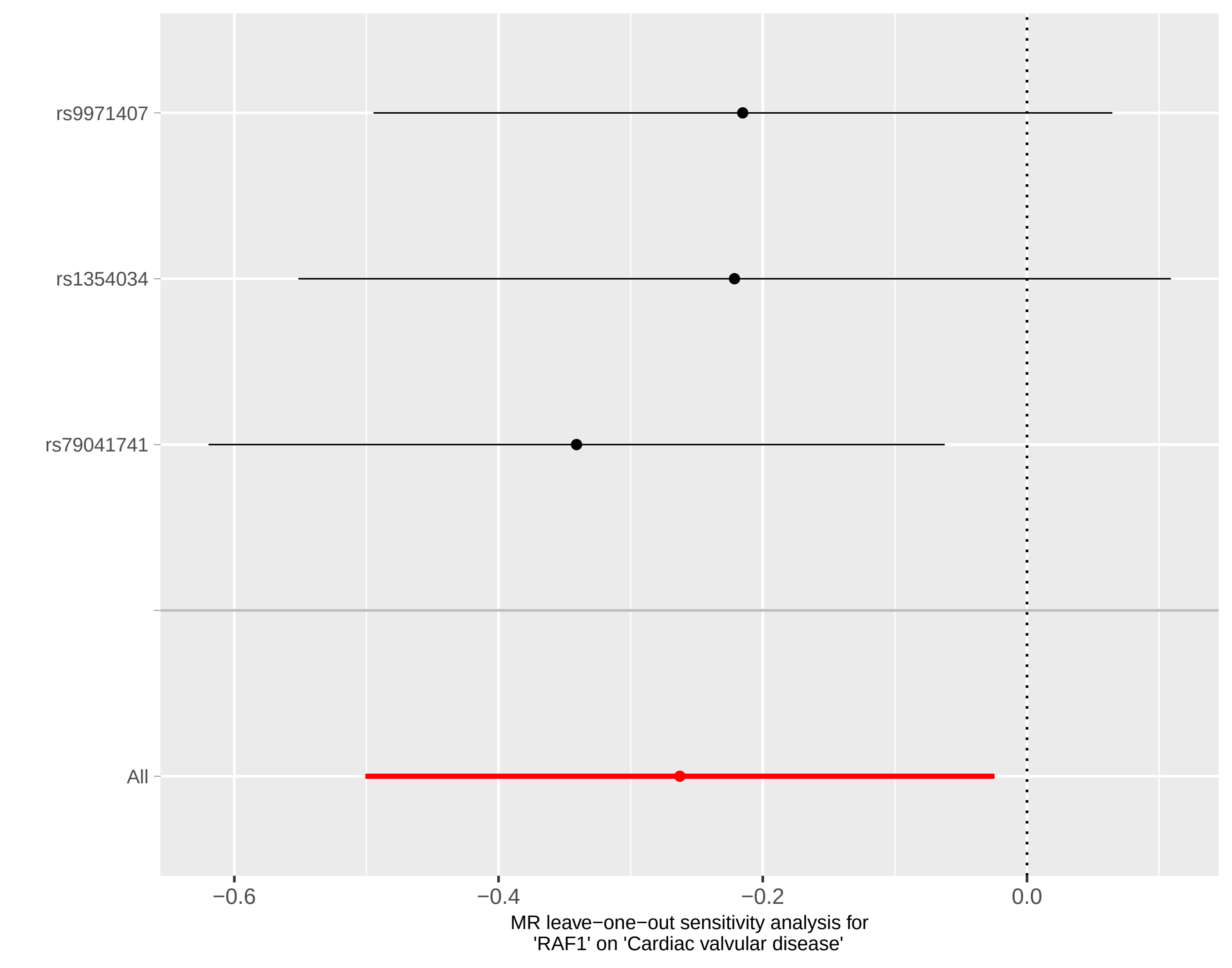

rs9971407
rs1354034
rs79041741
All
−0.6
−0.4
−0.2
0.0
MR leave−one−out sensitivity analysis for
'RAF1' on 'Cardiac valvular disease'

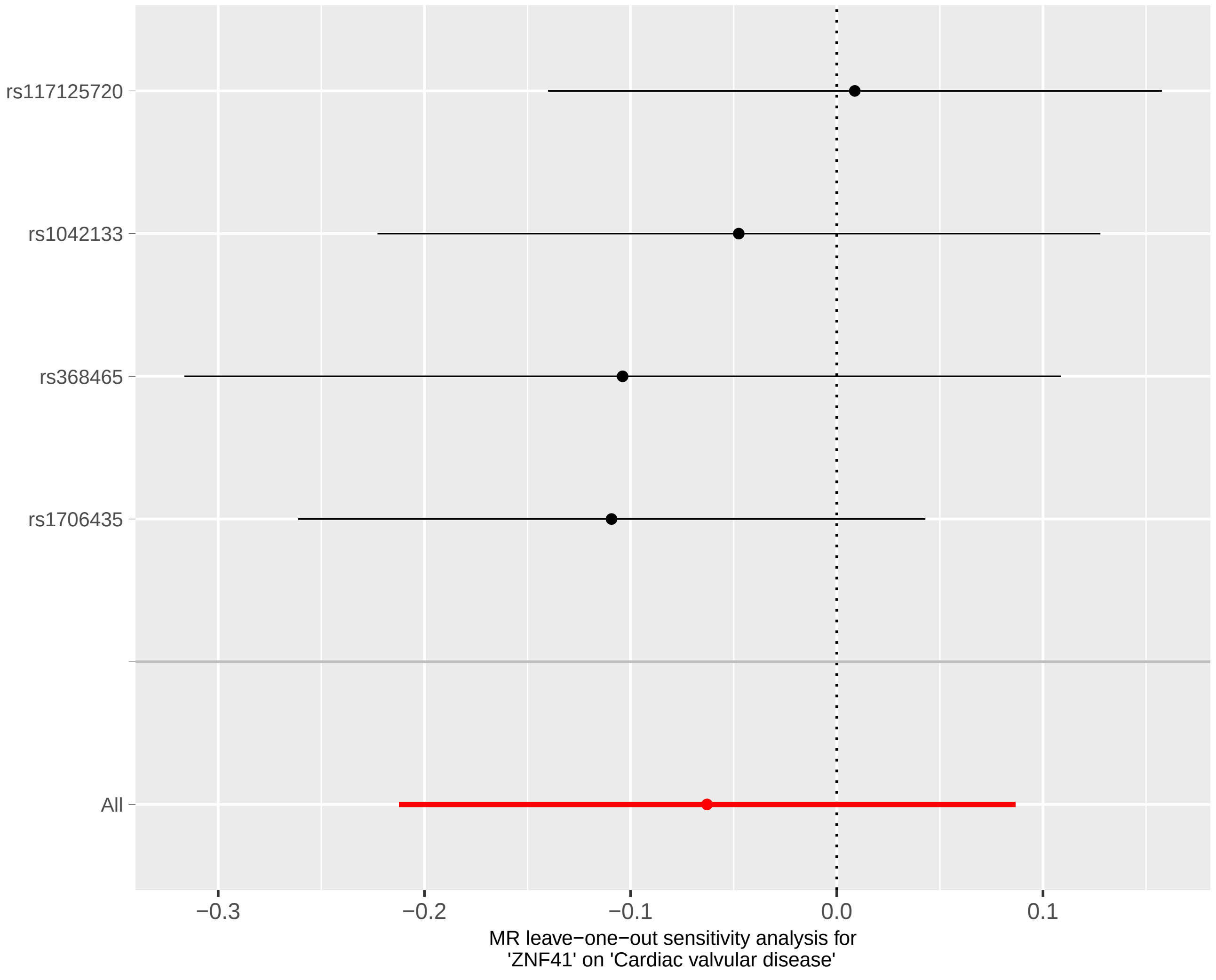

rs117125720
rs1042133
rs368465
rs1706435
All
−0.3
−0.2
−0.1
0.0
0.1
MR leave−one−out sensitivity analysis for
'ZNF41' on 'Cardiac valvular disease'

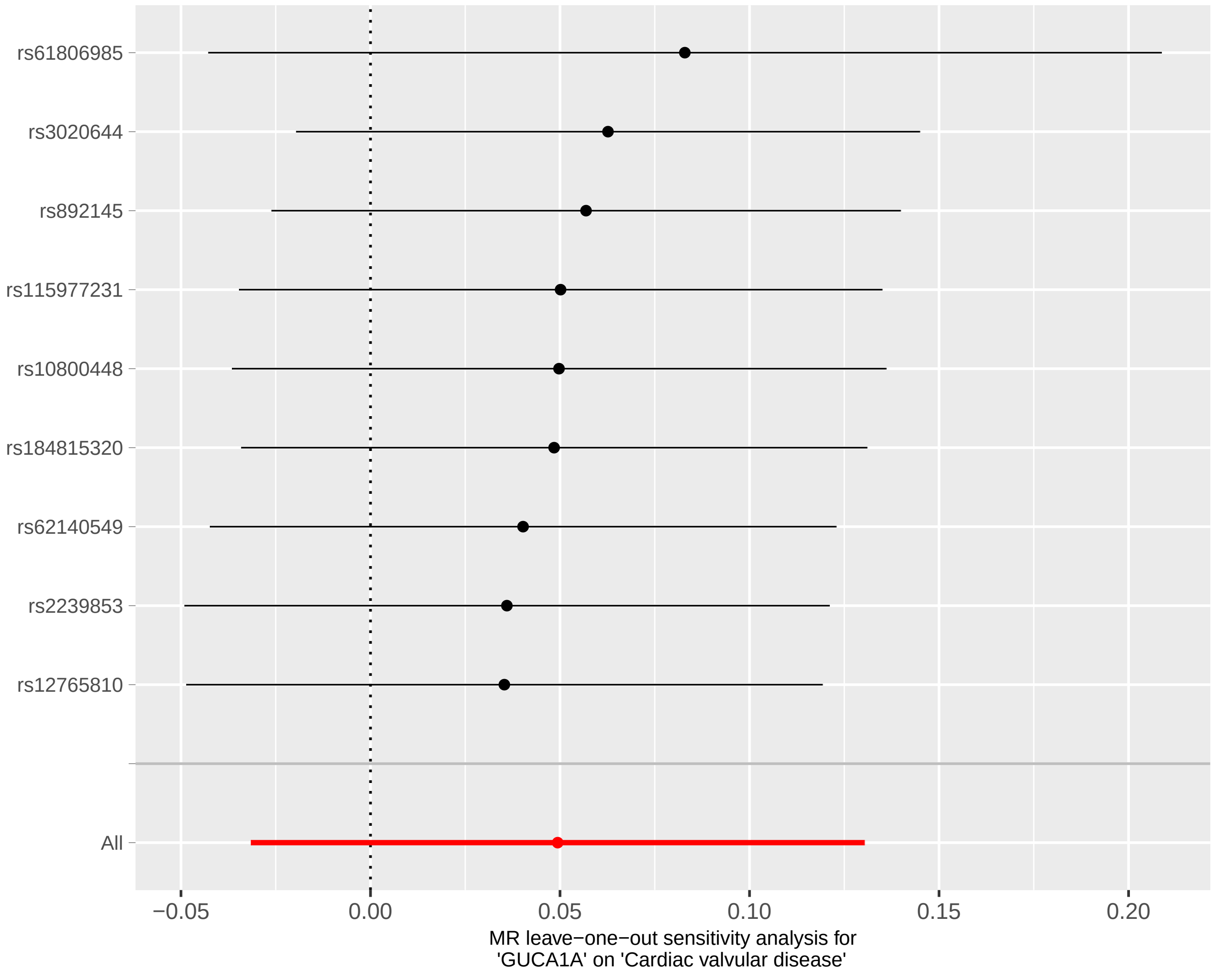

rs61806985
rs3020644
rs892145
rs115977231
rs10800448
rs184815320
rs62140549
rs2239853
rs12765810
All
−0.05
0.00
0.05
0.10
0.15
0.20
MR leave−one−out sensitivity analysis for
'GUCA1A' on 'Cardiac valvular disease'

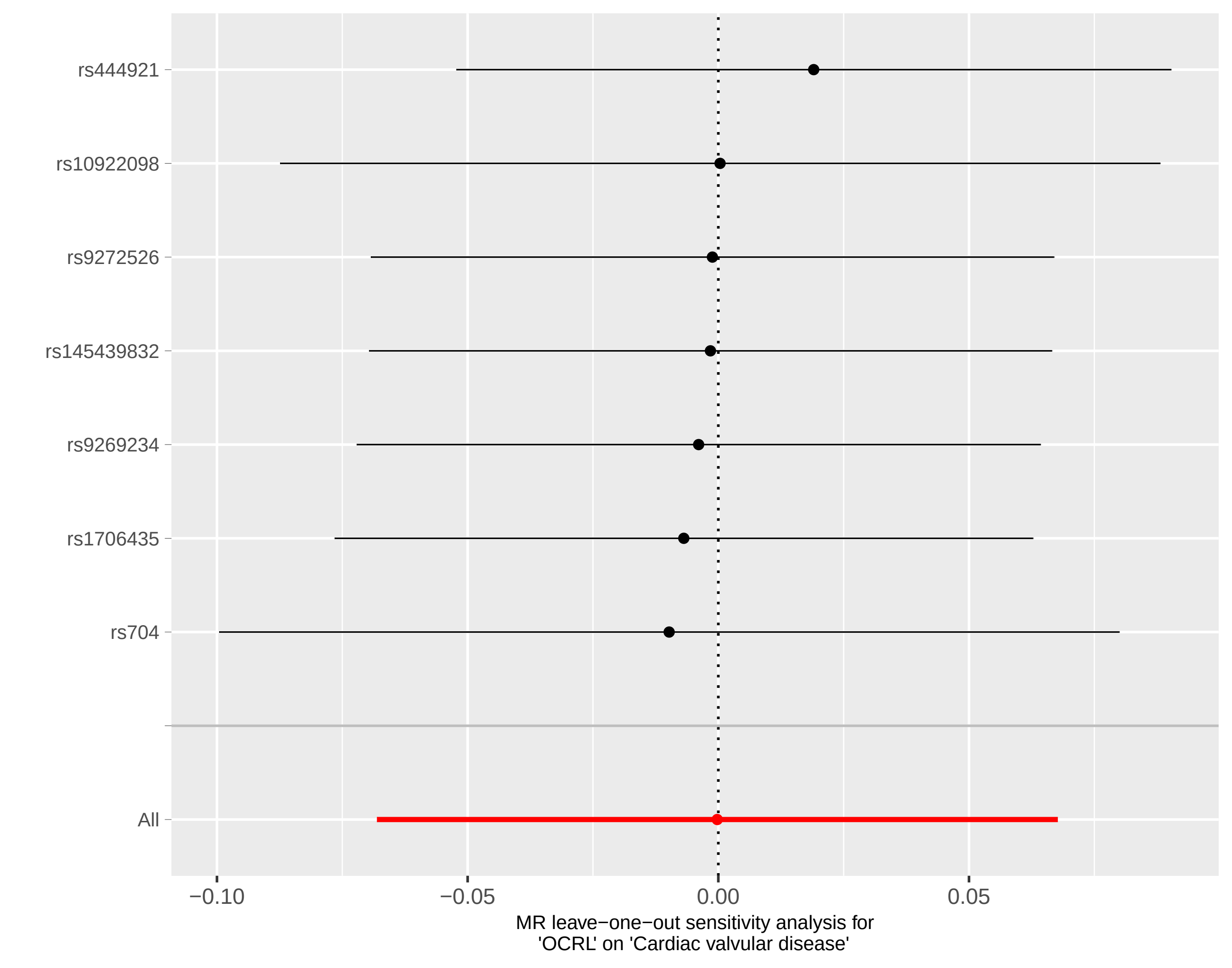
rs444921
rs10922098
rs9272526
rs145439832
rs9269234
rs1706435
rs704
All
−0.10
−0.05
0.00
0.05
MR leave−one−out sensitivity analysis for
'OCRL' on 'Cardiac valvular disease'

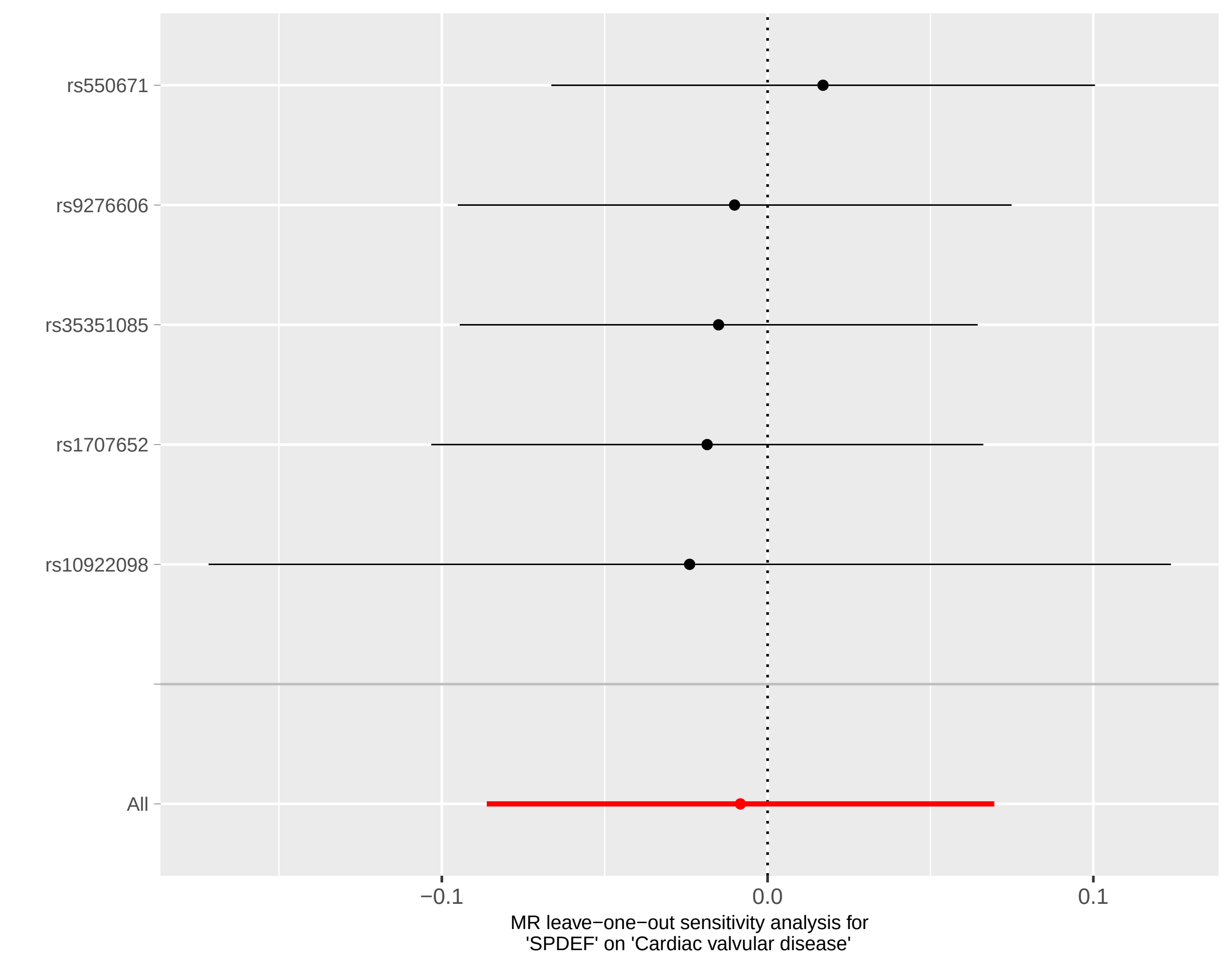
rs550671
rs9276606
rs35351085
rs1707652
rs10922098
All
−0.1
0.0
0.1
MR leave−one−out sensitivity analysis for
'SPDEF' on 'Cardiac valvular disease'

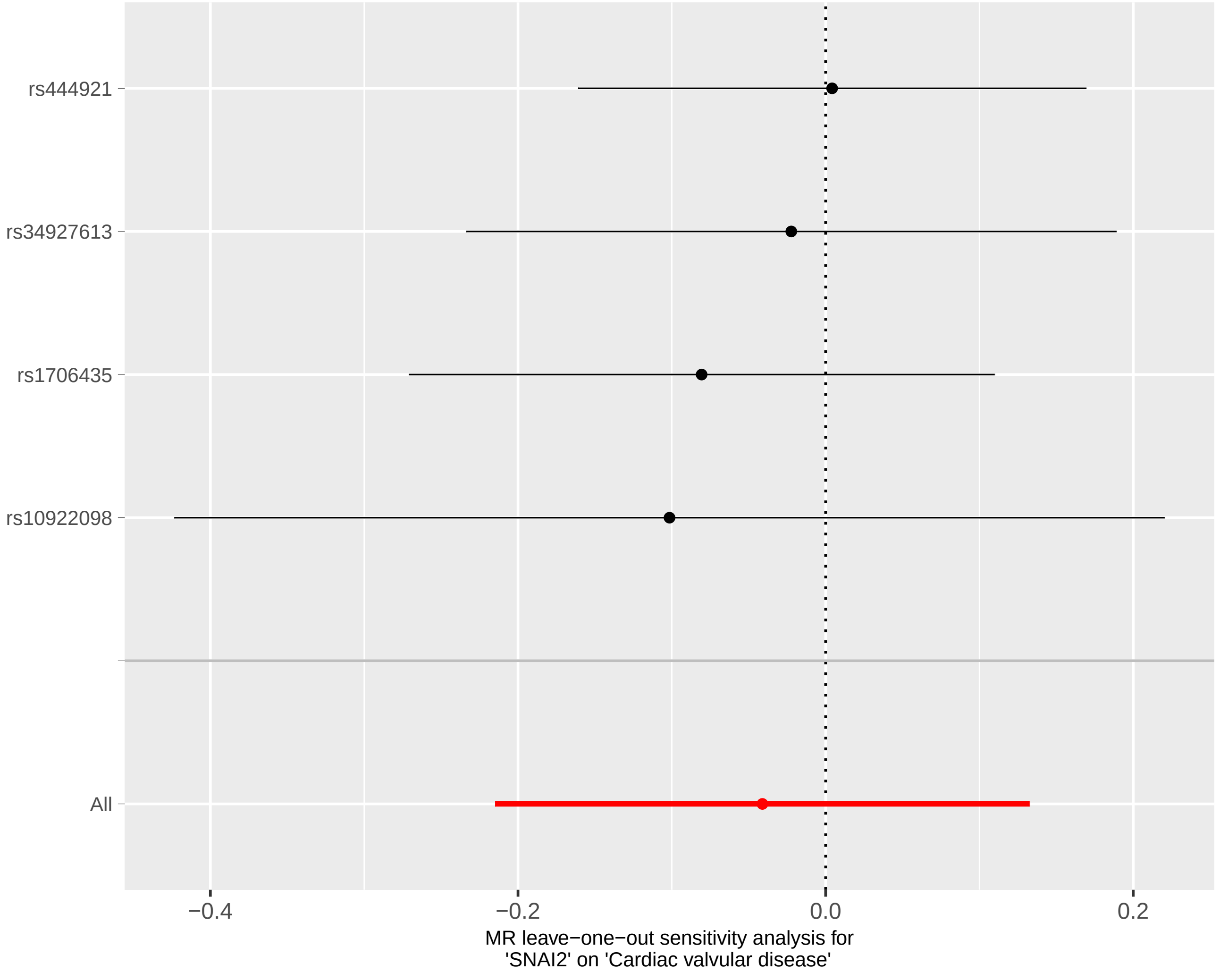
rs444921
rs34927613
rs1706435
rs10922098
All
−0.4
−0.2
0.0
0.2
MR leave−one−out sensitivity analysis for
'SNAI2' on 'Cardiac valvular disease'

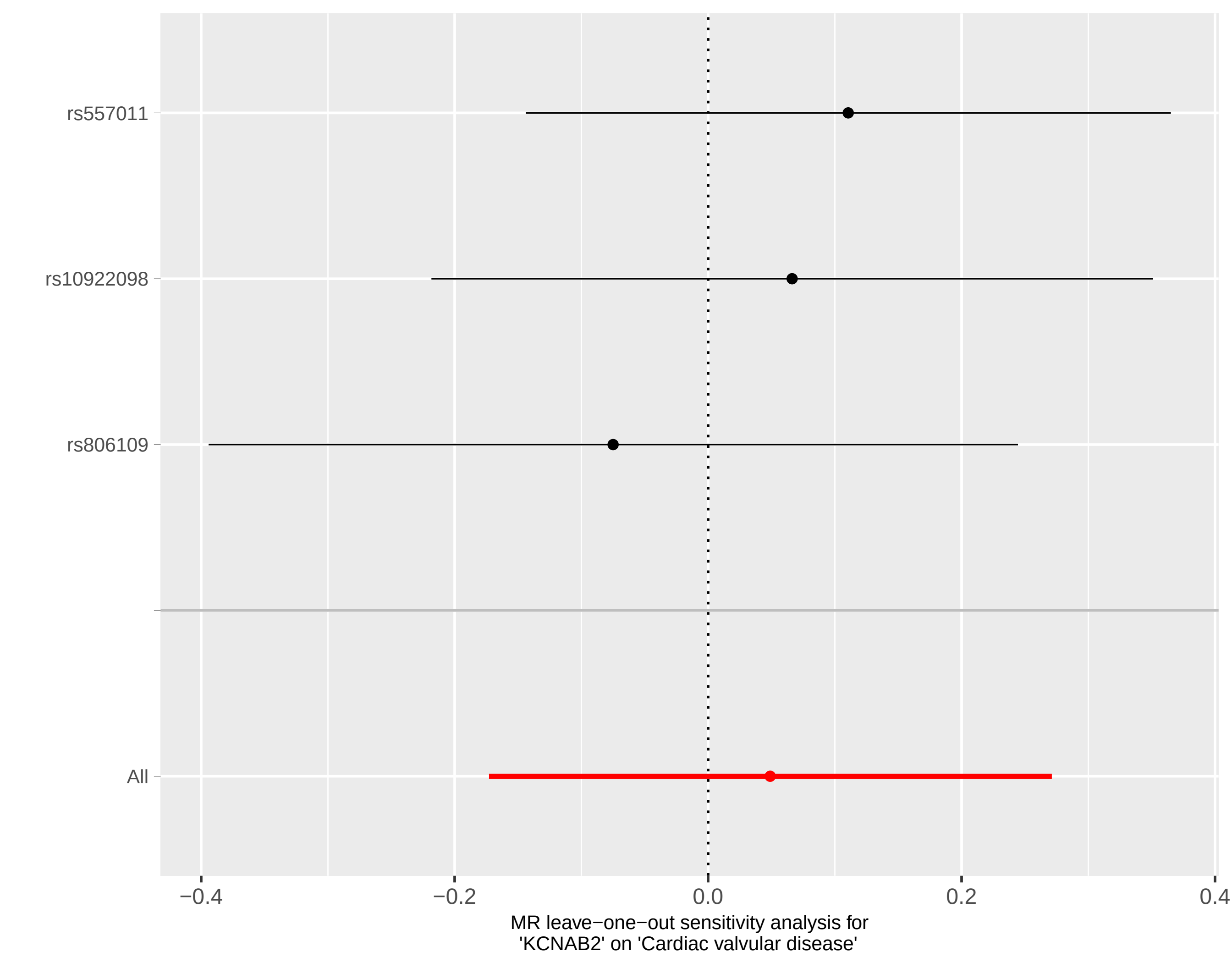

rs557011
rs10922098
rs806109
All
−0.4
−0.2
0.0
0.2
0.4
MR leave−one−out sensitivity analysis for
'KCNAB2' on 'Cardiac valvular disease'

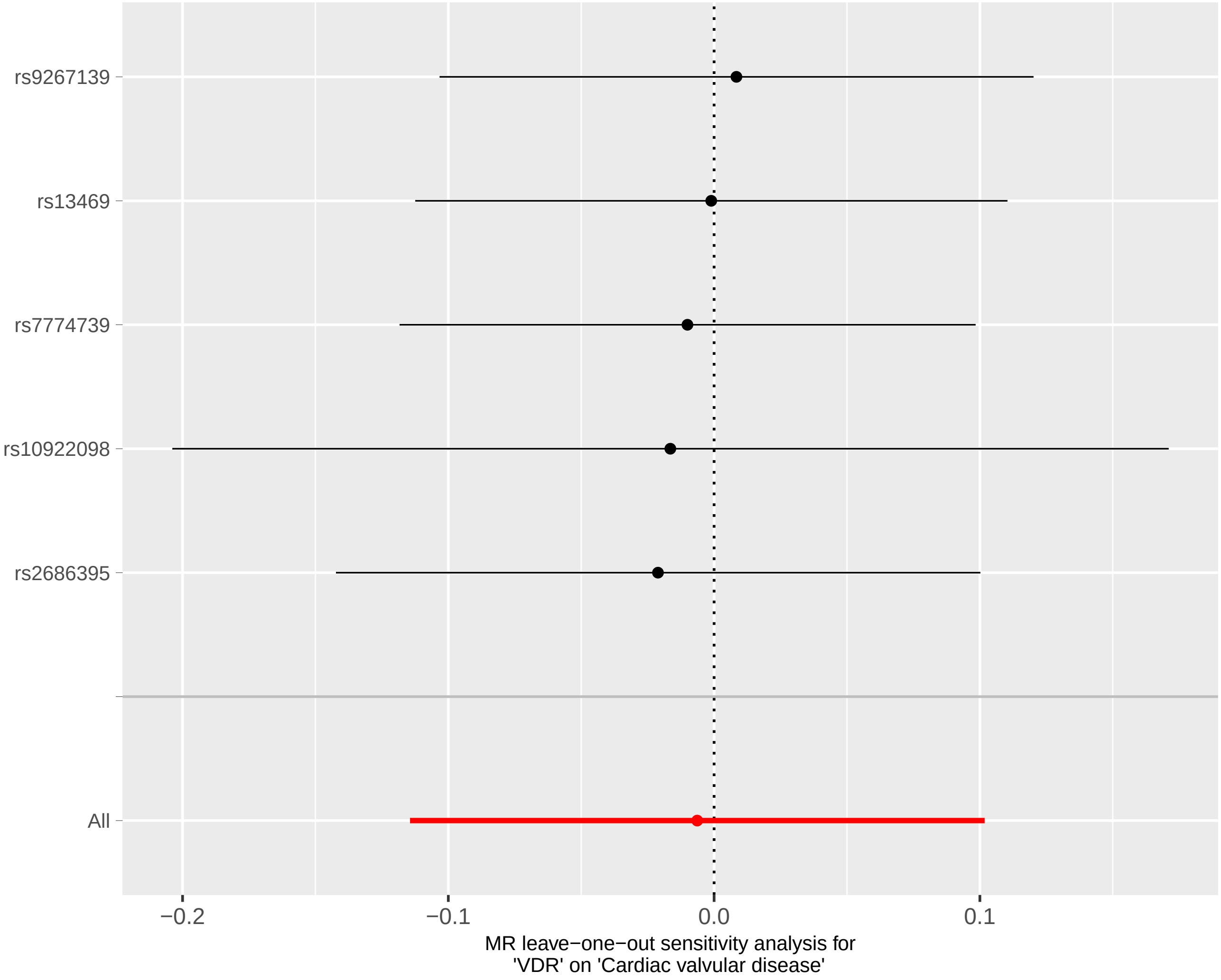

rs9267139
rs13469
rs7774739
rs10922098
rs2686395
All
−0.2
−0.1
0.0
0.1
MR leave−one−out sensitivity analysis for
'VDR' on 'Cardiac valvular disease'

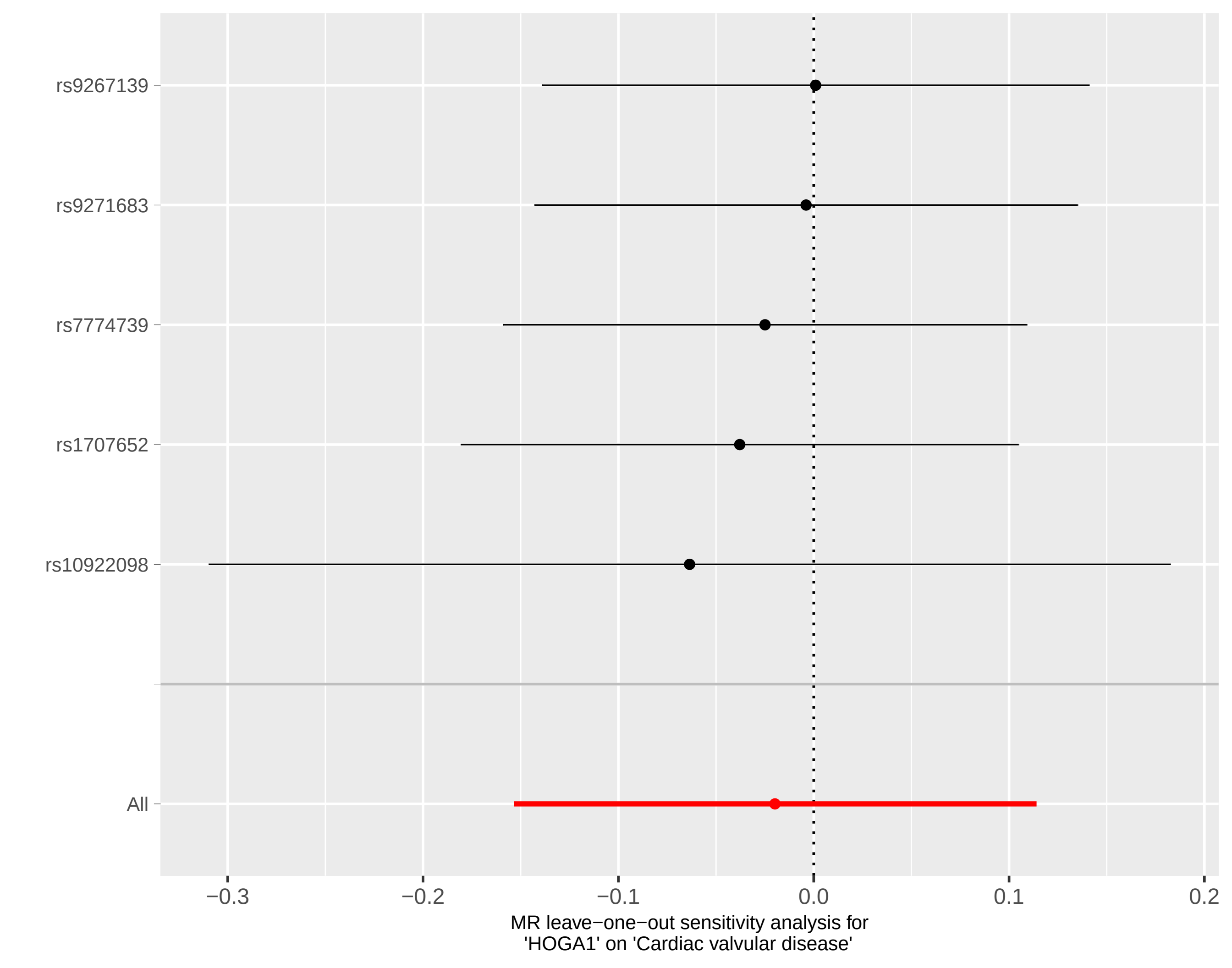
rs9267139
rs9271683
rs7774739
rs1707652
rs10922098
All
−0.3
−0.2
−0.1
0.0
0.1
0.2
MR leave−one−out sensitivity analysis for
'HOGA1' on 'Cardiac valvular disease'

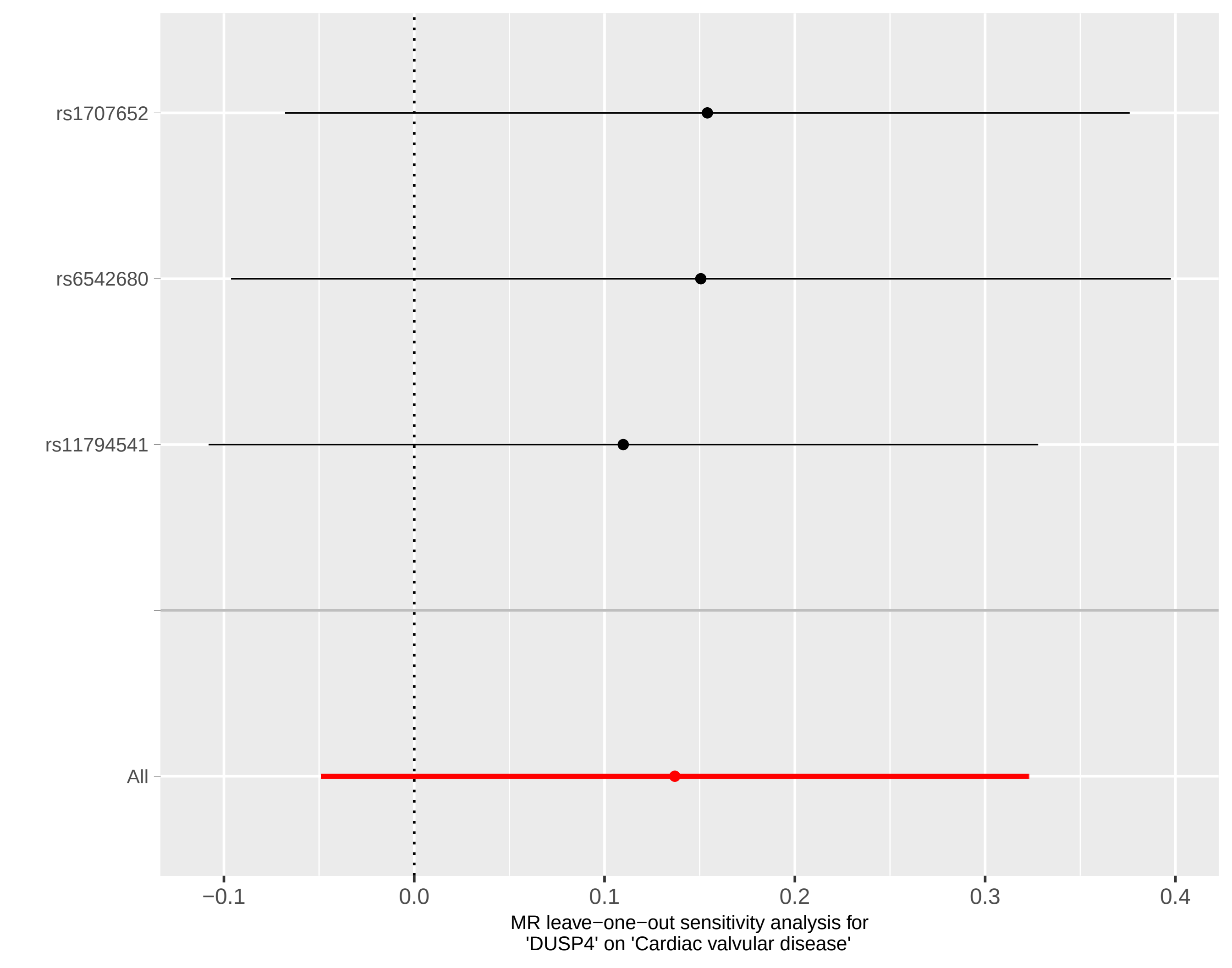
rs1707652
rs6542680
rs11794541
All
−0.1
0.0
0.1
0.2
0.3
0.4
MR leave−one−out sensitivity analysis for
'DUSP4' on 'Cardiac valvular disease'

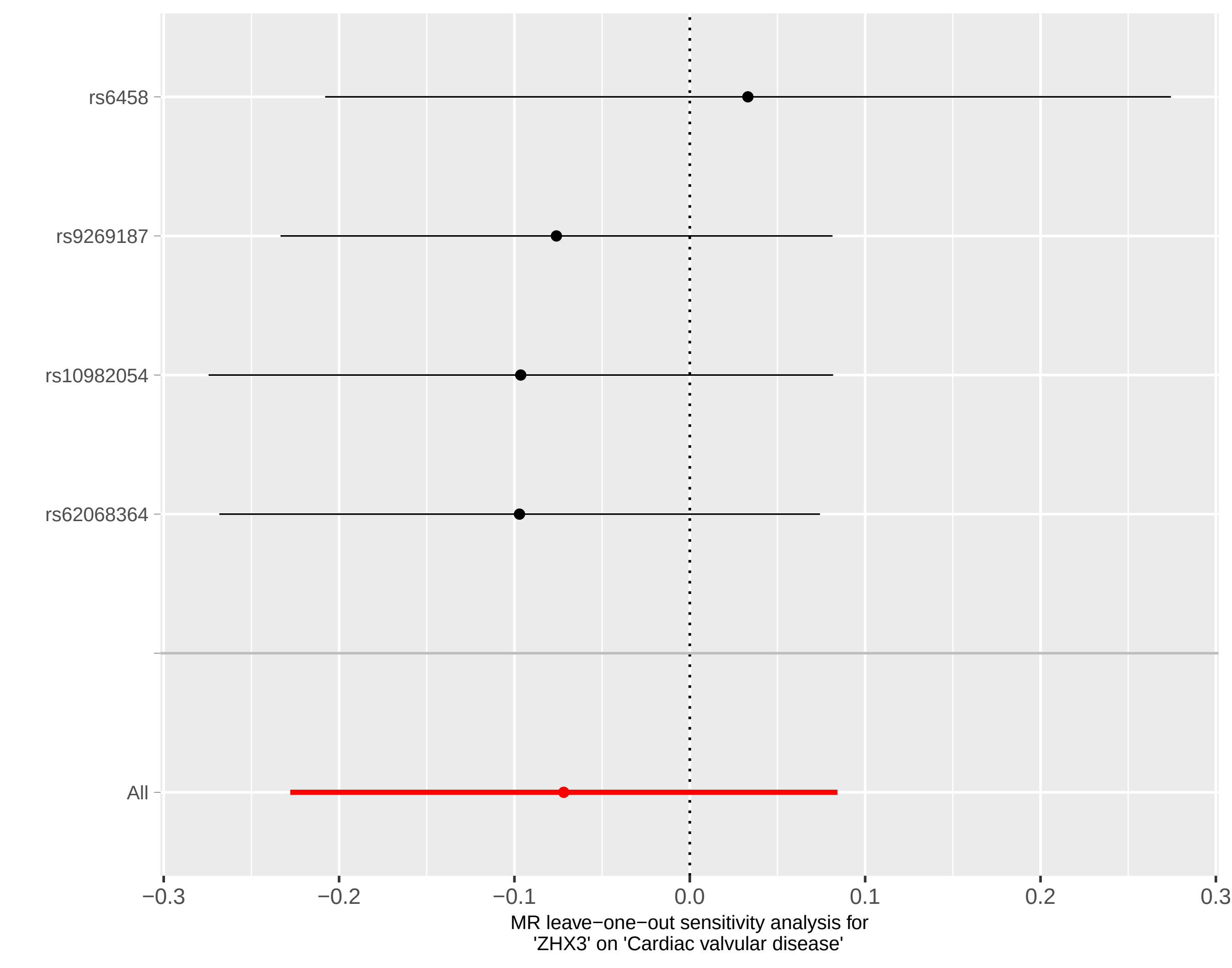

rs6458
rs9269187
rs10982054
rs62068364
All
−0.3
−0.2
−0.1
0.0
0.1
0.2
0.3
MR leave−one−out sensitivity analysis for
'ZHX3' on 'Cardiac valvular disease'

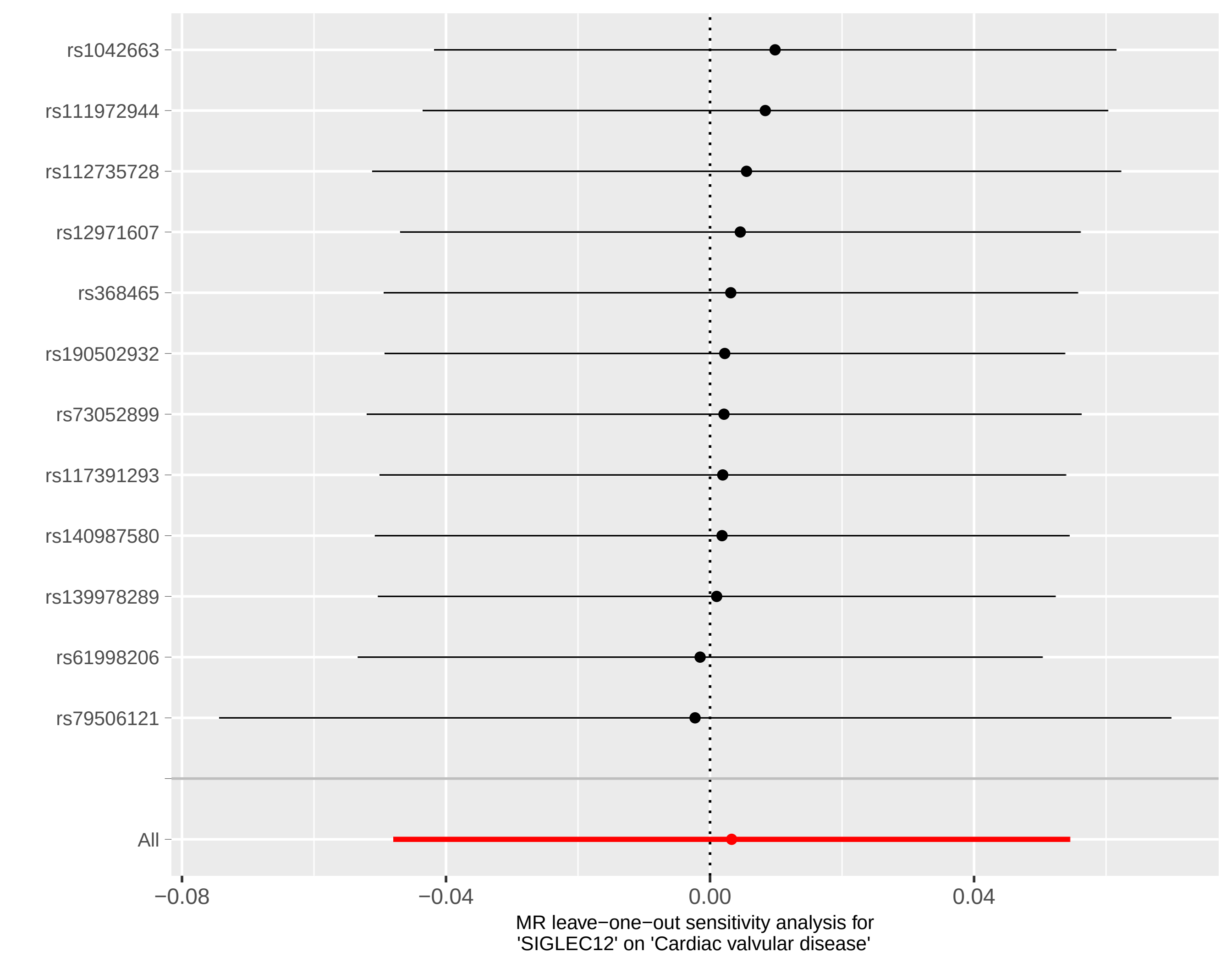

rs1042663
rs111972944
rs112735728
rs12971607
rs368465
rs190502932
rs73052899
rs117391293
rs140987580
rs139978289
rs61998206
rs79506121
All
−0.08
−0.04
0.00
0.04
MR leave−one−out sensitivity analysis for
'SIGLEC12' on 'Cardiac valvular disease'

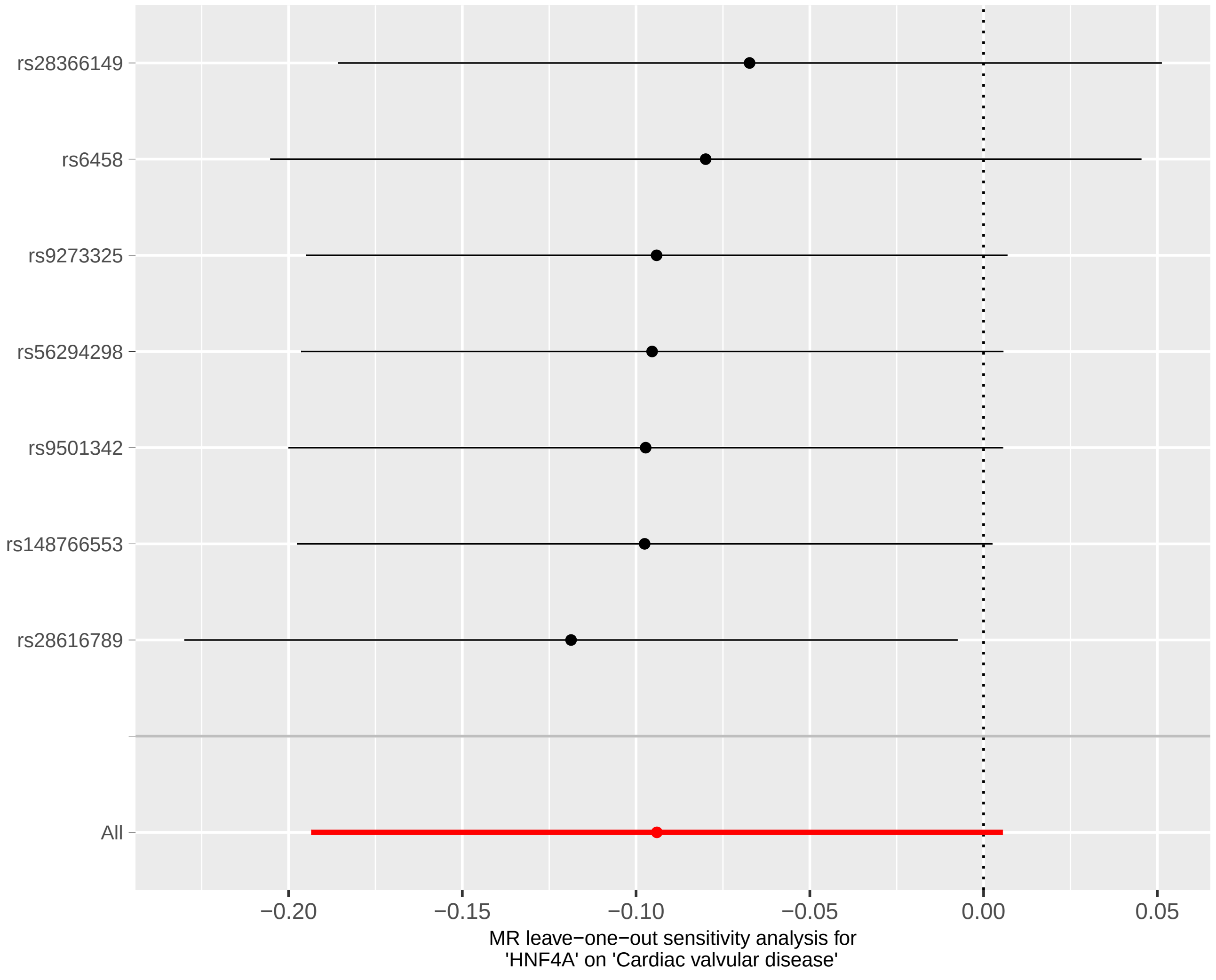
rs28366149
rs6458
rs9273325
rs56294298
rs9501342
rs148766553
rs28616789
All
−0.20
−0.15
−0.10
−0.05
0.00
0.05
MR leave−one−out sensitivity analysis for
'HNF4A' on 'Cardiac valvular disease'

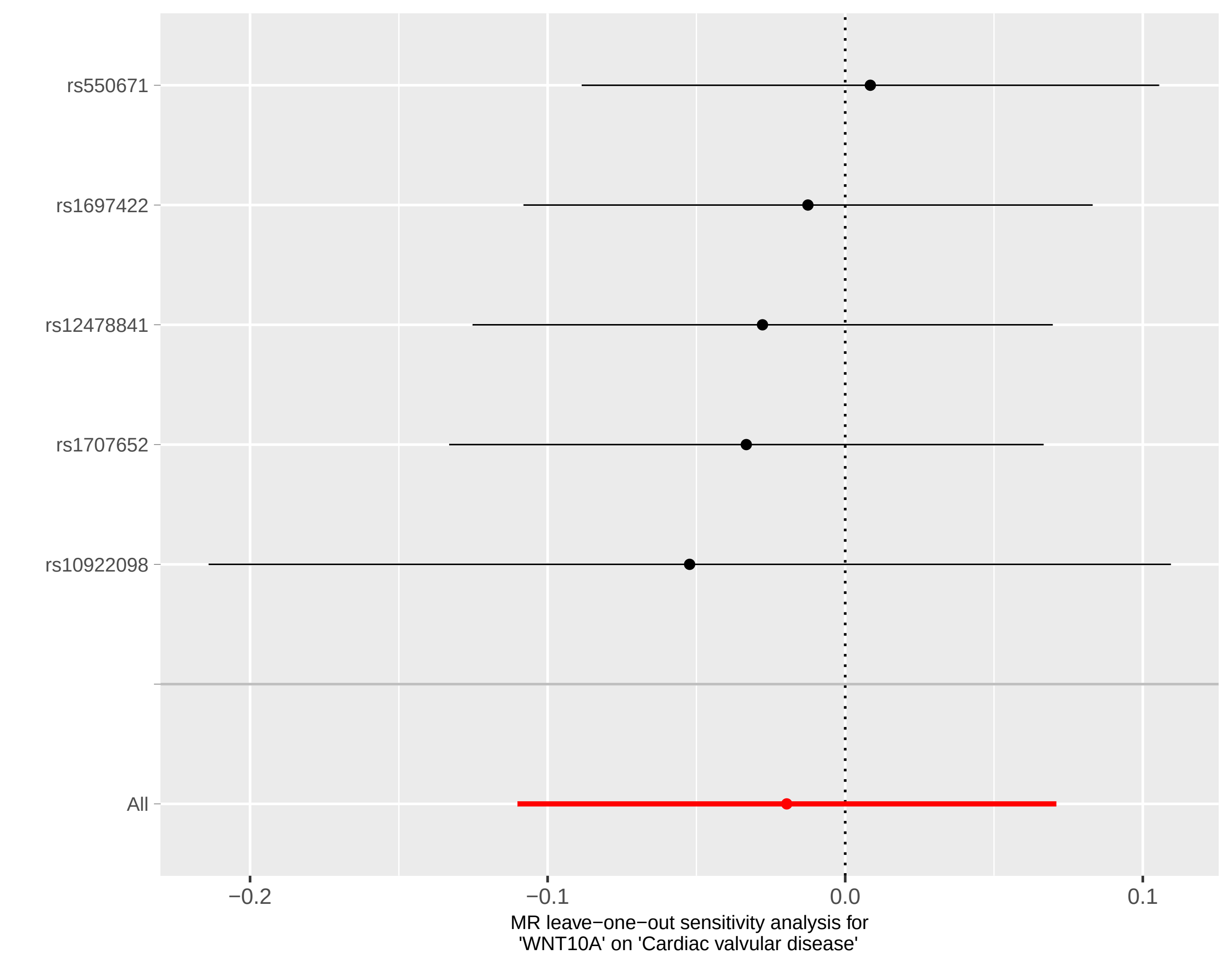
rs550671
rs1697422
rs12478841
rs1707652
rs10922098
All
−0.2
−0.1
0.0
0.1
MR leave−one−out sensitivity analysis for
'WNT10A' on 'Cardiac valvular disease'

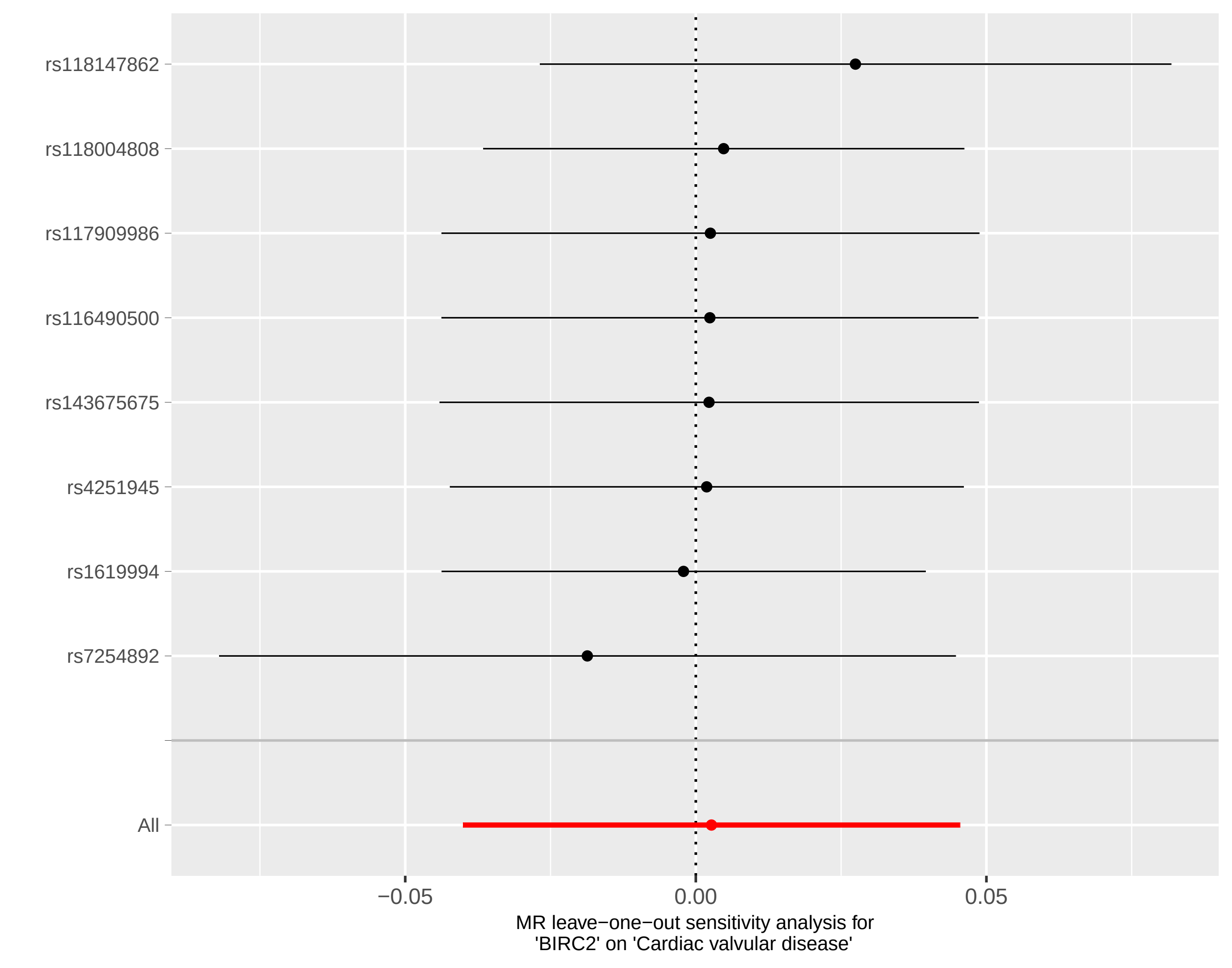
rs118147862
rs118004808
rs117909986
rs116490500
rs143675675
rs4251945
rs1619994
rs7254892
All
−0.05
0.00
0.05
MR leave−one−out sensitivity analysis for
'BIRC2' on 'Cardiac valvular disease'

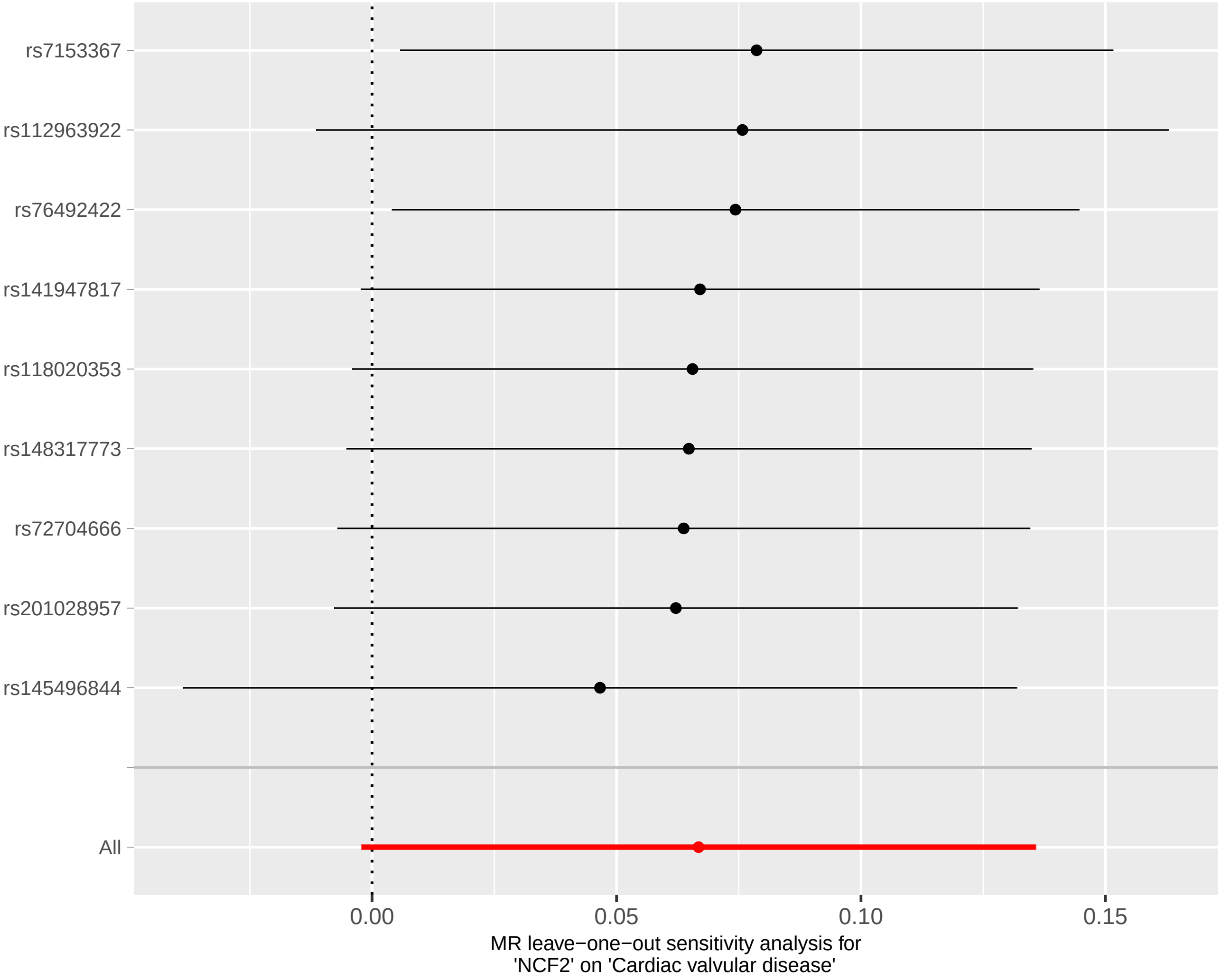

rs7153367
rs112963922
rs76492422
rs141947817
rs118020353
rs148317773
rs72704666
rs201028957
rs145496844
All
0.00
0.05
0.10
0.15
MR leave−one−out sensitivity analysis for
'NCF2' on 'Cardiac valvular disease'

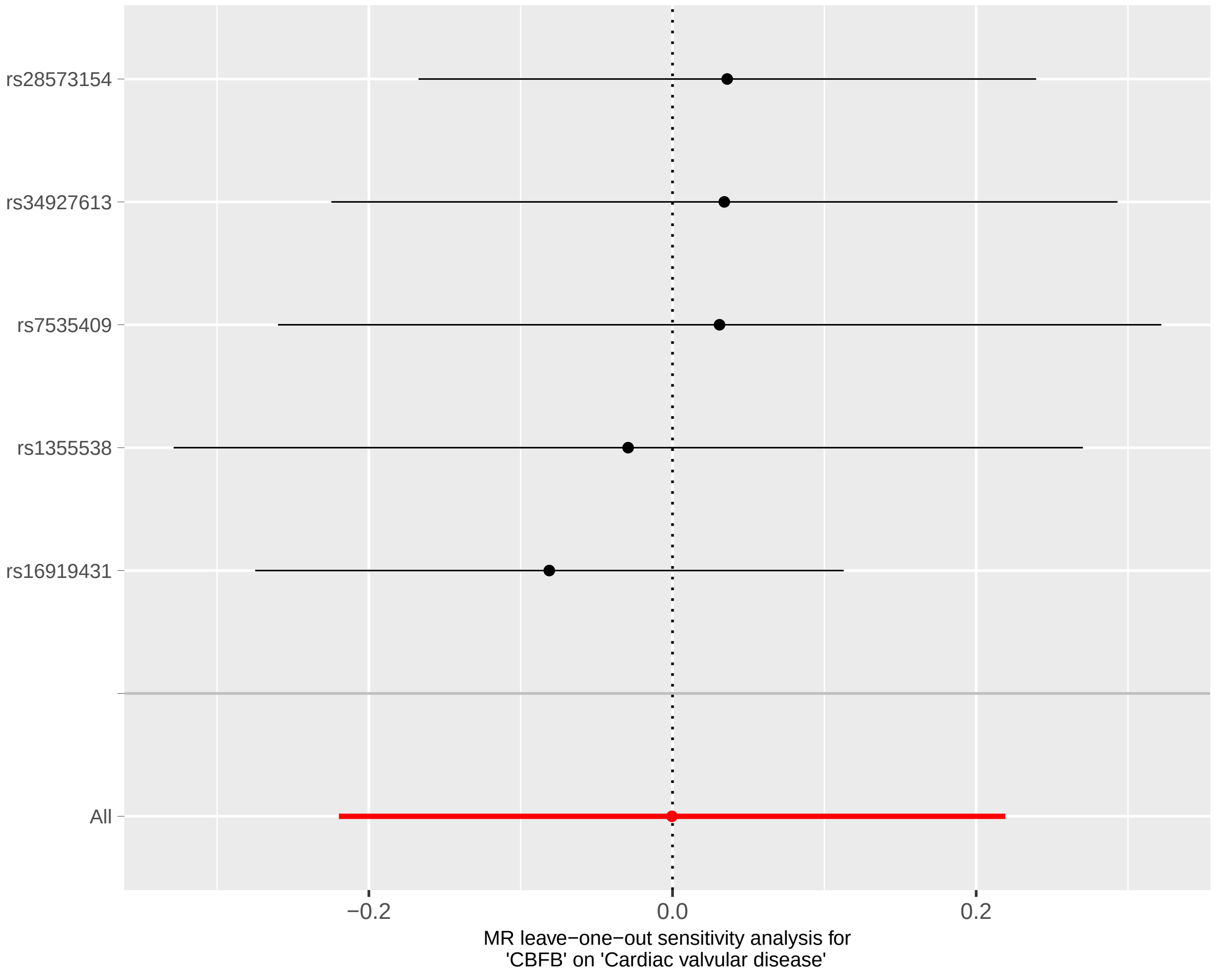
rs28573154
rs34927613
rs7535409
rs1355538
rs16919431
All
−0.2
0.0
0.2
MR leave−one−out sensitivity analysis for
'CBFB' on 'Cardiac valvular disease'

Insufficient number of SNPs

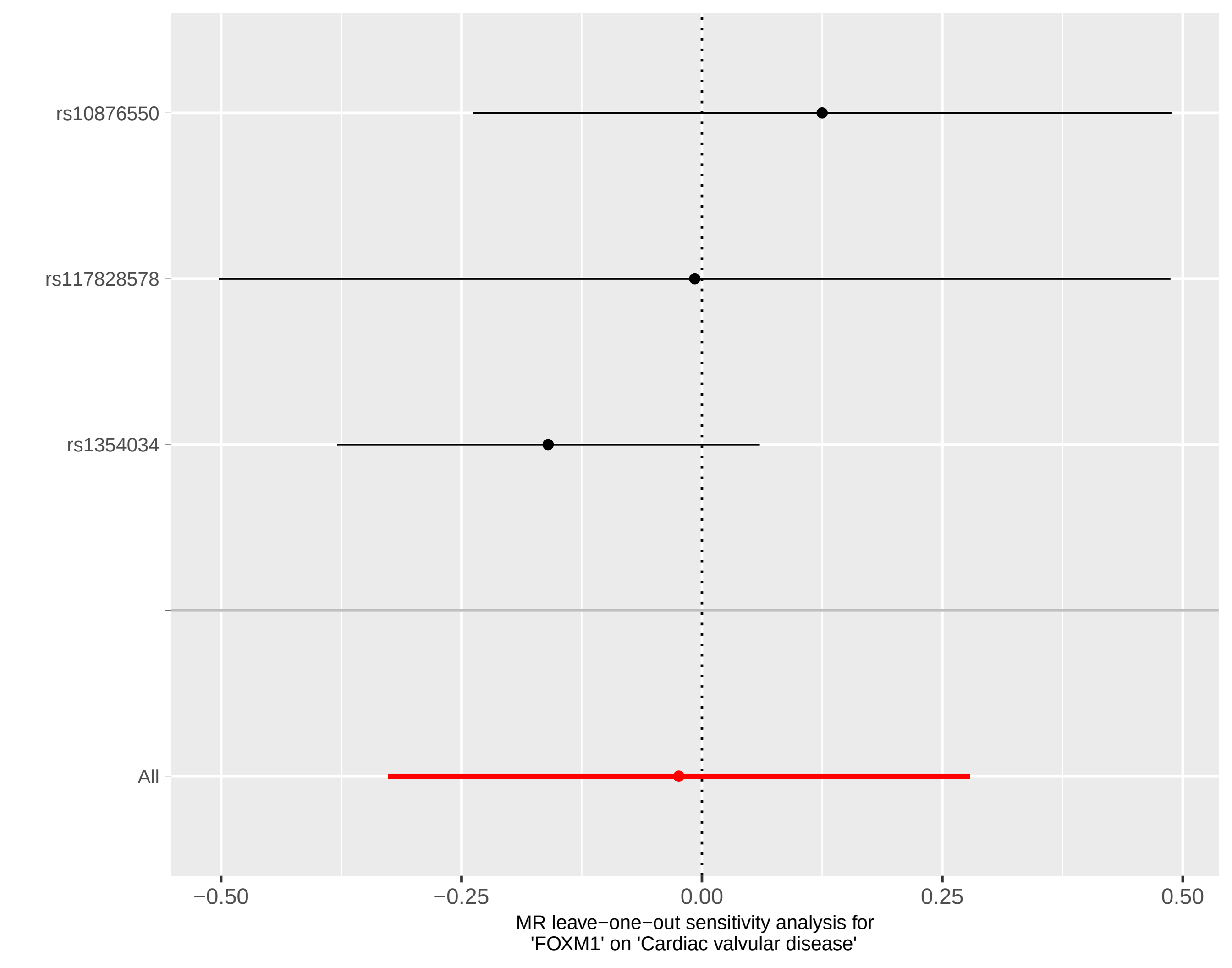

rs10876550
rs117828578
rs1354034
All
−0.50
−0.25
0.00
0.25
0.50
MR leave−one−out sensitivity analysis for
'FOXM1' on 'Cardiac valvular disease'

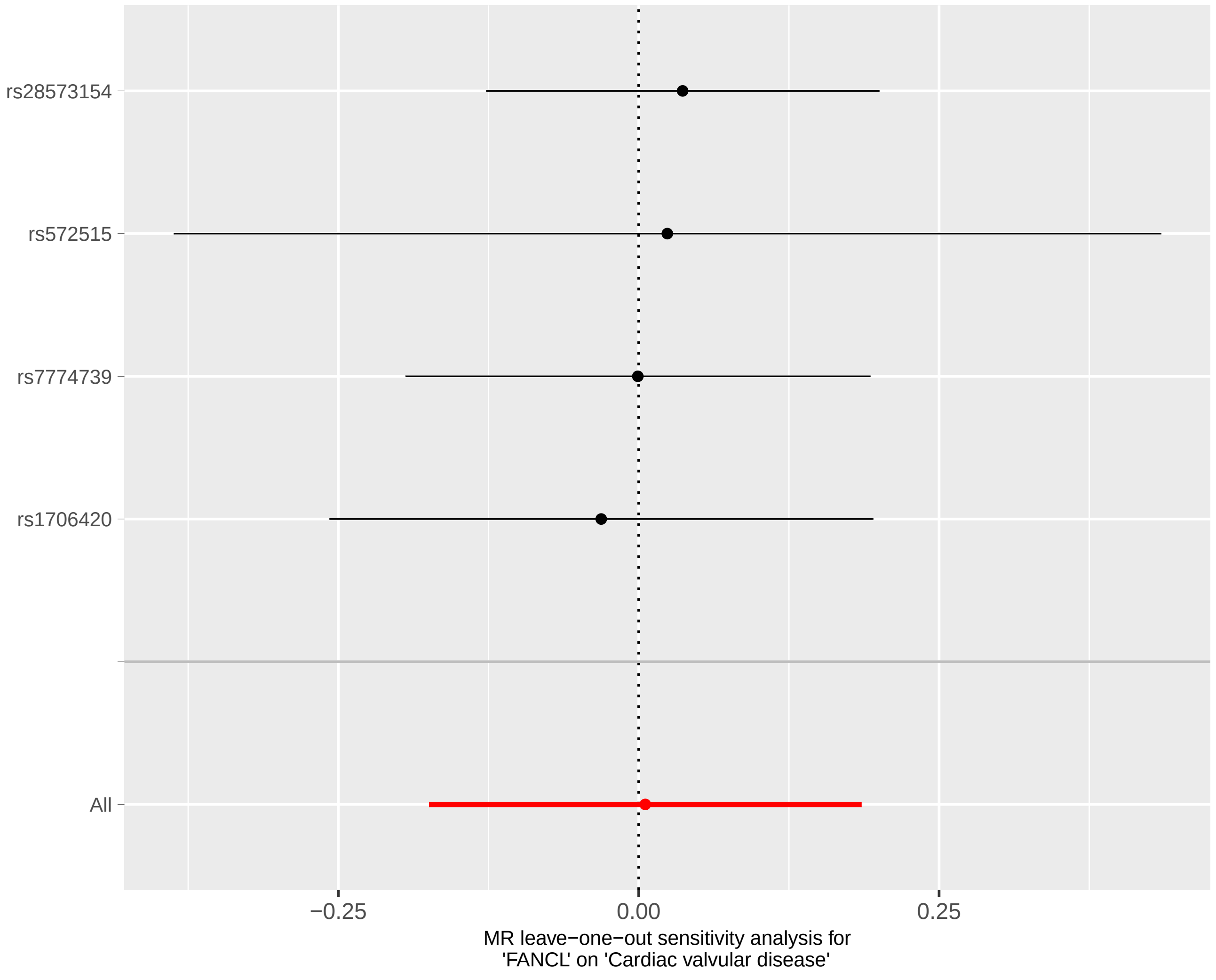

rs28573154
rs572515
rs7774739
rs1706420
All
−0.25
0.00
0.25
MR leave−one−out sensitivity analysis for
'FANCL' on 'Cardiac valvular disease'

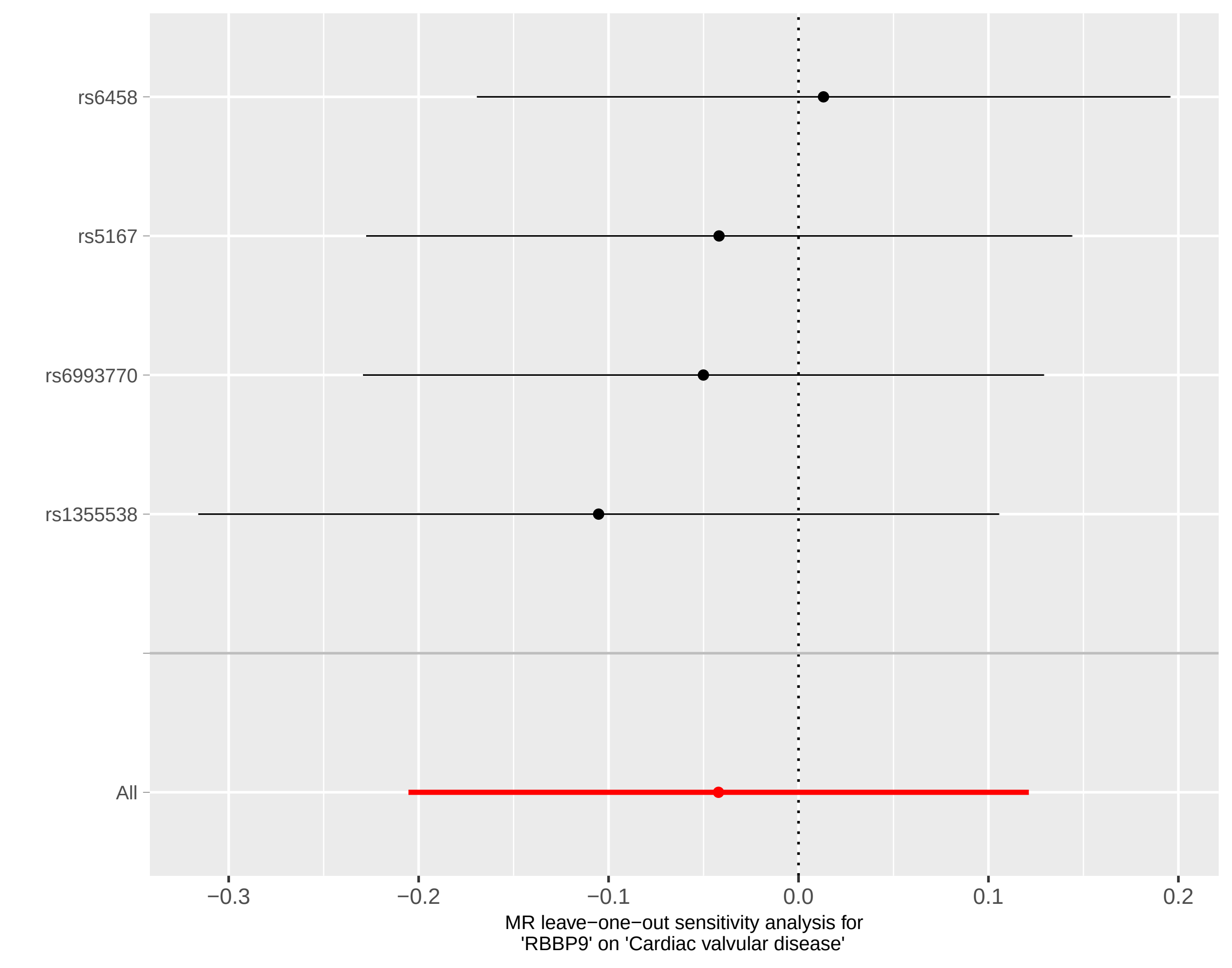
rs6458
rs5167
rs6993770
rs1355538
All
−0.3
−0.2
−0.1
0.0
0.1
0.2
MR leave−one−out sensitivity analysis for
'RBBP9' on 'Cardiac valvular disease'

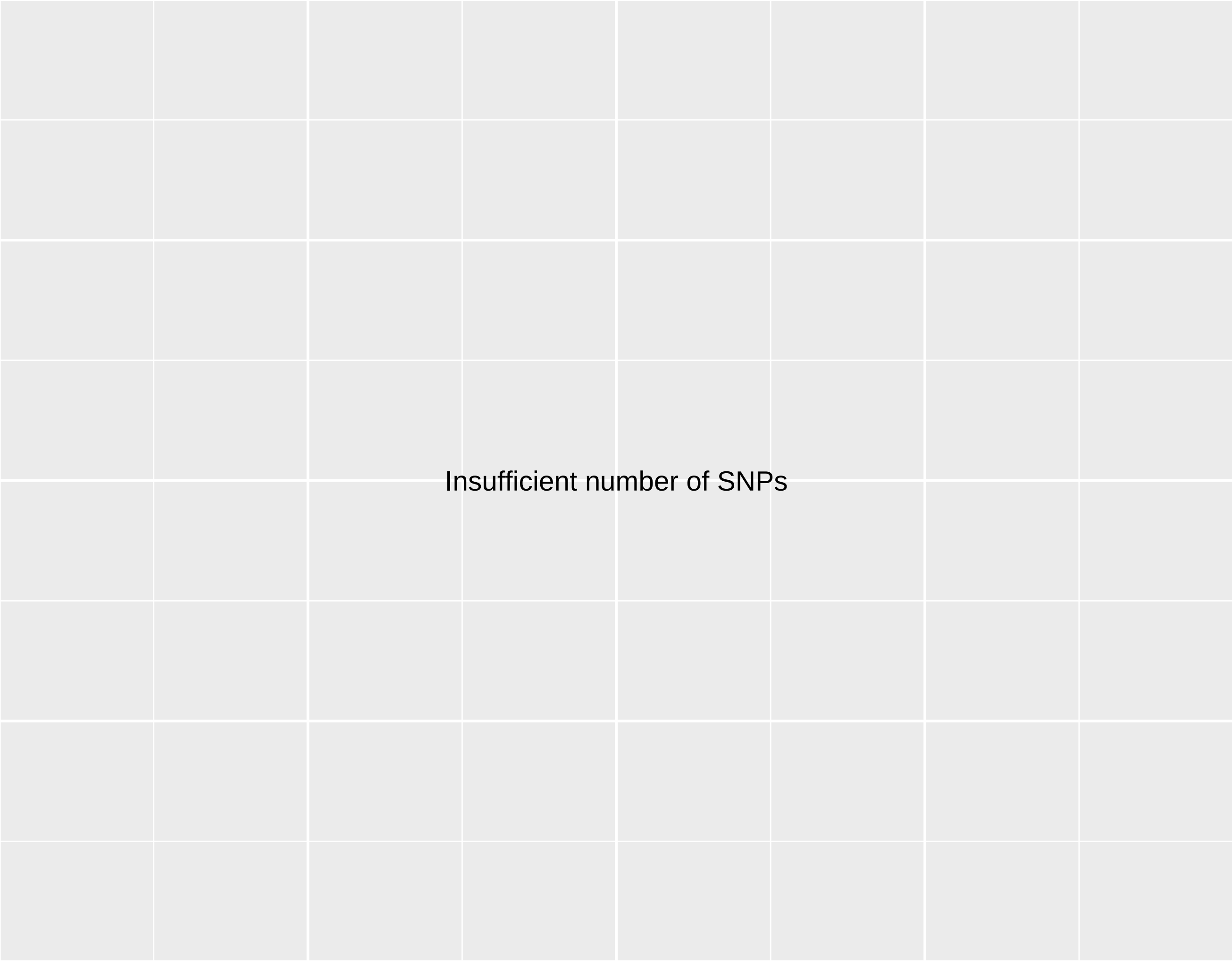
Insufficient number of SNPs

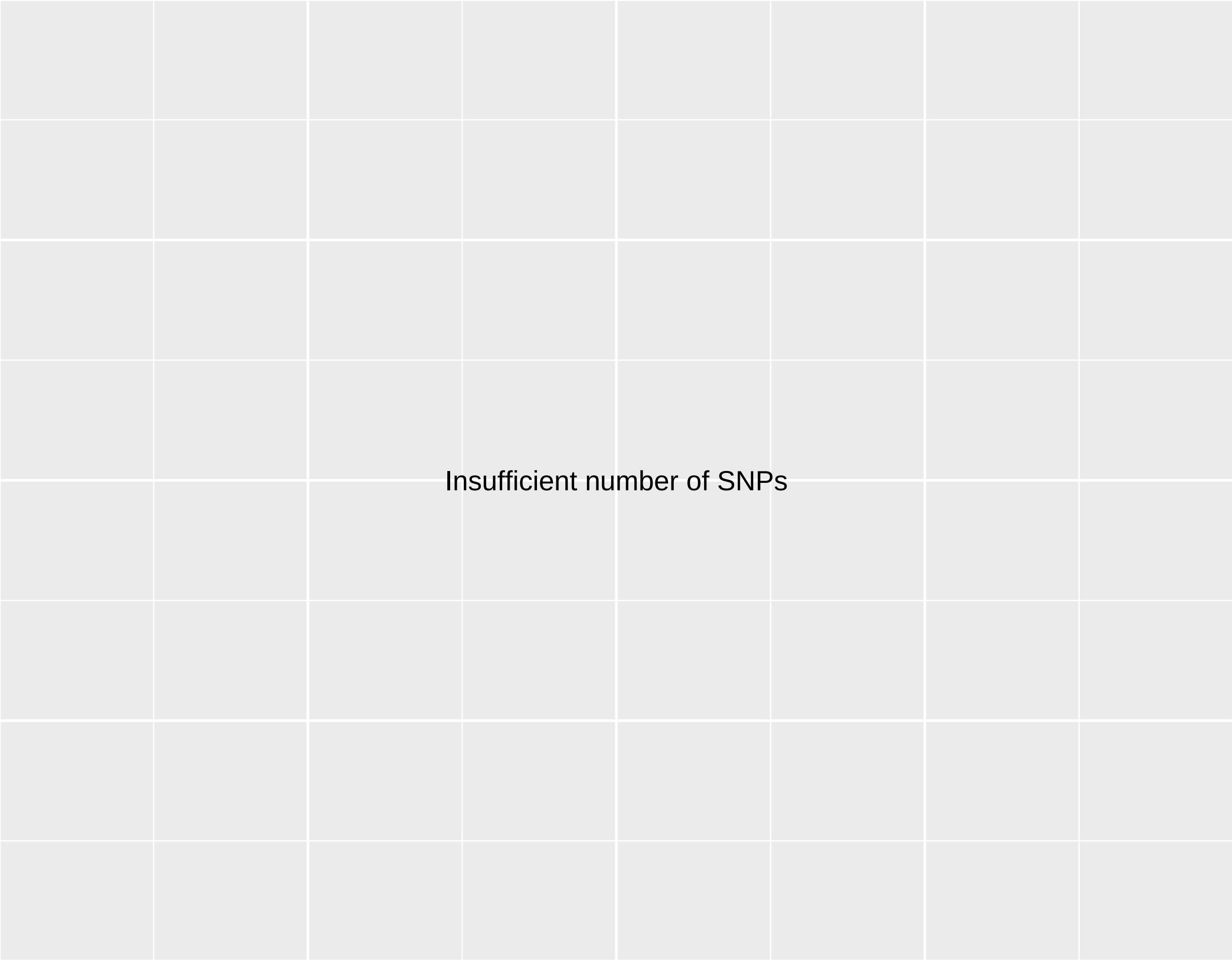
Insufficient number of SNPs

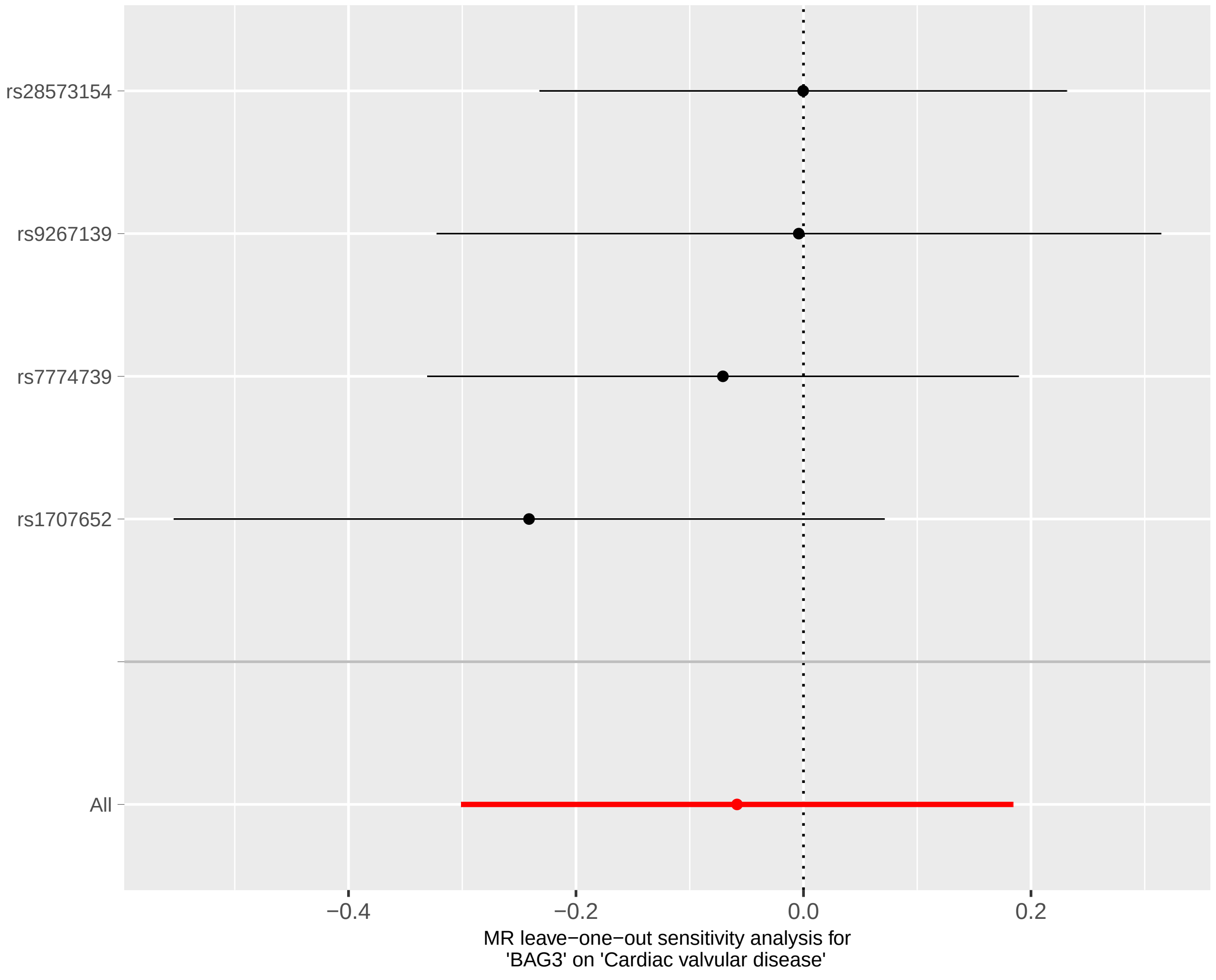
rs28573154
rs9267139
rs7774739
rs1707652
All
−0.4
−0.2
0.0
0.2
MR leave−one−out sensitivity analysis for
'BAG3' on 'Cardiac valvular disease'

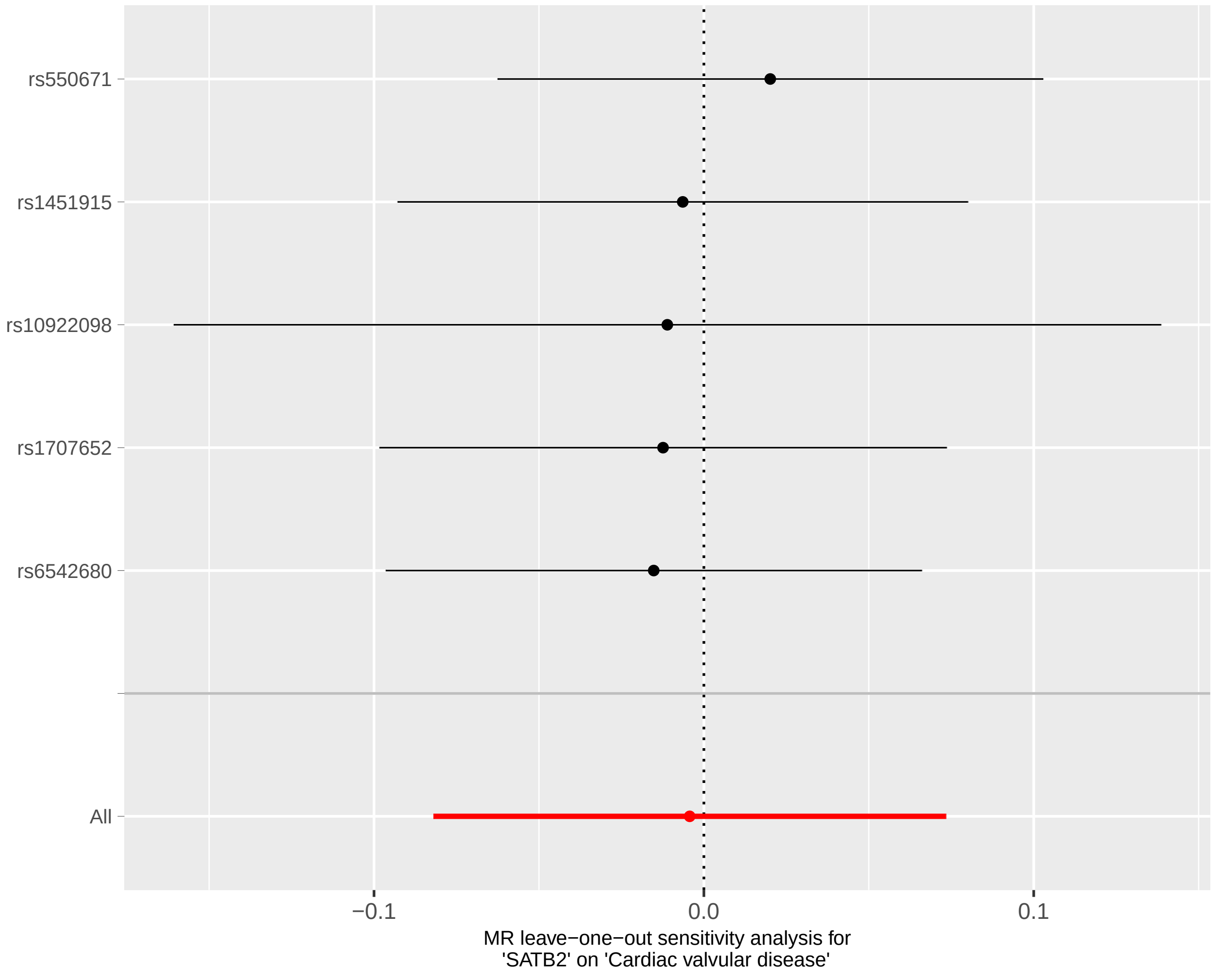
rs550671
rs1451915
rs10922098
rs1707652
rs6542680
All
−0.1
0.0
0.1
MR leave−one−out sensitivity analysis for
'SATB2' on 'Cardiac valvular disease'

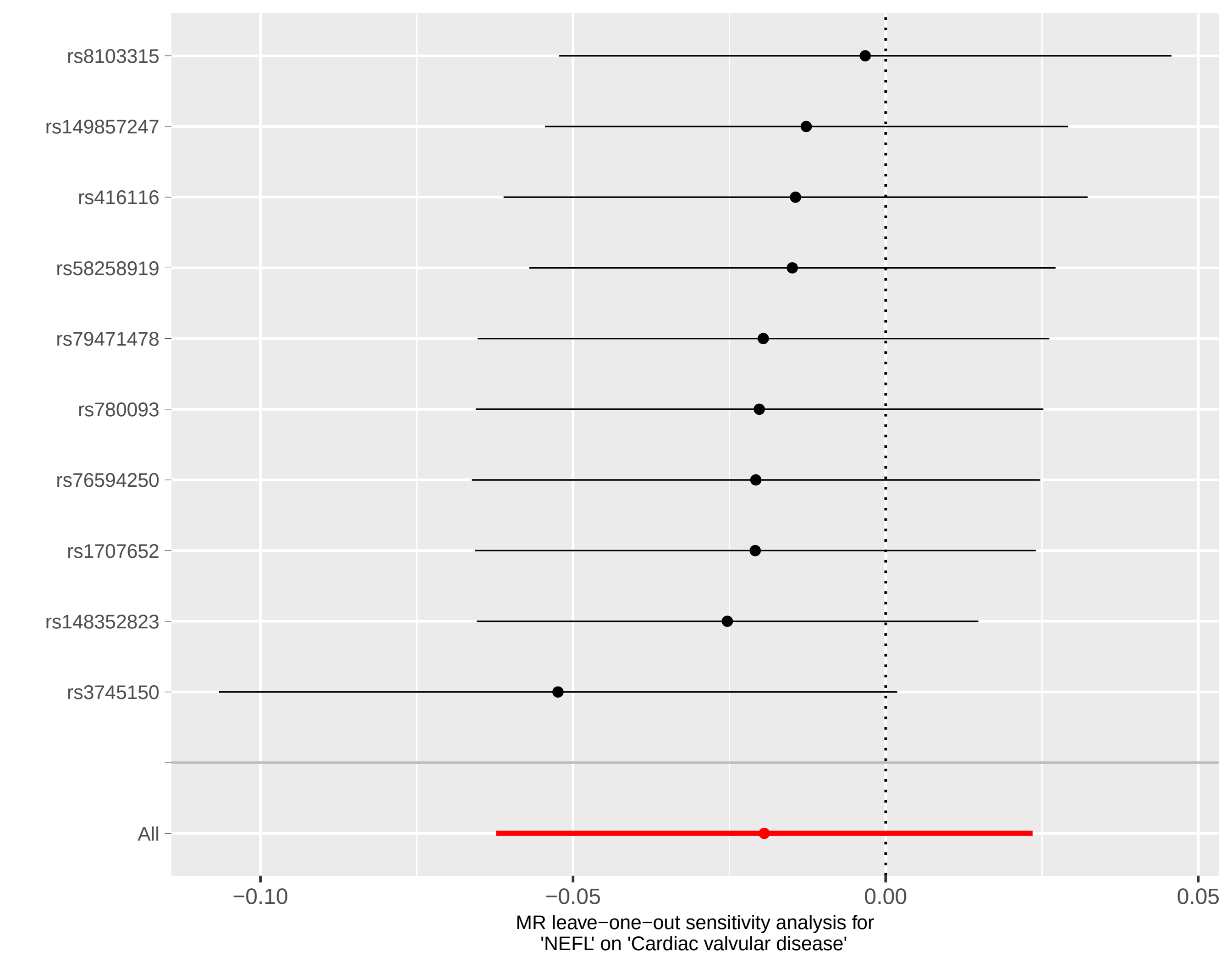

rs8103315
rs149857247
rs416116
rs58258919
rs79471478
rs780093
rs76594250
rs1707652
rs148352823
rs3745150
All
−0.10
−0.05
0.00
0.05
MR leave−one−out sensitivity analysis for
'NEFL' on 'Cardiac valvular disease'

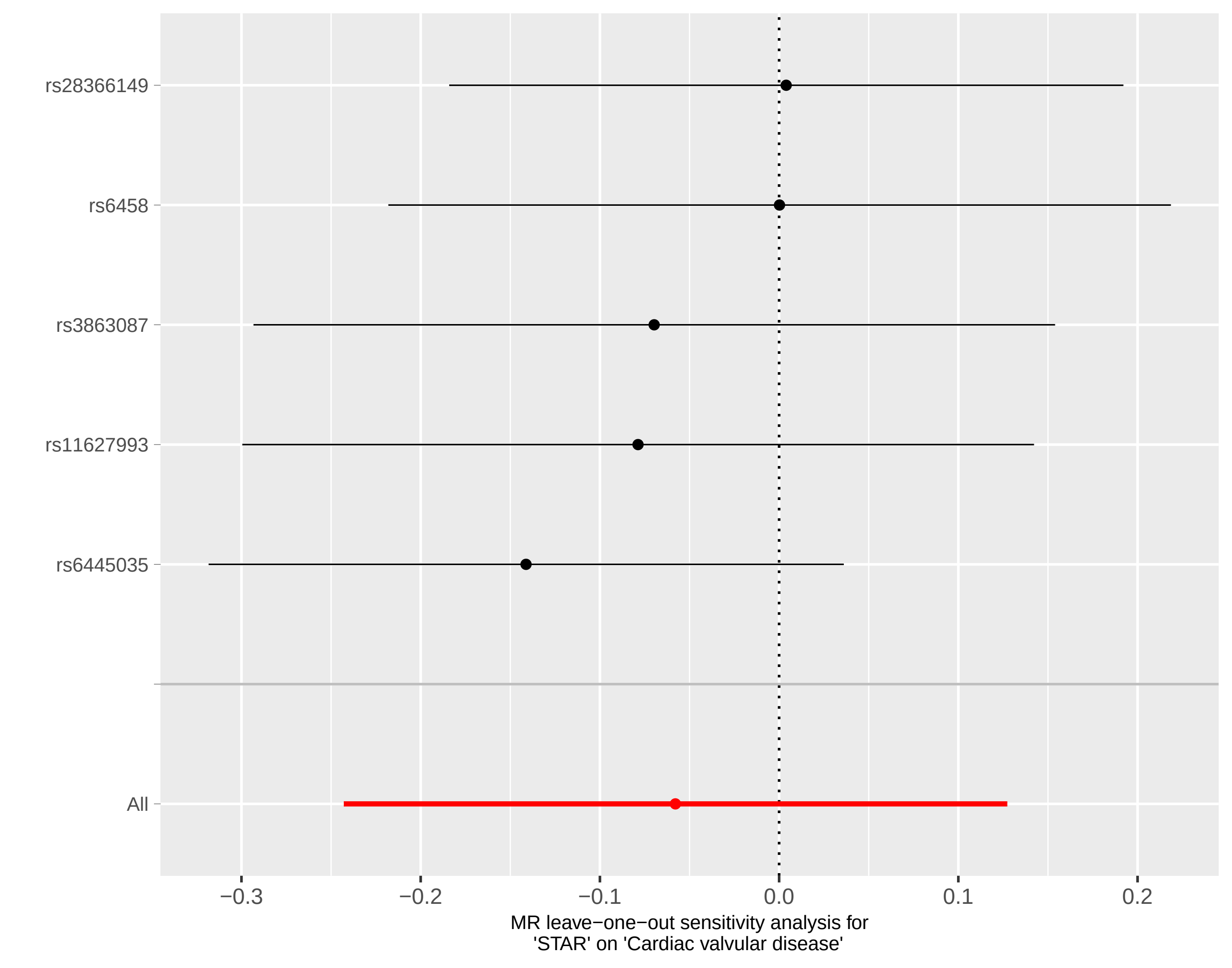
rs28366149
rs6458
rs3863087
rs11627993
rs6445035
All
−0.3
−0.2
−0.1
0.0
0.1
0.2
MR leave−one−out sensitivity analysis for
'STAR' on 'Cardiac valvular disease'

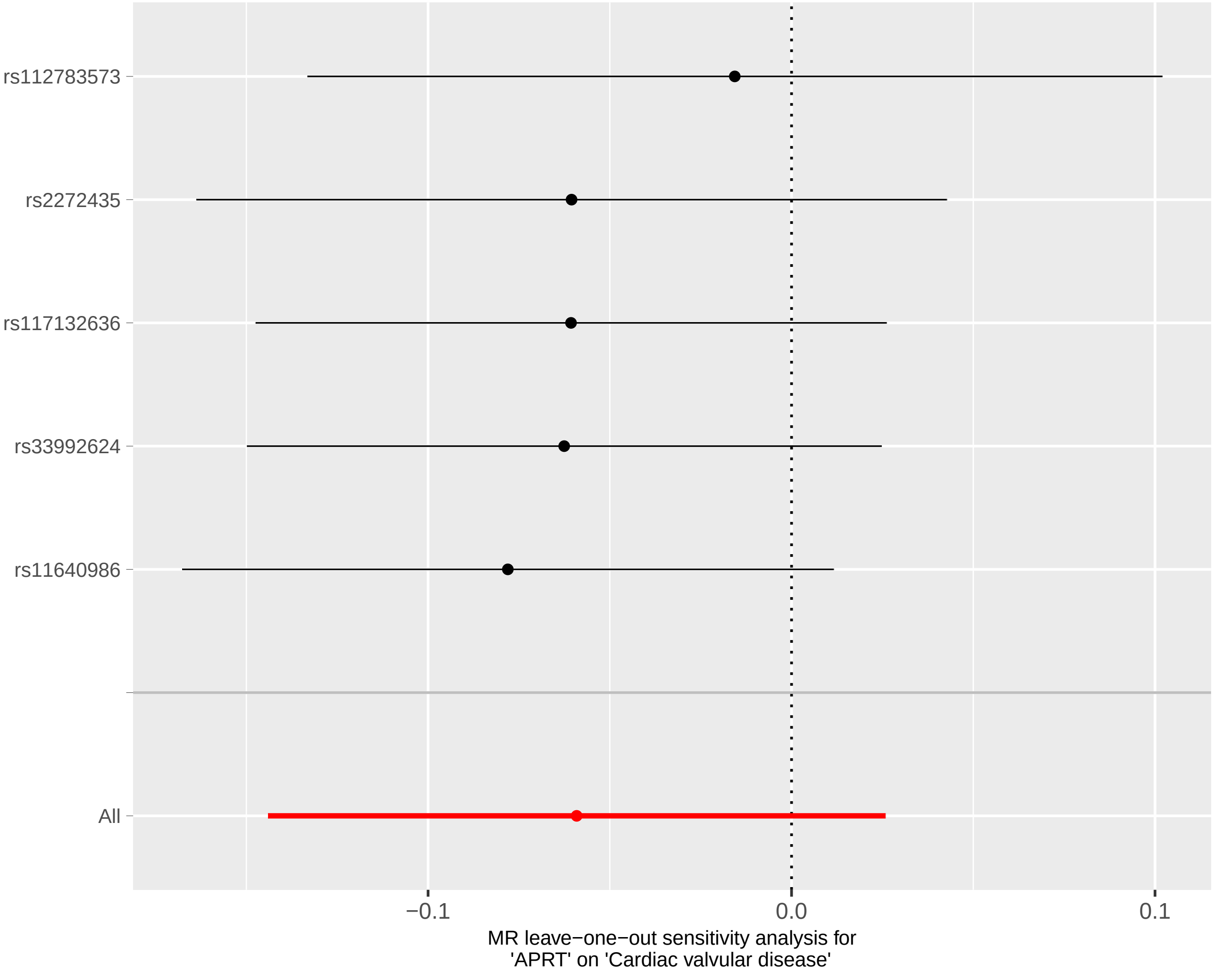
rs112783573
rs2272435
rs117132636
rs33992624
rs11640986
All
−0.1
0.0
0.1
MR leave−one−out sensitivity analysis for
'APRT' on 'Cardiac valvular disease'

Insufficient number of SNPs

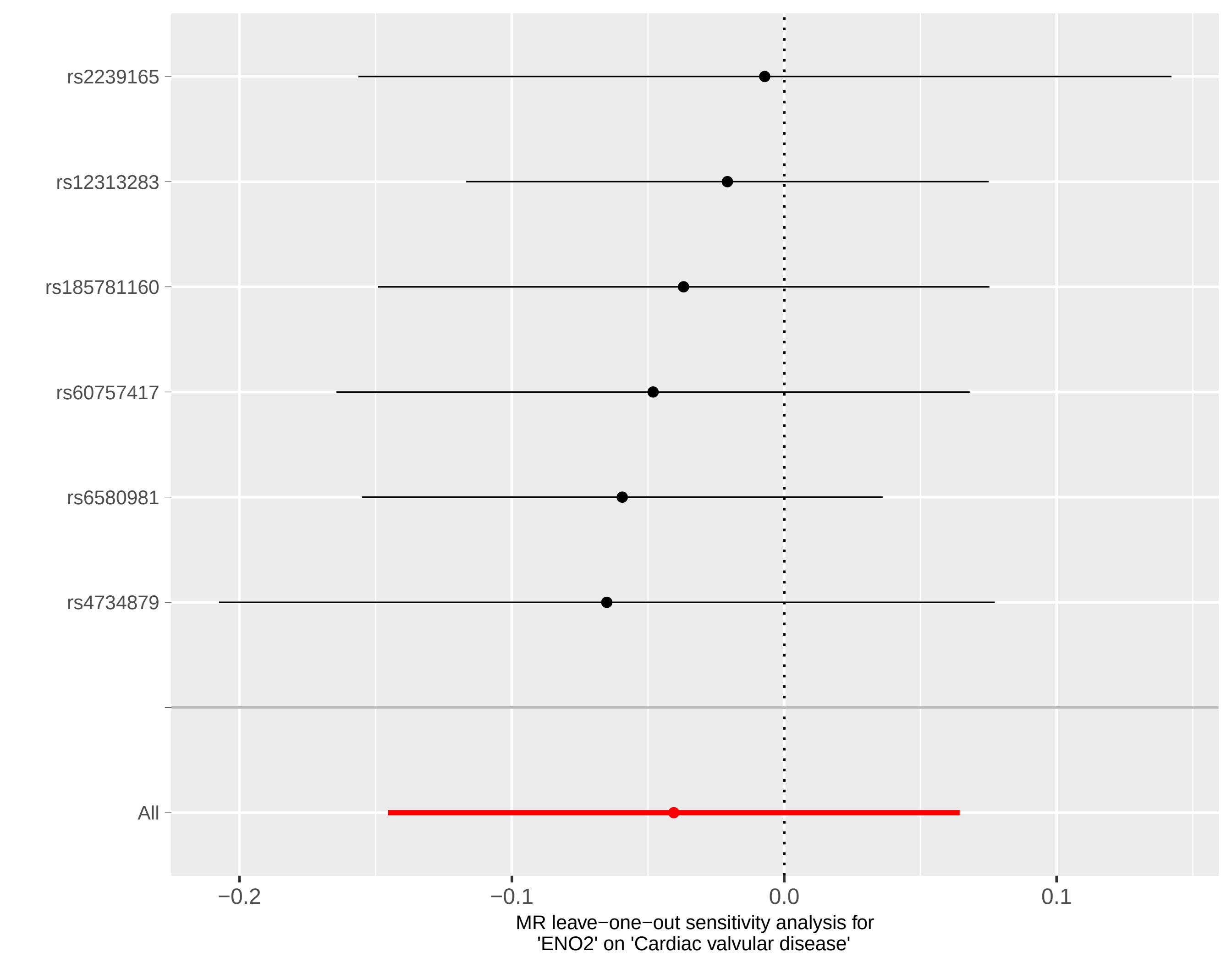
rs2239165
rs12313283
rs185781160
rs60757417
rs6580981
rs4734879
All
−0.2
−0.1
0.0
0.1
MR leave−one−out sensitivity analysis for
'ENO2' on 'Cardiac valvular disease'

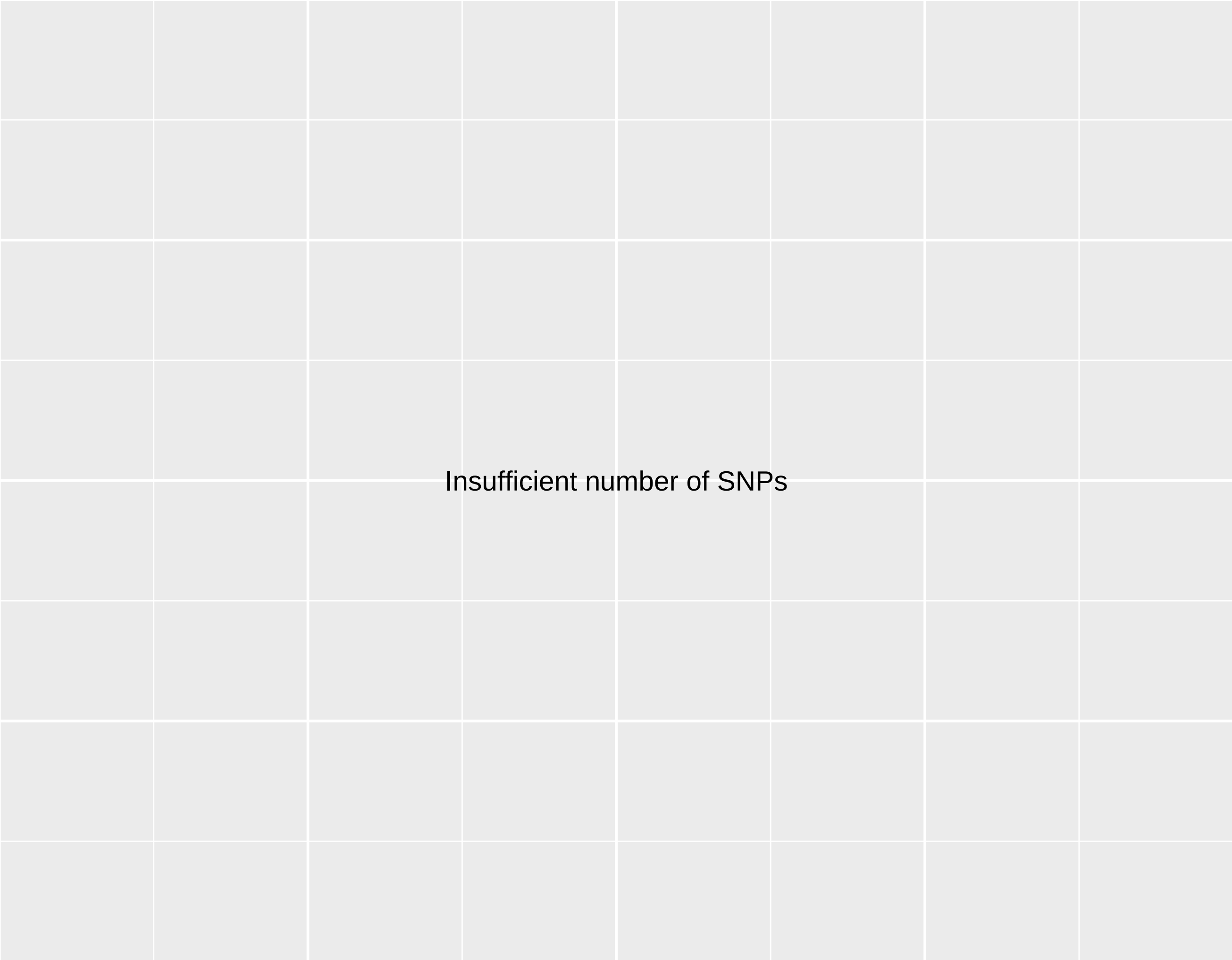
Insufficient number of SNPs

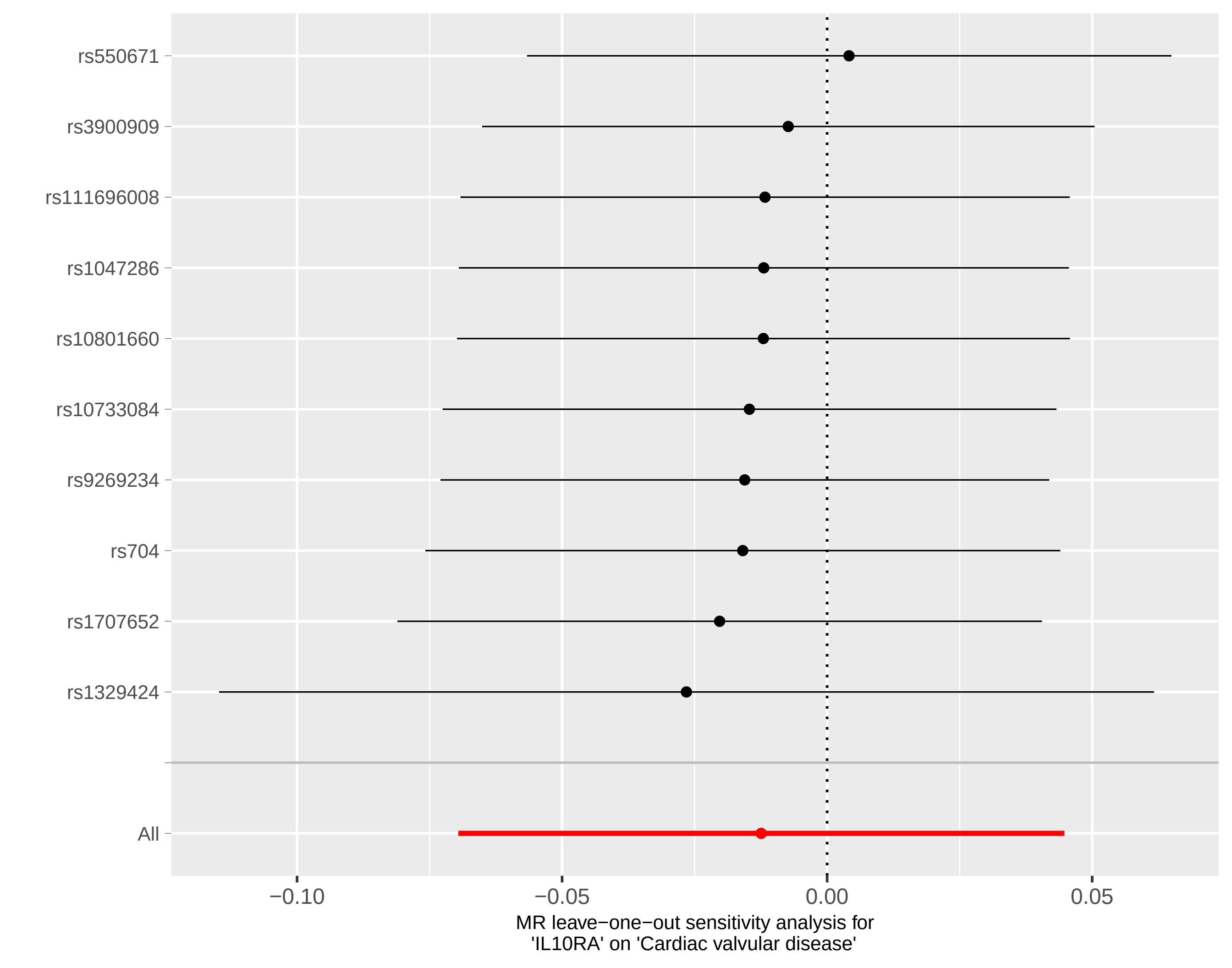

rs550671
rs3900909
rs111696008
rs1047286
rs10801660
rs10733084
rs9269234
rs704
rs1707652
rs1329424
All
−0.10
−0.05
0.00
0.05
MR leave−one−out sensitivity analysis for
'IL10RA' on 'Cardiac valvular disease'

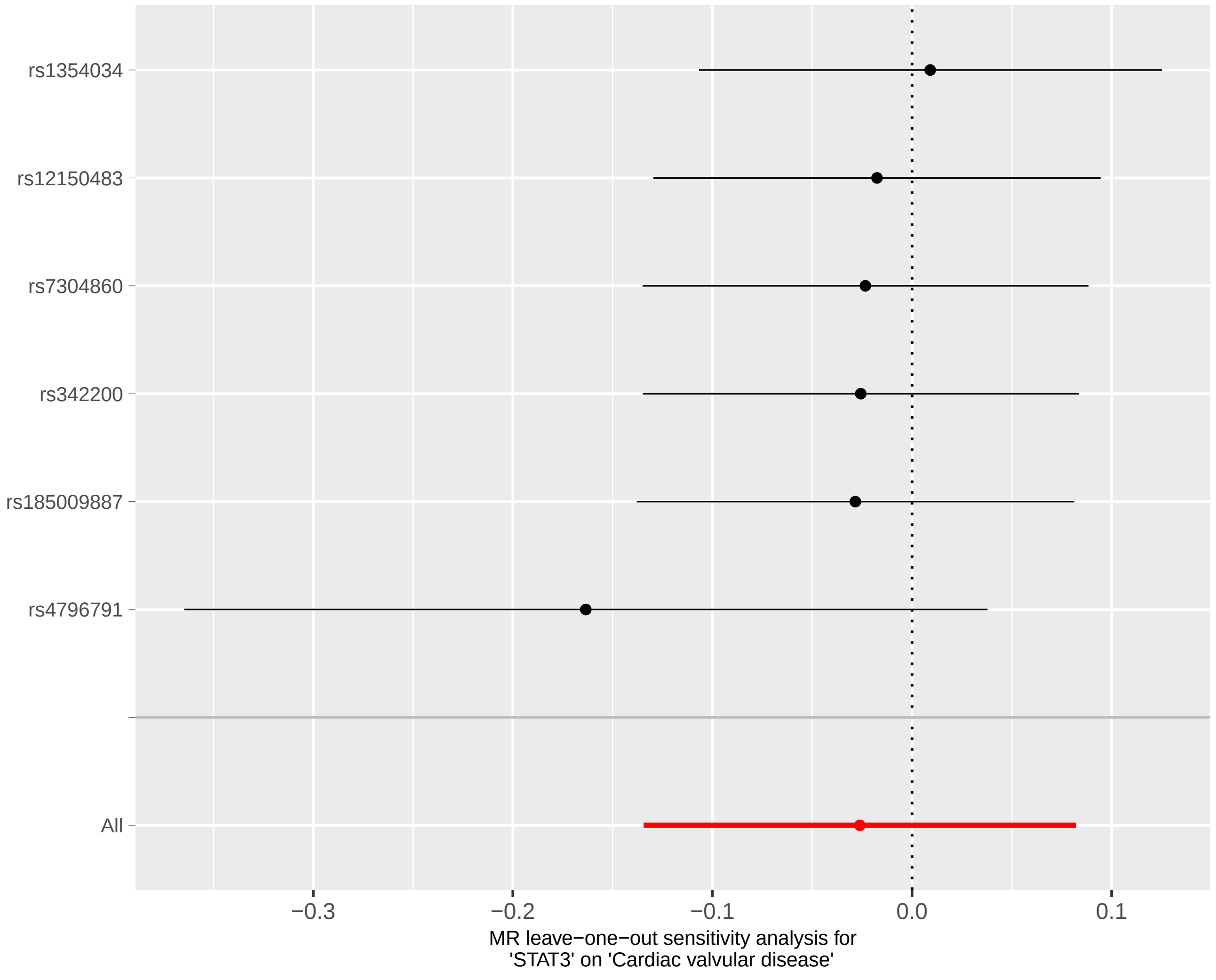

rs1354034
rs12150483
rs7304860
rs342200
rs185009887
rs4796791
All
−0.3
−0.2
−0.1
0.0
0.1
MR leave−one−out sensitivity analysis for
'STAT3' on 'Cardiac valvular disease'

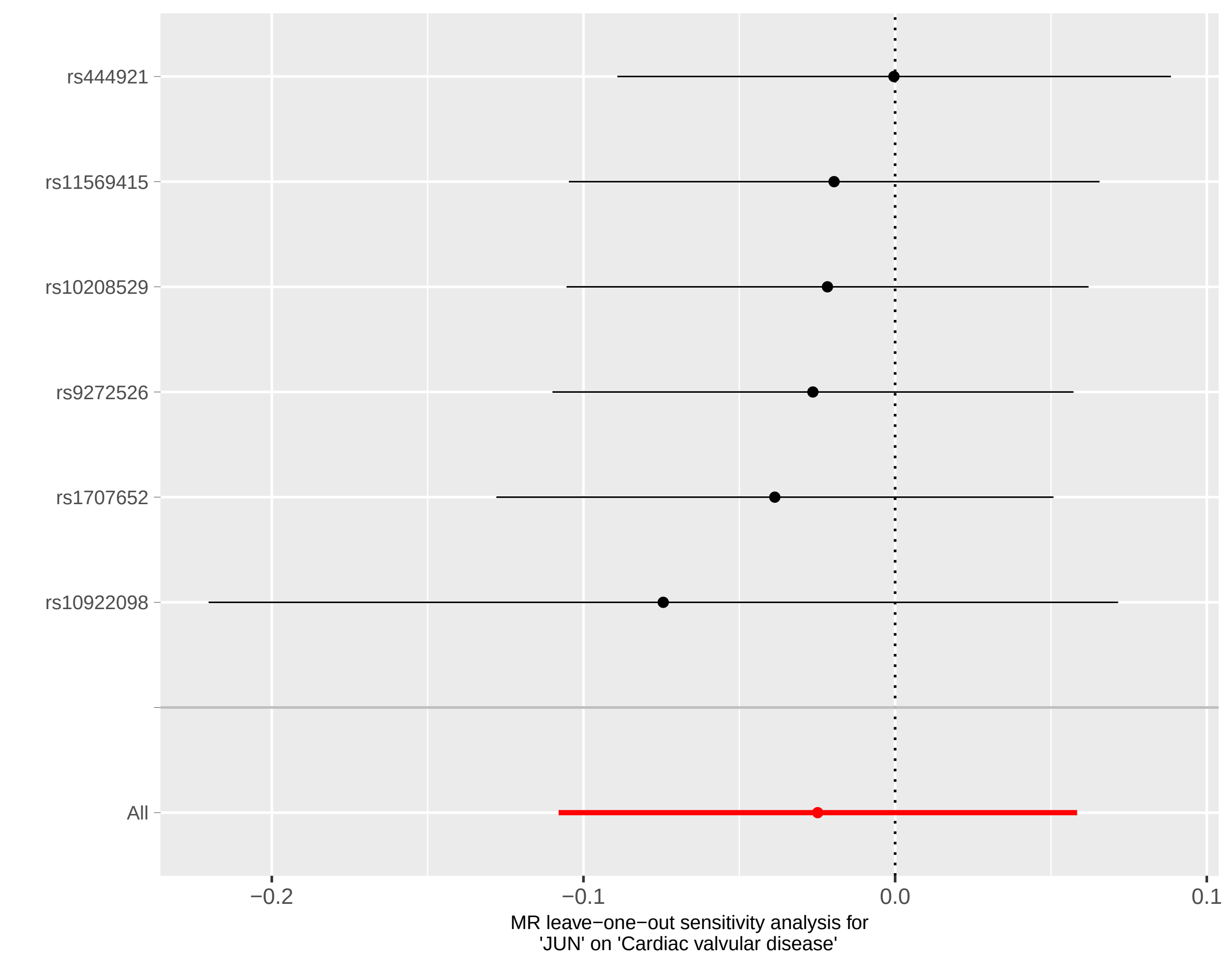
rs444921
rs11569415
rs10208529
rs9272526
rs1707652
rs10922098
All
−0.2
−0.1
0.0
0.1
MR leave−one−out sensitivity analysis for
'JUN' on 'Cardiac valvular disease'

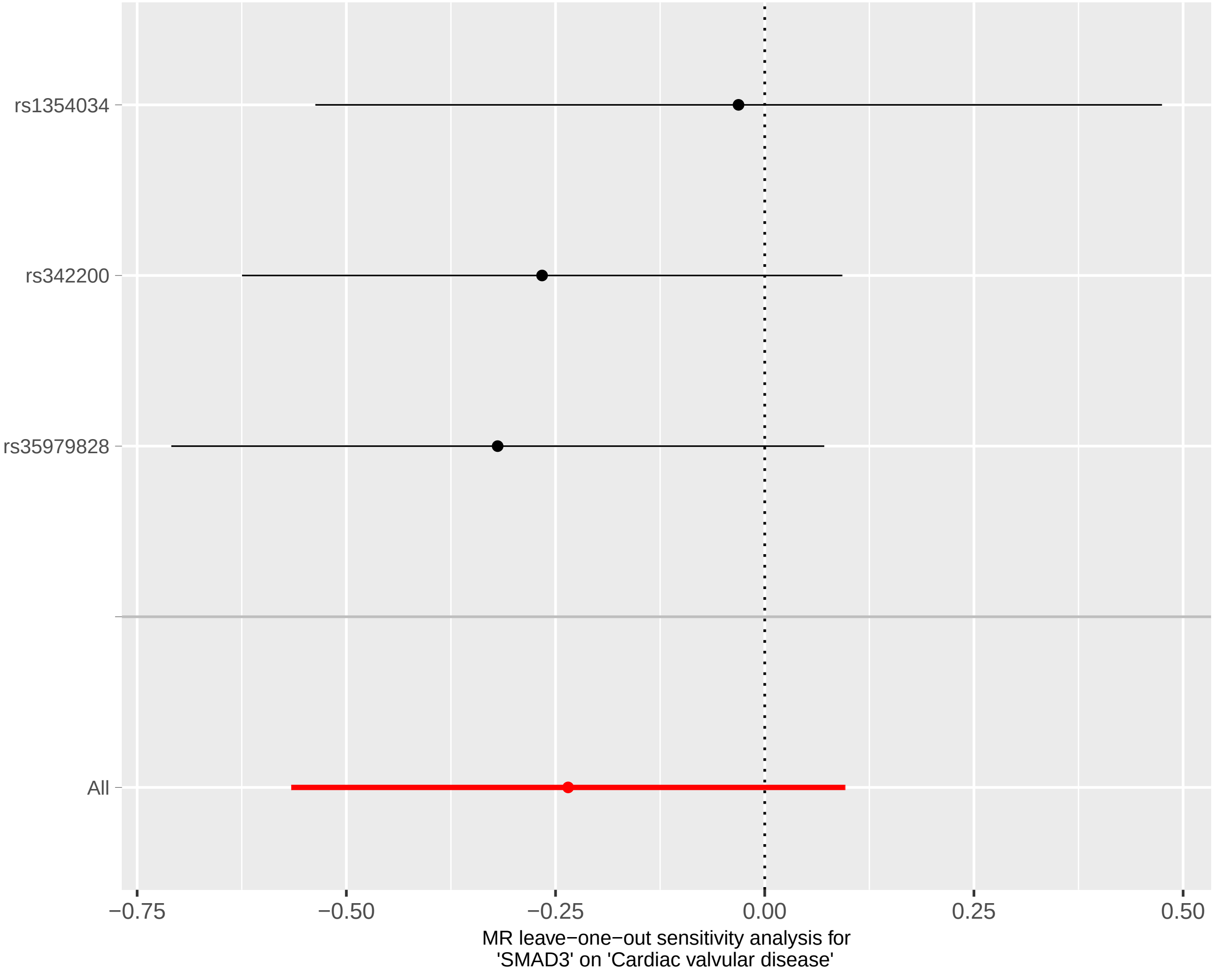

rs1354034
rs342200
rs35979828
All
−0.75
−0.50
−0.25
0.00
0.25
0.50
MR leave−one−out sensitivity analysis for
'SMAD3' on 'Cardiac valvular disease'

Insufficient number of SNPs

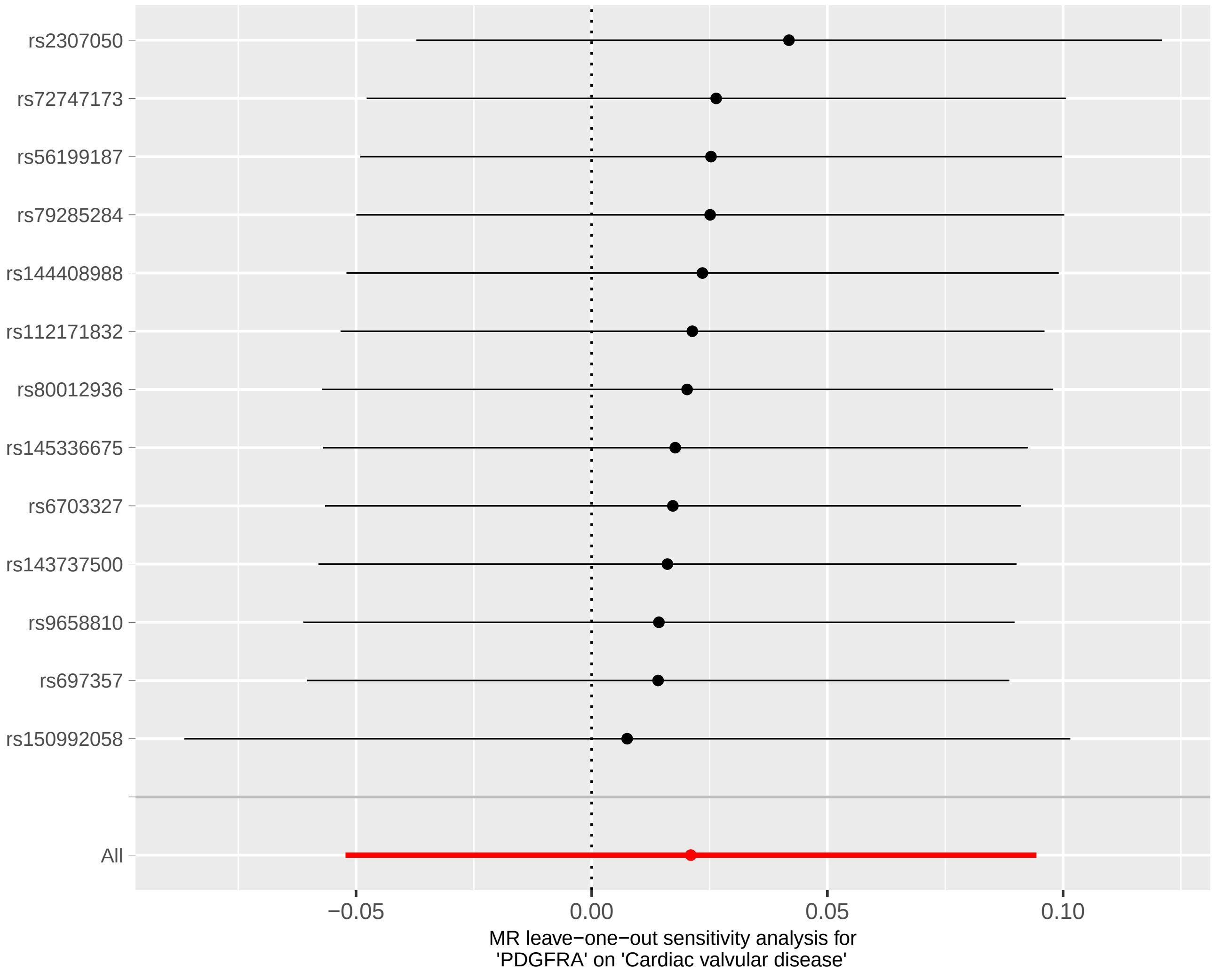
rs2307050
rs72747173
rs56199187
rs79285284
rs144408988
rs112171832
rs80012936
rs145336675
rs6703327
rs143737500
rs9658810
rs697357
rs150992058
All
−0.05
0.00
0.05
0.10
MR leave−one−out sensitivity analysis for
'PDGFRA' on 'Cardiac valvular disease'

Insufficient number of SNPs

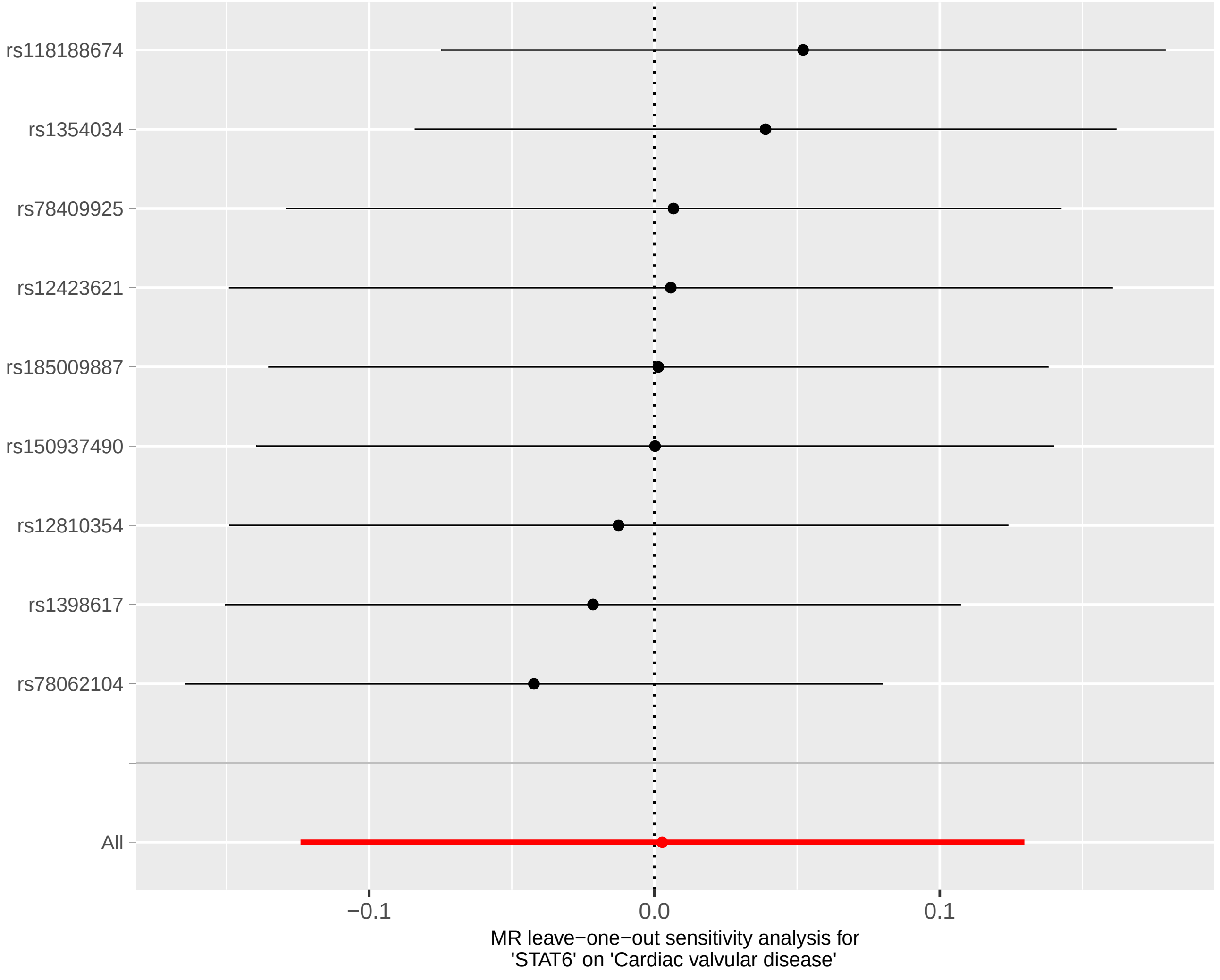

rs118188674
rs1354034
rs78409925
rs12423621
rs185009887
rs150937490
rs12810354
rs1398617
rs78062104
All
−0.1
0.0
0.1
MR leave−one−out sensitivity analysis for
'STAT6' on 'Cardiac valvular disease'

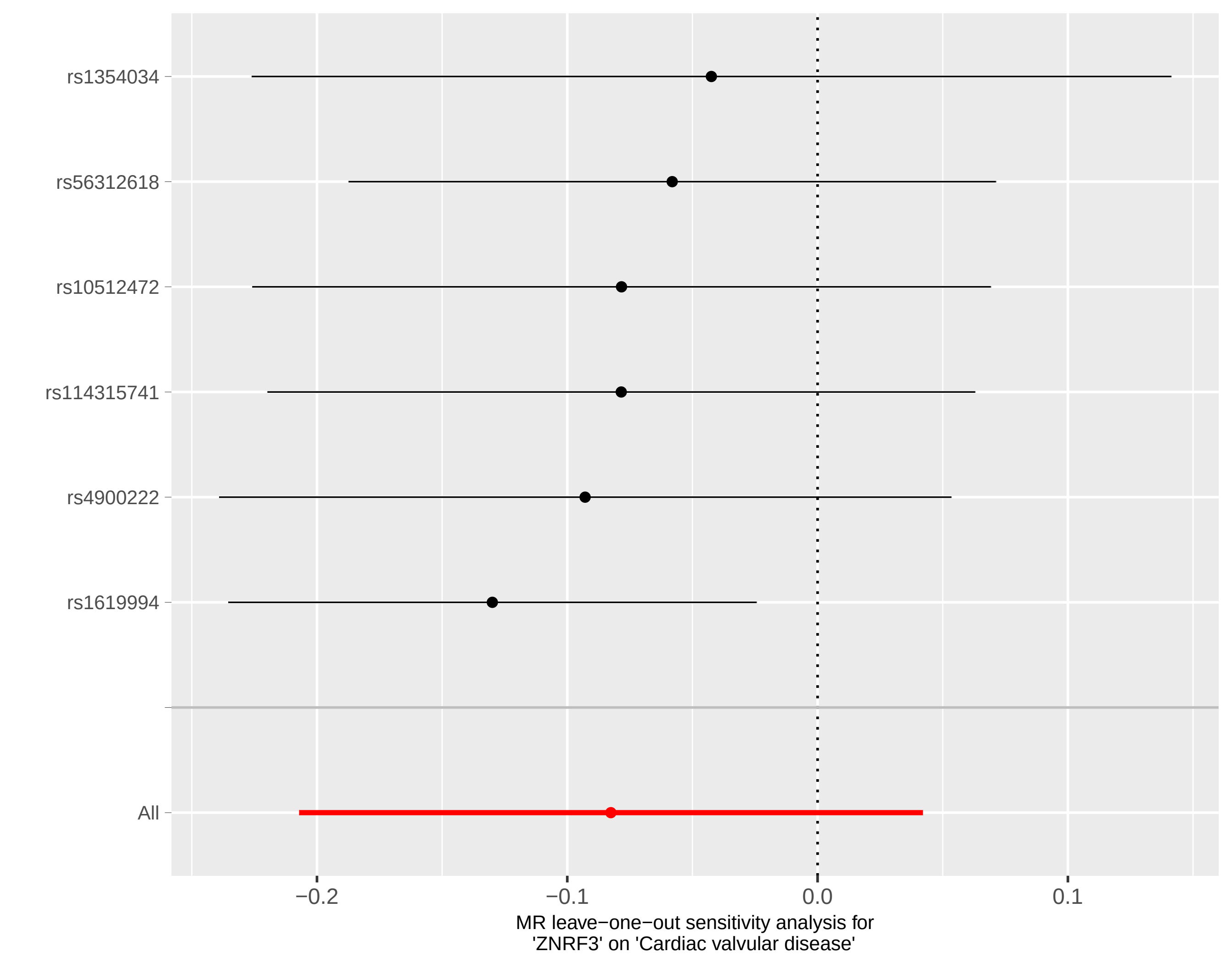
rs1354034
rs56312618
rs10512472
rs114315741
rs4900222
rs1619994
All
−0.2
−0.1
0.0
0.1
MR leave−one−out sensitivity analysis for
'ZNRF3' on 'Cardiac valvular disease'

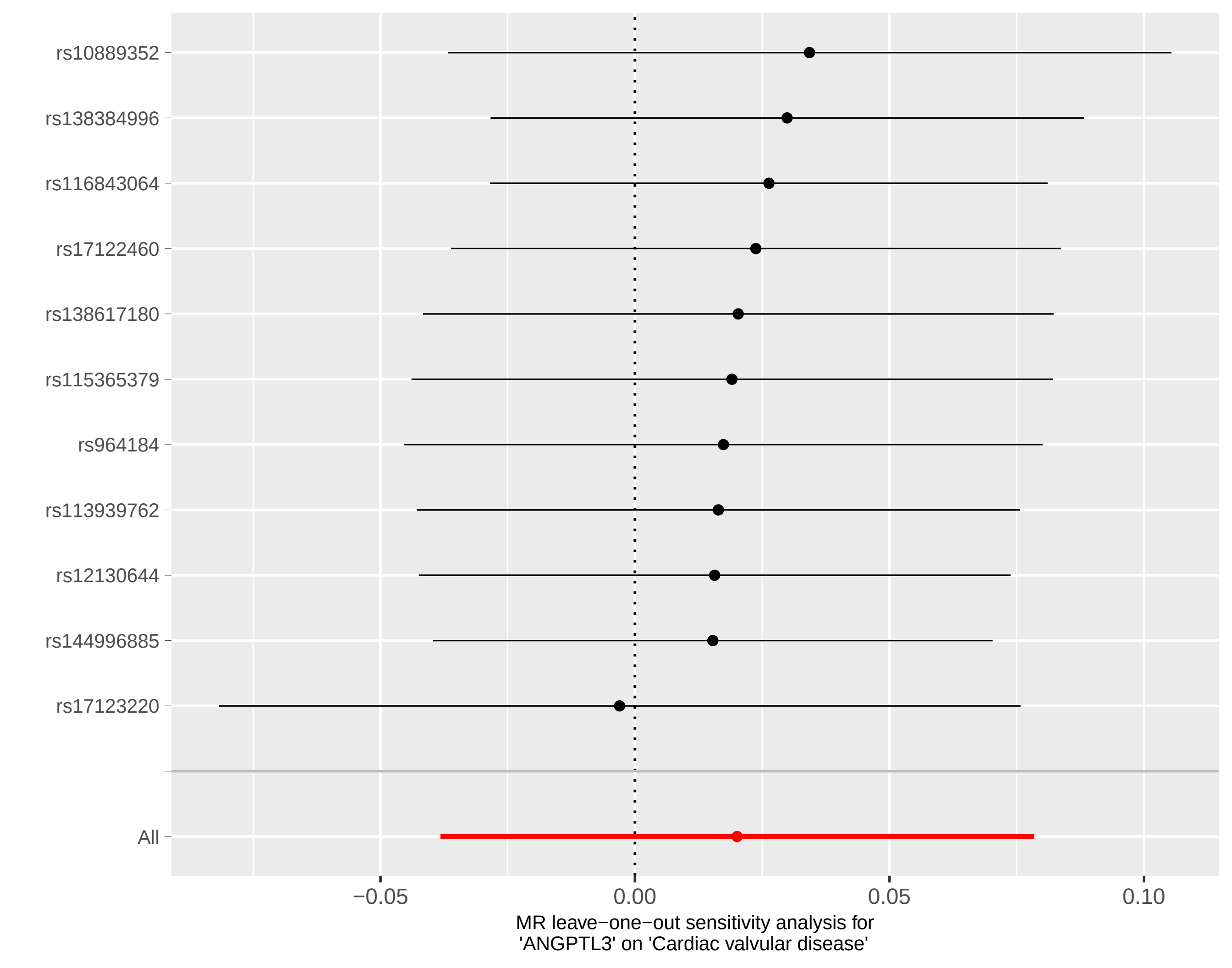
rs10889352
rs138384996
rs116843064
rs17122460
rs138617180
rs115365379
rs964184
rs113939762
rs12130644
rs144996885
rs17123220
All
−0.05
0.00
0.05
0.10
MR leave−one−out sensitivity analysis for
'ANGPTL3' on 'Cardiac valvular disease'

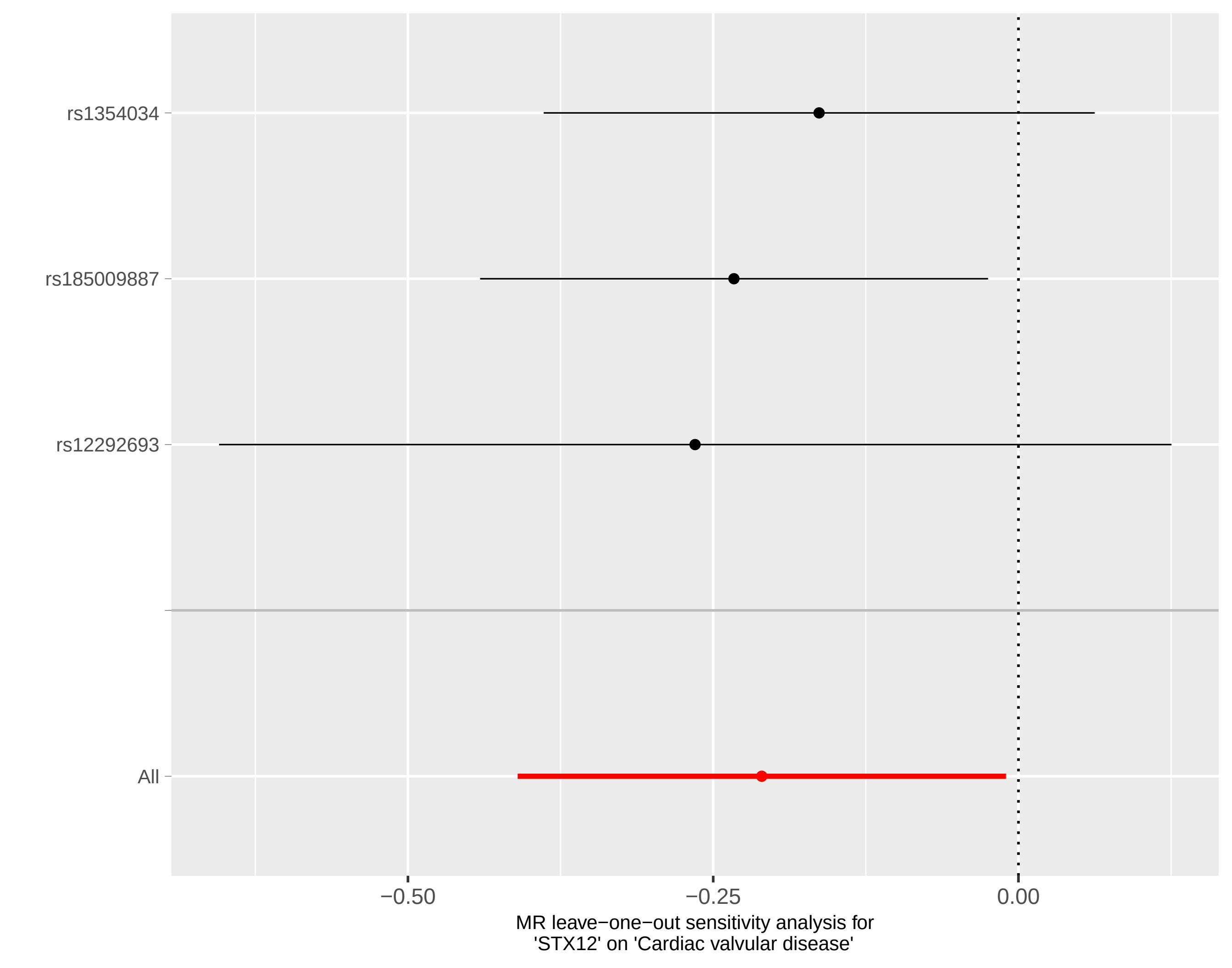
rs1354034
rs185009887
rs12292693
All
−0.50
−0.25
0.00
MR leave−one−out sensitivity analysis for
'STX12' on 'Cardiac valvular disease'

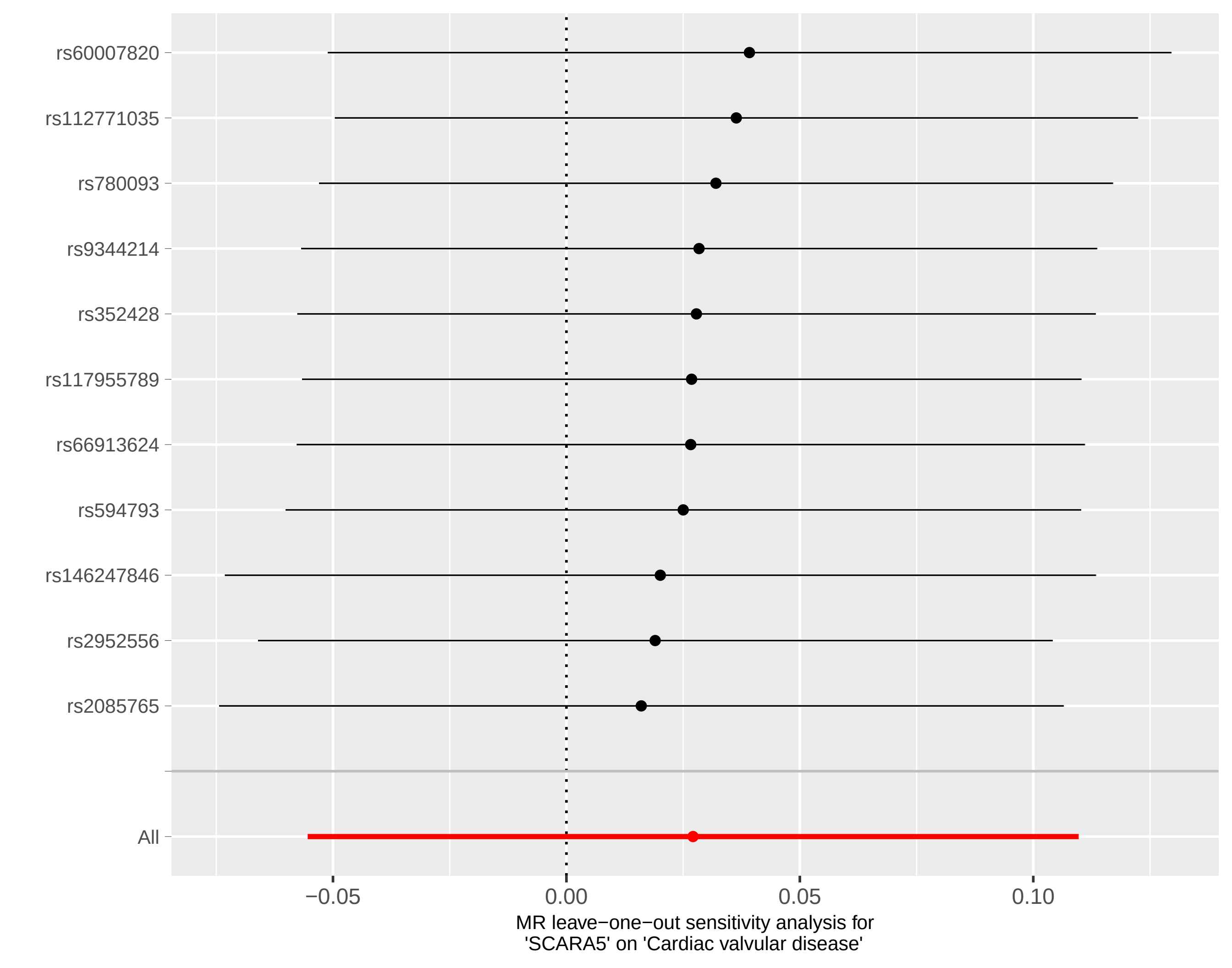
rs60007820
rs112771035
rs780093
rs9344214
rs352428
rs117955789
rs66913624
rs594793
rs146247846
rs2952556
rs2085765
All
−0.05
0.00
0.05
0.10
MR leave−one−out sensitivity analysis for
'SCARA5' on 'Cardiac valvular disease'

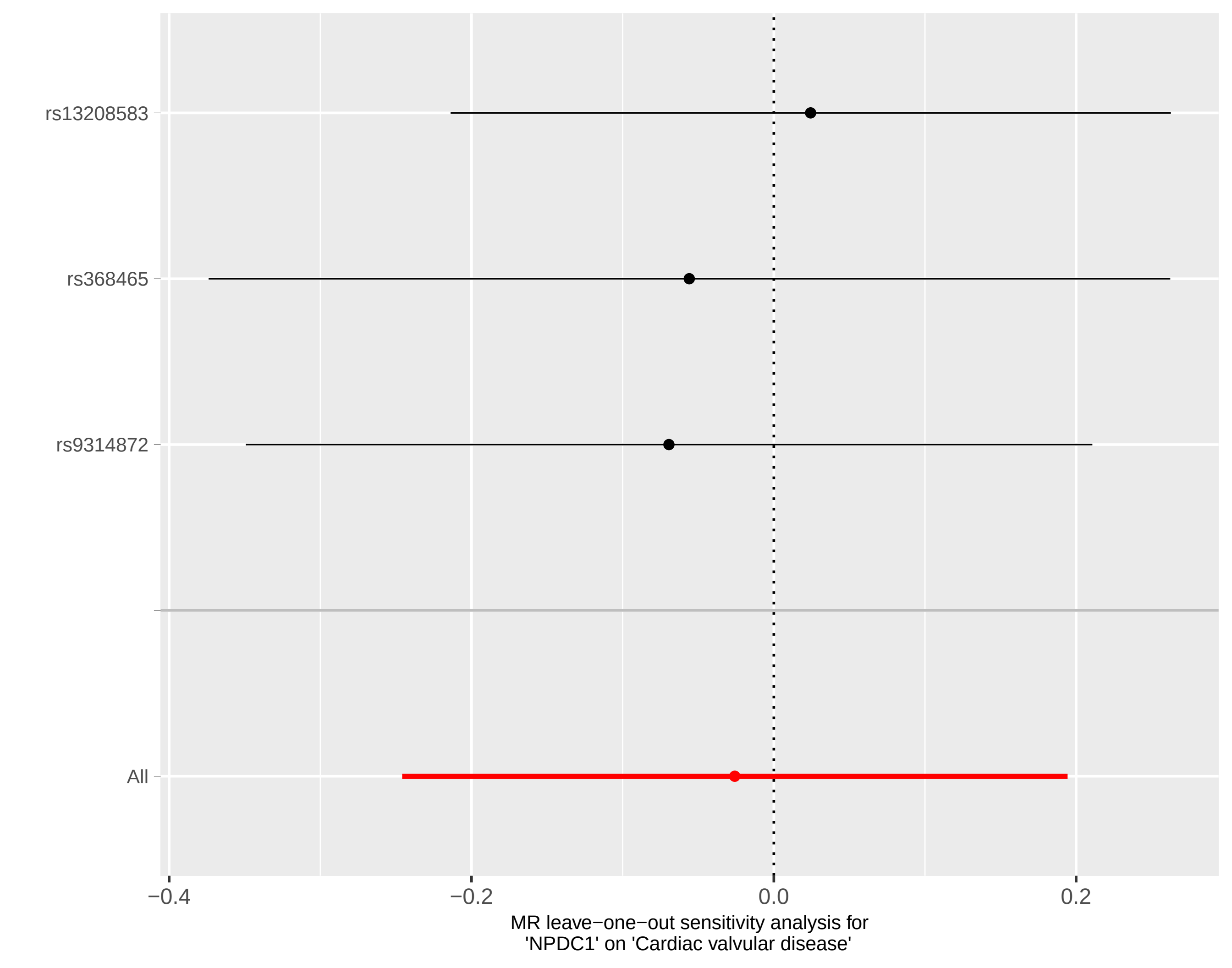
rs13208583
rs368465
rs9314872
All
−0.4
−0.2
0.0
0.2
MR leave−one−out sensitivity analysis for
'NPDC1' on 'Cardiac valvular disease'

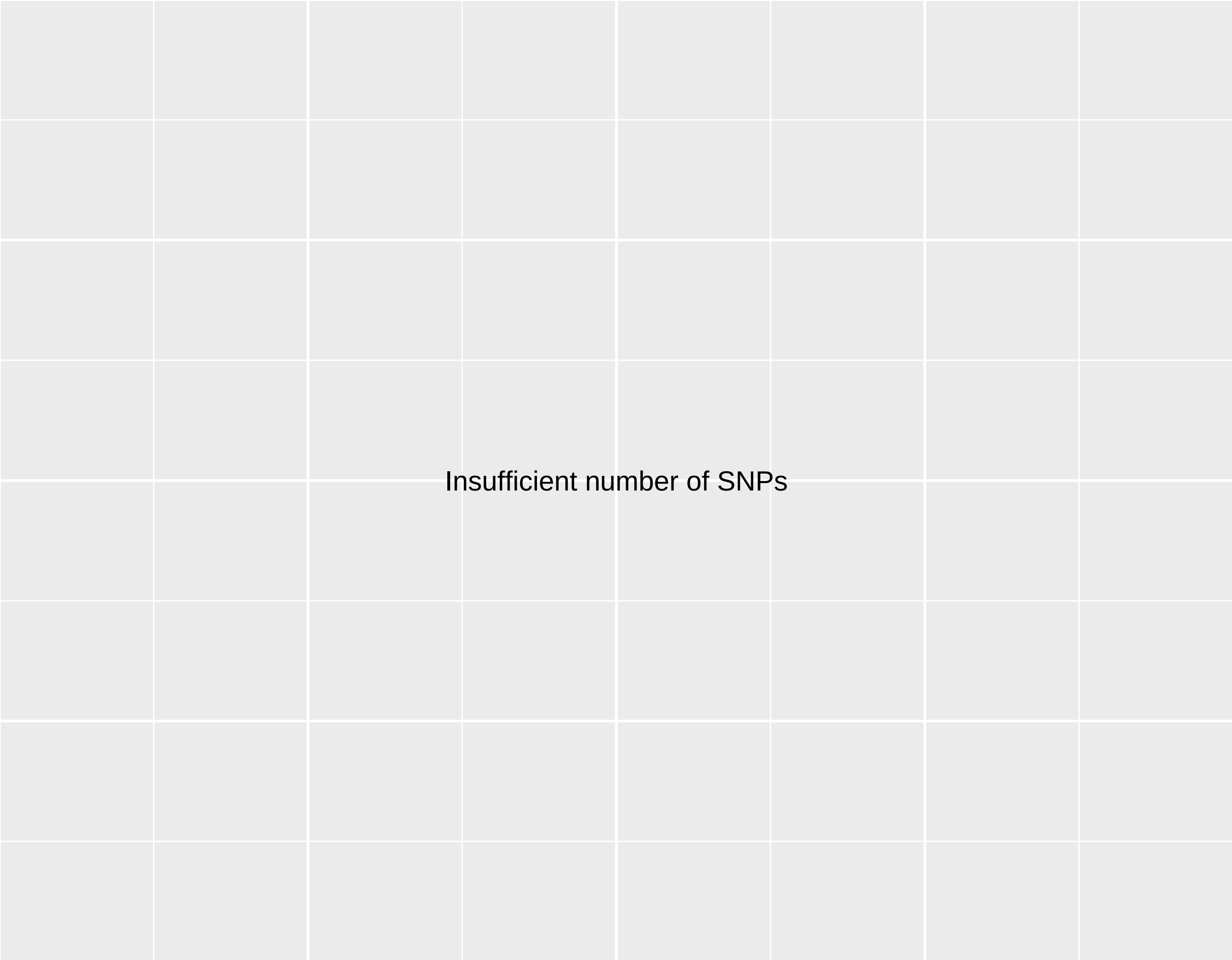
Insufficient number of SNPs

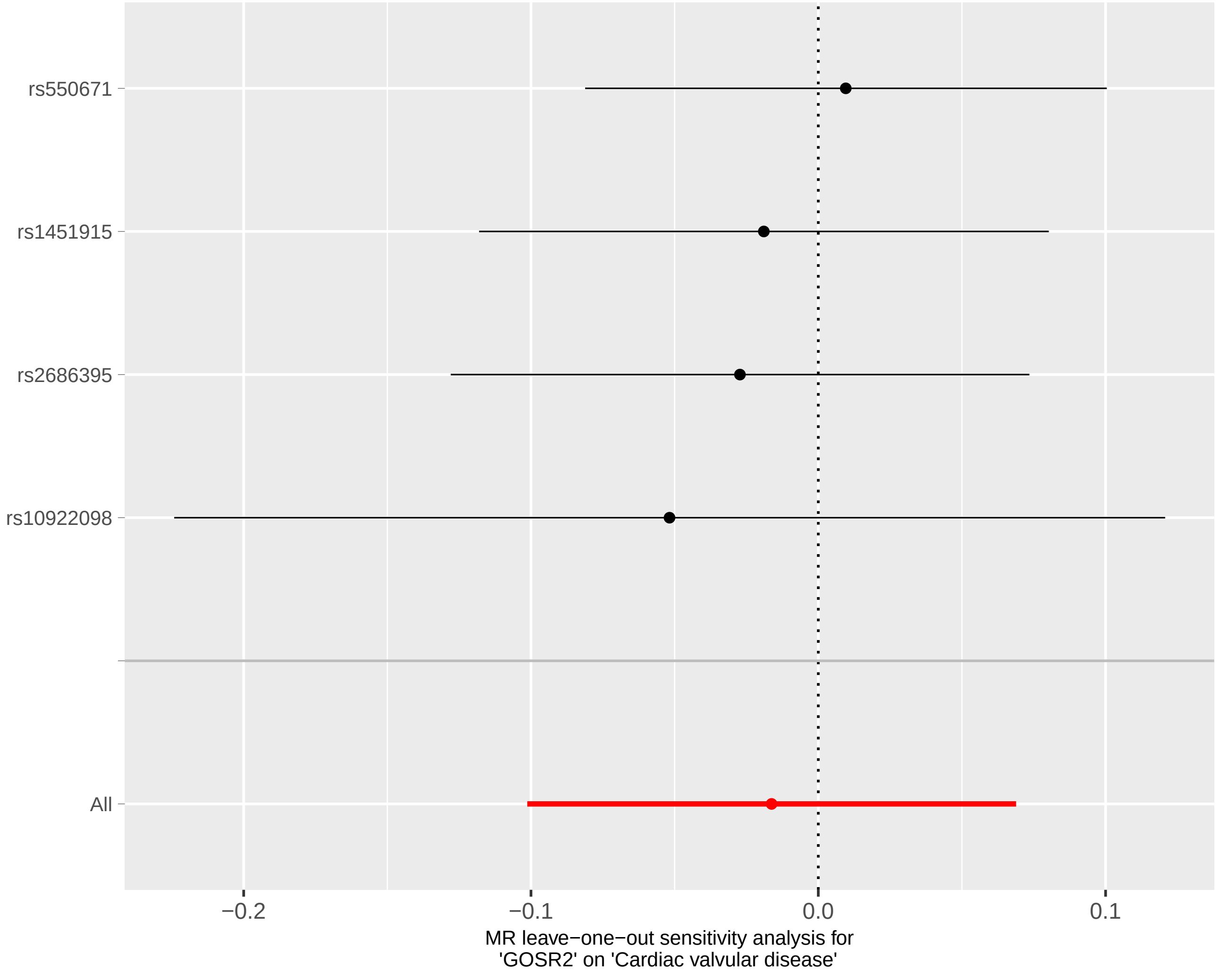

rs550671
rs1451915
rs2686395
rs10922098
All
−0.2
−0.1
0.0
0.1
MR leave−one−out sensitivity analysis for
'GOSR2' on 'Cardiac valvular disease'

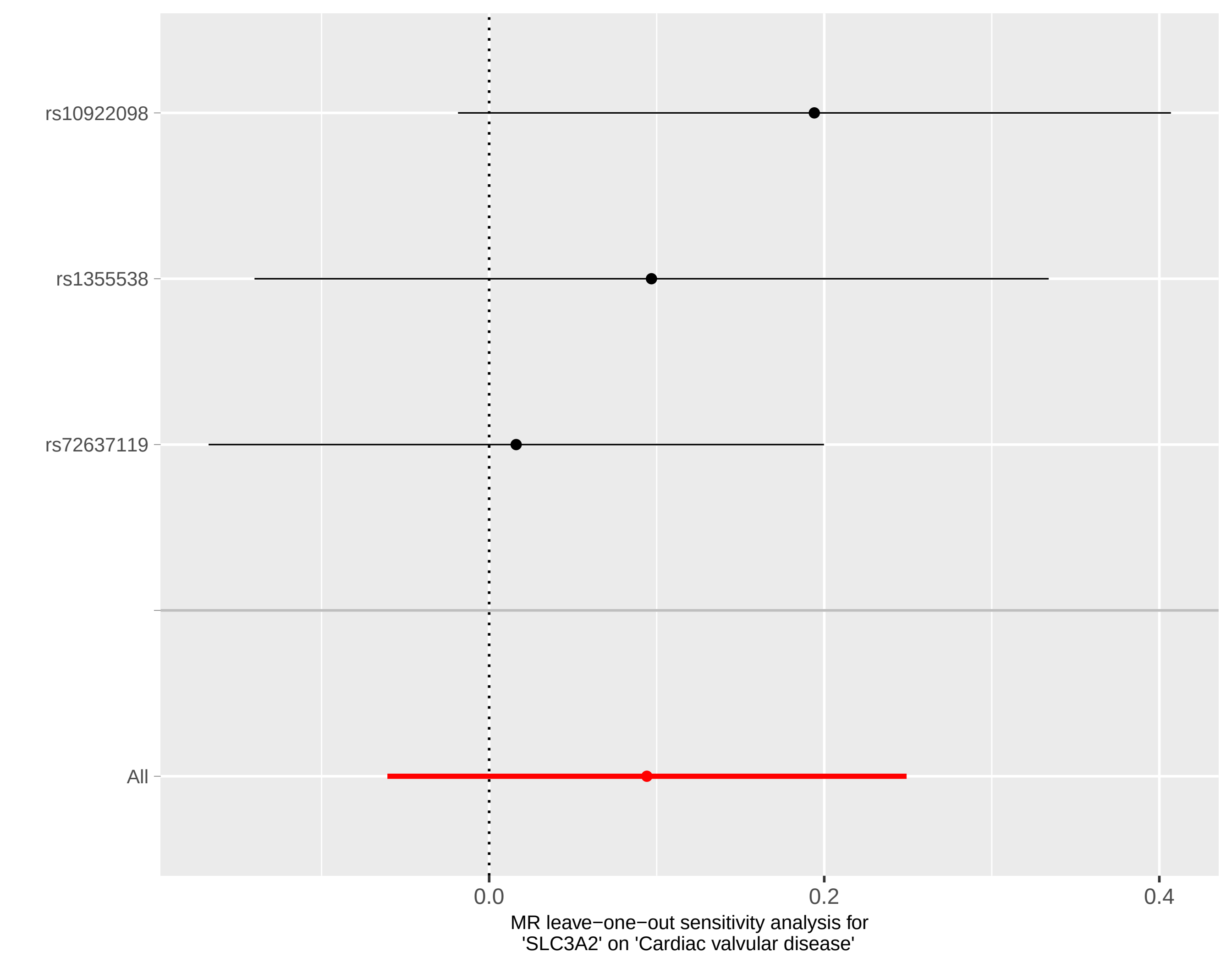
rs10922098
rs1355538
rs72637119
All
0.0
0.2
0.4
MR leave−one−out sensitivity analysis for
'SLC3A2' on 'Cardiac valvular disease'

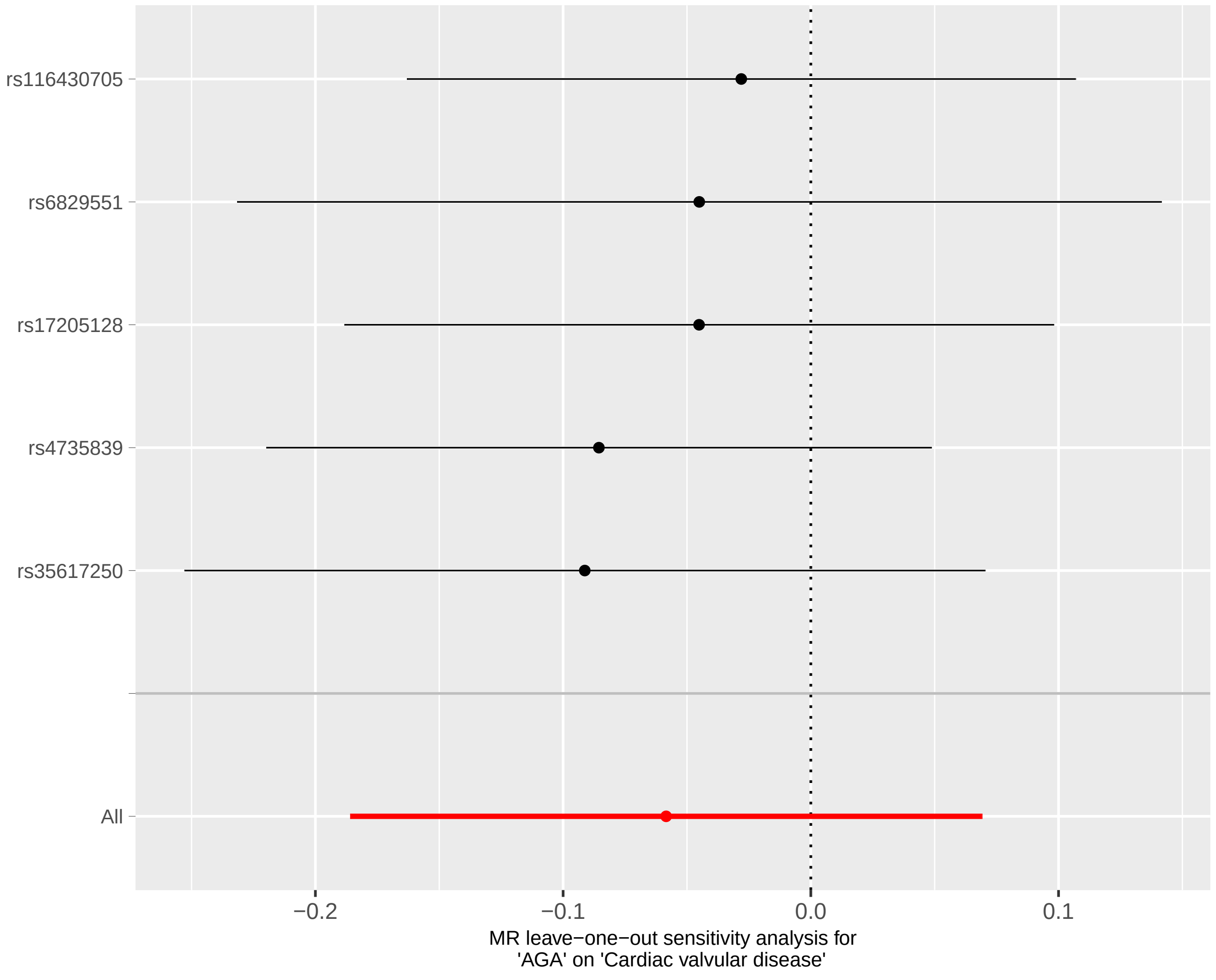

rs116430705
rs6829551
rs17205128
rs4735839
rs35617250
All
−0.2
−0.1
0.0
0.1
MR leave−one−out sensitivity analysis for
'AGA' on 'Cardiac valvular disease'

Insufficient number of SNPs

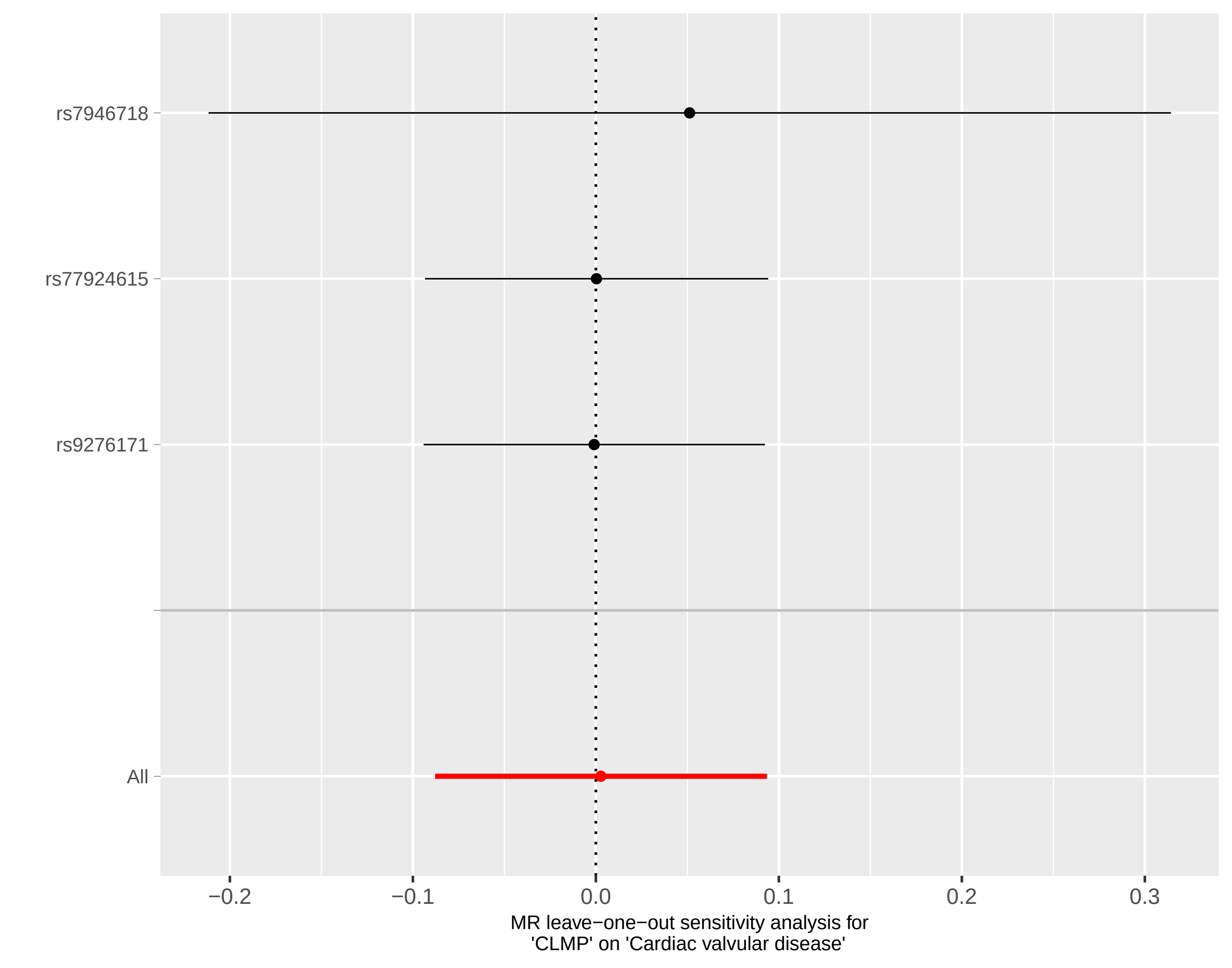

rs7946718
rs77924615
rs9276171
All
−0.2
−0.1
0.0
0.1
0.2
0.3
MR leave−one−out sensitivity analysis for
'CLMP' on 'Cardiac valvular disease'

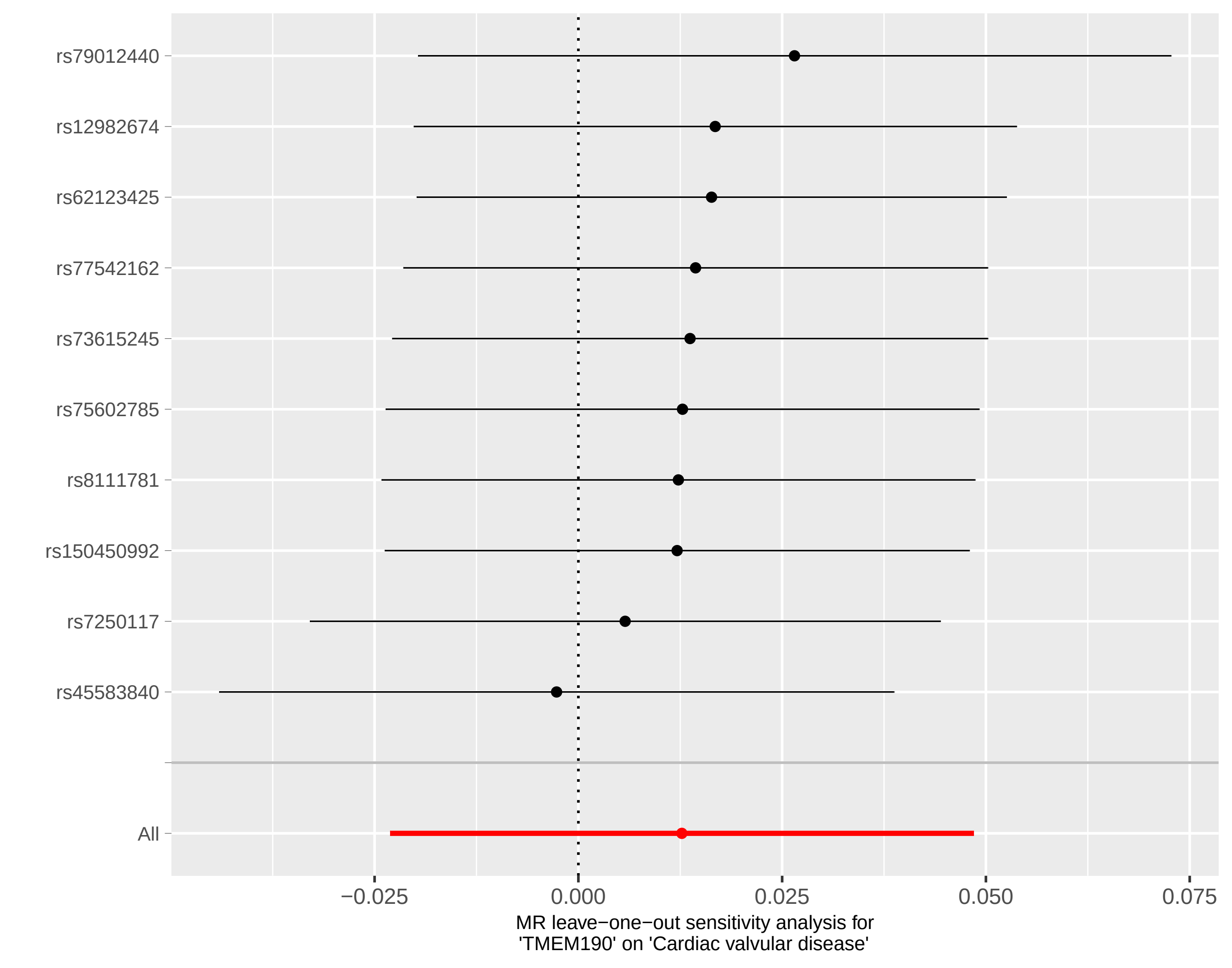
rs79012440
rs12982674
rs62123425
rs77542162
rs73615245
rs75602785
rs8111781
rs150450992
rs7250117
rs45583840
All
−0.025
0.000
0.025
0.050
0.075
MR leave−one−out sensitivity analysis for
'TMEM190' on 'Cardiac valvular disease'

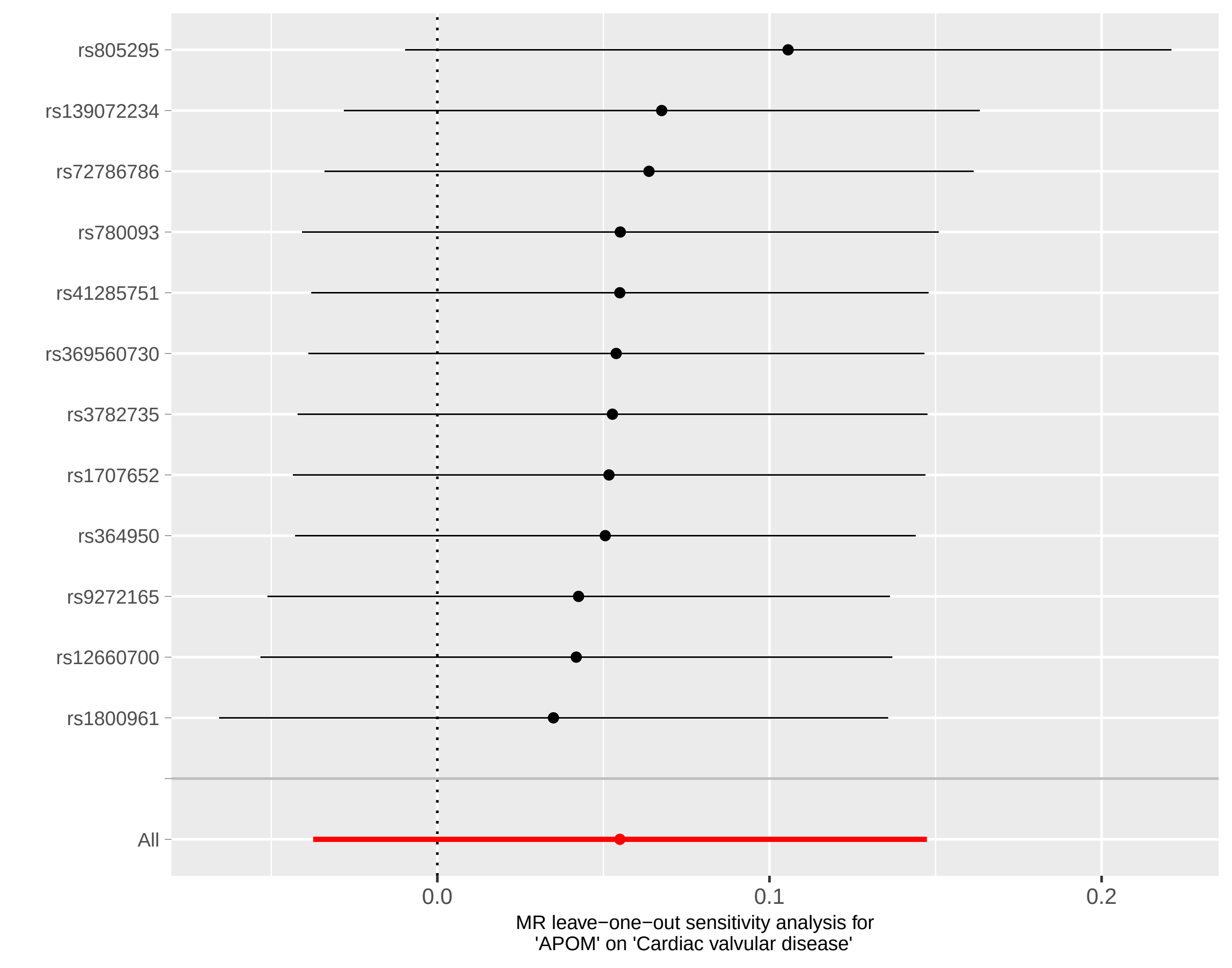

rs805295
rs139072234
rs72786786
rs780093
rs41285751
rs369560730
rs3782735
rs1707652
rs364950
rs9272165
rs12660700
rs1800961
All
0.0
0.1
0.2
MR leave−one−out sensitivity analysis for
'APOM' on 'Cardiac valvular disease'

Insufficient number of SNPs

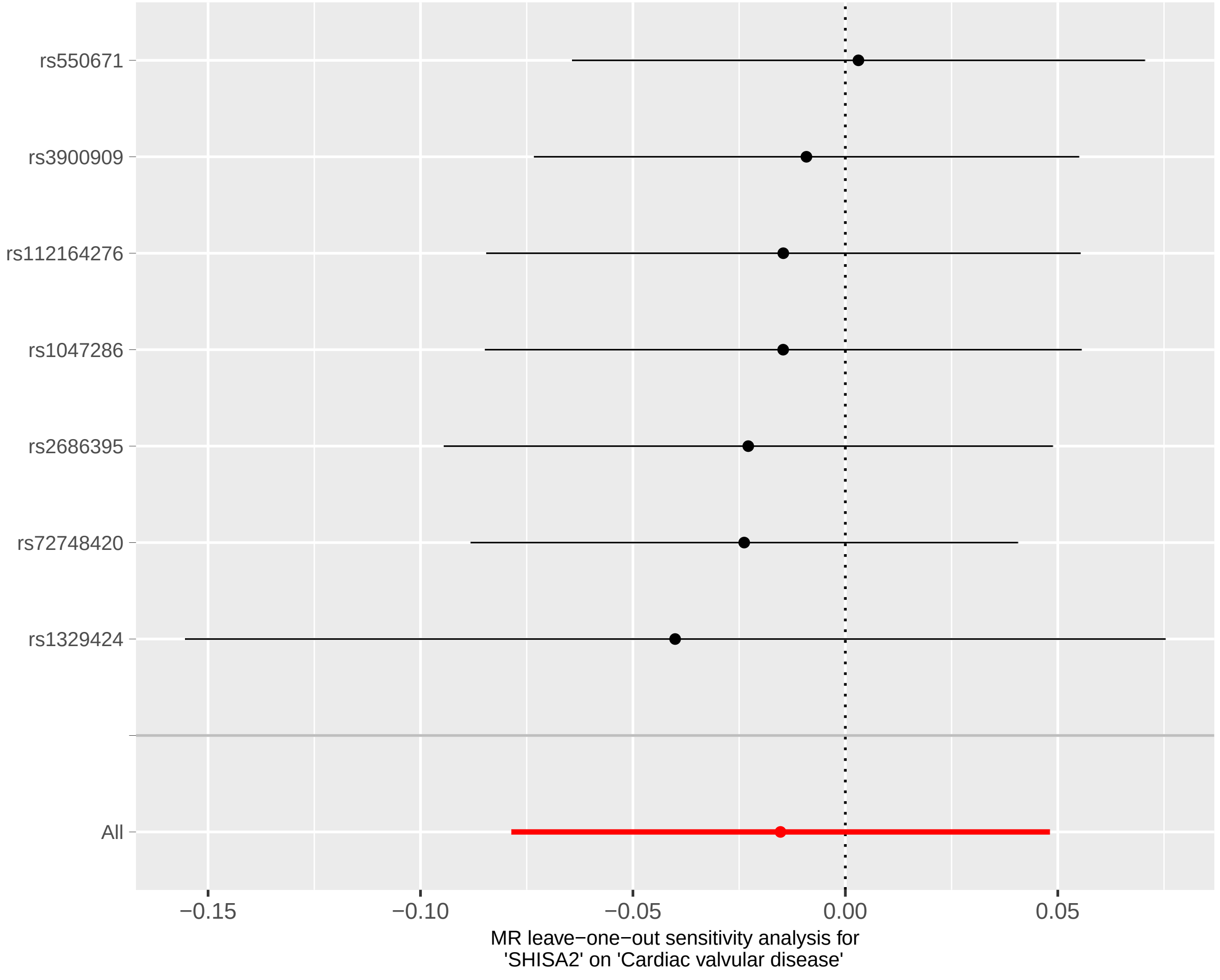
rs550671
rs3900909
rs112164276
rs1047286
rs2686395
rs72748420
rs1329424
All
−0.15
−0.10
−0.05
0.00
0.05
MR leave−one−out sensitivity analysis for
'SHISA2' on 'Cardiac valvular disease'

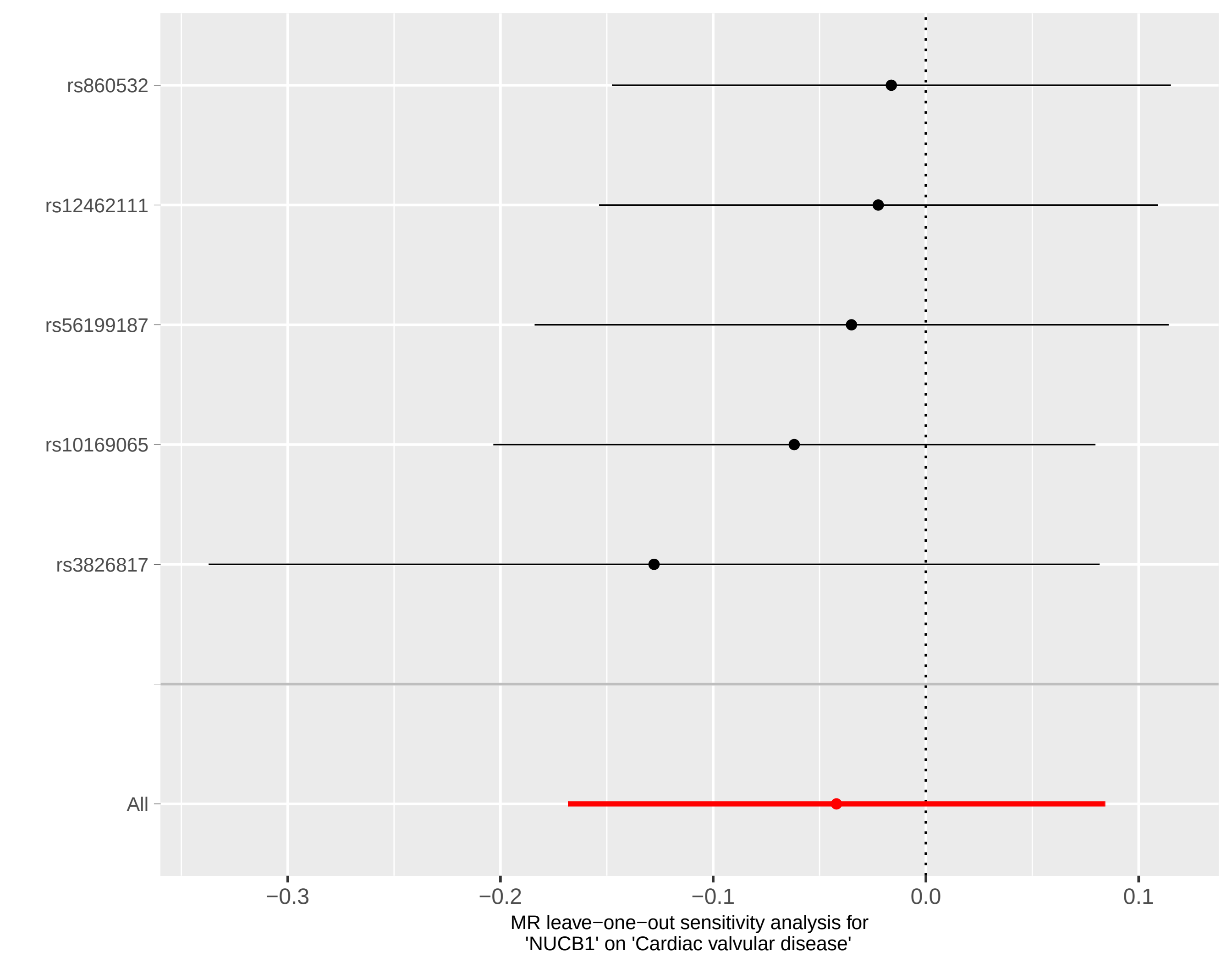

rs860532
rs12462111
rs56199187
rs10169065
rs3826817
All
−0.3
−0.2
−0.1
0.0
0.1
MR leave−one−out sensitivity analysis for
'NUCB1' on 'Cardiac valvular disease'

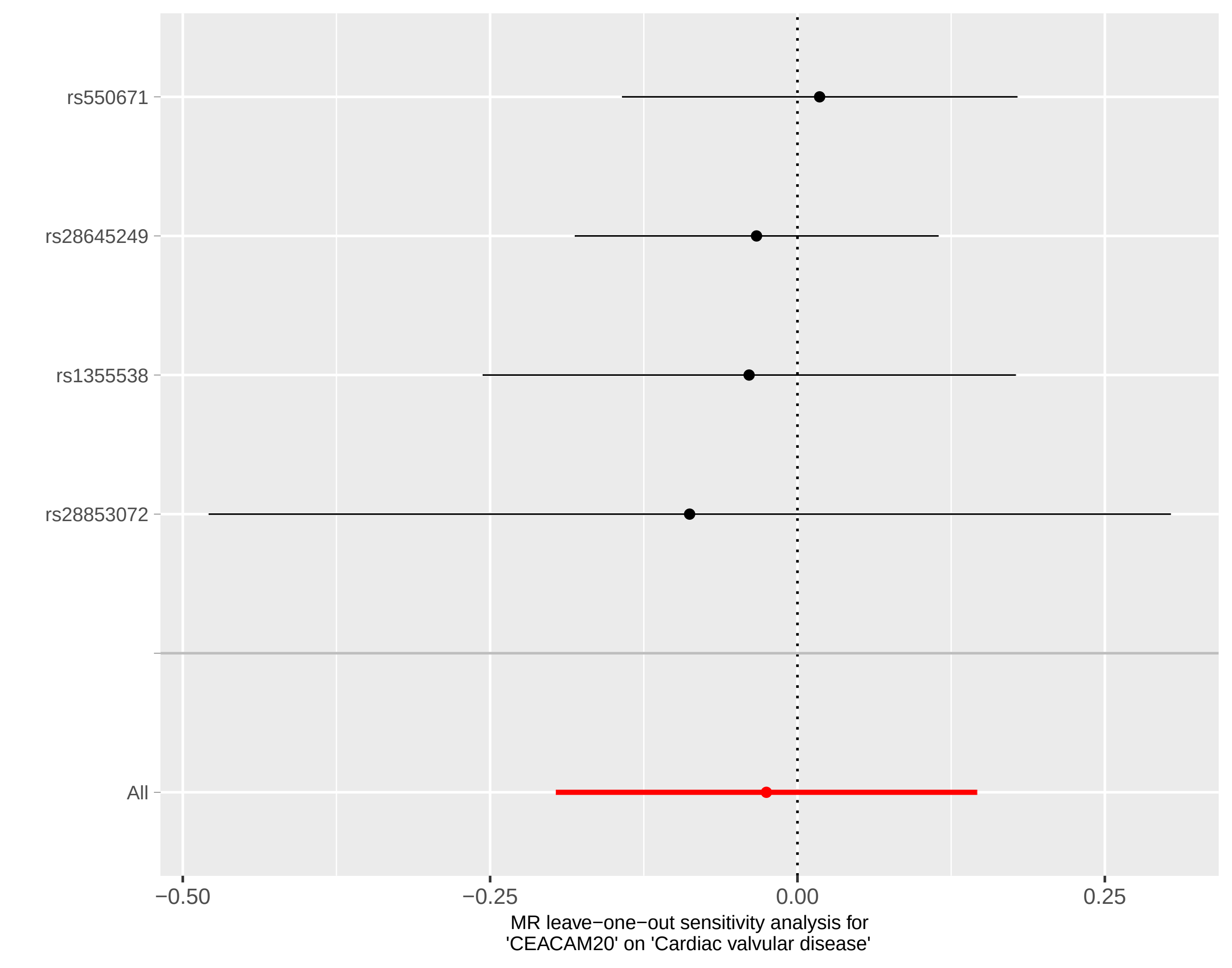

rs550671
rs28645249
rs1355538
rs28853072
All
−0.50
−0.25
0.00
0.25
MR leave−one−out sensitivity analysis for
'CEACAM20' on 'Cardiac valvular disease'

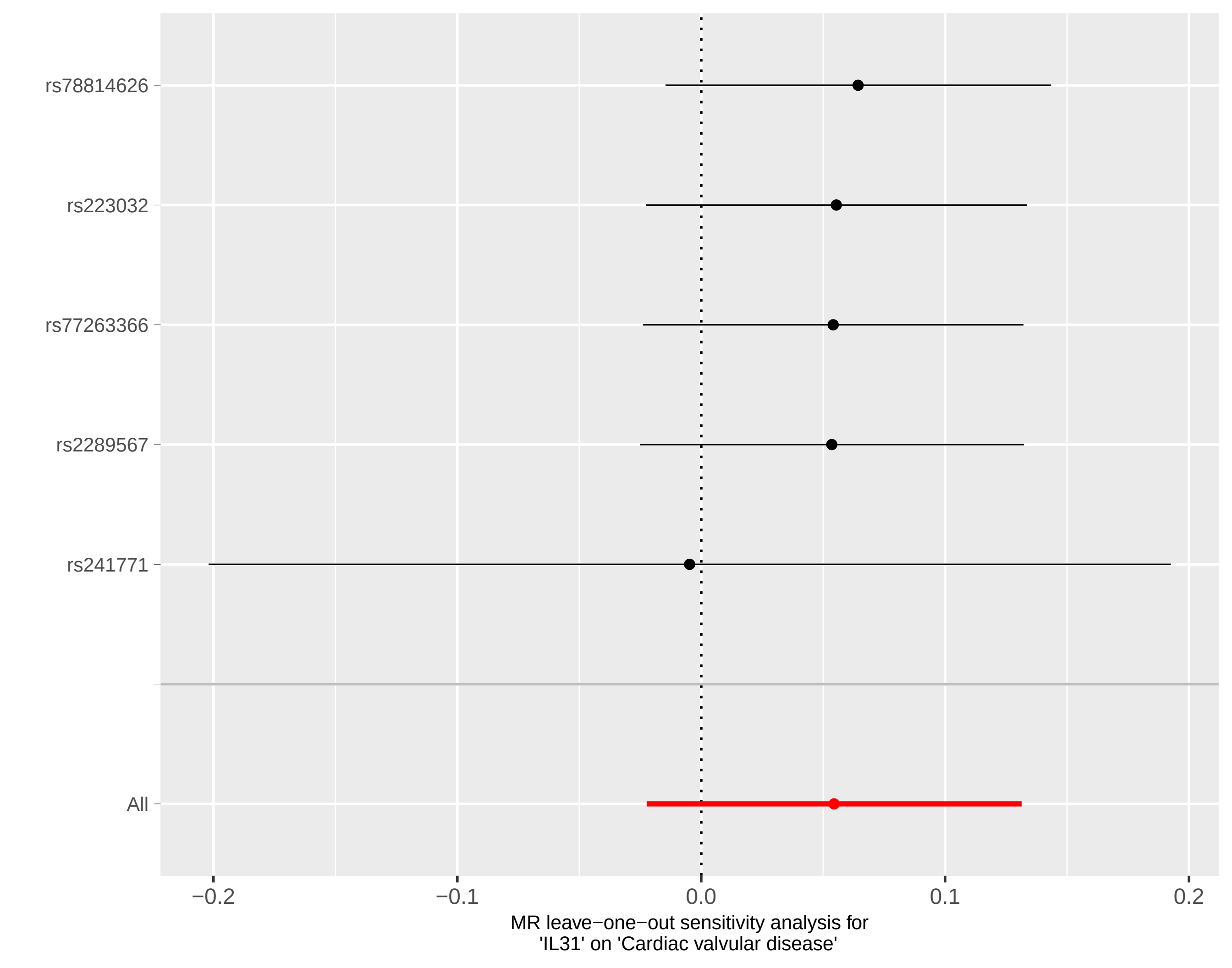
rs78814626
rs223032
rs77263366
rs2289567
rs241771
All
−0.2
−0.1
0.0
0.1
0.2
MR leave−one−out sensitivity analysis for
'IL31' on 'Cardiac valvular disease'

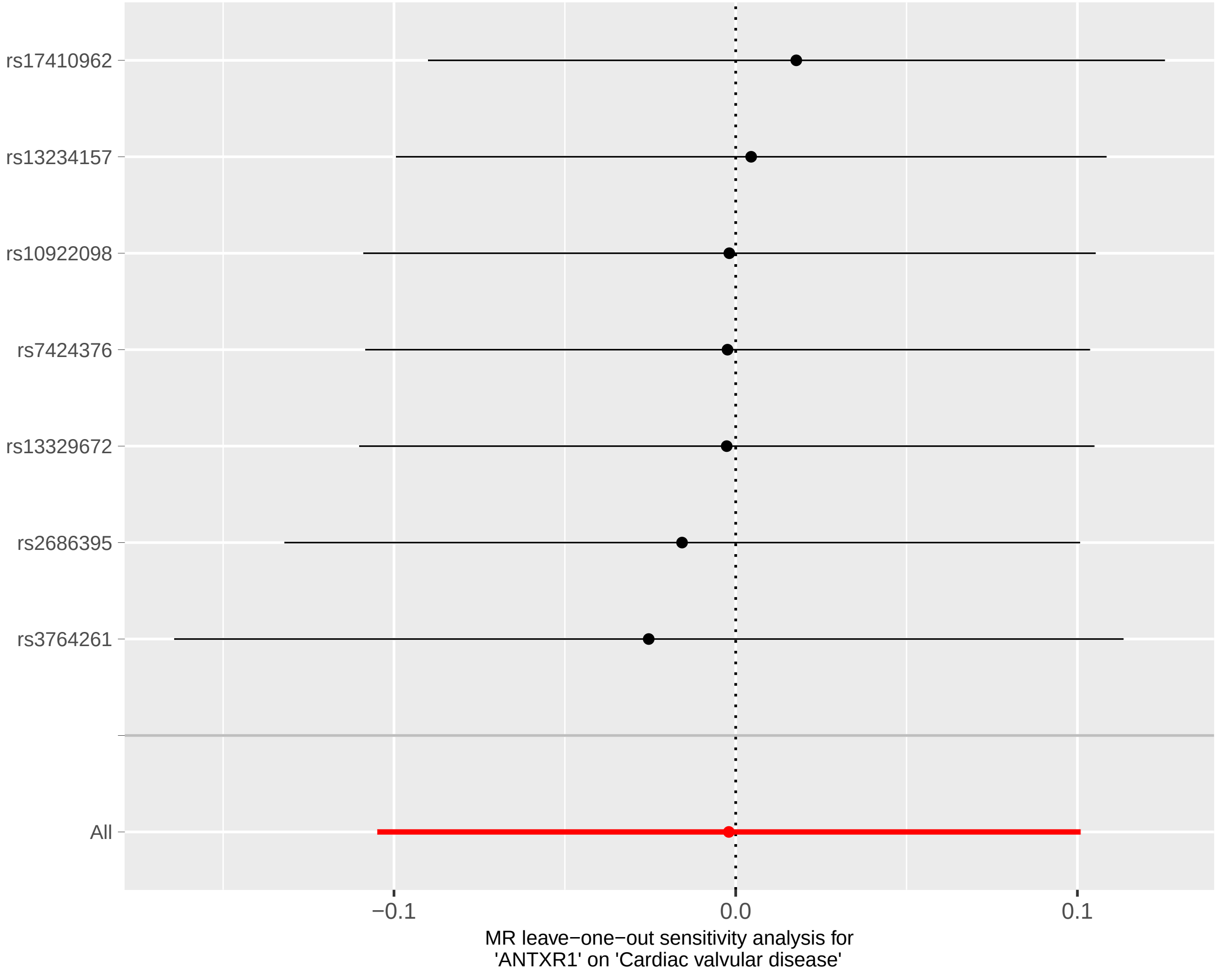
rs17410962
rs13234157
rs10922098
rs7424376
rs13329672
rs2686395
rs3764261
All
−0.1
0.0
0.1
MR leave−one−out sensitivity analysis for
'ANTXR1' on 'Cardiac valvular disease'

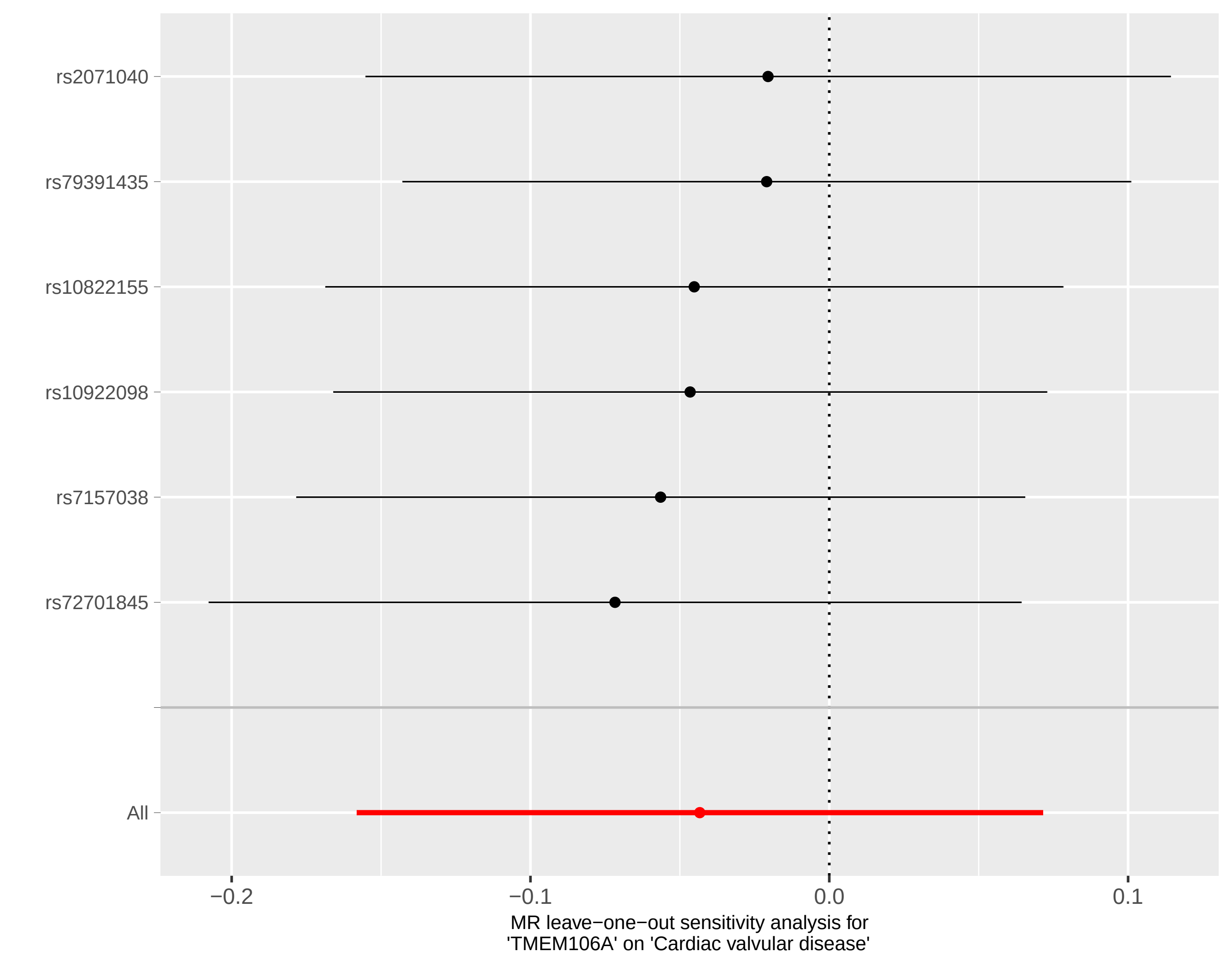

rs2071040
rs79391435
rs10822155
rs10922098
rs7157038
rs72701845
All
−0.2
−0.1
0.0
0.1
MR leave−one−out sensitivity analysis for
'TMEM106A' on 'Cardiac valvular disease'

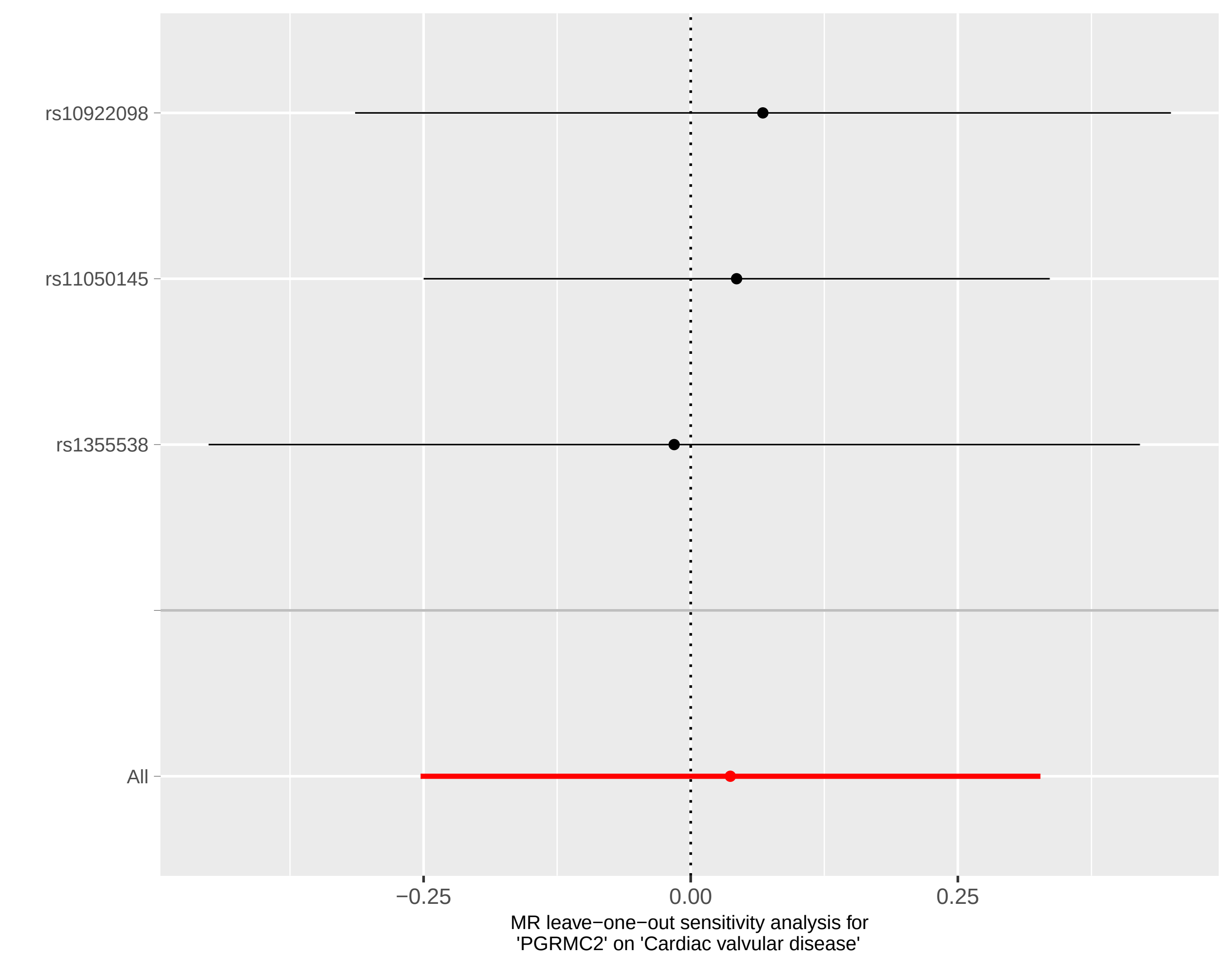

rs10922098
rs11050145
rs1355538
All
−0.25
0.00
0.25
MR leave−one−out sensitivity analysis for
'PGRMC2' on 'Cardiac valvular disease'

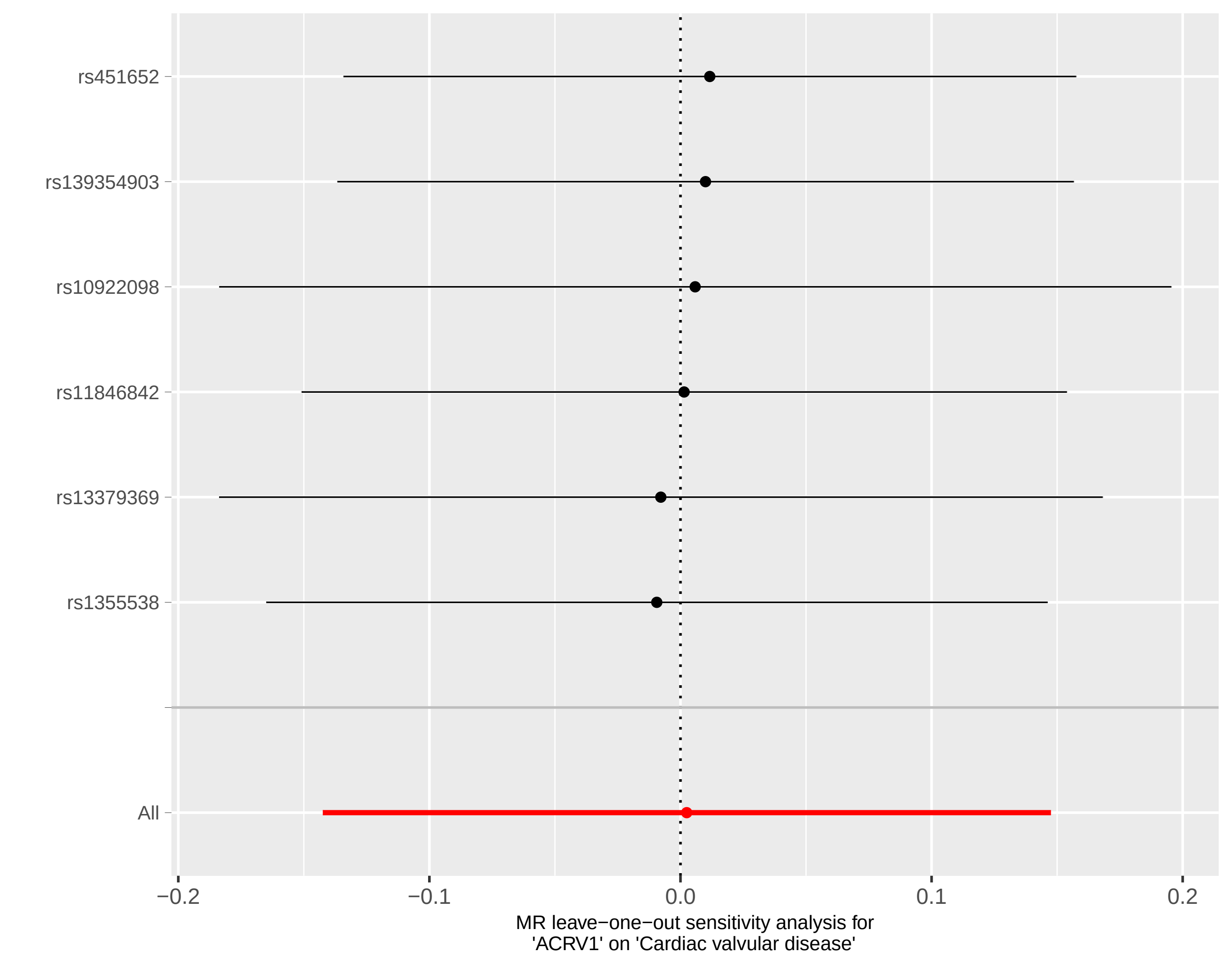
rs451652
rs139354903
rs10922098
rs11846842
rs13379369
rs1355538
All
−0.2
−0.1
0.0
0.1
0.2
MR leave−one−out sensitivity analysis for
'ACRV1' on 'Cardiac valvular disease'

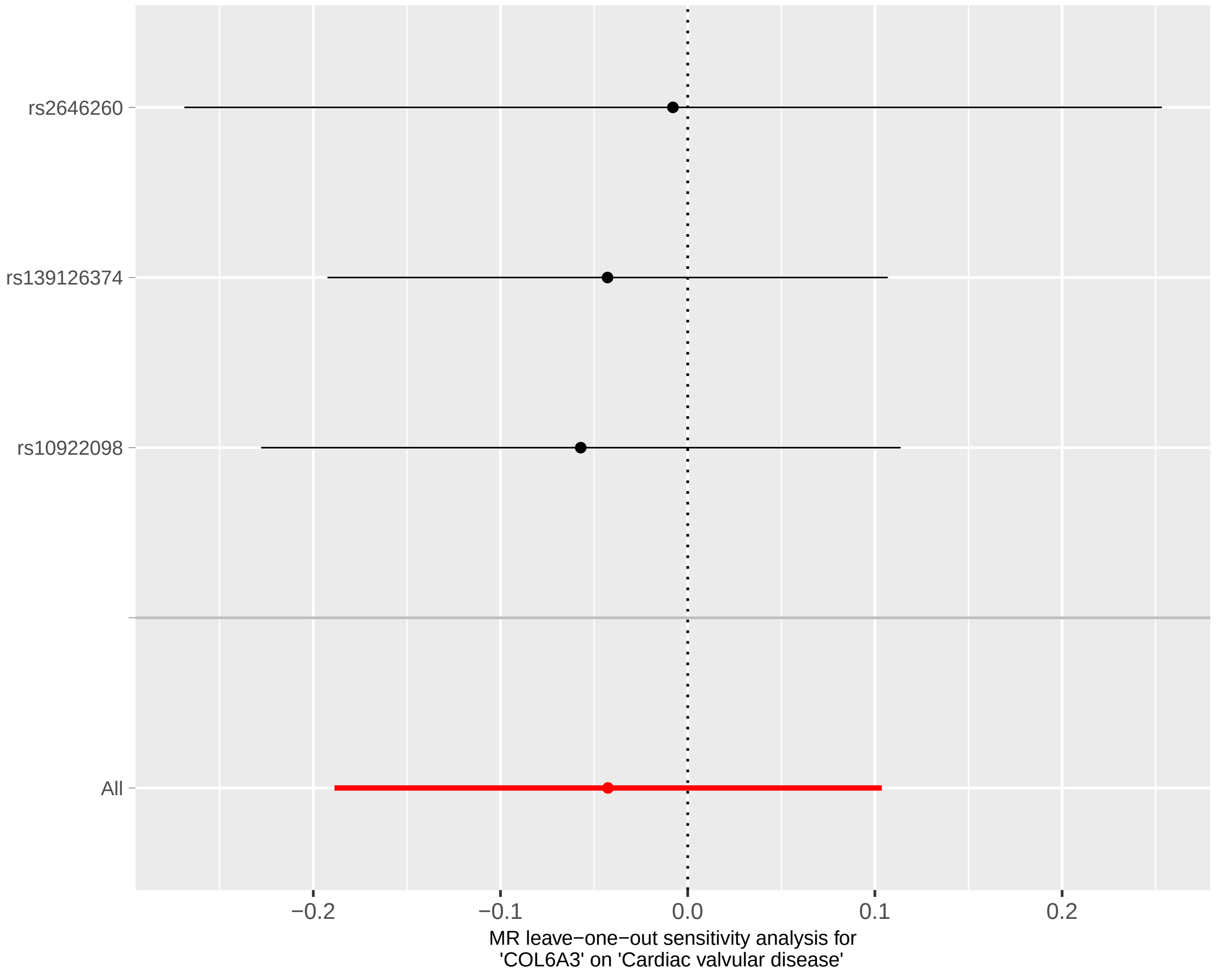
rs2646260
rs139126374
rs10922098
All
−0.2
−0.1
0.0
0.1
0.2
MR leave−one−out sensitivity analysis for
'COL6A3' on 'Cardiac valvular disease'

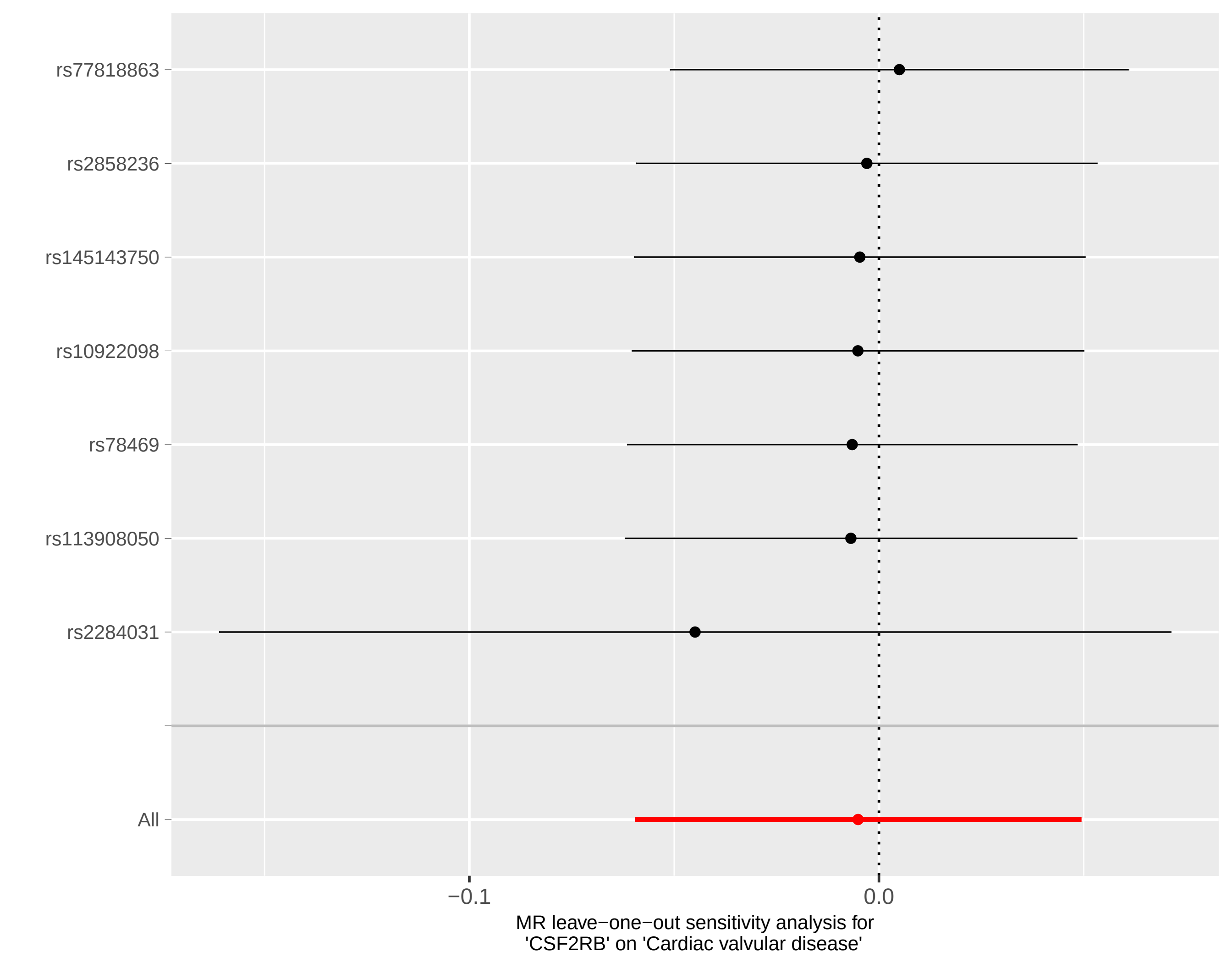
rs77818863
rs2858236
rs145143750
rs10922098
rs78469
rs113908050
rs2284031
All
−0.1
0.0
MR leave−one−out sensitivity analysis for
'CSF2RB' on 'Cardiac valvular disease'

Insufficient number of SNPs

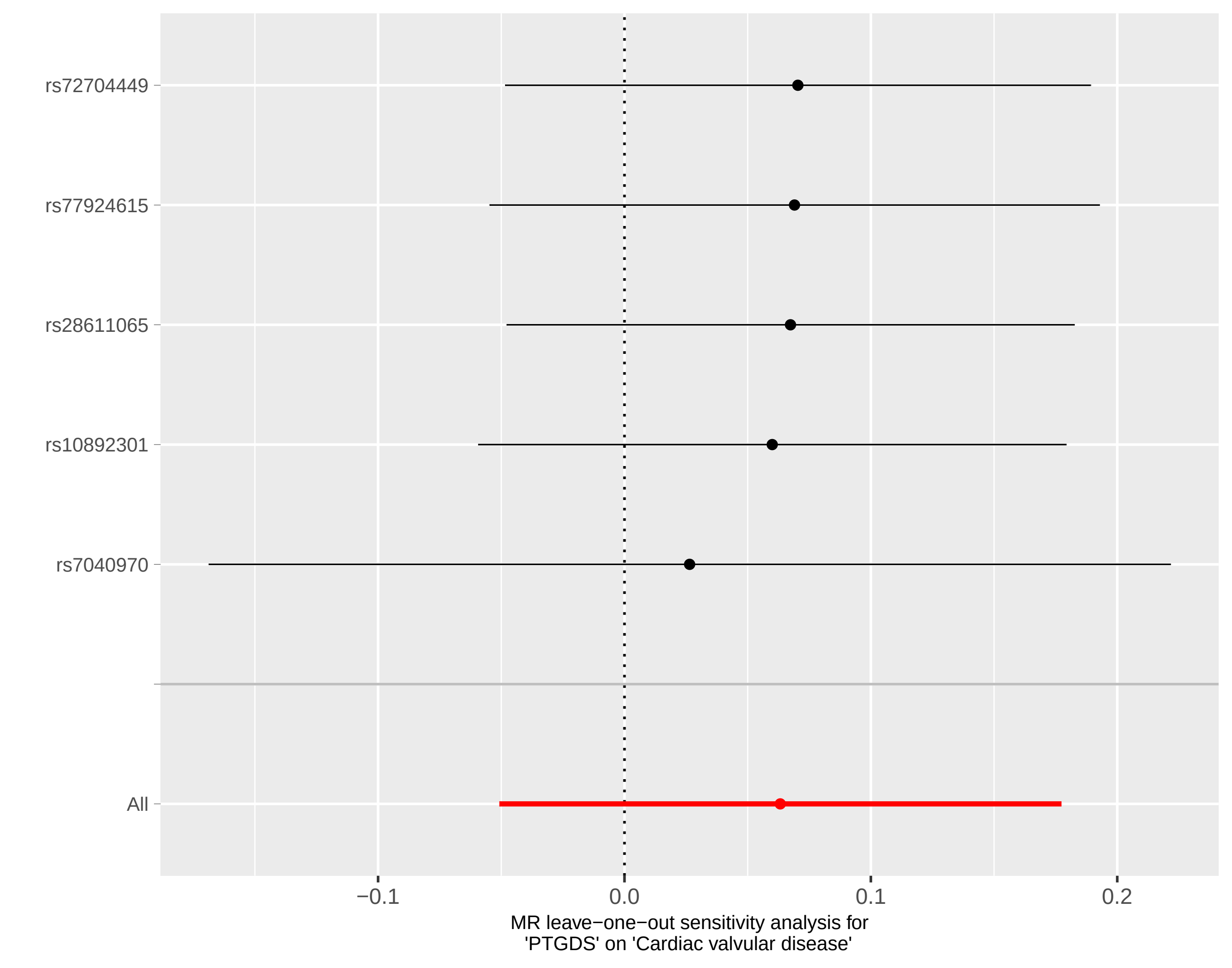
rs72704449
rs77924615
rs28611065
rs10892301
rs7040970
All
−0.1
0.0
0.1
0.2
MR leave−one−out sensitivity analysis for
'PTGDS' on 'Cardiac valvular disease'

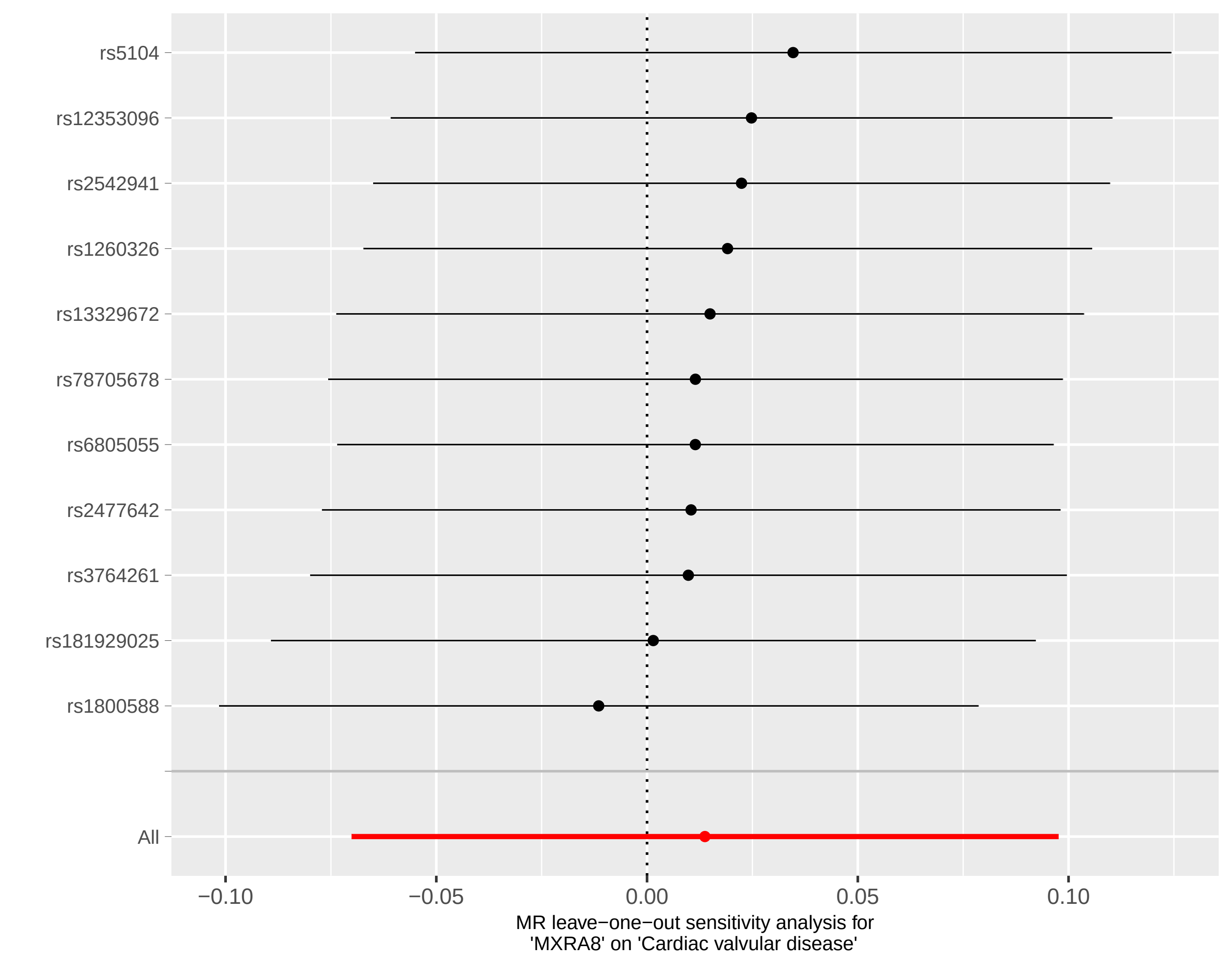
rs5104
rs12353096
rs2542941
rs1260326
rs13329672
rs78705678
rs6805055
rs2477642
rs3764261
rs181929025
rs1800588
All
−0.10
−0.05
0.00
0.05
0.10
MR leave−one−out sensitivity analysis for
'MXRA8' on 'Cardiac valvular disease'

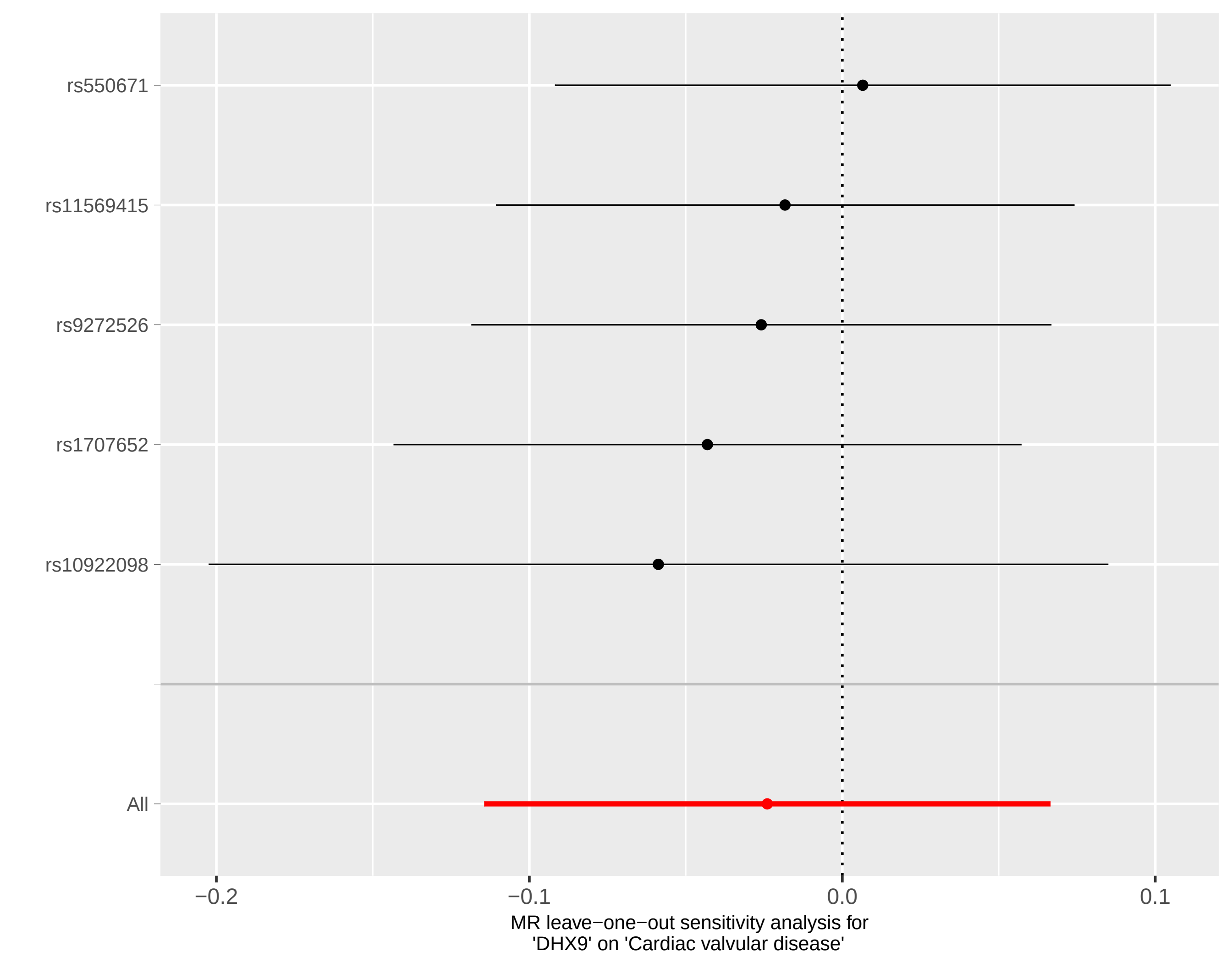
rs550671
rs11569415
rs9272526
rs1707652
rs10922098
All
−0.2
−0.1
0.0
0.1
MR leave−one−out sensitivity analysis for
'DHX9' on 'Cardiac valvular disease'

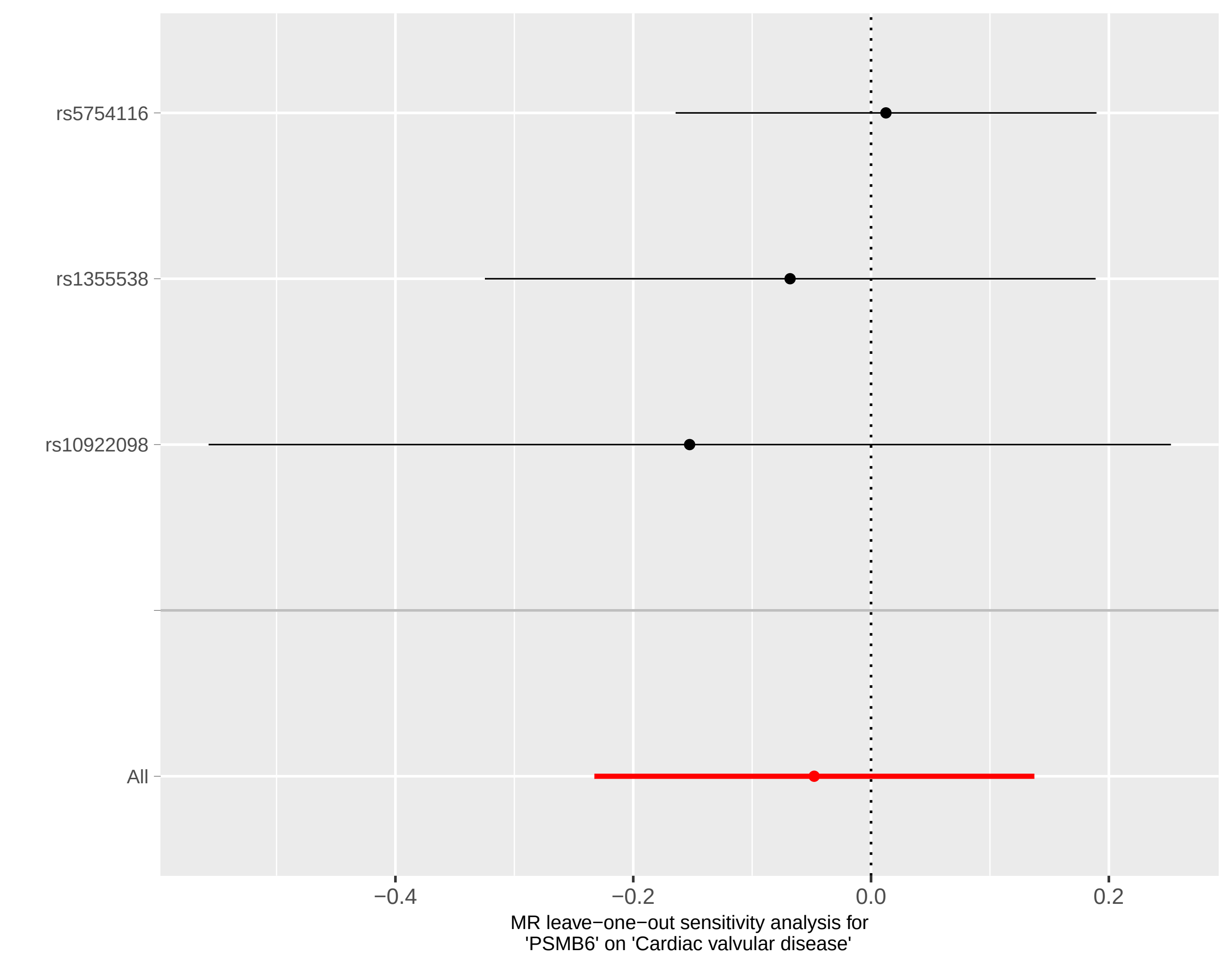
rs5754116
rs1355538
rs10922098
All
−0.4
−0.2
0.0
0.2
MR leave−one−out sensitivity analysis for
'PSMB6' on 'Cardiac valvular disease'

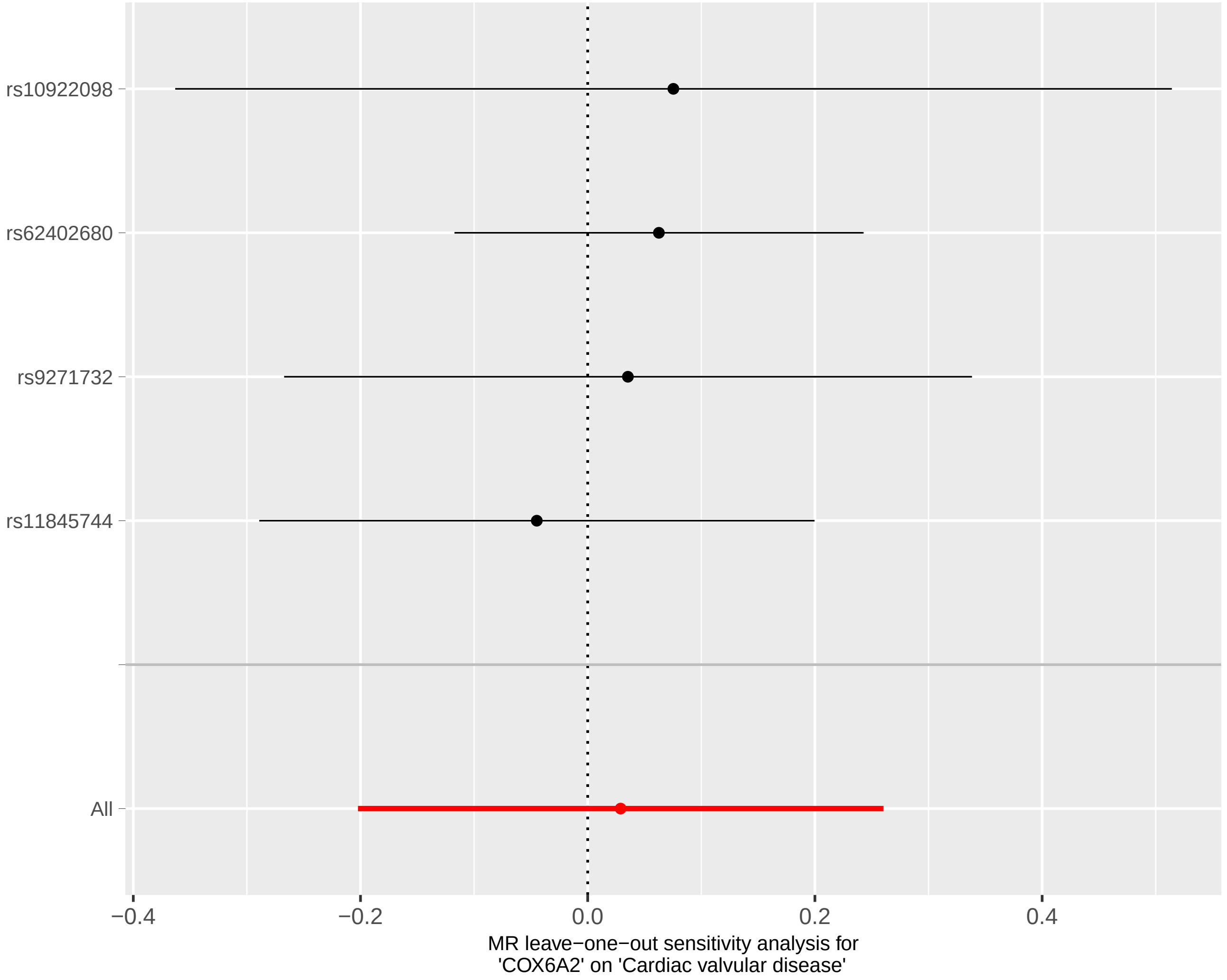
rs10922098
rs62402680
rs9271732
rs11845744
All
−0.4
−0.2
0.0
0.2
0.4
MR leave−one−out sensitivity analysis for
'COX6A2' on 'Cardiac valvular disease'

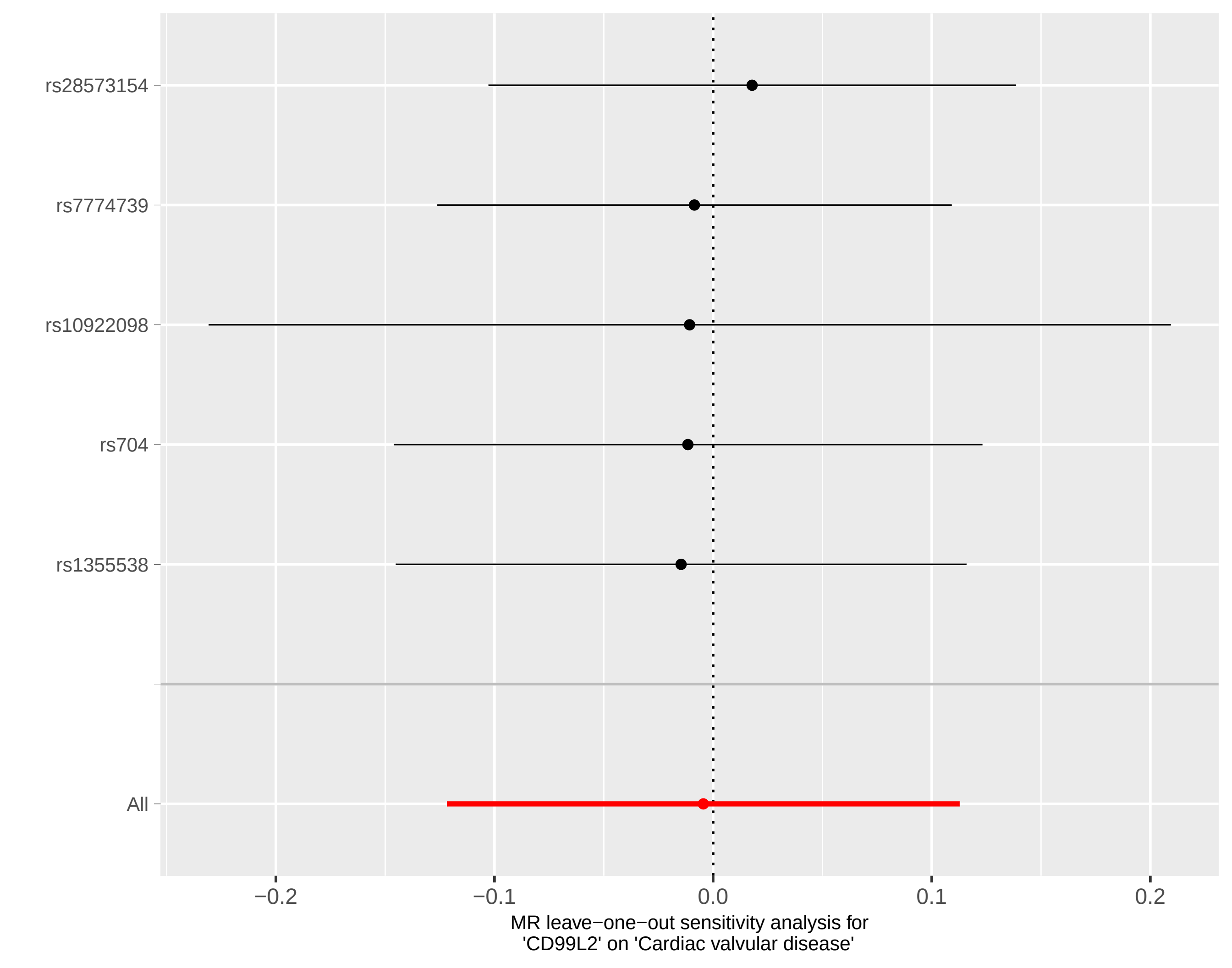

rs28573154
rs7774739
rs10922098
rs704
rs1355538
All
−0.2
−0.1
0.0
0.1
0.2
MR leave−one−out sensitivity analysis for
'CD99L2' on 'Cardiac valvular disease'

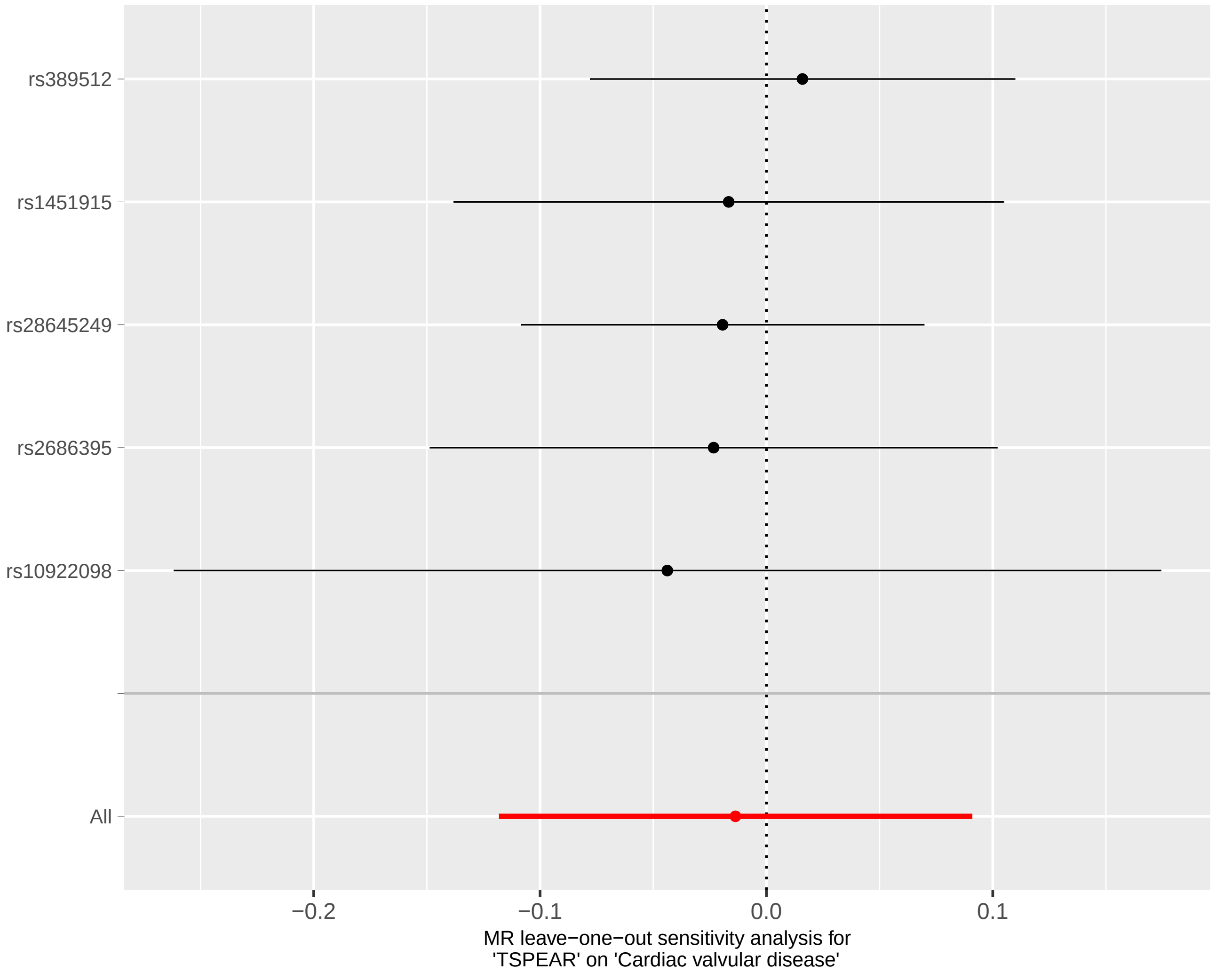
rs389512
rs1451915
rs28645249
rs2686395
rs10922098
All
−0.2
−0.1
0.0
0.1
MR leave−one−out sensitivity analysis for
'TSPEAR' on 'Cardiac valvular disease'

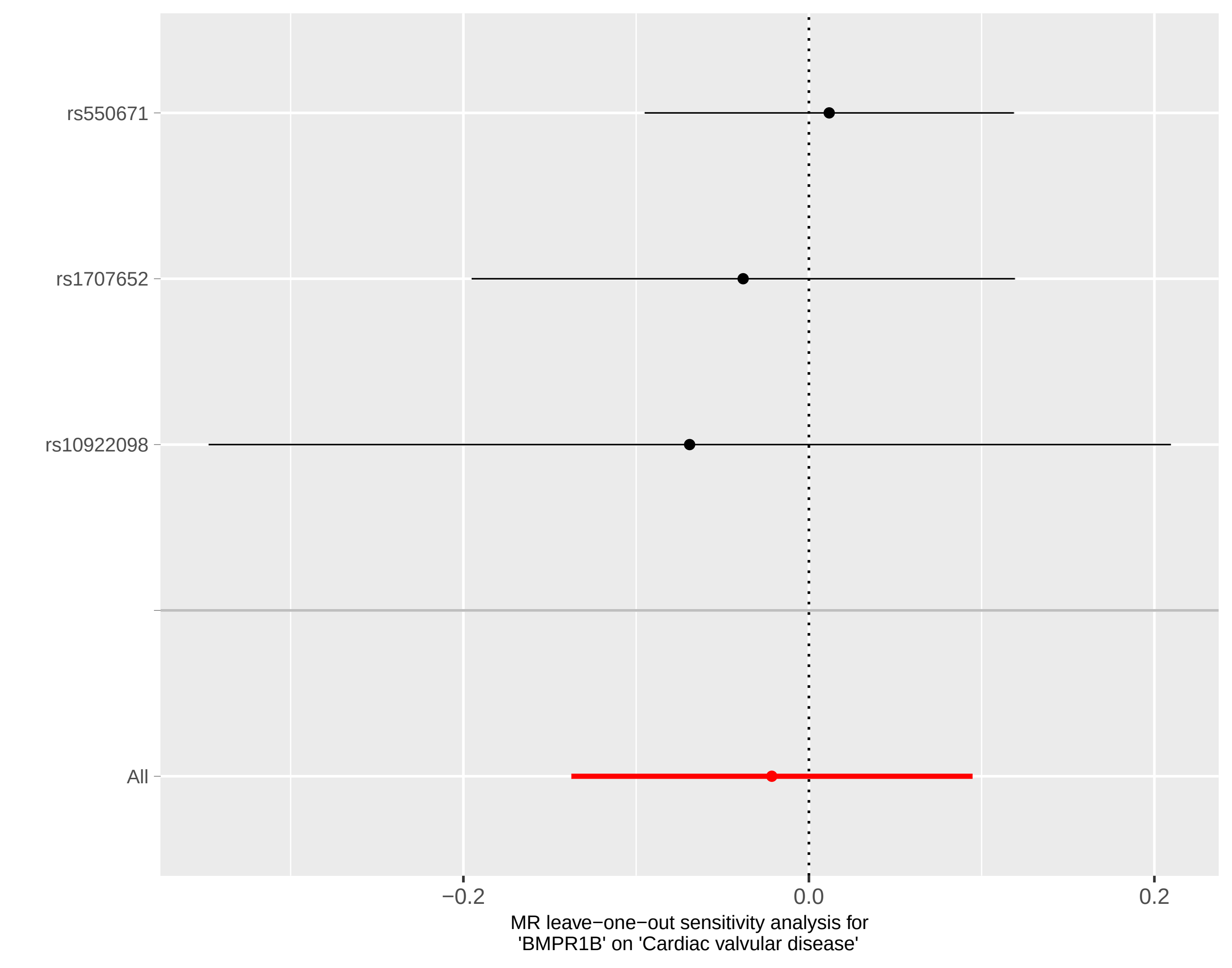

rs550671
rs1707652
rs10922098
All
−0.2
0.0
0.2
MR leave−one−out sensitivity analysis for
'BMPR1B' on 'Cardiac valvular disease'

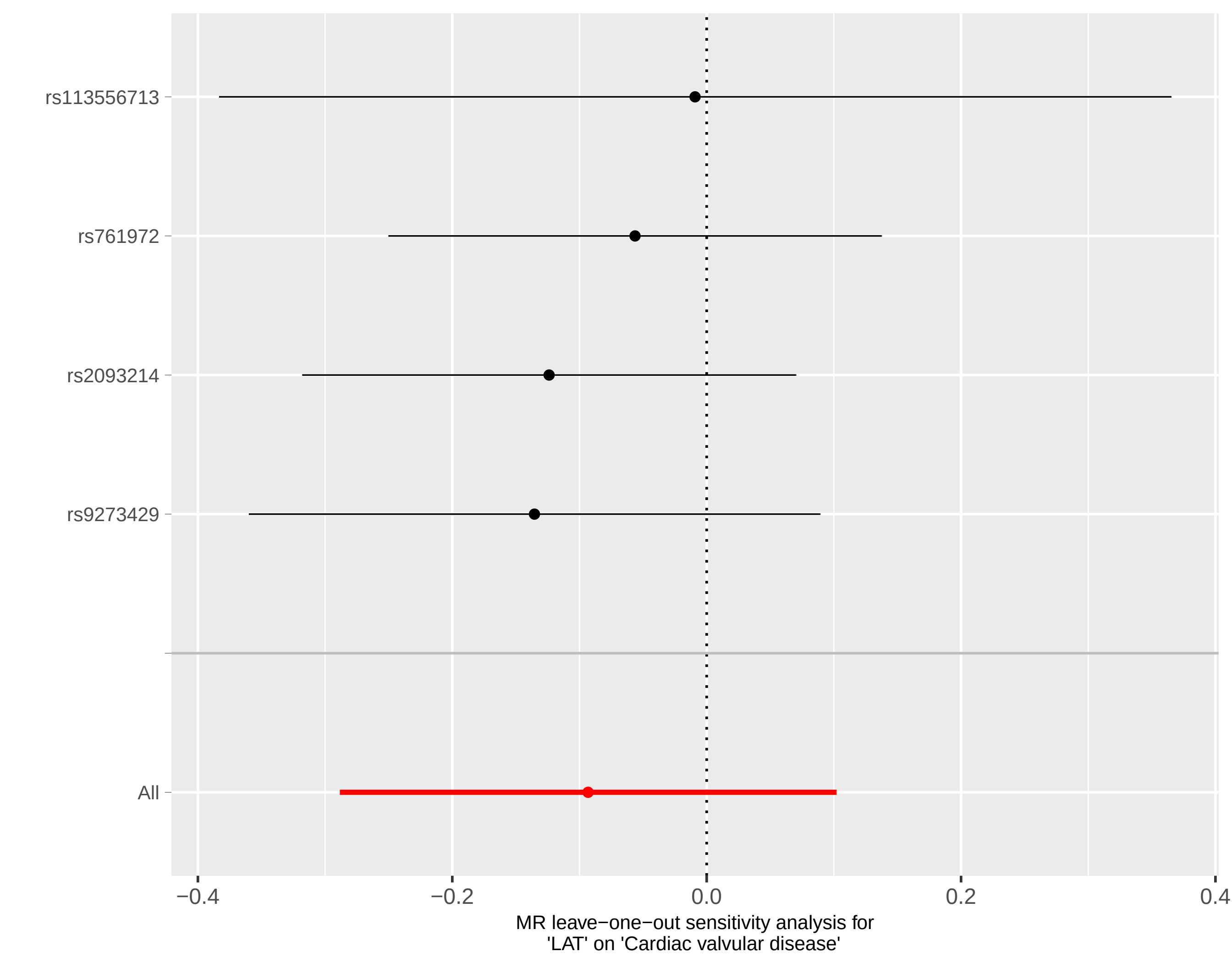
rs113556713
rs761972
rs2093214
rs9273429
All
−0.4
−0.2
0.0
0.2
0.4
MR leave−one−out sensitivity analysis for
'LAT' on 'Cardiac valvular disease'

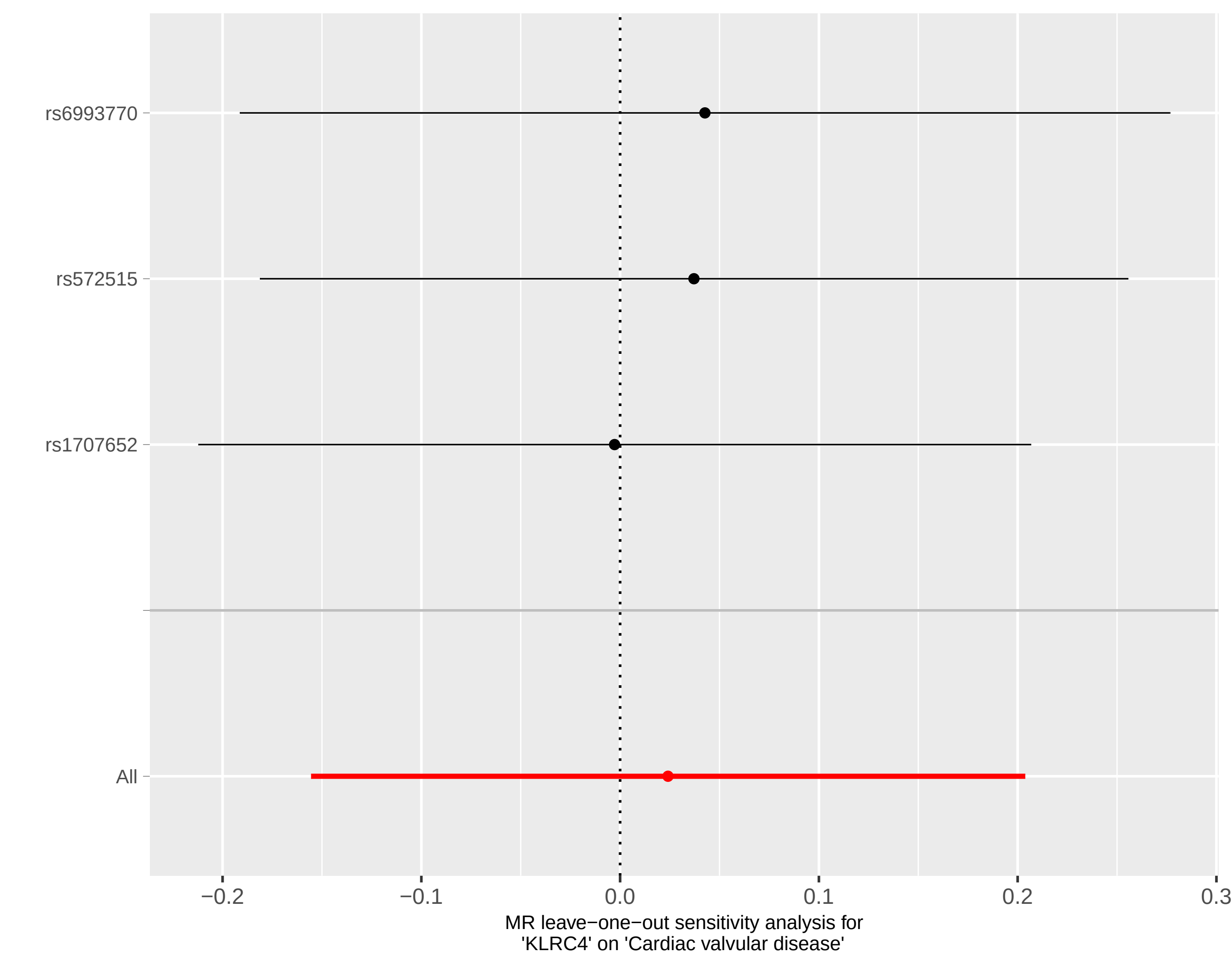

rs6993770
rs572515
rs1707652
All
−0.2
−0.1
0.0
0.1
0.2
0.3
MR leave−one−out sensitivity analysis for
'KLRC4' on 'Cardiac valvular disease'

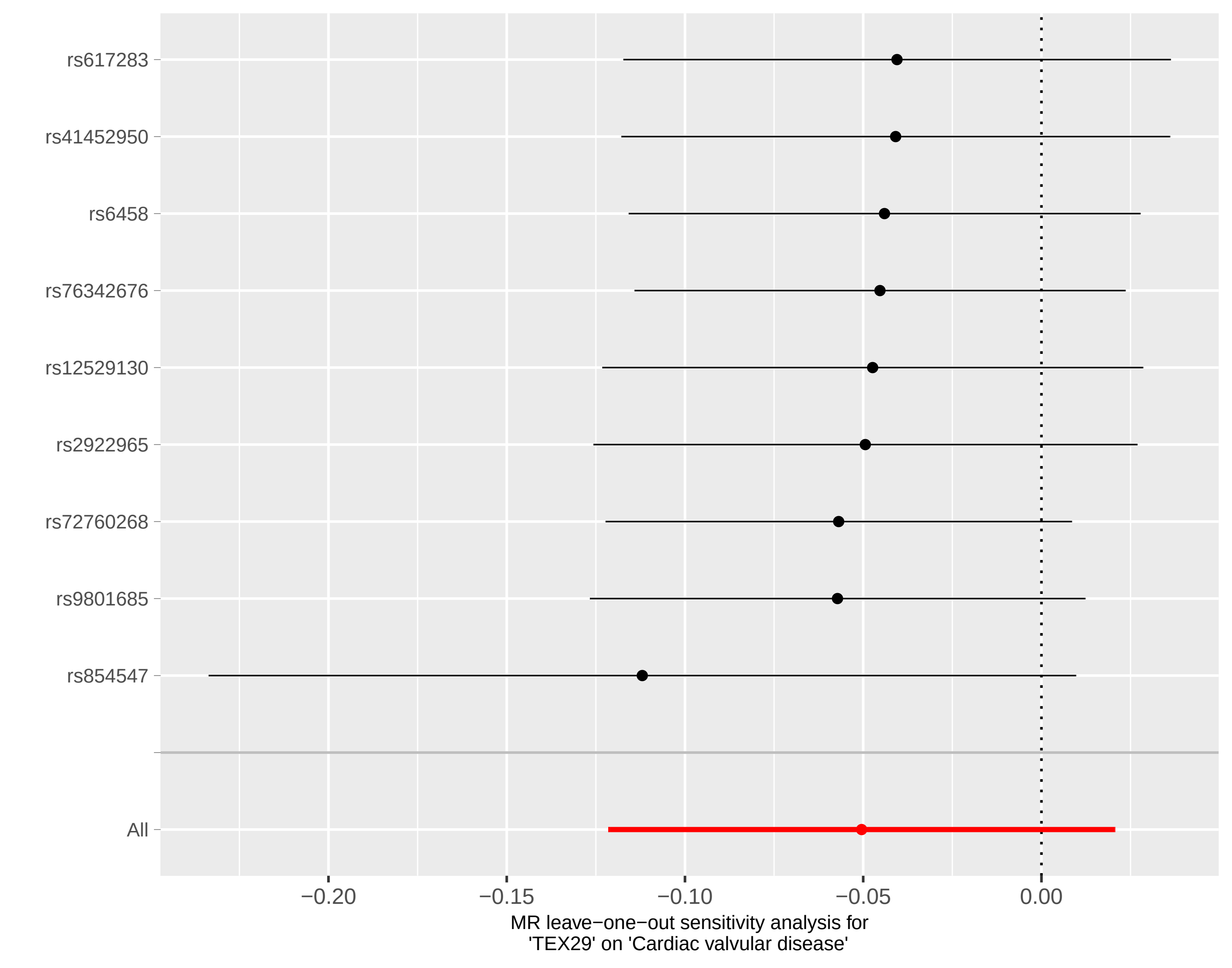
rs617283
rs41452950
rs6458
rs76342676
rs12529130
rs2922965
rs72760268
rs9801685
rs854547
All
−0.20
−0.15
−0.10
−0.05
0.00
MR leave−one−out sensitivity analysis for
'TEX29' on 'Cardiac valvular disease'

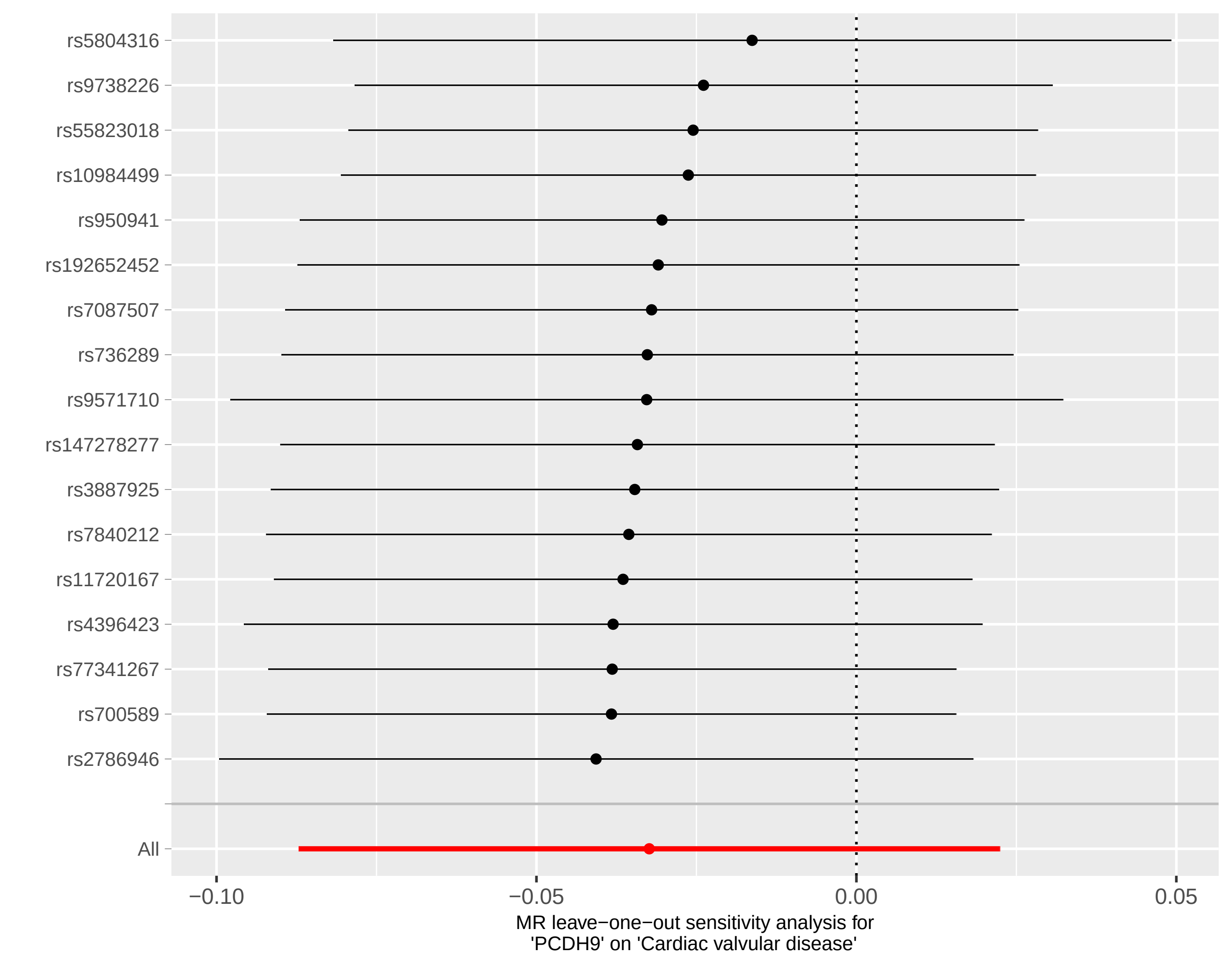
rs5804316
rs9738226
rs55823018
rs10984499
rs950941
rs192652452
rs7087507
rs736289
rs9571710
rs147278277
rs3887925
rs7840212
rs11720167
rs4396423
rs77341267
rs700589
rs2786946
All
−0.10
−0.05
0.00
0.05
MR leave−one−out sensitivity analysis for
'PCDH9' on 'Cardiac valvular disease'

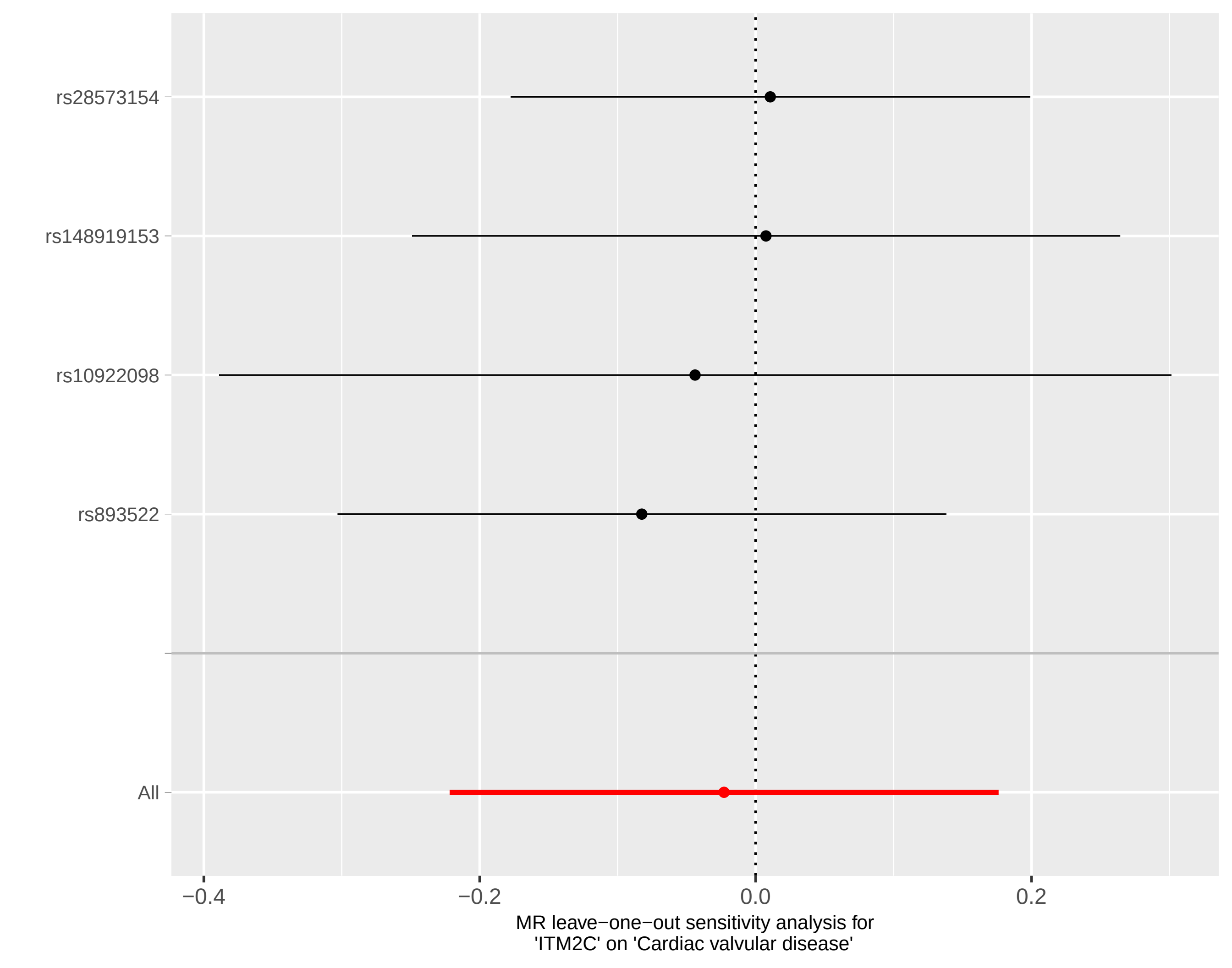

rs28573154
rs148919153
rs10922098
rs893522
All
−0.4
−0.2
0.0
0.2
MR leave−one−out sensitivity analysis for
'ITM2C' on 'Cardiac valvular disease'

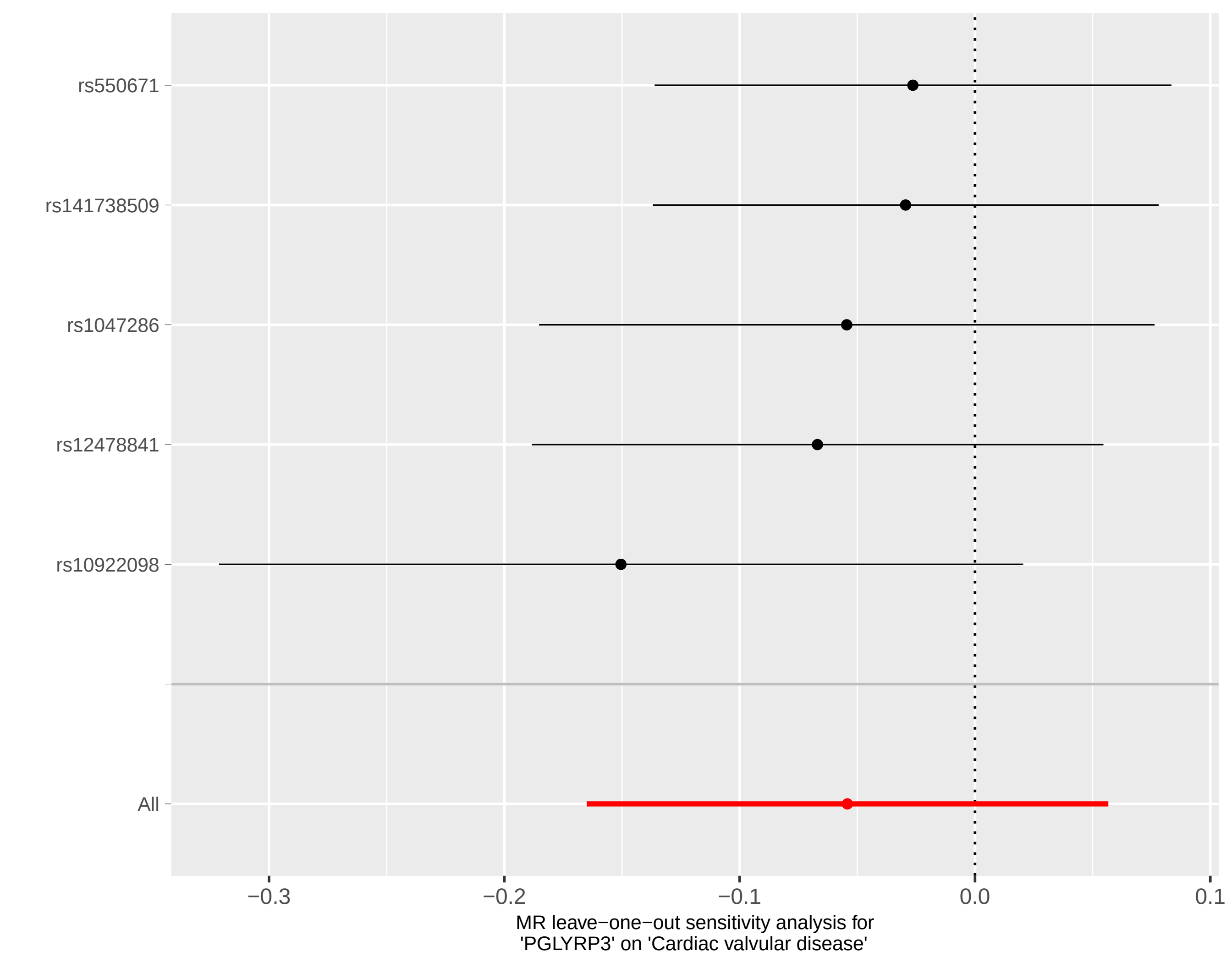
rs550671
rs141738509
rs1047286
rs12478841
rs10922098
All
−0.3
−0.2
−0.1
0.0
0.1
MR leave−one−out sensitivity analysis for
'PGLYRP3' on 'Cardiac valvular disease'

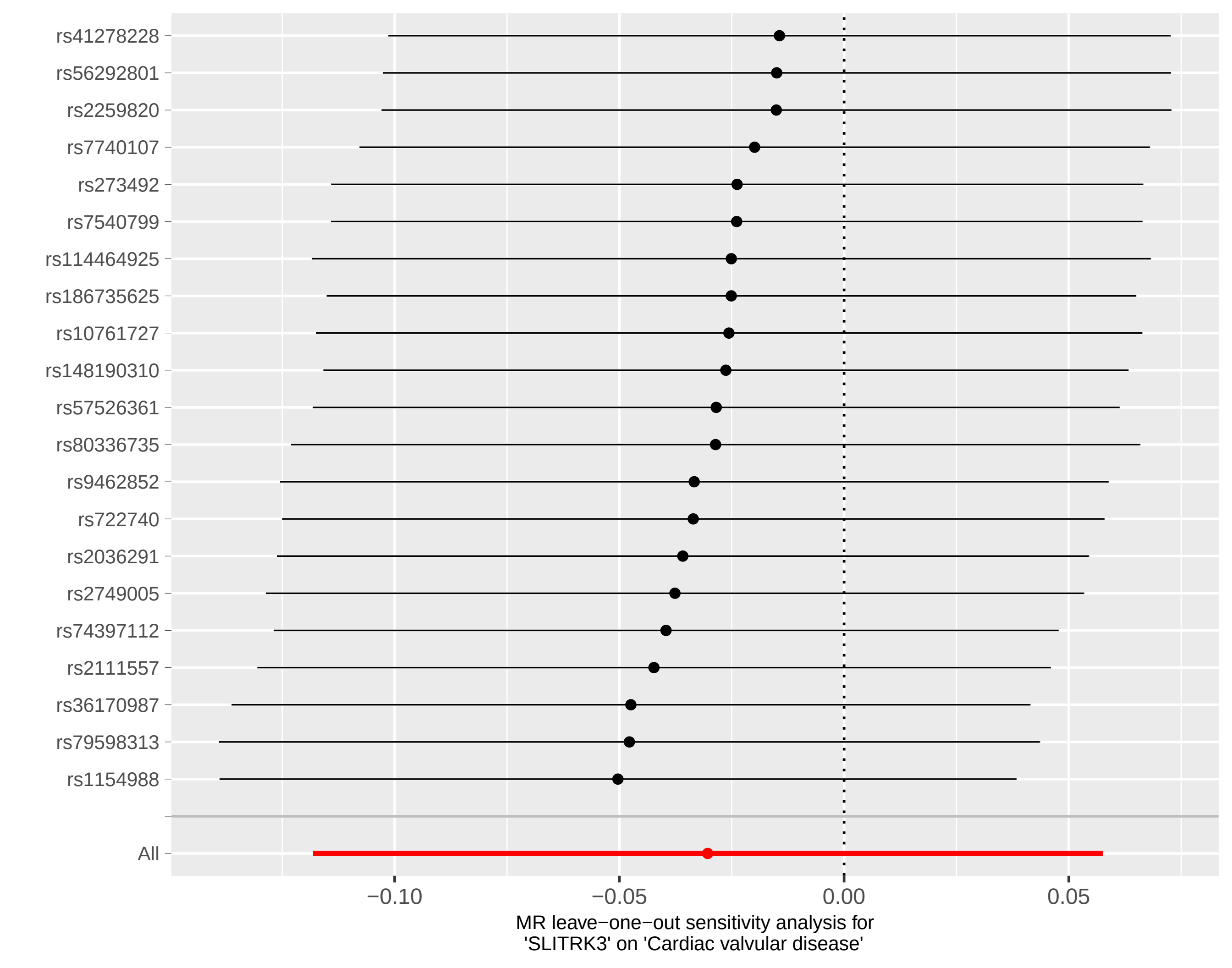
rs41278228
rs56292801
rs2259820
rs7740107
rs273492
rs7540799
rs114464925
rs186735625
rs10761727
rs148190310
rs57526361
rs80336735
rs9462852
rs722740
rs2036291
rs2749005
rs74397112
rs2111557
rs36170987
rs79598313
rs1154988
All
−0.10
−0.05
0.00
0.05
MR leave−one−out sensitivity analysis for
'SLITRK3' on 'Cardiac valvular disease'

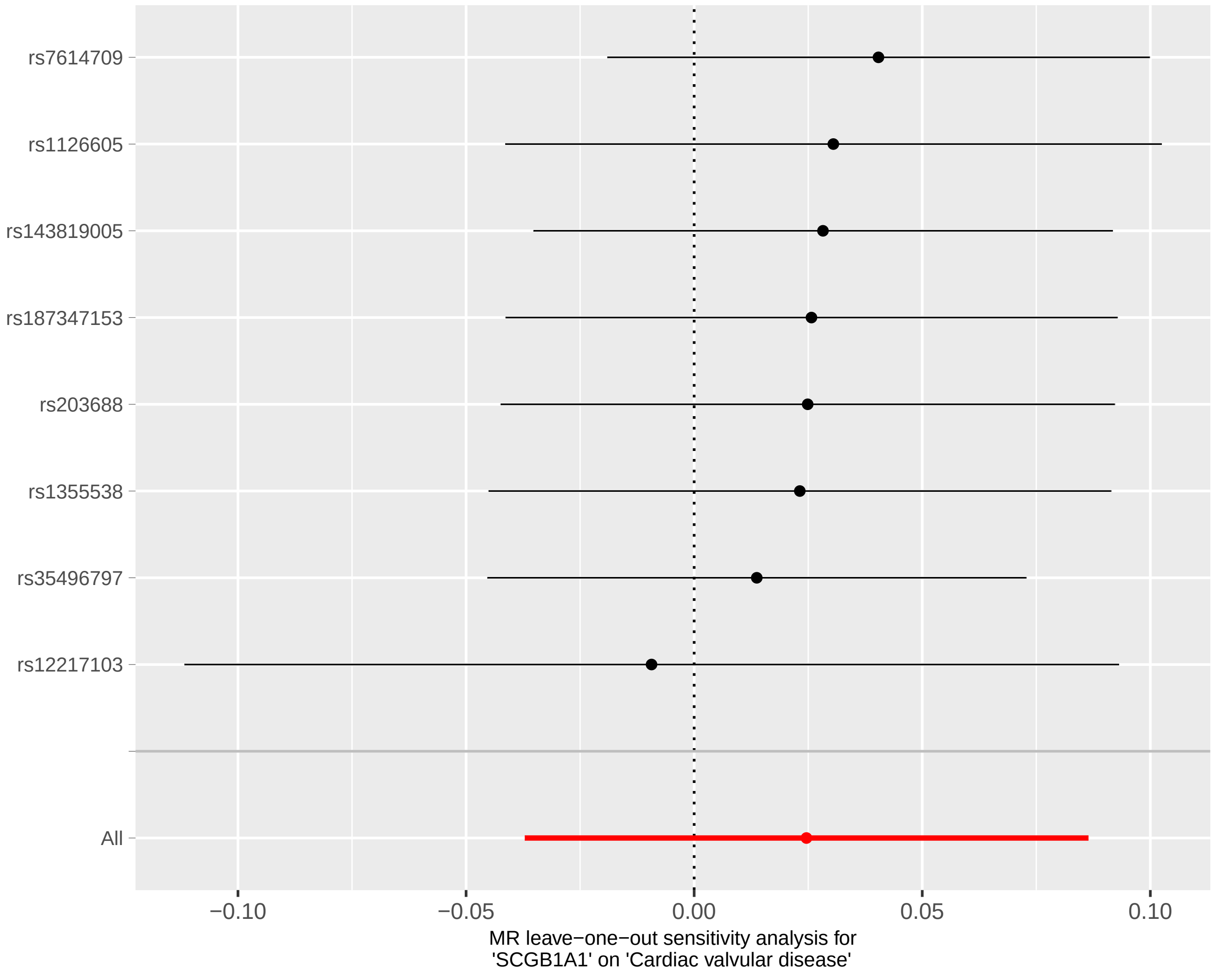

rs7614709
rs1126605
rs143819005
rs187347153
rs203688
rs1355538
rs35496797
rs12217103
All
−0.10
−0.05
0.00
0.05
0.10
MR leave−one−out sensitivity analysis for
'SCGB1A1' on 'Cardiac valvular disease'

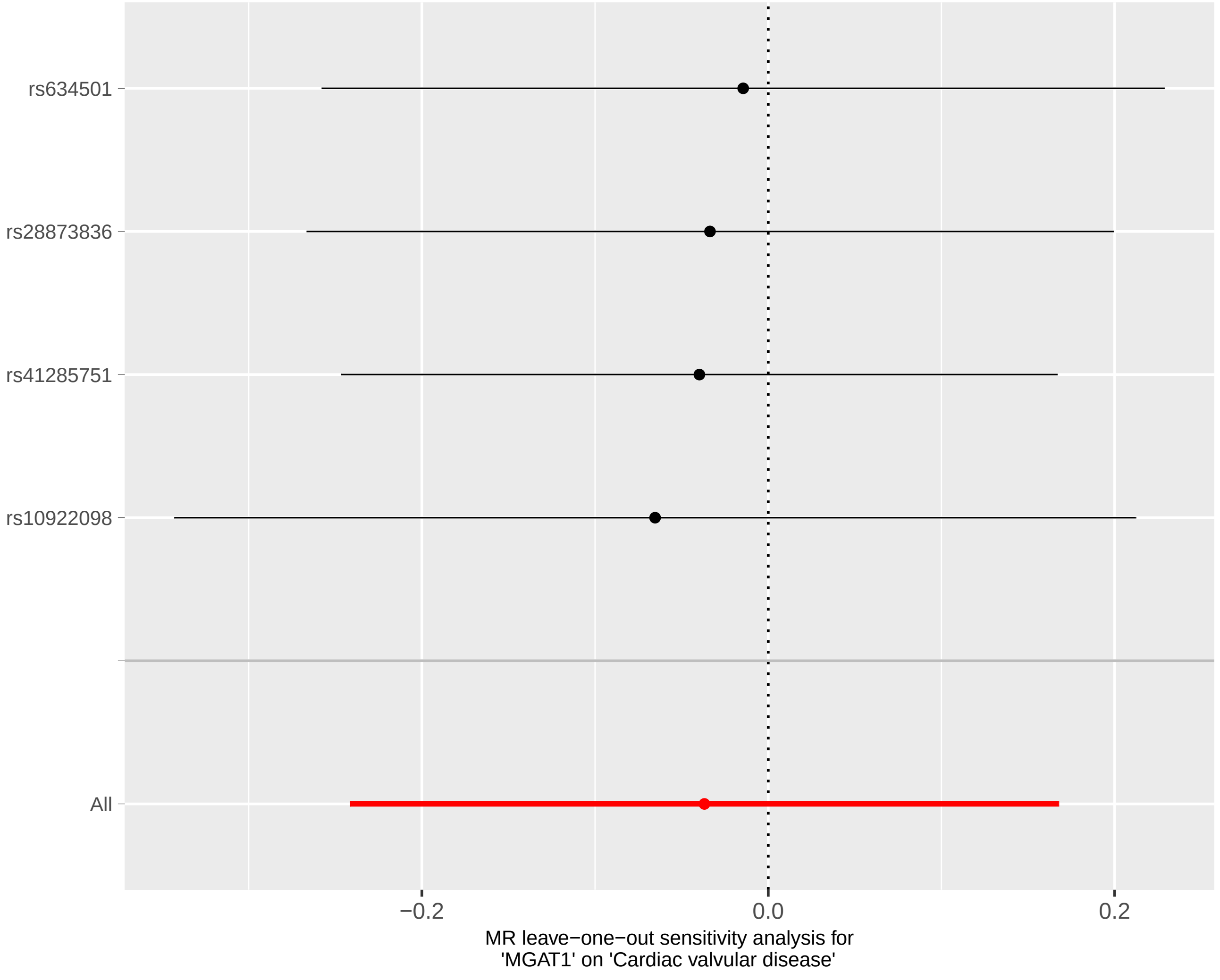

rs634501
rs28873836
rs41285751
rs10922098
All
−0.2
0.0
0.2
MR leave−one−out sensitivity analysis for
'MGAT1' on 'Cardiac valvular disease'

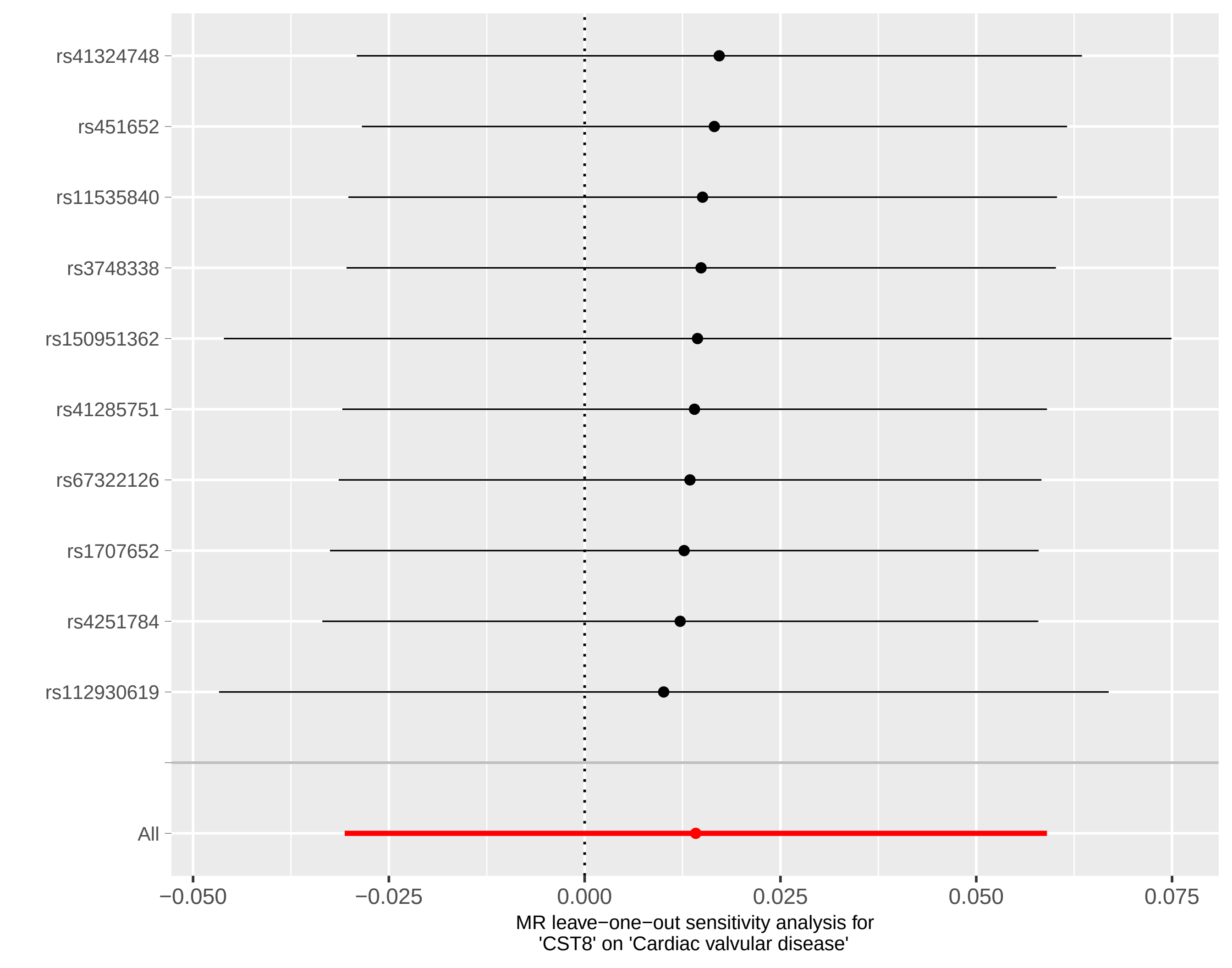

rs41324748
rs451652
rs11535840
rs3748338
rs150951362
rs41285751
rs67322126
rs1707652
rs4251784
rs112930619
All
−0.050
−0.025
0.000
0.025
0.050
0.075
MR leave−one−out sensitivity analysis for
'CST8' on 'Cardiac valvular disease'

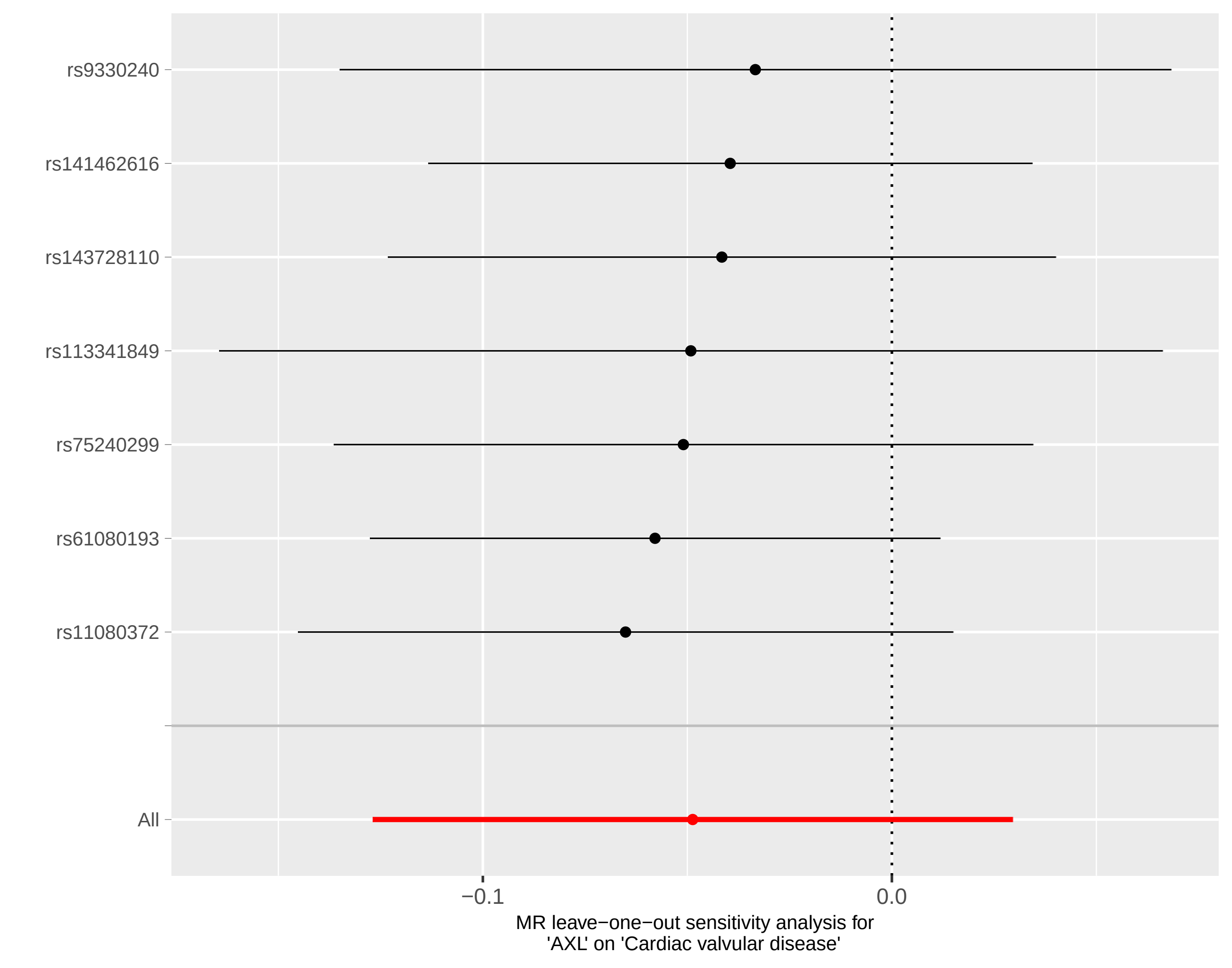

rs9330240
rs141462616
rs143728110
rs113341849
rs75240299
rs61080193
rs11080372
All
−0.1
0.0
MR leave−one−out sensitivity analysis for
'AXL' on 'Cardiac valvular disease'

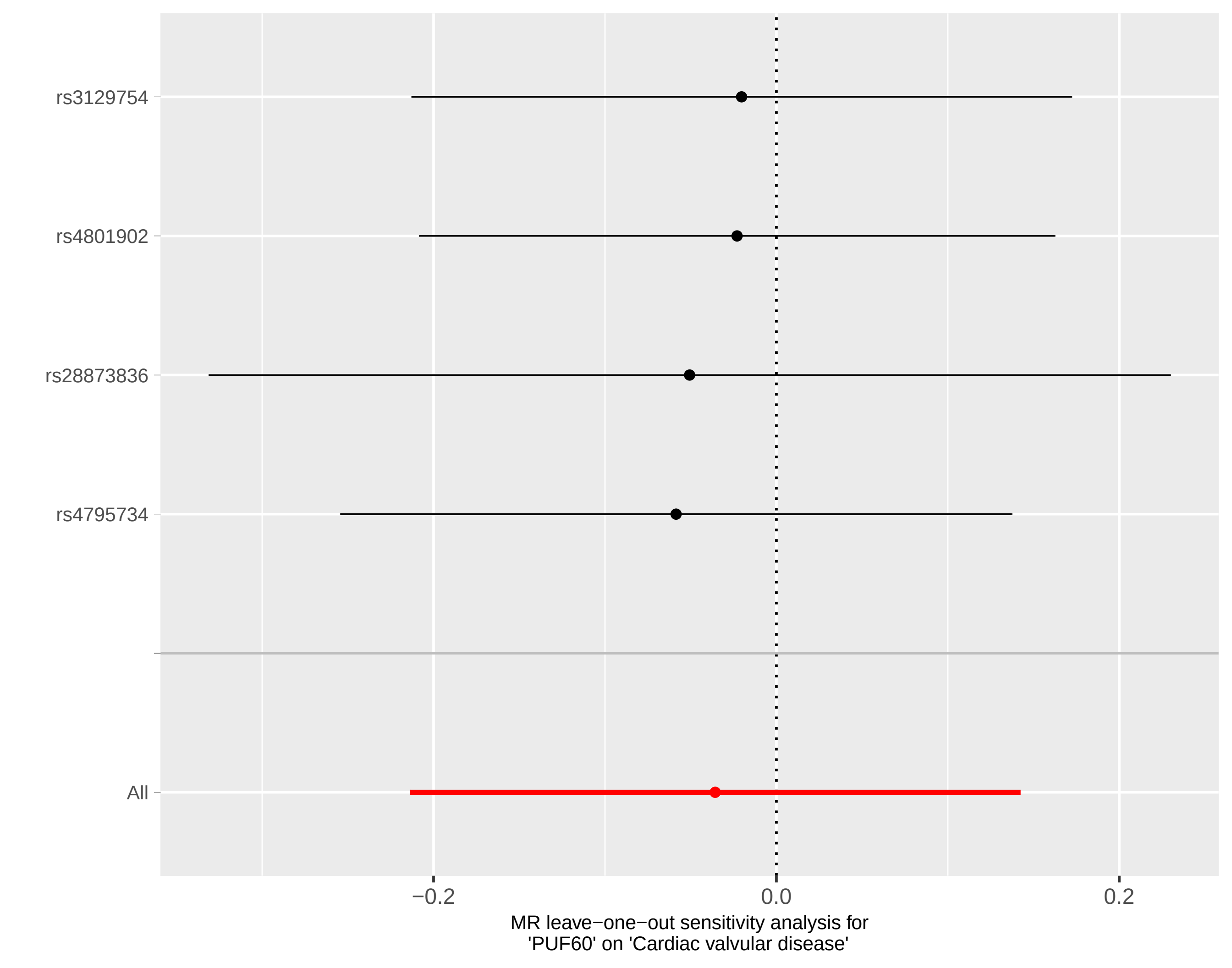
rs3129754
rs4801902
rs28873836
rs4795734
All
−0.2
0.0
0.2
MR leave−one−out sensitivity analysis for
'PUF60' on 'Cardiac valvular disease'

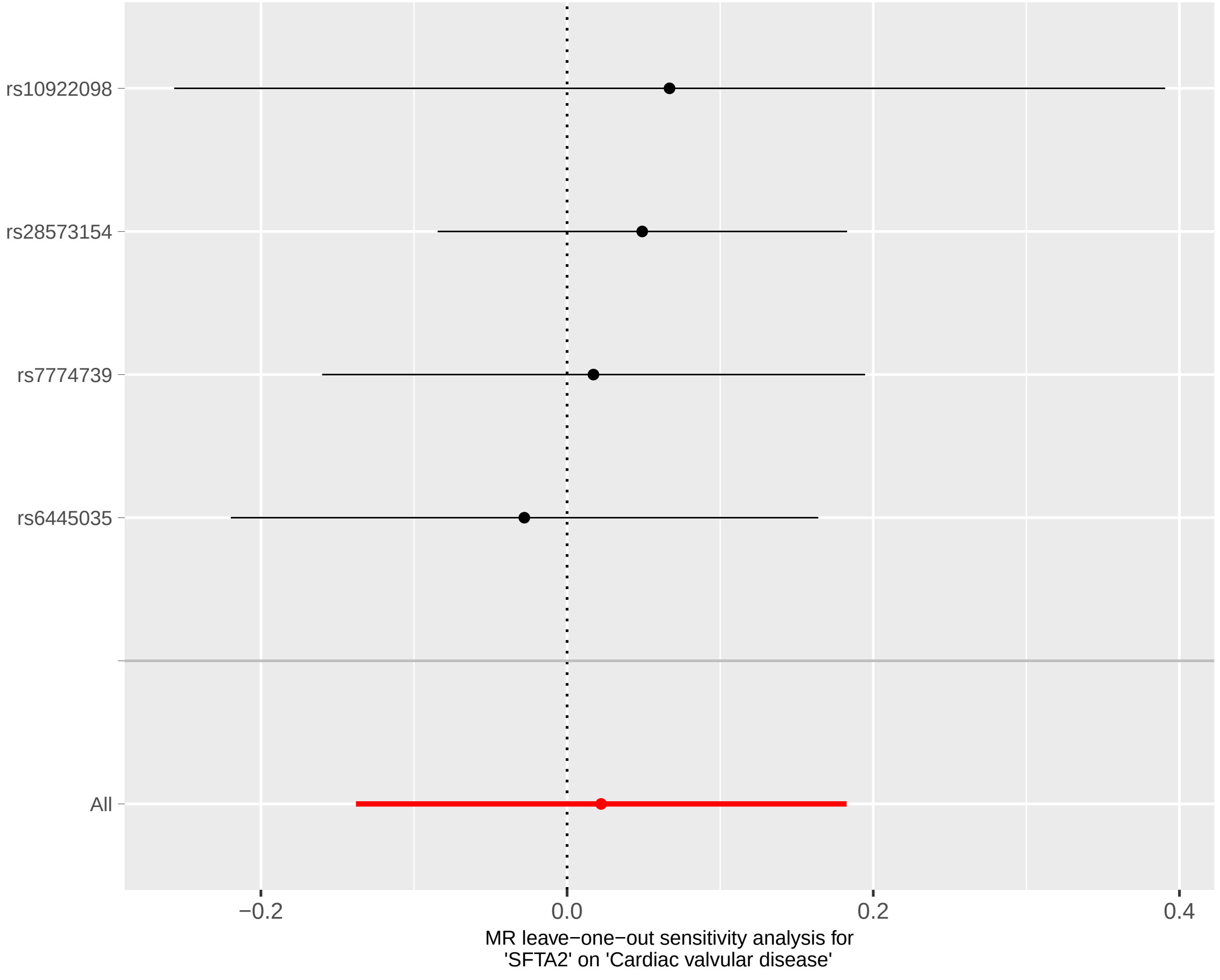

rs10922098
rs28573154
rs7774739
rs6445035
All
−0.2
0.0
0.2
0.4
MR leave−one−out sensitivity analysis for
'SFTA2' on 'Cardiac valvular disease'

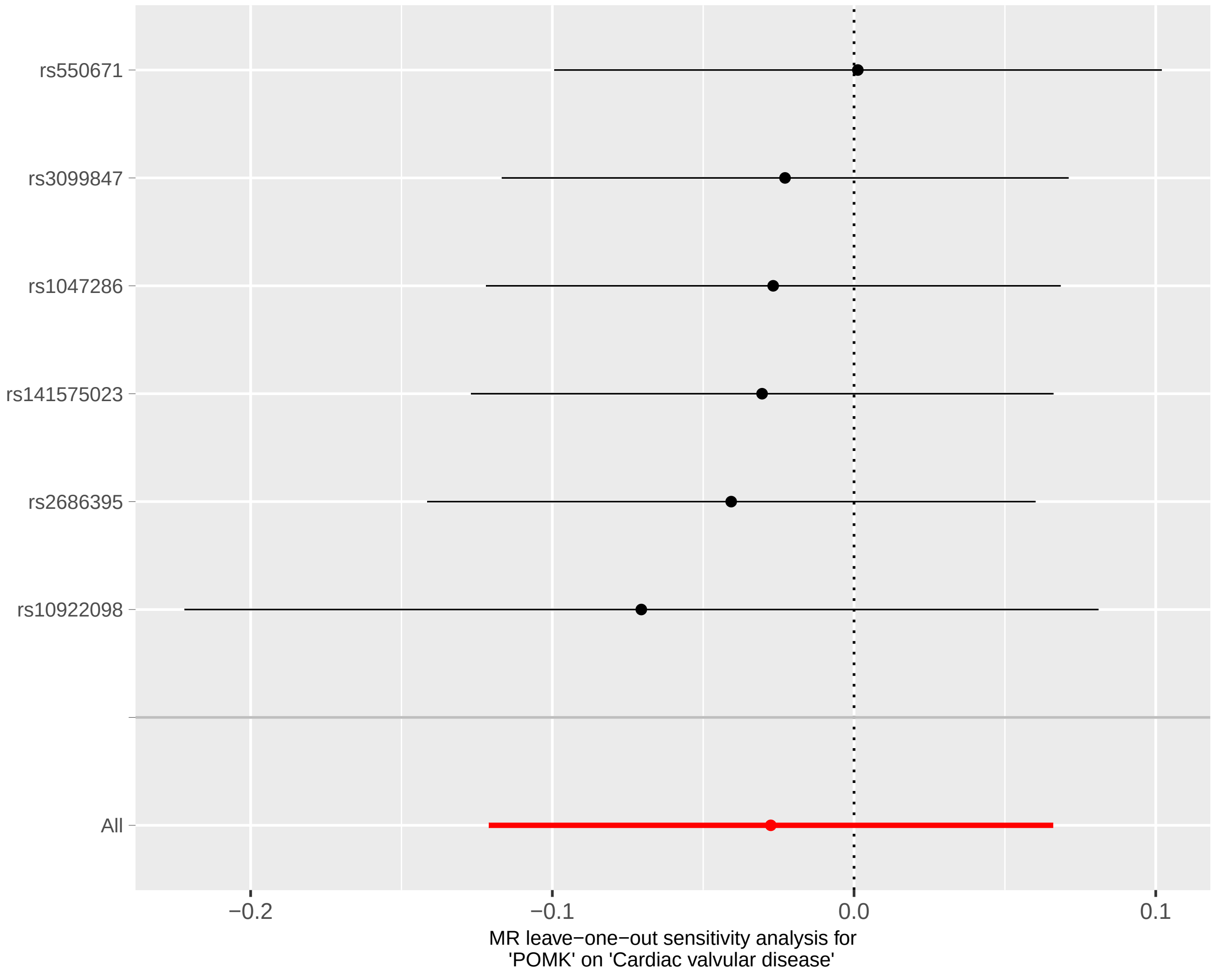
rs550671
rs3099847
rs1047286
rs141575023
rs2686395
rs10922098
All
−0.2
−0.1
0.0
0.1
MR leave−one−out sensitivity analysis for
'POMK' on 'Cardiac valvular disease'

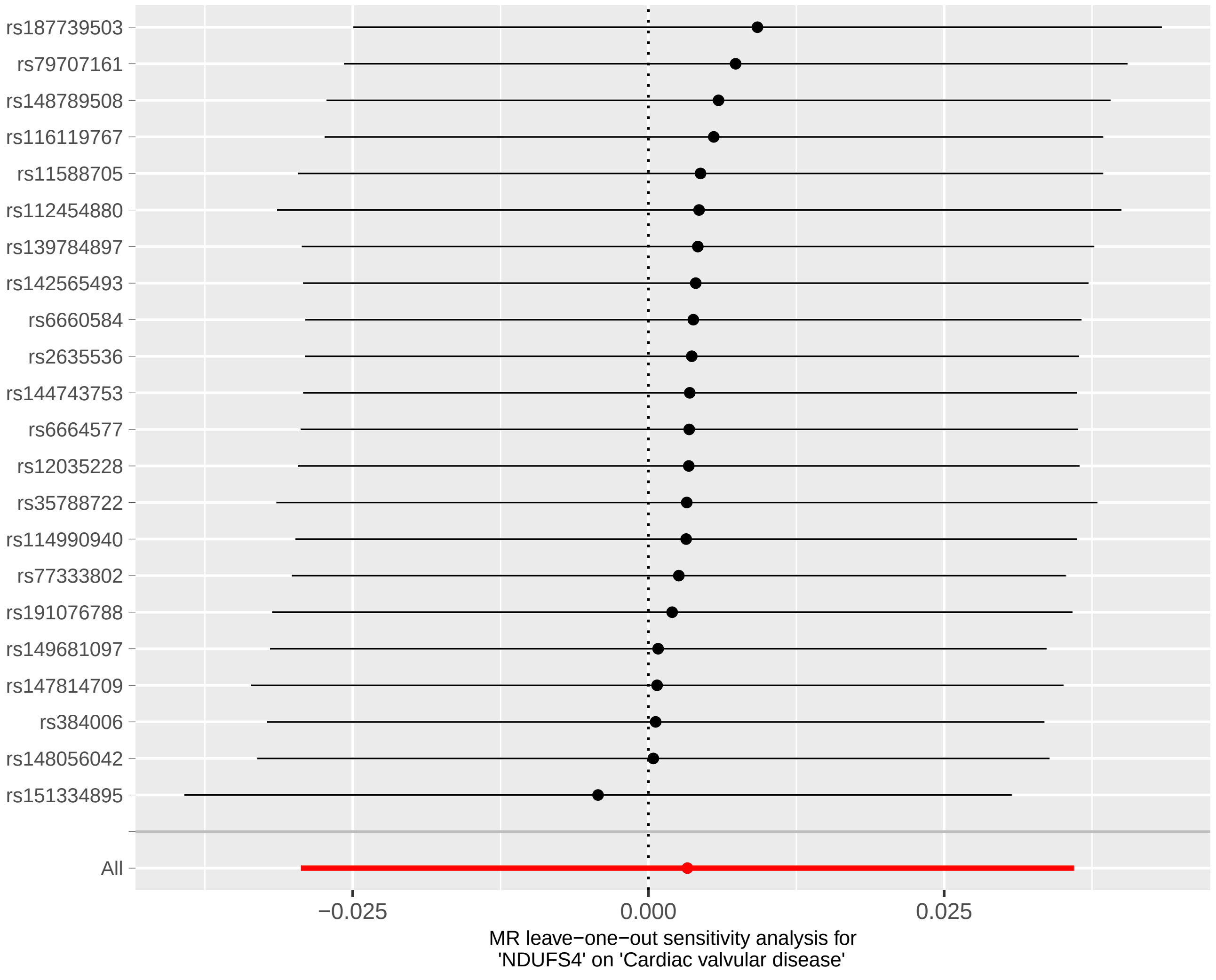
rs187739503
rs79707161
rs148789508
rs116119767
rs11588705
rs112454880
rs139784897
rs142565493
rs6660584
rs2635536
rs144743753
rs6664577
rs12035228
rs35788722
rs114990940
rs77333802
rs191076788
rs149681097
rs147814709
rs384006
rs148056042
rs151334895
All
−0.025
0.000
0.025
MR leave−one−out sensitivity analysis for
'NDUFS4' on 'Cardiac valvular disease'

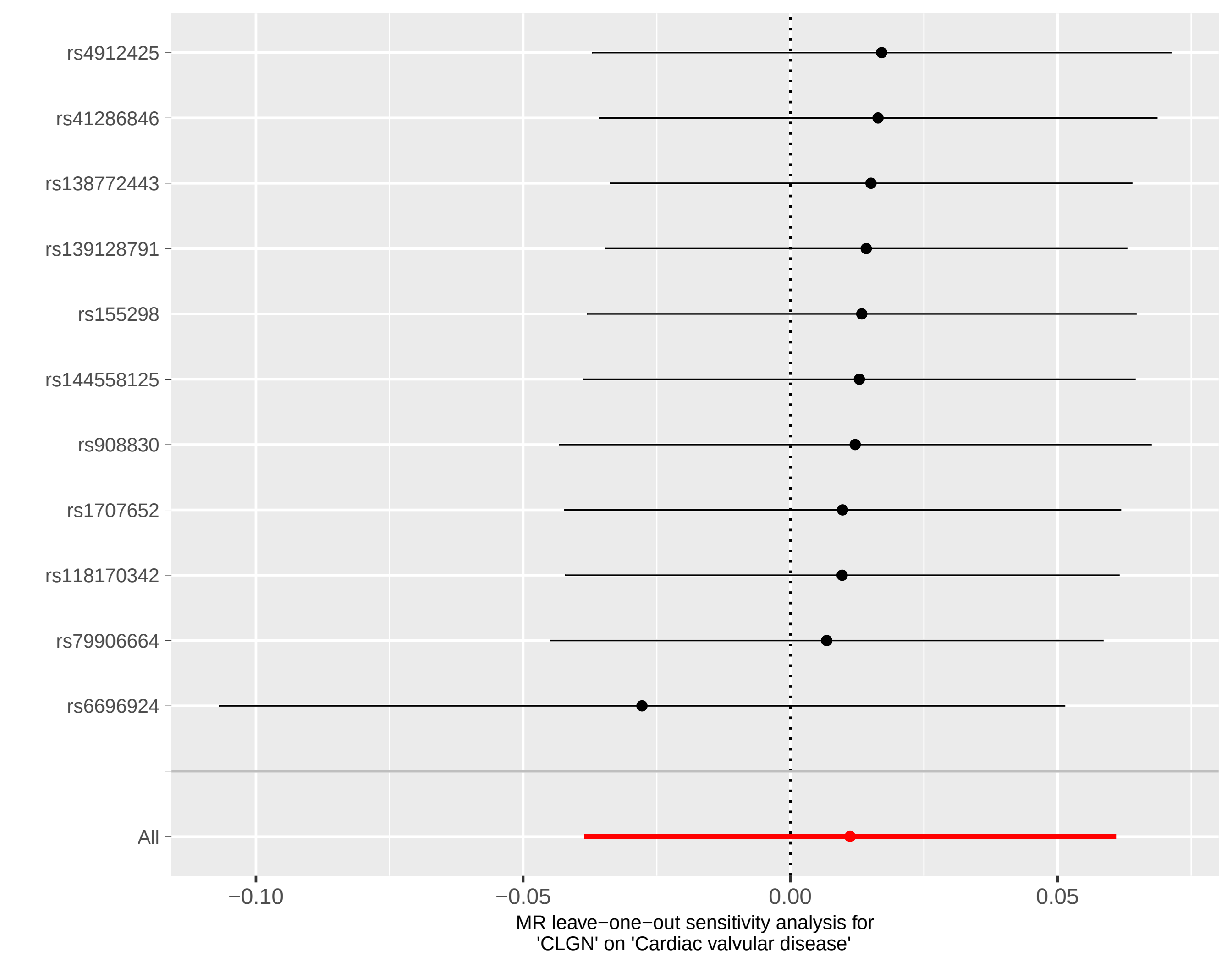

rs4912425
rs41286846
rs138772443
rs139128791
rs155298
rs144558125
rs908830
rs1707652
rs118170342
rs79906664
rs6696924
All
−0.10
−0.05
0.00
0.05
MR leave−one−out sensitivity analysis for
'CLGN' on 'Cardiac valvular disease'

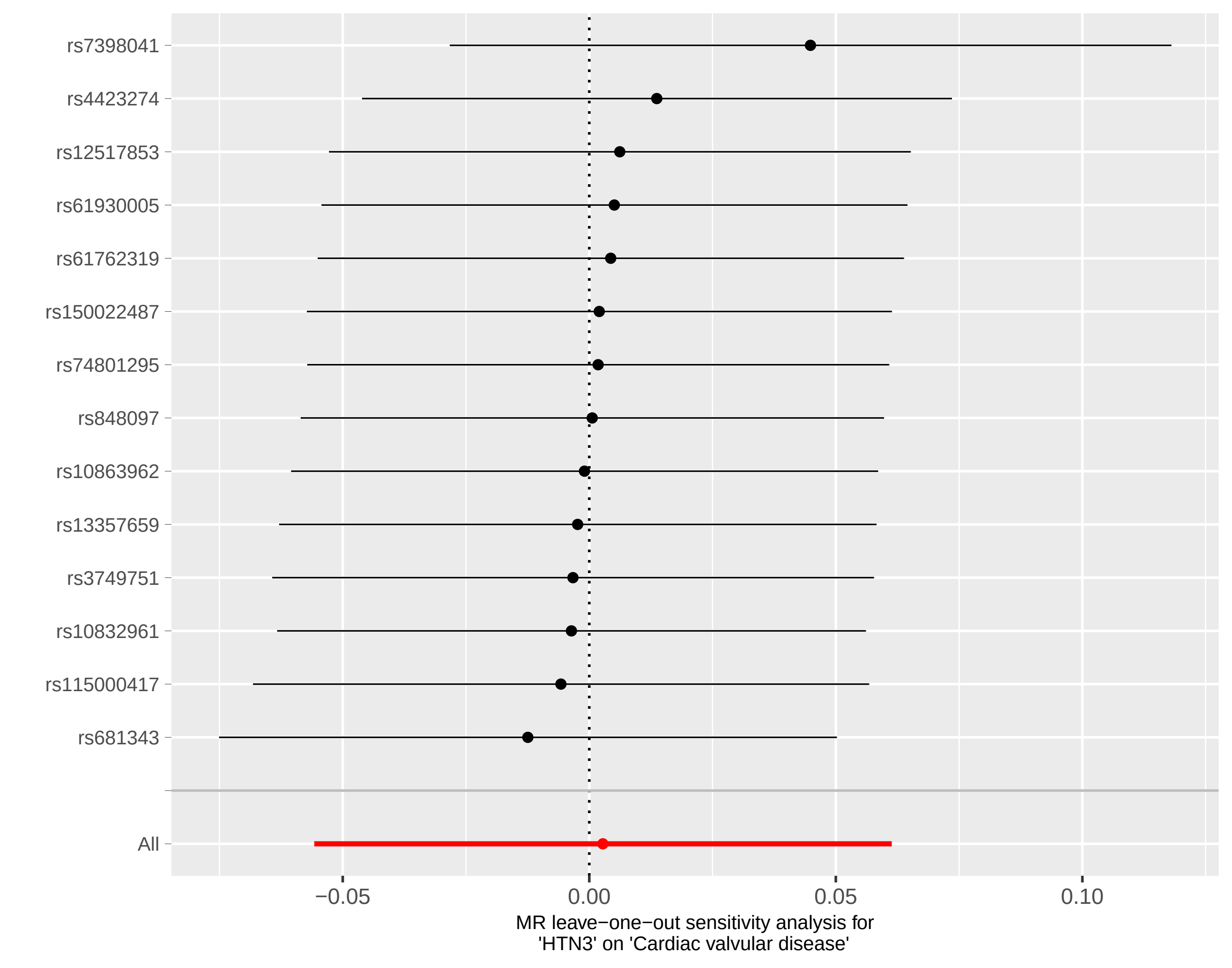
rs7398041
rs4423274
rs12517853
rs61930005
rs61762319
rs150022487
rs74801295
rs848097
rs10863962
rs13357659
rs3749751
rs10832961
rs115000417
rs681343
All
−0.05
0.00
0.05
0.10
MR leave−one−out sensitivity analysis for
'HTN3' on 'Cardiac valvular disease'

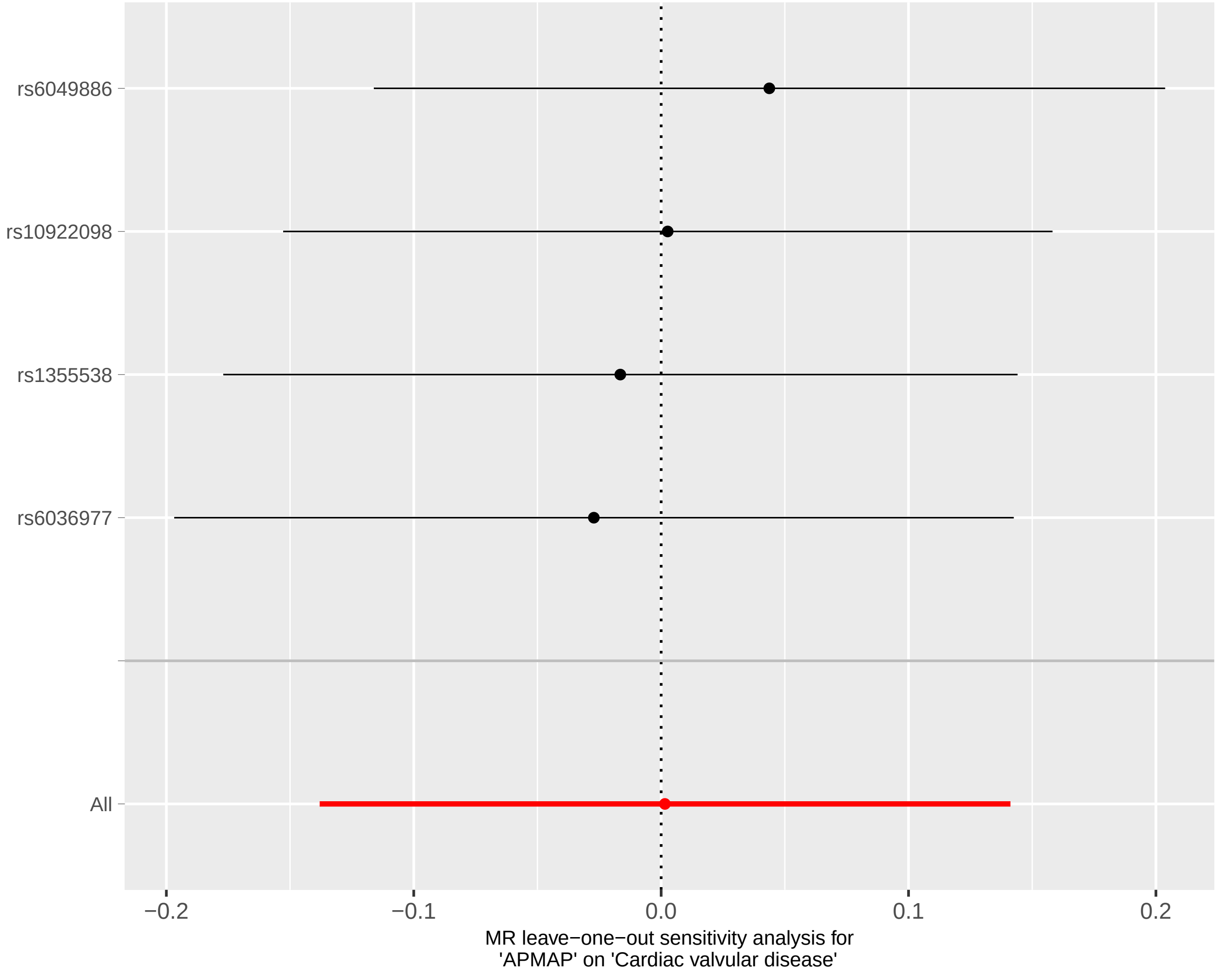

rs6049886
rs10922098
rs1355538
rs6036977
All
−0.2
−0.1
0.0
0.1
0.2
MR leave−one−out sensitivity analysis for
'APMAP' on 'Cardiac valvular disease'

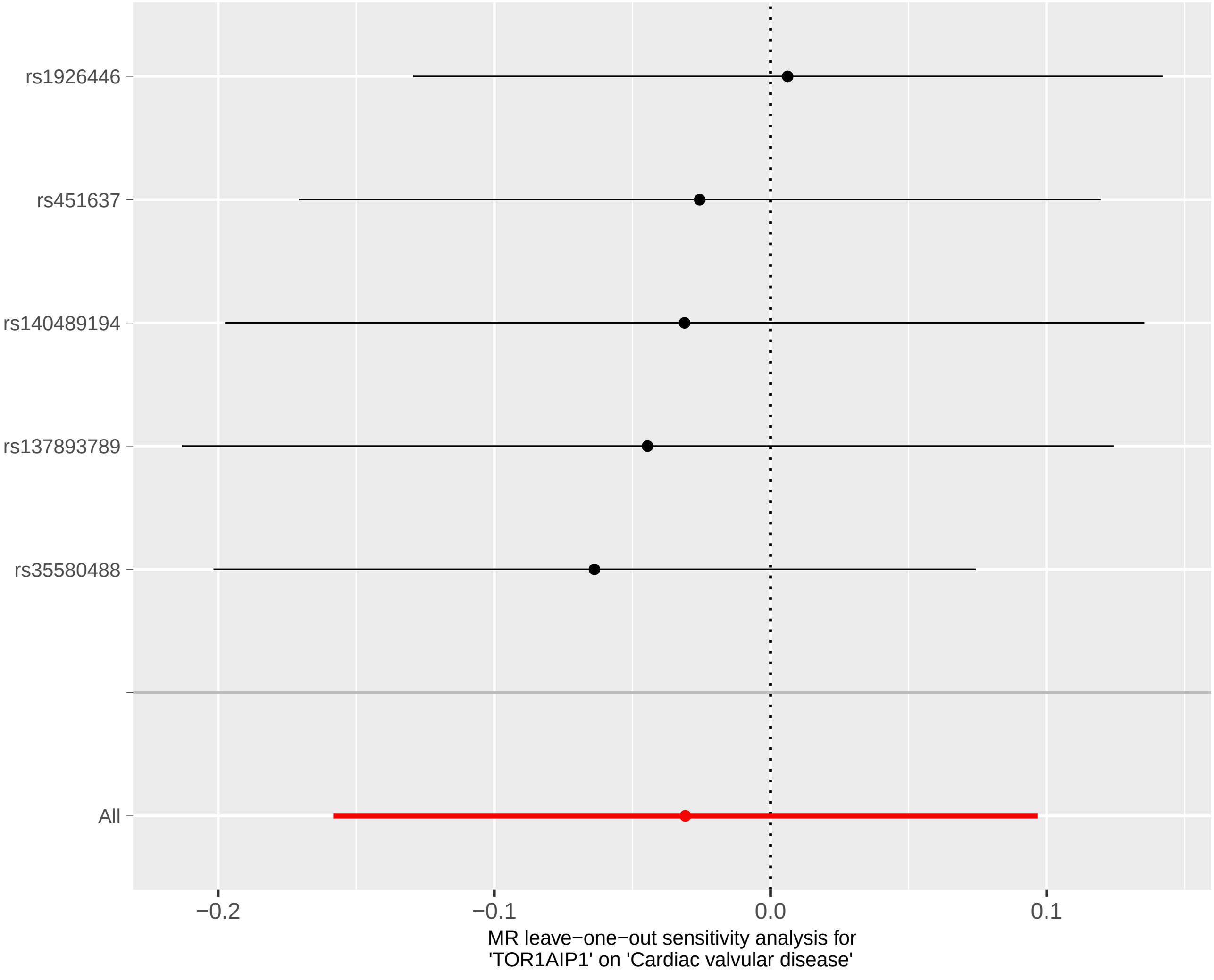

rs1926446
rs451637
rs140489194
rs137893789
rs35580488
All
−0.2
−0.1
0.0
0.1
MR leave−one−out sensitivity analysis for
'TOR1AIP1' on 'Cardiac valvular disease'

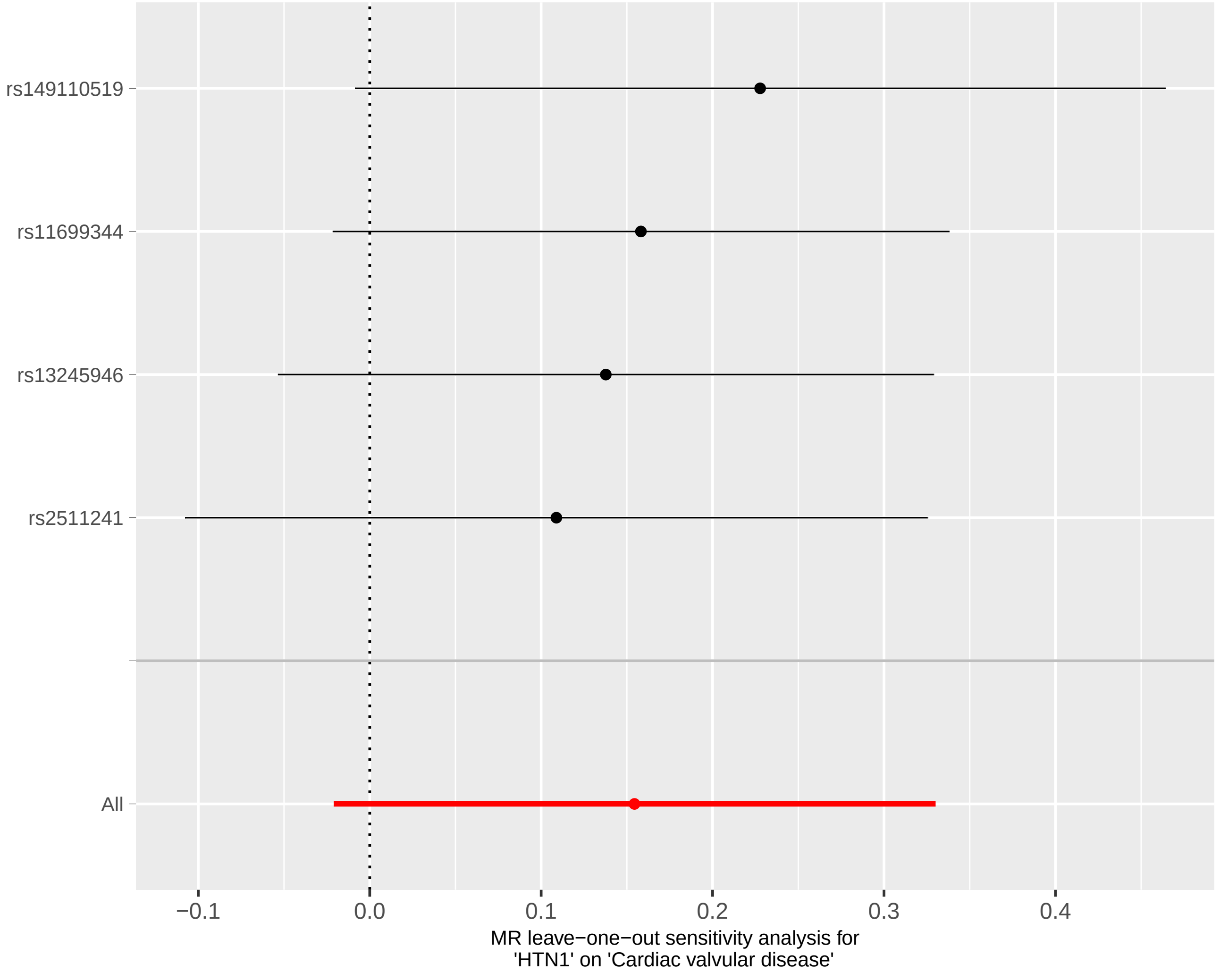
rs149110519
rs11699344
rs13245946
rs2511241
All
−0.1
0.0
0.1
0.2
0.3
0.4
MR leave−one−out sensitivity analysis for
'HTN1' on 'Cardiac valvular disease'

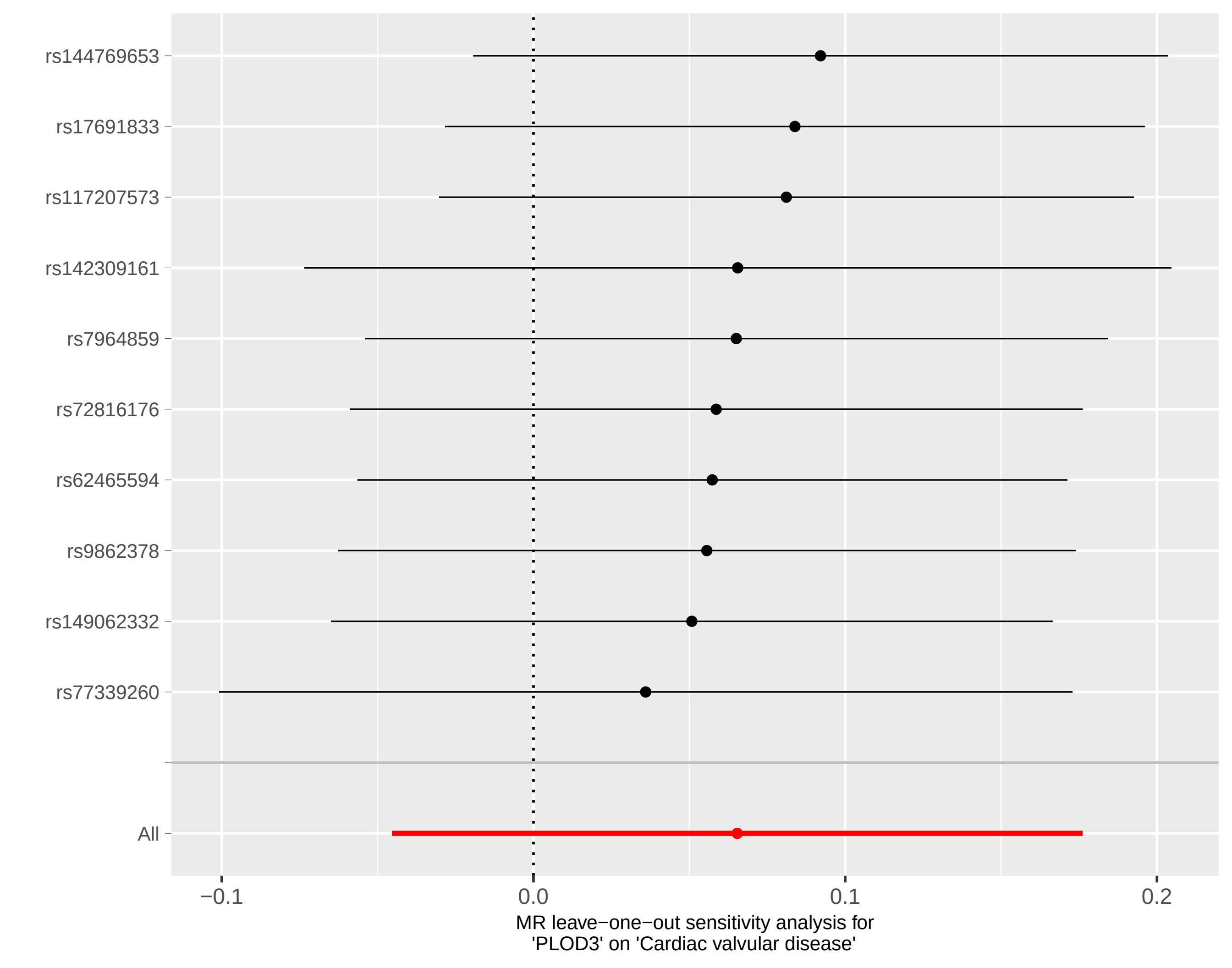
rs144769653
rs17691833
rs117207573
rs142309161
rs7964859
rs72816176
rs62465594
rs9862378
rs149062332
rs77339260
All
−0.1
0.0
0.1
0.2
MR leave−one−out sensitivity analysis for
'PLOD3' on 'Cardiac valvular disease'

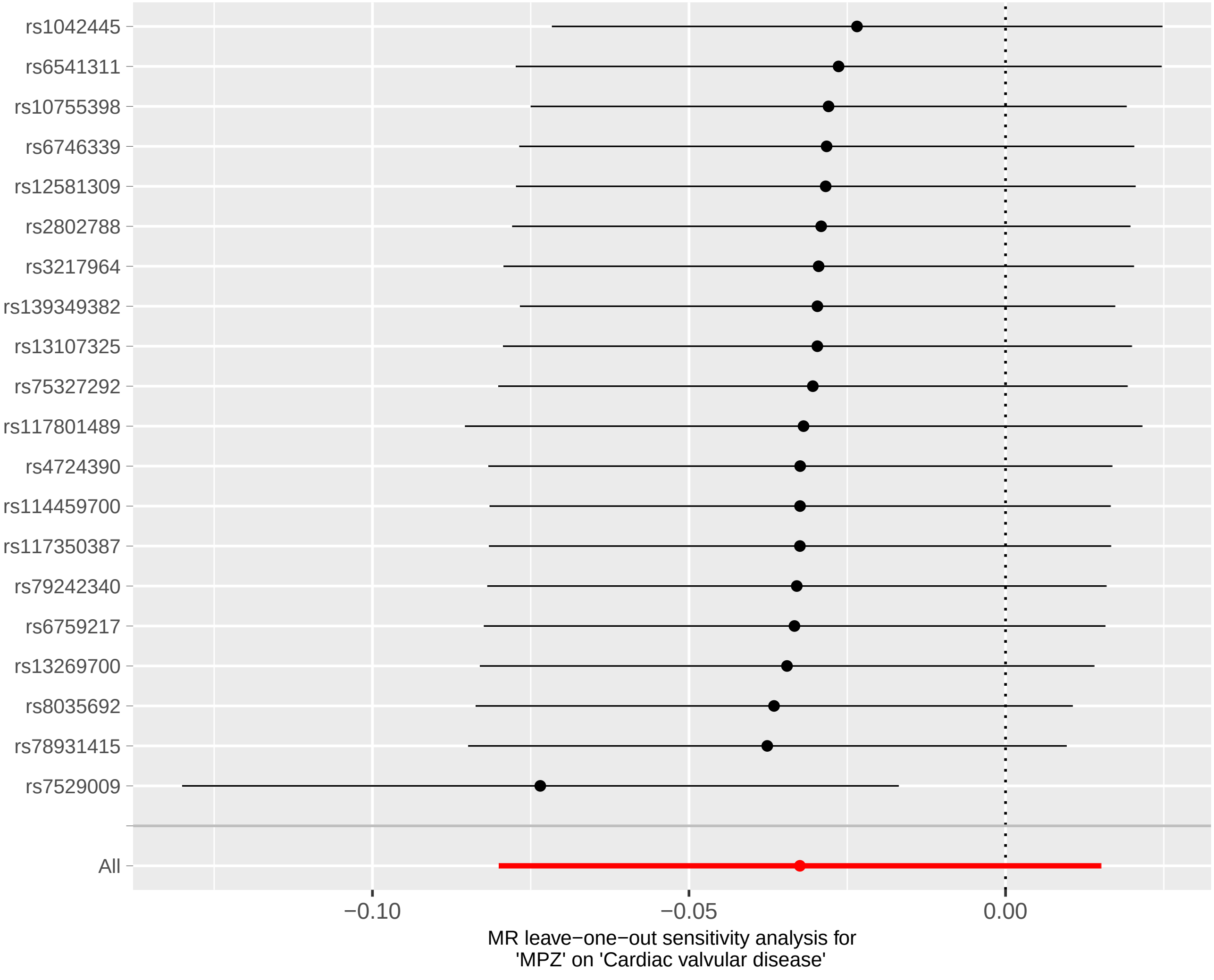
rs1042445
rs6541311
rs10755398
rs6746339
rs12581309
rs2802788
rs3217964
rs139349382
rs13107325
rs75327292
rs117801489
rs4724390
rs114459700
rs117350387
rs79242340
rs6759217
rs13269700
rs8035692
rs78931415
rs7529009
All
−0.10
−0.05
0.00
MR leave−one−out sensitivity analysis for
'MPZ' on 'Cardiac valvular disease'

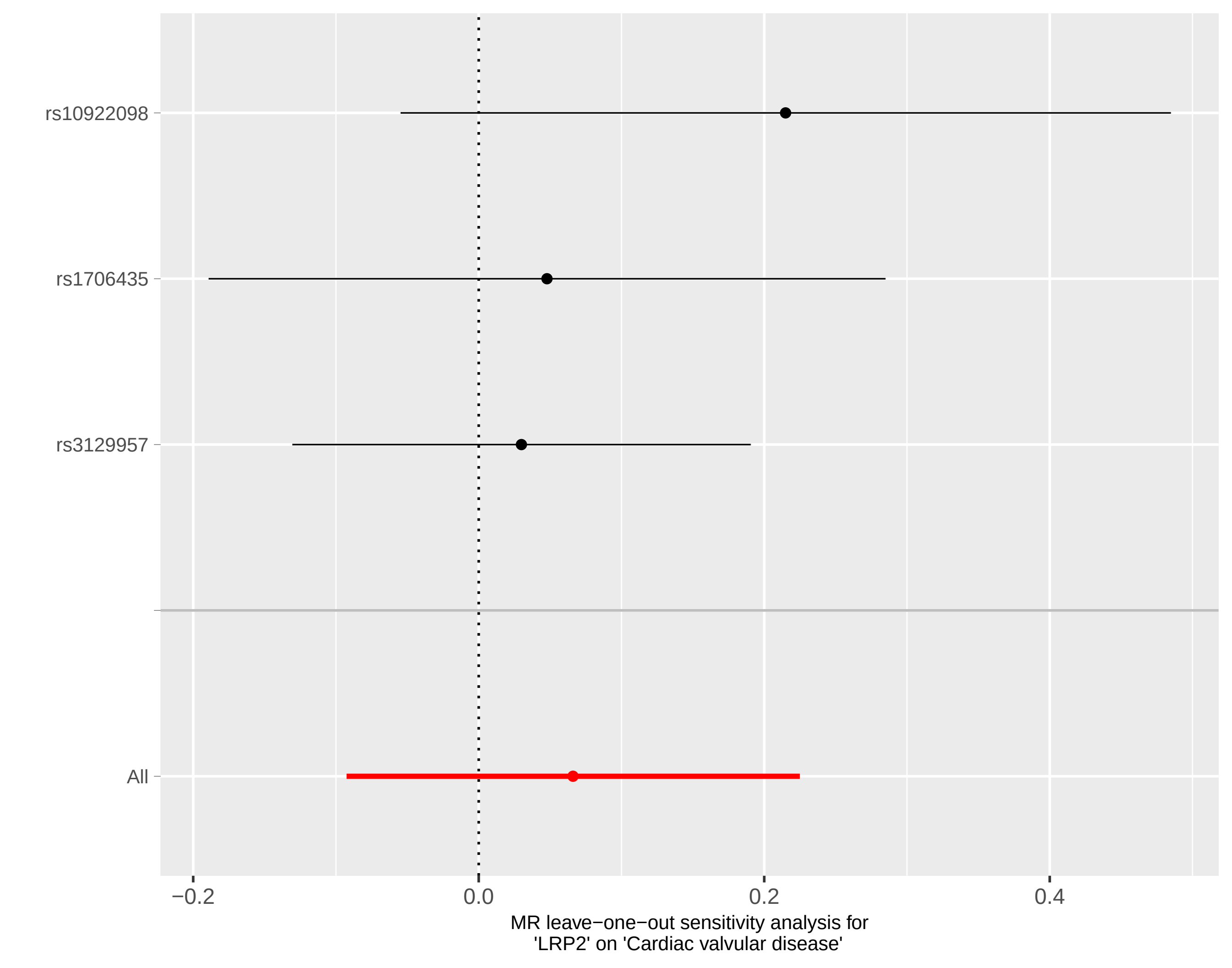
rs10922098
rs1706435
rs3129957
All
−0.2
0.0
0.2
0.4
MR leave−one−out sensitivity analysis for
'LRP2' on 'Cardiac valvular disease'

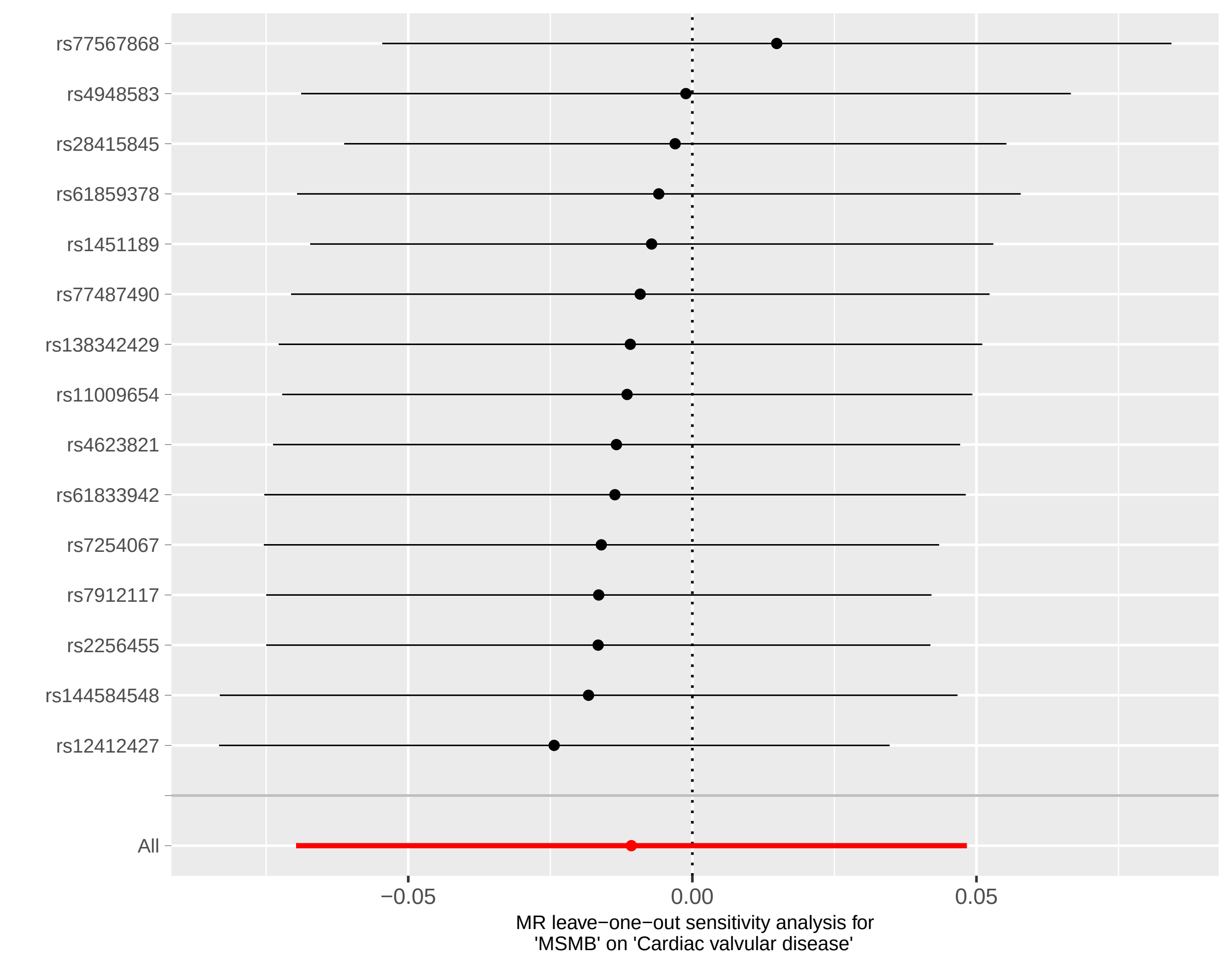

rs77567868
rs4948583
rs28415845
rs61859378
rs1451189
rs77487490
rs138342429
rs11009654
rs4623821
rs61833942
rs7254067
rs7912117
rs2256455
rs144584548
rs12412427
All
−0.05
0.00
0.05
MR leave−one−out sensitivity analysis for
'MSMB' on 'Cardiac valvular disease'

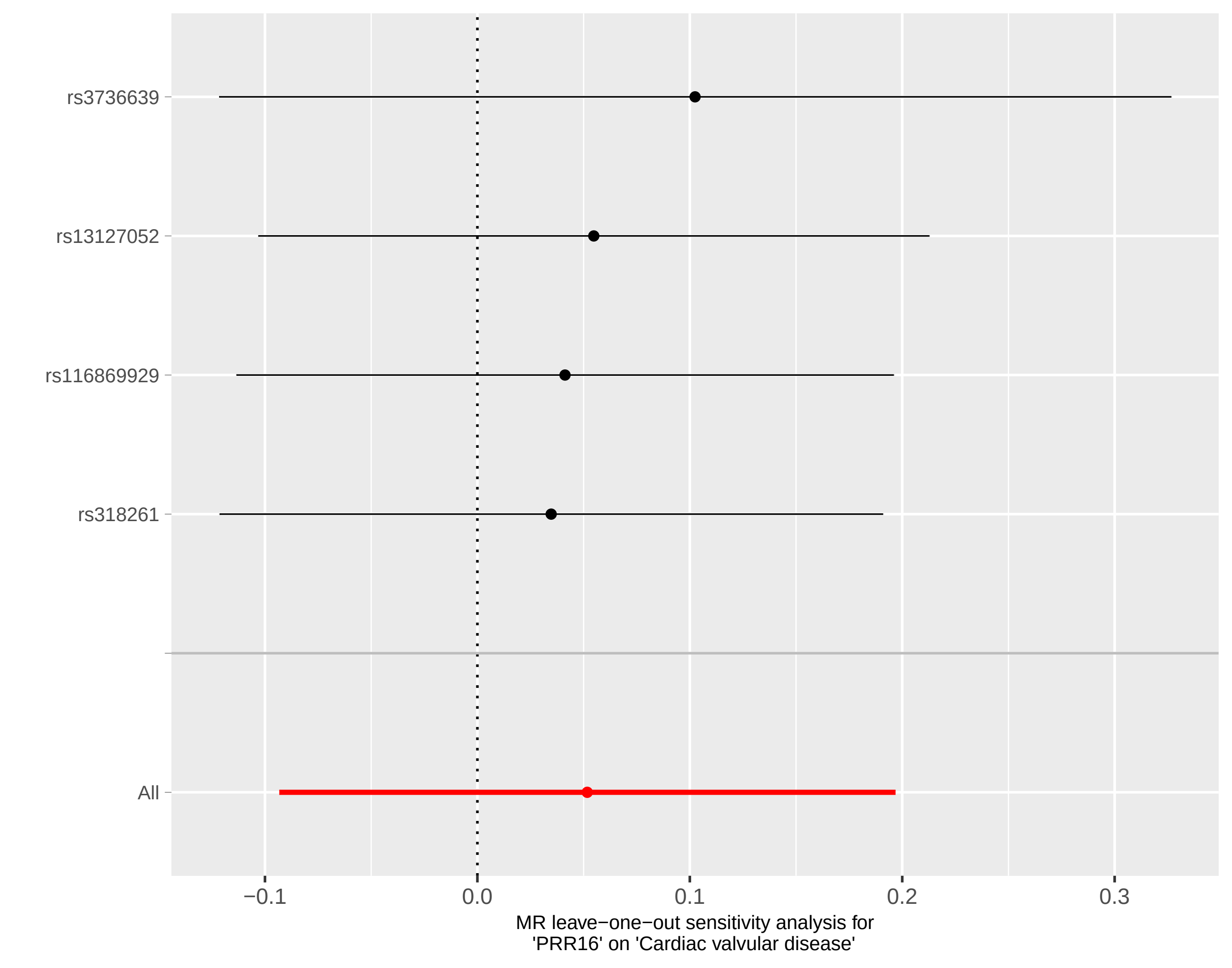
rs3736639
rs13127052
rs116869929
rs318261
All
−0.1
0.0
0.1
0.2
0.3
MR leave−one−out sensitivity analysis for
'PRR16' on 'Cardiac valvular disease'

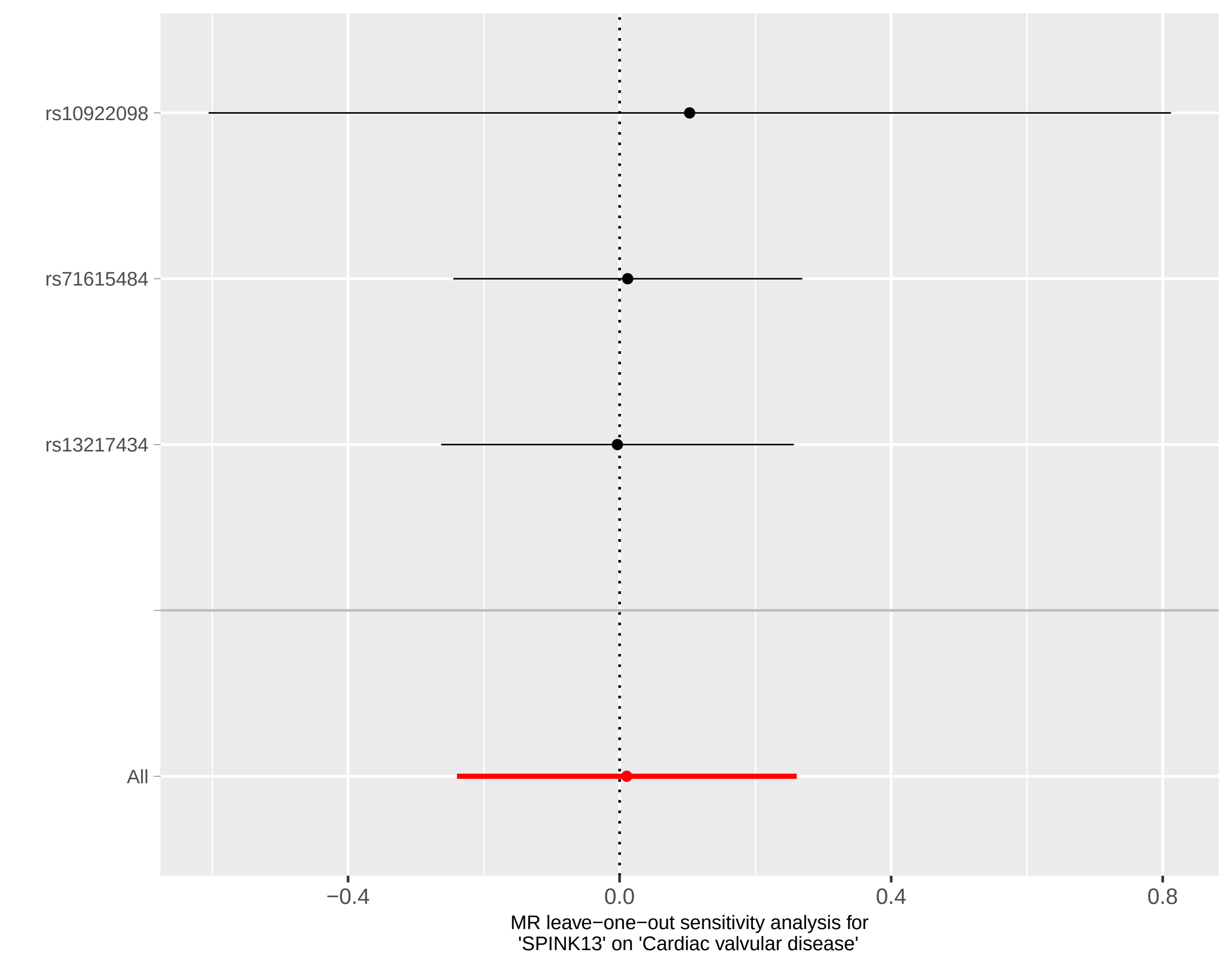
rs10922098
rs71615484
rs13217434
All
−0.4
0.0
0.4
0.8
MR leave−one−out sensitivity analysis for
'SPINK13' on 'Cardiac valvular disease'

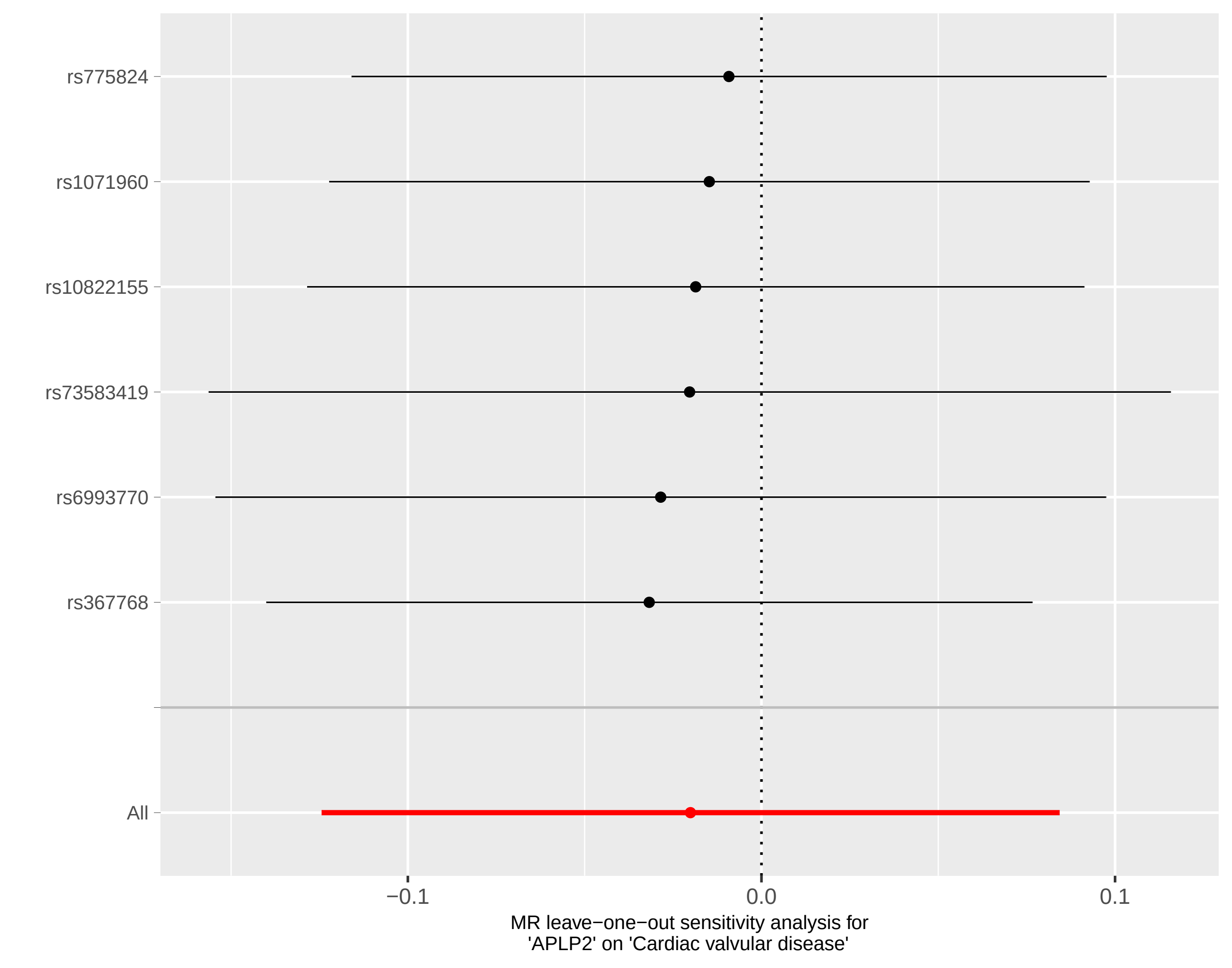
rs775824
rs1071960
rs10822155
rs73583419
rs6993770
rs367768
All
−0.1
0.0
0.1
MR leave−one−out sensitivity analysis for
'APLP2' on 'Cardiac valvular disease'

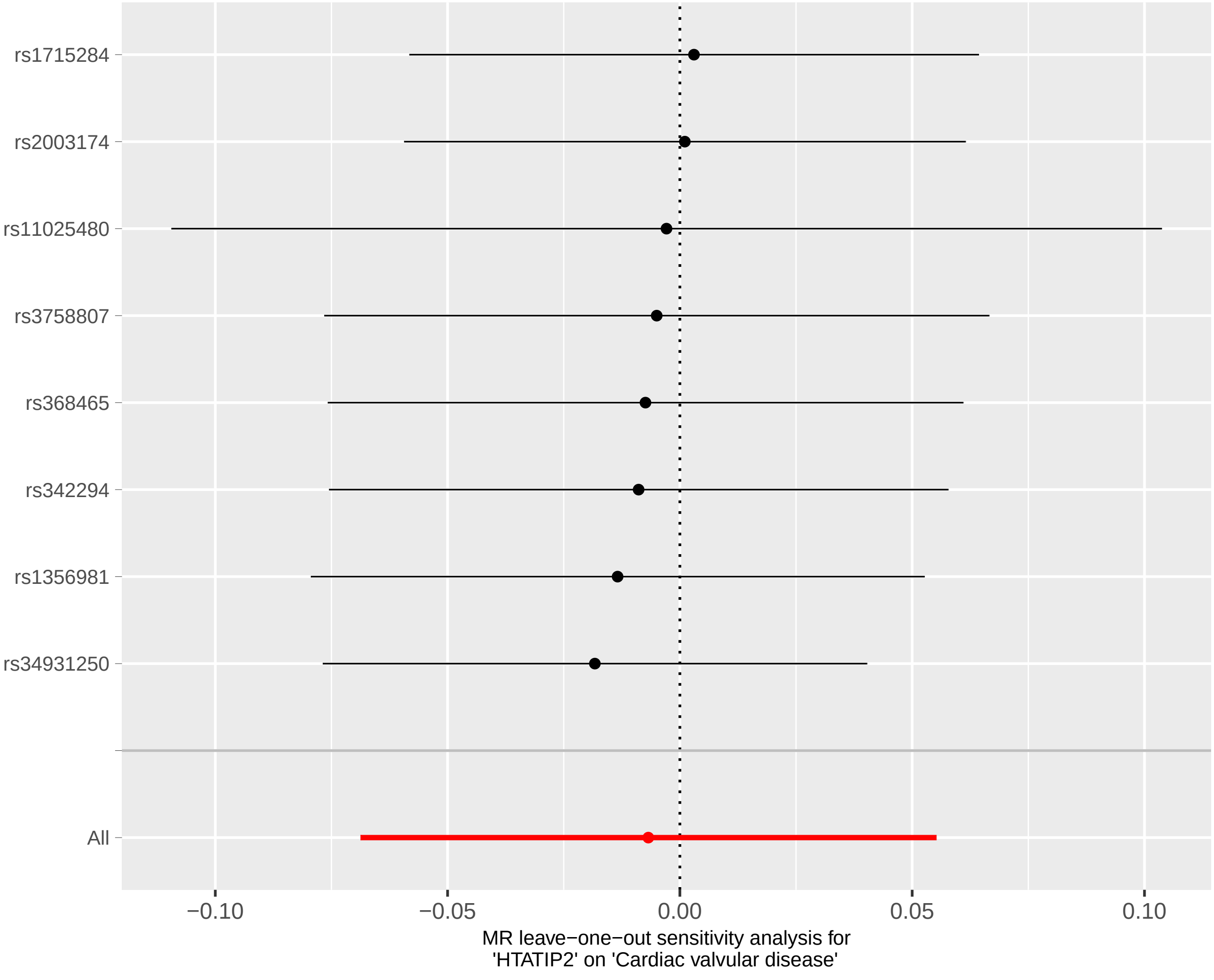

rs1715284
rs2003174
rs11025480
rs3758807
rs368465
rs342294
rs1356981
rs34931250
All
−0.10
−0.05
0.00
0.05
0.10
MR leave−one−out sensitivity analysis for
'HTATIP2' on 'Cardiac valvular disease'

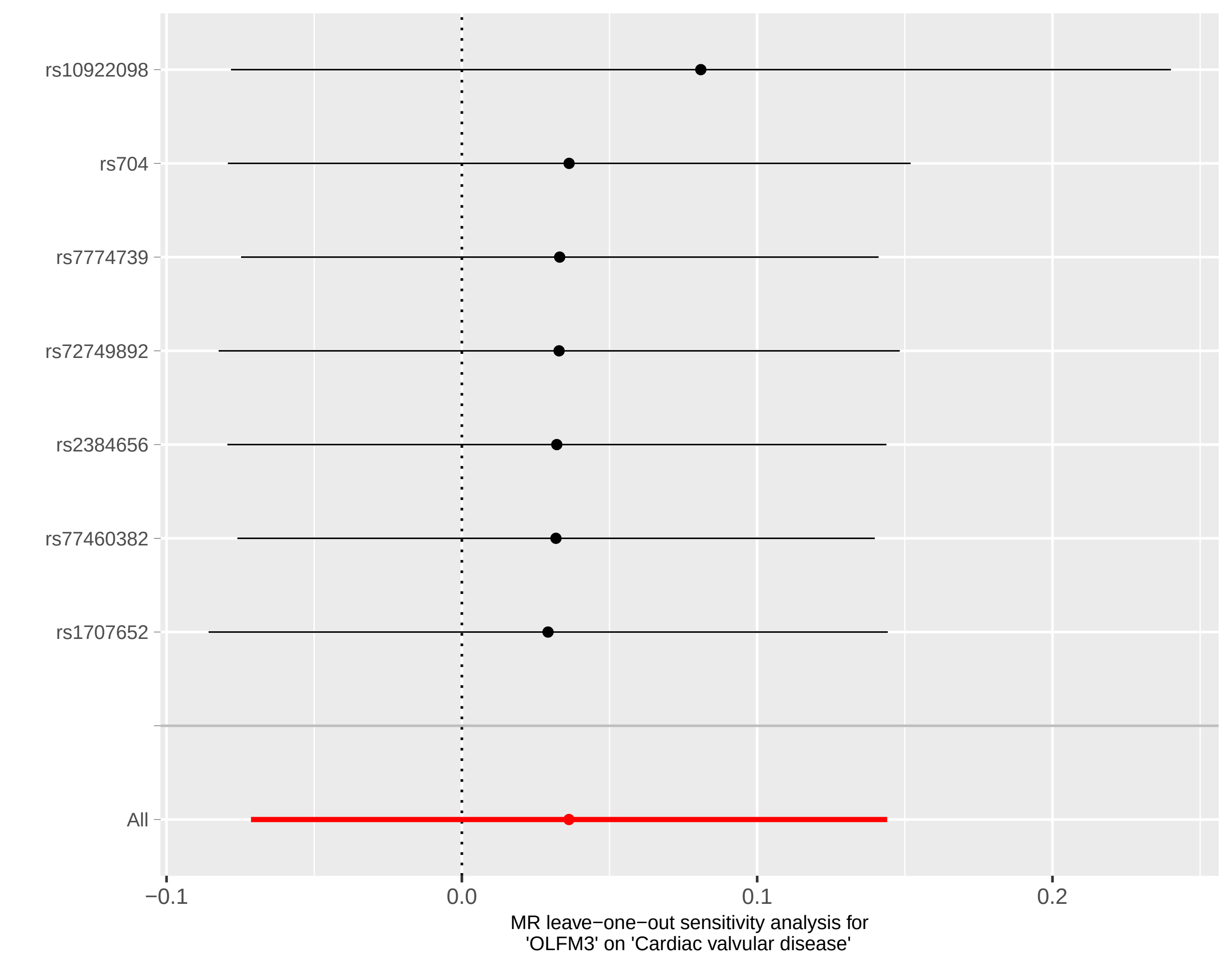

rs10922098
rs704
rs7774739
rs72749892
rs2384656
rs77460382
rs1707652
All
−0.1
0.0
0.1
0.2
MR leave−one−out sensitivity analysis for
'OLFM3' on 'Cardiac valvular disease'

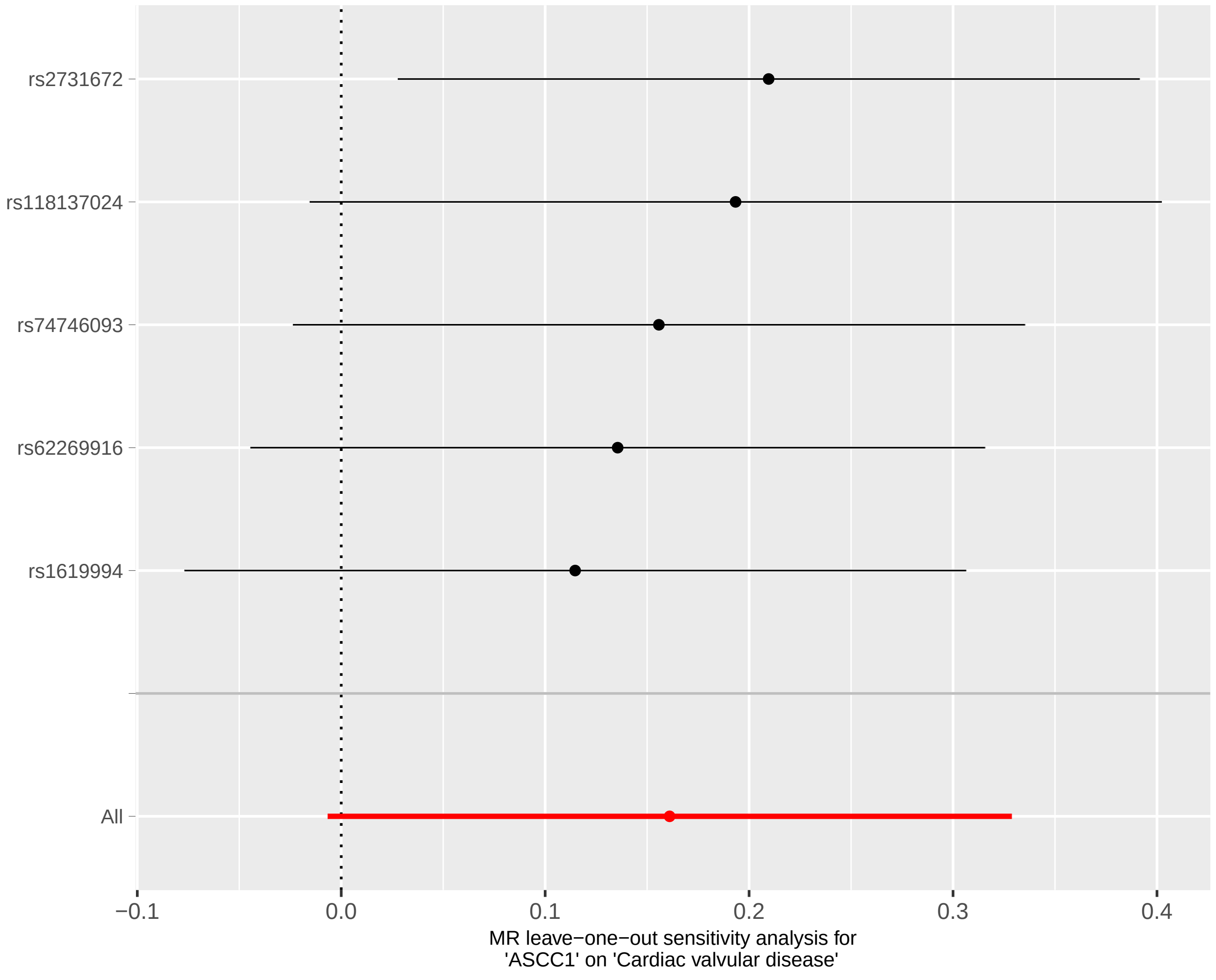
rs2731672
rs118137024
rs74746093
rs62269916
rs1619994
All
−0.1
0.0
0.1
0.2
0.3
0.4
MR leave−one−out sensitivity analysis for
'ASCC1' on 'Cardiac valvular disease'

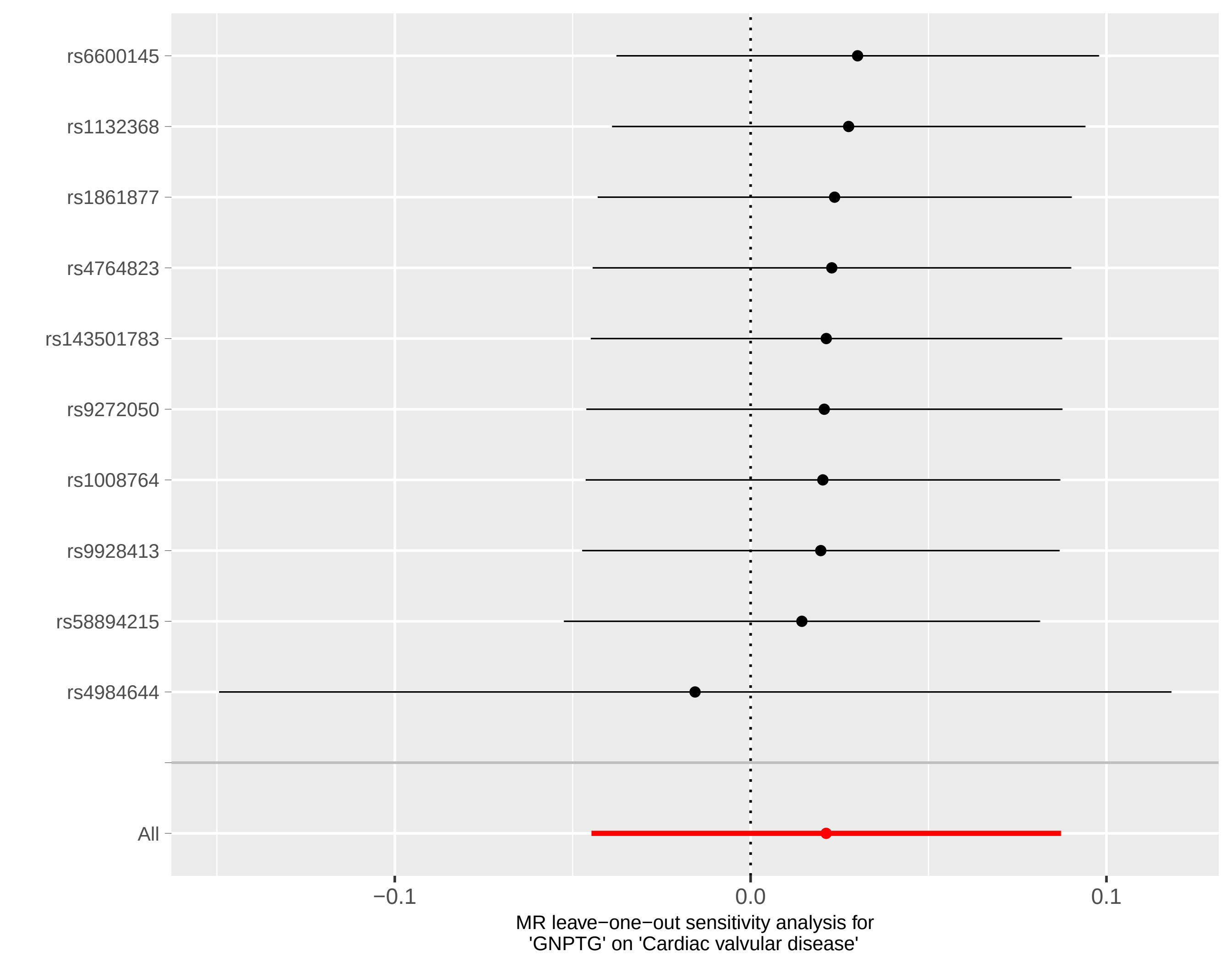
rs6600145
rs1132368
rs1861877
rs4764823
rs143501783
rs9272050
rs1008764
rs9928413
rs58894215
rs4984644
All
−0.1
0.0
0.1
MR leave−one−out sensitivity analysis for
'GNPTG' on 'Cardiac valvular disease'

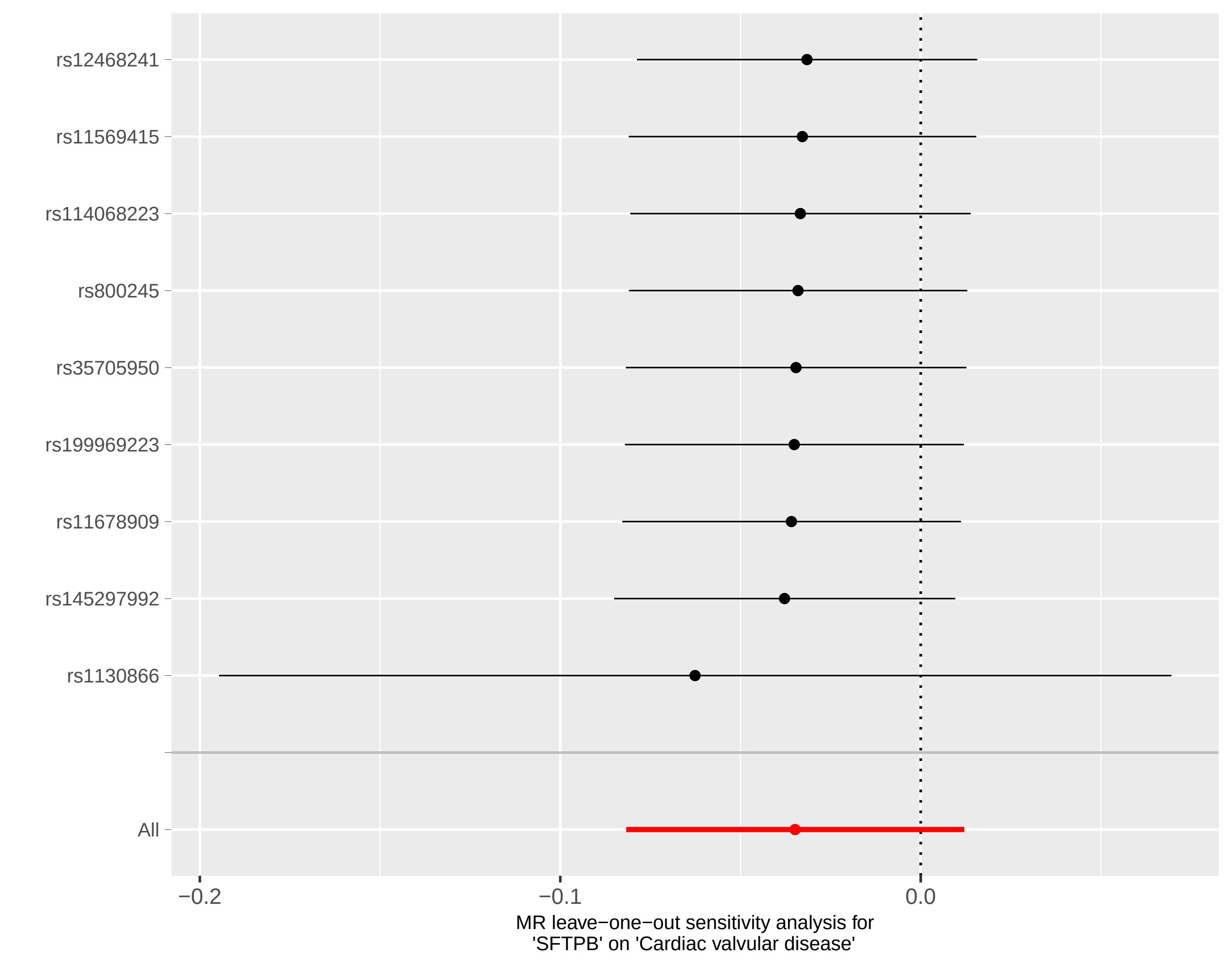

rs12468241
rs11569415
rs114068223
rs800245
rs35705950
rs199969223
rs11678909
rs145297992
rs1130866
All
−0.2
−0.1
0.0
MR leave−one−out sensitivity analysis for
'SFTPB' on 'Cardiac valvular disease'

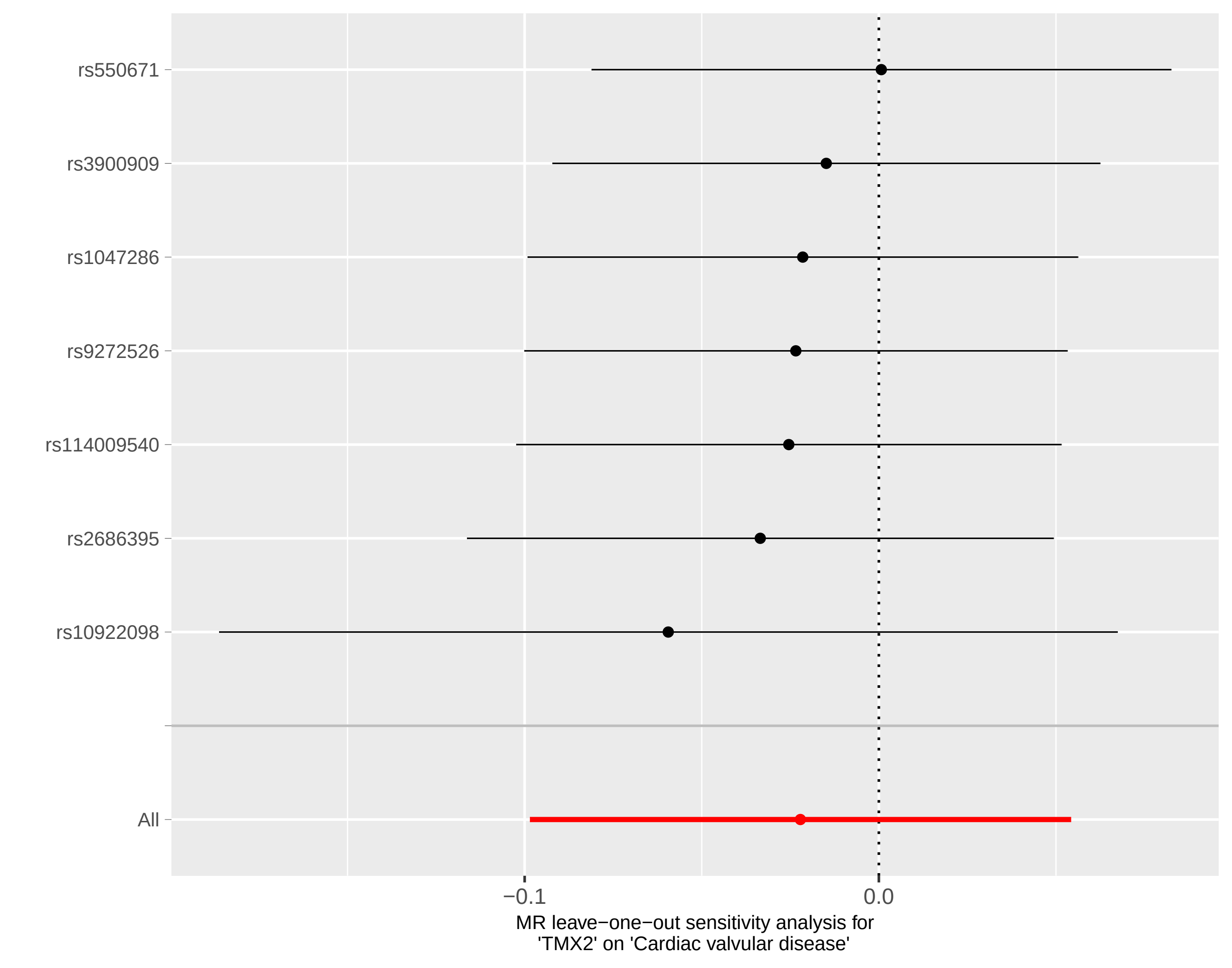

rs550671
rs3900909
rs1047286
rs9272526
rs114009540
rs2686395
rs10922098
All
−0.1
0.0
MR leave−one−out sensitivity analysis for
'TMX2' on 'Cardiac valvular disease'

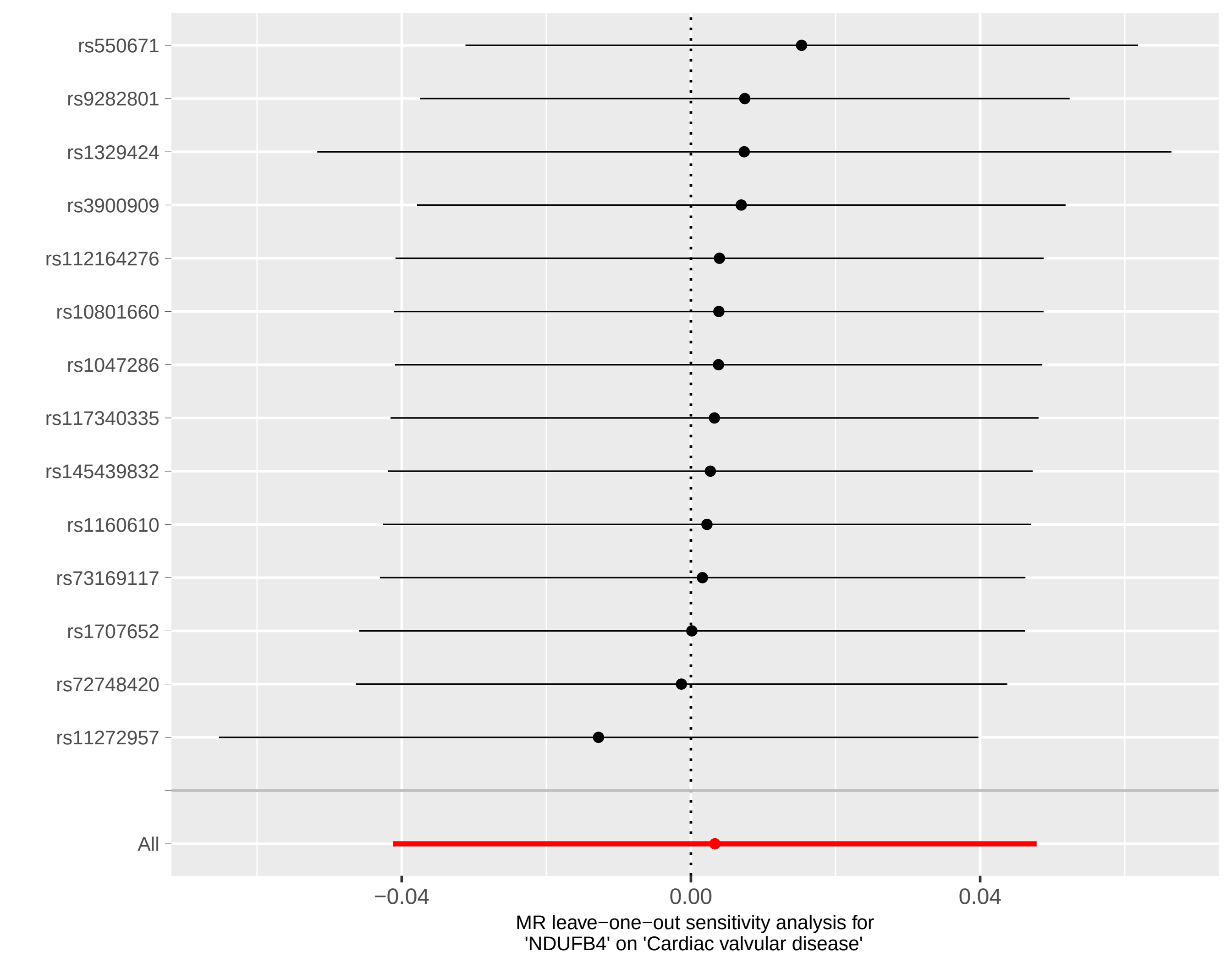
rs550671
rs9282801
rs1329424
rs3900909
rs112164276
rs10801660
rs1047286
rs117340335
rs145439832
rs1160610
rs73169117
rs1707652
rs72748420
rs11272957
All
−0.04
0.00
0.04
MR leave−one−out sensitivity analysis for
'NDUFB4' on 'Cardiac valvular disease'

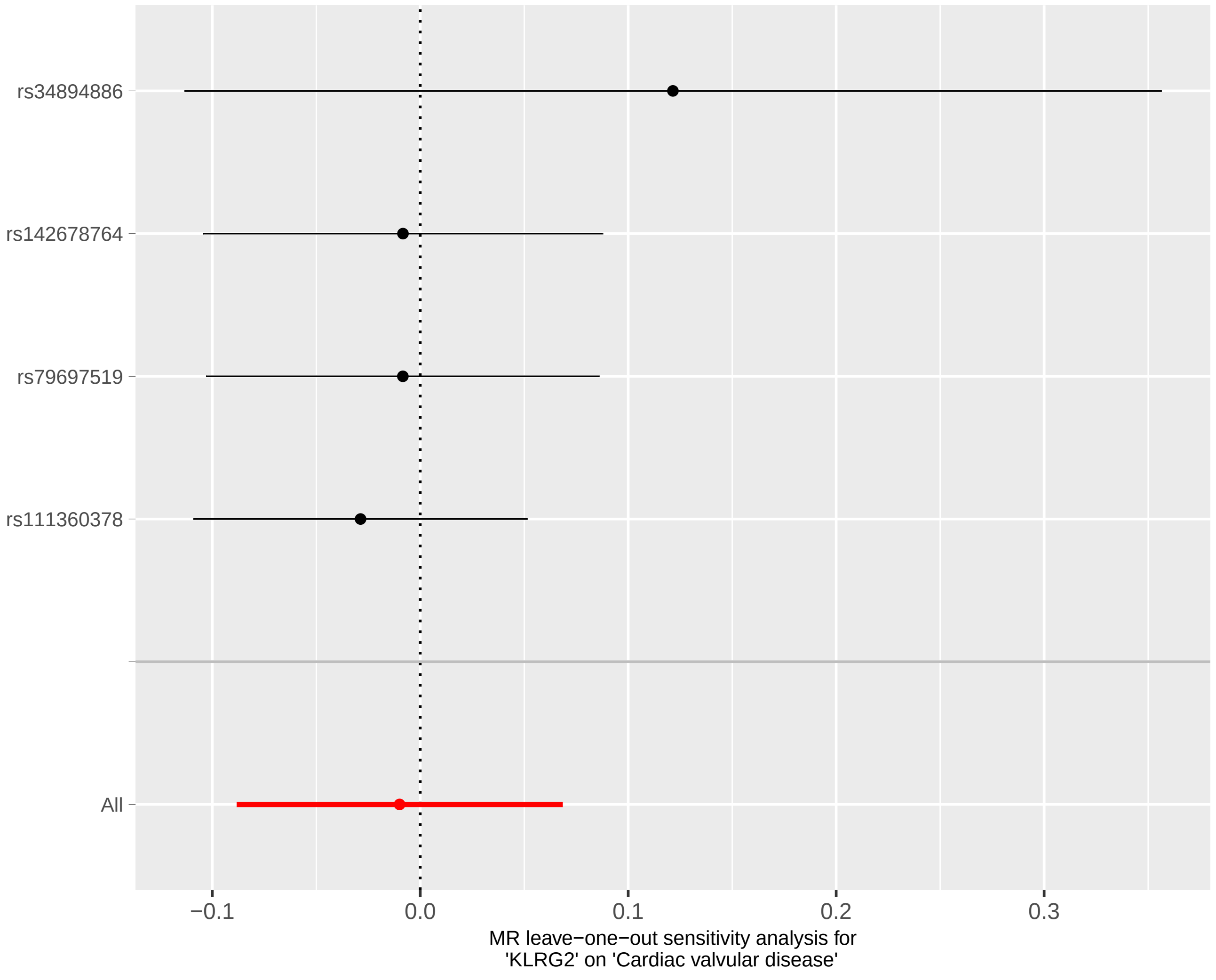
rs34894886
rs142678764
rs79697519
rs111360378
All
−0.1
0.0
0.1
0.2
0.3
MR leave−one−out sensitivity analysis for
'KLRG2' on 'Cardiac valvular disease'

Insufficient number of SNPs

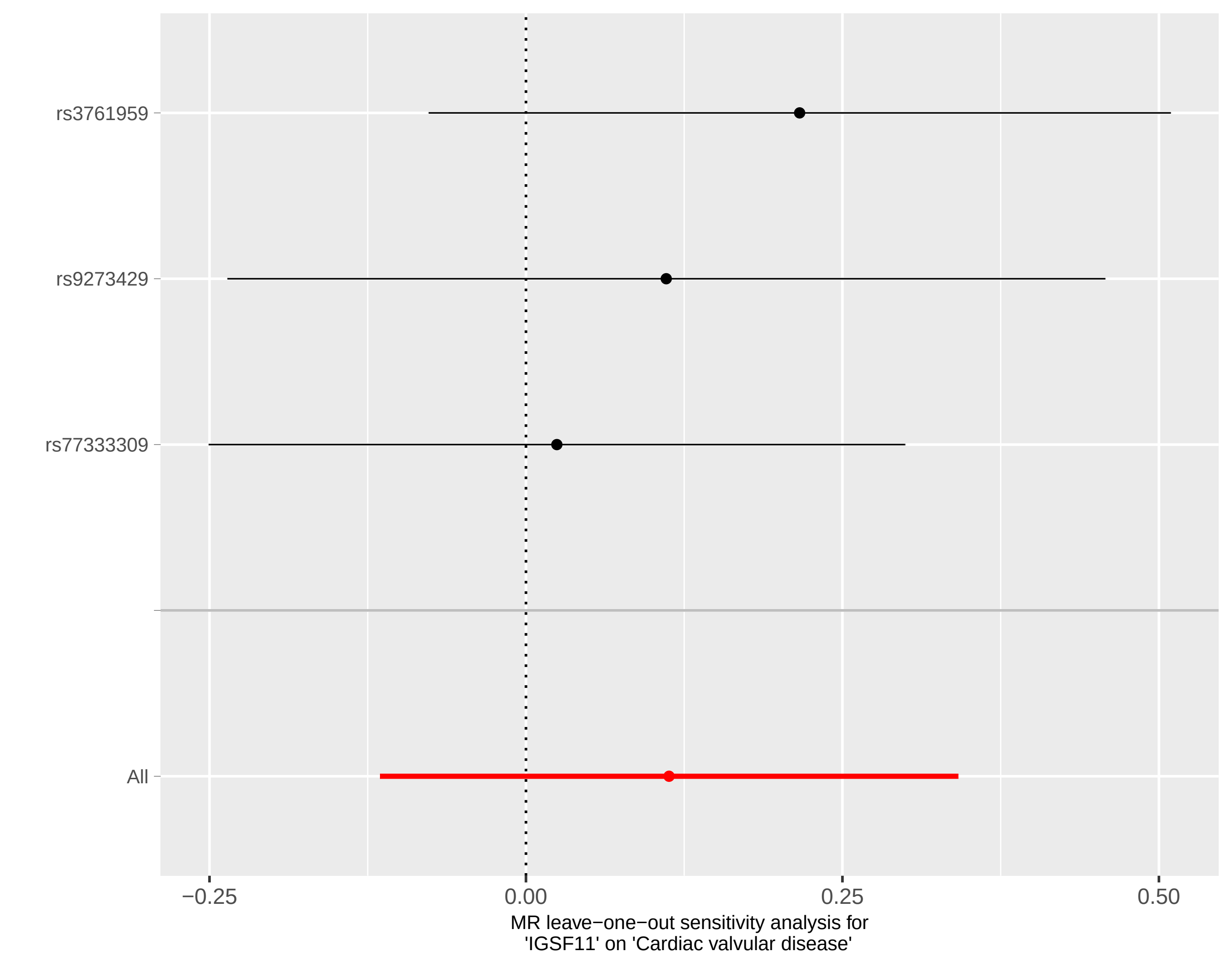
rs3761959
rs9273429
rs77333309
All
−0.25
0.00
0.25
0.50
MR leave−one−out sensitivity analysis for
'IGSF11' on 'Cardiac valvular disease'

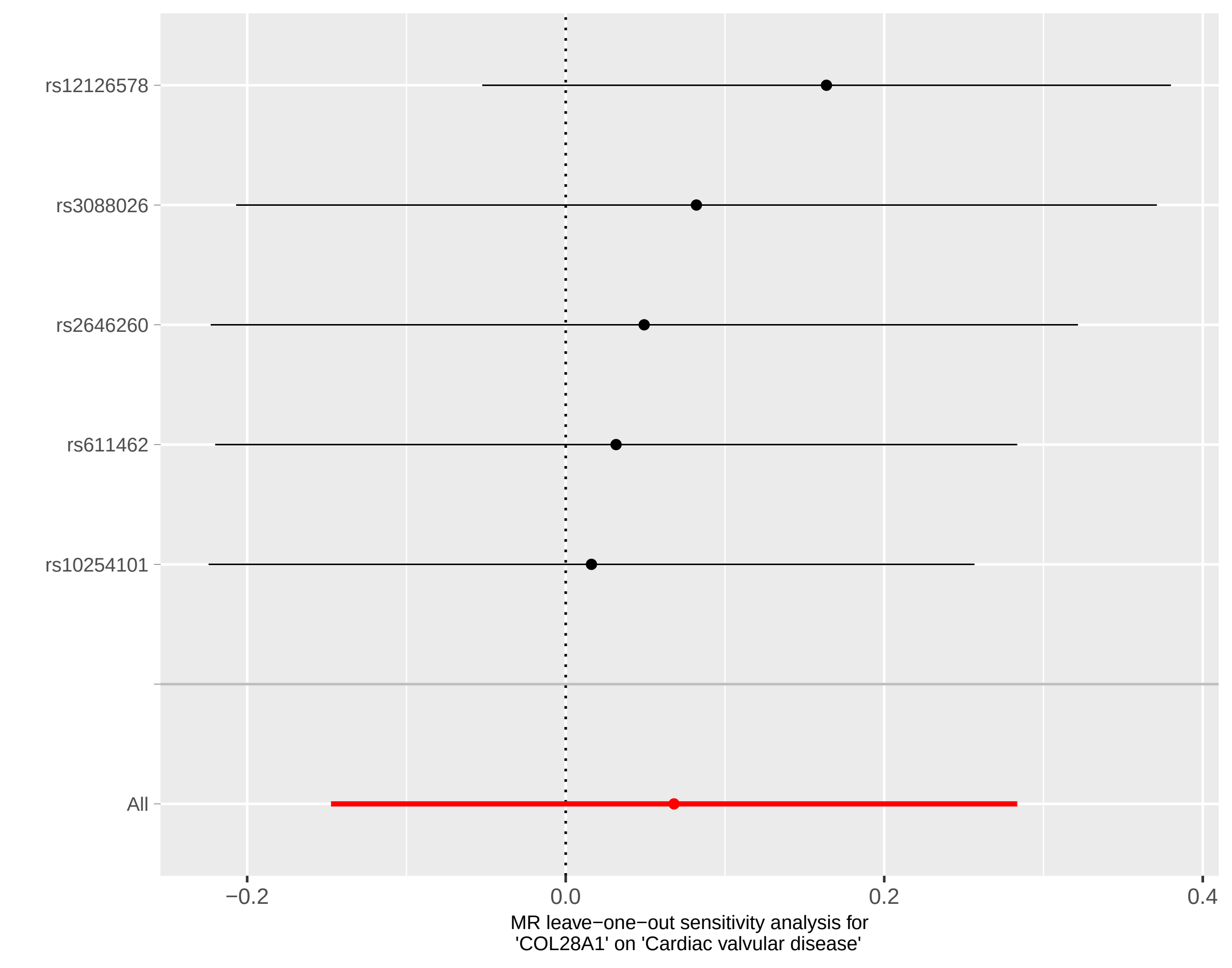
rs12126578
rs3088026
rs2646260
rs611462
rs10254101
All
−0.2
0.0
0.2
0.4
MR leave−one−out sensitivity analysis for
'COL28A1' on 'Cardiac valvular disease'

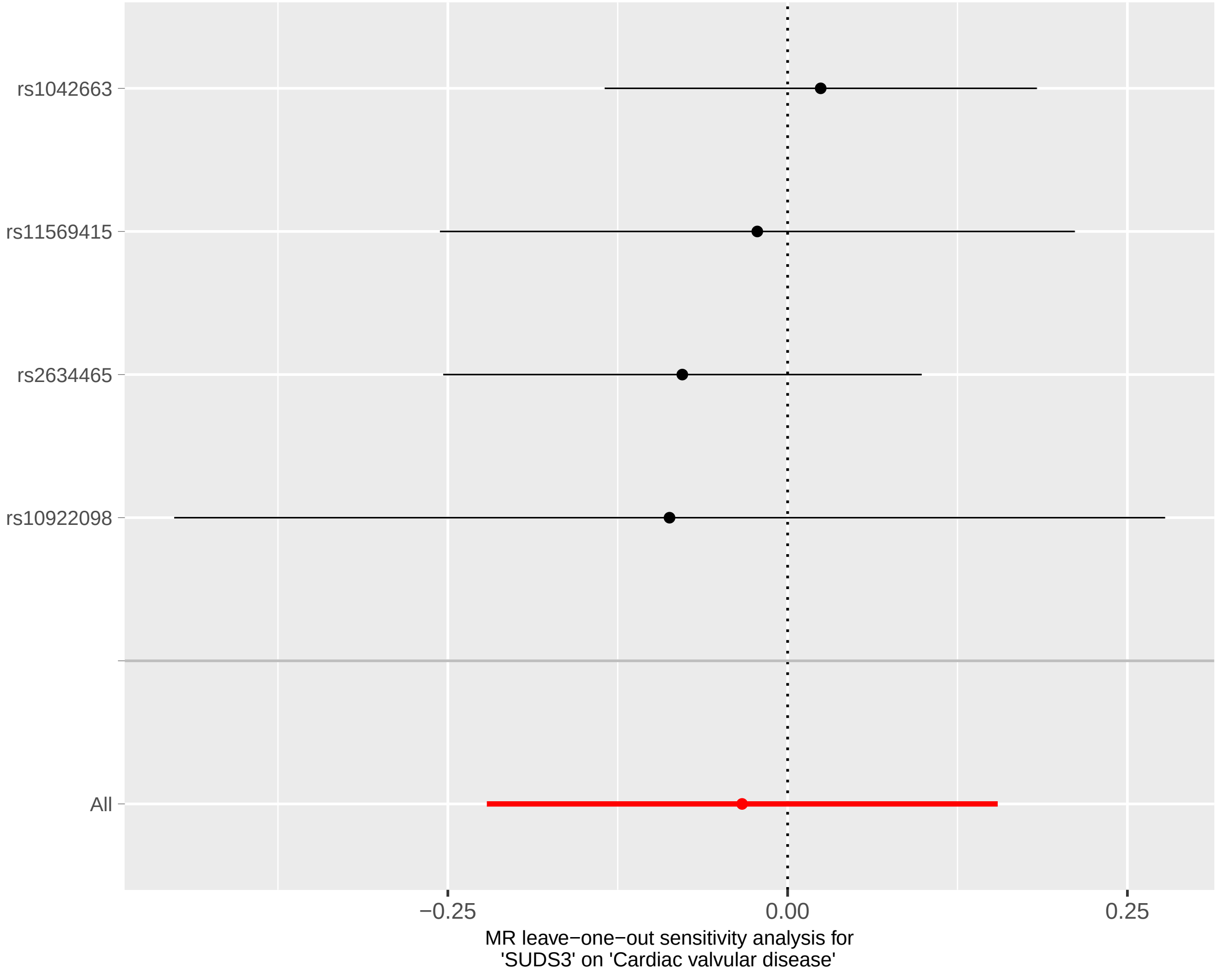
rs1042663
rs11569415
rs2634465
rs10922098
All
−0.25
0.00
0.25
MR leave−one−out sensitivity analysis for
'SUDS3' on 'Cardiac valvular disease'

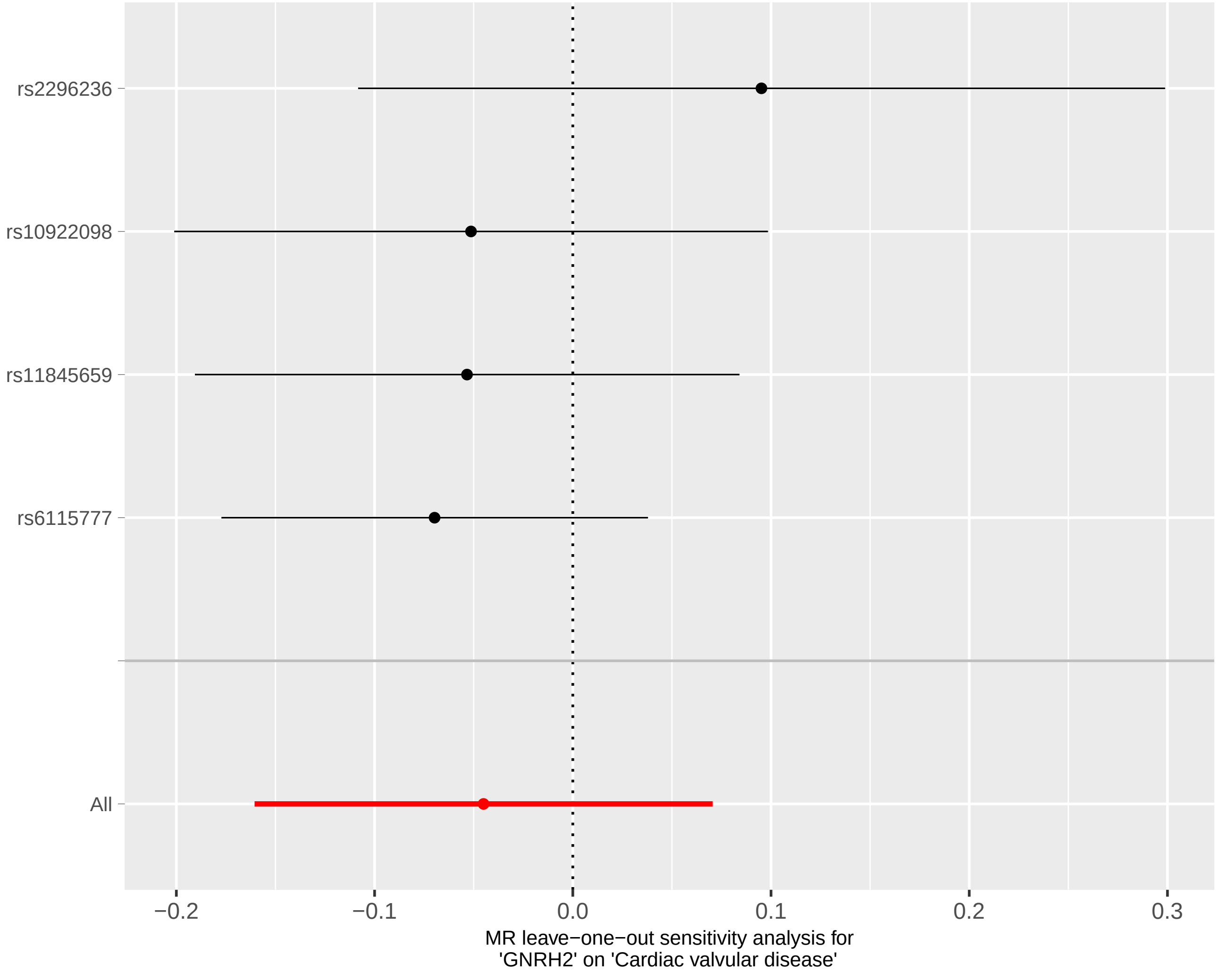

rs2296236
rs10922098
rs11845659
rs6115777
All
−0.2
−0.1
0.0
0.1
0.2
0.3
MR leave−one−out sensitivity analysis for
'GNRH2' on 'Cardiac valvular disease'

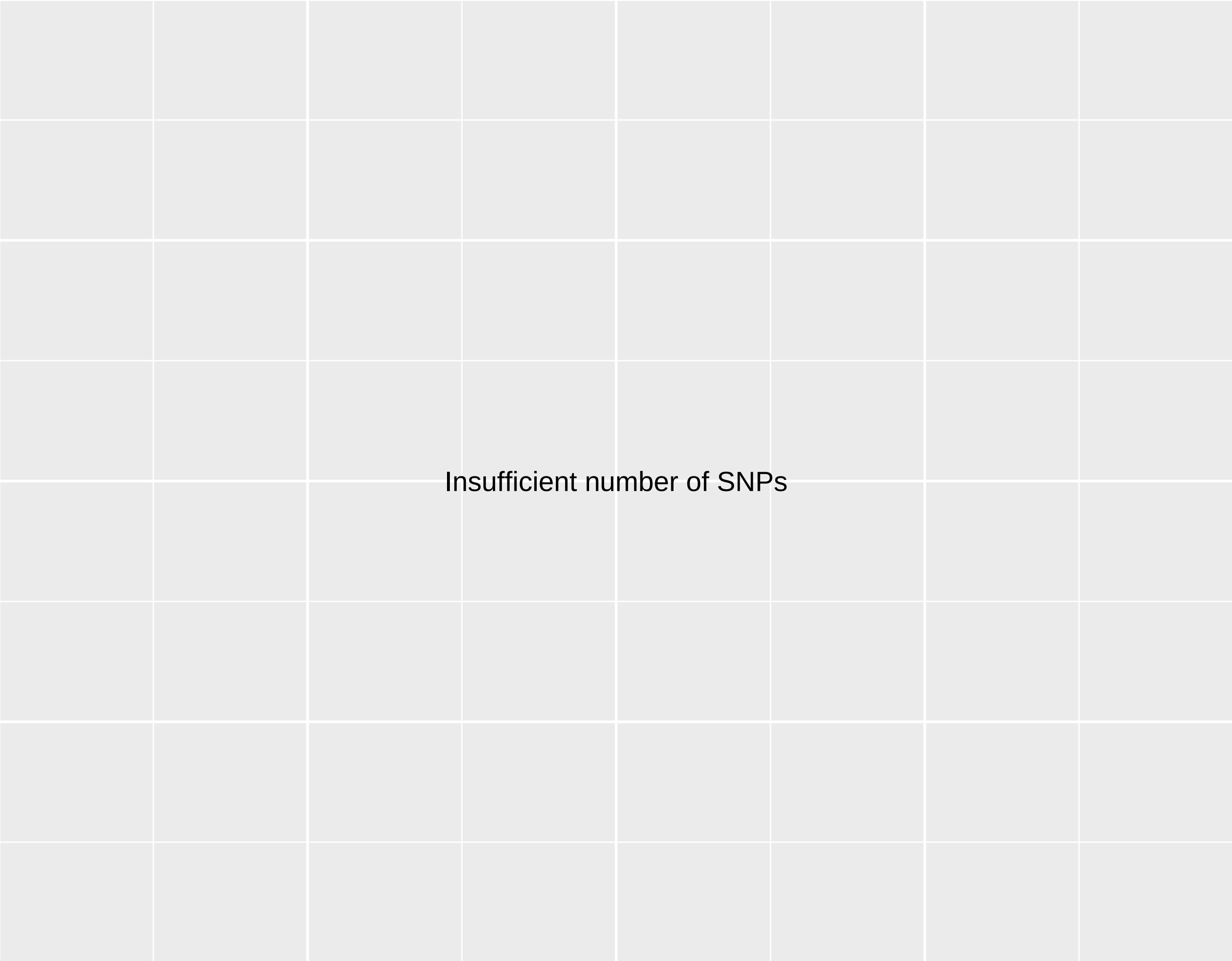
Insufficient number of SNPs

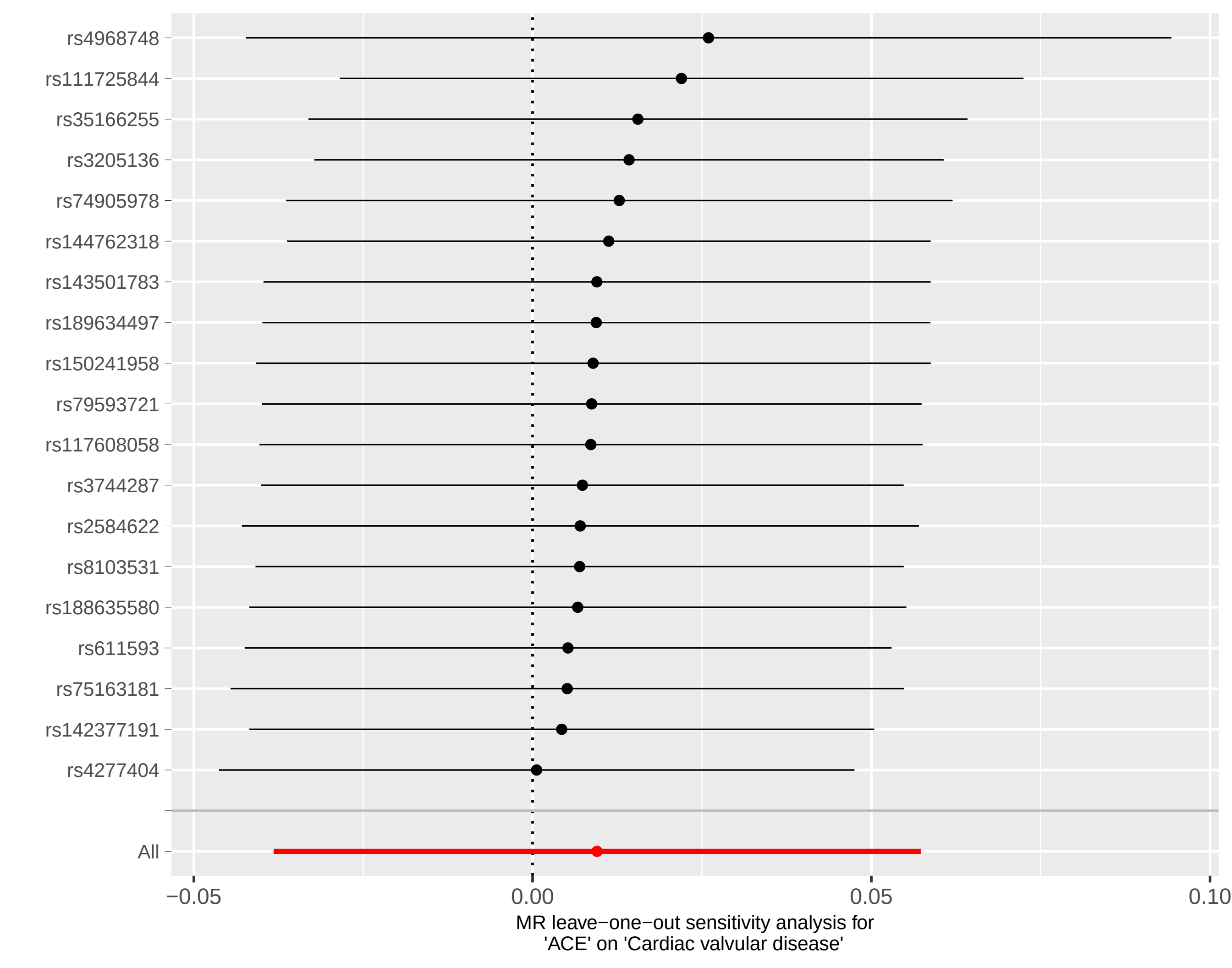
rs4968748
rs111725844
rs35166255
rs3205136
rs74905978
rs144762318
rs143501783
rs189634497
rs150241958
rs79593721
rs117608058
rs3744287
rs2584622
rs8103531
rs188635580
rs611593
rs75163181
rs142377191
rs4277404
All
−0.05
0.00
0.05
0.10
MR leave−one−out sensitivity analysis for
'ACE' on 'Cardiac valvular disease'

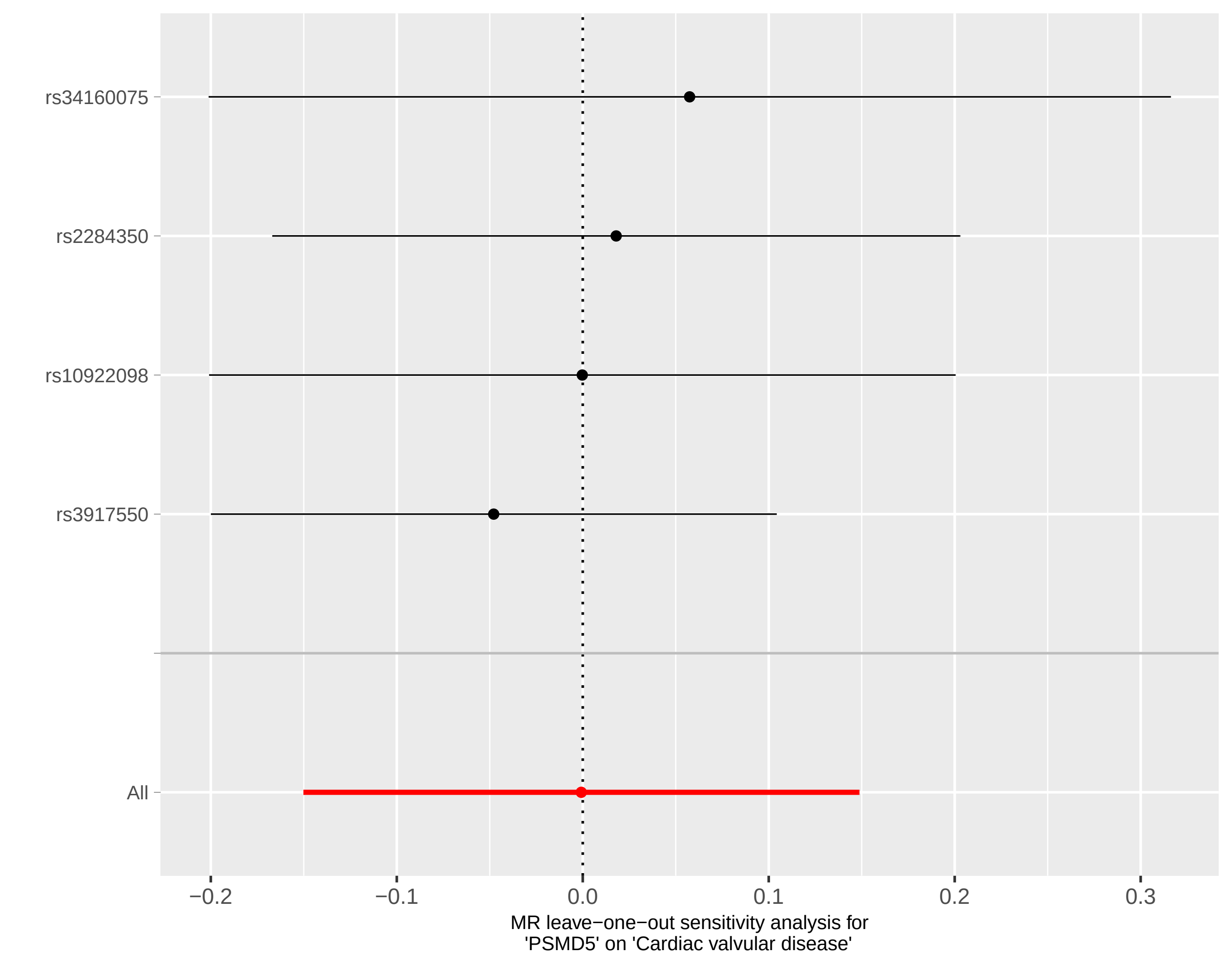
rs34160075
rs2284350
rs10922098
rs3917550
All
−0.2
−0.1
0.0
0.1
0.2
0.3
MR leave−one−out sensitivity analysis for
'PSMD5' on 'Cardiac valvular disease'

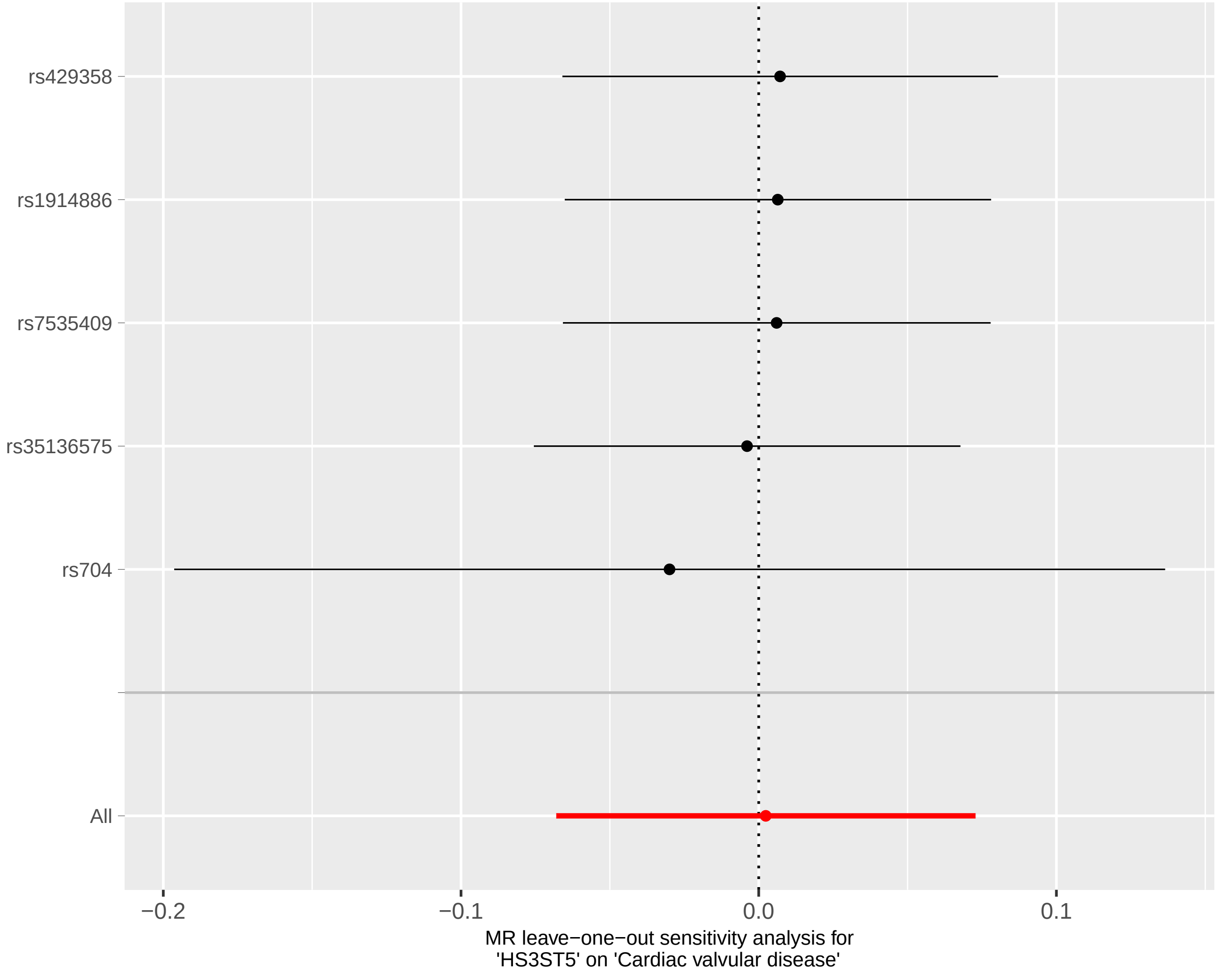

rs429358
rs1914886
rs7535409
rs35136575
rs704
All
−0.2
−0.1
0.0
0.1
MR leave−one−out sensitivity analysis for
'HS3ST5' on 'Cardiac valvular disease'

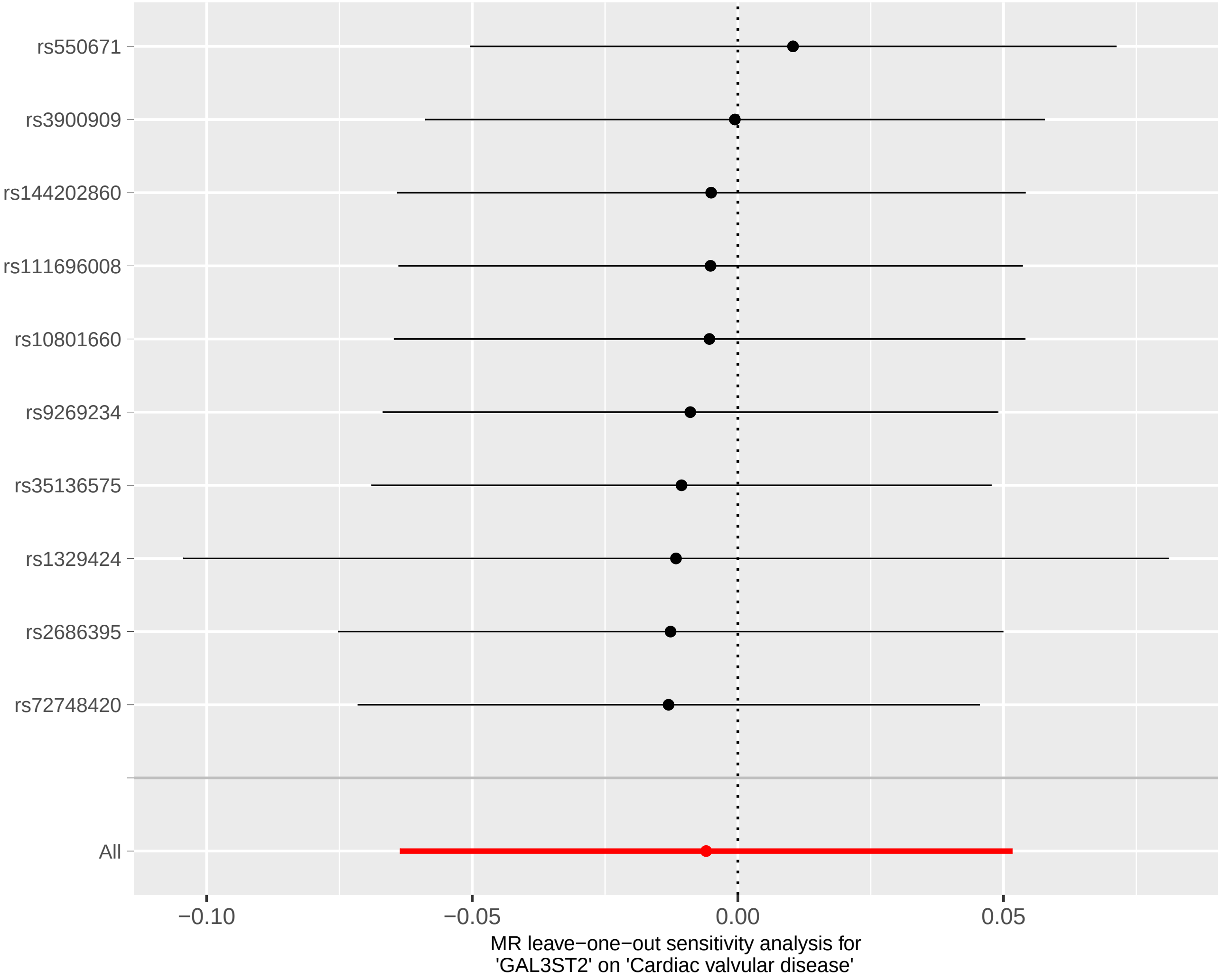
rs550671
rs3900909
rs144202860
rs111696008
rs10801660
rs9269234
rs35136575
rs1329424
rs2686395
rs72748420
All
−0.10
−0.05
0.00
0.05
MR leave−one−out sensitivity analysis for
'GAL3ST2' on 'Cardiac valvular disease'

Insufficient number of SNPs

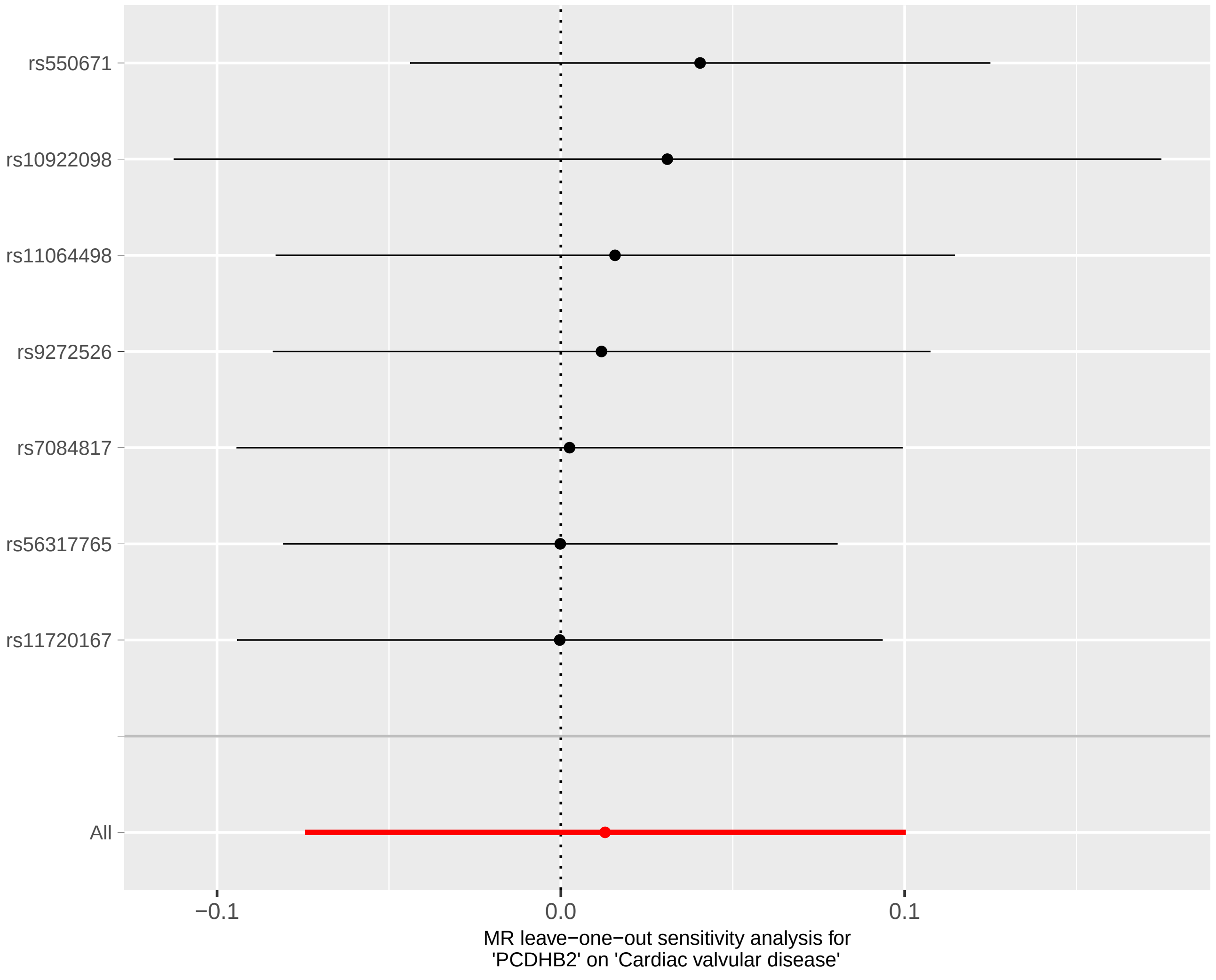
rs550671
rs10922098
rs11064498
rs9272526
rs7084817
rs56317765
rs11720167
All
−0.1
0.0
0.1
MR leave−one−out sensitivity analysis for
'PCDHB2' on 'Cardiac valvular disease'

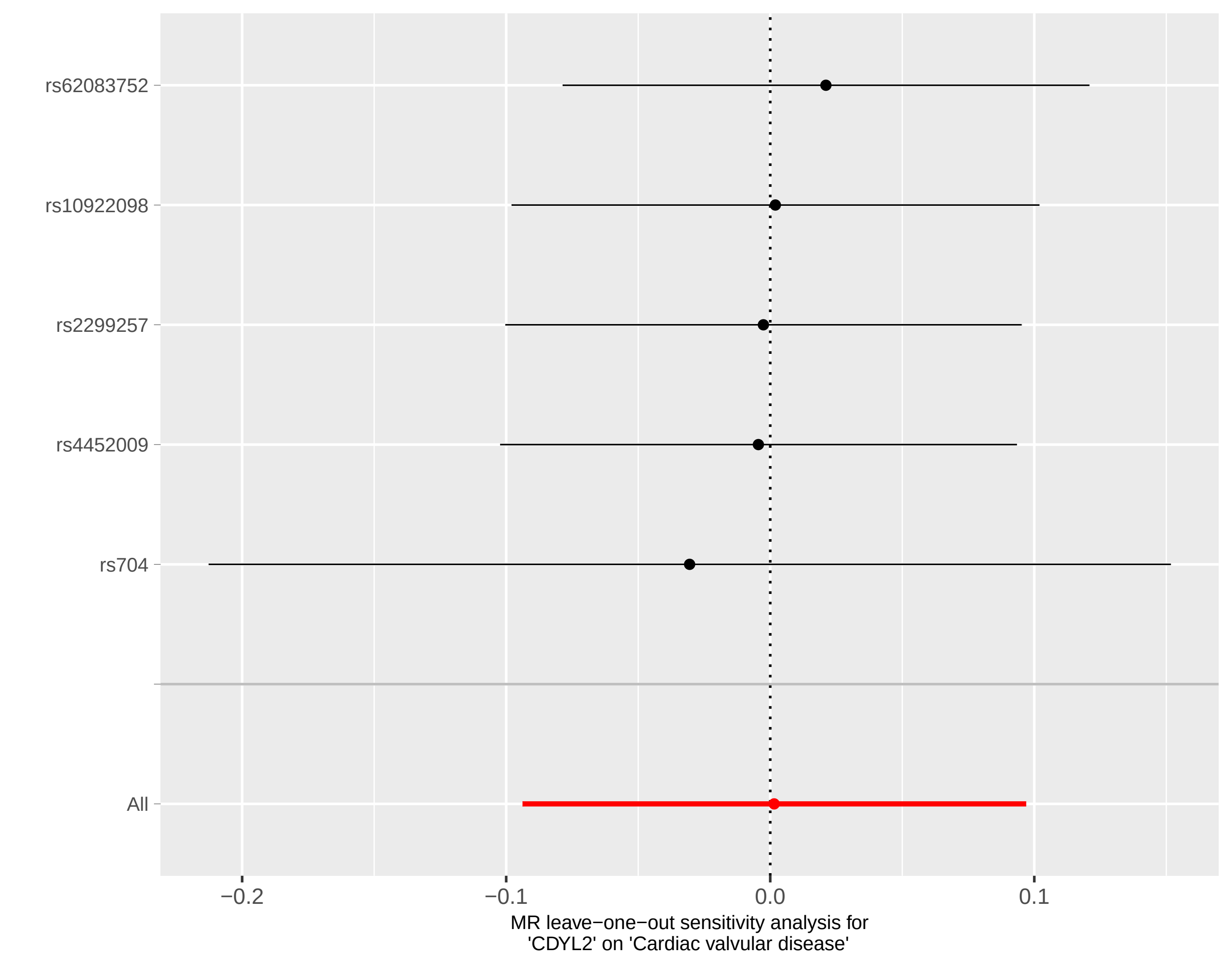

rs62083752
rs10922098
rs2299257
rs4452009
rs704
All
−0.2
−0.1
0.0
0.1
MR leave−one−out sensitivity analysis for
'CDYL2' on 'Cardiac valvular disease'

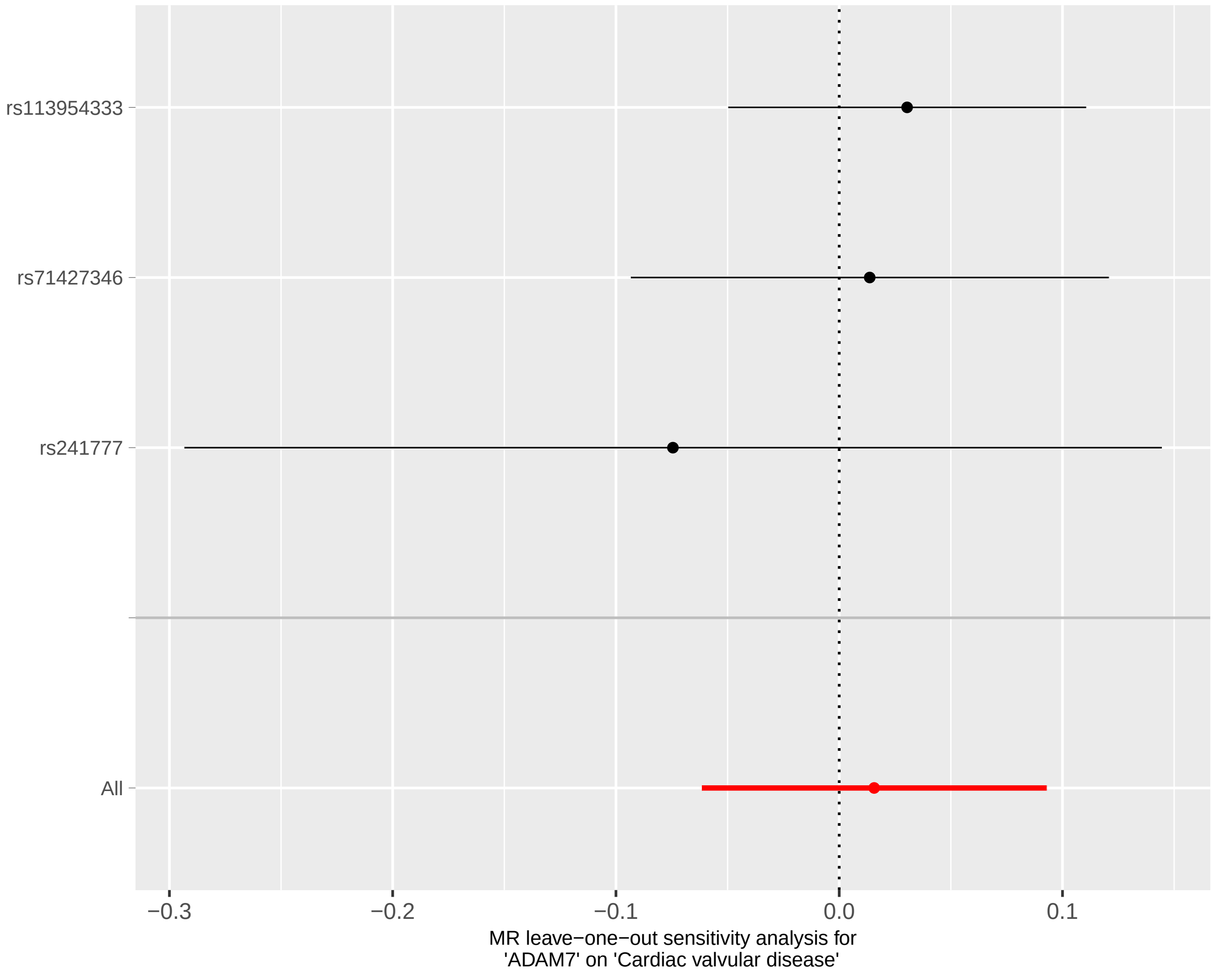
rs113954333
rs71427346
rs241777
All
−0.3
−0.2
−0.1
0.0
0.1
MR leave−one−out sensitivity analysis for
'ADAM7' on 'Cardiac valvular disease'

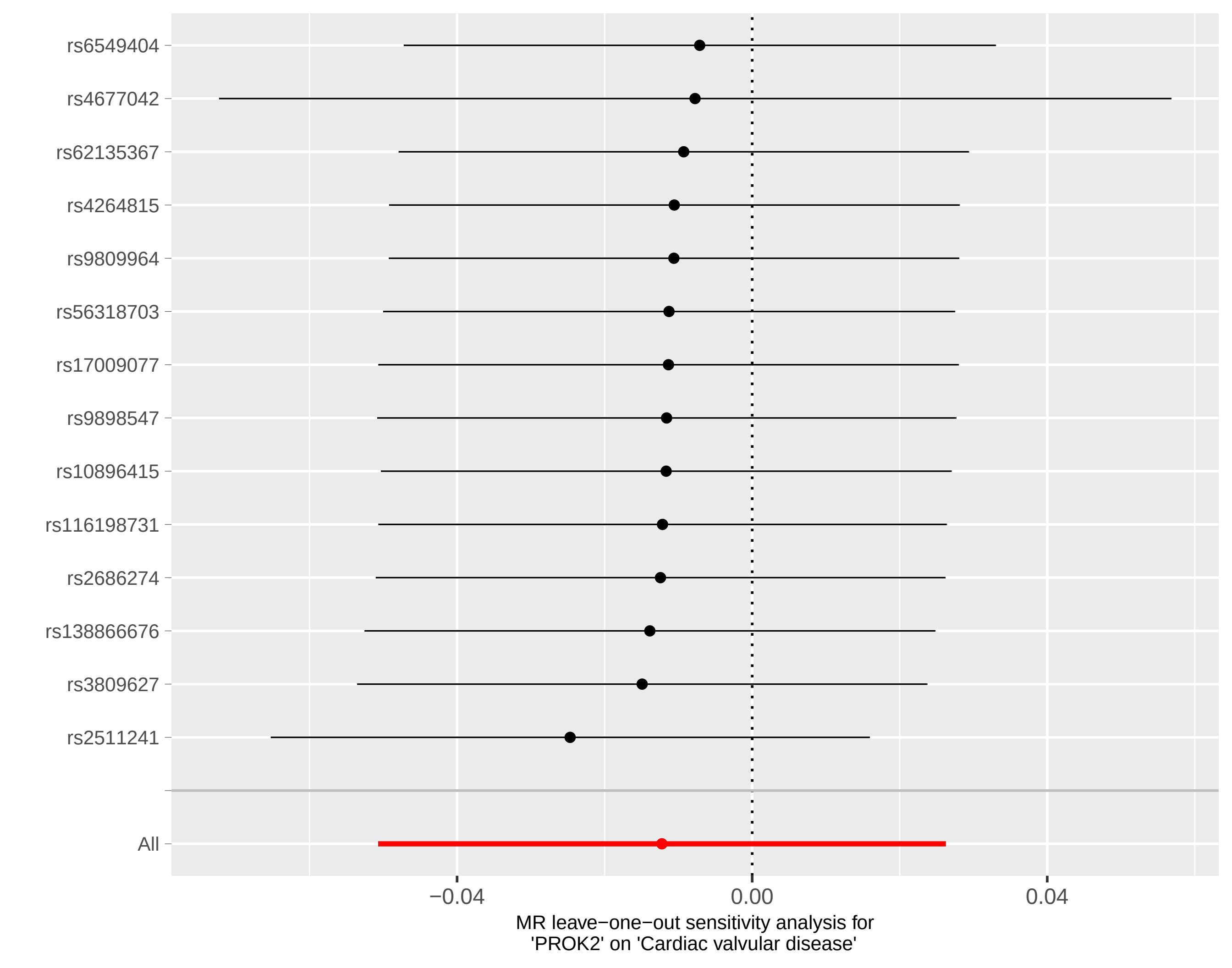
rs6549404
rs4677042
rs62135367
rs4264815
rs9809964
rs56318703
rs17009077
rs9898547
rs10896415
rs116198731
rs2686274
rs138866676
rs3809627
rs2511241
All
−0.04
0.00
0.04
MR leave−one−out sensitivity analysis for
'PROK2' on 'Cardiac valvular disease'

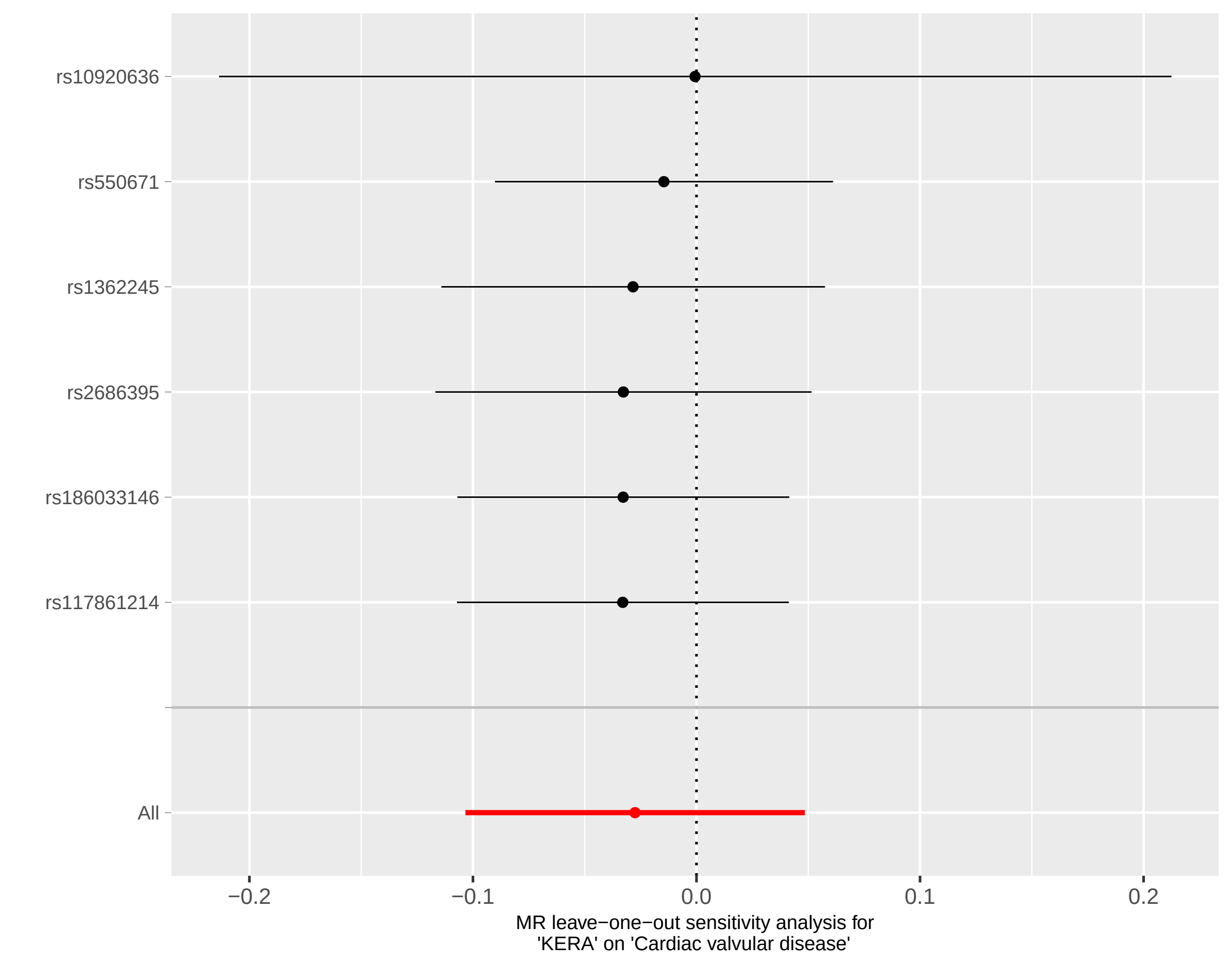
rs10920636
rs550671
rs1362245
rs2686395
rs186033146
rs117861214
All
−0.2
−0.1
0.0
0.1
0.2
MR leave−one−out sensitivity analysis for
'KERA' on 'Cardiac valvular disease'

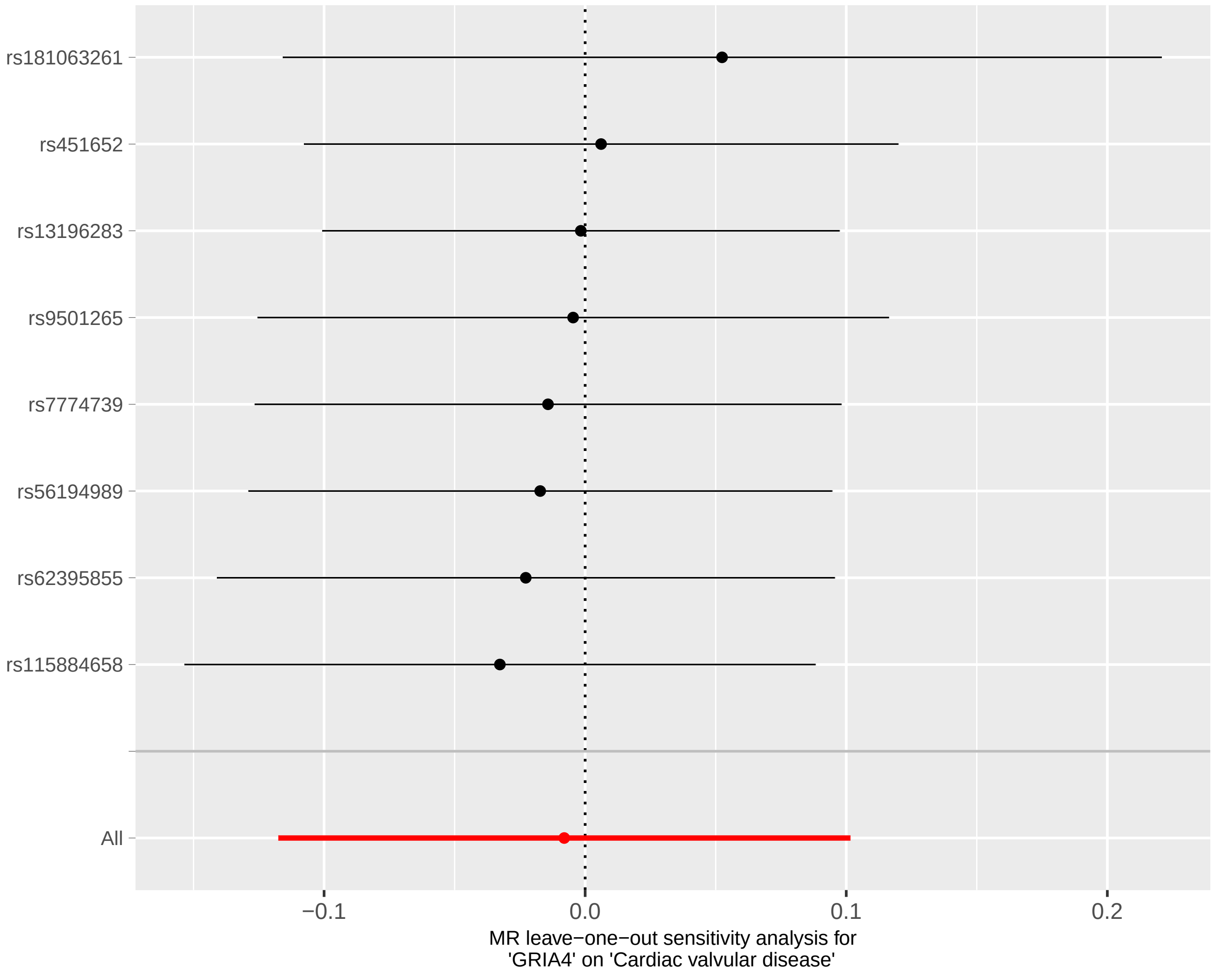
rs181063261
rs451652
rs13196283
rs9501265
rs7774739
rs56194989
rs62395855
rs115884658
All
−0.1
0.0
0.1
0.2
MR leave−one−out sensitivity analysis for
'GRIA4' on 'Cardiac valvular disease'

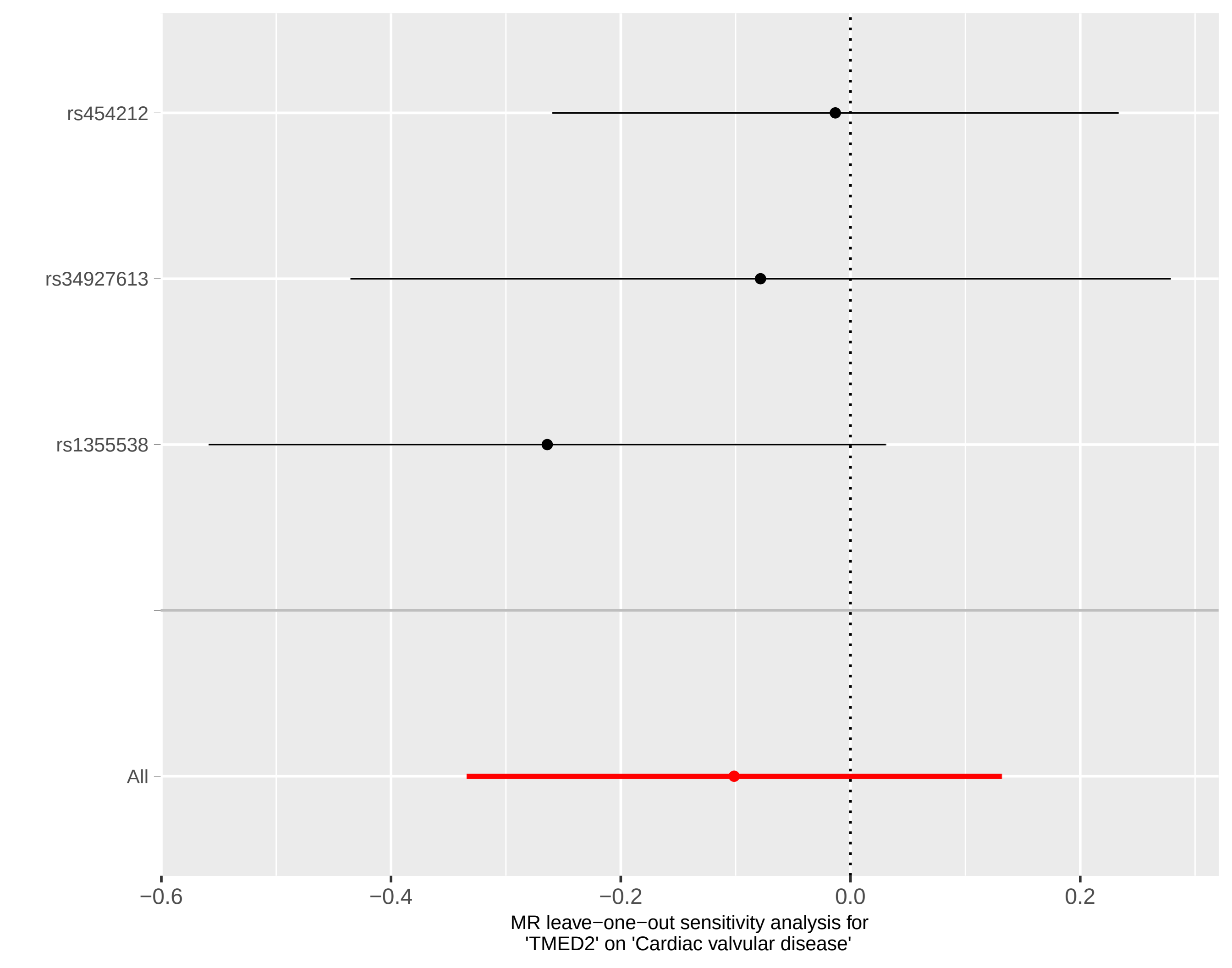

rs454212
rs34927613
rs1355538
All
−0.6
−0.4
−0.2
0.0
0.2
MR leave−one−out sensitivity analysis for
'TMED2' on 'Cardiac valvular disease'

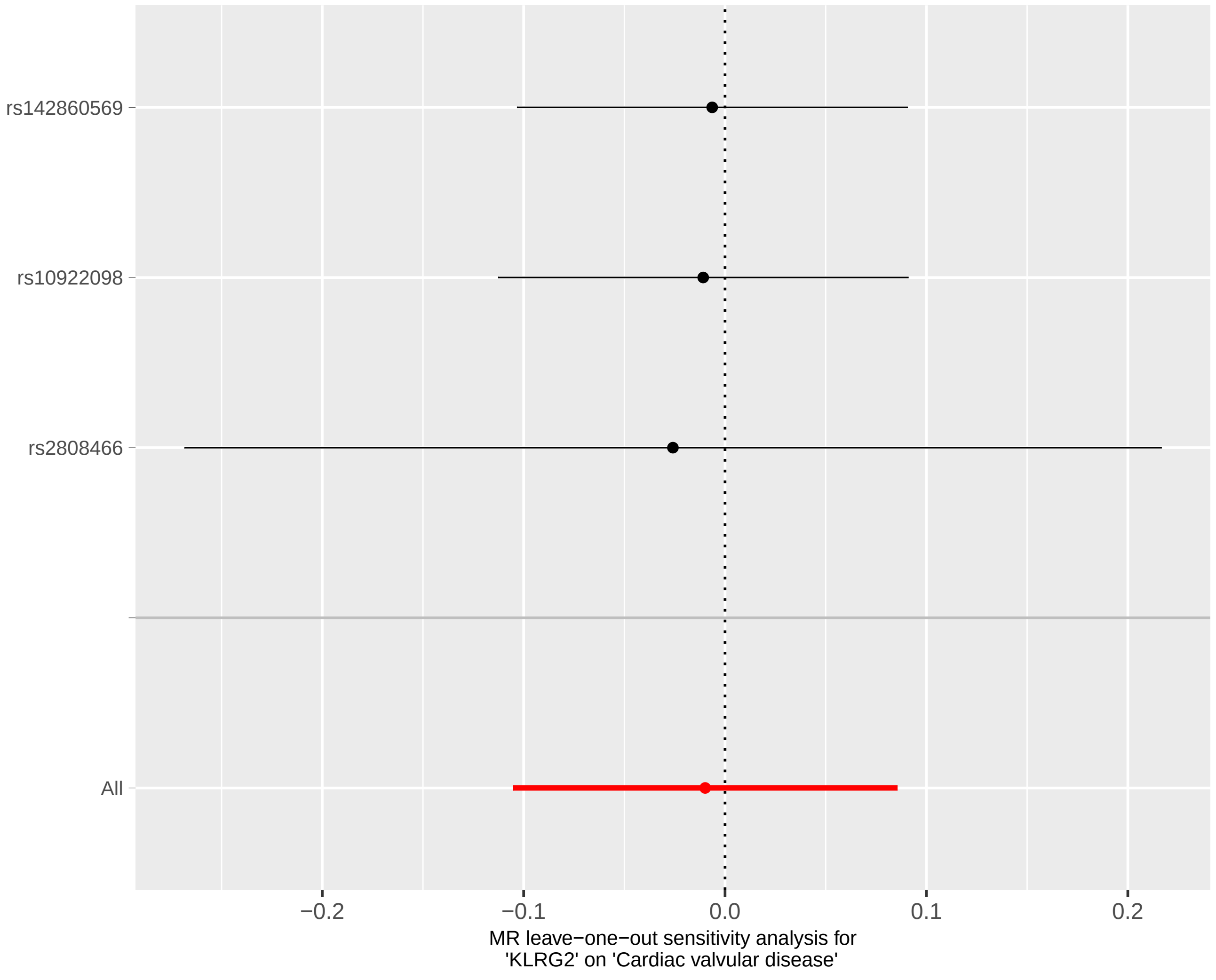
rs142860569
rs10922098
rs2808466
All
−0.2
−0.1
0.0
0.1
0.2
MR leave−one−out sensitivity analysis for
'KLRG2' on 'Cardiac valvular disease'

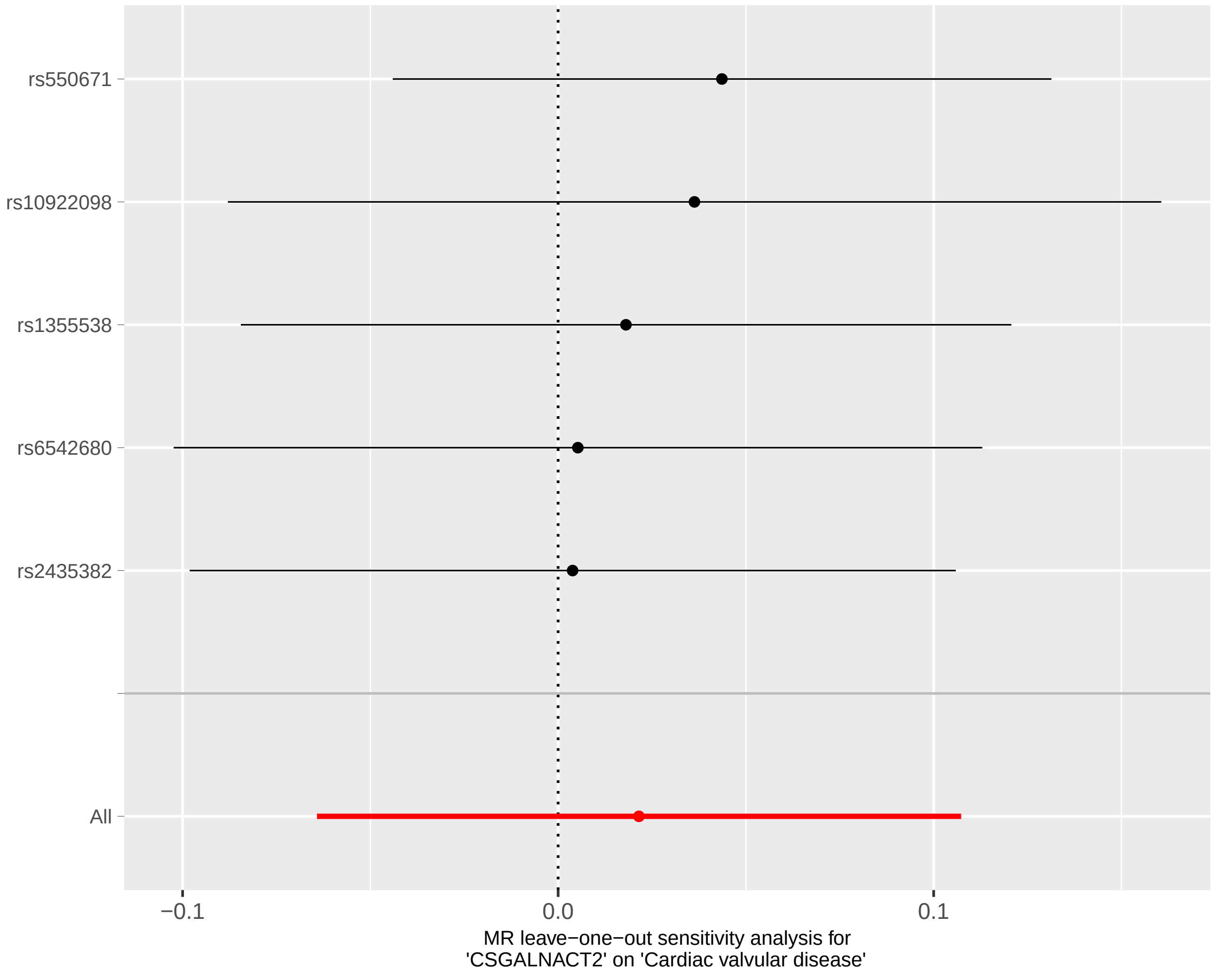
rs550671
rs10922098
rs1355538
rs6542680
rs2435382
All
−0.1
0.0
0.1
MR leave−one−out sensitivity analysis for
'CSGALNACT2' on 'Cardiac valvular disease'

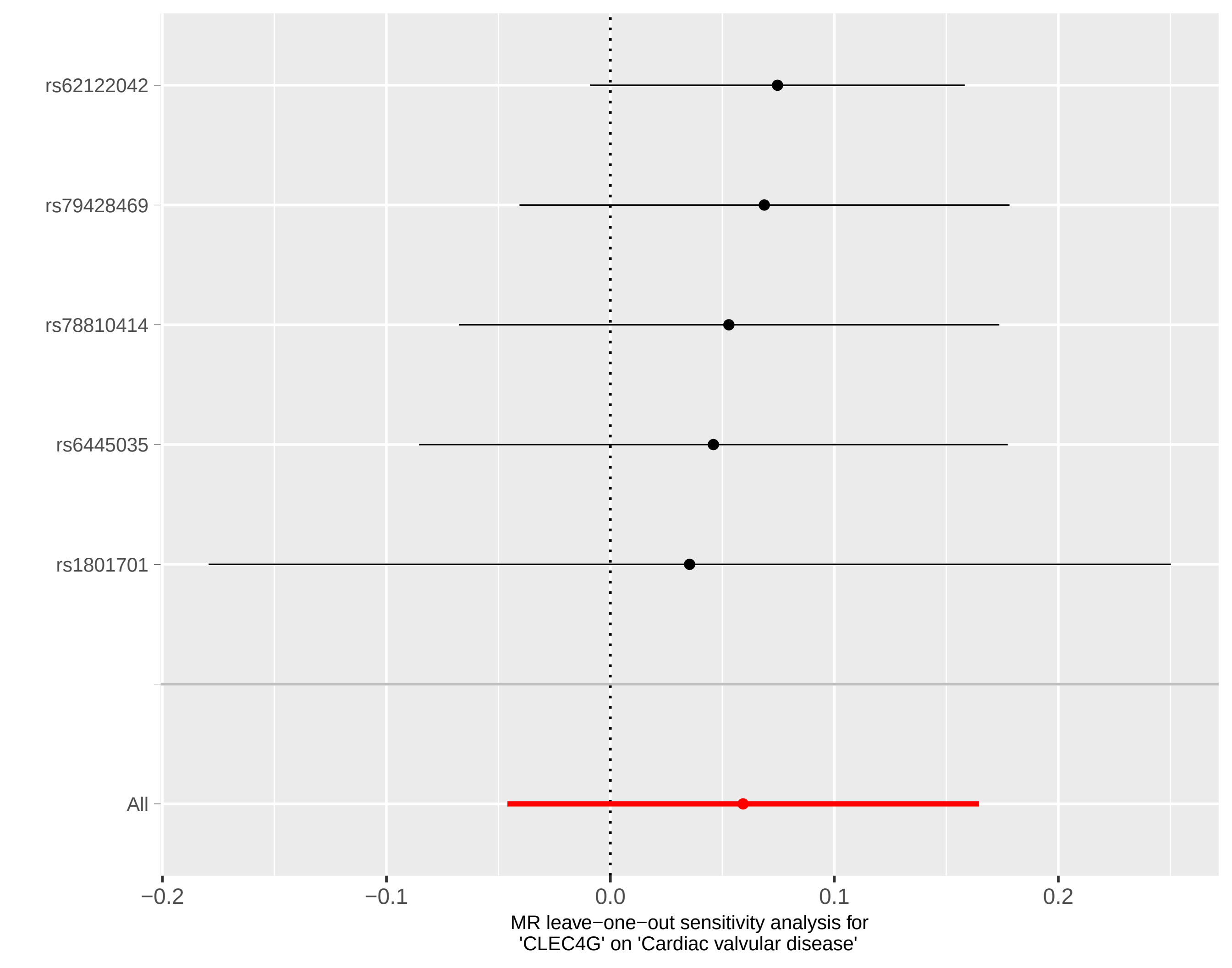
rs62122042
rs79428469
rs78810414
rs6445035
rs1801701
All
−0.2
−0.1
0.0
0.1
0.2
MR leave−one−out sensitivity analysis for
'CLEC4G' on 'Cardiac valvular disease'

Insufficient number of SNPs

Insufficient number of SNPs

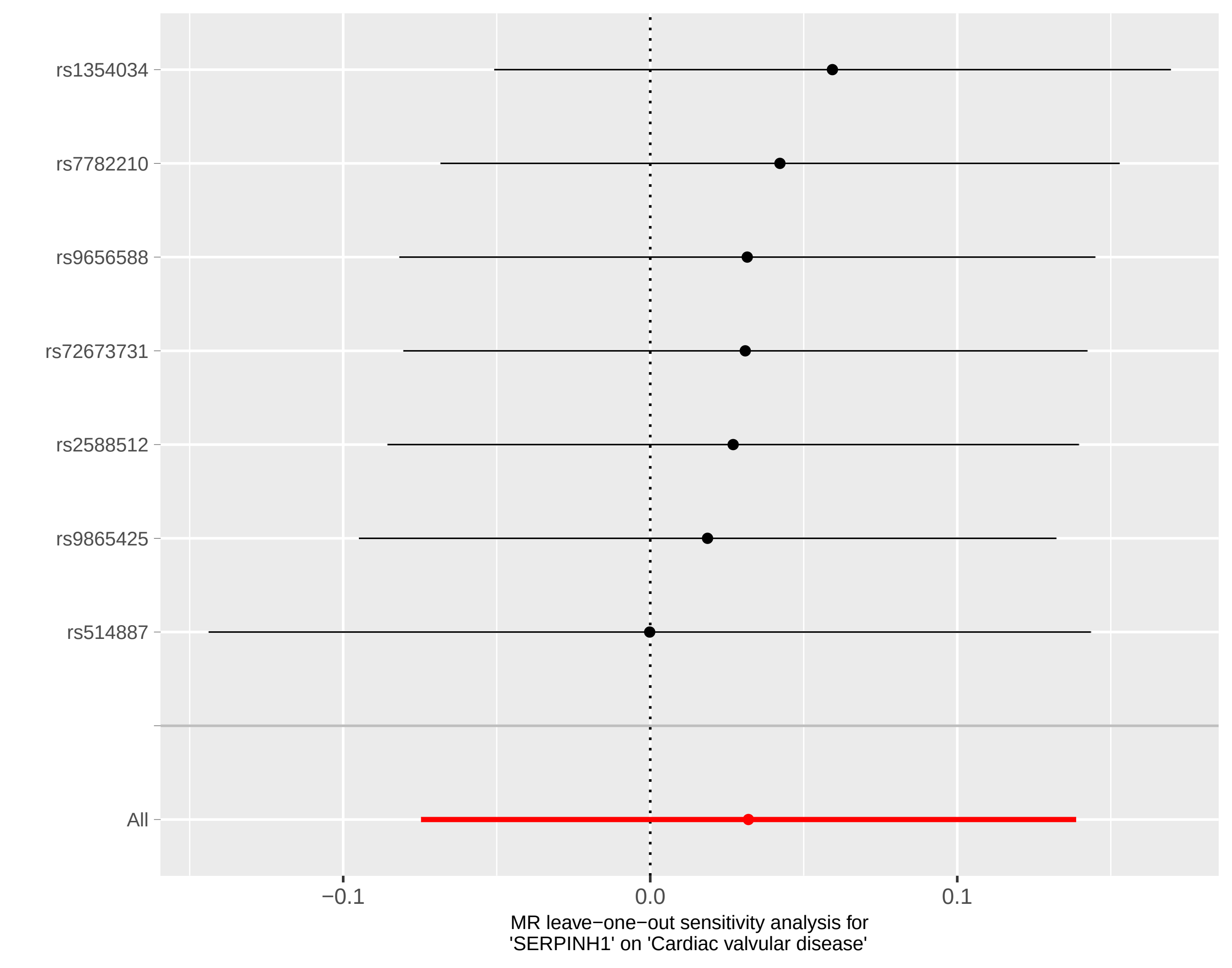
rs1354034
rs7782210
rs9656588
rs72673731
rs2588512
rs9865425
rs514887
All
−0.1
0.0
0.1
MR leave−one−out sensitivity analysis for
'SERPINH1' on 'Cardiac valvular disease'

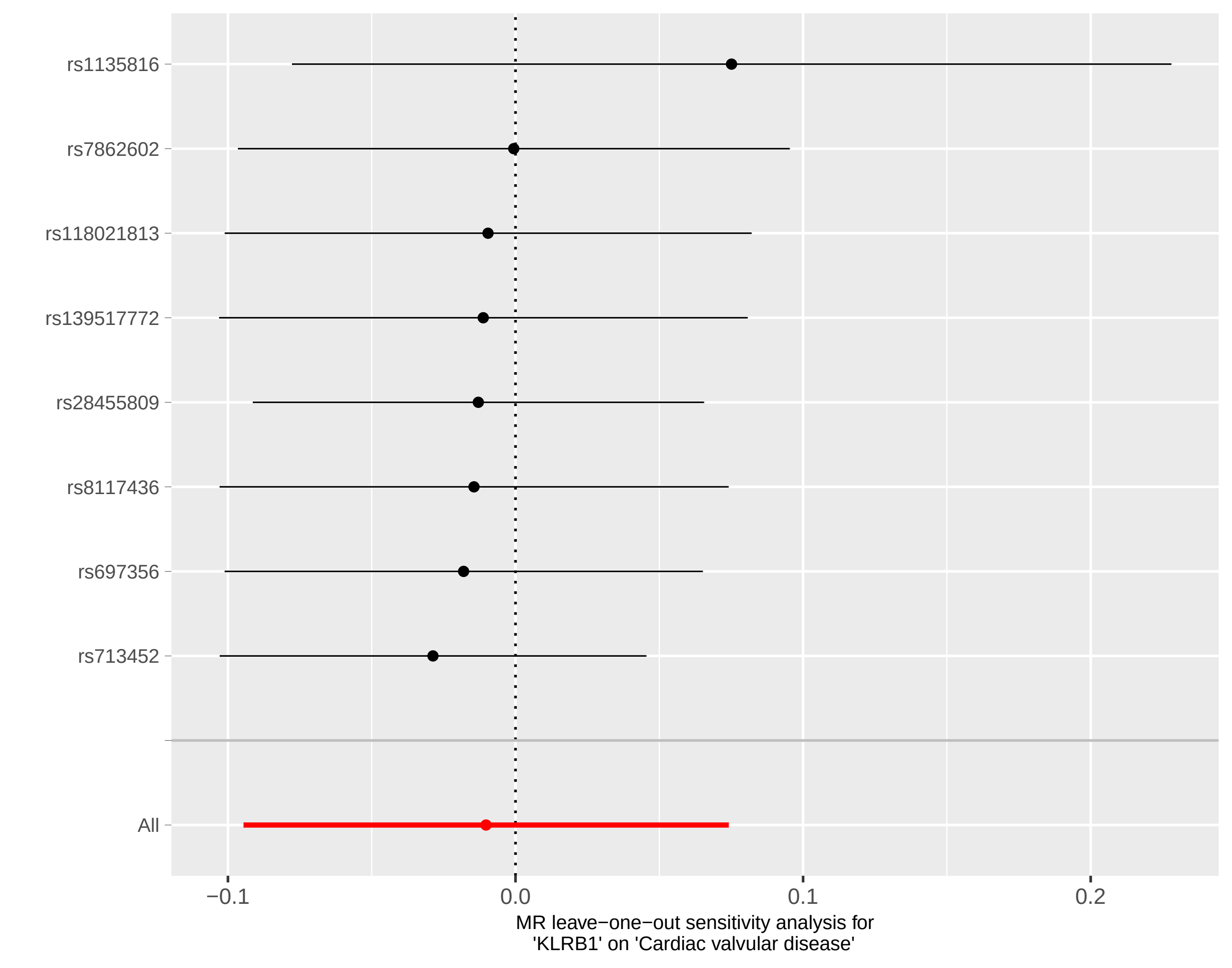
rs1135816
rs7862602
rs118021813
rs139517772
rs28455809
rs8117436
rs697356
rs713452
All
−0.1
0.0
0.1
0.2
MR leave−one−out sensitivity analysis for
'KLRB1' on 'Cardiac valvular disease'

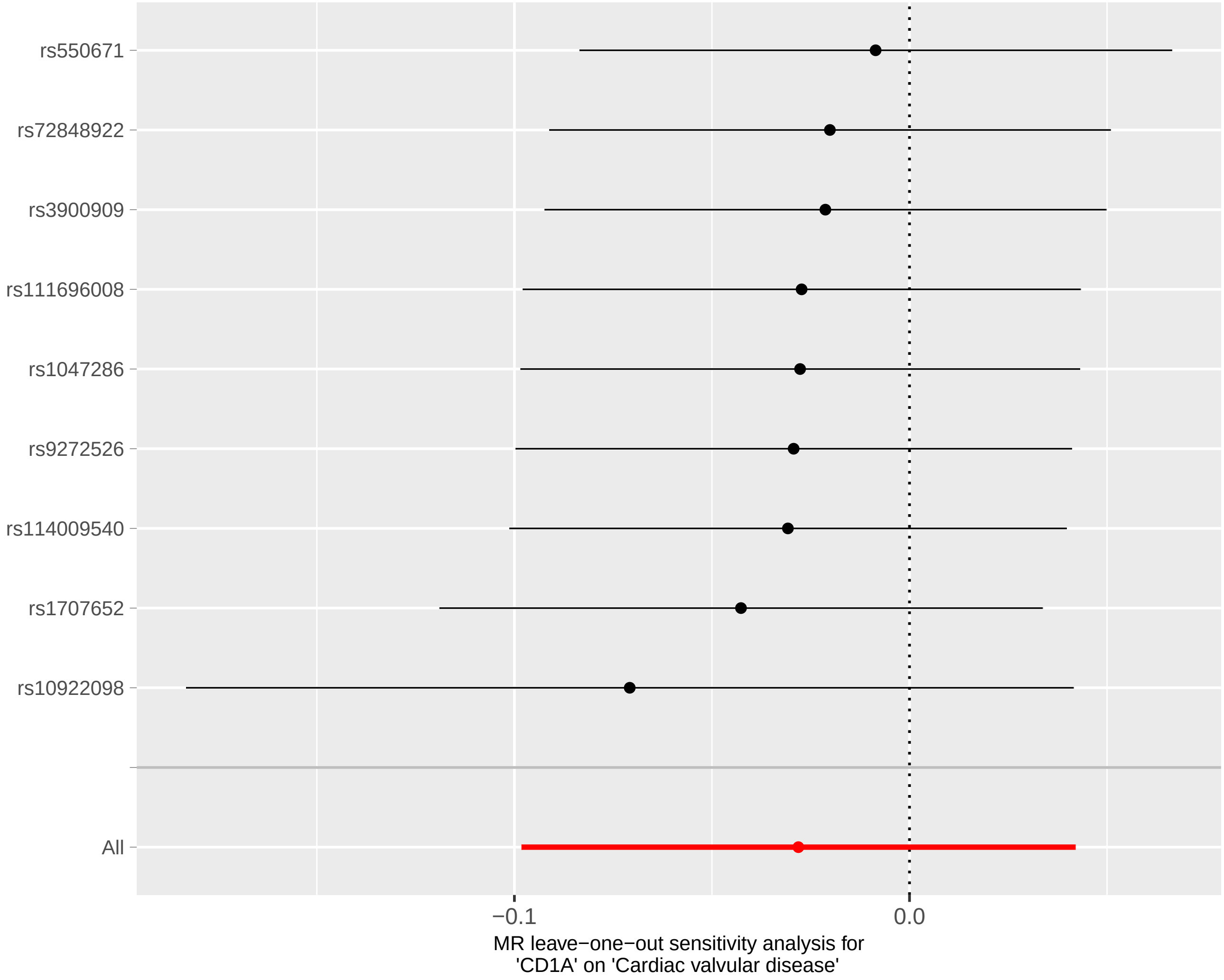

rs550671
rs72848922
rs3900909
rs111696008
rs1047286
rs9272526
rs114009540
rs1707652
rs10922098
All
−0.1
0.0
MR leave−one−out sensitivity analysis for
'CD1A' on 'Cardiac valvular disease'

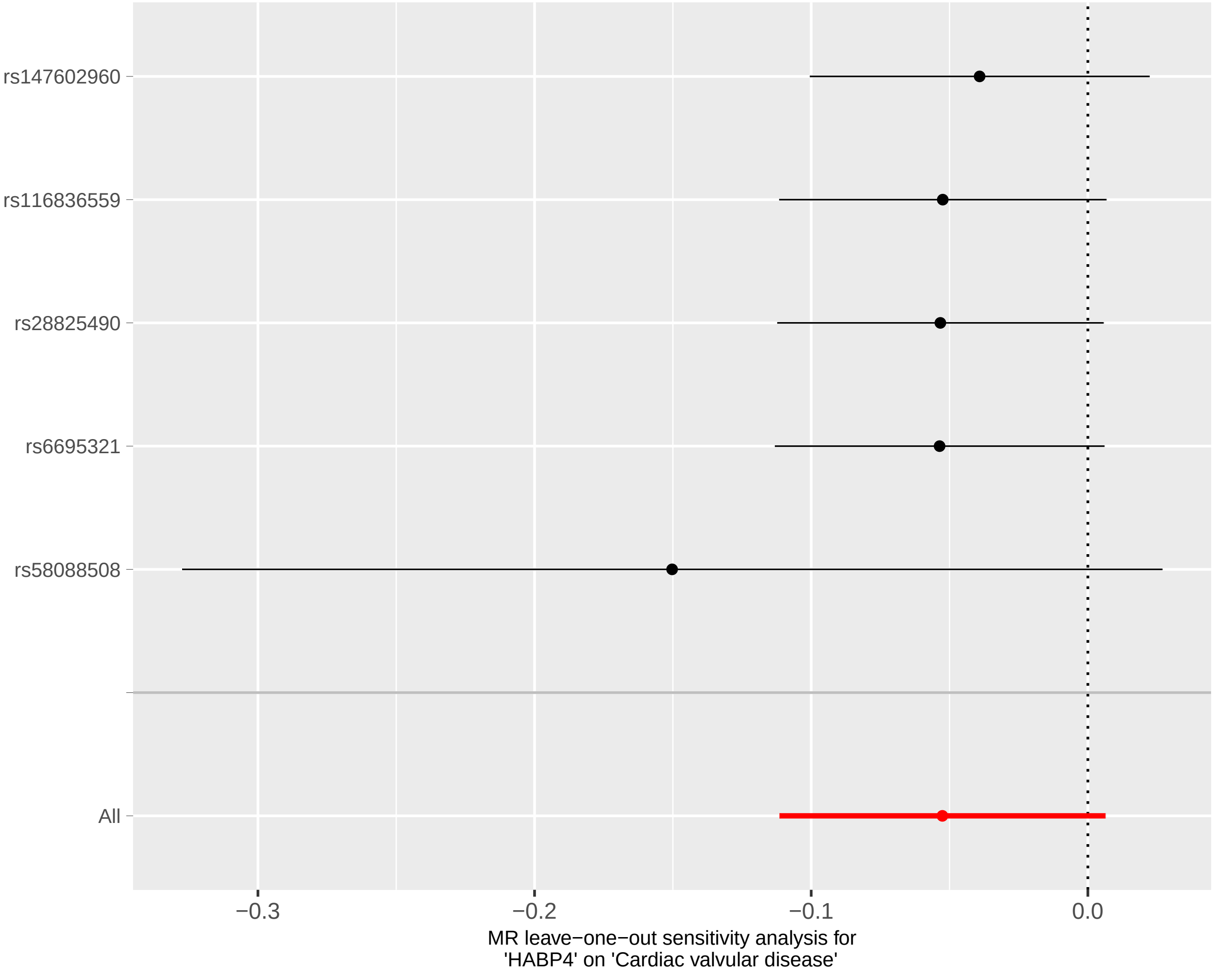

rs147602960
rs116836559
rs28825490
rs6695321
rs58088508
All
−0.3
−0.2
−0.1
0.0
MR leave−one−out sensitivity analysis for
'HABP4' on 'Cardiac valvular disease'

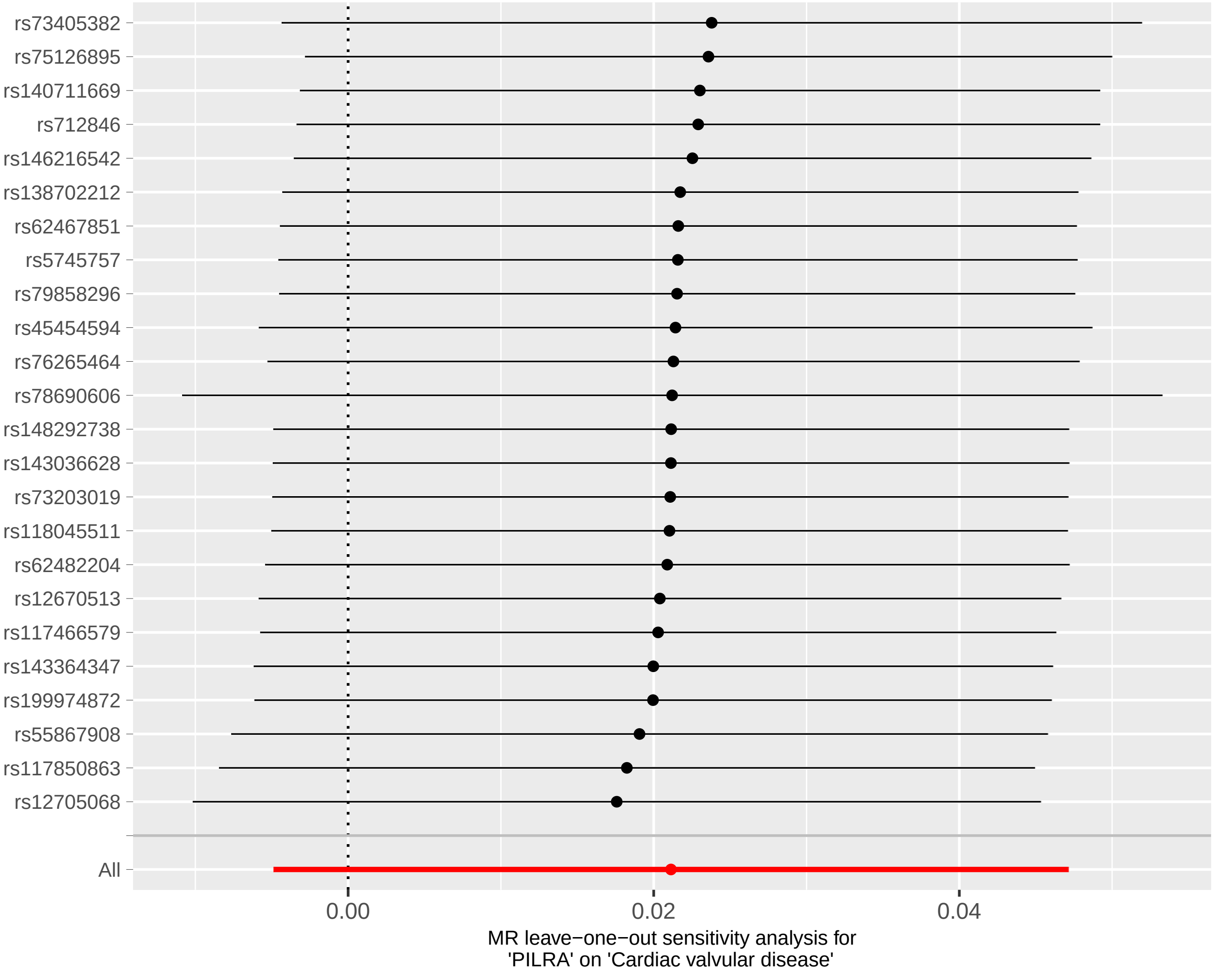
rs73405382
rs75126895
rs140711669
rs712846
rs146216542
rs138702212
rs62467851
rs5745757
rs79858296
rs45454594
rs76265464
rs78690606
rs148292738
rs143036628
rs73203019
rs118045511
rs62482204
rs12670513
rs117466579
rs143364347
rs199974872
rs55867908
rs117850863
rs12705068
All
0.00
0.02
0.04
MR leave−one−out sensitivity analysis for
'PILRA' on 'Cardiac valvular disease'

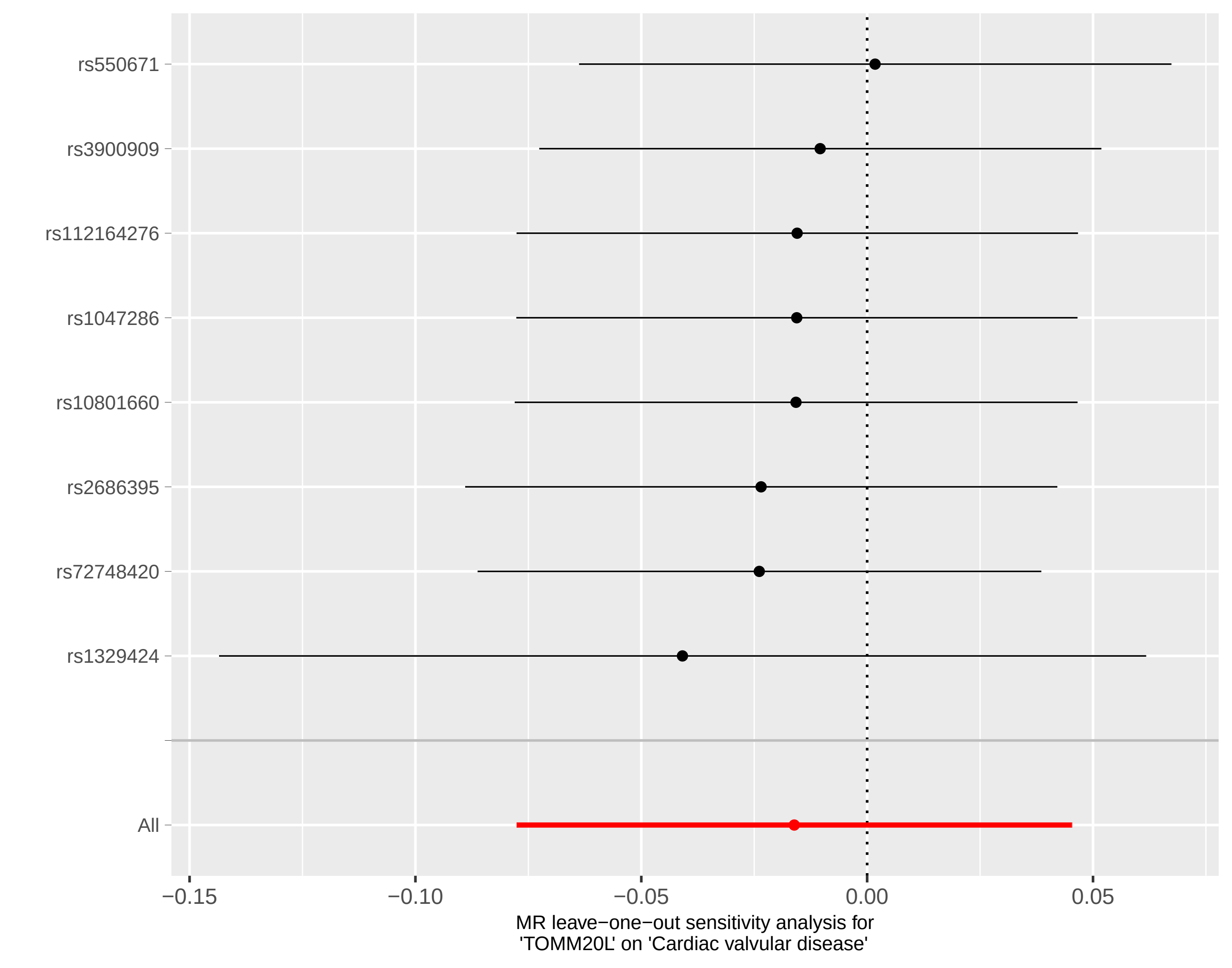
rs550671
rs3900909
rs112164276
rs1047286
rs10801660
rs2686395
rs72748420
rs1329424
All
−0.15
−0.10
−0.05
0.00
0.05
MR leave−one−out sensitivity analysis for
'TOMM20L' on 'Cardiac valvular disease'

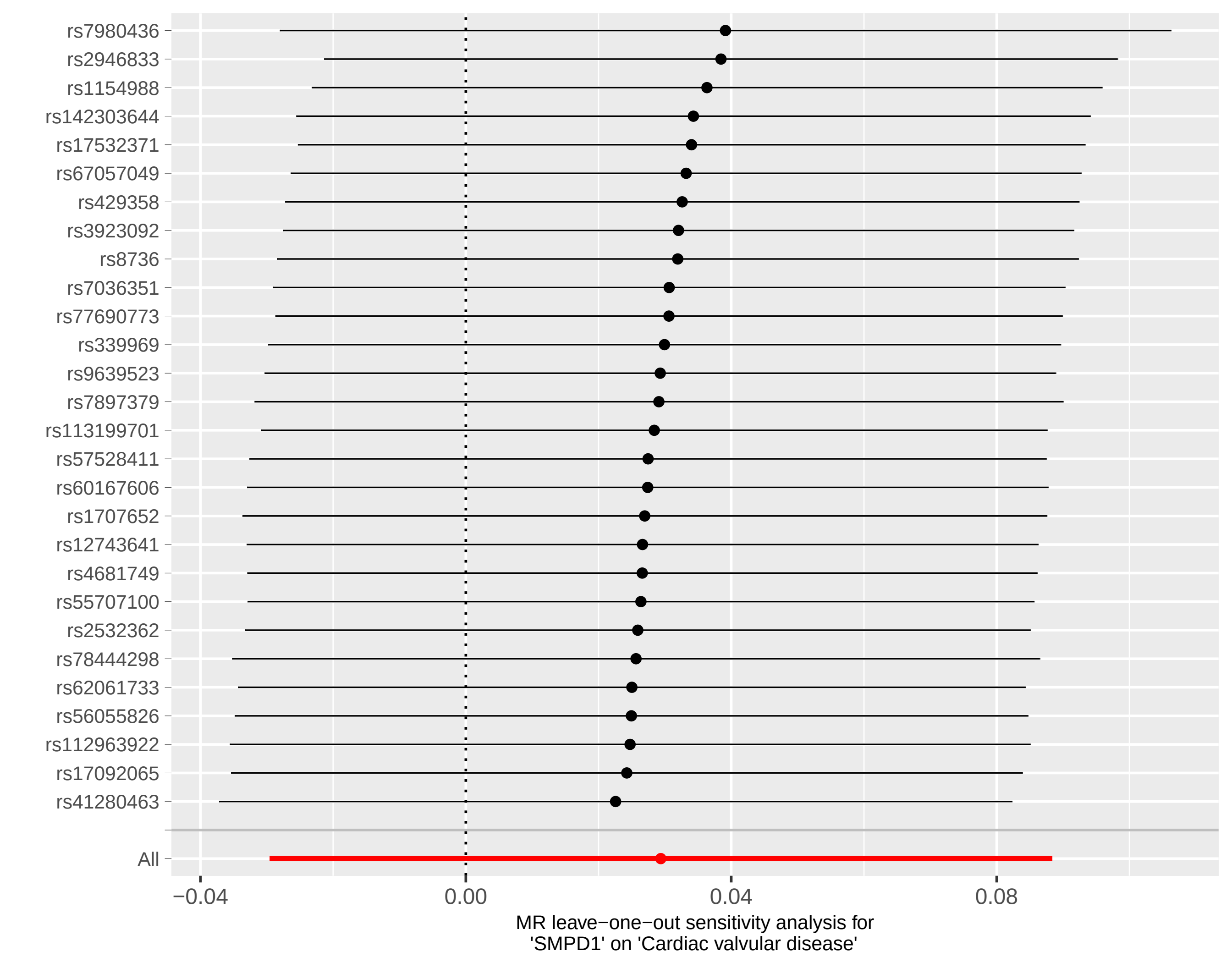
rs7980436
rs2946833
rs1154988
rs142303644
rs17532371
rs67057049
rs429358
rs3923092
rs8736
rs7036351
rs77690773
rs339969
rs9639523
rs7897379
rs113199701
rs57528411
rs60167606
rs1707652
rs12743641
rs4681749
rs55707100
rs2532362
rs78444298
rs62061733
rs56055826
rs112963922
rs17092065
rs41280463
All
−0.04
0.00
0.04
0.08
MR leave−one−out sensitivity analysis for
'SMPD1' on 'Cardiac valvular disease'

Insufficient number of SNPs

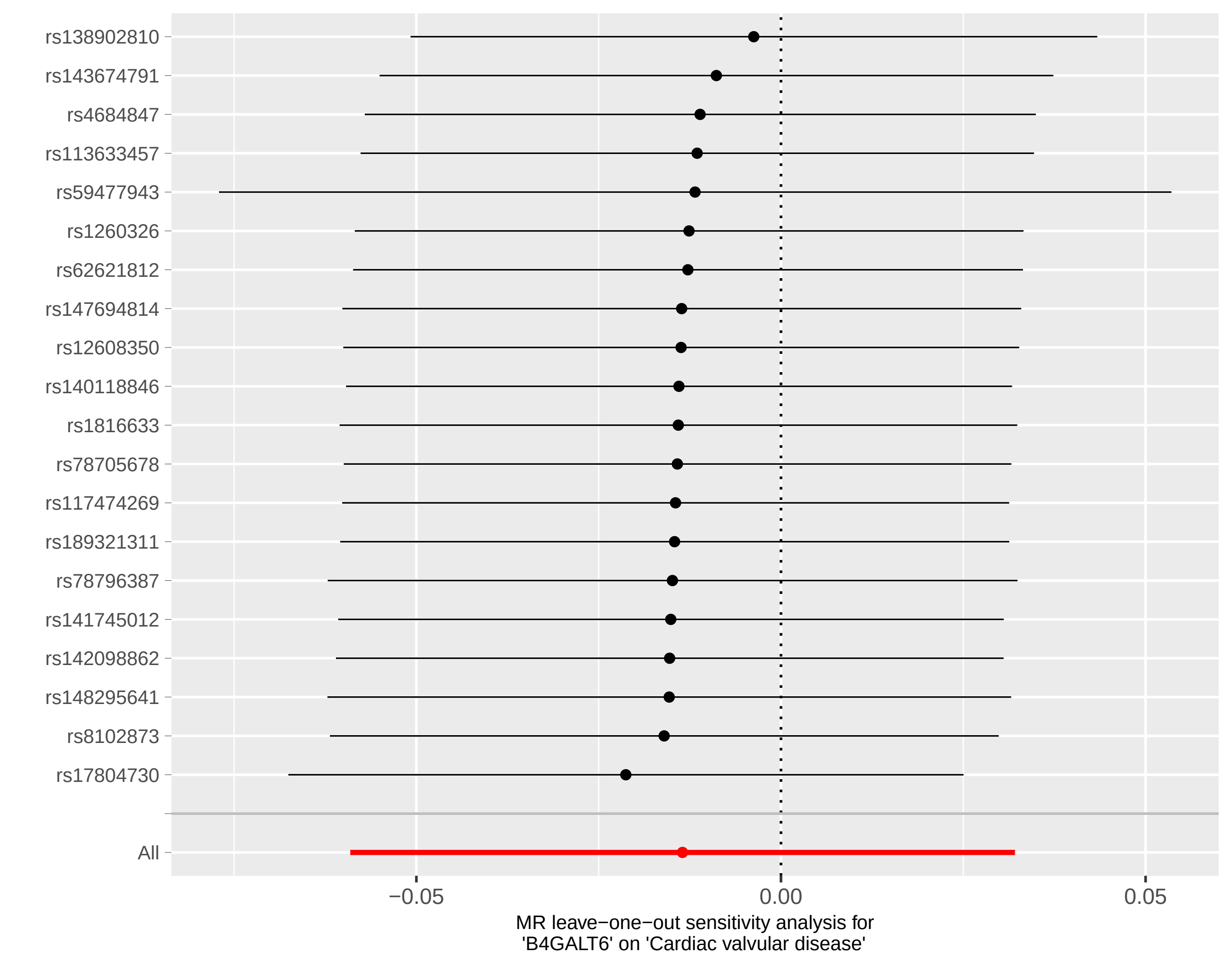
rs138902810
rs143674791
rs4684847
rs113633457
rs59477943
rs1260326
rs62621812
rs147694814
rs12608350
rs140118846
rs1816633
rs78705678
rs117474269
rs189321311
rs78796387
rs141745012
rs142098862
rs148295641
rs8102873
rs17804730
All
−0.05
0.00
0.05
MR leave−one−out sensitivity analysis for
'B4GALT6' on 'Cardiac valvular disease'

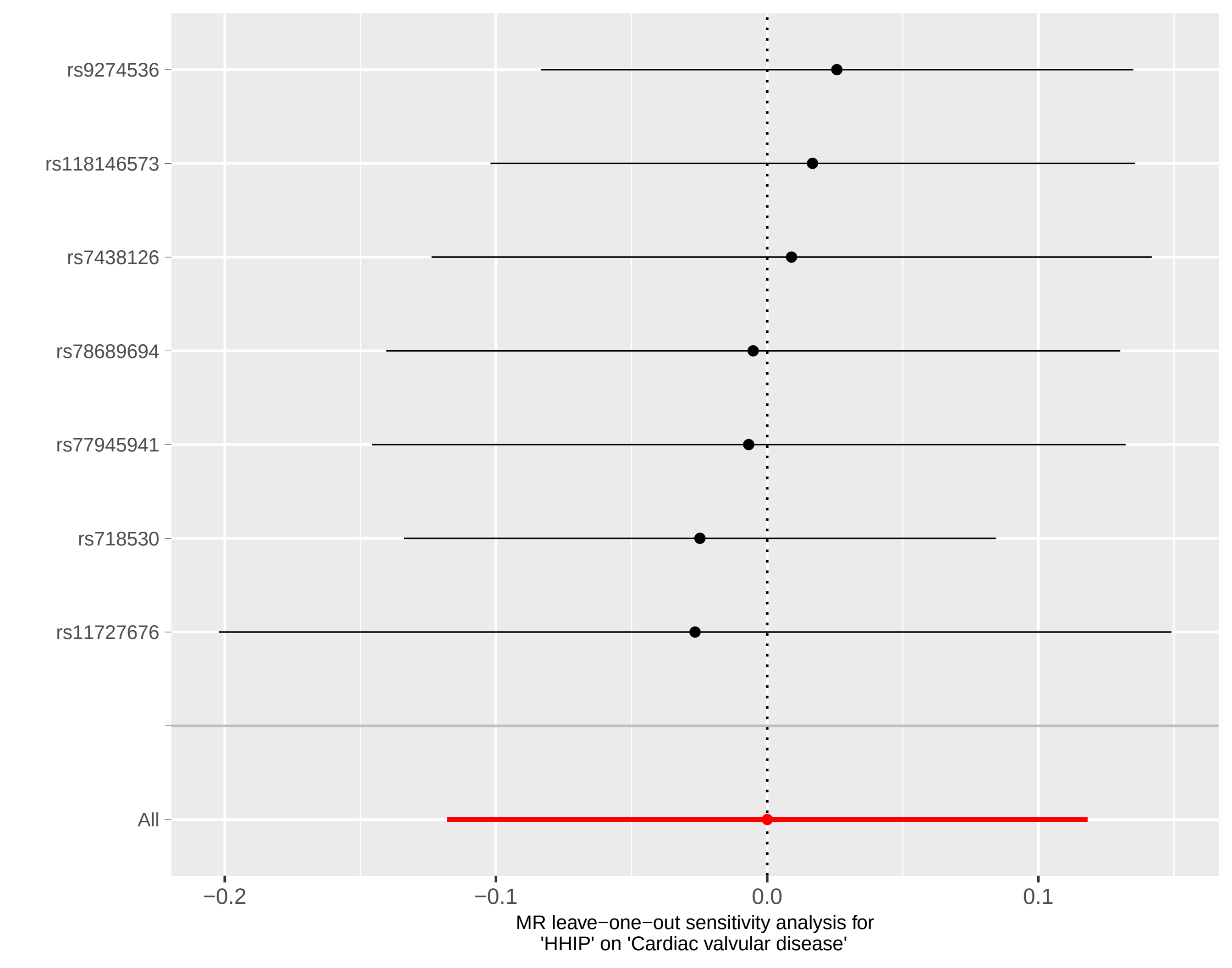

rs9274536
rs118146573
rs7438126
rs78689694
rs77945941
rs718530
rs11727676
All
−0.2
−0.1
0.0
0.1
MR leave−one−out sensitivity analysis for
'HHIP' on 'Cardiac valvular disease'

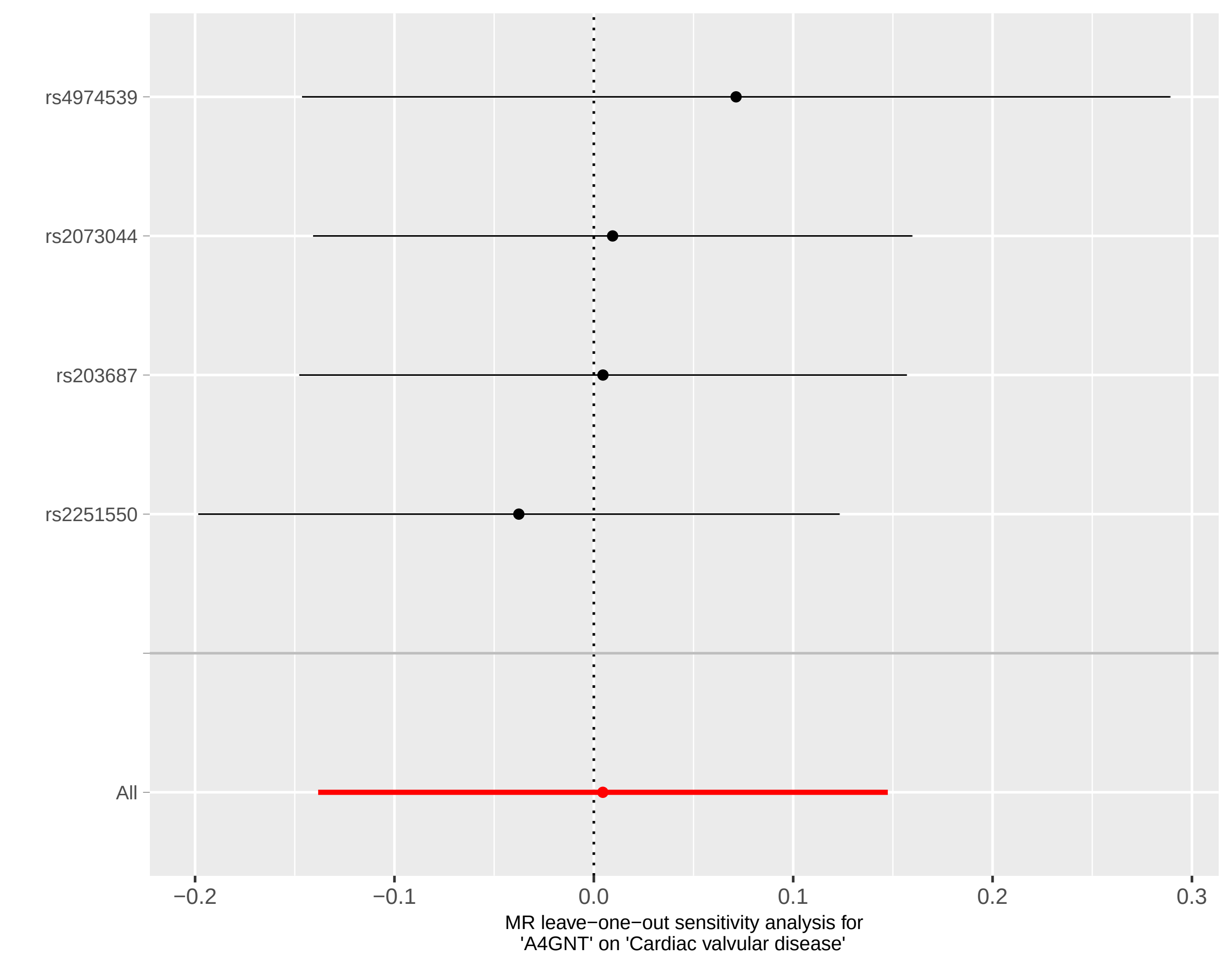
rs4974539
rs2073044
rs203687
rs2251550
All
−0.2
−0.1
0.0
0.1
0.2
0.3
MR leave−one−out sensitivity analysis for
'A4GNT' on 'Cardiac valvular disease'

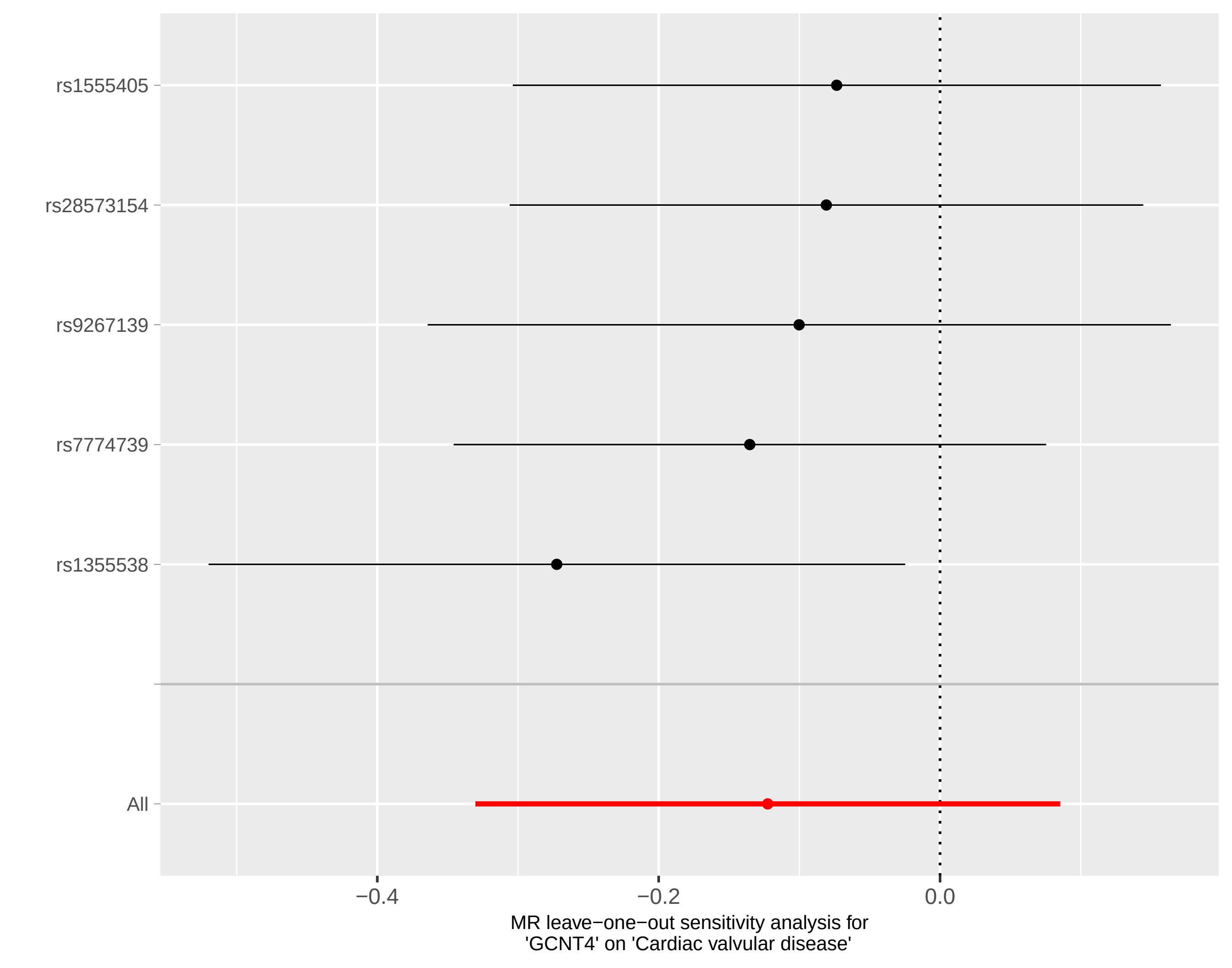

rs1555405
rs28573154
rs9267139
rs7774739
rs1355538
All
−0.4
−0.2
0.0
MR leave−one−out sensitivity analysis for
'GCNT4' on 'Cardiac valvular disease'

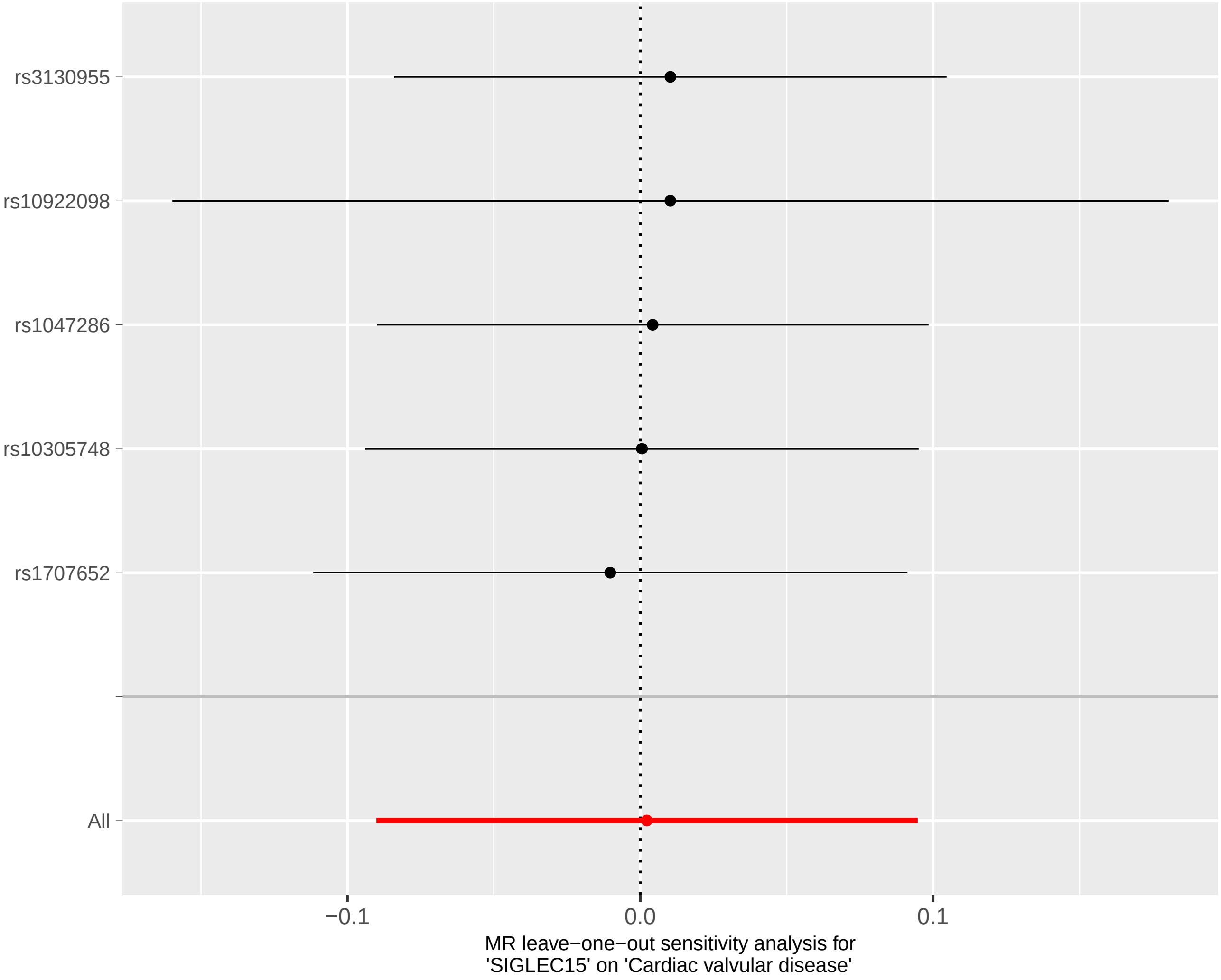

rs3130955
rs10922098
rs1047286
rs10305748
rs1707652
All
−0.1
0.0
0.1
MR leave−one−out sensitivity analysis for
'SIGLEC15' on 'Cardiac valvular disease'

Insufficient number of SNPs

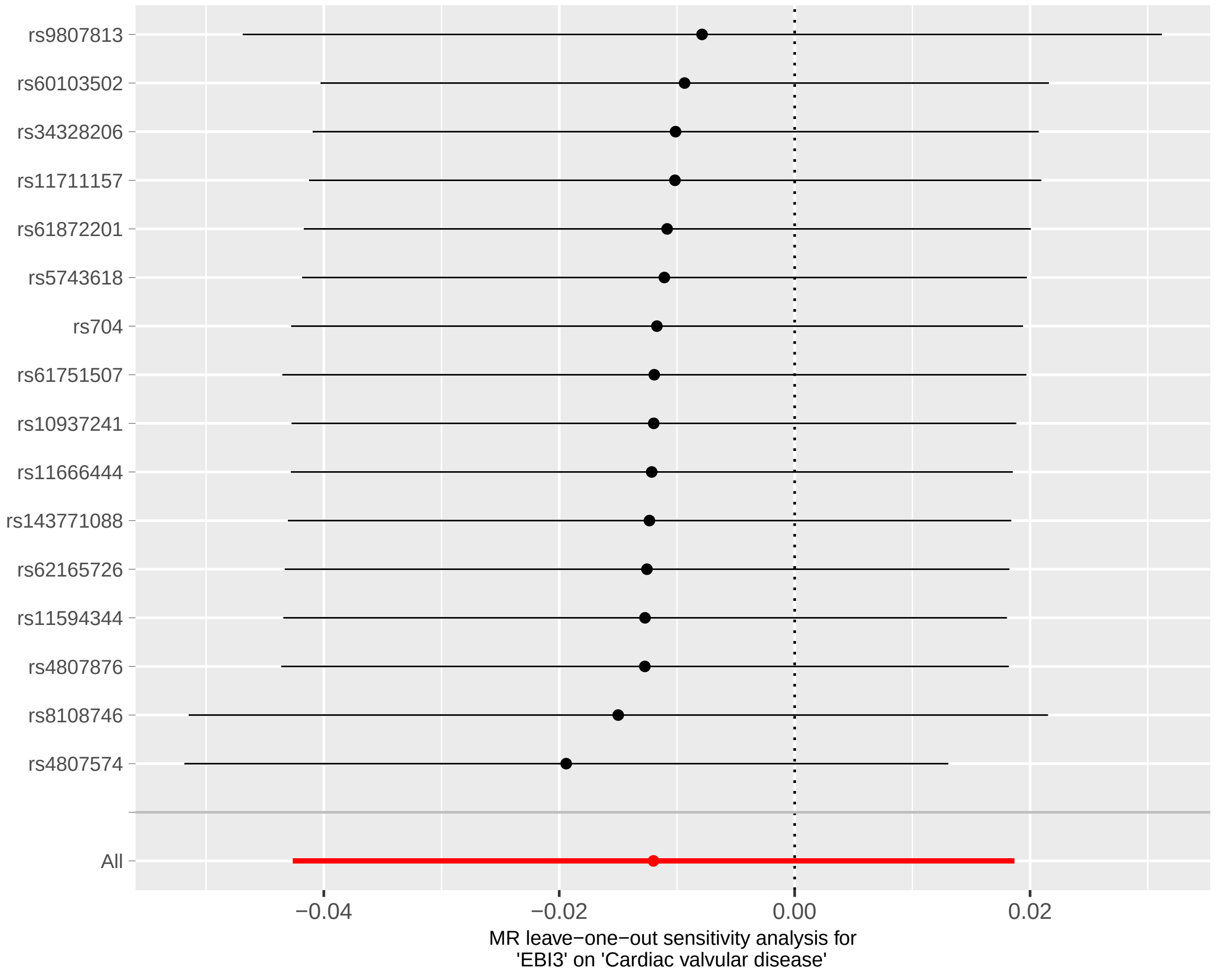

rs9807813
rs60103502
rs34328206
rs11711157
rs61872201
rs5743618
rs704
rs61751507
rs10937241
rs11666444
rs143771088
rs62165726
rs11594344
rs4807876
rs8108746
rs4807574
All
−0.04
−0.02
0.00
0.02
MR leave−one−out sensitivity analysis for
'EBI3' on 'Cardiac valvular disease'

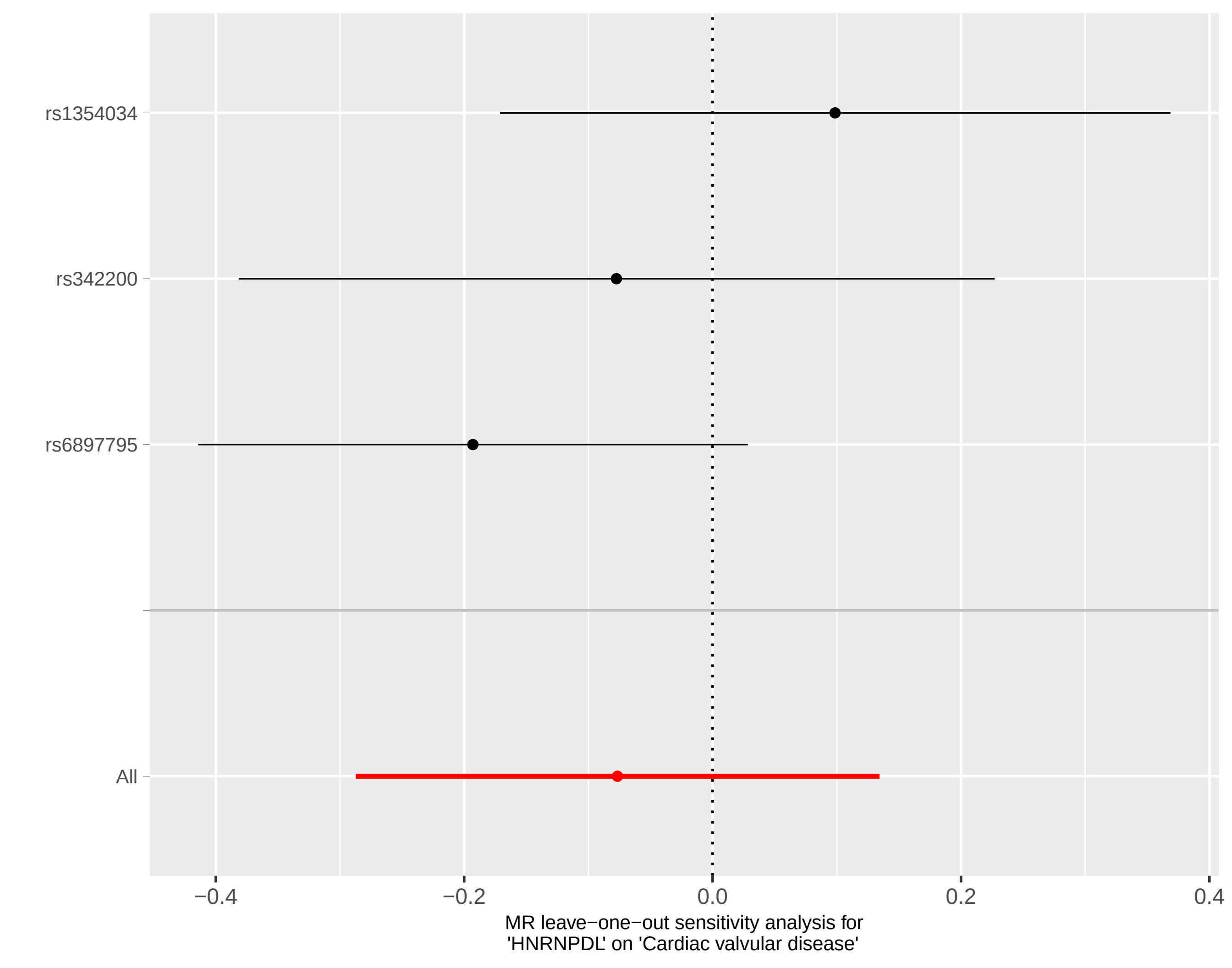
rs1354034
rs342200
rs6897795
All
−0.4
−0.2
0.0
0.2
0.4
MR leave−one−out sensitivity analysis for
'HNRNPDL' on 'Cardiac valvular disease'

Insufficient number of SNPs

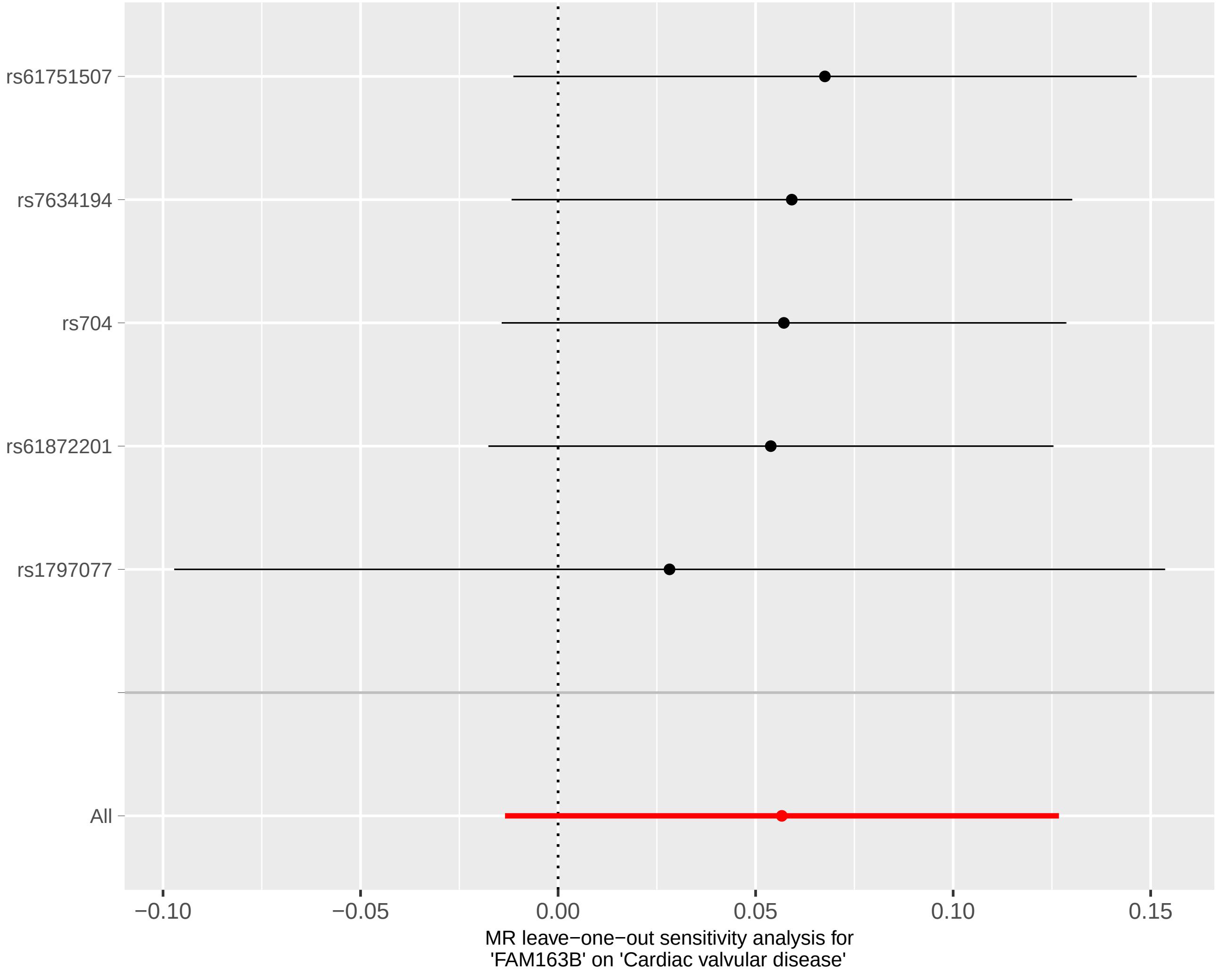

rs61751507
rs7634194
rs704
rs61872201
rs1797077
All
−0.10
−0.05
0.00
0.05
0.10
0.15
MR leave−one−out sensitivity analysis for
'FAM163B' on 'Cardiac valvular disease'

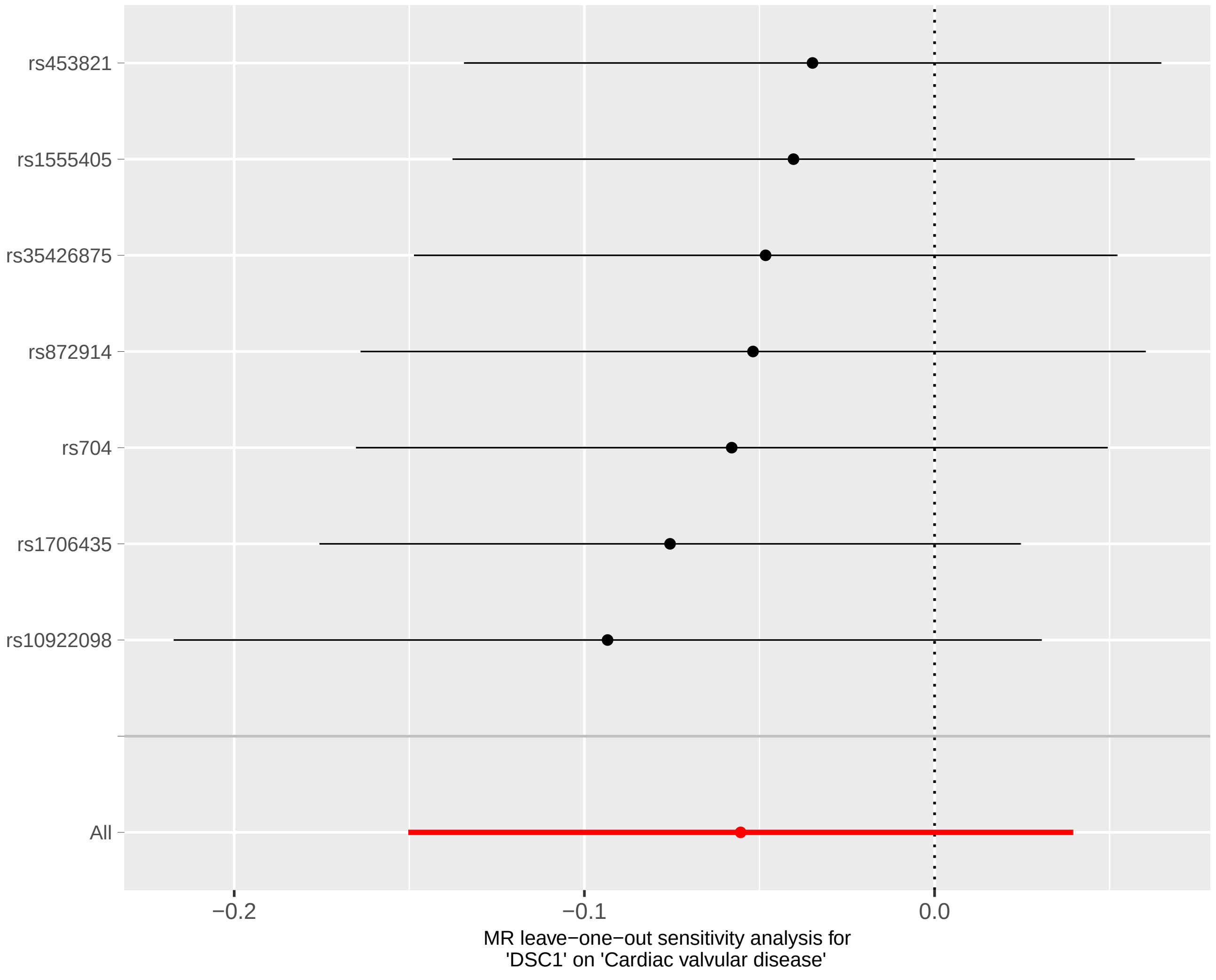
rs453821
rs1555405
rs35426875
rs872914
rs704
rs1706435
rs10922098
All
−0.2
−0.1
0.0
MR leave−one−out sensitivity analysis for
'DSC1' on 'Cardiac valvular disease'

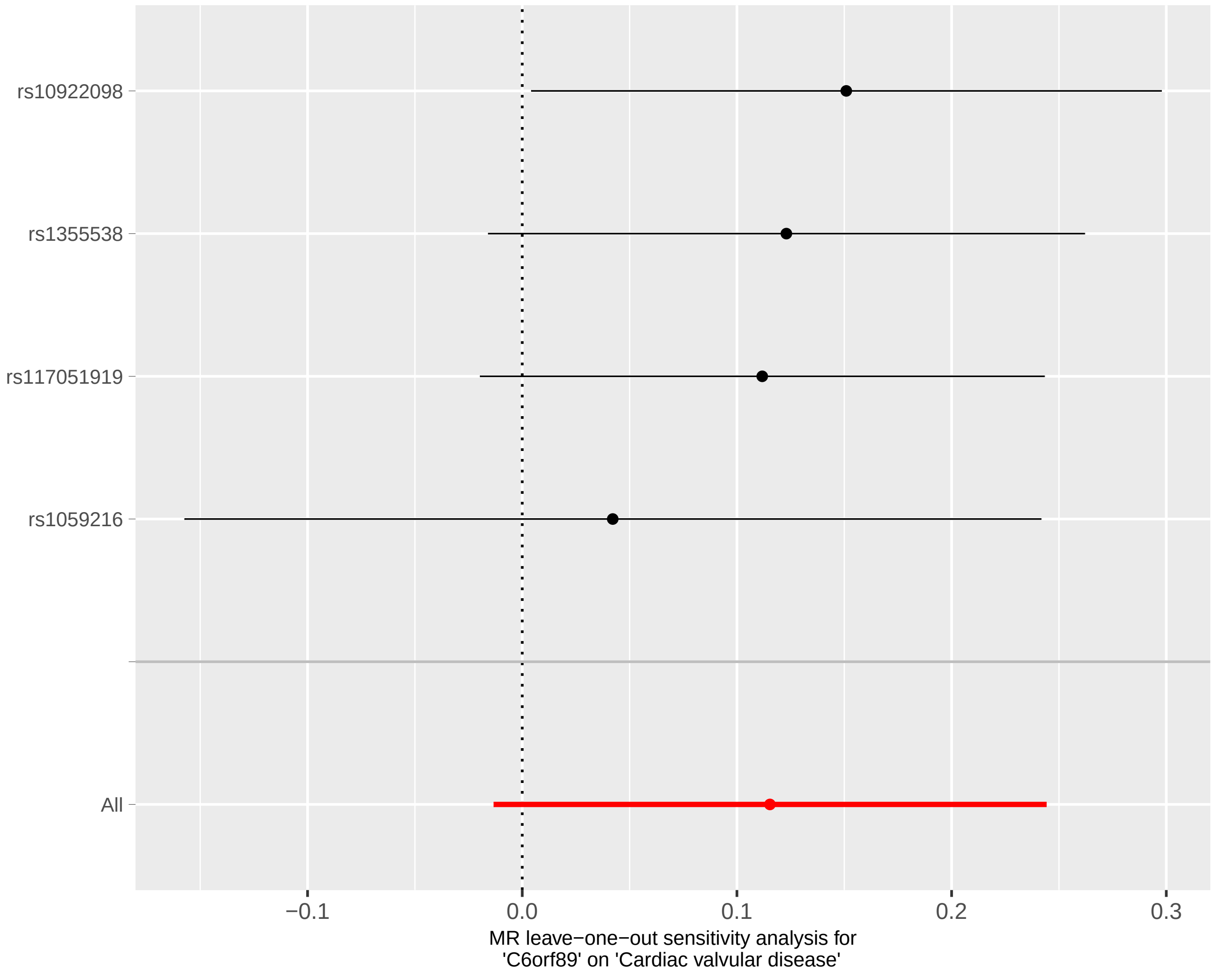
rs10922098
rs1355538
rs117051919
rs1059216
All
−0.1
0.0
0.1
0.2
0.3
MR leave−one−out sensitivity analysis for
'C6orf89' on 'Cardiac valvular disease'

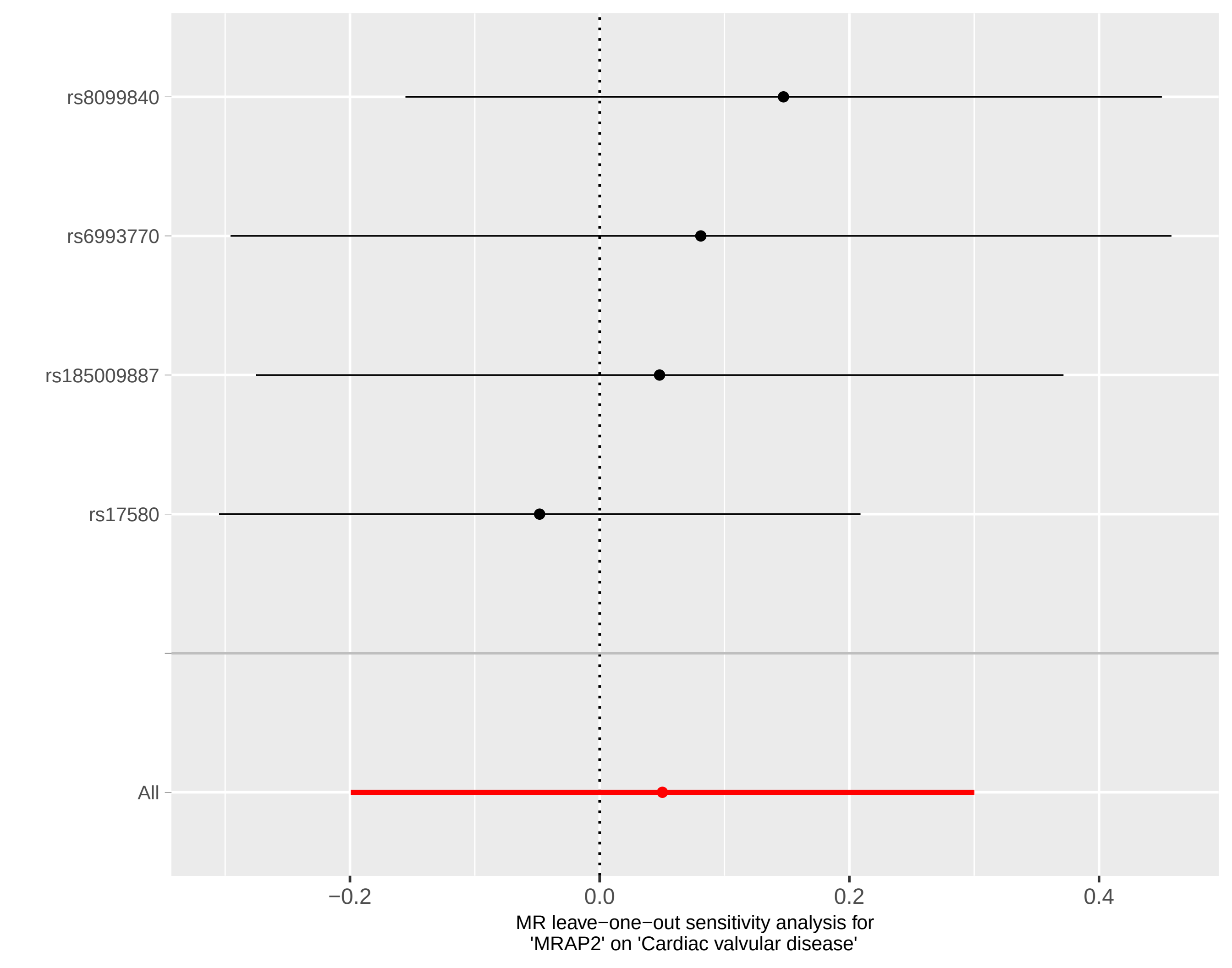
rs8099840
rs6993770
rs185009887
rs17580
All
−0.2
0.0
0.2
0.4
MR leave−one−out sensitivity analysis for
'MRAP2' on 'Cardiac valvular disease'

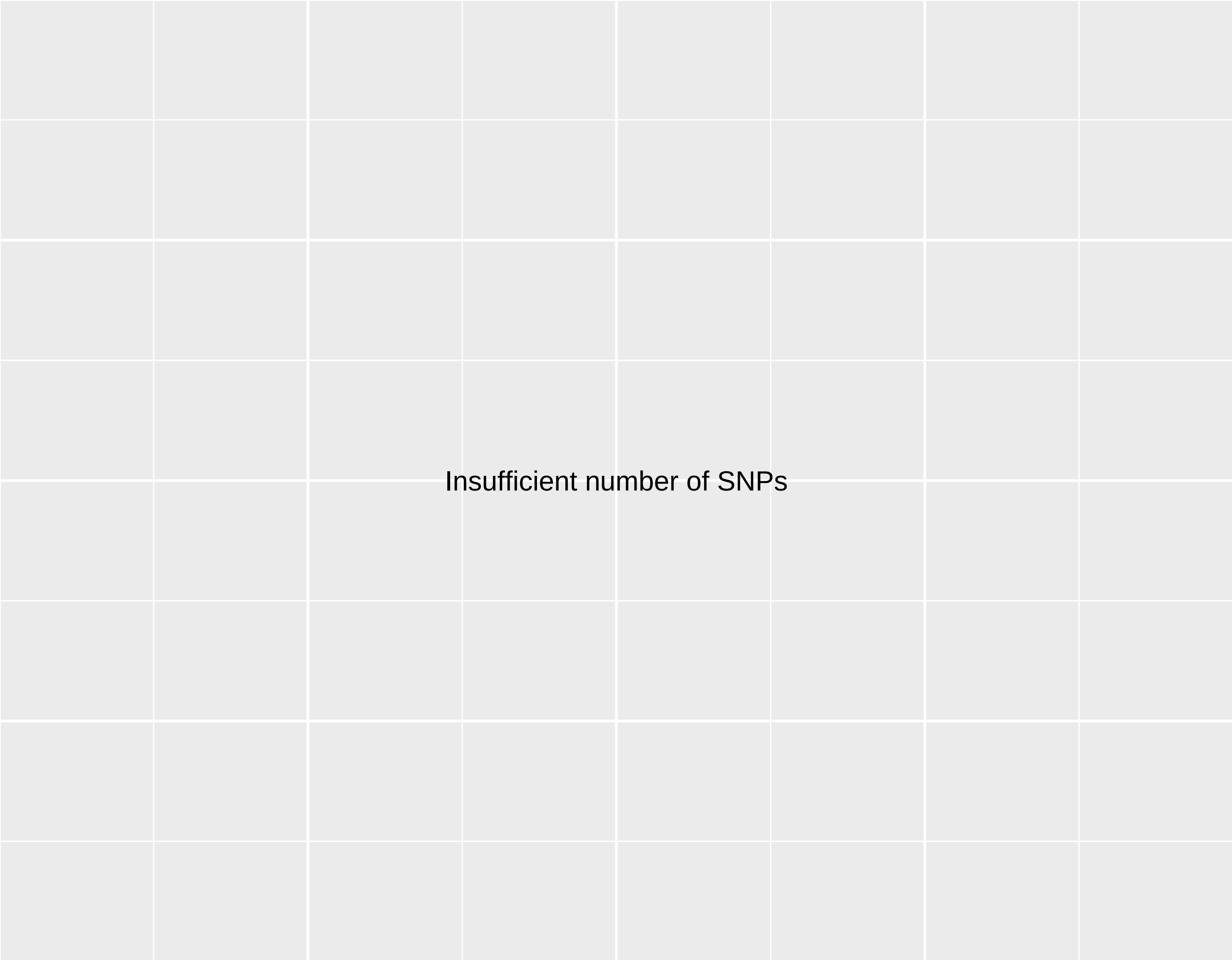
Insufficient number of SNPs

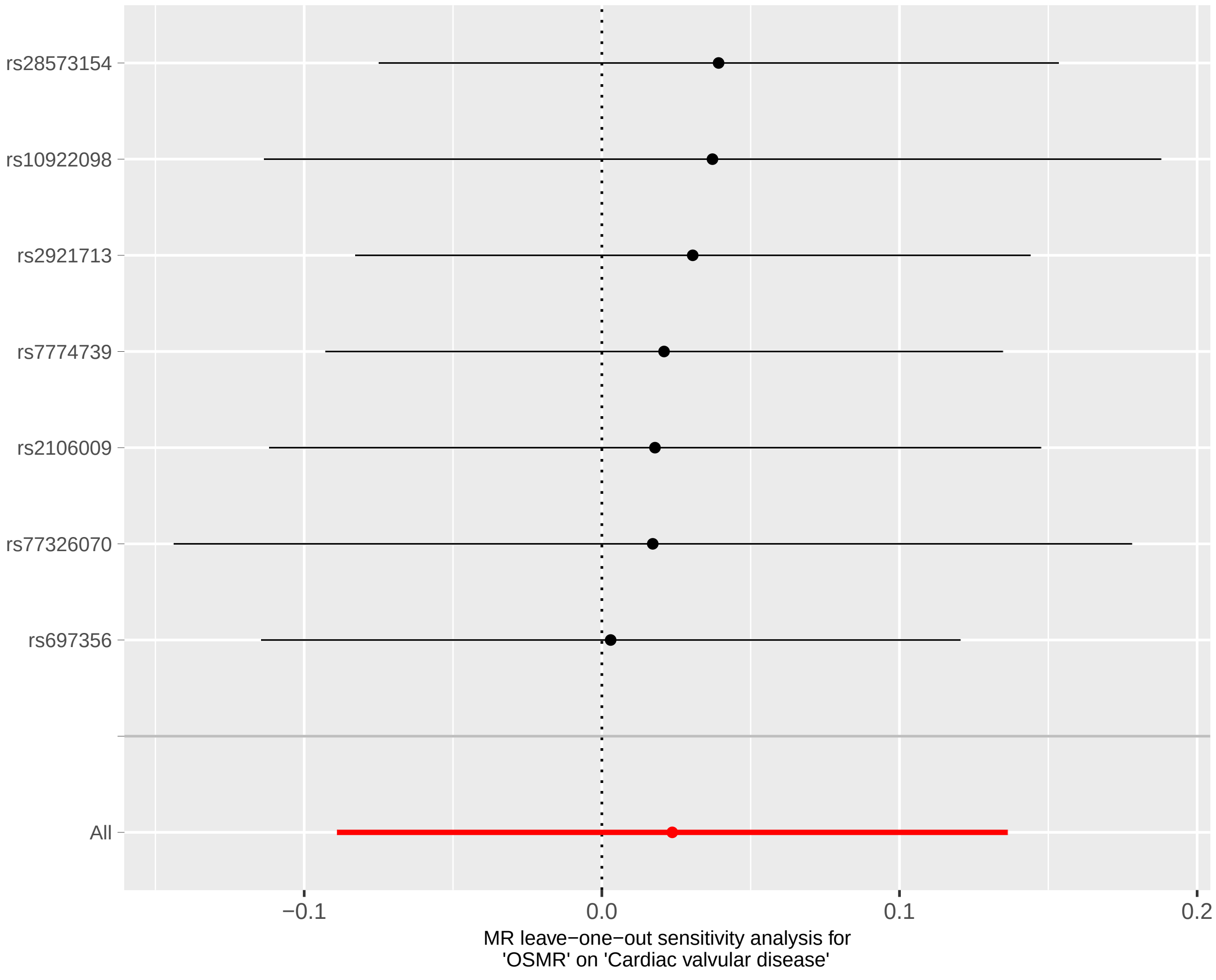

rs28573154
rs10922098
rs2921713
rs7774739
rs2106009
rs77326070
rs697356
All
−0.1
0.0
0.1
0.2
MR leave−one−out sensitivity analysis for
'OSMR' on 'Cardiac valvular disease'

Insufficient number of SNPs

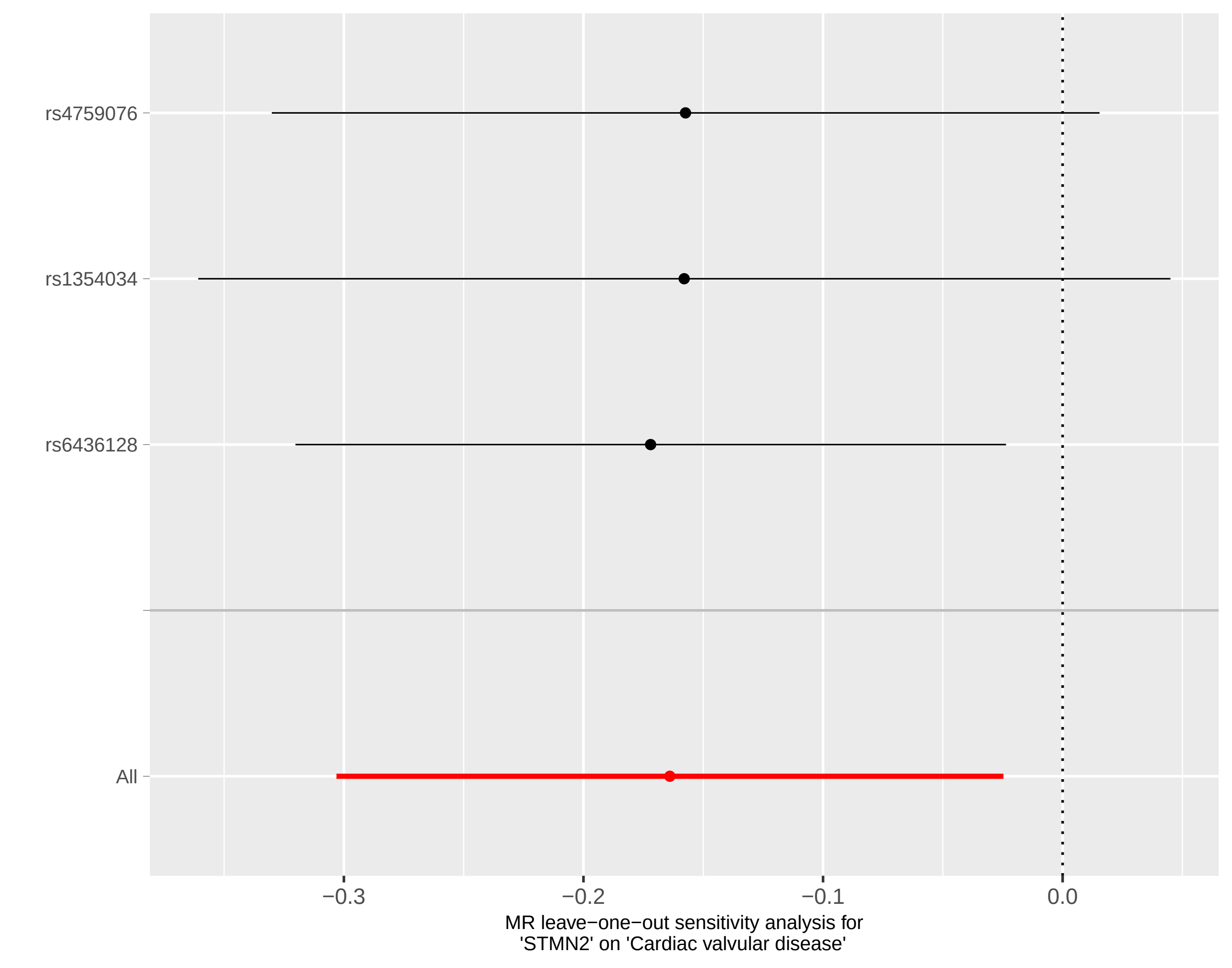

rs4759076
rs1354034
rs6436128
All
−0.3
−0.2
−0.1
0.0
MR leave−one−out sensitivity analysis for
'STMN2' on 'Cardiac valvular disease'

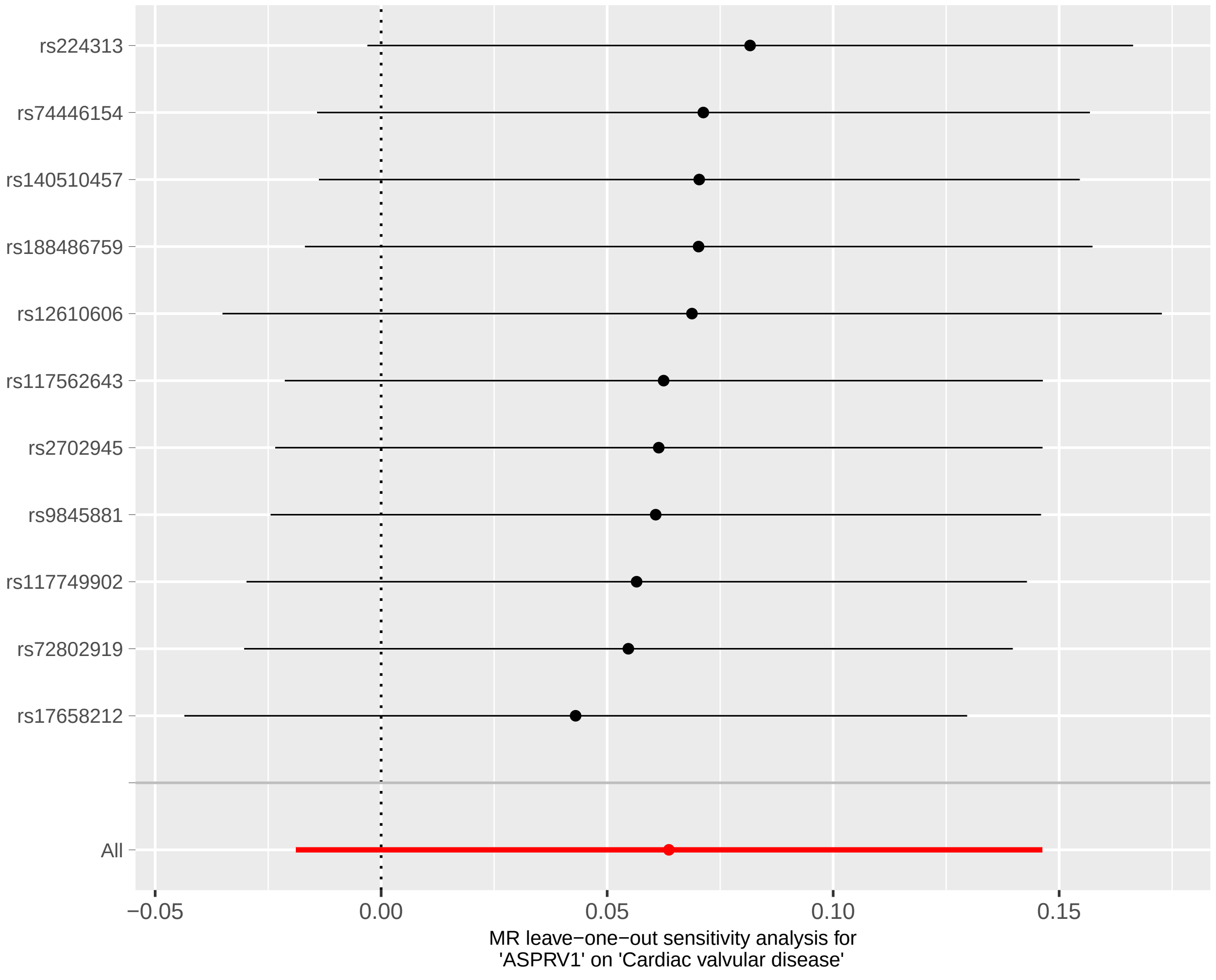
rs224313
rs74446154
rs140510457
rs188486759
rs12610606
rs117562643
rs2702945
rs9845881
rs117749902
rs72802919
rs17658212
All
−0.05
0.00
0.05
0.10
0.15
MR leave−one−out sensitivity analysis for
'ASPRV1' on 'Cardiac valvular disease'

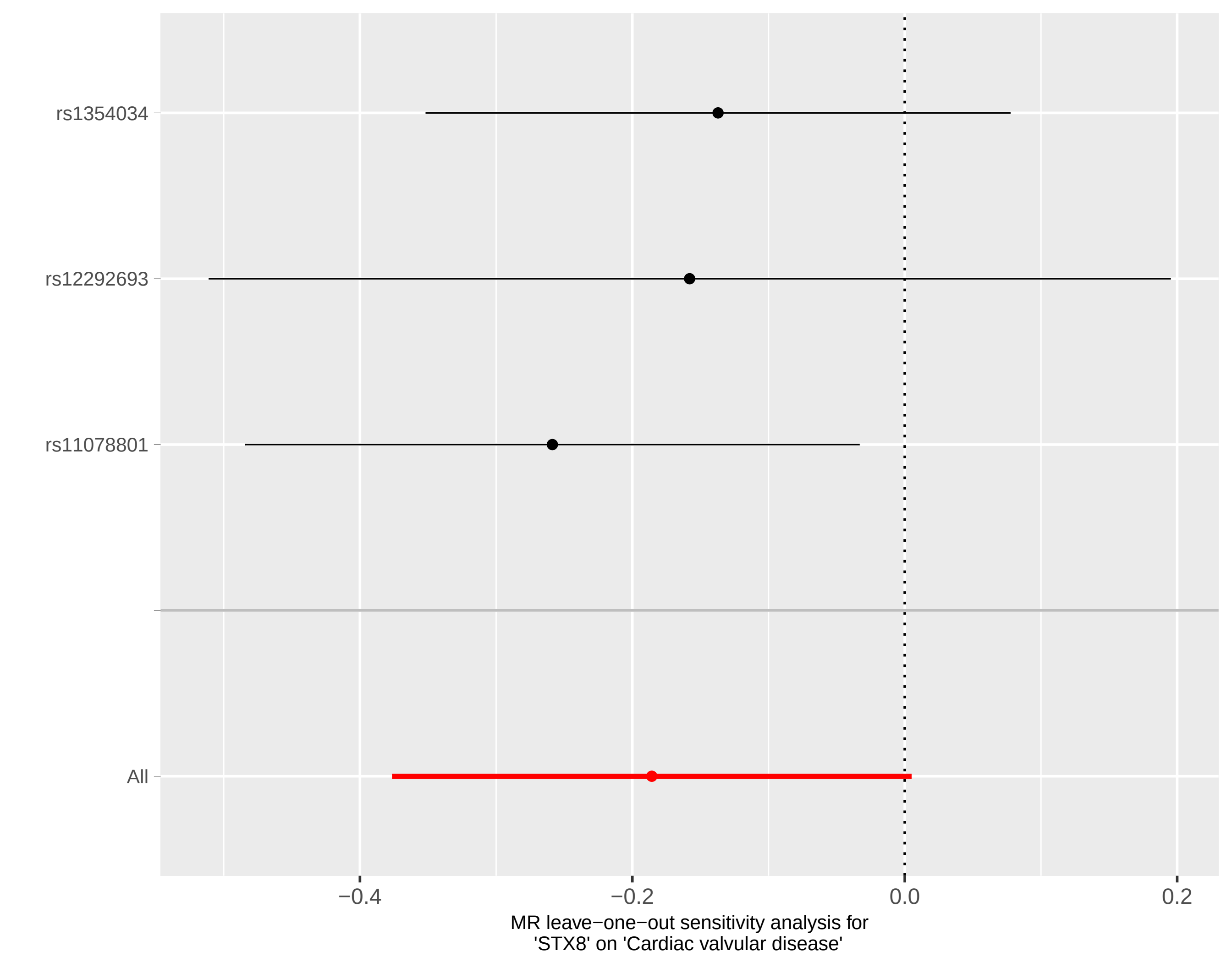
rs1354034
rs12292693
rs11078801
All
−0.4
−0.2
0.0
0.2
MR leave−one−out sensitivity analysis for
'STX8' on 'Cardiac valvular disease'

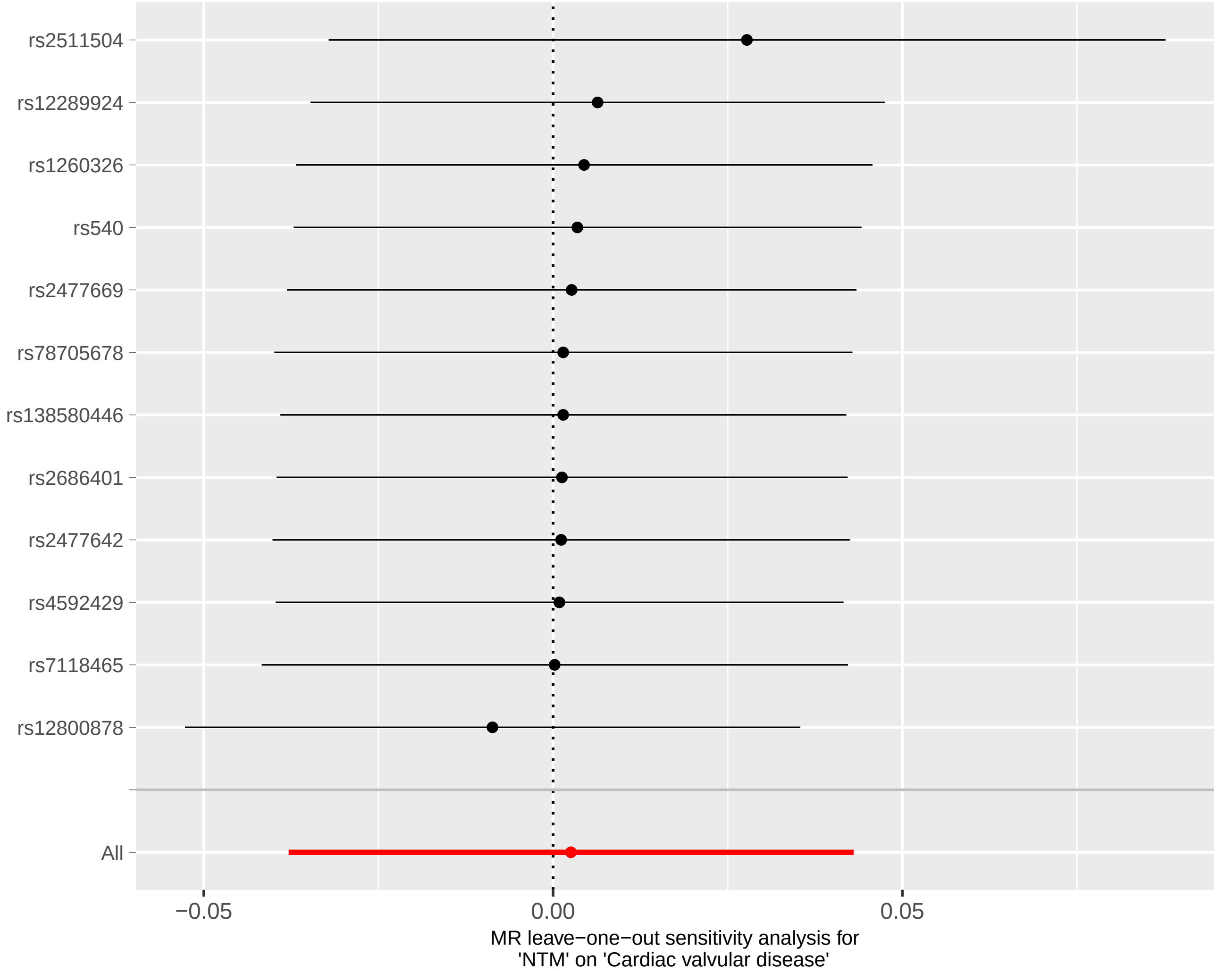
rs2511504
rs12289924
rs1260326
rs540
rs2477669
rs78705678
rs138580446
rs2686401
rs2477642
rs4592429
rs7118465
rs12800878
All
−0.05
0.00
0.05
MR leave−one−out sensitivity analysis for
'NTM' on 'Cardiac valvular disease'

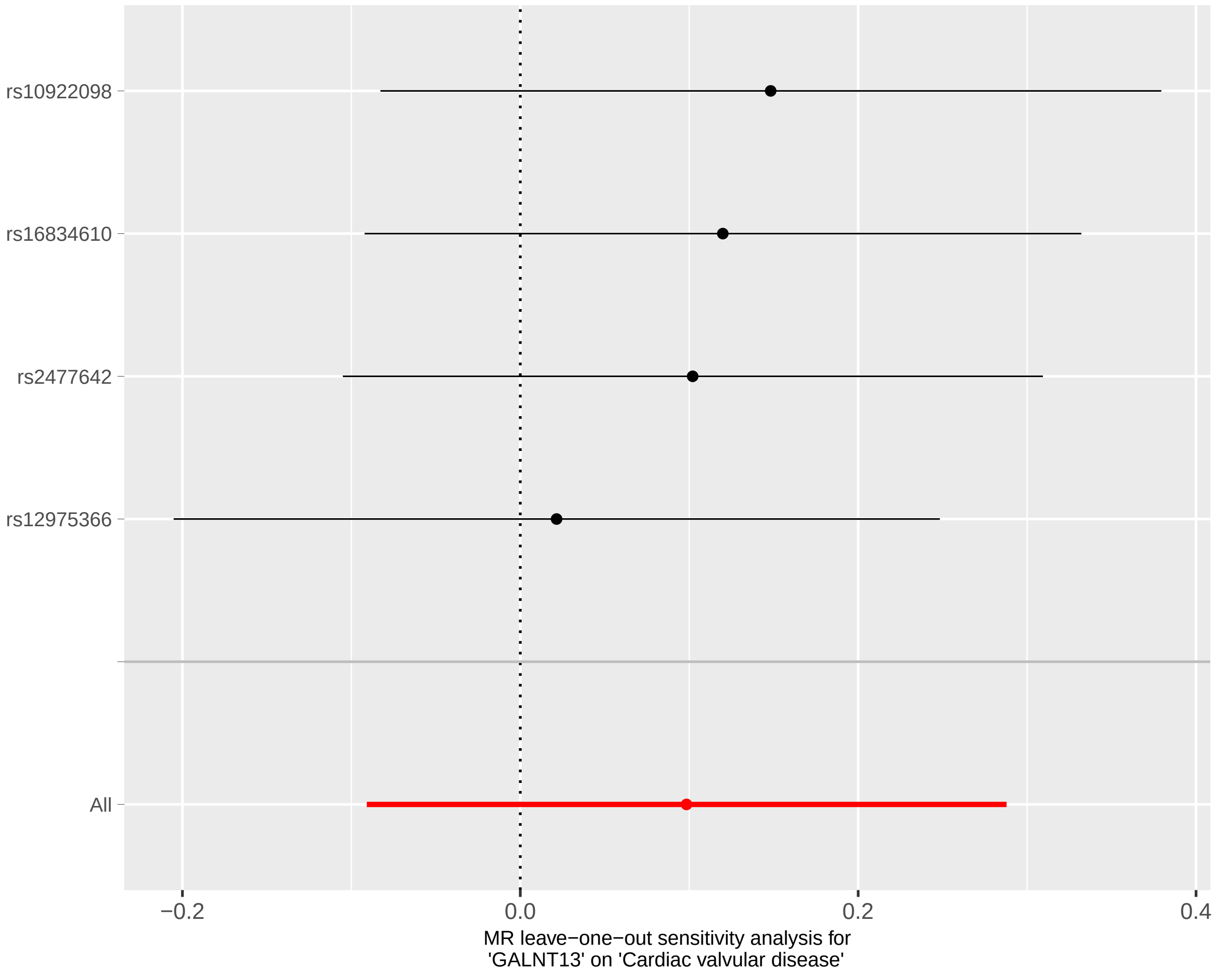

rs10922098
rs16834610
rs2477642
rs12975366
All
−0.2
0.0
0.2
0.4
MR leave−one−out sensitivity analysis for
'GALNT13' on 'Cardiac valvular disease'

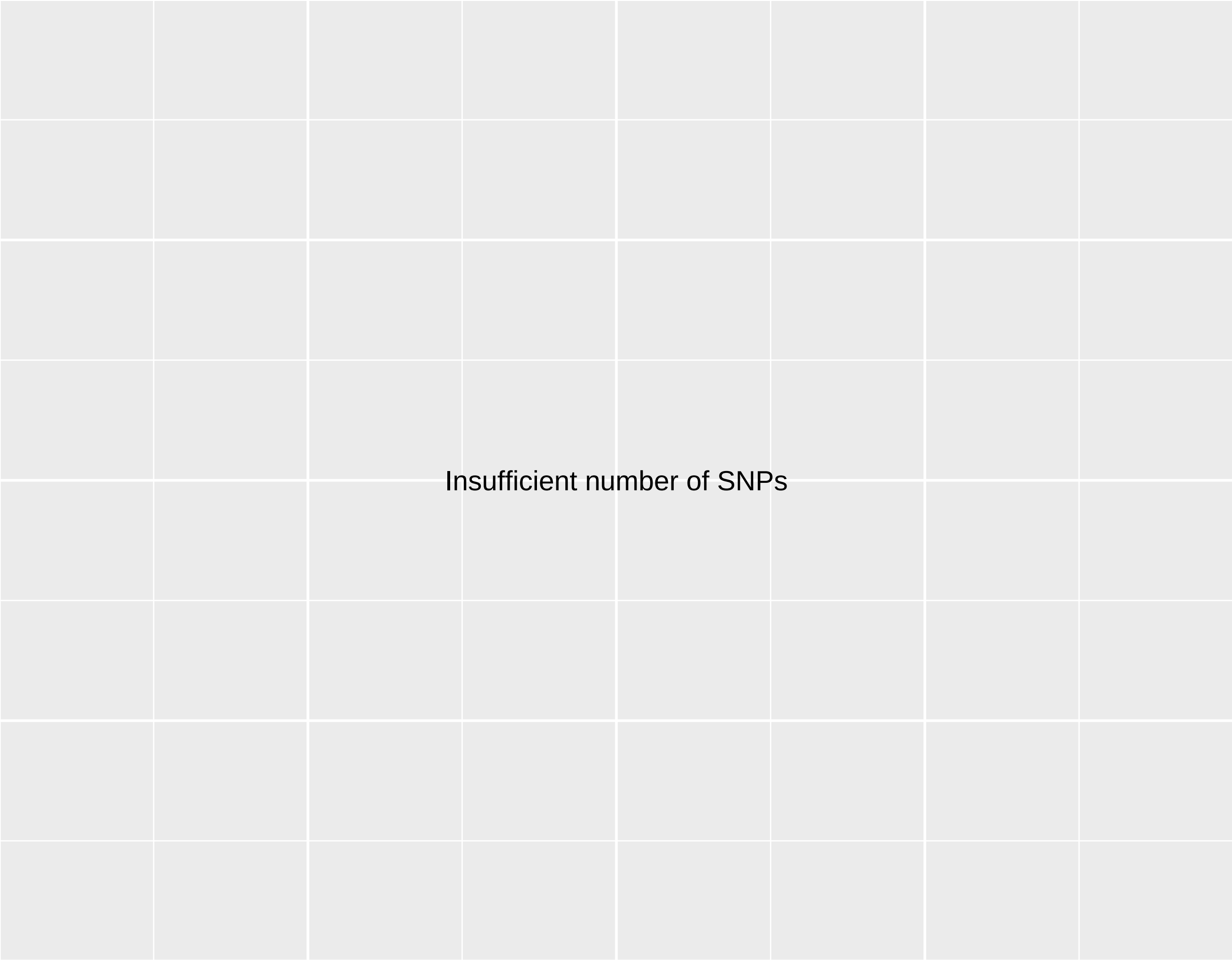
Insufficient number of SNPs

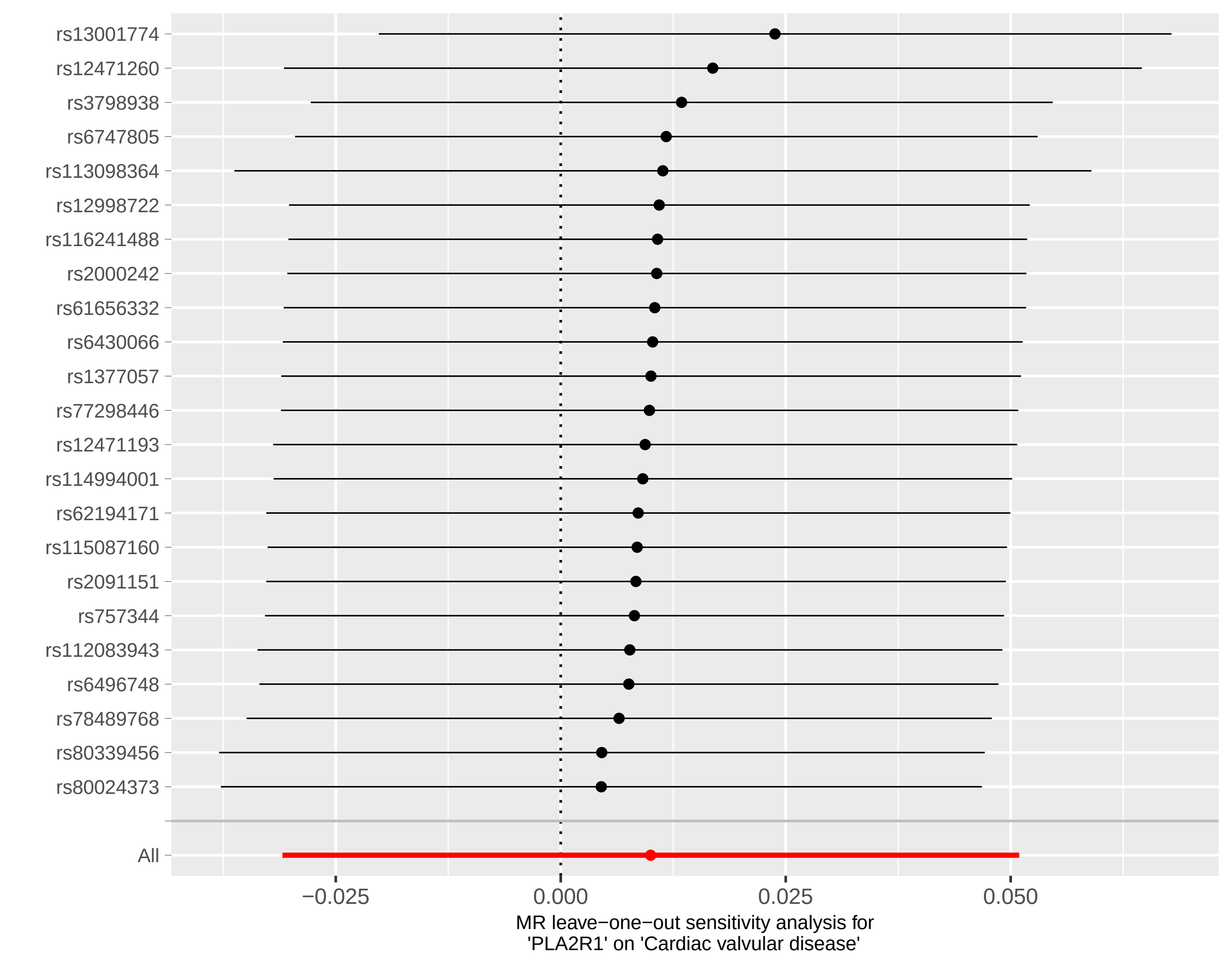
rs13001774
rs12471260
rs3798938
rs6747805
rs113098364
rs12998722
rs116241488
rs2000242
rs61656332
rs6430066
rs1377057
rs77298446
rs12471193
rs114994001
rs62194171
rs115087160
rs2091151
rs757344
rs112083943
rs6496748
rs78489768
rs80339456
rs80024373
All
−0.025
0.000
0.025
0.050
MR leave−one−out sensitivity analysis for
'PLA2R1' on 'Cardiac valvular disease'

Insufficient number of SNPs

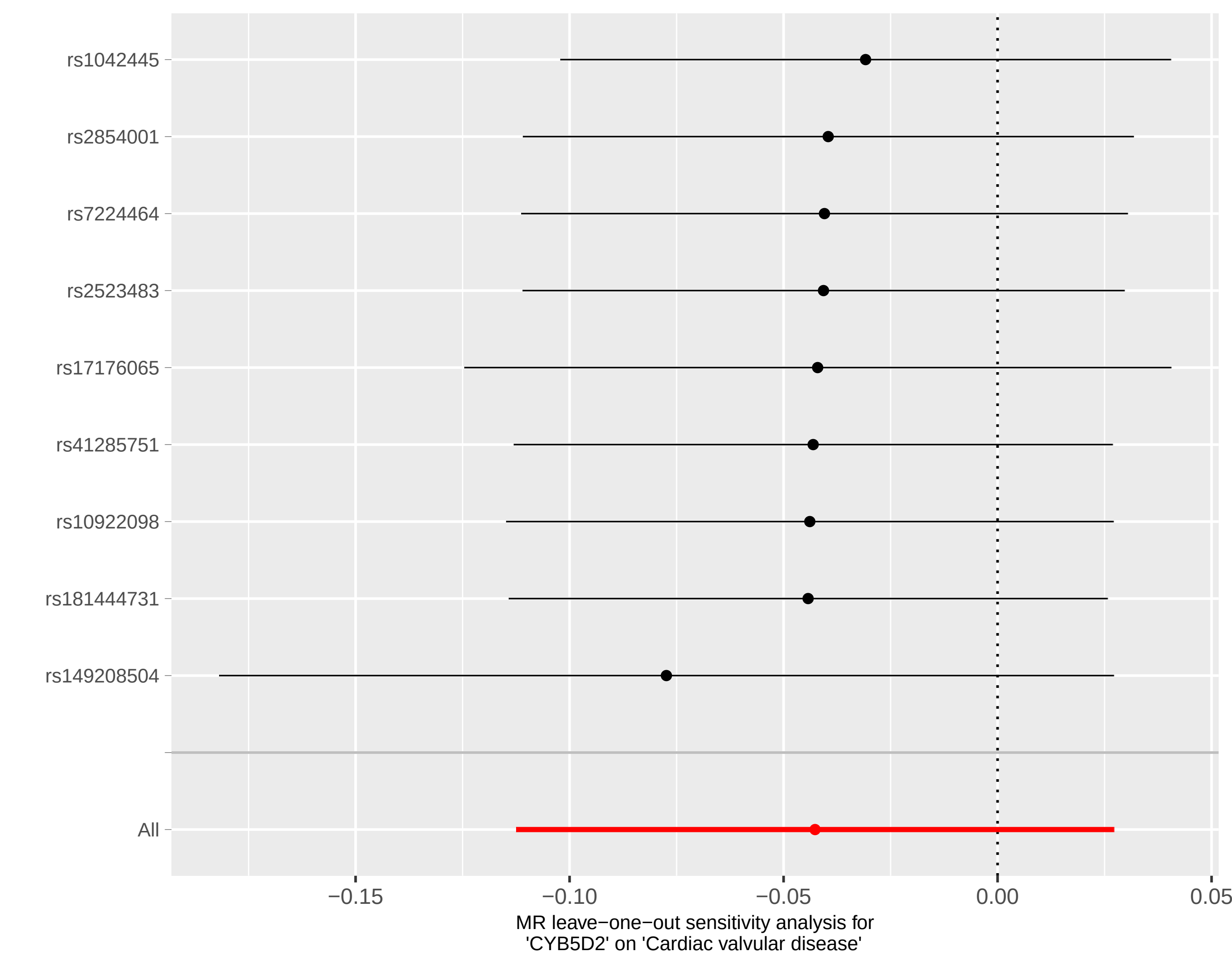
rs1042445
rs2854001
rs7224464
rs2523483
rs17176065
rs41285751
rs10922098
rs181444731
rs149208504
All
−0.15
−0.10
−0.05
0.00
0.05
MR leave−one−out sensitivity analysis for
'CYB5D2' on 'Cardiac valvular disease'

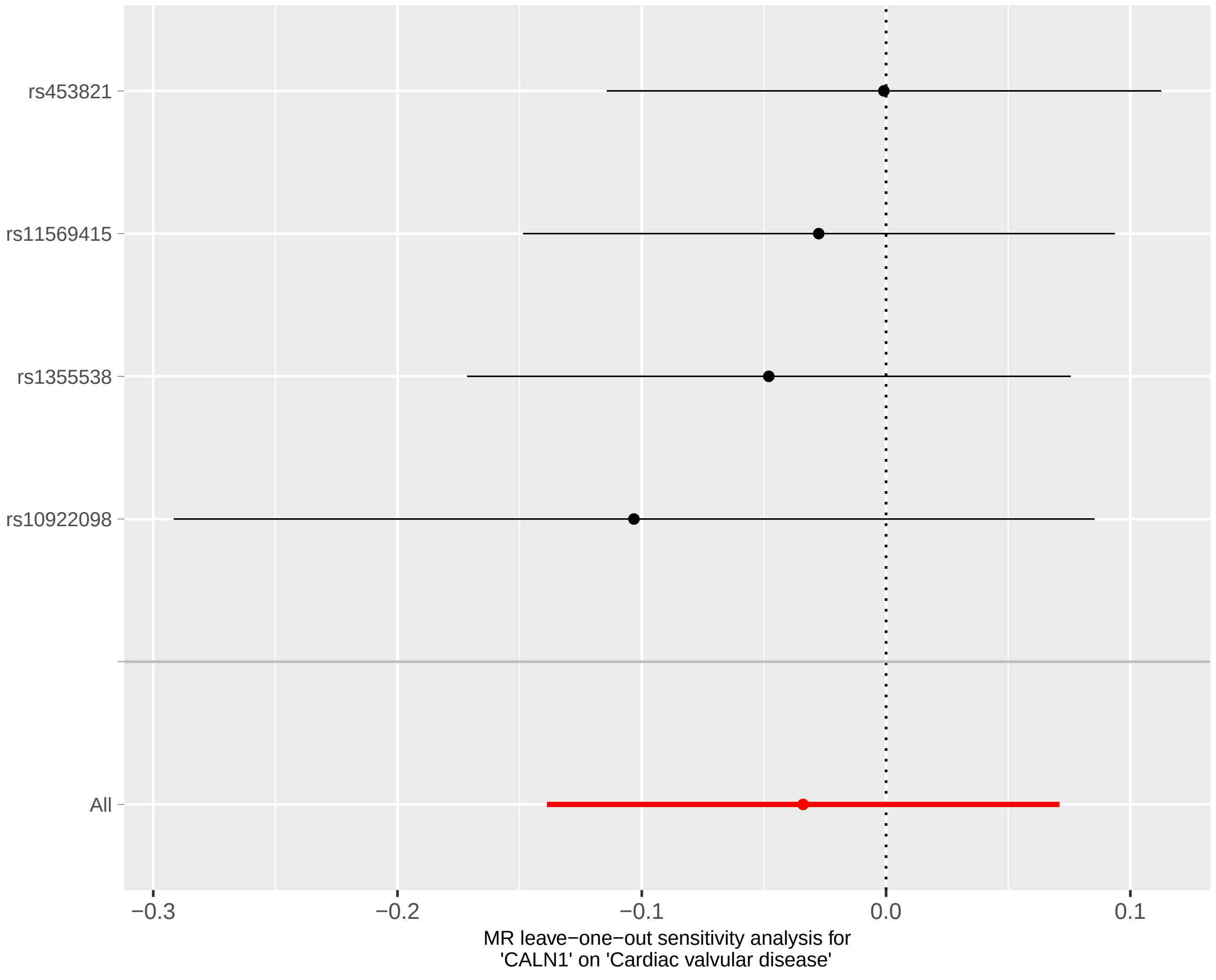
rs453821
rs11569415
rs1355538
rs10922098
All
−0.3
−0.2
−0.1
0.0
0.1
MR leave−one−out sensitivity analysis for
'CALN1' on 'Cardiac valvular disease'

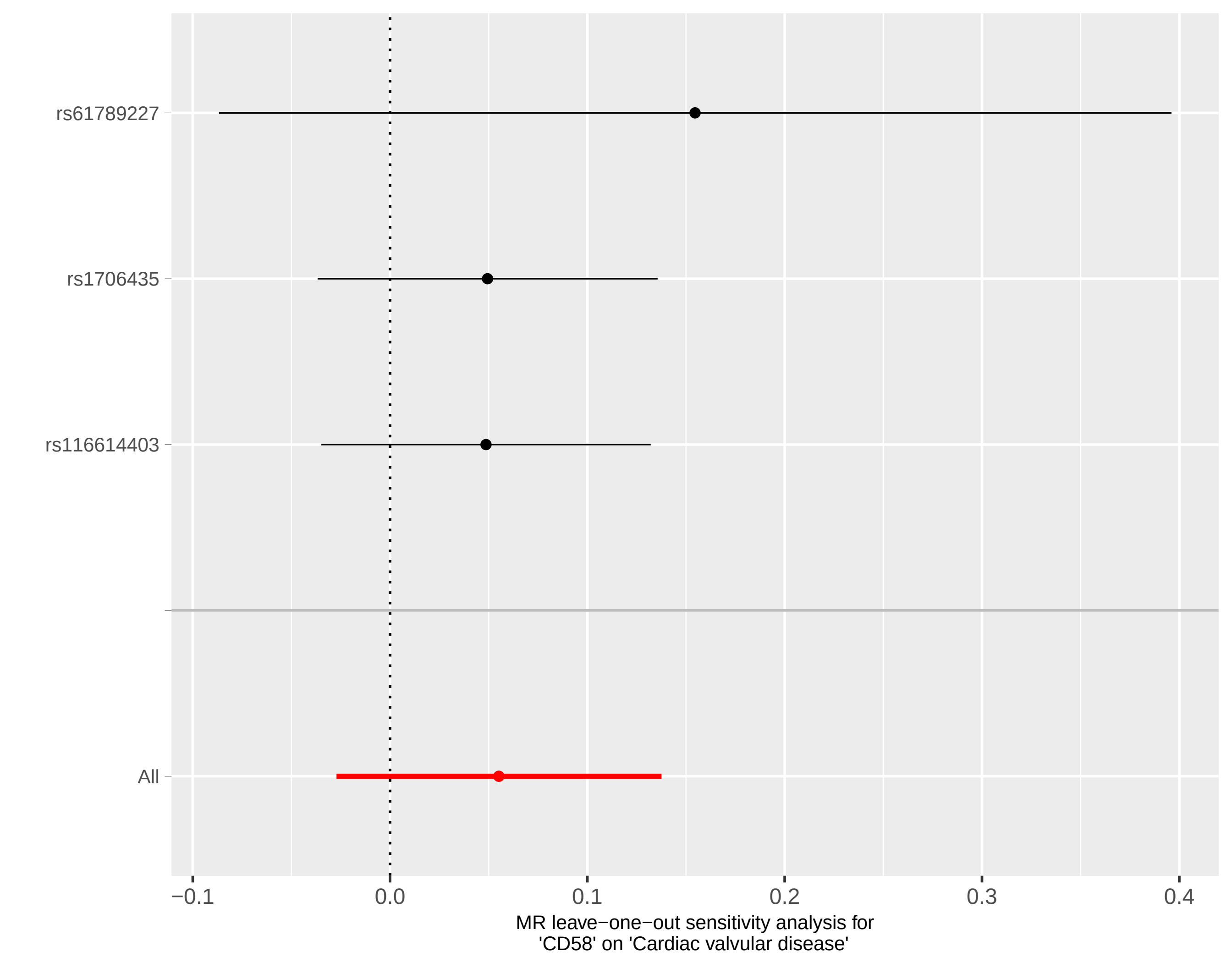

rs61789227
rs1706435
rs116614403
All
−0.1
0.0
0.1
0.2
0.3
0.4
MR leave−one−out sensitivity analysis for
'CD58' on 'Cardiac valvular disease'

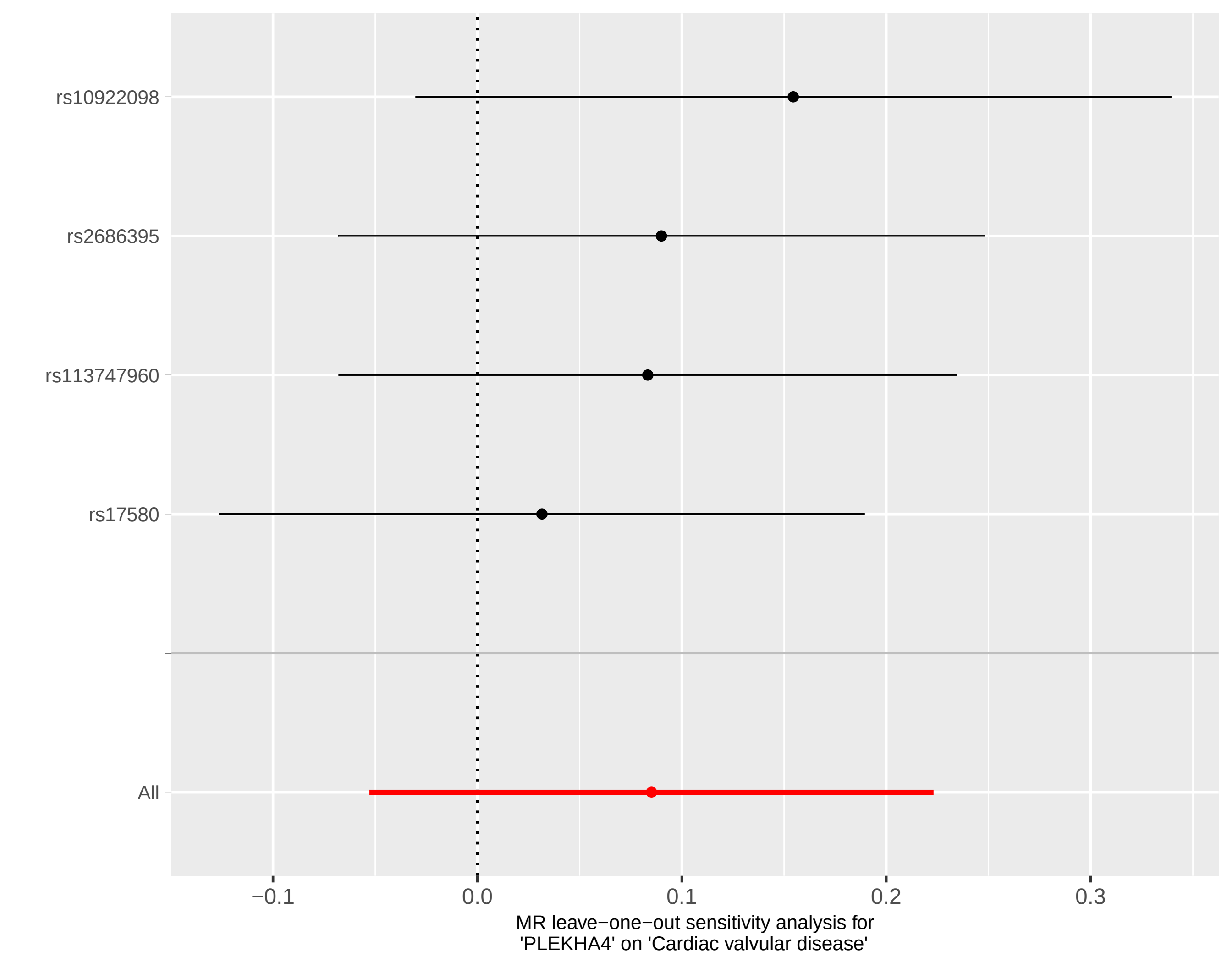
rs10922098
rs2686395
rs113747960
rs17580
All
−0.1
0.0
0.1
0.2
0.3
MR leave−one−out sensitivity analysis for
'PLEKHA4' on 'Cardiac valvular disease'

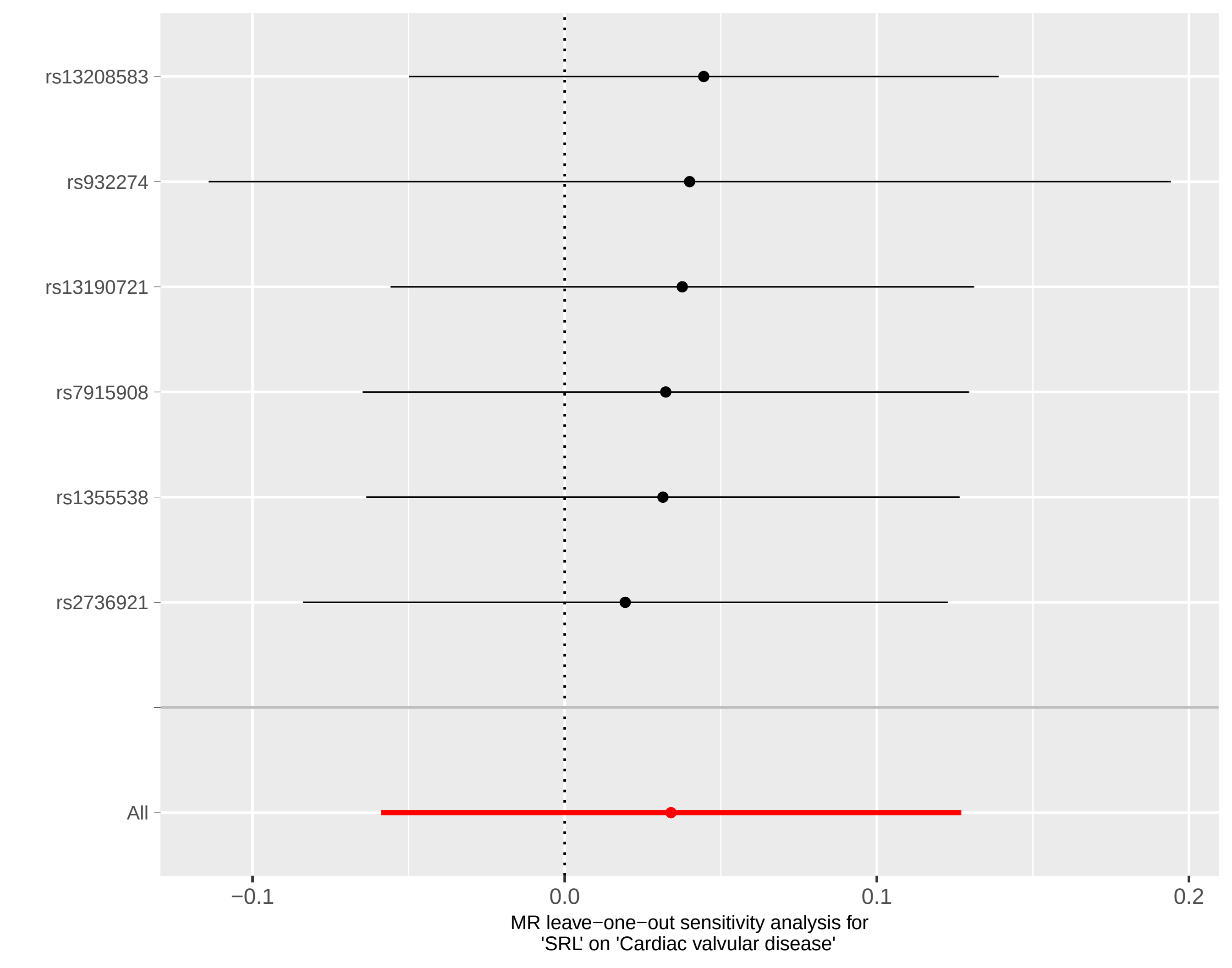

rs13208583
rs932274
rs13190721
rs7915908
rs1355538
rs2736921
All
−0.1
0.0
0.1
0.2
MR leave−one−out sensitivity analysis for
'SRL' on 'Cardiac valvular disease'

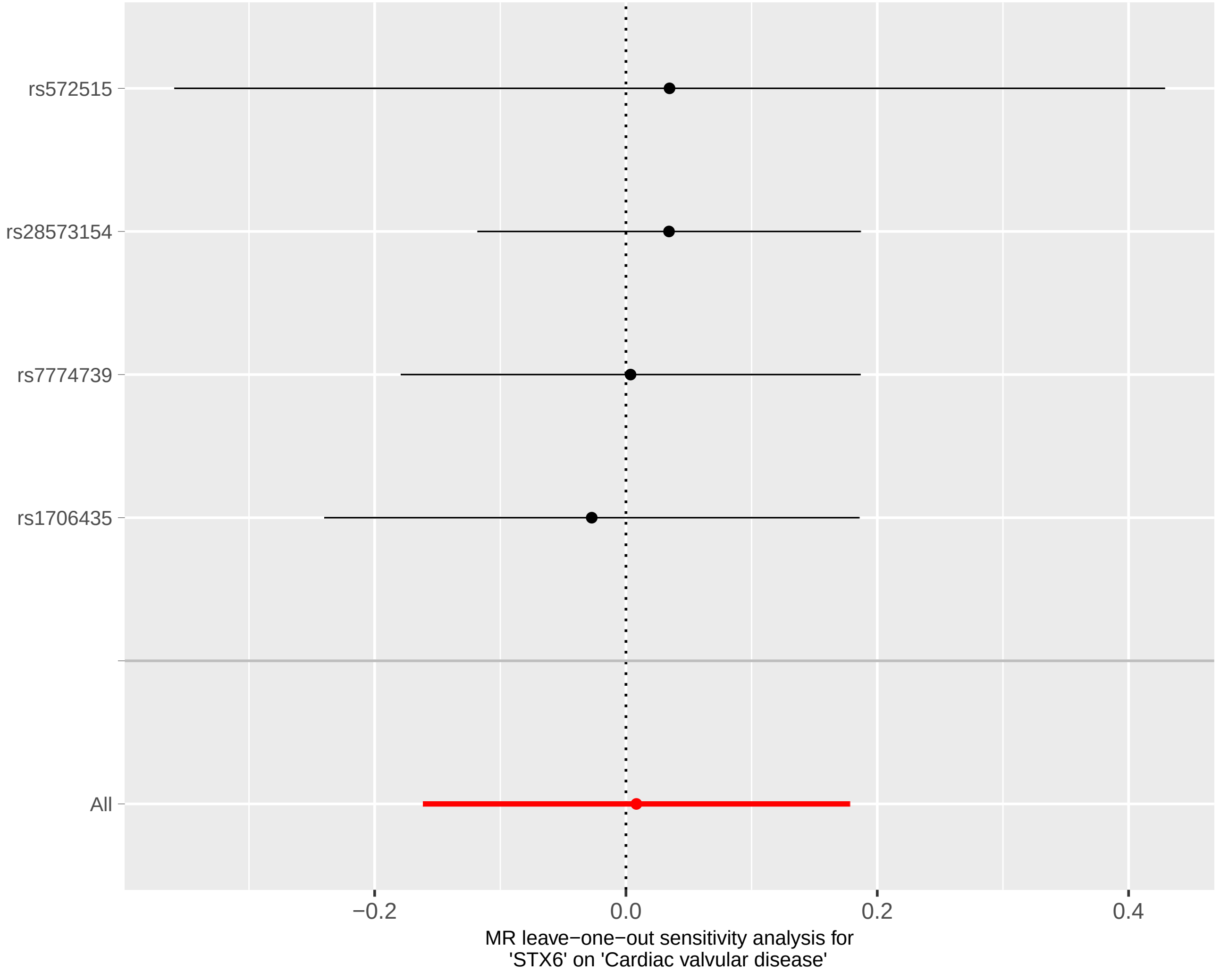
rs572515
rs28573154
rs7774739
rs1706435
All
−0.2
0.0
0.2
0.4
MR leave−one−out sensitivity analysis for
'STX6' on 'Cardiac valvular disease'

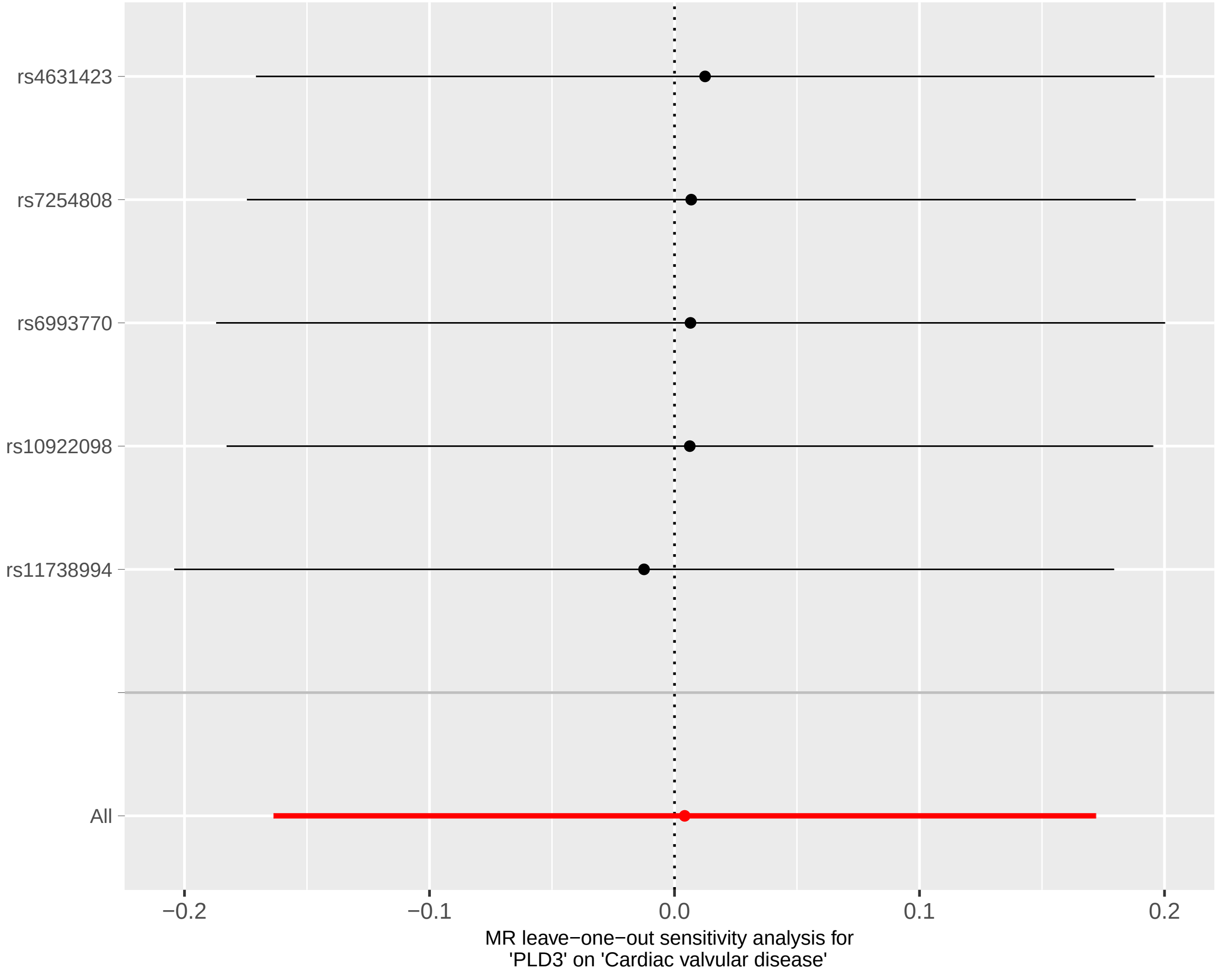
rs4631423
rs7254808
rs6993770
rs10922098
rs11738994
All
−0.2
−0.1
0.0
0.1
0.2
MR leave−one−out sensitivity analysis for
'PLD3' on 'Cardiac valvular disease'

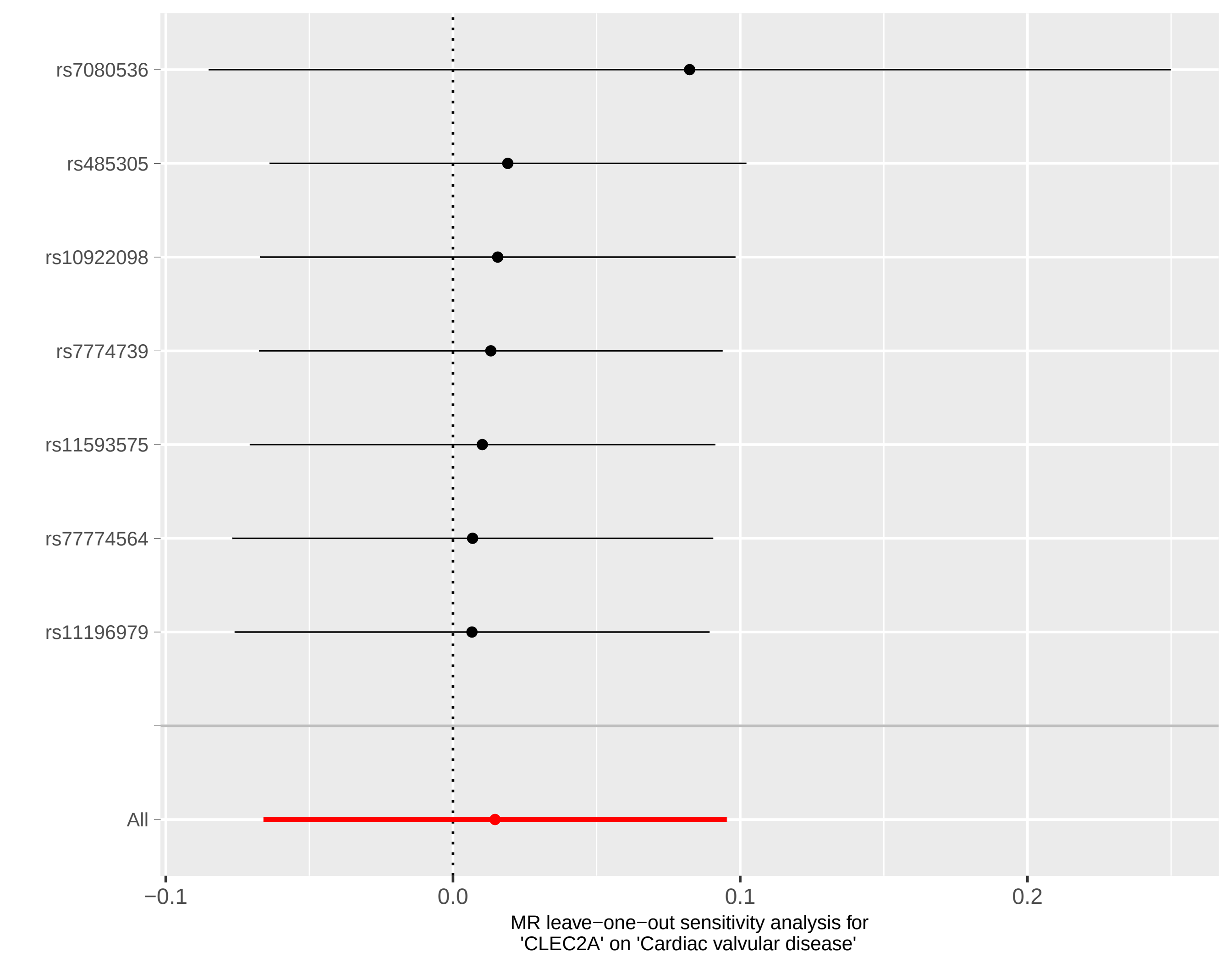

rs7080536
rs485305
rs10922098
rs7774739
rs11593575
rs77774564
rs11196979
All
−0.1
0.0
0.1
0.2
MR leave−one−out sensitivity analysis for
'CLEC2A' on 'Cardiac valvular disease'

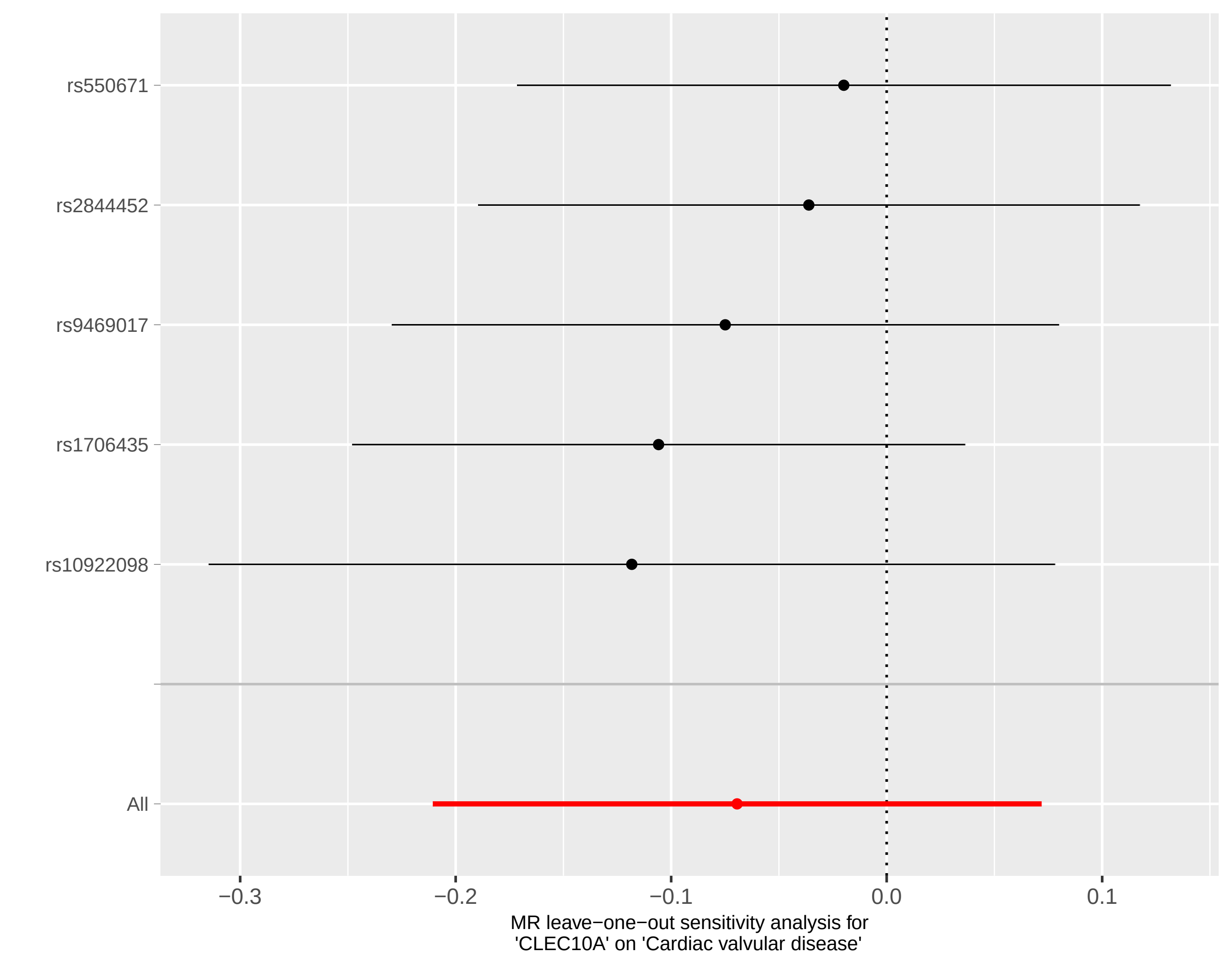
rs550671
rs2844452
rs9469017
rs1706435
rs10922098
All
−0.3
−0.2
−0.1
0.0
0.1
MR leave−one−out sensitivity analysis for
'CLEC10A' on 'Cardiac valvular disease'

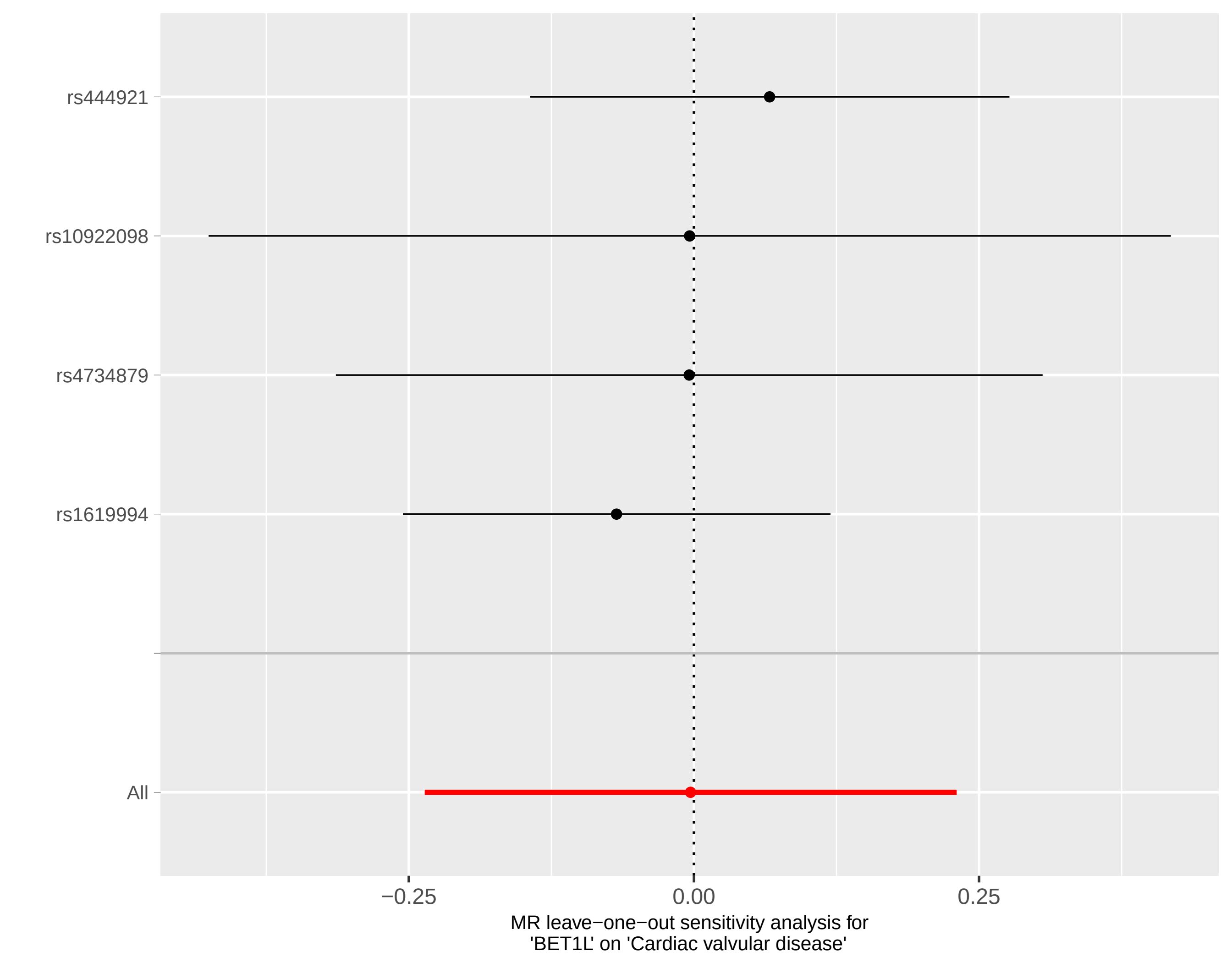
rs444921
rs10922098
rs4734879
rs1619994
All
−0.25
0.00
0.25
MR leave−one−out sensitivity analysis for
'BET1L' on 'Cardiac valvular disease'

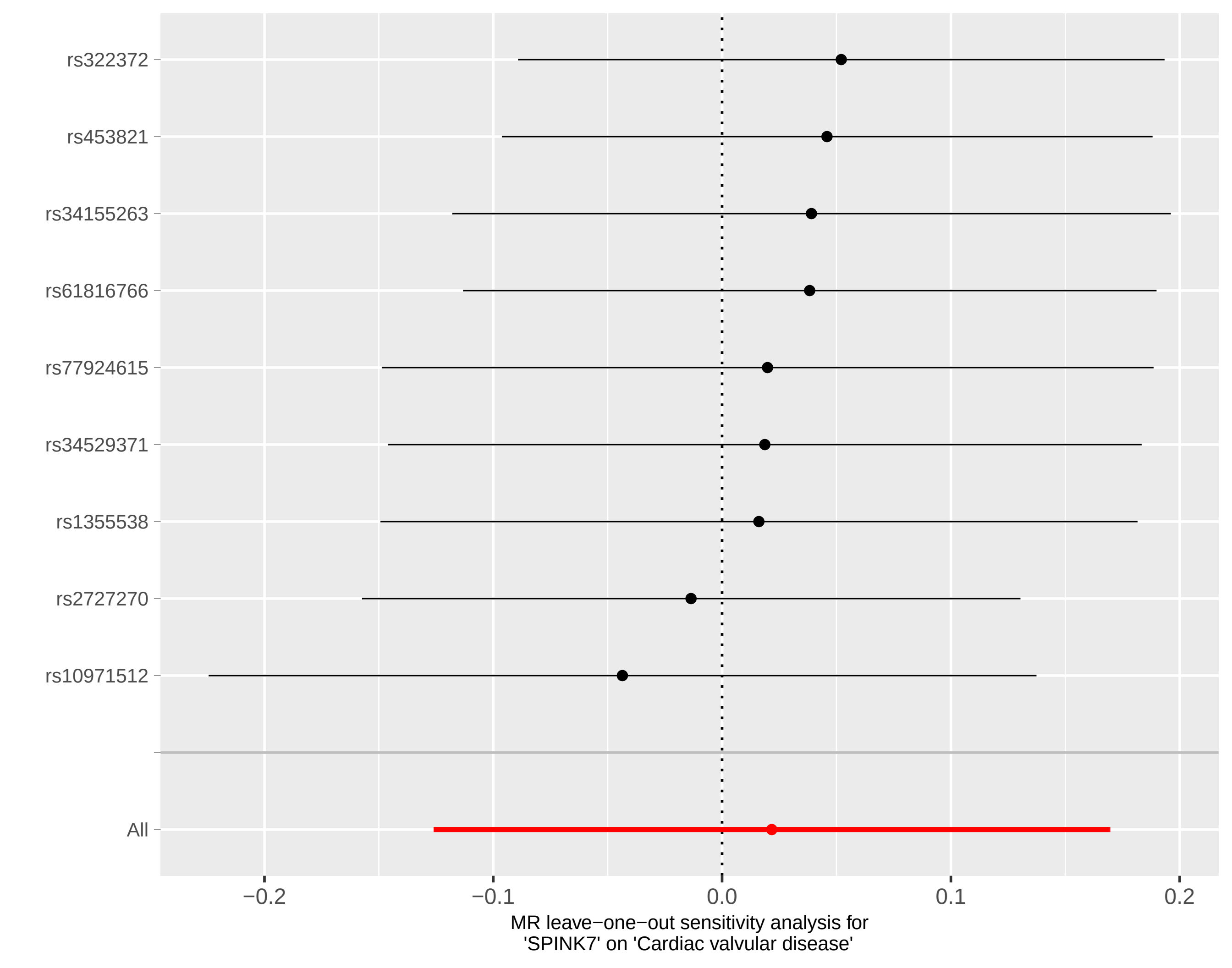
rs322372
rs453821
rs34155263
rs61816766
rs77924615
rs34529371
rs1355538
rs2727270
rs10971512
All
−0.2
−0.1
0.0
0.1
0.2
MR leave−one−out sensitivity analysis for
'SPINK7' on 'Cardiac valvular disease'

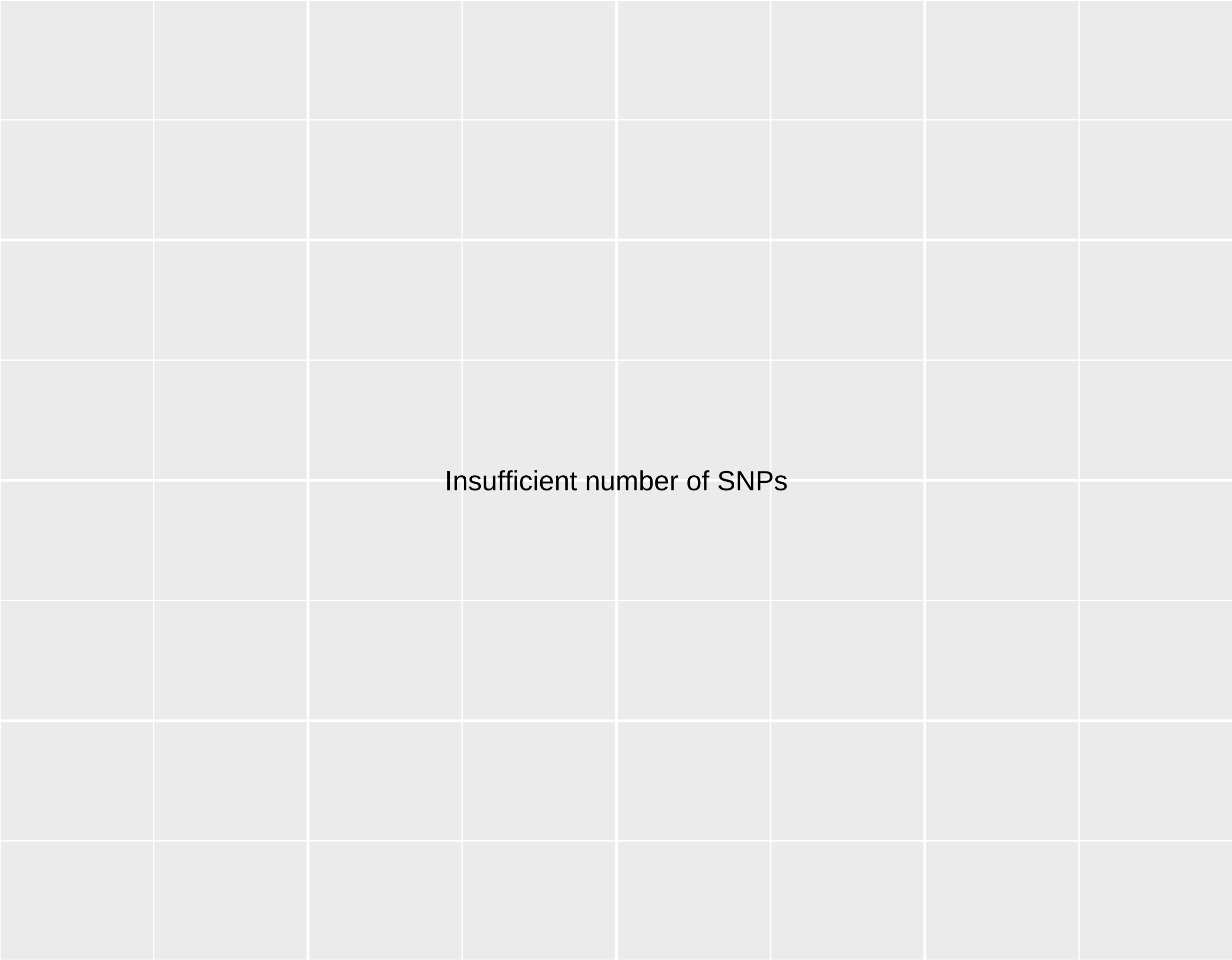
Insufficient number of SNPs

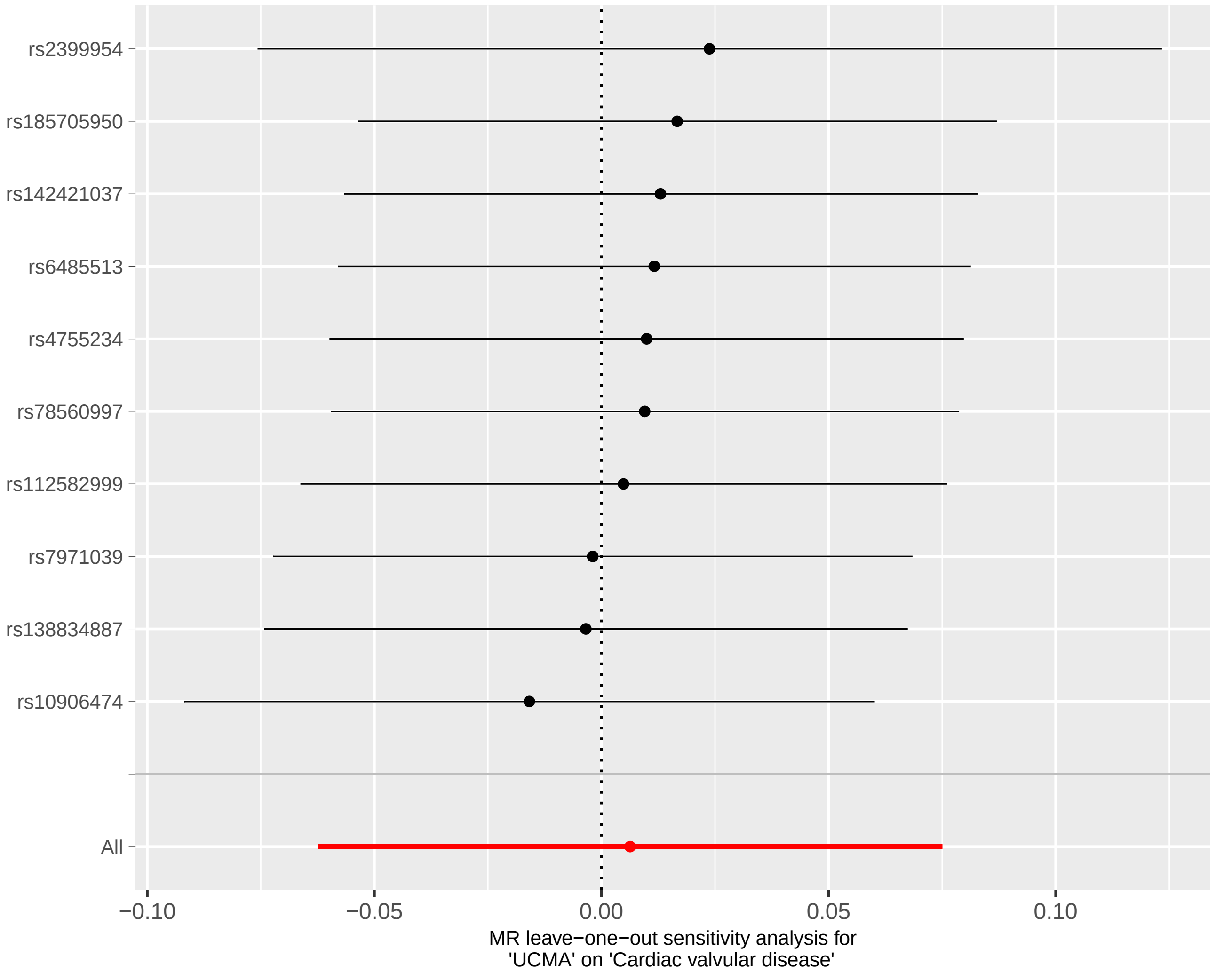
rs2399954
rs185705950
rs142421037
rs6485513
rs4755234
rs78560997
rs112582999
rs7971039
rs138834887
rs10906474
All
−0.10
−0.05
0.00
0.05
0.10
MR leave−one−out sensitivity analysis for
'UCMA' on 'Cardiac valvular disease'

Insufficient number of SNPs

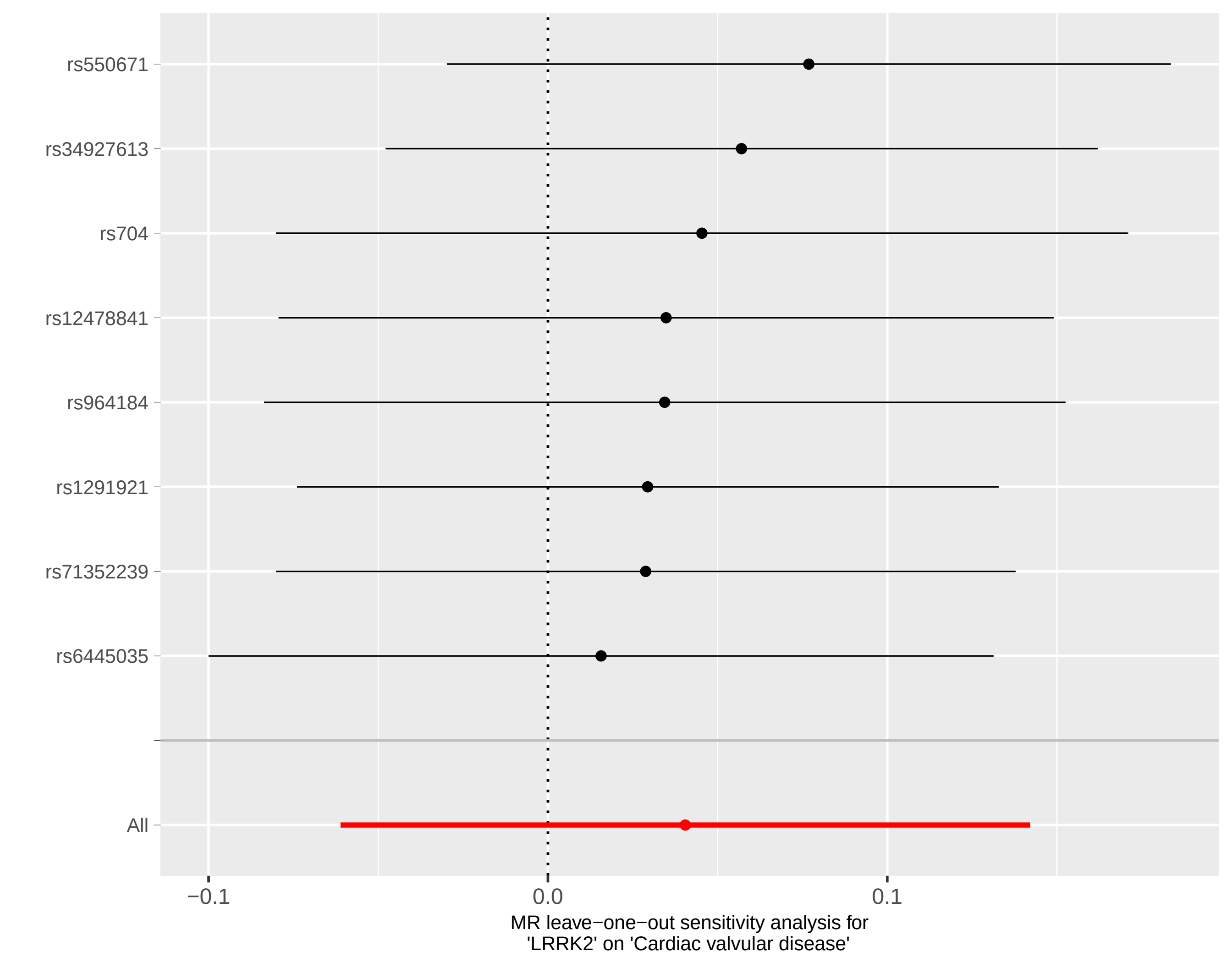
rs550671
rs34927613
rs704
rs12478841
rs964184
rs1291921
rs71352239
rs6445035
All
−0.1
0.0
0.1
MR leave−one−out sensitivity analysis for
'LRRK2' on 'Cardiac valvular disease'

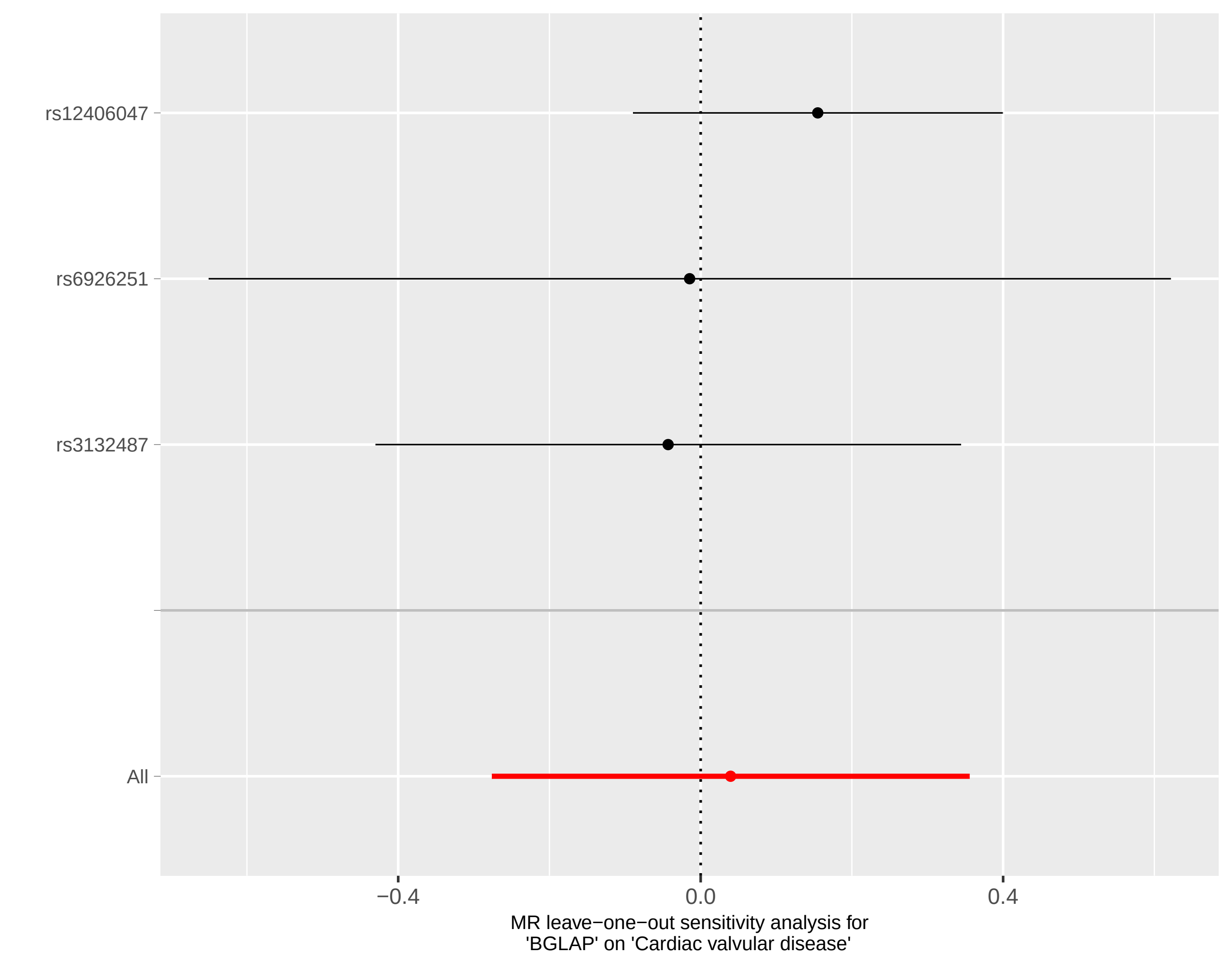
rs12406047
rs6926251
rs3132487
All
−0.4
0.0
0.4
MR leave−one−out sensitivity analysis for
'BGLAP' on 'Cardiac valvular disease'

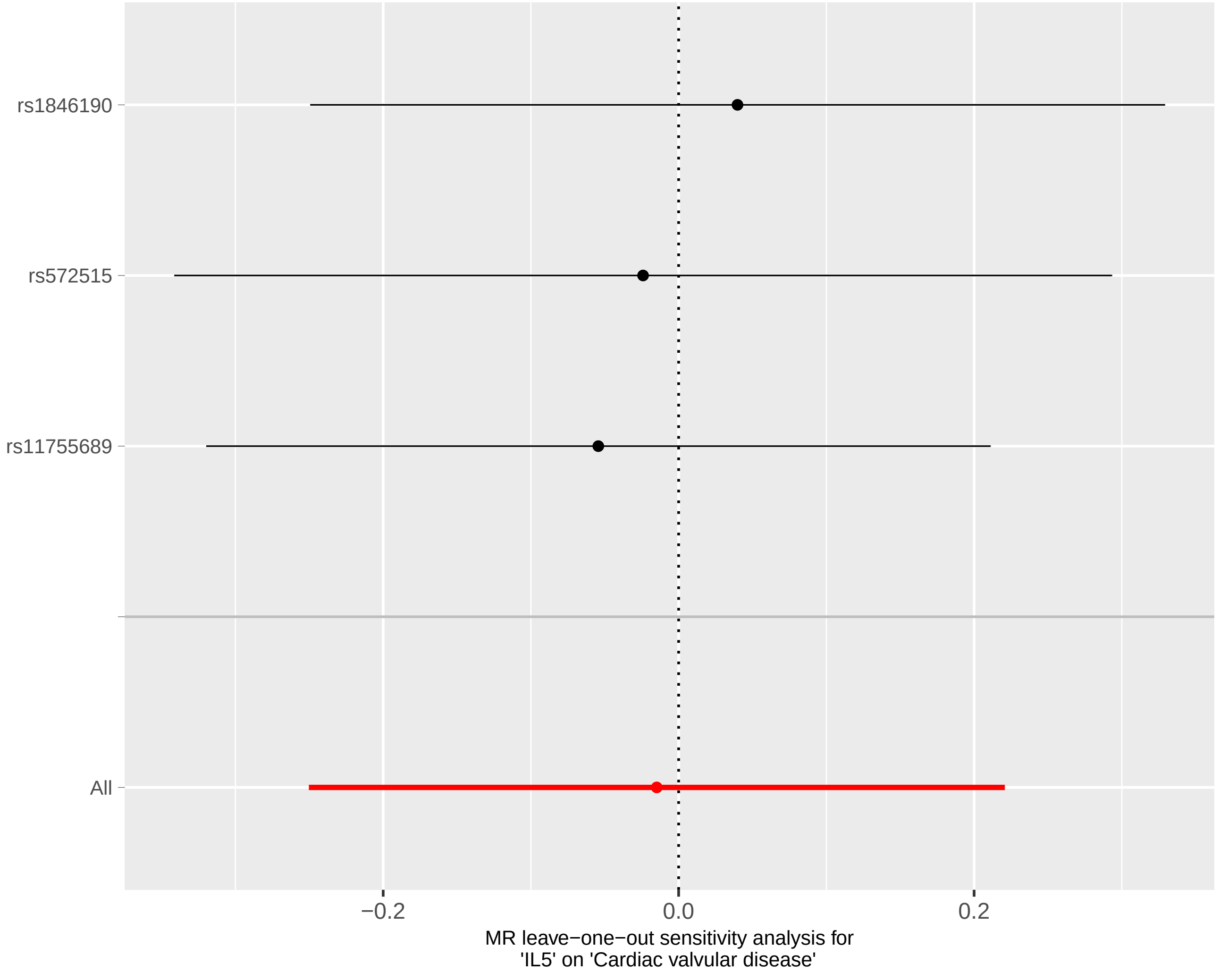
rs1846190
rs572515
rs11755689
All
−0.2
0.0
0.2
MR leave−one−out sensitivity analysis for
'IL5' on 'Cardiac valvular disease'

Insufficient number of SNPs

Insufficient number of SNPs

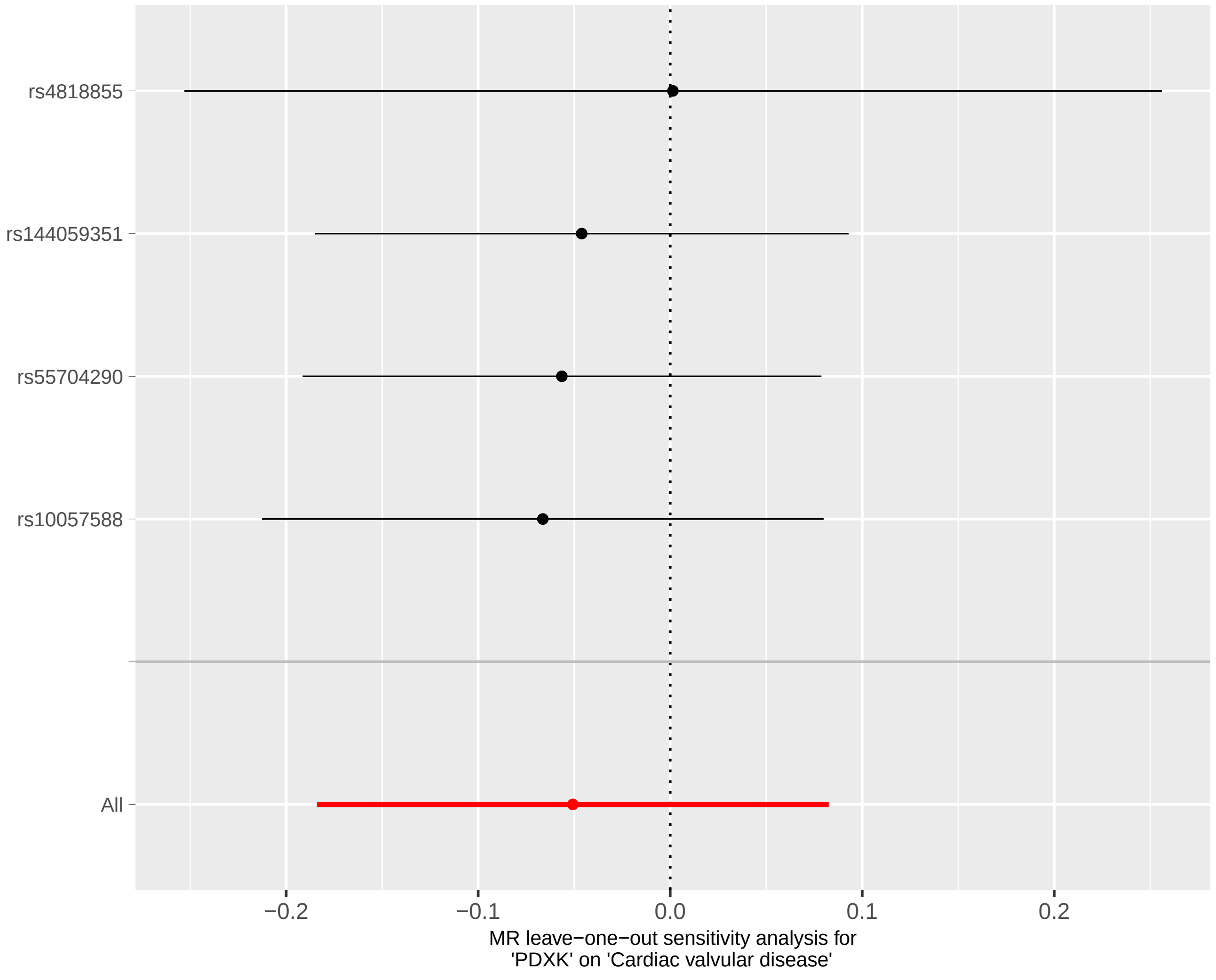

rs4818855
rs144059351
rs55704290
rs10057588
All
−0.2
−0.1
0.0
0.1
0.2
MR leave−one−out sensitivity analysis for
'PDXK' on 'Cardiac valvular disease'

Insufficient number of SNPs

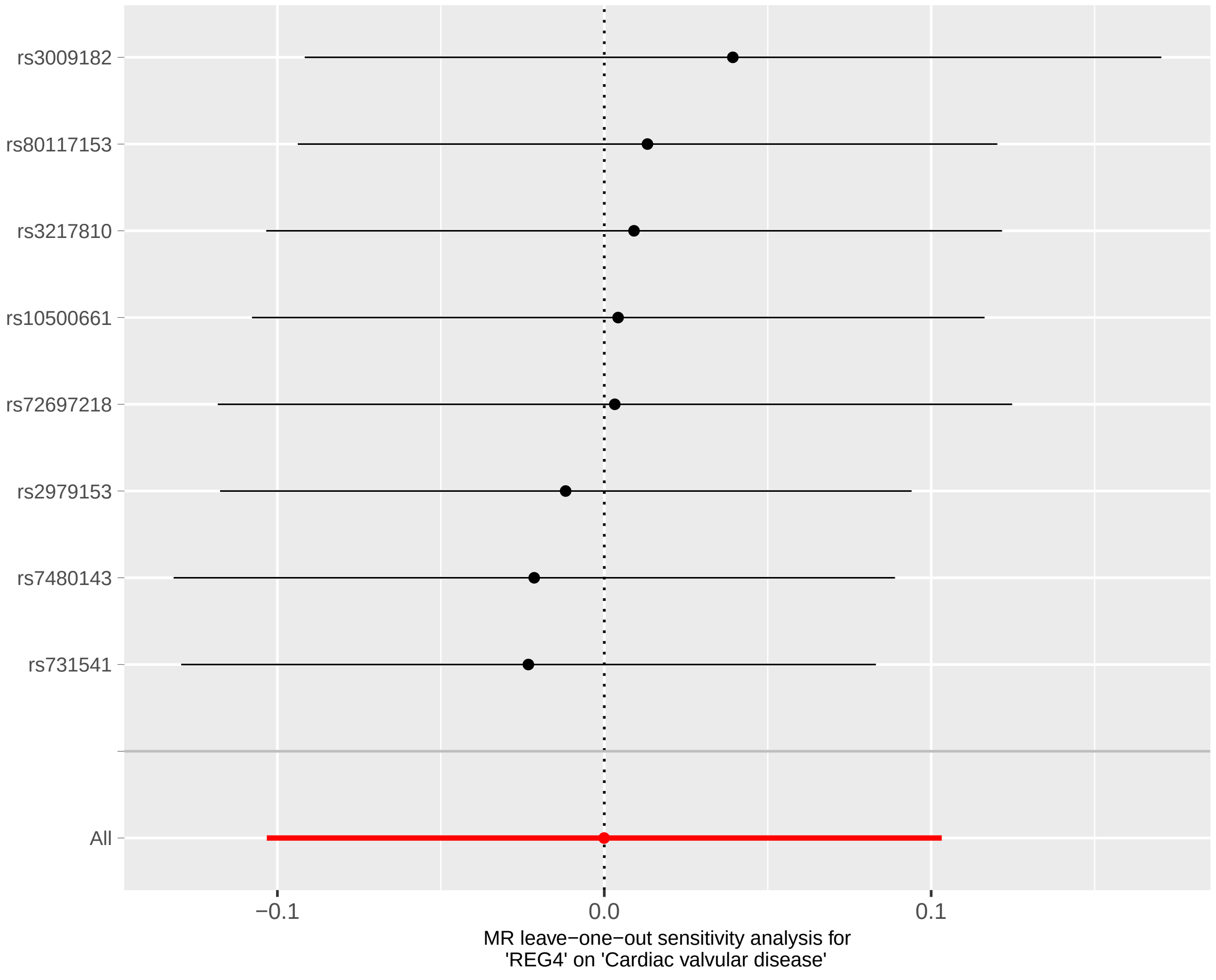
rs3009182
rs80117153
rs3217810
rs10500661
rs72697218
rs2979153
rs7480143
rs731541
All
−0.1
0.0
0.1
MR leave−one−out sensitivity analysis for
'REG4' on 'Cardiac valvular disease'

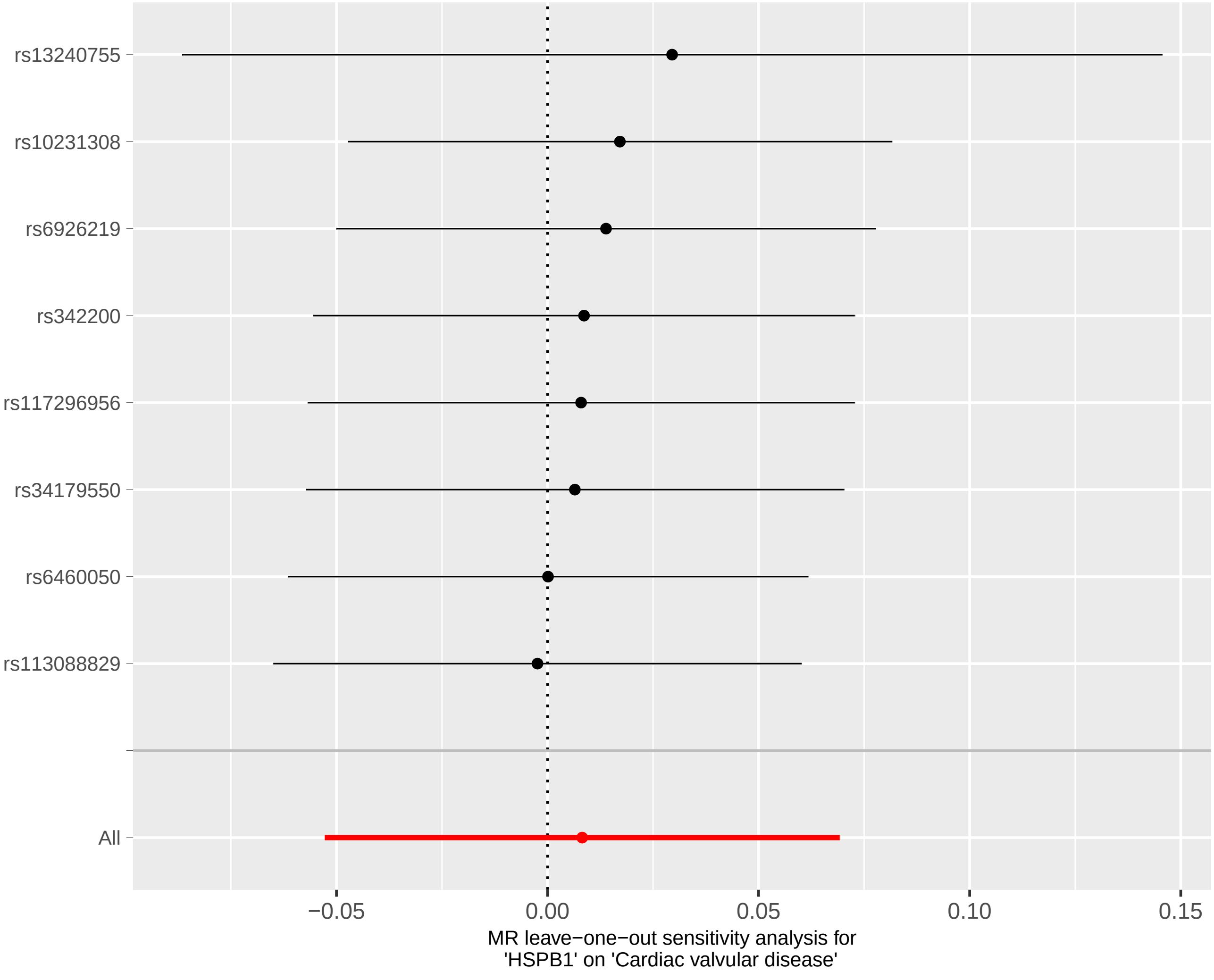

rs13240755
rs10231308
rs6926219
rs342200
rs117296956
rs34179550
rs6460050
rs113088829
All
−0.05
0.00
0.05
0.10
0.15
MR leave−one−out sensitivity analysis for
'HSPB1' on 'Cardiac valvular disease'

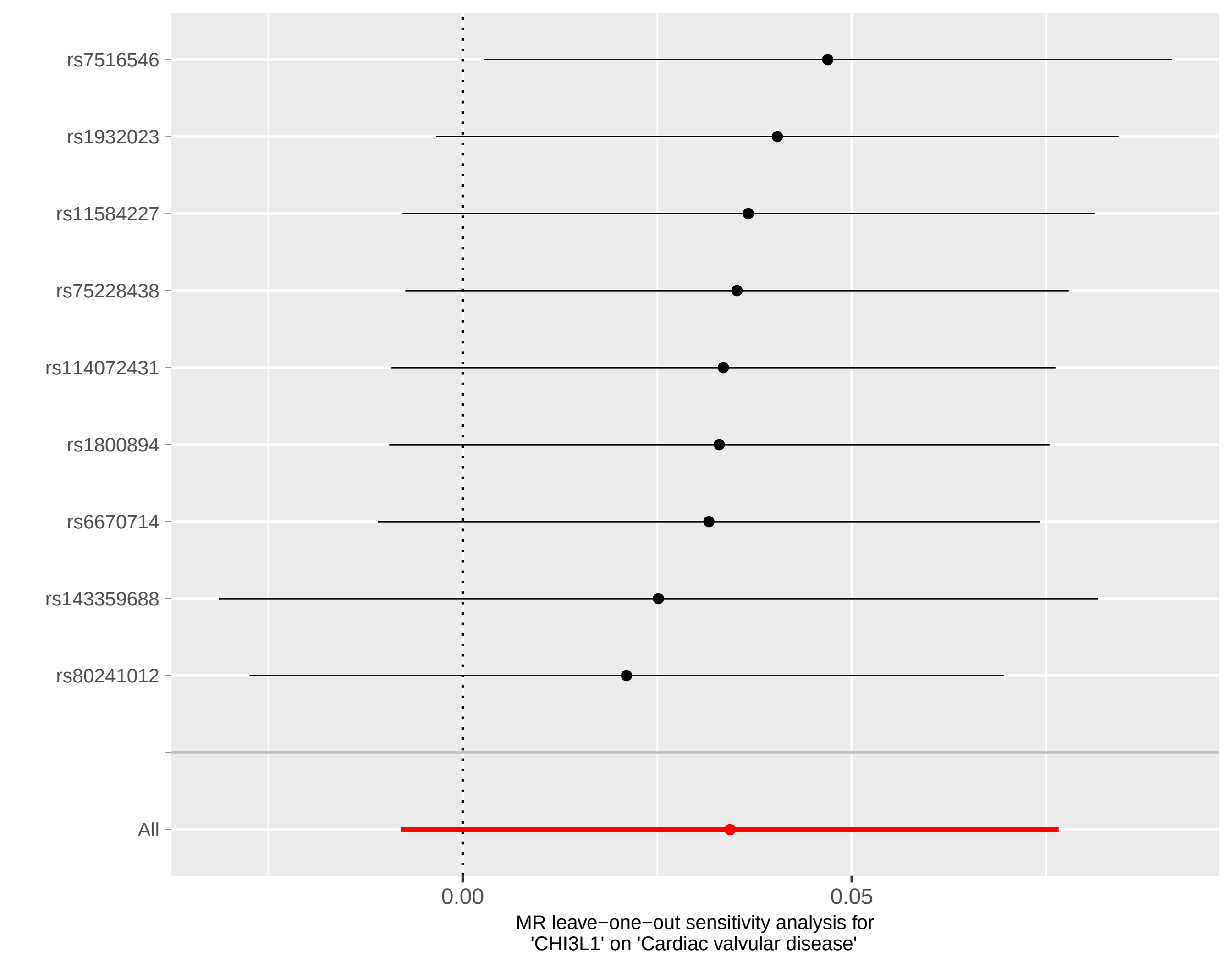

rs7516546
rs1932023
rs11584227
rs75228438
rs114072431
rs1800894
rs6670714
rs143359688
rs80241012
All
0.00
0.05
MR leave−one−out sensitivity analysis for
'CHI3L1' on 'Cardiac valvular disease'

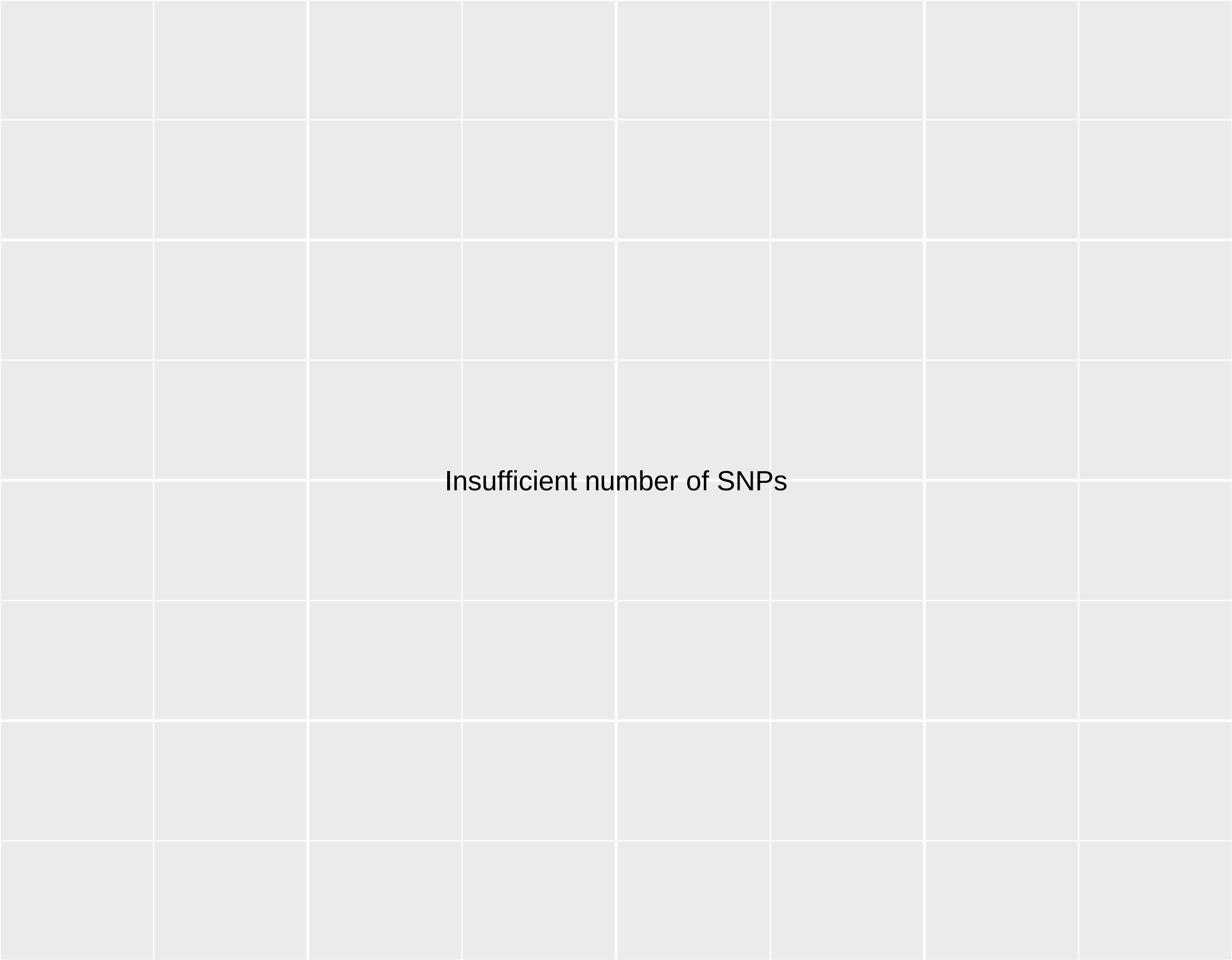
Insufficient number of SNPs

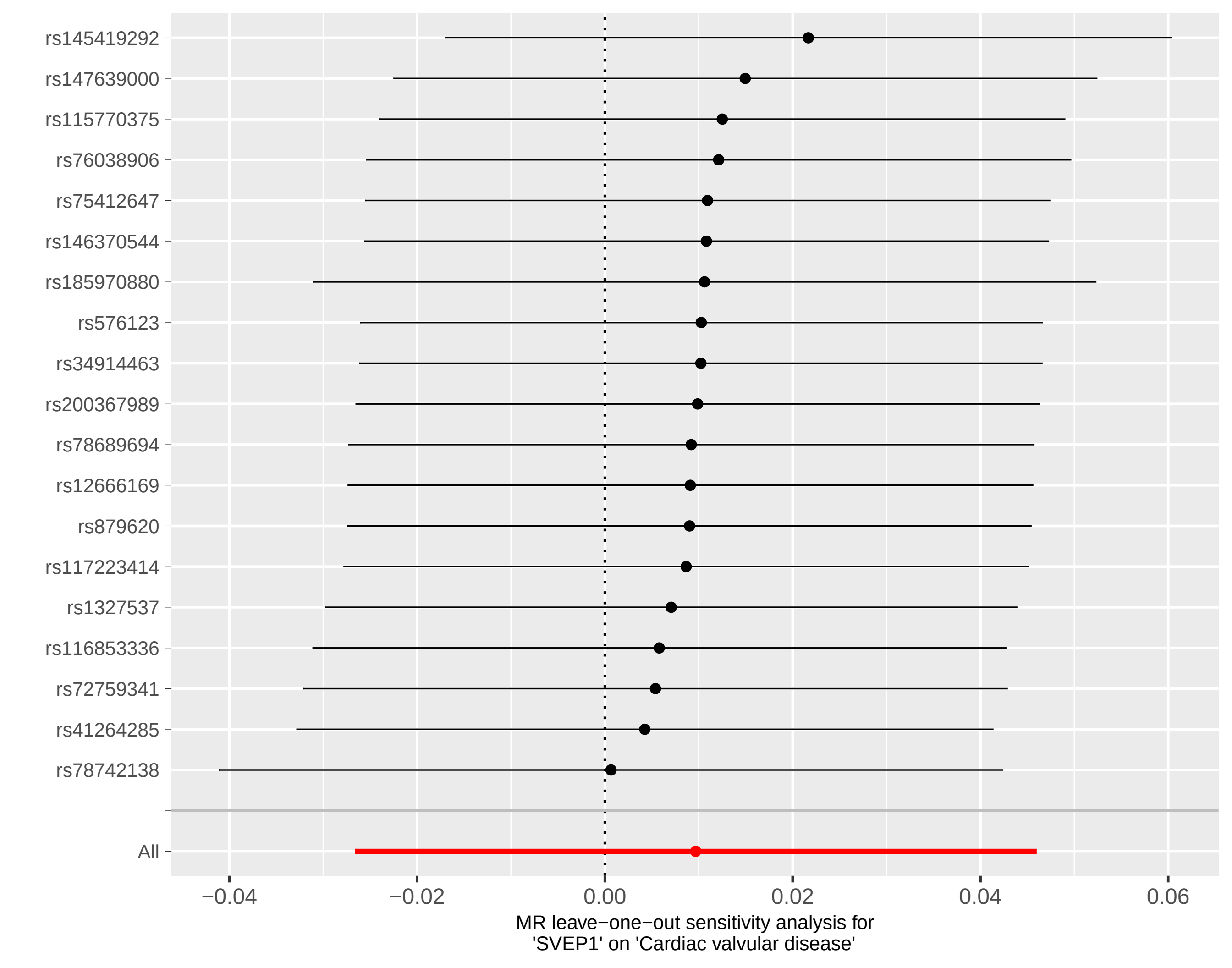
rs145419292
rs147639000
rs115770375
rs76038906
rs75412647
rs146370544
rs185970880
rs576123
rs34914463
rs200367989
rs78689694
rs12666169
rs879620
rs117223414
rs1327537
rs116853336
rs72759341
rs41264285
rs78742138
All
−0.04
−0.02
0.00
0.02
0.04
0.06
MR leave−one−out sensitivity analysis for
'SVEP1' on 'Cardiac valvular disease'

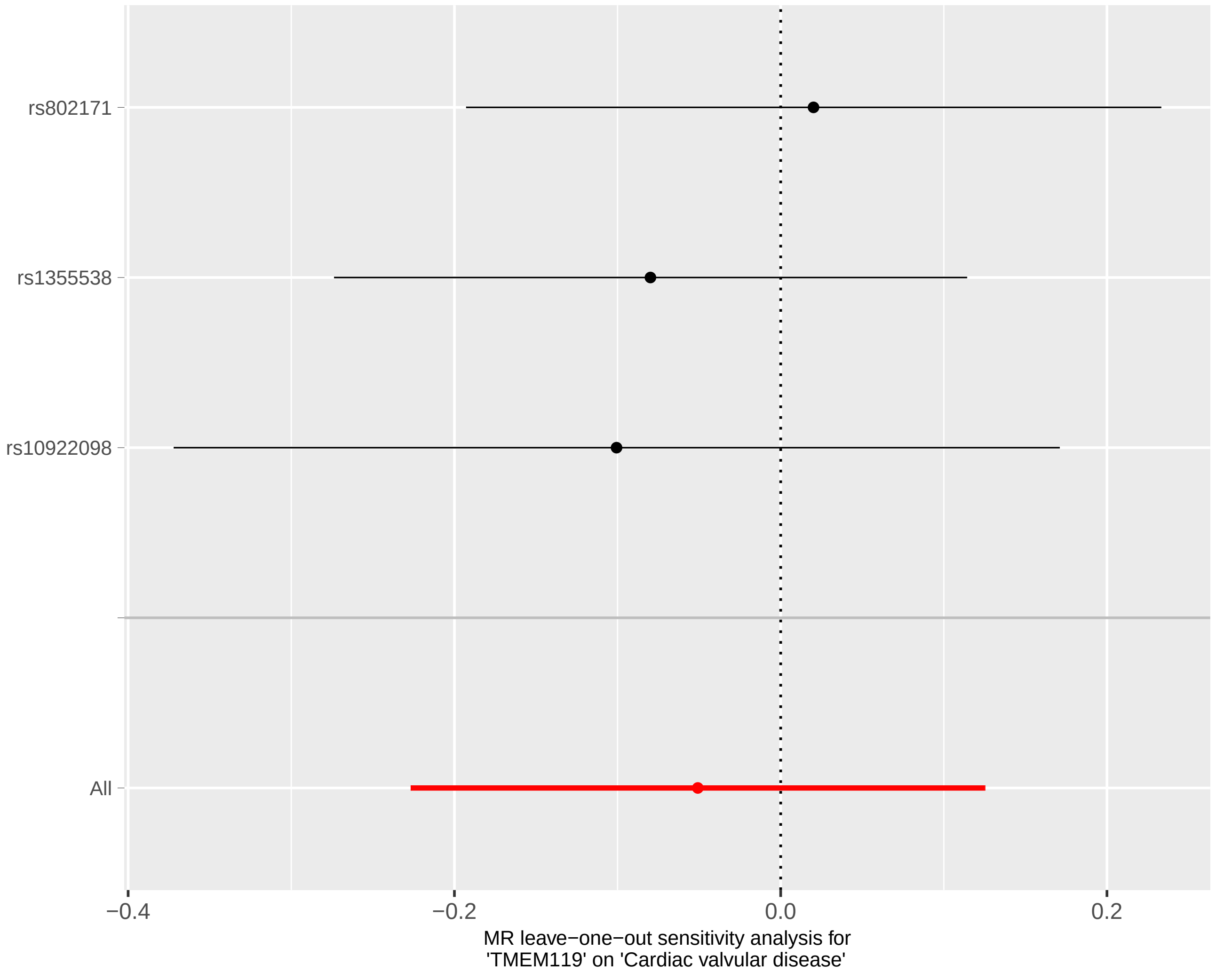

rs802171
rs1355538
rs10922098
All
−0.4
−0.2
0.0
0.2
MR leave−one−out sensitivity analysis for
'TMEM119' on 'Cardiac valvular disease'

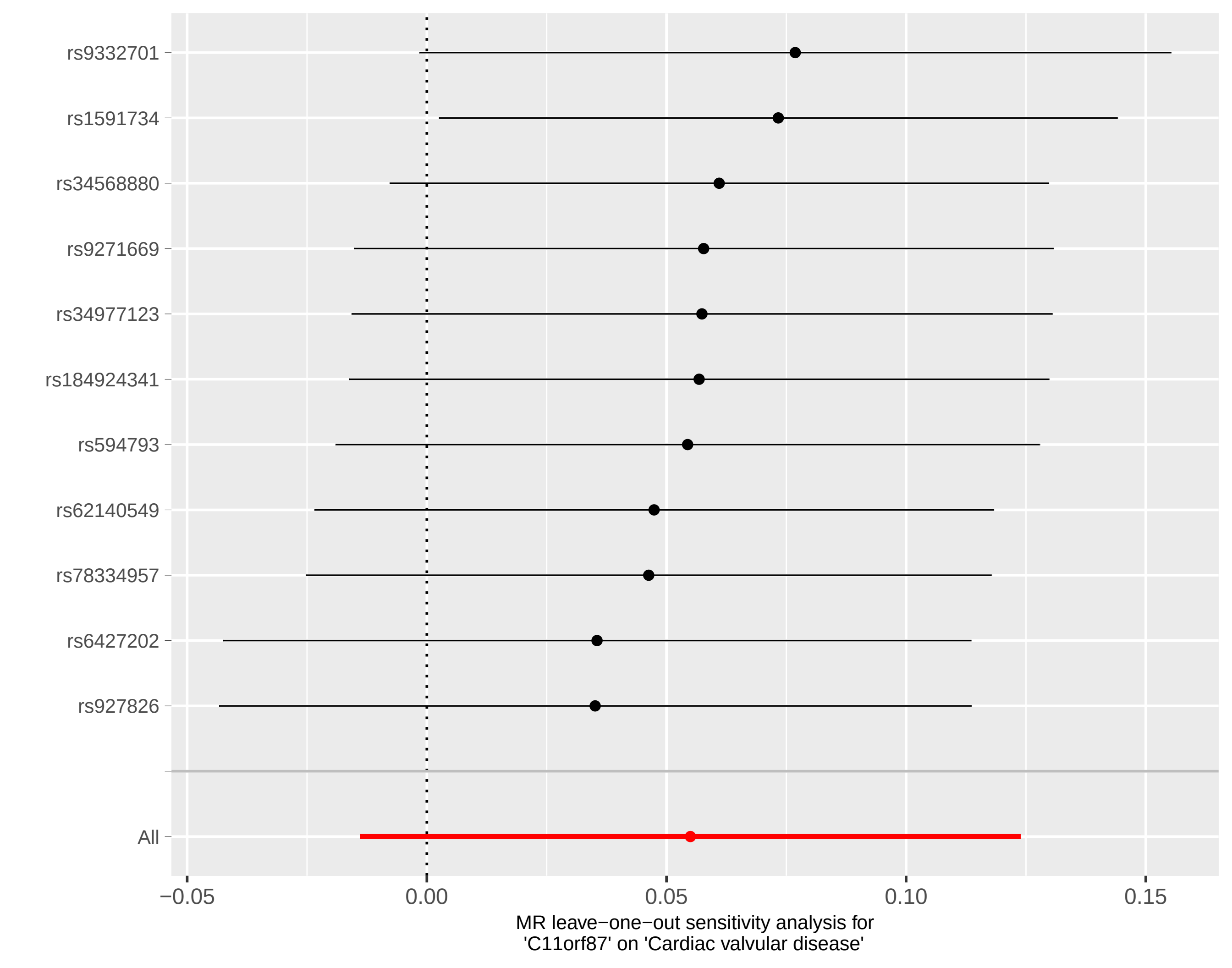
rs9332701
rs1591734
rs34568880
rs9271669
rs34977123
rs184924341
rs594793
rs62140549
rs78334957
rs6427202
rs927826
All
−0.05
0.00
0.05
0.10
0.15
MR leave−one−out sensitivity analysis for
'C11orf87' on 'Cardiac valvular disease'

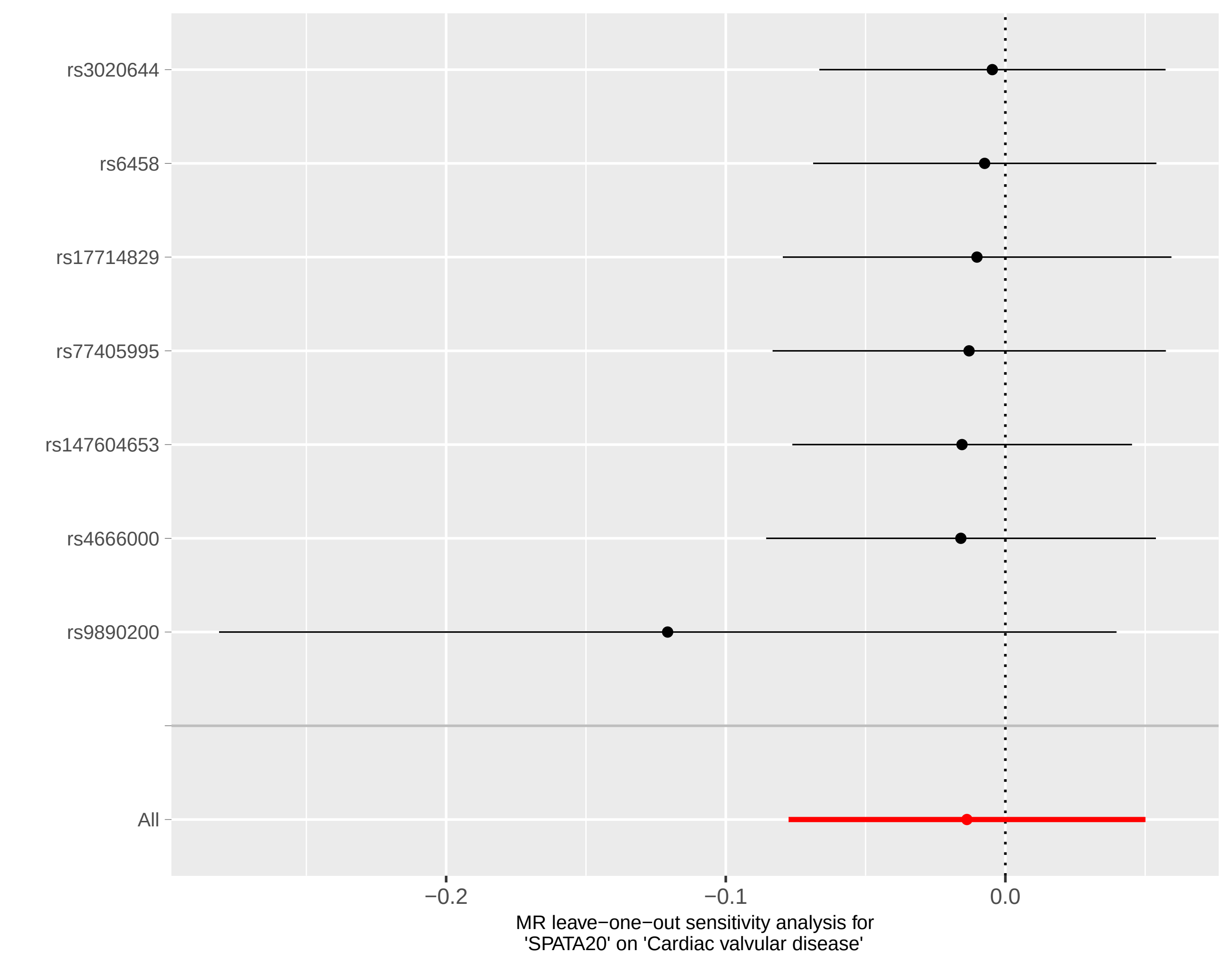

rs3020644
rs6458
rs17714829
rs77405995
rs147604653
rs4666000
rs9890200
All
−0.2
−0.1
0.0
MR leave−one−out sensitivity analysis for
'SPATA20' on 'Cardiac valvular disease'

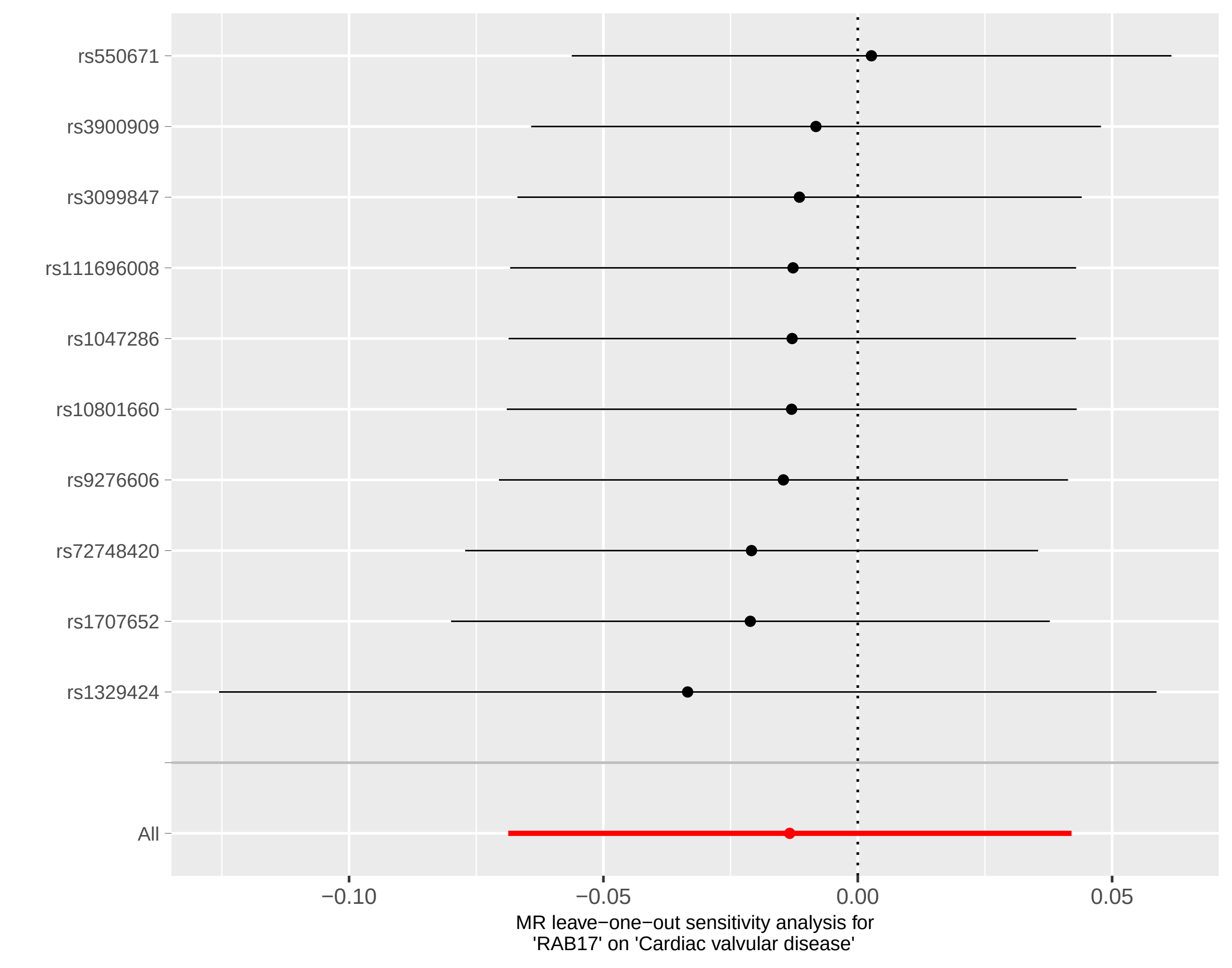
rs550671
rs3900909
rs3099847
rs111696008
rs1047286
rs10801660
rs9276606
rs72748420
rs1707652
rs1329424
All
−0.10
−0.05
0.00
0.05
MR leave−one−out sensitivity analysis for
'RAB17' on 'Cardiac valvular disease'

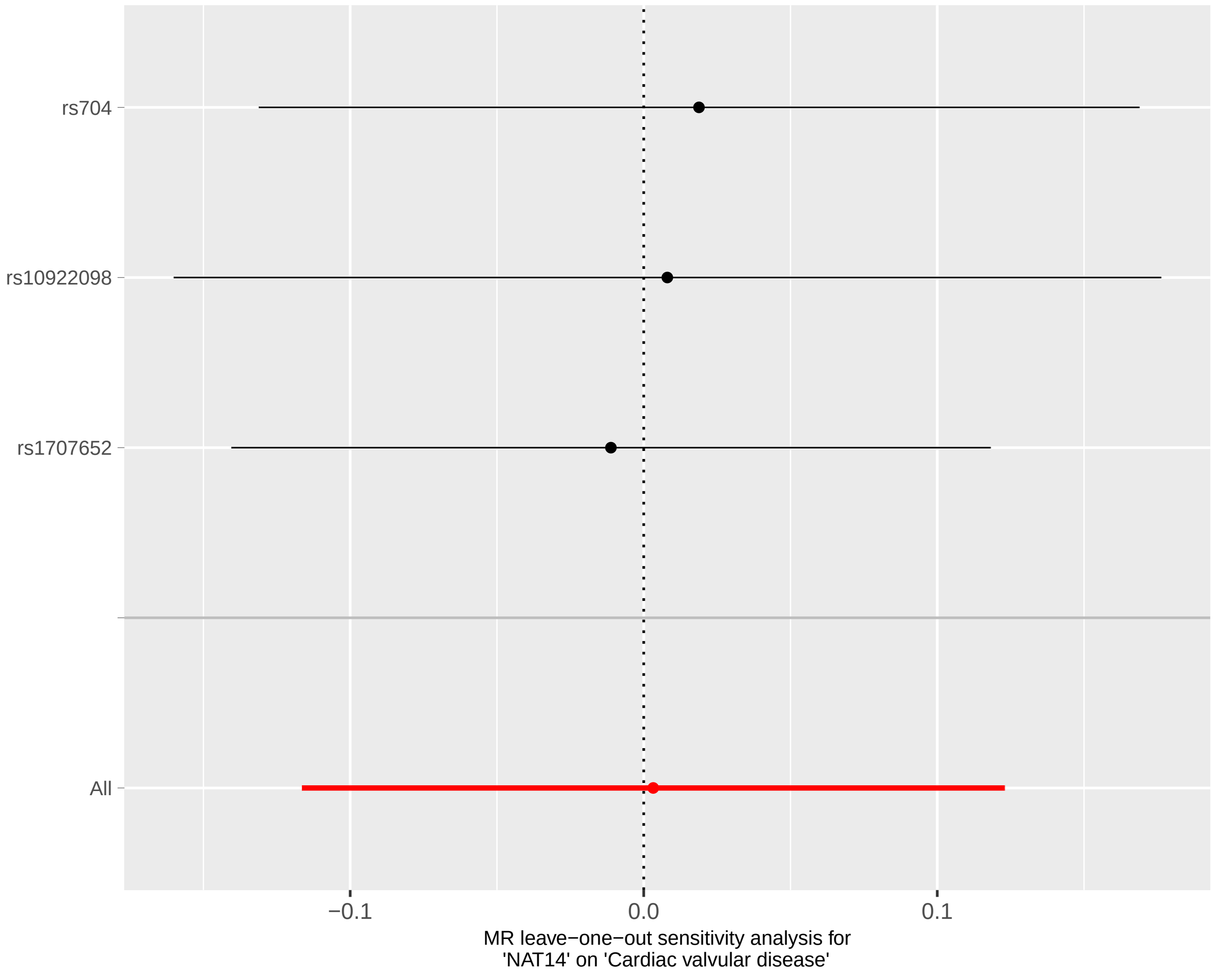
rs704
rs10922098
rs1707652
All
−0.1
0.0
0.1
MR leave−one−out sensitivity analysis for
'NAT14' on 'Cardiac valvular disease'

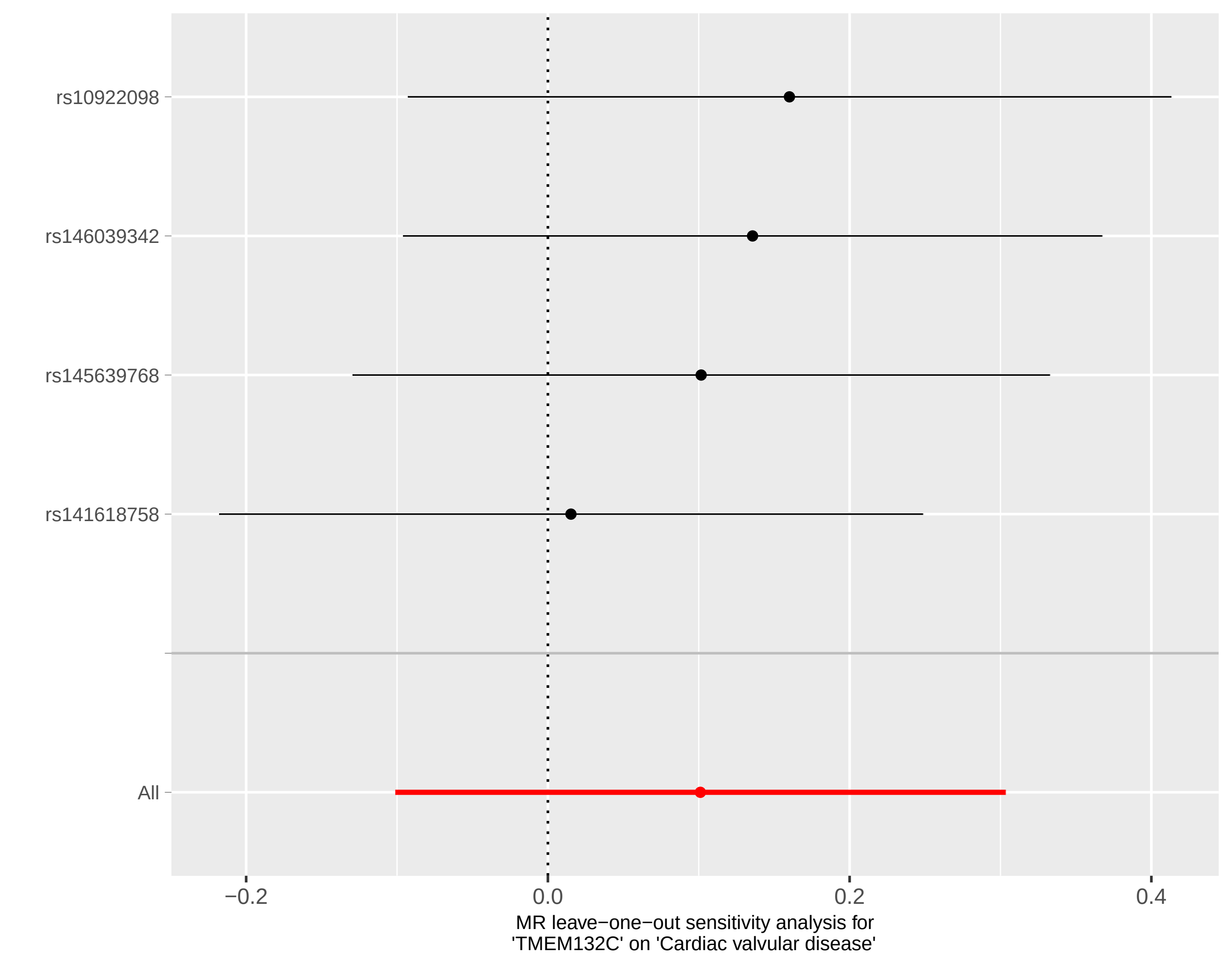
rs10922098
rs146039342
rs145639768
rs141618758
All
−0.2
0.0
0.2
0.4
MR leave−one−out sensitivity analysis for
'TMEM132C' on 'Cardiac valvular disease'

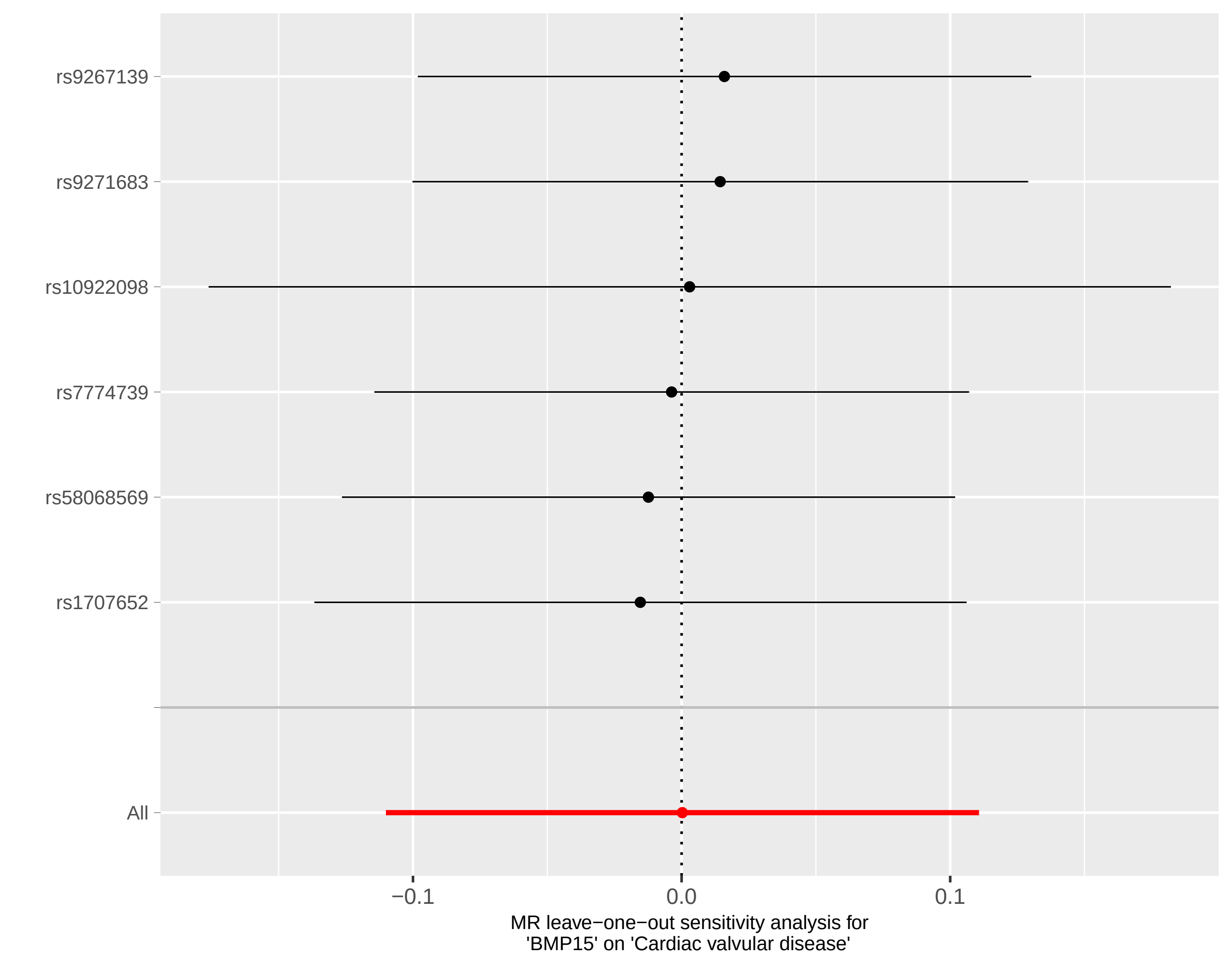
rs9267139
rs9271683
rs10922098
rs7774739
rs58068569
rs1707652
All
−0.1
0.0
0.1
MR leave−one−out sensitivity analysis for
'BMP15' on 'Cardiac valvular disease'

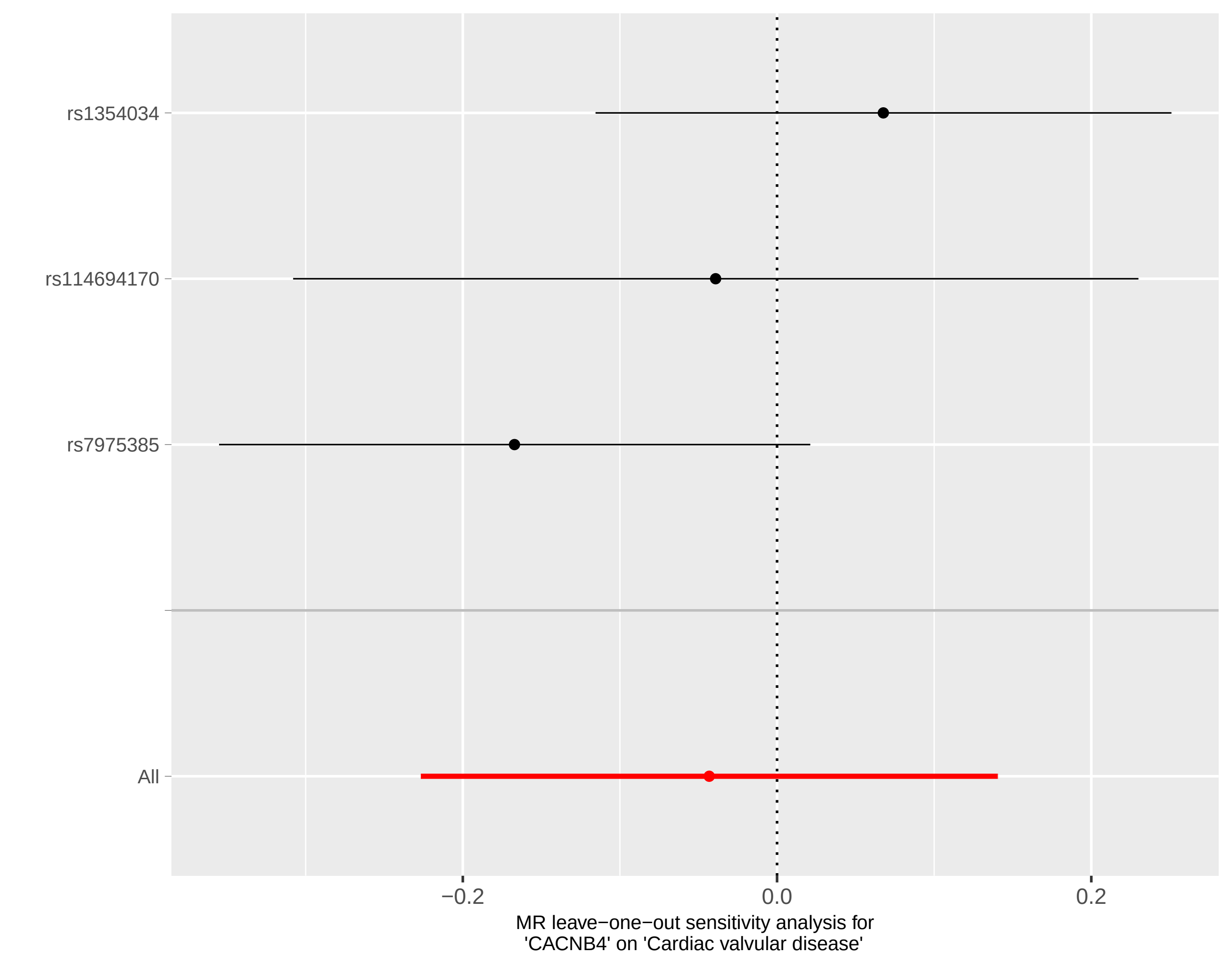
rs1354034
rs114694170
rs7975385
All
−0.2
0.0
0.2
MR leave−one−out sensitivity analysis for
'CACNB4' on 'Cardiac valvular disease'

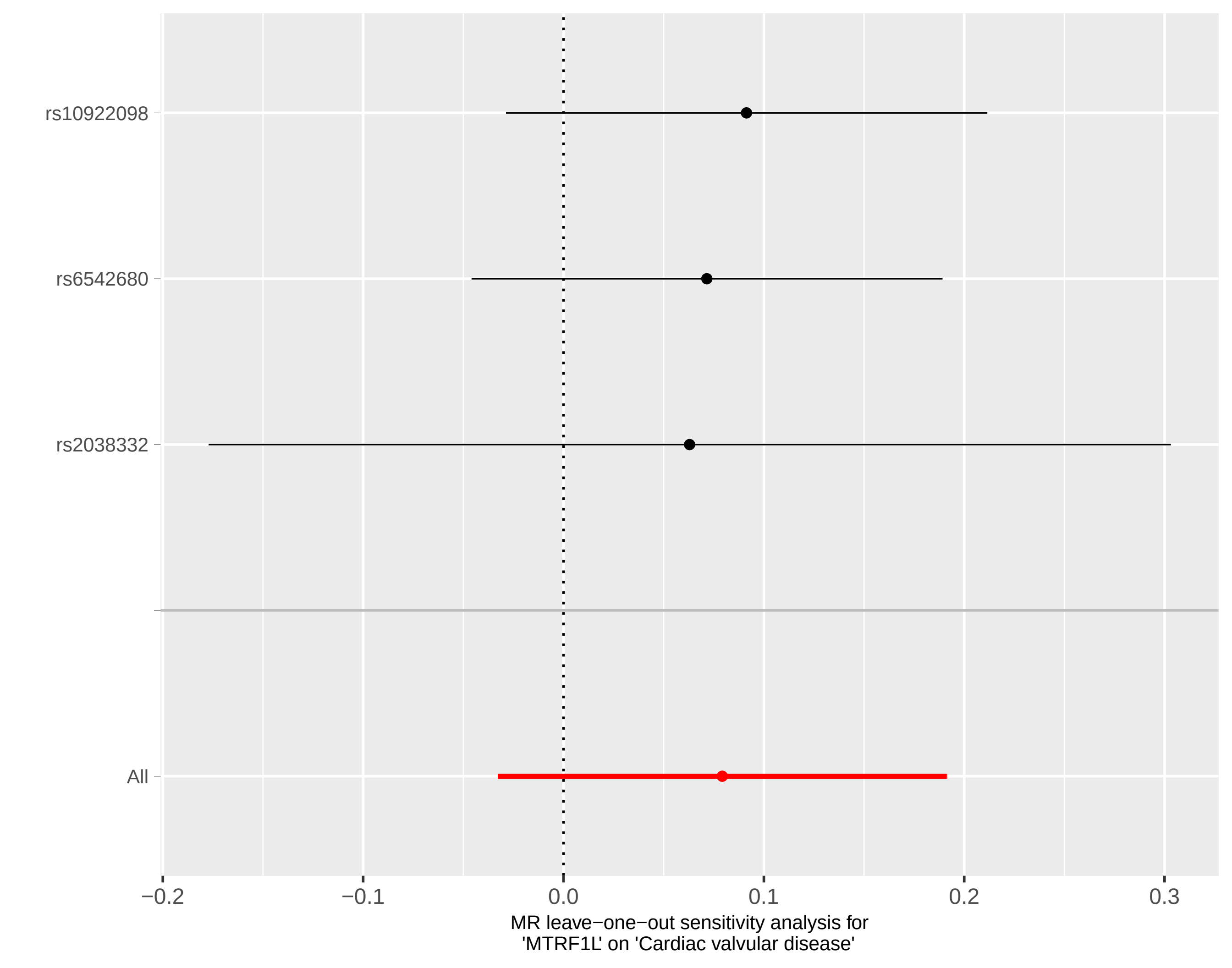
rs10922098
rs6542680
rs2038332
All
−0.2
−0.1
0.0
0.1
0.2
0.3
MR leave−one−out sensitivity analysis for
'MTRF1L' on 'Cardiac valvular disease'

Insufficient number of SNPs

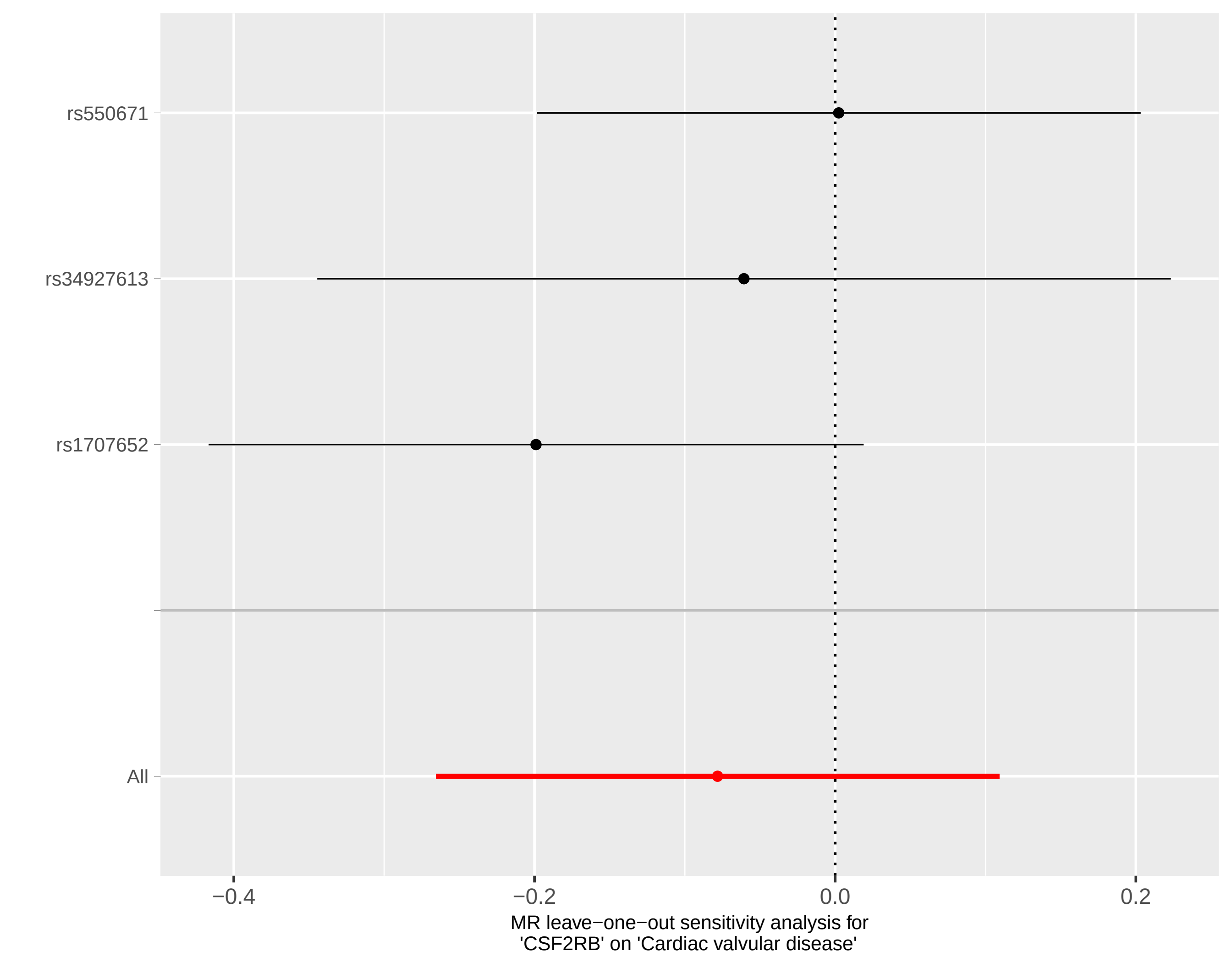
rs550671
rs34927613
rs1707652
All
−0.4
−0.2
0.0
0.2
MR leave−one−out sensitivity analysis for
'CSF2RB' on 'Cardiac valvular disease'

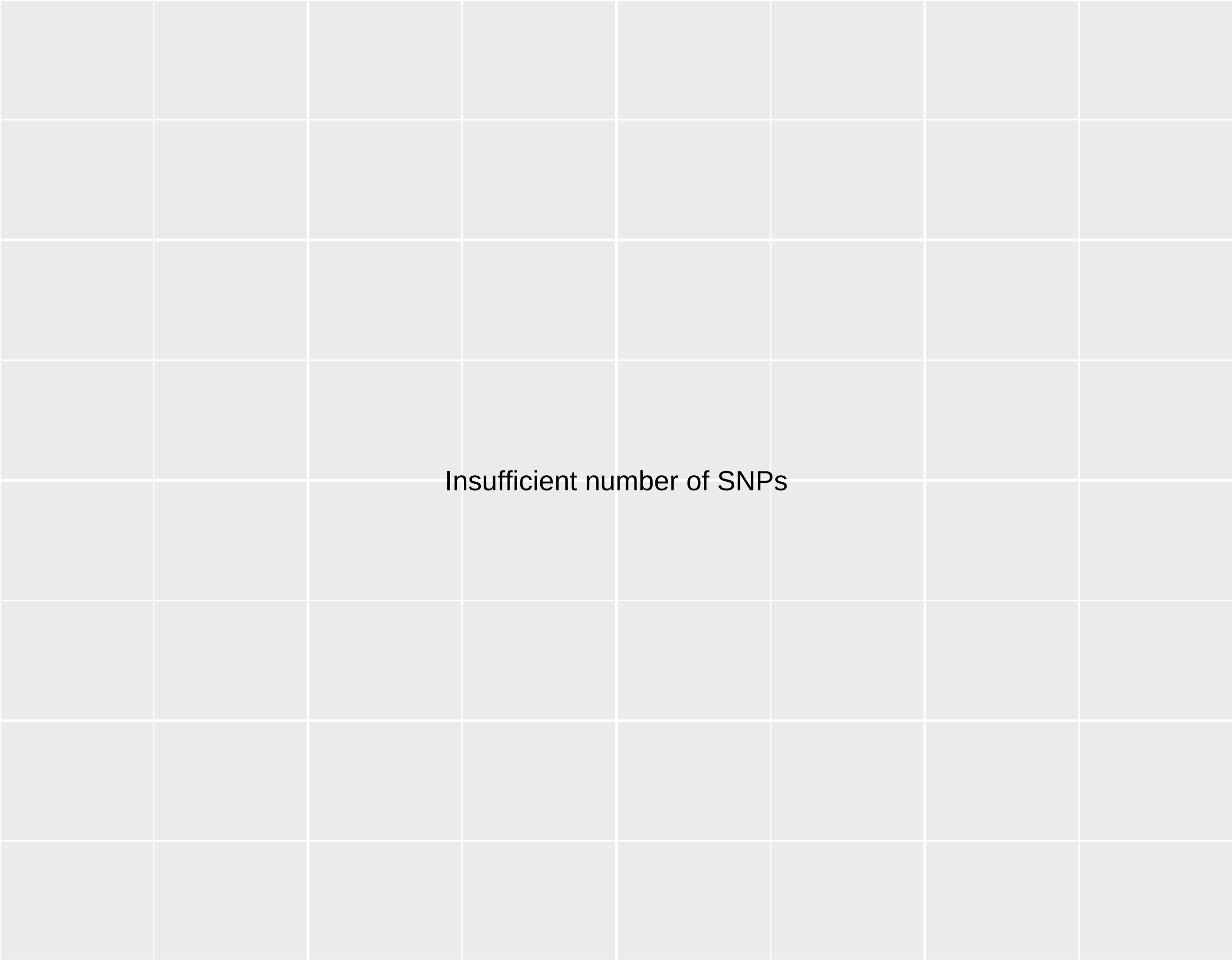
Insufficient number of SNPs

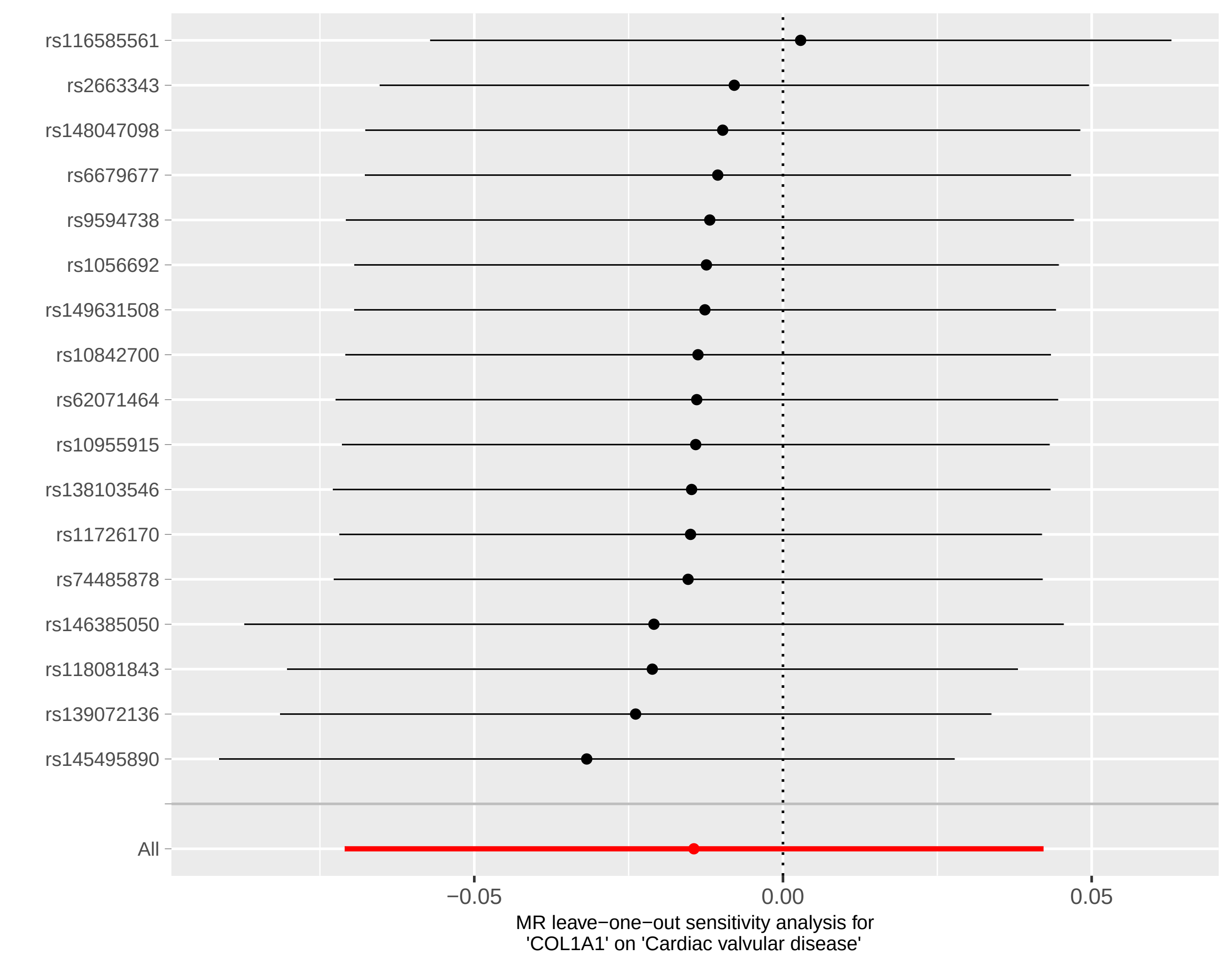

rs116585561
rs2663343
rs148047098
rs6679677
rs9594738
rs1056692
rs149631508
rs10842700
rs62071464
rs10955915
rs138103546
rs11726170
rs74485878
rs146385050
rs118081843
rs139072136
rs145495890
All
−0.05
0.00
0.05
MR leave−one−out sensitivity analysis for
'COL1A1' on 'Cardiac valvular disease'

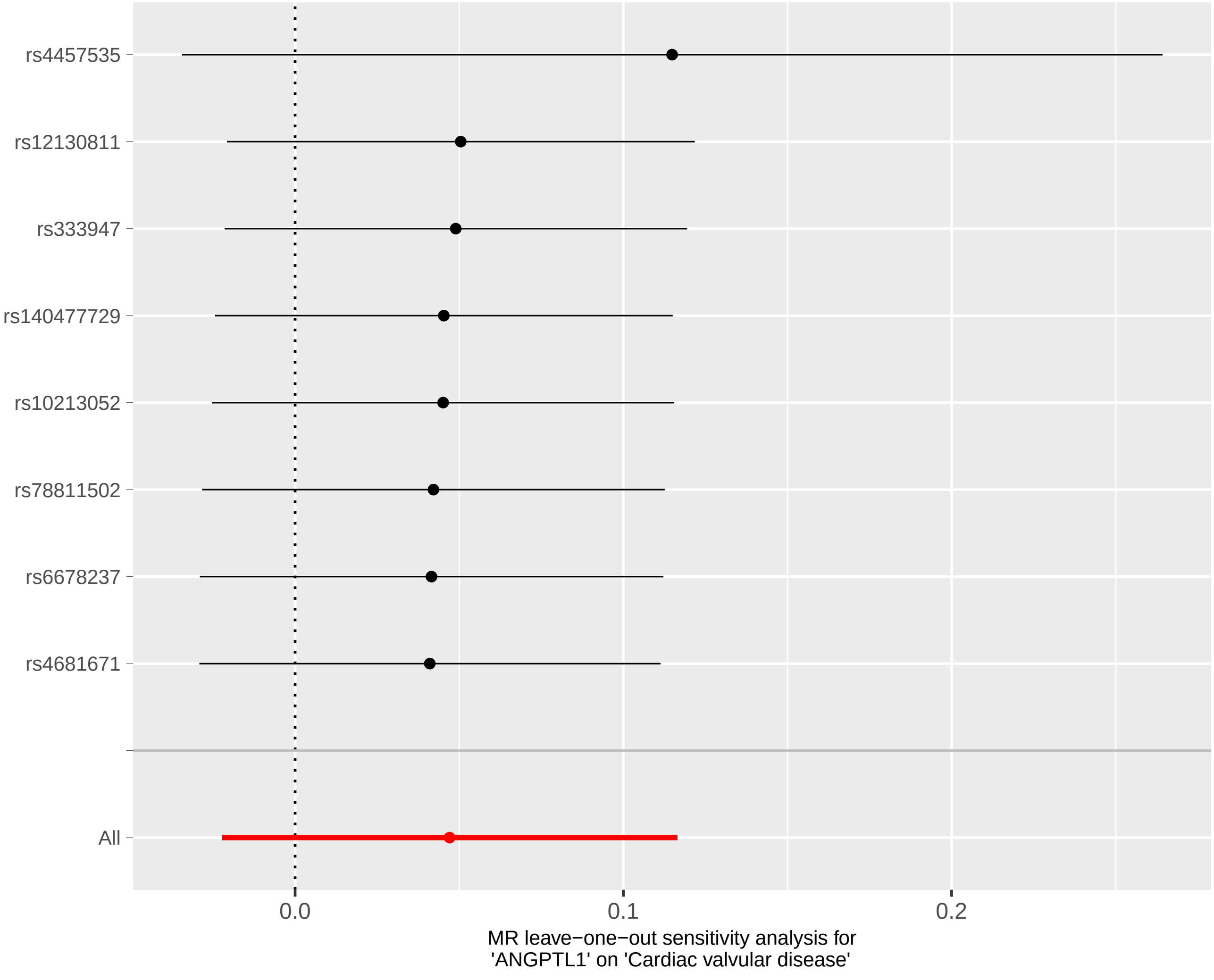

rs4457535
rs12130811
rs333947
rs140477729
rs10213052
rs78811502
rs6678237
rs4681671
All
0.0
0.1
0.2
MR leave−one−out sensitivity analysis for
'ANGPTL1' on 'Cardiac valvular disease'

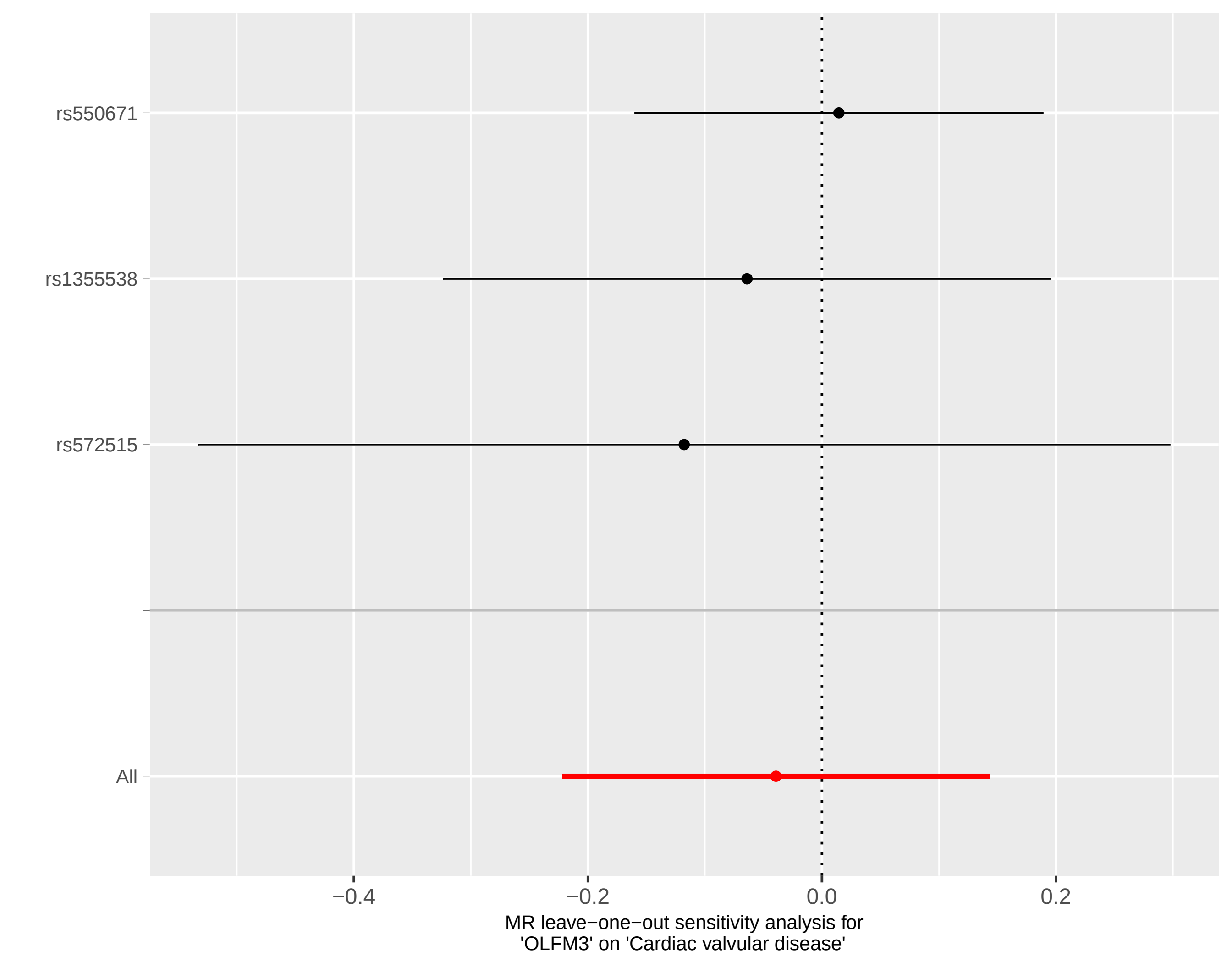

rs550671
rs1355538
rs572515
All
−0.4
−0.2
0.0
0.2
MR leave−one−out sensitivity analysis for
'OLFM3' on 'Cardiac valvular disease'

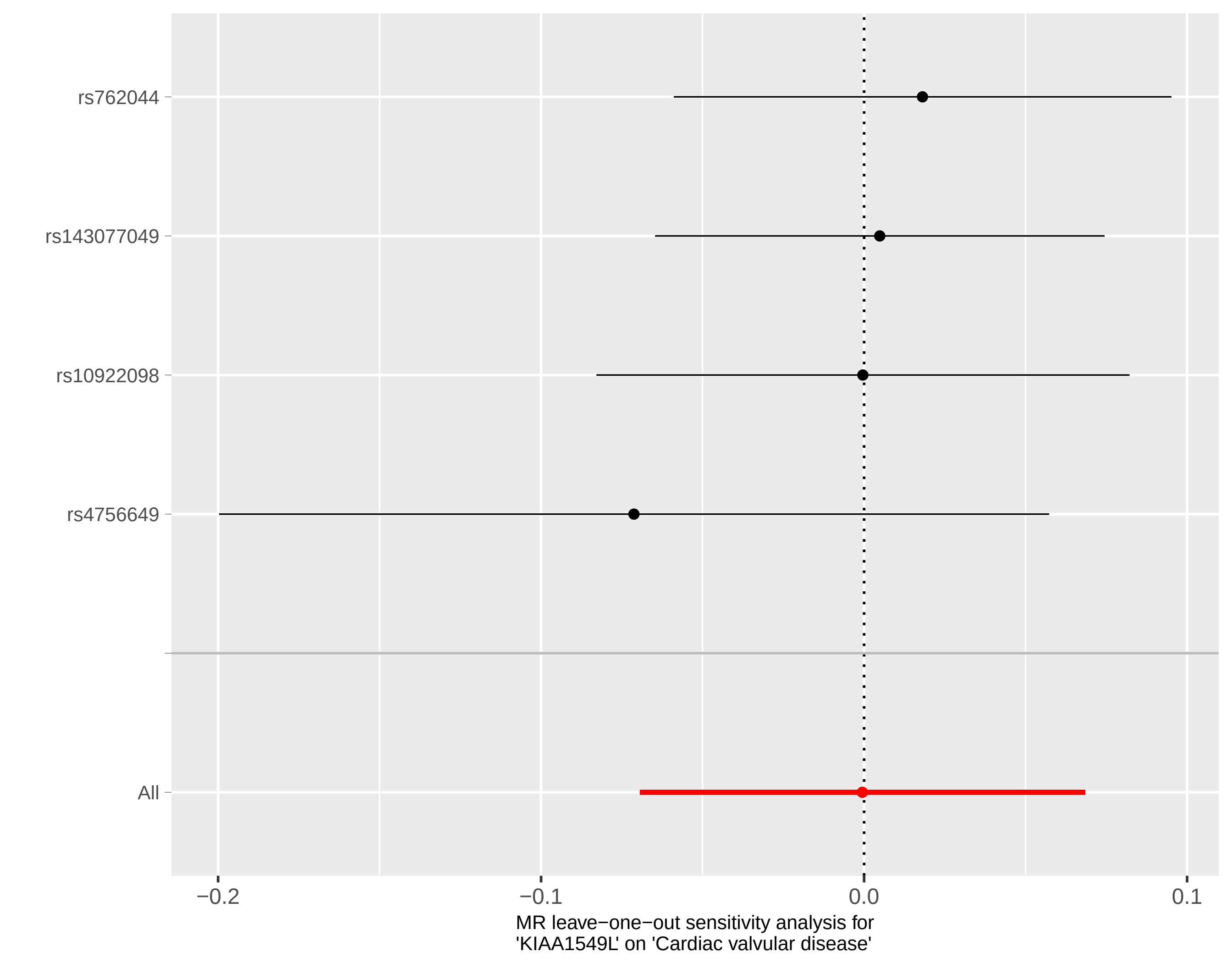
rs762044
rs143077049
rs10922098
rs4756649
All
−0.2
−0.1
0.0
0.1
MR leave−one−out sensitivity analysis for
'KIAA1549L' on 'Cardiac valvular disease'

Insufficient number of SNPs

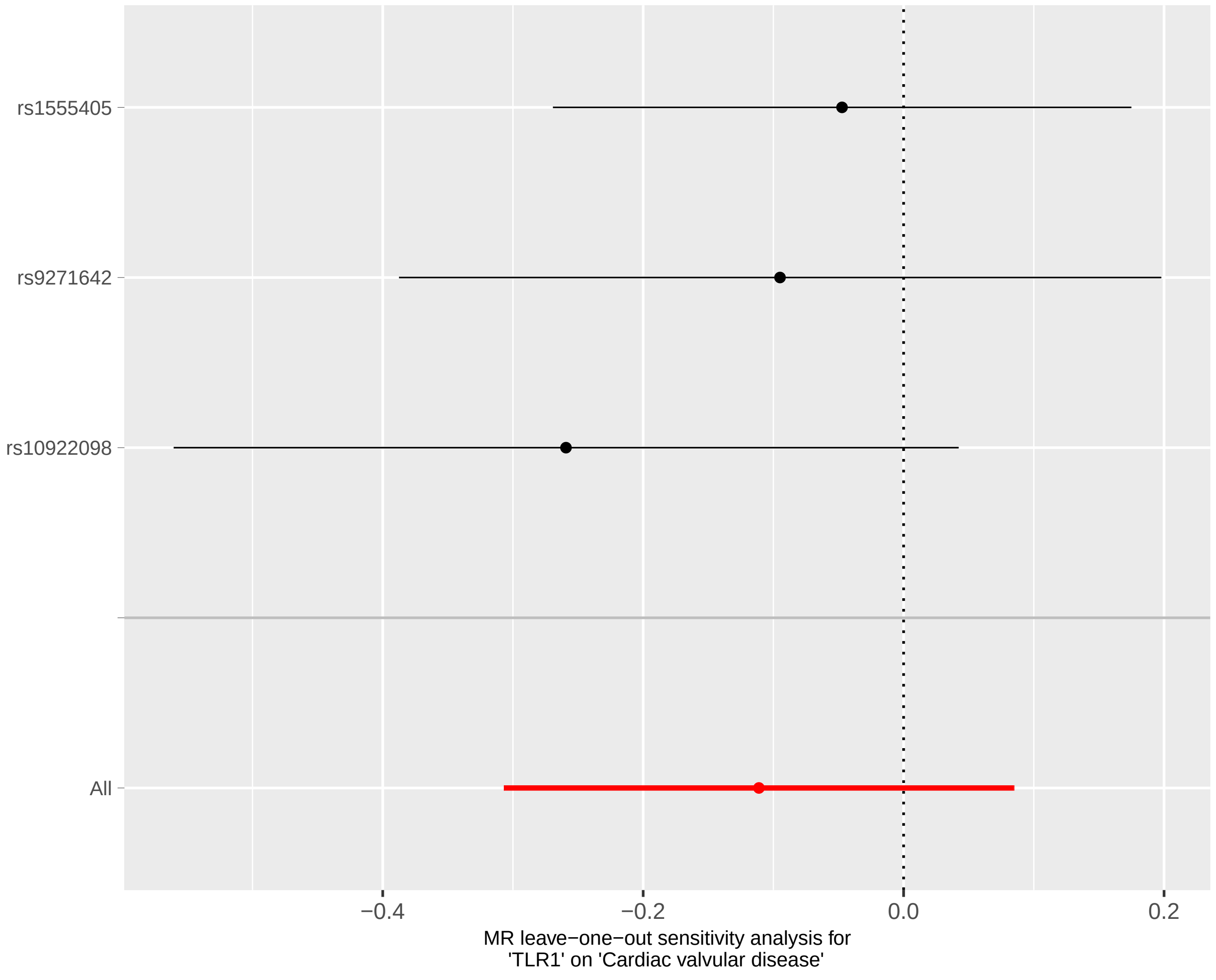
rs1555405
rs9271642
rs10922098
All
−0.4
−0.2
0.0
0.2
MR leave−one−out sensitivity analysis for
'TLR1' on 'Cardiac valvular disease'

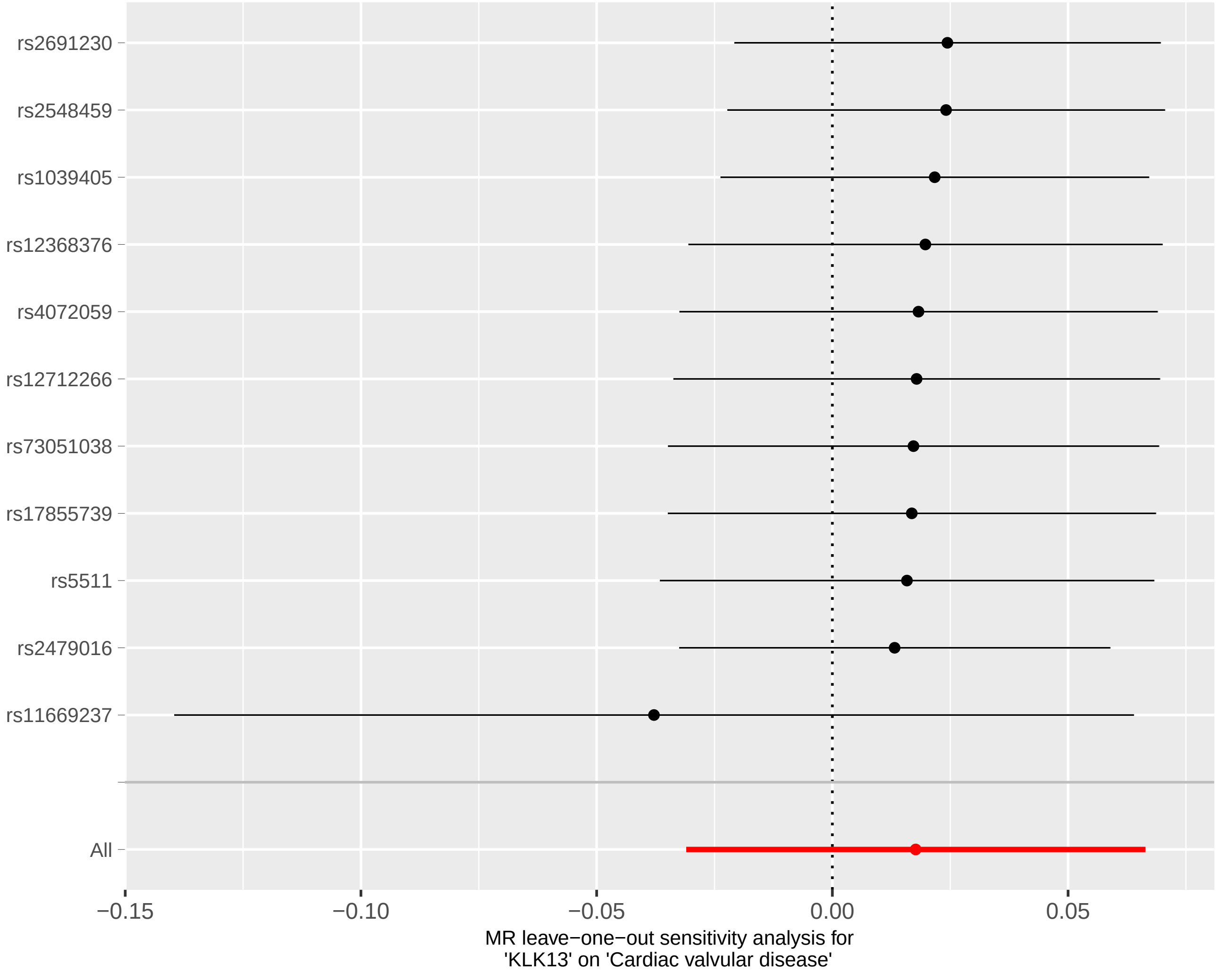
rs2691230
rs2548459
rs1039405
rs12368376
rs4072059
rs12712266
rs73051038
rs17855739
rs5511
rs2479016
rs11669237
All
−0.15
−0.10
−0.05
0.00
0.05
MR leave−one−out sensitivity analysis for
'KLK13' on 'Cardiac valvular disease'

Insufficient number of SNPs

Insufficient number of SNPs

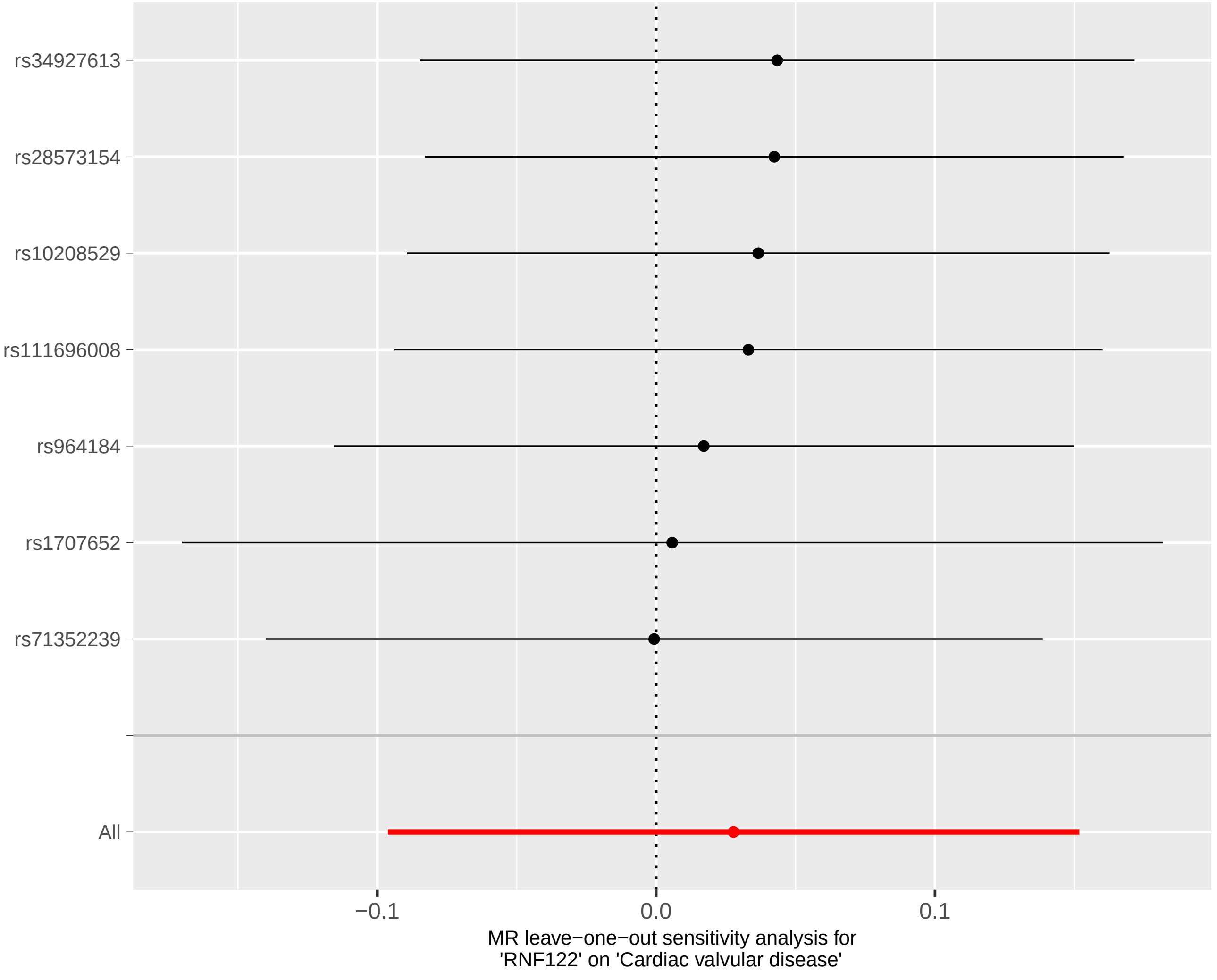

rs34927613
rs28573154
rs10208529
rs111696008
rs964184
rs1707652
rs71352239
All
−0.1
0.0
0.1
MR leave−one−out sensitivity analysis for
'RNF122' on 'Cardiac valvular disease'

Insufficient number of SNPs

Insufficient number of SNPs

Insufficient number of SNPs

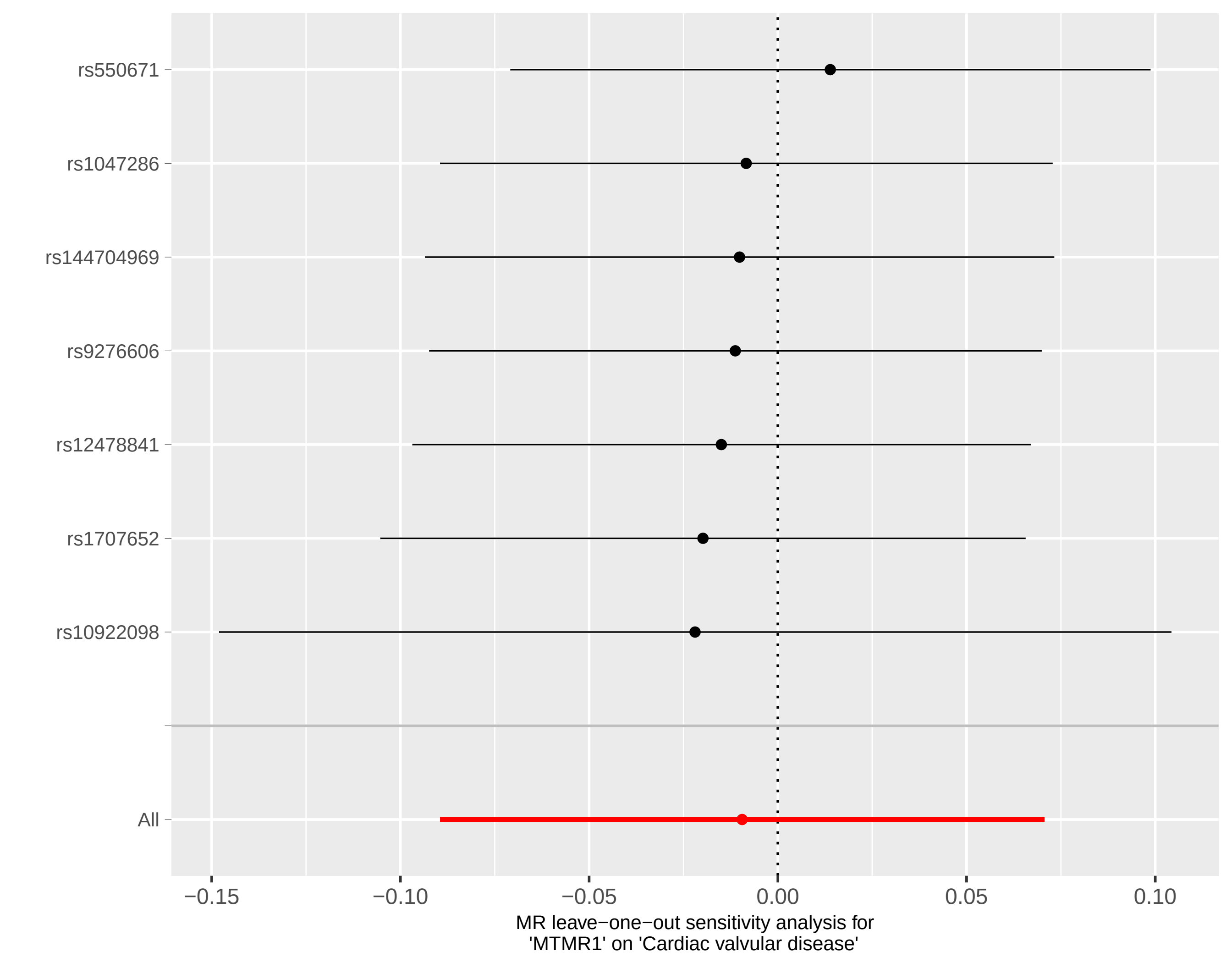
rs550671
rs1047286
rs144704969
rs9276606
rs12478841
rs1707652
rs10922098
All
−0.15
−0.10
−0.05
0.00
0.05
0.10
MR leave−one−out sensitivity analysis for
'MTMR1' on 'Cardiac valvular disease'

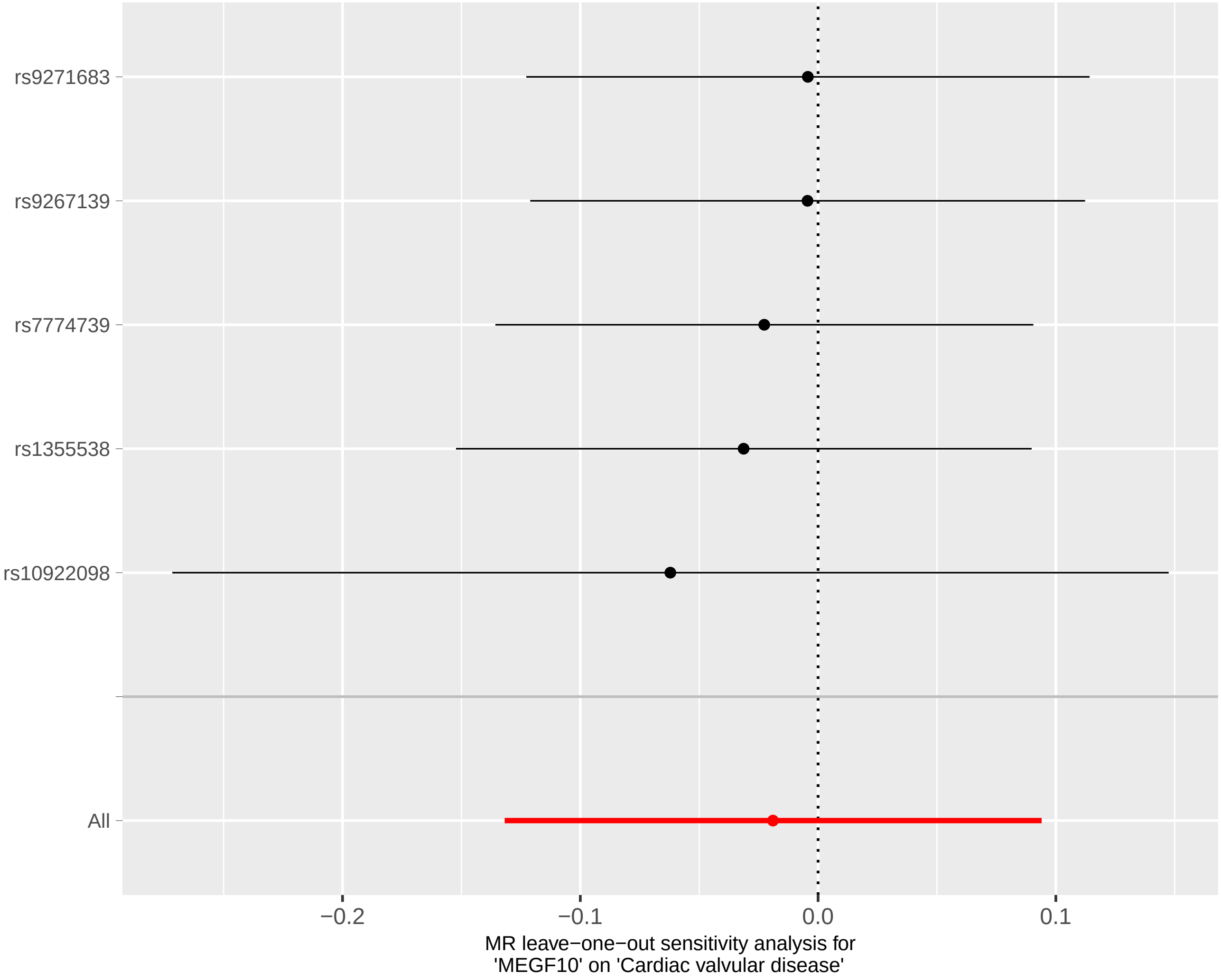

rs9271683
rs9267139
rs7774739
rs1355538
rs10922098
All
−0.2
−0.1
0.0
0.1
MR leave−one−out sensitivity analysis for
'MEGF10' on 'Cardiac valvular disease'

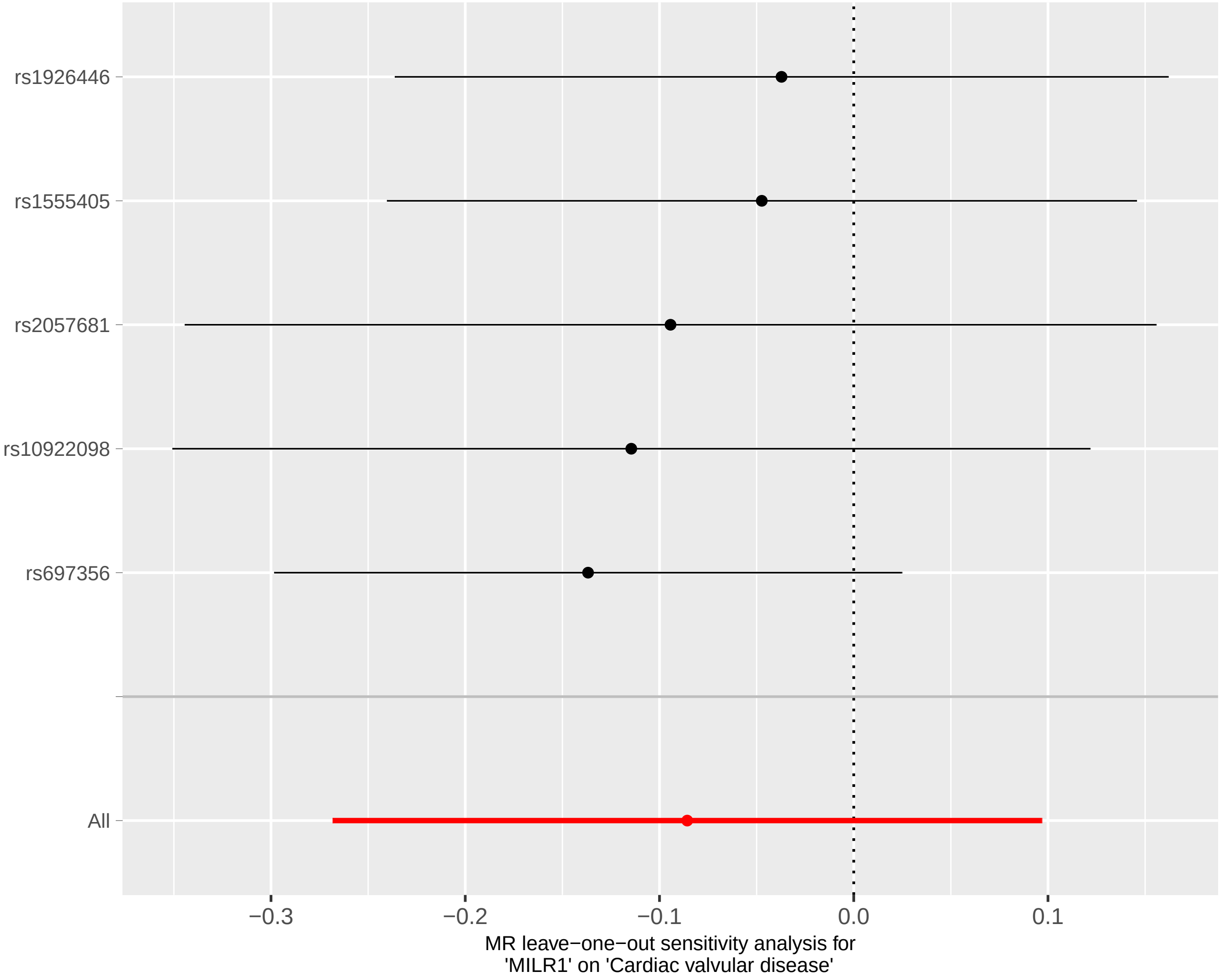
rs1926446
rs1555405
rs2057681
rs10922098
rs697356
All
−0.3
−0.2
−0.1
0.0
0.1
MR leave−one−out sensitivity analysis for
'MILR1' on 'Cardiac valvular disease'

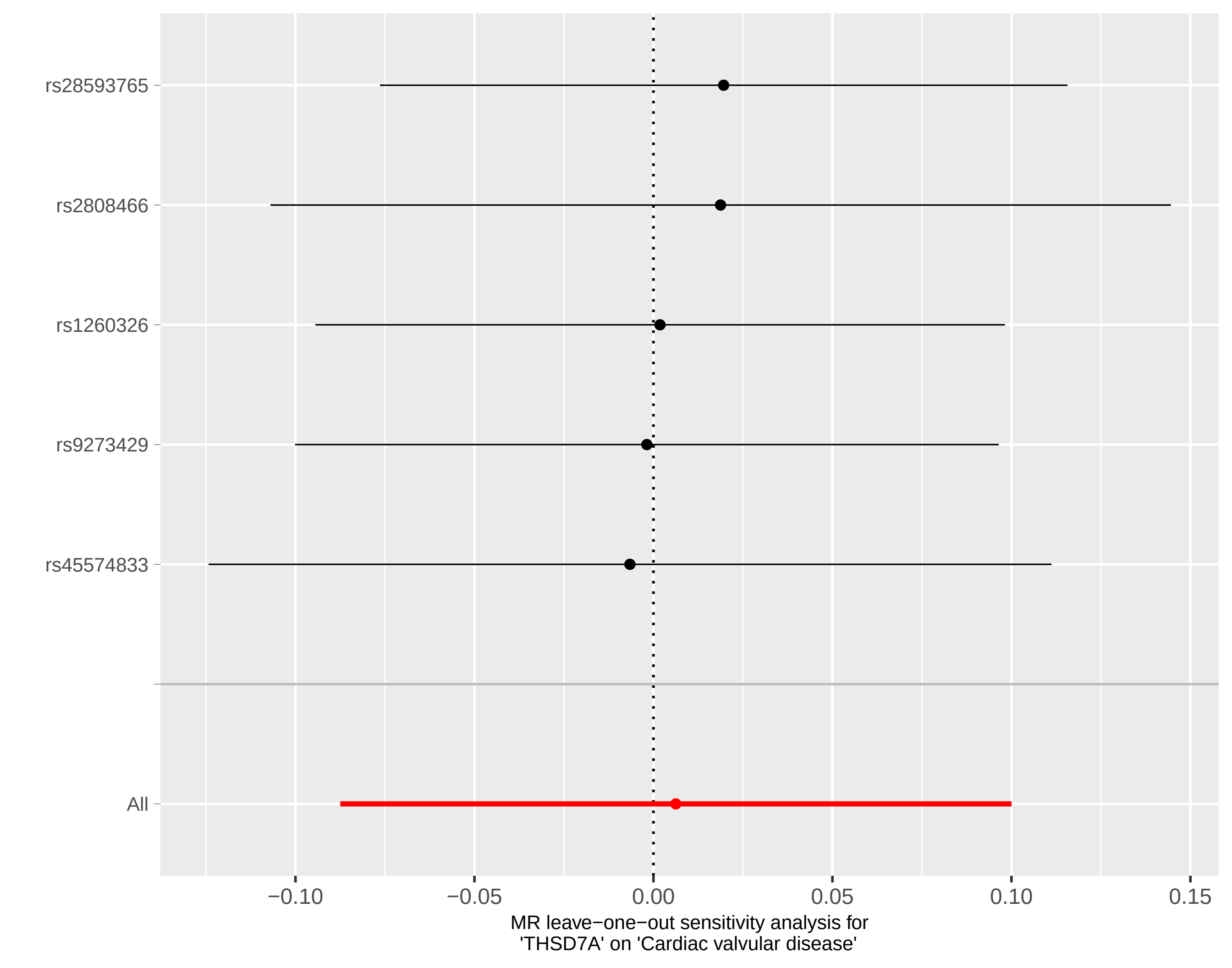

rs28593765
rs2808466
rs1260326
rs9273429
rs45574833
All
−0.10
−0.05
0.00
0.05
0.10
0.15
MR leave−one−out sensitivity analysis for
'THSD7A' on 'Cardiac valvular disease'

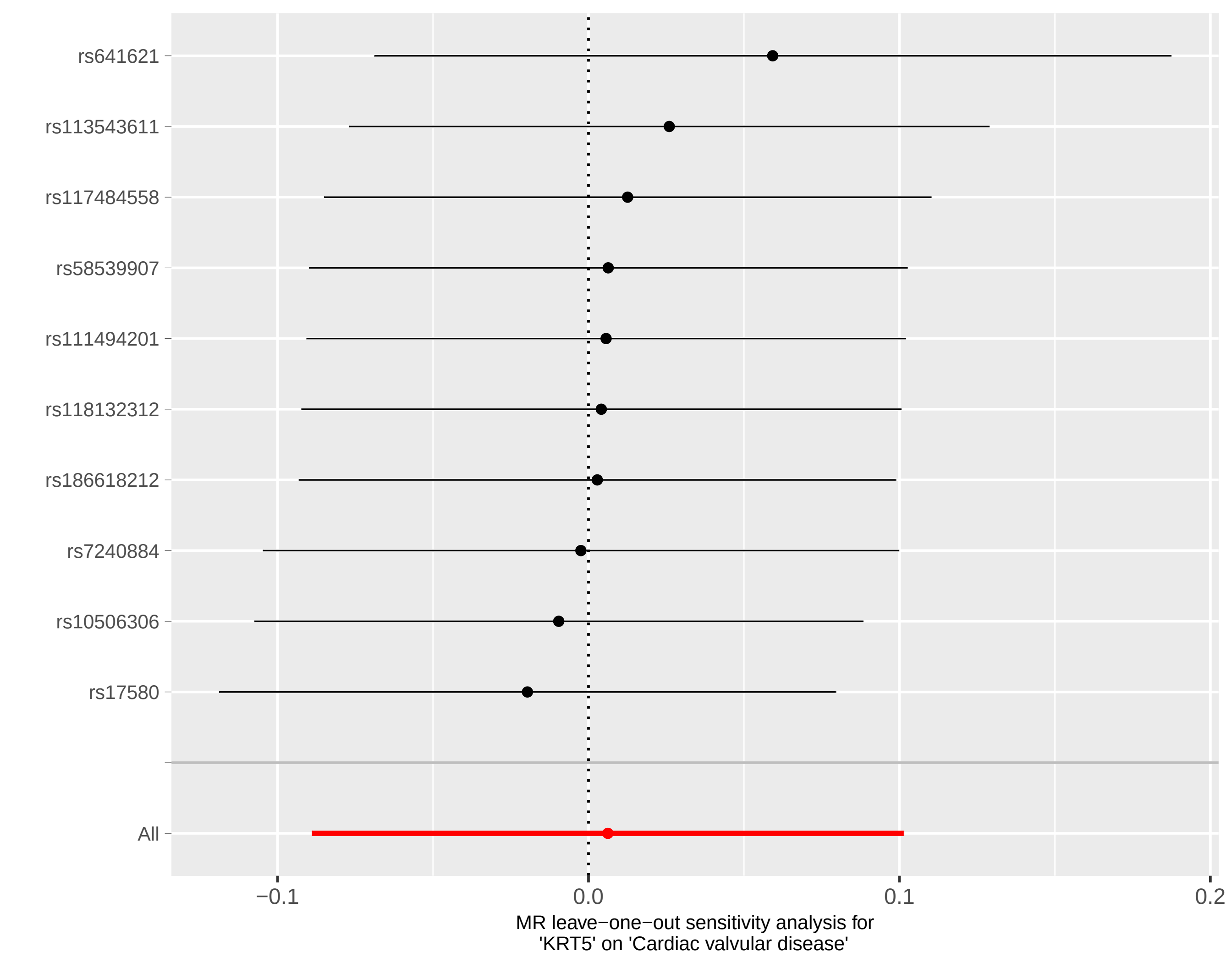
rs641621
rs113543611
rs117484558
rs58539907
rs111494201
rs118132312
rs186618212
rs7240884
rs10506306
rs17580
All
−0.1
0.0
0.1
0.2
MR leave−one−out sensitivity analysis for
'KRT5' on 'Cardiac valvular disease'

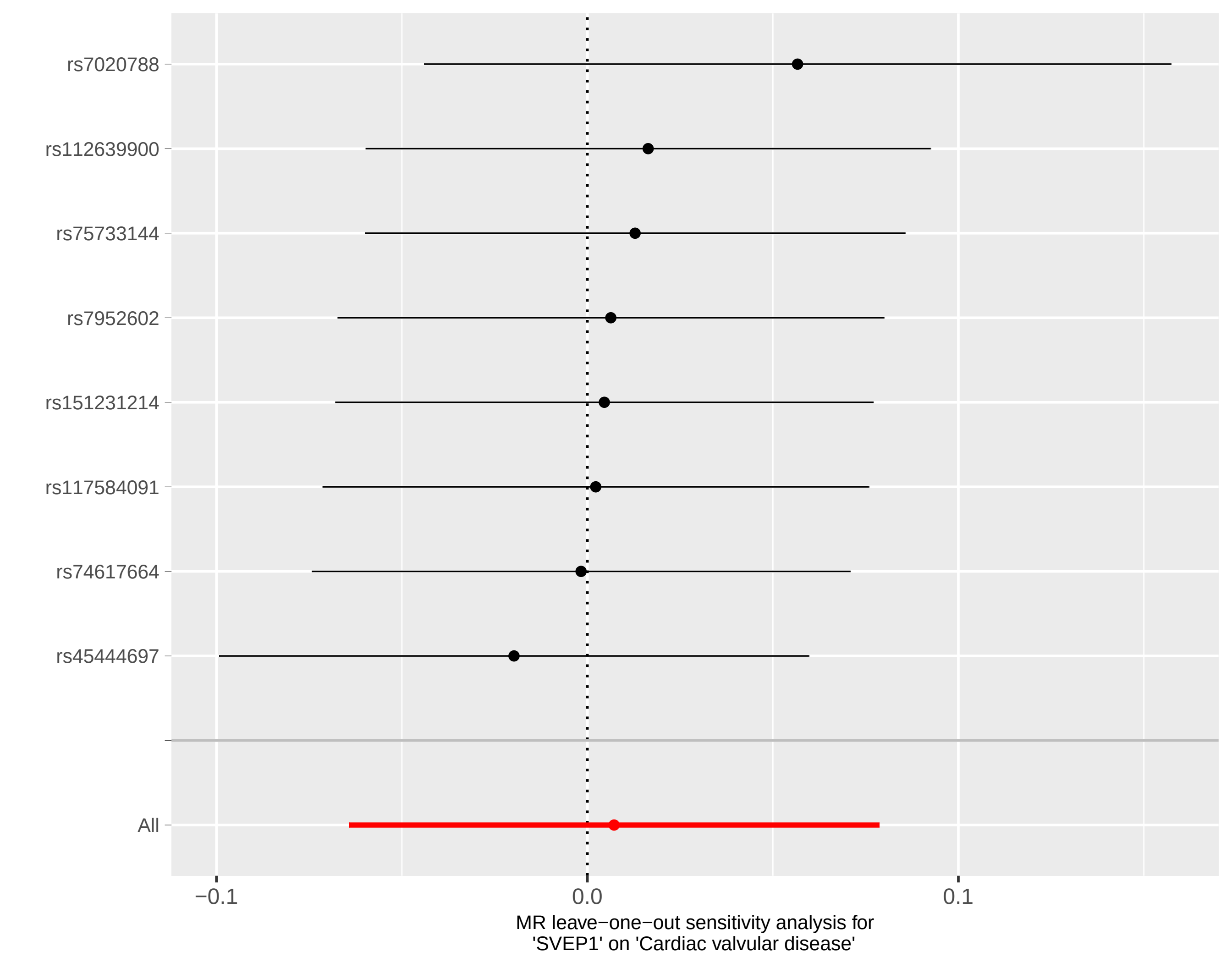
rs7020788
rs112639900
rs75733144
rs7952602
rs151231214
rs117584091
rs74617664
rs45444697
All
−0.1
0.0
0.1
MR leave−one−out sensitivity analysis for
'SVEP1' on 'Cardiac valvular disease'

Insufficient number of SNPs

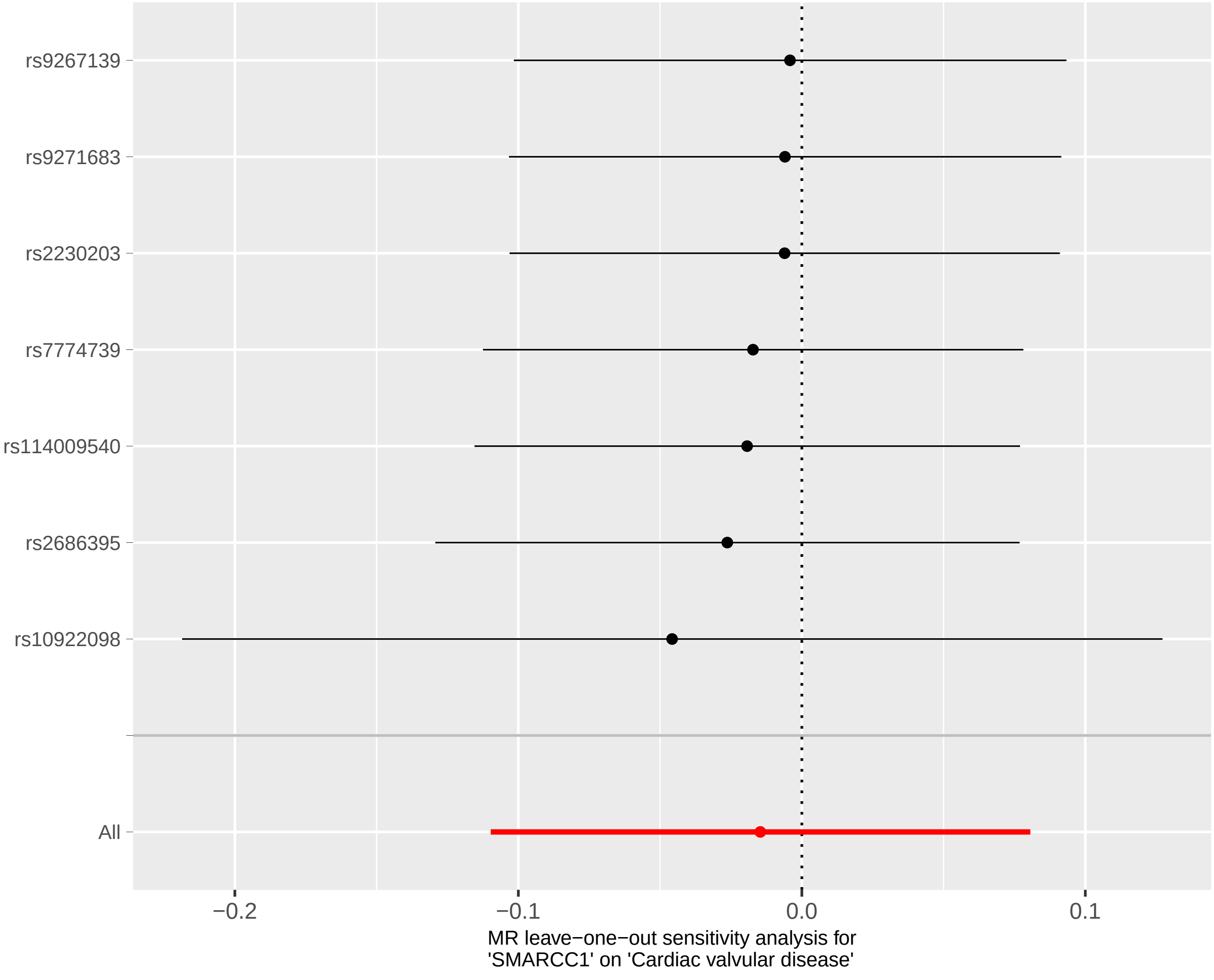
rs9267139
rs9271683
rs2230203
rs7774739
rs114009540
rs2686395
rs10922098
All
−0.2
−0.1
0.0
0.1
MR leave−one−out sensitivity analysis for
'SMARCC1' on 'Cardiac valvular disease'

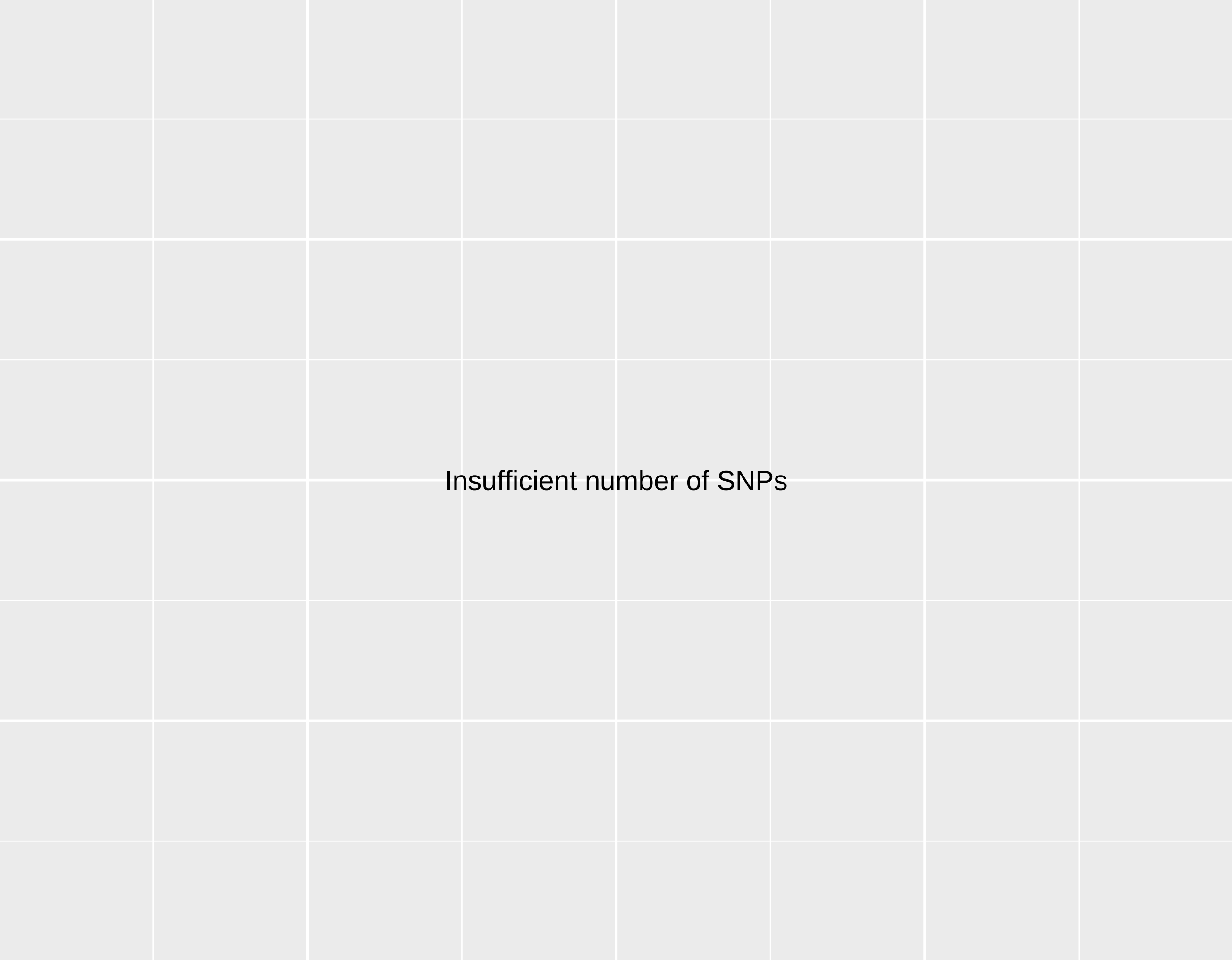
Insufficient number of SNPs

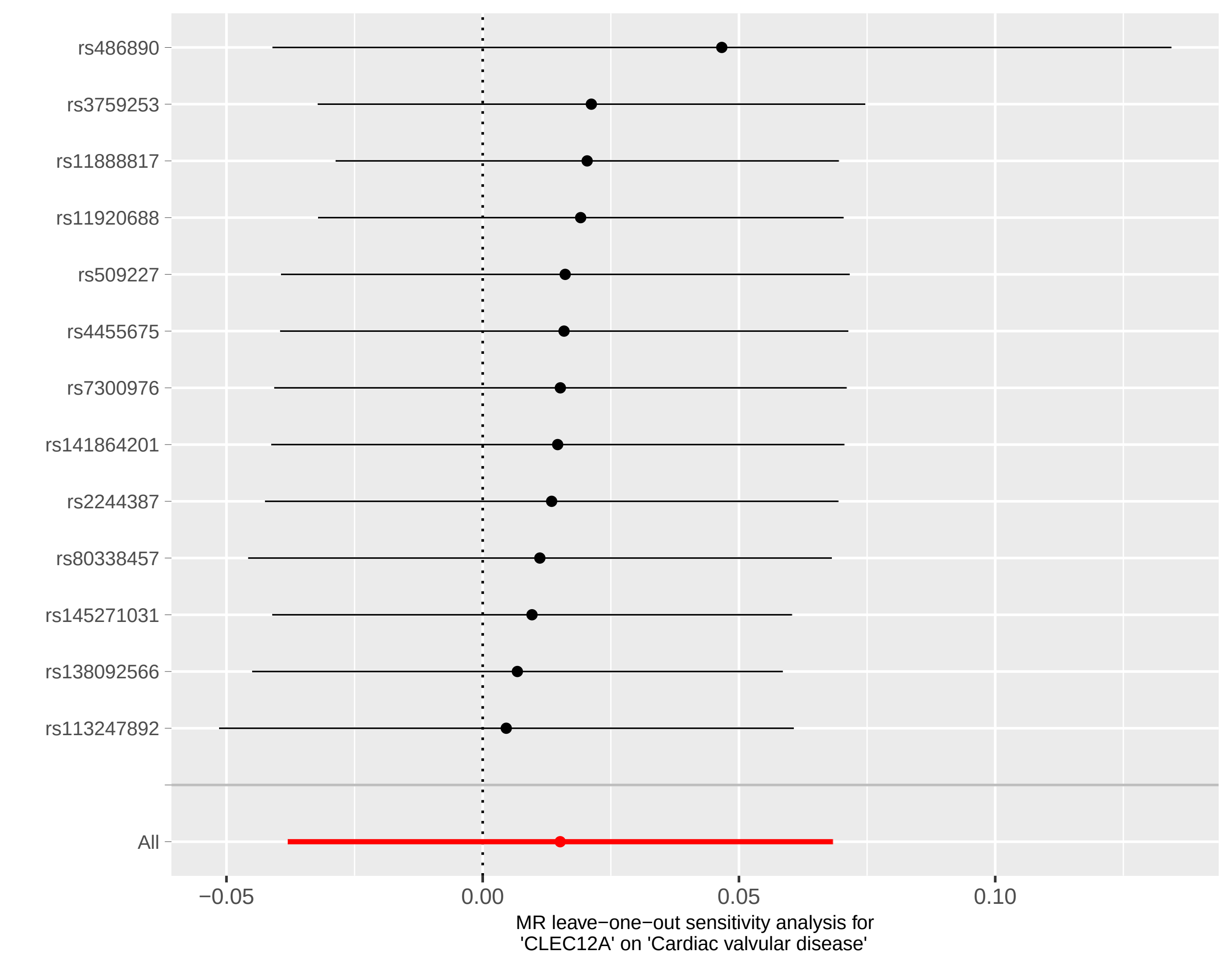

rs486890
rs3759253
rs11888817
rs11920688
rs509227
rs4455675
rs7300976
rs141864201
rs2244387
rs80338457
rs145271031
rs138092566
rs113247892
All
−0.05
0.00
0.05
0.10
MR leave−one−out sensitivity analysis for
'CLEC12A' on 'Cardiac valvular disease'

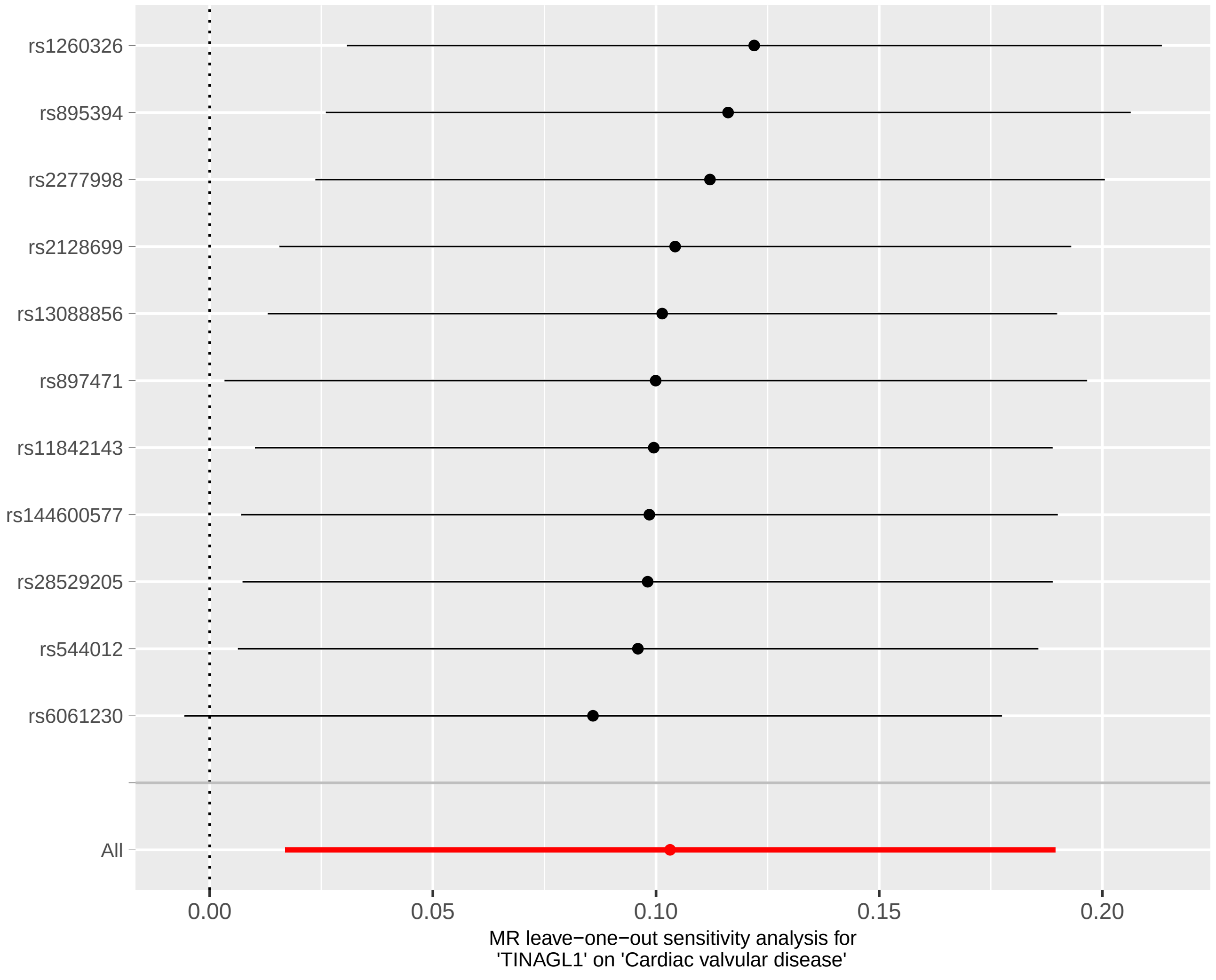
rs1260326
rs895394
rs2277998
rs2128699
rs13088856
rs897471
rs11842143
rs144600577
rs28529205
rs544012
rs6061230
All
0.00
0.05
0.10
0.15
0.20
MR leave−one−out sensitivity analysis for
'TINAGL1' on 'Cardiac valvular disease'

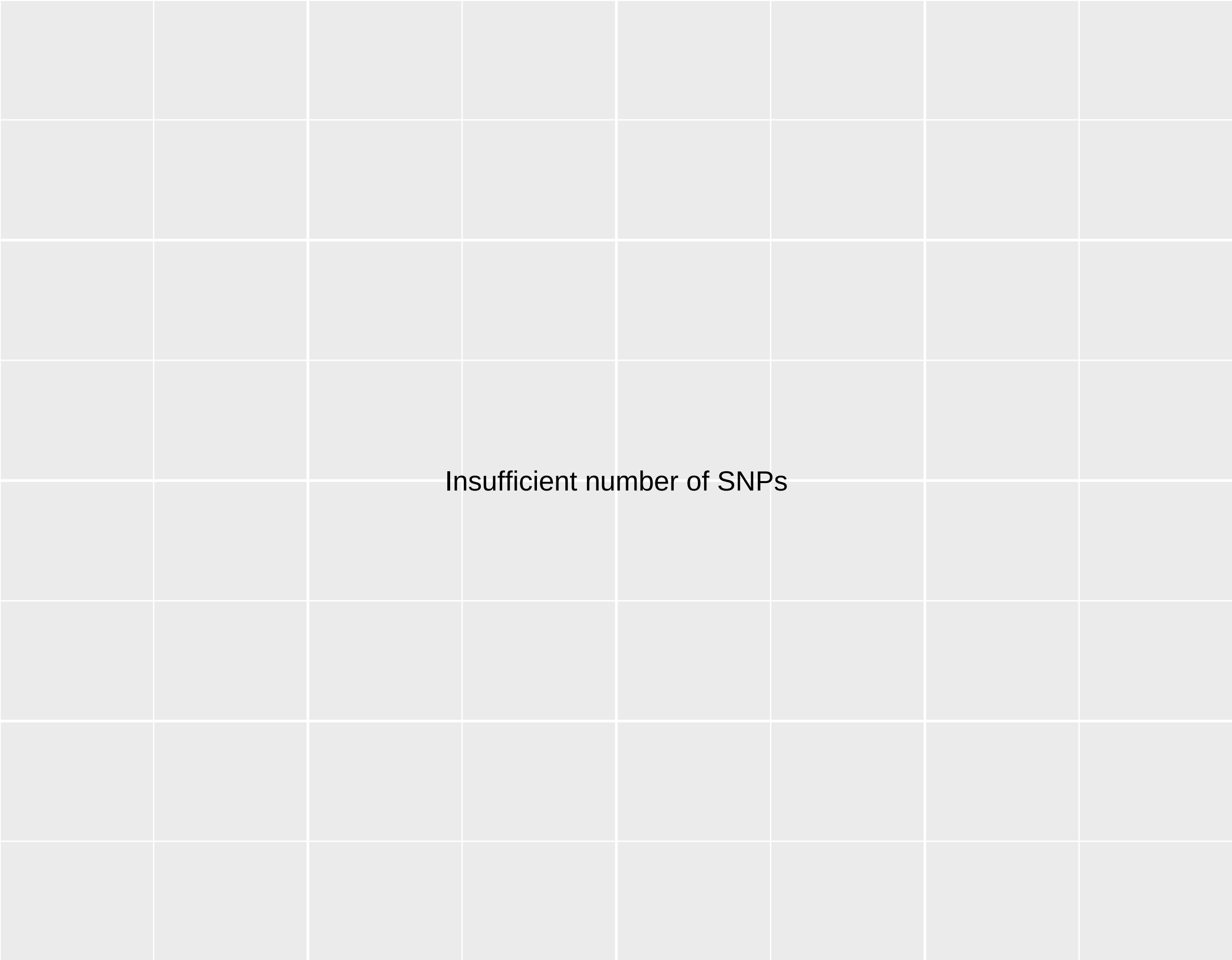
Insufficient number of SNPs

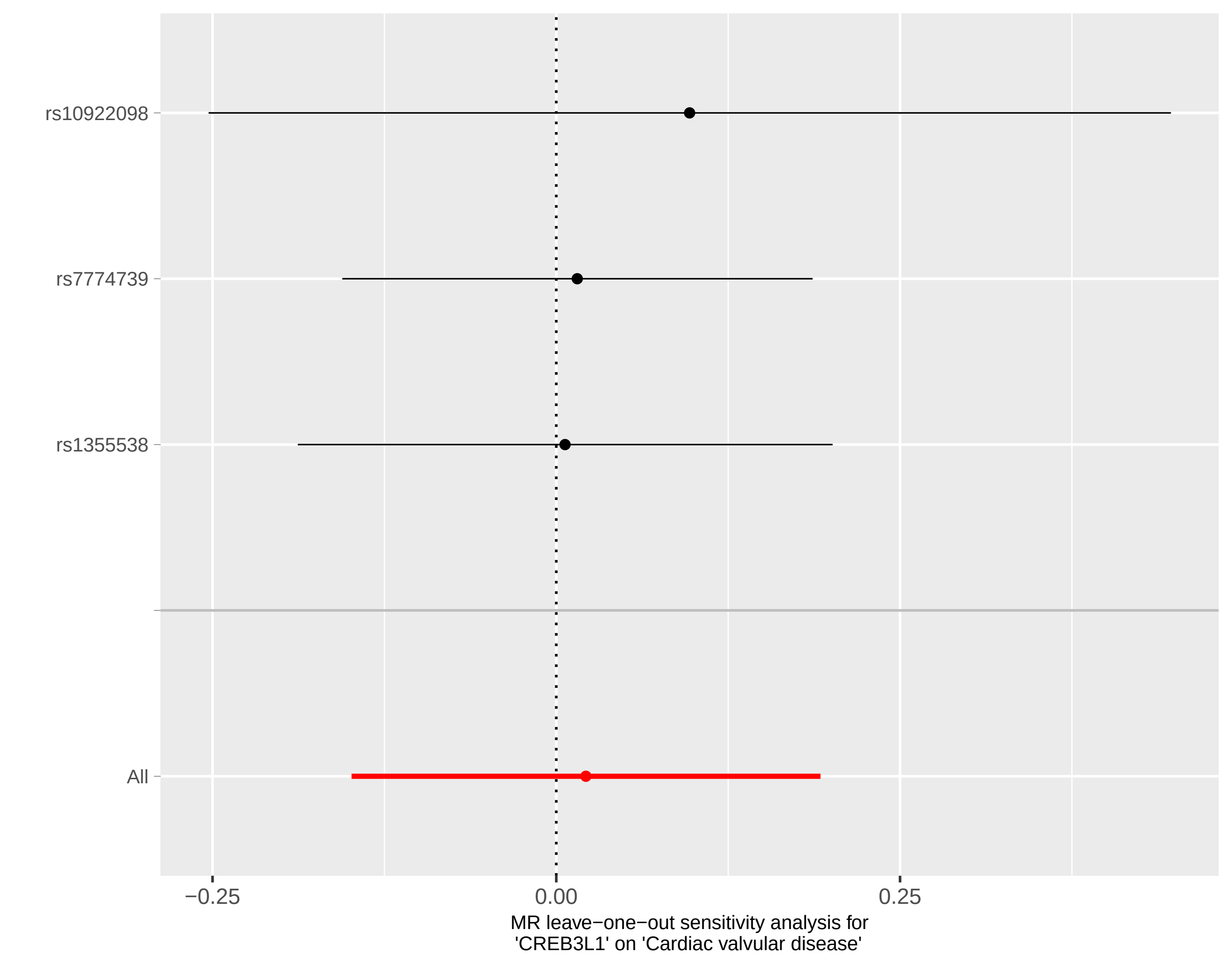

rs10922098
rs7774739
rs1355538
All
−0.25
0.00
0.25
MR leave−one−out sensitivity analysis for
'CREB3L1' on 'Cardiac valvular disease'

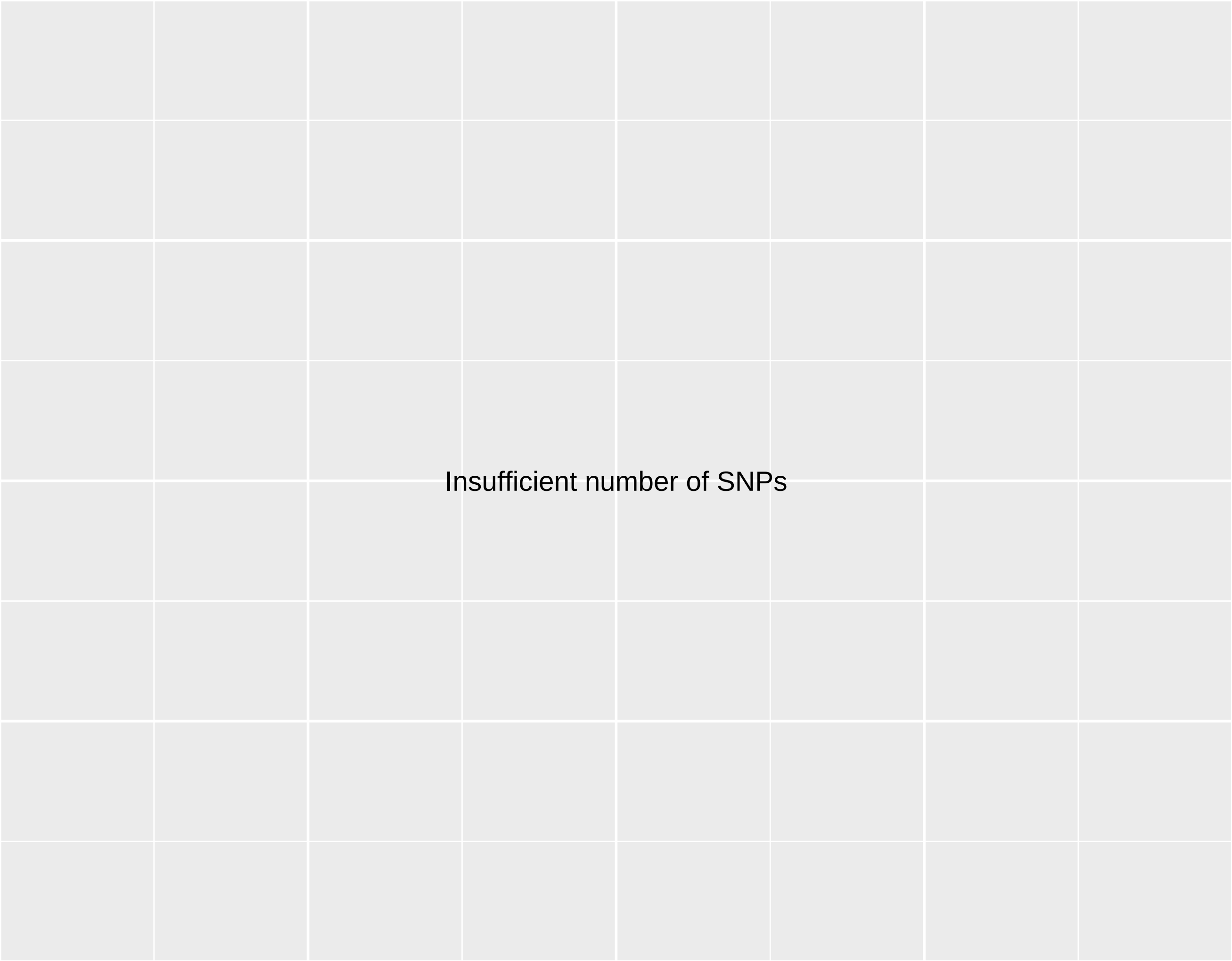
Insufficient number of SNPs

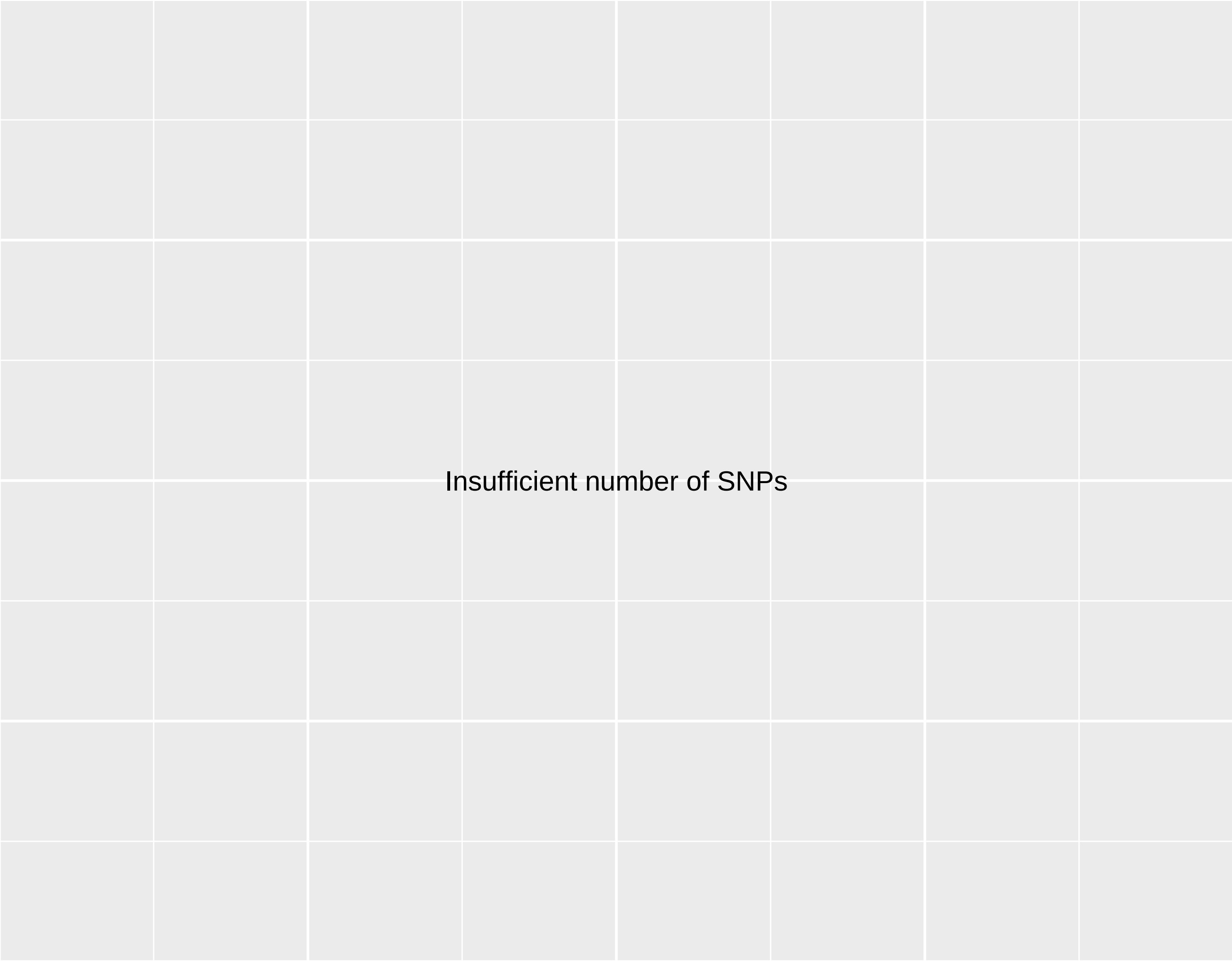
Insufficient number of SNPs

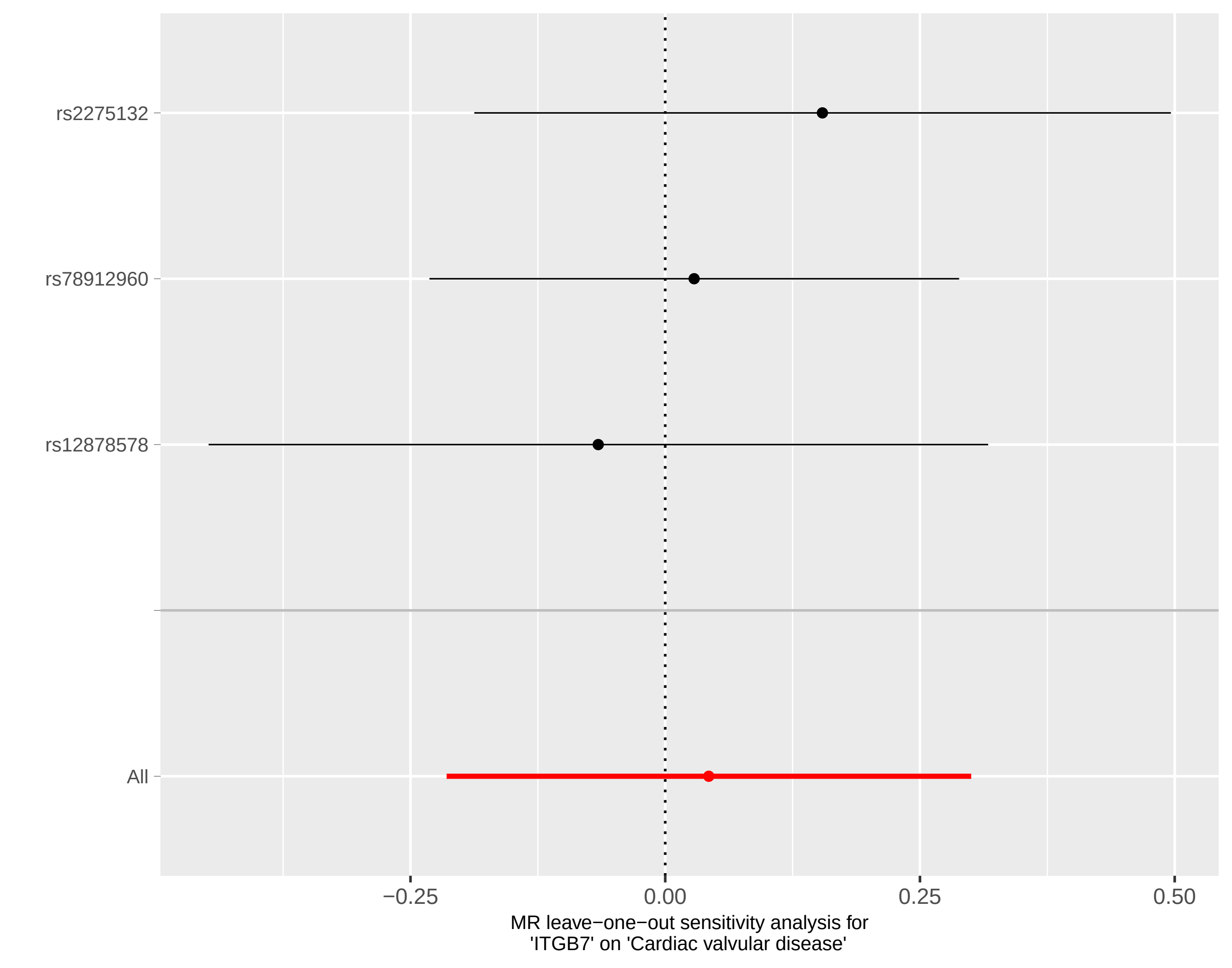
rs2275132
rs78912960
rs12878578
All
−0.25
0.00
0.25
0.50
MR leave−one−out sensitivity analysis for
'ITGB7' on 'Cardiac valvular disease'

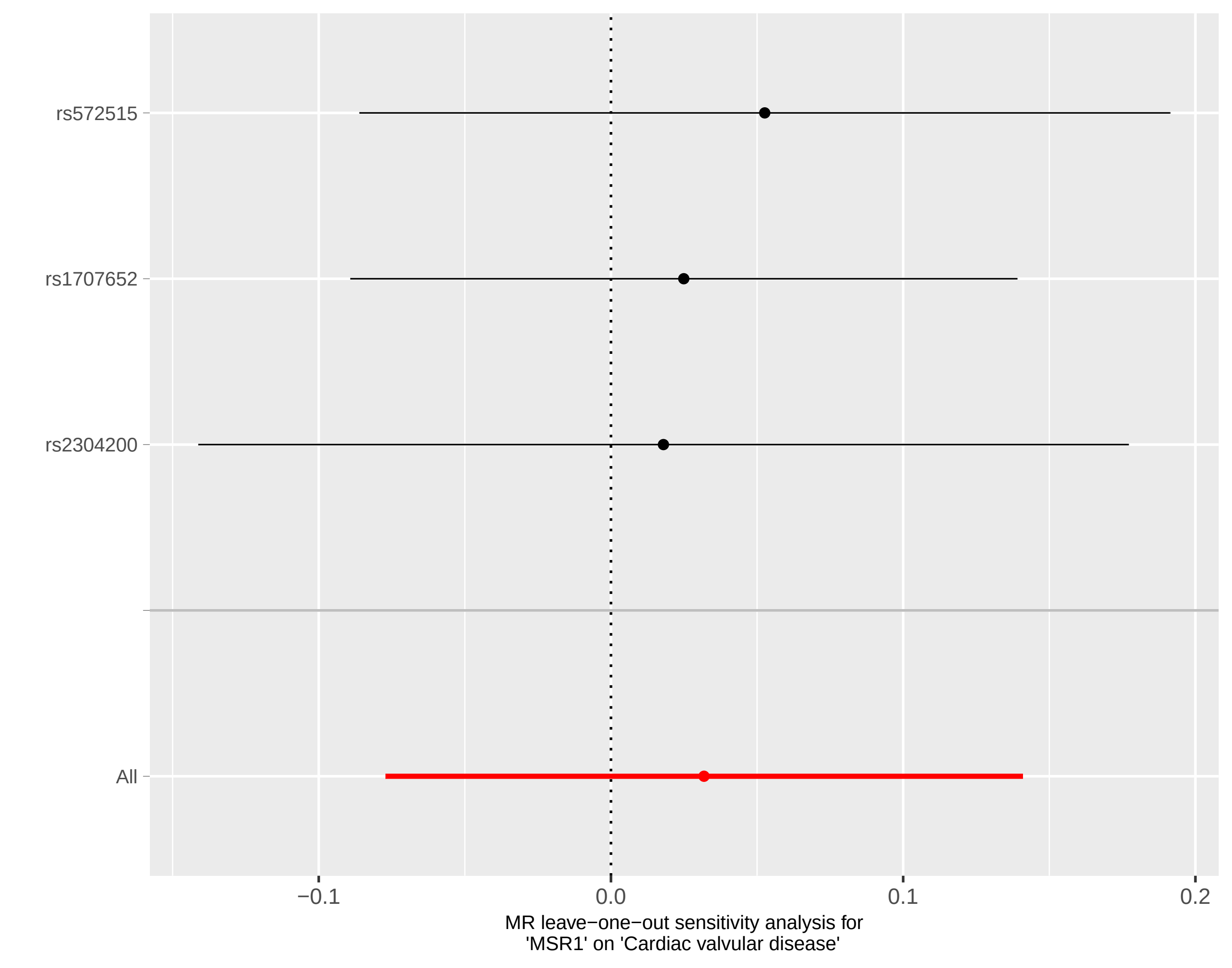

rs572515
rs1707652
rs2304200
All
−0.1
0.0
0.1
0.2
MR leave−one−out sensitivity analysis for
'MSR1' on 'Cardiac valvular disease'

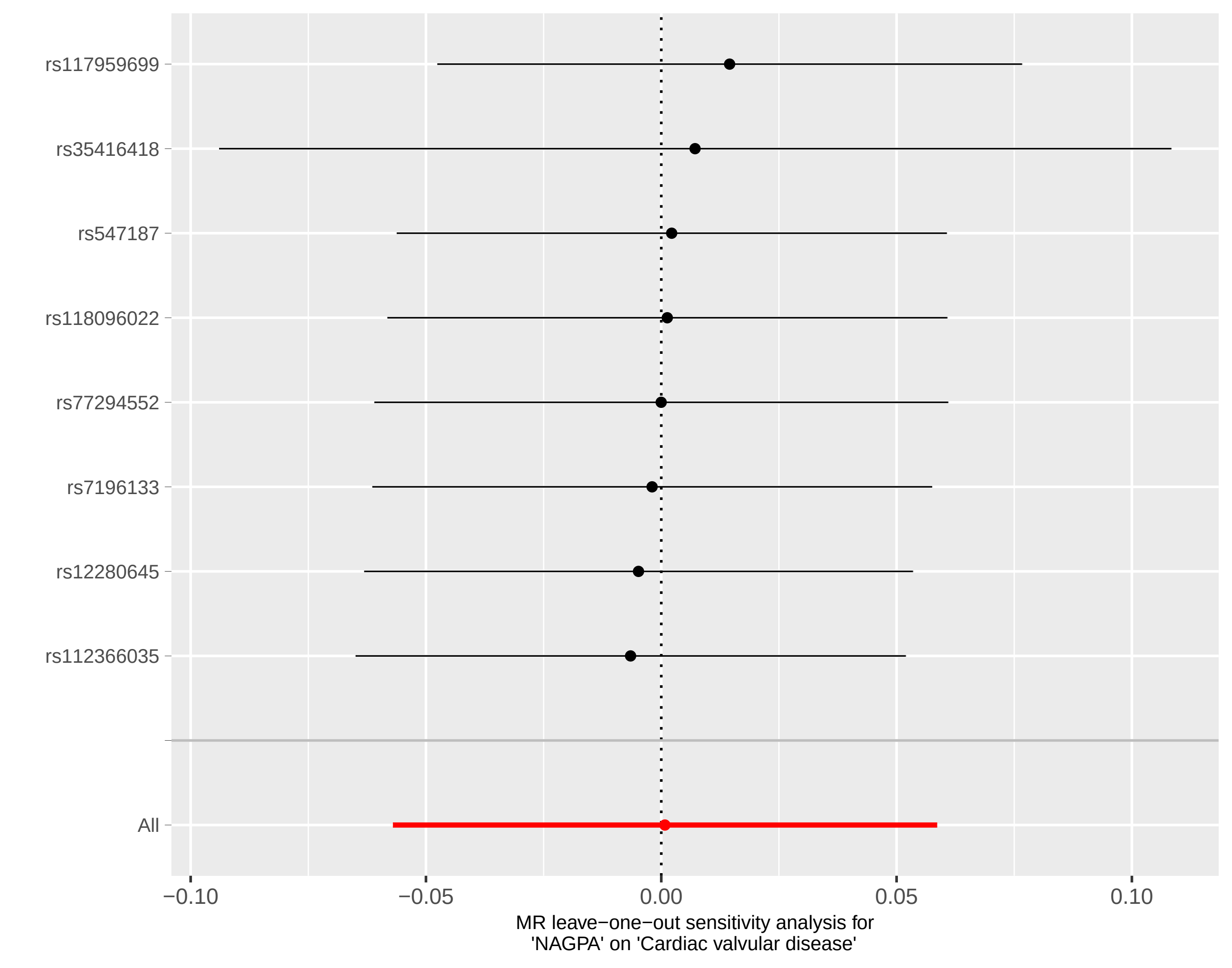
rs117959699
rs35416418
rs547187
rs118096022
rs77294552
rs7196133
rs12280645
rs112366035
All
−0.10
−0.05
0.00
0.05
0.10
MR leave−one−out sensitivity analysis for
'NAGPA' on 'Cardiac valvular disease'

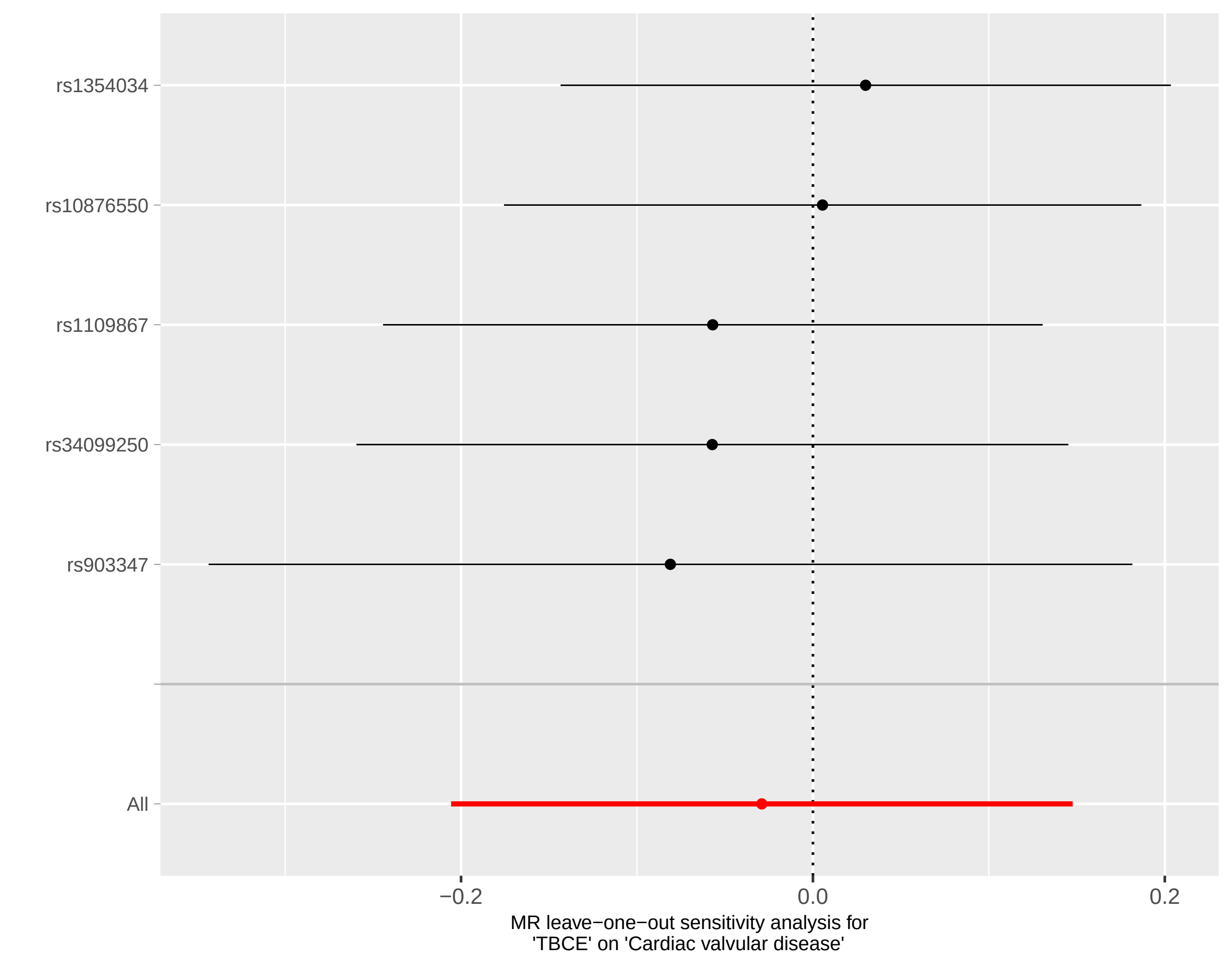
rs1354034
rs10876550
rs1109867
rs34099250
rs903347
All
−0.2
0.0
0.2
MR leave−one−out sensitivity analysis for
'TBCE' on 'Cardiac valvular disease'

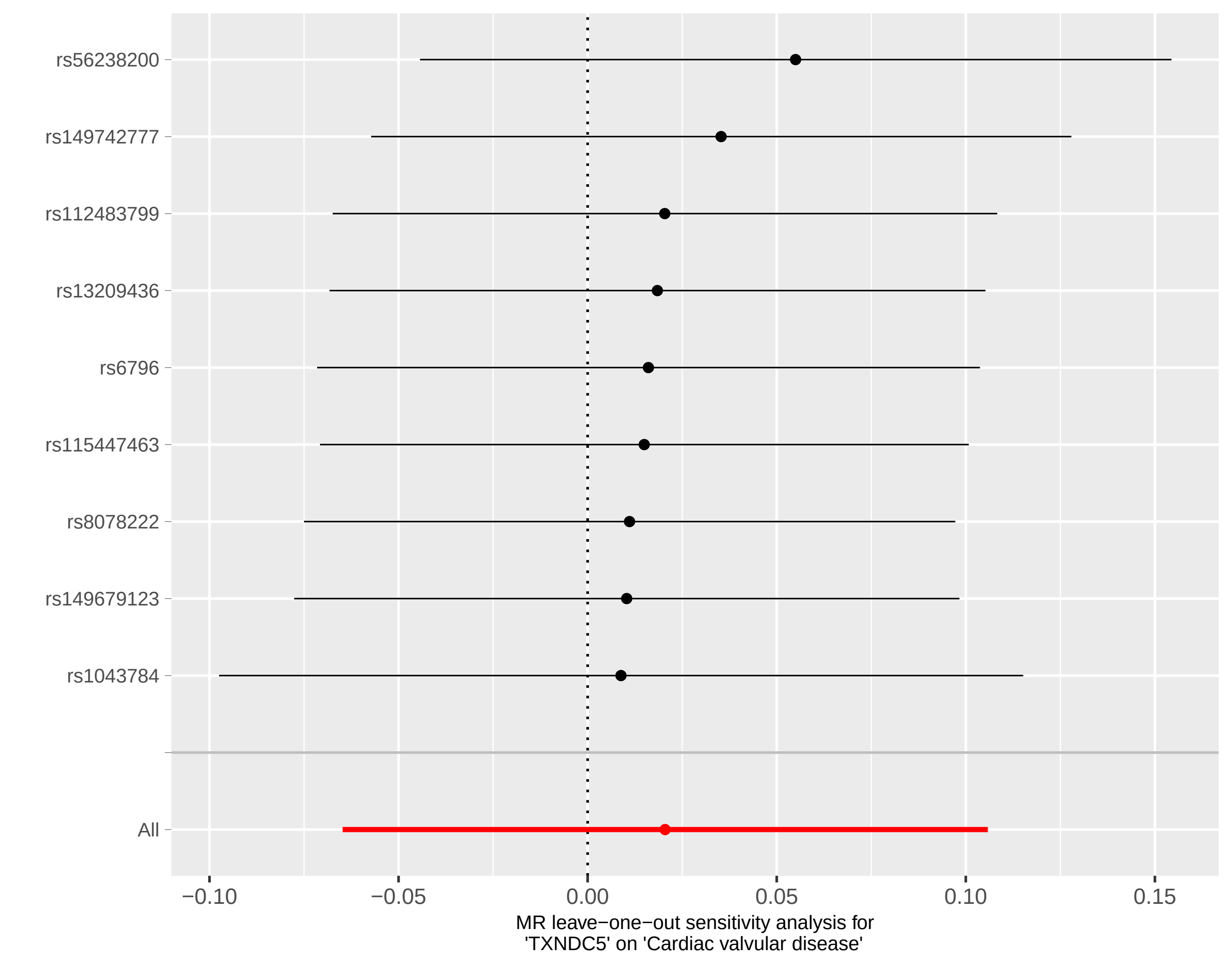
rs56238200
rs149742777
rs112483799
rs13209436
rs6796
rs115447463
rs8078222
rs149679123
rs1043784
All
−0.10
−0.05
0.00
0.05
0.10
0.15
MR leave−one−out sensitivity analysis for
'TXNDC5' on 'Cardiac valvular disease'

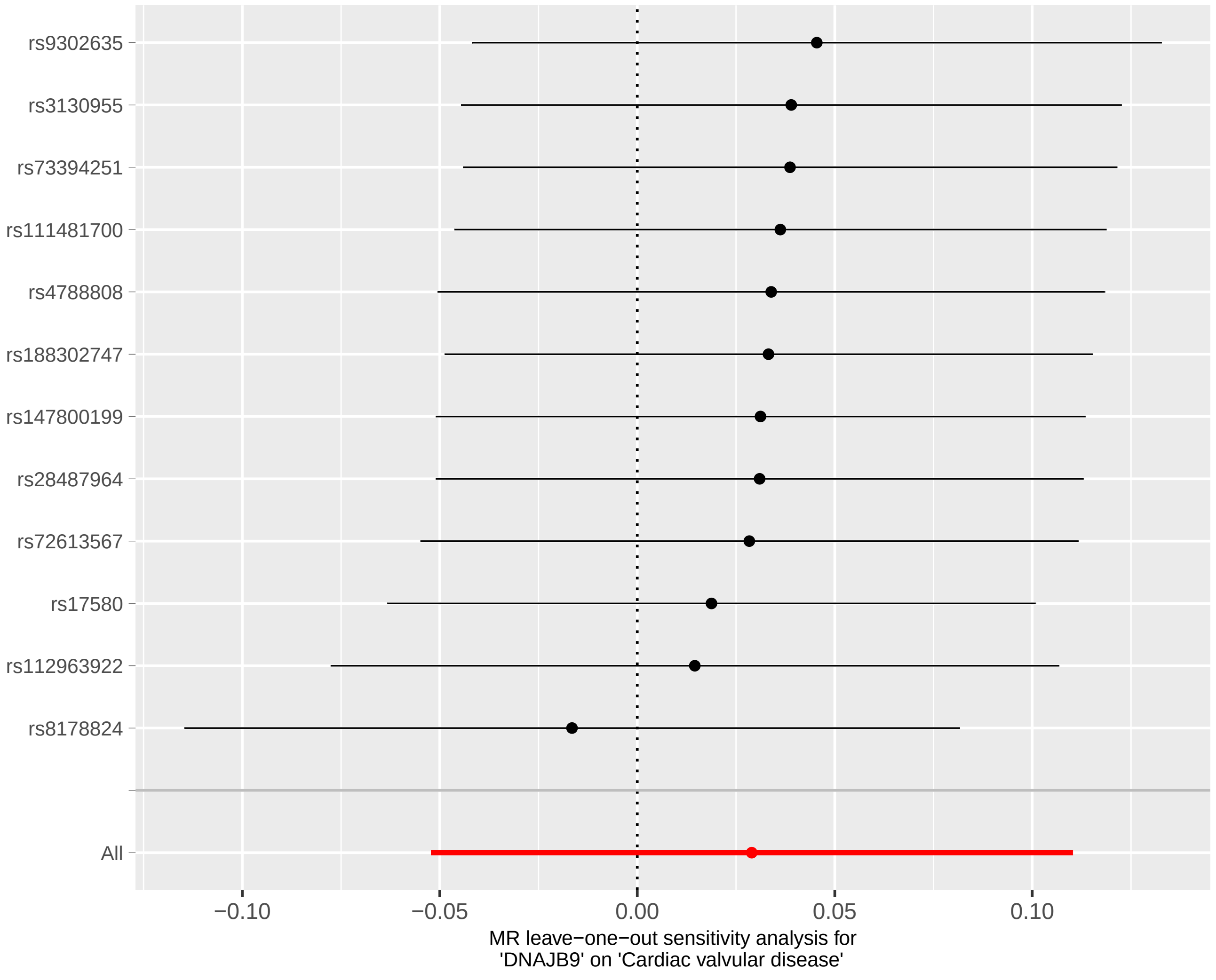

rs9302635
rs3130955
rs73394251
rs111481700
rs4788808
rs188302747
rs147800199
rs28487964
rs72613567
rs17580
rs112963922
rs8178824
All
−0.10
−0.05
0.00
0.05
0.10
MR leave−one−out sensitivity analysis for
'DNAJB9' on 'Cardiac valvular disease'

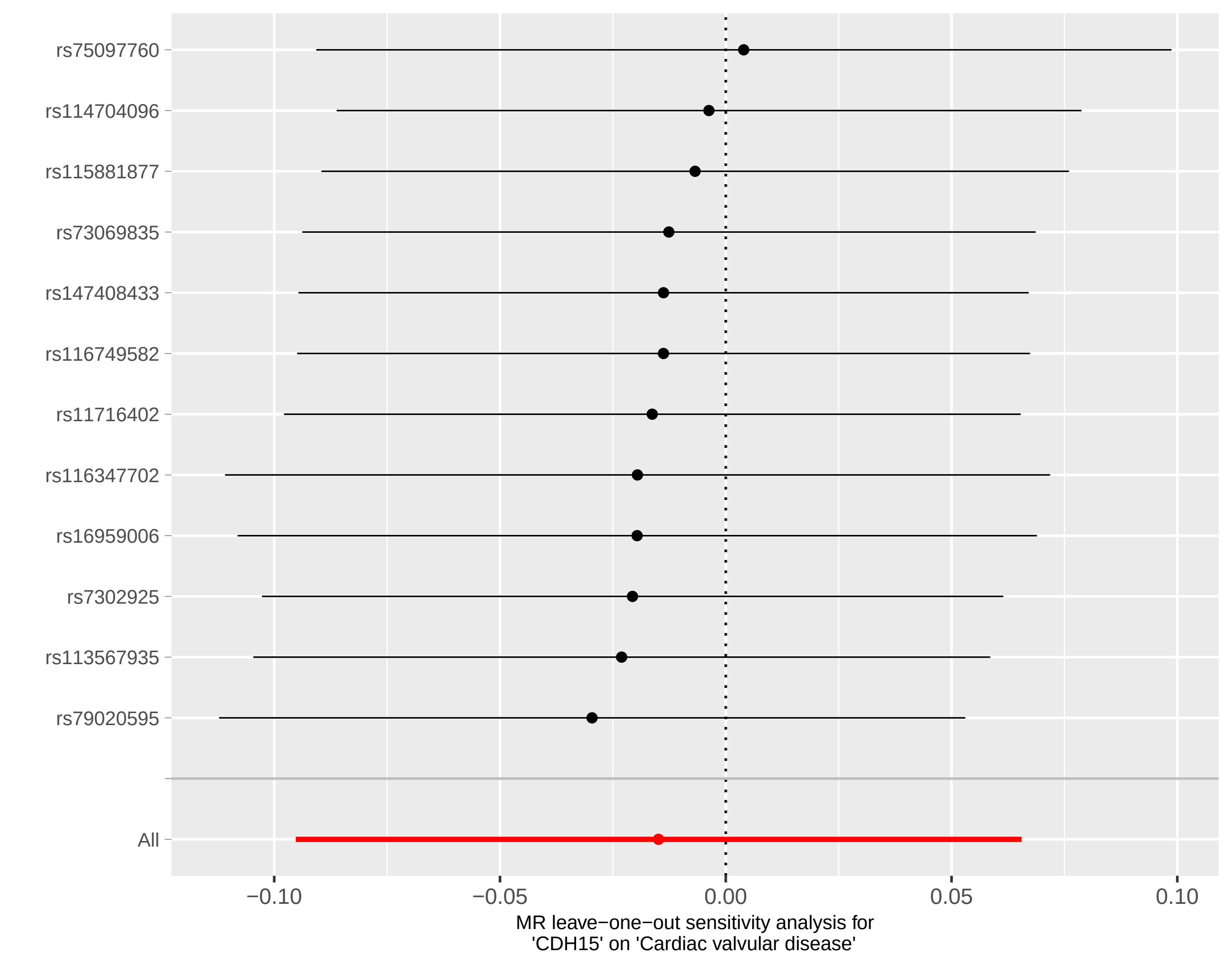

rs75097760
rs114704096
rs115881877
rs73069835
rs147408433
rs116749582
rs11716402
rs116347702
rs16959006
rs7302925
rs113567935
rs79020595
All
−0.10
−0.05
0.00
0.05
0.10
MR leave−one−out sensitivity analysis for
'CDH15' on 'Cardiac valvular disease'

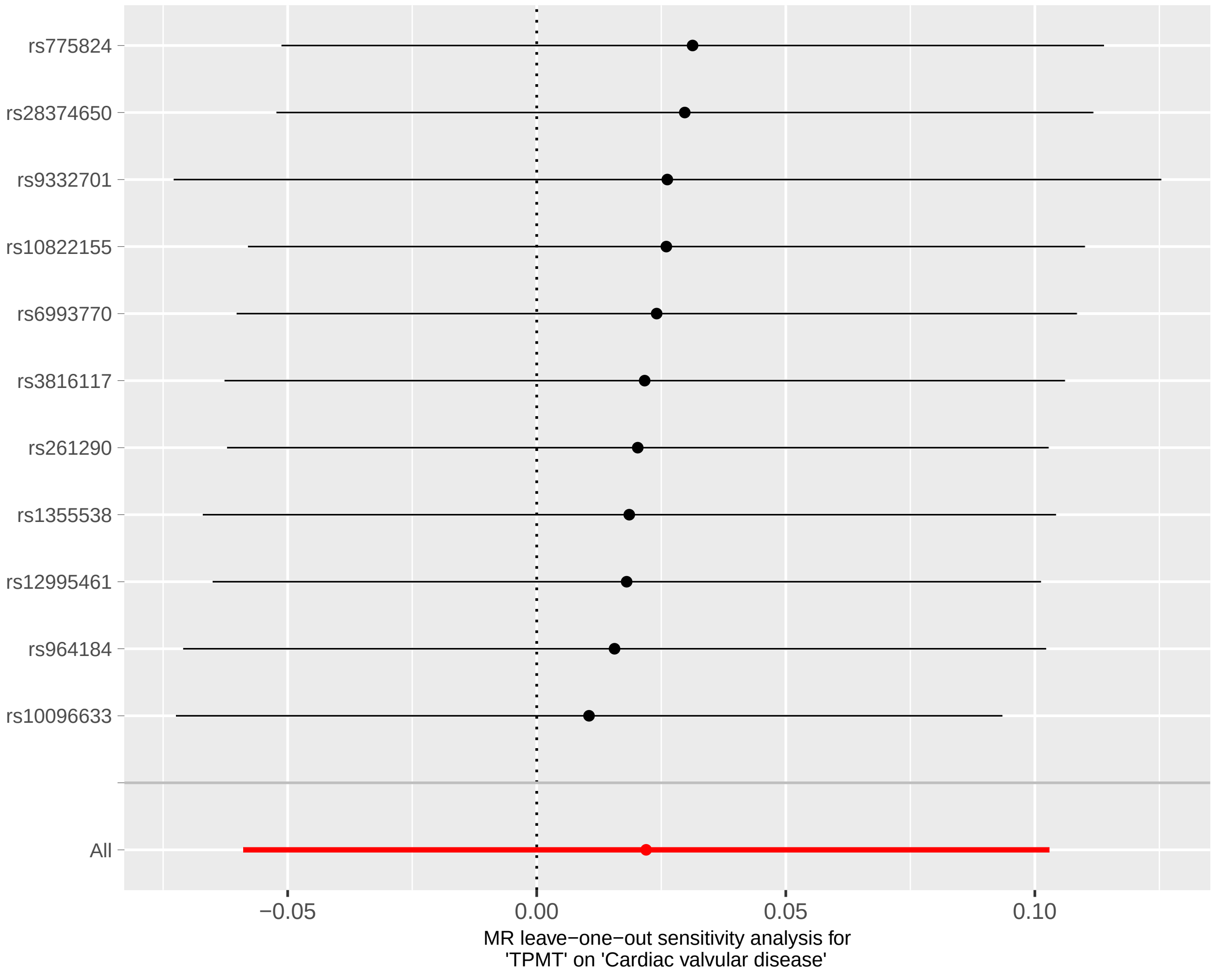
rs775824
rs28374650
rs9332701
rs10822155
rs6993770
rs3816117
rs261290
rs1355538
rs12995461
rs964184
rs10096633
All
−0.05
0.00
0.05
0.10
MR leave−one−out sensitivity analysis for
'TPMT' on 'Cardiac valvular disease'

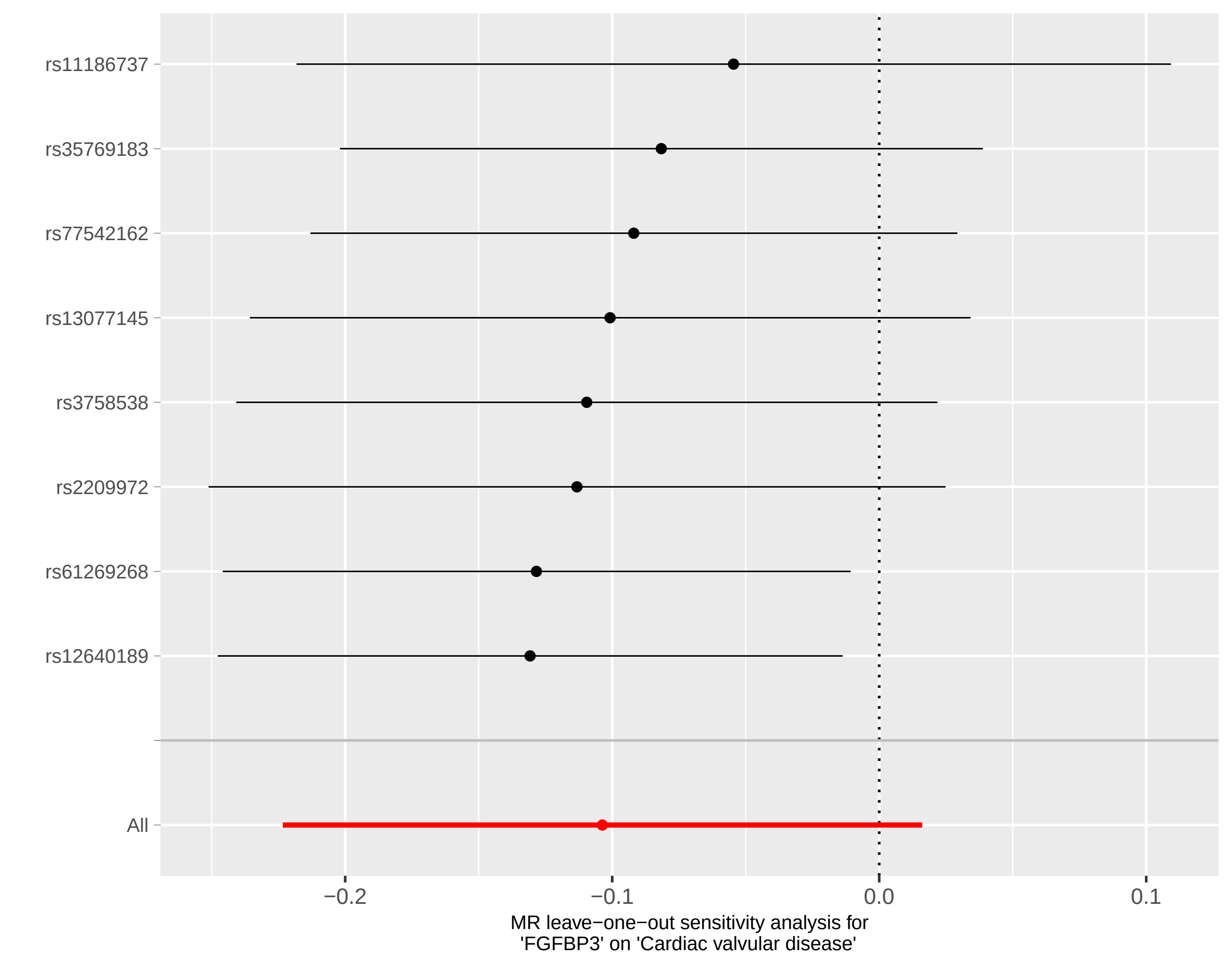

rs11186737
rs35769183
rs77542162
rs13077145
rs3758538
rs2209972
rs61269268
rs12640189
All
−0.2
−0.1
0.0
0.1
MR leave−one−out sensitivity analysis for
'FGFBP3' on 'Cardiac valvular disease'

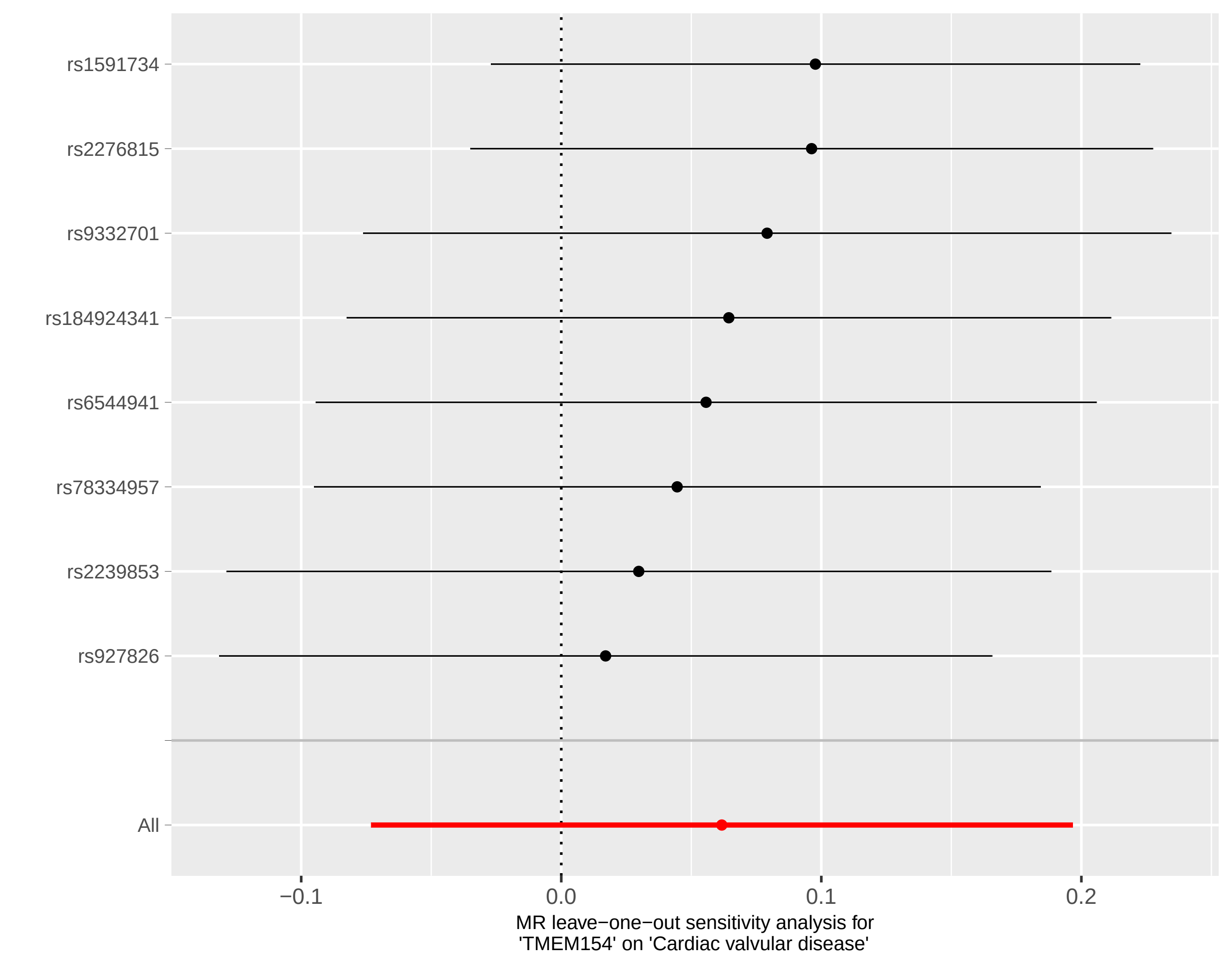
rs1591734
rs2276815
rs9332701
rs184924341
rs6544941
rs78334957
rs2239853
rs927826
All
−0.1
0.0
0.1
0.2
MR leave−one−out sensitivity analysis for
'TMEM154' on 'Cardiac valvular disease'

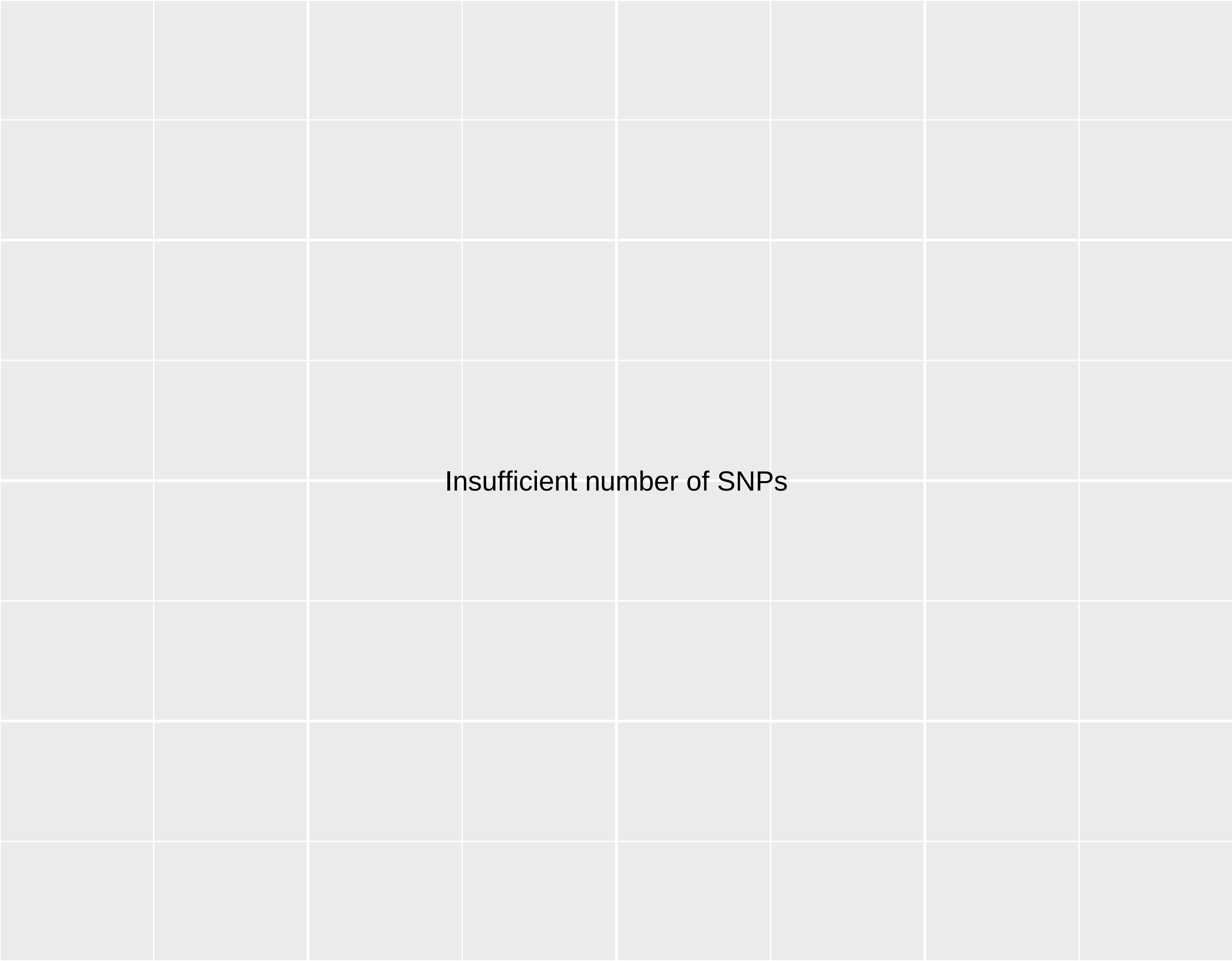
Insufficient number of SNPs

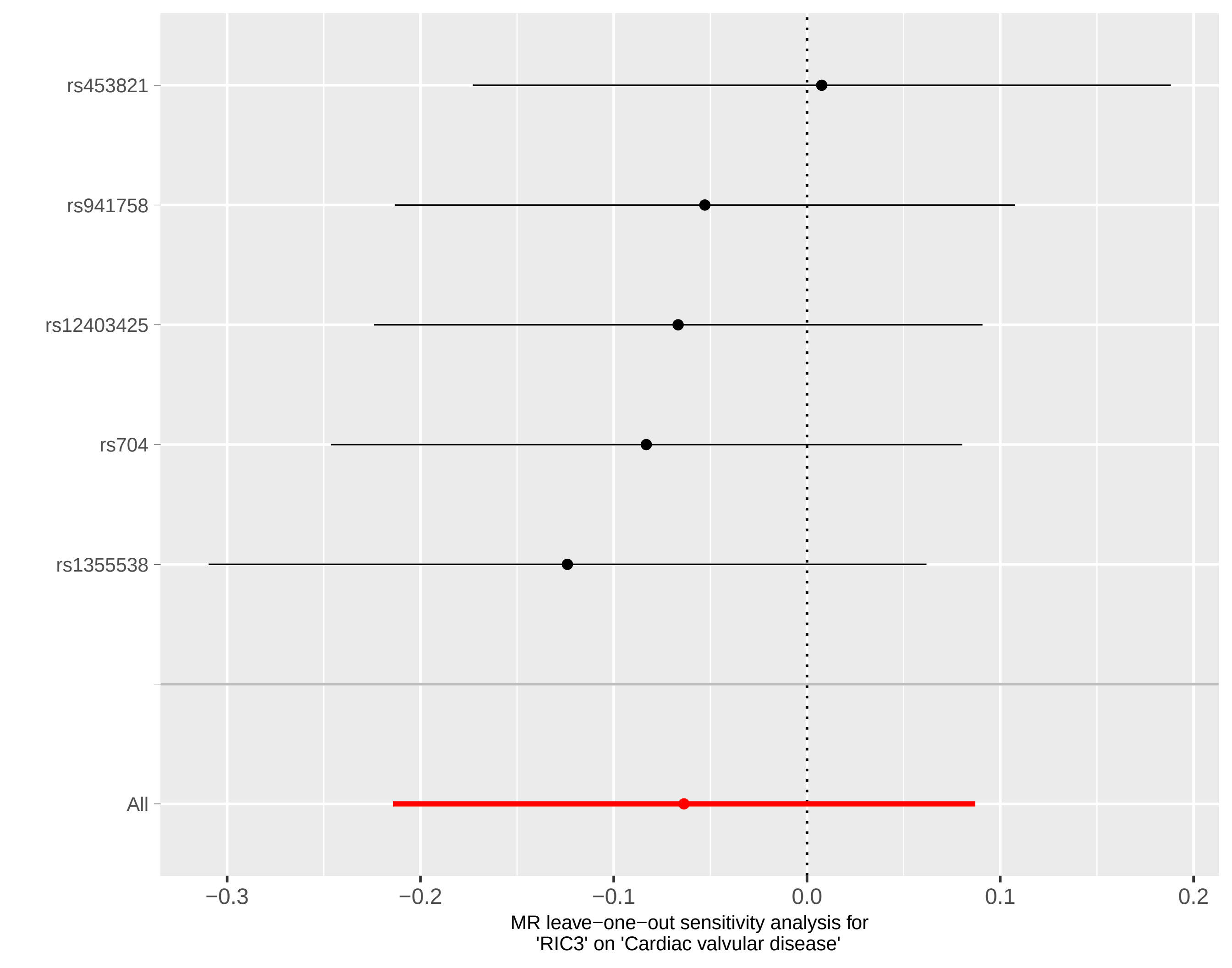

rs453821
rs941758
rs12403425
rs704
rs1355538
All
−0.3
−0.2
−0.1
0.0
0.1
0.2
MR leave−one−out sensitivity analysis for
'RIC3' on 'Cardiac valvular disease'

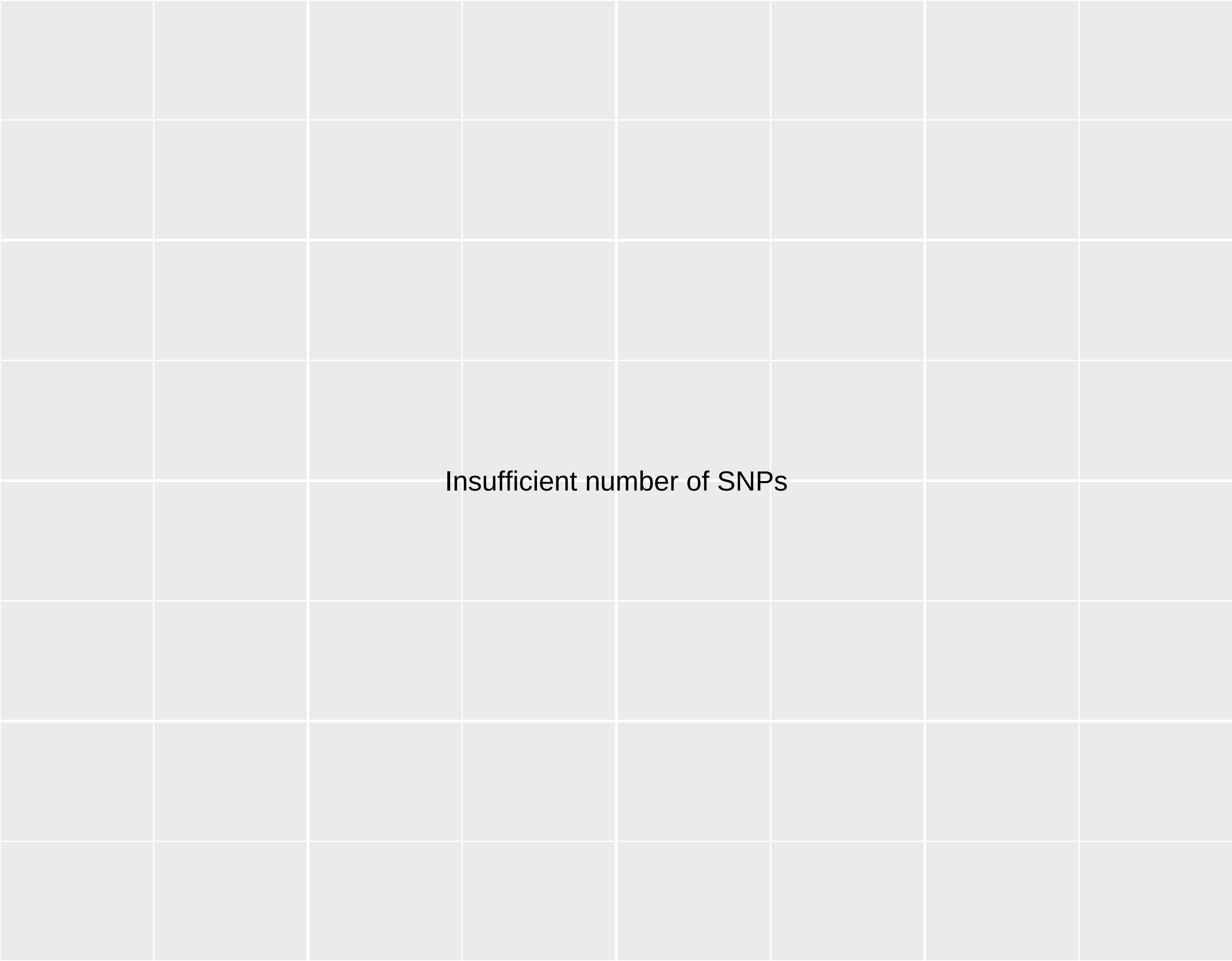
Insufficient number of SNPs

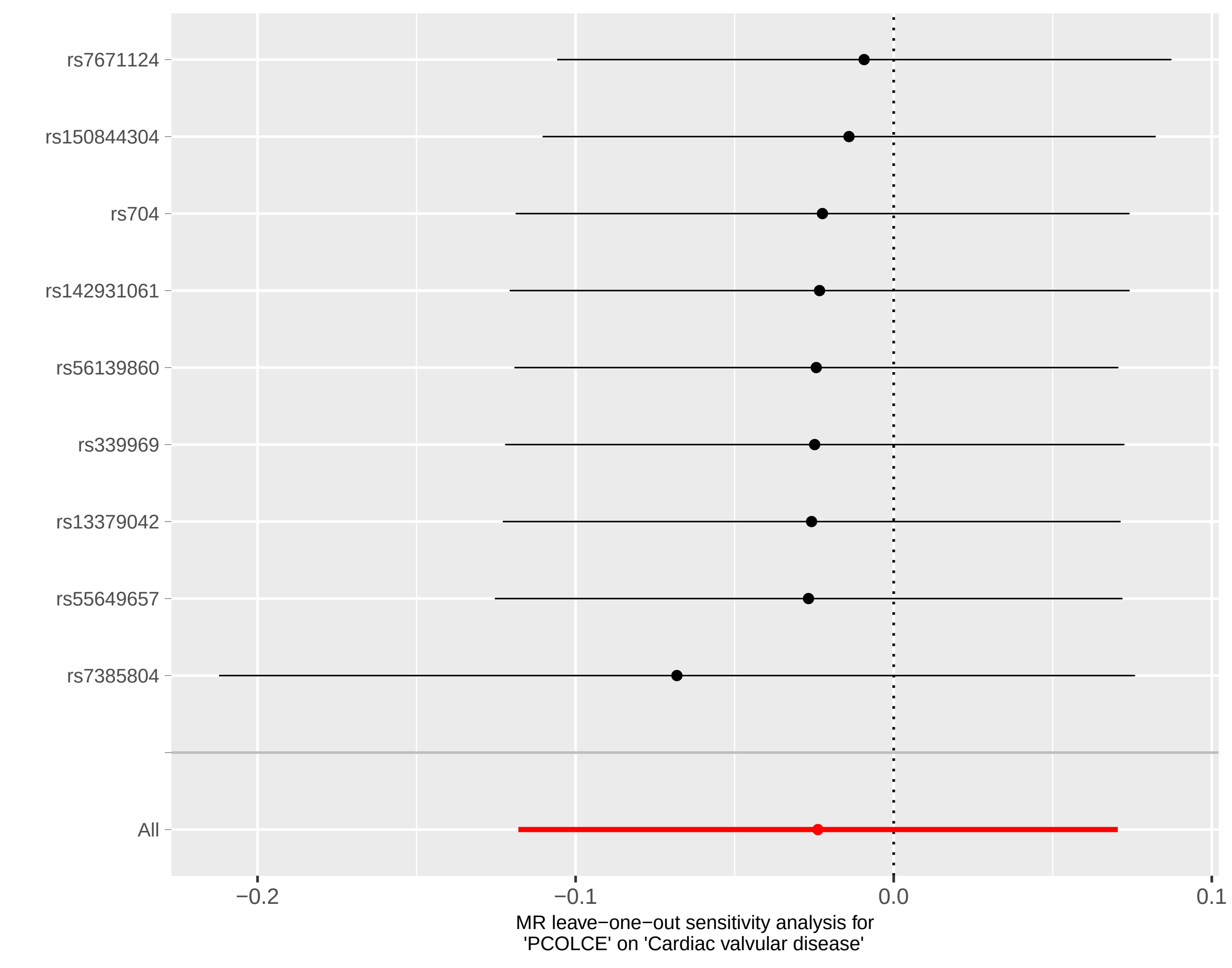
rs7671124
rs150844304
rs704
rs142931061
rs56139860
rs339969
rs13379042
rs55649657
rs7385804
All
−0.2
−0.1
0.0
0.1
MR leave−one−out sensitivity analysis for
'PCOLCE' on 'Cardiac valvular disease'

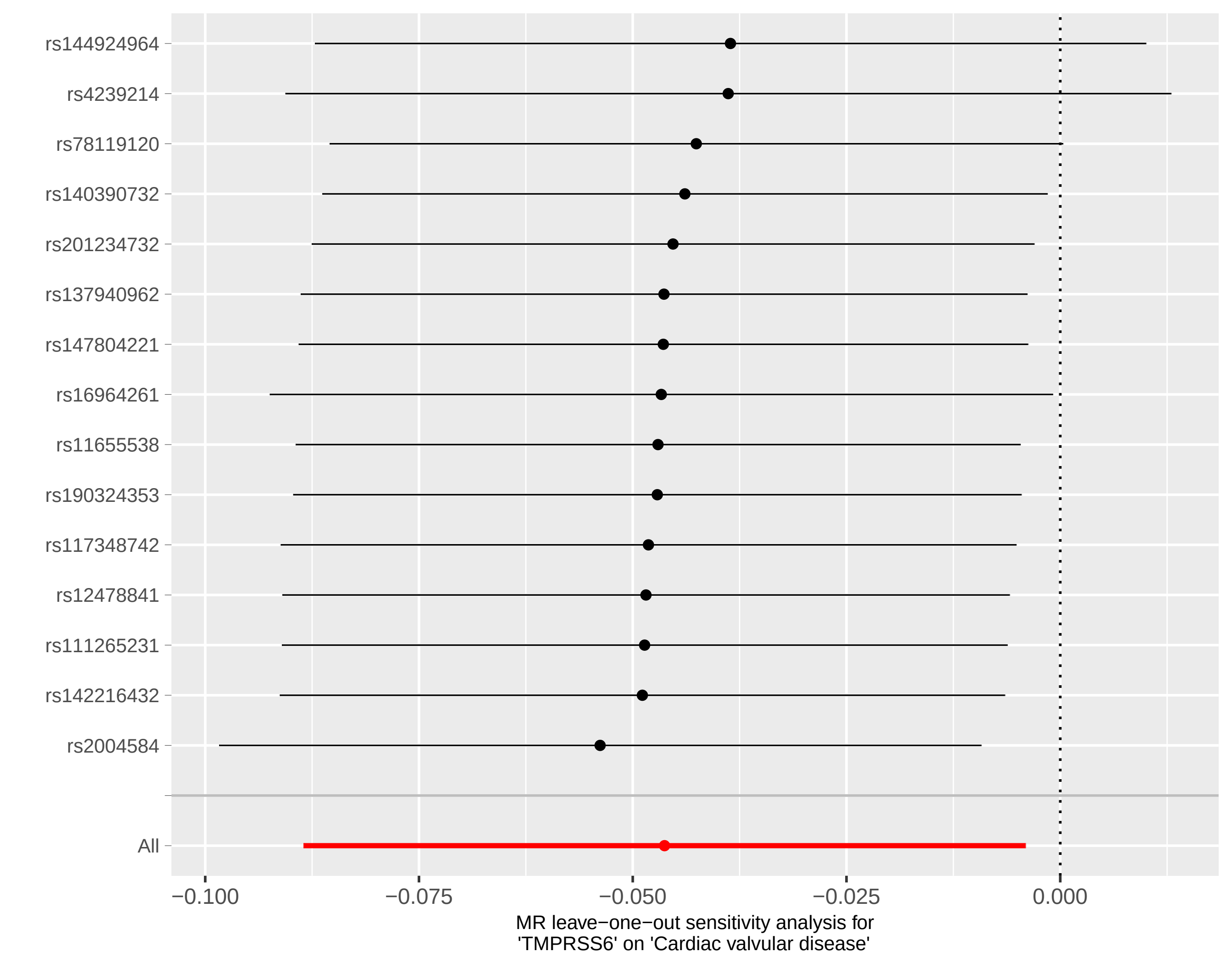

rs144924964
rs4239214
rs78119120
rs140390732
rs201234732
rs137940962
rs147804221
rs16964261
rs11655538
rs190324353
rs117348742
rs12478841
rs111265231
rs142216432
rs2004584
All
−0.100
−0.075
−0.050
−0.025
0.000
MR leave−one−out sensitivity analysis for
'TMPRSS6' on 'Cardiac valvular disease'

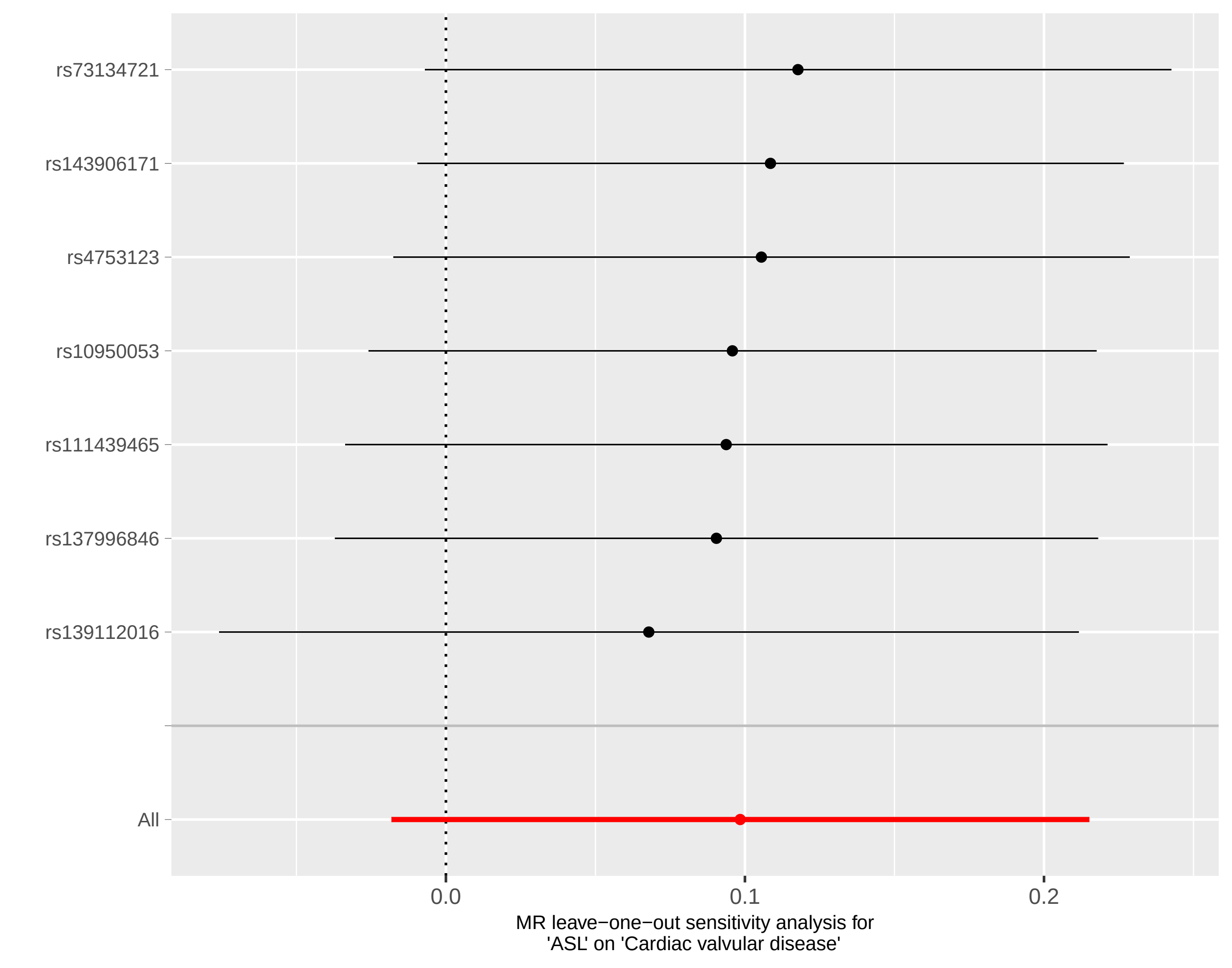
rs73134721
rs143906171
rs4753123
rs10950053
rs111439465
rs137996846
rs139112016
All
0.0
0.1
0.2
MR leave−one−out sensitivity analysis for
'ASL' on 'Cardiac valvular disease'

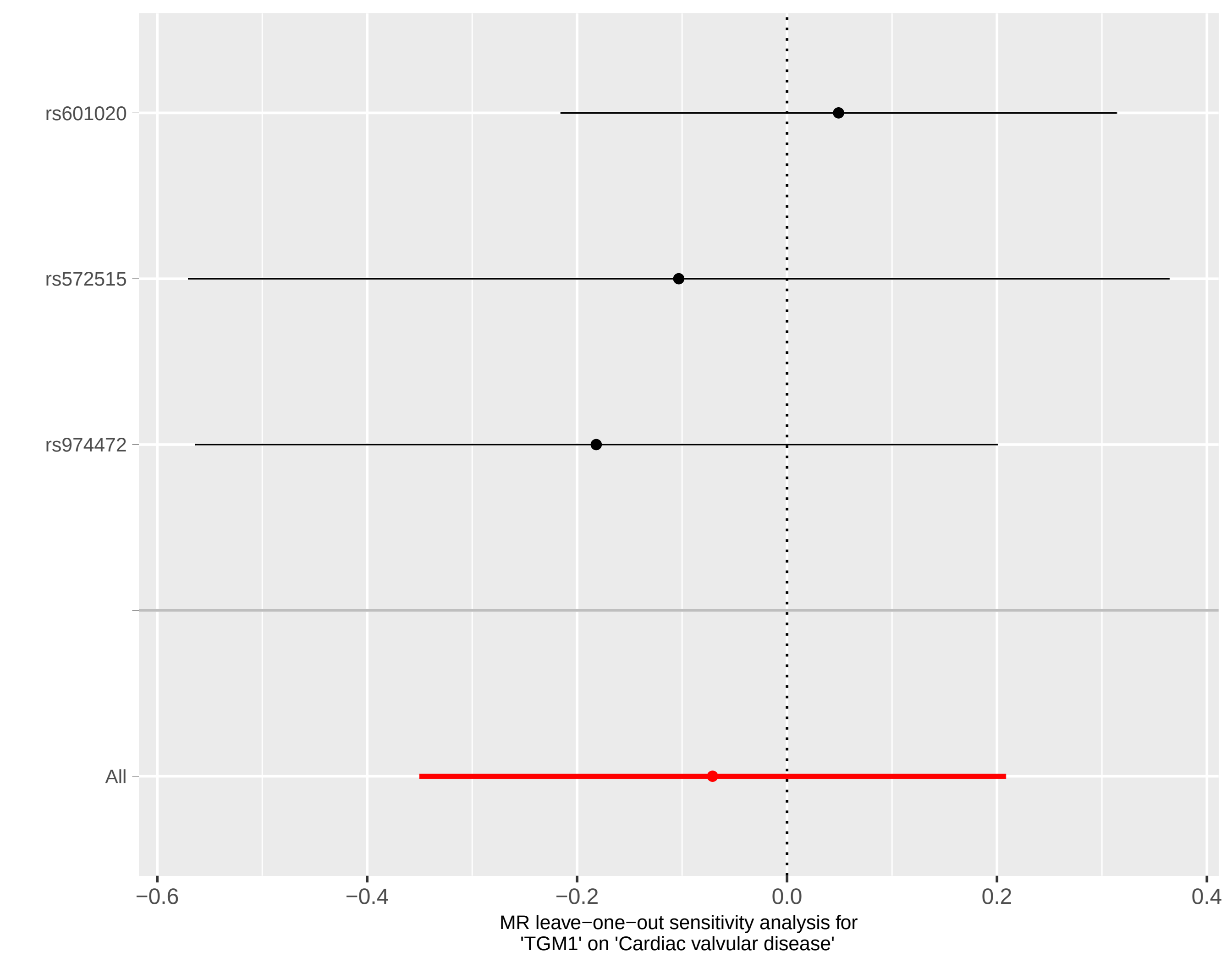

rs601020
rs572515
rs974472
All
−0.6
−0.4
−0.2
0.0
0.2
0.4
MR leave−one−out sensitivity analysis for
'TGM1' on 'Cardiac valvular disease'

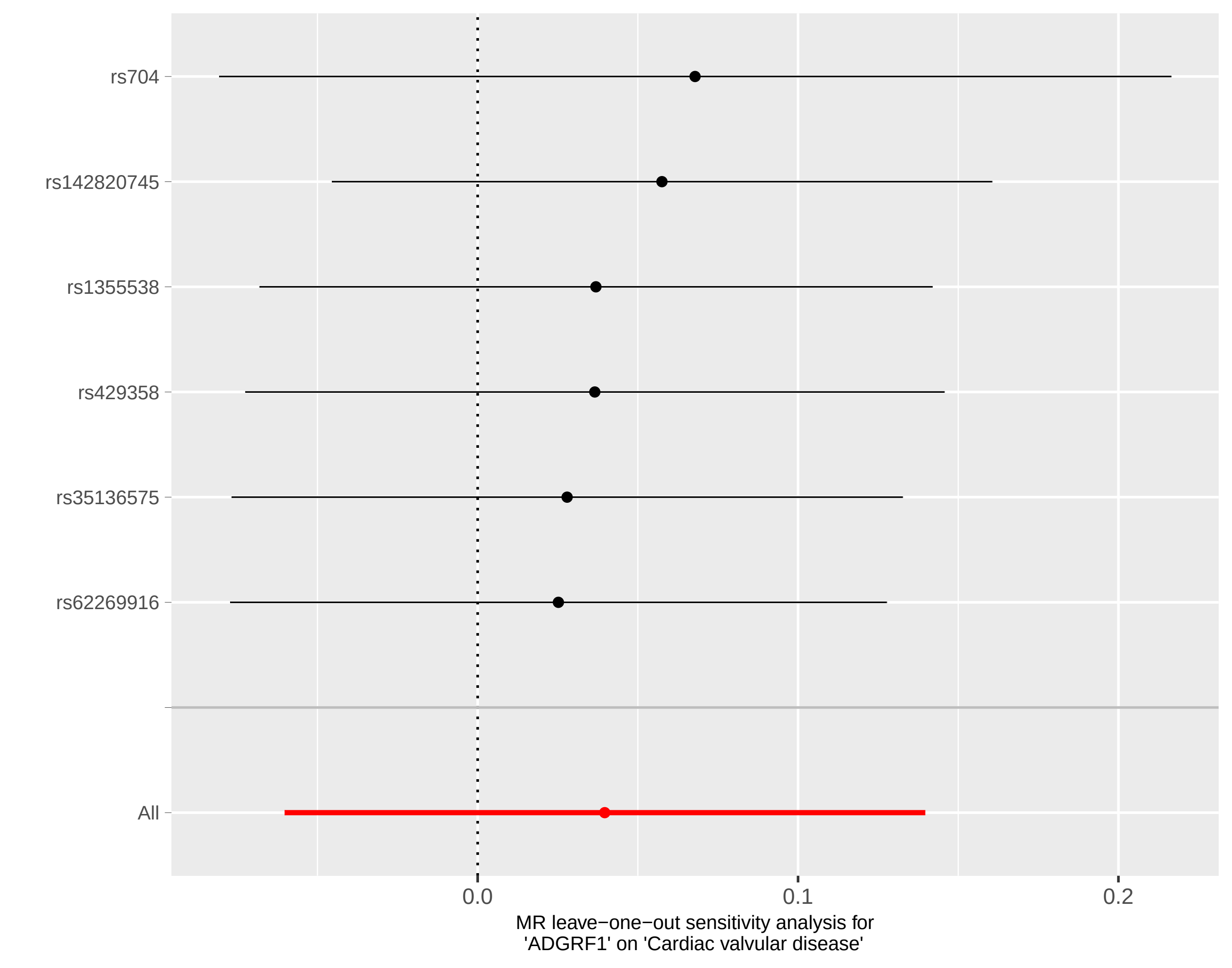

rs704
rs142820745
rs1355538
rs429358
rs35136575
rs62269916
All
0.0
0.1
0.2
MR leave−one−out sensitivity analysis for
'ADGRF1' on 'Cardiac valvular disease'

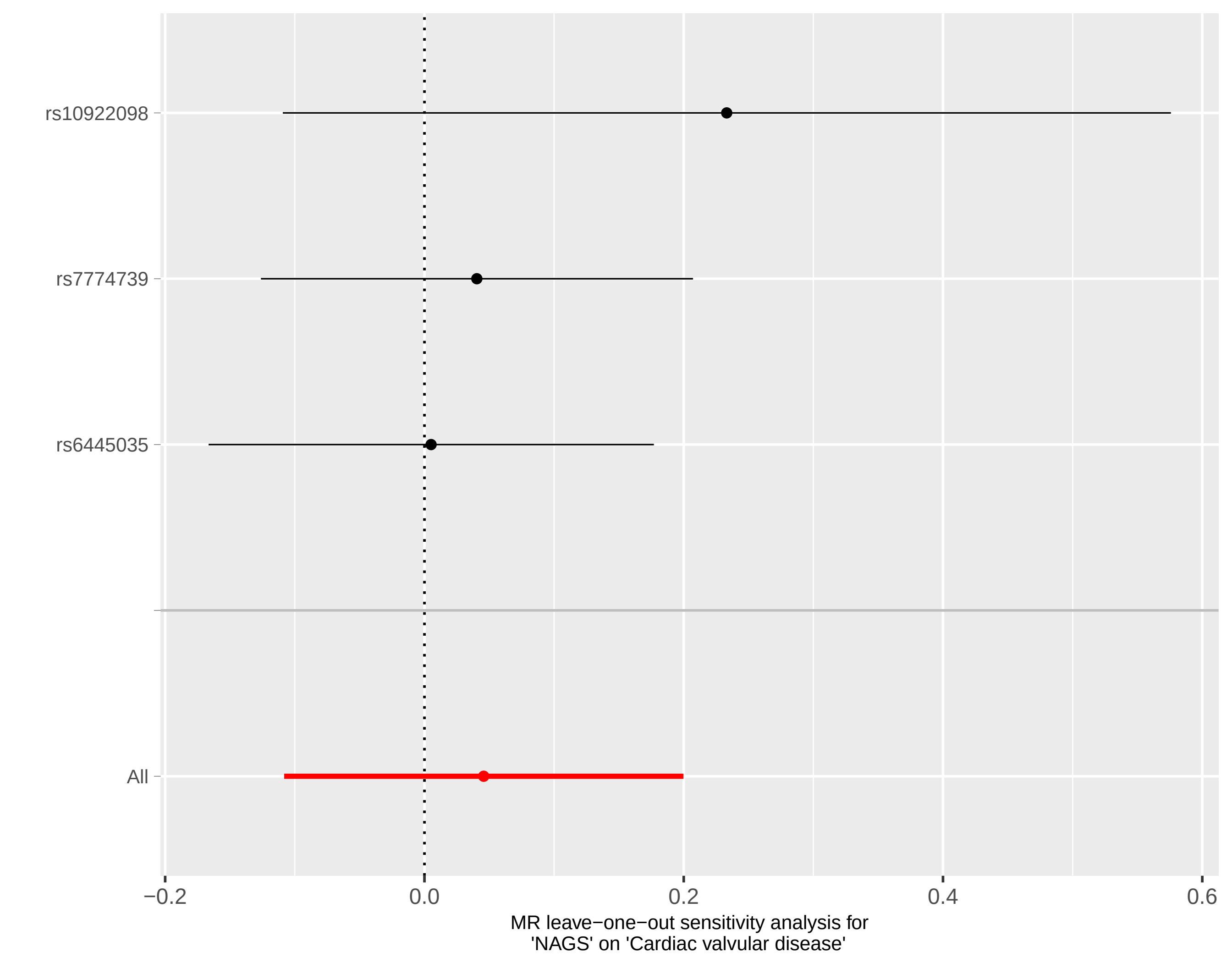

rs10922098
rs7774739
rs6445035
All
−0.2
0.0
0.2
0.4
0.6
MR leave−one−out sensitivity analysis for
'NAGS' on 'Cardiac valvular disease'

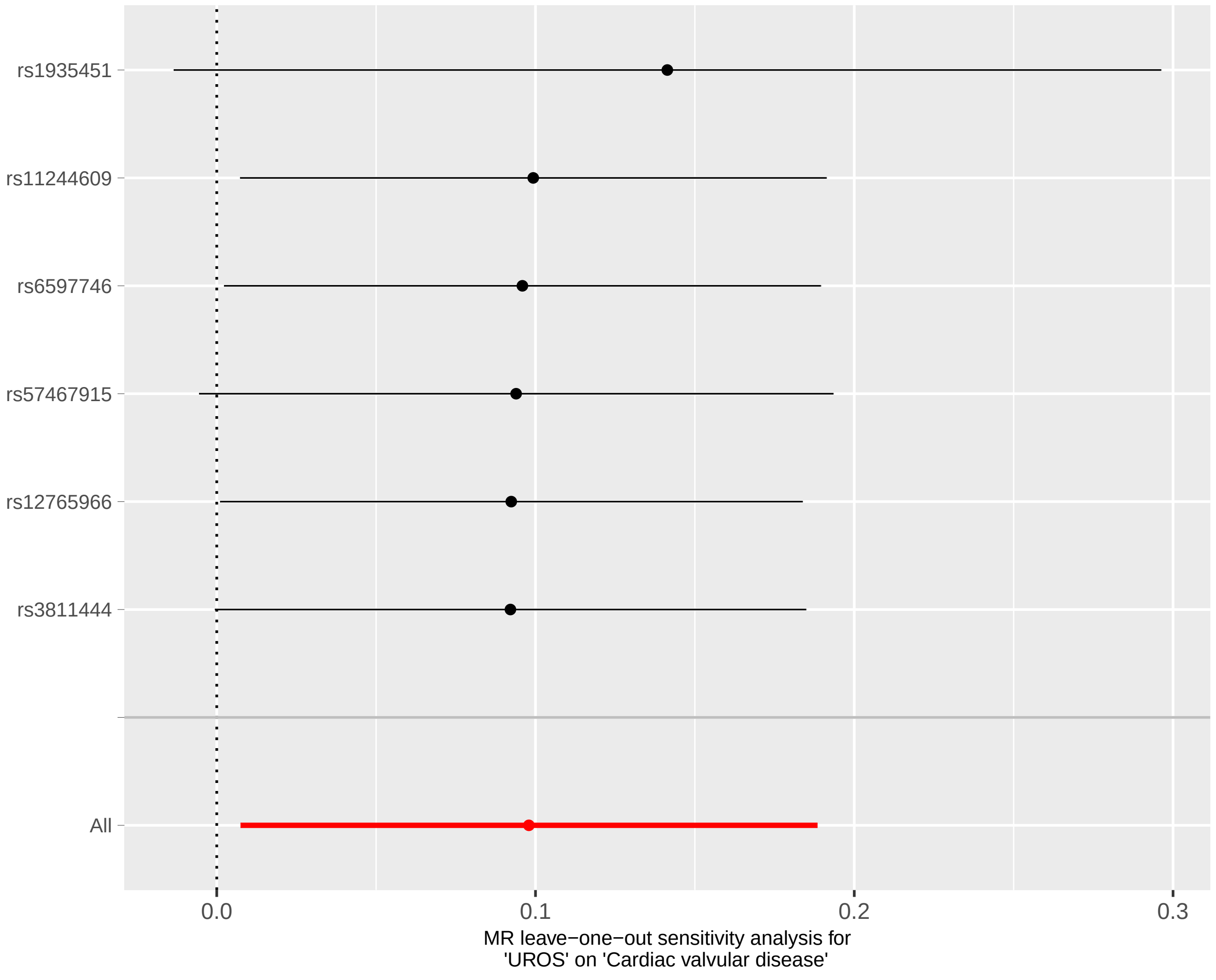
rs1935451
rs11244609
rs6597746
rs57467915
rs12765966
rs3811444
All
0.0
0.1
0.2
0.3
MR leave−one−out sensitivity analysis for
'UROS' on 'Cardiac valvular disease'

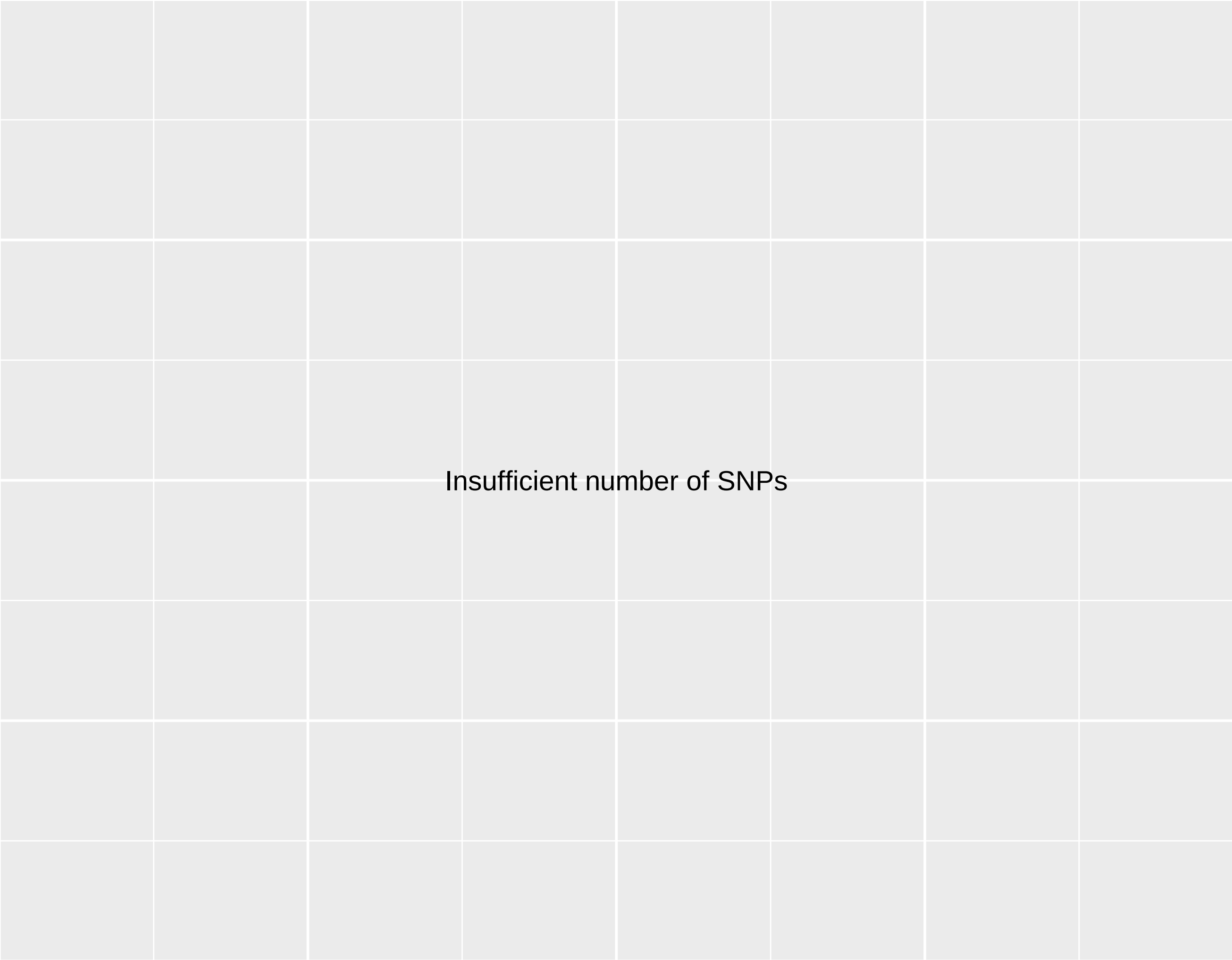
Insufficient number of SNPs

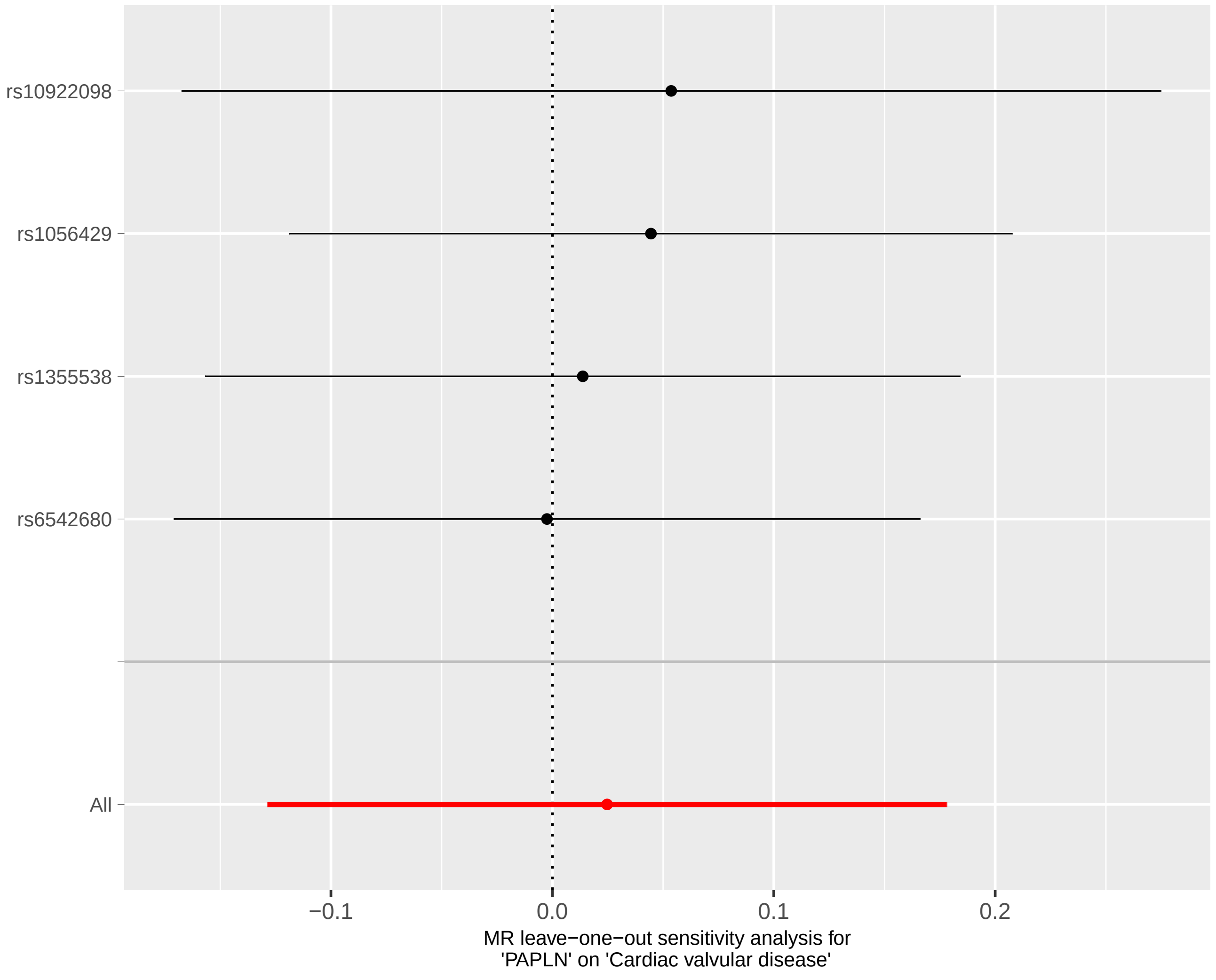
rs10922098
rs1056429
rs1355538
rs6542680
All
−0.1
0.0
0.1
0.2
MR leave−one−out sensitivity analysis for
'PAPLN' on 'Cardiac valvular disease'

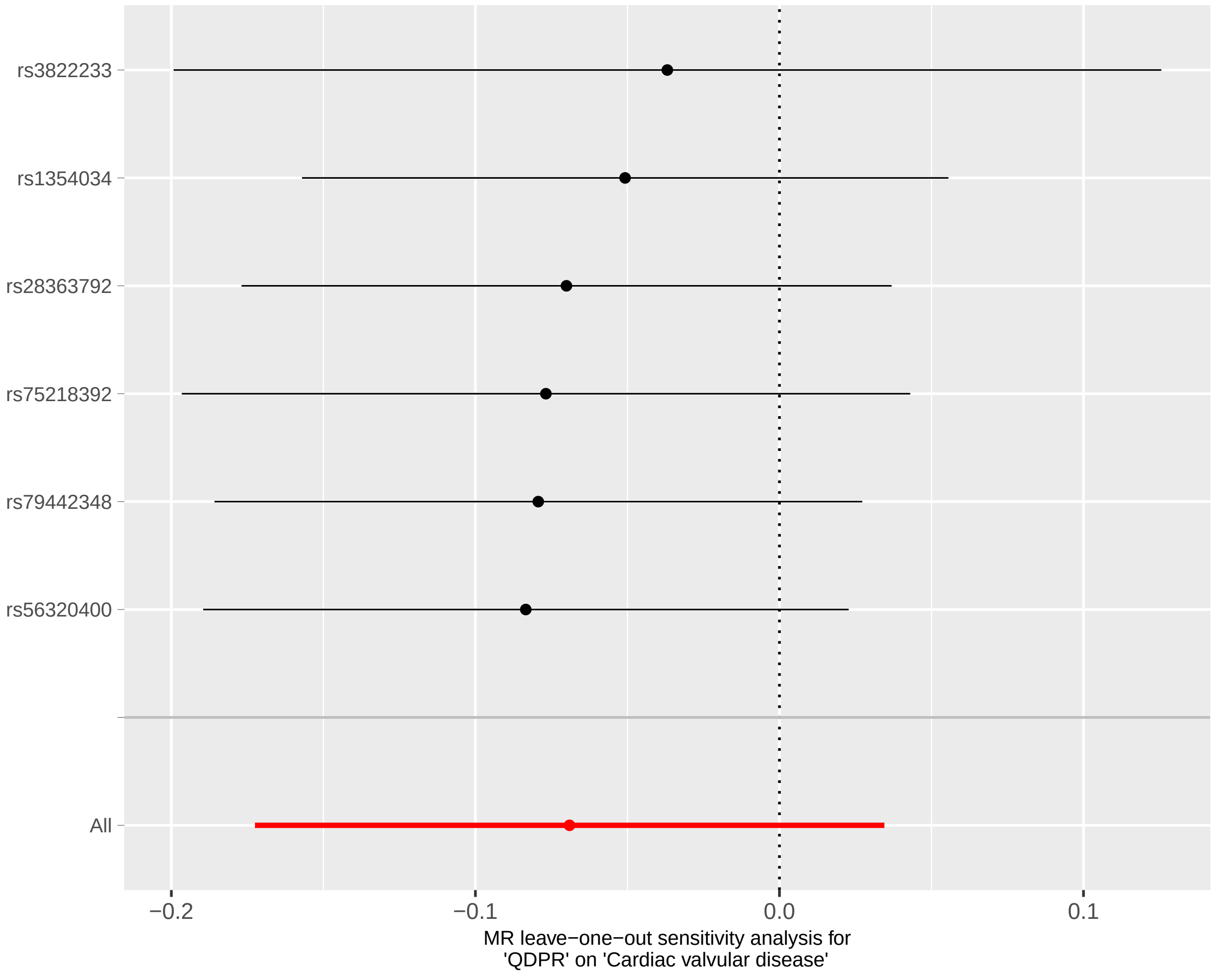
rs3822233
rs1354034
rs28363792
rs75218392
rs79442348
rs56320400
All
−0.2
−0.1
0.0
0.1
MR leave−one−out sensitivity analysis for
'QDPR' on 'Cardiac valvular disease'

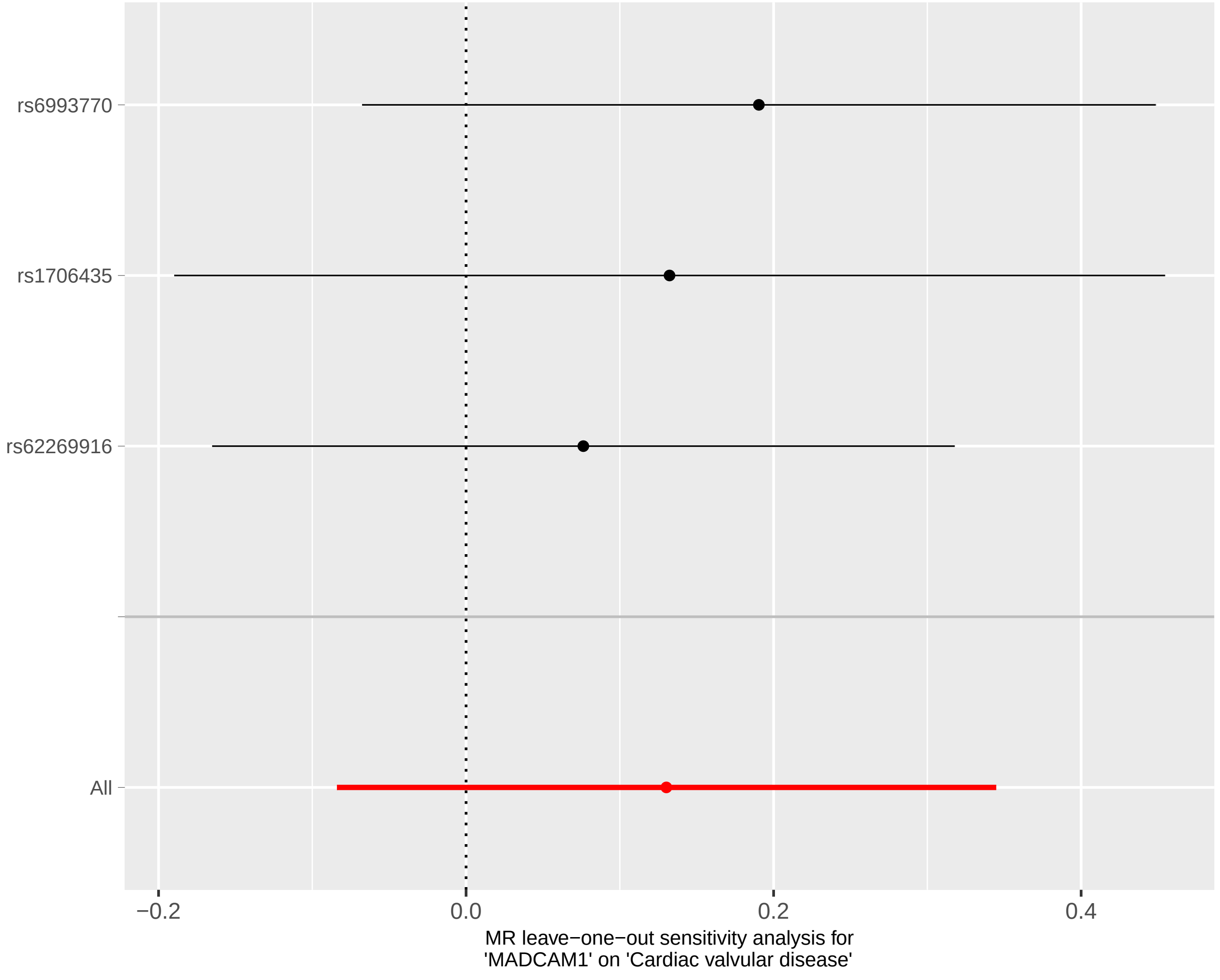
rs6993770
rs1706435
rs62269916
All
−0.2
0.0
0.2
0.4
MR leave−one−out sensitivity analysis for
'MADCAM1' on 'Cardiac valvular disease'

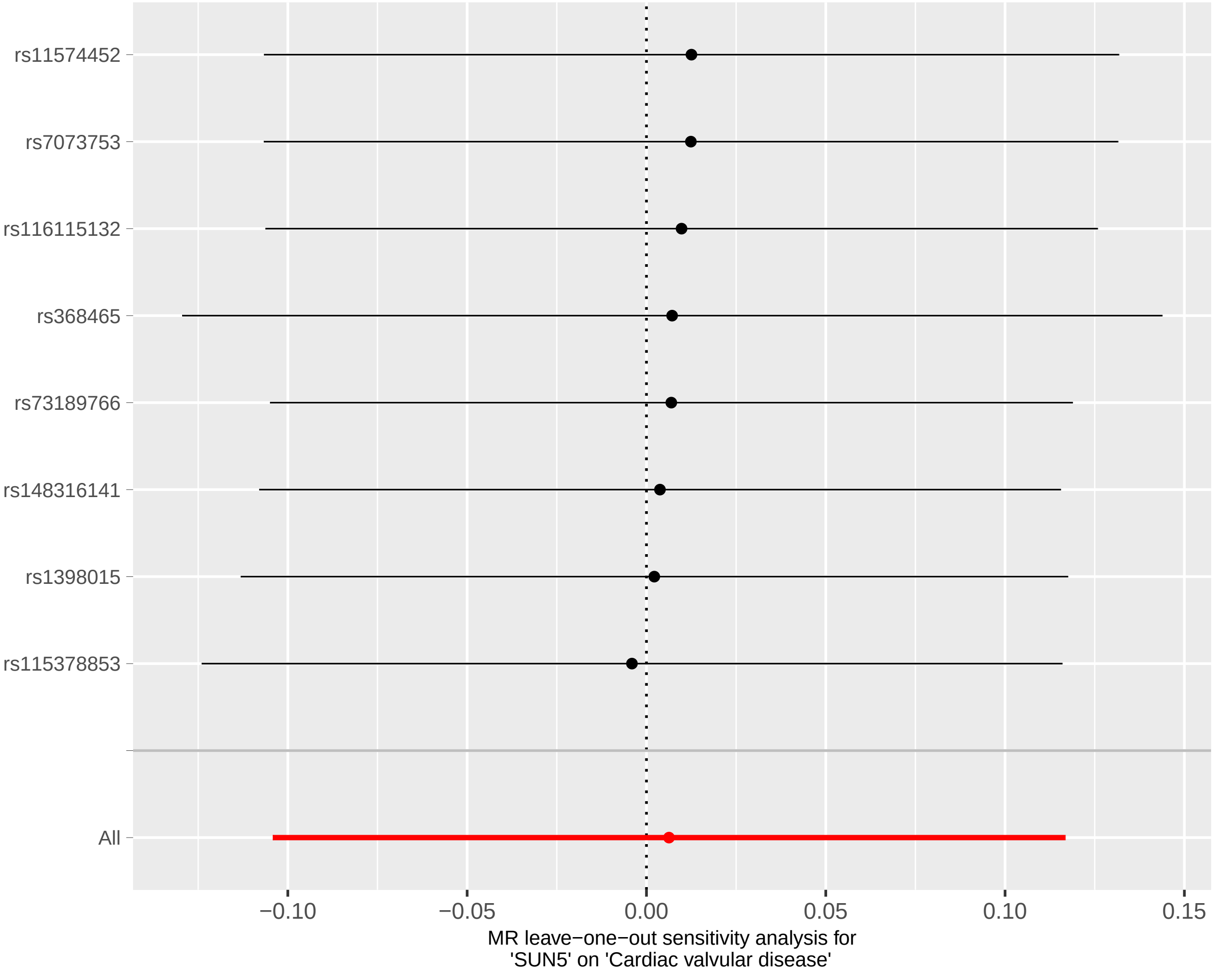
rs11574452
rs7073753
rs116115132
rs368465
rs73189766
rs148316141
rs1398015
rs115378853
All
−0.10
−0.05
0.00
0.05
0.10
0.15
MR leave−one−out sensitivity analysis for
'SUN5' on 'Cardiac valvular disease'

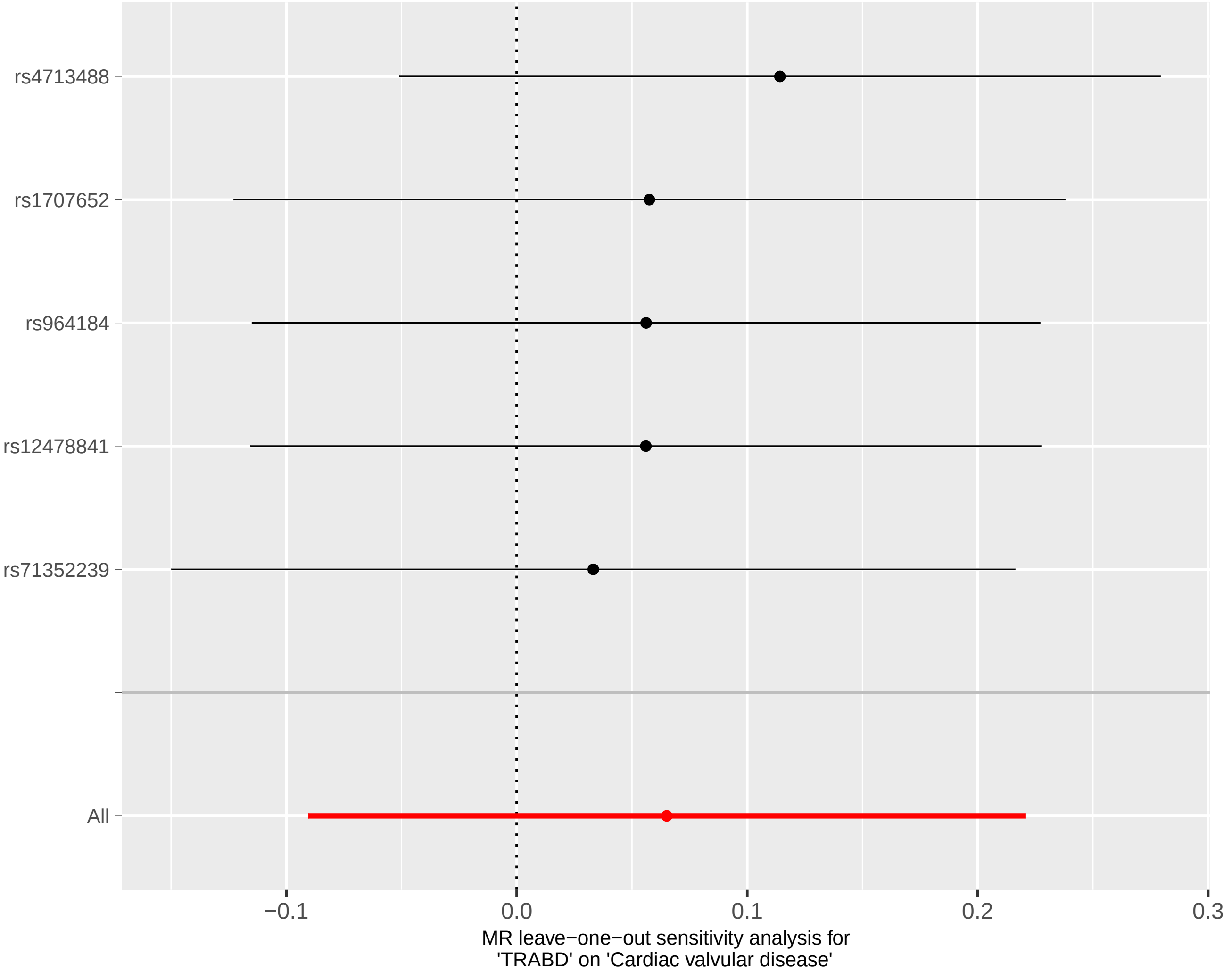
rs4713488
rs1707652
rs964184
rs12478841
rs71352239
All
−0.1
0.0
0.1
0.2
0.3
MR leave−one−out sensitivity analysis for
'TRABD' on 'Cardiac valvular disease'

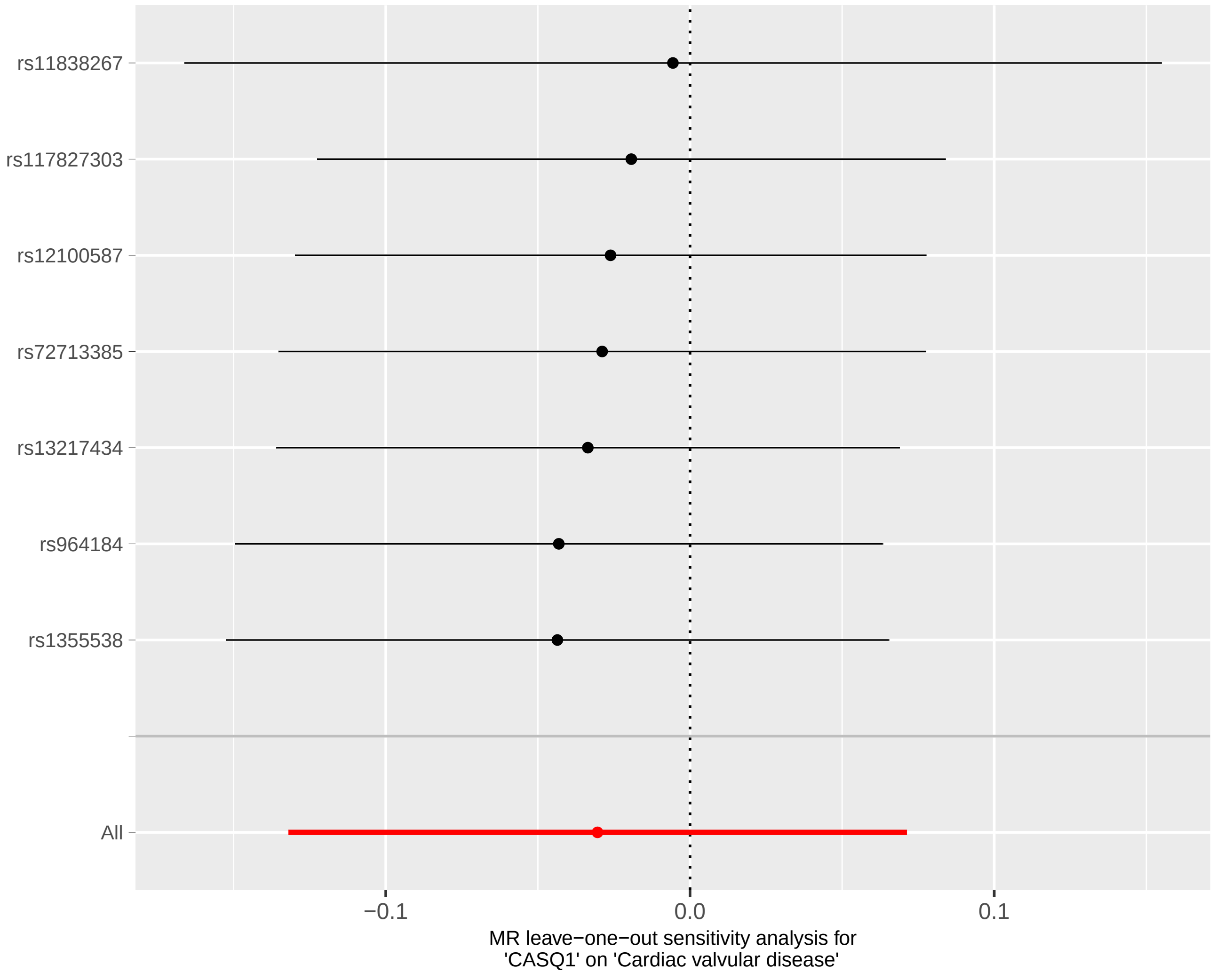

rs11838267
rs117827303
rs12100587
rs72713385
rs13217434
rs964184
rs1355538
All
−0.1
0.0
0.1
MR leave−one−out sensitivity analysis for
'CASQ1' on 'Cardiac valvular disease'

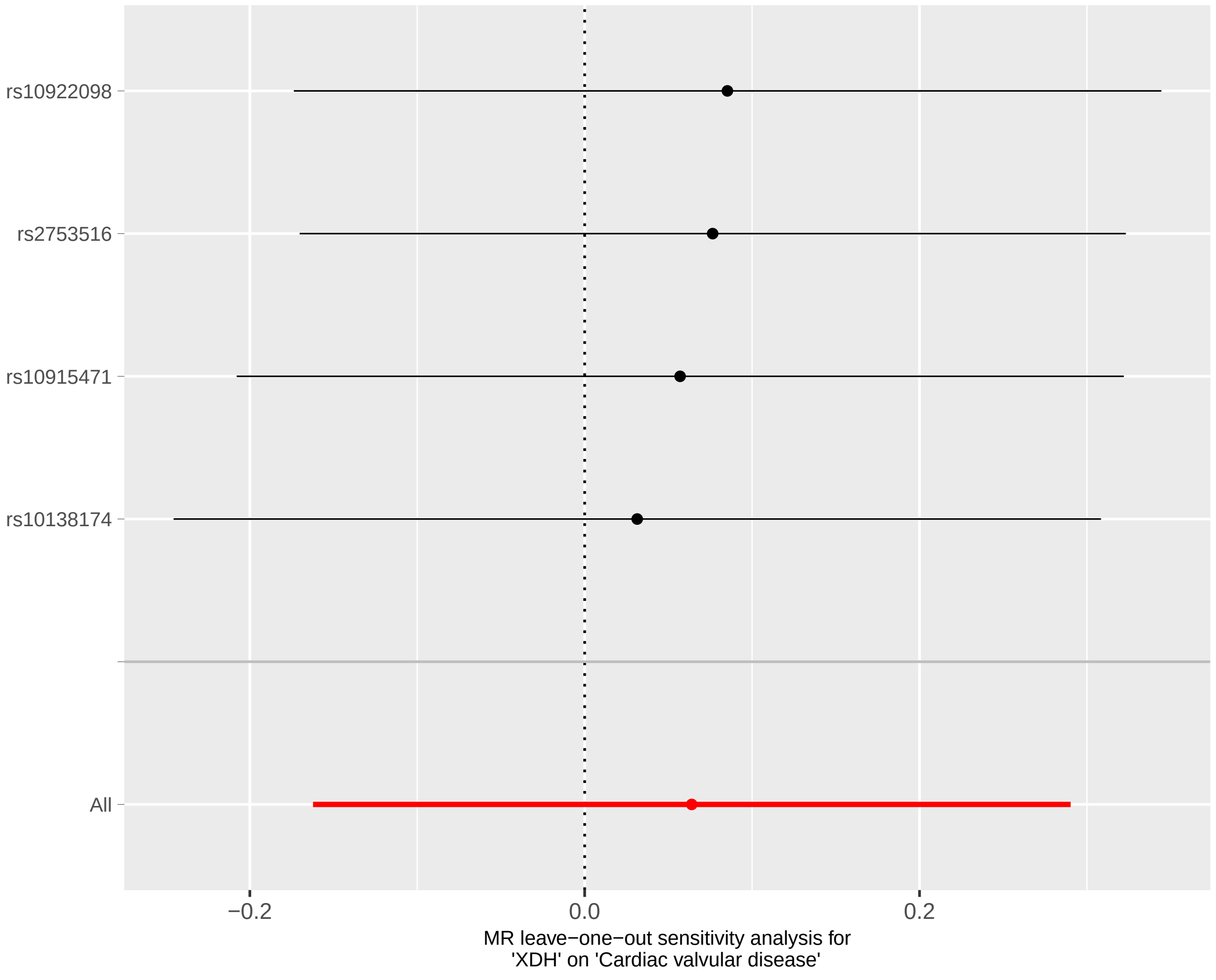

rs10922098
rs2753516
rs10915471
rs10138174
All
−0.2
0.0
0.2
MR leave−one−out sensitivity analysis for
'XDH' on 'Cardiac valvular disease'

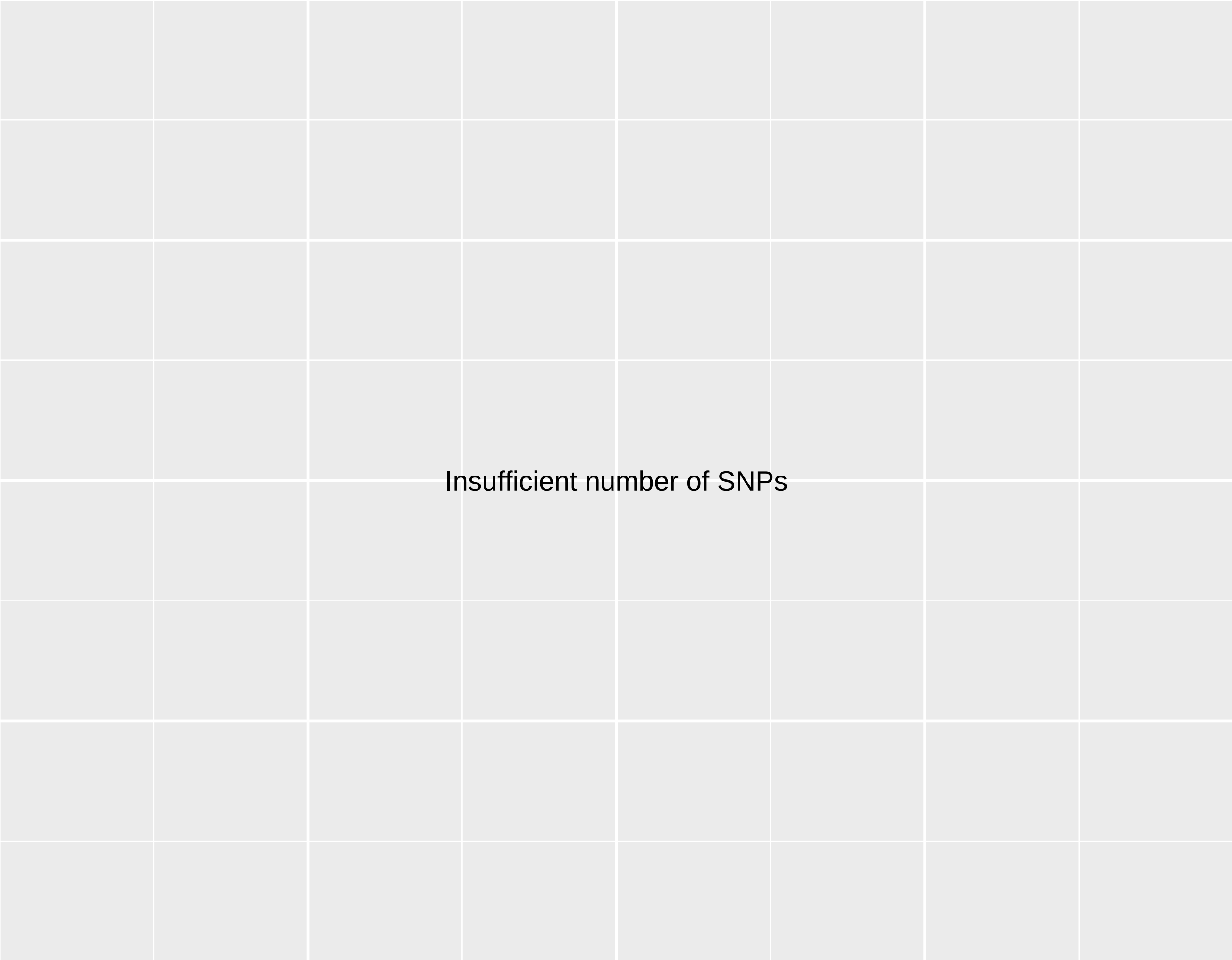
Insufficient number of SNPs

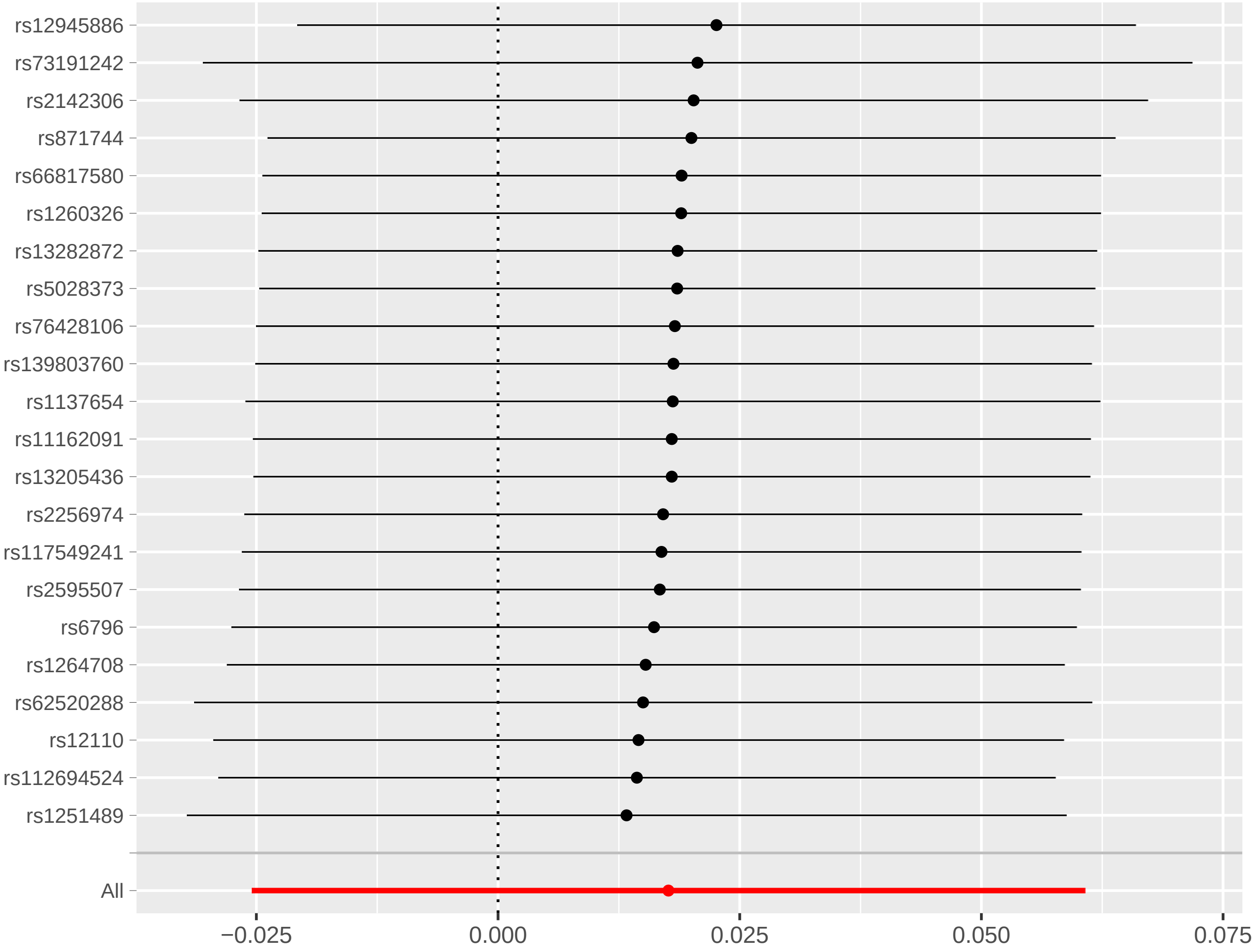

MR leave−one−out sensitivity analysis for
'SELPLG' on 'Cardiac valvular disease'

Insufficient number of SNPs

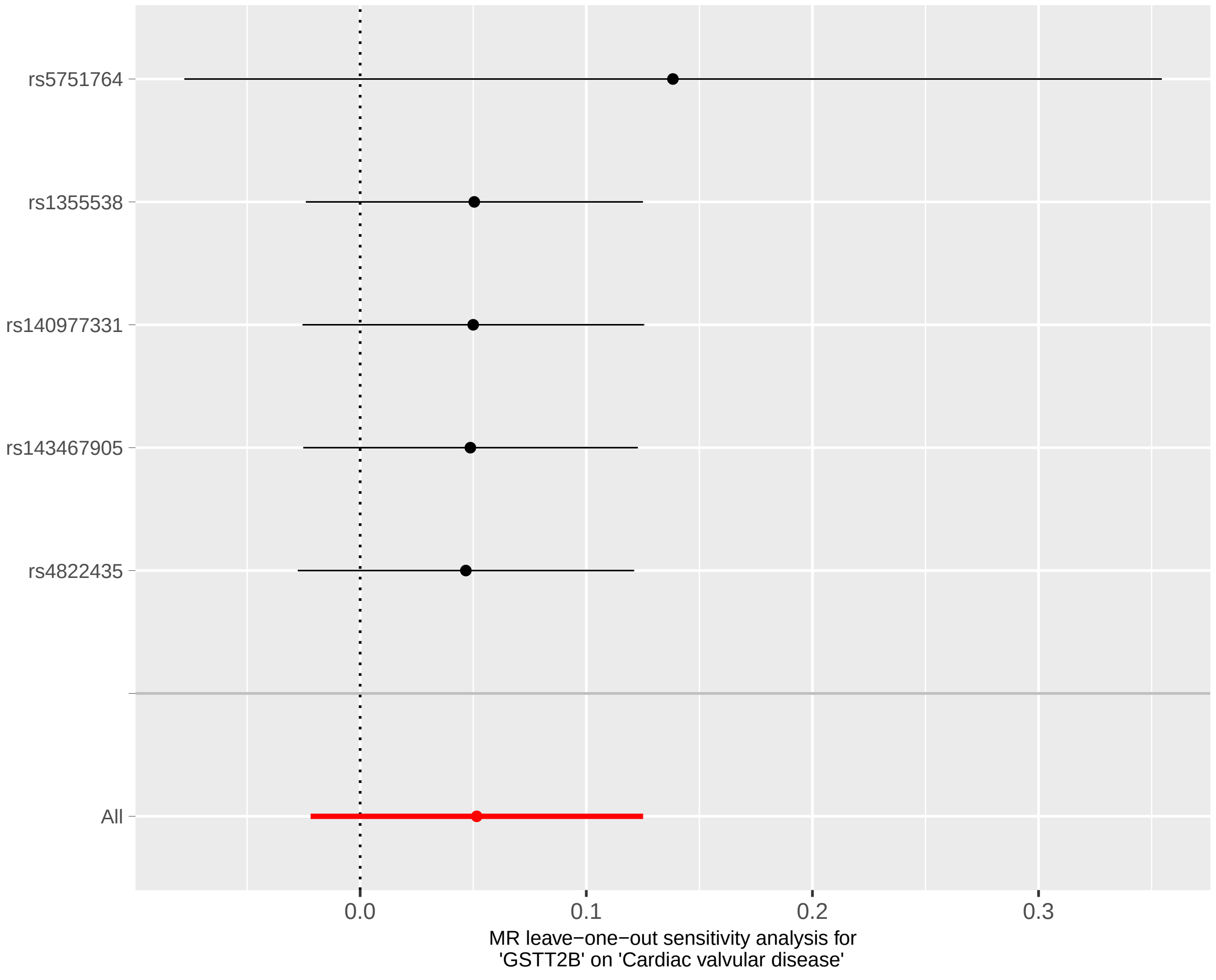
rs5751764
rs1355538
rs140977331
rs143467905
rs4822435
All
0.0
0.1
0.2
0.3
MR leave−one−out sensitivity analysis for
'GSTT2B' on 'Cardiac valvular disease'

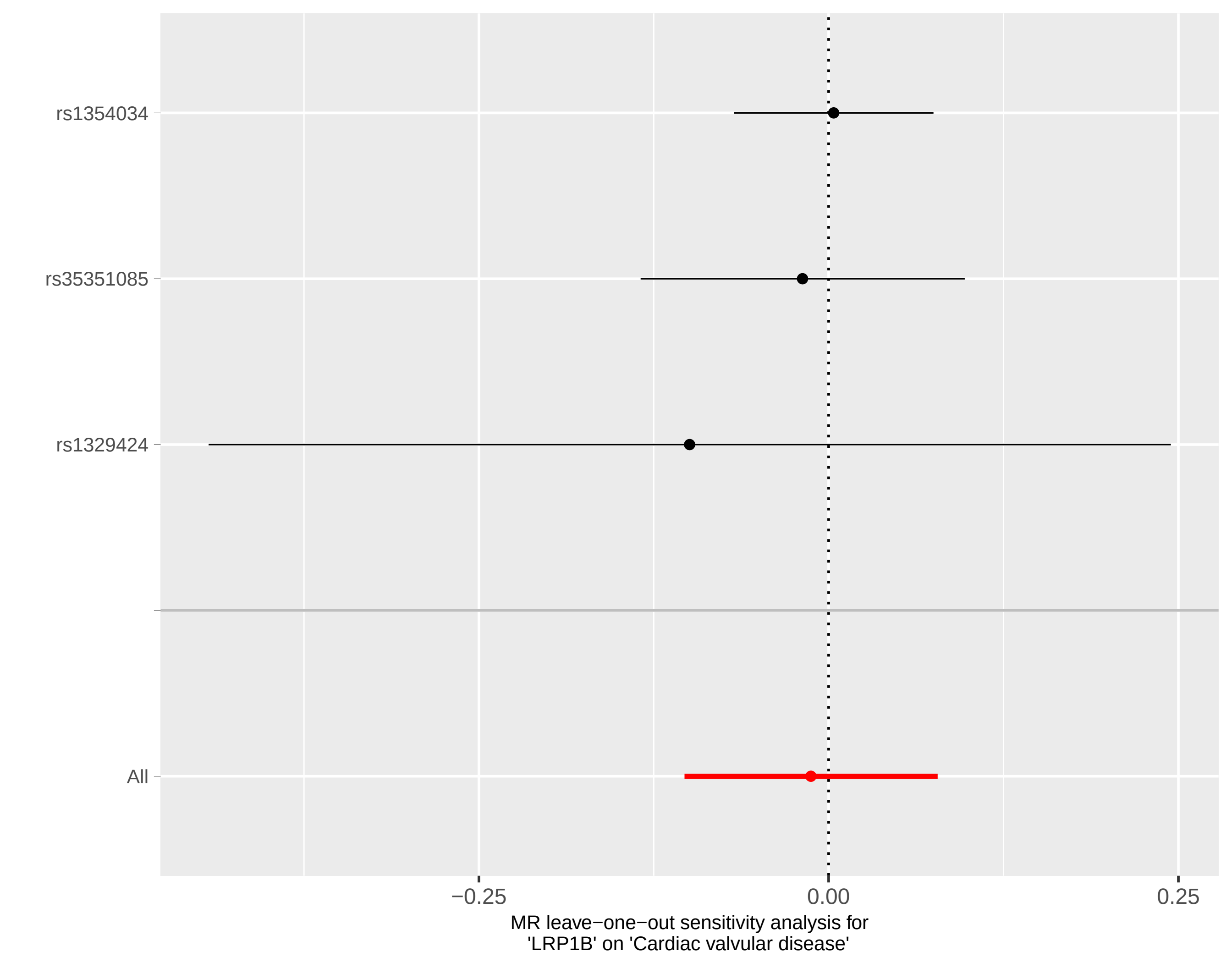
rs1354034
rs35351085
rs1329424
All
−0.25
0.00
0.25
MR leave−one−out sensitivity analysis for
'LRP1B' on 'Cardiac valvular disease'

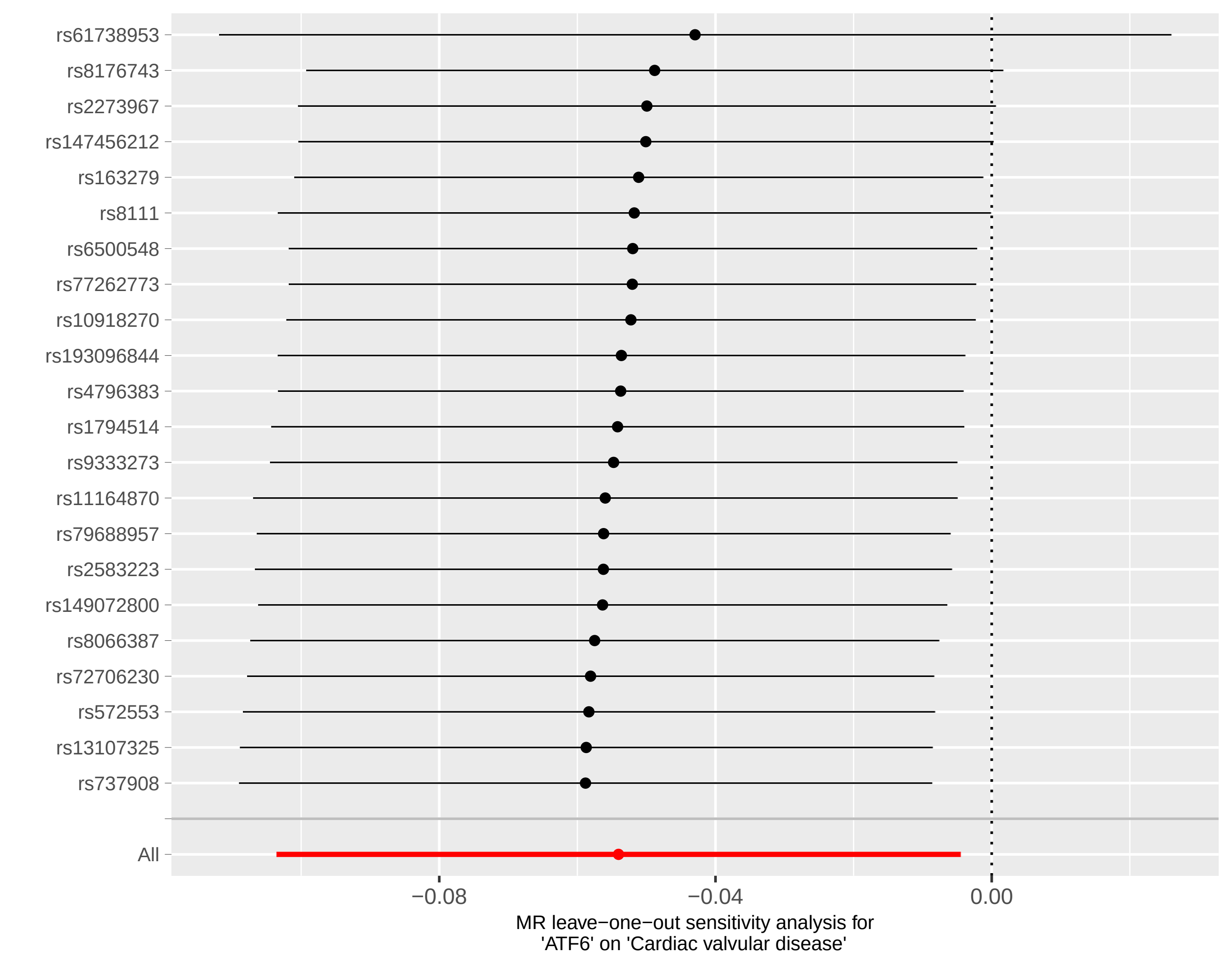
rs61738953
rs8176743
rs2273967
rs147456212
rs163279
rs8111
rs6500548
rs77262773
rs10918270
rs193096844
rs4796383
rs1794514
rs9333273
rs11164870
rs79688957
rs2583223
rs149072800
rs8066387
rs72706230
rs572553
rs13107325
rs737908
All
−0.08
−0.04
0.00
MR leave−one−out sensitivity analysis for
'ATF6' on 'Cardiac valvular disease'

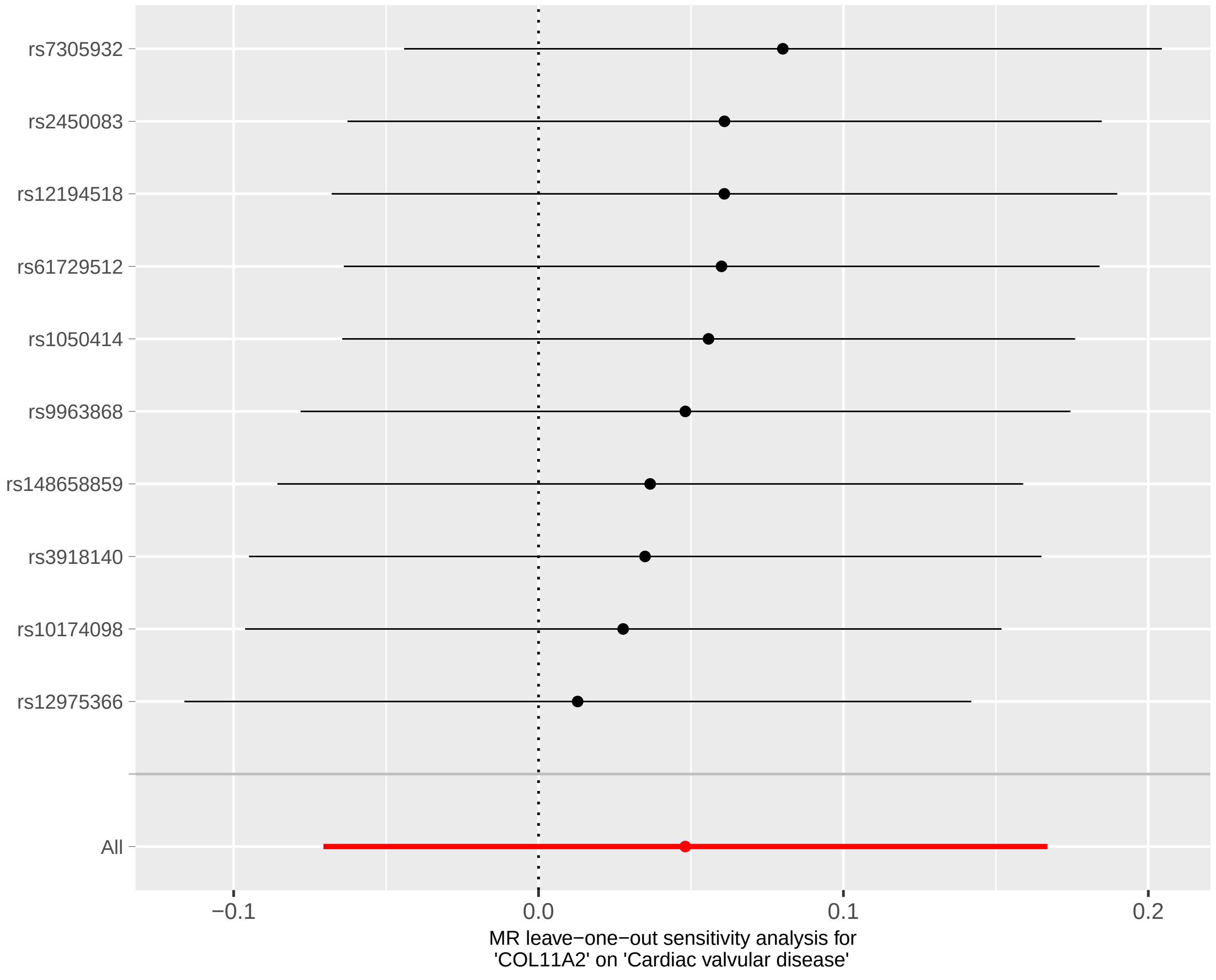
rs7305932
rs2450083
rs12194518
rs61729512
rs1050414
rs9963868
rs148658859
rs3918140
rs10174098
rs12975366
All
−0.1
0.0
0.1
0.2
MR leave−one−out sensitivity analysis for
'COL11A2' on 'Cardiac valvular disease'

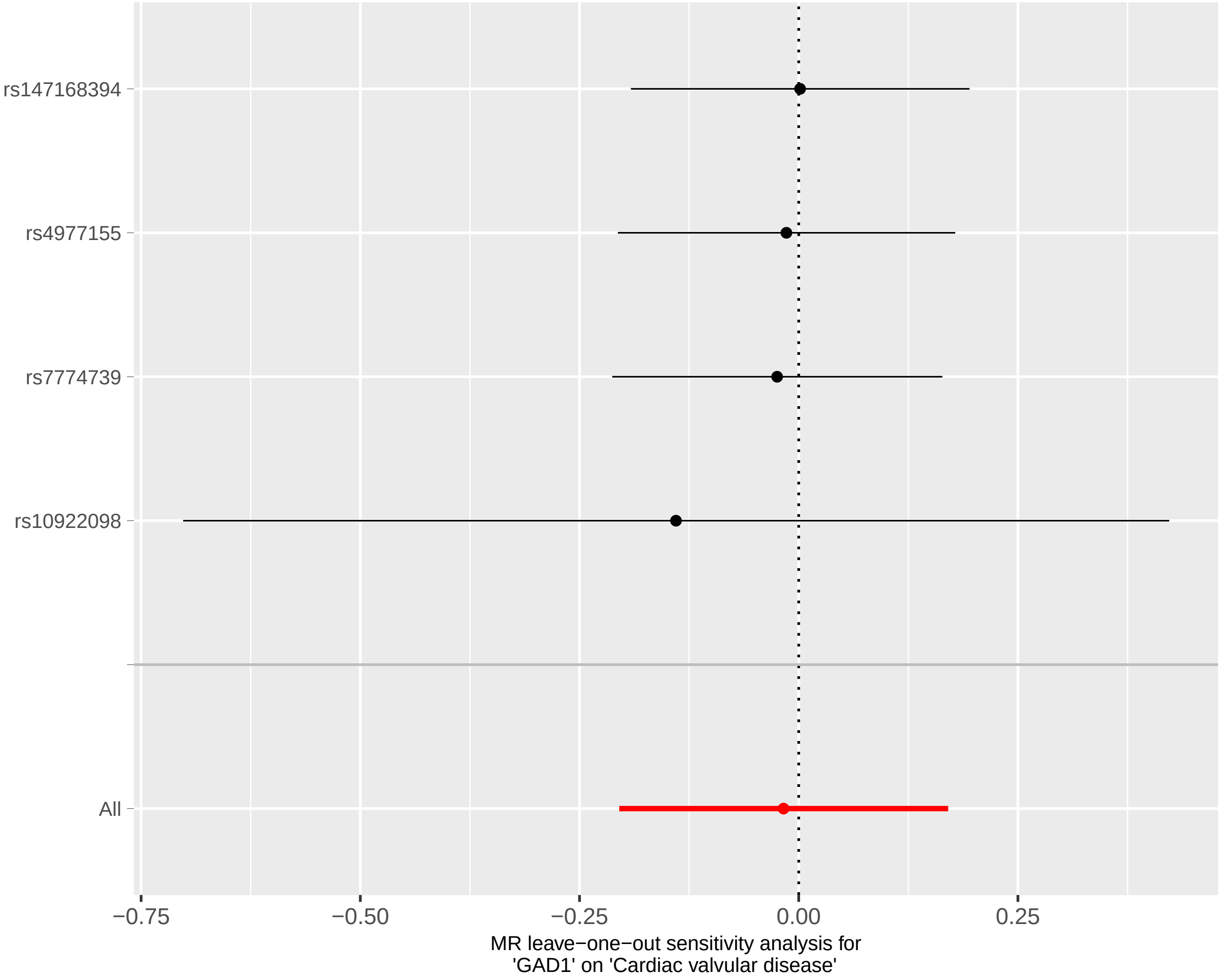
rs147168394
rs4977155
rs7774739
rs10922098
All
−0.75
−0.50
−0.25
0.00
0.25
MR leave−one−out sensitivity analysis for
'GAD1' on 'Cardiac valvular disease'

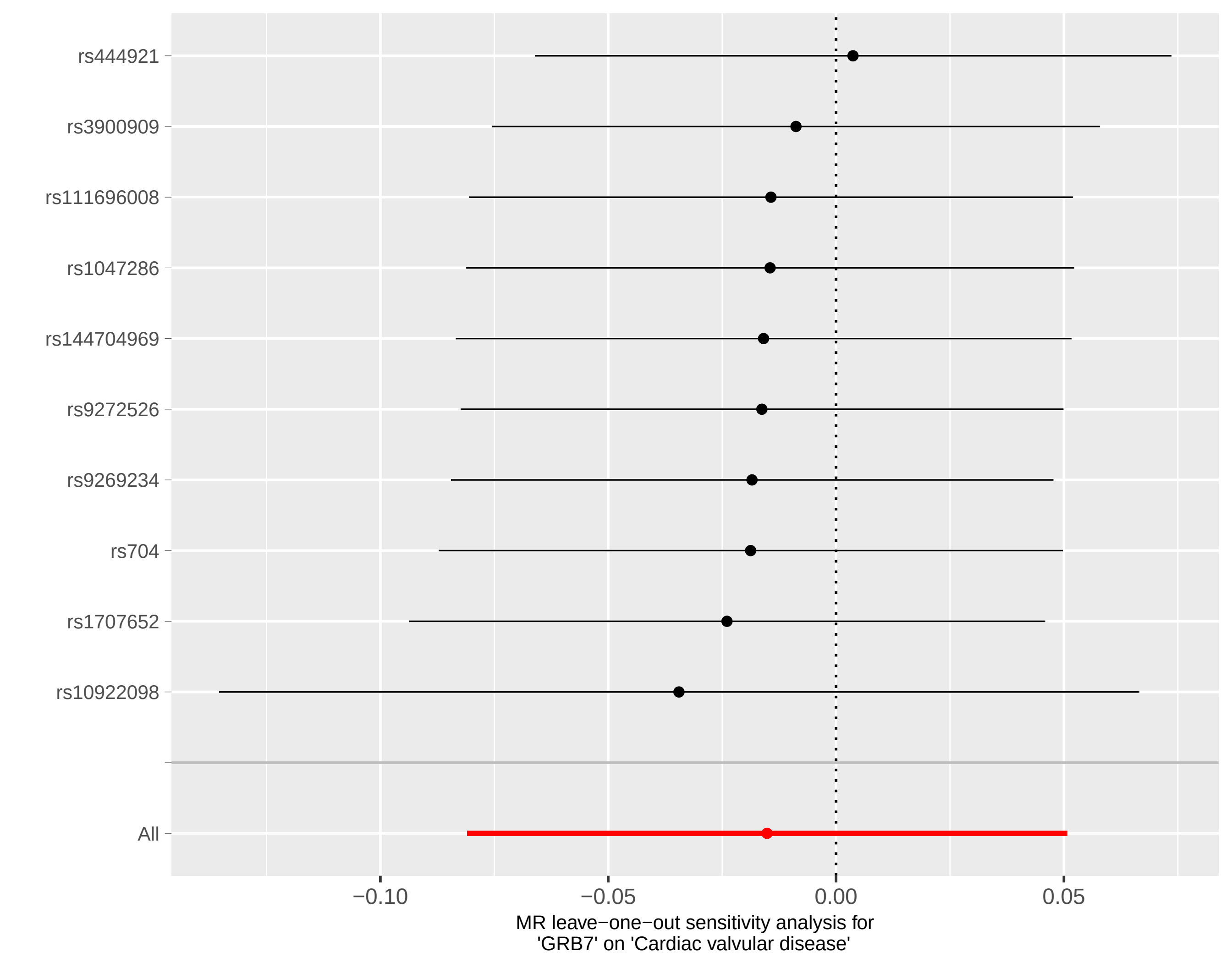

rs444921
rs3900909
rs111696008
rs1047286
rs144704969
rs9272526
rs9269234
rs704
rs1707652
rs10922098
All
−0.10
−0.05
0.00
0.05
MR leave−one−out sensitivity analysis for
'GRB7' on 'Cardiac valvular disease'

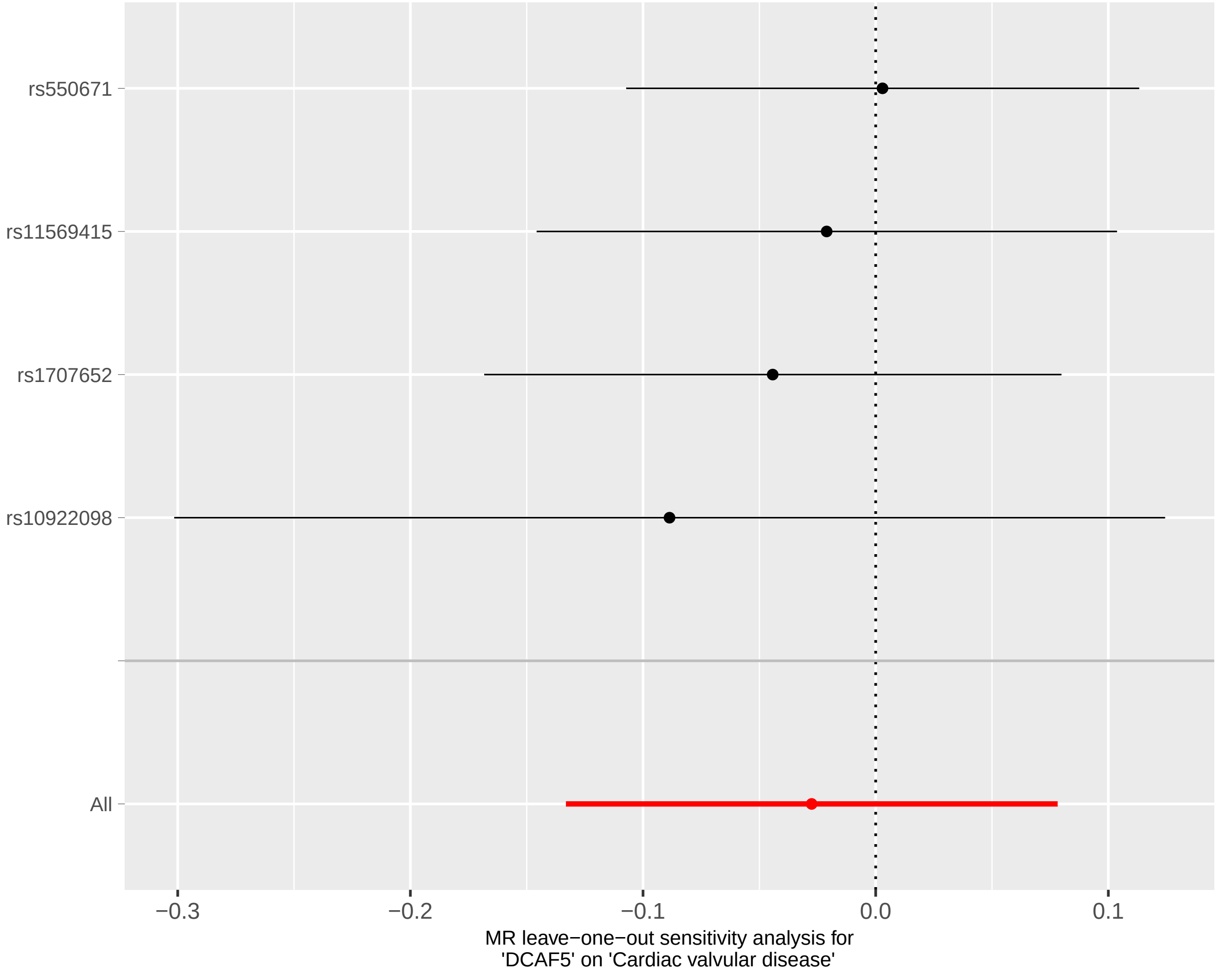
rs550671
rs11569415
rs1707652
rs10922098
All
−0.3
−0.2
−0.1
0.0
0.1
MR leave−one−out sensitivity analysis for
'DCAF5' on 'Cardiac valvular disease'

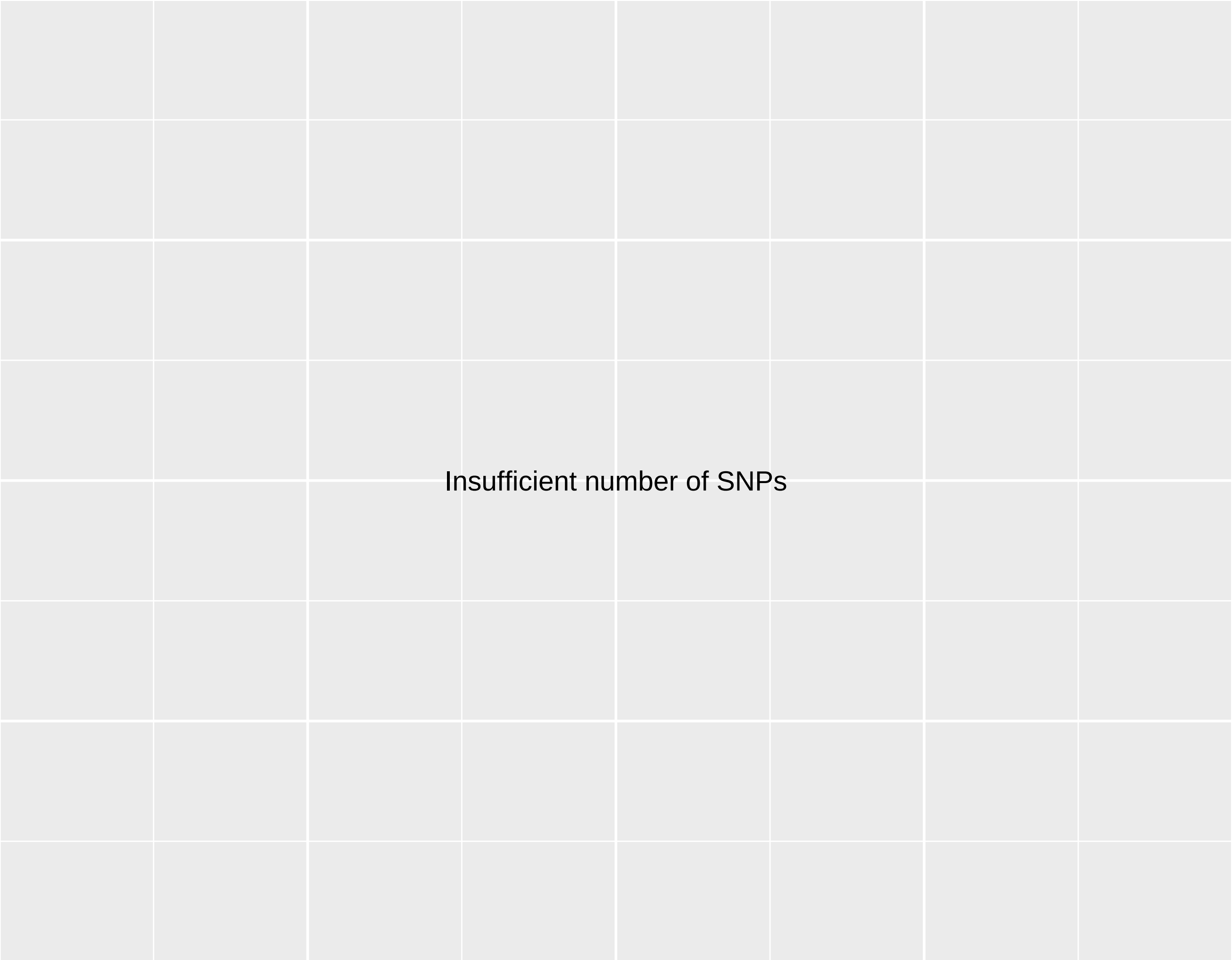
Insufficient number of SNPs

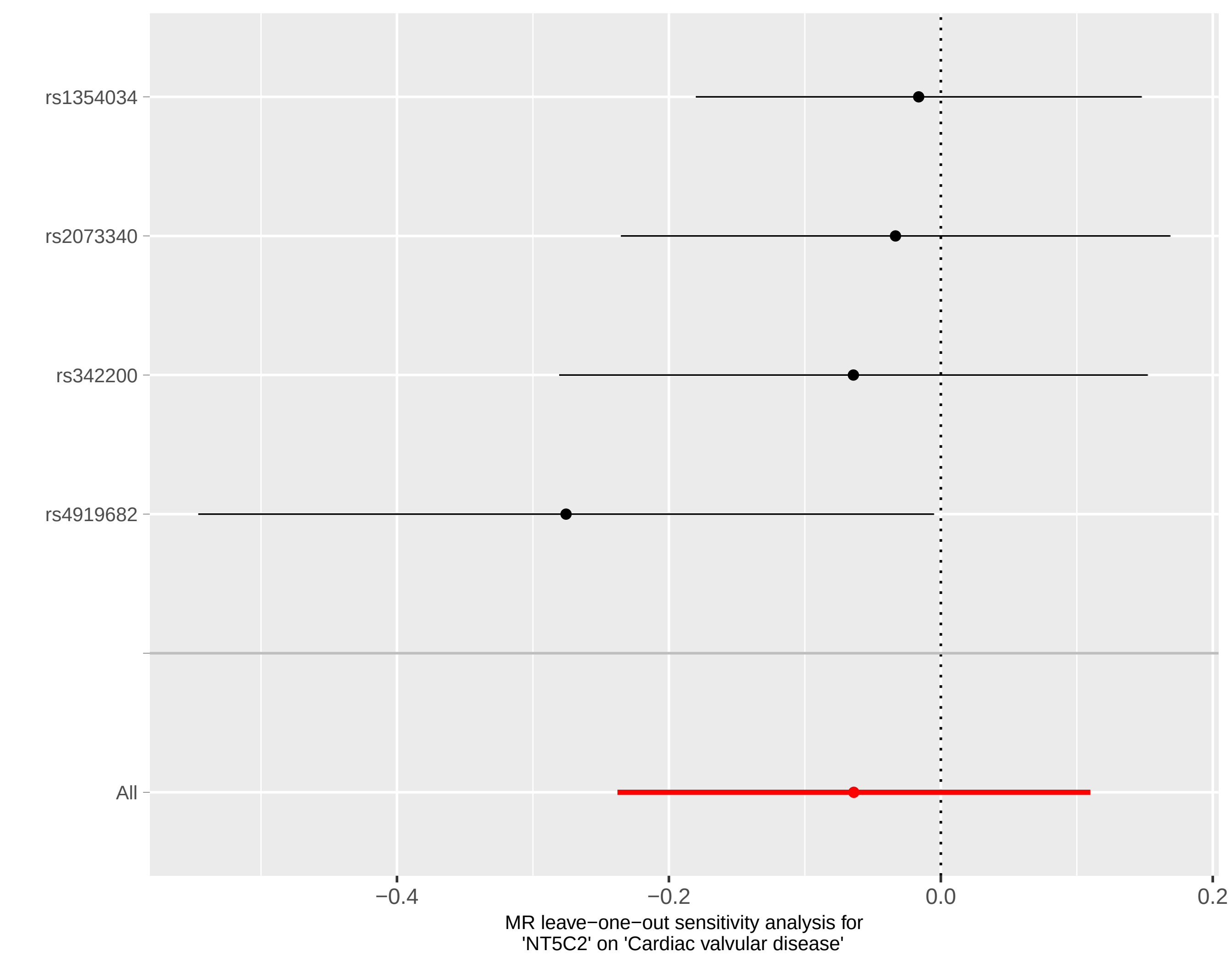

rs1354034
rs2073340
rs342200
rs4919682
All
−0.4
−0.2
0.0
0.2
MR leave−one−out sensitivity analysis for
'NT5C2' on 'Cardiac valvular disease'

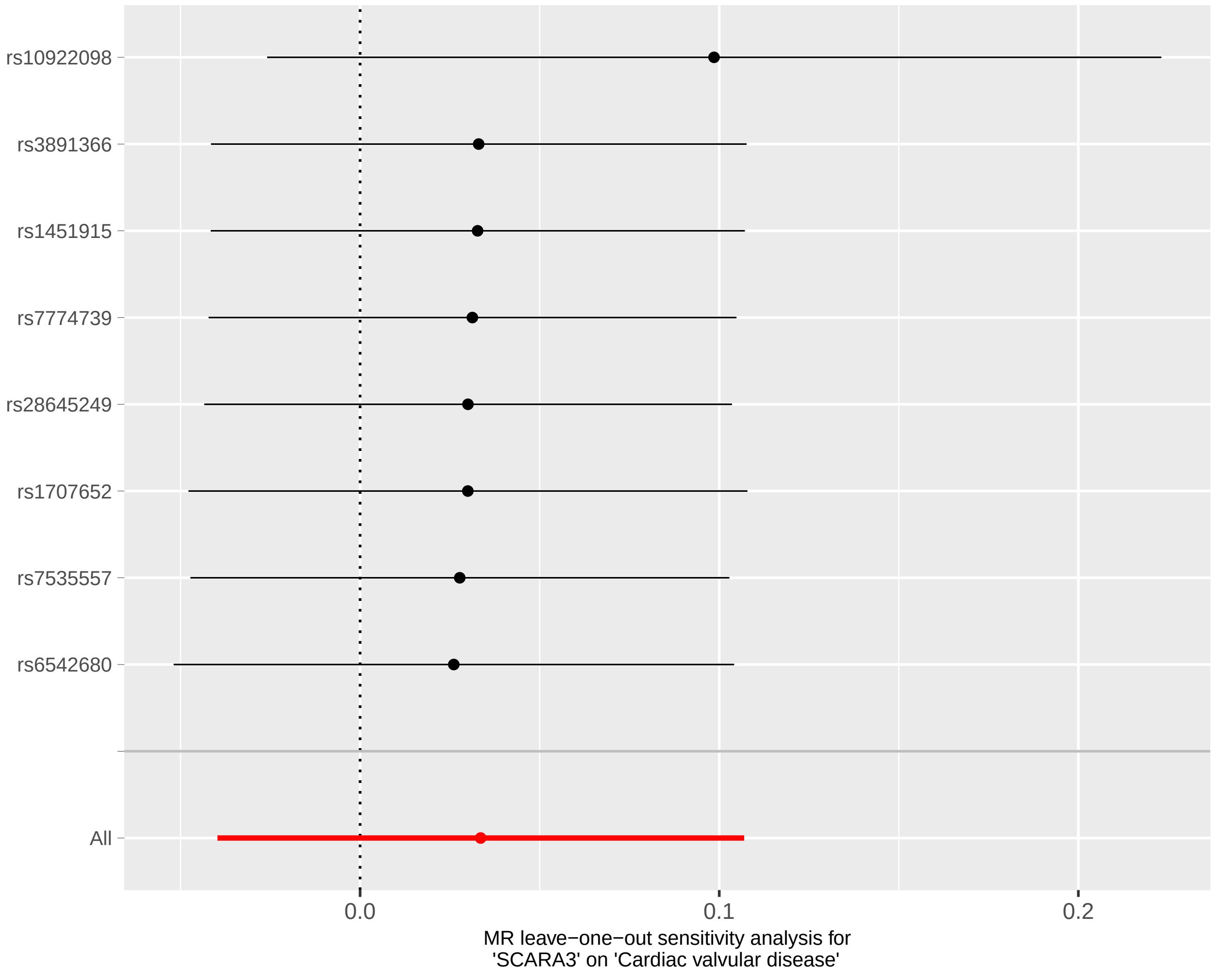
rs10922098
rs3891366
rs1451915
rs7774739
rs28645249
rs1707652
rs7535557
rs6542680
All
0.0
0.1
0.2
MR leave−one−out sensitivity analysis for
'SCARA3' on 'Cardiac valvular disease'

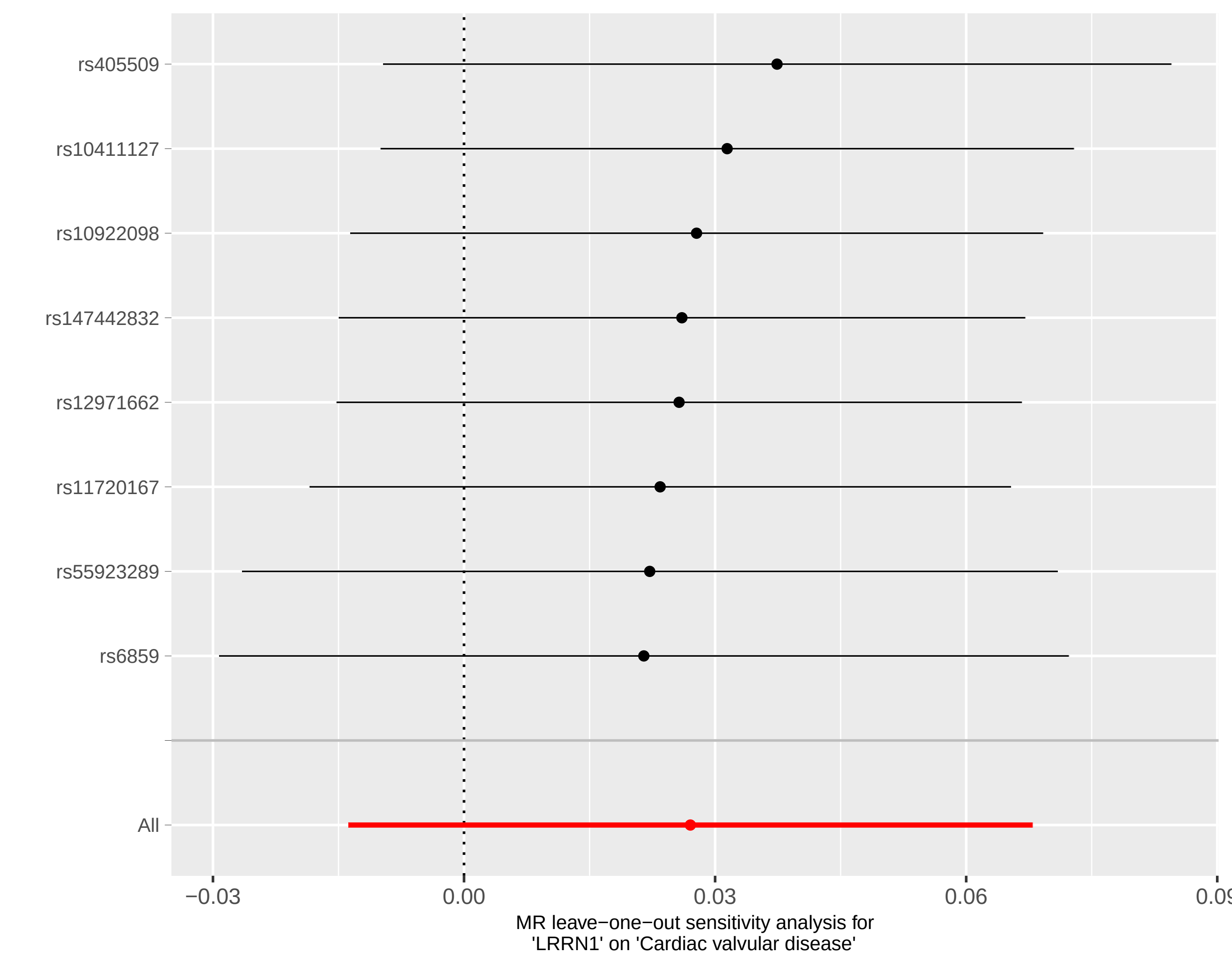
rs405509
rs10411127
rs10922098
rs147442832
rs12971662
rs11720167
rs55923289
rs6859
All
−0.03
0.00
0.03
0.06
MR leave−one−out sensitivity analysis for
'LRRN1' on 'Cardiac valvular disease'

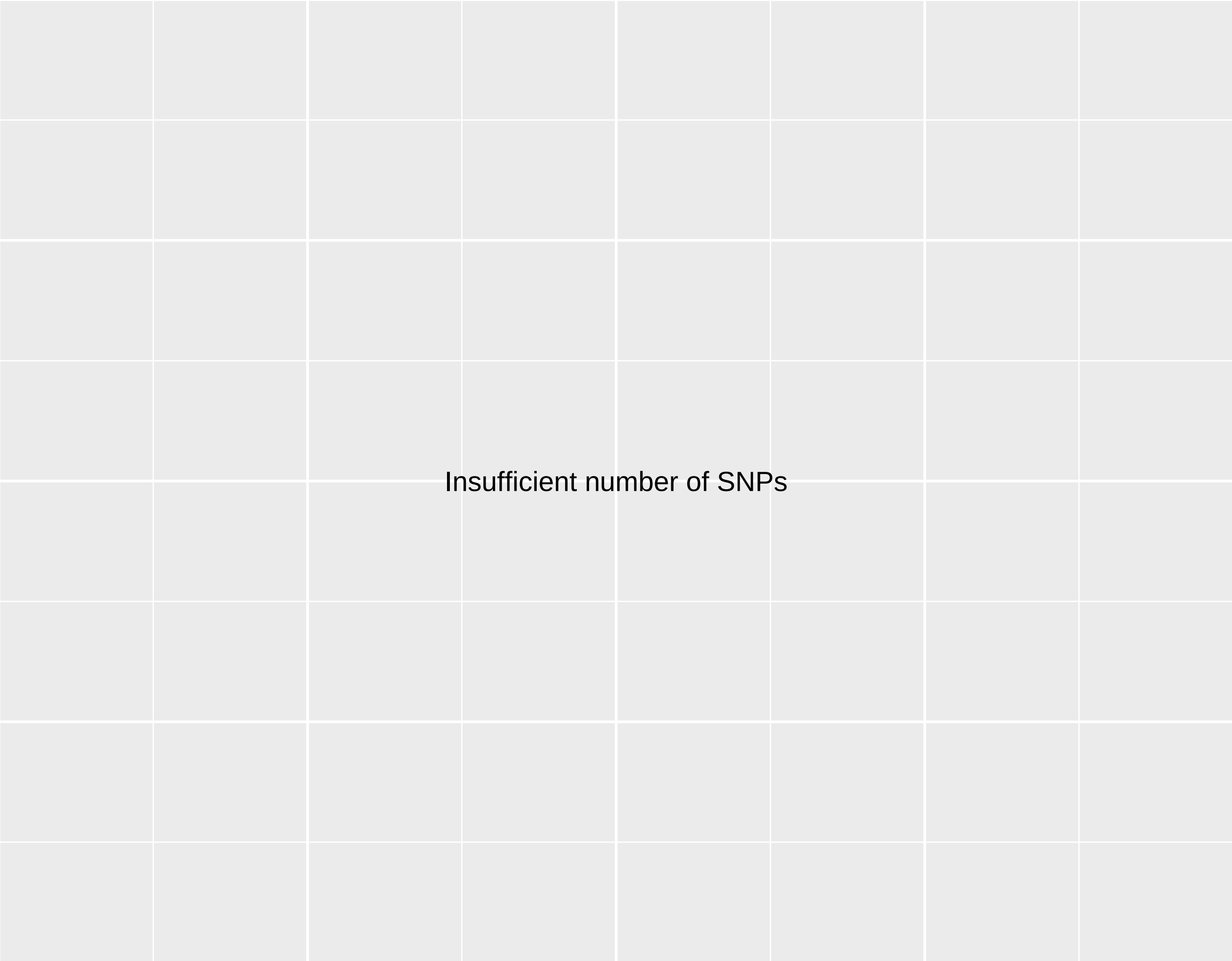
Insufficient number of SNPs

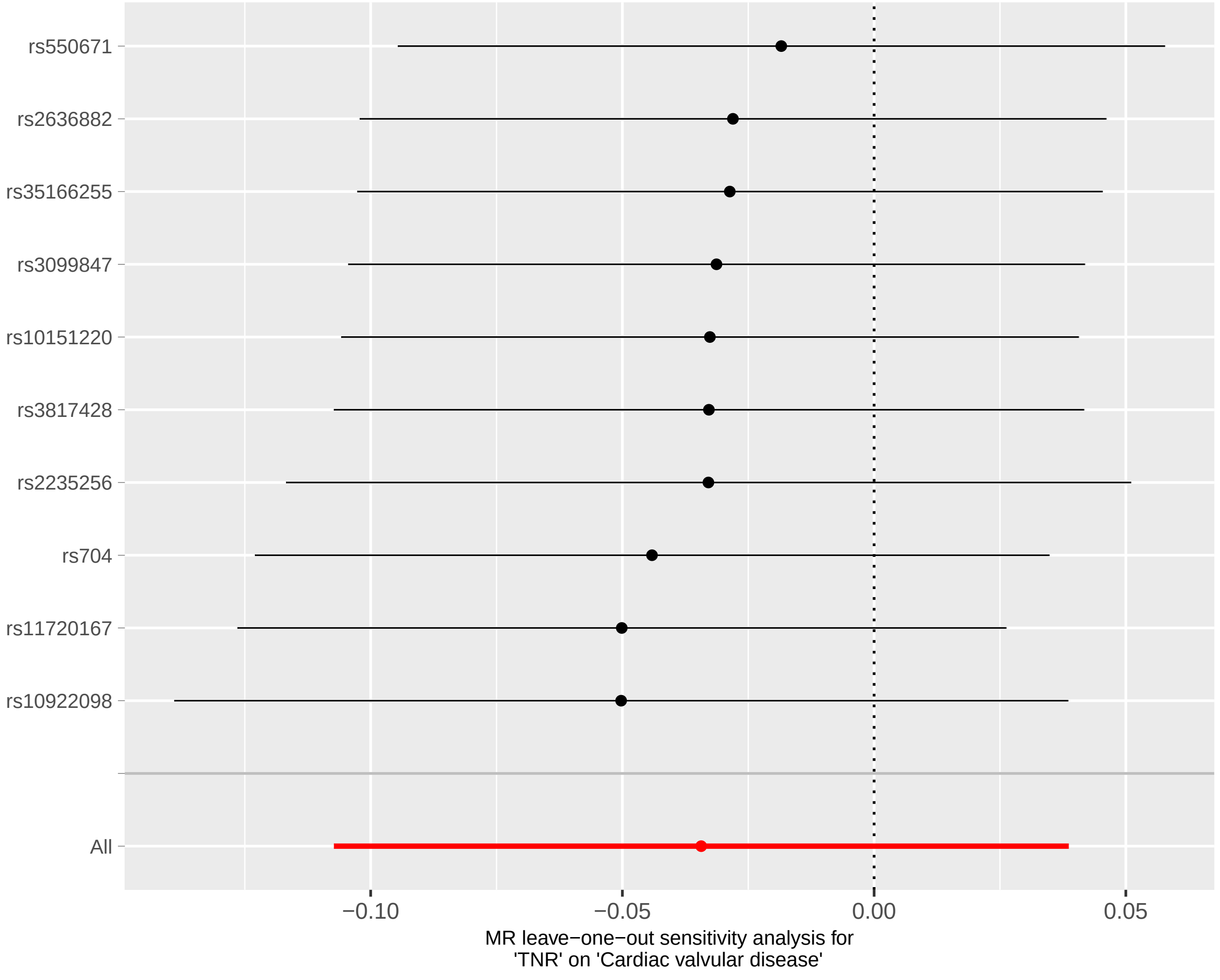
rs550671
rs2636882
rs35166255
rs3099847
rs10151220
rs3817428
rs2235256
rs704
rs11720167
rs10922098
All
−0.10
−0.05
0.00
0.05
MR leave−one−out sensitivity analysis for
'TNR' on 'Cardiac valvular disease'

Insufficient number of SNPs

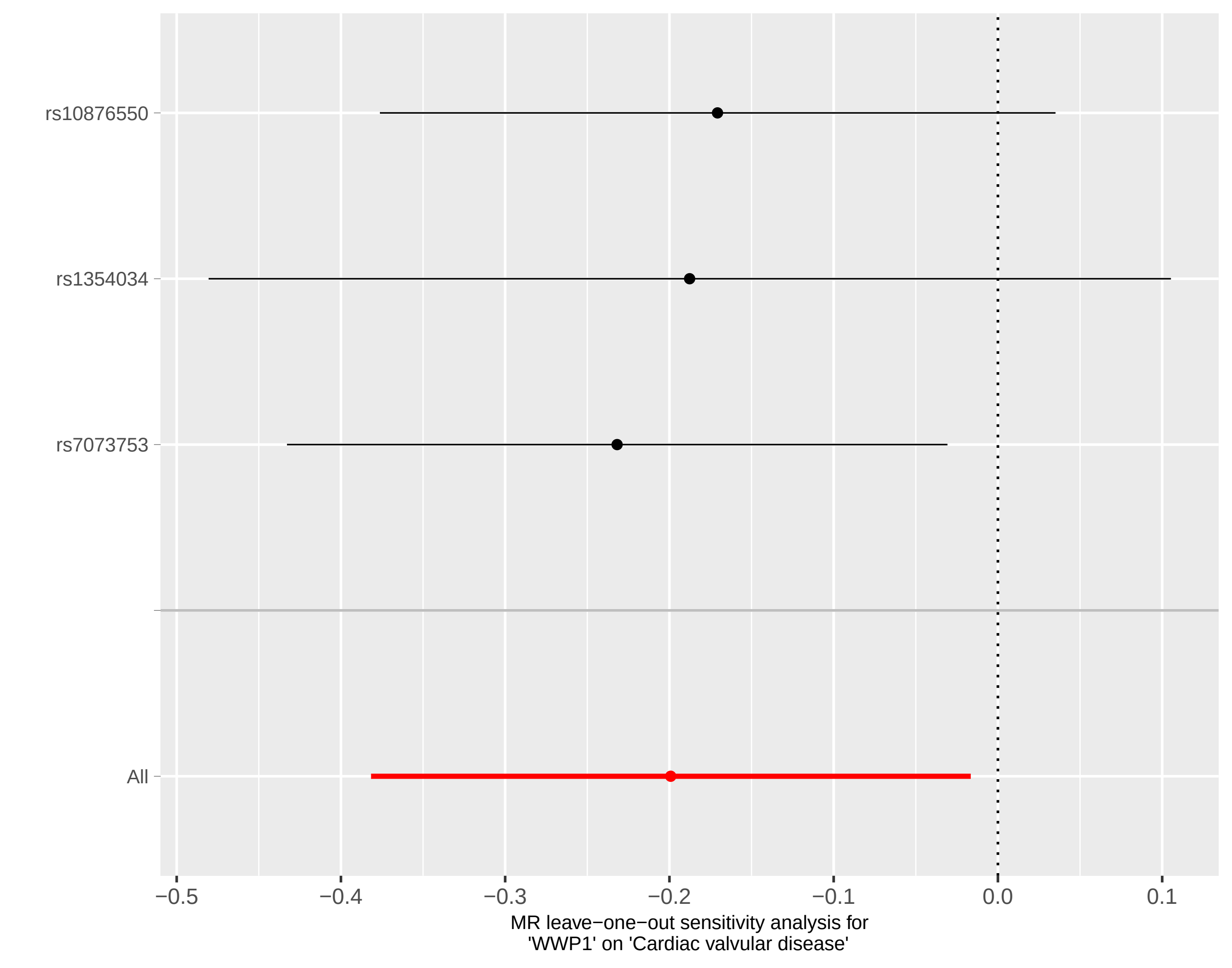
rs10876550
rs1354034
rs7073753
All
−0.5
−0.4
−0.3
−0.2
−0.1
0.0
0.1
MR leave−one−out sensitivity analysis for
'WWP1' on 'Cardiac valvular disease'

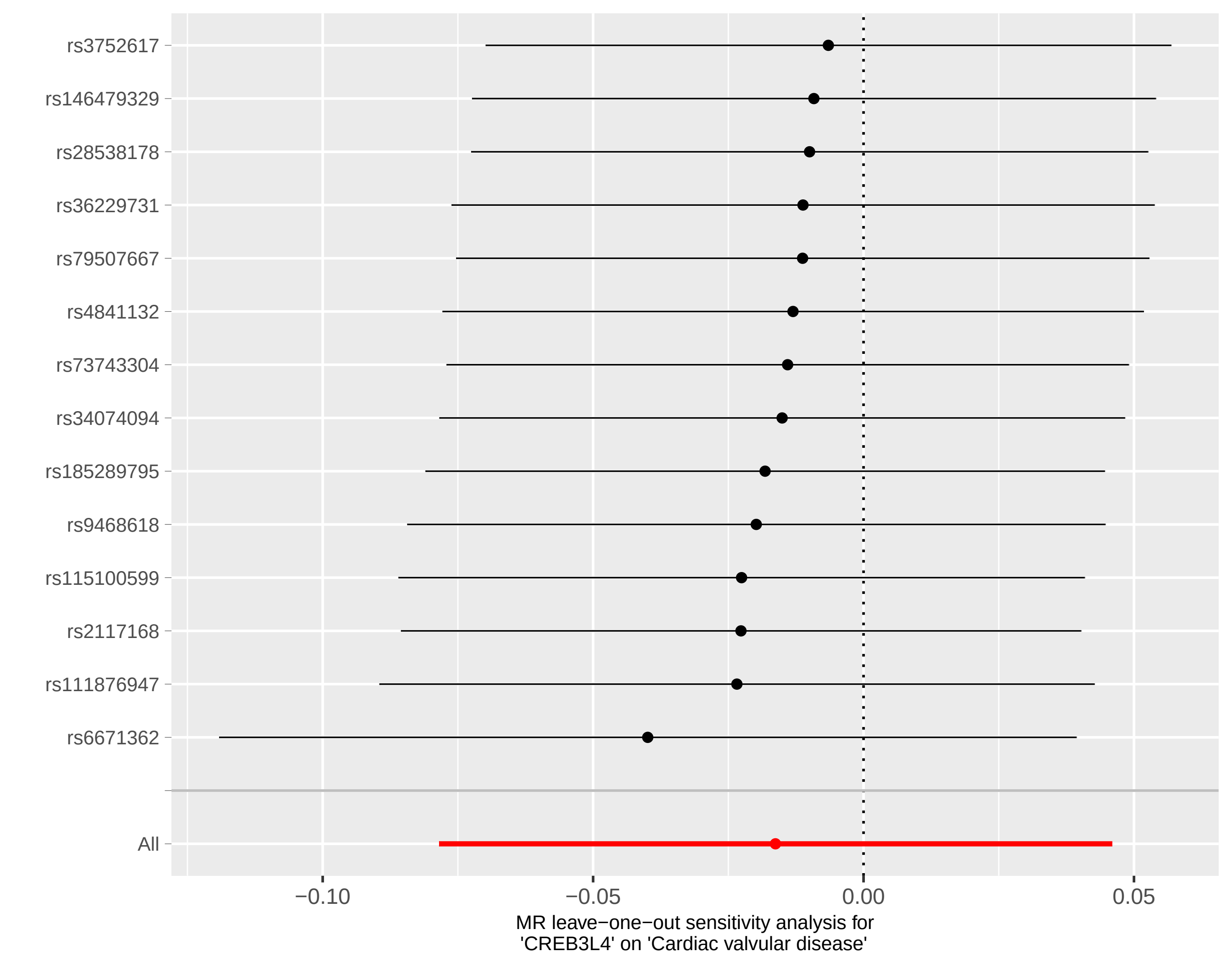
rs3752617
rs146479329
rs28538178
rs36229731
rs79507667
rs4841132
rs73743304
rs34074094
rs185289795
rs9468618
rs115100599
rs2117168
rs111876947
rs6671362
All
−0.10
−0.05
0.00
0.05
MR leave−one−out sensitivity analysis for
'CREB3L4' on 'Cardiac valvular disease'

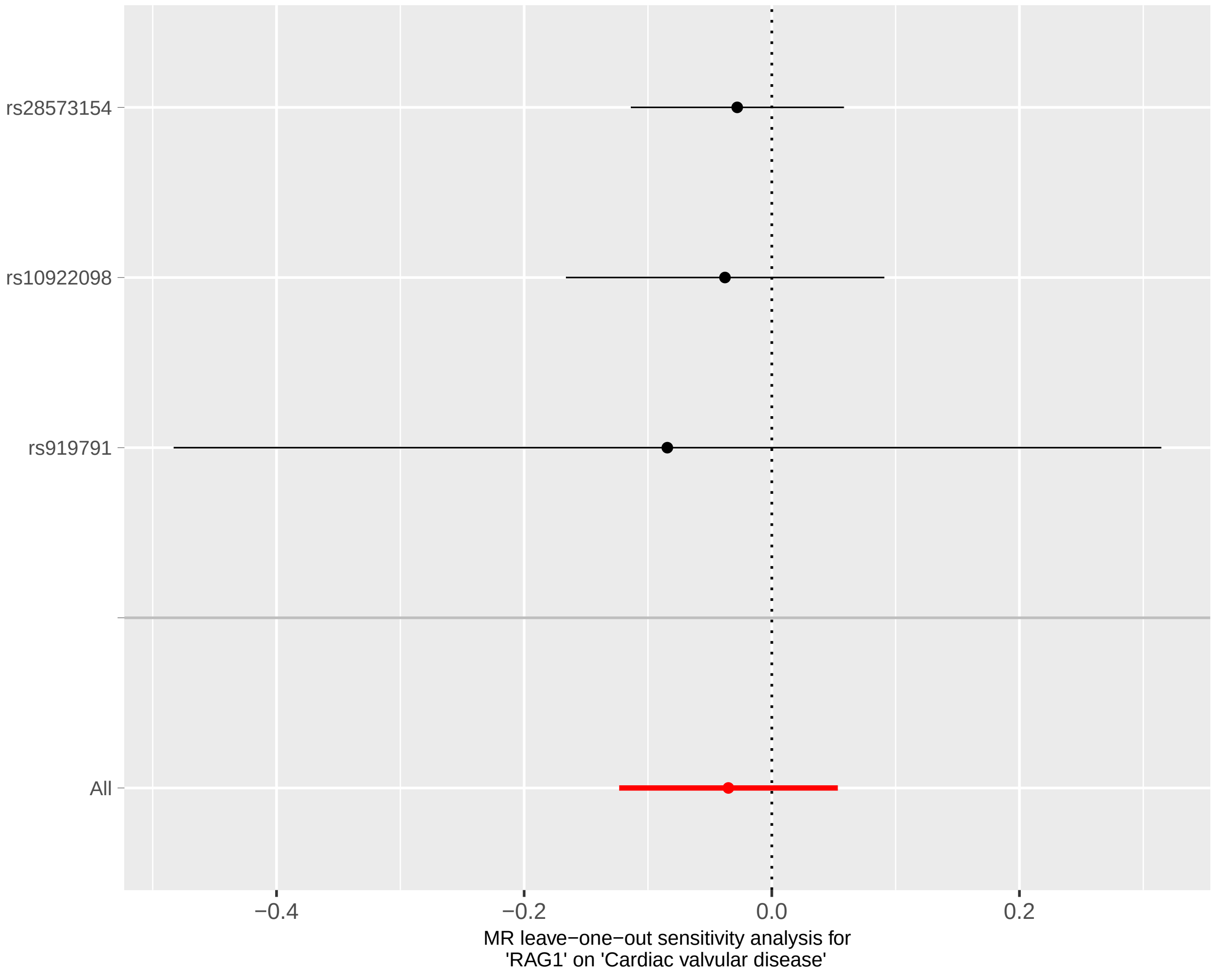
rs28573154
rs10922098
rs919791
All
−0.4
−0.2
0.0
0.2
MR leave−one−out sensitivity analysis for
'RAG1' on 'Cardiac valvular disease'

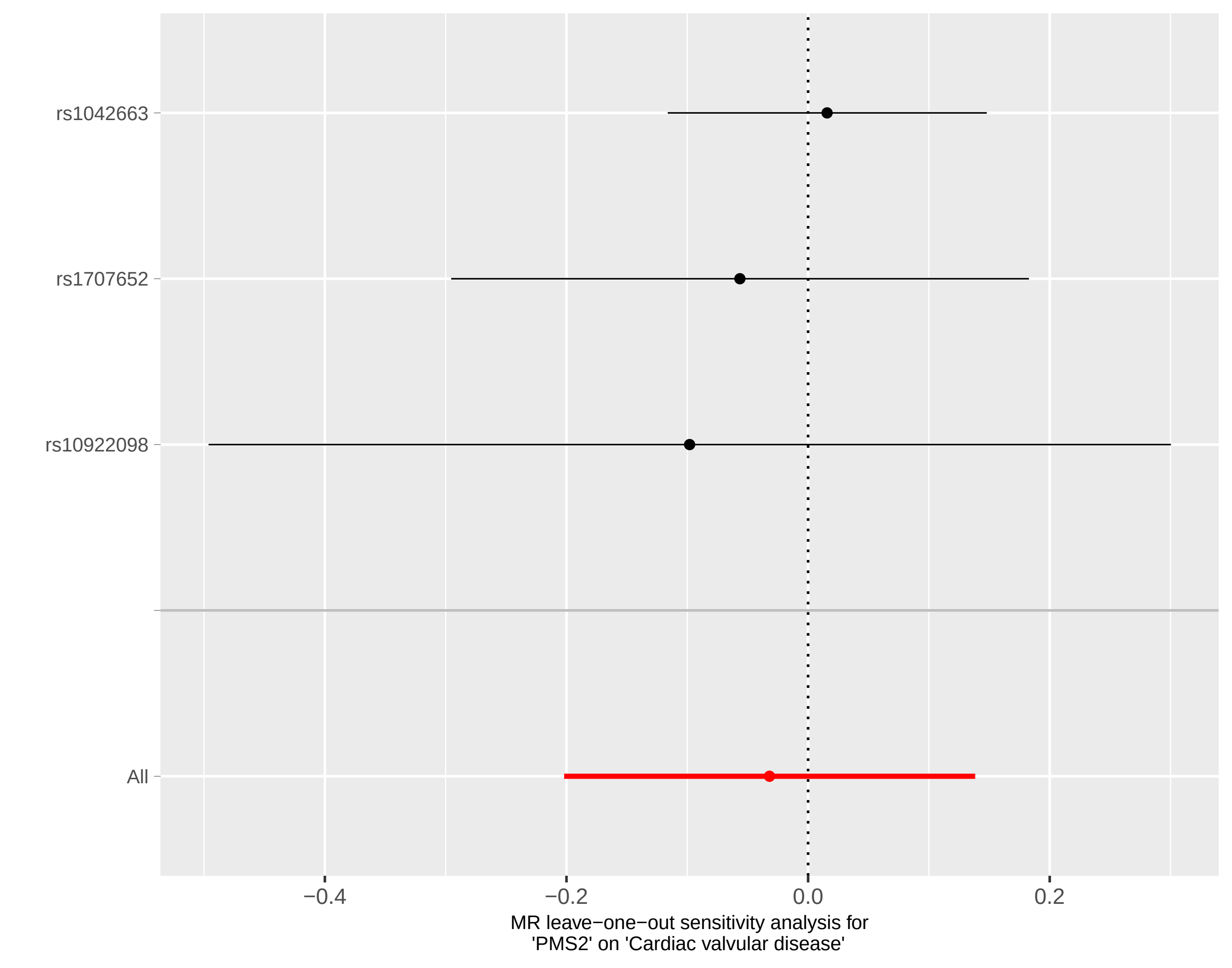

rs1042663
rs1707652
rs10922098
All
−0.4
−0.2
0.0
0.2
MR leave−one−out sensitivity analysis for
'PMS2' on 'Cardiac valvular disease'

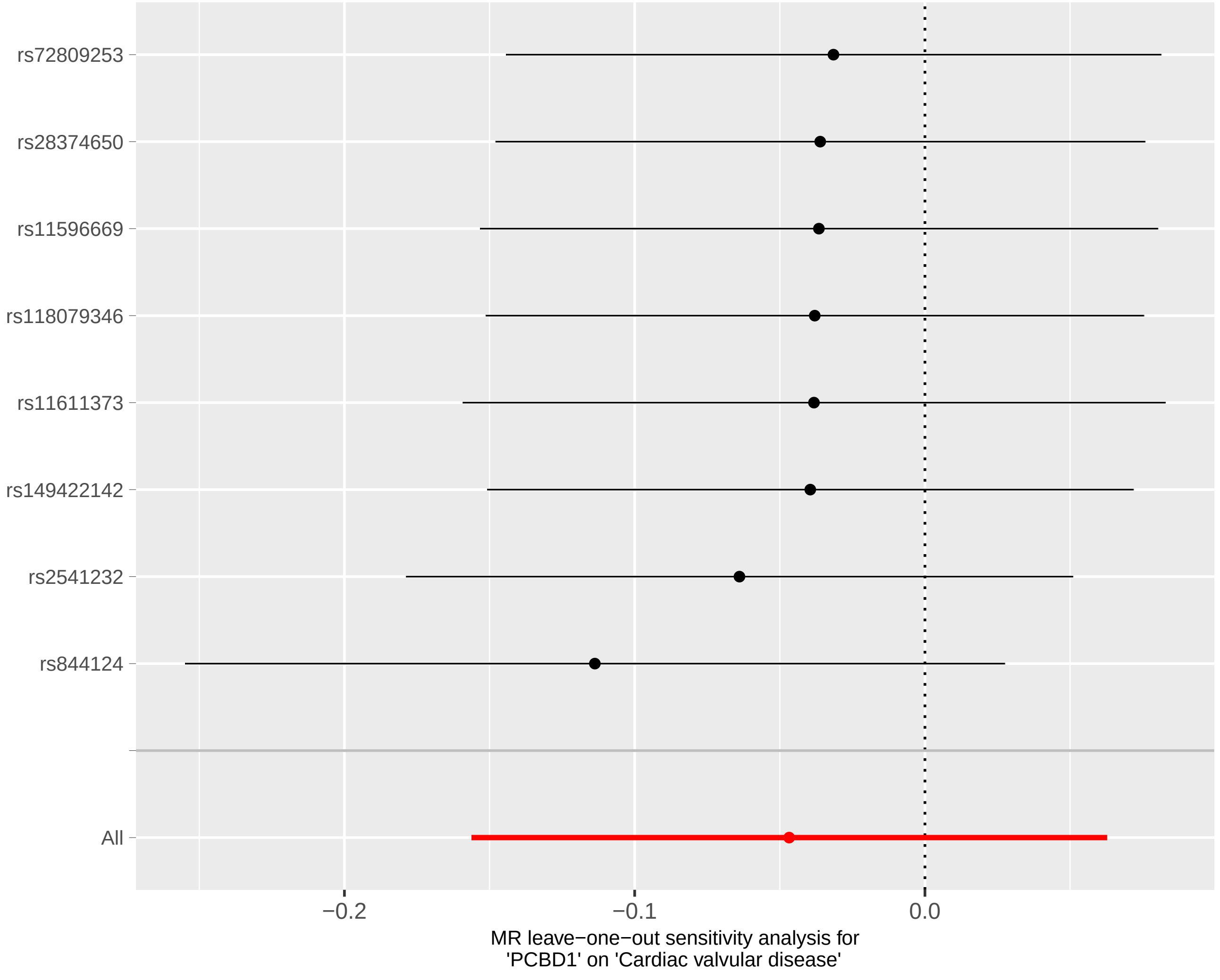

rs72809253
rs28374650
rs11596669
rs118079346
rs11611373
rs149422142
rs2541232
rs844124
All
−0.2
−0.1
0.0
MR leave−one−out sensitivity analysis for
'PCBD1' on 'Cardiac valvular disease'

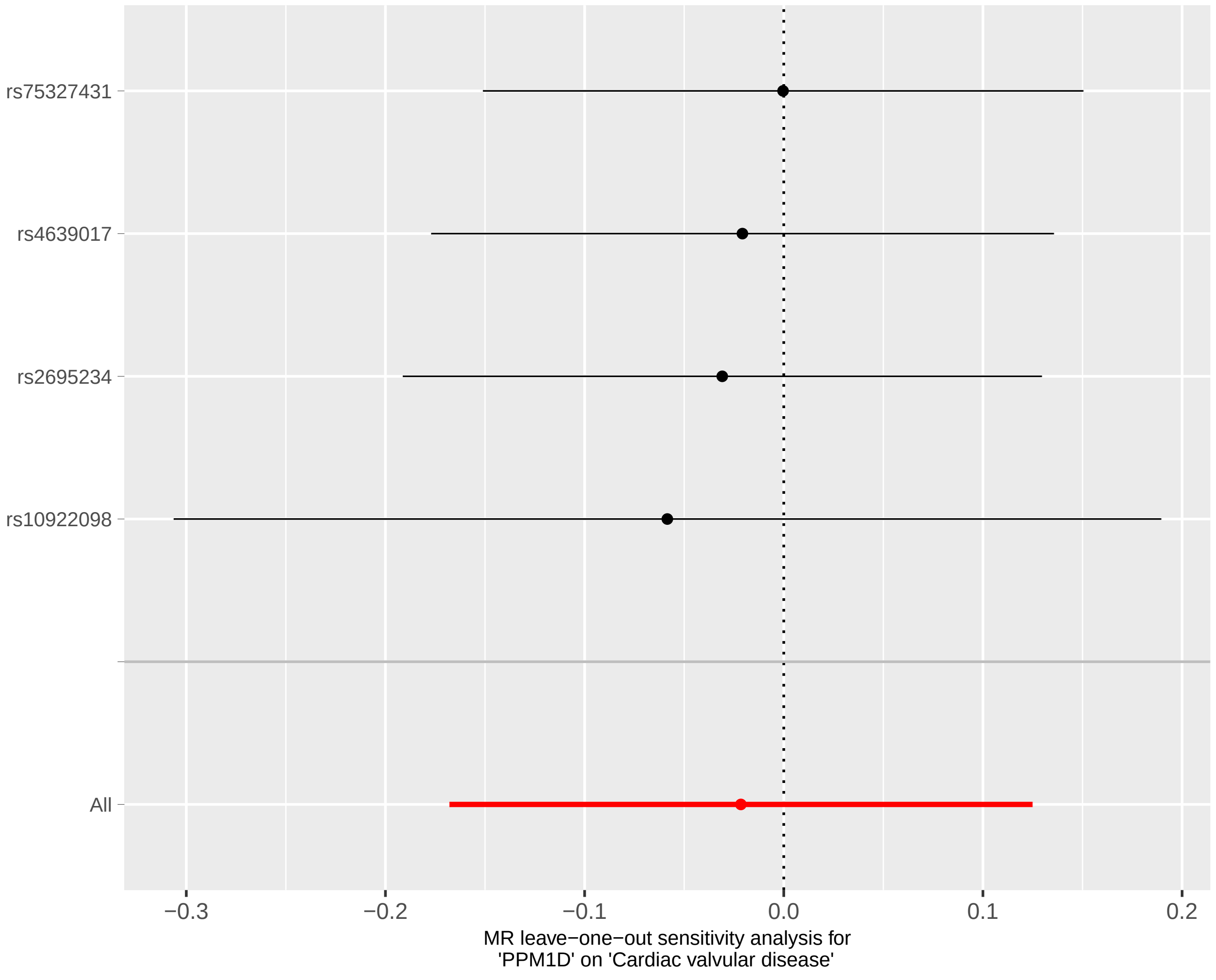
rs75327431
rs4639017
rs2695234
rs10922098
All
−0.3
−0.2
−0.1
0.0
0.1
0.2
MR leave−one−out sensitivity analysis for
'PPM1D' on 'Cardiac valvular disease'

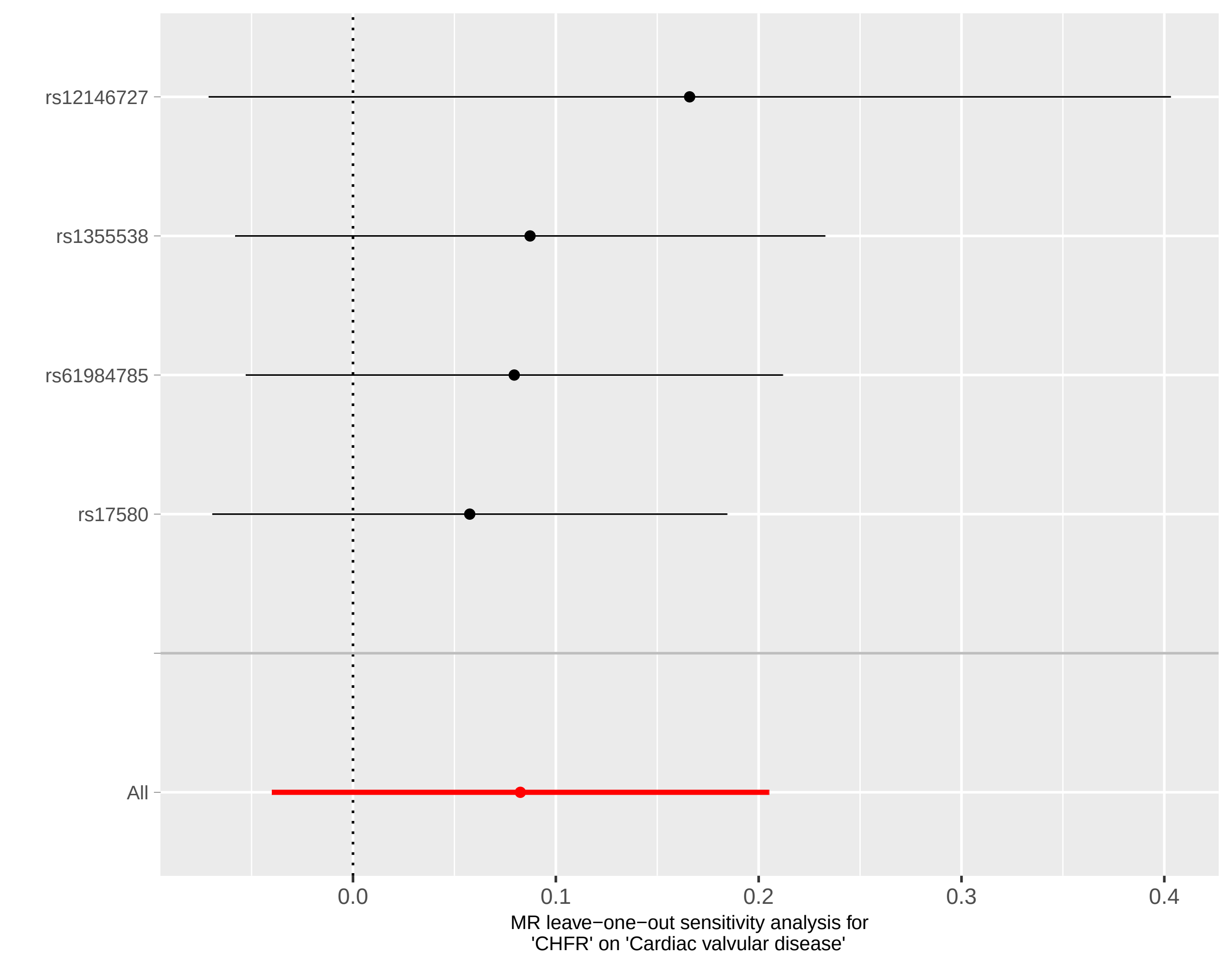

rs12146727
rs1355538
rs61984785
rs17580
All
0.0
0.1
0.2
0.3
0.4
MR leave−one−out sensitivity analysis for
'CHFR' on 'Cardiac valvular disease'

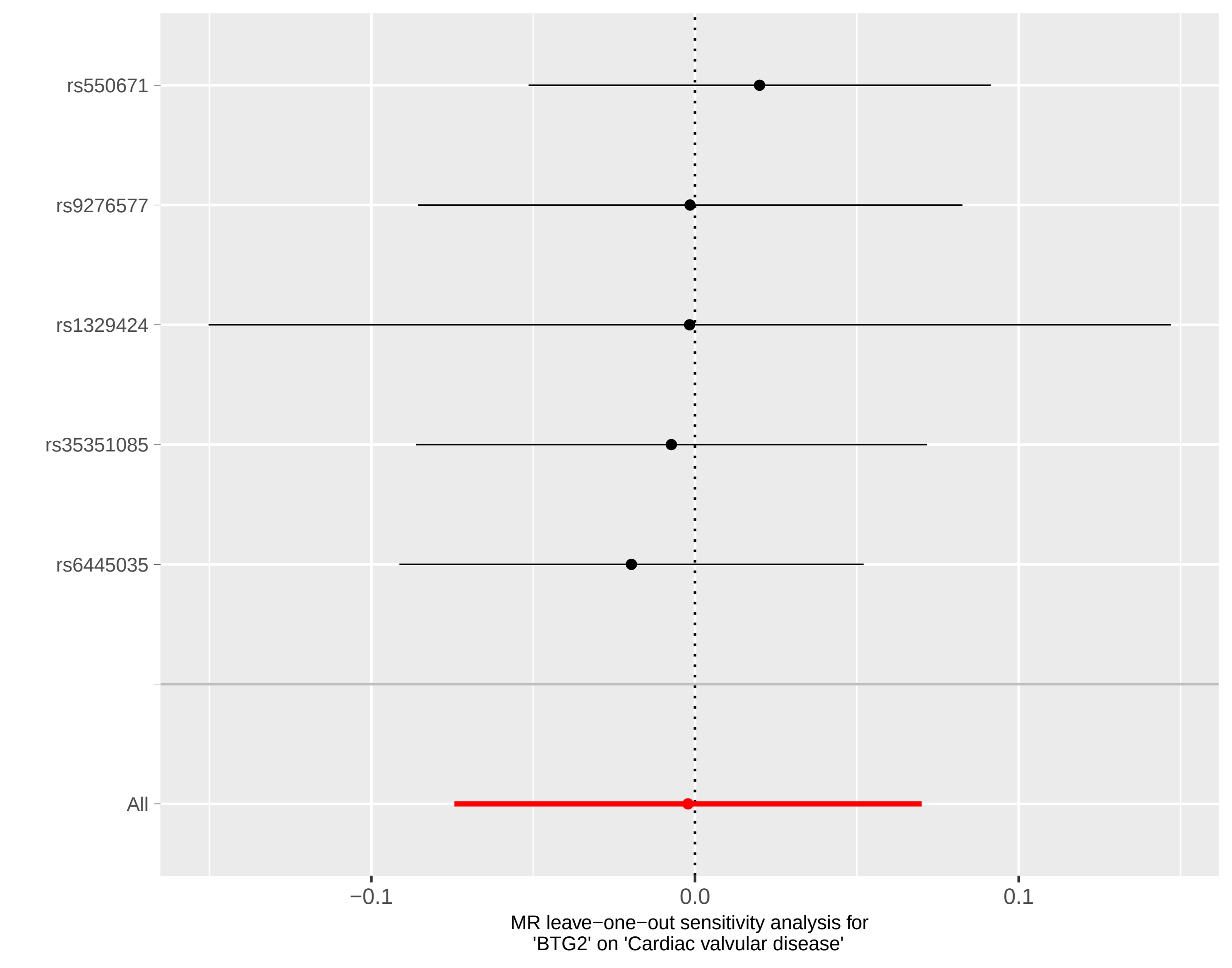

rs550671
rs9276577
rs1329424
rs35351085
rs6445035
All
−0.1
0.0
0.1
MR leave−one−out sensitivity analysis for
'BTG2' on 'Cardiac valvular disease'

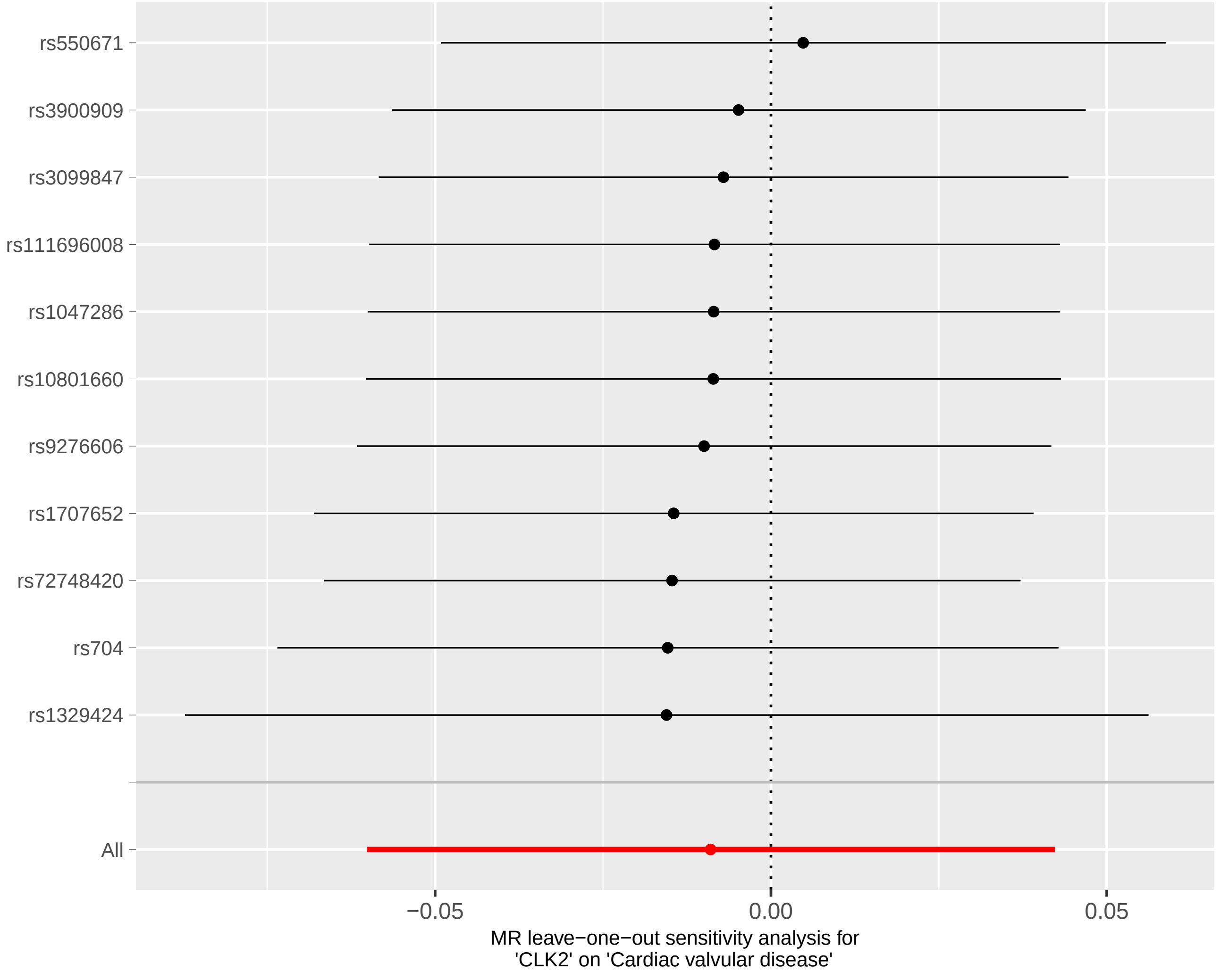
rs550671
rs3900909
rs3099847
rs111696008
rs1047286
rs10801660
rs9276606
rs1707652
rs72748420
rs704
rs1329424
All
−0.05
0.00
0.05
MR leave−one−out sensitivity analysis for
'CLK2' on 'Cardiac valvular disease'

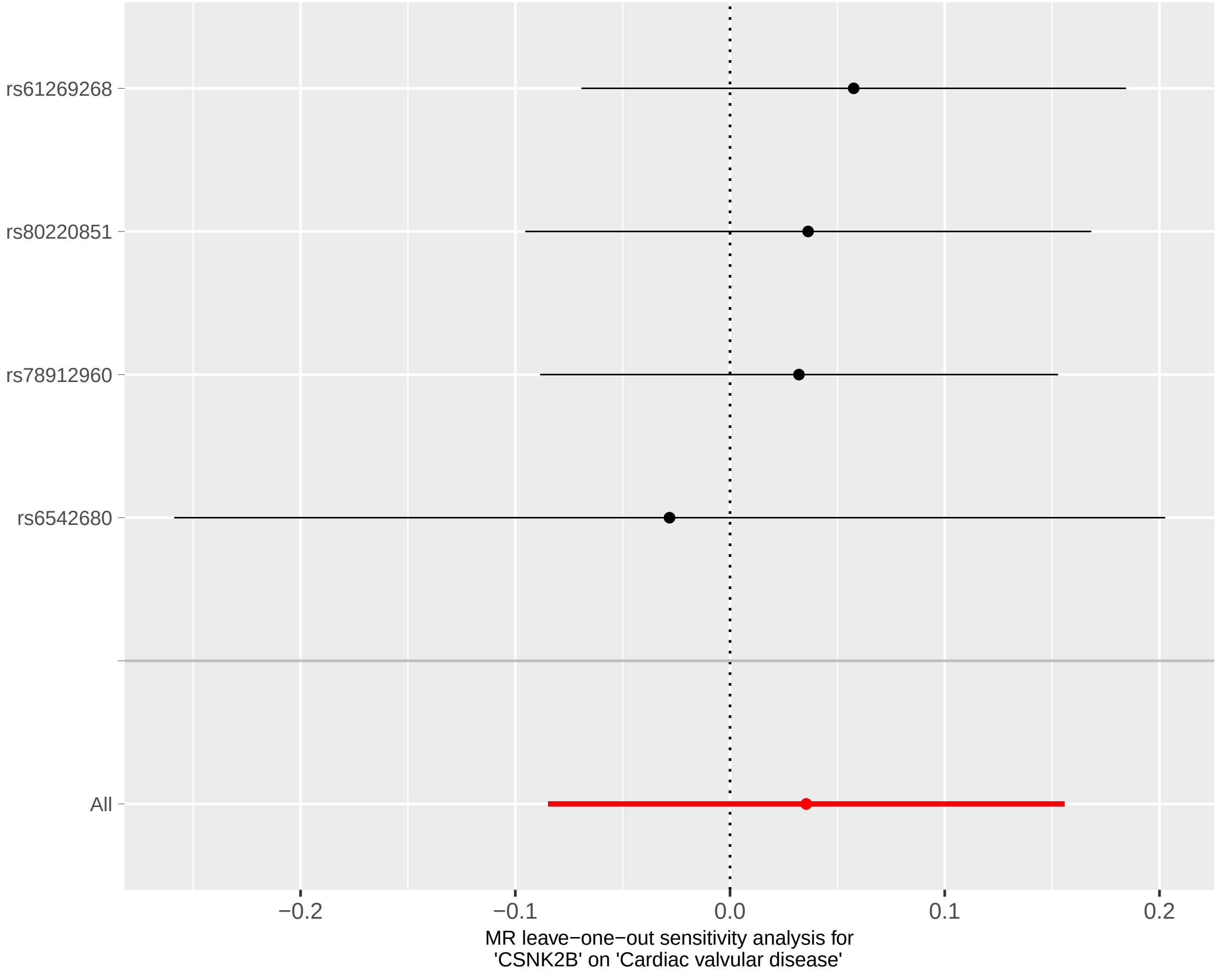
rs61269268
rs80220851
rs78912960
rs6542680
All
−0.2
−0.1
0.0
0.1
0.2
MR leave−one−out sensitivity analysis for
'CSNK2B' on 'Cardiac valvular disease'

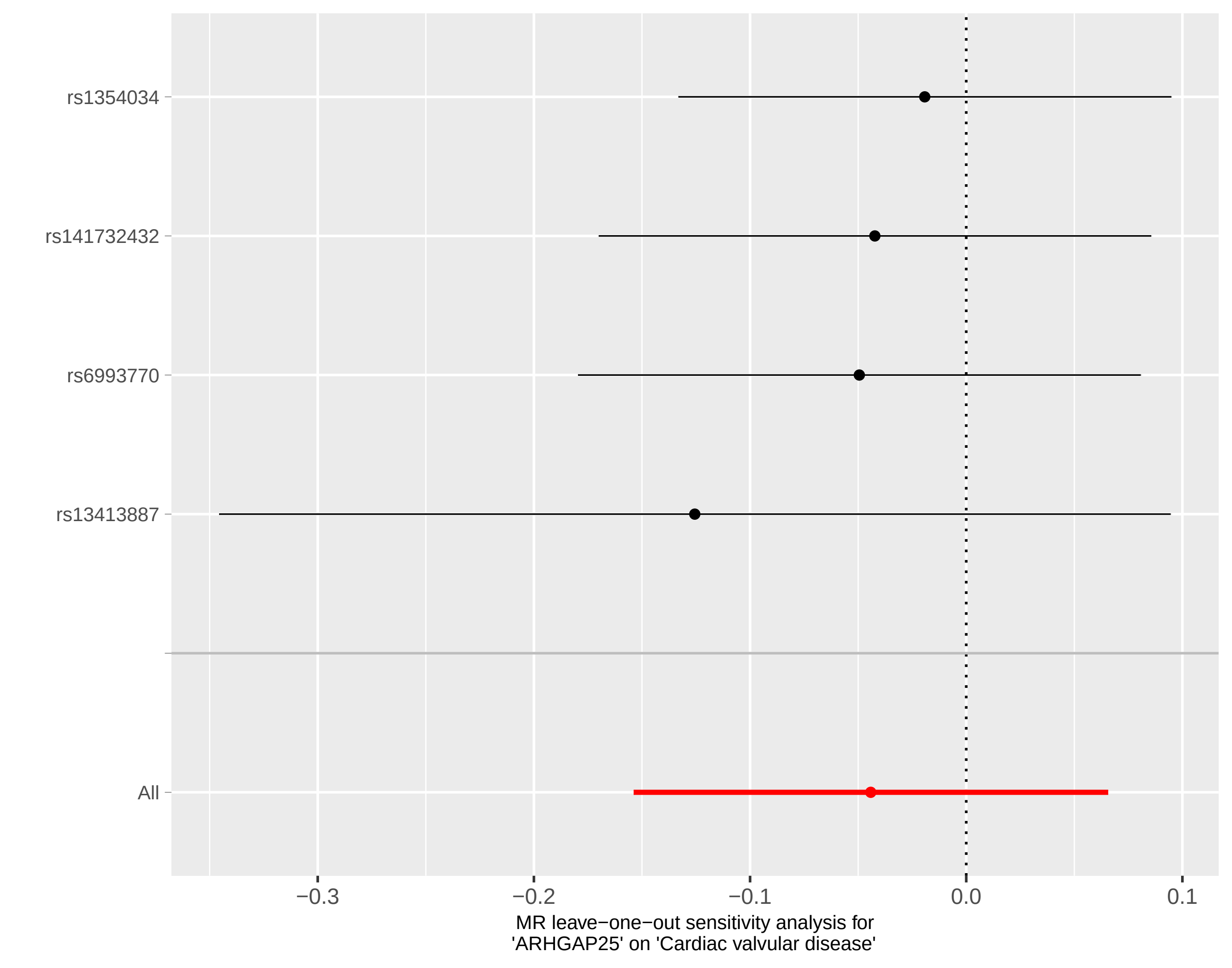
rs1354034
rs141732432
rs6993770
rs13413887
All
−0.3
−0.2
−0.1
0.0
0.1
MR leave−one−out sensitivity analysis for
'ARHGAP25' on 'Cardiac valvular disease'

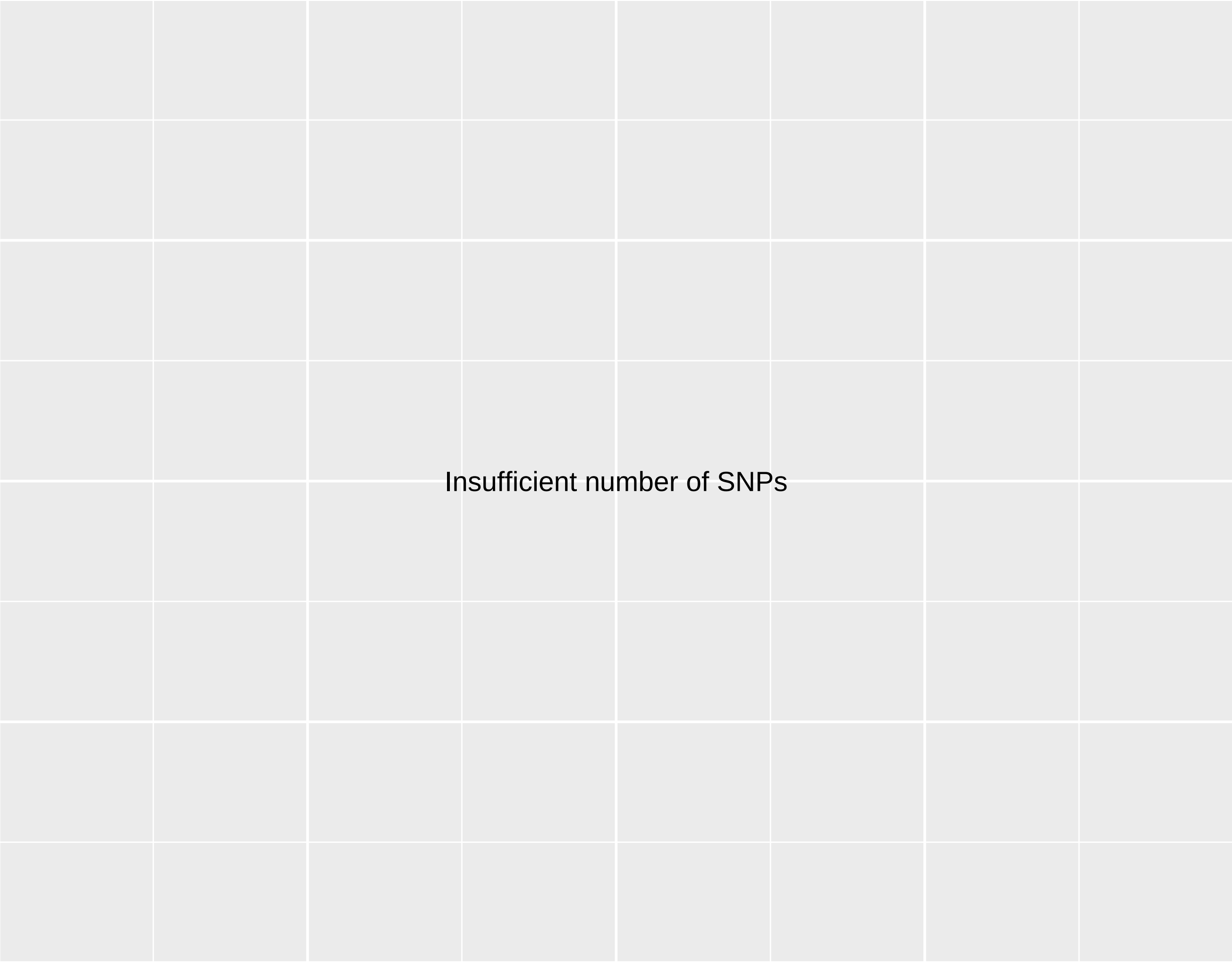
Insufficient number of SNPs

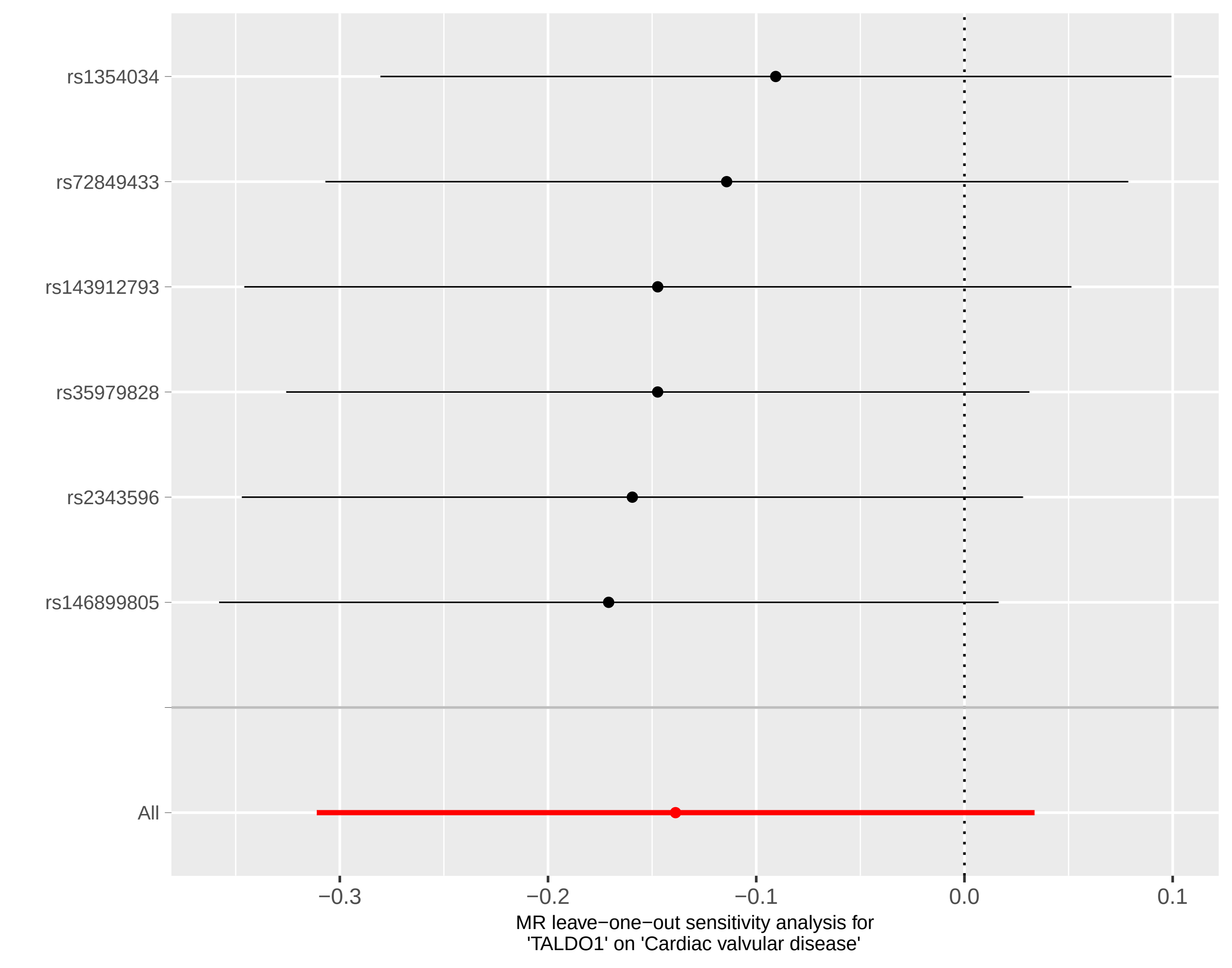
rs1354034
rs72849433
rs143912793
rs35979828
rs2343596
rs146899805
All
−0.3
−0.2
−0.1
0.0
0.1
MR leave−one−out sensitivity analysis for
'TALDO1' on 'Cardiac valvular disease'

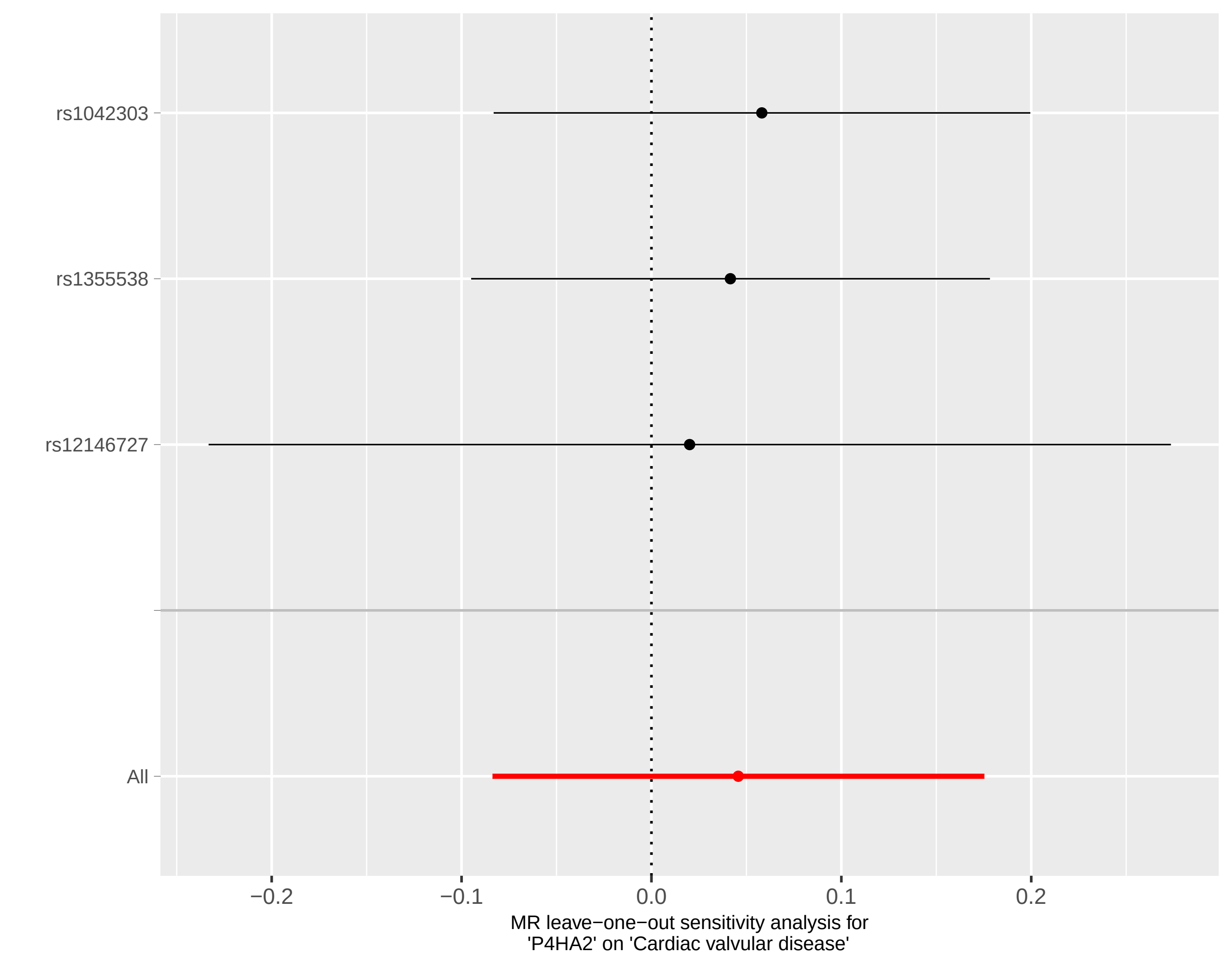

rs1042303
rs1355538
rs12146727
All
−0.2
−0.1
0.0
0.1
0.2
MR leave−one−out sensitivity analysis for
'P4HA2' on 'Cardiac valvular disease'

Insufficient number of SNPs

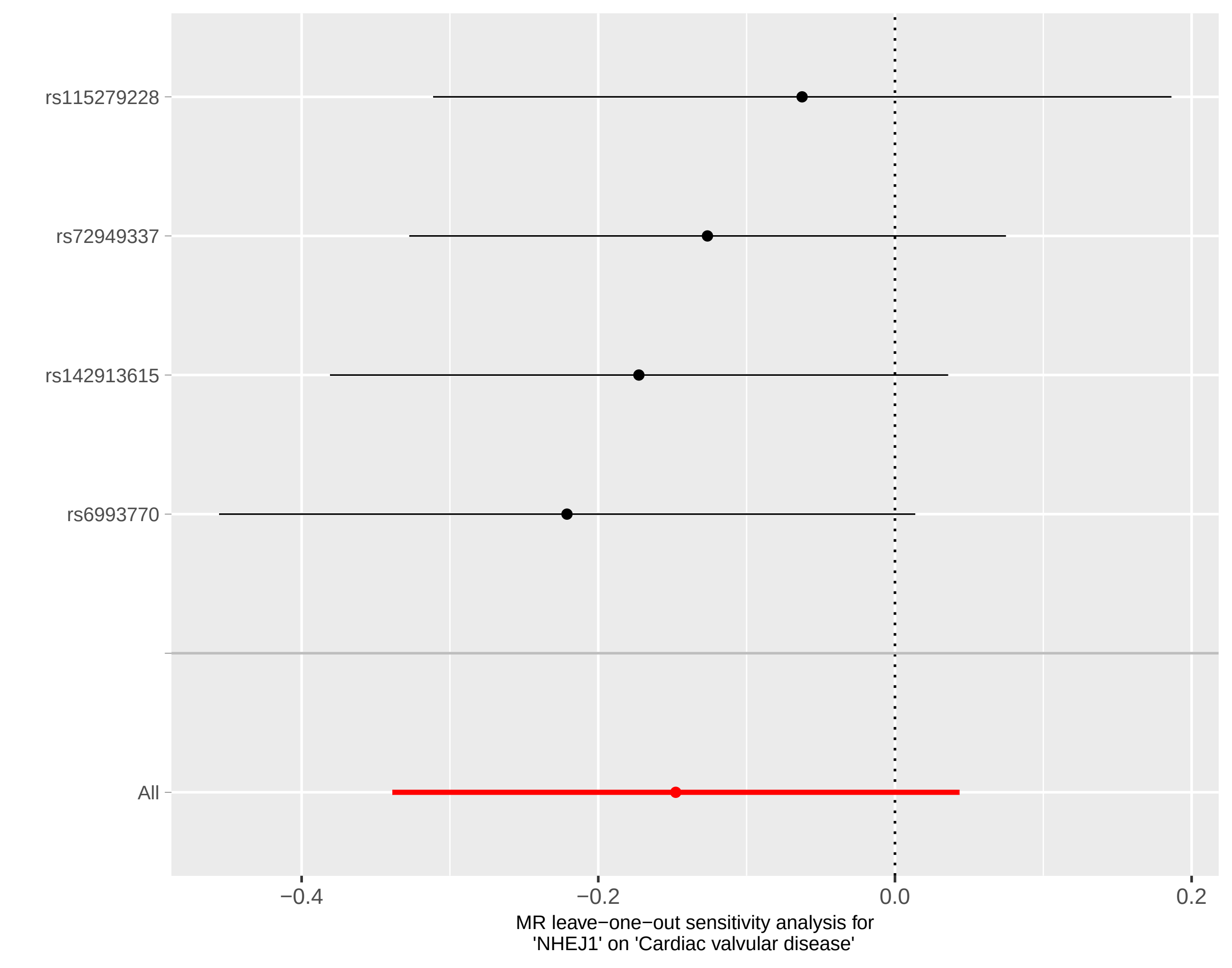
rs115279228
rs72949337
rs142913615
rs6993770
All
−0.4
−0.2
0.0
0.2
MR leave−one−out sensitivity analysis for
'NHEJ1' on 'Cardiac valvular disease'

Insufficient number of SNPs

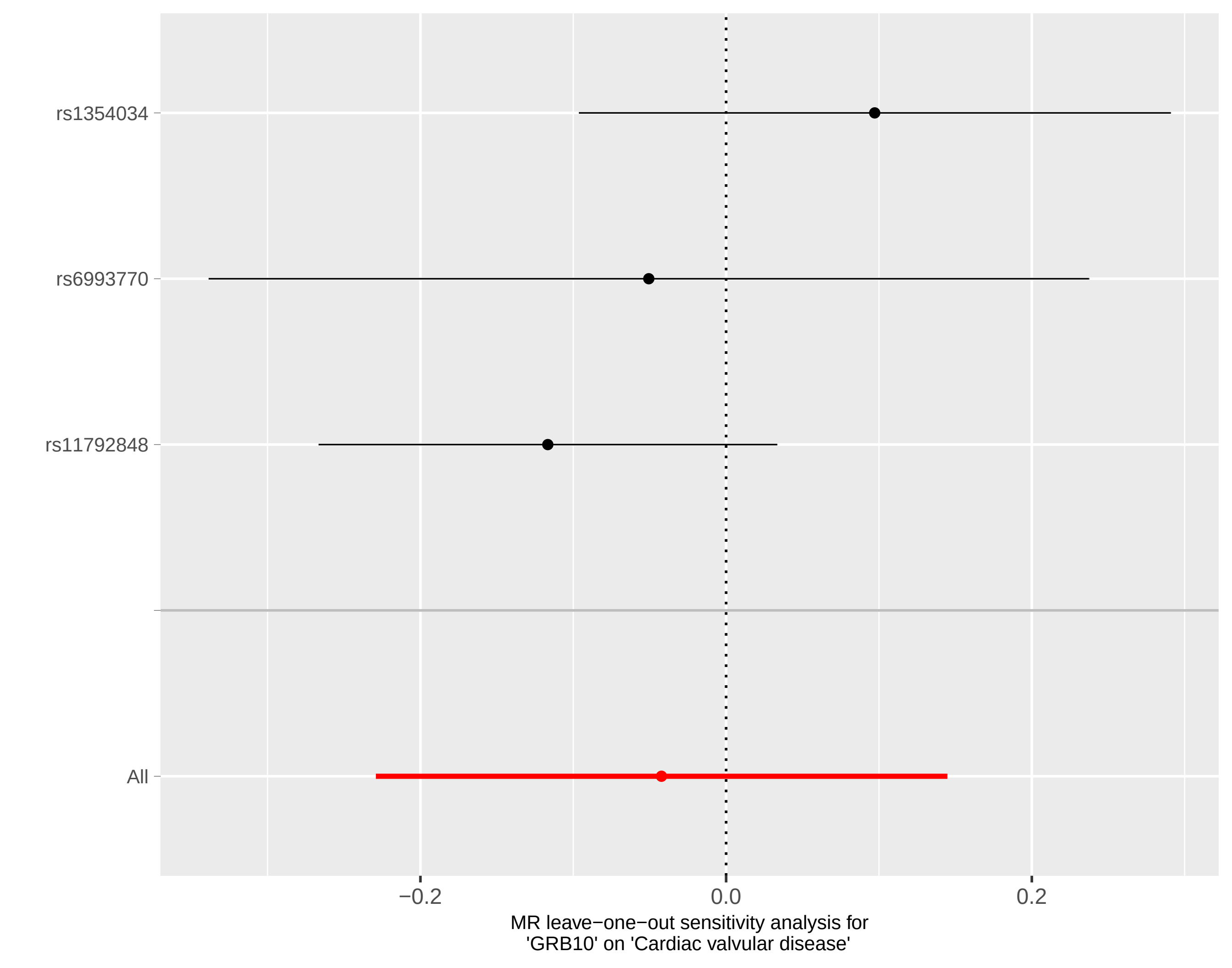
rs1354034
rs6993770
rs11792848
All
−0.2
0.0
0.2
MR leave−one−out sensitivity analysis for
'GRB10' on 'Cardiac valvular disease'

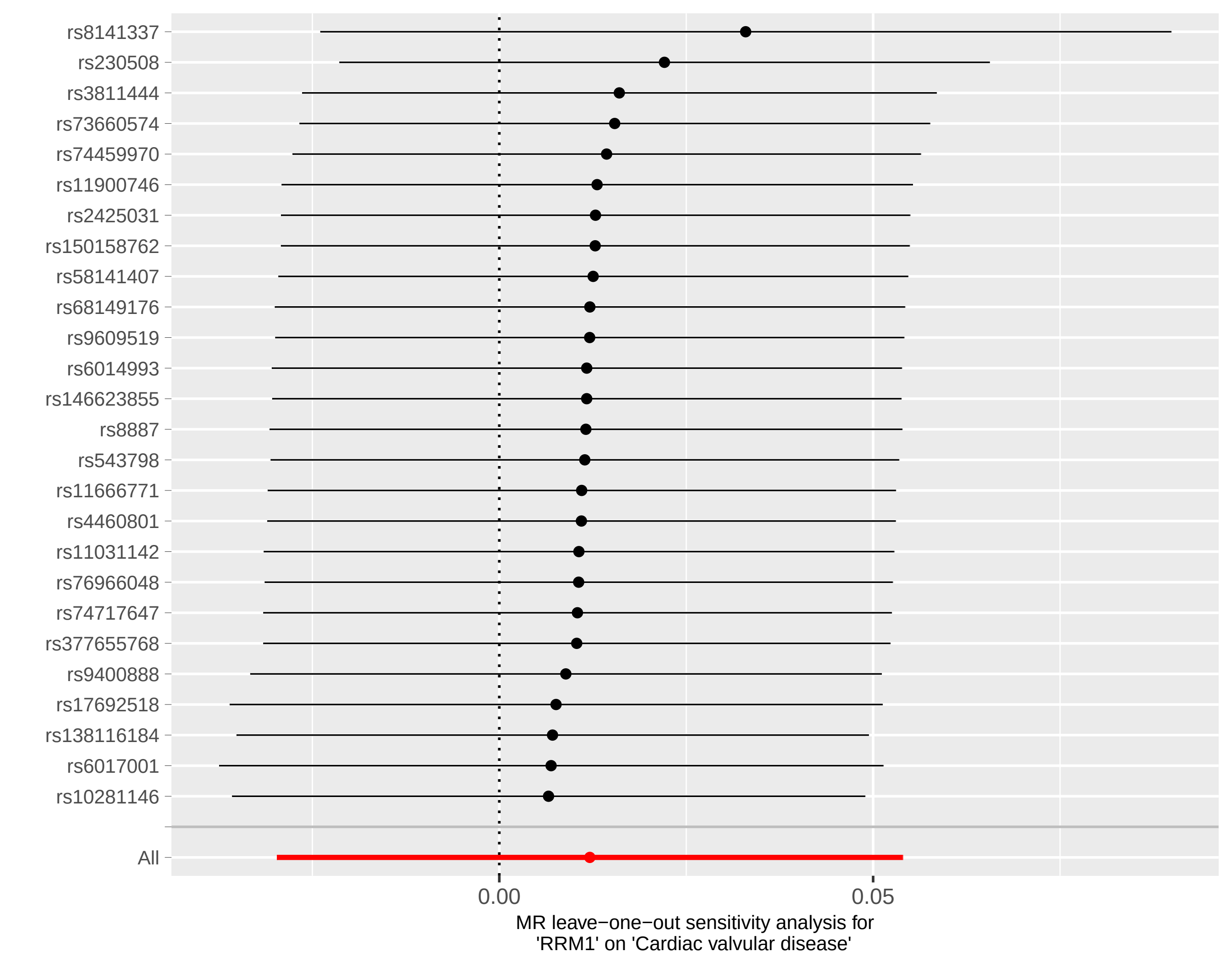
rs8141337
rs230508
rs3811444
rs73660574
rs74459970
rs11900746
rs2425031
rs150158762
rs58141407
rs68149176
rs9609519
rs6014993
rs146623855
rs8887
rs543798
rs11666771
rs4460801
rs11031142
rs76966048
rs74717647
rs377655768
rs9400888
rs17692518
rs138116184
rs6017001
rs10281146
All
0.00
0.05
MR leave−one−out sensitivity analysis for
'RRM1' on 'Cardiac valvular disease'

Insufficient number of SNPs

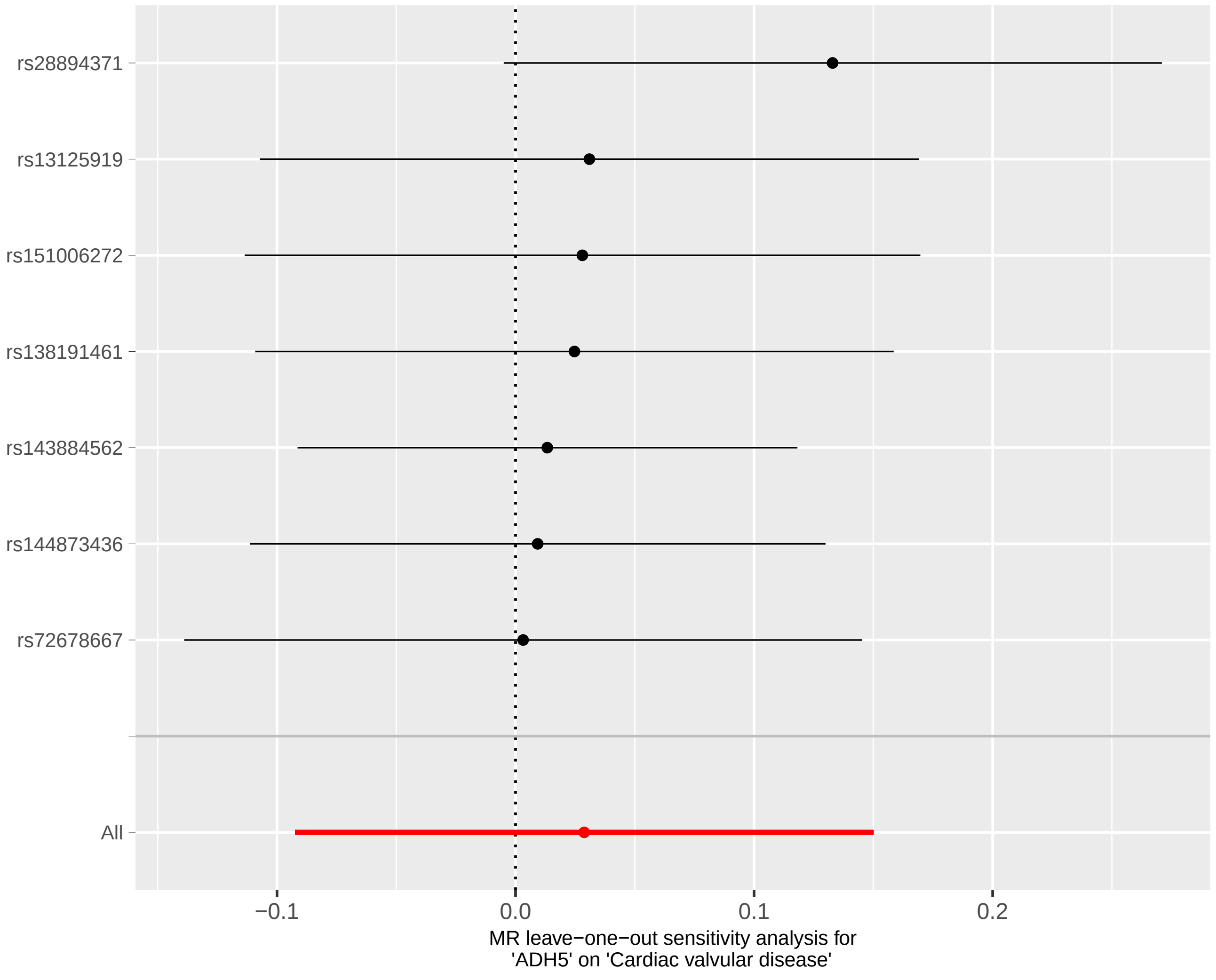
rs28894371
rs13125919
rs151006272
rs138191461
rs143884562
rs144873436
rs72678667
All
−0.1
0.0
0.1
0.2
MR leave−one−out sensitivity analysis for
'ADH5' on 'Cardiac valvular disease'

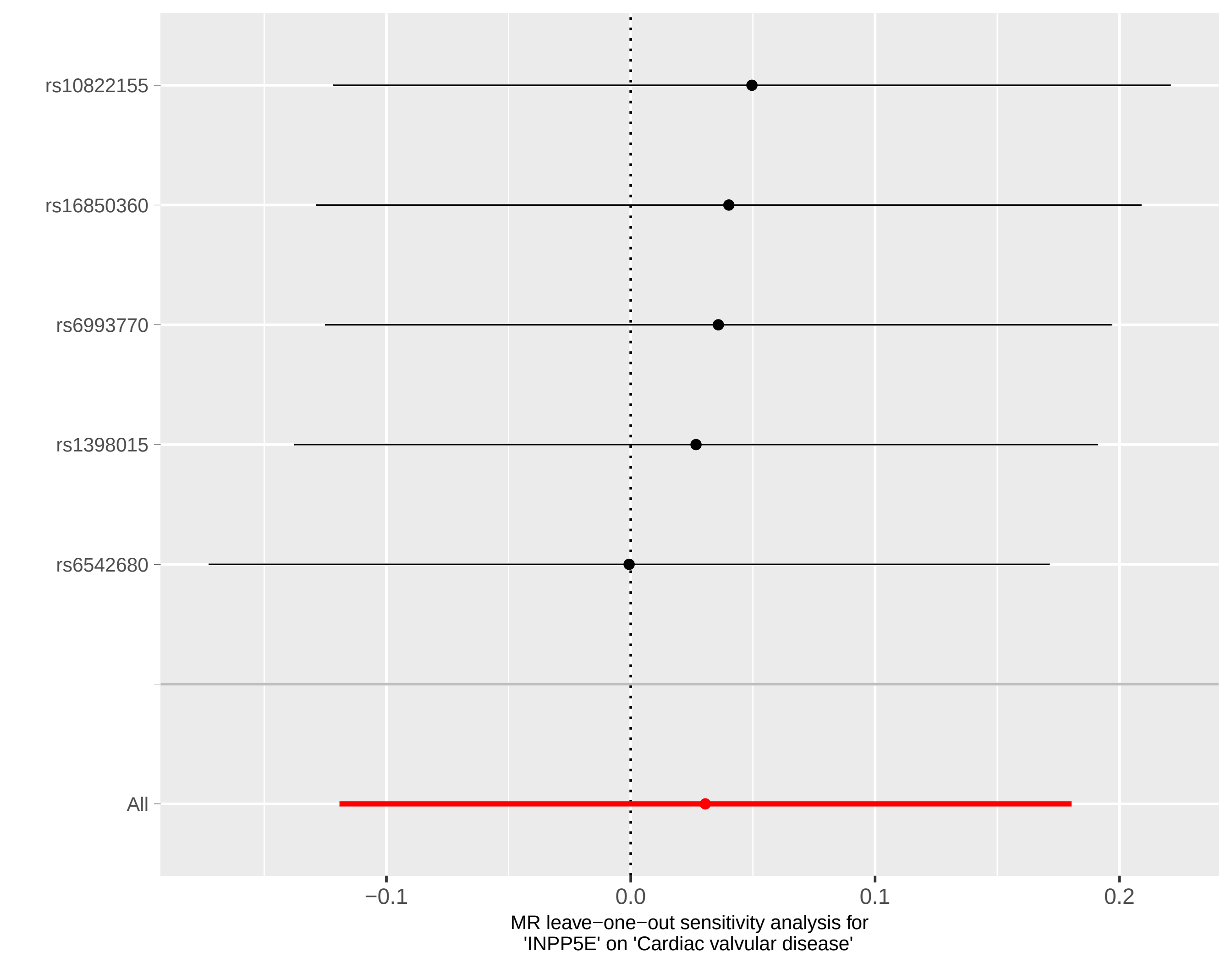
rs10822155
rs16850360
rs6993770
rs1398015
rs6542680
All
−0.1
0.0
0.1
0.2
MR leave−one−out sensitivity analysis for
'INPP5E' on 'Cardiac valvular disease'

Insufficient number of SNPs

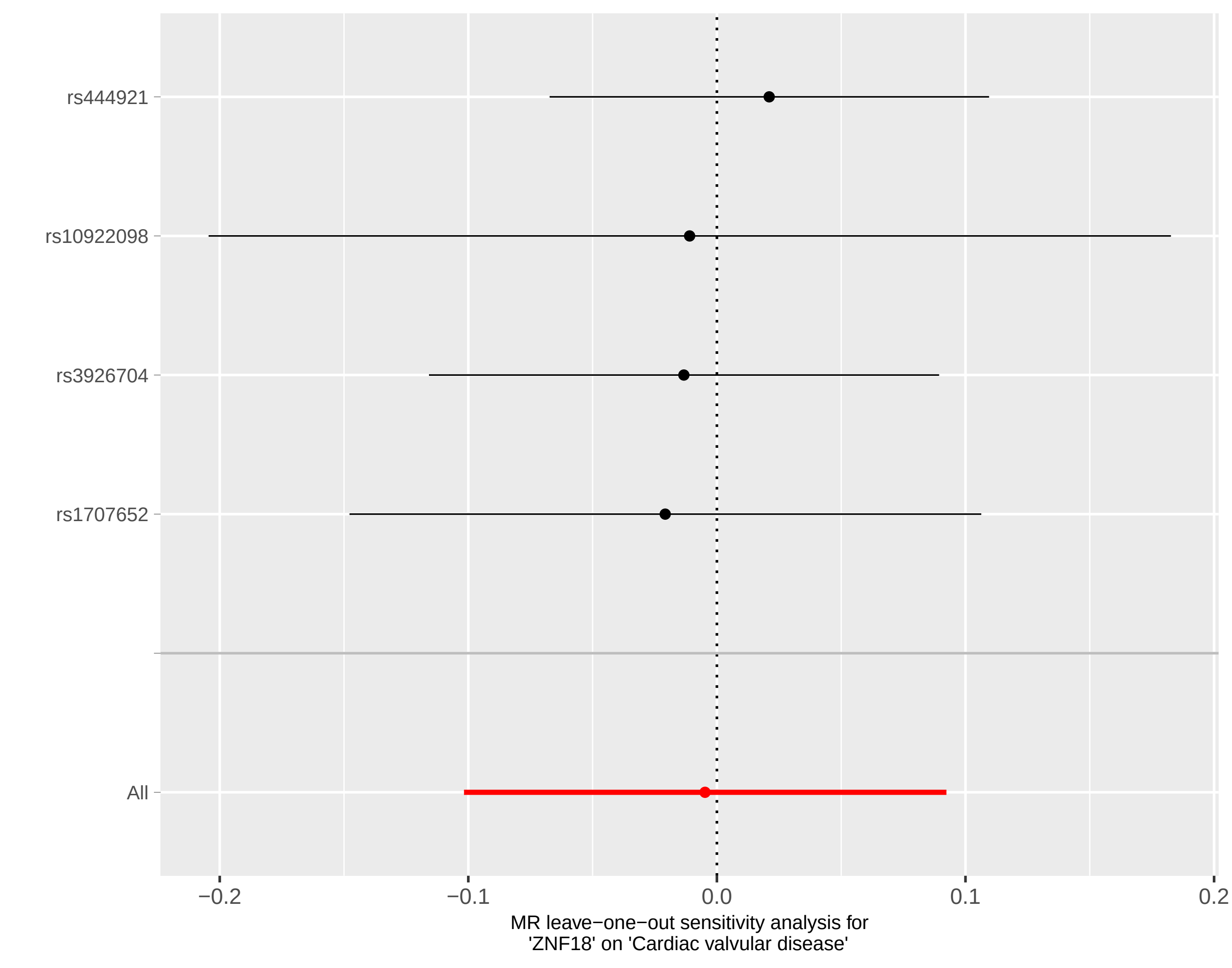
rs444921
rs10922098
rs3926704
rs1707652
All
−0.2
−0.1
0.0
0.1
0.2
MR leave−one−out sensitivity analysis for
'ZNF18' on 'Cardiac valvular disease'

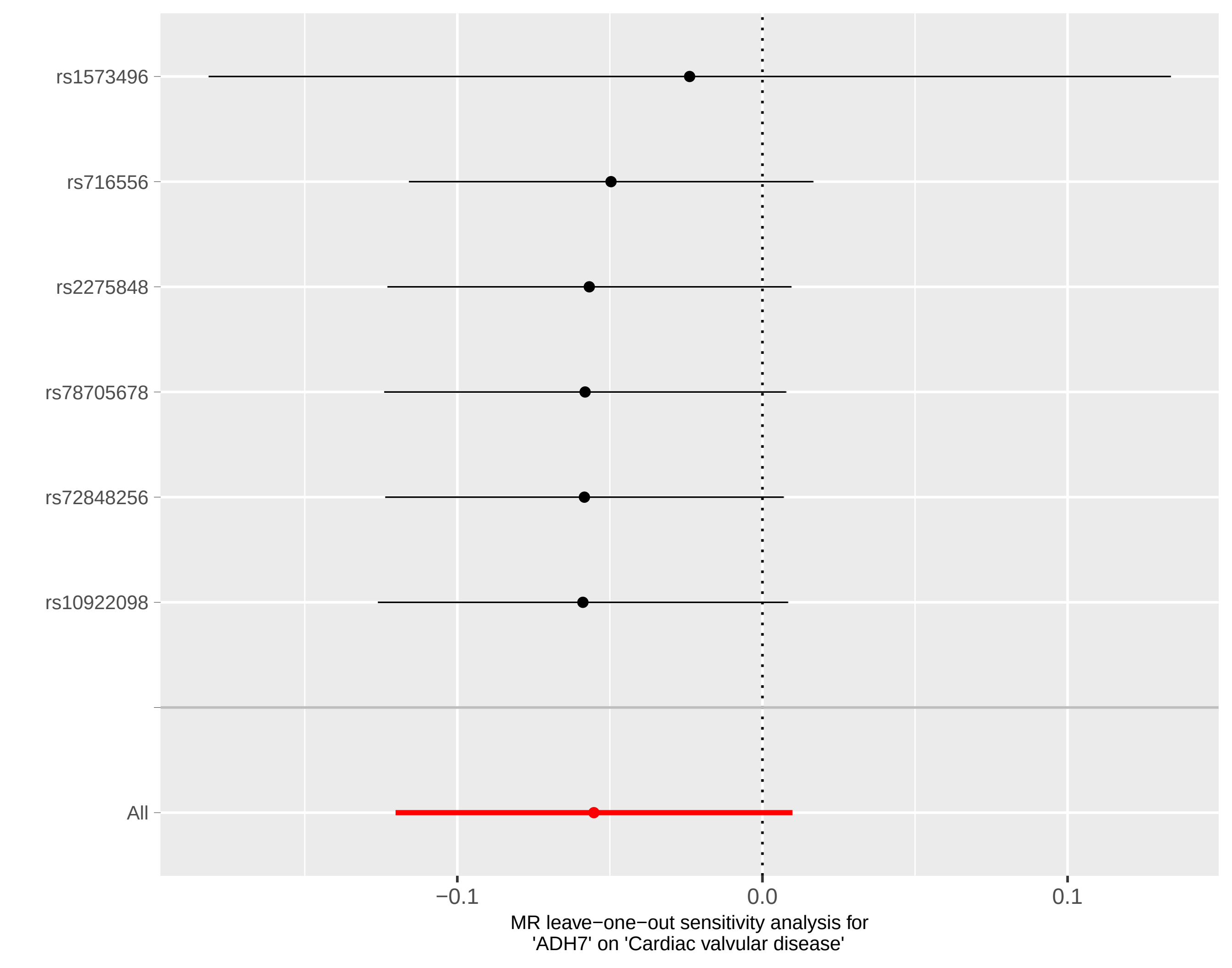

rs1573496
rs716556
rs2275848
rs78705678
rs72848256
rs10922098
All
−0.1
0.0
0.1
MR leave−one−out sensitivity analysis for
'ADH7' on 'Cardiac valvular disease'

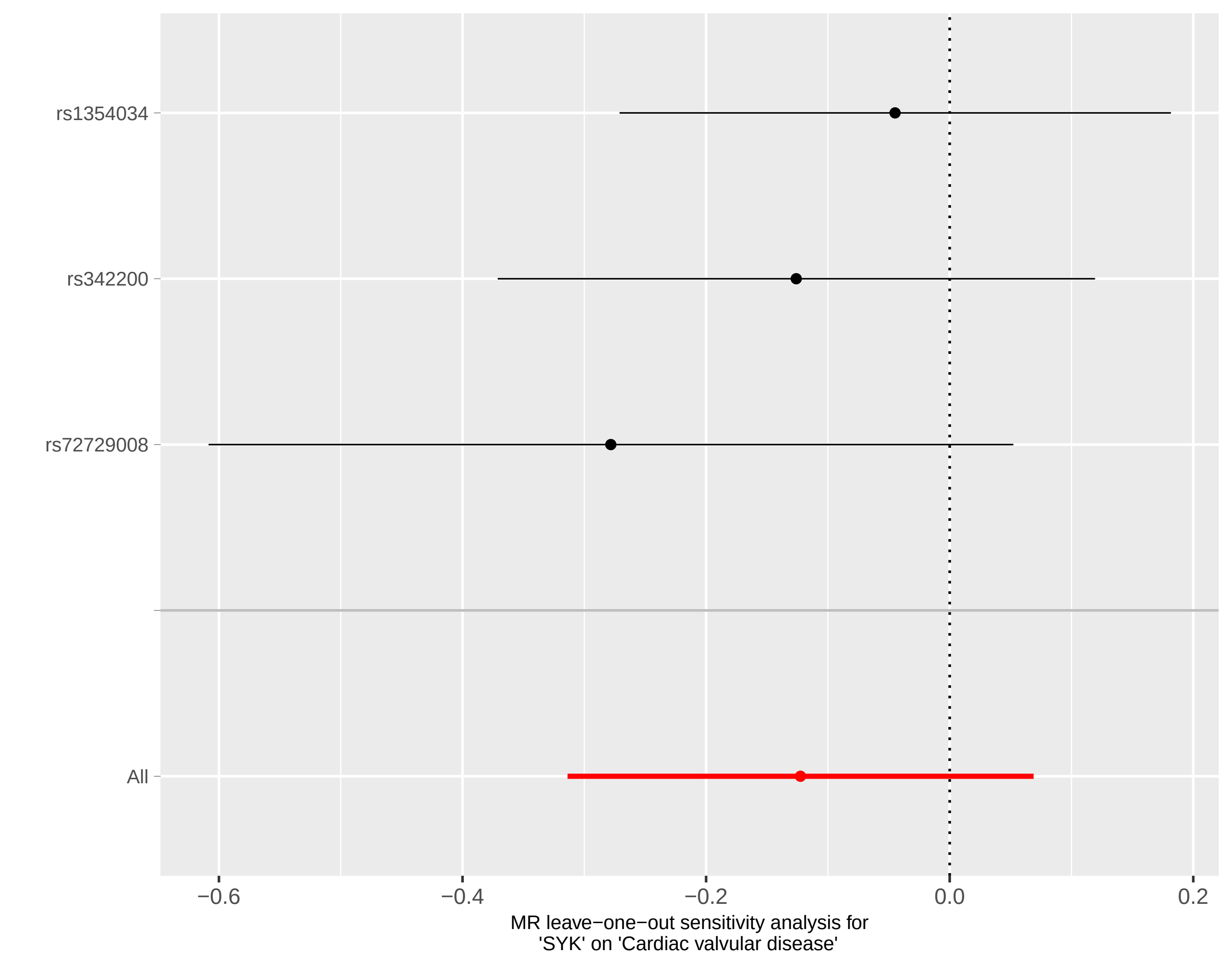
rs1354034
rs342200
rs72729008
All
−0.6
−0.4
−0.2
0.0
0.2
MR leave−one−out sensitivity analysis for
'SYK' on 'Cardiac valvular disease'

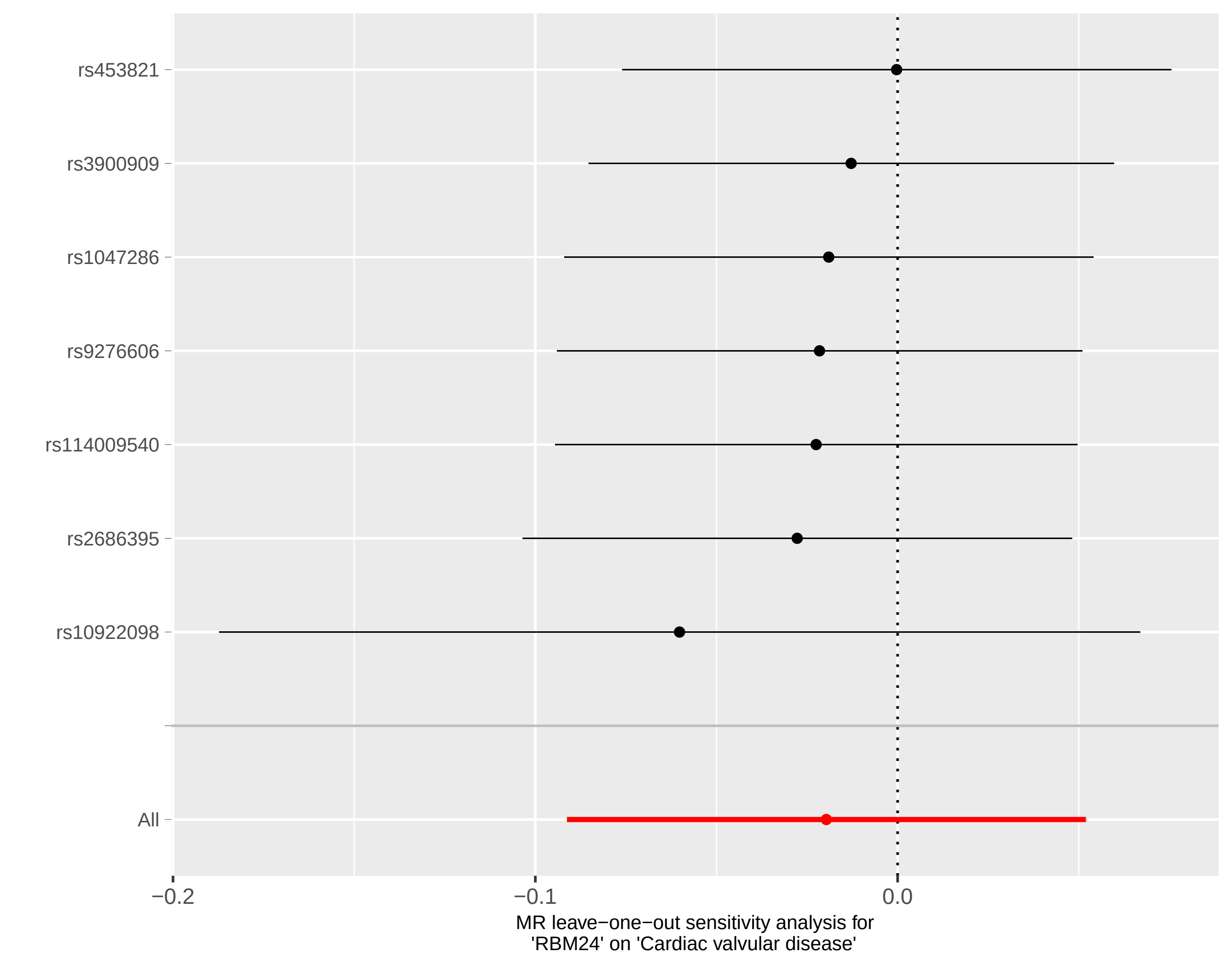
rs453821
rs3900909
rs1047286
rs9276606
rs114009540
rs2686395
rs10922098
All
−0.2
−0.1
0.0
MR leave−one−out sensitivity analysis for
'RBM24' on 'Cardiac valvular disease'

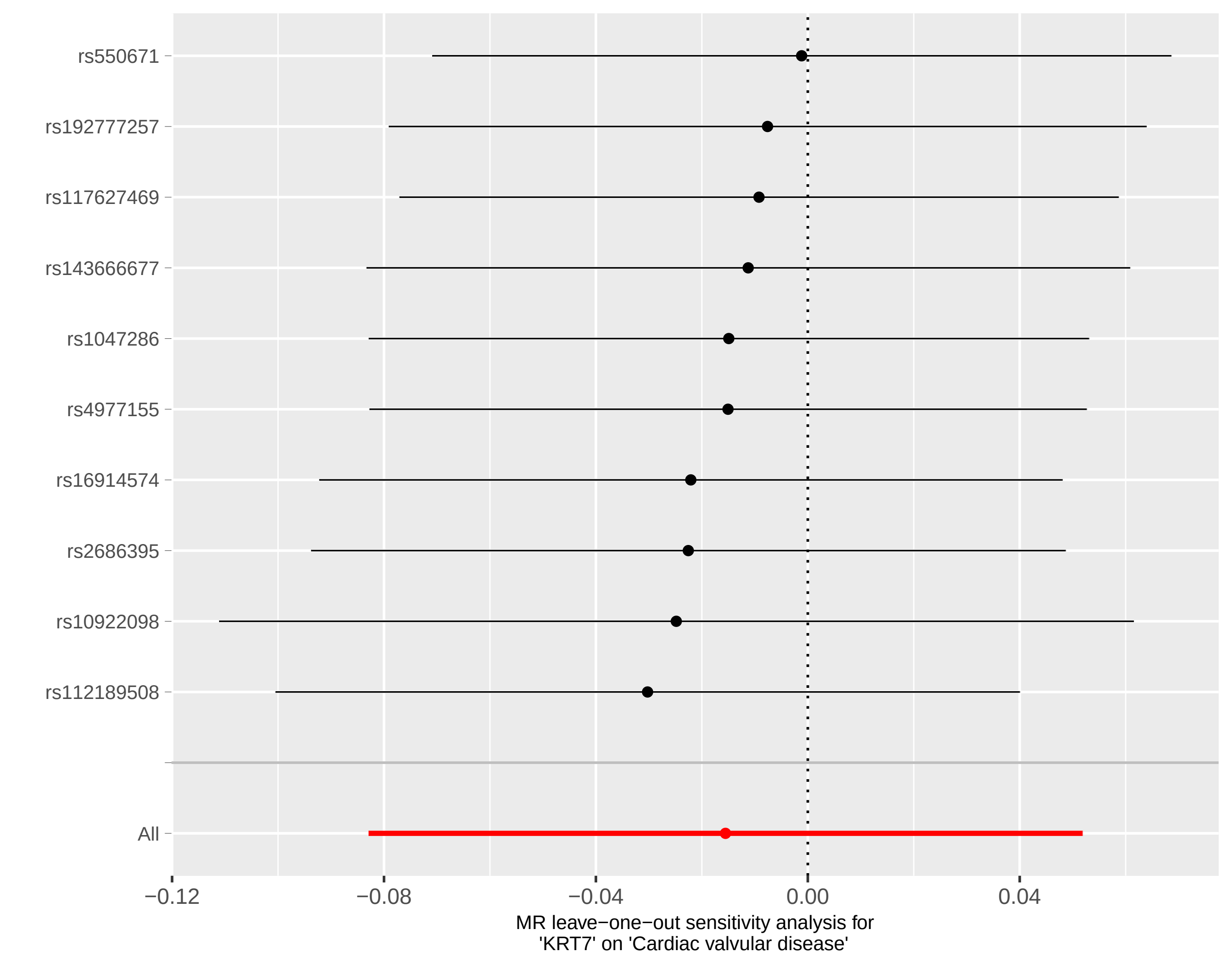

rs550671
rs192777257
rs117627469
rs143666677
rs1047286
rs4977155
rs16914574
rs2686395
rs10922098
rs112189508
All
−0.12
−0.08
−0.04
0.00
0.04
MR leave−one−out sensitivity analysis for
'KRT7' on 'Cardiac valvular disease'

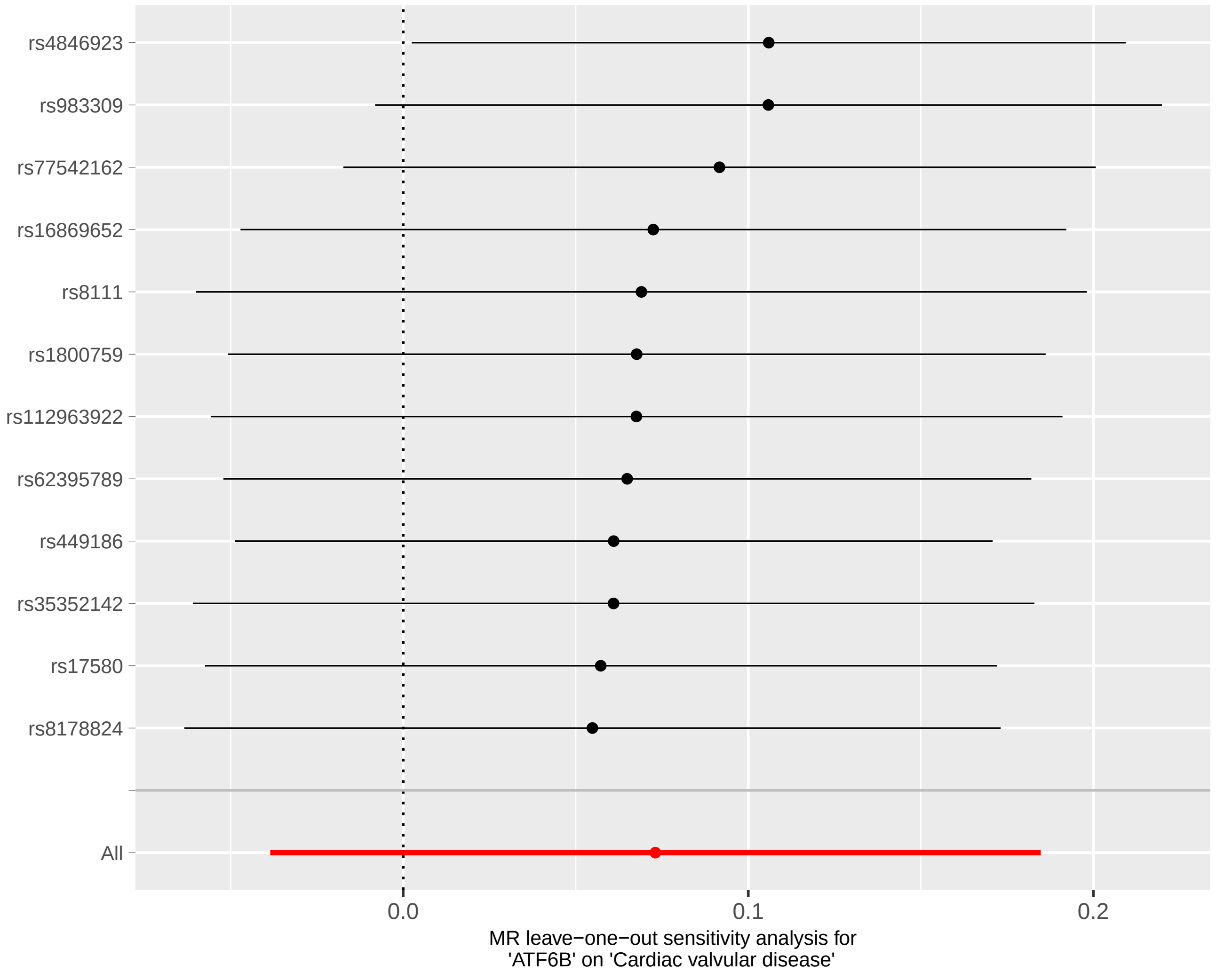
rs4846923
rs983309
rs77542162
rs16869652
rs8111
rs1800759
rs112963922
rs62395789
rs449186
rs35352142
rs17580
rs8178824
All
0.0
0.1
0.2
MR leave−one−out sensitivity analysis for
'ATF6B' on 'Cardiac valvular disease'

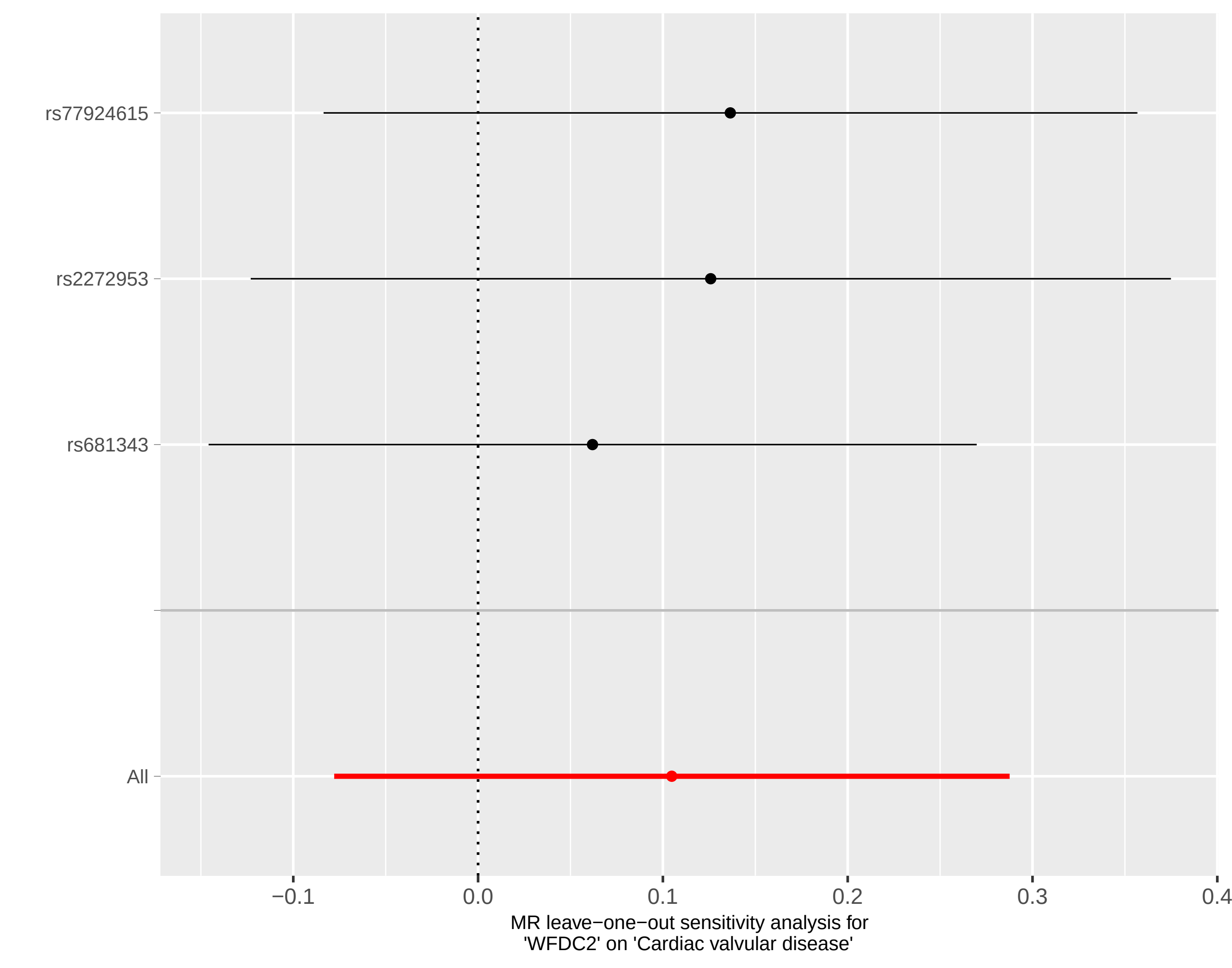
rs77924615
rs2272953
rs681343
All
−0.1
0.0
0.1
0.2
0.3
0.4
MR leave−one−out sensitivity analysis for
'WFDC2' on 'Cardiac valvular disease'

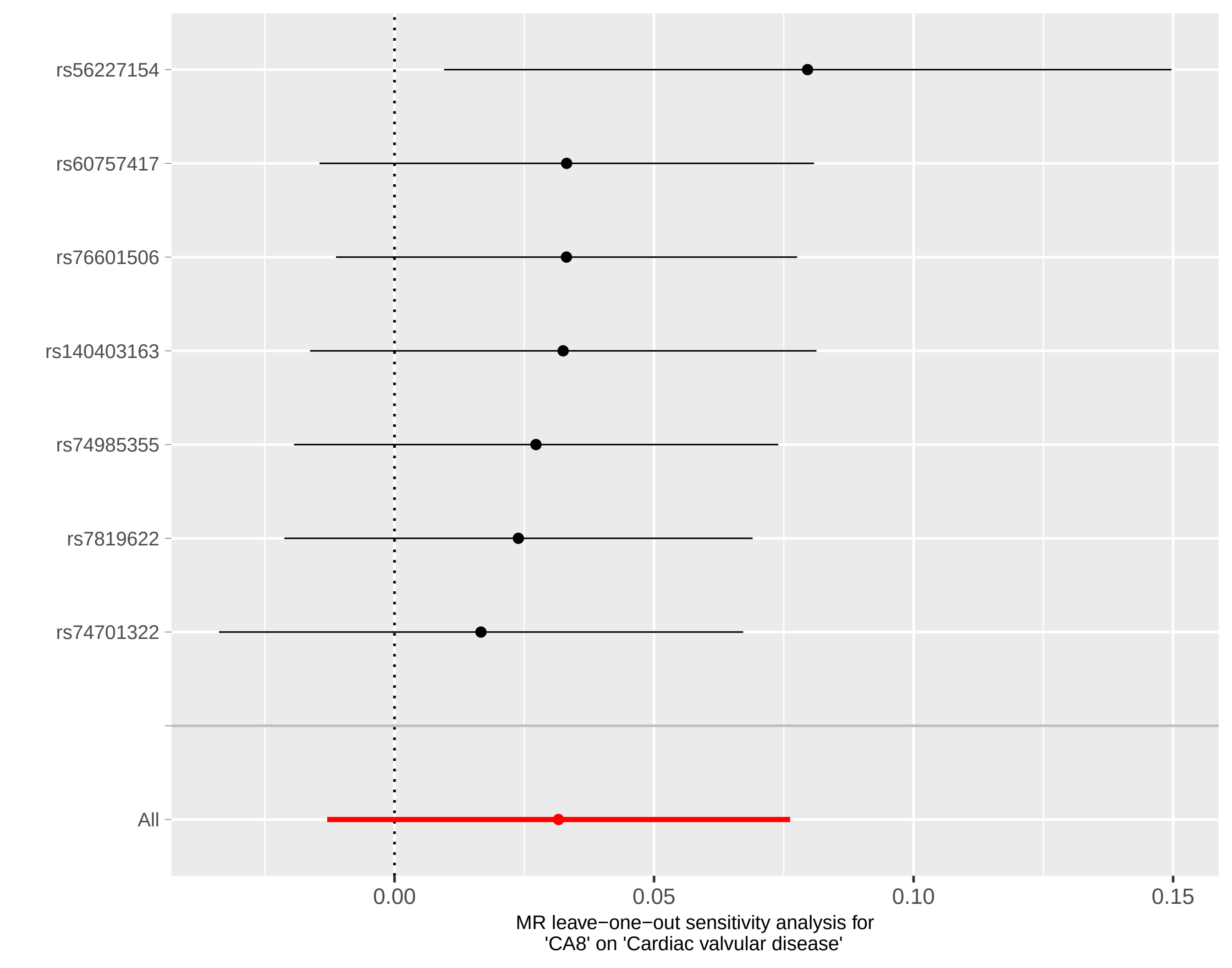
rs56227154
rs60757417
rs76601506
rs140403163
rs74985355
rs7819622
rs74701322
All
0.00
0.05
0.10
0.15
MR leave−one−out sensitivity analysis for
'CA8' on 'Cardiac valvular disease'

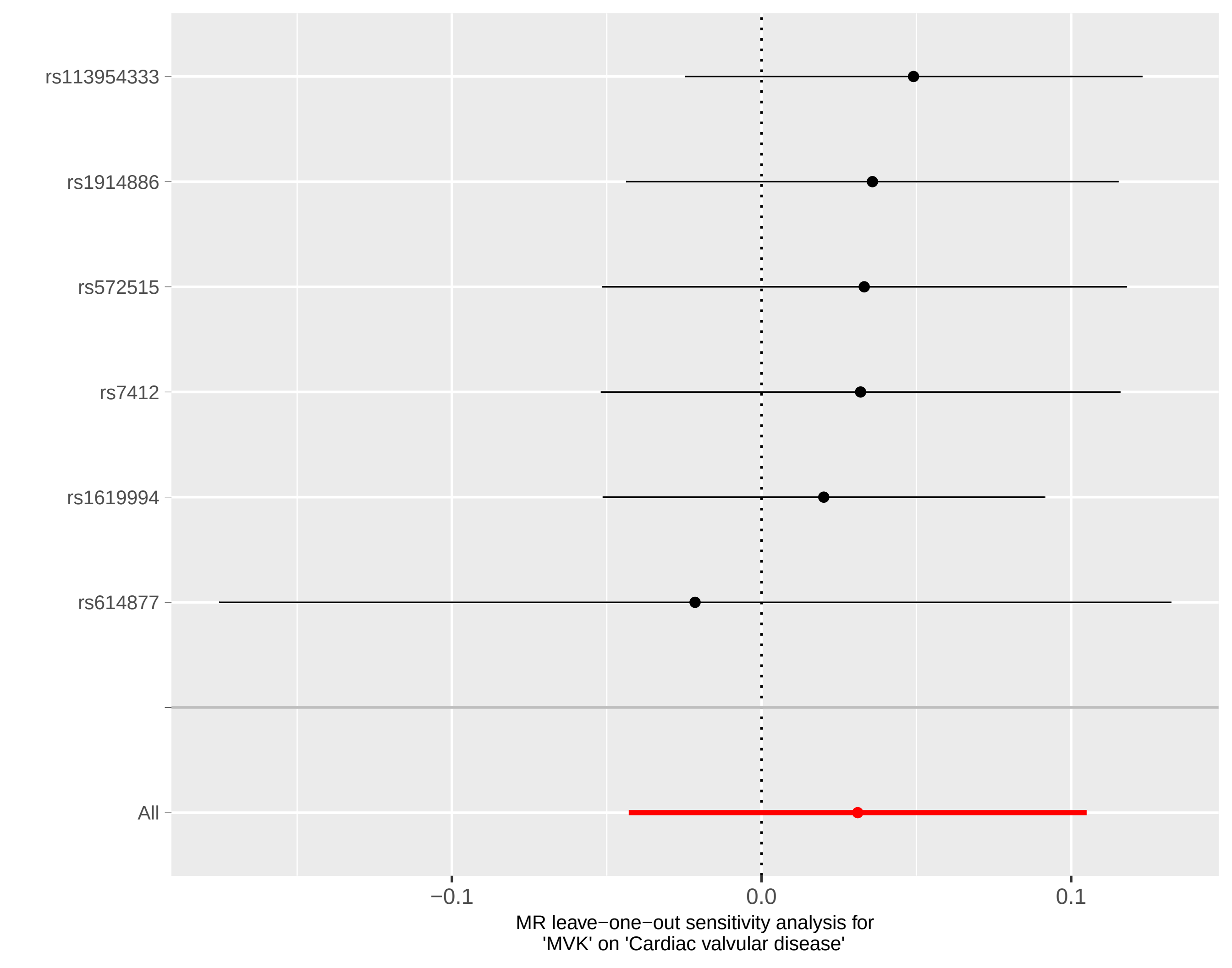

rs113954333
rs1914886
rs572515
rs7412
rs1619994
rs614877
All
−0.1
0.0
0.1
MR leave−one−out sensitivity analysis for
'MVK' on 'Cardiac valvular disease'

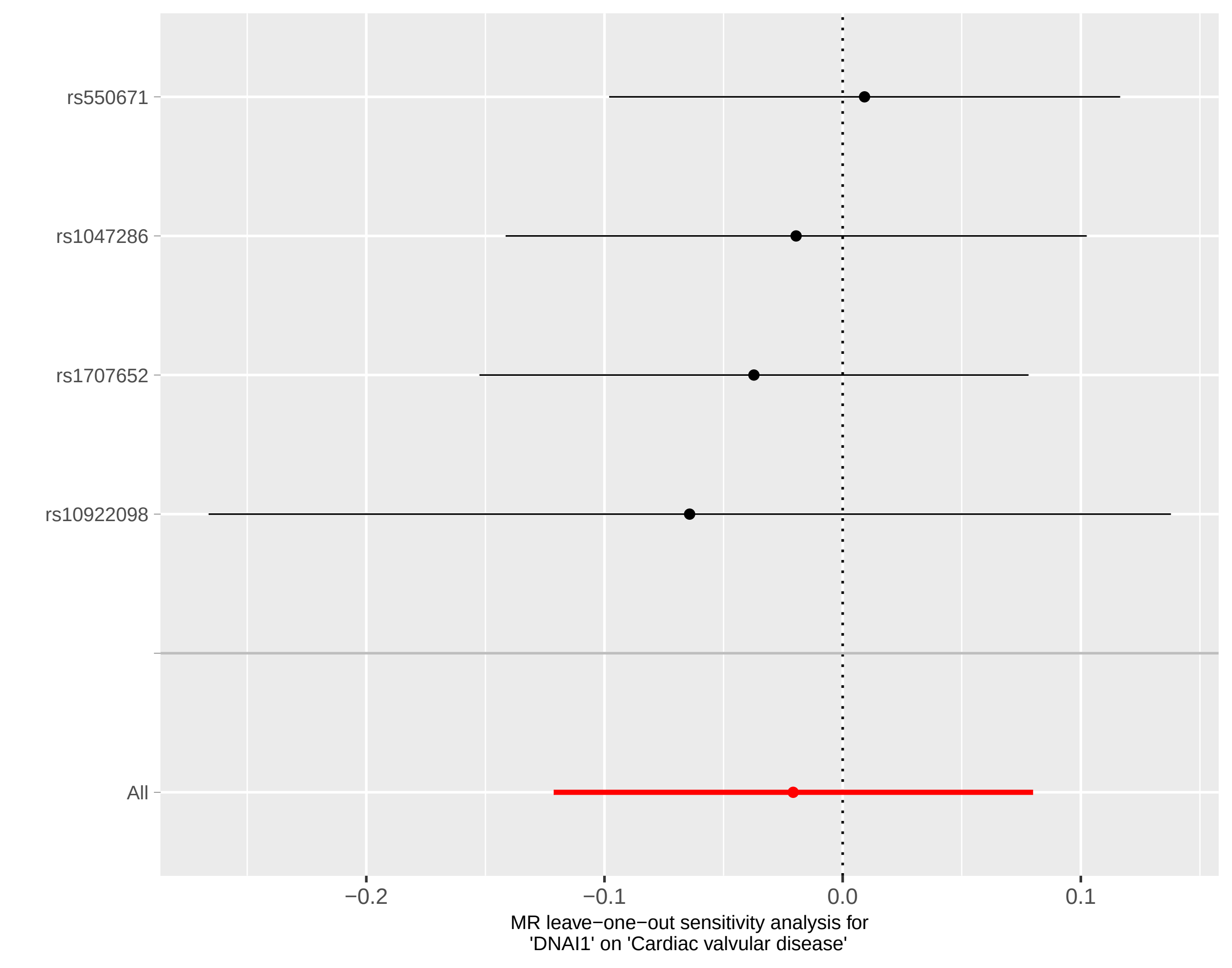
rs550671
rs1047286
rs1707652
rs10922098
All
−0.2
−0.1
0.0
0.1
MR leave−one−out sensitivity analysis for
'DNAI1' on 'Cardiac valvular disease'

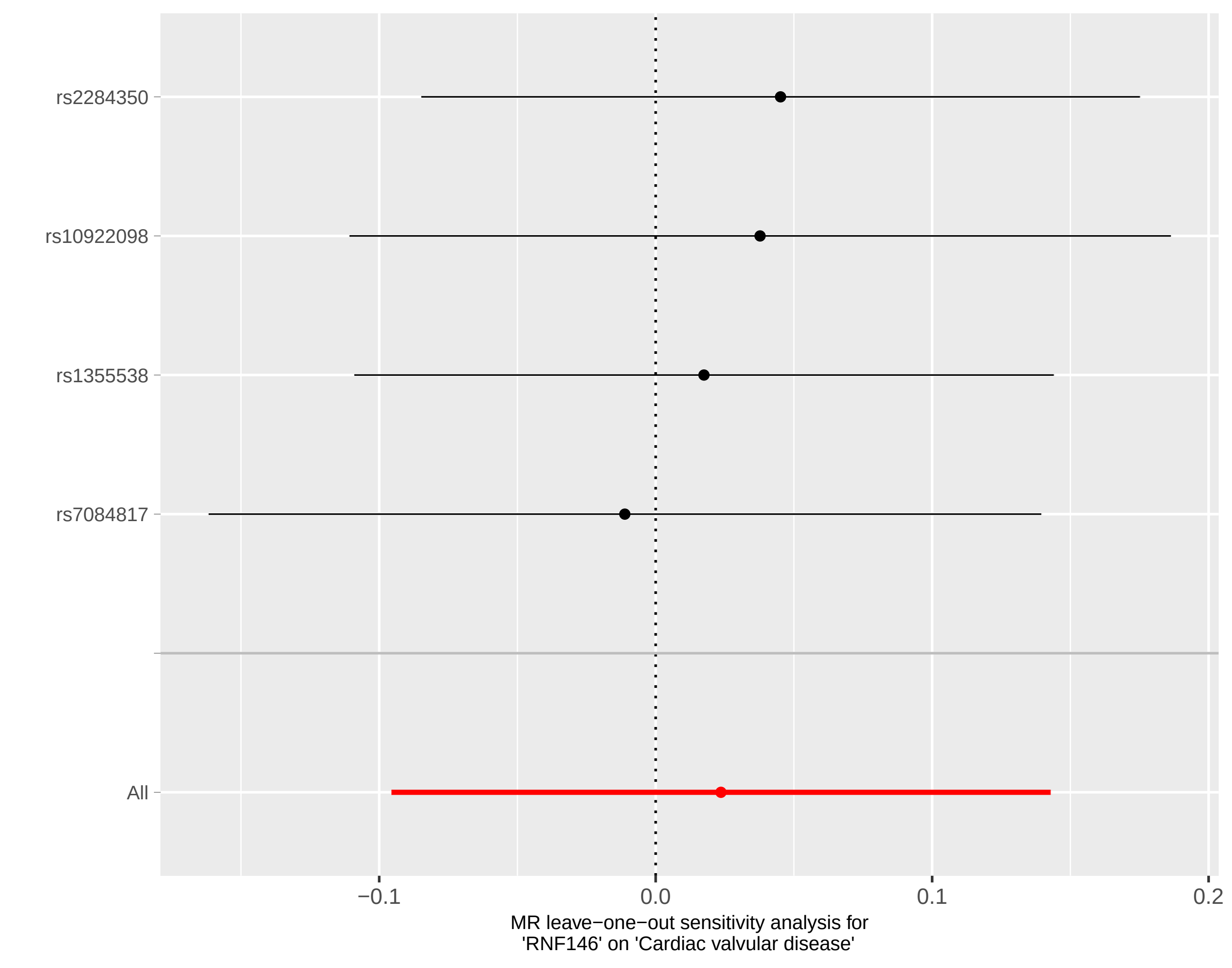
rs2284350
rs10922098
rs1355538
rs7084817
All
−0.1
0.0
0.1
0.2
MR leave−one−out sensitivity analysis for
'RNF146' on 'Cardiac valvular disease'

Insufficient number of SNPs

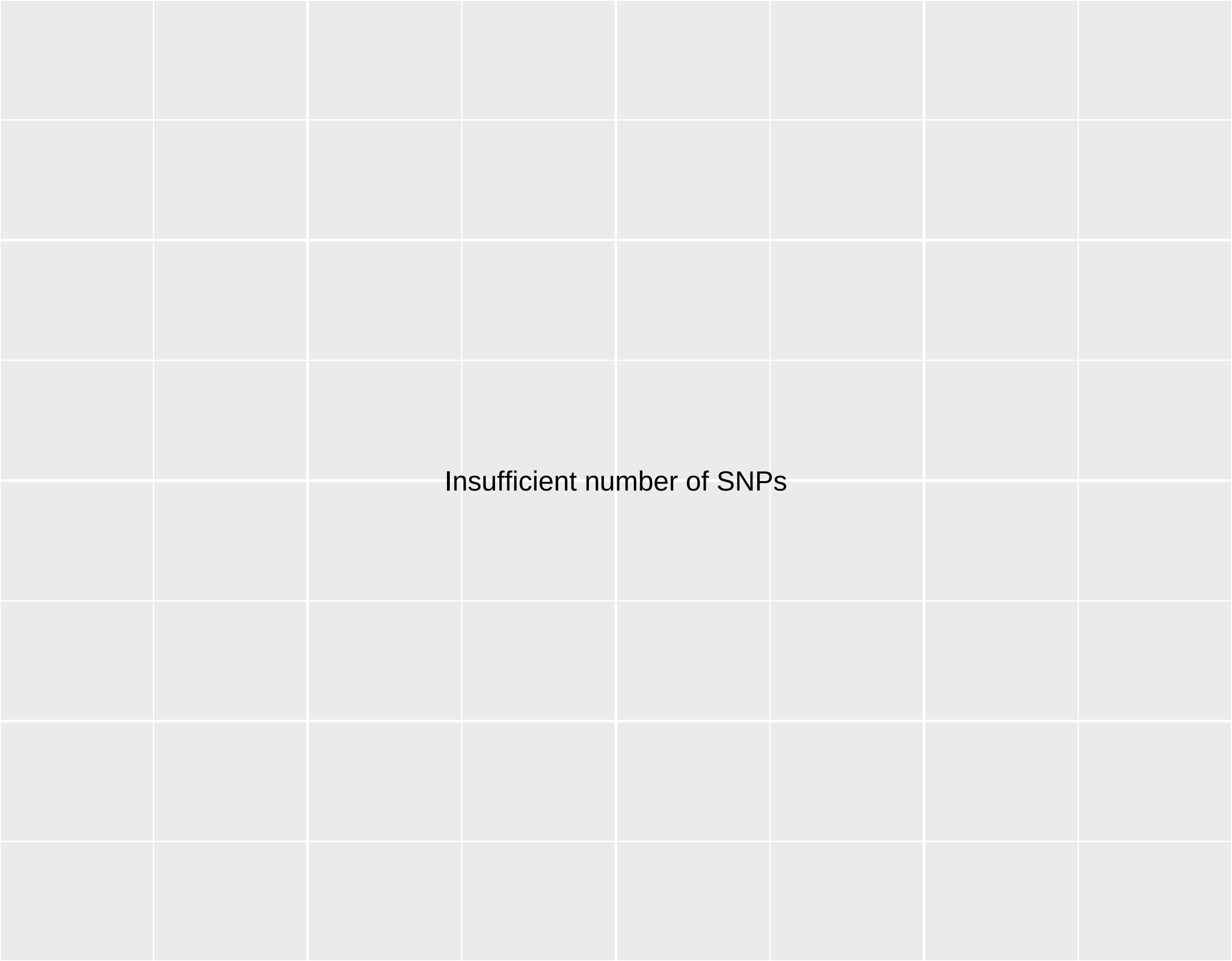
Insufficient number of SNPs

Insufficient number of SNPs

Insufficient number of SNPs

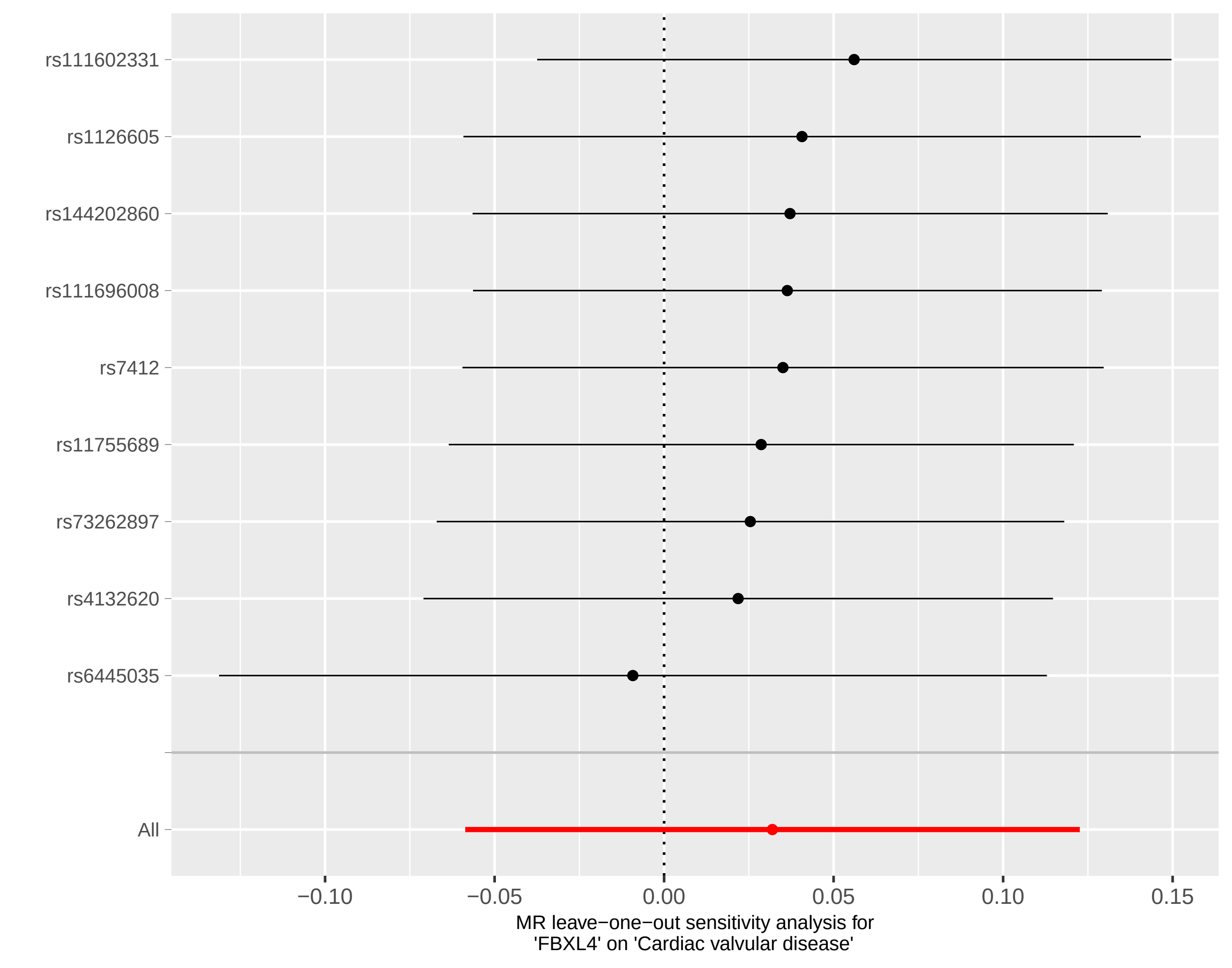

rs111602331
rs1126605
rs144202860
rs111696008
rs7412
rs11755689
rs73262897
rs4132620
rs6445035
All
−0.10
−0.05
0.00
0.05
0.10
0.15
MR leave−one−out sensitivity analysis for
'FBXL4' on 'Cardiac valvular disease'

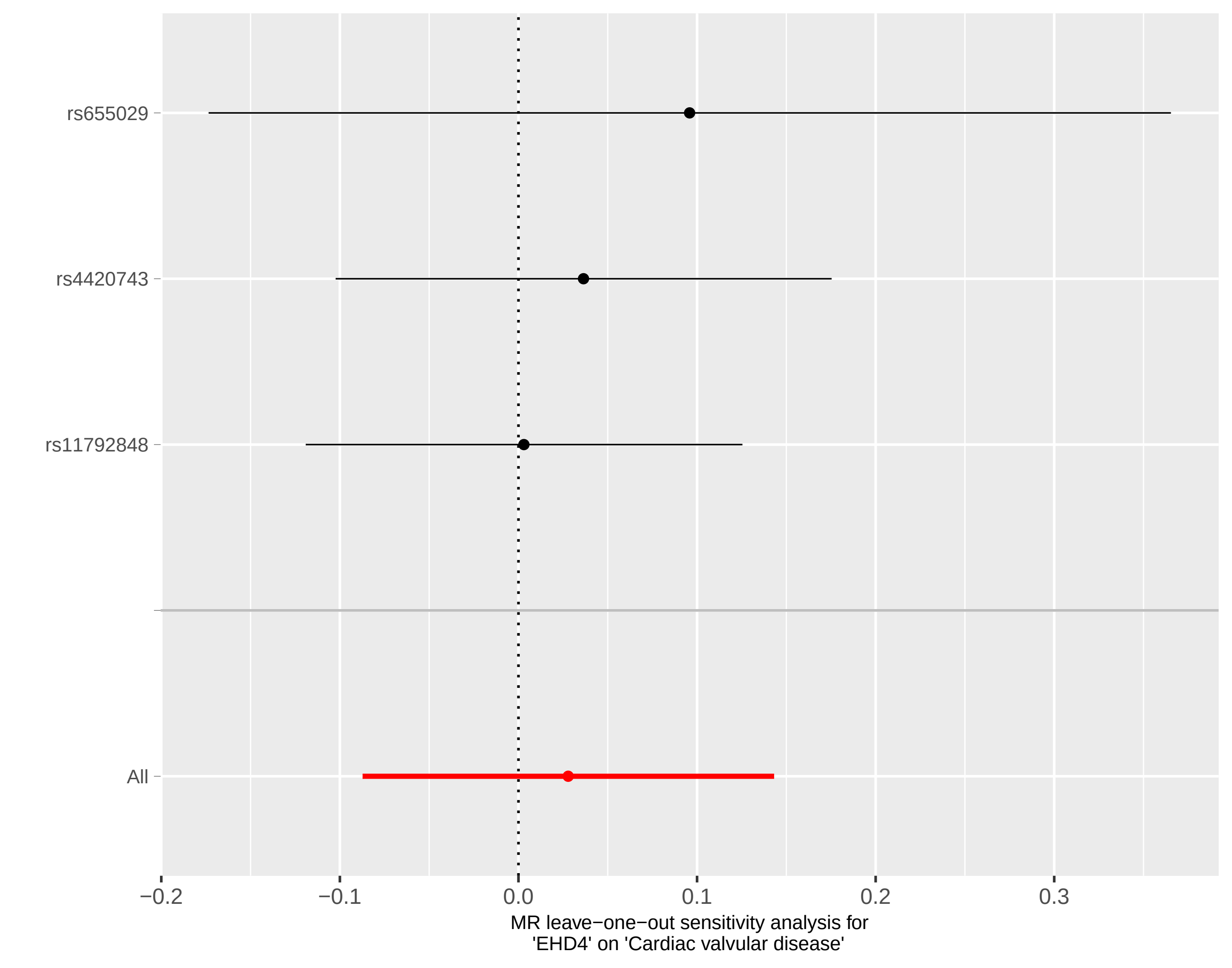

rs655029
rs4420743
rs11792848
All
−0.2
−0.1
0.0
0.1
0.2
0.3
MR leave−one−out sensitivity analysis for
'EHD4' on 'Cardiac valvular disease'

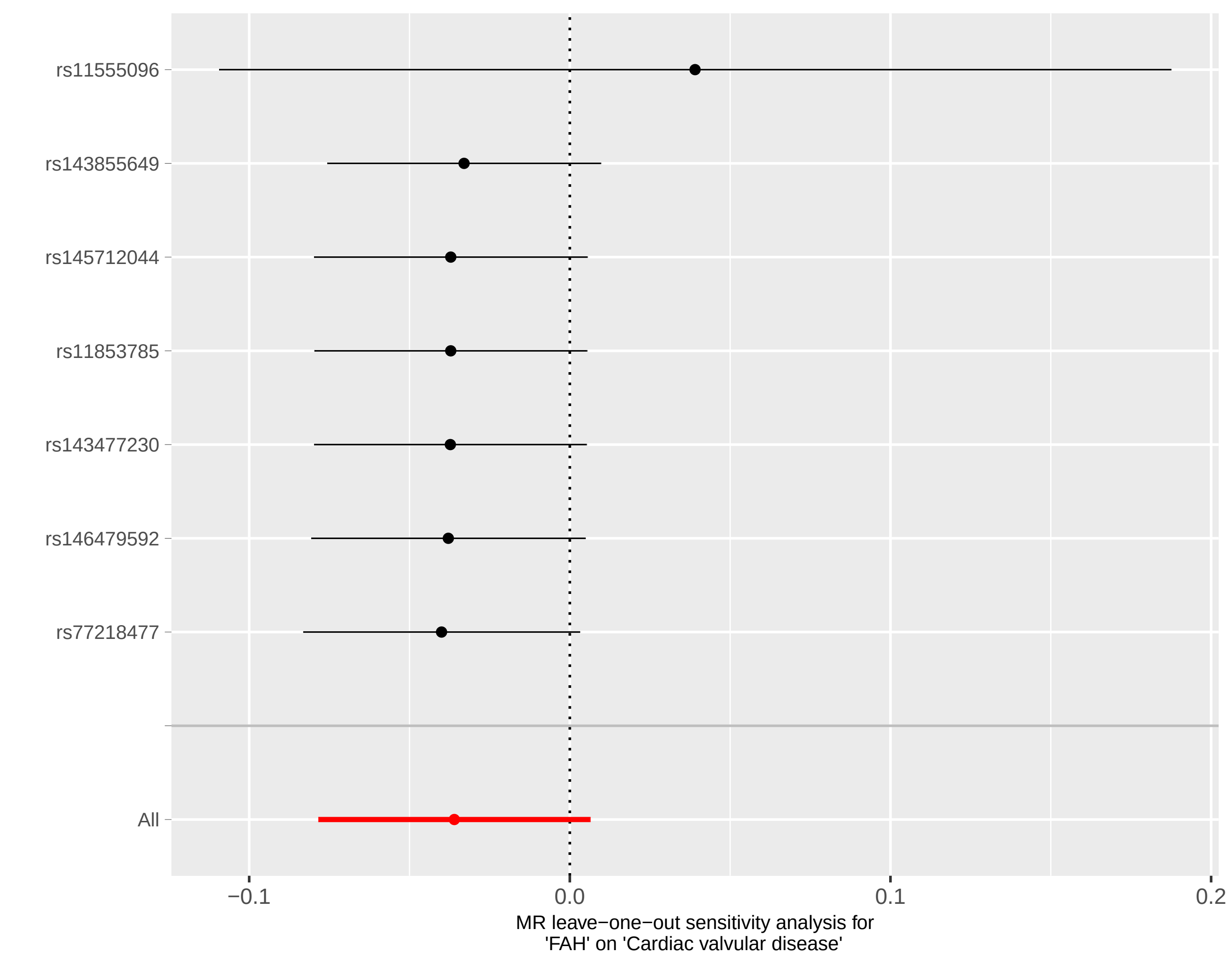
rs11555096
rs143855649
rs145712044
rs11853785
rs143477230
rs146479592
rs77218477
All
−0.1
0.0
0.1
0.2
MR leave−one−out sensitivity analysis for
'FAH' on 'Cardiac valvular disease'

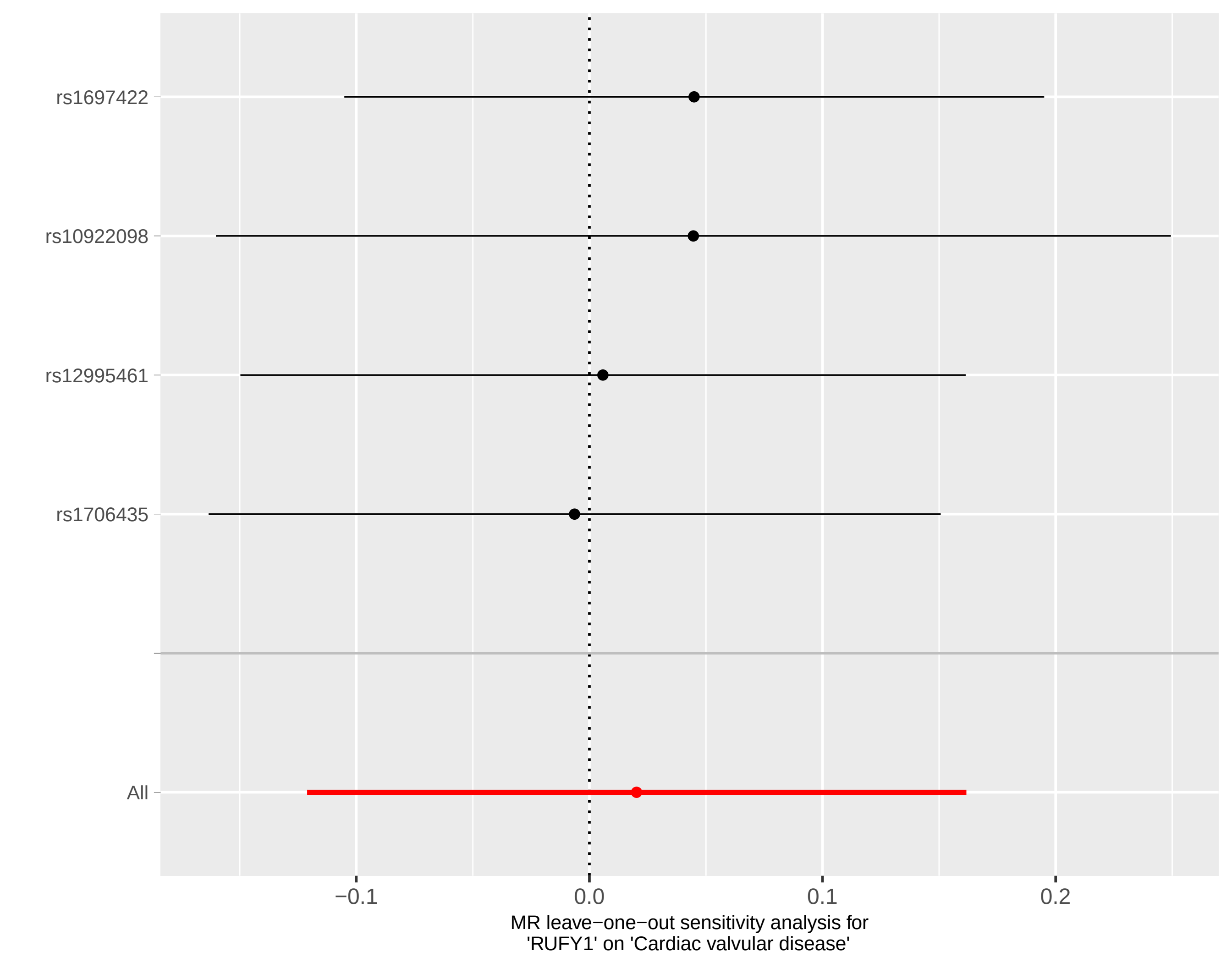
rs1697422
rs10922098
rs12995461
rs1706435
All
−0.1
0.0
0.1
0.2
MR leave−one−out sensitivity analysis for
'RUFY1' on 'Cardiac valvular disease'

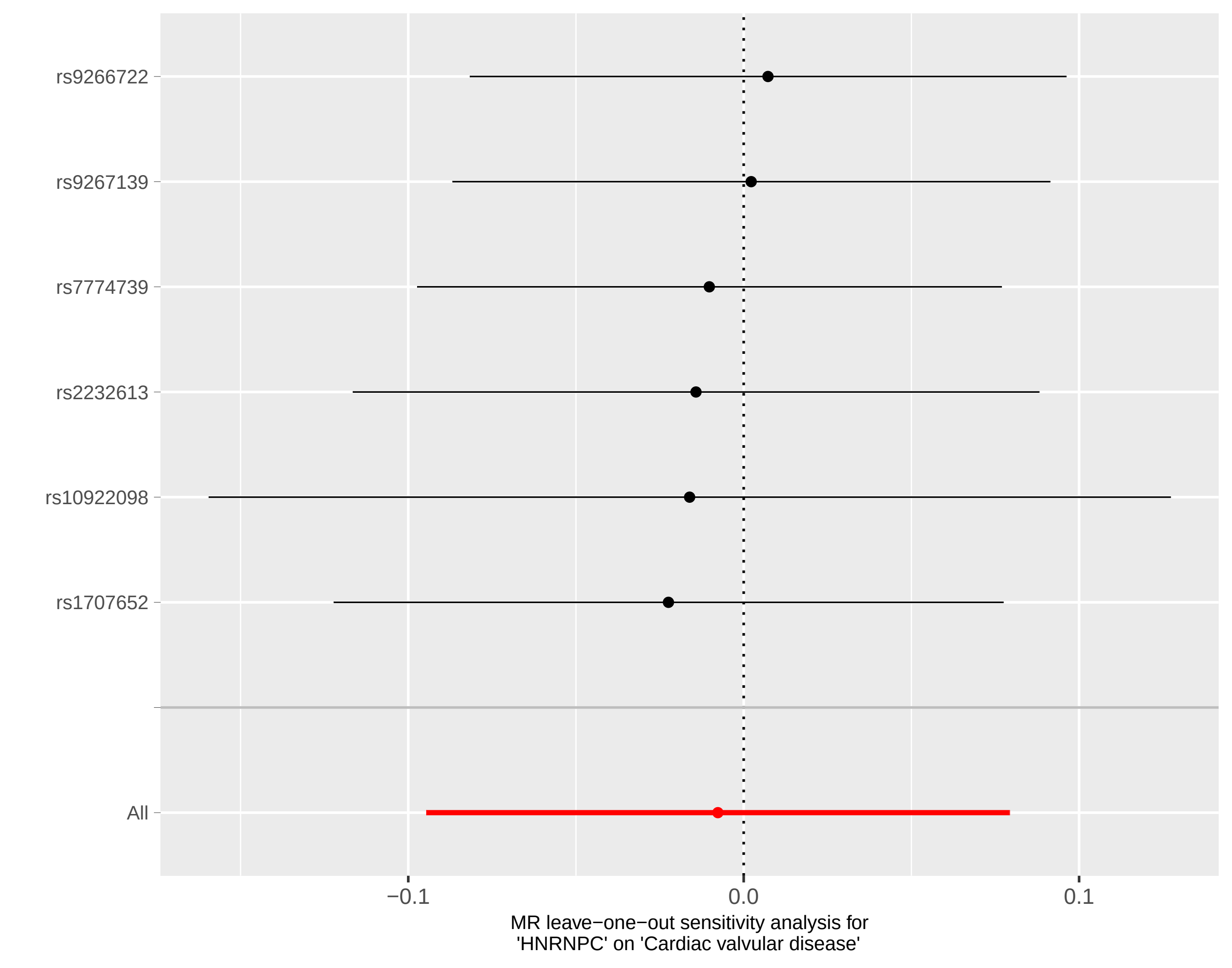
rs9266722
rs9267139
rs7774739
rs2232613
rs10922098
rs1707652
All
−0.1
0.0
0.1
MR leave−one−out sensitivity analysis for
'HNRNPC' on 'Cardiac valvular disease'

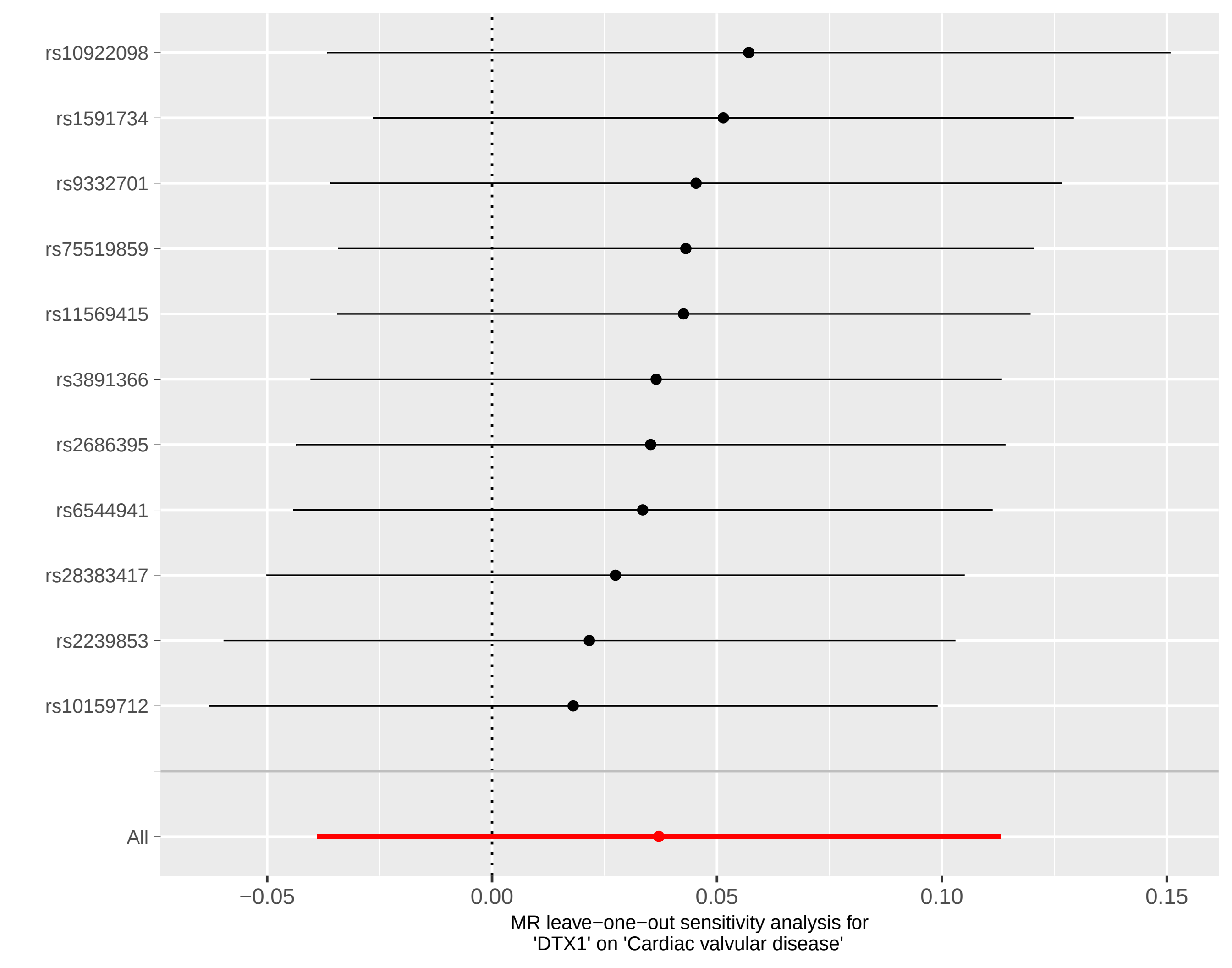
rs10922098
rs1591734
rs9332701
rs75519859
rs11569415
rs3891366
rs2686395
rs6544941
rs28383417
rs2239853
rs10159712
All
−0.05
0.00
0.05
0.10
0.15
MR leave−one−out sensitivity analysis for
'DTX1' on 'Cardiac valvular disease'

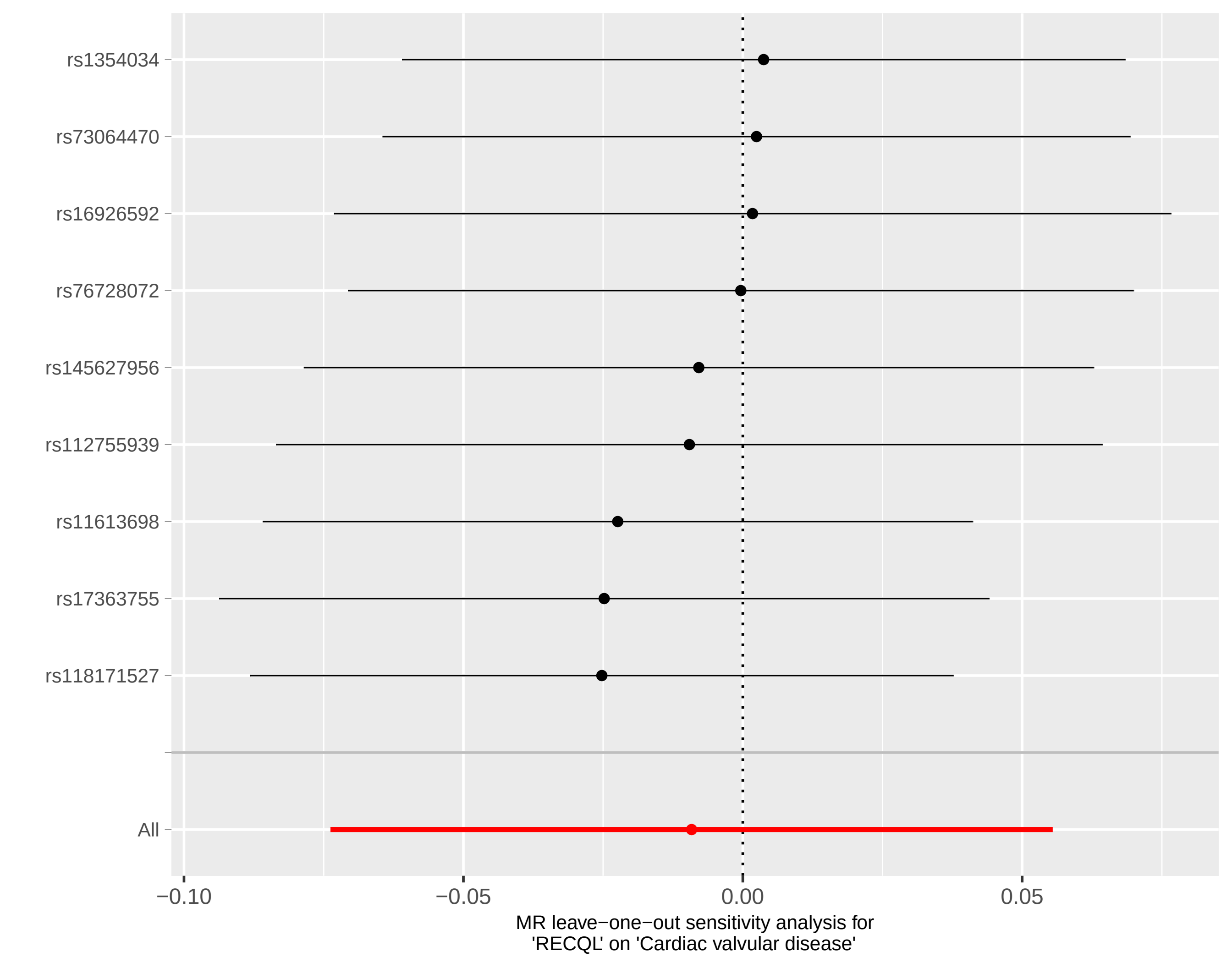
rs1354034
rs73064470
rs16926592
rs76728072
rs145627956
rs112755939
rs11613698
rs17363755
rs118171527
All
−0.10
−0.05
0.00
0.05
MR leave−one−out sensitivity analysis for
'RECQL' on 'Cardiac valvular disease'

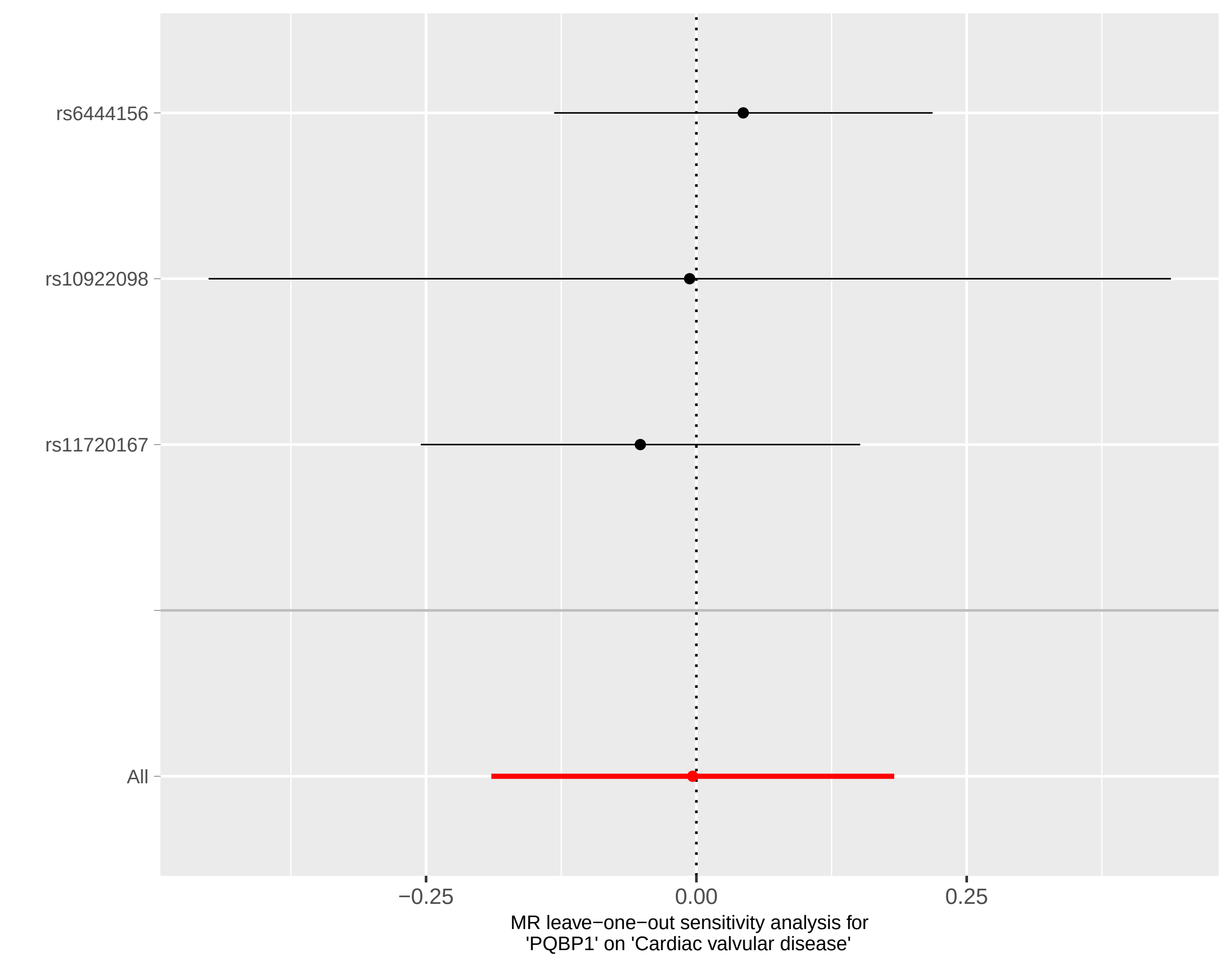
rs6444156
rs10922098
rs11720167
All
−0.25
0.00
0.25
MR leave−one−out sensitivity analysis for
'PQBP1' on 'Cardiac valvular disease'

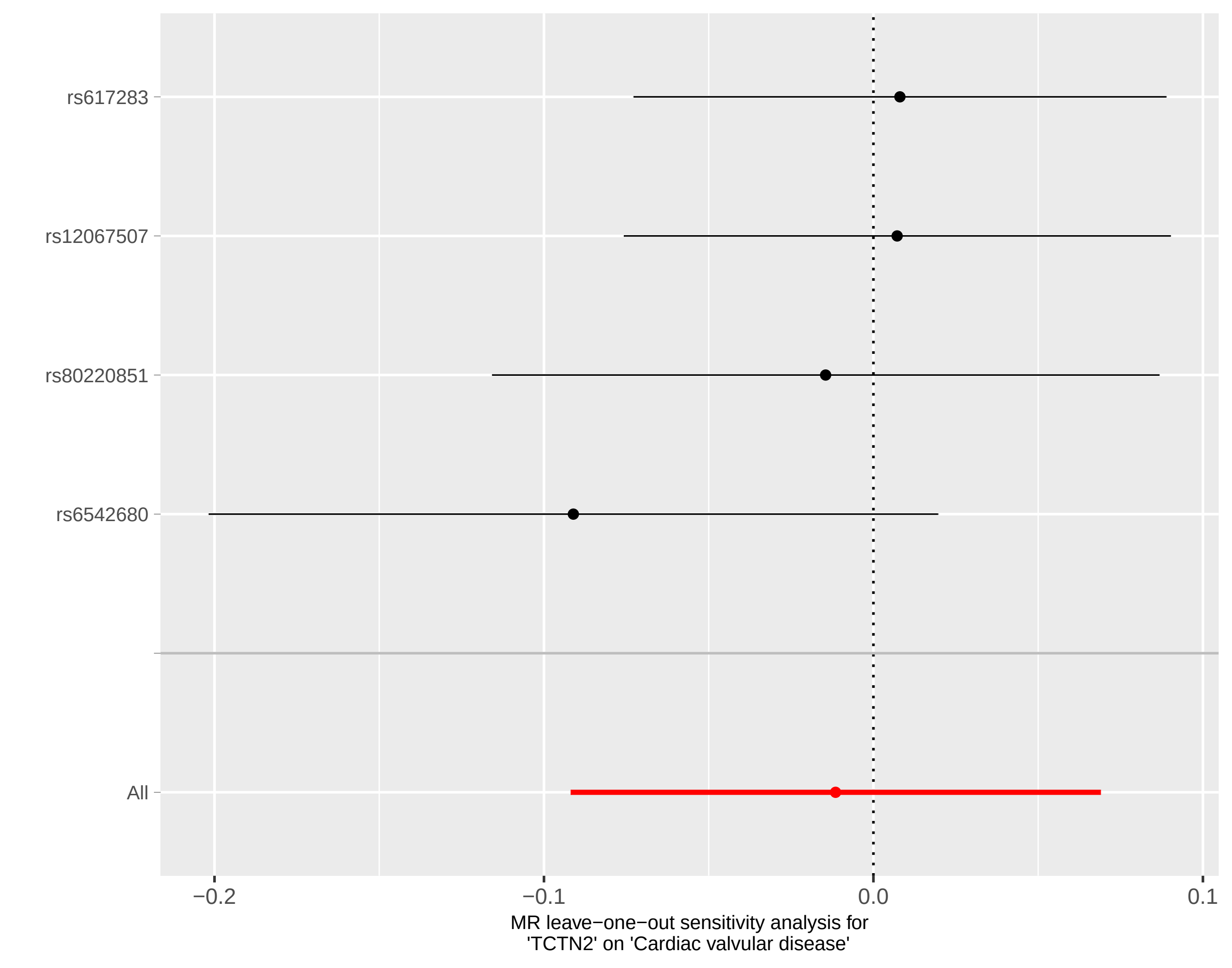

rs617283
rs12067507
rs80220851
rs6542680
All
−0.2
−0.1
0.0
0.1
MR leave−one−out sensitivity analysis for
'TCTN2' on 'Cardiac valvular disease'

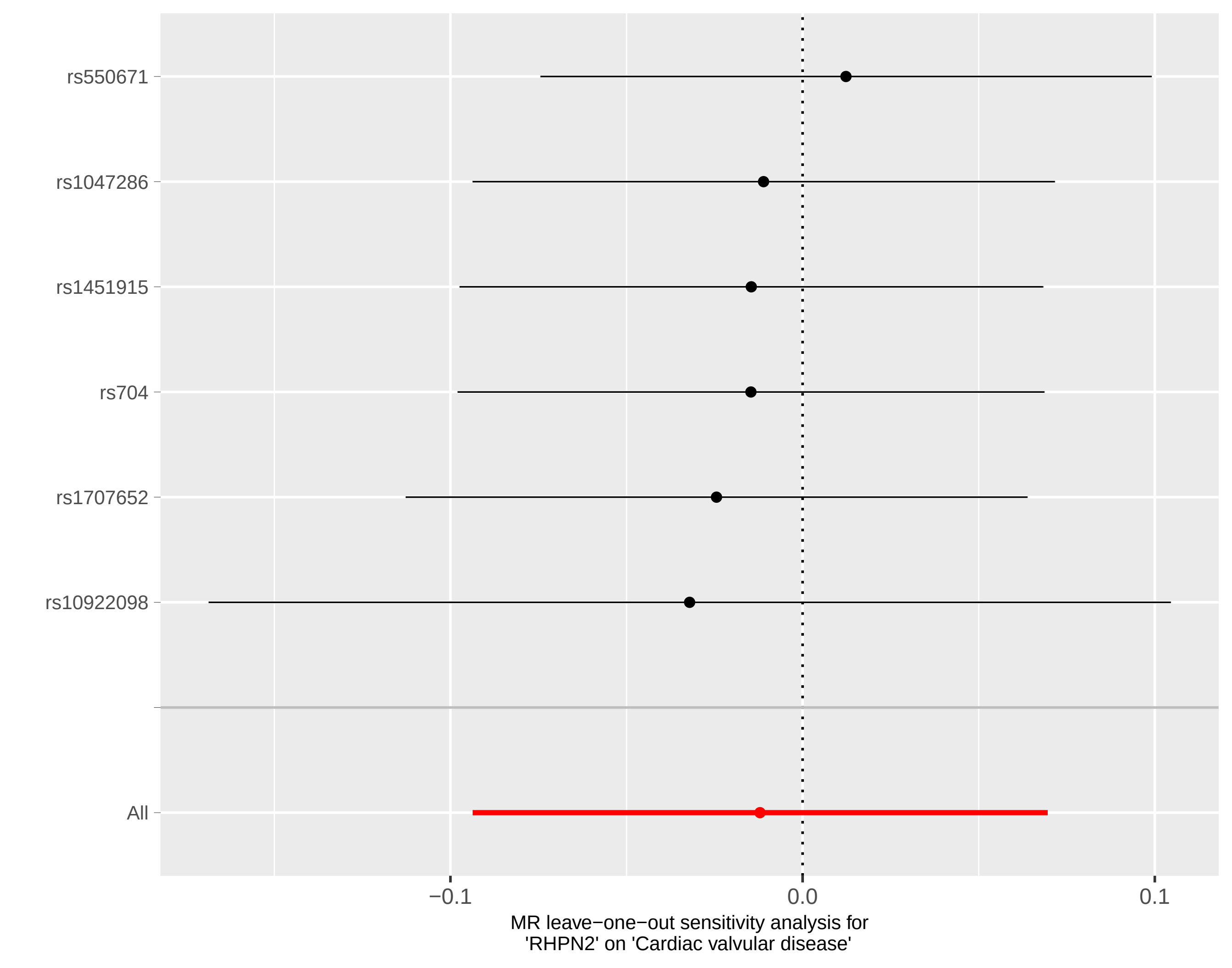
rs550671
rs1047286
rs1451915
rs704
rs1707652
rs10922098
All
−0.1
0.0
0.1
MR leave−one−out sensitivity analysis for
'RHPN2' on 'Cardiac valvular disease'

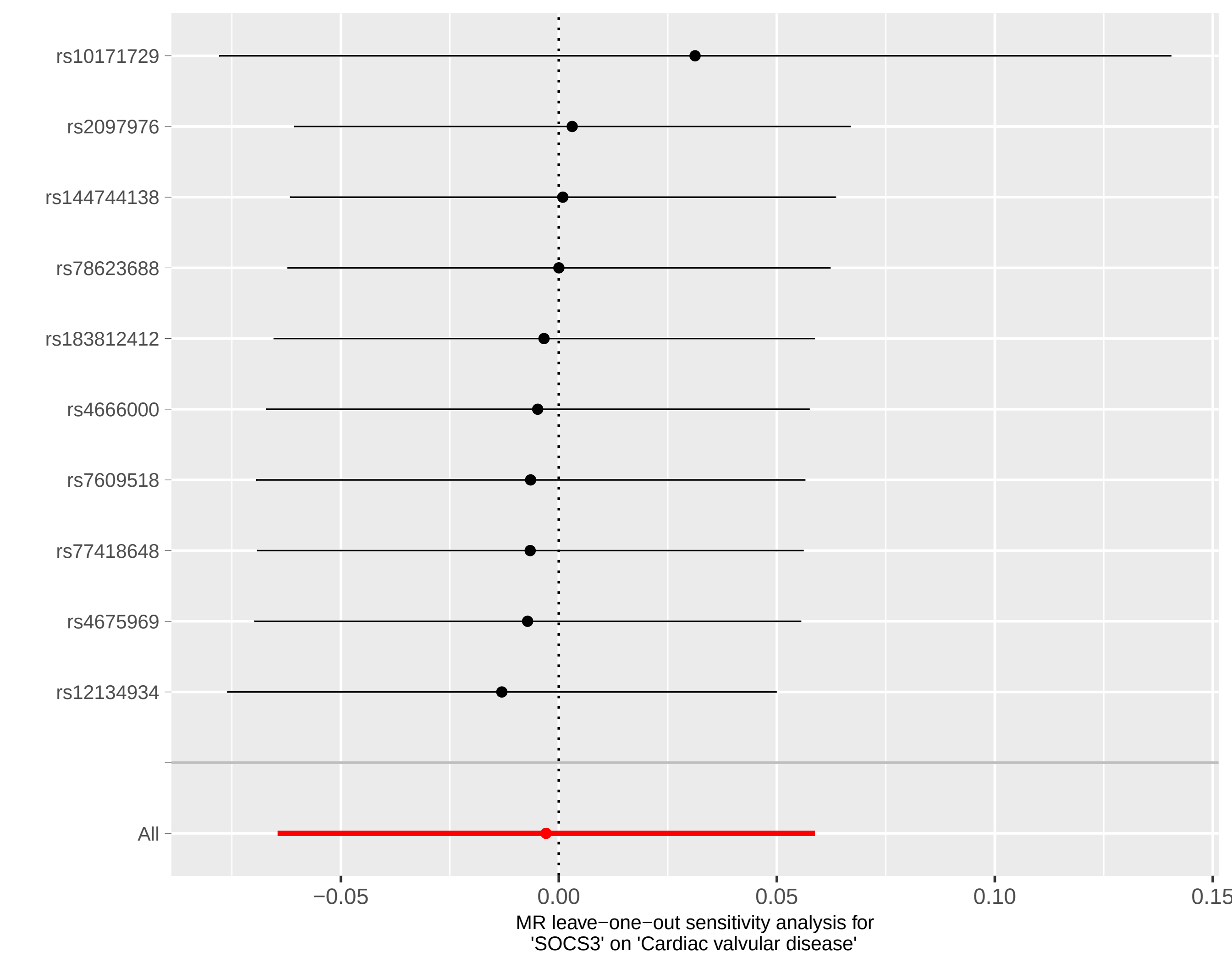
rs10171729
rs2097976
rs144744138
rs78623688
rs183812412
rs4666000
rs7609518
rs77418648
rs4675969
rs12134934
All
−0.05
0.00
0.05
0.10
0.15
MR leave−one−out sensitivity analysis for
'SOCS3' on 'Cardiac valvular disease'

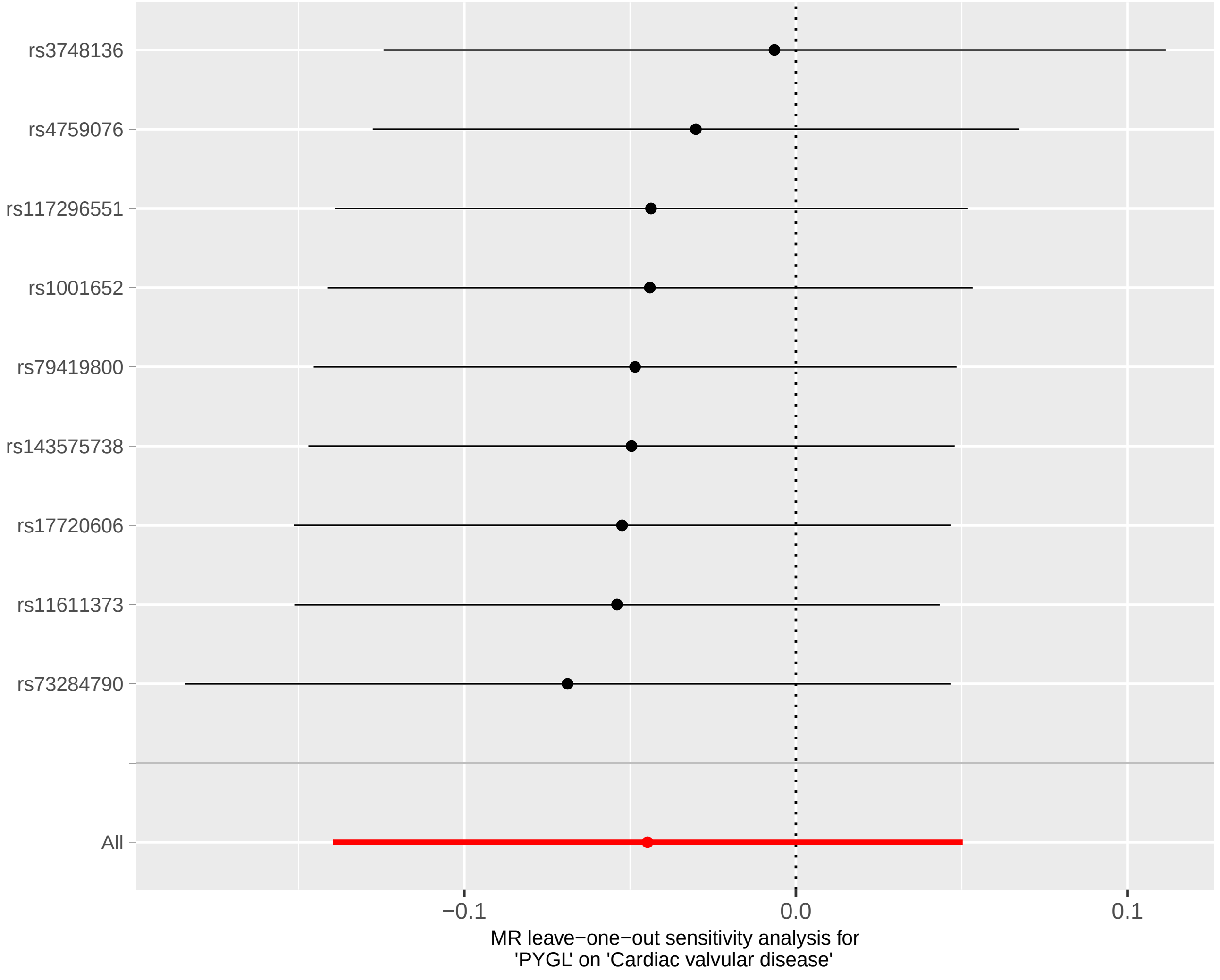
rs3748136
rs4759076
rs117296551
rs1001652
rs79419800
rs143575738
rs17720606
rs11611373
rs73284790
All
−0.1
0.0
0.1
MR leave−one−out sensitivity analysis for
'PYGL' on 'Cardiac valvular disease'

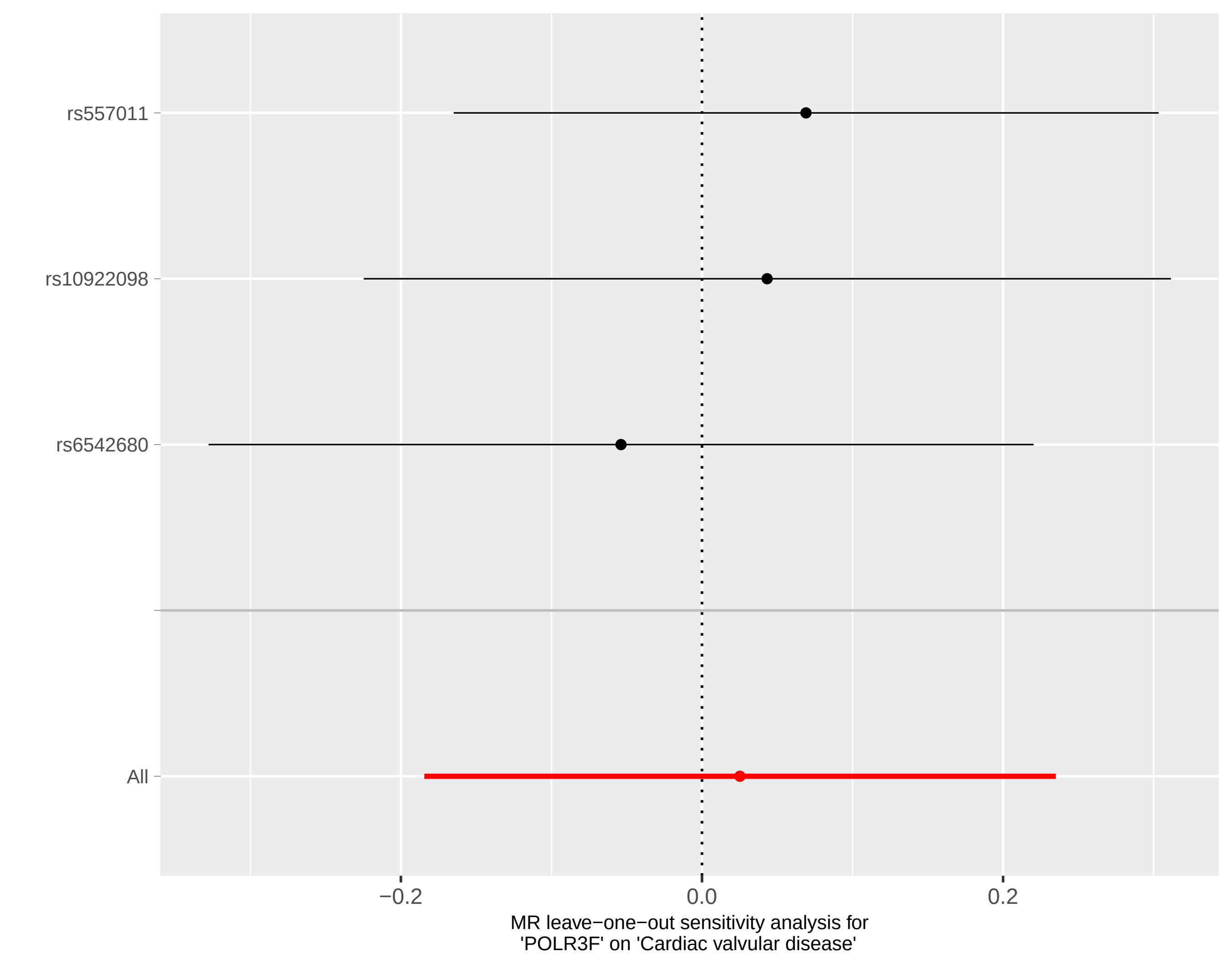
rs557011
rs10922098
rs6542680
All
−0.2
0.0
0.2
MR leave−one−out sensitivity analysis for
'POLR3F' on 'Cardiac valvular disease'

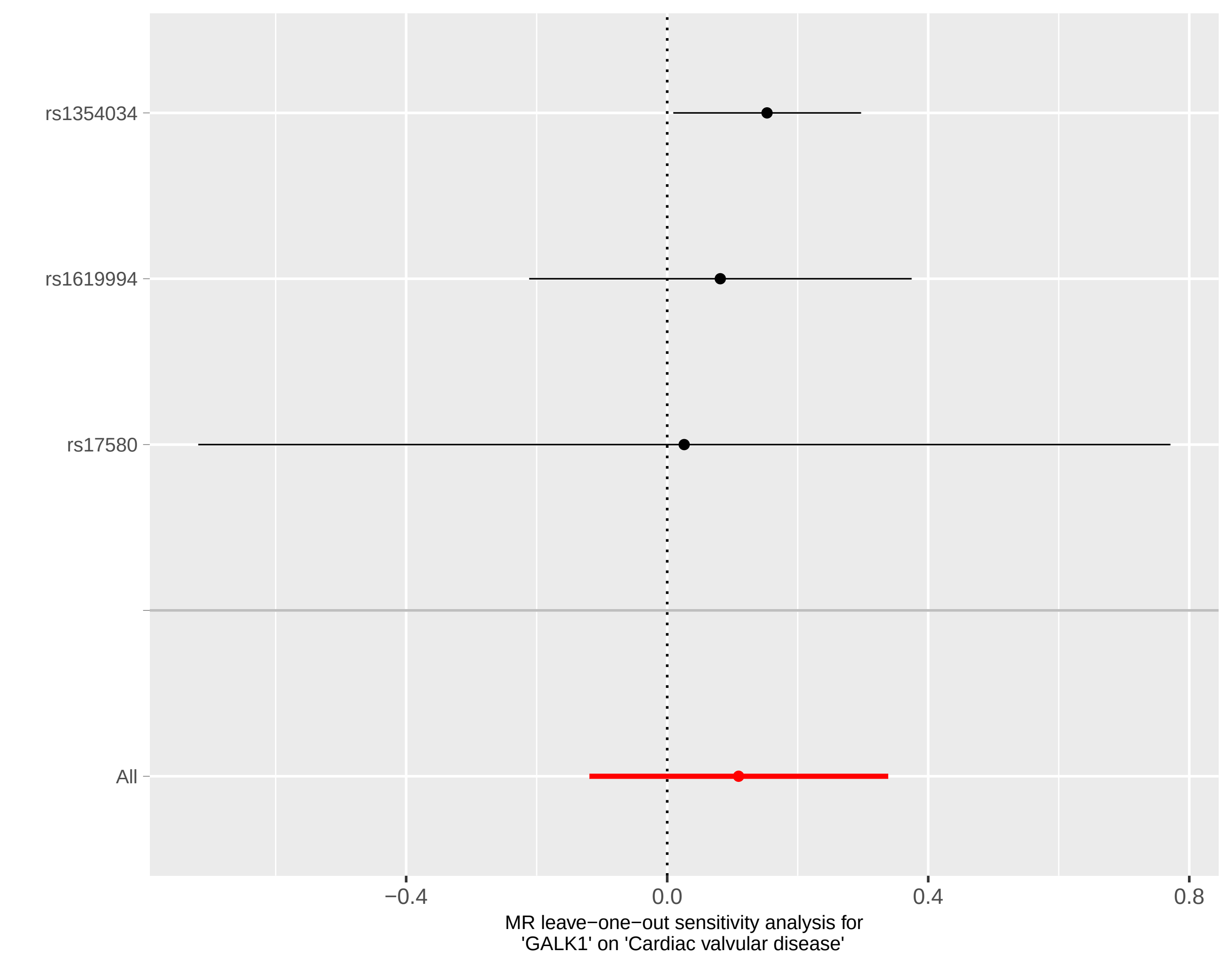
rs1354034
rs1619994
rs17580
All
−0.4
0.0
0.4
0.8
MR leave−one−out sensitivity analysis for
'GALK1' on 'Cardiac valvular disease'

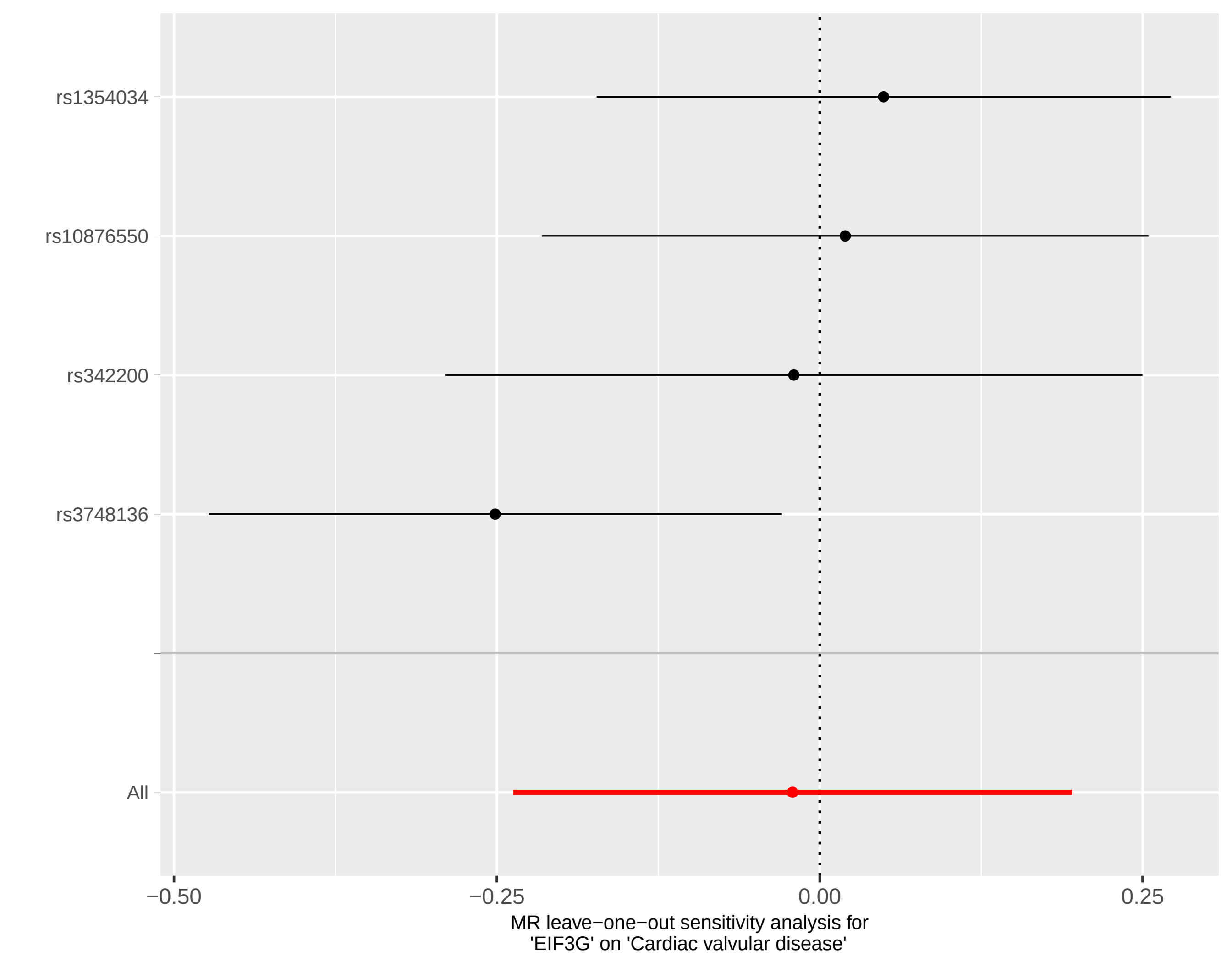
rs1354034
rs10876550
rs342200
rs3748136
All
−0.50
−0.25
0.00
0.25
MR leave−one−out sensitivity analysis for
'EIF3G' on 'Cardiac valvular disease'

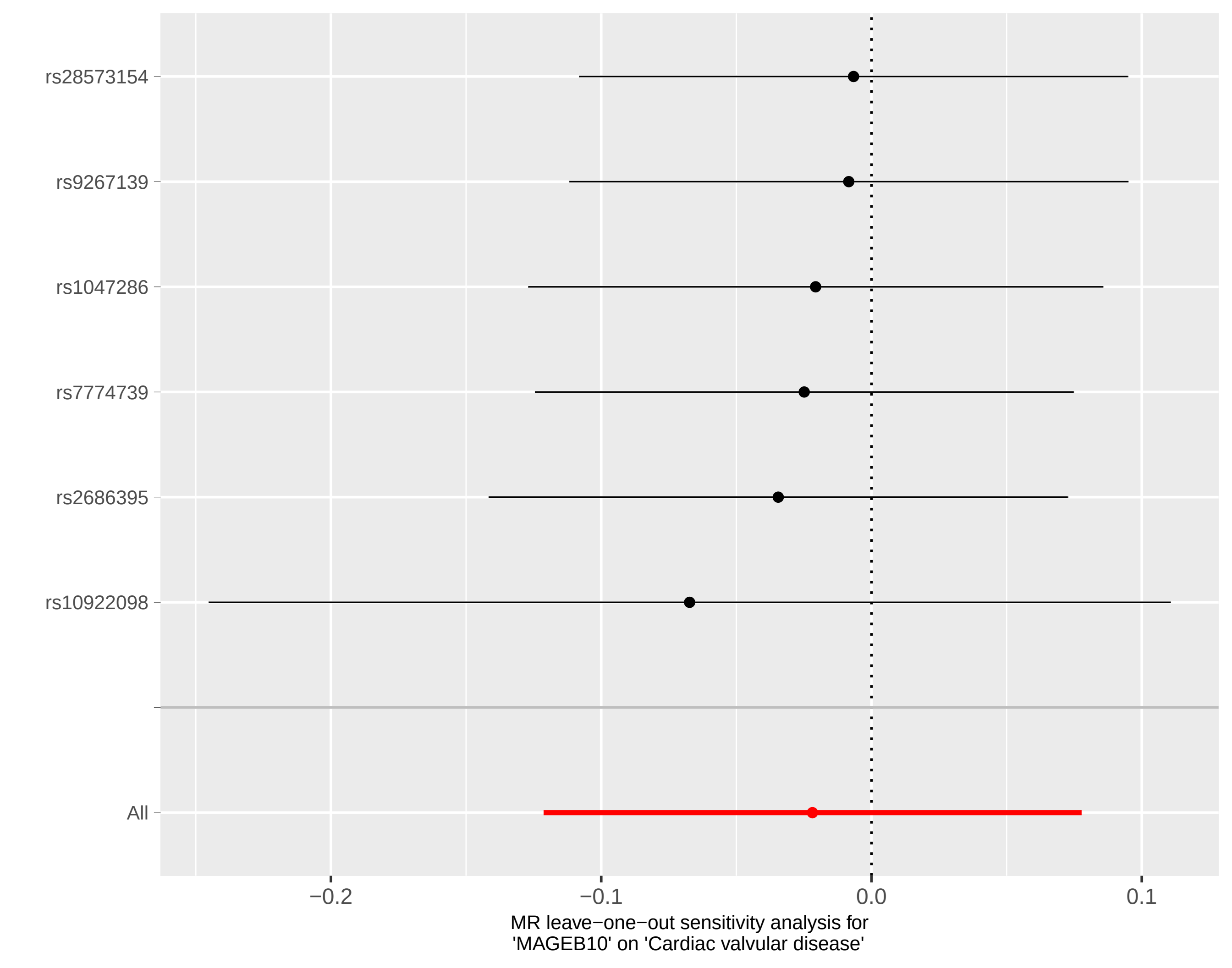

rs28573154
rs9267139
rs1047286
rs7774739
rs2686395
rs10922098
All
−0.2
−0.1
0.0
0.1
MR leave−one−out sensitivity analysis for
'MAGEB10' on 'Cardiac valvular disease'

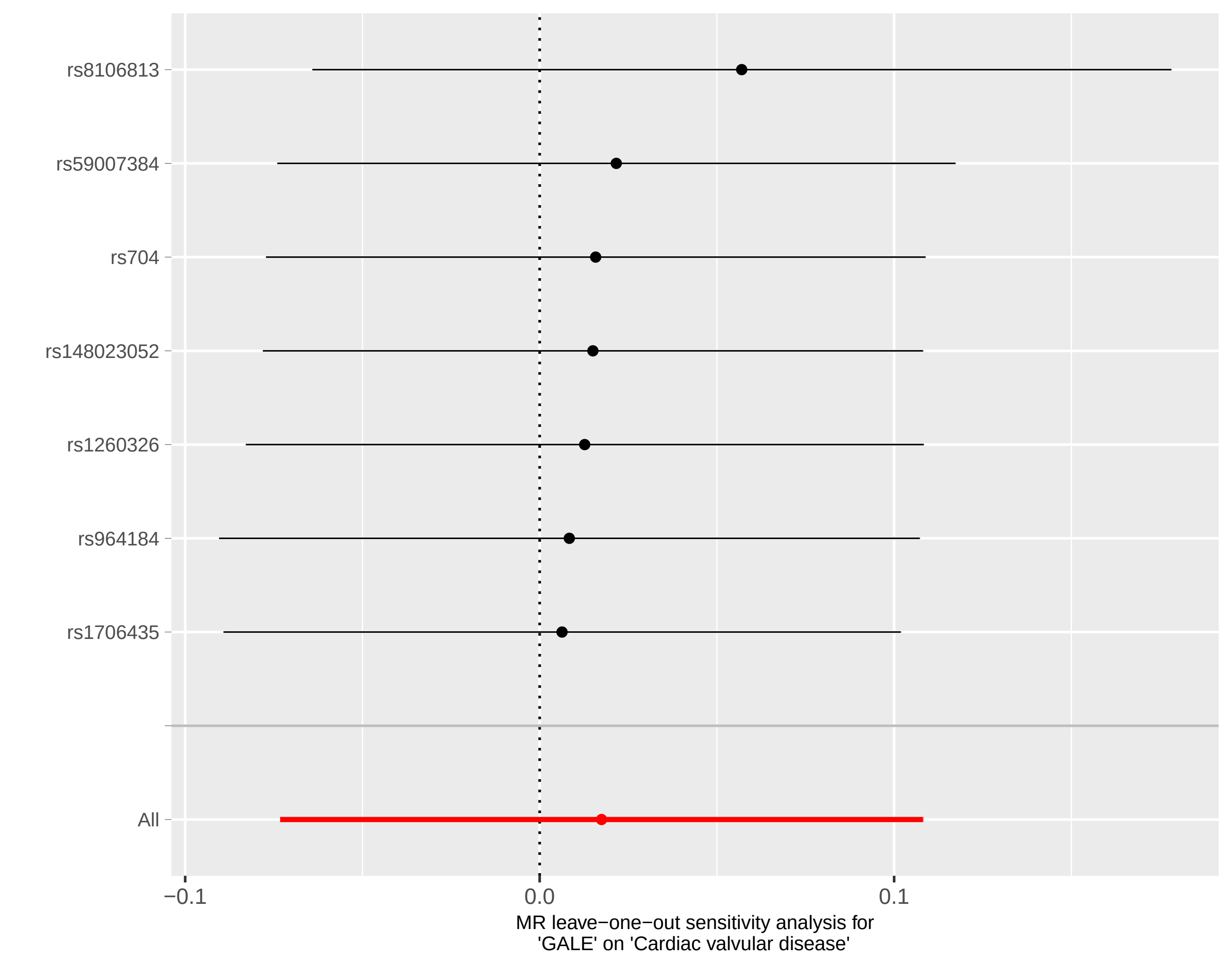
rs8106813
rs59007384
rs704
rs148023052
rs1260326
rs964184
rs1706435
All
−0.1
0.0
0.1
MR leave−one−out sensitivity analysis for
'GALE' on 'Cardiac valvular disease'

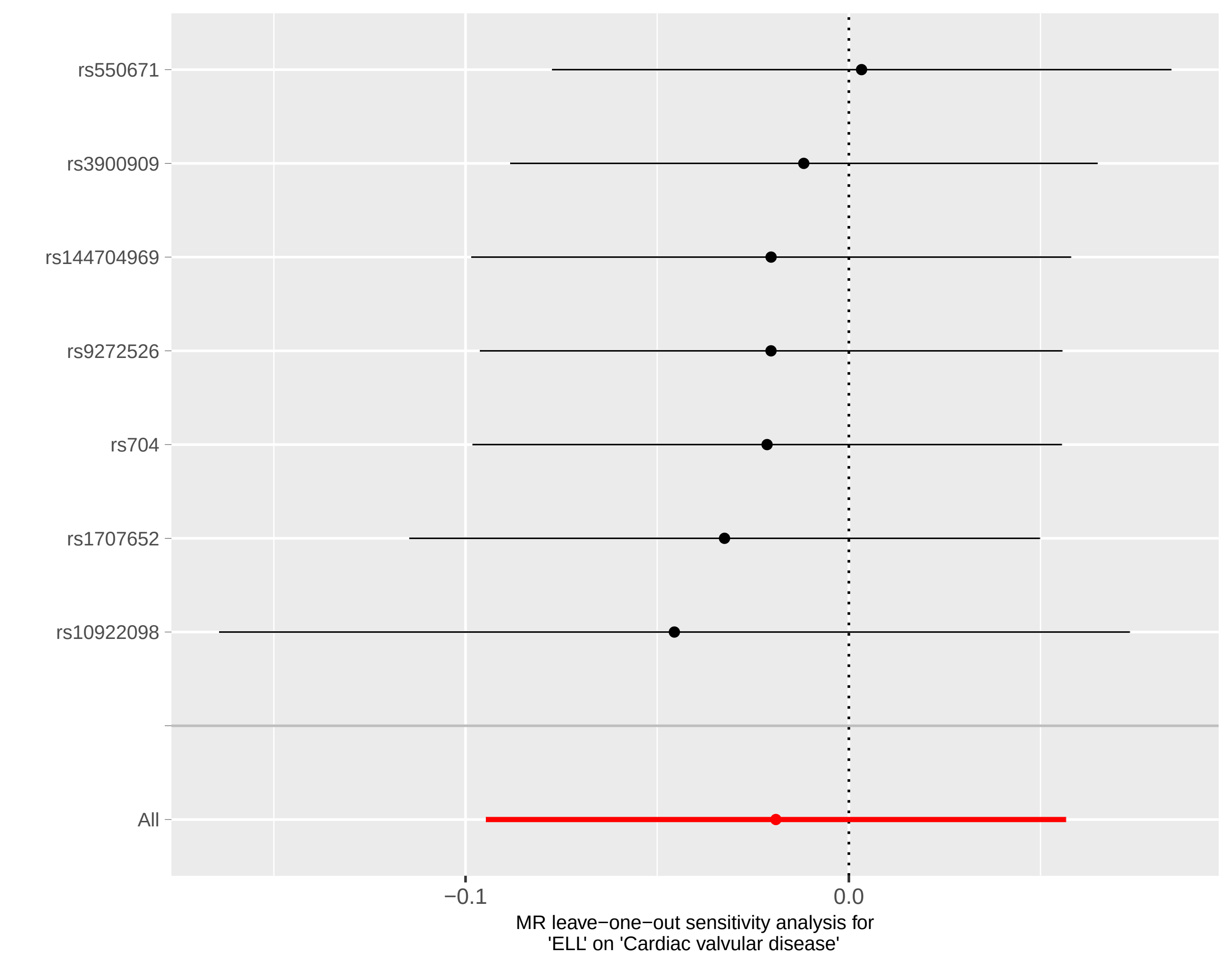

rs550671
rs3900909
rs144704969
rs9272526
rs704
rs1707652
rs10922098
All
−0.1
0.0
MR leave−one−out sensitivity analysis for
'ELL' on 'Cardiac valvular disease'

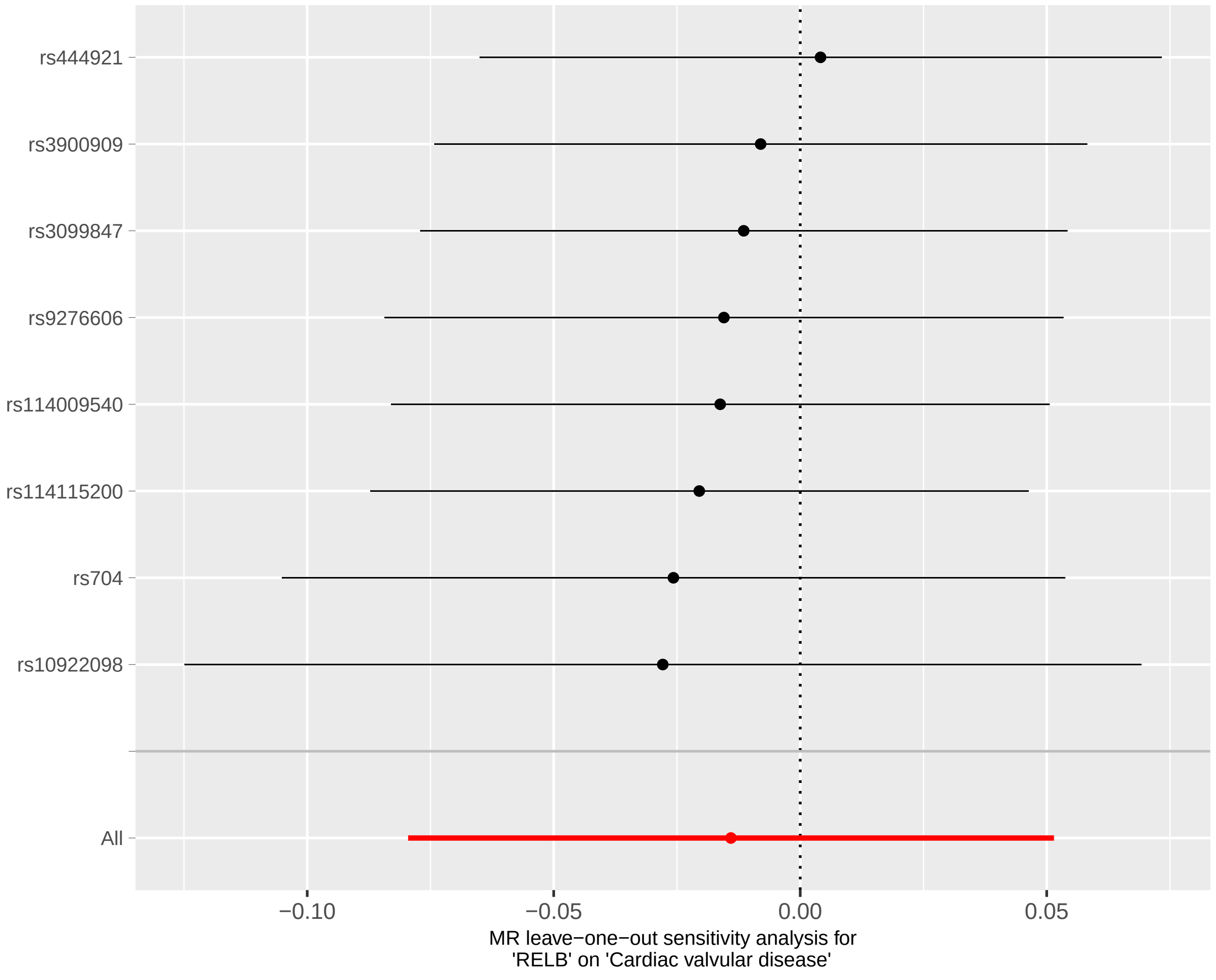

rs444921
rs3900909
rs3099847
rs9276606
rs114009540
rs114115200
rs704
rs10922098
All
−0.10
−0.05
0.00
0.05
MR leave−one−out sensitivity analysis for
'RELB' on 'Cardiac valvular disease'

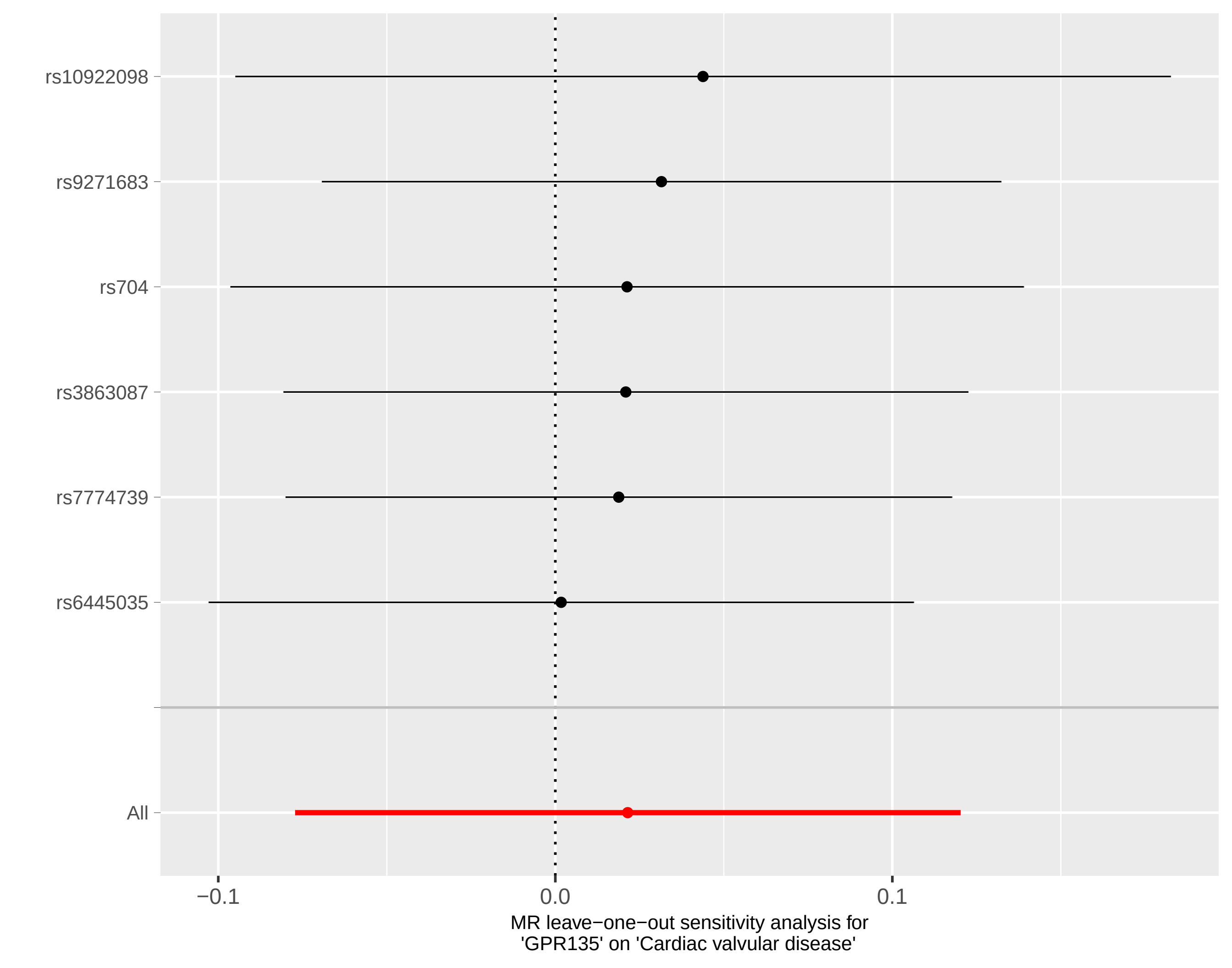

rs10922098
rs9271683
rs704
rs3863087
rs7774739
rs6445035
All
−0.1
0.0
0.1
MR leave−one−out sensitivity analysis for
'GPR135' on 'Cardiac valvular disease'

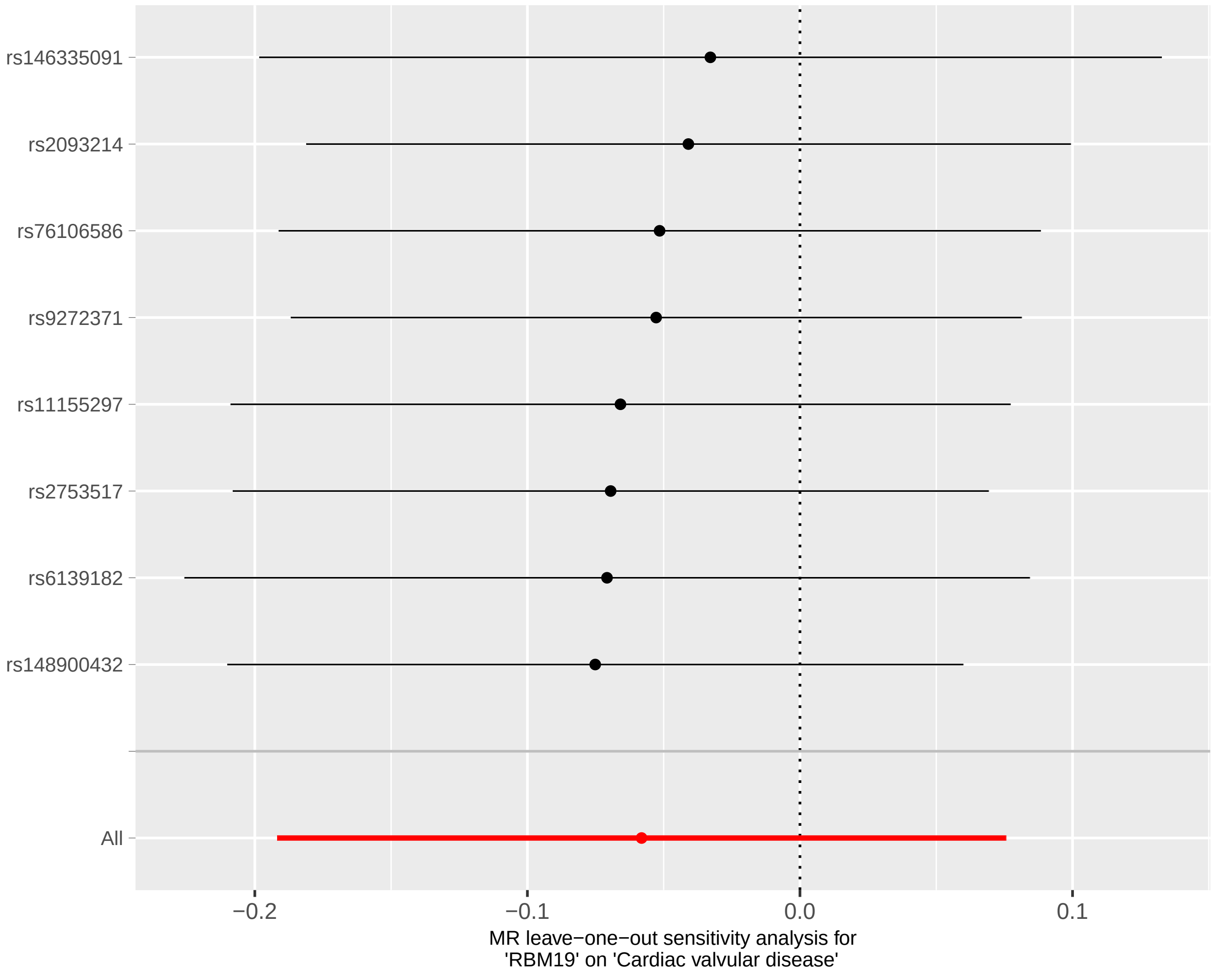
rs146335091
rs2093214
rs76106586
rs9272371
rs11155297
rs2753517
rs6139182
rs148900432
All
−0.2
−0.1
0.0
0.1
MR leave−one−out sensitivity analysis for
'RBM19' on 'Cardiac valvular disease'

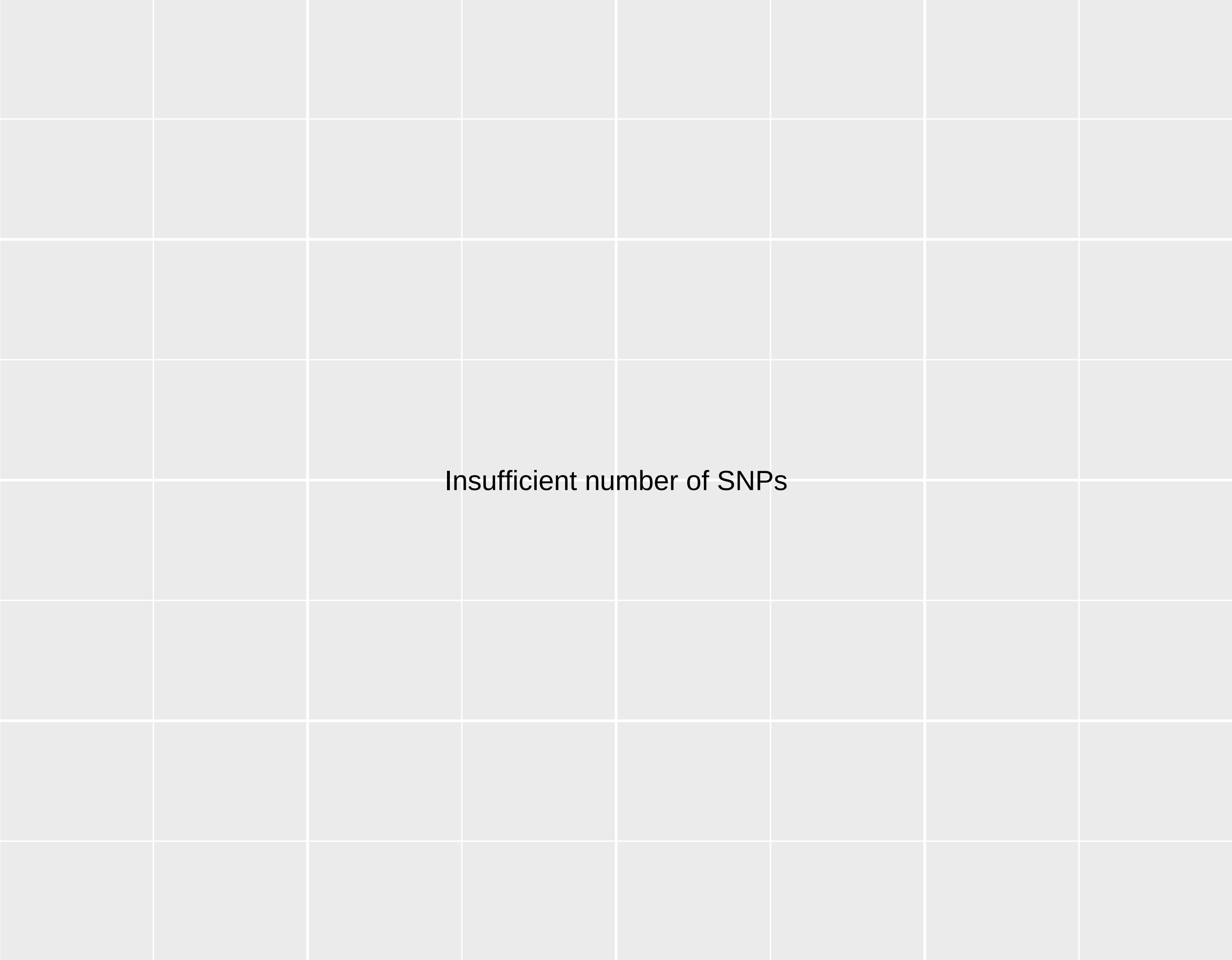
Insufficient number of SNPs

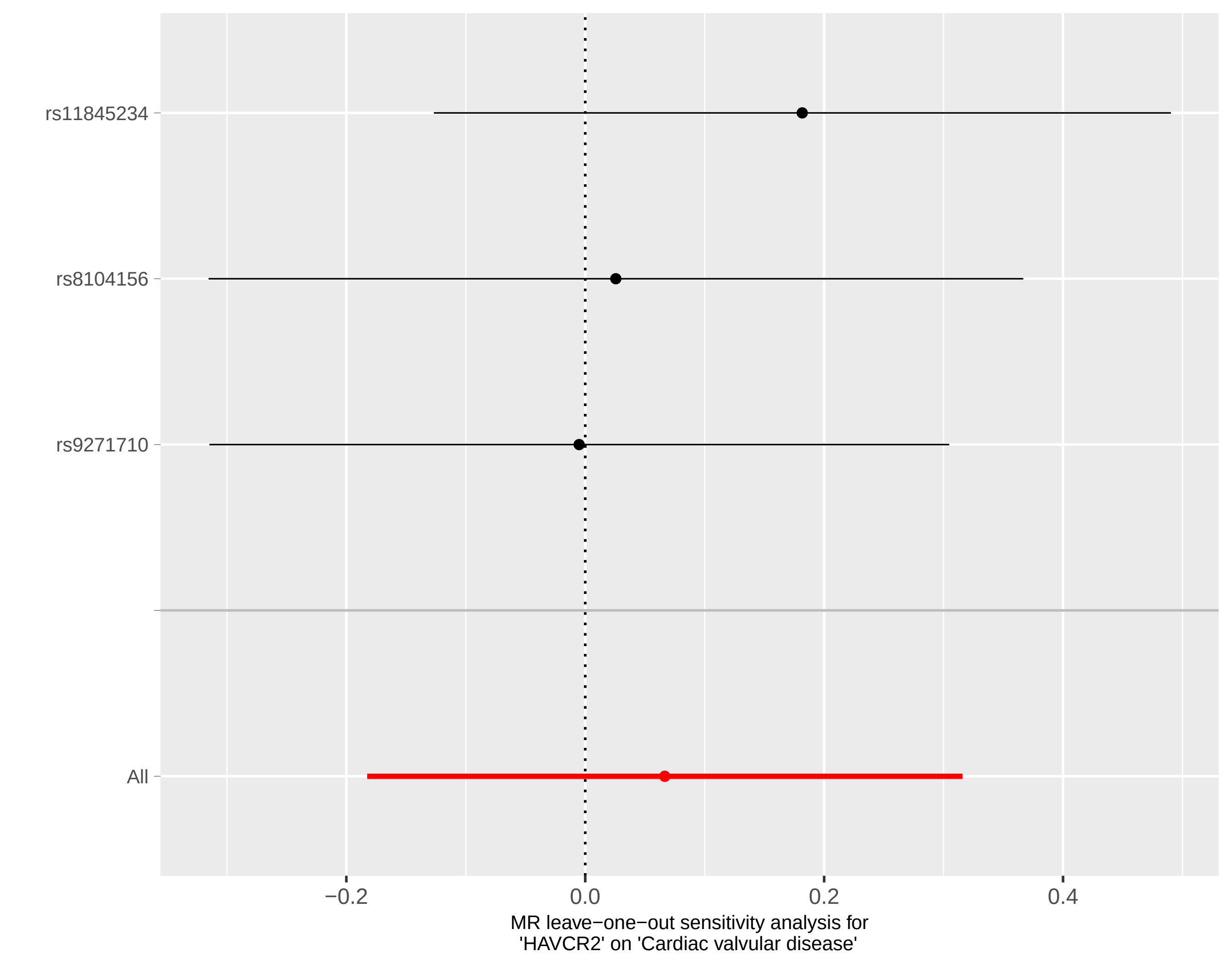
rs11845234
rs8104156
rs9271710
All
−0.2
0.0
0.2
0.4
MR leave−one−out sensitivity analysis for
'HAVCR2' on 'Cardiac valvular disease'

Insufficient number of SNPs

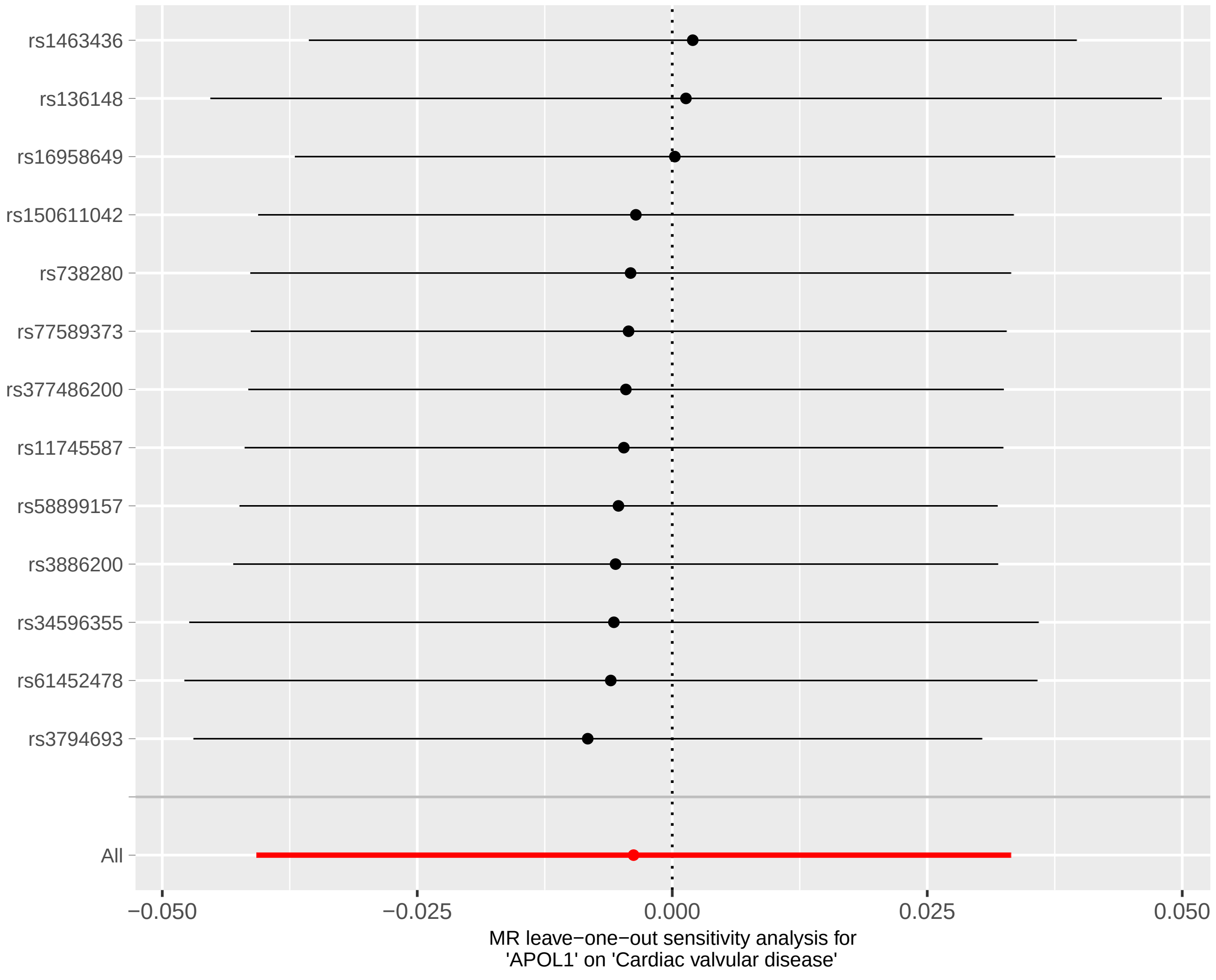
rs1463436
rs136148
rs16958649
rs150611042
rs738280
rs77589373
rs377486200
rs11745587
rs58899157
rs3886200
rs34596355
rs61452478
rs3794693
All
−0.050
−0.025
0.000
0.025
0.050
MR leave−one−out sensitivity analysis for
'APOL1' on 'Cardiac valvular disease'

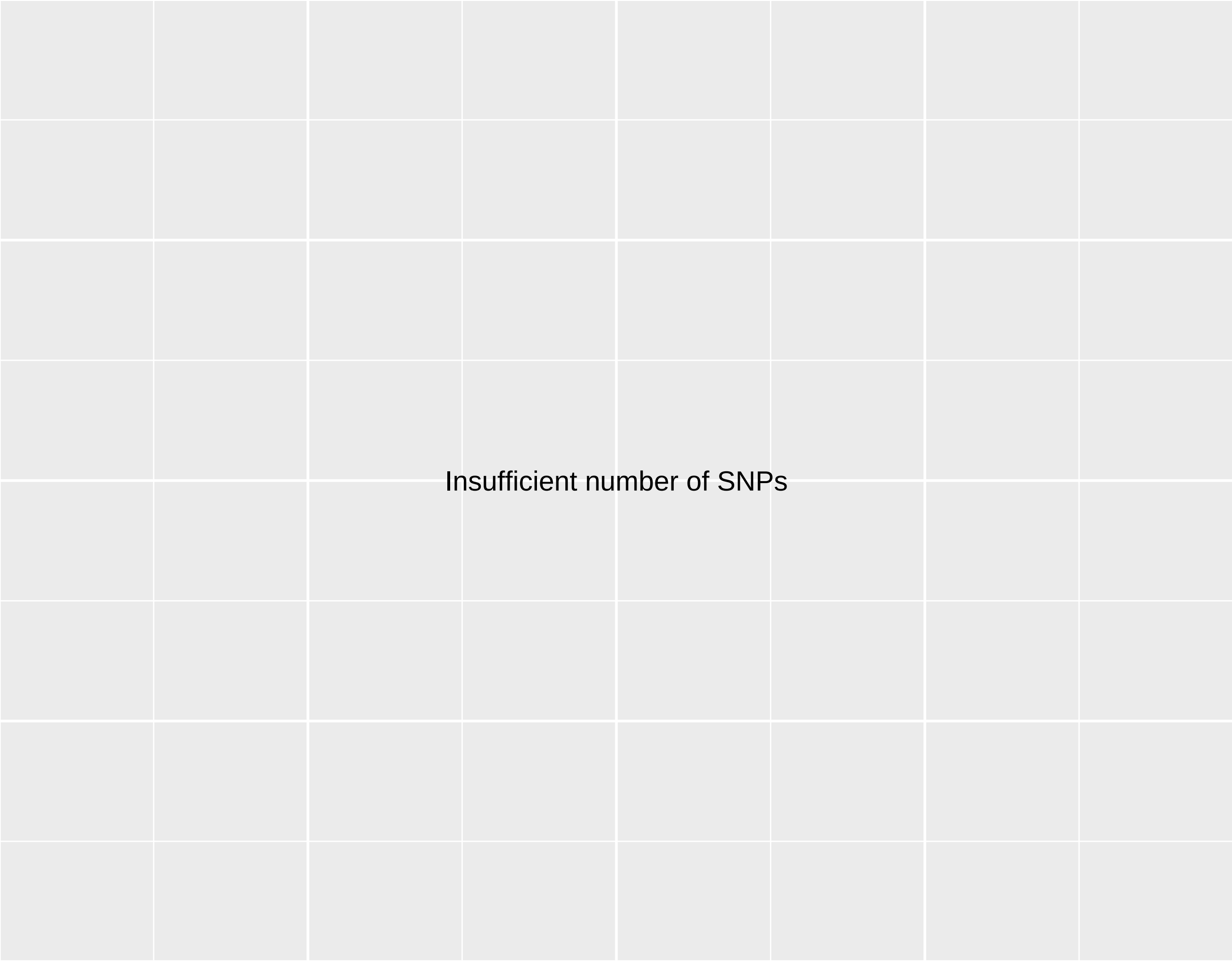
Insufficient number of SNPs

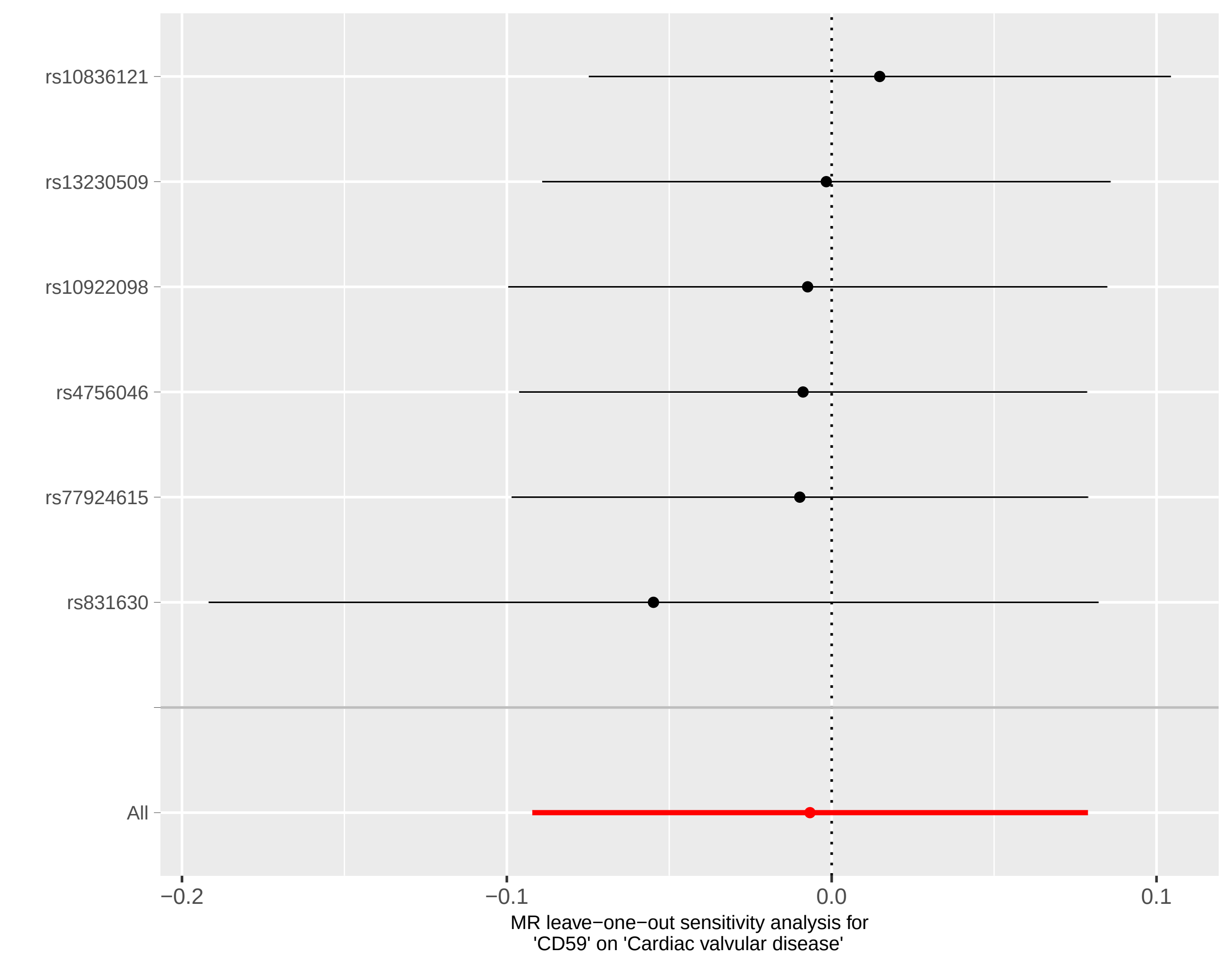

rs10836121
rs13230509
rs10922098
rs4756046
rs77924615
rs831630
All
−0.2
−0.1
0.0
0.1
MR leave−one−out sensitivity analysis for
'CD59' on 'Cardiac valvular disease'

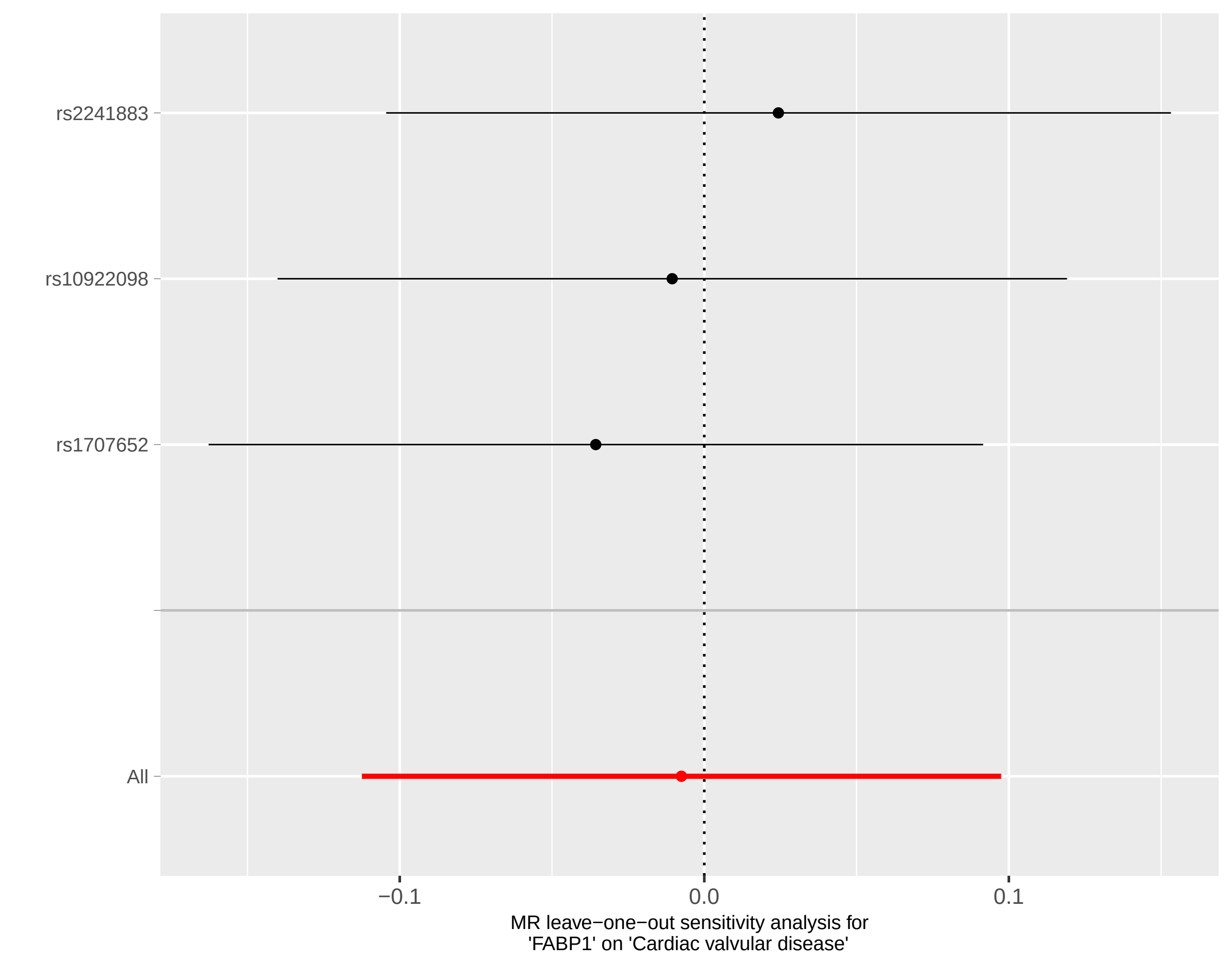
rs2241883
rs10922098
rs1707652
All
−0.1
0.0
0.1
MR leave−one−out sensitivity analysis for
'FABP1' on 'Cardiac valvular disease'

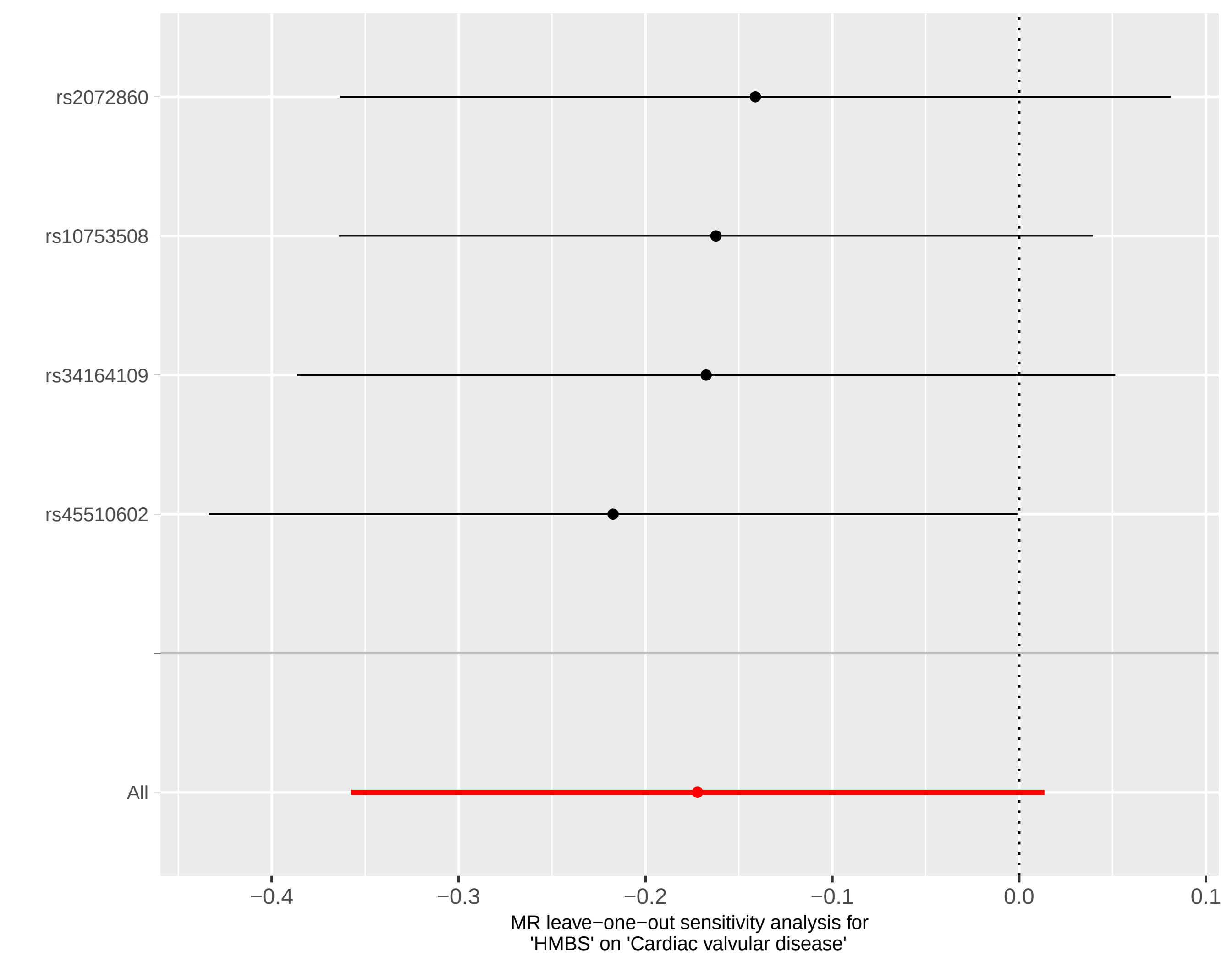

rs2072860
rs10753508
rs34164109
rs45510602
All
−0.4
−0.3
−0.2
−0.1
0.0
0.1
MR leave−one−out sensitivity analysis for
'HMBS' on 'Cardiac valvular disease'

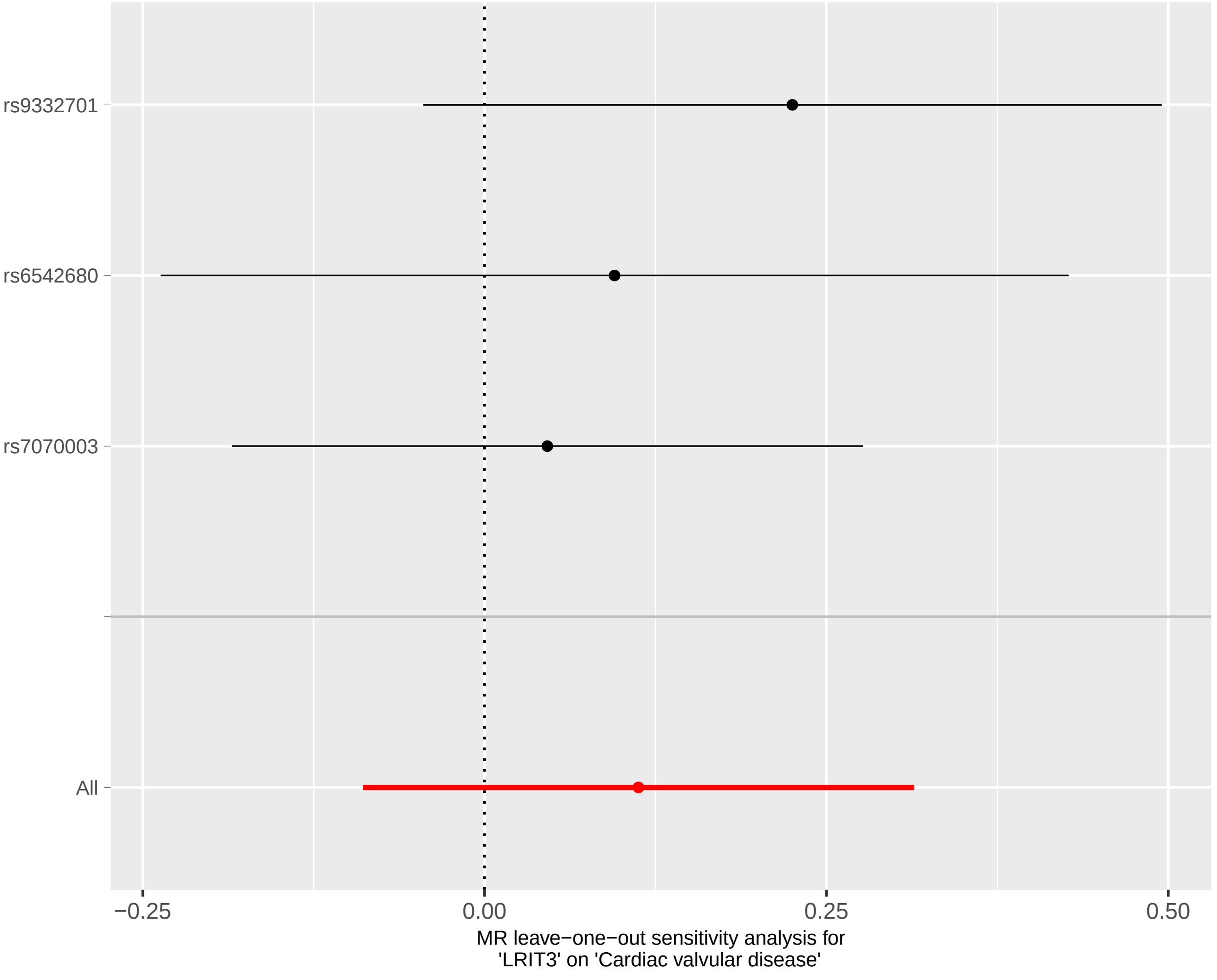
rs9332701
rs6542680
rs7070003
All
−0.25
0.00
0.25
0.50
MR leave−one−out sensitivity analysis for
'LRIT3' on 'Cardiac valvular disease'

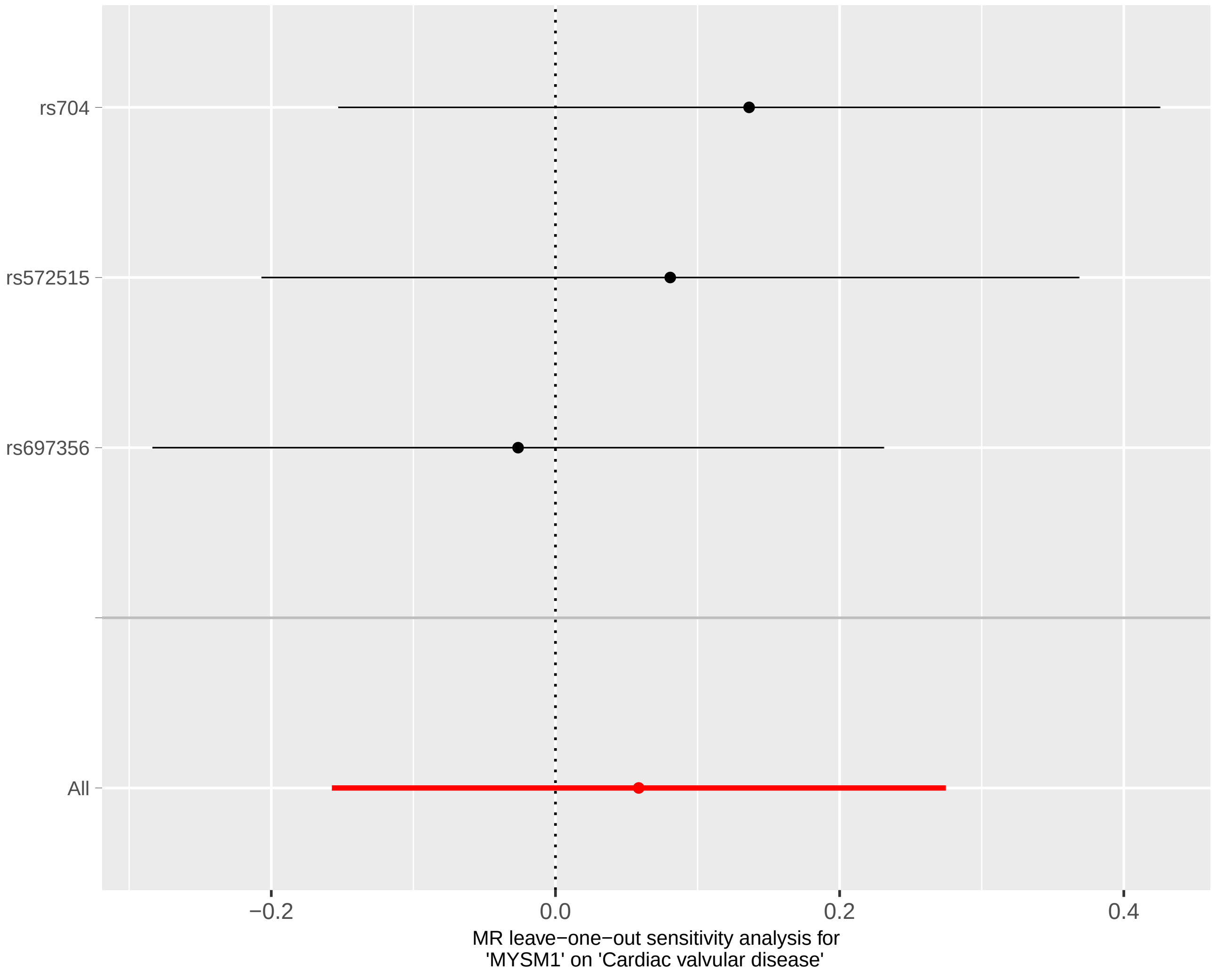

rs704
rs572515
rs697356
All
−0.2
0.0
0.2
0.4
MR leave−one−out sensitivity analysis for
'MYSM1' on 'Cardiac valvular disease'

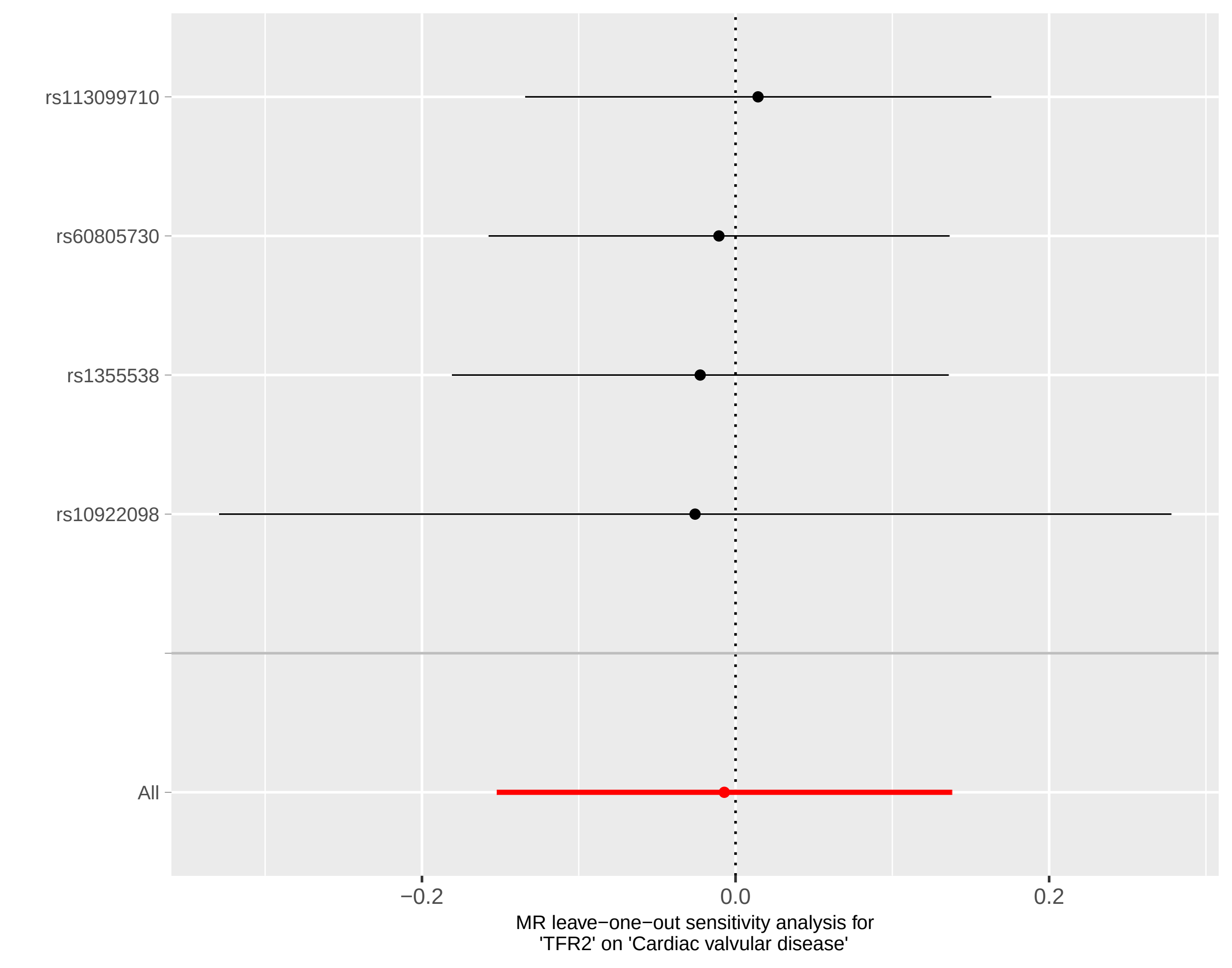
rs113099710
rs60805730
rs1355538
rs10922098
All
−0.2
0.0
0.2
MR leave−one−out sensitivity analysis for
'TFR2' on 'Cardiac valvular disease'

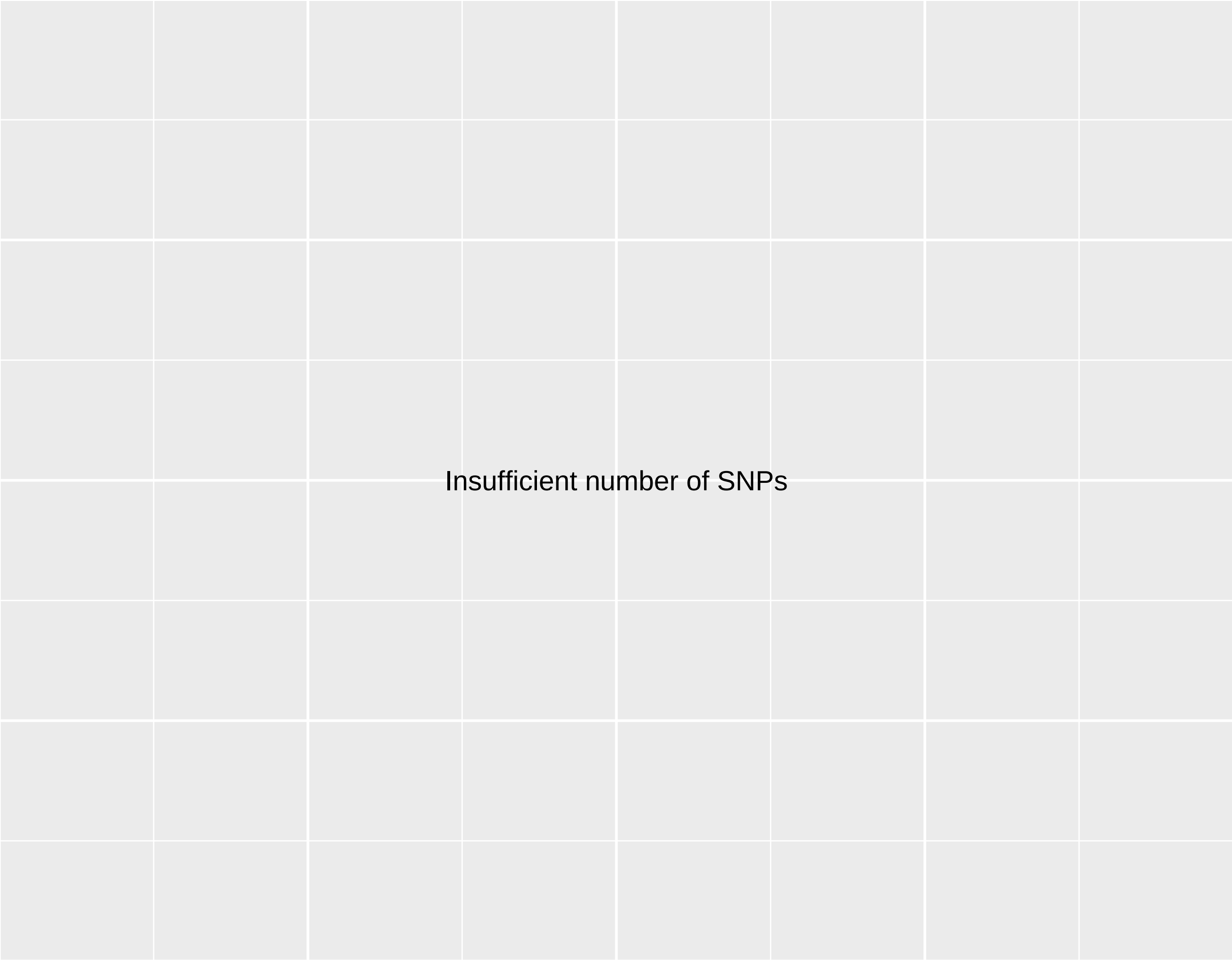
Insufficient number of SNPs

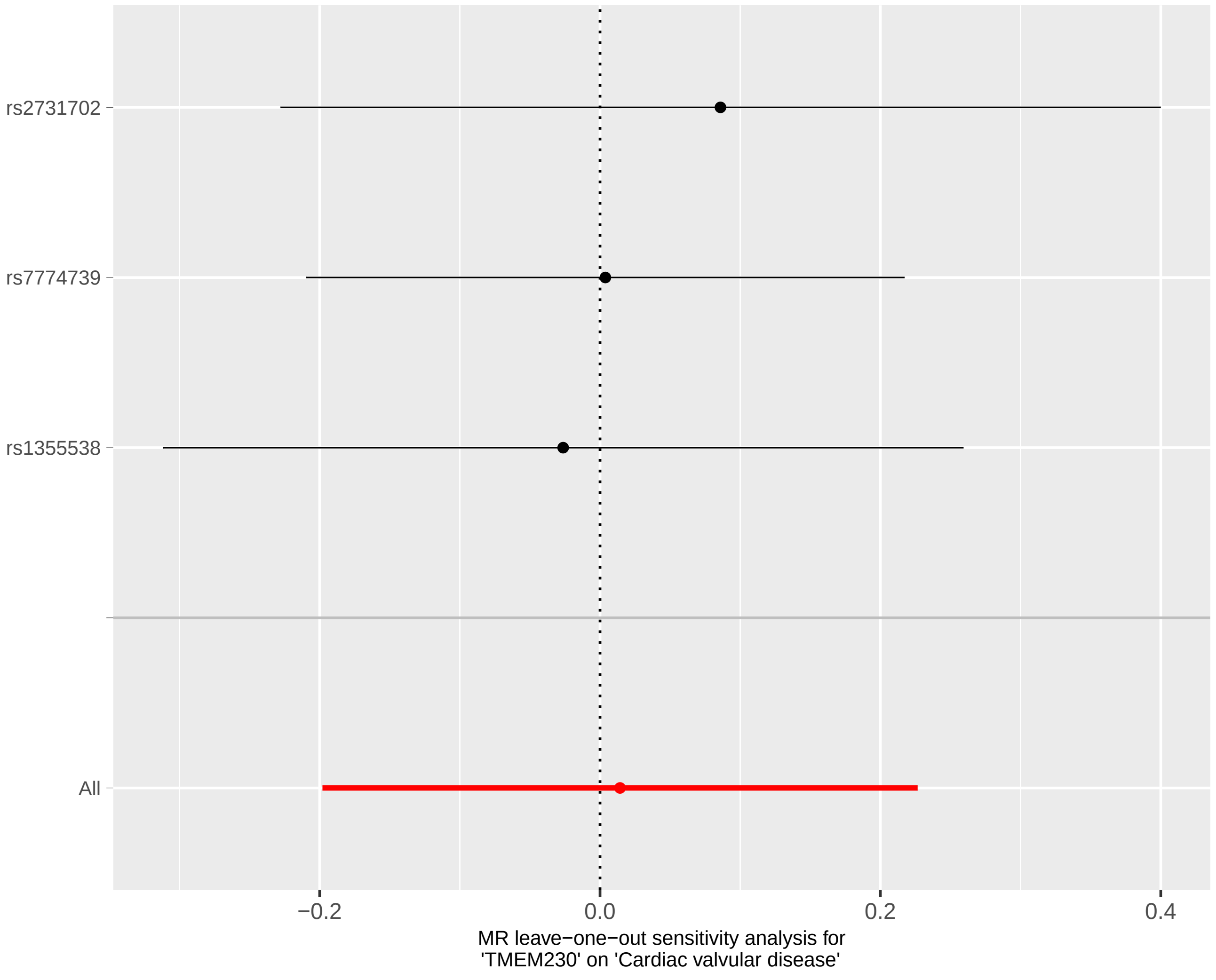
rs2731702
rs7774739
rs1355538
All
−0.2
0.0
0.2
0.4
MR leave−one−out sensitivity analysis for
'TMEM230' on 'Cardiac valvular disease'

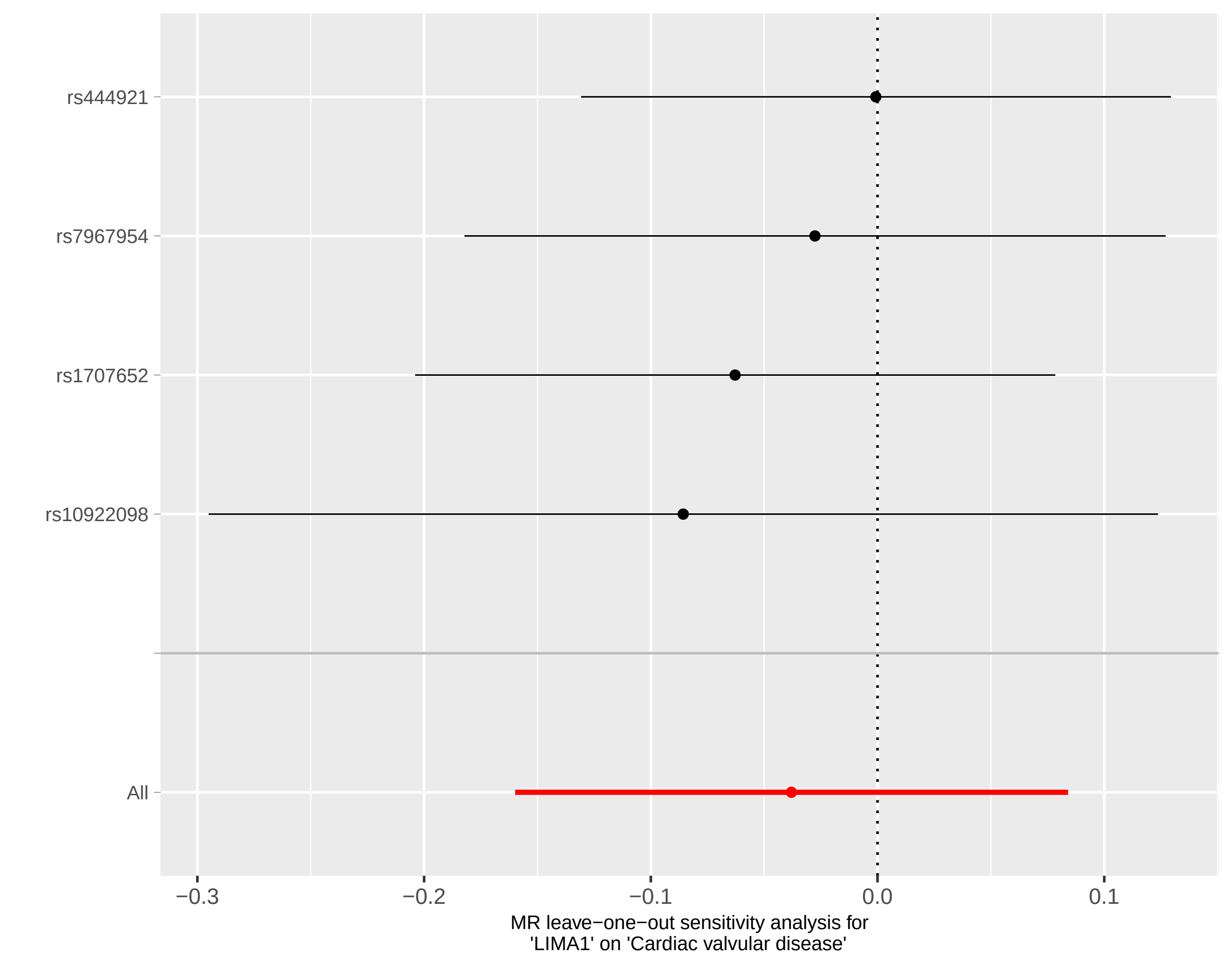

rs444921
rs7967954
rs1707652
rs10922098
All
−0.3
−0.2
−0.1
0.0
0.1
MR leave−one−out sensitivity analysis for
'LIMA1' on 'Cardiac valvular disease'

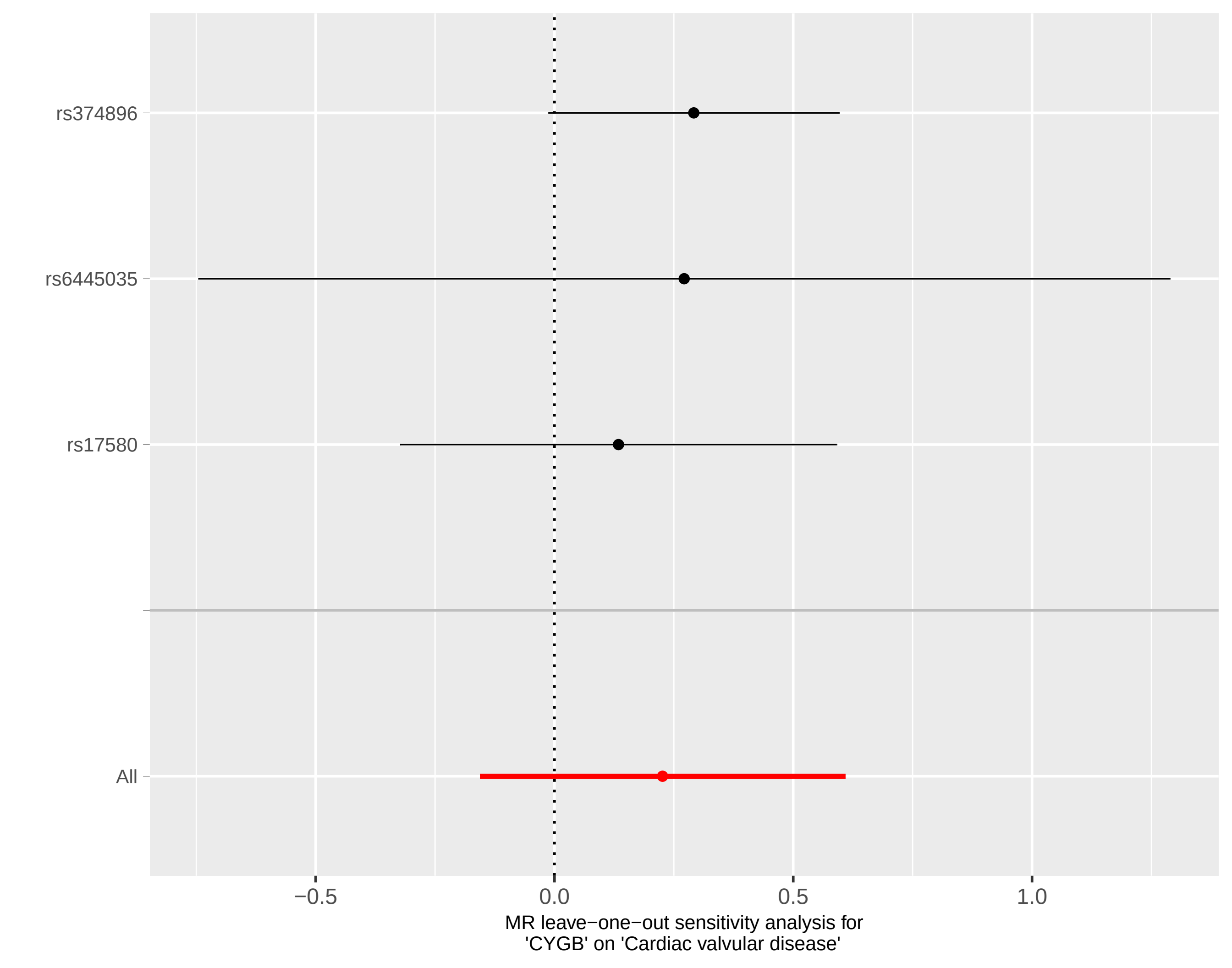
rs374896
rs6445035
rs17580
All
−0.5
0.0
0.5
1.0
MR leave−one−out sensitivity analysis for
'CYGB' on 'Cardiac valvular disease'

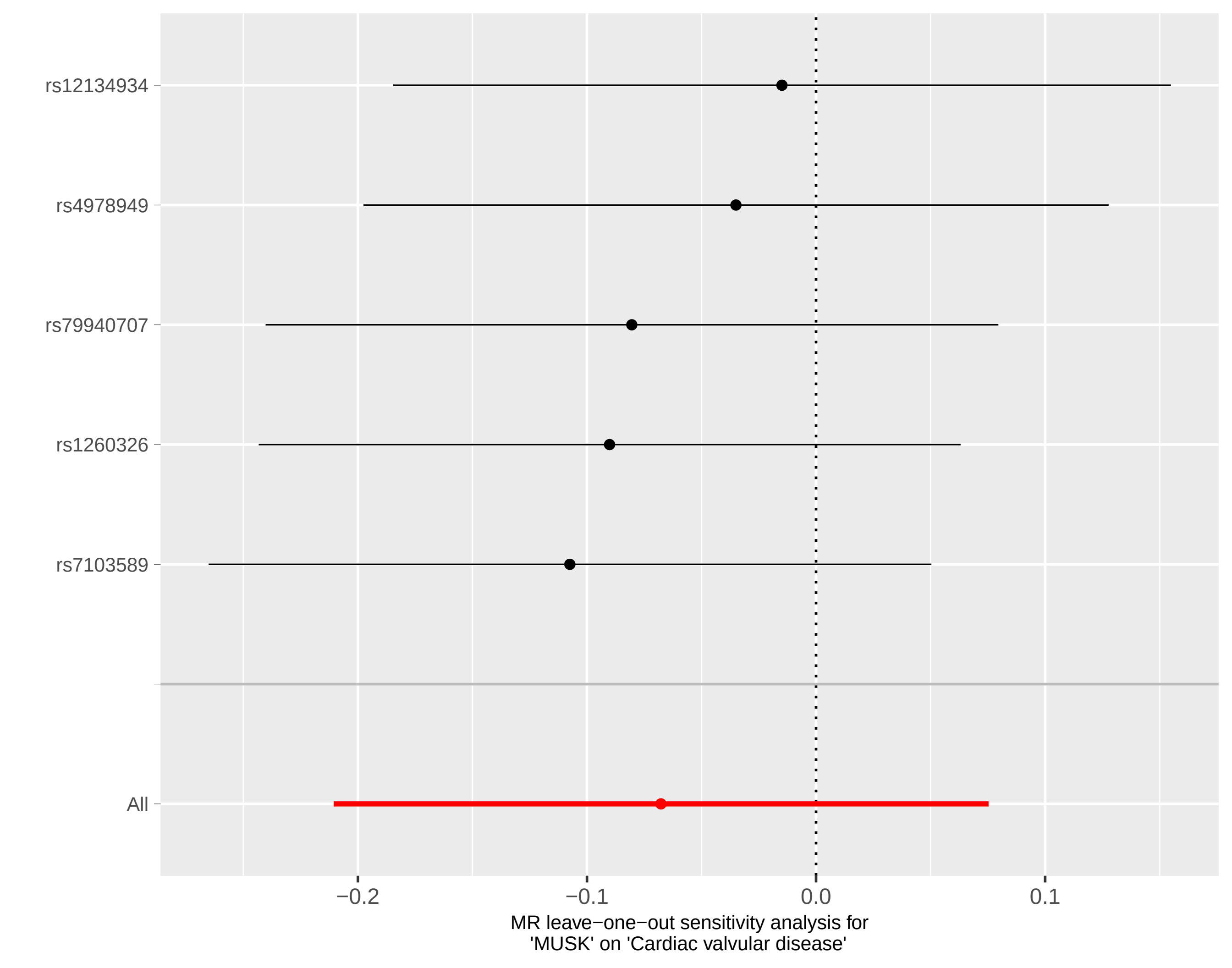
rs12134934
rs4978949
rs79940707
rs1260326
rs7103589
All
−0.2
−0.1
0.0
0.1
MR leave−one−out sensitivity analysis for
'MUSK' on 'Cardiac valvular disease'

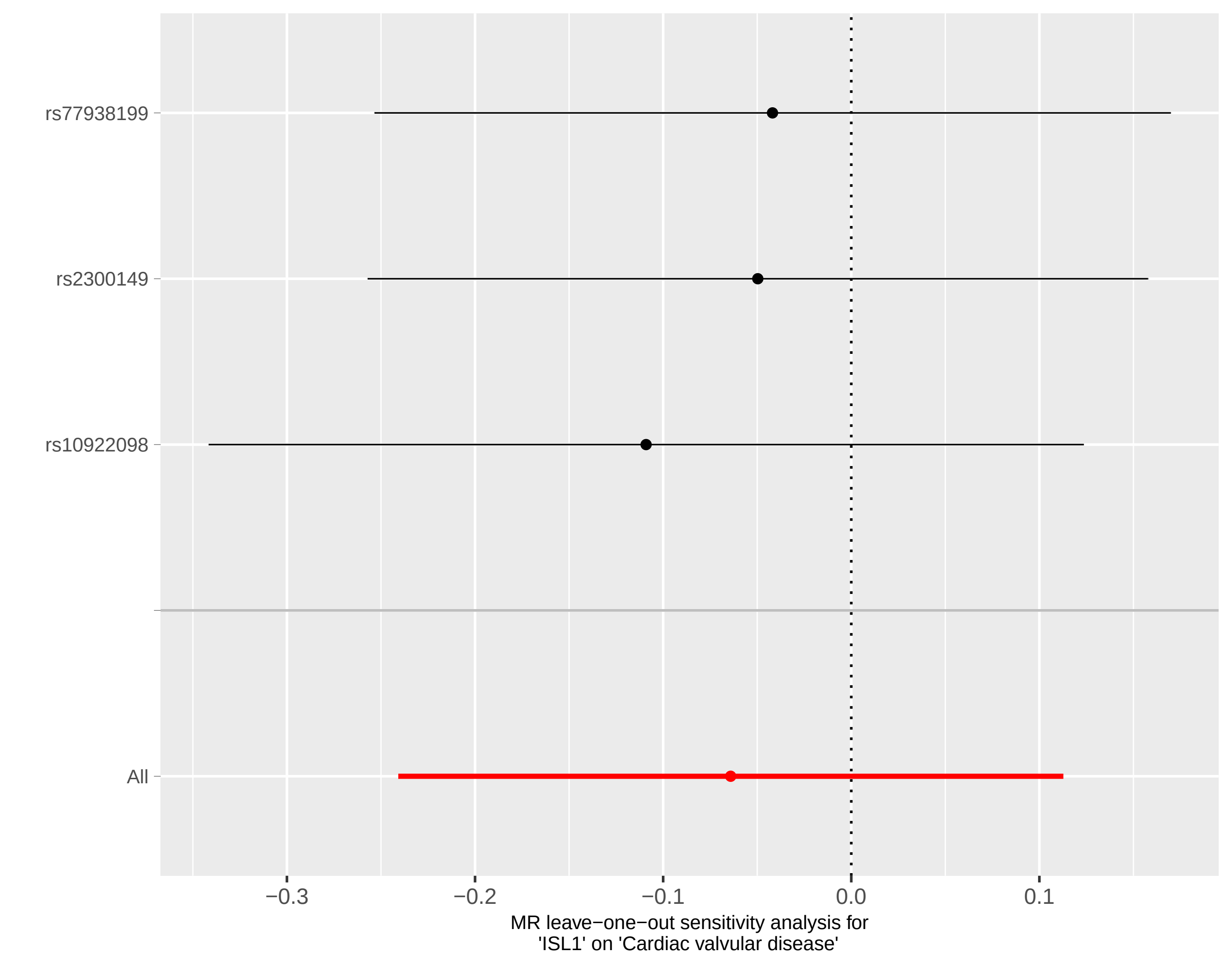
rs77938199
rs2300149
rs10922098
All
−0.3
−0.2
−0.1
0.0
0.1
MR leave−one−out sensitivity analysis for
'ISL1' on 'Cardiac valvular disease'

Insufficient number of SNPs

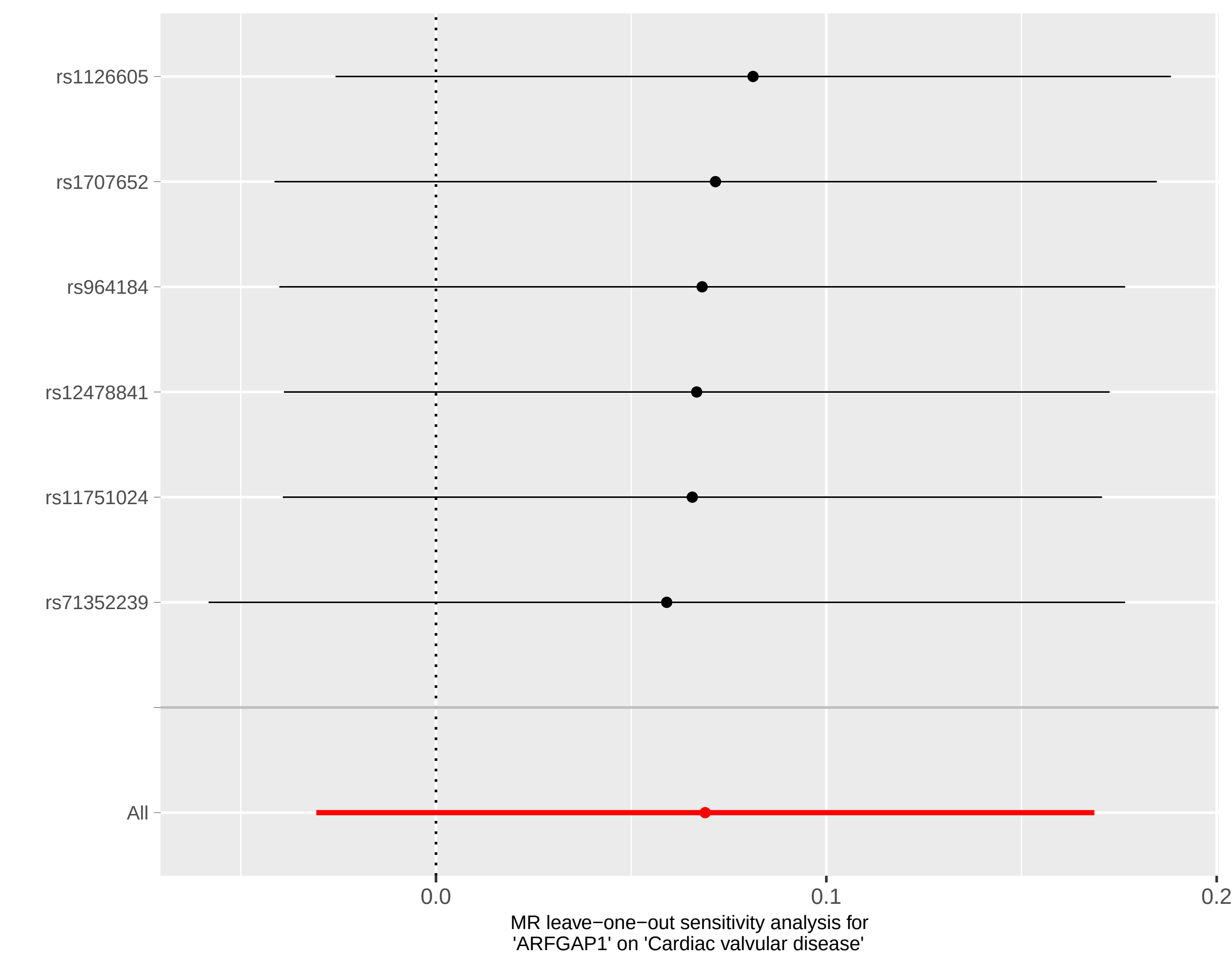
rs1126605
rs1707652
rs964184
rs12478841
rs11751024
rs71352239
All
0.0
0.1
0.2
MR leave−one−out sensitivity analysis for
'ARFGAP1' on 'Cardiac valvular disease'

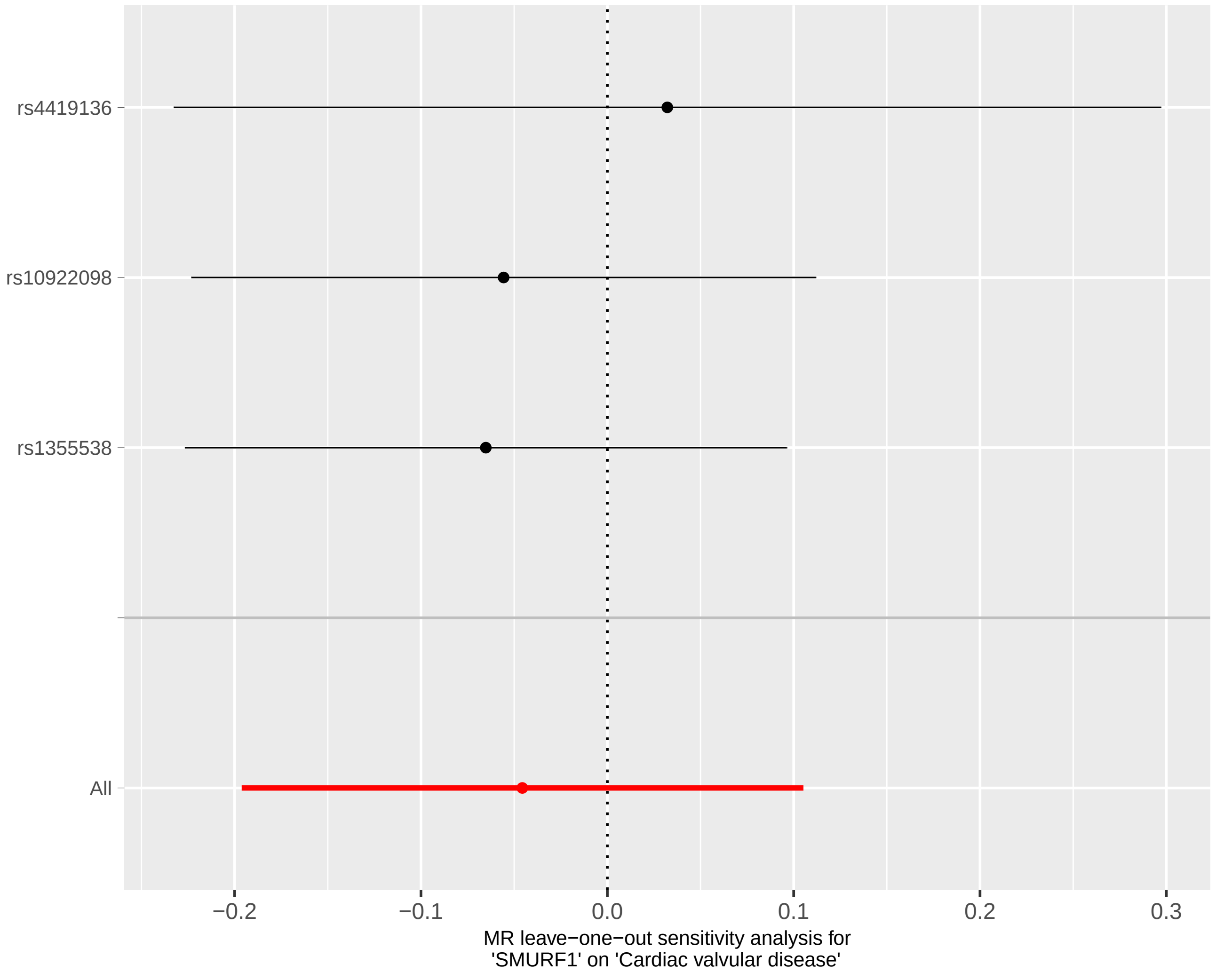
rs4419136
rs10922098
rs1355538
All
−0.2
−0.1
0.0
0.1
0.2
0.3
MR leave−one−out sensitivity analysis for
'SMURF1' on 'Cardiac valvular disease'

Insufficient number of SNPs

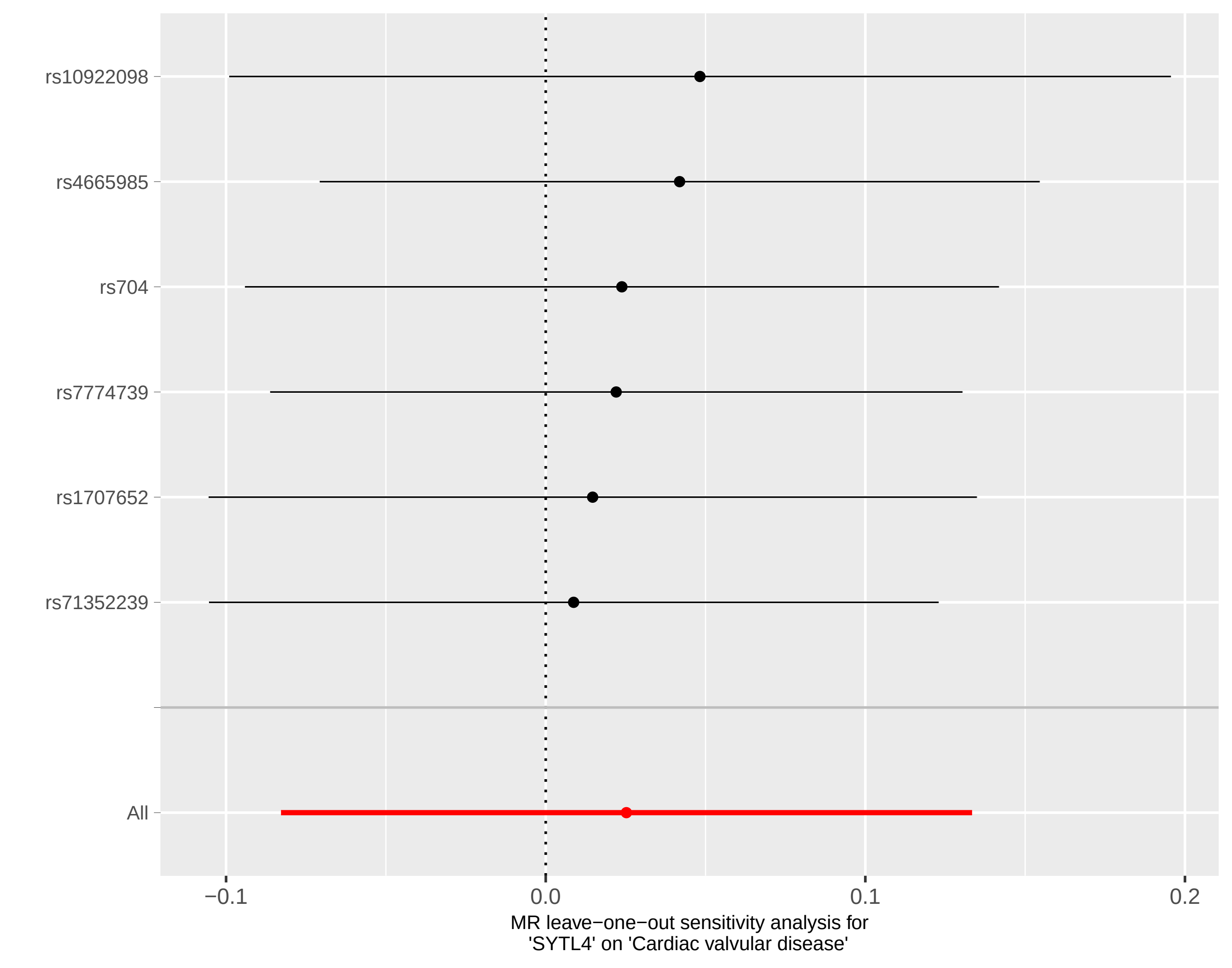
rs10922098
rs4665985
rs704
rs7774739
rs1707652
rs71352239
All
−0.1
0.0
0.1
0.2
MR leave−one−out sensitivity analysis for
'SYTL4' on 'Cardiac valvular disease'

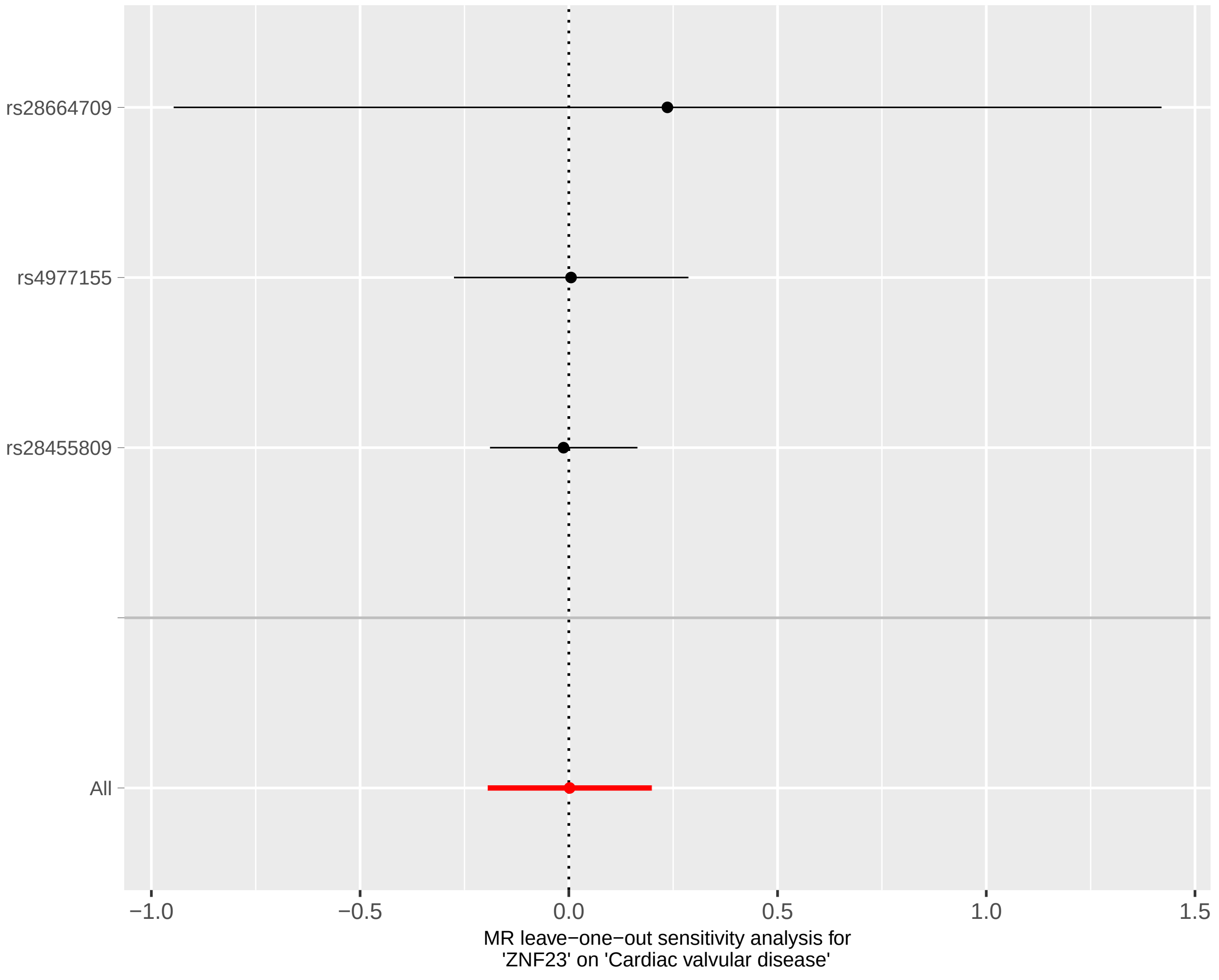

rs28664709
rs4977155
rs28455809
All
−1.0
−0.5
0.0
0.5
1.0
1.5
MR leave−one−out sensitivity analysis for
'ZNF23' on 'Cardiac valvular disease'

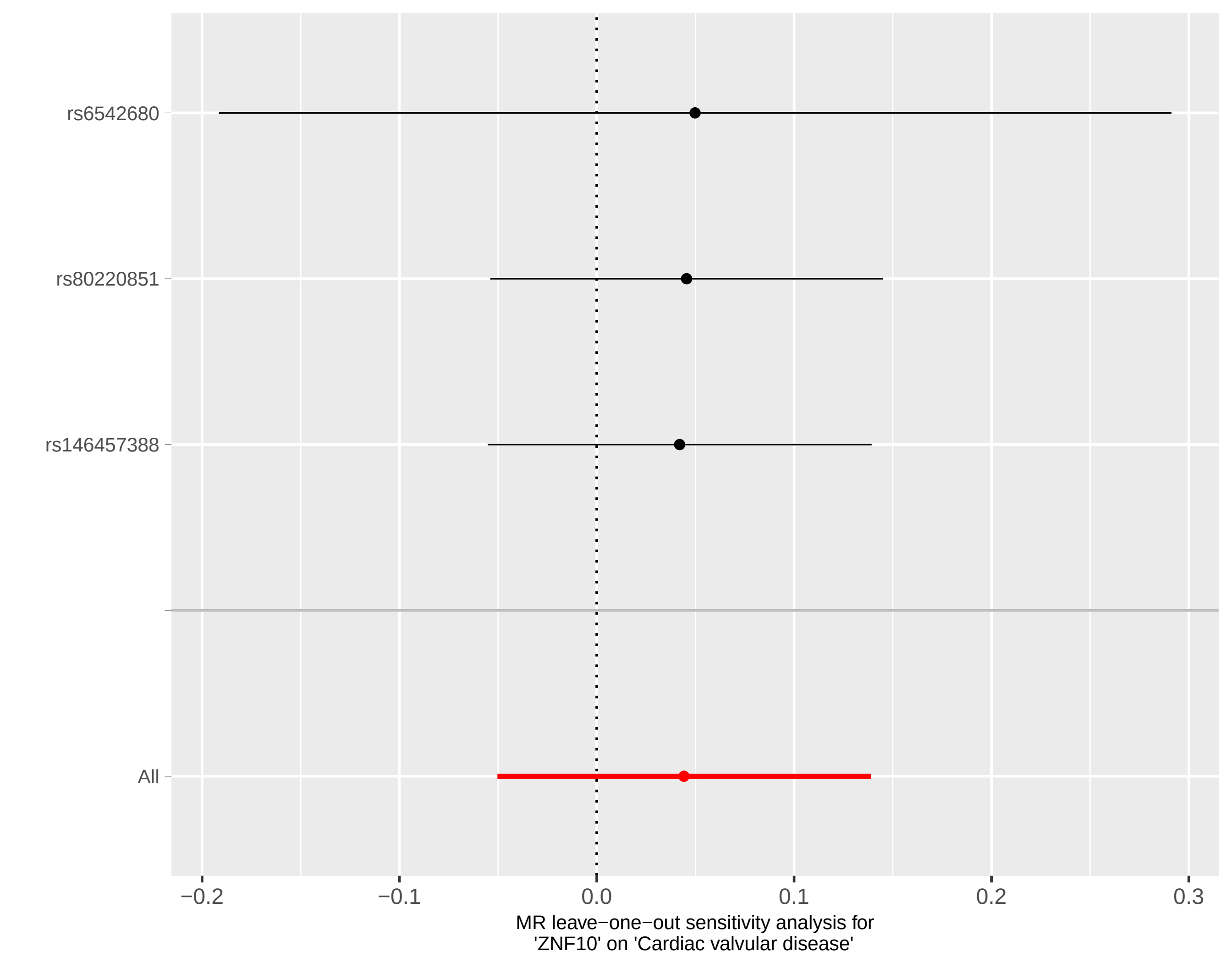
rs6542680
rs80220851
rs146457388
All
−0.2
−0.1
0.0
0.1
0.2
0.3
MR leave−one−out sensitivity analysis for
'ZNF10' on 'Cardiac valvular disease'

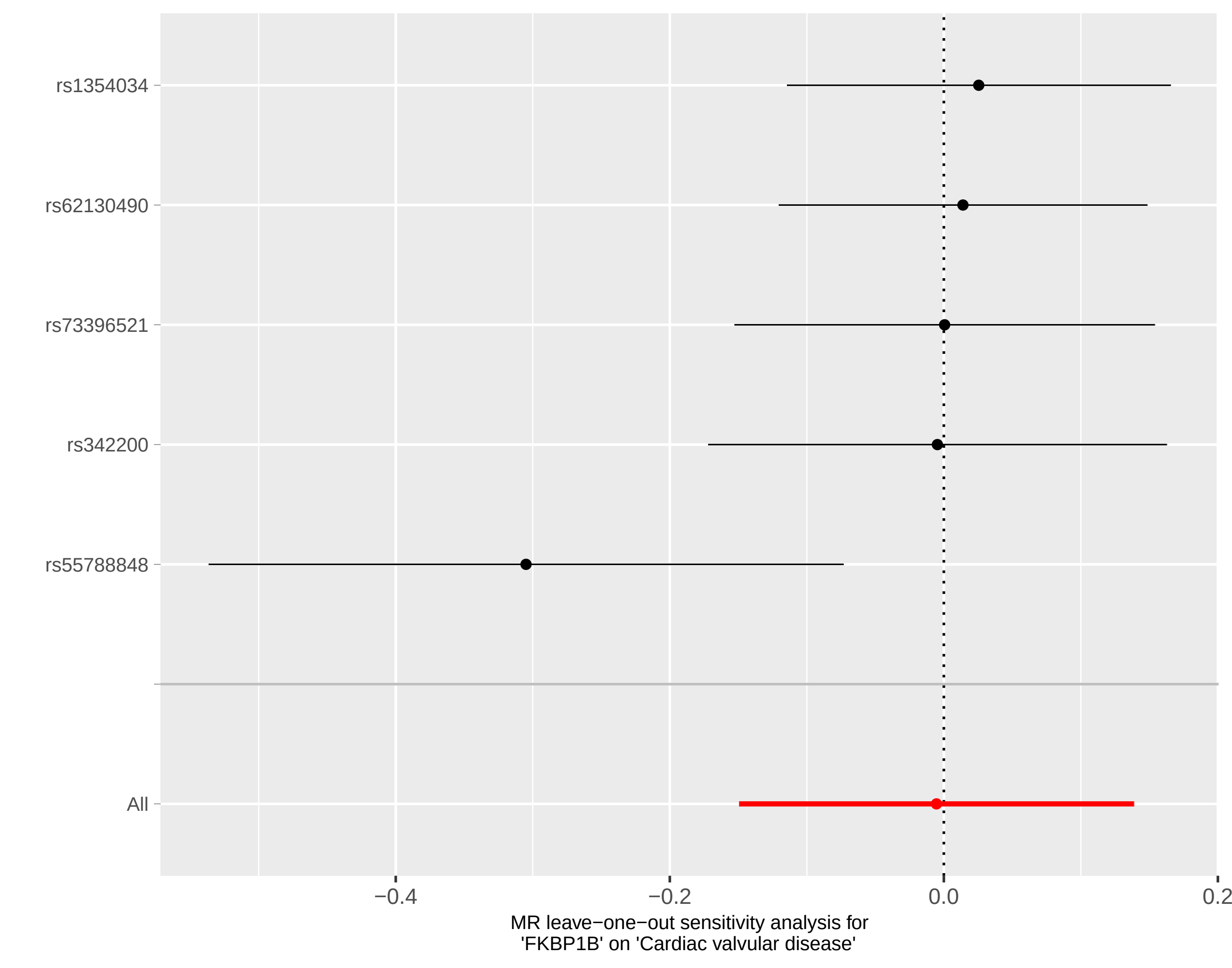
rs1354034
rs62130490
rs73396521
rs342200
rs55788848
All
−0.4
−0.2
0.0
0.2
MR leave−one−out sensitivity analysis for
'FKBP1B' on 'Cardiac valvular disease'

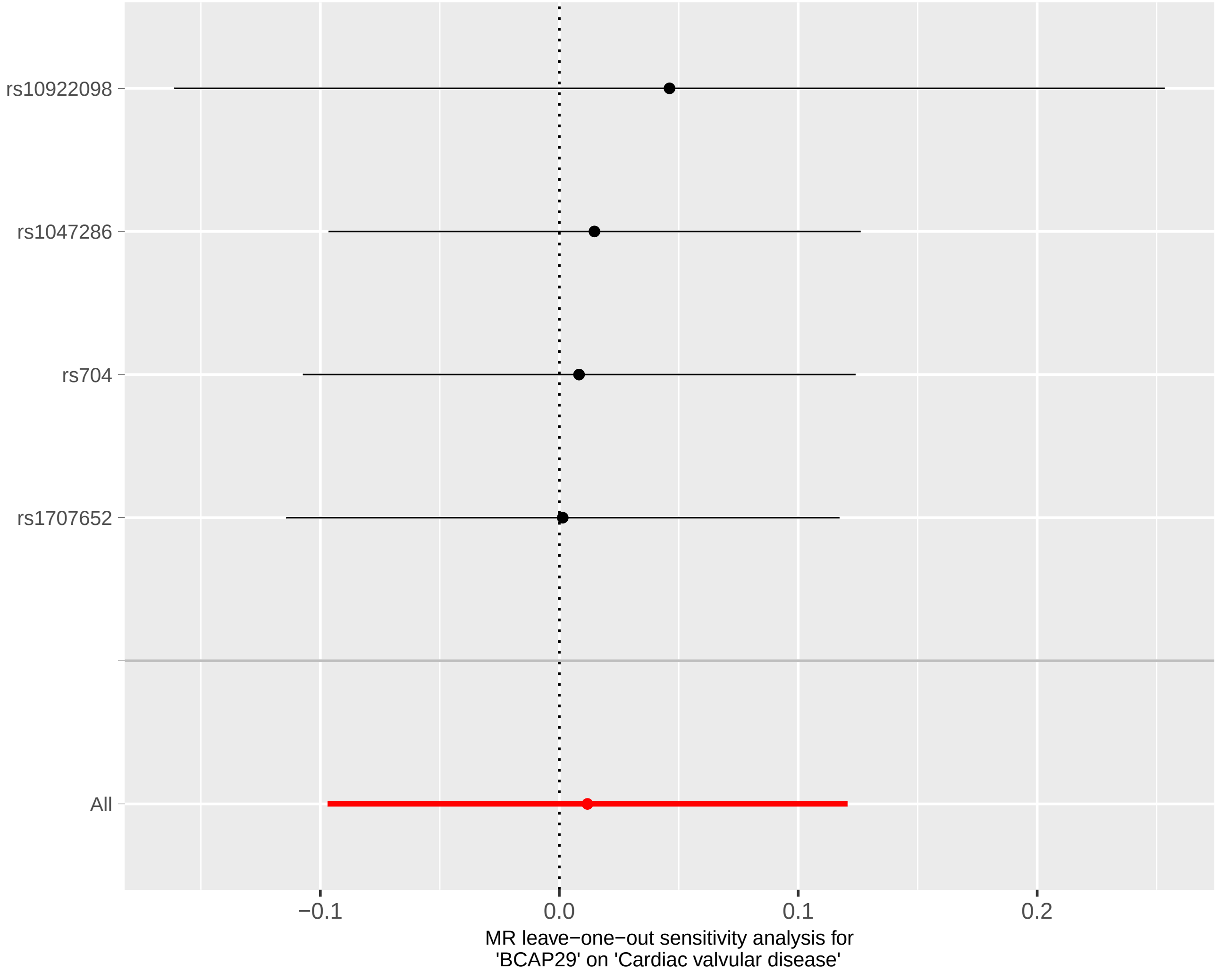
rs10922098
rs1047286
rs704
rs1707652
All
−0.1
0.0
0.1
0.2
MR leave−one−out sensitivity analysis for
'BCAP29' on 'Cardiac valvular disease'

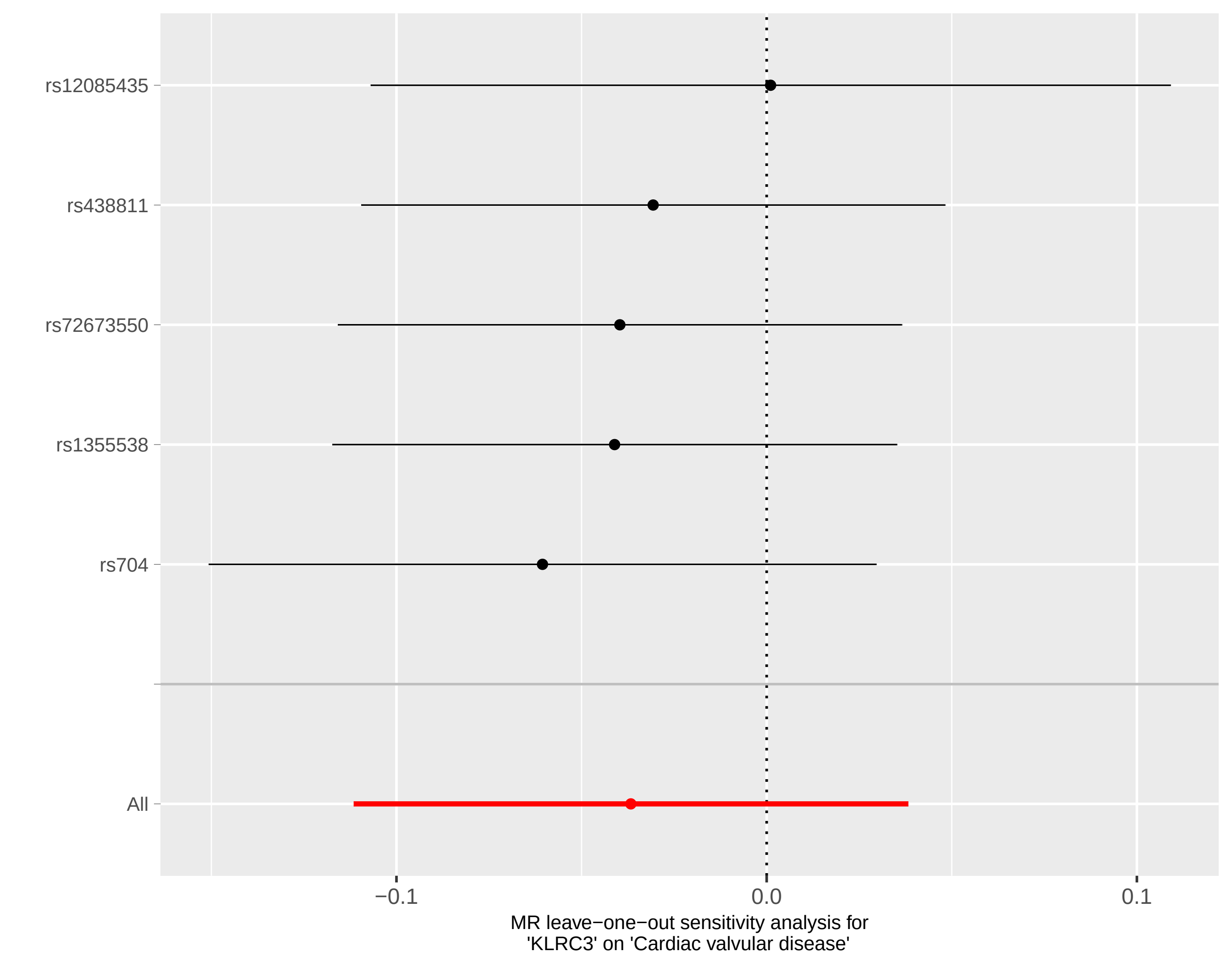

rs12085435
rs438811
rs72673550
rs1355538
rs704
All
−0.1
0.0
0.1
MR leave−one−out sensitivity analysis for
'KLRC3' on 'Cardiac valvular disease'

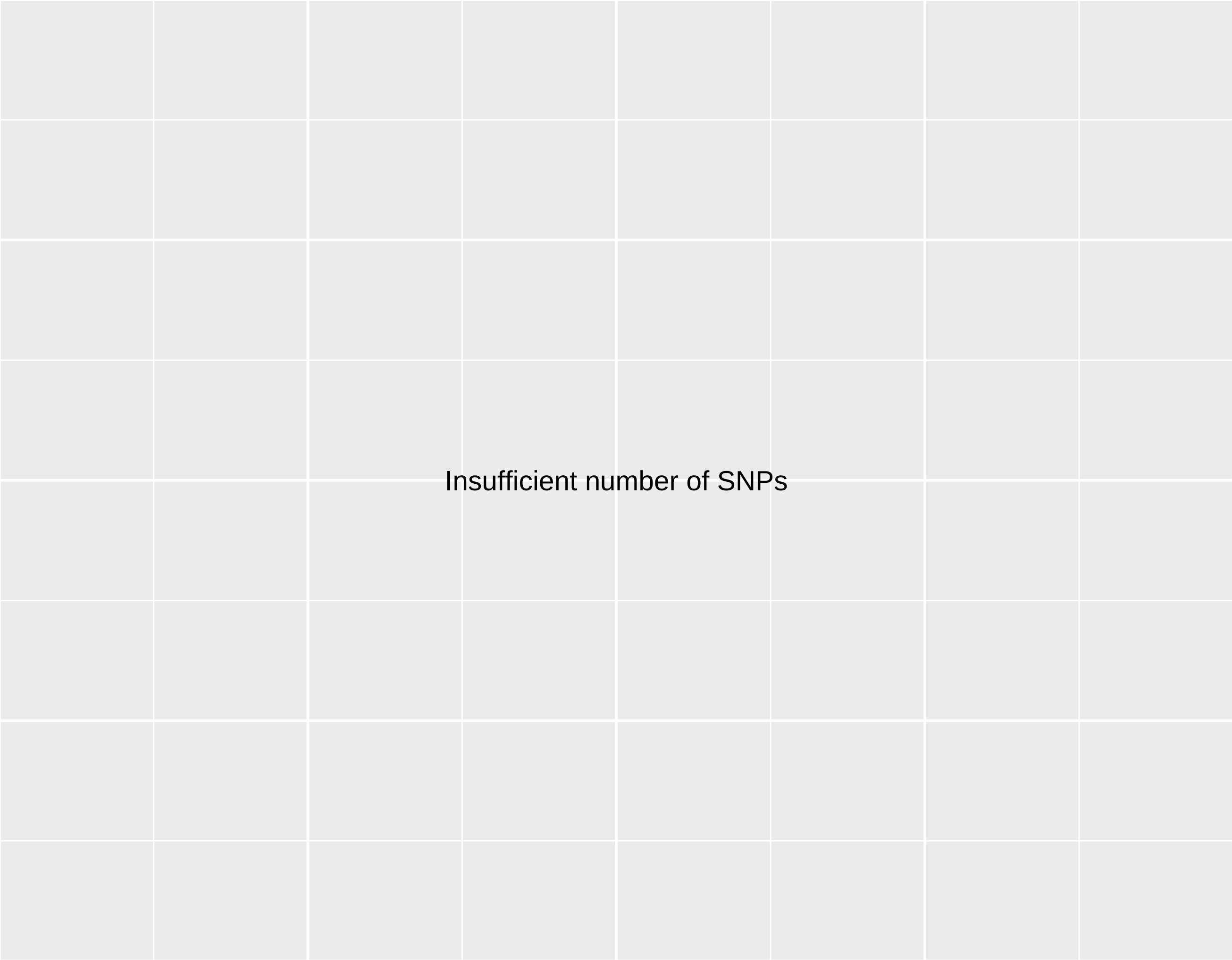
Insufficient number of SNPs

Insufficient number of SNPs

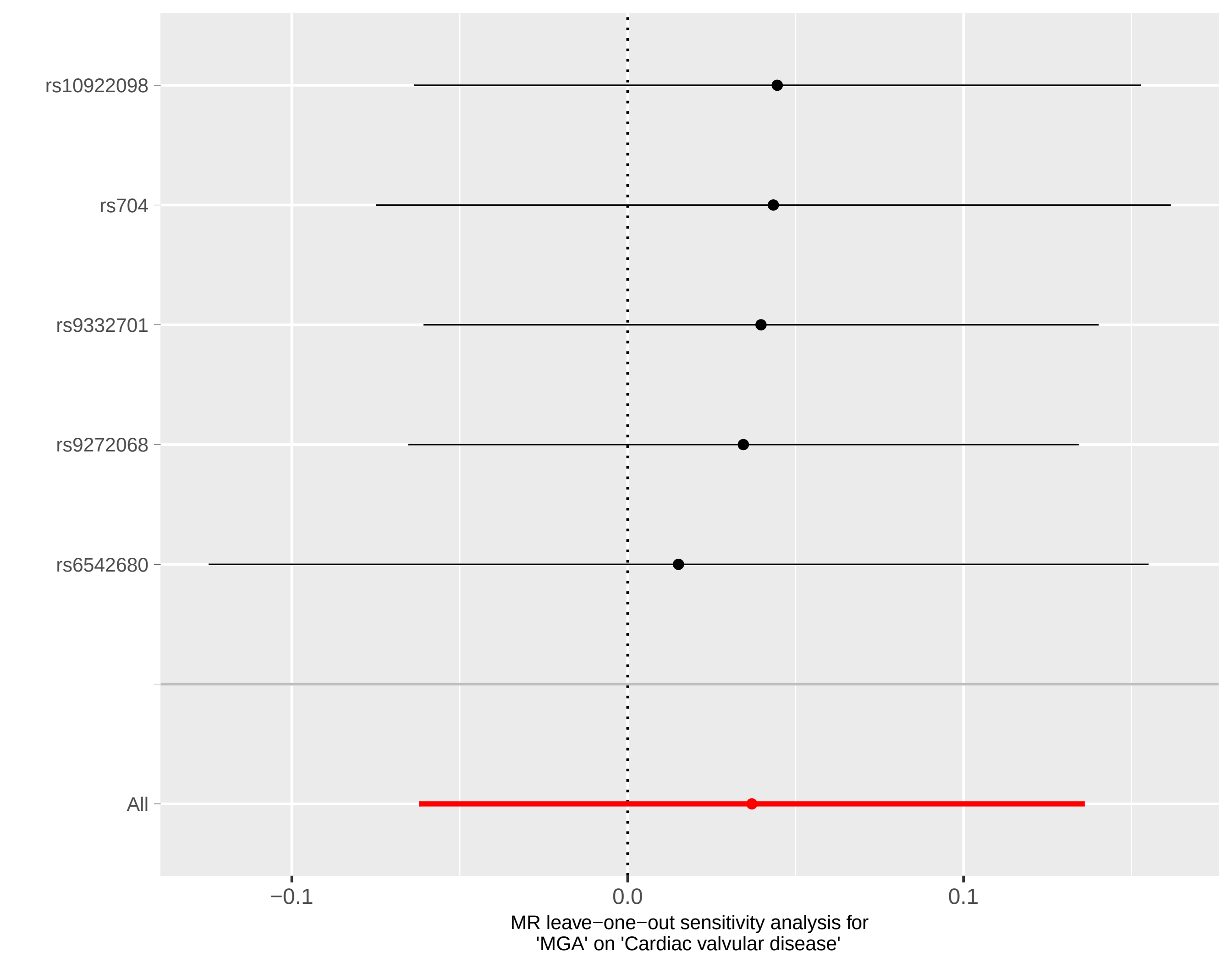
rs10922098
rs704
rs9332701
rs9272068
rs6542680
All
−0.1
0.0
0.1
MR leave−one−out sensitivity analysis for
'MGA' on 'Cardiac valvular disease'

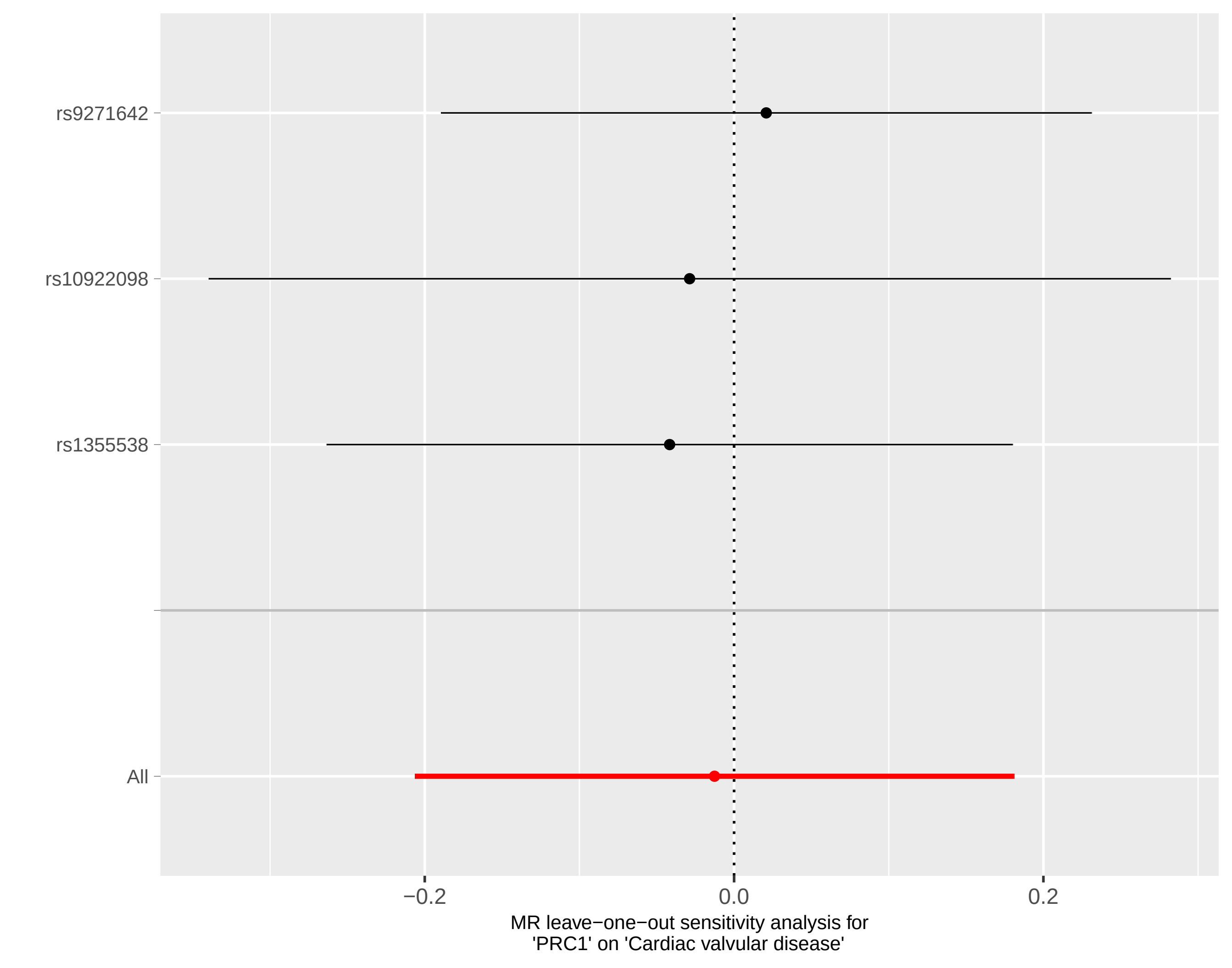

rs9271642
rs10922098
rs1355538
All
−0.2
0.0
0.2
MR leave−one−out sensitivity analysis for
'PRC1' on 'Cardiac valvular disease'

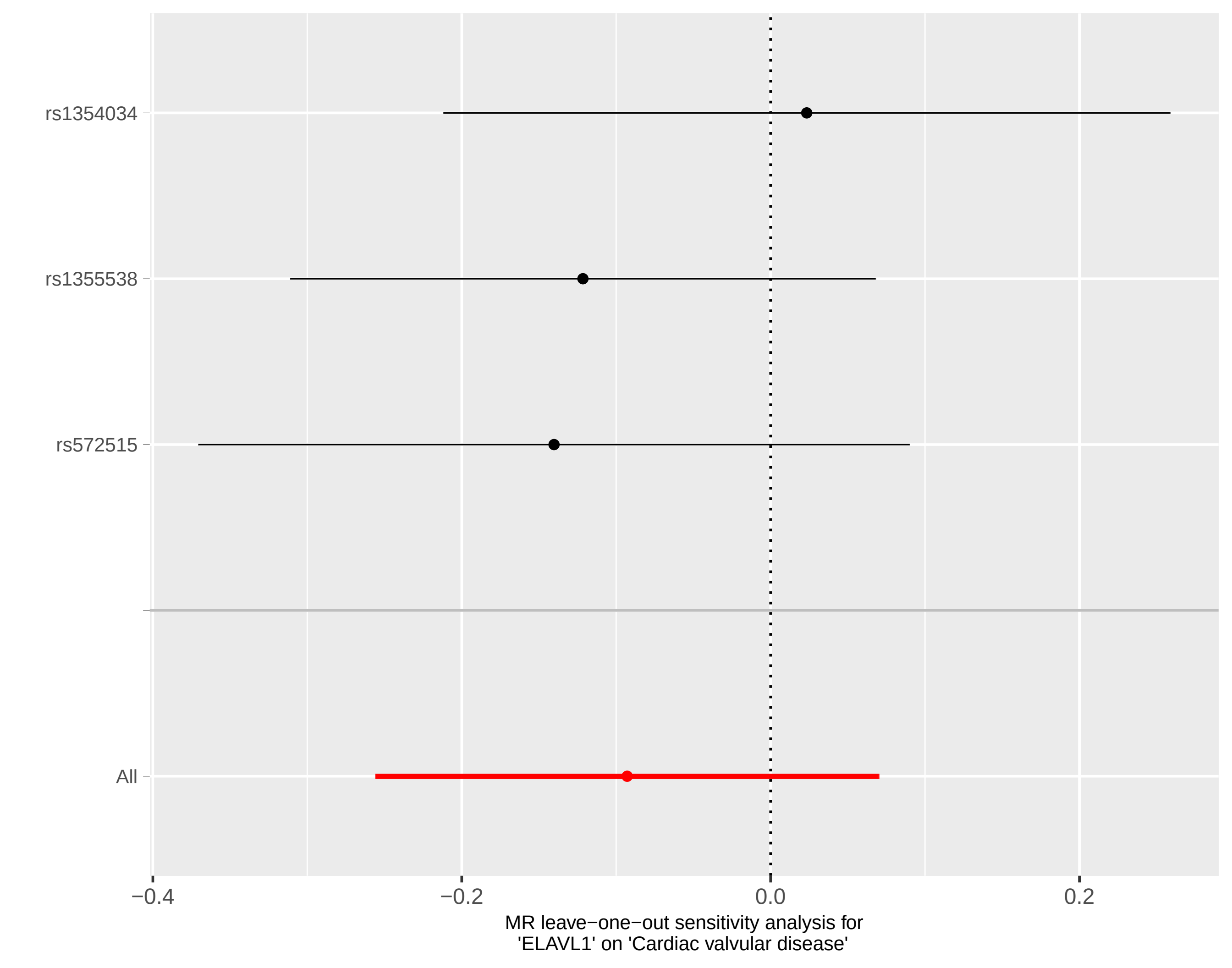
rs1354034
rs1355538
rs572515
All
−0.4
−0.2
0.0
0.2
MR leave−one−out sensitivity analysis for
'ELAVL1' on 'Cardiac valvular disease'

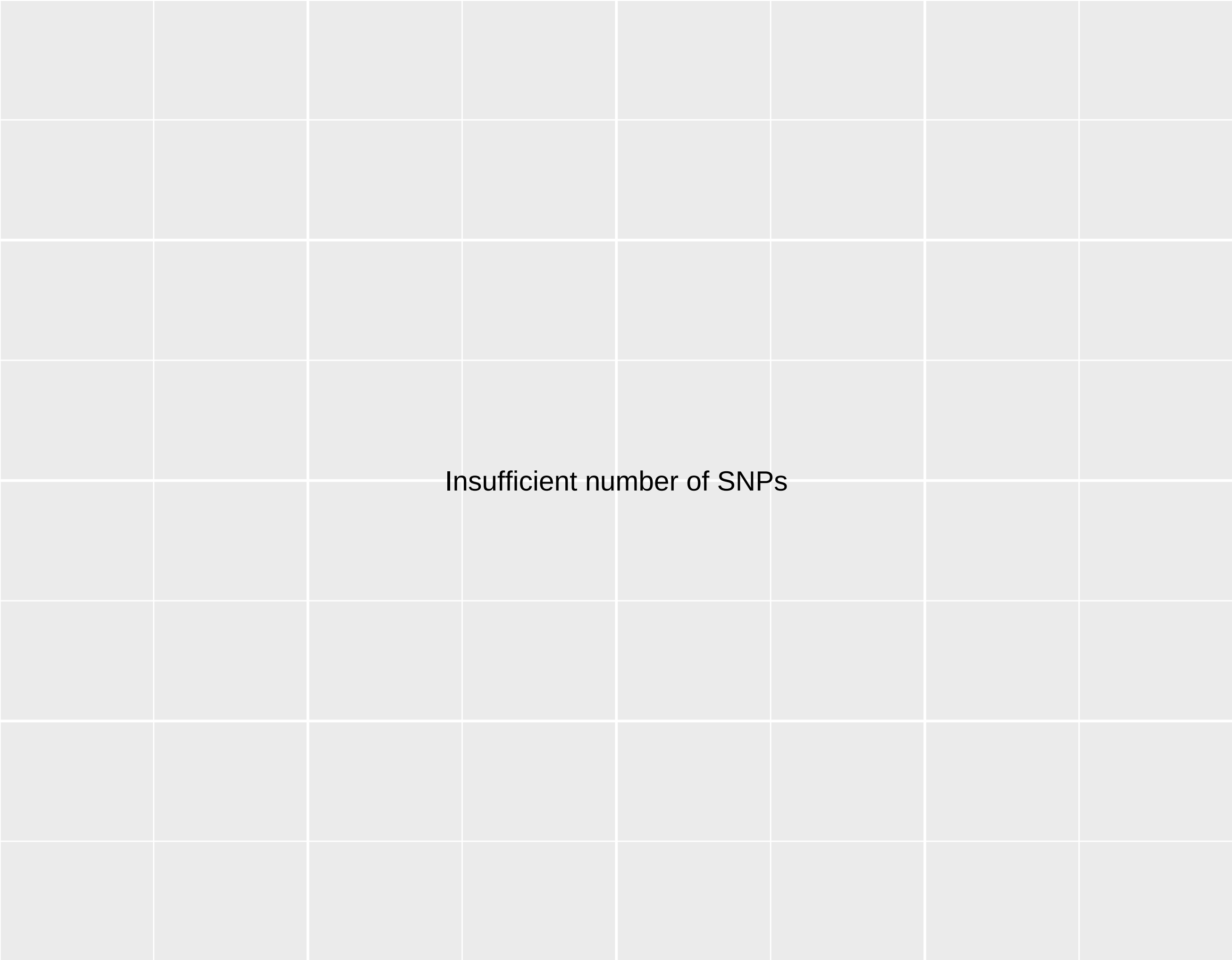
Insufficient number of SNPs

Insufficient number of SNPs

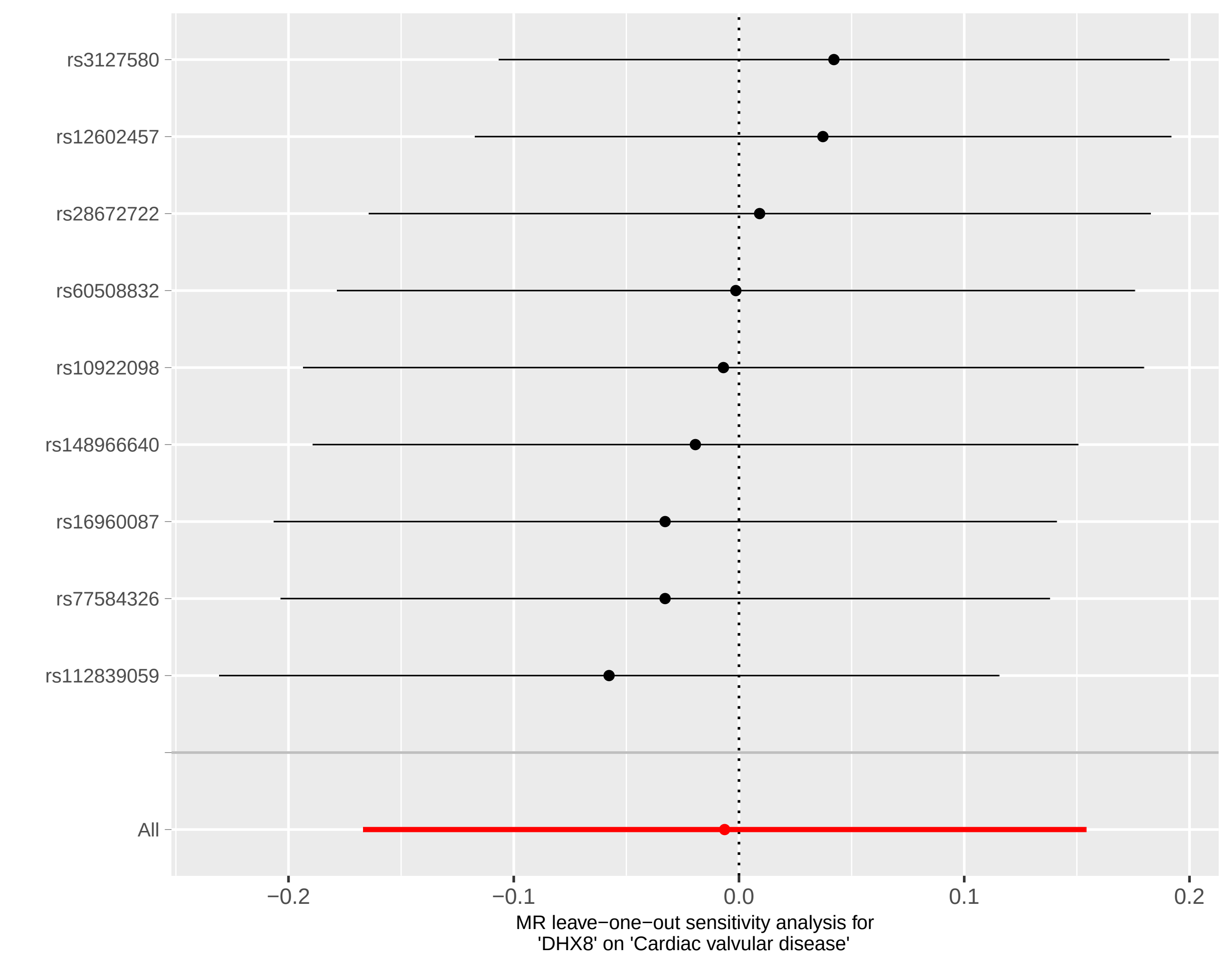
rs3127580
rs12602457
rs28672722
rs60508832
rs10922098
rs148966640
rs16960087
rs77584326
rs112839059
All
−0.2
−0.1
0.0
0.1
0.2
MR leave−one−out sensitivity analysis for
'DHX8' on 'Cardiac valvular disease'

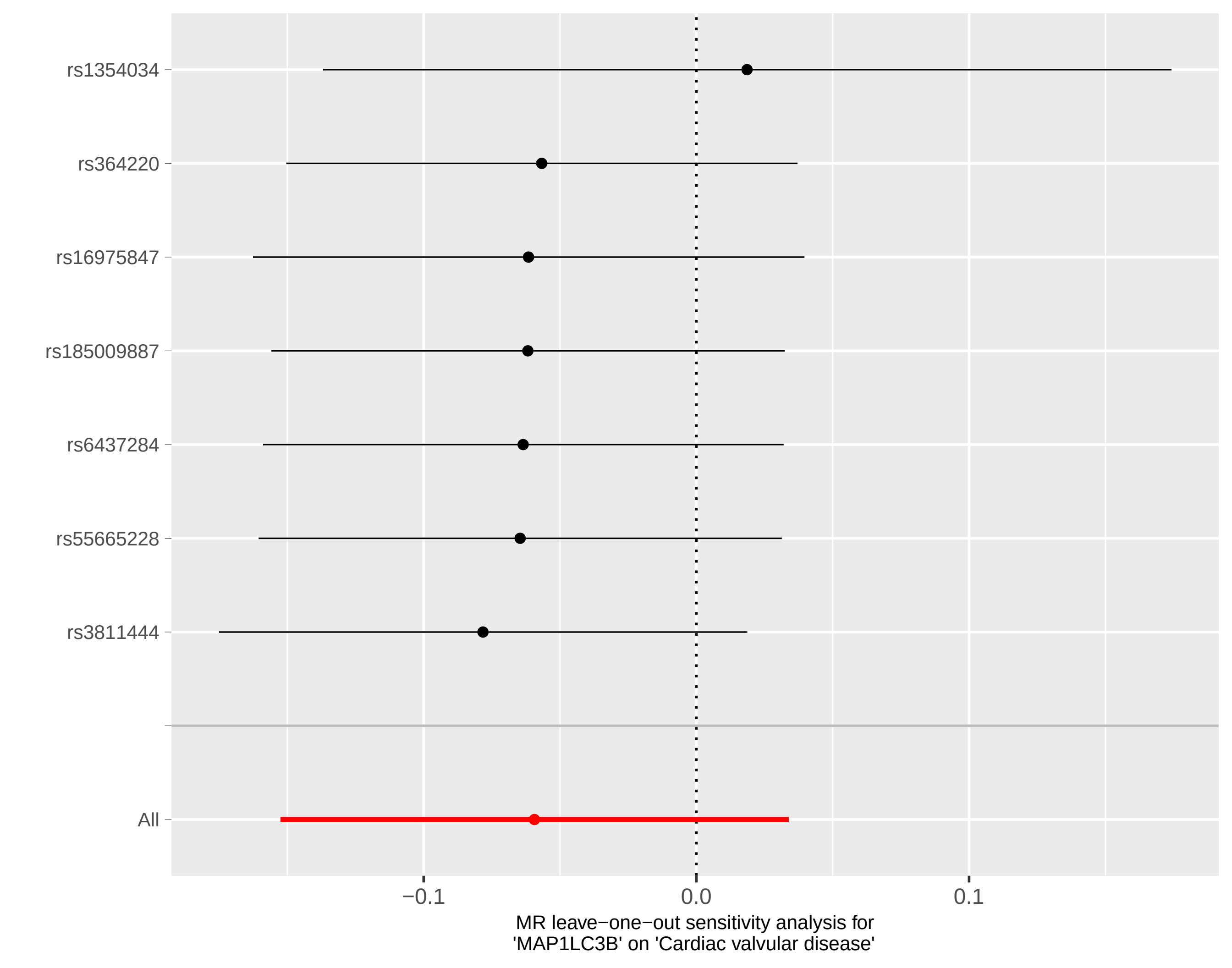

rs1354034
rs364220
rs16975847
rs185009887
rs6437284
rs55665228
rs3811444
All
−0.1
0.0
0.1
MR leave−one−out sensitivity analysis for
'MAP1LC3B' on 'Cardiac valvular disease'

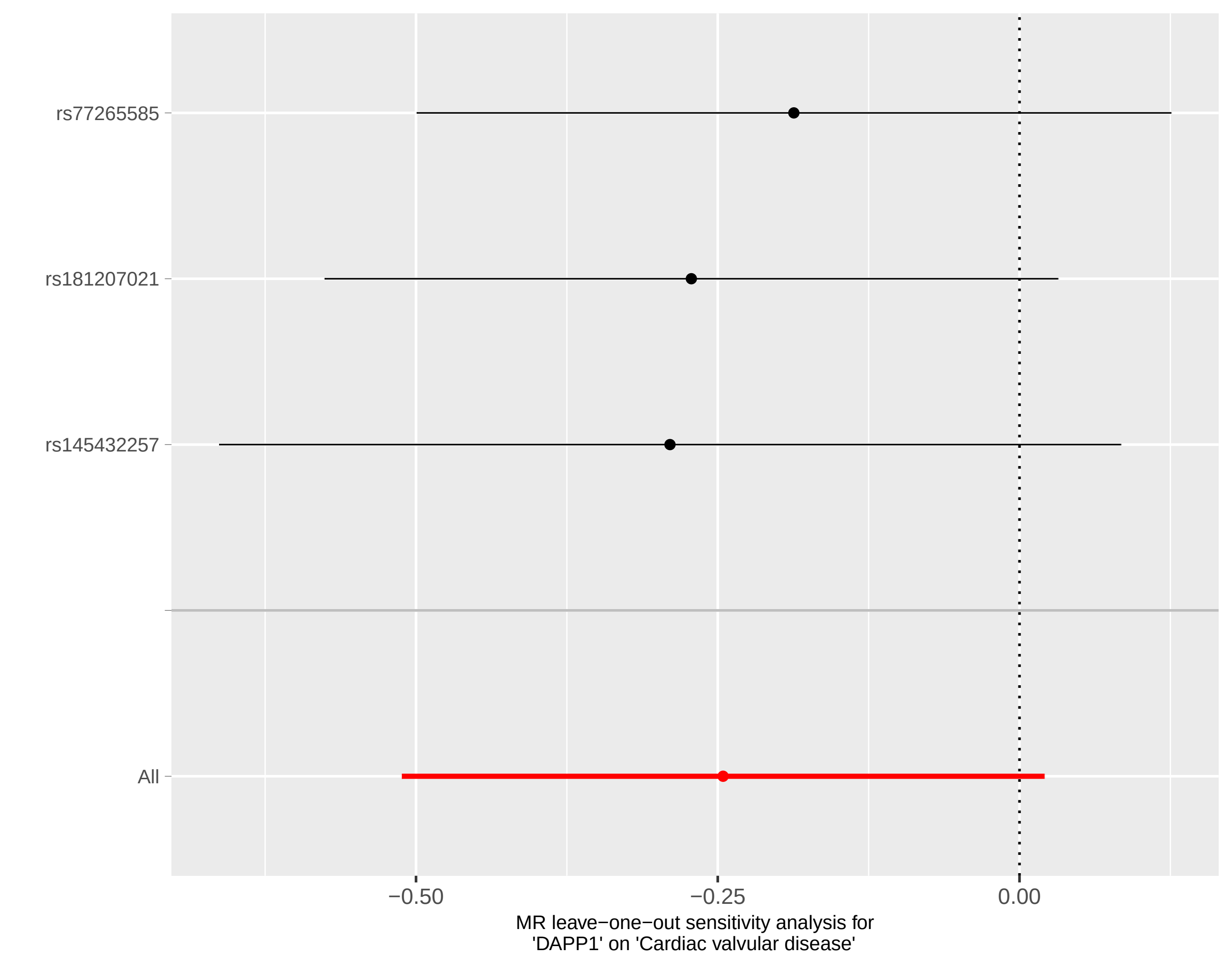
rs77265585
rs181207021
rs145432257
All
−0.50
−0.25
0.00
MR leave−one−out sensitivity analysis for
'DAPP1' on 'Cardiac valvular disease'

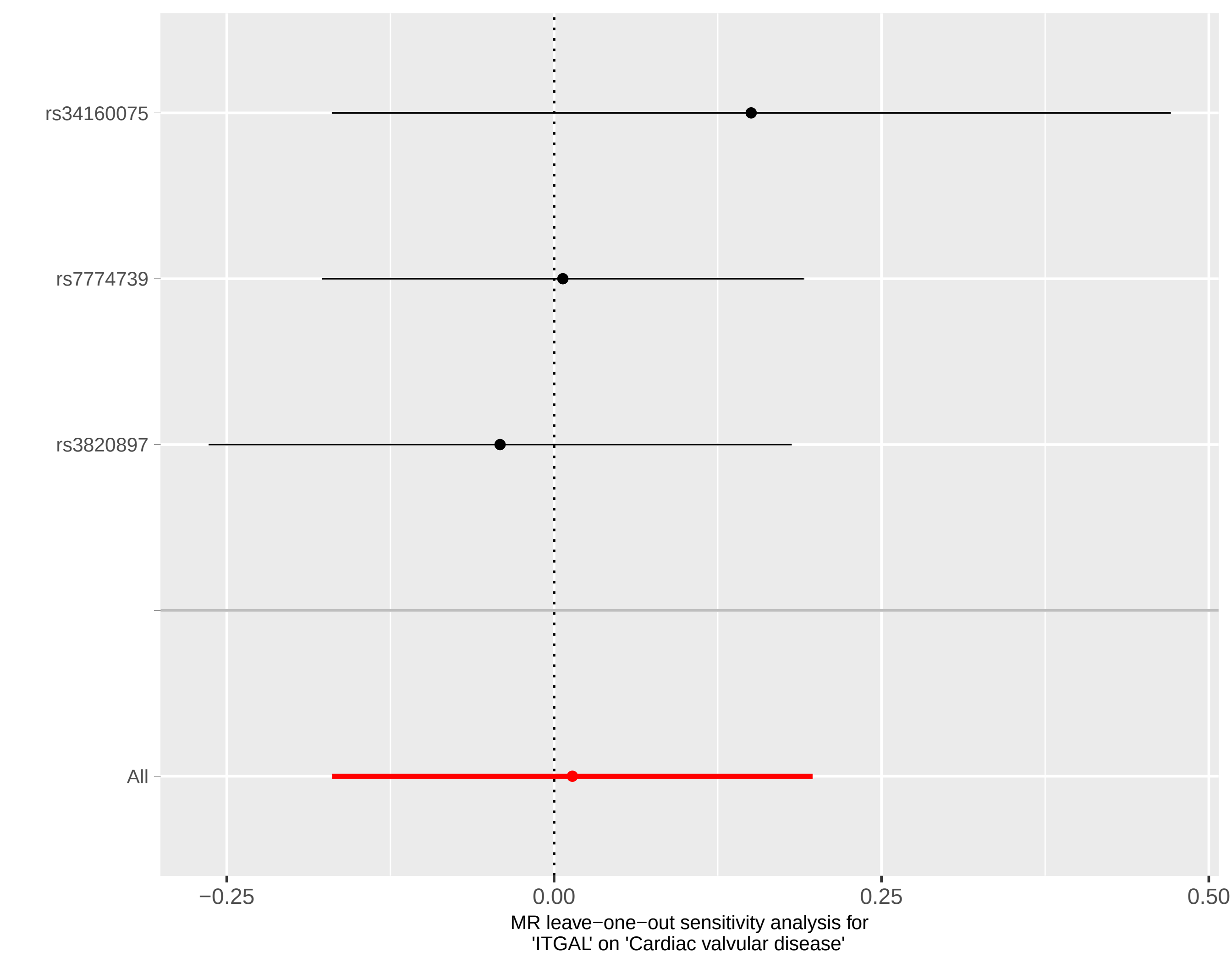
rs34160075
rs7774739
rs3820897
All
−0.25
0.00
0.25
0.50
MR leave−one−out sensitivity analysis for
'ITGAL' on 'Cardiac valvular disease'

Insufficient number of SNPs

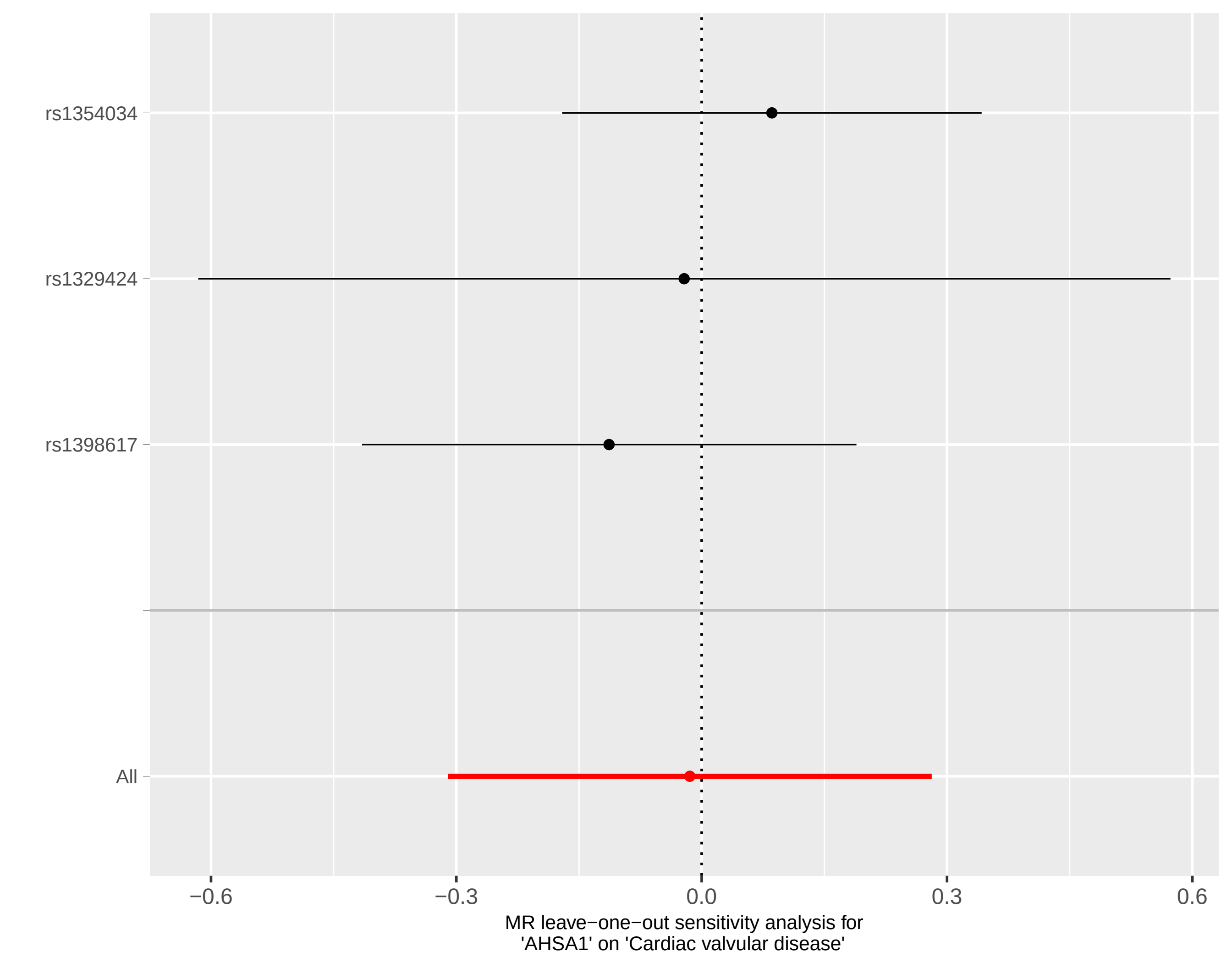
rs1354034
rs1329424
rs1398617
All
−0.6
−0.3
0.0
0.3
0.6
MR leave−one−out sensitivity analysis for
'AHSA1' on 'Cardiac valvular disease'

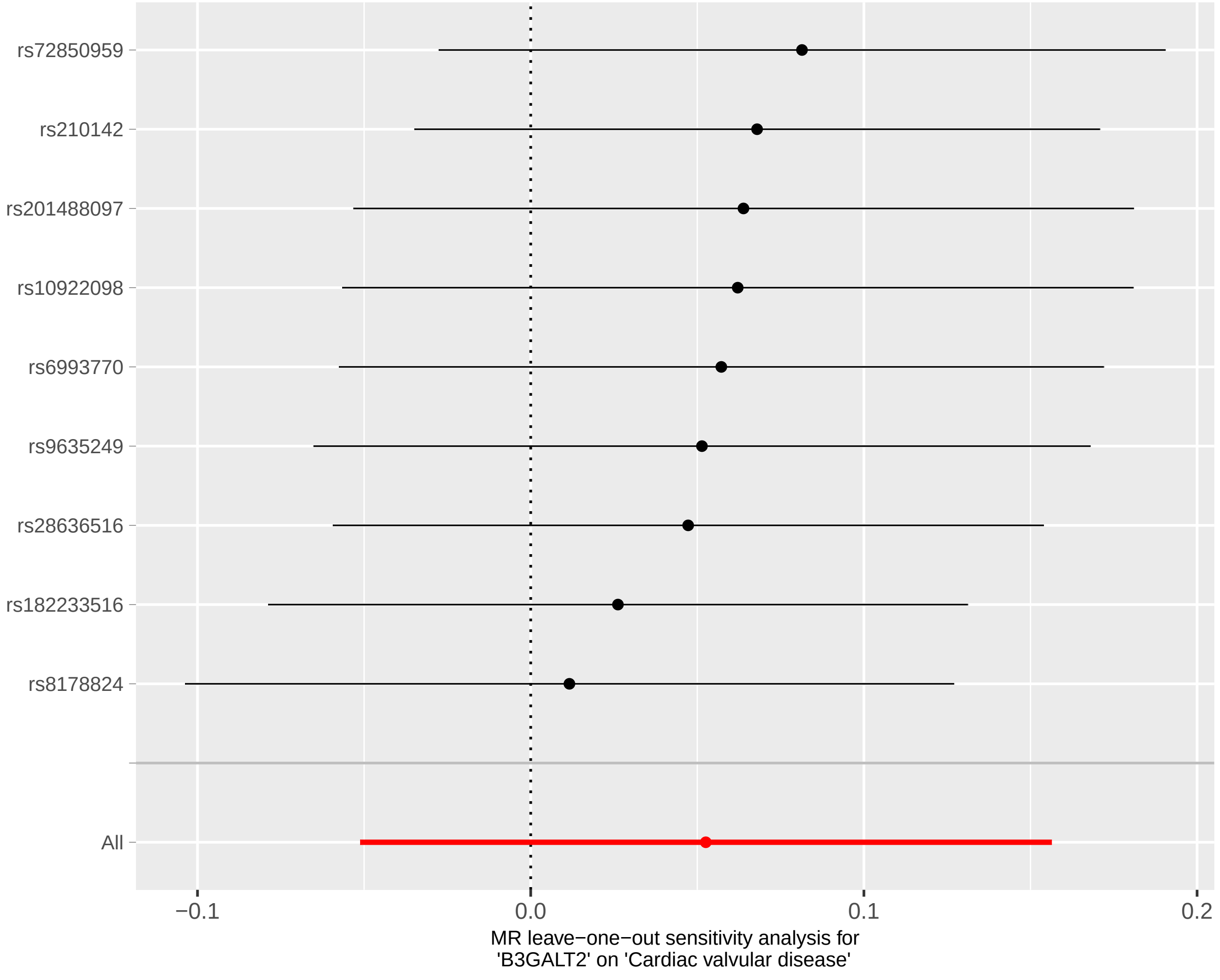
rs72850959
rs210142
rs201488097
rs10922098
rs6993770
rs9635249
rs28636516
rs182233516
rs8178824
All
−0.1
0.0
0.1
0.2
MR leave−one−out sensitivity analysis for
'B3GALT2' on 'Cardiac valvular disease'

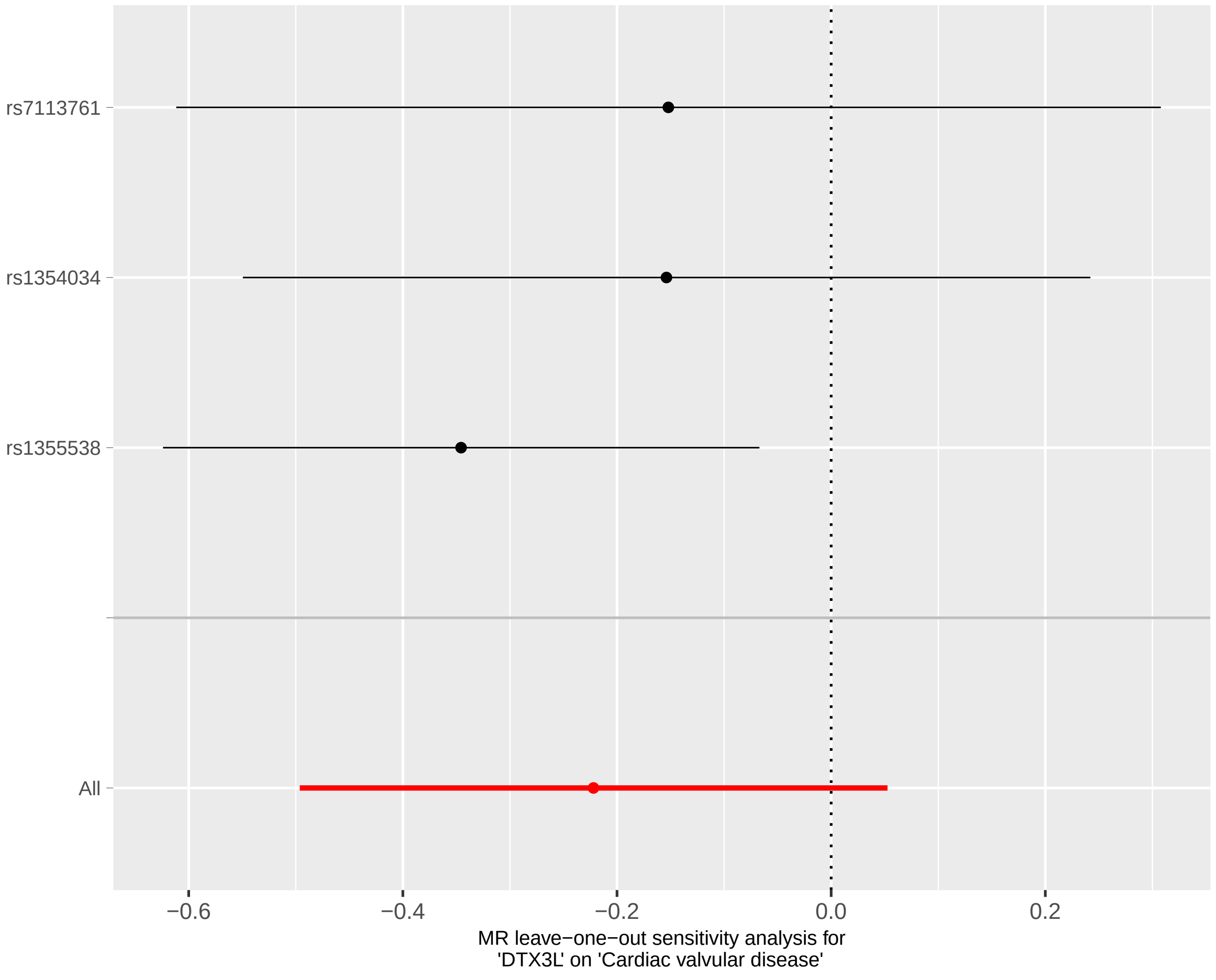

rs7113761
rs1354034
rs1355538
All
−0.6
−0.4
−0.2
0.0
0.2
MR leave−one−out sensitivity analysis for
'DTX3L' on 'Cardiac valvular disease'

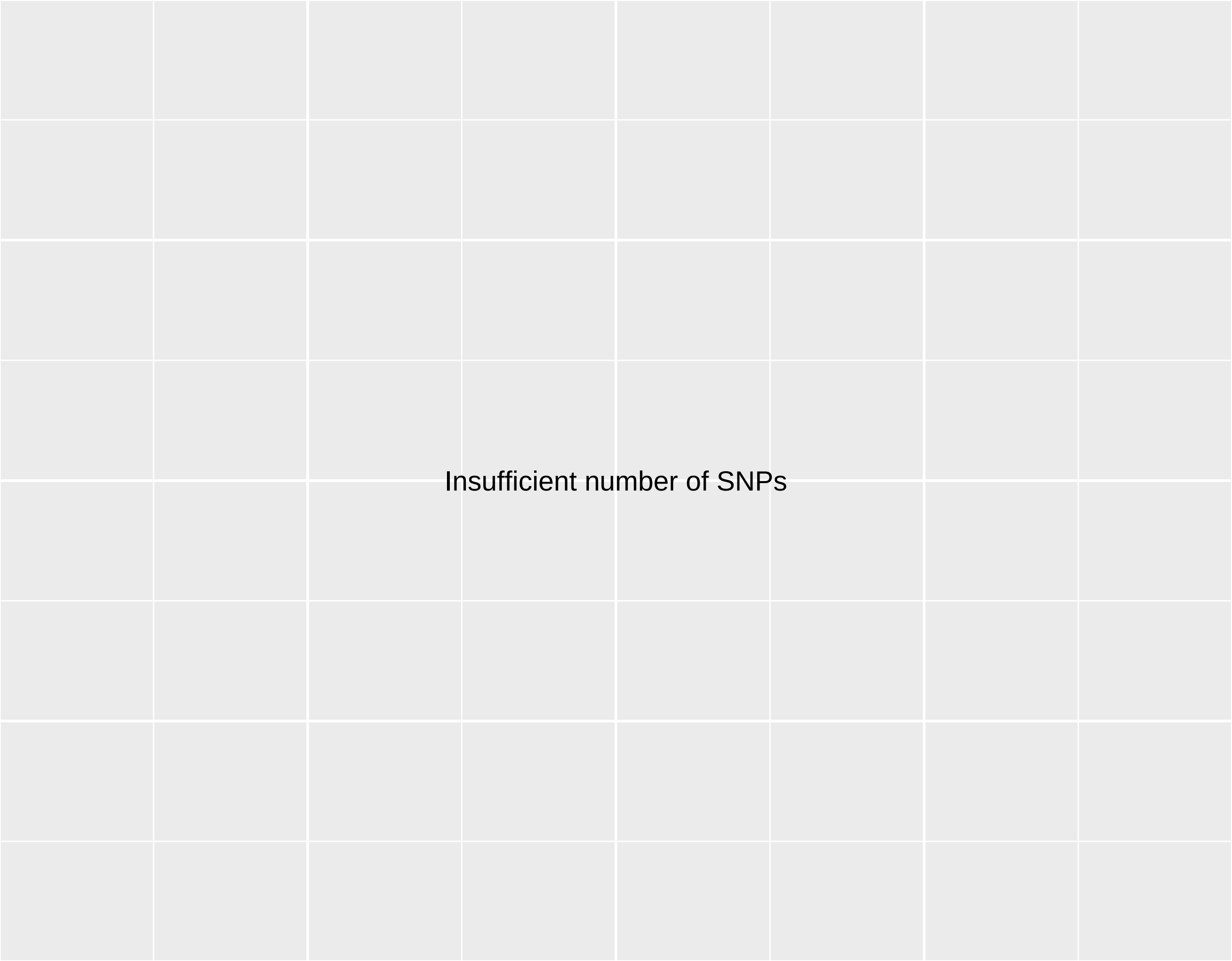
Insufficient number of SNPs

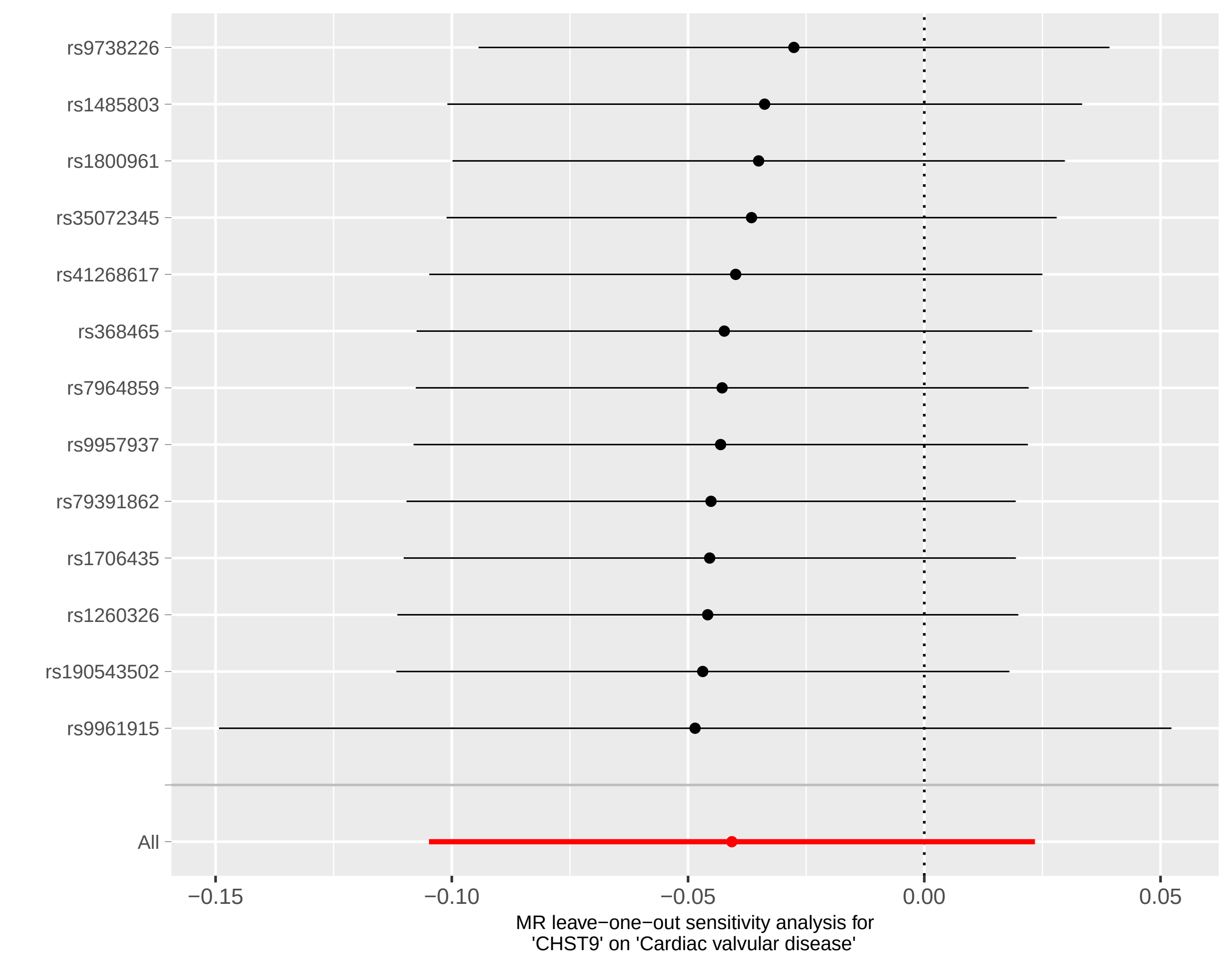
rs9738226
rs1485803
rs1800961
rs35072345
rs41268617
rs368465
rs7964859
rs9957937
rs79391862
rs1706435
rs1260326
rs190543502
rs9961915
All
−0.15
−0.10
−0.05
0.00
0.05
MR leave−one−out sensitivity analysis for
'CHST9' on 'Cardiac valvular disease'

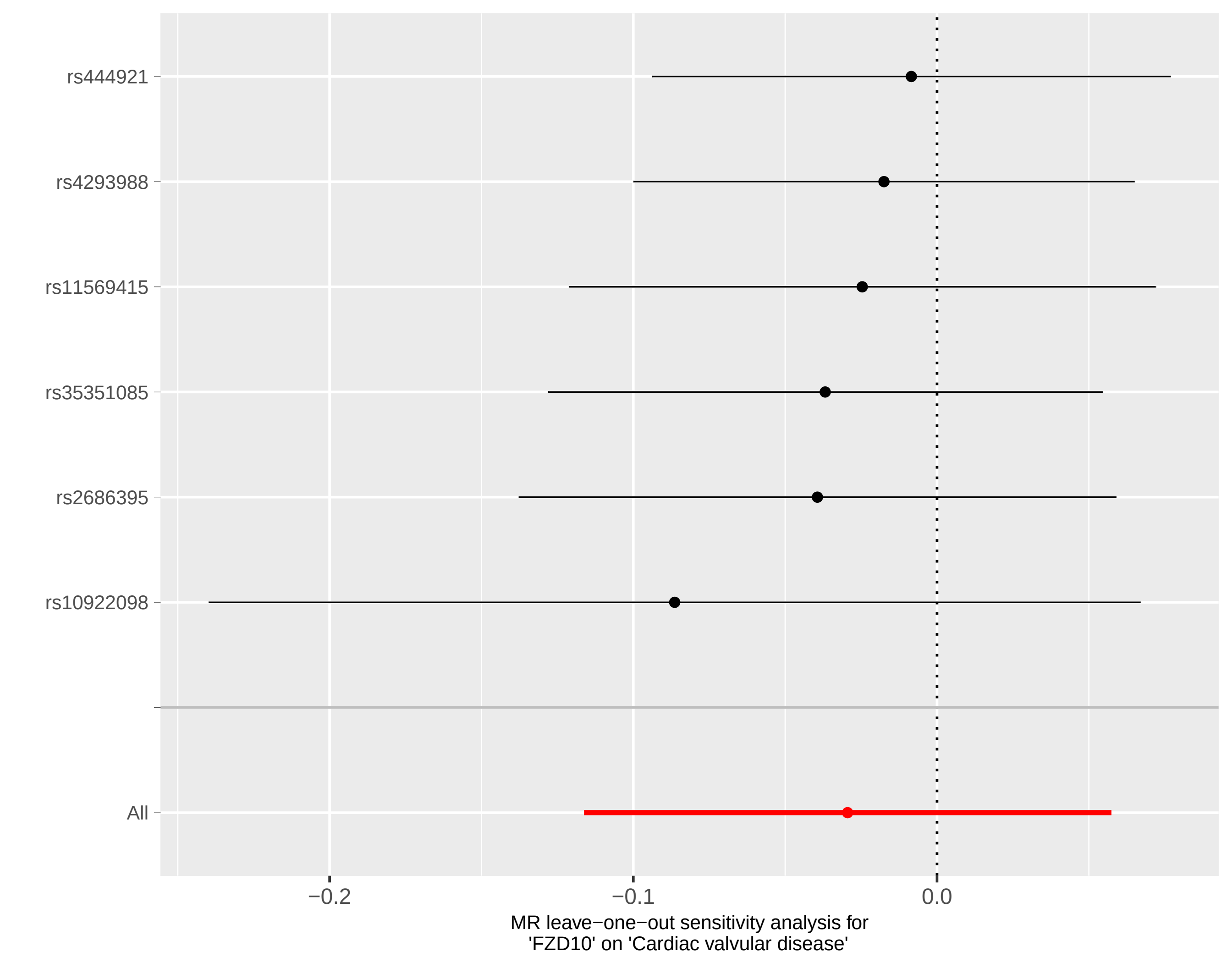
rs444921
rs4293988
rs11569415
rs35351085
rs2686395
rs10922098
All
−0.2
−0.1
0.0
MR leave−one−out sensitivity analysis for
'FZD10' on 'Cardiac valvular disease'

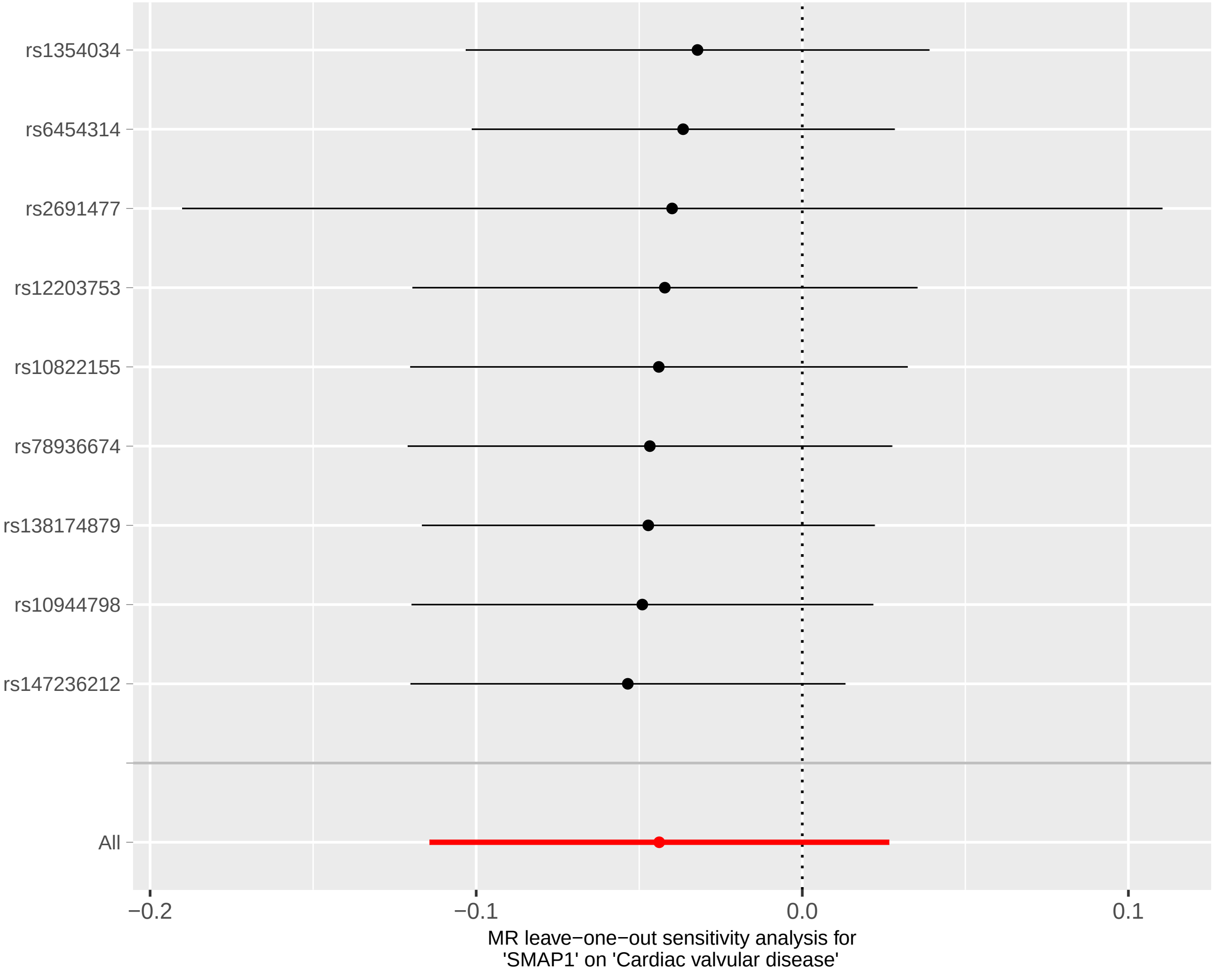
rs1354034
rs6454314
rs2691477
rs12203753
rs10822155
rs78936674
rs138174879
rs10944798
rs147236212
All
−0.2
−0.1
0.0
0.1
MR leave−one−out sensitivity analysis for
'SMAP1' on 'Cardiac valvular disease'

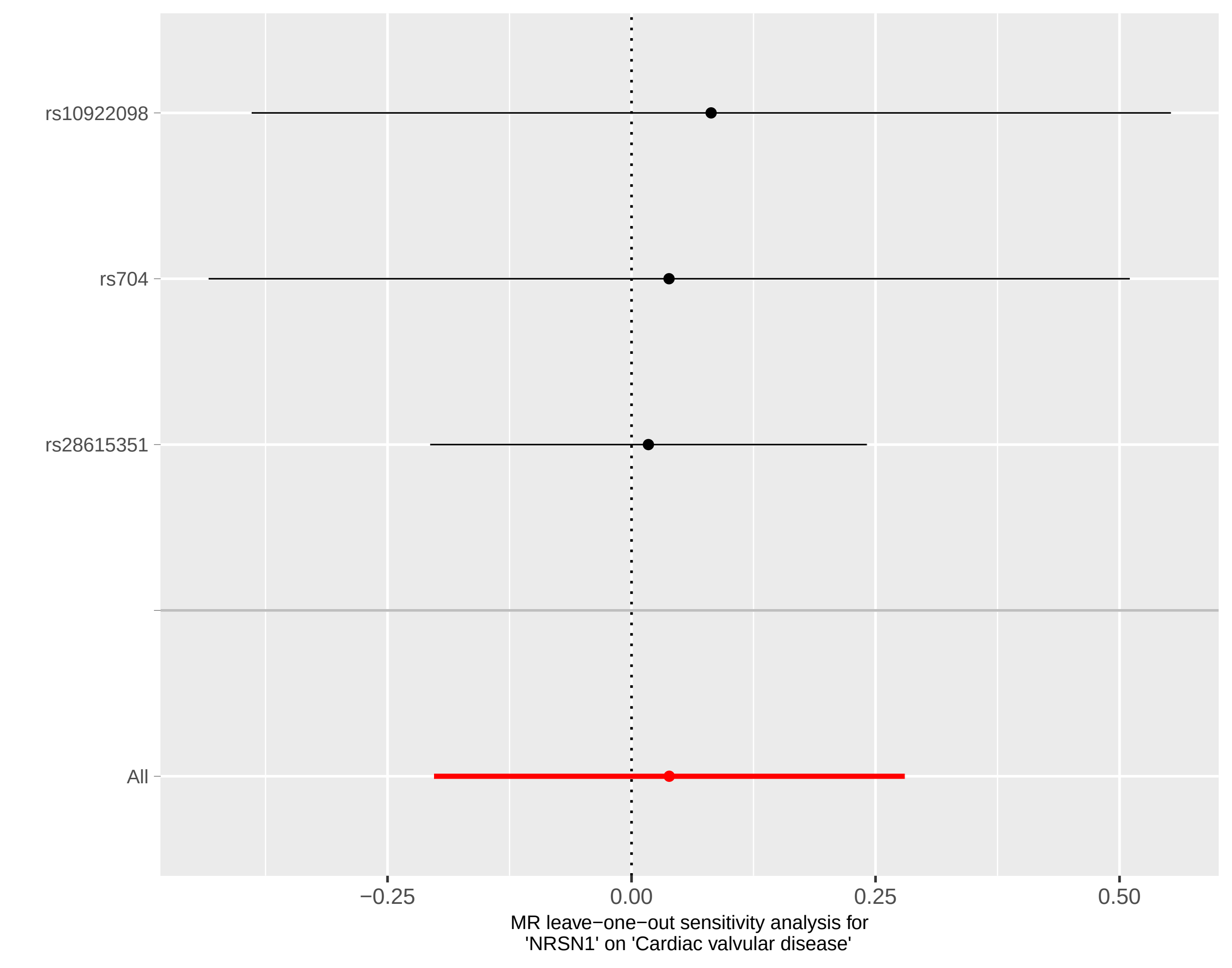

rs10922098
rs704
rs28615351
All
−0.25
0.00
0.25
0.50
MR leave−one−out sensitivity analysis for
'NRSN1' on 'Cardiac valvular disease'

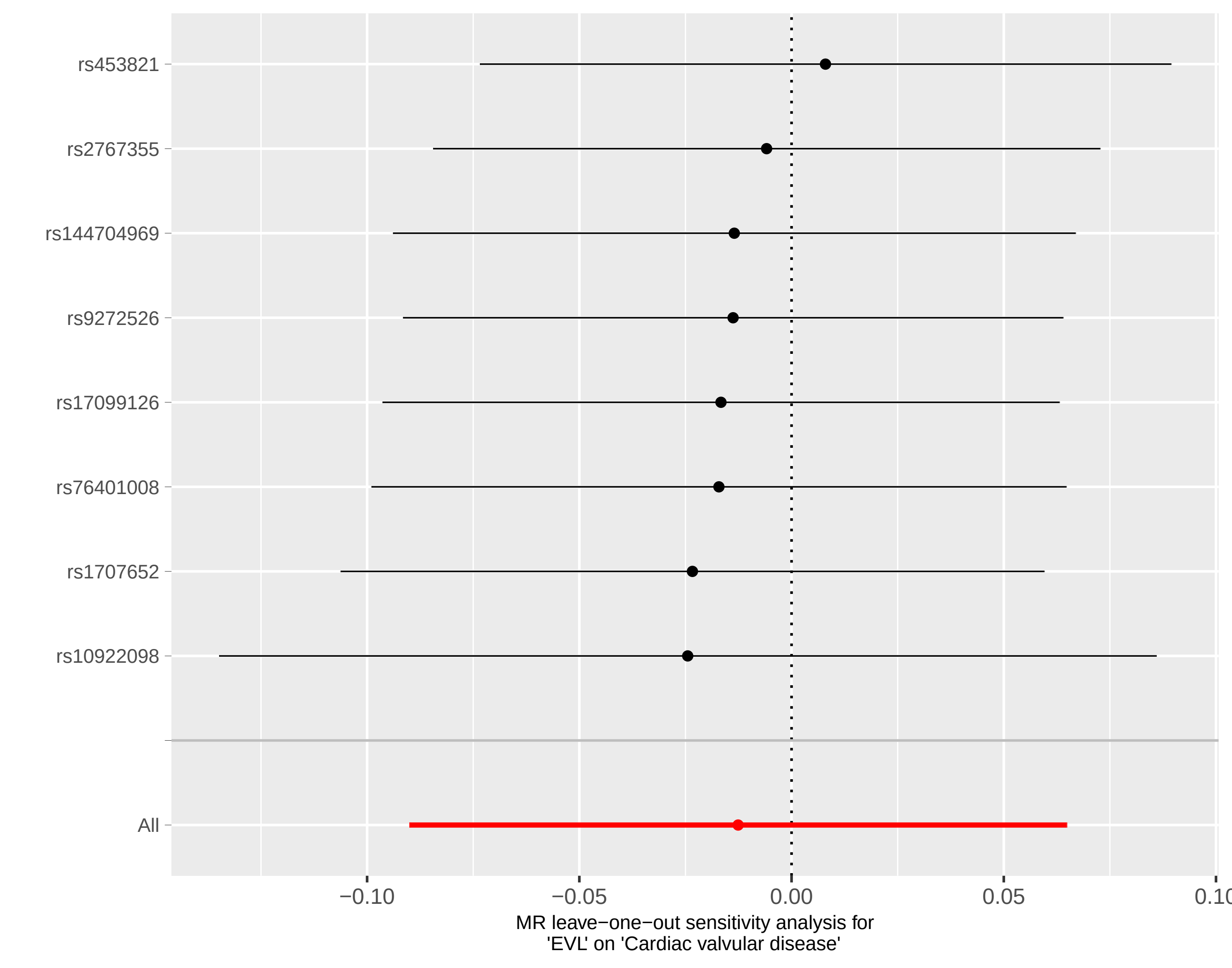

rs453821
rs2767355
rs144704969
rs9272526
rs17099126
rs76401008
rs1707652
rs10922098
All
−0.10
−0.05
0.00
0.05
0.10
MR leave−one−out sensitivity analysis for
'EVL' on 'Cardiac valvular disease'

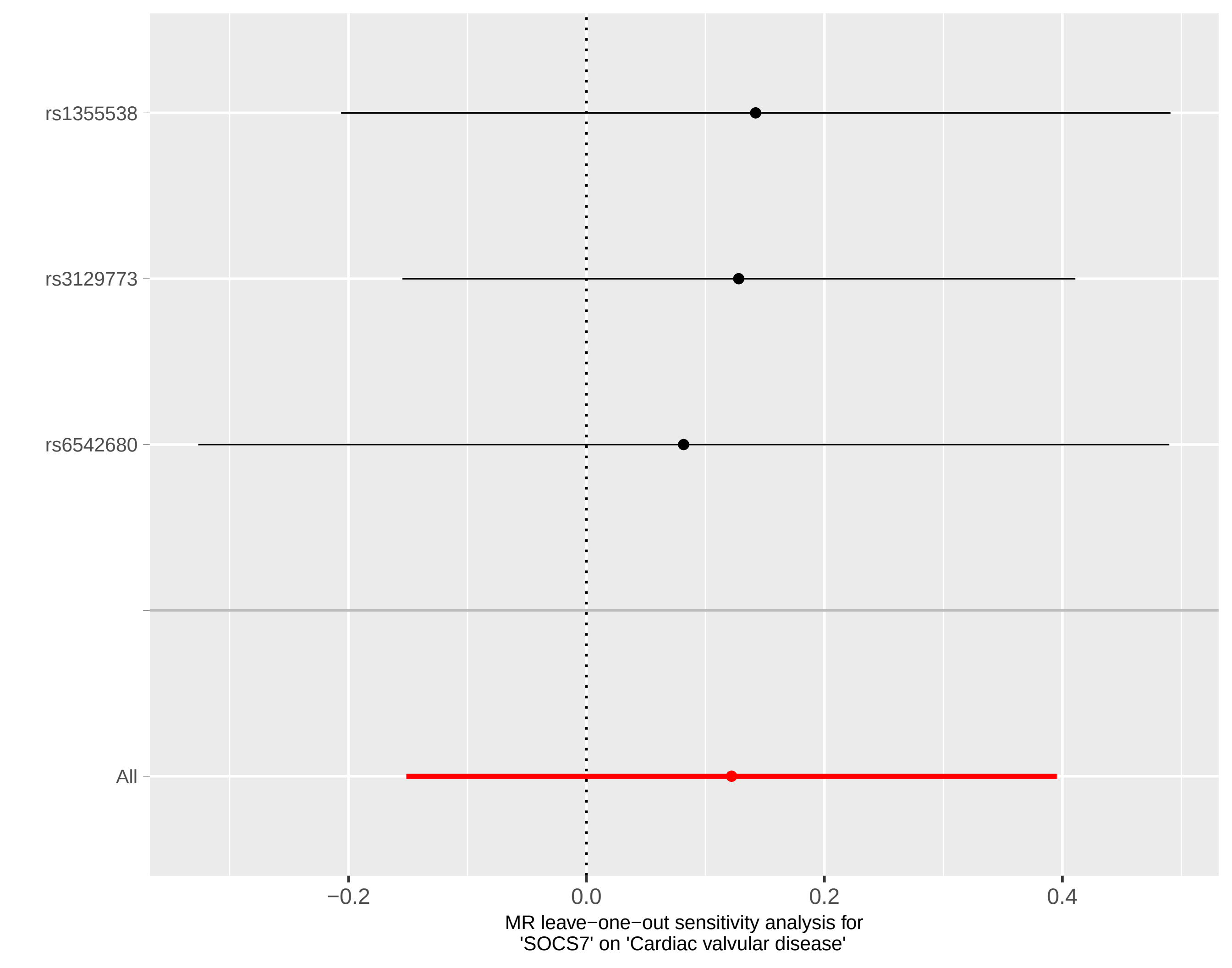
rs1355538
rs3129773
rs6542680
All
−0.2
0.0
0.2
0.4
MR leave−one−out sensitivity analysis for
'SOCS7' on 'Cardiac valvular disease'

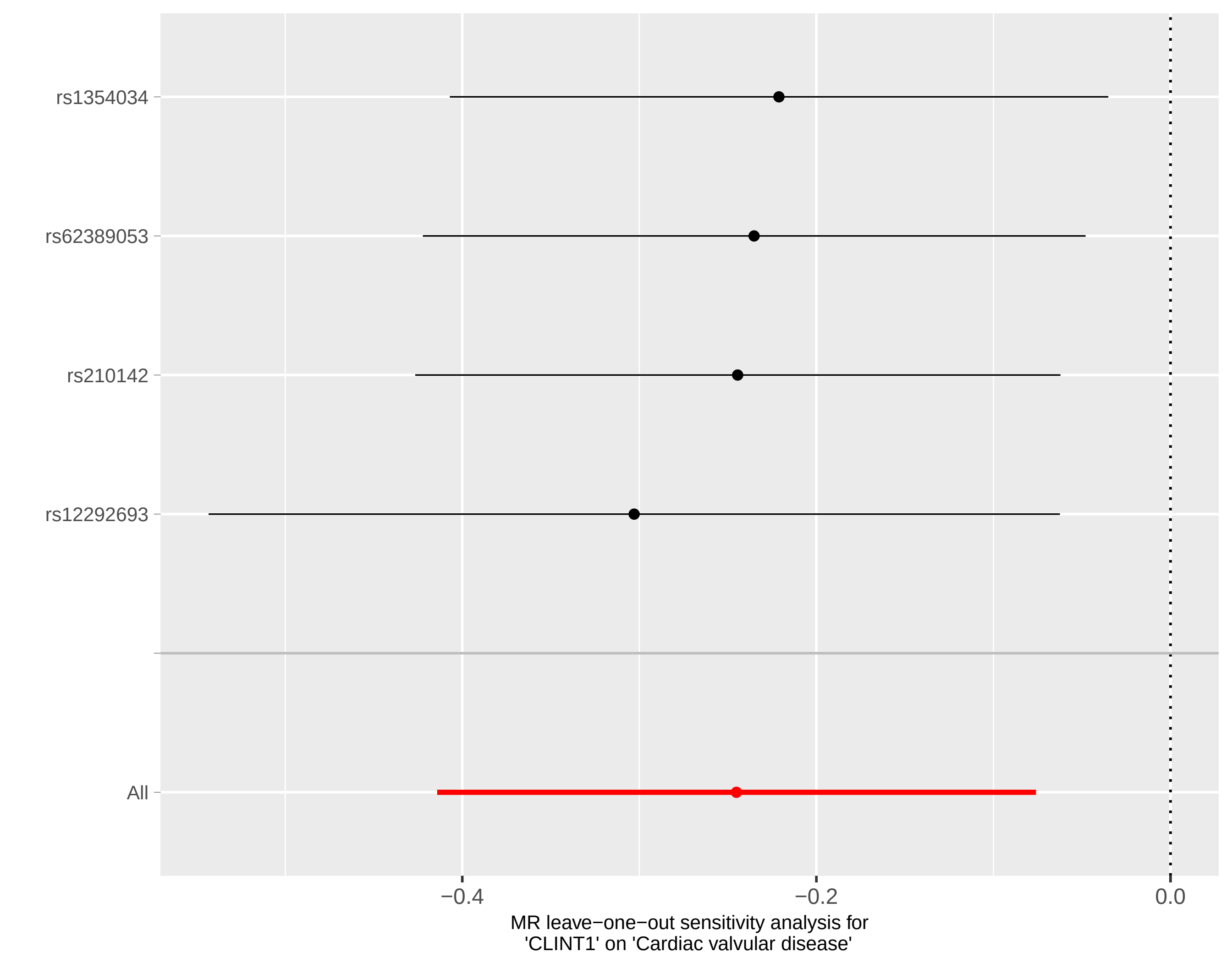
rs1354034
rs62389053
rs210142
rs12292693
All
−0.4
−0.2
0.0
MR leave−one−out sensitivity analysis for
'CLINT1' on 'Cardiac valvular disease'

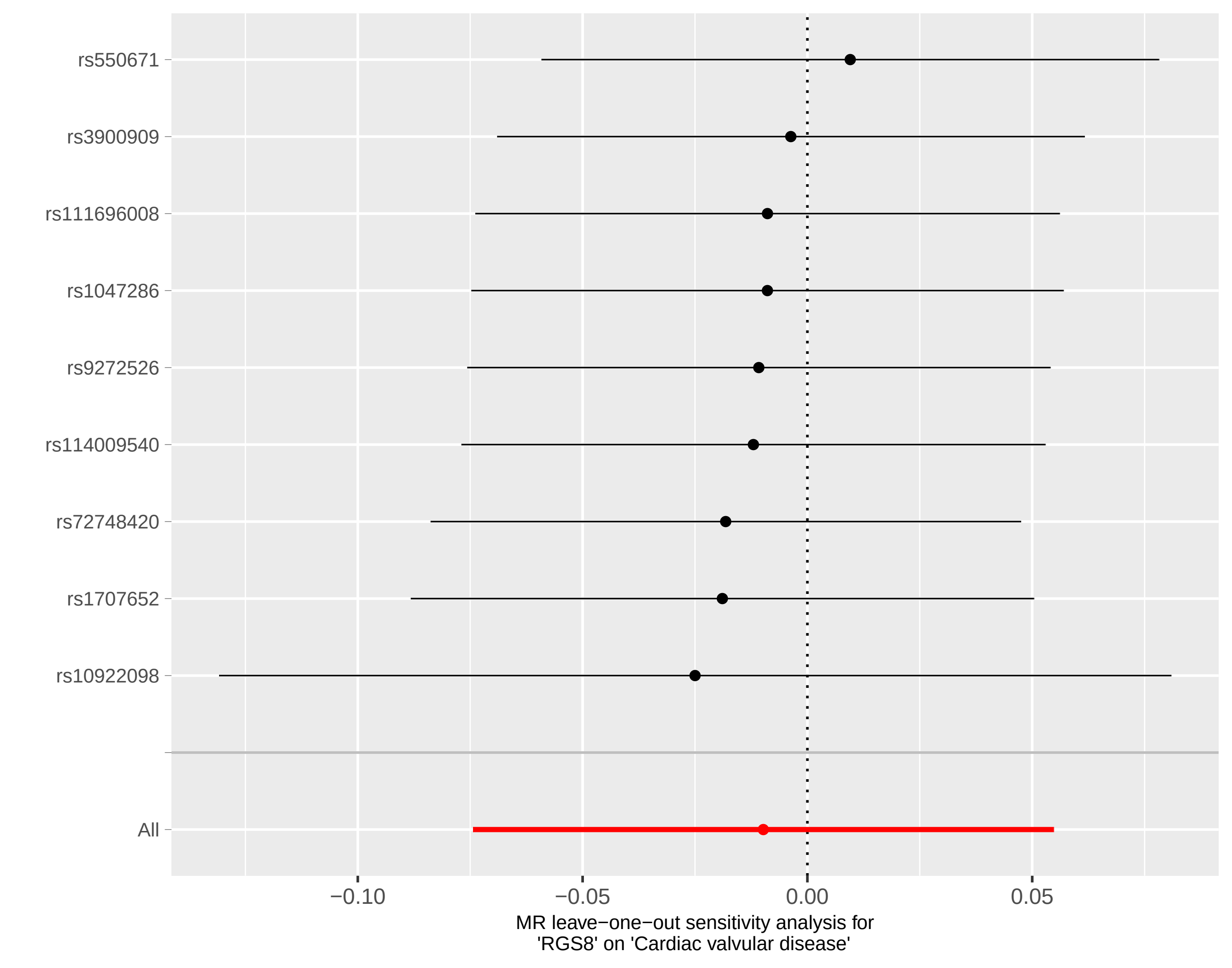

rs550671
rs3900909
rs111696008
rs1047286
rs9272526
rs114009540
rs72748420
rs1707652
rs10922098
All
−0.10
−0.05
0.00
0.05
MR leave−one−out sensitivity analysis for
'RGS8' on 'Cardiac valvular disease'

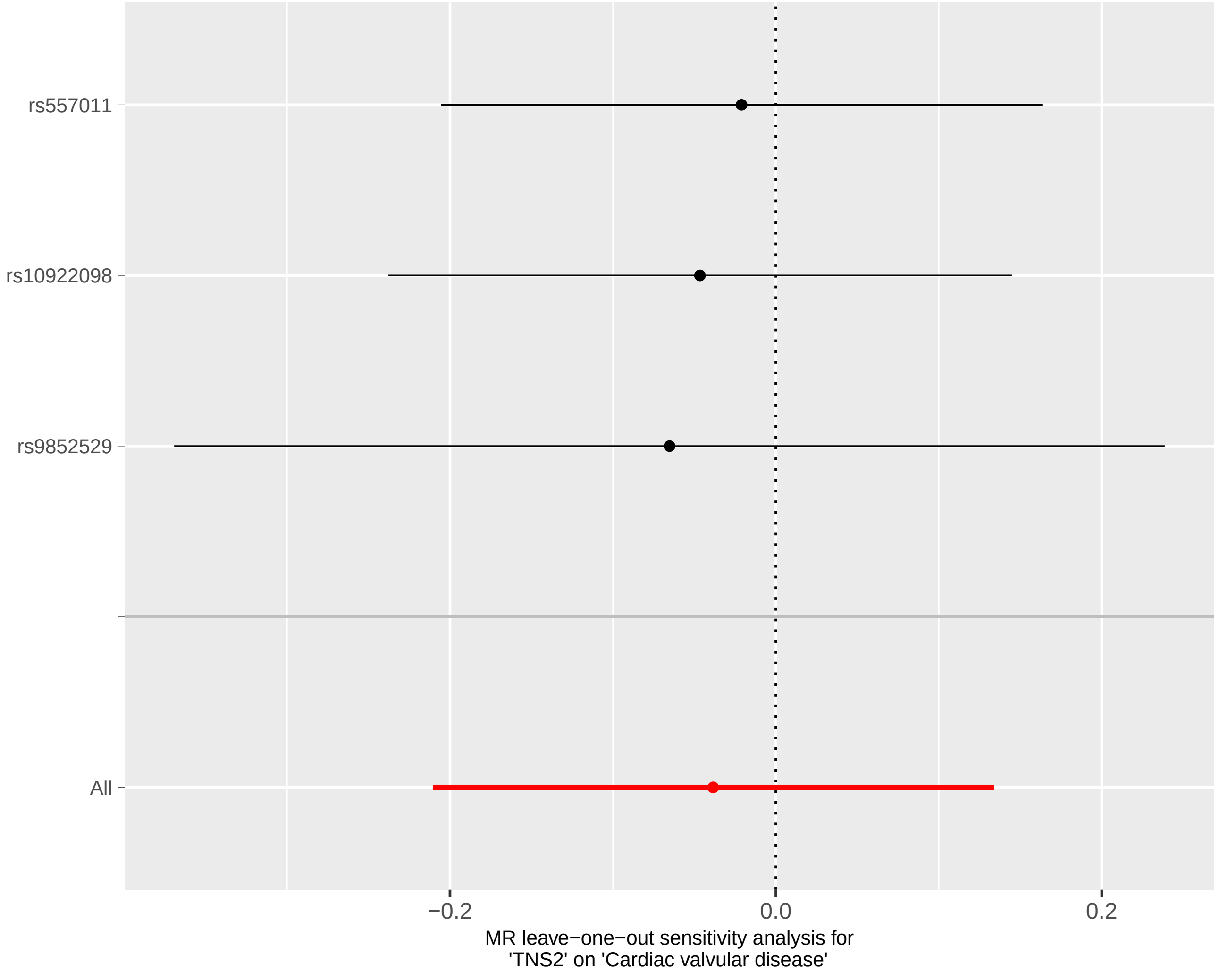

rs557011
rs10922098
rs9852529
All
−0.2
0.0
0.2
MR leave−one−out sensitivity analysis for
'TNS2' on 'Cardiac valvular disease'

Insufficient number of SNPs

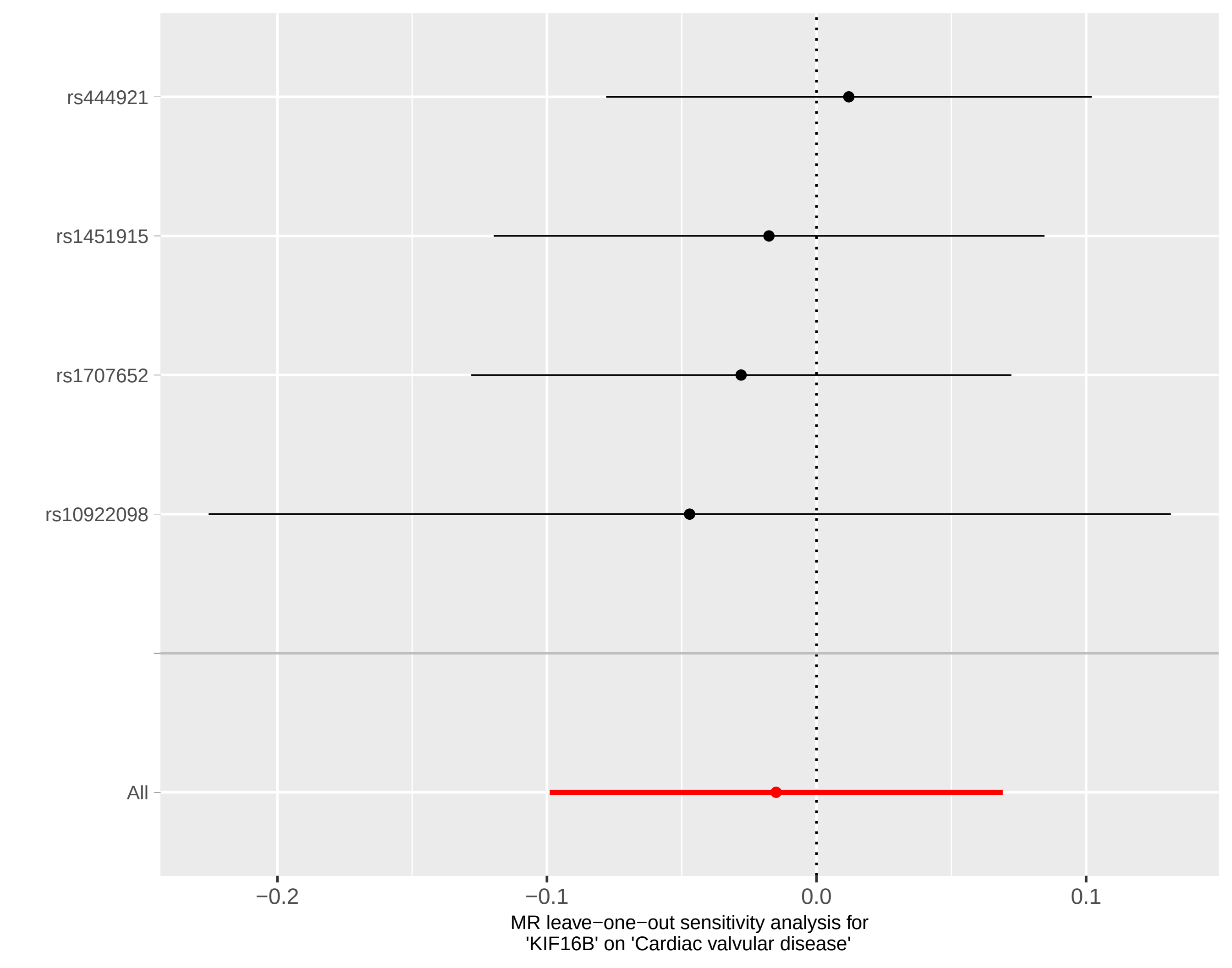

rs444921
rs1451915
rs1707652
rs10922098
All
−0.2
−0.1
0.0
0.1
MR leave−one−out sensitivity analysis for
'KIF16B' on 'Cardiac valvular disease'

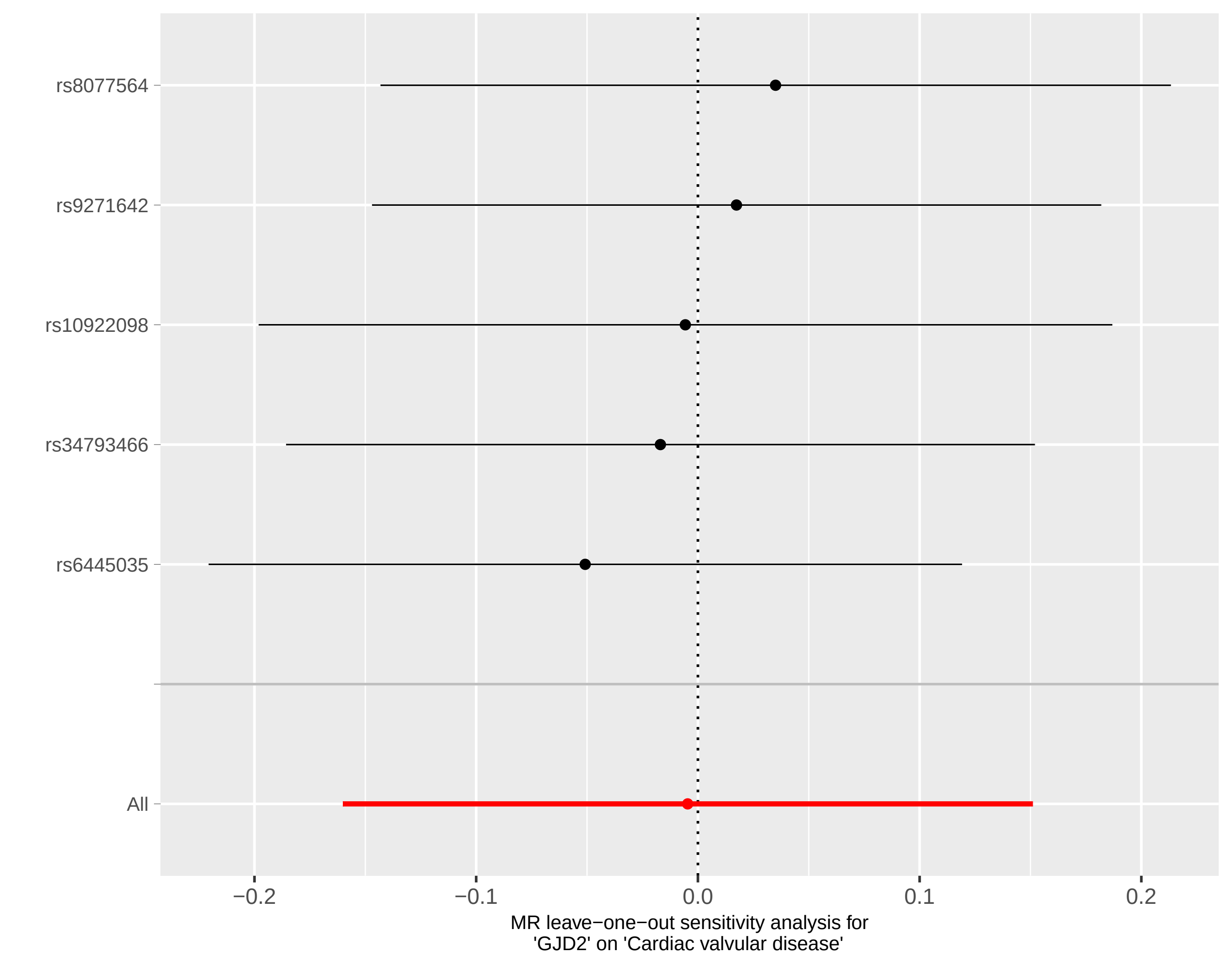
rs8077564
rs9271642
rs10922098
rs34793466
rs6445035
All
−0.2
−0.1
0.0
0.1
0.2
MR leave−one−out sensitivity analysis for
'GJD2' on 'Cardiac valvular disease'

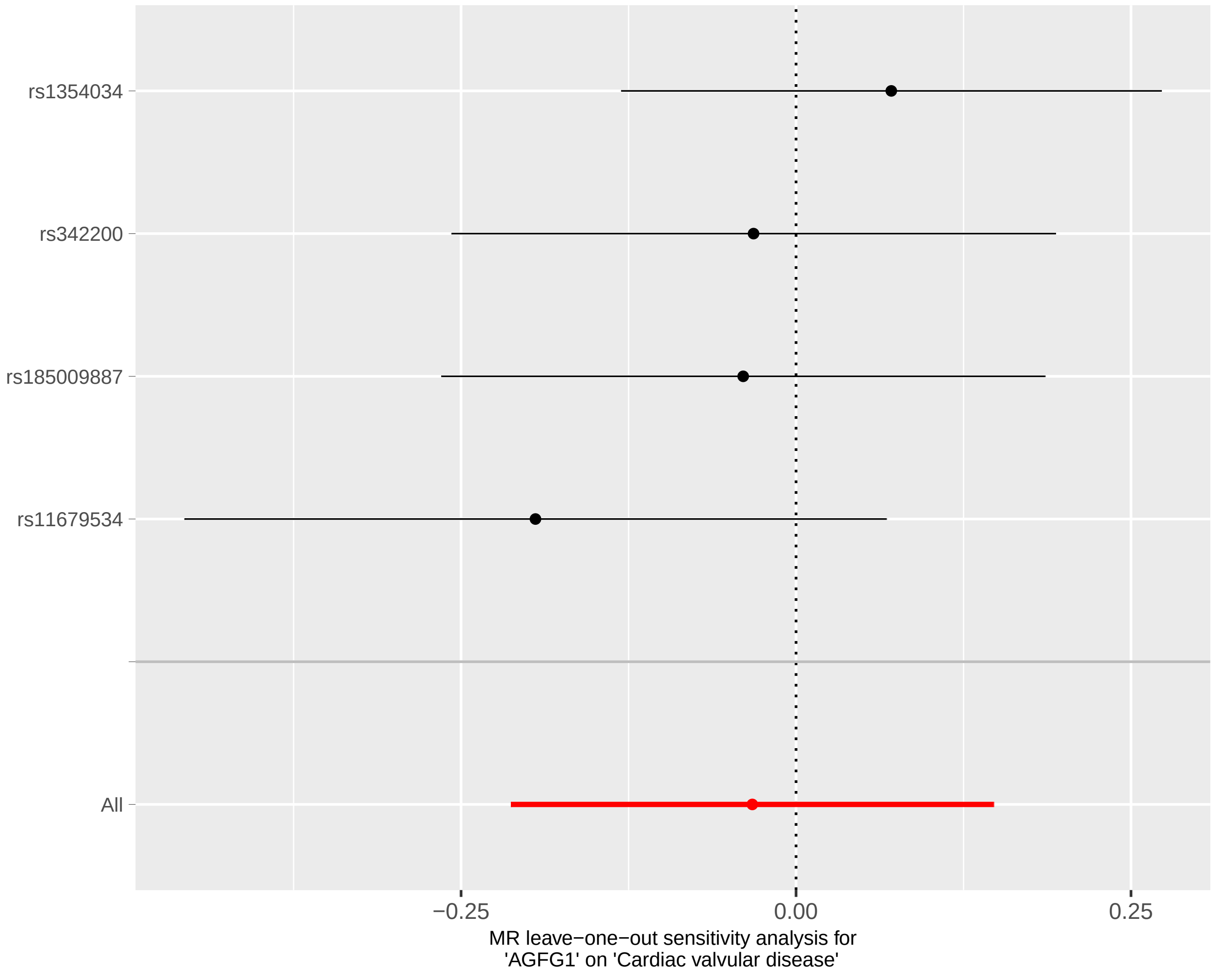
rs1354034
rs342200
rs185009887
rs11679534
All
−0.25
0.00
0.25
MR leave−one−out sensitivity analysis for
'AGFG1' on 'Cardiac valvular disease'

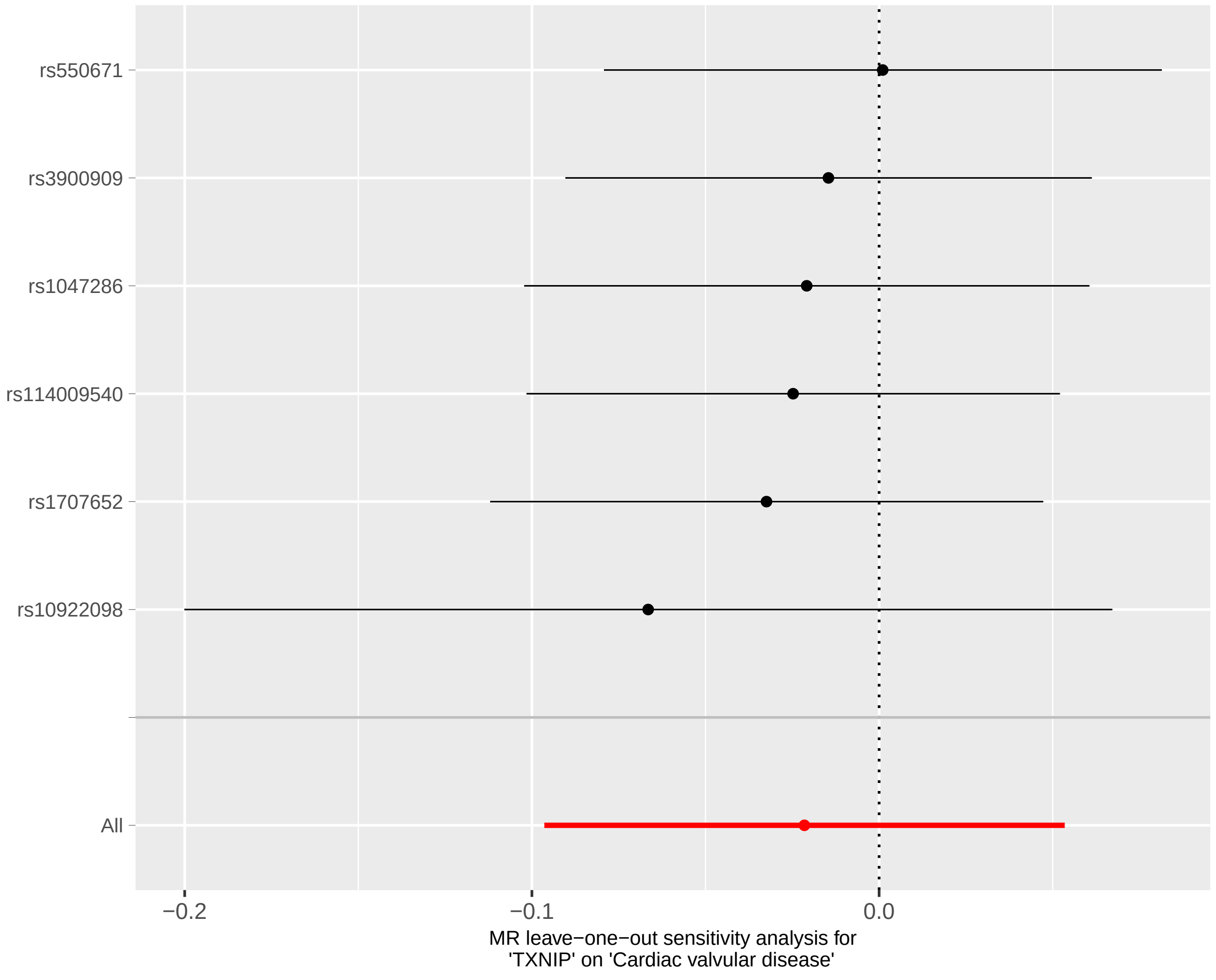

rs550671
rs3900909
rs1047286
rs114009540
rs1707652
rs10922098
All
−0.2
−0.1
0.0
MR leave−one−out sensitivity analysis for
'TXNIP' on 'Cardiac valvular disease'

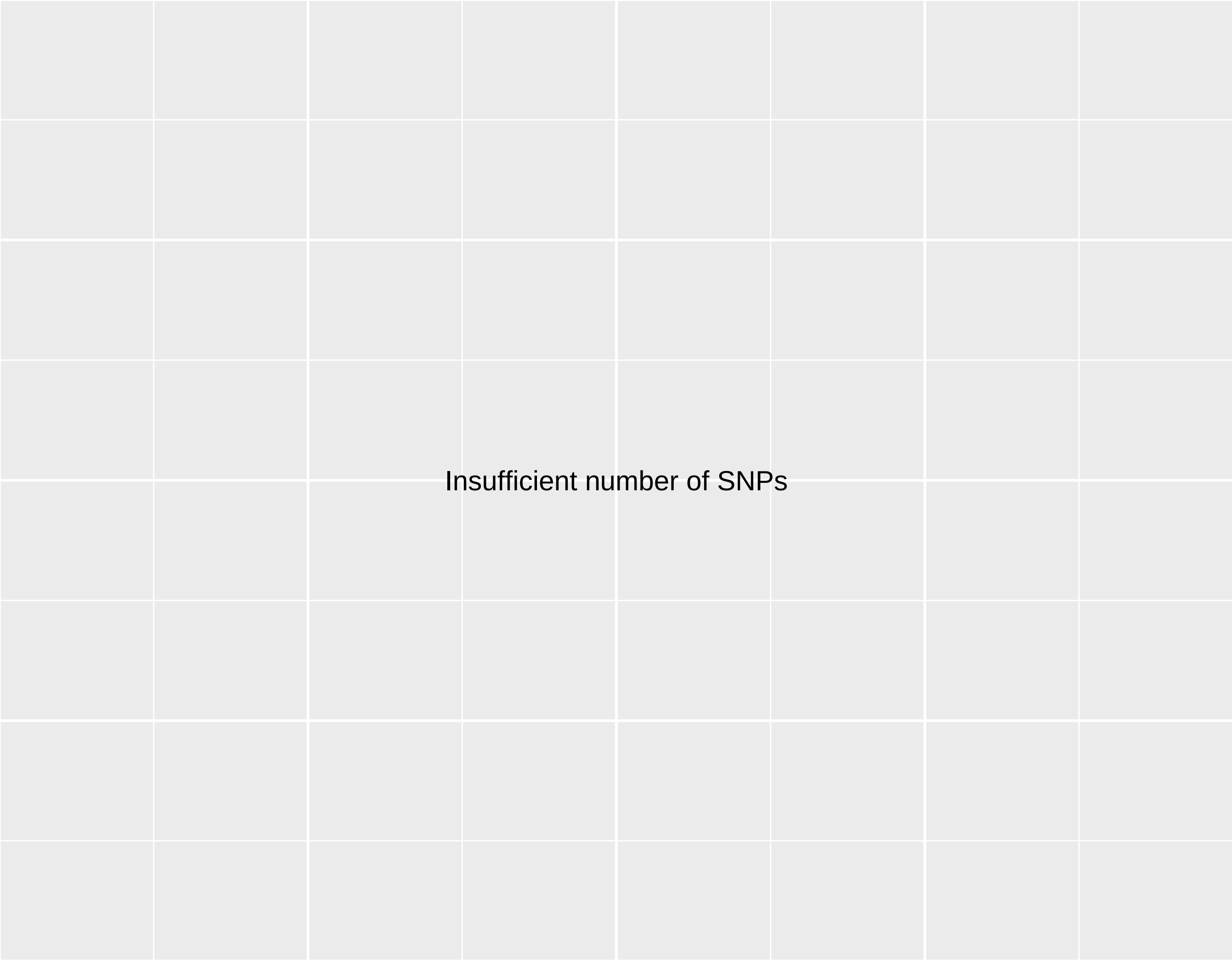
Insufficient number of SNPs

Insufficient number of SNPs

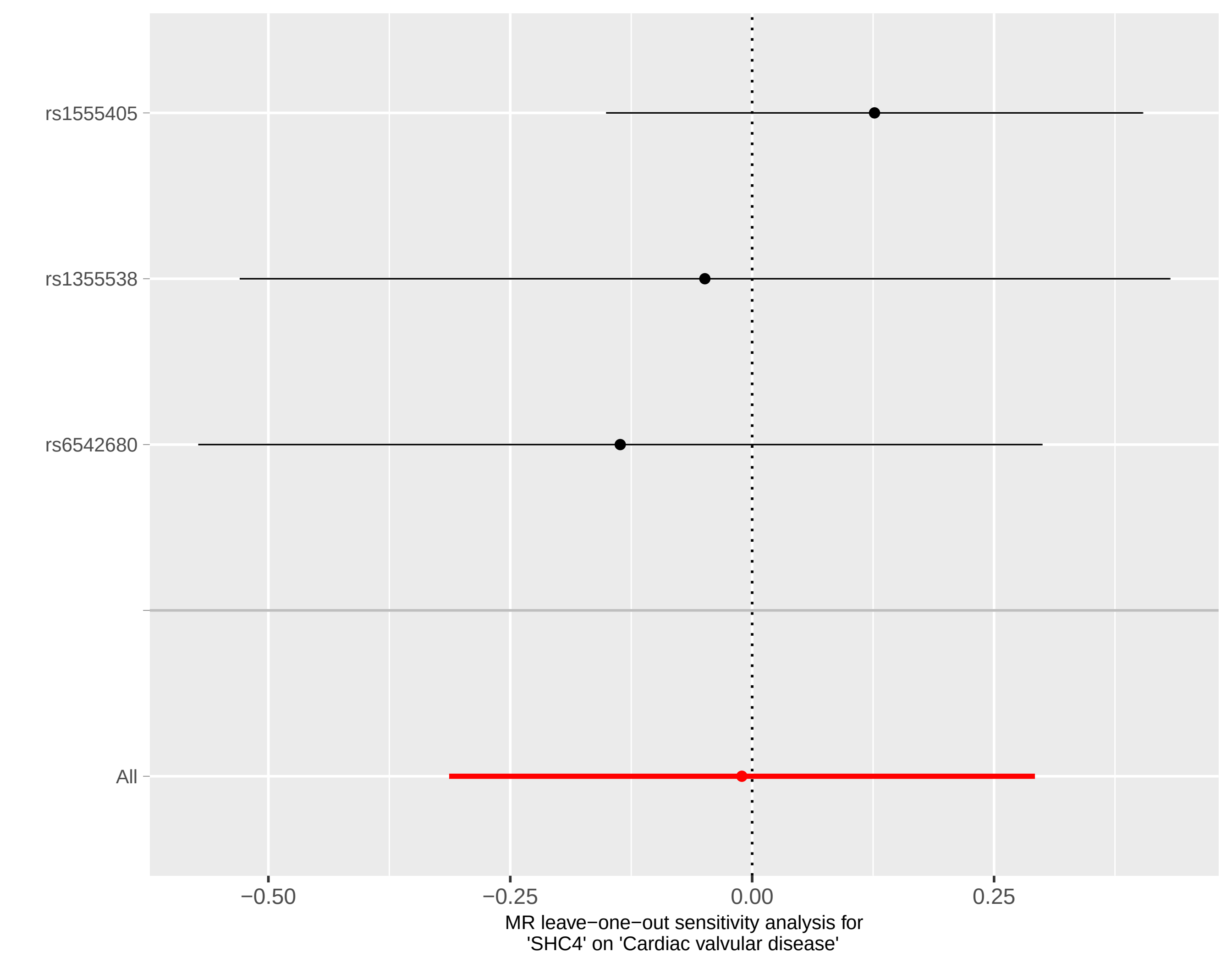
rs1555405
rs1355538
rs6542680
All
−0.50
−0.25
0.00
0.25
MR leave−one−out sensitivity analysis for
'SHC4' on 'Cardiac valvular disease'

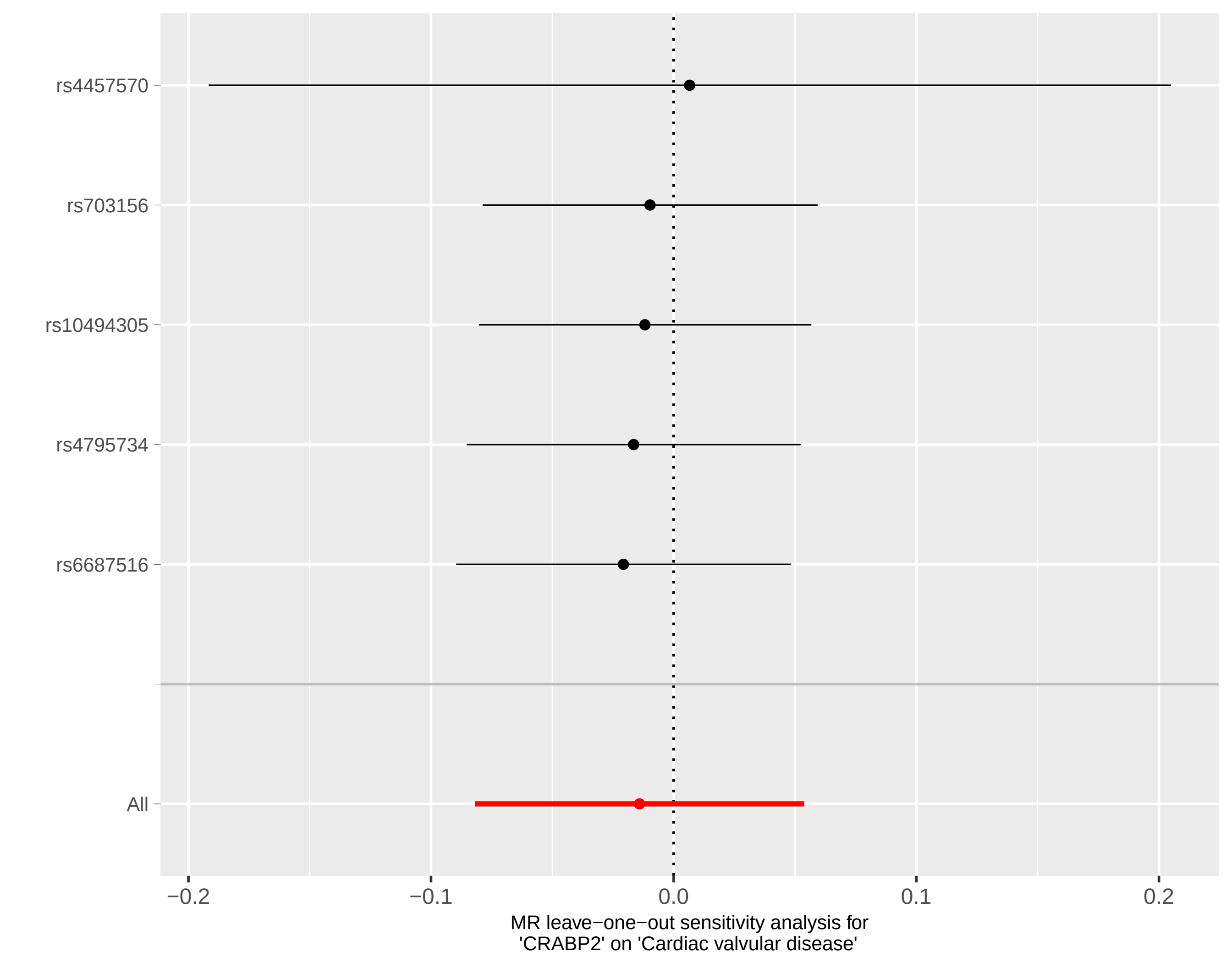

rs4457570
rs703156
rs10494305
rs4795734
rs6687516
All
−0.2
−0.1
0.0
0.1
0.2
MR leave−one−out sensitivity analysis for
'CRABP2' on 'Cardiac valvular disease'

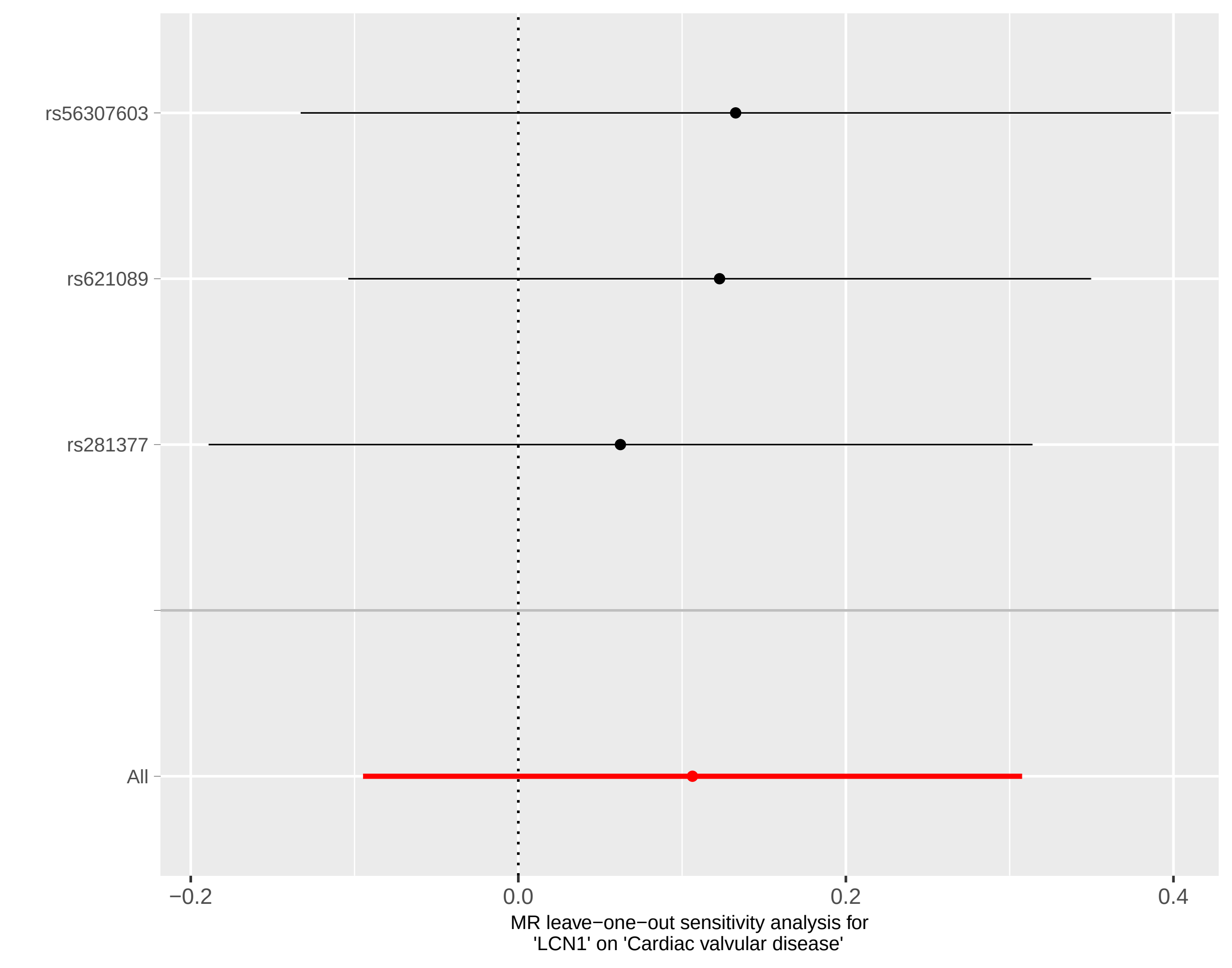
rs56307603
rs621089
rs281377
All
−0.2
0.0
0.2
0.4
MR leave−one−out sensitivity analysis for
'LCN1' on 'Cardiac valvular disease'

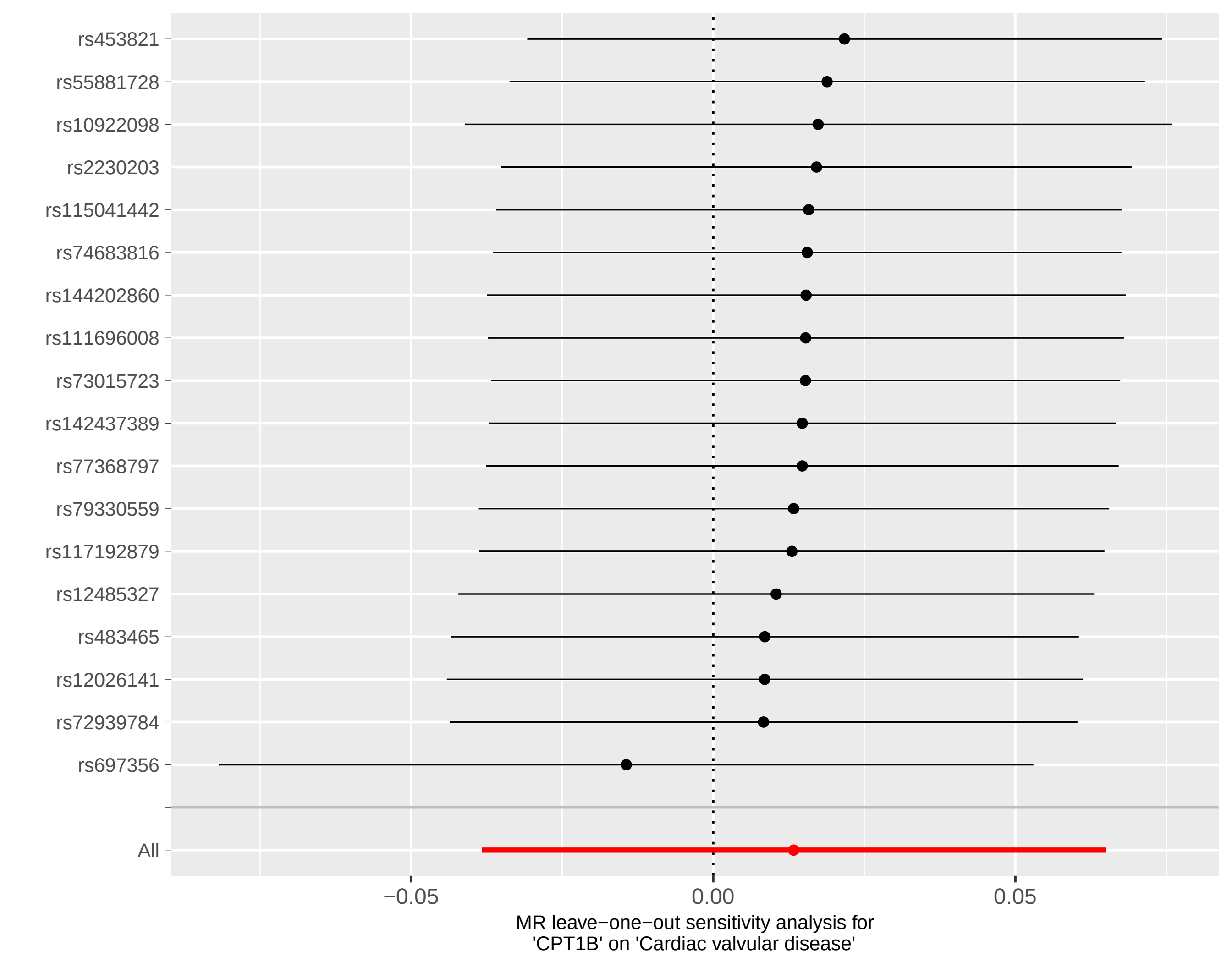

rs453821
rs55881728
rs10922098
rs2230203
rs115041442
rs74683816
rs144202860
rs111696008
rs73015723
rs142437389
rs77368797
rs79330559
rs117192879
rs12485327
rs483465
rs12026141
rs72939784
rs697356
All
−0.05
0.00
0.05
MR leave−one−out sensitivity analysis for
'CPT1B' on 'Cardiac valvular disease'

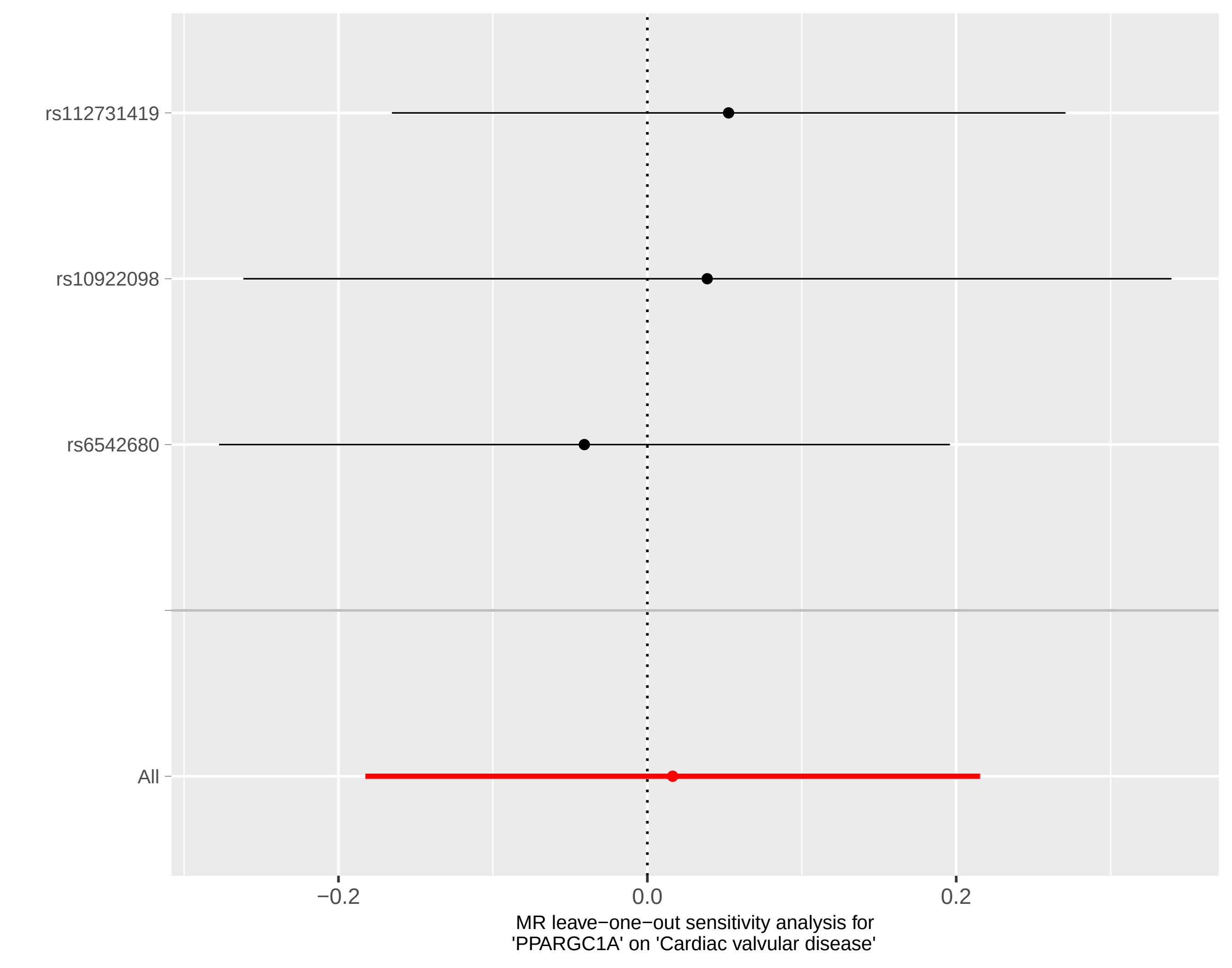
rs112731419
rs10922098
rs6542680
All
−0.2
0.0
0.2
MR leave−one−out sensitivity analysis for
'PPARGC1A' on 'Cardiac valvular disease'

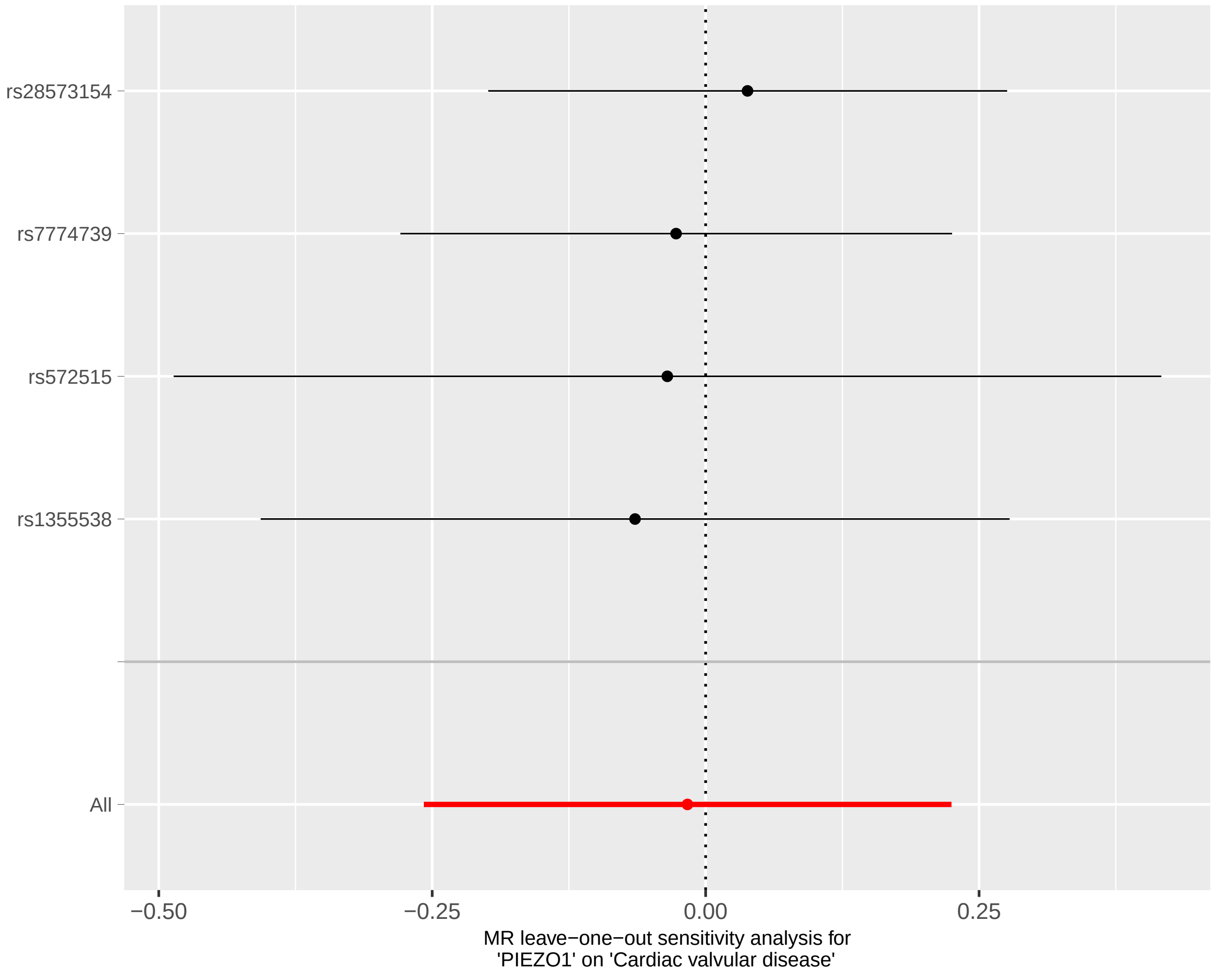
rs28573154
rs7774739
rs572515
rs1355538
All
−0.50
−0.25
0.00
0.25
MR leave−one−out sensitivity analysis for
'PIEZO1' on 'Cardiac valvular disease'

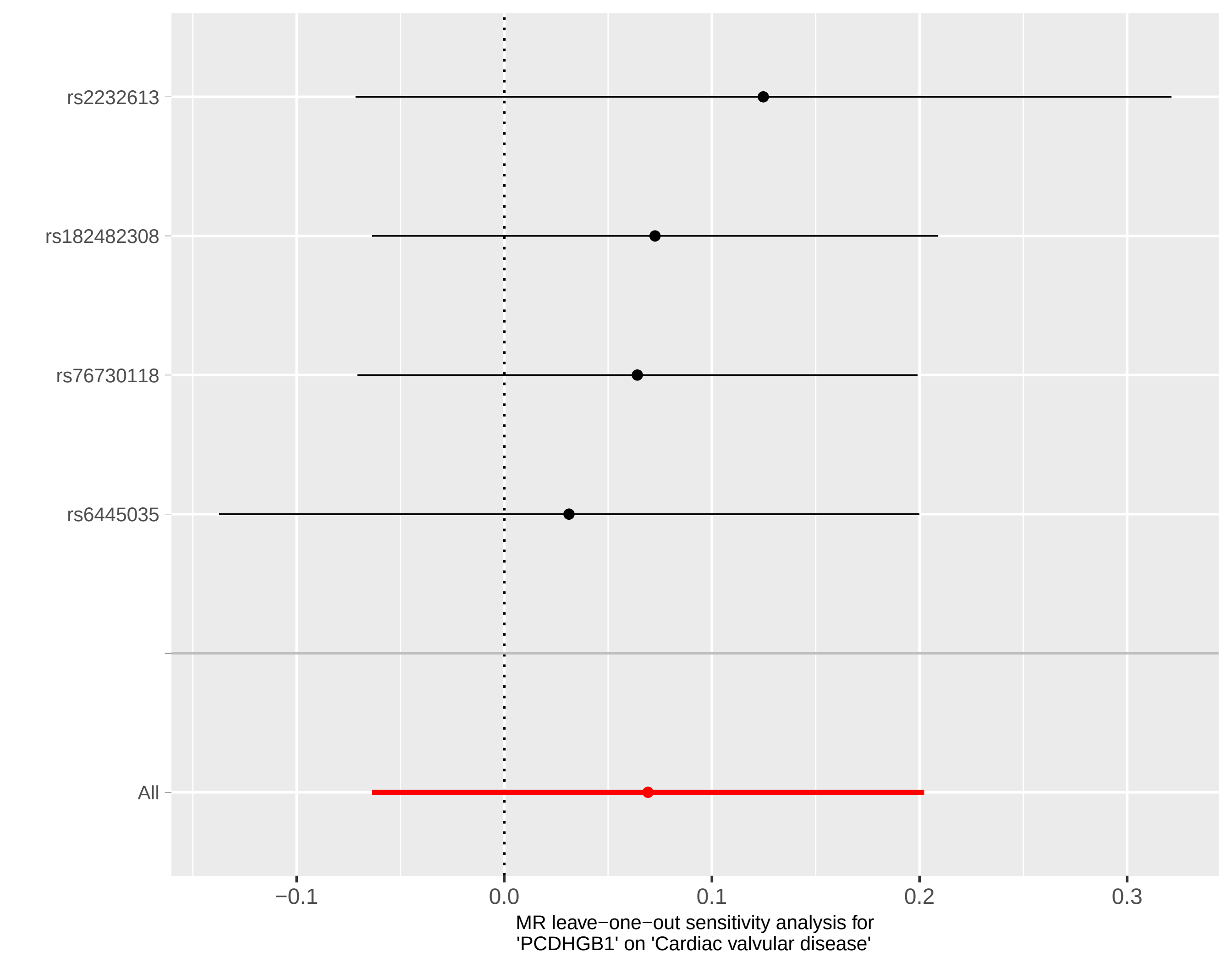
rs2232613
rs182482308
rs76730118
rs6445035
All
−0.1
0.0
0.1
0.2
0.3
MR leave−one−out sensitivity analysis for
'PCDHGB1' on 'Cardiac valvular disease'

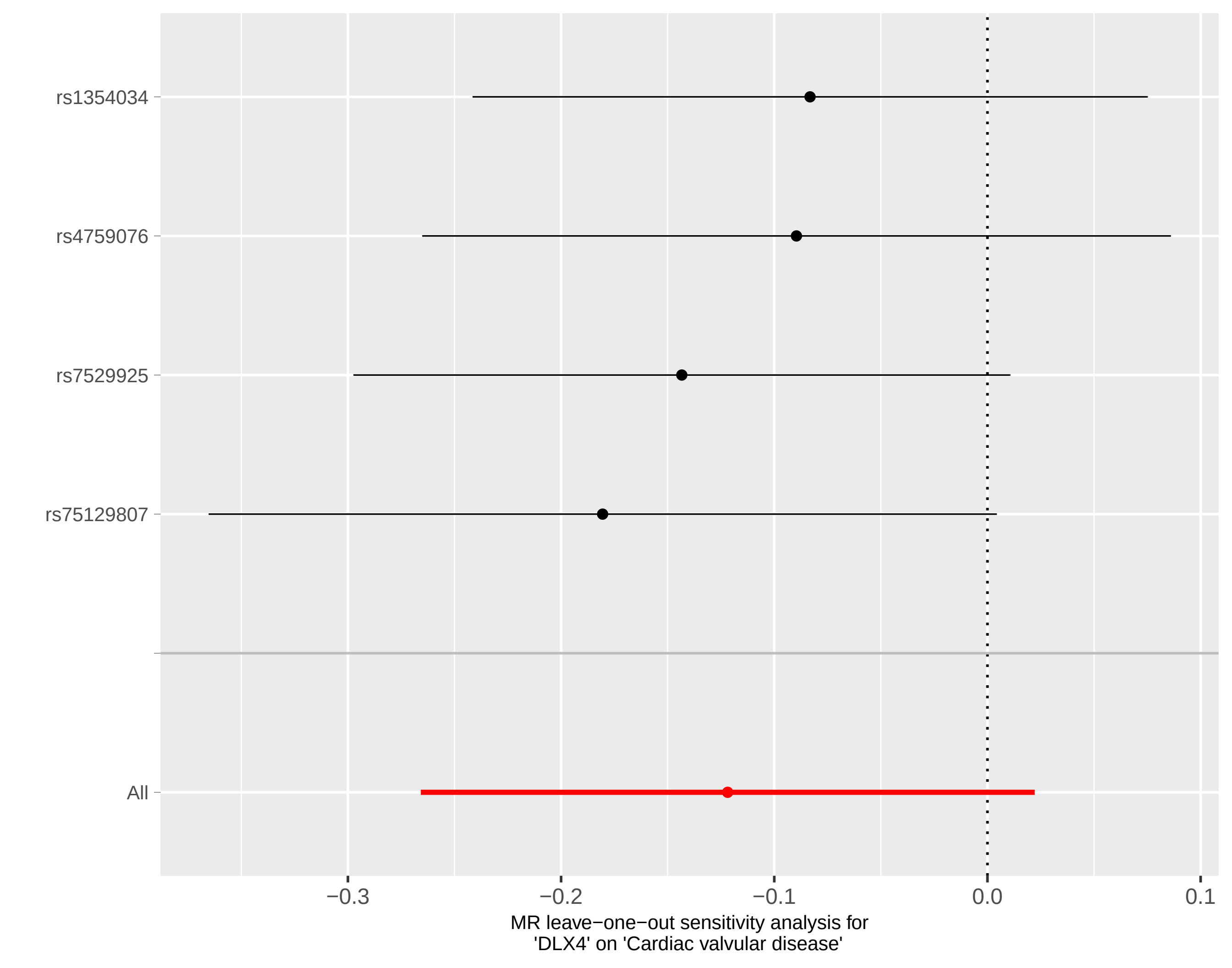

rs1354034
rs4759076
rs7529925
rs75129807
All
−0.3
−0.2
−0.1
0.0
0.1
MR leave−one−out sensitivity analysis for
'DLX4' on 'Cardiac valvular disease'

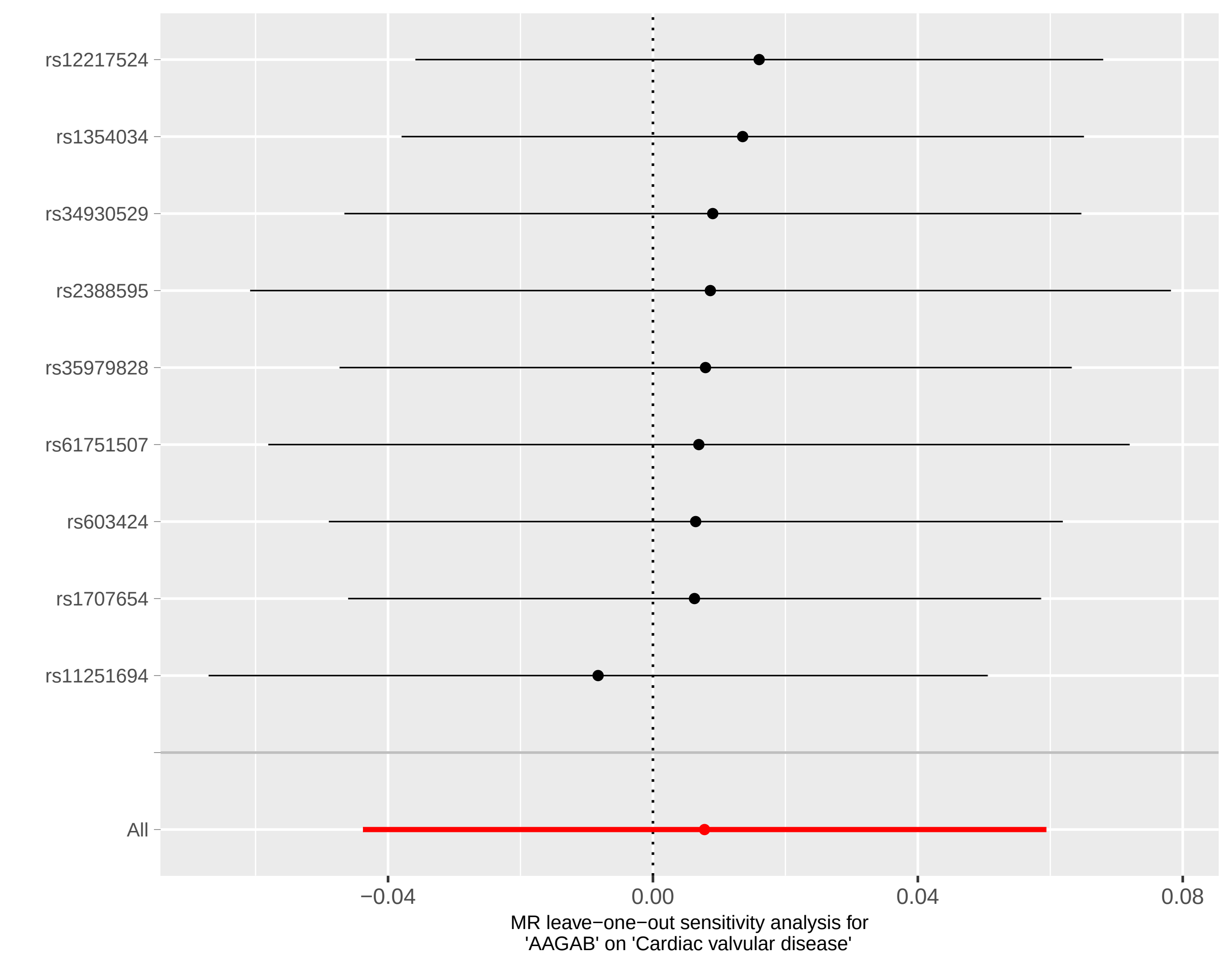
rs12217524
rs1354034
rs34930529
rs2388595
rs35979828
rs61751507
rs603424
rs1707654
rs11251694
All
−0.04
0.00
0.04
0.08
MR leave−one−out sensitivity analysis for
'AAGAB' on 'Cardiac valvular disease'

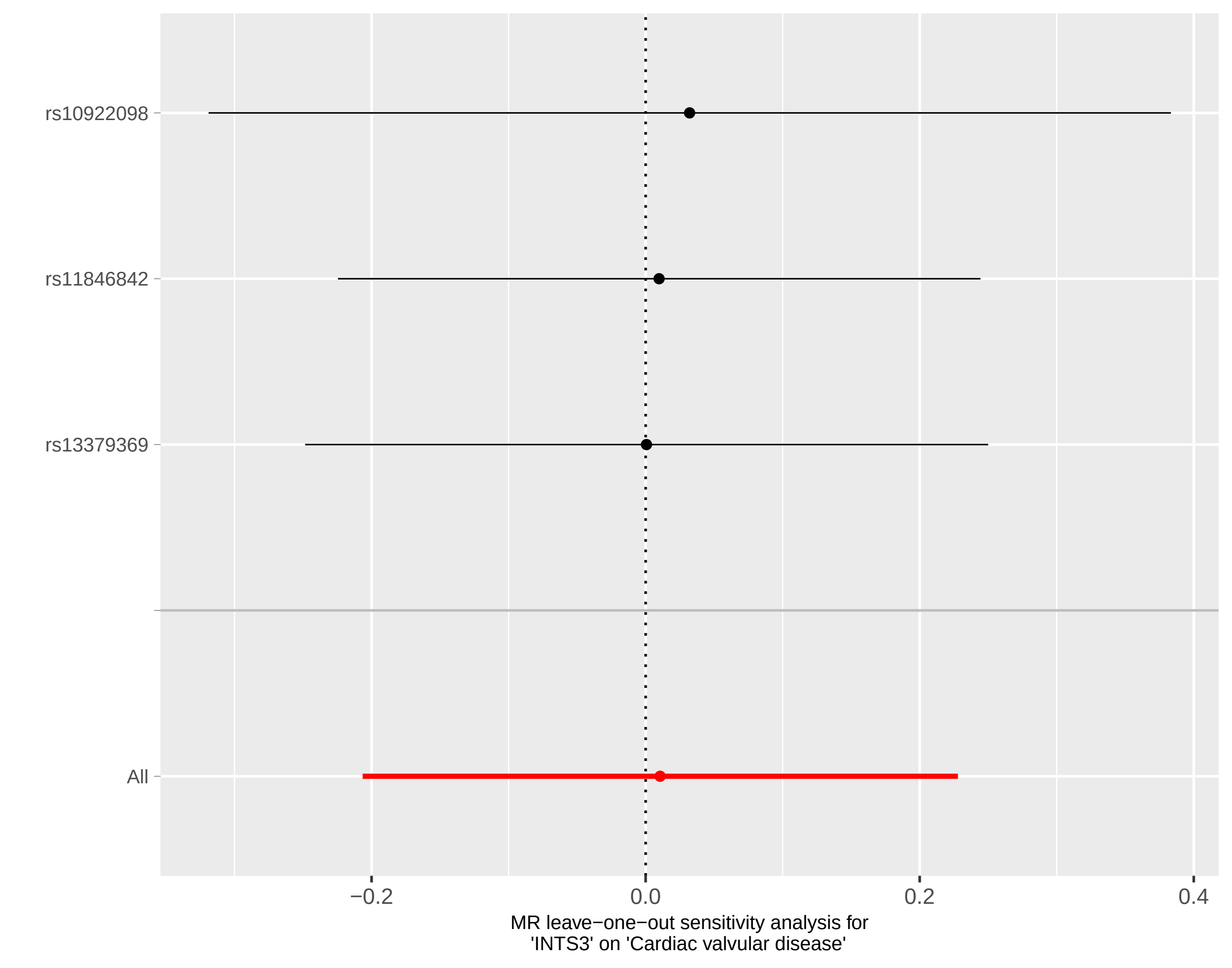
rs10922098
rs11846842
rs13379369
All
−0.2
0.0
0.2
0.4
MR leave−one−out sensitivity analysis for
'INTS3' on 'Cardiac valvular disease'

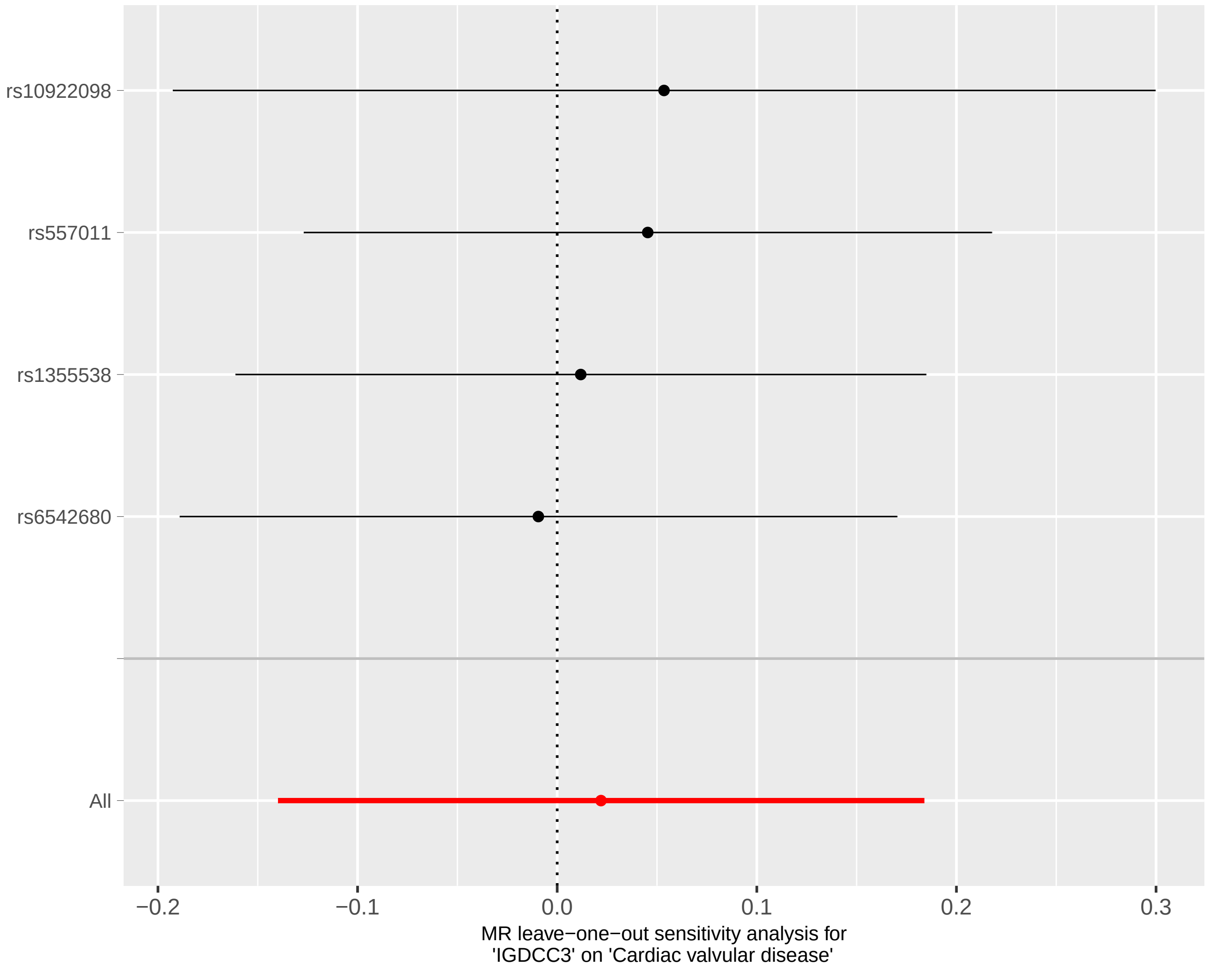
rs10922098
rs557011
rs1355538
rs6542680
All
−0.2
−0.1
0.0
0.1
0.2
0.3
MR leave−one−out sensitivity analysis for
'IGDCC3' on 'Cardiac valvular disease'

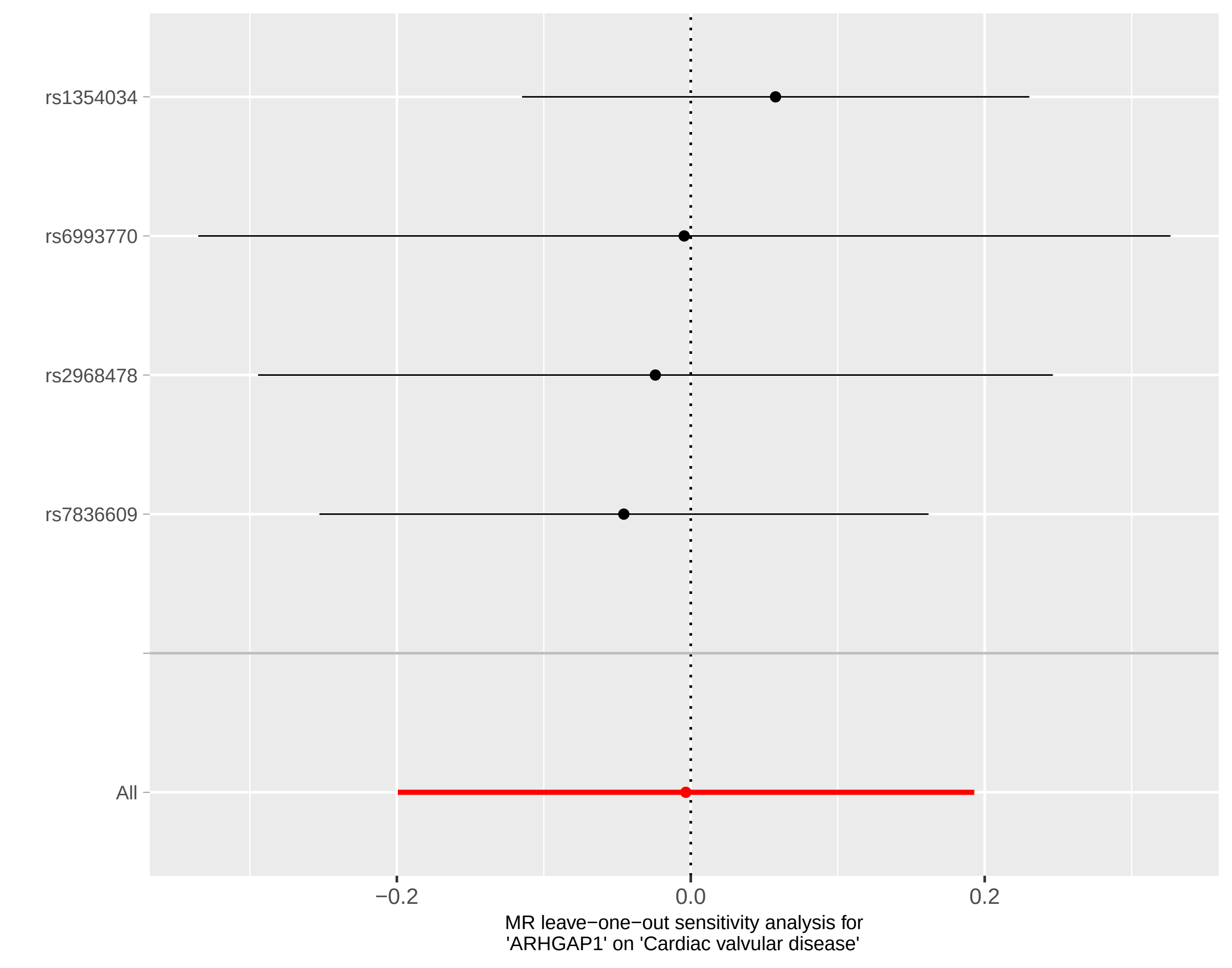
rs1354034
rs6993770
rs2968478
rs7836609
All
−0.2
0.0
0.2
MR leave−one−out sensitivity analysis for
'ARHGAP1' on 'Cardiac valvular disease'

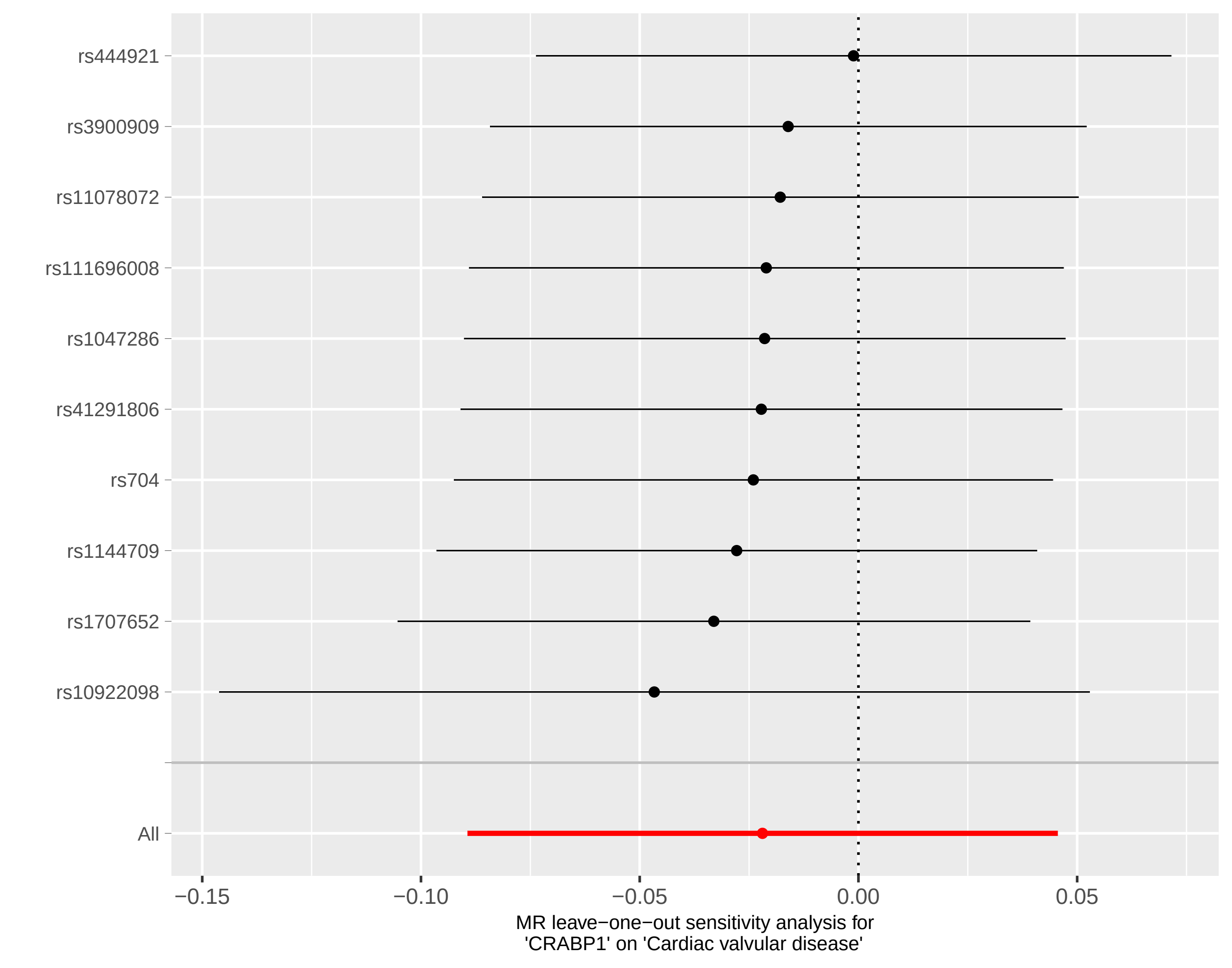

rs444921
rs3900909
rs11078072
rs111696008
rs1047286
rs41291806
rs704
rs1144709
rs1707652
rs10922098
All
−0.15
−0.10
−0.05
0.00
0.05
MR leave−one−out sensitivity analysis for
'CRABP1' on 'Cardiac valvular disease'

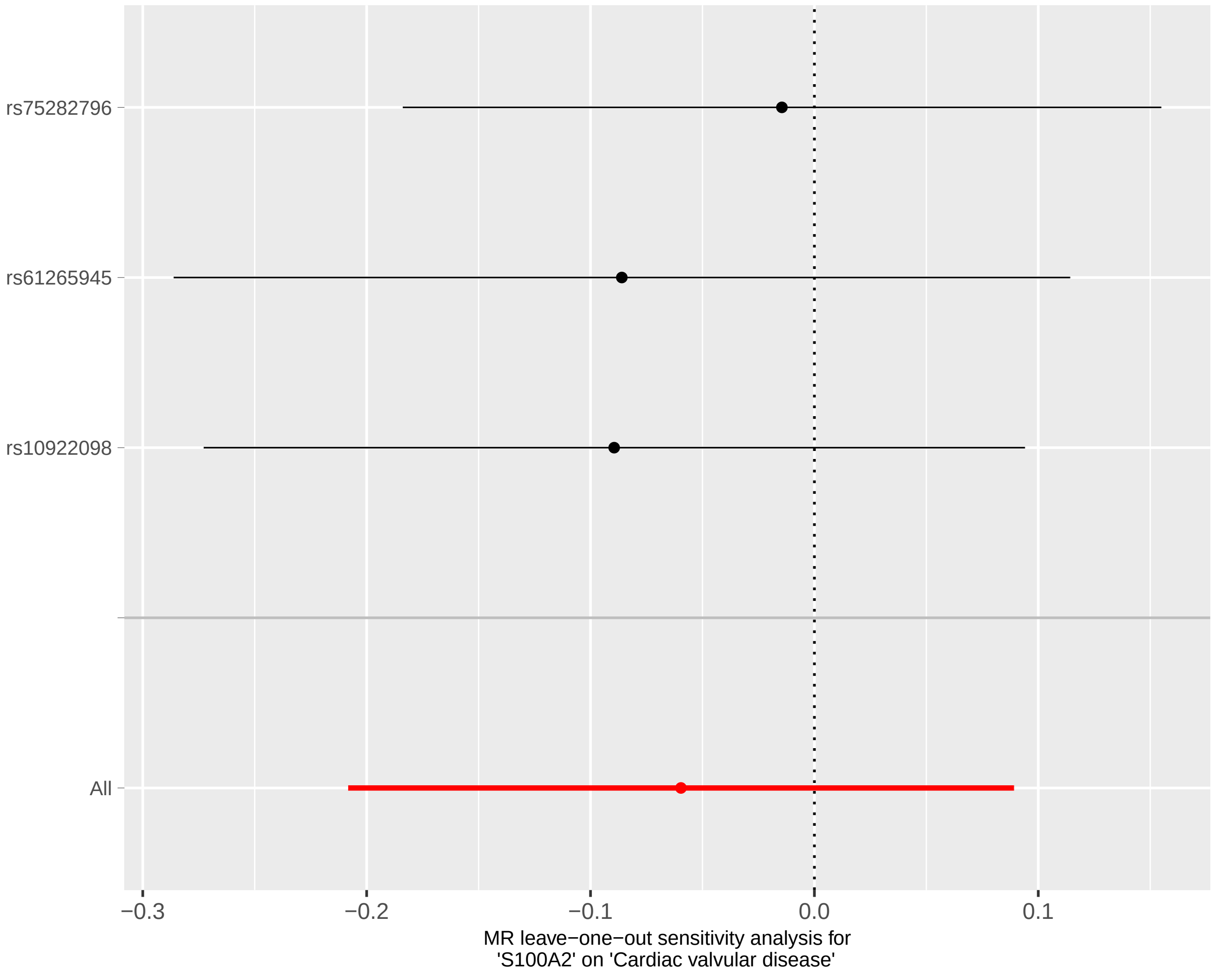
rs75282796
rs61265945
rs10922098
All
−0.3
−0.2
−0.1
0.0
0.1
MR leave−one−out sensitivity analysis for
'S100A2' on 'Cardiac valvular disease'

Insufficient number of SNPs

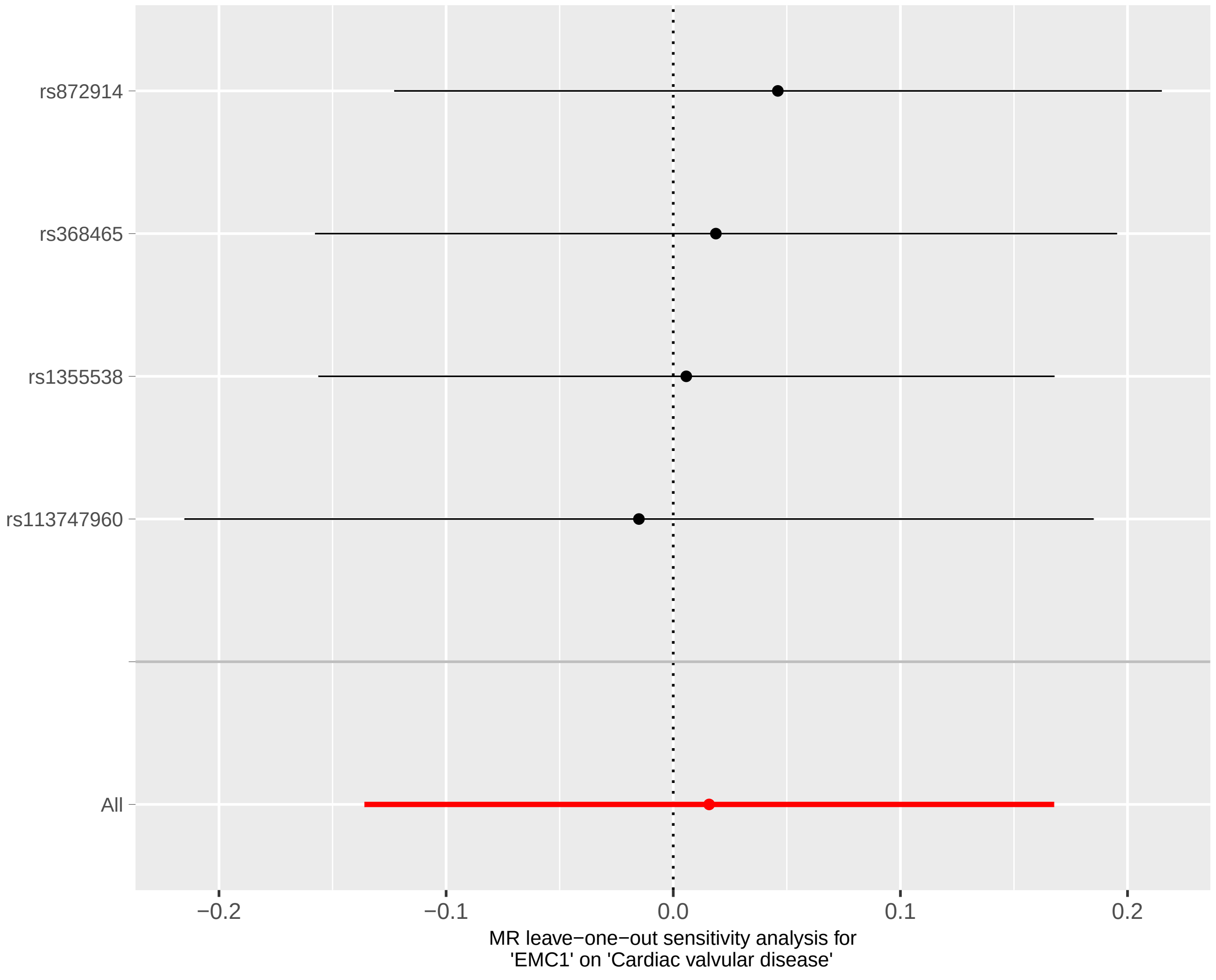
rs872914
rs368465
rs1355538
rs113747960
All
−0.2
−0.1
0.0
0.1
0.2
MR leave−one−out sensitivity analysis for
'EMC1' on 'Cardiac valvular disease'

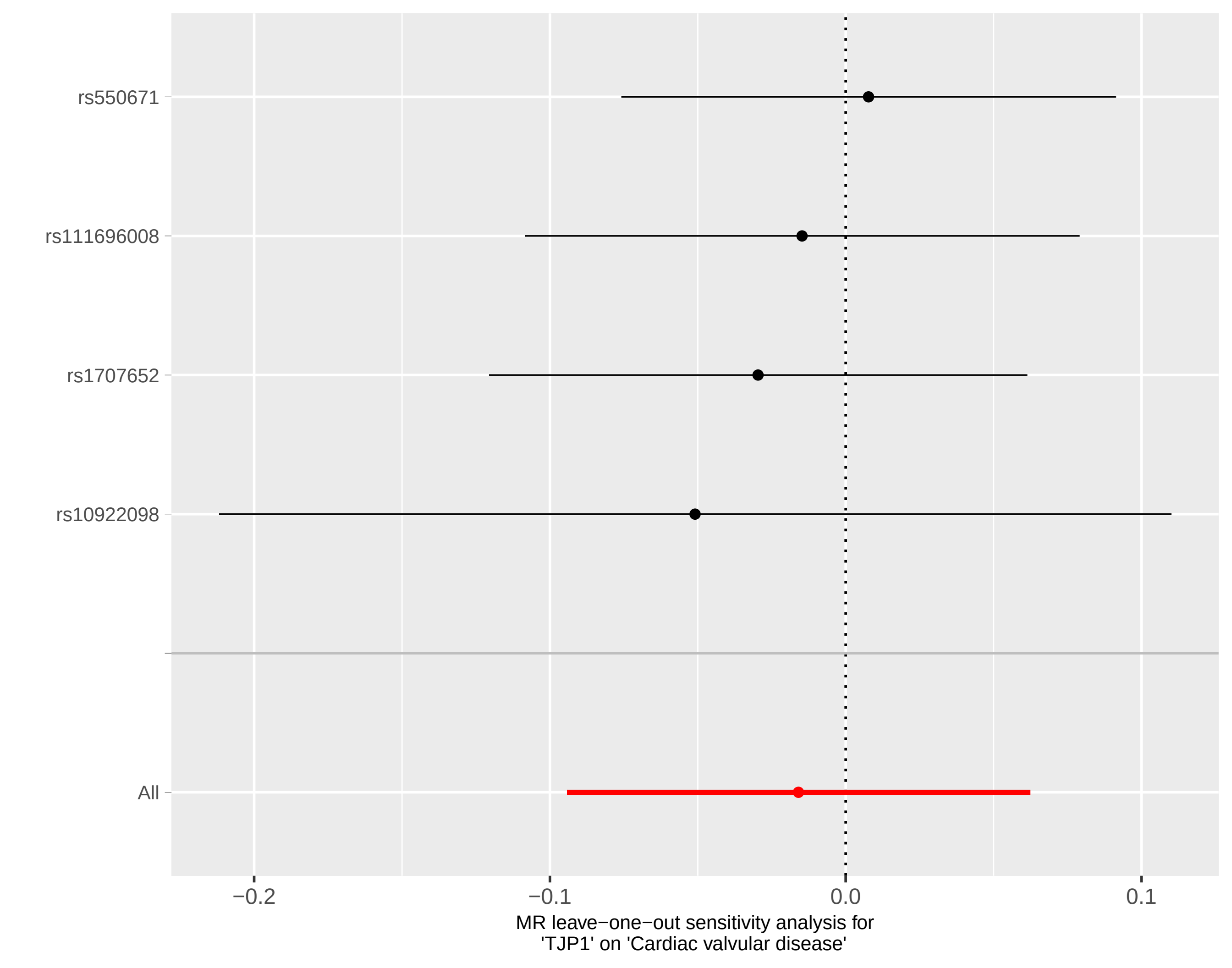
rs550671
rs111696008
rs1707652
rs10922098
All
−0.2
−0.1
0.0
0.1
MR leave−one−out sensitivity analysis for
'TJP1' on 'Cardiac valvular disease'

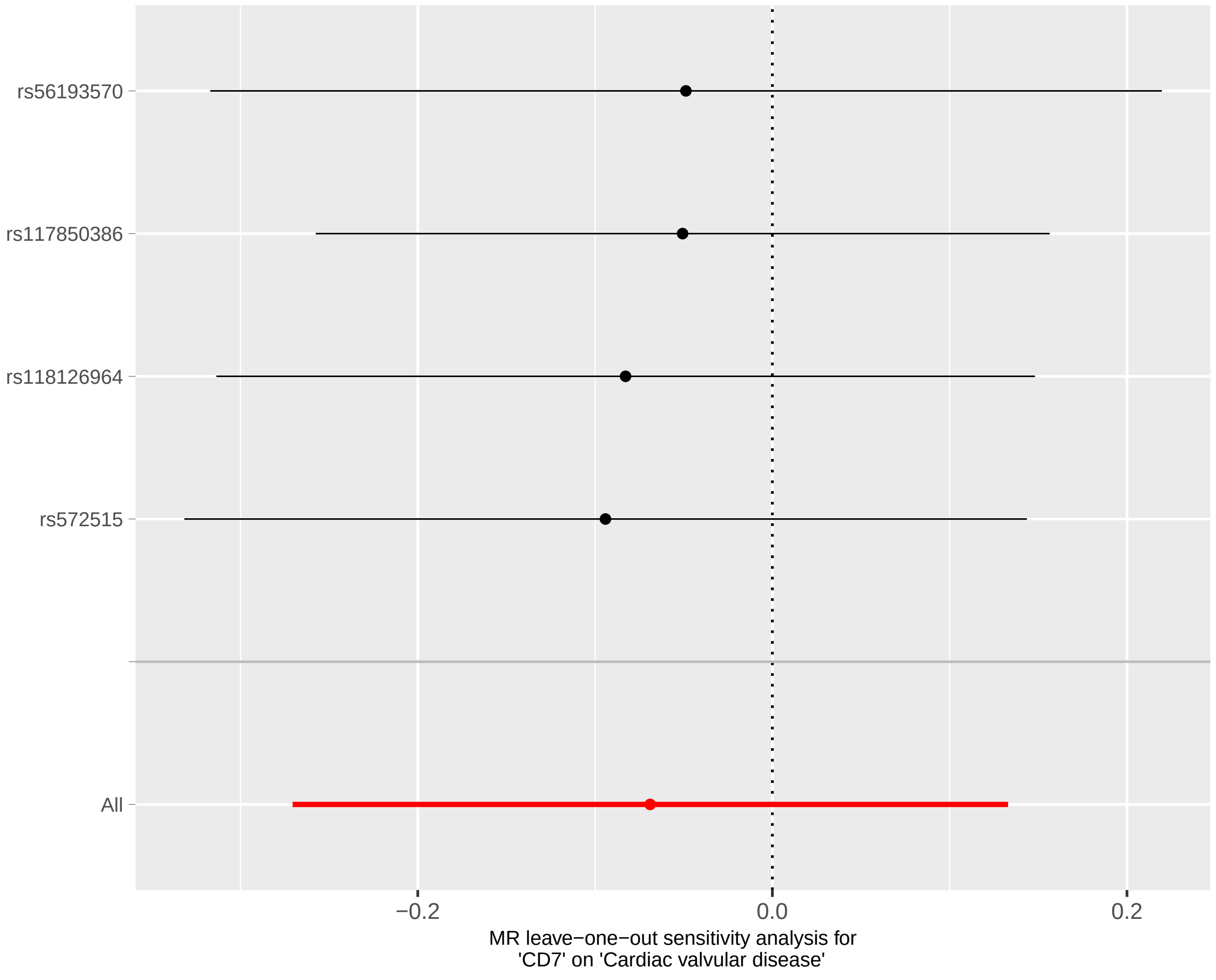
rs56193570
rs117850386
rs118126964
rs572515
All
−0.2
0.0
0.2
MR leave−one−out sensitivity analysis for
'CD7' on 'Cardiac valvular disease'

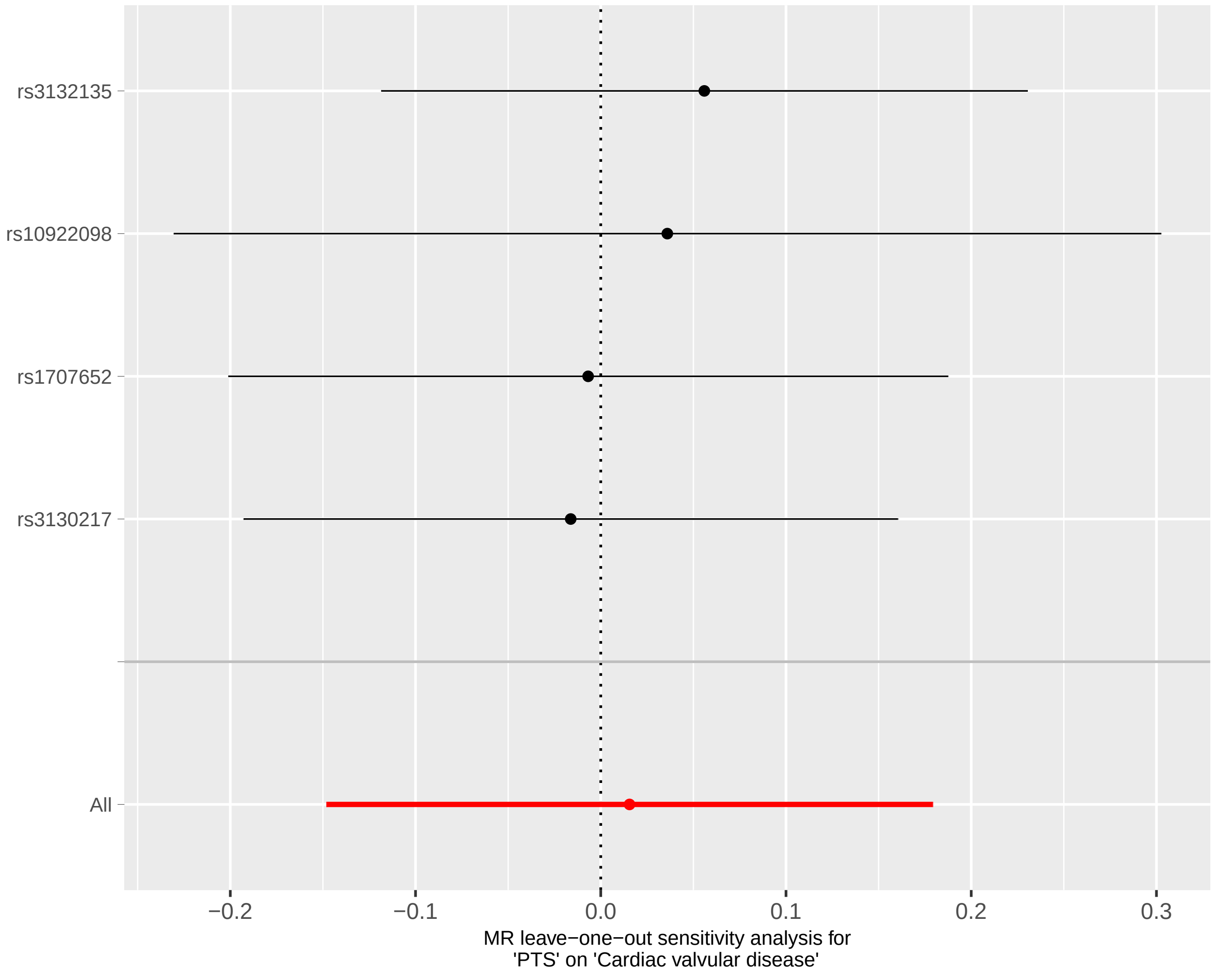
rs3132135
rs10922098
rs1707652
rs3130217
All
−0.2
−0.1
0.0
0.1
0.2
0.3
MR leave−one−out sensitivity analysis for
'PTS' on 'Cardiac valvular disease'

Insufficient number of SNPs

Insufficient number of SNPs

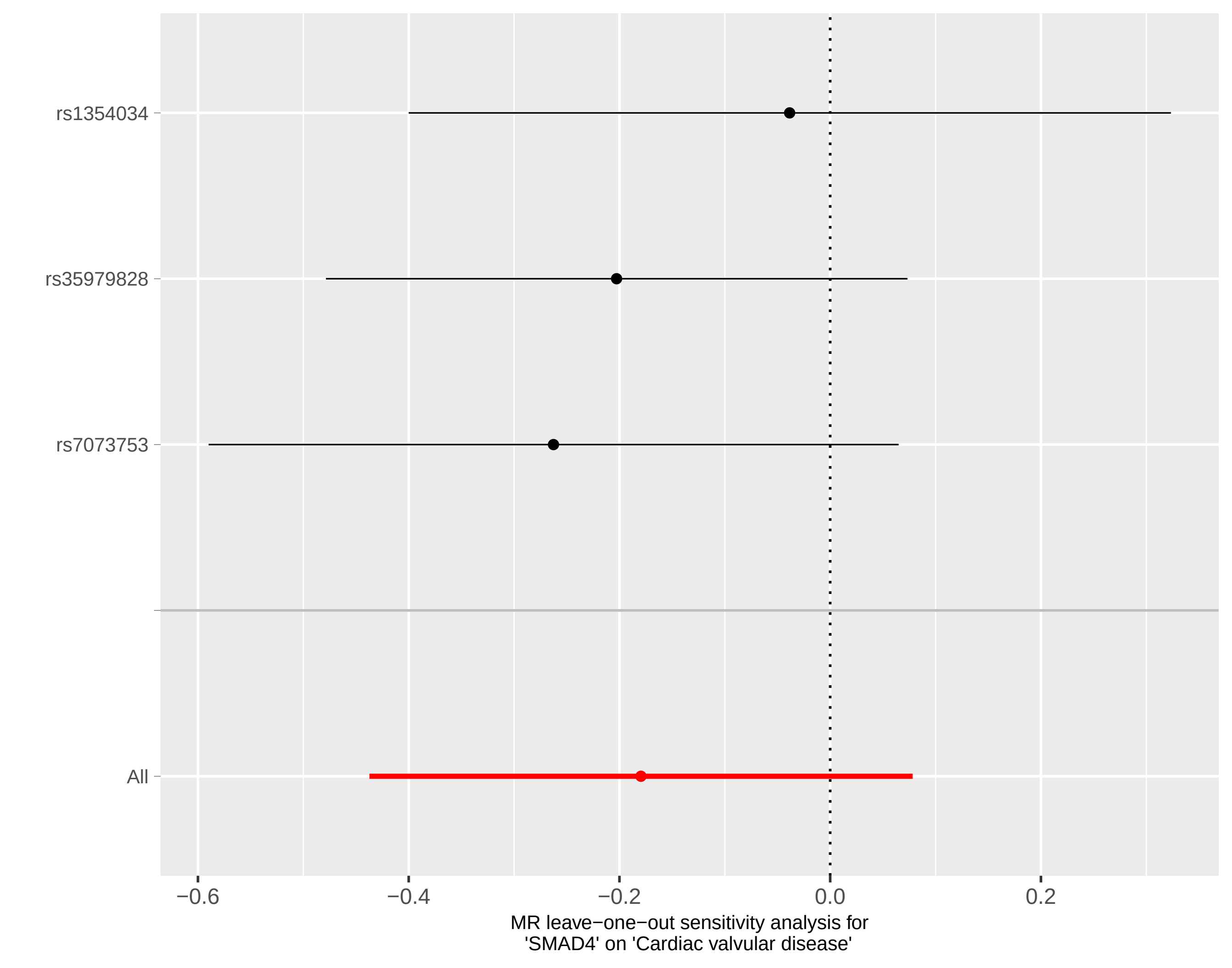

rs1354034
rs35979828
rs7073753
All
−0.6
−0.4
−0.2
0.0
0.2
MR leave−one−out sensitivity analysis for
'SMAD4' on 'Cardiac valvular disease'

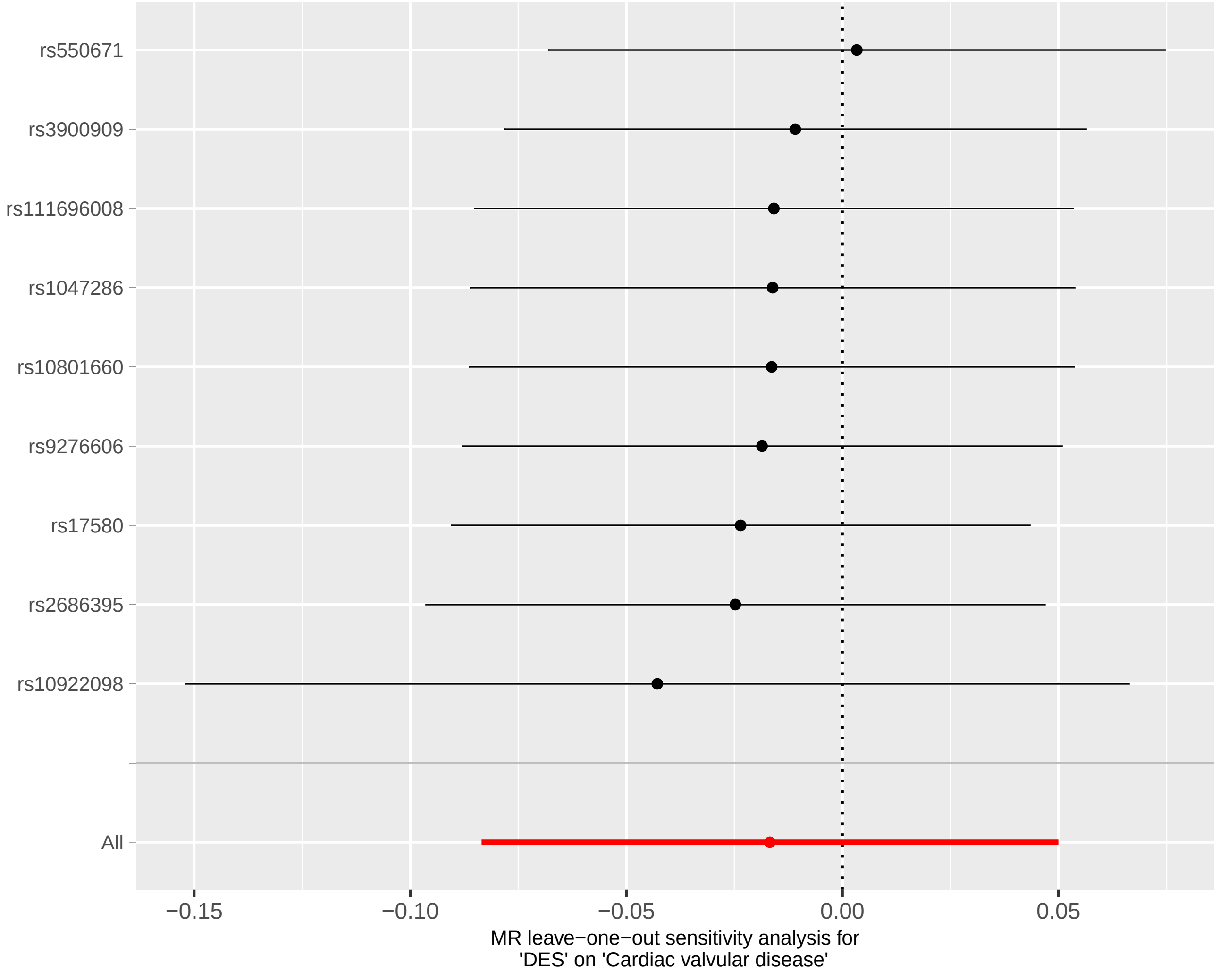
rs550671
rs3900909
rs111696008
rs1047286
rs10801660
rs9276606
rs17580
rs2686395
rs10922098
All
−0.15
−0.10
−0.05
0.00
0.05
MR leave−one−out sensitivity analysis for
'DES' on 'Cardiac valvular disease'

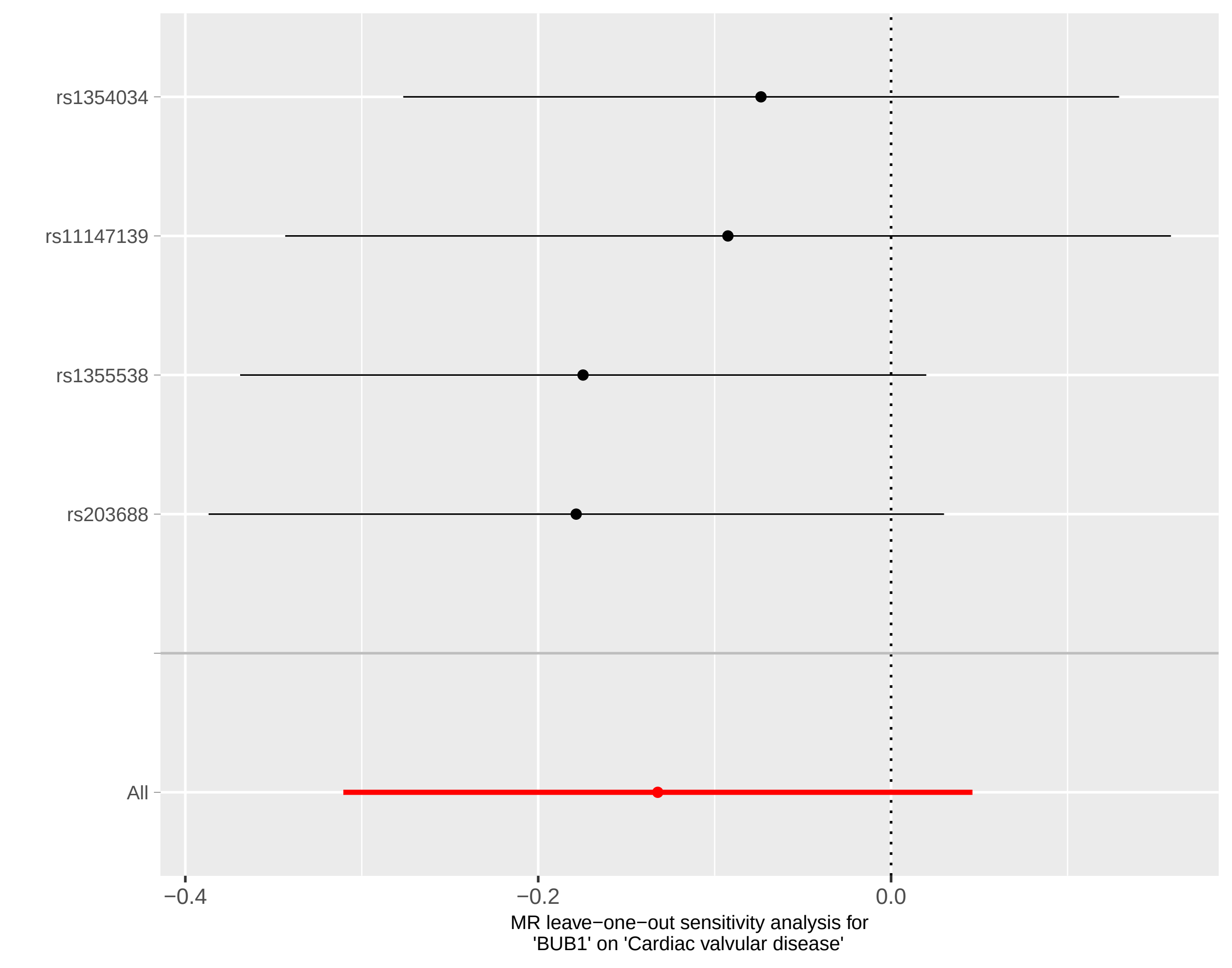
rs1354034
rs11147139
rs1355538
rs203688
All
−0.4
−0.2
0.0
MR leave−one−out sensitivity analysis for
'BUB1' on 'Cardiac valvular disease'

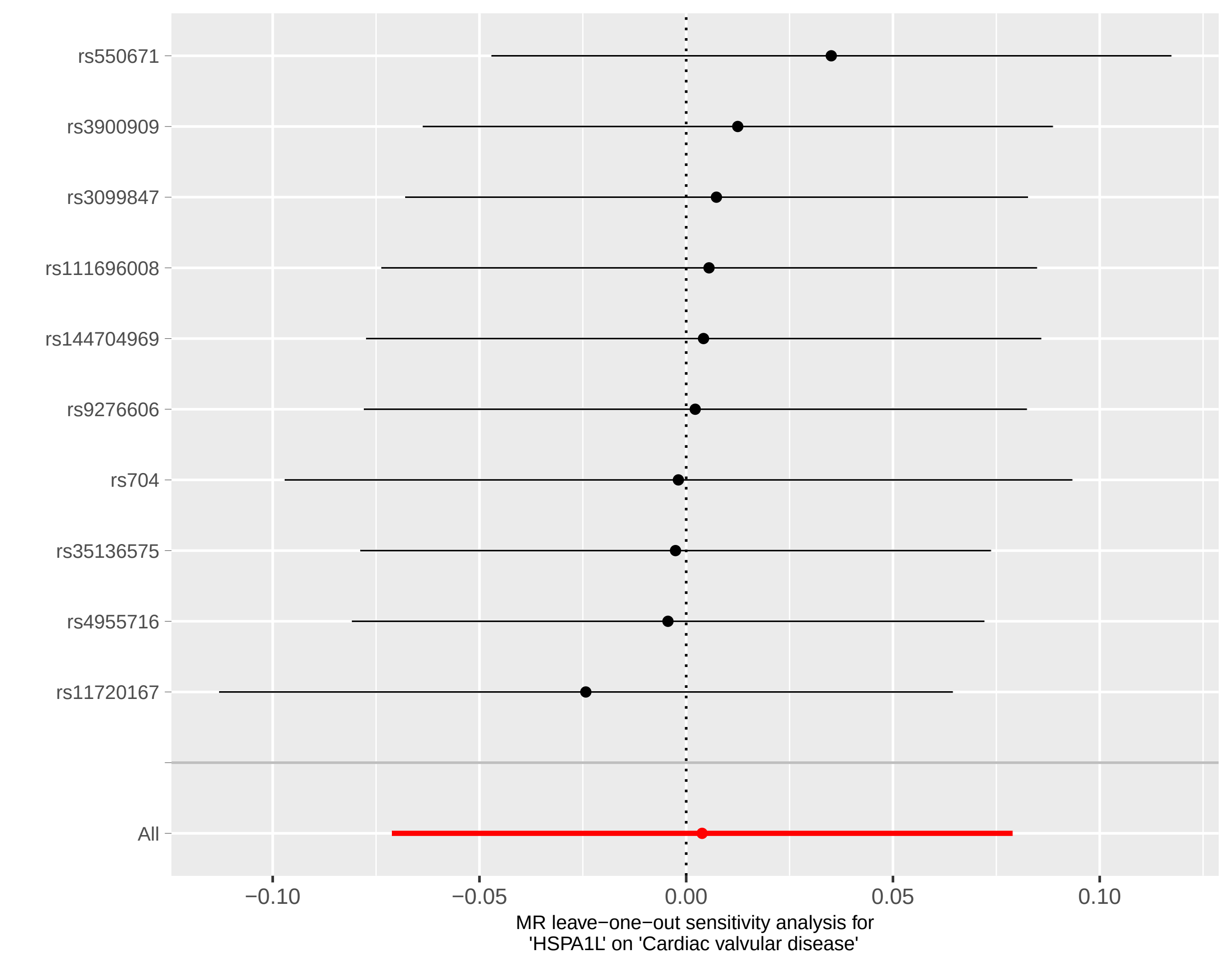

rs550671
rs3900909
rs3099847
rs111696008
rs144704969
rs9276606
rs704
rs35136575
rs4955716
rs11720167
All
−0.10
−0.05
0.00
0.05
0.10
MR leave−one−out sensitivity analysis for
'HSPA1L' on 'Cardiac valvular disease'

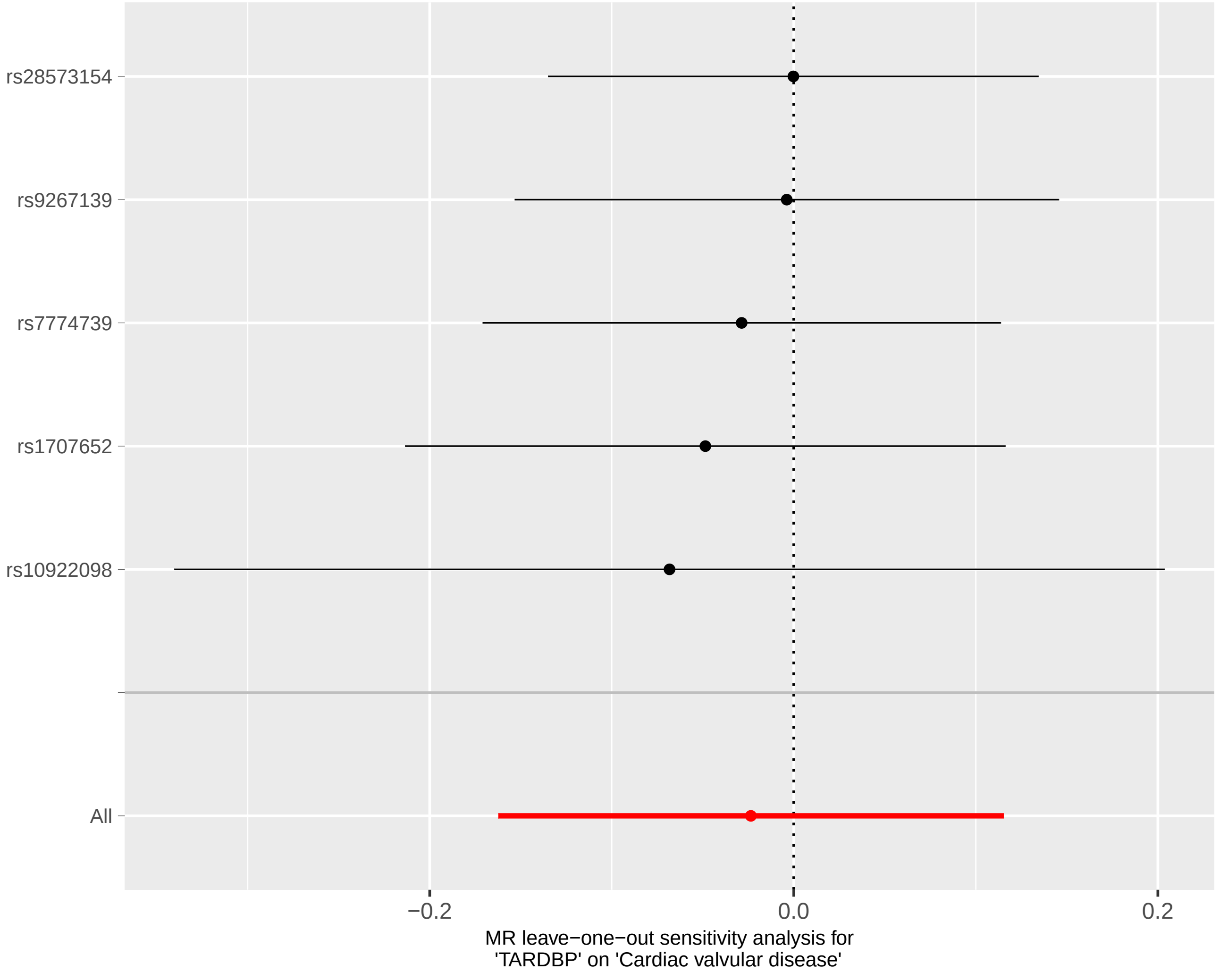
rs28573154
rs9267139
rs7774739
rs1707652
rs10922098
All
−0.2
0.0
0.2
MR leave−one−out sensitivity analysis for
'TARDBP' on 'Cardiac valvular disease'

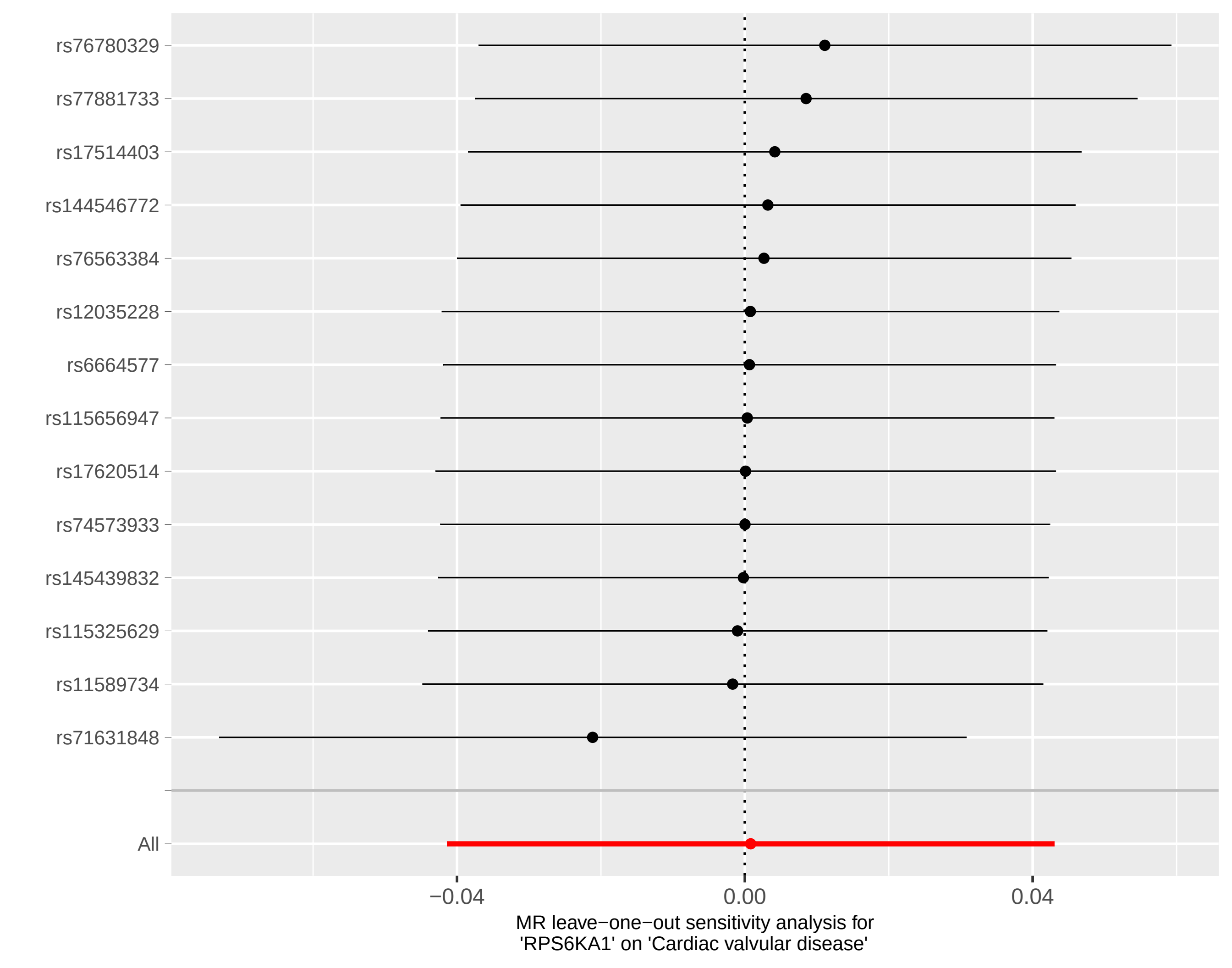
rs76780329
rs77881733
rs17514403
rs144546772
rs76563384
rs12035228
rs6664577
rs115656947
rs17620514
rs74573933
rs145439832
rs115325629
rs11589734
rs71631848
All
−0.04
0.00
0.04
MR leave−one−out sensitivity analysis for
'RPS6KA1' on 'Cardiac valvular disease'

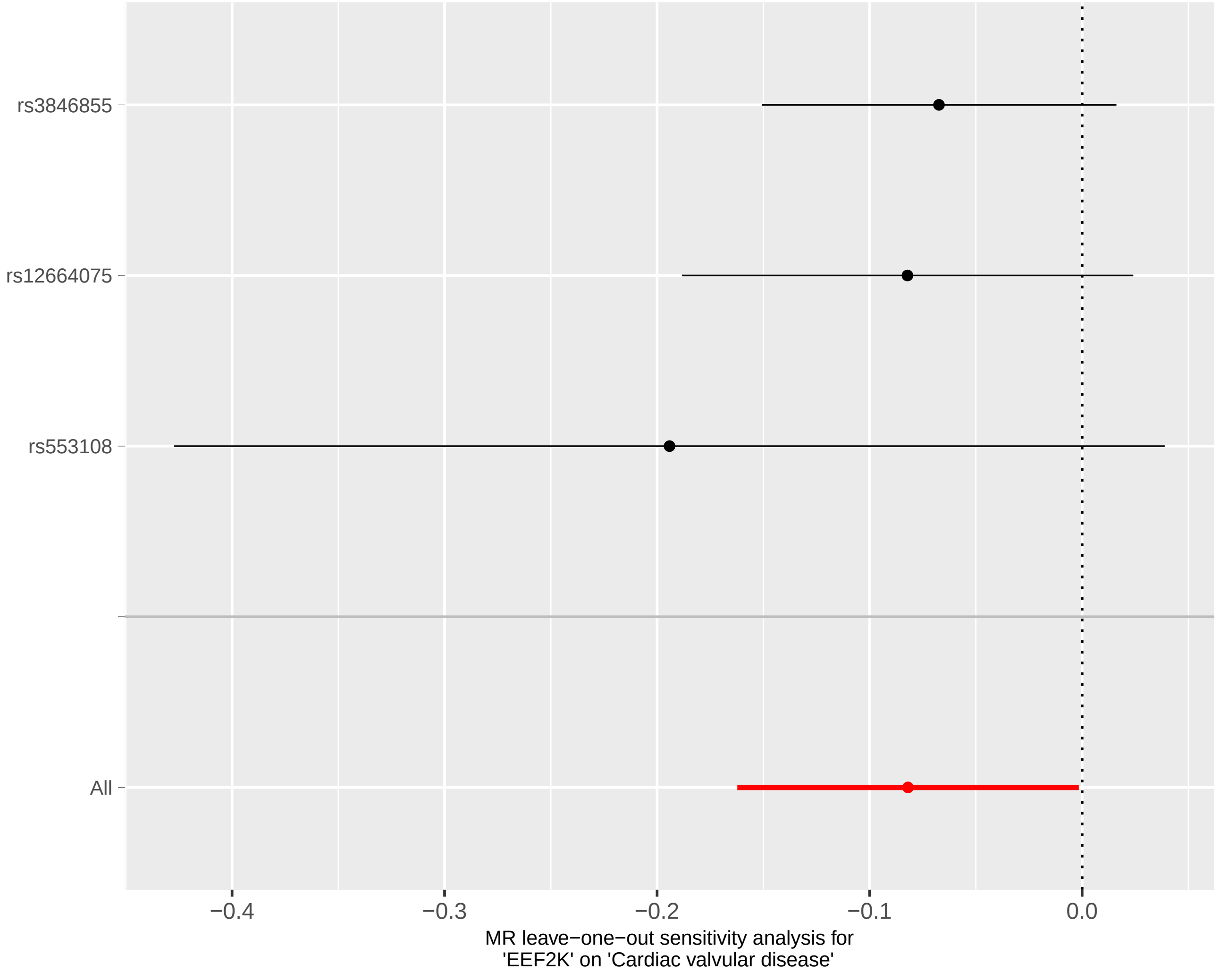

rs3846855
rs12664075
rs553108
All
−0.4
−0.3
−0.2
−0.1
0.0
MR leave−one−out sensitivity analysis for
'EEF2K' on 'Cardiac valvular disease'

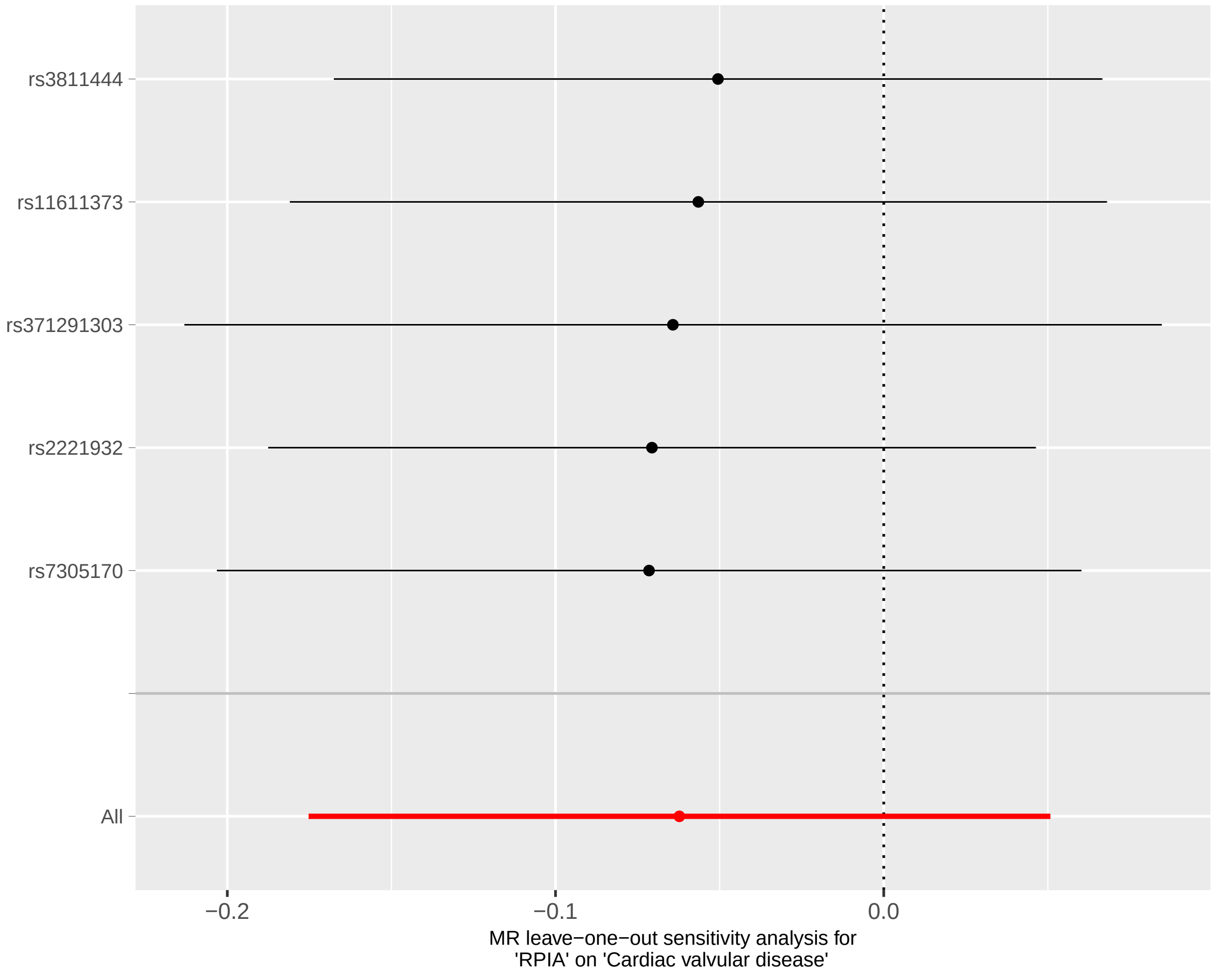

rs3811444
rs11611373
rs371291303
rs2221932
rs7305170
All
−0.2
−0.1
0.0
MR leave−one−out sensitivity analysis for
'RPIA' on 'Cardiac valvular disease'

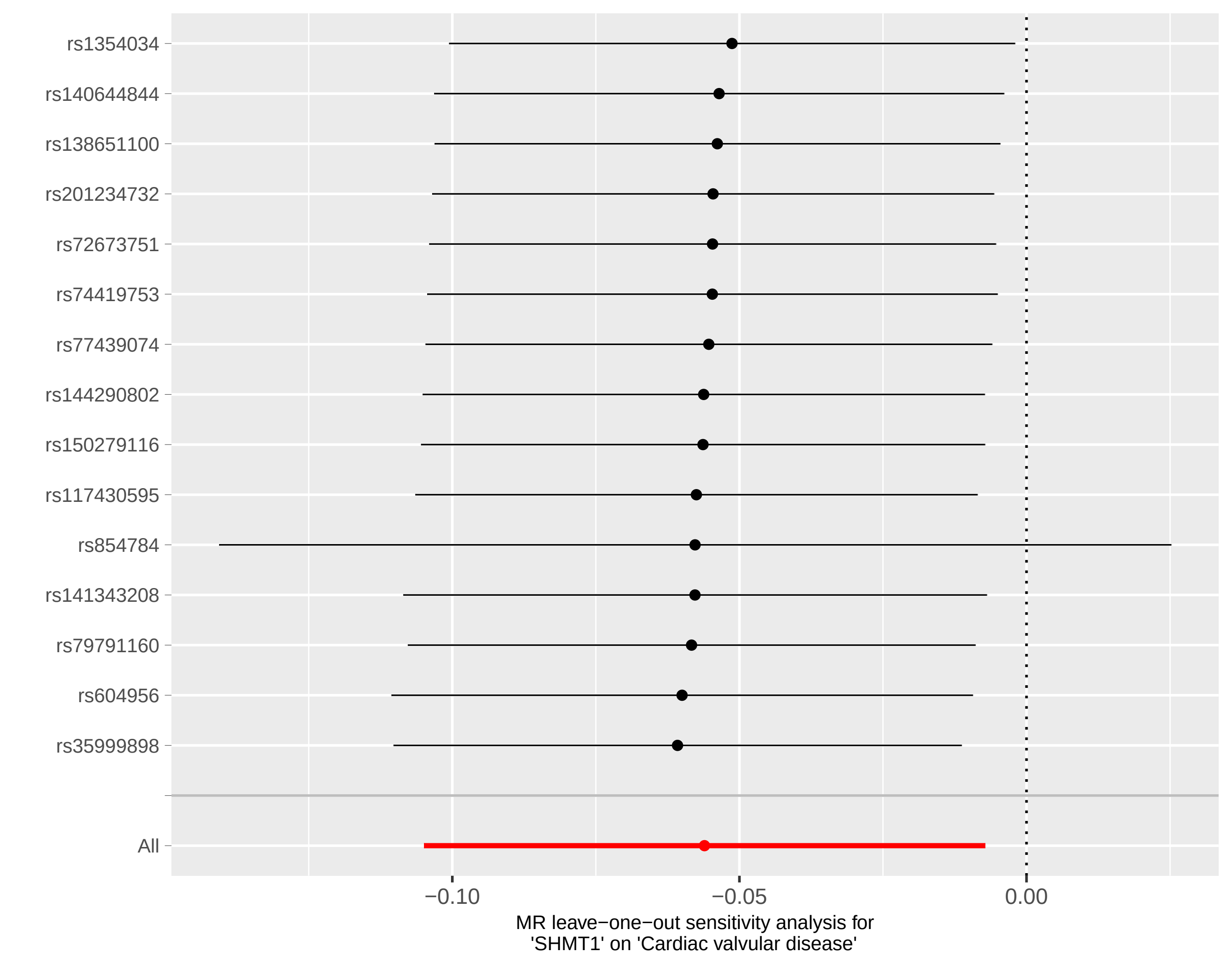

rs1354034
rs140644844
rs138651100
rs201234732
rs72673751
rs74419753
rs77439074
rs144290802
rs150279116
rs117430595
rs854784
rs141343208
rs79791160
rs604956
rs35999898
All
−0.10
−0.05
0.00
MR leave−one−out sensitivity analysis for
'SHMT1' on 'Cardiac valvular disease'

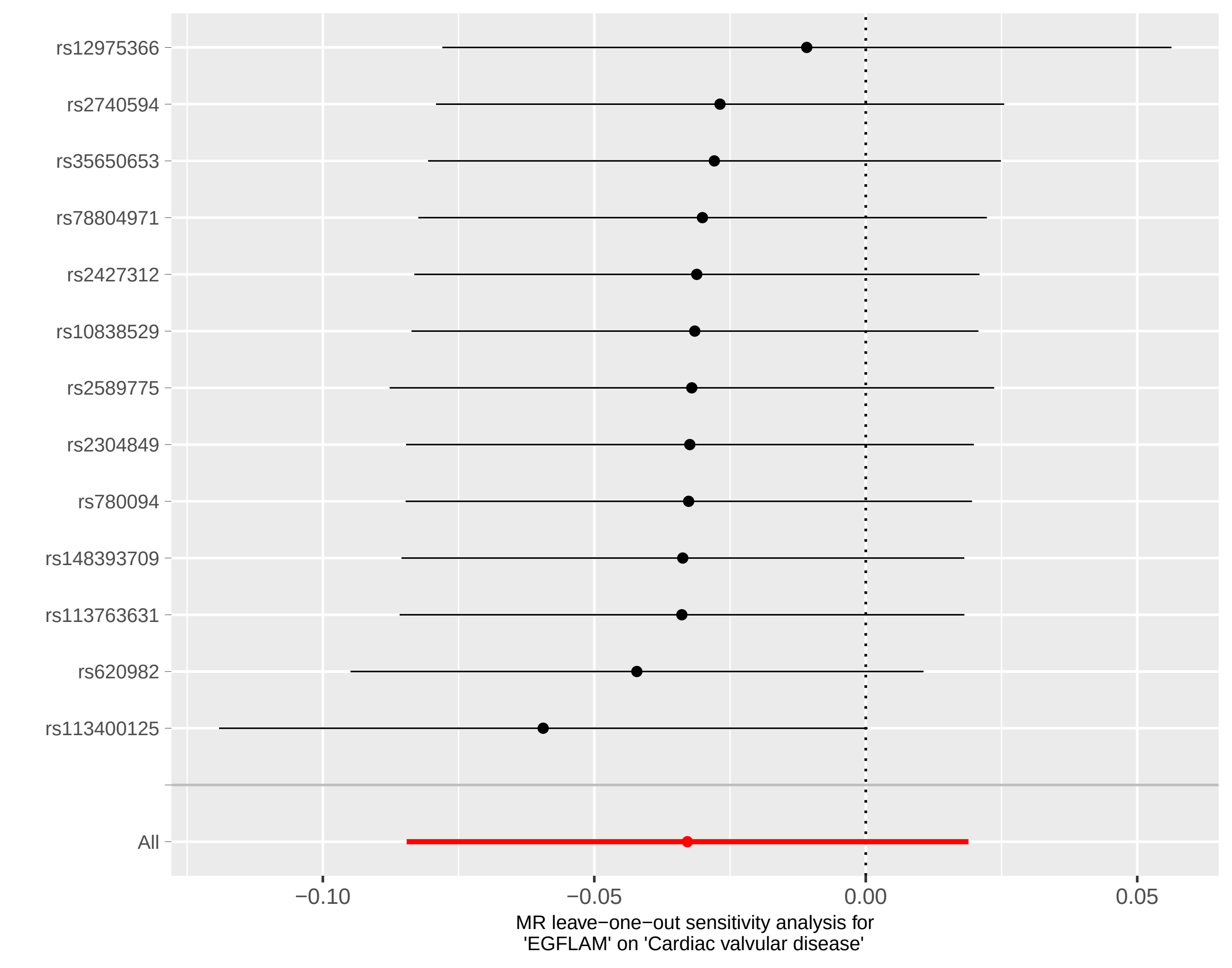

rs12975366
rs2740594
rs35650653
rs78804971
rs2427312
rs10838529
rs2589775
rs2304849
rs780094
rs148393709
rs113763631
rs620982
rs113400125
All
−0.10
−0.05
0.00
0.05
MR leave−one−out sensitivity analysis for
'EGFLAM' on 'Cardiac valvular disease'

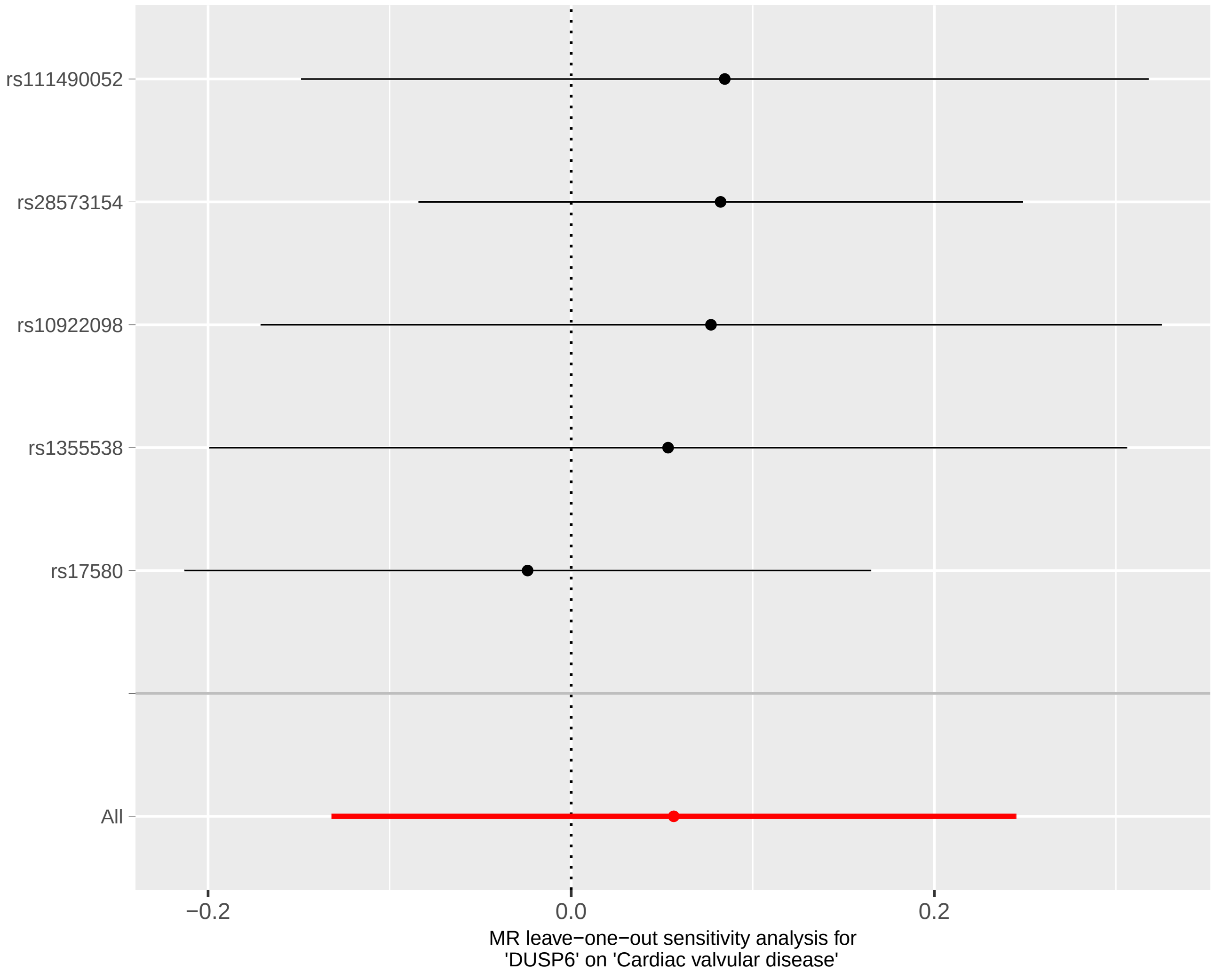
rs111490052
rs28573154
rs10922098
rs1355538
rs17580
All
−0.2
0.0
0.2
MR leave−one−out sensitivity analysis for
'DUSP6' on 'Cardiac valvular disease'

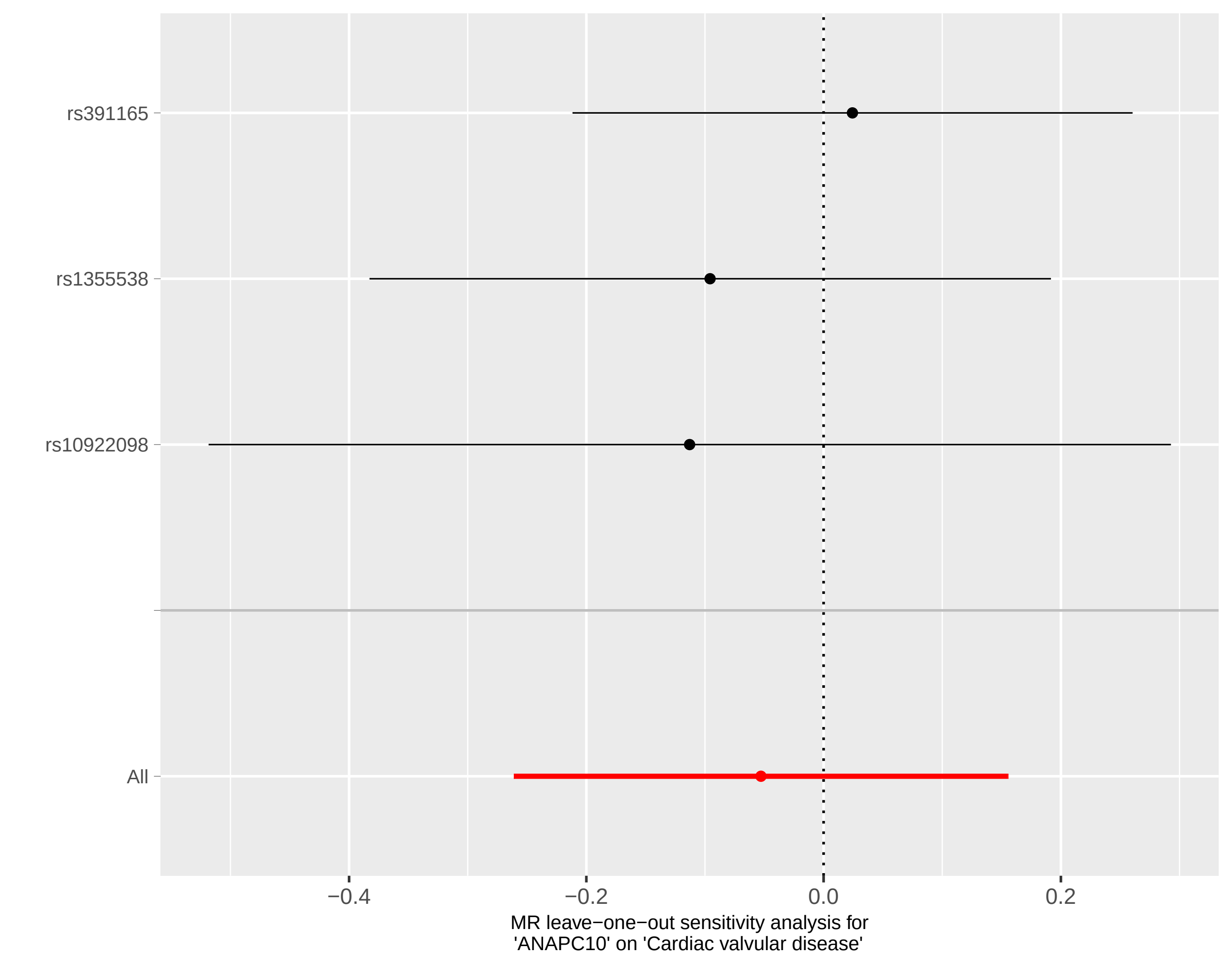
rs391165
rs1355538
rs10922098
All
−0.4
−0.2
0.0
0.2
MR leave−one−out sensitivity analysis for
'ANAPC10' on 'Cardiac valvular disease'

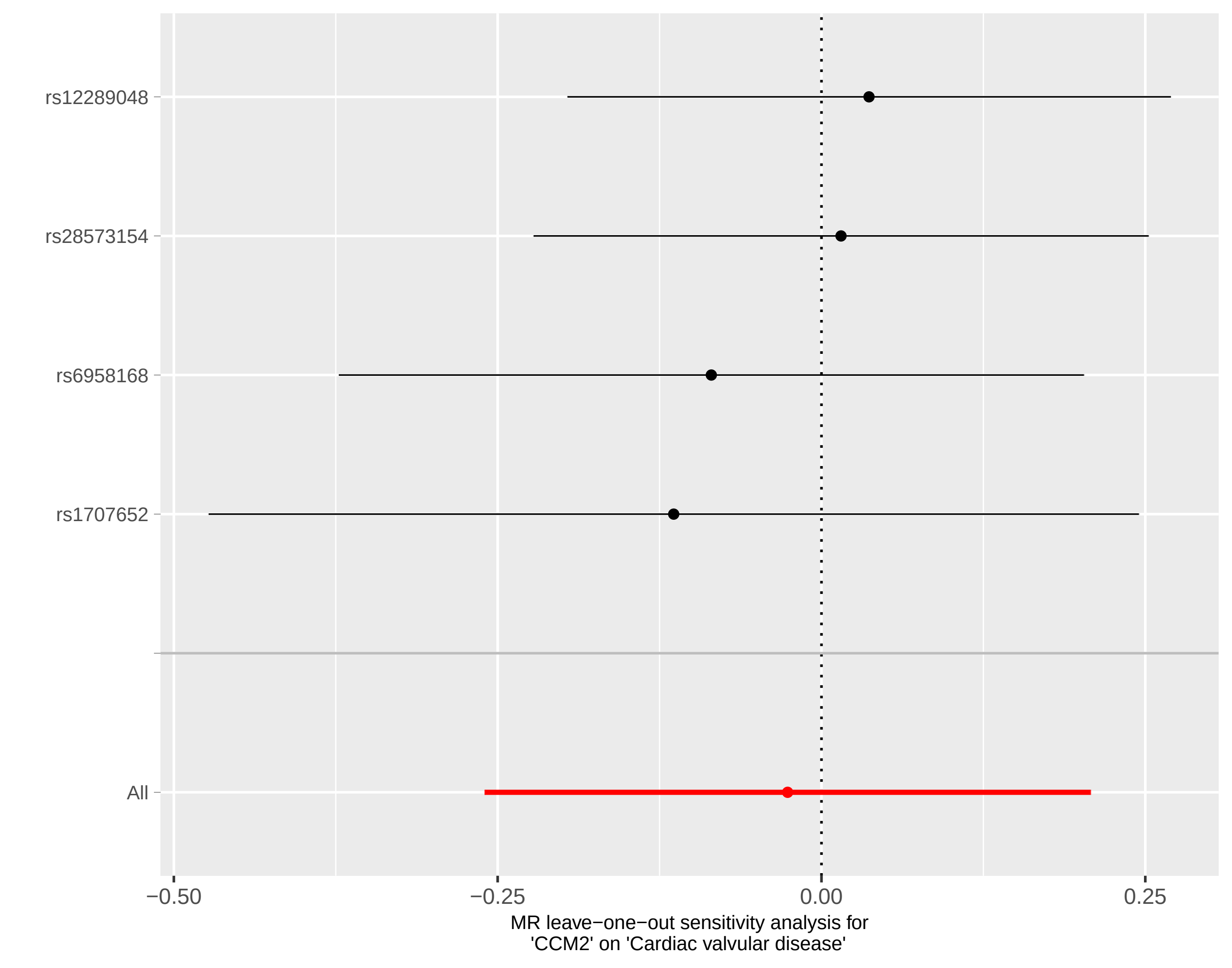
rs12289048
rs28573154
rs6958168
rs1707652
All
−0.50
−0.25
0.00
0.25
MR leave−one−out sensitivity analysis for
'CCM2' on 'Cardiac valvular disease'

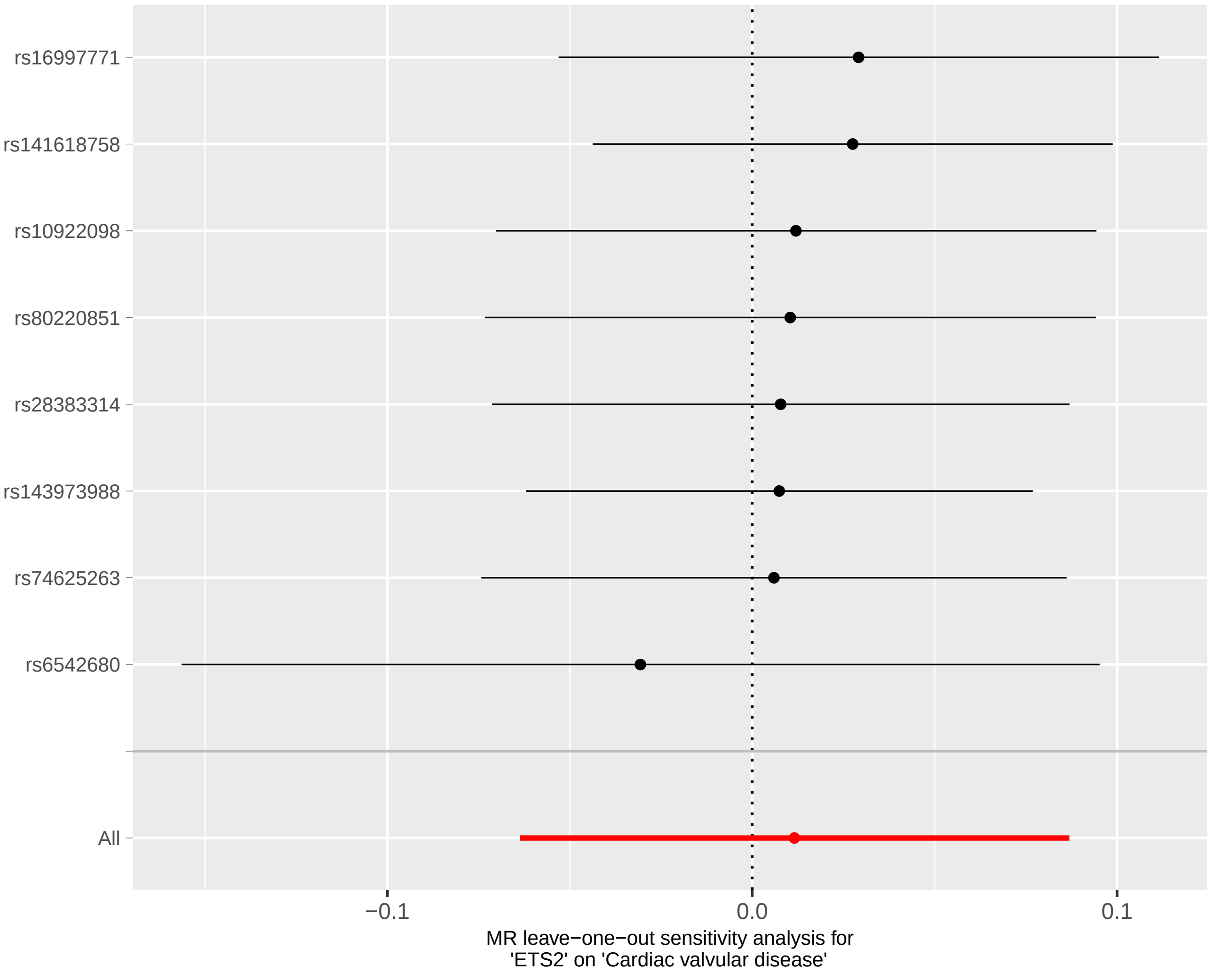
rs16997771
rs141618758
rs10922098
rs80220851
rs28383314
rs143973988
rs74625263
rs6542680
All
−0.1
0.0
0.1
MR leave−one−out sensitivity analysis for
'ETS2' on 'Cardiac valvular disease'

Insufficient number of SNPs

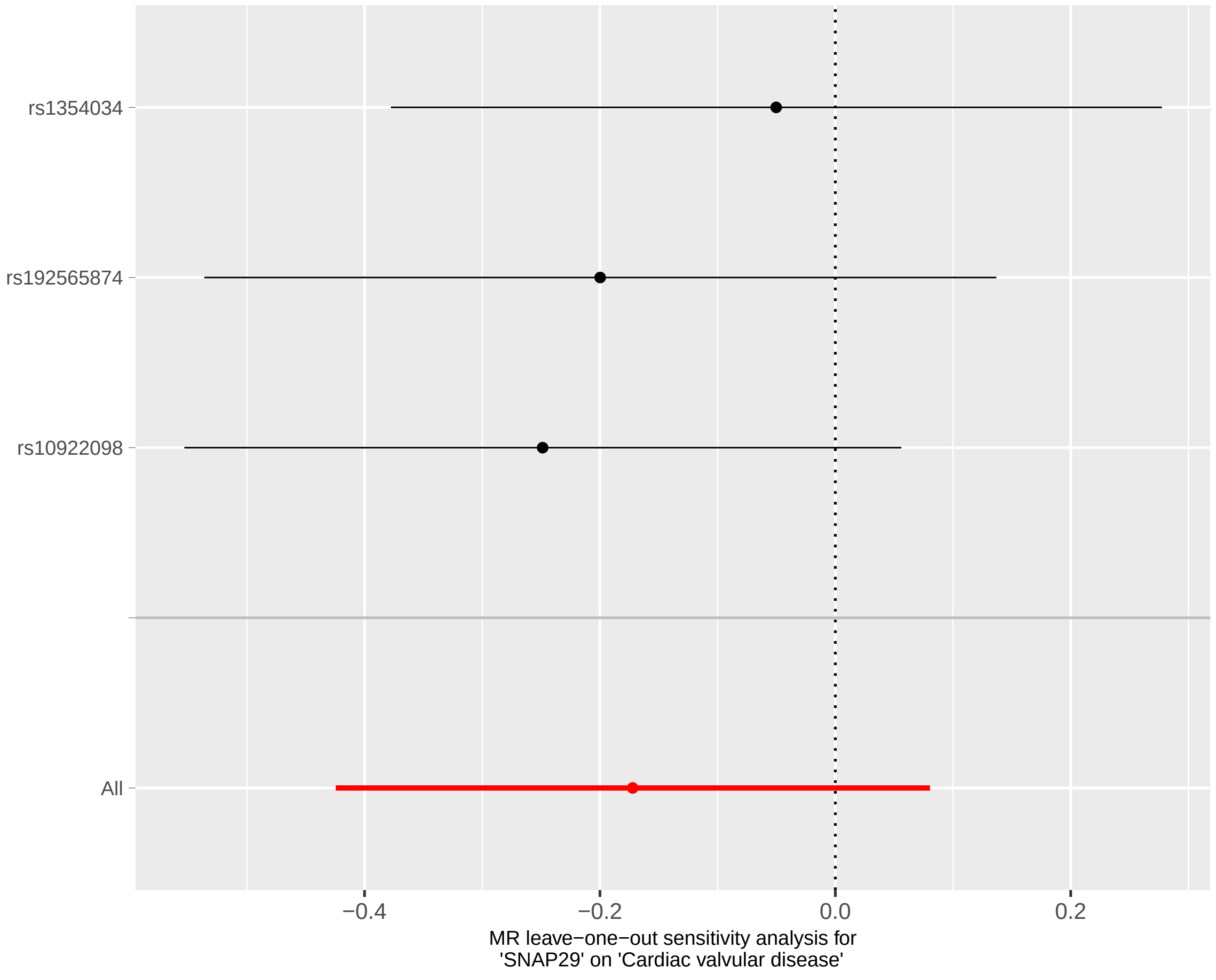
rs1354034
rs192565874
rs10922098
All
−0.4
−0.2
0.0
0.2
MR leave−one−out sensitivity analysis for
'SNAP29' on 'Cardiac valvular disease'

Insufficient number of SNPs

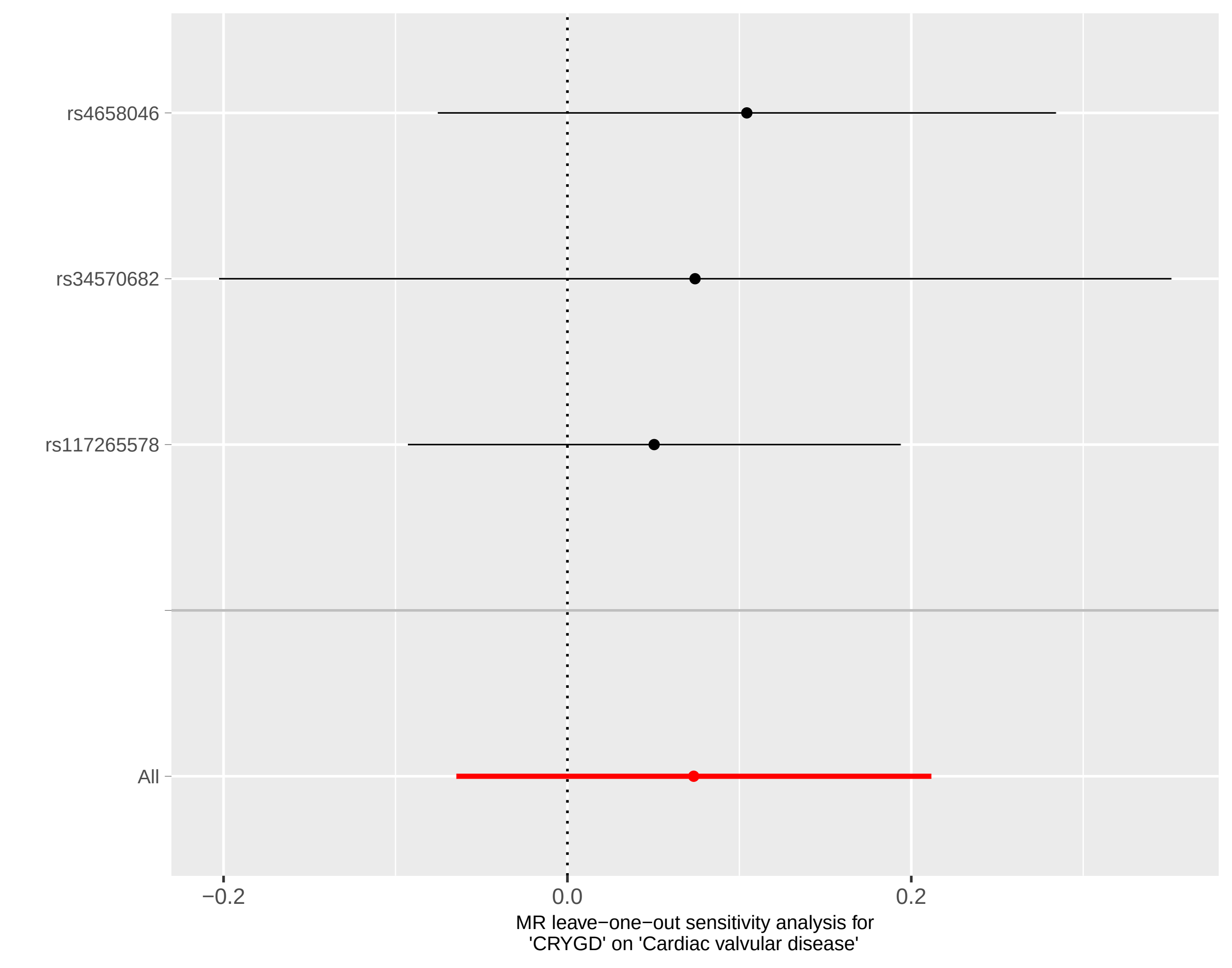
rs4658046
rs34570682
rs117265578
All
−0.2
0.0
0.2
MR leave−one−out sensitivity analysis for
'CRYGD' on 'Cardiac valvular disease'

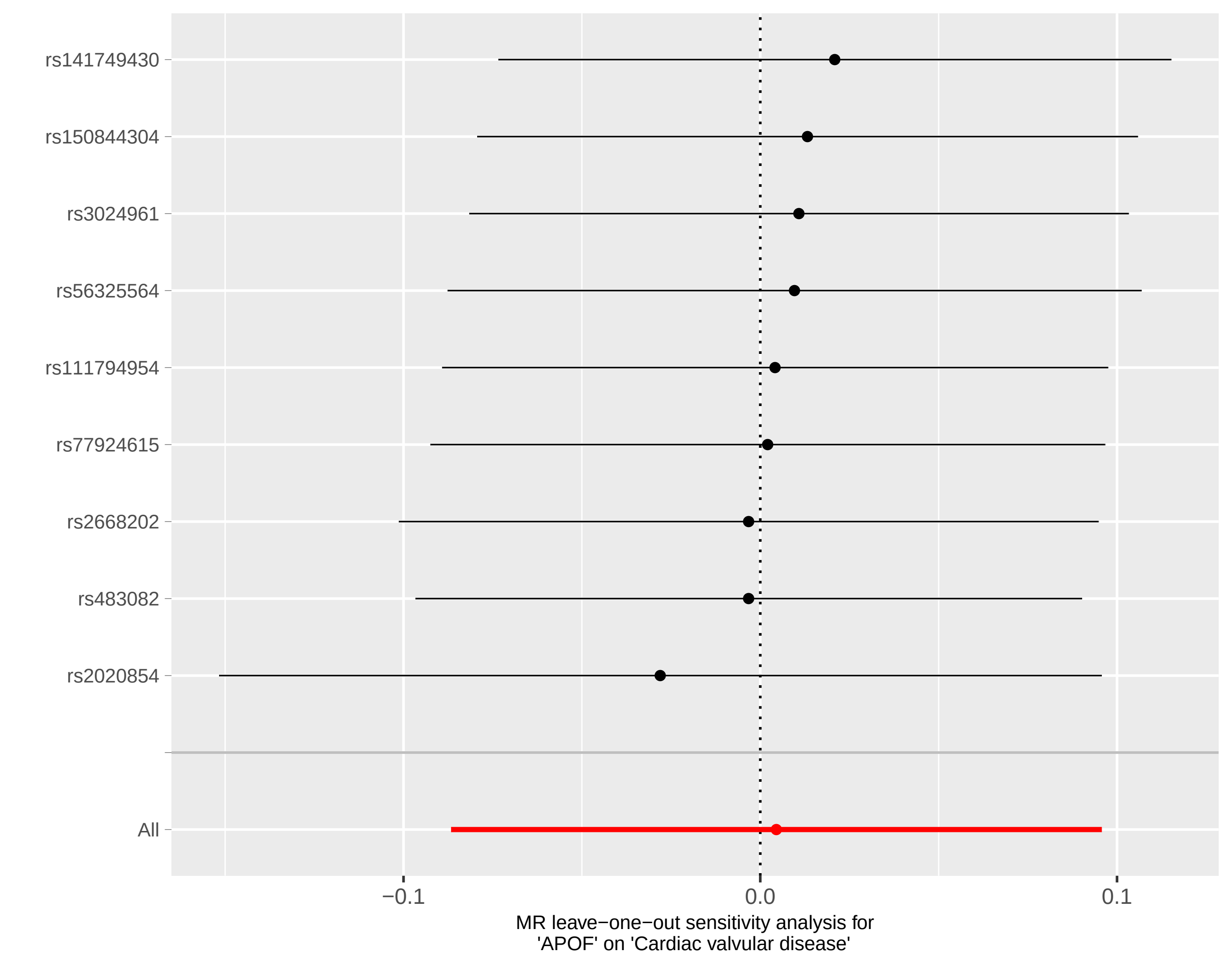

rs141749430
rs150844304
rs3024961
rs56325564
rs111794954
rs77924615
rs2668202
rs483082
rs2020854
All
−0.1
0.0
0.1
MR leave−one−out sensitivity analysis for
'APOF' on 'Cardiac valvular disease'

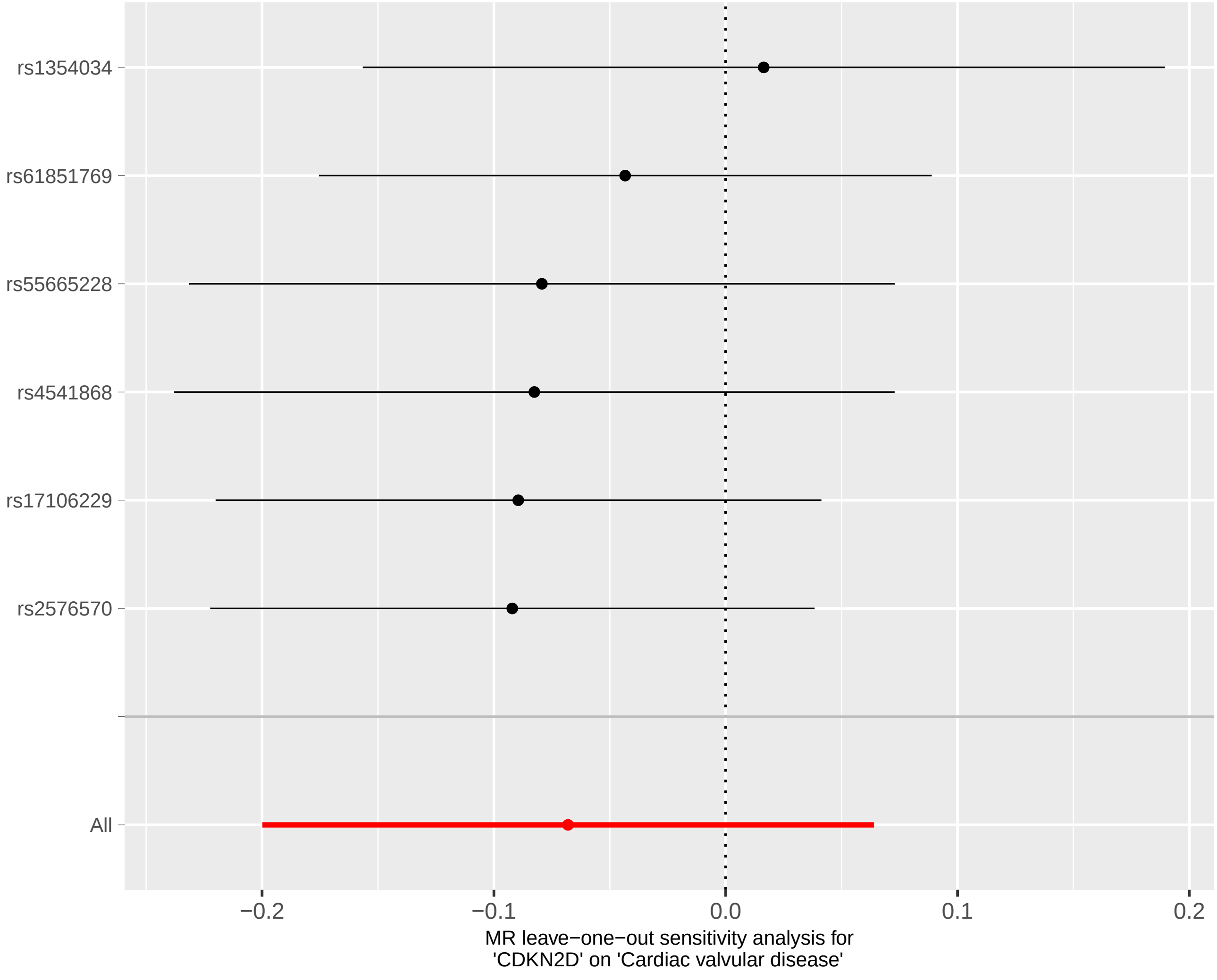

rs1354034
rs61851769
rs55665228
rs4541868
rs17106229
rs2576570
All
−0.2
−0.1
0.0
0.1
0.2
MR leave−one−out sensitivity analysis for
'CDKN2D' on 'Cardiac valvular disease'

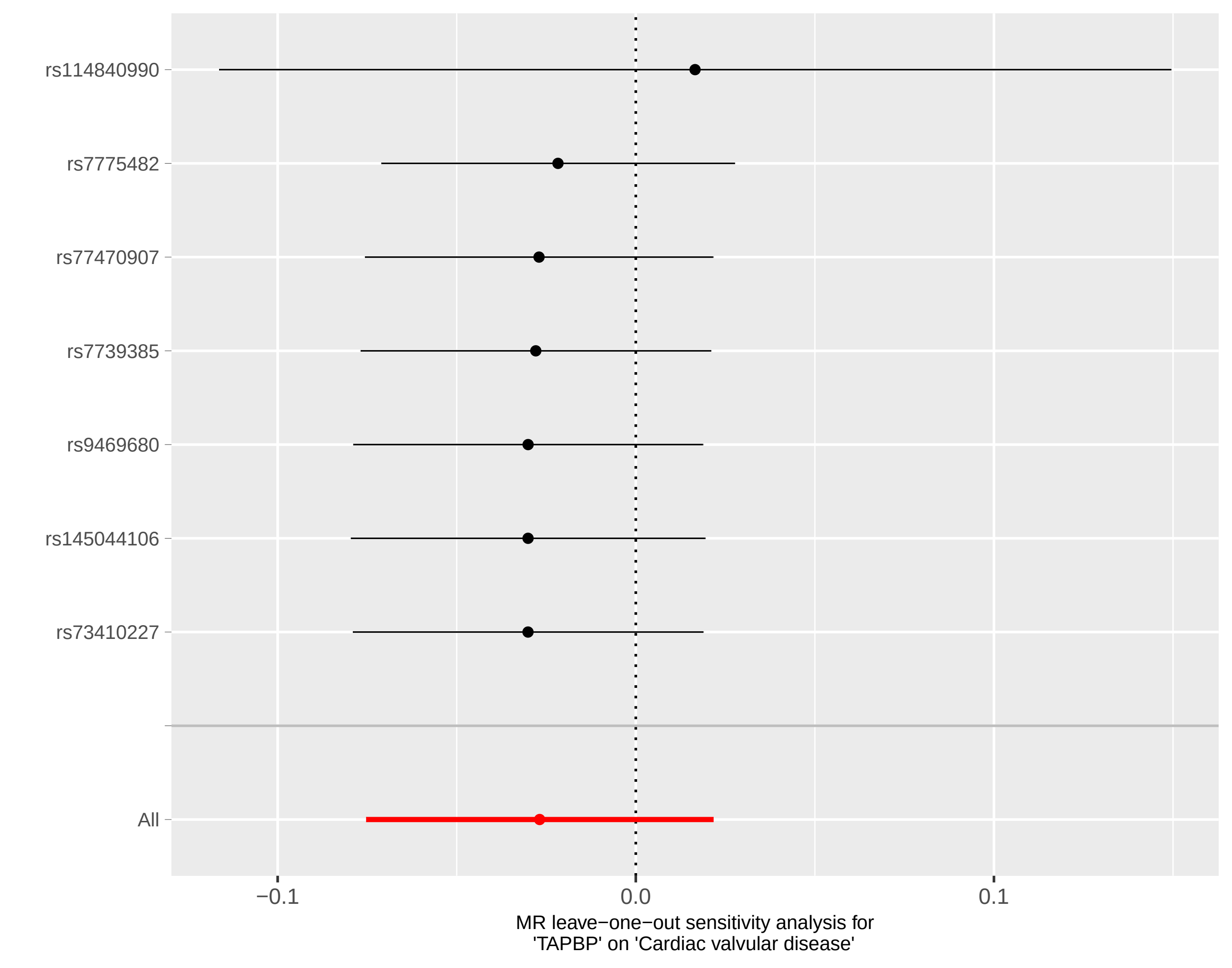

rs114840990
rs7775482
rs77470907
rs7739385
rs9469680
rs145044106
rs73410227
All
−0.1
0.0
0.1
MR leave−one−out sensitivity analysis for
'TAPBP' on 'Cardiac valvular disease'

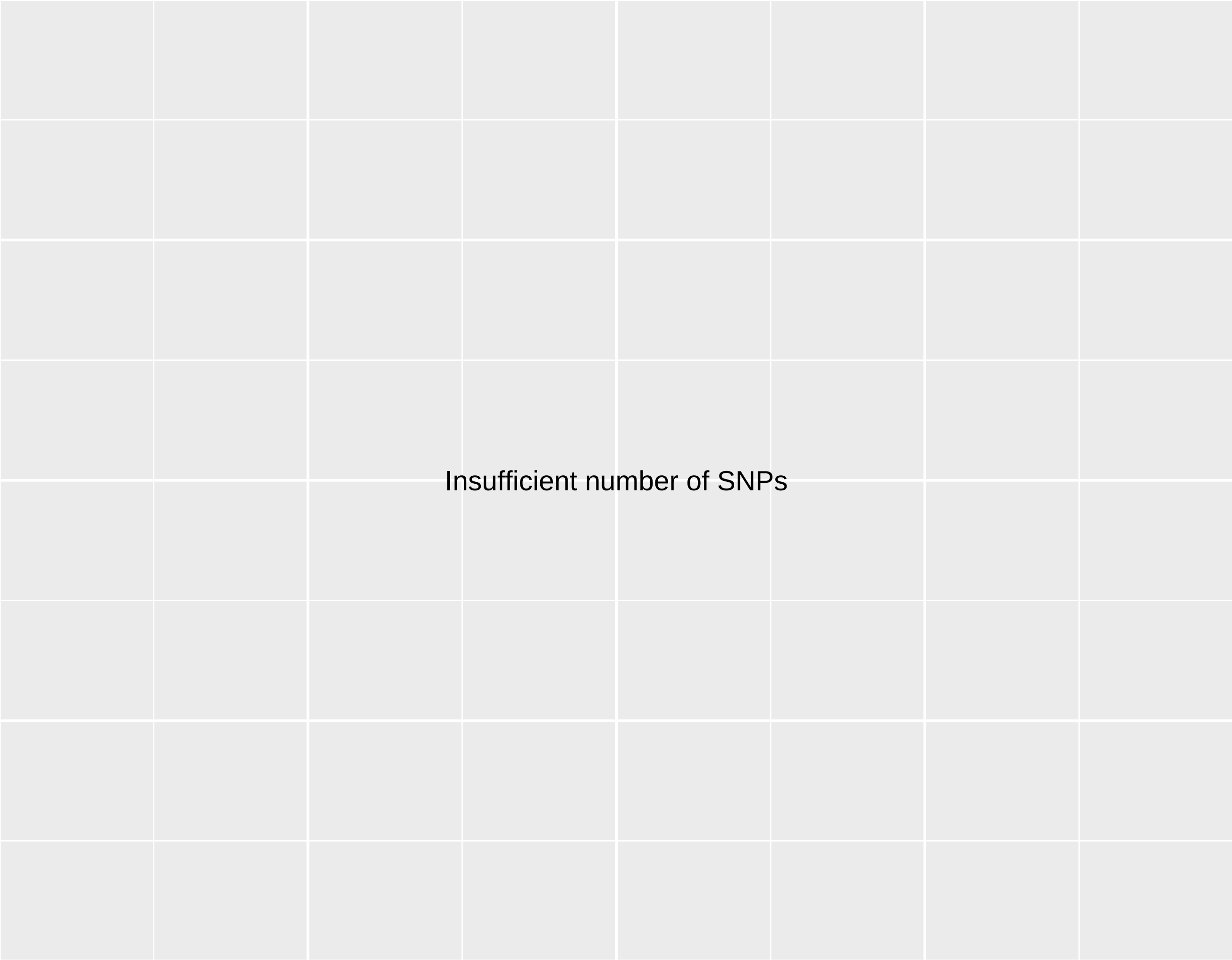
Insufficient number of SNPs

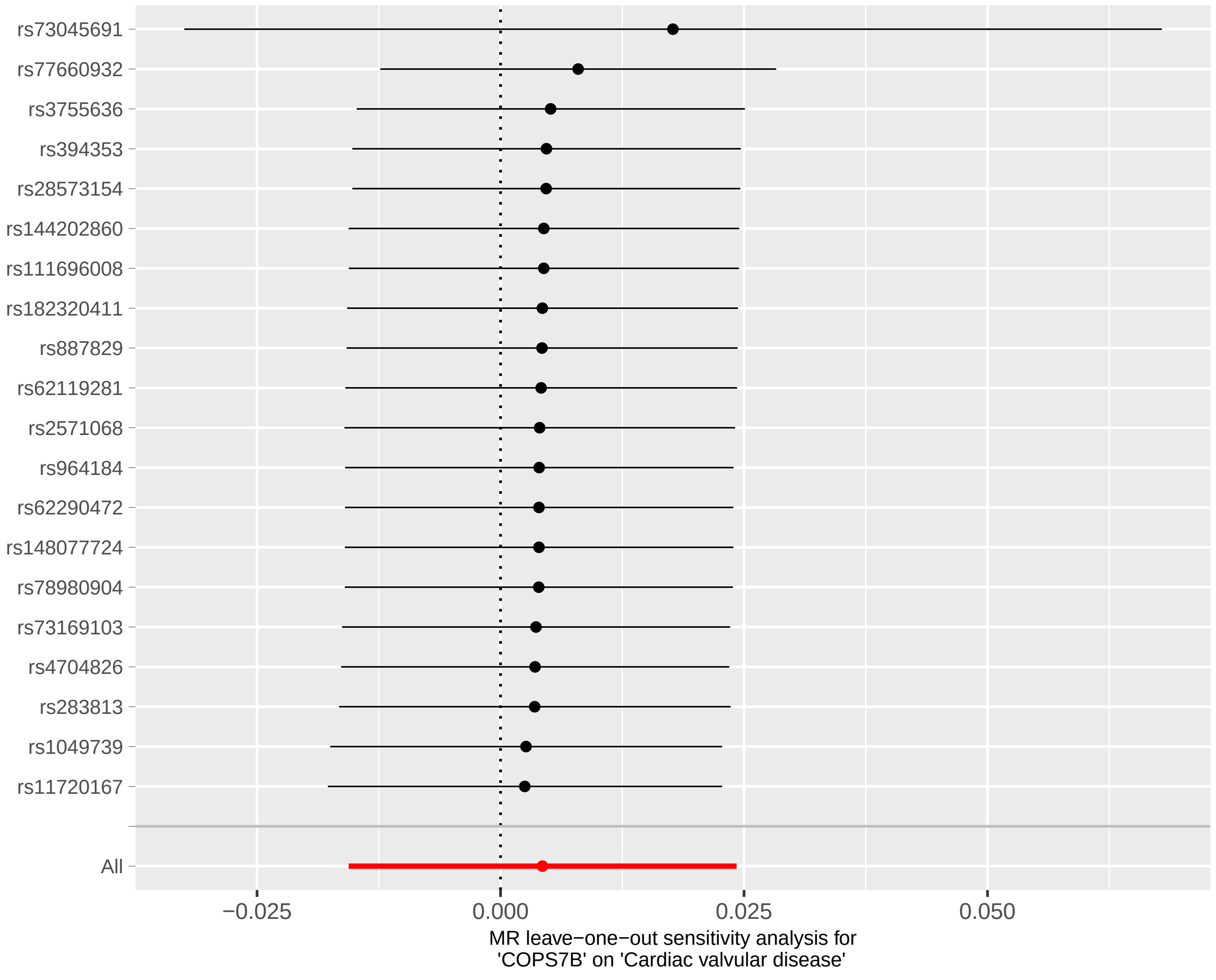
rs73045691
rs77660932
rs3755636
rs394353
rs28573154
rs144202860
rs111696008
rs182320411
rs887829
rs62119281
rs2571068
rs964184
rs62290472
rs148077724
rs78980904
rs73169103
rs4704826
rs283813
rs1049739
rs11720167
All
−0.025
0.000
0.025
0.050
MR leave−one−out sensitivity analysis for
'COPS7B' on 'Cardiac valvular disease'

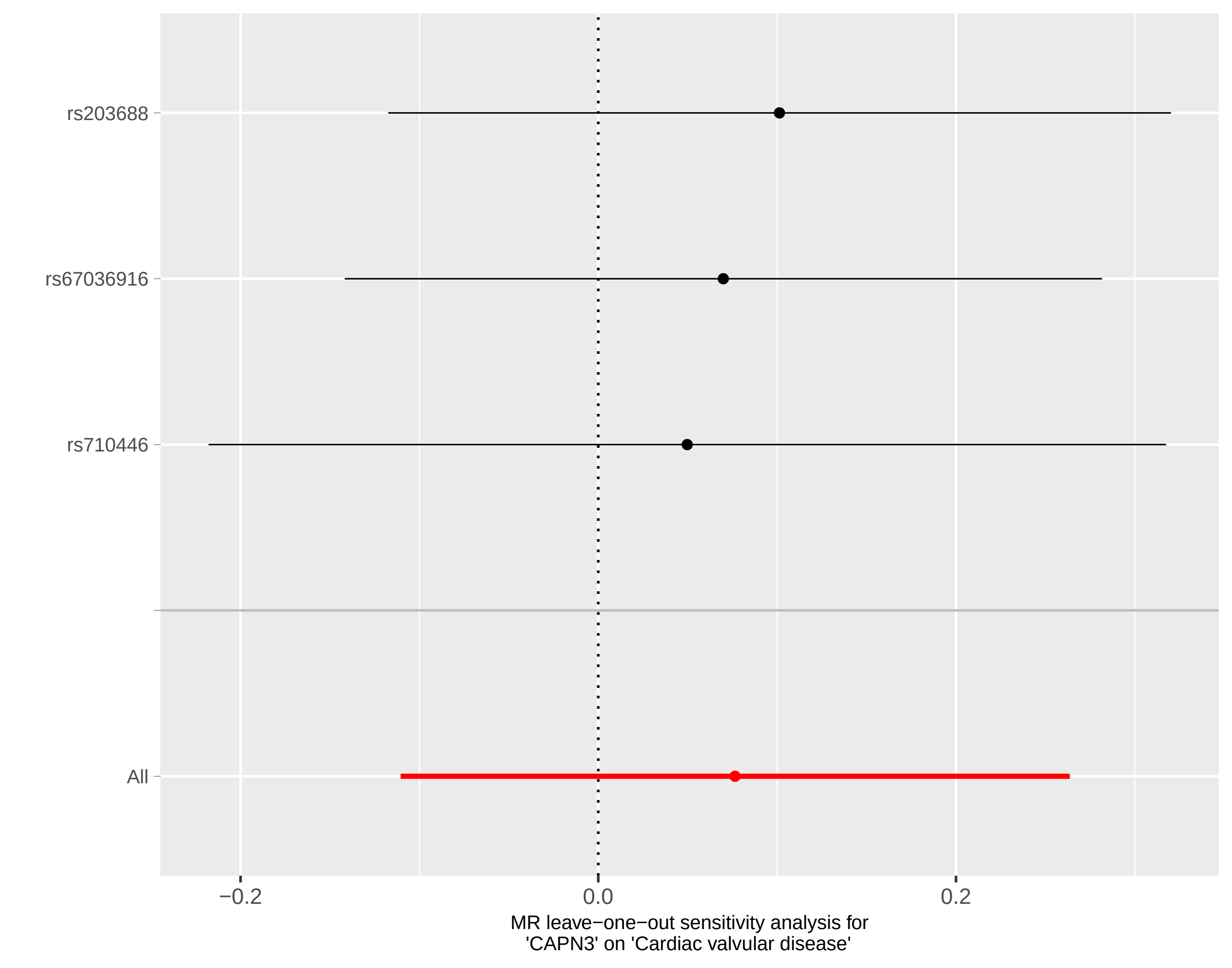

rs203688
rs67036916
rs710446
All
−0.2
0.0
0.2
MR leave−one−out sensitivity analysis for
'CAPN3' on 'Cardiac valvular disease'

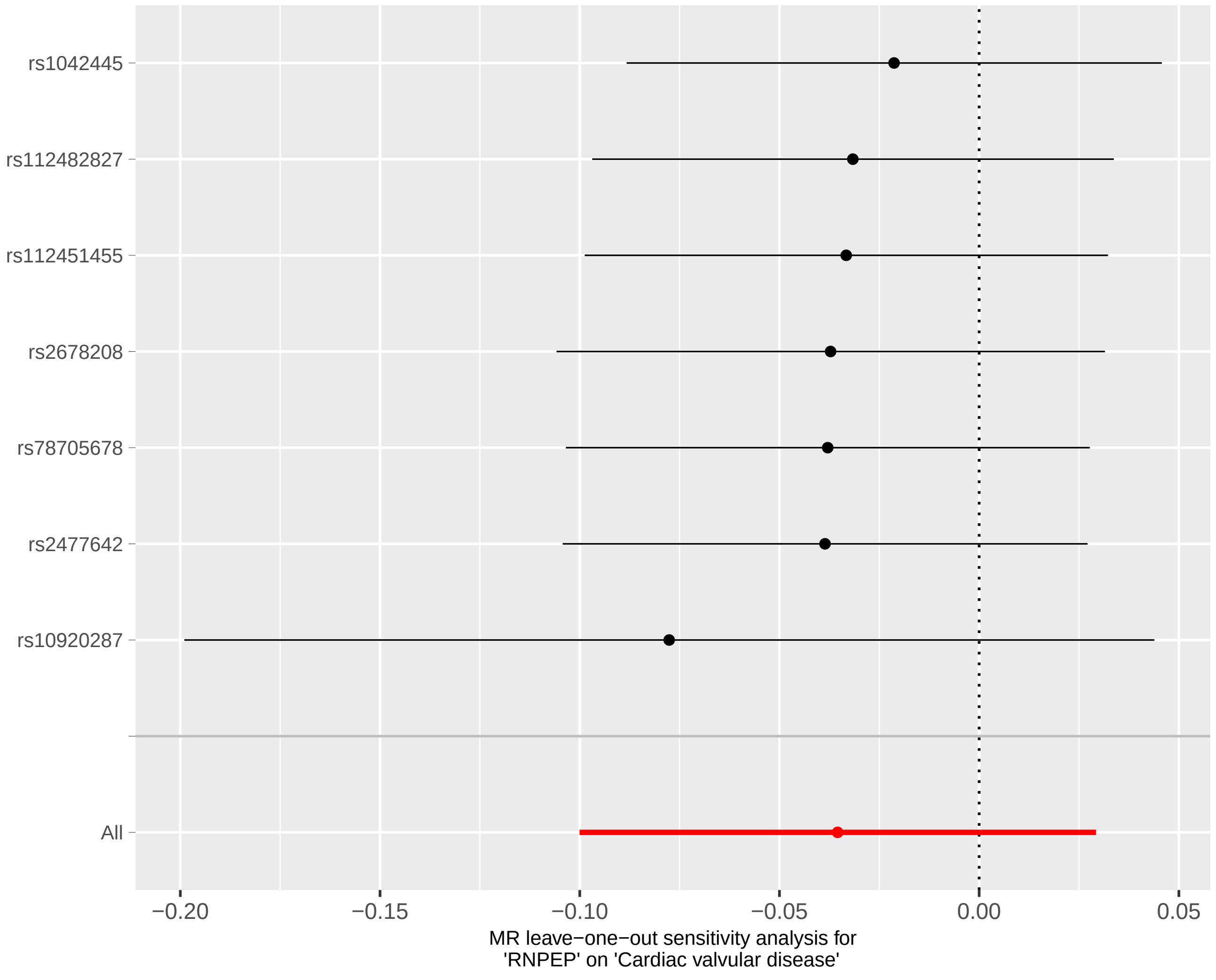
rs1042445
rs112482827
rs112451455
rs2678208
rs78705678
rs2477642
rs10920287
All
−0.20
−0.15
−0.10
−0.05
0.00
0.05
MR leave−one−out sensitivity analysis for
'RNPEP' on 'Cardiac valvular disease'

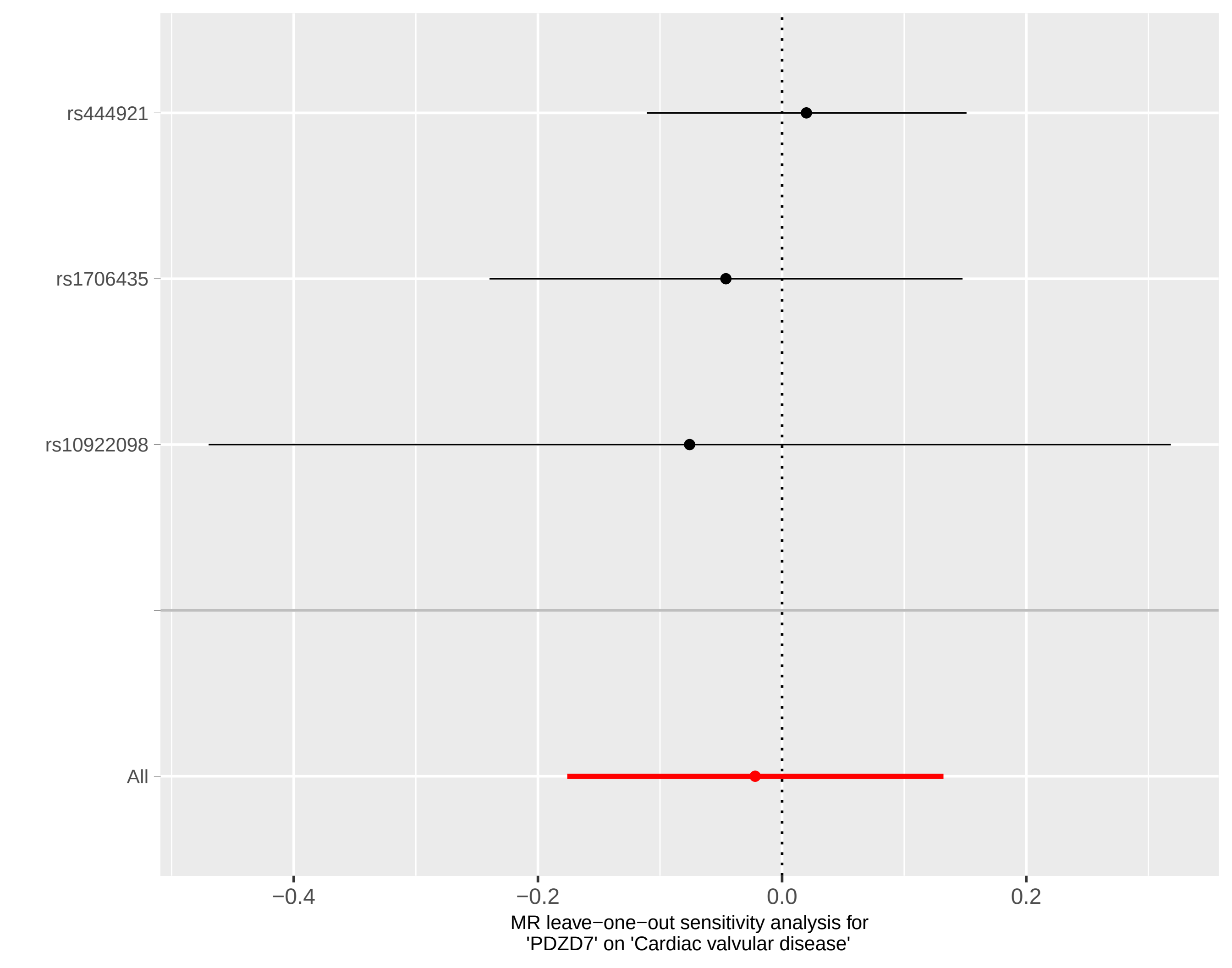
rs444921
rs1706435
rs10922098
All
−0.4
−0.2
0.0
0.2
MR leave−one−out sensitivity analysis for
'PDZD7' on 'Cardiac valvular disease'

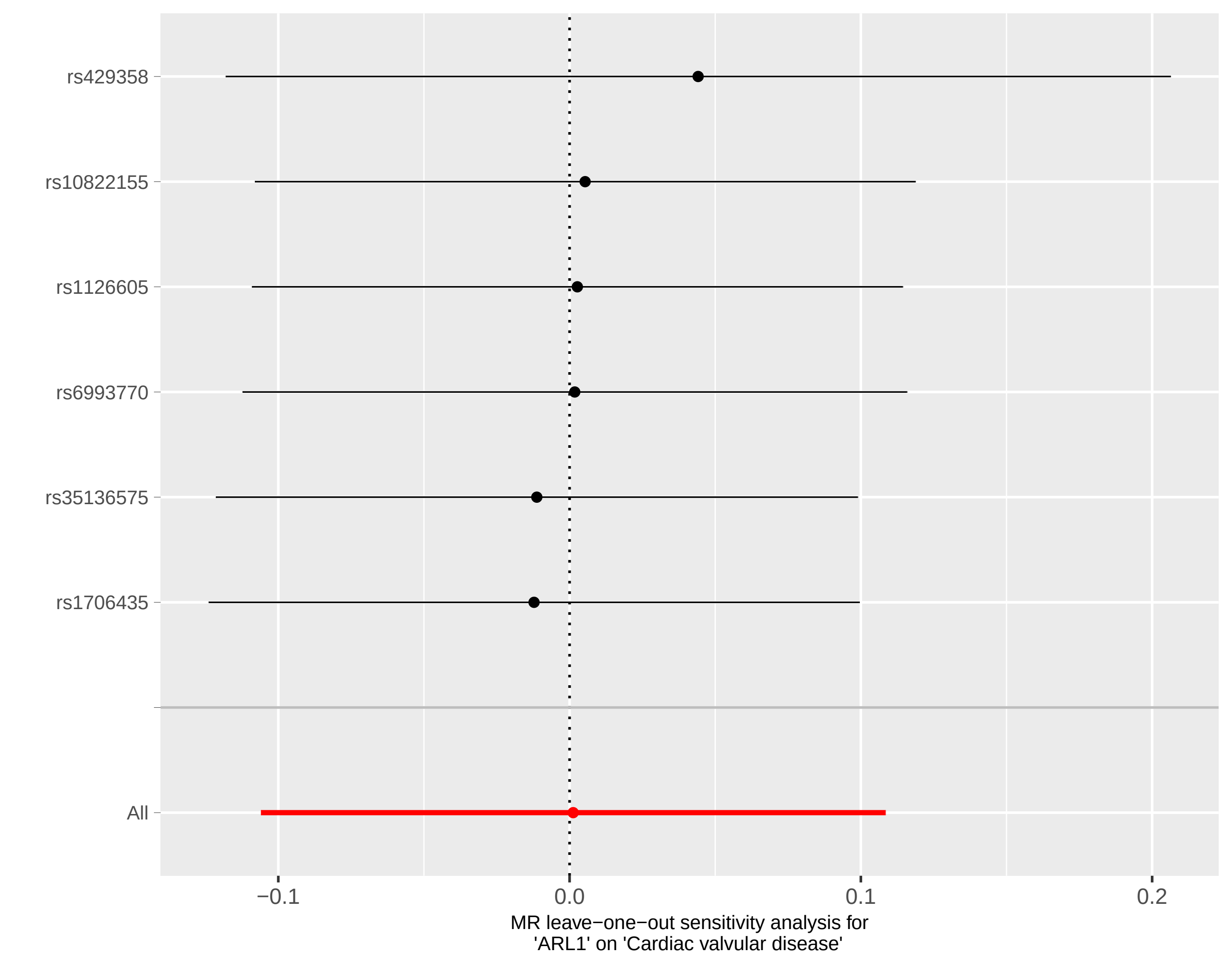
rs429358
rs10822155
rs1126605
rs6993770
rs35136575
rs1706435
All
−0.1
0.0
0.1
0.2
MR leave−one−out sensitivity analysis for
'ARL1' on 'Cardiac valvular disease'

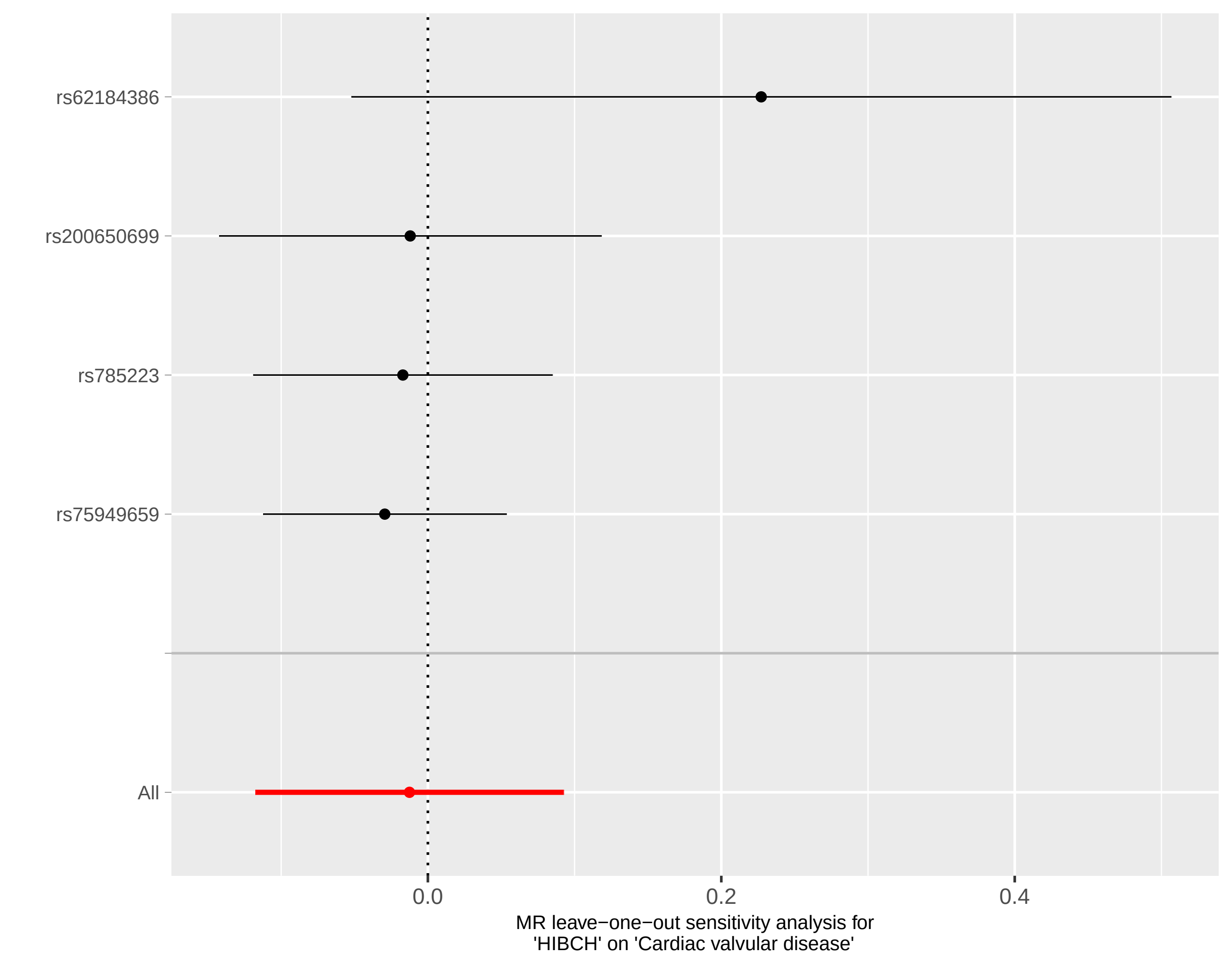
rs62184386
rs200650699
rs785223
rs75949659
All
0.0
0.2
0.4
MR leave−one−out sensitivity analysis for
'HIBCH' on 'Cardiac valvular disease'

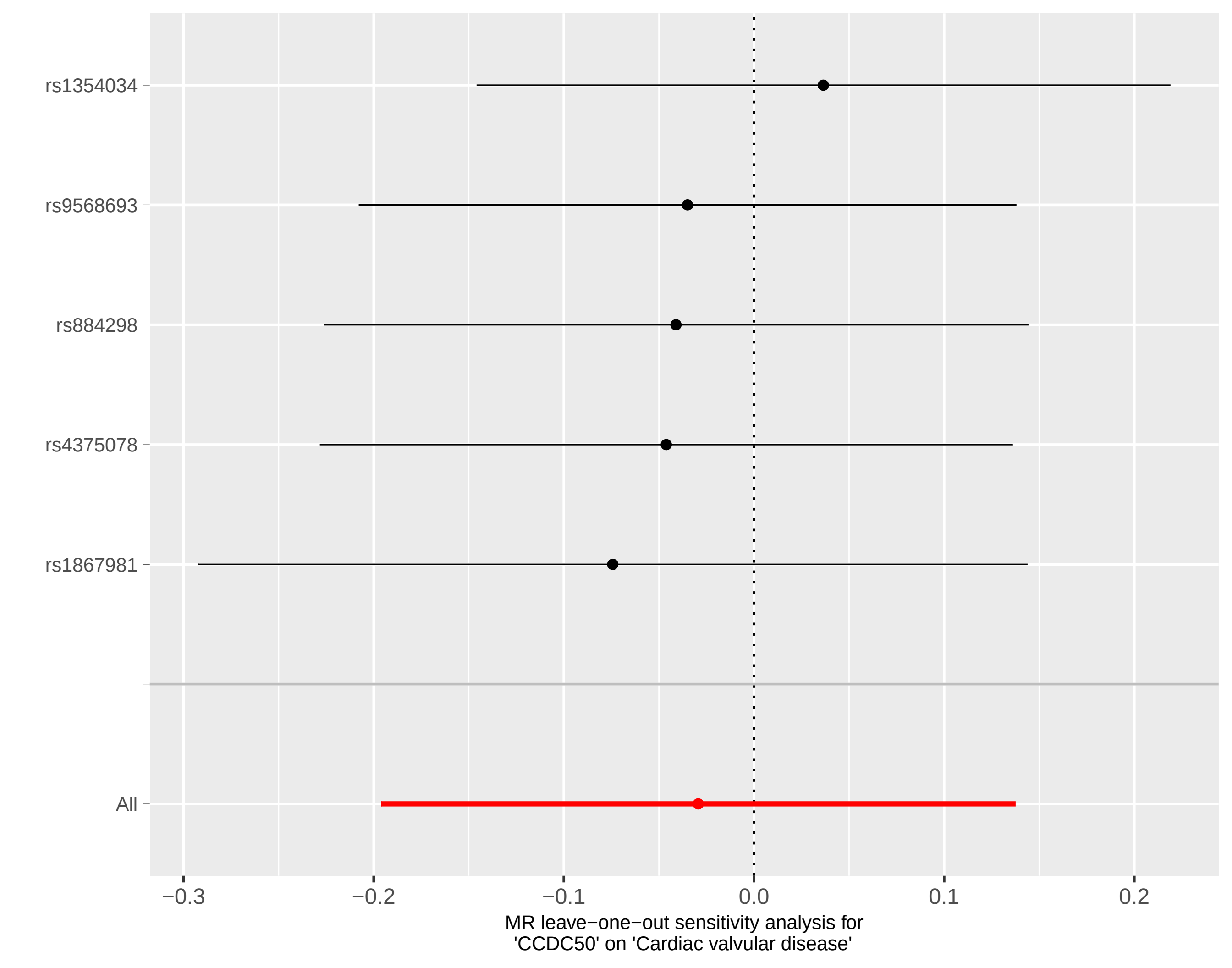

rs1354034
rs9568693
rs884298
rs4375078
rs1867981
All
−0.3
−0.2
−0.1
0.0
0.1
0.2
MR leave−one−out sensitivity analysis for
'CCDC50' on 'Cardiac valvular disease'

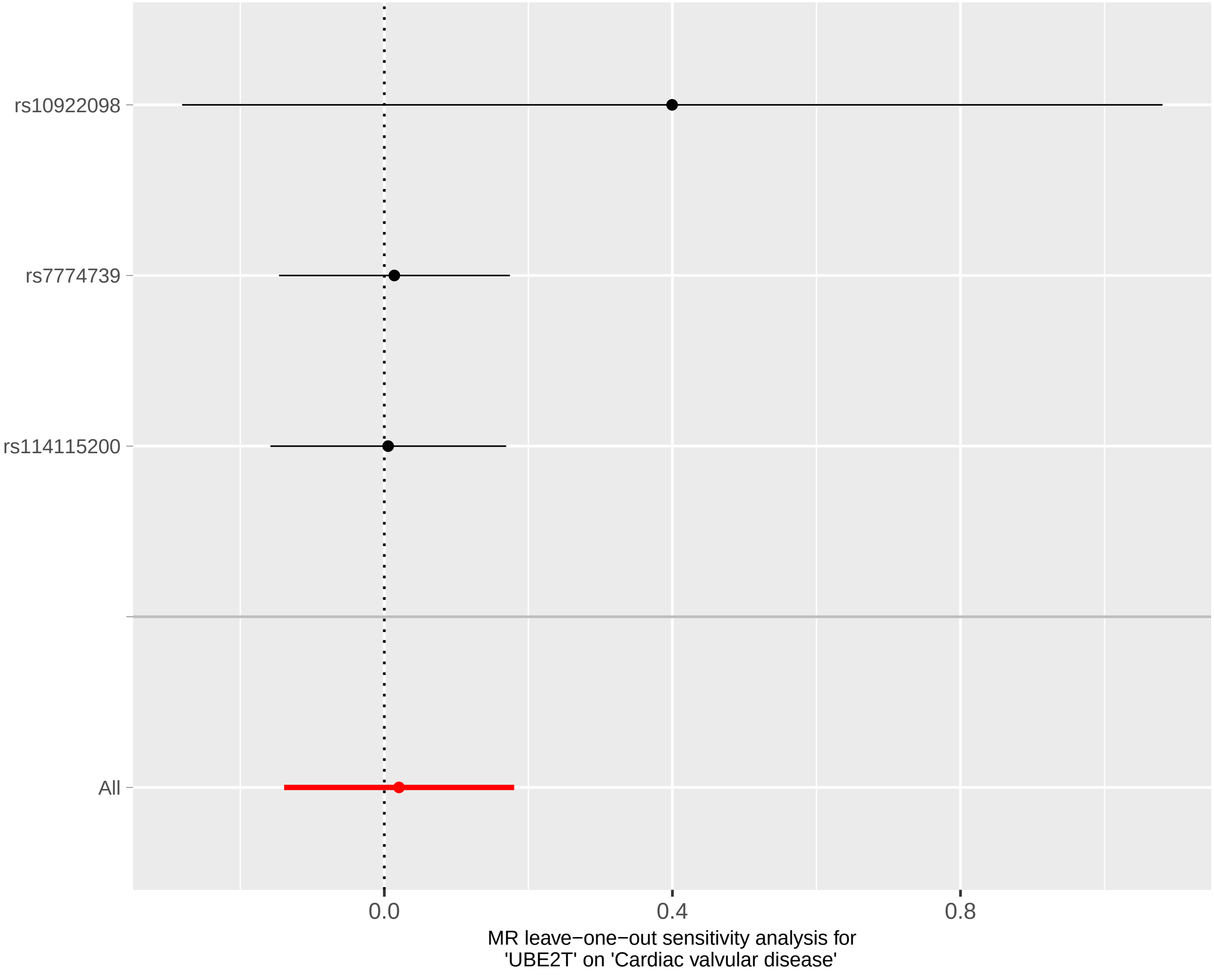
rs10922098
rs7774739
rs114115200
All
0.0
0.4
0.8
MR leave−one−out sensitivity analysis for
'UBE2T' on 'Cardiac valvular disease'

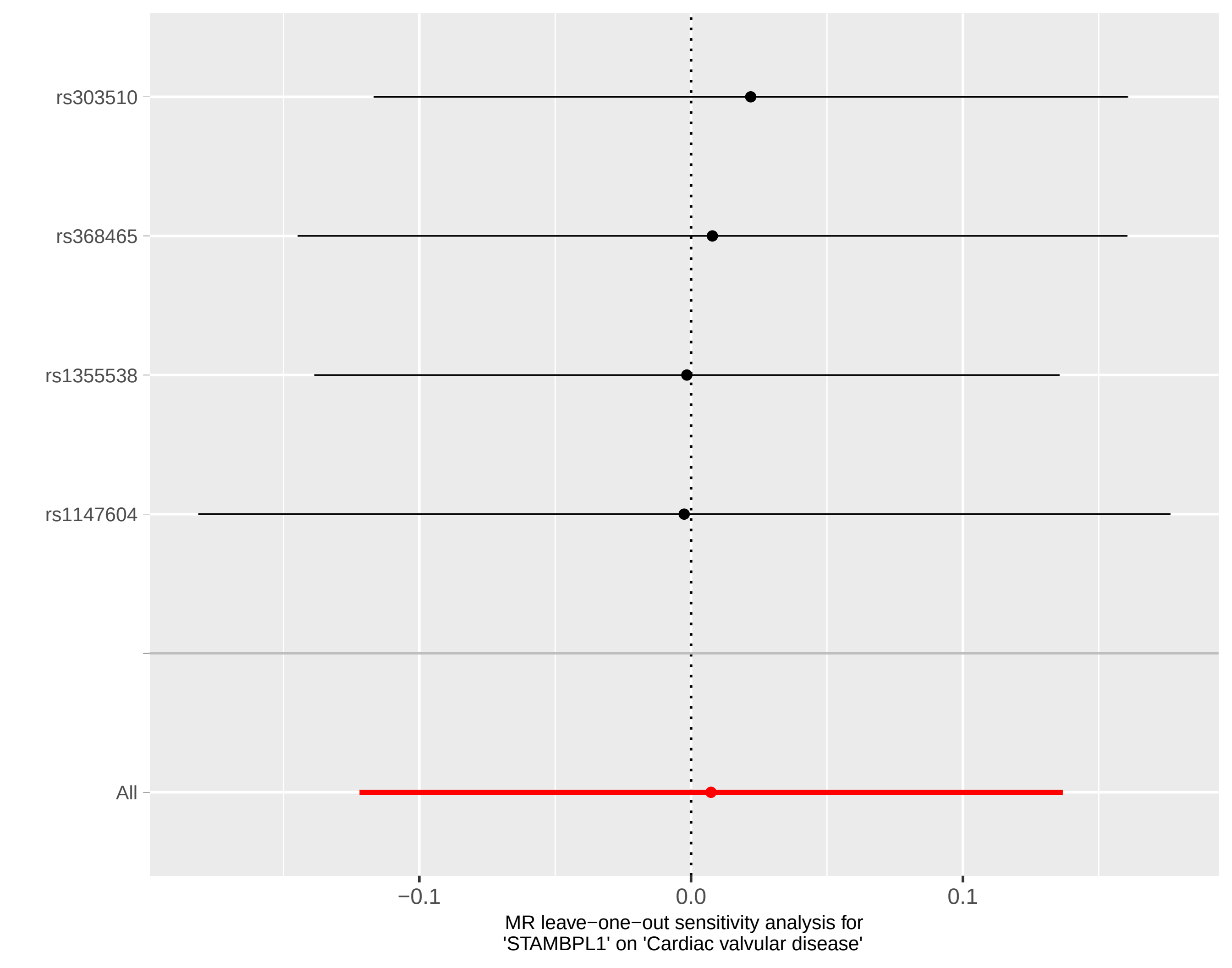
rs303510
rs368465
rs1355538
rs1147604
All
−0.1
0.0
0.1
MR leave−one−out sensitivity analysis for
'STAMBPL1' on 'Cardiac valvular disease'

Insufficient number of SNPs

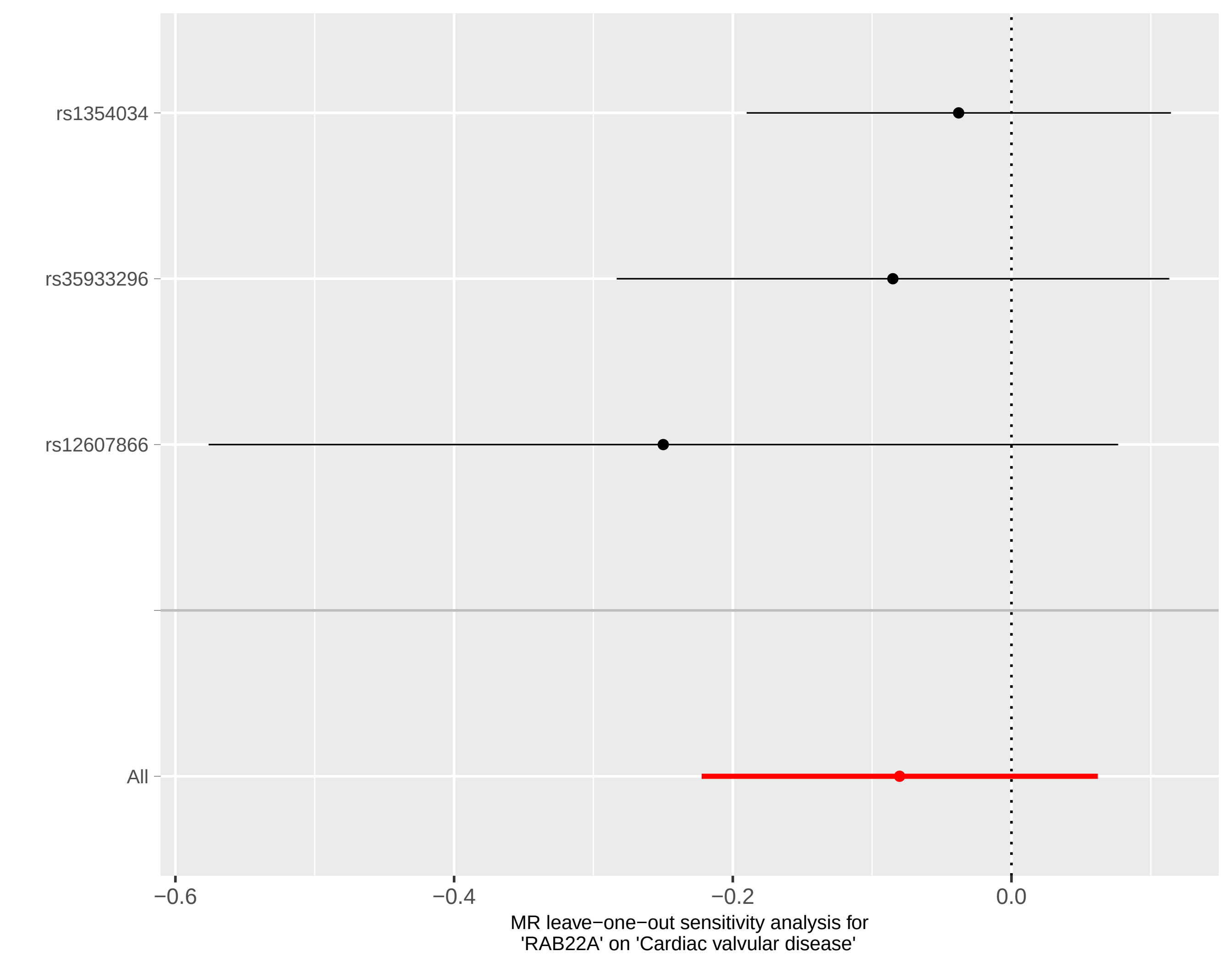
rs1354034
rs35933296
rs12607866
All
−0.6
−0.4
−0.2
0.0
MR leave−one−out sensitivity analysis for
'RAB22A' on 'Cardiac valvular disease'

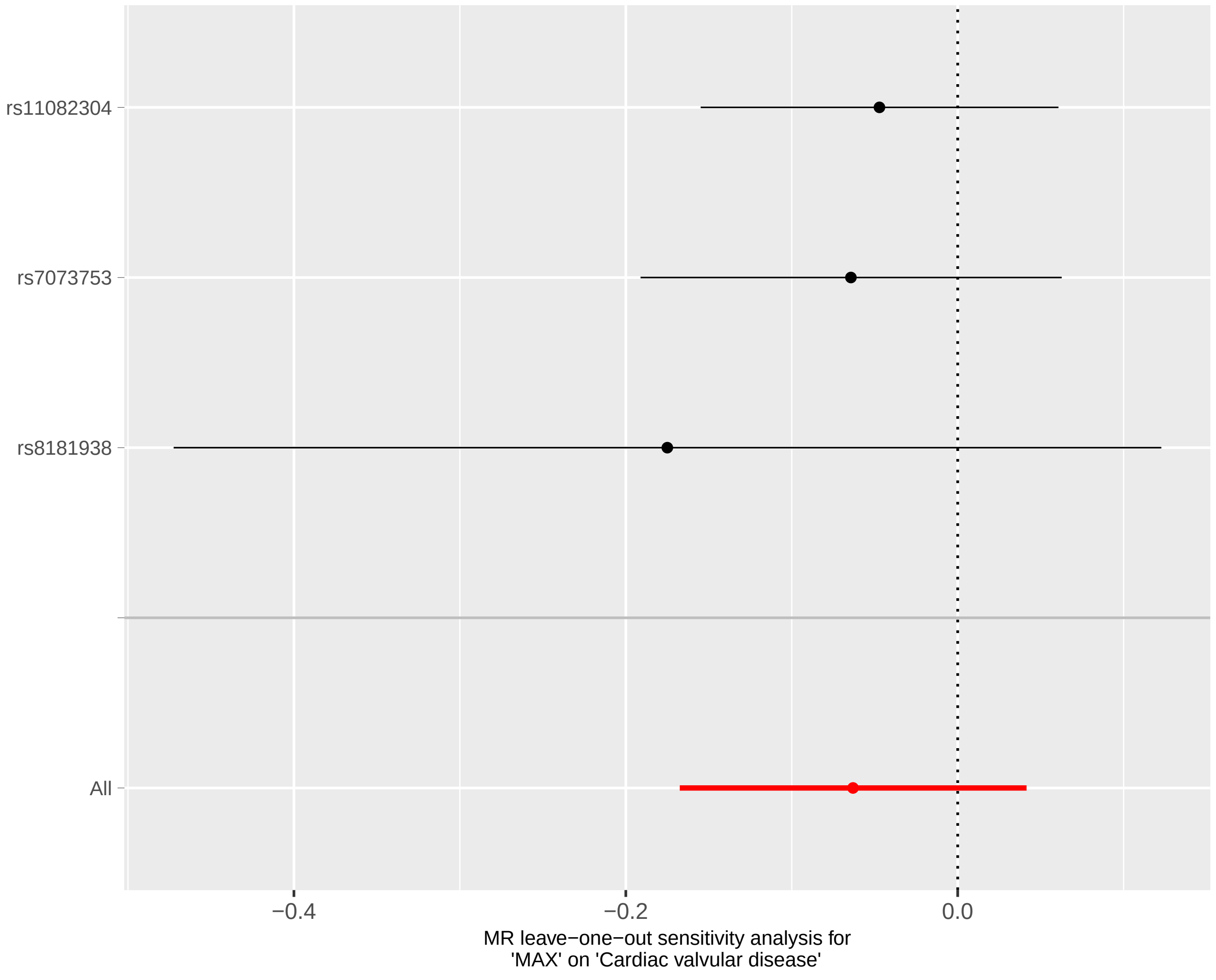
rs11082304
rs7073753
rs8181938
All
−0.4
−0.2
0.0
MR leave−one−out sensitivity analysis for
'MAX' on 'Cardiac valvular disease'

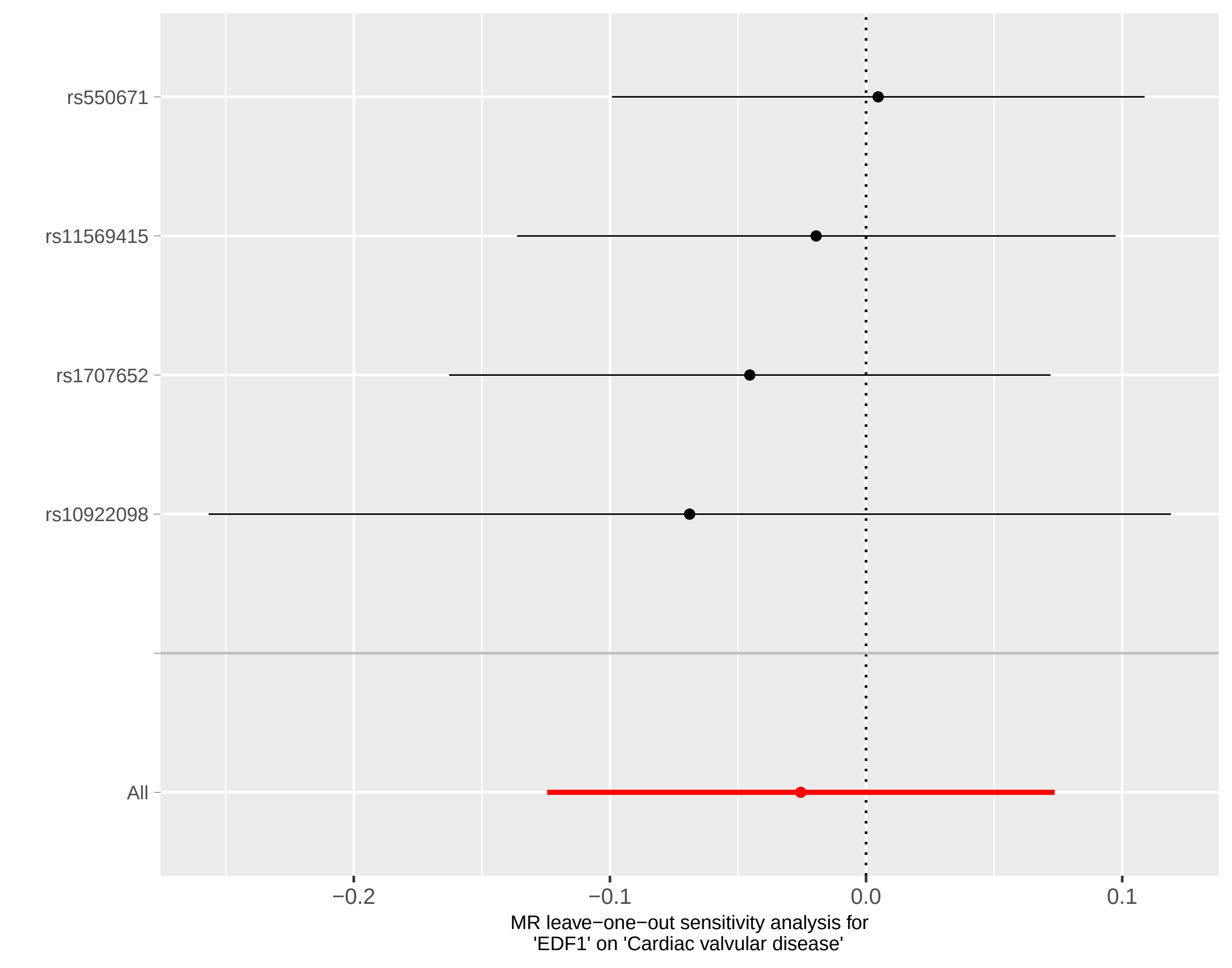

rs550671
rs11569415
rs1707652
rs10922098
All
−0.2
−0.1
0.0
0.1
MR leave−one−out sensitivity analysis for
'EDF1' on 'Cardiac valvular disease'

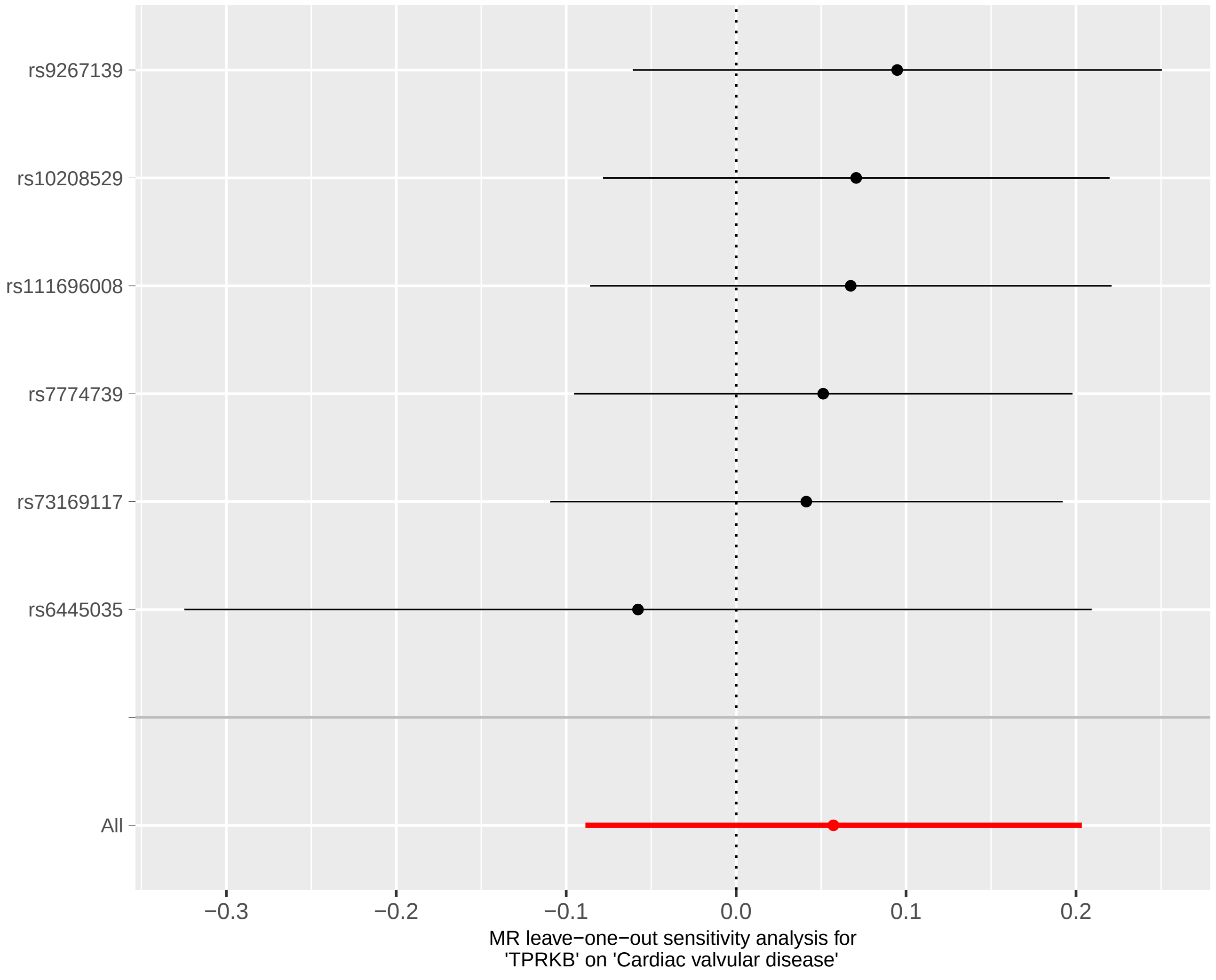

rs9267139
rs10208529
rs111696008
rs7774739
rs73169117
rs6445035
All
−0.3
−0.2
−0.1
0.0
0.1
0.2
MR leave−one−out sensitivity analysis for
'TPRKB' on 'Cardiac valvular disease'

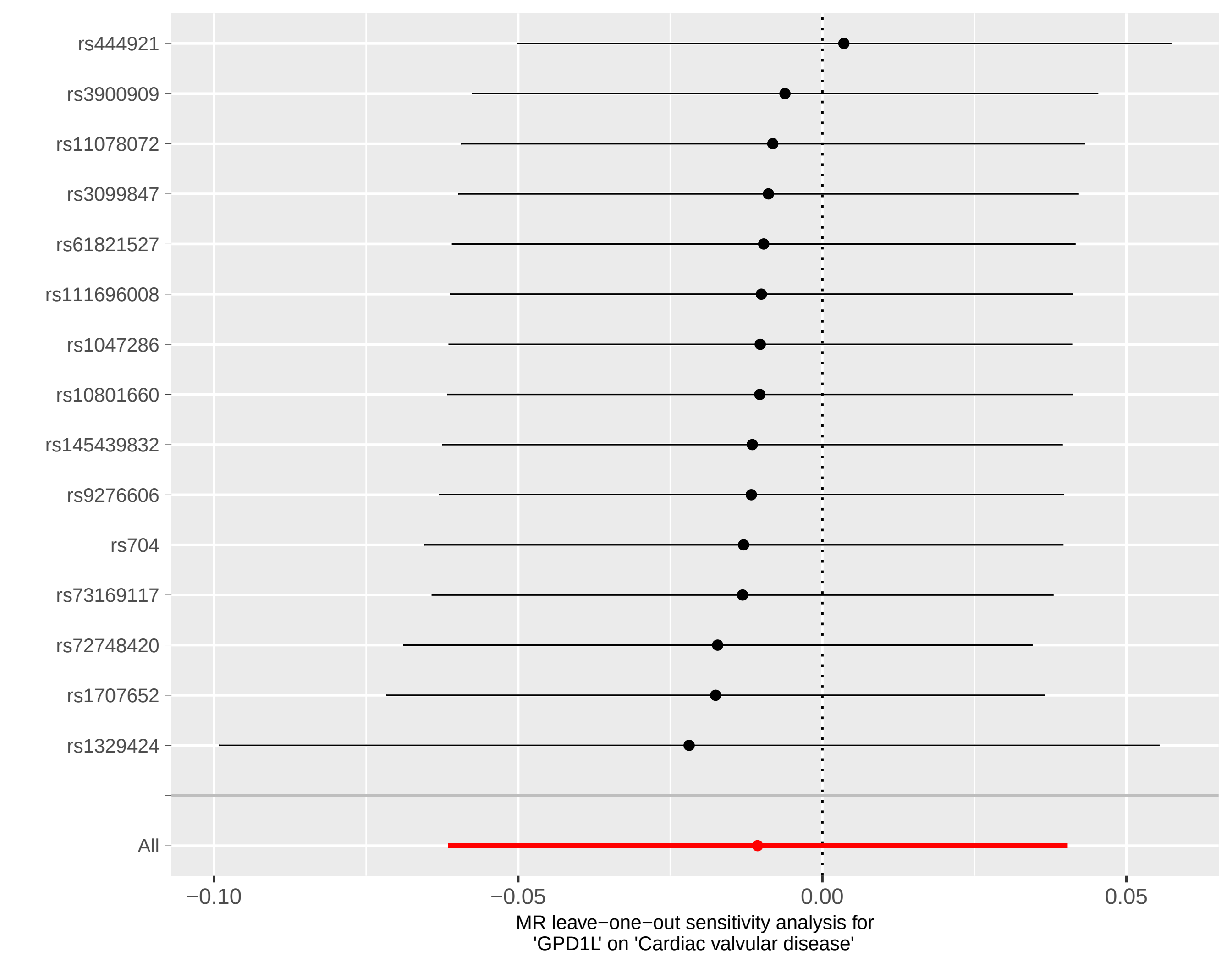

rs444921
rs3900909
rs11078072
rs3099847
rs61821527
rs111696008
rs1047286
rs10801660
rs145439832
rs9276606
rs704
rs73169117
rs72748420
rs1707652
rs1329424
All
−0.10
−0.05
0.00
0.05
MR leave−one−out sensitivity analysis for
'GPD1L' on 'Cardiac valvular disease'

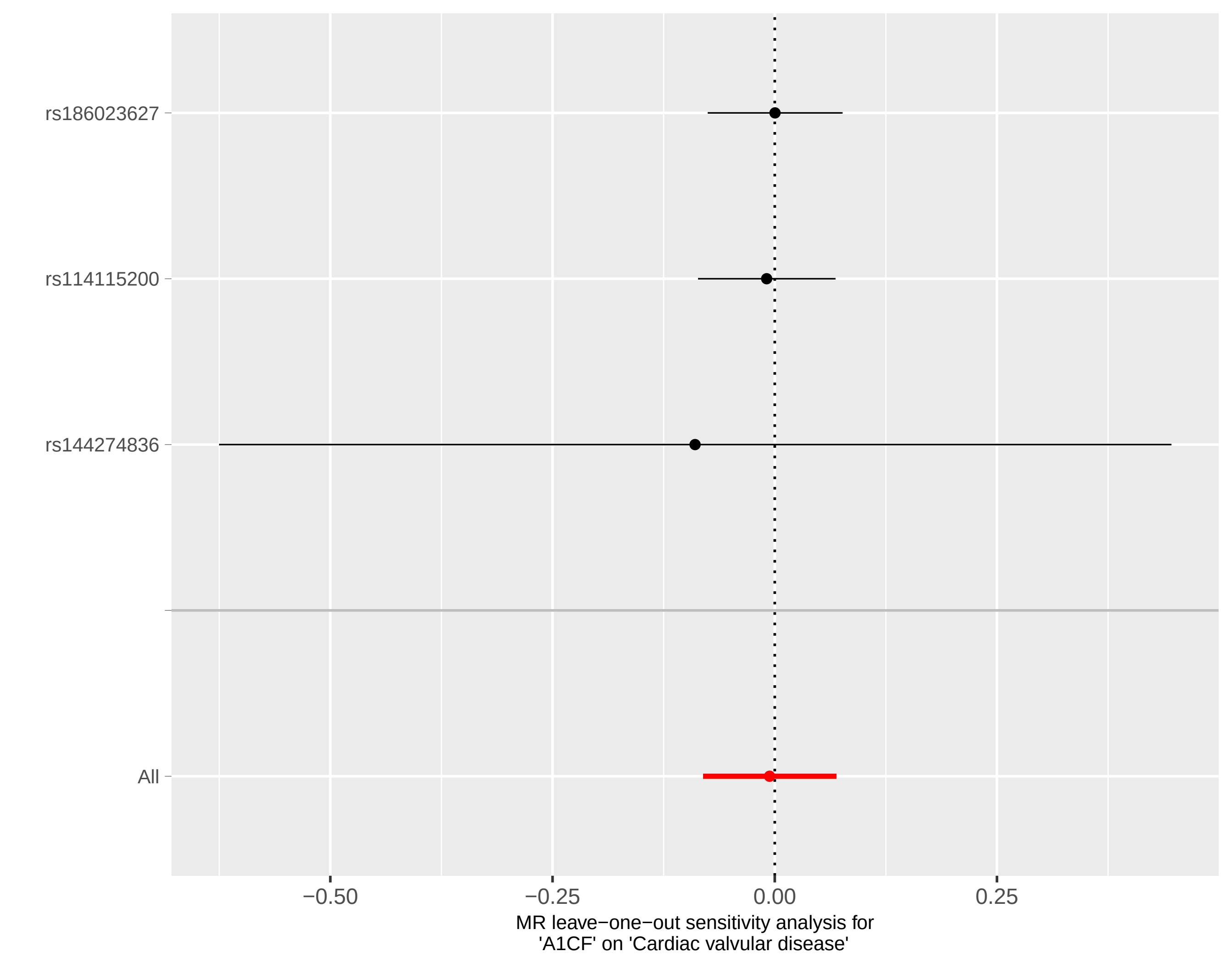

rs186023627
rs114115200
rs144274836
All
−0.50
−0.25
0.00
0.25
MR leave−one−out sensitivity analysis for
'A1CF' on 'Cardiac valvular disease'

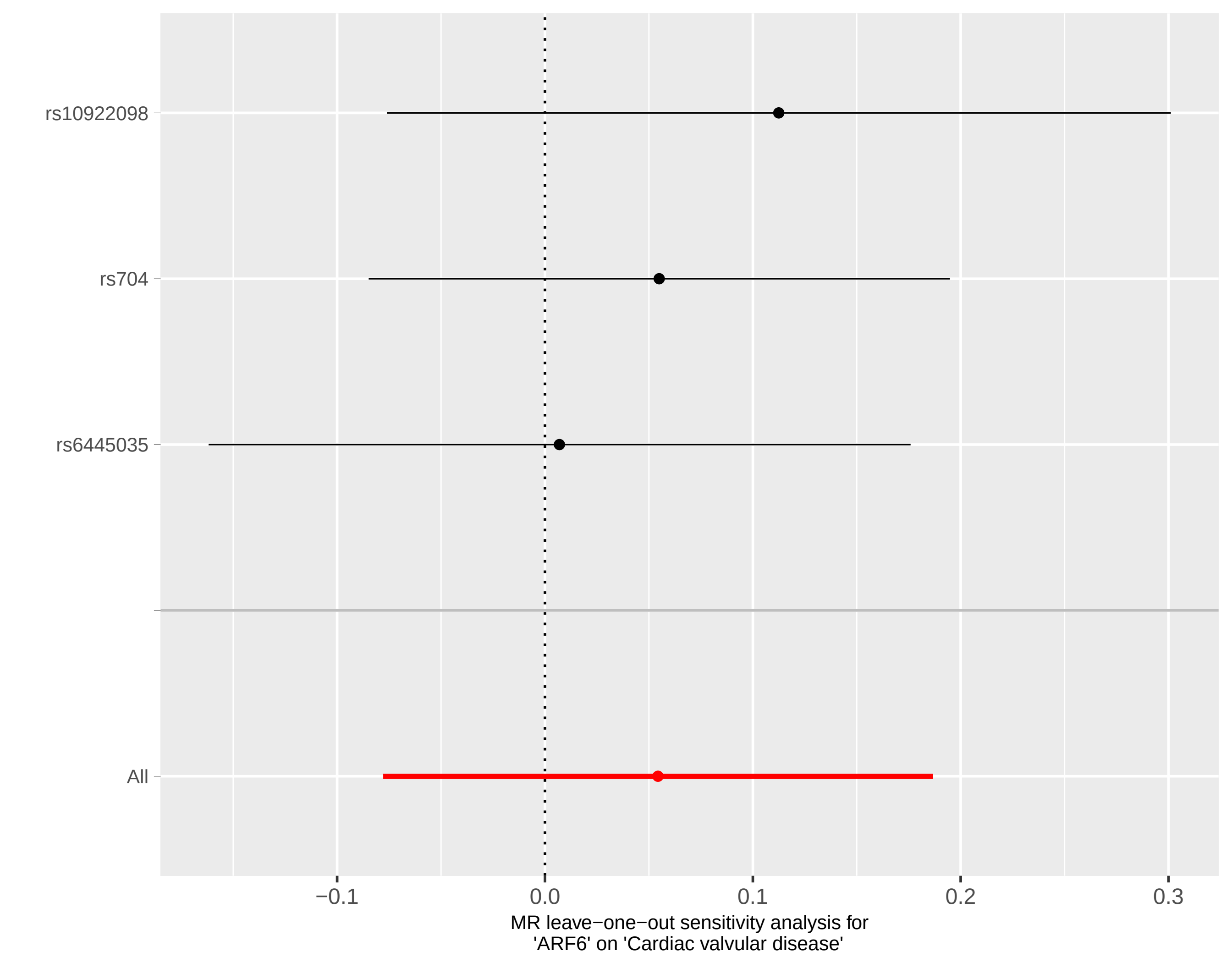
rs10922098
rs704
rs6445035
All
−0.1
0.0
0.1
0.2
0.3
MR leave−one−out sensitivity analysis for
'ARF6' on 'Cardiac valvular disease'

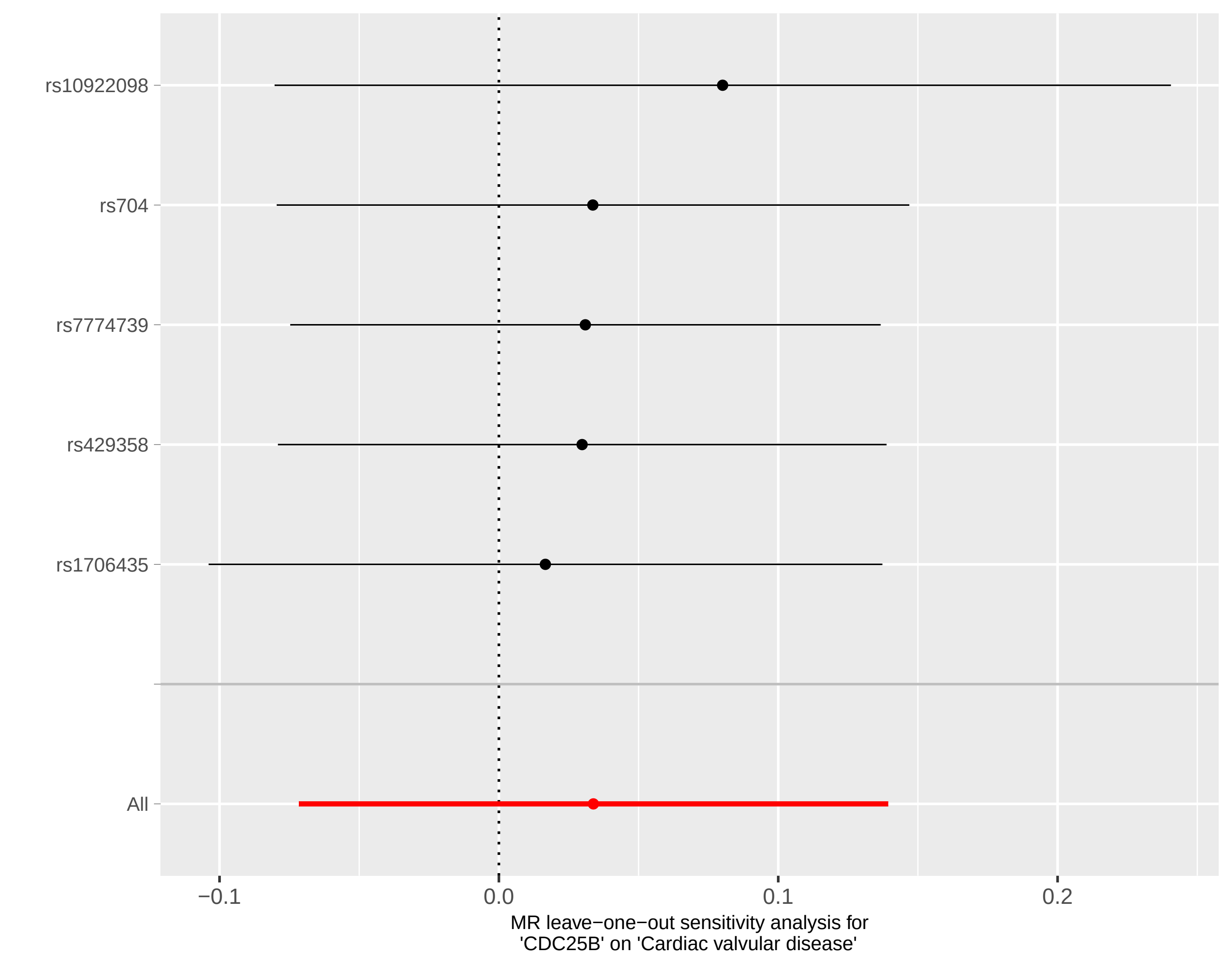

rs10922098
rs704
rs7774739
rs429358
rs1706435
All
−0.1
0.0
0.1
0.2
MR leave−one−out sensitivity analysis for
'CDC25B' on 'Cardiac valvular disease'

Insufficient number of SNPs

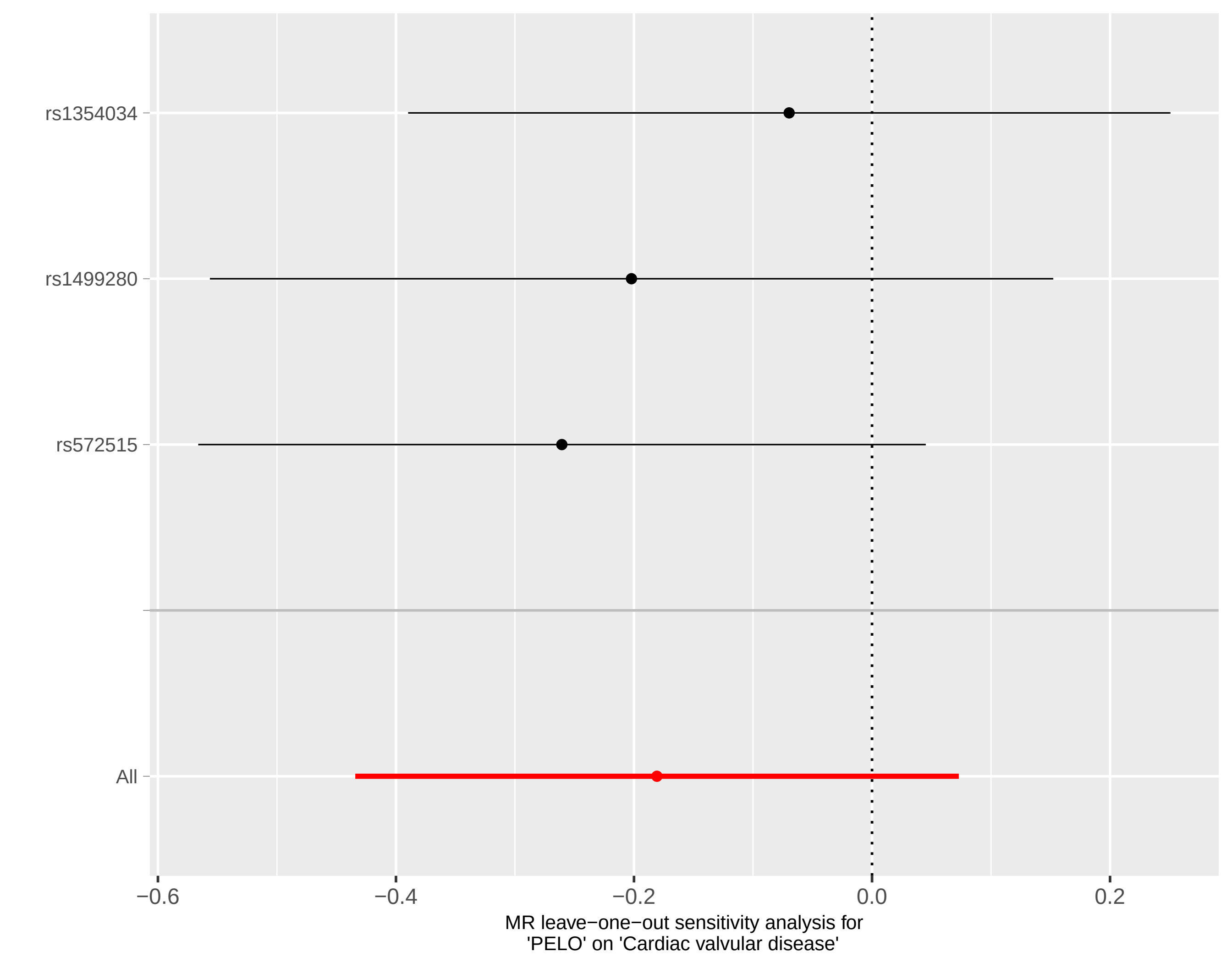
rs1354034
rs1499280
rs572515
All
−0.6
−0.4
−0.2
0.0
0.2
MR leave−one−out sensitivity analysis for
'PELO' on 'Cardiac valvular disease'

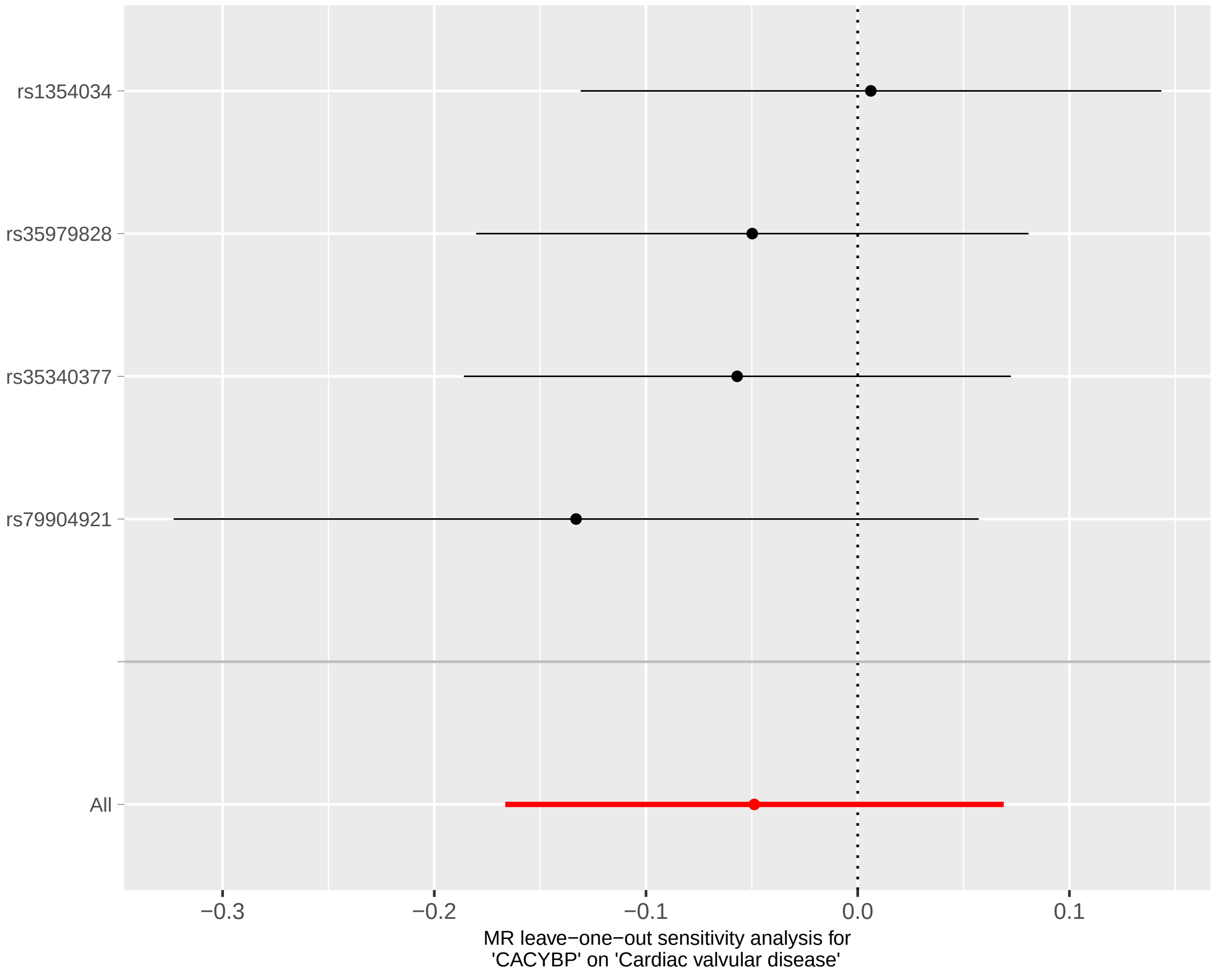
rs1354034
rs35979828
rs35340377
rs79904921
All
−0.3
−0.2
−0.1
0.0
0.1
MR leave−one−out sensitivity analysis for
'CACYBP' on 'Cardiac valvular disease'

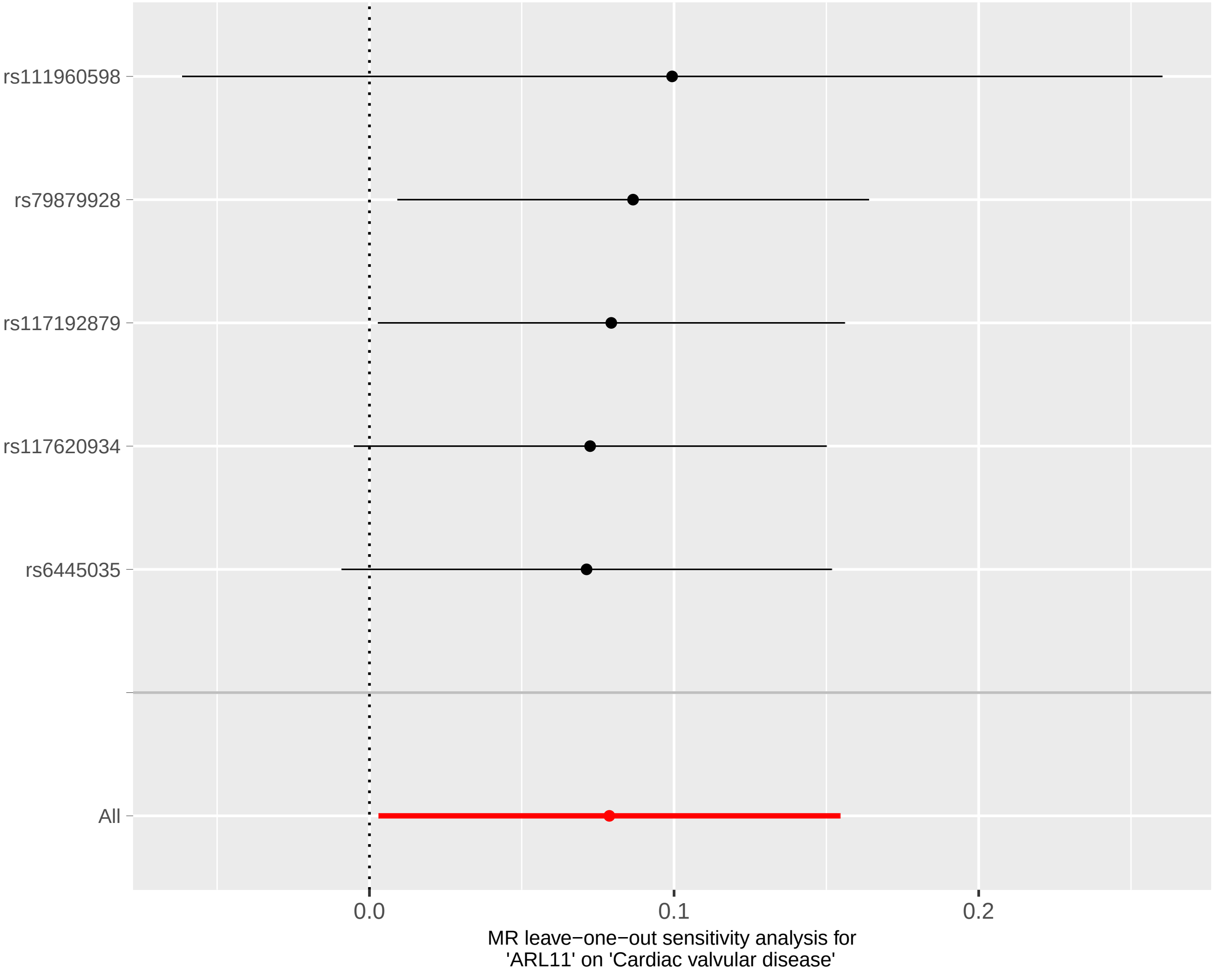
rs111960598
rs79879928
rs117192879
rs117620934
rs6445035
All
0.0
0.1
0.2
MR leave−one−out sensitivity analysis for
'ARL11' on 'Cardiac valvular disease'

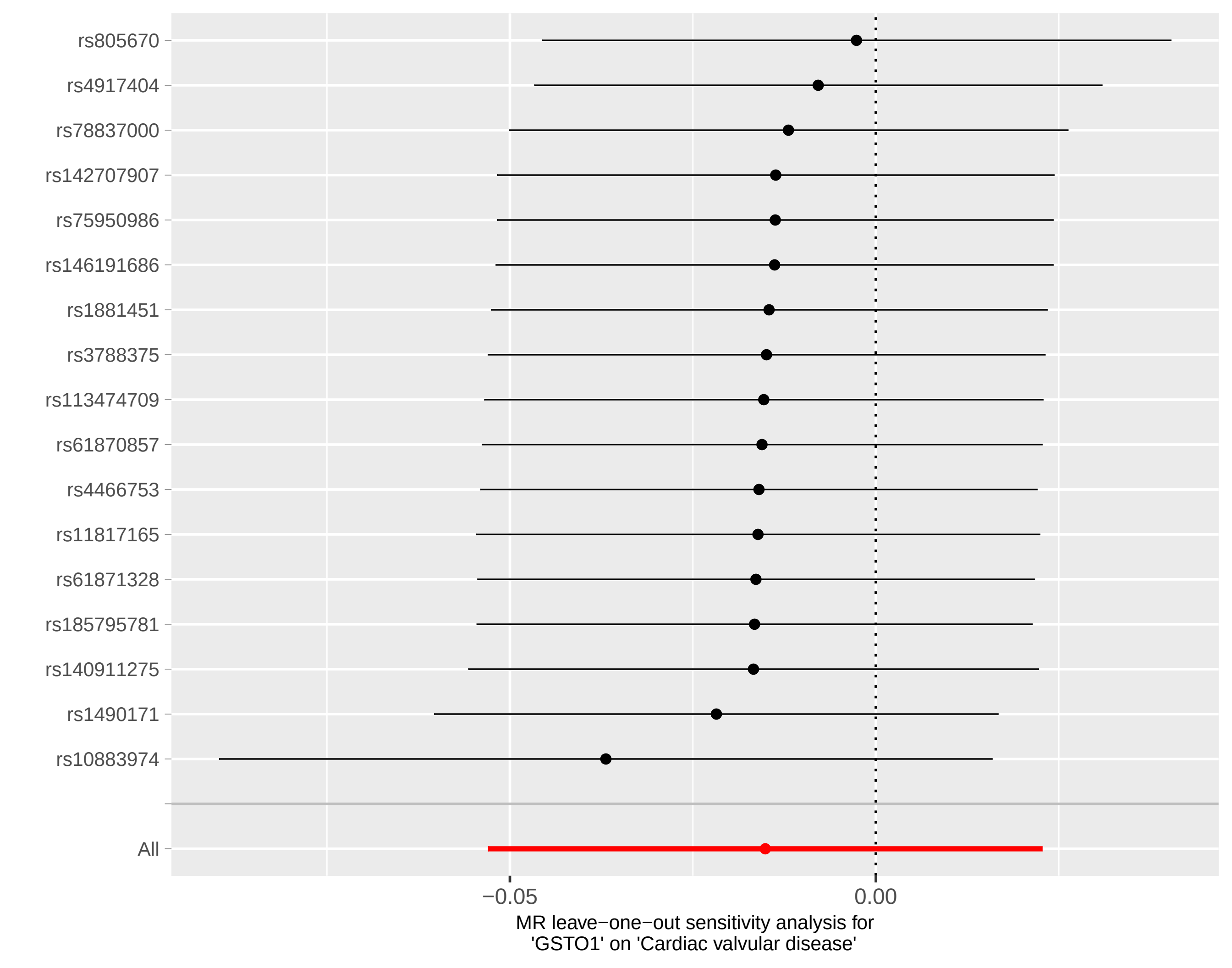
rs805670
rs4917404
rs78837000
rs142707907
rs75950986
rs146191686
rs1881451
rs3788375
rs113474709
rs61870857
rs4466753
rs11817165
rs61871328
rs185795781
rs140911275
rs1490171
rs10883974
All
−0.05
0.00
MR leave−one−out sensitivity analysis for
'GSTO1' on 'Cardiac valvular disease'

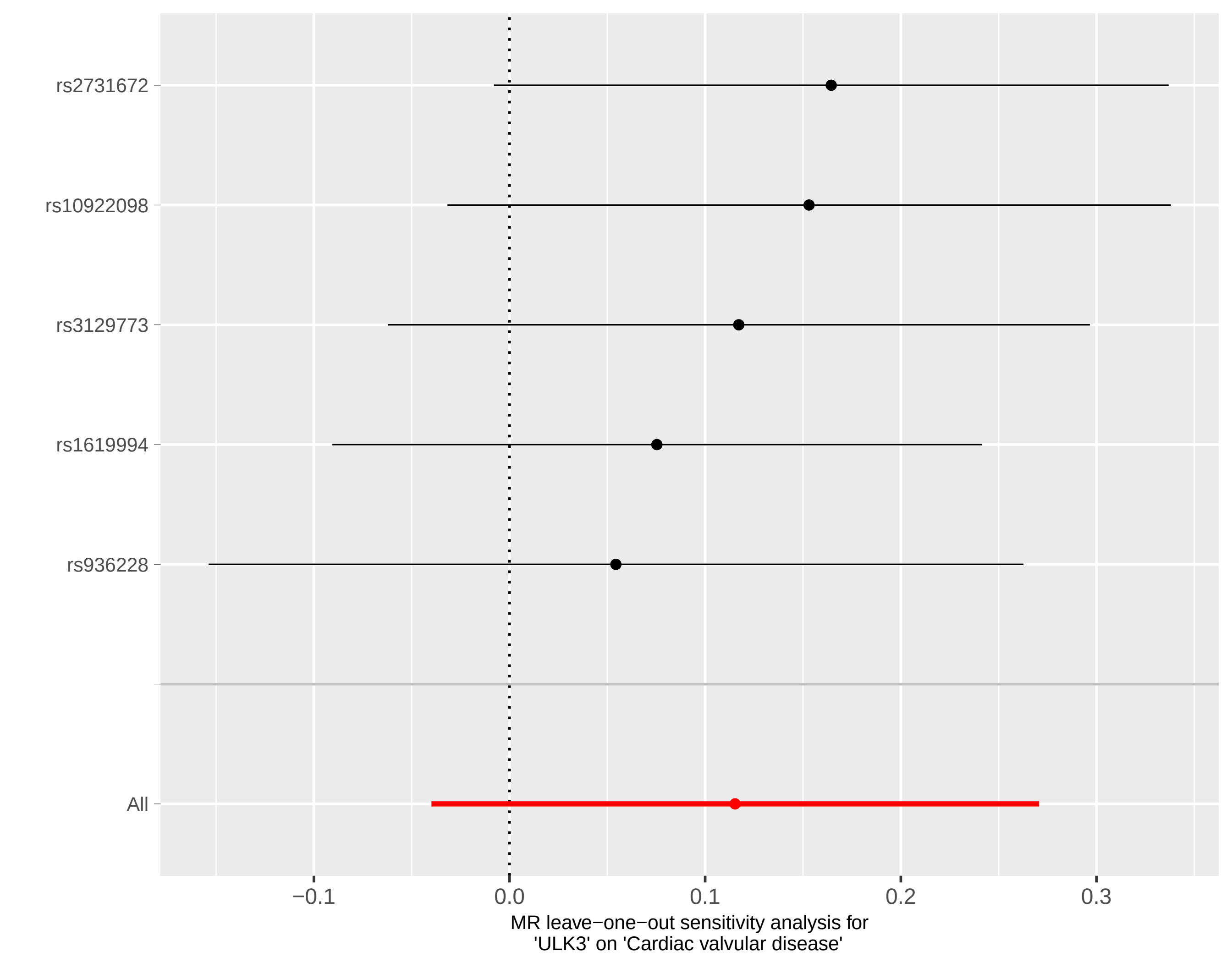
rs2731672
rs10922098
rs3129773
rs1619994
rs936228
All
−0.1
0.0
0.1
0.2
0.3
MR leave−one−out sensitivity analysis for
'ULK3' on 'Cardiac valvular disease'

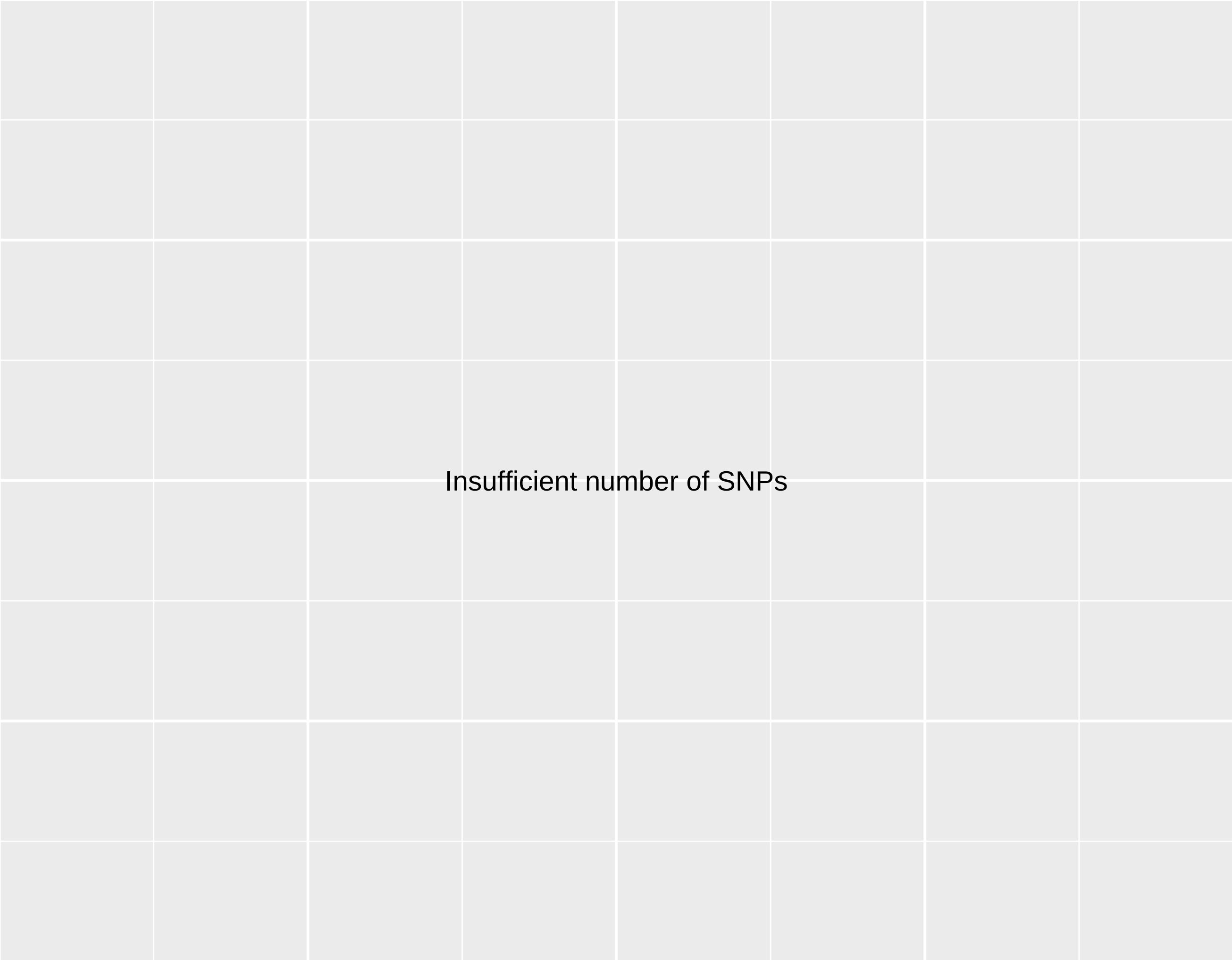
Insufficient number of SNPs

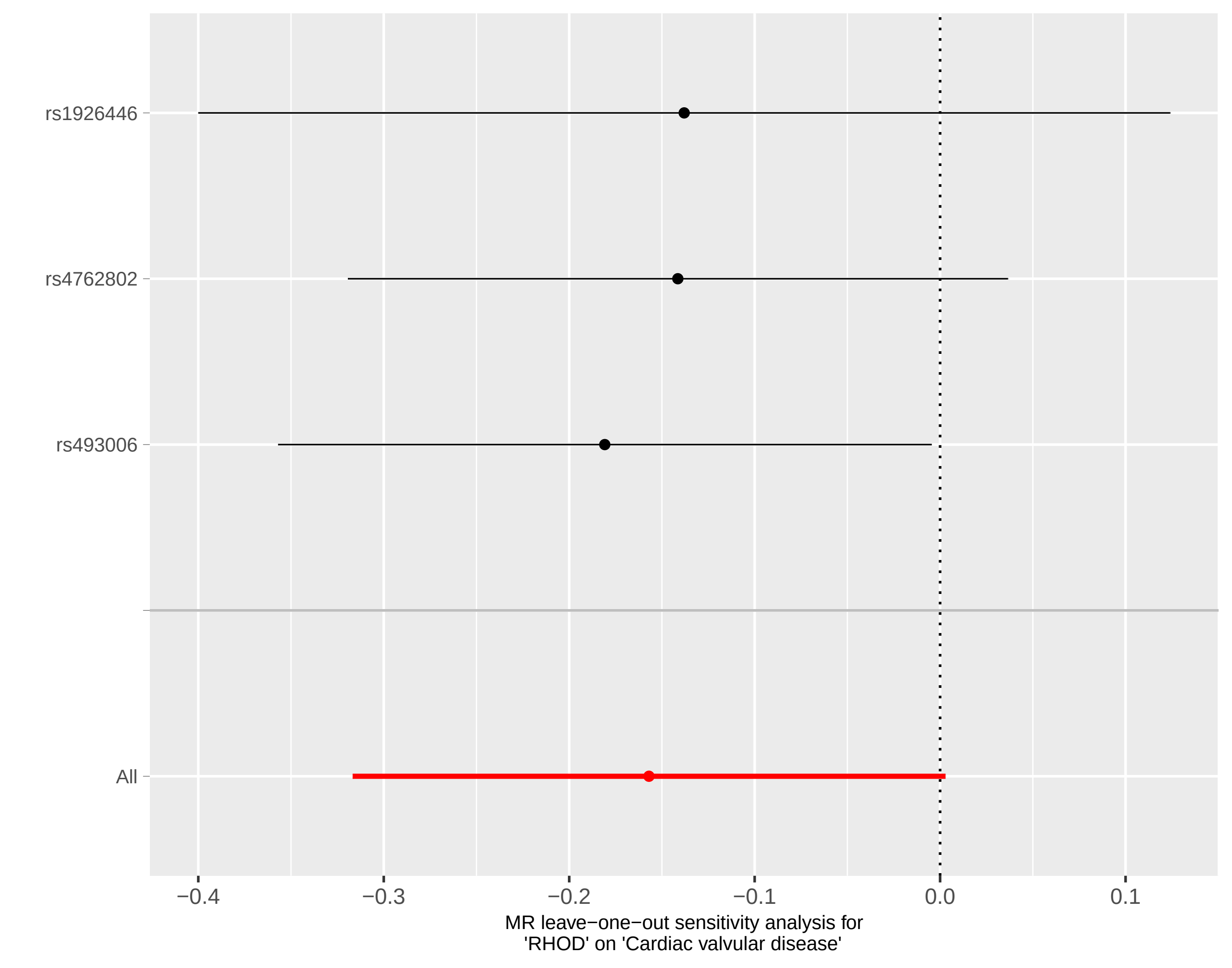
rs1926446
rs4762802
rs493006
All
−0.4
−0.3
−0.2
−0.1
0.0
0.1
MR leave−one−out sensitivity analysis for
'RHOD' on 'Cardiac valvular disease'

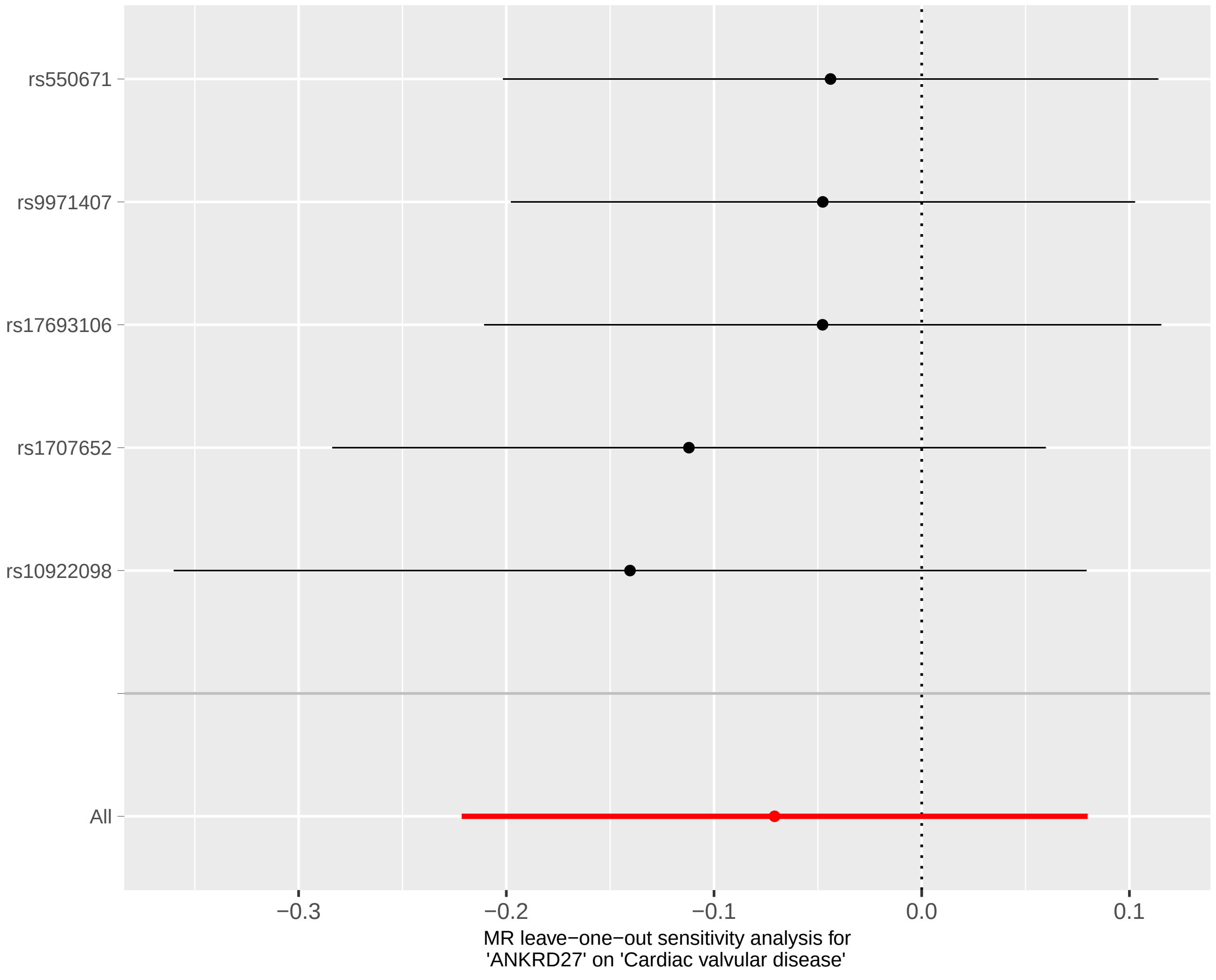
rs550671
rs9971407
rs17693106
rs1707652
rs10922098
All
−0.3
−0.2
−0.1
0.0
0.1
MR leave−one−out sensitivity analysis for
'ANKRD27' on 'Cardiac valvular disease'

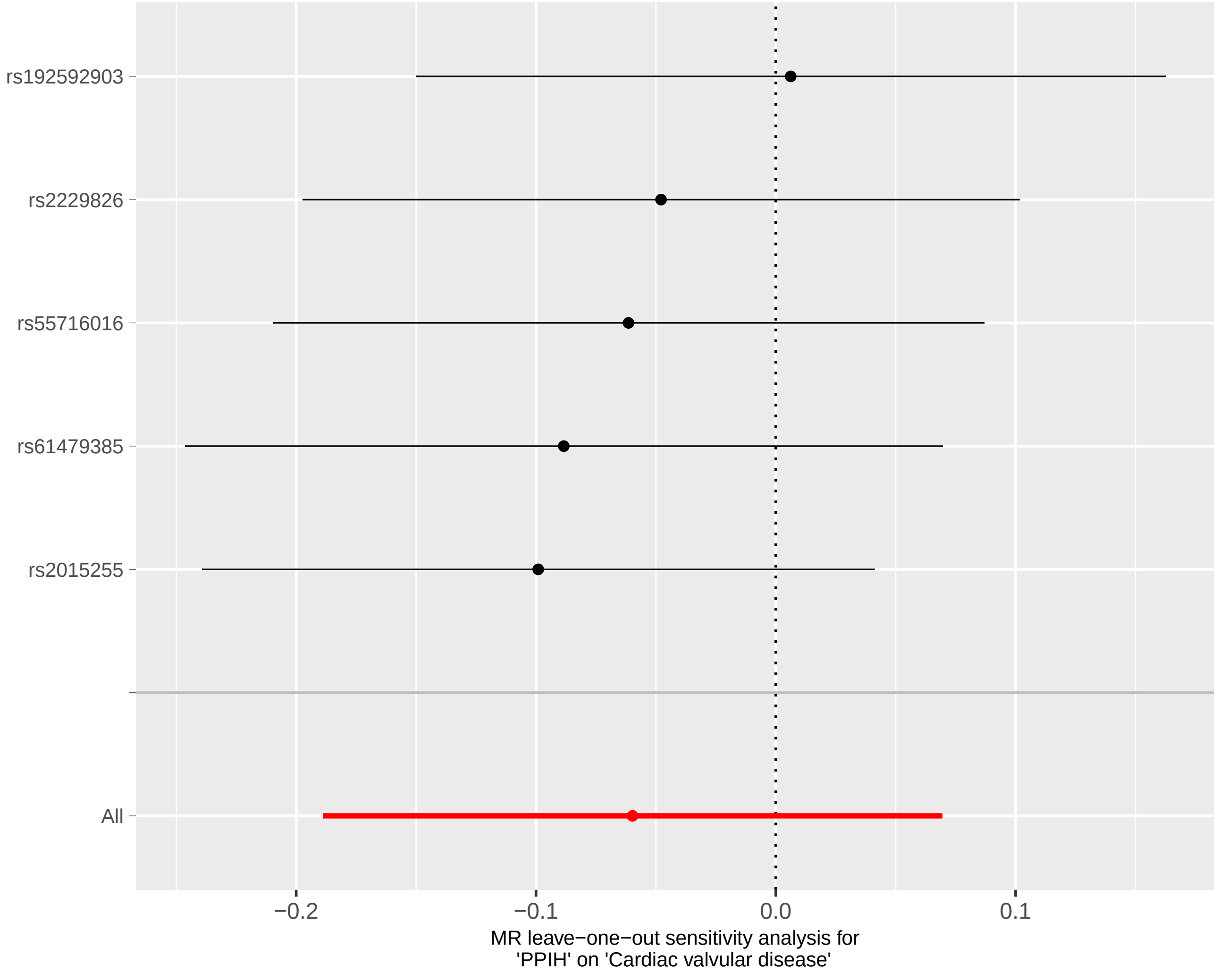
rs192592903
rs2229826
rs55716016
rs61479385
rs2015255
All
−0.2
−0.1
0.0
0.1
MR leave−one−out sensitivity analysis for
'PPIH' on 'Cardiac valvular disease'

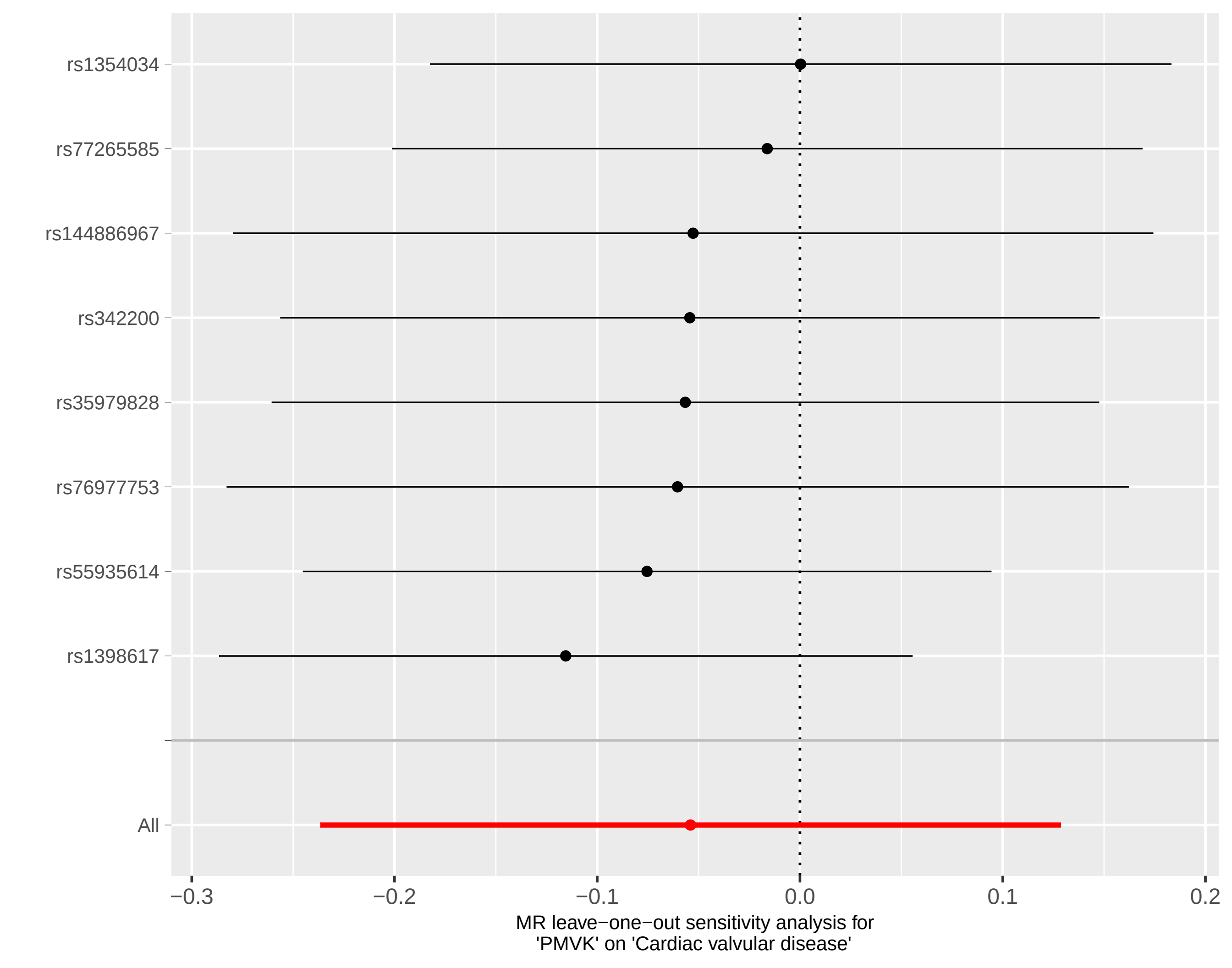
rs1354034
rs77265585
rs144886967
rs342200
rs35979828
rs76977753
rs55935614
rs1398617
All
−0.3
−0.2
−0.1
0.0
0.1
0.2
MR leave−one−out sensitivity analysis for
'PMVK' on 'Cardiac valvular disease'

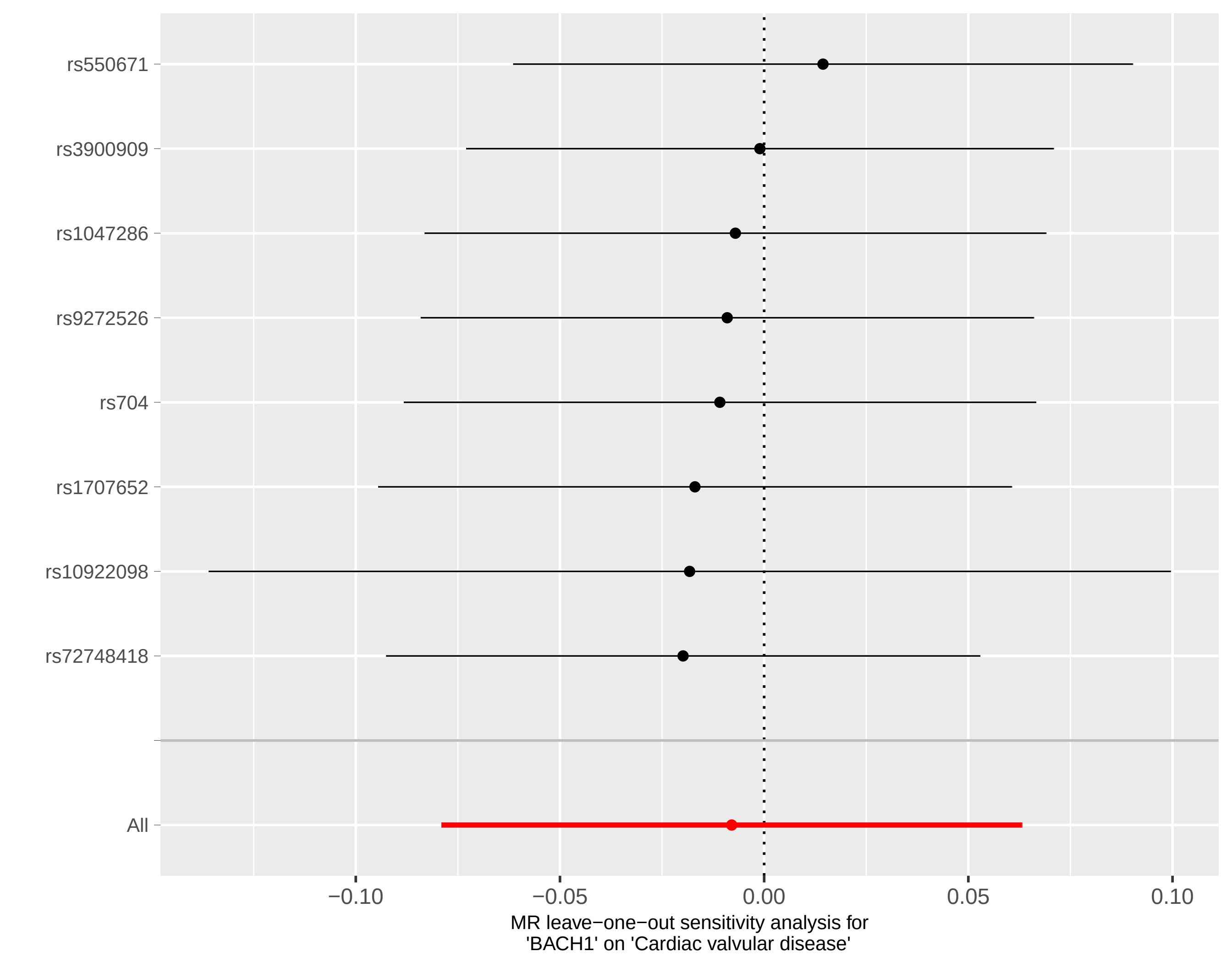
rs550671
rs3900909
rs1047286
rs9272526
rs704
rs1707652
rs10922098
rs72748418
All
−0.10
−0.05
0.00
0.05
0.10
MR leave−one−out sensitivity analysis for
'BACH1' on 'Cardiac valvular disease'

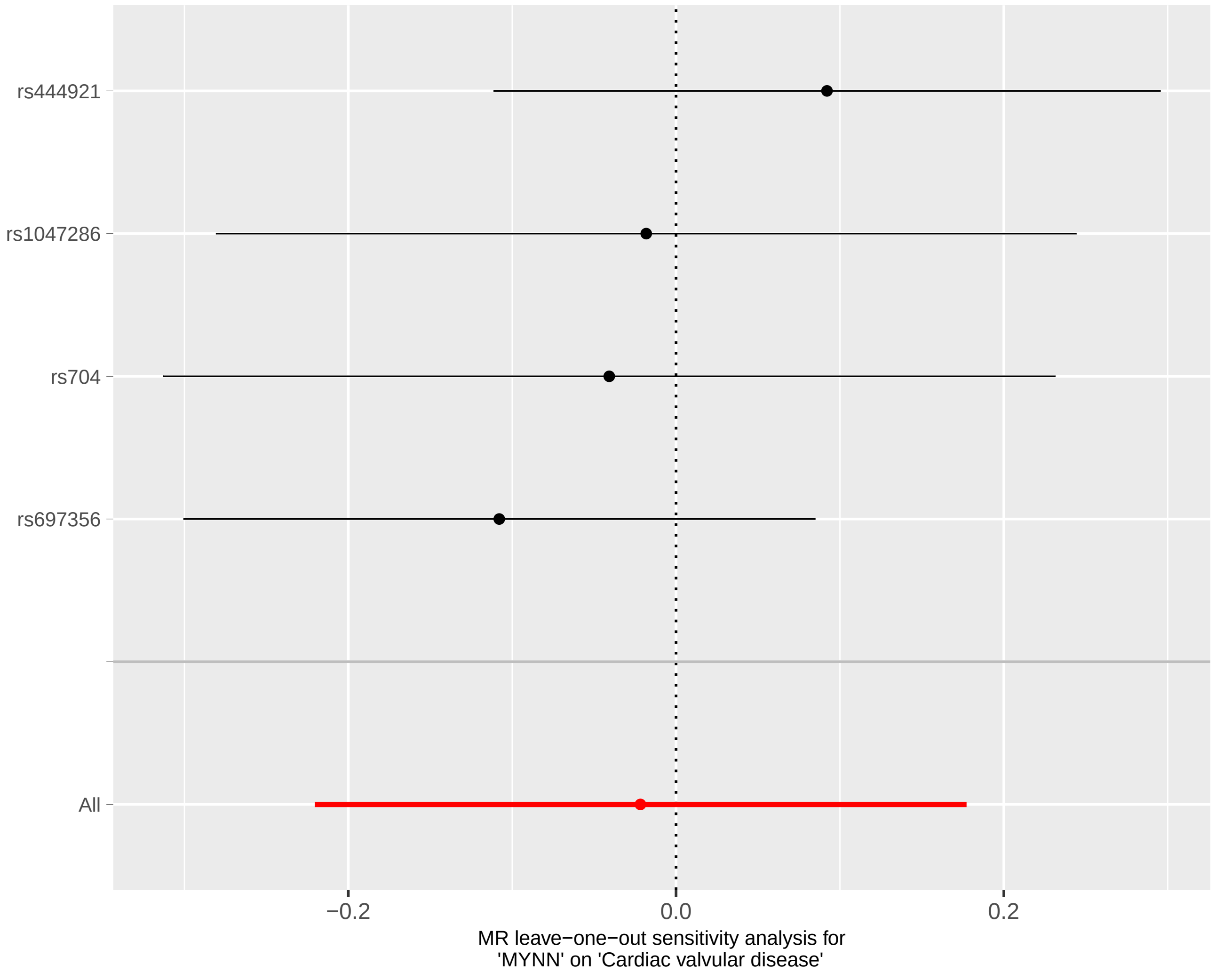
rs444921
rs1047286
rs704
rs697356
All
−0.2
0.0
0.2
MR leave−one−out sensitivity analysis for
'MYNN' on 'Cardiac valvular disease'

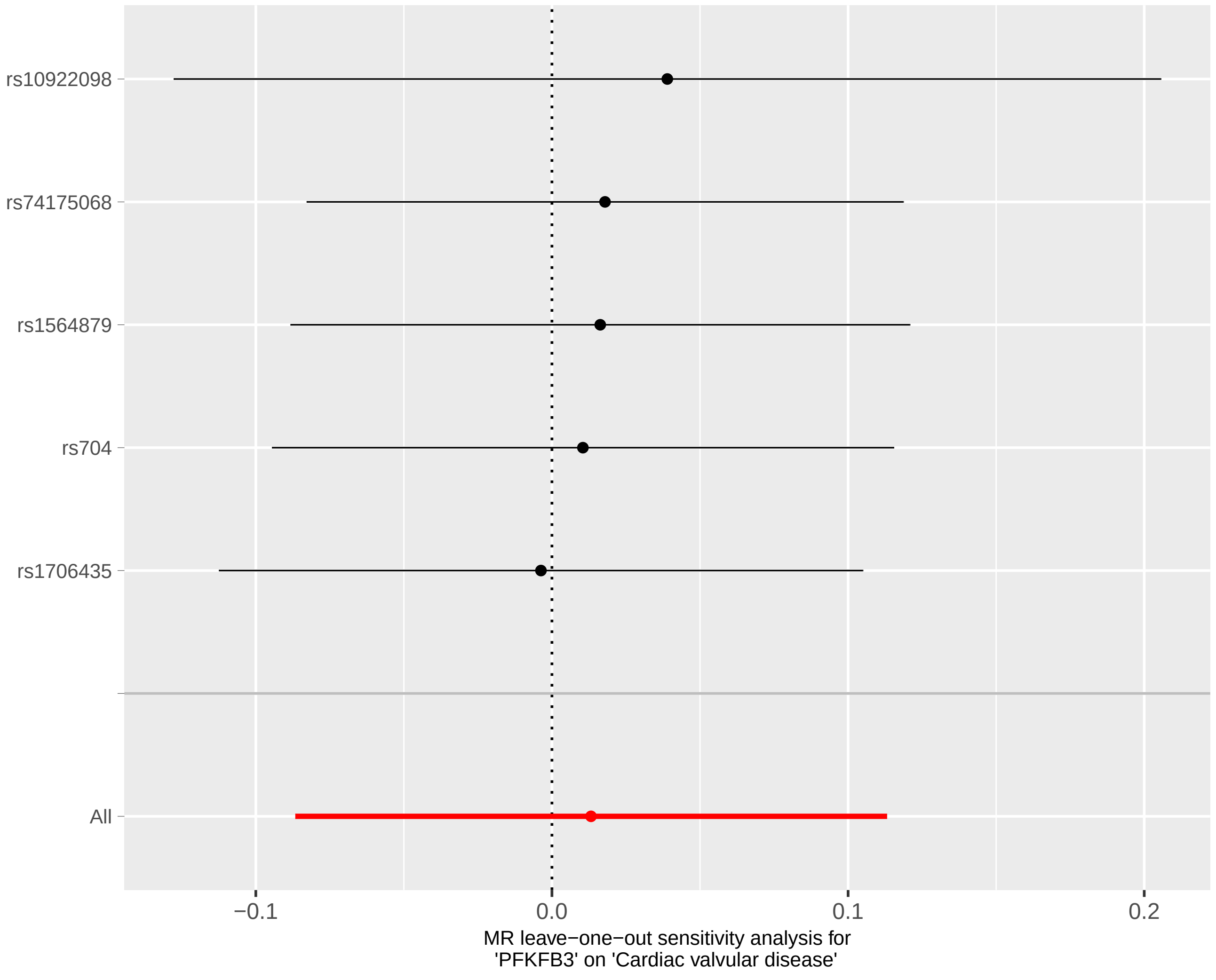

rs10922098
rs74175068
rs1564879
rs704
rs1706435
All
−0.1
0.0
0.1
0.2
MR leave−one−out sensitivity analysis for
'PFKFB3' on 'Cardiac valvular disease'

Insufficient number of SNPs

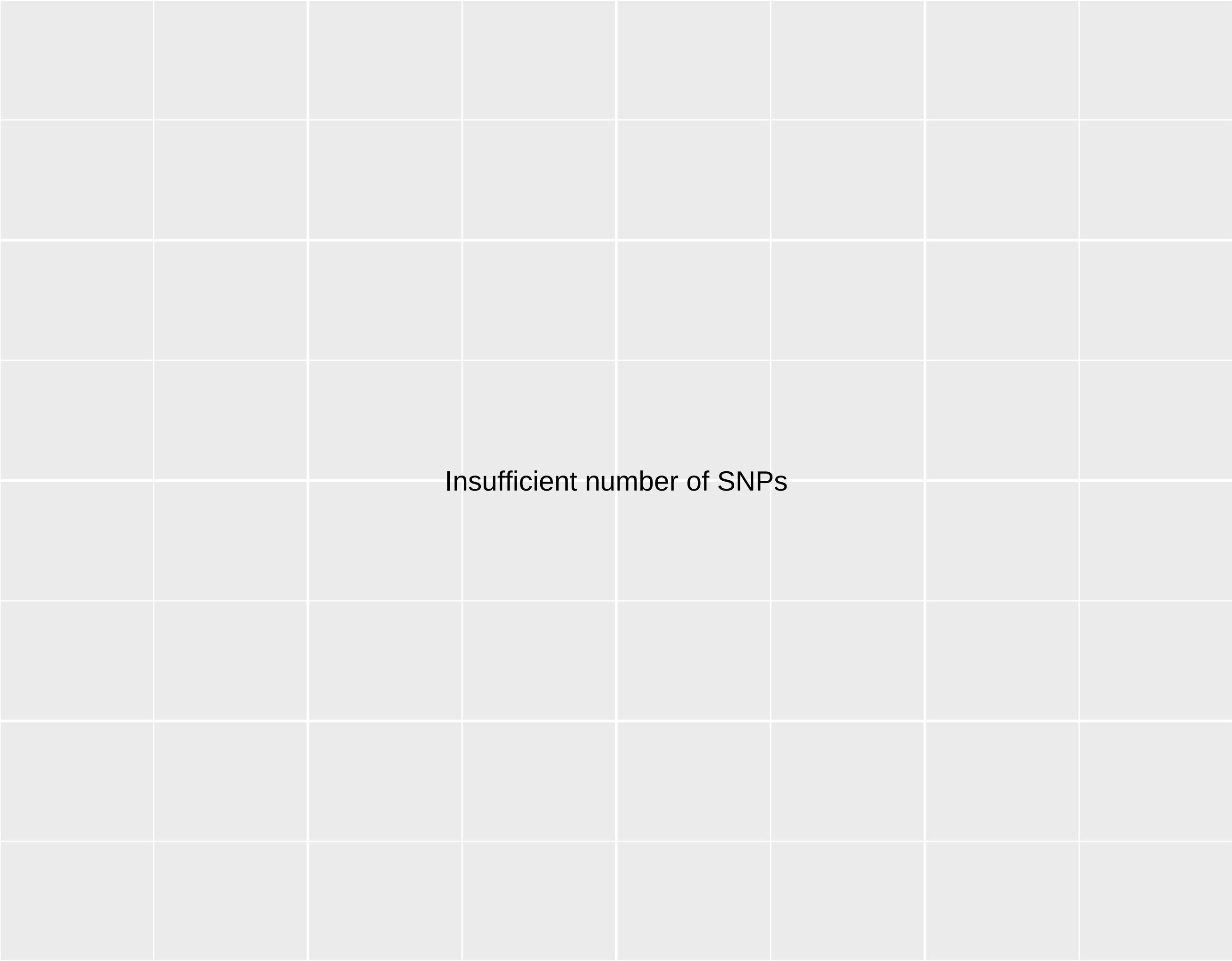
Insufficient number of SNPs

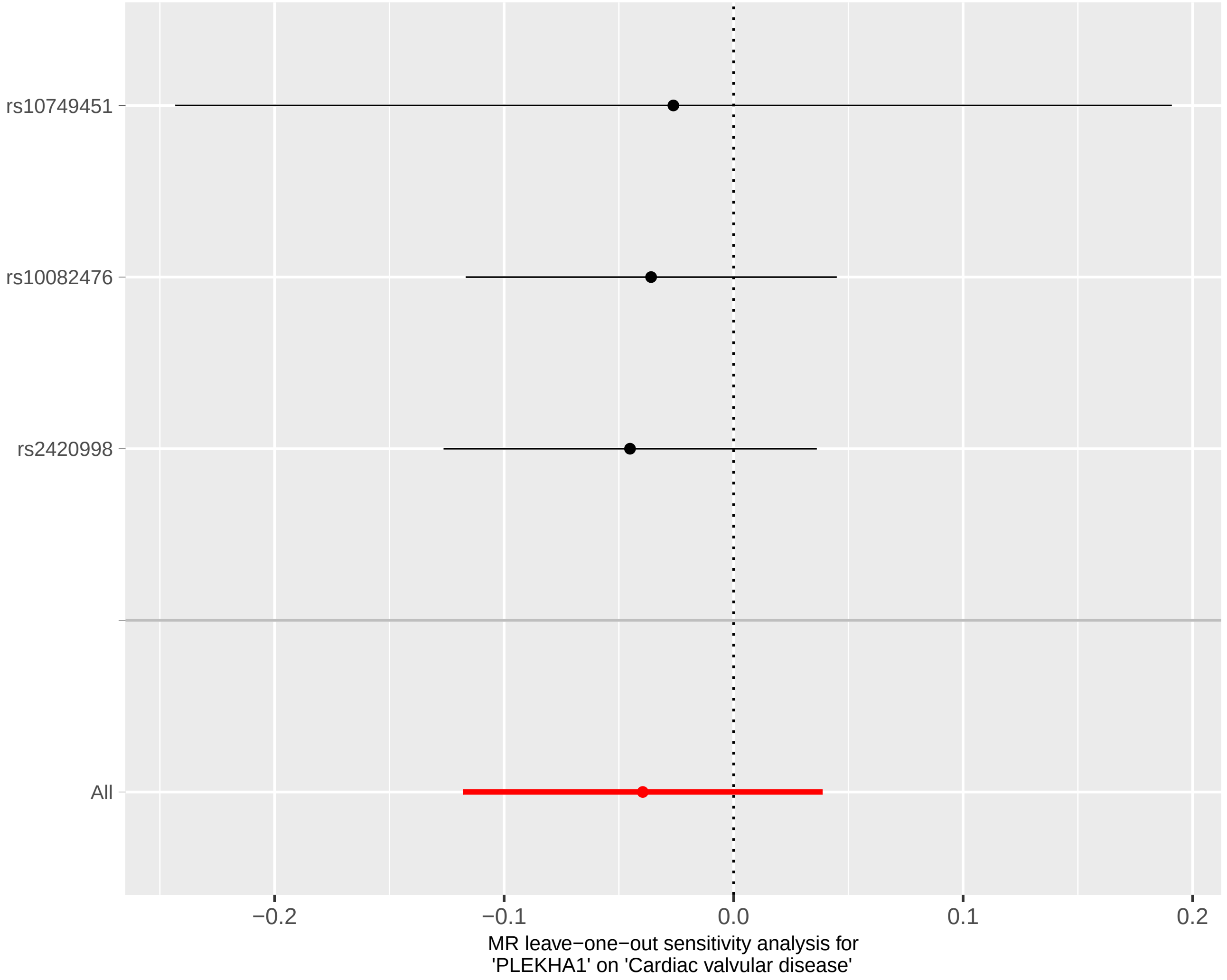

rs10749451
rs10082476
rs2420998
All
−0.2
−0.1
0.0
0.1
0.2
MR leave−one−out sensitivity analysis for
'PLEKHA1' on 'Cardiac valvular disease'

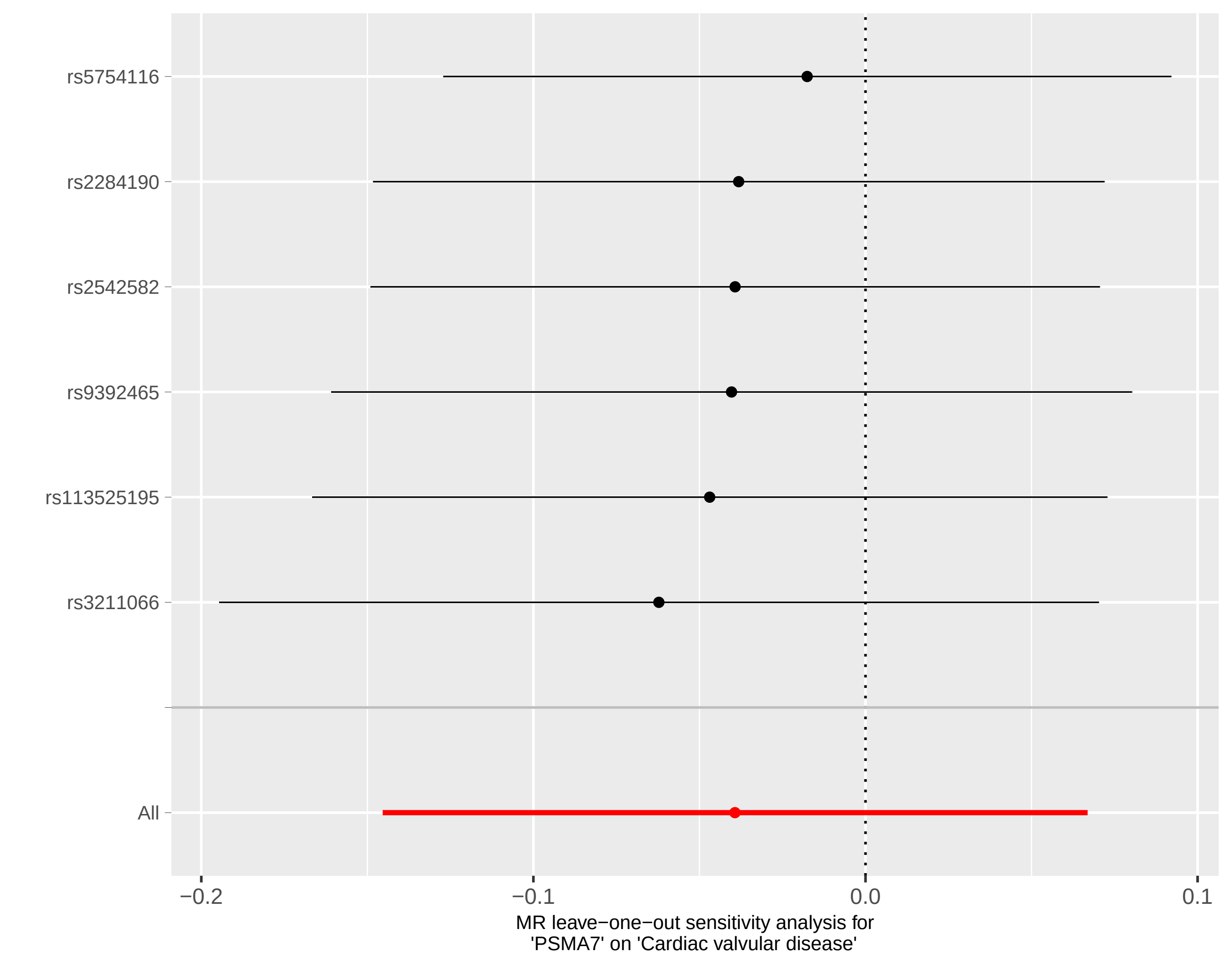

rs5754116
rs2284190
rs2542582
rs9392465
rs113525195
rs3211066
All
−0.2
−0.1
0.0
0.1
MR leave−one−out sensitivity analysis for
'PSMA7' on 'Cardiac valvular disease'

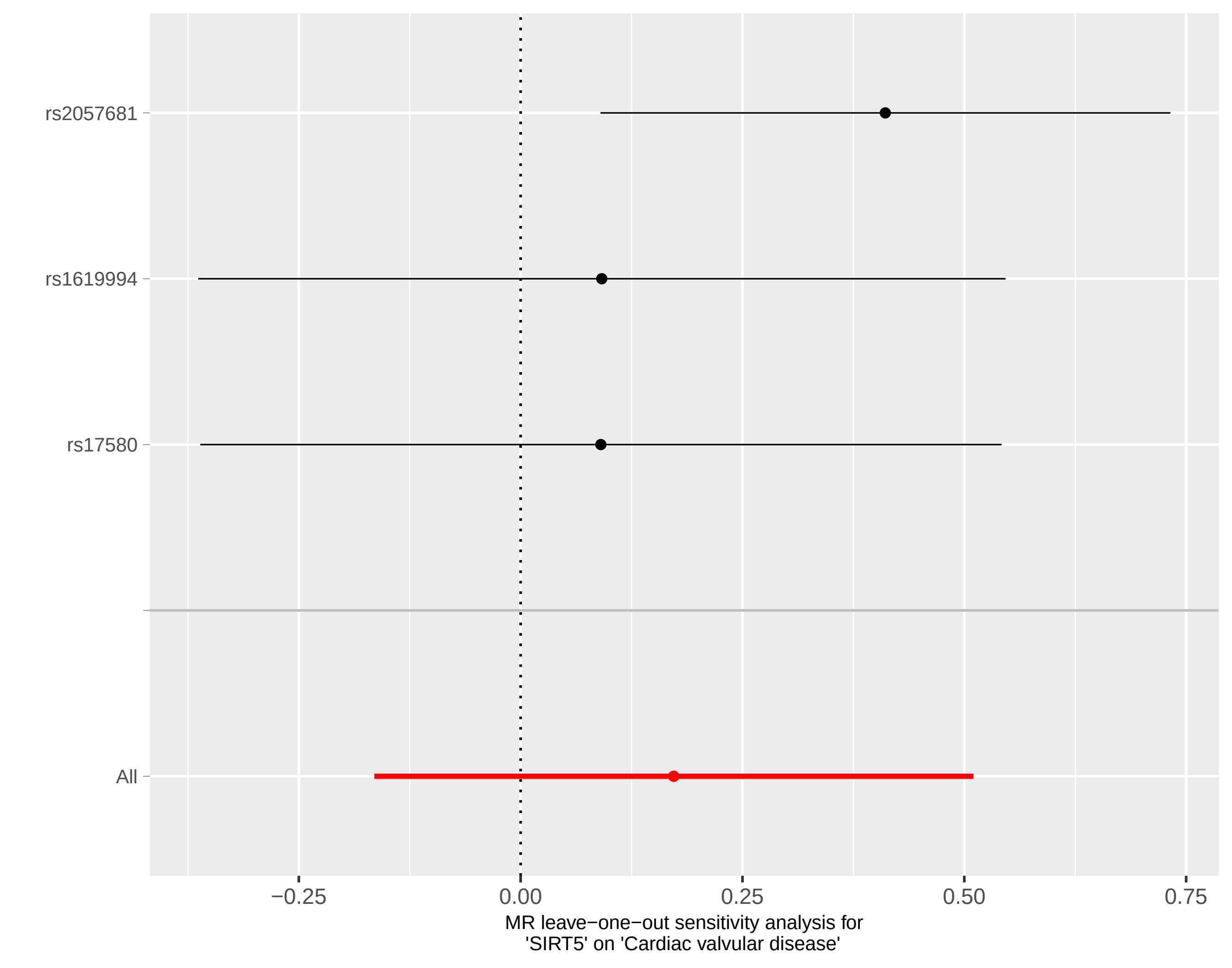
rs2057681
rs1619994
rs17580
All
−0.25
0.00
0.25
0.50
0.75
MR leave−one−out sensitivity analysis for
'SIRT5' on 'Cardiac valvular disease'

Insufficient number of SNPs

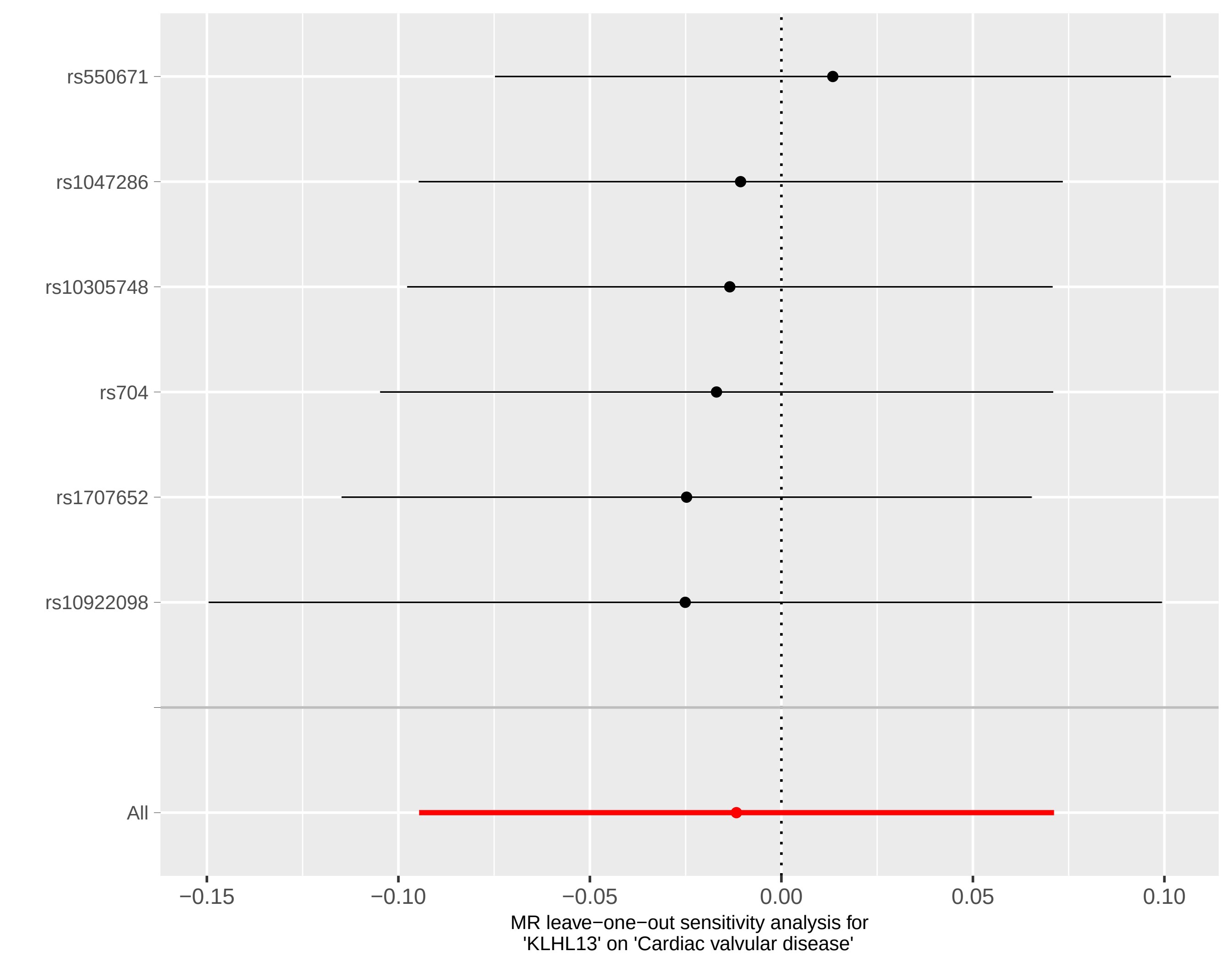
rs550671
rs1047286
rs10305748
rs704
rs1707652
rs10922098
All
−0.15
−0.10
−0.05
0.00
0.05
0.10
MR leave−one−out sensitivity analysis for
'KLHL13' on 'Cardiac valvular disease'

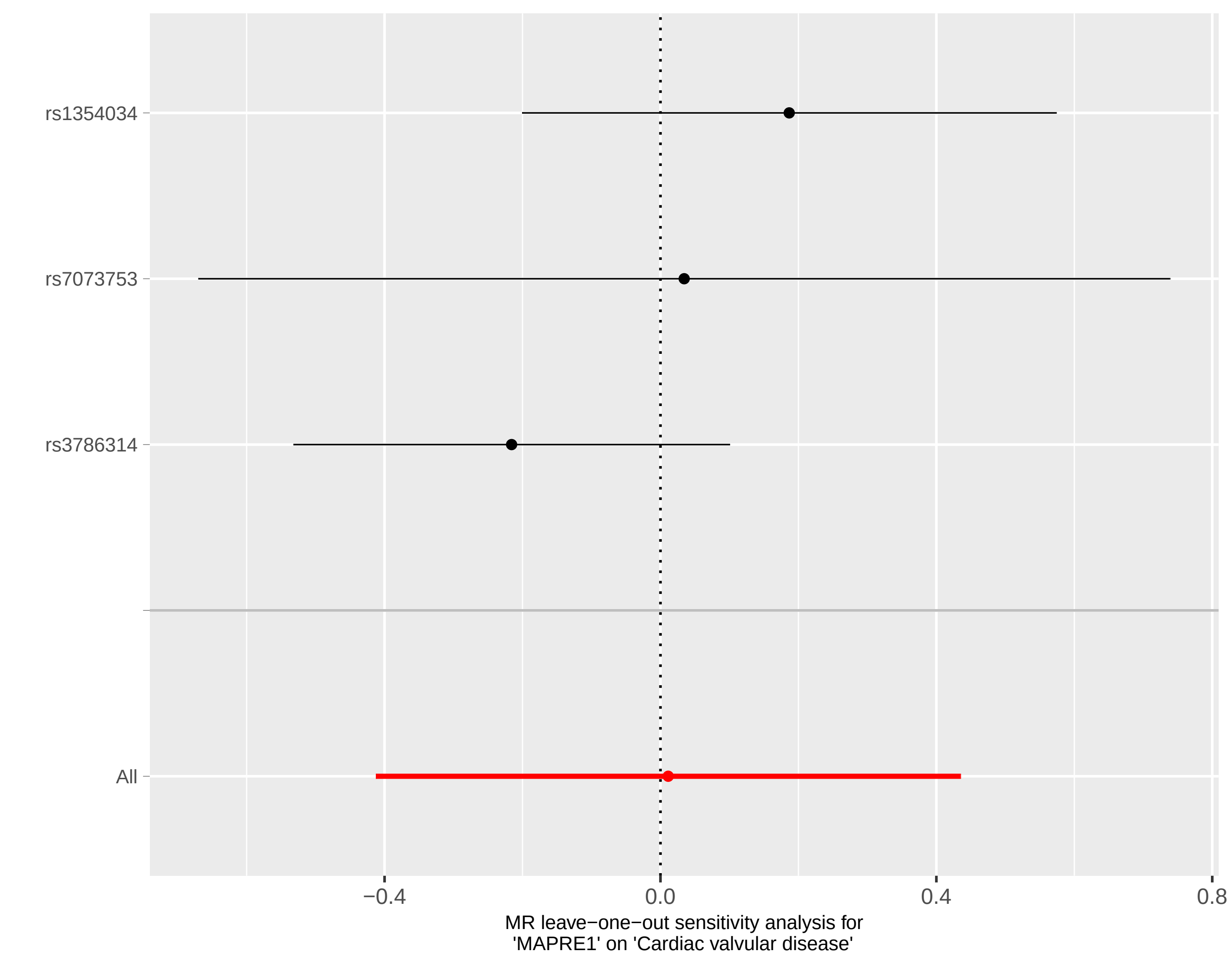
rs1354034
rs7073753
rs3786314
All
−0.4
0.0
0.4
0.8
MR leave−one−out sensitivity analysis for
'MAPRE1' on 'Cardiac valvular disease'

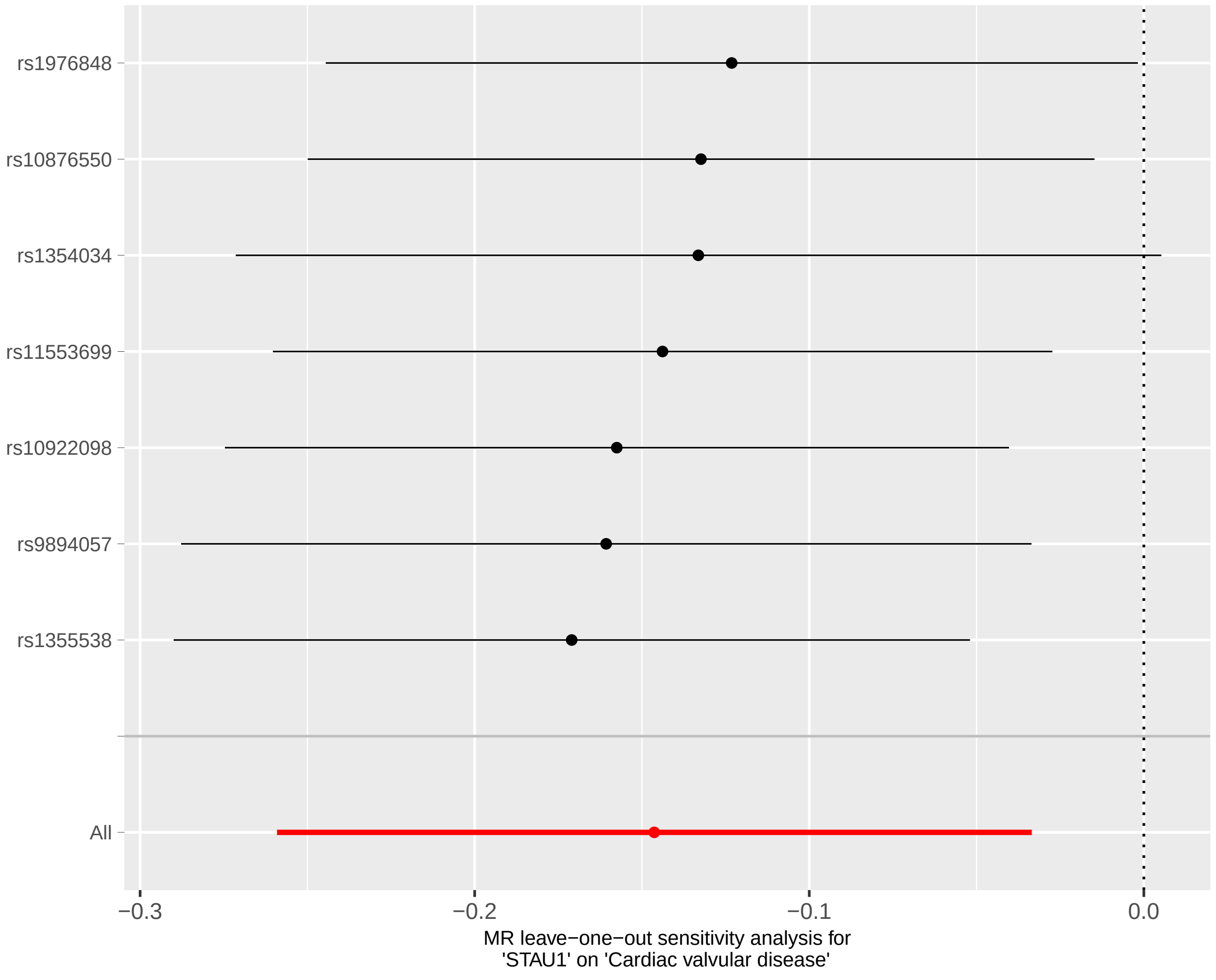
rs1976848
rs10876550
rs1354034
rs11553699
rs10922098
rs9894057
rs1355538
All
−0.3
−0.2
−0.1
0.0
MR leave−one−out sensitivity analysis for
'STAU1' on 'Cardiac valvular disease'

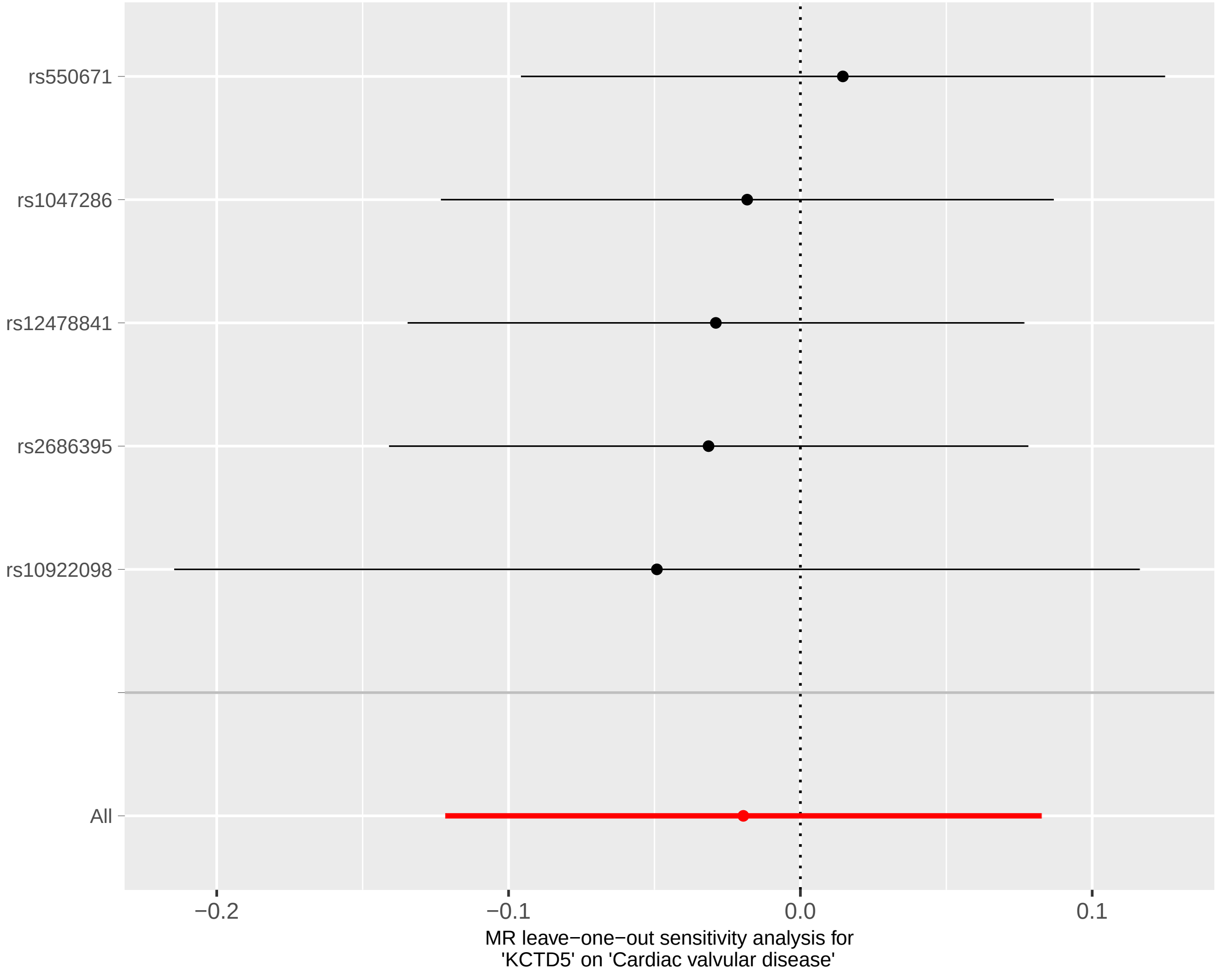
rs550671
rs1047286
rs12478841
rs2686395
rs10922098
All
−0.2
−0.1
0.0
0.1
MR leave−one−out sensitivity analysis for
'KCTD5' on 'Cardiac valvular disease'

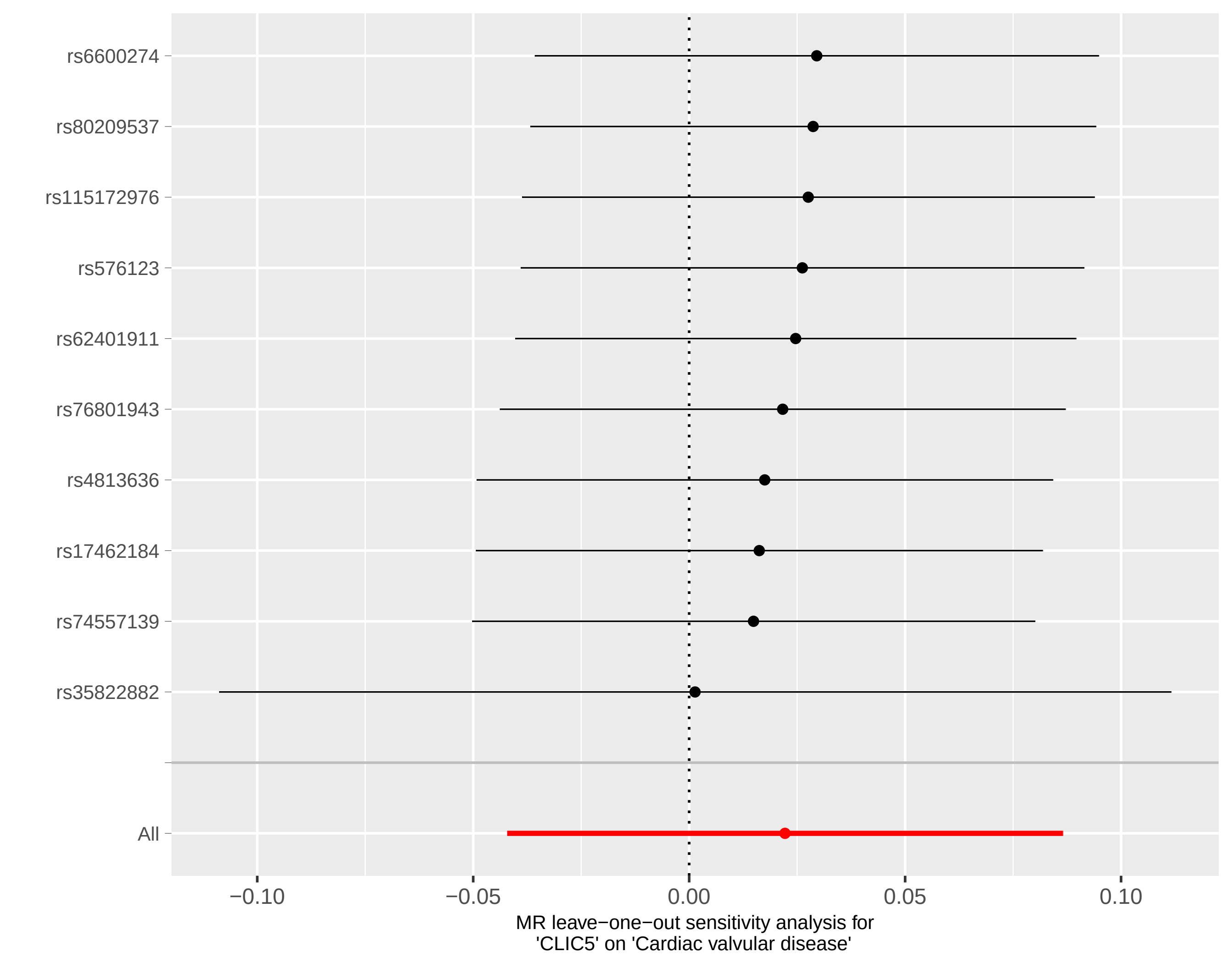
rs6600274
rs80209537
rs115172976
rs576123
rs62401911
rs76801943
rs4813636
rs17462184
rs74557139
rs35822882
All
−0.10
−0.05
0.00
0.05
0.10
MR leave−one−out sensitivity analysis for
'CLIC5' on 'Cardiac valvular disease'

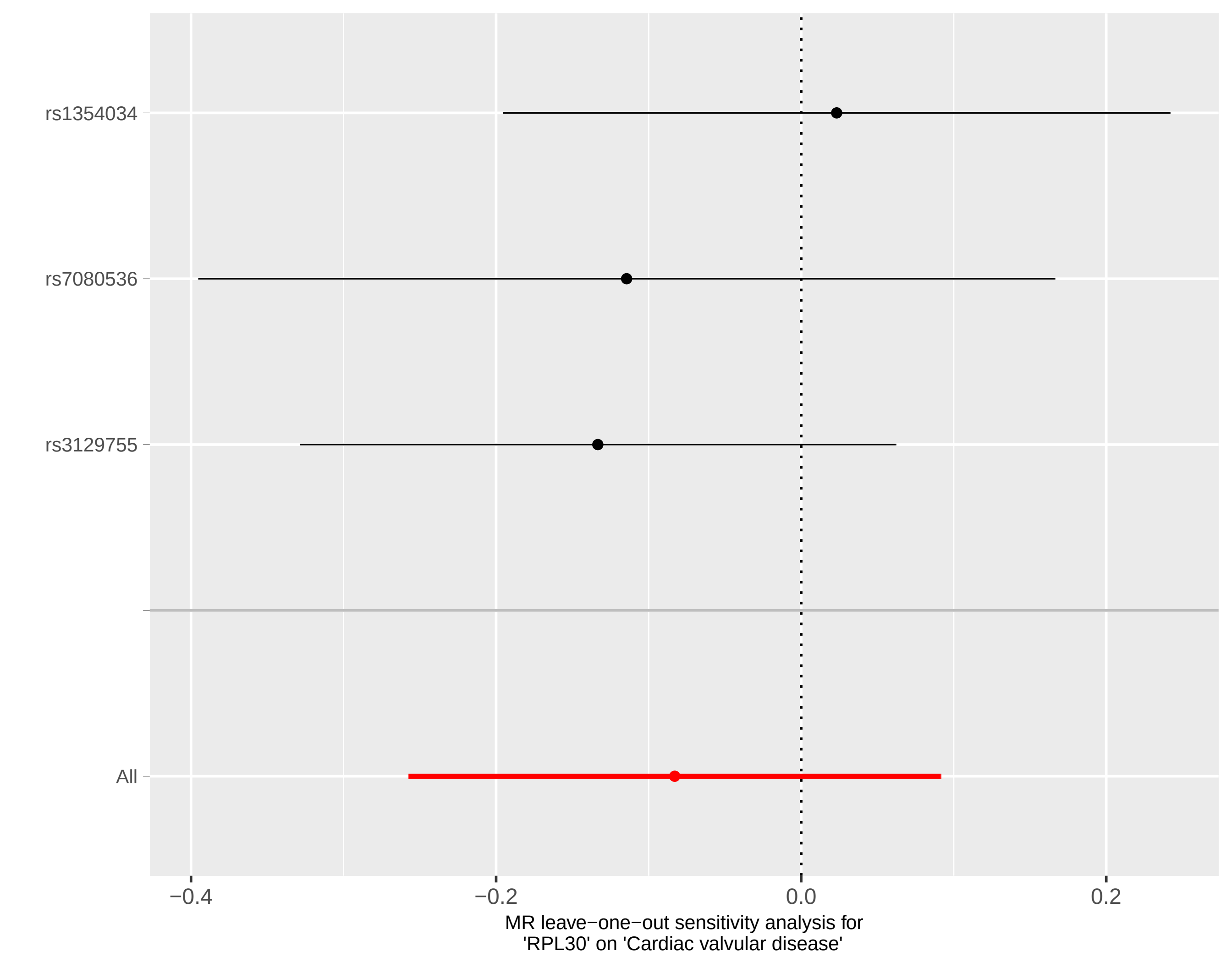

rs1354034
rs7080536
rs3129755
All
−0.4
−0.2
0.0
0.2
MR leave−one−out sensitivity analysis for
'RPL30' on 'Cardiac valvular disease'

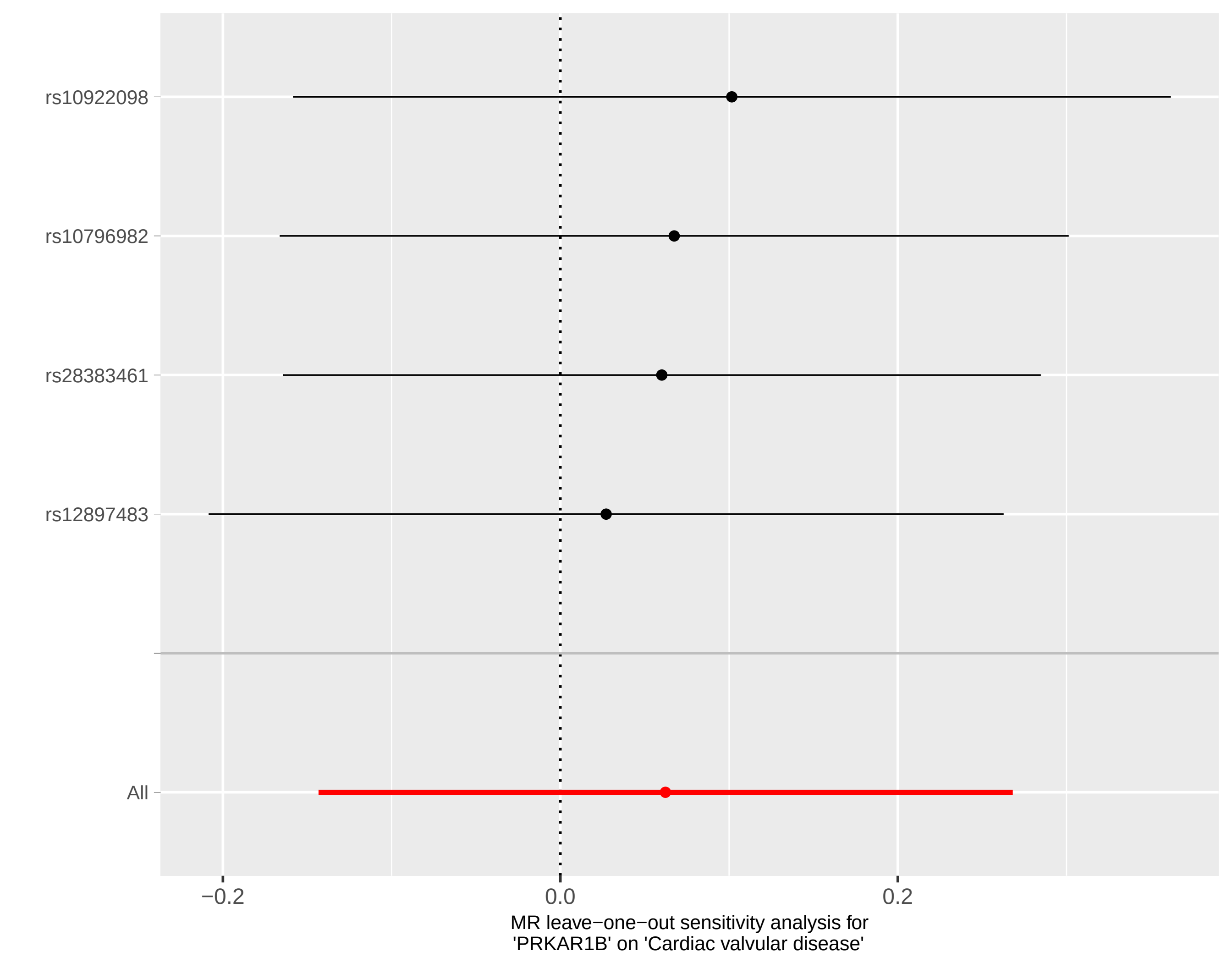
rs10922098
rs10796982
rs28383461
rs12897483
All
−0.2
0.0
0.2
MR leave−one−out sensitivity analysis for
'PRKAR1B' on 'Cardiac valvular disease'

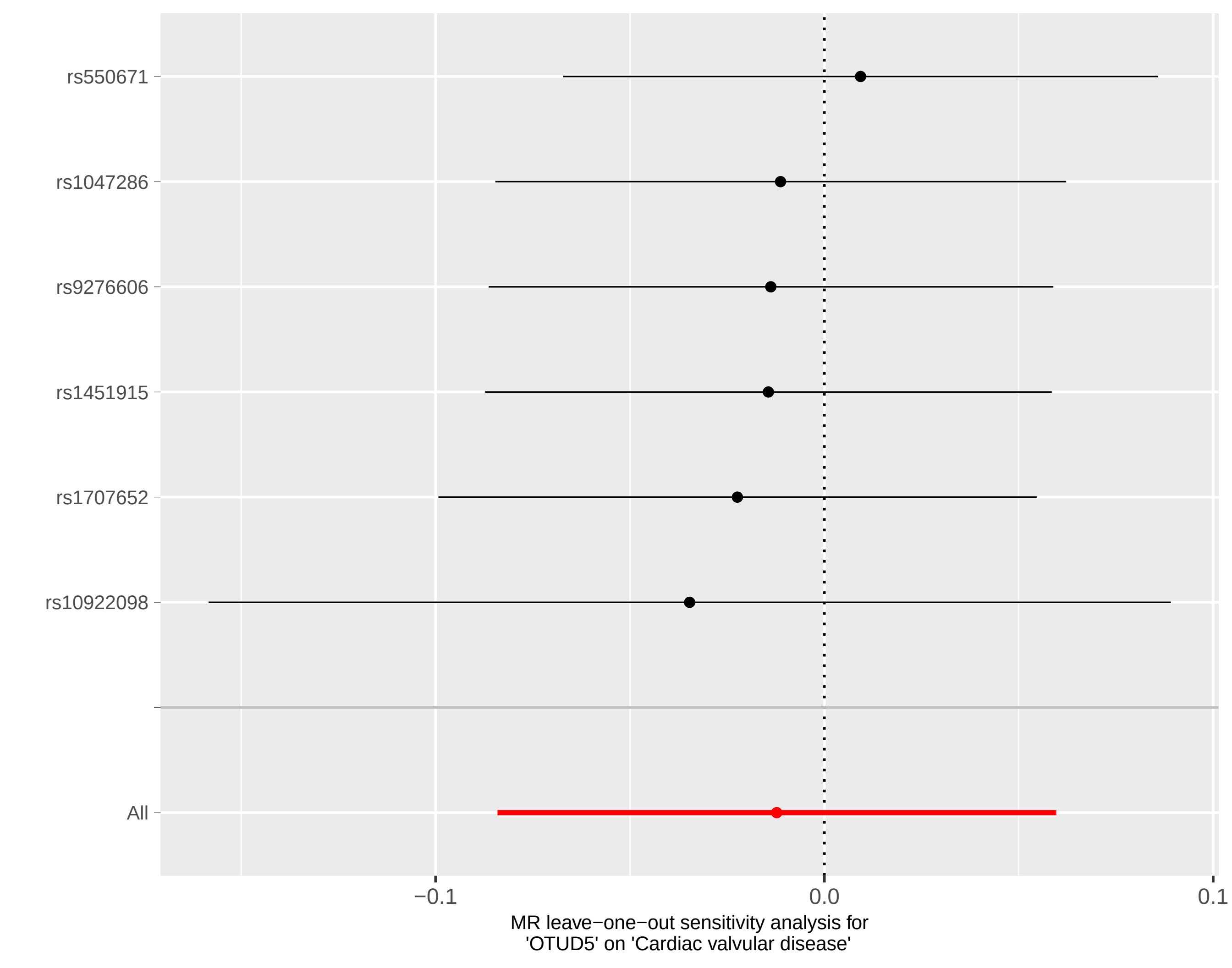

rs550671
rs1047286
rs9276606
rs1451915
rs1707652
rs10922098
All
−0.1
0.0
0.1
MR leave−one−out sensitivity analysis for
'OTUD5' on 'Cardiac valvular disease'

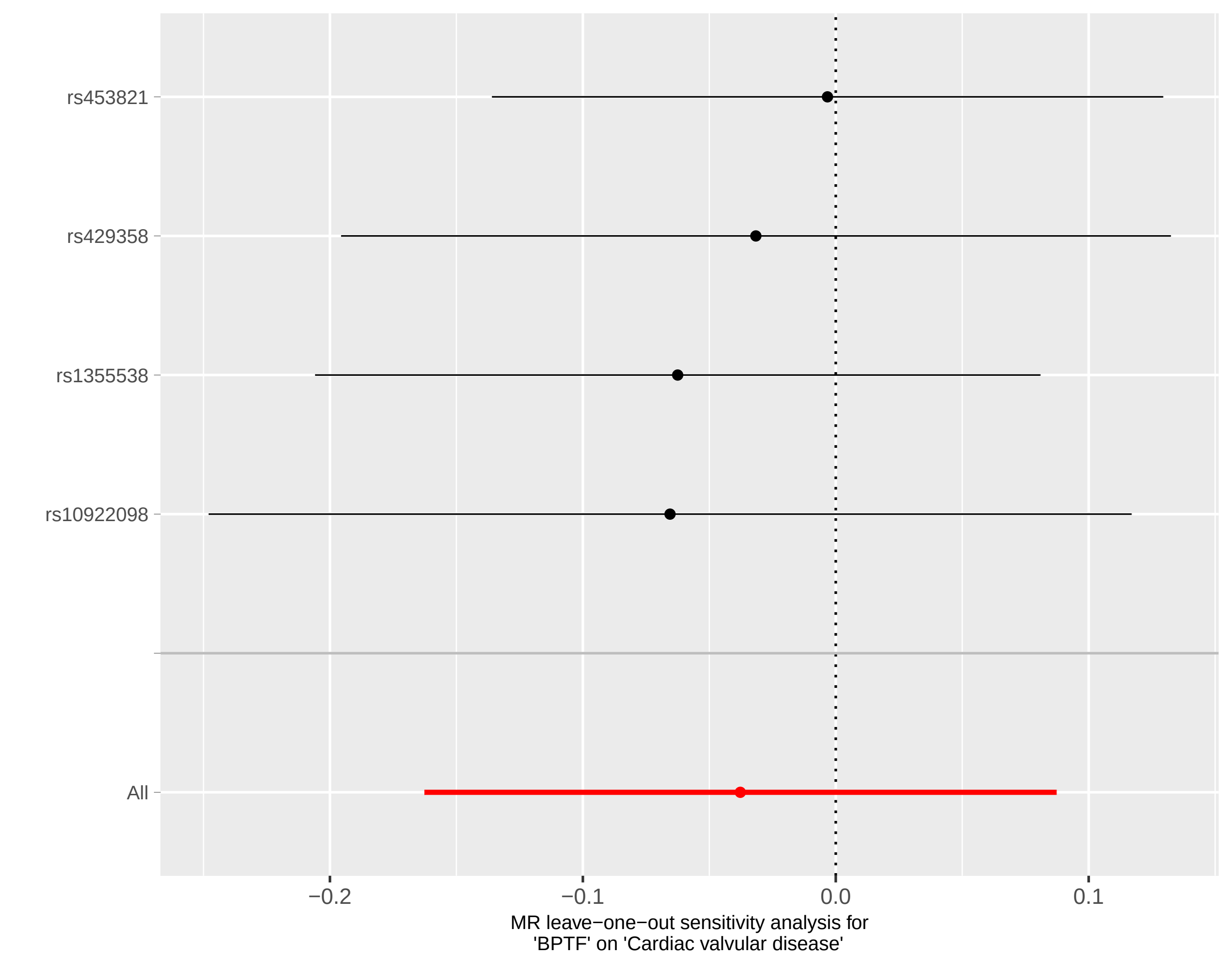
rs453821
rs429358
rs1355538
rs10922098
All
−0.2
−0.1
0.0
0.1
MR leave−one−out sensitivity analysis for
'BPTF' on 'Cardiac valvular disease'

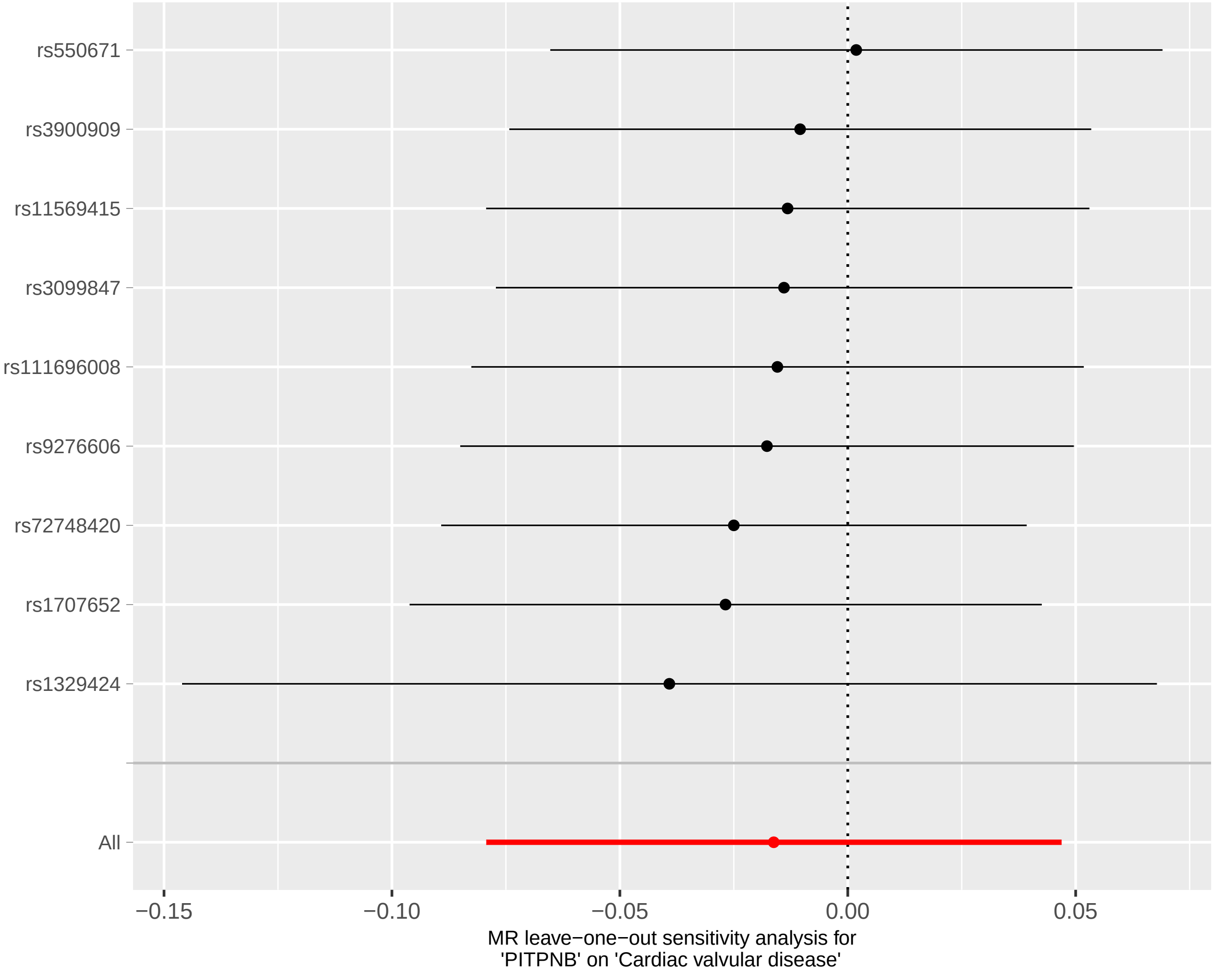
rs550671
rs3900909
rs11569415
rs3099847
rs111696008
rs9276606
rs72748420
rs1707652
rs1329424
All
−0.15
−0.10
−0.05
0.00
0.05
MR leave−one−out sensitivity analysis for
'PITPNB' on 'Cardiac valvular disease'

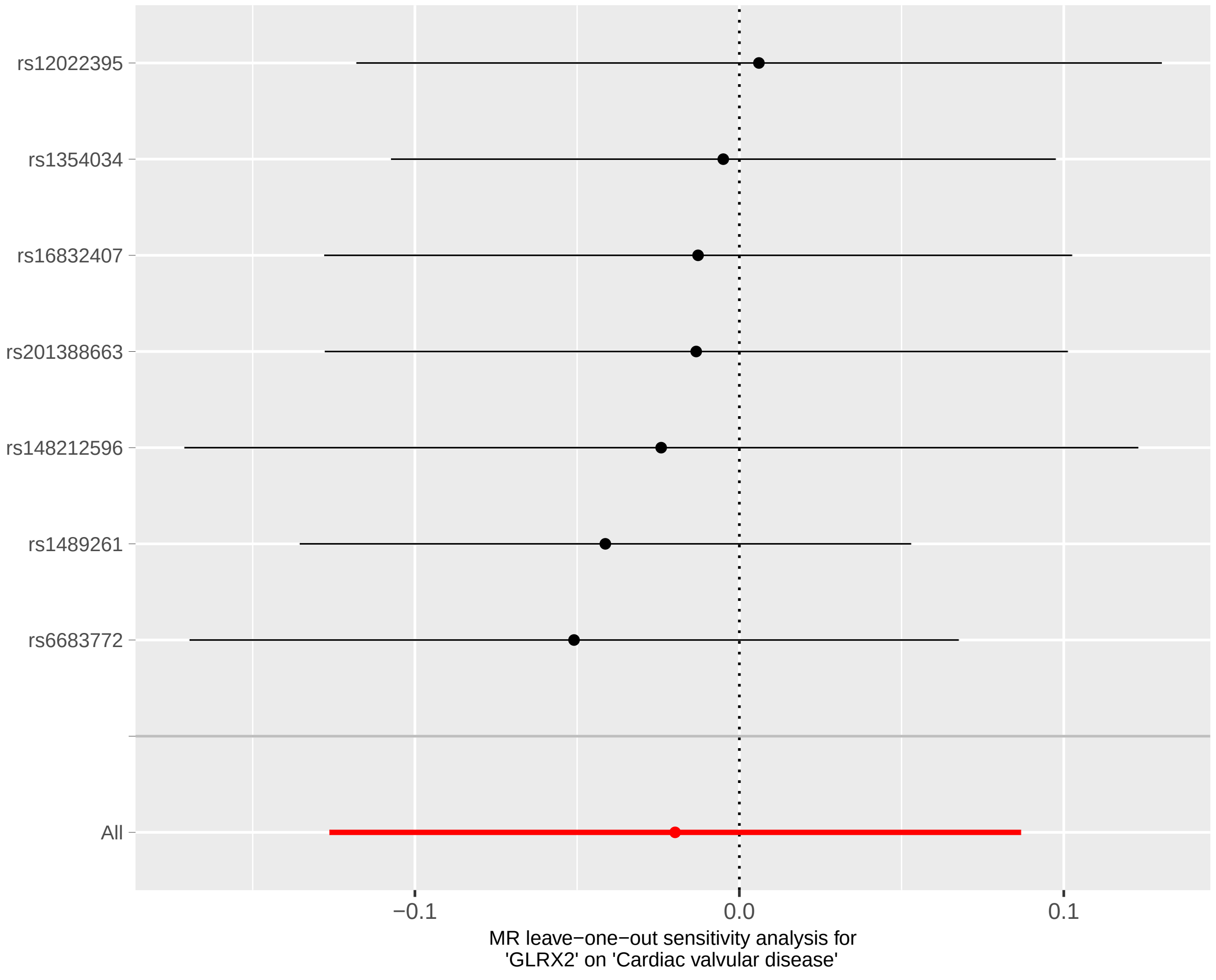
rs12022395
rs1354034
rs16832407
rs201388663
rs148212596
rs1489261
rs6683772
All
−0.1
0.0
0.1
MR leave−one−out sensitivity analysis for
'GLRX2' on 'Cardiac valvular disease'

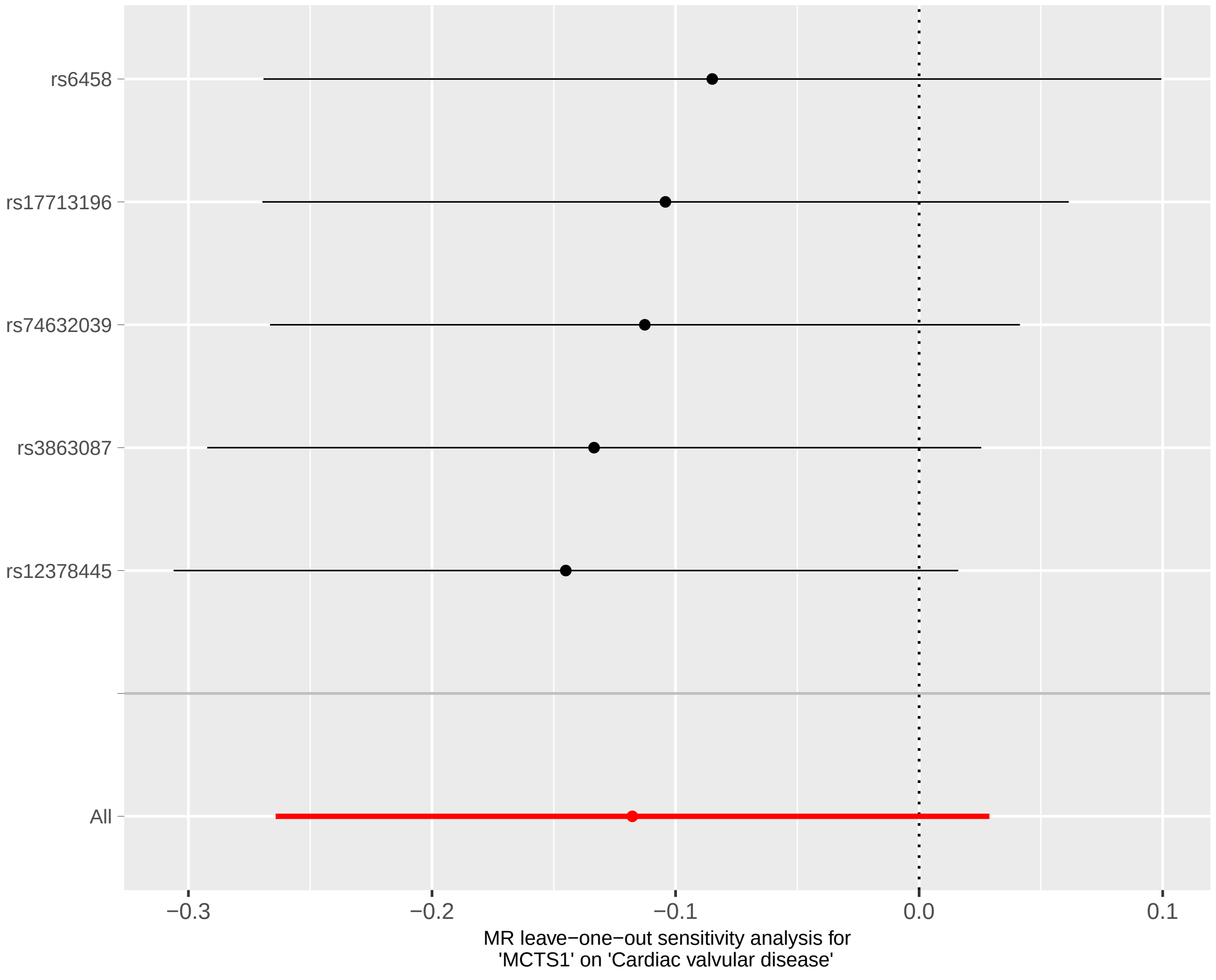

rs6458
rs17713196
rs74632039
rs3863087
rs12378445
All
−0.3
−0.2
−0.1
0.0
0.1
MR leave−one−out sensitivity analysis for
'MCTS1' on 'Cardiac valvular disease'

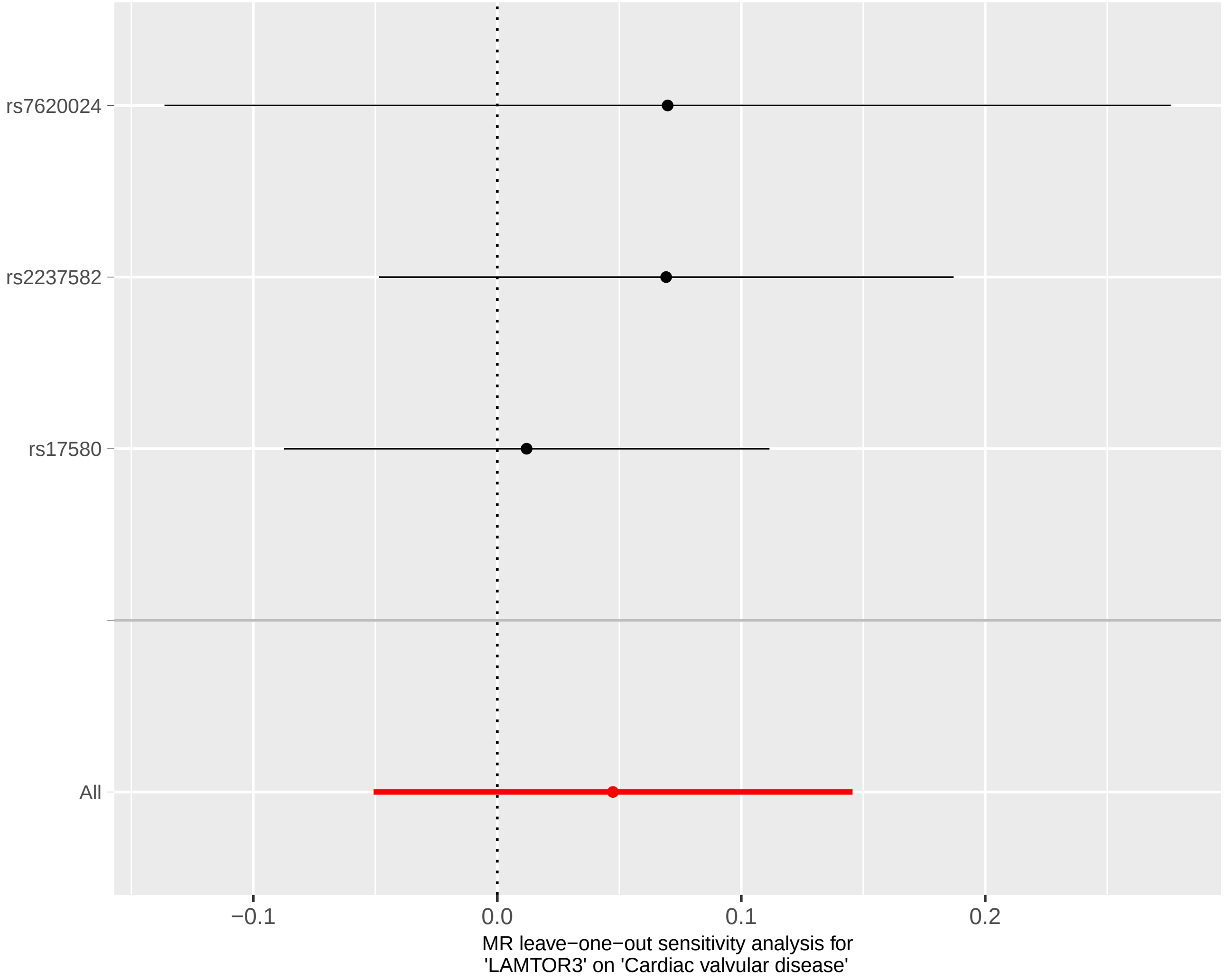

rs7620024
rs2237582
rs17580
All
−0.1
0.0
0.1
0.2
MR leave−one−out sensitivity analysis for
'LAMTOR3' on 'Cardiac valvular disease'

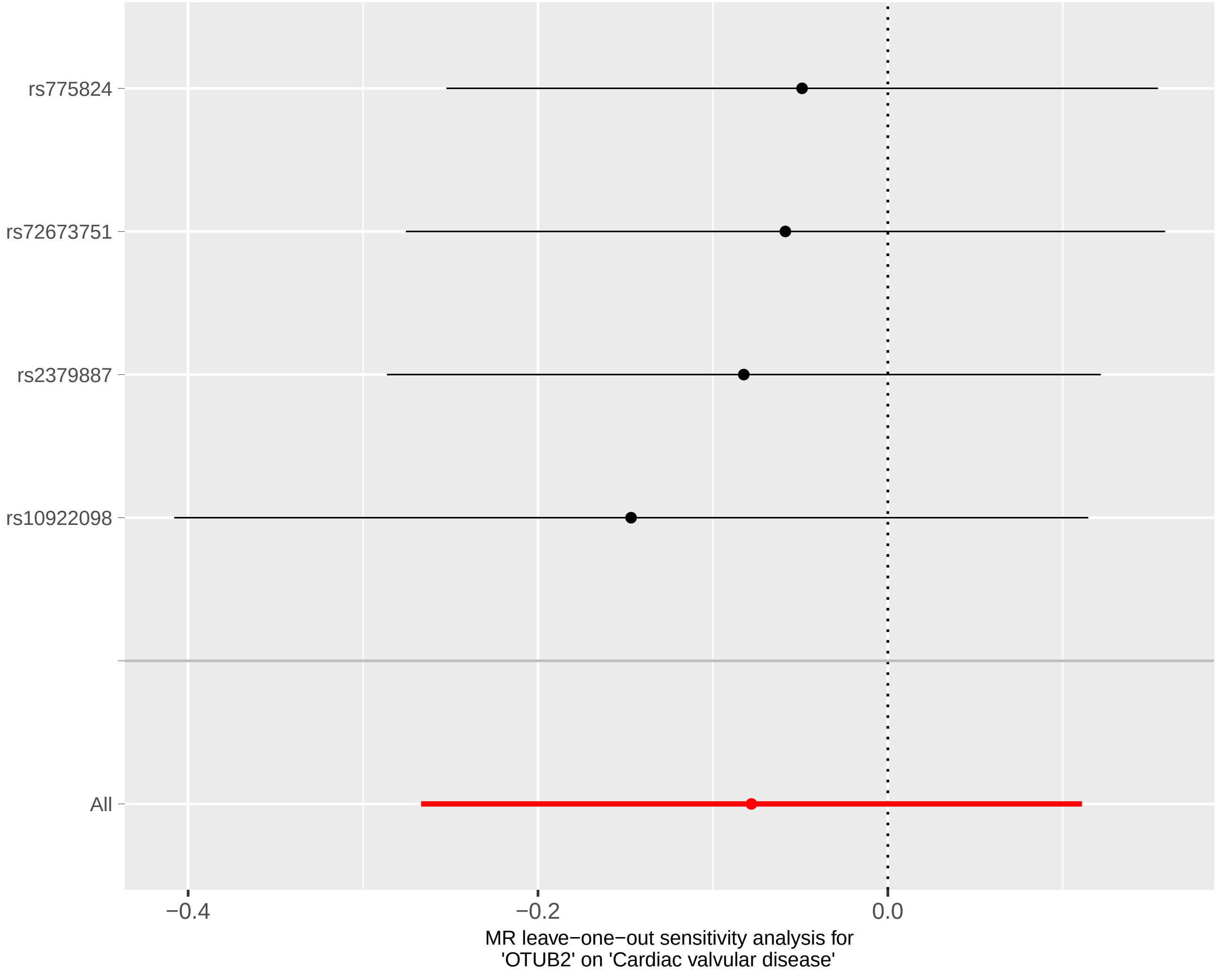

rs775824
rs72673751
rs2379887
rs10922098
All
−0.4
−0.2
0.0
MR leave−one−out sensitivity analysis for
'OTUB2' on 'Cardiac valvular disease'

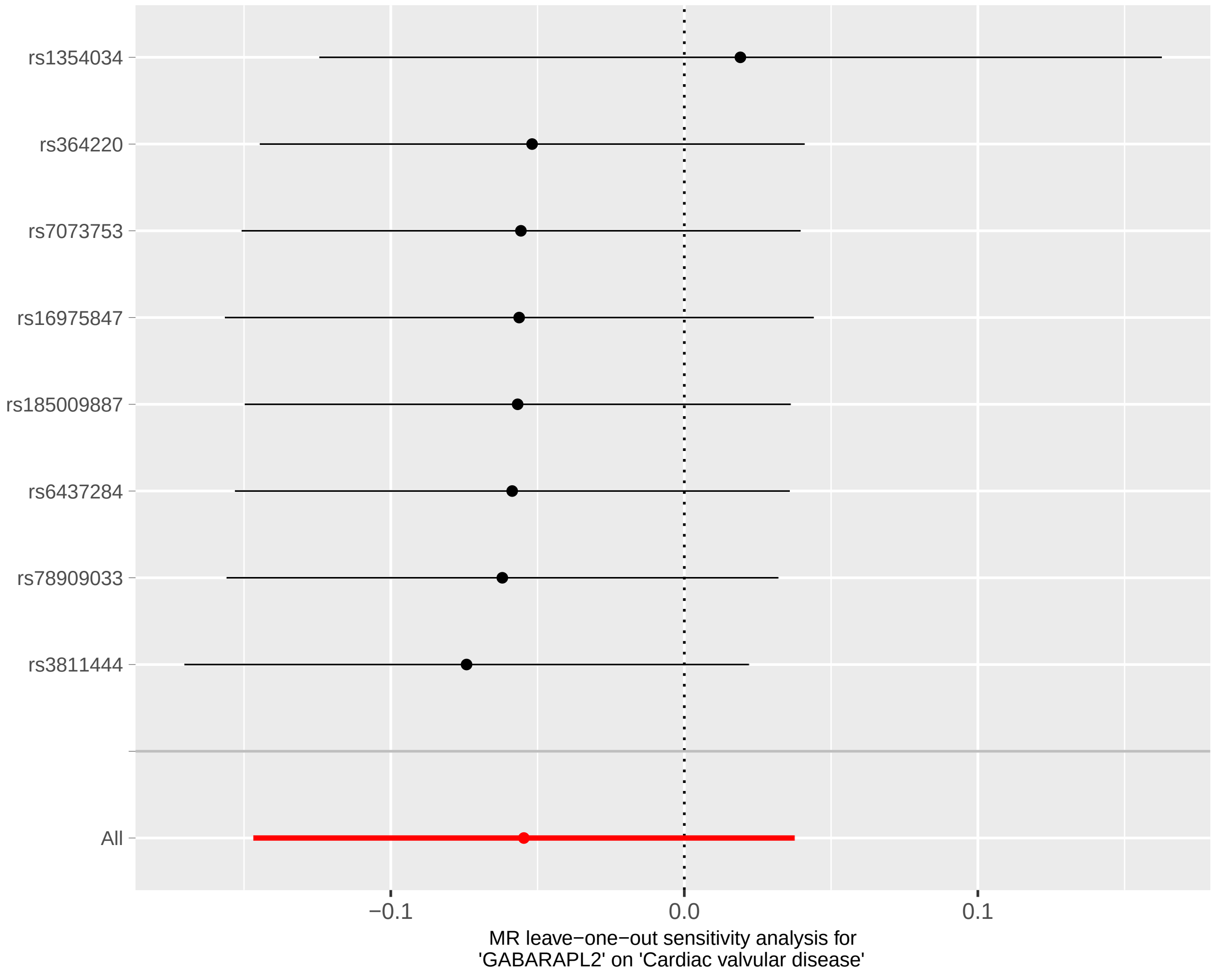
rs1354034
rs364220
rs7073753
rs16975847
rs185009887
rs6437284
rs78909033
rs3811444
All
−0.1
0.0
0.1
MR leave−one−out sensitivity analysis for
'GABARAPL2' on 'Cardiac valvular disease'

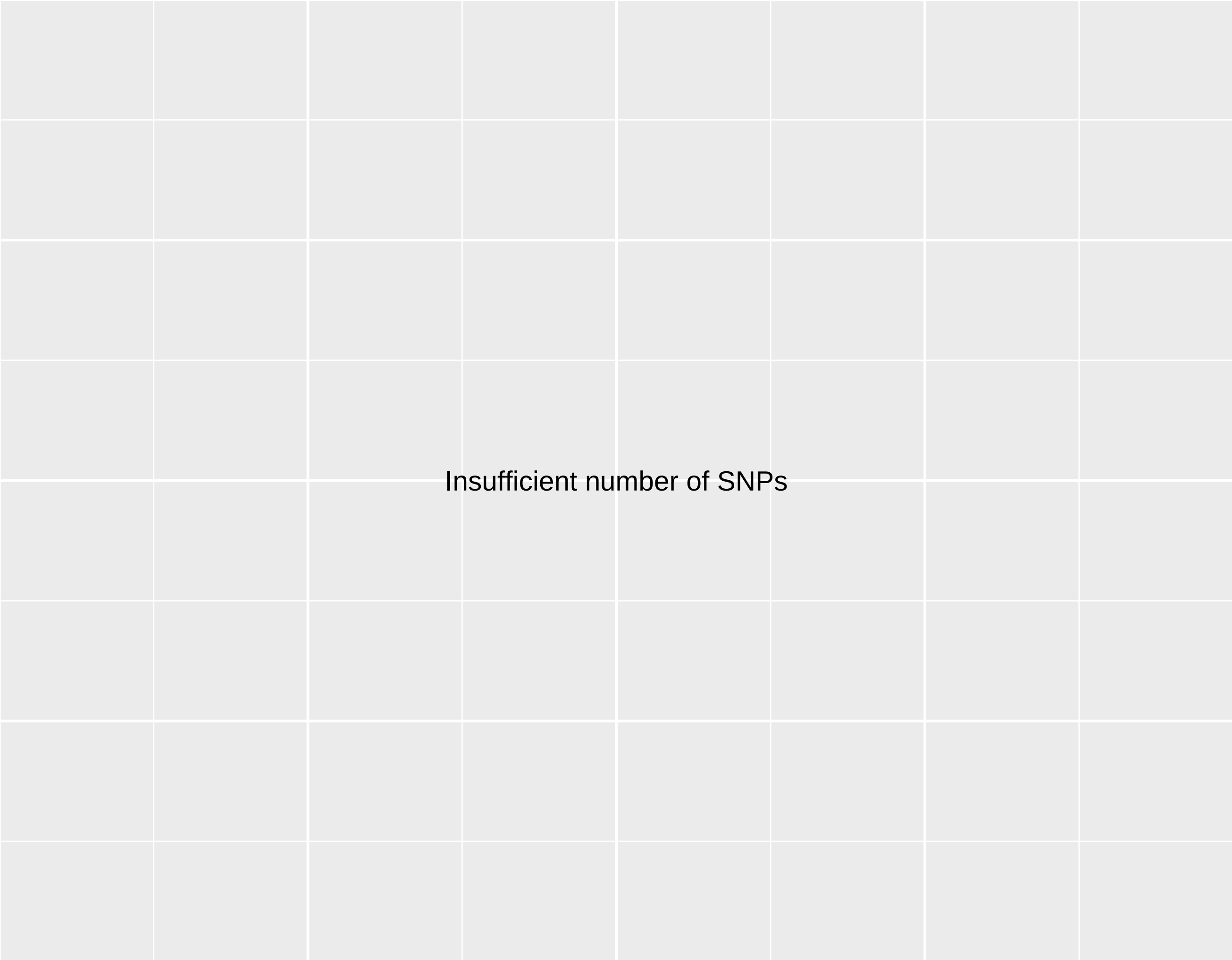
Insufficient number of SNPs

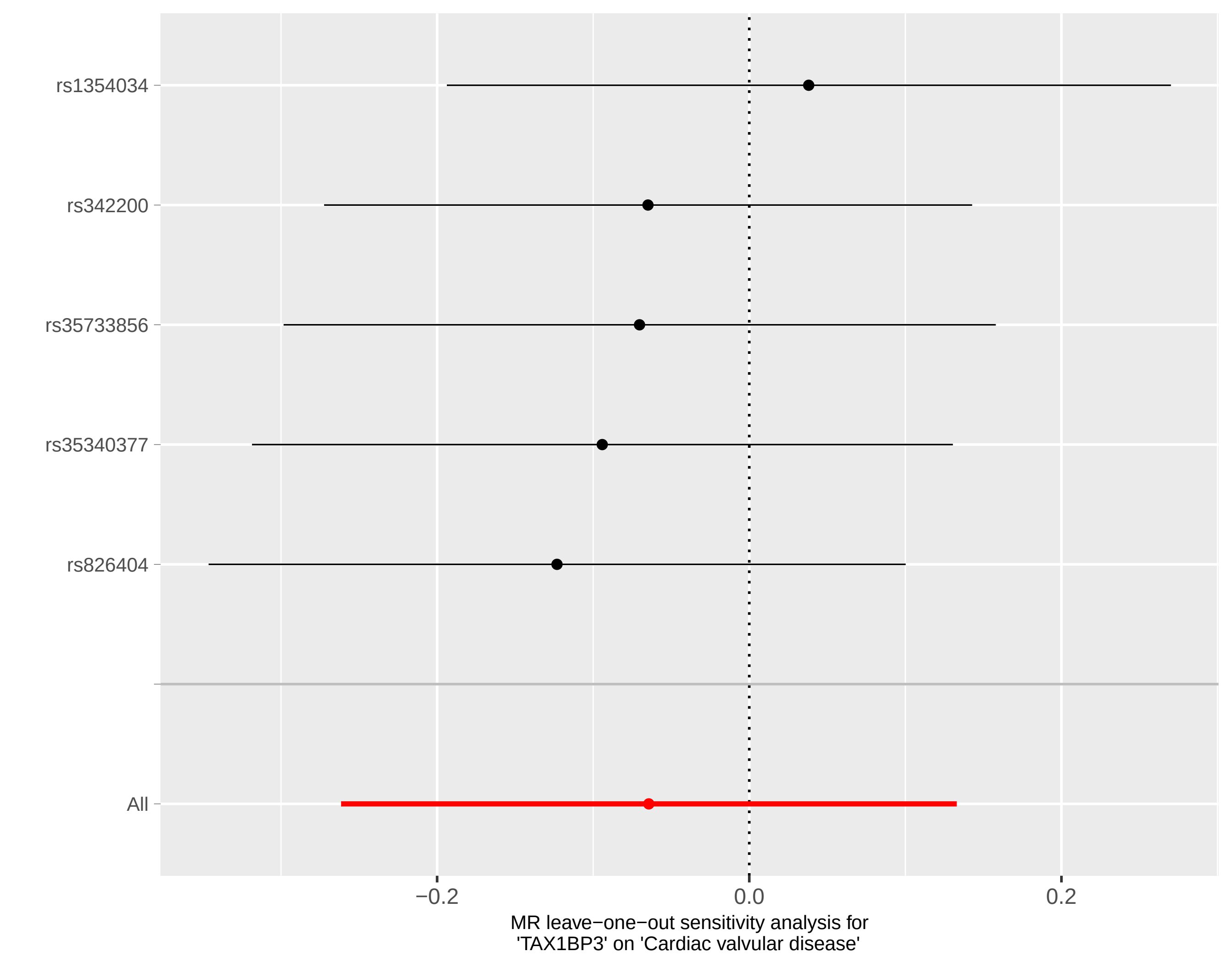
rs1354034
rs342200
rs35733856
rs35340377
rs826404
All
−0.2
0.0
0.2
MR leave−one−out sensitivity analysis for
'TAX1BP3' on 'Cardiac valvular disease'

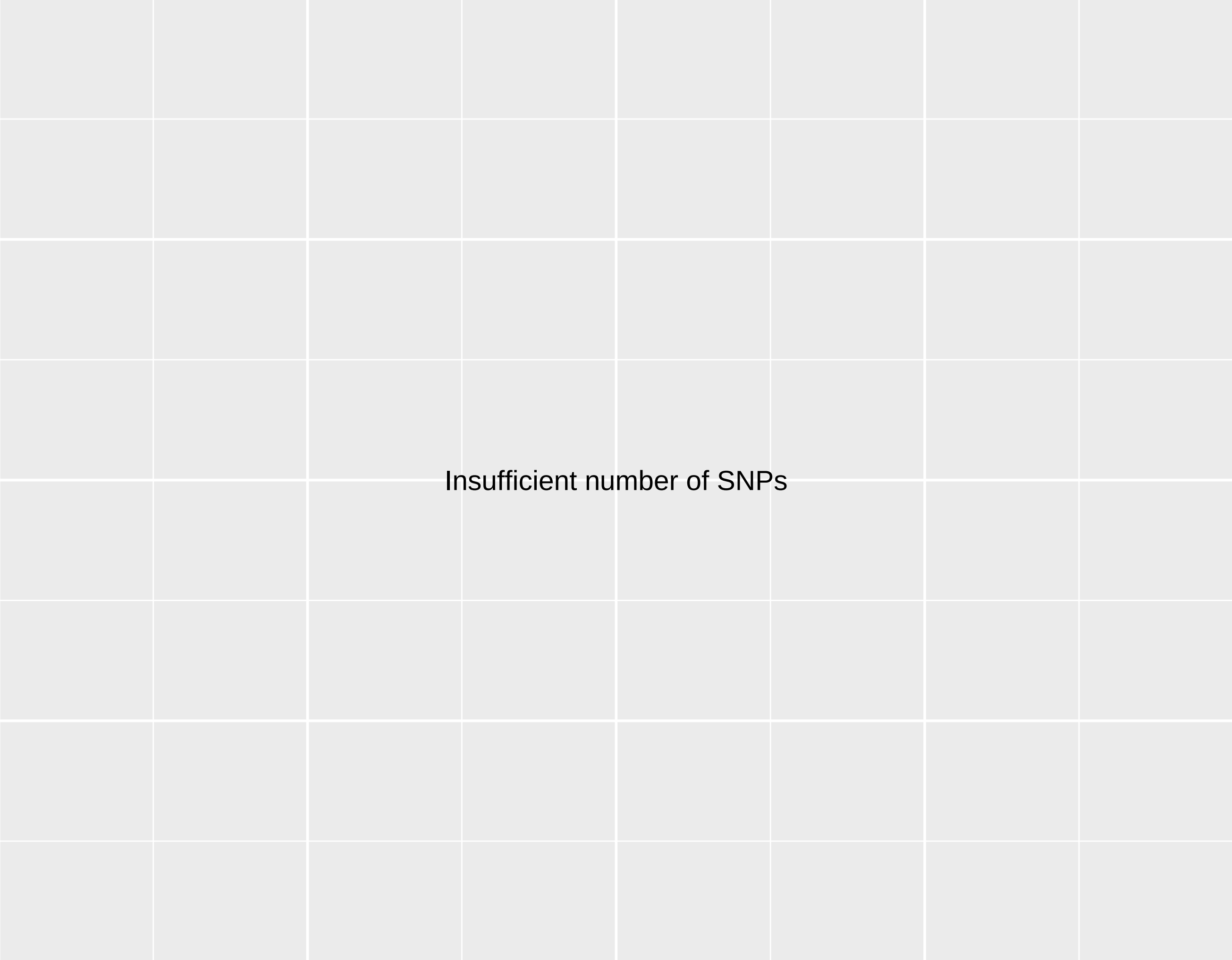
Insufficient number of SNPs

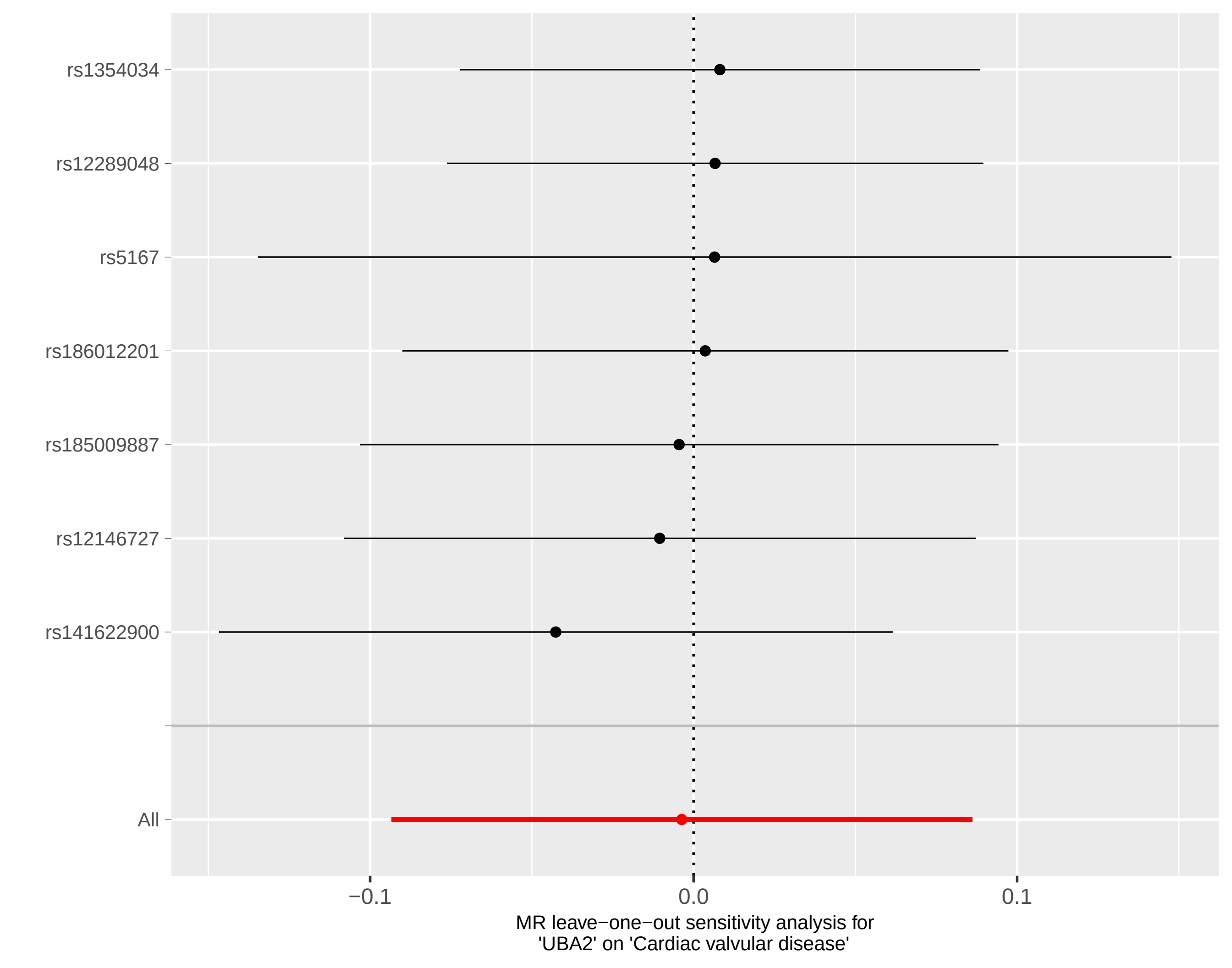

rs1354034
rs12289048
rs5167
rs186012201
rs185009887
rs12146727
rs141622900
All
−0.1
0.0
0.1
MR leave−one−out sensitivity analysis for
'UBA2' on 'Cardiac valvular disease'

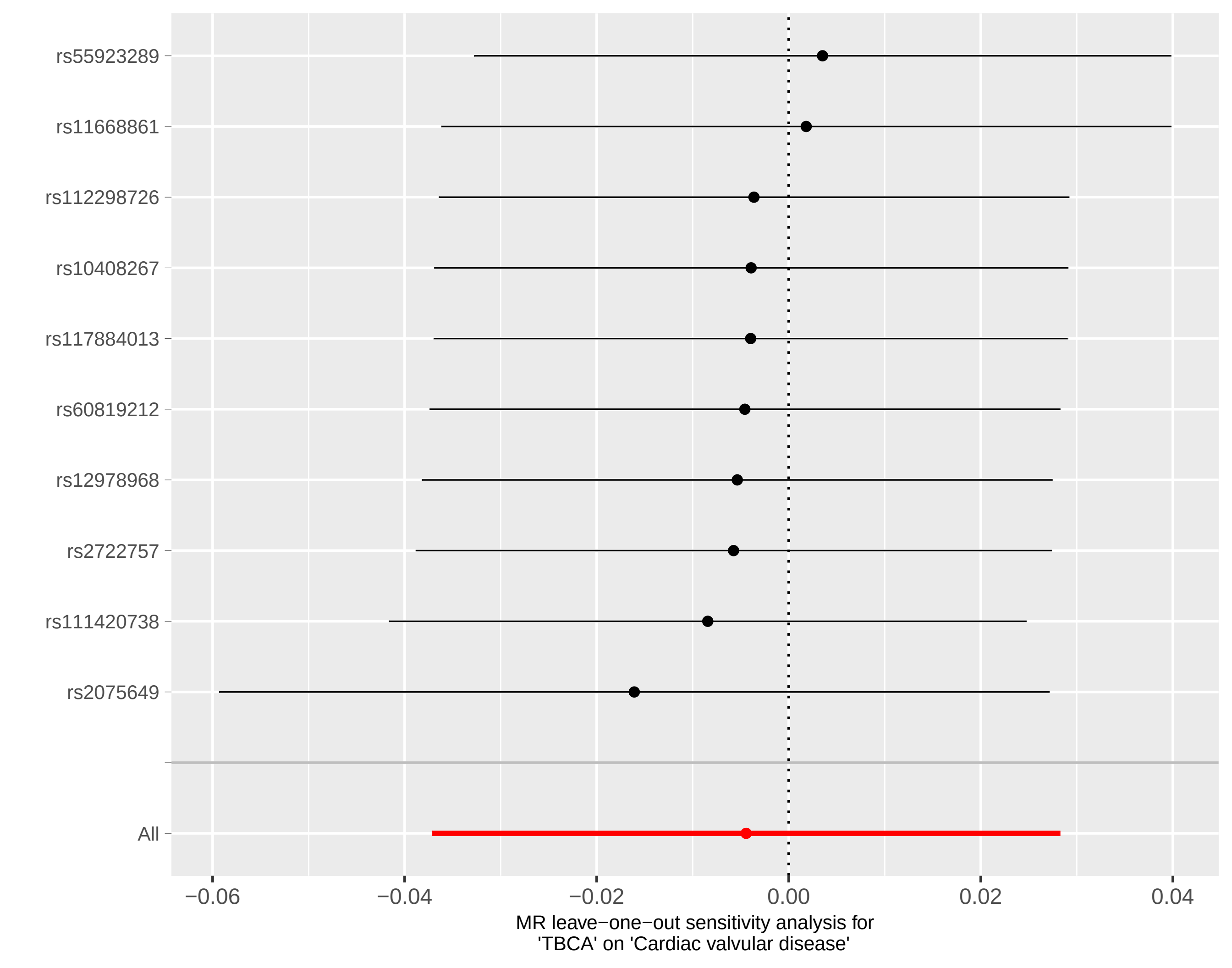

rs55923289
rs11668861
rs112298726
rs10408267
rs117884013
rs60819212
rs12978968
rs2722757
rs111420738
rs2075649
All
−0.06
−0.04
−0.02
0.00
0.02
0.04
MR leave−one−out sensitivity analysis for
'TBCA' on 'Cardiac valvular disease'

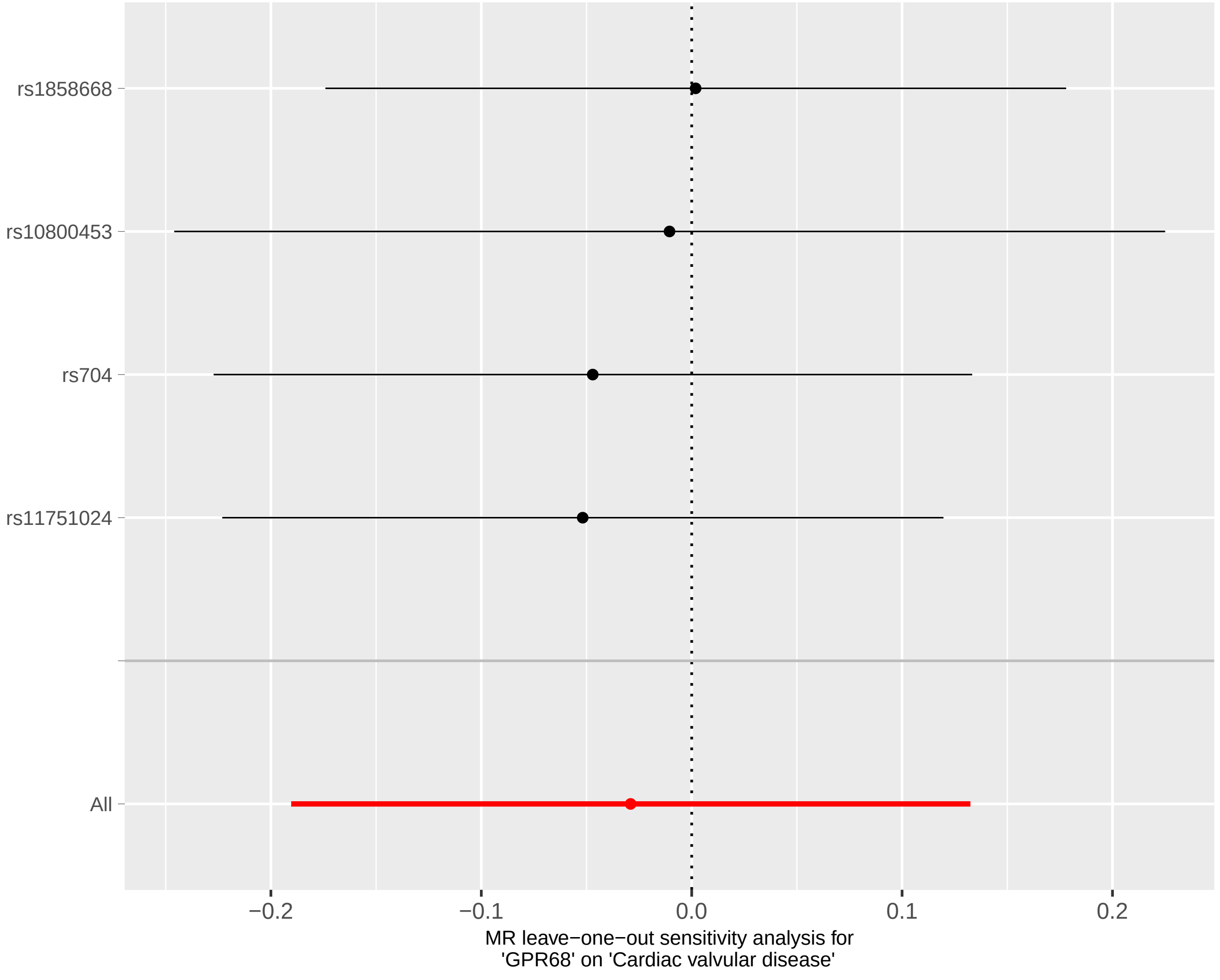

rs1858668
rs10800453
rs704
rs11751024
All
−0.2
−0.1
0.0
0.1
0.2
MR leave−one−out sensitivity analysis for
'GPR68' on 'Cardiac valvular disease'

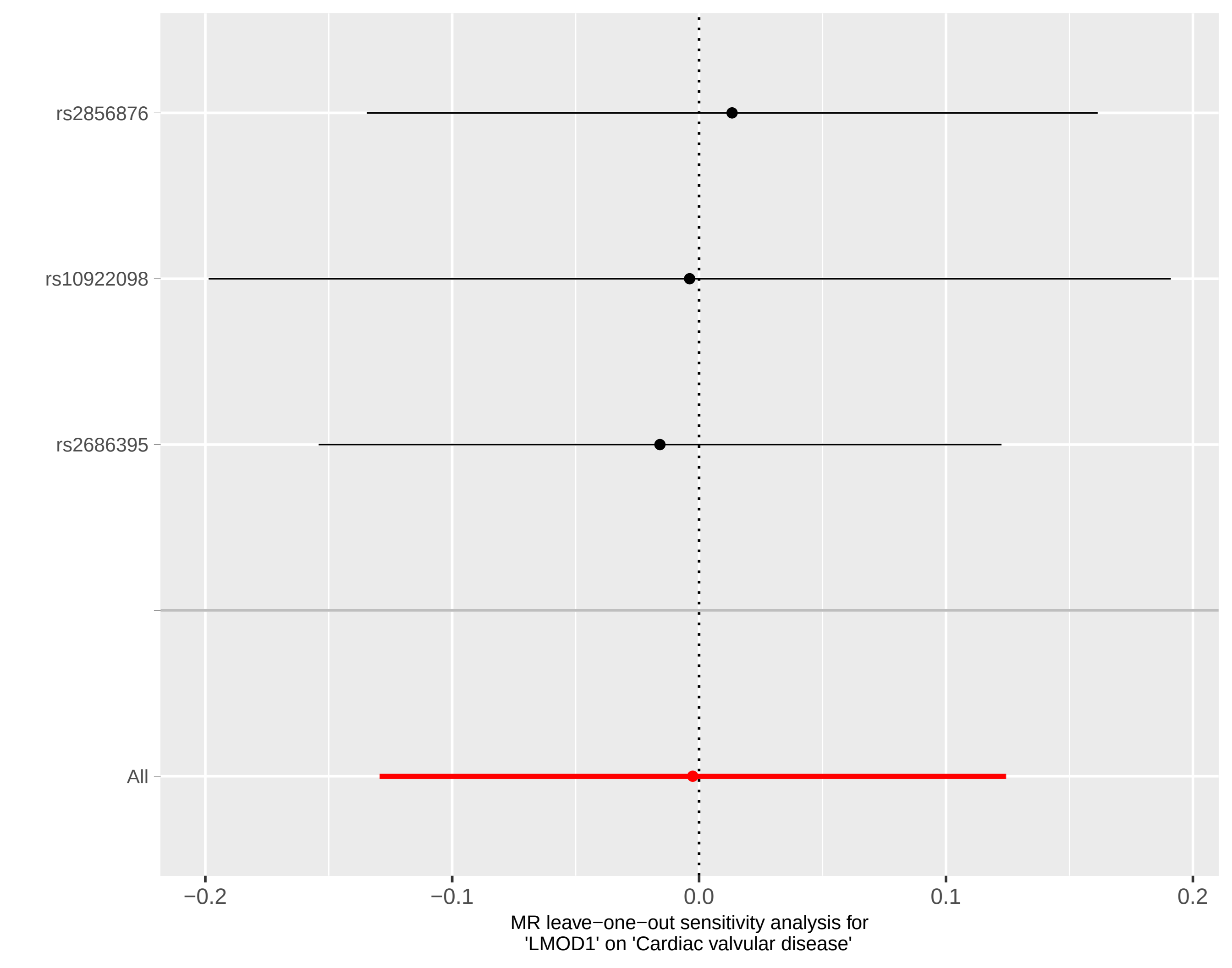
rs2856876
rs10922098
rs2686395
All
−0.2
−0.1
0.0
0.1
0.2
MR leave−one−out sensitivity analysis for
'LMOD1' on 'Cardiac valvular disease'

Insufficient number of SNPs

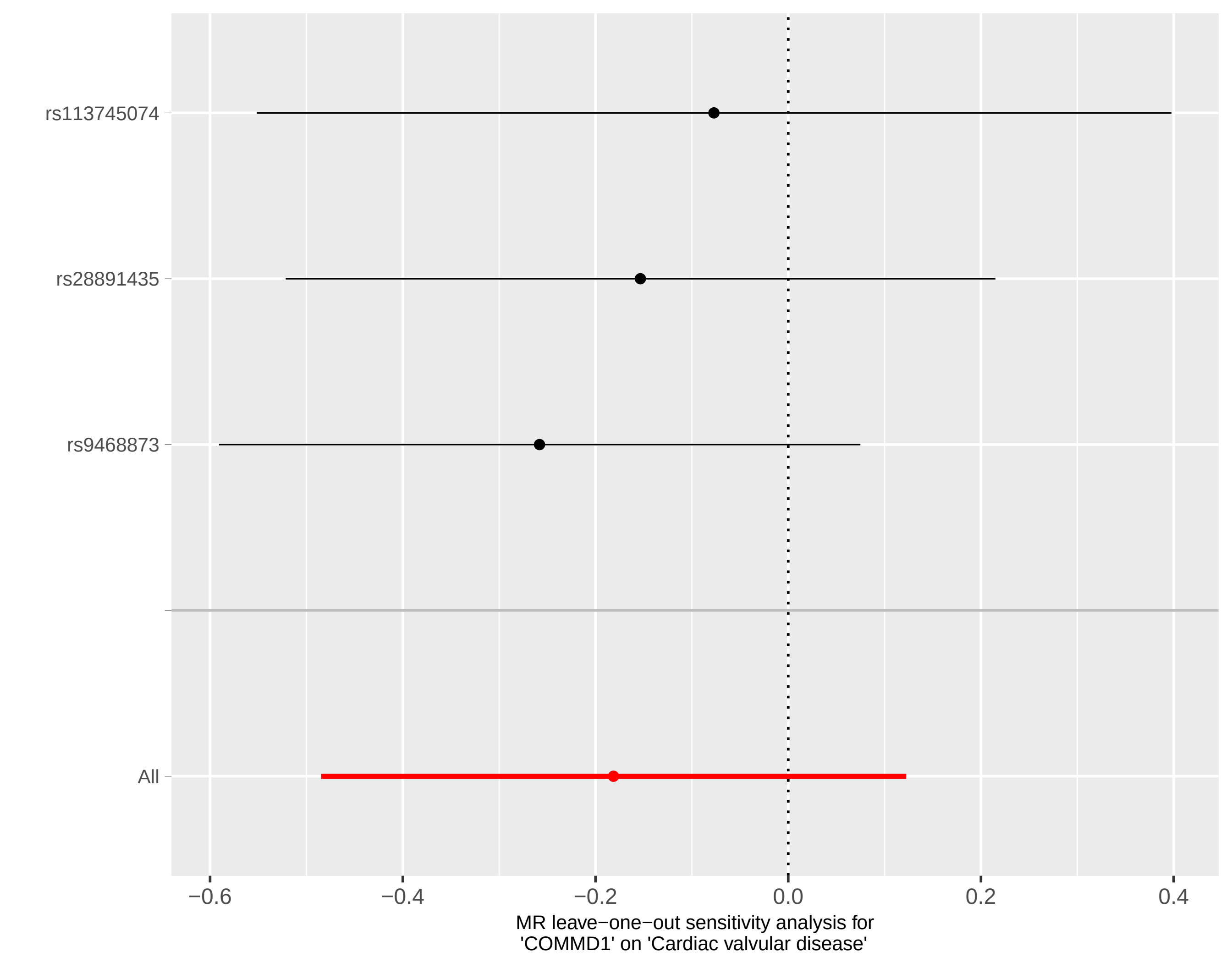
rs113745074
rs28891435
rs9468873
All
−0.6
−0.4
−0.2
0.0
0.2
0.4
MR leave−one−out sensitivity analysis for
'COMMD1' on 'Cardiac valvular disease'

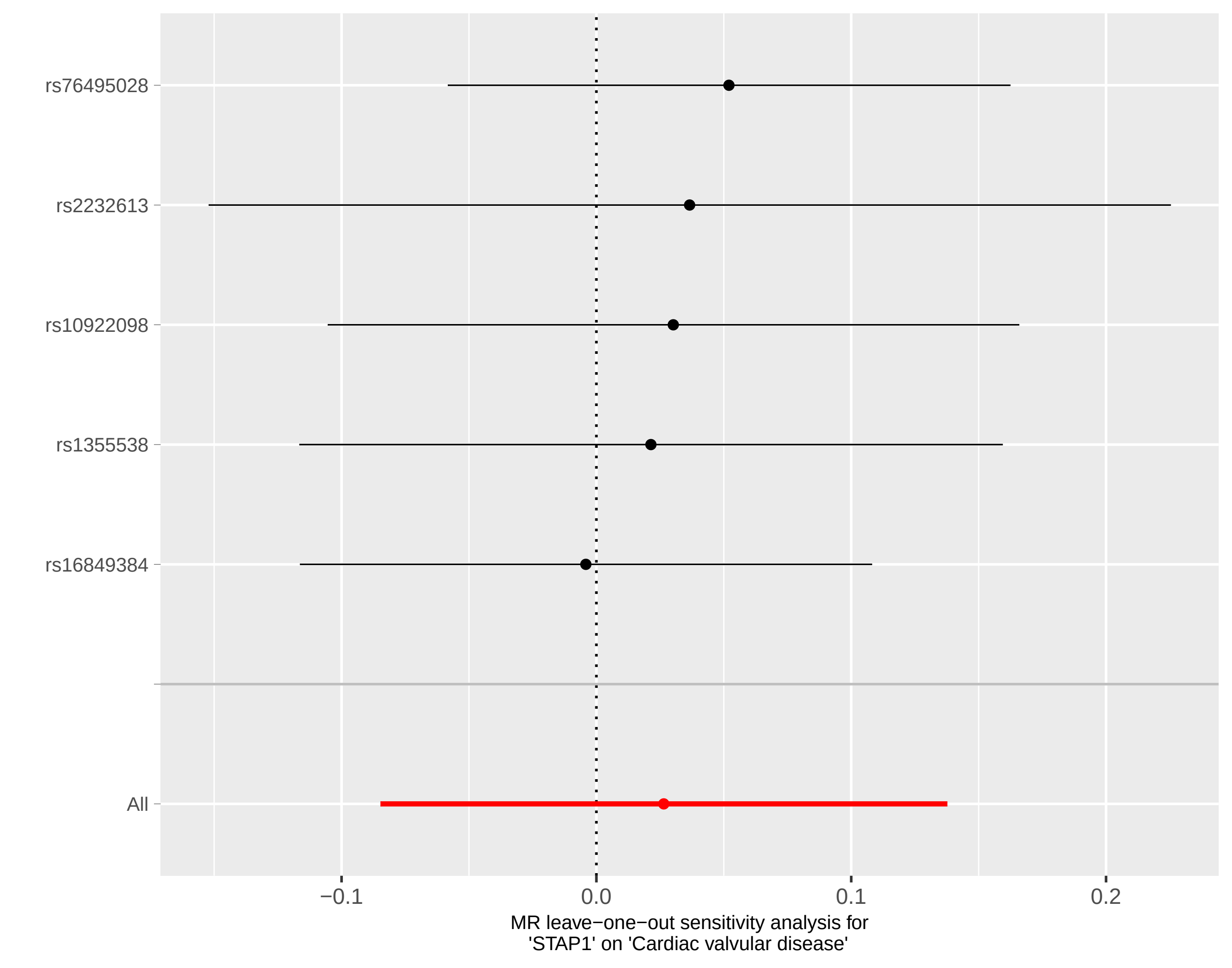
rs76495028
rs2232613
rs10922098
rs1355538
rs16849384
All
−0.1
0.0
0.1
0.2
MR leave−one−out sensitivity analysis for
'STAP1' on 'Cardiac valvular disease'

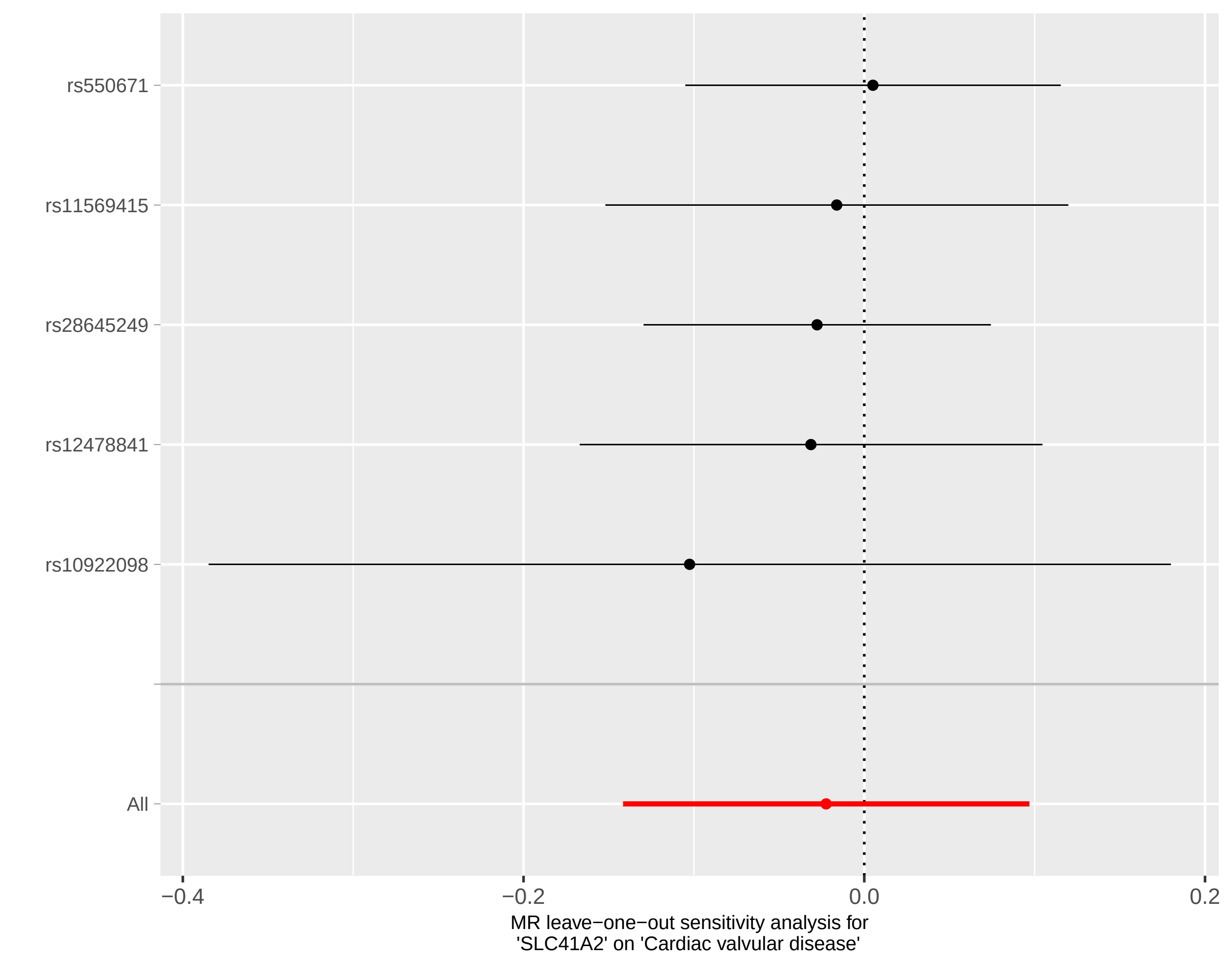

rs550671
rs11569415
rs28645249
rs12478841
rs10922098
All
−0.4
−0.2
0.0
0.2
MR leave−one−out sensitivity analysis for
'SLC41A2' on 'Cardiac valvular disease'

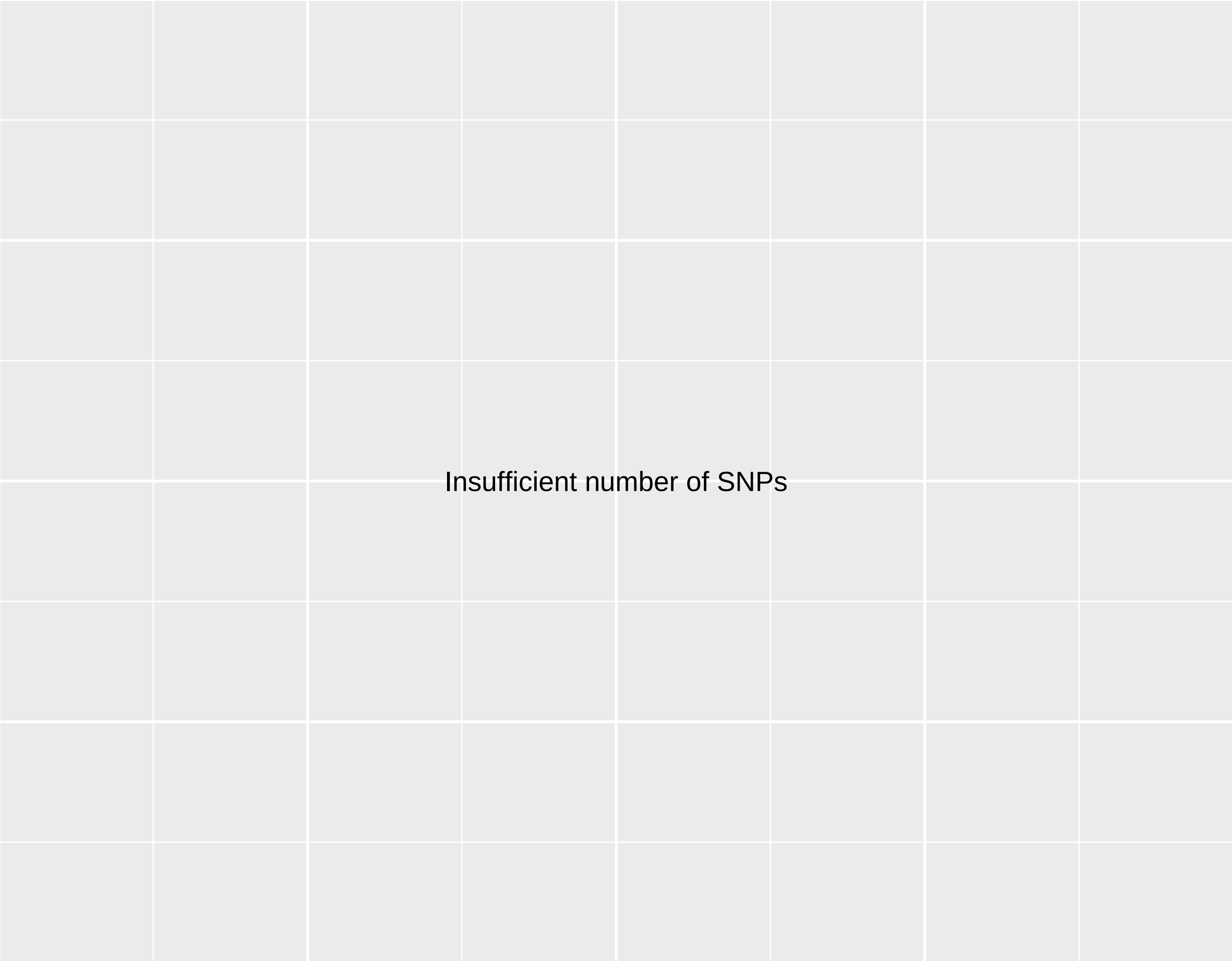
Insufficient number of SNPs

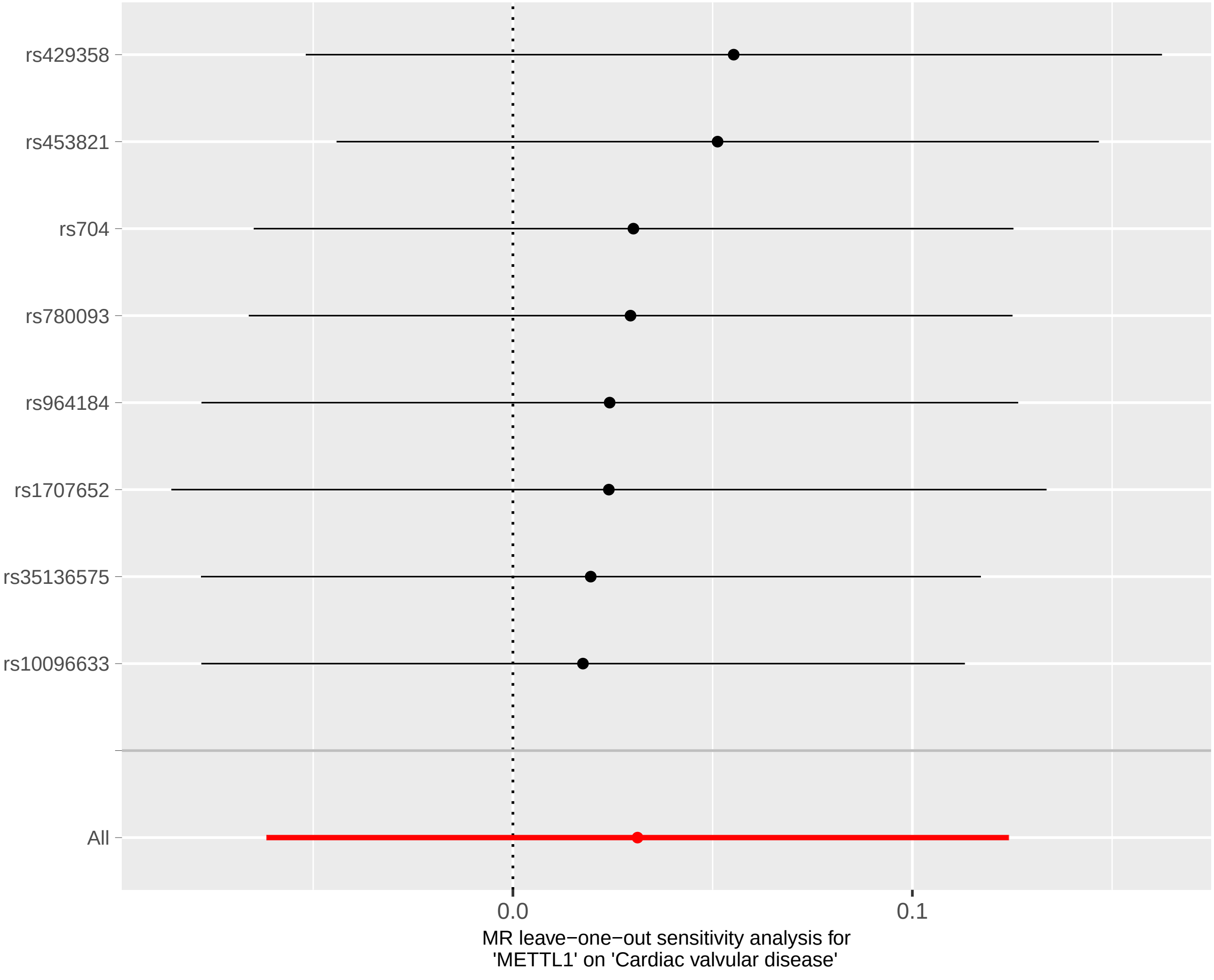

rs429358
rs453821
rs704
rs780093
rs964184
rs1707652
rs35136575
rs10096633
All
0.0
0.1
MR leave−one−out sensitivity analysis for
'METTL1' on 'Cardiac valvular disease'

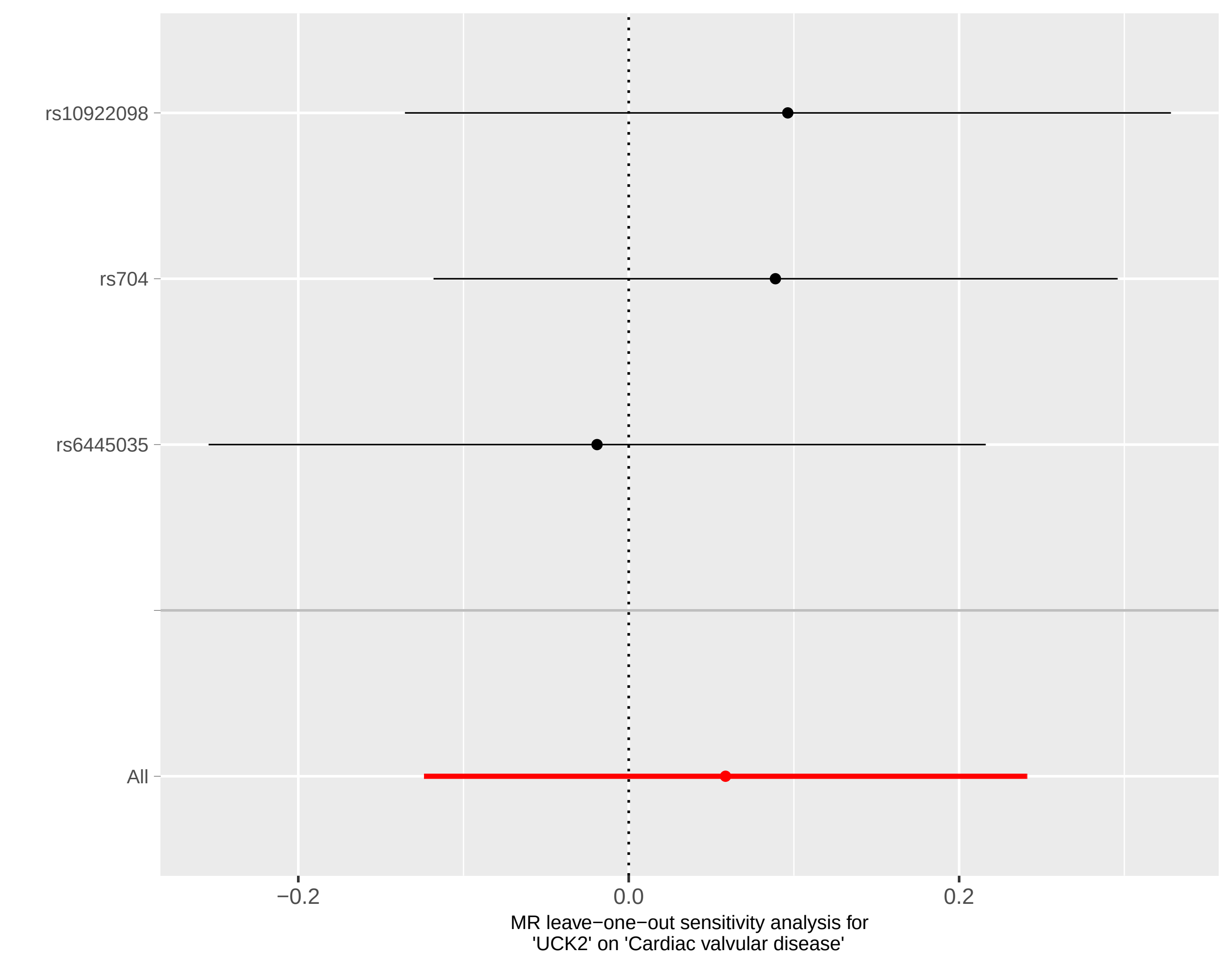
rs10922098
rs704
rs6445035
All
−0.2
0.0
0.2
MR leave−one−out sensitivity analysis for
'UCK2' on 'Cardiac valvular disease'

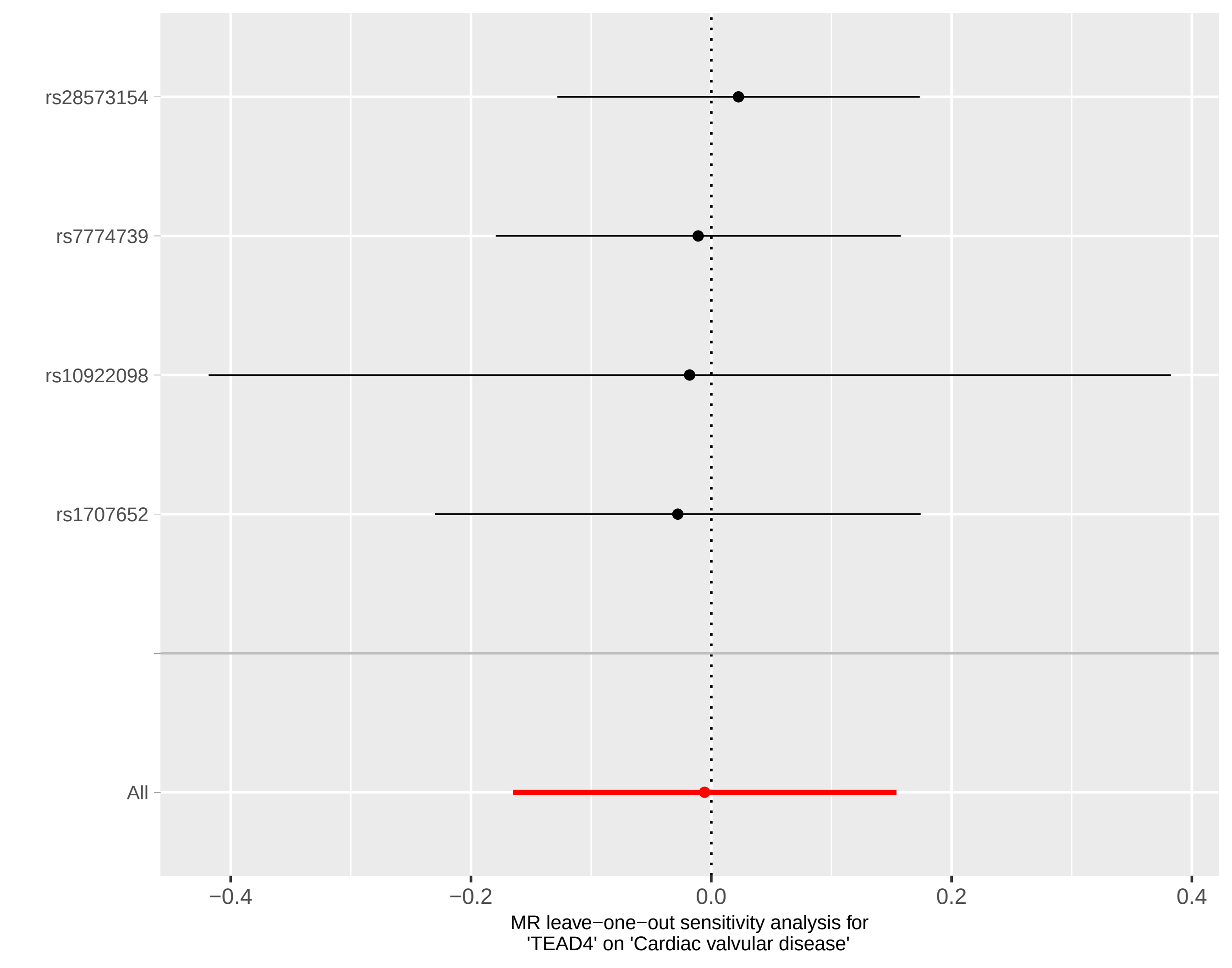
rs28573154
rs7774739
rs10922098
rs1707652
All
−0.4
−0.2
0.0
0.2
0.4
MR leave−one−out sensitivity analysis for
'TEAD4' on 'Cardiac valvular disease'

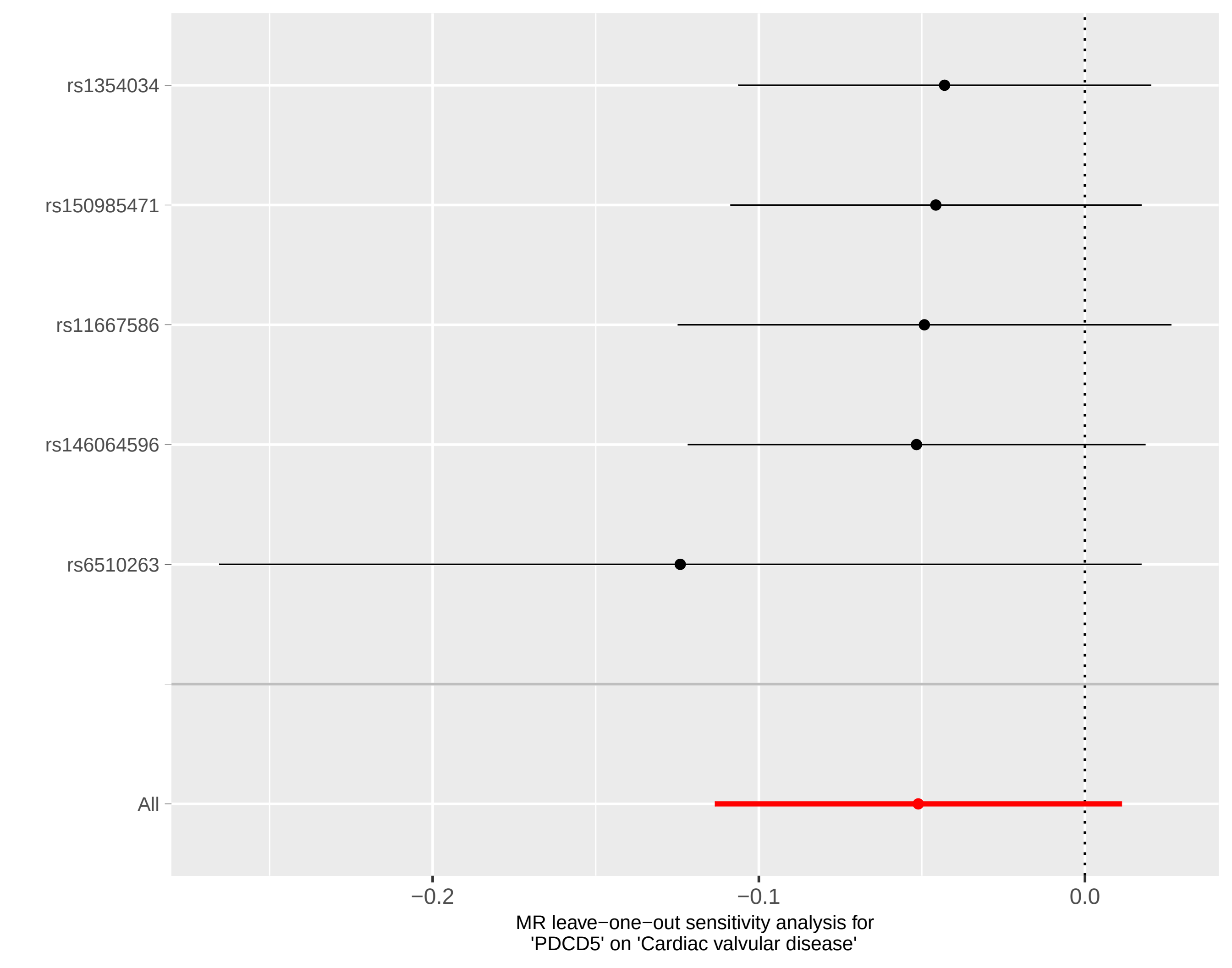

rs1354034
rs150985471
rs11667586
rs146064596
rs6510263
All
−0.2
−0.1
0.0
MR leave−one−out sensitivity analysis for
'PDCD5' on 'Cardiac valvular disease'

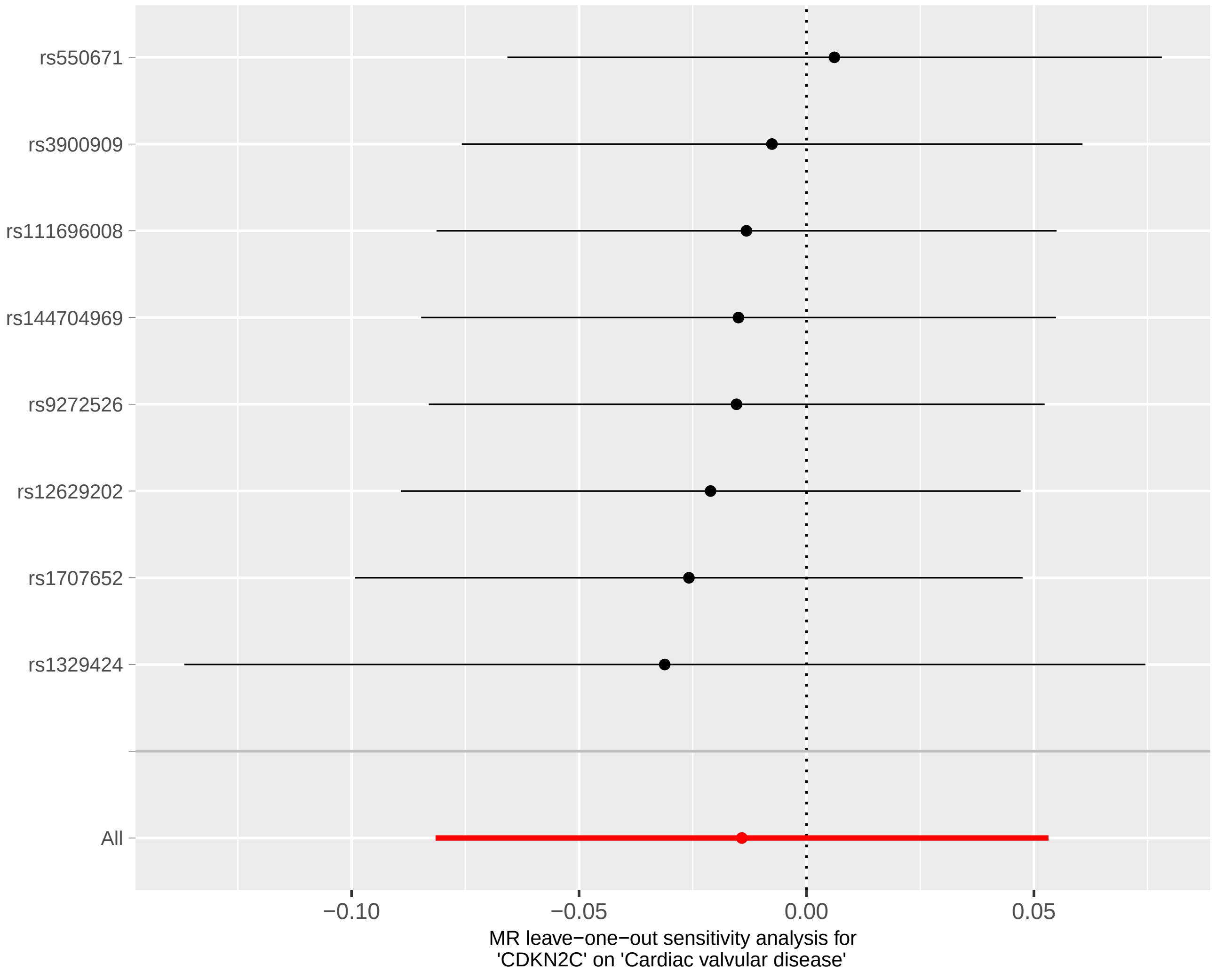

rs550671
rs3900909
rs111696008
rs144704969
rs9272526
rs12629202
rs1707652
rs1329424
All
−0.10
−0.05
0.00
0.05
MR leave−one−out sensitivity analysis for
'CDKN2C' on 'Cardiac valvular disease'

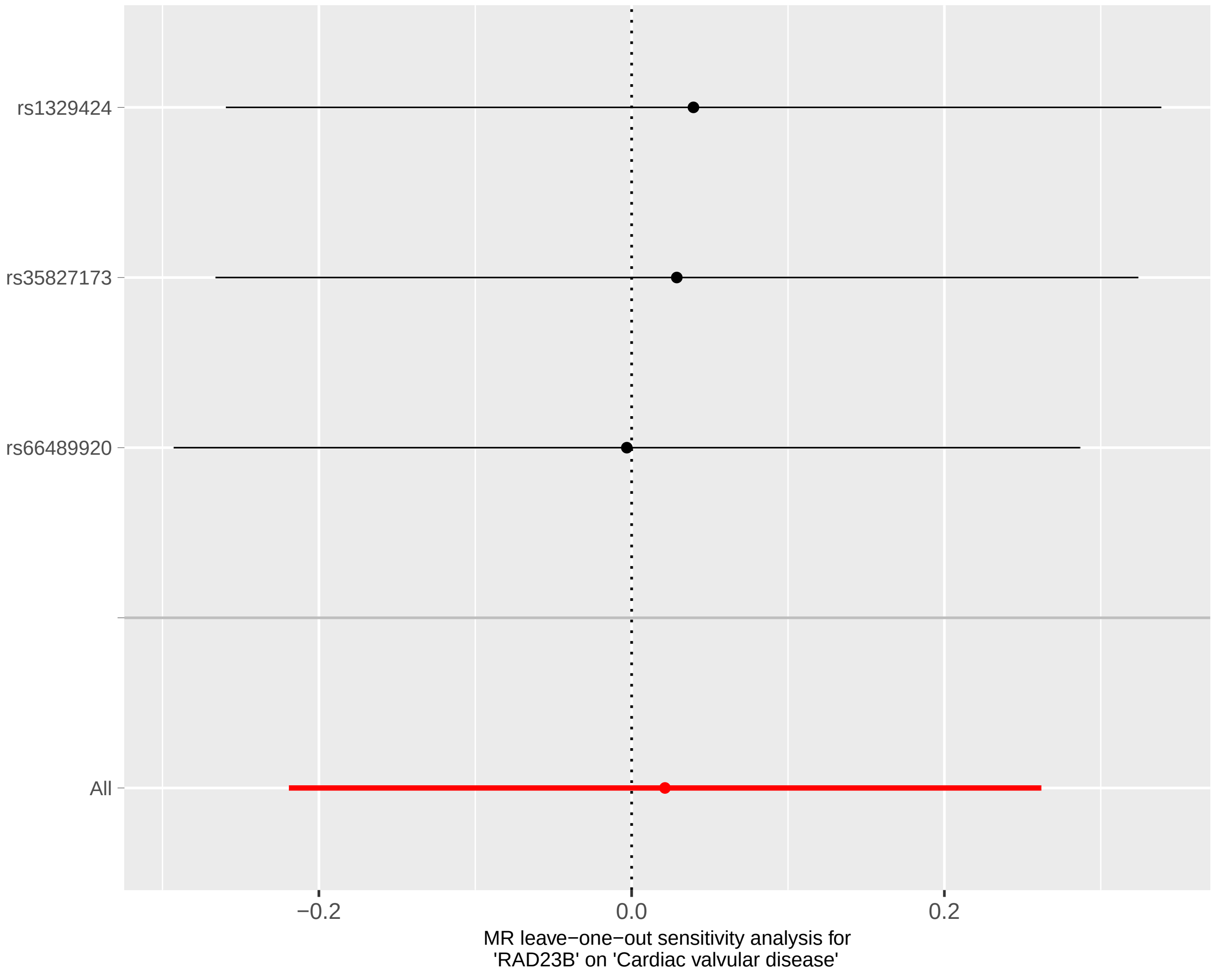
rs1329424
rs35827173
rs66489920
All
−0.2
0.0
0.2
MR leave−one−out sensitivity analysis for
'RAD23B' on 'Cardiac valvular disease'

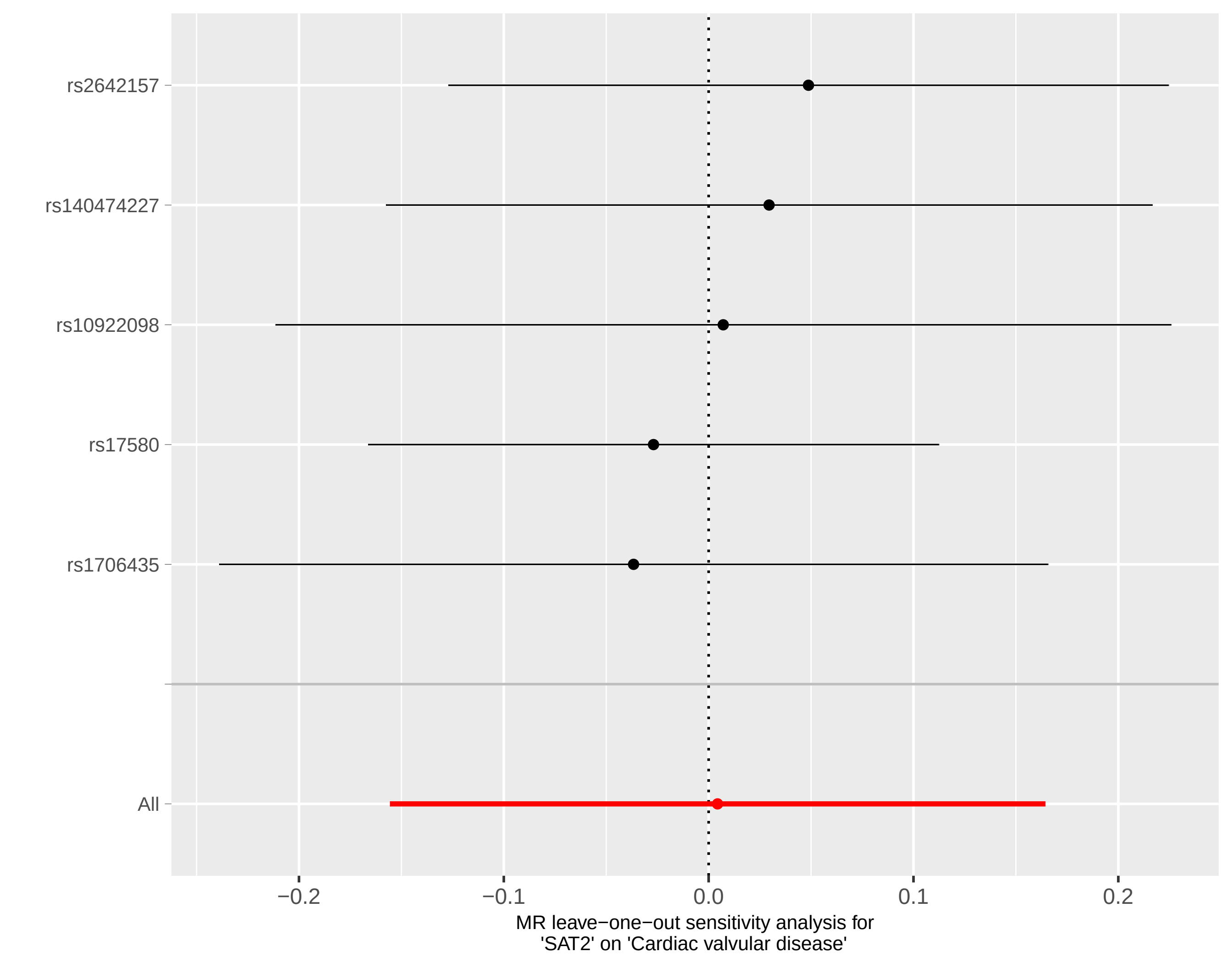
rs2642157
rs140474227
rs10922098
rs17580
rs1706435
All
−0.2
−0.1
0.0
0.1
0.2
MR leave−one−out sensitivity analysis for
'SAT2' on 'Cardiac valvular disease'

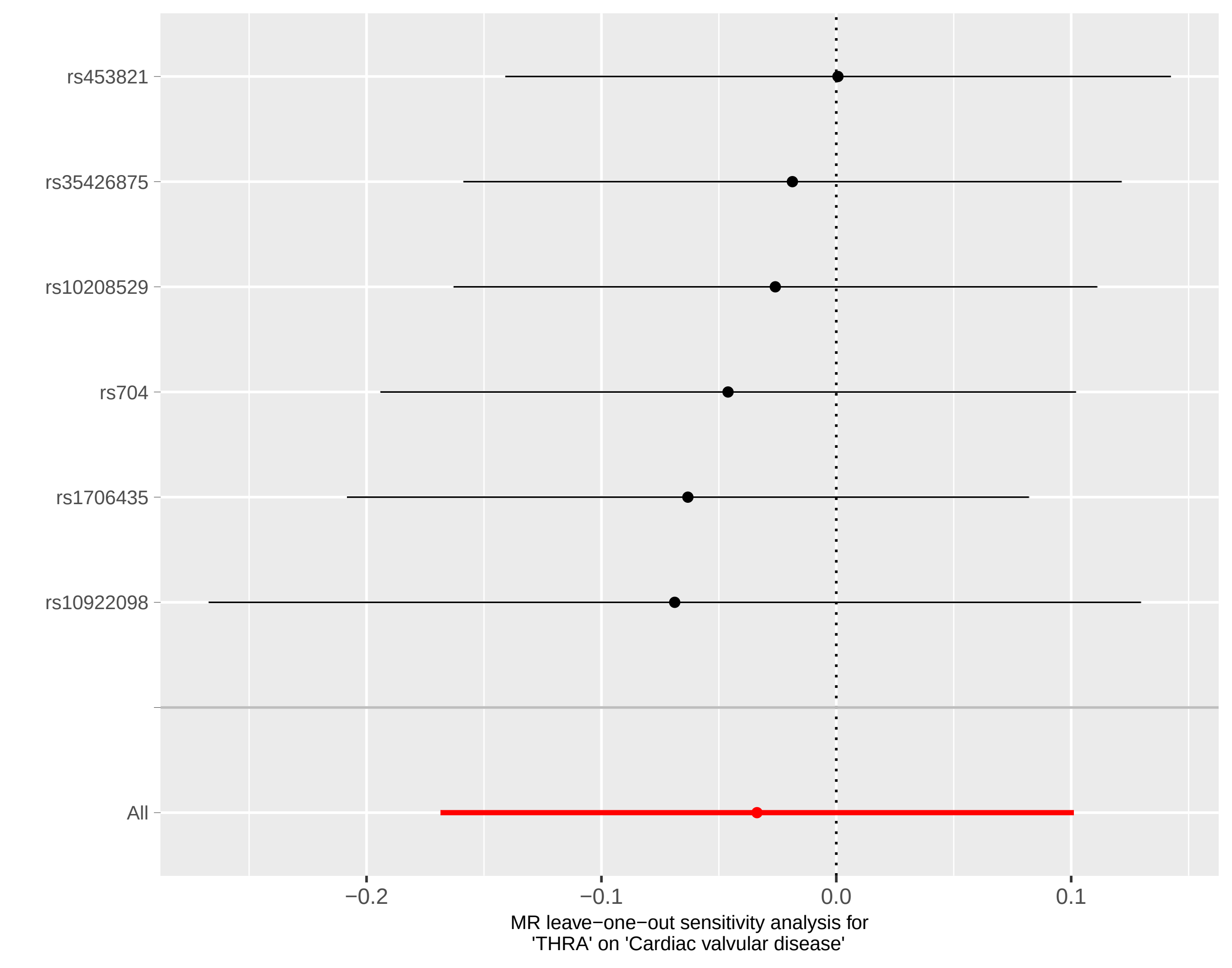
rs453821
rs35426875
rs10208529
rs704
rs1706435
rs10922098
All
−0.2
−0.1
0.0
0.1
MR leave−one−out sensitivity analysis for
'THRA' on 'Cardiac valvular disease'

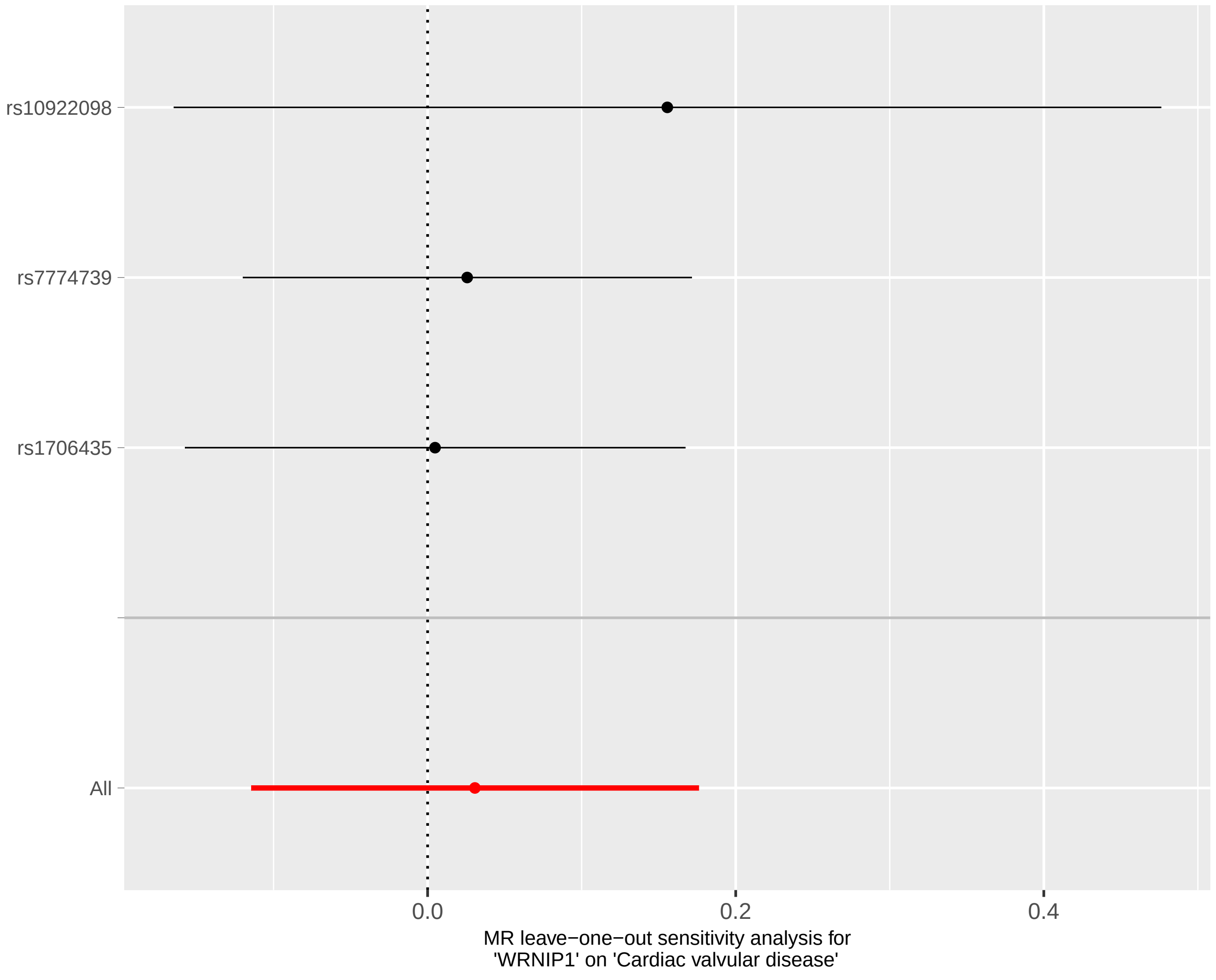
rs10922098
rs7774739
rs1706435
All
0.0
0.2
0.4
MR leave−one−out sensitivity analysis for
'WRNIP1' on 'Cardiac valvular disease'

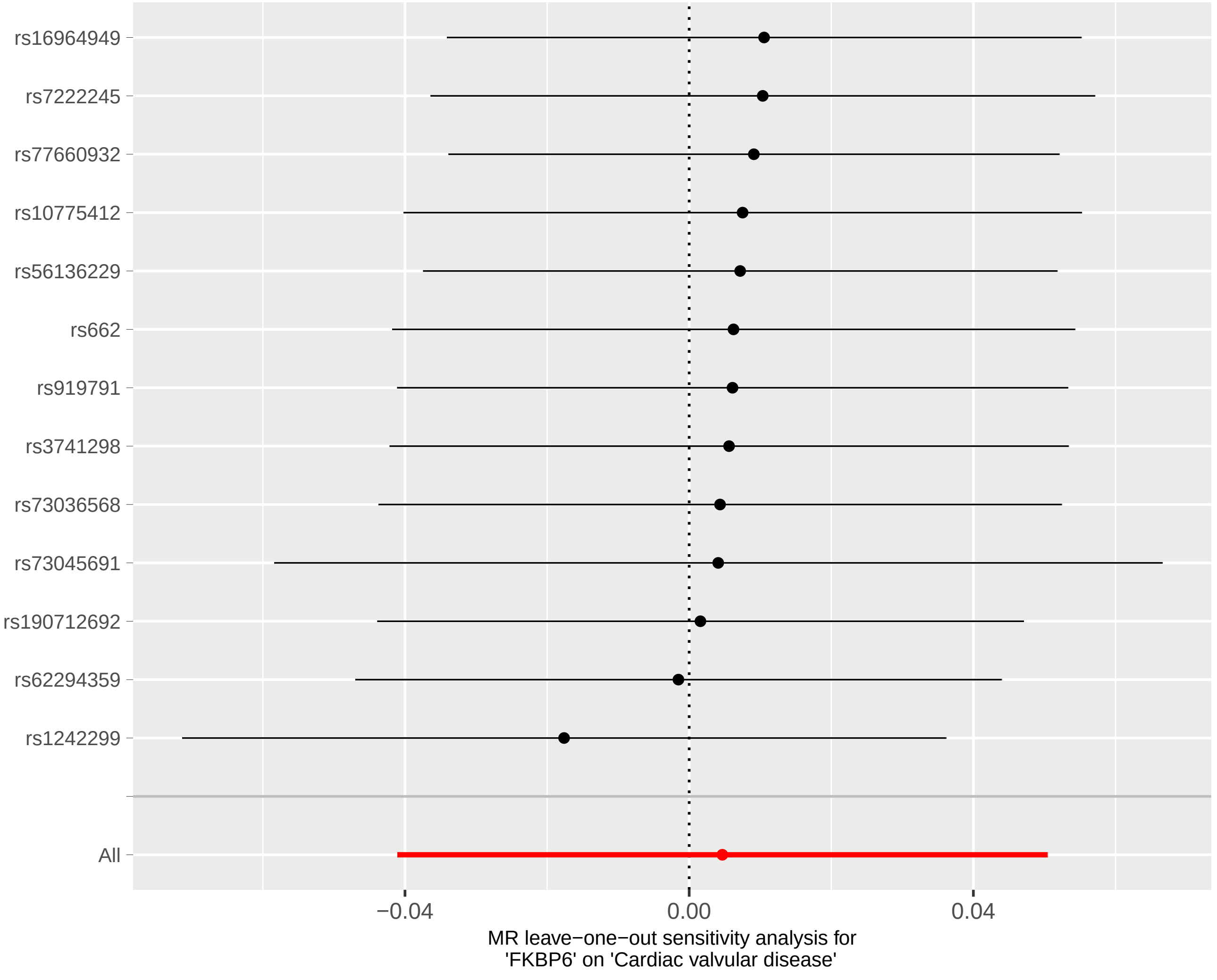
rs16964949
rs7222245
rs77660932
rs10775412
rs56136229
rs662
rs919791
rs3741298
rs73036568
rs73045691
rs190712692
rs62294359
rs1242299
All
−0.04
0.00
0.04
MR leave−one−out sensitivity analysis for
'FKBP6' on 'Cardiac valvular disease'

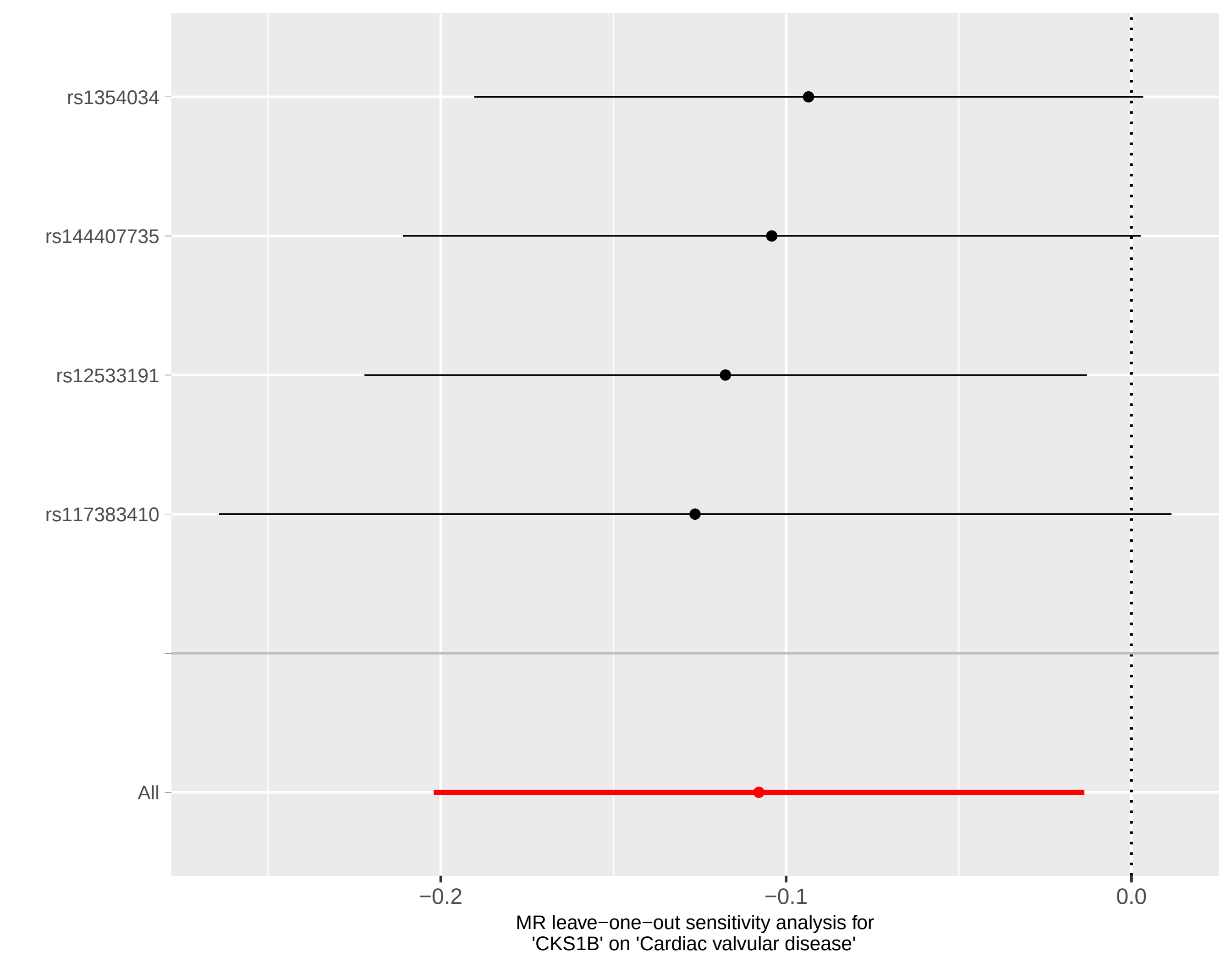
rs1354034
rs144407735
rs12533191
rs117383410
All
−0.2
−0.1
0.0
MR leave−one−out sensitivity analysis for
'CKS1B' on 'Cardiac valvular disease'

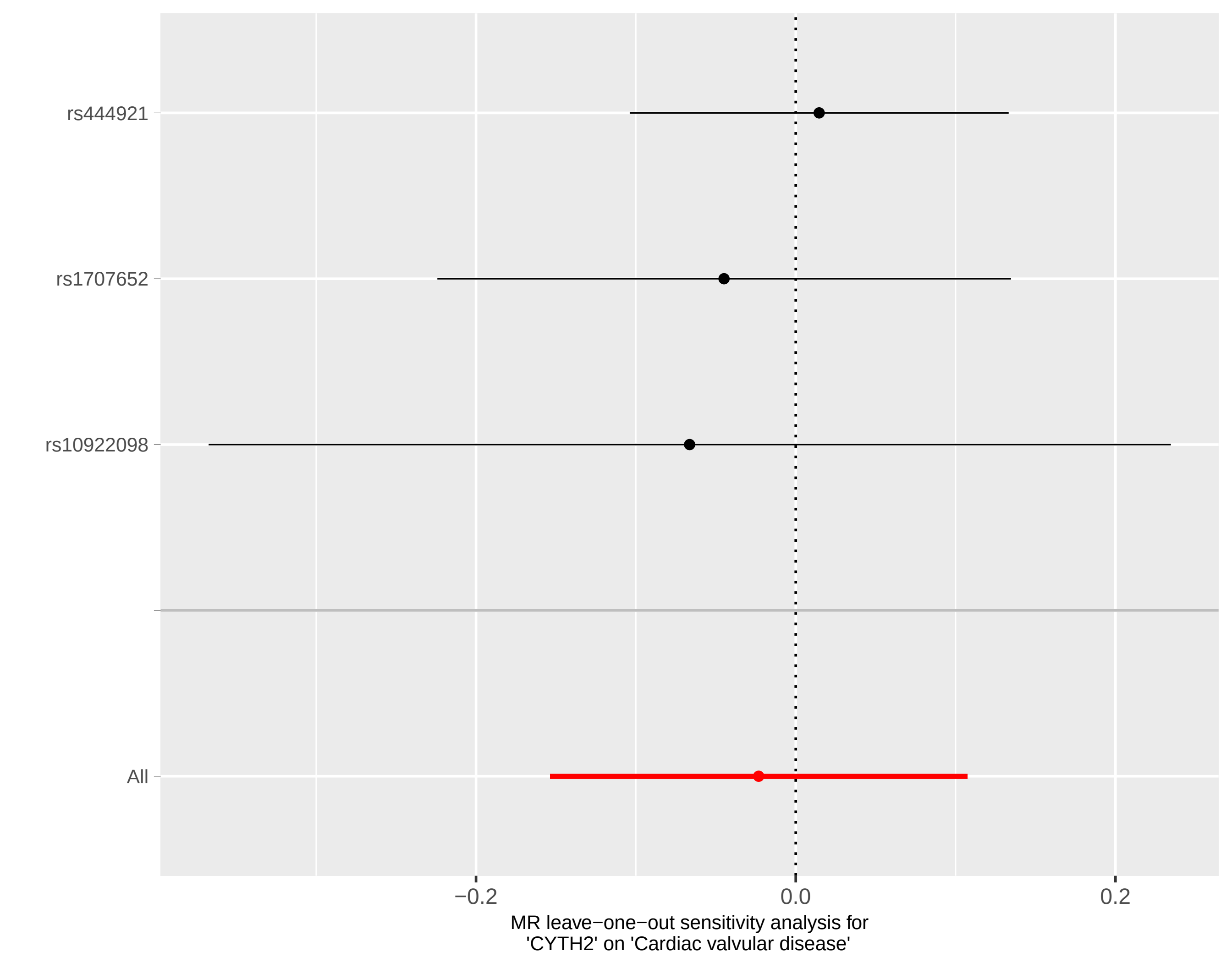
rs444921
rs1707652
rs10922098
All
−0.2
0.0
0.2
MR leave−one−out sensitivity analysis for
'CYTH2' on 'Cardiac valvular disease'

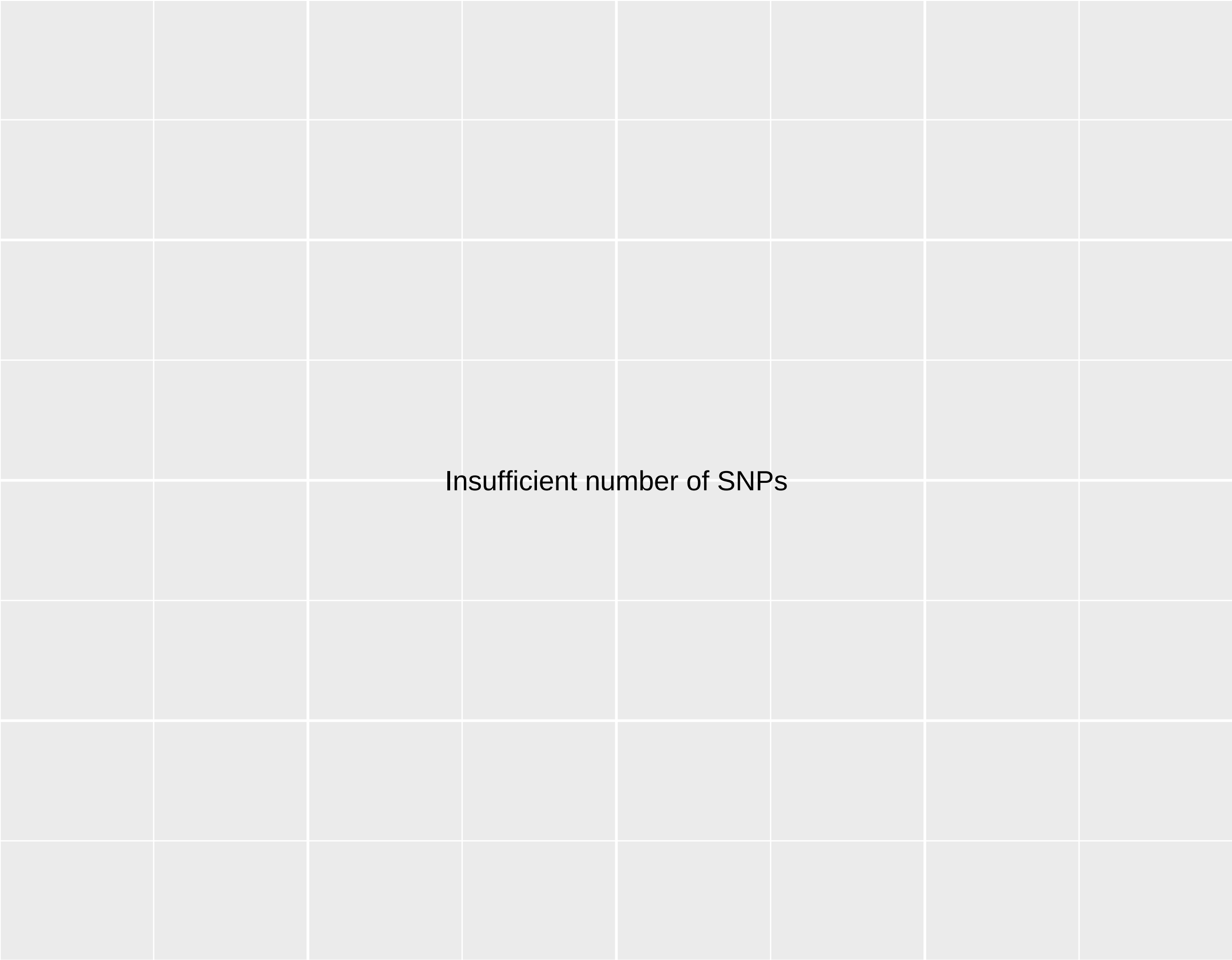
Insufficient number of SNPs

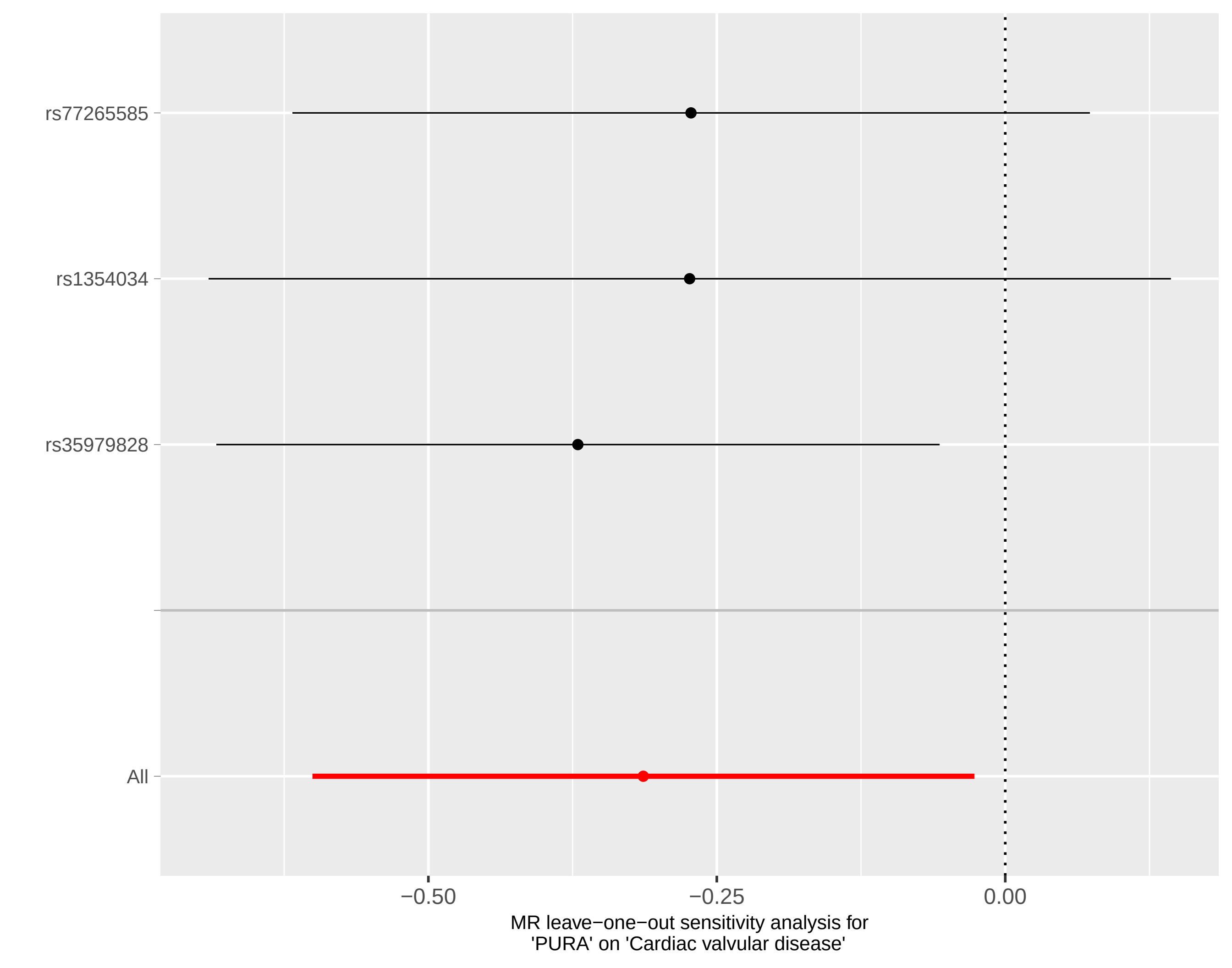

rs77265585
rs1354034
rs35979828
All
−0.50
−0.25
0.00
MR leave−one−out sensitivity analysis for
'PURA' on 'Cardiac valvular disease'

Insufficient number of SNPs

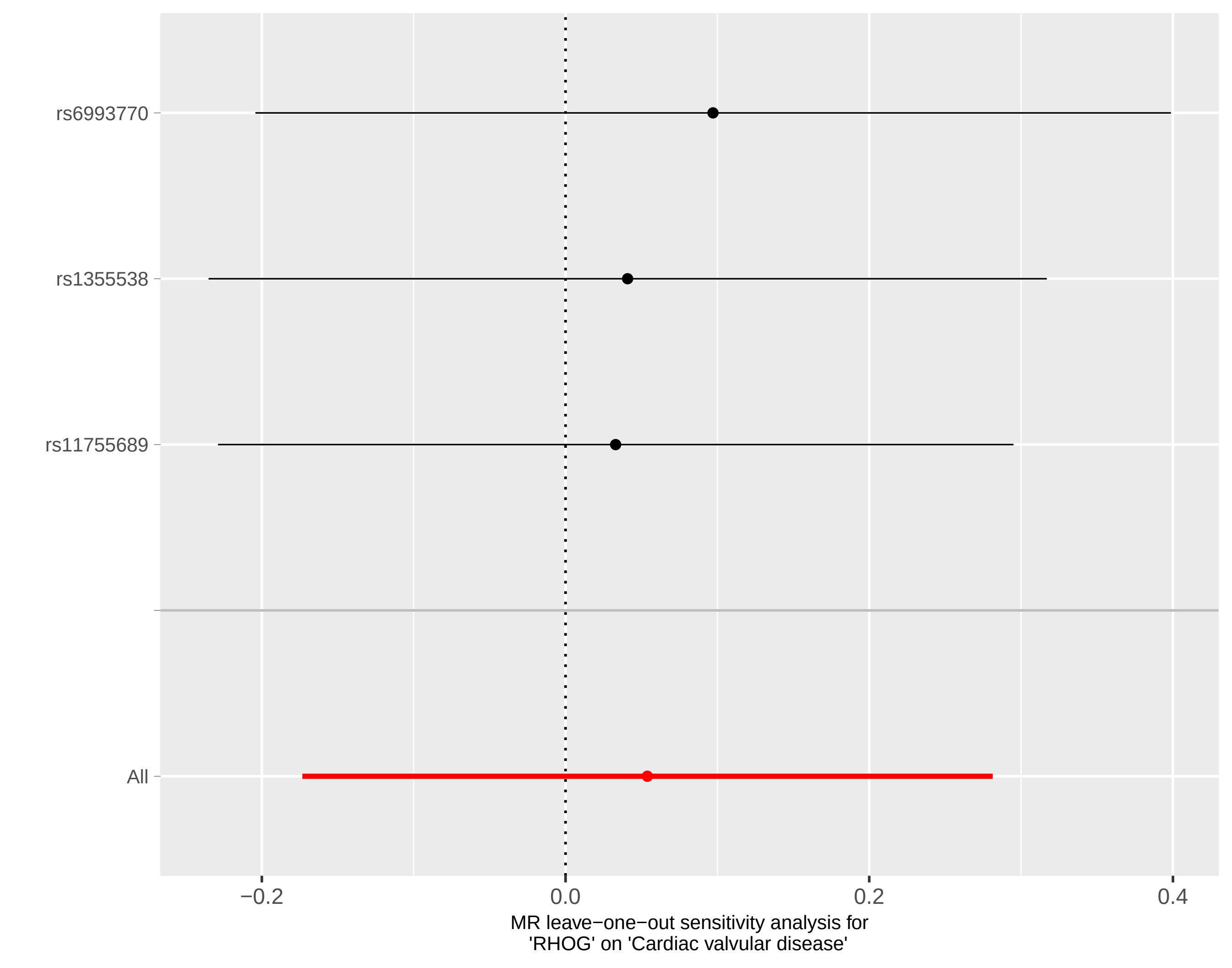

rs6993770
rs1355538
rs11755689
All
−0.2
0.0
0.2
0.4
MR leave−one−out sensitivity analysis for
'RHOG' on 'Cardiac valvular disease'

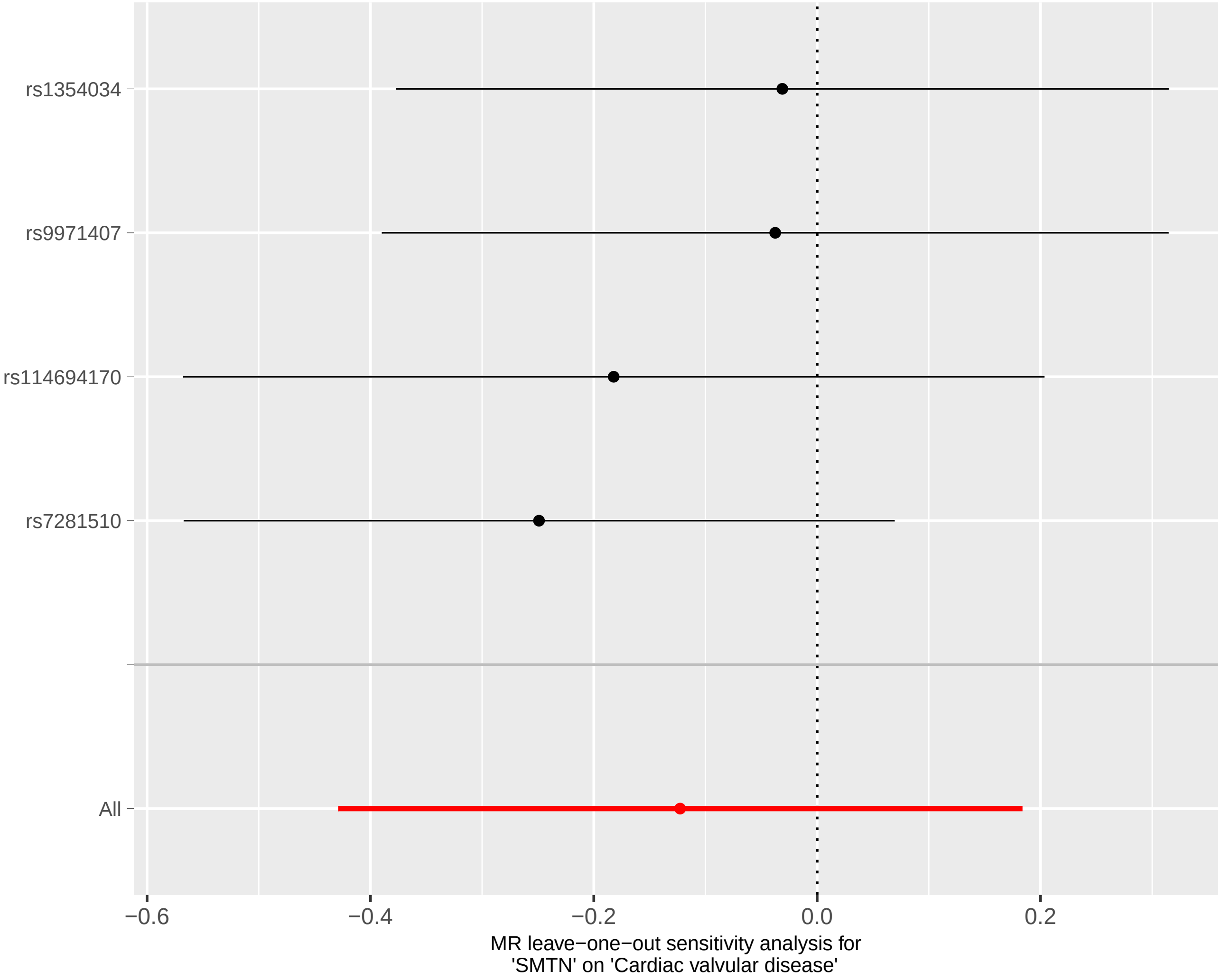
rs1354034
rs9971407
rs114694170
rs7281510
All
−0.6
−0.4
−0.2
0.0
0.2
MR leave−one−out sensitivity analysis for
'SMTN' on 'Cardiac valvular disease'

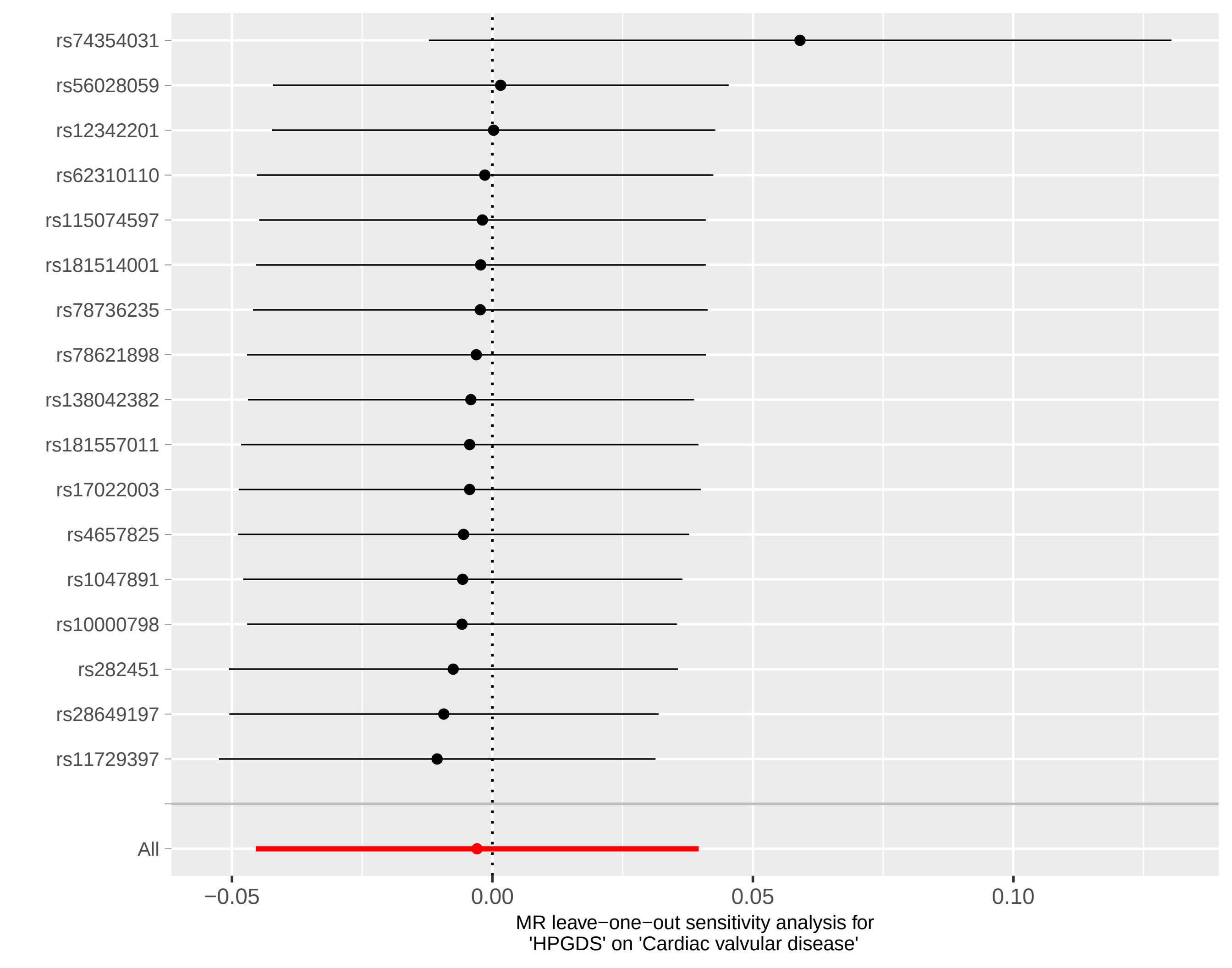
rs74354031
rs56028059
rs12342201
rs62310110
rs115074597
rs181514001
rs78736235
rs78621898
rs138042382
rs181557011
rs17022003
rs4657825
rs1047891
rs10000798
rs282451
rs28649197
rs11729397
All
−0.05
0.00
0.05
0.10
MR leave−one−out sensitivity analysis for
'HPGDS' on 'Cardiac valvular disease'

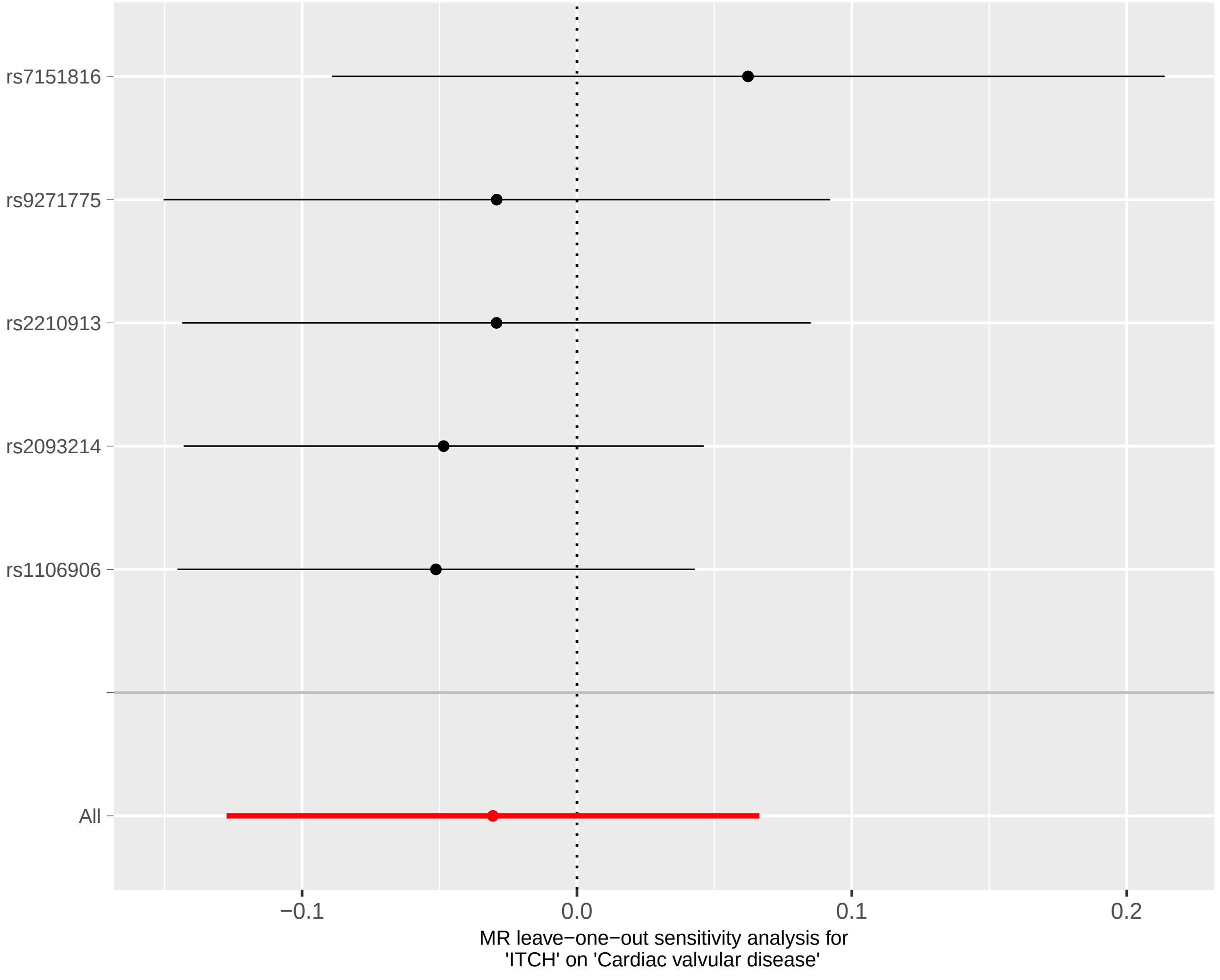
rs7151816
rs9271775
rs2210913
rs2093214
rs1106906
All
−0.1
0.0
0.1
0.2
MR leave−one−out sensitivity analysis for
'ITCH' on 'Cardiac valvular disease'

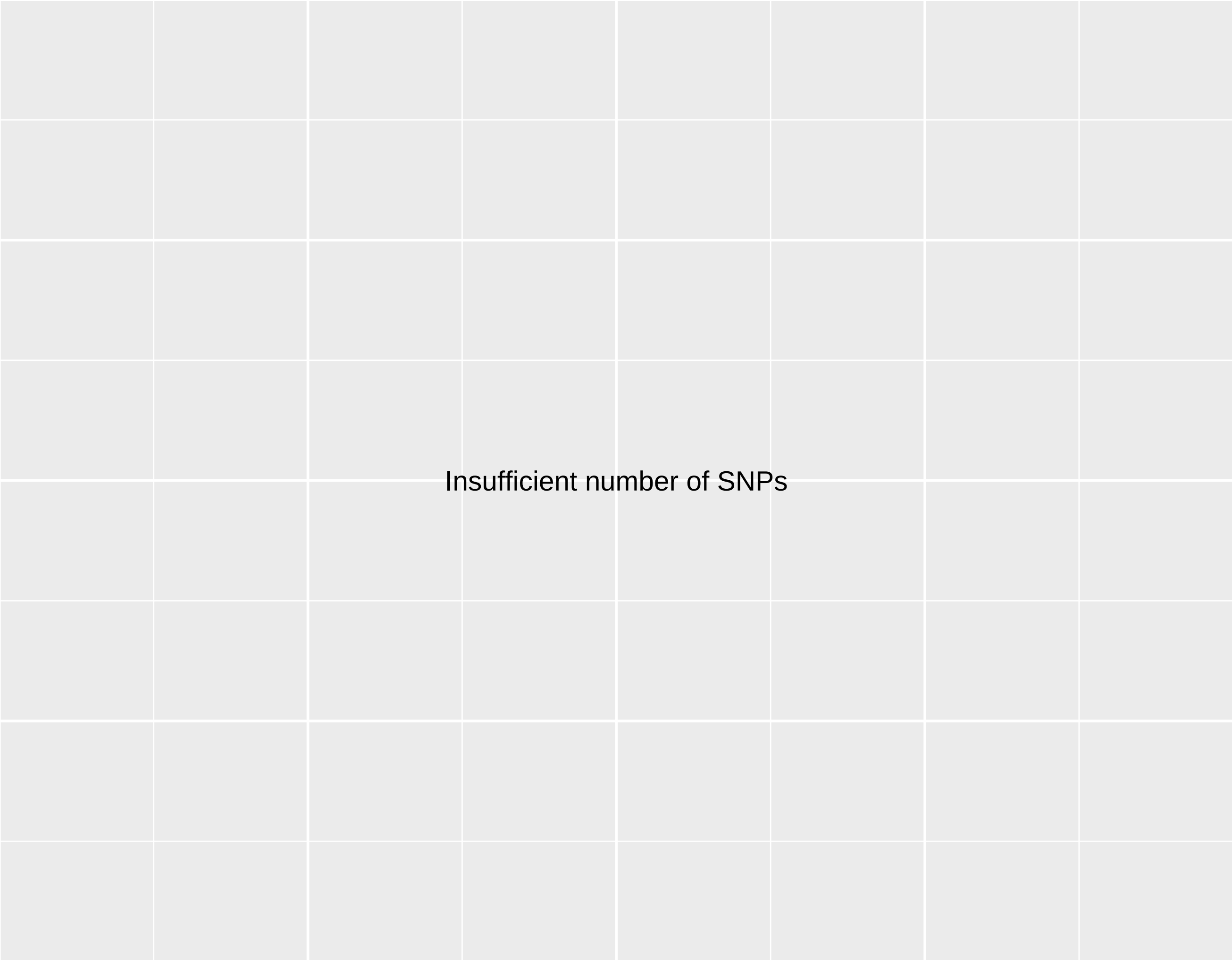
Insufficient number of SNPs

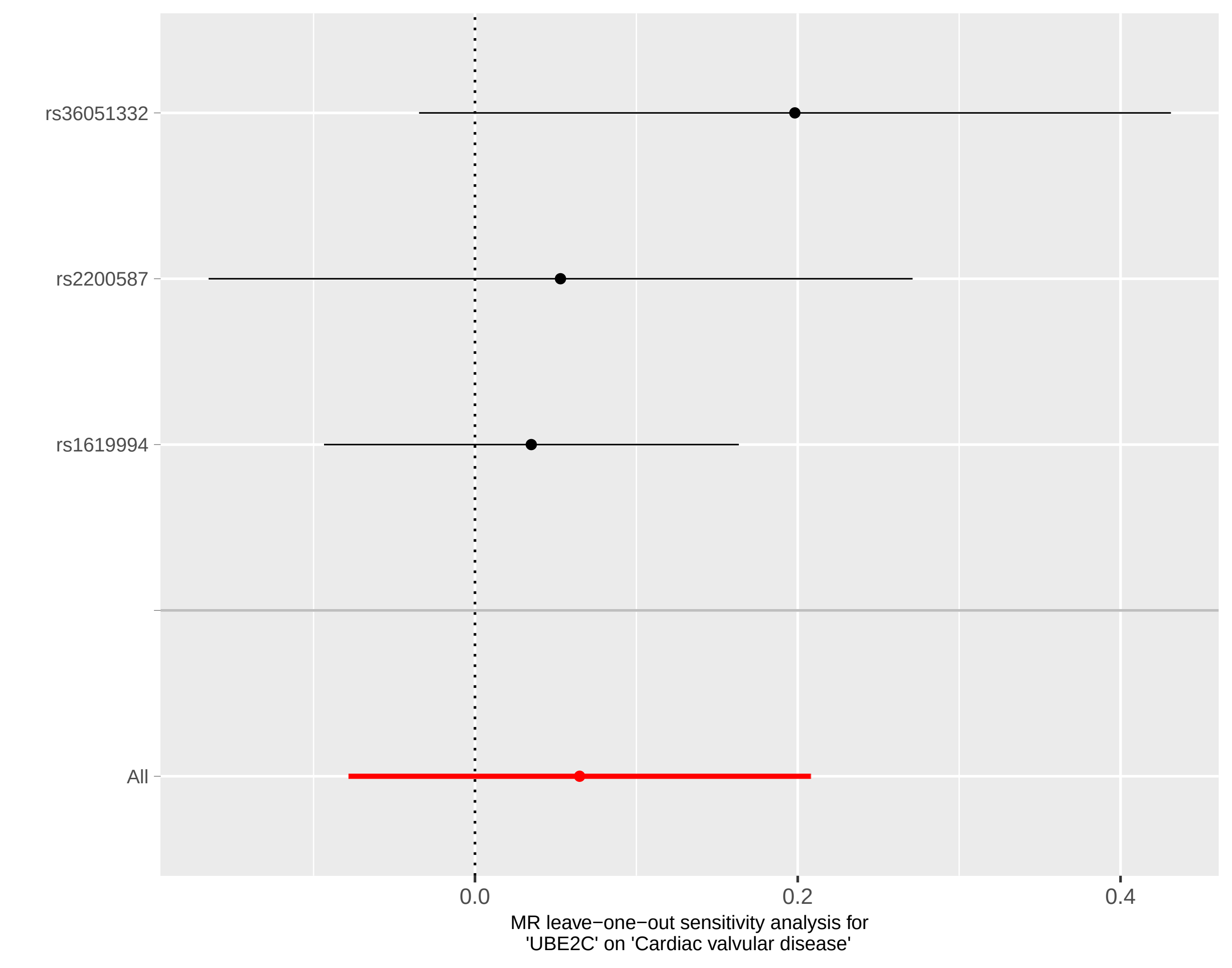

rs36051332
rs2200587
rs1619994
All
0.0
0.2
0.4
MR leave−one−out sensitivity analysis for
'UBE2C' on 'Cardiac valvular disease'

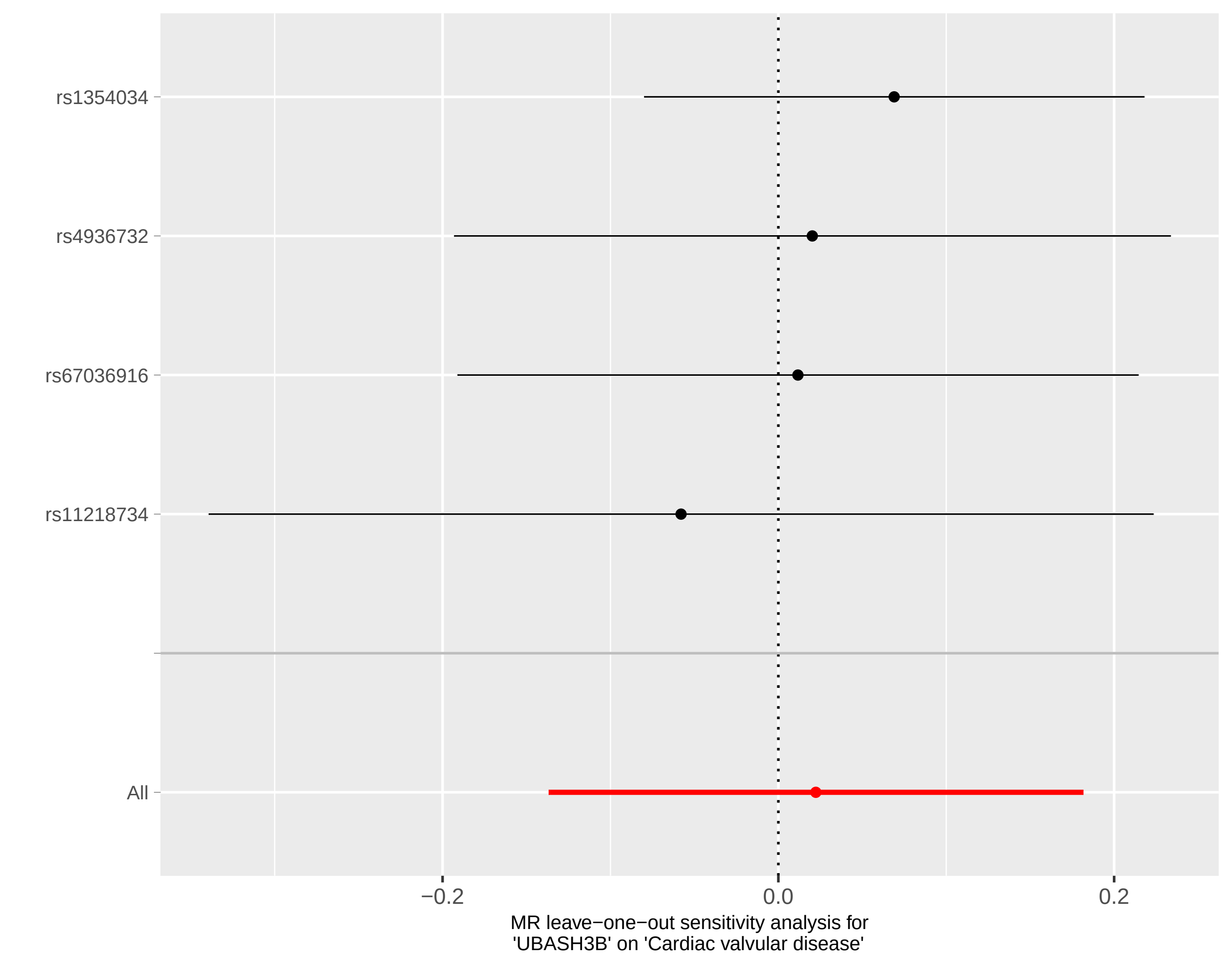
rs1354034
rs4936732
rs67036916
rs11218734
All
−0.2
0.0
0.2
MR leave−one−out sensitivity analysis for
'UBASH3B' on 'Cardiac valvular disease'

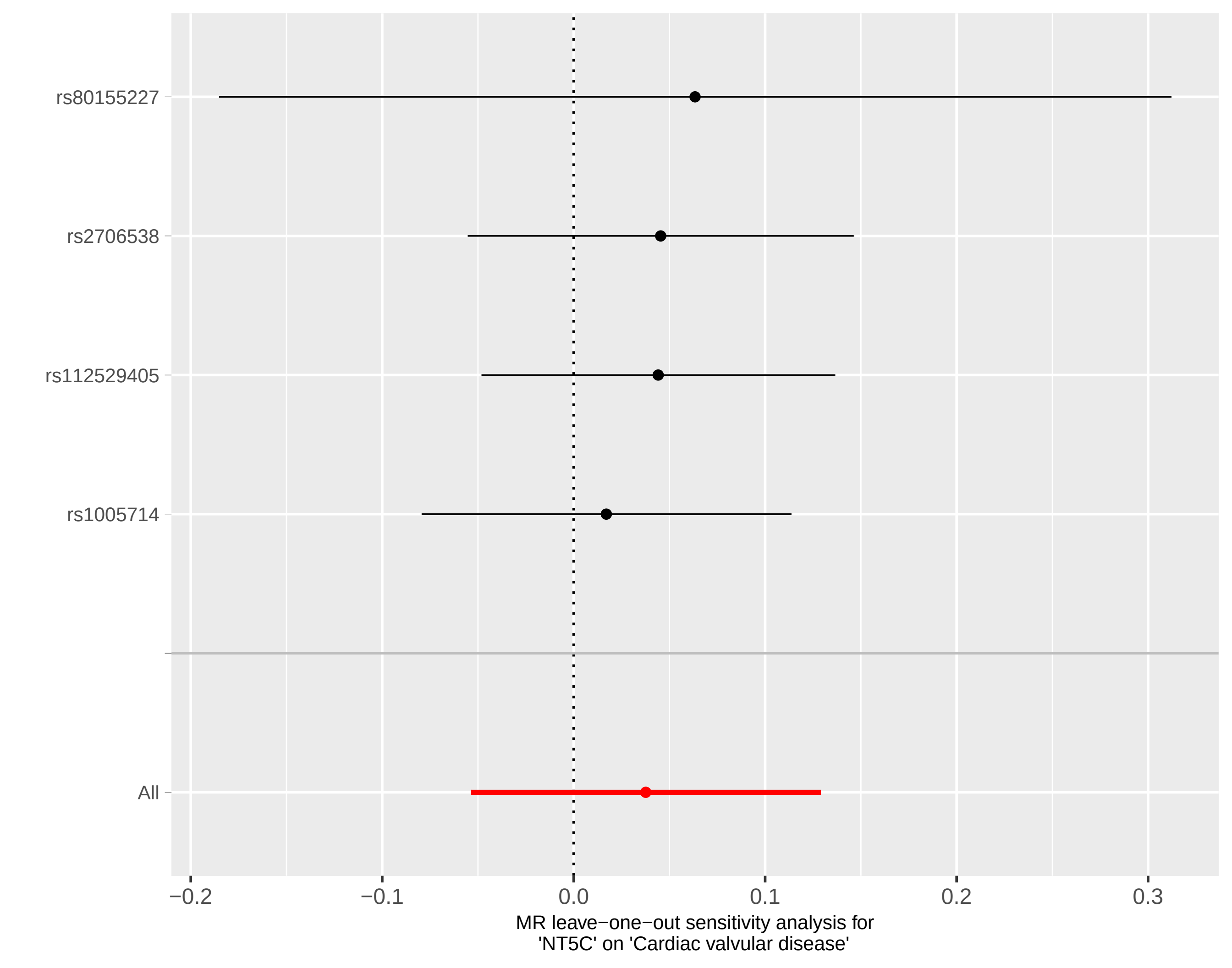
rs80155227
rs2706538
rs112529405
rs1005714
All
−0.2
−0.1
0.0
0.1
0.2
0.3
MR leave−one−out sensitivity analysis for
'NT5C' on 'Cardiac valvular disease'

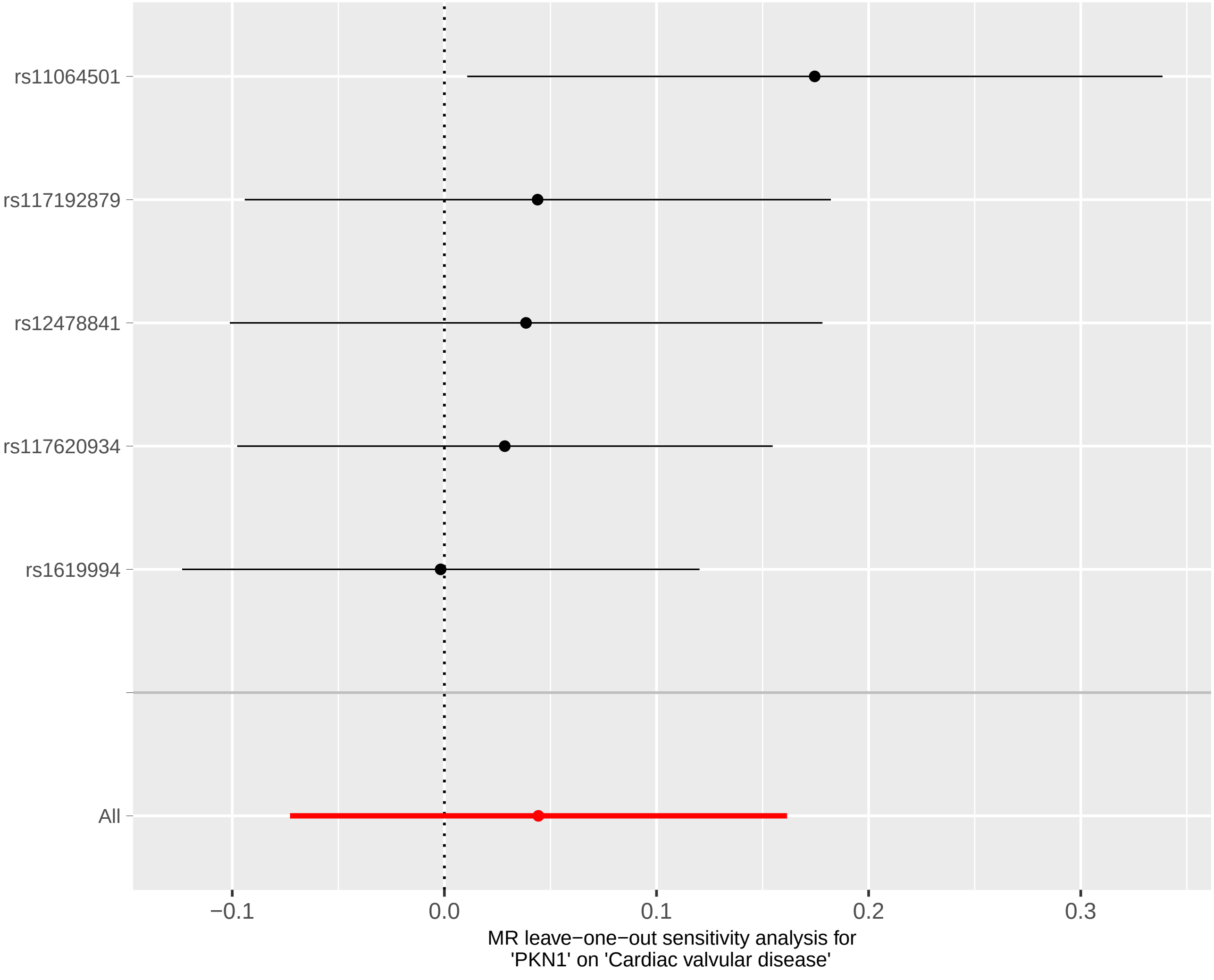
rs11064501
rs117192879
rs12478841
rs117620934
rs1619994
All
−0.1
0.0
0.1
0.2
0.3
MR leave−one−out sensitivity analysis for
'PKN1' on 'Cardiac valvular disease'

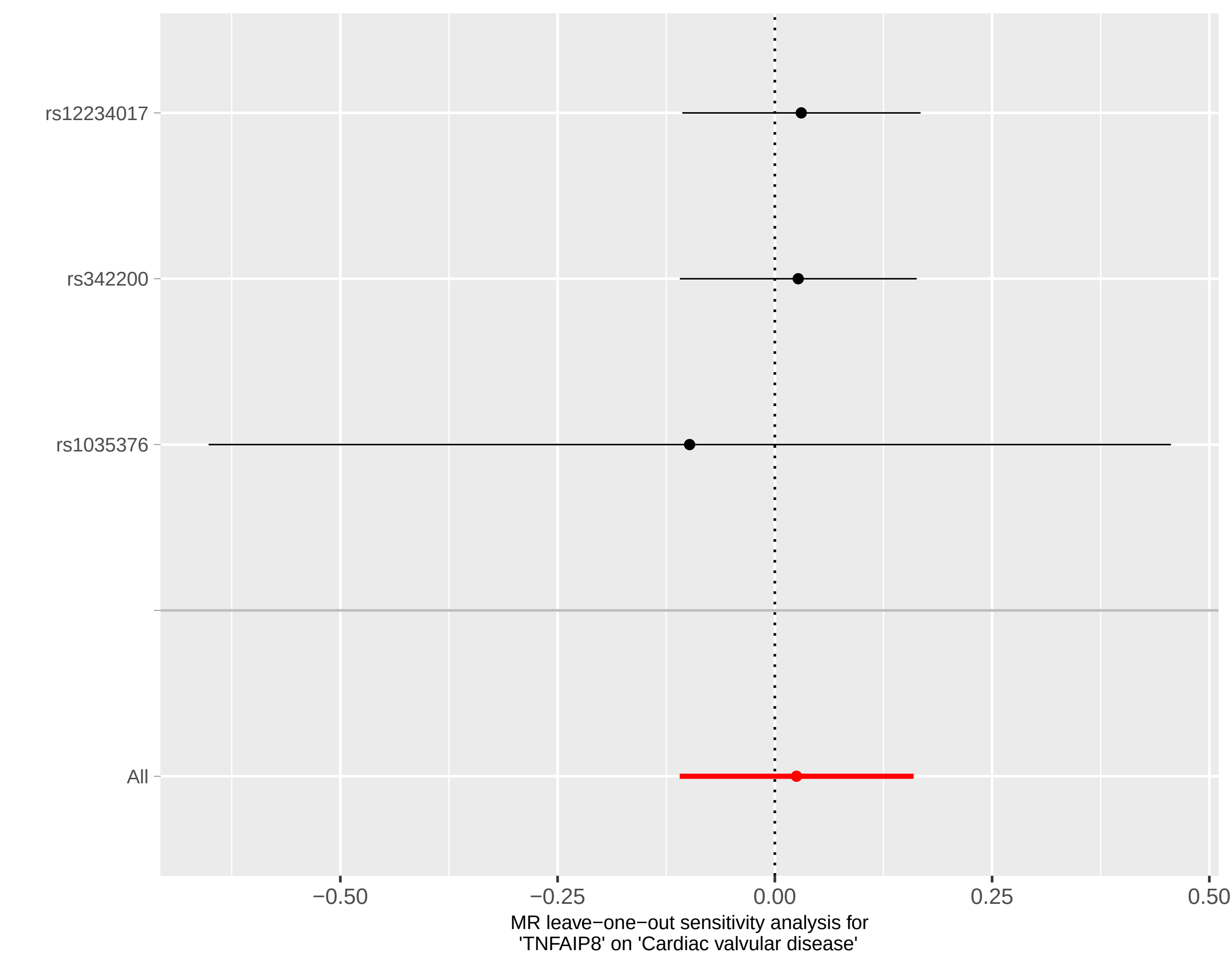
rs12234017
rs342200
rs1035376
All
−0.50
−0.25
0.00
0.25
0.50
MR leave−one−out sensitivity analysis for
'TNFAIP8' on 'Cardiac valvular disease'

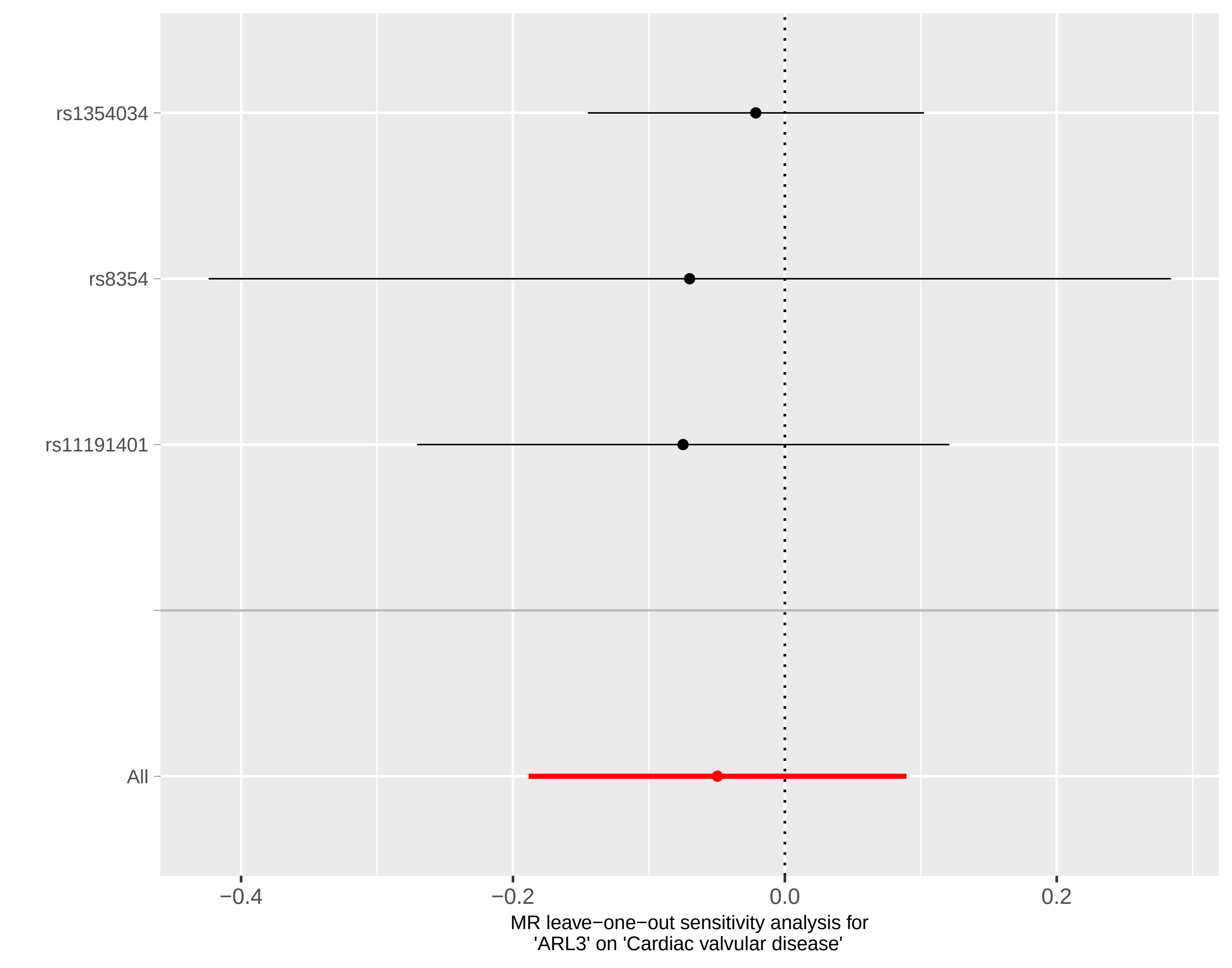
rs1354034
rs8354
rs11191401
All
−0.4
−0.2
0.0
0.2
MR leave−one−out sensitivity analysis for
'ARL3' on 'Cardiac valvular disease'

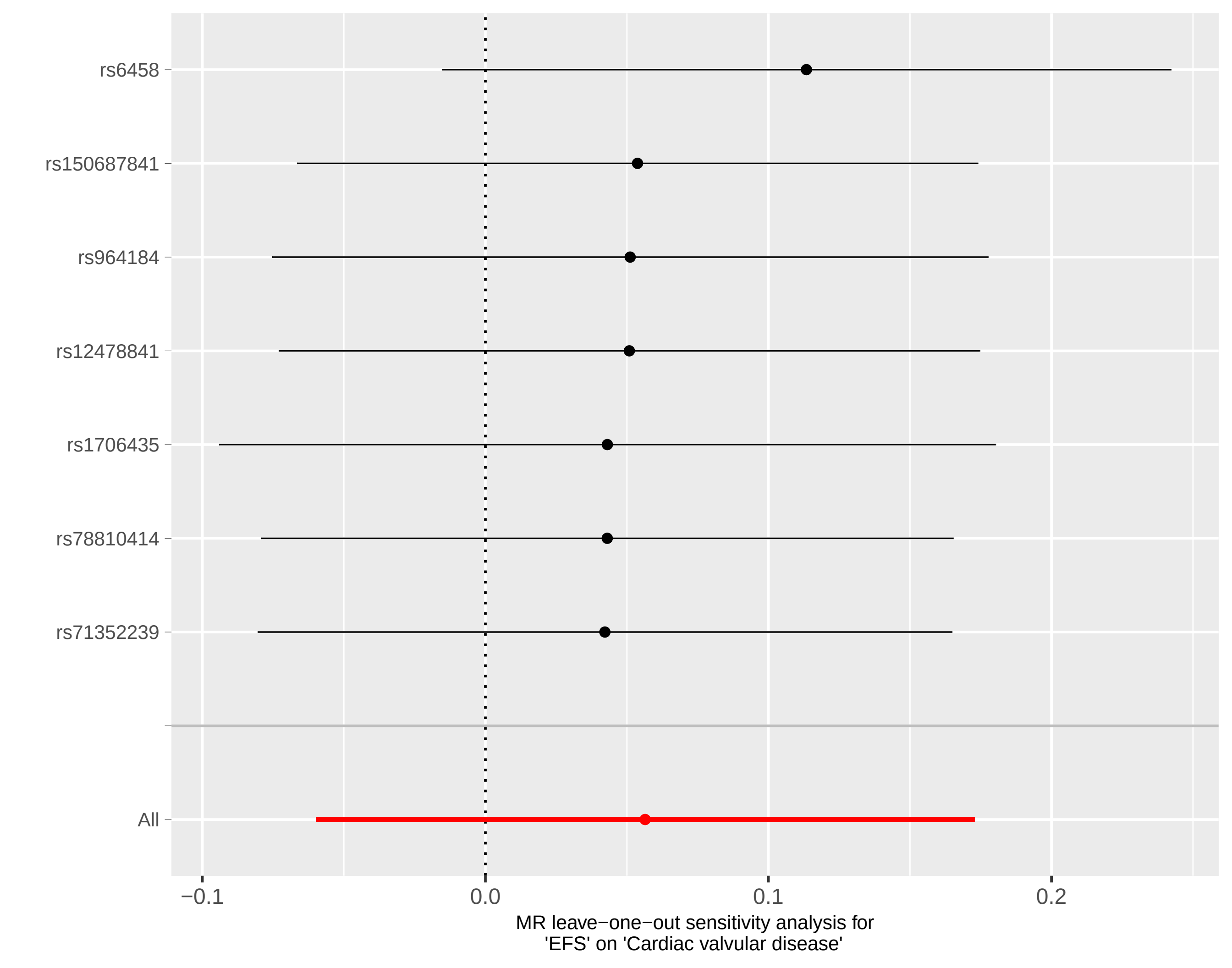
rs6458
rs150687841
rs964184
rs12478841
rs1706435
rs78810414
rs71352239
All
−0.1
0.0
0.1
0.2
MR leave−one−out sensitivity analysis for
'EFS' on 'Cardiac valvular disease'

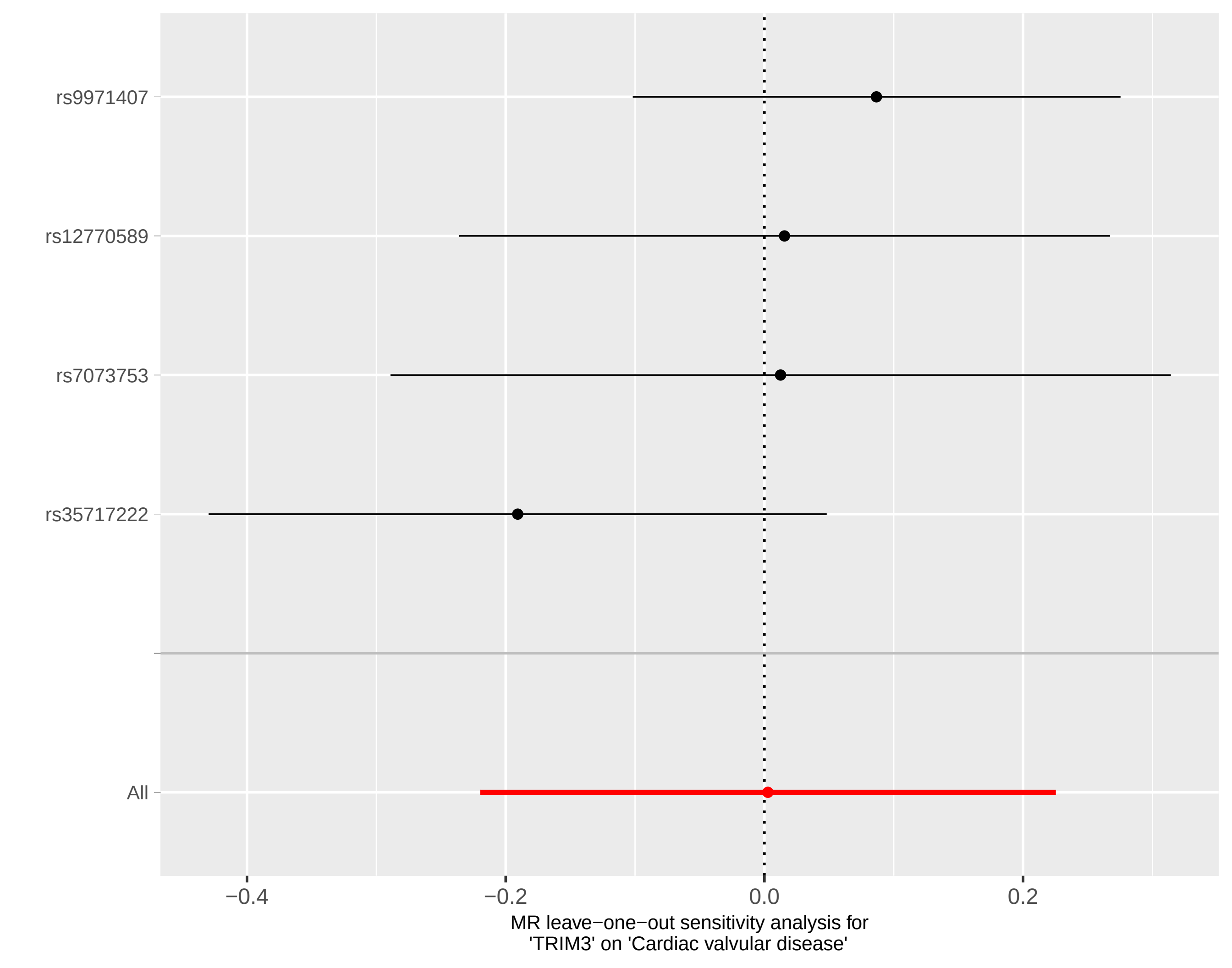

rs9971407
rs12770589
rs7073753
rs35717222
All
−0.4
−0.2
0.0
0.2
MR leave−one−out sensitivity analysis for
'TRIM3' on 'Cardiac valvular disease'

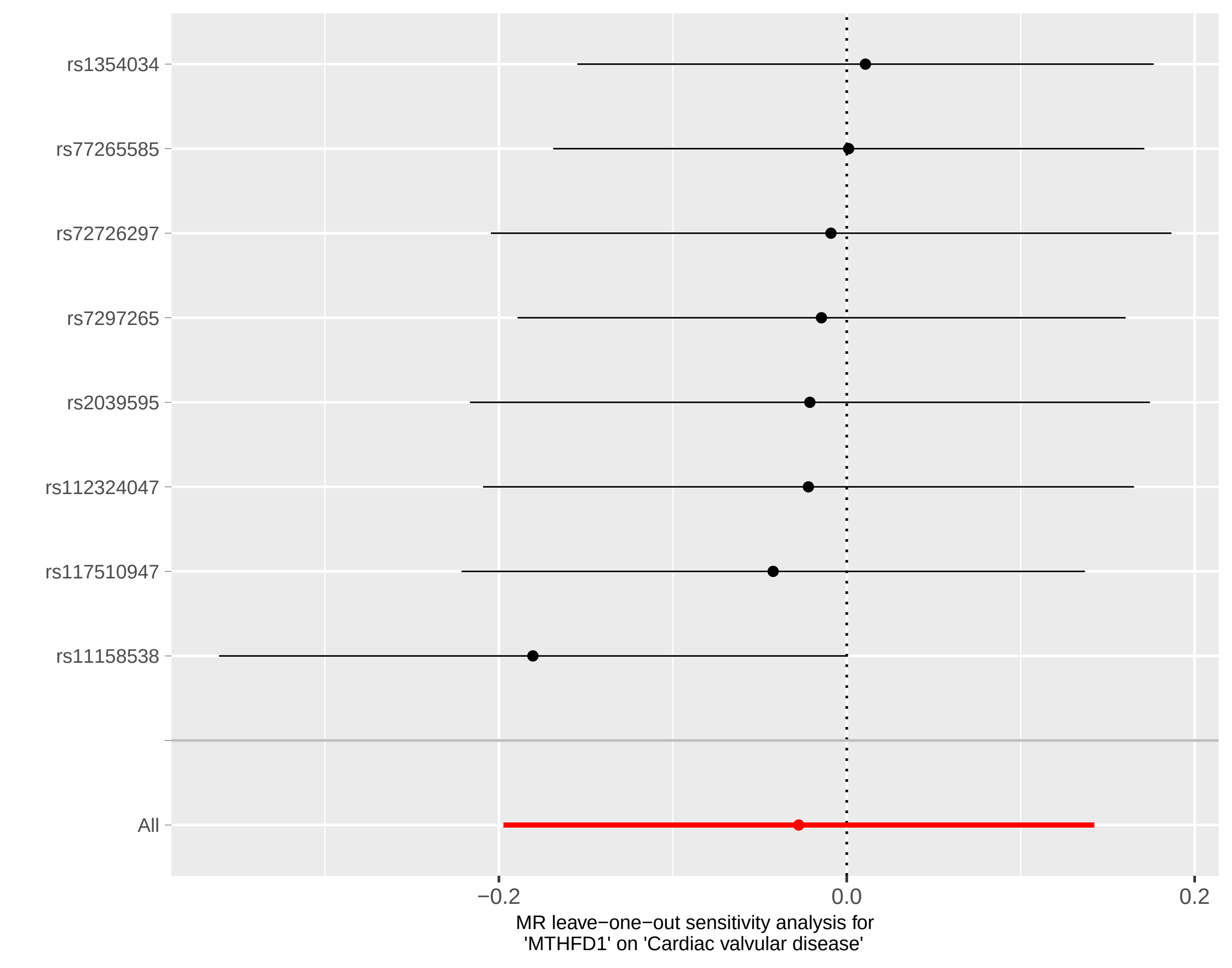
rs1354034
rs77265585
rs72726297
rs7297265
rs2039595
rs112324047
rs117510947
rs11158538
All
−0.2
0.0
0.2
MR leave−one−out sensitivity analysis for
'MTHFD1' on 'Cardiac valvular disease'

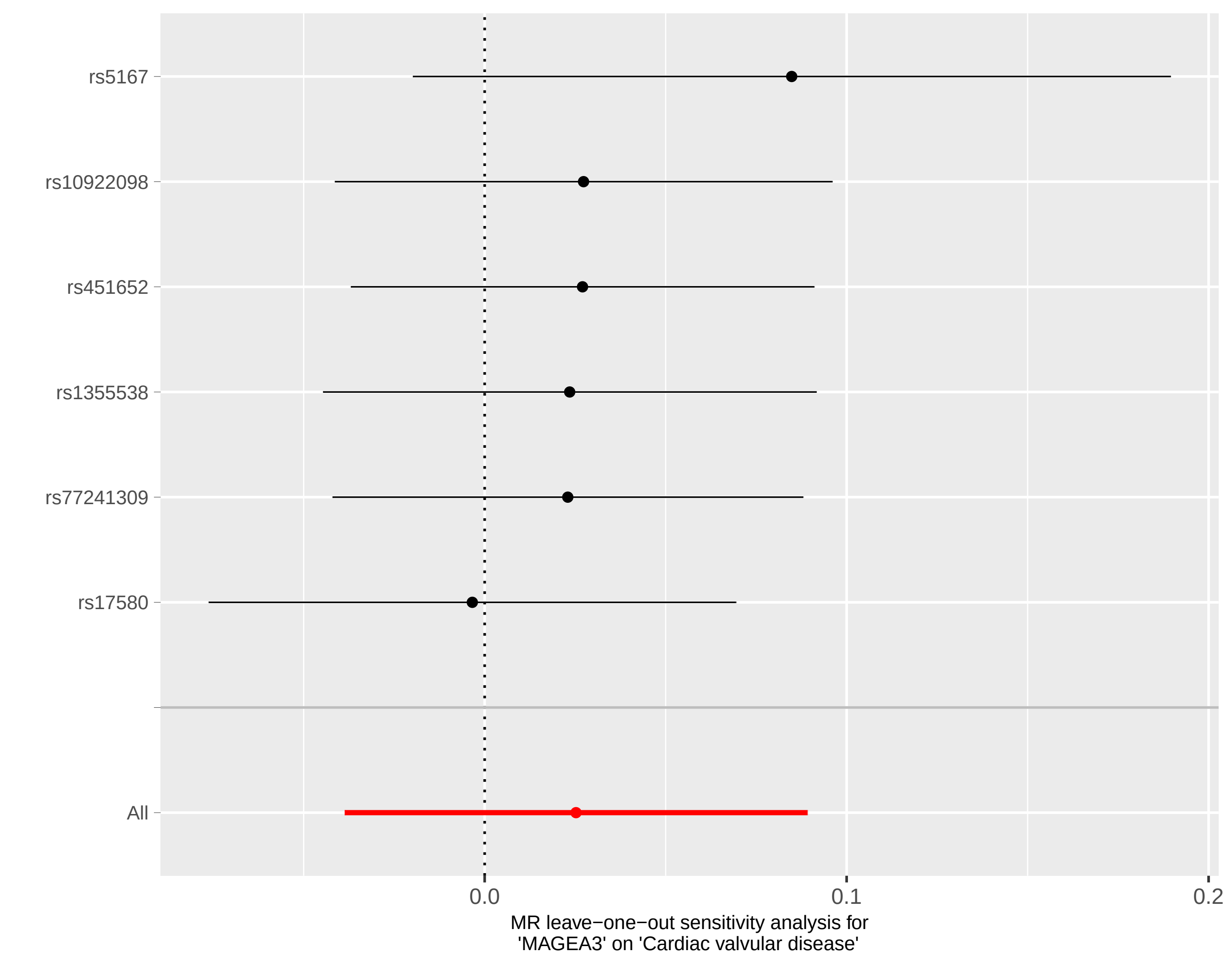

rs5167
rs10922098
rs451652
rs1355538
rs77241309
rs17580
All
0.0
0.1
0.2
MR leave−one−out sensitivity analysis for
'MAGEA3' on 'Cardiac valvular disease'

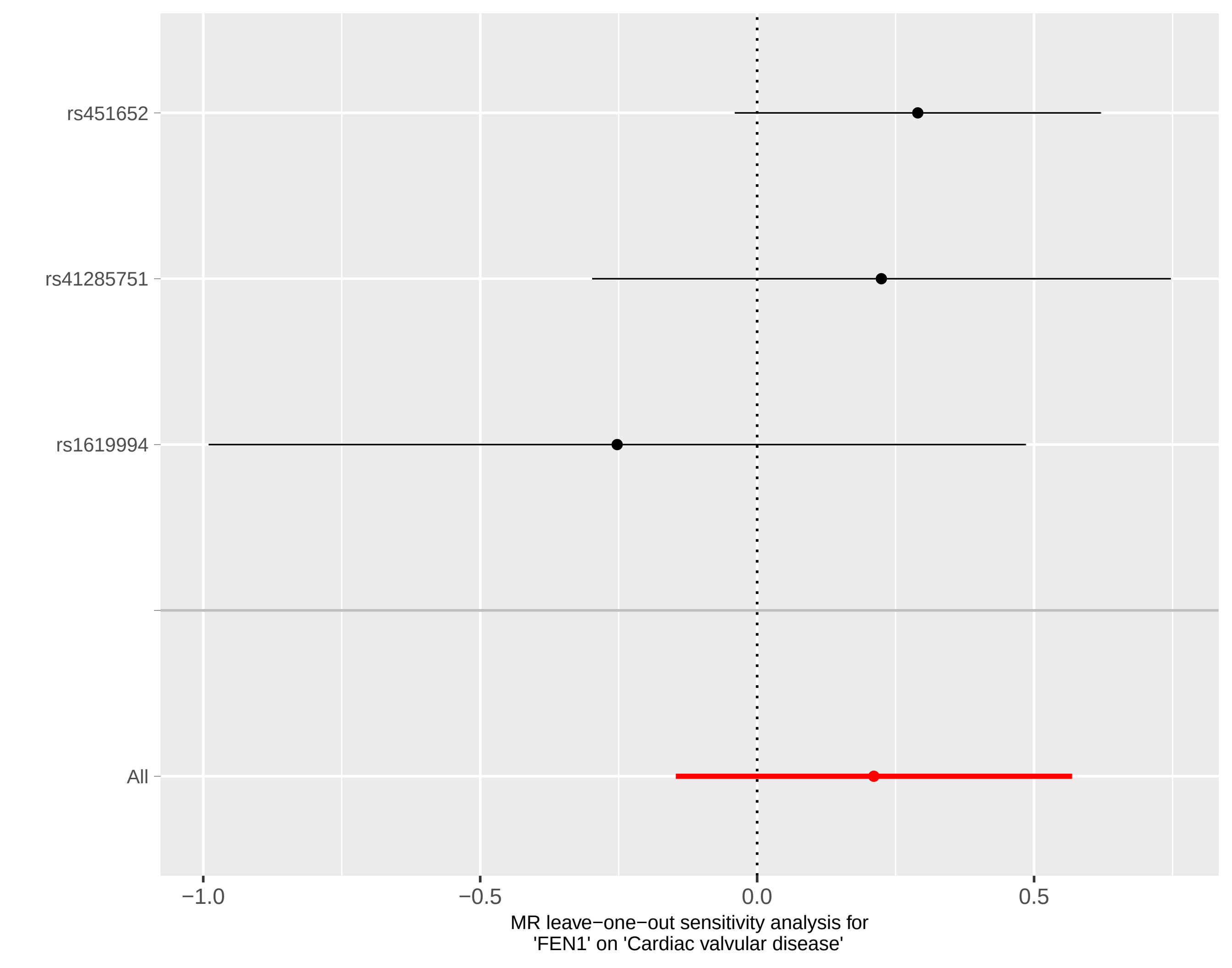
rs451652
rs41285751
rs1619994
All
−1.0
−0.5
0.0
0.5
MR leave−one−out sensitivity analysis for
'FEN1' on 'Cardiac valvular disease'

Insufficient number of SNPs

Insufficient number of SNPs

Insufficient number of SNPs

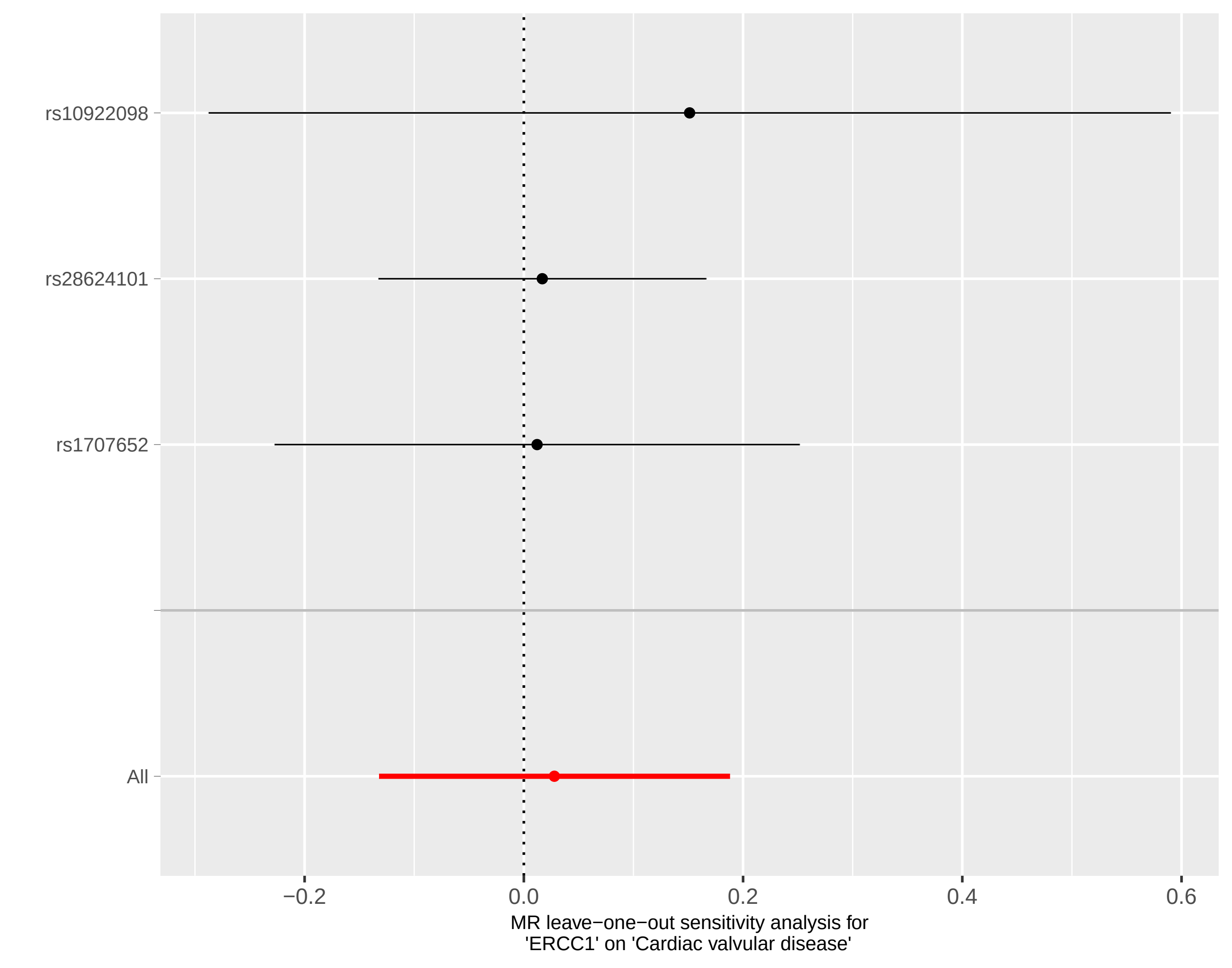
rs10922098
rs28624101
rs1707652
All
−0.2
0.0
0.2
0.4
0.6
MR leave−one−out sensitivity analysis for
'ERCC1' on 'Cardiac valvular disease'

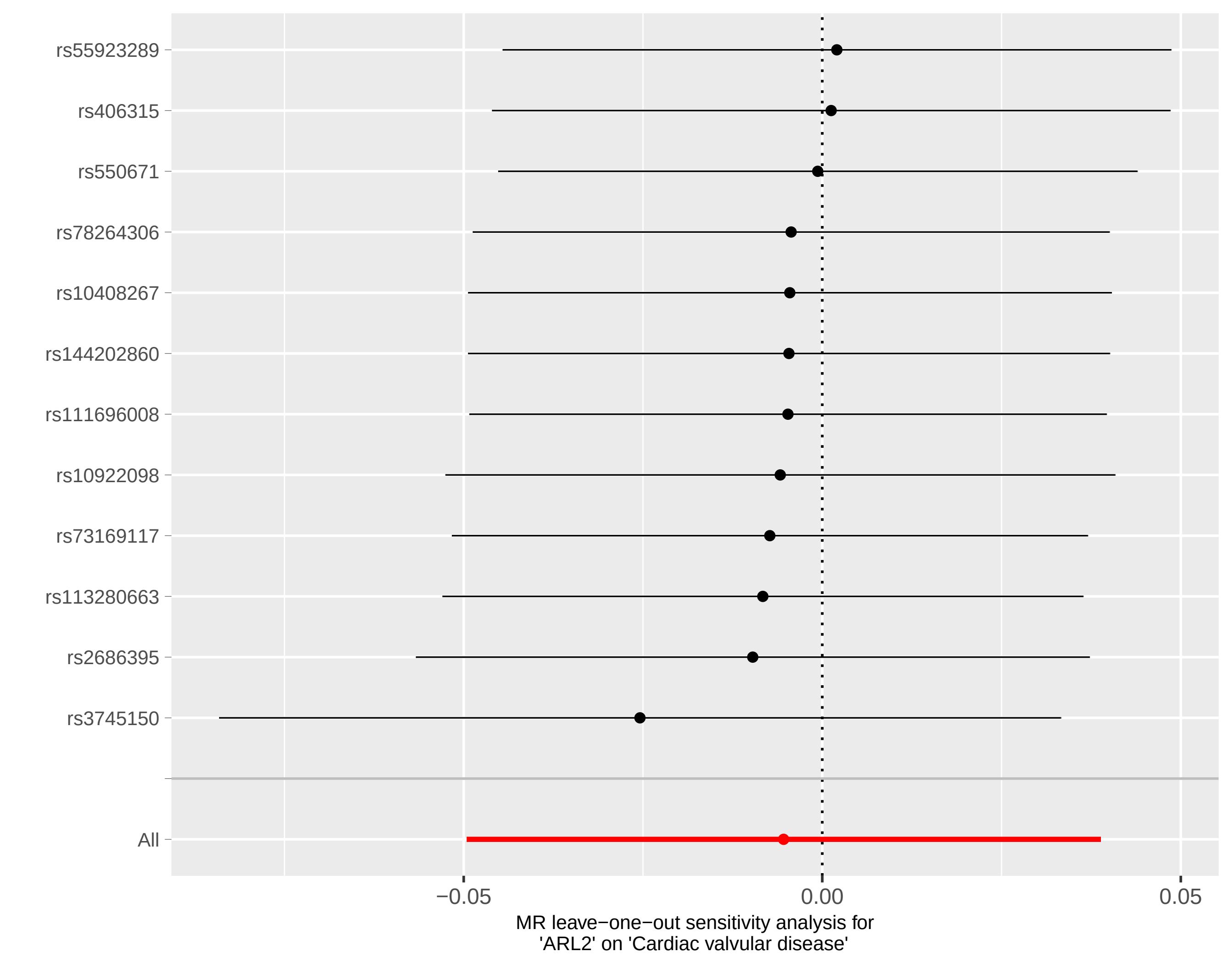

rs55923289
rs406315
rs550671
rs78264306
rs10408267
rs144202860
rs111696008
rs10922098
rs73169117
rs113280663
rs2686395
rs3745150
All
−0.05
0.00
0.05
MR leave−one−out sensitivity analysis for
'ARL2' on 'Cardiac valvular disease'

Insufficient number of SNPs

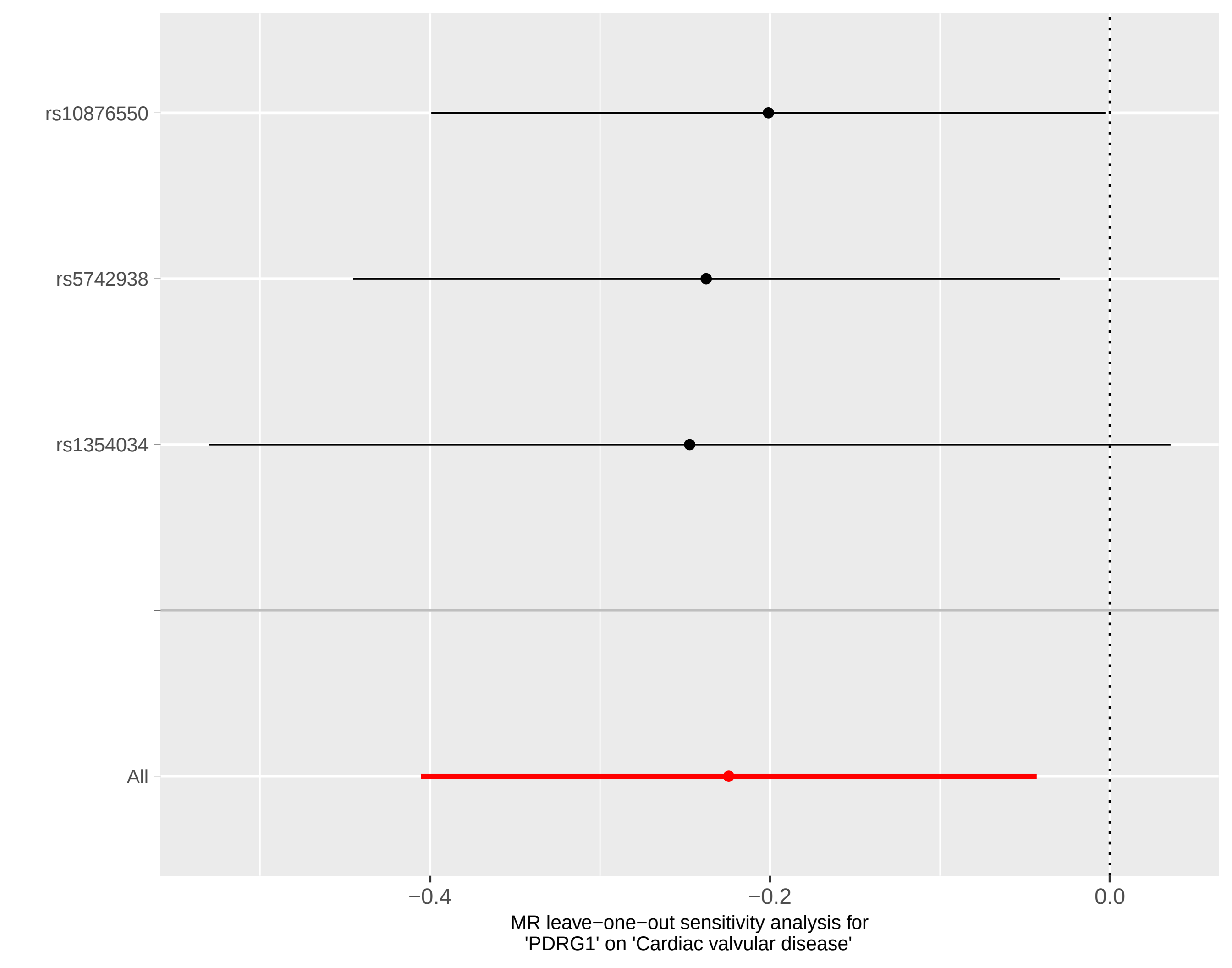
rs10876550
rs5742938
rs1354034
All
−0.4
−0.2
0.0
MR leave−one−out sensitivity analysis for
'PDRG1' on 'Cardiac valvular disease'

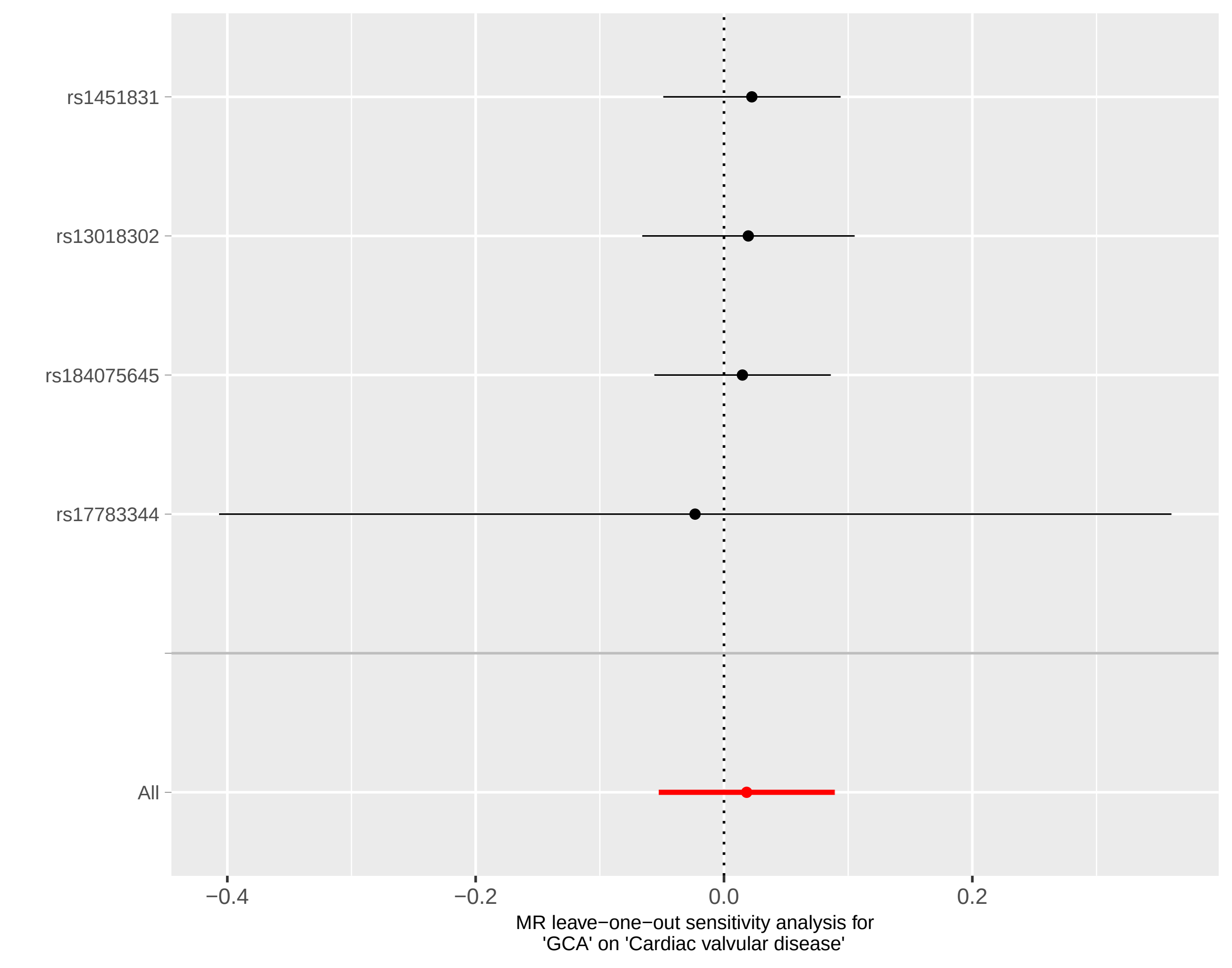
rs1451831
rs13018302
rs184075645
rs17783344
All
−0.4
−0.2
0.0
0.2
MR leave−one−out sensitivity analysis for
'GCA' on 'Cardiac valvular disease'

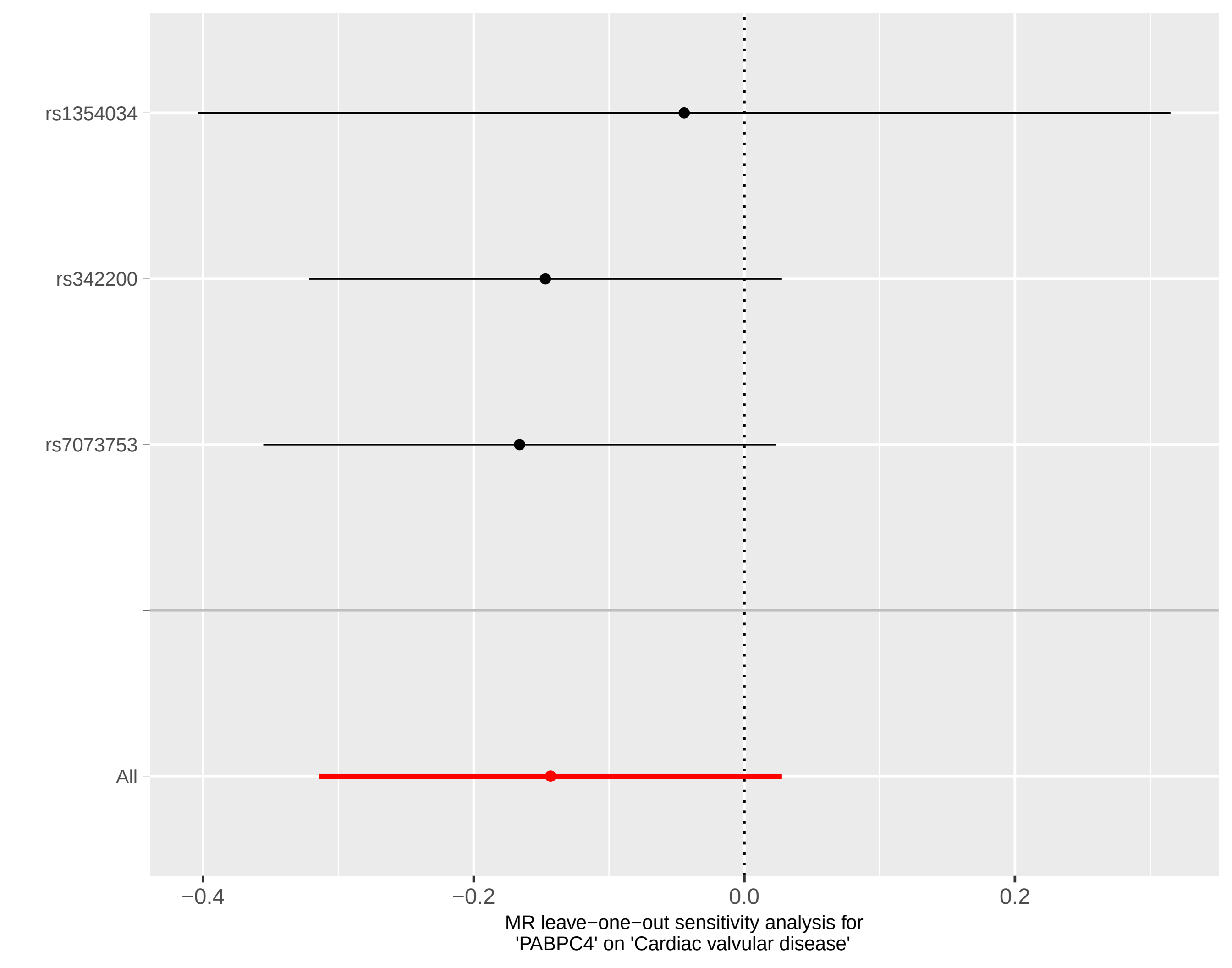
rs1354034
rs342200
rs7073753
All
−0.4
−0.2
0.0
0.2
MR leave−one−out sensitivity analysis for
'PABPC4' on 'Cardiac valvular disease'

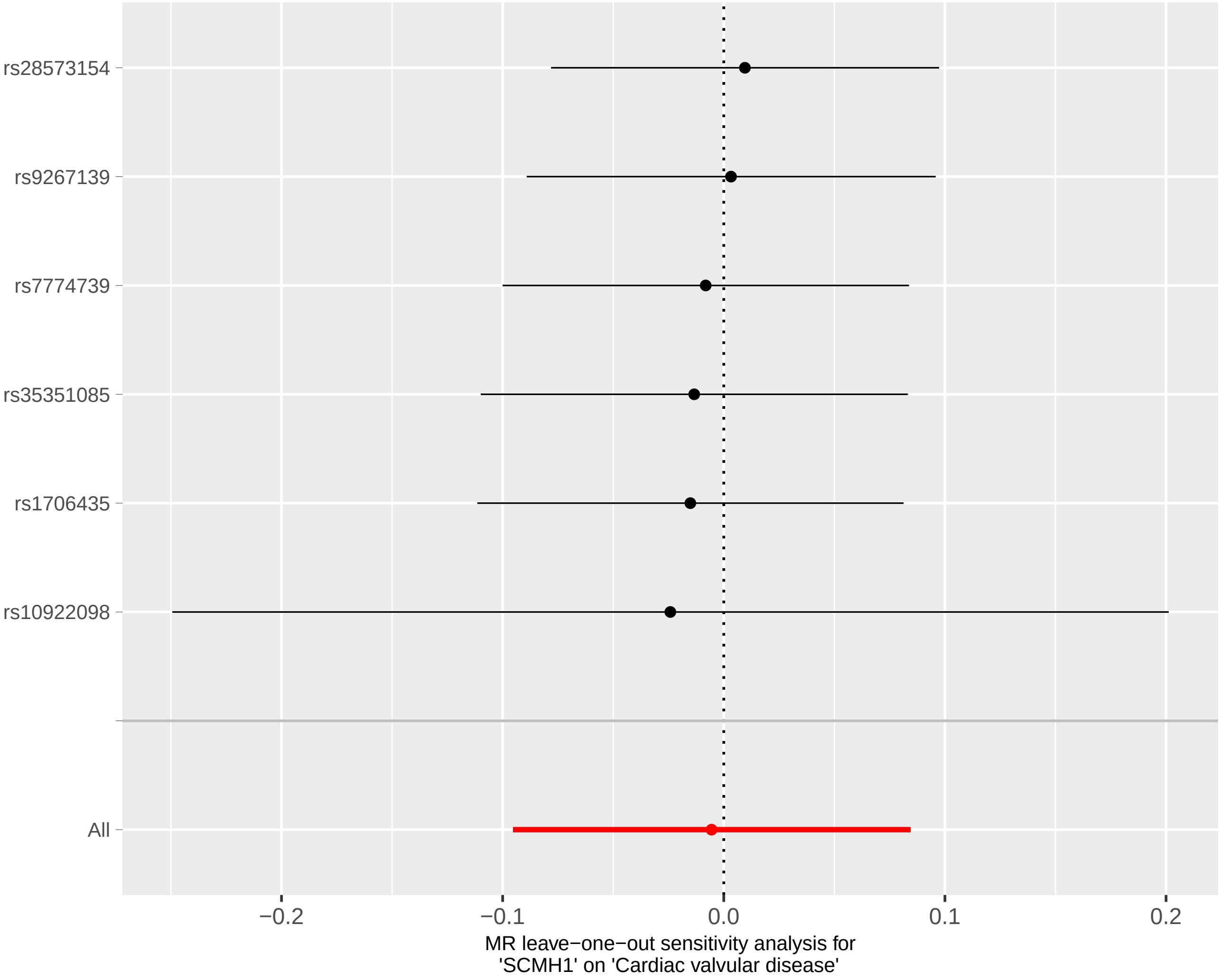
rs28573154
rs9267139
rs7774739
rs35351085
rs1706435
rs10922098
All
−0.2
−0.1
0.0
0.1
0.2
MR leave−one−out sensitivity analysis for
'SCMH1' on 'Cardiac valvular disease'

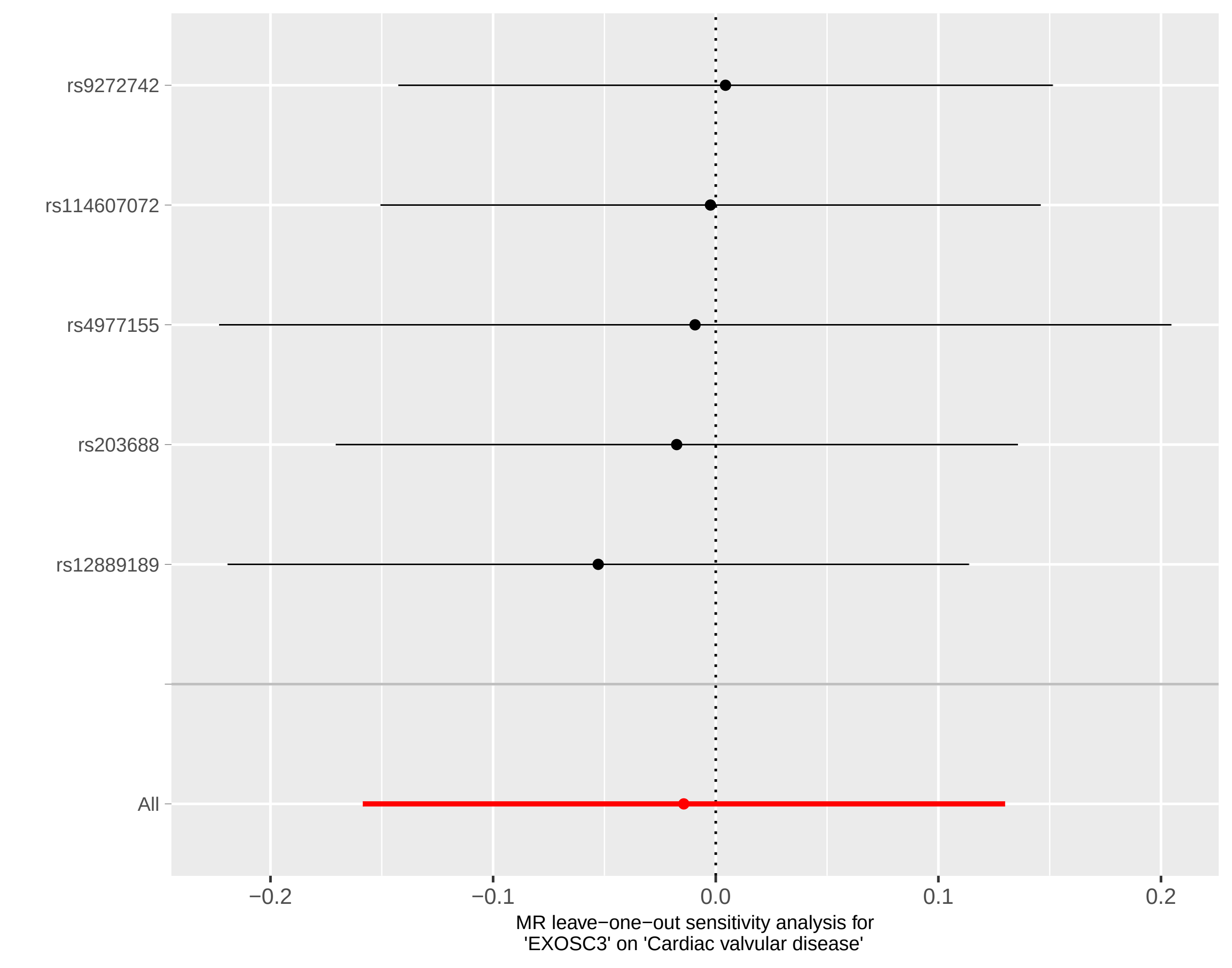
rs9272742
rs114607072
rs4977155
rs203688
rs12889189
All
−0.2
−0.1
0.0
0.1
0.2
MR leave−one−out sensitivity analysis for
'EXOSC3' on 'Cardiac valvular disease'

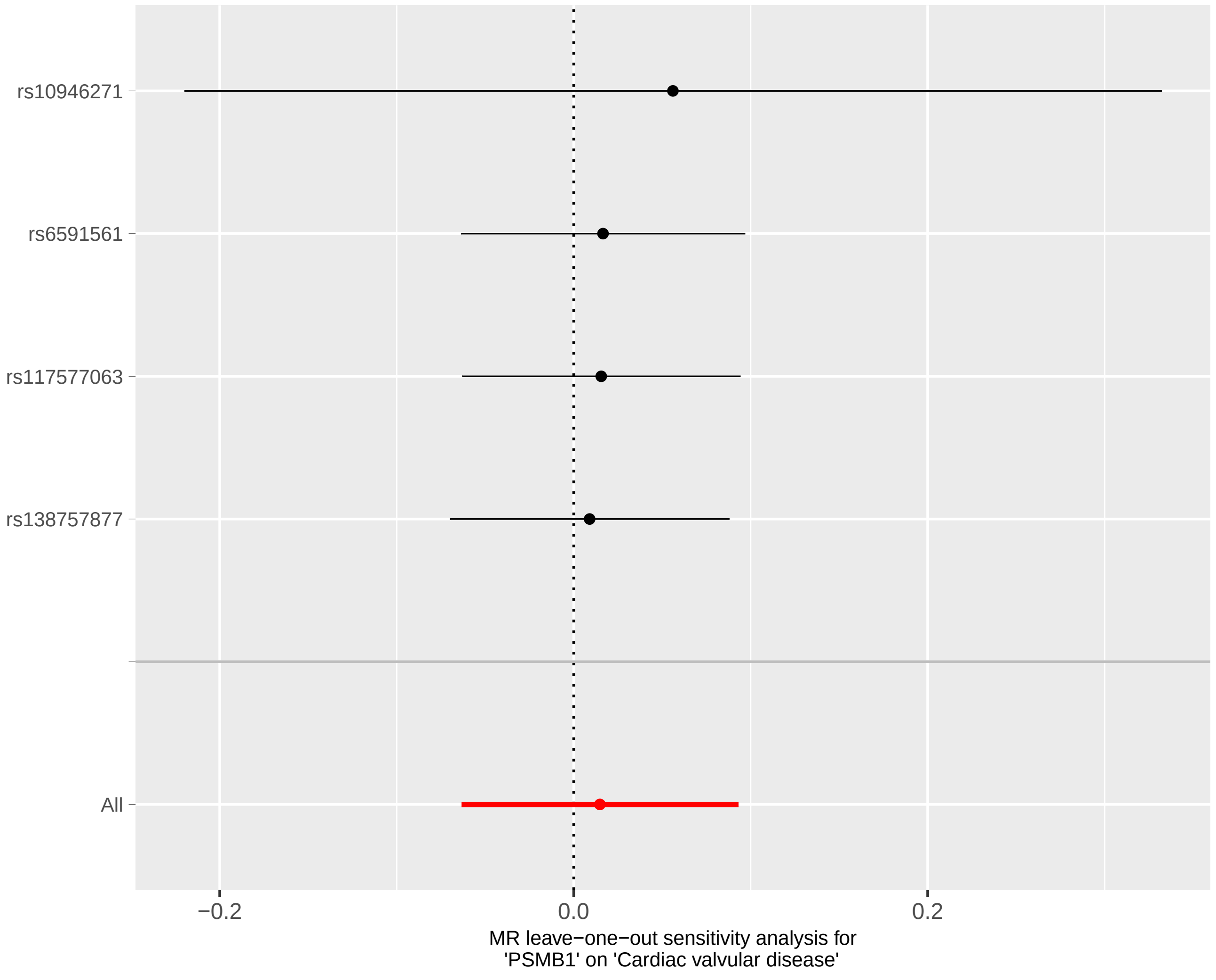
rs10946271
rs6591561
rs117577063
rs138757877
All
−0.2
0.0
0.2
MR leave−one−out sensitivity analysis for
'PSMB1' on 'Cardiac valvular disease'

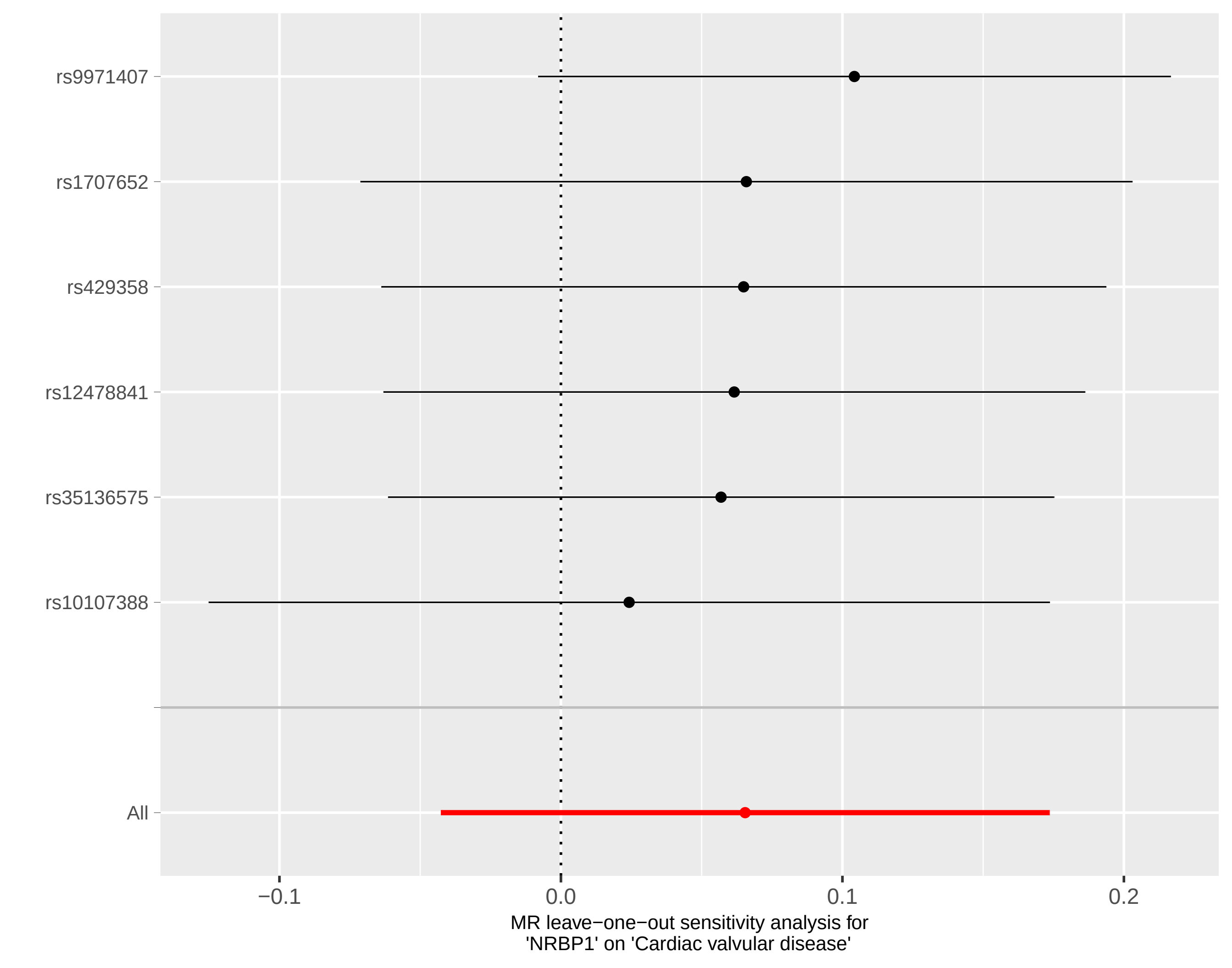
rs9971407
rs1707652
rs429358
rs12478841
rs35136575
rs10107388
All
−0.1
0.0
0.1
0.2
MR leave−one−out sensitivity analysis for
'NRBP1' on 'Cardiac valvular disease'

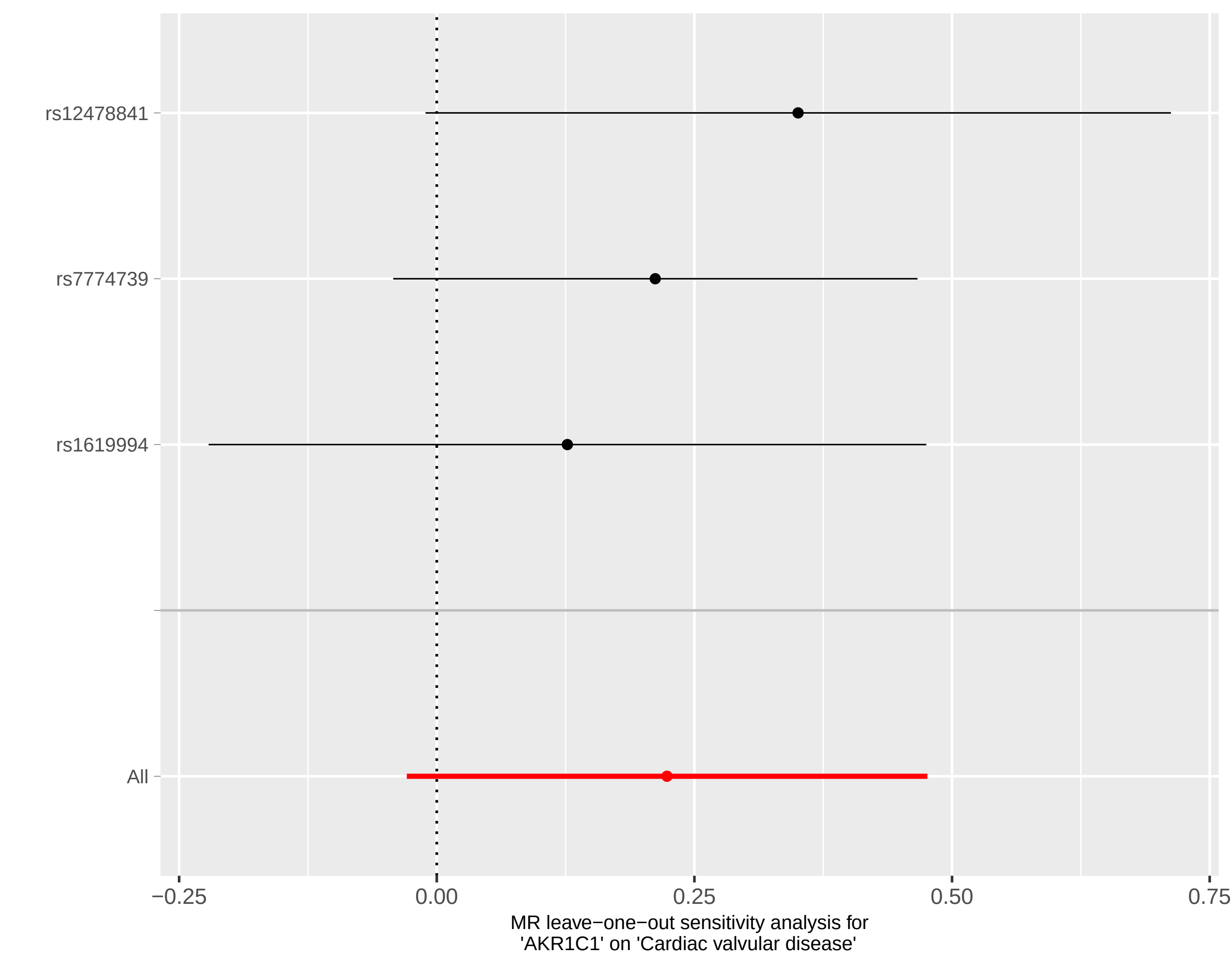
rs12478841
rs7774739
rs1619994
All
−0.25
0.00
0.25
0.50
0.75
MR leave−one−out sensitivity analysis for
'AKR1C1' on 'Cardiac valvular disease'

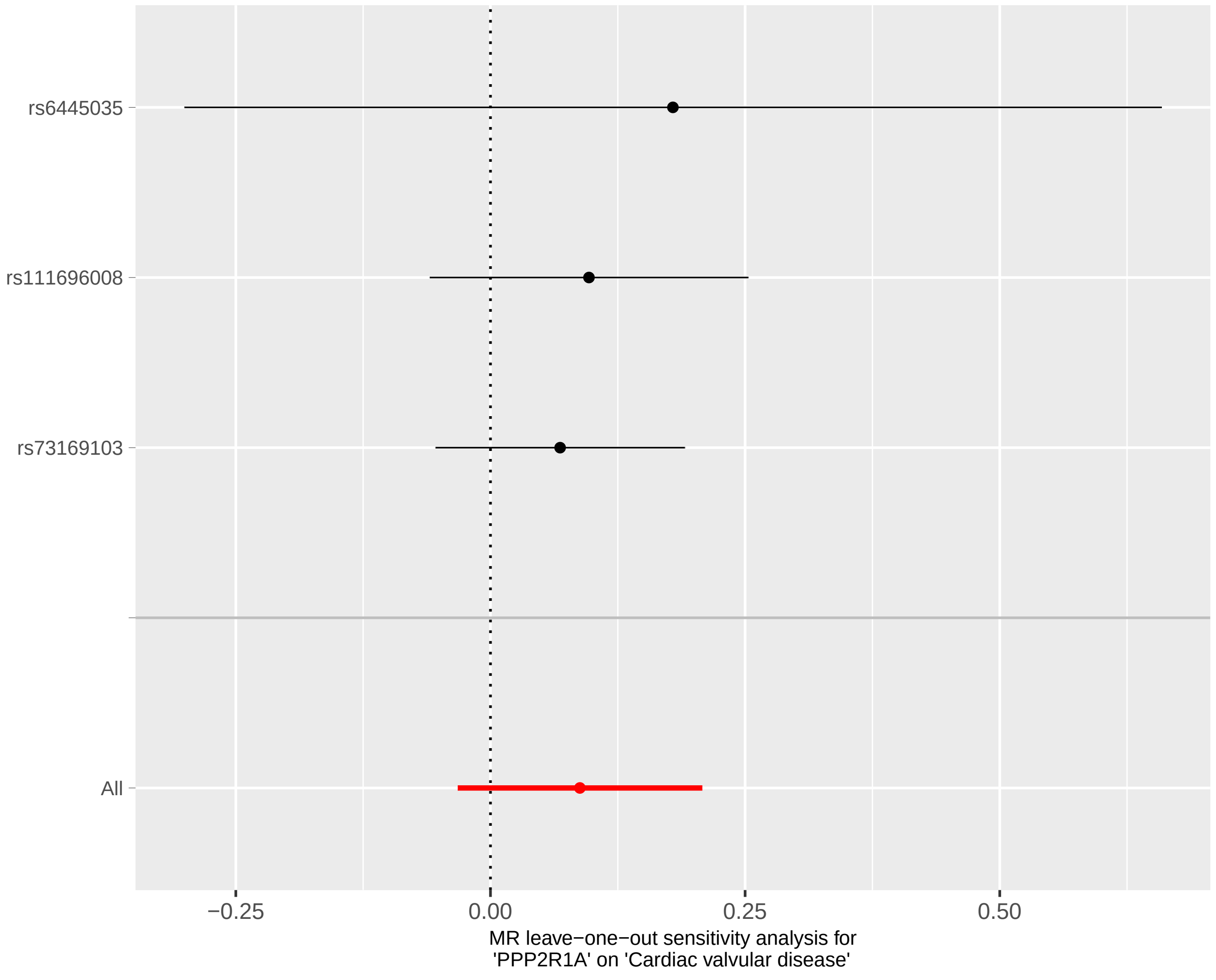

rs6445035
rs111696008
rs73169103
All
−0.25
0.00
0.25
0.50
MR leave−one−out sensitivity analysis for
'PPP2R1A' on 'Cardiac valvular disease'

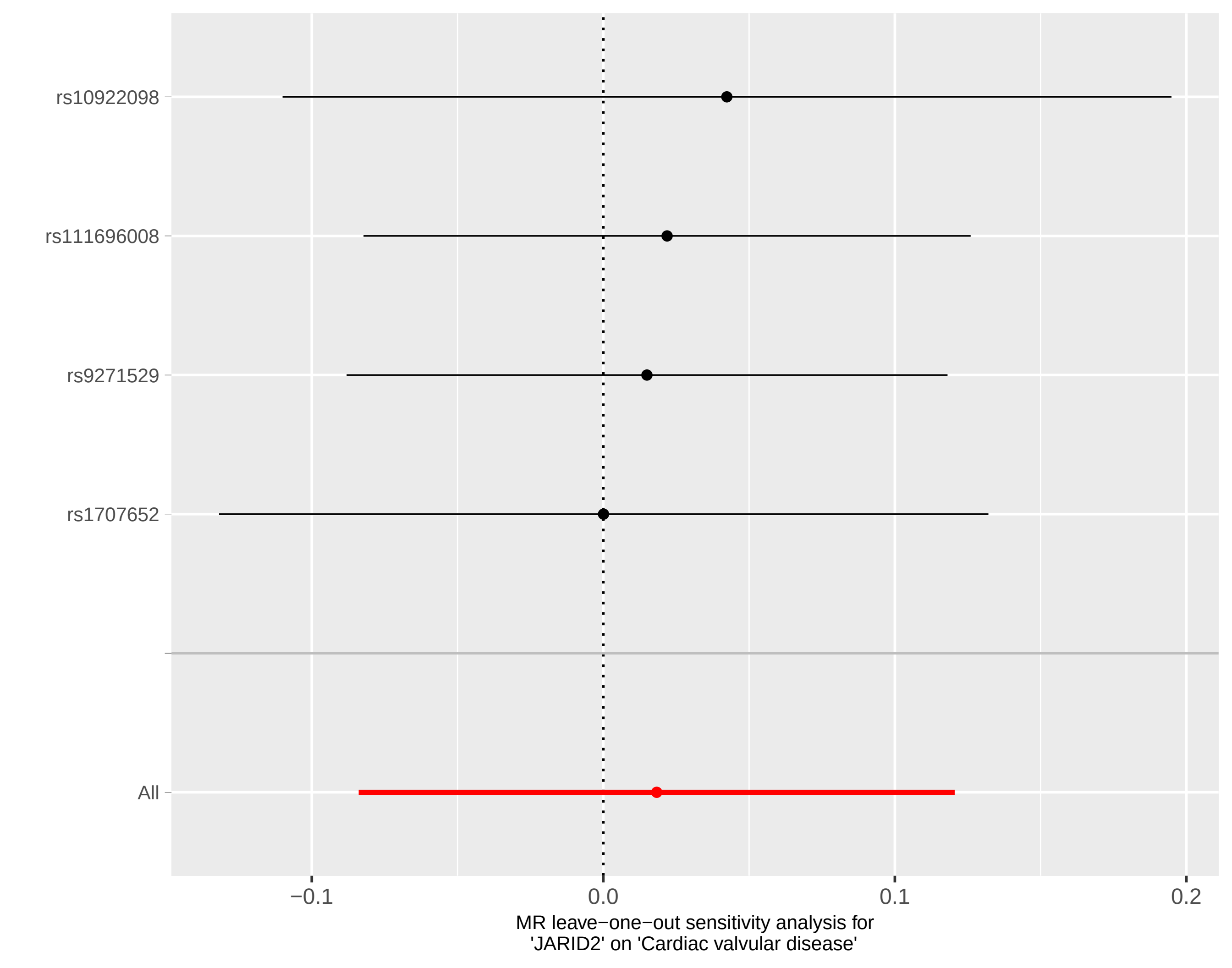

rs10922098
rs111696008
rs9271529
rs1707652
All
−0.1
0.0
0.1
0.2
MR leave−one−out sensitivity analysis for
'JARID2' on 'Cardiac valvular disease'

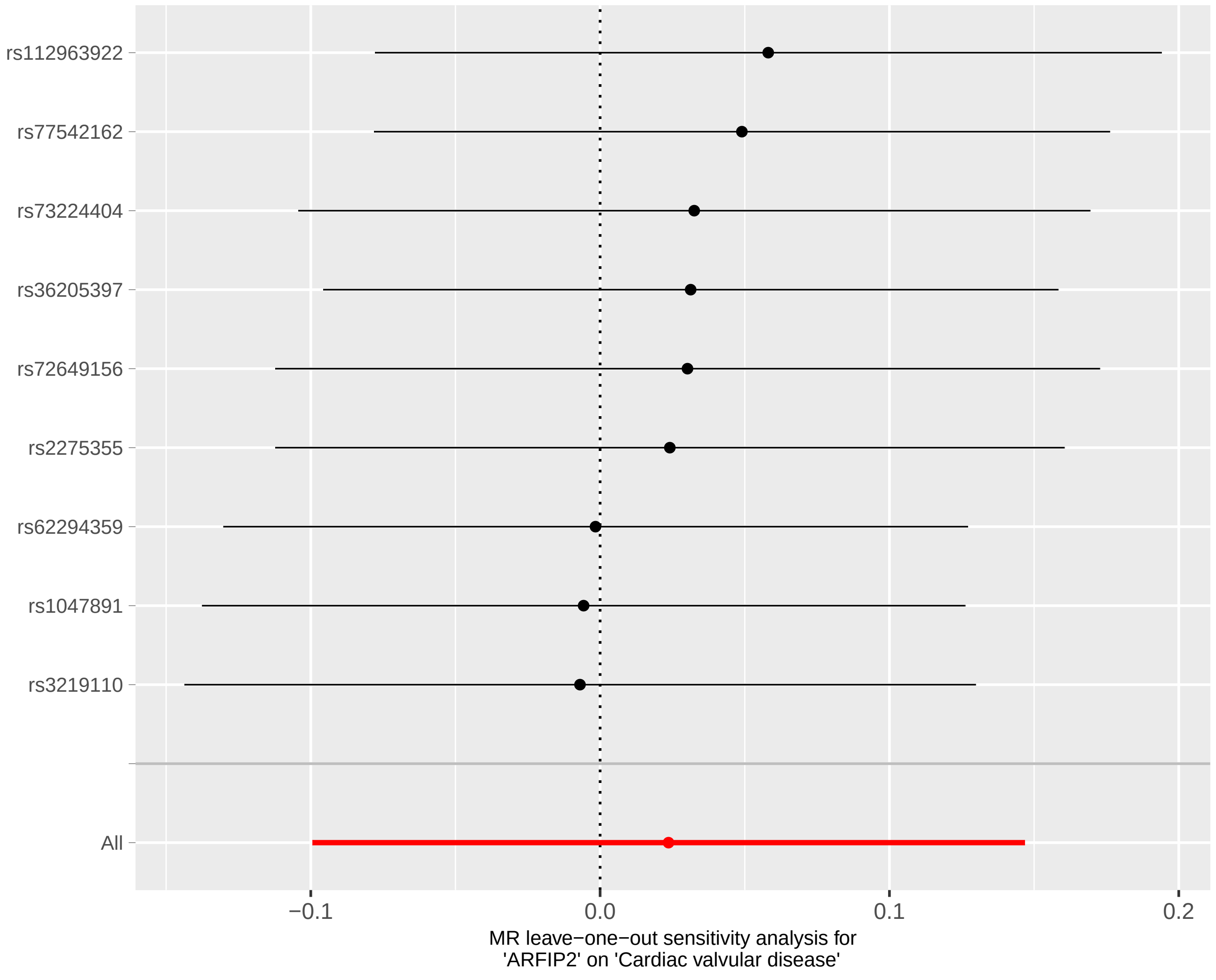
rs112963922
rs77542162
rs73224404
rs36205397
rs72649156
rs2275355
rs62294359
rs1047891
rs3219110
All
−0.1
0.0
0.1
0.2
MR leave−one−out sensitivity analysis for
'ARFIP2' on 'Cardiac valvular disease'

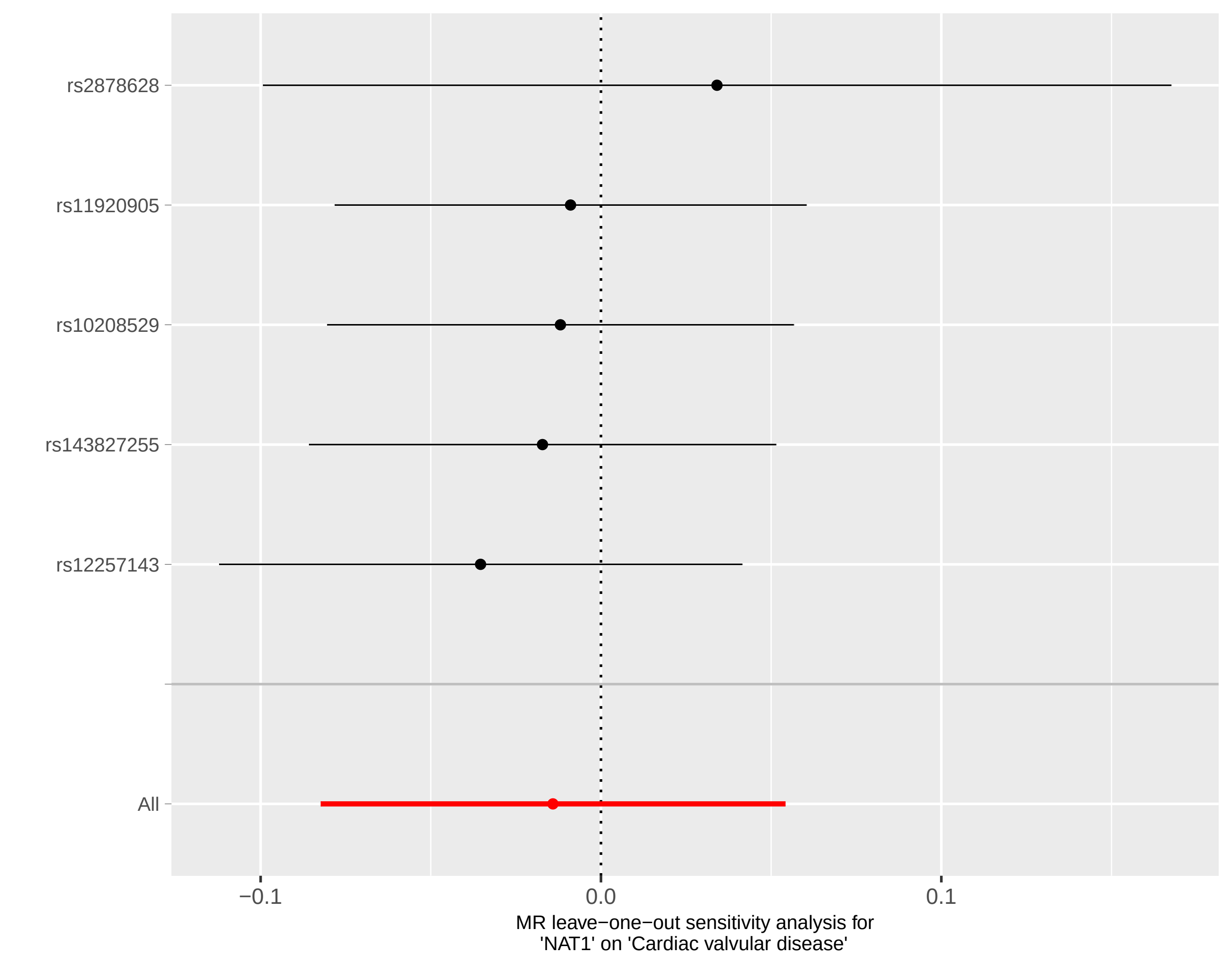

rs2878628
rs11920905
rs10208529
rs143827255
rs12257143
All
−0.1
0.0
0.1
MR leave−one−out sensitivity analysis for
'NAT1' on 'Cardiac valvular disease'

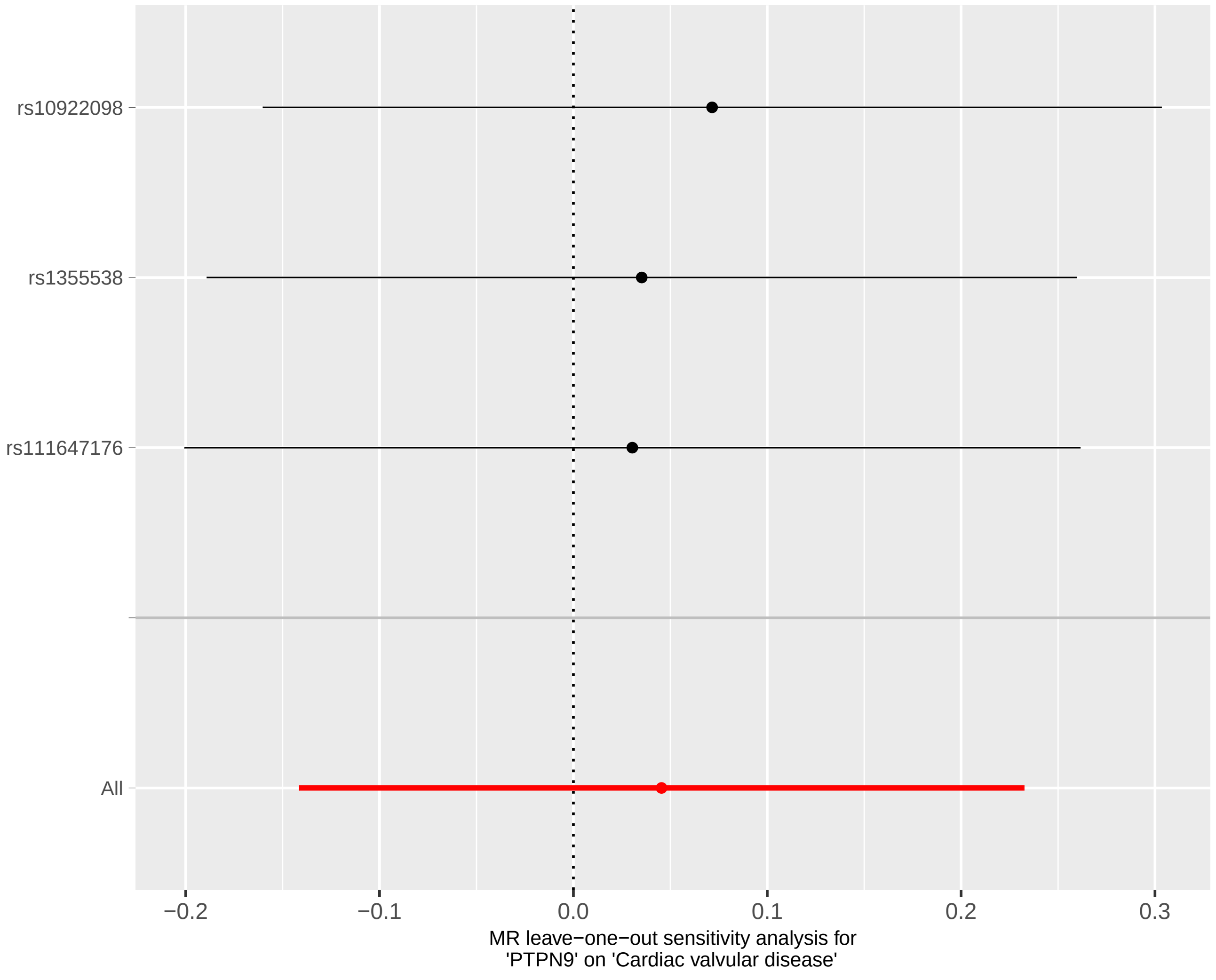
rs10922098
rs1355538
rs111647176
All
−0.2
−0.1
0.0
0.1
0.2
0.3
MR leave−one−out sensitivity analysis for
'PTPN9' on 'Cardiac valvular disease'

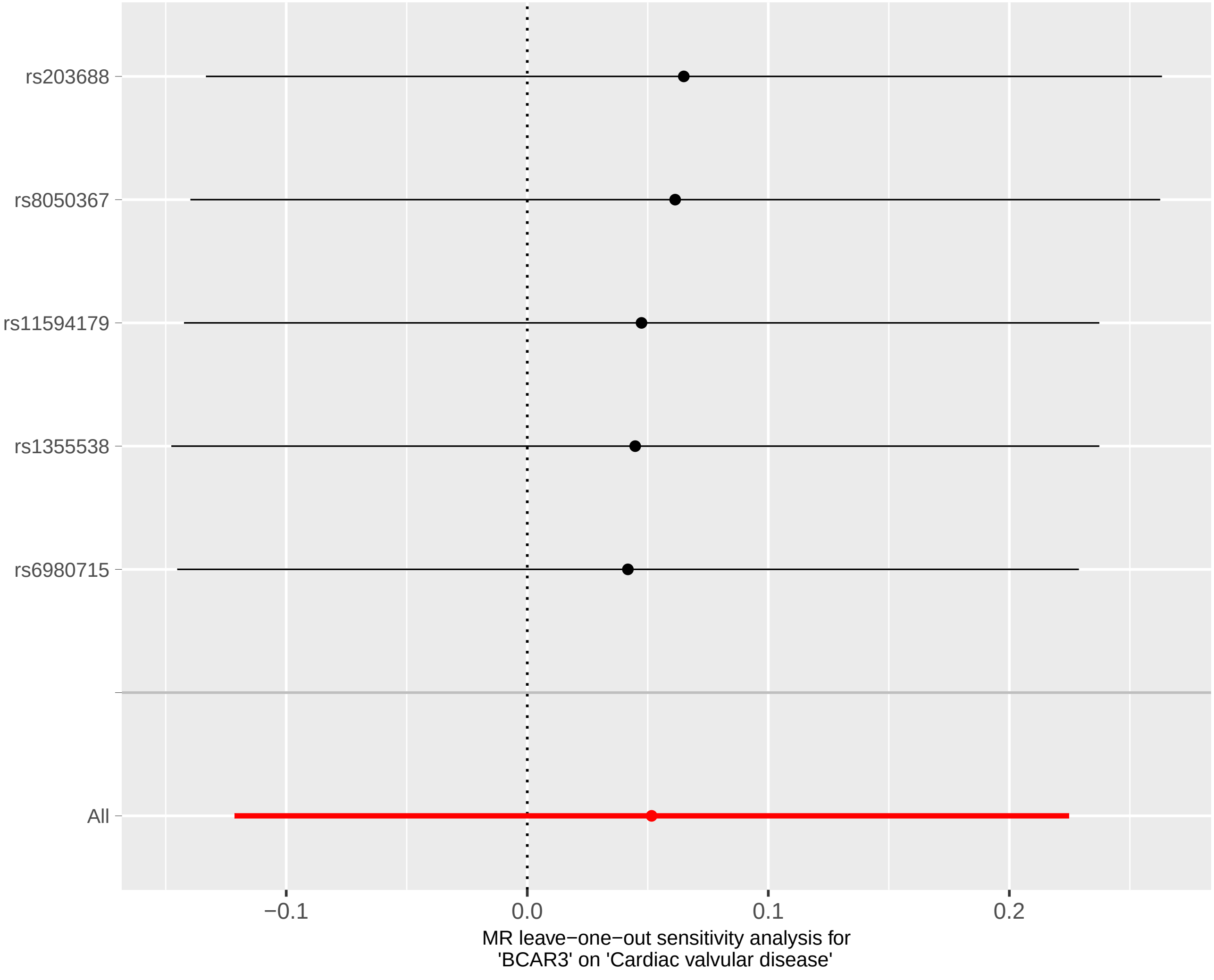
rs203688
rs8050367
rs11594179
rs1355538
rs6980715
All
−0.1
0.0
0.1
0.2
MR leave−one−out sensitivity analysis for
'BCAR3' on 'Cardiac valvular disease'

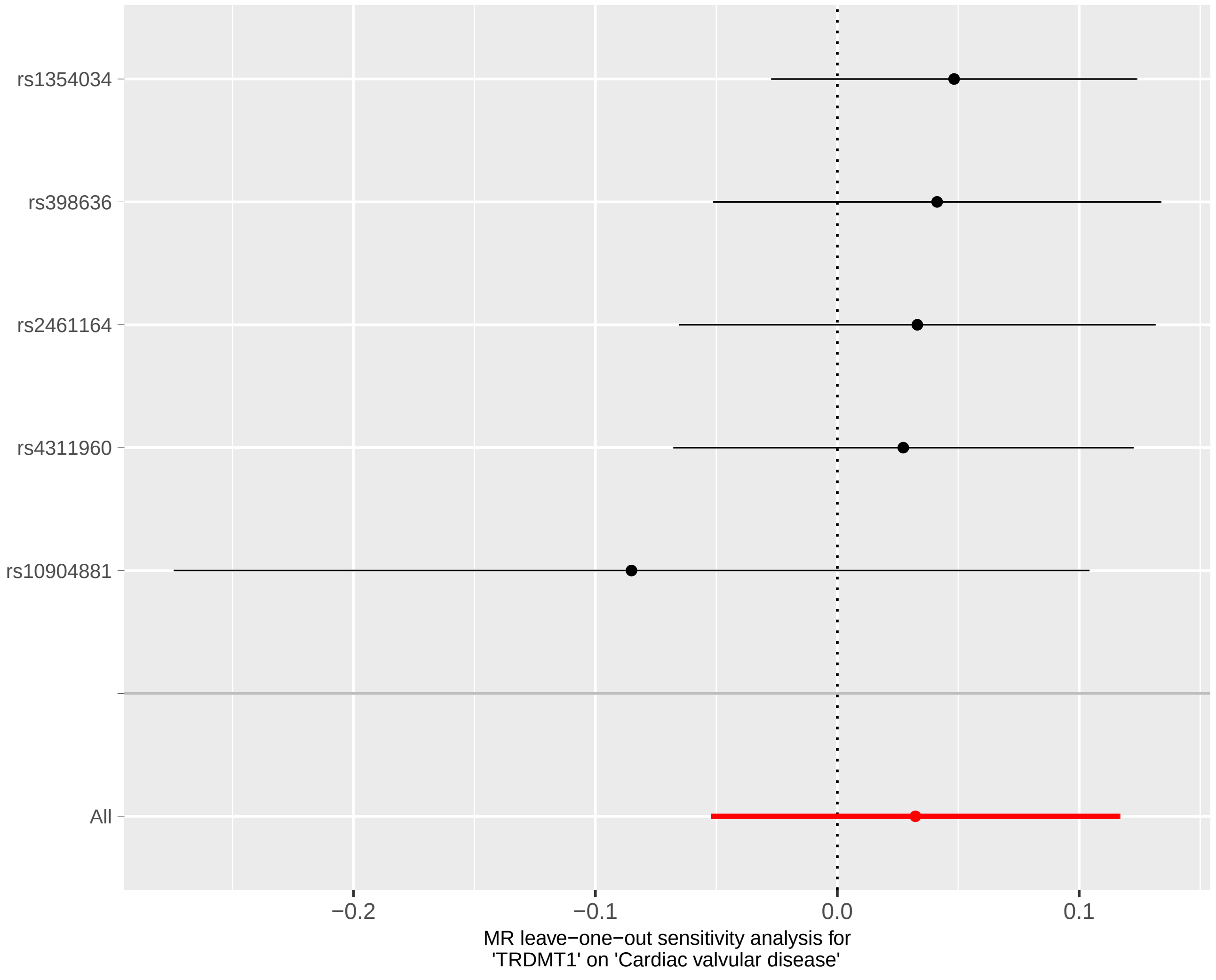

rs1354034
rs398636
rs2461164
rs4311960
rs10904881
All
−0.2
−0.1
0.0
0.1
MR leave−one−out sensitivity analysis for
'TRDMT1' on 'Cardiac valvular disease'

Insufficient number of SNPs

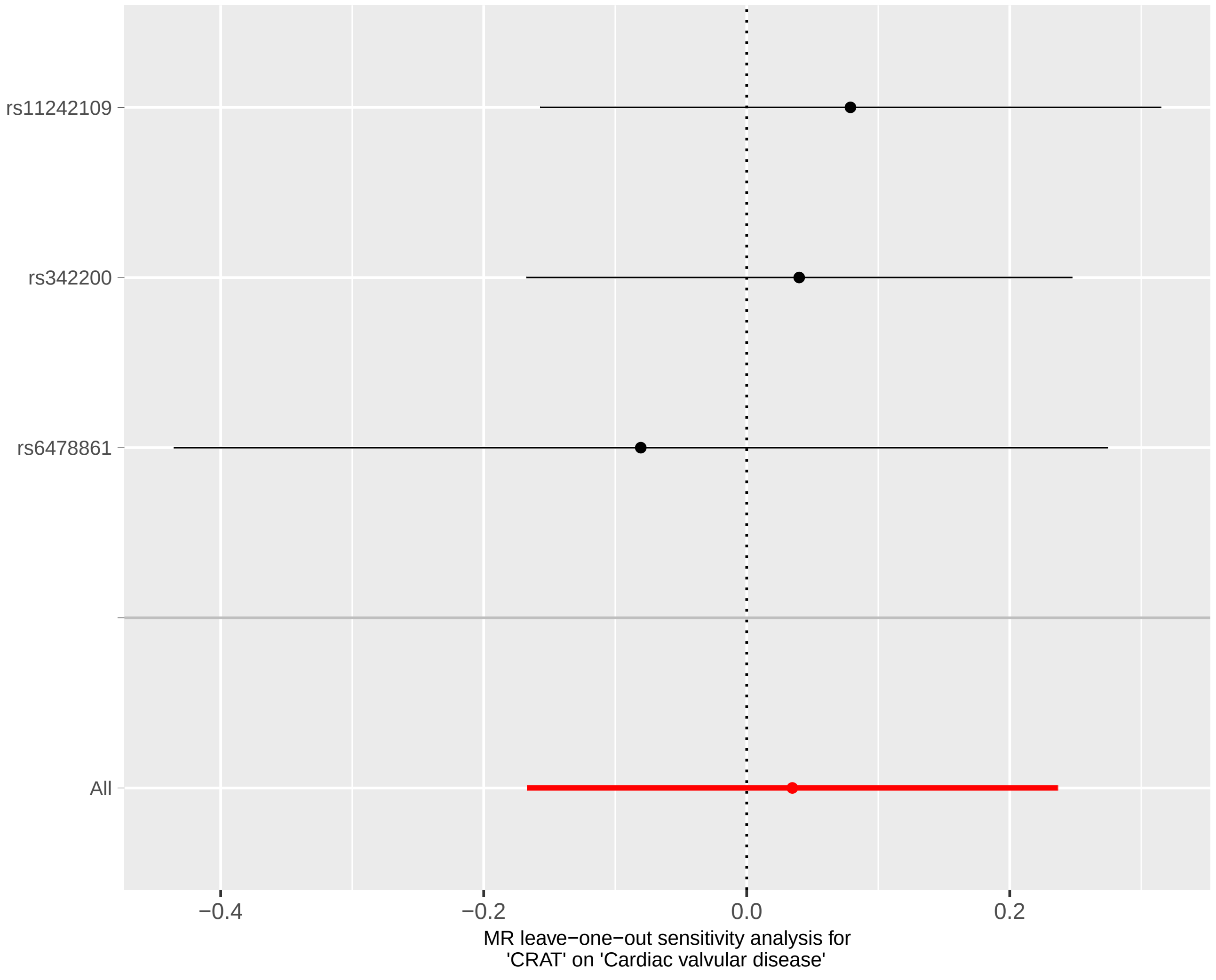

rs11242109
rs342200
rs6478861
All
−0.4
−0.2
0.0
0.2
MR leave−one−out sensitivity analysis for
'CRAT' on 'Cardiac valvular disease'

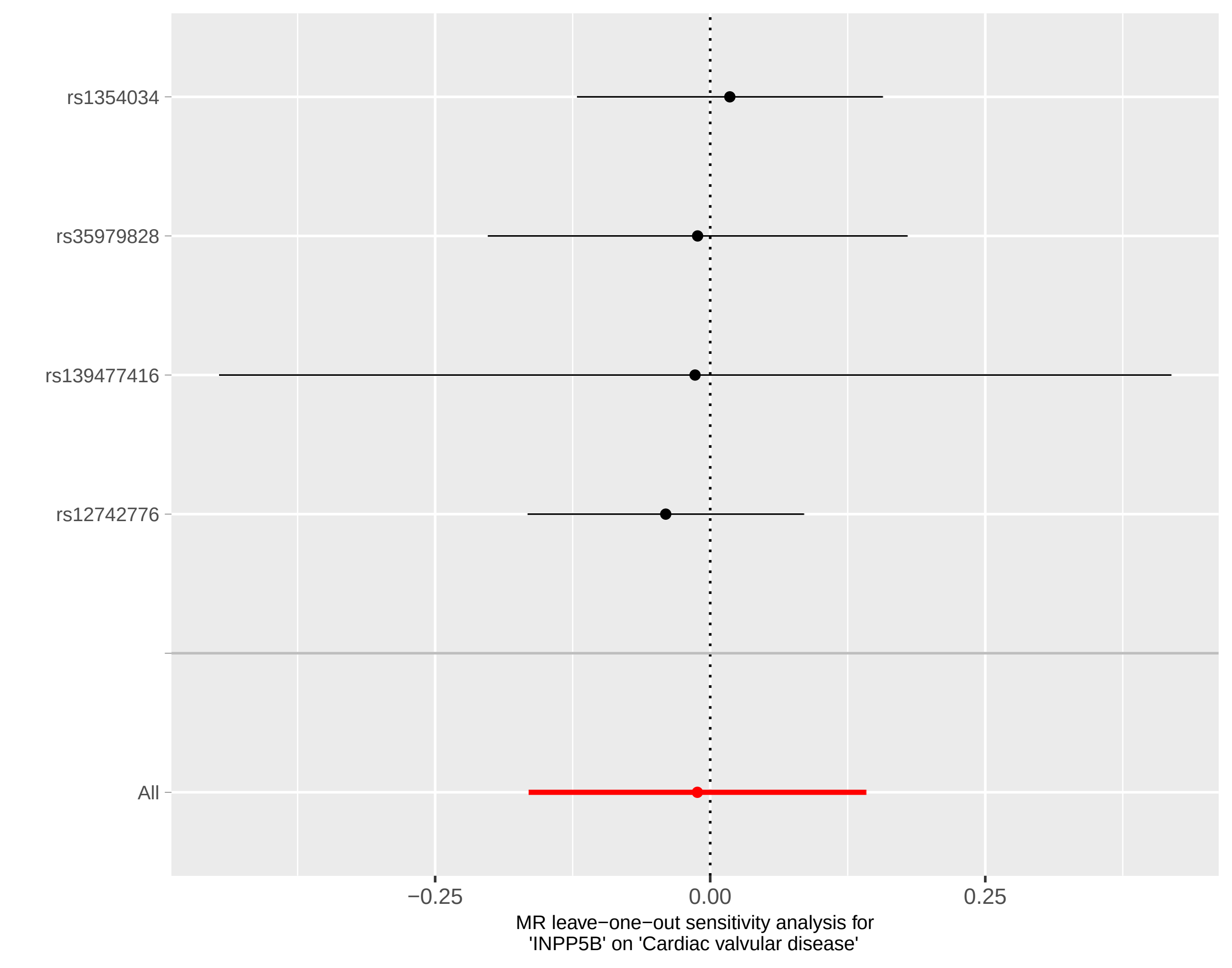
rs1354034
rs35979828
rs139477416
rs12742776
All
−0.25
0.00
0.25
MR leave−one−out sensitivity analysis for
'INPP5B' on 'Cardiac valvular disease'

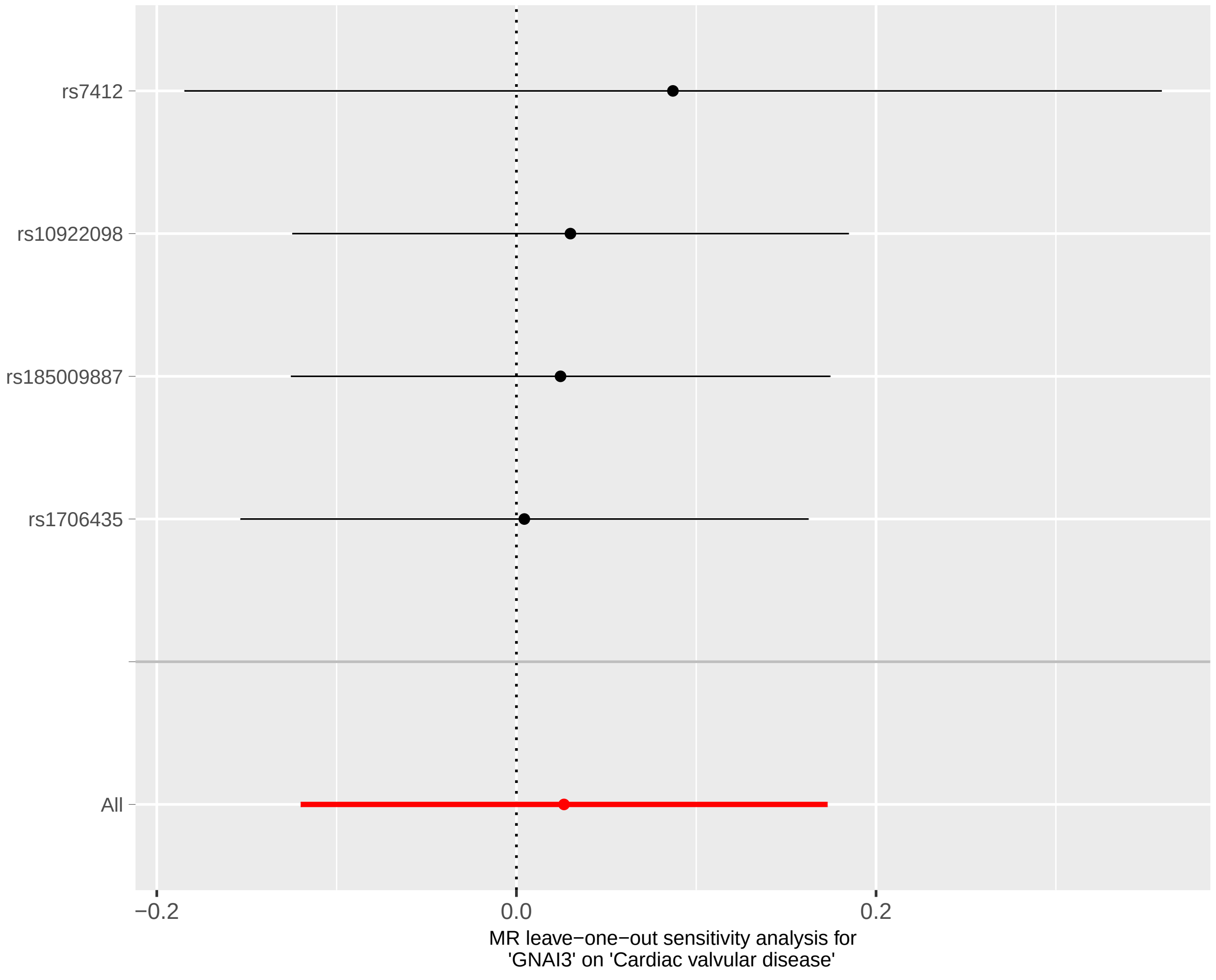

rs7412
rs10922098
rs185009887
rs1706435
All
−0.2
0.0
0.2
MR leave−one−out sensitivity analysis for
'GNAI3' on 'Cardiac valvular disease'

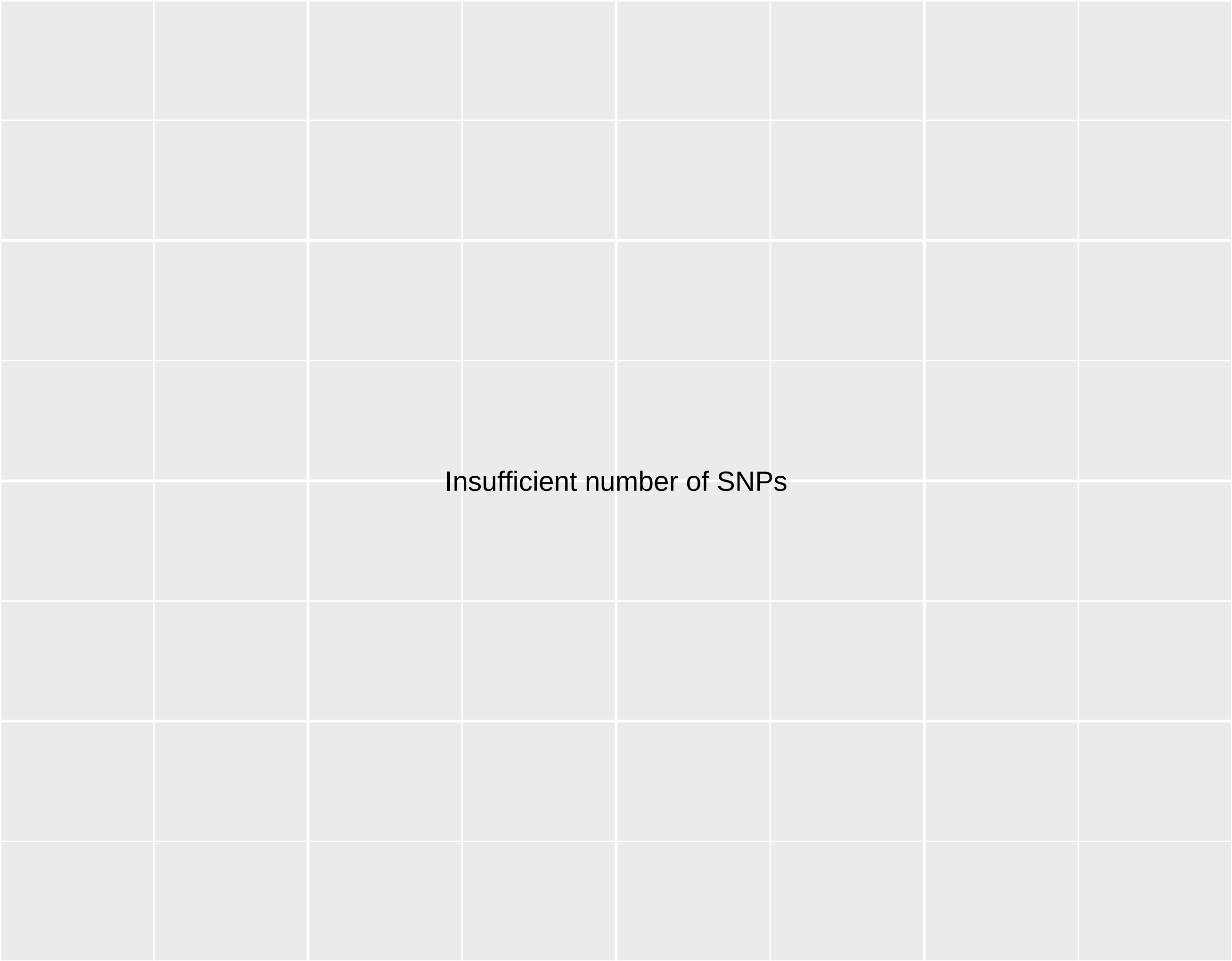
Insufficient number of SNPs

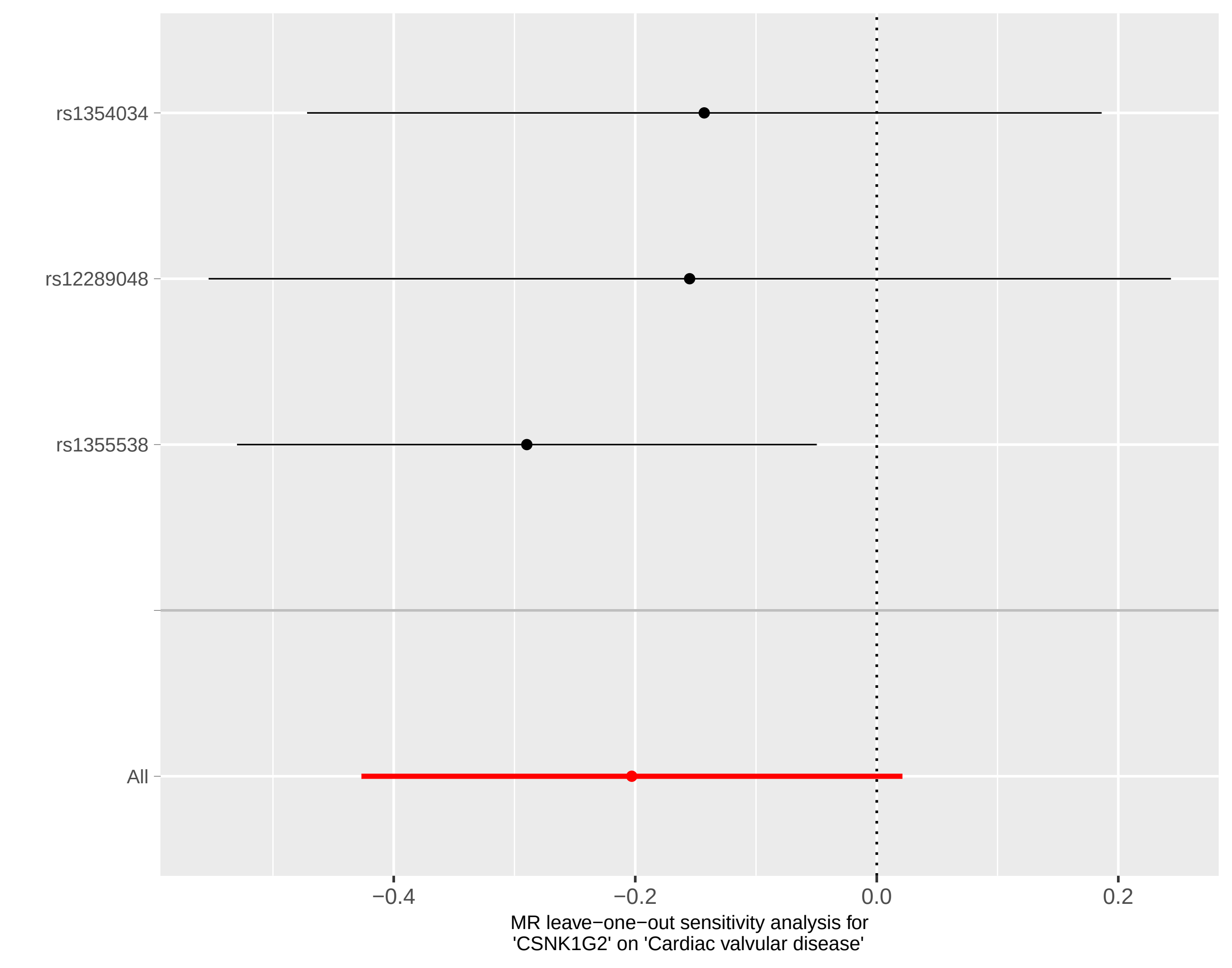
rs1354034
rs12289048
rs1355538
All
−0.4
−0.2
0.0
0.2
MR leave−one−out sensitivity analysis for
'CSNK1G2' on 'Cardiac valvular disease'

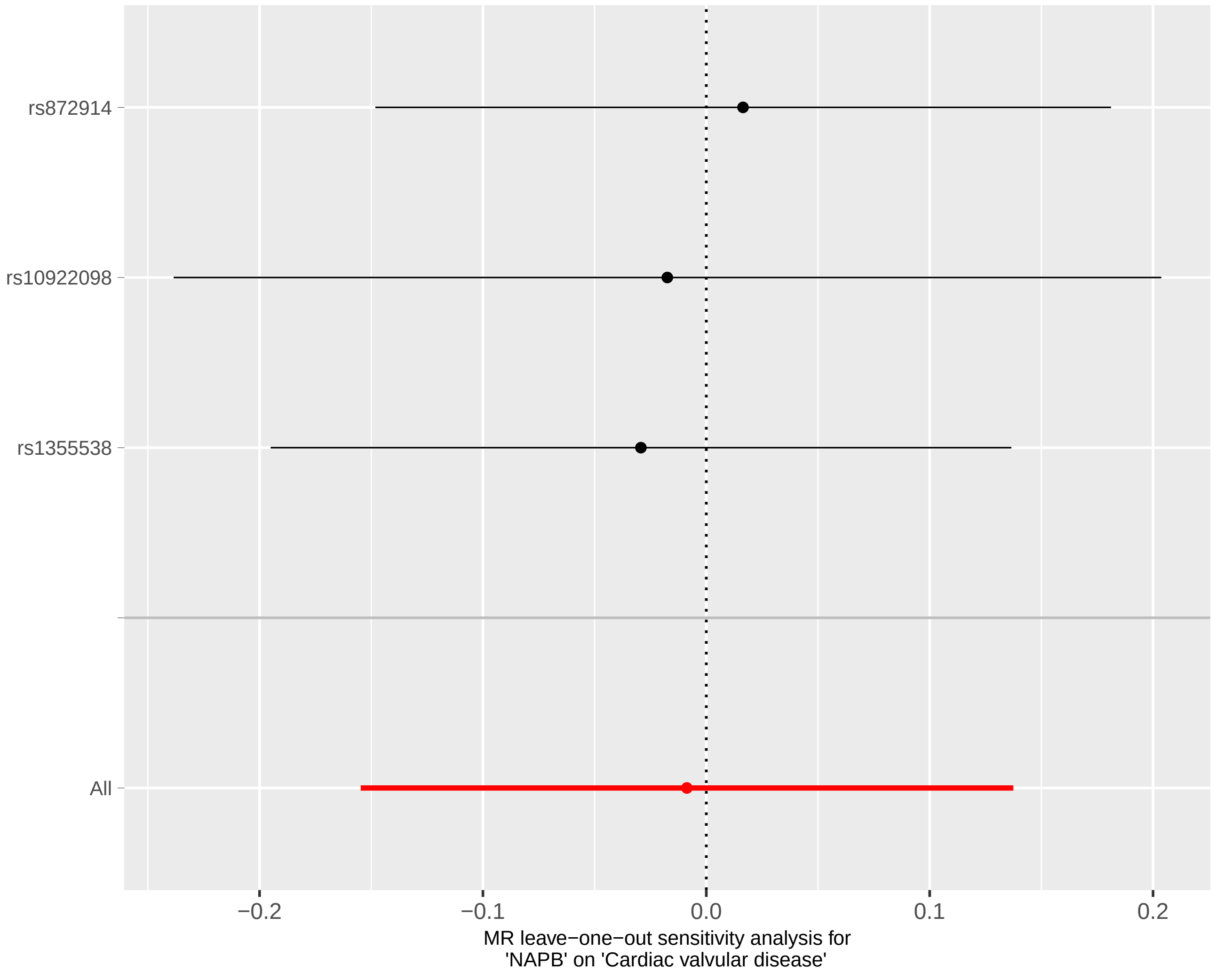
rs872914
rs10922098
rs1355538
All
−0.2
−0.1
0.0
0.1
0.2
MR leave−one−out sensitivity analysis for
'NAPB' on 'Cardiac valvular disease'

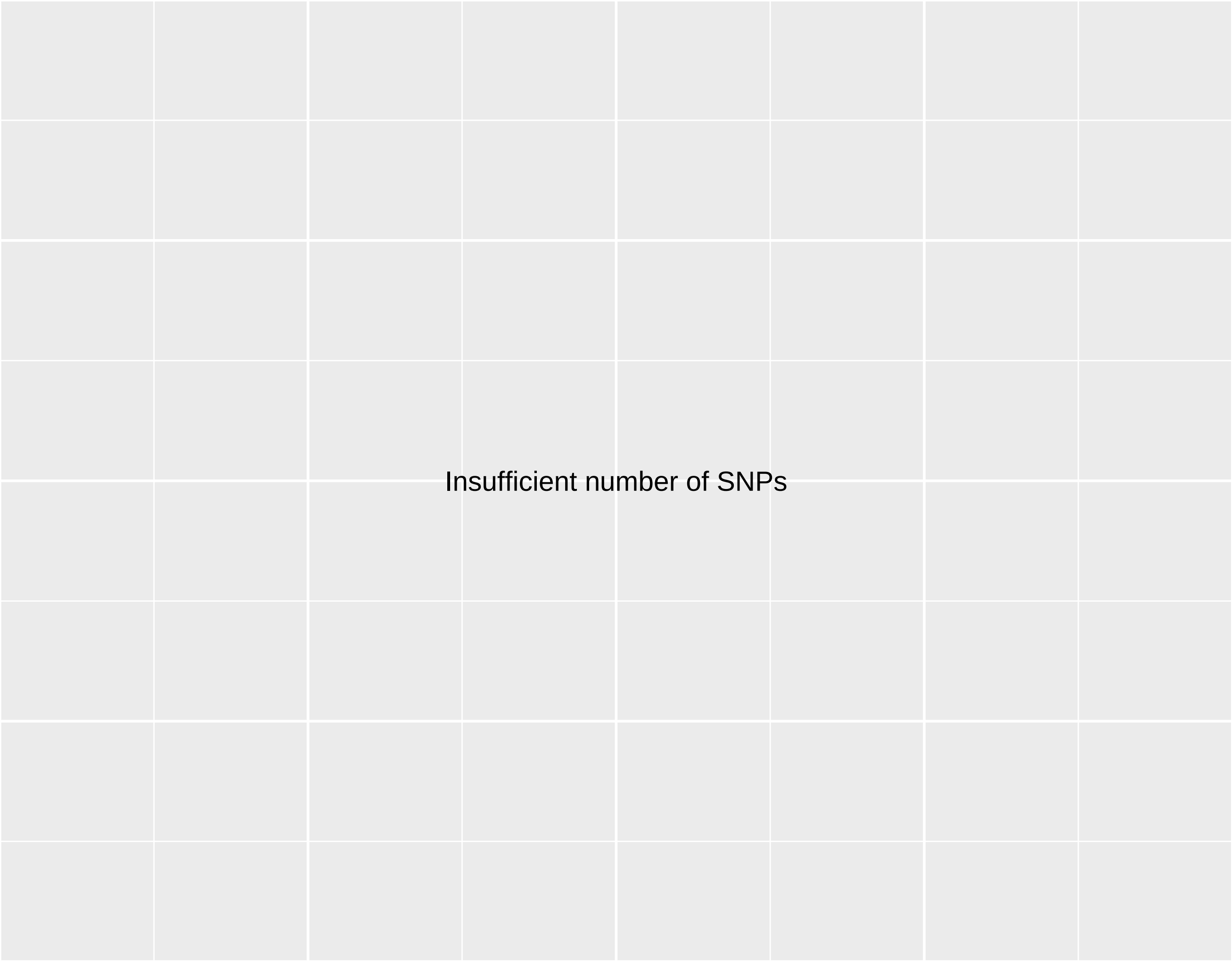
Insufficient number of SNPs

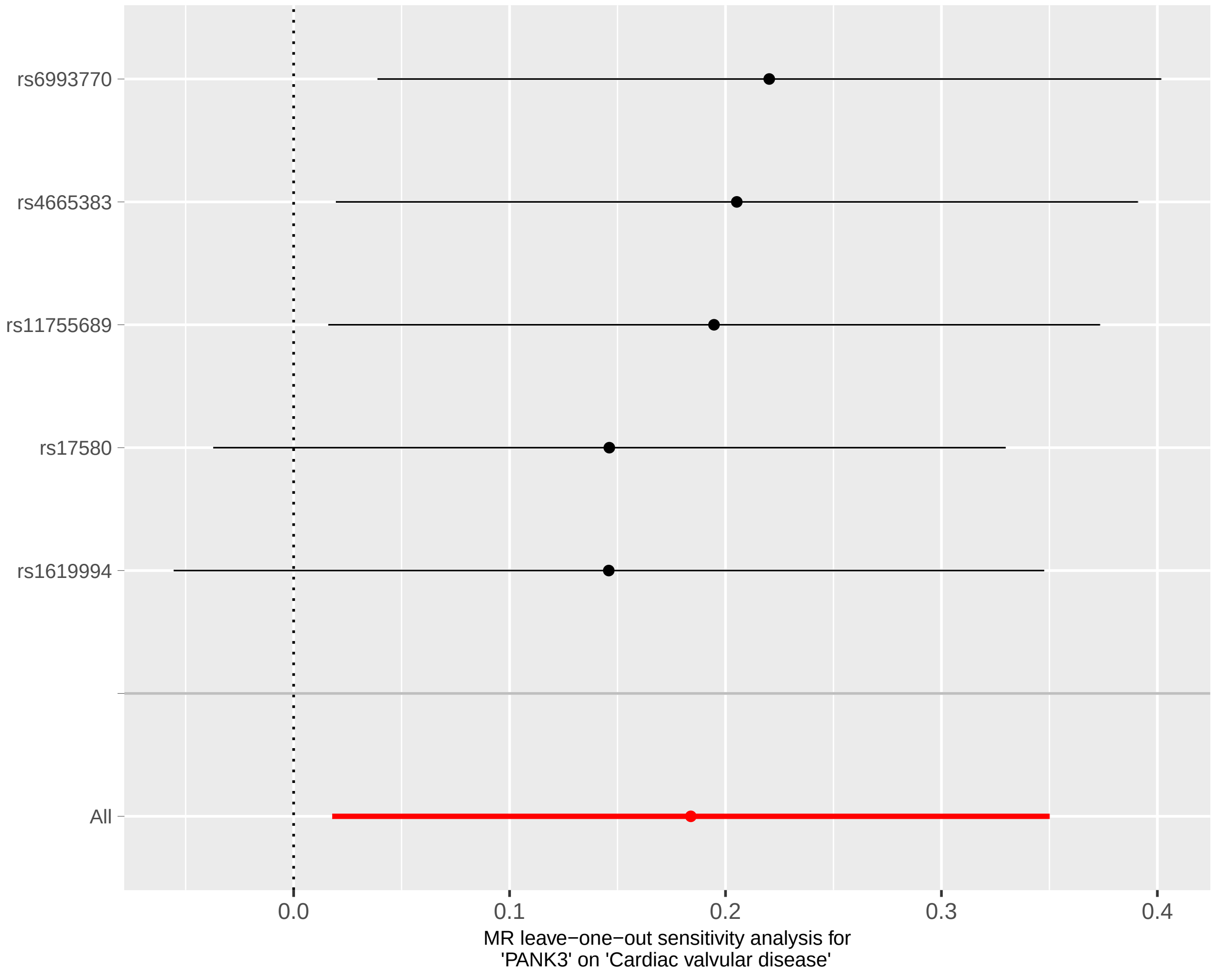

rs6993770
rs4665383
rs11755689
rs17580
rs1619994
All
0.0
0.1
0.2
0.3
0.4
MR leave−one−out sensitivity analysis for
'PANK3' on 'Cardiac valvular disease'

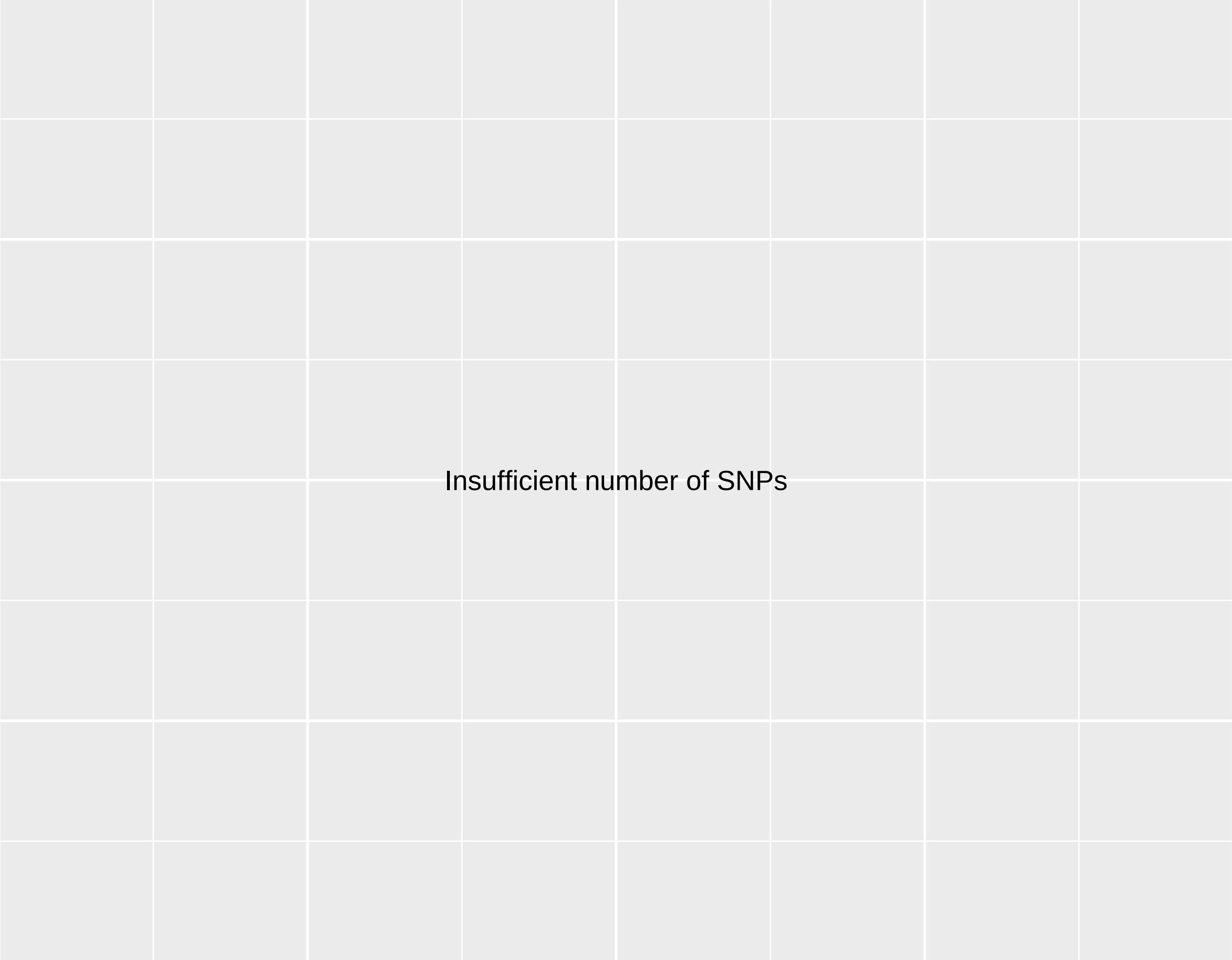
Insufficient number of SNPs

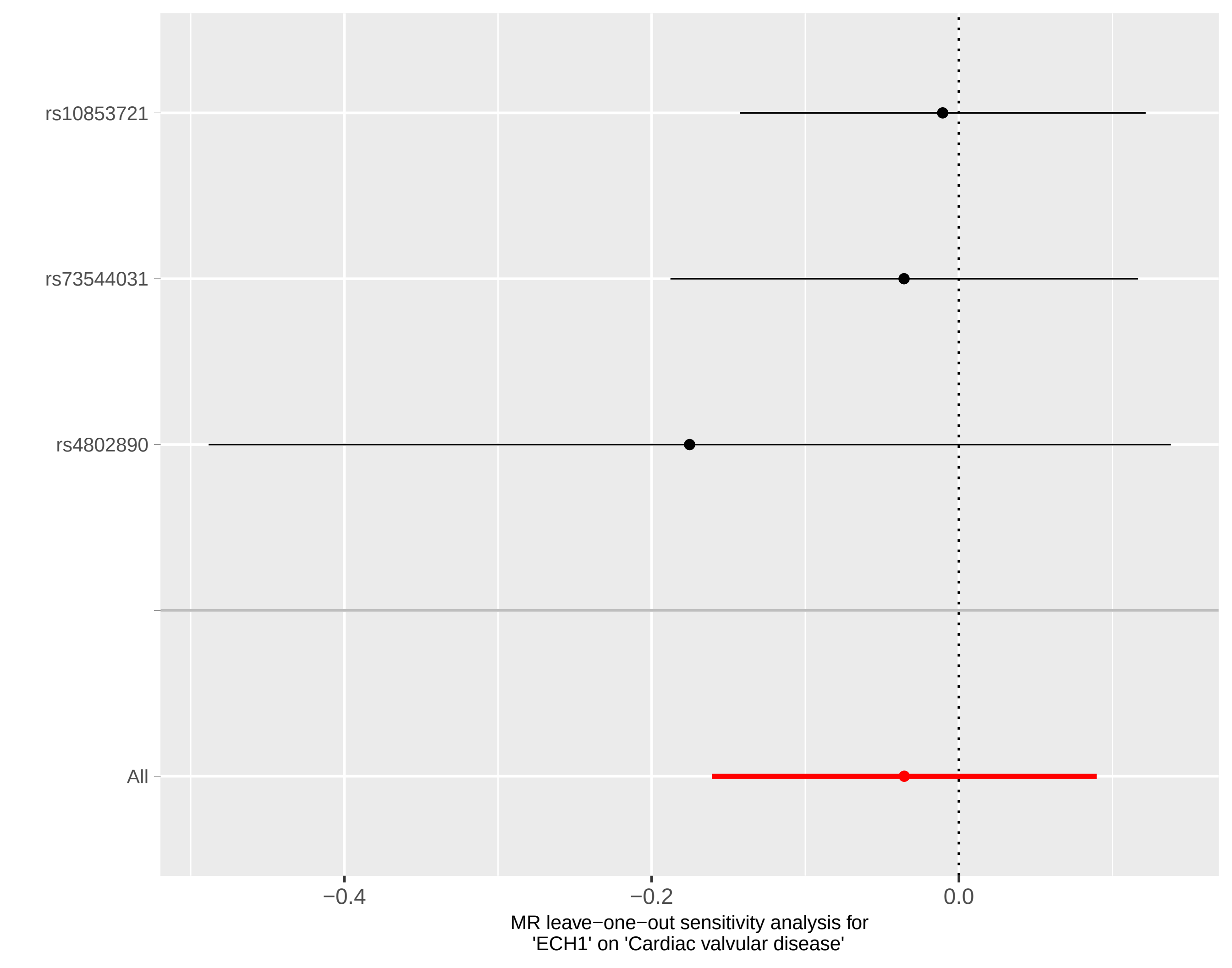
rs10853721
rs73544031
rs4802890
All
−0.4
−0.2
0.0
MR leave−one−out sensitivity analysis for
'ECH1' on 'Cardiac valvular disease'

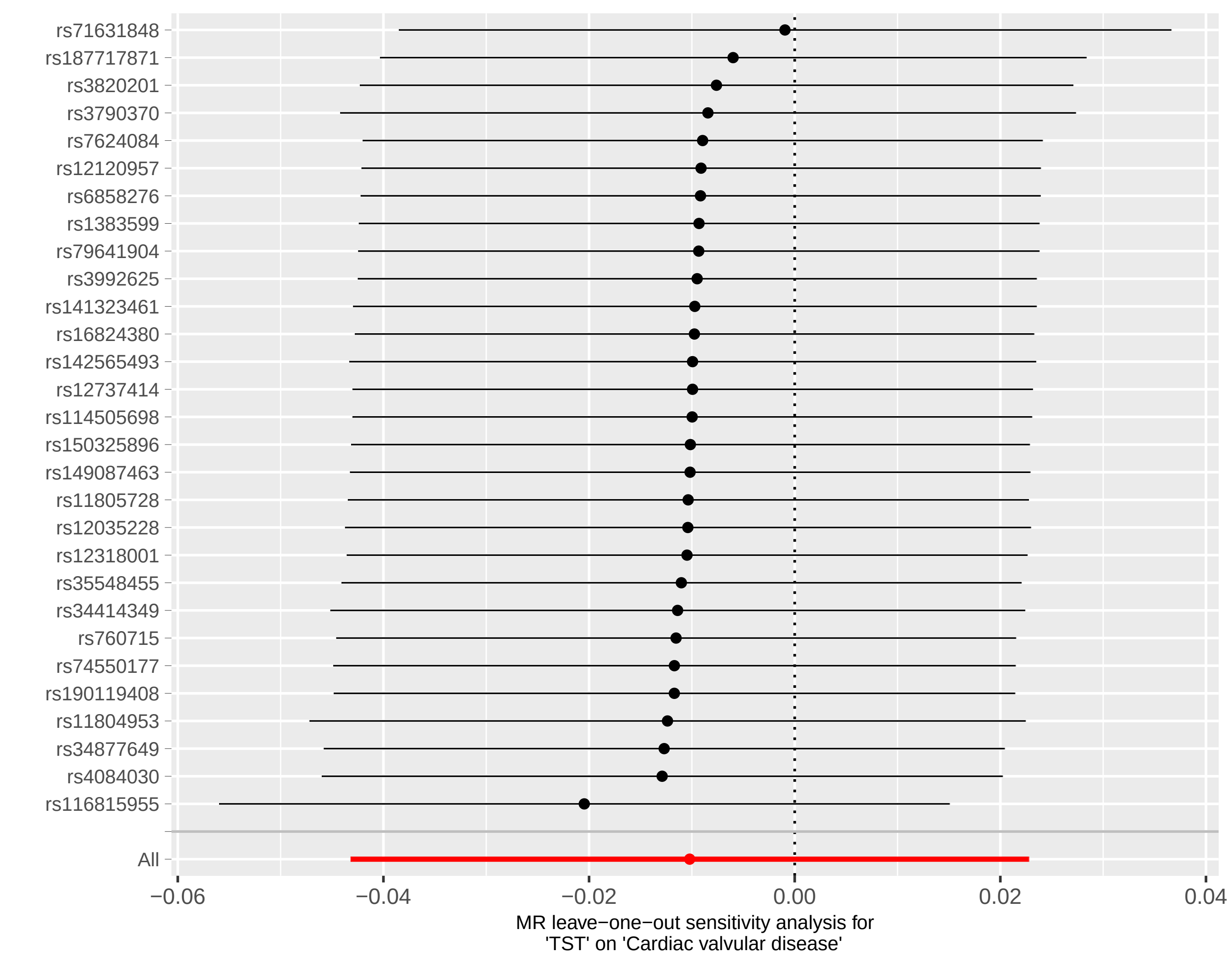

rs71631848
rs187717871
rs3820201
rs3790370
rs7624084
rs12120957
rs6858276
rs1383599
rs79641904
rs3992625
rs141323461
rs16824380
rs142565493
rs12737414
rs114505698
rs150325896
rs149087463
rs11805728
rs12035228
rs12318001
rs35548455
rs34414349
rs760715
rs74550177
rs190119408
rs11804953
rs34877649
rs4084030
rs116815955
All
−0.06
−0.04
−0.02
0.00
0.02
0.04
MR leave−one−out sensitivity analysis for
'TST' on 'Cardiac valvular disease'

Insufficient number of SNPs

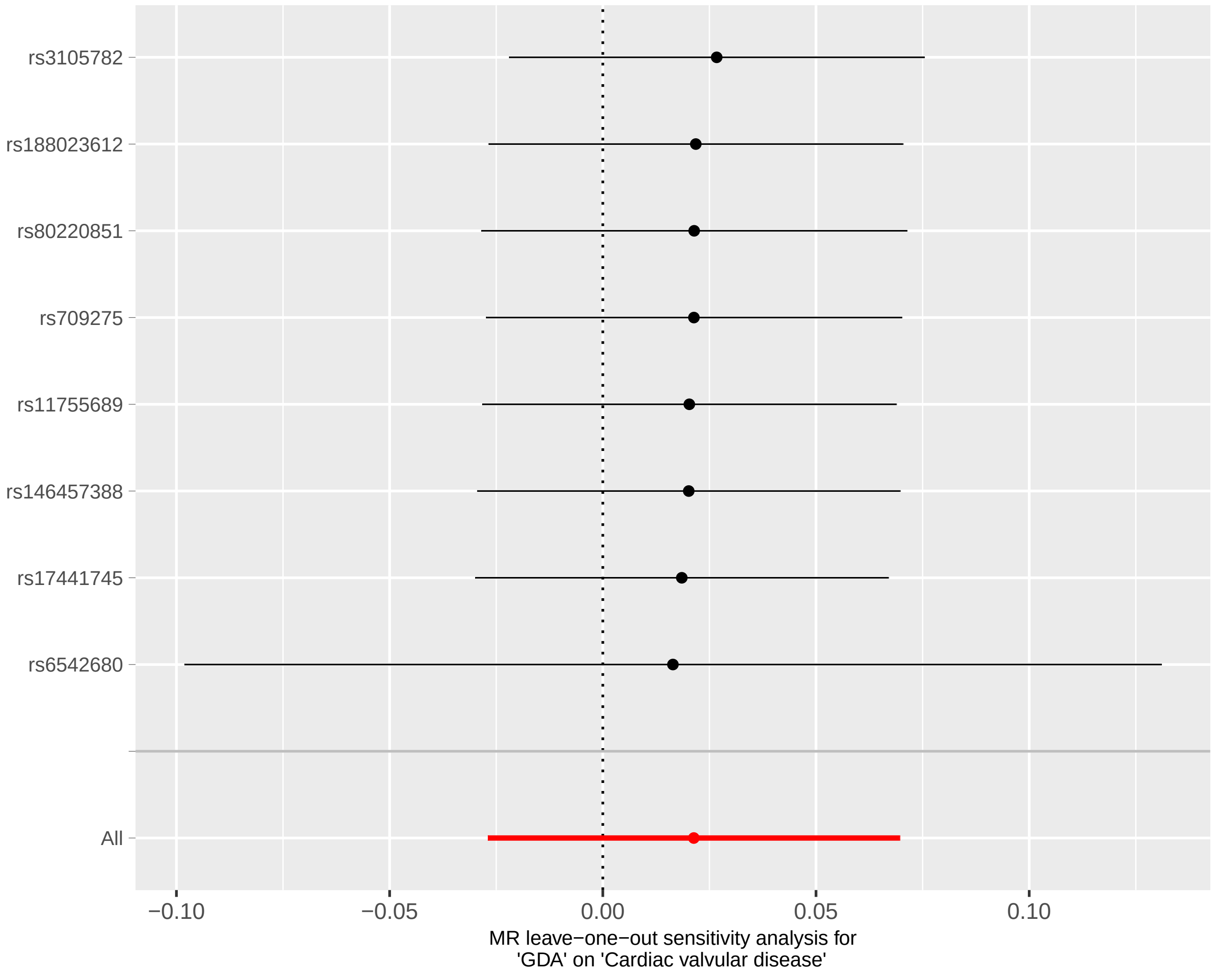

rs3105782
rs188023612
rs80220851
rs709275
rs11755689
rs146457388
rs17441745
rs6542680
All
−0.10
−0.05
0.00
0.05
0.10
MR leave−one−out sensitivity analysis for
'GDA' on 'Cardiac valvular disease'

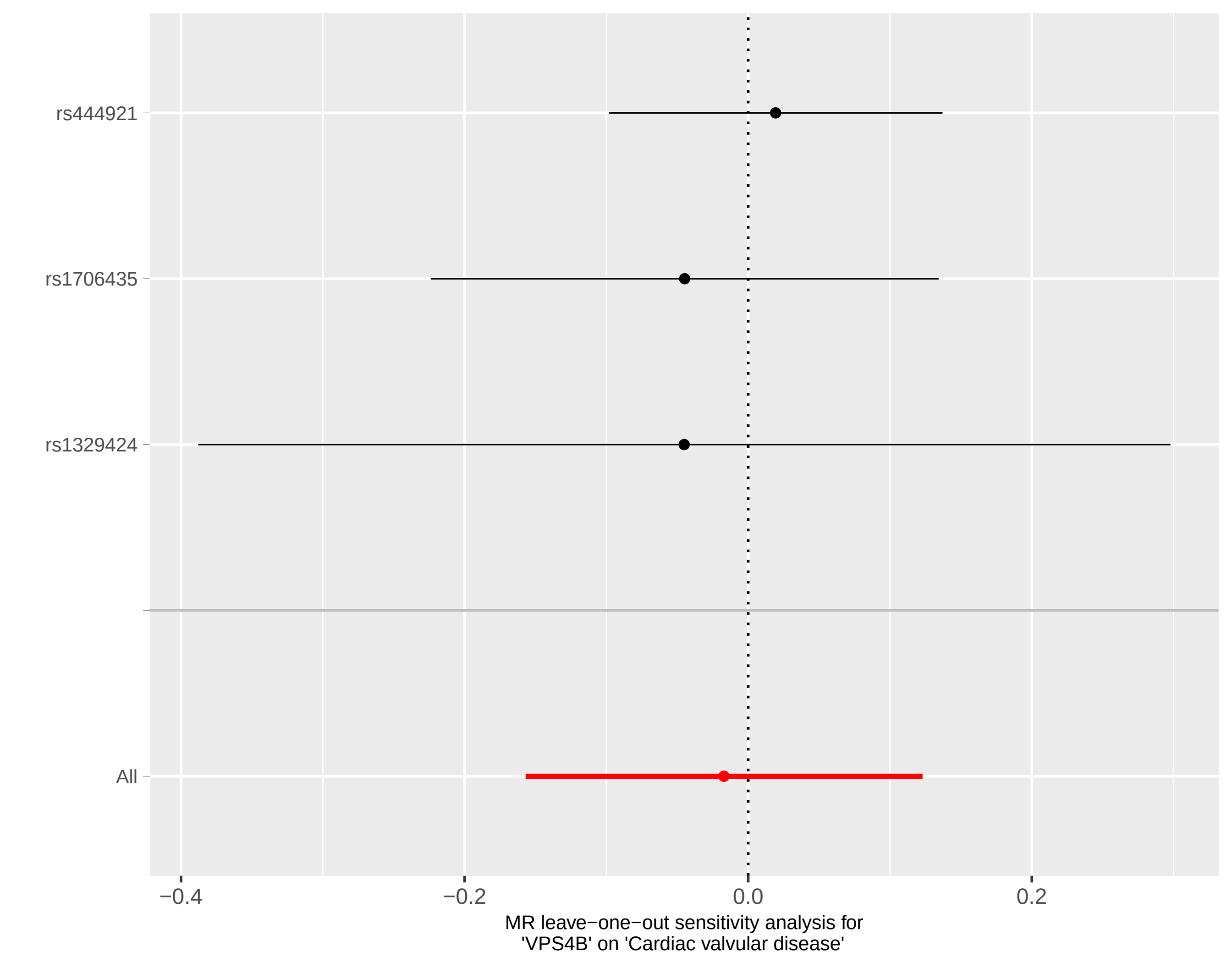
rs444921
rs1706435
rs1329424
All
−0.4
−0.2
0.0
0.2
MR leave−one−out sensitivity analysis for
'VPS4B' on 'Cardiac valvular disease'

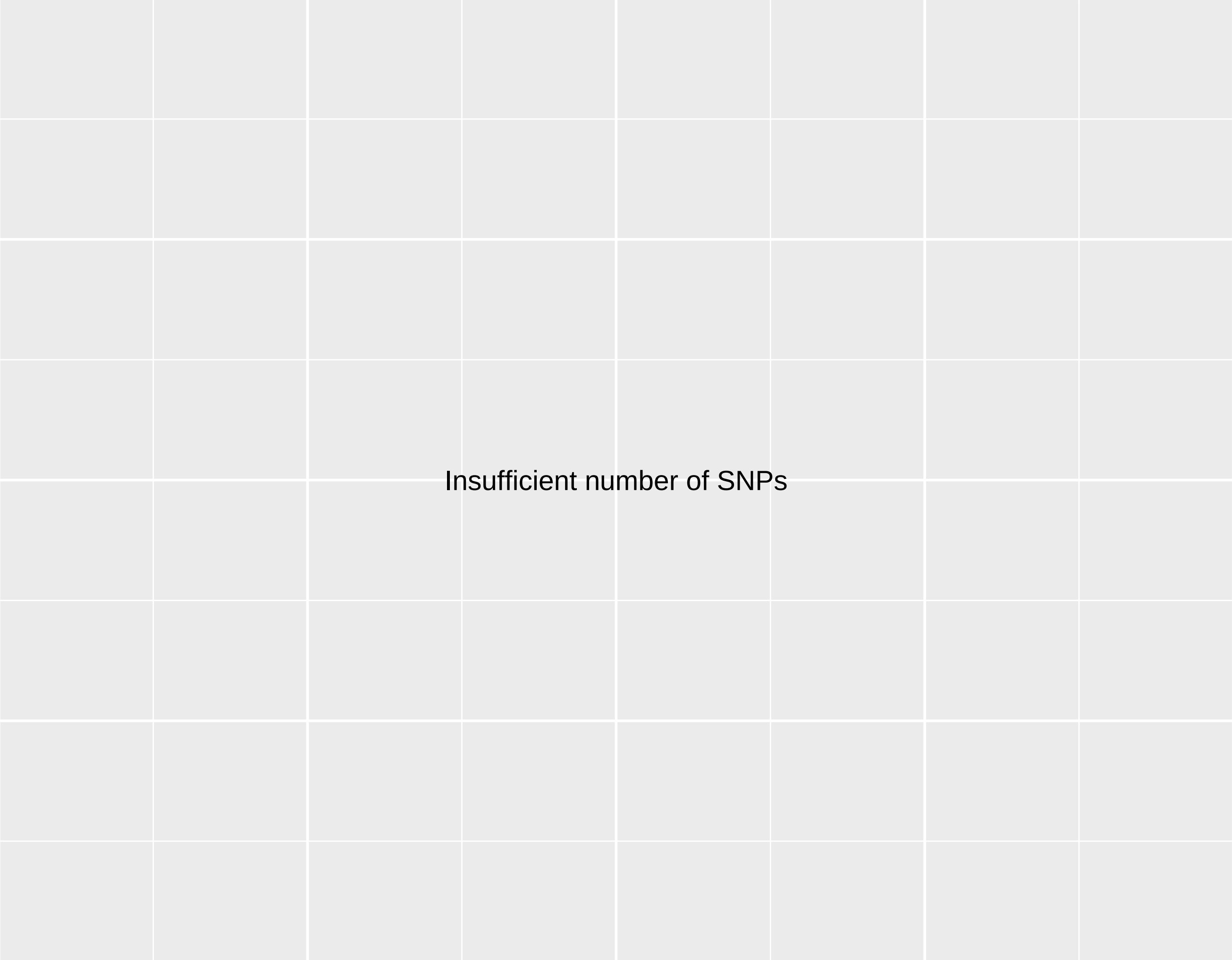
Insufficient number of SNPs

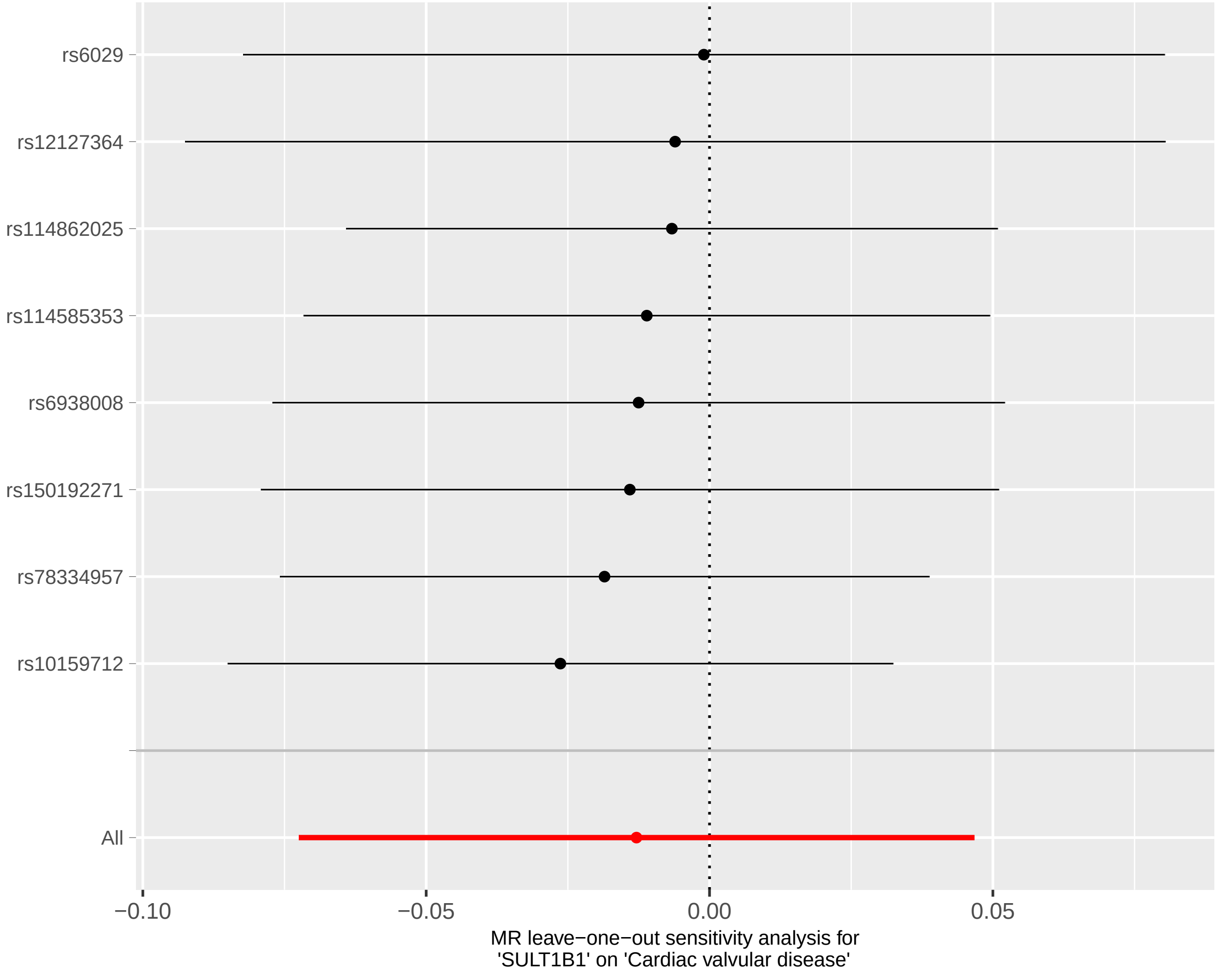

rs6029
rs12127364
rs114862025
rs114585353
rs6938008
rs150192271
rs78334957
rs10159712
All
−0.10
−0.05
0.00
0.05
MR leave−one−out sensitivity analysis for
'SULT1B1' on 'Cardiac valvular disease'

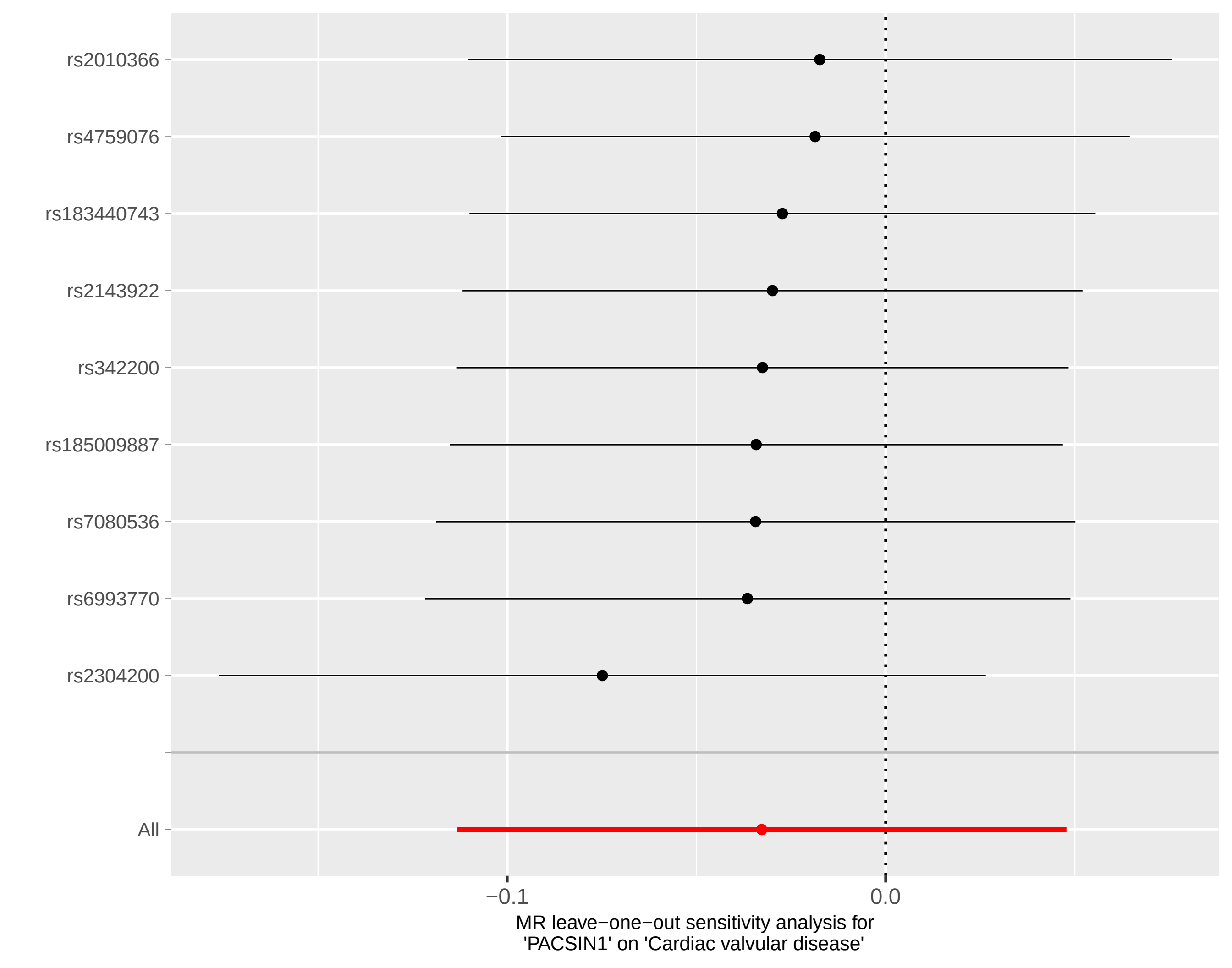
rs2010366
rs4759076
rs183440743
rs2143922
rs342200
rs185009887
rs7080536
rs6993770
rs2304200
All
−0.1
0.0
MR leave−one−out sensitivity analysis for
'PACSIN1' on 'Cardiac valvular disease'

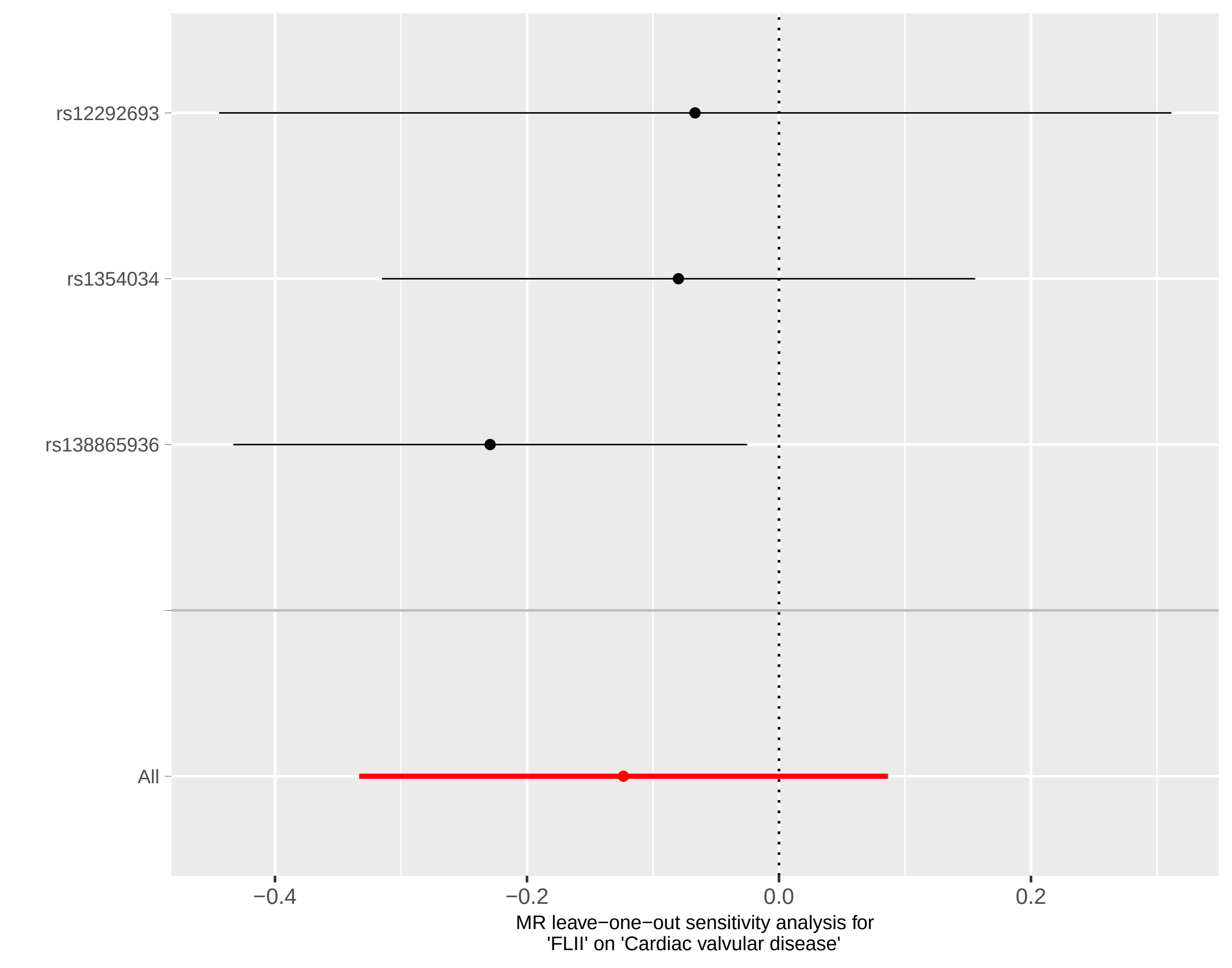
rs12292693
rs1354034
rs138865936
All
−0.4
−0.2
0.0
0.2
MR leave−one−out sensitivity analysis for
'FLII' on 'Cardiac valvular disease'

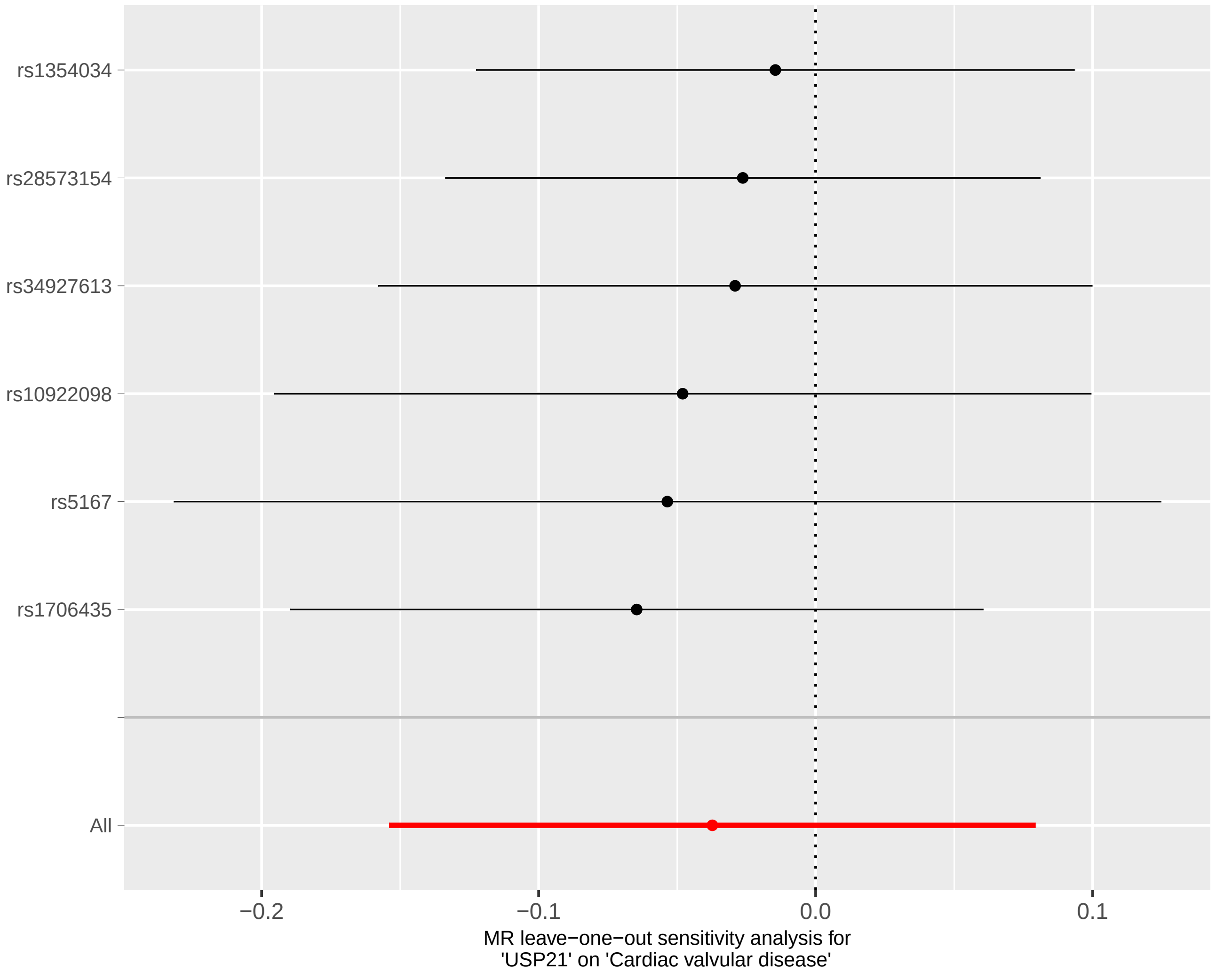
rs1354034
rs28573154
rs34927613
rs10922098
rs5167
rs1706435
All
−0.2
−0.1
0.0
0.1
MR leave−one−out sensitivity analysis for
'USP21' on 'Cardiac valvular disease'

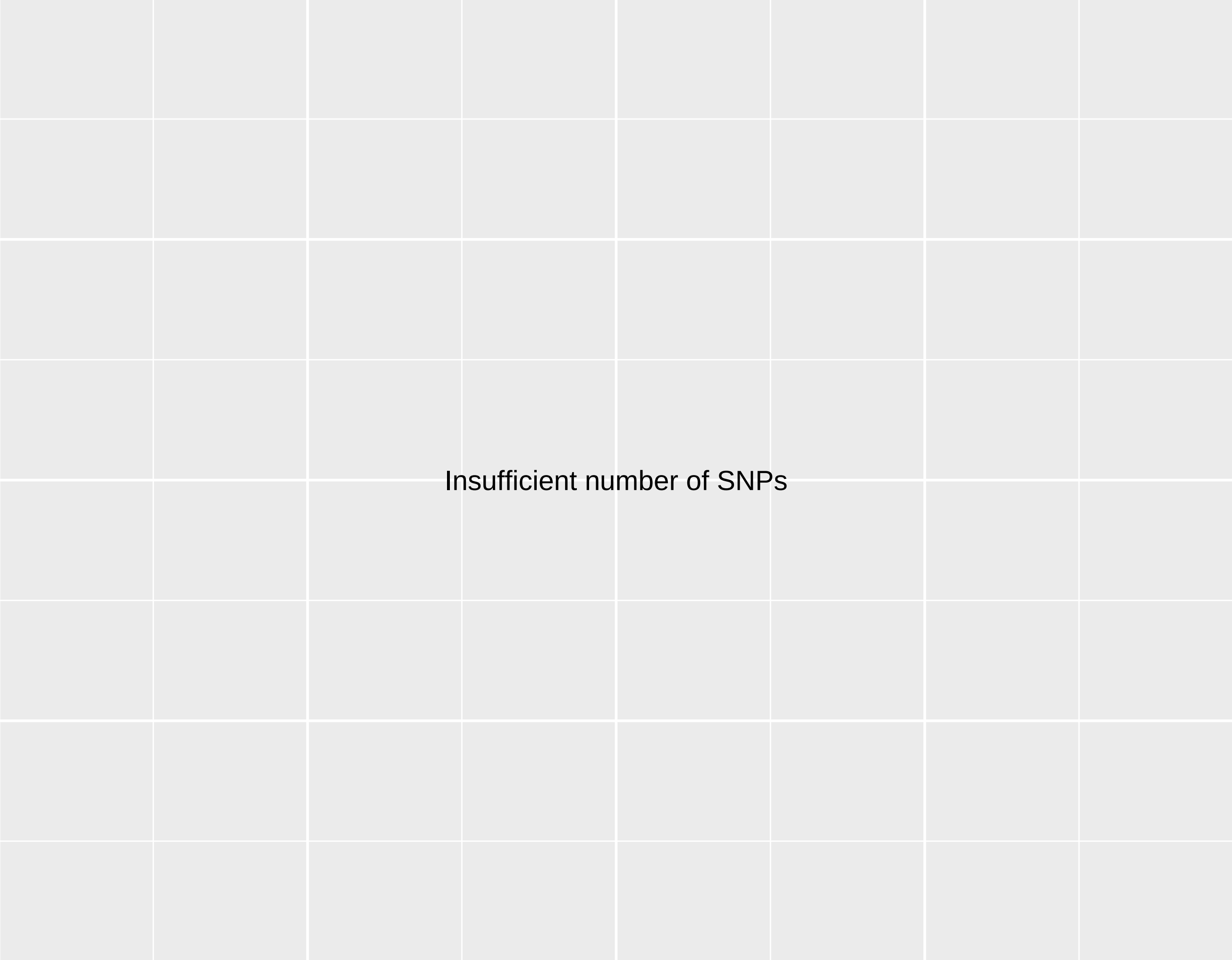
Insufficient number of SNPs

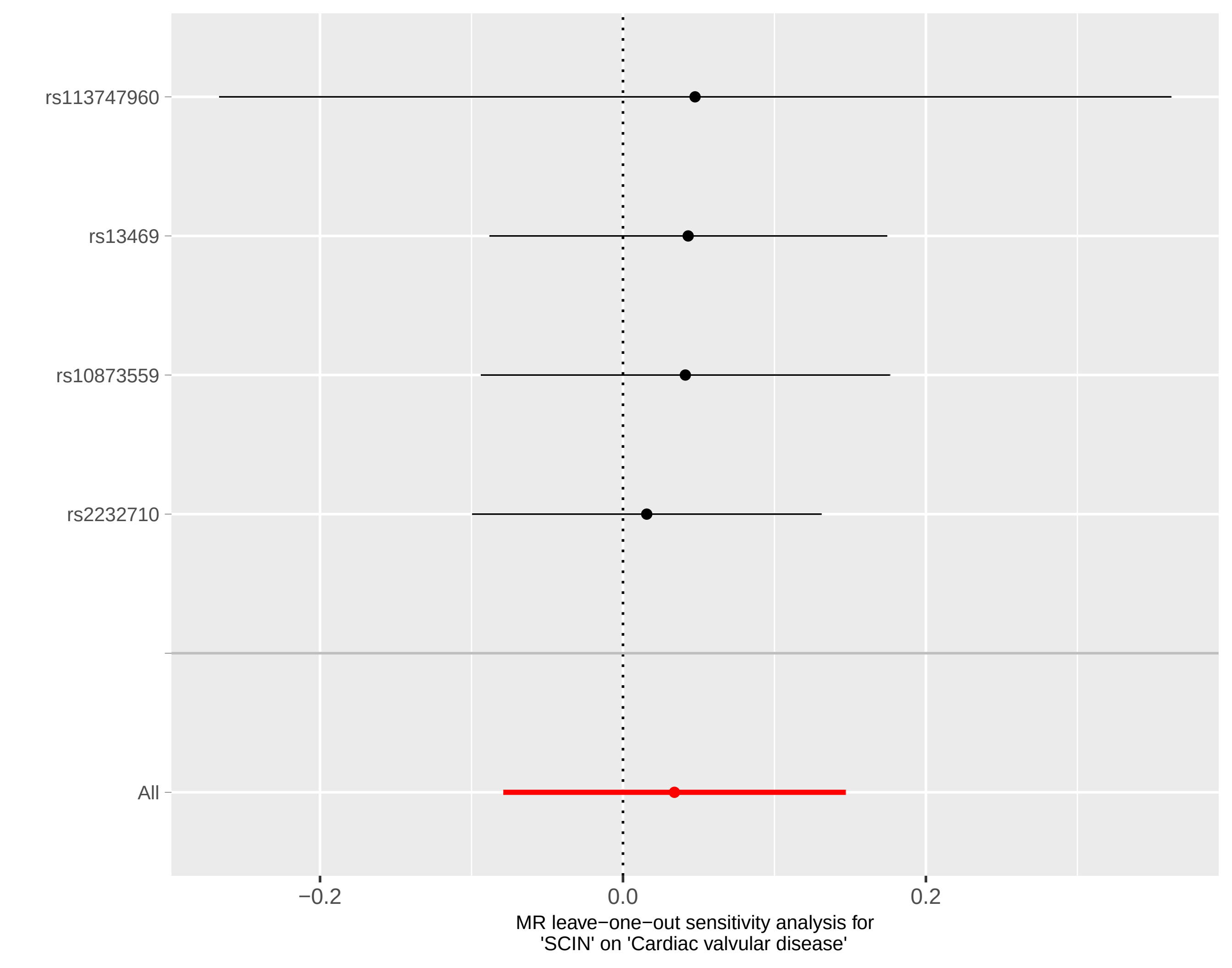
rs113747960
rs13469
rs10873559
rs2232710
All
−0.2
0.0
0.2
MR leave−one−out sensitivity analysis for
'SCIN' on 'Cardiac valvular disease'

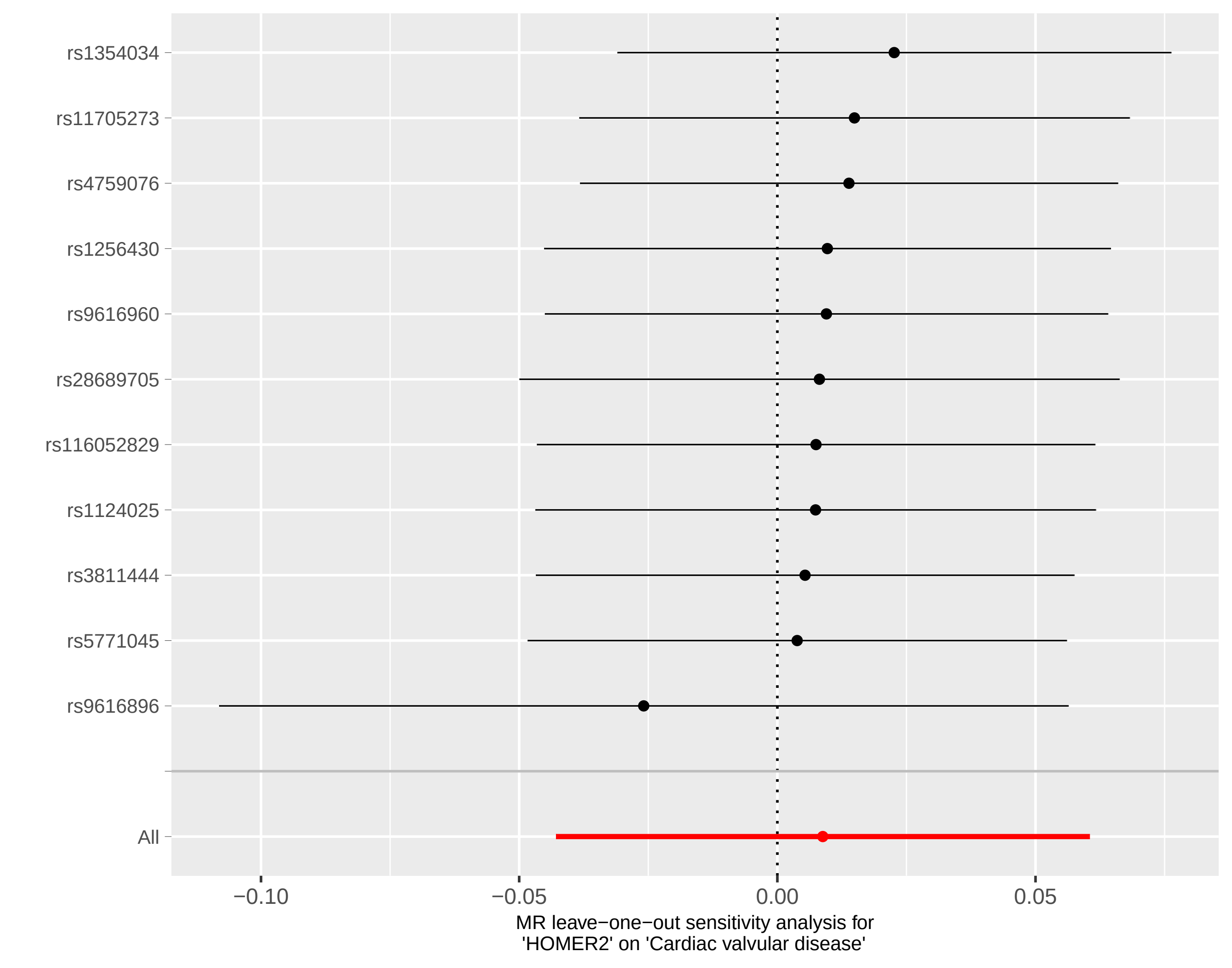
rs1354034
rs11705273
rs4759076
rs1256430
rs9616960
rs28689705
rs116052829
rs1124025
rs3811444
rs5771045
rs9616896
All
−0.10
−0.05
0.00
0.05
MR leave−one−out sensitivity analysis for
'HOMER2' on 'Cardiac valvular disease'

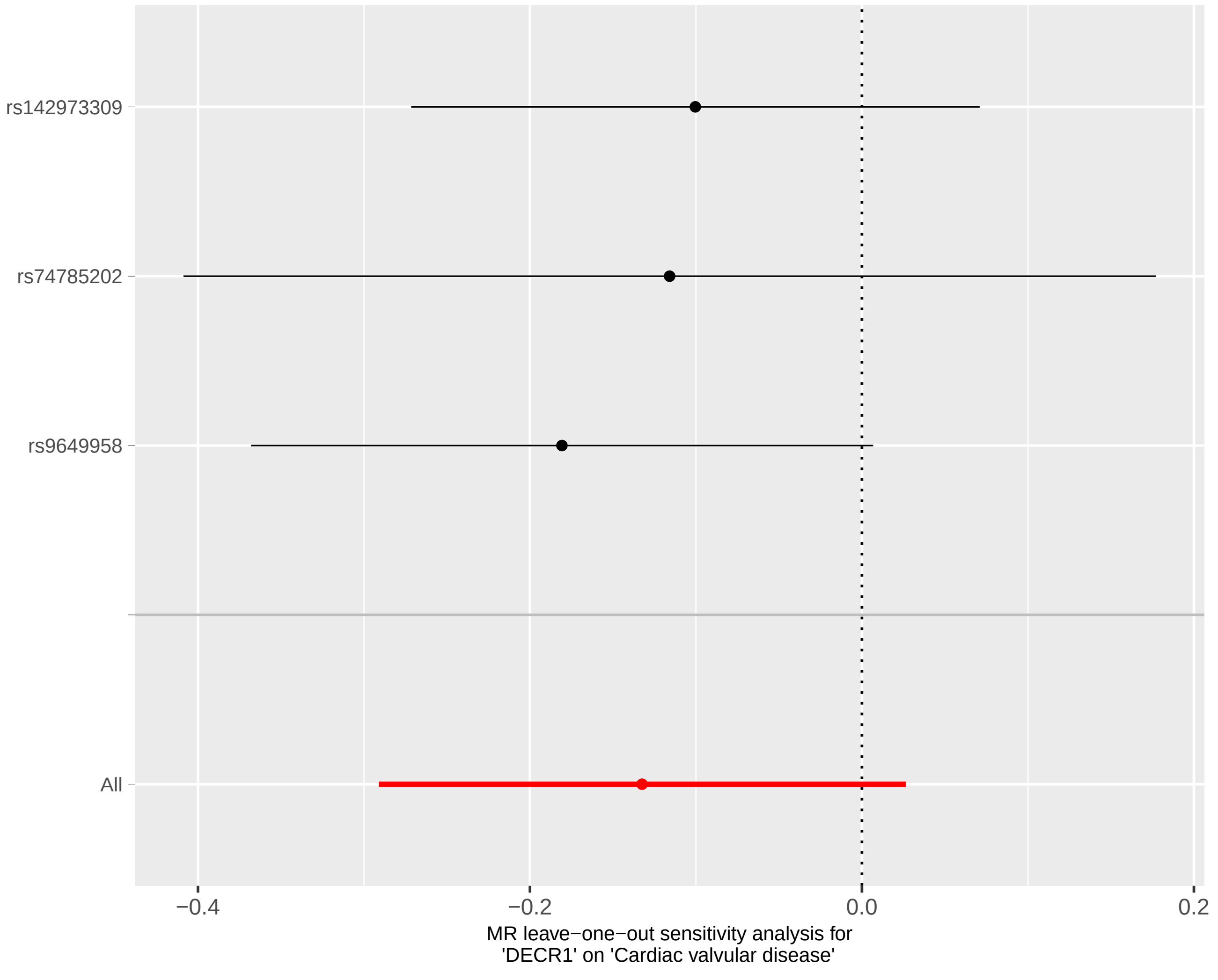
rs142973309
rs74785202
rs9649958
All
−0.4
−0.2
0.0
0.2
MR leave−one−out sensitivity analysis for
'DECR1' on 'Cardiac valvular disease'

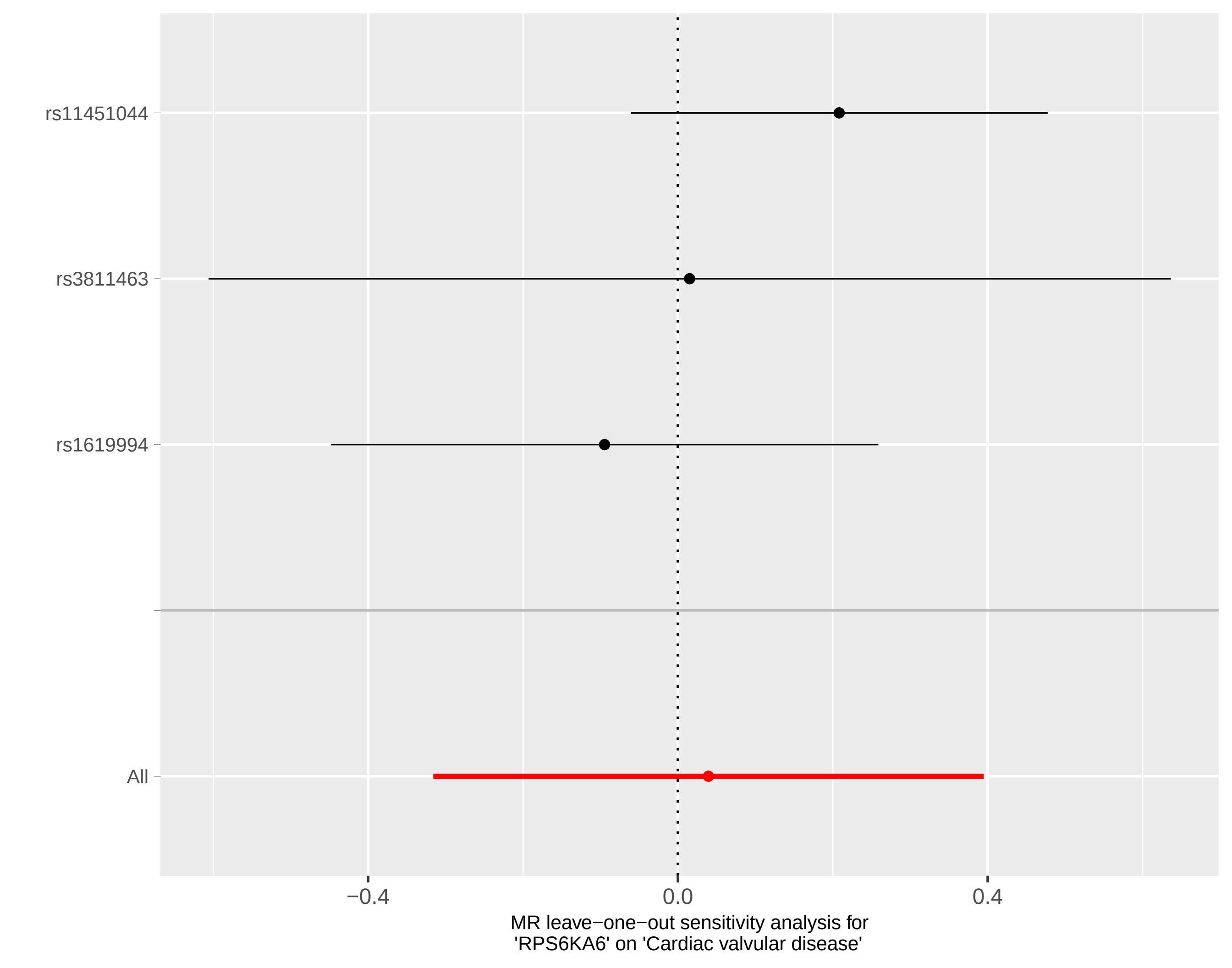
rs11451044
rs3811463
rs1619994
All
−0.4
0.0
0.4
MR leave−one−out sensitivity analysis for
'RPS6KA6' on 'Cardiac valvular disease'

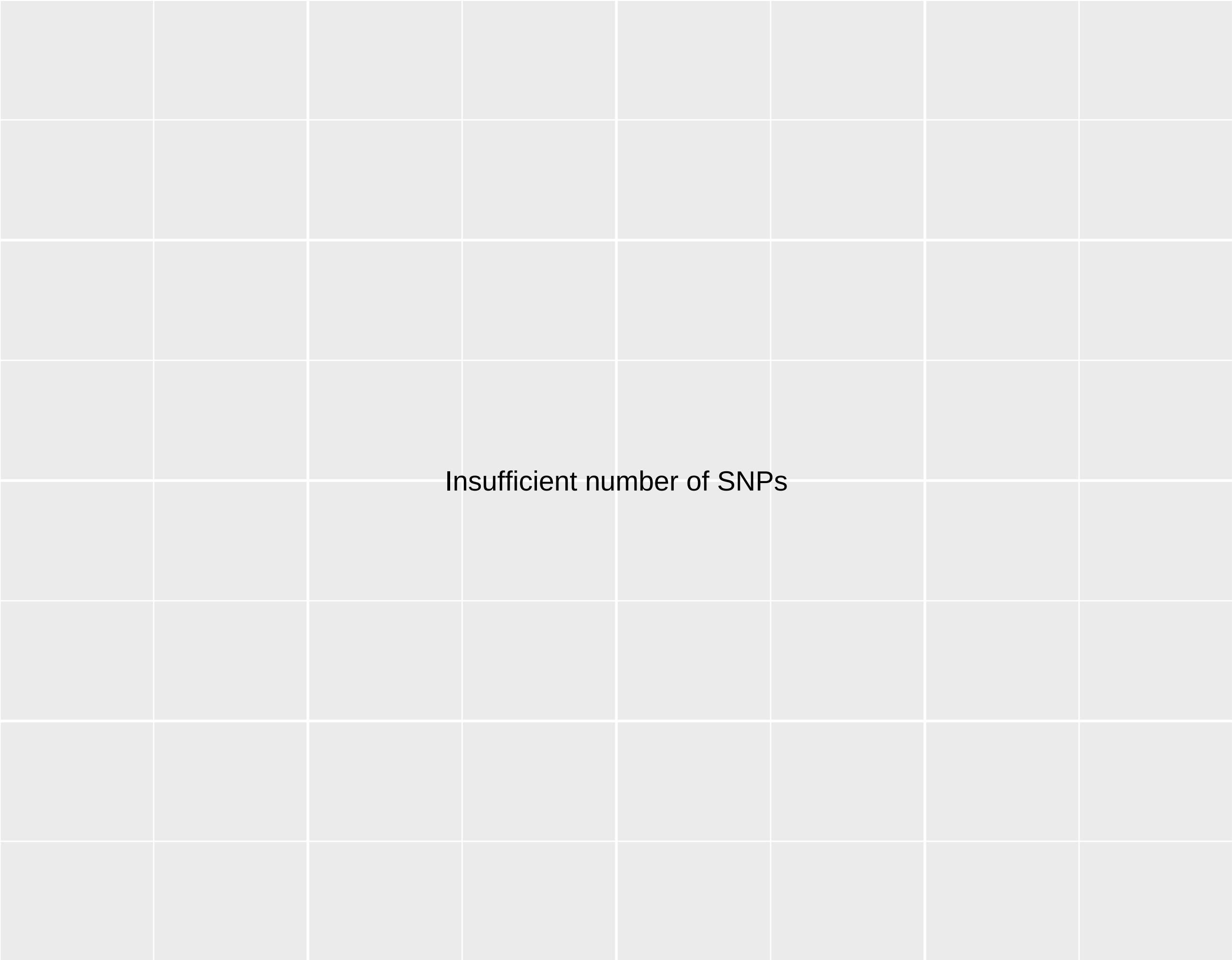
Insufficient number of SNPs

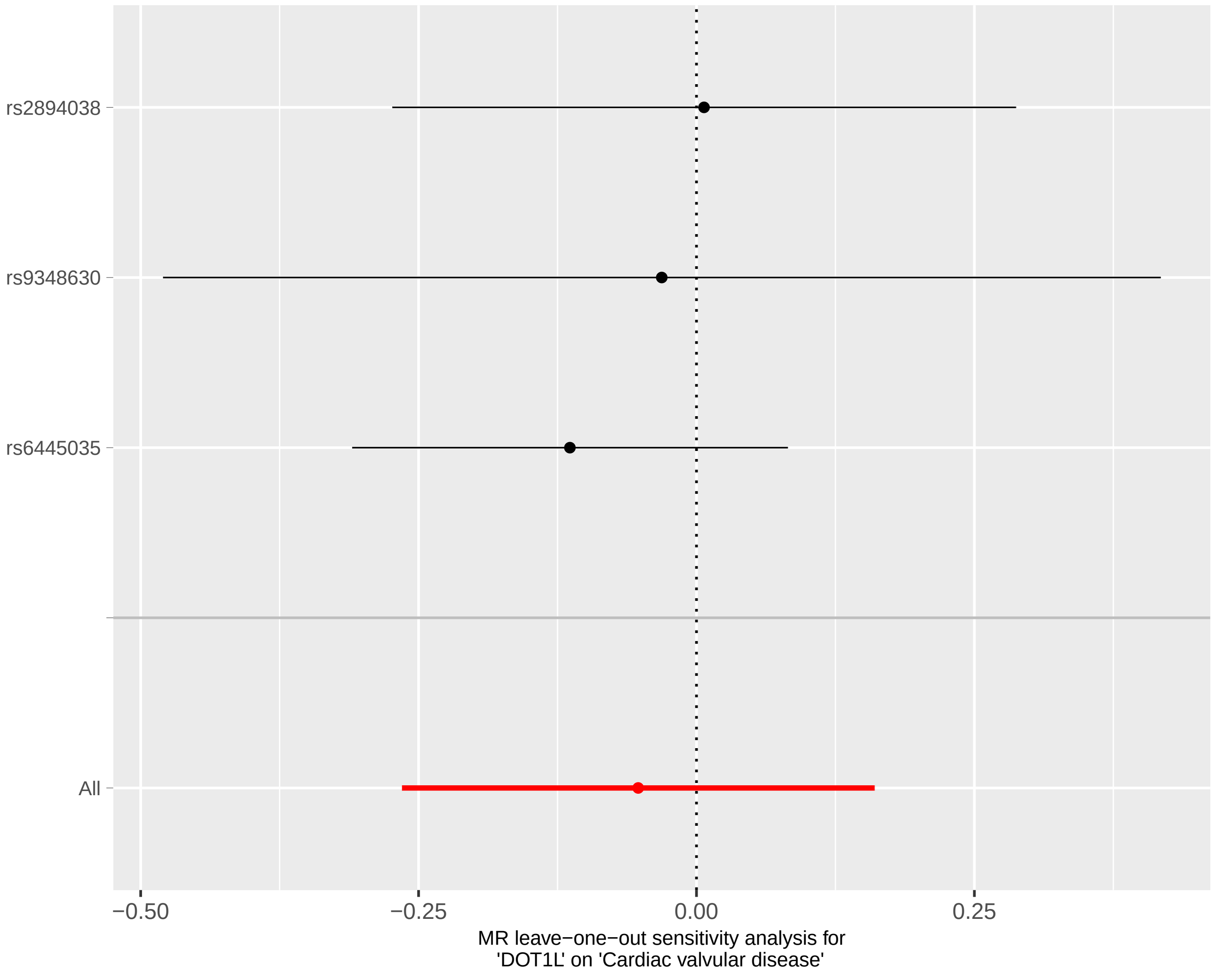

rs2894038
rs9348630
rs6445035
All
−0.50
−0.25
0.00
0.25
MR leave−one−out sensitivity analysis for
'DOT1L' on 'Cardiac valvular disease'

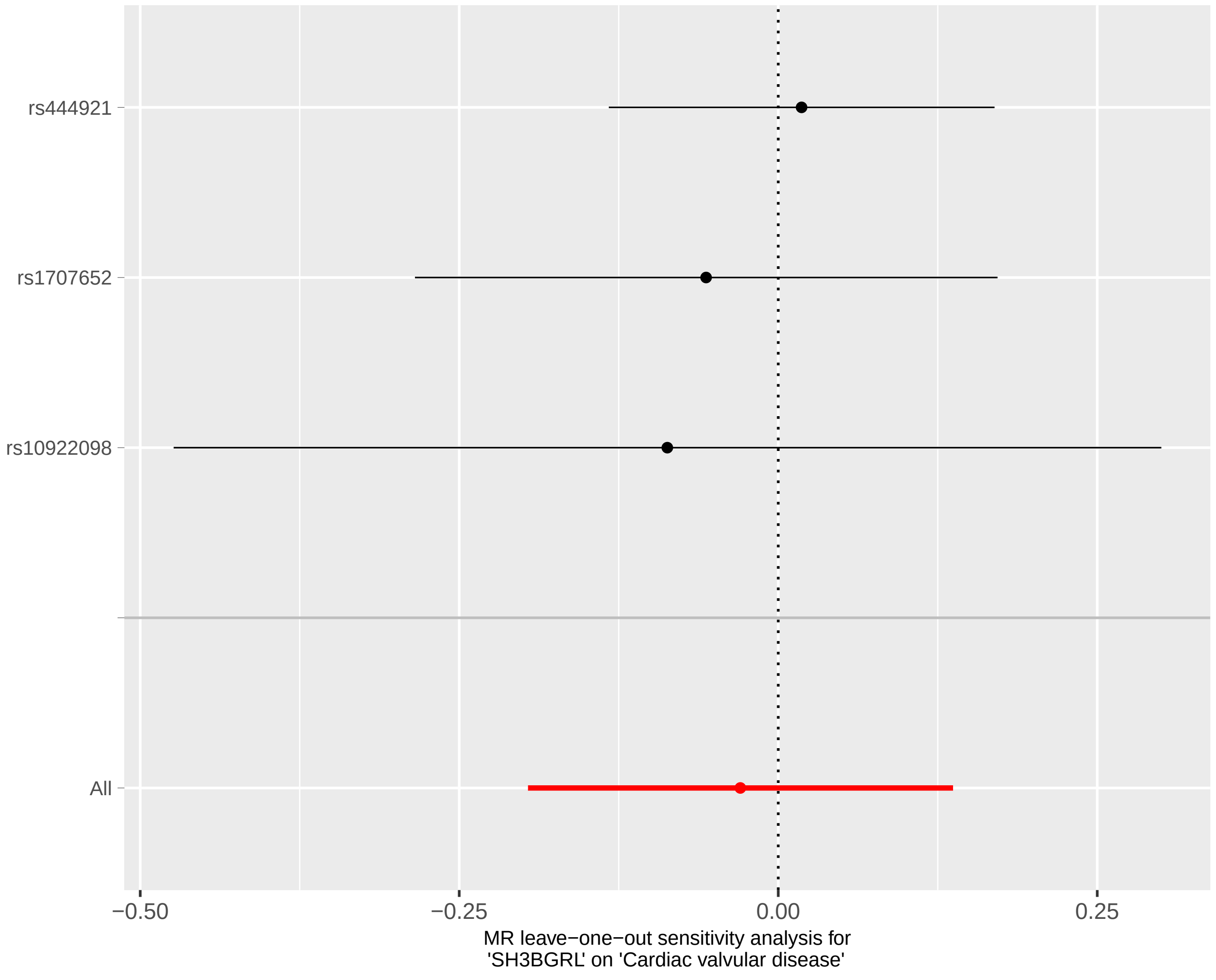
rs444921
rs1707652
rs10922098
All
−0.50
−0.25
0.00
0.25
MR leave−one−out sensitivity analysis for
'SH3BGRL' on 'Cardiac valvular disease'

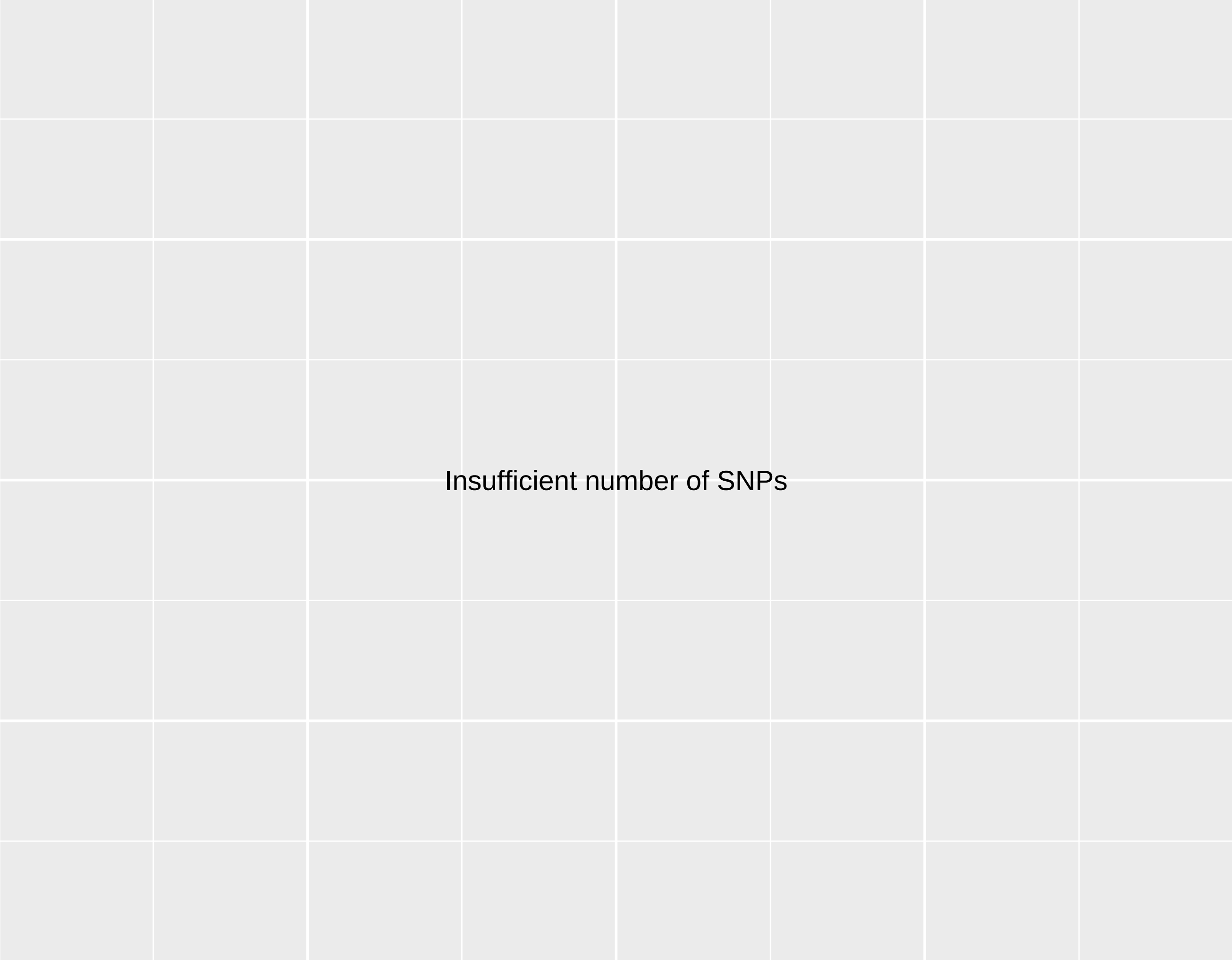
Insufficient number of SNPs

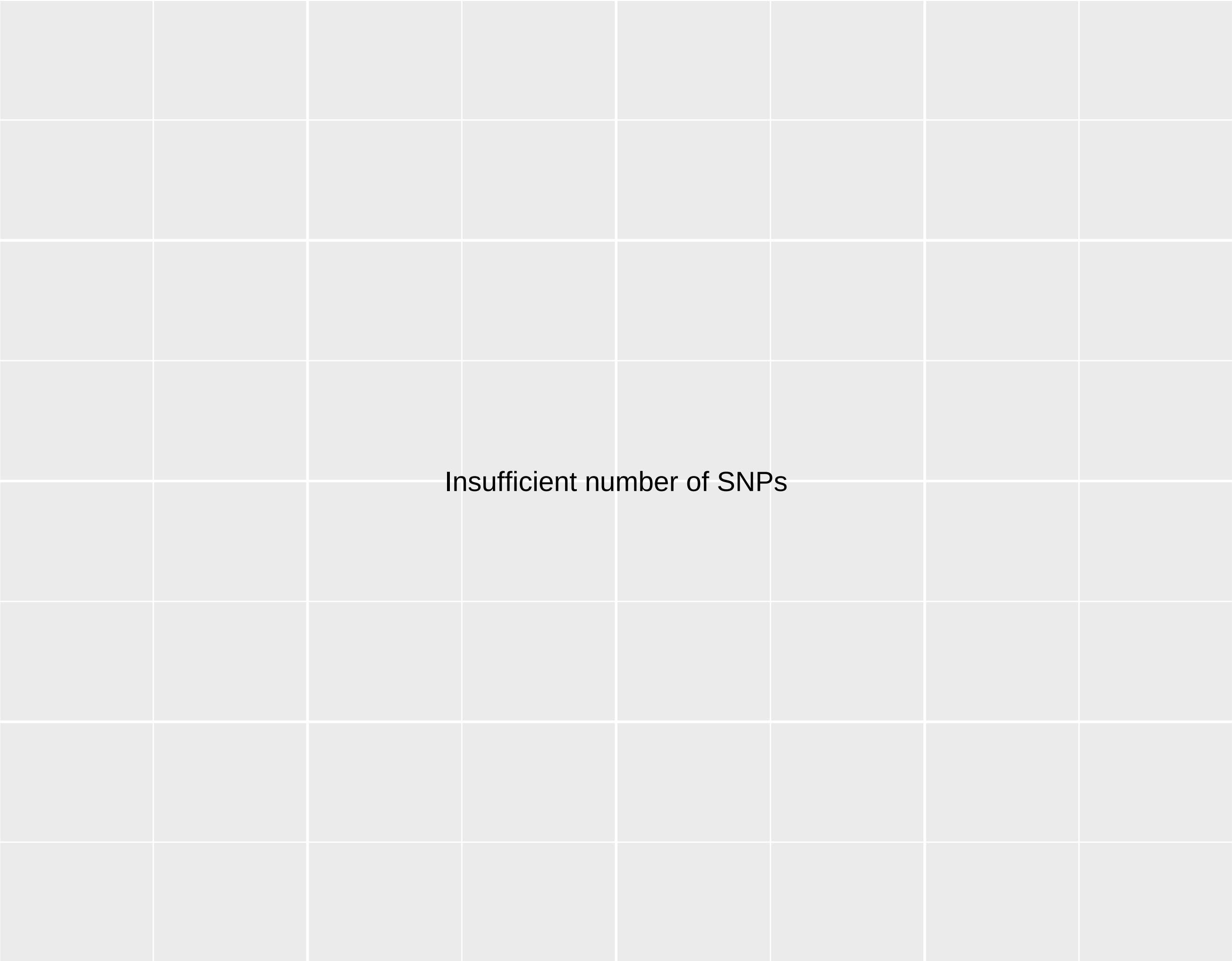
Insufficient number of SNPs

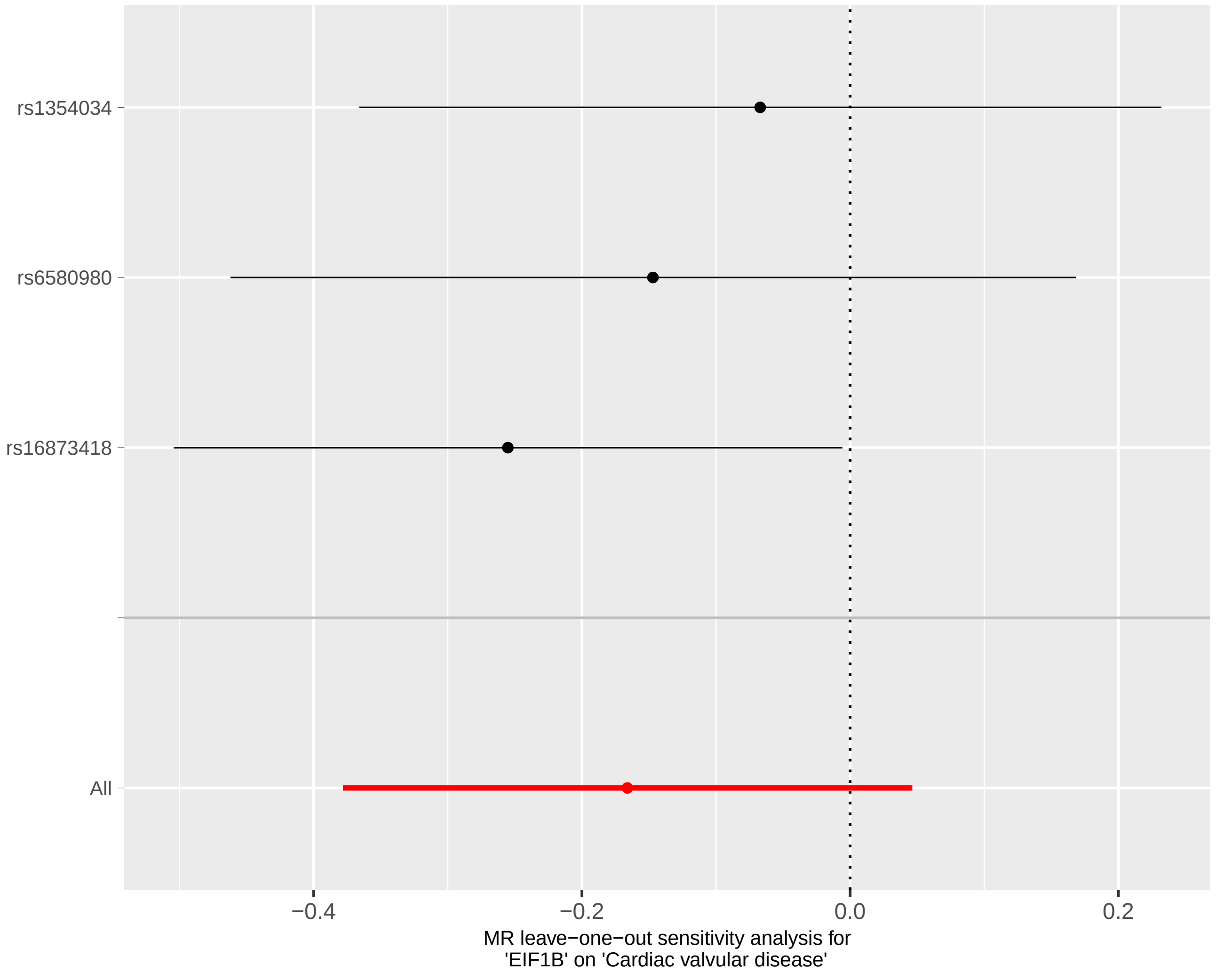
rs1354034
rs6580980
rs16873418
All
−0.4
−0.2
0.0
0.2
MR leave−one−out sensitivity analysis for
'EIF1B' on 'Cardiac valvular disease'

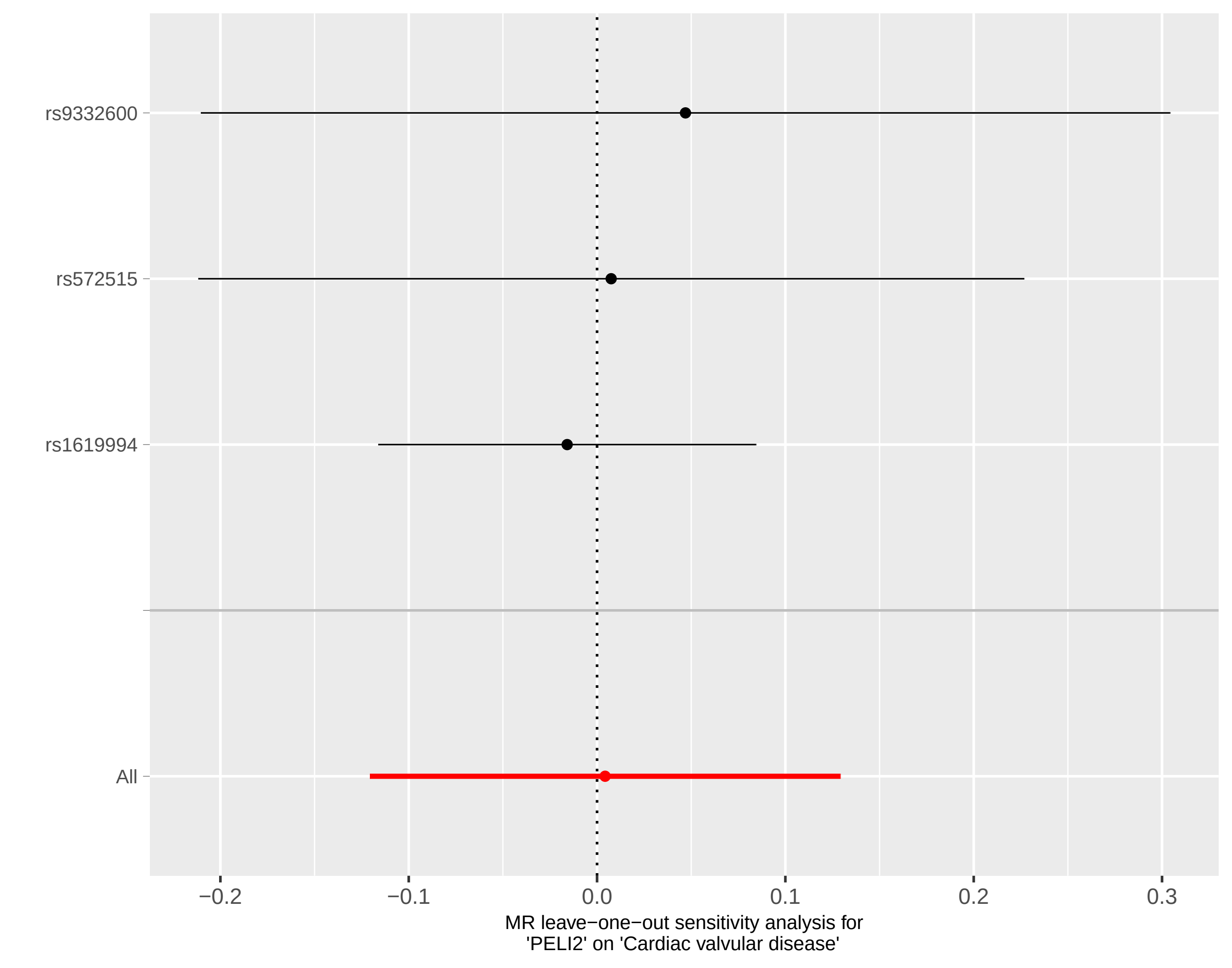
rs9332600
rs572515
rs1619994
All
−0.2
−0.1
0.0
0.1
0.2
0.3
MR leave−one−out sensitivity analysis for
'PELI2' on 'Cardiac valvular disease'

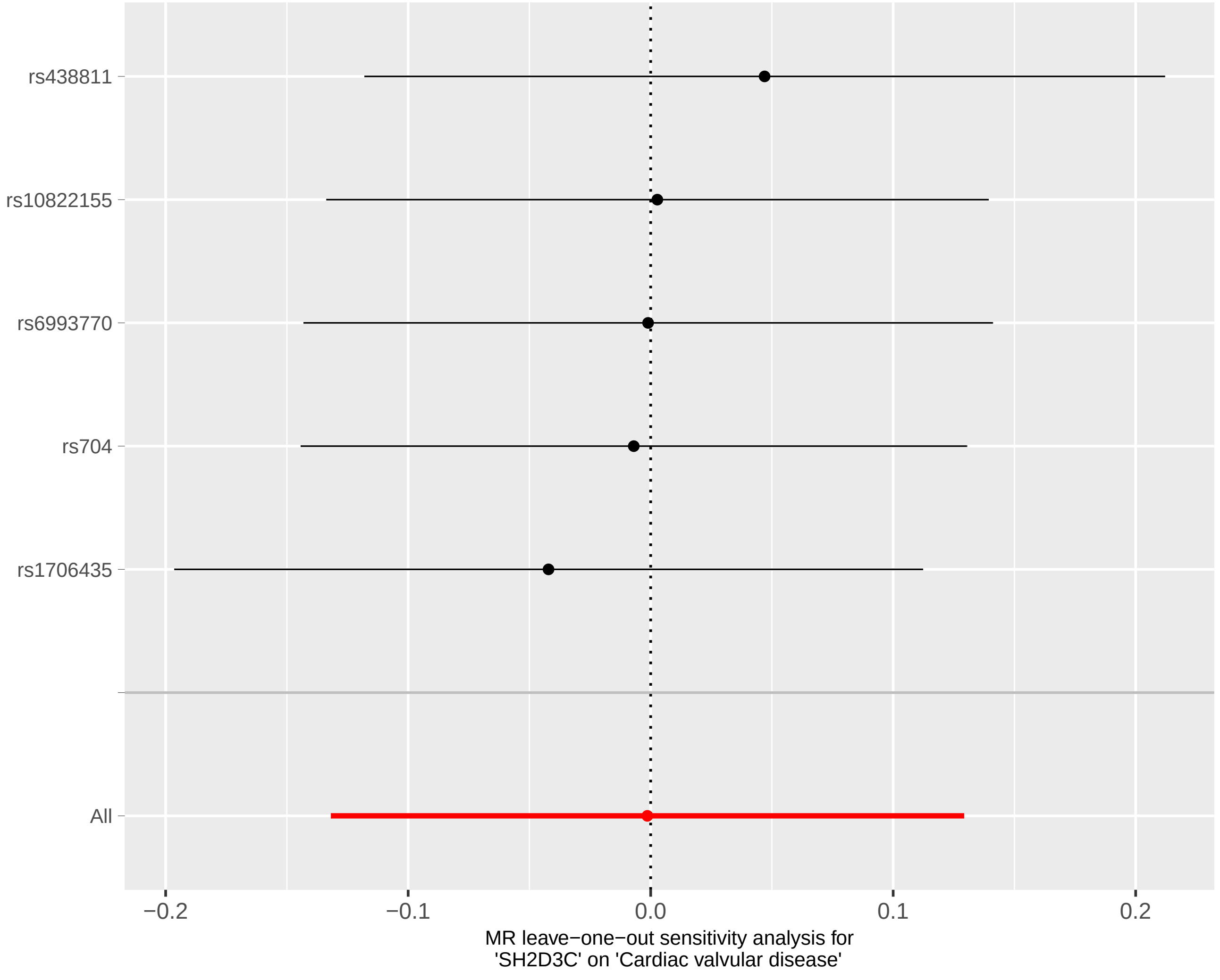
rs438811
rs10822155
rs6993770
rs704
rs1706435
All
−0.2
−0.1
0.0
0.1
0.2
MR leave−one−out sensitivity analysis for
'SH2D3C' on 'Cardiac valvular disease'

Insufficient number of SNPs

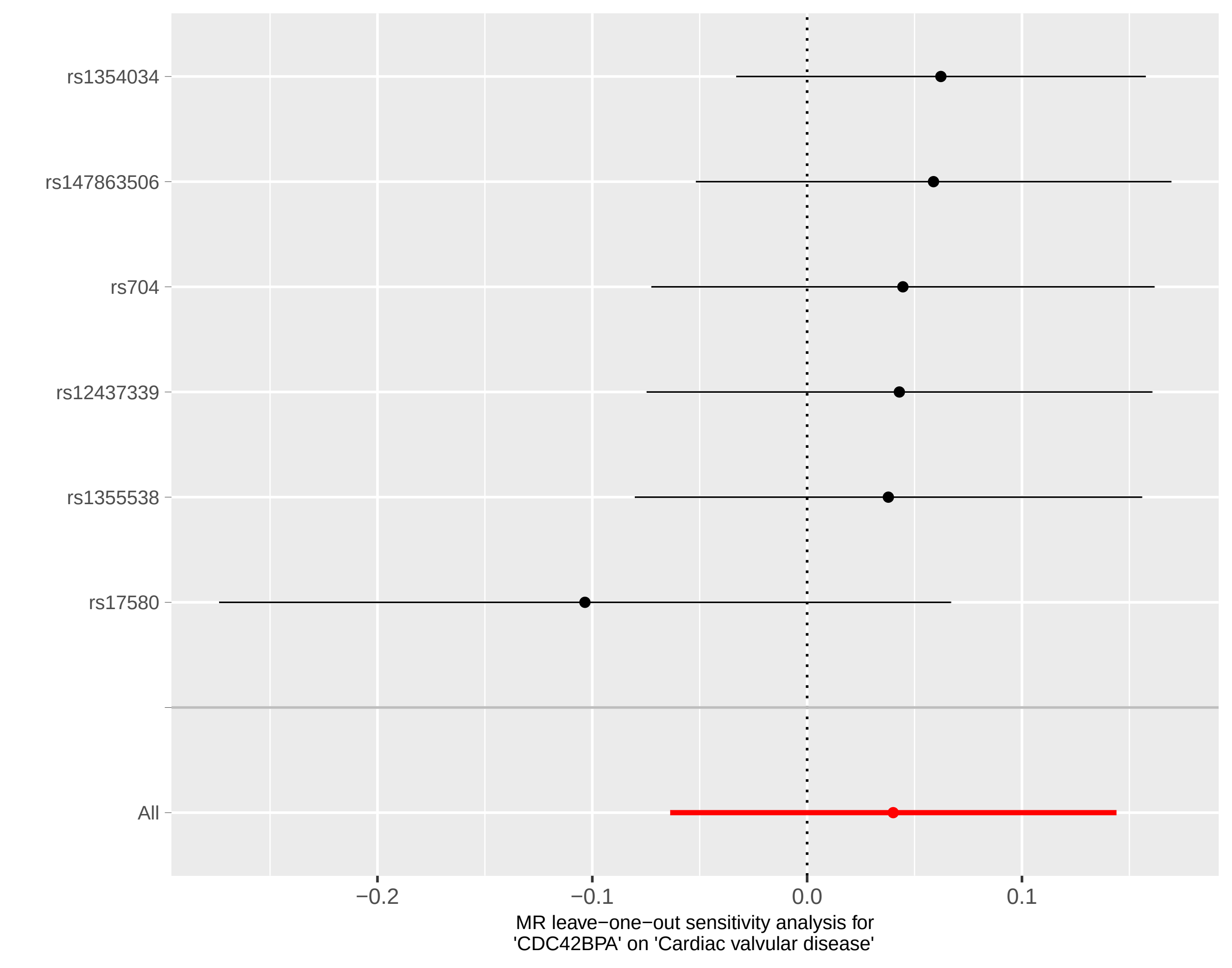
rs1354034
rs147863506
rs704
rs12437339
rs1355538
rs17580
All
−0.2
−0.1
0.0
0.1
MR leave−one−out sensitivity analysis for
'CDC42BPA' on 'Cardiac valvular disease'

Insufficient number of SNPs

Insufficient number of SNPs

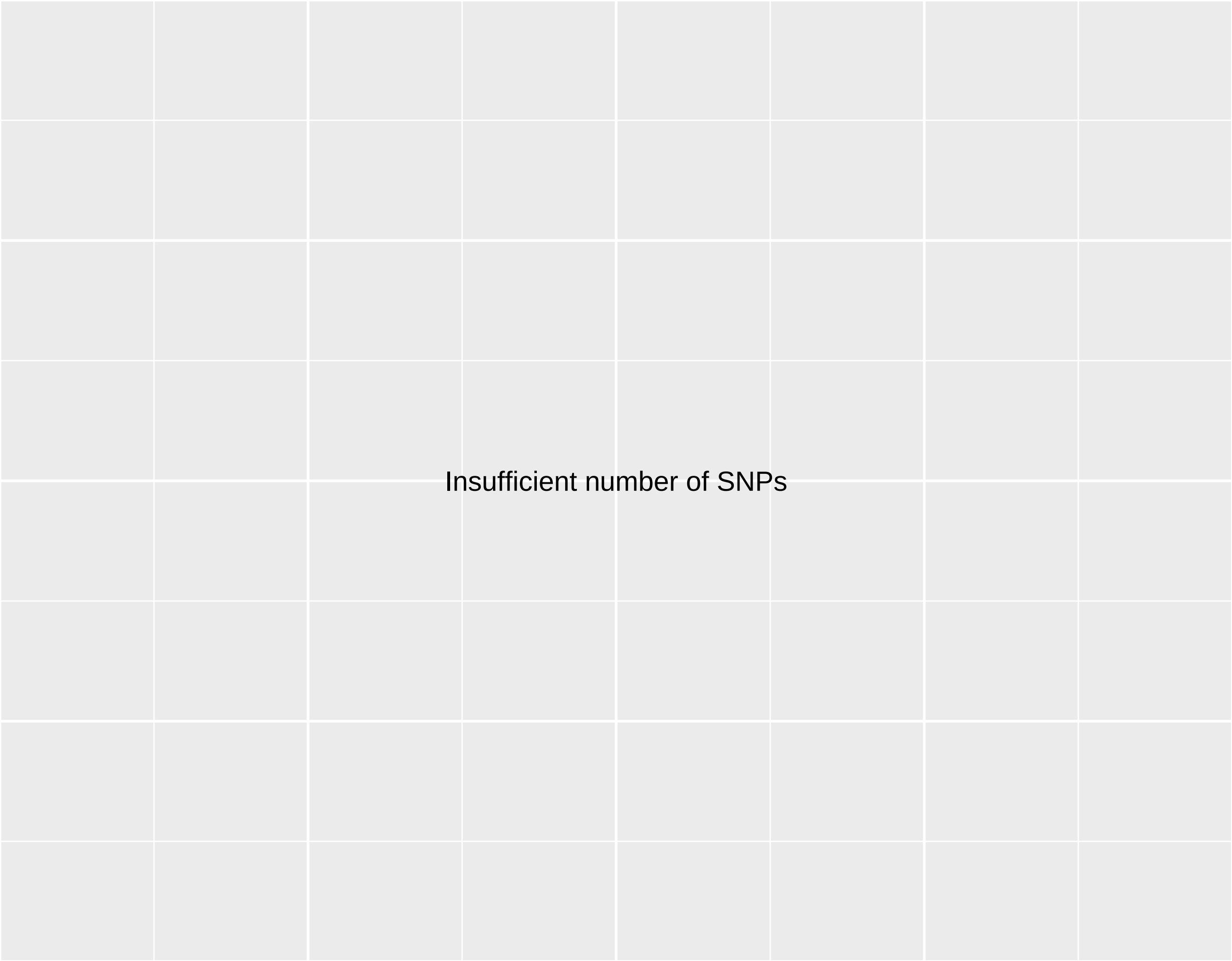
Insufficient number of SNPs

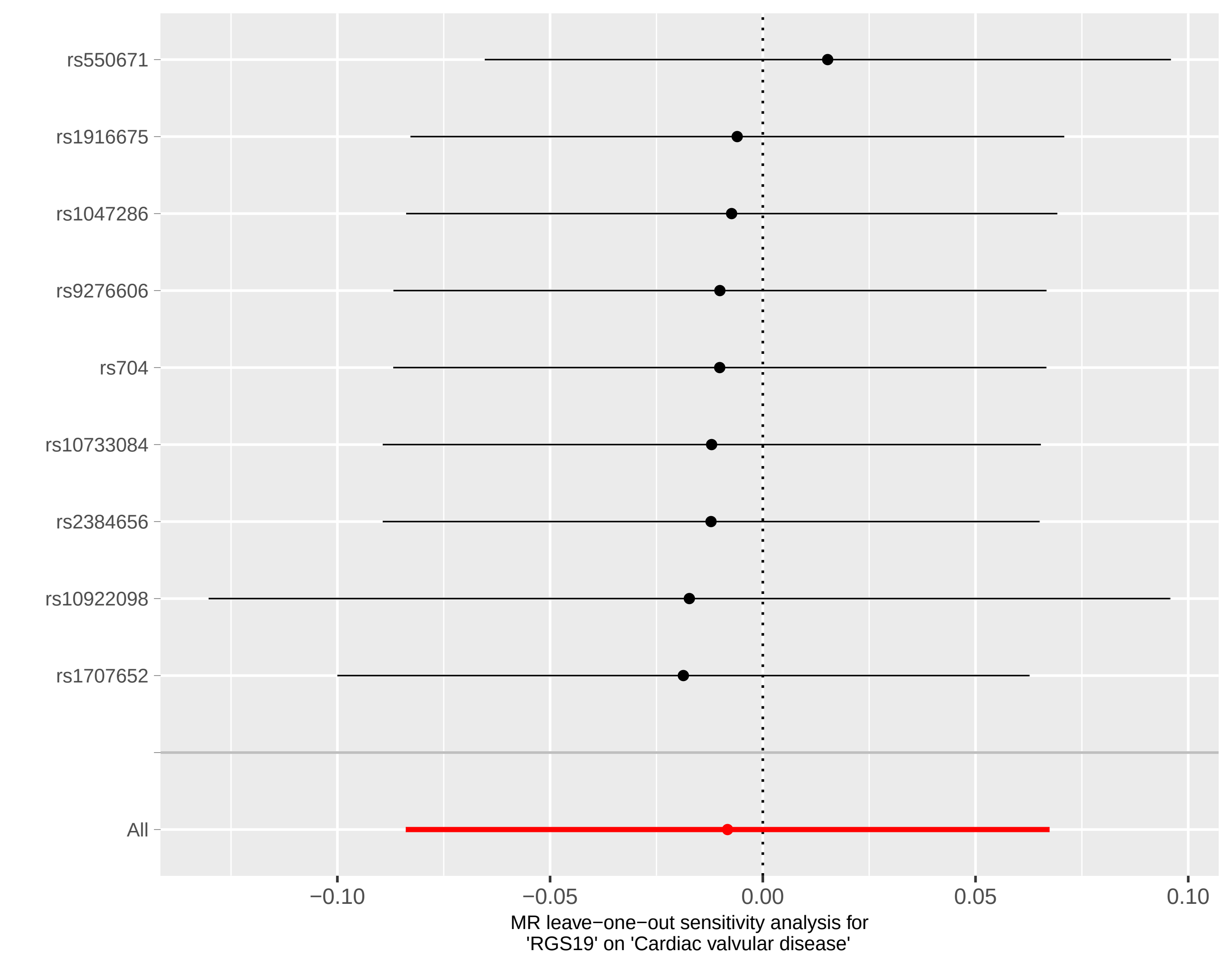
rs550671
rs1916675
rs1047286
rs9276606
rs704
rs10733084
rs2384656
rs10922098
rs1707652
All
−0.10
−0.05
0.00
0.05
0.10
MR leave−one−out sensitivity analysis for
'RGS19' on 'Cardiac valvular disease'

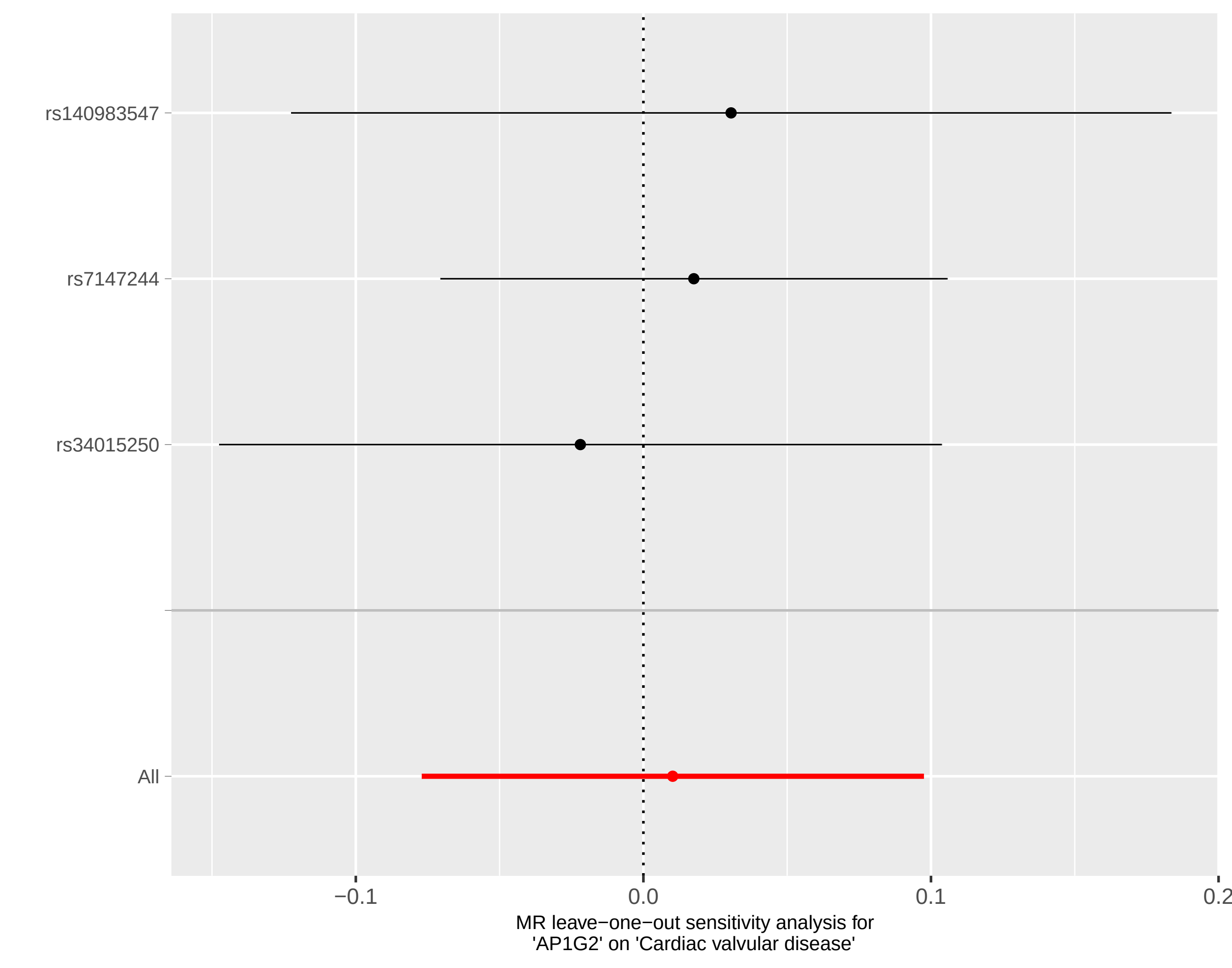

rs140983547
rs7147244
rs34015250
All
−0.1
0.0
0.1
0.2
MR leave−one−out sensitivity analysis for
'AP1G2' on 'Cardiac valvular disease'

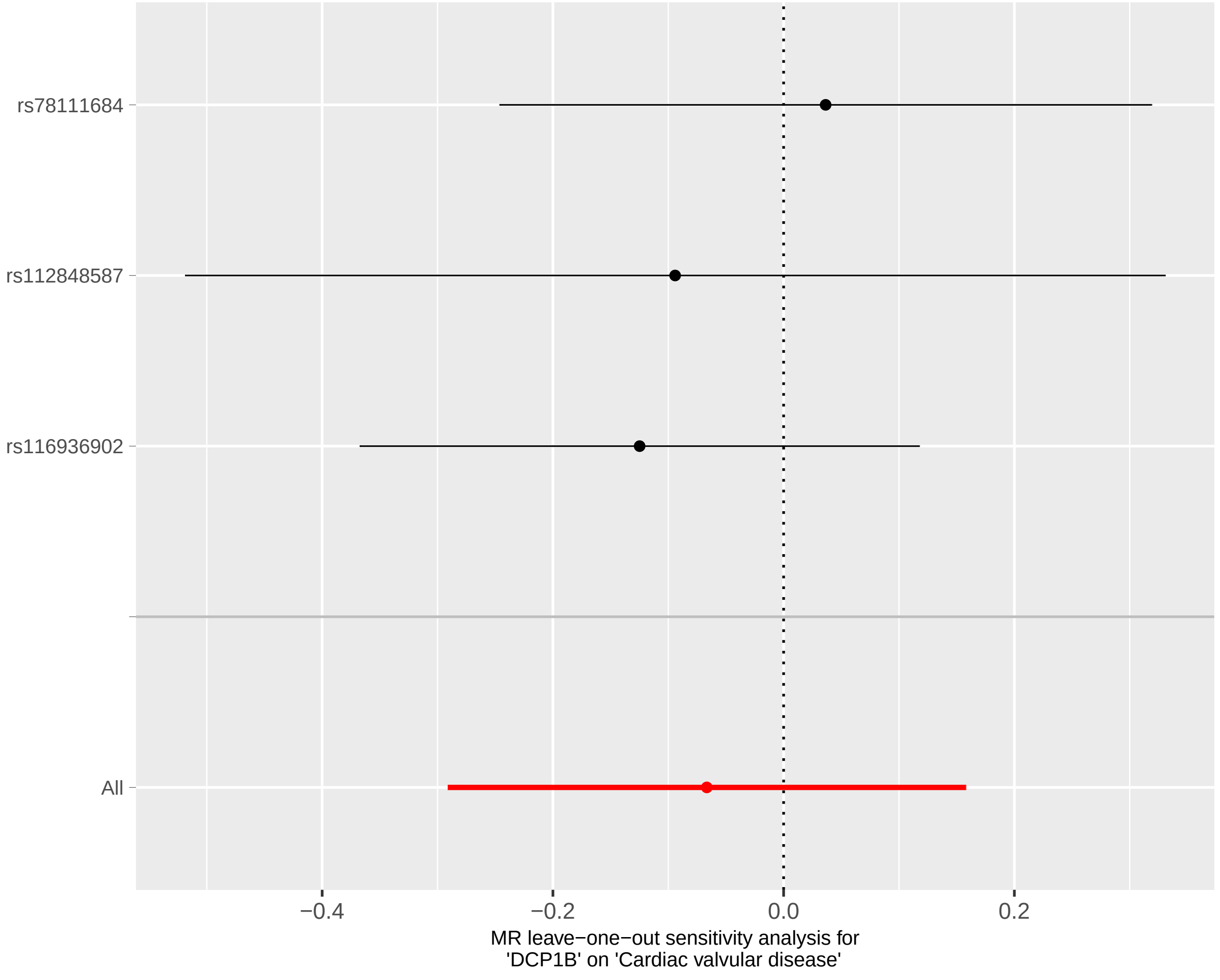

rs78111684
rs112848587
rs116936902
All
−0.4
−0.2
0.0
0.2
MR leave−one−out sensitivity analysis for
'DCP1B' on 'Cardiac valvular disease'

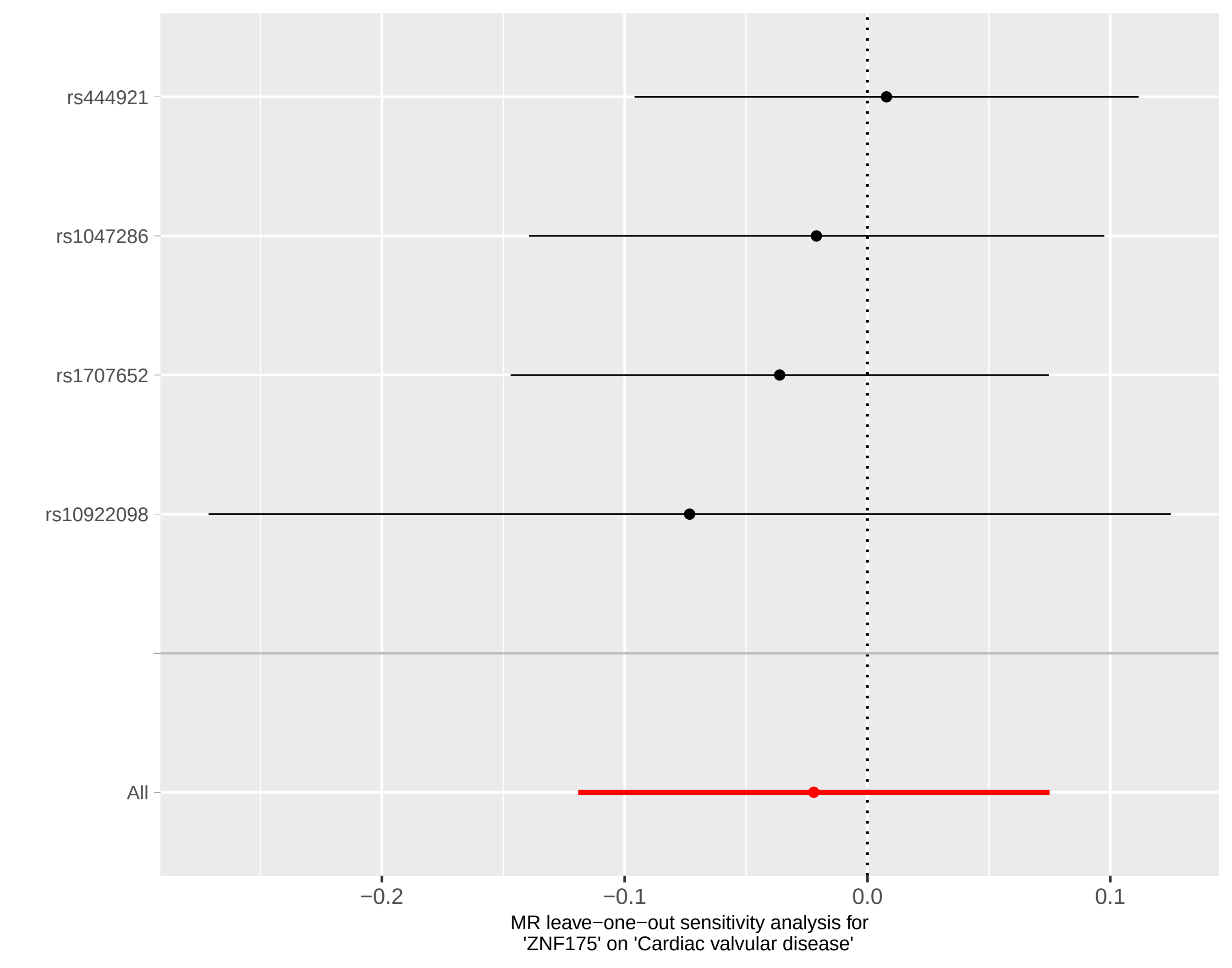
rs444921
rs1047286
rs1707652
rs10922098
All
−0.2
−0.1
0.0
0.1
MR leave−one−out sensitivity analysis for
'ZNF175' on 'Cardiac valvular disease'

Insufficient number of SNPs

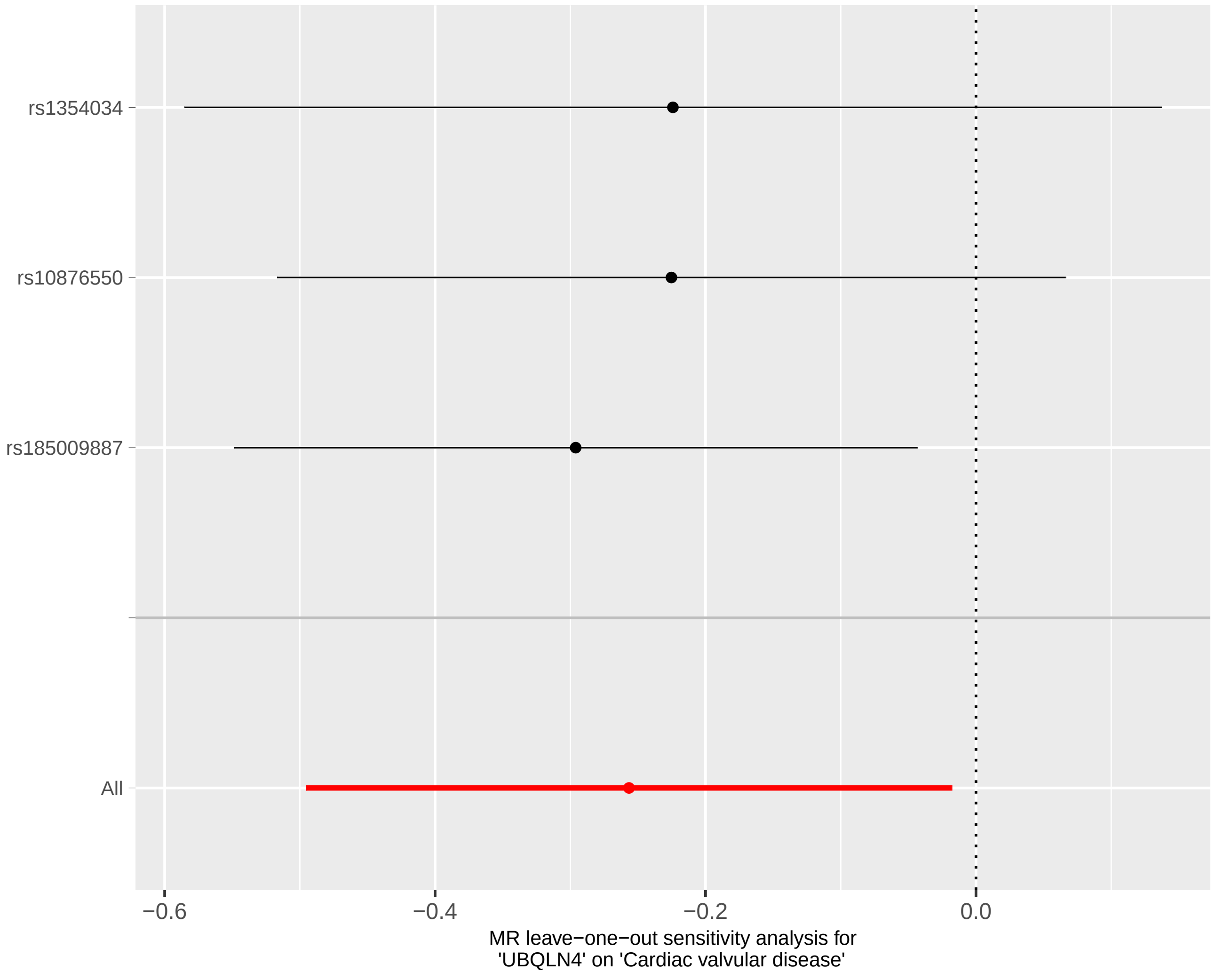

rs1354034
rs10876550
rs185009887
All
−0.6
−0.4
−0.2
0.0
MR leave−one−out sensitivity analysis for
'UBQLN4' on 'Cardiac valvular disease'

Insufficient number of SNPs

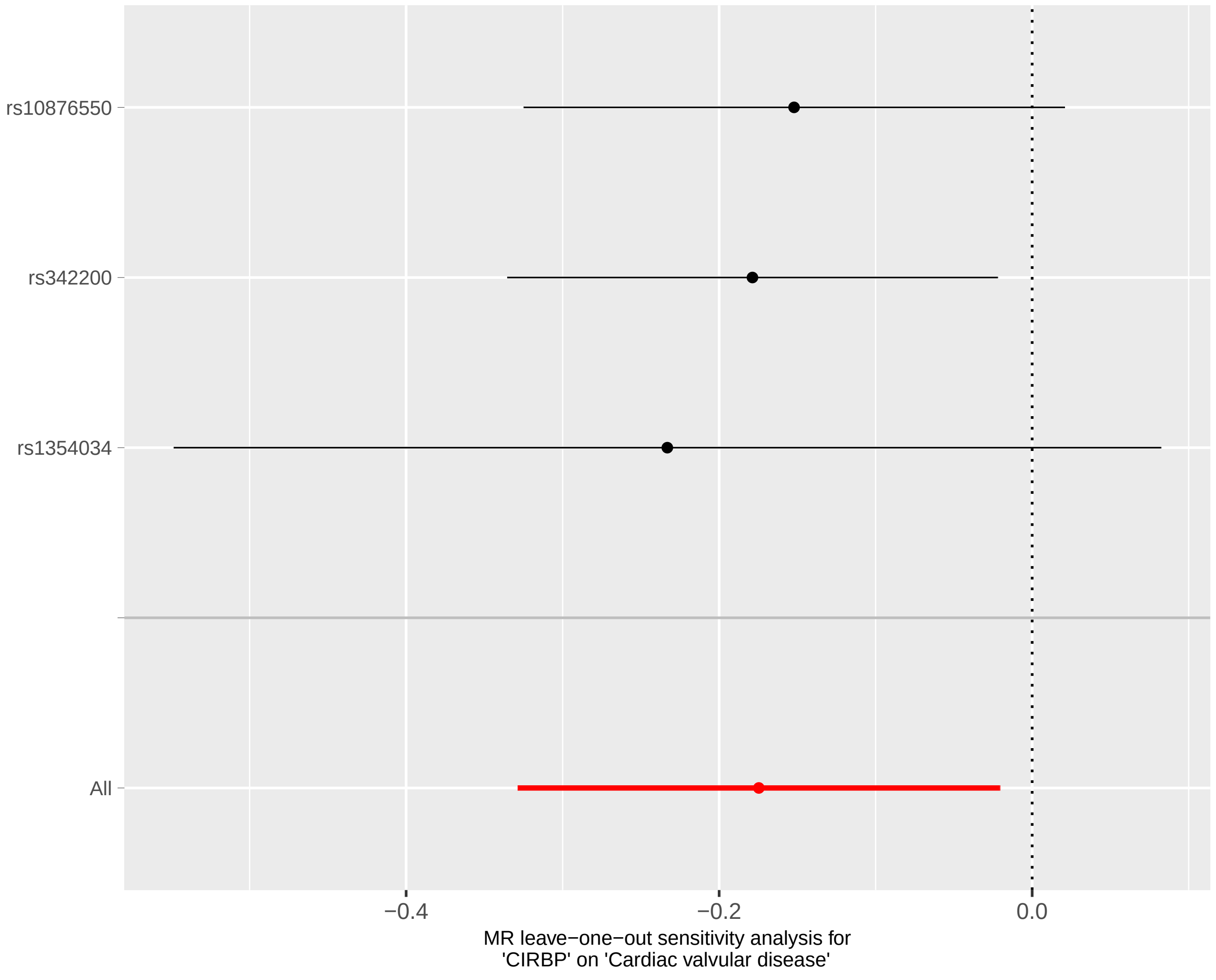
rs10876550
rs342200
rs1354034
All
−0.4
−0.2
0.0
MR leave−one−out sensitivity analysis for
'CIRBP' on 'Cardiac valvular disease'

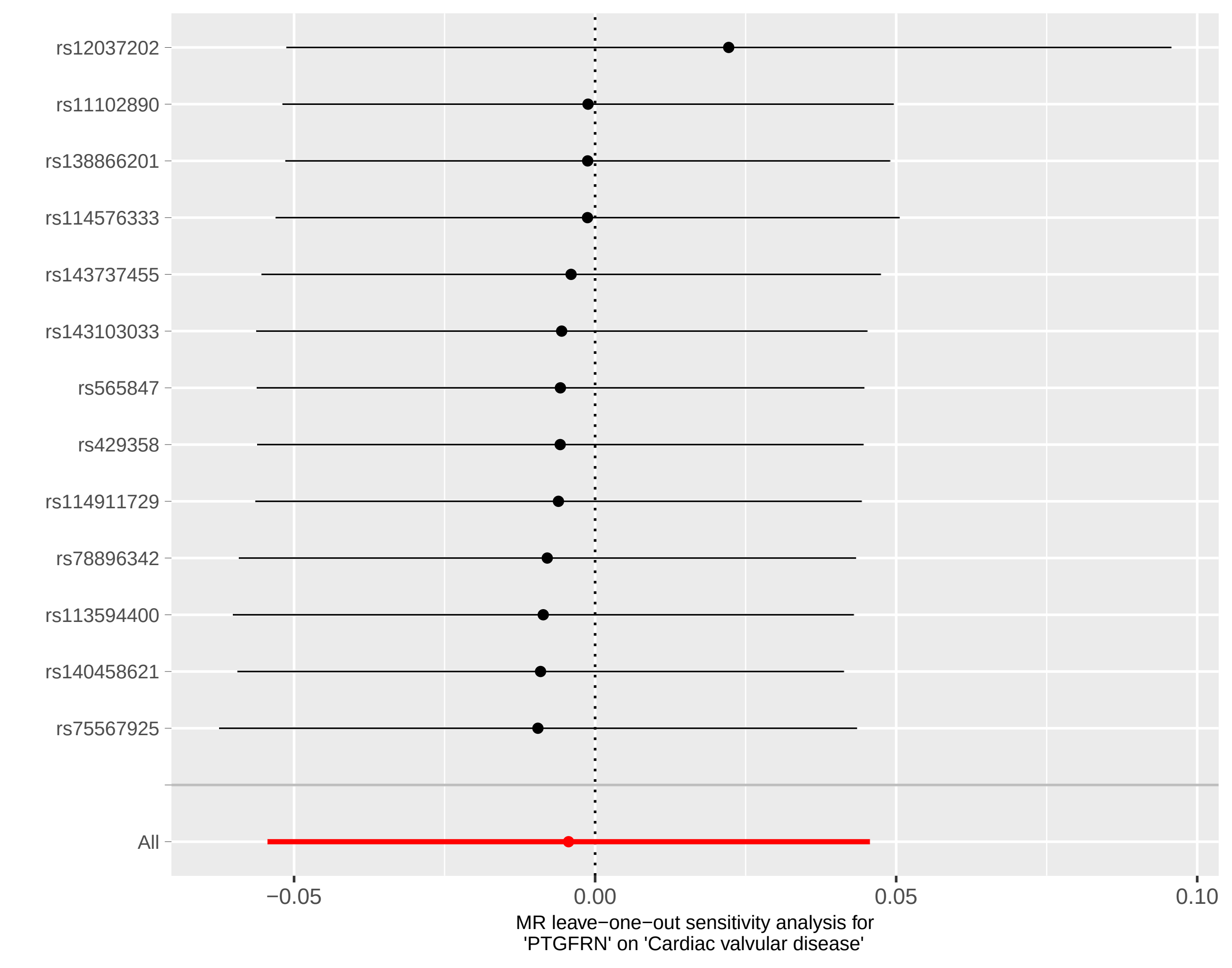
rs12037202
rs11102890
rs138866201
rs114576333
rs143737455
rs143103033
rs565847
rs429358
rs114911729
rs78896342
rs113594400
rs140458621
rs75567925
All
−0.05
0.00
0.05
0.10
MR leave−one−out sensitivity analysis for
'PTGFRN' on 'Cardiac valvular disease'

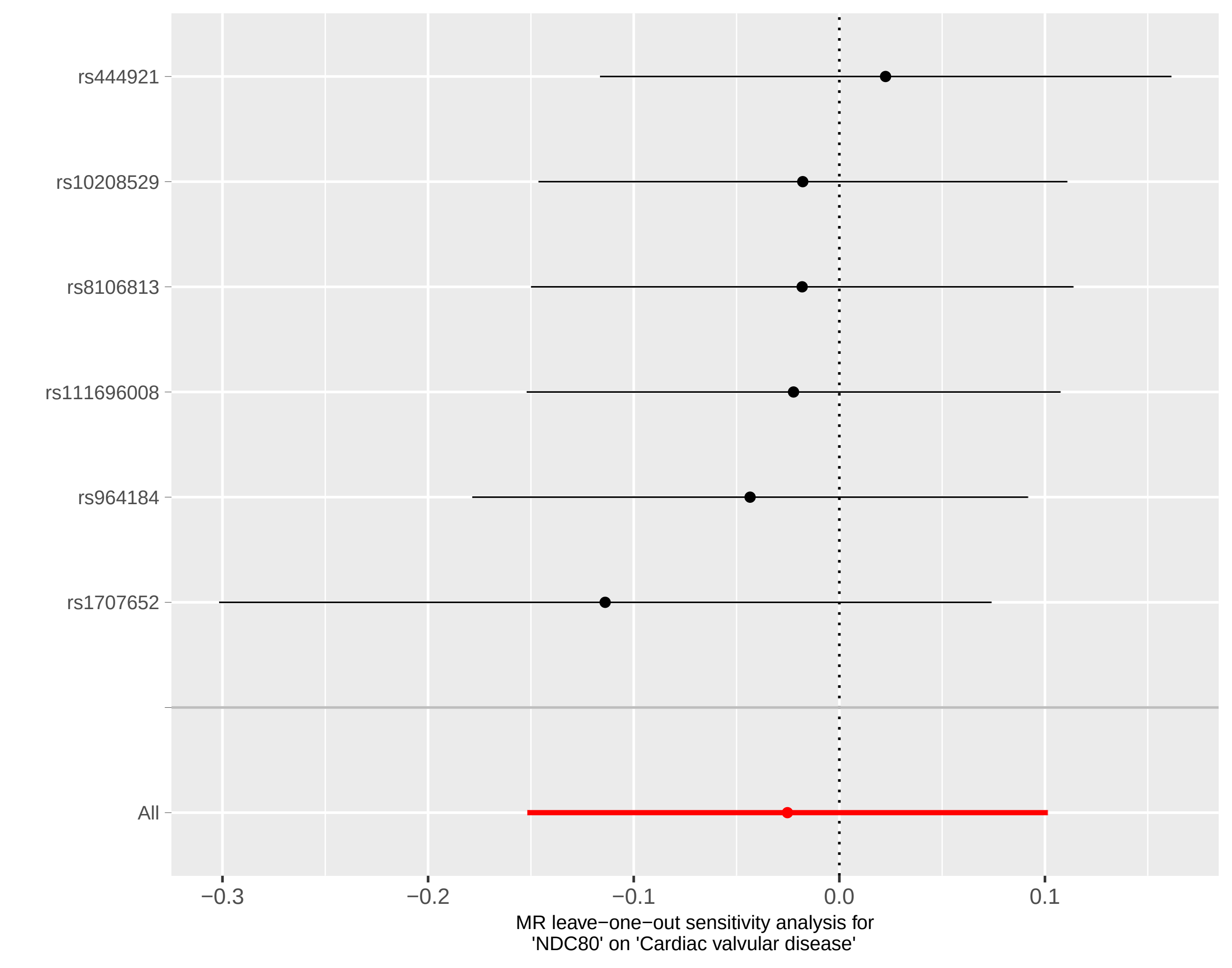

rs444921
rs10208529
rs8106813
rs111696008
rs964184
rs1707652
All
−0.3
−0.2
−0.1
0.0
0.1
MR leave−one−out sensitivity analysis for
'NDC80' on 'Cardiac valvular disease'

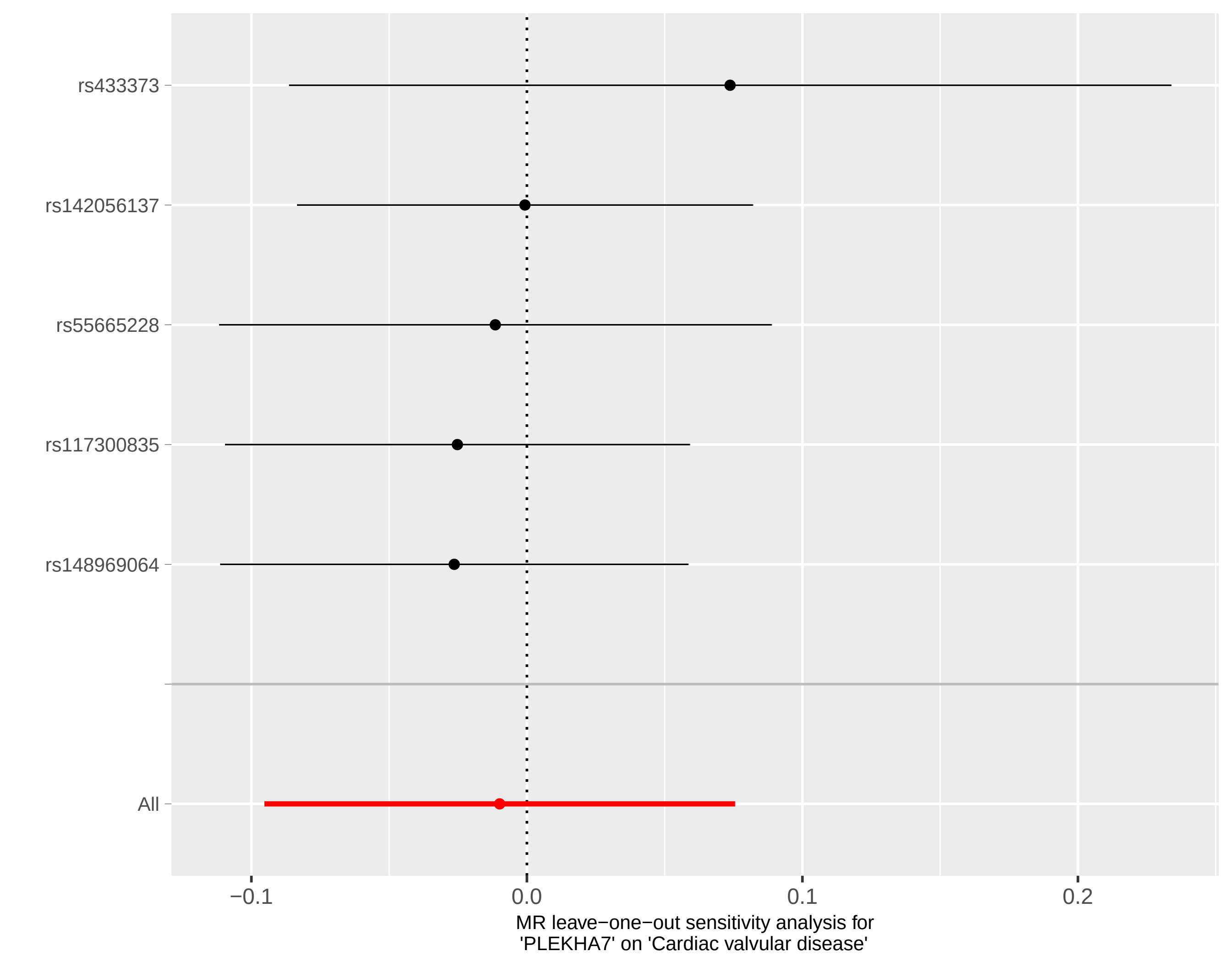
rs433373
rs142056137
rs55665228
rs117300835
rs148969064
All
−0.1
0.0
0.1
0.2
MR leave−one−out sensitivity analysis for
'PLEKHA7' on 'Cardiac valvular disease'

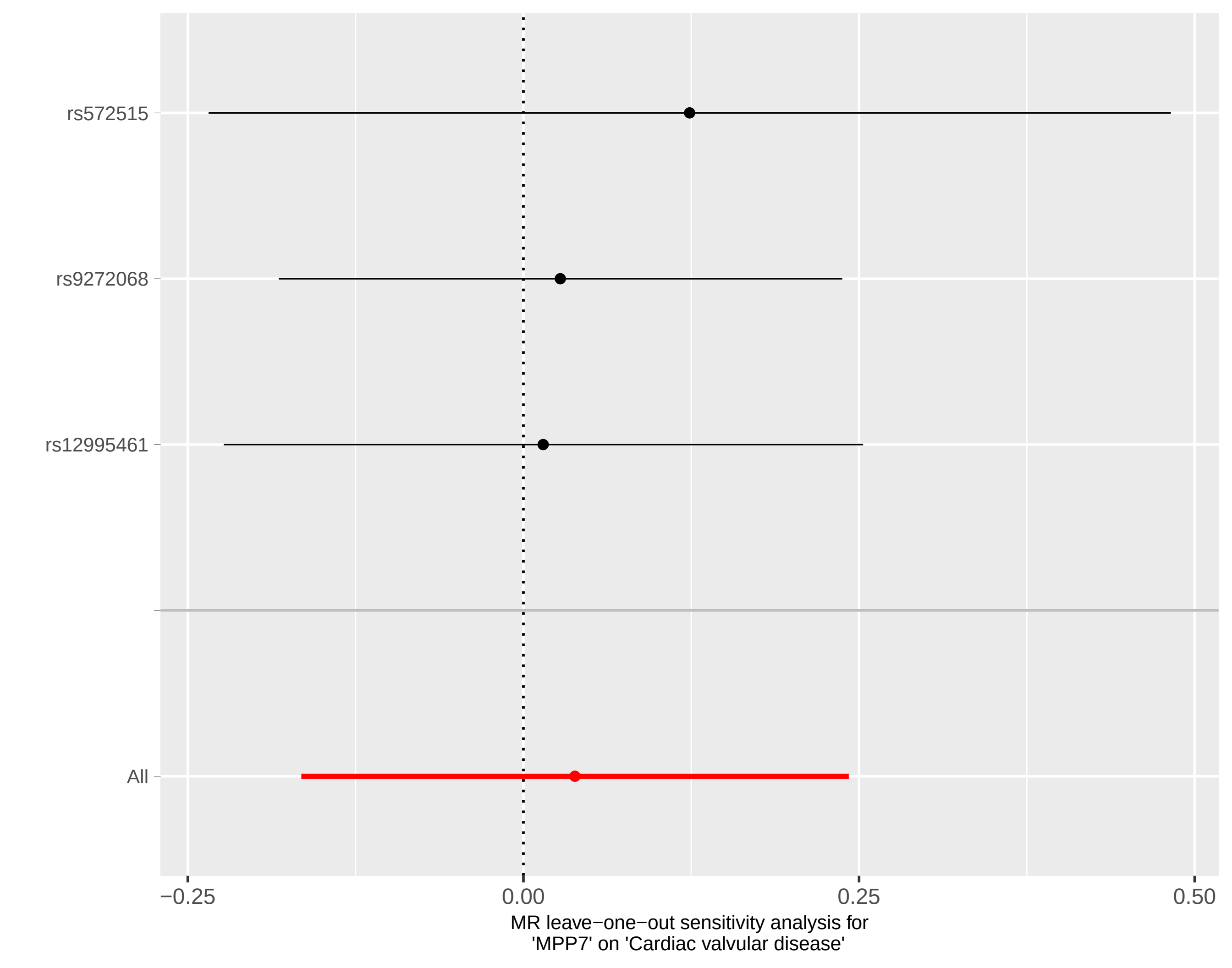

rs572515
rs9272068
rs12995461
All
−0.25
0.00
0.25
0.50
MR leave−one−out sensitivity analysis for
'MPP7' on 'Cardiac valvular disease'

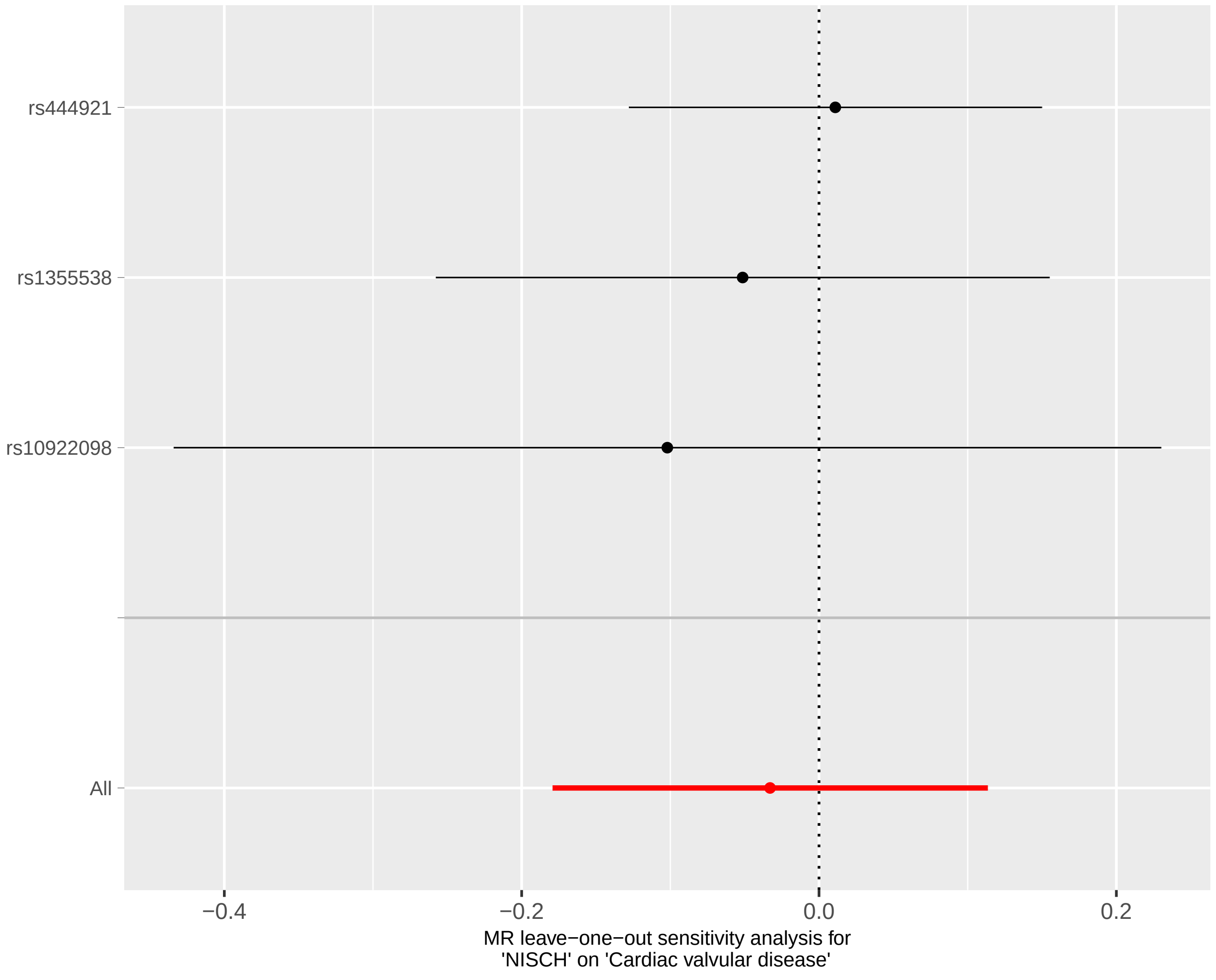
rs444921
rs1355538
rs10922098
All
−0.4
−0.2
0.0
0.2
MR leave−one−out sensitivity analysis for
'NISCH' on 'Cardiac valvular disease'

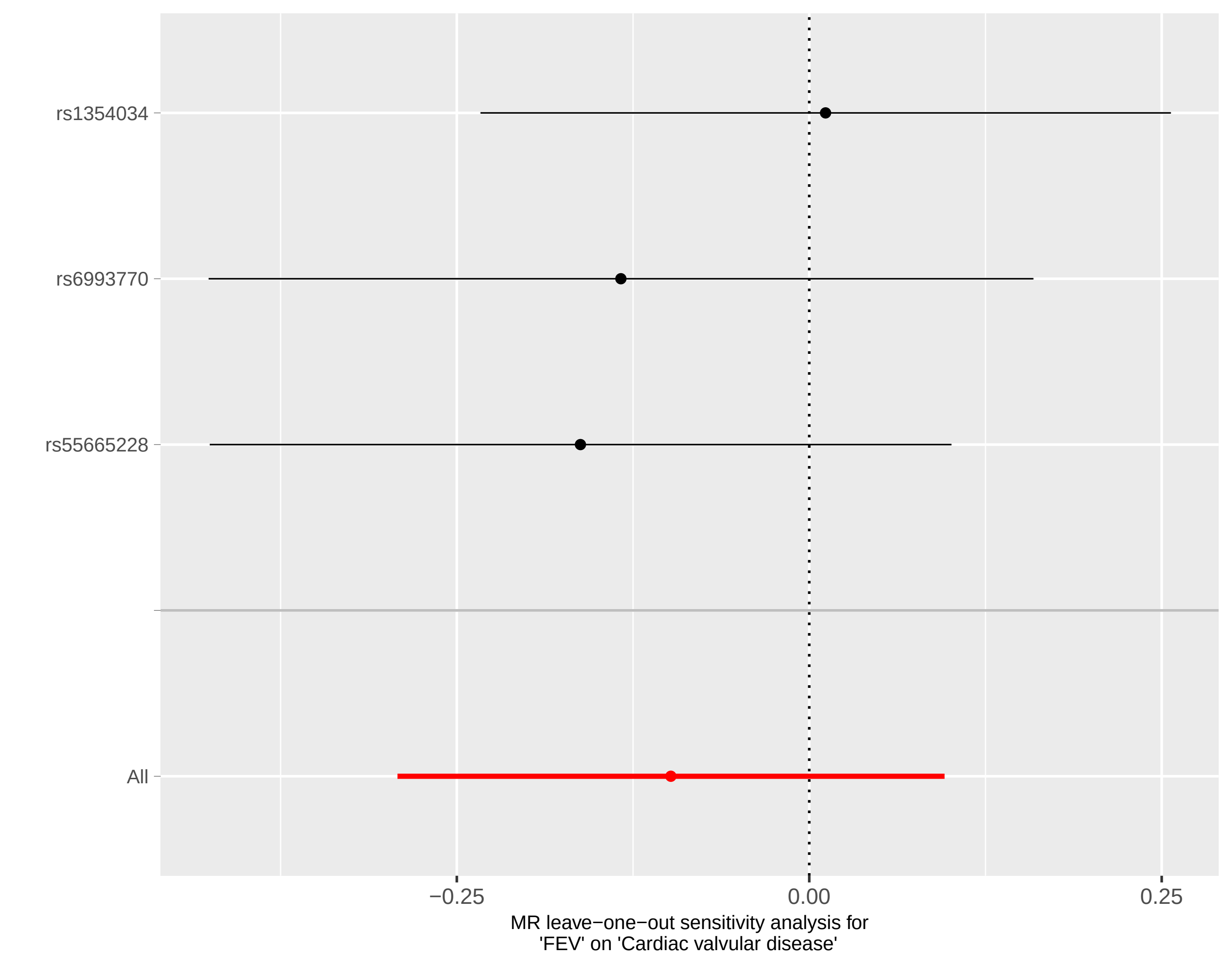
rs1354034
rs6993770
rs55665228
All
−0.25
0.00
0.25
MR leave−one−out sensitivity analysis for
'FEV' on 'Cardiac valvular disease'

Insufficient number of SNPs

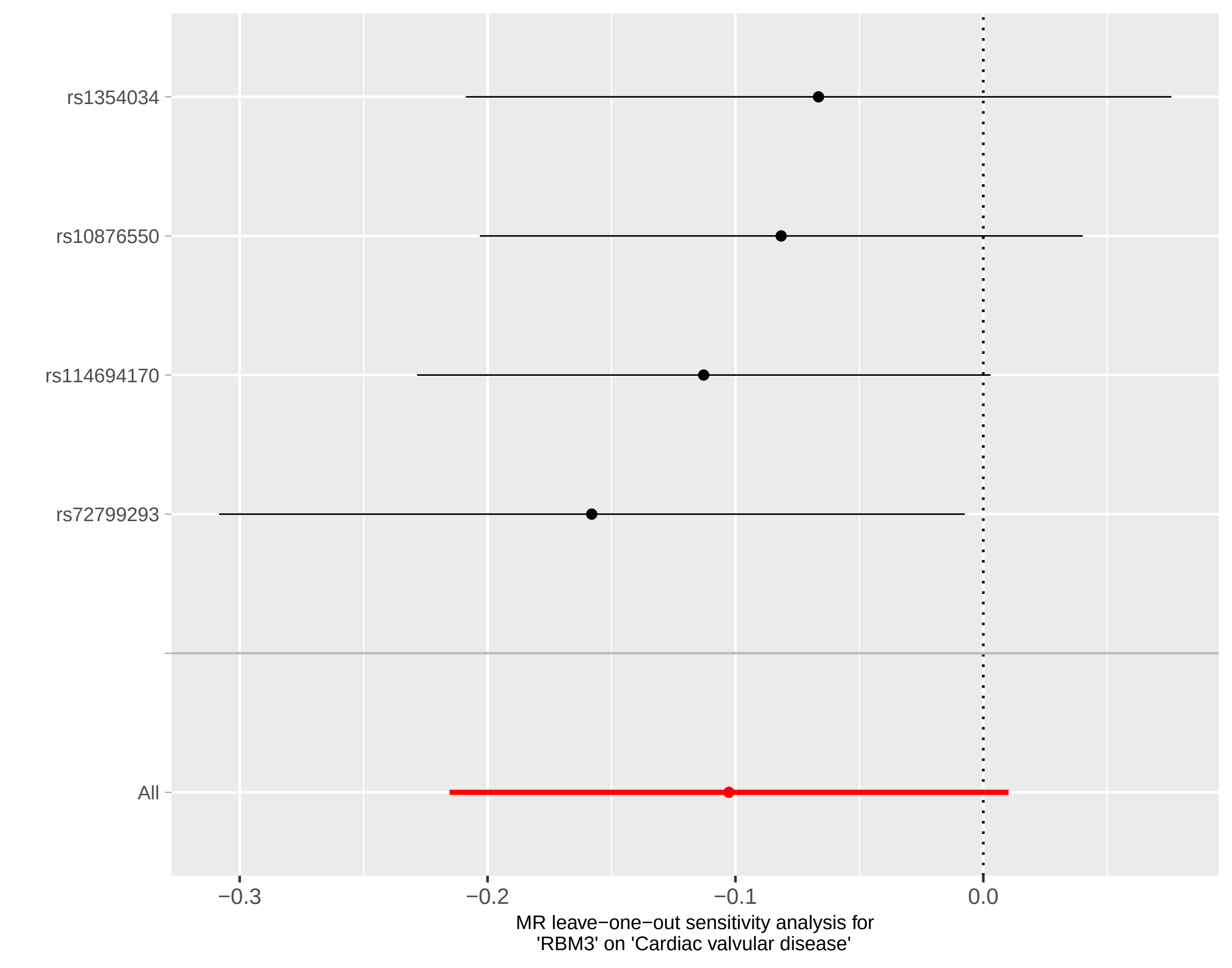
rs1354034
rs10876550
rs114694170
rs72799293
All
−0.3
−0.2
−0.1
0.0
MR leave−one−out sensitivity analysis for
'RBM3' on 'Cardiac valvular disease'

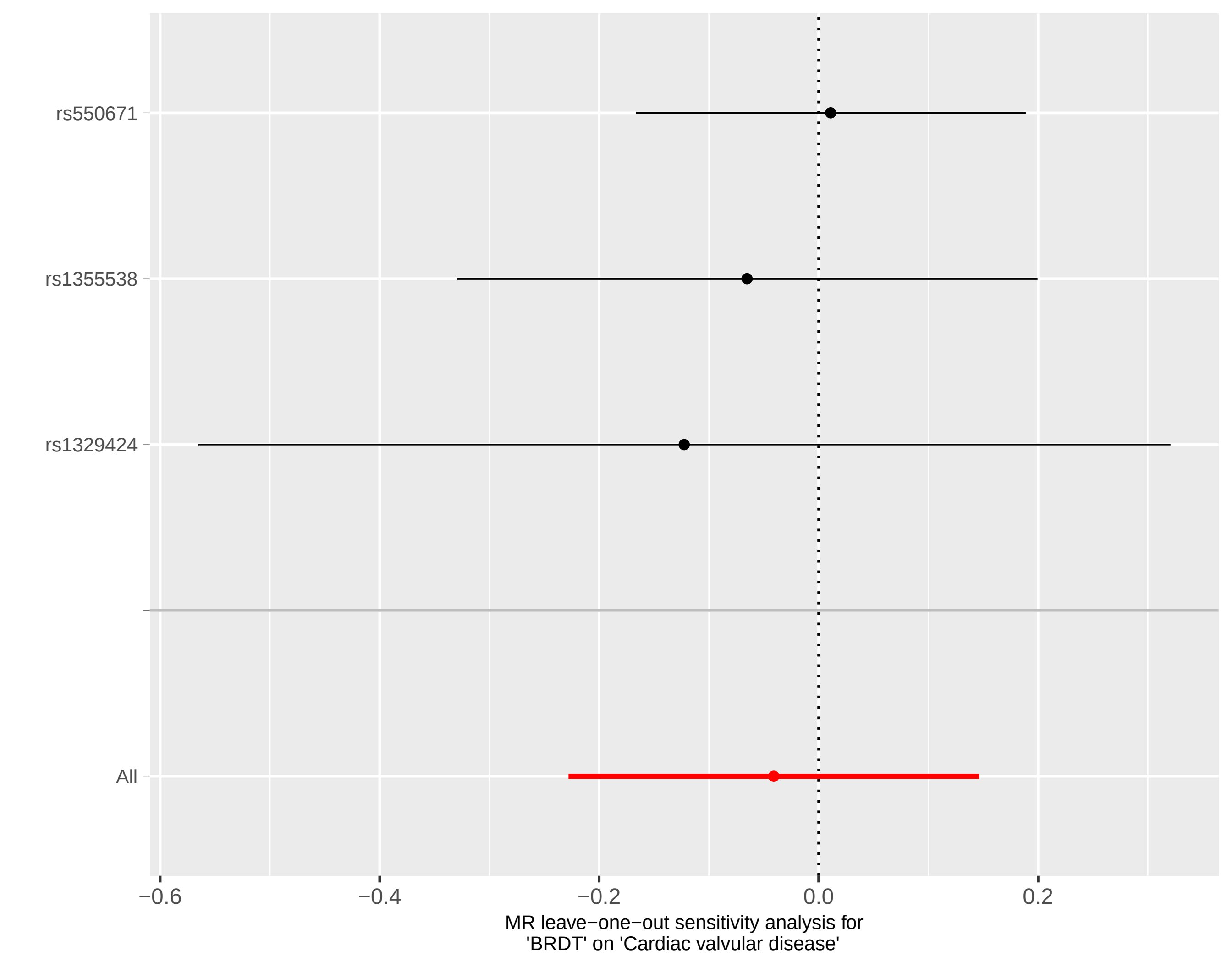
rs550671
rs1355538
rs1329424
All
−0.6
−0.4
−0.2
0.0
0.2
MR leave−one−out sensitivity analysis for
'BRDT' on 'Cardiac valvular disease'

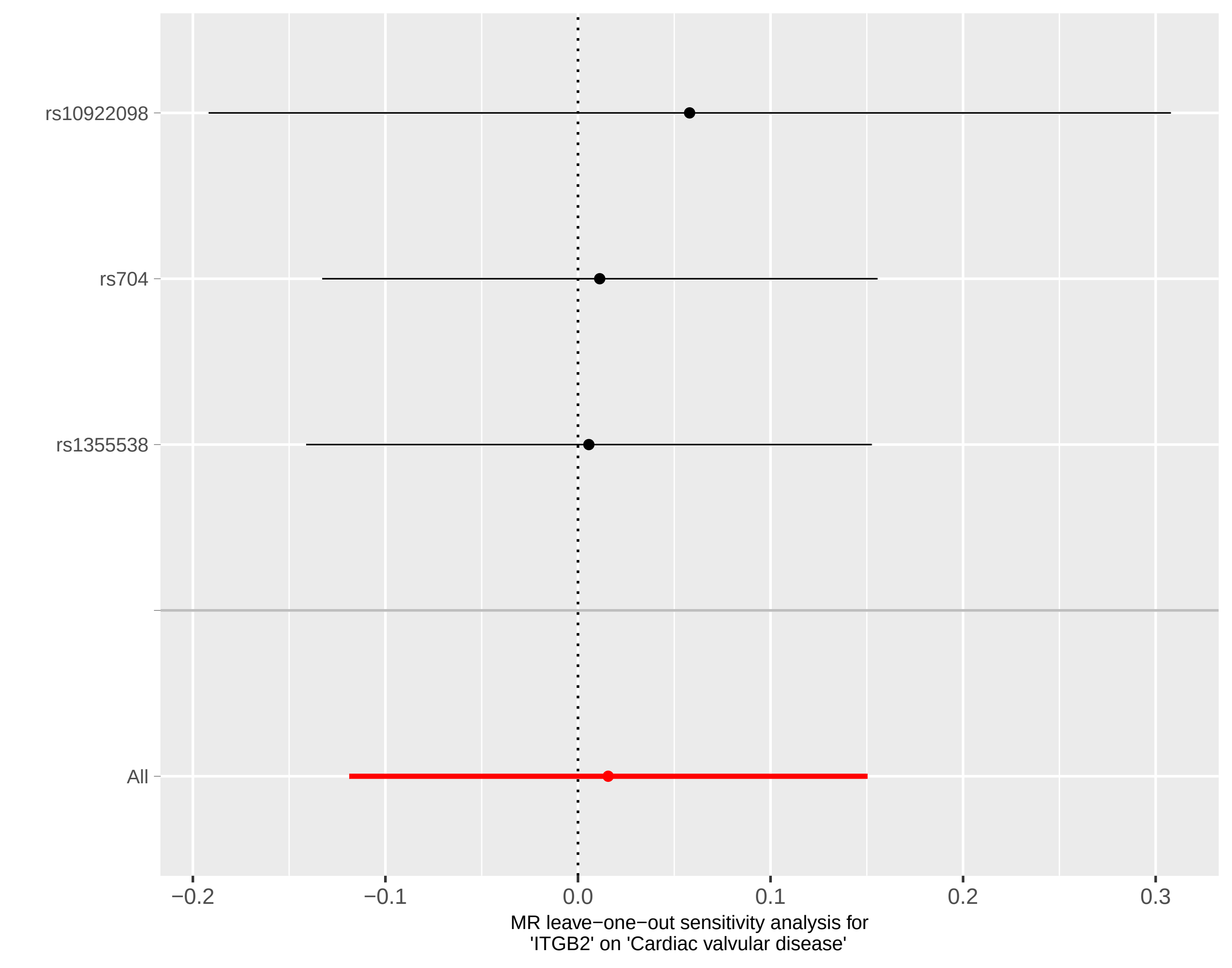
rs10922098
rs704
rs1355538
All
−0.2
−0.1
0.0
0.1
0.2
0.3
MR leave−one−out sensitivity analysis for
'ITGB2' on 'Cardiac valvular disease'

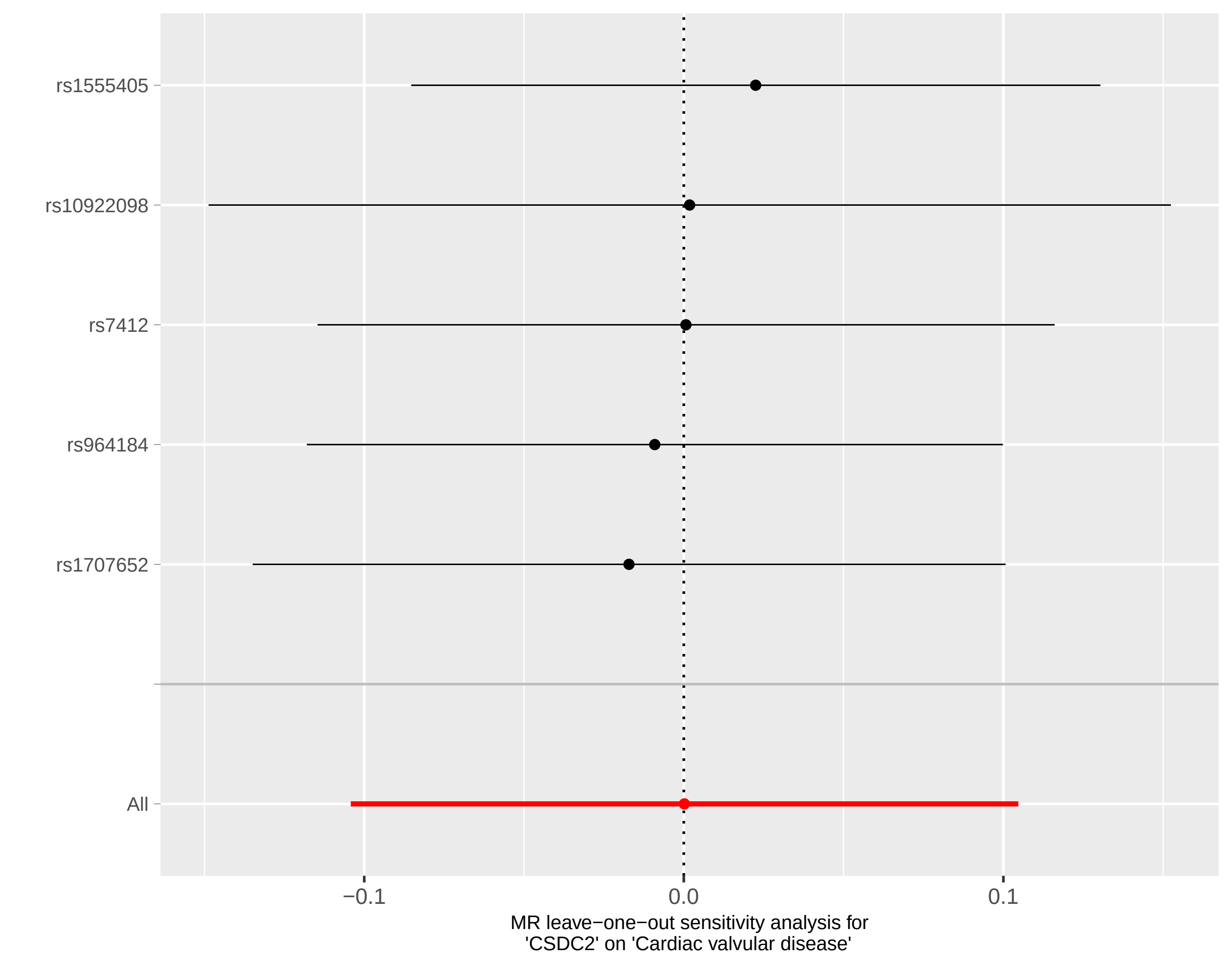
rs1555405
rs10922098
rs7412
rs964184
rs1707652
All
−0.1
0.0
0.1
MR leave−one−out sensitivity analysis for
'CSDC2' on 'Cardiac valvular disease'

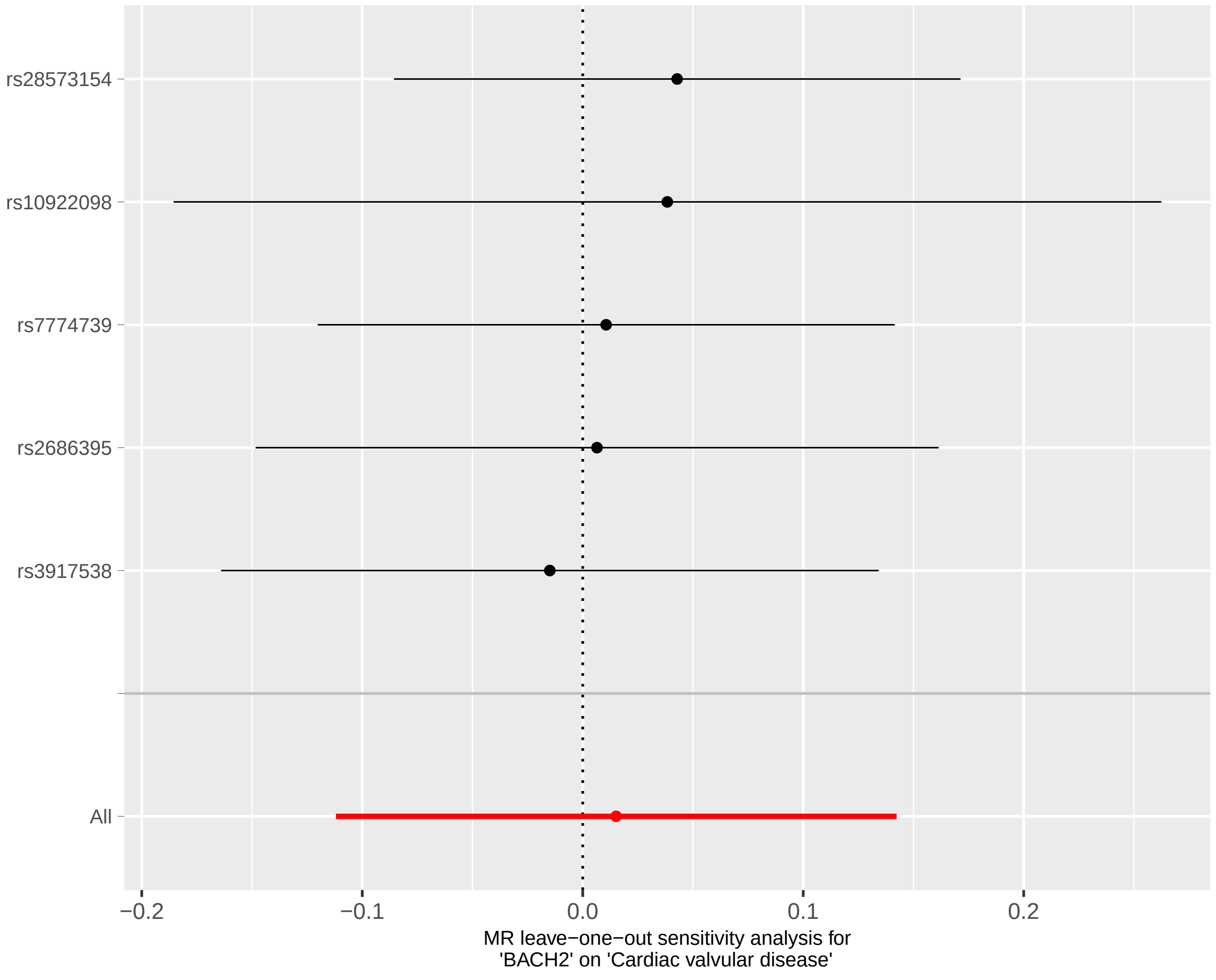
rs28573154
rs10922098
rs7774739
rs2686395
rs3917538
All
−0.2
−0.1
0.0
0.1
0.2
MR leave−one−out sensitivity analysis for
'BACH2' on 'Cardiac valvular disease'

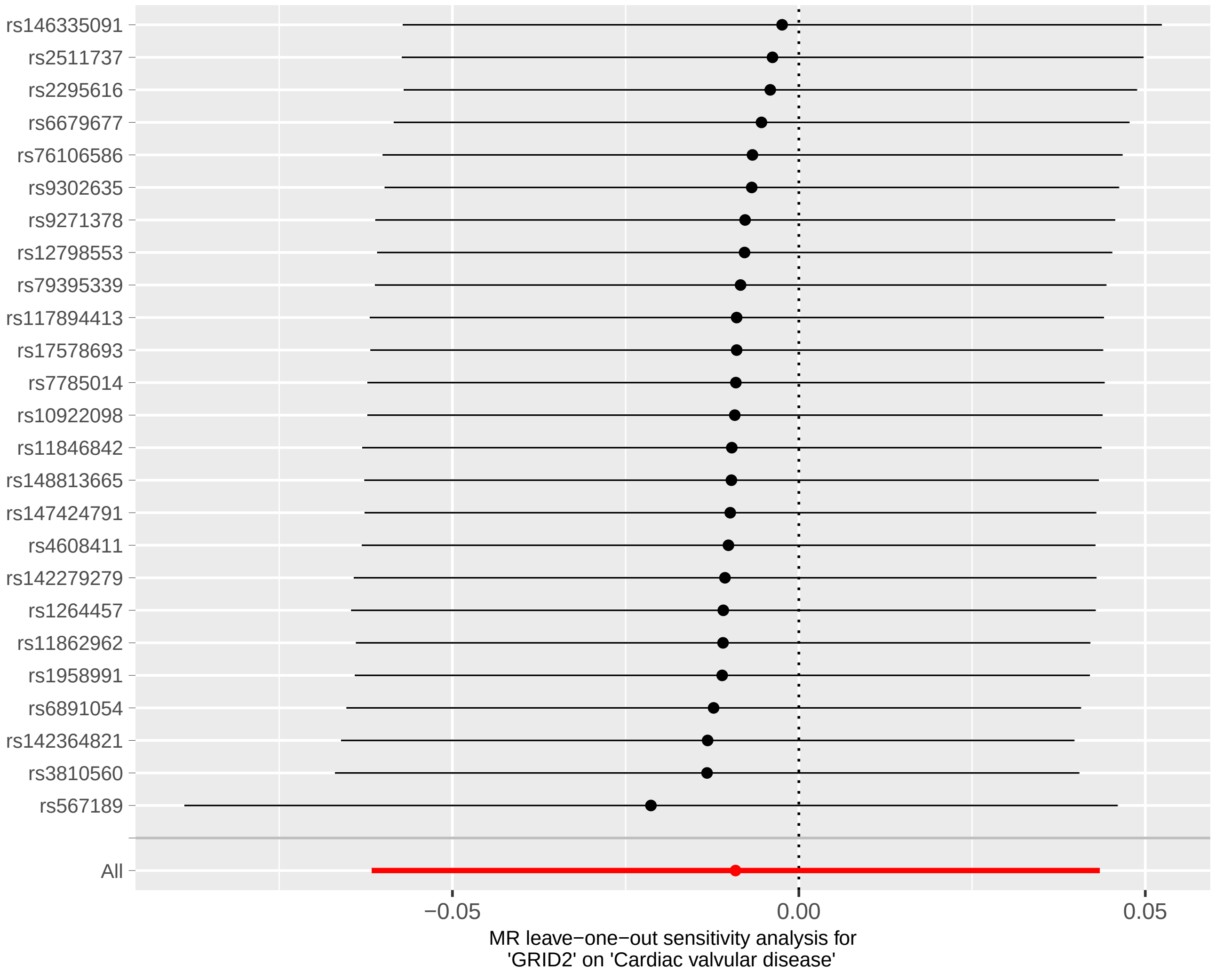

rs146335091
rs2511737
rs2295616
rs6679677
rs76106586
rs9302635
rs9271378
rs12798553
rs79395339
rs117894413
rs17578693
rs7785014
rs10922098
rs11846842
rs148813665
rs147424791
rs4608411
rs142279279
rs1264457
rs11862962
rs1958991
rs6891054
rs142364821
rs3810560
rs567189
All
−0.05
0.00
0.05
MR leave−one−out sensitivity analysis for
'GRID2' on 'Cardiac valvular disease'

Insufficient number of SNPs

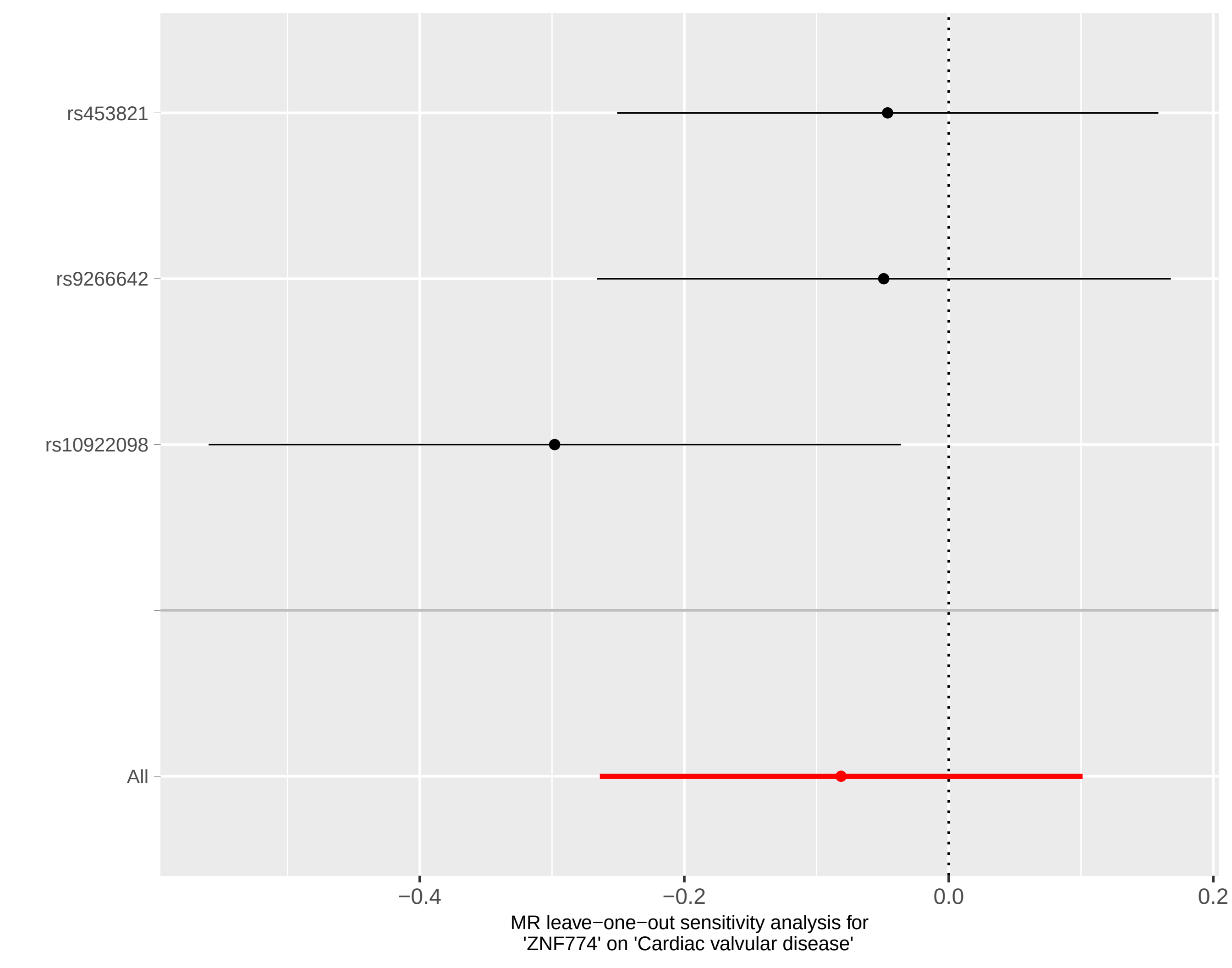
rs453821
rs9266642
rs10922098
All
−0.4
−0.2
0.0
0.2
MR leave−one−out sensitivity analysis for
'ZNF774' on 'Cardiac valvular disease'

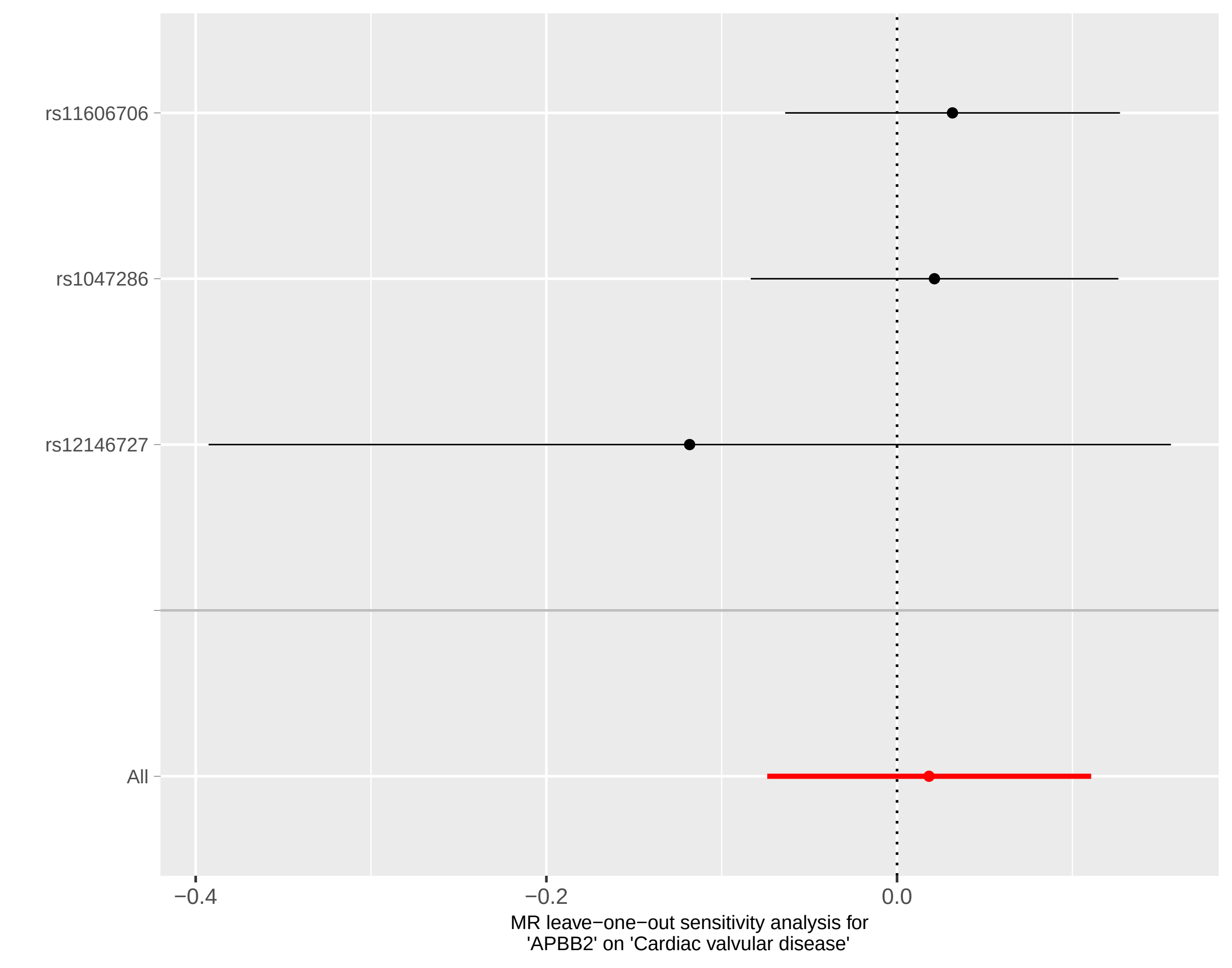
rs11606706
rs1047286
rs12146727
All
−0.4
−0.2
0.0
MR leave−one−out sensitivity analysis for
'APBB2' on 'Cardiac valvular disease'

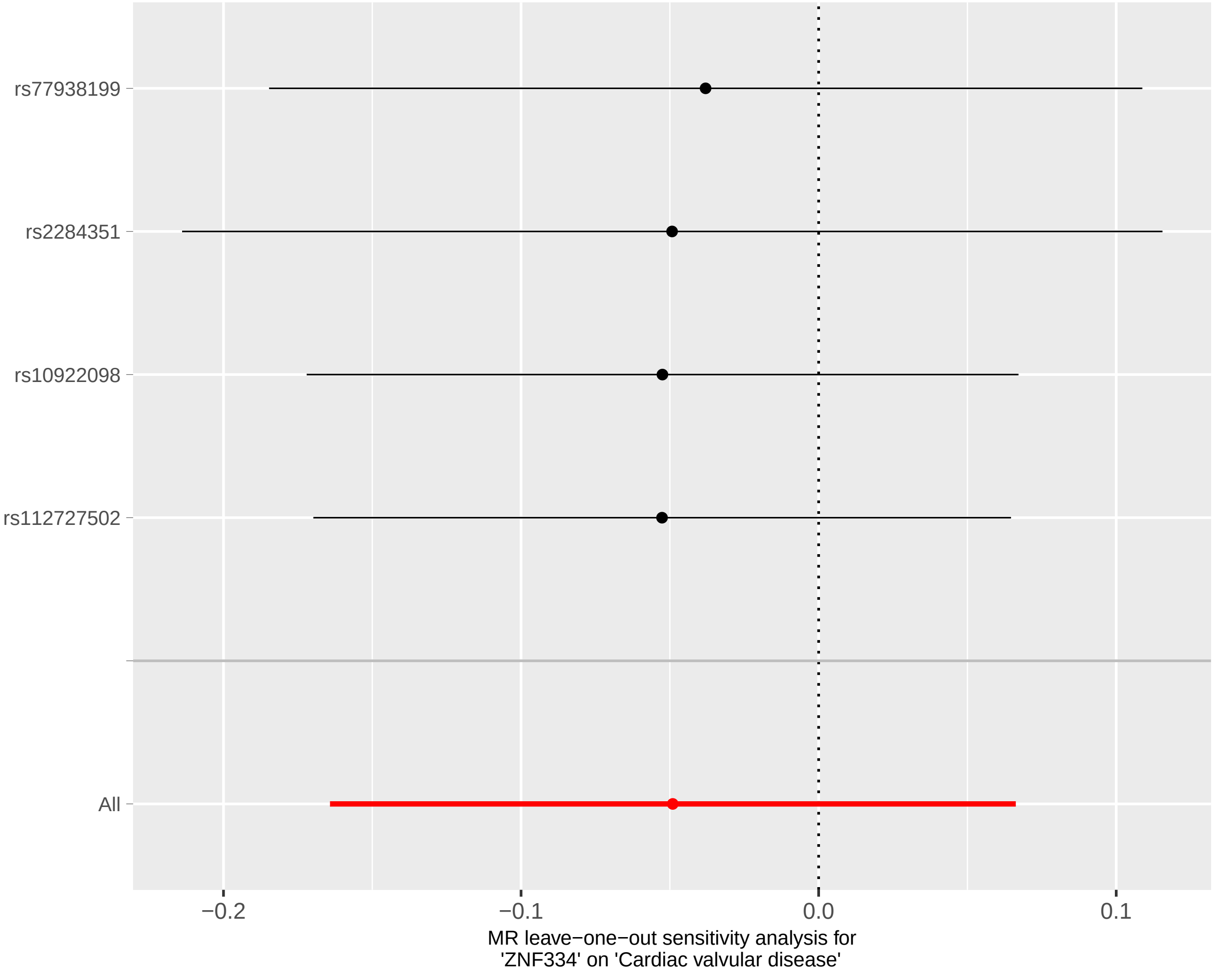
rs77938199
rs2284351
rs10922098
rs112727502
All
−0.2
−0.1
0.0
0.1
MR leave−one−out sensitivity analysis for
'ZNF334' on 'Cardiac valvular disease'

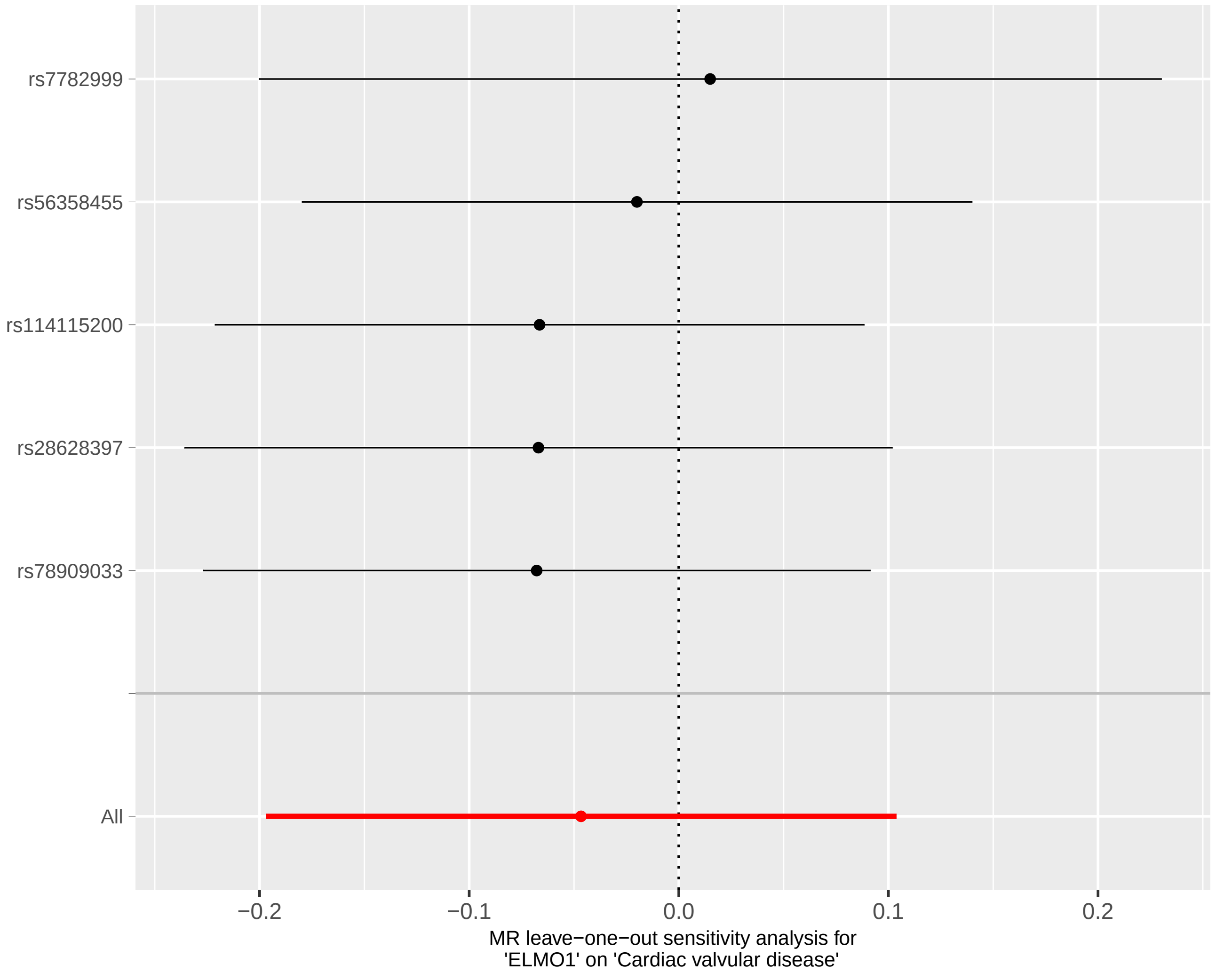
rs7782999
rs56358455
rs114115200
rs28628397
rs78909033
All
−0.2
−0.1
0.0
0.1
0.2
MR leave−one−out sensitivity analysis for
'ELMO1' on 'Cardiac valvular disease'

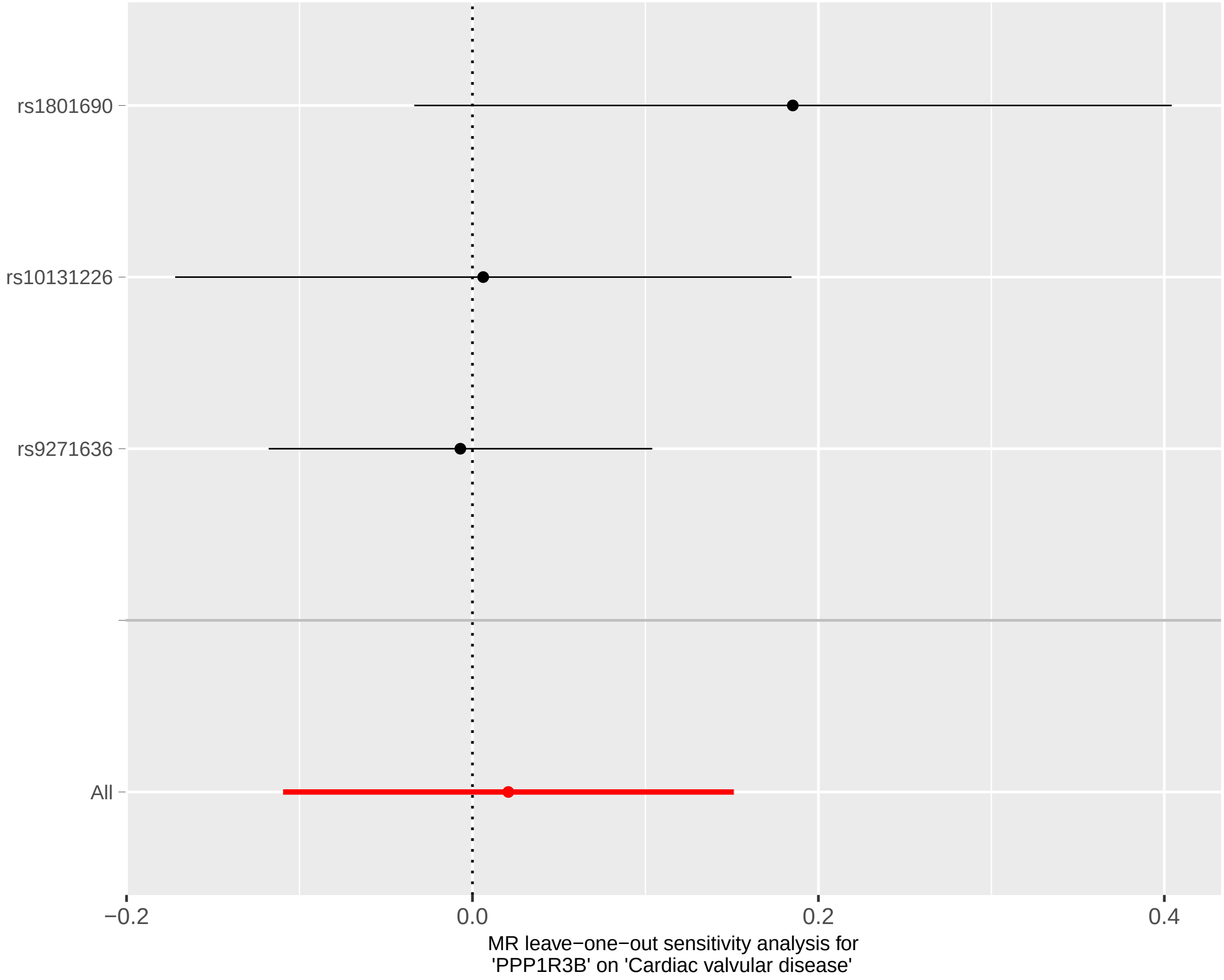

rs1801690
rs10131226
rs9271636
All
−0.2
0.0
0.2
0.4
MR leave−one−out sensitivity analysis for
'PPP1R3B' on 'Cardiac valvular disease'

Insufficient number of SNPs

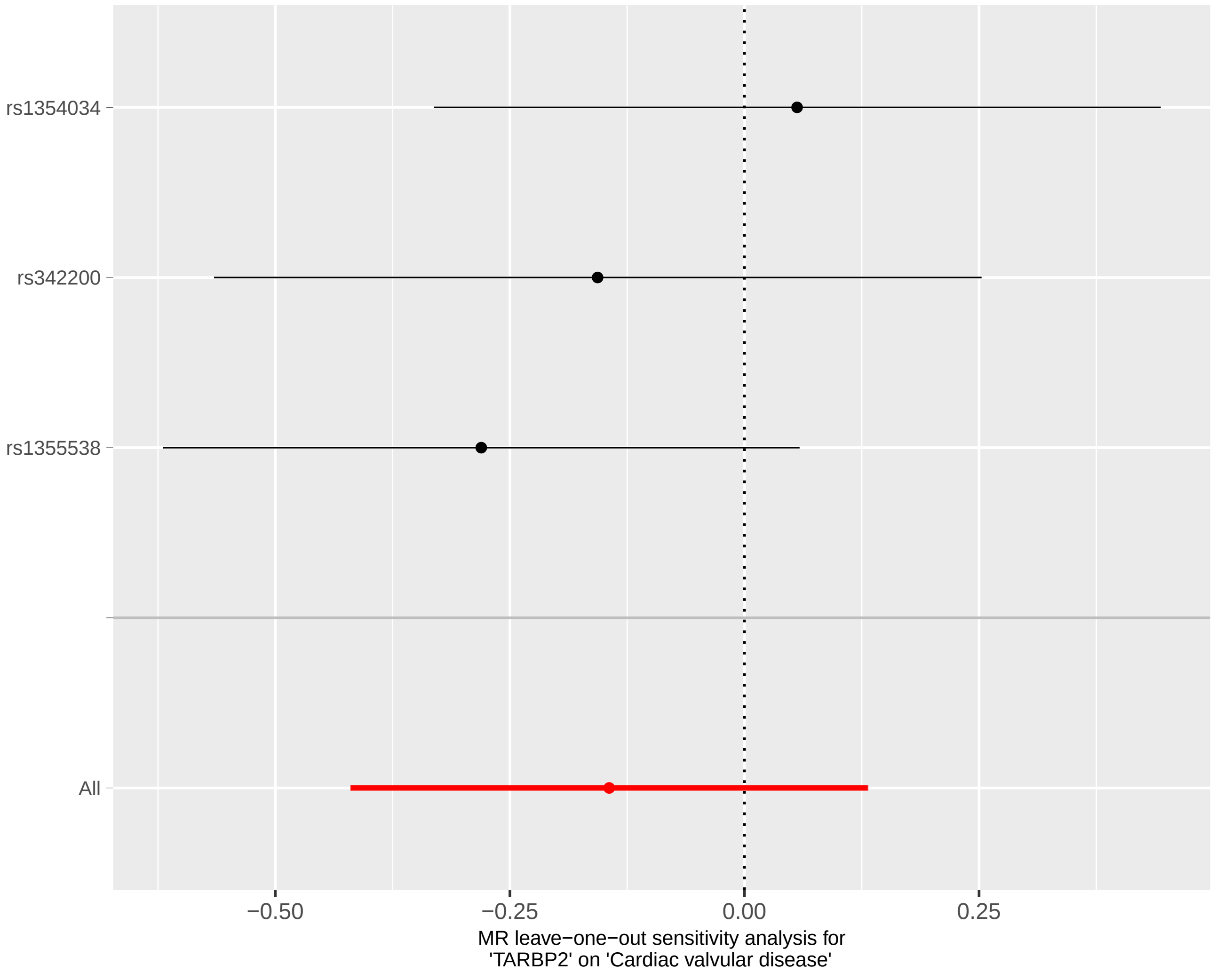
rs1354034
rs342200
rs1355538
All
−0.50
−0.25
0.00
0.25
MR leave−one−out sensitivity analysis for
'TARBP2' on 'Cardiac valvular disease'

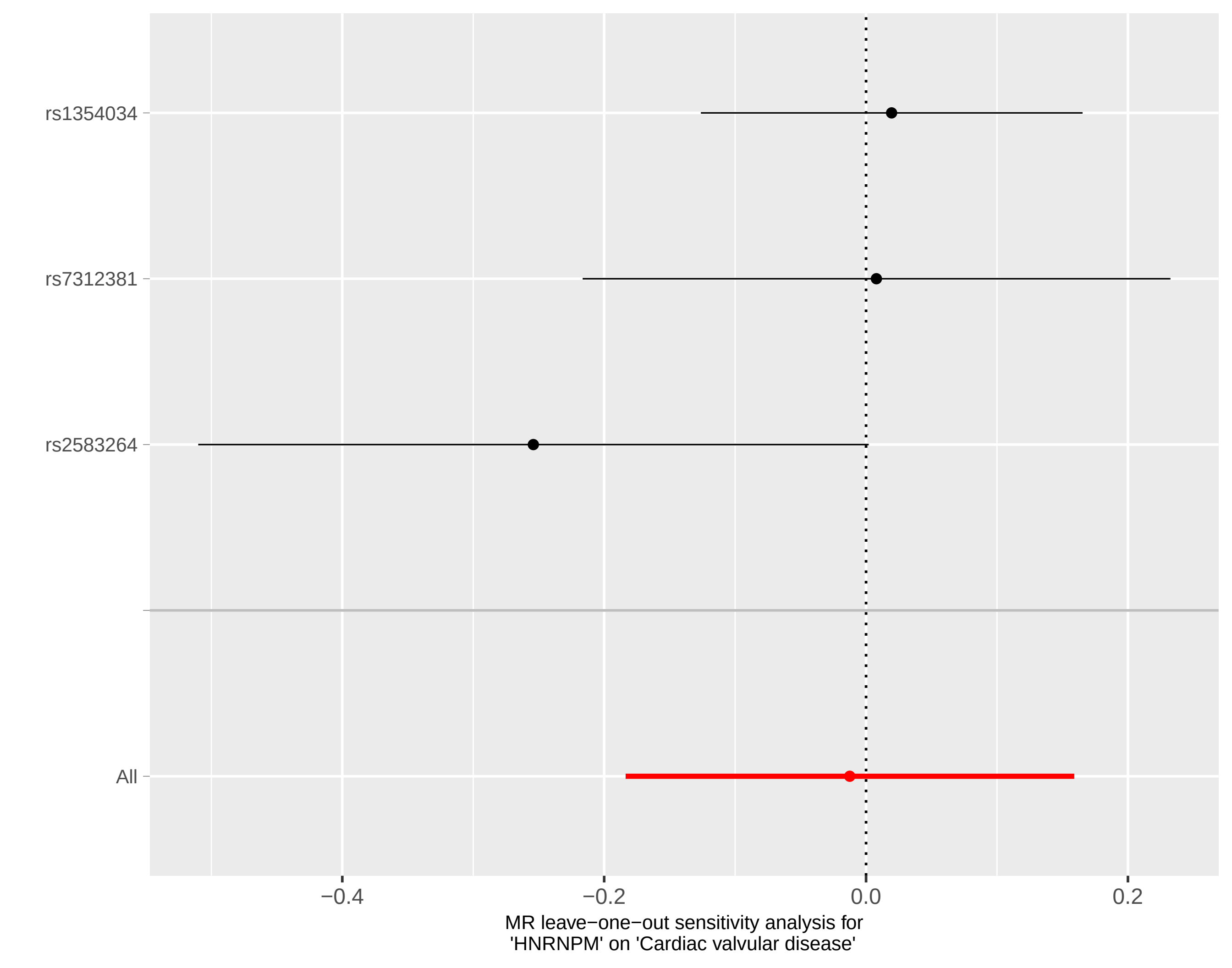

rs1354034
rs7312381
rs2583264
All
−0.4
−0.2
0.0
0.2
MR leave−one−out sensitivity analysis for
'HNRNPM' on 'Cardiac valvular disease'

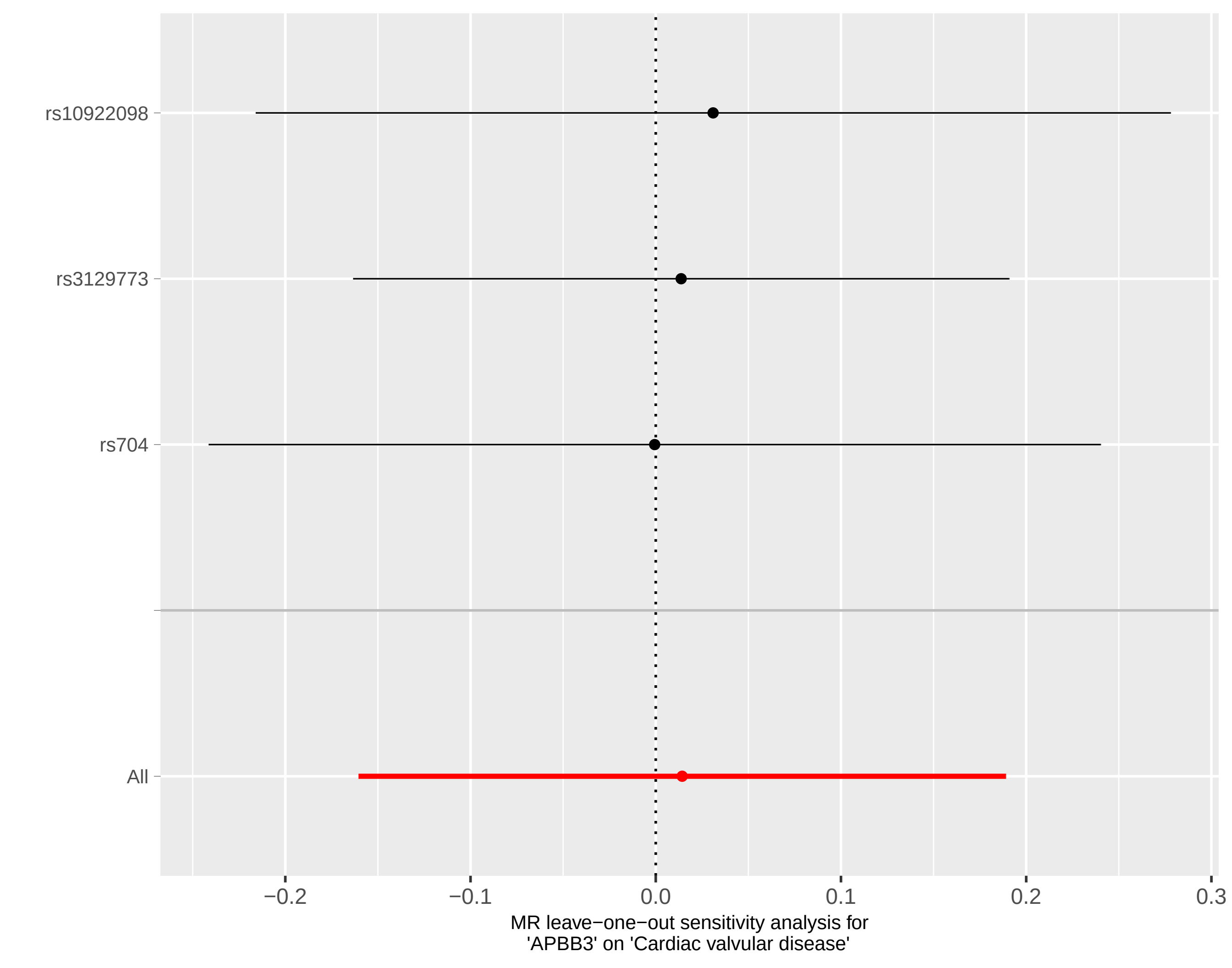
rs10922098
rs3129773
rs704
All
−0.2
−0.1
0.0
0.1
0.2
0.3
MR leave−one−out sensitivity analysis for
'APBB3' on 'Cardiac valvular disease'

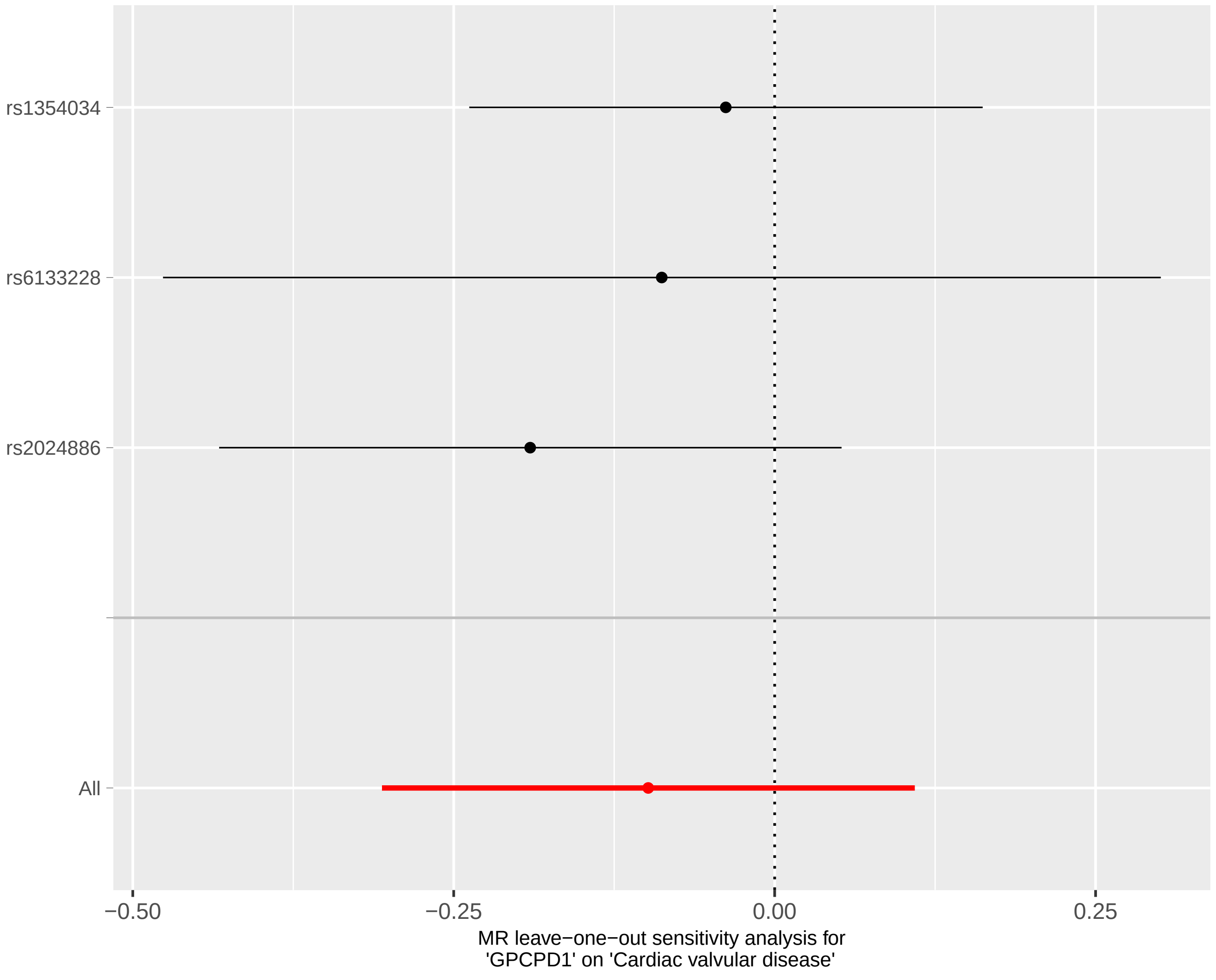
rs1354034
rs6133228
rs2024886
All
−0.50
−0.25
0.00
0.25
MR leave−one−out sensitivity analysis for
'GPCPD1' on 'Cardiac valvular disease'

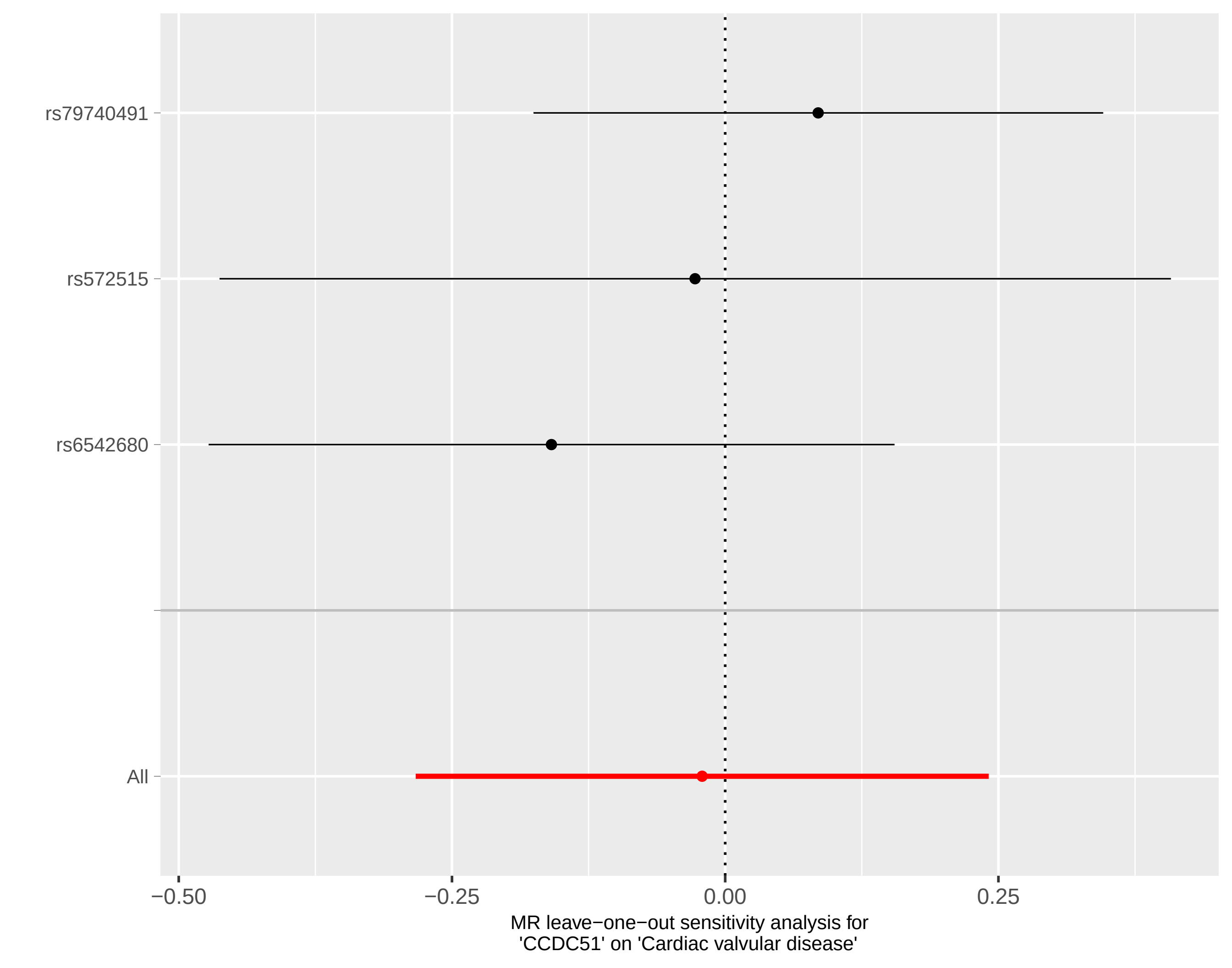

rs79740491
rs572515
rs6542680
All
−0.50
−0.25
0.00
0.25
MR leave−one−out sensitivity analysis for
'CCDC51' on 'Cardiac valvular disease'

Insufficient number of SNPs

Insufficient number of SNPs

Insufficient number of SNPs

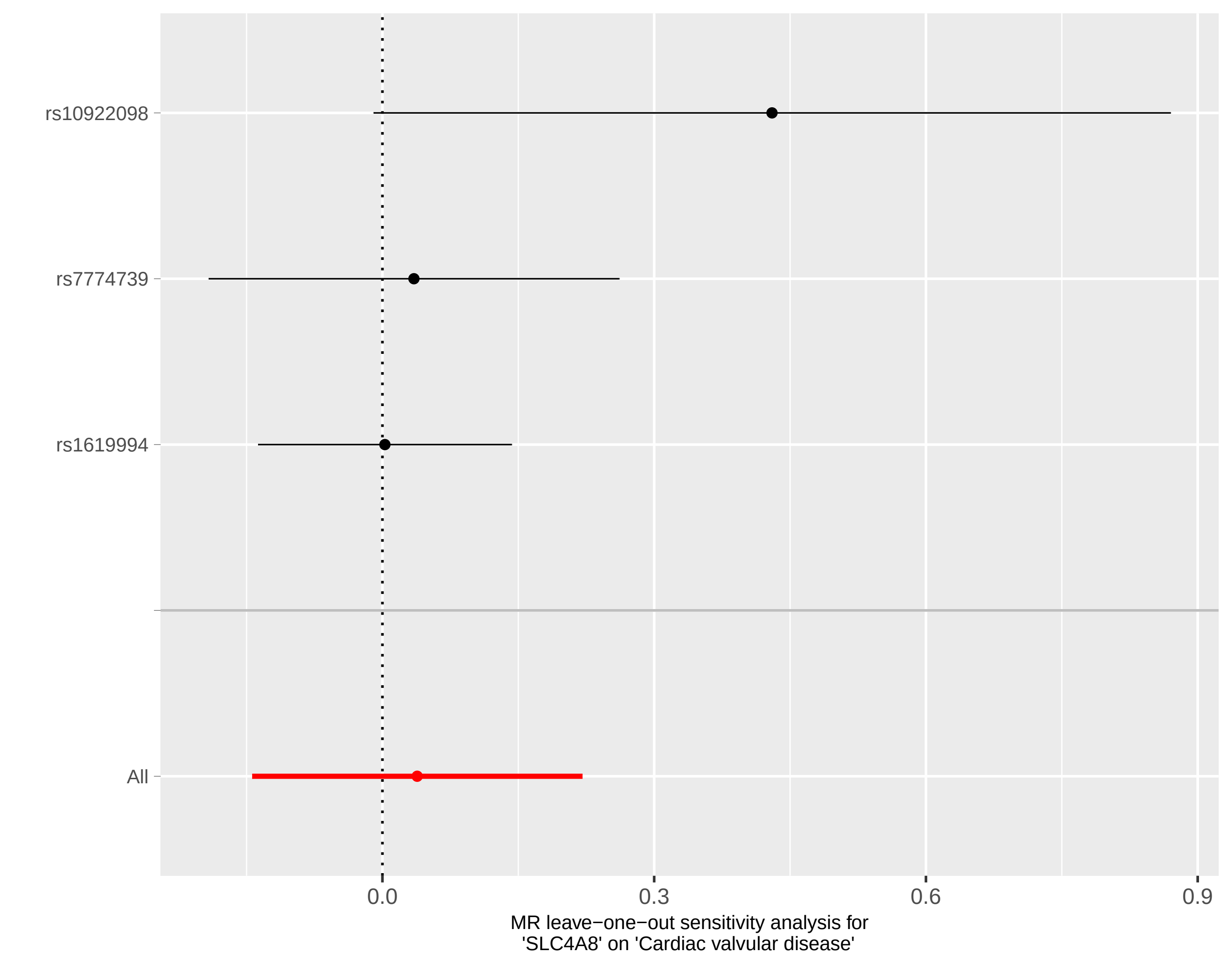
rs10922098
rs7774739
rs1619994
All
0.0
0.3
0.6
0.9
MR leave−one−out sensitivity analysis for
'SLC4A8' on 'Cardiac valvular disease'

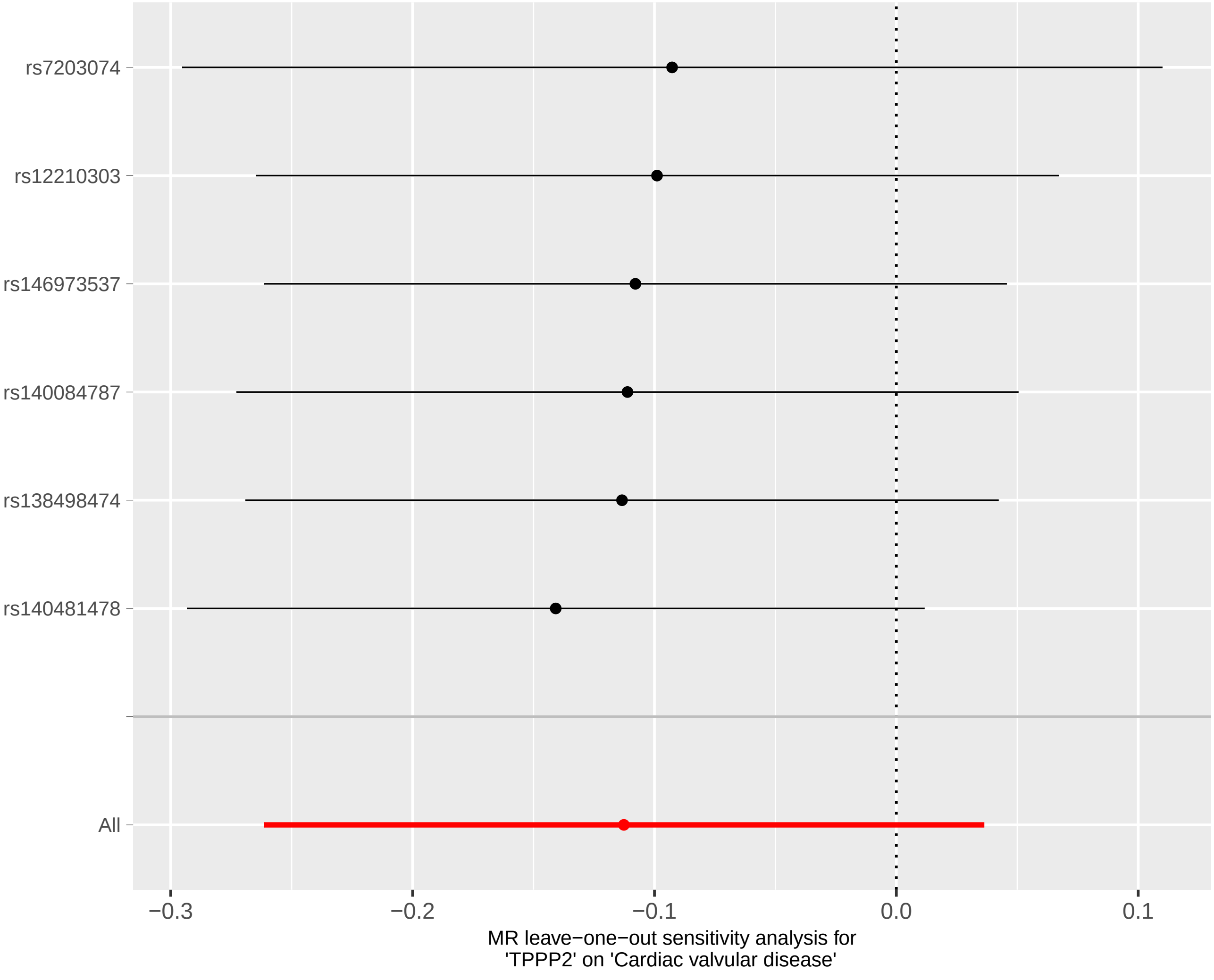
rs7203074
rs12210303
rs146973537
rs140084787
rs138498474
rs140481478
All
−0.3
−0.2
−0.1
0.0
0.1
MR leave−one−out sensitivity analysis for
'TPPP2' on 'Cardiac valvular disease'

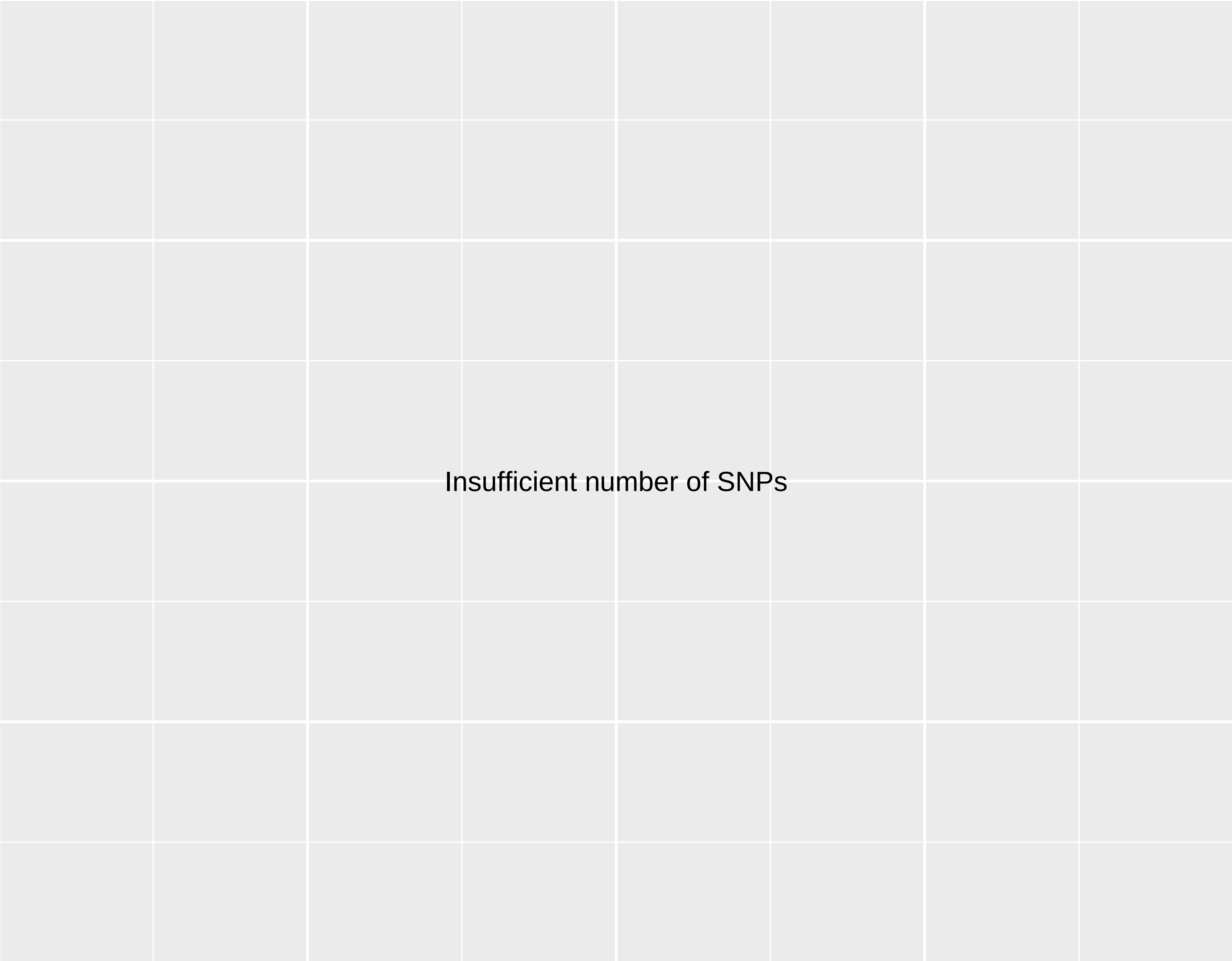
Insufficient number of SNPs

Insufficient number of SNPs

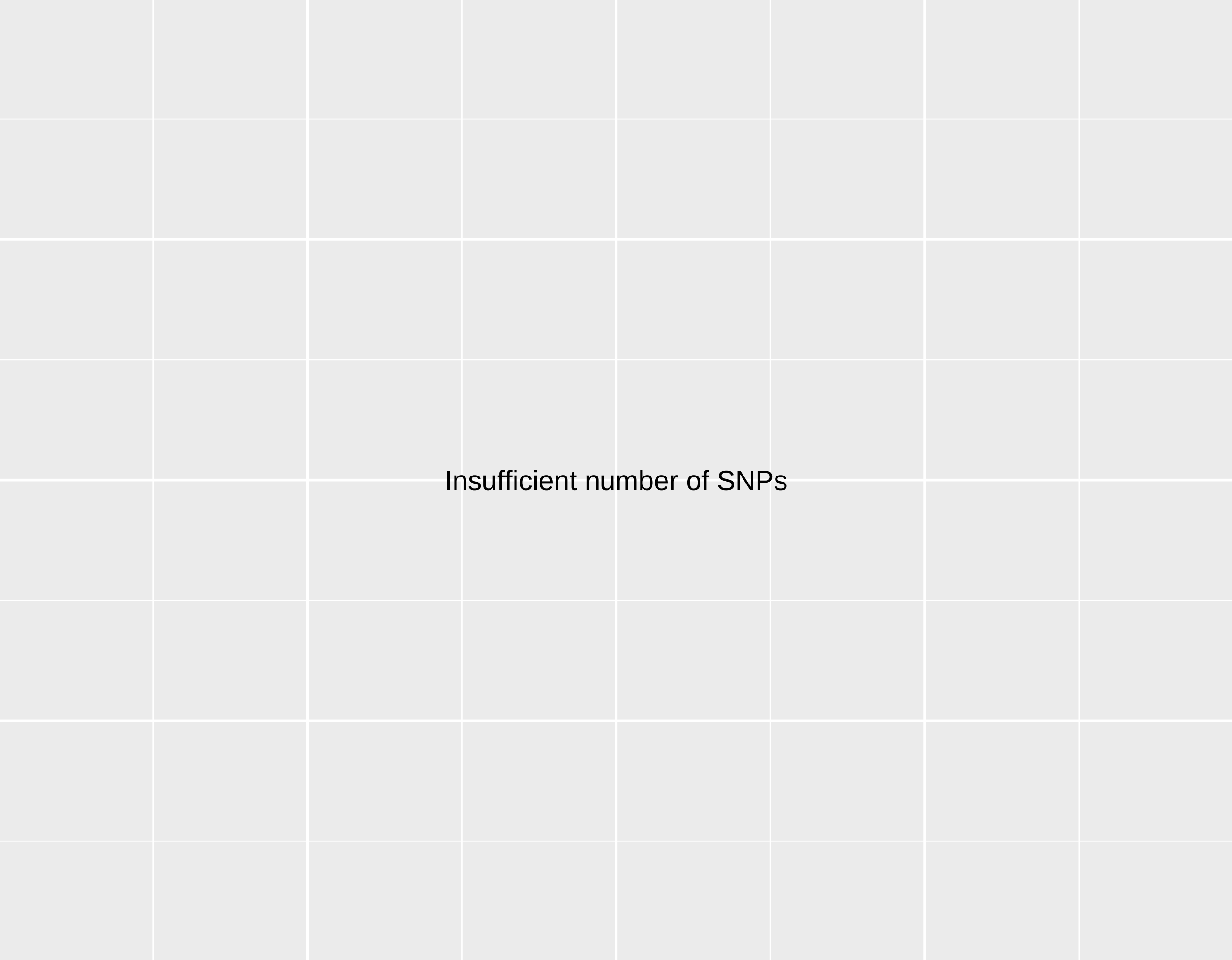
Insufficient number of SNPs

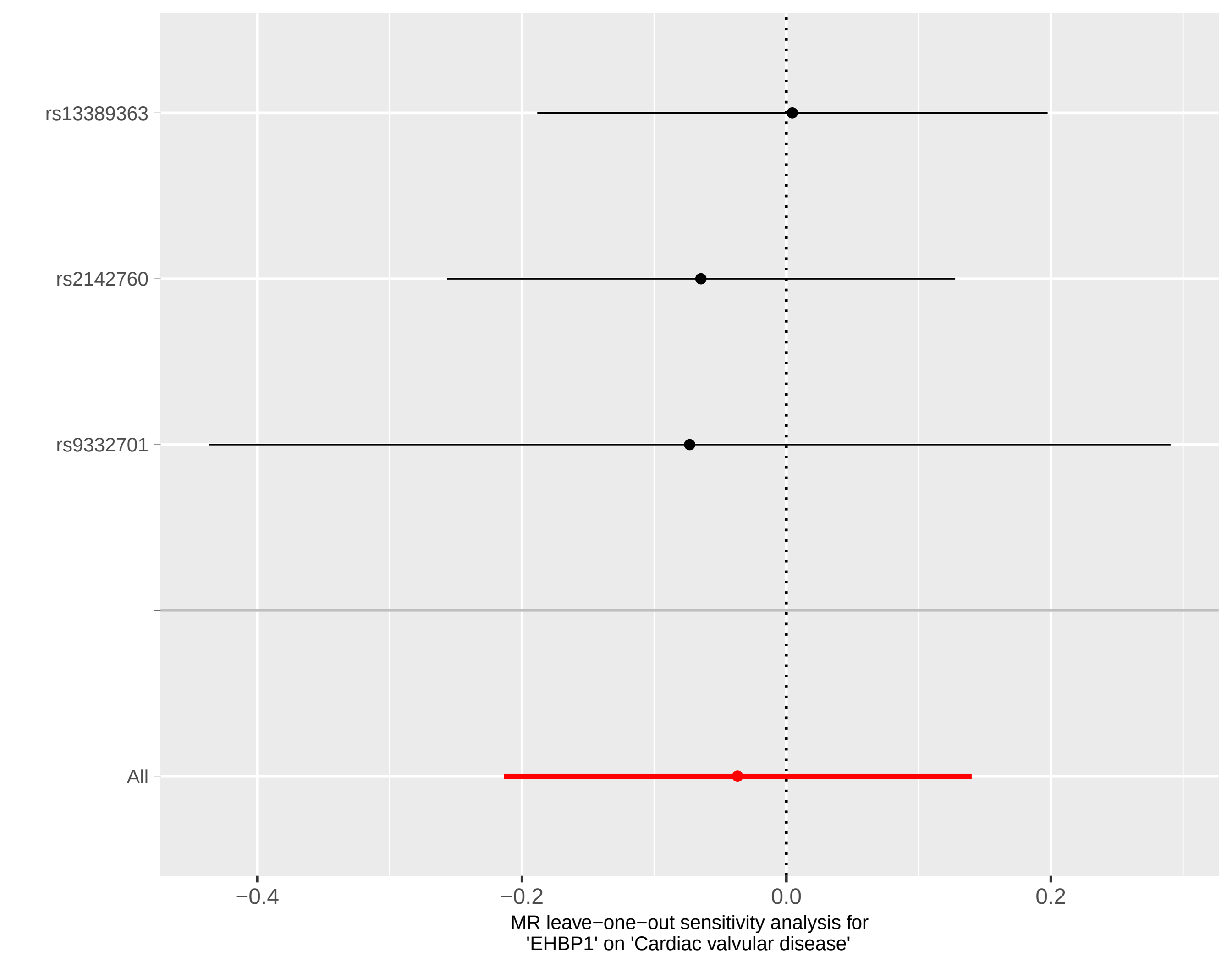

rs13389363
rs2142760
rs9332701
All
−0.4
−0.2
0.0
0.2
MR leave−one−out sensitivity analysis for
'EHBP1' on 'Cardiac valvular disease'

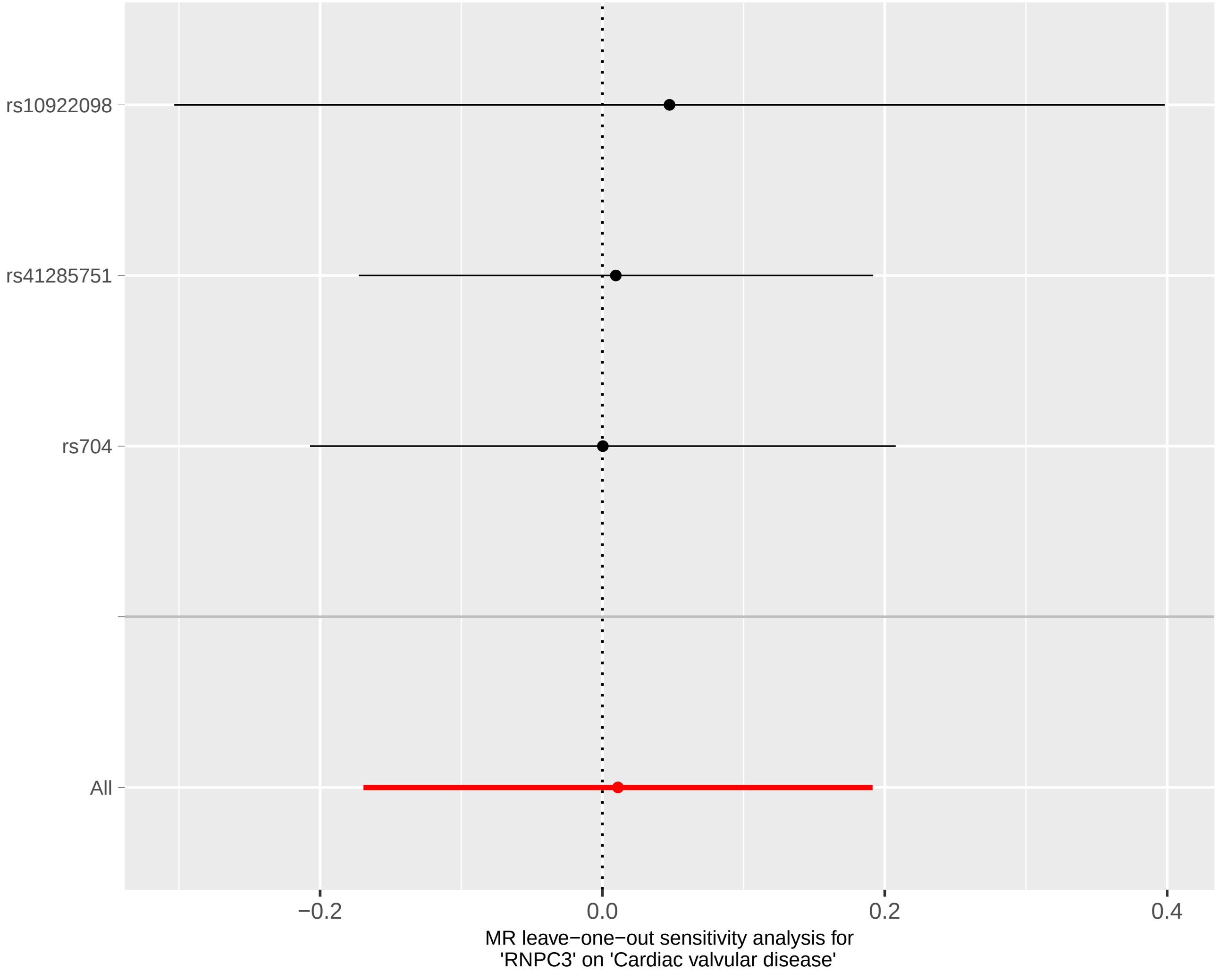
rs10922098
rs41285751
rs704
All
−0.2
0.0
0.2
0.4
MR leave−one−out sensitivity analysis for
'RNPC3' on 'Cardiac valvular disease'

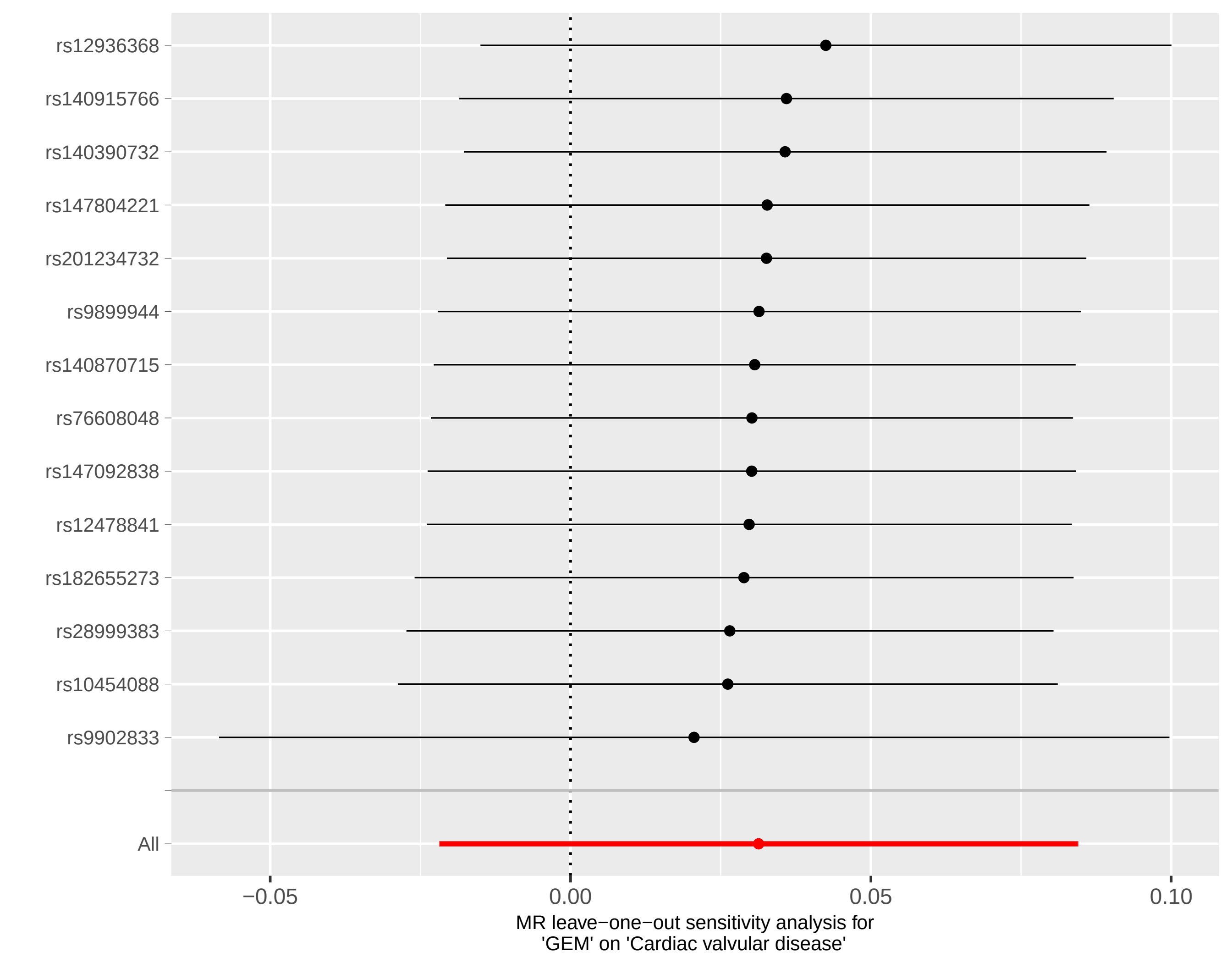
rs12936368
rs140915766
rs140390732
rs147804221
rs201234732
rs9899944
rs140870715
rs76608048
rs147092838
rs12478841
rs182655273
rs28999383
rs10454088
rs9902833
All
−0.05
0.00
0.05
0.10
MR leave−one−out sensitivity analysis for
'GEM' on 'Cardiac valvular disease'

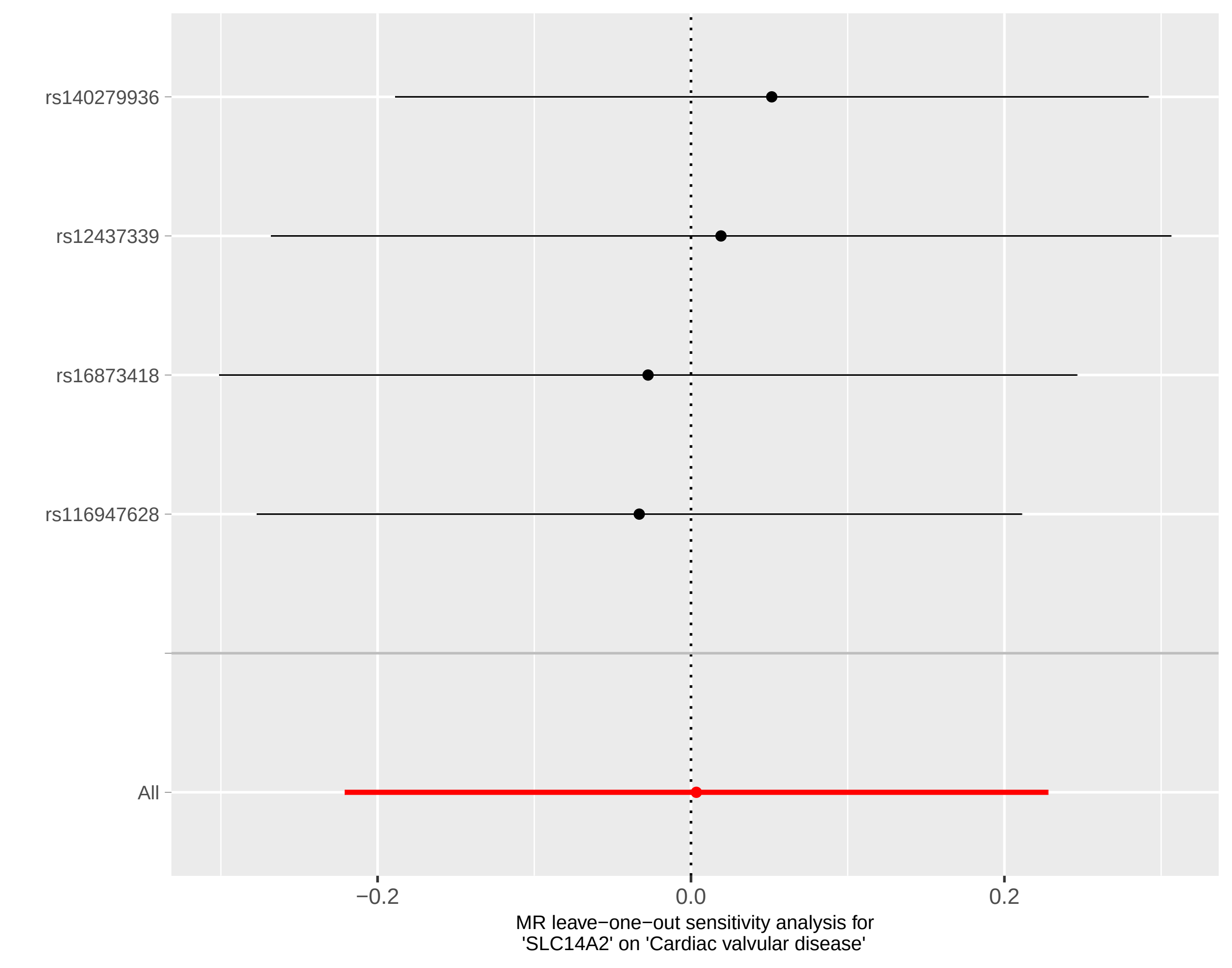
rs140279936
rs12437339
rs16873418
rs116947628
All
−0.2
0.0
0.2
MR leave−one−out sensitivity analysis for
'SLC14A2' on 'Cardiac valvular disease'

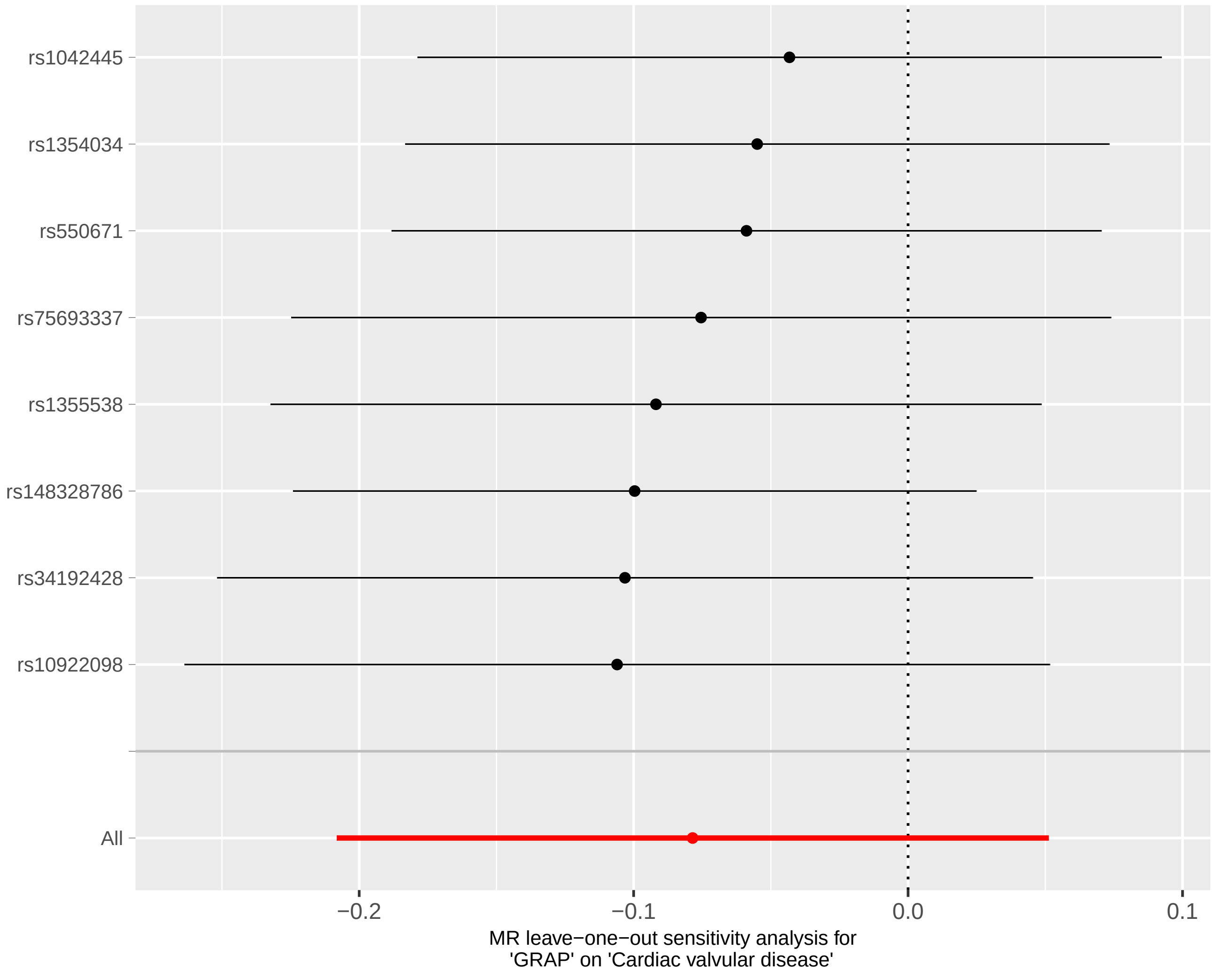
rs1042445
rs1354034
rs550671
rs75693337
rs1355538
rs148328786
rs34192428
rs10922098
All
−0.2
−0.1
0.0
0.1
MR leave−one−out sensitivity analysis for
'GRAP' on 'Cardiac valvular disease'

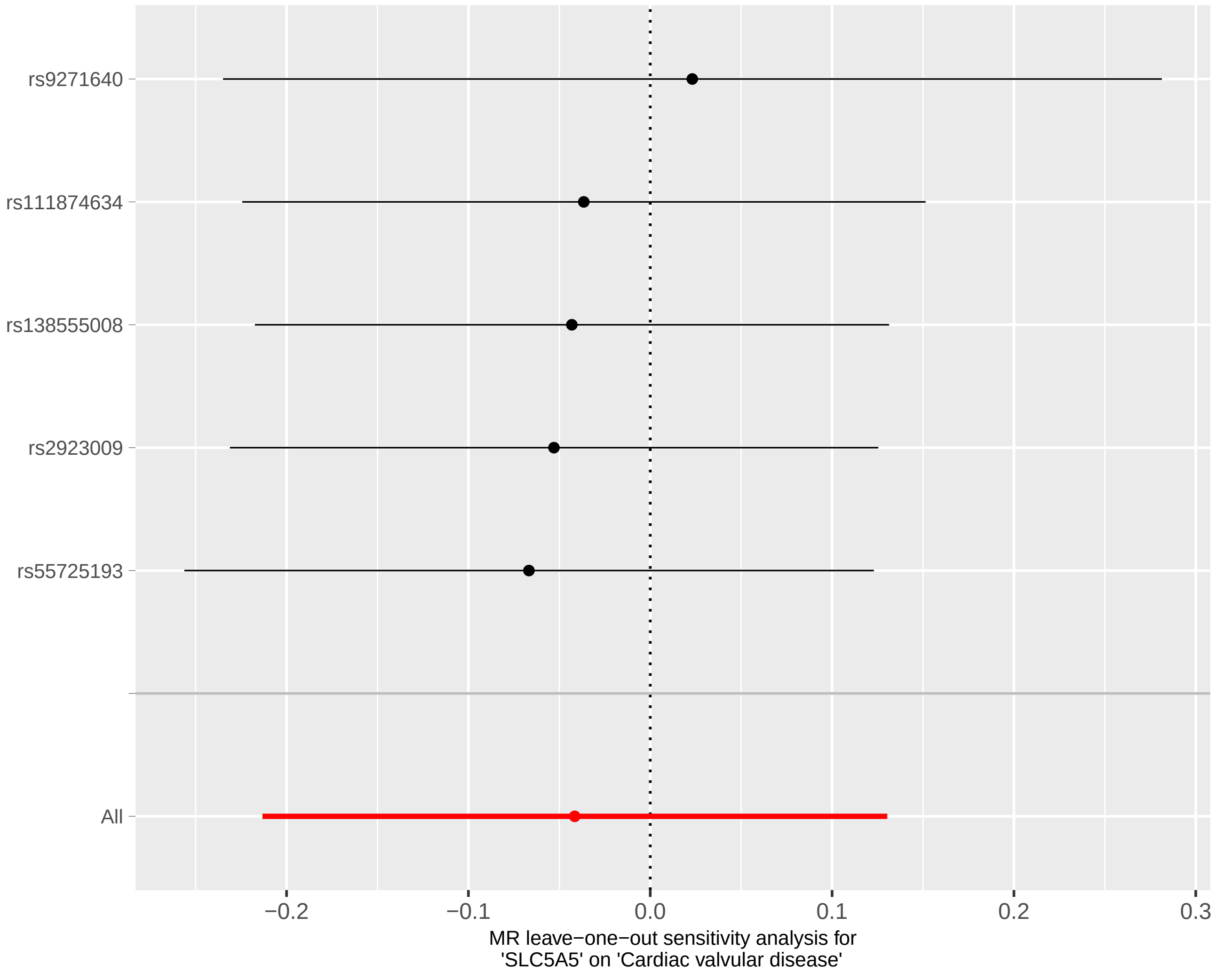
rs9271640
rs111874634
rs138555008
rs2923009
rs55725193
All
−0.2
−0.1
0.0
0.1
0.2
0.3
MR leave−one−out sensitivity analysis for
'SLC5A5' on 'Cardiac valvular disease'

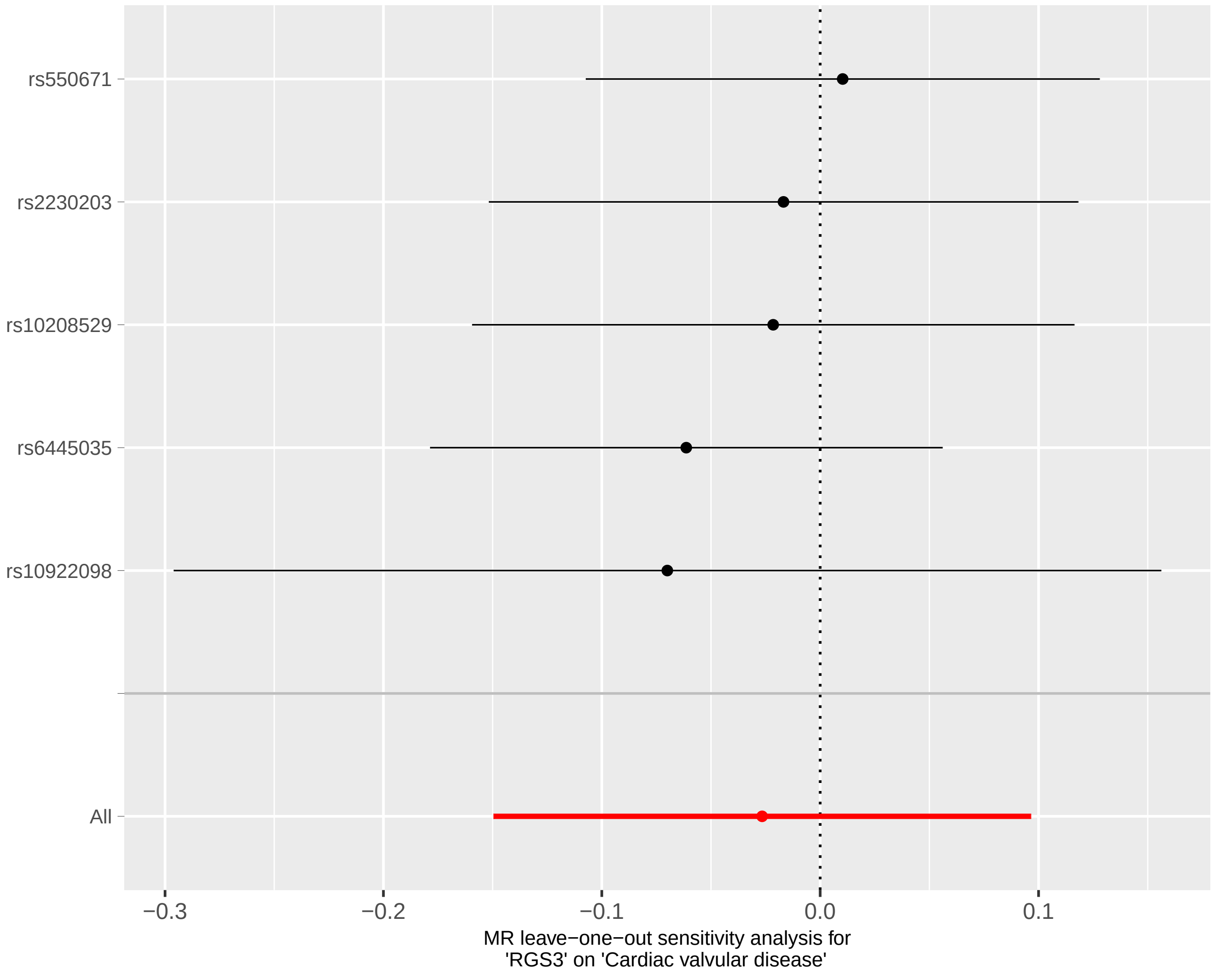
rs550671
rs2230203
rs10208529
rs6445035
rs10922098
All
−0.3
−0.2
−0.1
0.0
0.1
MR leave−one−out sensitivity analysis for
'RGS3' on 'Cardiac valvular disease'

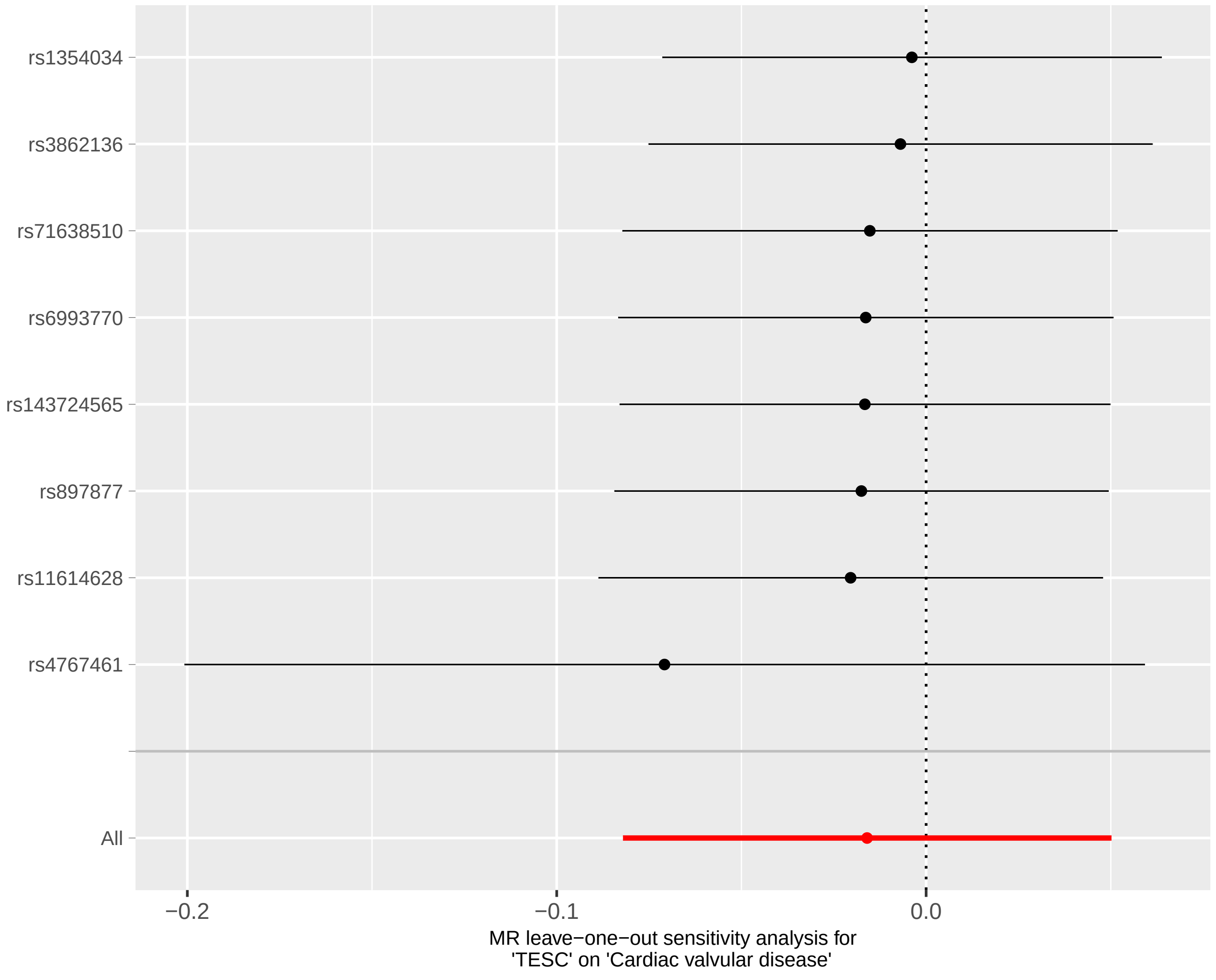
rs1354034
rs3862136
rs71638510
rs6993770
rs143724565
rs897877
rs11614628
rs4767461
All
−0.2
−0.1
0.0
MR leave−one−out sensitivity analysis for
'TESC' on 'Cardiac valvular disease'

Insufficient number of SNPs

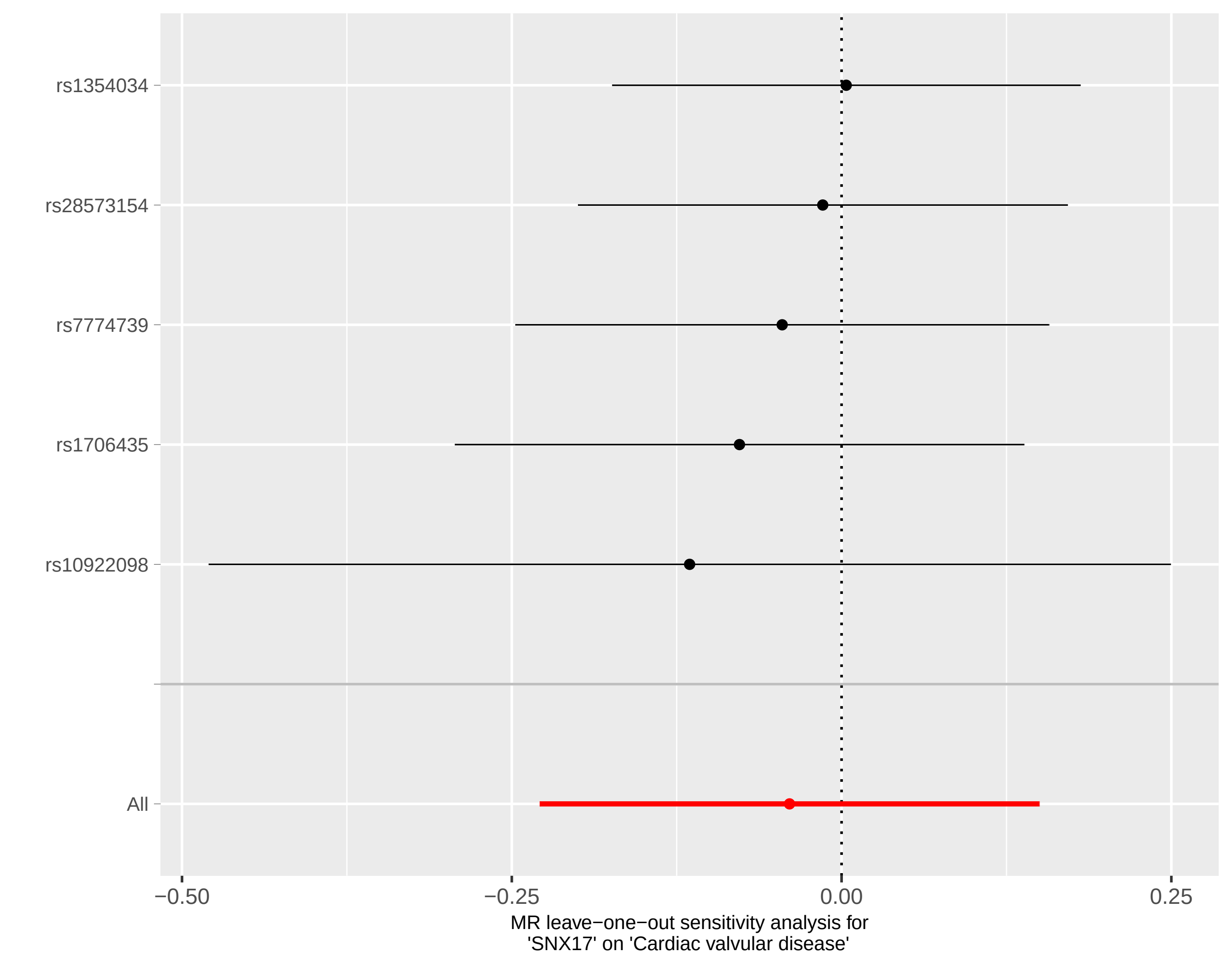
rs1354034
rs28573154
rs7774739
rs1706435
rs10922098
All
−0.50
−0.25
0.00
0.25
MR leave−one−out sensitivity analysis for
'SNX17' on 'Cardiac valvular disease'

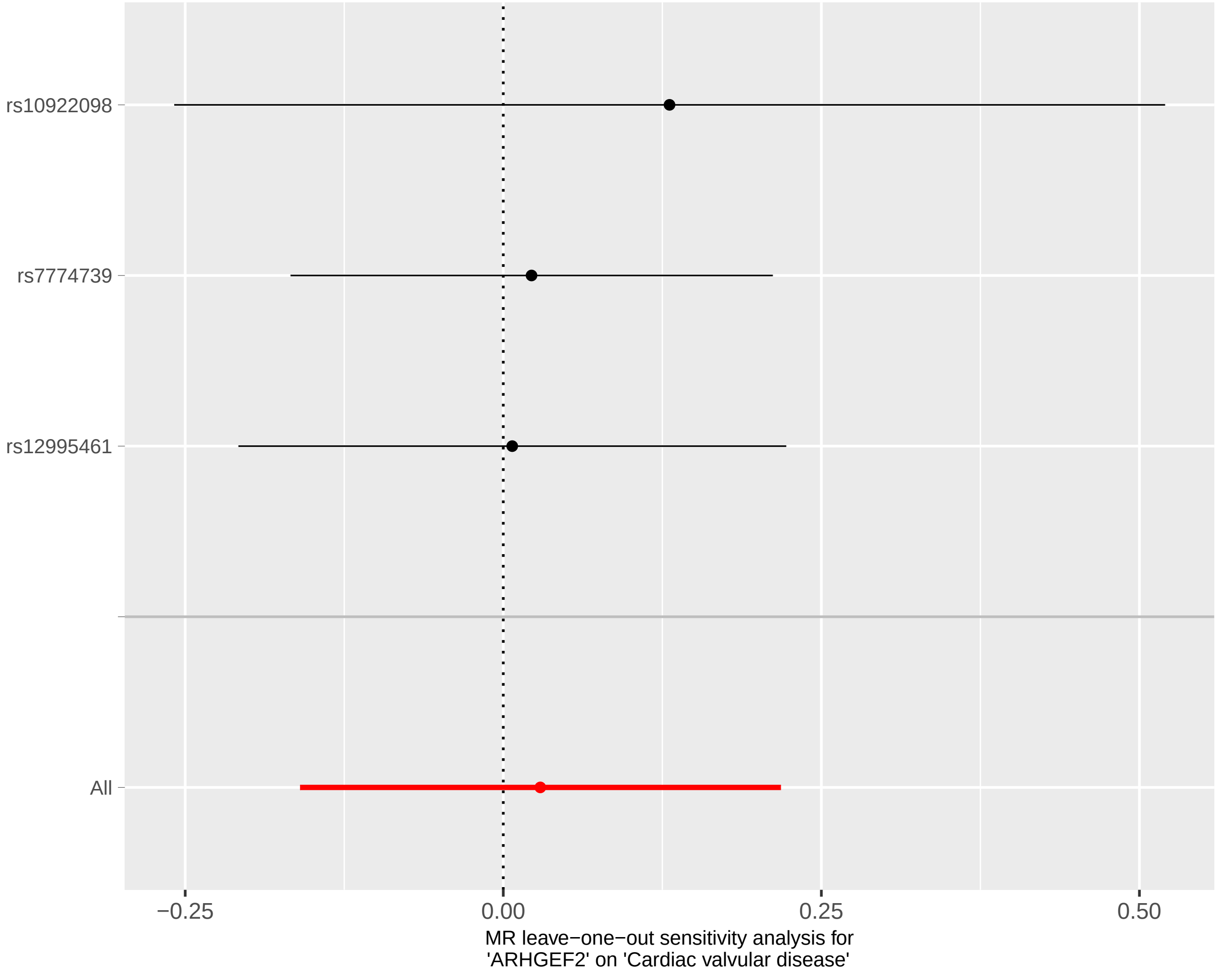

rs10922098
rs7774739
rs12995461
All
−0.25
0.00
0.25
0.50
MR leave−one−out sensitivity analysis for
'ARHGEF2' on 'Cardiac valvular disease'

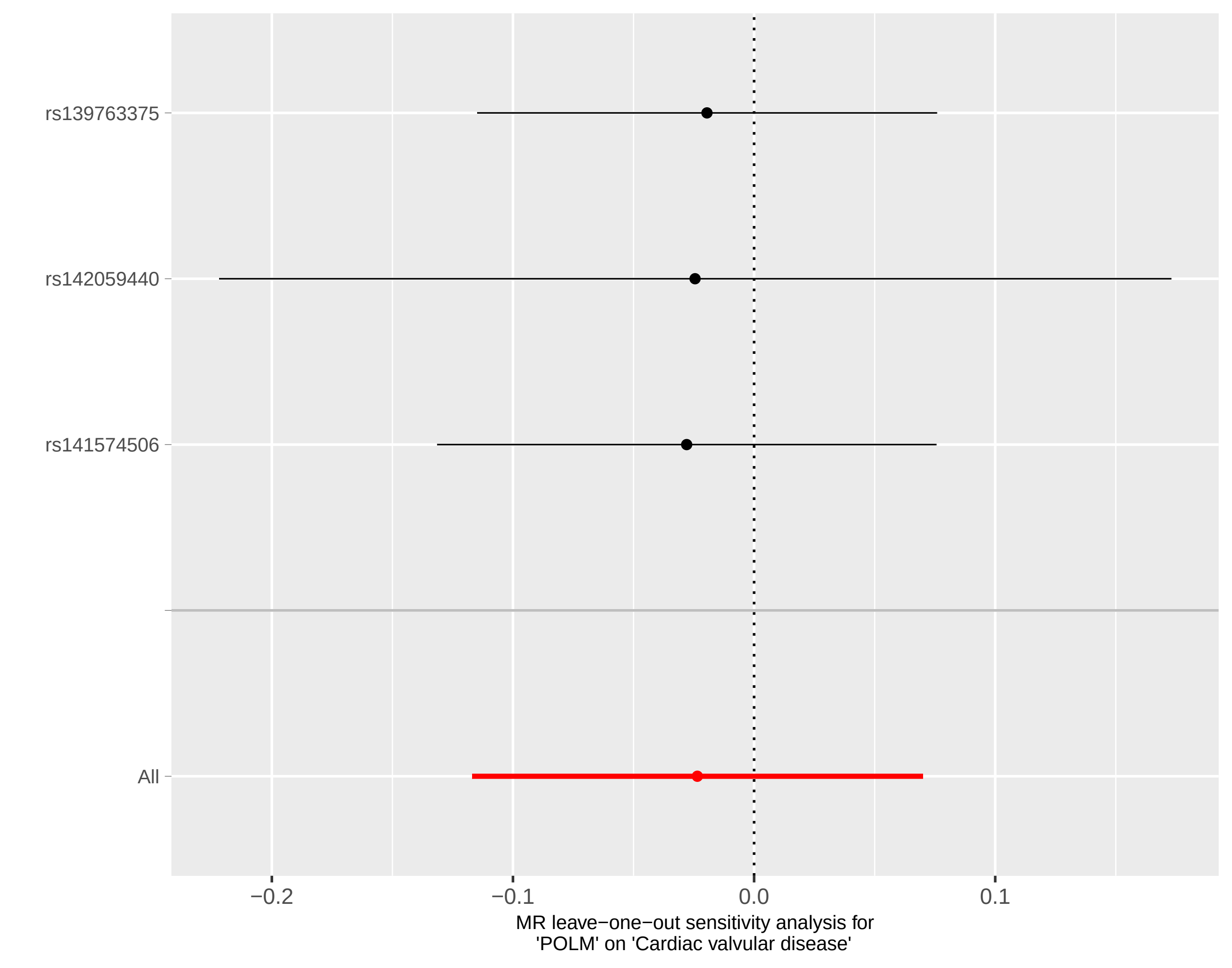
rs139763375
rs142059440
rs141574506
All
−0.2
−0.1
0.0
0.1
MR leave−one−out sensitivity analysis for
'POLM' on 'Cardiac valvular disease'

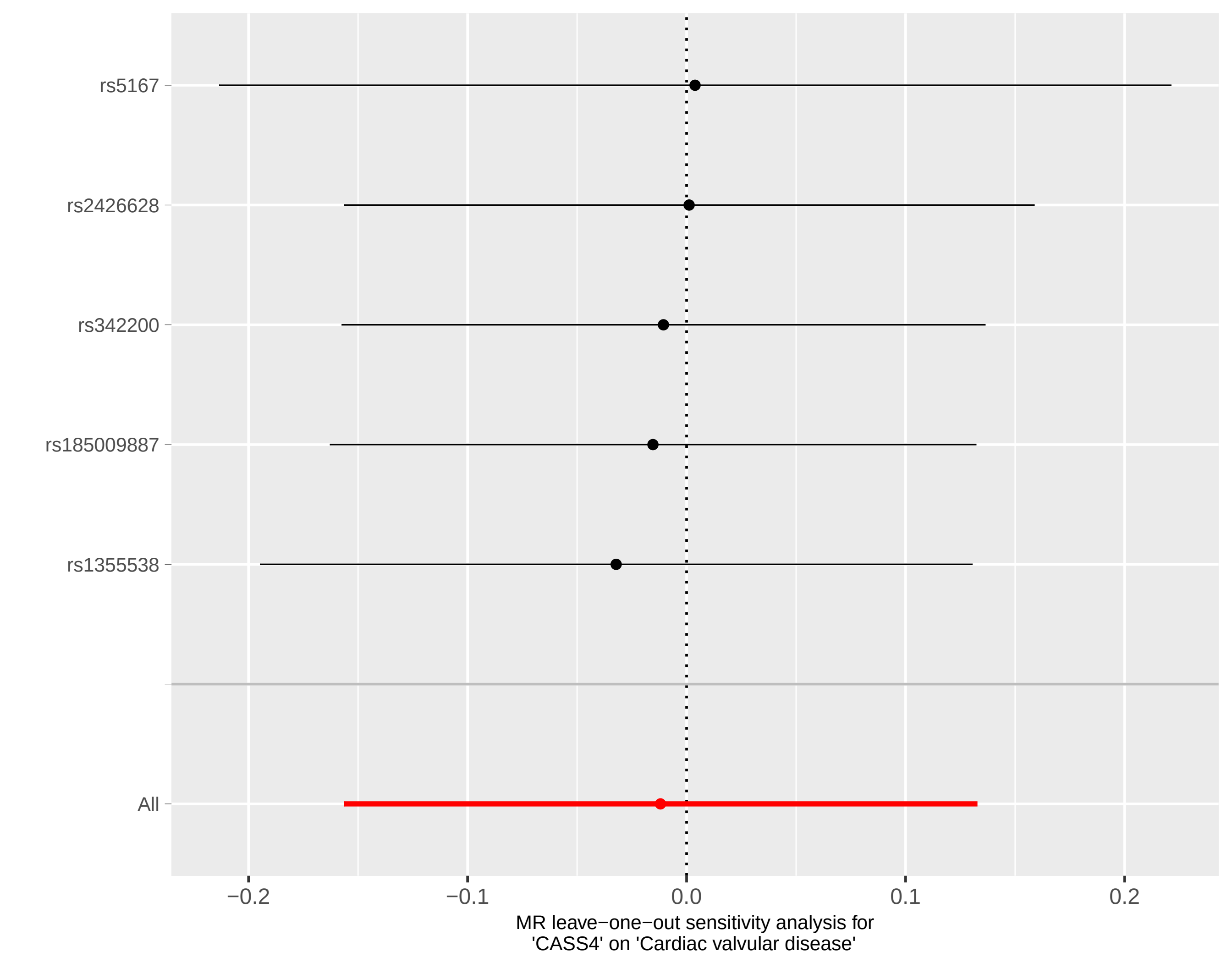
rs5167
rs2426628
rs342200
rs185009887
rs1355538
All
−0.2
−0.1
0.0
0.1
0.2
MR leave−one−out sensitivity analysis for
'CASS4' on 'Cardiac valvular disease'

Insufficient number of SNPs

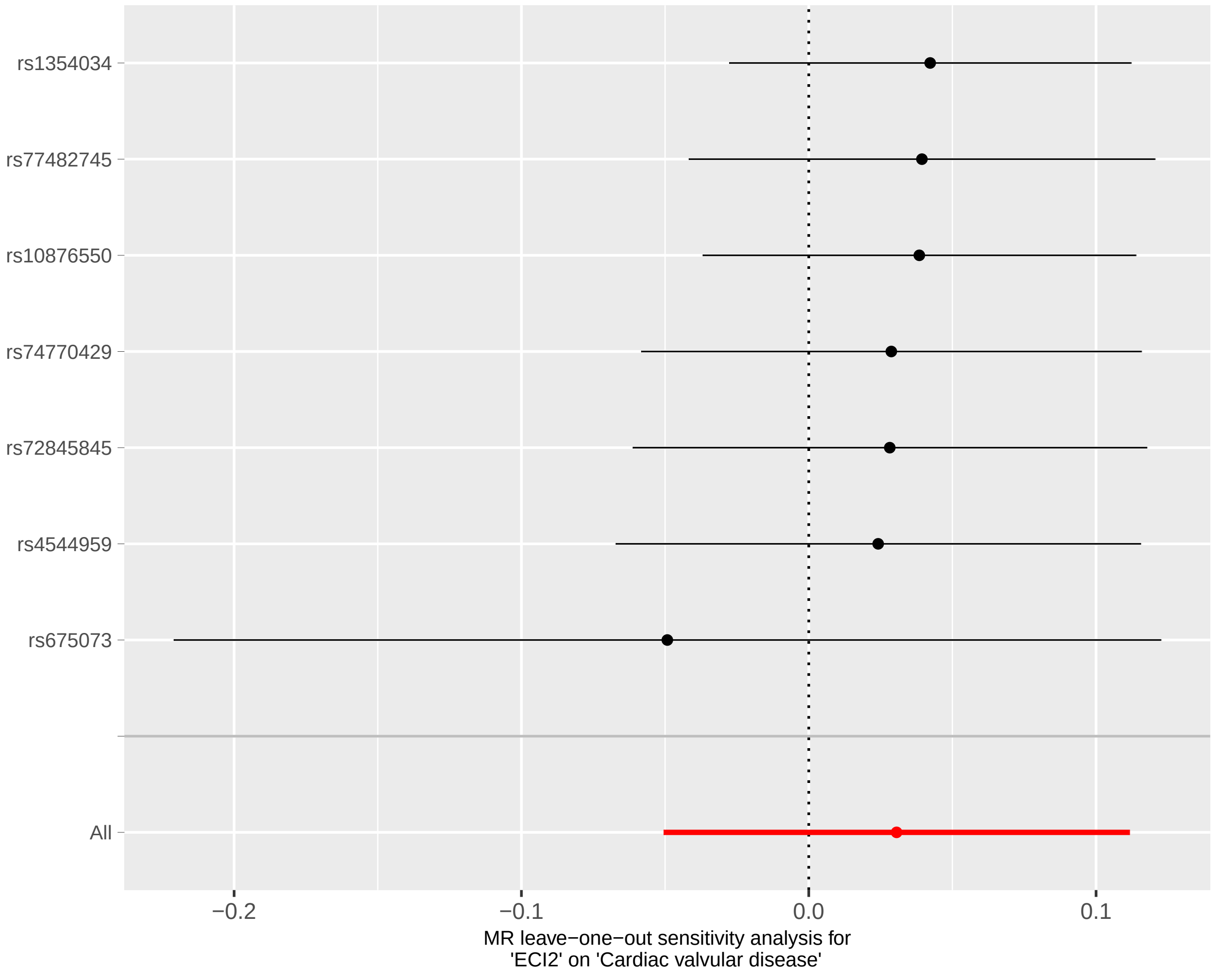
rs1354034
rs77482745
rs10876550
rs74770429
rs72845845
rs4544959
rs675073
All
−0.2
−0.1
0.0
0.1
MR leave−one−out sensitivity analysis for
'ECI2' on 'Cardiac valvular disease'

Insufficient number of SNPs

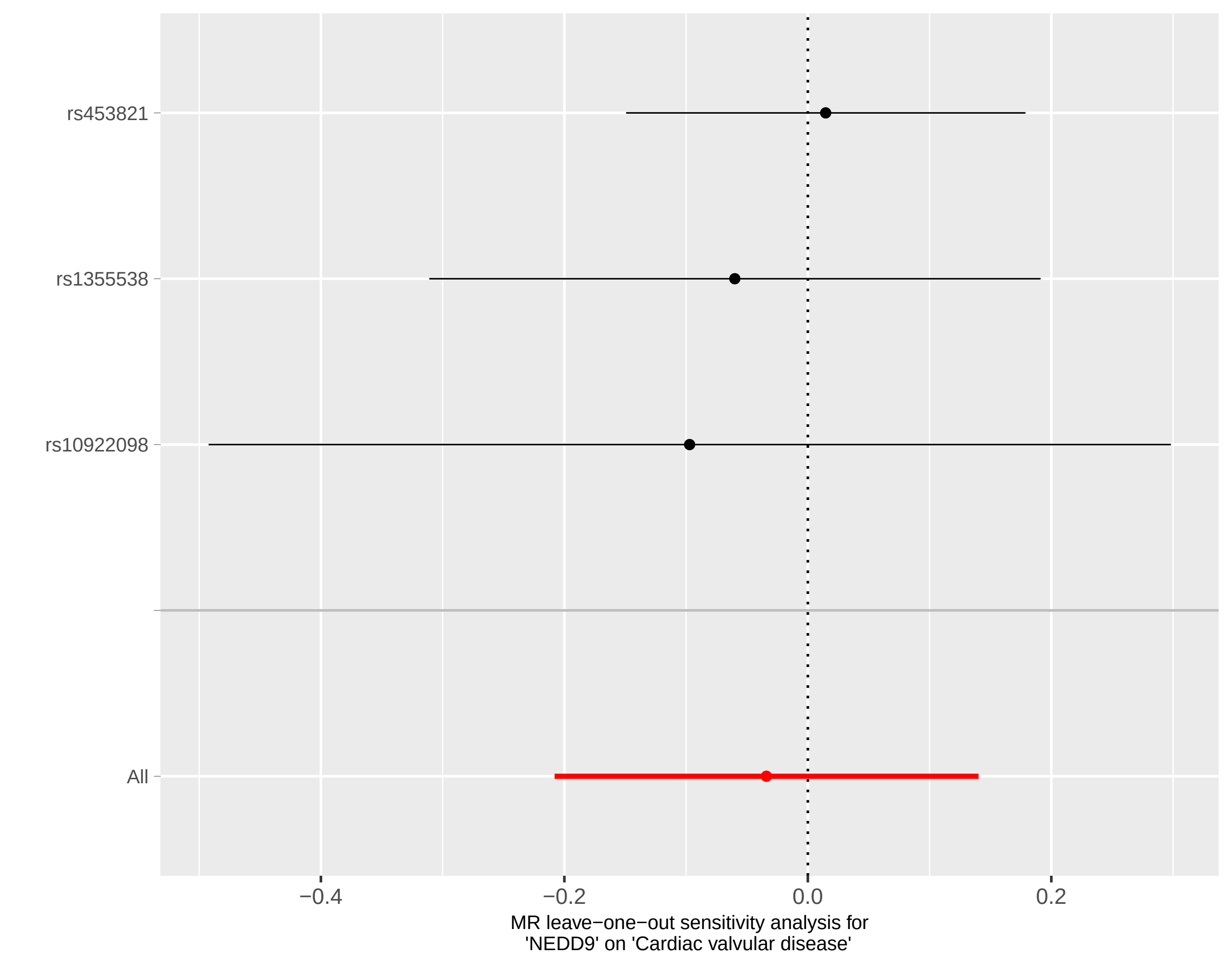

rs453821
rs1355538
rs10922098
All
−0.4
−0.2
0.0
0.2
MR leave−one−out sensitivity analysis for
'NEDD9' on 'Cardiac valvular disease'

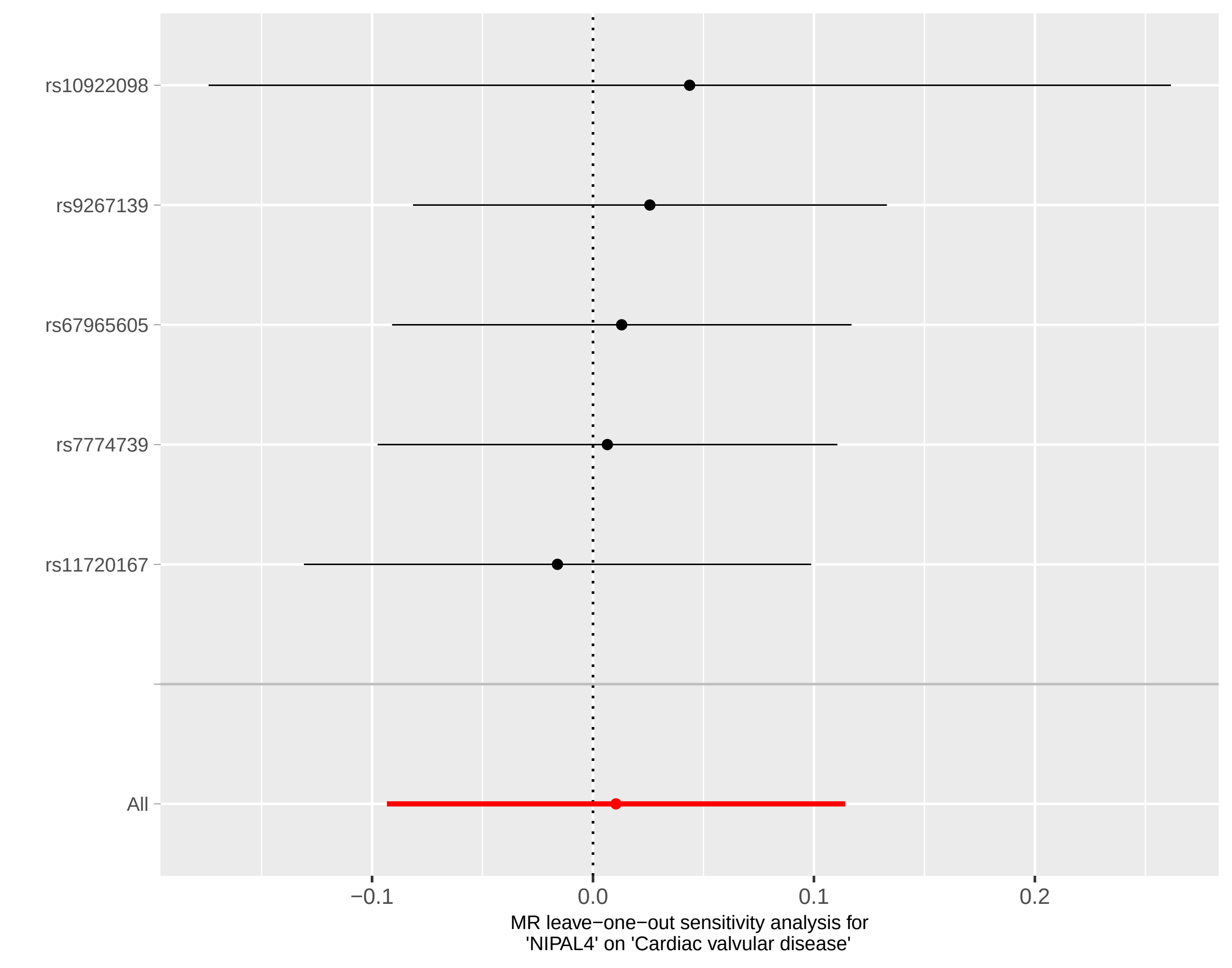
rs10922098
rs9267139
rs67965605
rs7774739
rs11720167
All
−0.1
0.0
0.1
0.2
MR leave−one−out sensitivity analysis for
'NIPAL4' on 'Cardiac valvular disease'

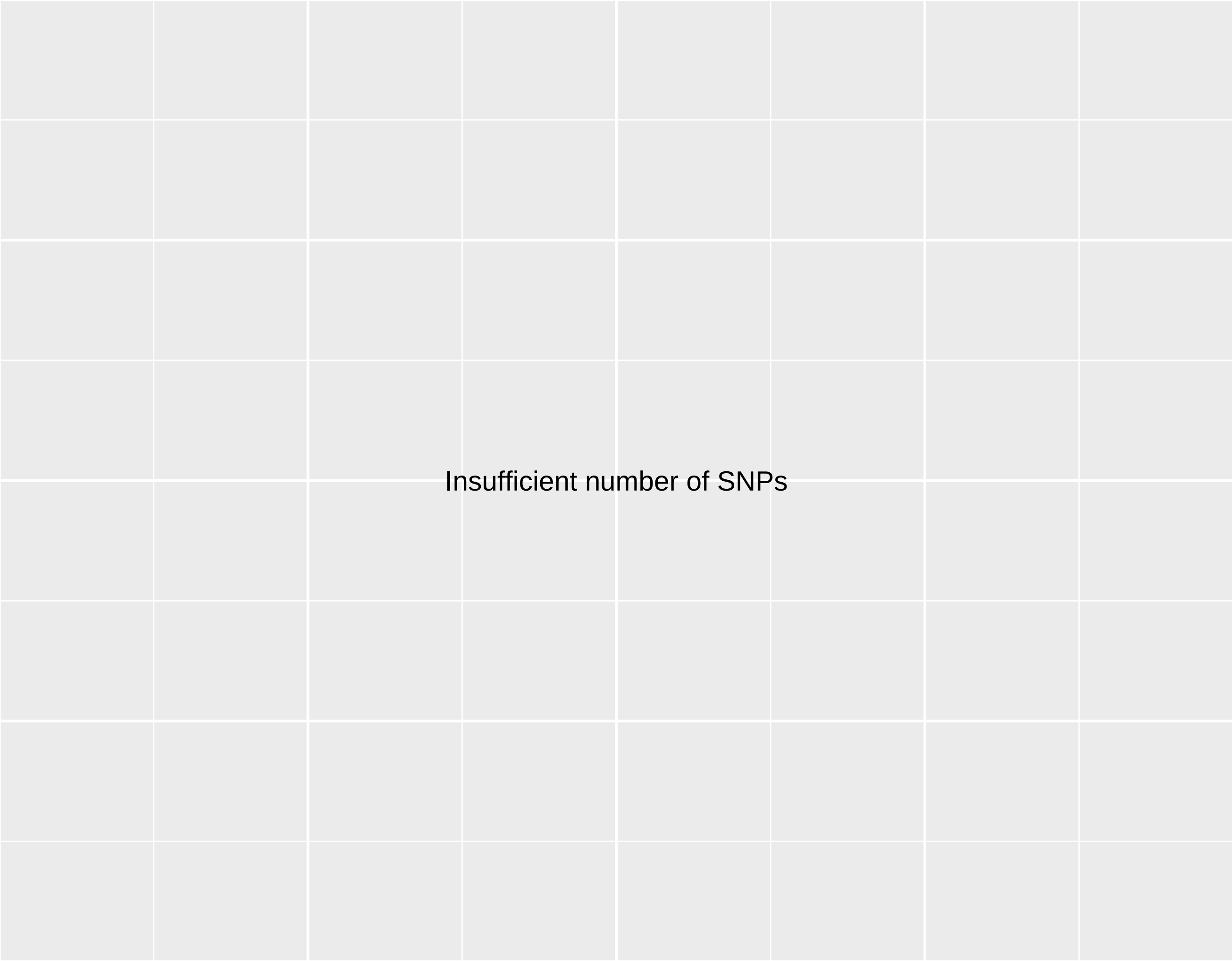
Insufficient number of SNPs

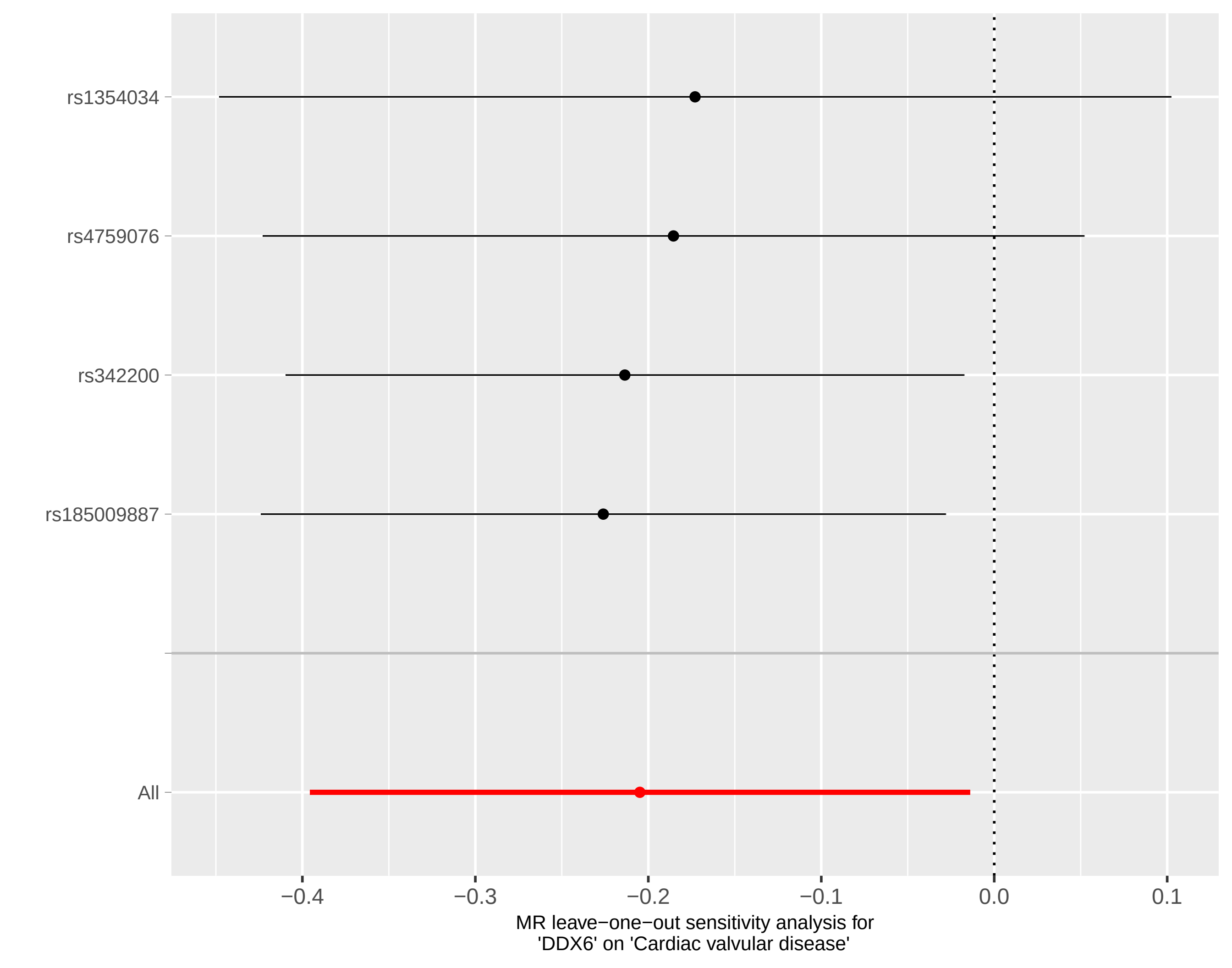
rs1354034
rs4759076
rs342200
rs185009887
All
−0.4
−0.3
−0.2
−0.1
0.0
0.1
MR leave−one−out sensitivity analysis for
'DDX6' on 'Cardiac valvular disease'

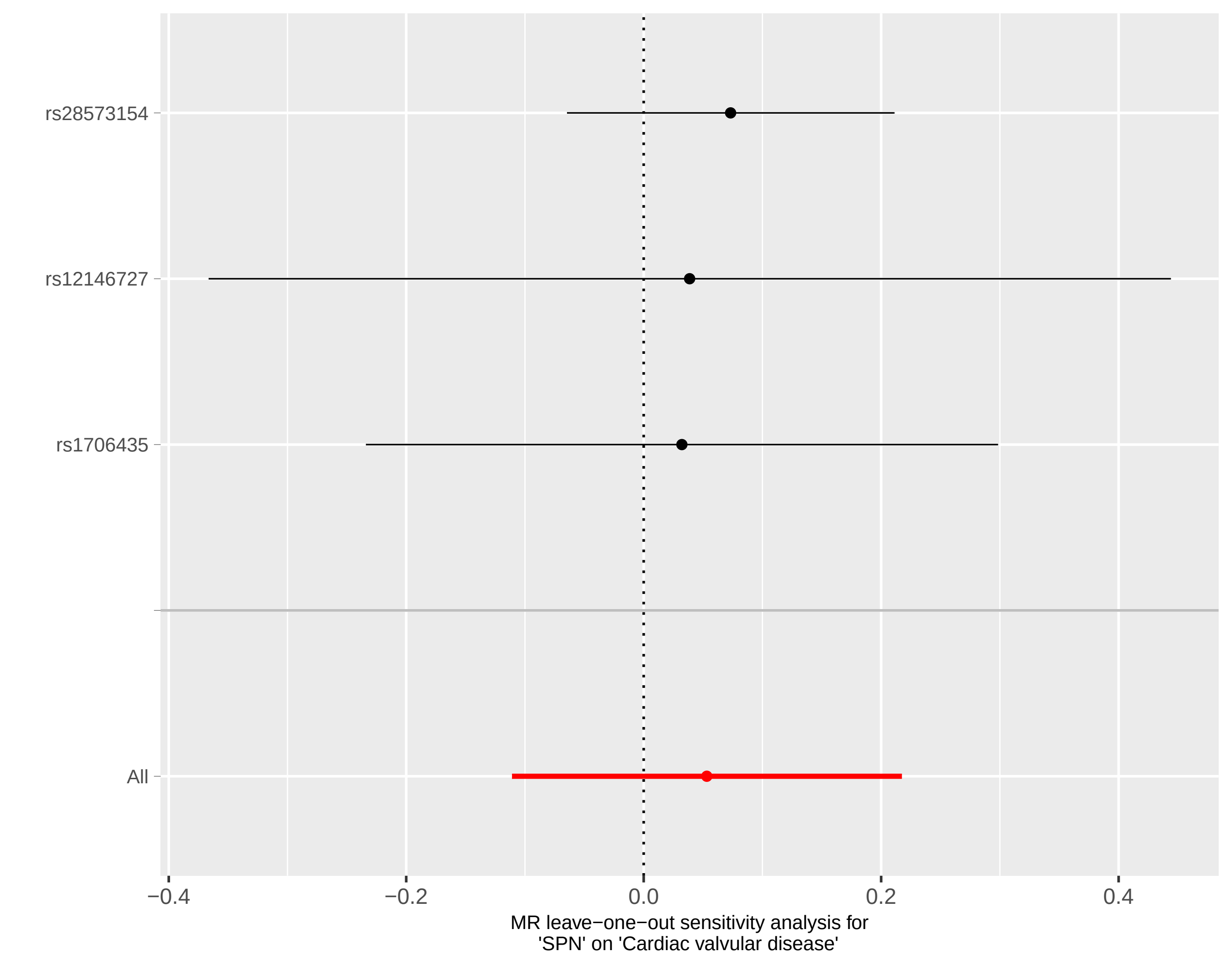
rs28573154
rs12146727
rs1706435
All
−0.4
−0.2
0.0
0.2
0.4
MR leave−one−out sensitivity analysis for
'SPN' on 'Cardiac valvular disease'

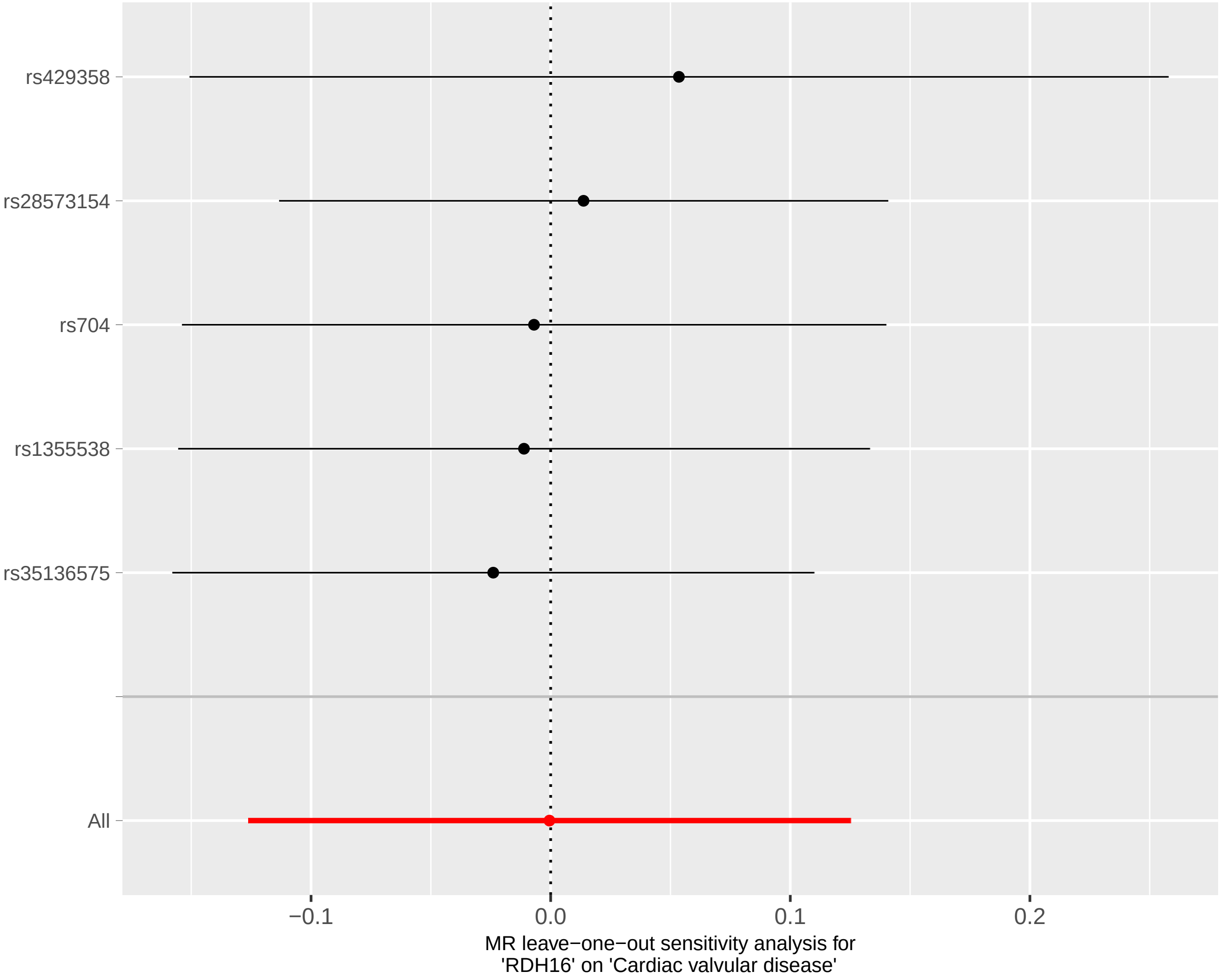

rs429358
rs28573154
rs704
rs1355538
rs35136575
All
−0.1
0.0
0.1
0.2
MR leave−one−out sensitivity analysis for
'RDH16' on 'Cardiac valvular disease'

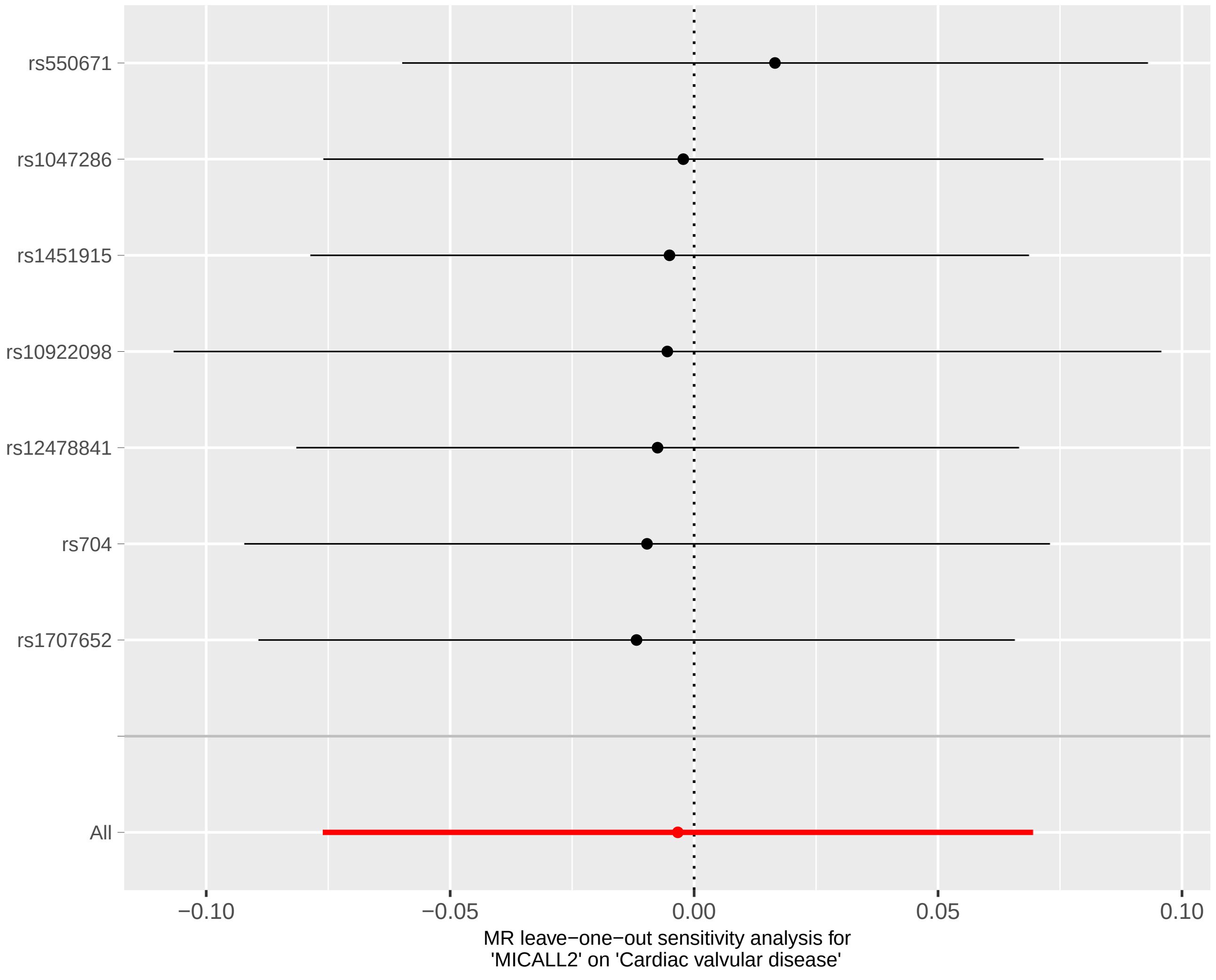
rs550671
rs1047286
rs1451915
rs10922098
rs12478841
rs704
rs1707652
All
−0.10
−0.05
0.00
0.05
0.10
MR leave−one−out sensitivity analysis for
'MICALL2' on 'Cardiac valvular disease'

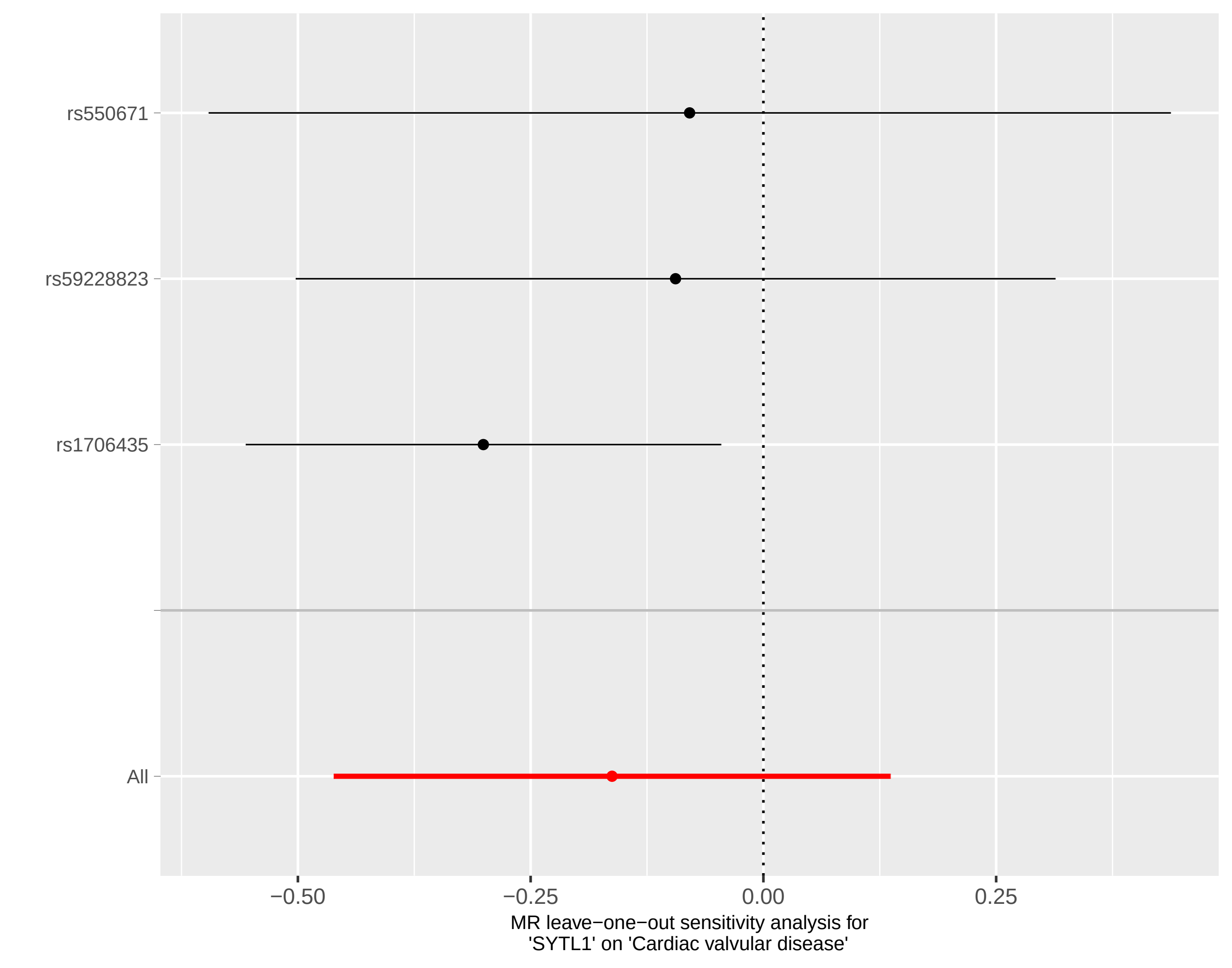
rs550671
rs59228823
rs1706435
All
−0.50
−0.25
0.00
0.25
MR leave−one−out sensitivity analysis for
'SYTL1' on 'Cardiac valvular disease'

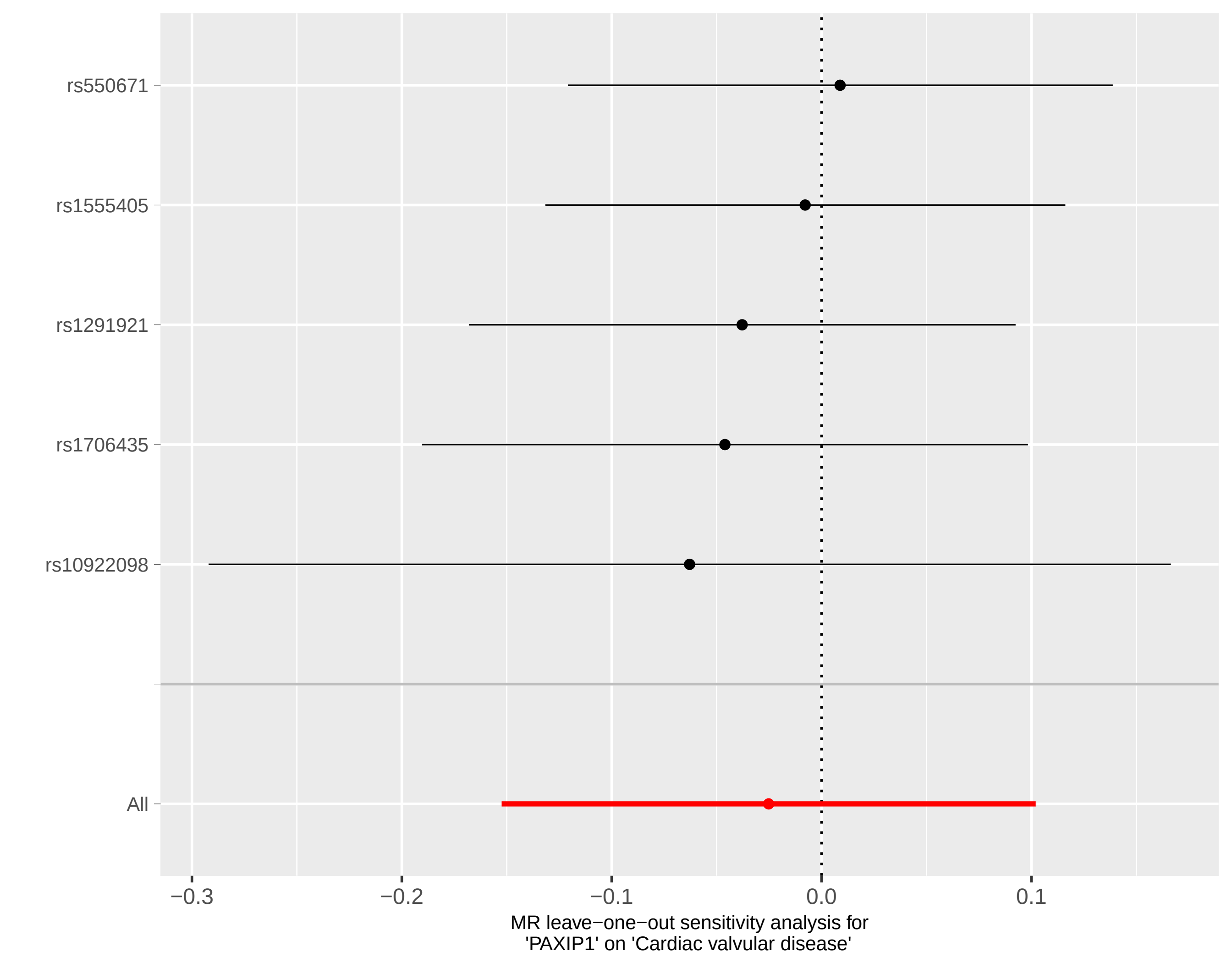

rs550671
rs1555405
rs1291921
rs1706435
rs10922098
All
−0.3
−0.2
−0.1
0.0
0.1
MR leave−one−out sensitivity analysis for
'PAXIP1' on 'Cardiac valvular disease'

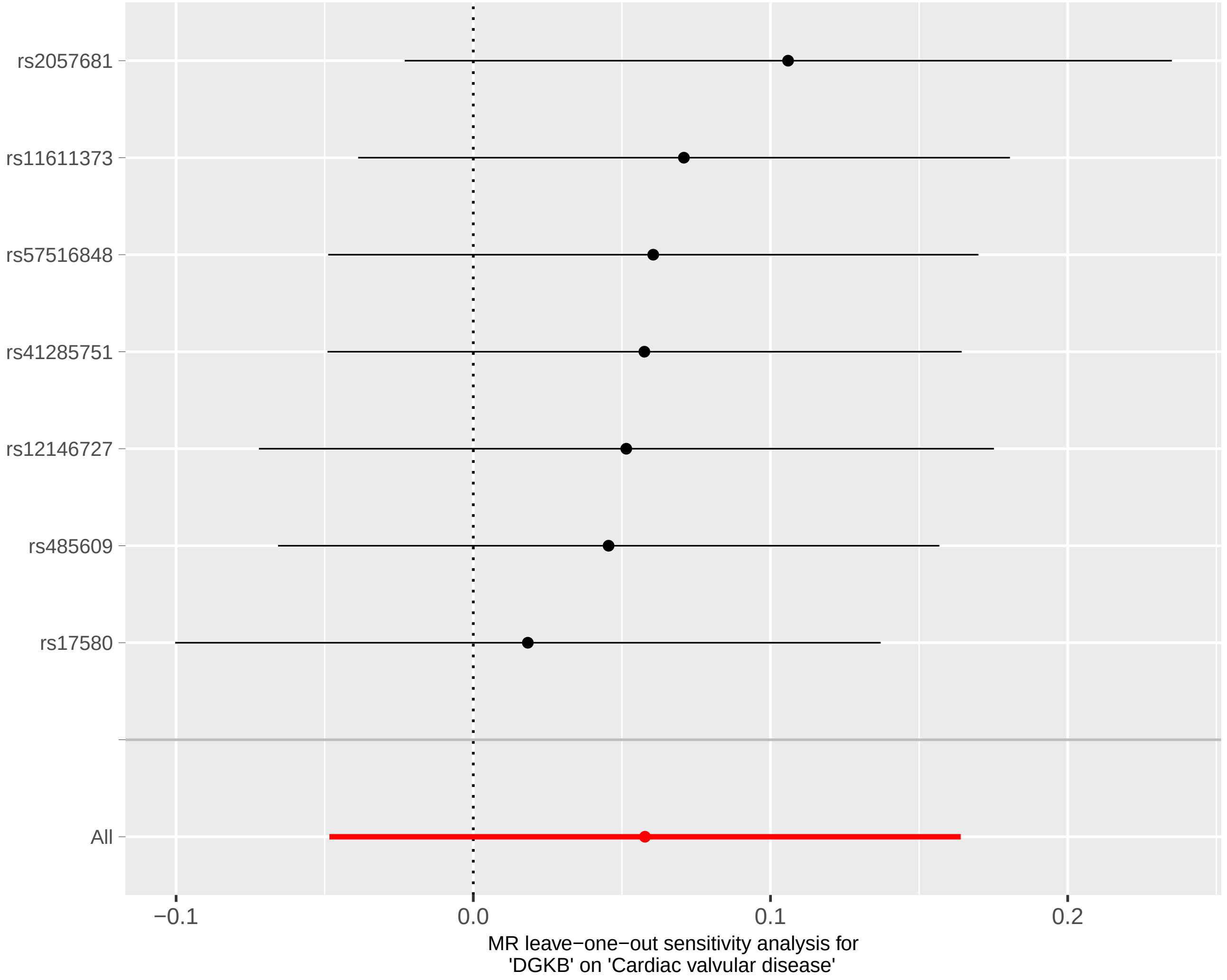
rs2057681
rs11611373
rs57516848
rs41285751
rs12146727
rs485609
rs17580
All
−0.1
0.0
0.1
0.2
MR leave−one−out sensitivity analysis for
'DGKB' on 'Cardiac valvular disease'

Insufficient number of SNPs

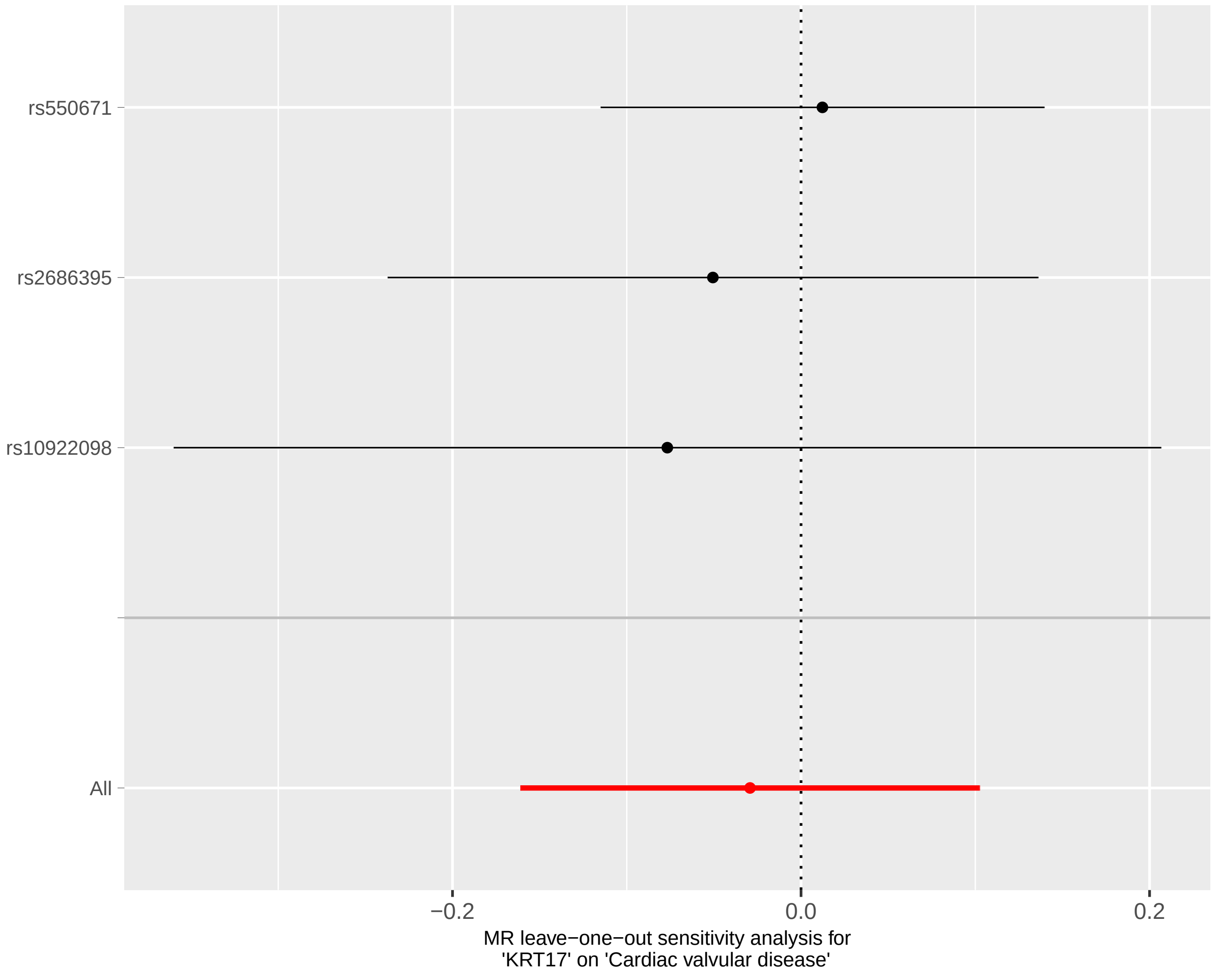
rs550671
rs2686395
rs10922098
All
−0.2
0.0
0.2
MR leave−one−out sensitivity analysis for
'KRT17' on 'Cardiac valvular disease'

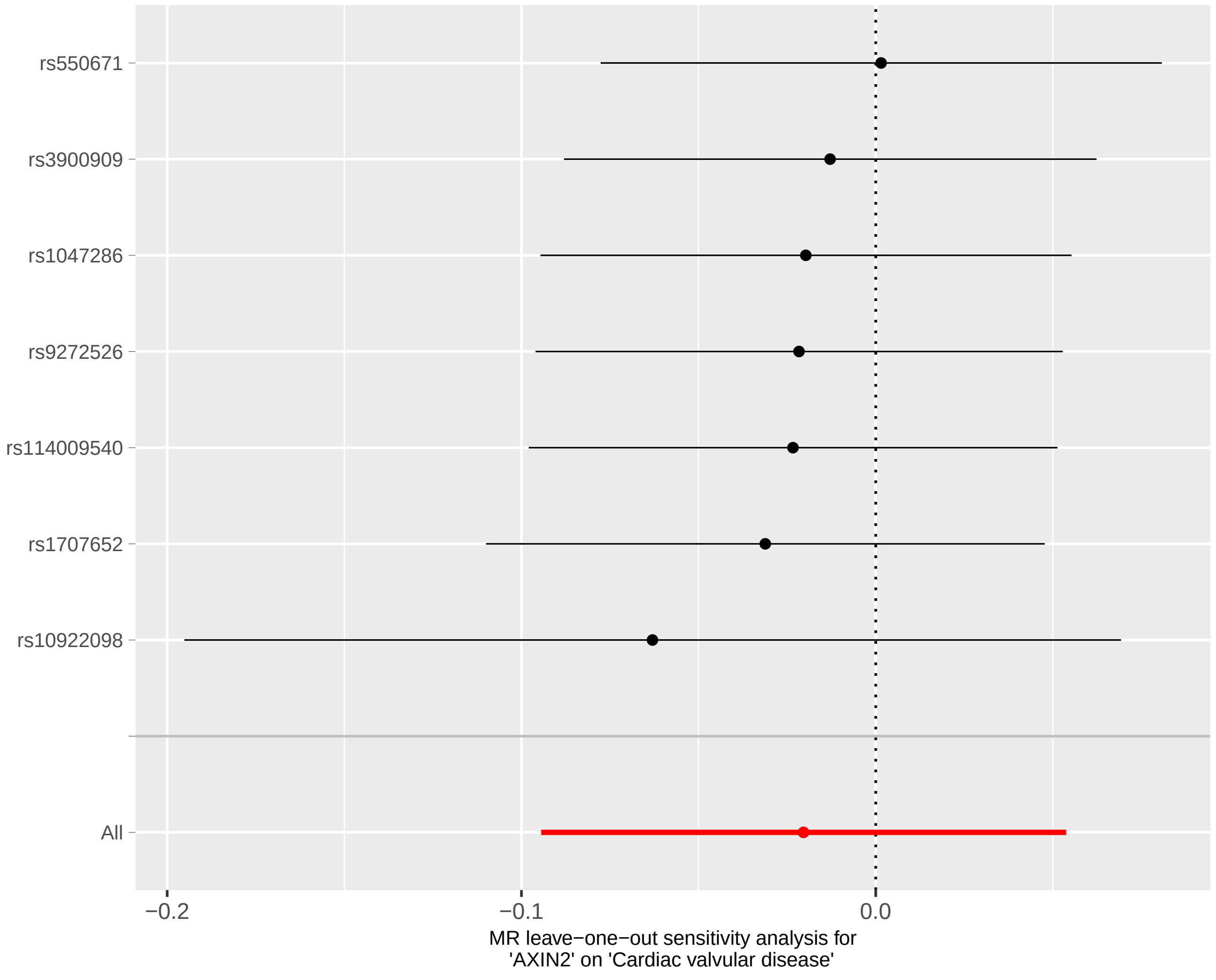
rs550671
rs3900909
rs1047286
rs9272526
rs114009540
rs1707652
rs10922098
All
−0.2
−0.1
0.0
MR leave−one−out sensitivity analysis for
'AXIN2' on 'Cardiac valvular disease'

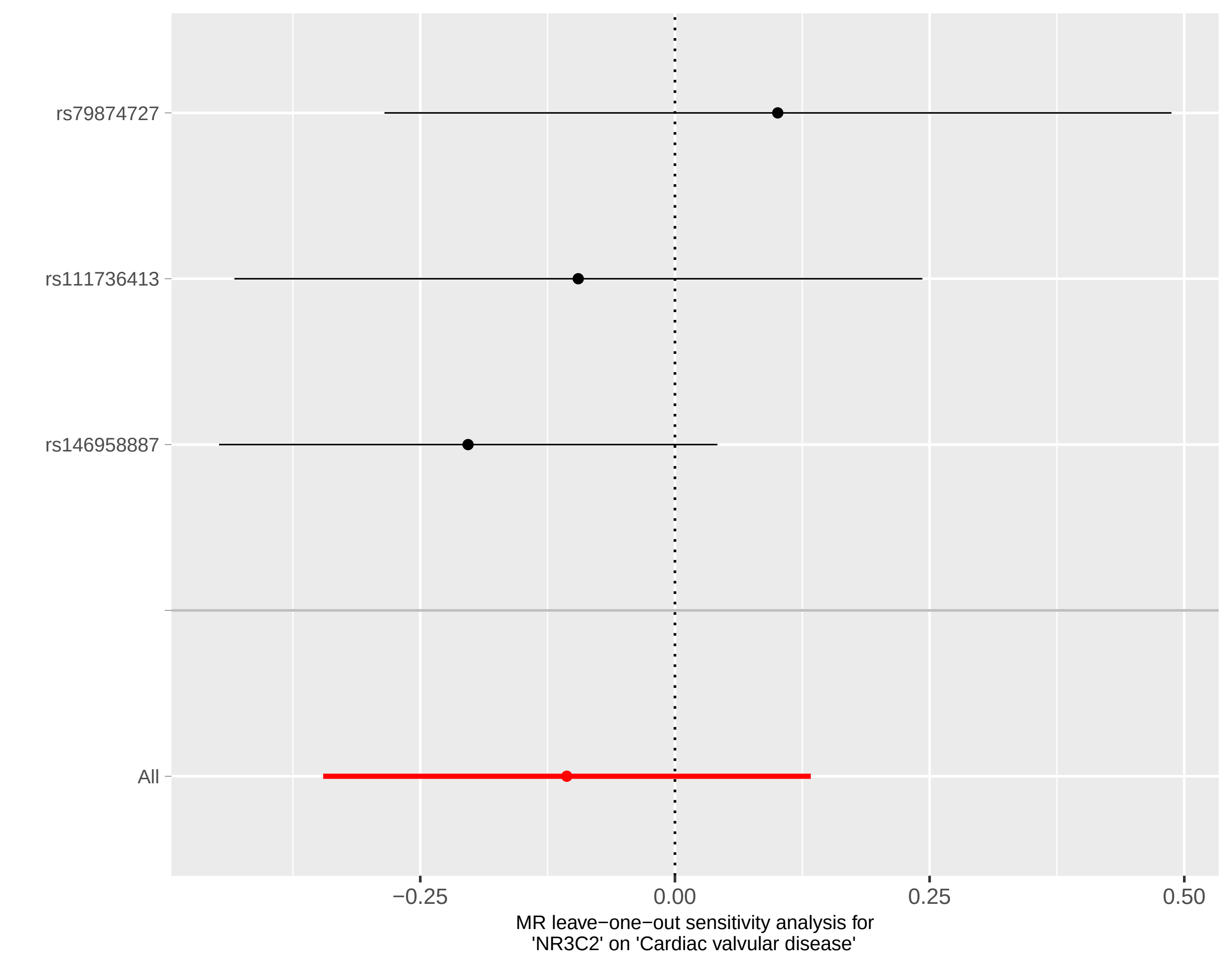
rs79874727
rs111736413
rs146958887
All
−0.25
0.00
0.25
0.50
MR leave−one−out sensitivity analysis for
'NR3C2' on 'Cardiac valvular disease'

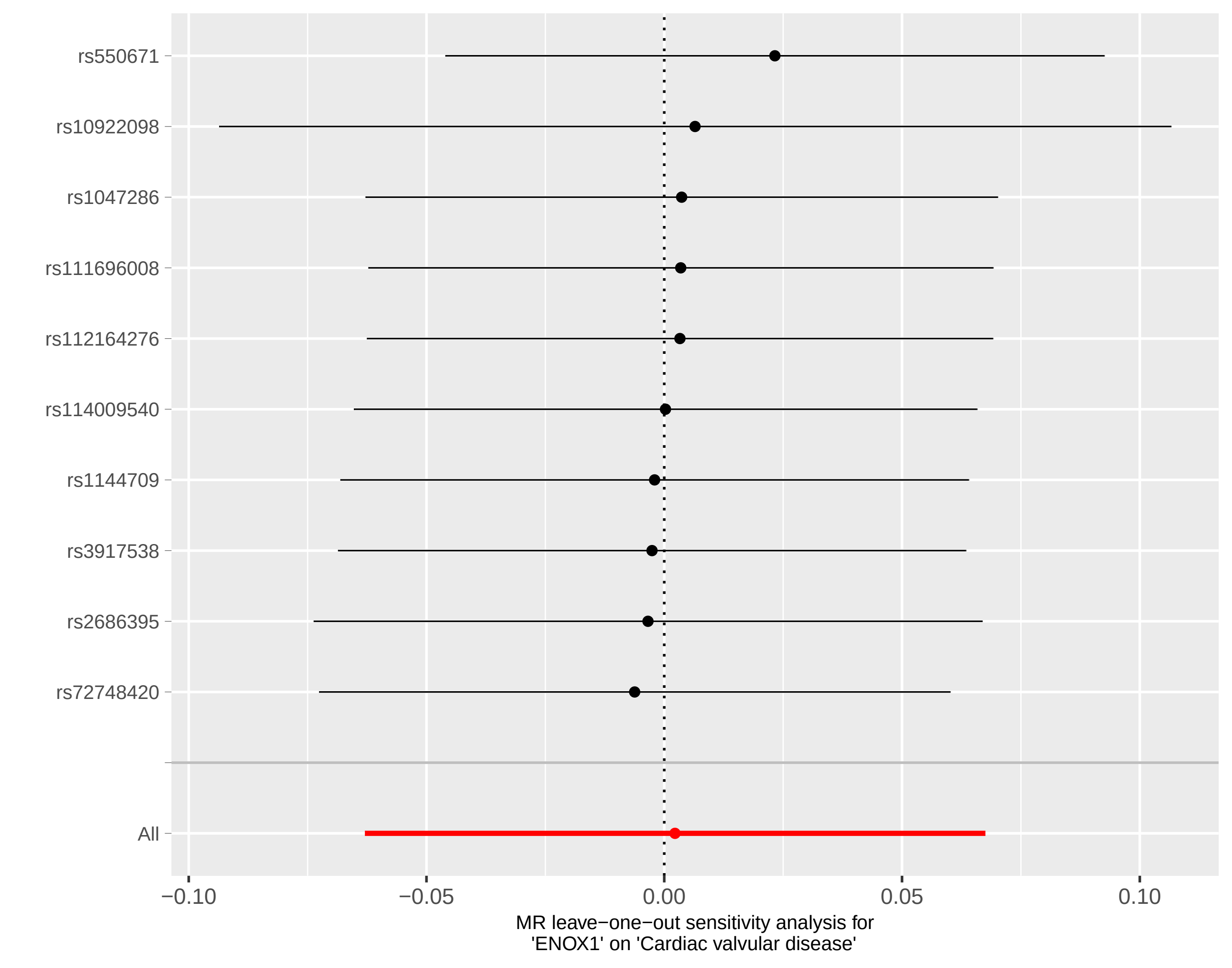
rs550671
rs10922098
rs1047286
rs111696008
rs112164276
rs114009540
rs1144709
rs3917538
rs2686395
rs72748420
All
−0.10
−0.05
0.00
0.05
0.10
MR leave−one−out sensitivity analysis for
'ENOX1' on 'Cardiac valvular disease'

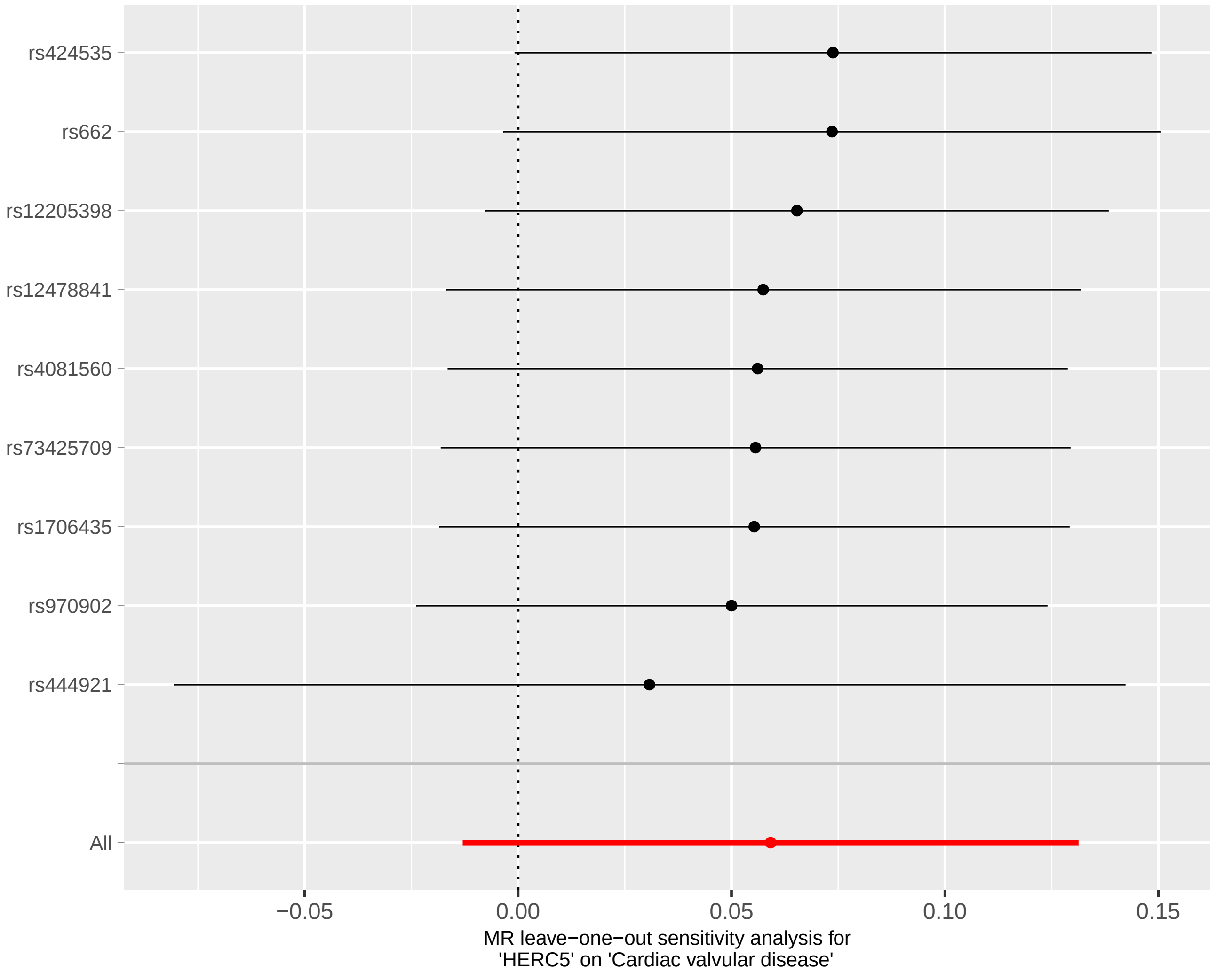
rs424535
rs662
rs12205398
rs12478841
rs4081560
rs73425709
rs1706435
rs970902
rs444921
All
−0.05
0.00
0.05
0.10
0.15
MR leave−one−out sensitivity analysis for
'HERC5' on 'Cardiac valvular disease'

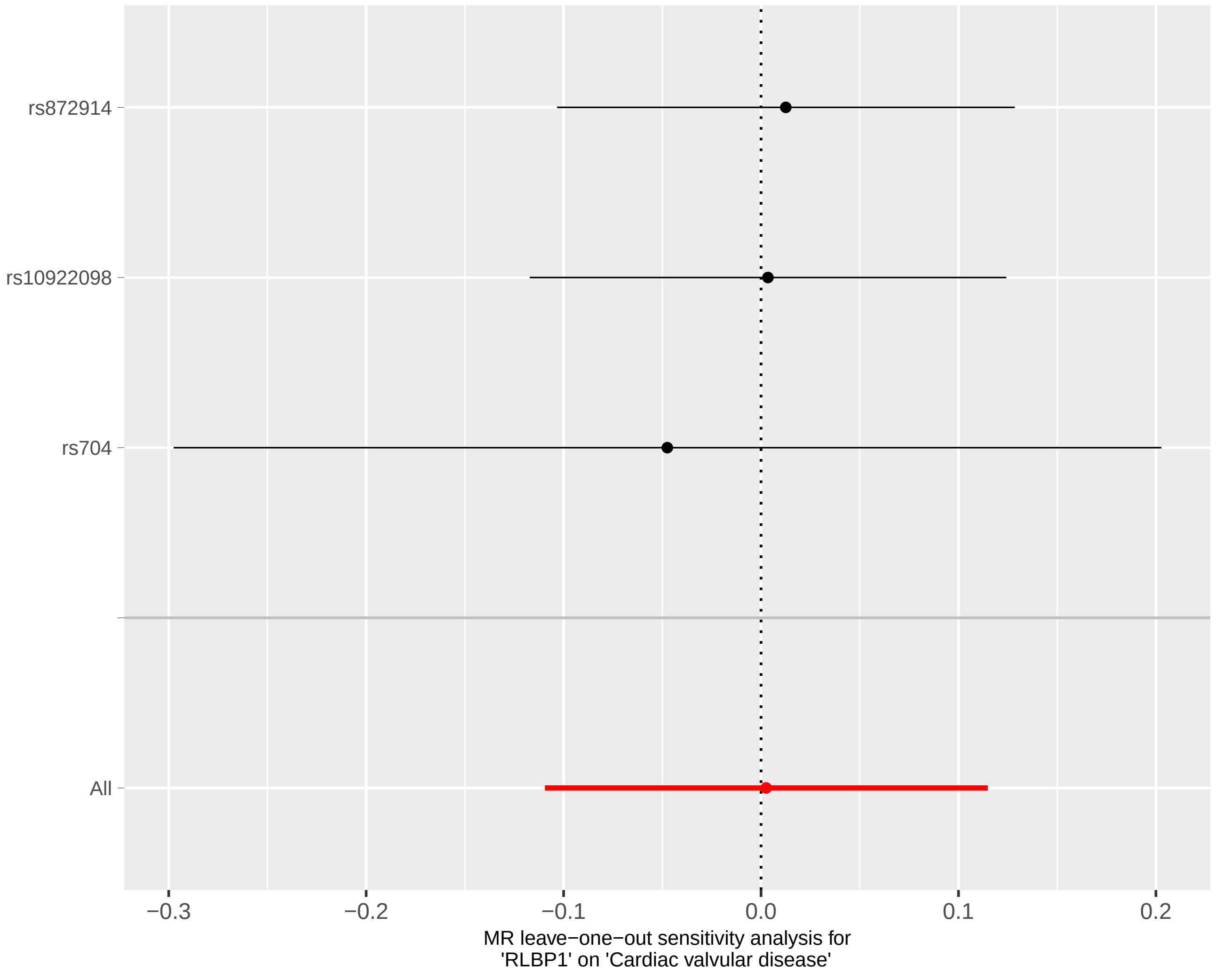
rs872914
rs10922098
rs704
All
−0.3
−0.2
−0.1
0.0
0.1
0.2
MR leave−one−out sensitivity analysis for
'RLBP1' on 'Cardiac valvular disease'

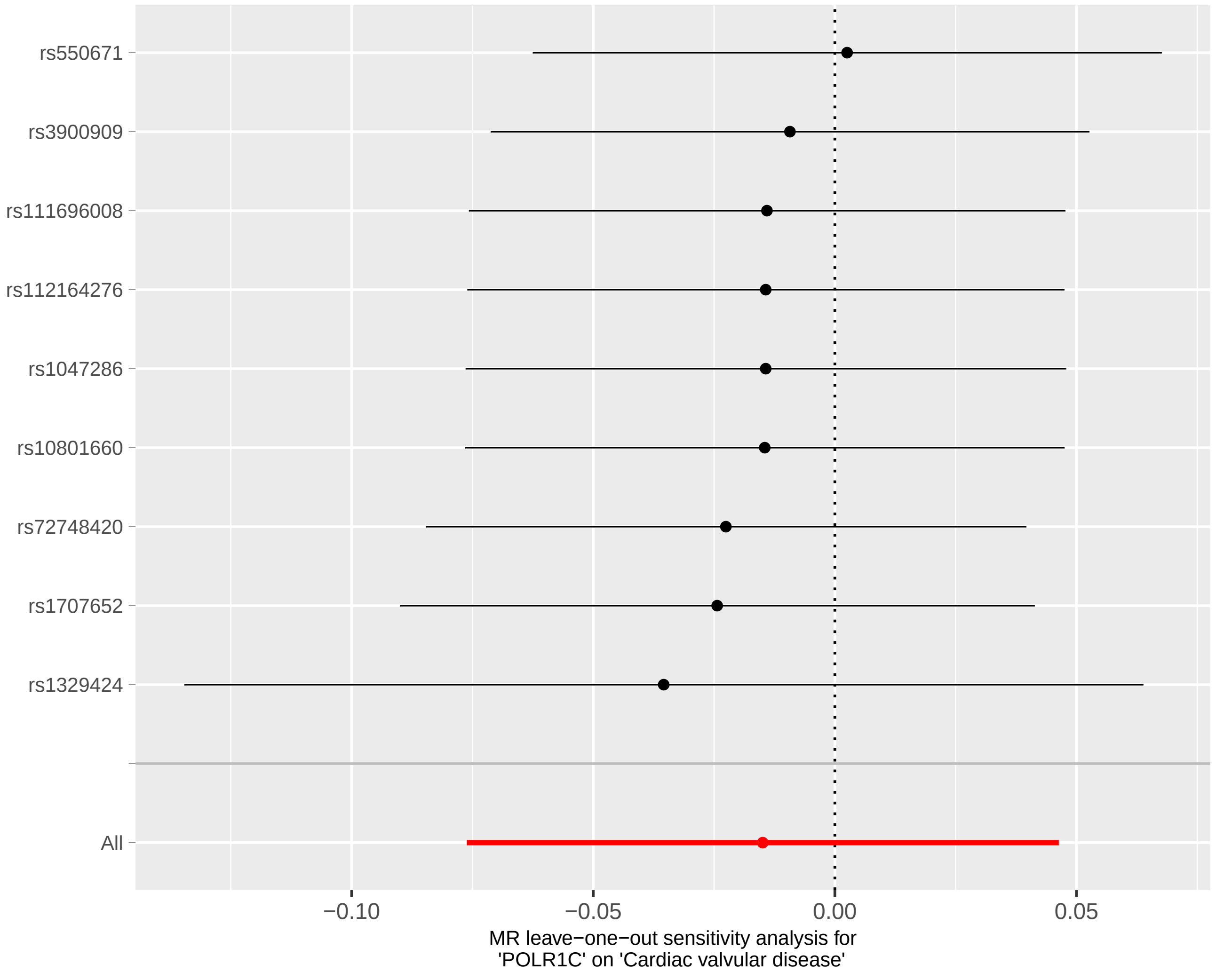
rs550671
rs3900909
rs111696008
rs112164276
rs1047286
rs10801660
rs72748420
rs1707652
rs1329424
All
−0.10
−0.05
0.00
0.05
MR leave−one−out sensitivity analysis for
'POLR1C' on 'Cardiac valvular disease'

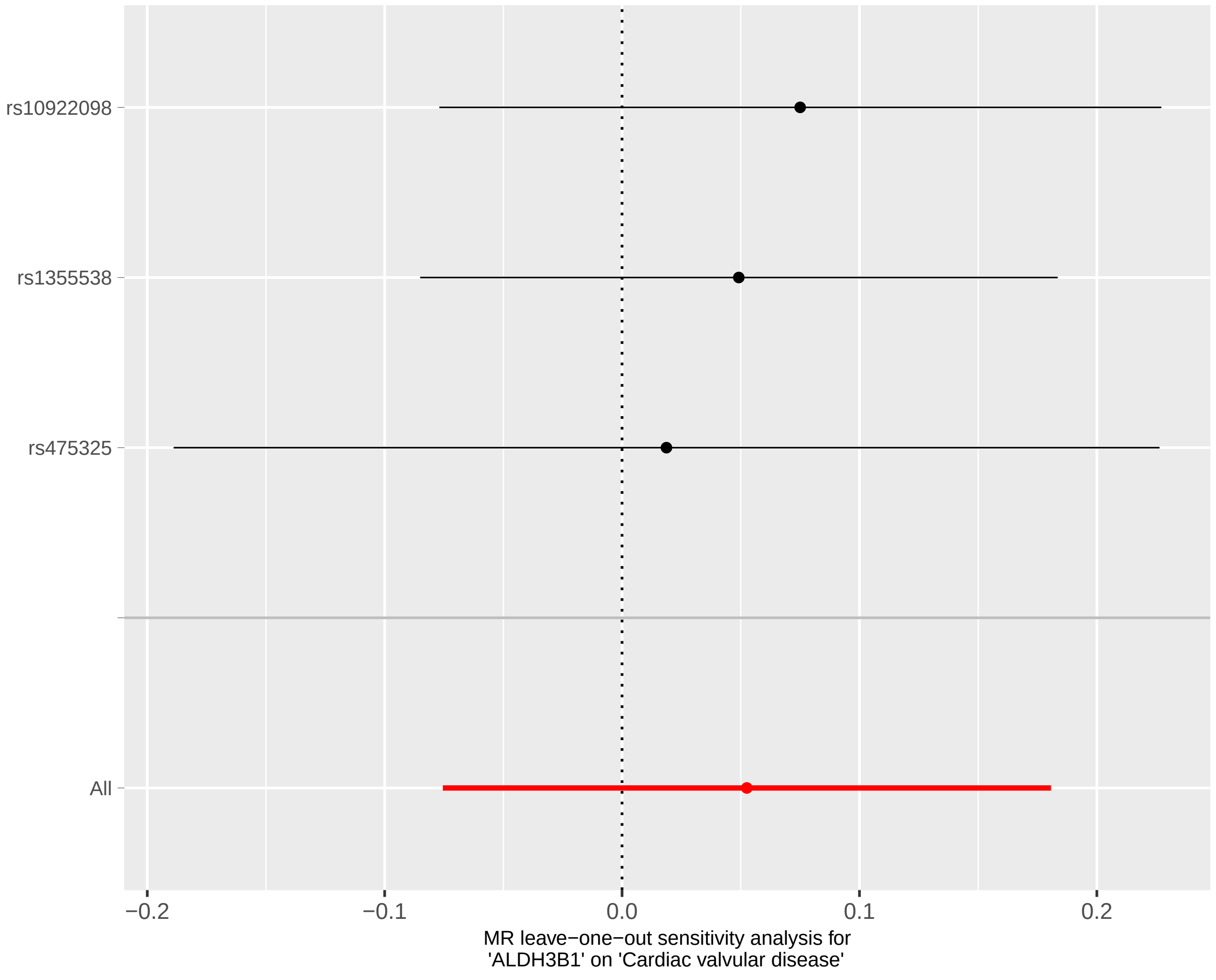
rs10922098
rs1355538
rs475325
All
−0.2
−0.1
0.0
0.1
0.2
MR leave−one−out sensitivity analysis for
'ALDH3B1' on 'Cardiac valvular disease'

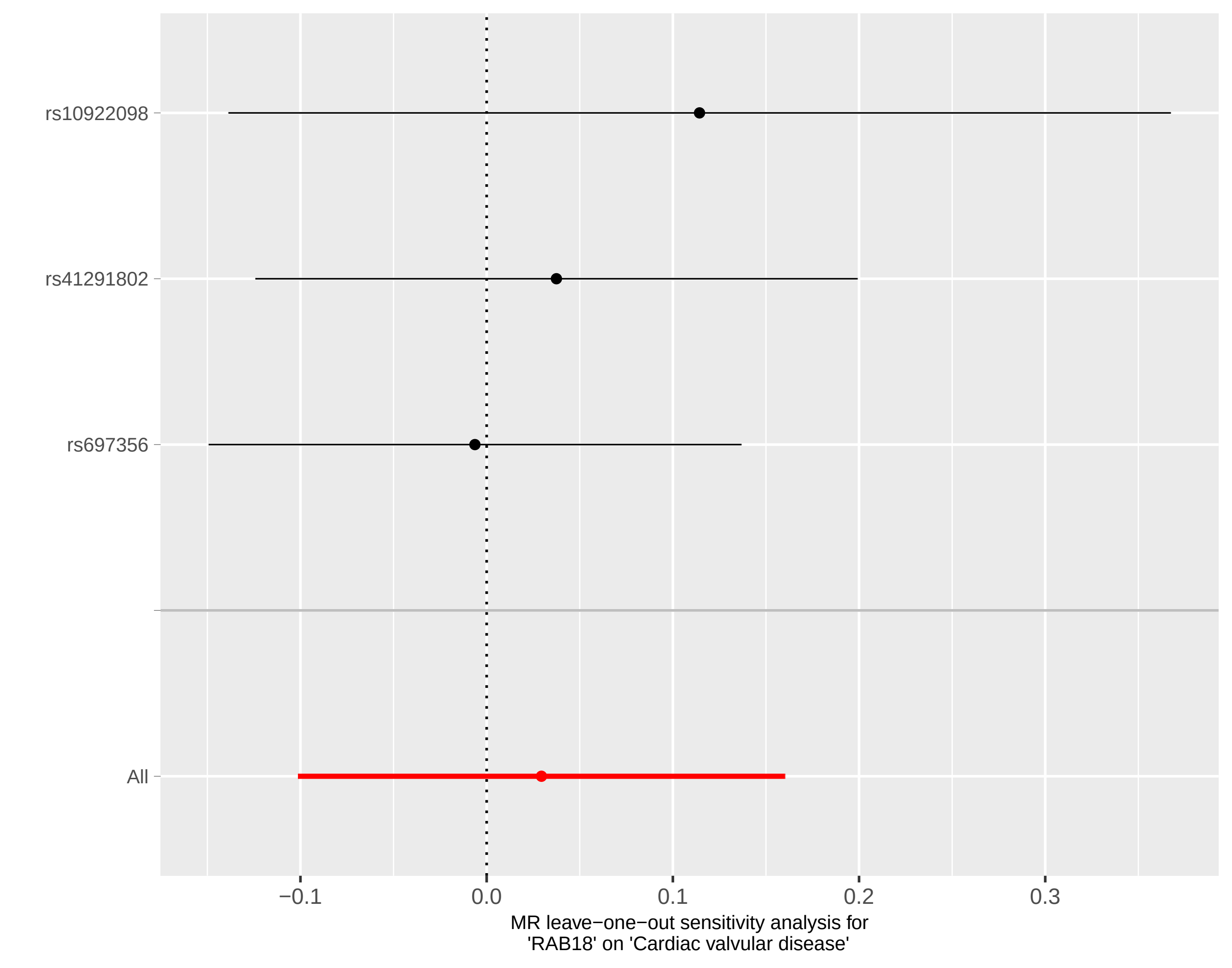

rs10922098
rs41291802
rs697356
All
−0.1
0.0
0.1
0.2
0.3
MR leave−one−out sensitivity analysis for
'RAB18' on 'Cardiac valvular disease'

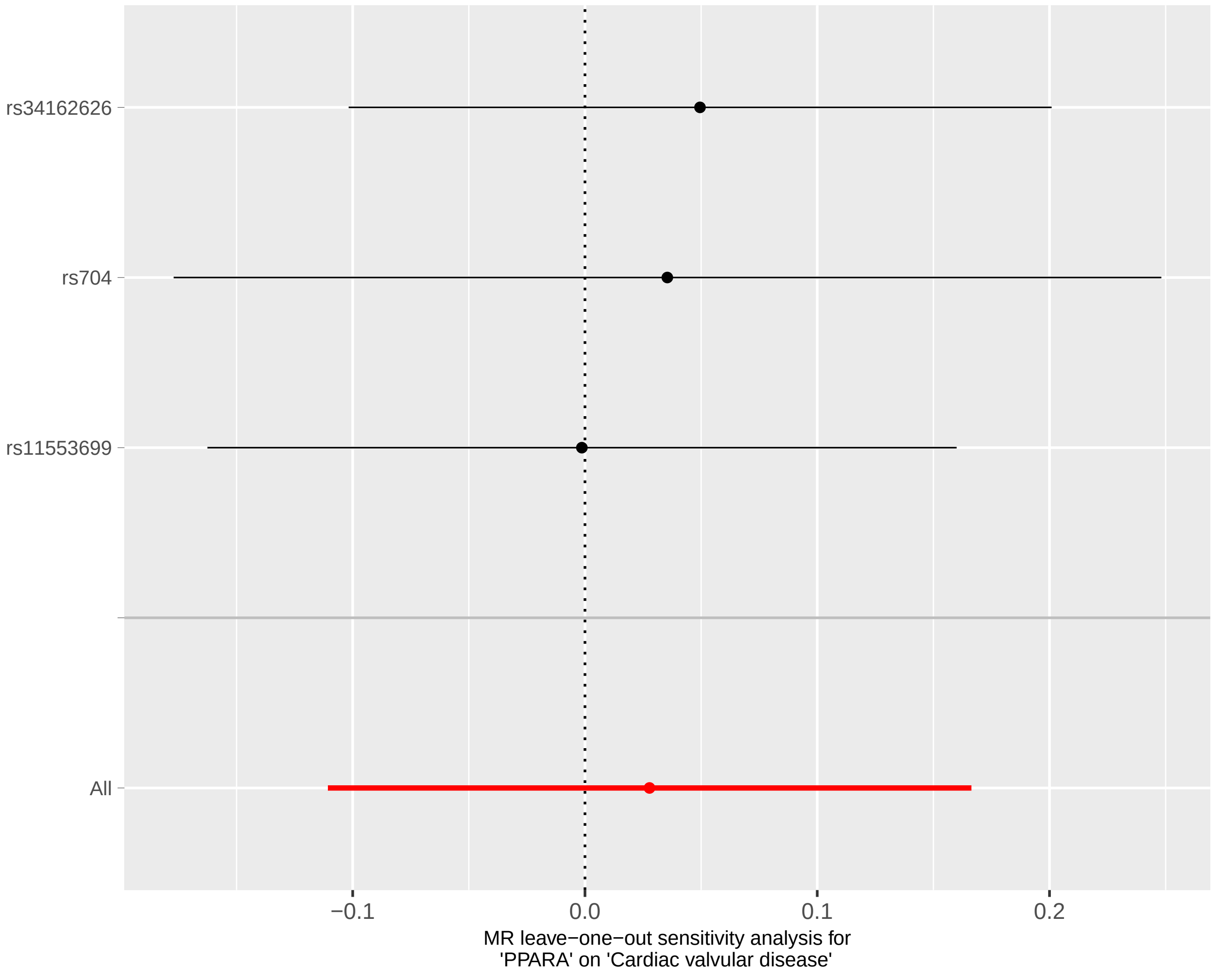
rs34162626
rs704
rs11553699
All
−0.1
0.0
0.1
0.2
MR leave−one−out sensitivity analysis for
'PPARA' on 'Cardiac valvular disease'

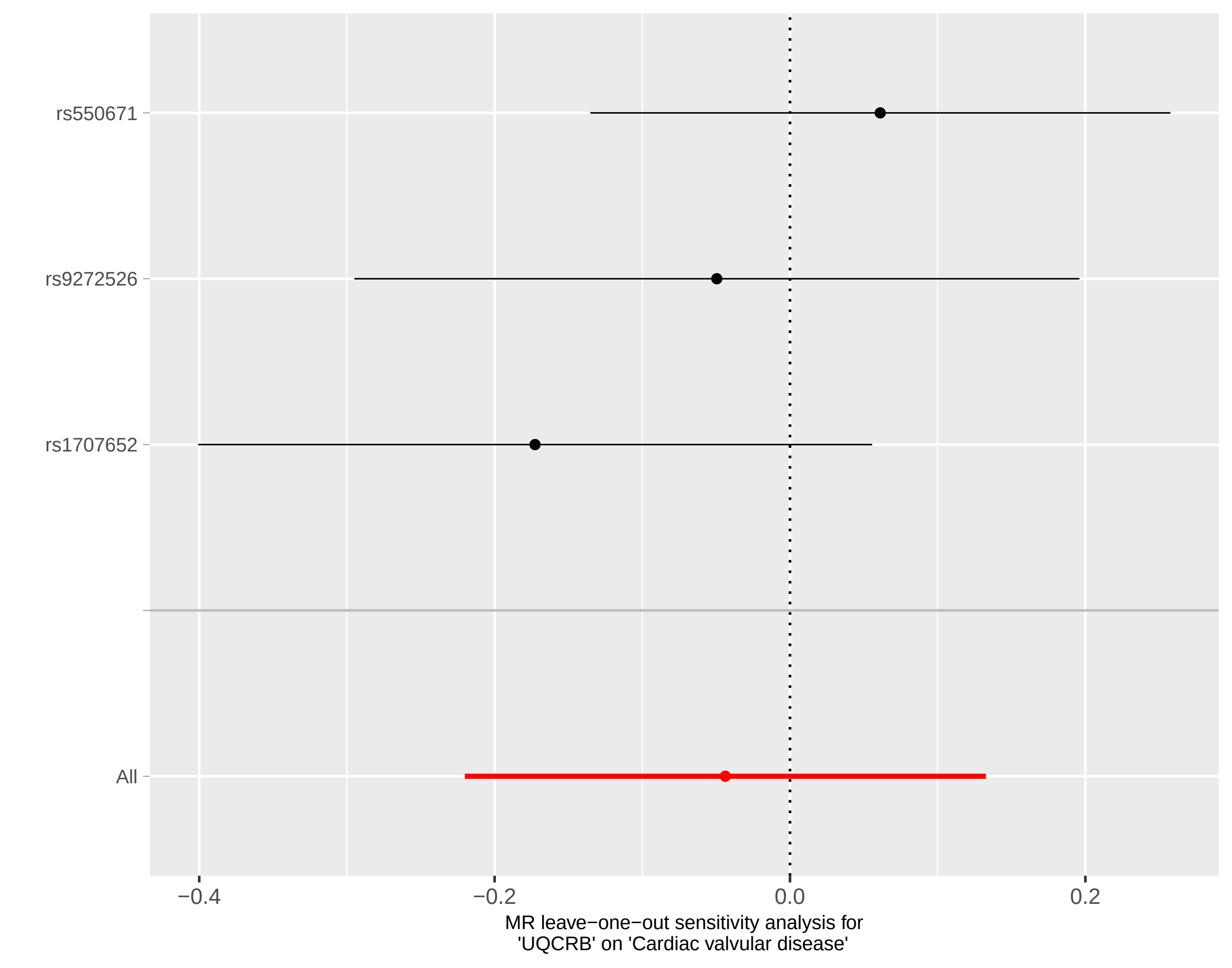
rs550671
rs9272526
rs1707652
All
−0.4
−0.2
0.0
0.2
MR leave−one−out sensitivity analysis for
'UQCRB' on 'Cardiac valvular disease'

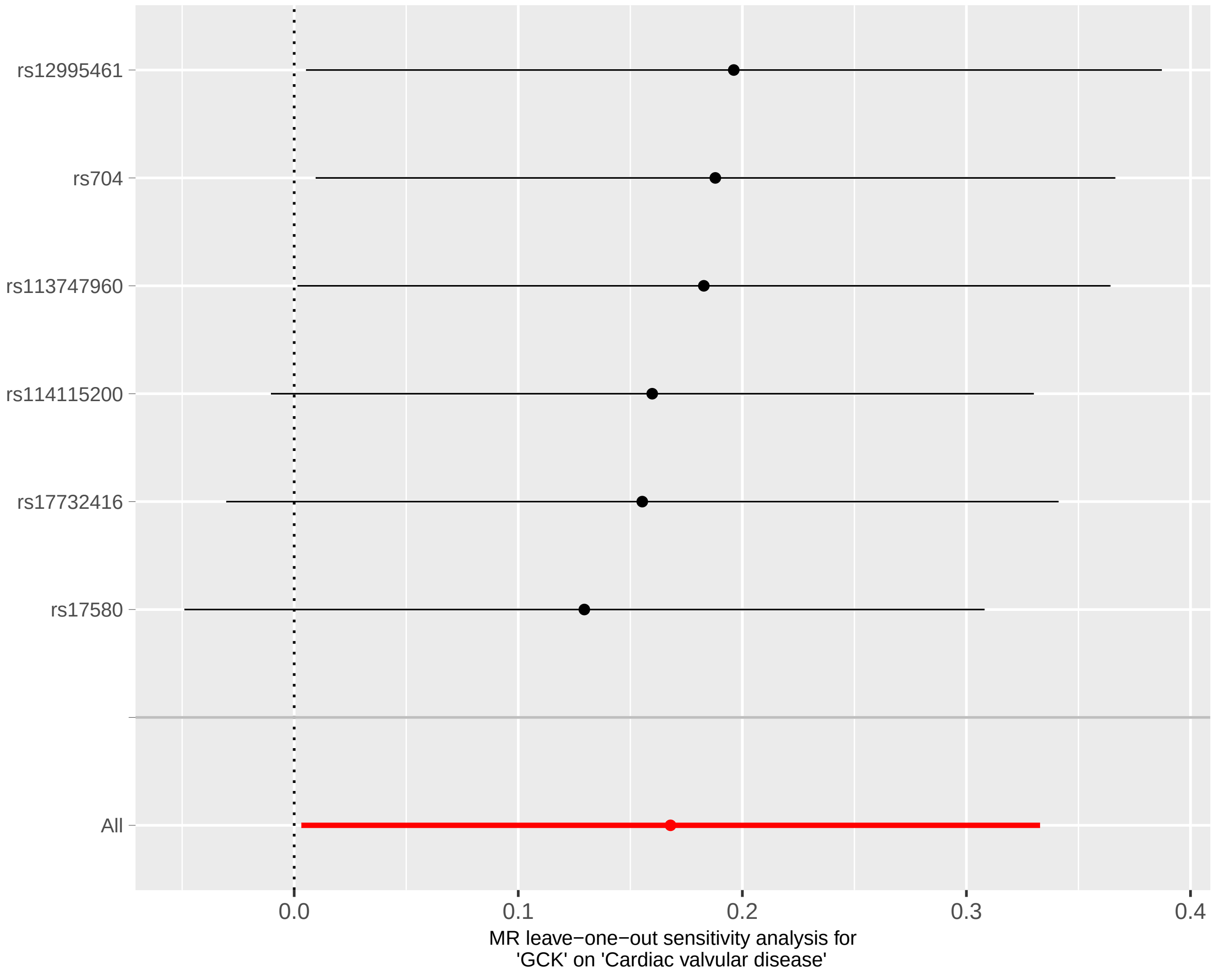
rs12995461
rs704
rs113747960
rs114115200
rs17732416
rs17580
All
0.0
0.1
0.2
0.3
0.4
MR leave−one−out sensitivity analysis for
'GCK' on 'Cardiac valvular disease'

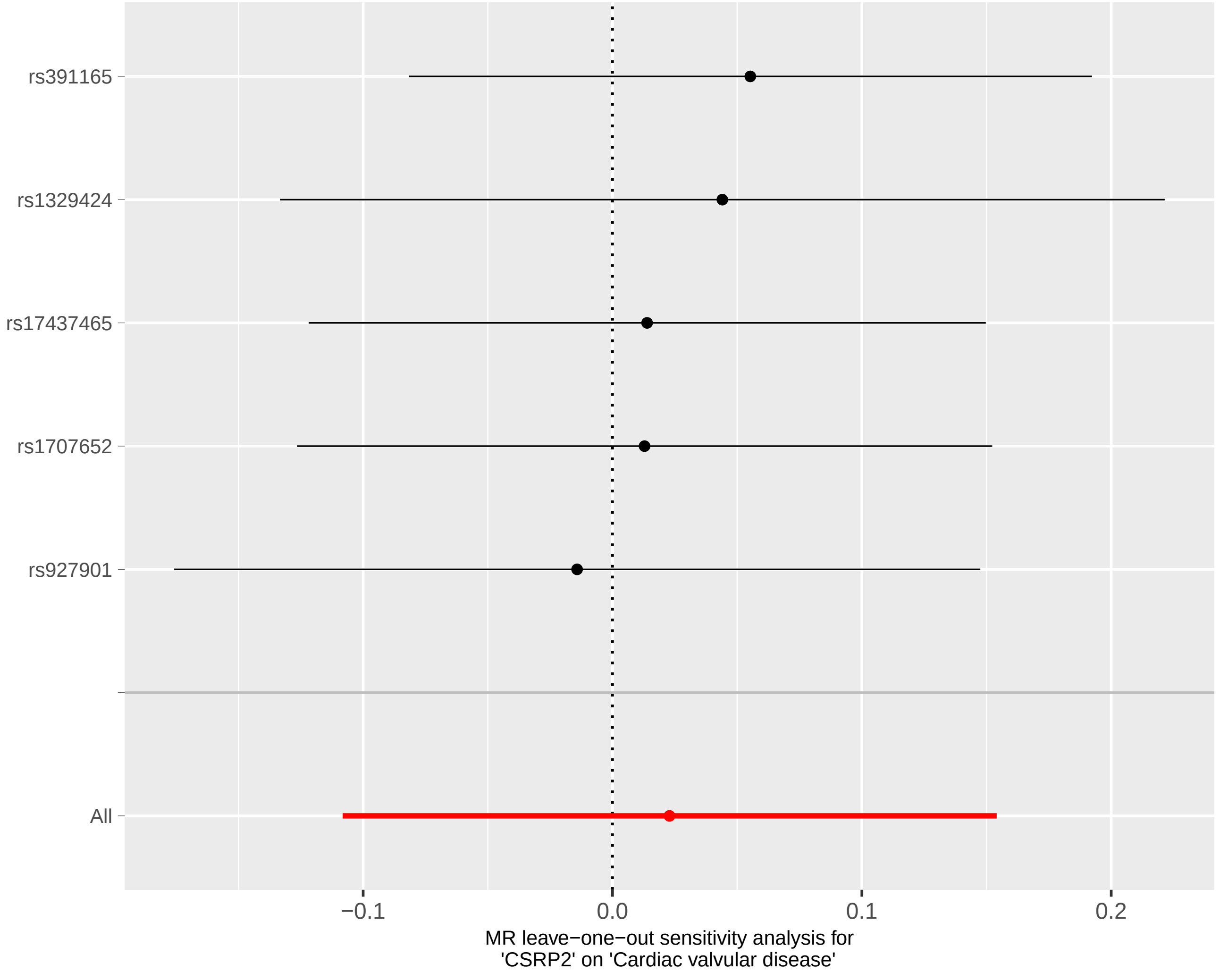
rs391165
rs1329424
rs17437465
rs1707652
rs927901
All
−0.1
0.0
0.1
0.2
MR leave−one−out sensitivity analysis for
'CSRP2' on 'Cardiac valvular disease'

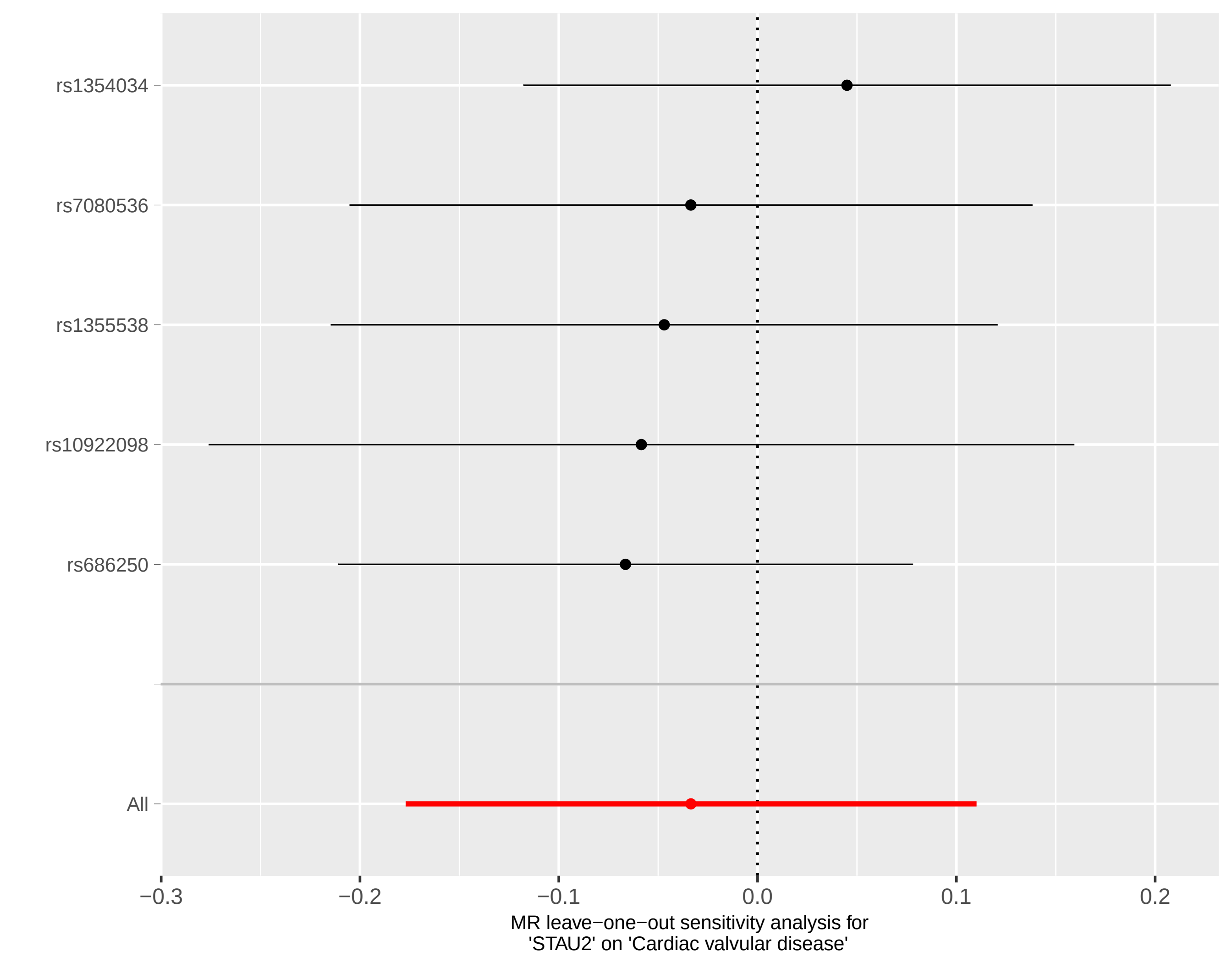

rs1354034
rs7080536
rs1355538
rs10922098
rs686250
All
−0.3
−0.2
−0.1
0.0
0.1
0.2
MR leave−one−out sensitivity analysis for
'STAU2' on 'Cardiac valvular disease'

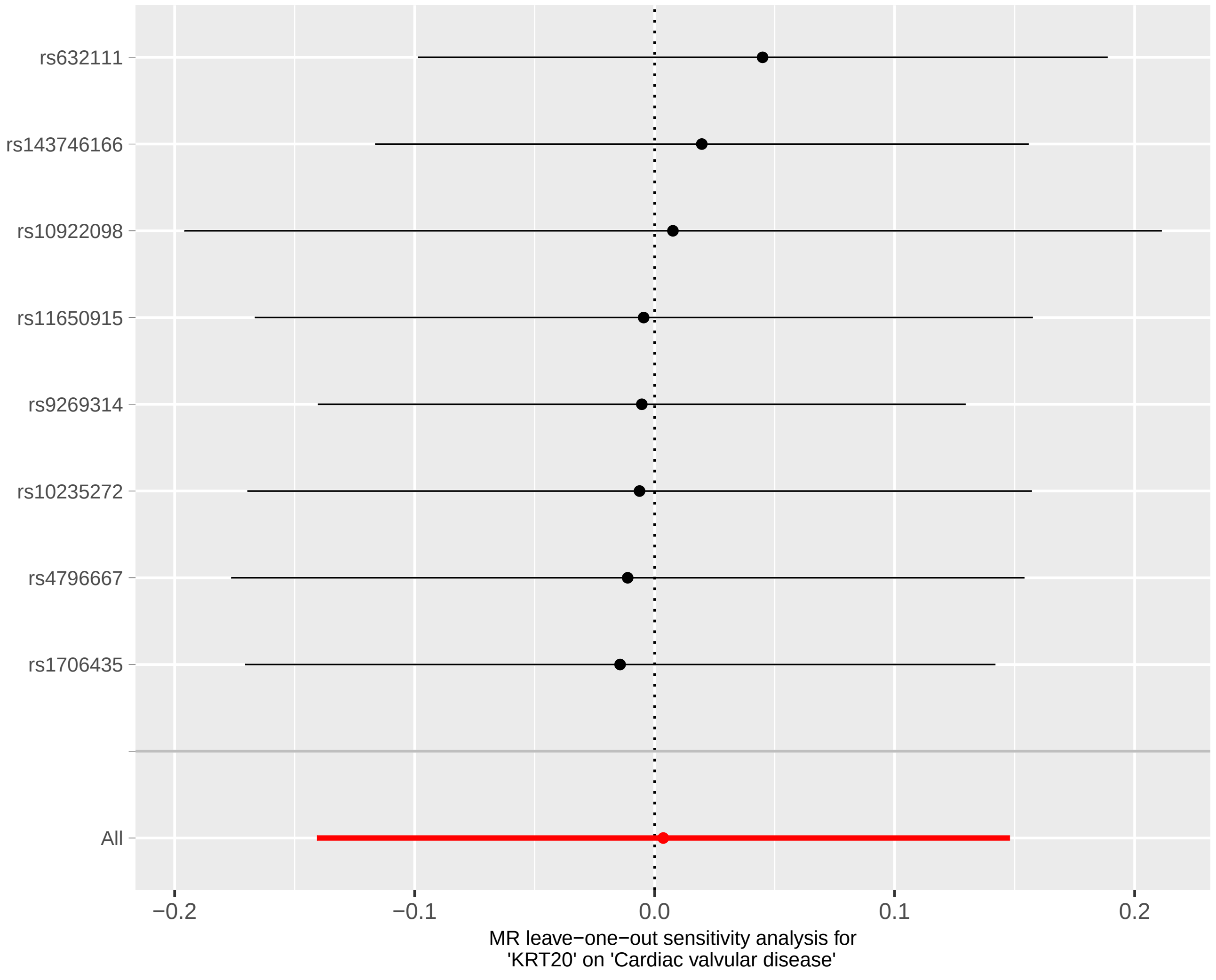
rs632111
rs143746166
rs10922098
rs11650915
rs9269314
rs10235272
rs4796667
rs1706435
All
−0.2
−0.1
0.0
0.1
0.2
MR leave−one−out sensitivity analysis for
'KRT20' on 'Cardiac valvular disease'

Insufficient number of SNPs

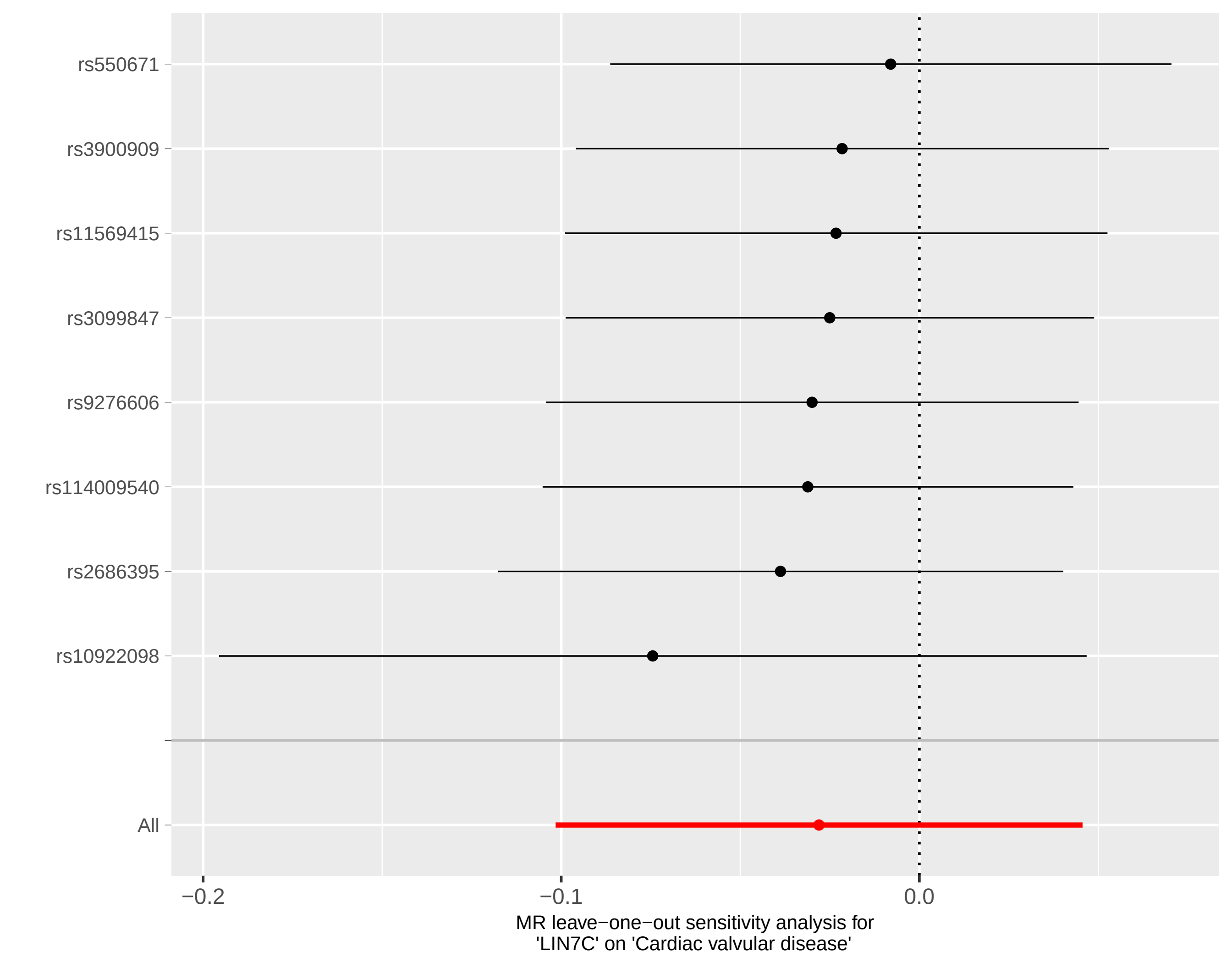
rs550671
rs3900909
rs11569415
rs3099847
rs9276606
rs114009540
rs2686395
rs10922098
All
−0.2
−0.1
0.0
MR leave−one−out sensitivity analysis for
'LIN7C' on 'Cardiac valvular disease'

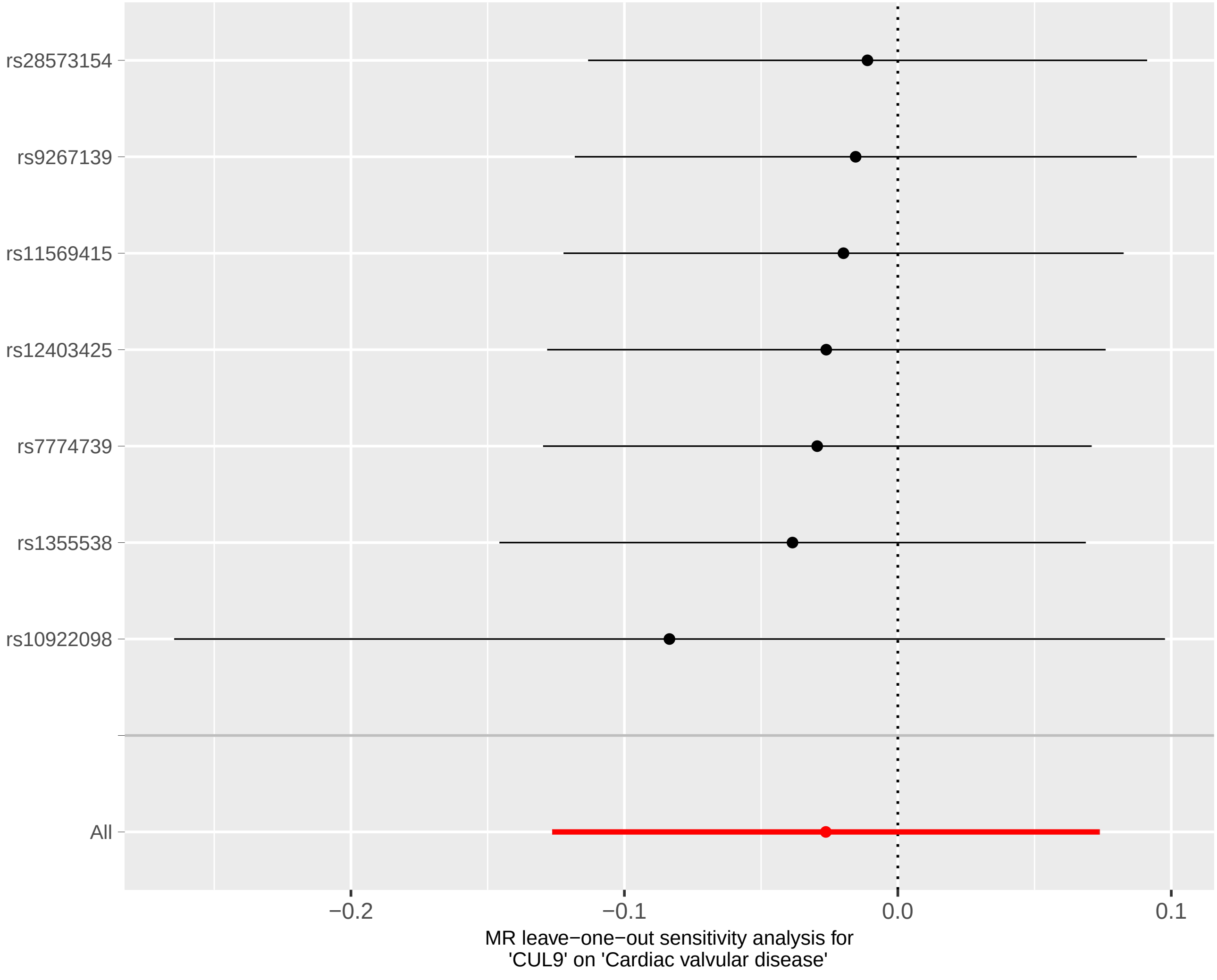
rs28573154
rs9267139
rs11569415
rs12403425
rs7774739
rs1355538
rs10922098
All
−0.2
−0.1
0.0
0.1
MR leave−one−out sensitivity analysis for
'CUL9' on 'Cardiac valvular disease'

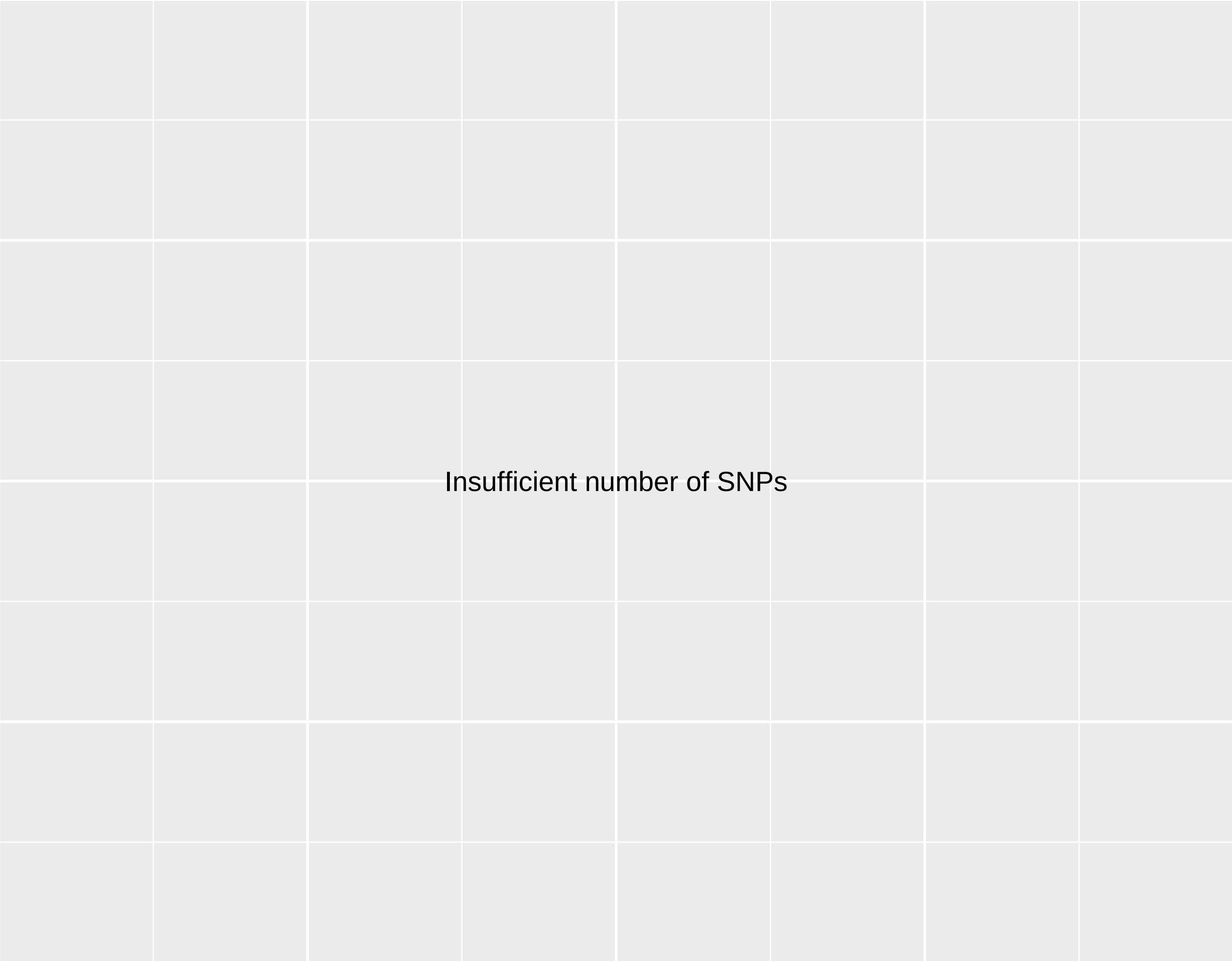
Insufficient number of SNPs

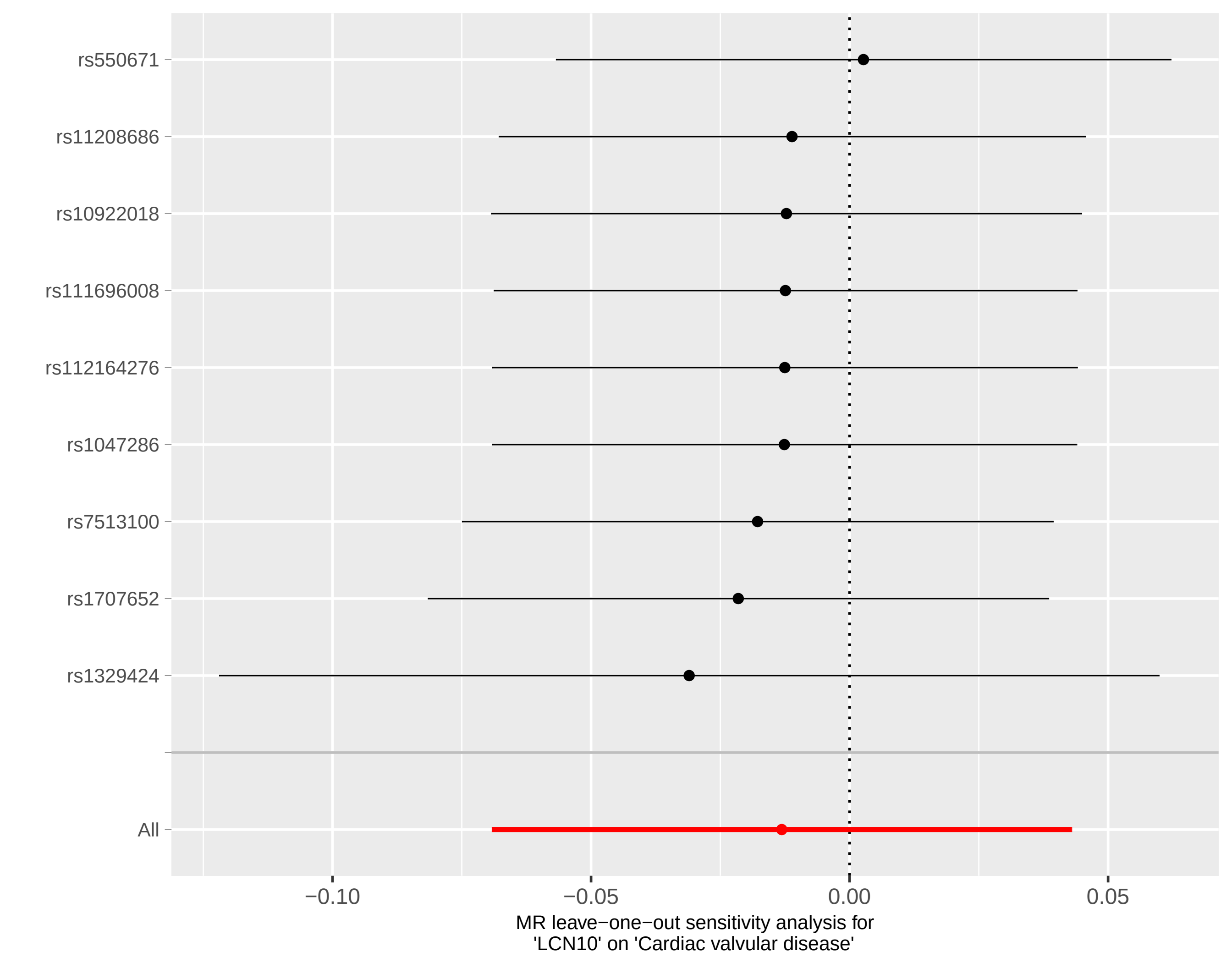
rs550671
rs11208686
rs10922018
rs111696008
rs112164276
rs1047286
rs7513100
rs1707652
rs1329424
All
−0.10
−0.05
0.00
0.05
MR leave−one−out sensitivity analysis for
'LCN10' on 'Cardiac valvular disease'

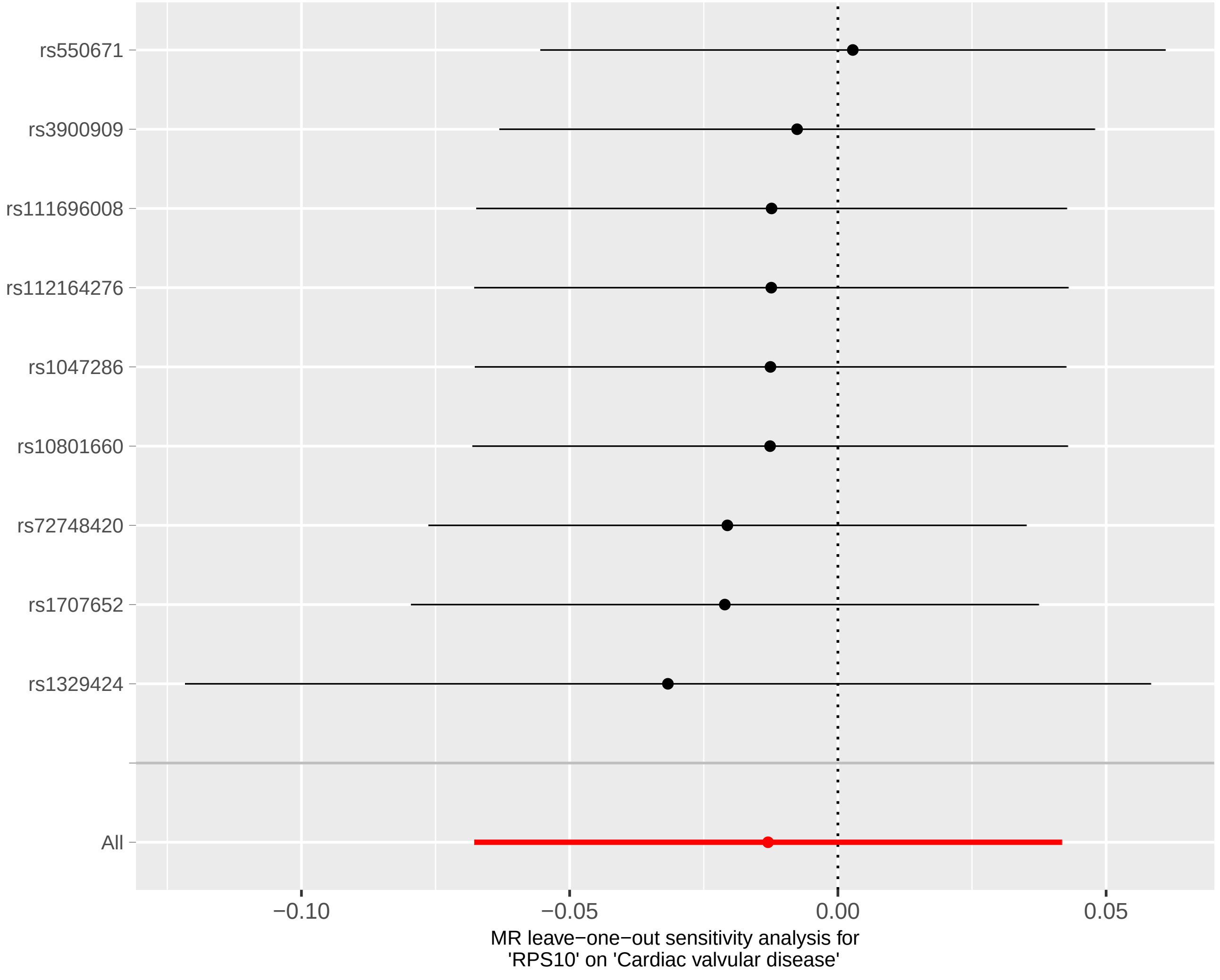

rs550671
rs3900909
rs111696008
rs112164276
rs1047286
rs10801660
rs72748420
rs1707652
rs1329424
All
−0.10
−0.05
0.00
0.05
MR leave−one−out sensitivity analysis for
'RPS10' on 'Cardiac valvular disease'

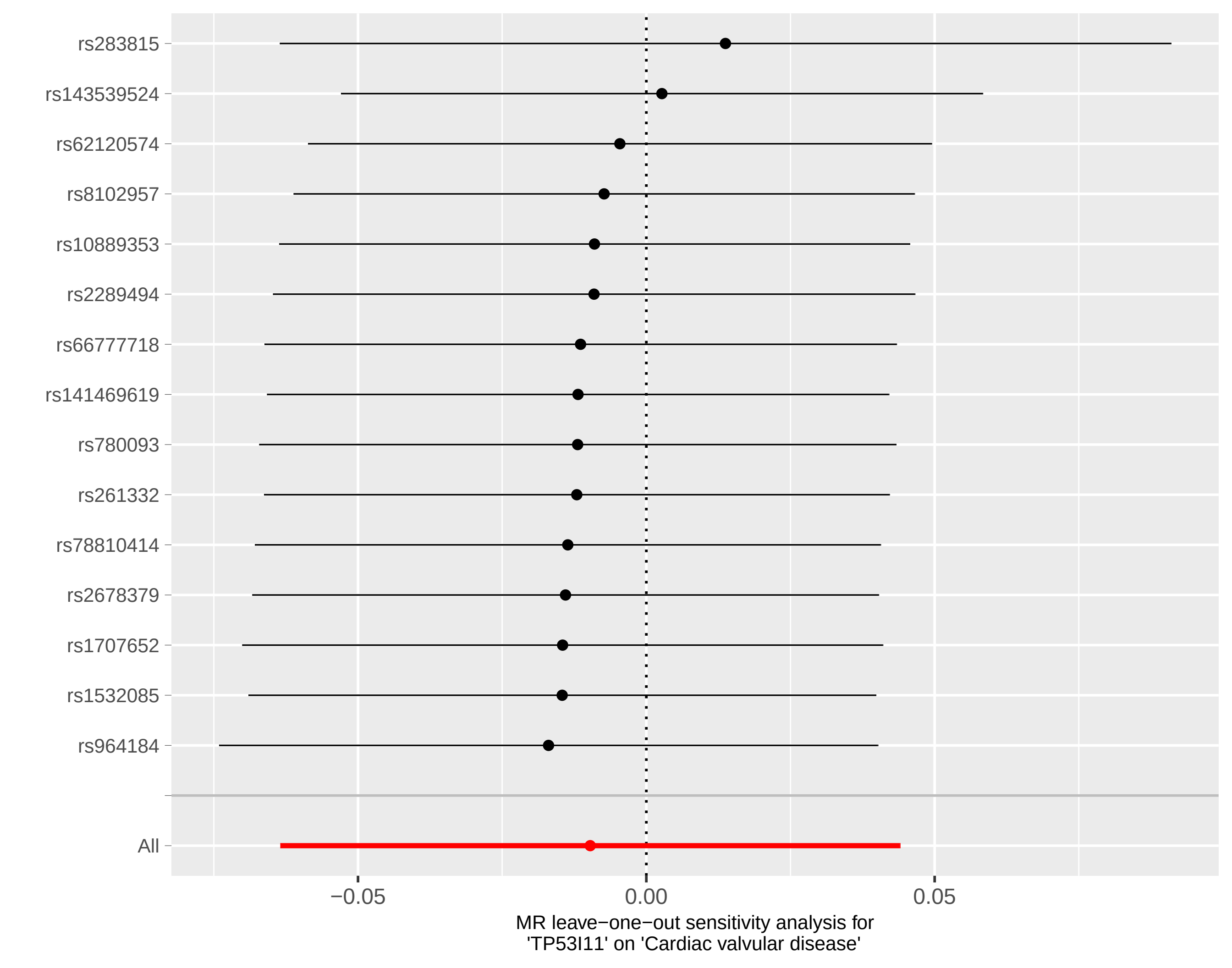
rs283815
rs143539524
rs62120574
rs8102957
rs10889353
rs2289494
rs66777718
rs141469619
rs780093
rs261332
rs78810414
rs2678379
rs1707652
rs1532085
rs964184
All
−0.05
0.00
0.05
MR leave−one−out sensitivity analysis for
'TP53I11' on 'Cardiac valvular disease'

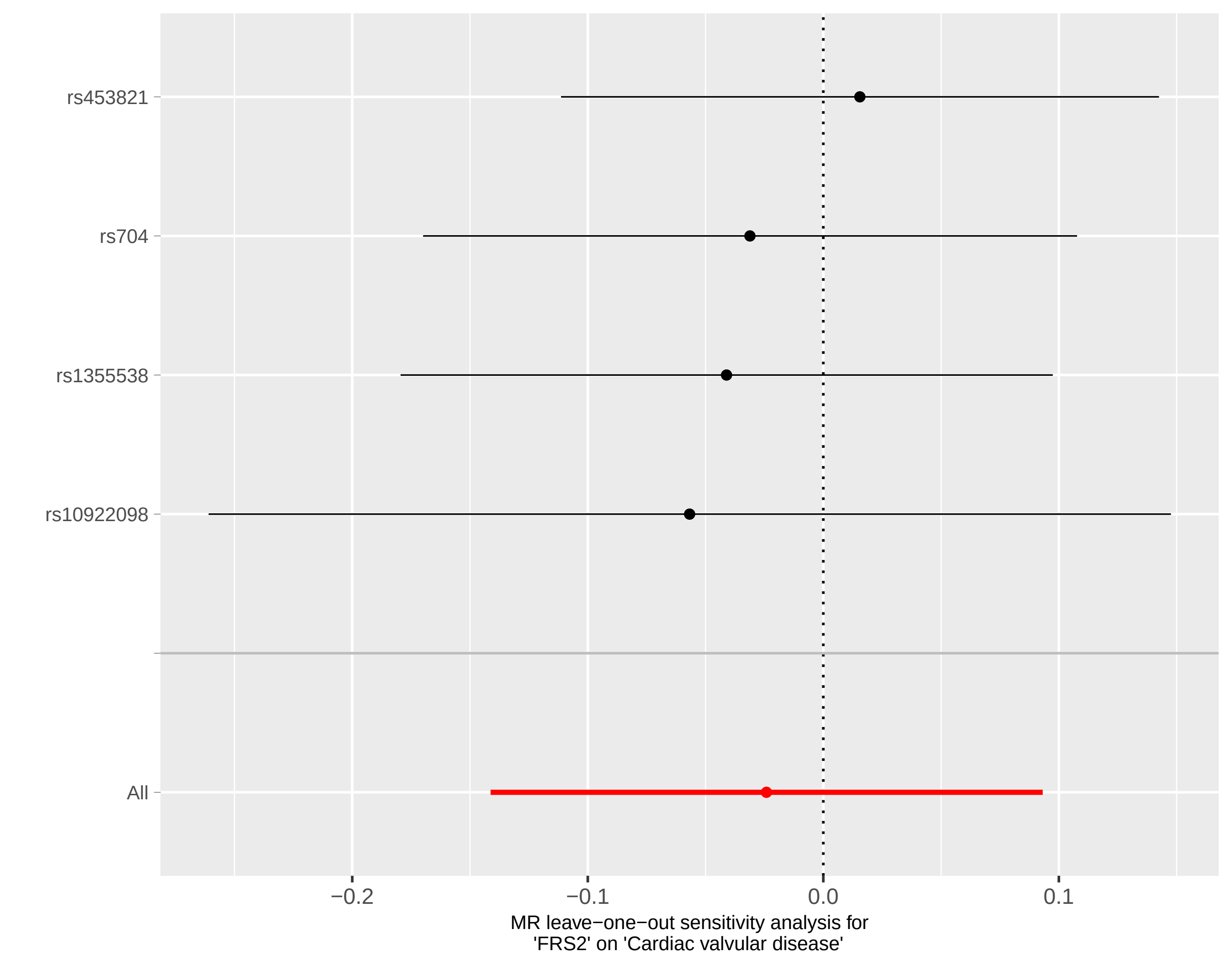

rs453821
rs704
rs1355538
rs10922098
All
−0.2
−0.1
0.0
0.1
MR leave−one−out sensitivity analysis for
'FRS2' on 'Cardiac valvular disease'

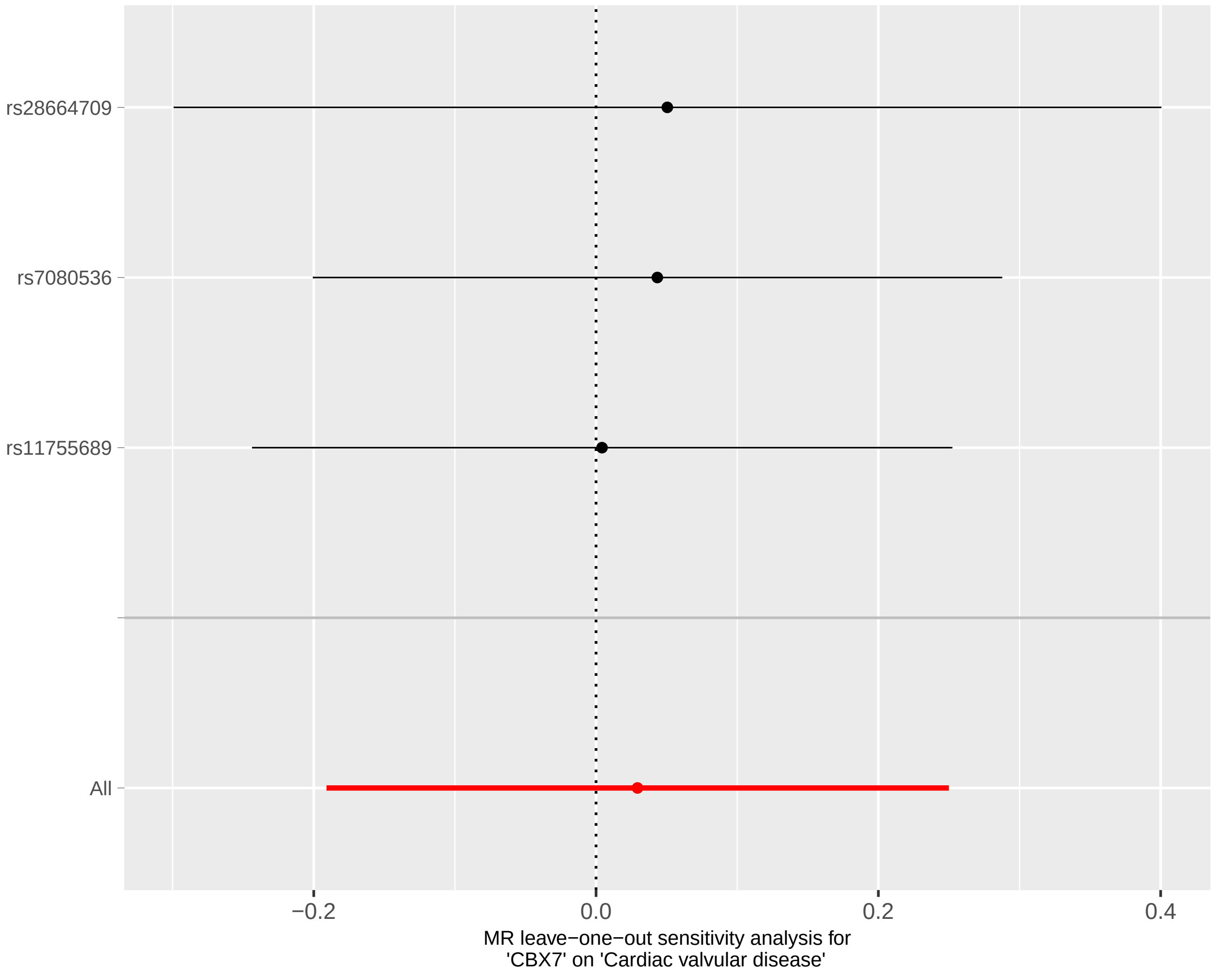
rs28664709
rs7080536
rs11755689
All
−0.2
0.0
0.2
0.4
MR leave−one−out sensitivity analysis for
'CBX7' on 'Cardiac valvular disease'

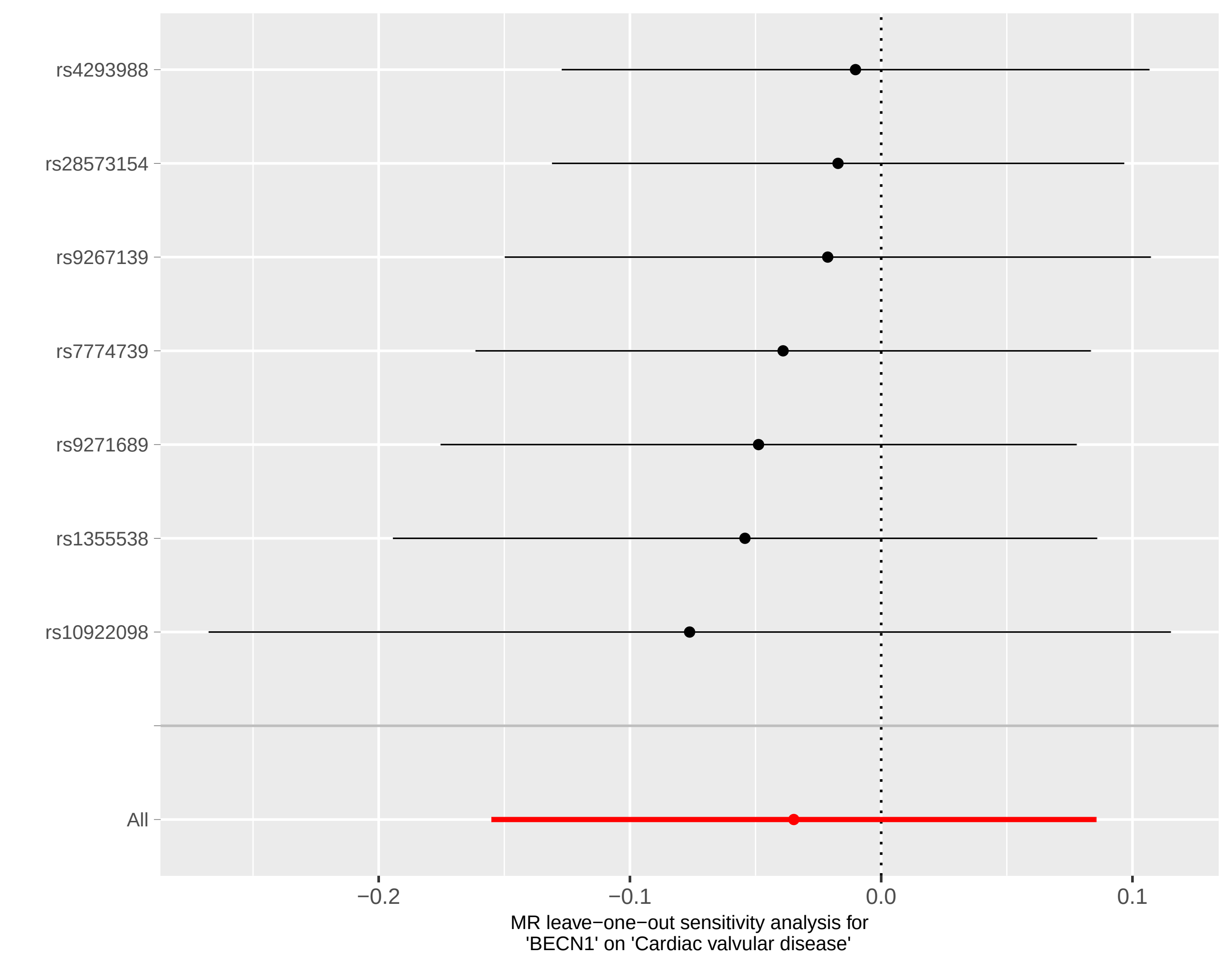

rs4293988
rs28573154
rs9267139
rs7774739
rs9271689
rs1355538
rs10922098
All
−0.2
−0.1
0.0
0.1
MR leave−one−out sensitivity analysis for
'BECN1' on 'Cardiac valvular disease'

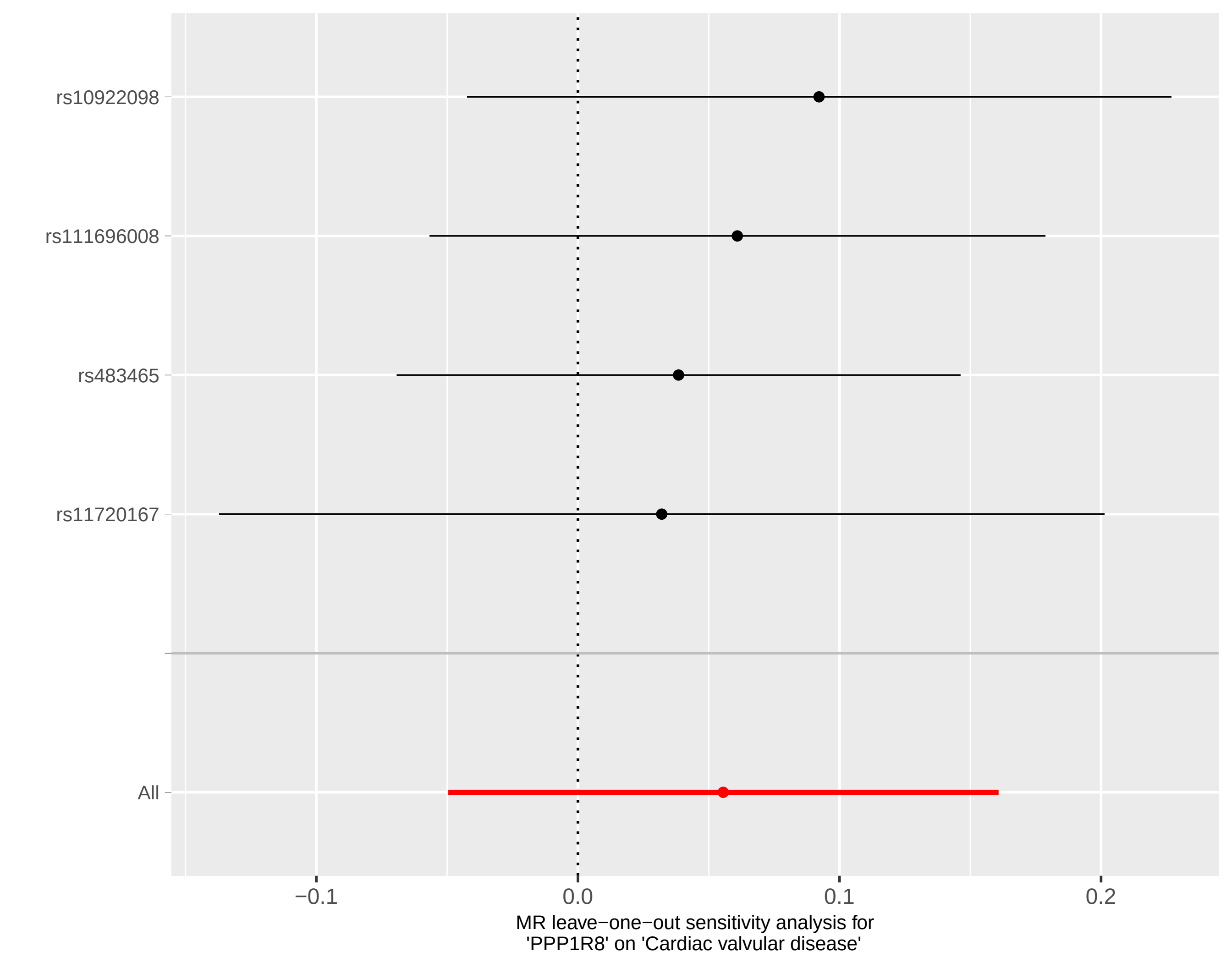

rs10922098
rs111696008
rs483465
rs11720167
All
−0.1
0.0
0.1
0.2
MR leave−one−out sensitivity analysis for
'PPP1R8' on 'Cardiac valvular disease'

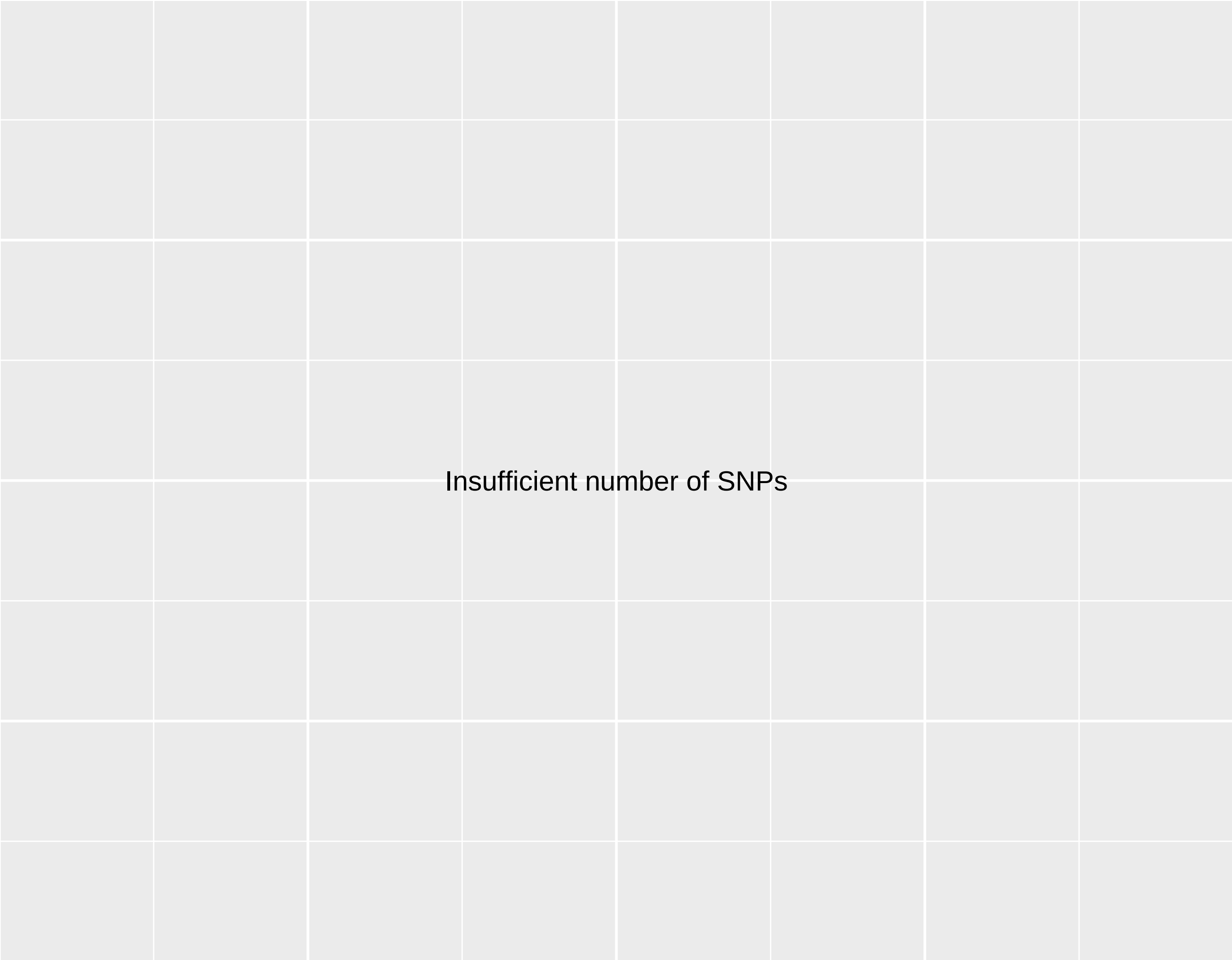
Insufficient number of SNPs

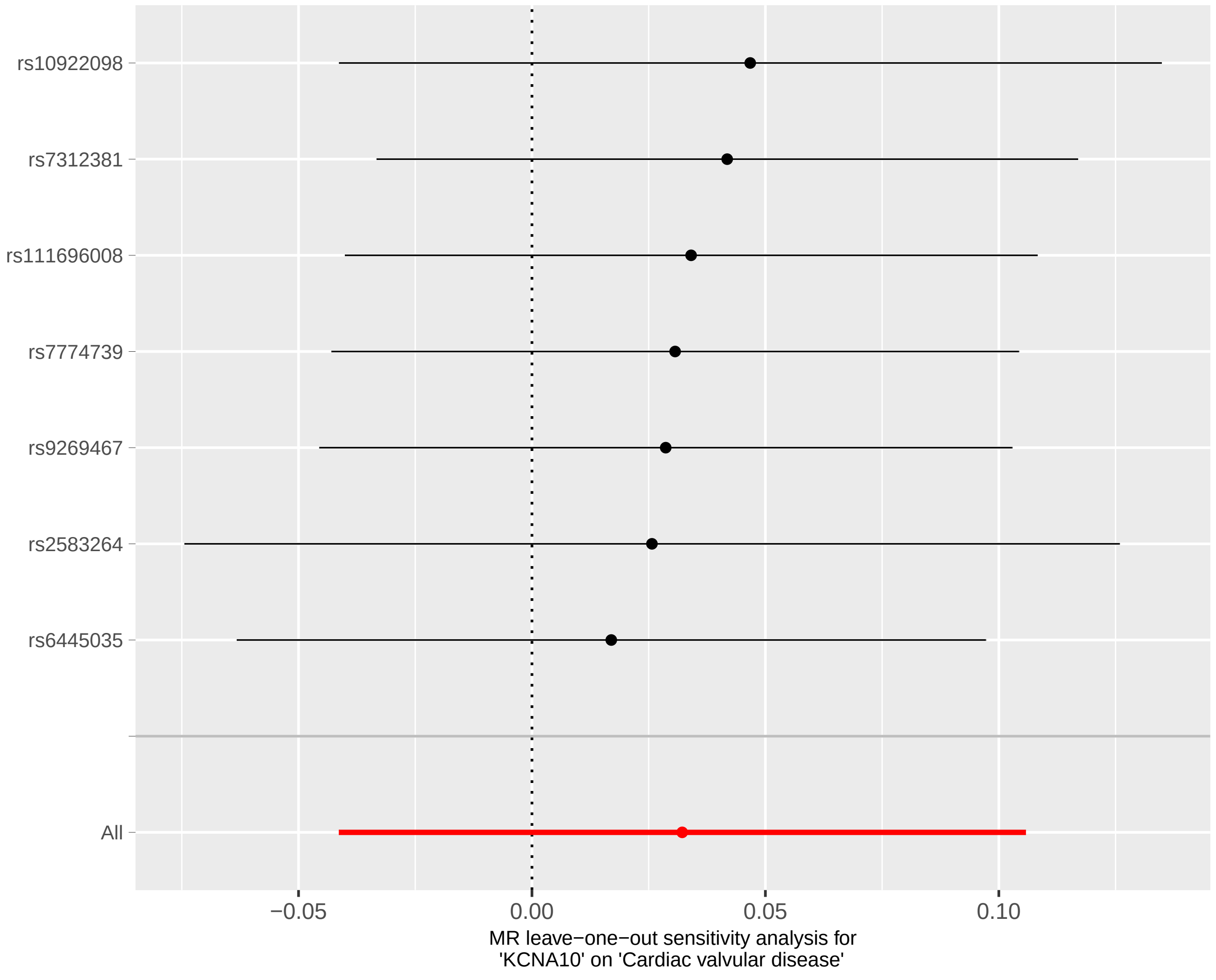
rs10922098
rs7312381
rs111696008
rs7774739
rs9269467
rs2583264
rs6445035
All
−0.05
0.00
0.05
0.10
MR leave−one−out sensitivity analysis for
'KCNA10' on 'Cardiac valvular disease'

Insufficient number of SNPs

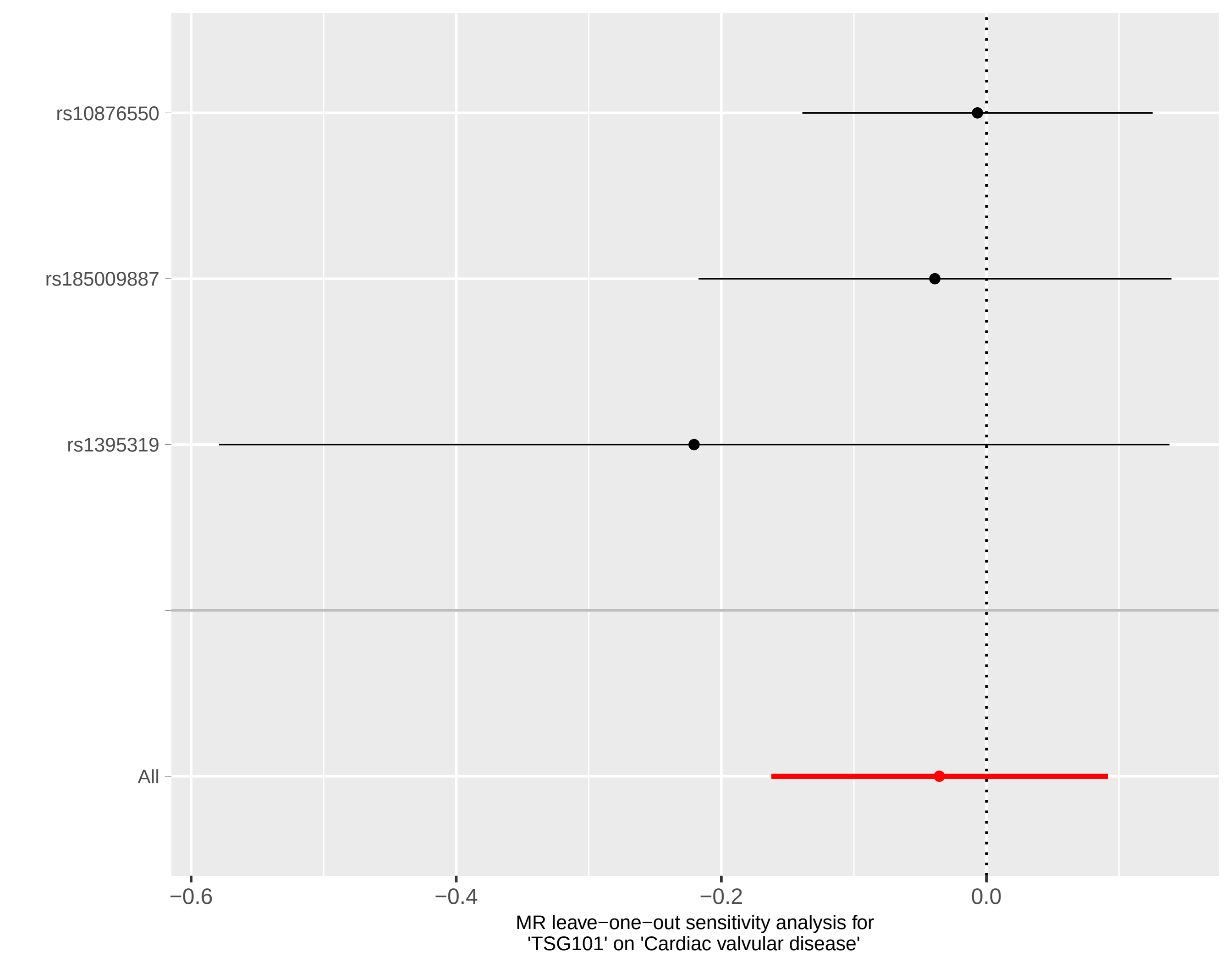
rs10876550
rs185009887
rs1395319
All
−0.6
−0.4
−0.2
0.0
MR leave−one−out sensitivity analysis for
'TSG101' on 'Cardiac valvular disease'

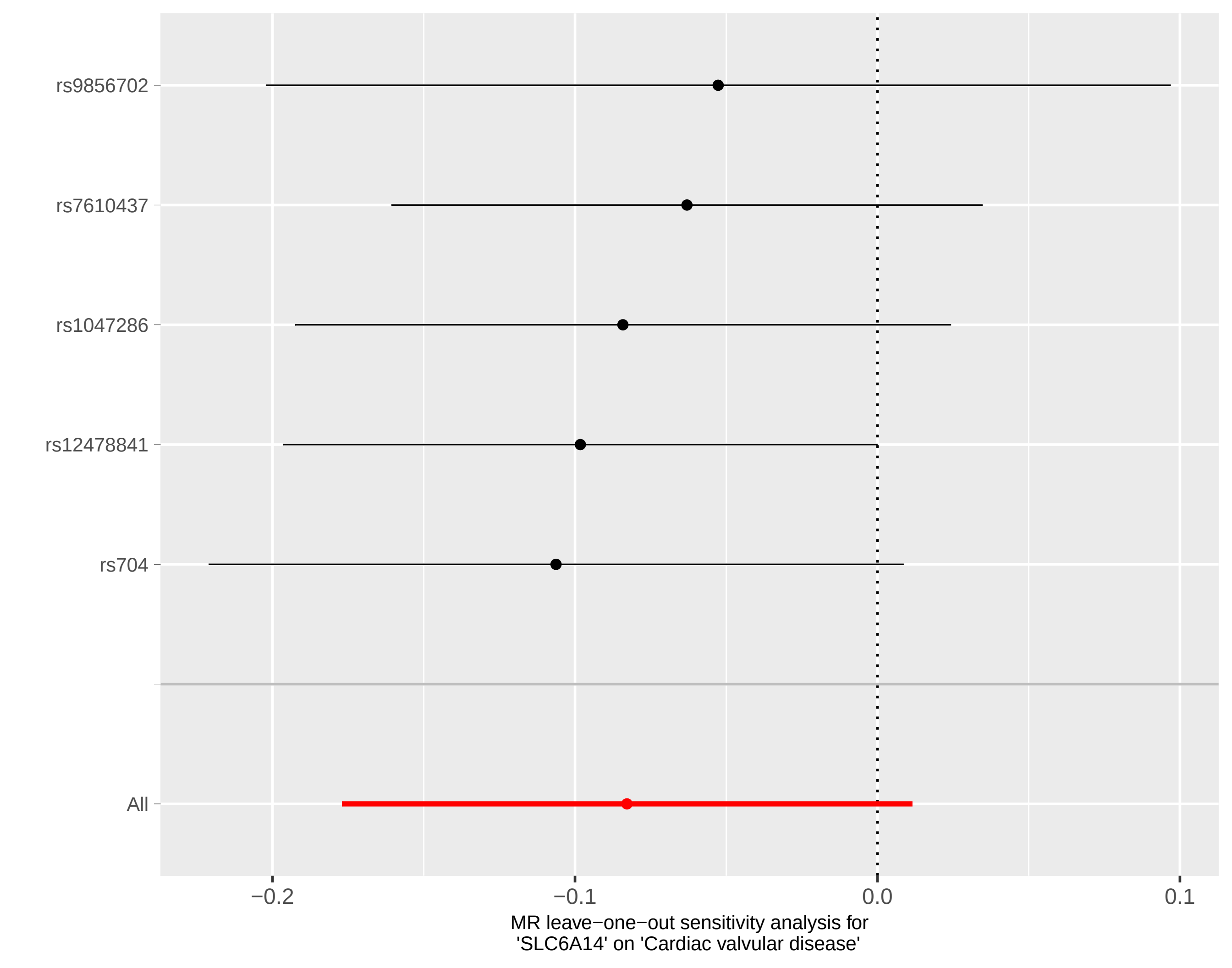
rs9856702
rs7610437
rs1047286
rs12478841
rs704
All
−0.2
−0.1
0.0
0.1
MR leave−one−out sensitivity analysis for
'SLC6A14' on 'Cardiac valvular disease'

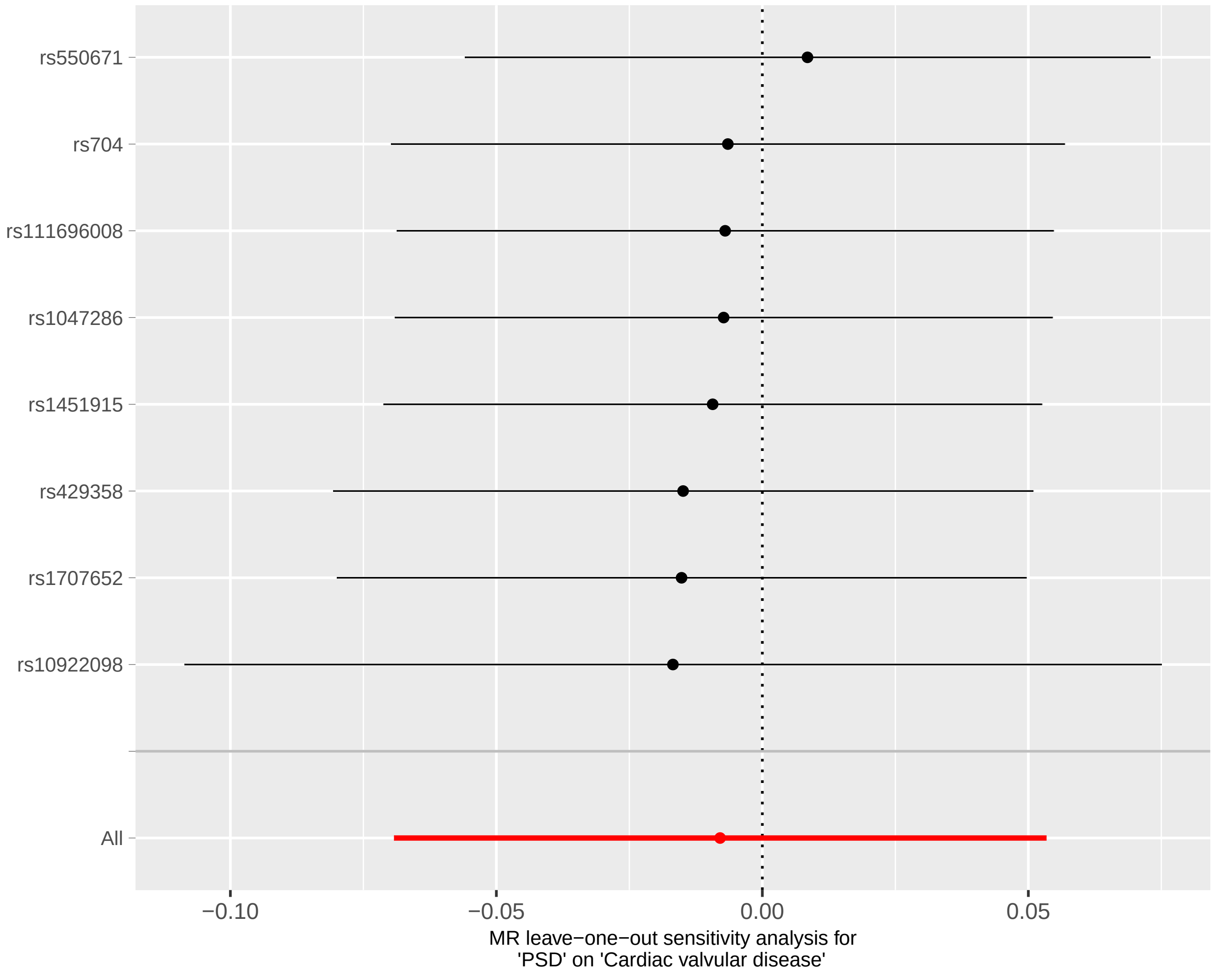
rs550671
rs704
rs111696008
rs1047286
rs1451915
rs429358
rs1707652
rs10922098
All
−0.10
−0.05
0.00
0.05
MR leave−one−out sensitivity analysis for
'PSD' on 'Cardiac valvular disease'

Insufficient number of SNPs

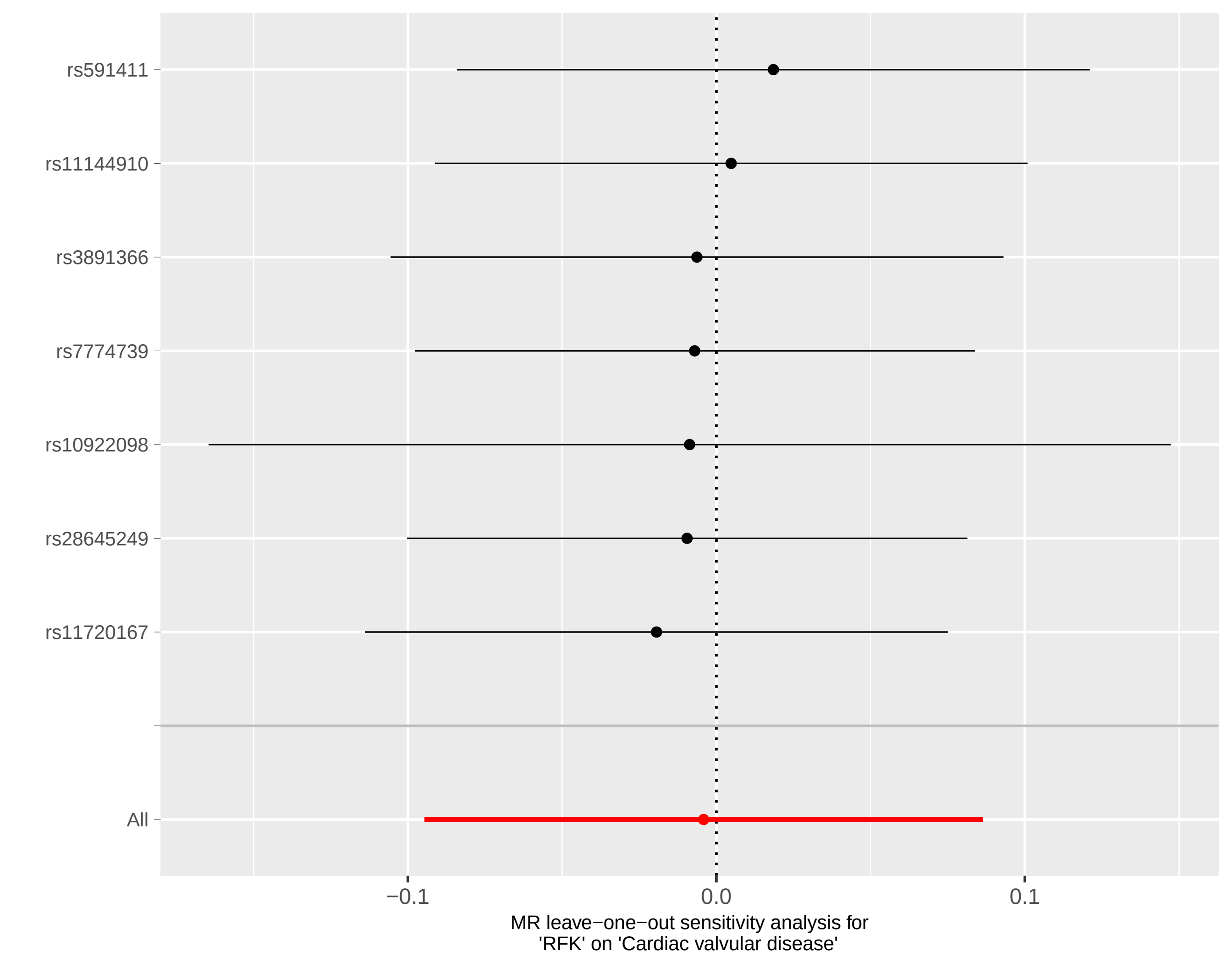
rs591411
rs11144910
rs3891366
rs7774739
rs10922098
rs28645249
rs11720167
All
−0.1
0.0
0.1
MR leave−one−out sensitivity analysis for
'RFK' on 'Cardiac valvular disease'

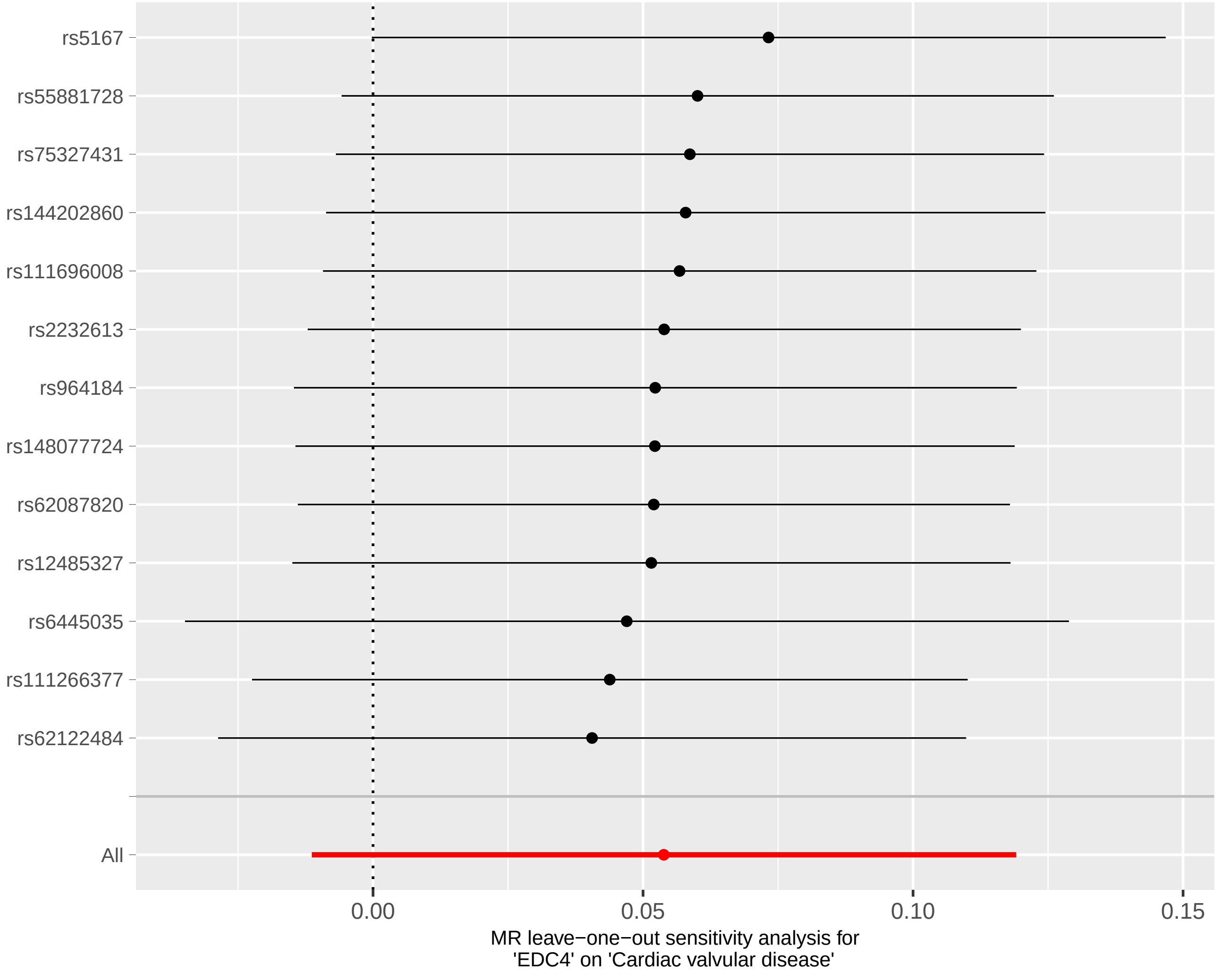
rs5167
rs55881728
rs75327431
rs144202860
rs111696008
rs2232613
rs964184
rs148077724
rs62087820
rs12485327
rs6445035
rs111266377
rs62122484
All
0.00
0.05
0.10
0.15
MR leave−one−out sensitivity analysis for
'EDC4' on 'Cardiac valvular disease'

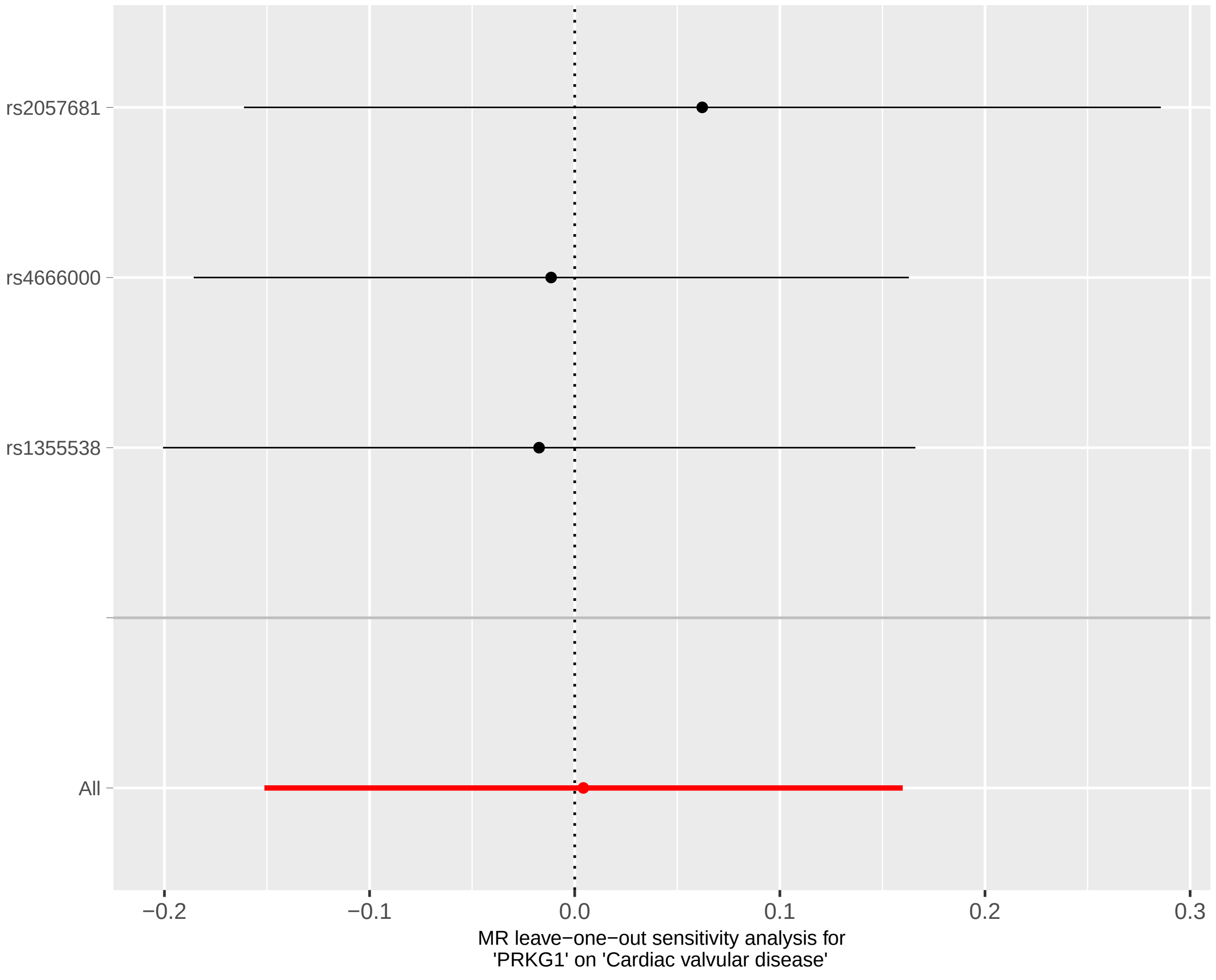

rs2057681
rs4666000
rs1355538
All
−0.2
−0.1
0.0
0.1
0.2
0.3
MR leave−one−out sensitivity analysis for
'PRKG1' on 'Cardiac valvular disease'

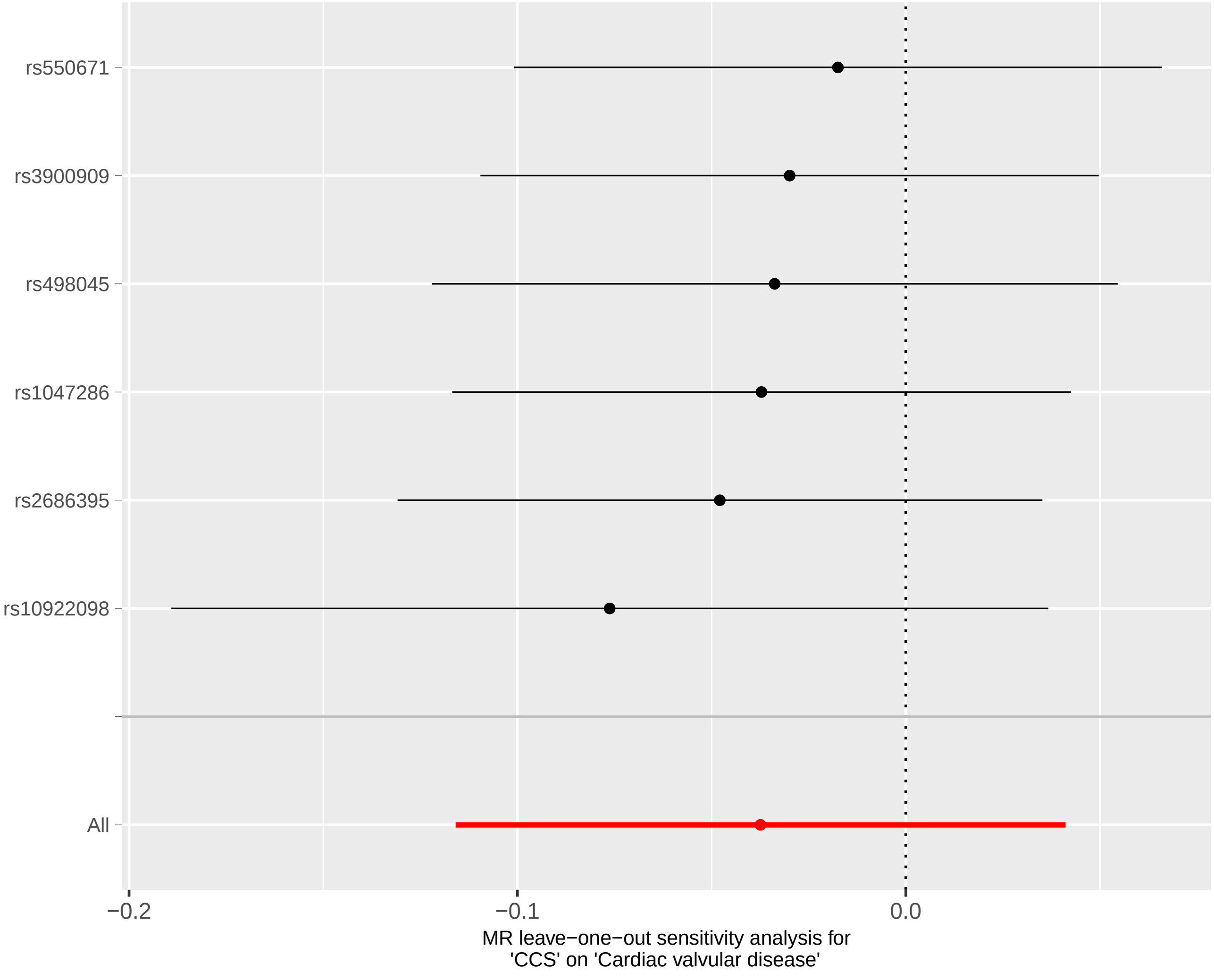

rs550671
rs3900909
rs498045
rs1047286
rs2686395
rs10922098
All
−0.2
−0.1
0.0
MR leave−one−out sensitivity analysis for
'CCS' on 'Cardiac valvular disease'

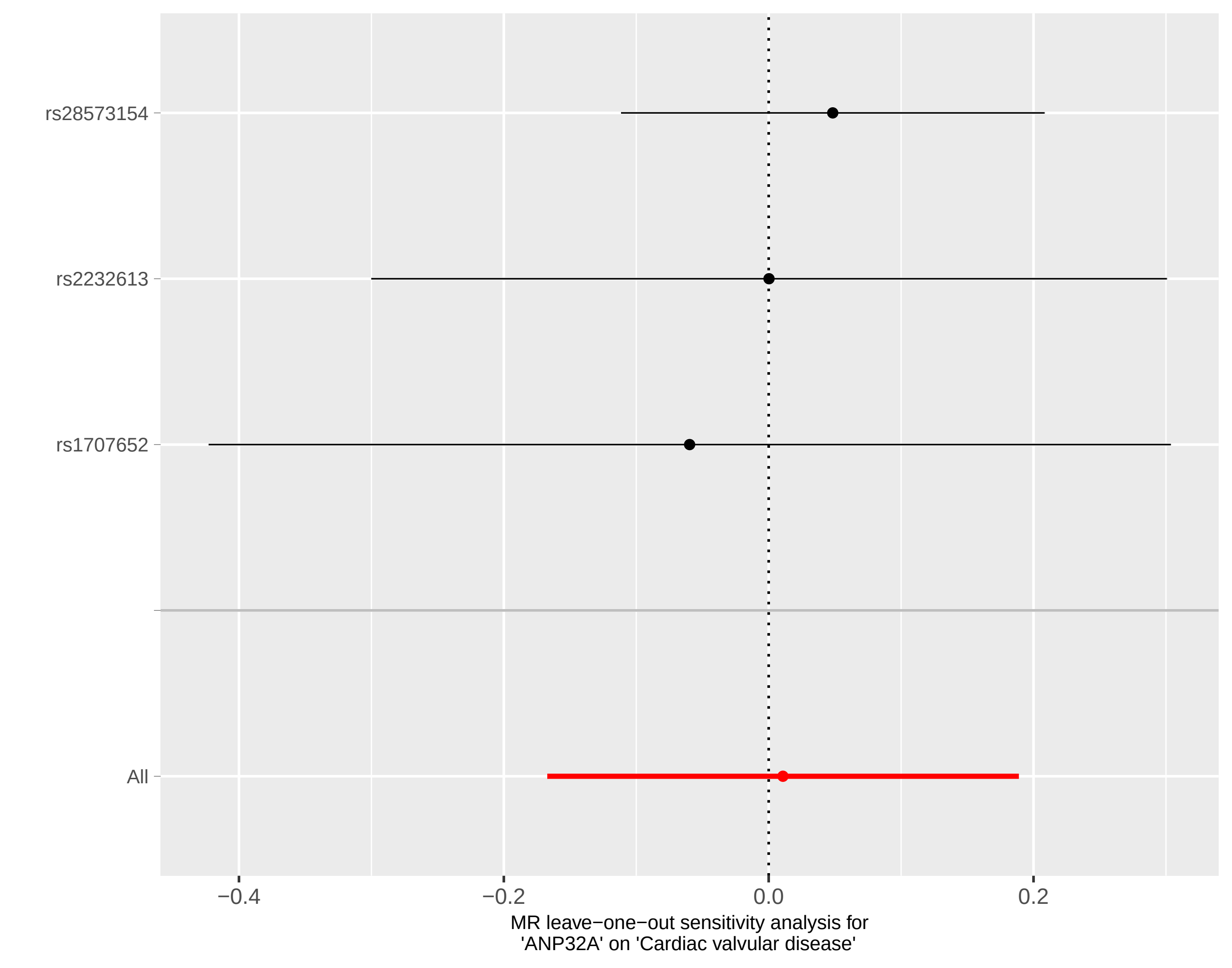

rs28573154
rs2232613
rs1707652
All
−0.4
−0.2
0.0
0.2
MR leave−one−out sensitivity analysis for
'ANP32A' on 'Cardiac valvular disease'

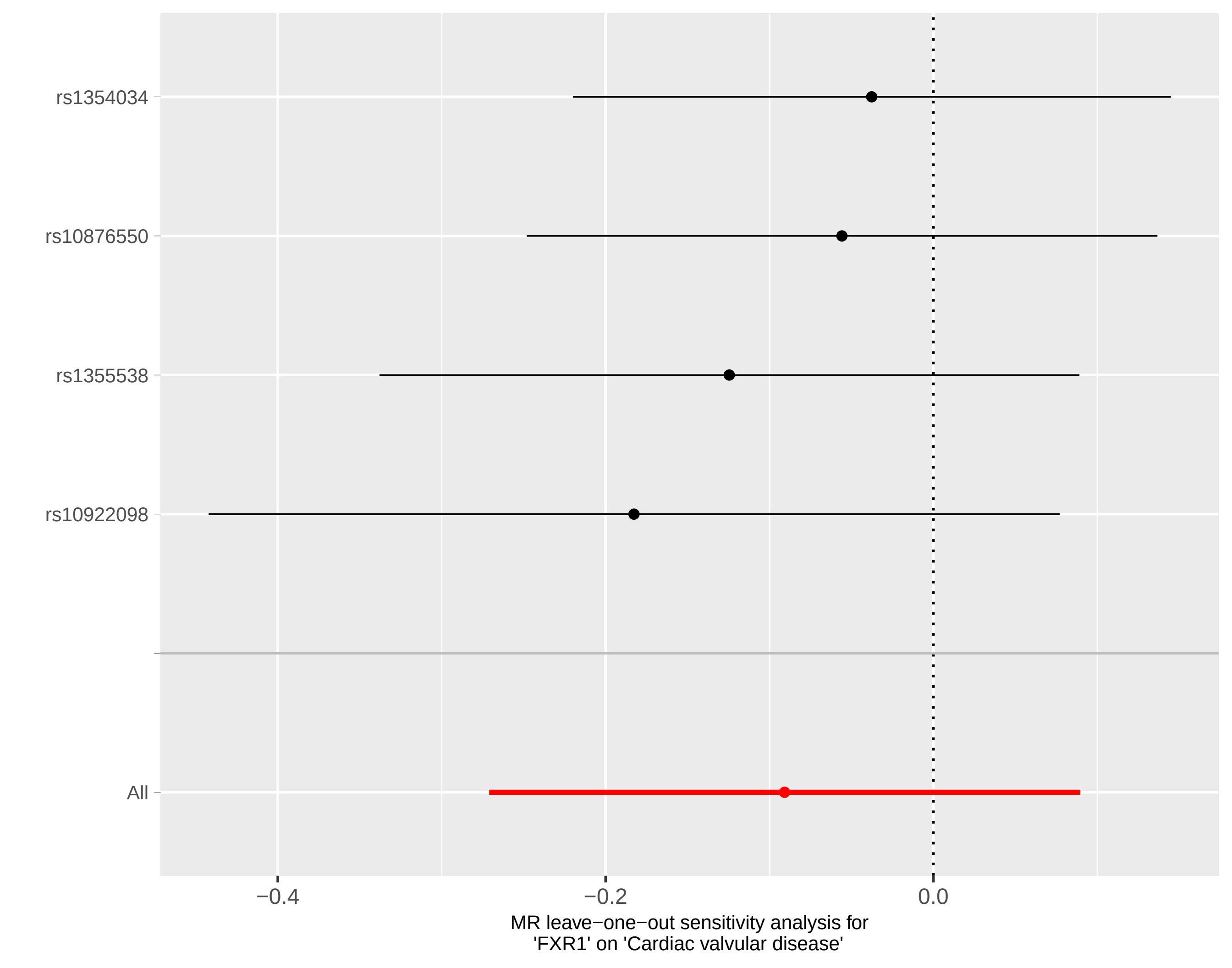
rs1354034
rs10876550
rs1355538
rs10922098
All
−0.4
−0.2
0.0
MR leave−one−out sensitivity analysis for
'FXR1' on 'Cardiac valvular disease'

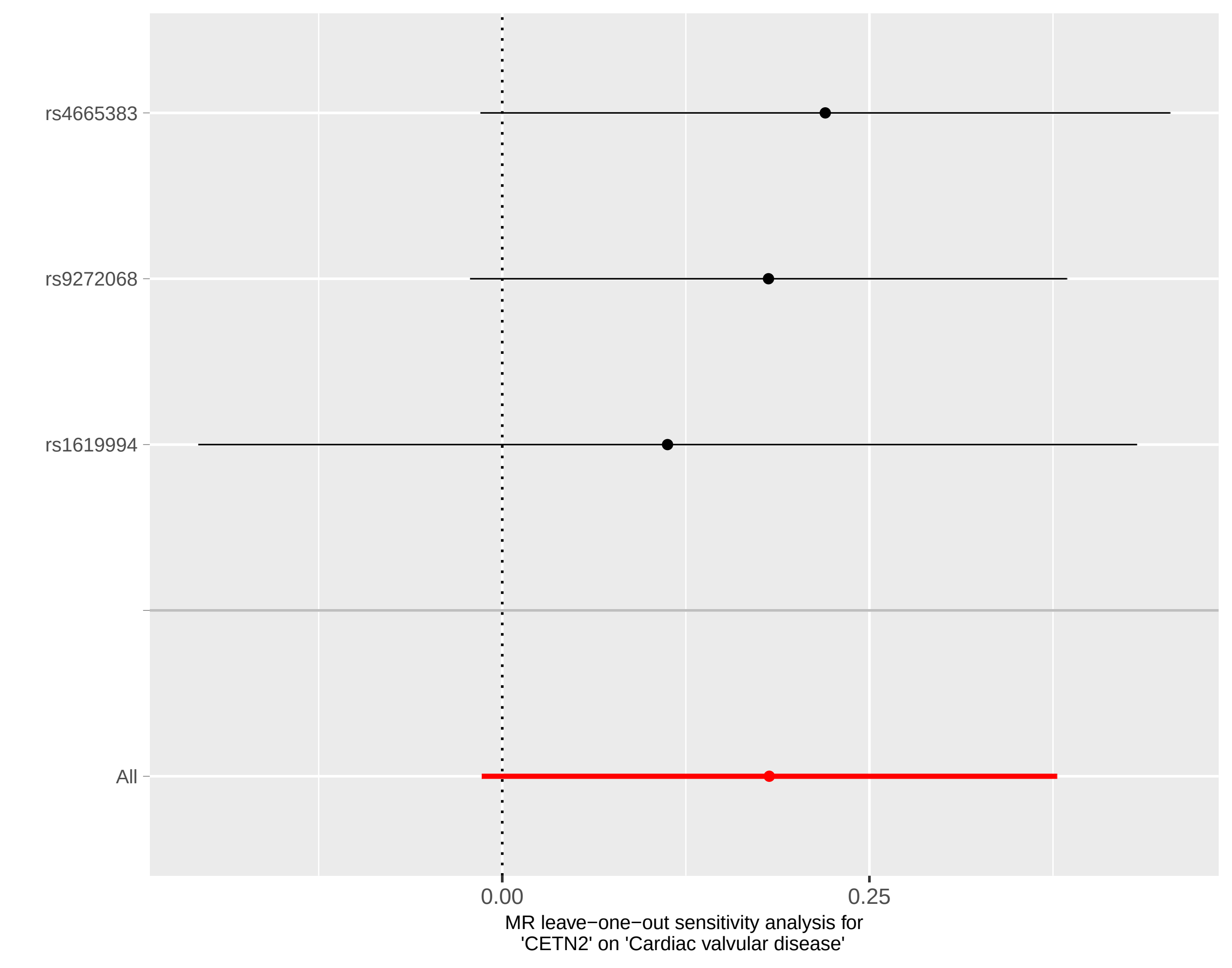

rs4665383
rs9272068
rs1619994
All
0.00
0.25
MR leave−one−out sensitivity analysis for
'CETN2' on 'Cardiac valvular disease'

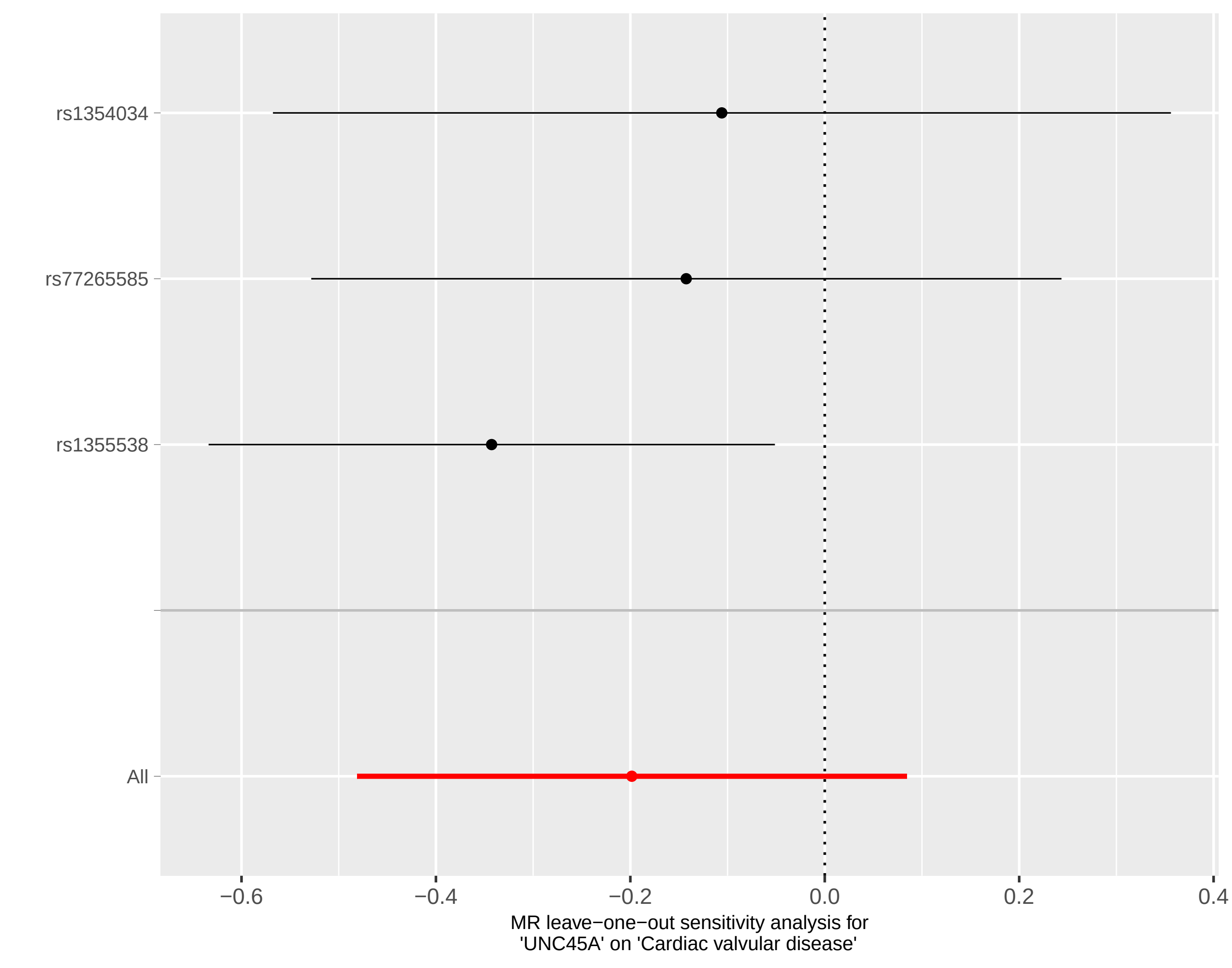
rs1354034
rs77265585
rs1355538
All
−0.6
−0.4
−0.2
0.0
0.2
0.4
MR leave−one−out sensitivity analysis for
'UNC45A' on 'Cardiac valvular disease'

Insufficient number of SNPs

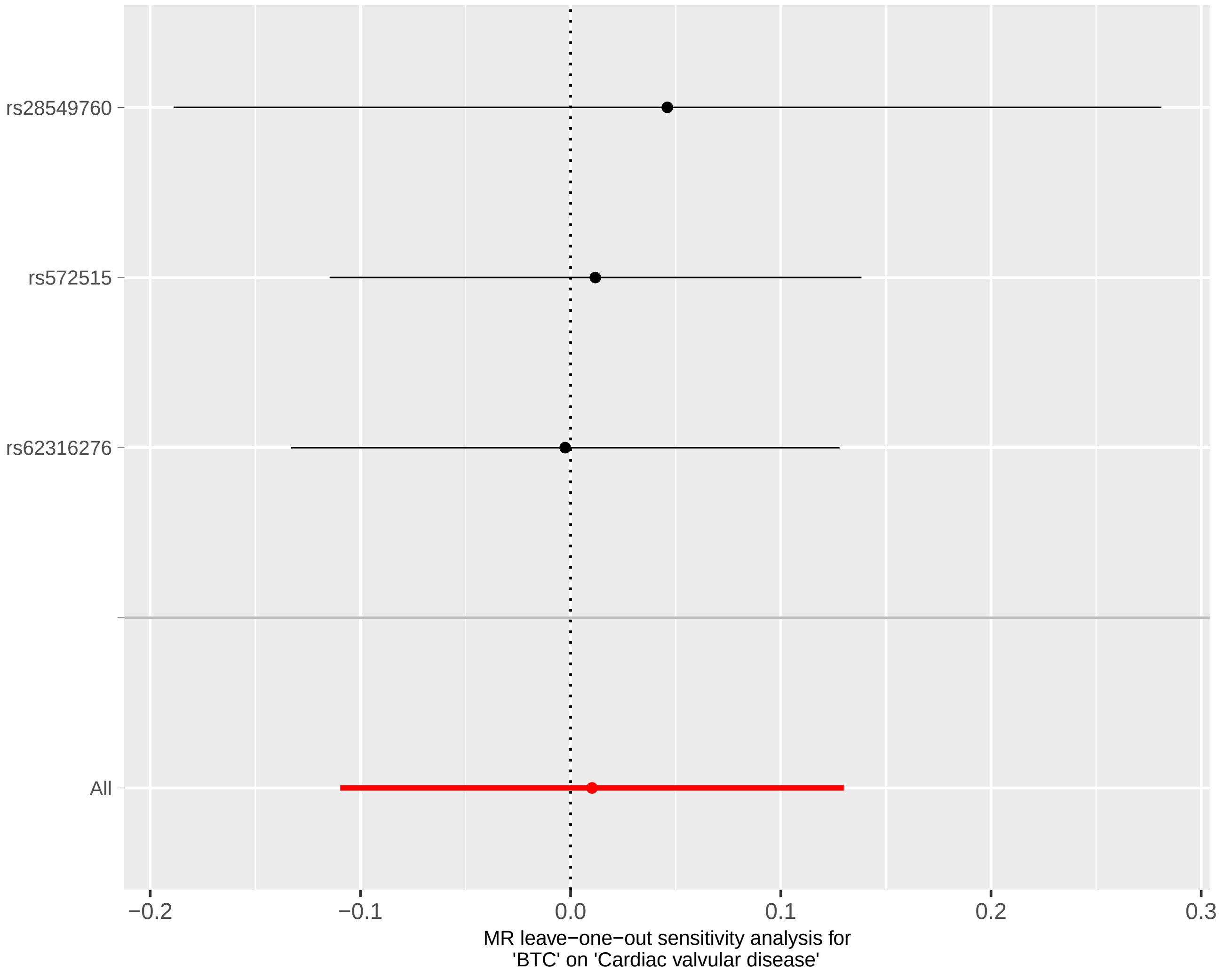

rs28549760
rs572515
rs62316276
All
−0.2
−0.1
0.0
0.1
0.2
0.3
MR leave−one−out sensitivity analysis for
'BTC' on 'Cardiac valvular disease'

Insufficient number of SNPs

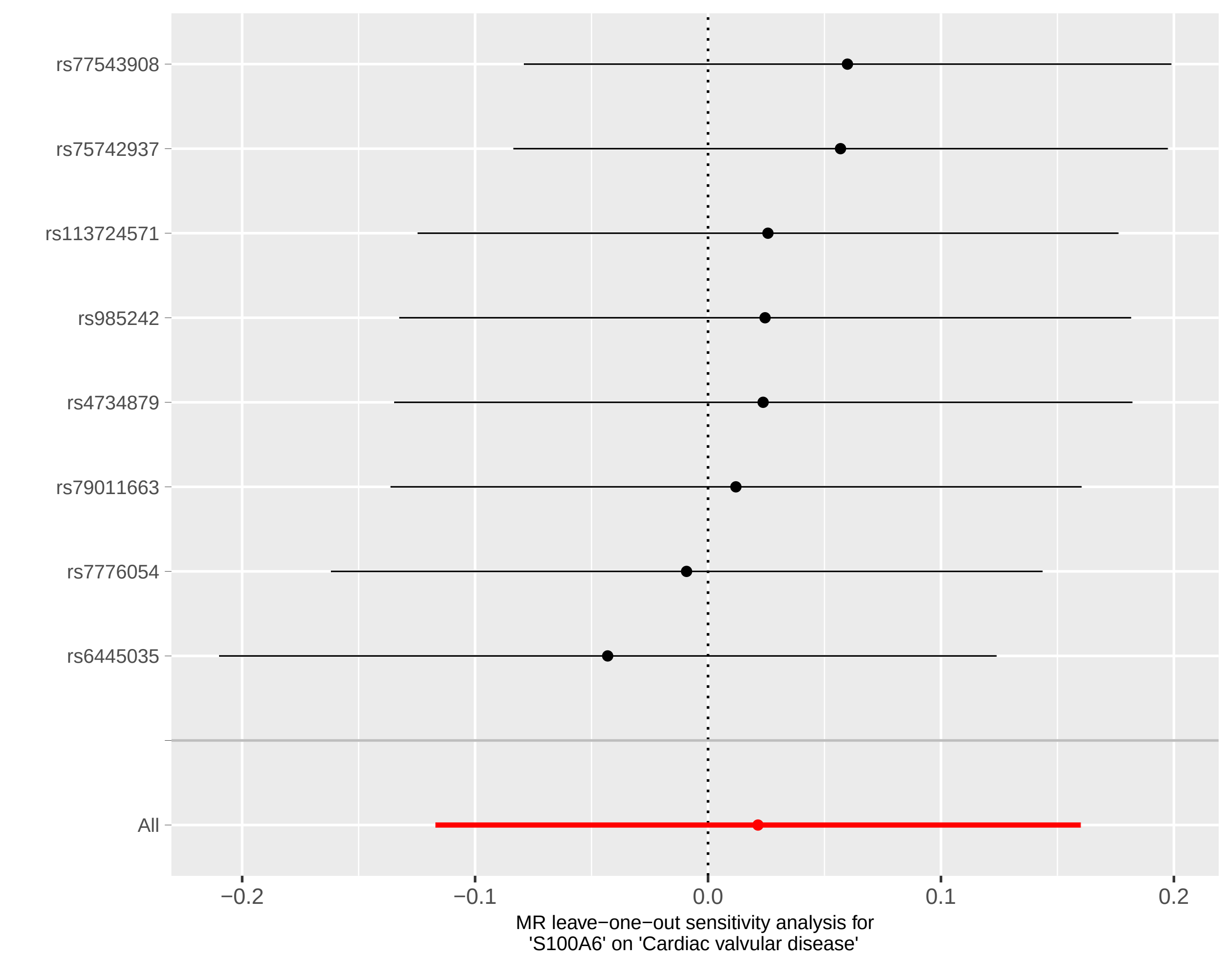
rs77543908
rs75742937
rs113724571
rs985242
rs4734879
rs79011663
rs7776054
rs6445035
All
−0.2
−0.1
0.0
0.1
0.2
MR leave−one−out sensitivity analysis for
'S100A6' on 'Cardiac valvular disease'

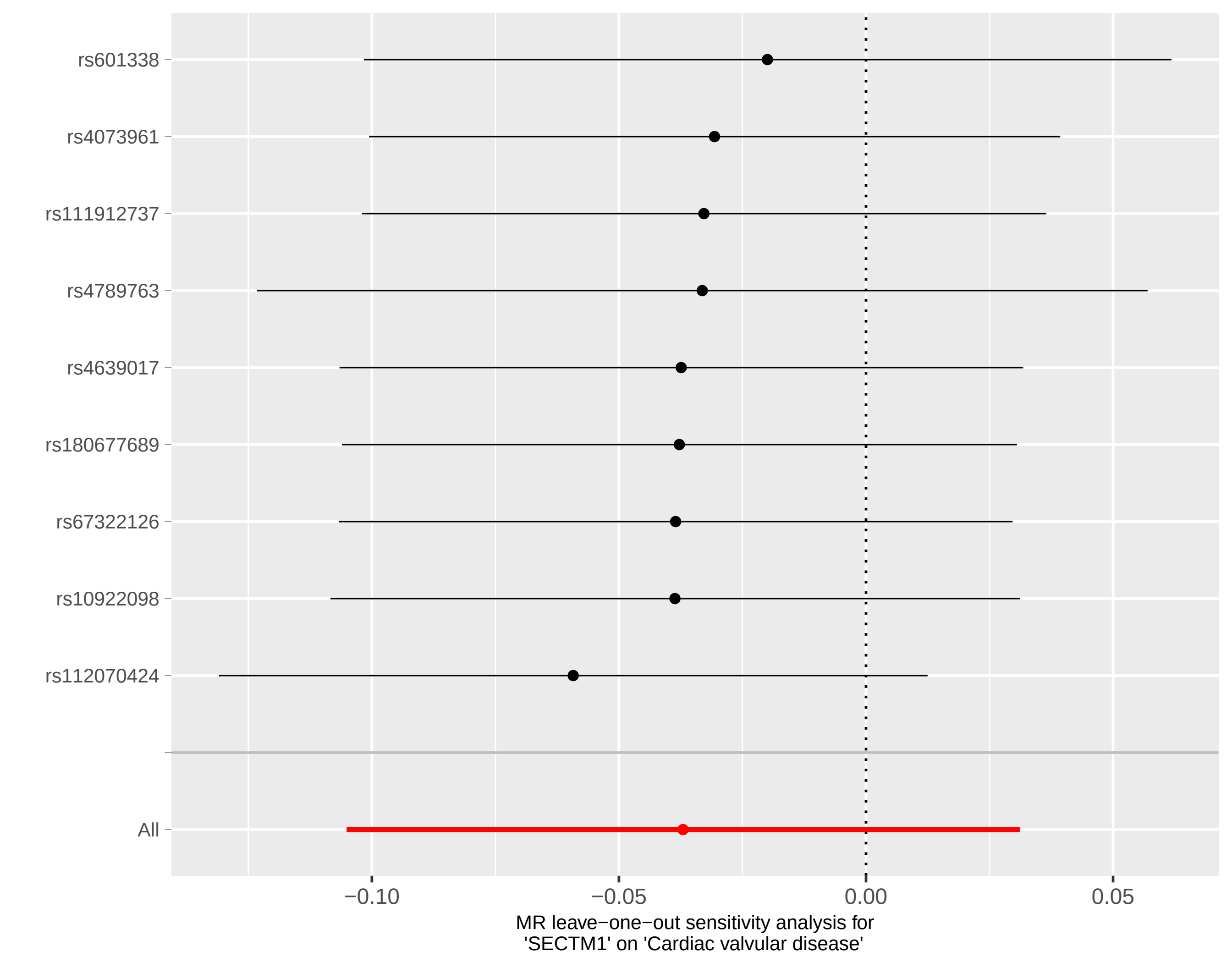
rs601338
rs4073961
rs111912737
rs4789763
rs4639017
rs180677689
rs67322126
rs10922098
rs112070424
All
−0.10
−0.05
0.00
0.05
MR leave−one−out sensitivity analysis for
'SECTM1' on 'Cardiac valvular disease'

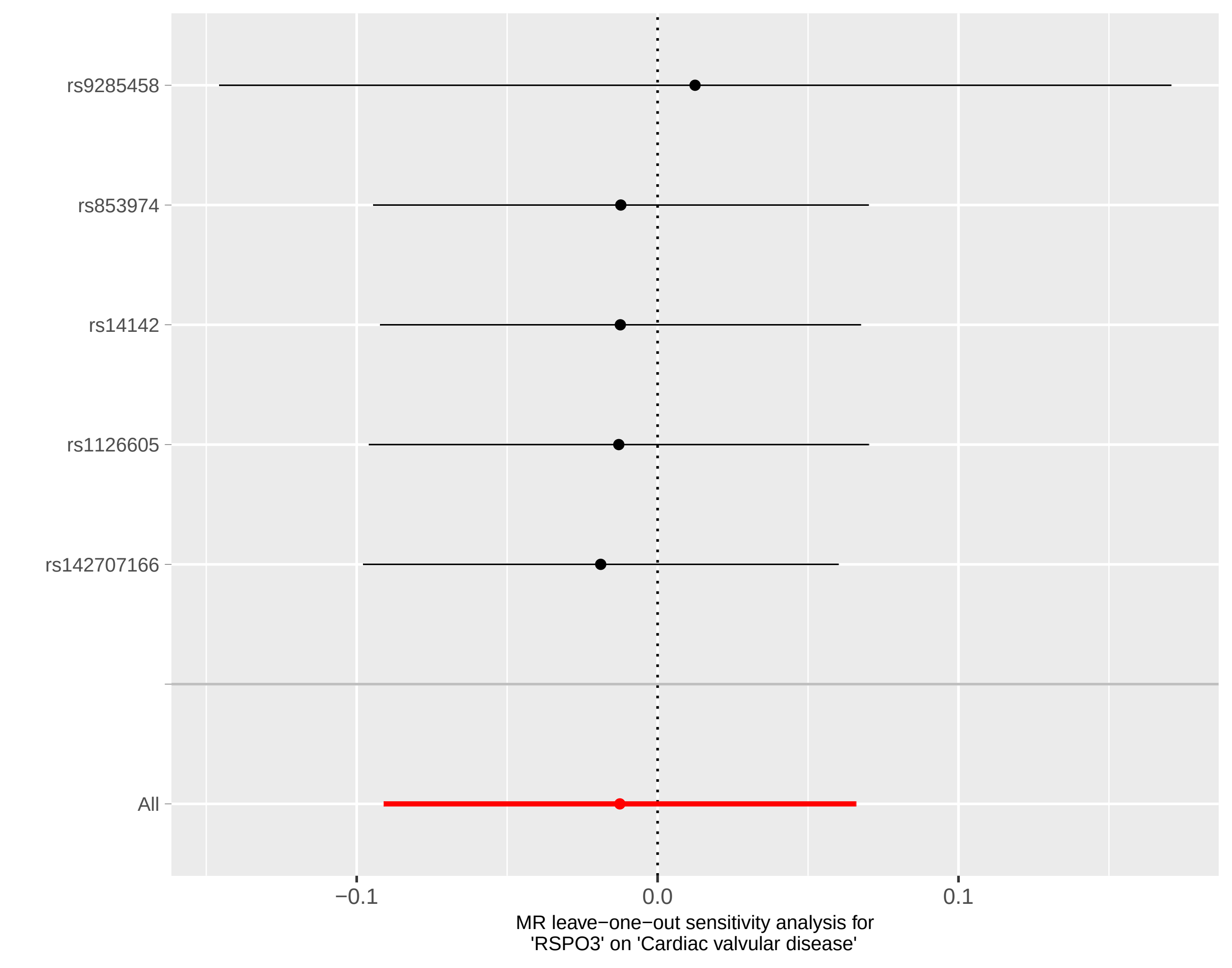
rs9285458
rs853974
rs14142
rs1126605
rs142707166
All
−0.1
0.0
0.1
MR leave−one−out sensitivity analysis for
'RSPO3' on 'Cardiac valvular disease'

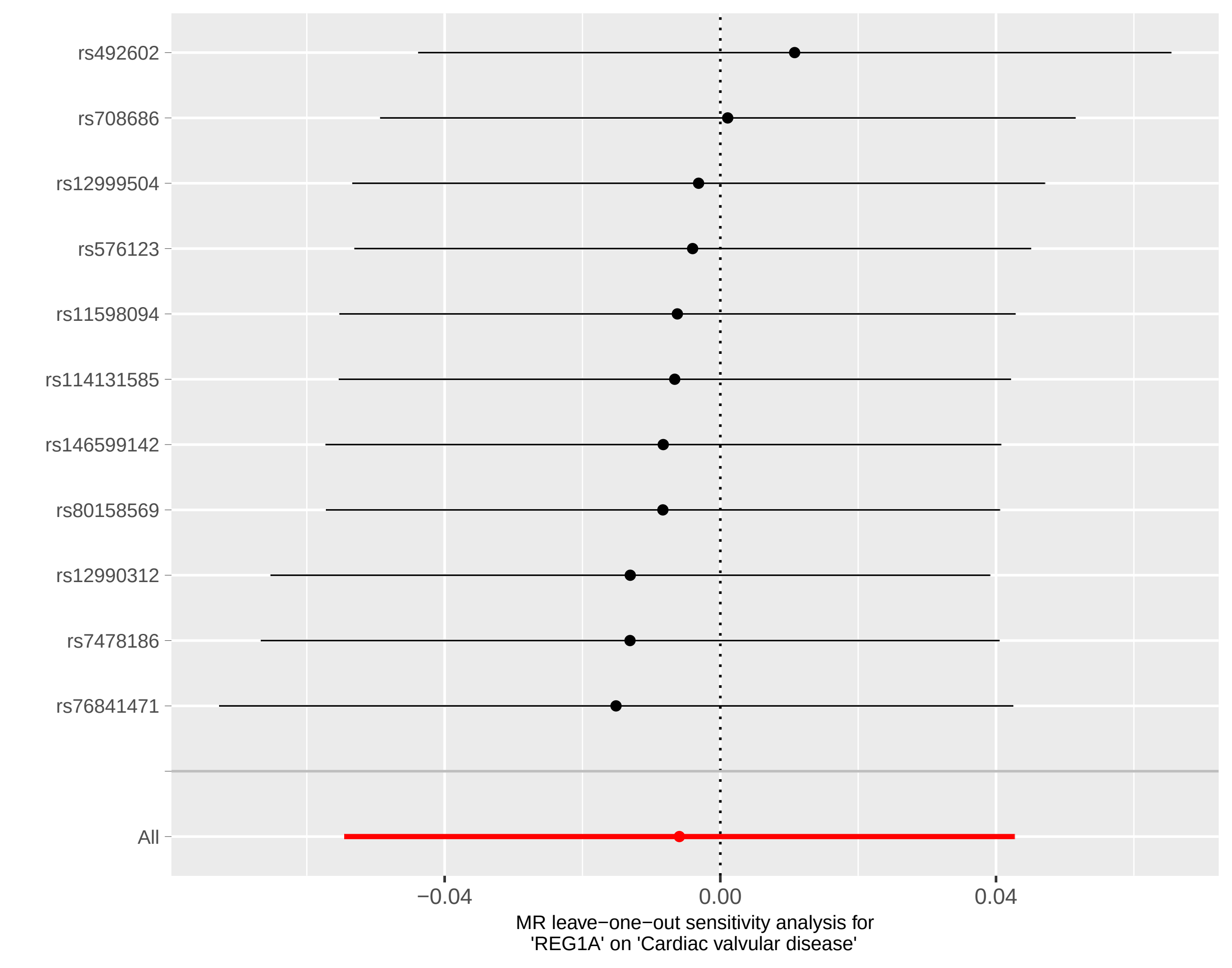

rs492602
rs708686
rs12999504
rs576123
rs11598094
rs114131585
rs146599142
rs80158569
rs12990312
rs7478186
rs76841471
All
−0.04
0.00
0.04
MR leave−one−out sensitivity analysis for
'REG1A' on 'Cardiac valvular disease'

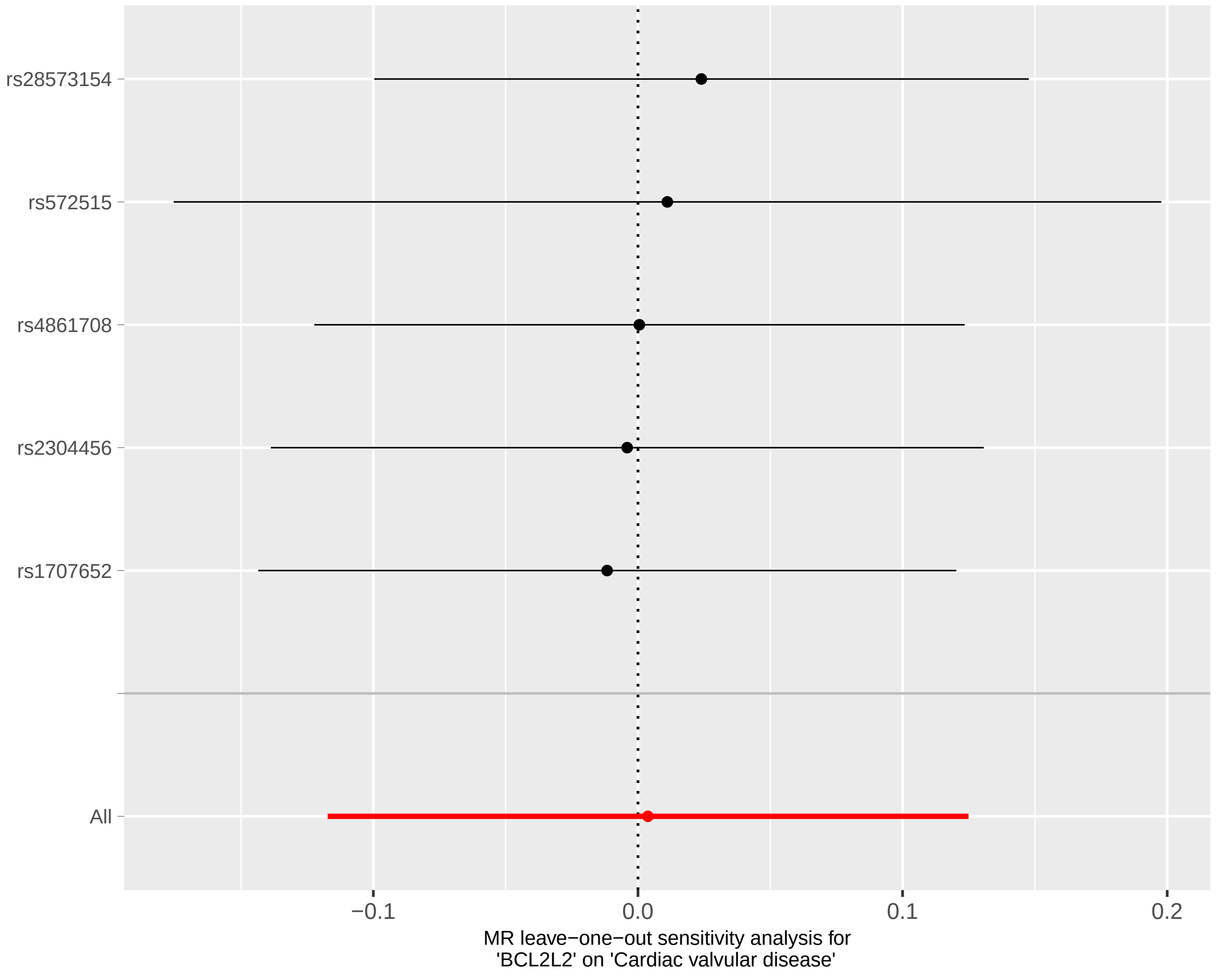

rs28573154
rs572515
rs4861708
rs2304456
rs1707652
All
−0.1
0.0
0.1
0.2
MR leave−one−out sensitivity analysis for
'BCL2L2' on 'Cardiac valvular disease'

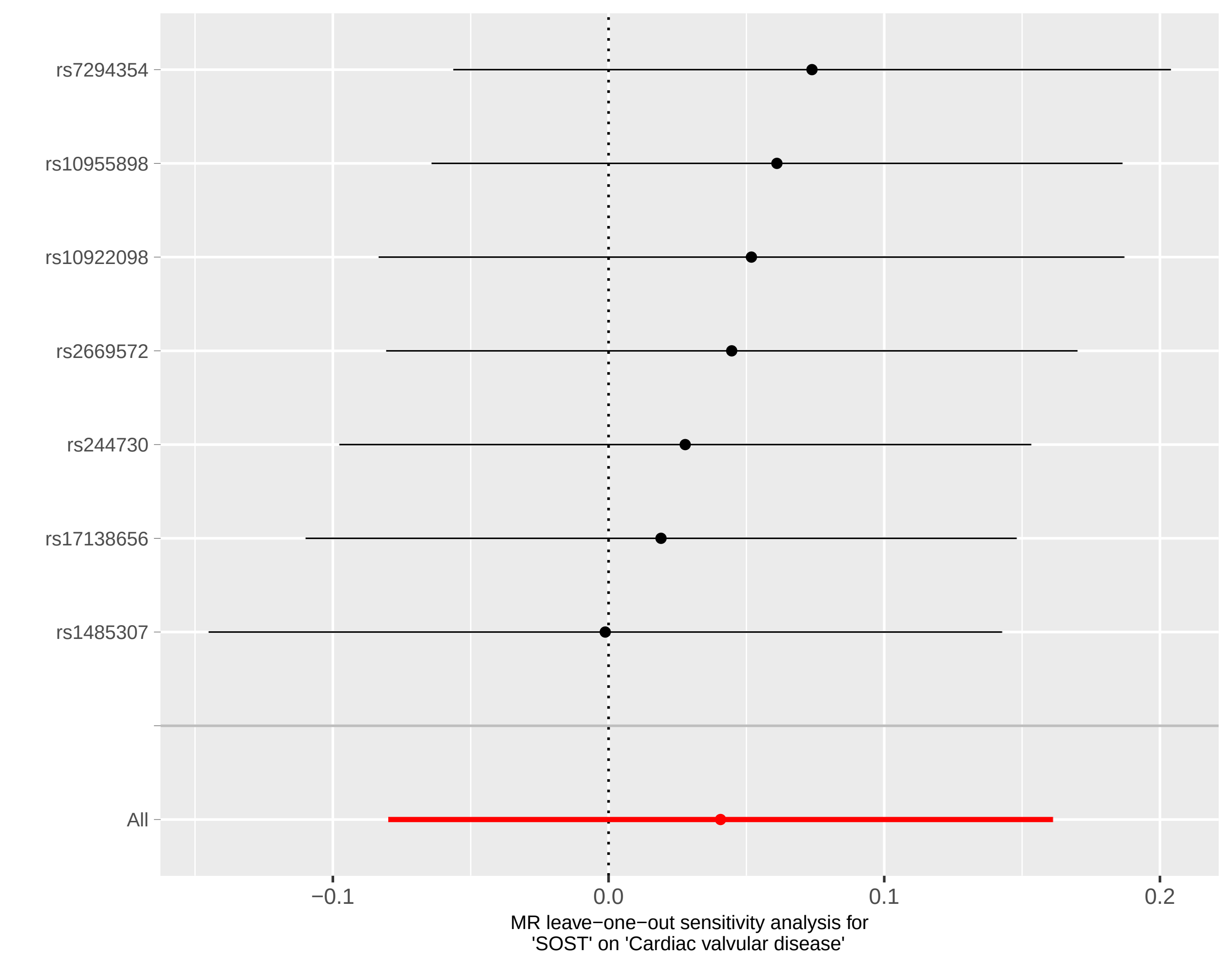
rs7294354
rs10955898
rs10922098
rs2669572
rs244730
rs17138656
rs1485307
All
−0.1
0.0
0.1
0.2
MR leave−one−out sensitivity analysis for
'SOST' on 'Cardiac valvular disease'

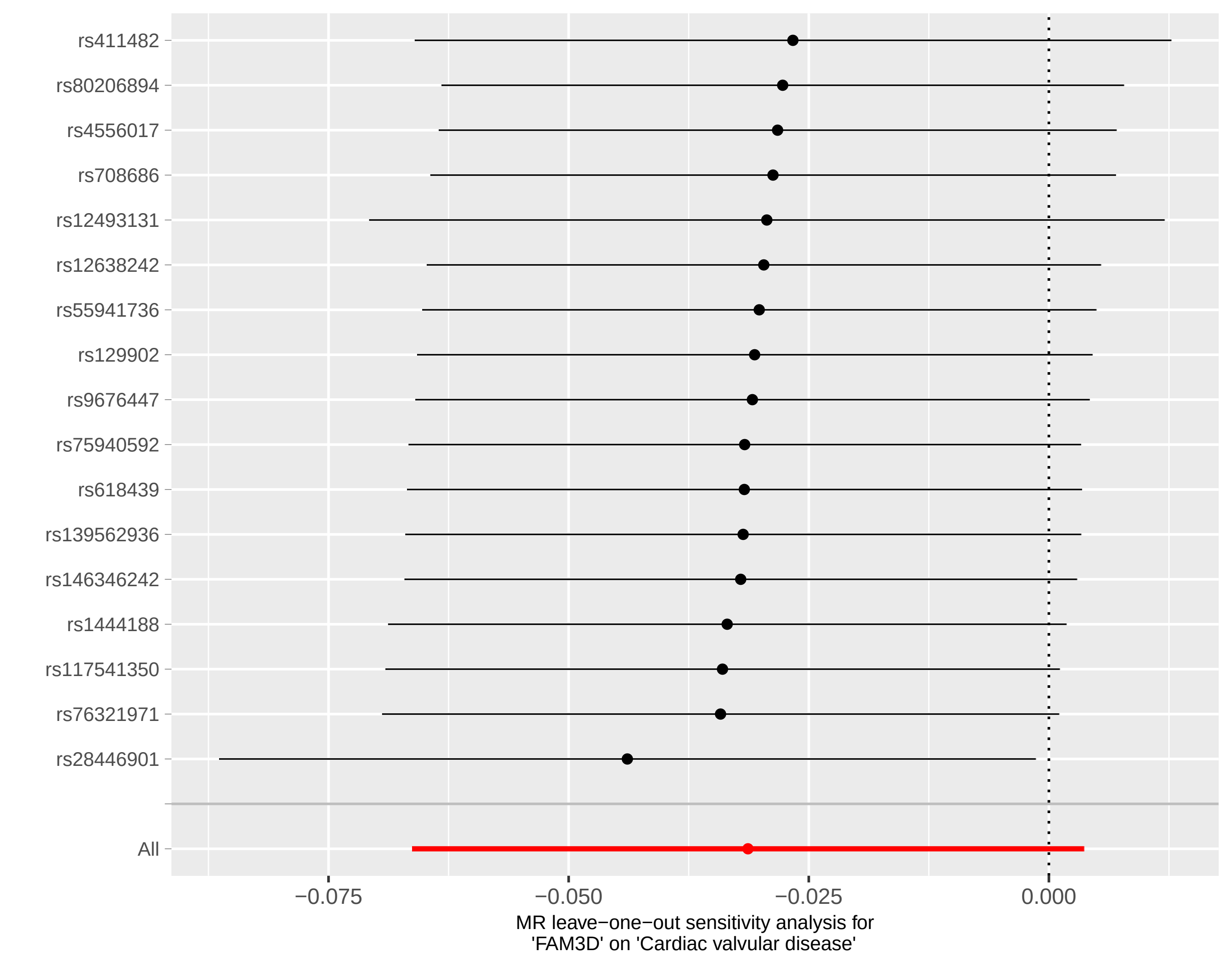

rs411482
rs80206894
rs4556017
rs708686
rs12493131
rs12638242
rs55941736
rs129902
rs9676447
rs75940592
rs618439
rs139562936
rs146346242
rs1444188
rs117541350
rs76321971
rs28446901
All
−0.075
−0.050
−0.025
0.000
MR leave−one−out sensitivity analysis for
'FAM3D' on 'Cardiac valvular disease'

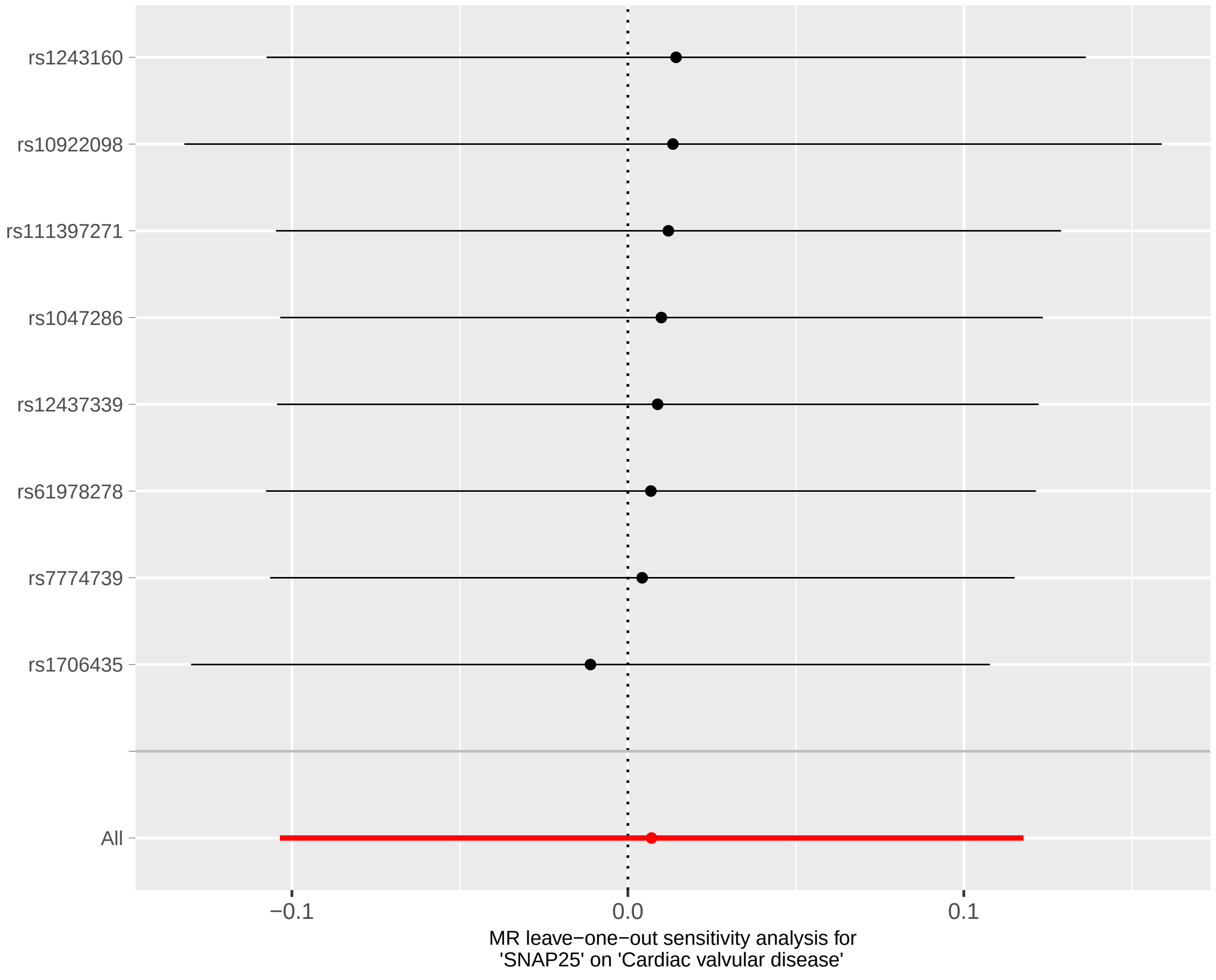
rs1243160
rs10922098
rs111397271
rs1047286
rs12437339
rs61978278
rs7774739
rs1706435
All
−0.1
0.0
0.1
MR leave−one−out sensitivity analysis for
'SNAP25' on 'Cardiac valvular disease'

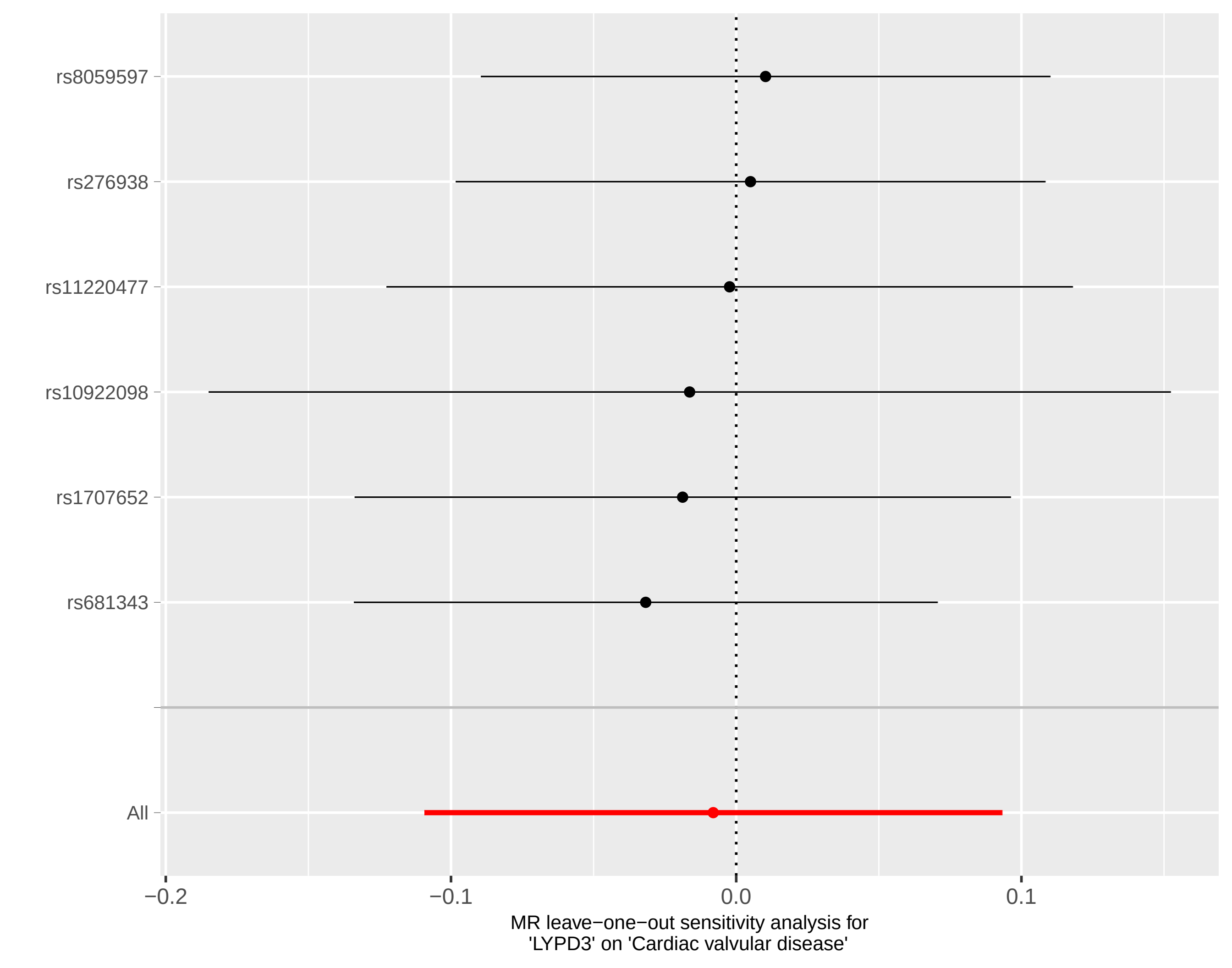
rs8059597
rs276938
rs11220477
rs10922098
rs1707652
rs681343
All
−0.2
−0.1
0.0
0.1
MR leave−one−out sensitivity analysis for
'LYPD3' on 'Cardiac valvular disease'

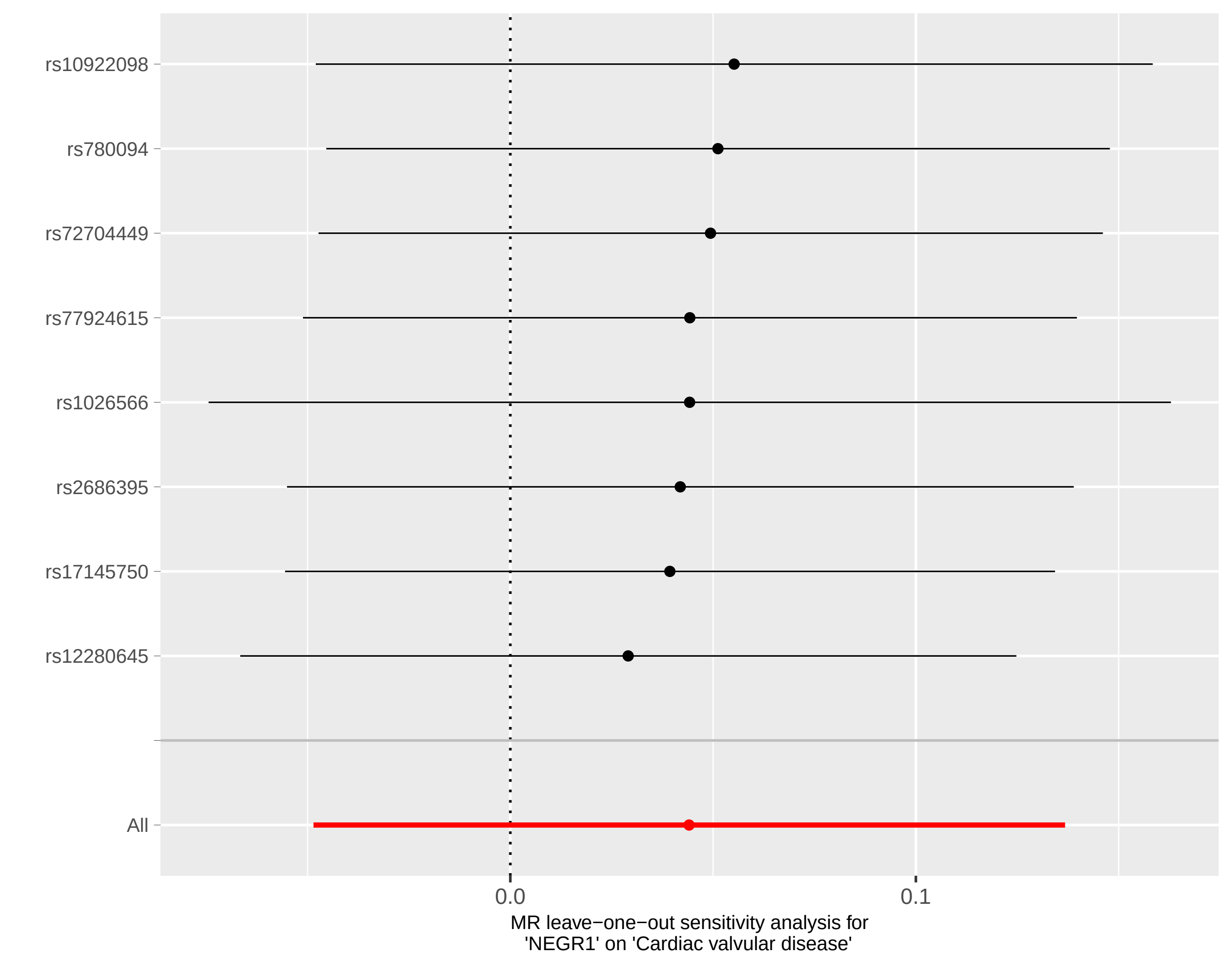

rs10922098
rs780094
rs72704449
rs77924615
rs1026566
rs2686395
rs17145750
rs12280645
All
0.0
0.1
MR leave−one−out sensitivity analysis for
'NEGR1' on 'Cardiac valvular disease'

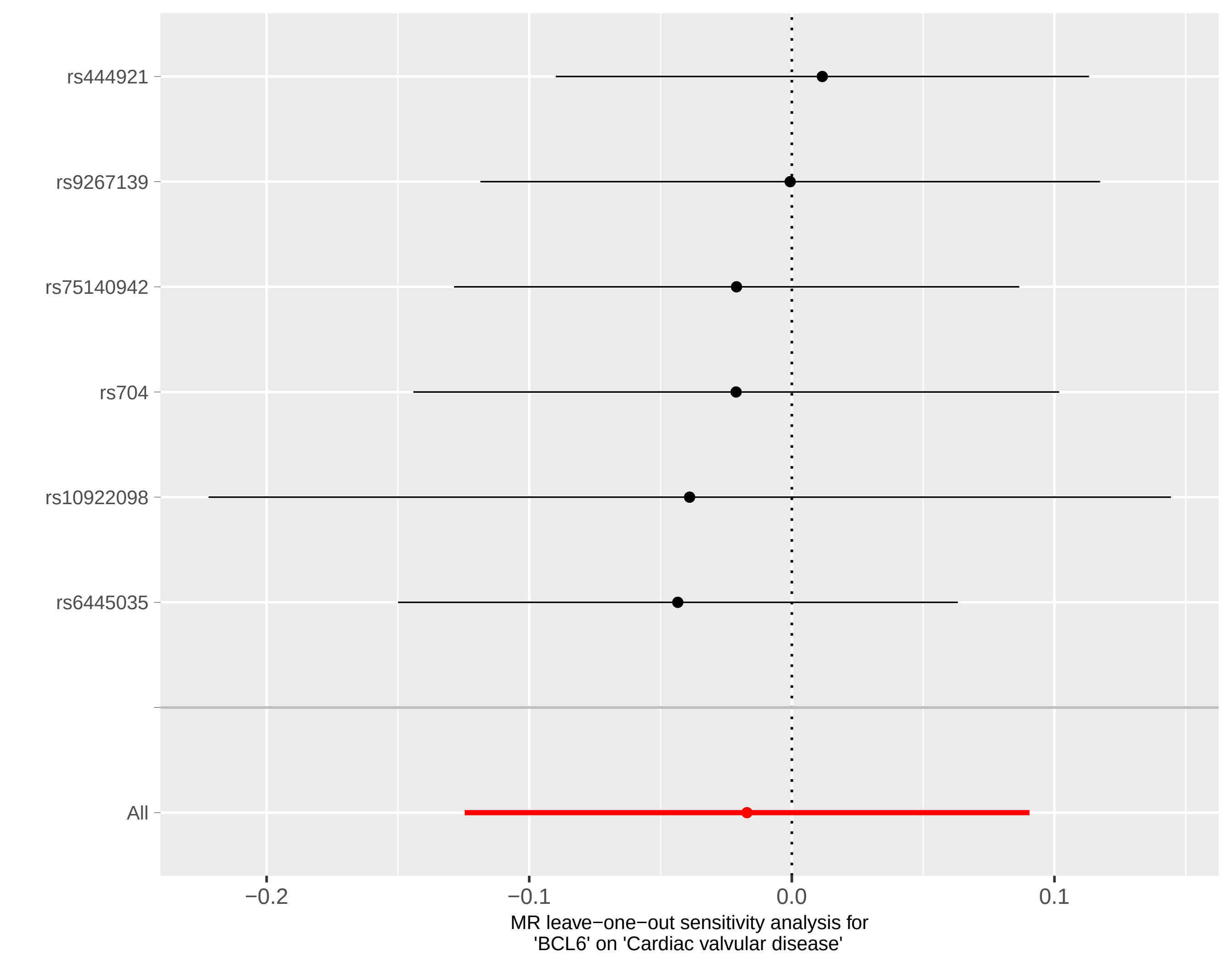
rs444921
rs9267139
rs75140942
rs704
rs10922098
rs6445035
All
−0.2
−0.1
0.0
0.1
MR leave−one−out sensitivity analysis for
'BCL6' on 'Cardiac valvular disease'

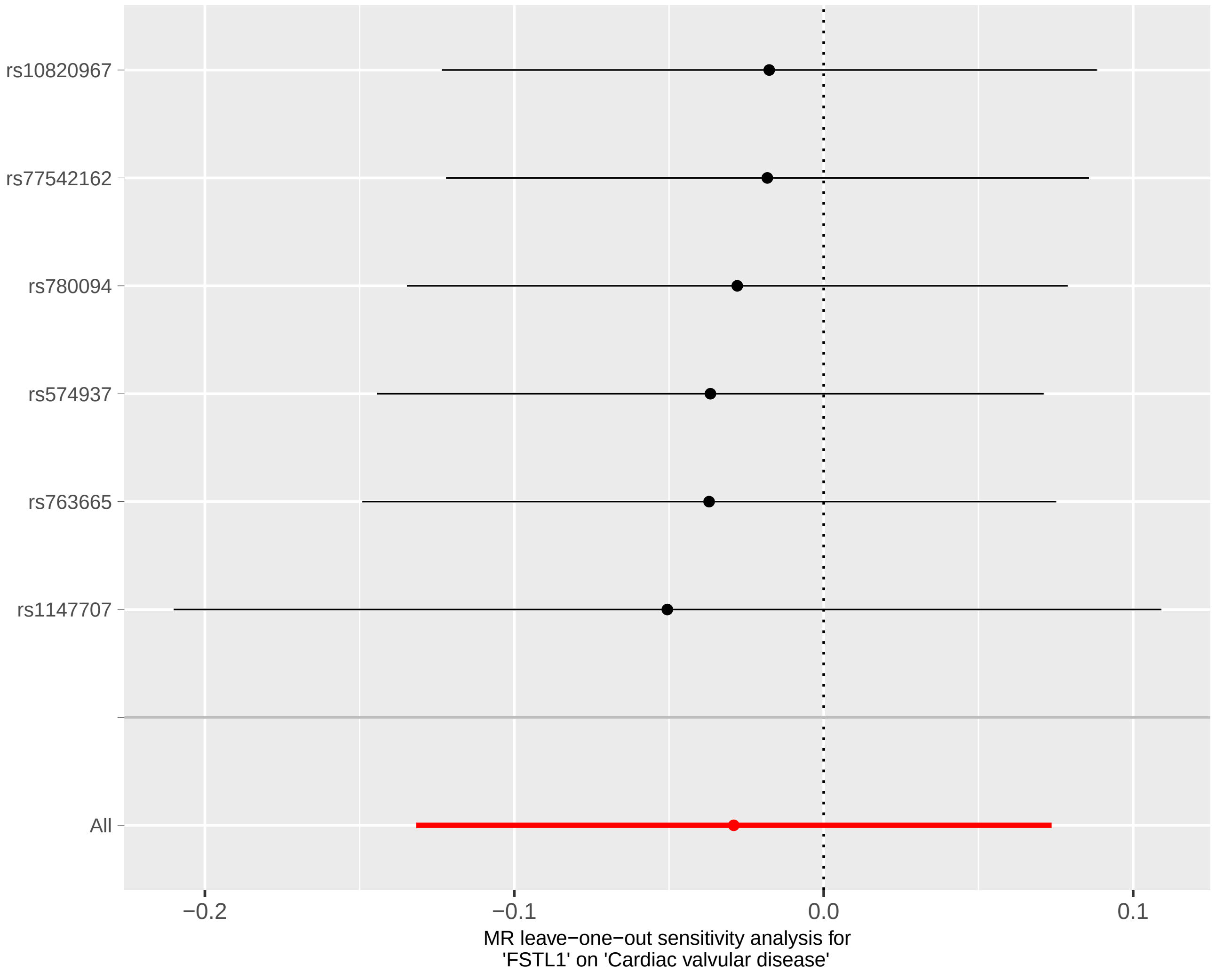
rs10820967
rs77542162
rs780094
rs574937
rs763665
rs1147707
All
−0.2
−0.1
0.0
0.1
MR leave−one−out sensitivity analysis for
'FSTL1' on 'Cardiac valvular disease'

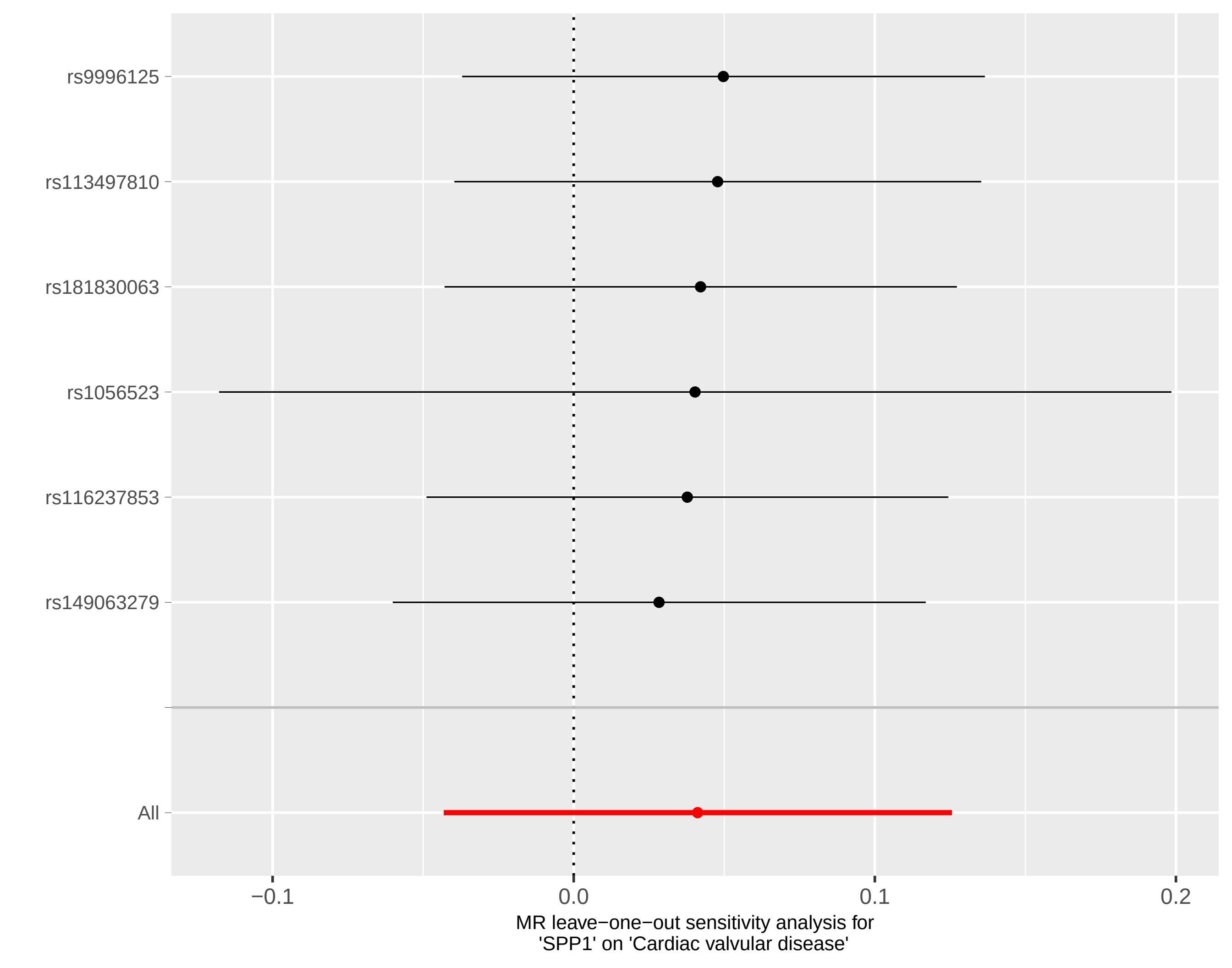
rs9996125
rs113497810
rs181830063
rs1056523
rs116237853
rs149063279
All
−0.1
0.0
0.1
0.2
MR leave−one−out sensitivity analysis for
'SPP1' on 'Cardiac valvular disease'

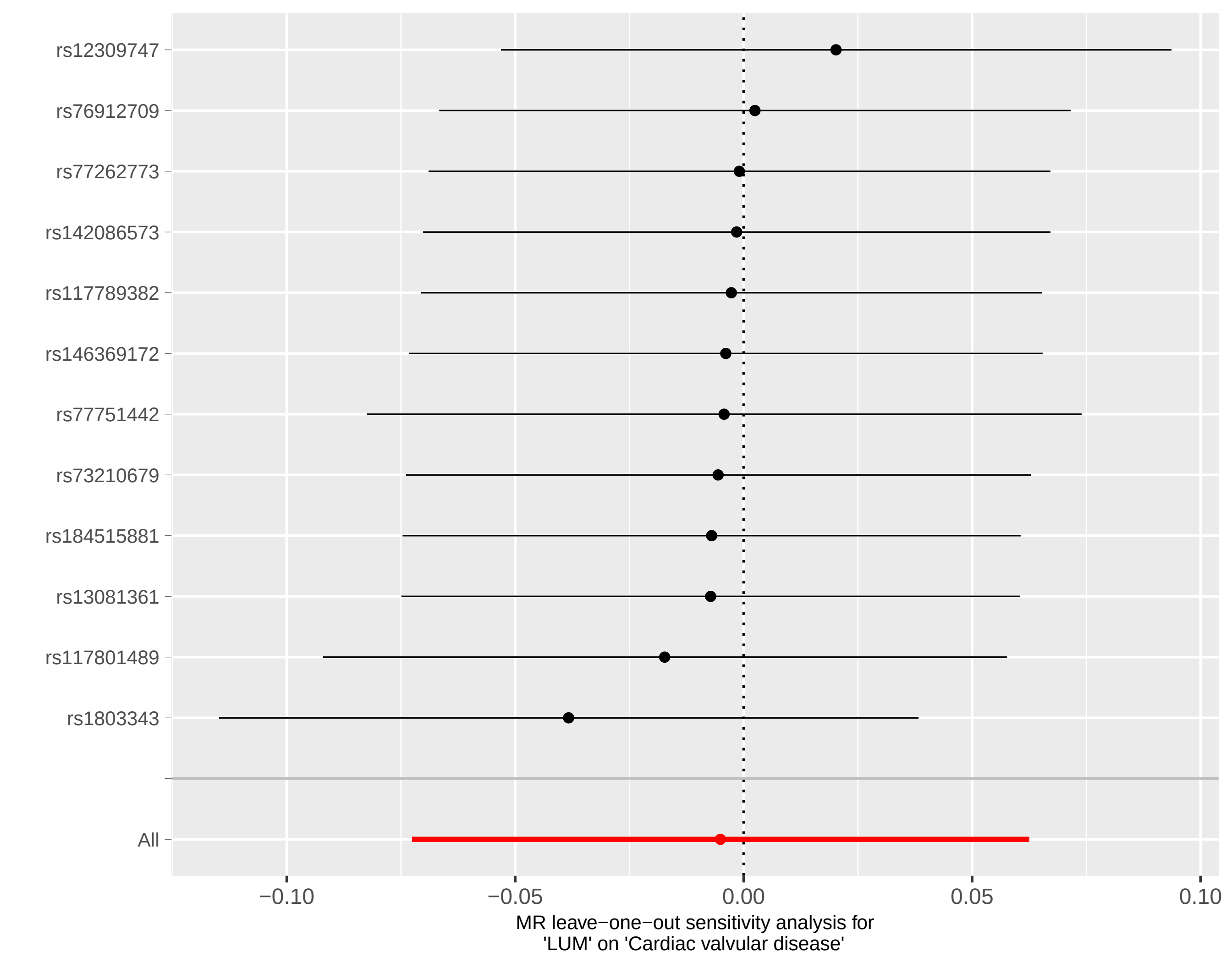
rs12309747
rs76912709
rs77262773
rs142086573
rs117789382
rs146369172
rs77751442
rs73210679
rs184515881
rs13081361
rs117801489
rs1803343
All
−0.10
−0.05
0.00
0.05
0.10
MR leave−one−out sensitivity analysis for
'LUM' on 'Cardiac valvular disease'

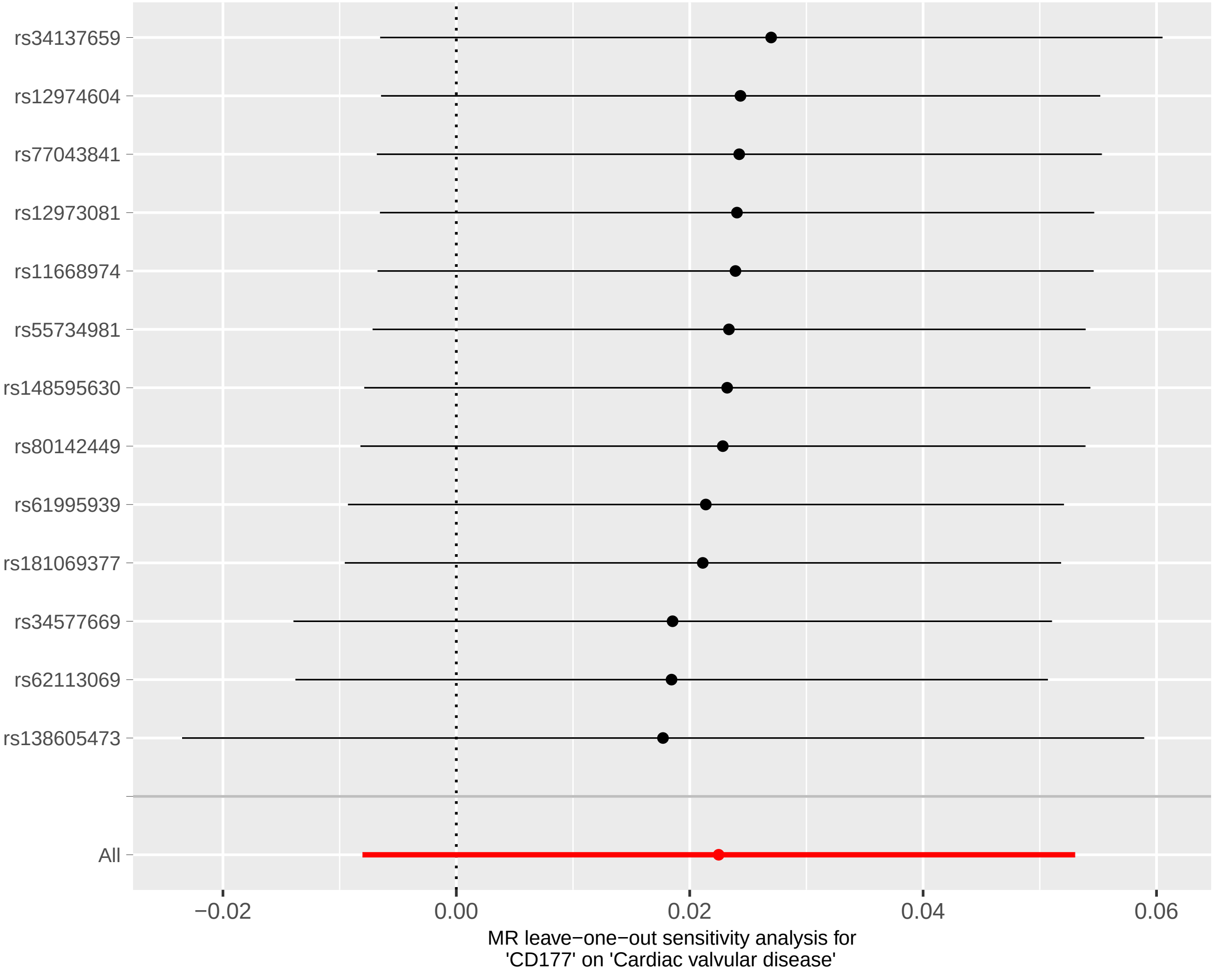
rs34137659
rs12974604
rs77043841
rs12973081
rs11668974
rs55734981
rs148595630
rs80142449
rs61995939
rs181069377
rs34577669
rs62113069
rs138605473
All
−0.02
0.00
0.02
0.04
0.06
MR leave−one−out sensitivity analysis for
'CD177' on 'Cardiac valvular disease'

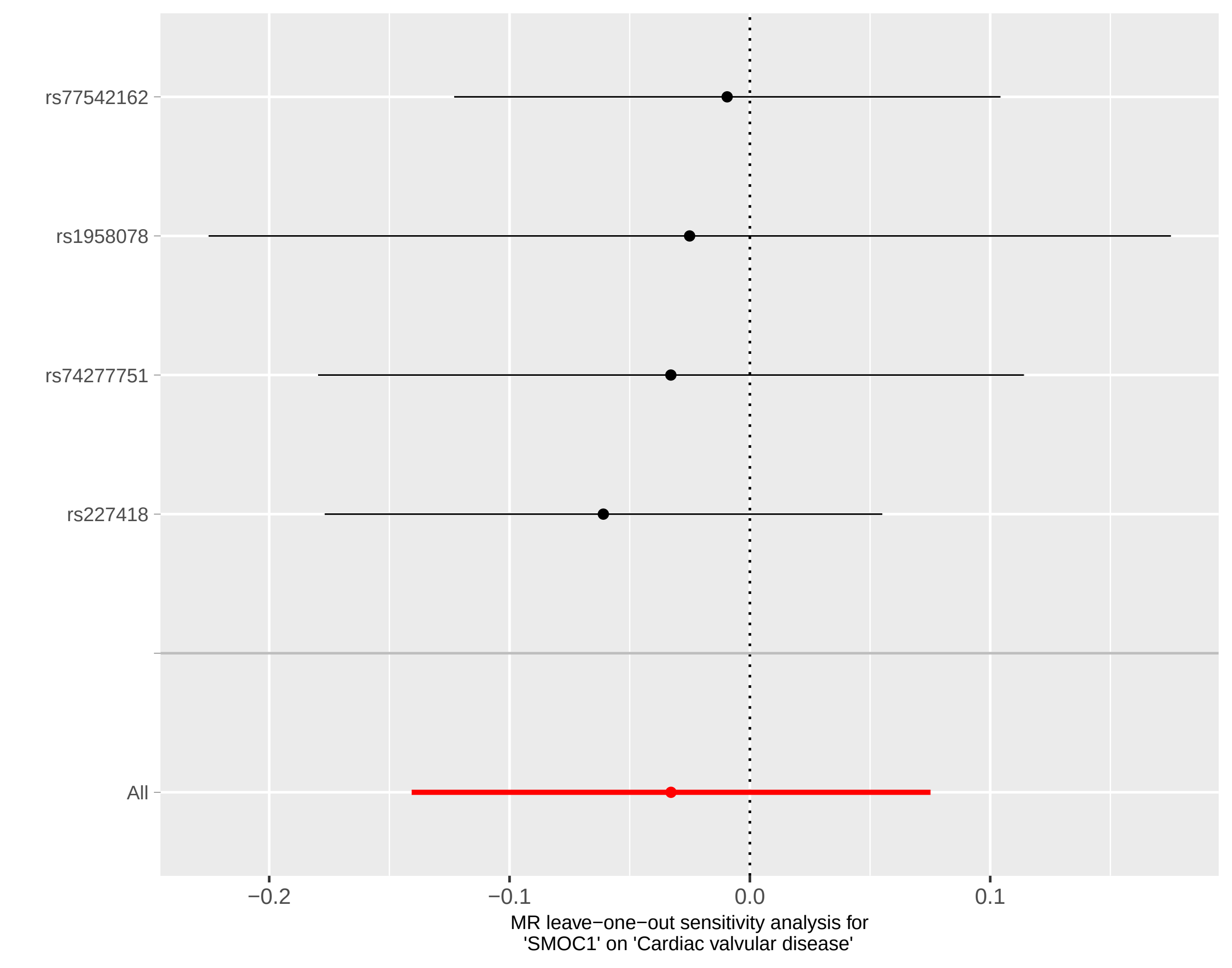
rs77542162
rs1958078
rs74277751
rs227418
All
−0.2
−0.1
0.0
0.1
MR leave−one−out sensitivity analysis for
'SMOC1' on 'Cardiac valvular disease'

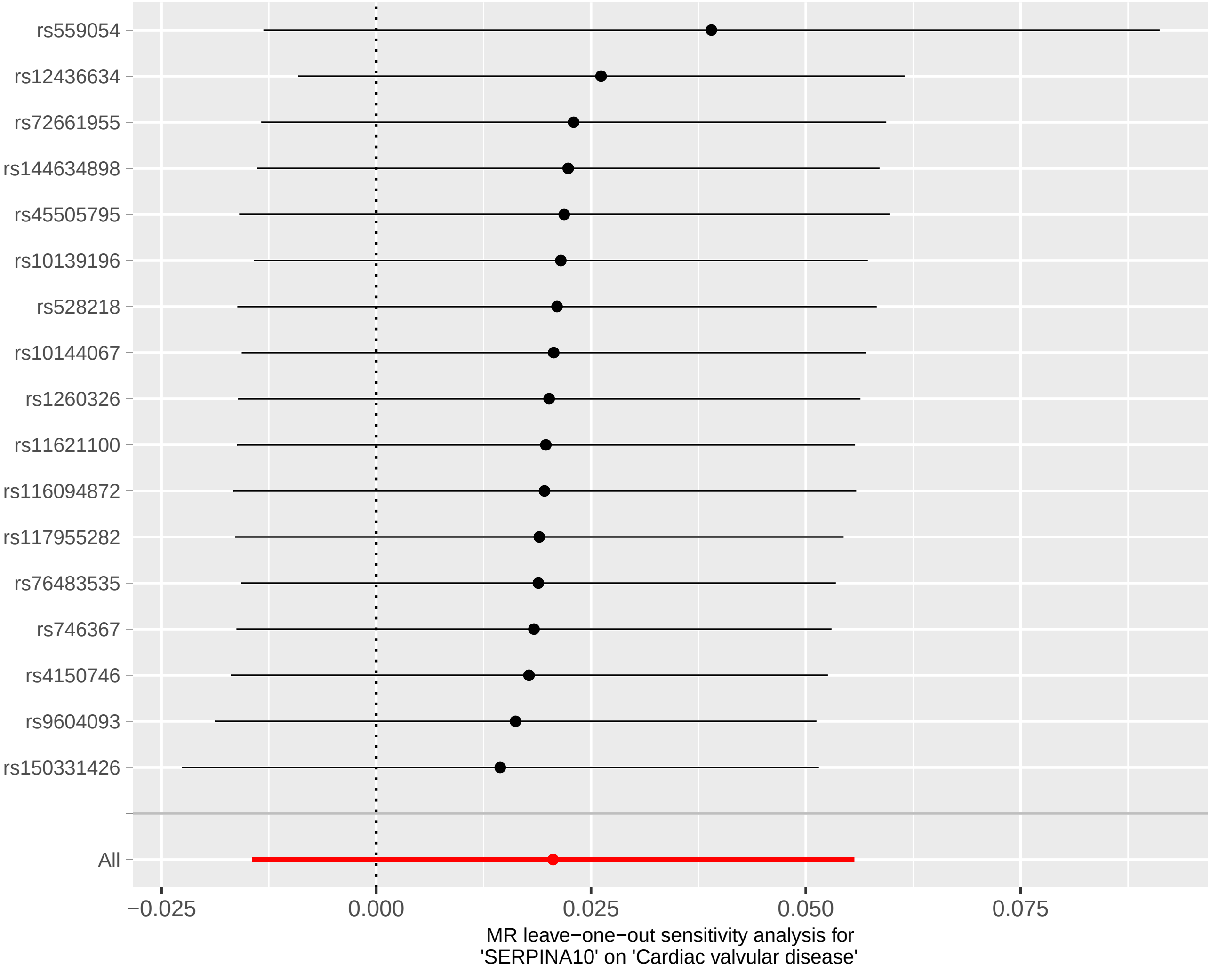
rs559054
rs12436634
rs72661955
rs144634898
rs45505795
rs10139196
rs528218
rs10144067
rs1260326
rs11621100
rs116094872
rs117955282
rs76483535
rs746367
rs4150746
rs9604093
rs150331426
All
−0.025
0.000
0.025
0.050
0.075
MR leave−one−out sensitivity analysis for
'SERPINA10' on 'Cardiac valvular disease'

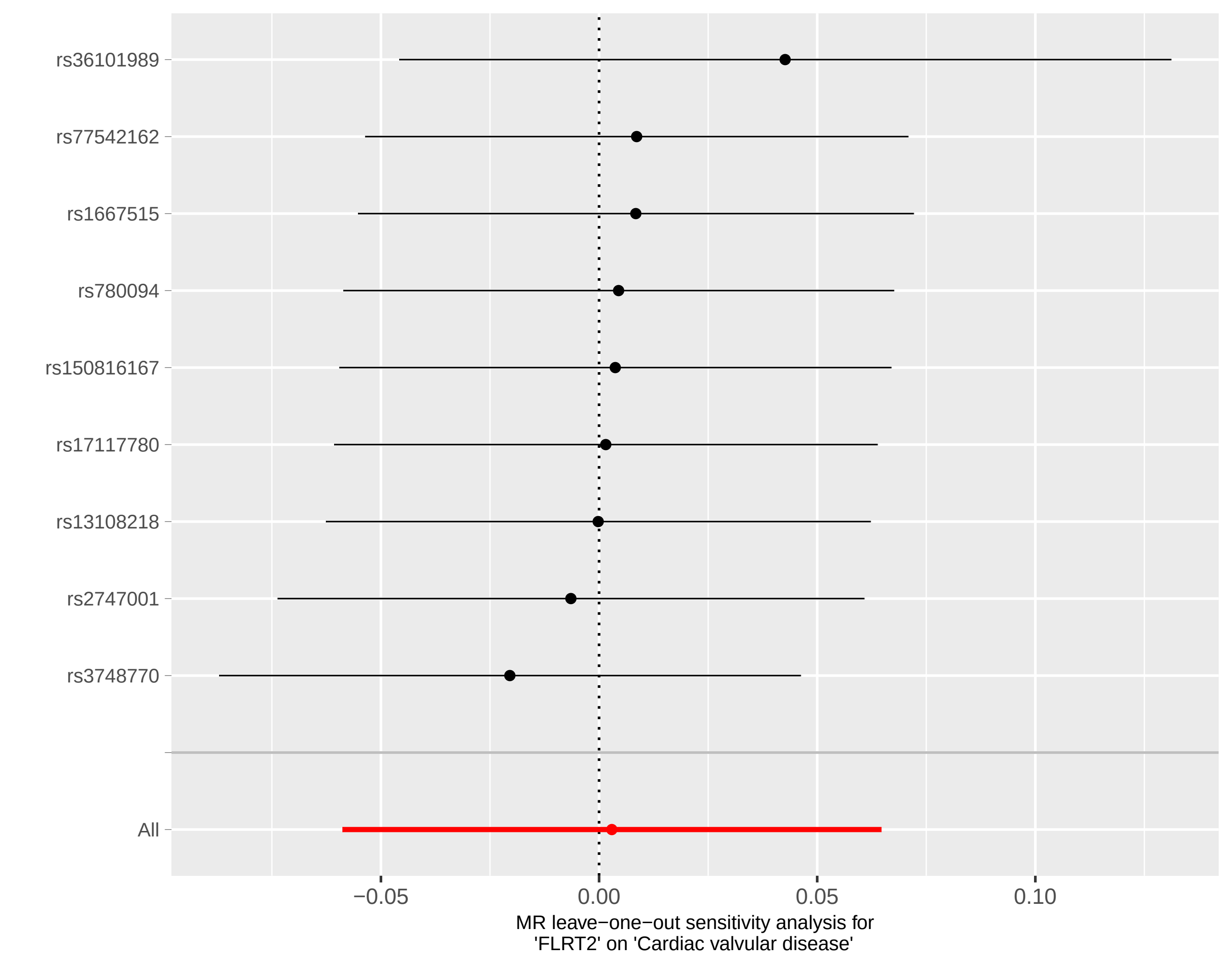

rs36101989
rs77542162
rs1667515
rs780094
rs150816167
rs17117780
rs13108218
rs2747001
rs3748770
All
−0.05
0.00
0.05
0.10
MR leave−one−out sensitivity analysis for
'FLRT2' on 'Cardiac valvular disease'

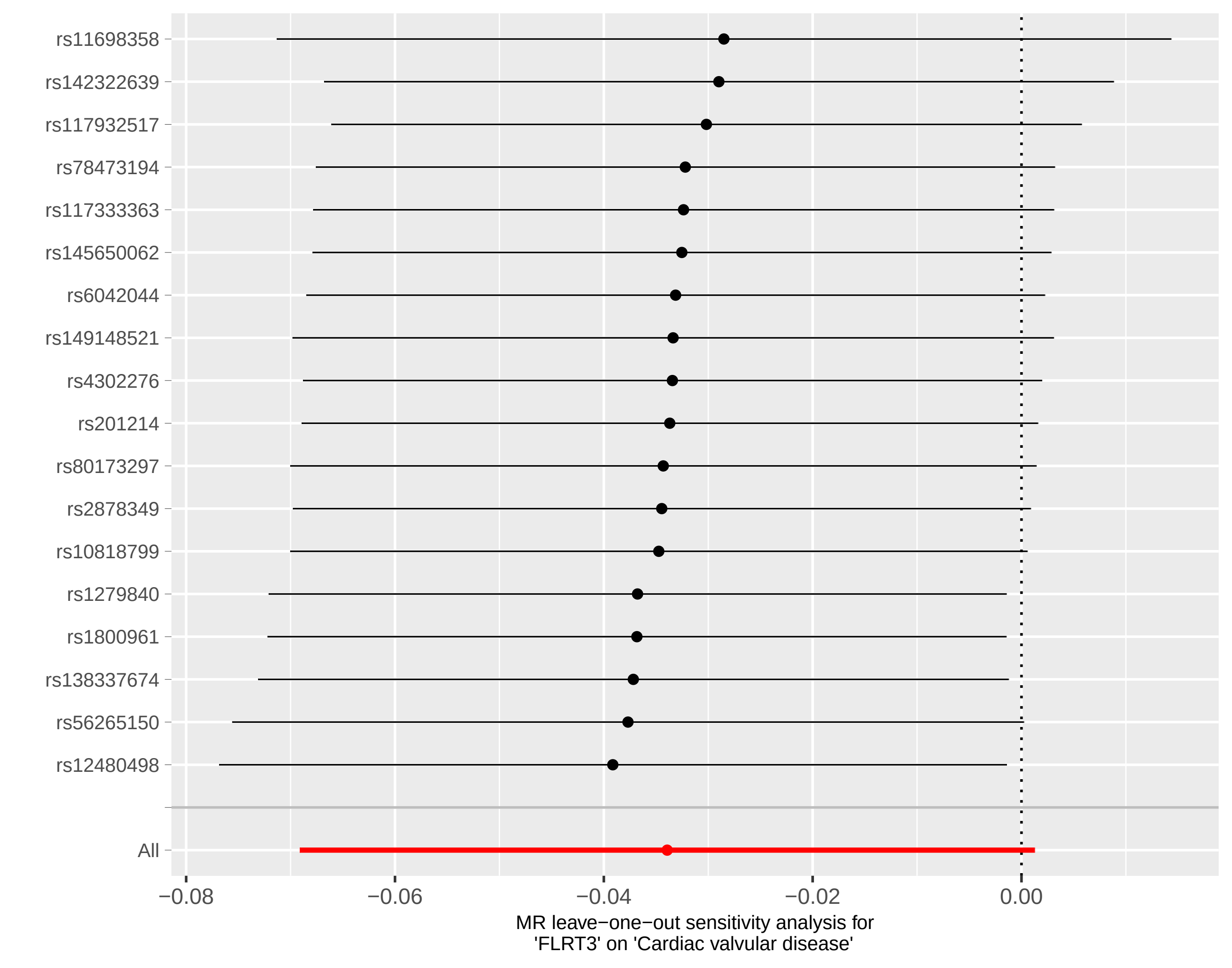
rs11698358
rs142322639
rs117932517
rs78473194
rs117333363
rs145650062
rs6042044
rs149148521
rs4302276
rs201214
rs80173297
rs2878349
rs10818799
rs1279840
rs1800961
rs138337674
rs56265150
rs12480498
All
−0.08
−0.06
−0.04
−0.02
0.00
MR leave−one−out sensitivity analysis for
'FLRT3' on 'Cardiac valvular disease'

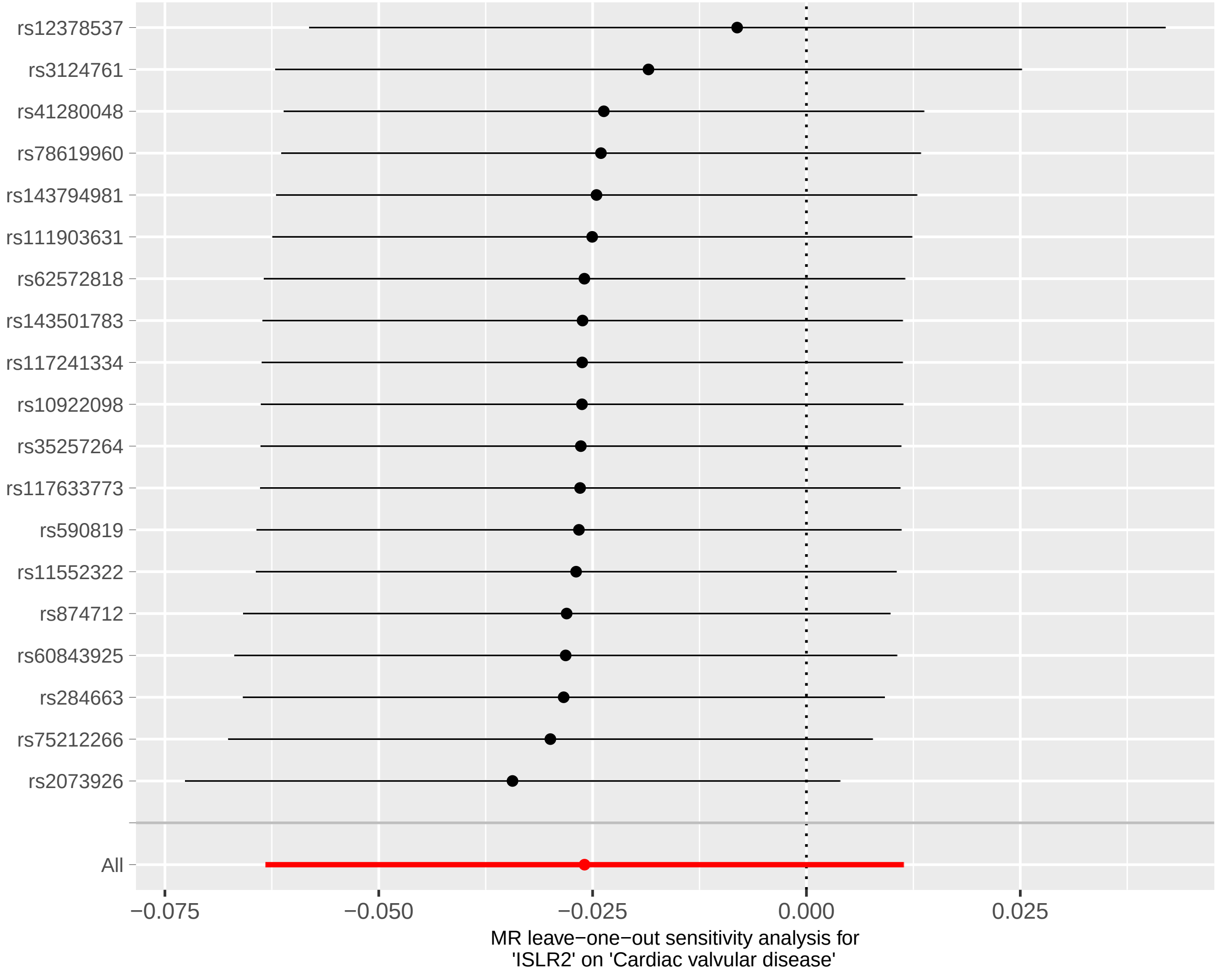
rs12378537
rs3124761
rs41280048
rs78619960
rs143794981
rs111903631
rs62572818
rs143501783
rs117241334
rs10922098
rs35257264
rs117633773
rs590819
rs11552322
rs874712
rs60843925
rs284663
rs75212266
rs2073926
All
−0.075
−0.050
−0.025
0.000
0.025
MR leave−one−out sensitivity analysis for
'ISLR2' on 'Cardiac valvular disease'

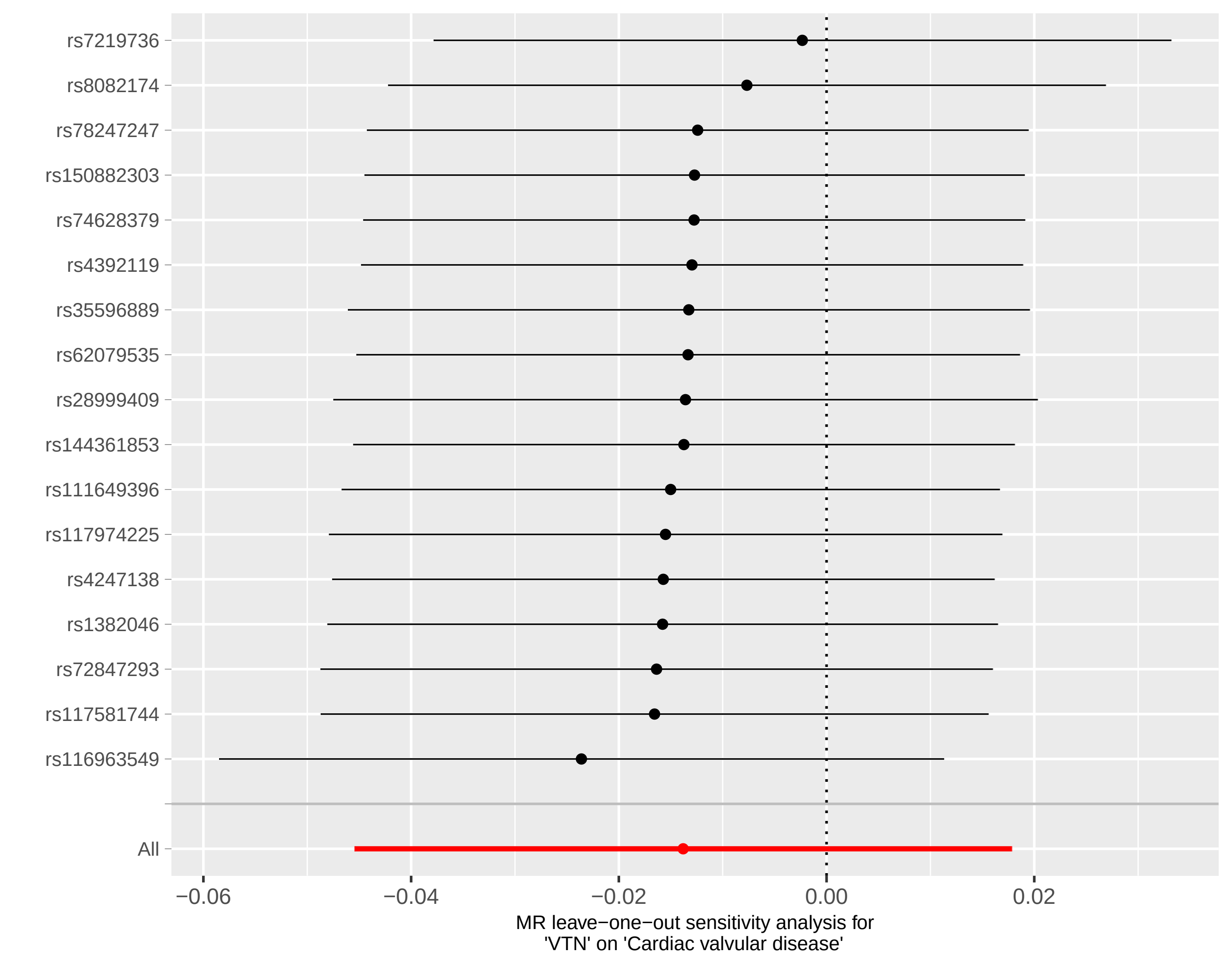
rs7219736
rs8082174
rs78247247
rs150882303
rs74628379
rs4392119
rs35596889
rs62079535
rs28999409
rs144361853
rs111649396
rs117974225
rs4247138
rs1382046
rs72847293
rs117581744
rs116963549
All
−0.06
−0.04
−0.02
0.00
0.02
MR leave−one−out sensitivity analysis for
'VTN' on 'Cardiac valvular disease'

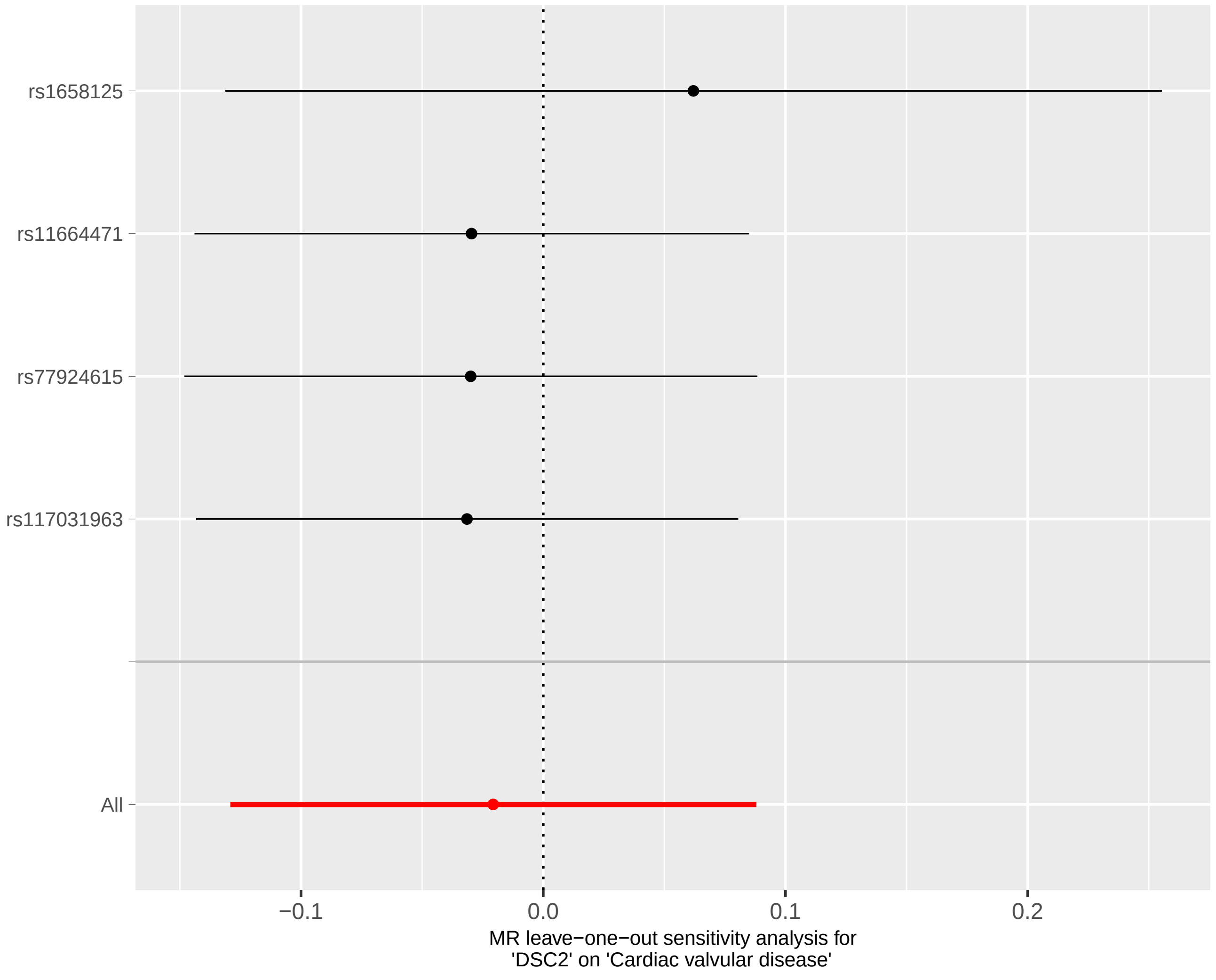
rs1658125
rs11664471
rs77924615
rs117031963
All
−0.1
0.0
0.1
0.2
MR leave−one−out sensitivity analysis for
'DSC2' on 'Cardiac valvular disease'

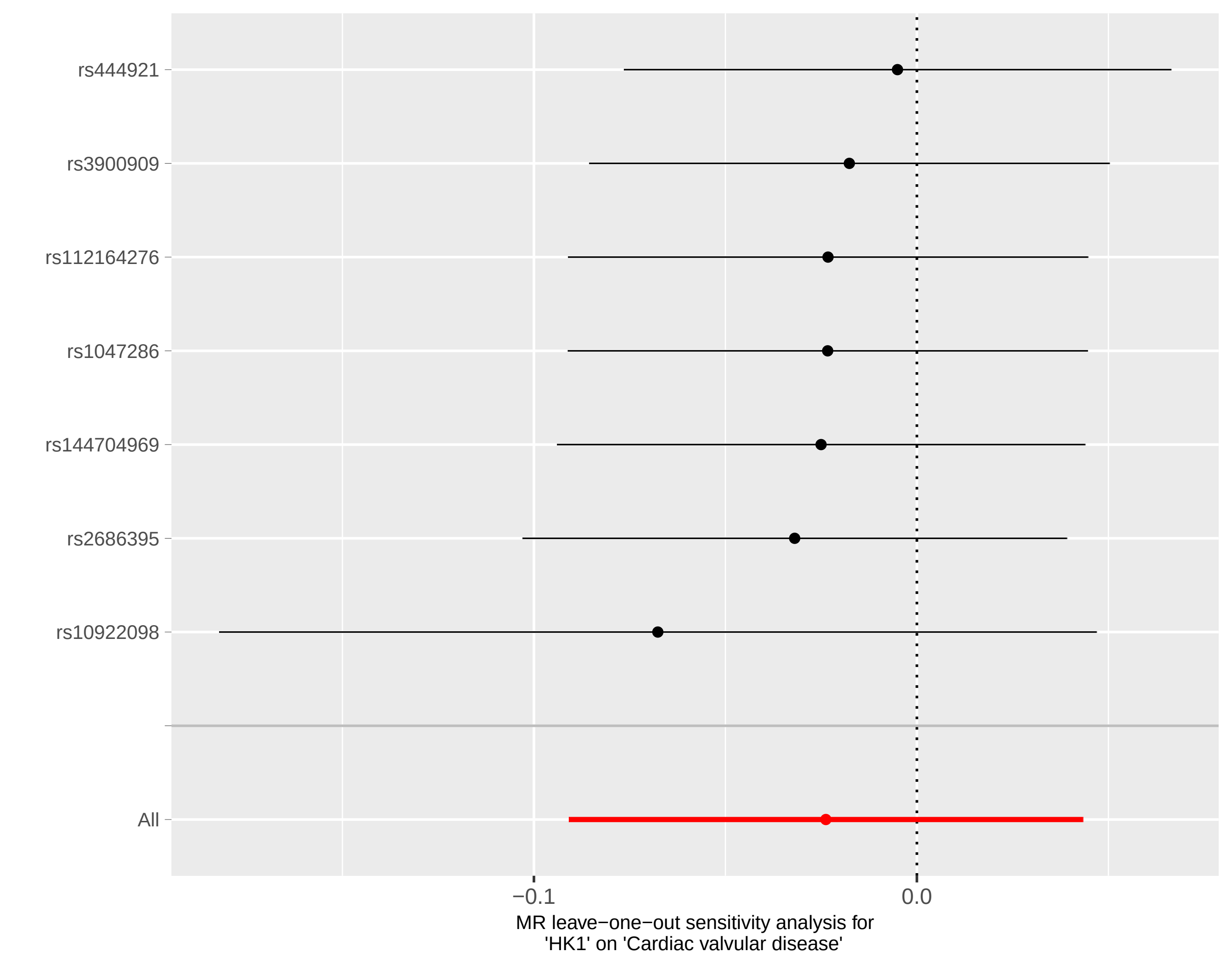

rs444921
rs3900909
rs112164276
rs1047286
rs144704969
rs2686395
rs10922098
All
−0.1
0.0
MR leave−one−out sensitivity analysis for
'HK1' on 'Cardiac valvular disease'

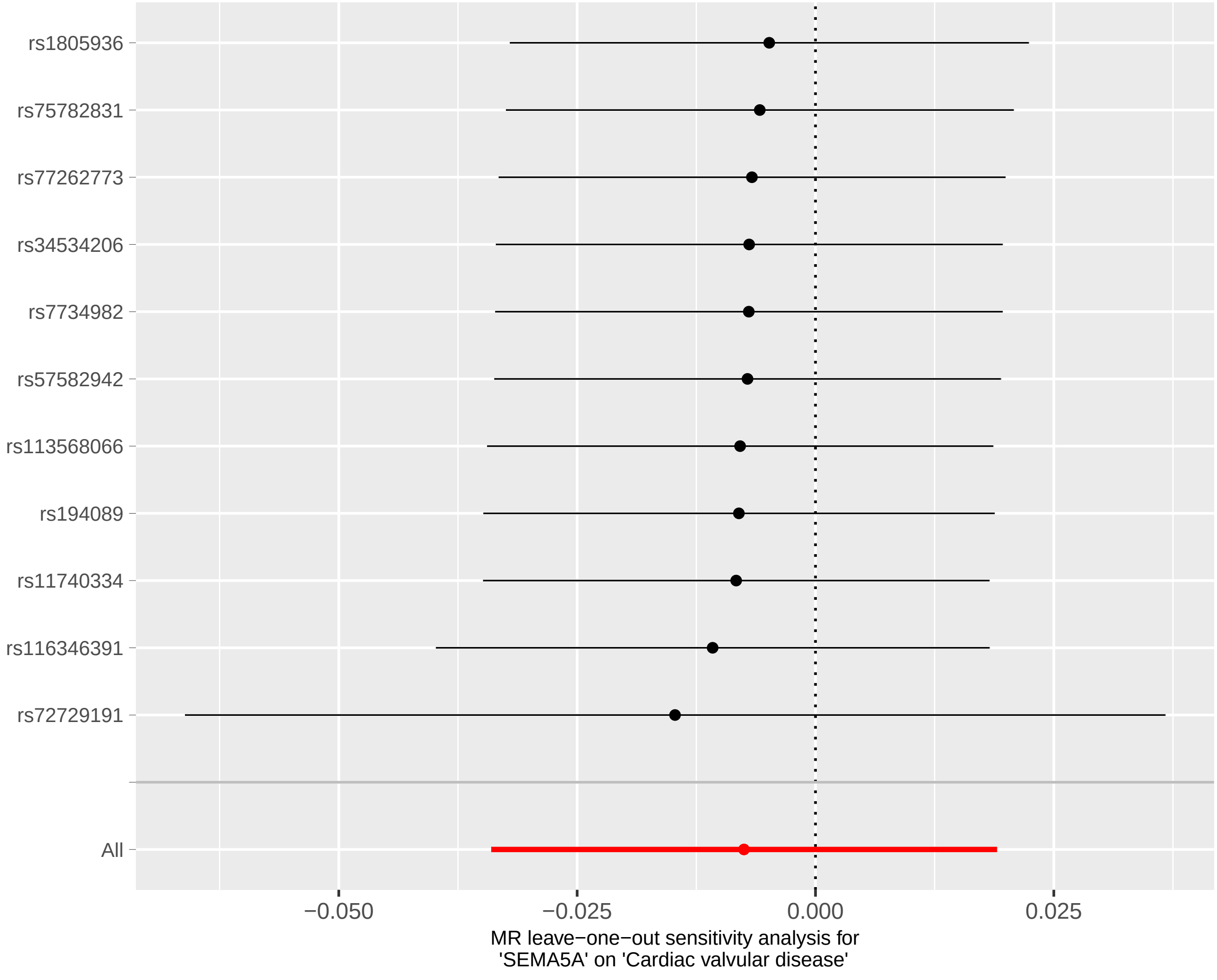
rs1805936
rs75782831
rs77262773
rs34534206
rs7734982
rs57582942
rs113568066
rs194089
rs11740334
rs116346391
rs72729191
All
−0.050
−0.025
0.000
0.025
MR leave−one−out sensitivity analysis for
'SEMA5A' on 'Cardiac valvular disease'

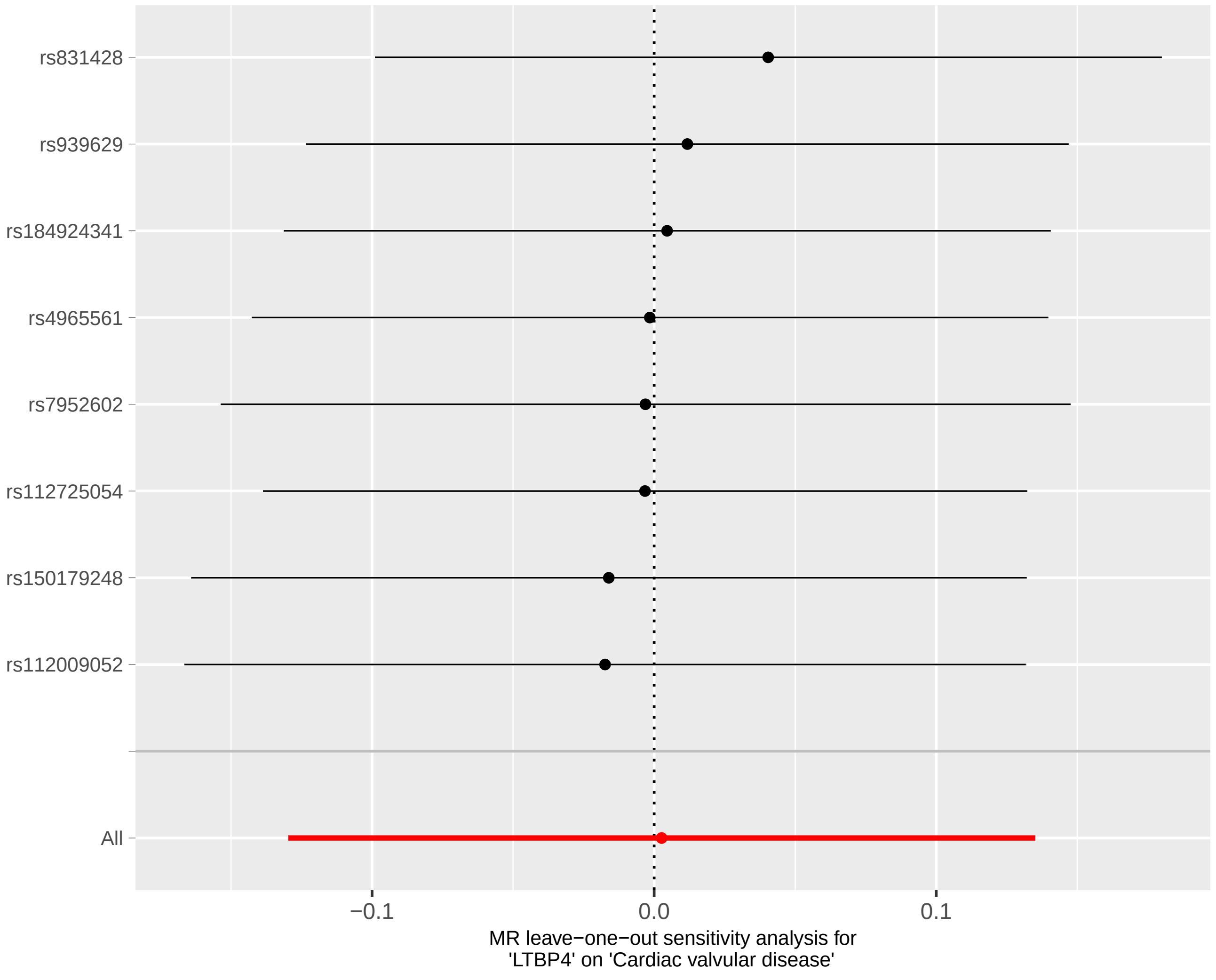
rs831428
rs939629
rs184924341
rs4965561
rs7952602
rs112725054
rs150179248
rs112009052
All
−0.1
0.0
0.1
MR leave−one−out sensitivity analysis for
'LTBP4' on 'Cardiac valvular disease'

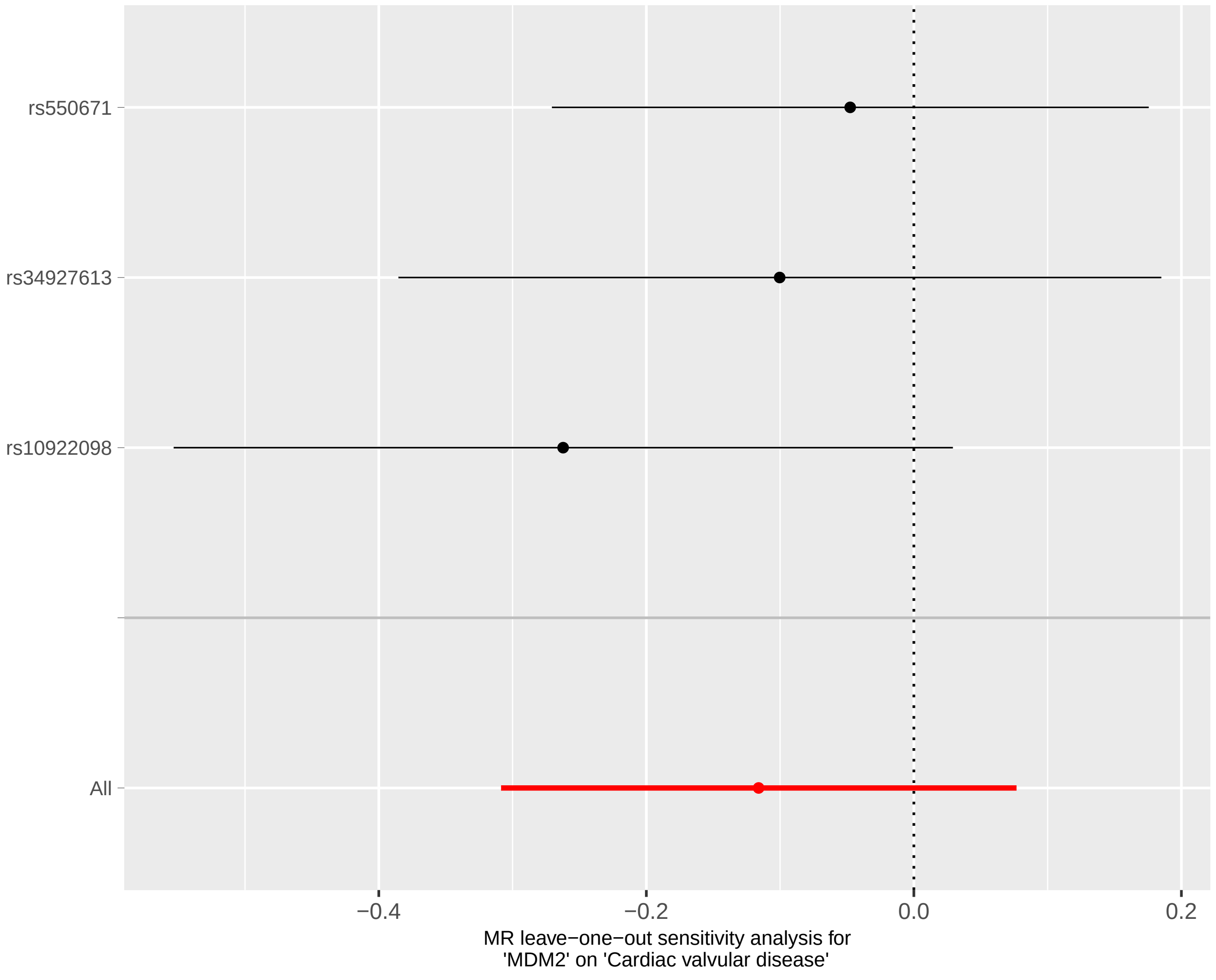

rs550671
rs34927613
rs10922098
All
−0.4
−0.2
0.0
0.2
MR leave−one−out sensitivity analysis for
'MDM2' on 'Cardiac valvular disease'

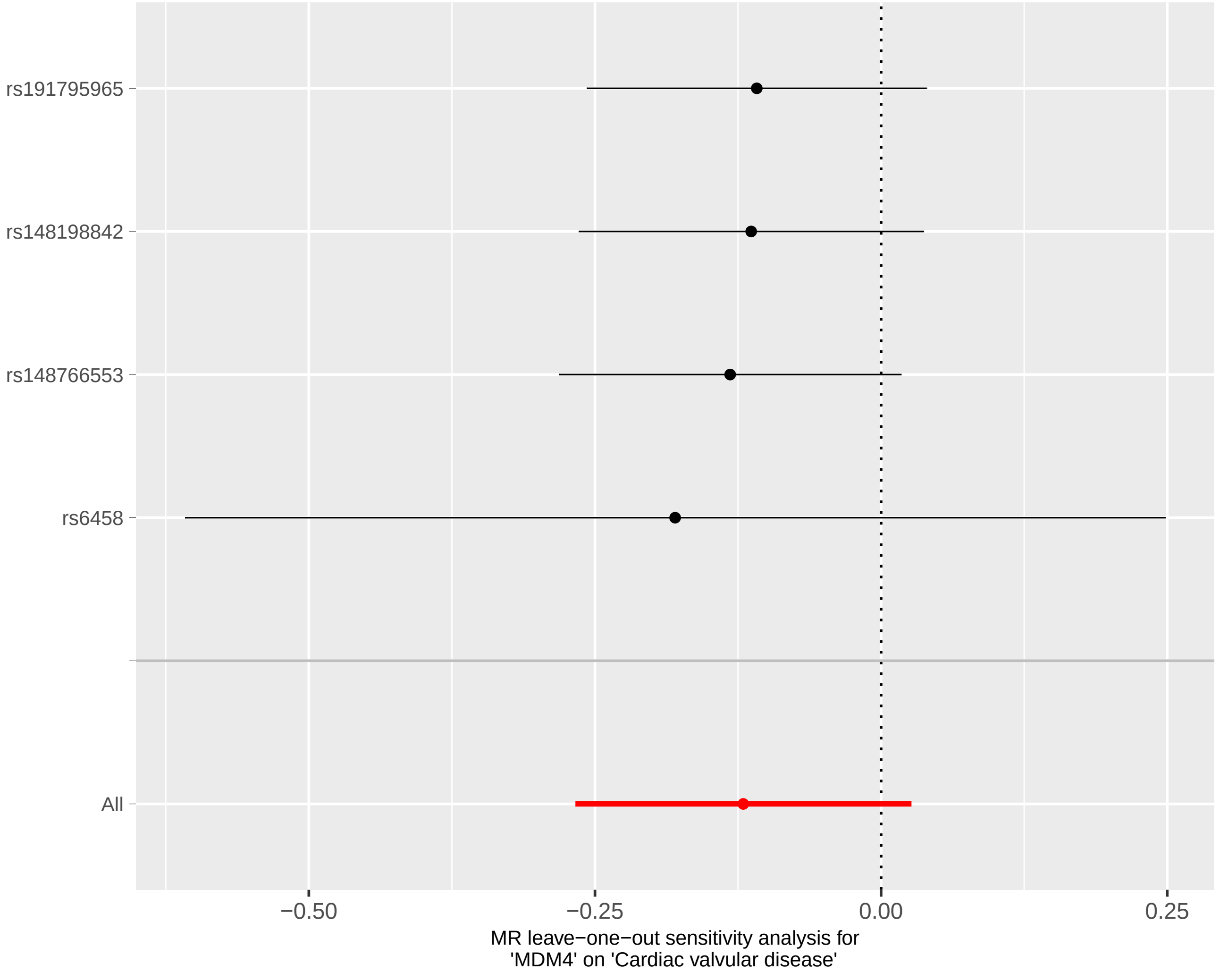
rs191795965
rs148198842
rs148766553
rs6458
All
−0.50
−0.25
0.00
0.25
MR leave−one−out sensitivity analysis for
'MDM4' on 'Cardiac valvular disease'

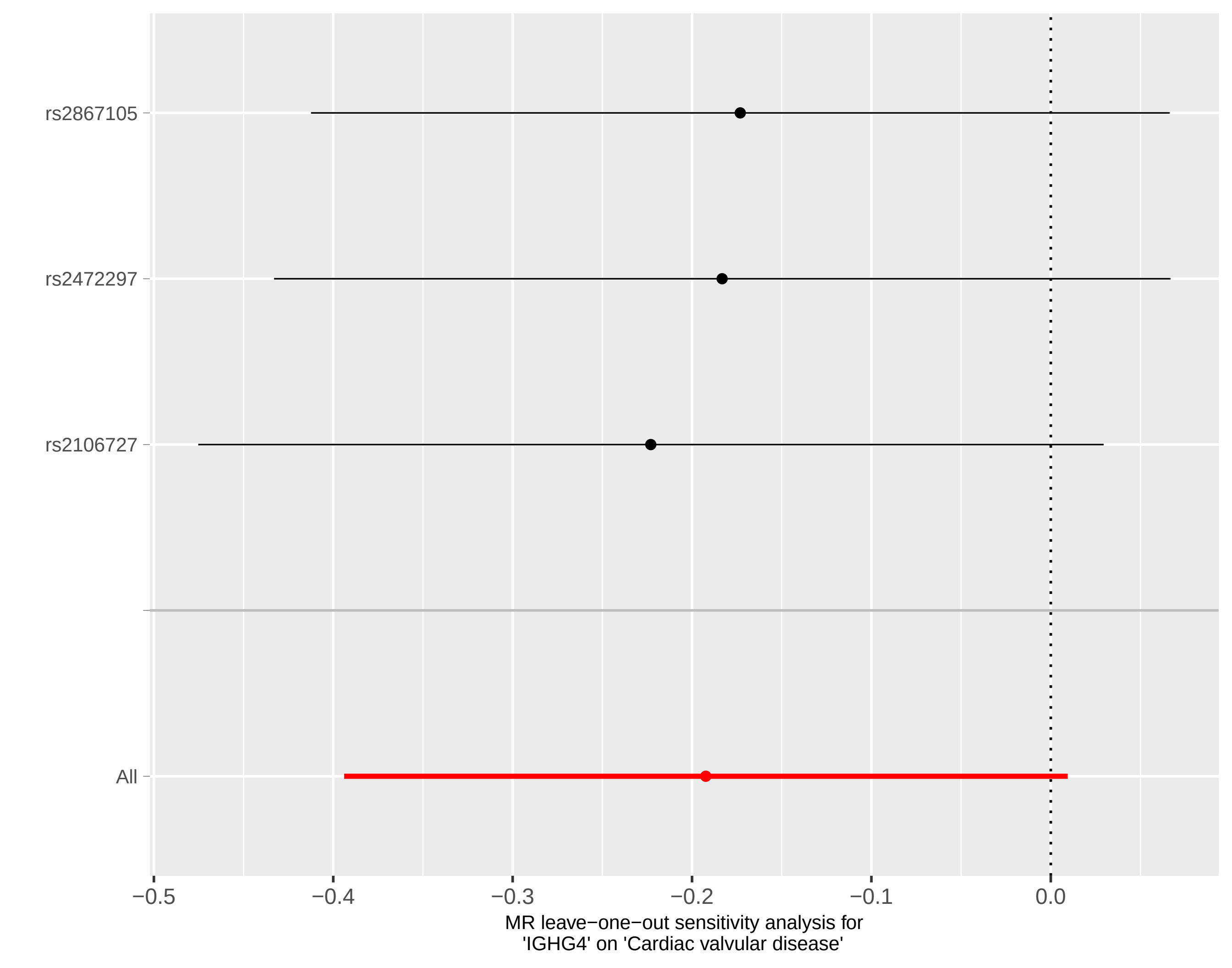
rs2867105
rs2472297
rs2106727
All
−0.5
−0.4
−0.3
−0.2
−0.1
0.0
MR leave−one−out sensitivity analysis for
'IGHG4' on 'Cardiac valvular disease'

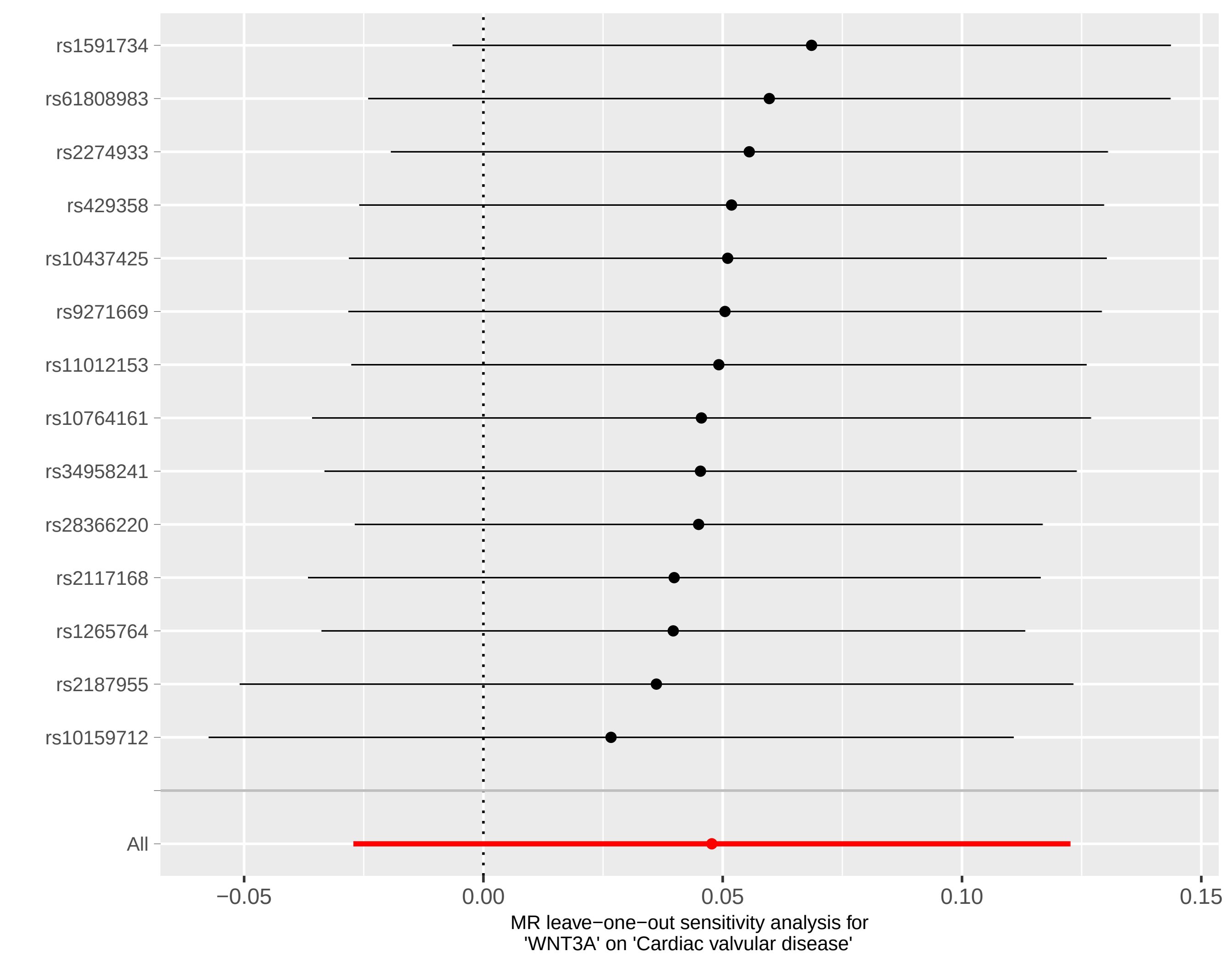
rs1591734
rs61808983
rs2274933
rs429358
rs10437425
rs9271669
rs11012153
rs10764161
rs34958241
rs28366220
rs2117168
rs1265764
rs2187955
rs10159712
All
−0.05
0.00
0.05
0.10
0.15
MR leave−one−out sensitivity analysis for
'WNT3A' on 'Cardiac valvular disease'

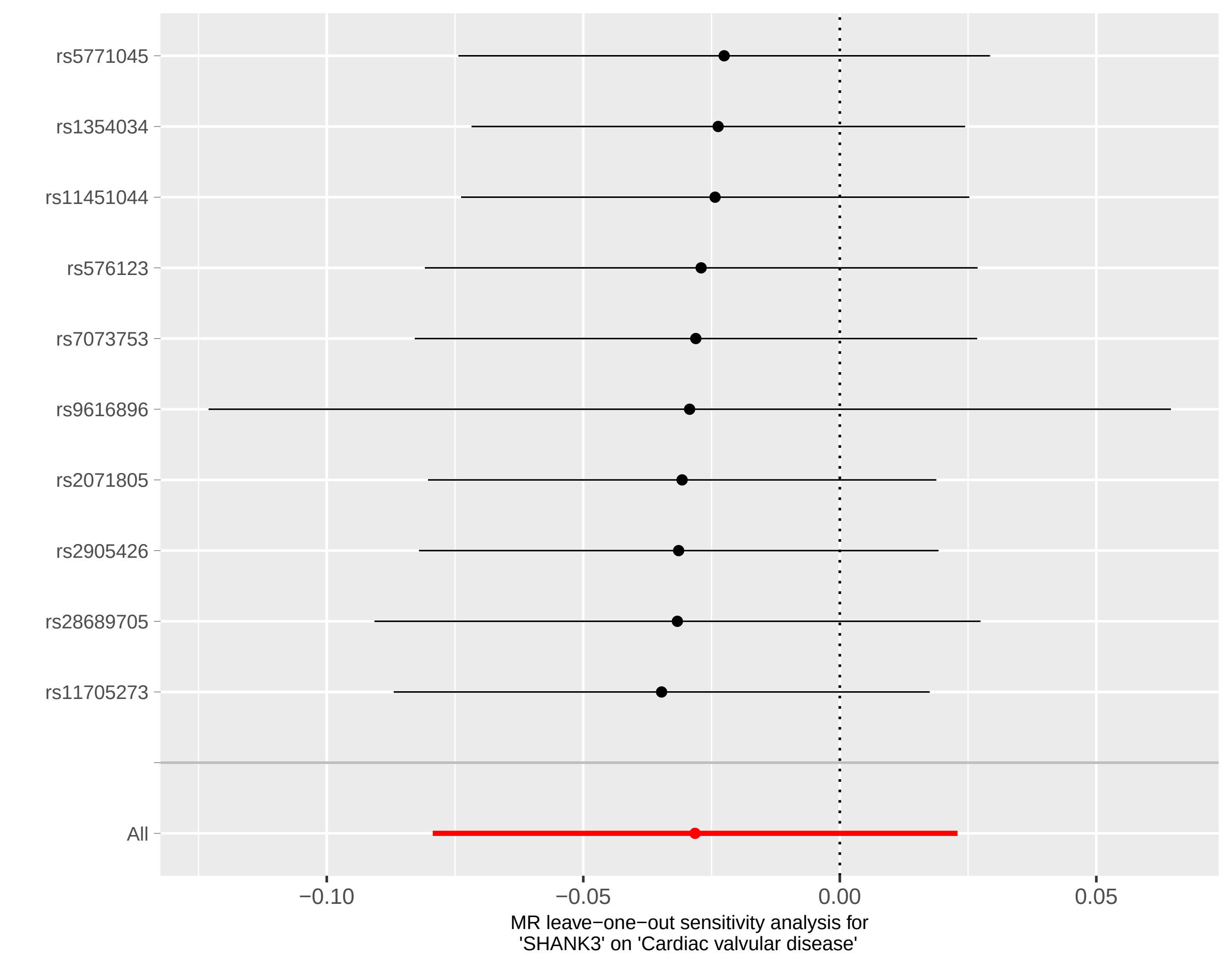

rs5771045
rs1354034
rs11451044
rs576123
rs7073753
rs9616896
rs2071805
rs2905426
rs28689705
rs11705273
All
−0.10
−0.05
0.00
0.05
MR leave−one−out sensitivity analysis for
'SHANK3' on 'Cardiac valvular disease'

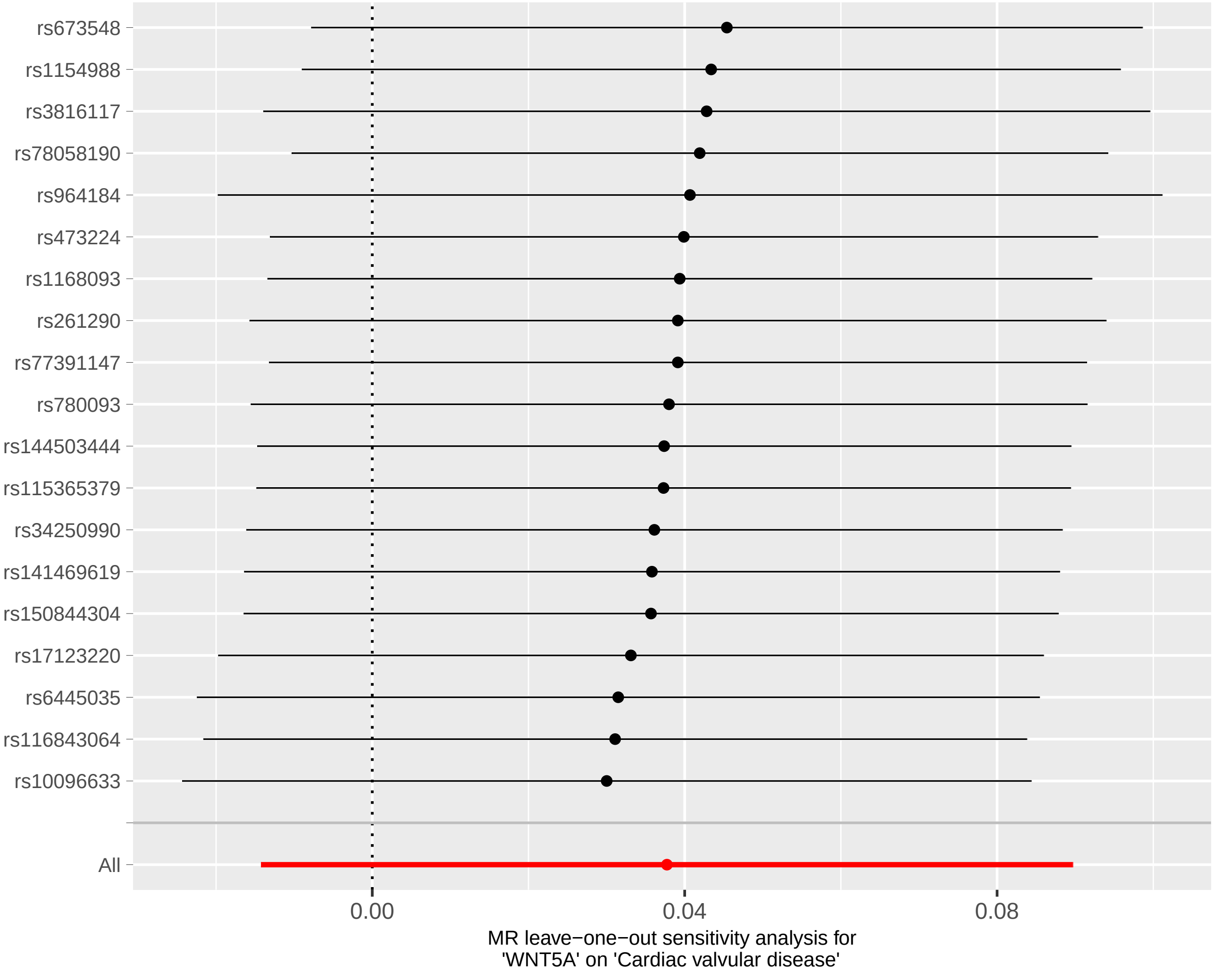
rs673548
rs1154988
rs3816117
rs78058190
rs964184
rs473224
rs1168093
rs261290
rs77391147
rs780093
rs144503444
rs115365379
rs34250990
rs141469619
rs150844304
rs17123220
rs6445035
rs116843064
rs10096633
All
0.00
0.04
0.08
MR leave−one−out sensitivity analysis for
'WNT5A' on 'Cardiac valvular disease'

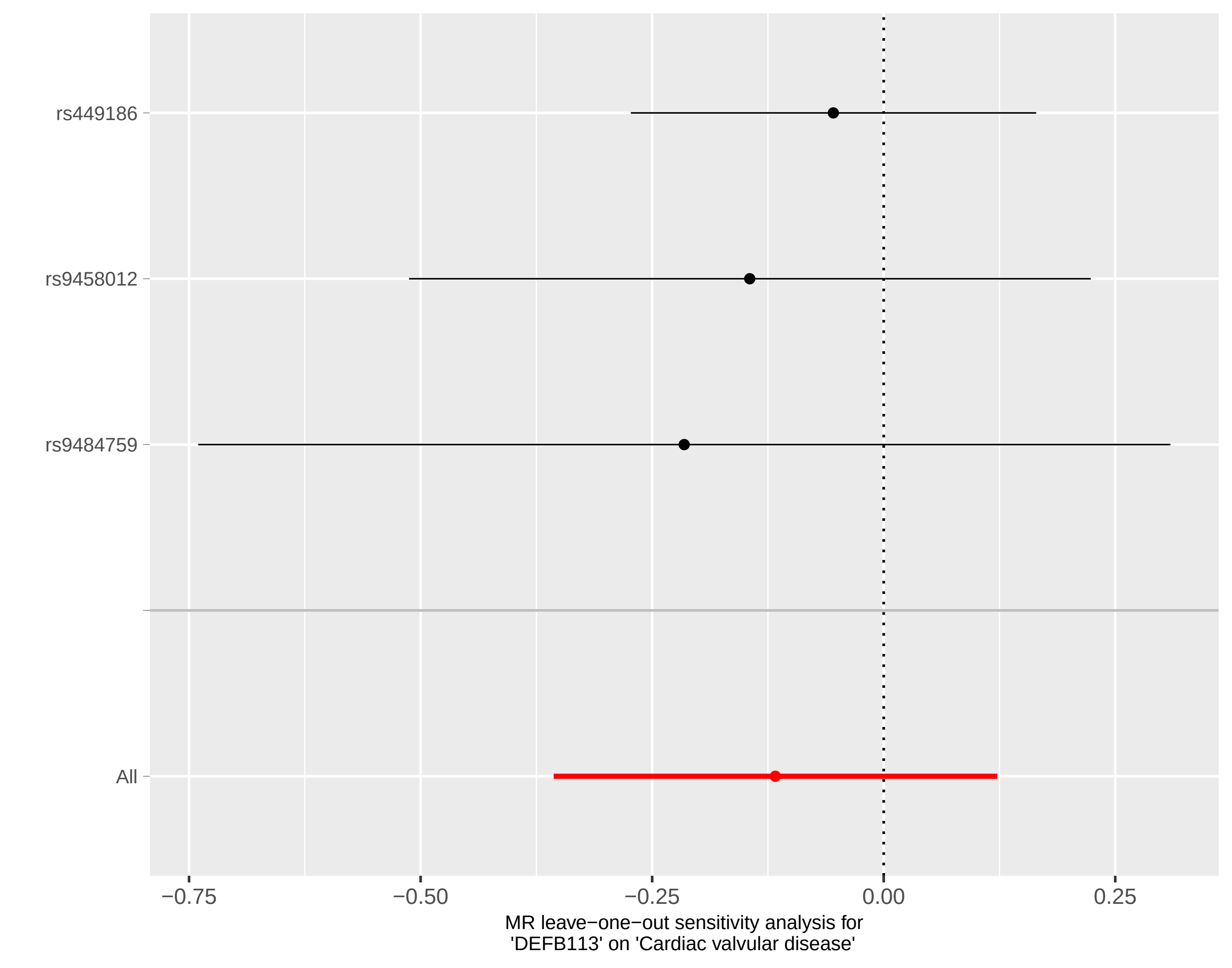
rs449186
rs9458012
rs9484759
All
−0.75
−0.50
−0.25
0.00
0.25
MR leave−one−out sensitivity analysis for
'DEFB113' on 'Cardiac valvular disease'

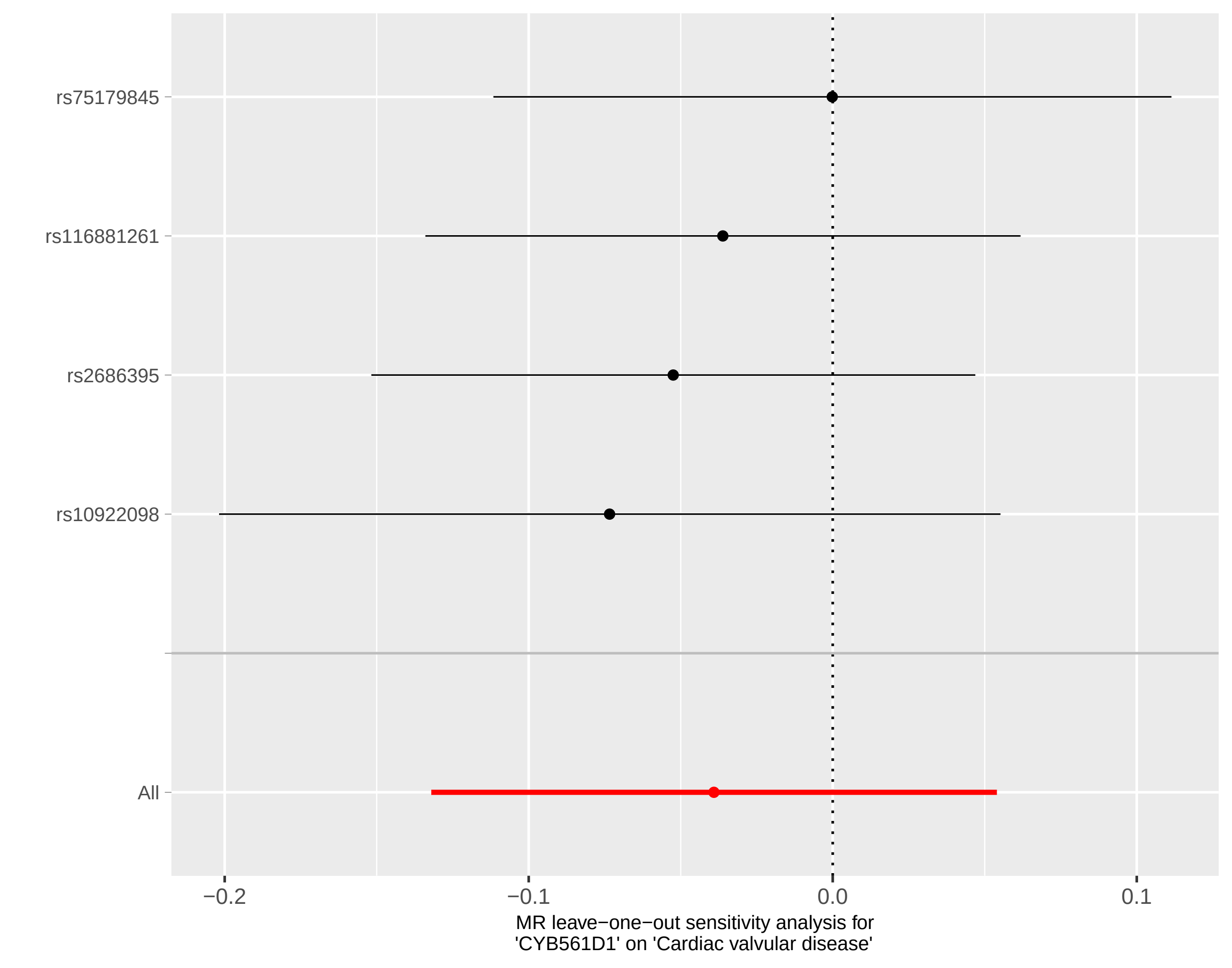
rs75179845
rs116881261
rs2686395
rs10922098
All
−0.2
−0.1
0.0
0.1
MR leave−one−out sensitivity analysis for
'CYB561D1' on 'Cardiac valvular disease'

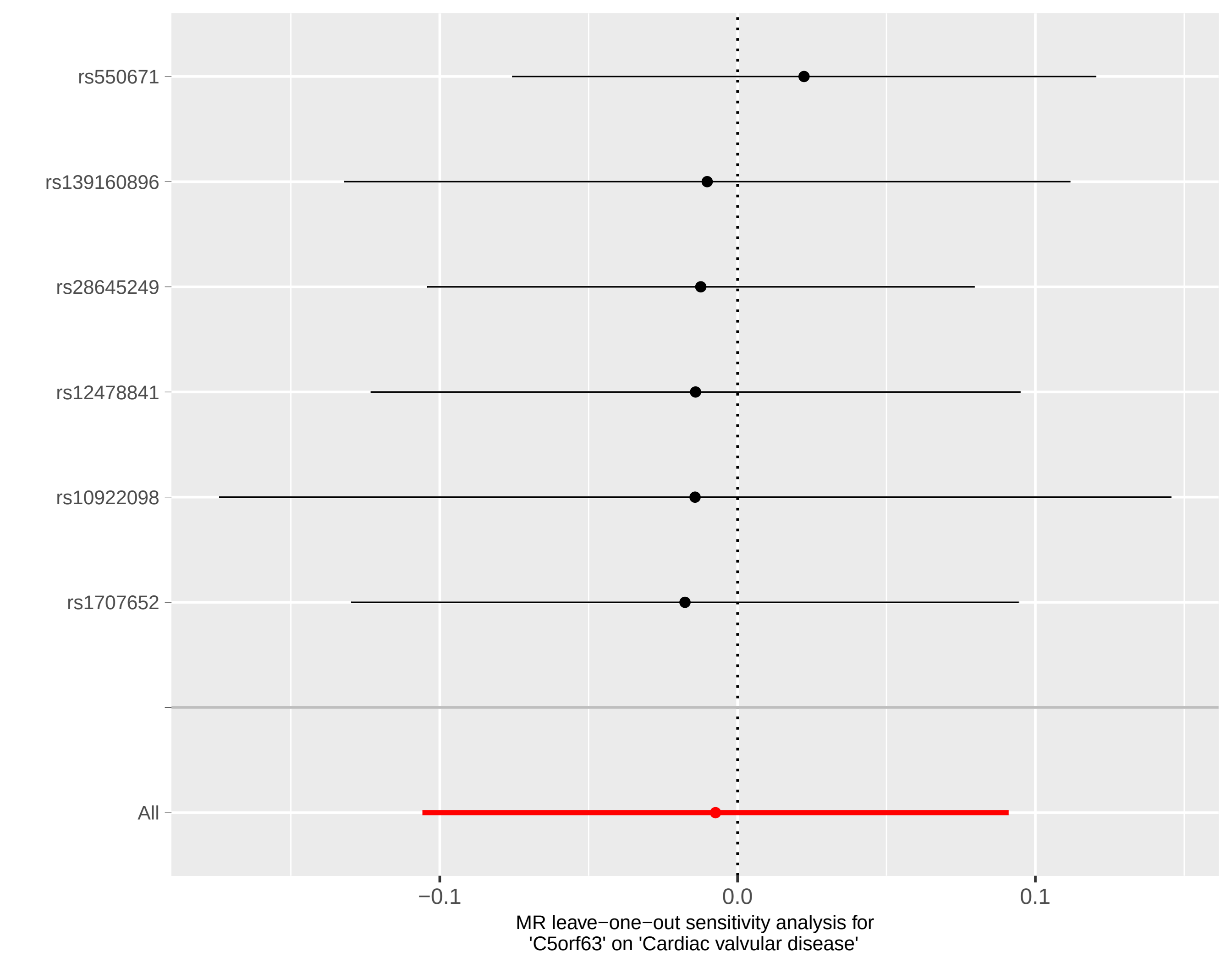
rs550671
rs139160896
rs28645249
rs12478841
rs10922098
rs1707652
All
−0.1
0.0
0.1
MR leave−one−out sensitivity analysis for
'C5orf63' on 'Cardiac valvular disease'

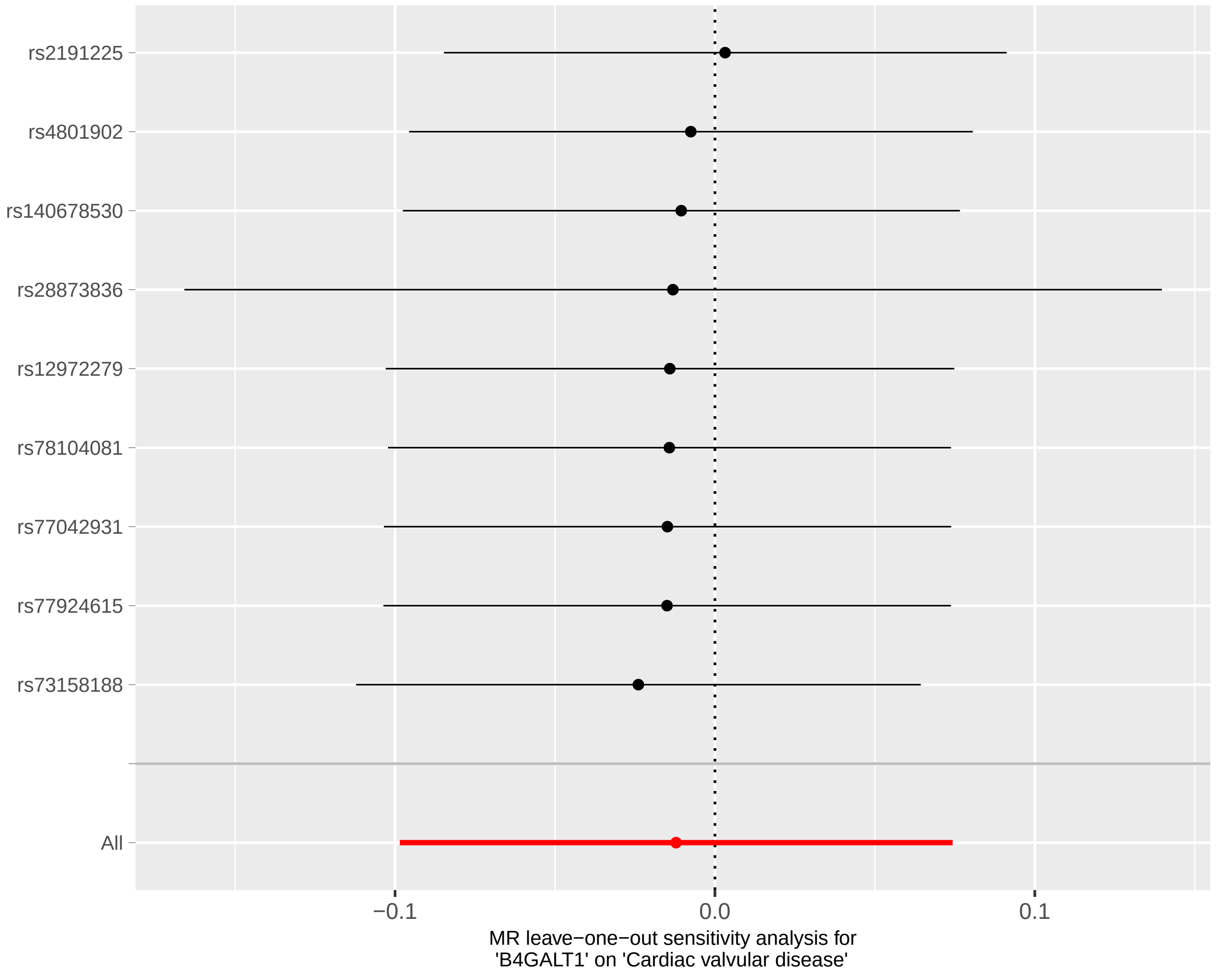
rs2191225
rs4801902
rs140678530
rs28873836
rs12972279
rs78104081
rs77042931
rs77924615
rs73158188
All
−0.1
0.0
0.1
MR leave−one−out sensitivity analysis for
'B4GALT1' on 'Cardiac valvular disease'

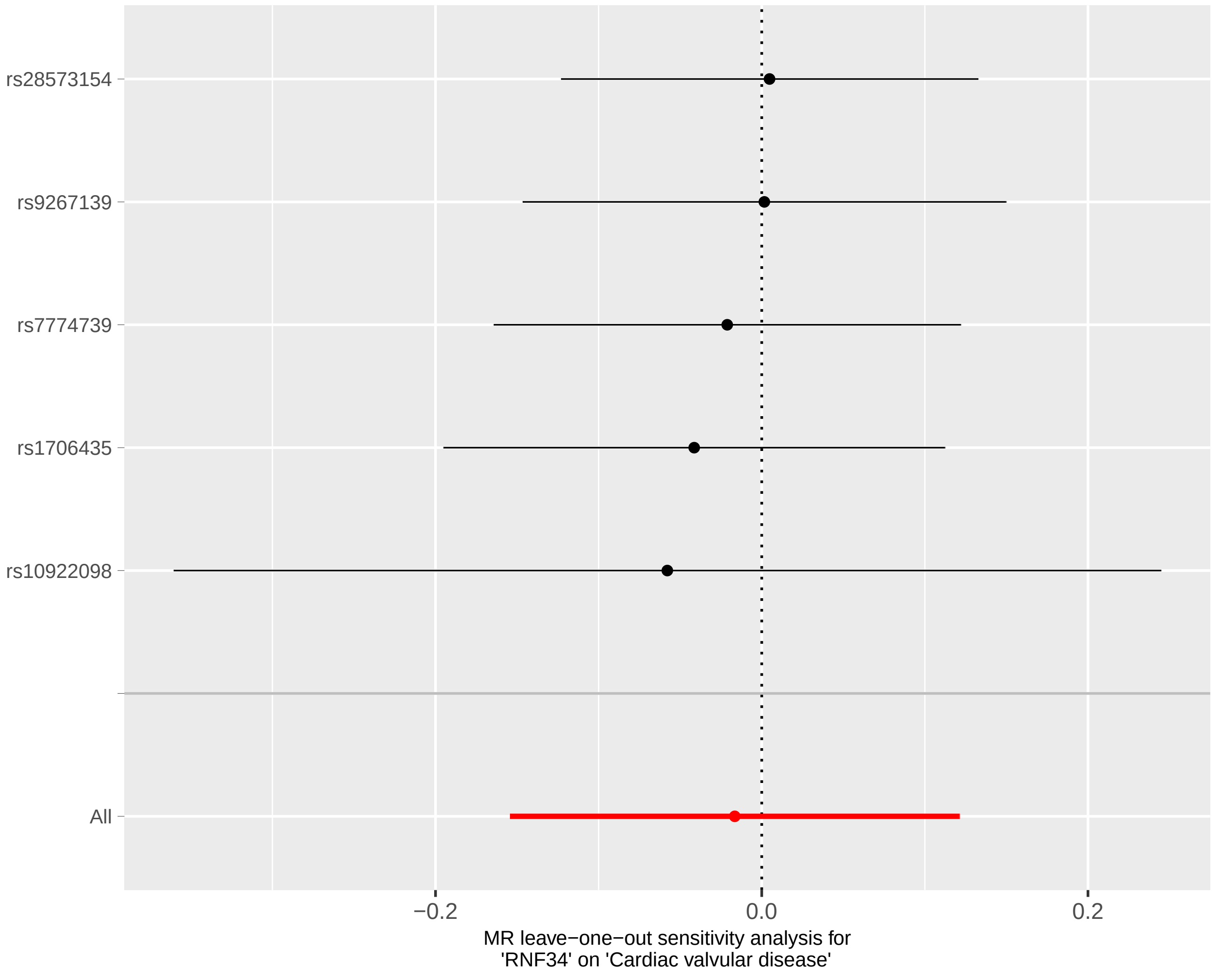
rs28573154
rs9267139
rs7774739
rs1706435
rs10922098
All
−0.2
0.0
0.2
MR leave−one−out sensitivity analysis for
'RNF34' on 'Cardiac valvular disease'

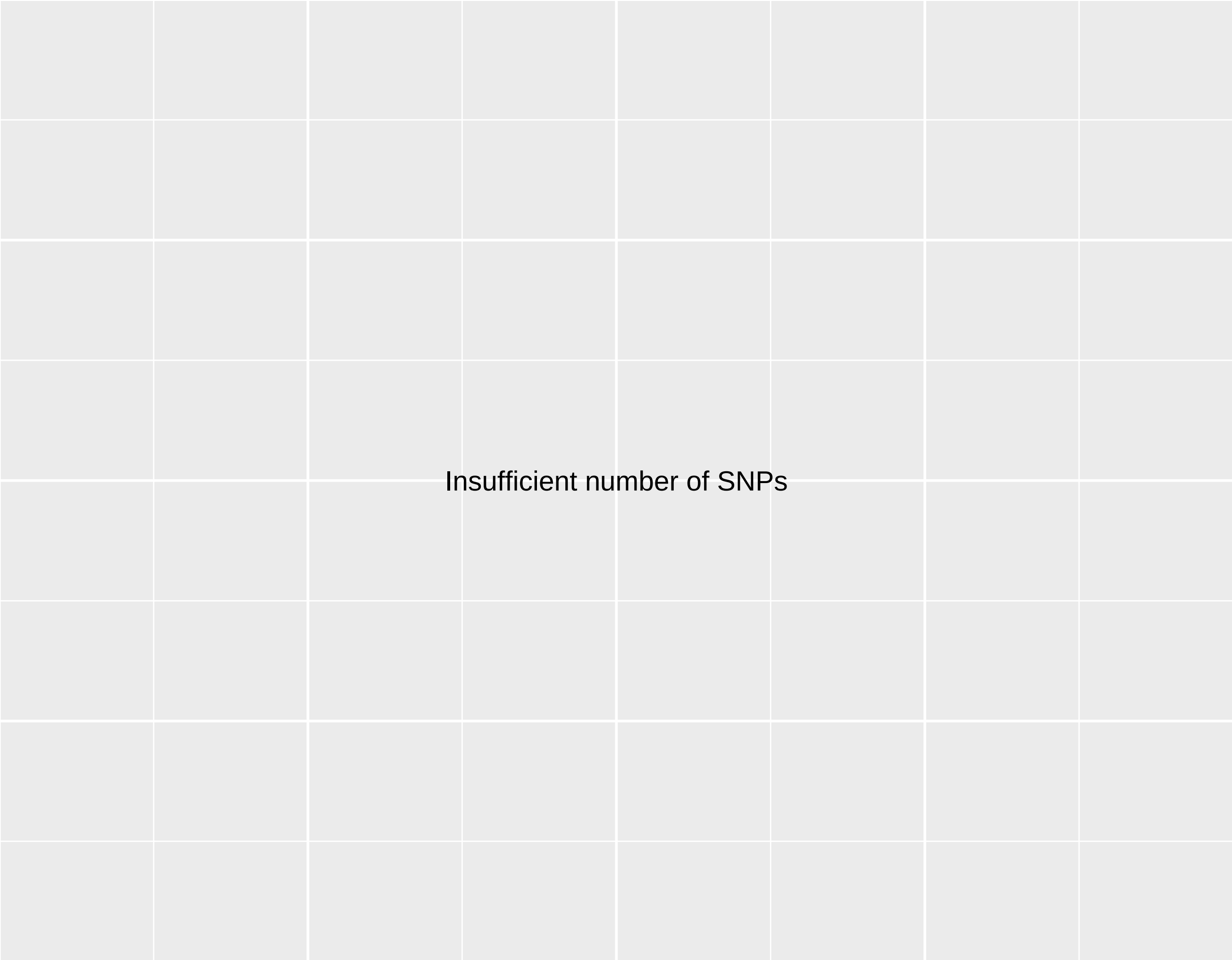
Insufficient number of SNPs

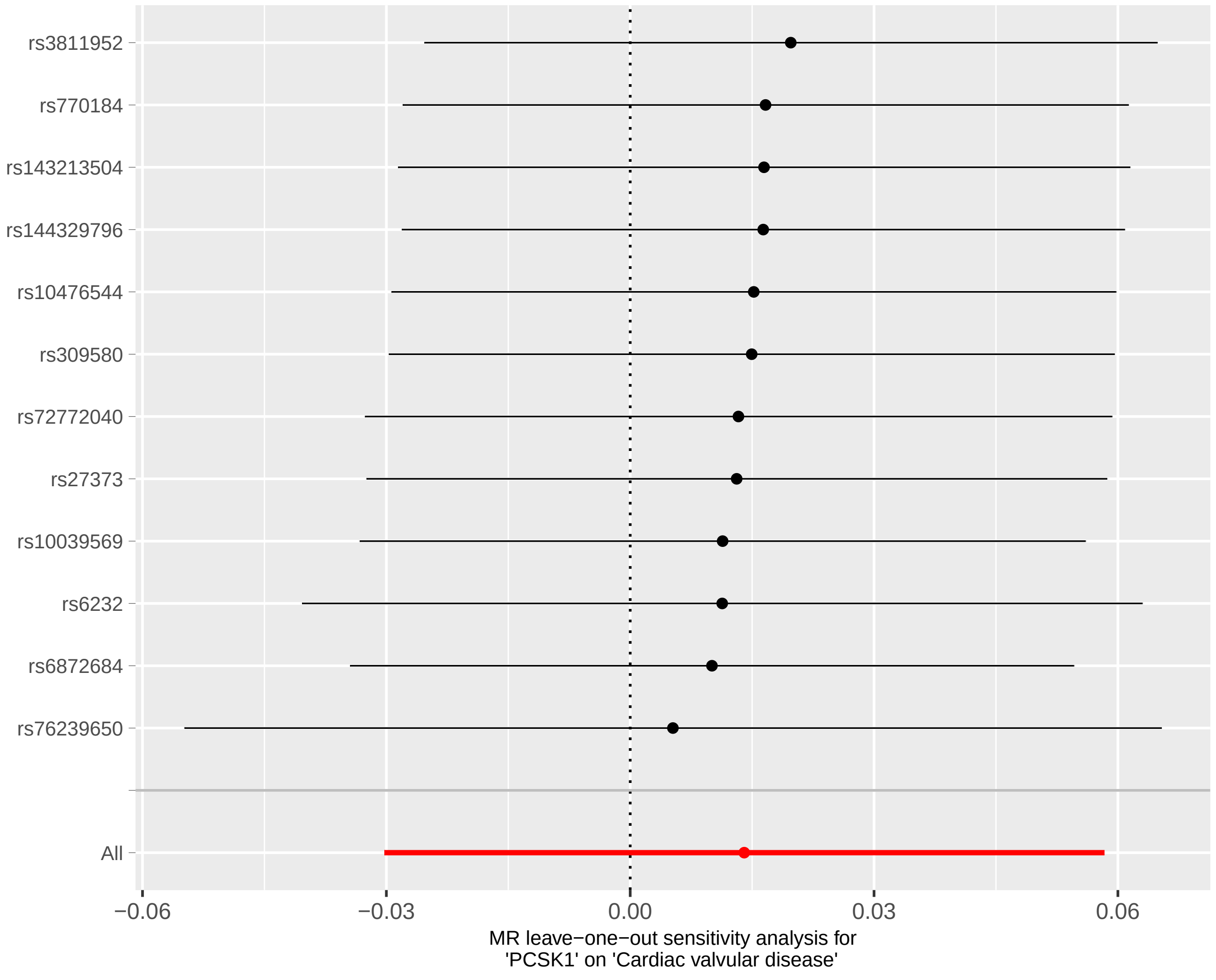
rs3811952
rs770184
rs143213504
rs144329796
rs10476544
rs309580
rs72772040
rs27373
rs10039569
rs6232
rs6872684
rs76239650
All
−0.06
−0.03
0.00
0.03
0.06
MR leave−one−out sensitivity analysis for
'PCSK1' on 'Cardiac valvular disease'

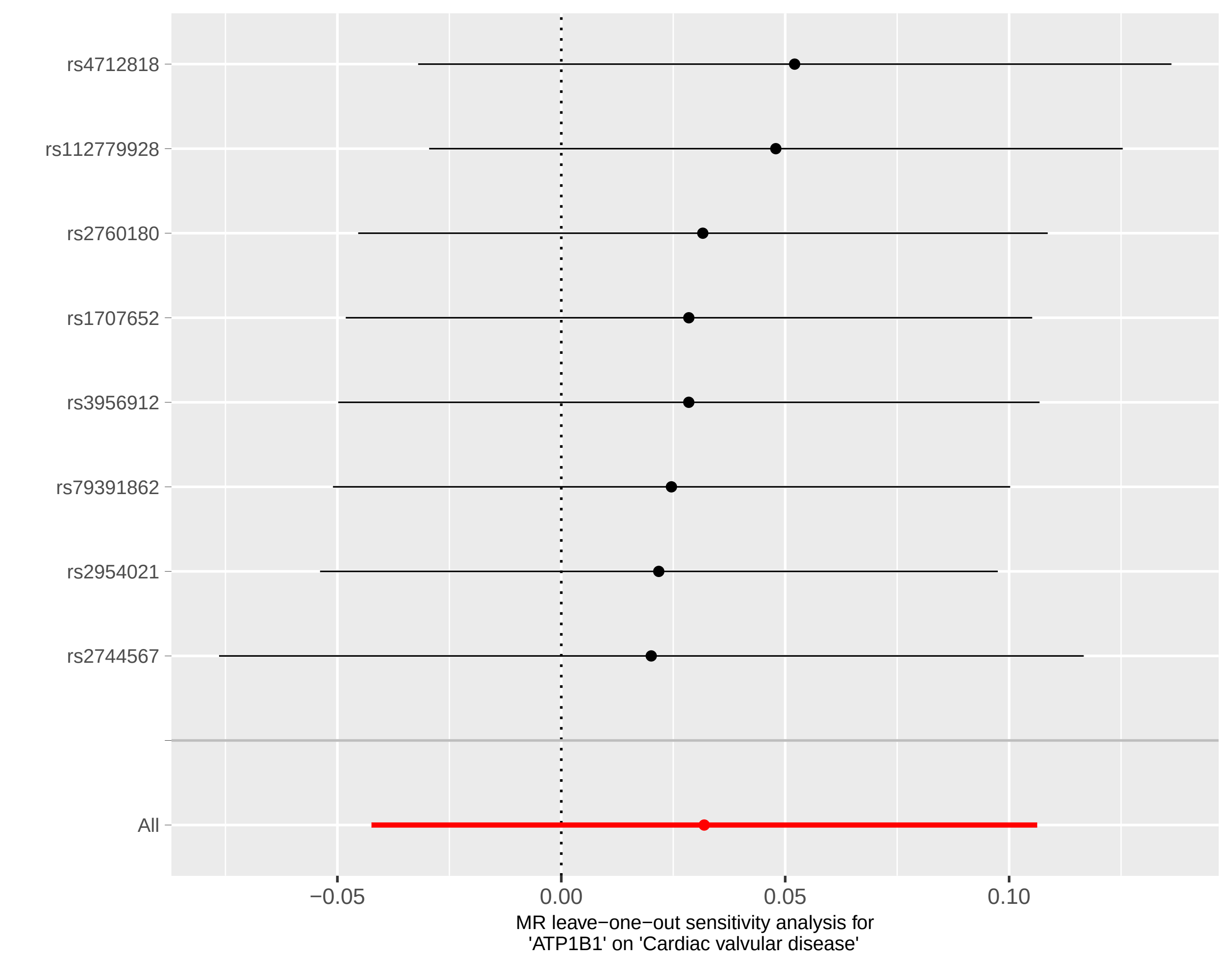
rs4712818
rs112779928
rs2760180
rs1707652
rs3956912
rs79391862
rs2954021
rs2744567
All
−0.05
0.00
0.05
0.10
MR leave−one−out sensitivity analysis for
'ATP1B1' on 'Cardiac valvular disease'

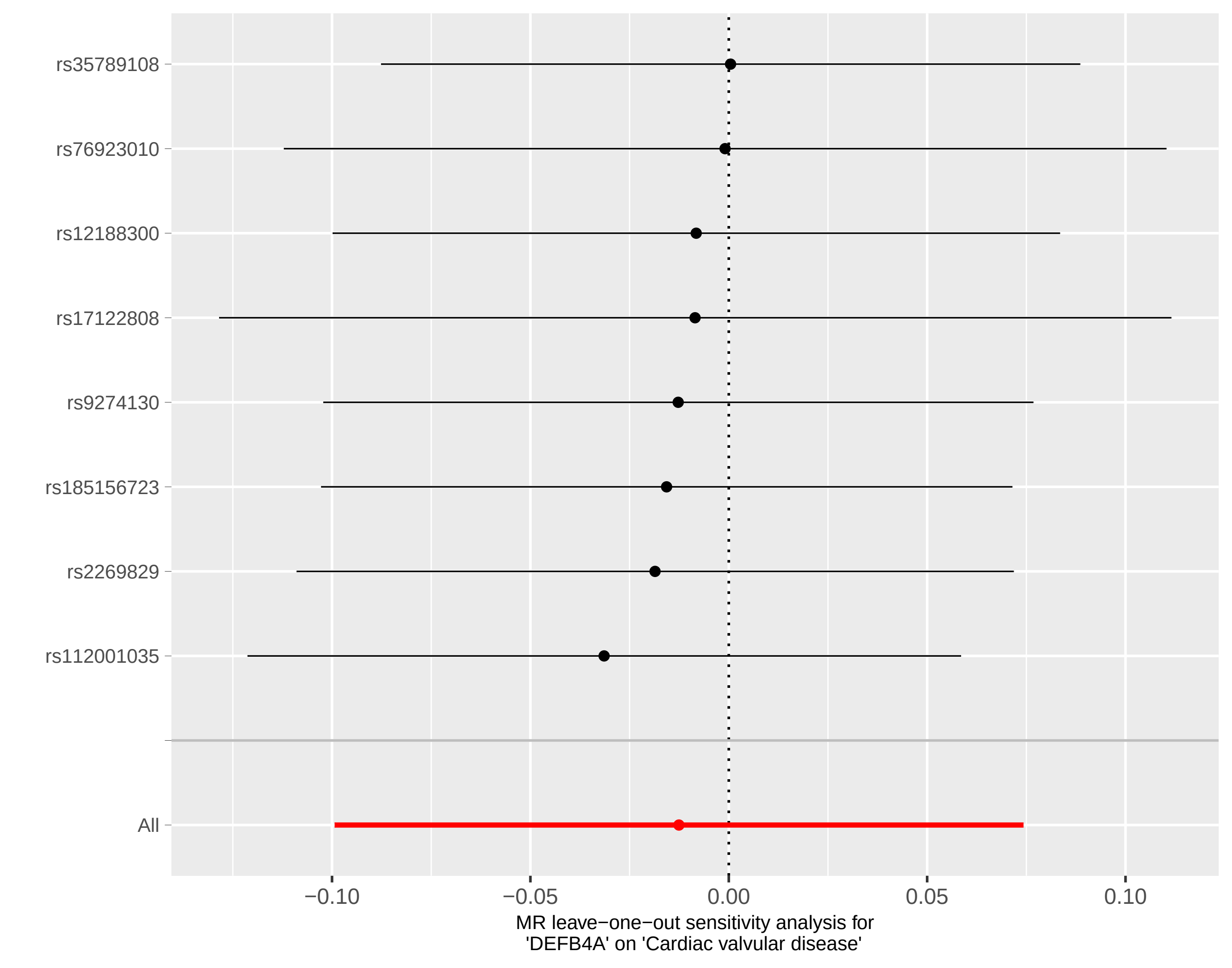
rs35789108
rs76923010
rs12188300
rs17122808
rs9274130
rs185156723
rs2269829
rs112001035
All
−0.10
−0.05
0.00
0.05
0.10
MR leave−one−out sensitivity analysis for
'DEFB4A' on 'Cardiac valvular disease'

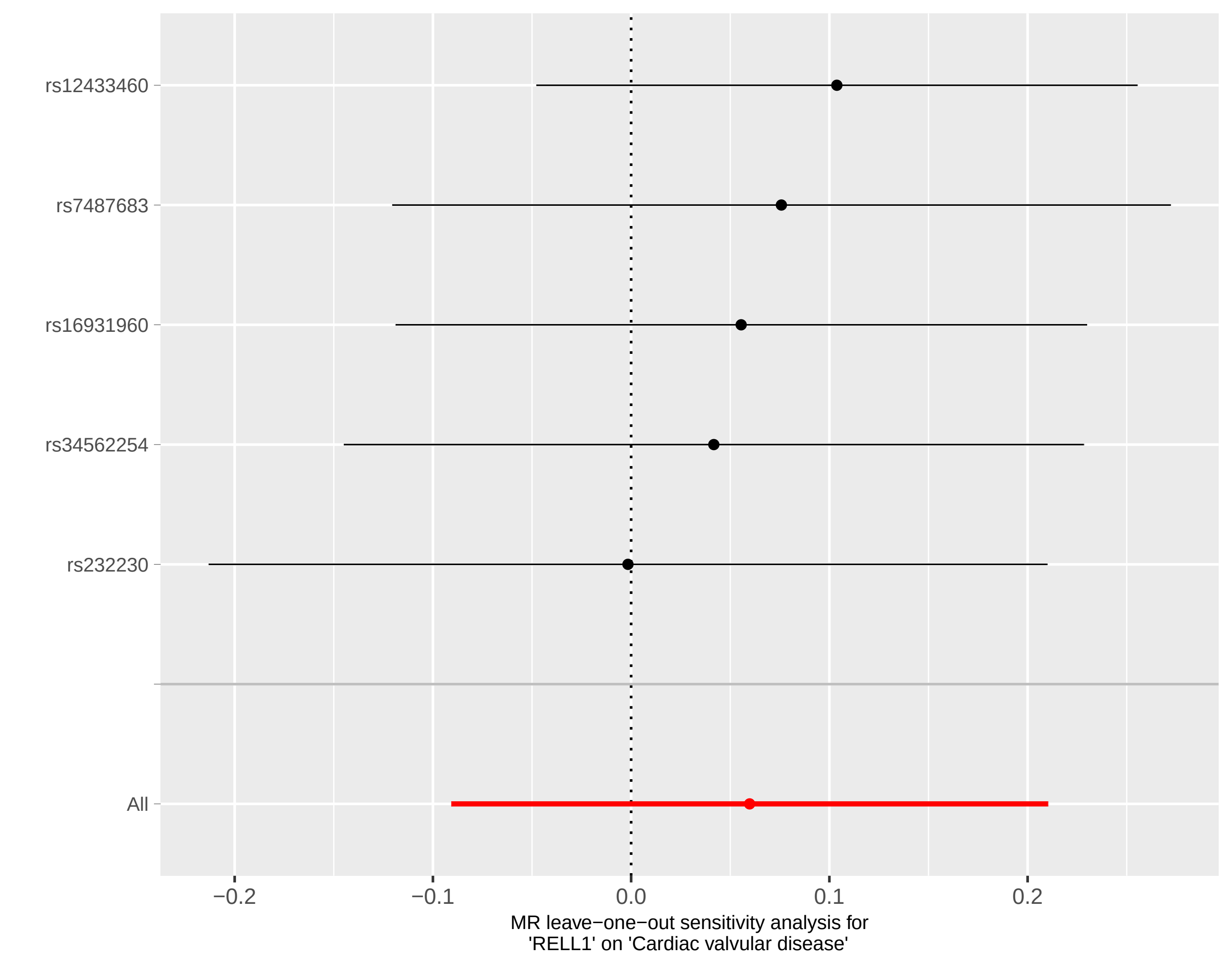
rs12433460
rs7487683
rs16931960
rs34562254
rs232230
All
−0.2
−0.1
0.0
0.1
0.2
MR leave−one−out sensitivity analysis for
'RELL1' on 'Cardiac valvular disease'

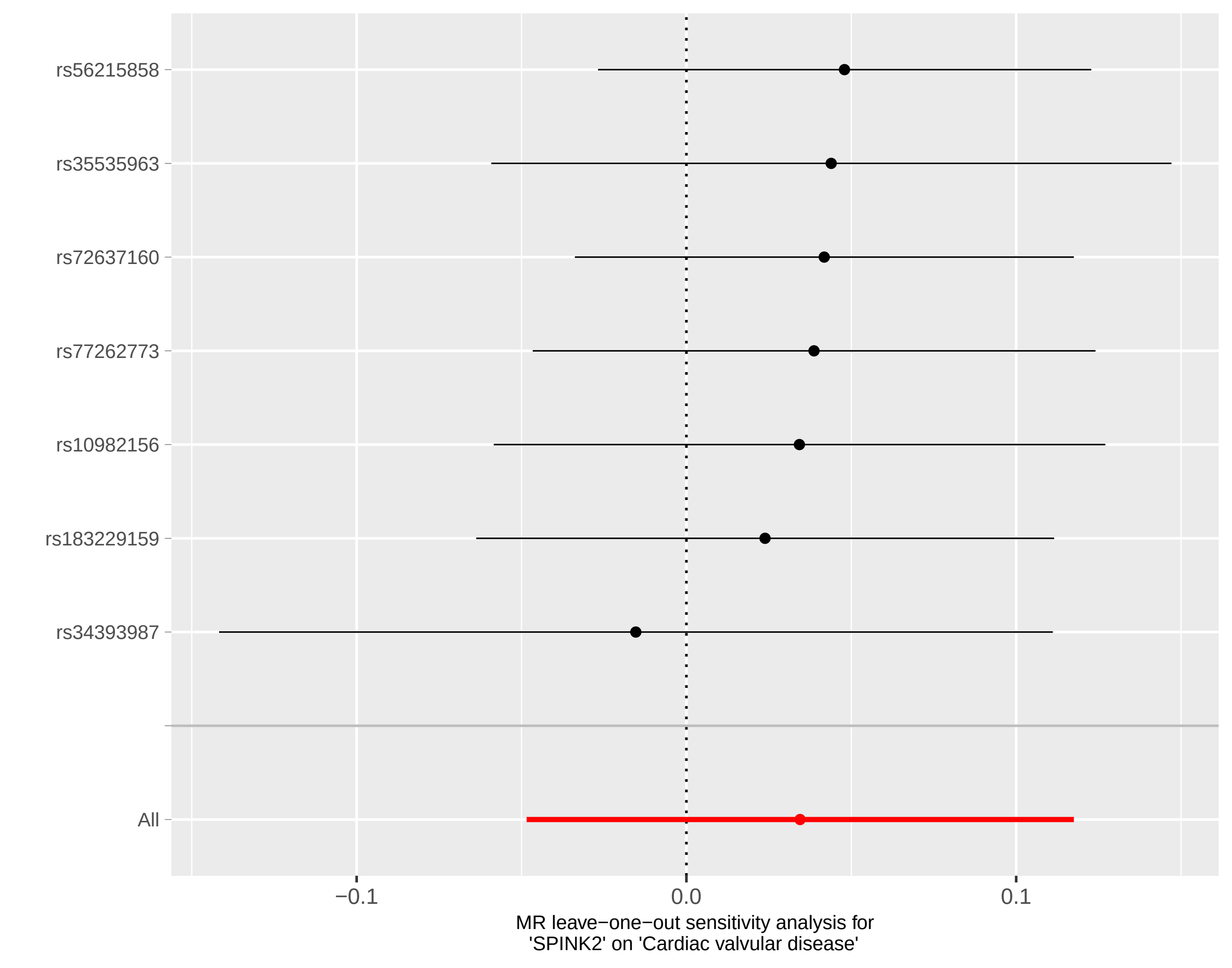

rs56215858
rs35535963
rs72637160
rs77262773
rs10982156
rs183229159
rs34393987
All
−0.1
0.0
0.1
MR leave−one−out sensitivity analysis for
'SPINK2' on 'Cardiac valvular disease'

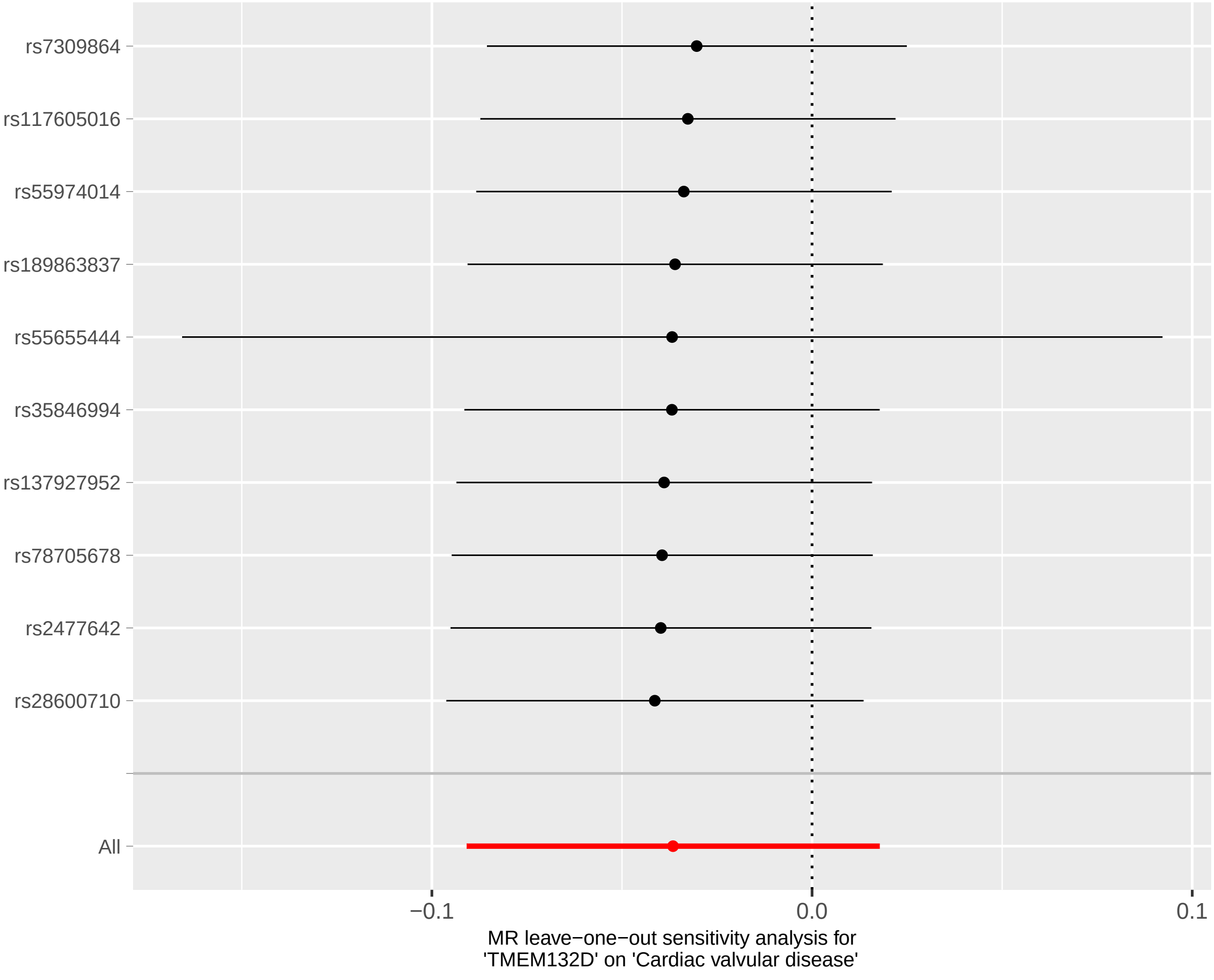
rs7309864
rs117605016
rs55974014
rs189863837
rs55655444
rs35846994
rs137927952
rs78705678
rs2477642
rs28600710
All
−0.1
0.0
0.1
MR leave−one−out sensitivity analysis for
'TMEM132D' on 'Cardiac valvular disease'

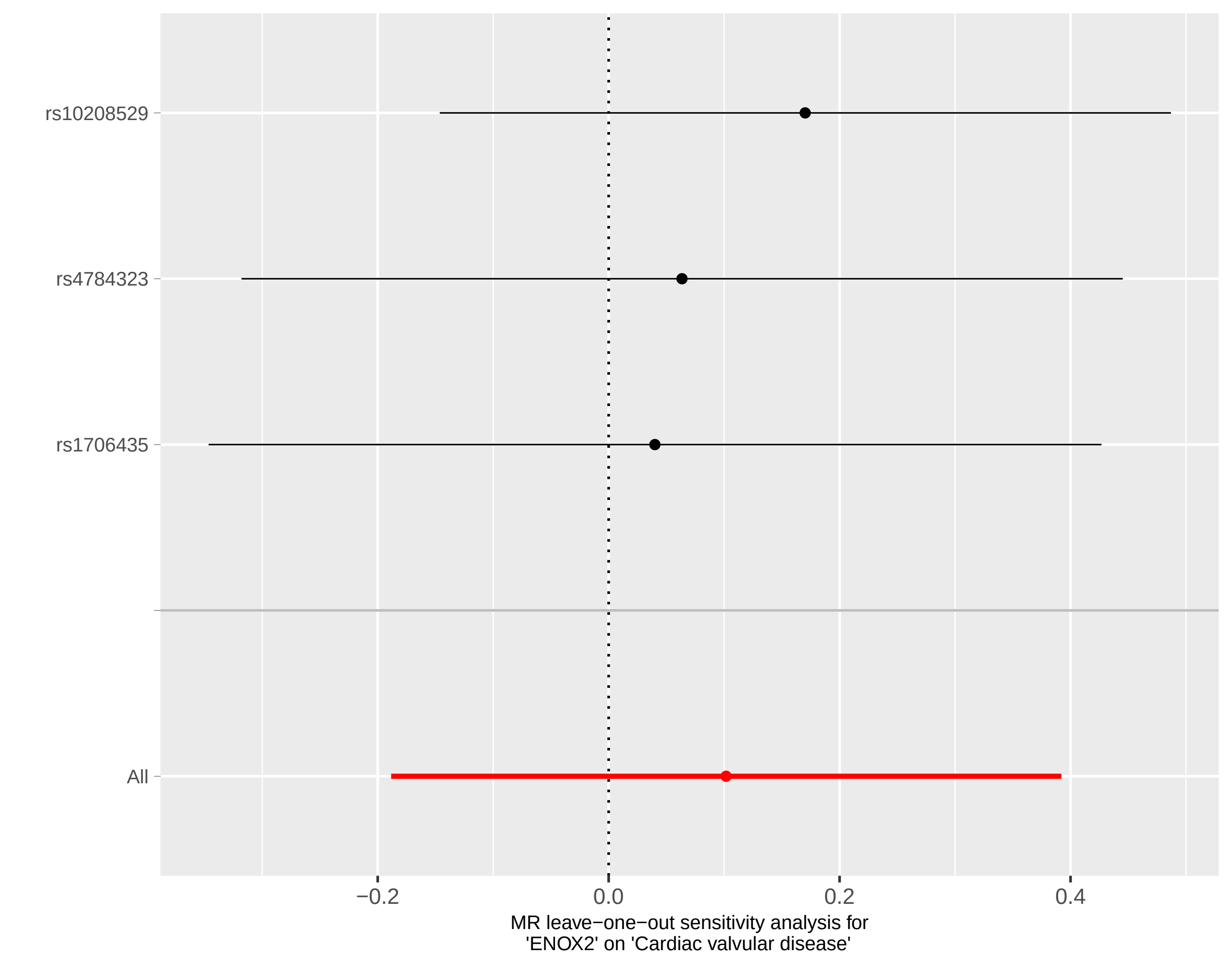

rs10208529
rs4784323
rs1706435
All
−0.2
0.0
0.2
0.4
MR leave−one−out sensitivity analysis for
'ENOX2' on 'Cardiac valvular disease'

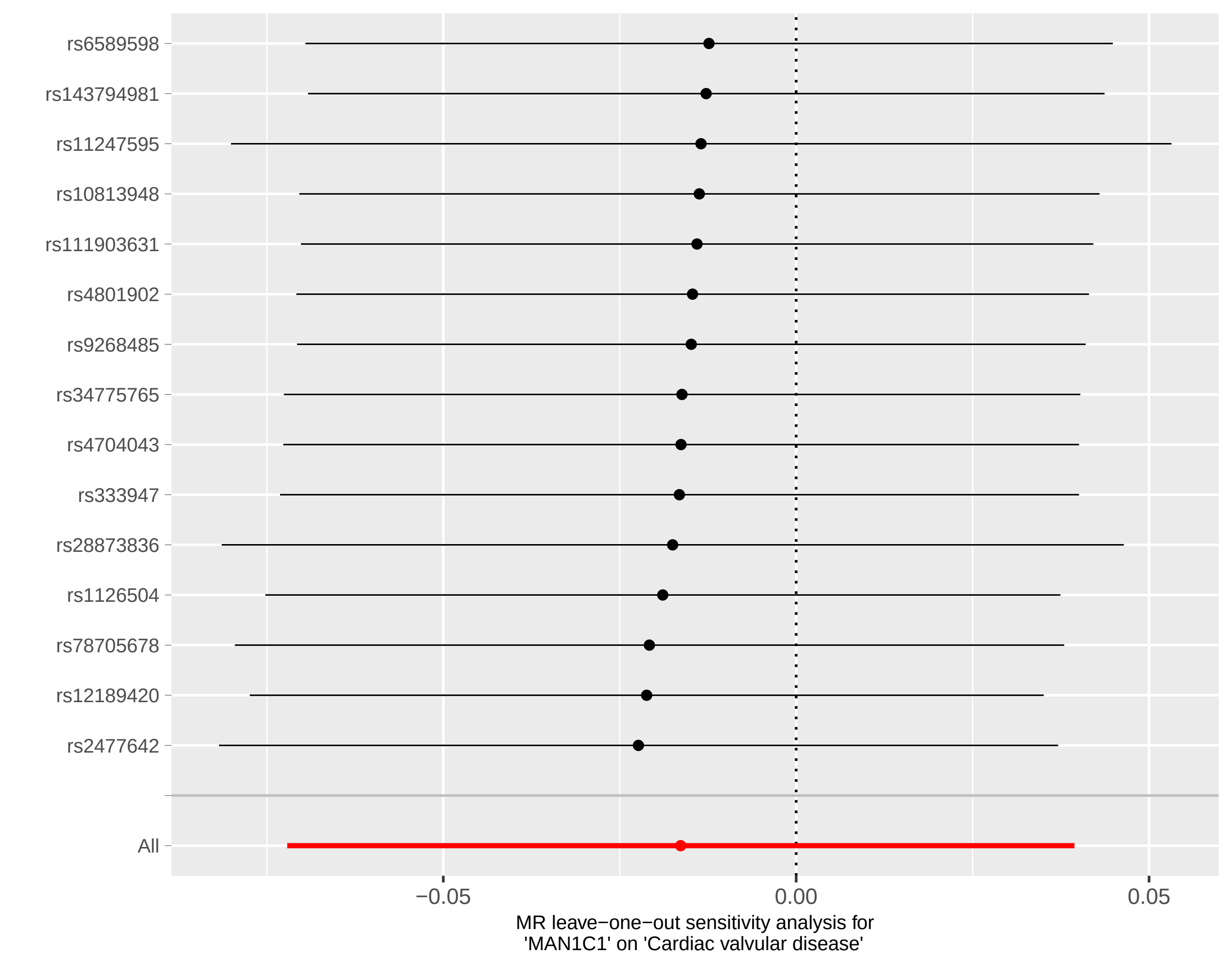
rs6589598
rs143794981
rs11247595
rs10813948
rs111903631
rs4801902
rs9268485
rs34775765
rs4704043
rs333947
rs28873836
rs1126504
rs78705678
rs12189420
rs2477642
All
−0.05
0.00
0.05
MR leave−one−out sensitivity analysis for
'MAN1C1' on 'Cardiac valvular disease'

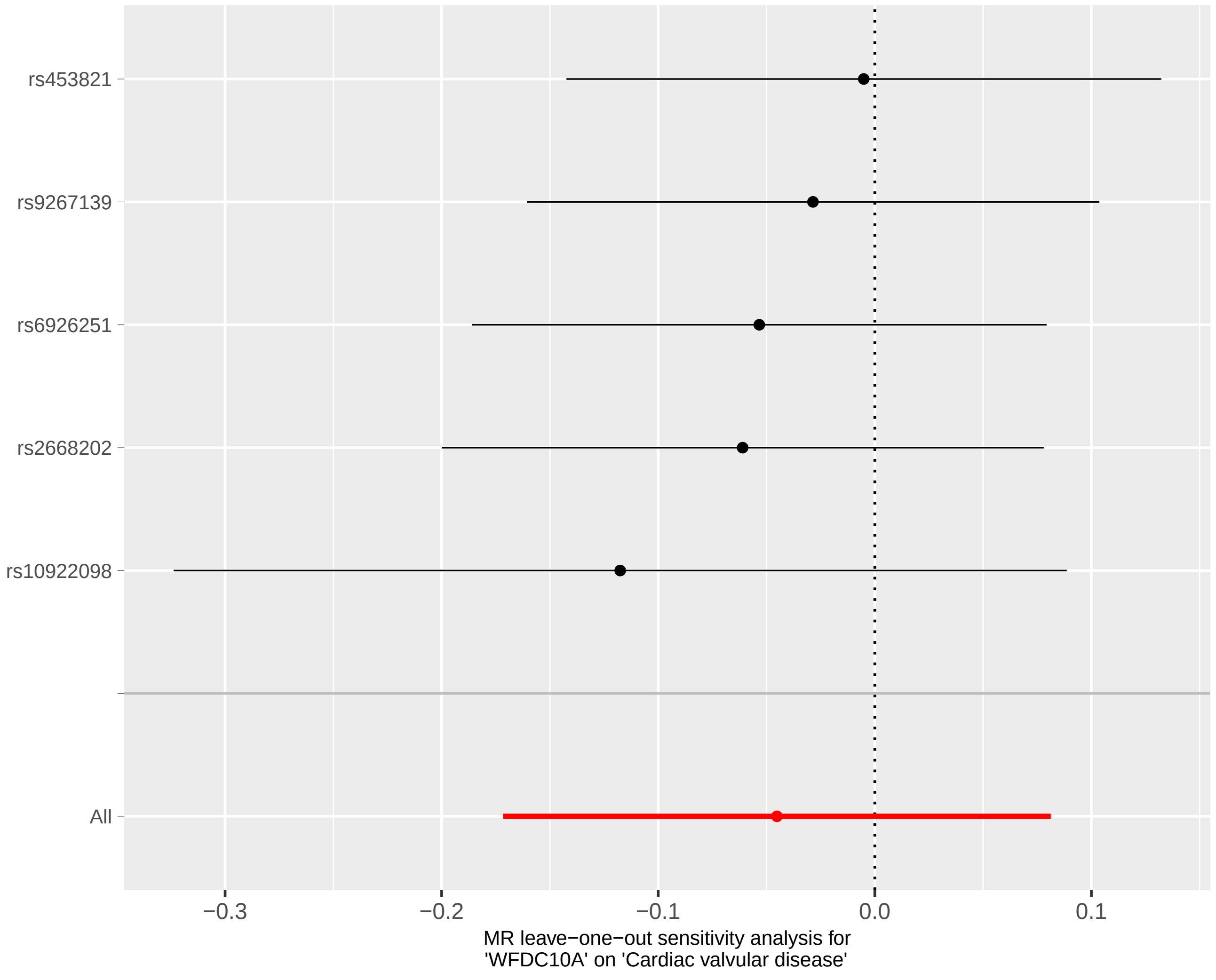
rs453821
rs9267139
rs6926251
rs2668202
rs10922098
All
−0.3
−0.2
−0.1
0.0
0.1
MR leave−one−out sensitivity analysis for
'WFDC10A' on 'Cardiac valvular disease'

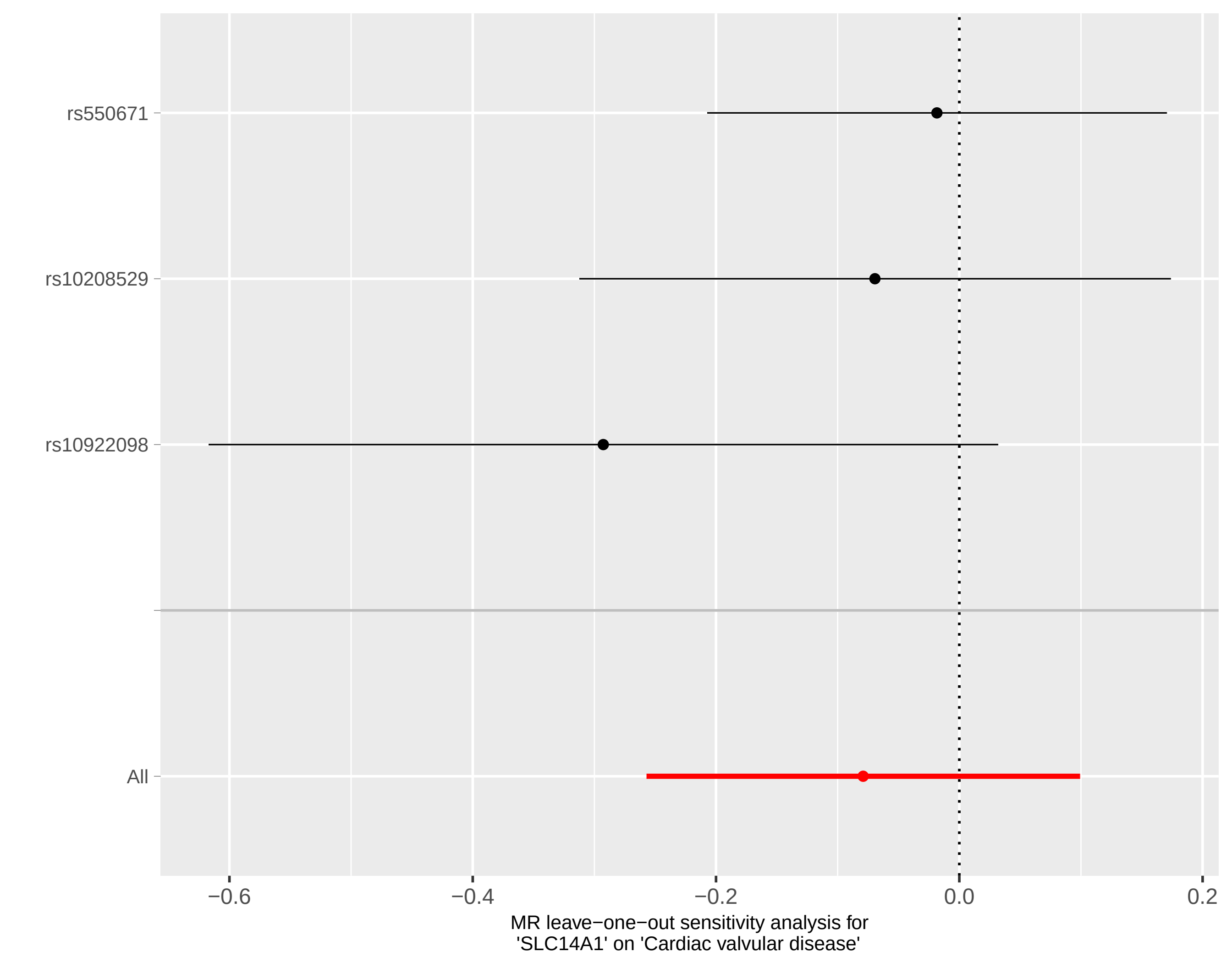
rs550671
rs10208529
rs10922098
All
−0.6
−0.4
−0.2
0.0
0.2
MR leave−one−out sensitivity analysis for
'SLC14A1' on 'Cardiac valvular disease'

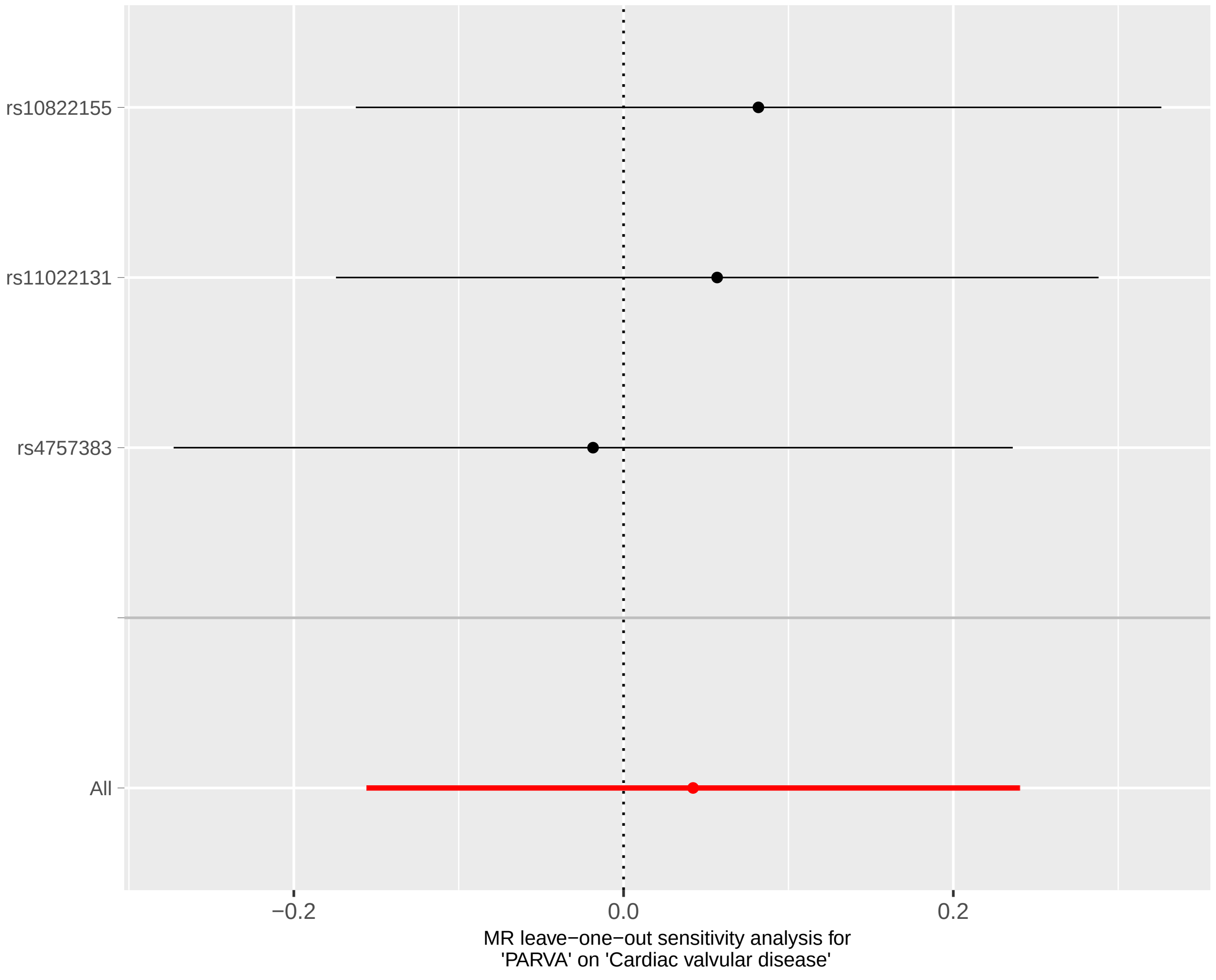
rs10822155
rs11022131
rs4757383
All
−0.2
0.0
0.2
MR leave−one−out sensitivity analysis for
'PARVA' on 'Cardiac valvular disease'

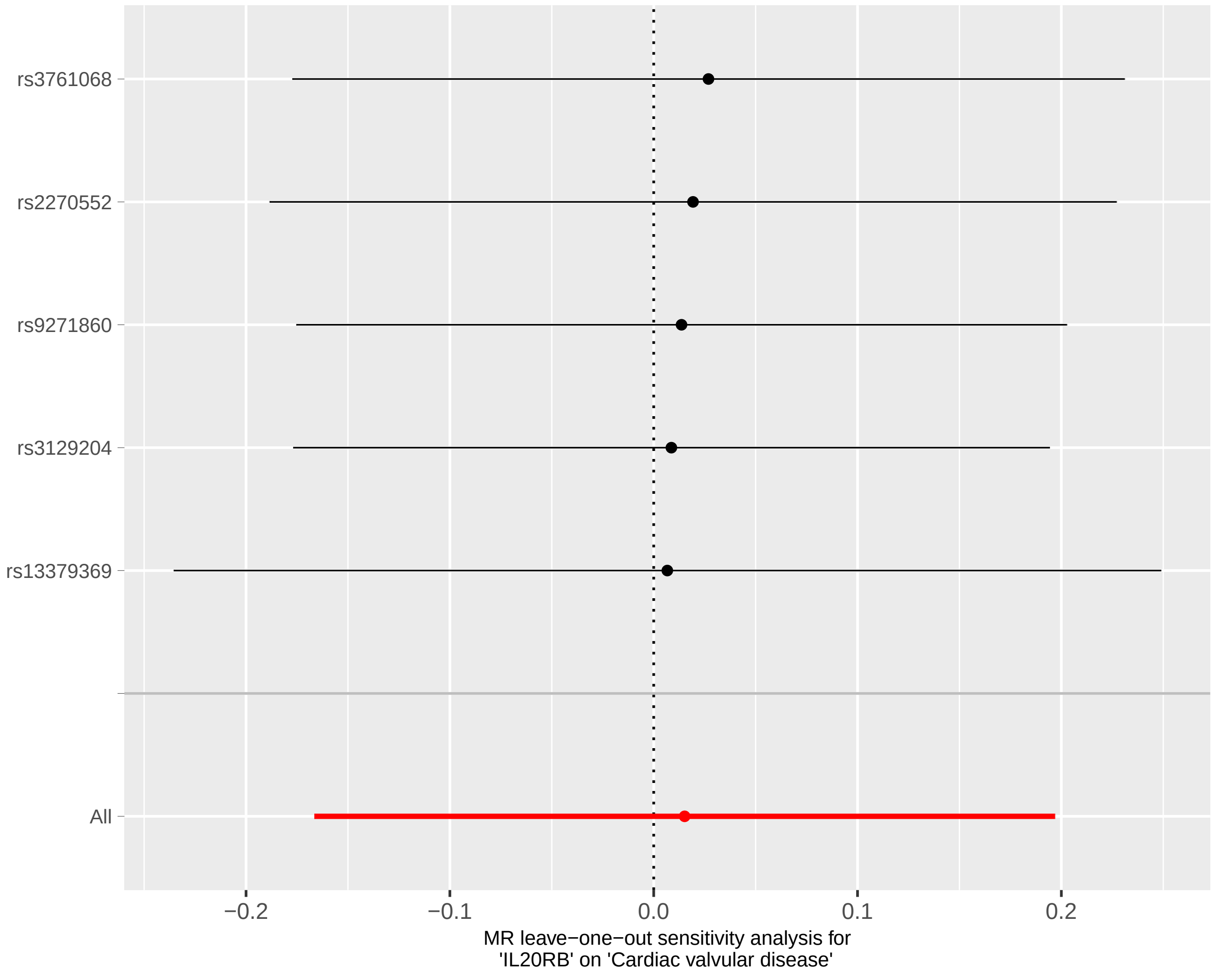
rs3761068
rs2270552
rs9271860
rs3129204
rs13379369
All
−0.2
−0.1
0.0
0.1
0.2
MR leave−one−out sensitivity analysis for
'IL20RB' on 'Cardiac valvular disease'

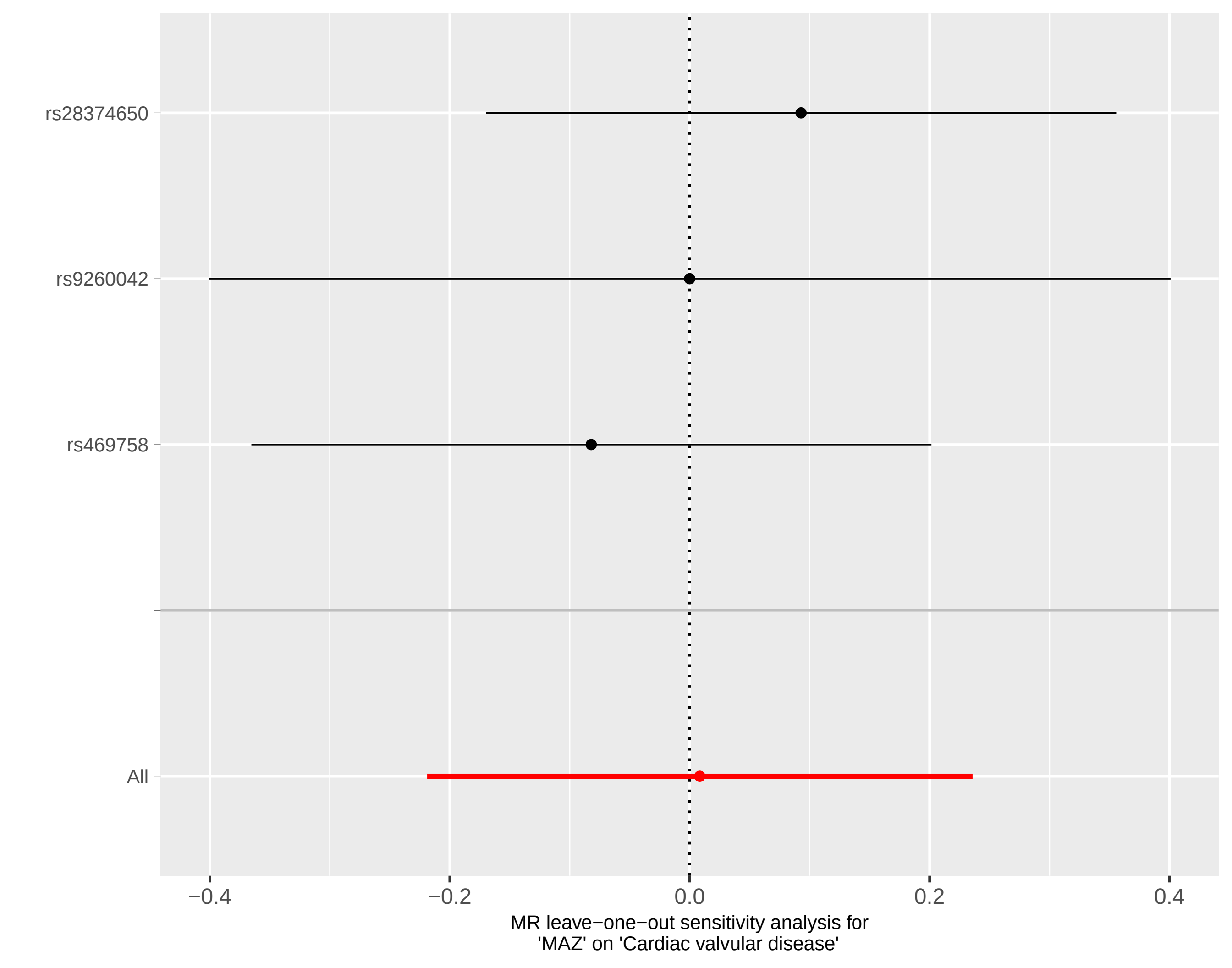
rs28374650
rs9260042
rs469758
All
−0.4
−0.2
0.0
0.2
0.4
MR leave−one−out sensitivity analysis for
'MAZ' on 'Cardiac valvular disease'

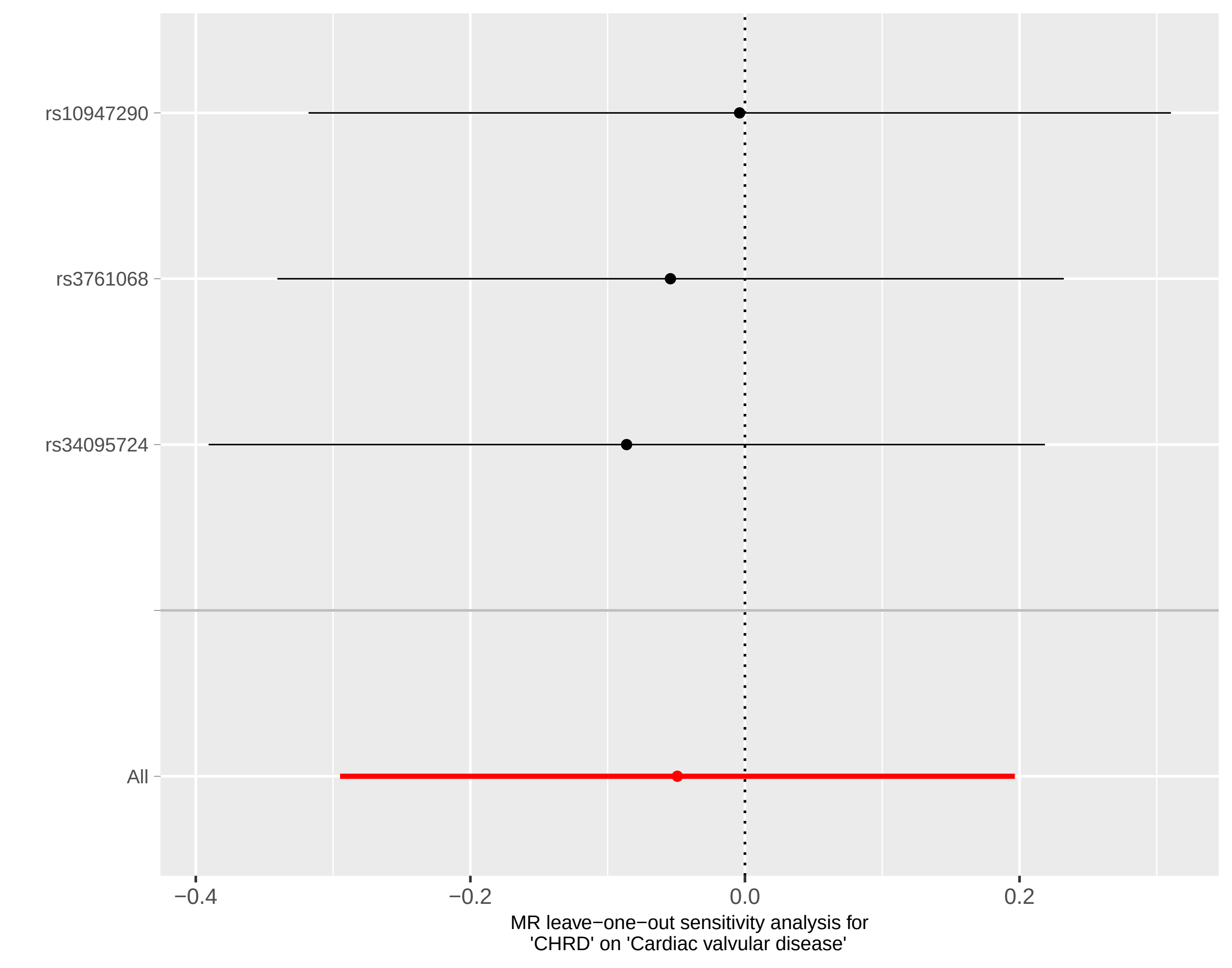

rs10947290
rs3761068
rs34095724
All
−0.4
−0.2
0.0
0.2
MR leave−one−out sensitivity analysis for
'CHRD' on 'Cardiac valvular disease'

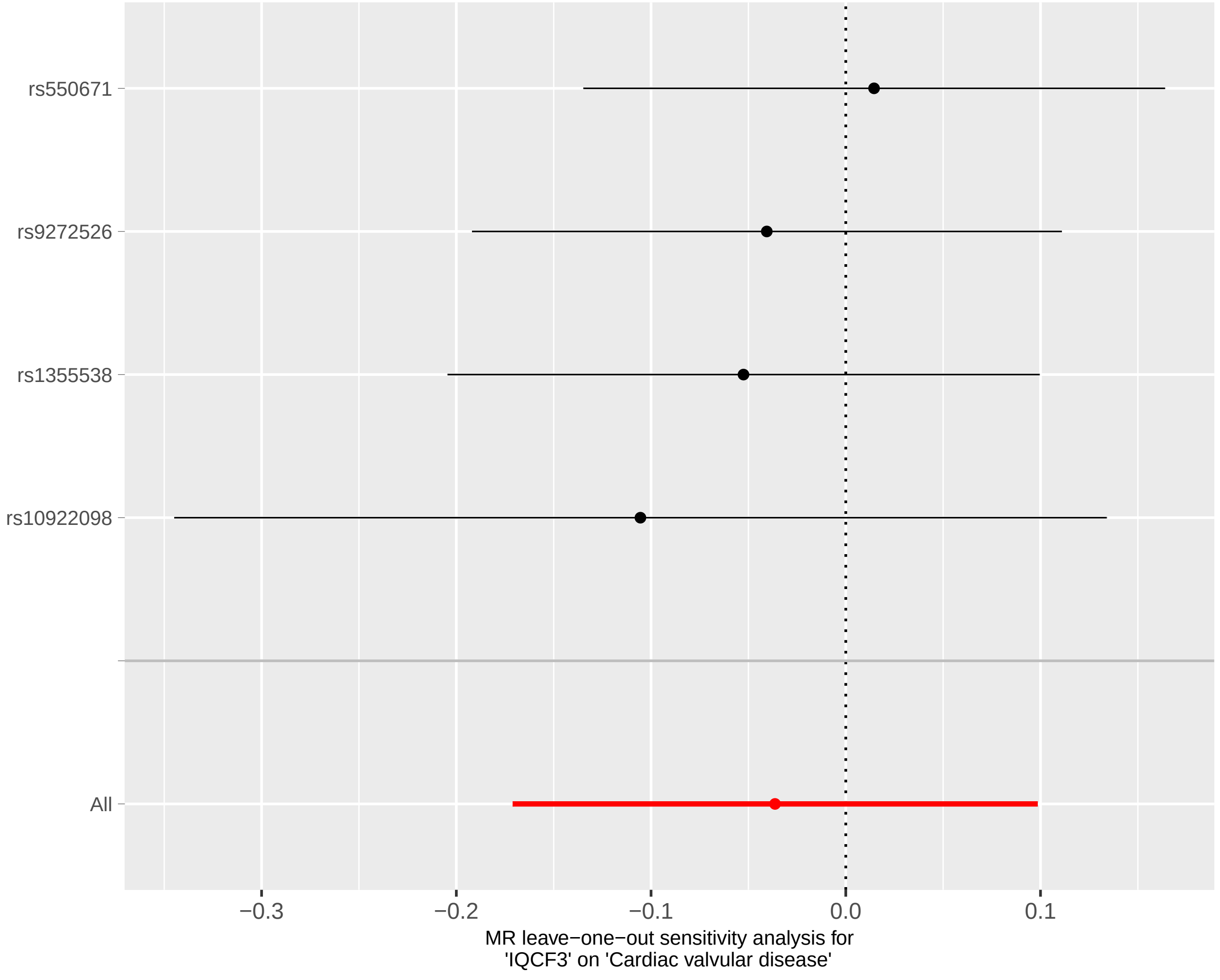

rs550671
rs9272526
rs1355538
rs10922098
All
−0.3
−0.2
−0.1
0.0
0.1
MR leave−one−out sensitivity analysis for
'IQCF3' on 'Cardiac valvular disease'

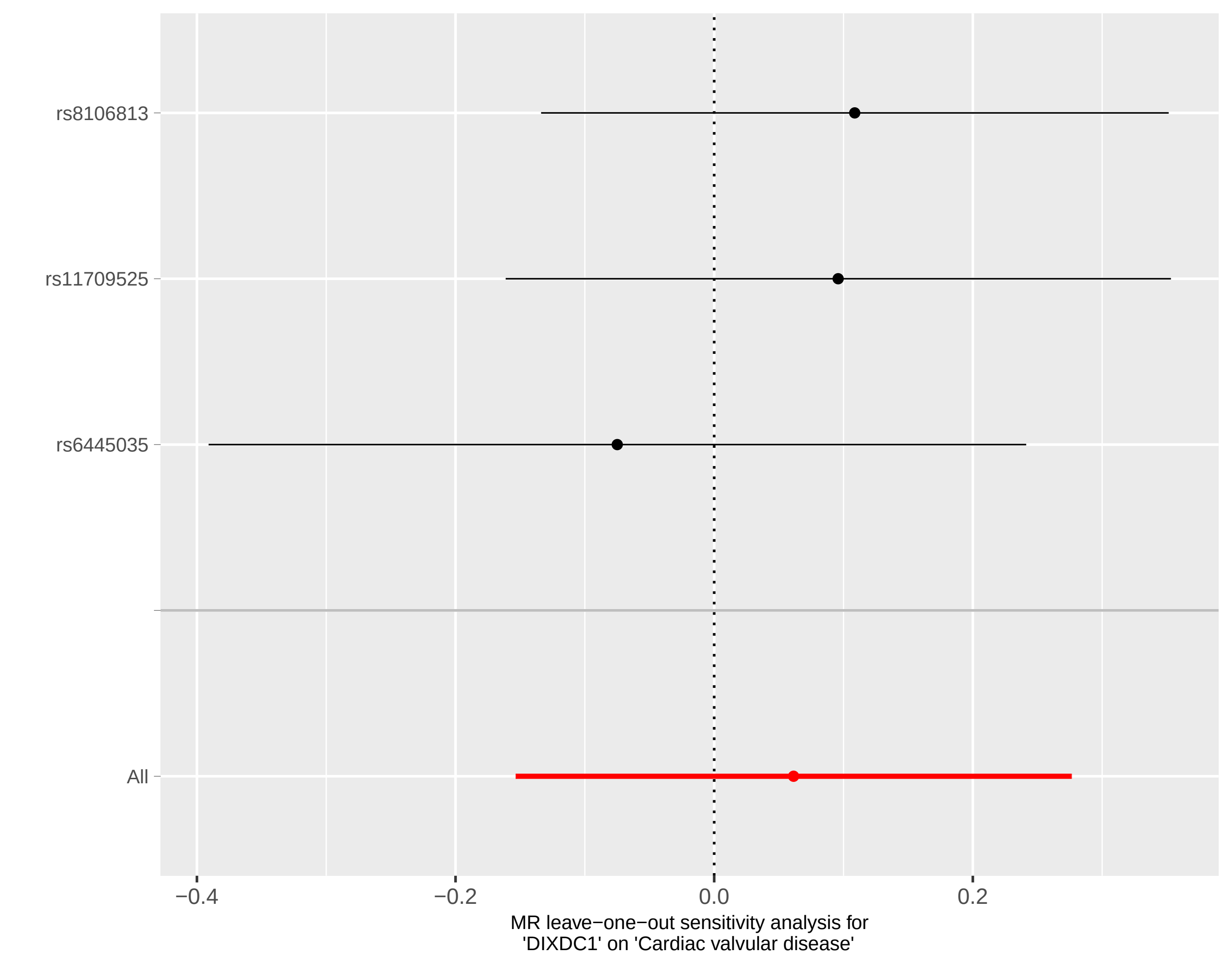
rs8106813
rs11709525
rs6445035
All
−0.4
−0.2
0.0
0.2
MR leave−one−out sensitivity analysis for
'DIXDC1' on 'Cardiac valvular disease'

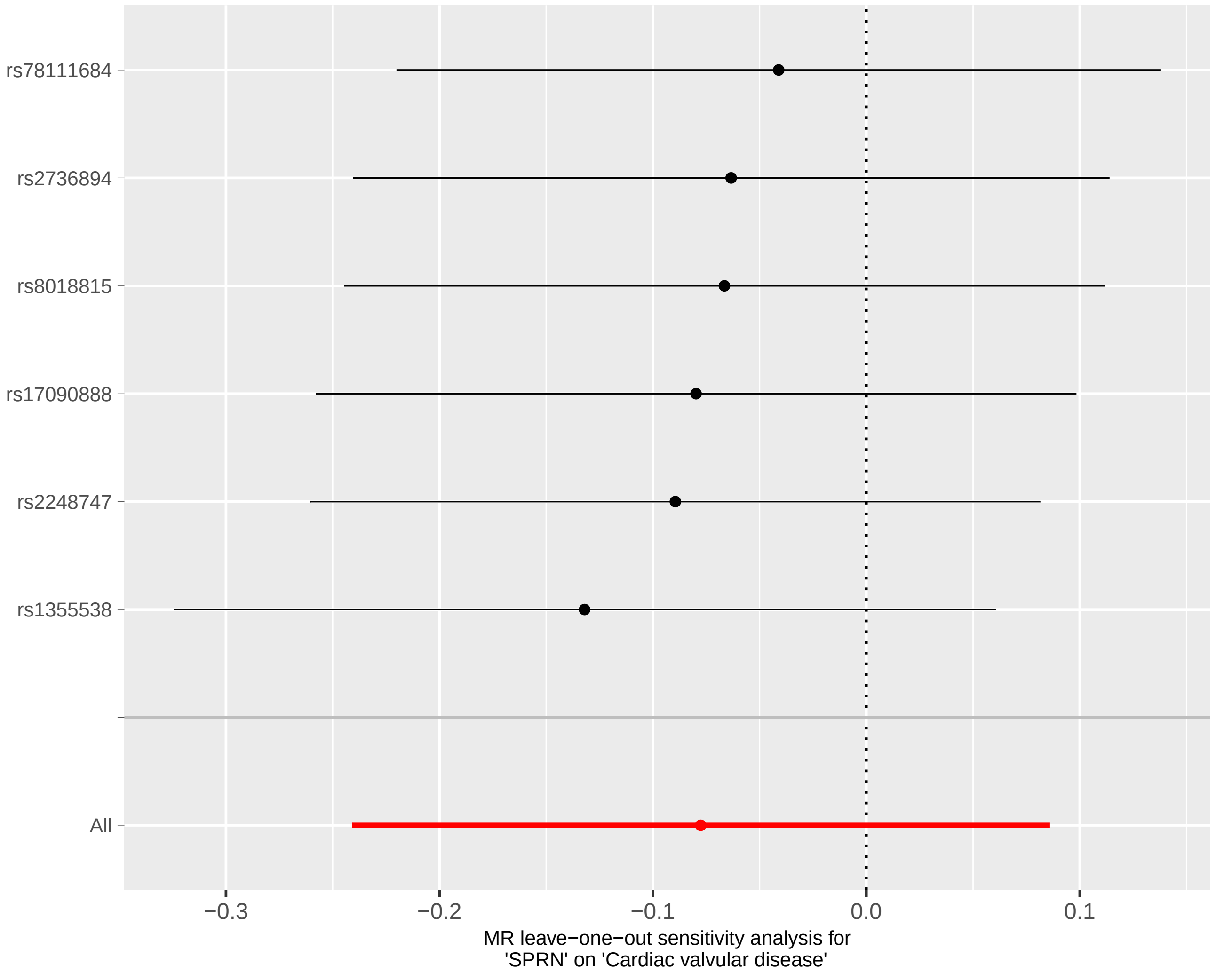
rs78111684
rs2736894
rs8018815
rs17090888
rs2248747
rs1355538
All
−0.3
−0.2
−0.1
0.0
0.1
MR leave−one−out sensitivity analysis for
'SPRN' on 'Cardiac valvular disease'

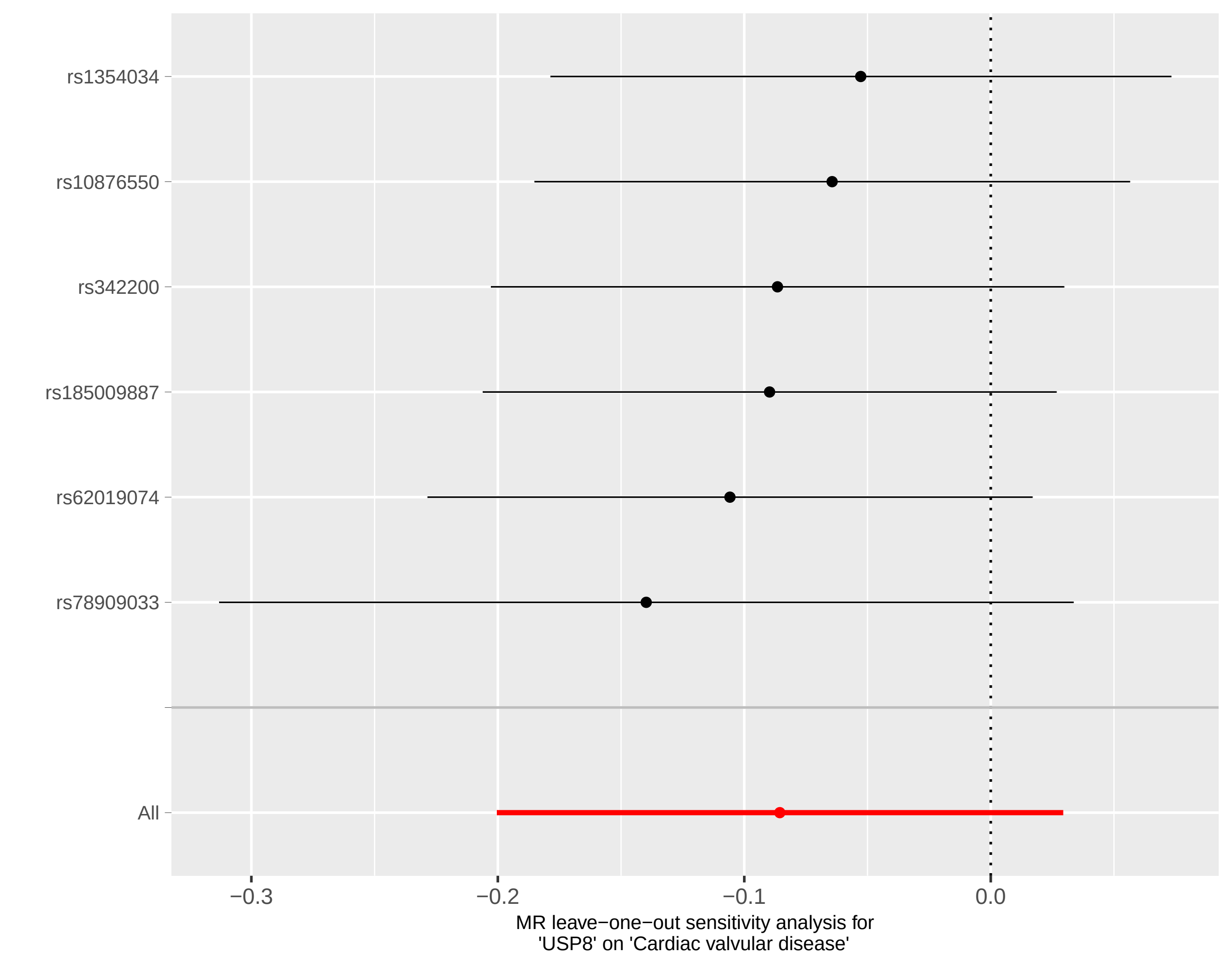
rs1354034
rs10876550
rs342200
rs185009887
rs62019074
rs78909033
All
−0.3
−0.2
−0.1
0.0
MR leave−one−out sensitivity analysis for
'USP8' on 'Cardiac valvular disease'

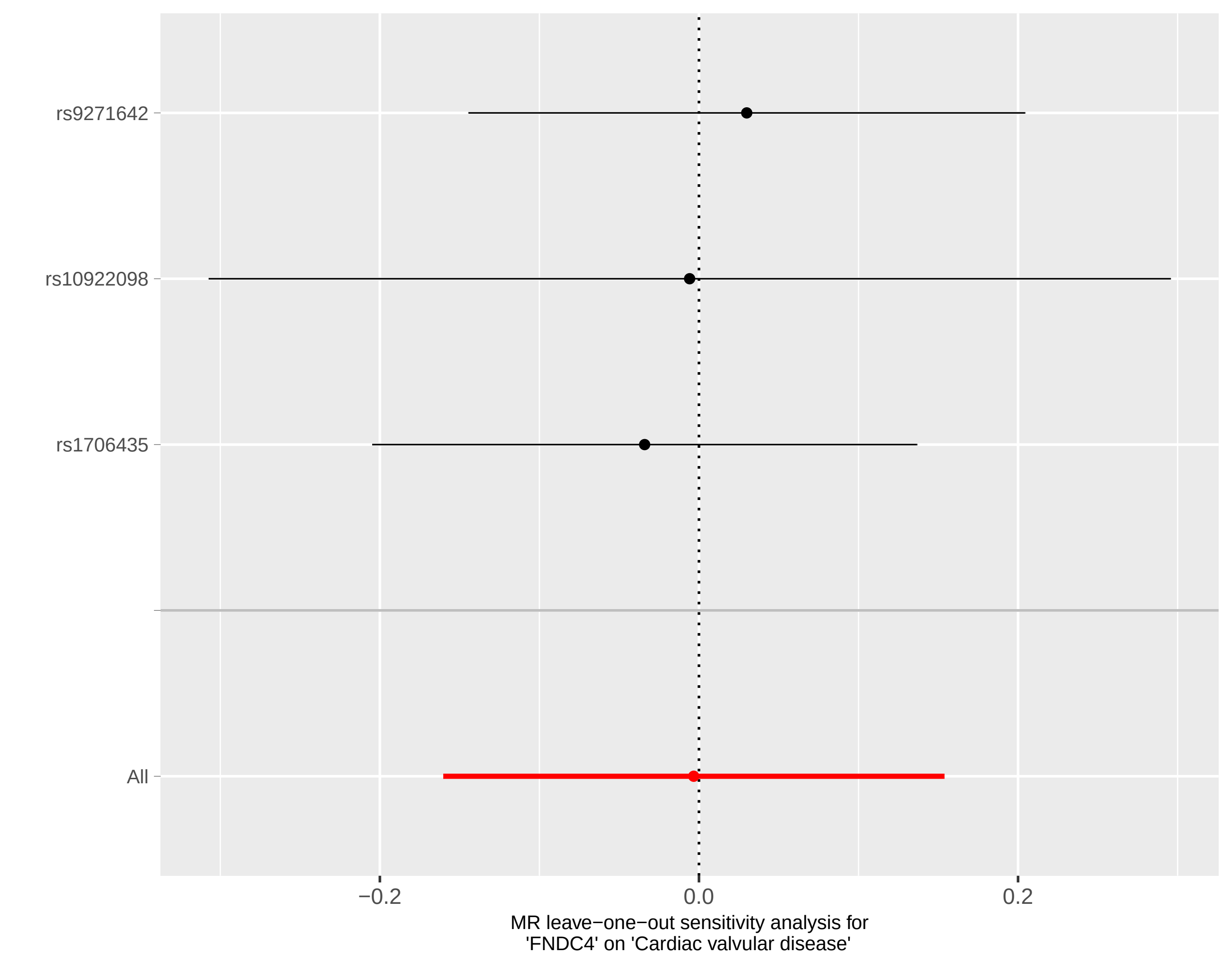
rs9271642
rs10922098
rs1706435
All
−0.2
0.0
0.2
MR leave−one−out sensitivity analysis for
'FNDC4' on 'Cardiac valvular disease'

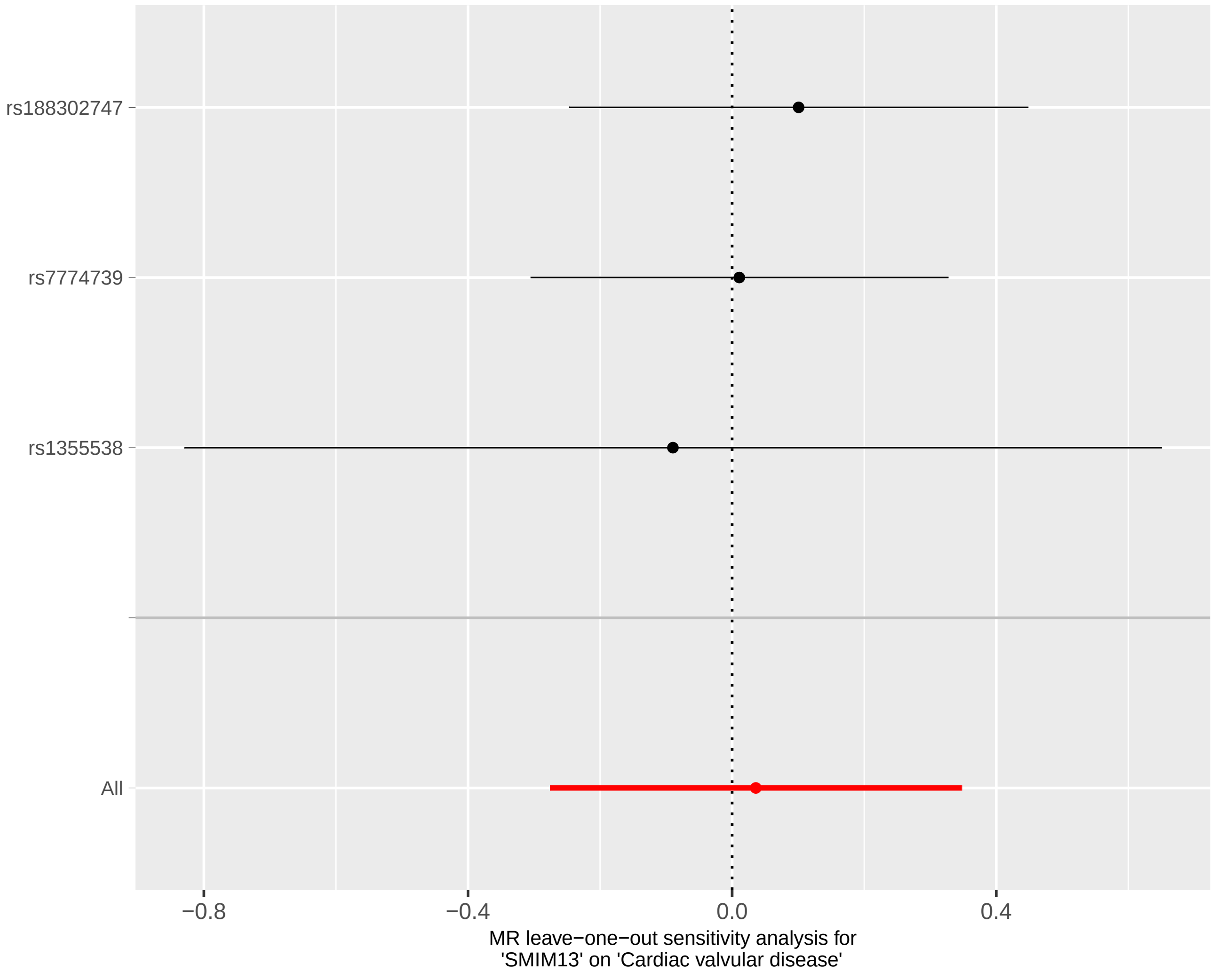
rs188302747
rs7774739
rs1355538
All
−0.8
−0.4
0.0
0.4
MR leave−one−out sensitivity analysis for
'SMIM13' on 'Cardiac valvular disease'

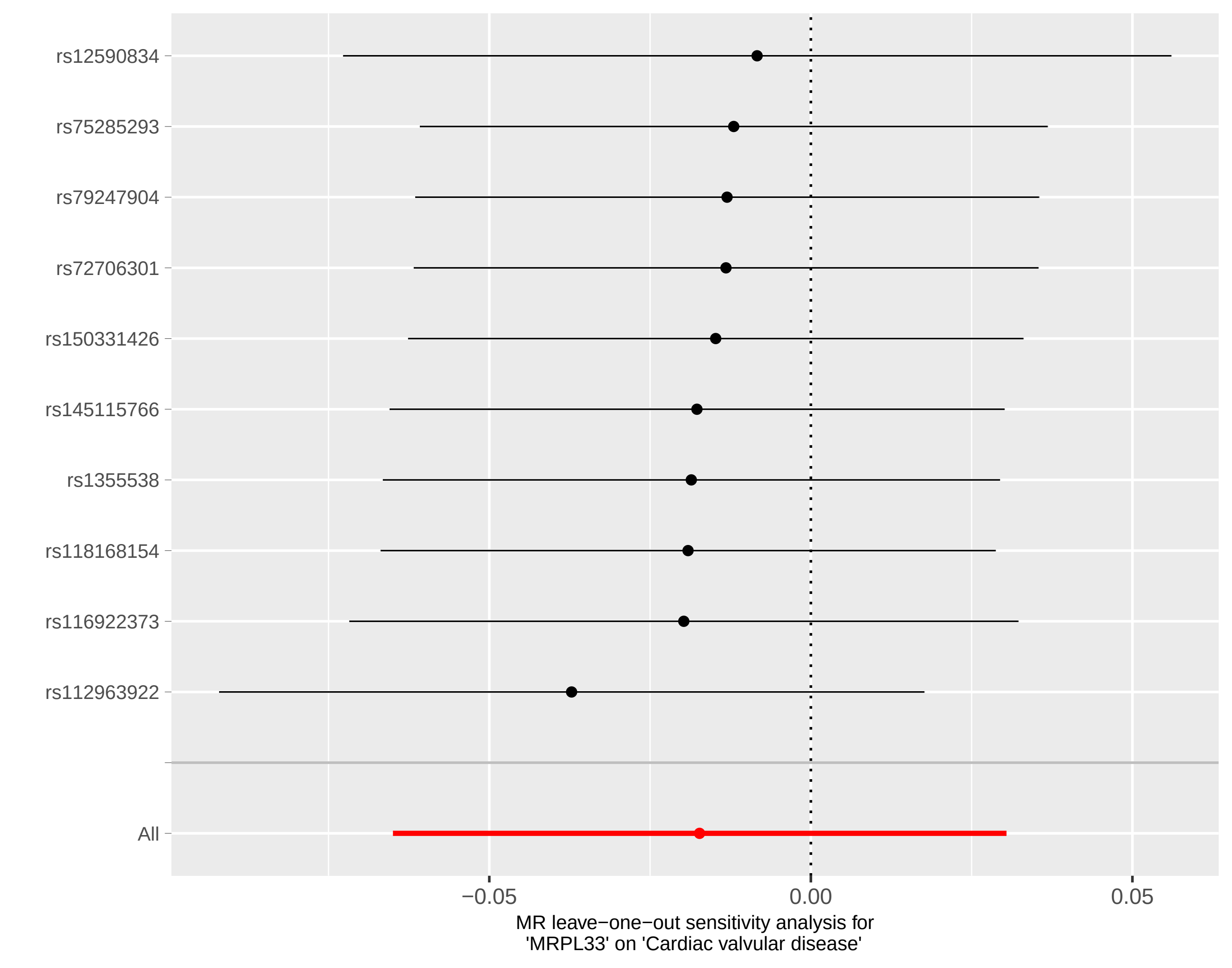
rs12590834
rs75285293
rs79247904
rs72706301
rs150331426
rs145115766
rs1355538
rs118168154
rs116922373
rs112963922
All
−0.05
0.00
0.05
MR leave−one−out sensitivity analysis for
'MRPL33' on 'Cardiac valvular disease'

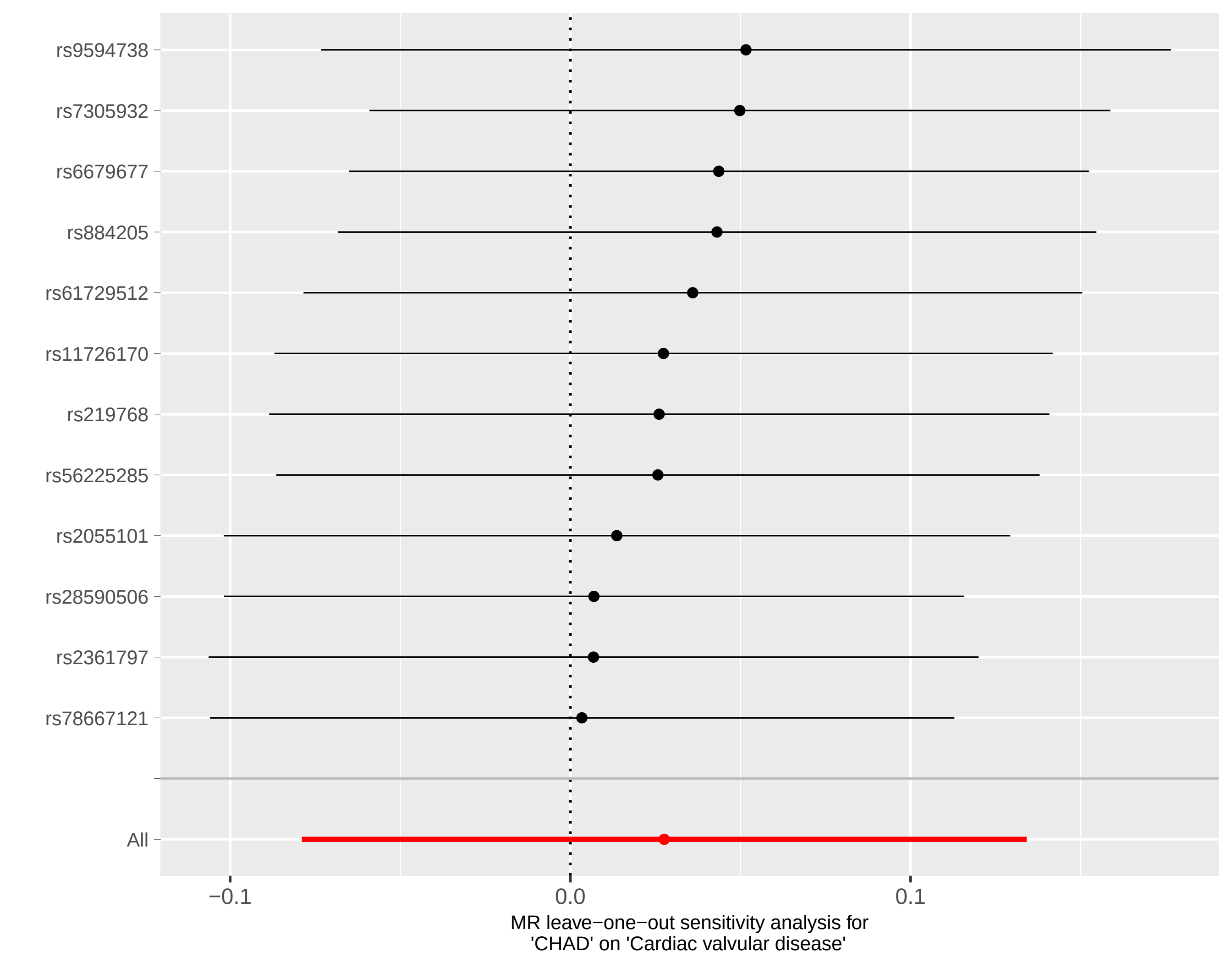
rs9594738
rs7305932
rs6679677
rs884205
rs61729512
rs11726170
rs219768
rs56225285
rs2055101
rs28590506
rs2361797
rs78667121
All
−0.1
0.0
0.1
MR leave−one−out sensitivity analysis for
'CHAD' on 'Cardiac valvular disease'

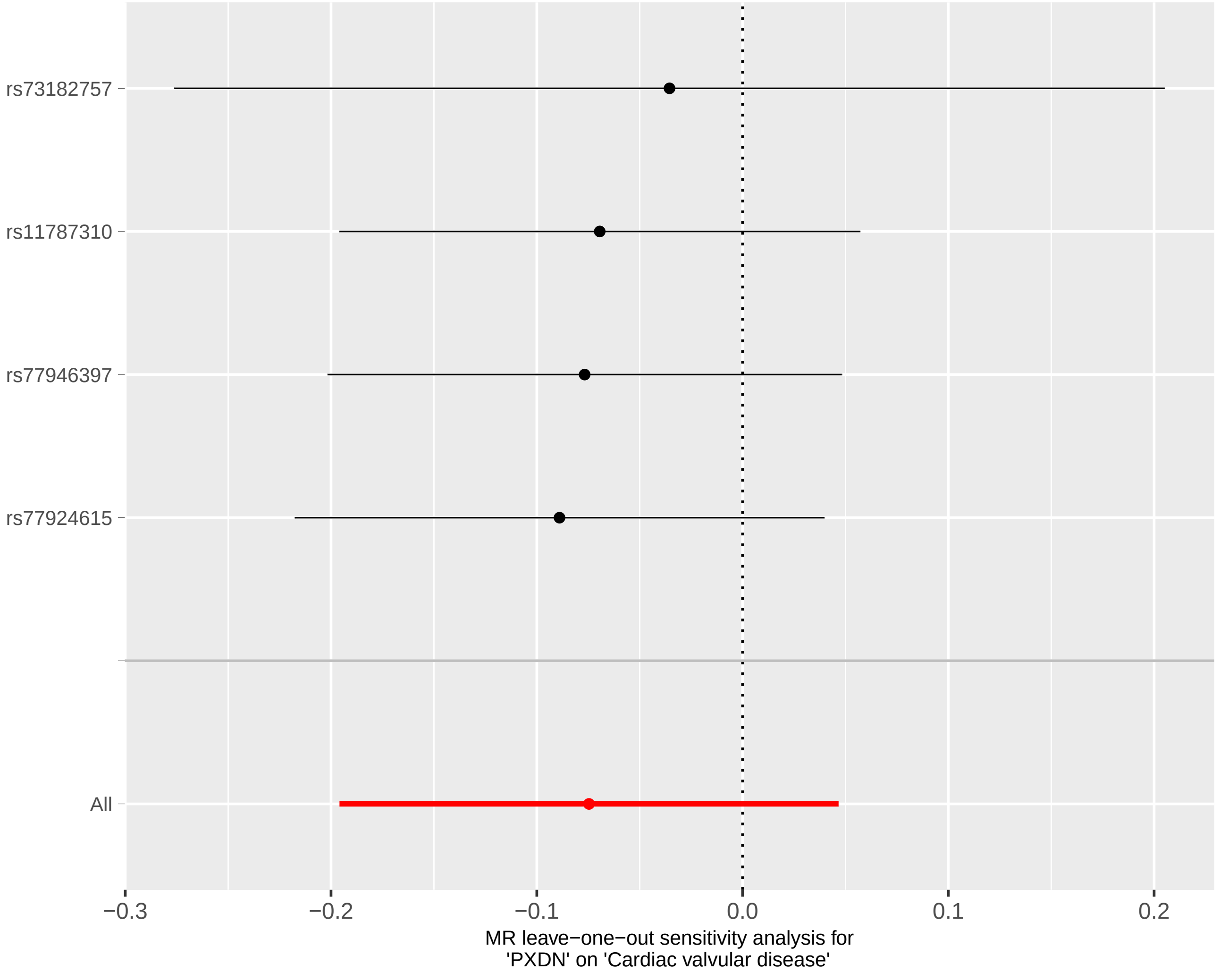

rs73182757
rs11787310
rs77946397
rs77924615
All
−0.3
−0.2
−0.1
0.0
0.1
0.2
MR leave−one−out sensitivity analysis for
'PXDN' on 'Cardiac valvular disease'

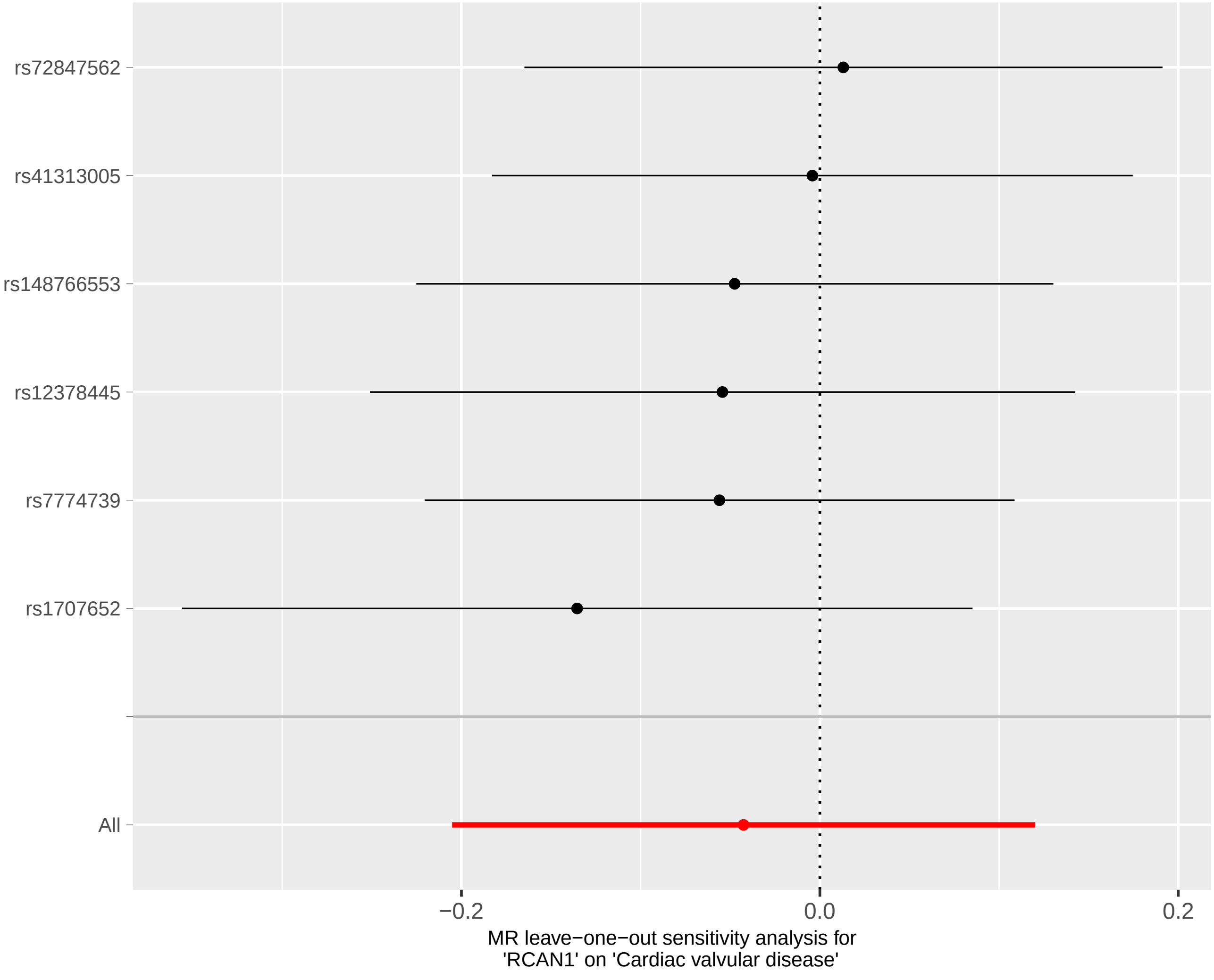
rs72847562
rs41313005
rs148766553
rs12378445
rs7774739
rs1707652
All
−0.2
0.0
0.2
MR leave−one−out sensitivity analysis for
'RCAN1' on 'Cardiac valvular disease'

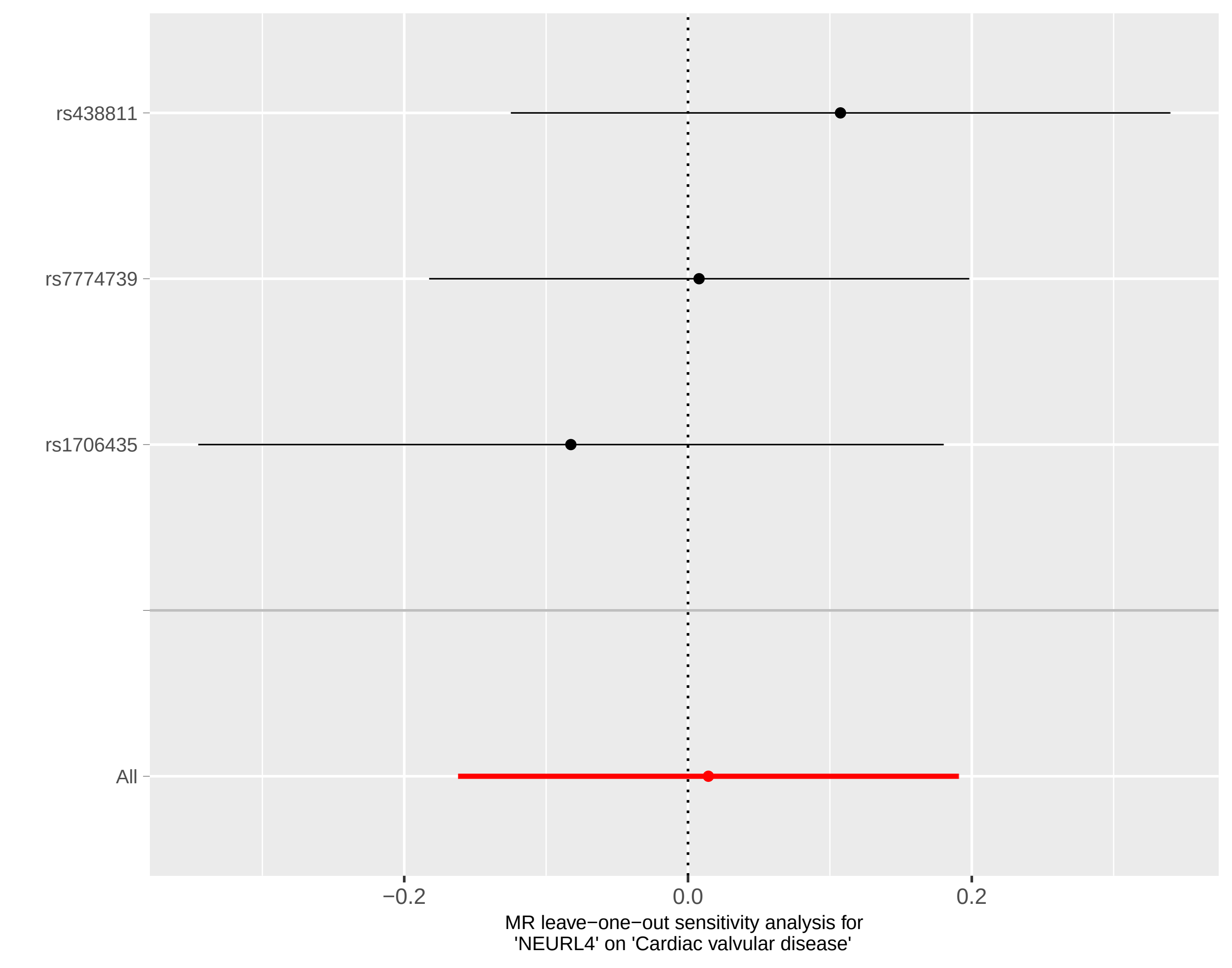

rs438811
rs7774739
rs1706435
All
−0.2
0.0
0.2
MR leave−one−out sensitivity analysis for
'NEURL4' on 'Cardiac valvular disease'

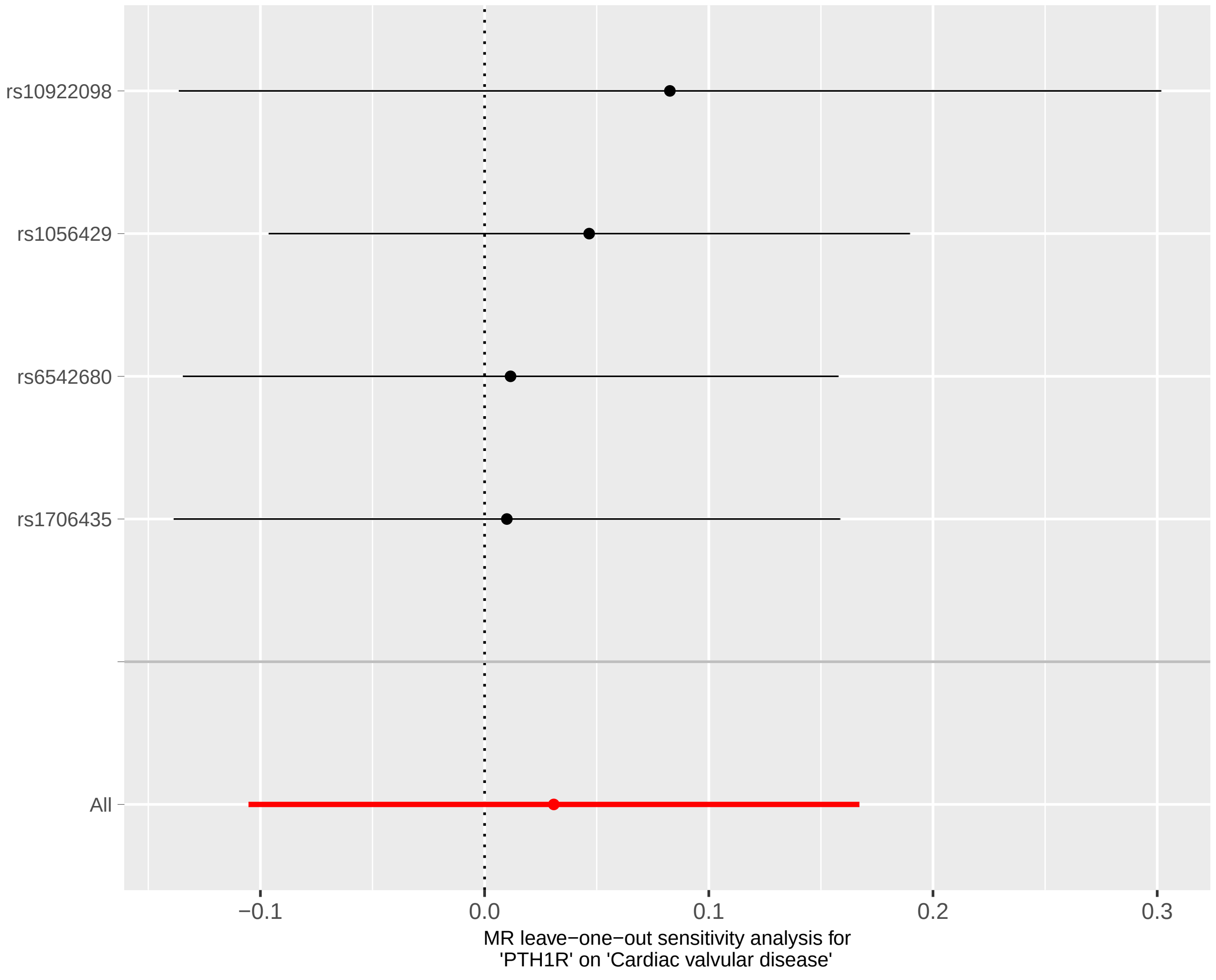

rs10922098
rs1056429
rs6542680
rs1706435
All
−0.1
0.0
0.1
0.2
0.3
MR leave−one−out sensitivity analysis for
'PTH1R' on 'Cardiac valvular disease'

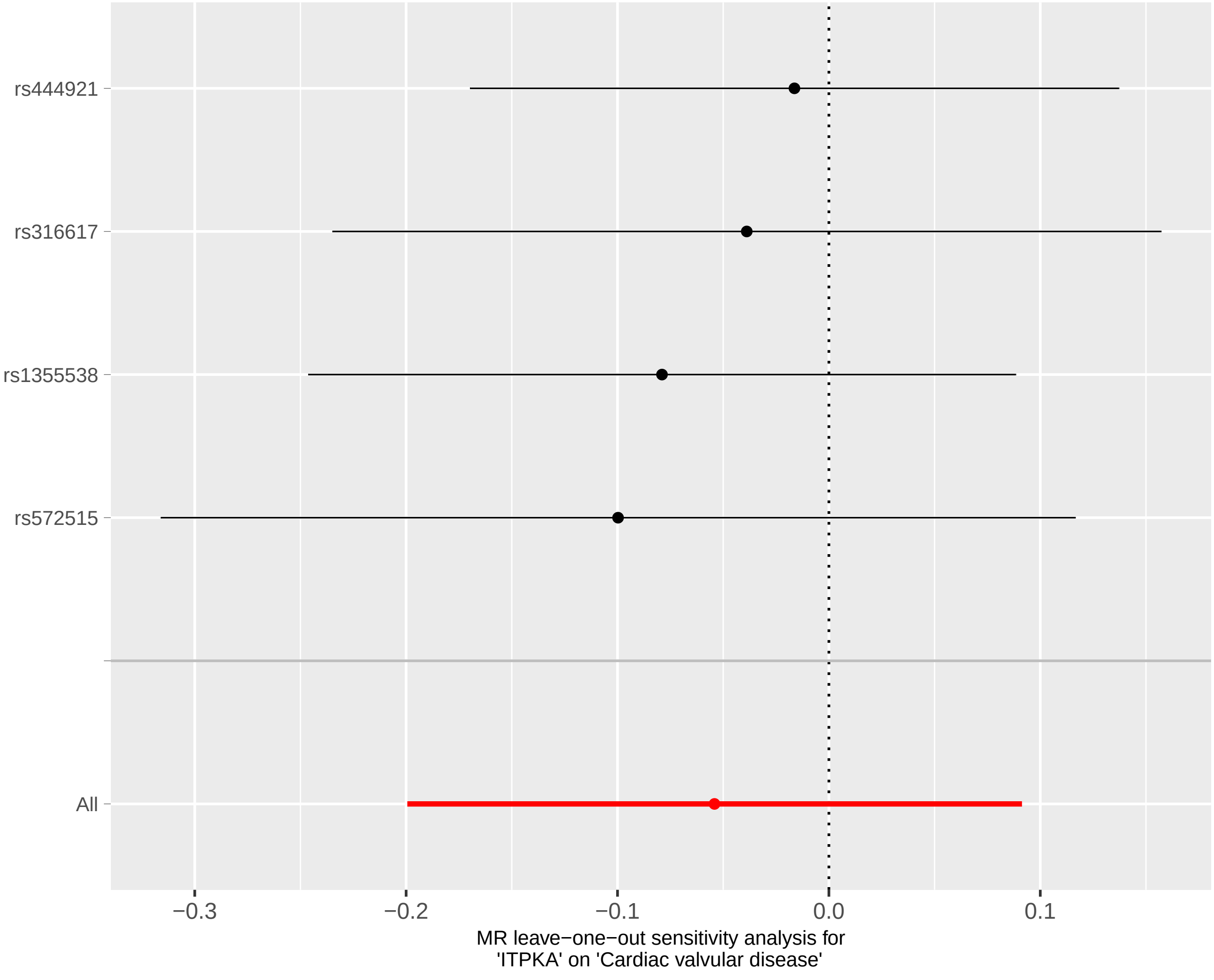
rs444921
rs316617
rs1355538
rs572515
All
−0.3
−0.2
−0.1
0.0
0.1
MR leave−one−out sensitivity analysis for
'ITPKA' on 'Cardiac valvular disease'

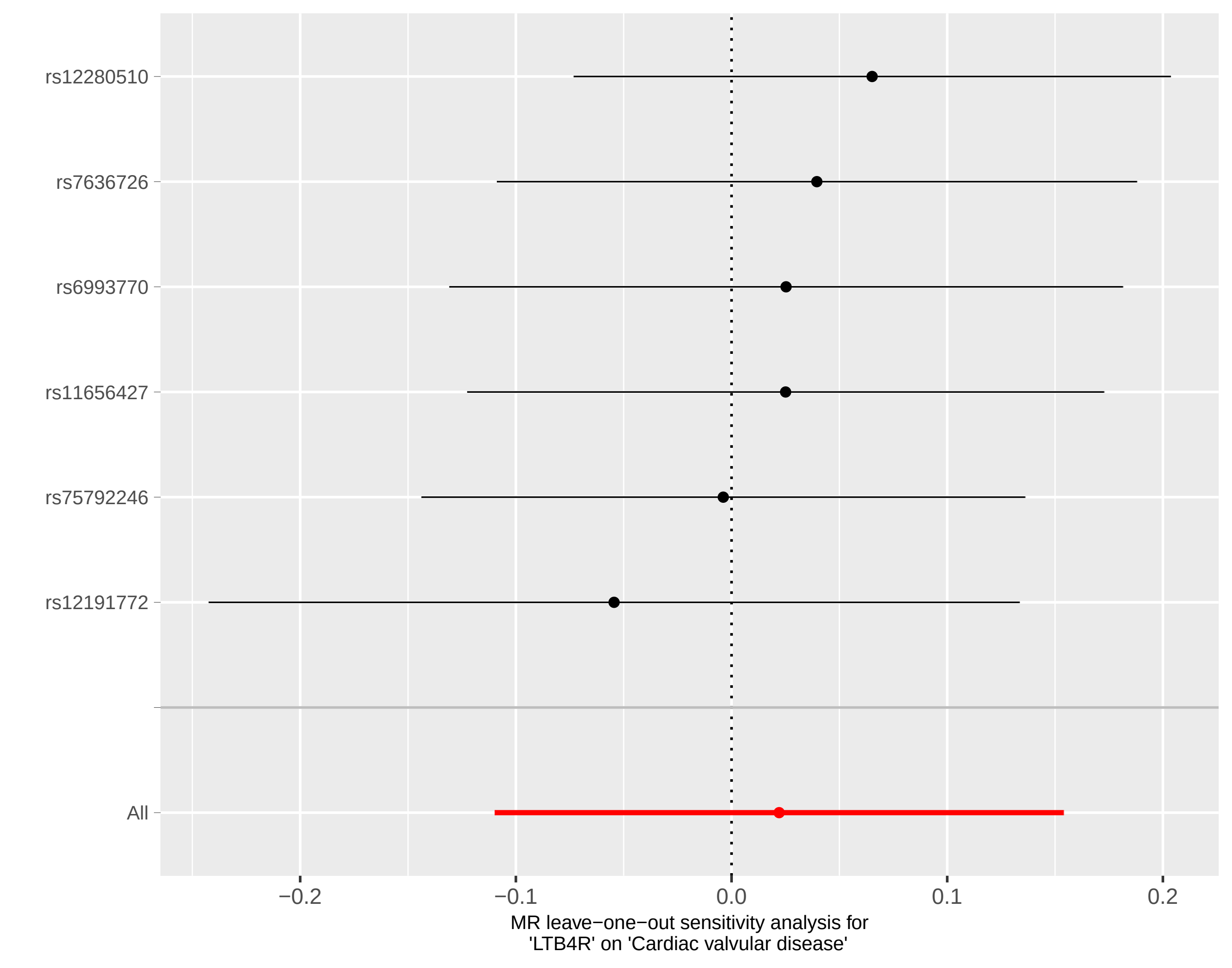
rs12280510
rs7636726
rs6993770
rs11656427
rs75792246
rs12191772
All
−0.2
−0.1
0.0
0.1
0.2
MR leave−one−out sensitivity analysis for
'LTB4R' on 'Cardiac valvular disease'

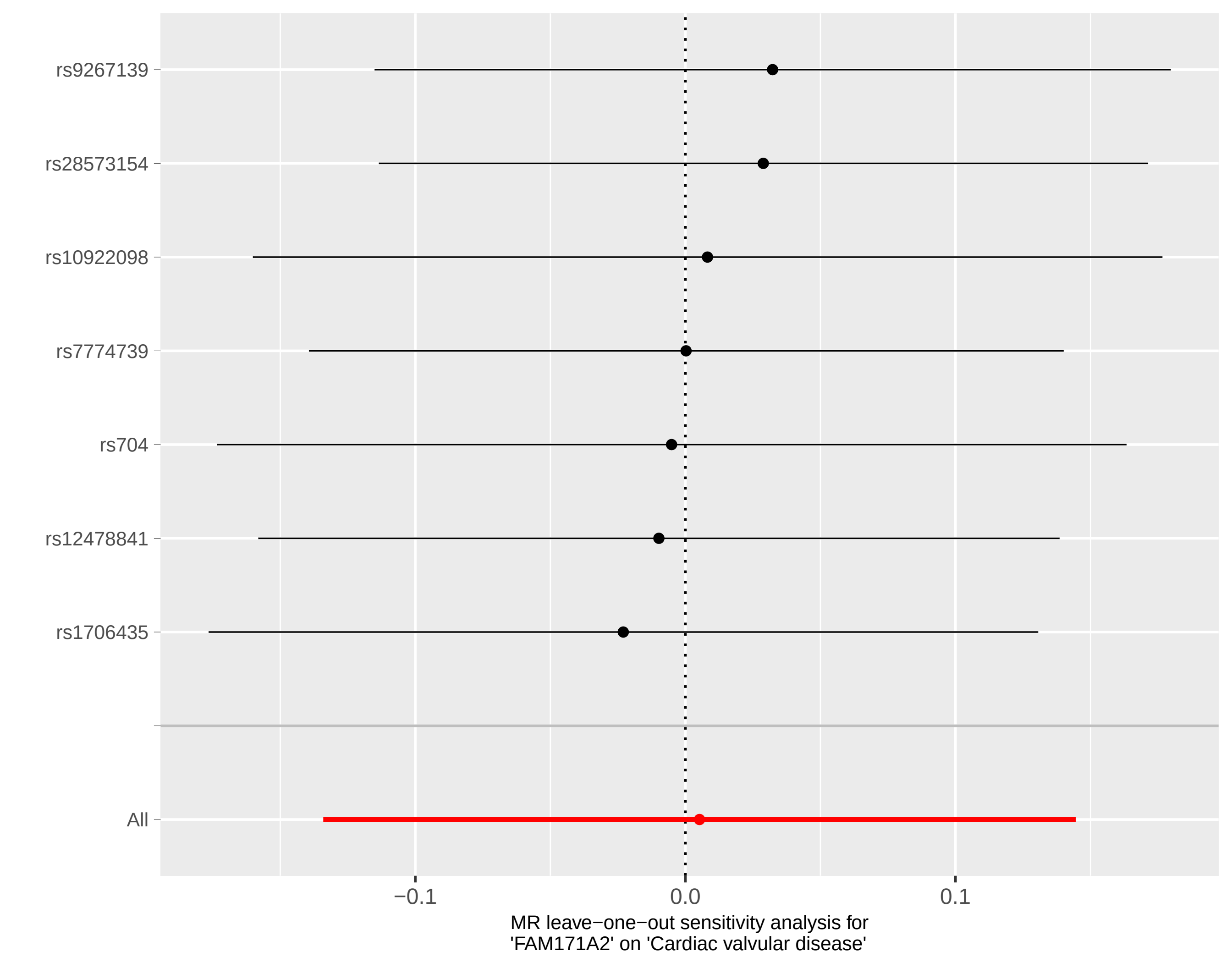

rs9267139
rs28573154
rs10922098
rs7774739
rs704
rs12478841
rs1706435
All
−0.1
0.0
0.1
MR leave−one−out sensitivity analysis for
'FAM171A2' on 'Cardiac valvular disease'

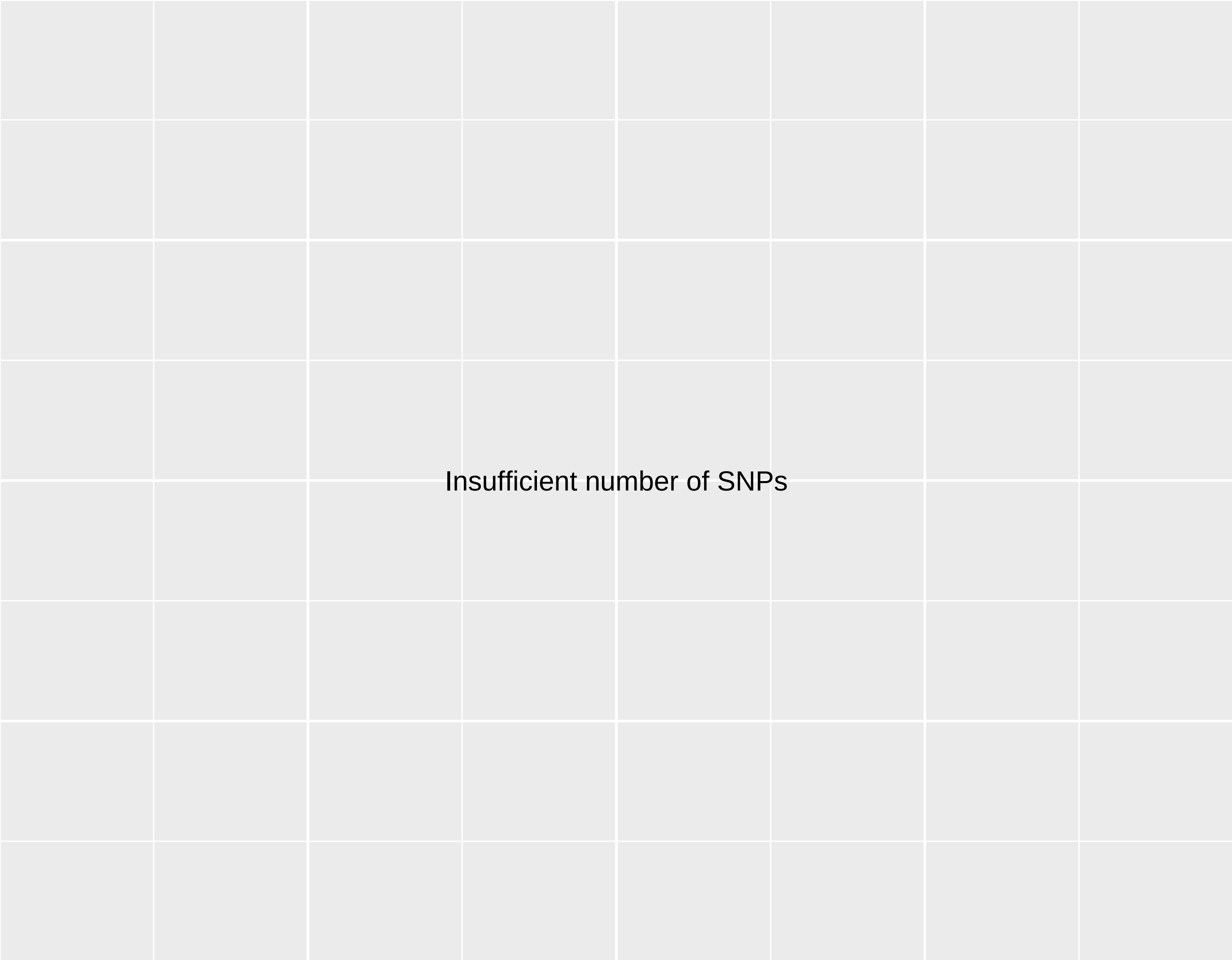
Insufficient number of SNPs

Insufficient number of SNPs

Insufficient number of SNPs

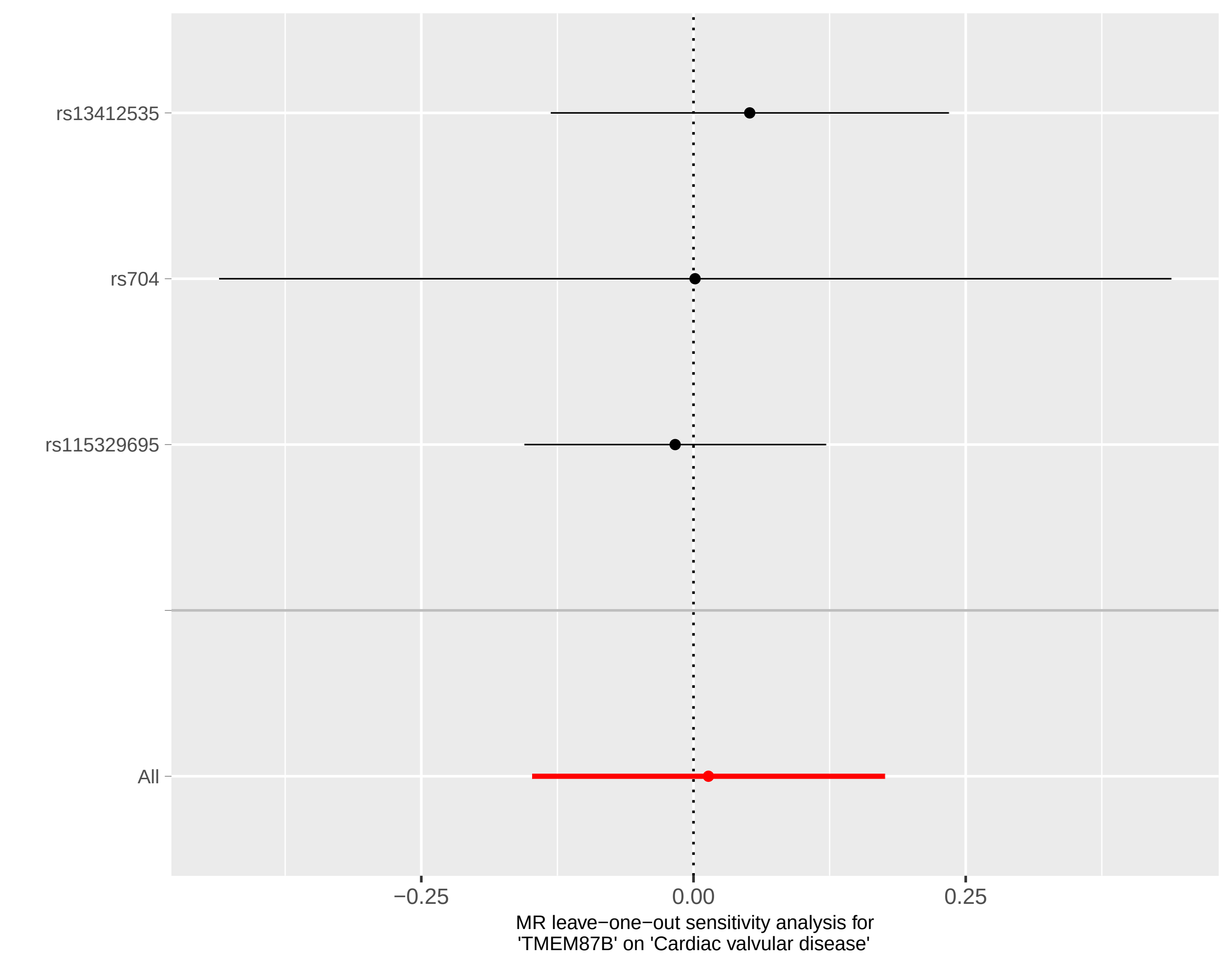
rs13412535
rs704
rs115329695
All
−0.25
0.00
0.25
MR leave−one−out sensitivity analysis for
'TMEM87B' on 'Cardiac valvular disease'

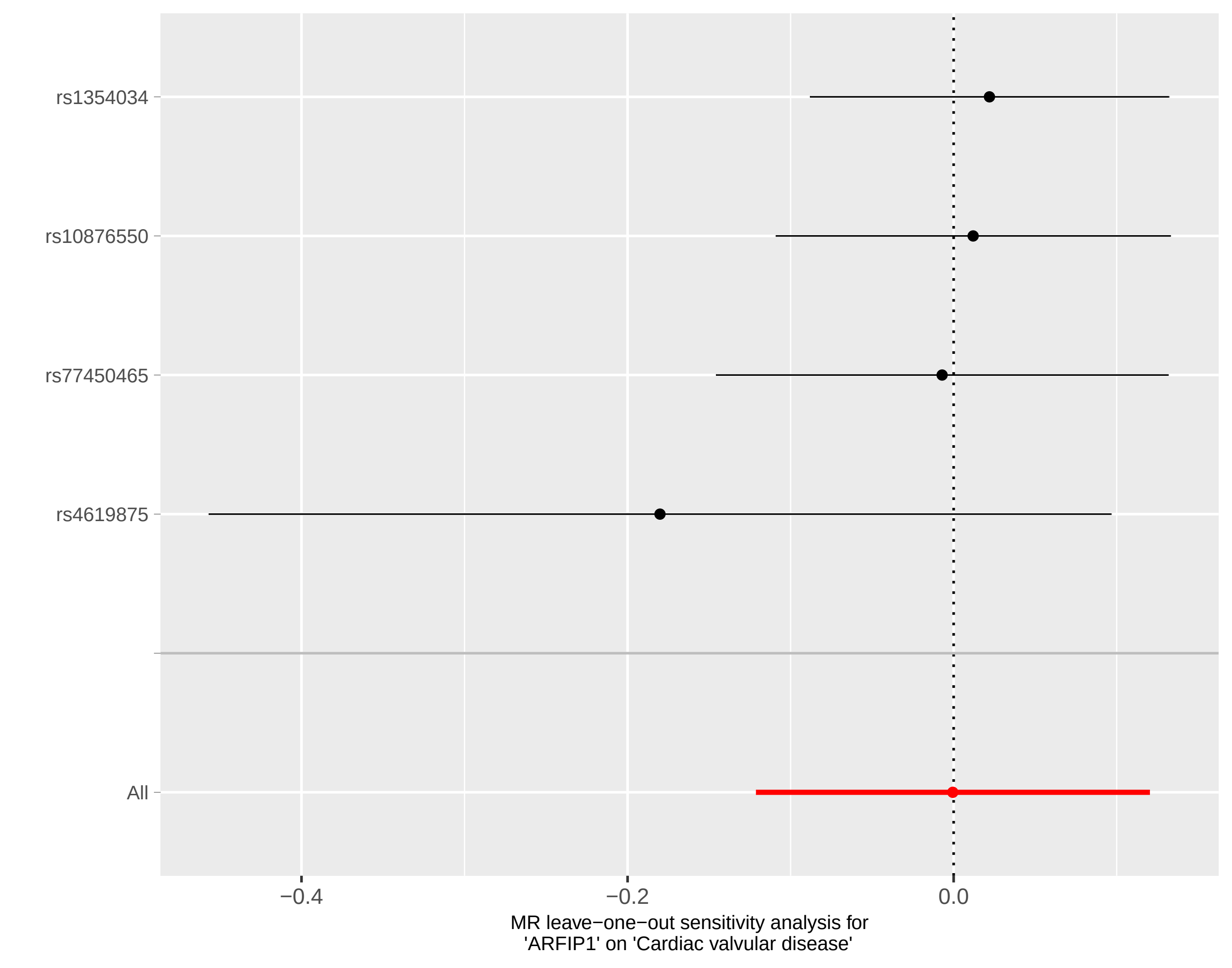

rs1354034
rs10876550
rs77450465
rs4619875
All
−0.4
−0.2
0.0
MR leave−one−out sensitivity analysis for
'ARFIP1' on 'Cardiac valvular disease'

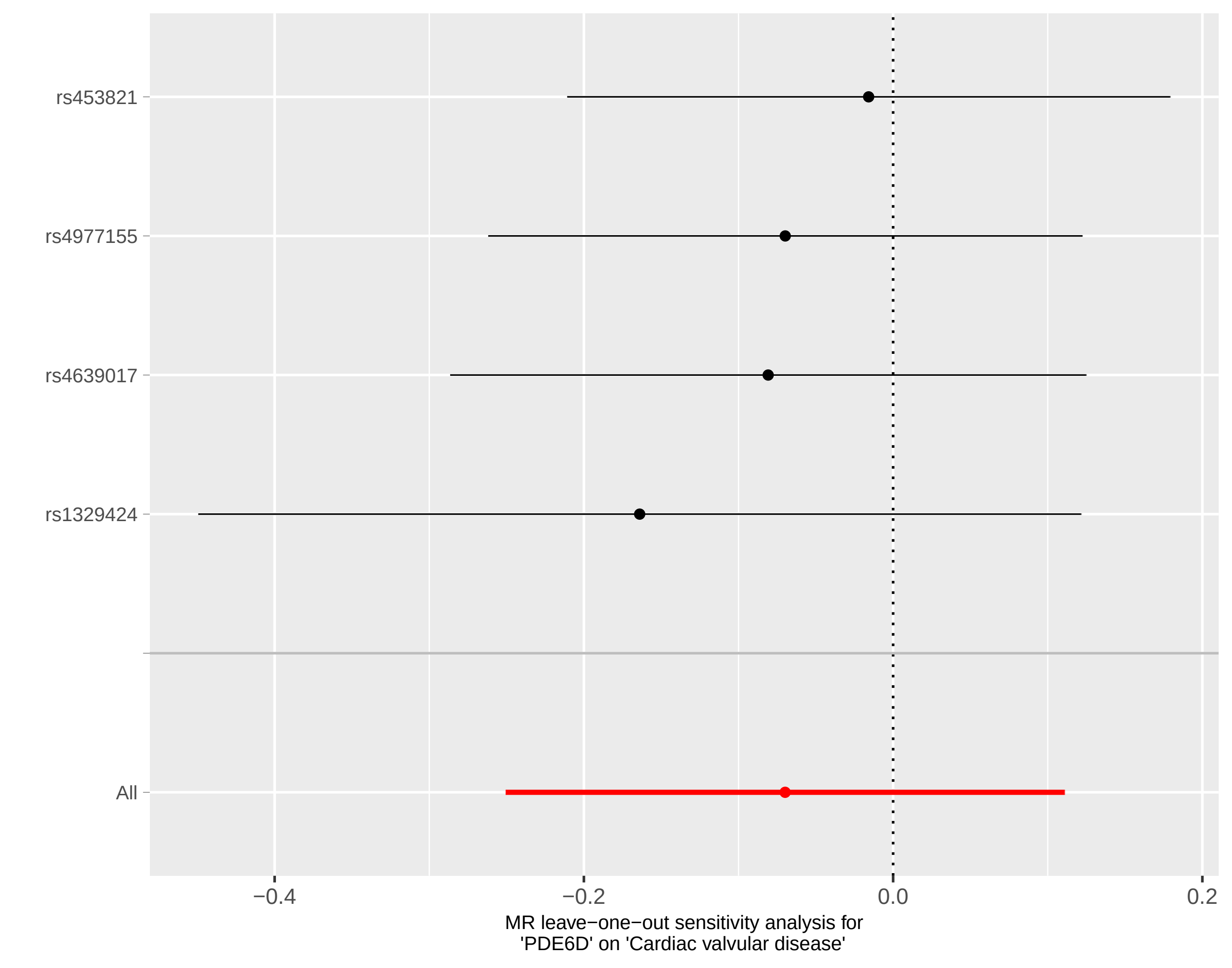
rs453821
rs4977155
rs4639017
rs1329424
All
−0.4
−0.2
0.0
0.2
MR leave−one−out sensitivity analysis for
'PDE6D' on 'Cardiac valvular disease'

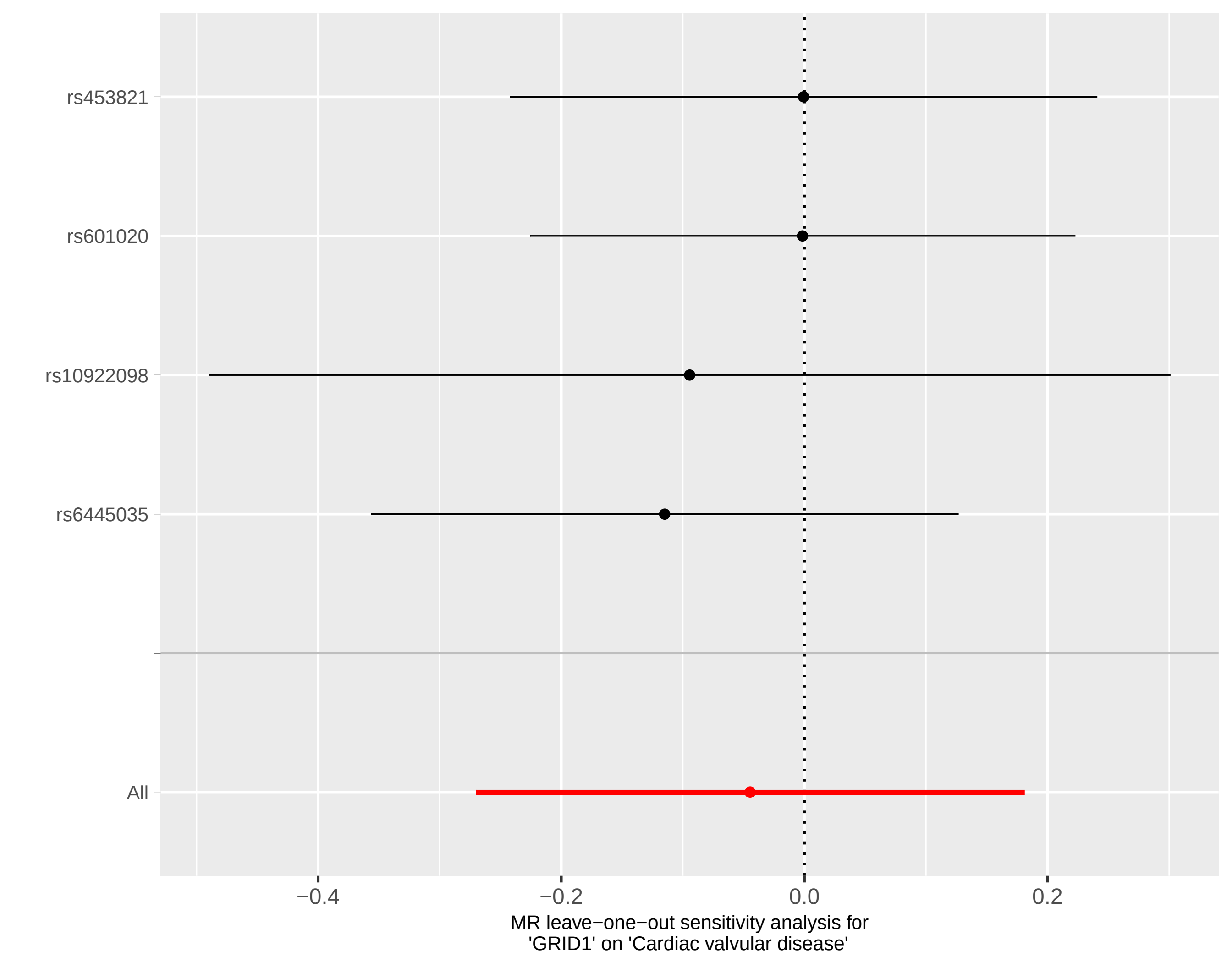

rs453821
rs601020
rs10922098
rs6445035
All
−0.4
−0.2
0.0
0.2
MR leave−one−out sensitivity analysis for
'GRID1' on 'Cardiac valvular disease'

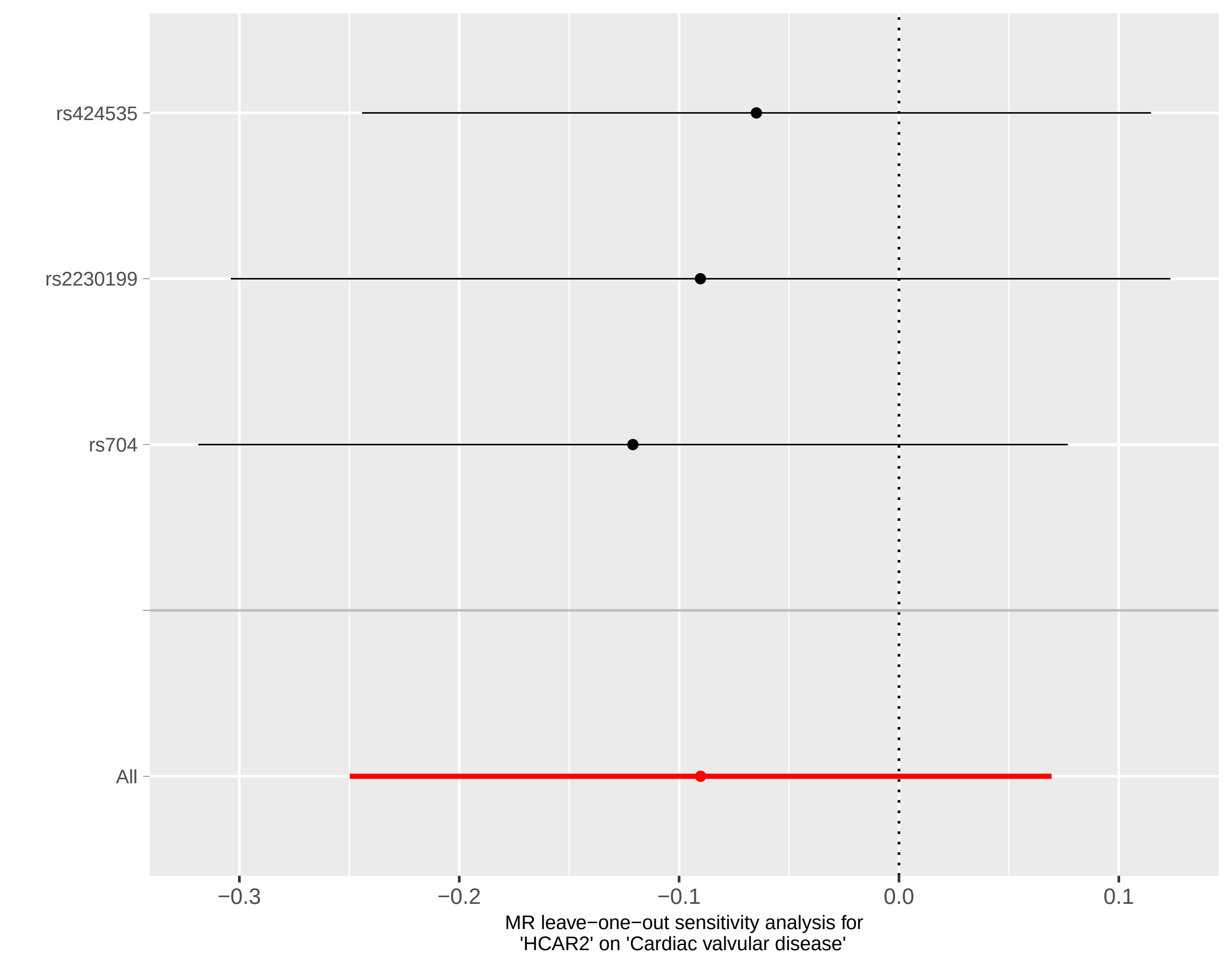
rs424535
rs2230199
rs704
All
−0.3
−0.2
−0.1
0.0
0.1
MR leave−one−out sensitivity analysis for
'HCAR2' on 'Cardiac valvular disease'

Insufficient number of SNPs

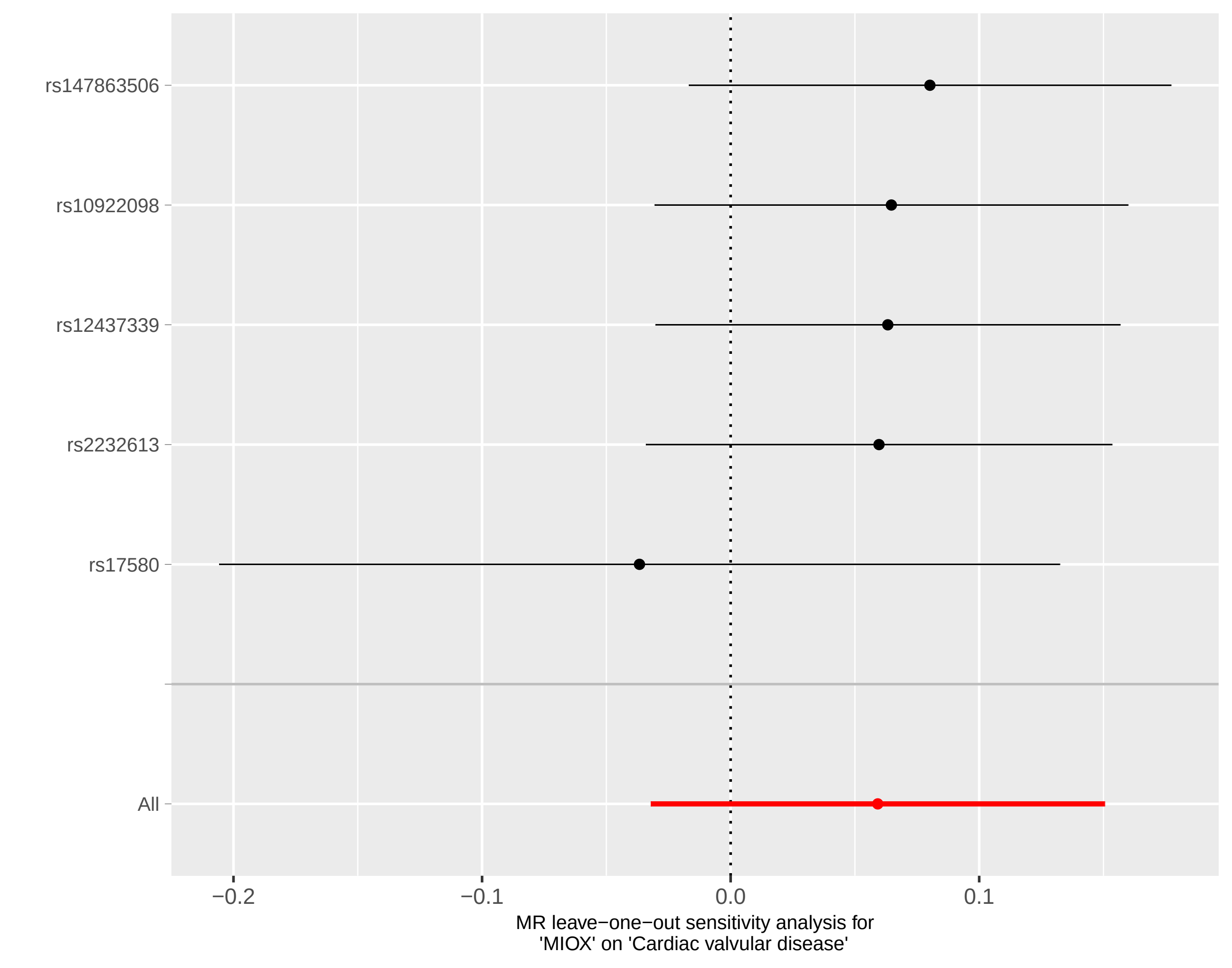

rs147863506
rs10922098
rs12437339
rs2232613
rs17580
All
−0.2
−0.1
0.0
0.1
MR leave−one−out sensitivity analysis for
'MIOX' on 'Cardiac valvular disease'

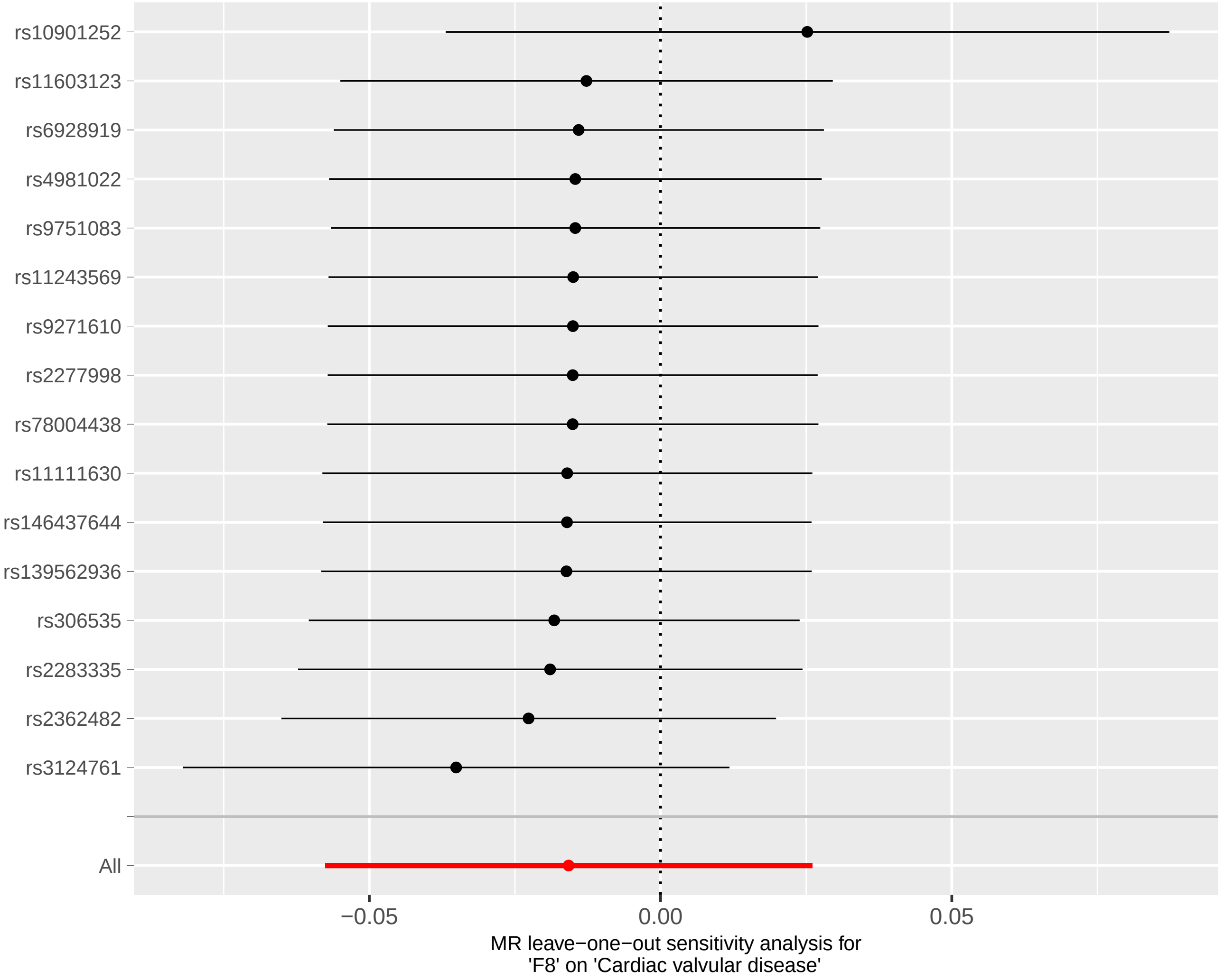

rs10901252
rs11603123
rs6928919
rs4981022
rs9751083
rs11243569
rs9271610
rs2277998
rs78004438
rs11111630
rs146437644
rs139562936
rs306535
rs2283335
rs2362482
rs3124761
All
−0.05
0.00
0.05
MR leave−one−out sensitivity analysis for
'F8' on 'Cardiac valvular disease'

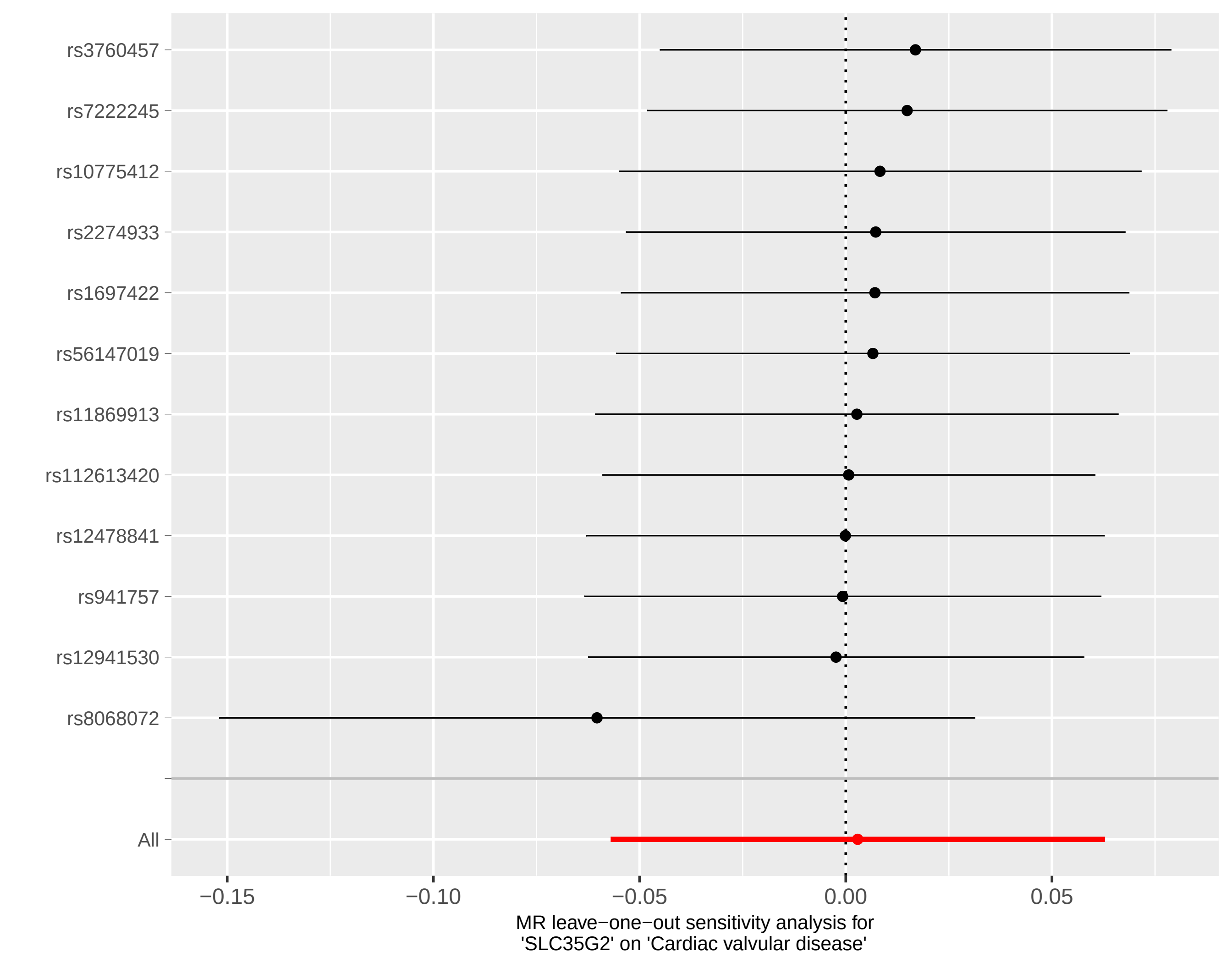

rs3760457
rs7222245
rs10775412
rs2274933
rs1697422
rs56147019
rs11869913
rs112613420
rs12478841
rs941757
rs12941530
rs8068072
All
−0.15
−0.10
−0.05
0.00
0.05
MR leave−one−out sensitivity analysis for
'SLC35G2' on 'Cardiac valvular disease'

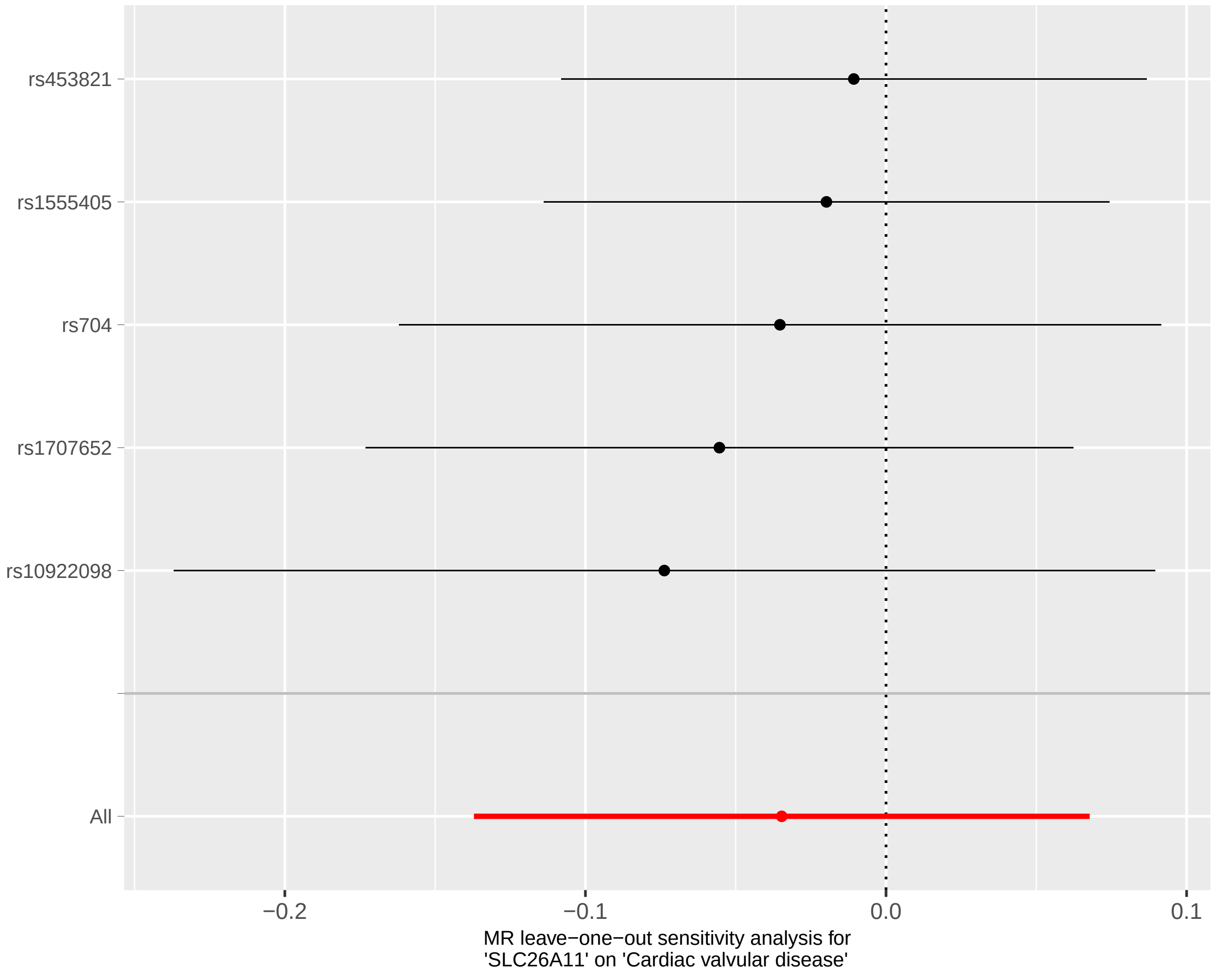
rs453821
rs1555405
rs704
rs1707652
rs10922098
All
−0.2
−0.1
0.0
0.1
MR leave−one−out sensitivity analysis for
'SLC26A11' on 'Cardiac valvular disease'

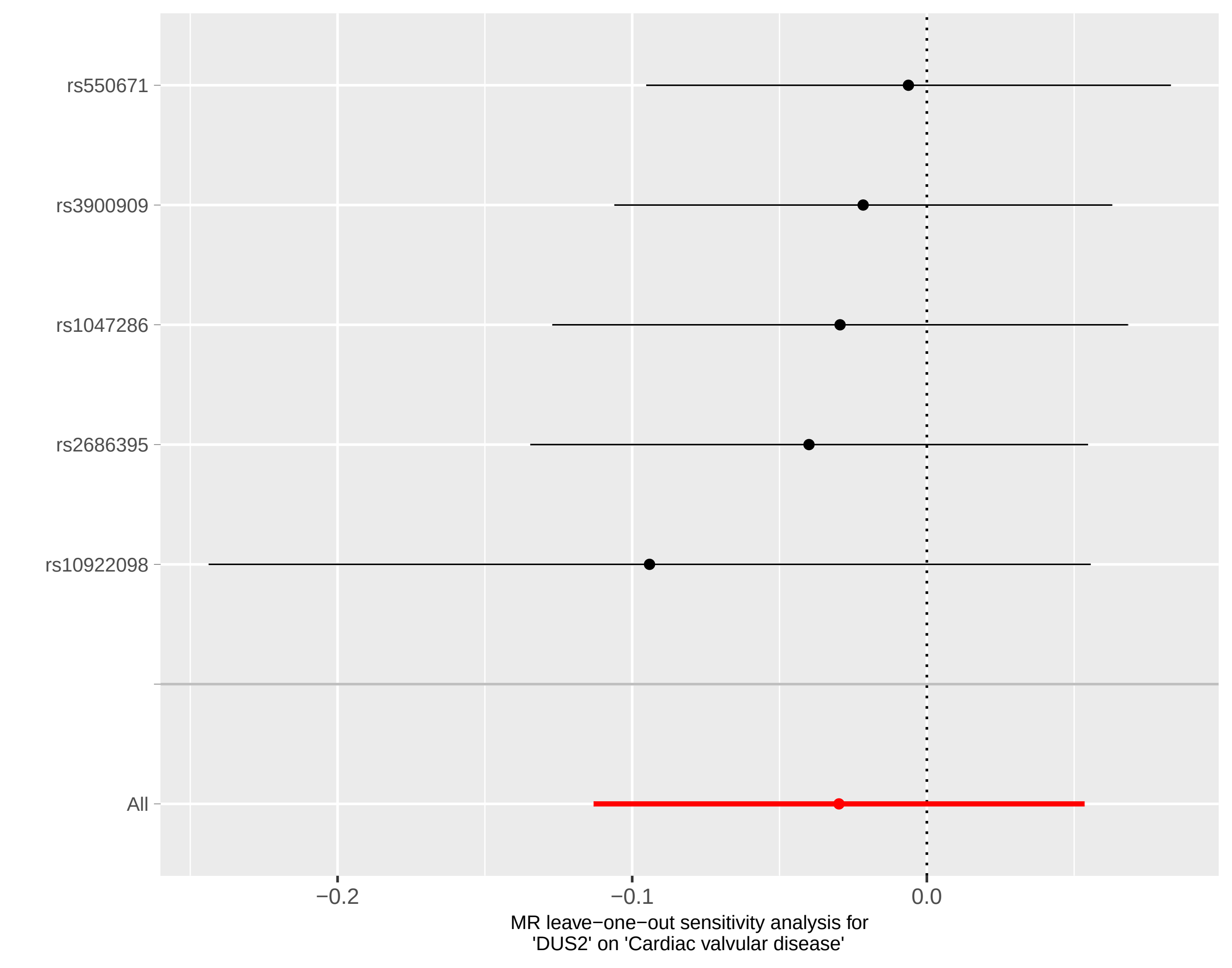

rs550671
rs3900909
rs1047286
rs2686395
rs10922098
All
−0.2
−0.1
0.0
MR leave−one−out sensitivity analysis for
'DUS2' on 'Cardiac valvular disease'

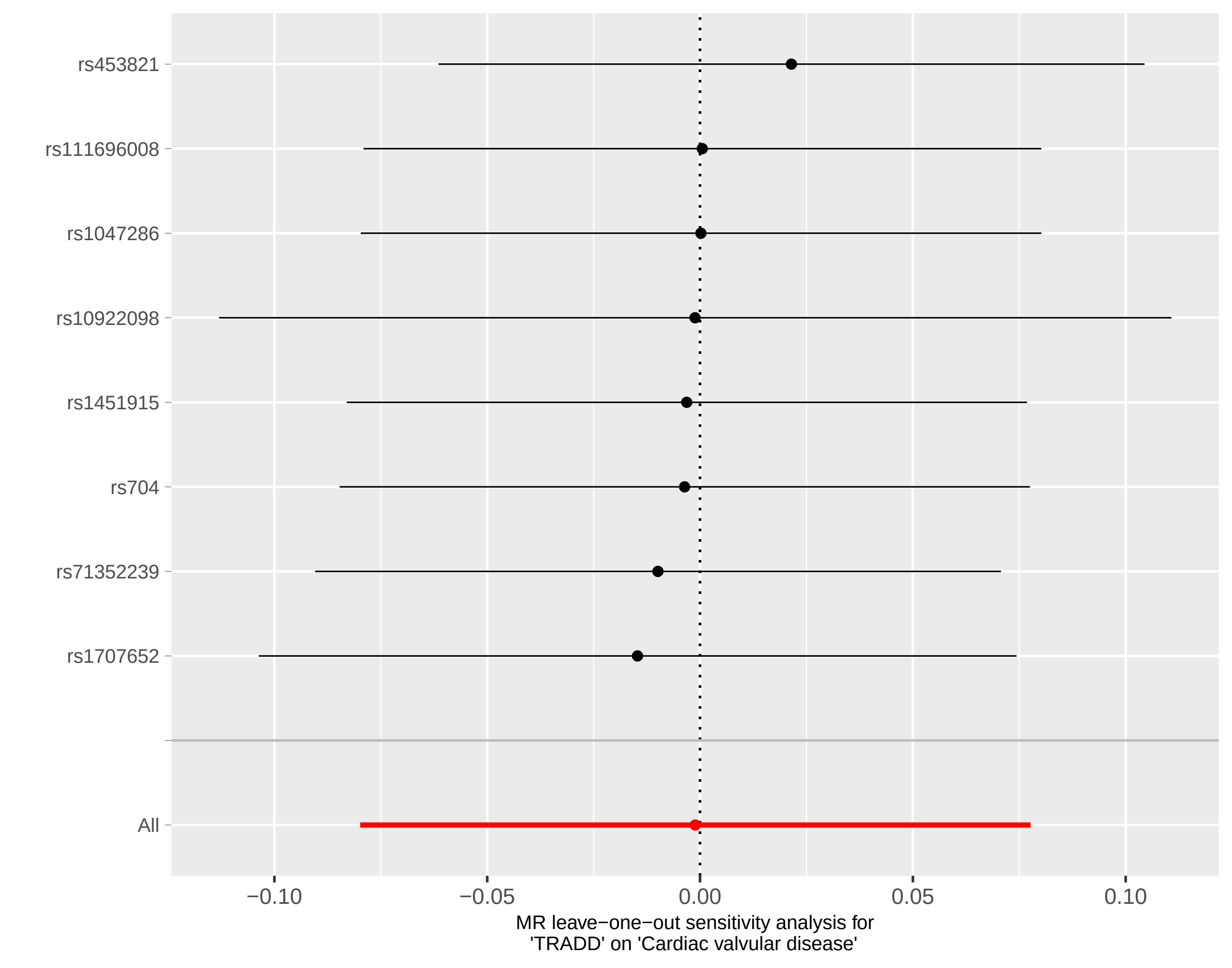

rs453821
rs111696008
rs1047286
rs10922098
rs1451915
rs704
rs71352239
rs1707652
All
−0.10
−0.05
0.00
0.05
0.10
MR leave−one−out sensitivity analysis for
'TRADD' on 'Cardiac valvular disease'

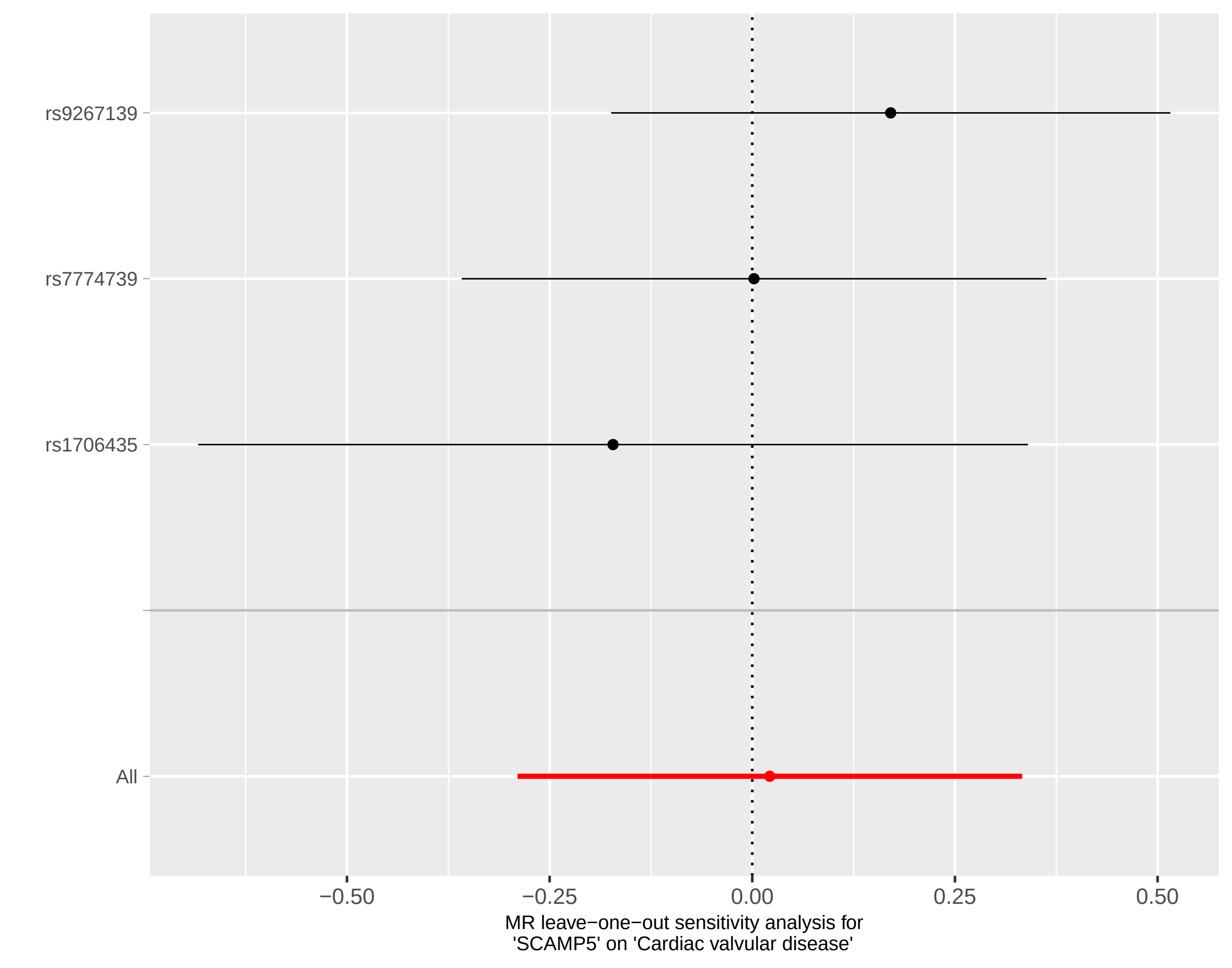
rs9267139
rs7774739
rs1706435
All
−0.50
−0.25
0.00
0.25
0.50
MR leave−one−out sensitivity analysis for
'SCAMP5' on 'Cardiac valvular disease'

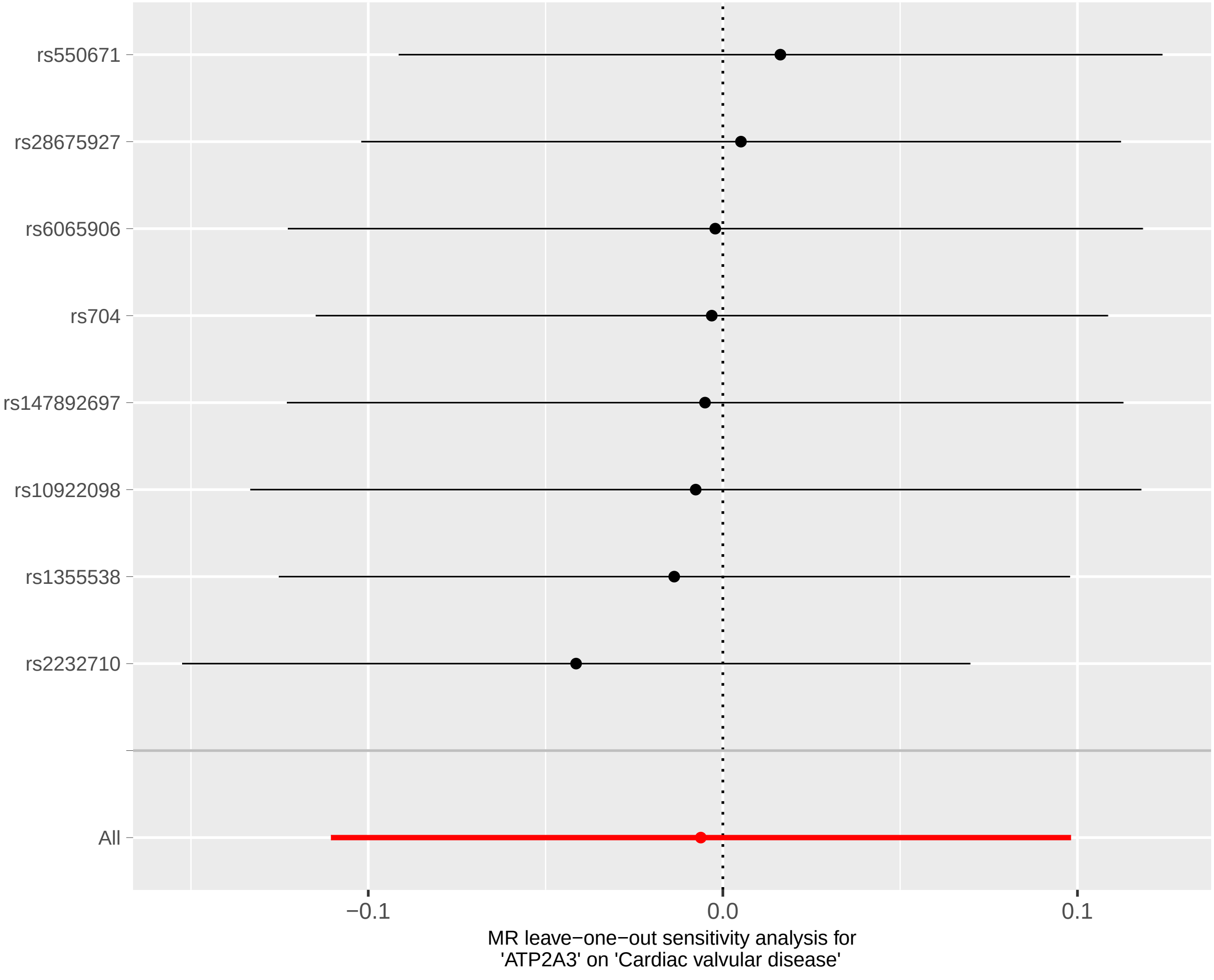

rs550671
rs28675927
rs6065906
rs704
rs147892697
rs10922098
rs1355538
rs2232710
All
−0.1
0.0
0.1
MR leave−one−out sensitivity analysis for
'ATP2A3' on 'Cardiac valvular disease'

Insufficient number of SNPs

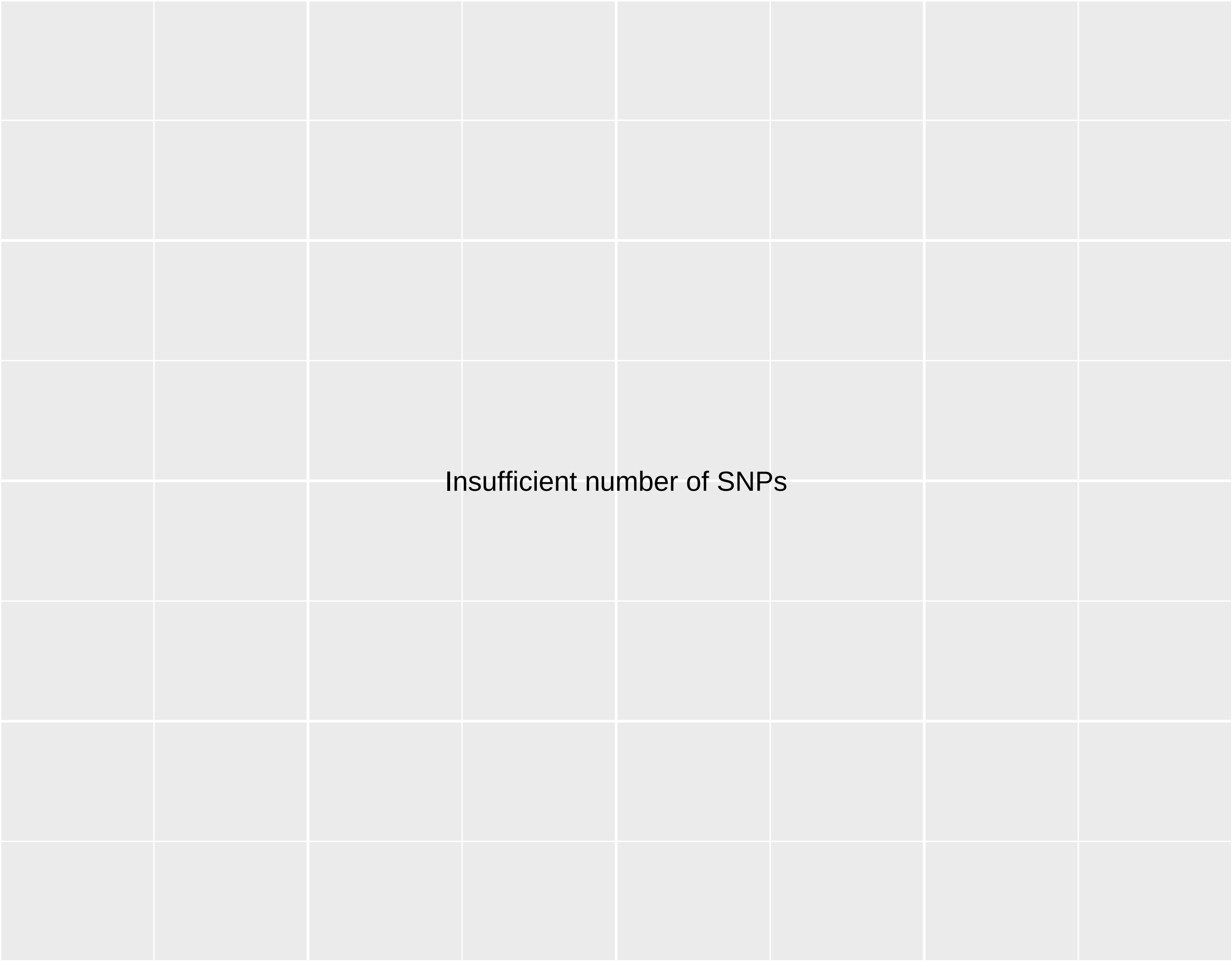
Insufficient number of SNPs

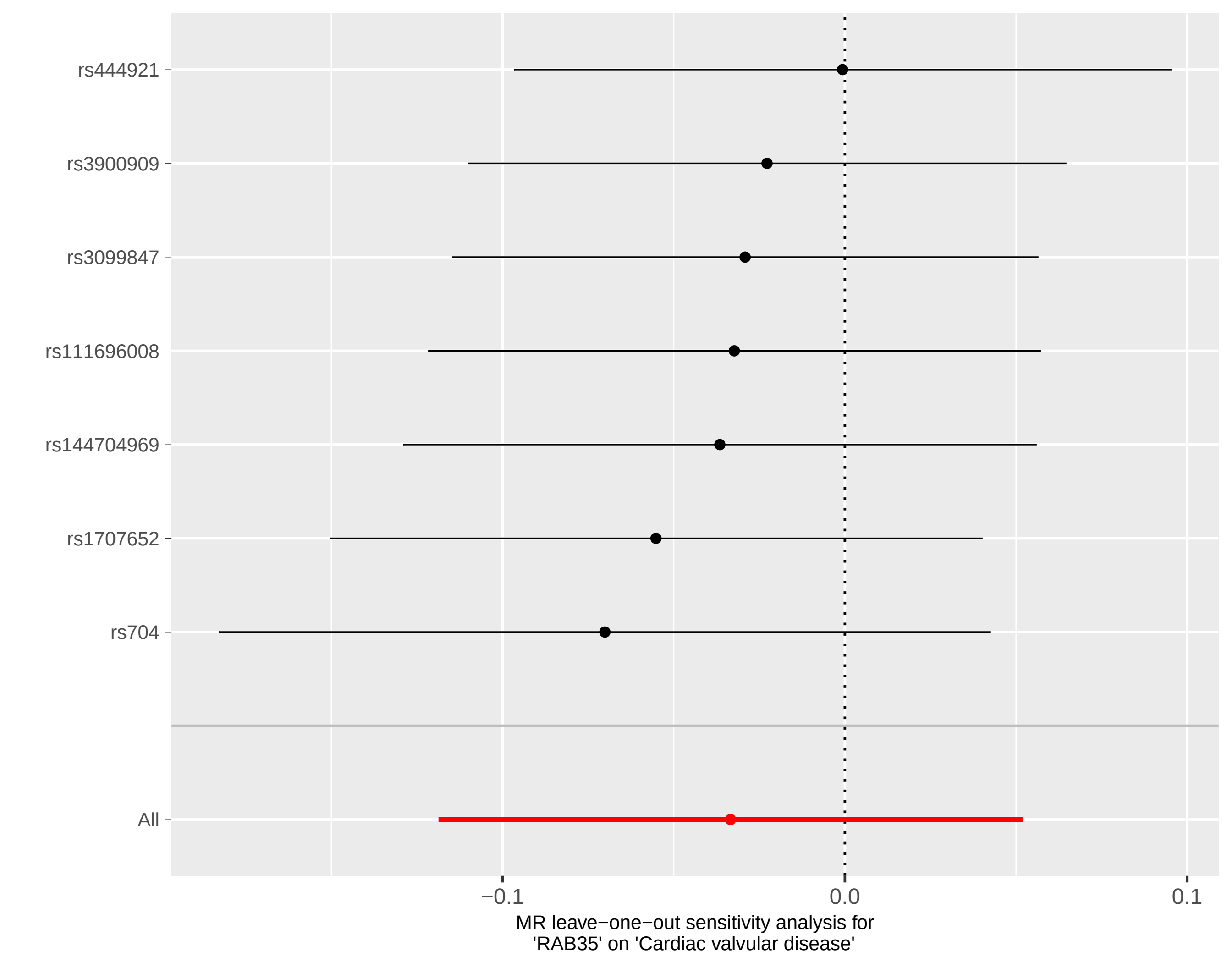

rs444921
rs3900909
rs3099847
rs111696008
rs144704969
rs1707652
rs704
All
−0.1
0.0
0.1
MR leave−one−out sensitivity analysis for
'RAB35' on 'Cardiac valvular disease'

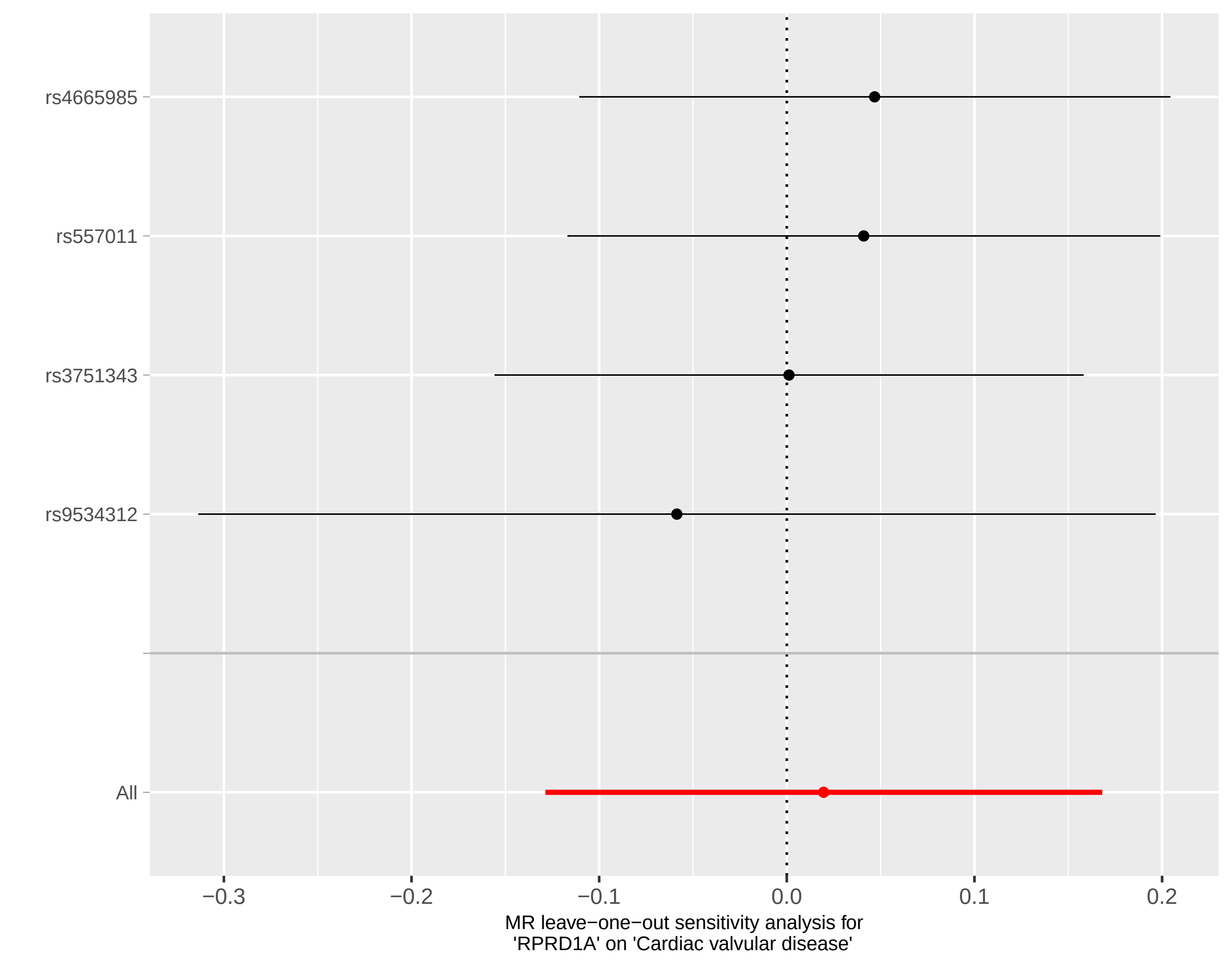
rs4665985
rs557011
rs3751343
rs9534312
All
−0.3
−0.2
−0.1
0.0
0.1
0.2
MR leave−one−out sensitivity analysis for
'RPRD1A' on 'Cardiac valvular disease'

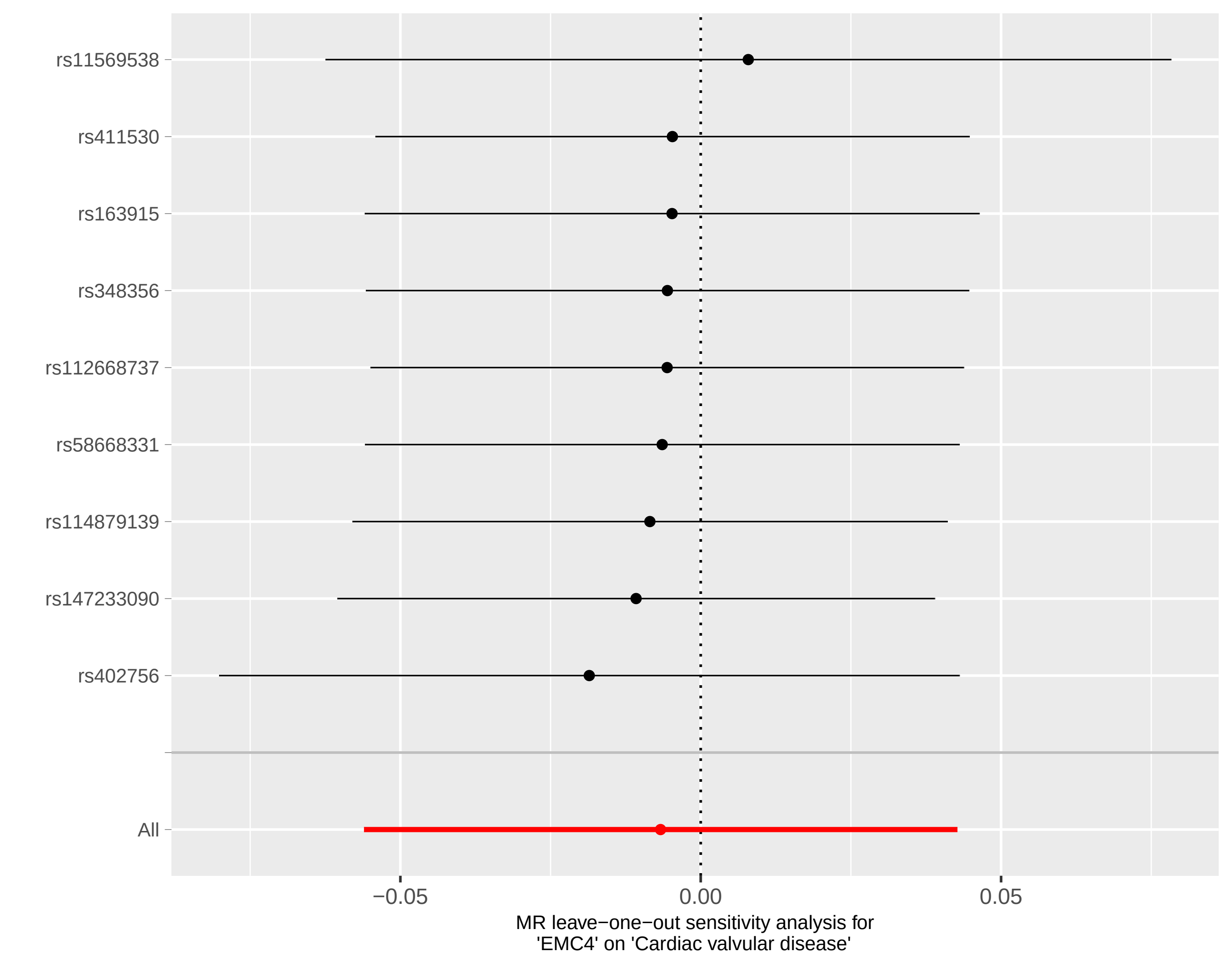
rs11569538
rs411530
rs163915
rs348356
rs112668737
rs58668331
rs114879139
rs147233090
rs402756
All
−0.05
0.00
0.05
MR leave−one−out sensitivity analysis for
'EMC4' on 'Cardiac valvular disease'

Insufficient number of SNPs

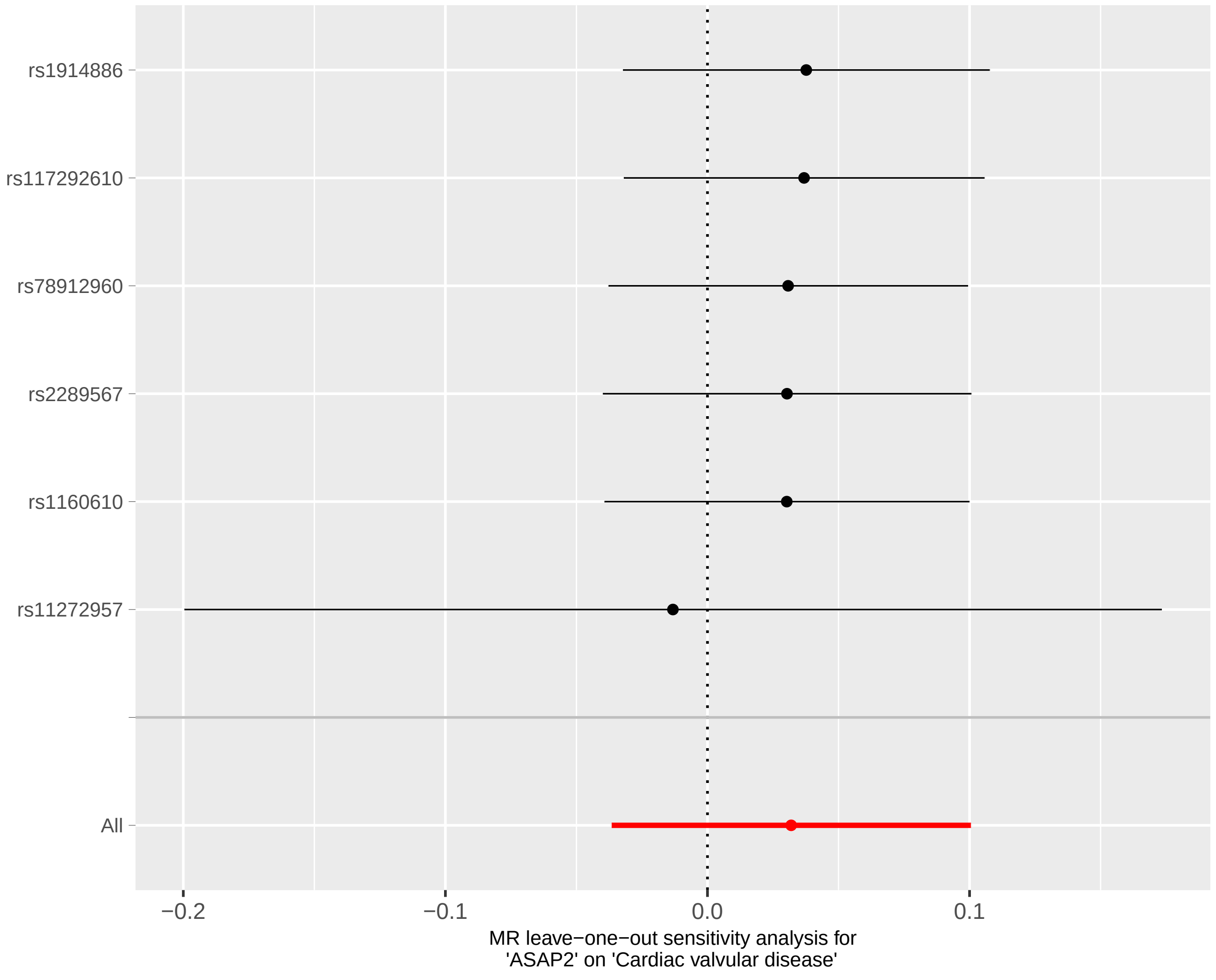

rs1914886
rs117292610
rs78912960
rs2289567
rs1160610
rs11272957
All
−0.2
−0.1
0.0
0.1
MR leave−one−out sensitivity analysis for
'ASAP2' on 'Cardiac valvular disease'

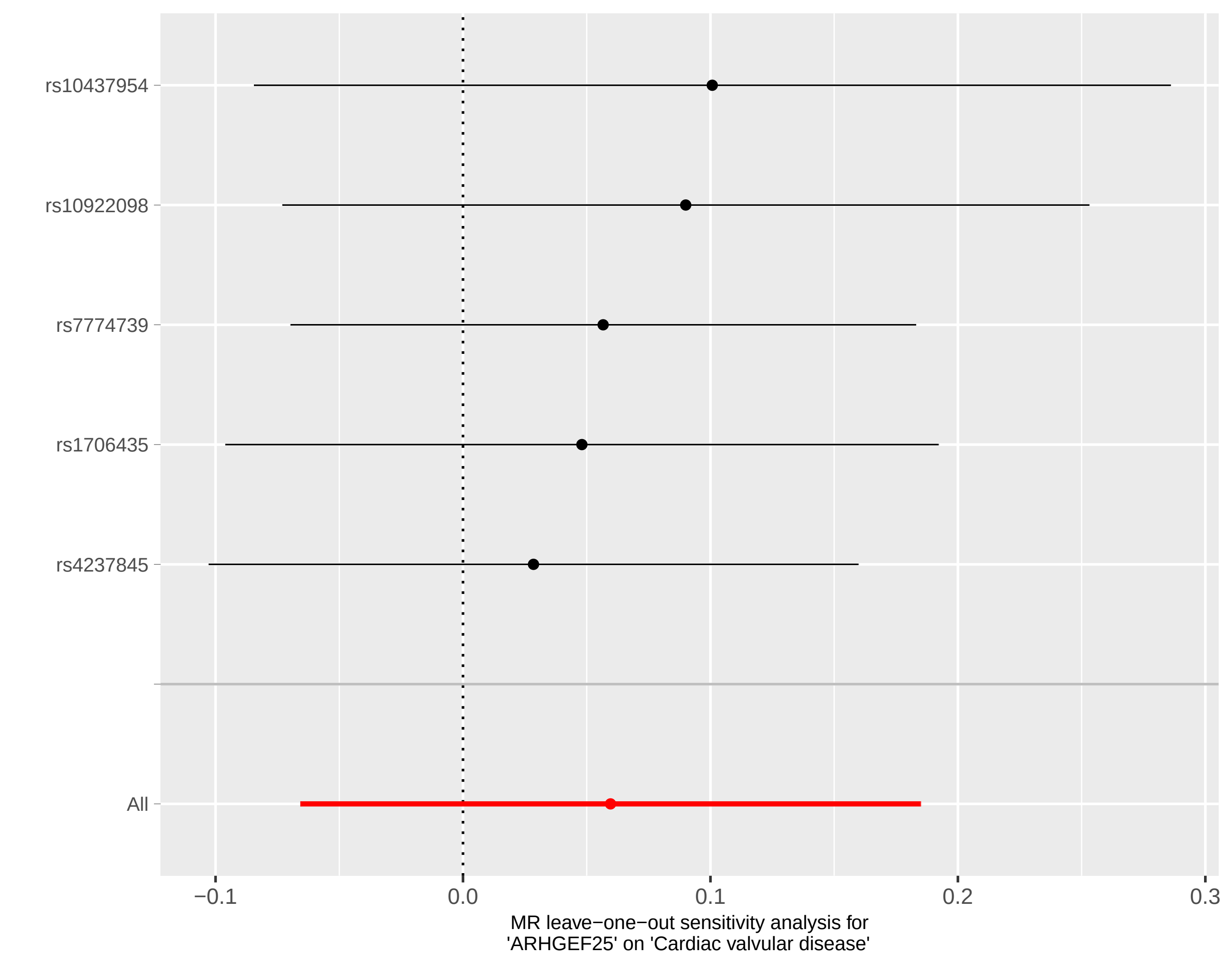

rs10437954
rs10922098
rs7774739
rs1706435
rs4237845
All
−0.1
0.0
0.1
0.2
0.3
MR leave−one−out sensitivity analysis for
'ARHGEF25' on 'Cardiac valvular disease'

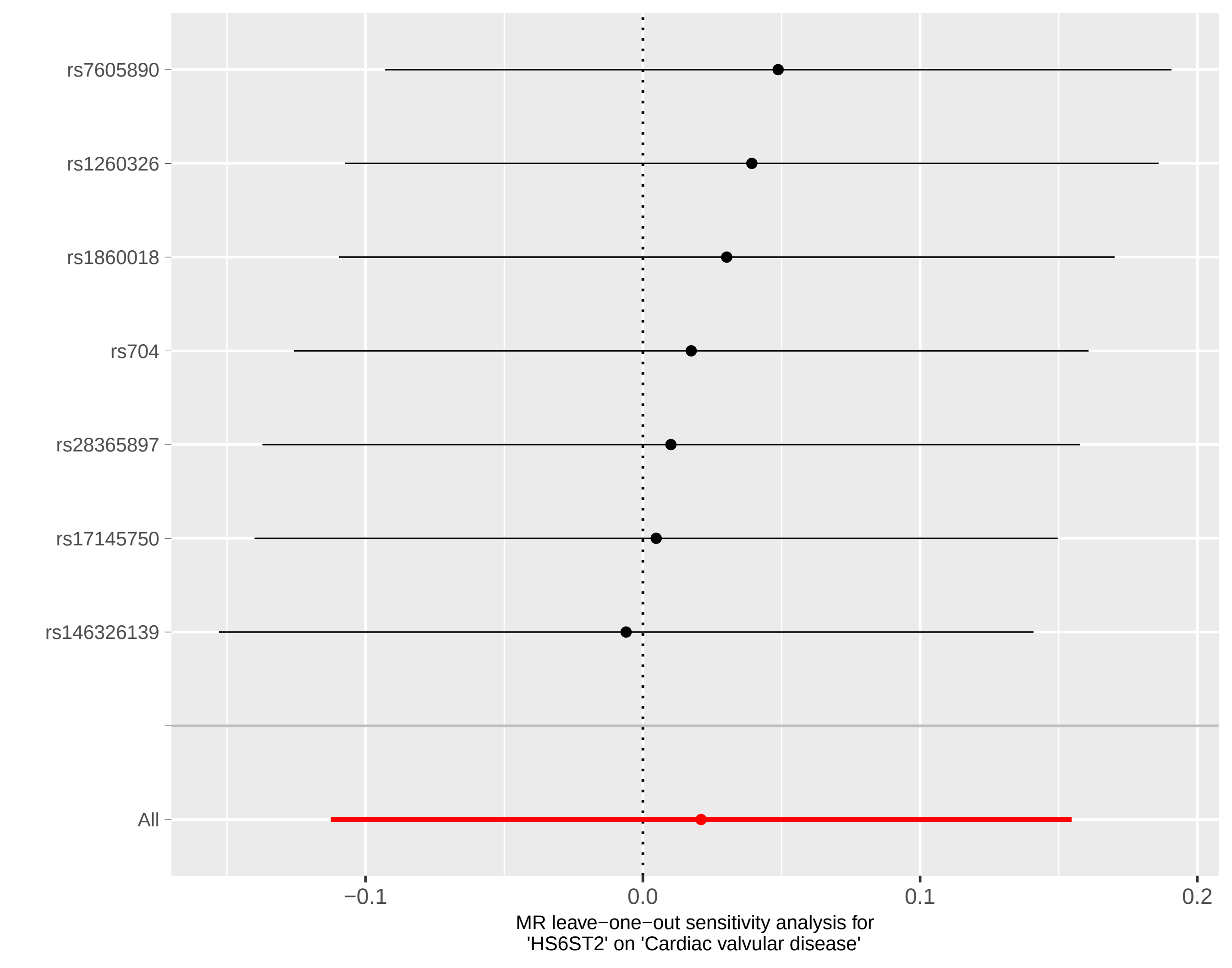
rs7605890
rs1260326
rs1860018
rs704
rs28365897
rs17145750
rs146326139
All
−0.1
0.0
0.1
0.2
MR leave−one−out sensitivity analysis for
'HS6ST2' on 'Cardiac valvular disease'

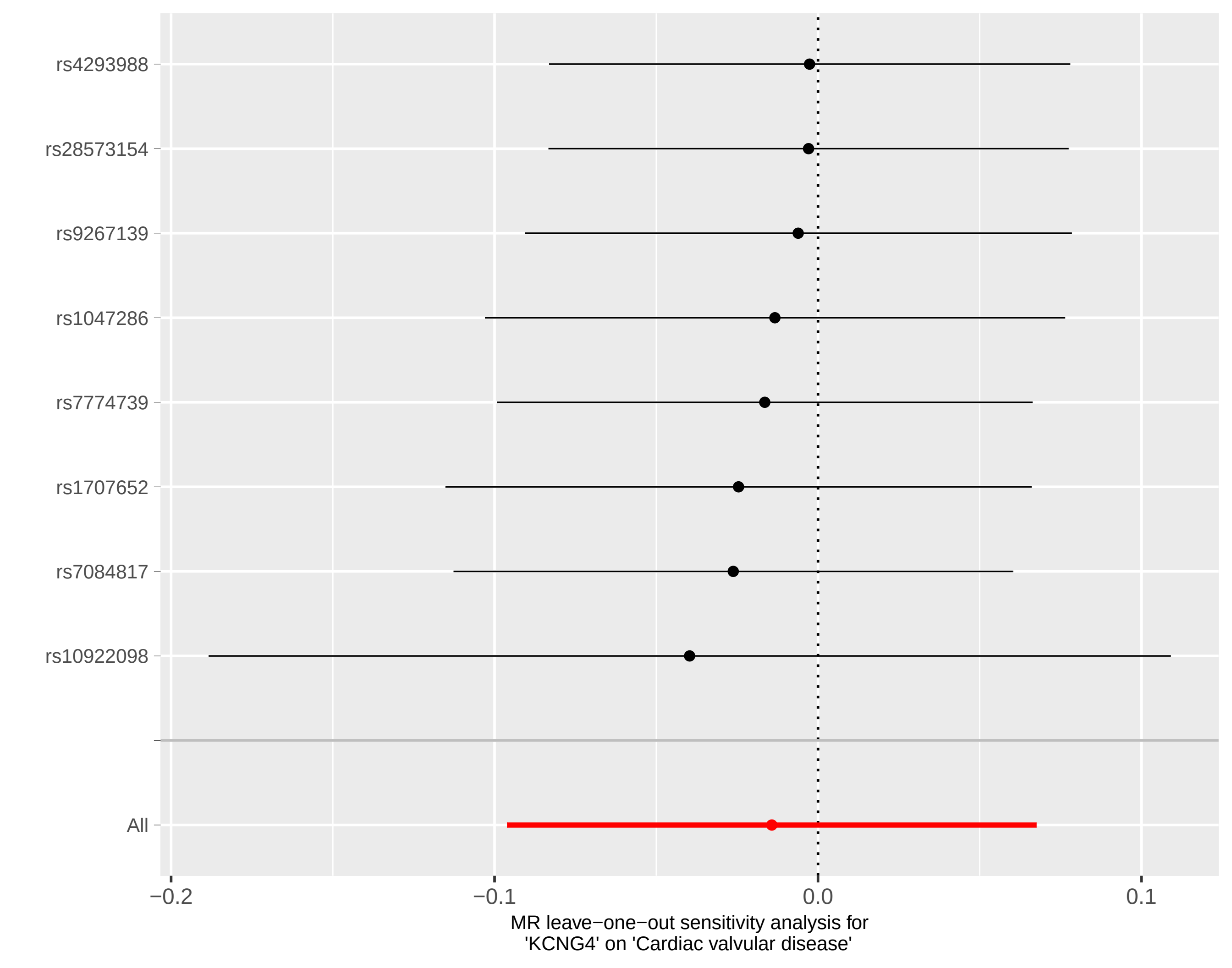
rs4293988
rs28573154
rs9267139
rs1047286
rs7774739
rs1707652
rs7084817
rs10922098
All
−0.2
−0.1
0.0
0.1
MR leave−one−out sensitivity analysis for
'KCNG4' on 'Cardiac valvular disease'

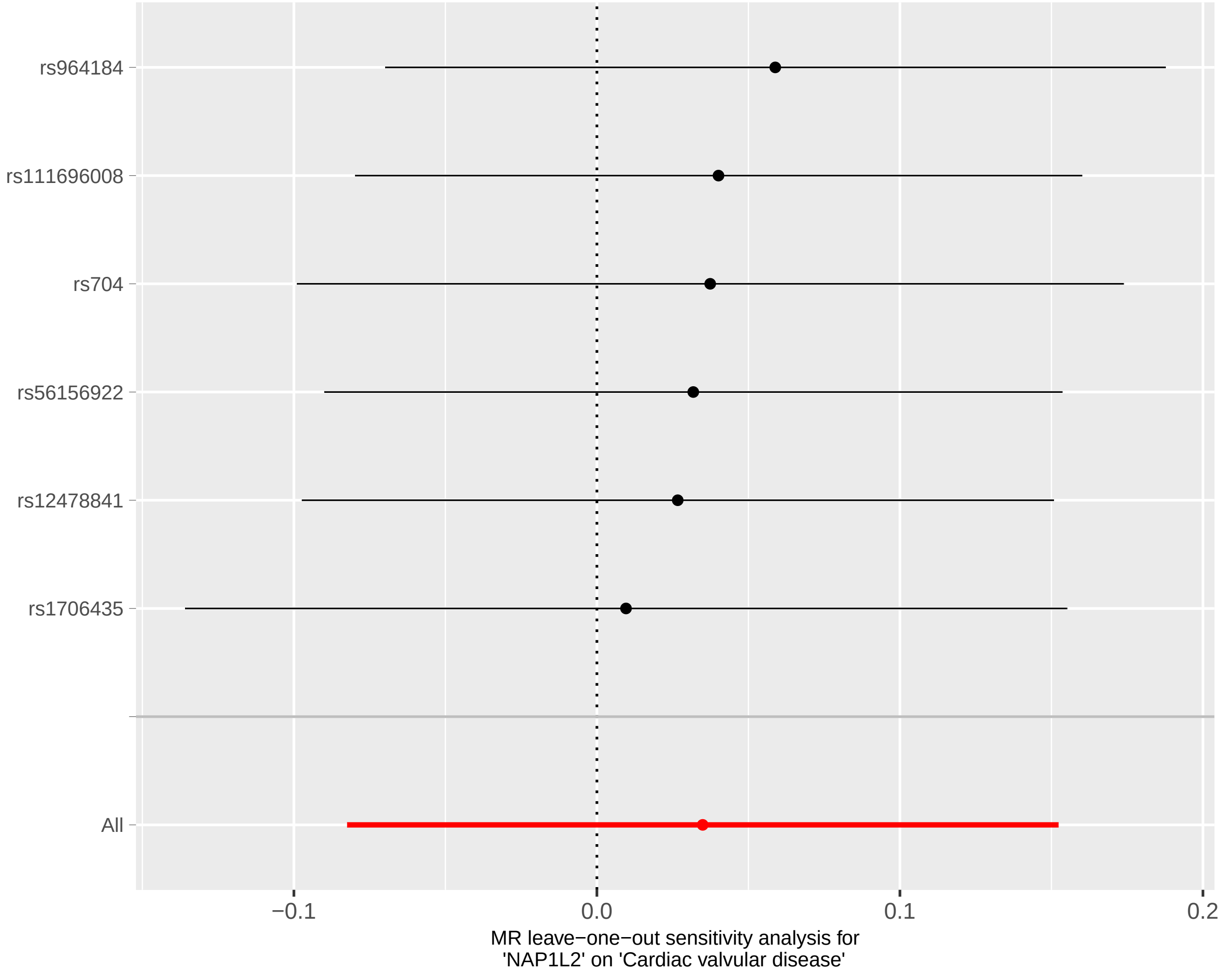
rs964184
rs111696008
rs704
rs56156922
rs12478841
rs1706435
All
−0.1
0.0
0.1
0.2
MR leave−one−out sensitivity analysis for
'NAP1L2' on 'Cardiac valvular disease'

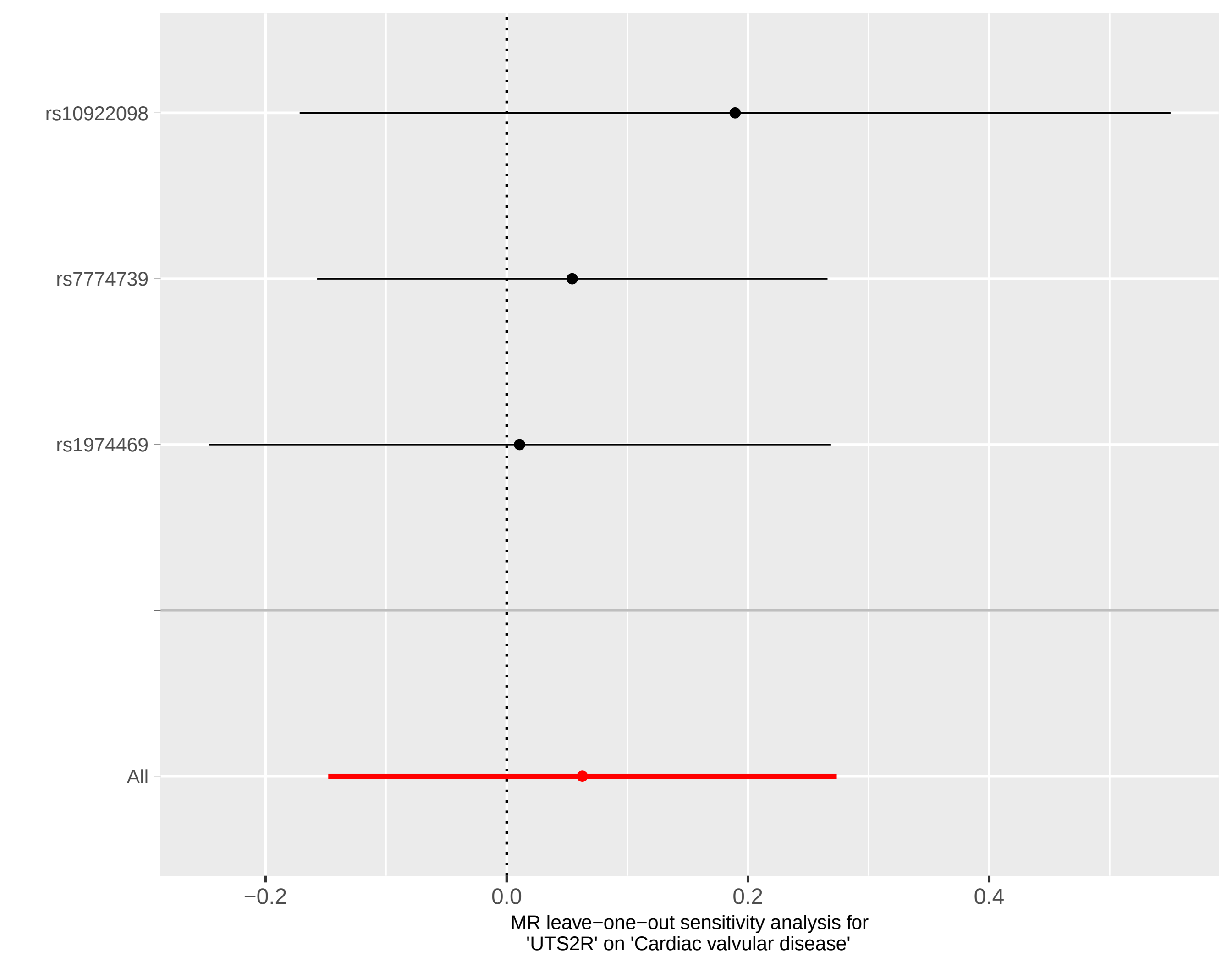
rs10922098
rs7774739
rs1974469
All
−0.2
0.0
0.2
0.4
MR leave−one−out sensitivity analysis for
'UTS2R' on 'Cardiac valvular disease'

Insufficient number of SNPs

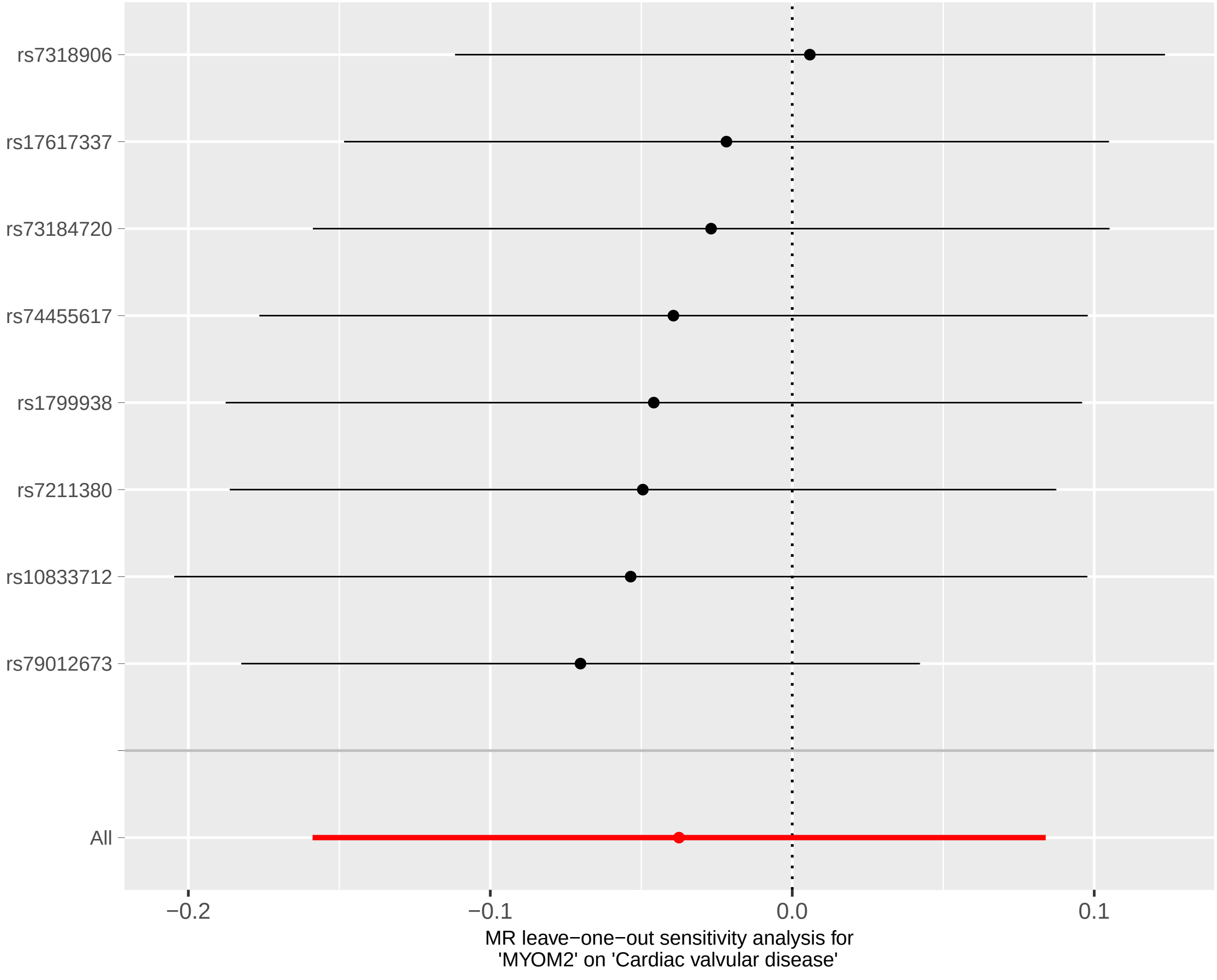

rs7318906
rs17617337
rs73184720
rs74455617
rs1799938
rs7211380
rs10833712
rs79012673
All
−0.2
−0.1
0.0
0.1
MR leave−one−out sensitivity analysis for
'MYOM2' on 'Cardiac valvular disease'

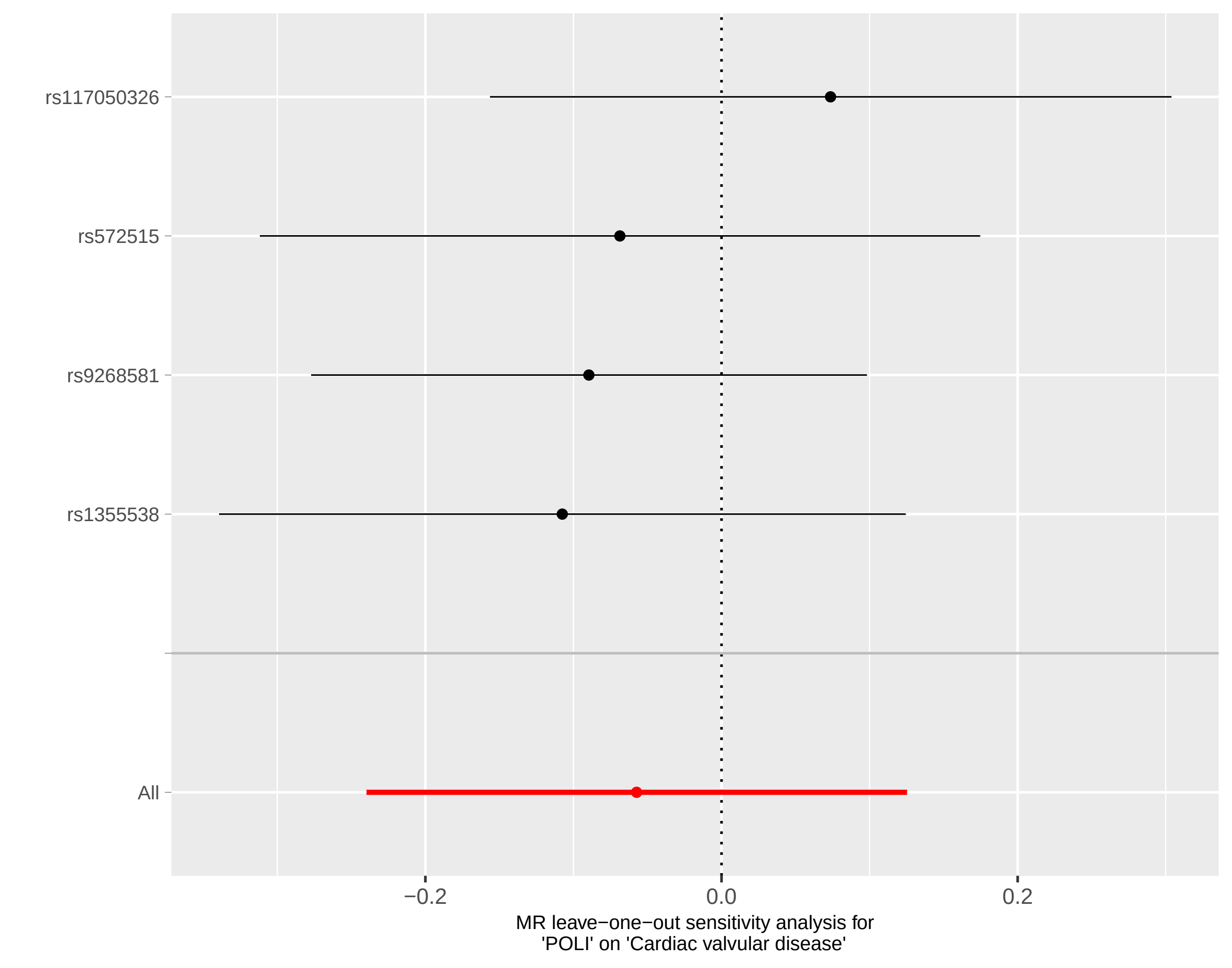
rs117050326
rs572515
rs9268581
rs1355538
All
−0.2
0.0
0.2
MR leave−one−out sensitivity analysis for
'POLI' on 'Cardiac valvular disease'

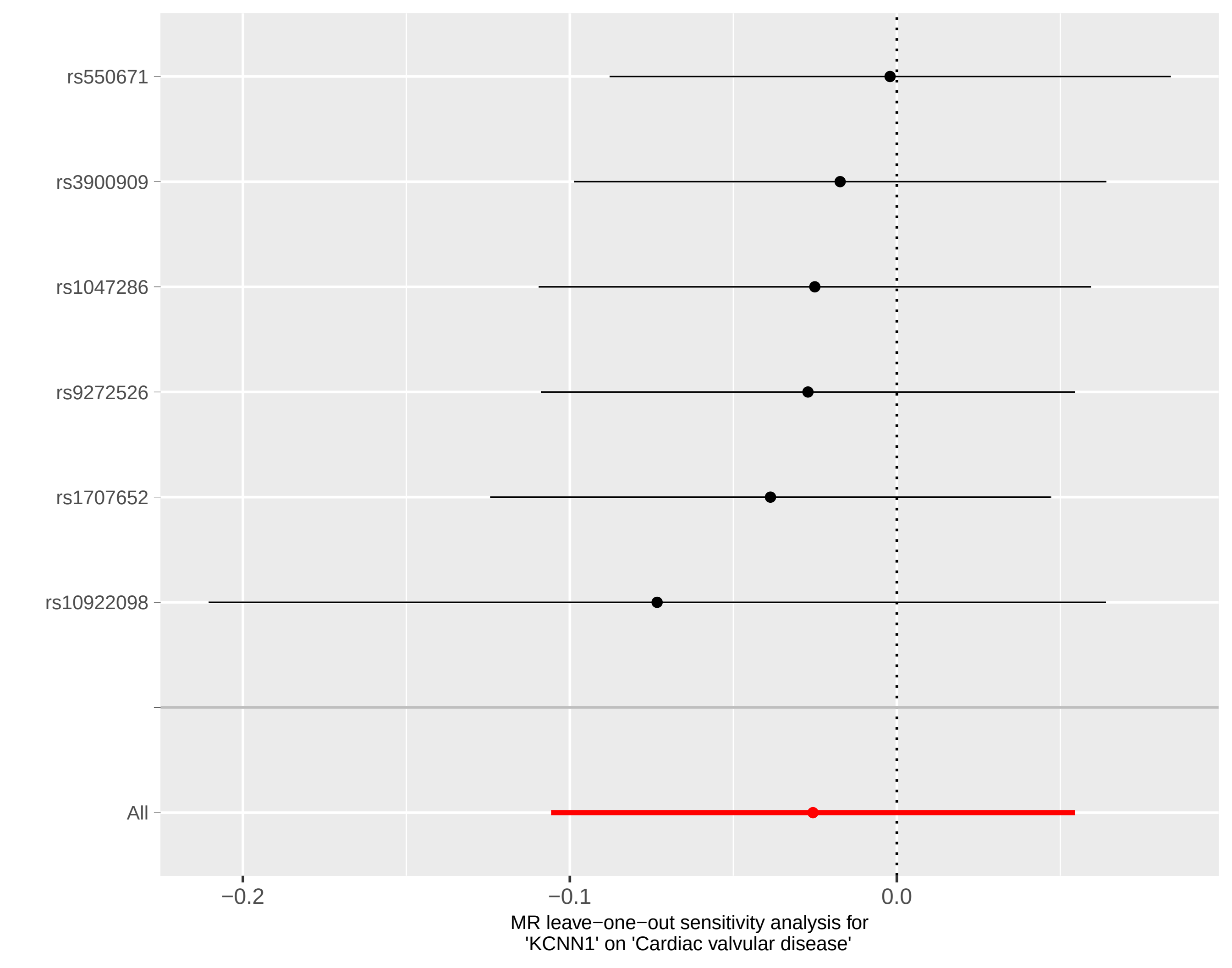

rs550671
rs3900909
rs1047286
rs9272526
rs1707652
rs10922098
All
−0.2
−0.1
0.0
MR leave−one−out sensitivity analysis for
'KCNN1' on 'Cardiac valvular disease'

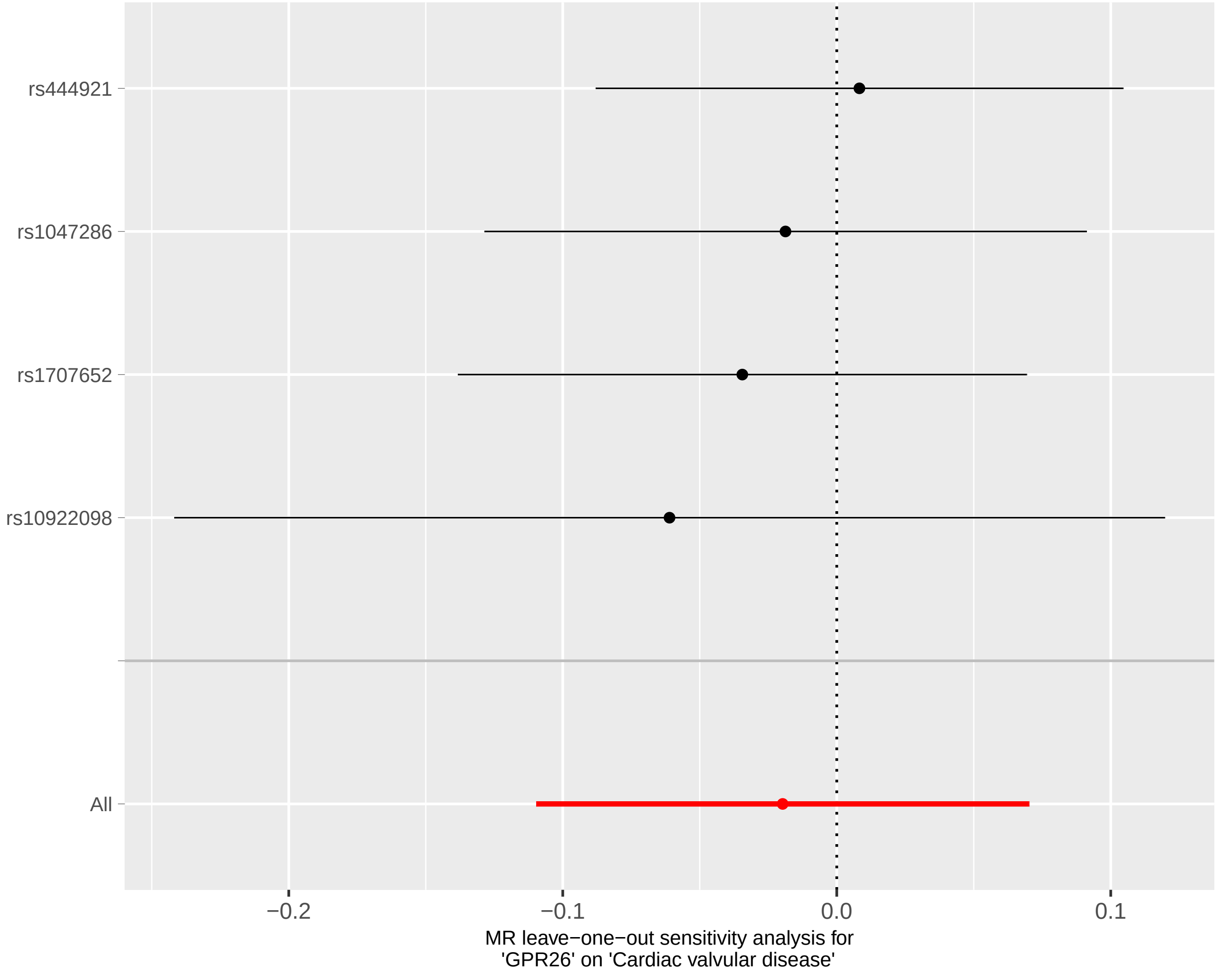
rs444921
rs1047286
rs1707652
rs10922098
All
−0.2
−0.1
0.0
0.1
MR leave−one−out sensitivity analysis for
'GPR26' on 'Cardiac valvular disease'

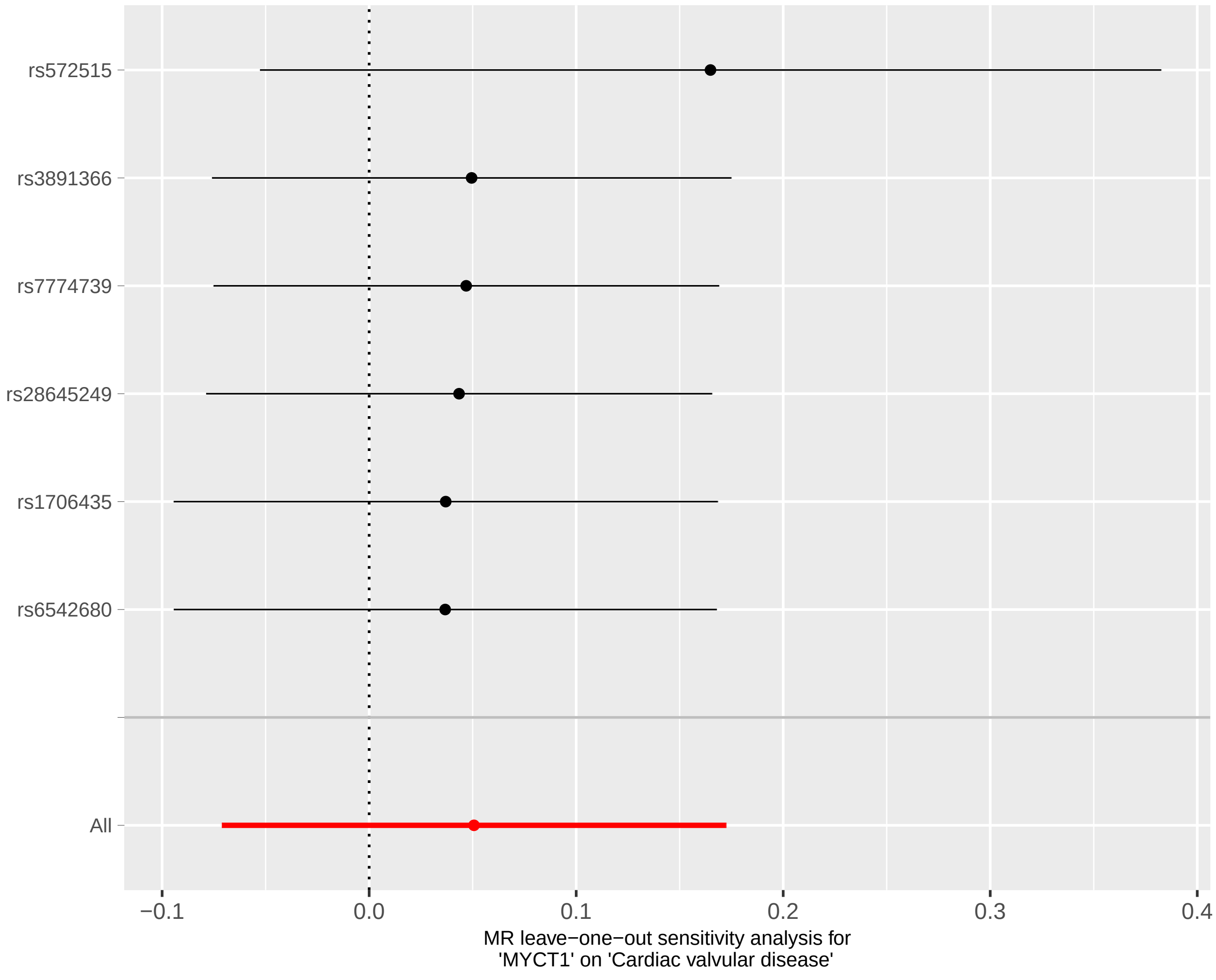
rs572515
rs3891366
rs7774739
rs28645249
rs1706435
rs6542680
All
−0.1
0.0
0.1
0.2
0.3
0.4
MR leave−one−out sensitivity analysis for
'MYCT1' on 'Cardiac valvular disease'

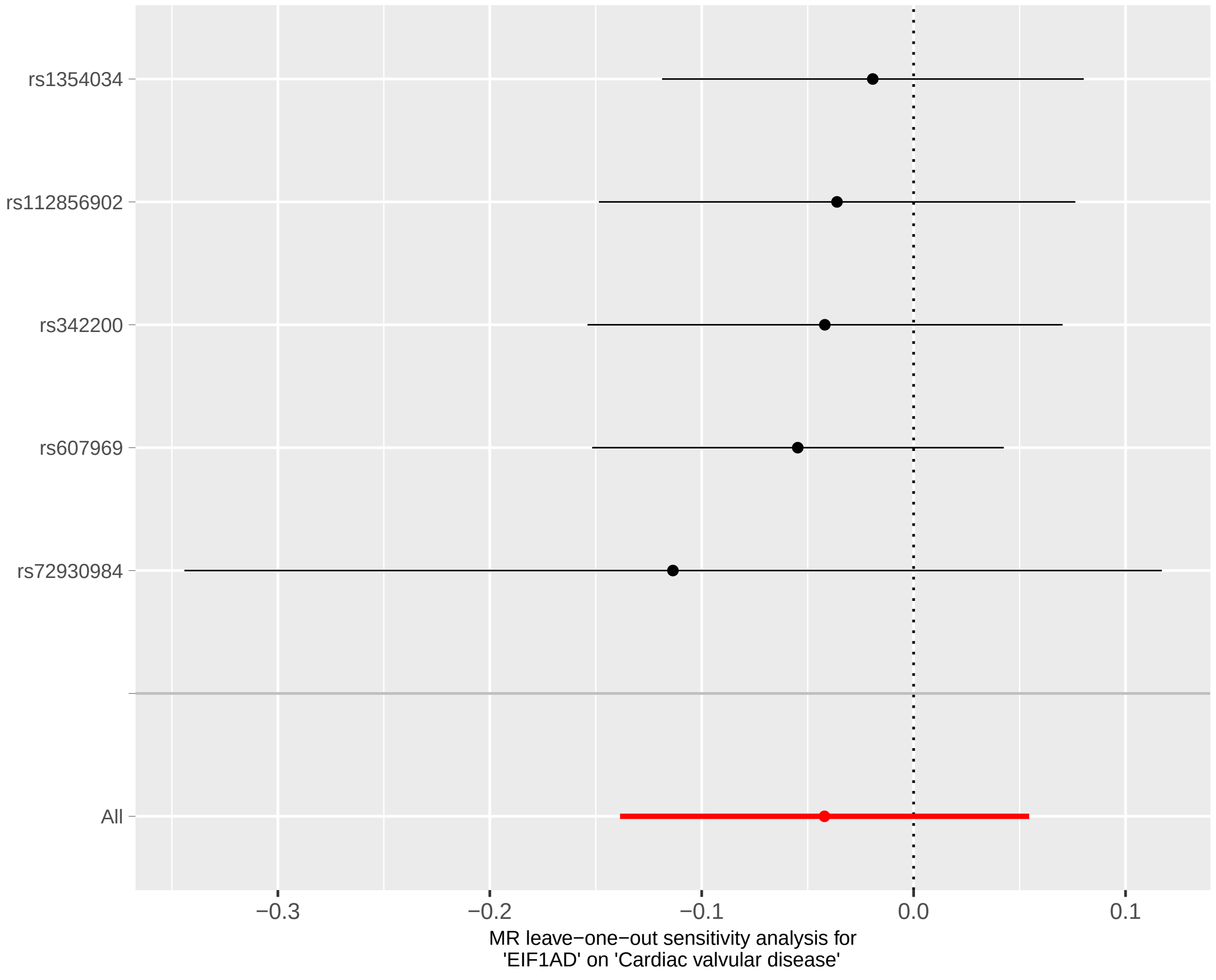

rs1354034
rs112856902
rs342200
rs607969
rs72930984
All
−0.3
−0.2
−0.1
0.0
0.1
MR leave−one−out sensitivity analysis for
'EIF1AD' on 'Cardiac valvular disease'

Insufficient number of SNPs

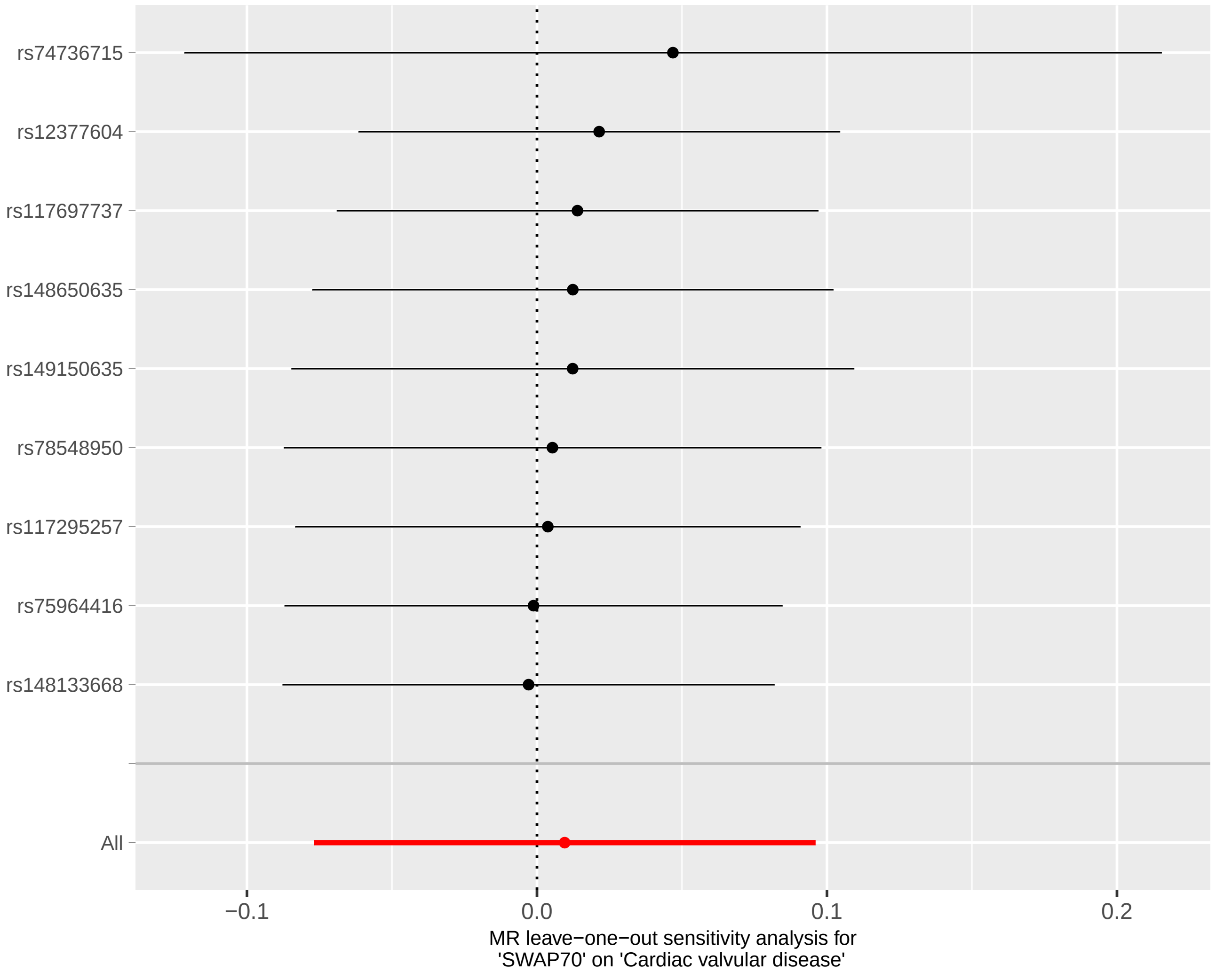
rs74736715
rs12377604
rs117697737
rs148650635
rs149150635
rs78548950
rs117295257
rs75964416
rs148133668
All
−0.1
0.0
0.1
0.2
MR leave−one−out sensitivity analysis for
'SWAP70' on 'Cardiac valvular disease'

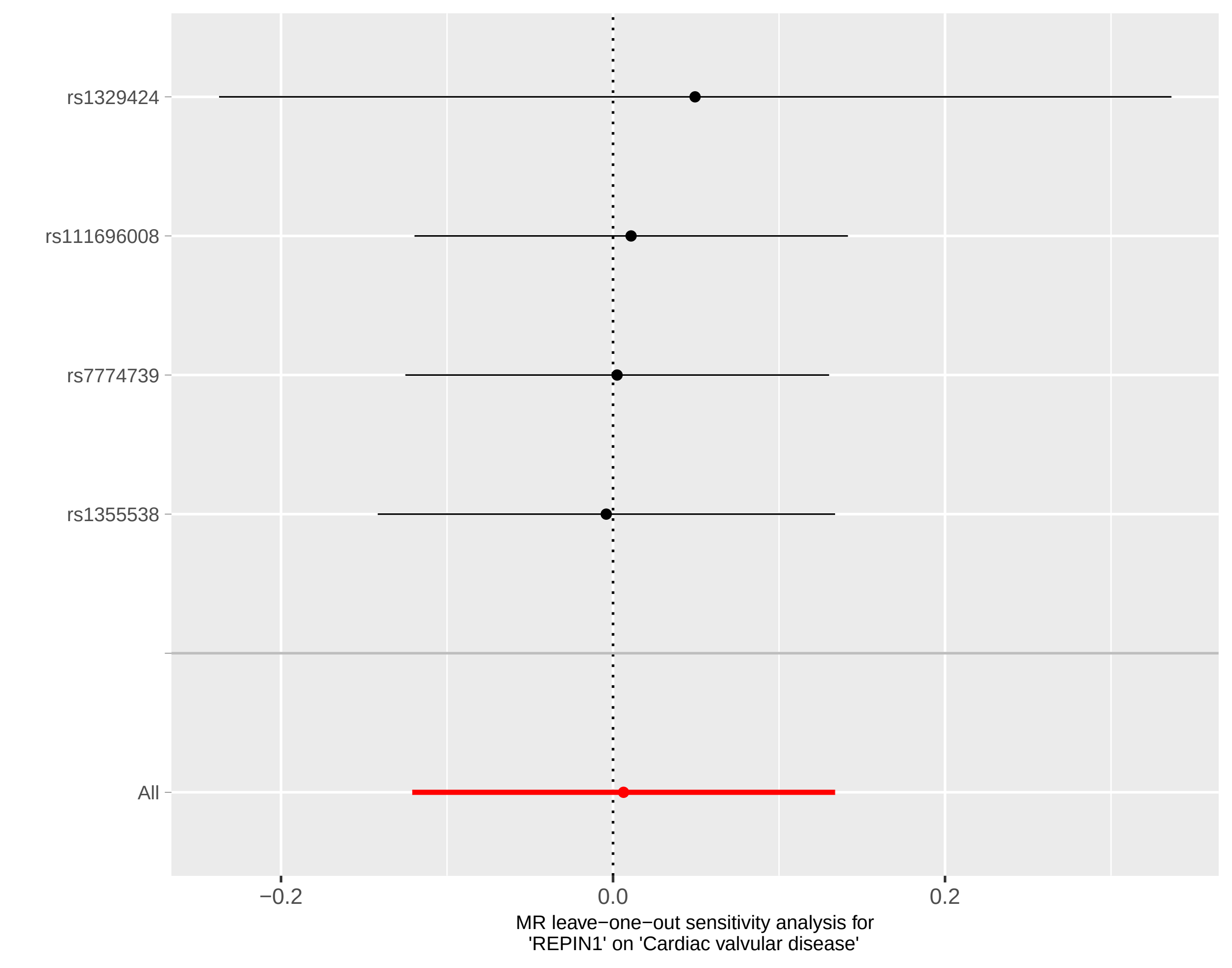
rs1329424
rs111696008
rs7774739
rs1355538
All
−0.2
0.0
0.2
MR leave−one−out sensitivity analysis for
'REPIN1' on 'Cardiac valvular disease'

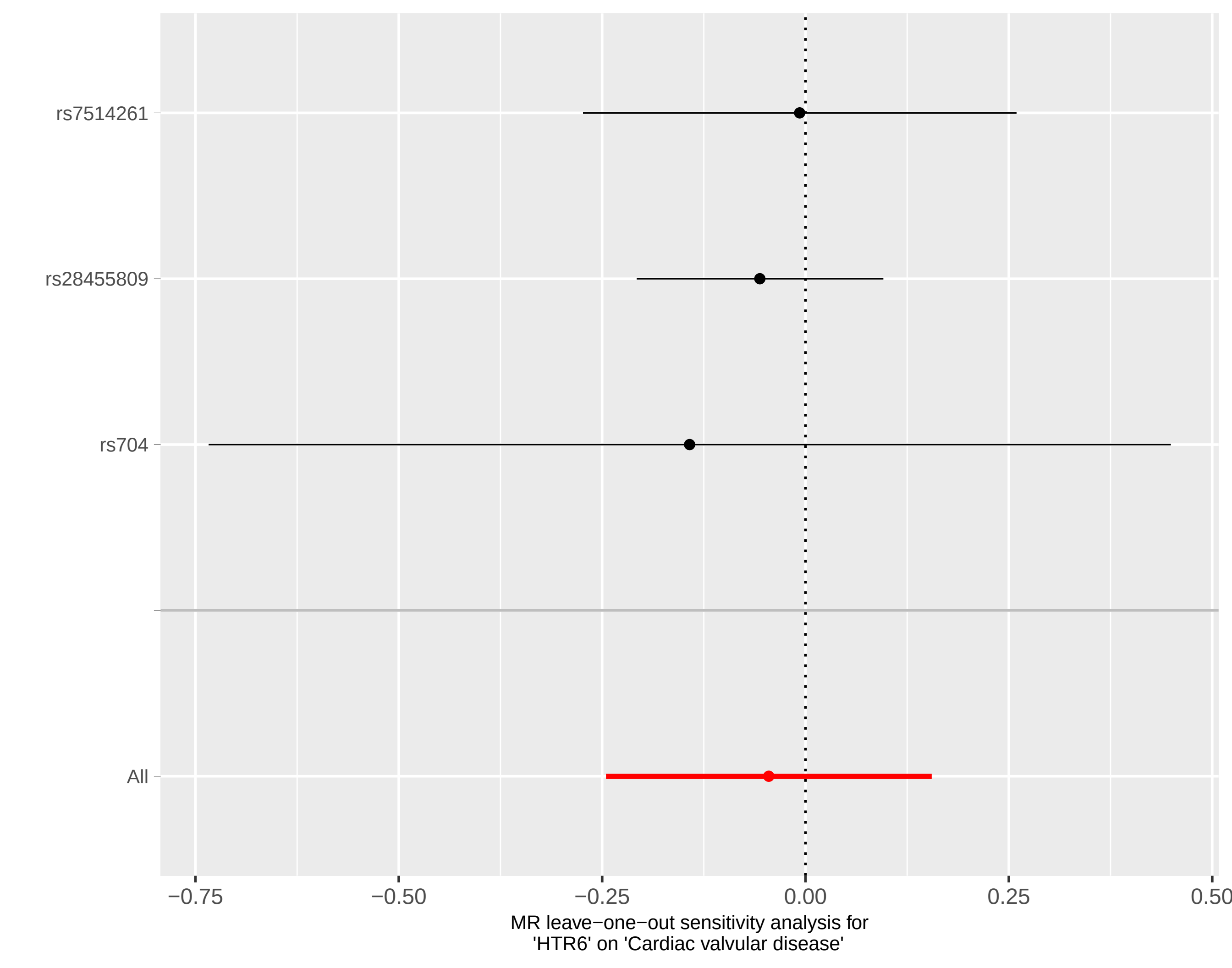
rs7514261
rs28455809
rs704
All
−0.75
−0.50
−0.25
0.00
0.25
0.50
MR leave−one−out sensitivity analysis for
'HTR6' on 'Cardiac valvular disease'

Insufficient number of SNPs

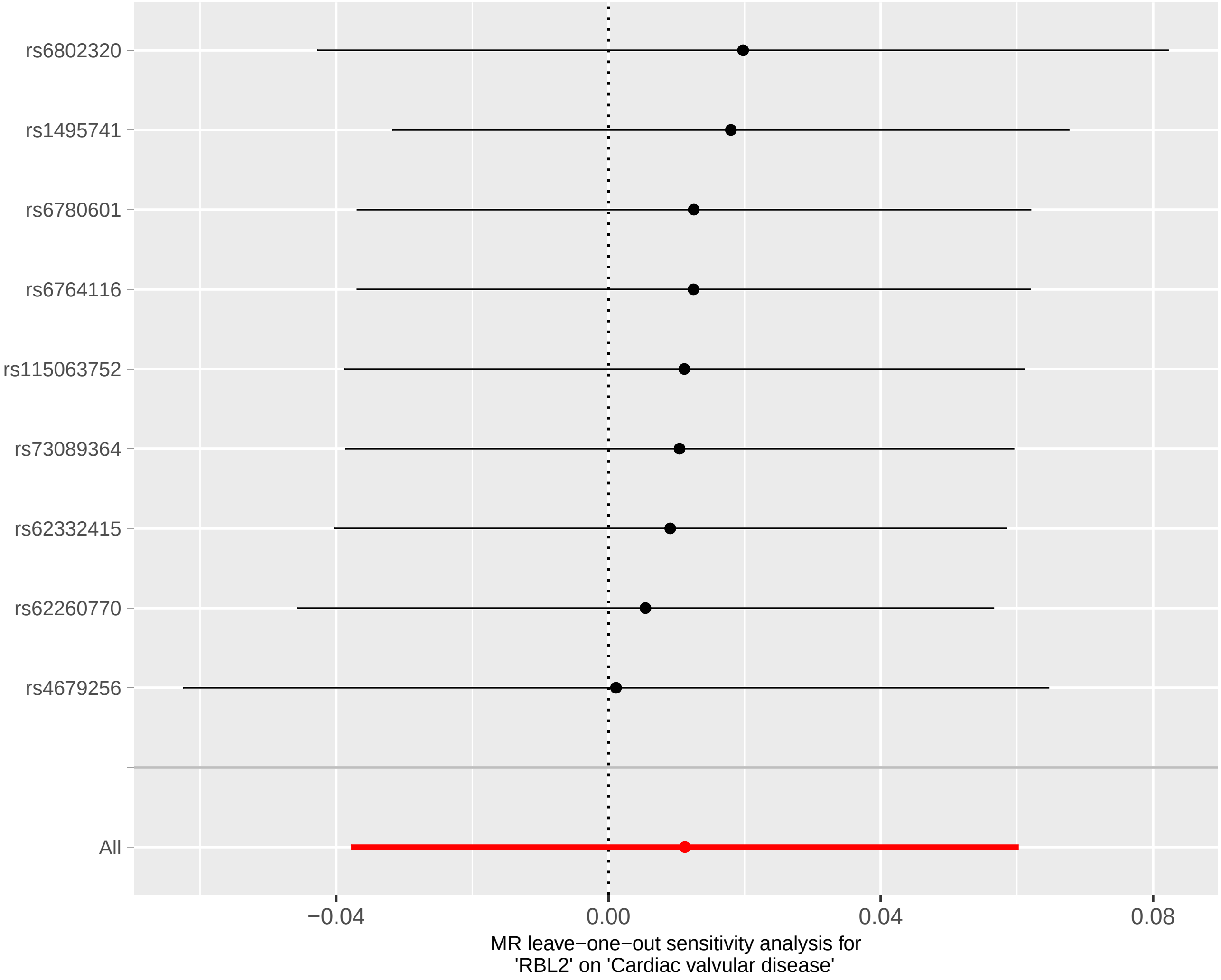
rs6802320
rs1495741
rs6780601
rs6764116
rs115063752
rs73089364
rs62332415
rs62260770
rs4679256
All
−0.04
0.00
0.04
0.08
MR leave−one−out sensitivity analysis for
'RBL2' on 'Cardiac valvular disease'

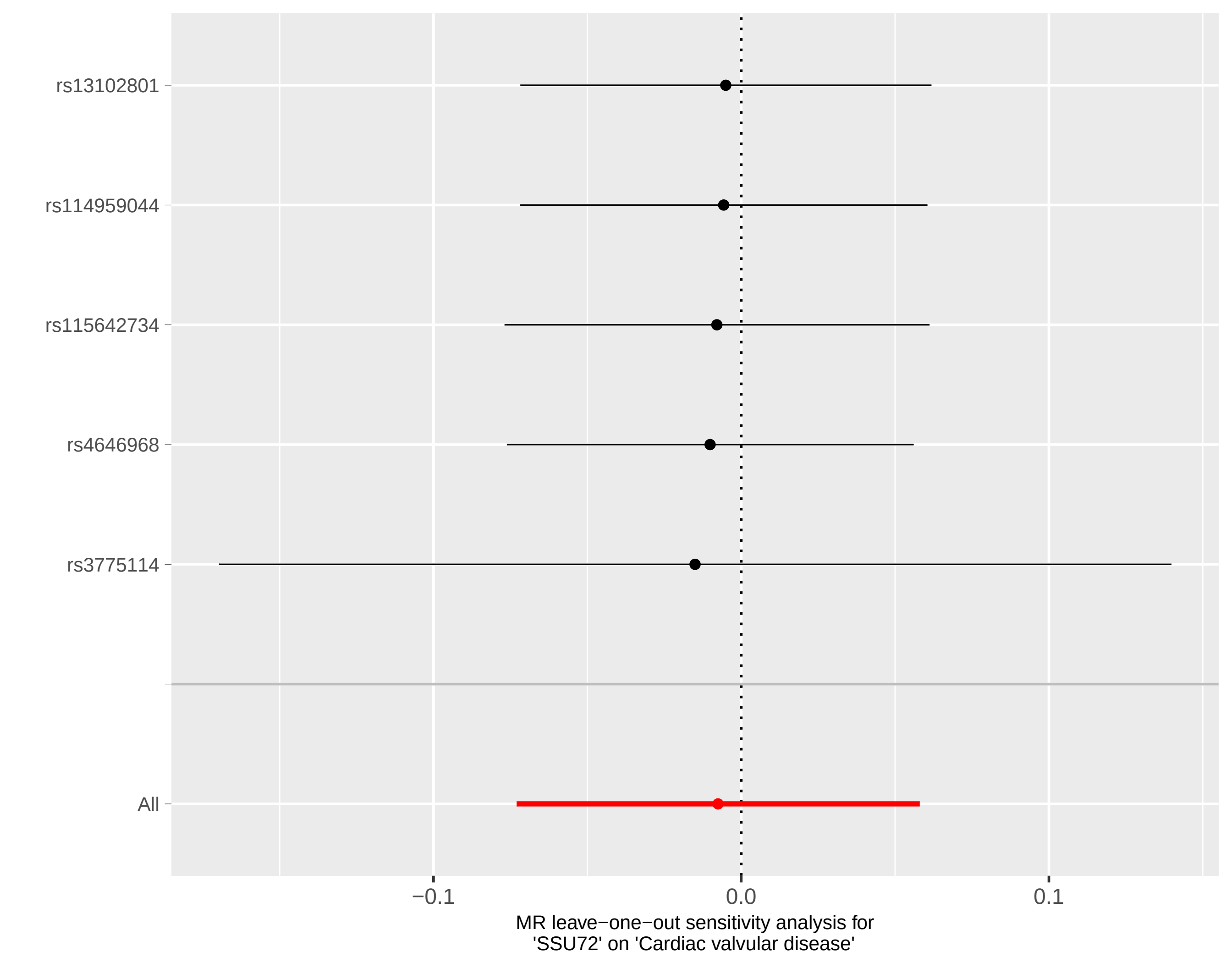
rs13102801
rs114959044
rs115642734
rs4646968
rs3775114
All
−0.1
0.0
0.1
MR leave−one−out sensitivity analysis for
'SSU72' on 'Cardiac valvular disease'

Insufficient number of SNPs

Insufficient number of SNPs

Insufficient number of SNPs

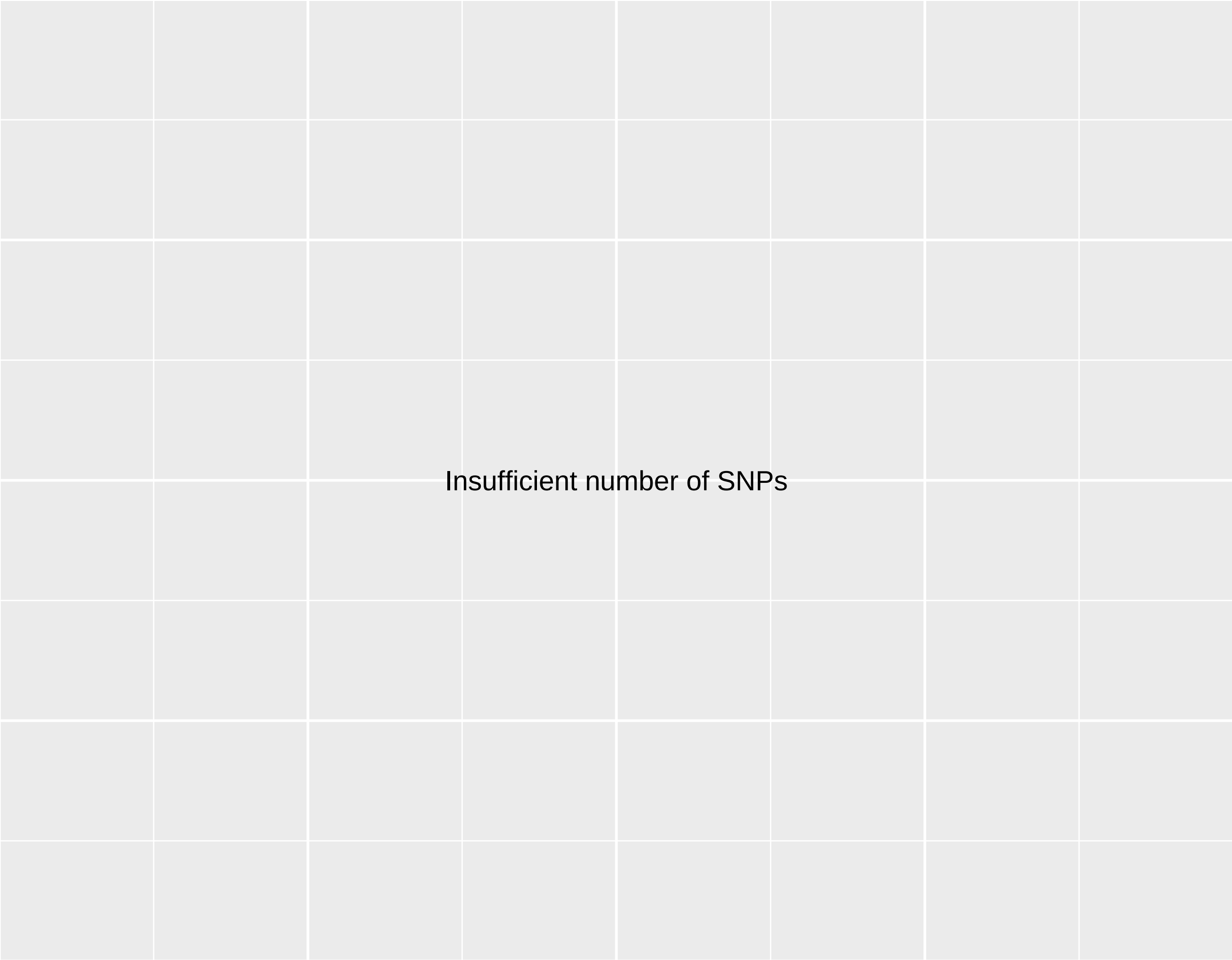
Insufficient number of SNPs

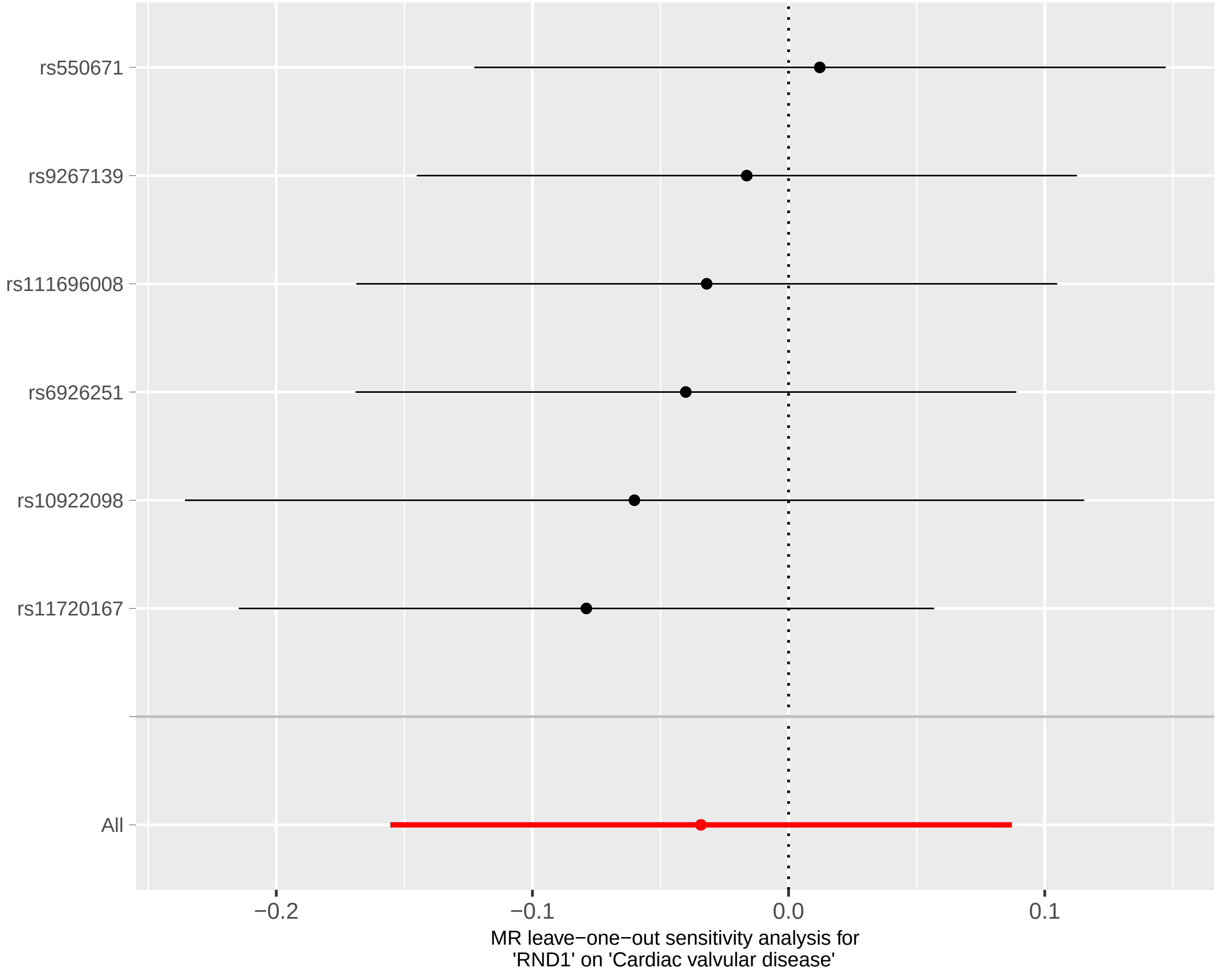

rs550671
rs9267139
rs111696008
rs6926251
rs10922098
rs11720167
All
−0.2
−0.1
0.0
0.1
MR leave−one−out sensitivity analysis for
'RND1' on 'Cardiac valvular disease'

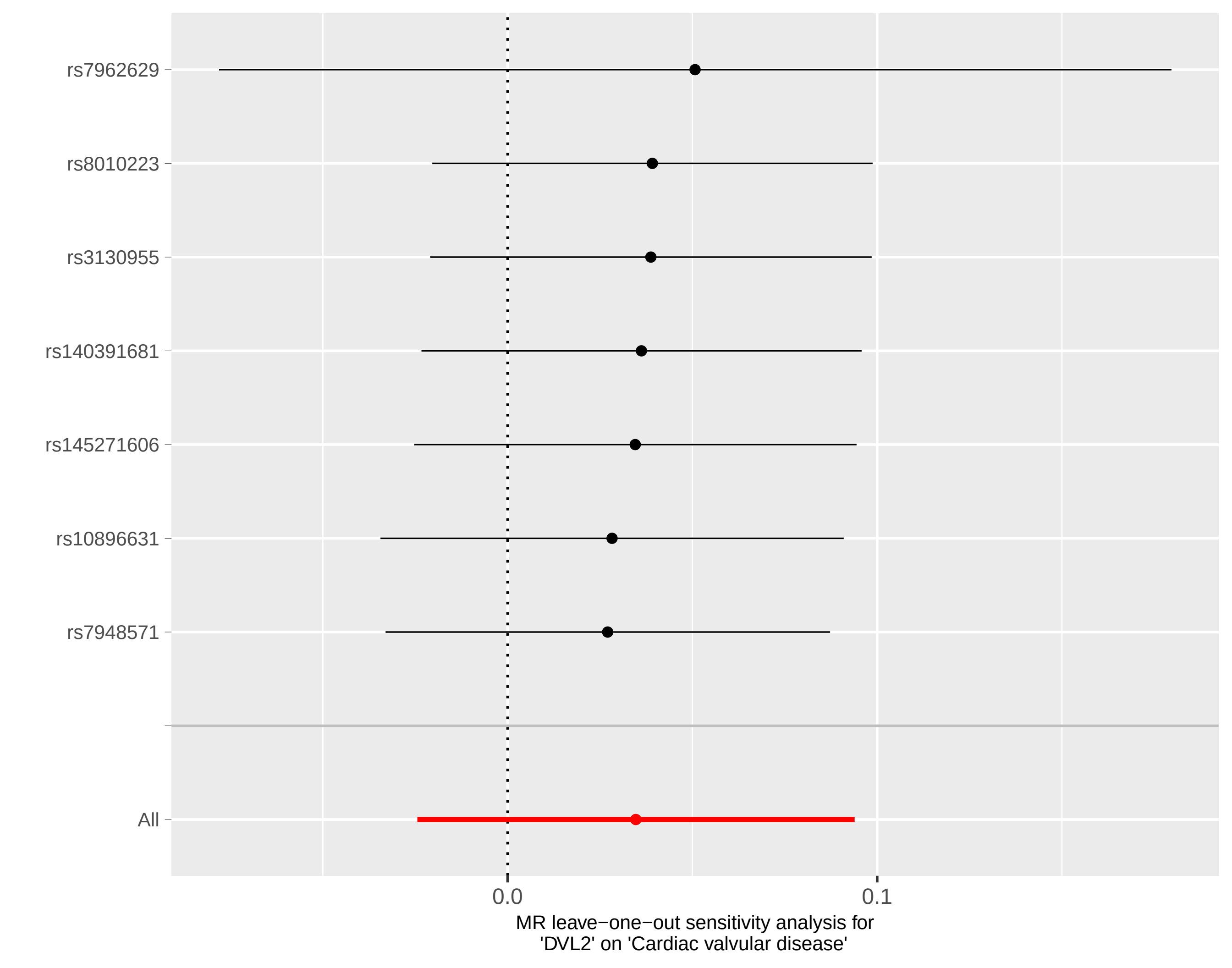

rs7962629
rs8010223
rs3130955
rs140391681
rs145271606
rs10896631
rs7948571
All
0.0
0.1
MR leave−one−out sensitivity analysis for
'DVL2' on 'Cardiac valvular disease'

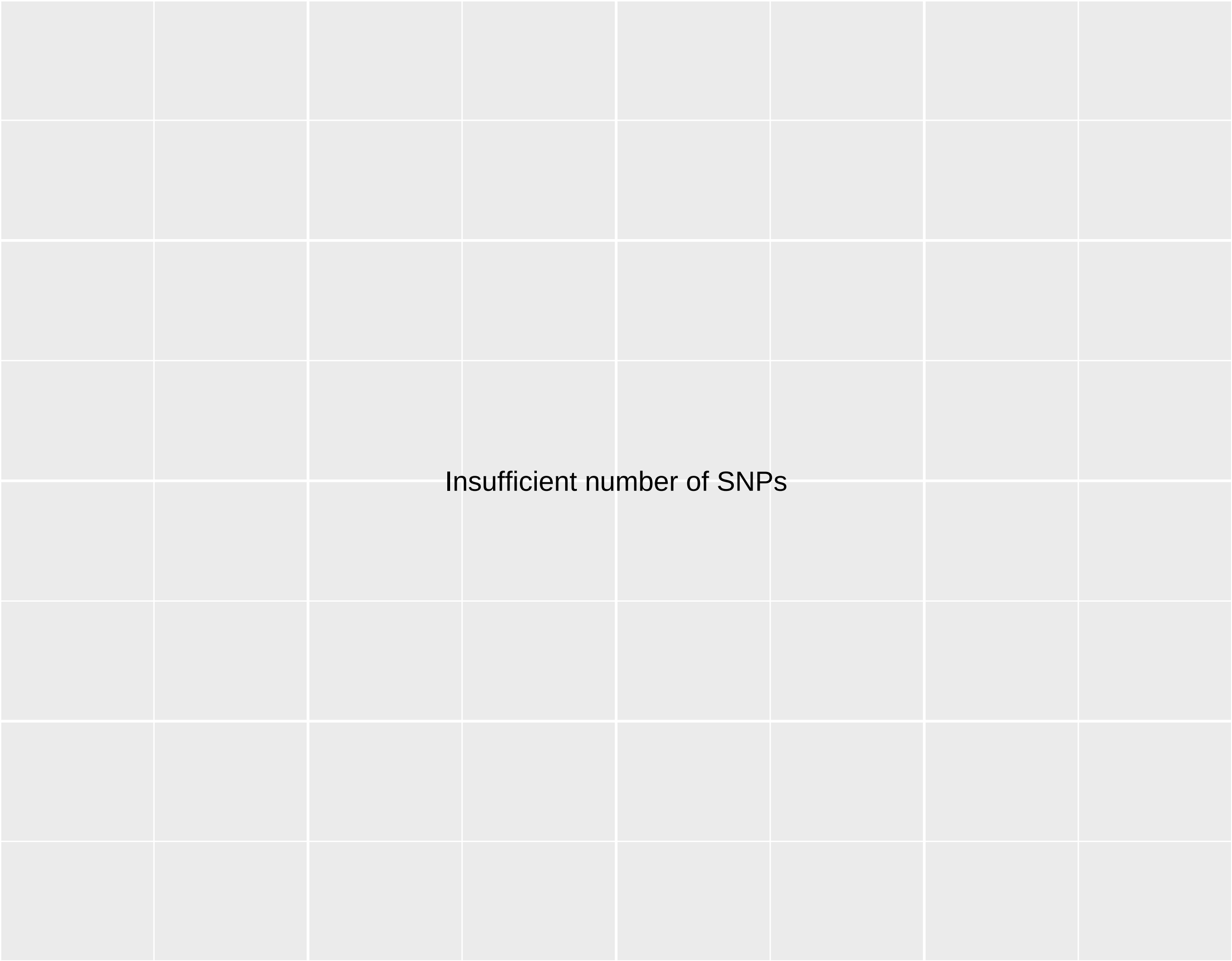
Insufficient number of SNPs

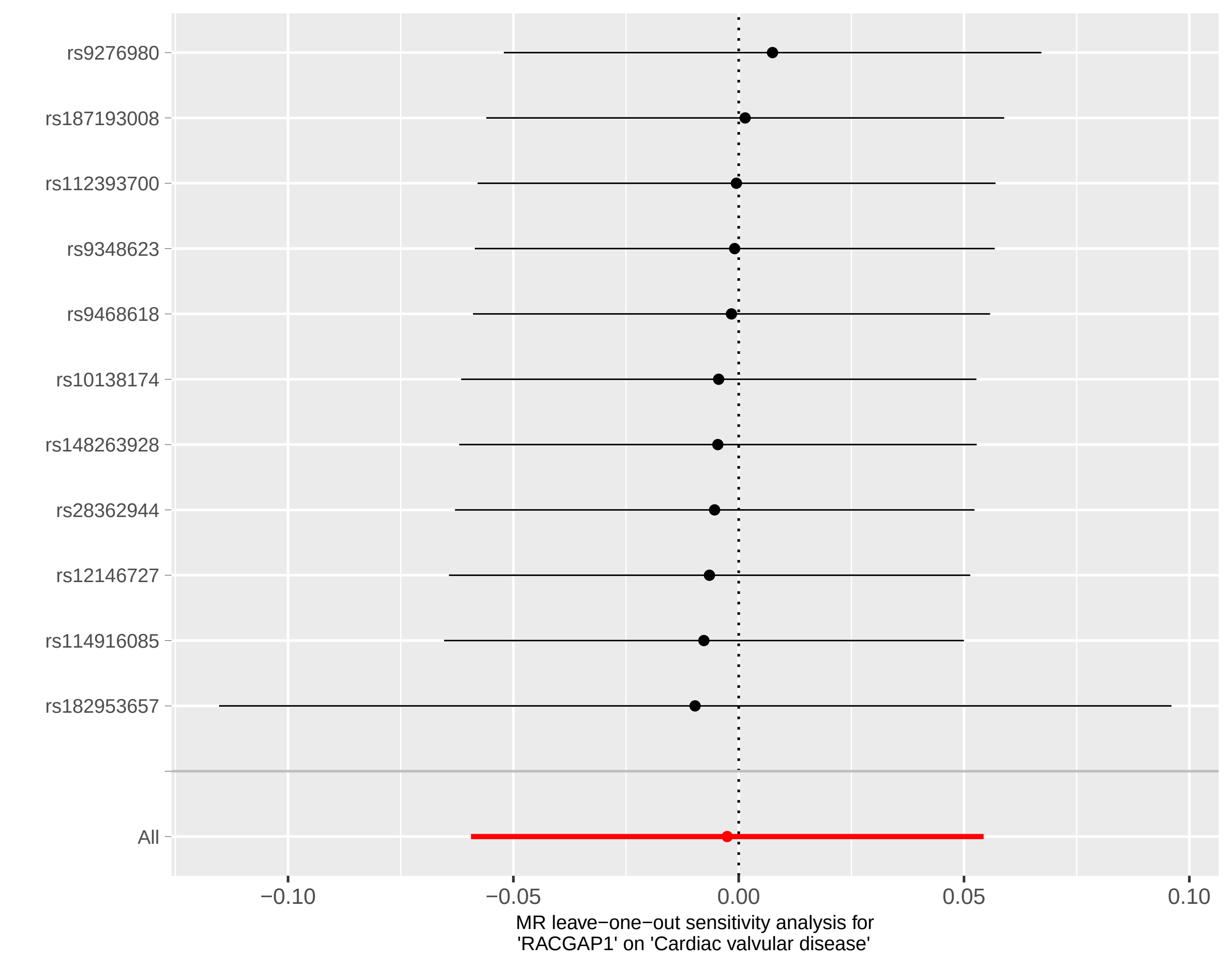

rs9276980
rs187193008
rs112393700
rs9348623
rs9468618
rs10138174
rs148263928
rs28362944
rs12146727
rs114916085
rs182953657
All
−0.10
−0.05
0.00
0.05
0.10
MR leave−one−out sensitivity analysis for
'RACGAP1' on 'Cardiac valvular disease'

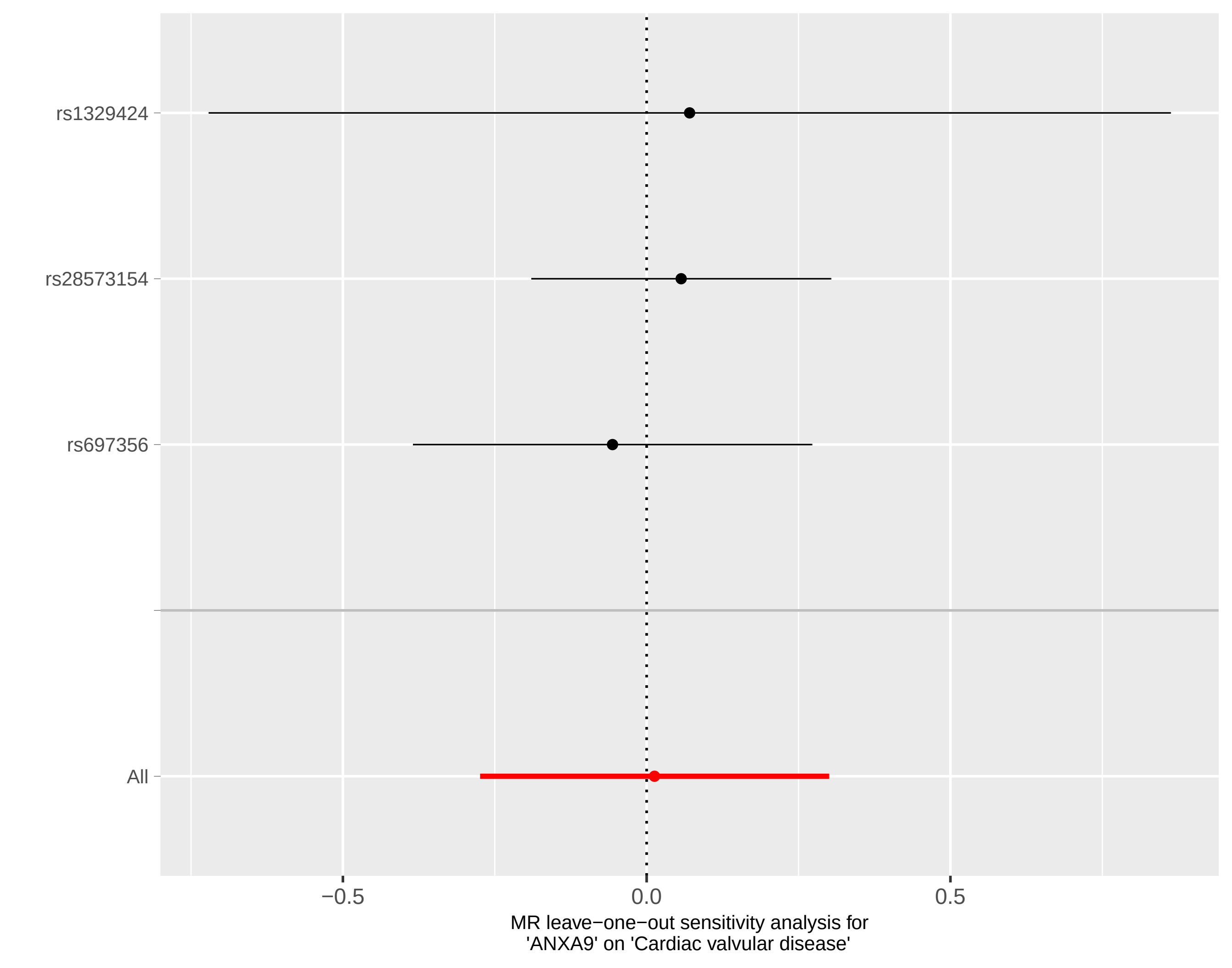

rs1329424
rs28573154
rs697356
All
−0.5
0.0
0.5
MR leave−one−out sensitivity analysis for
'ANXA9' on 'Cardiac valvular disease'

Insufficient number of SNPs

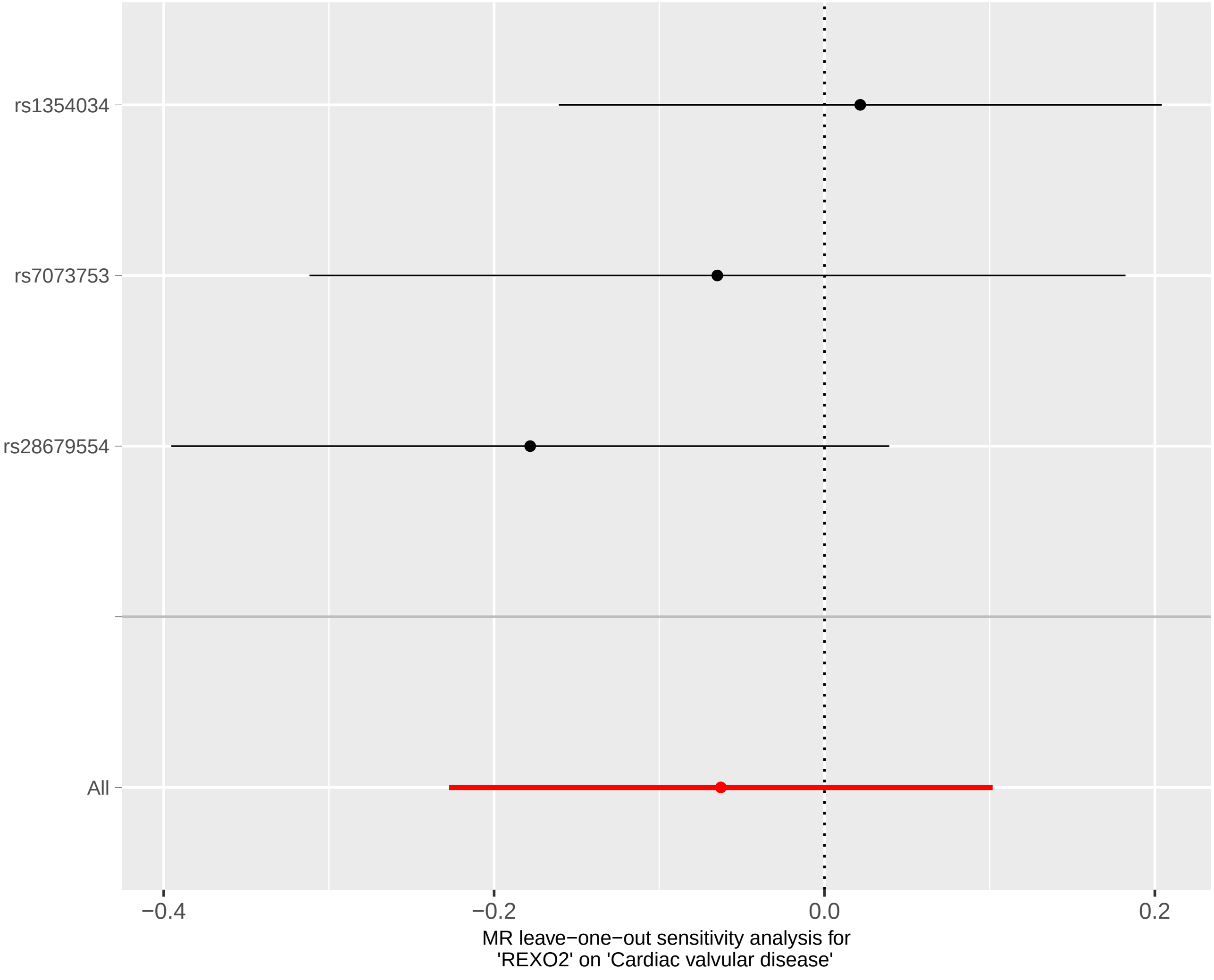
rs1354034
rs7073753
rs28679554
All
−0.4
−0.2
0.0
0.2
MR leave−one−out sensitivity analysis for
'REXO2' on 'Cardiac valvular disease'

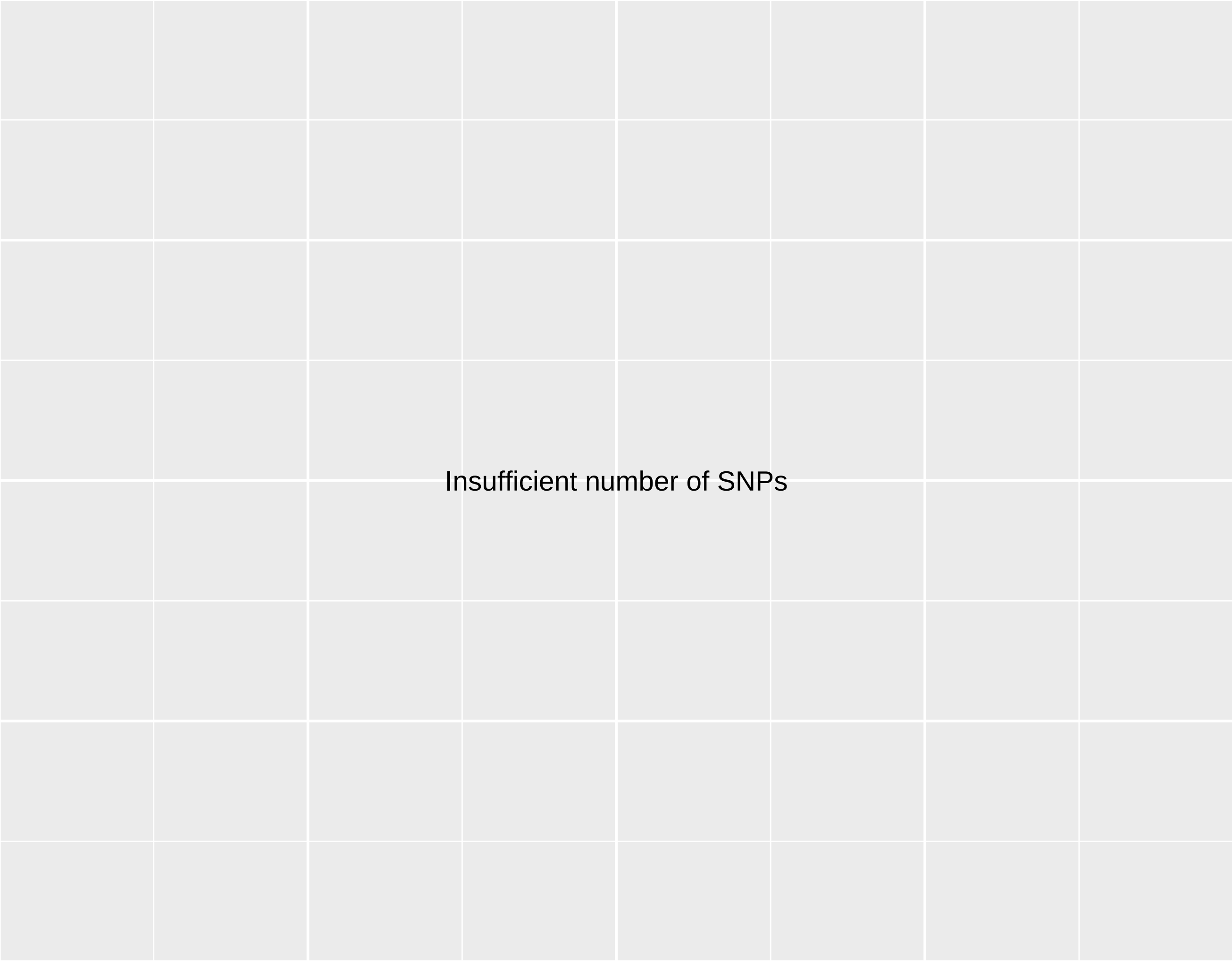
Insufficient number of SNPs

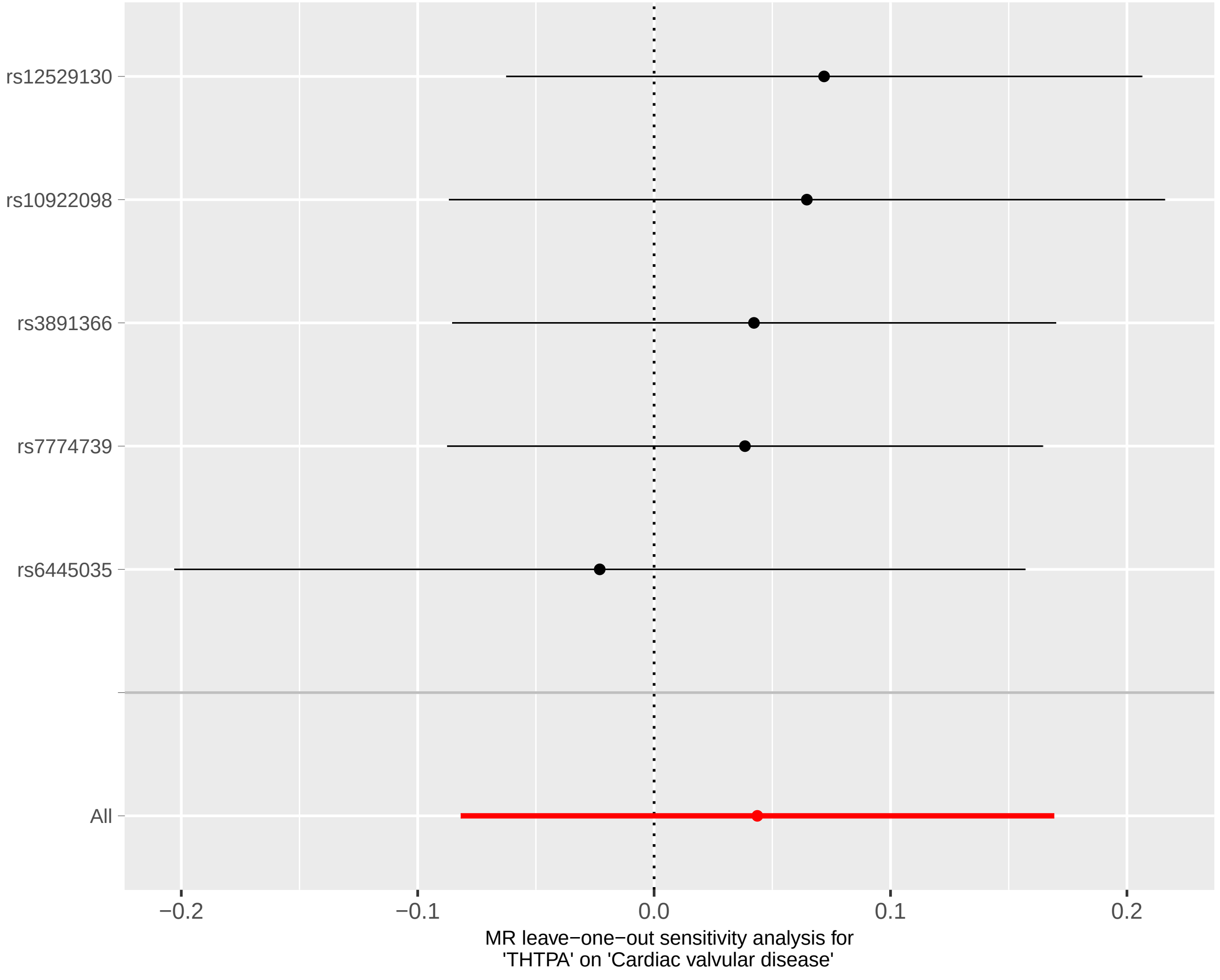
rs12529130
rs10922098
rs3891366
rs7774739
rs6445035
All
−0.2
−0.1
0.0
0.1
0.2
MR leave−one−out sensitivity analysis for
'THTPA' on 'Cardiac valvular disease'

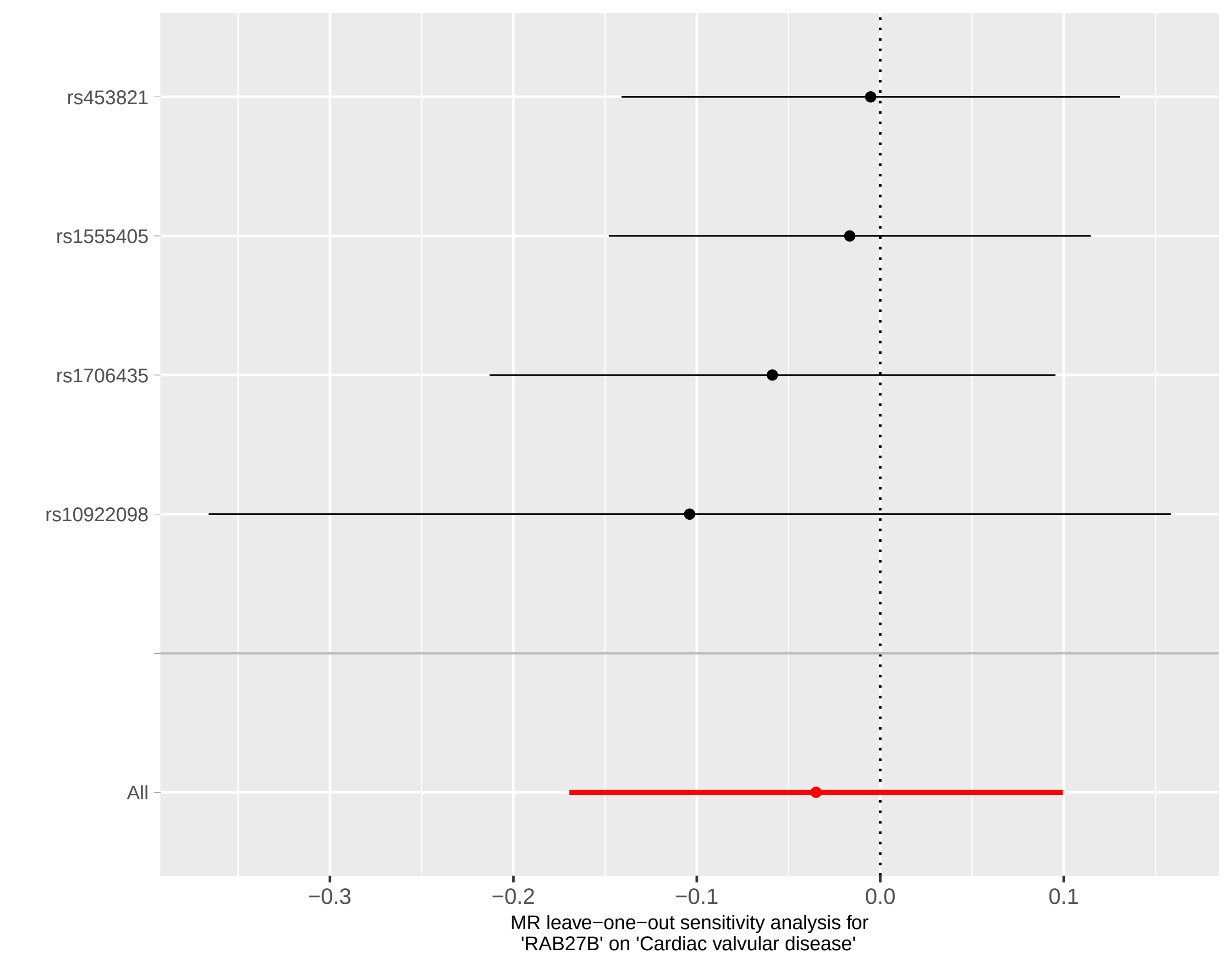
rs453821
rs1555405
rs1706435
rs10922098
All
−0.3
−0.2
−0.1
0.0
0.1
MR leave−one−out sensitivity analysis for
'RAB27B' on 'Cardiac valvular disease'

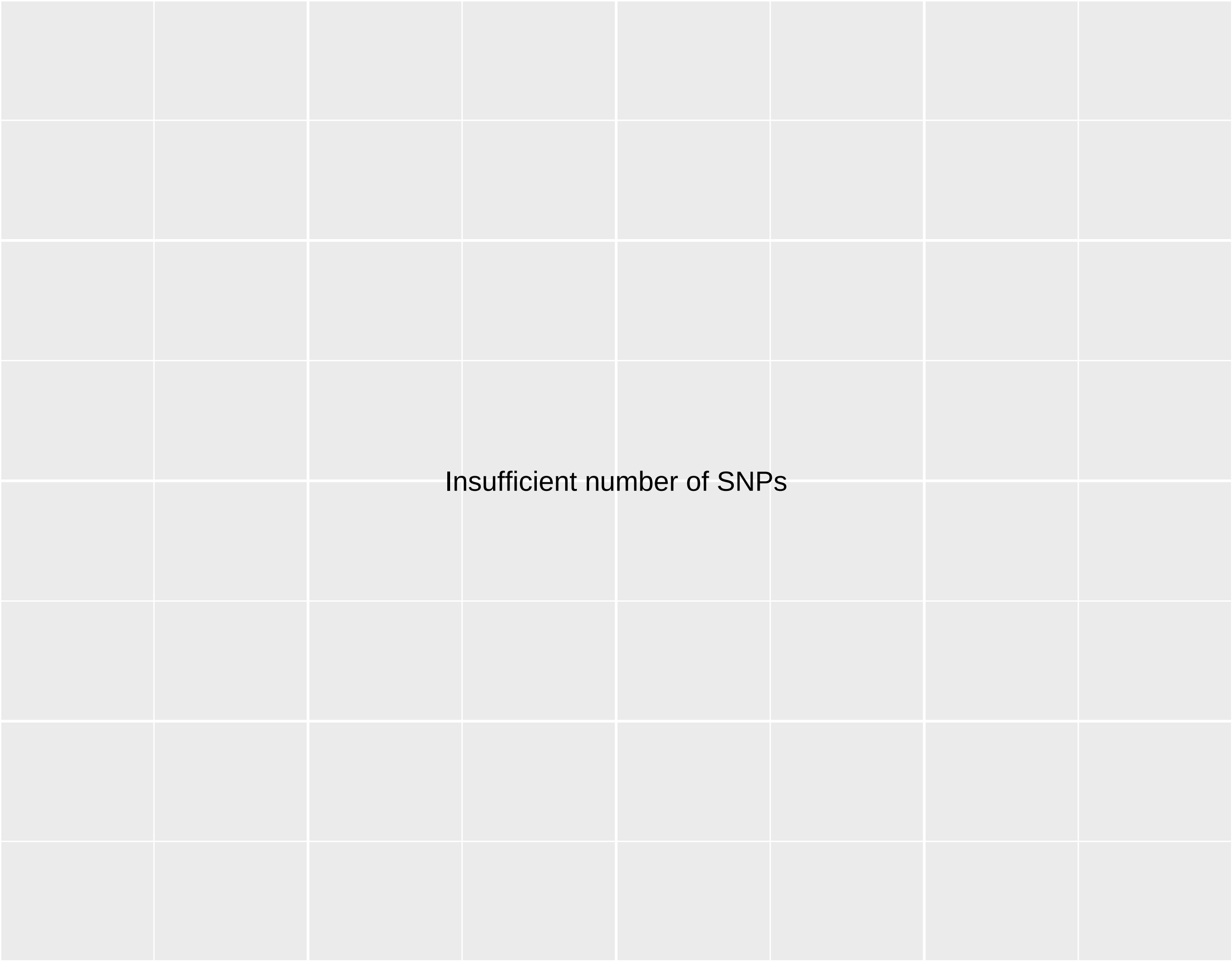
Insufficient number of SNPs

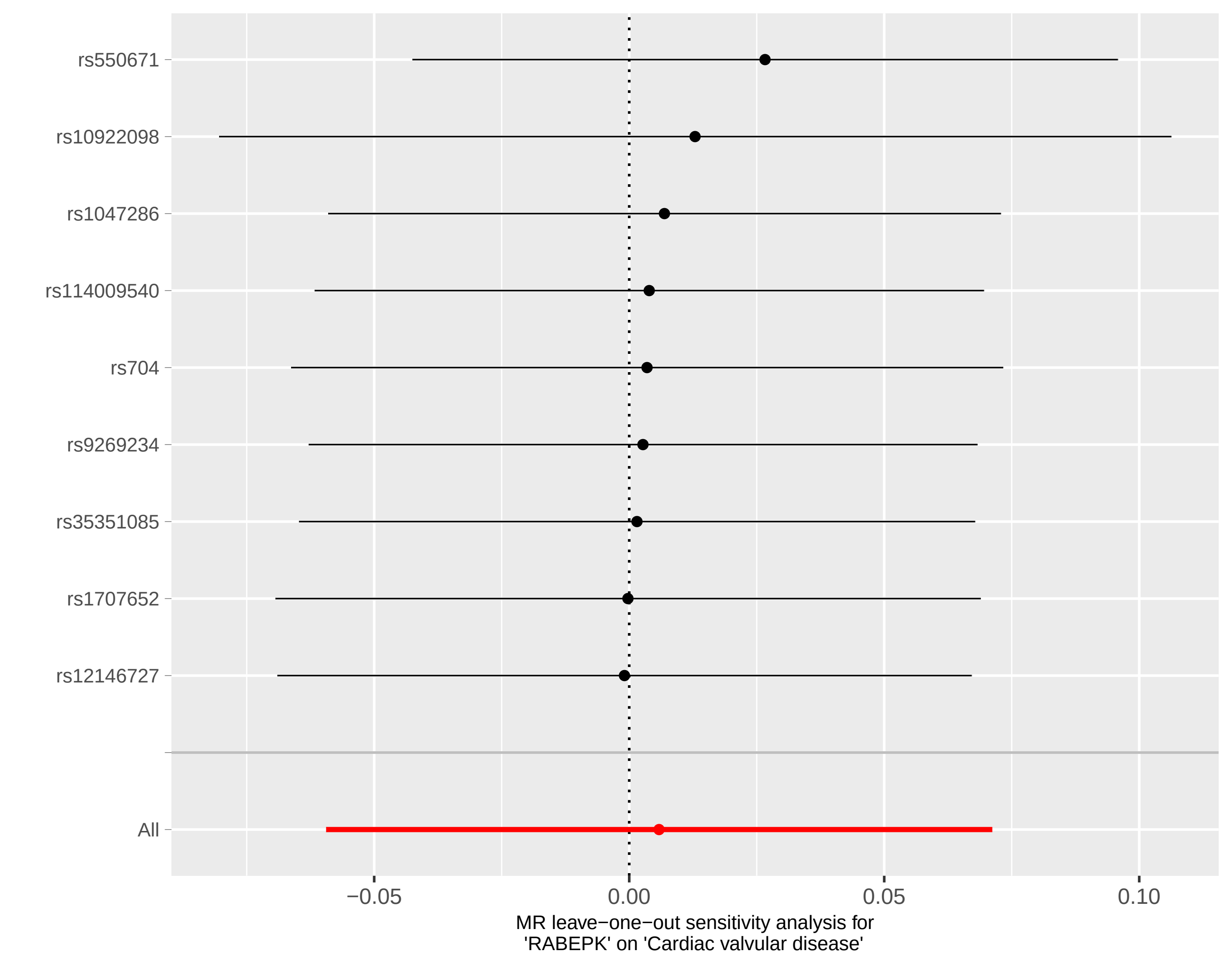
rs550671
rs10922098
rs1047286
rs114009540
rs704
rs9269234
rs35351085
rs1707652
rs12146727
All
−0.05
0.00
0.05
0.10
MR leave−one−out sensitivity analysis for
'RABEPK' on 'Cardiac valvular disease'

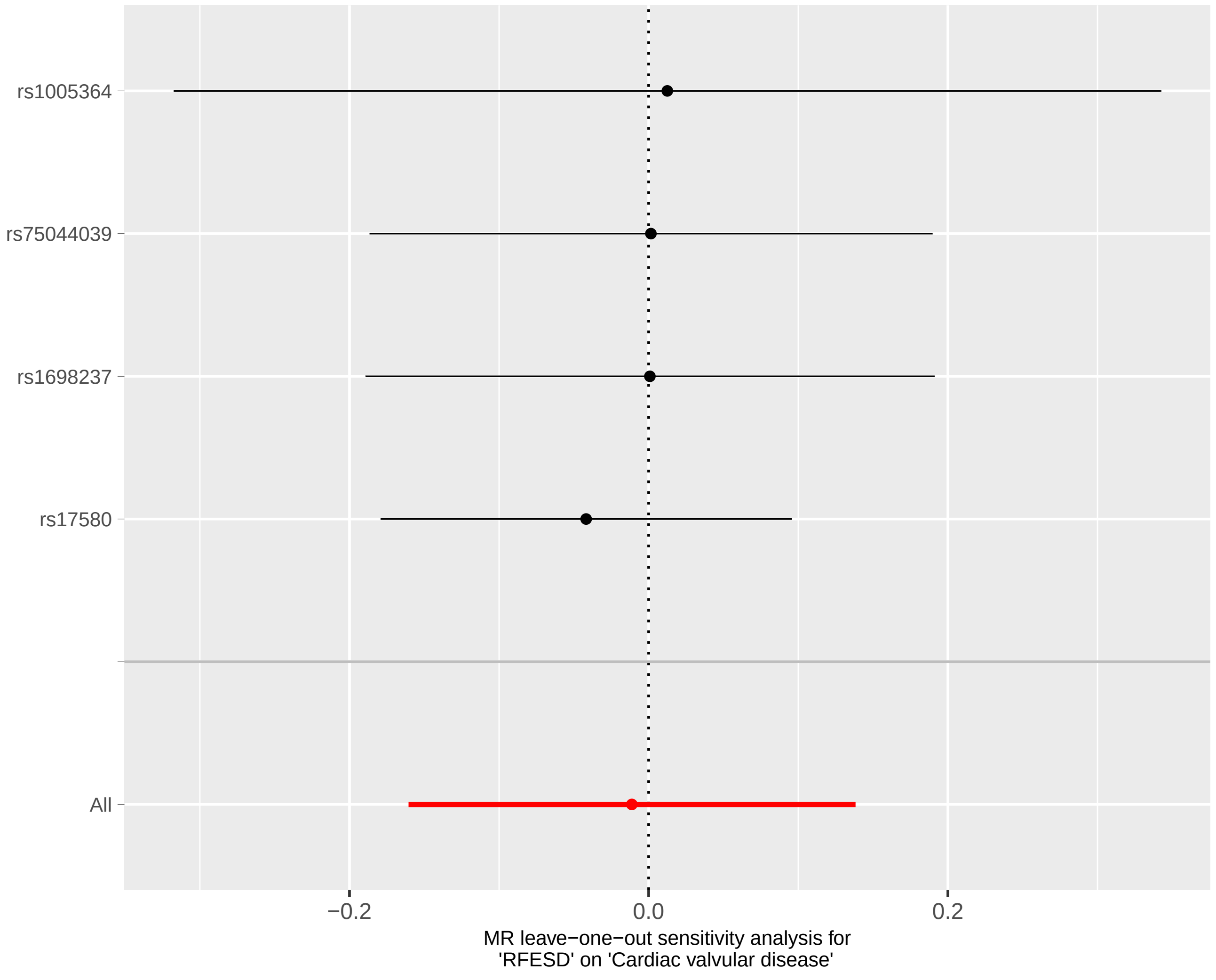
rs1005364
rs75044039
rs1698237
rs17580
All
−0.2
0.0
0.2
MR leave−one−out sensitivity analysis for
'RFESD' on 'Cardiac valvular disease'

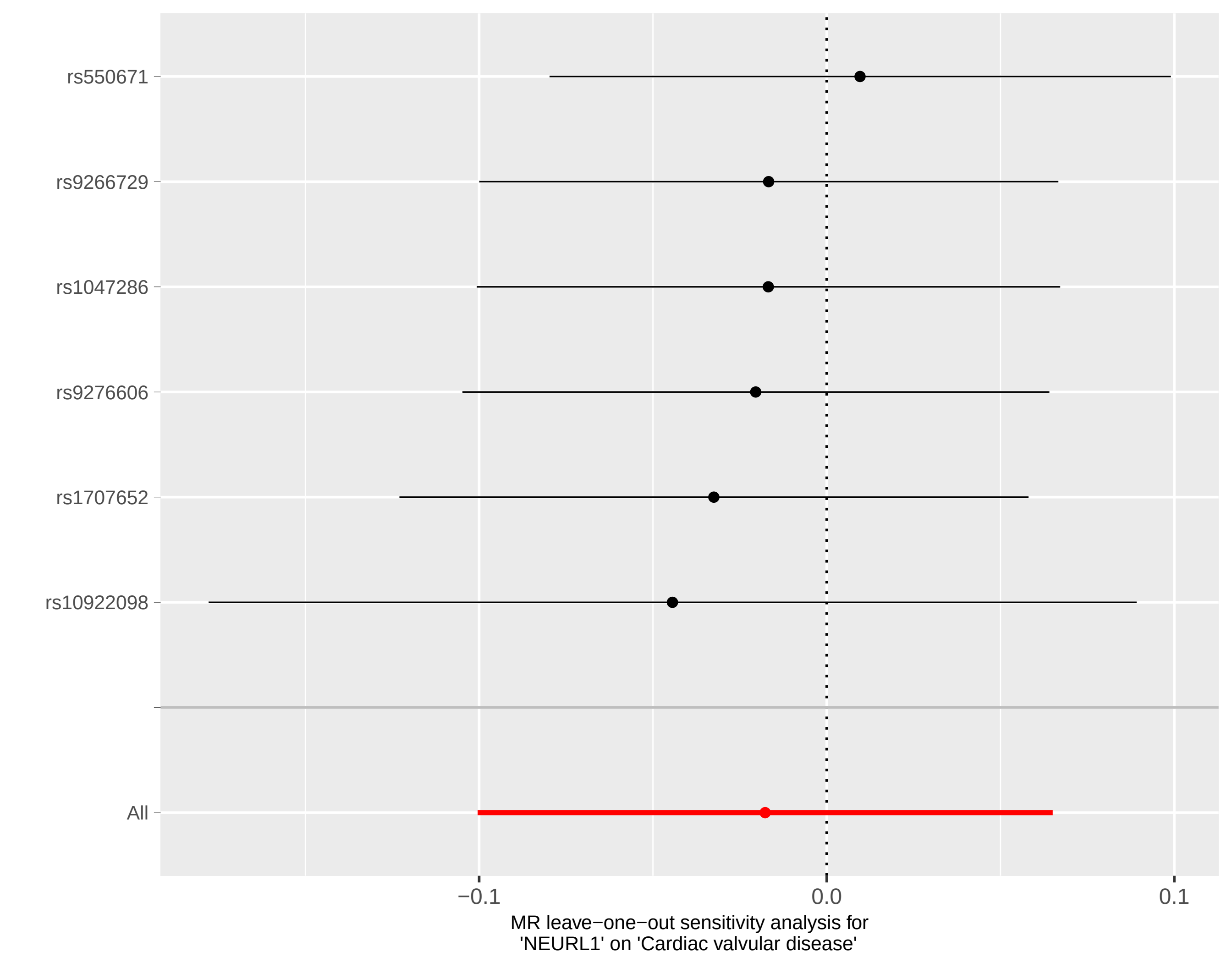
rs550671
rs9266729
rs1047286
rs9276606
rs1707652
rs10922098
All
−0.1
0.0
0.1
MR leave−one−out sensitivity analysis for
'NEURL1' on 'Cardiac valvular disease'

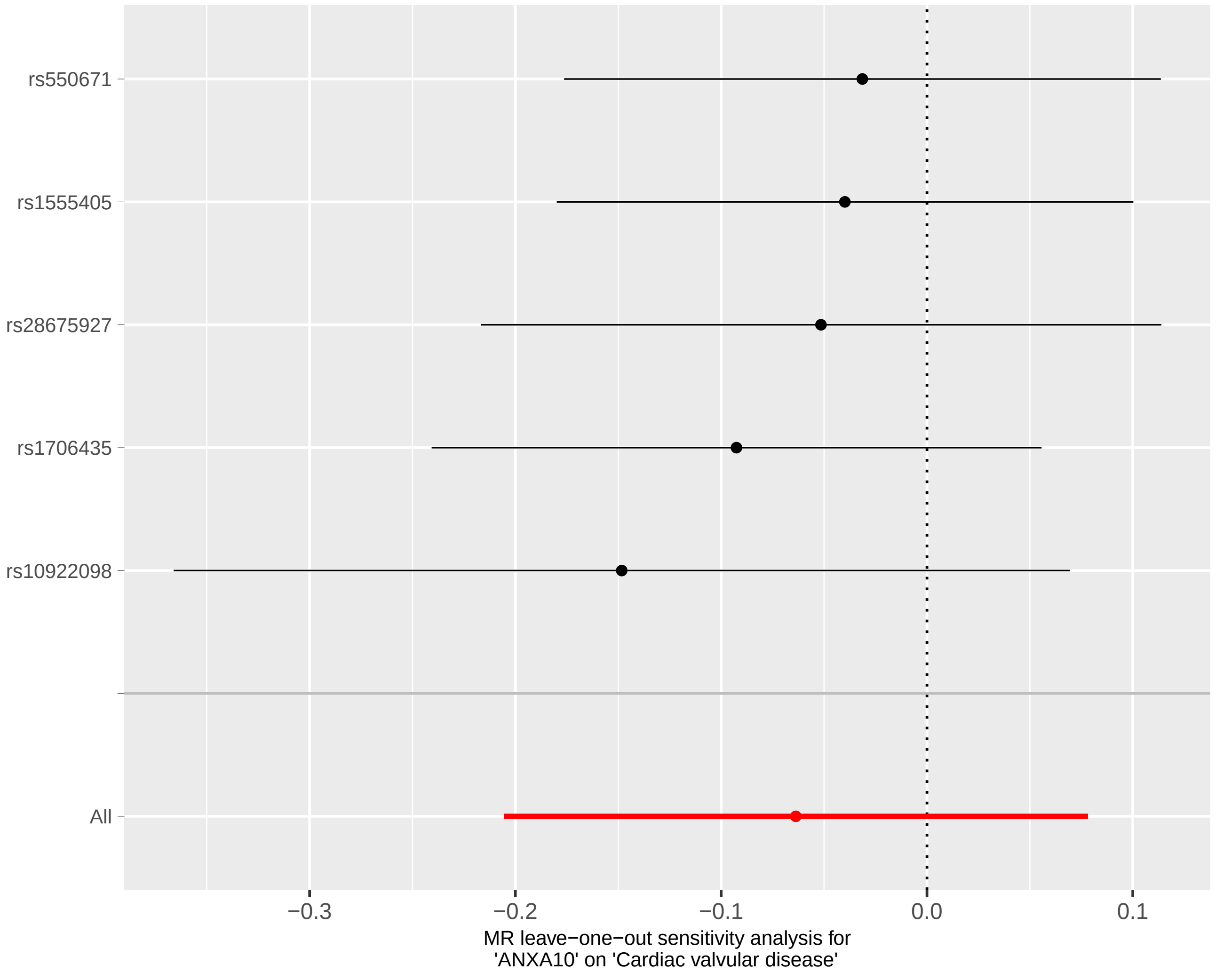
rs550671
rs1555405
rs28675927
rs1706435
rs10922098
All
−0.3
−0.2
−0.1
0.0
0.1
MR leave−one−out sensitivity analysis for
'ANXA10' on 'Cardiac valvular disease'

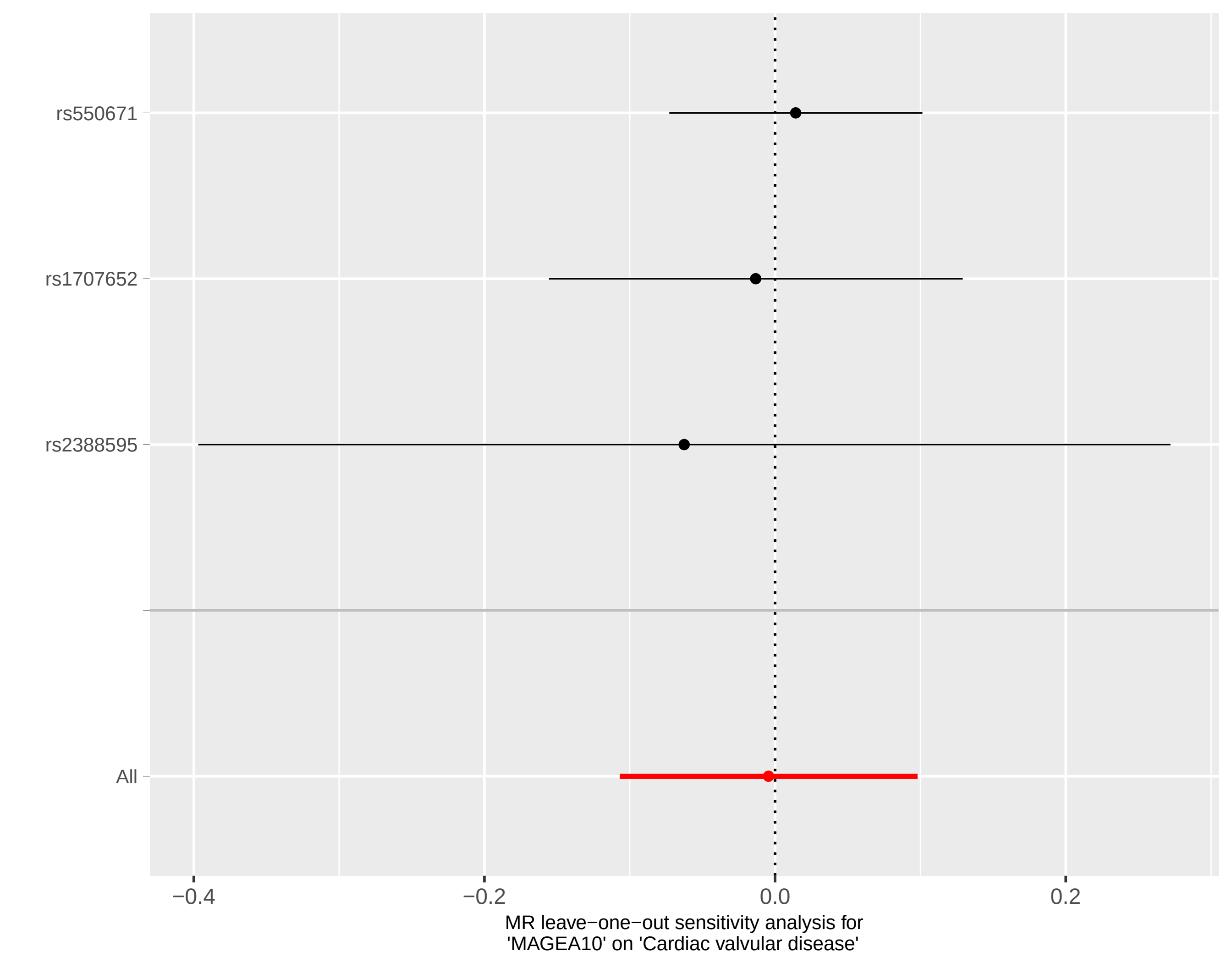

rs550671
rs1707652
rs2388595
All
−0.4
−0.2
0.0
0.2
MR leave−one−out sensitivity analysis for
'MAGEA10' on 'Cardiac valvular disease'

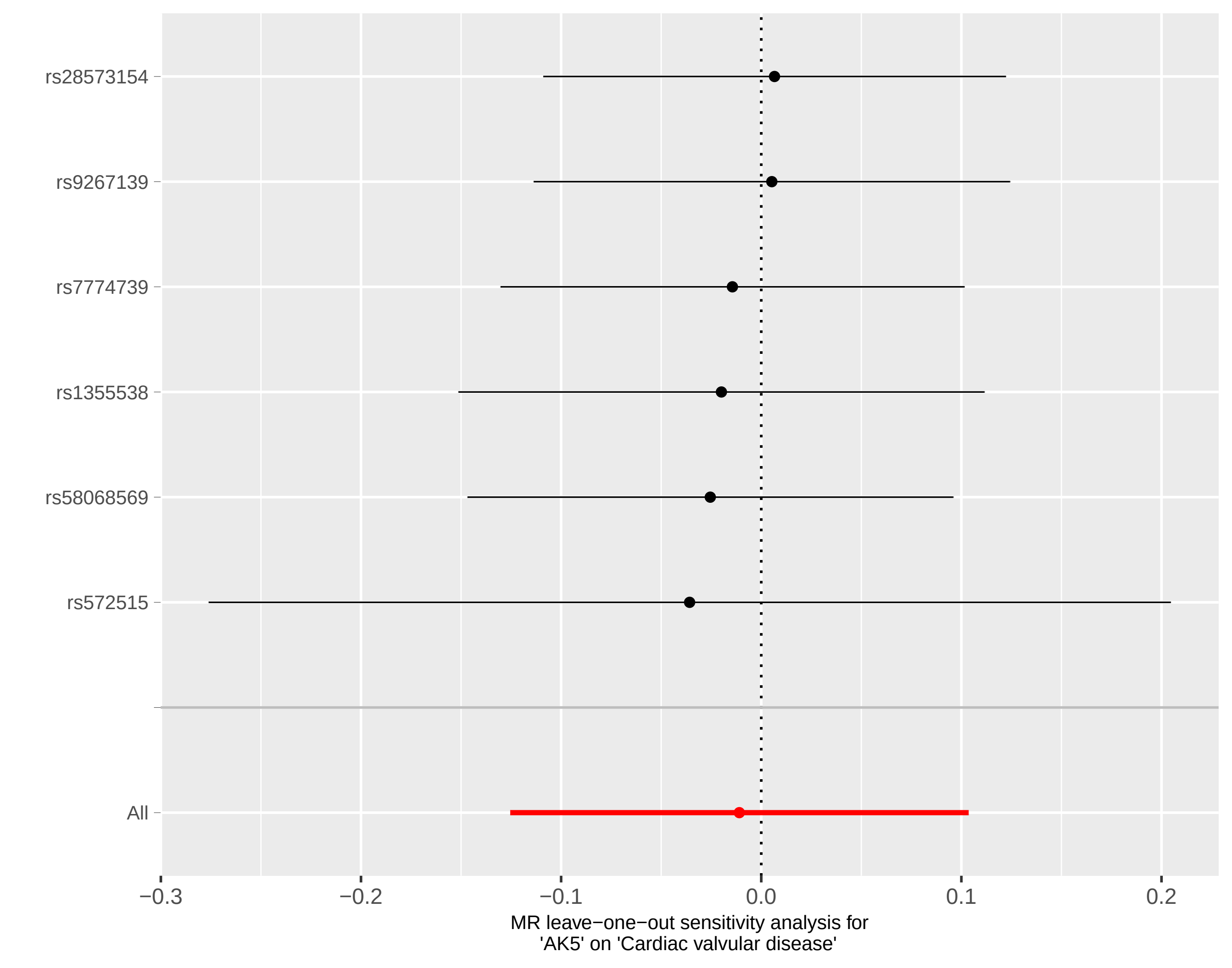

rs28573154
rs9267139
rs7774739
rs1355538
rs58068569
rs572515
All
−0.3
−0.2
−0.1
0.0
0.1
0.2
MR leave−one−out sensitivity analysis for
'AK5' on 'Cardiac valvular disease'

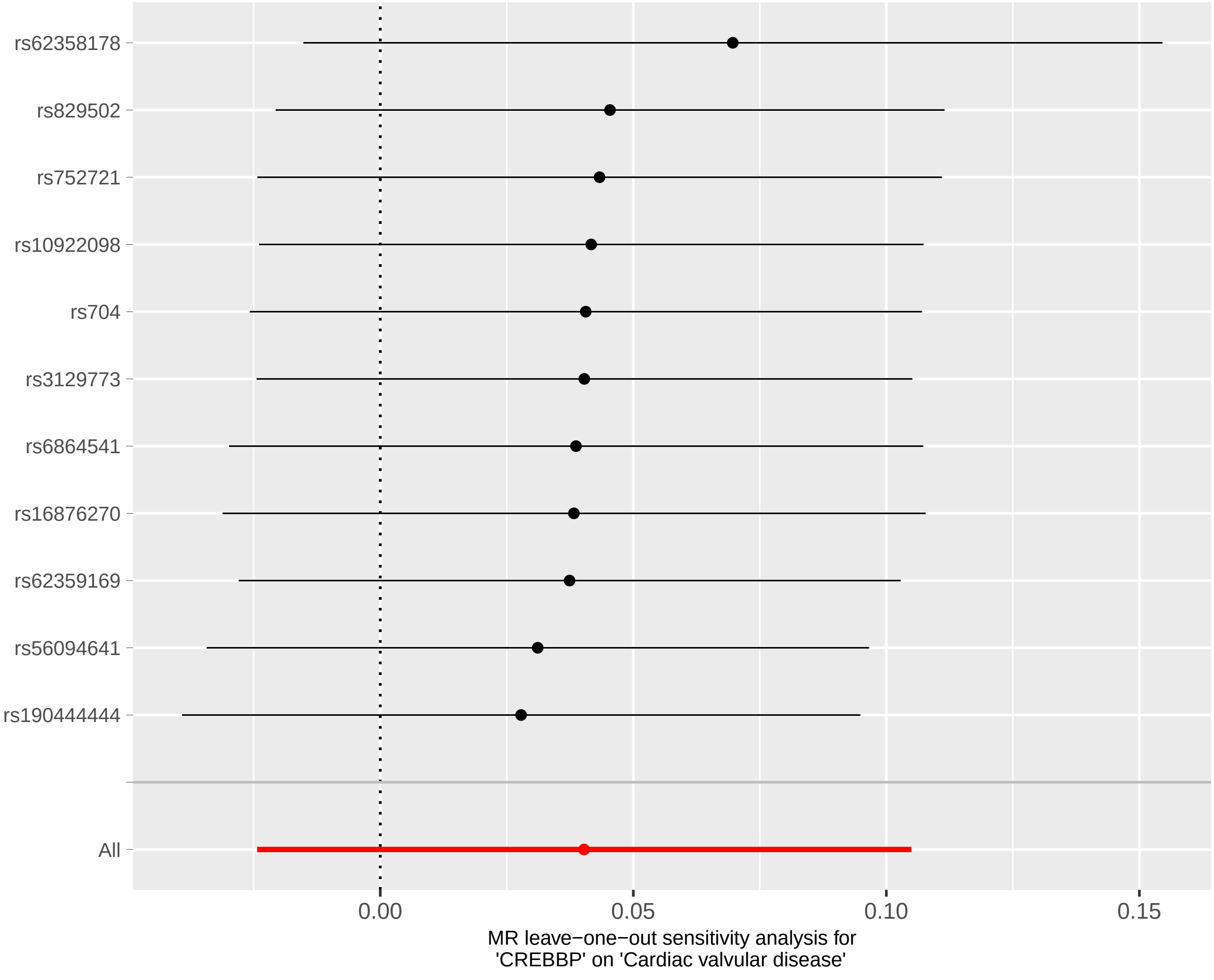
rs62358178
rs829502
rs752721
rs10922098
rs704
rs3129773
rs6864541
rs16876270
rs62359169
rs56094641
rs190444444
All
0.00
0.05
0.10
0.15
MR leave−one−out sensitivity analysis for
'CREBBP' on 'Cardiac valvular disease'

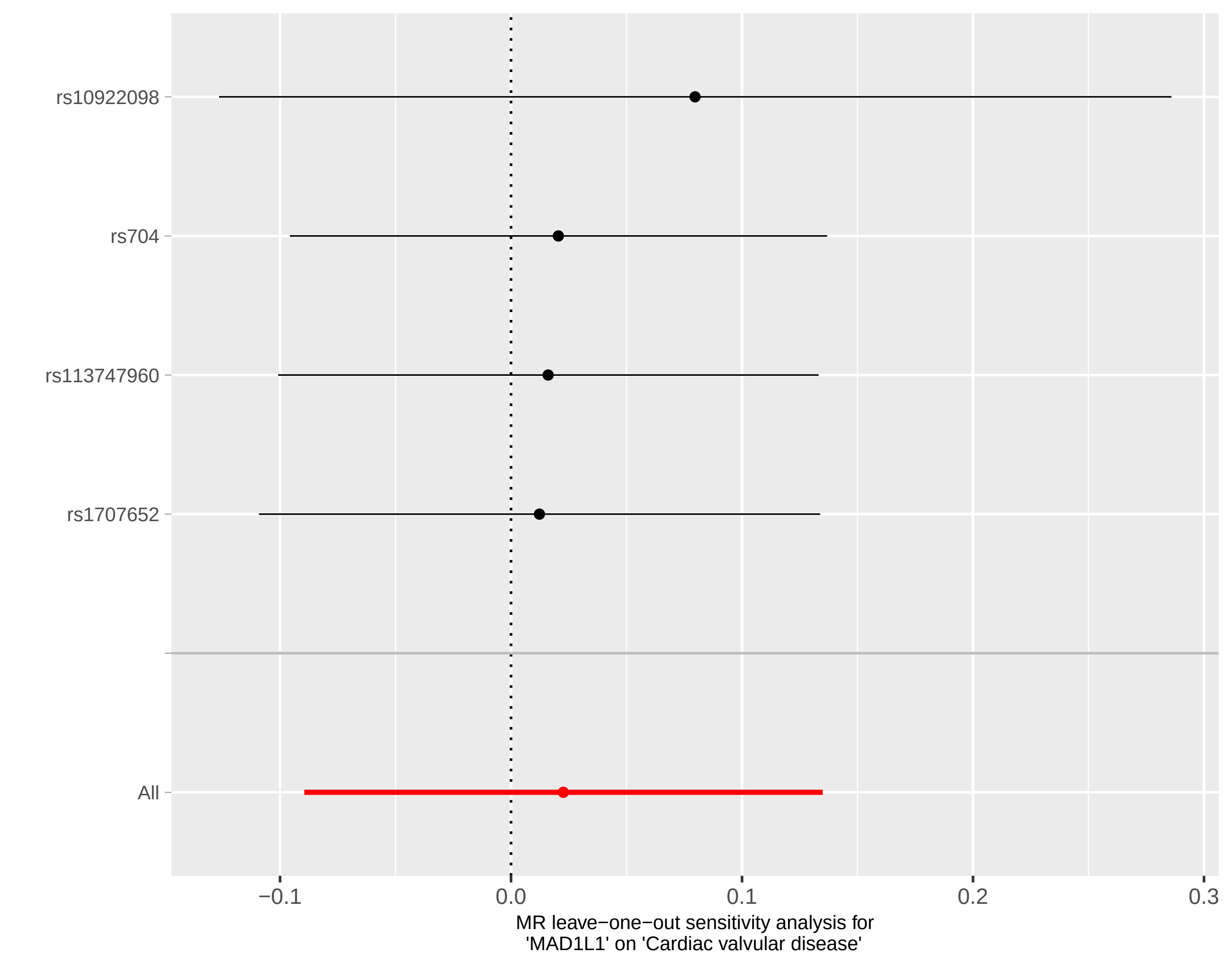
rs10922098
rs704
rs113747960
rs1707652
All
−0.1
0.0
0.1
0.2
0.3
MR leave−one−out sensitivity analysis for
'MAD1L1' on 'Cardiac valvular disease'

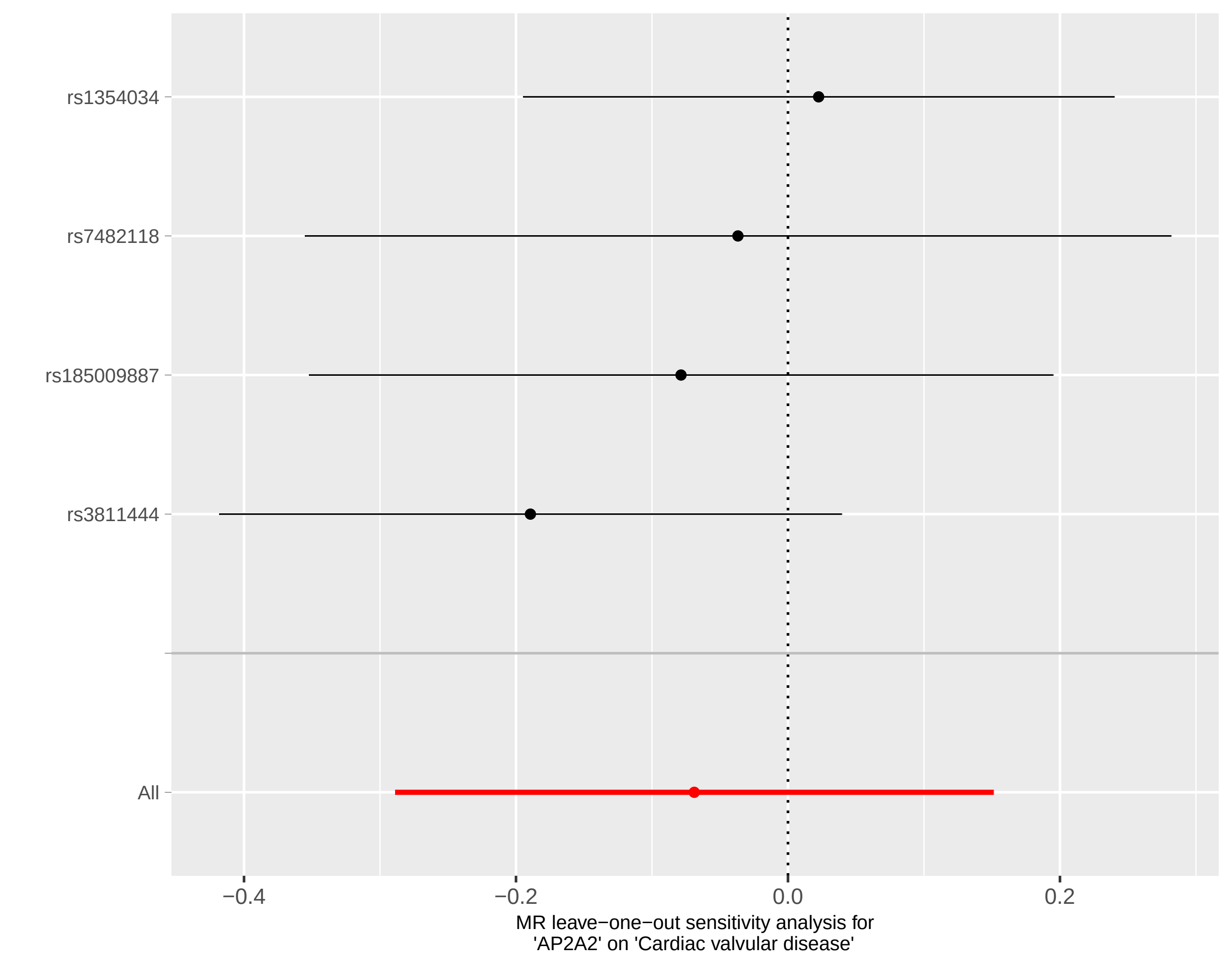
rs1354034
rs7482118
rs185009887
rs3811444
All
−0.4
−0.2
0.0
0.2
MR leave−one−out sensitivity analysis for
'AP2A2' on 'Cardiac valvular disease'

Insufficient number of SNPs

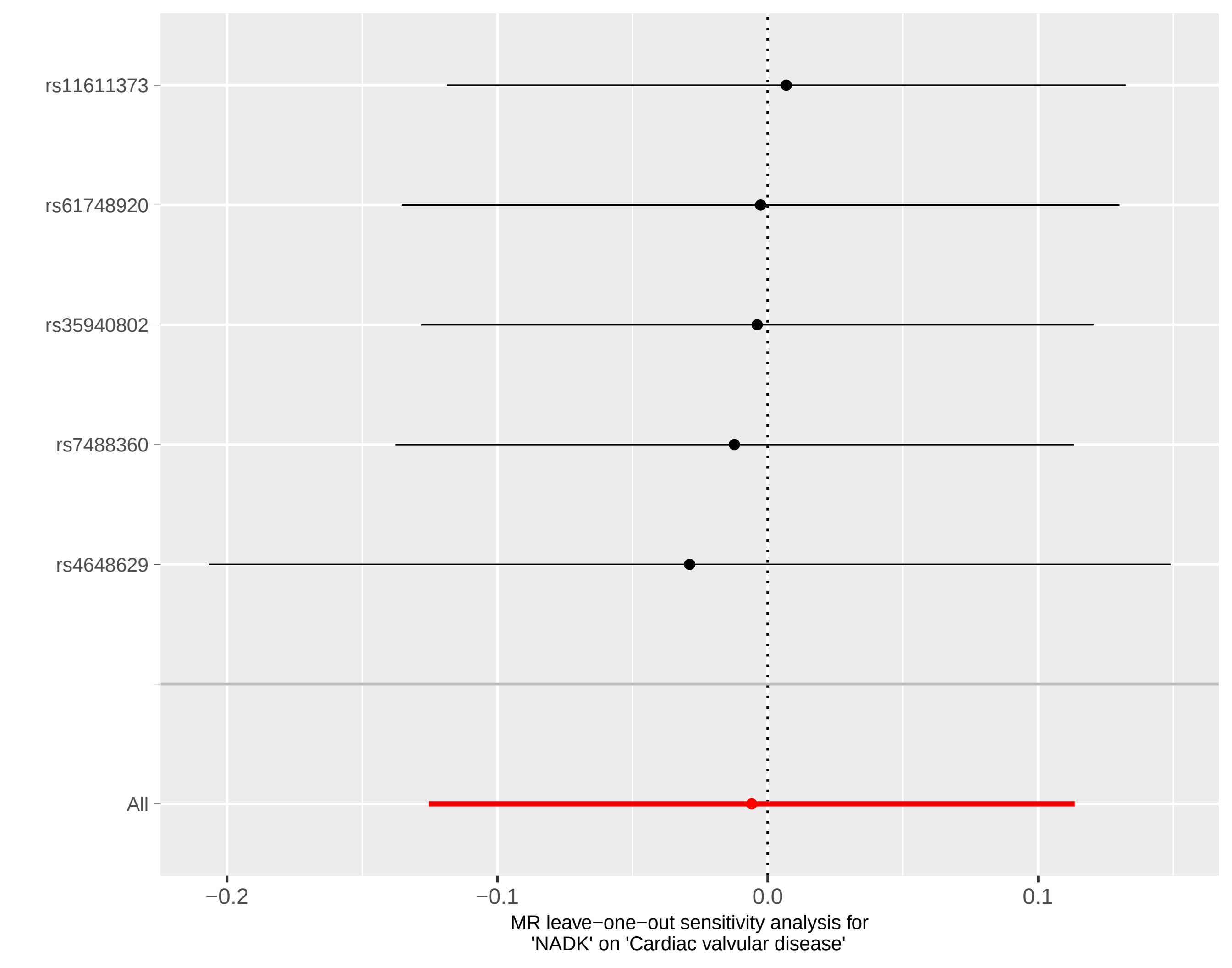
rs11611373
rs61748920
rs35940802
rs7488360
rs4648629
All
−0.2
−0.1
0.0
0.1
MR leave−one−out sensitivity analysis for
'NADK' on 'Cardiac valvular disease'

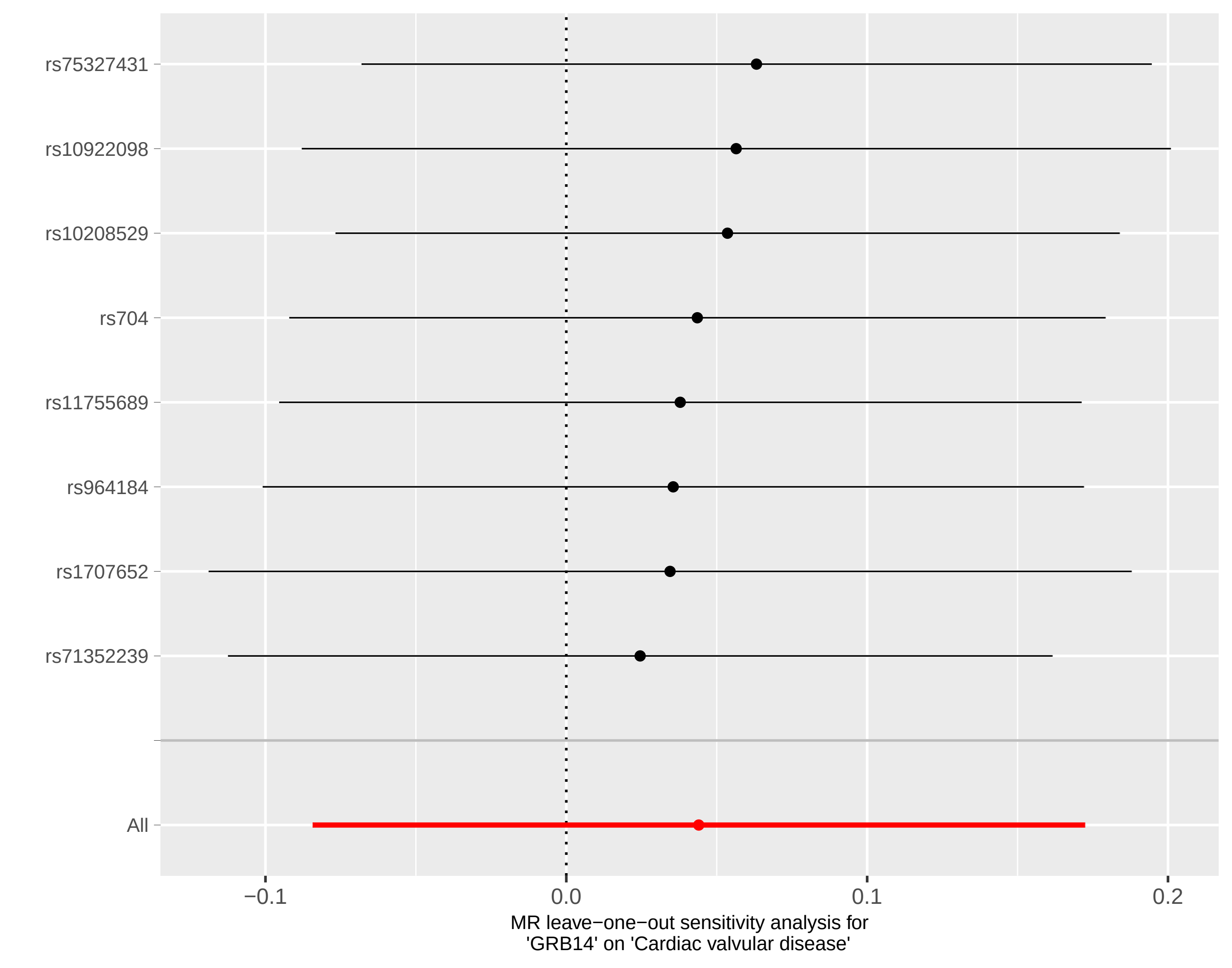
rs75327431
rs10922098
rs10208529
rs704
rs11755689
rs964184
rs1707652
rs71352239
All
−0.1
0.0
0.1
0.2
MR leave−one−out sensitivity analysis for
'GRB14' on 'Cardiac valvular disease'

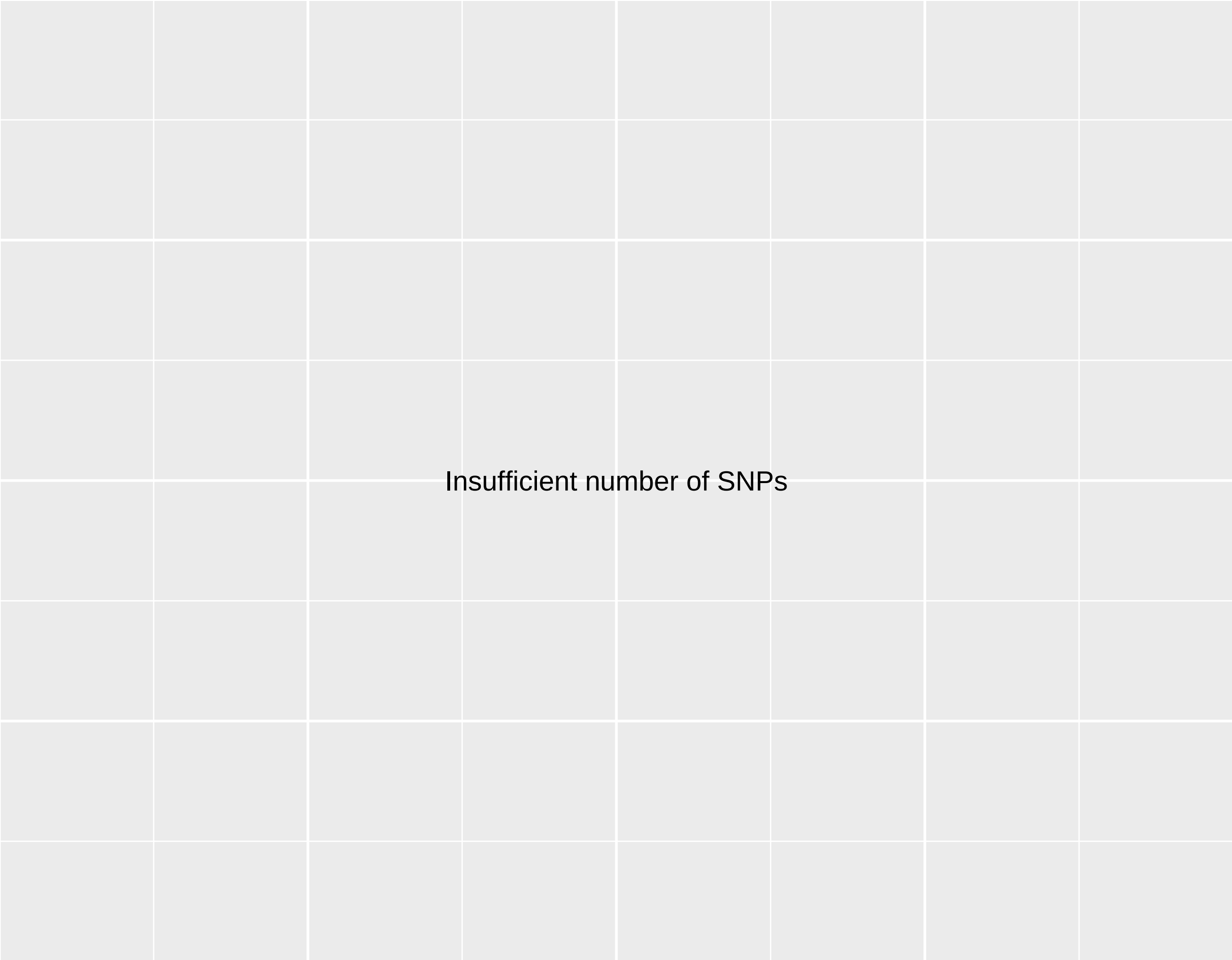
Insufficient number of SNPs

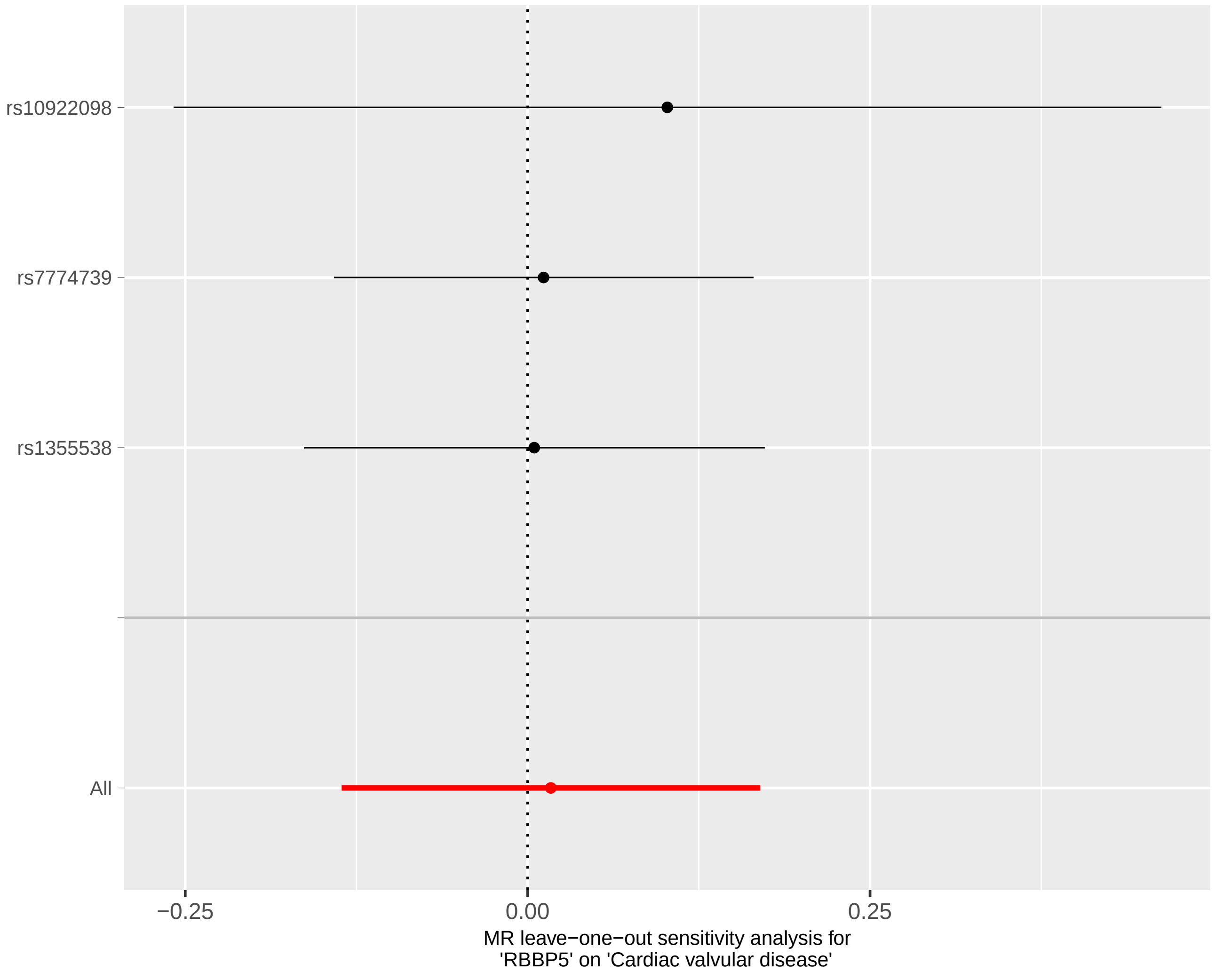
rs10922098
rs7774739
rs1355538
All
−0.25
0.00
0.25
MR leave−one−out sensitivity analysis for
'RBBP5' on 'Cardiac valvular disease'

Insufficient number of SNPs

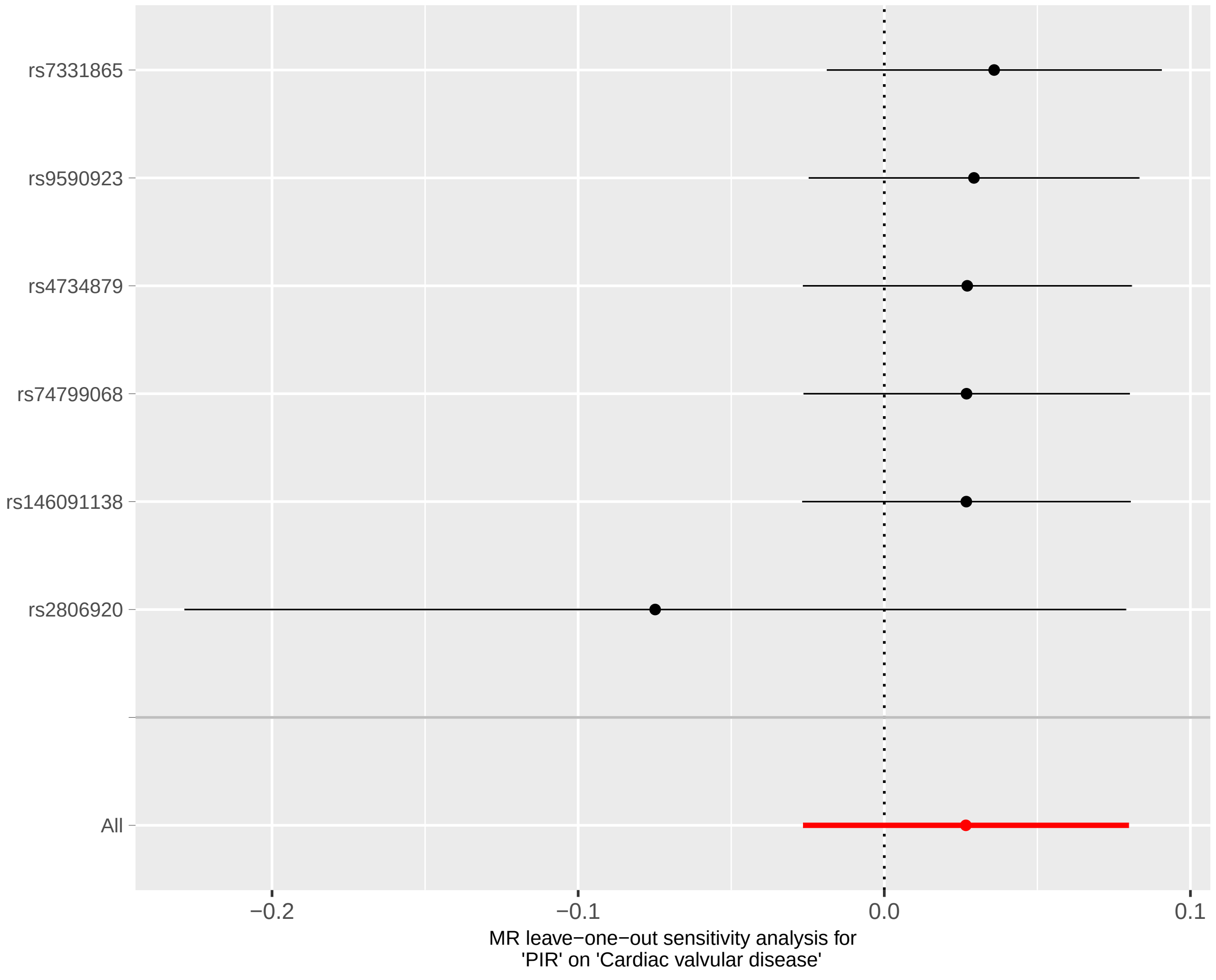

rs7331865
rs9590923
rs4734879
rs74799068
rs146091138
rs2806920
All
−0.2
−0.1
0.0
0.1
MR leave−one−out sensitivity analysis for
'PIR' on 'Cardiac valvular disease'

Insufficient number of SNPs

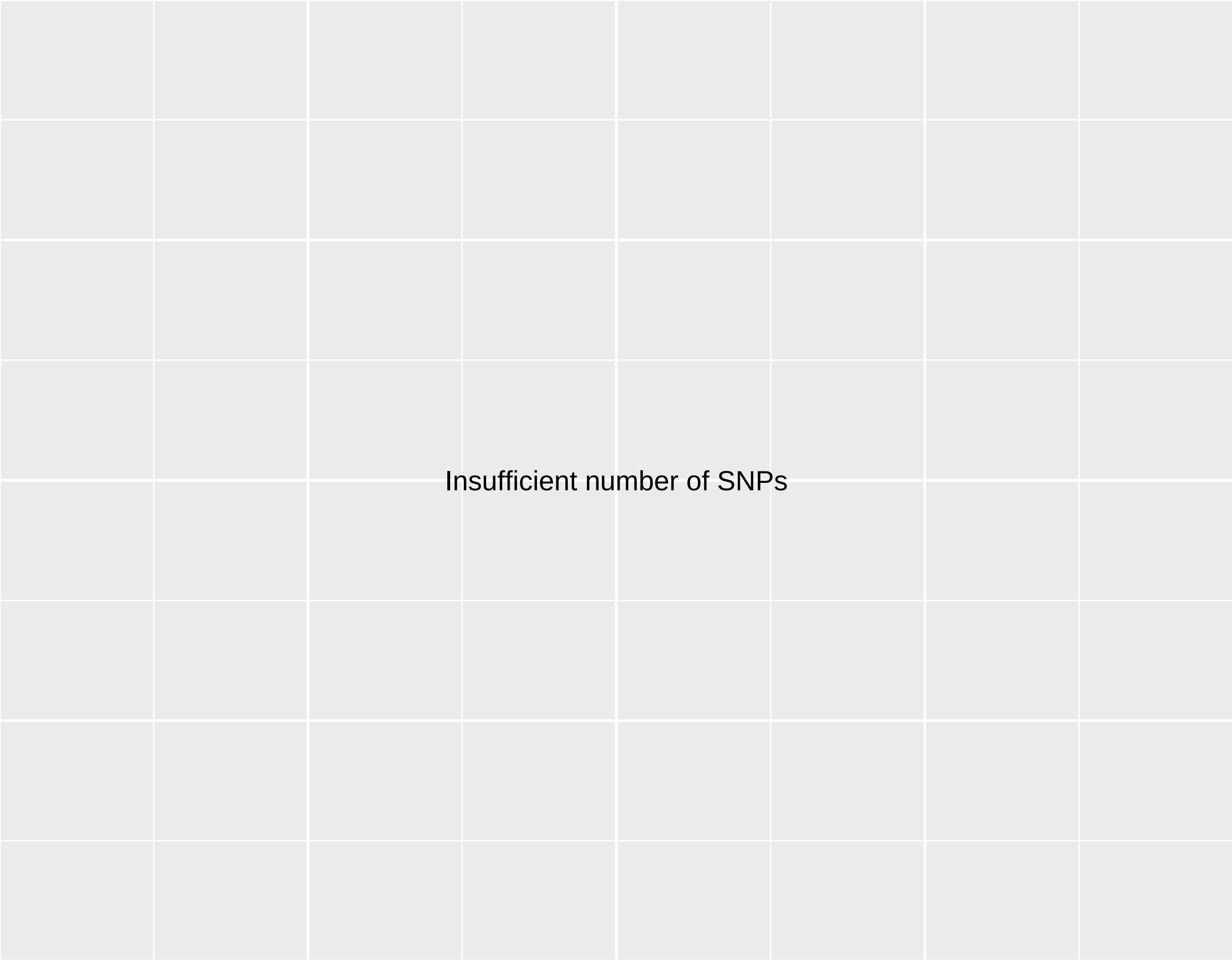
Insufficient number of SNPs

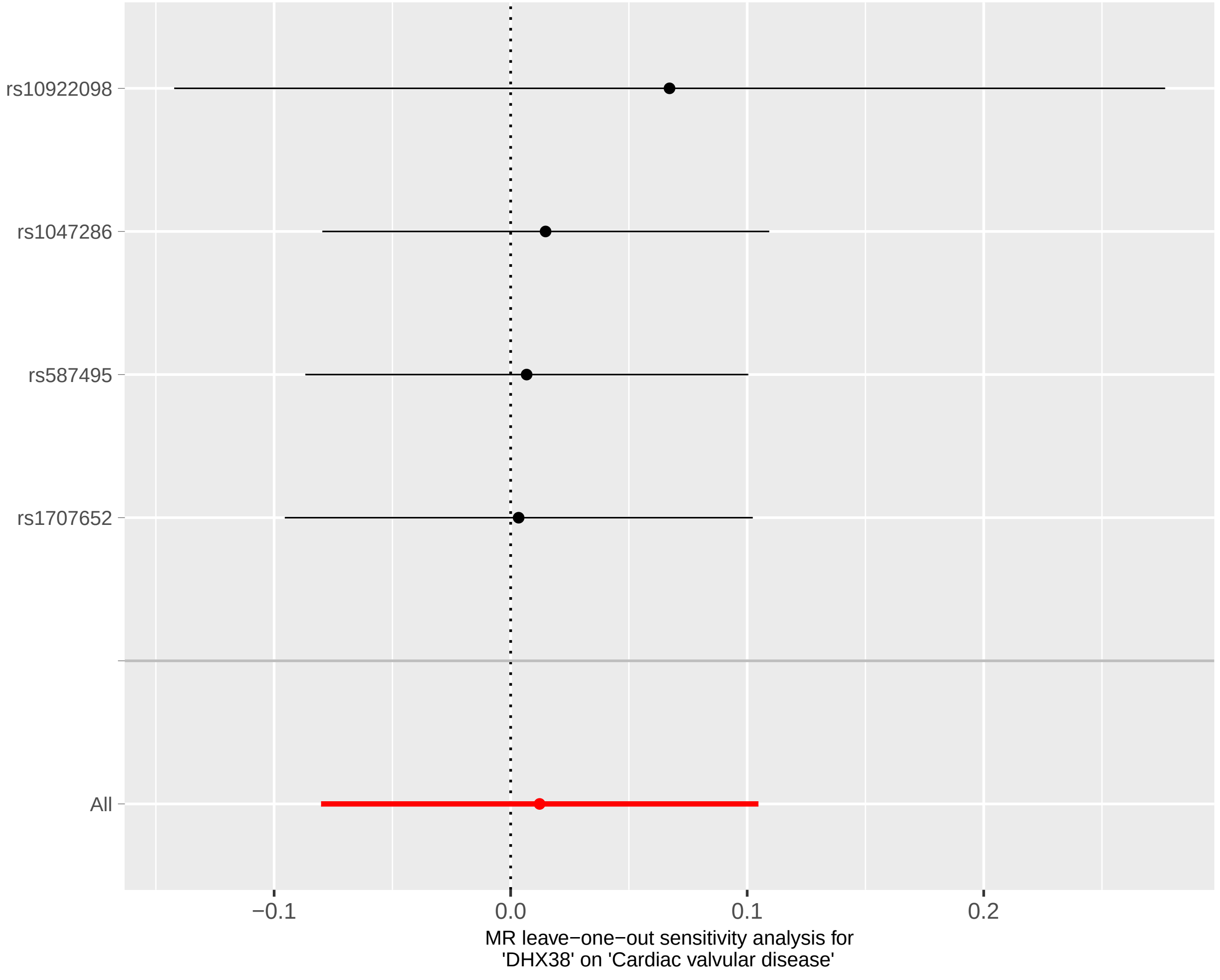
rs10922098
rs1047286
rs587495
rs1707652
All
−0.1
0.0
0.1
0.2
MR leave−one−out sensitivity analysis for
'DHX38' on 'Cardiac valvular disease'

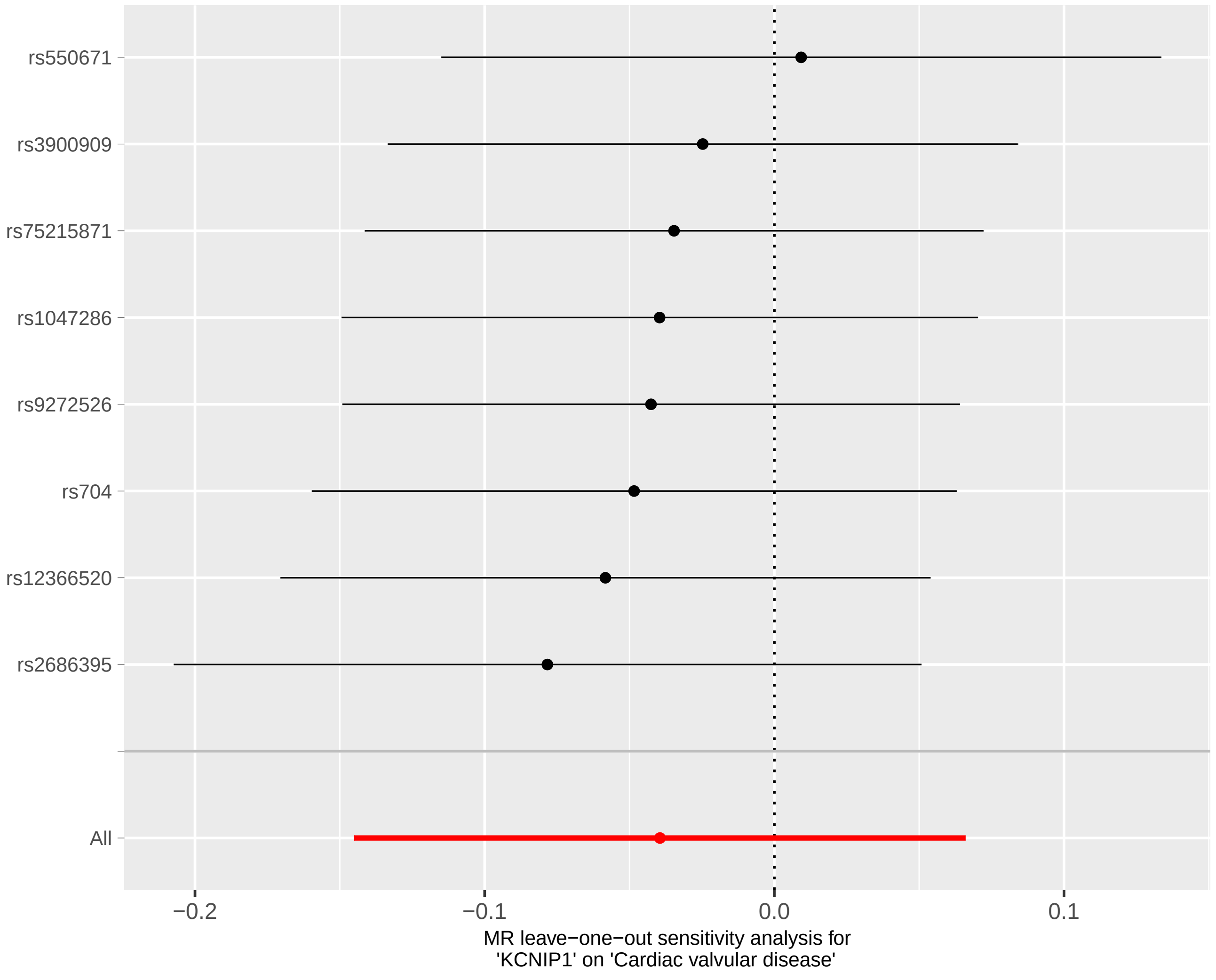
rs550671
rs3900909
rs75215871
rs1047286
rs9272526
rs704
rs12366520
rs2686395
All
−0.2
−0.1
0.0
0.1
MR leave−one−out sensitivity analysis for
'KCNIP1' on 'Cardiac valvular disease'

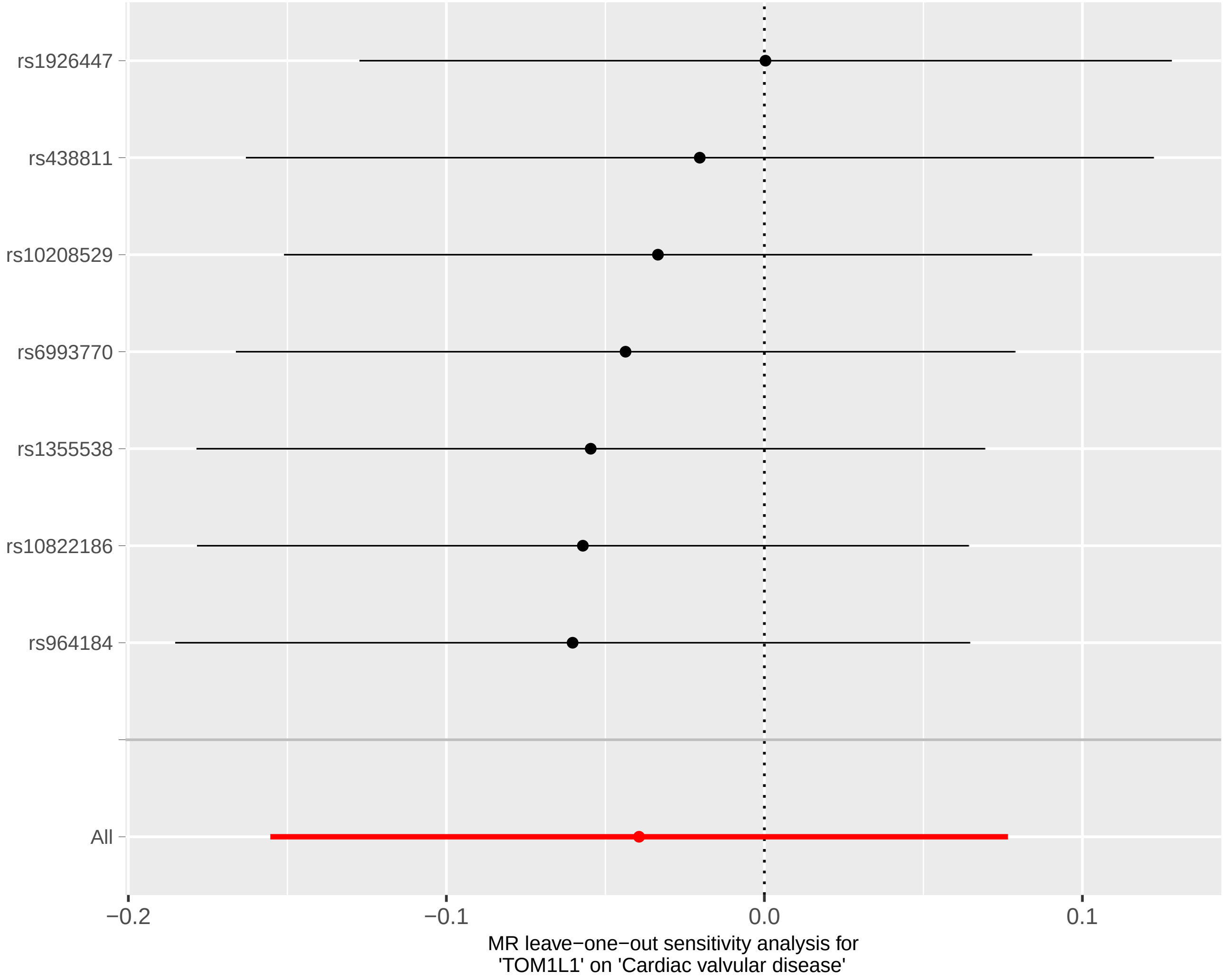

rs1926447
rs438811
rs10208529
rs6993770
rs1355538
rs10822186
rs964184
All
−0.2
−0.1
0.0
0.1
MR leave−one−out sensitivity analysis for
'TOM1L1' on 'Cardiac valvular disease'

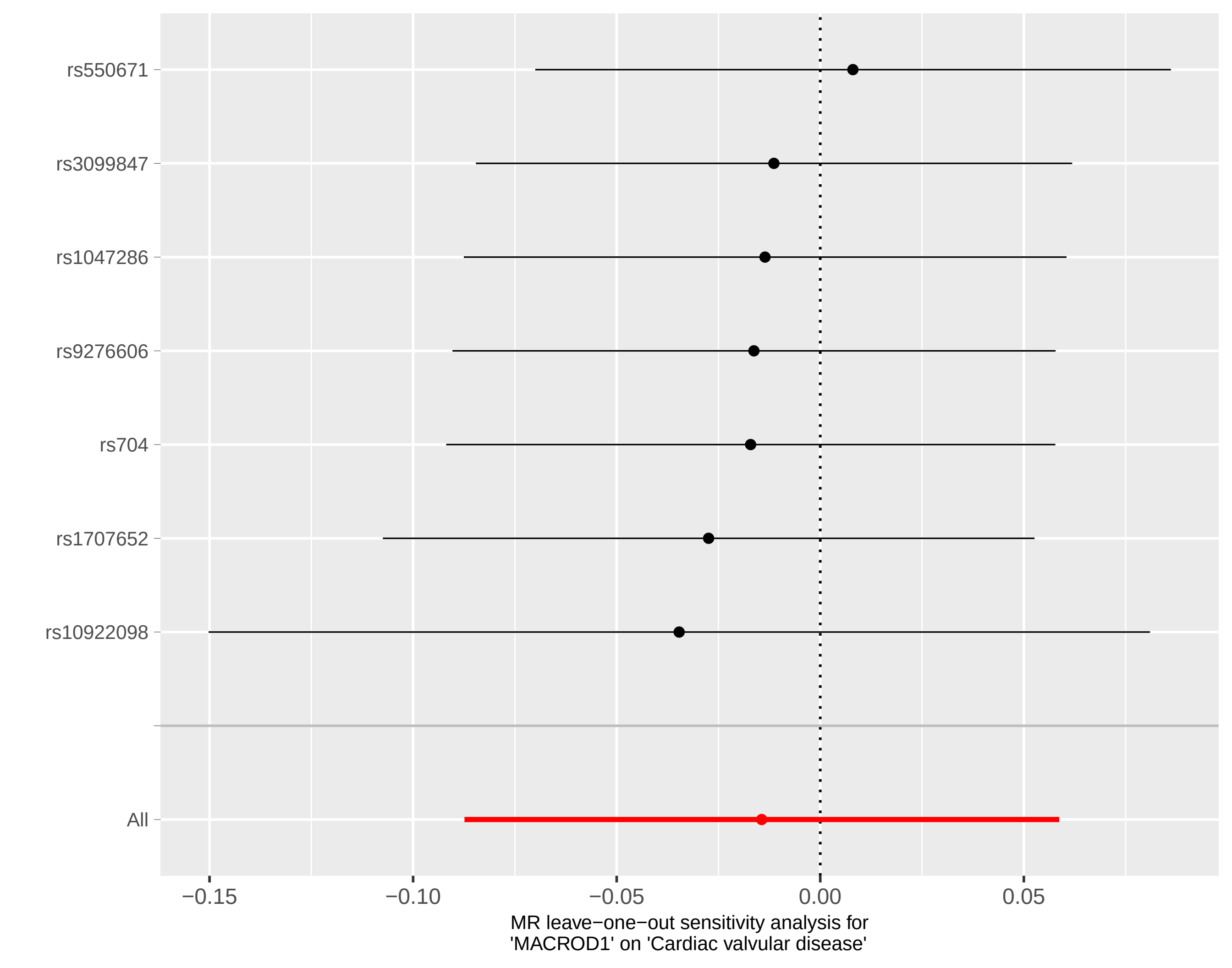
rs550671
rs3099847
rs1047286
rs9276606
rs704
rs1707652
rs10922098
All
−0.15
−0.10
−0.05
0.00
0.05
MR leave−one−out sensitivity analysis for
'MACROD1' on 'Cardiac valvular disease'

Insufficient number of SNPs

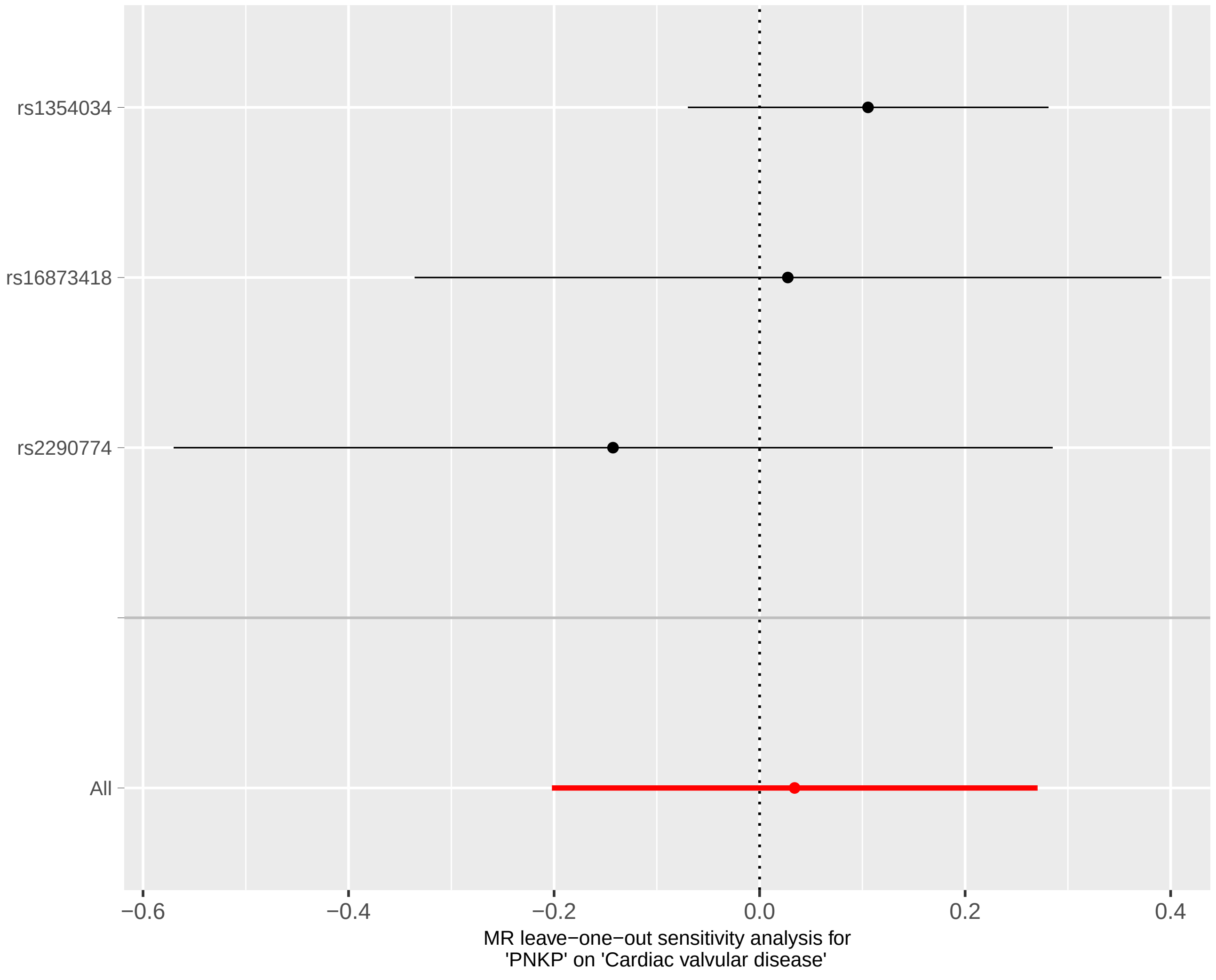
rs1354034
rs16873418
rs2290774
All
−0.6
−0.4
−0.2
0.0
0.2
0.4
MR leave−one−out sensitivity analysis for
'PNKP' on 'Cardiac valvular disease'

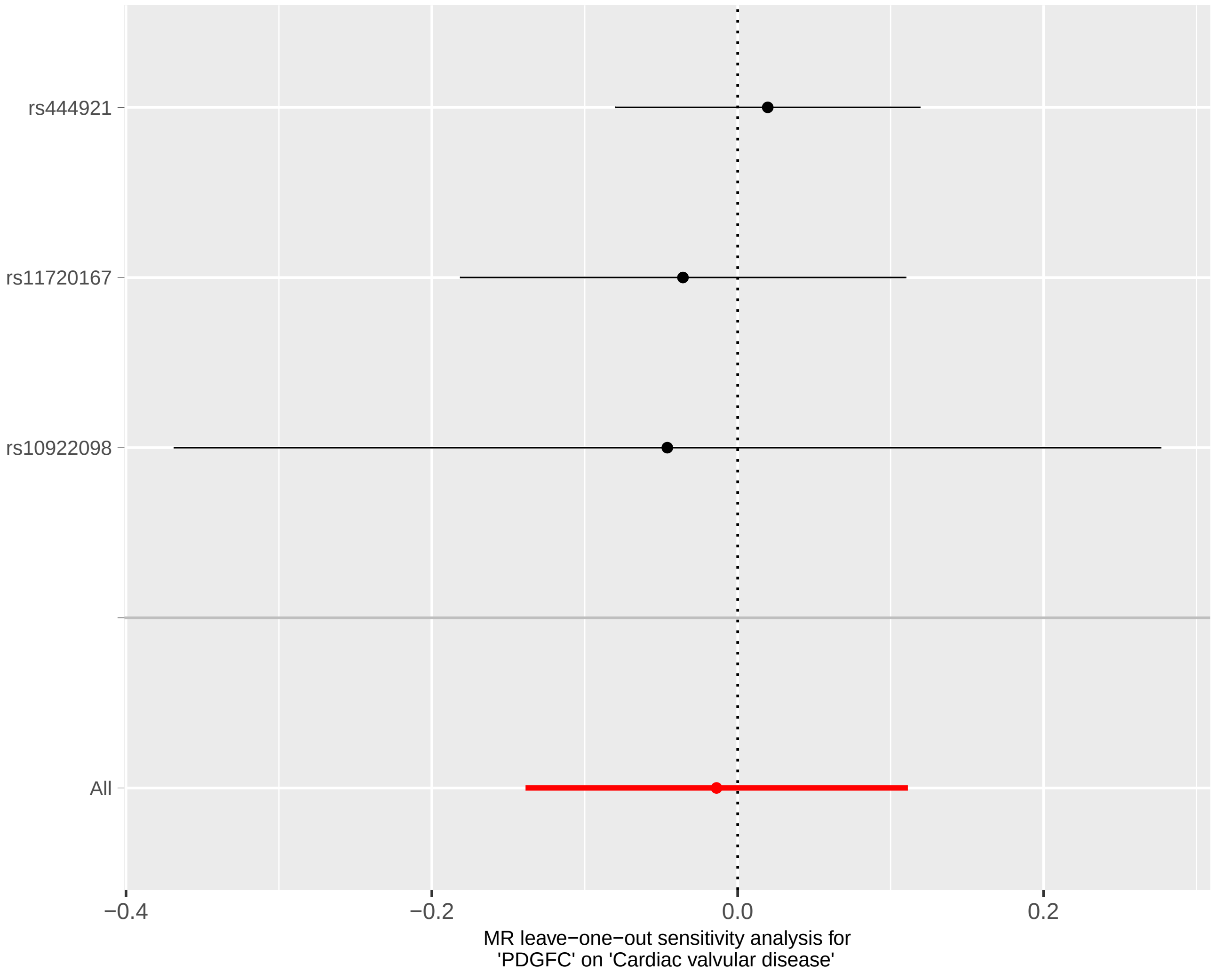

rs444921
rs11720167
rs10922098
All
−0.4
−0.2
0.0
0.2
MR leave−one−out sensitivity analysis for
'PDGFC' on 'Cardiac valvular disease'

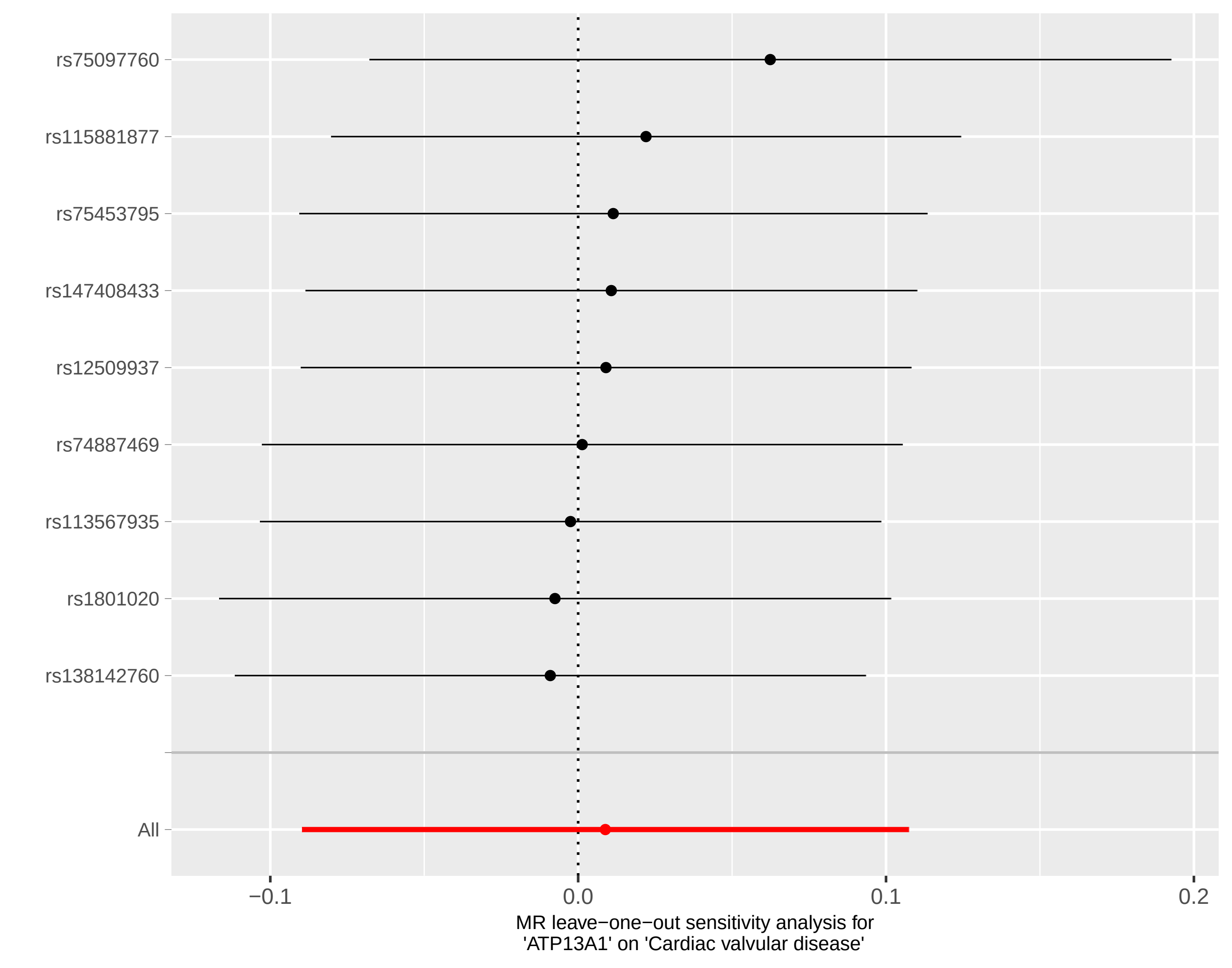
rs75097760
rs115881877
rs75453795
rs147408433
rs12509937
rs74887469
rs113567935
rs1801020
rs138142760
All
−0.1
0.0
0.1
0.2
MR leave−one−out sensitivity analysis for
'ATP13A1' on 'Cardiac valvular disease'

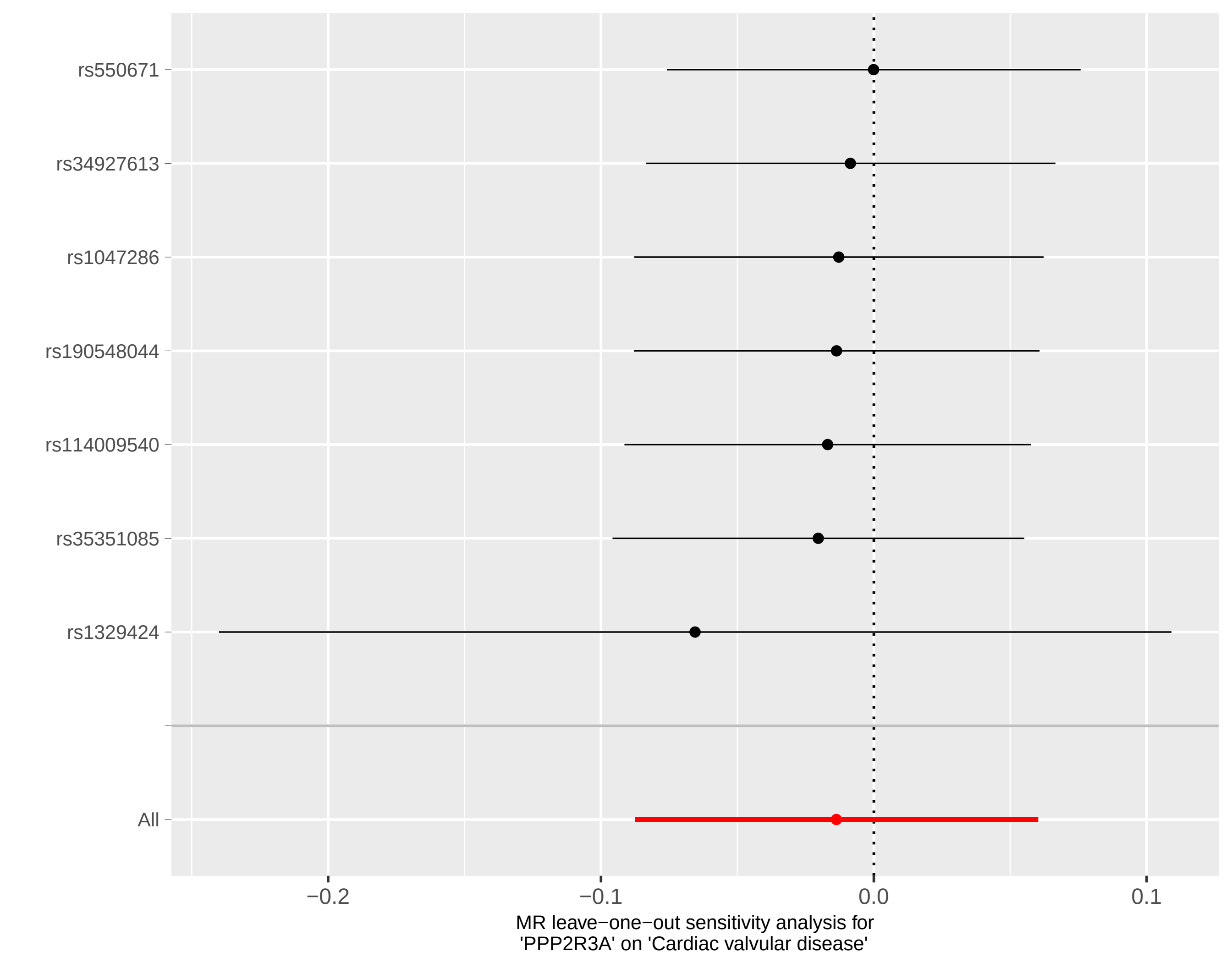
rs550671
rs34927613
rs1047286
rs190548044
rs114009540
rs35351085
rs1329424
All
−0.2
−0.1
0.0
0.1
MR leave−one−out sensitivity analysis for
'PPP2R3A' on 'Cardiac valvular disease'

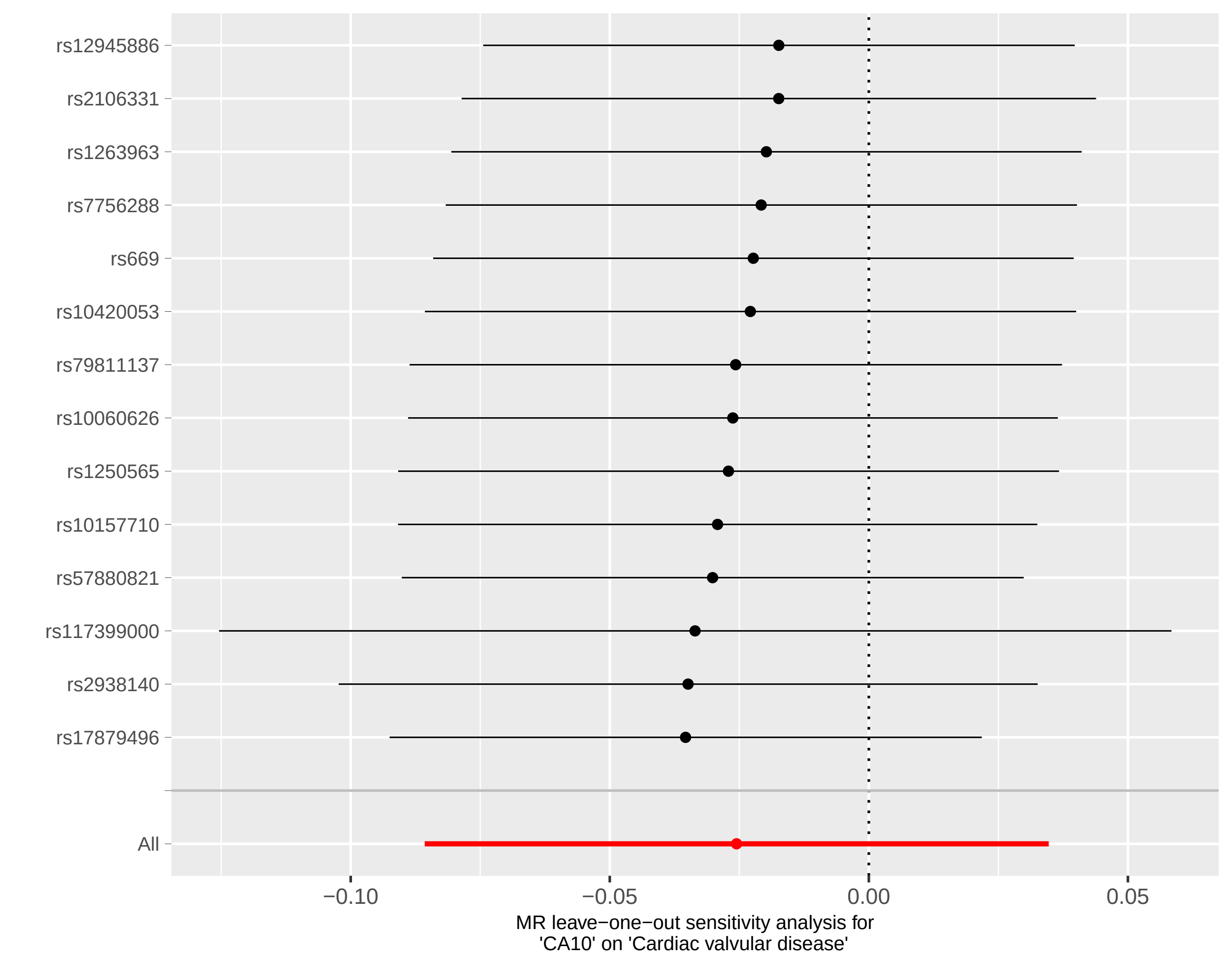
rs12945886
rs2106331
rs1263963
rs7756288
rs669
rs10420053
rs79811137
rs10060626
rs1250565
rs10157710
rs57880821
rs117399000
rs2938140
rs17879496
All
−0.10
−0.05
0.00
0.05
MR leave−one−out sensitivity analysis for
'CA10' on 'Cardiac valvular disease'

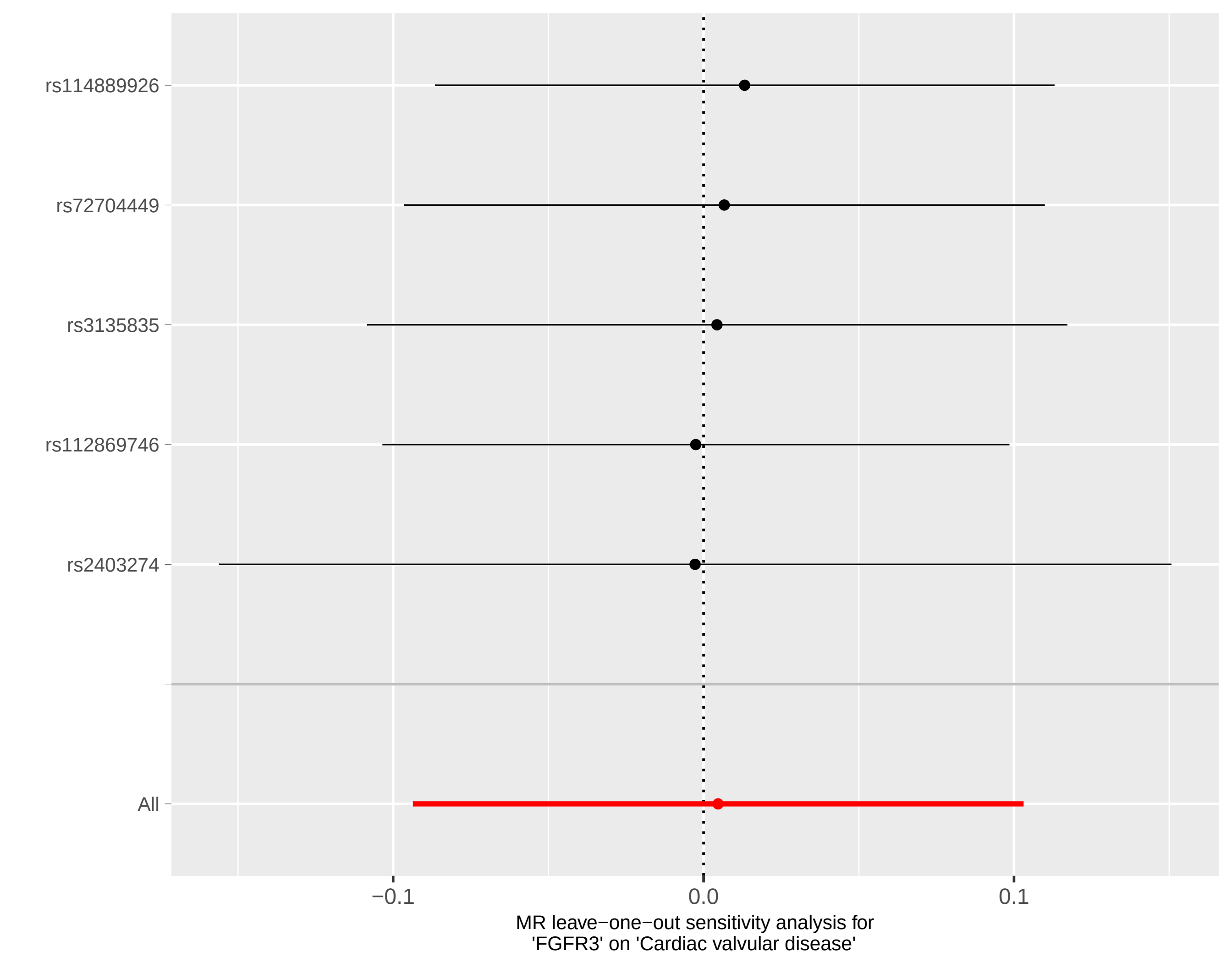
rs114889926
rs72704449
rs3135835
rs112869746
rs2403274
All
−0.1
0.0
0.1
MR leave−one−out sensitivity analysis for
'FGFR3' on 'Cardiac valvular disease'

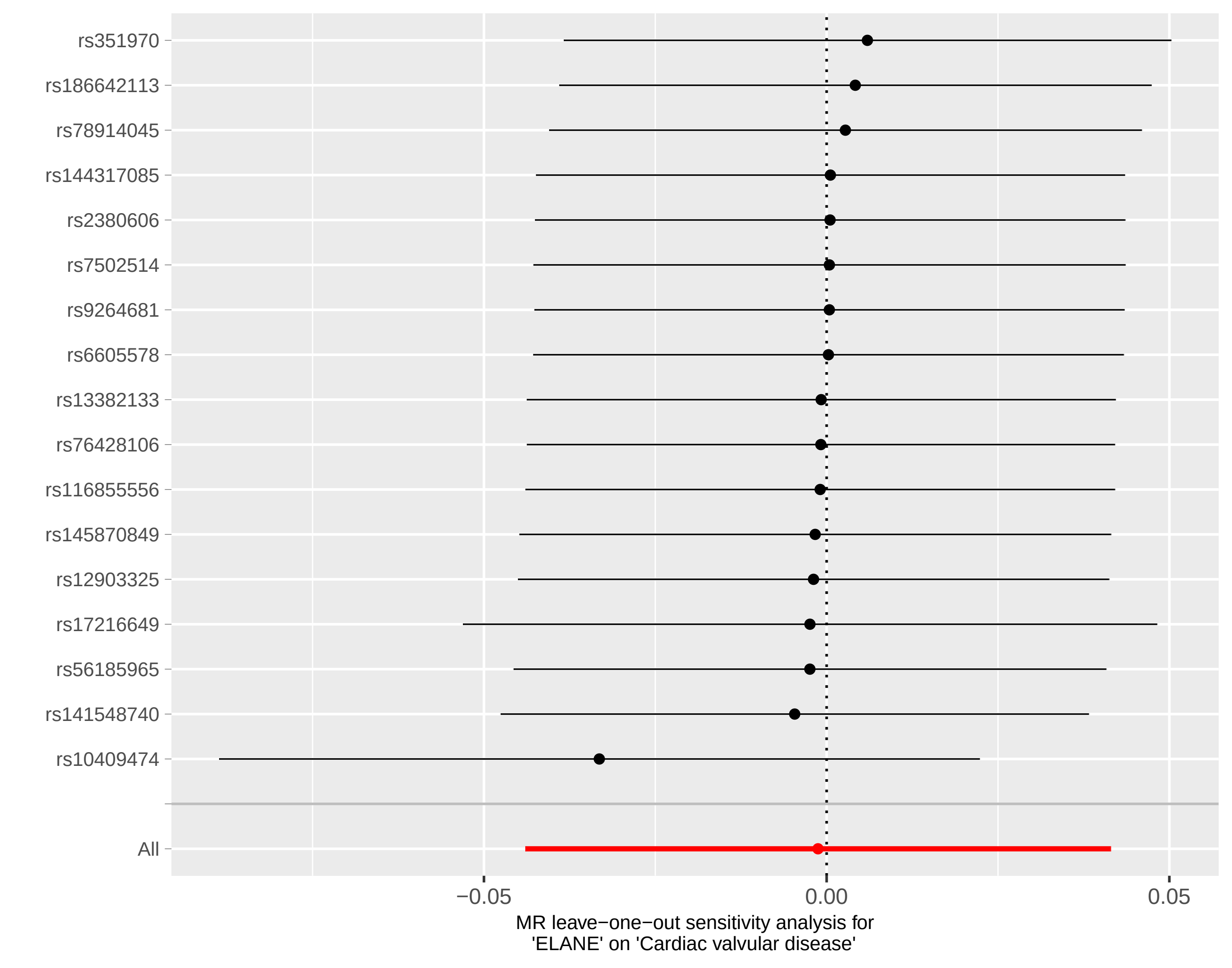

rs351970
rs186642113
rs78914045
rs144317085
rs2380606
rs7502514
rs9264681
rs6605578
rs13382133
rs76428106
rs116855556
rs145870849
rs12903325
rs17216649
rs56185965
rs141548740
rs10409474
All
−0.05
0.00
0.05
MR leave−one−out sensitivity analysis for
'ELANE' on 'Cardiac valvular disease'

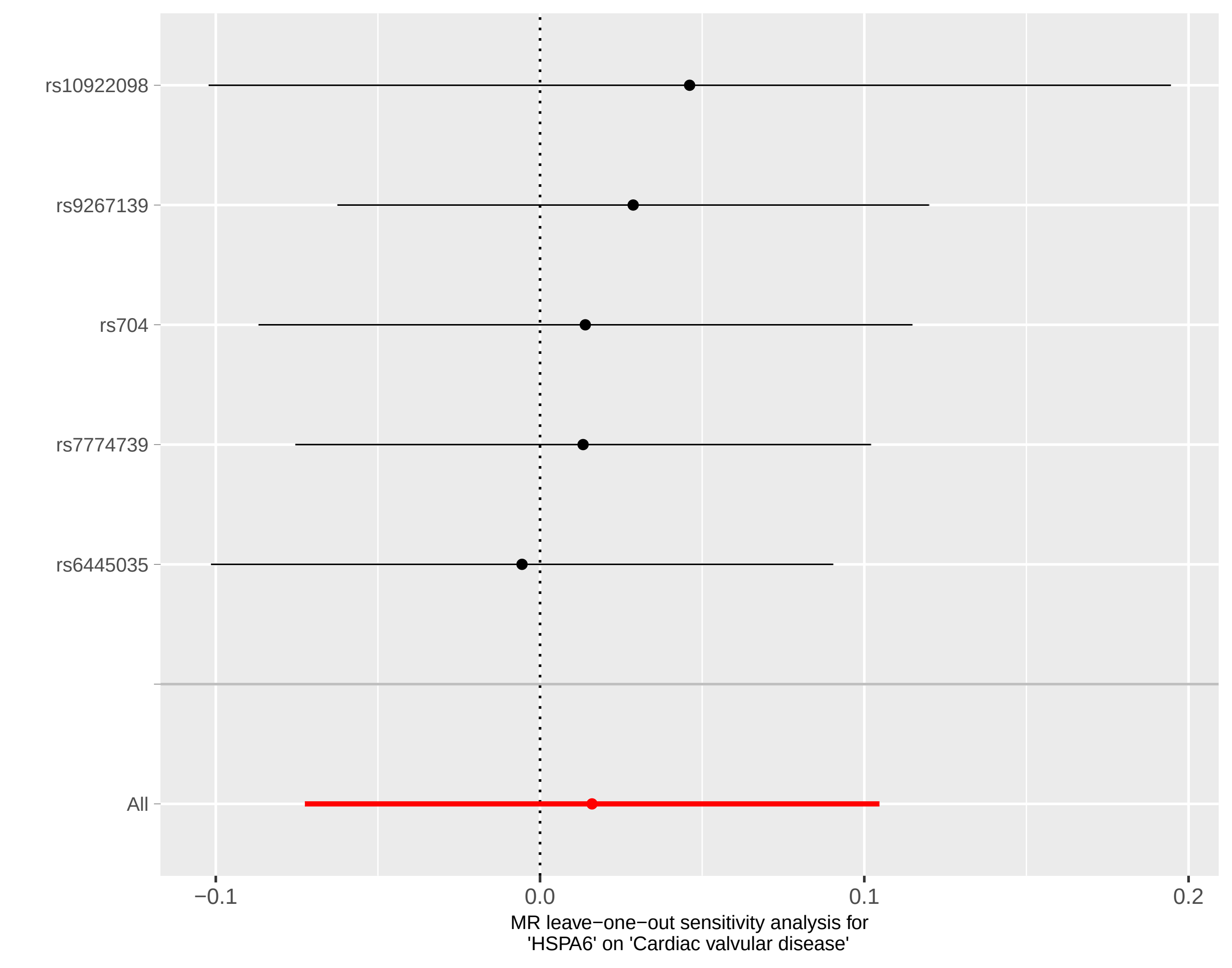
rs10922098
rs9267139
rs704
rs7774739
rs6445035
All
−0.1
0.0
0.1
0.2
MR leave−one−out sensitivity analysis for
'HSPA6' on 'Cardiac valvular disease'

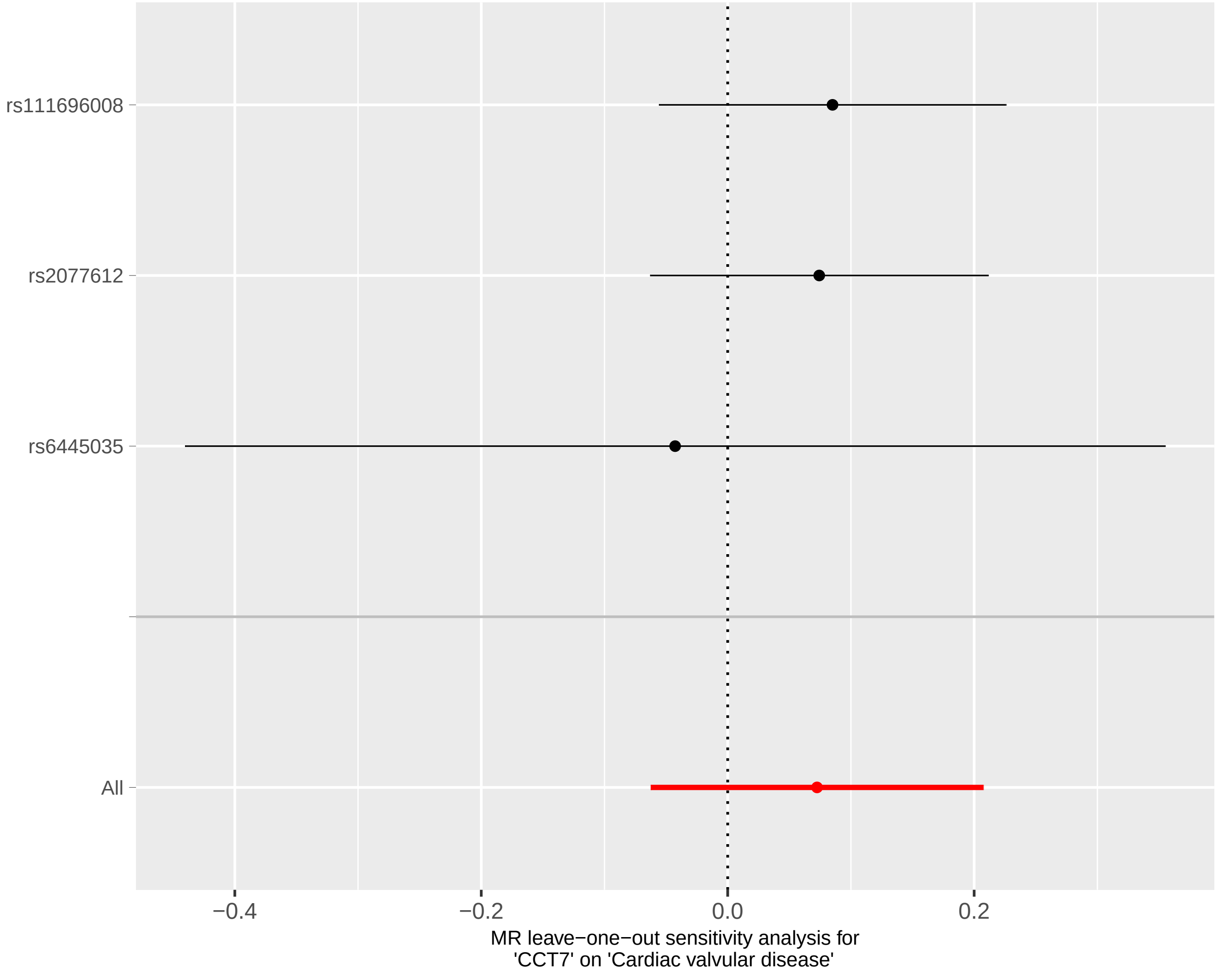
rs111696008
rs2077612
rs6445035
All
−0.4
−0.2
0.0
0.2
MR leave−one−out sensitivity analysis for
'CCT7' on 'Cardiac valvular disease'

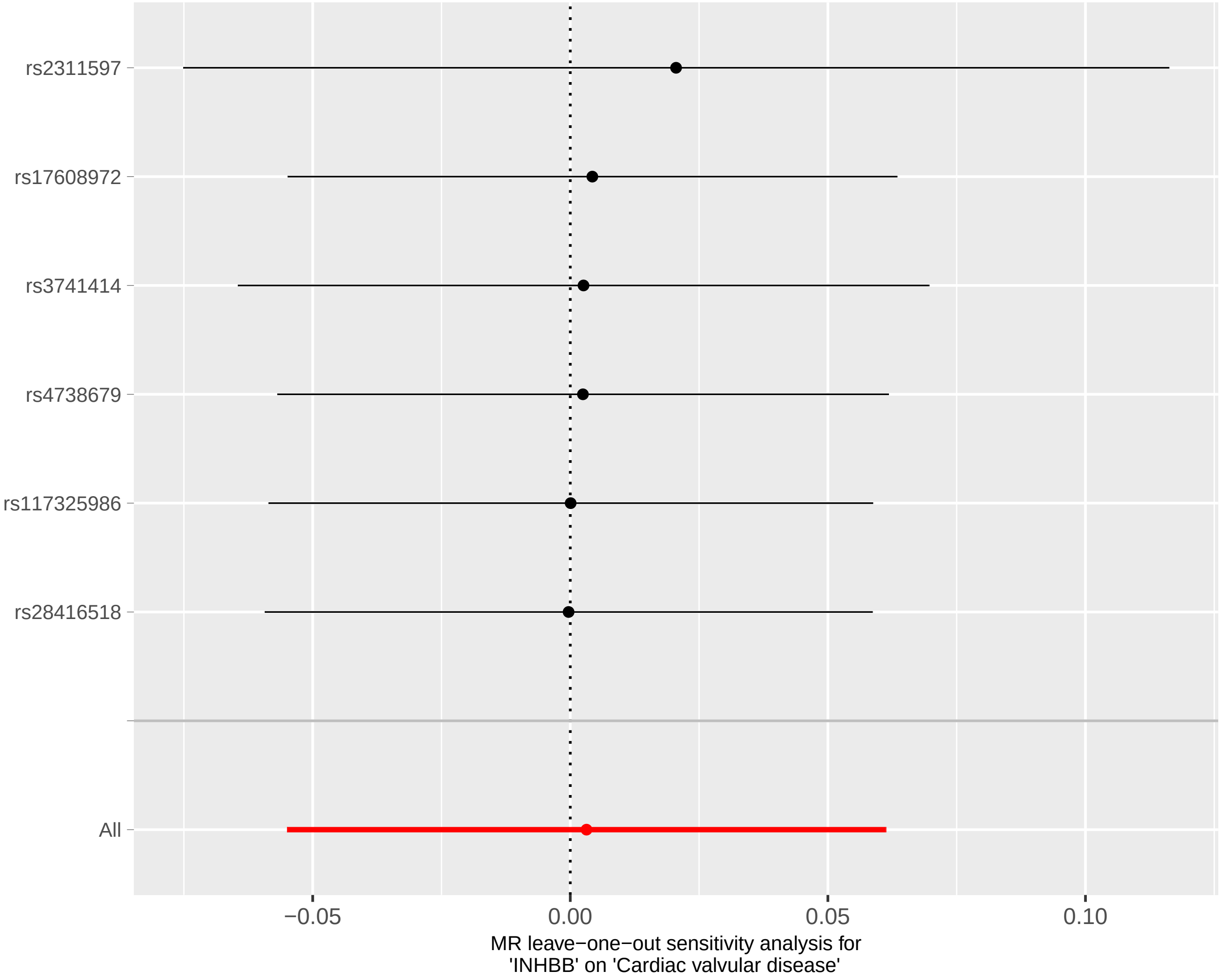
rs2311597
rs17608972
rs3741414
rs4738679
rs117325986
rs28416518
All
−0.05
0.00
0.05
0.10
MR leave−one−out sensitivity analysis for
'INHBB' on 'Cardiac valvular disease'

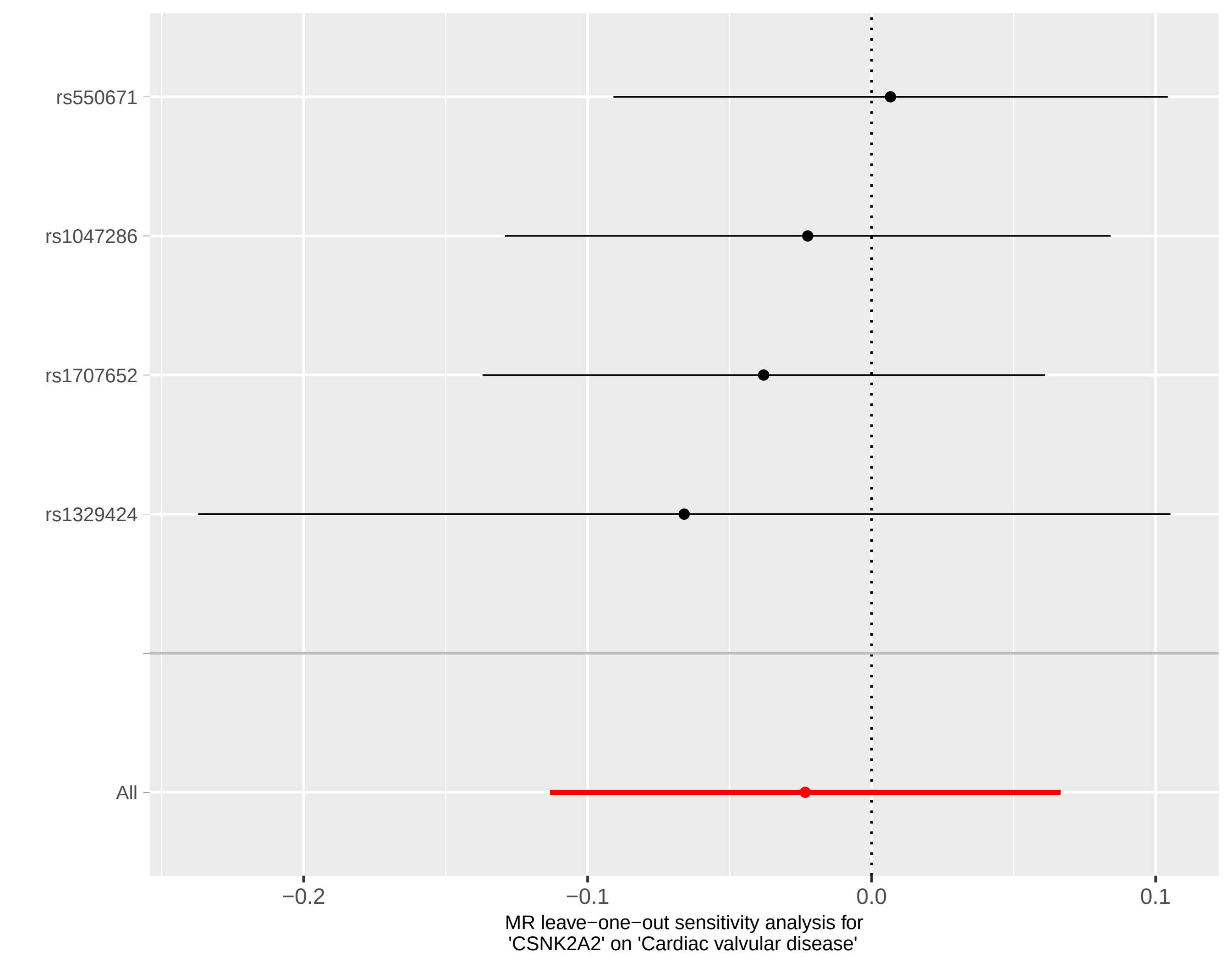

rs550671
rs1047286
rs1707652
rs1329424
All
−0.2
−0.1
0.0
0.1
MR leave−one−out sensitivity analysis for
'CSNK2A2' on 'Cardiac valvular disease'

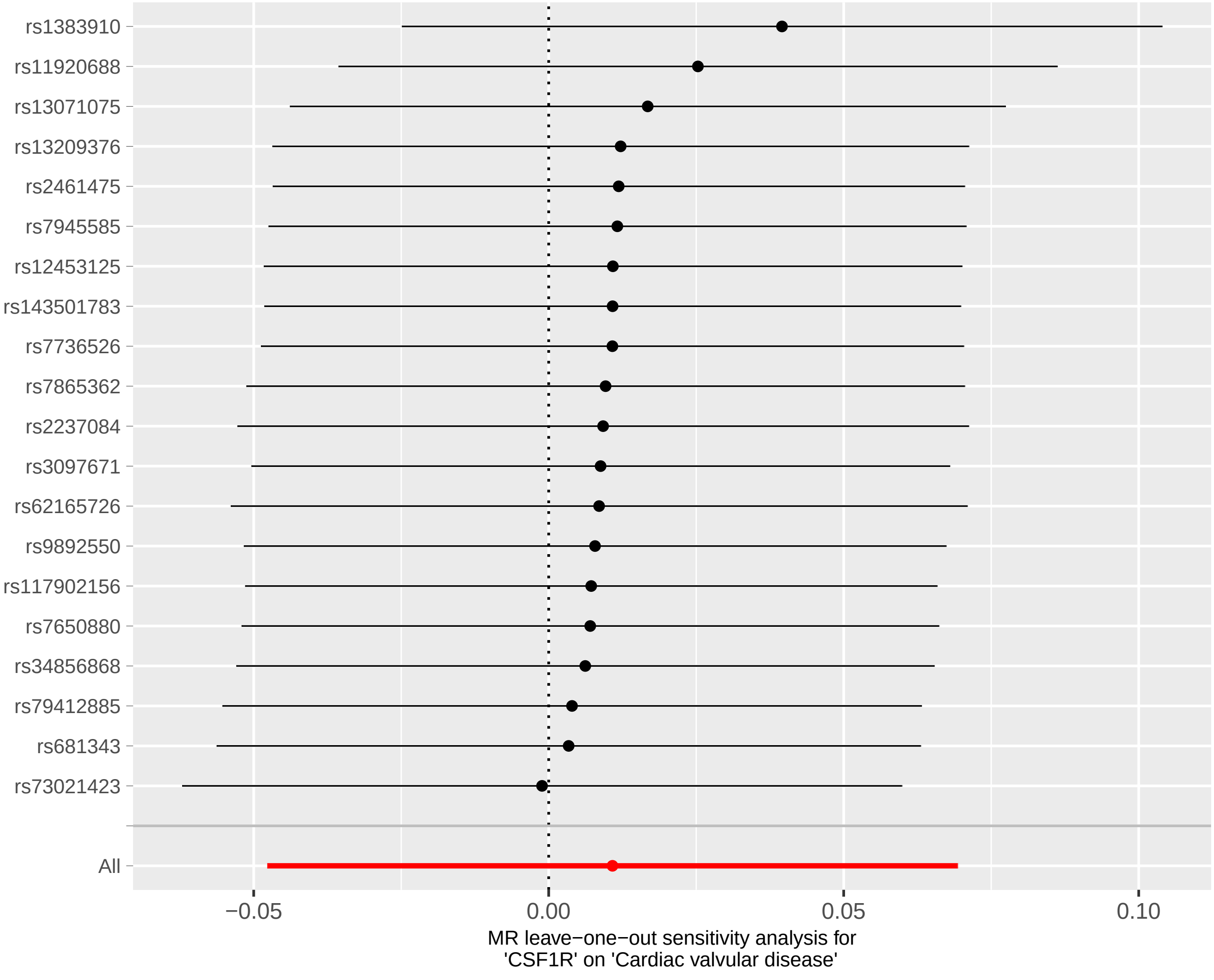
rs1383910
rs11920688
rs13071075
rs13209376
rs2461475
rs7945585
rs12453125
rs143501783
rs7736526
rs7865362
rs2237084
rs3097671
rs62165726
rs9892550
rs117902156
rs7650880
rs34856868
rs79412885
rs681343
rs73021423
All
−0.05
0.00
0.05
0.10
MR leave−one−out sensitivity analysis for
'CSF1R' on 'Cardiac valvular disease'

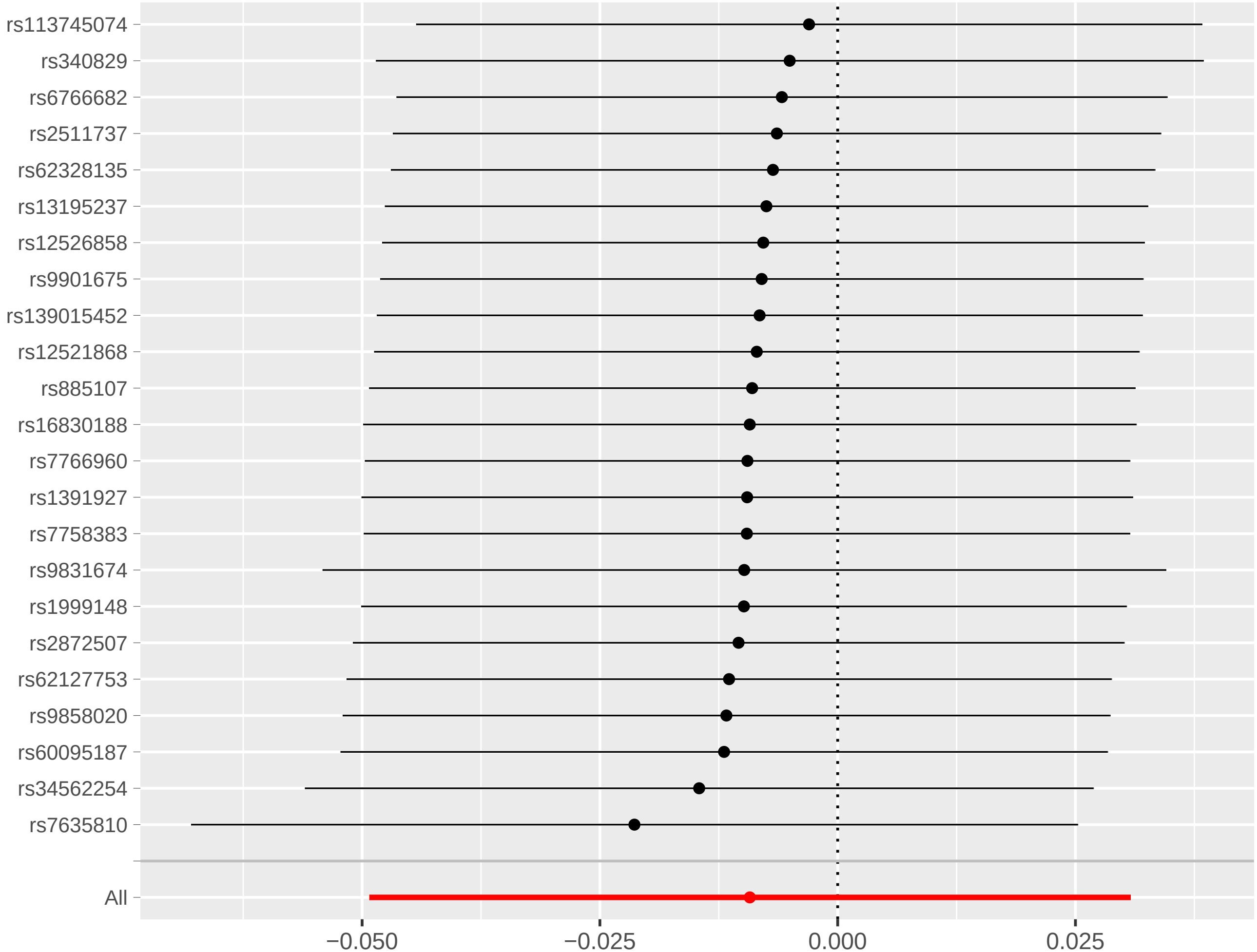

MR leave−one−out sensitivity analysis for
'IL5RA' on 'Cardiac valvular disease'

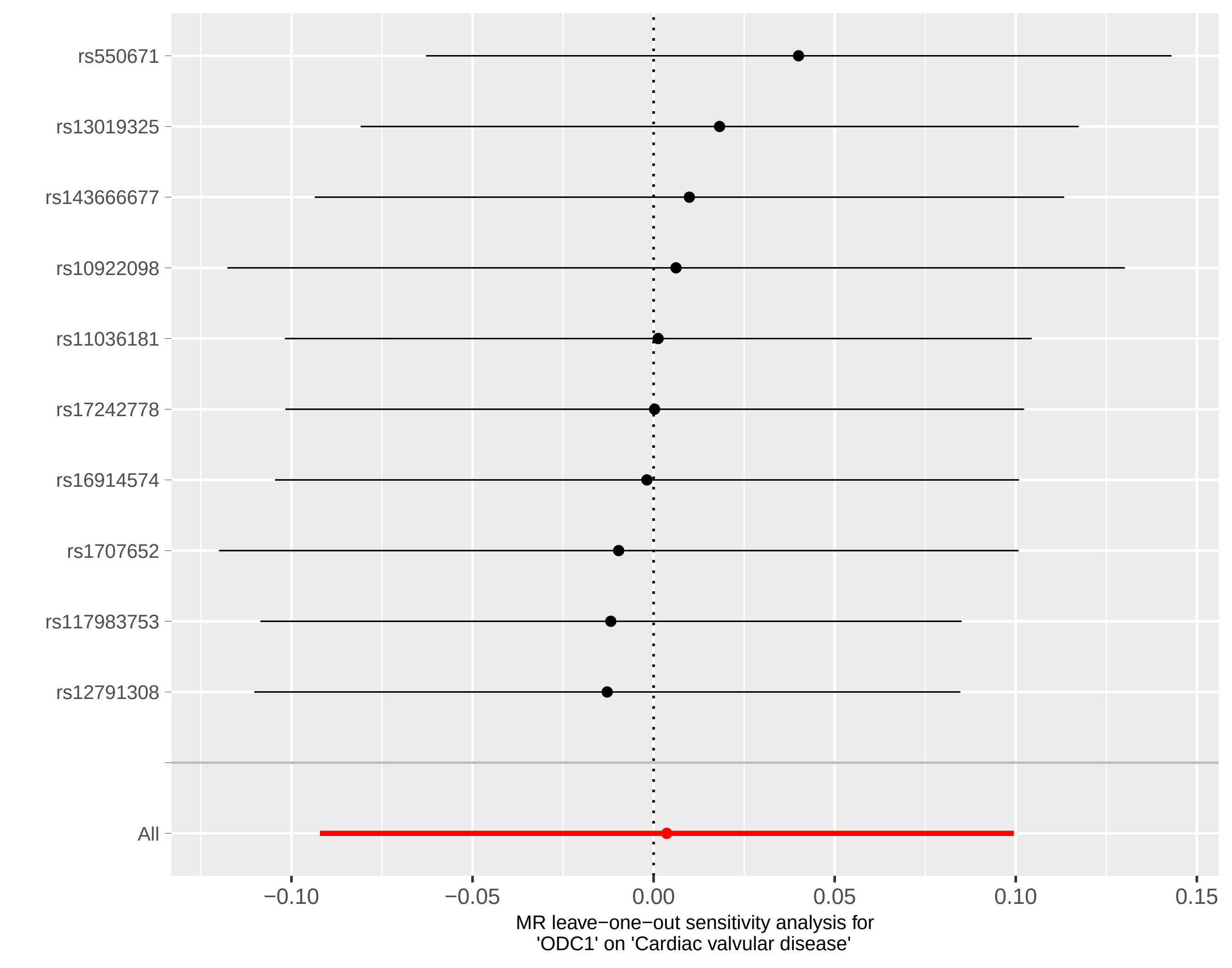

rs550671
rs13019325
rs143666677
rs10922098
rs11036181
rs17242778
rs16914574
rs1707652
rs117983753
rs12791308
All
−0.10
−0.05
0.00
0.05
0.10
0.15
MR leave−one−out sensitivity analysis for
'ODC1' on 'Cardiac valvular disease'

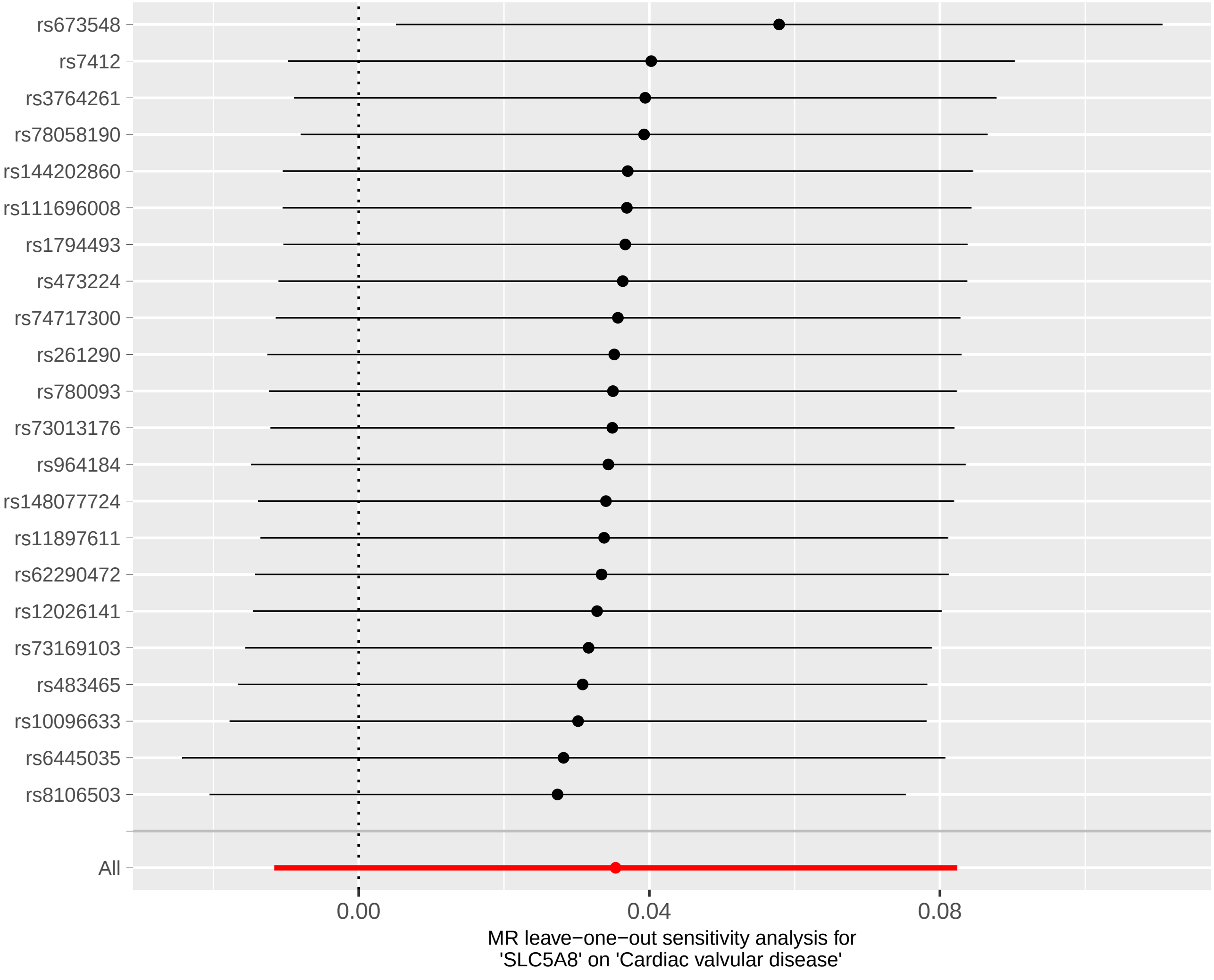

rs673548
rs7412
rs3764261
rs78058190
rs144202860
rs111696008
rs1794493
rs473224
rs74717300
rs261290
rs780093
rs73013176
rs964184
rs148077724
rs11897611
rs62290472
rs12026141
rs73169103
rs483465
rs10096633
rs6445035
rs8106503
All
0.00
0.04
0.08
MR leave−one−out sensitivity analysis for
'SLC5A8' on 'Cardiac valvular disease'

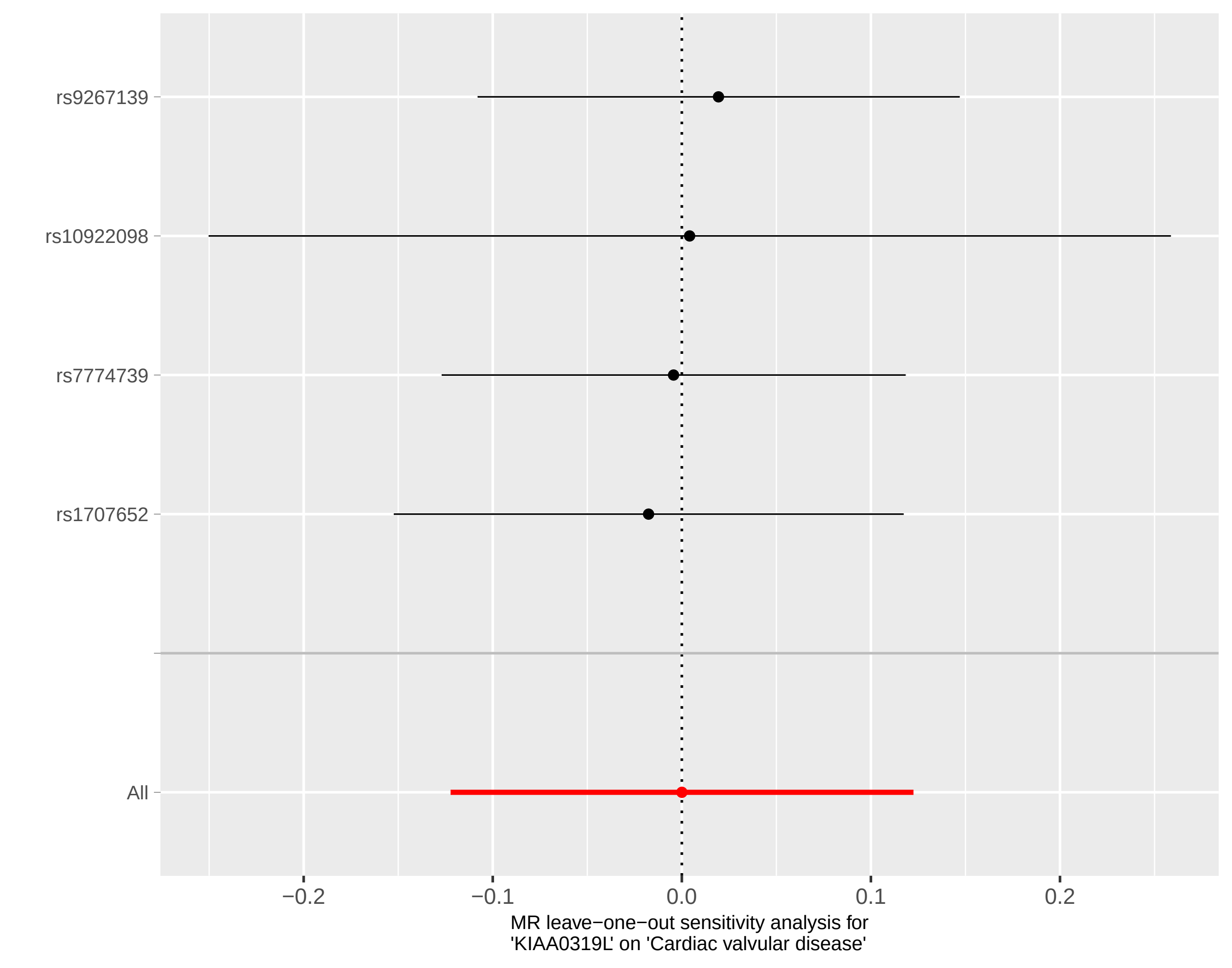
rs9267139
rs10922098
rs7774739
rs1707652
All
−0.2
−0.1
0.0
0.1
0.2
MR leave−one−out sensitivity analysis for
'KIAA0319L' on 'Cardiac valvular disease'

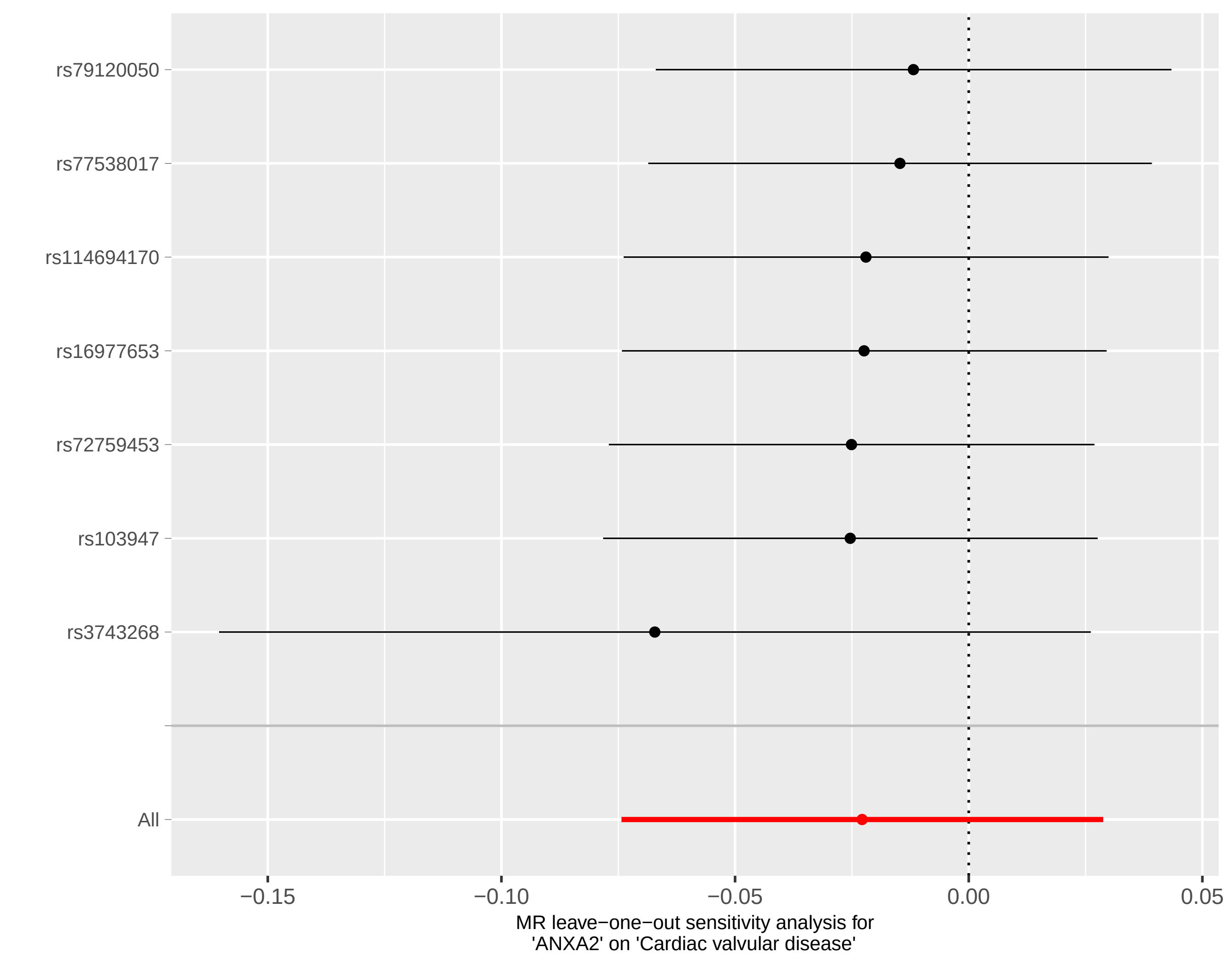

rs79120050
rs77538017
rs114694170
rs16977653
rs72759453
rs103947
rs3743268
All
−0.15
−0.10
−0.05
0.00
0.05
MR leave−one−out sensitivity analysis for
'ANXA2' on 'Cardiac valvular disease'

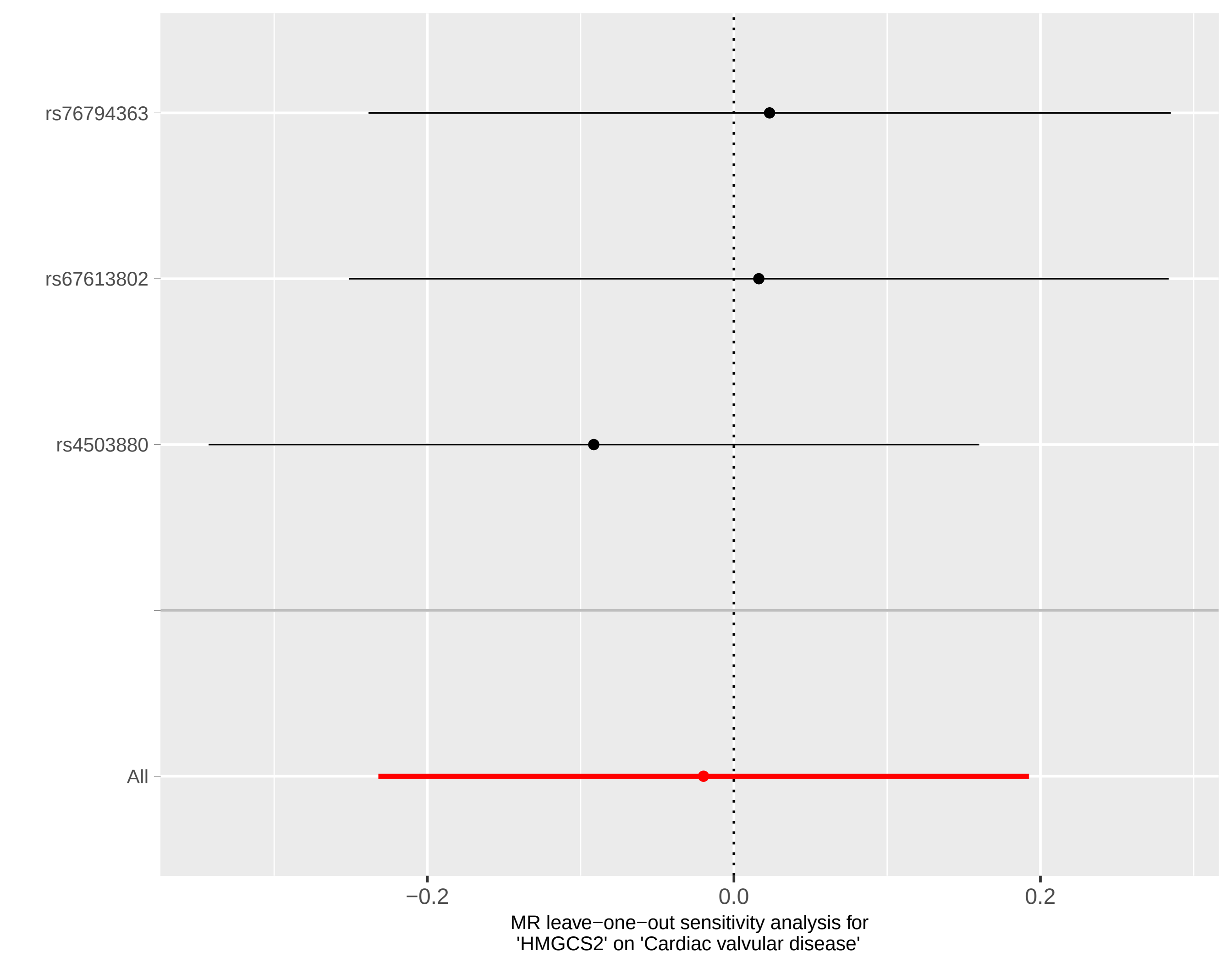
rs76794363
rs67613802
rs4503880
All
−0.2
0.0
0.2
MR leave−one−out sensitivity analysis for
'HMGCS2' on 'Cardiac valvular disease'

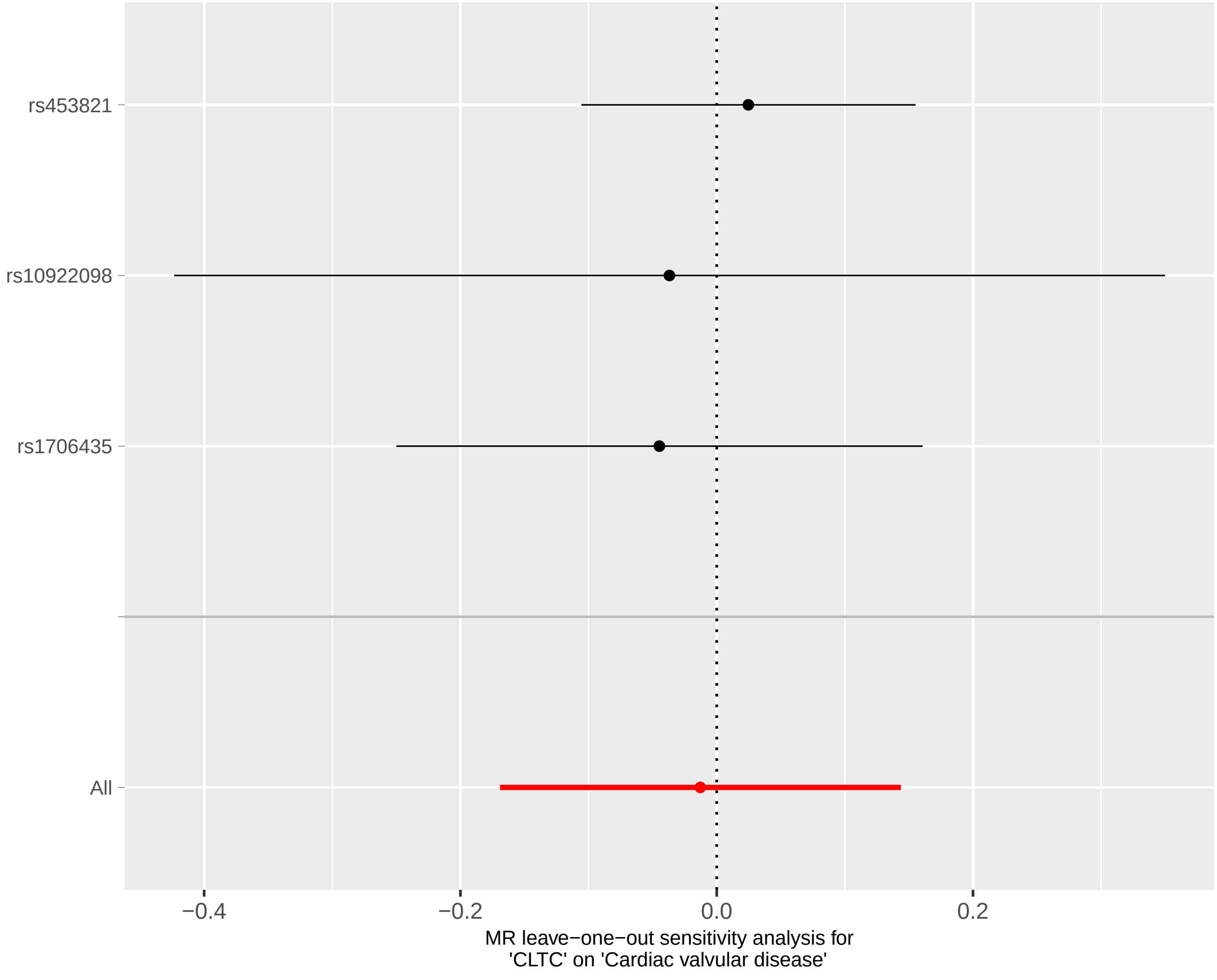

rs453821
rs10922098
rs1706435
All
−0.4
−0.2
0.0
0.2
MR leave−one−out sensitivity analysis for
'CLTC' on 'Cardiac valvular disease'

Insufficient number of SNPs

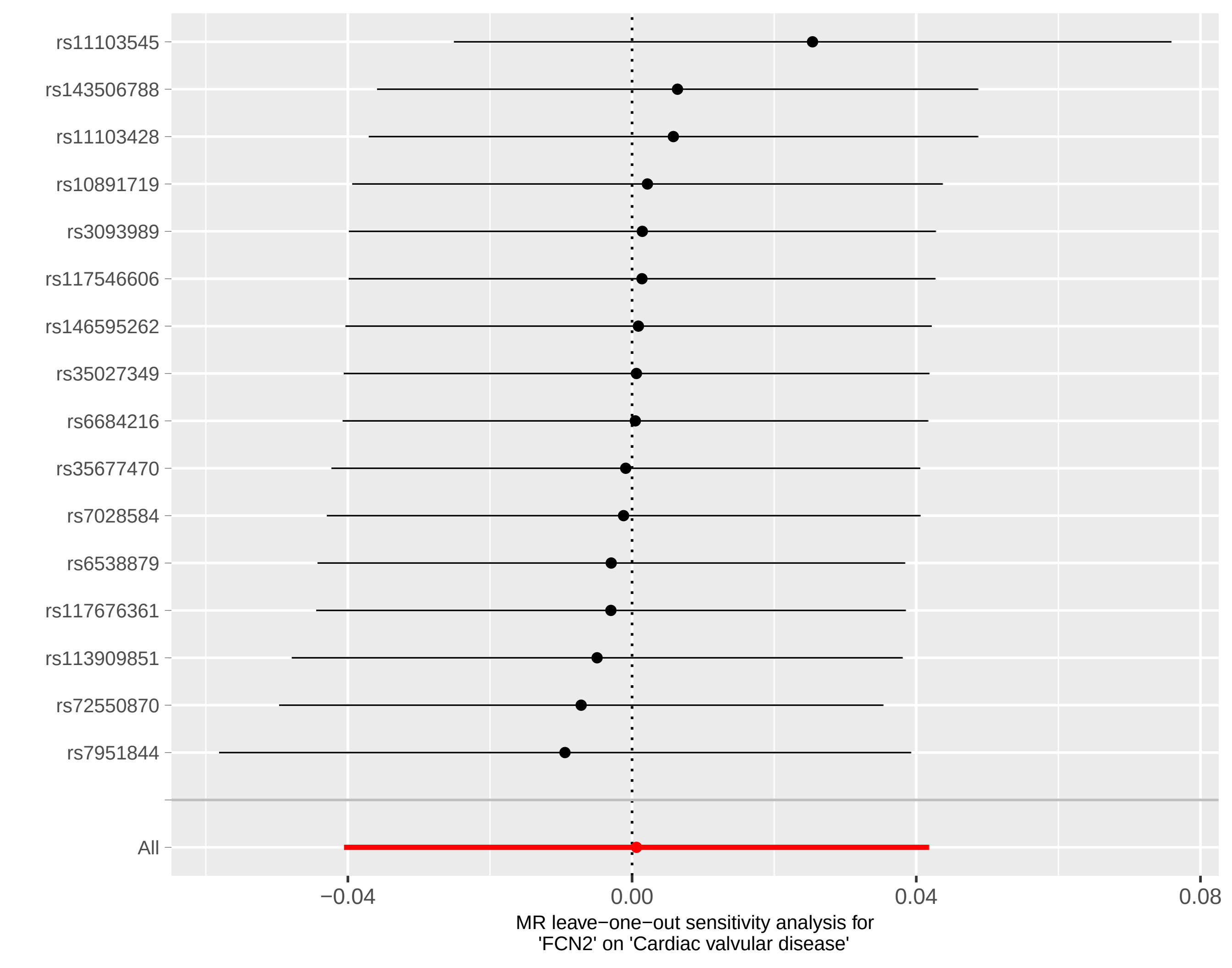
rs11103545
rs143506788
rs11103428
rs10891719
rs3093989
rs117546606
rs146595262
rs35027349
rs6684216
rs35677470
rs7028584
rs6538879
rs117676361
rs113909851
rs72550870
rs7951844
All
−0.04
0.00
0.04
0.08
MR leave−one−out sensitivity analysis for
'FCN2' on 'Cardiac valvular disease'

Insufficient number of SNPs

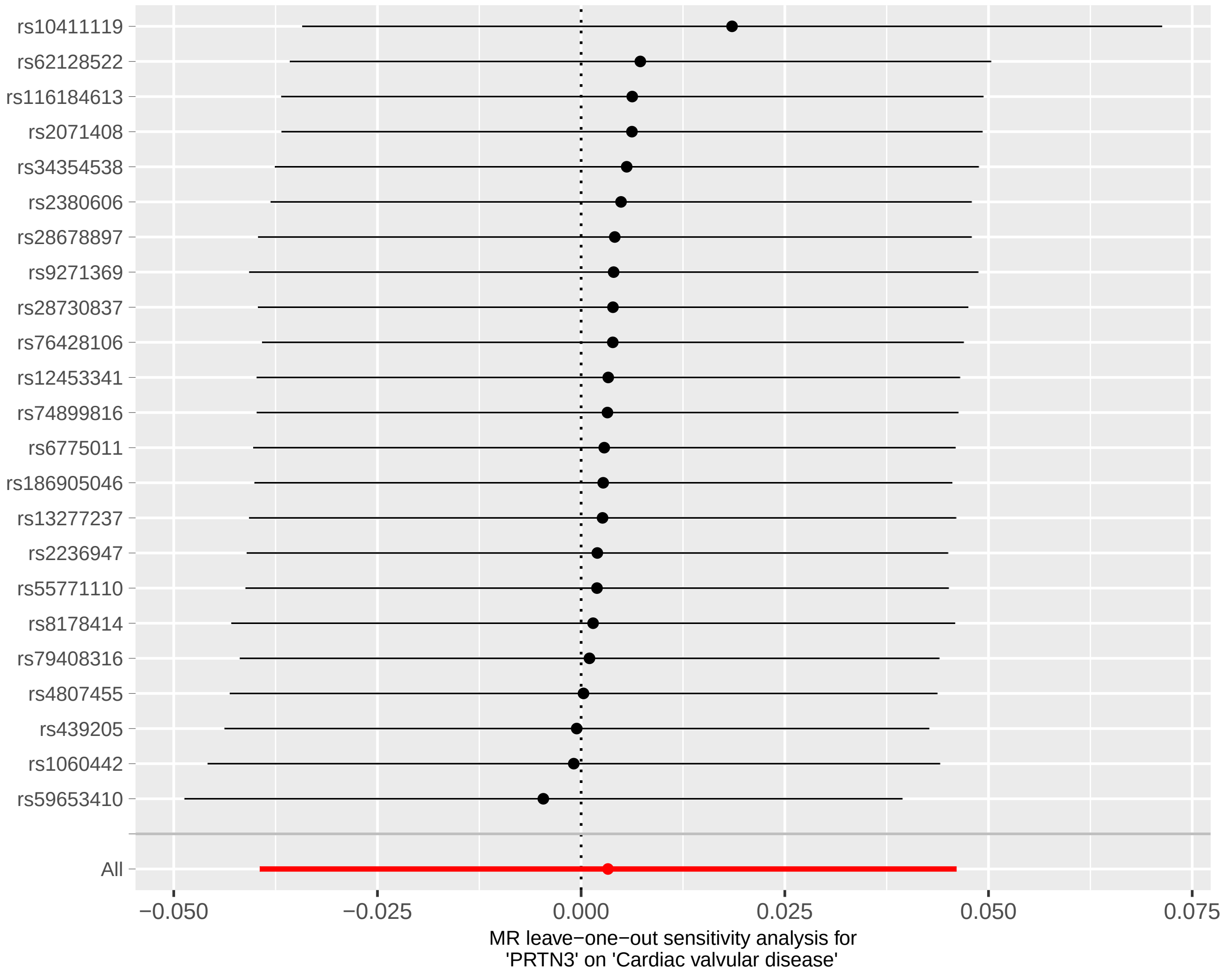

rs10411119
rs62128522
rs116184613
rs2071408
rs34354538
rs2380606
rs28678897
rs9271369
rs28730837
rs76428106
rs12453341
rs74899816
rs6775011
rs186905046
rs13277237
rs2236947
rs55771110
rs8178414
rs79408316
rs4807455
rs439205
rs1060442
rs59653410
All
−0.050
−0.025
0.000
0.025
0.050
0.075
MR leave−one−out sensitivity analysis for
'PRTN3' on 'Cardiac valvular disease'

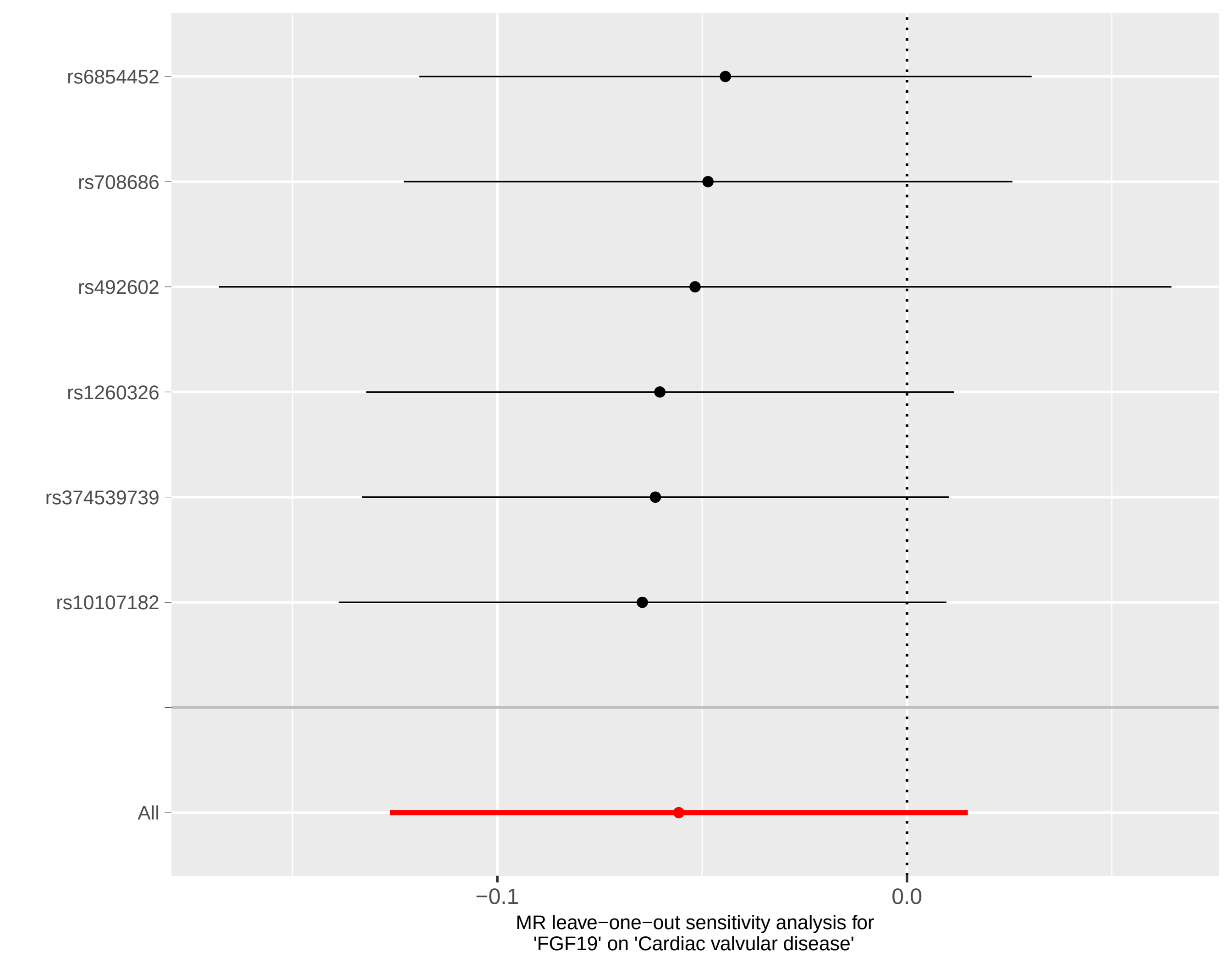
rs6854452
rs708686
rs492602
rs1260326
rs374539739
rs10107182
All
−0.1
0.0
MR leave−one−out sensitivity analysis for
'FGF19' on 'Cardiac valvular disease'

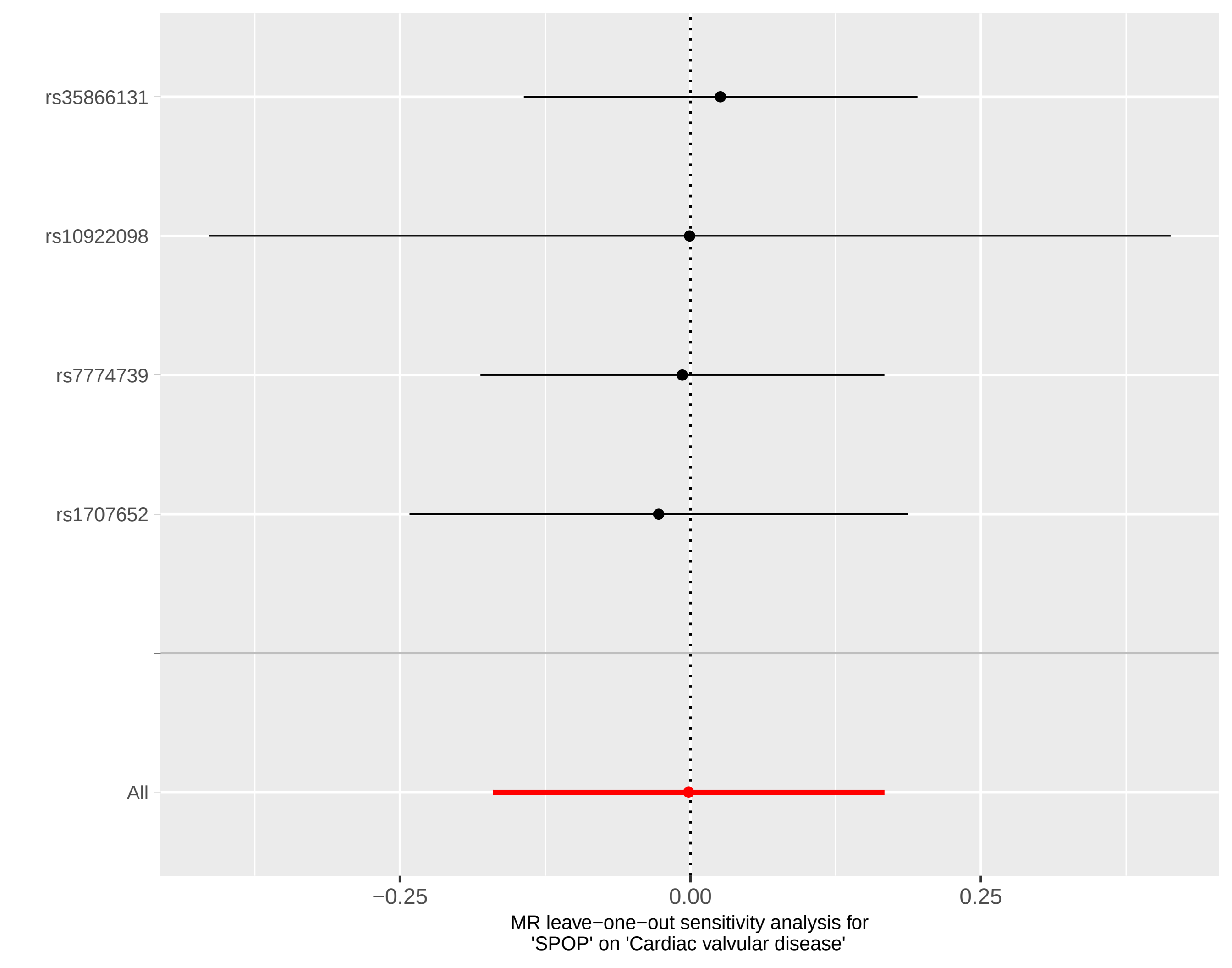

rs35866131
rs10922098
rs7774739
rs1707652
All
−0.25
0.00
0.25
MR leave−one−out sensitivity analysis for
'SPOP' on 'Cardiac valvular disease'

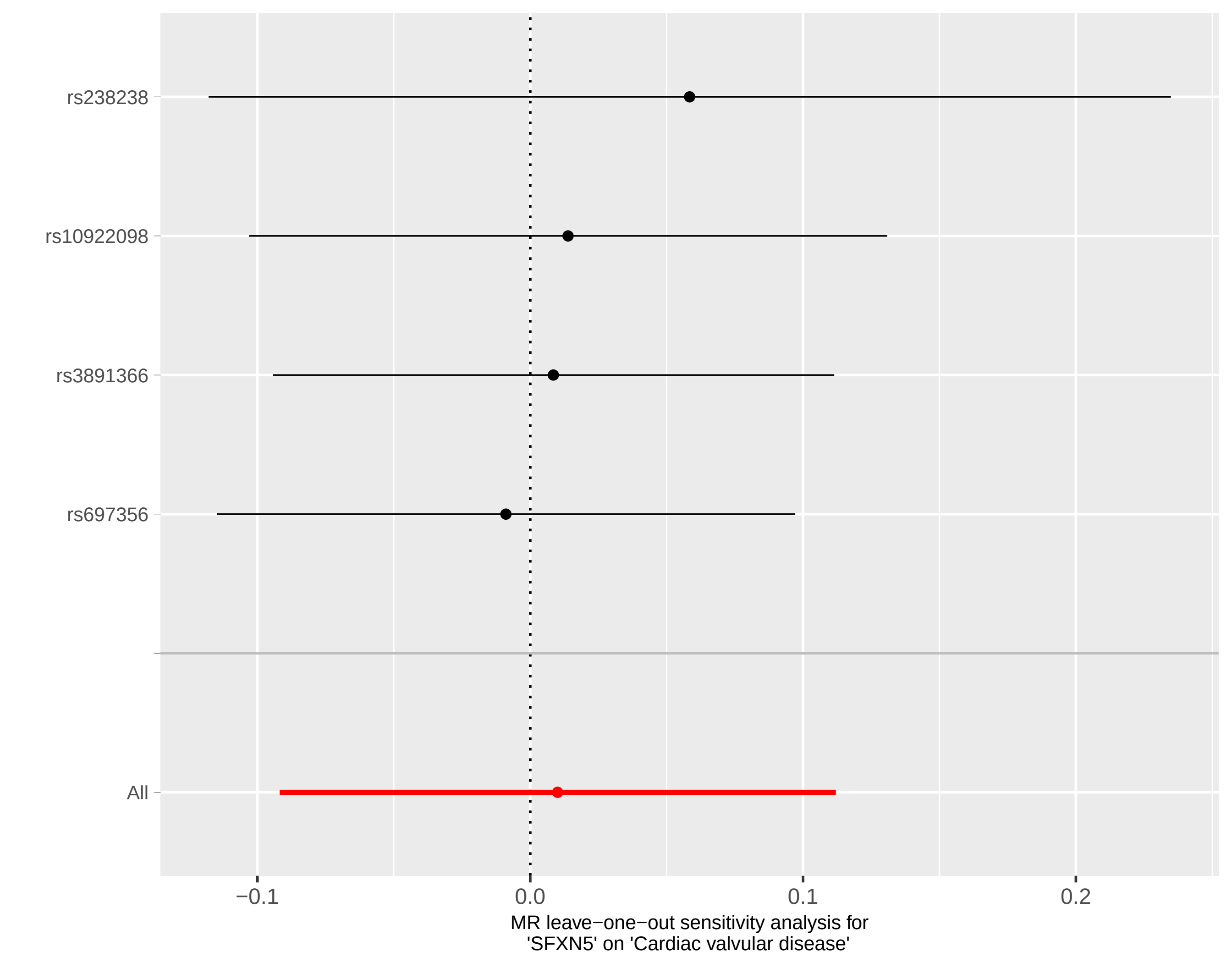

rs238238
rs10922098
rs3891366
rs697356
All
−0.1
0.0
0.1
0.2
MR leave−one−out sensitivity analysis for
'SFXN5' on 'Cardiac valvular disease'

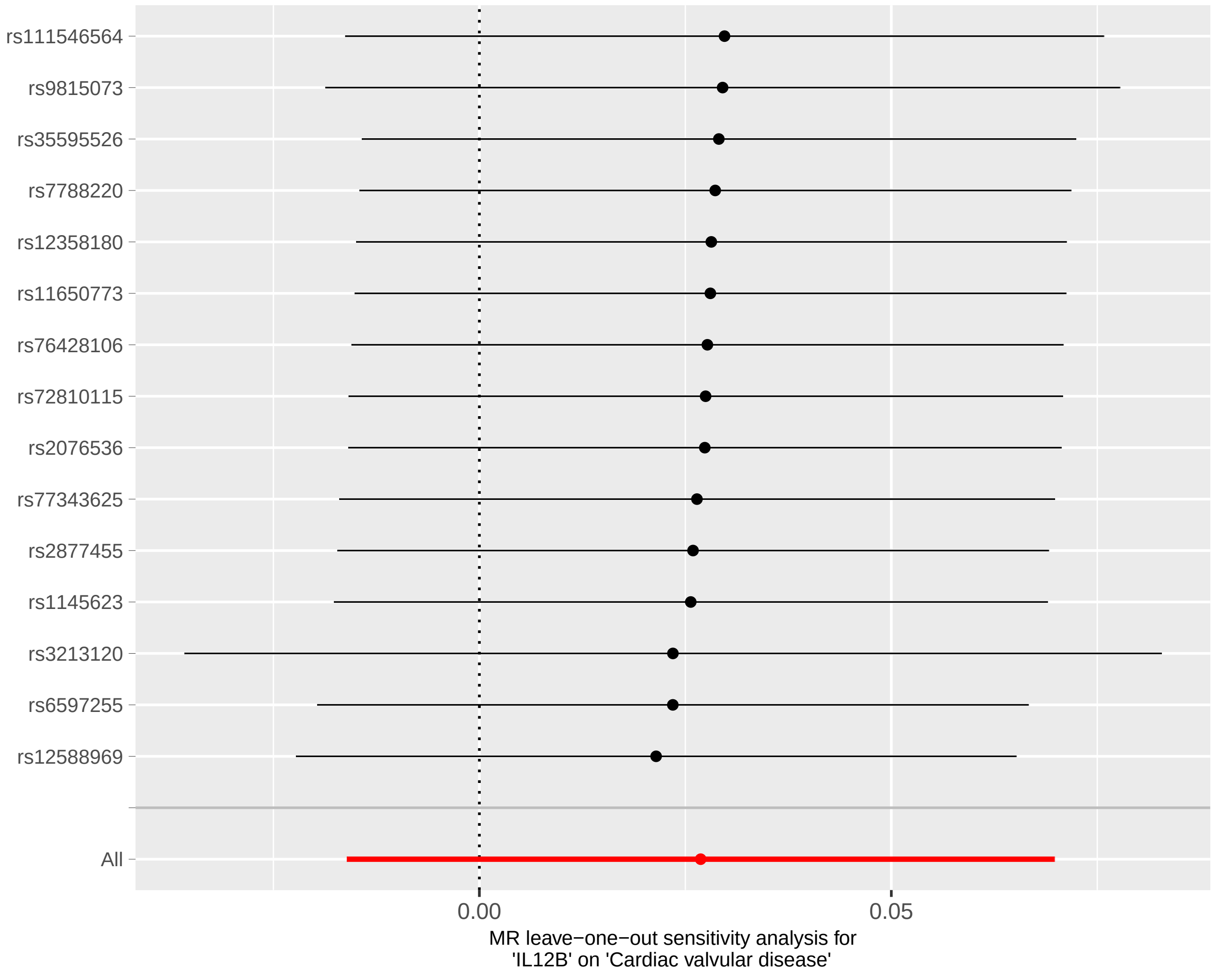
rs111546564
rs9815073
rs35595526
rs7788220
rs12358180
rs11650773
rs76428106
rs72810115
rs2076536
rs77343625
rs2877455
rs1145623
rs3213120
rs6597255
rs12588969
All
0.00
0.05
MR leave−one−out sensitivity analysis for
'IL12B' on 'Cardiac valvular disease'

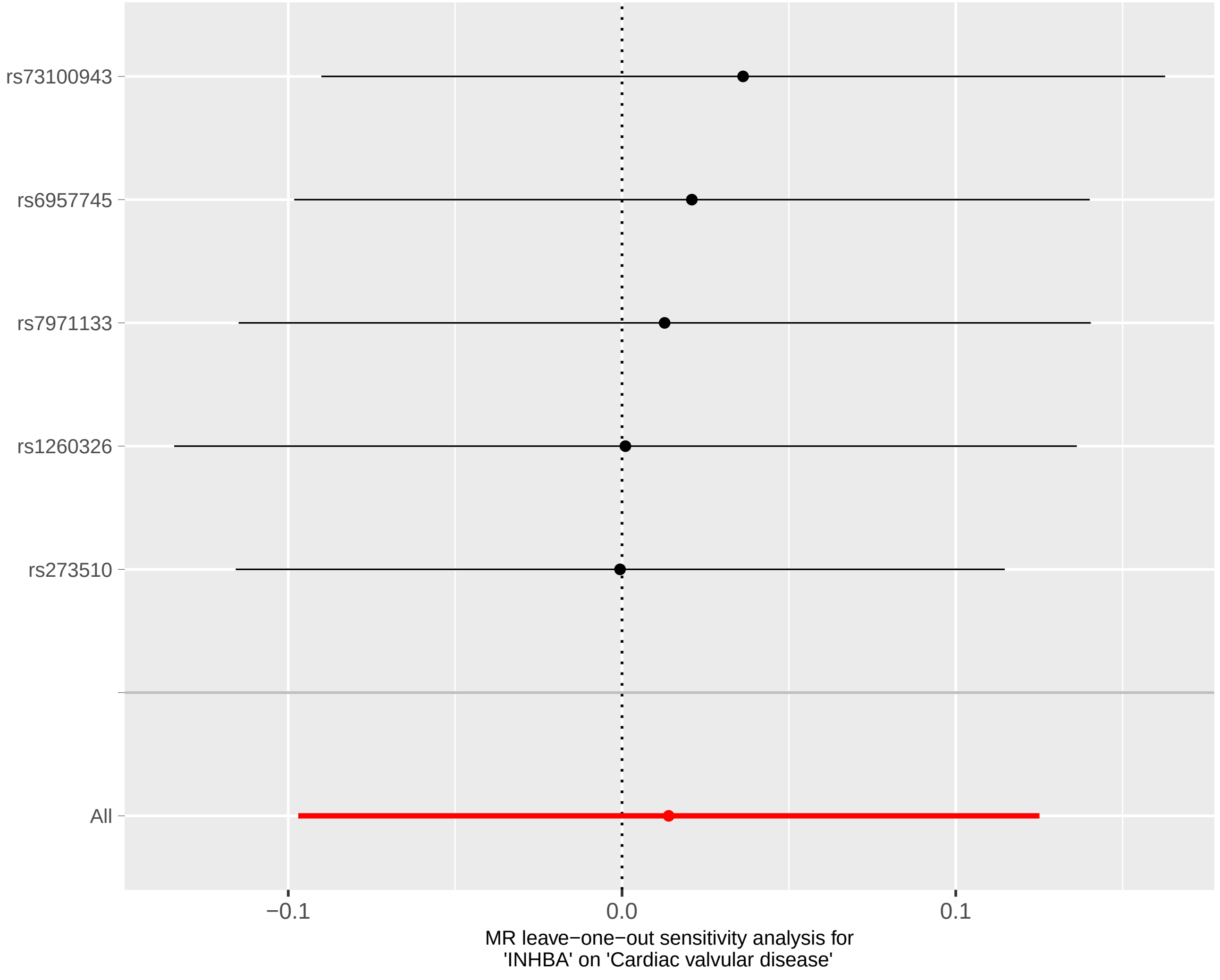
rs73100943
rs6957745
rs7971133
rs1260326
rs273510
All
−0.1
0.0
0.1
MR leave−one−out sensitivity analysis for
'INHBA' on 'Cardiac valvular disease'

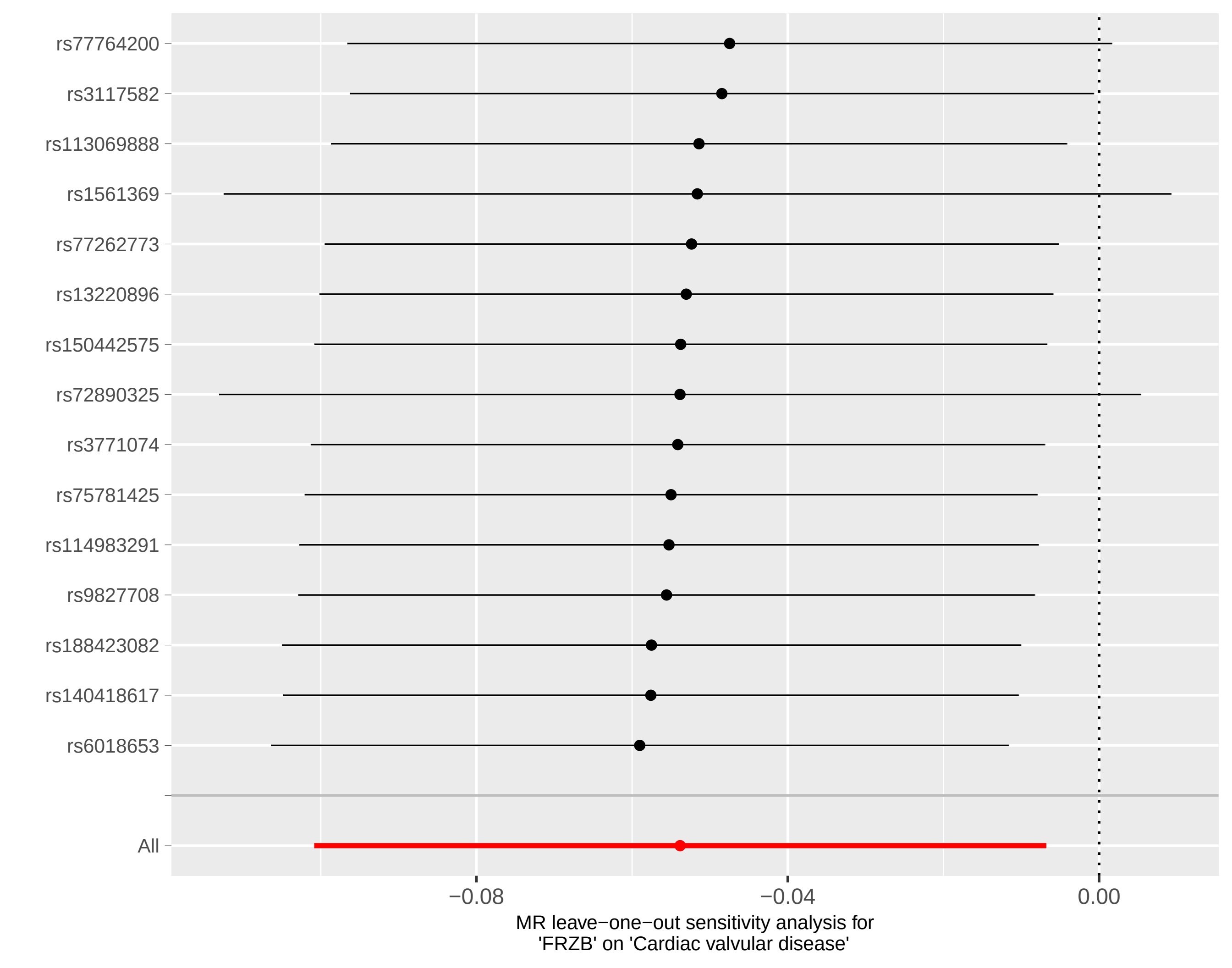
rs77764200
rs3117582
rs113069888
rs1561369
rs77262773
rs13220896
rs150442575
rs72890325
rs3771074
rs75781425
rs114983291
rs9827708
rs188423082
rs140418617
rs6018653
All
−0.08
−0.04
0.00
MR leave−one−out sensitivity analysis for
'FRZB' on 'Cardiac valvular disease'

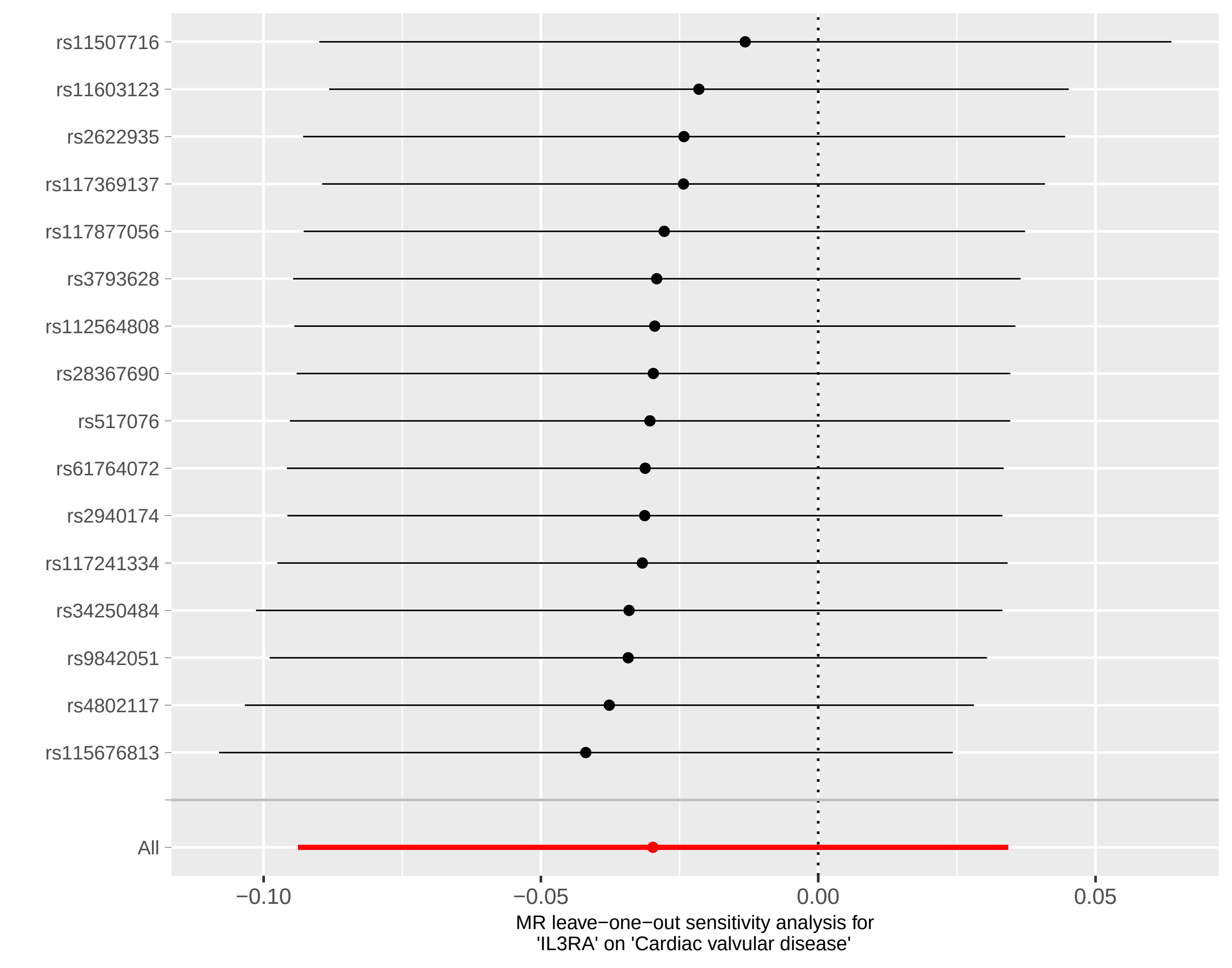
rs11507716
rs11603123
rs2622935
rs117369137
rs117877056
rs3793628
rs112564808
rs28367690
rs517076
rs61764072
rs2940174
rs117241334
rs34250484
rs9842051
rs4802117
rs115676813
All
−0.10
−0.05
0.00
0.05
MR leave−one−out sensitivity analysis for
'IL3RA' on 'Cardiac valvular disease'

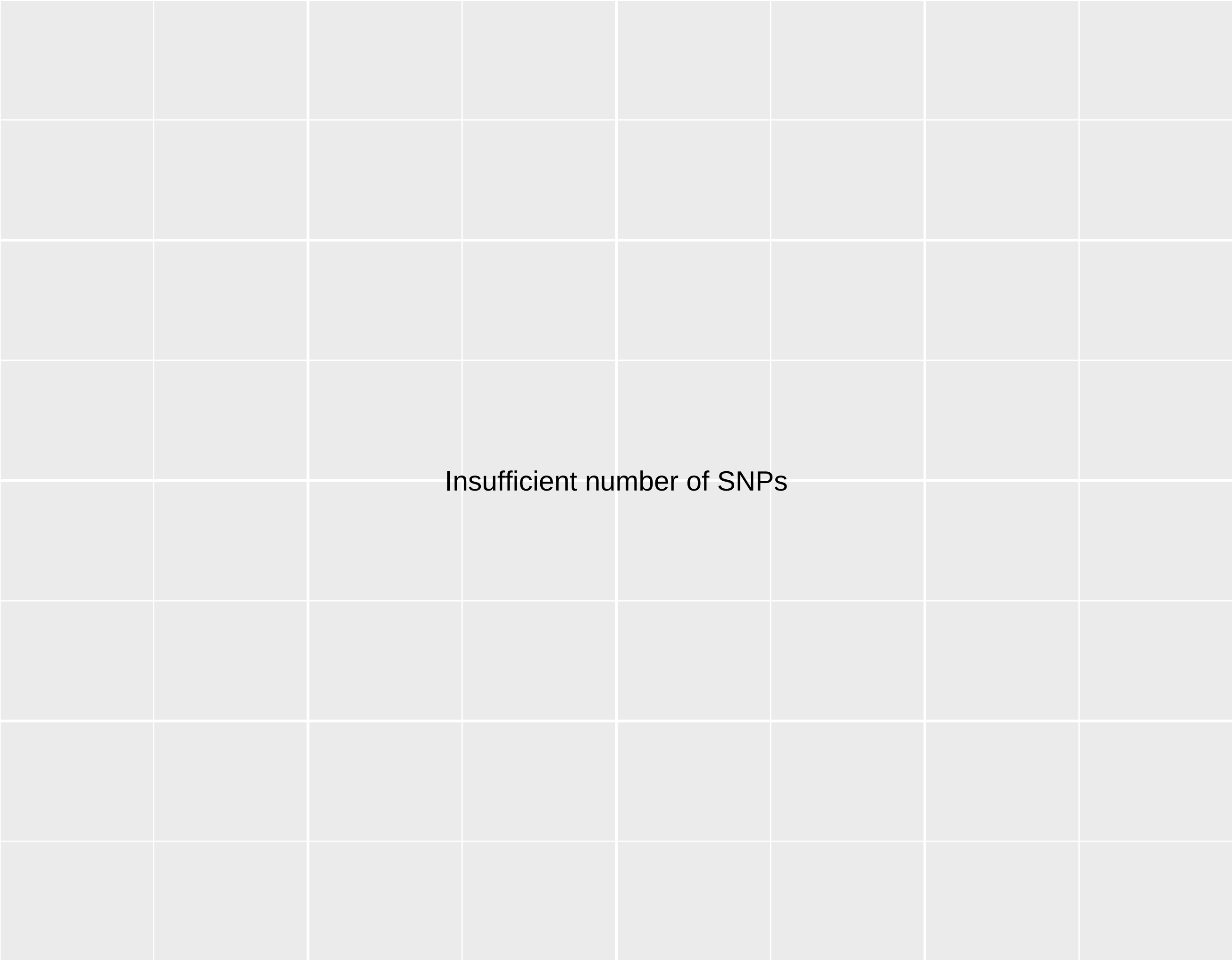
Insufficient number of SNPs

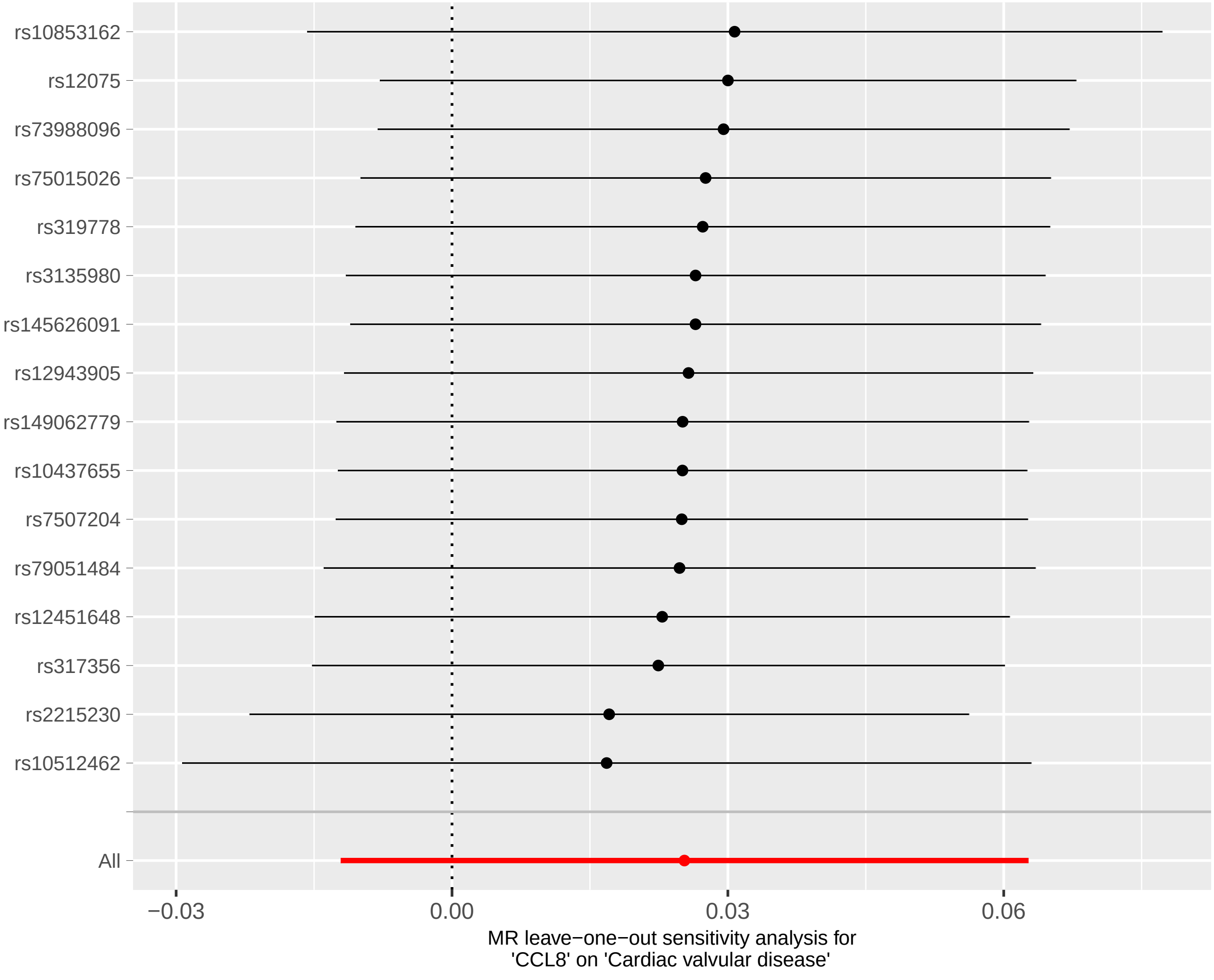
rs10853162
rs12075
rs73988096
rs75015026
rs319778
rs3135980
rs145626091
rs12943905
rs149062779
rs10437655
rs7507204
rs79051484
rs12451648
rs317356
rs2215230
rs10512462
All
−0.03
0.00
0.03
0.06
MR leave−one−out sensitivity analysis for
'CCL8' on 'Cardiac valvular disease'

Insufficient number of SNPs

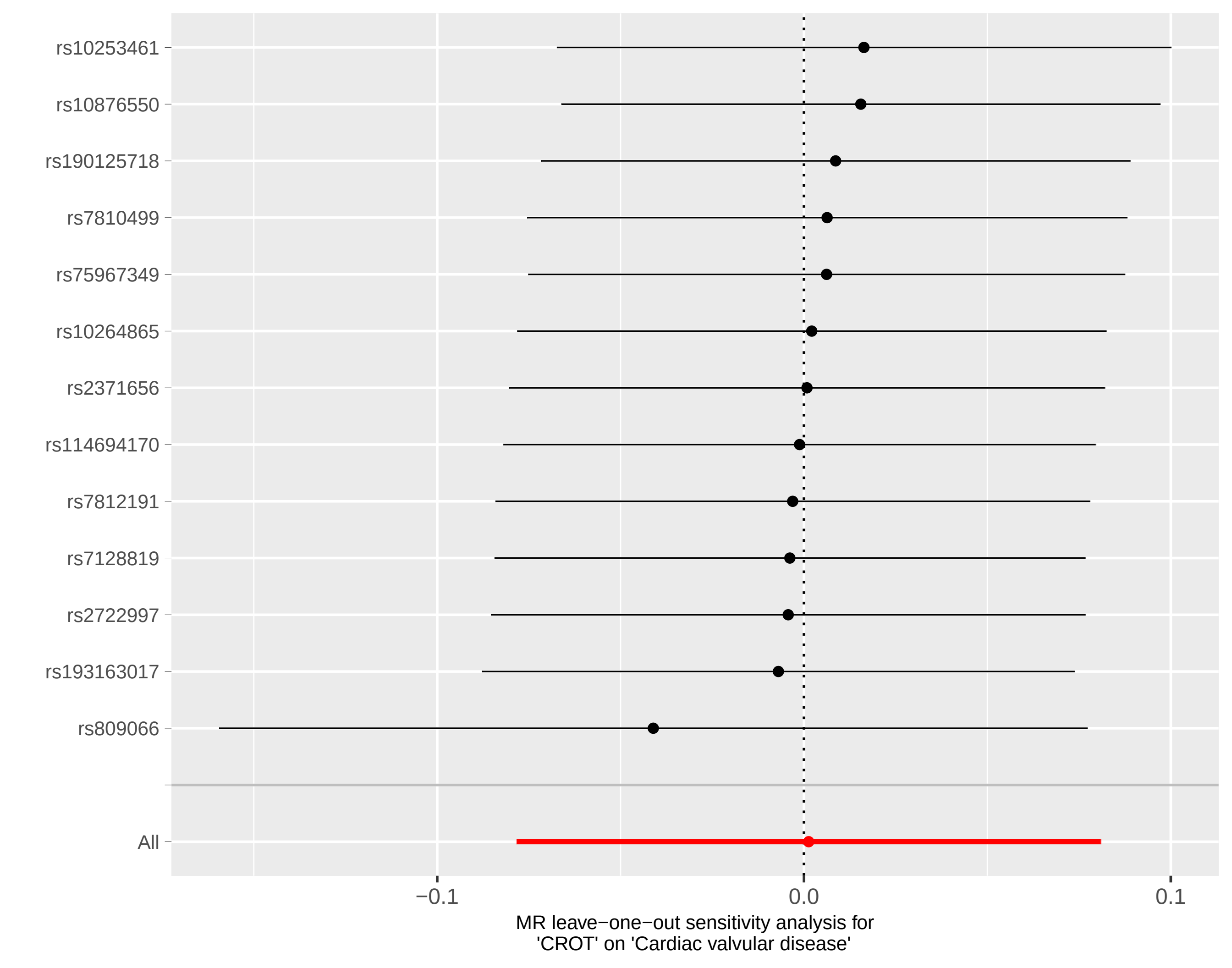
rs10253461
rs10876550
rs190125718
rs7810499
rs75967349
rs10264865
rs2371656
rs114694170
rs7812191
rs7128819
rs2722997
rs193163017
rs809066
All
−0.1
0.0
0.1
MR leave−one−out sensitivity analysis for
'CROT' on 'Cardiac valvular disease'

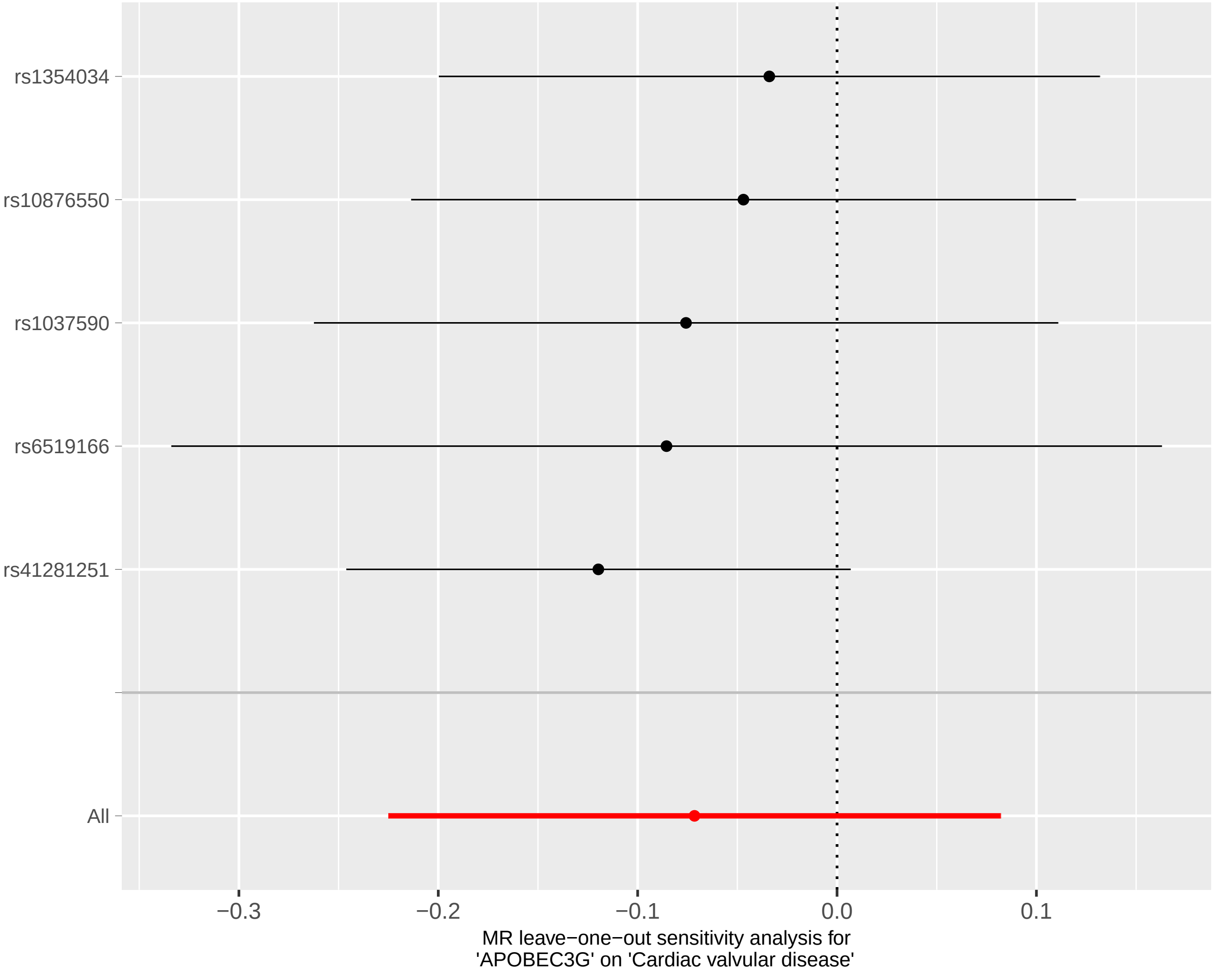

rs1354034
rs10876550
rs1037590
rs6519166
rs41281251
All
−0.3
−0.2
−0.1
0.0
0.1
MR leave−one−out sensitivity analysis for
'APOBEC3G' on 'Cardiac valvular disease'

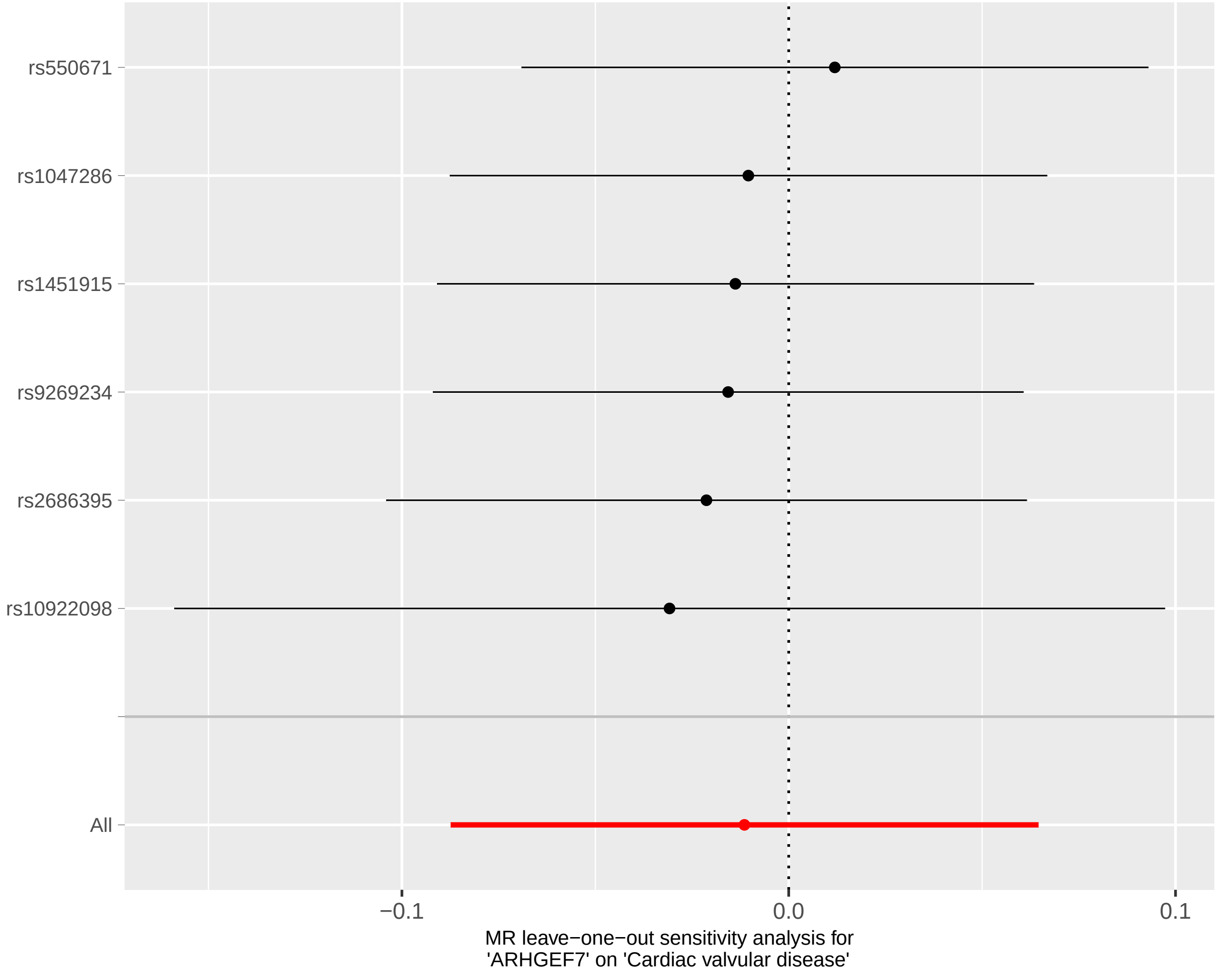

rs550671
rs1047286
rs1451915
rs9269234
rs2686395
rs10922098
All
−0.1
0.0
0.1
MR leave−one−out sensitivity analysis for
'ARHGEF7' on 'Cardiac valvular disease'

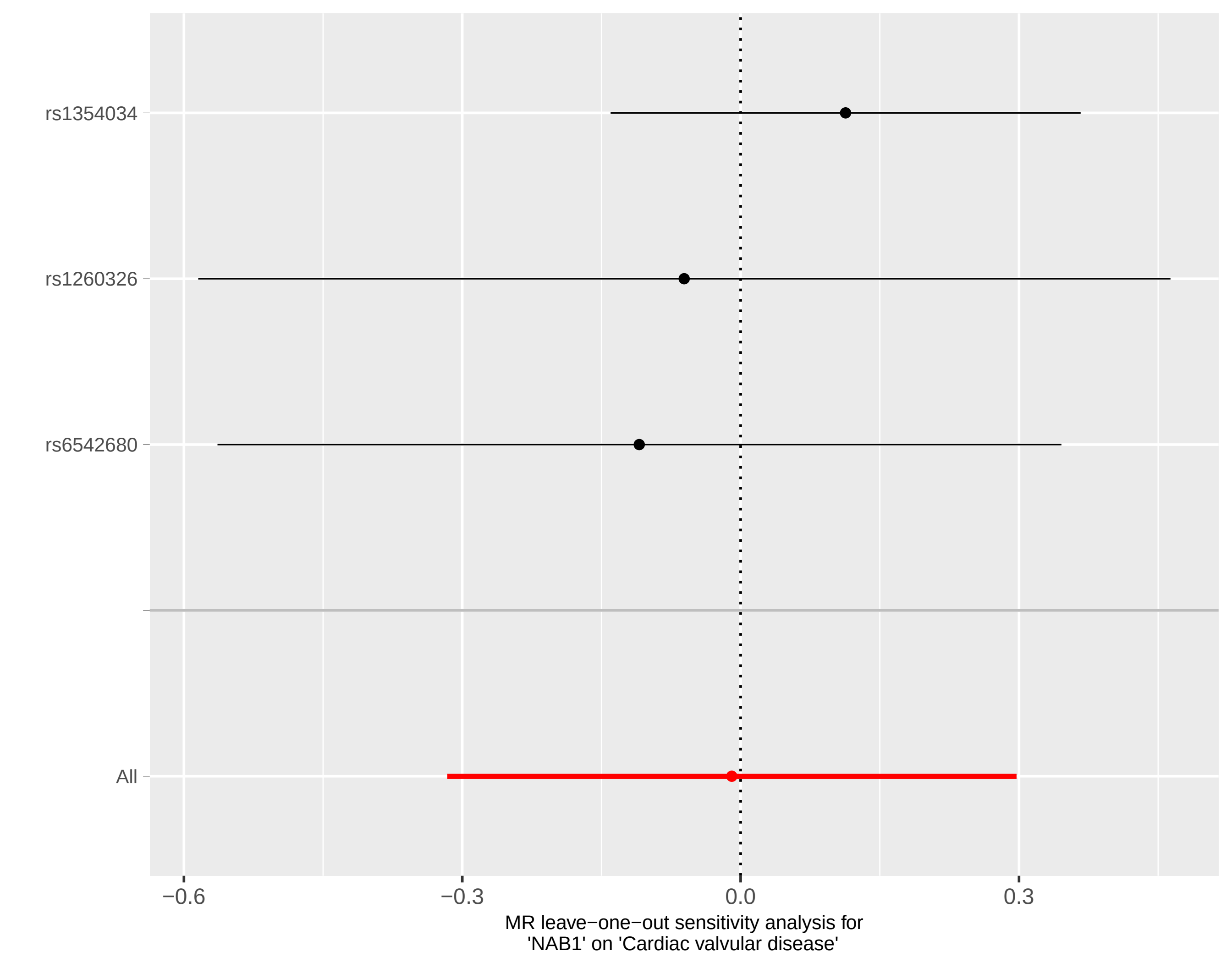
rs1354034
rs1260326
rs6542680
All
−0.6
−0.3
0.0
0.3
MR leave−one−out sensitivity analysis for
'NAB1' on 'Cardiac valvular disease'

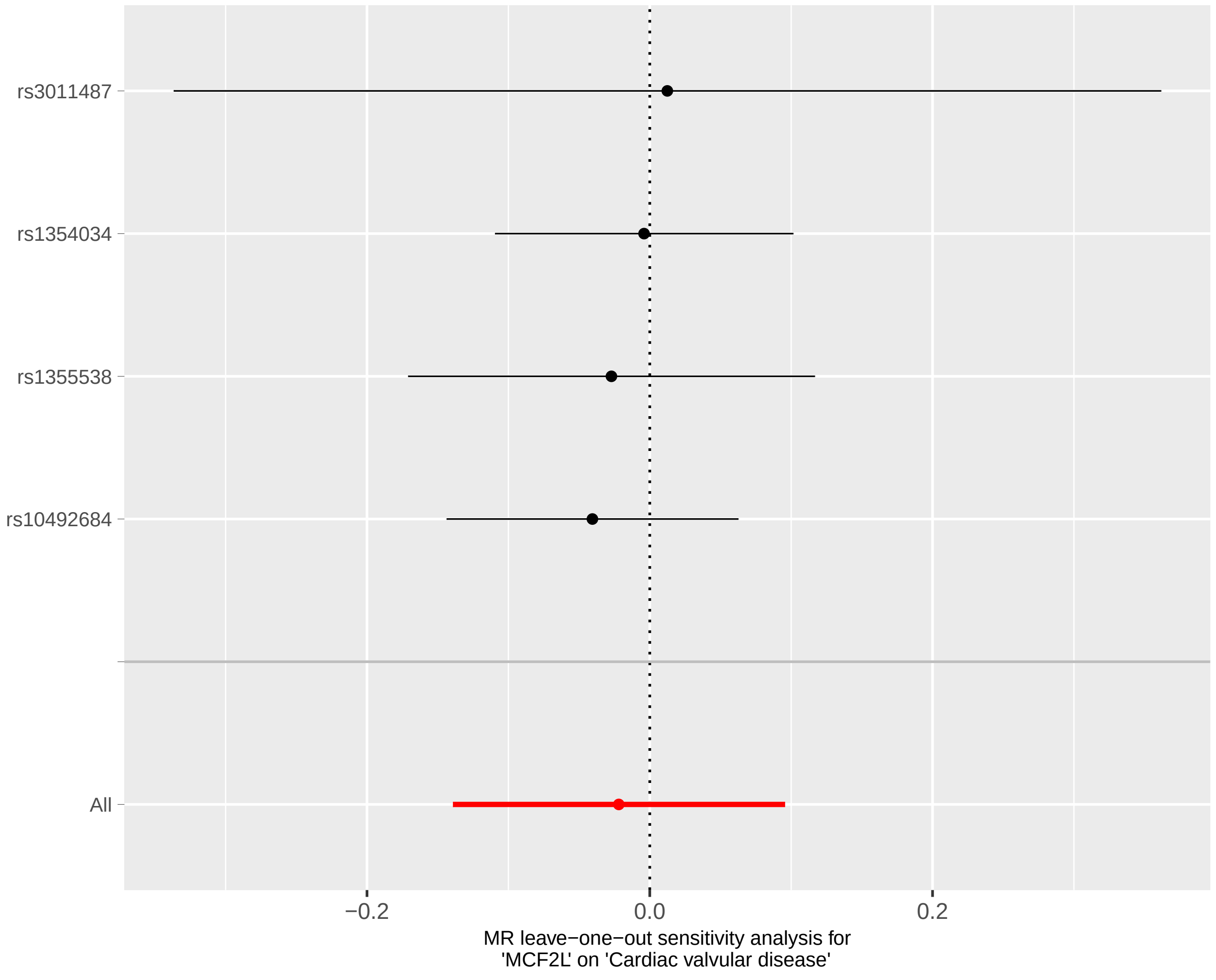

rs3011487
rs1354034
rs1355538
rs10492684
All
−0.2
0.0
0.2
MR leave−one−out sensitivity analysis for
'MCF2L' on 'Cardiac valvular disease'

Insufficient number of SNPs

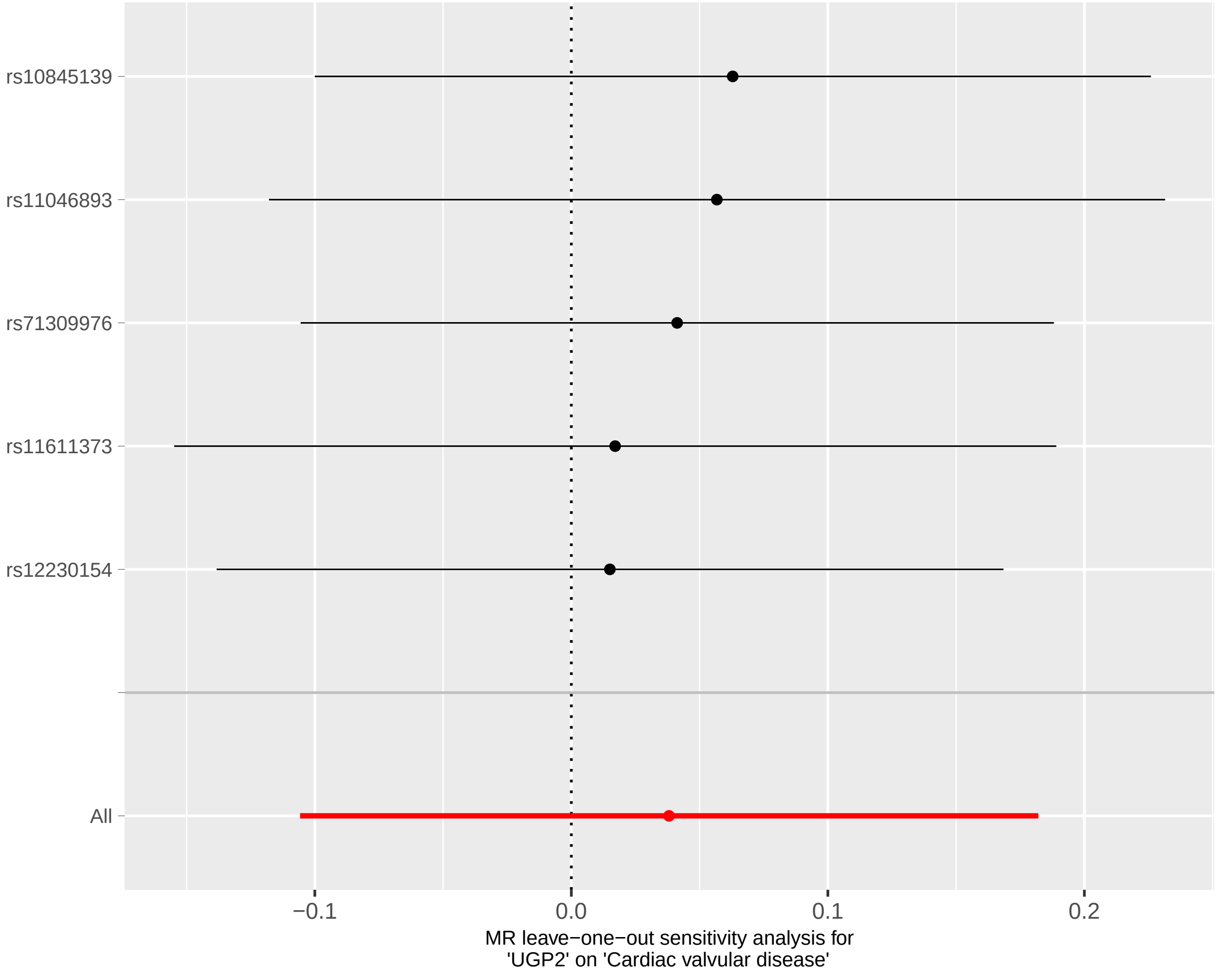

rs10845139
rs11046893
rs71309976
rs11611373
rs12230154
All
−0.1
0.0
0.1
0.2
MR leave−one−out sensitivity analysis for
'UGP2' on 'Cardiac valvular disease'

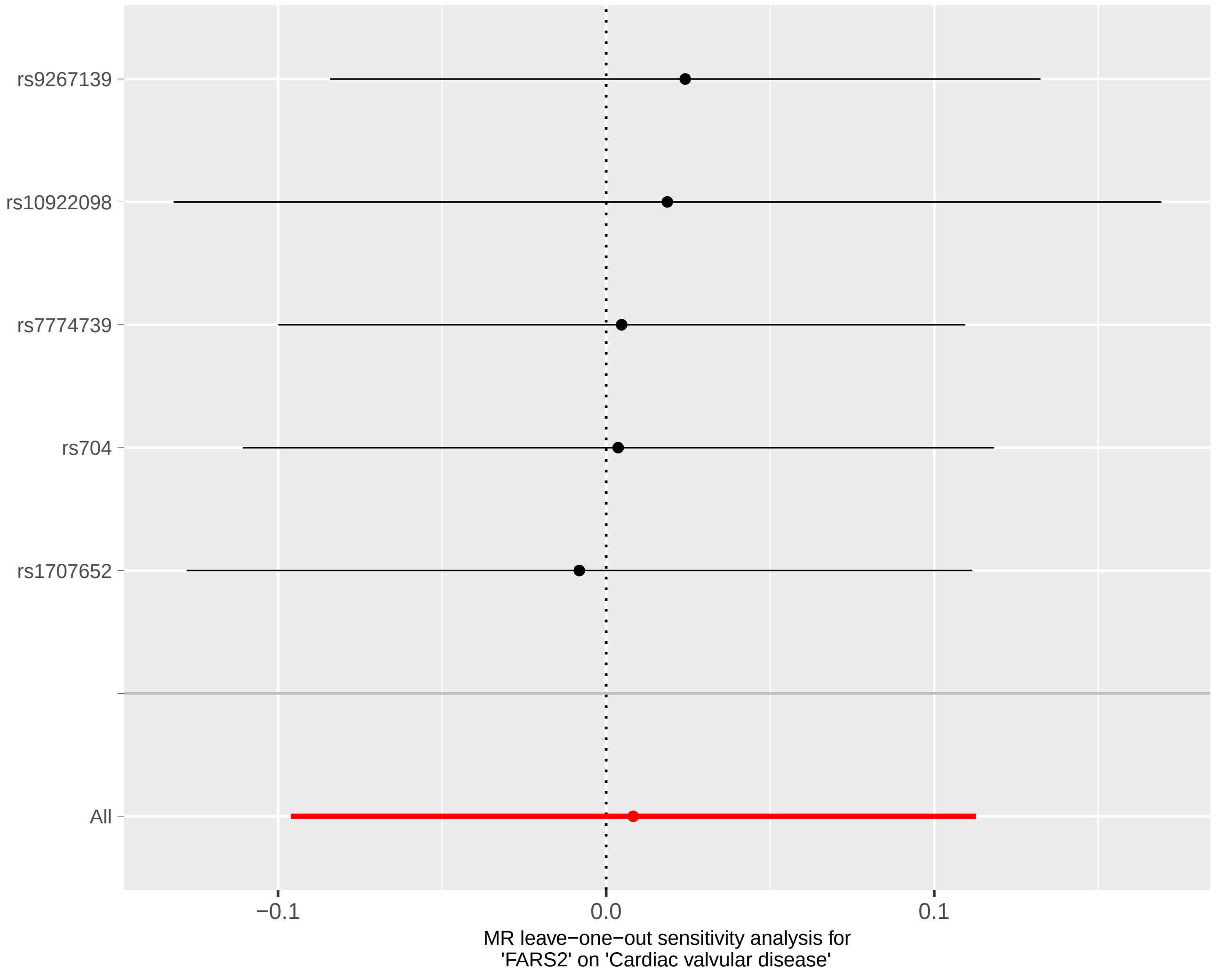
rs9267139
rs10922098
rs7774739
rs704
rs1707652
All
−0.1
0.0
0.1
MR leave−one−out sensitivity analysis for
'FARS2' on 'Cardiac valvular disease'

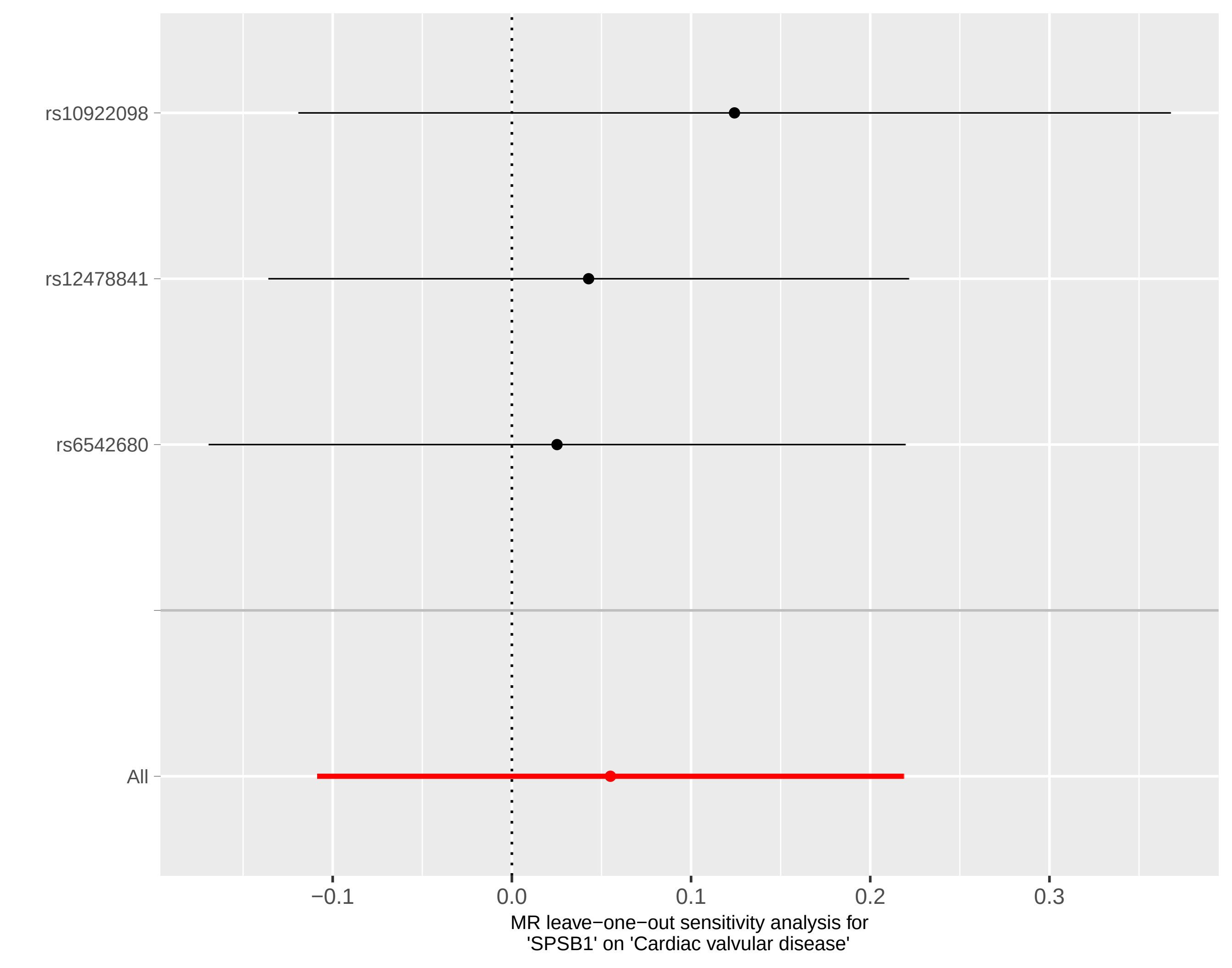
rs10922098
rs12478841
rs6542680
All
−0.1
0.0
0.1
0.2
0.3
MR leave−one−out sensitivity analysis for
'SPSB1' on 'Cardiac valvular disease'

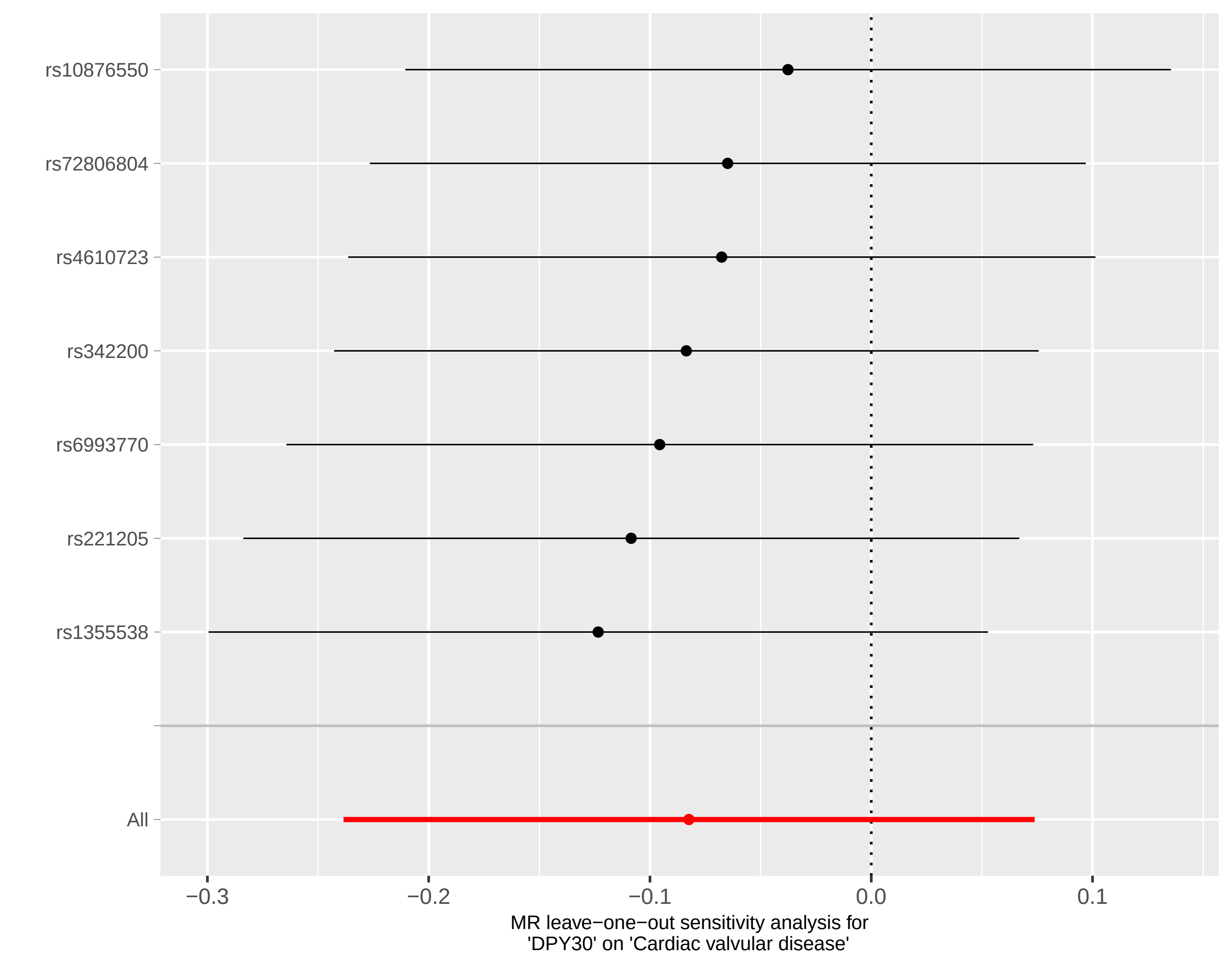

rs10876550
rs72806804
rs4610723
rs342200
rs6993770
rs221205
rs1355538
All
−0.3
−0.2
−0.1
0.0
0.1
MR leave−one−out sensitivity analysis for
'DPY30' on 'Cardiac valvular disease'

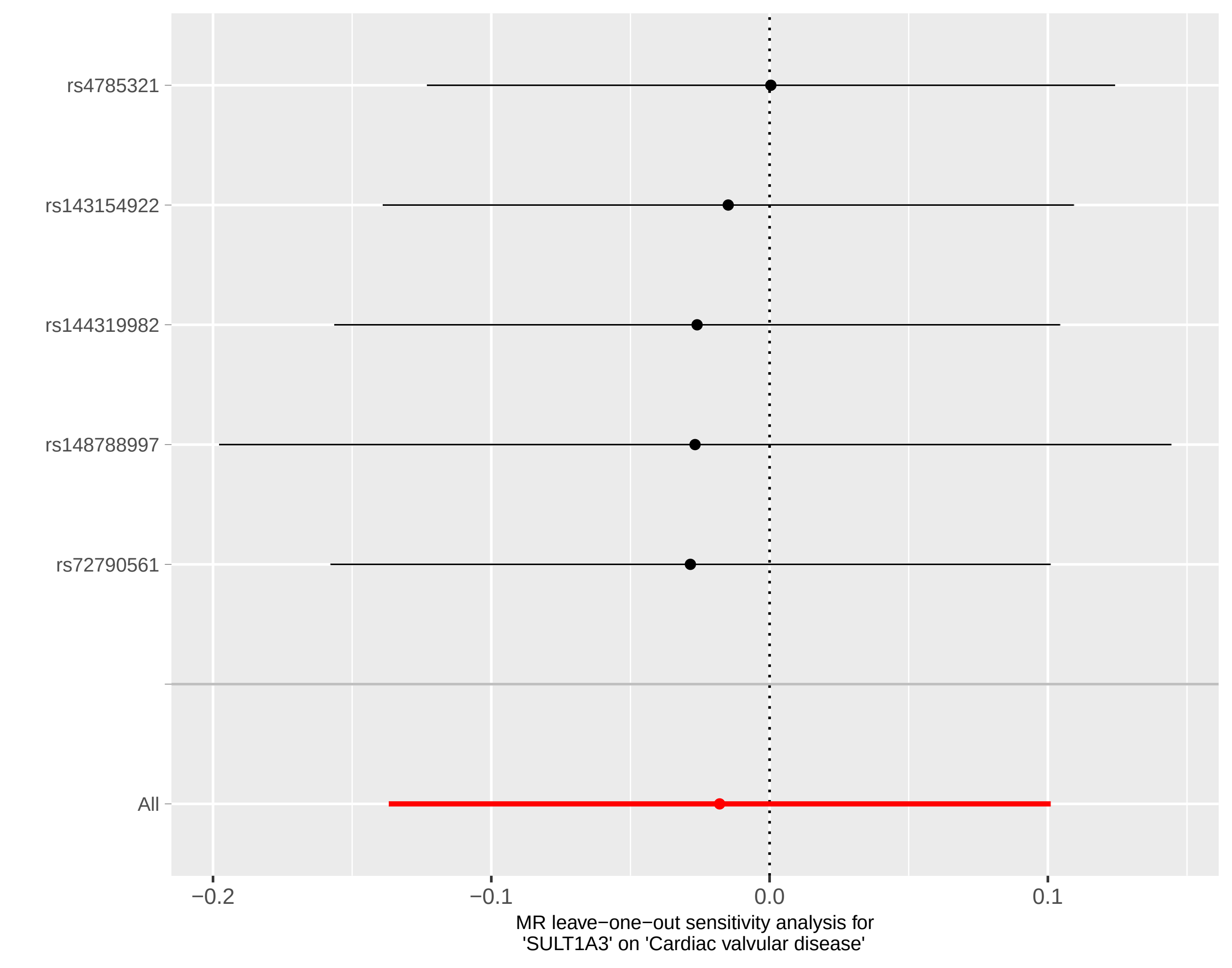
rs4785321
rs143154922
rs144319982
rs148788997
rs72790561
All
−0.2
−0.1
0.0
0.1
MR leave−one−out sensitivity analysis for
'SULT1A3' on 'Cardiac valvular disease'

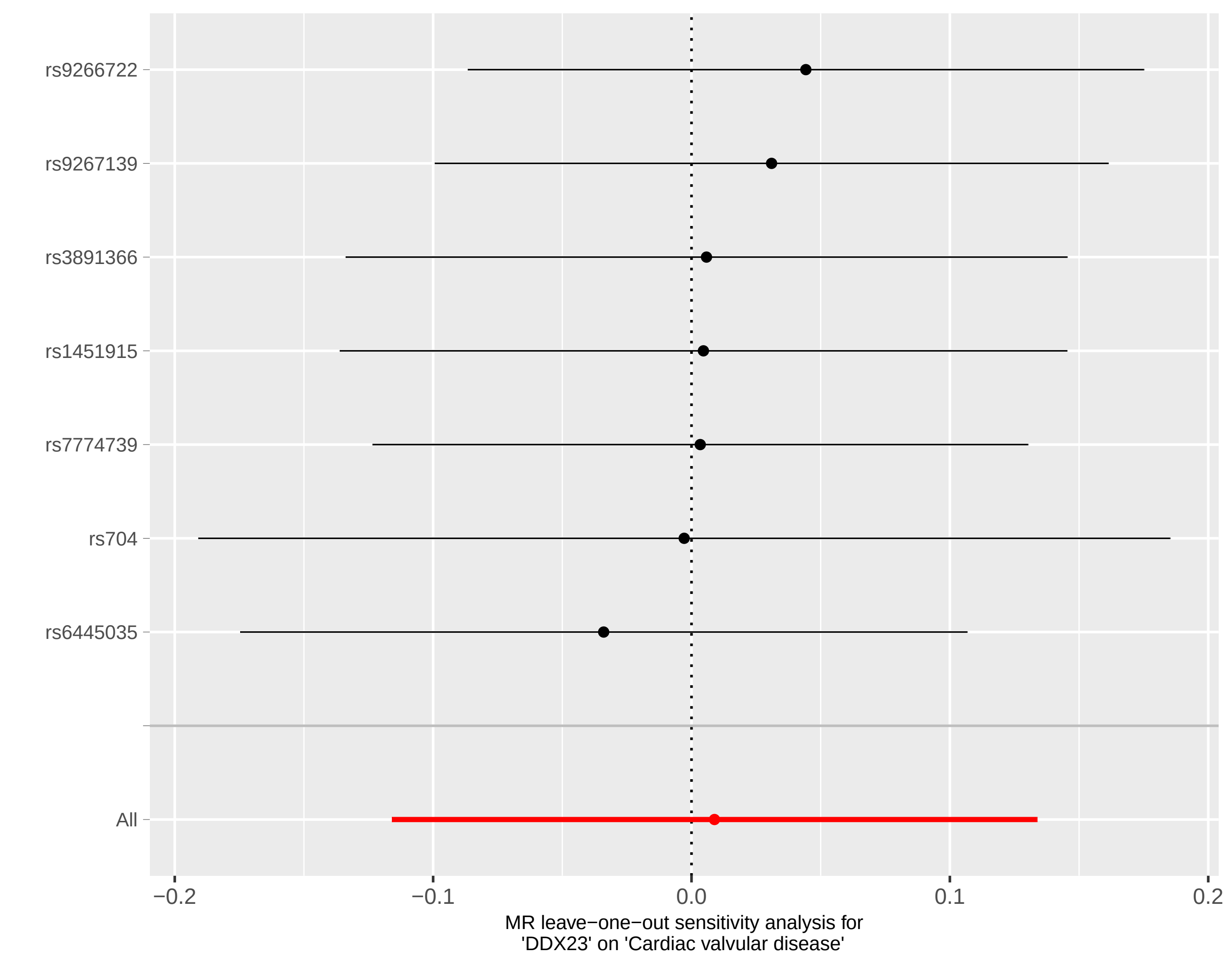

rs9266722
rs9267139
rs3891366
rs1451915
rs7774739
rs704
rs6445035
All
−0.2
−0.1
0.0
0.1
0.2
MR leave−one−out sensitivity analysis for
'DDX23' on 'Cardiac valvular disease'

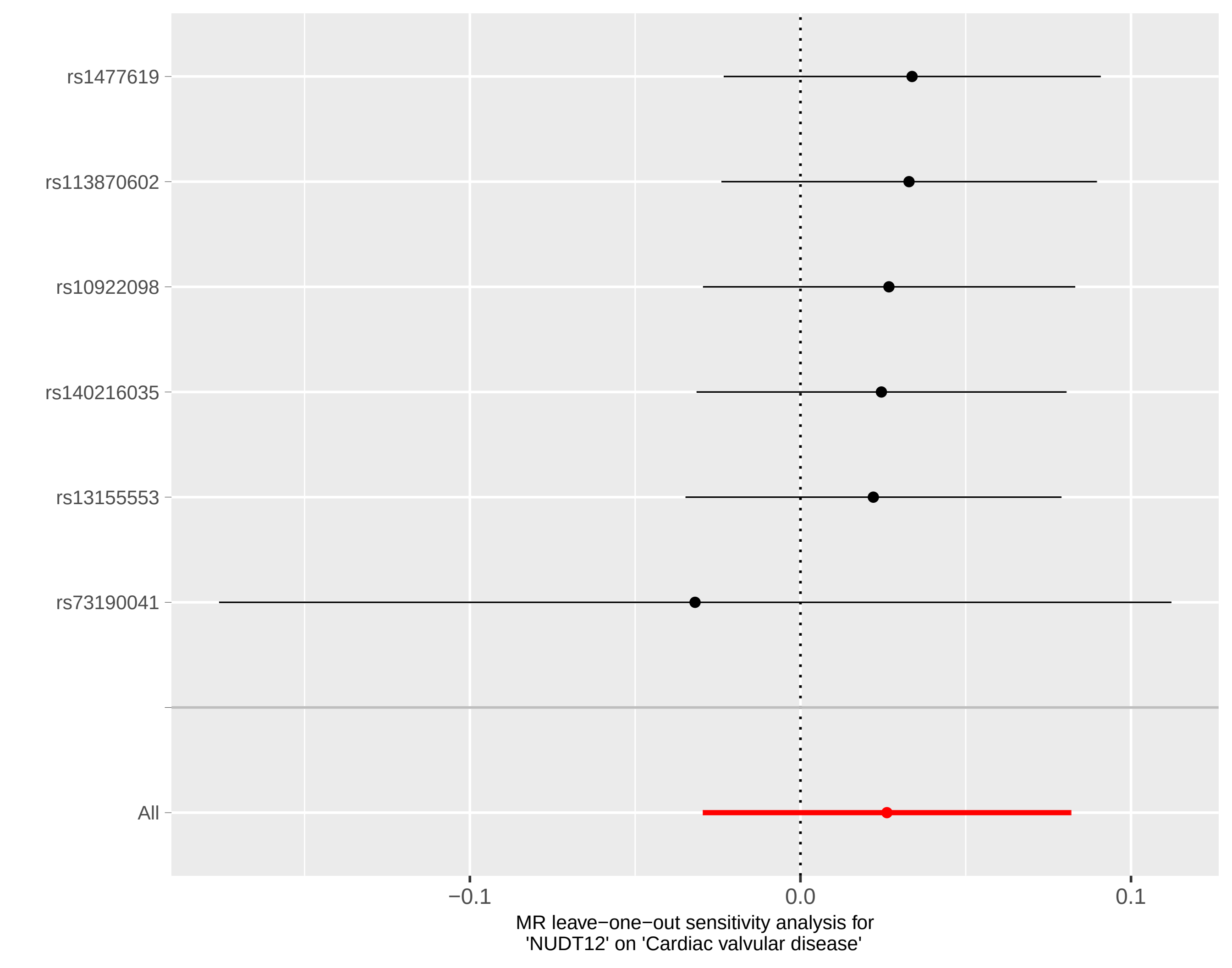

rs1477619
rs113870602
rs10922098
rs140216035
rs13155553
rs73190041
All
−0.1
0.0
0.1
MR leave−one−out sensitivity analysis for
'NUDT12' on 'Cardiac valvular disease'

Insufficient number of SNPs

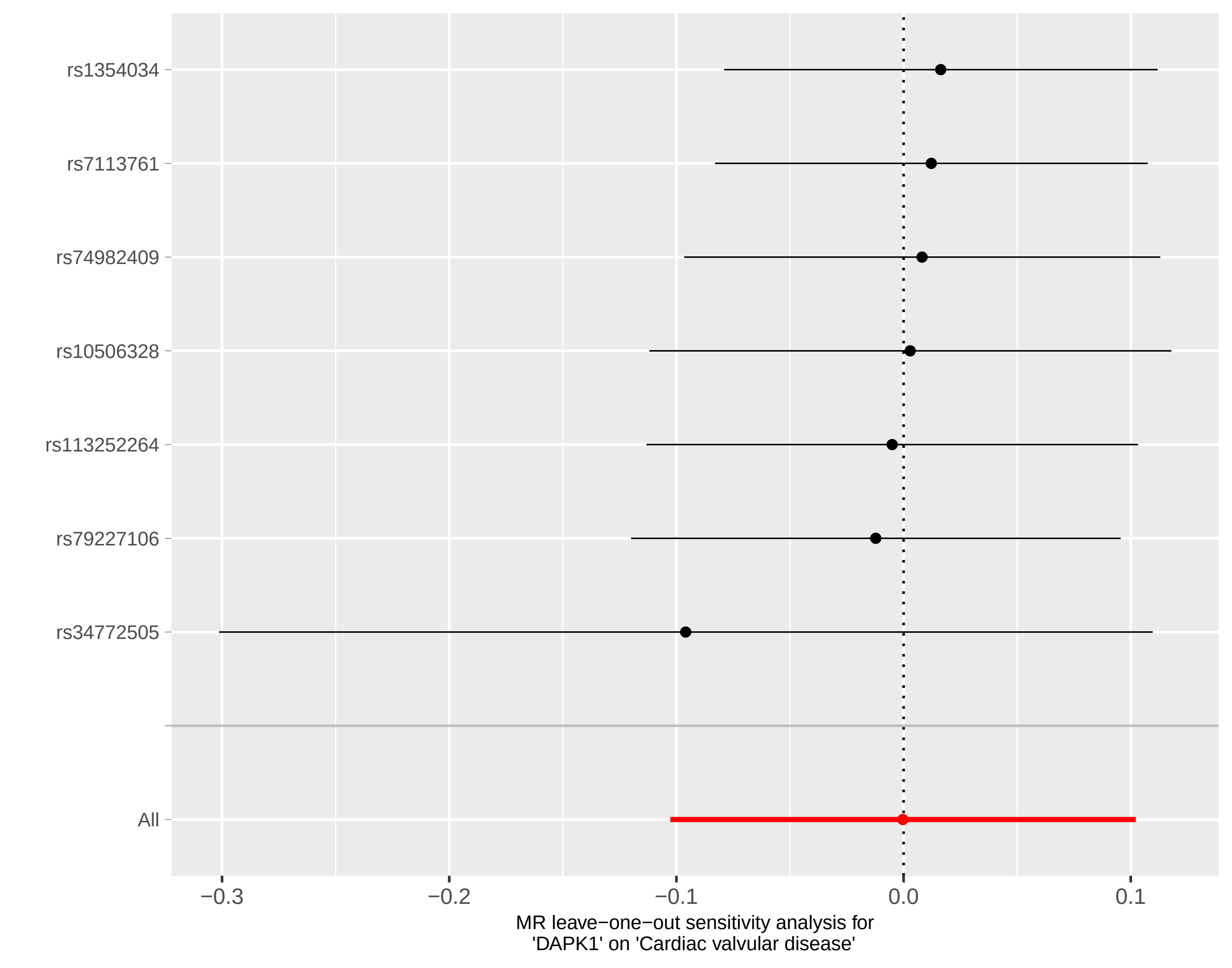

rs1354034
rs7113761
rs74982409
rs10506328
rs113252264
rs79227106
rs34772505
All
−0.3
−0.2
−0.1
0.0
0.1
MR leave−one−out sensitivity analysis for
'DAPK1' on 'Cardiac valvular disease'

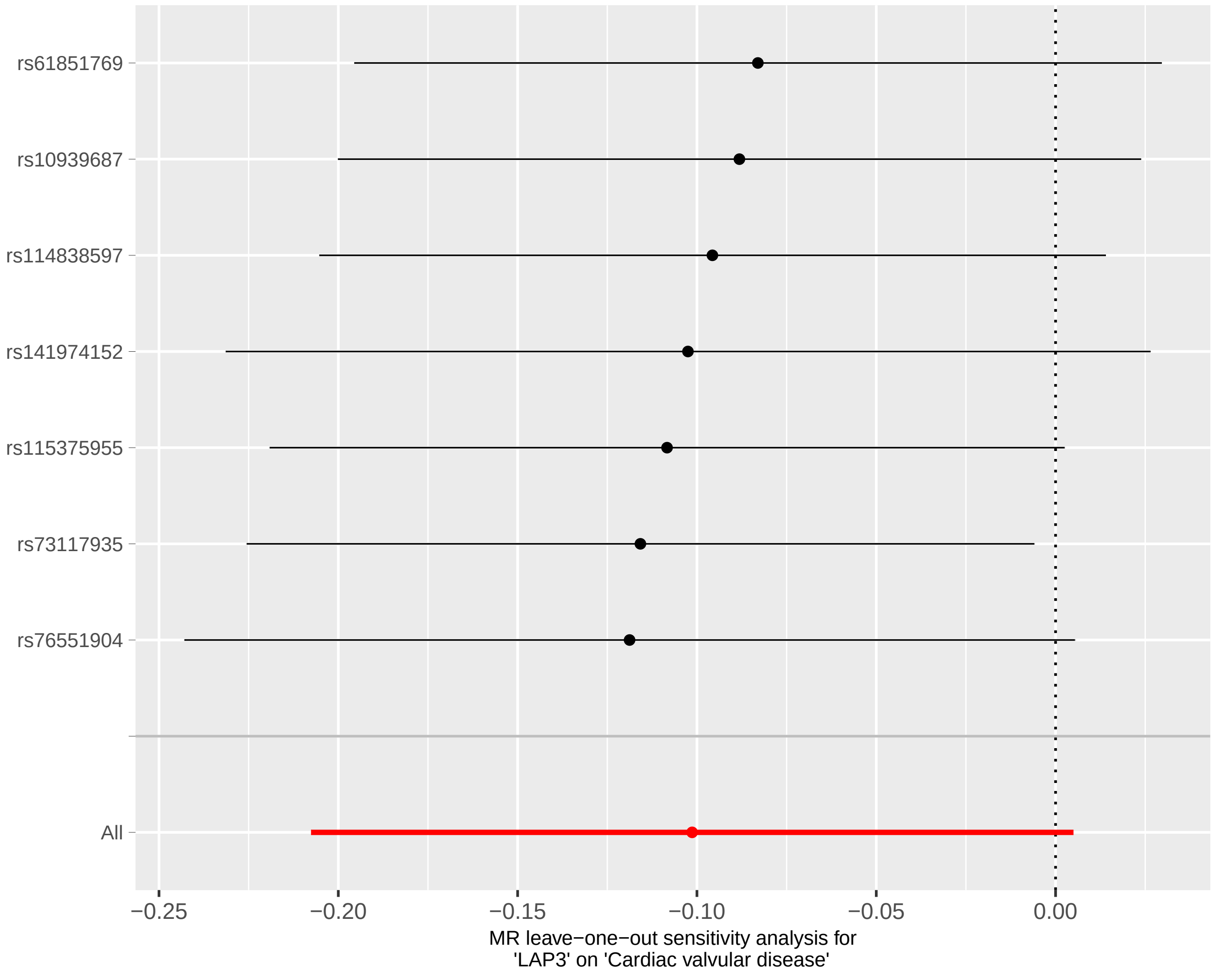
rs61851769
rs10939687
rs114838597
rs141974152
rs115375955
rs73117935
rs76551904
All
−0.25
−0.20
−0.15
−0.10
−0.05
0.00
MR leave−one−out sensitivity analysis for
'LAP3' on 'Cardiac valvular disease'

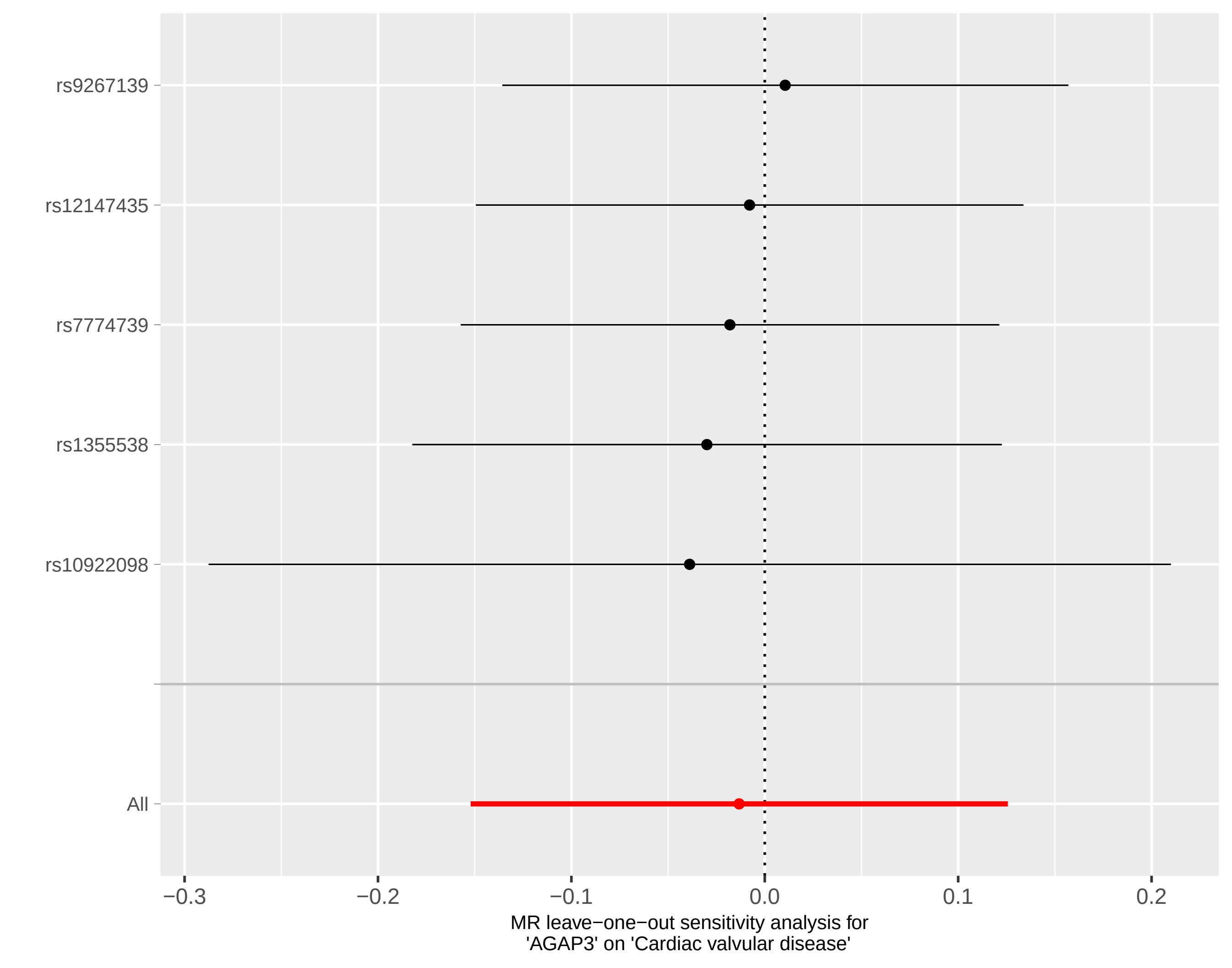
rs9267139
rs12147435
rs7774739
rs1355538
rs10922098
All
−0.3
−0.2
−0.1
0.0
0.1
0.2
MR leave−one−out sensitivity analysis for
'AGAP3' on 'Cardiac valvular disease'

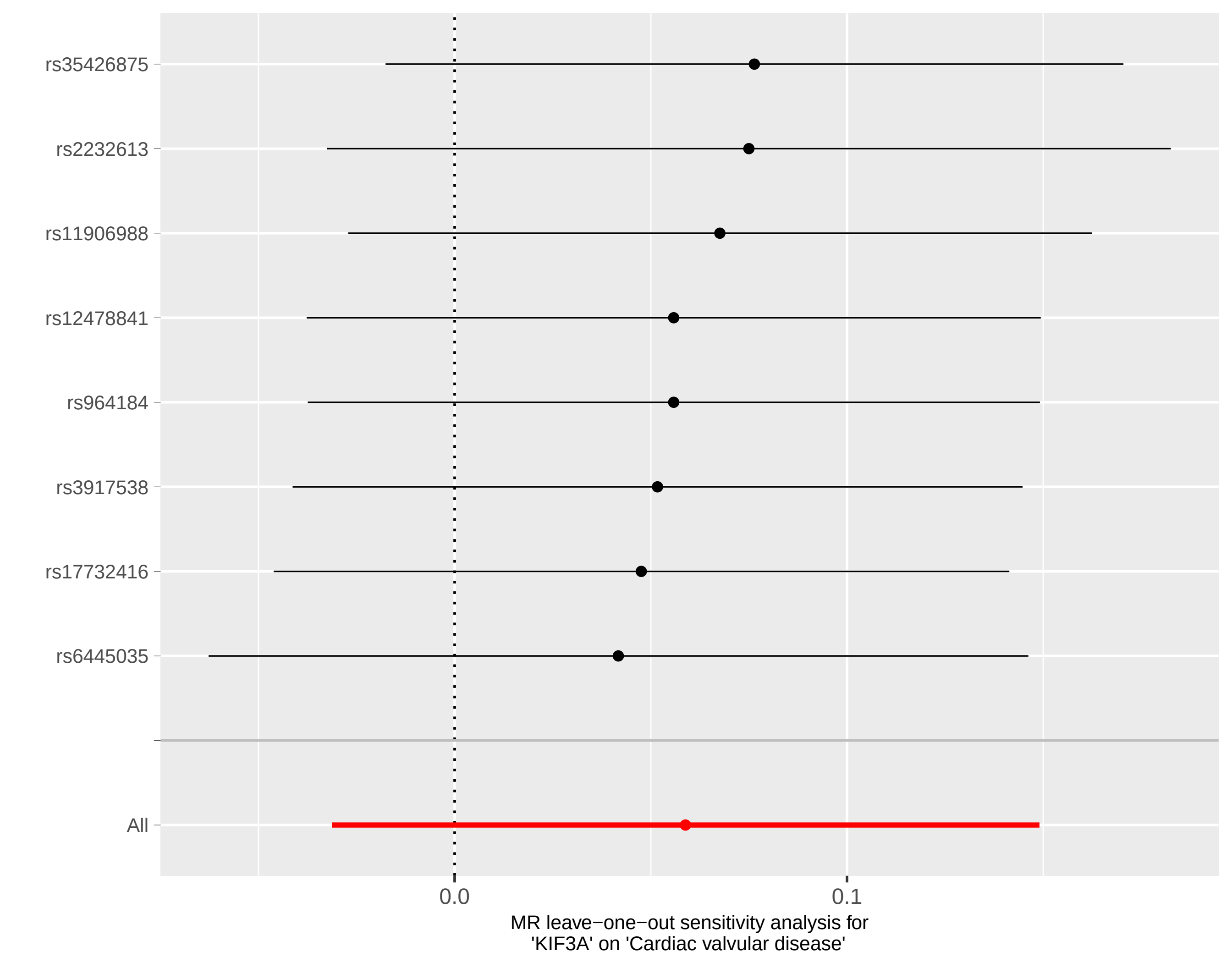

rs35426875
rs2232613
rs11906988
rs12478841
rs964184
rs3917538
rs17732416
rs6445035
All
0.0
0.1
MR leave−one−out sensitivity analysis for
'KIF3A' on 'Cardiac valvular disease'

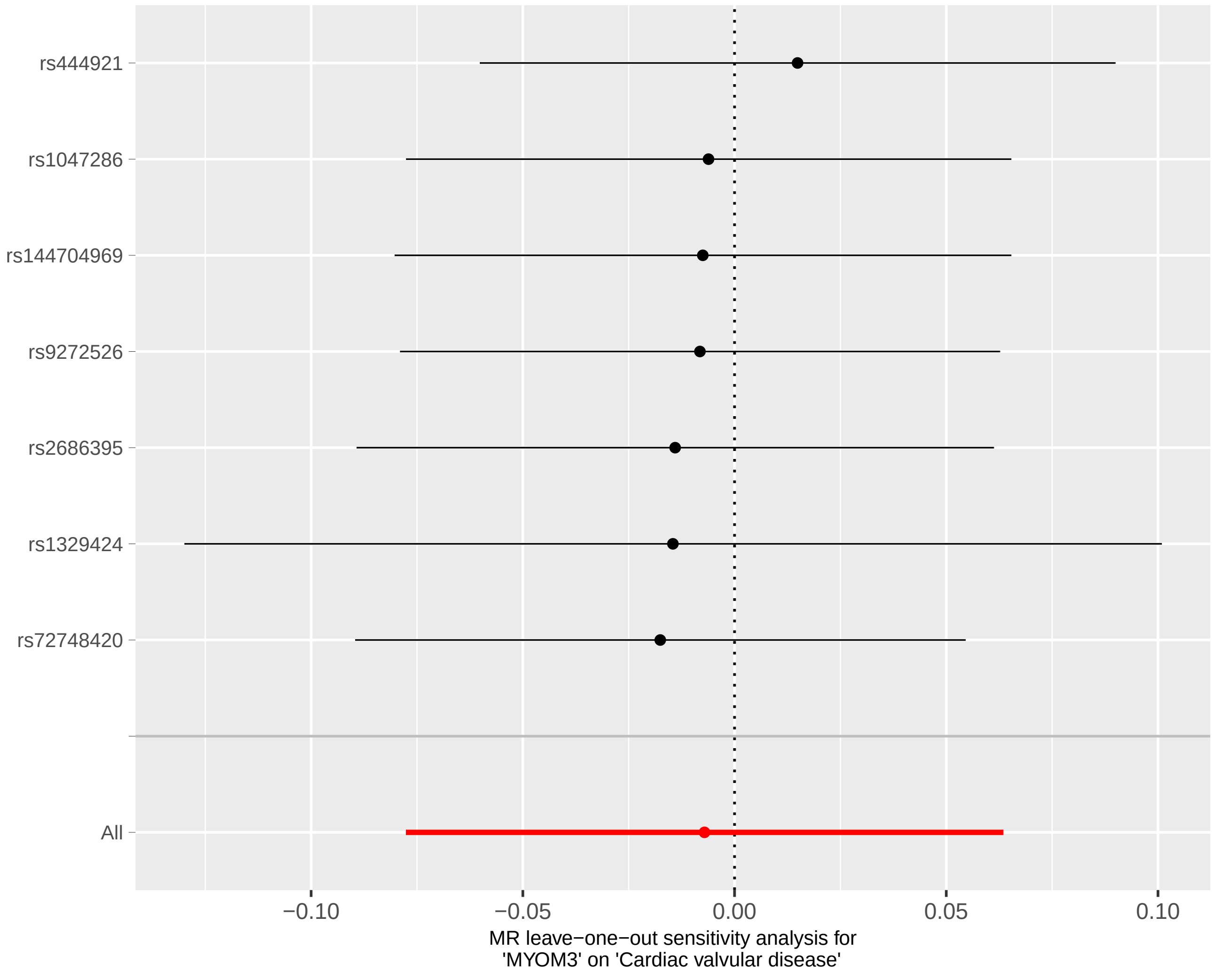
rs444921
rs1047286
rs144704969
rs9272526
rs2686395
rs1329424
rs72748420
All
−0.10
−0.05
0.00
0.05
0.10
MR leave−one−out sensitivity analysis for
'MYOM3' on 'Cardiac valvular disease'

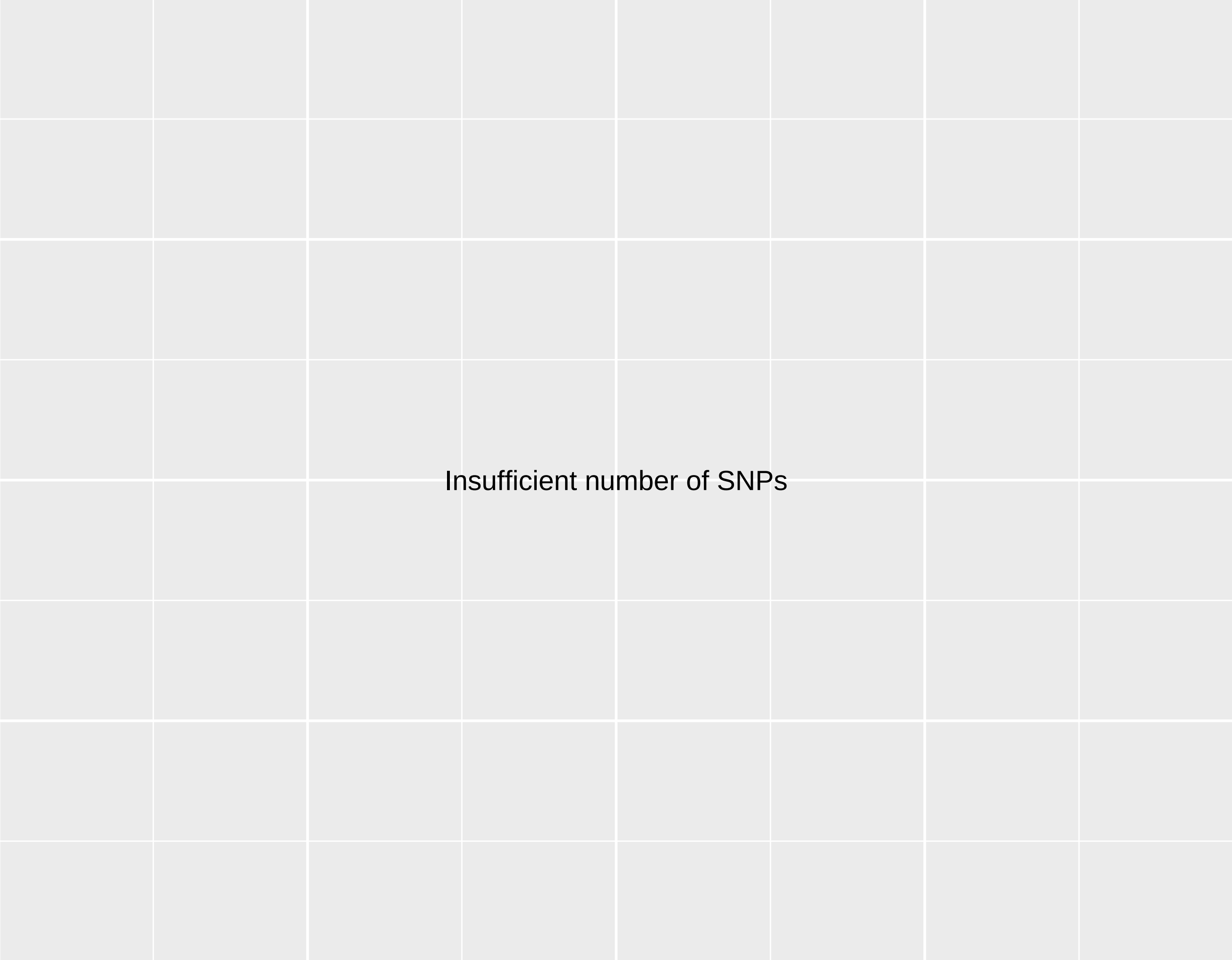
Insufficient number of SNPs

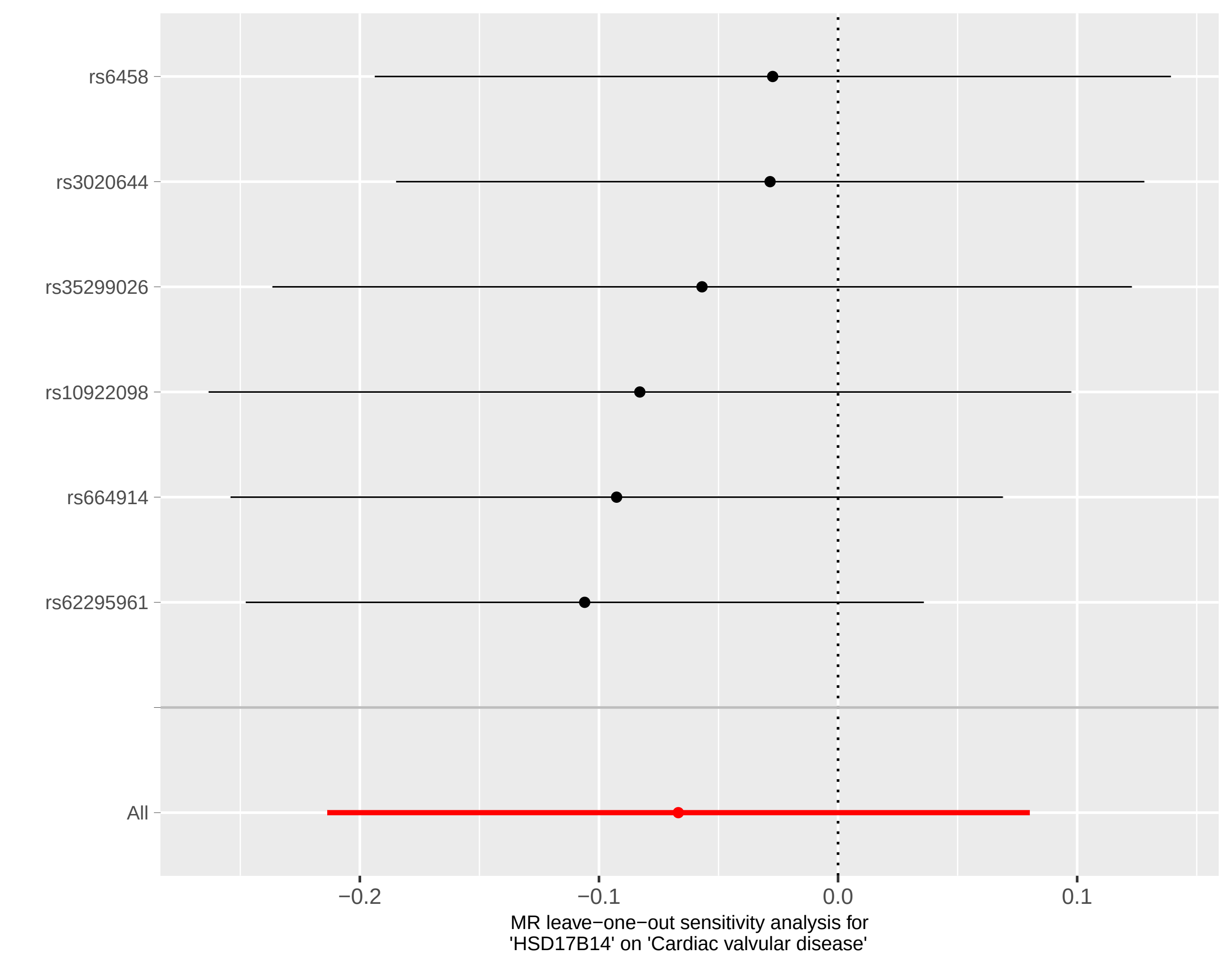

rs6458
rs3020644
rs35299026
rs10922098
rs664914
rs62295961
All
−0.2
−0.1
0.0
0.1
MR leave−one−out sensitivity analysis for
'HSD17B14' on 'Cardiac valvular disease'

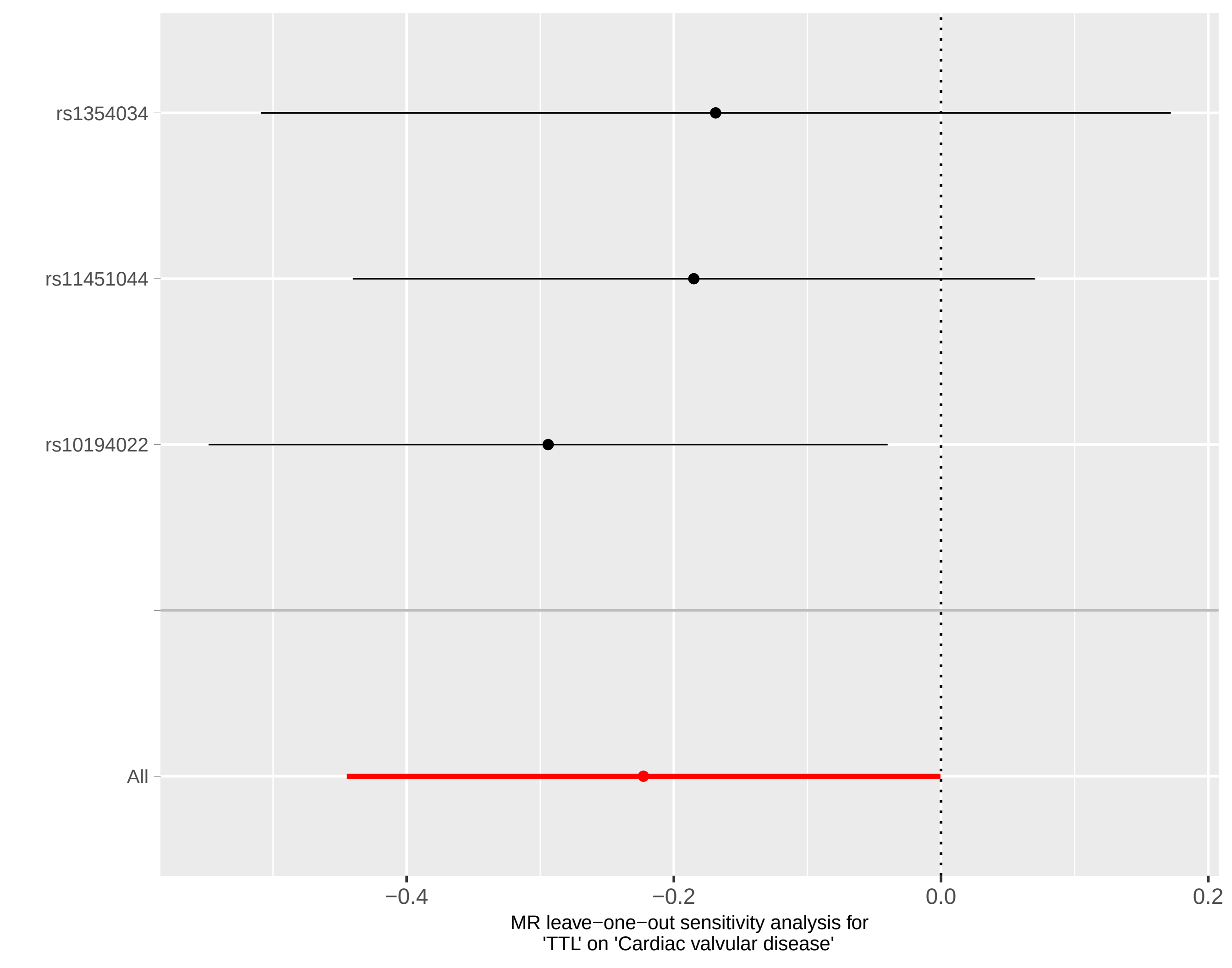

rs1354034
rs11451044
rs10194022
All
−0.4
−0.2
0.0
0.2
MR leave−one−out sensitivity analysis for
'TTL' on 'Cardiac valvular disease'

Insufficient number of SNPs

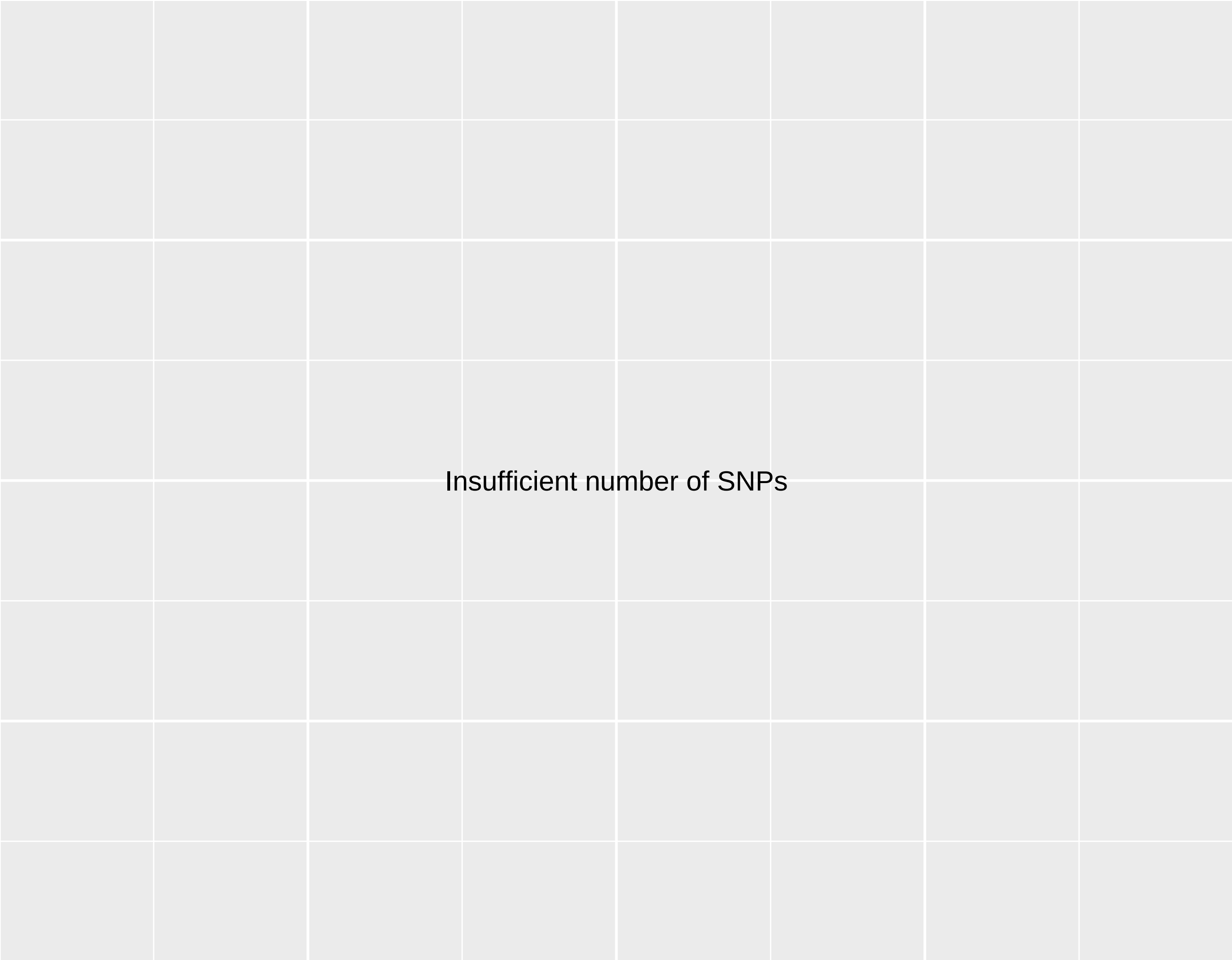
Insufficient number of SNPs

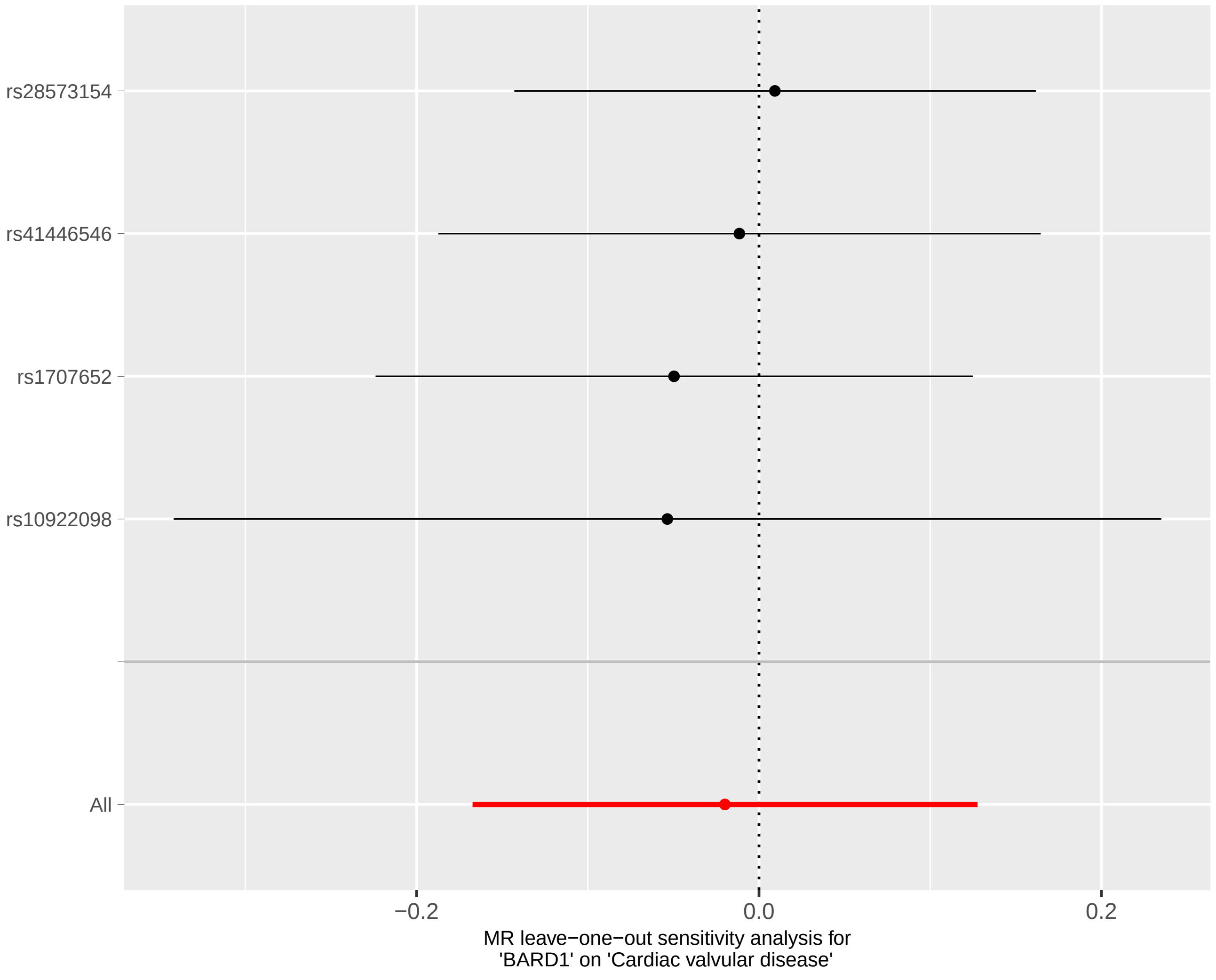
rs28573154
rs41446546
rs1707652
rs10922098
All
−0.2
0.0
0.2
MR leave−one−out sensitivity analysis for
'BARD1' on 'Cardiac valvular disease'

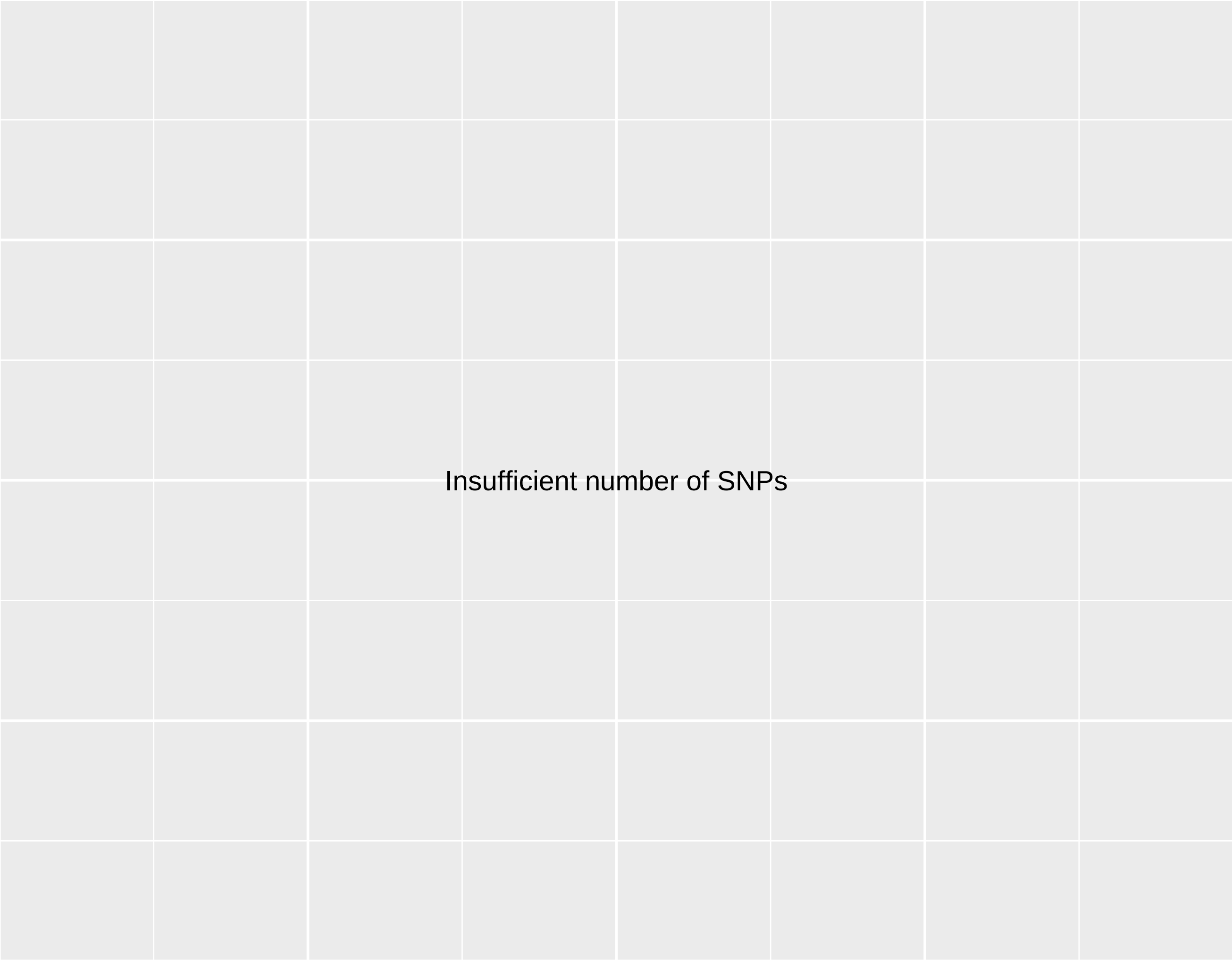
Insufficient number of SNPs

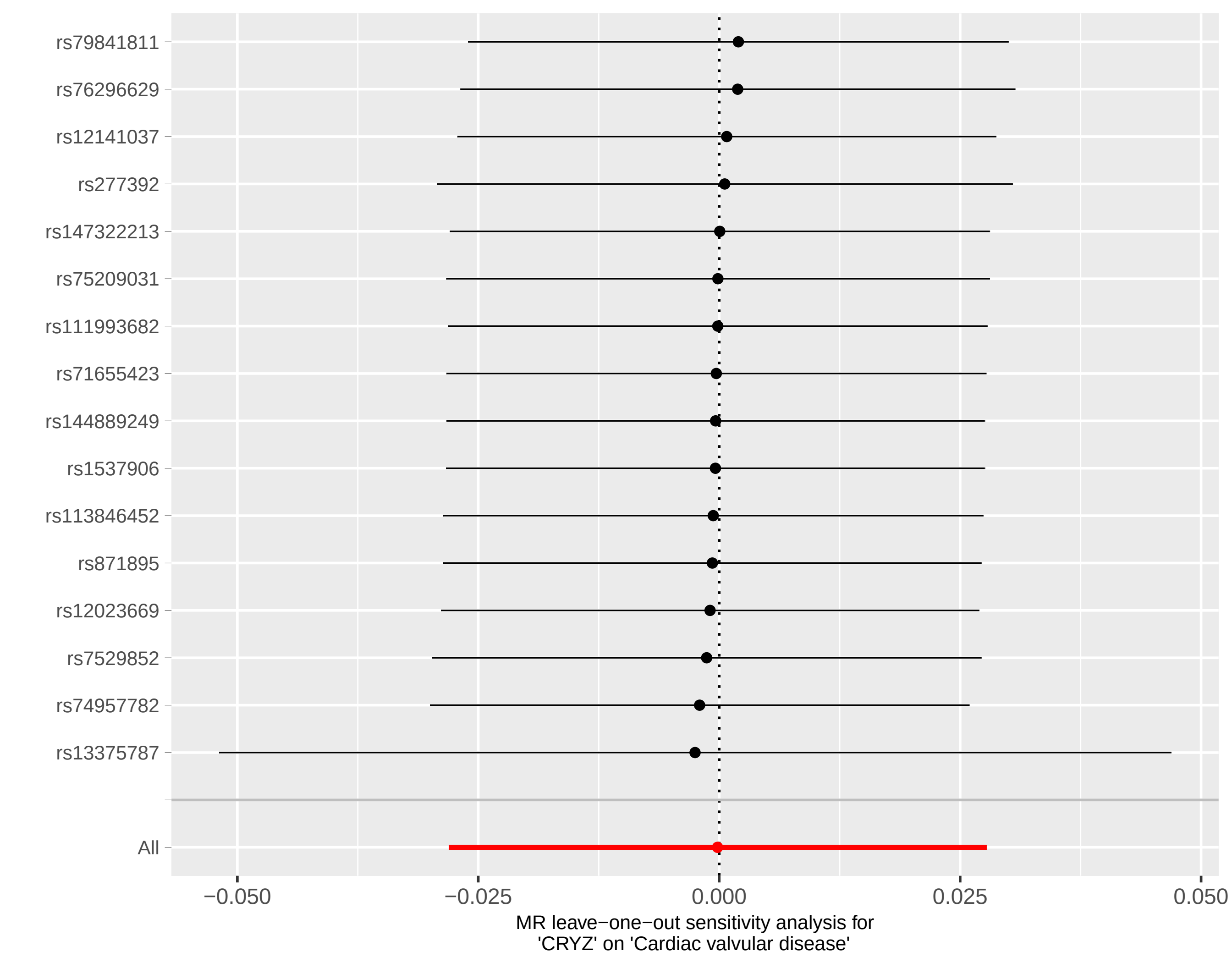
rs79841811
rs76296629
rs12141037
rs277392
rs147322213
rs75209031
rs111993682
rs71655423
rs144889249
rs1537906
rs113846452
rs871895
rs12023669
rs7529852
rs74957782
rs13375787
All
−0.050
−0.025
0.000
0.025
0.050
MR leave−one−out sensitivity analysis for
'CRYZ' on 'Cardiac valvular disease'

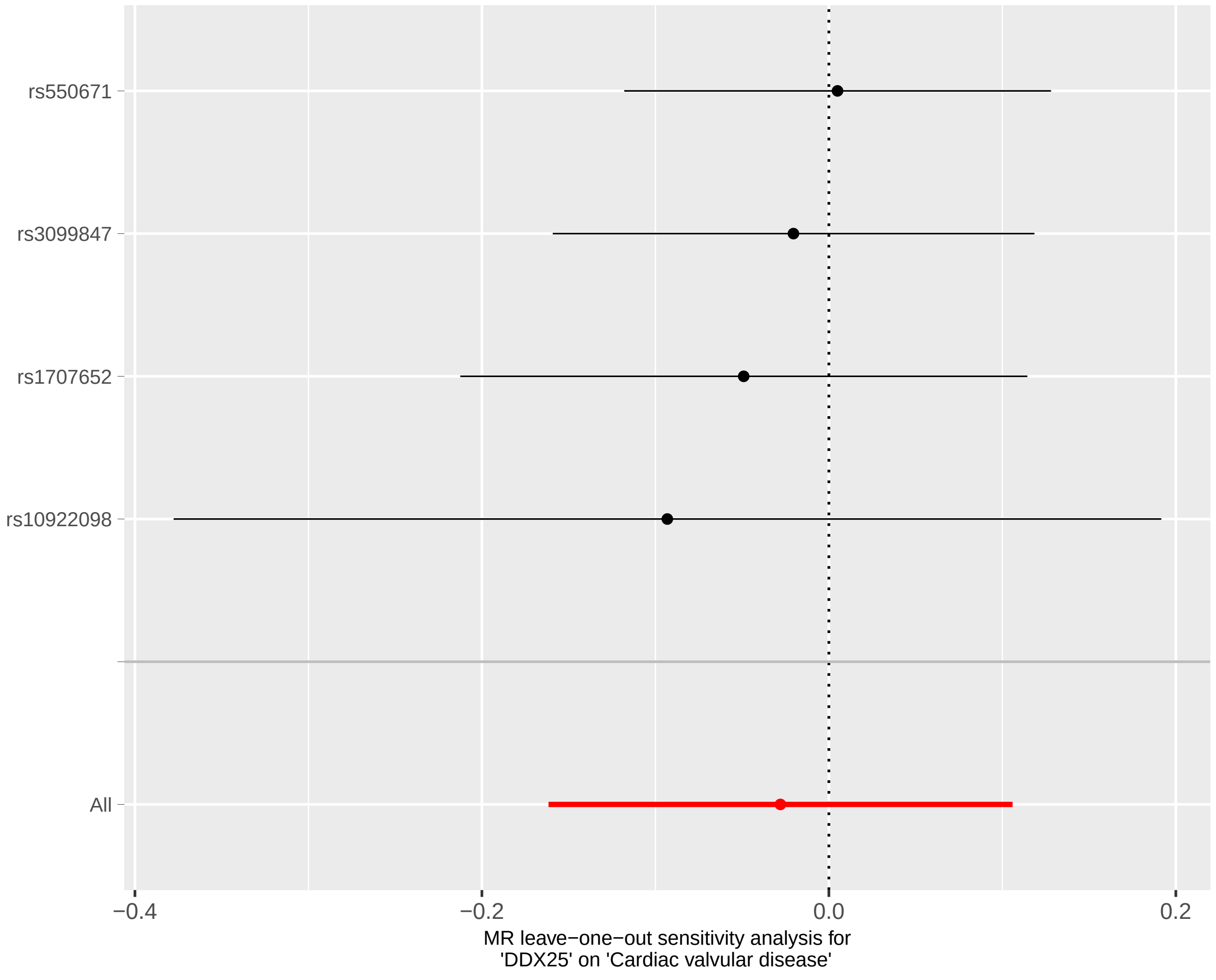

rs550671
rs3099847
rs1707652
rs10922098
All
−0.4
−0.2
0.0
0.2
MR leave−one−out sensitivity analysis for
'DDX25' on 'Cardiac valvular disease'

Insufficient number of SNPs

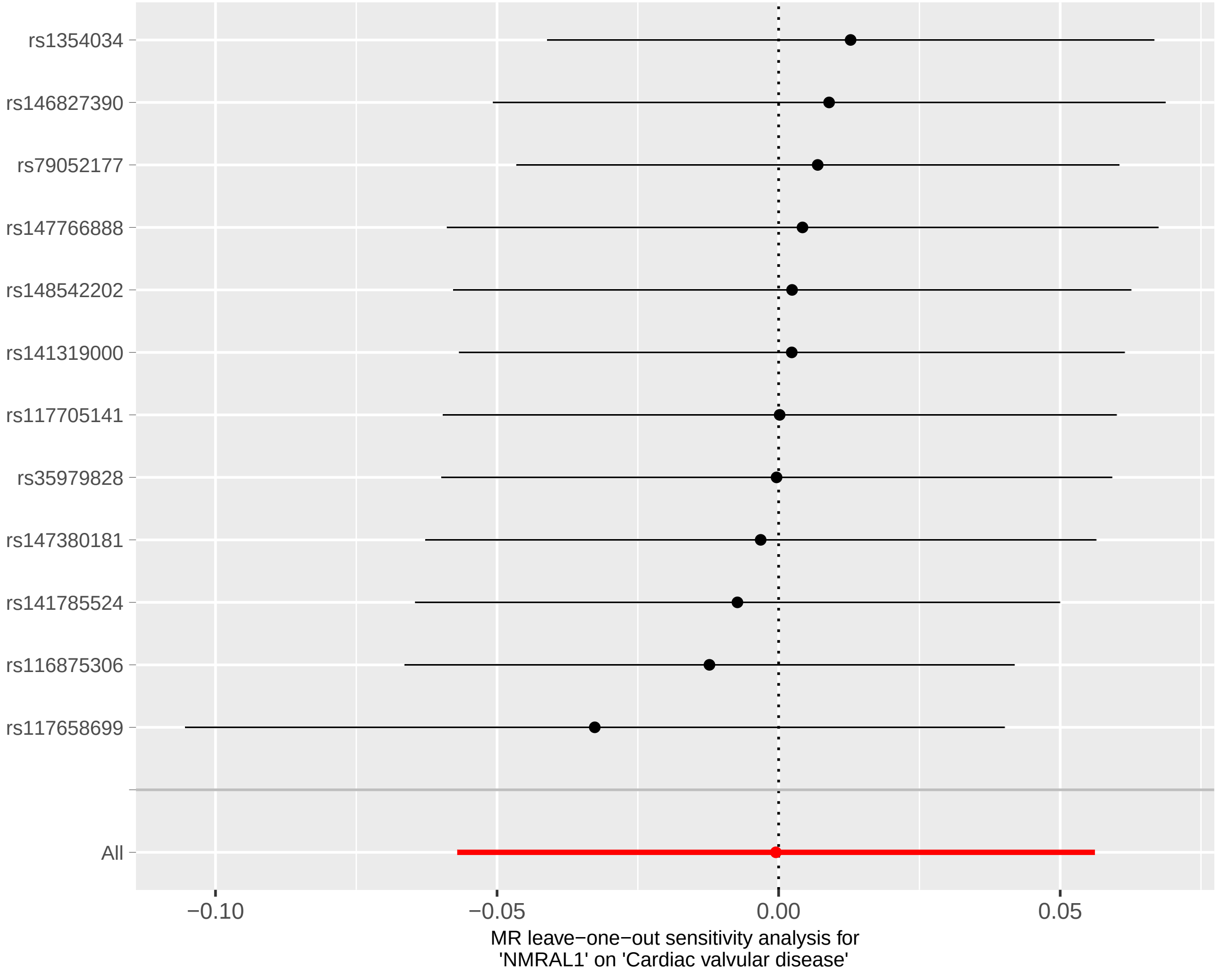
rs1354034
rs146827390
rs79052177
rs147766888
rs148542202
rs141319000
rs117705141
rs35979828
rs147380181
rs141785524
rs116875306
rs117658699
All
−0.10
−0.05
0.00
0.05
MR leave−one−out sensitivity analysis for
'NMRAL1' on 'Cardiac valvular disease'

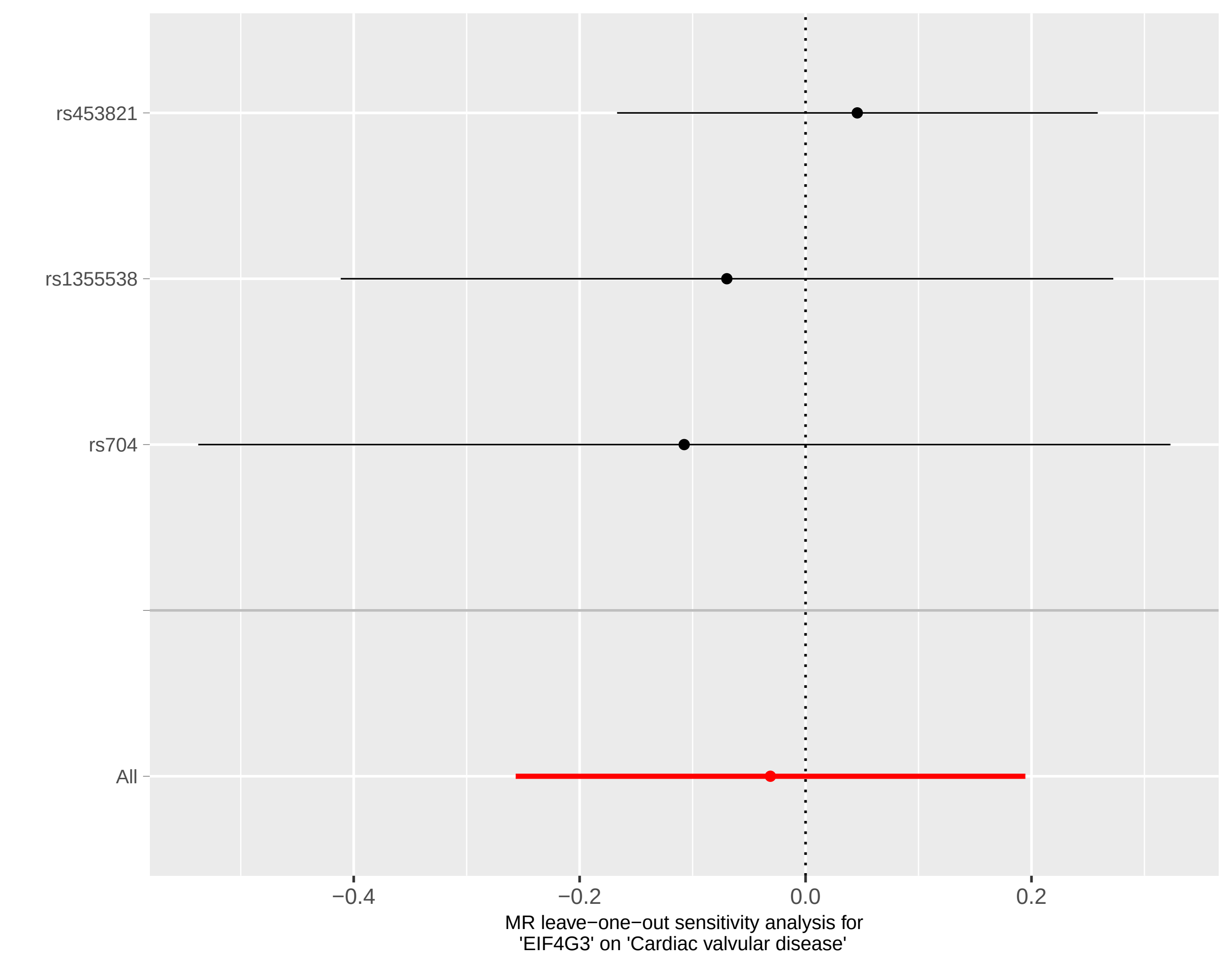
rs453821
rs1355538
rs704
All
−0.4
−0.2
0.0
0.2
MR leave−one−out sensitivity analysis for
'EIF4G3' on 'Cardiac valvular disease'

Insufficient number of SNPs

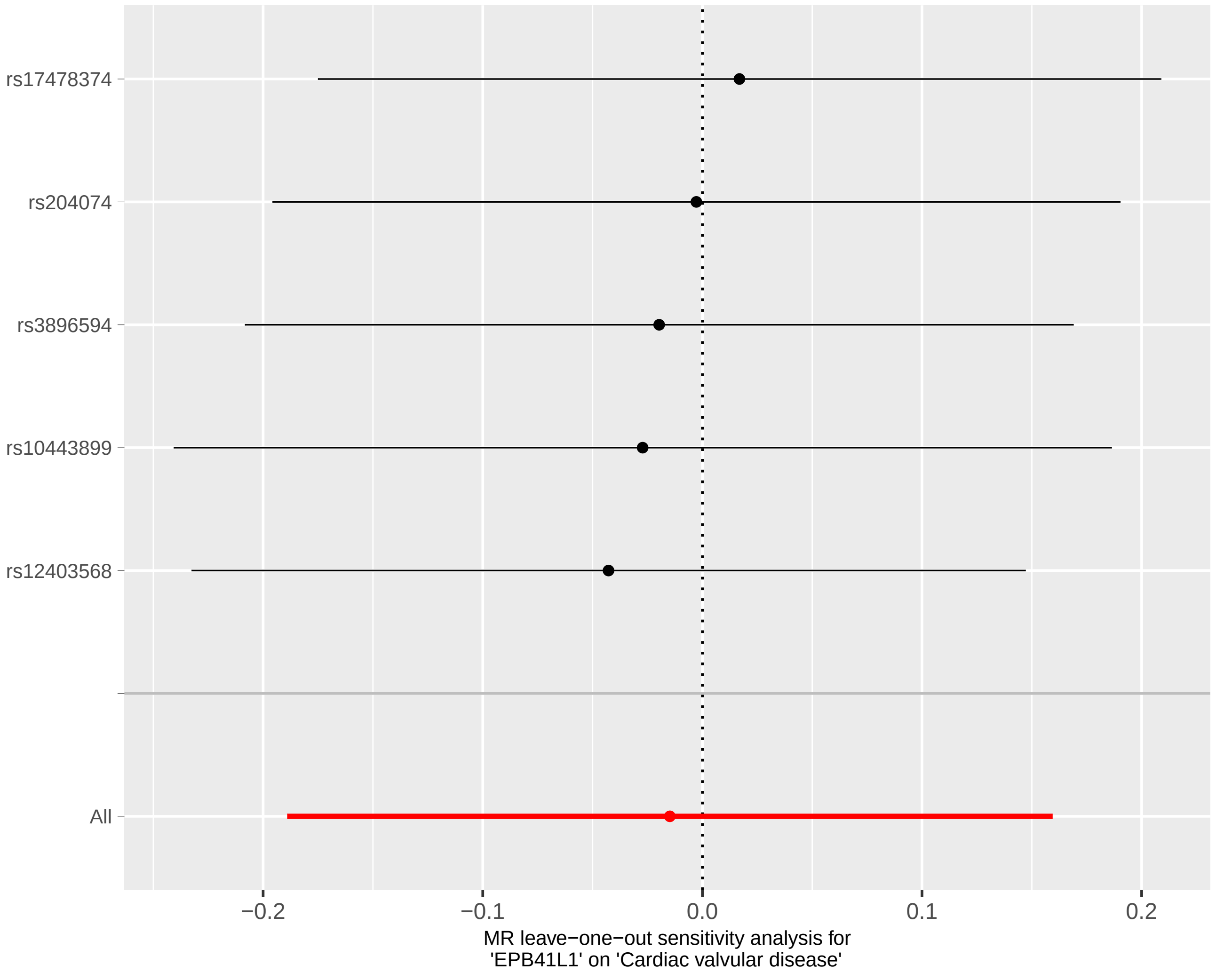

rs17478374
rs204074
rs3896594
rs10443899
rs12403568
All
−0.2
−0.1
0.0
0.1
0.2
MR leave−one−out sensitivity analysis for
'EPB41L1' on 'Cardiac valvular disease'

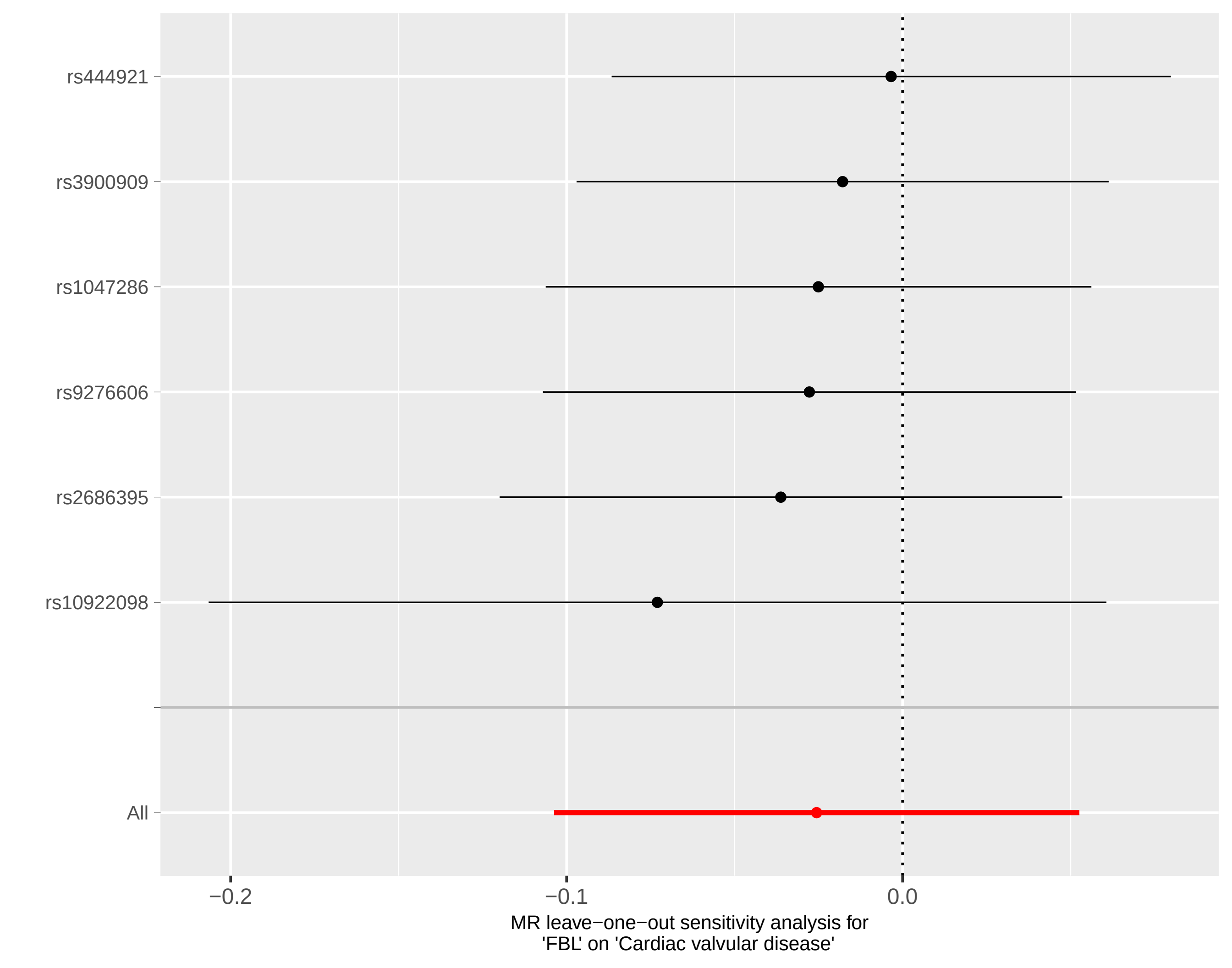
rs444921
rs3900909
rs1047286
rs9276606
rs2686395
rs10922098
All
−0.2
−0.1
0.0
MR leave−one−out sensitivity analysis for
'FBL' on 'Cardiac valvular disease'

Insufficient number of SNPs

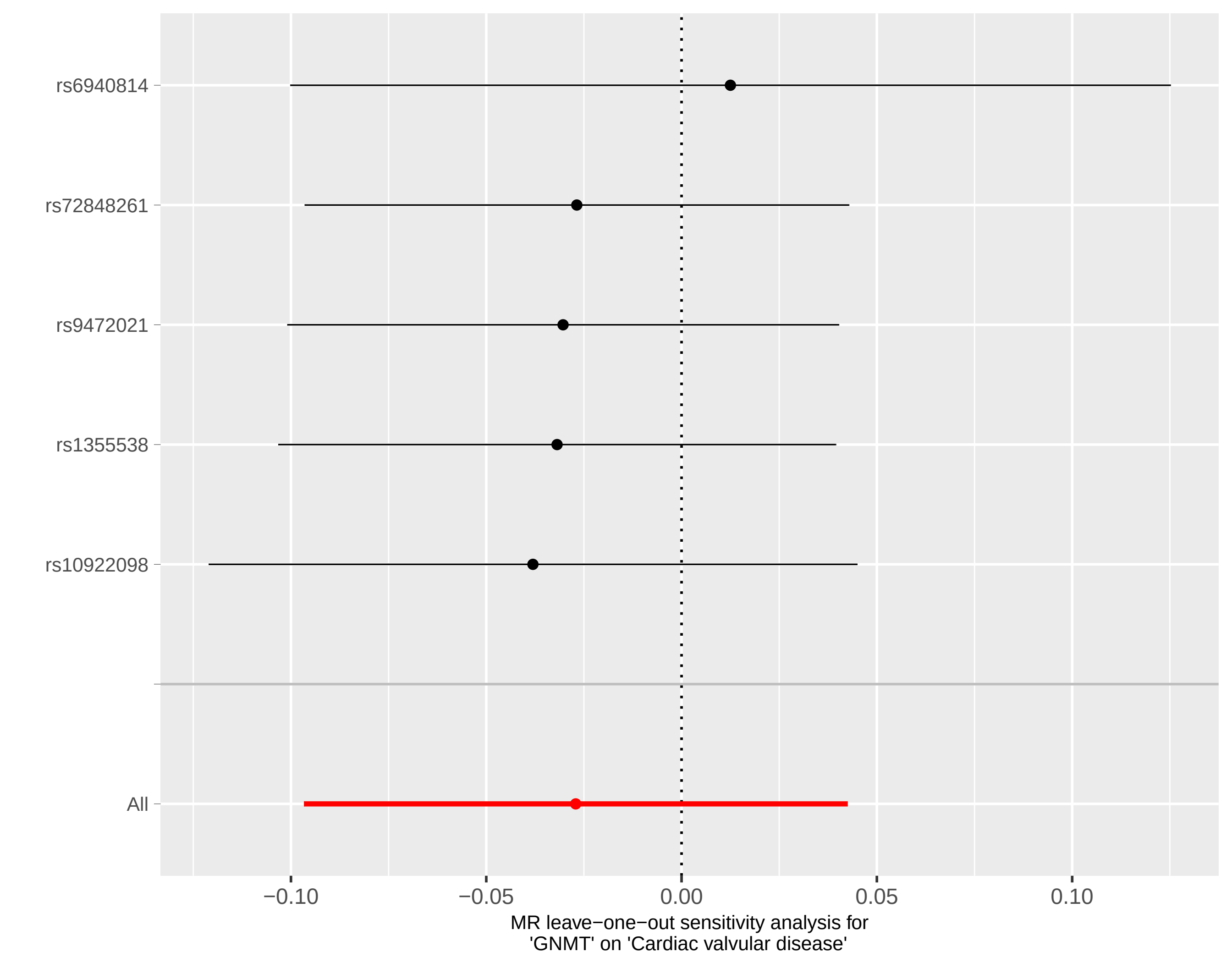
rs6940814
rs72848261
rs9472021
rs1355538
rs10922098
All
−0.10
−0.05
0.00
0.05
0.10
MR leave−one−out sensitivity analysis for
'GNMT' on 'Cardiac valvular disease'

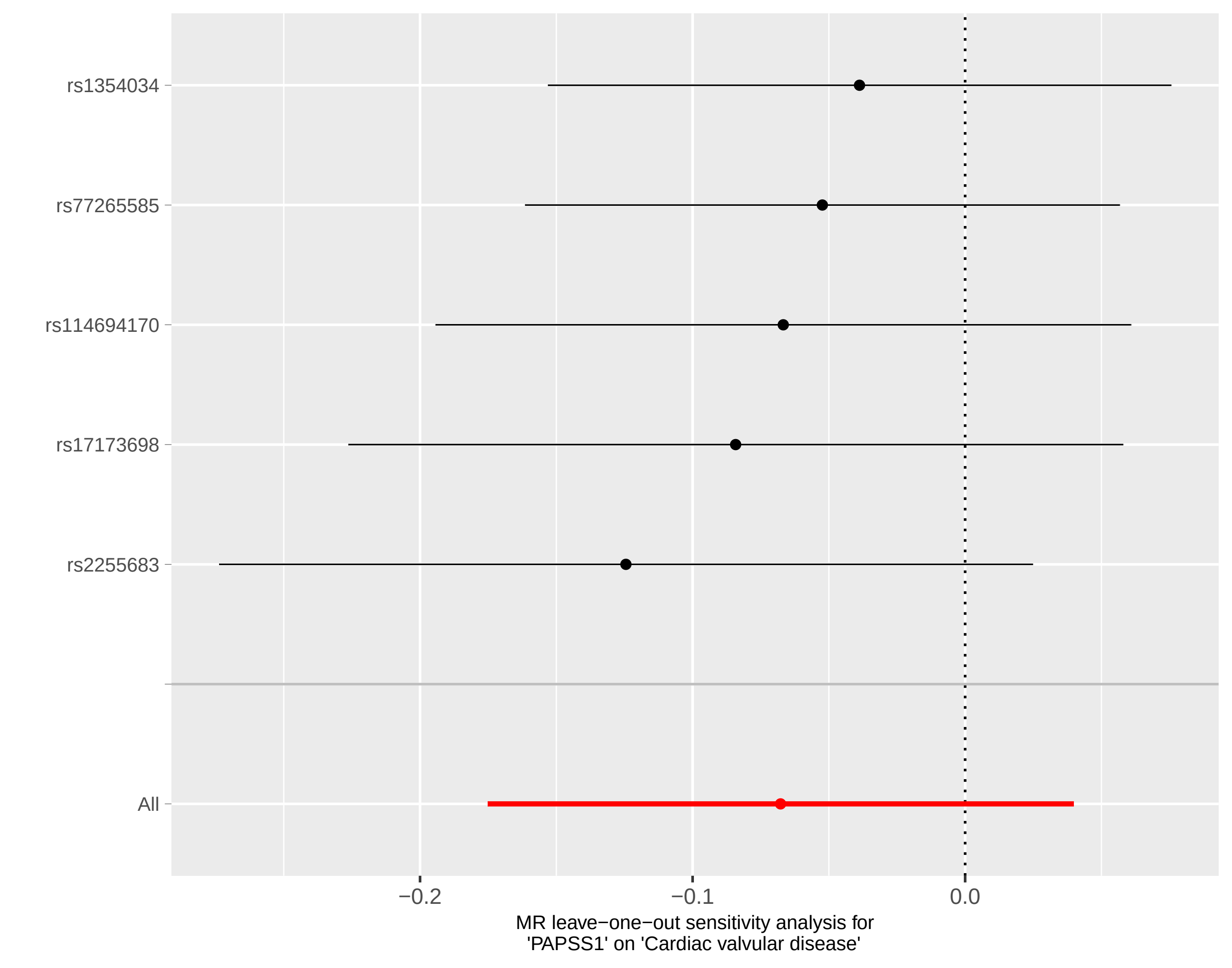

rs1354034
rs77265585
rs114694170
rs17173698
rs2255683
All
−0.2
−0.1
0.0
MR leave−one−out sensitivity analysis for
'PAPSS1' on 'Cardiac valvular disease'

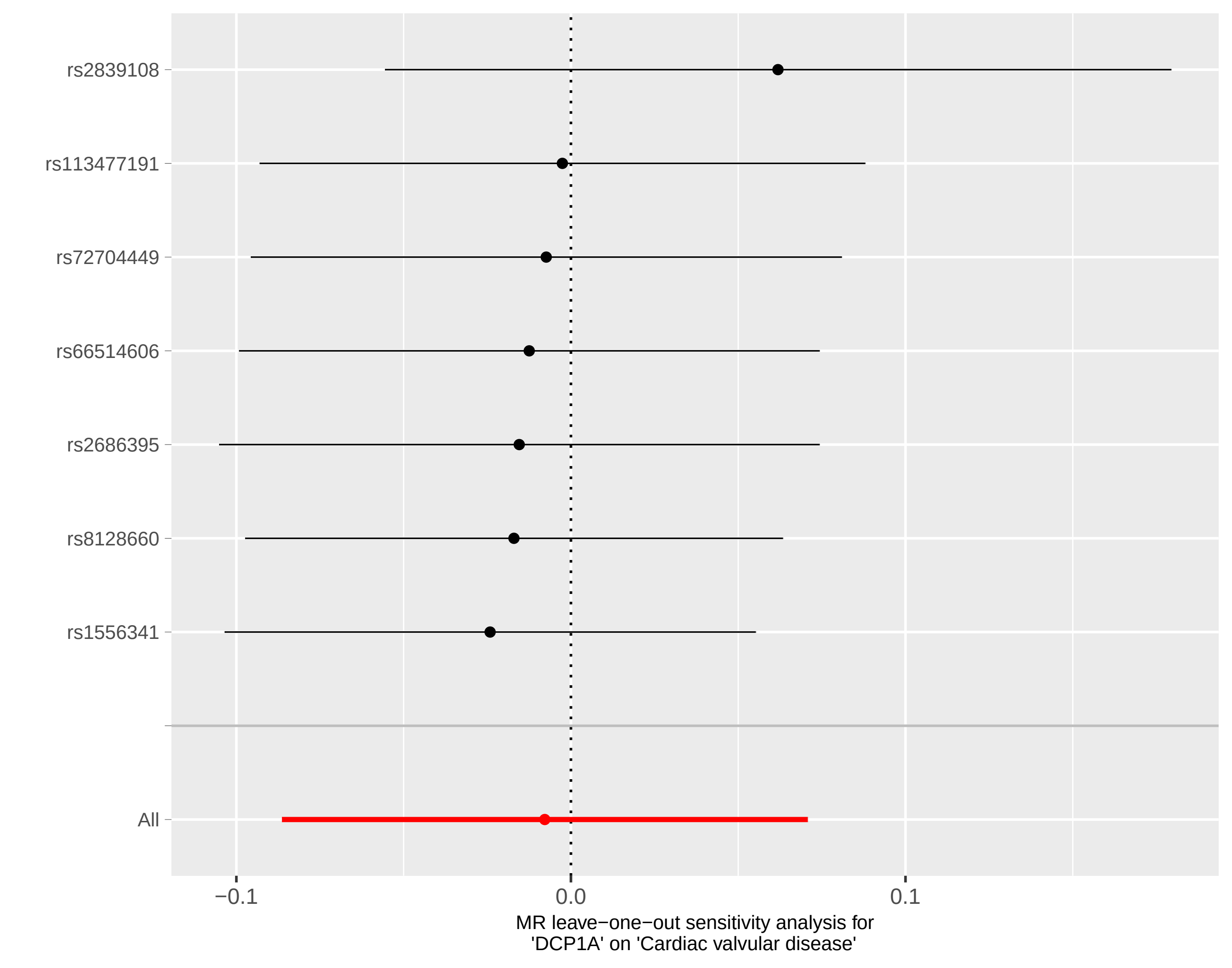
rs2839108
rs113477191
rs72704449
rs66514606
rs2686395
rs8128660
rs1556341
All
−0.1
0.0
0.1
MR leave−one−out sensitivity analysis for
'DCP1A' on 'Cardiac valvular disease'

Insufficient number of SNPs

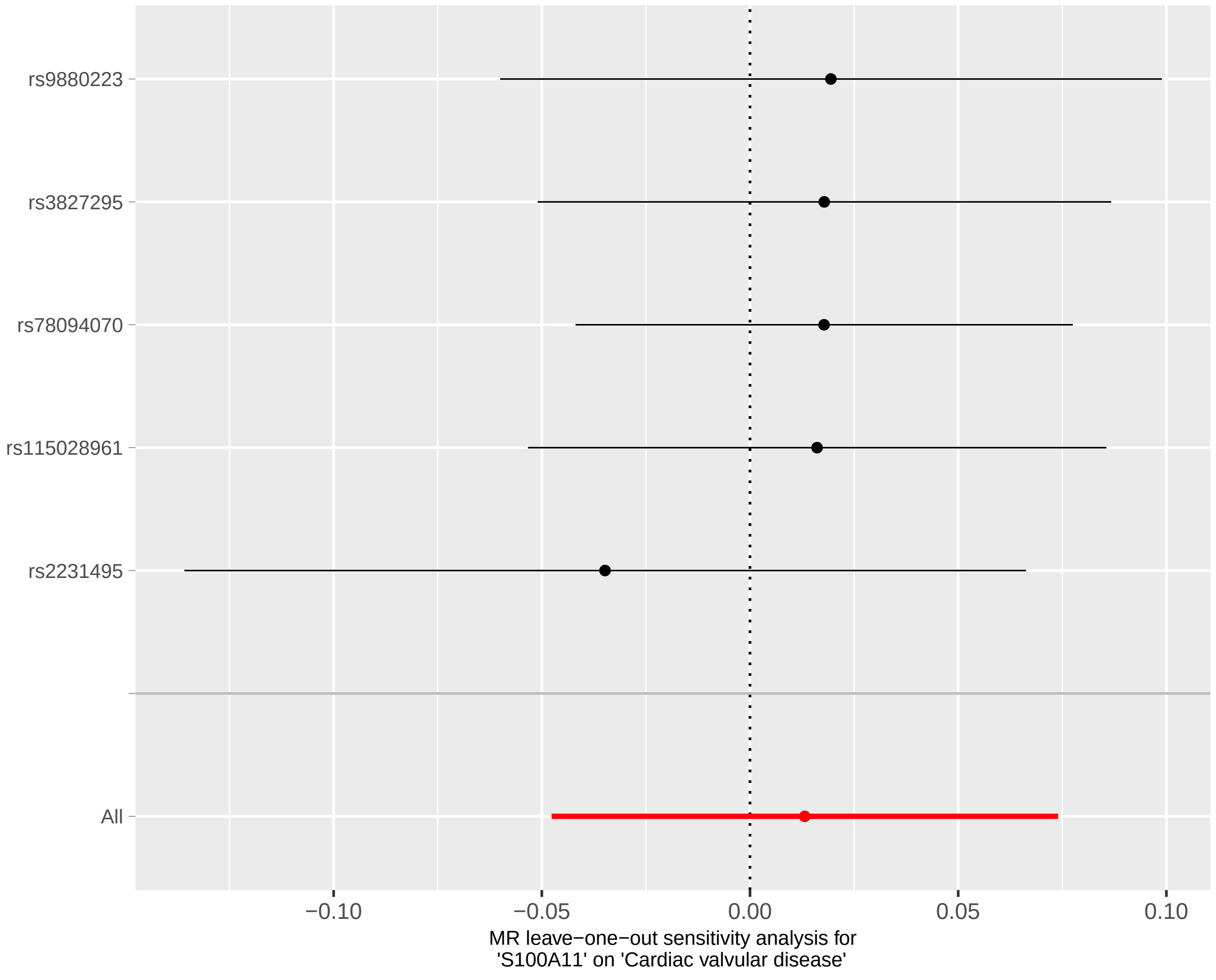
rs9880223
rs3827295
rs78094070
rs115028961
rs2231495
All
−0.10
−0.05
0.00
0.05
0.10
MR leave−one−out sensitivity analysis for
'S100A11' on 'Cardiac valvular disease'

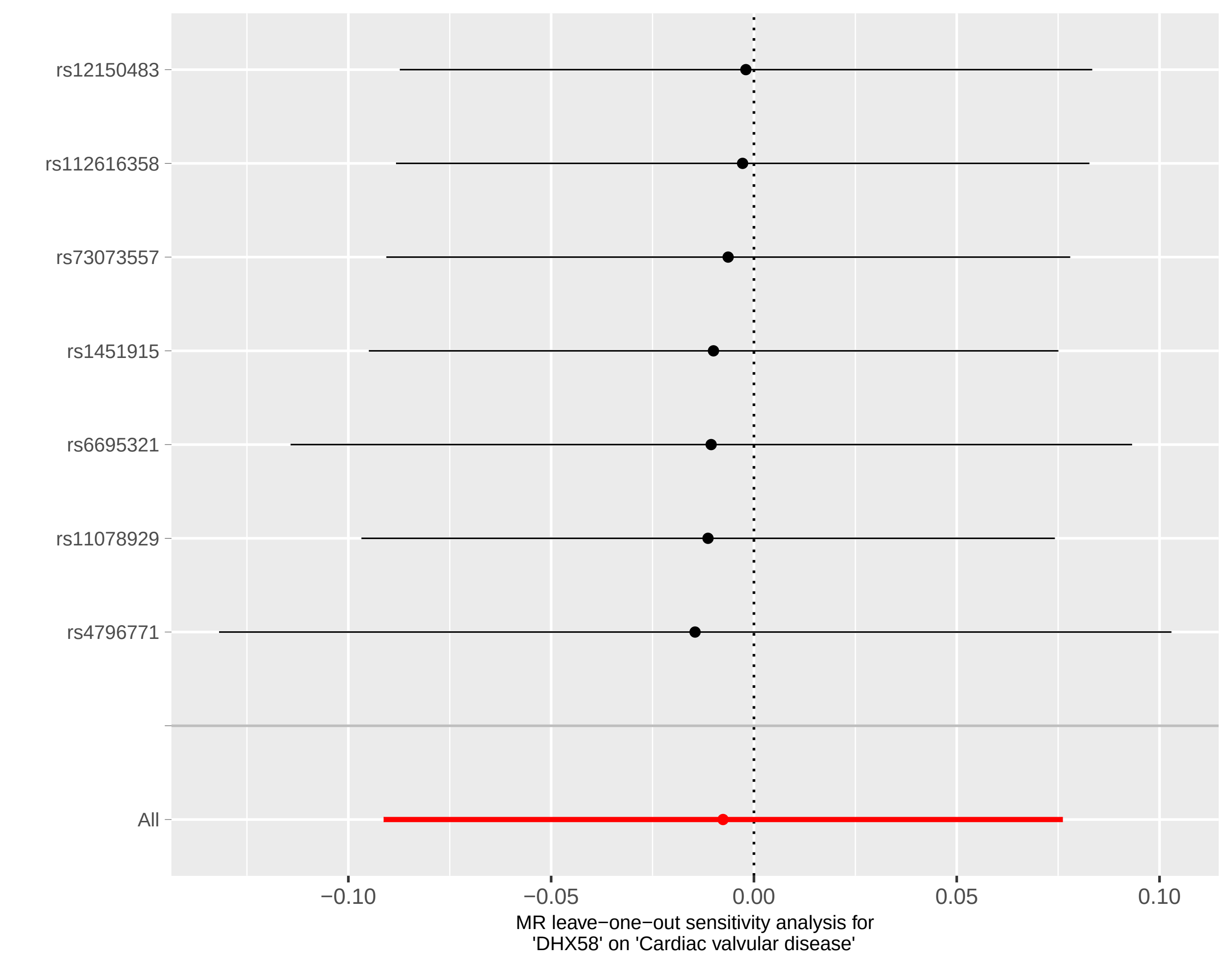
rs12150483
rs112616358
rs73073557
rs1451915
rs6695321
rs11078929
rs4796771
All
−0.10
−0.05
0.00
0.05
0.10
MR leave−one−out sensitivity analysis for
'DHX58' on 'Cardiac valvular disease'

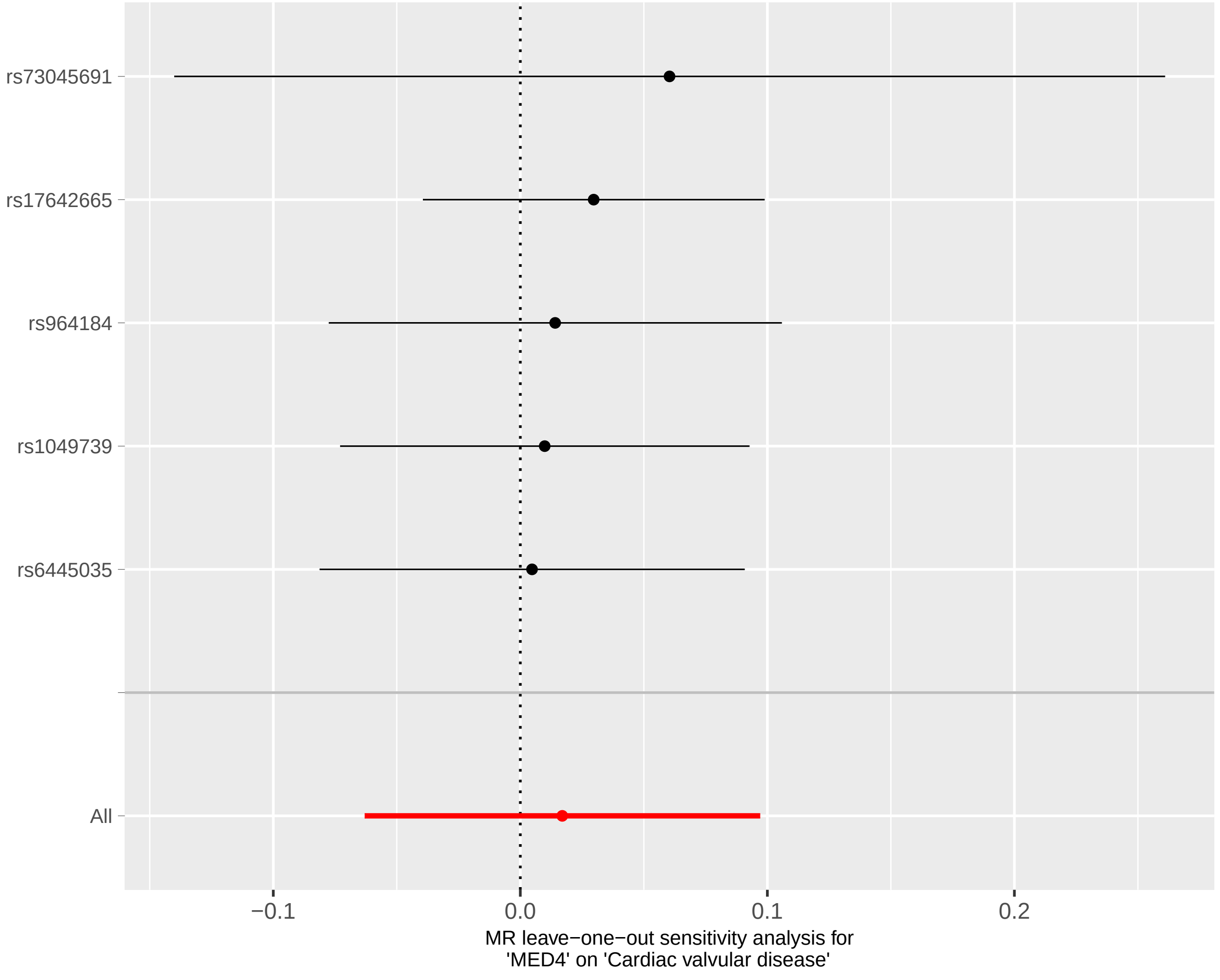
rs73045691
rs17642665
rs964184
rs1049739
rs6445035
All
−0.1
0.0
0.1
0.2
MR leave−one−out sensitivity analysis for
'MED4' on 'Cardiac valvular disease'

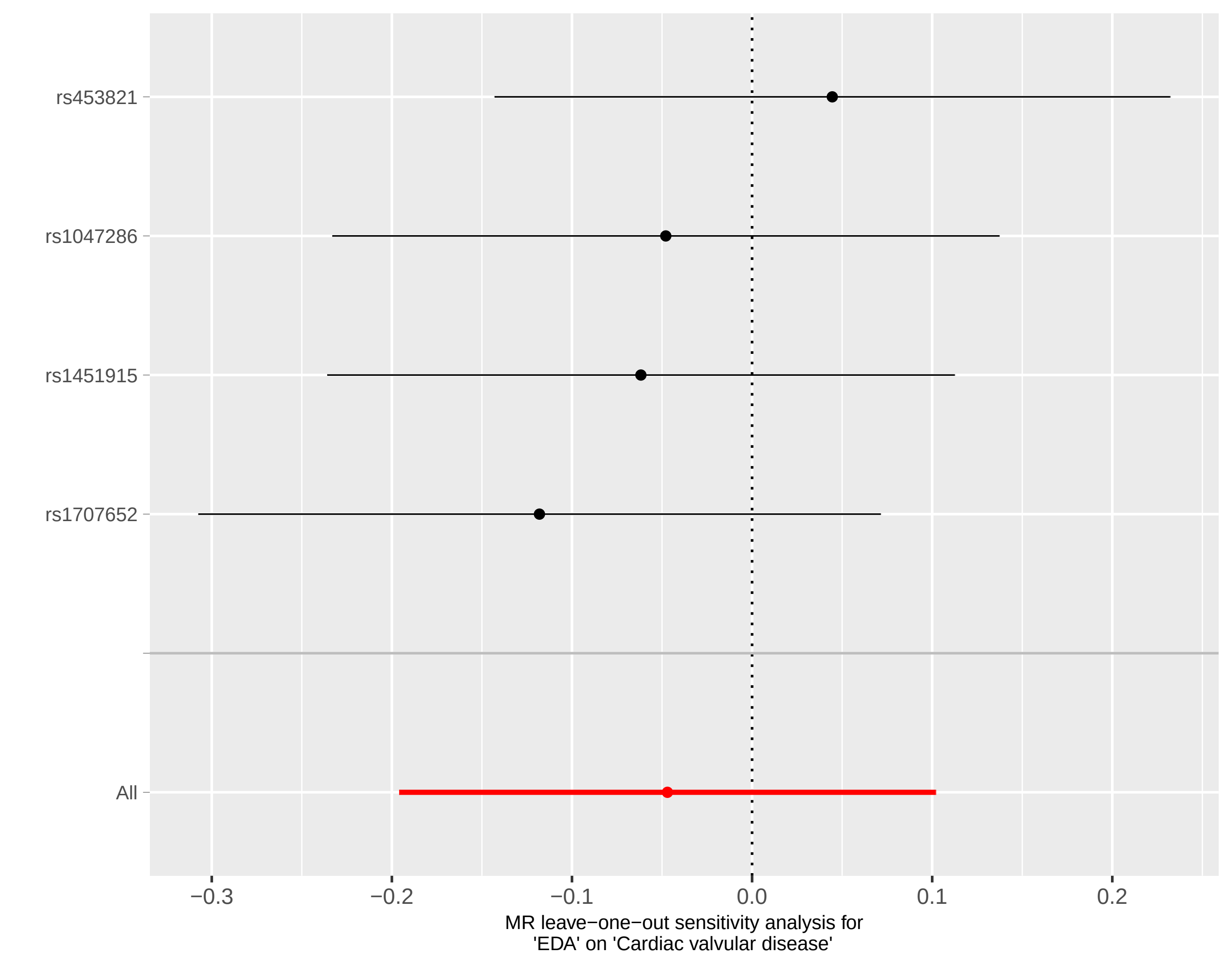
rs453821
rs1047286
rs1451915
rs1707652
All
−0.3
−0.2
−0.1
0.0
0.1
0.2
MR leave−one−out sensitivity analysis for
'EDA' on 'Cardiac valvular disease'

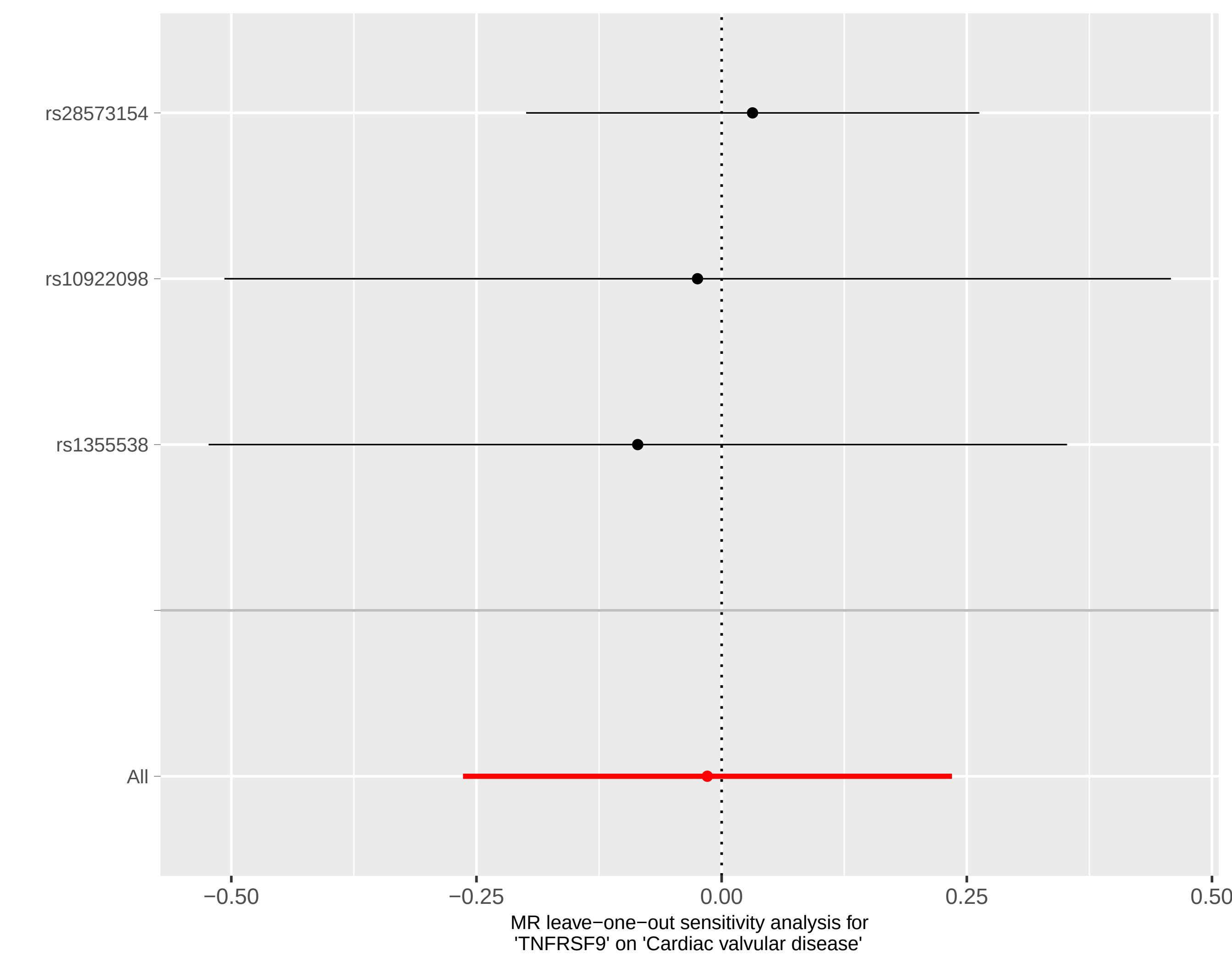

rs28573154
rs10922098
rs1355538
All
−0.50
−0.25
0.00
0.25
0.50
MR leave−one−out sensitivity analysis for
'TNFRSF9' on 'Cardiac valvular disease'

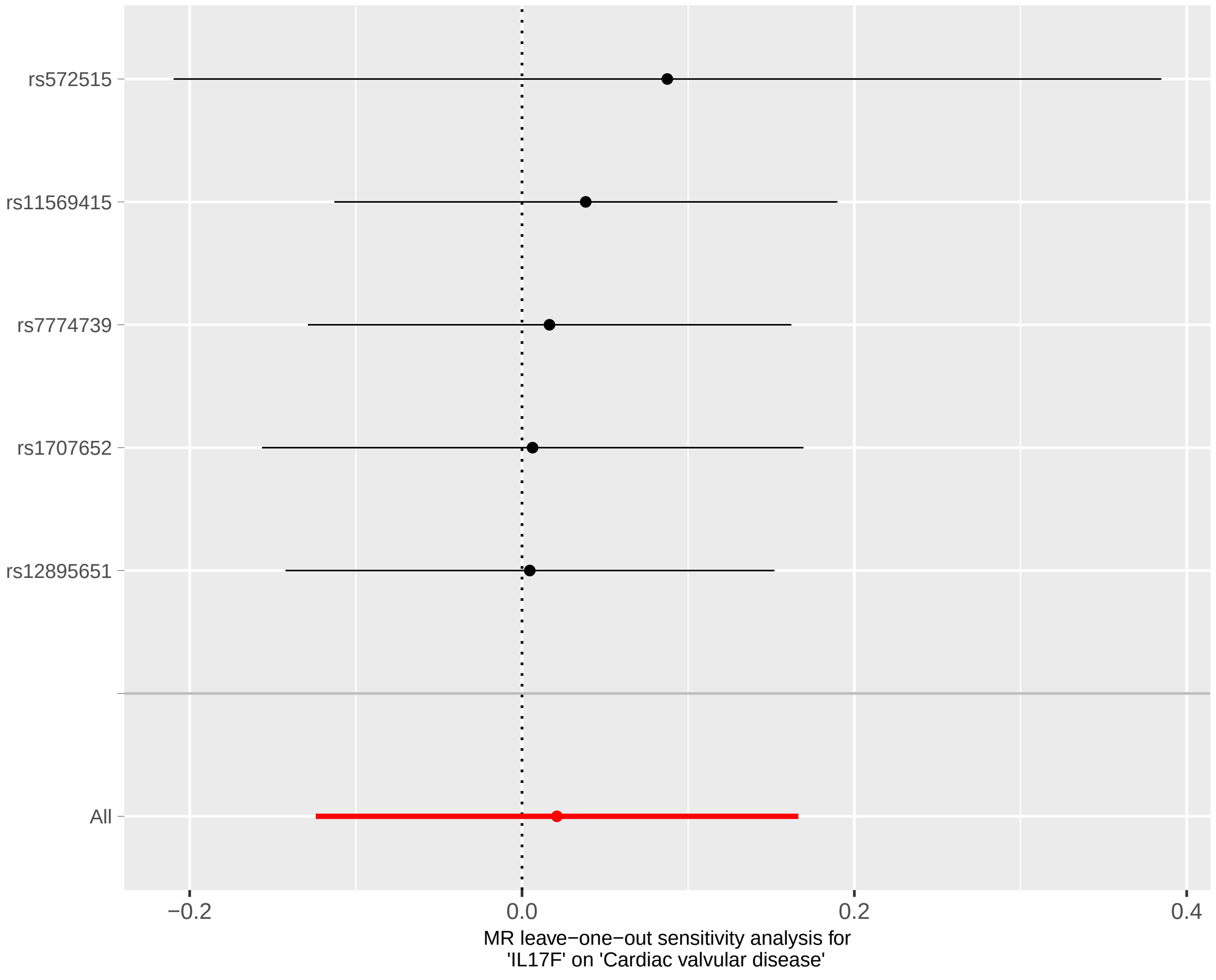

rs572515
rs11569415
rs7774739
rs1707652
rs12895651
All
−0.2
0.0
0.2
0.4
MR leave−one−out sensitivity analysis for
'IL17F' on 'Cardiac valvular disease'

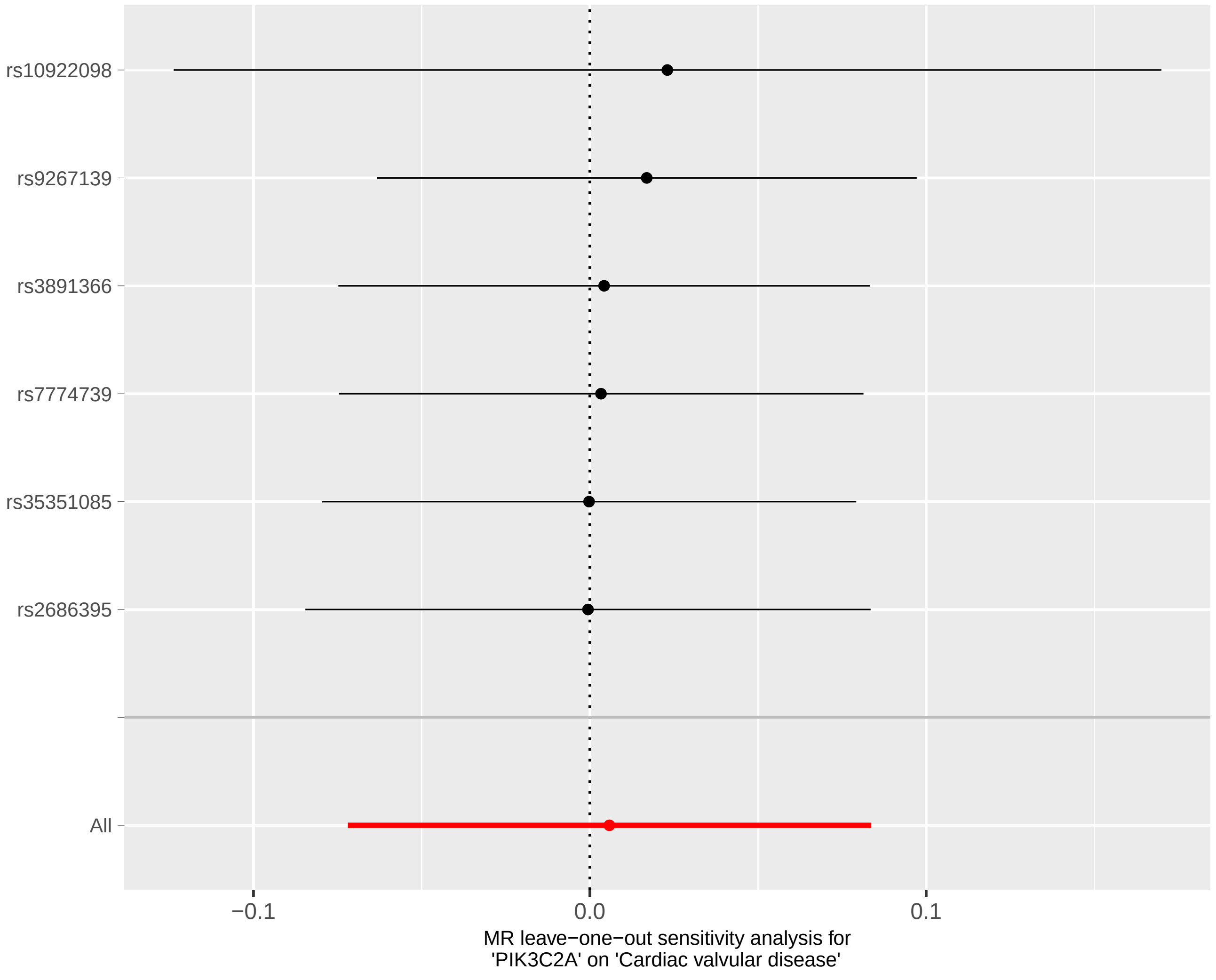
rs10922098
rs9267139
rs3891366
rs7774739
rs35351085
rs2686395
All
−0.1
0.0
0.1
MR leave−one−out sensitivity analysis for
'PIK3C2A' on 'Cardiac valvular disease'

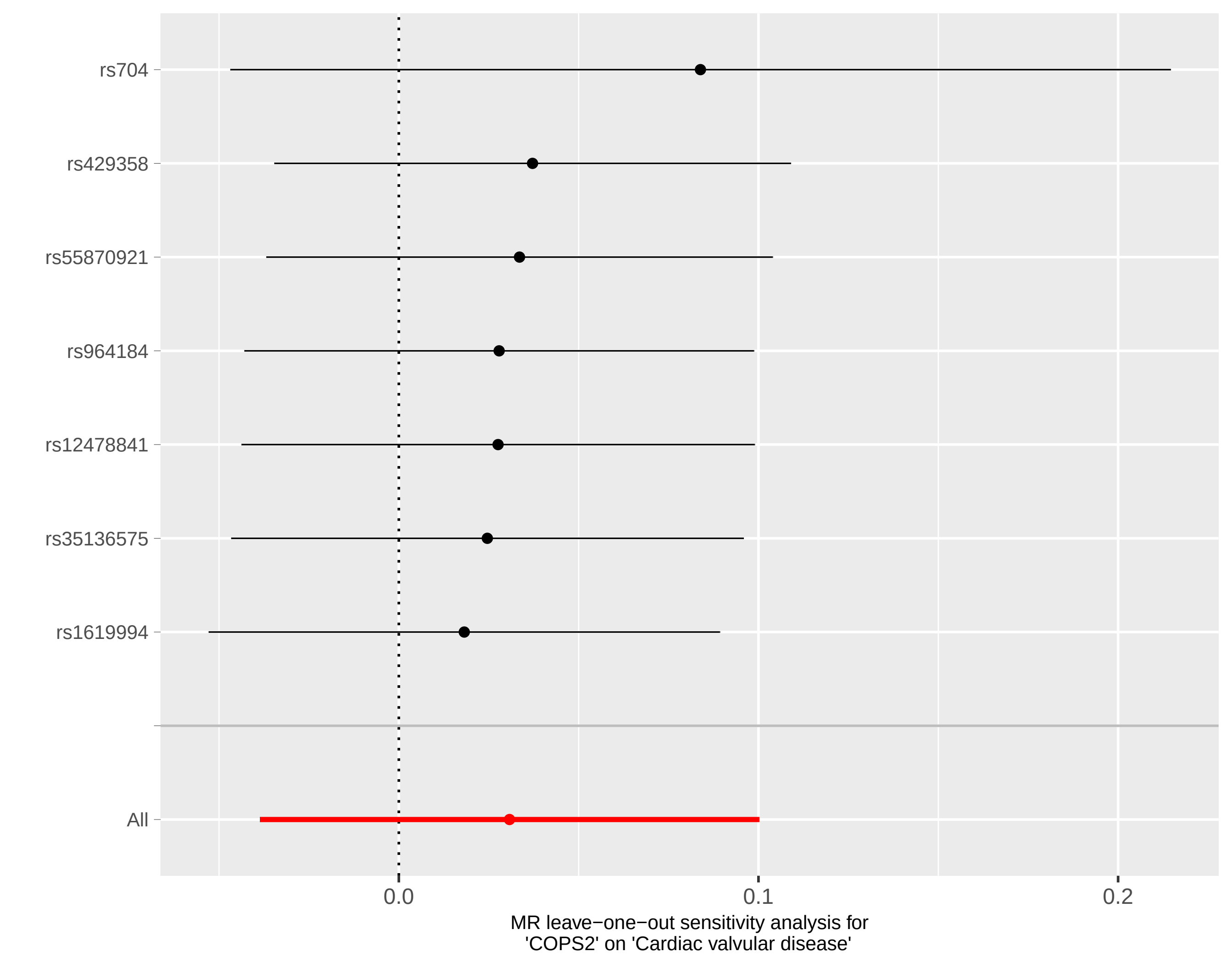
rs704
rs429358
rs55870921
rs964184
rs12478841
rs35136575
rs1619994
All
0.0
0.1
0.2
MR leave−one−out sensitivity analysis for
'COPS2' on 'Cardiac valvular disease'

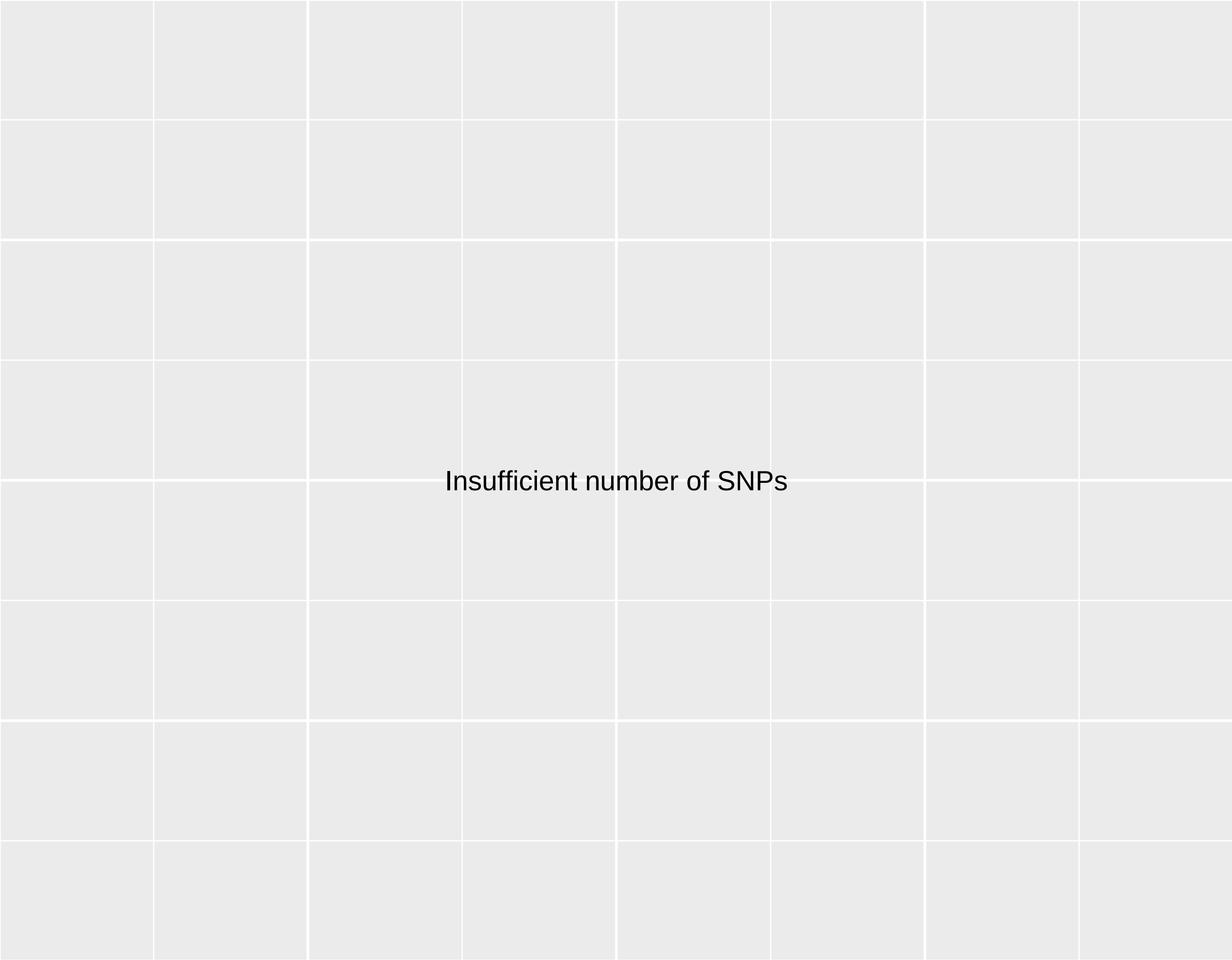
Insufficient number of SNPs

Insufficient number of SNPs

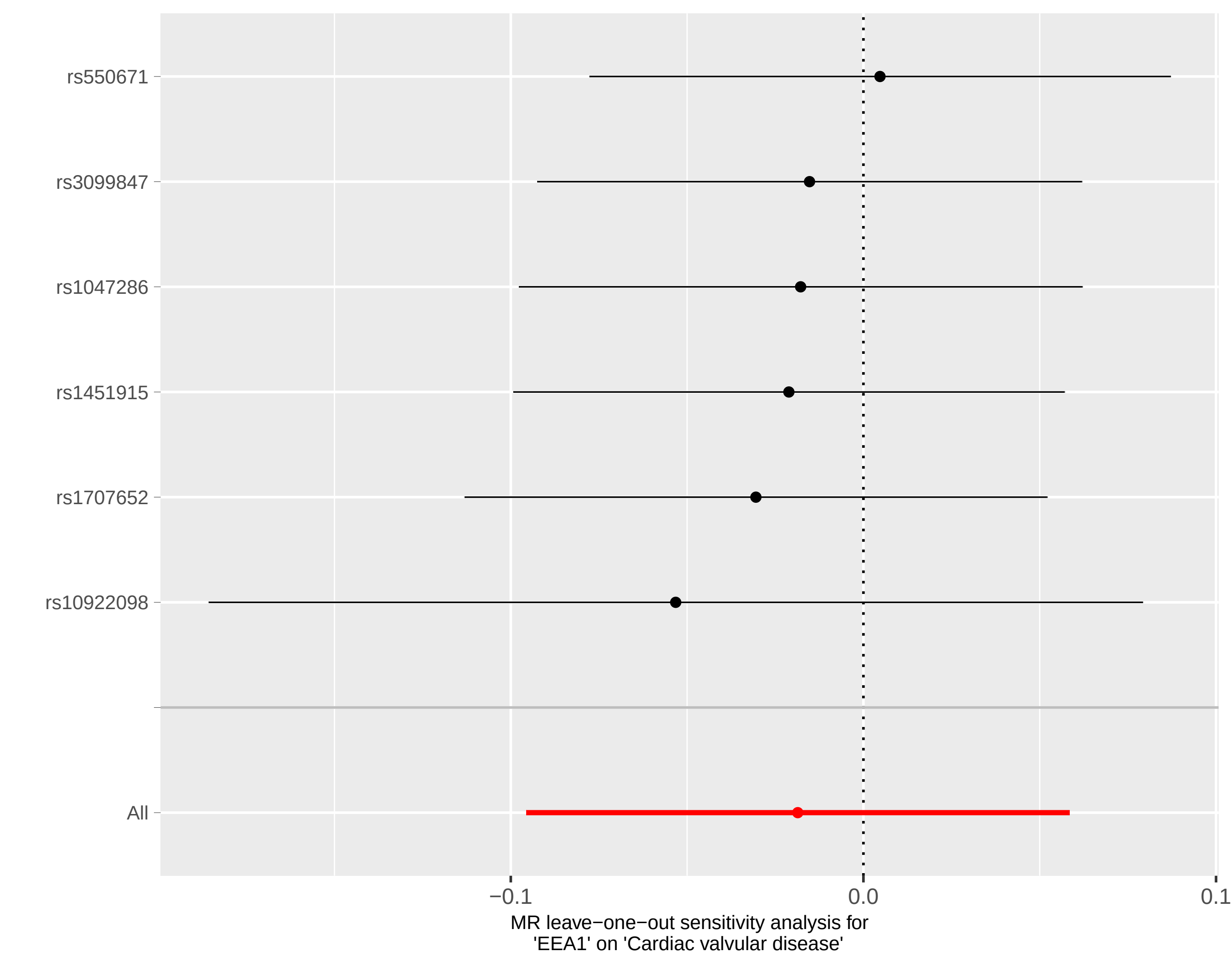
rs550671
rs3099847
rs1047286
rs1451915
rs1707652
rs10922098
All
−0.1
0.0
0.1
MR leave−one−out sensitivity analysis for
'EEA1' on 'Cardiac valvular disease'

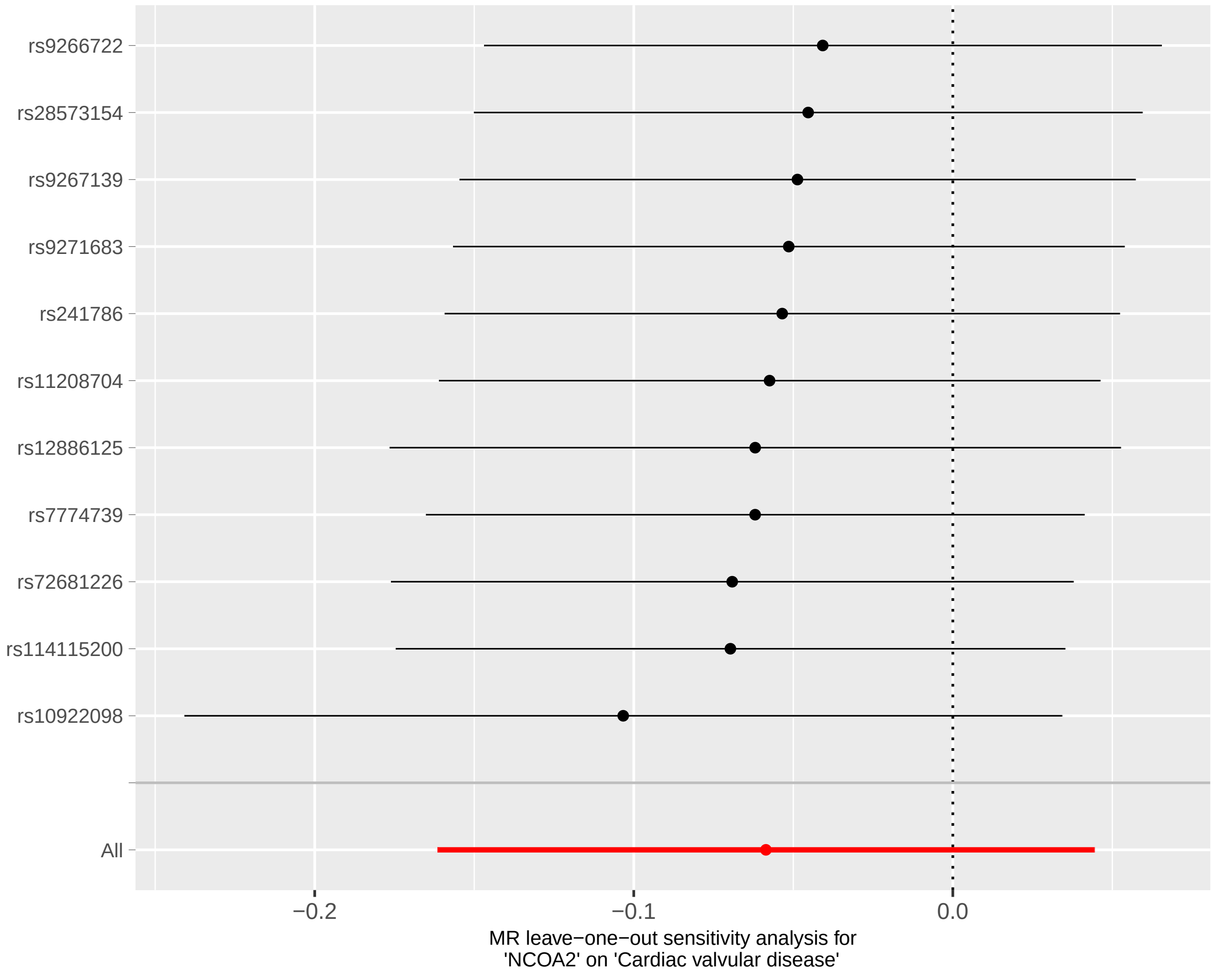
rs9266722
rs28573154
rs9267139
rs9271683
rs241786
rs11208704
rs12886125
rs7774739
rs72681226
rs114115200
rs10922098
All
−0.2
−0.1
0.0
MR leave−one−out sensitivity analysis for
'NCOA2' on 'Cardiac valvular disease'

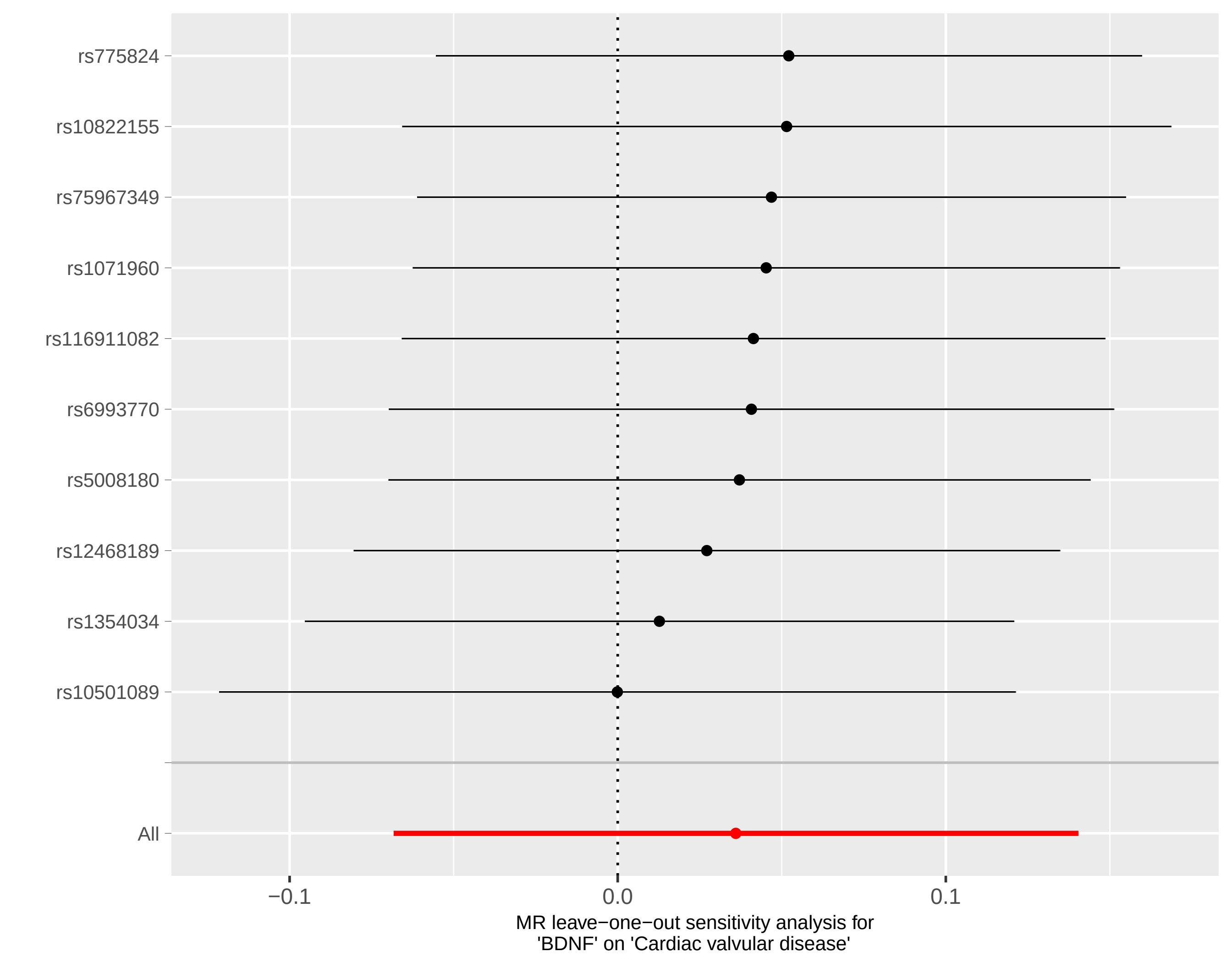
rs775824
rs10822155
rs75967349
rs1071960
rs116911082
rs6993770
rs5008180
rs12468189
rs1354034
rs10501089
All
−0.1
0.0
0.1
MR leave−one−out sensitivity analysis for
'BDNF' on 'Cardiac valvular disease'

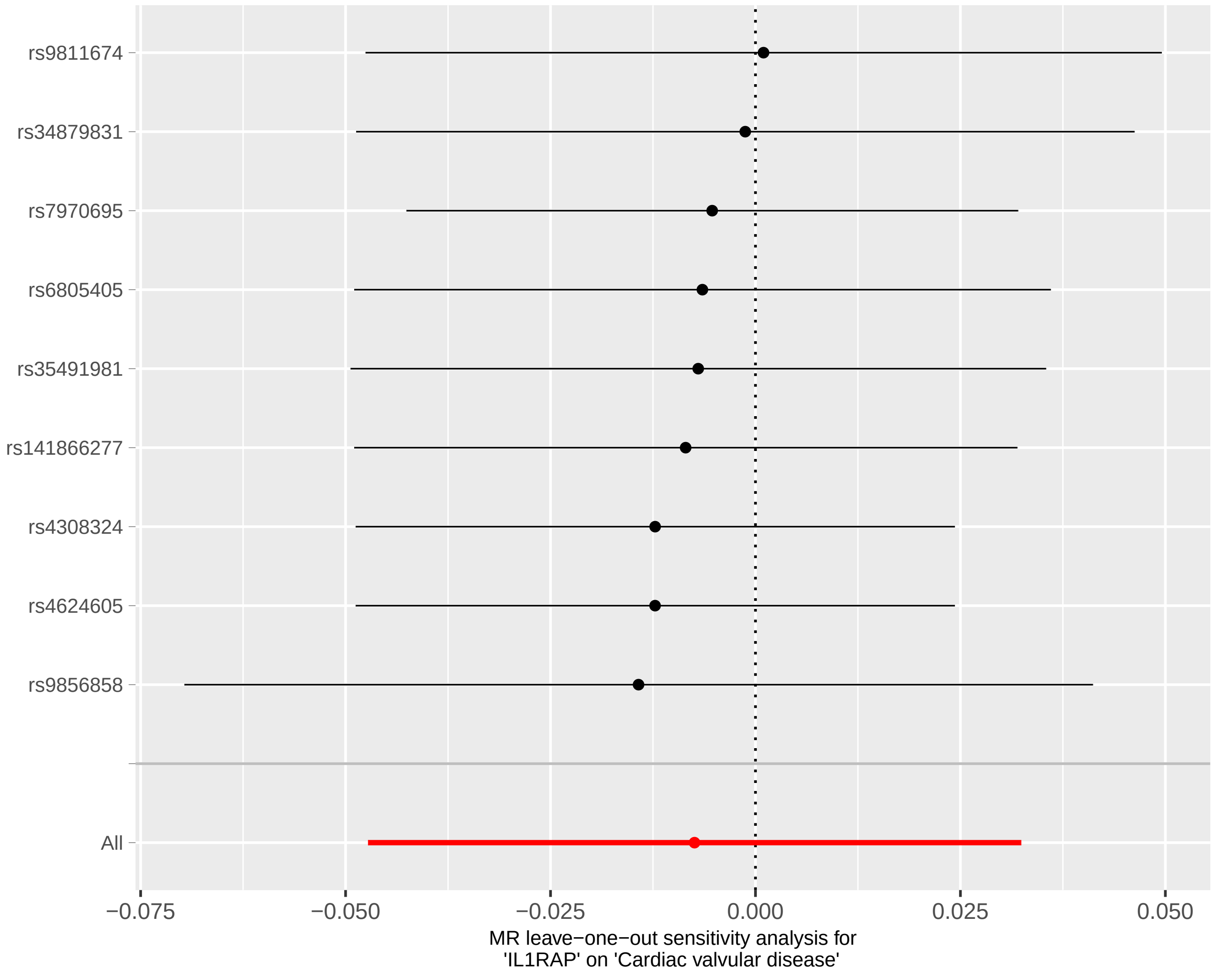
rs9811674
rs34879831
rs7970695
rs6805405
rs35491981
rs141866277
rs4308324
rs4624605
rs9856858
All
−0.075
−0.050
−0.025
0.000
0.025
0.050
MR leave−one−out sensitivity analysis for
'IL1RAP' on 'Cardiac valvular disease'

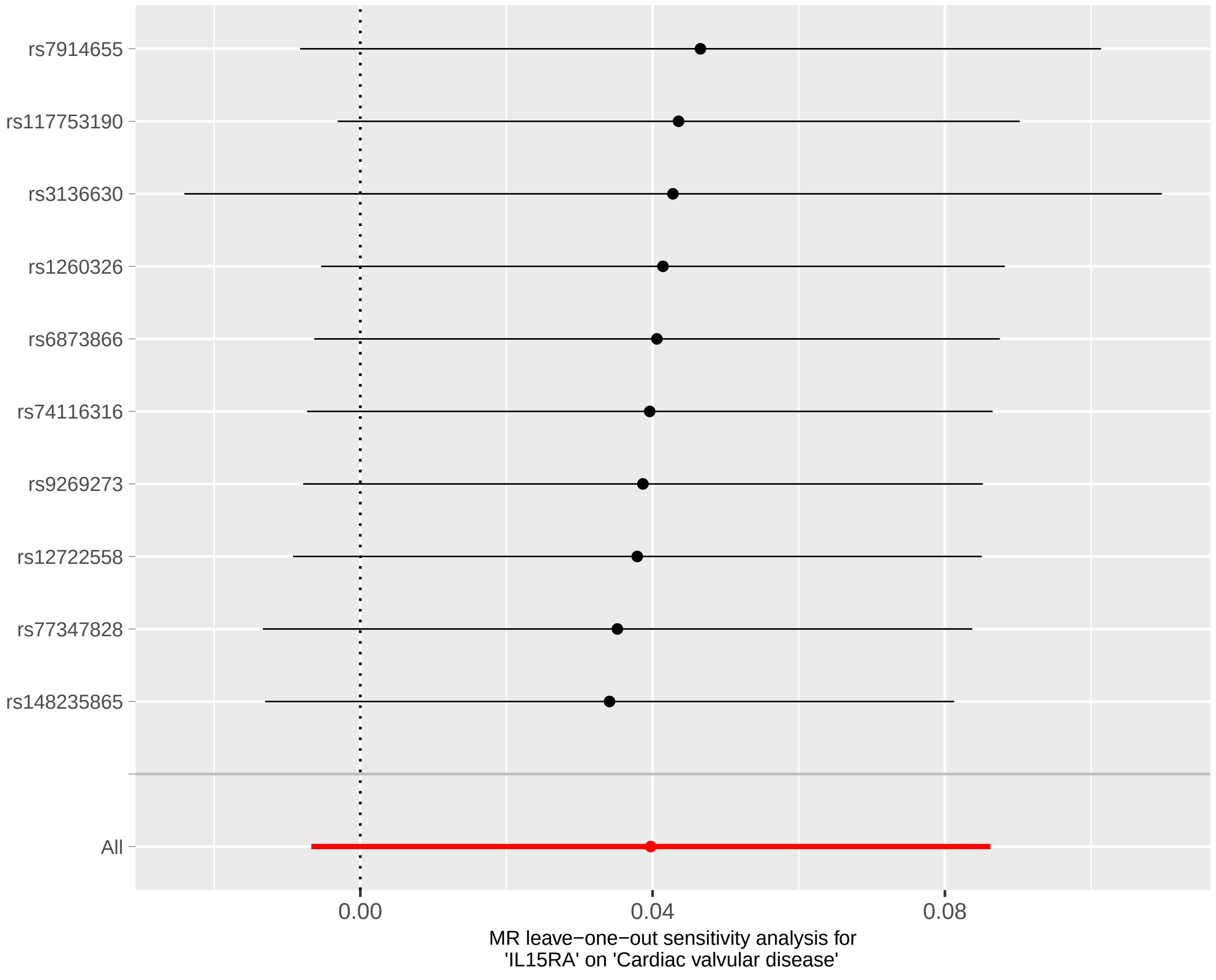

rs7914655
rs117753190
rs3136630
rs1260326
rs6873866
rs74116316
rs9269273
rs12722558
rs77347828
rs148235865
All
0.00
0.04
0.08
MR leave−one−out sensitivity analysis for
'IL15RA' on 'Cardiac valvular disease'

Insufficient number of SNPs

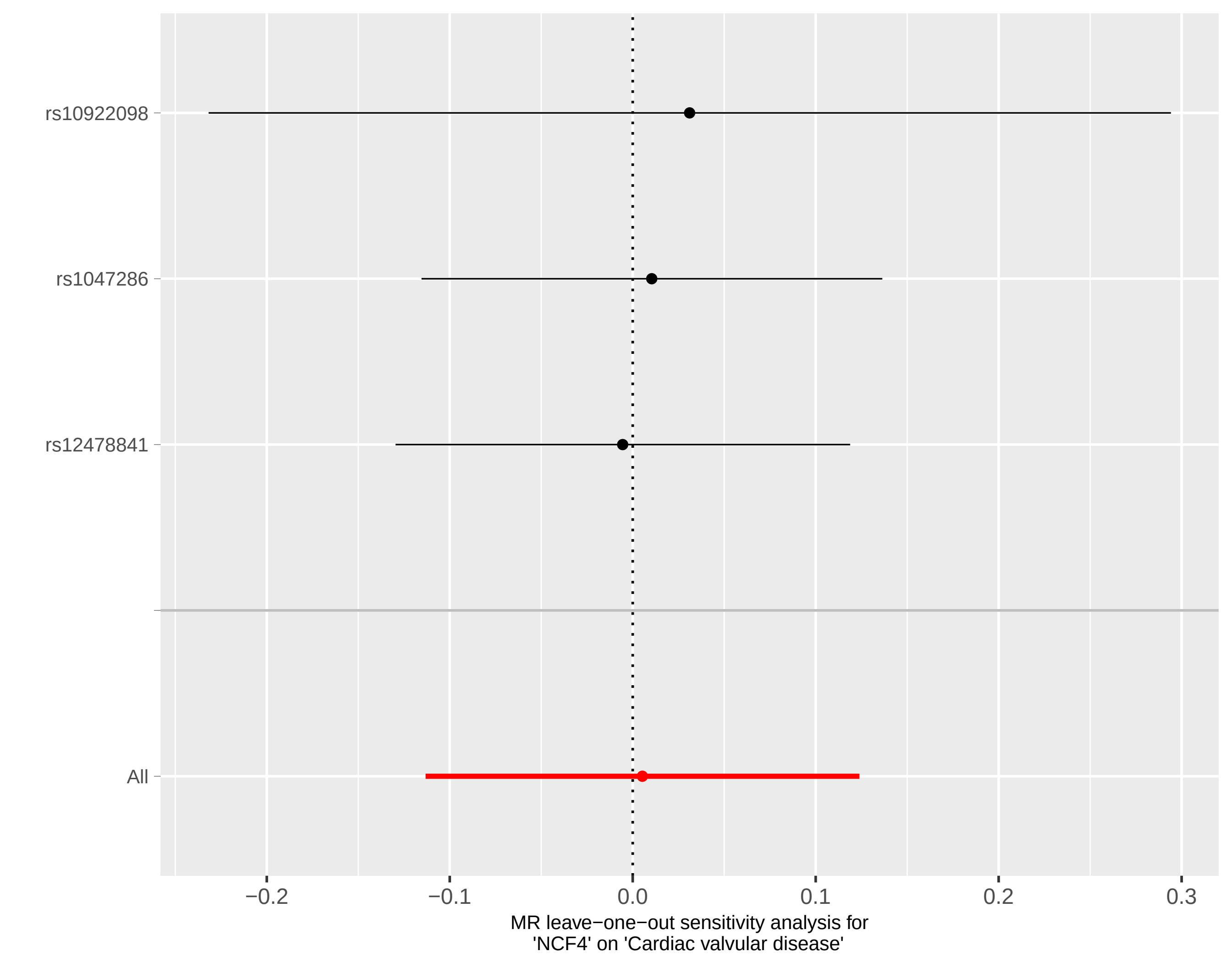

rs10922098
rs1047286
rs12478841
All
−0.2
−0.1
0.0
0.1
0.2
0.3
MR leave−one−out sensitivity analysis for
'NCF4' on 'Cardiac valvular disease'

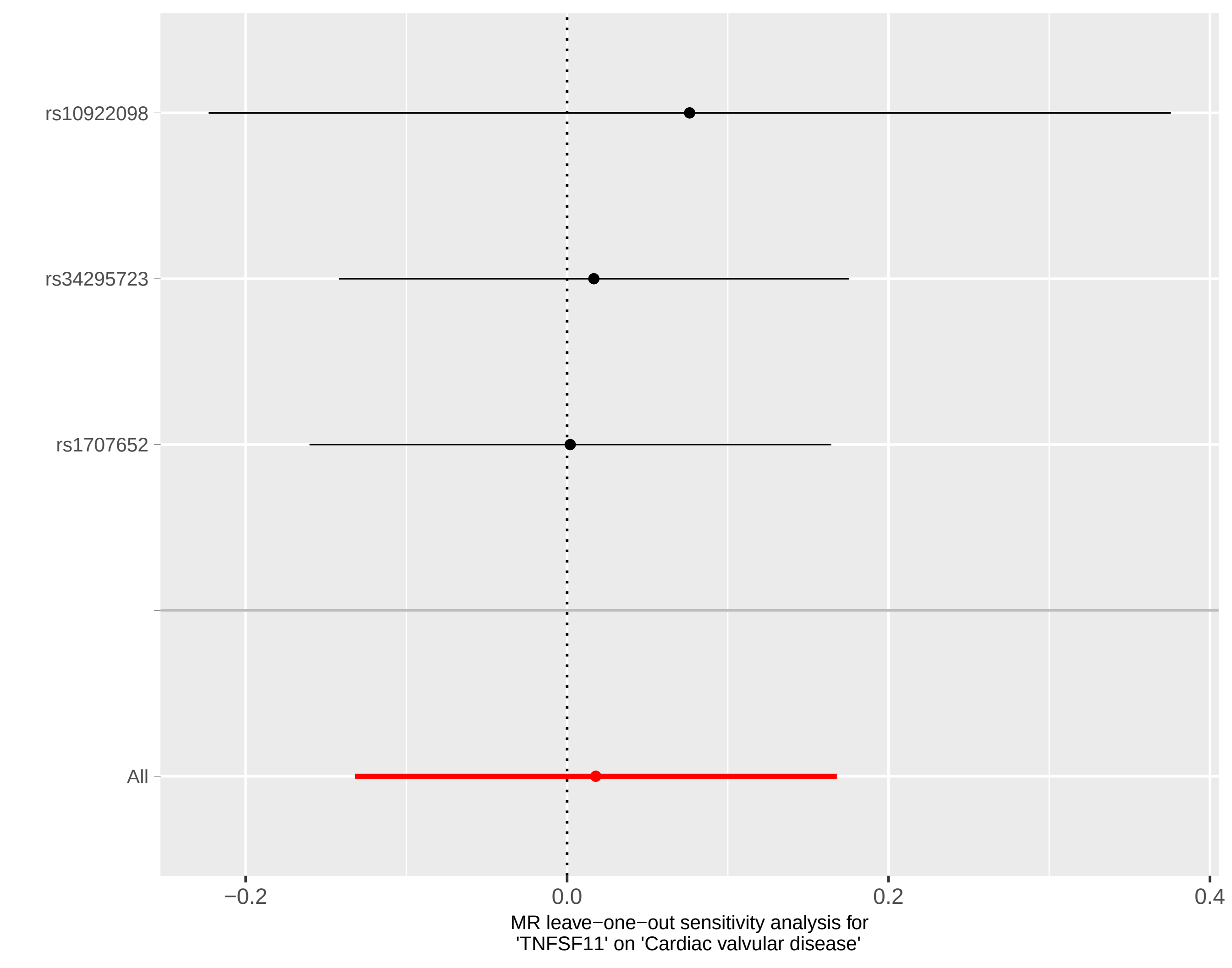
rs10922098
rs34295723
rs1707652
All
−0.2
0.0
0.2
0.4
MR leave−one−out sensitivity analysis for
'TNFSF11' on 'Cardiac valvular disease'

Insufficient number of SNPs

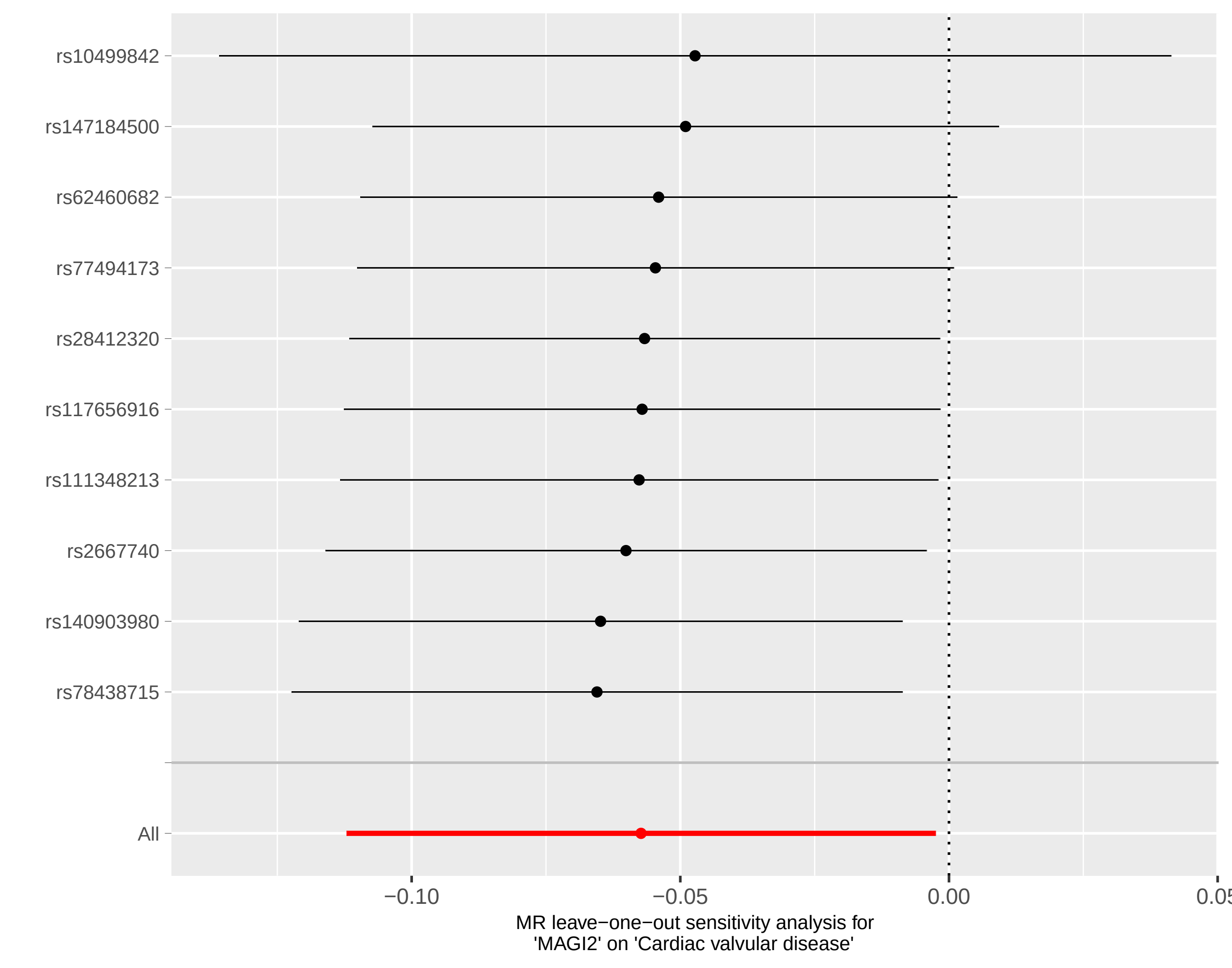
rs10499842
rs147184500
rs62460682
rs77494173
rs28412320
rs117656916
rs111348213
rs2667740
rs140903980
rs78438715
All
−0.10
−0.05
0.00
MR leave−one−out sensitivity analysis for
'MAGI2' on 'Cardiac valvular disease'

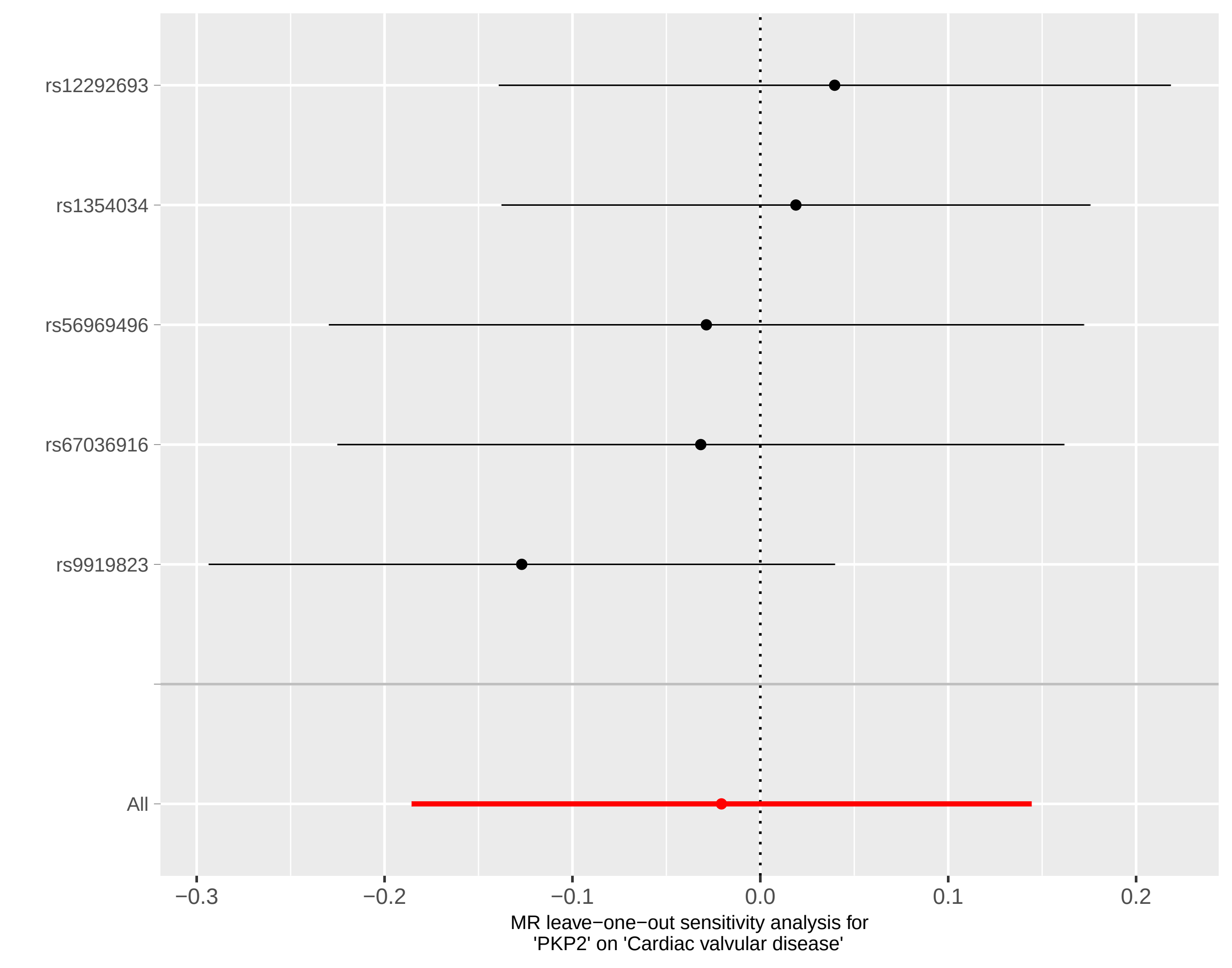
rs12292693
rs1354034
rs56969496
rs67036916
rs9919823
All
−0.3
−0.2
−0.1
0.0
0.1
0.2
MR leave−one−out sensitivity analysis for
'PKP2' on 'Cardiac valvular disease'

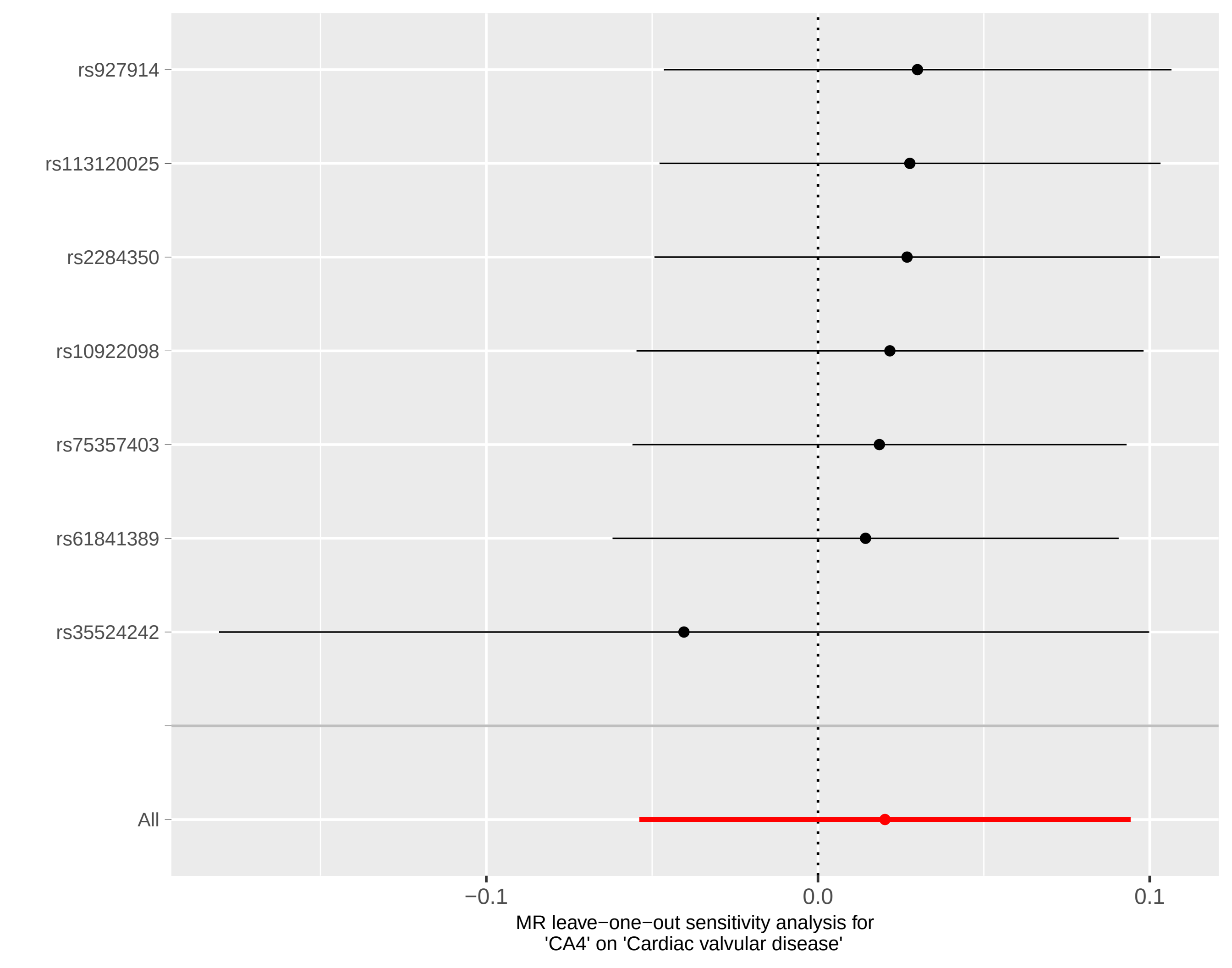
rs927914
rs113120025
rs2284350
rs10922098
rs75357403
rs61841389
rs35524242
All
−0.1
0.0
0.1
MR leave−one−out sensitivity analysis for
'CA4' on 'Cardiac valvular disease'

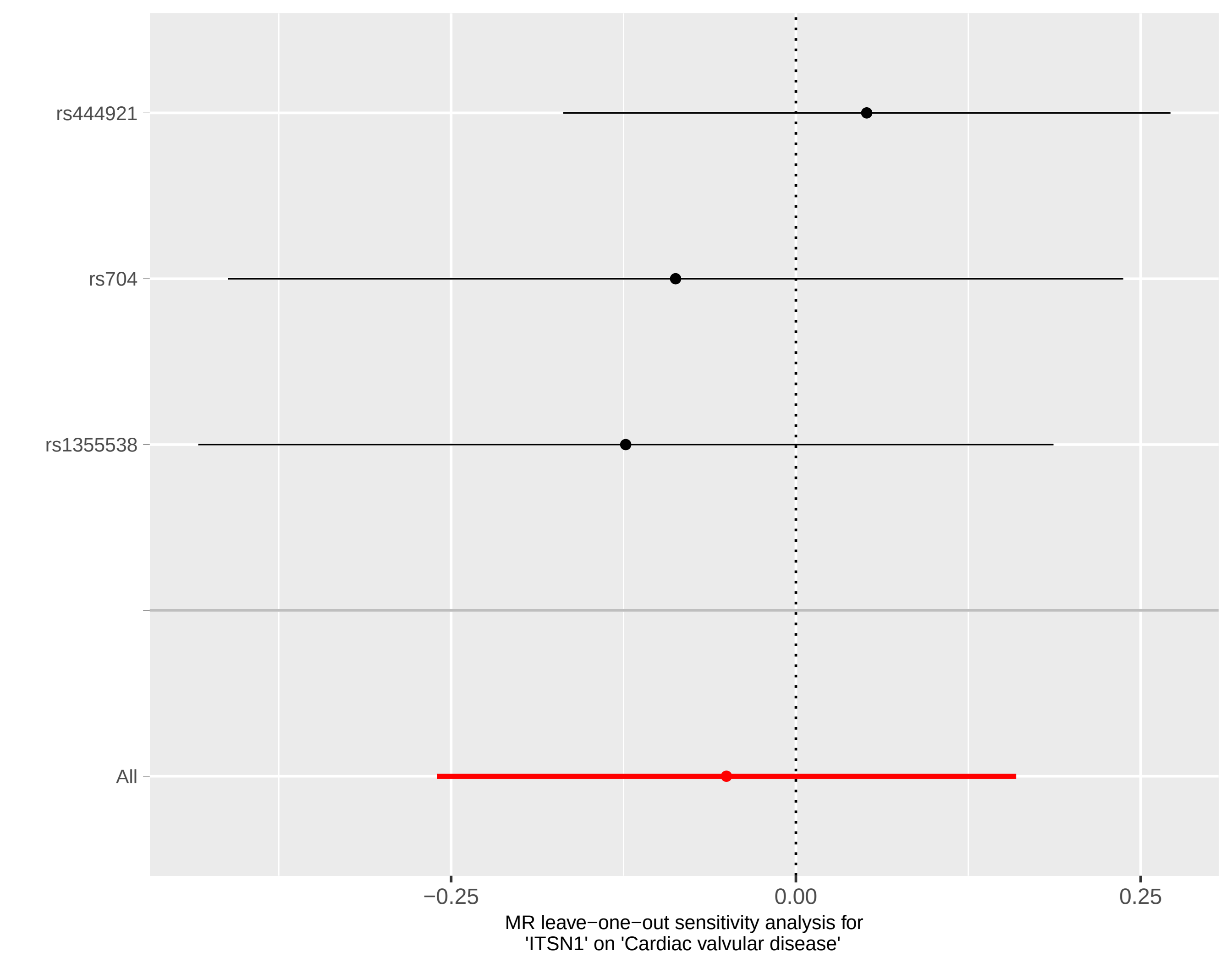

rs444921
rs704
rs1355538
All
−0.25
0.00
0.25
MR leave−one−out sensitivity analysis for
'ITSN1' on 'Cardiac valvular disease'

Insufficient number of SNPs

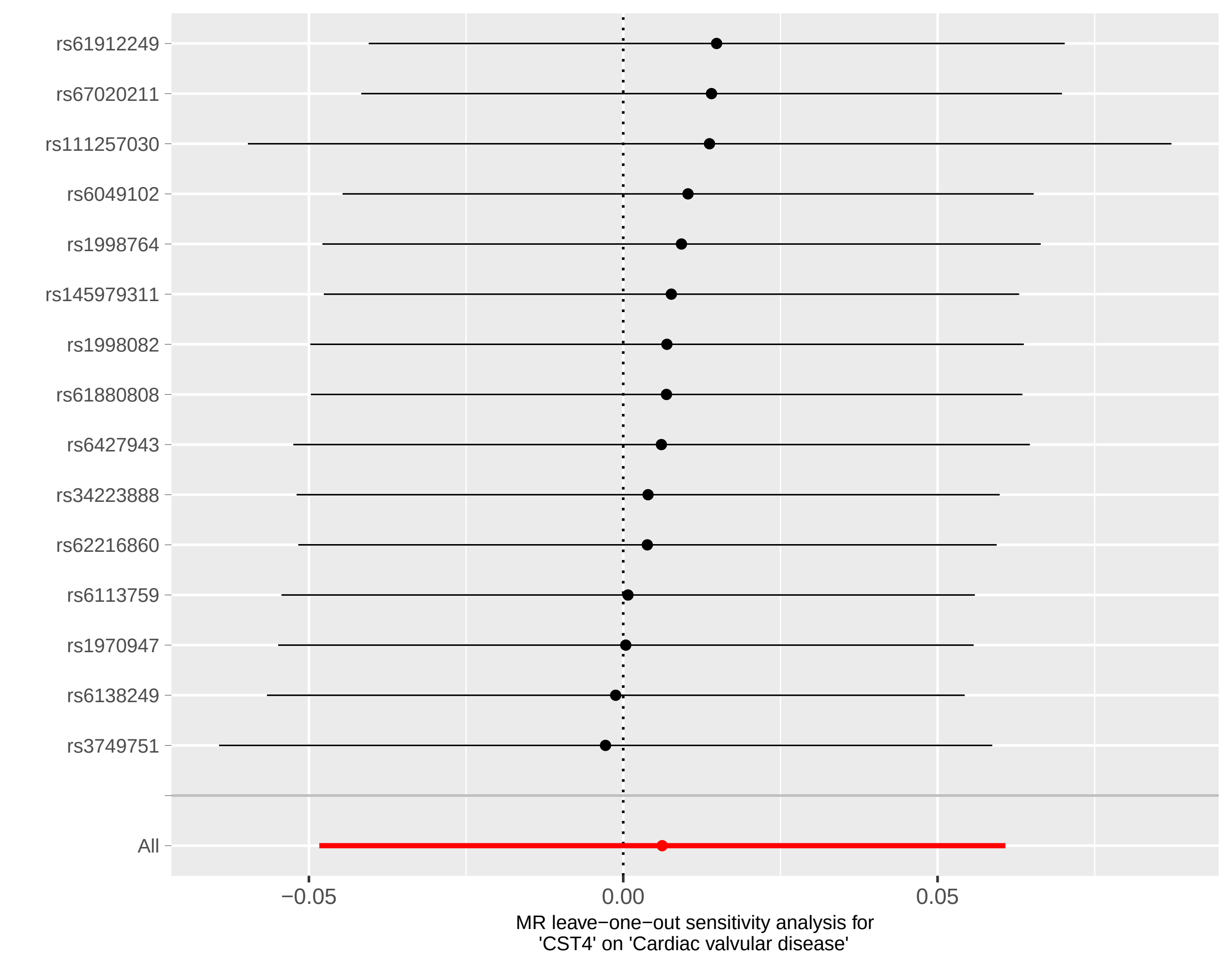
rs61912249
rs67020211
rs111257030
rs6049102
rs1998764
rs145979311
rs1998082
rs61880808
rs6427943
rs34223888
rs62216860
rs6113759
rs1970947
rs6138249
rs3749751
All
−0.05
0.00
0.05
MR leave−one−out sensitivity analysis for
'CST4' on 'Cardiac valvular disease'

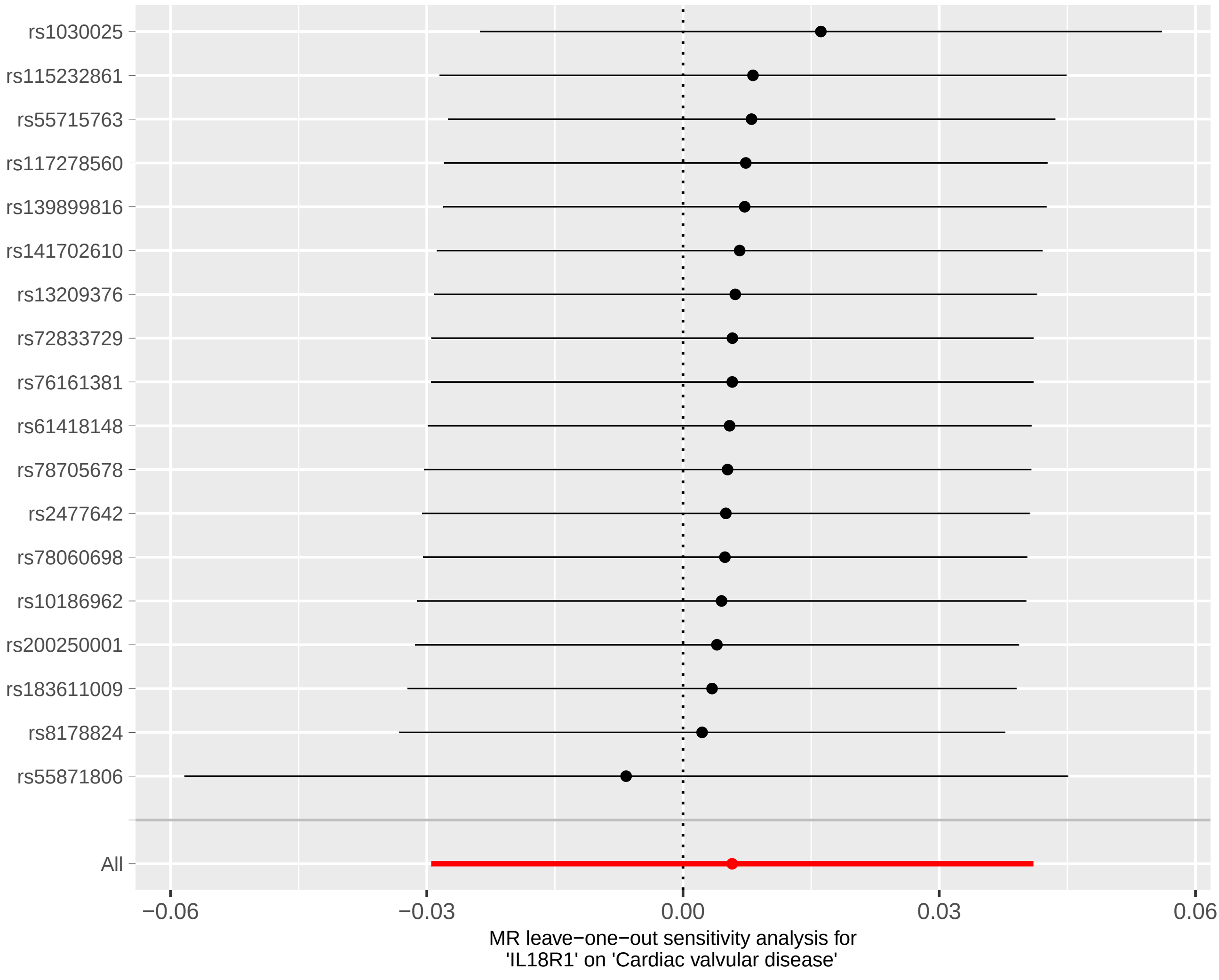
rs1030025
rs115232861
rs55715763
rs117278560
rs139899816
rs141702610
rs13209376
rs72833729
rs76161381
rs61418148
rs78705678
rs2477642
rs78060698
rs10186962
rs200250001
rs183611009
rs8178824
rs55871806
All
−0.06
−0.03
0.00
0.03
0.06
MR leave−one−out sensitivity analysis for
'IL18R1' on 'Cardiac valvular disease'

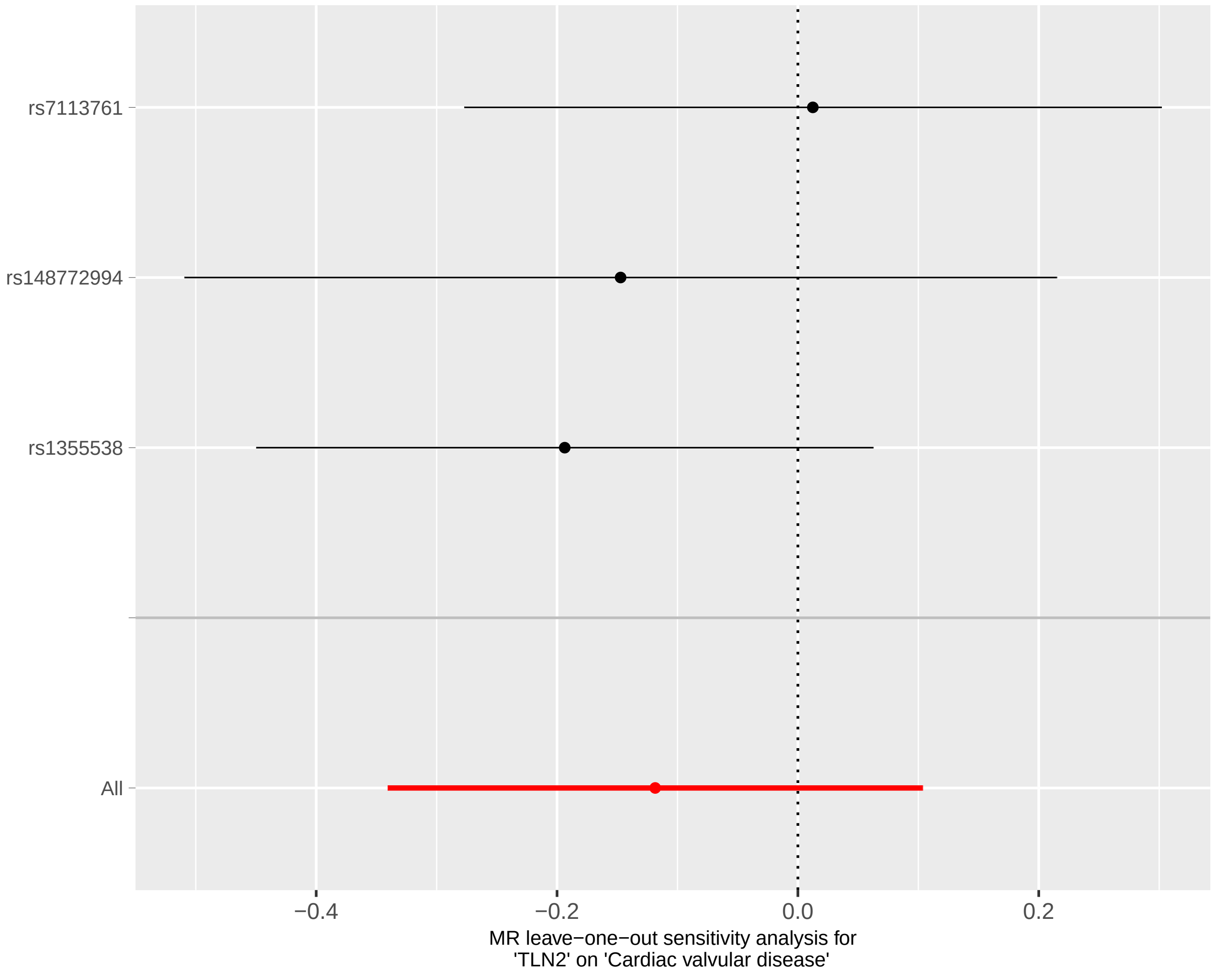

rs7113761
rs148772994
rs1355538
All
−0.4
−0.2
0.0
0.2
MR leave−one−out sensitivity analysis for
'TLN2' on 'Cardiac valvular disease'

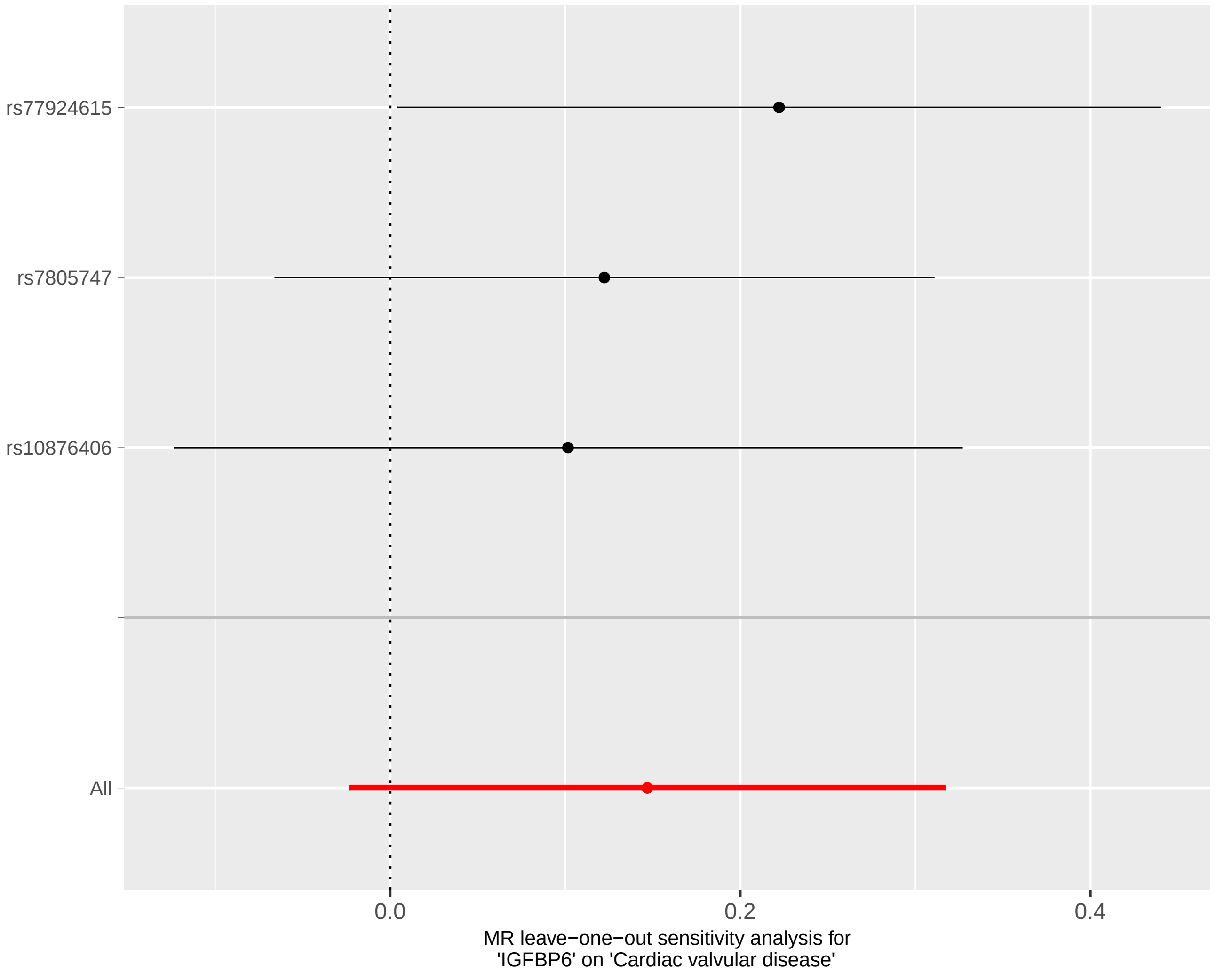

rs77924615
rs7805747
rs10876406
All
0.0
0.2
0.4
MR leave−one−out sensitivity analysis for
'IGFBP6' on 'Cardiac valvular disease'

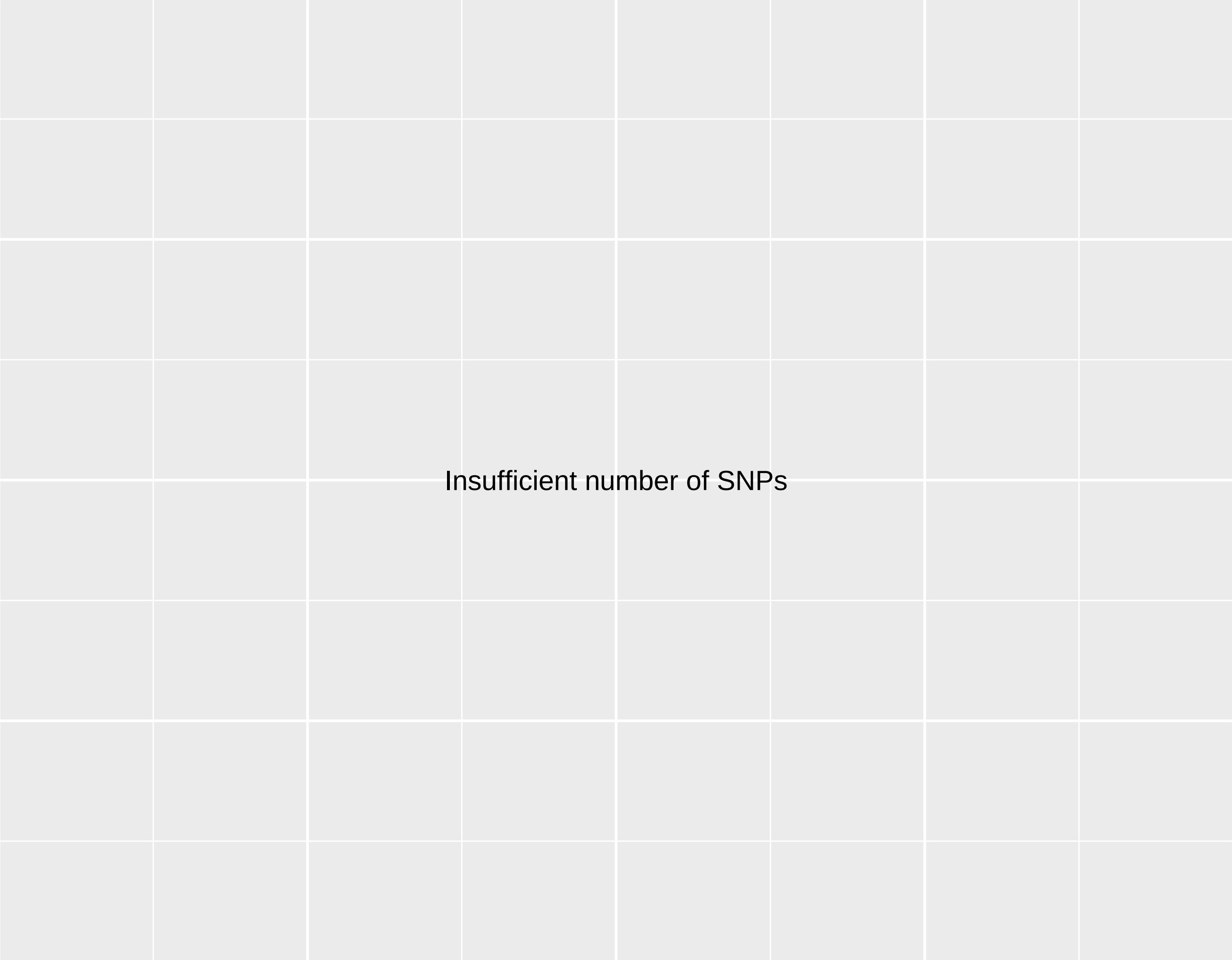
Insufficient number of SNPs

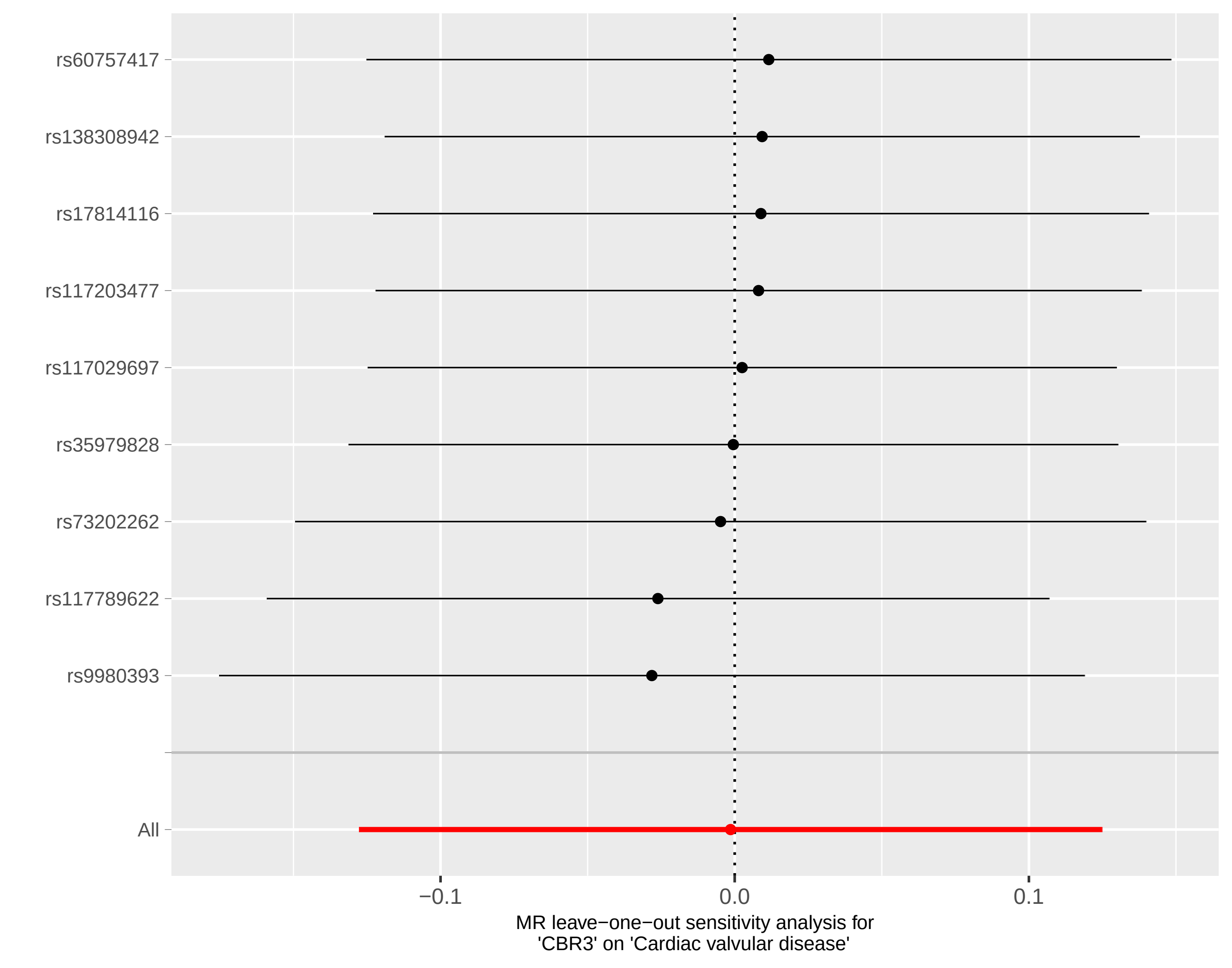
rs60757417
rs138308942
rs17814116
rs117203477
rs117029697
rs35979828
rs73202262
rs117789622
rs9980393
All
−0.1
0.0
0.1
MR leave−one−out sensitivity analysis for
'CBR3' on 'Cardiac valvular disease'

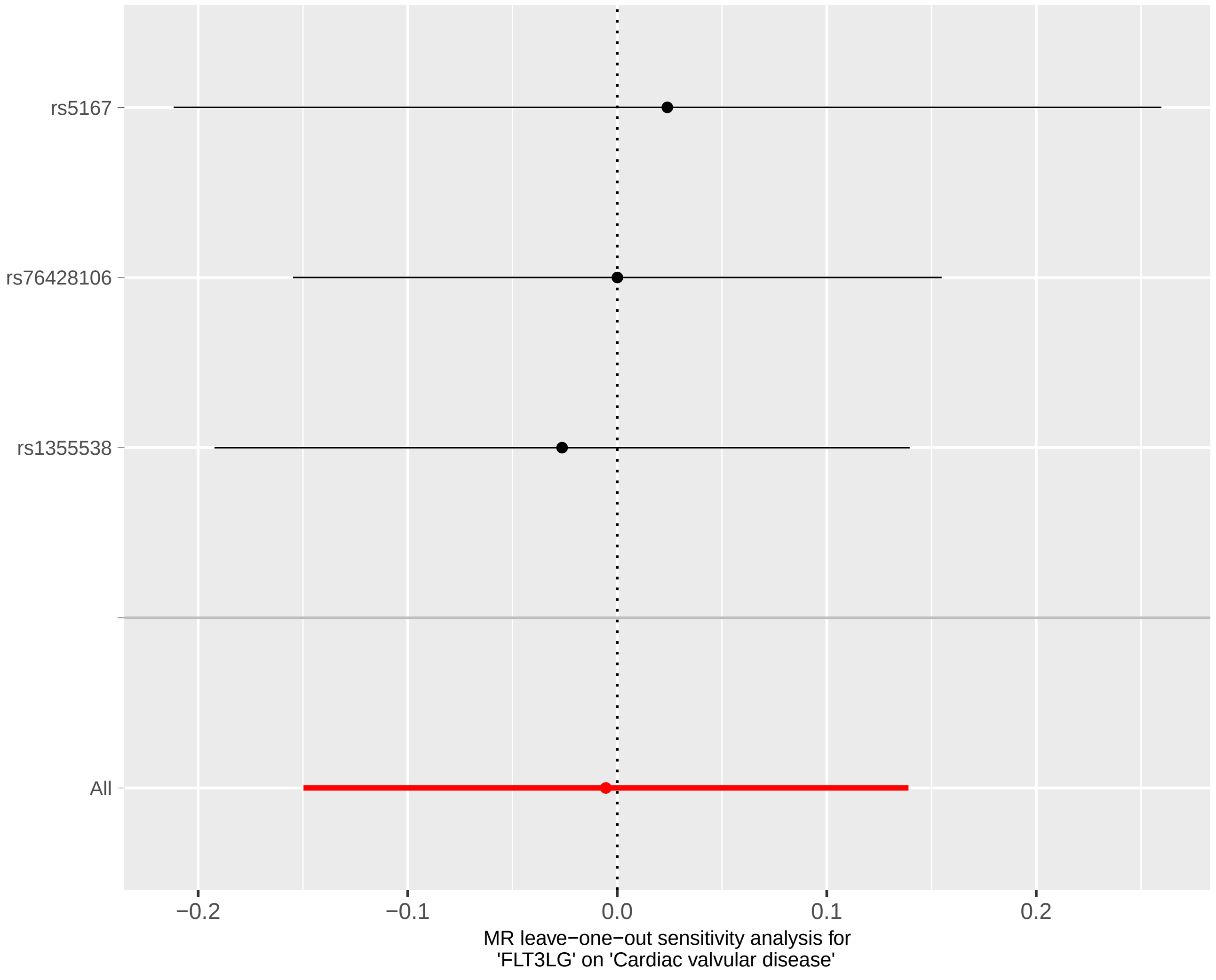
rs5167
rs76428106
rs1355538
All
−0.2
−0.1
0.0
0.1
0.2
MR leave−one−out sensitivity analysis for
'FLT3LG' on 'Cardiac valvular disease'

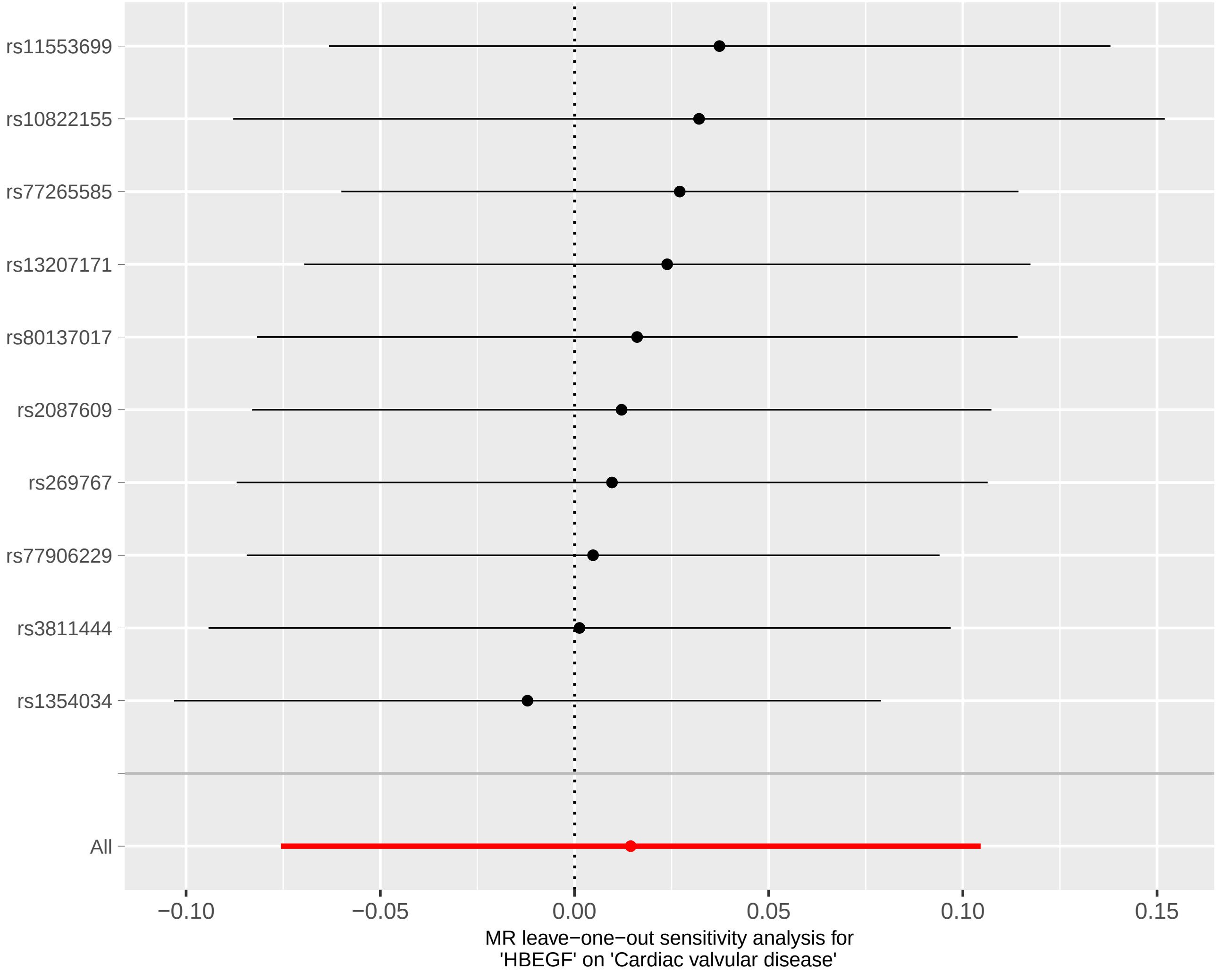
rs11553699
rs10822155
rs77265585
rs13207171
rs80137017
rs2087609
rs269767
rs77906229
rs3811444
rs1354034
All
−0.10
−0.05
0.00
0.05
0.10
0.15
MR leave−one−out sensitivity analysis for
'HBEGF' on 'Cardiac valvular disease'

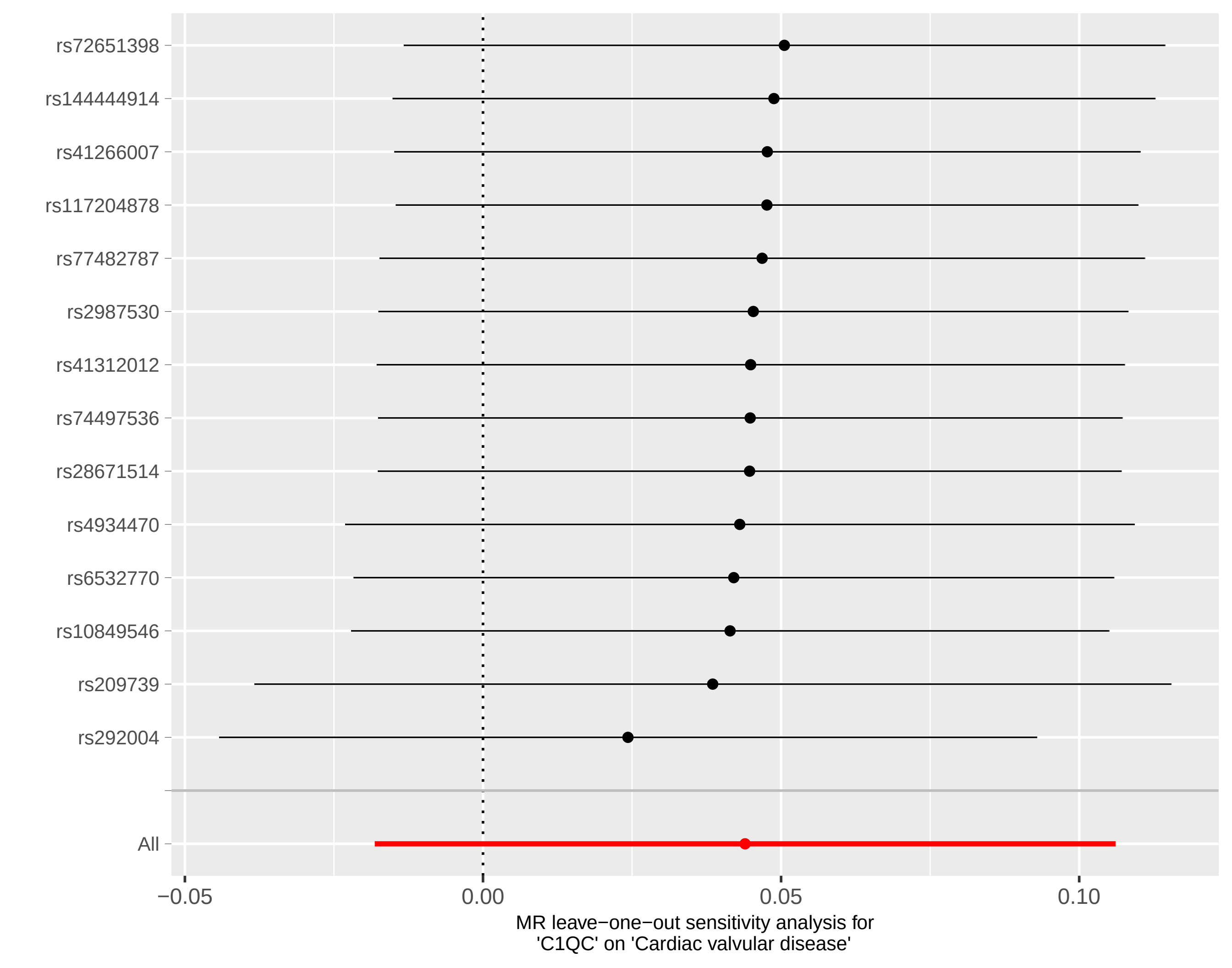
rs72651398
rs144444914
rs41266007
rs117204878
rs77482787
rs2987530
rs41312012
rs74497536
rs28671514
rs4934470
rs6532770
rs10849546
rs209739
rs292004
All
−0.05
0.00
0.05
0.10
MR leave−one−out sensitivity analysis for
'C1QC' on 'Cardiac valvular disease'

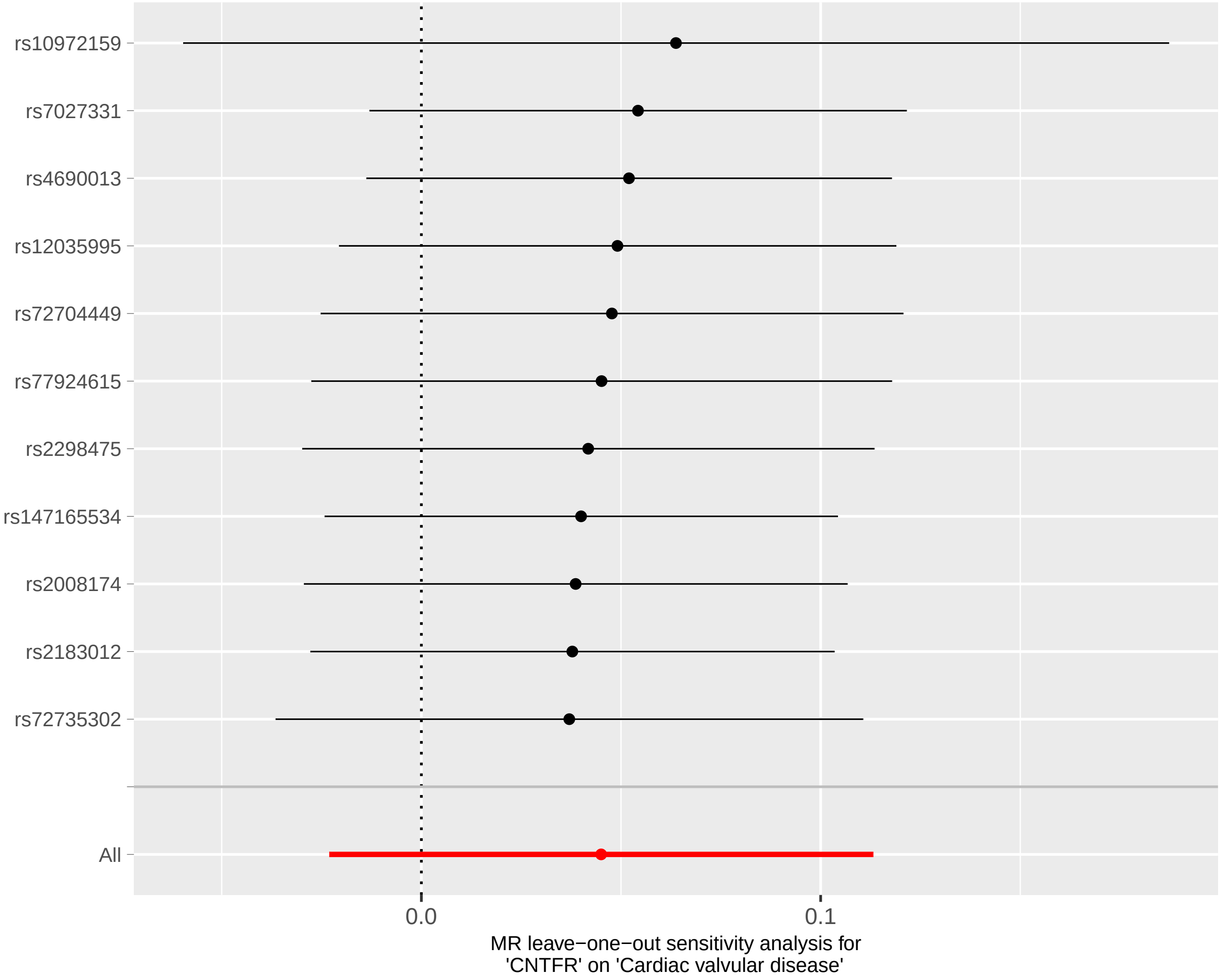
rs10972159
rs7027331
rs4690013
rs12035995
rs72704449
rs77924615
rs2298475
rs147165534
rs2008174
rs2183012
rs72735302
All
0.0
0.1
MR leave−one−out sensitivity analysis for
'CNTFR' on 'Cardiac valvular disease'

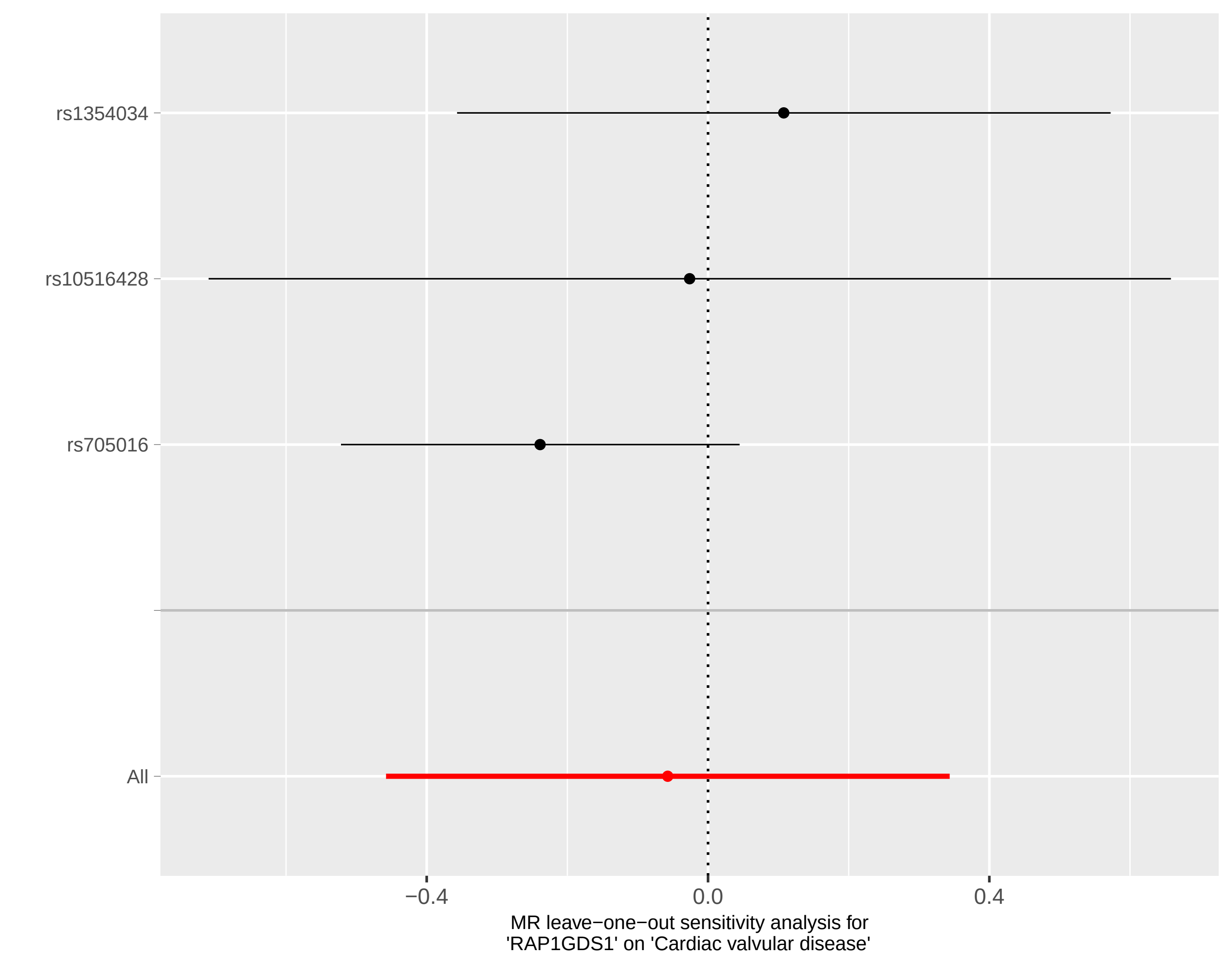

rs1354034
rs10516428
rs705016
All
−0.4
0.0
0.4
MR leave−one−out sensitivity analysis for
'RAP1GDS1' on 'Cardiac valvular disease'

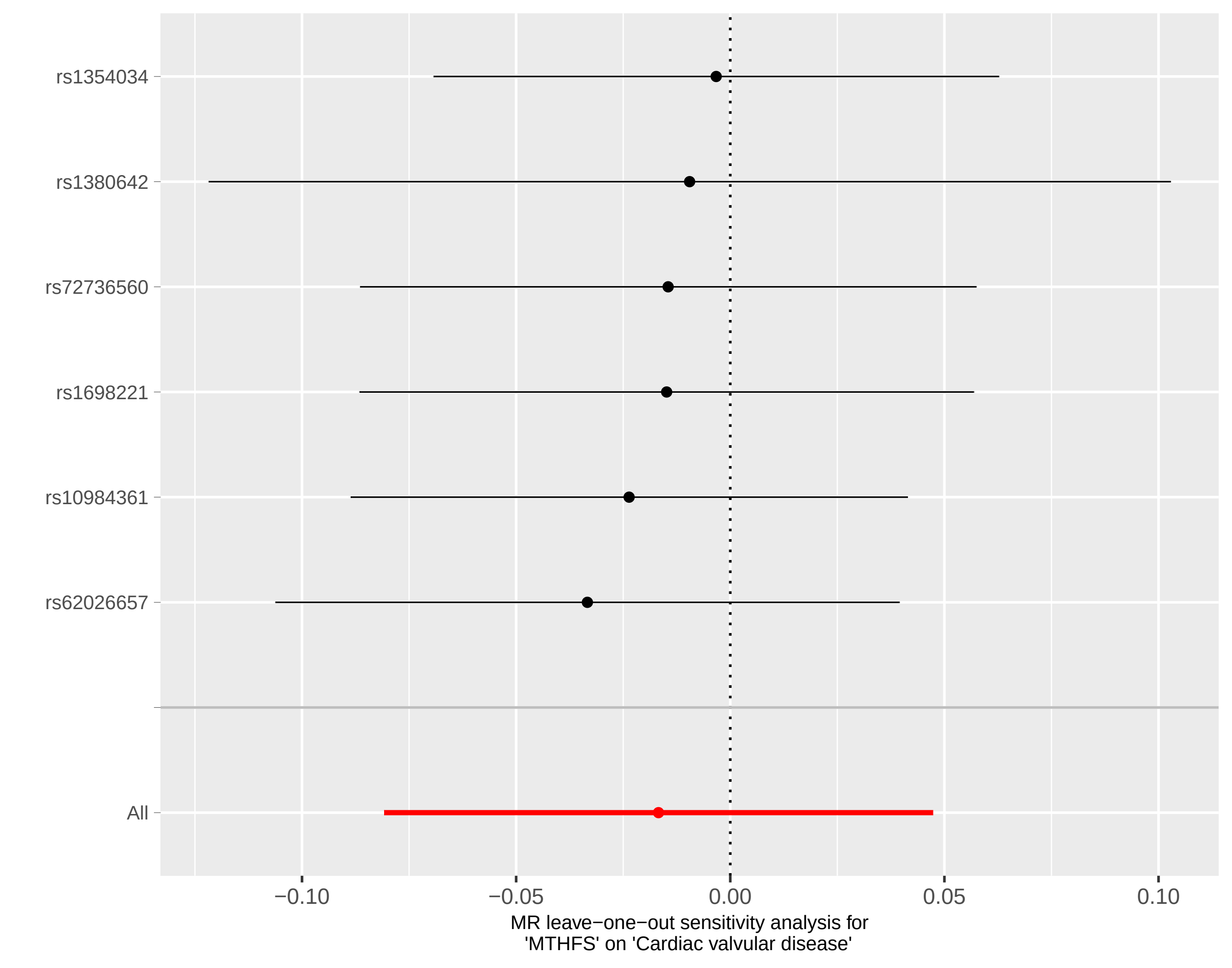
rs1354034
rs1380642
rs72736560
rs1698221
rs10984361
rs62026657
All
−0.10
−0.05
0.00
0.05
0.10
MR leave−one−out sensitivity analysis for
'MTHFS' on 'Cardiac valvular disease'

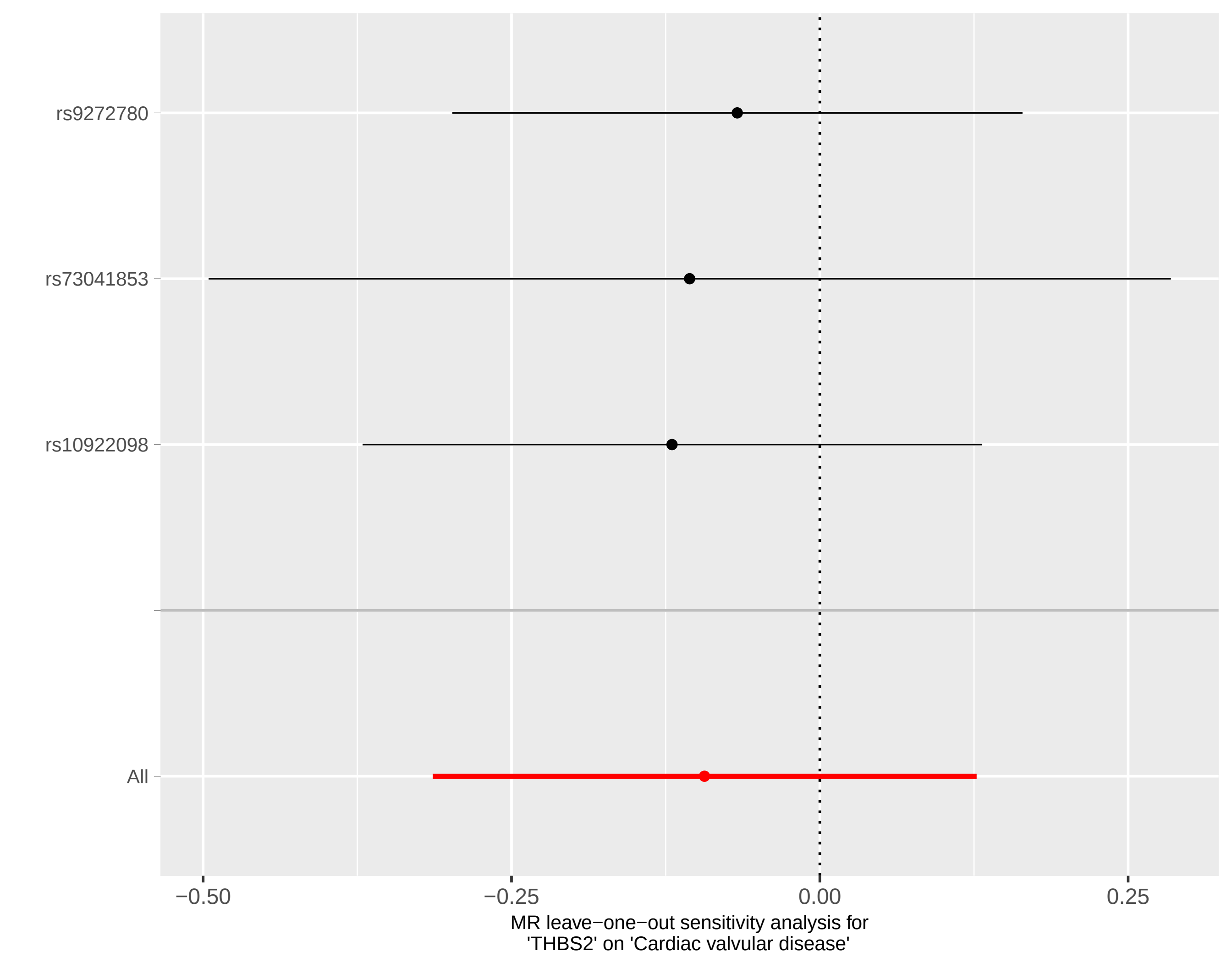
rs9272780
rs73041853
rs10922098
All
−0.50
−0.25
0.00
0.25
MR leave−one−out sensitivity analysis for
'THBS2' on 'Cardiac valvular disease'

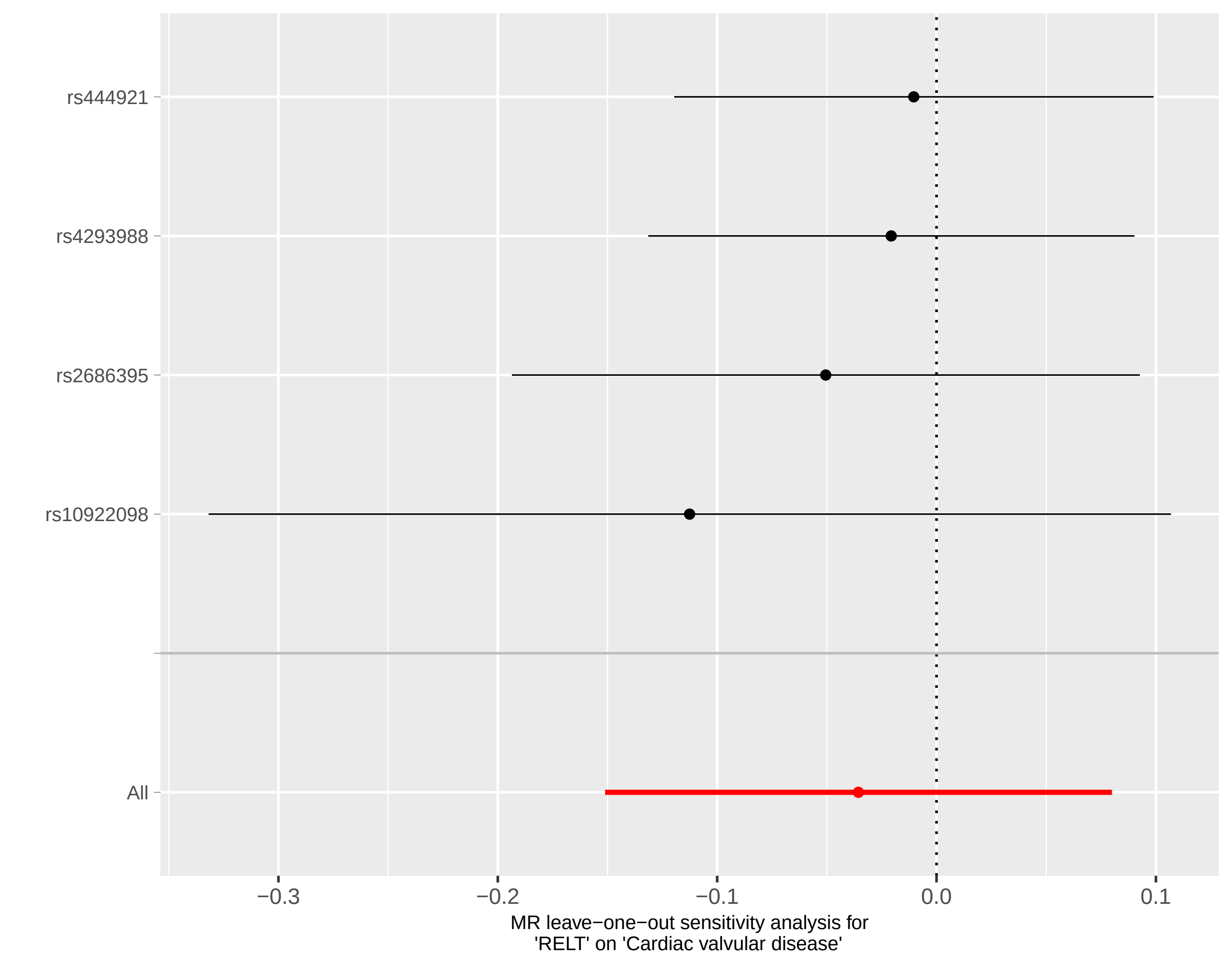
rs444921
rs4293988
rs2686395
rs10922098
All
−0.3
−0.2
−0.1
0.0
0.1
MR leave−one−out sensitivity analysis for
'RELT' on 'Cardiac valvular disease'

Insufficient number of SNPs

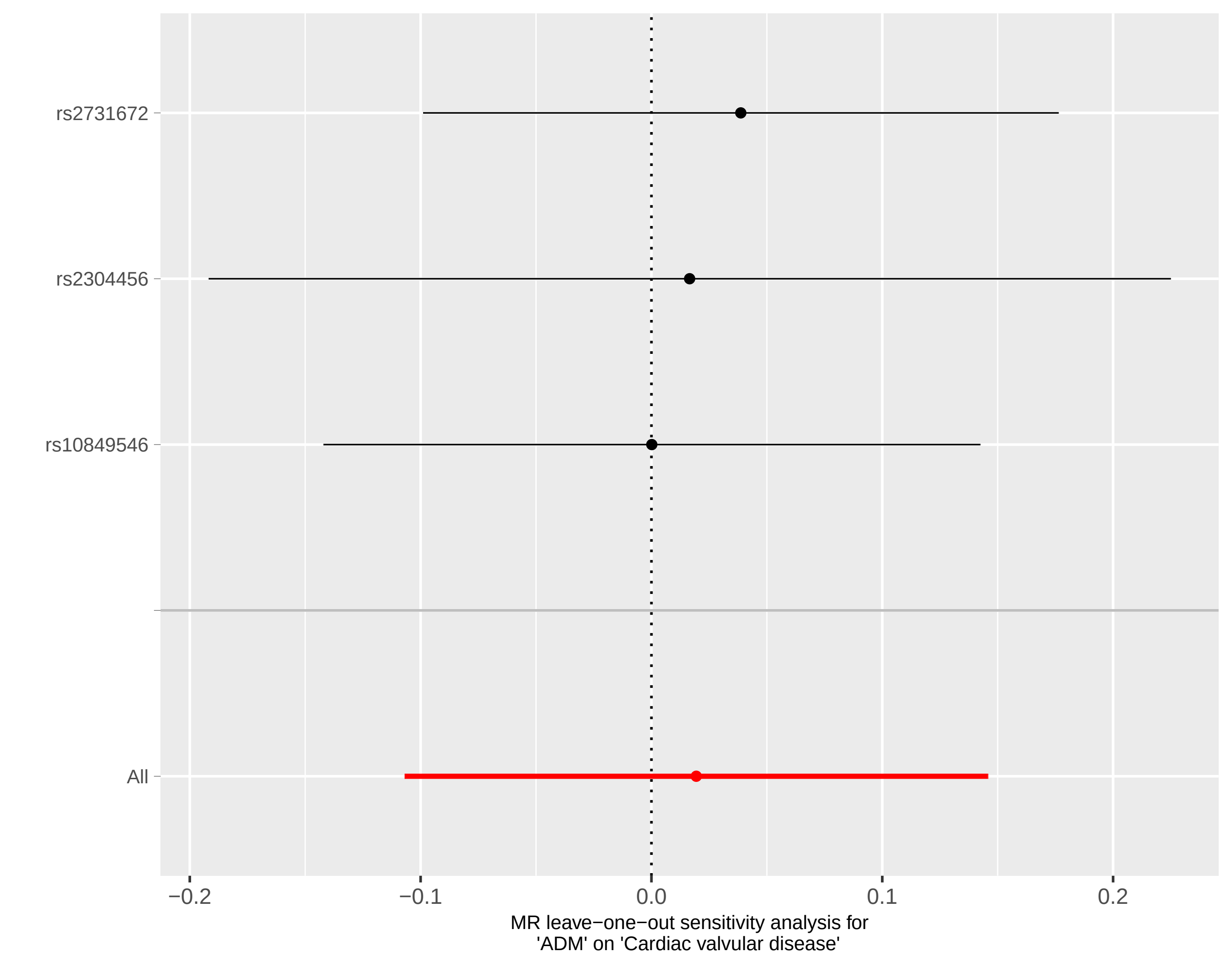

rs2731672
rs2304456
rs10849546
All
−0.2
−0.1
0.0
0.1
0.2
MR leave−one−out sensitivity analysis for
'ADM' on 'Cardiac valvular disease'

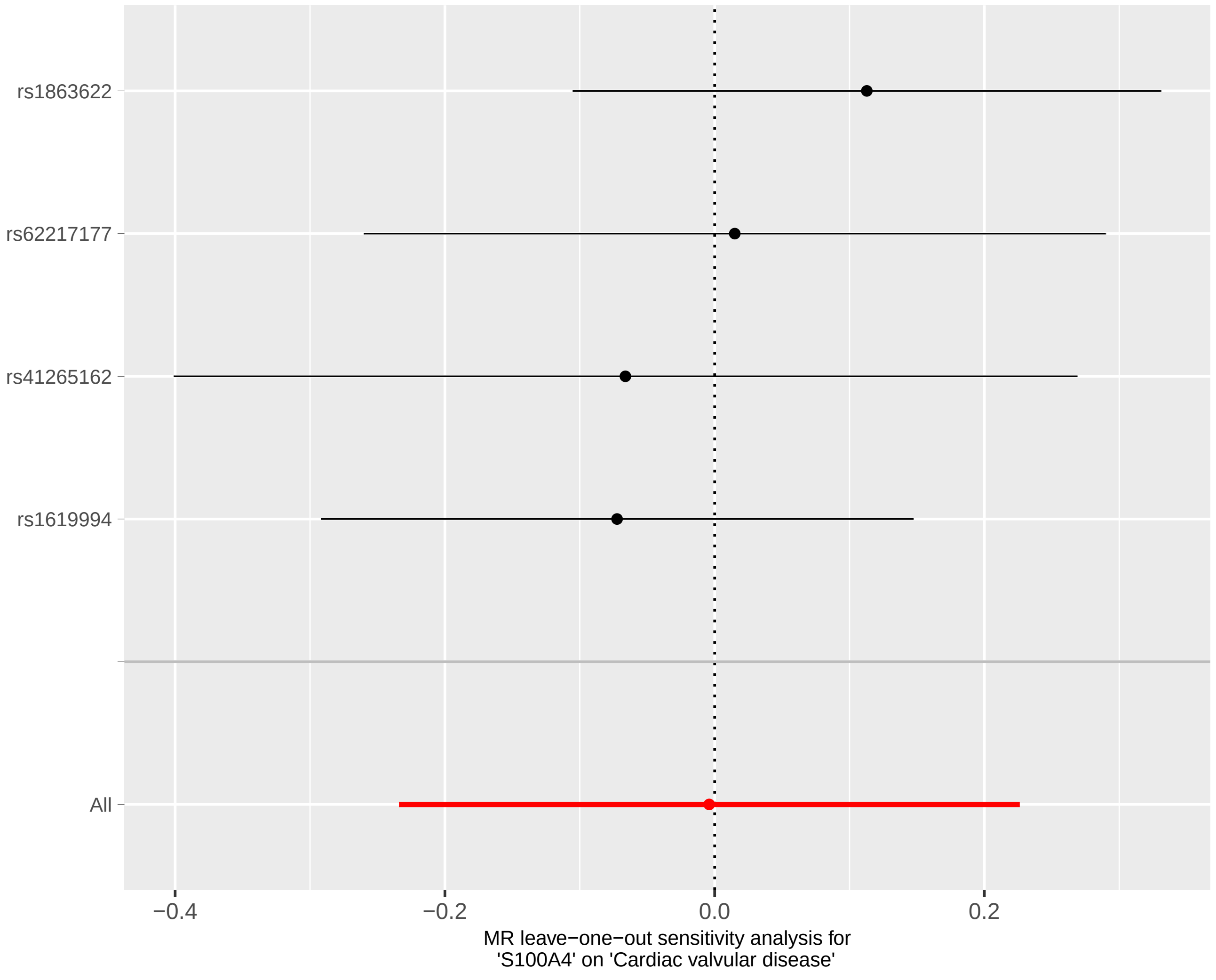
rs1863622
rs62217177
rs41265162
rs1619994
All
−0.4
−0.2
0.0
0.2
MR leave−one−out sensitivity analysis for
'S100A4' on 'Cardiac valvular disease'

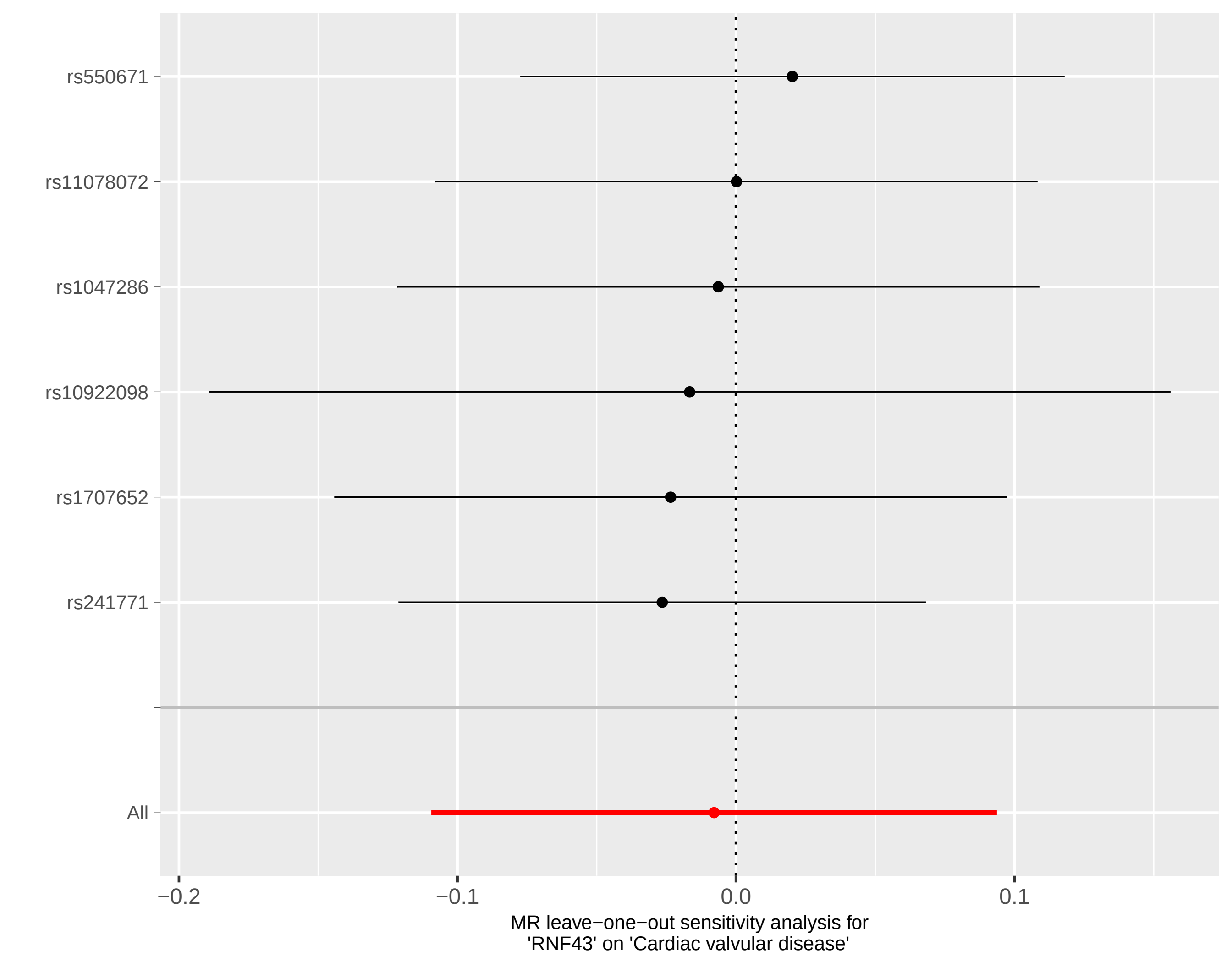
rs550671
rs11078072
rs1047286
rs10922098
rs1707652
rs241771
All
−0.2
−0.1
0.0
0.1
MR leave−one−out sensitivity analysis for
'RNF43' on 'Cardiac valvular disease'

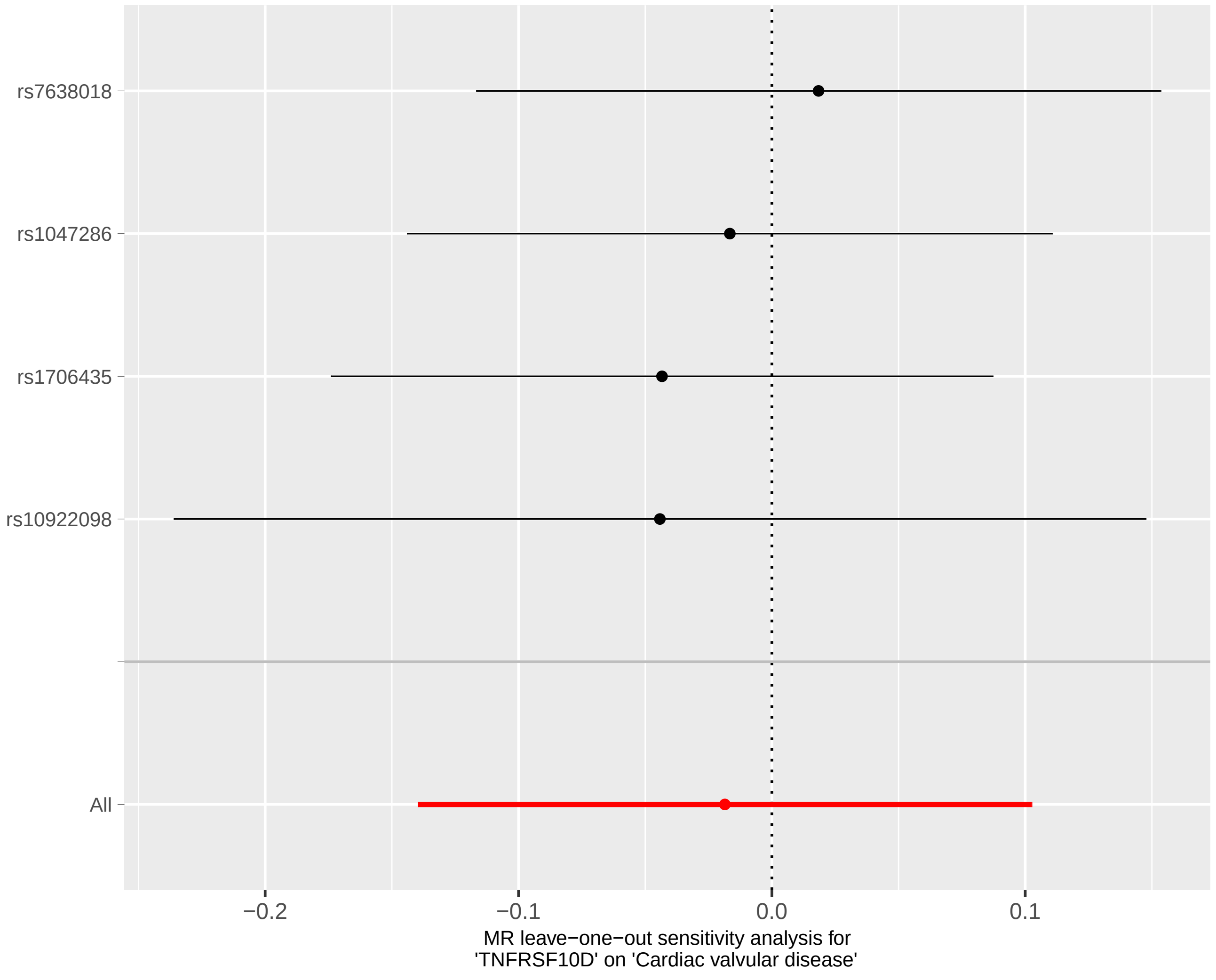
rs7638018
rs1047286
rs1706435
rs10922098
All
−0.2
−0.1
0.0
0.1
MR leave−one−out sensitivity analysis for
'TNFRSF10D' on 'Cardiac valvular disease'

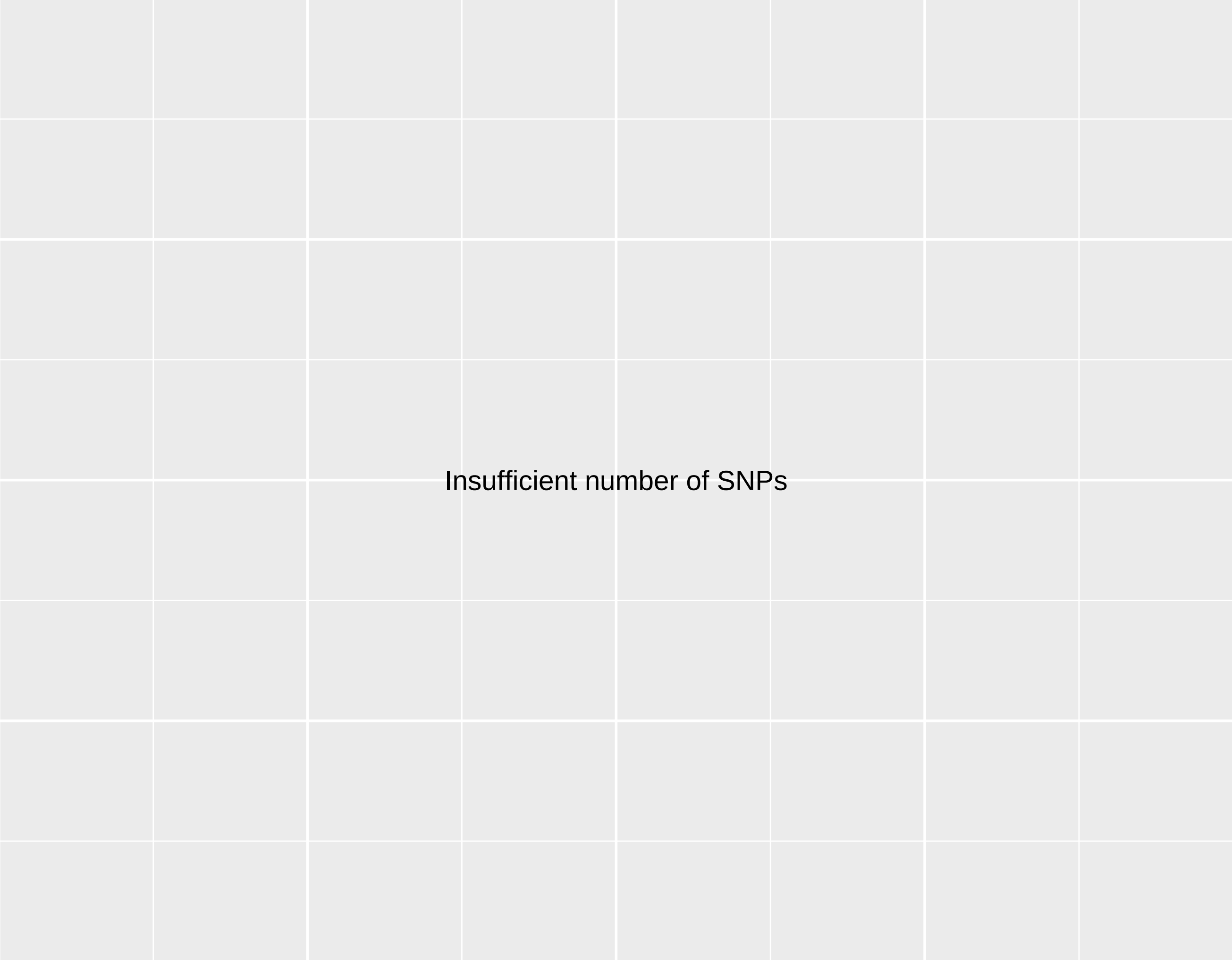
Insufficient number of SNPs

Insufficient number of SNPs

Insufficient number of SNPs

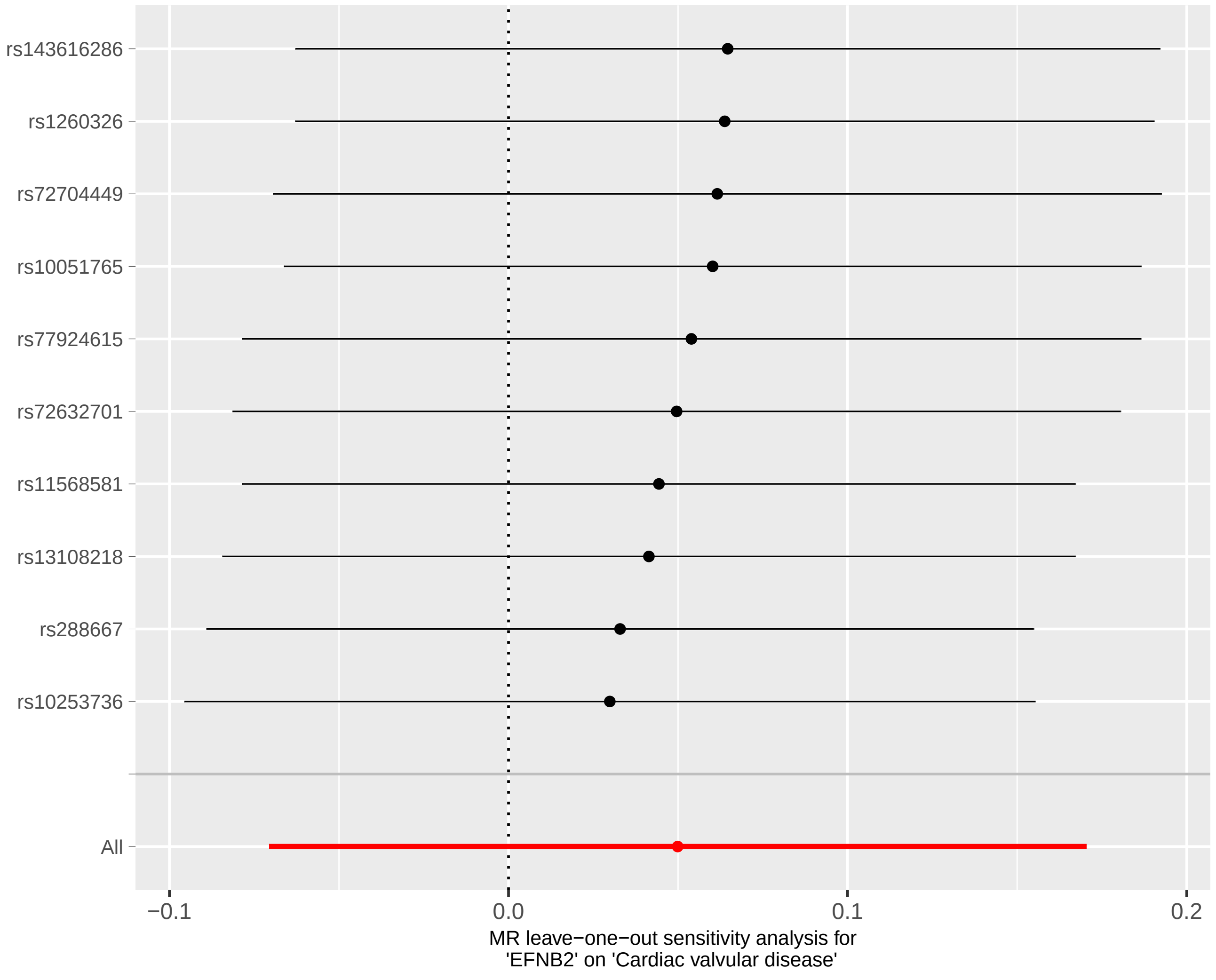

rs143616286
rs1260326
rs72704449
rs10051765
rs77924615
rs72632701
rs11568581
rs13108218
rs288667
rs10253736
All
−0.1
0.0
0.1
0.2
MR leave−one−out sensitivity analysis for
'EFNB2' on 'Cardiac valvular disease'

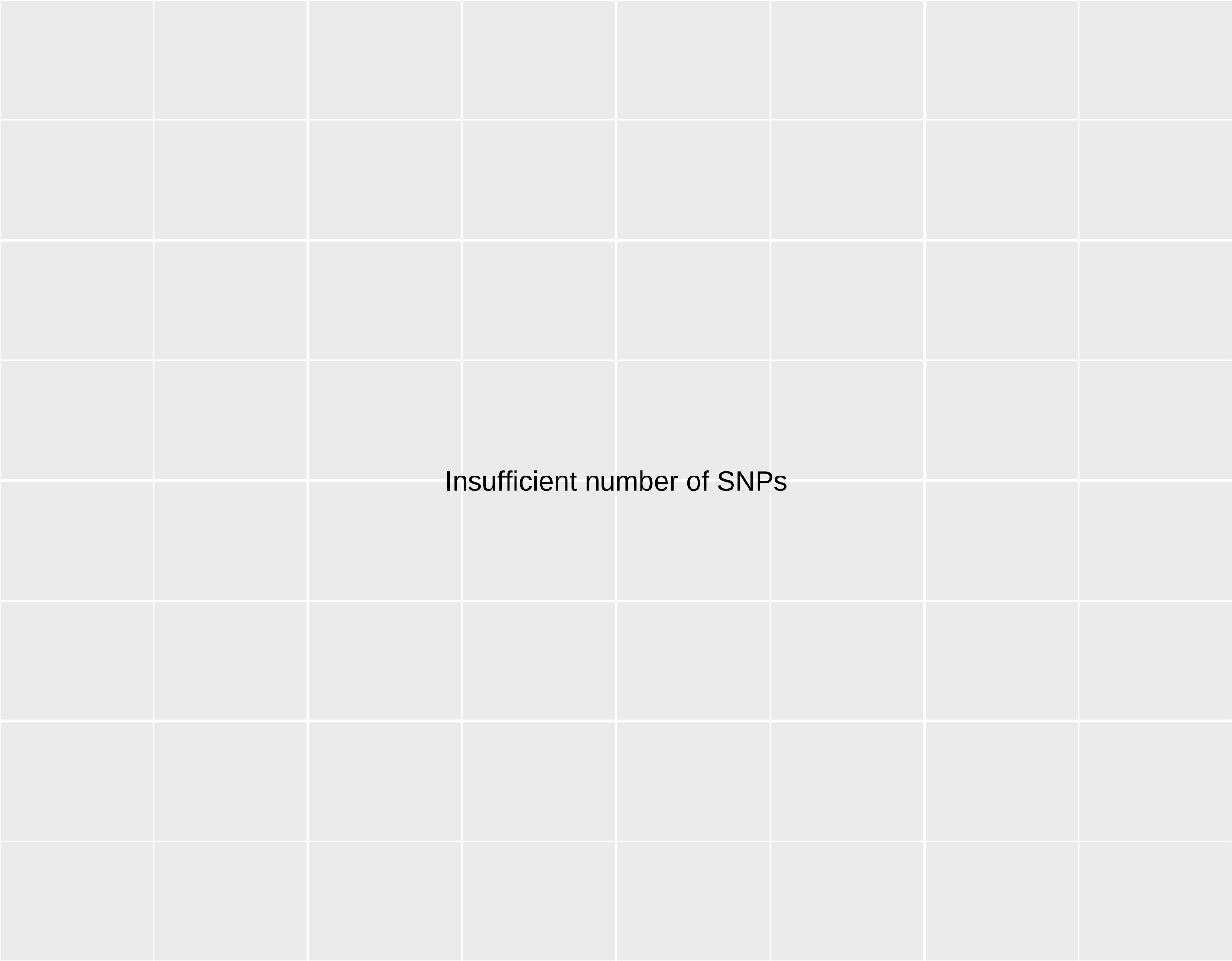
Insufficient number of SNPs

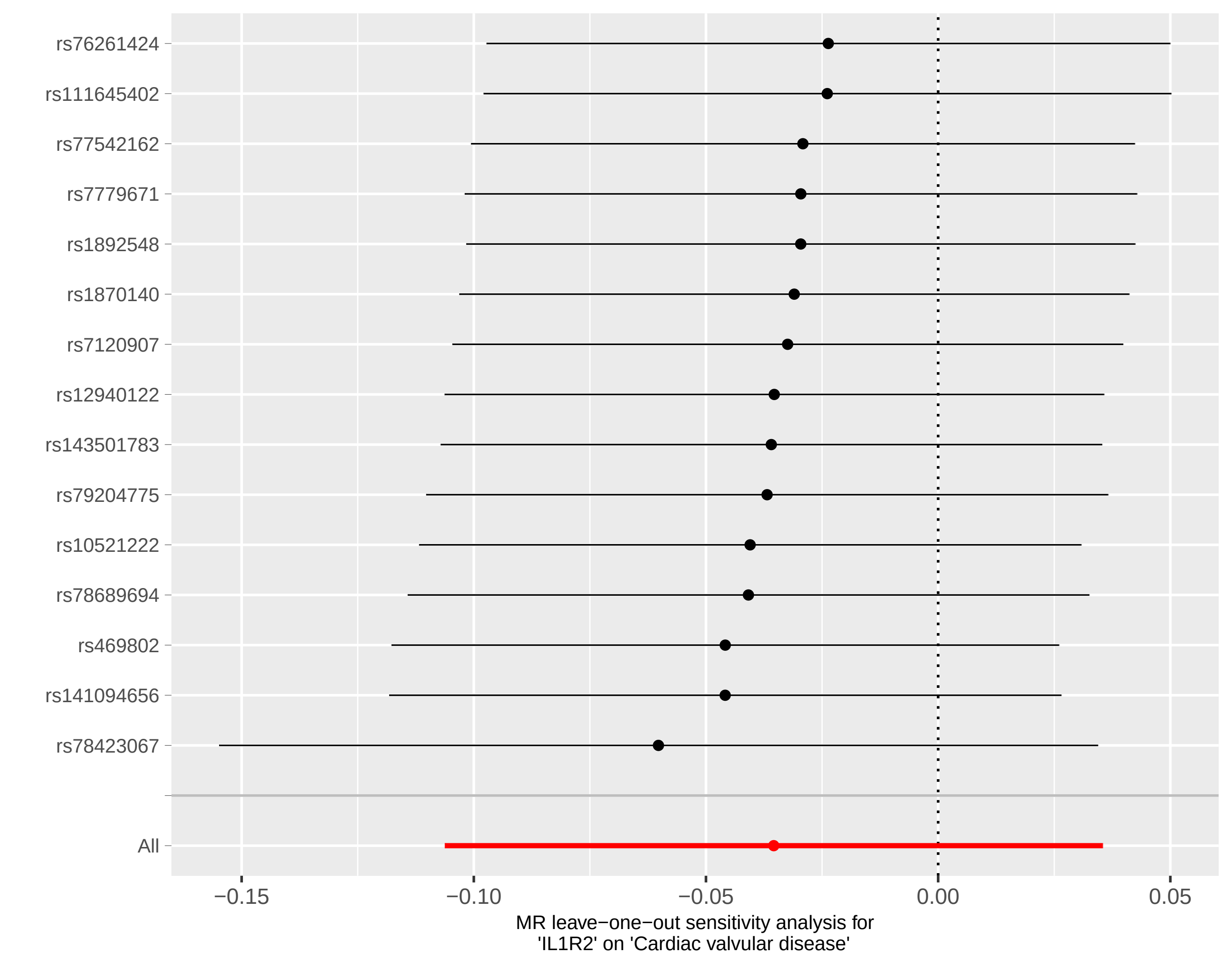
rs76261424
rs111645402
rs77542162
rs7779671
rs1892548
rs1870140
rs7120907
rs12940122
rs143501783
rs79204775
rs10521222
rs78689694
rs469802
rs141094656
rs78423067
All
−0.15
−0.10
−0.05
0.00
0.05
MR leave−one−out sensitivity analysis for
'IL1R2' on 'Cardiac valvular disease'

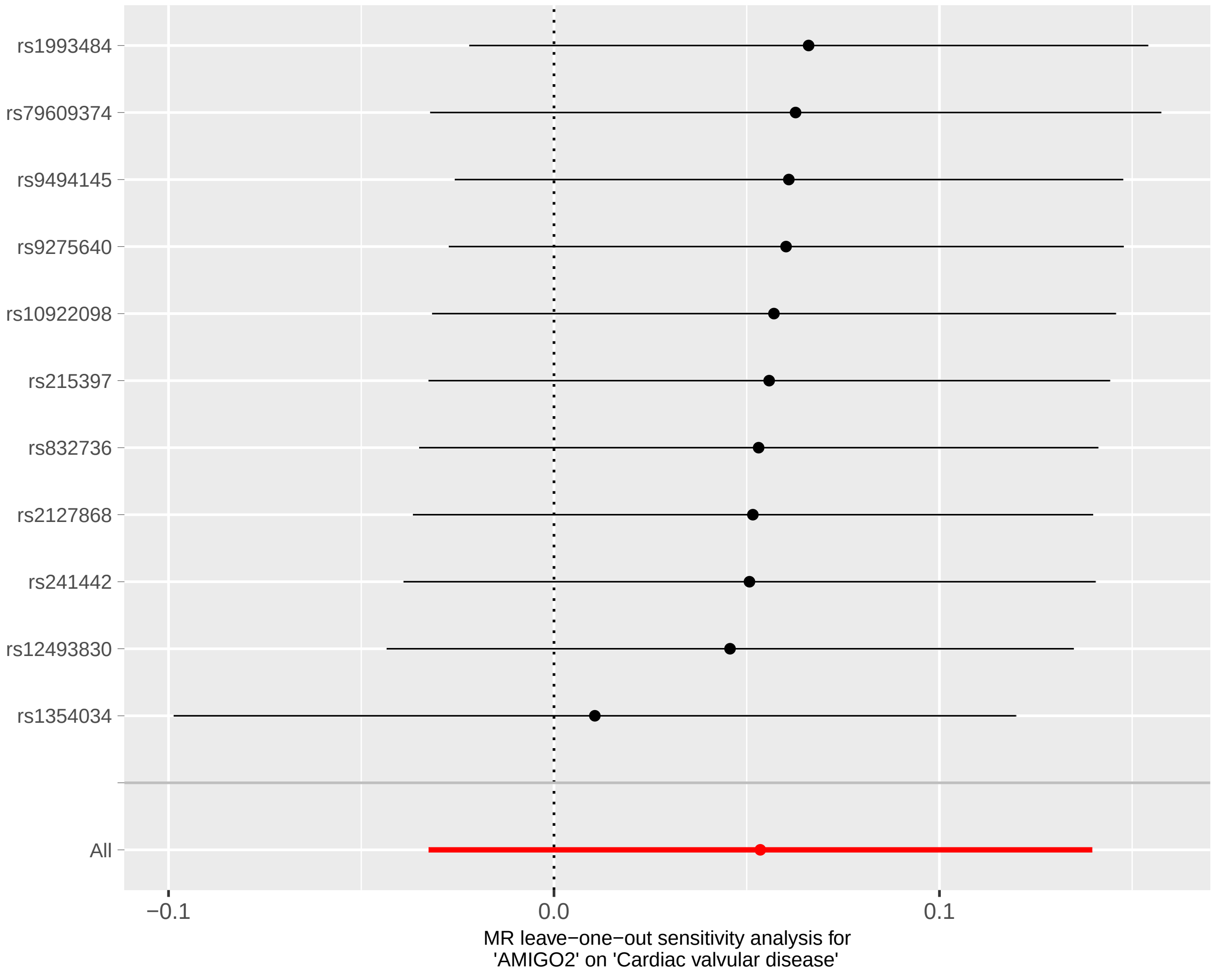
rs1993484
rs79609374
rs9494145
rs9275640
rs10922098
rs215397
rs832736
rs2127868
rs241442
rs12493830
rs1354034
All
−0.1
0.0
0.1
MR leave−one−out sensitivity analysis for
'AMIGO2' on 'Cardiac valvular disease'

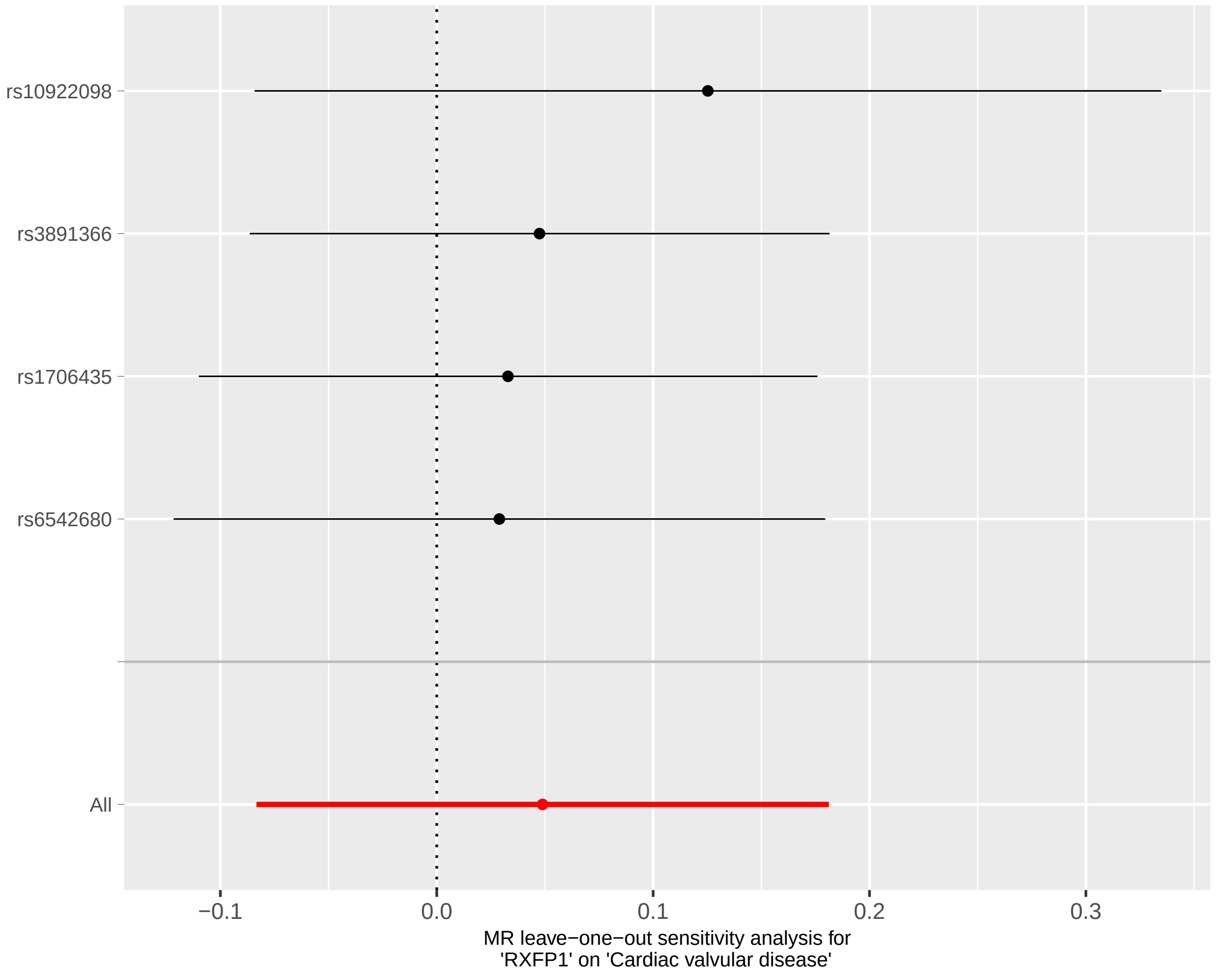
rs10922098
rs3891366
rs1706435
rs6542680
All
−0.1
0.0
0.1
0.2
0.3
MR leave−one−out sensitivity analysis for
'RXFP1' on 'Cardiac valvular disease'

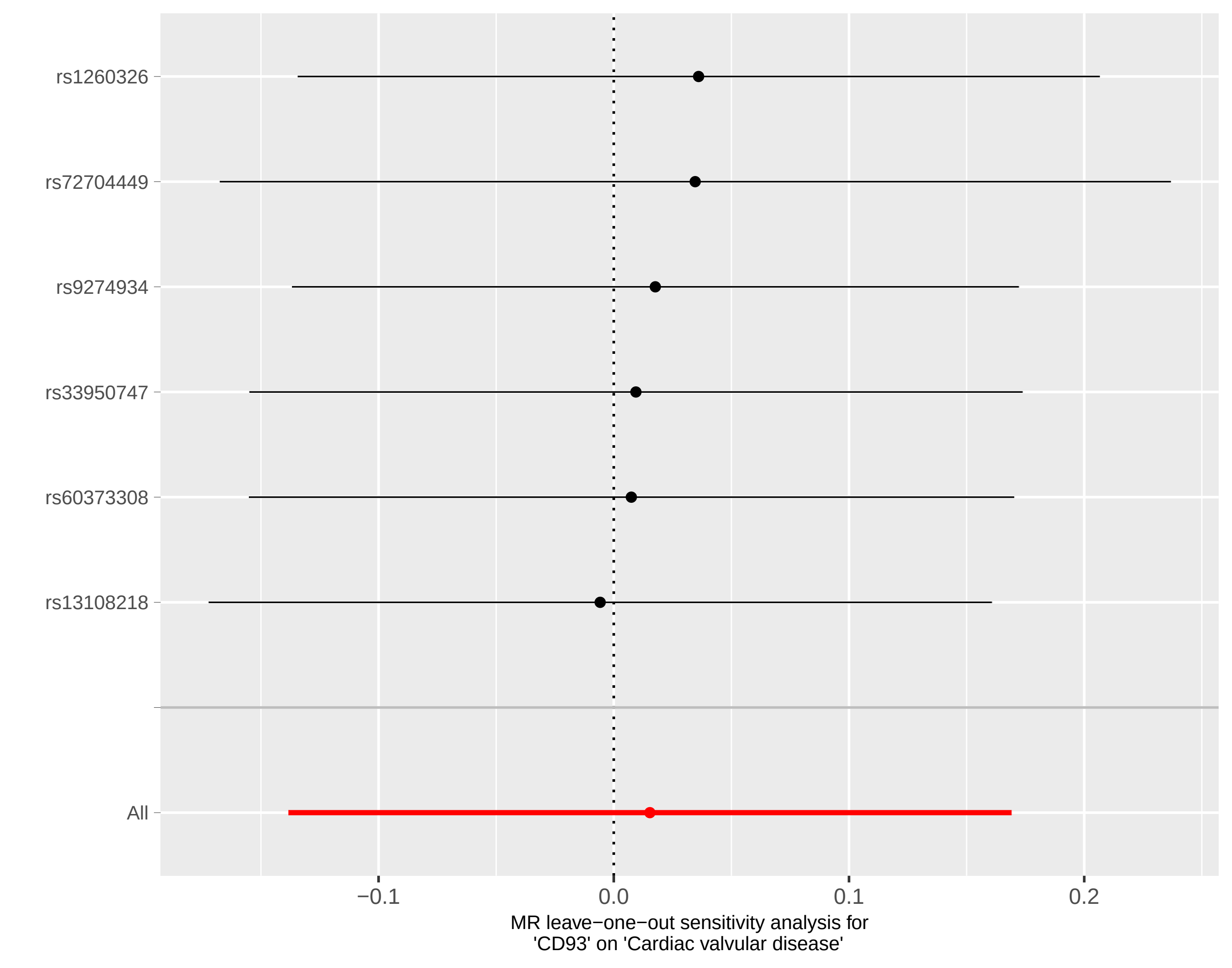

rs1260326
rs72704449
rs9274934
rs33950747
rs60373308
rs13108218
All
−0.1
0.0
0.1
0.2
MR leave−one−out sensitivity analysis for
'CD93' on 'Cardiac valvular disease'

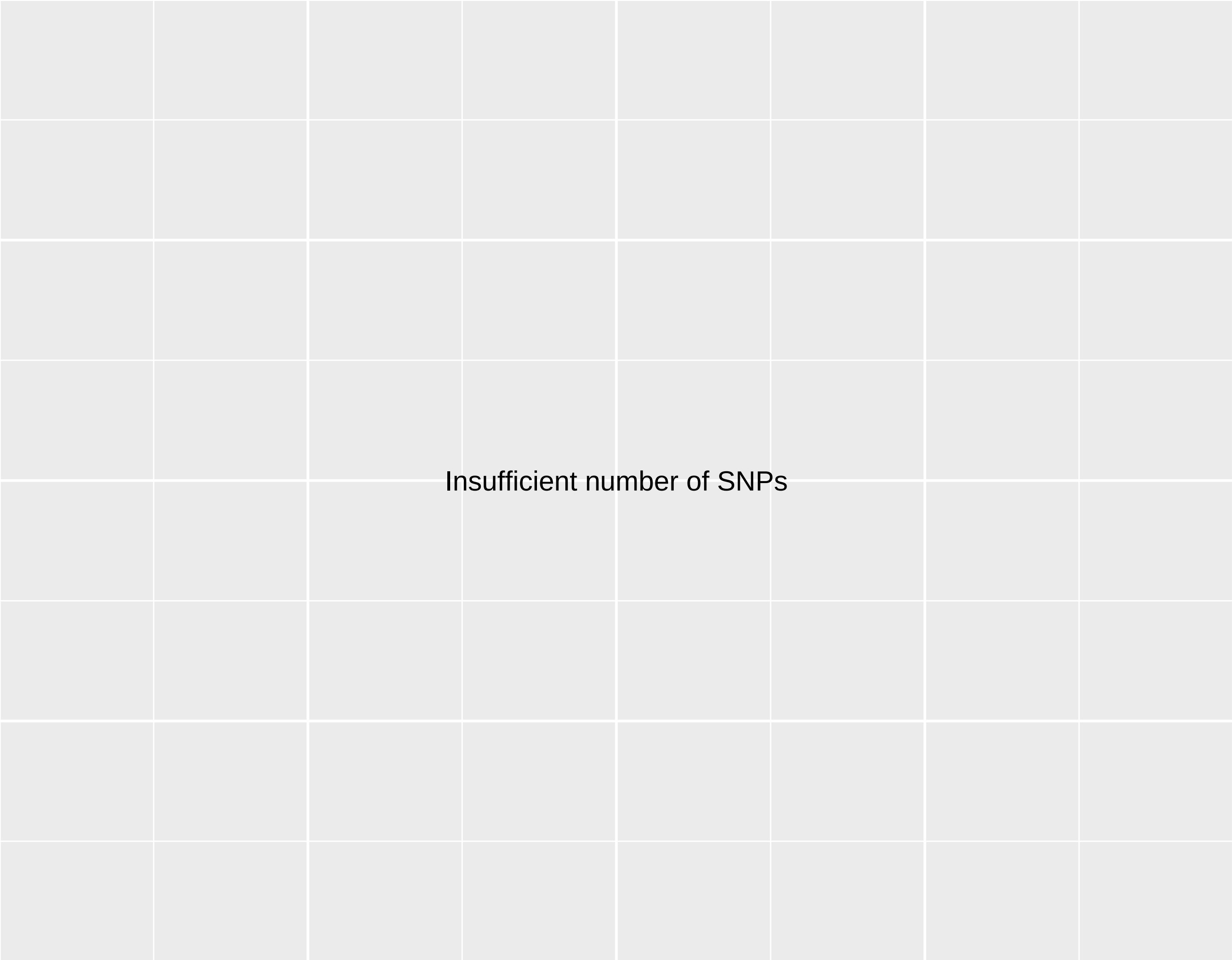
Insufficient number of SNPs

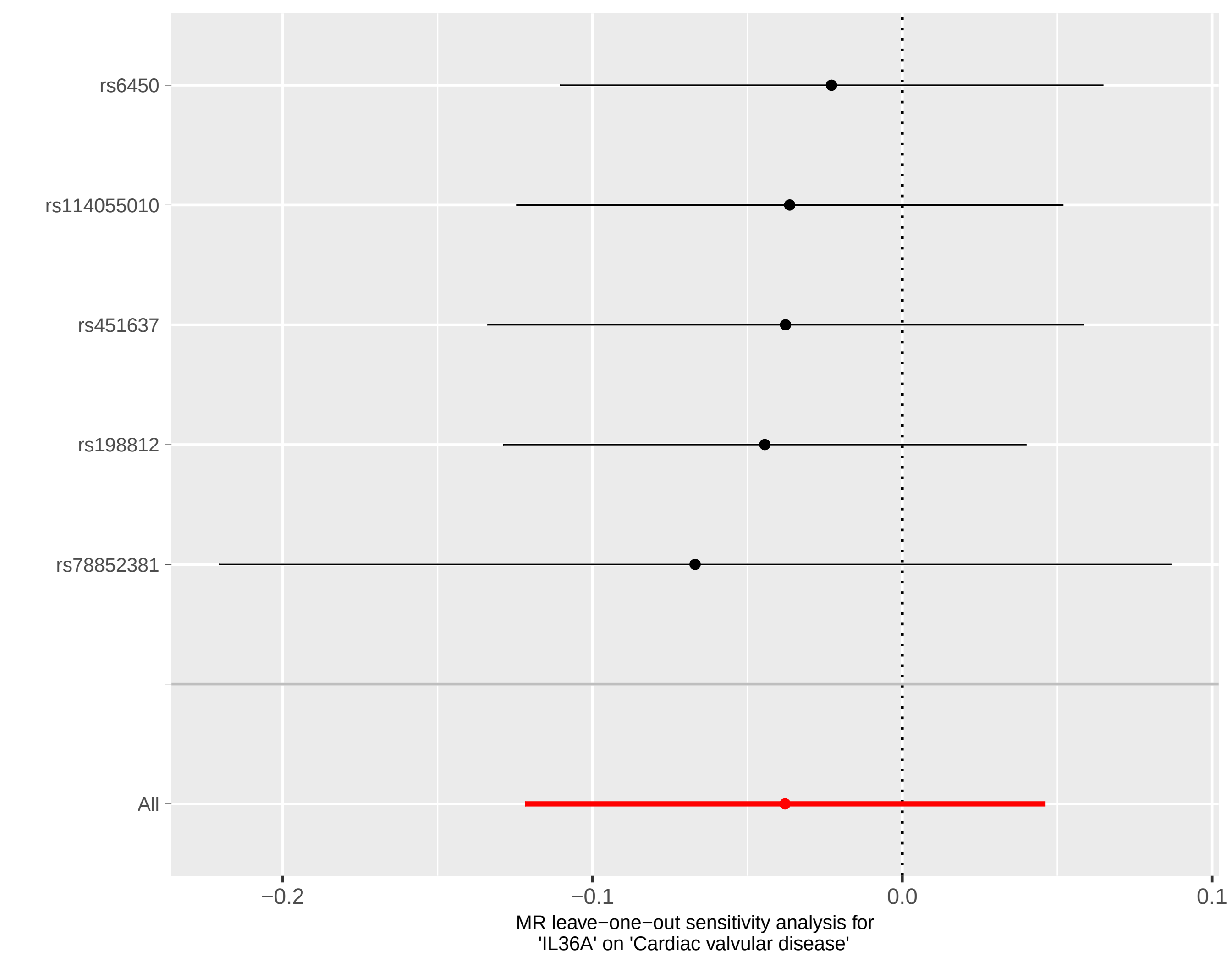
rs6450
rs114055010
rs451637
rs198812
rs78852381
All
−0.2
−0.1
0.0
0.1
MR leave−one−out sensitivity analysis for
'IL36A' on 'Cardiac valvular disease'

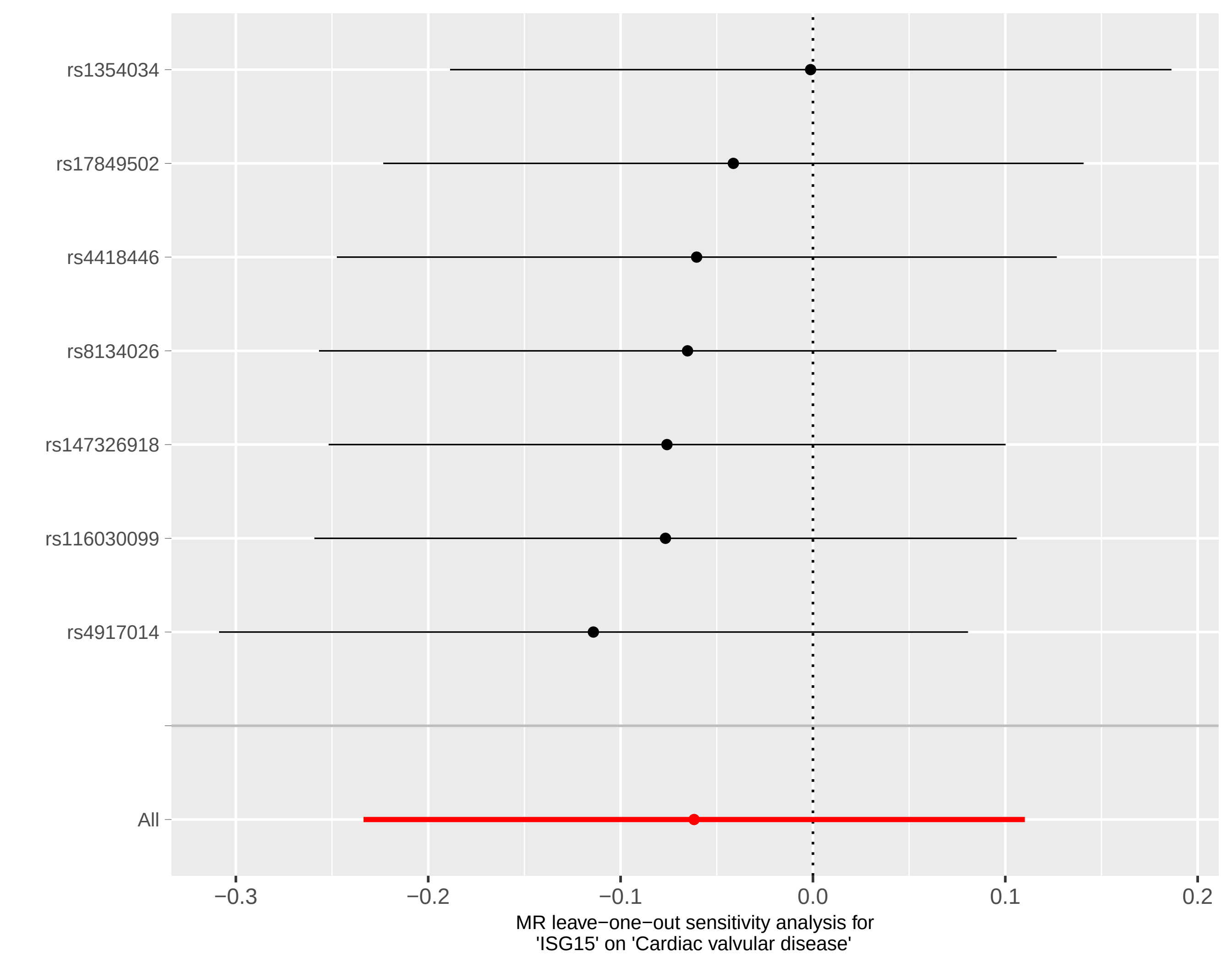
rs1354034
rs17849502
rs4418446
rs8134026
rs147326918
rs116030099
rs4917014
All
−0.3
−0.2
−0.1
0.0
0.1
0.2
MR leave−one−out sensitivity analysis for
'ISG15' on 'Cardiac valvular disease'

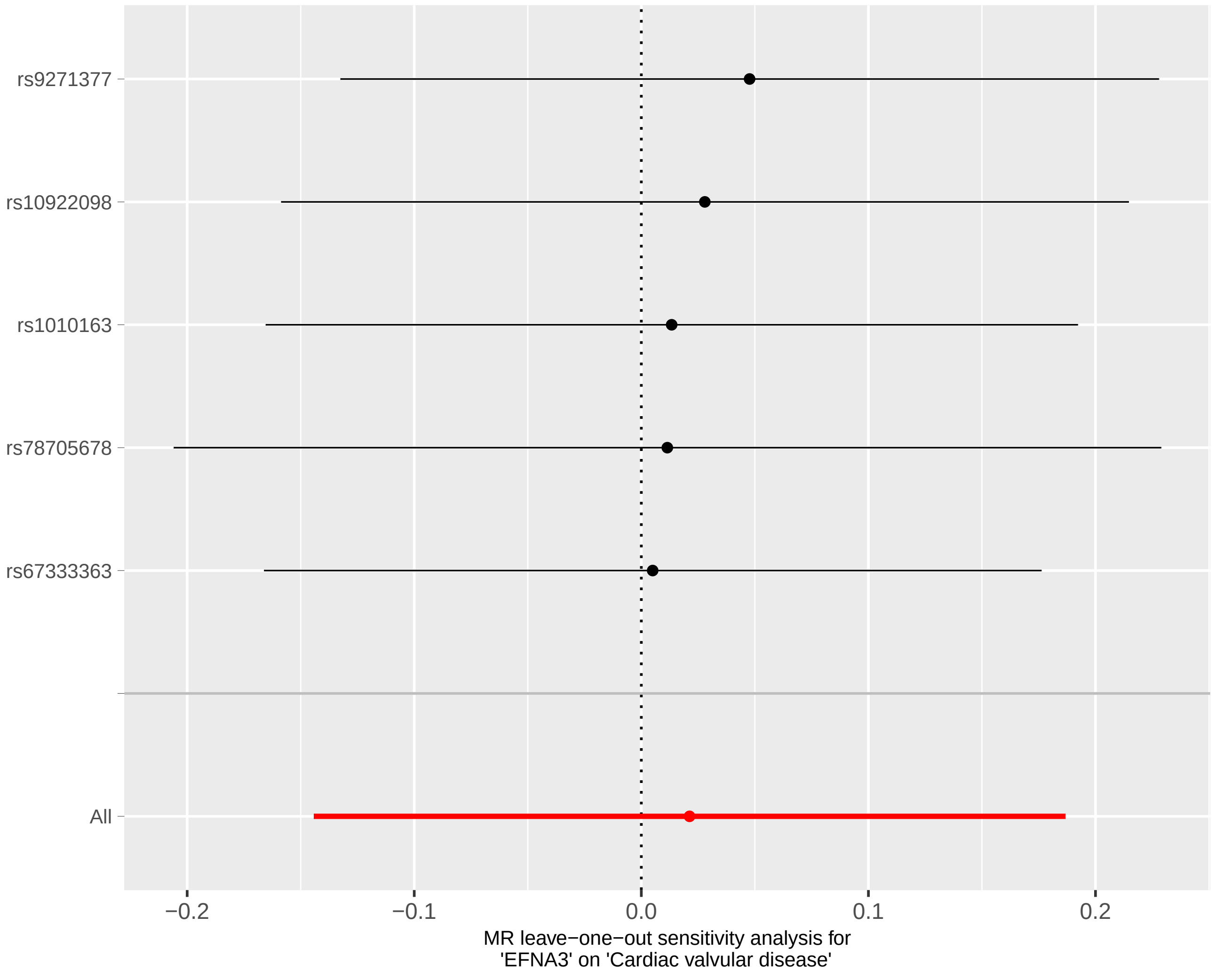
rs9271377
rs10922098
rs1010163
rs78705678
rs67333363
All
−0.2
−0.1
0.0
0.1
0.2
MR leave−one−out sensitivity analysis for
'EFNA3' on 'Cardiac valvular disease'

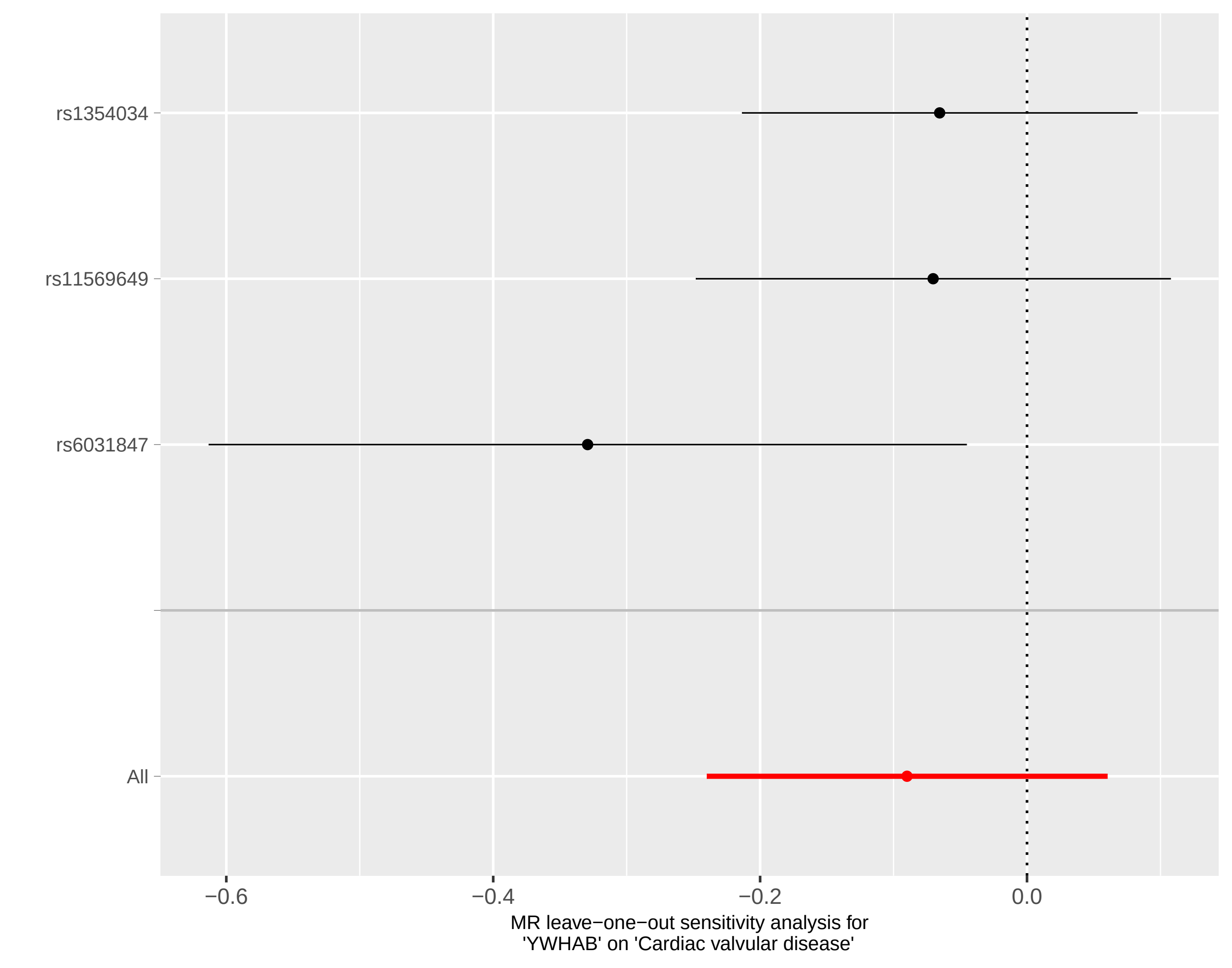
rs1354034
rs11569649
rs6031847
All
−0.6
−0.4
−0.2
0.0
MR leave−one−out sensitivity analysis for
'YWHAB' on 'Cardiac valvular disease'

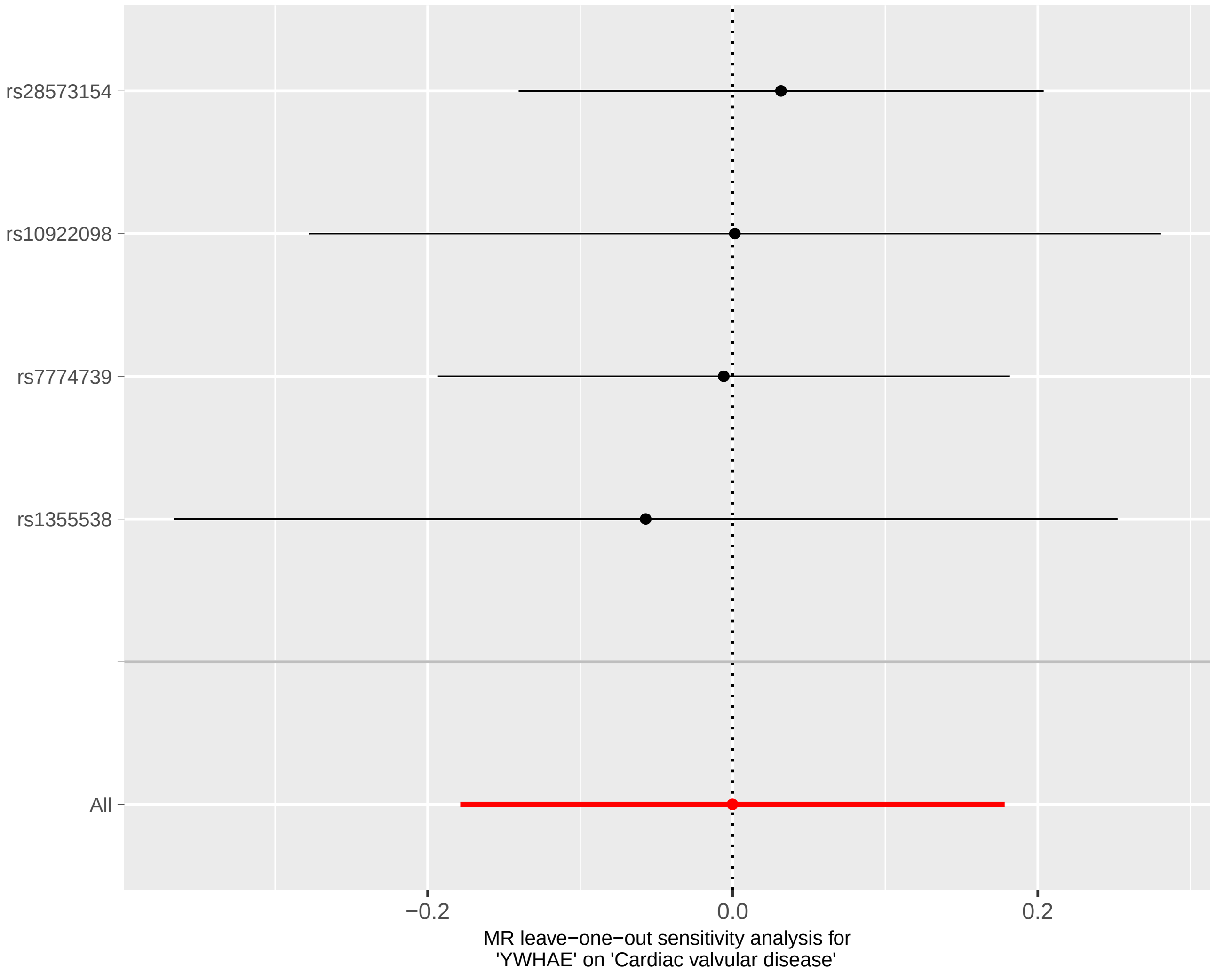
rs28573154
rs10922098
rs7774739
rs1355538
All
−0.2
0.0
0.2
MR leave−one−out sensitivity analysis for
'YWHAE' on 'Cardiac valvular disease'

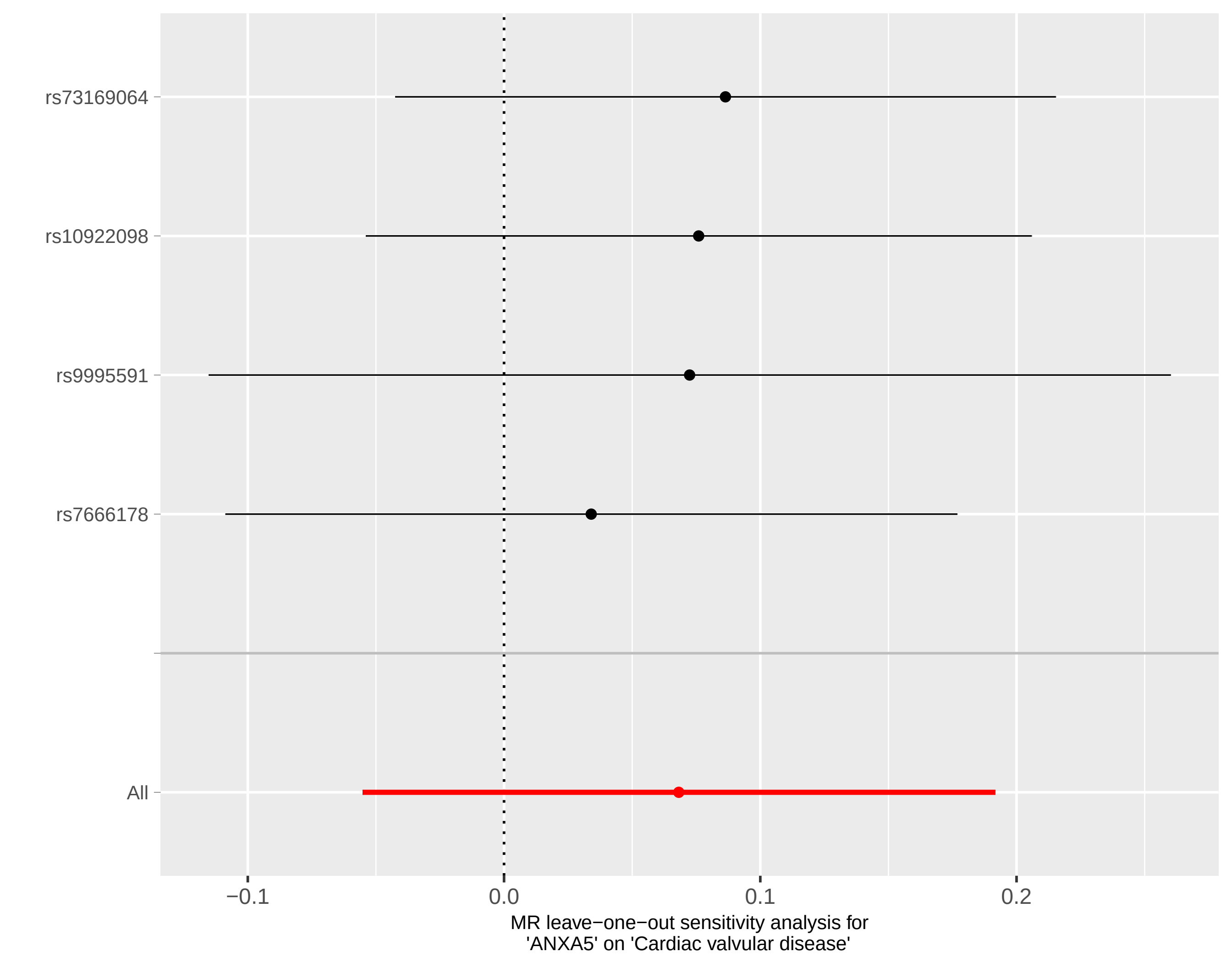

rs73169064
rs10922098
rs9995591
rs7666178
All
−0.1
0.0
0.1
0.2
MR leave−one−out sensitivity analysis for
'ANXA5' on 'Cardiac valvular disease'

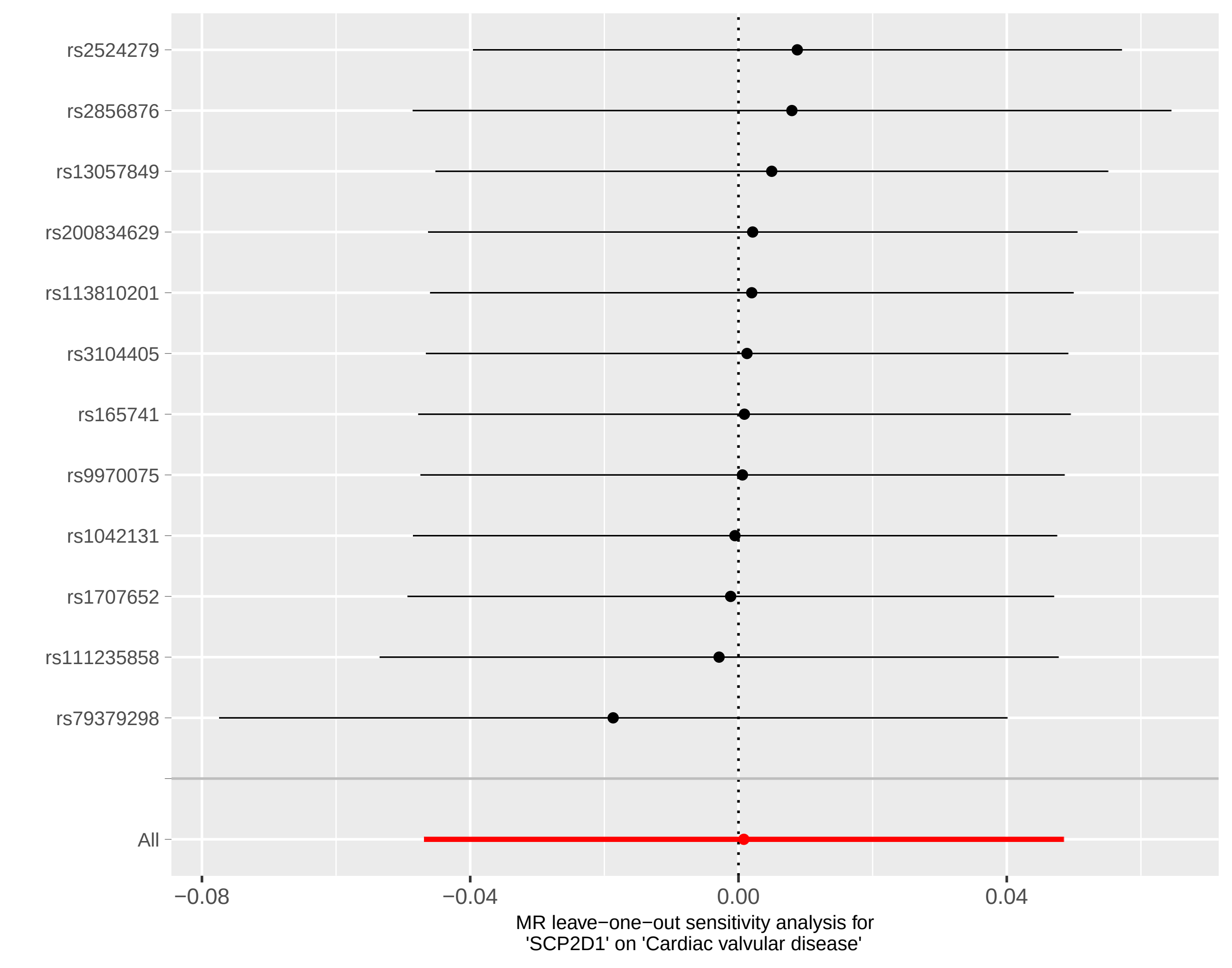
rs2524279
rs2856876
rs13057849
rs200834629
rs113810201
rs3104405
rs165741
rs9970075
rs1042131
rs1707652
rs111235858
rs79379298
All
−0.08
−0.04
0.00
0.04
MR leave−one−out sensitivity analysis for
'SCP2D1' on 'Cardiac valvular disease'

Insufficient number of SNPs

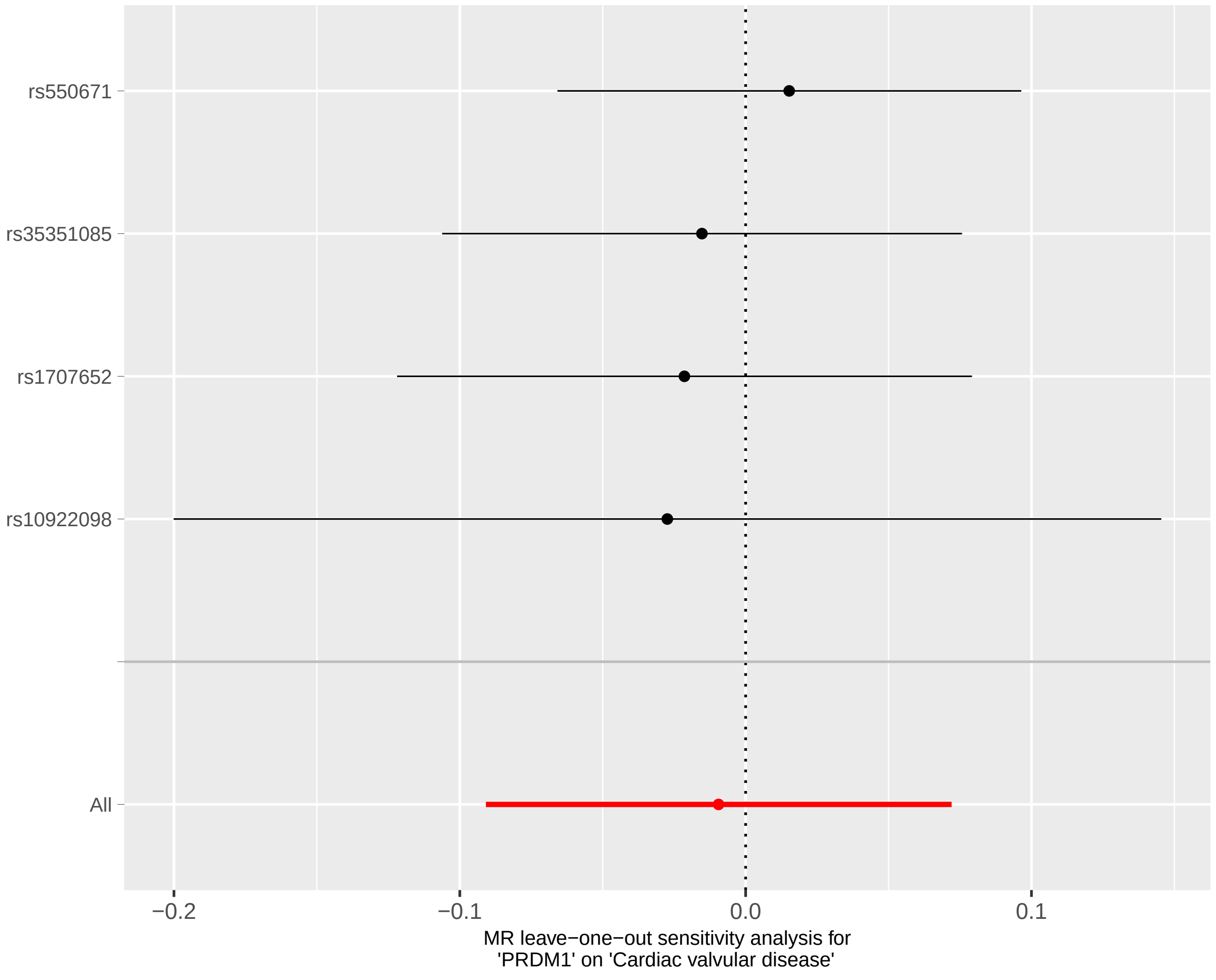
rs550671
rs35351085
rs1707652
rs10922098
All
−0.2
−0.1
0.0
0.1
MR leave−one−out sensitivity analysis for
'PRDM1' on 'Cardiac valvular disease'

Insufficient number of SNPs

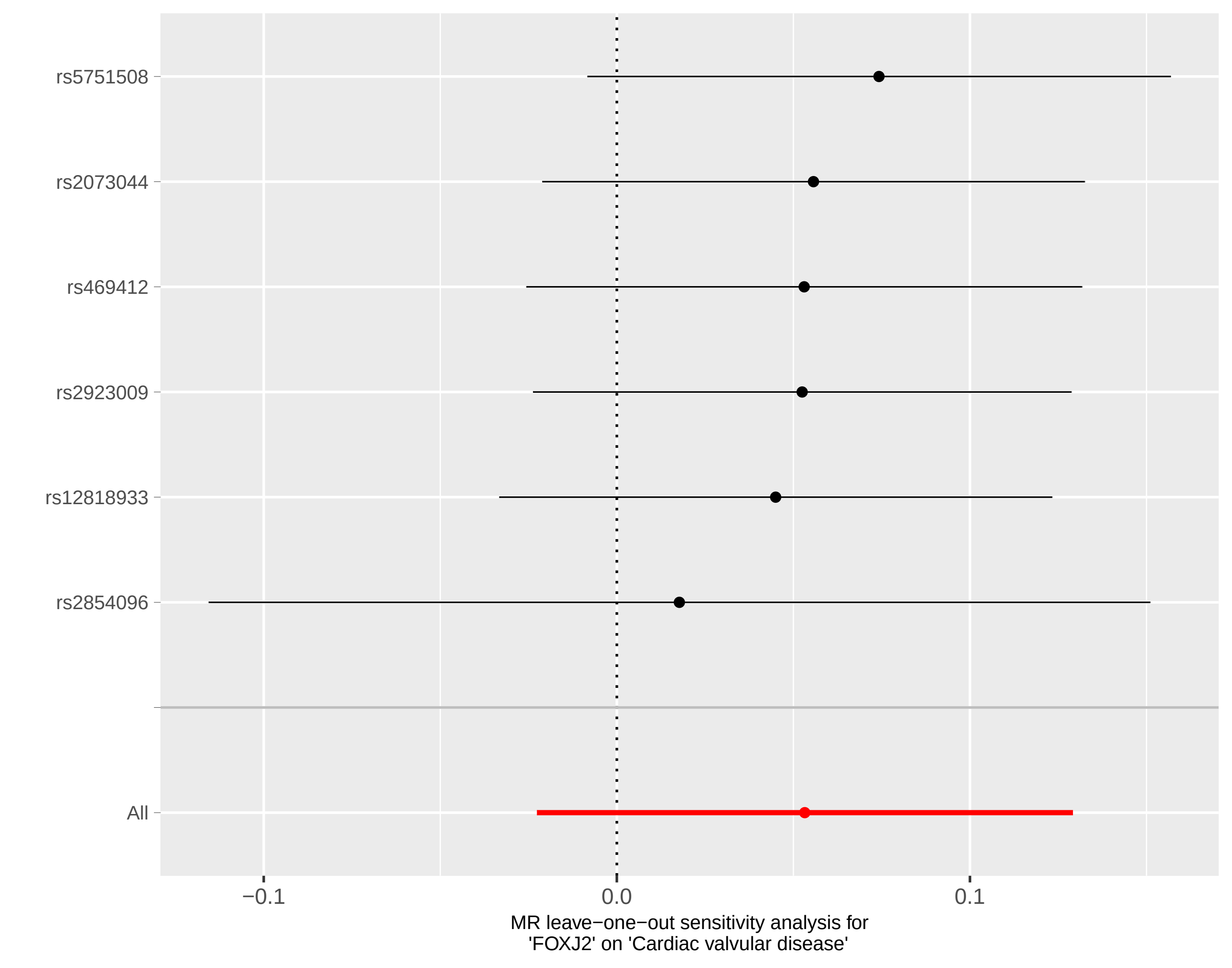
rs5751508
rs2073044
rs469412
rs2923009
rs12818933
rs2854096
All
−0.1
0.0
0.1
MR leave−one−out sensitivity analysis for
'FOXJ2' on 'Cardiac valvular disease'

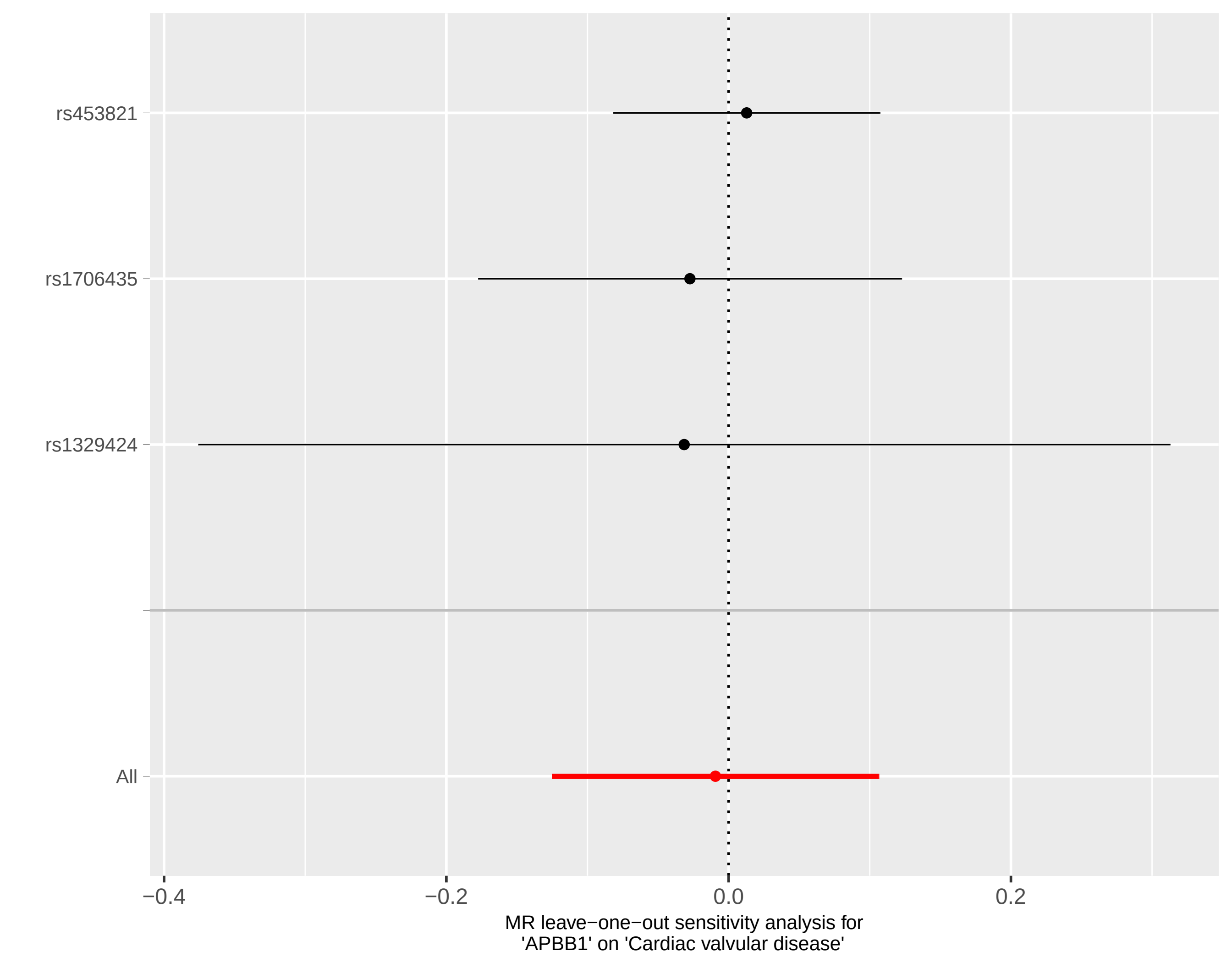
rs453821
rs1706435
rs1329424
All
−0.4
−0.2
0.0
0.2
MR leave−one−out sensitivity analysis for
'APBB1' on 'Cardiac valvular disease'

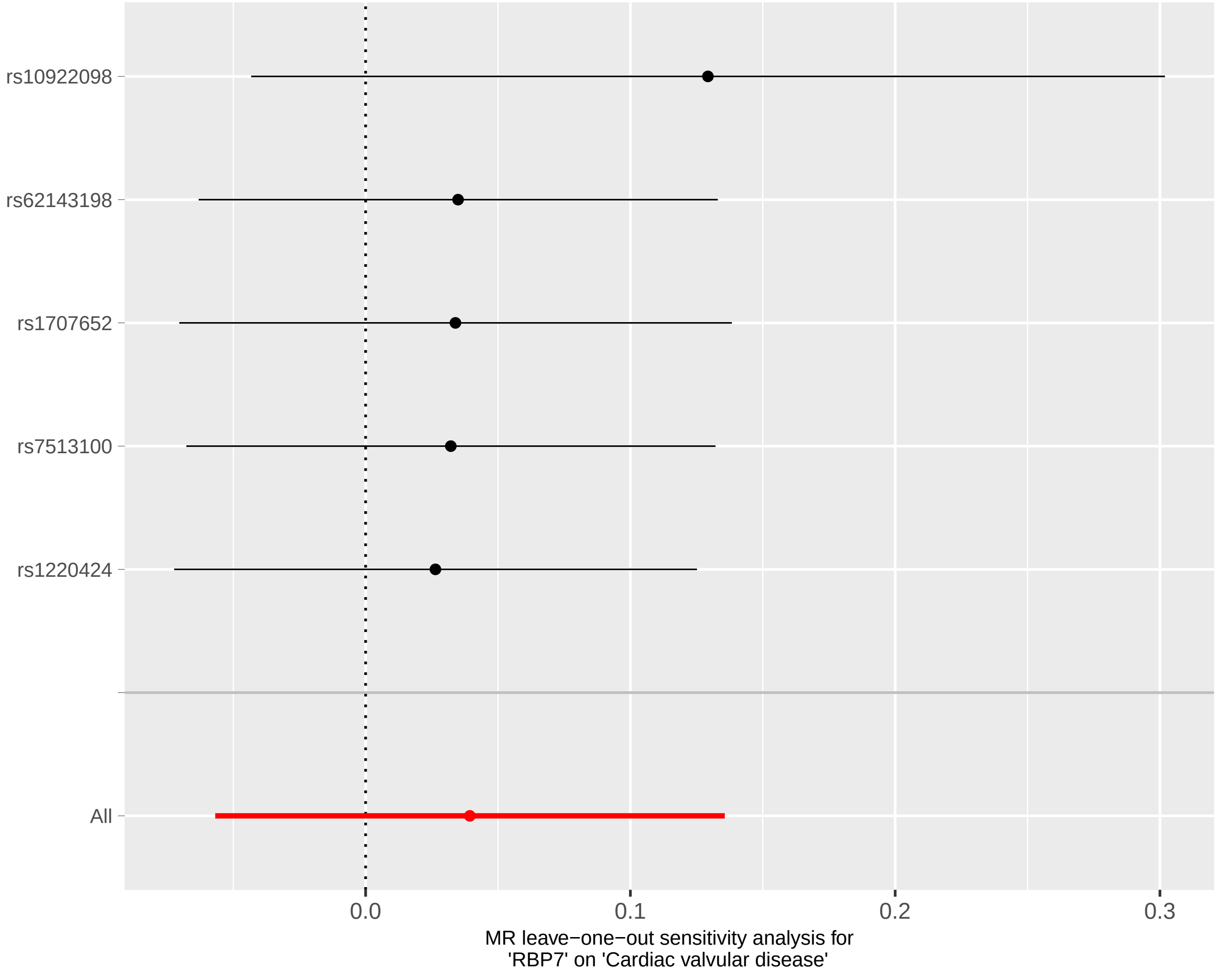

rs10922098
rs62143198
rs1707652
rs7513100
rs1220424
All
0.0
0.1
0.2
0.3
MR leave−one−out sensitivity analysis for
'RBP7' on 'Cardiac valvular disease'

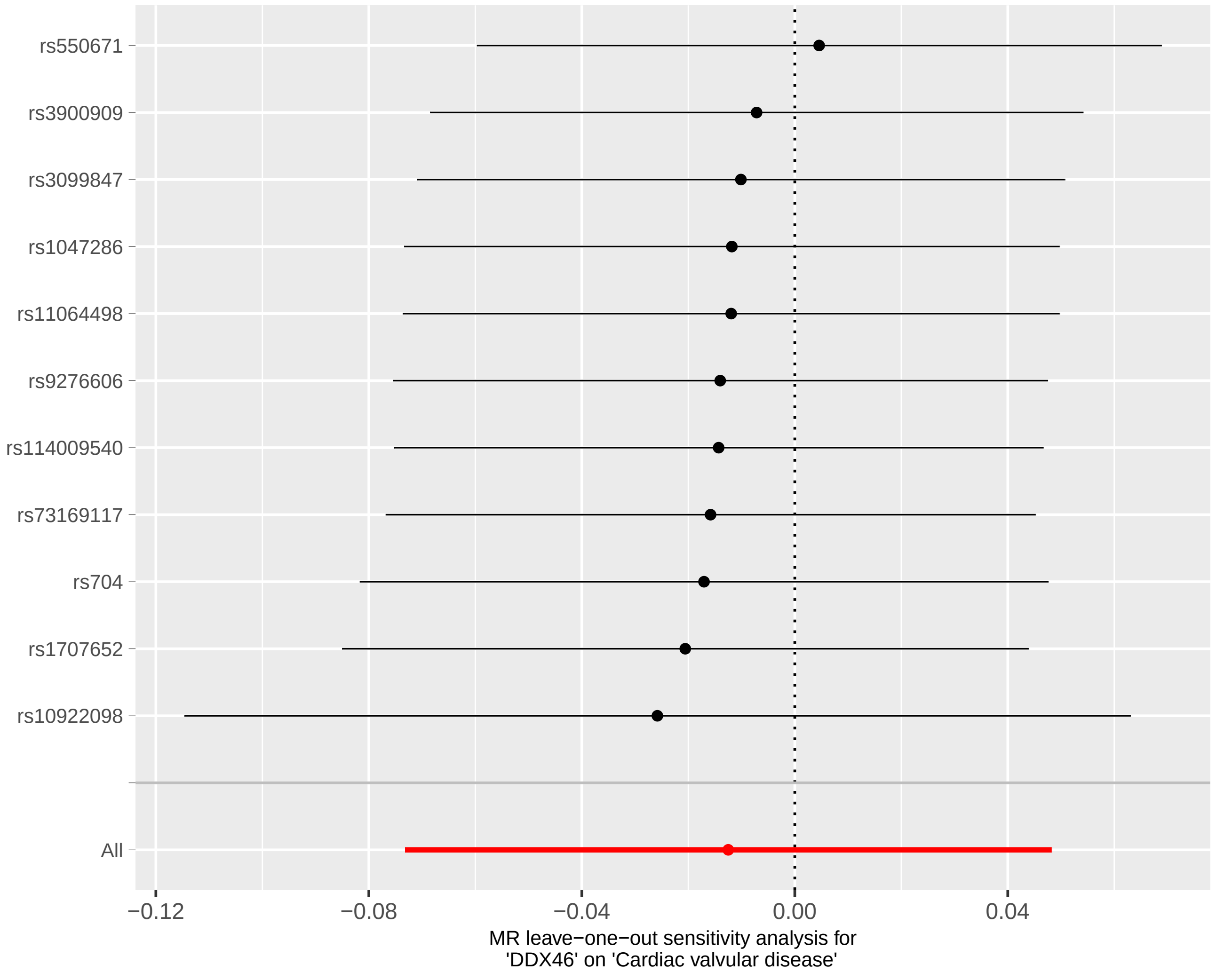

rs550671
rs3900909
rs3099847
rs1047286
rs11064498
rs9276606
rs114009540
rs73169117
rs704
rs1707652
rs10922098
All
−0.12
−0.08
−0.04
0.00
0.04
MR leave−one−out sensitivity analysis for
'DDX46' on 'Cardiac valvular disease'

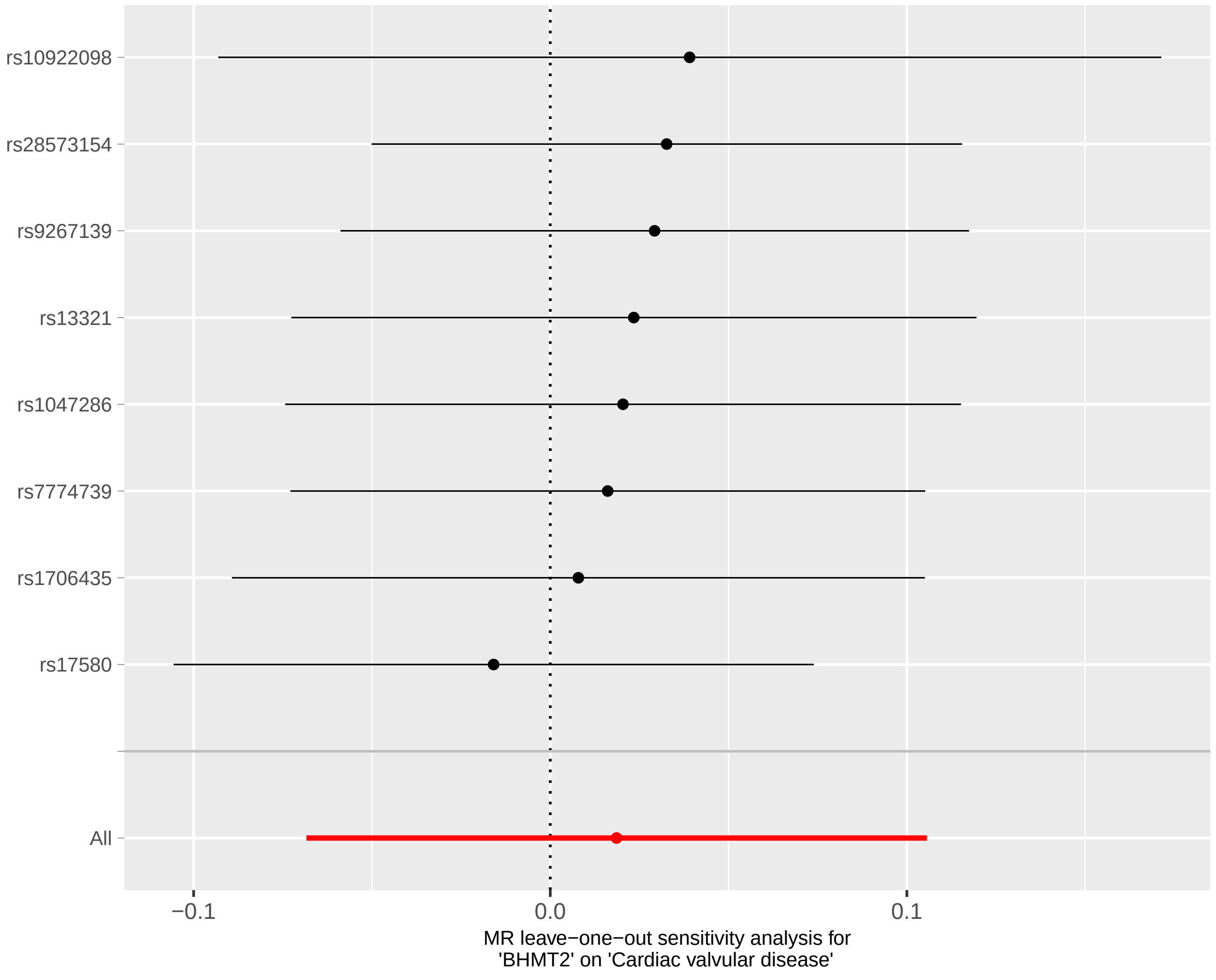
rs10922098
rs28573154
rs9267139
rs13321
rs1047286
rs7774739
rs1706435
rs17580
All
−0.1
0.0
0.1
MR leave−one−out sensitivity analysis for
'BHMT2' on 'Cardiac valvular disease'

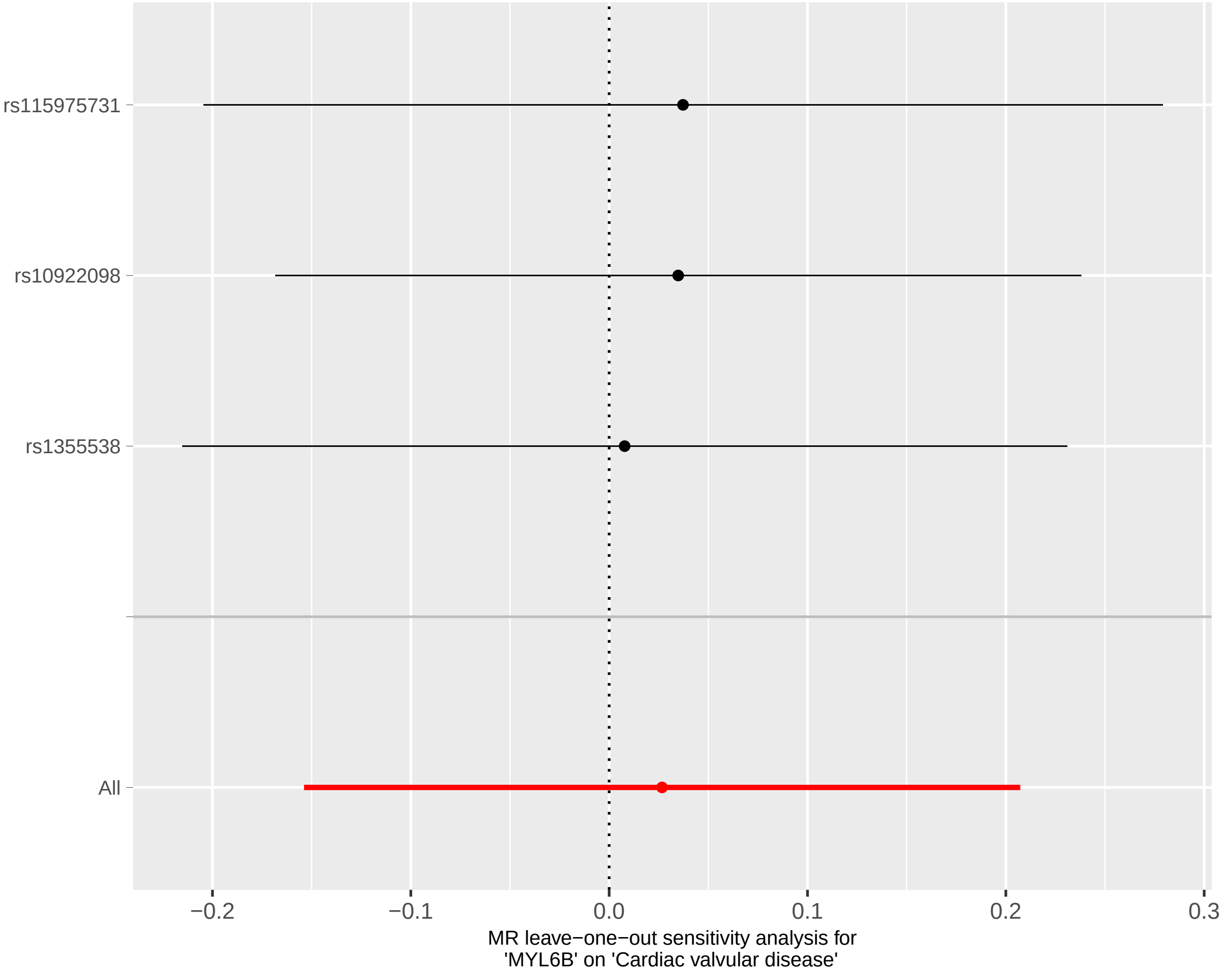
rs115975731
rs10922098
rs1355538
All
−0.2
−0.1
0.0
0.1
0.2
0.3
MR leave−one−out sensitivity analysis for
'MYL6B' on 'Cardiac valvular disease'

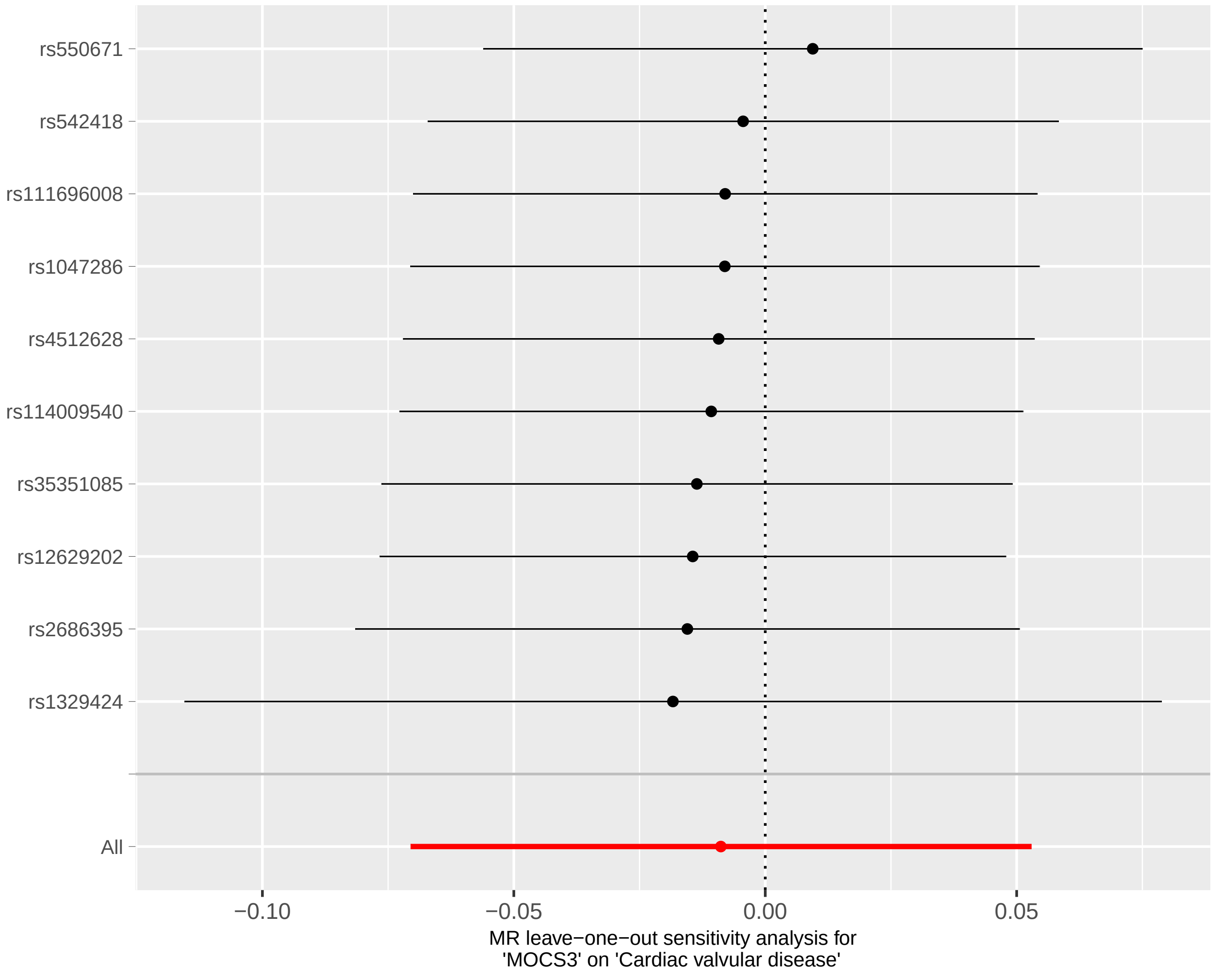
rs550671
rs542418
rs111696008
rs1047286
rs4512628
rs114009540
rs35351085
rs12629202
rs2686395
rs1329424
All
−0.10
−0.05
0.00
0.05
MR leave−one−out sensitivity analysis for
'MOCS3' on 'Cardiac valvular disease'

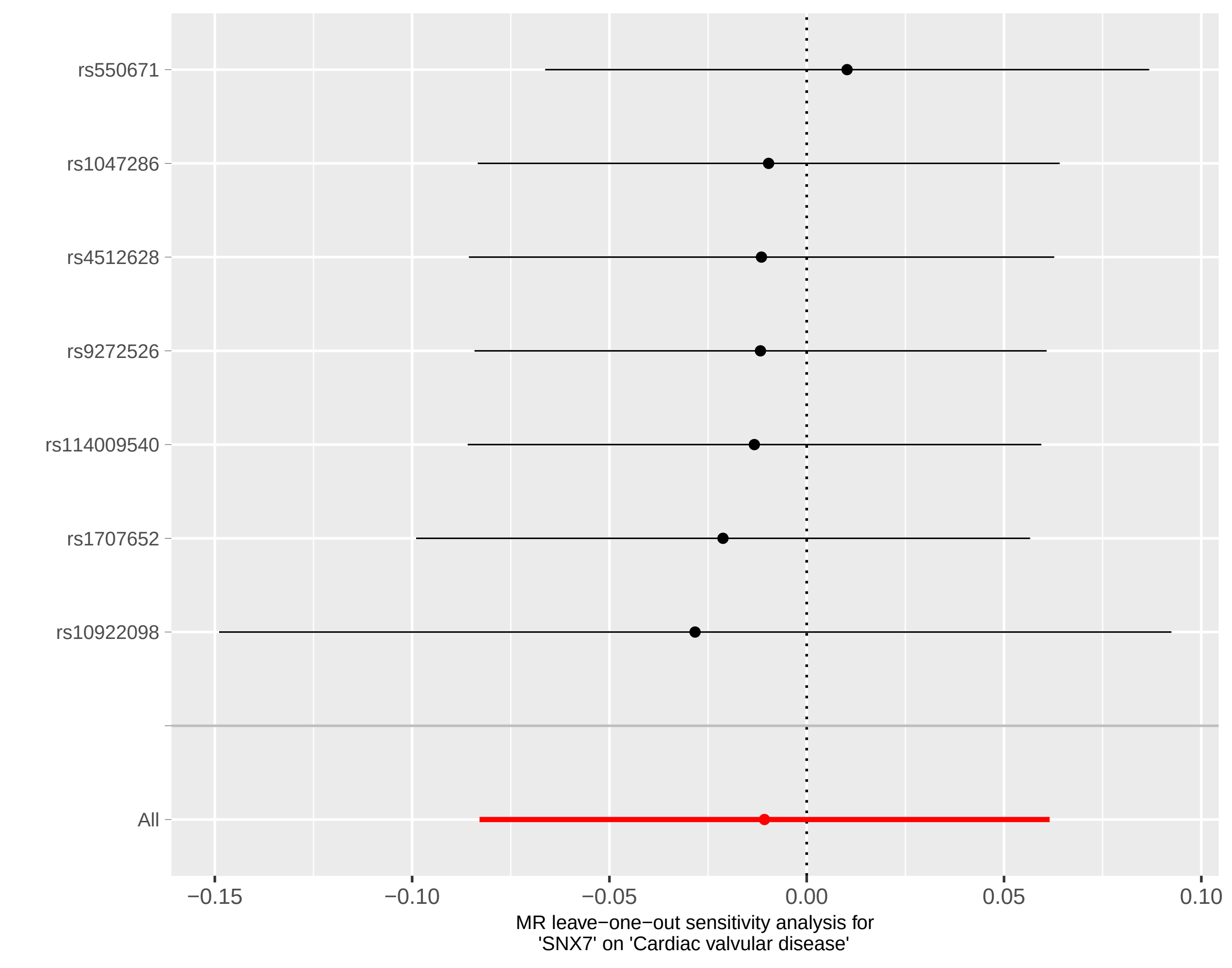

rs550671
rs1047286
rs4512628
rs9272526
rs114009540
rs1707652
rs10922098
All
−0.15
−0.10
−0.05
0.00
0.05
0.10
MR leave−one−out sensitivity analysis for
'SNX7' on 'Cardiac valvular disease'

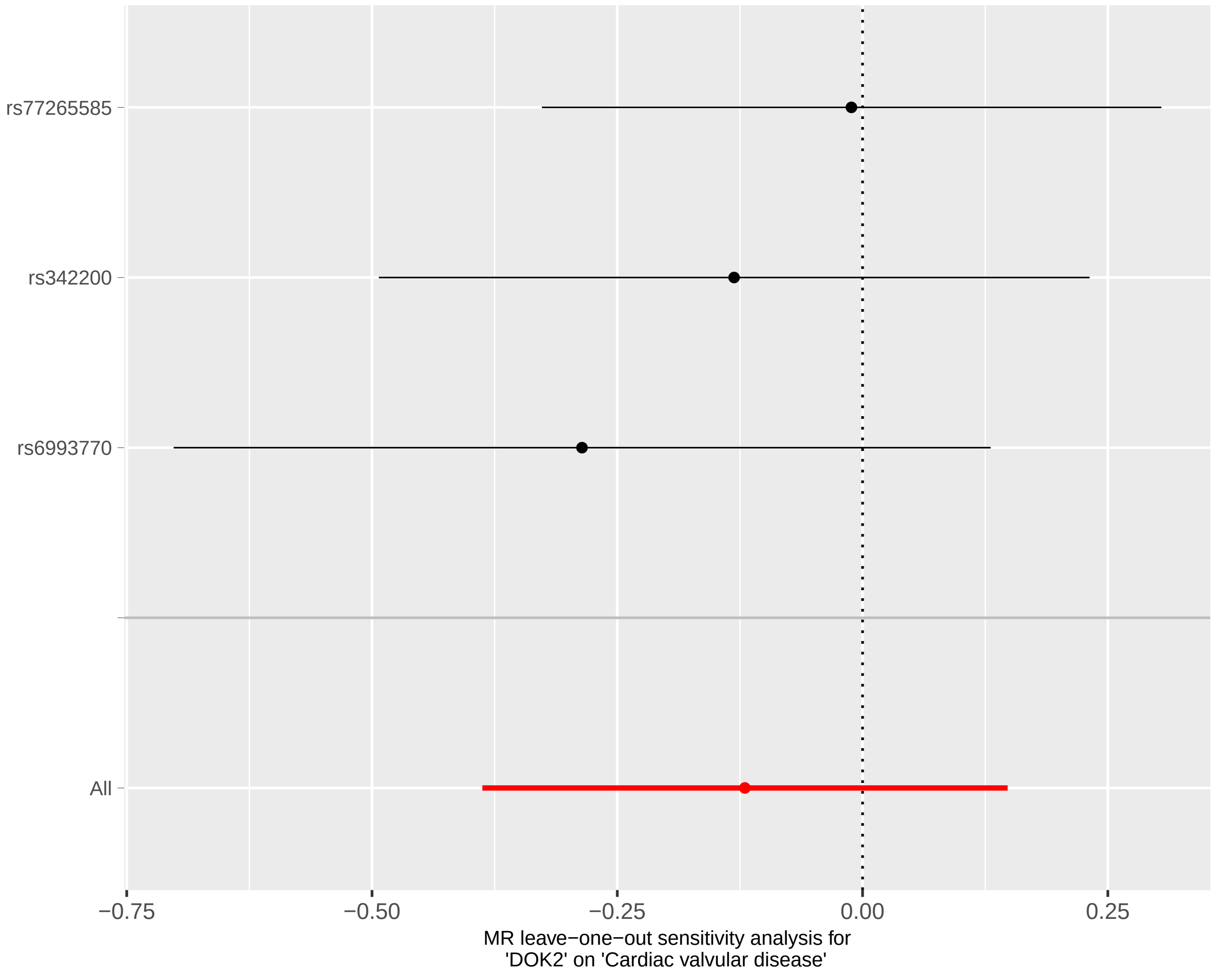
rs77265585
rs342200
rs6993770
All
−0.75
−0.50
−0.25
0.00
0.25
MR leave−one−out sensitivity analysis for
'DOK2' on 'Cardiac valvular disease'

Insufficient number of SNPs

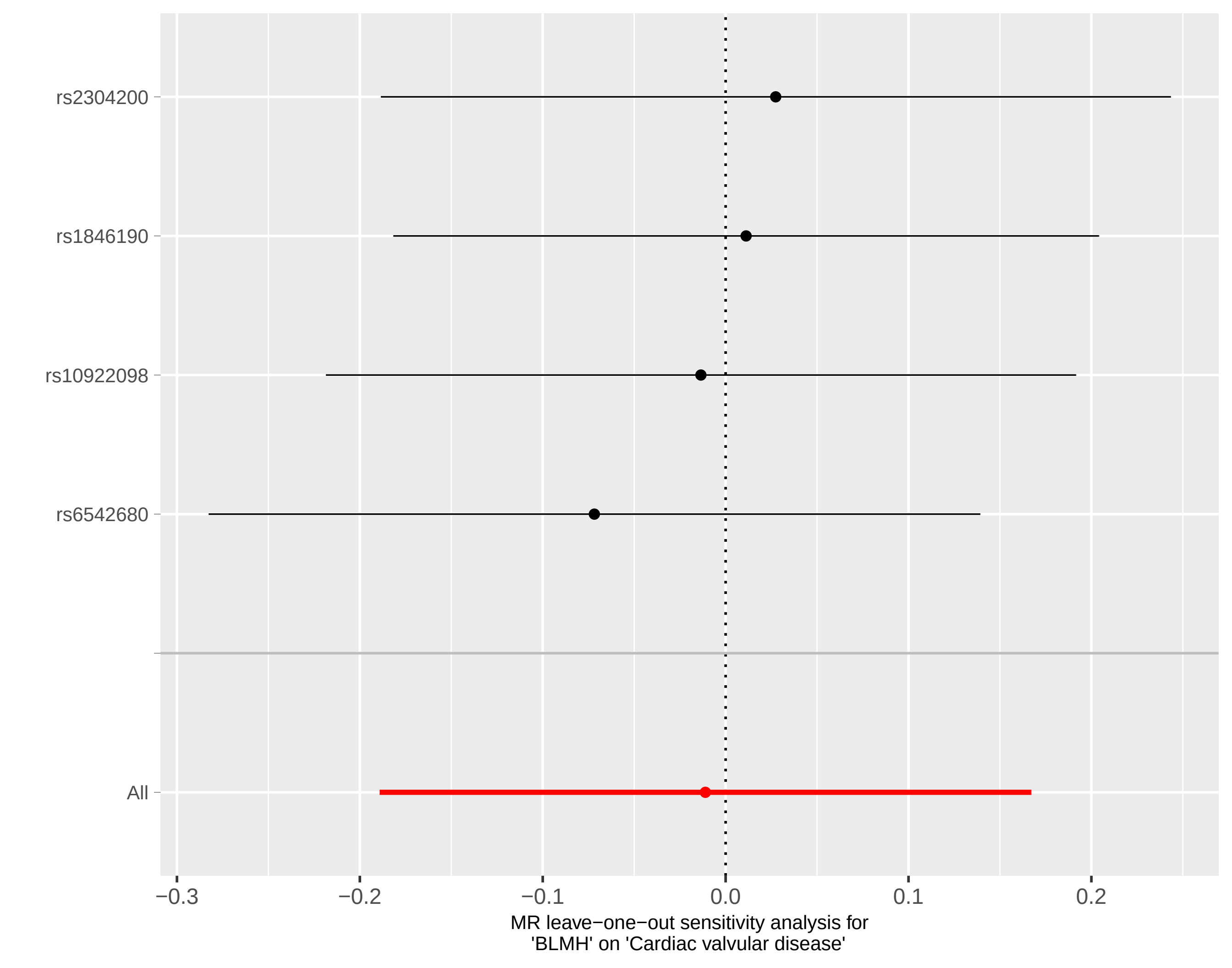

rs2304200
rs1846190
rs10922098
rs6542680
All
−0.3
−0.2
−0.1
0.0
0.1
0.2
MR leave−one−out sensitivity analysis for
'BLMH' on 'Cardiac valvular disease'

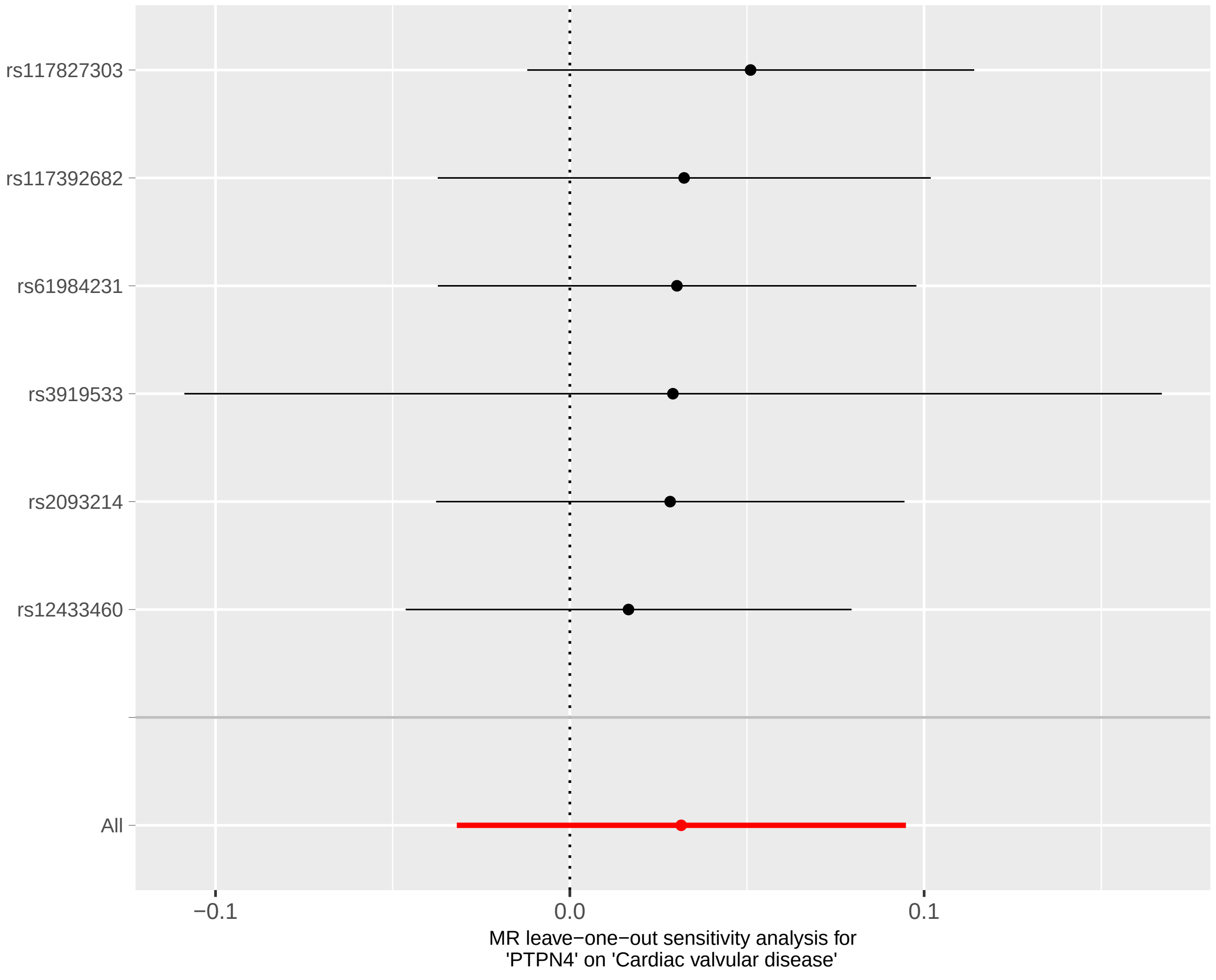
rs117827303
rs117392682
rs61984231
rs3919533
rs2093214
rs12433460
All
−0.1
0.0
0.1
MR leave−one−out sensitivity analysis for
'PTPN4' on 'Cardiac valvular disease'

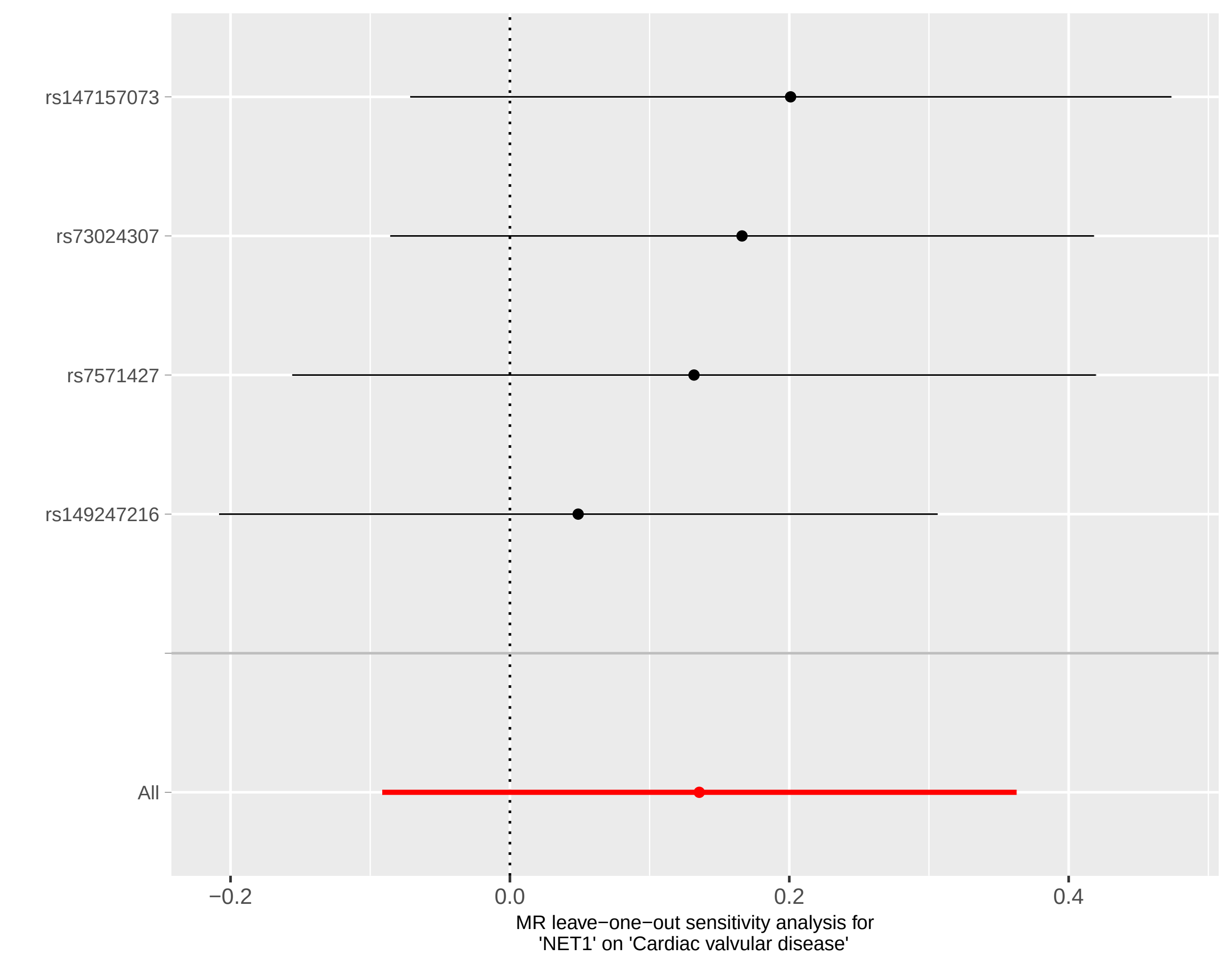
rs147157073
rs73024307
rs7571427
rs149247216
All
−0.2
0.0
0.2
0.4
MR leave−one−out sensitivity analysis for
'NET1' on 'Cardiac valvular disease'

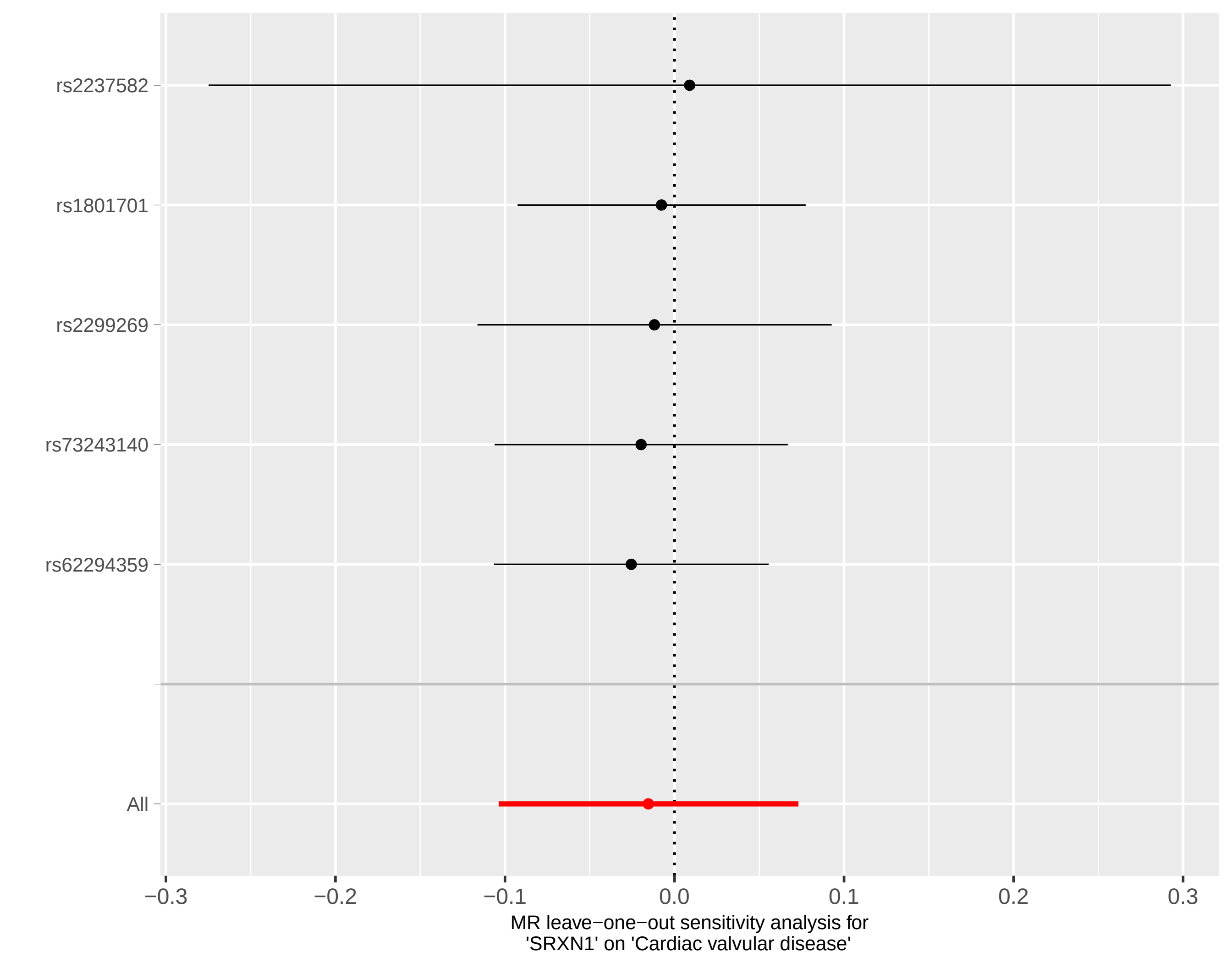

rs2237582
rs1801701
rs2299269
rs73243140
rs62294359
All
−0.3
−0.2
−0.1
0.0
0.1
0.2
0.3
MR leave−one−out sensitivity analysis for
'SRXN1' on 'Cardiac valvular disease'

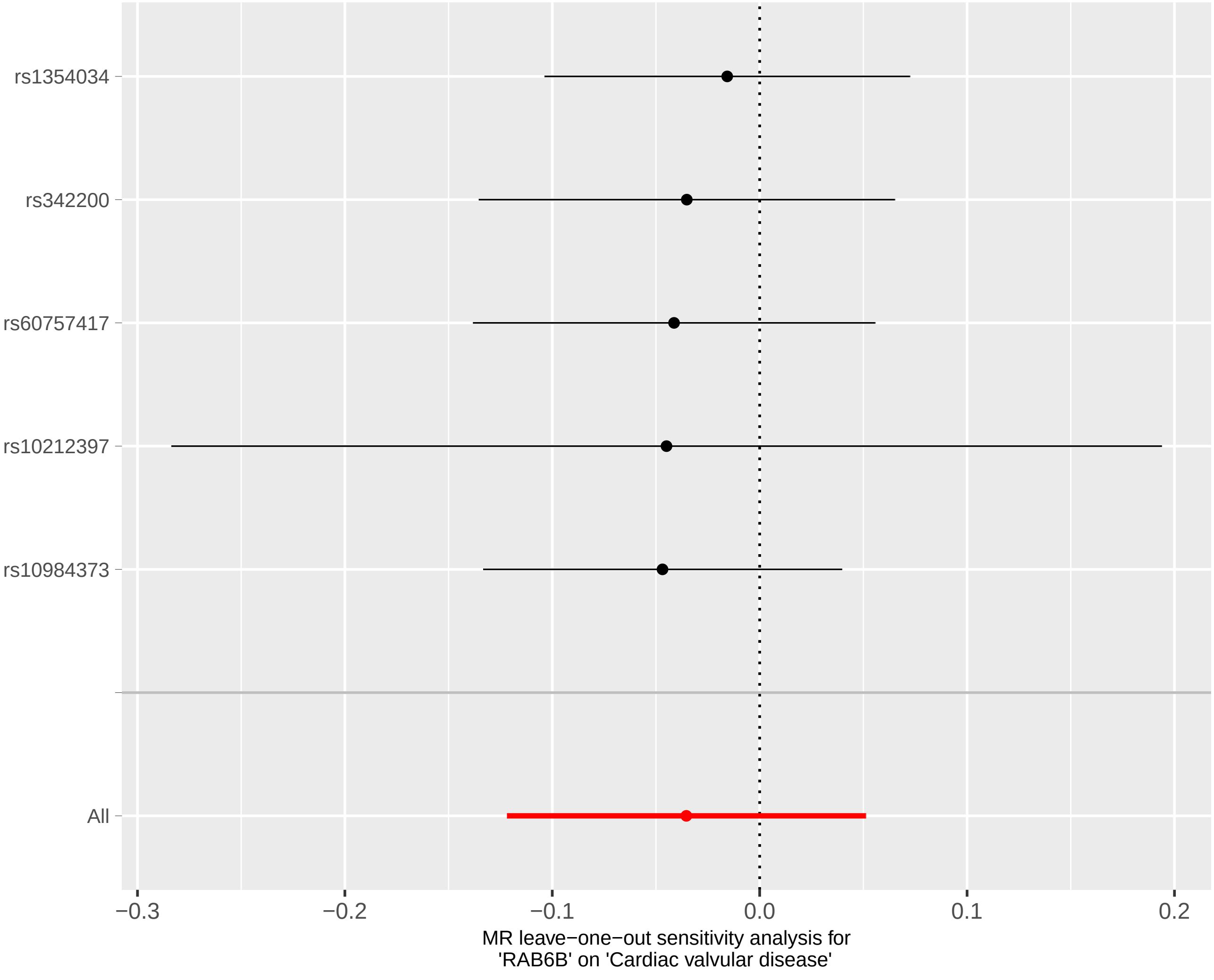
rs1354034
rs342200
rs60757417
rs10212397
rs10984373
All
−0.3
−0.2
−0.1
0.0
0.1
0.2
MR leave−one−out sensitivity analysis for
'RAB6B' on 'Cardiac valvular disease'

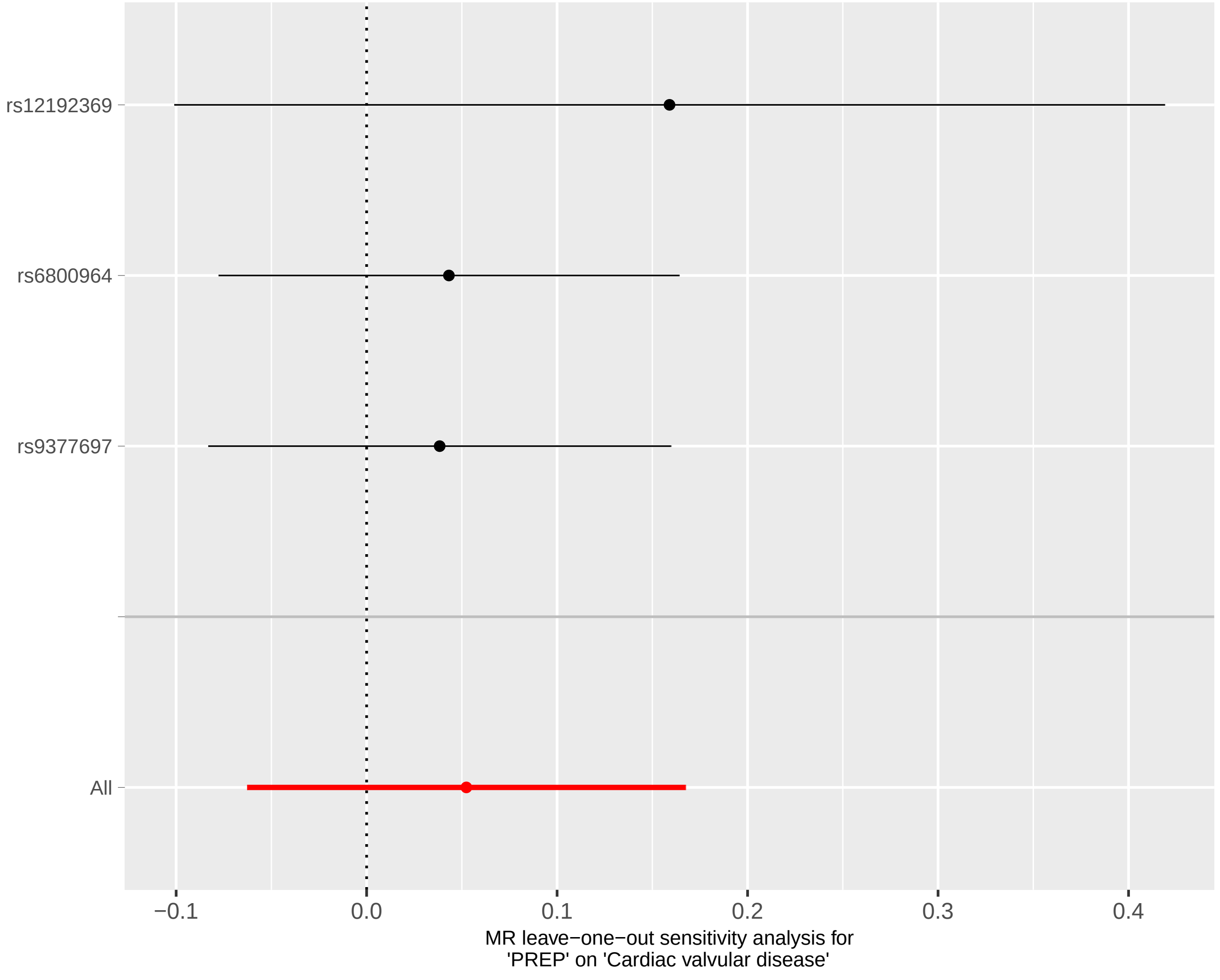
rs12192369
rs6800964
rs9377697
All
−0.1
0.0
0.1
0.2
0.3
0.4
MR leave−one−out sensitivity analysis for
'PREP' on 'Cardiac valvular disease'

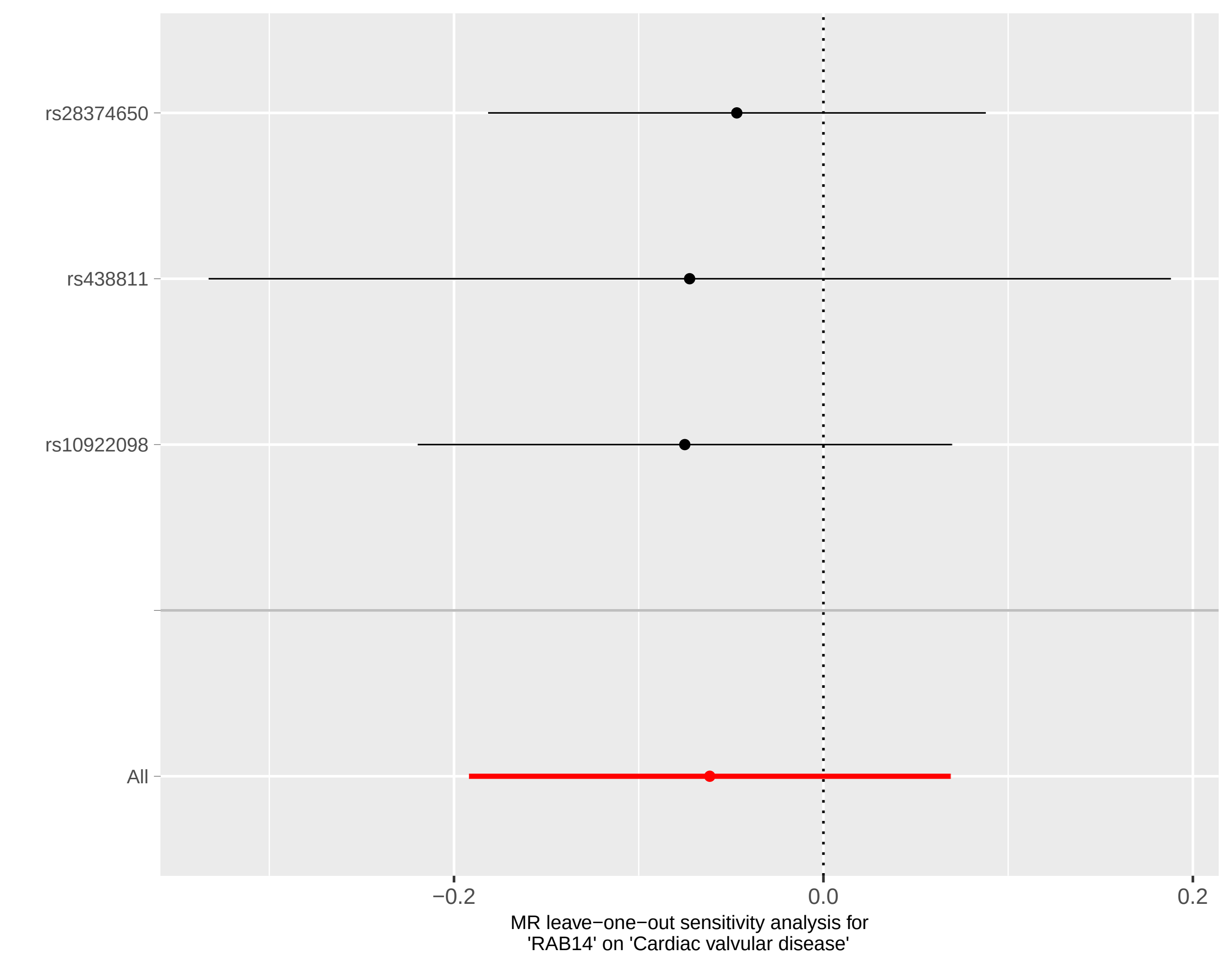

rs28374650
rs438811
rs10922098
All
−0.2
0.0
0.2
MR leave−one−out sensitivity analysis for
'RAB14' on 'Cardiac valvular disease'

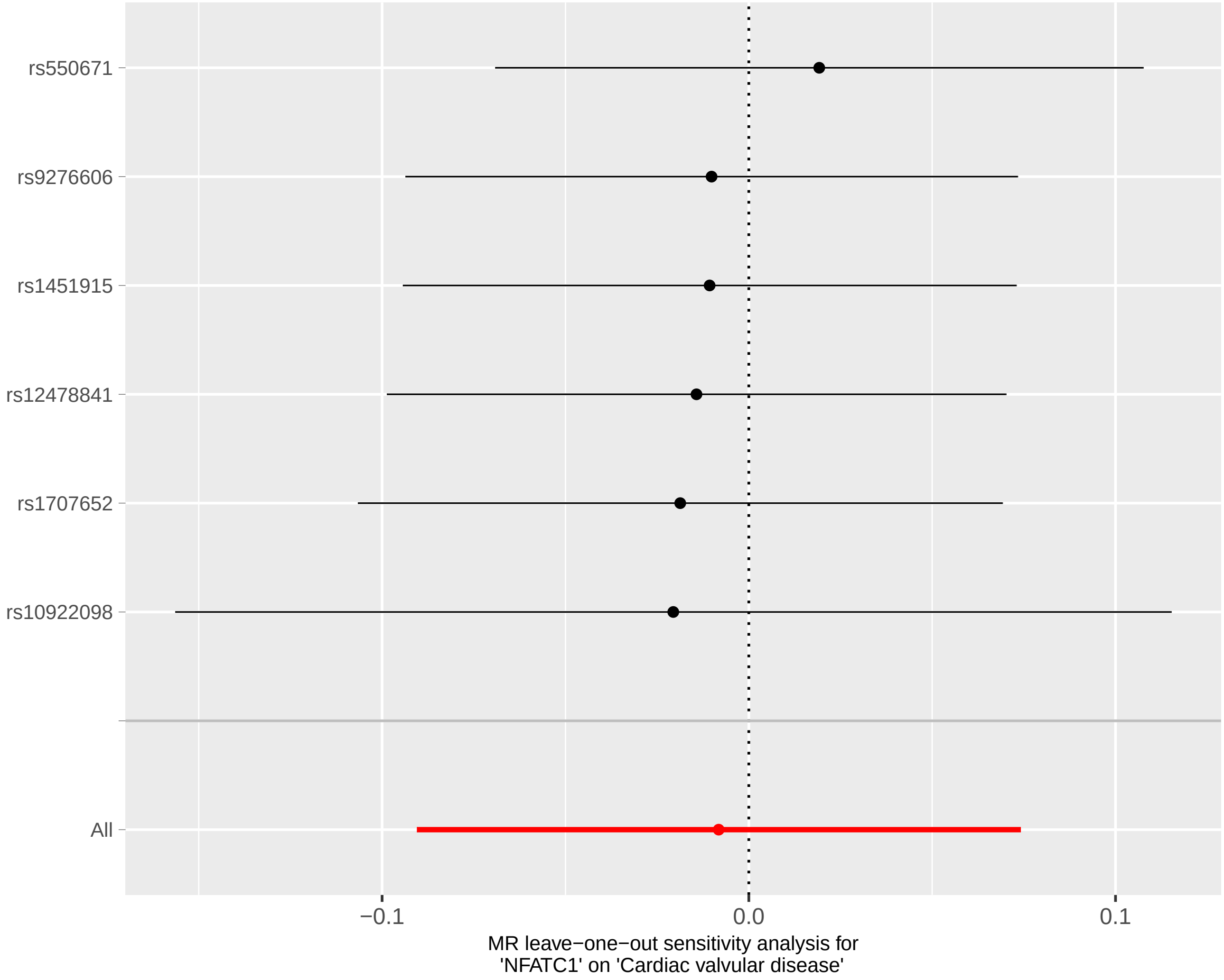
rs550671
rs9276606
rs1451915
rs12478841
rs1707652
rs10922098
All
−0.1
0.0
0.1
MR leave−one−out sensitivity analysis for
'NFATC1' on 'Cardiac valvular disease'

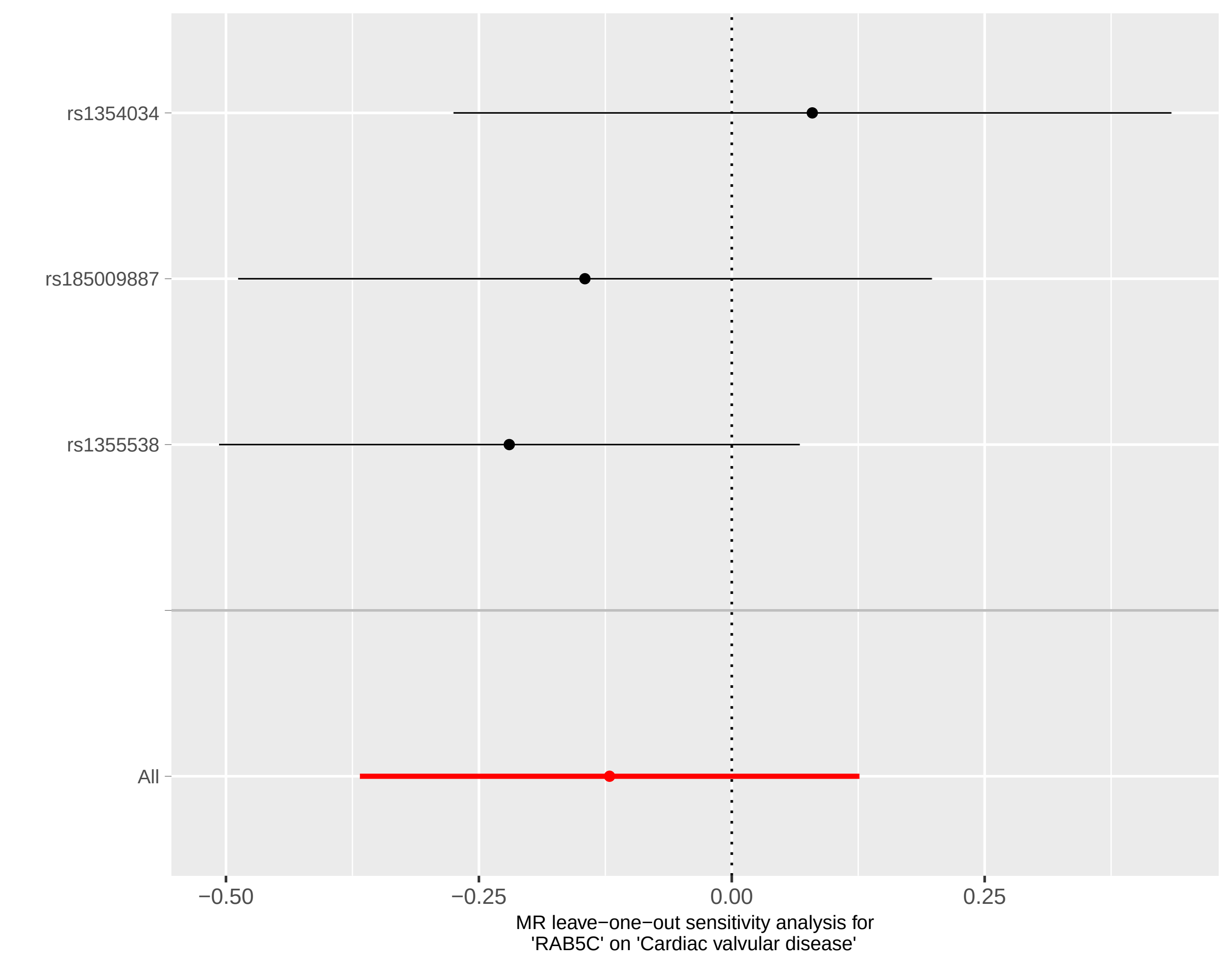
rs1354034
rs185009887
rs1355538
All
−0.50
−0.25
0.00
0.25
MR leave−one−out sensitivity analysis for
'RAB5C' on 'Cardiac valvular disease'

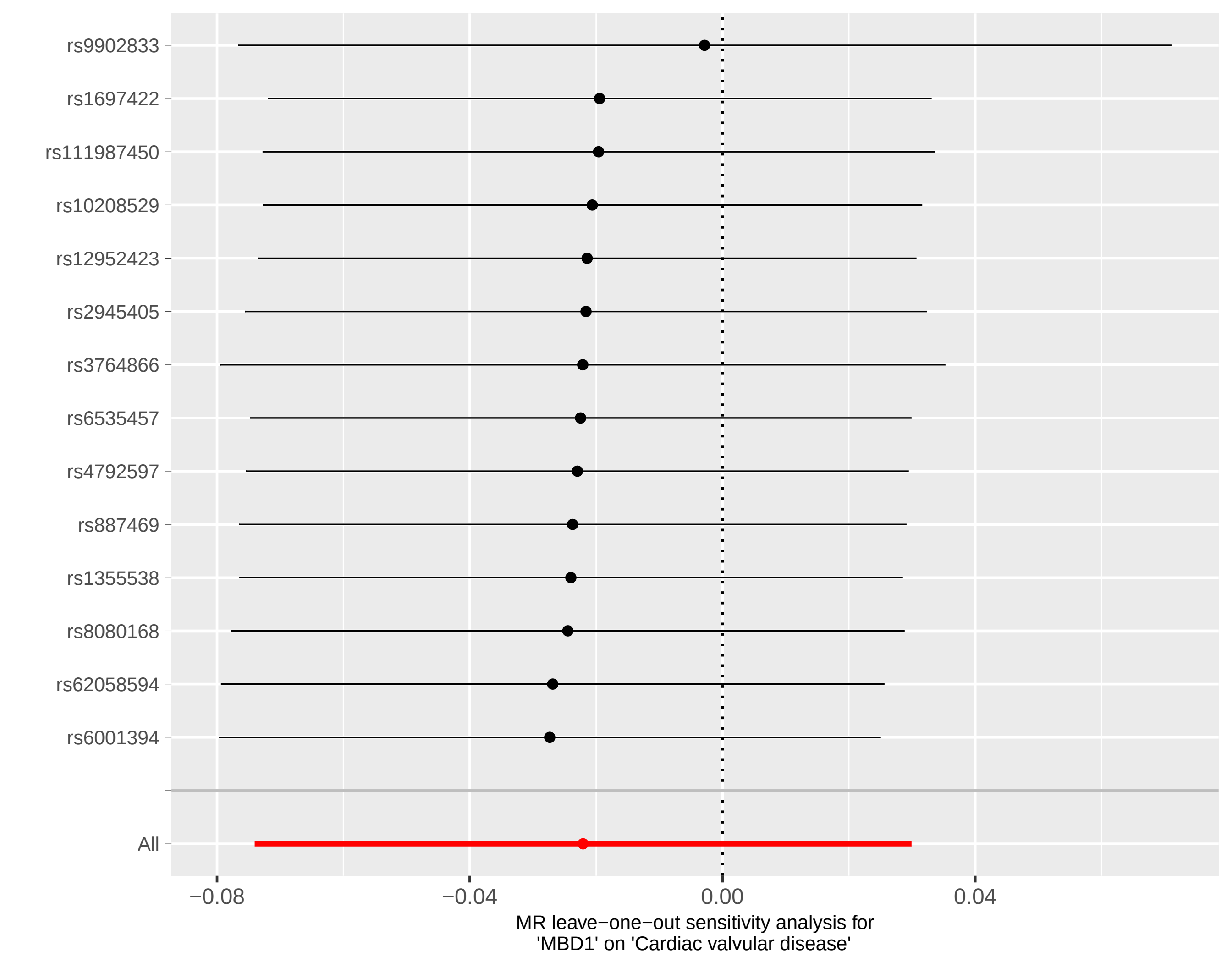
rs9902833
rs1697422
rs111987450
rs10208529
rs12952423
rs2945405
rs3764866
rs6535457
rs4792597
rs887469
rs1355538
rs8080168
rs62058594
rs6001394
All
−0.08
−0.04
0.00
0.04
MR leave−one−out sensitivity analysis for
'MBD1' on 'Cardiac valvular disease'

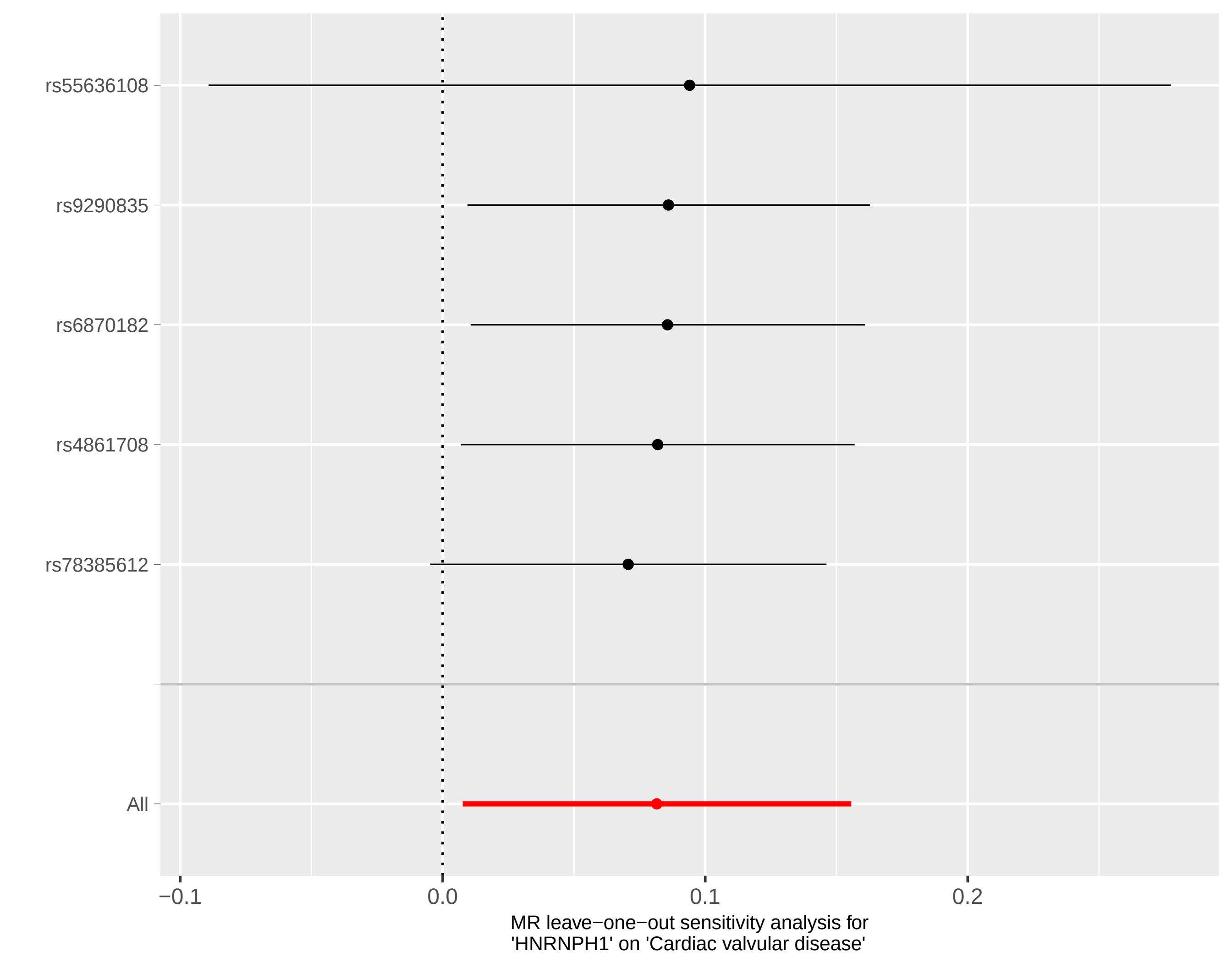
rs55636108
rs9290835
rs6870182
rs4861708
rs78385612
All
−0.1
0.0
0.1
0.2
MR leave−one−out sensitivity analysis for
'HNRNPH1' on 'Cardiac valvular disease'

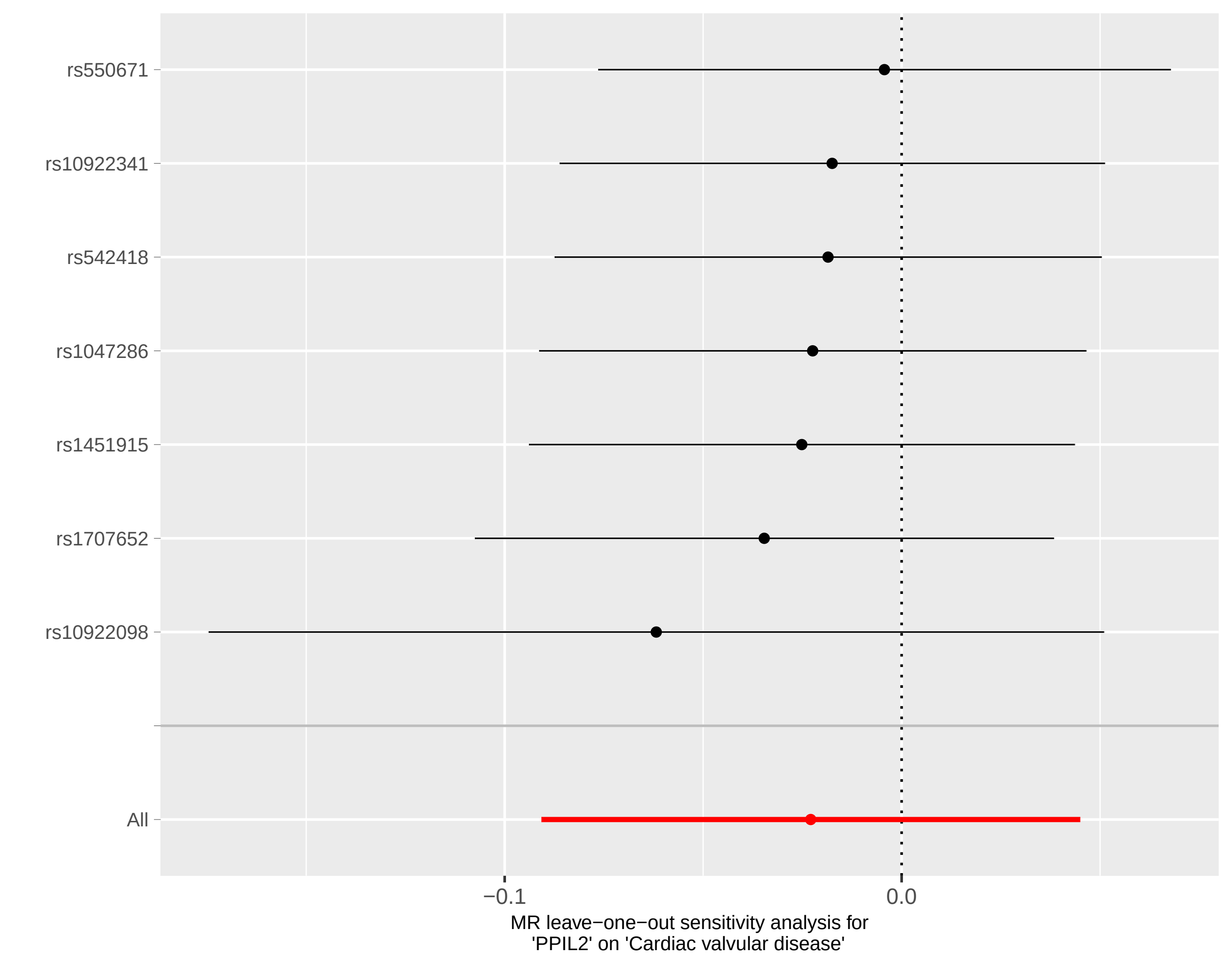
rs550671
rs10922341
rs542418
rs1047286
rs1451915
rs1707652
rs10922098
All
−0.1
0.0
MR leave−one−out sensitivity analysis for
'PPIL2' on 'Cardiac valvular disease'

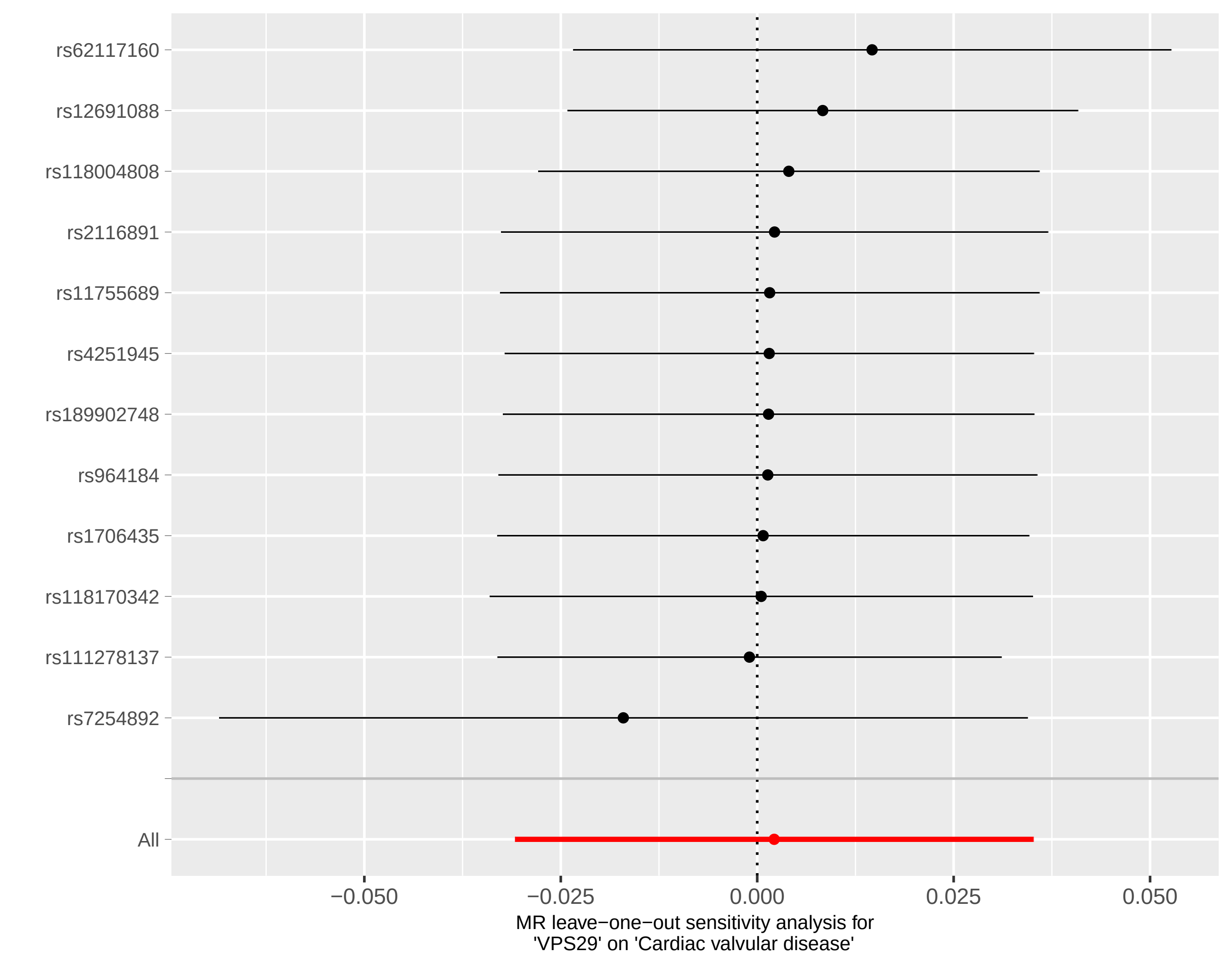
rs62117160
rs12691088
rs118004808
rs2116891
rs11755689
rs4251945
rs189902748
rs964184
rs1706435
rs118170342
rs111278137
rs7254892
All
−0.050
−0.025
0.000
0.025
0.050
MR leave−one−out sensitivity analysis for
'VPS29' on 'Cardiac valvular disease'

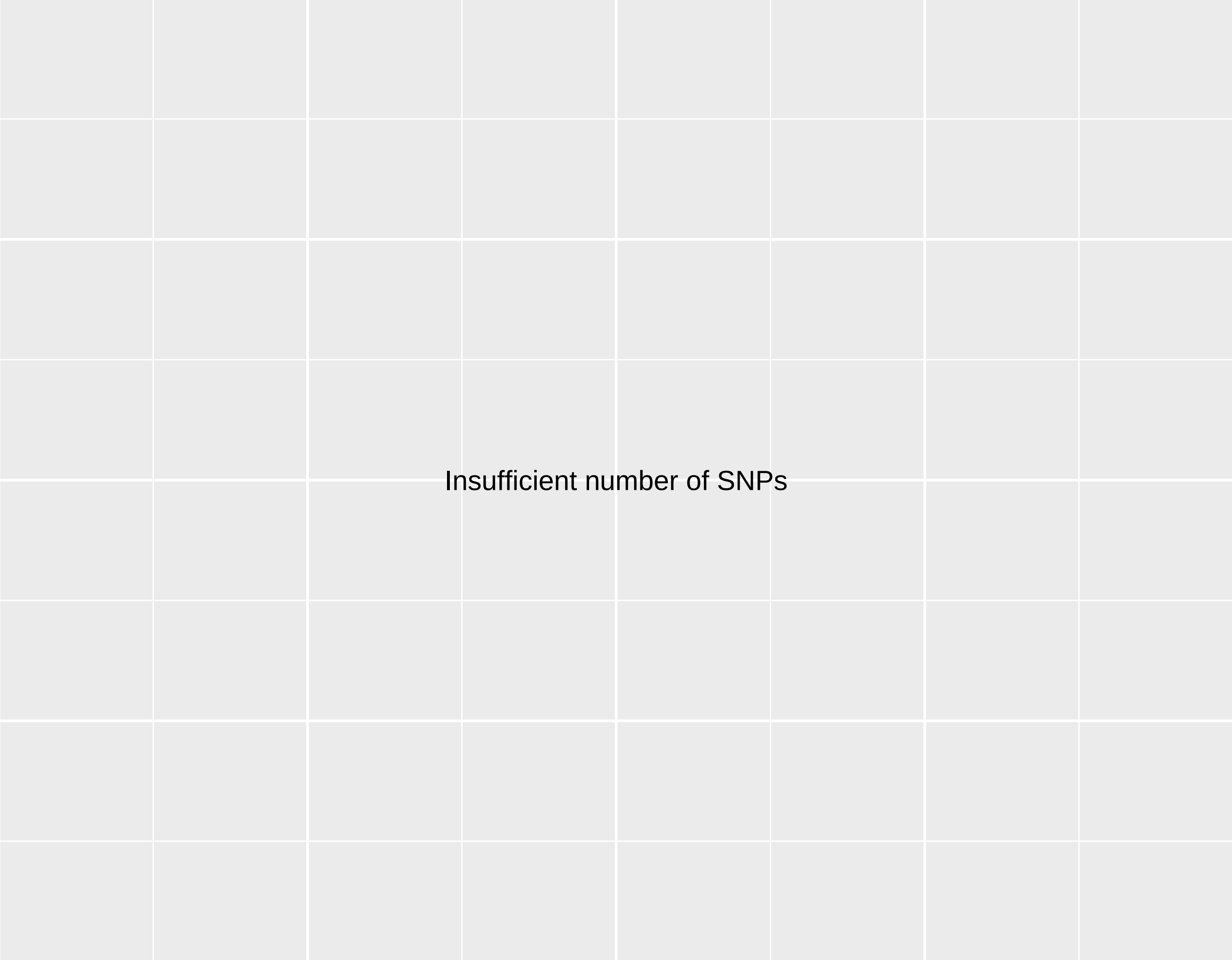
Insufficient number of SNPs

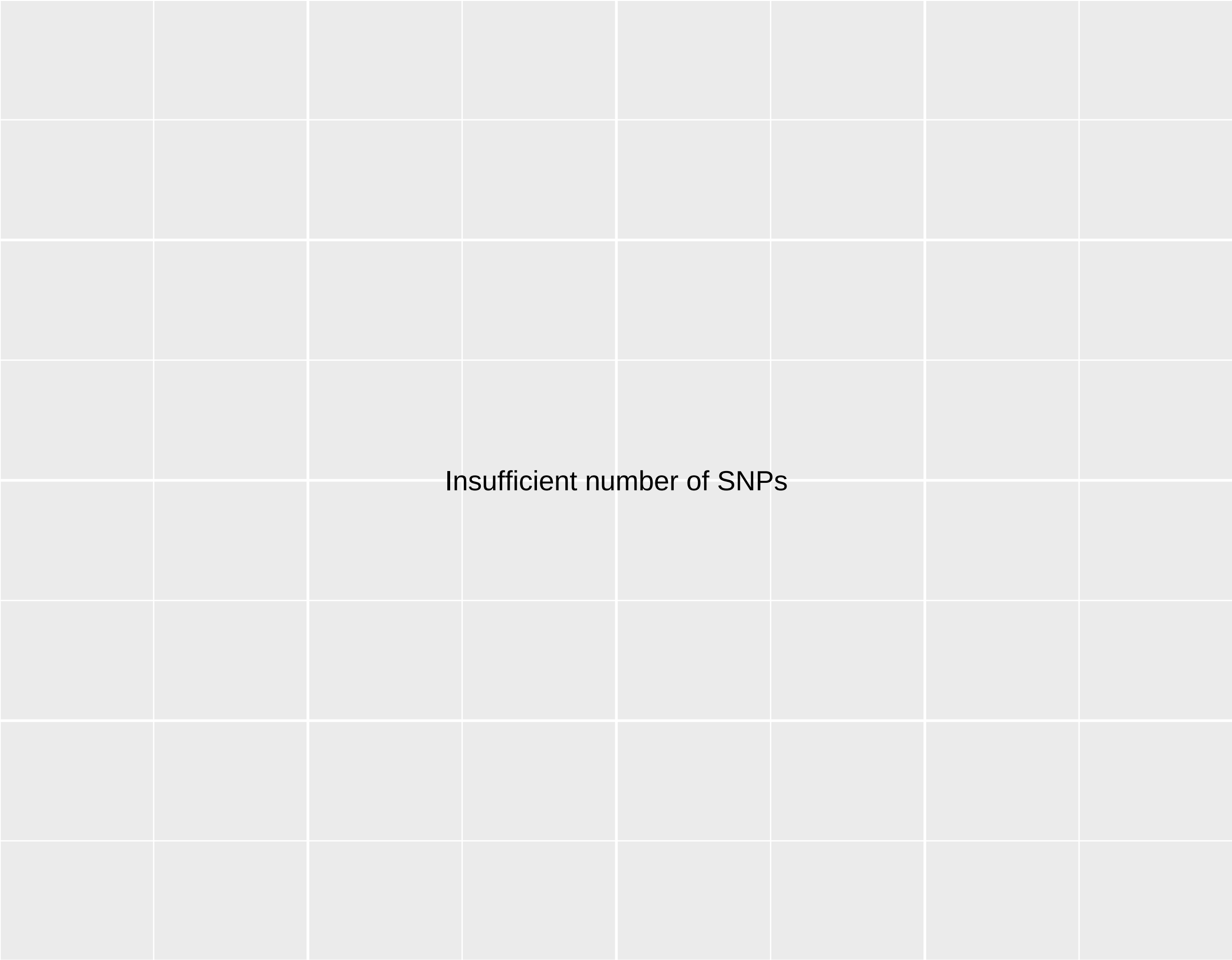
Insufficient number of SNPs

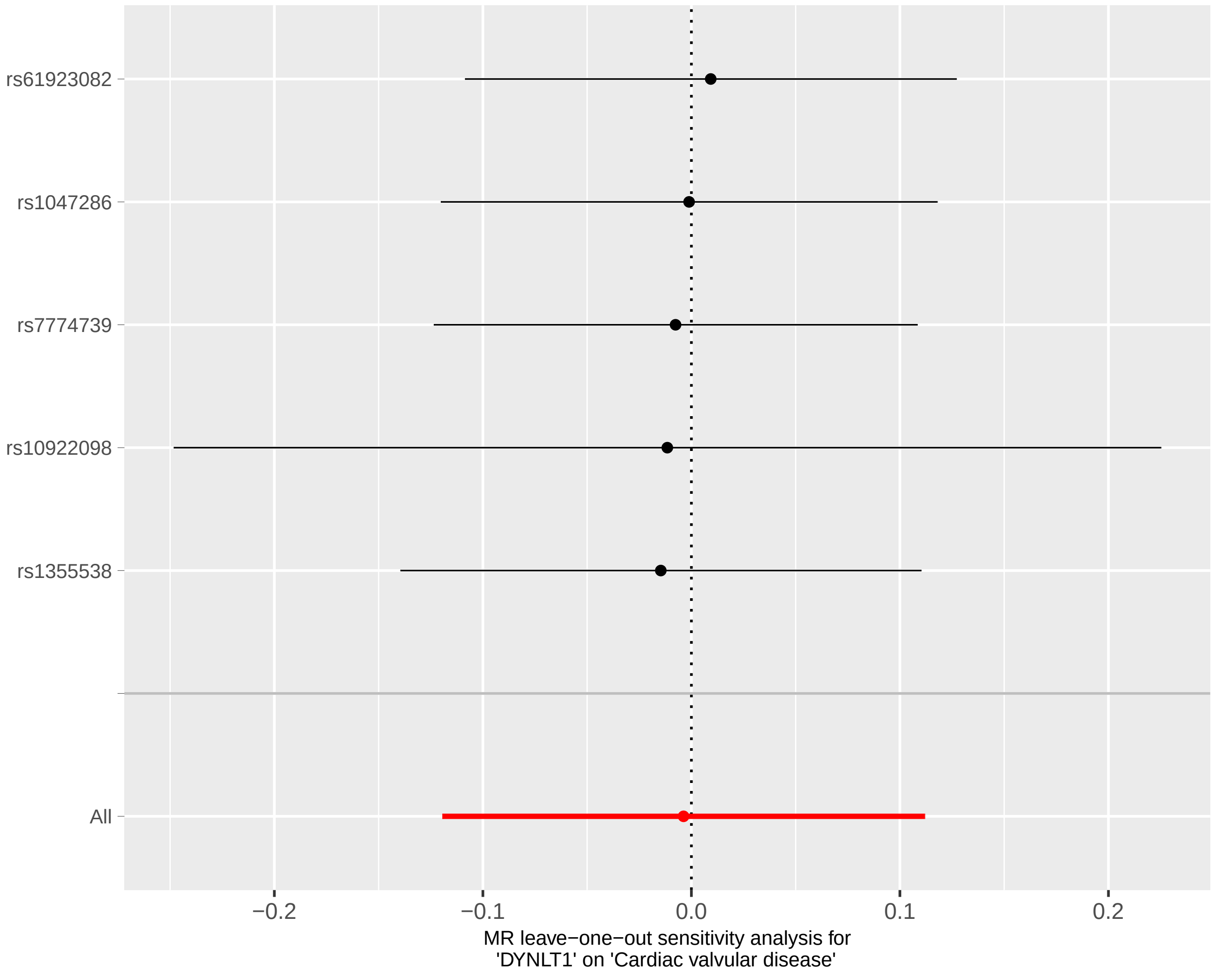
rs61923082
rs1047286
rs7774739
rs10922098
rs1355538
All
−0.2
−0.1
0.0
0.1
0.2
MR leave−one−out sensitivity analysis for
'DYNLT1' on 'Cardiac valvular disease'

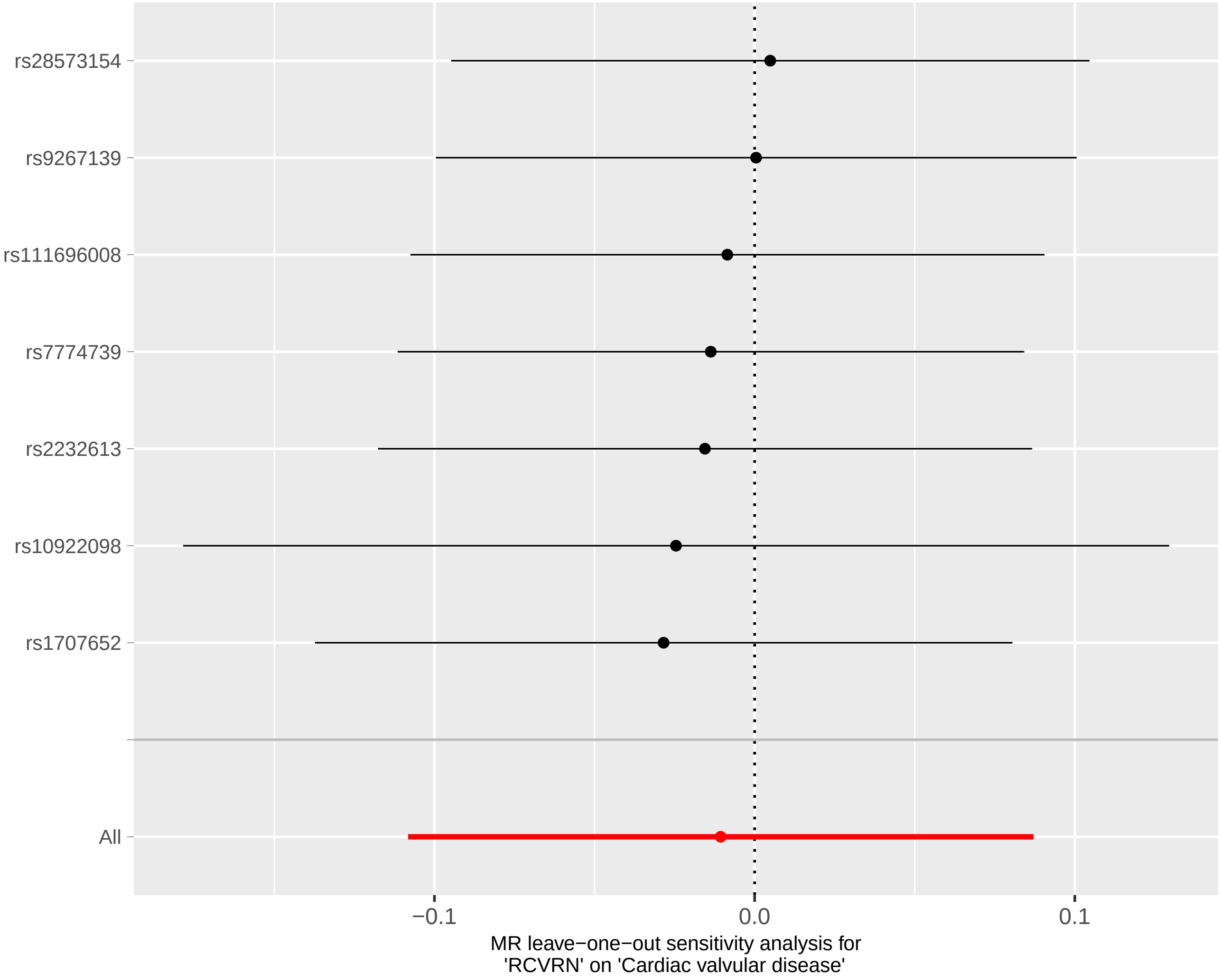

rs28573154
rs9267139
rs111696008
rs7774739
rs2232613
rs10922098
rs1707652
All
−0.1
0.0
0.1
MR leave−one−out sensitivity analysis for
'RCVRN' on 'Cardiac valvular disease'

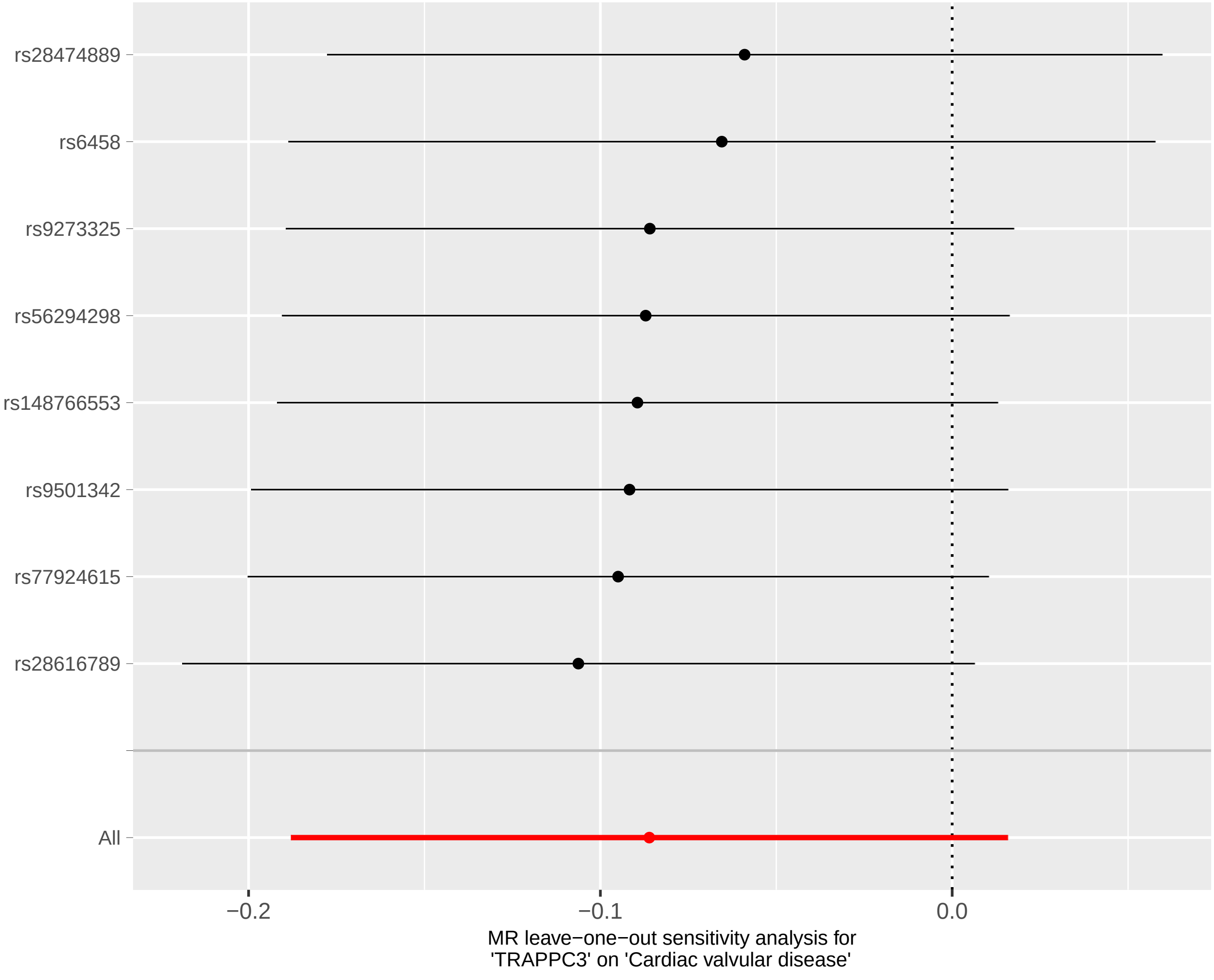
rs28474889
rs6458
rs9273325
rs56294298
rs148766553
rs9501342
rs77924615
rs28616789
All
−0.2
−0.1
0.0
MR leave−one−out sensitivity analysis for
'TRAPPC3' on 'Cardiac valvular disease'

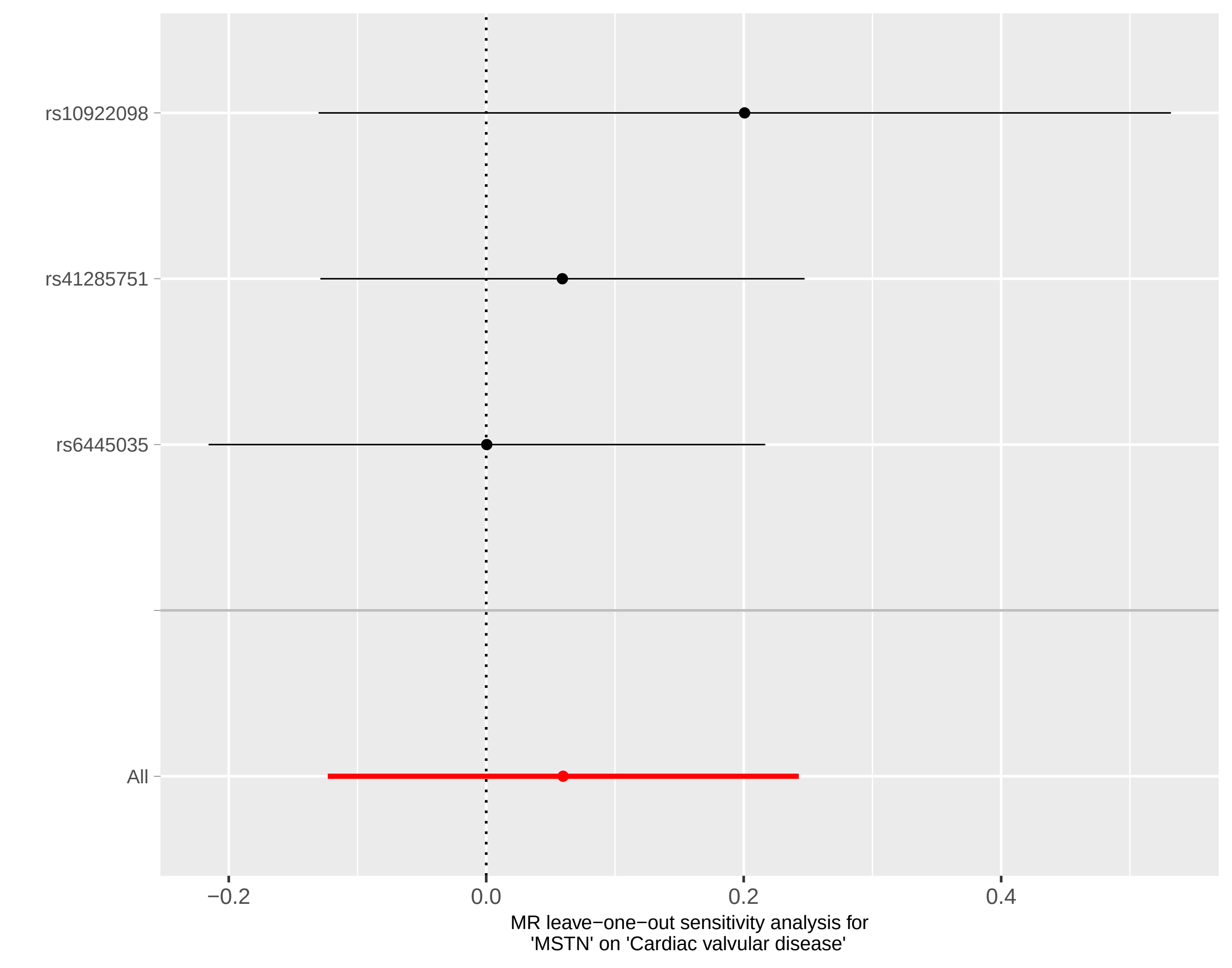
rs10922098
rs41285751
rs6445035
All
−0.2
0.0
0.2
0.4
MR leave−one−out sensitivity analysis for
'MSTN' on 'Cardiac valvular disease'

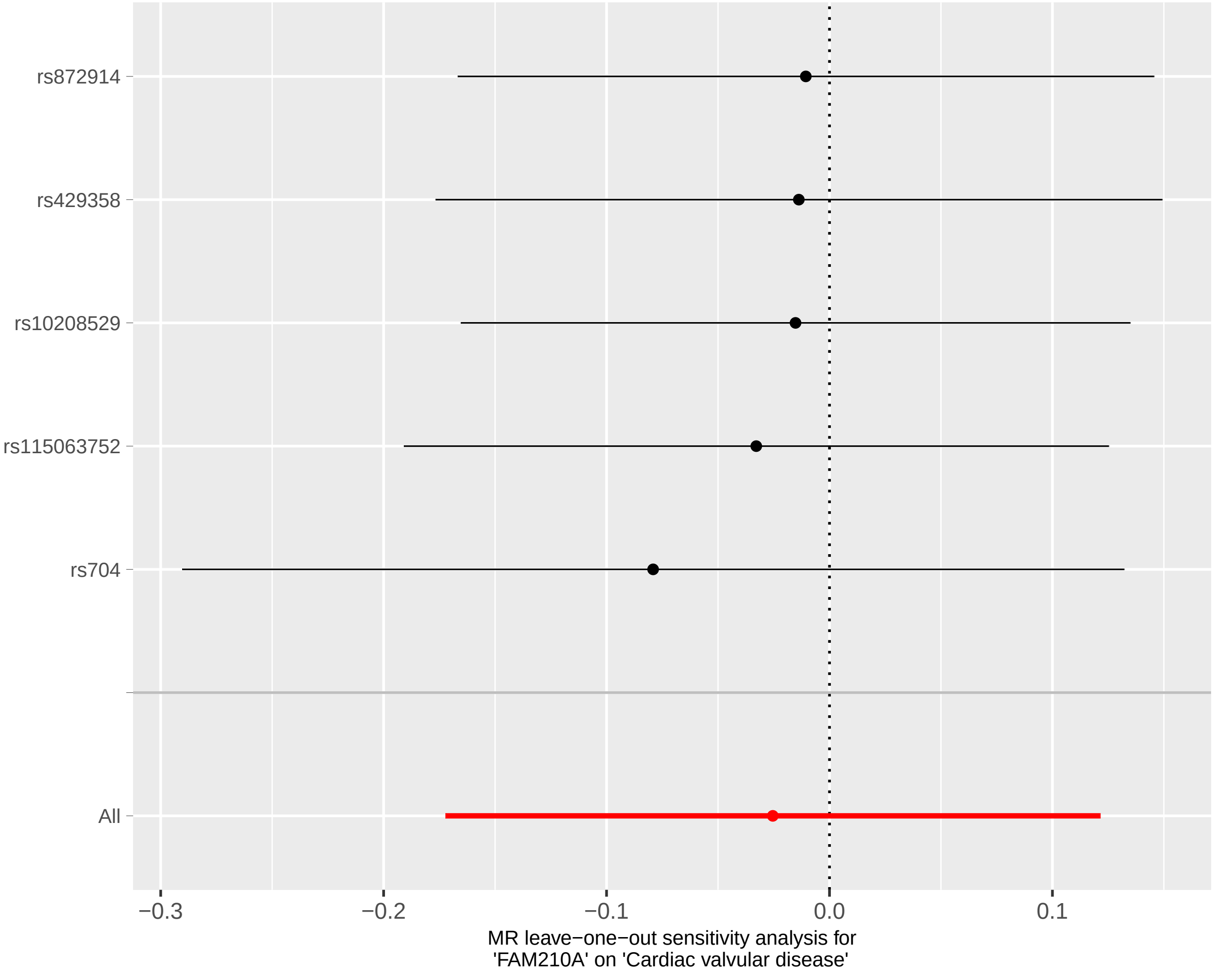

rs872914
rs429358
rs10208529
rs115063752
rs704
All
−0.3
−0.2
−0.1
0.0
0.1
MR leave−one−out sensitivity analysis for
'FAM210A' on 'Cardiac valvular disease'

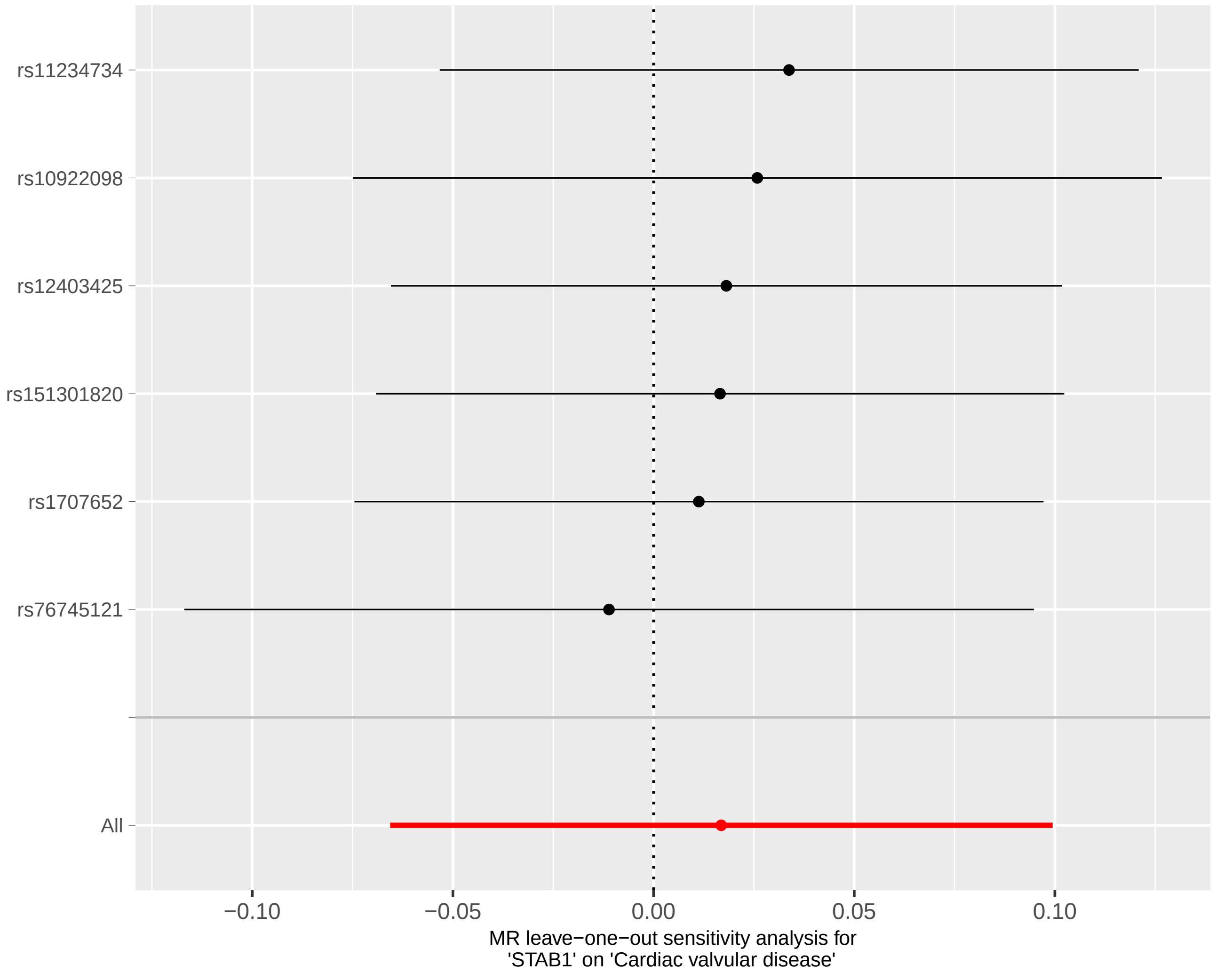
rs11234734
rs10922098
rs12403425
rs151301820
rs1707652
rs76745121
All
−0.10
−0.05
0.00
0.05
0.10
MR leave−one−out sensitivity analysis for
'STAB1' on 'Cardiac valvular disease'

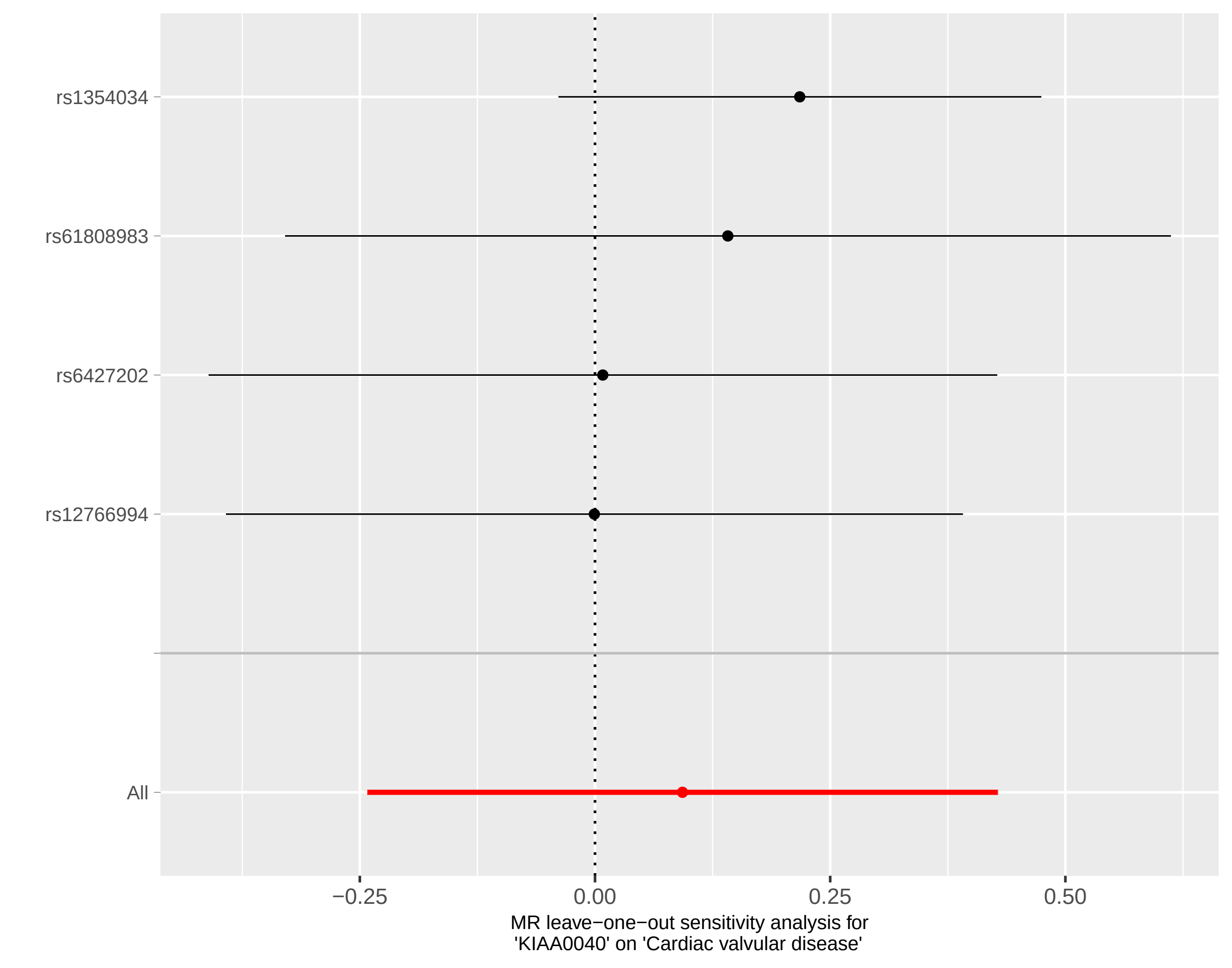

rs1354034
rs61808983
rs6427202
rs12766994
All
−0.25
0.00
0.25
0.50
MR leave−one−out sensitivity analysis for
'KIAA0040' on 'Cardiac valvular disease'

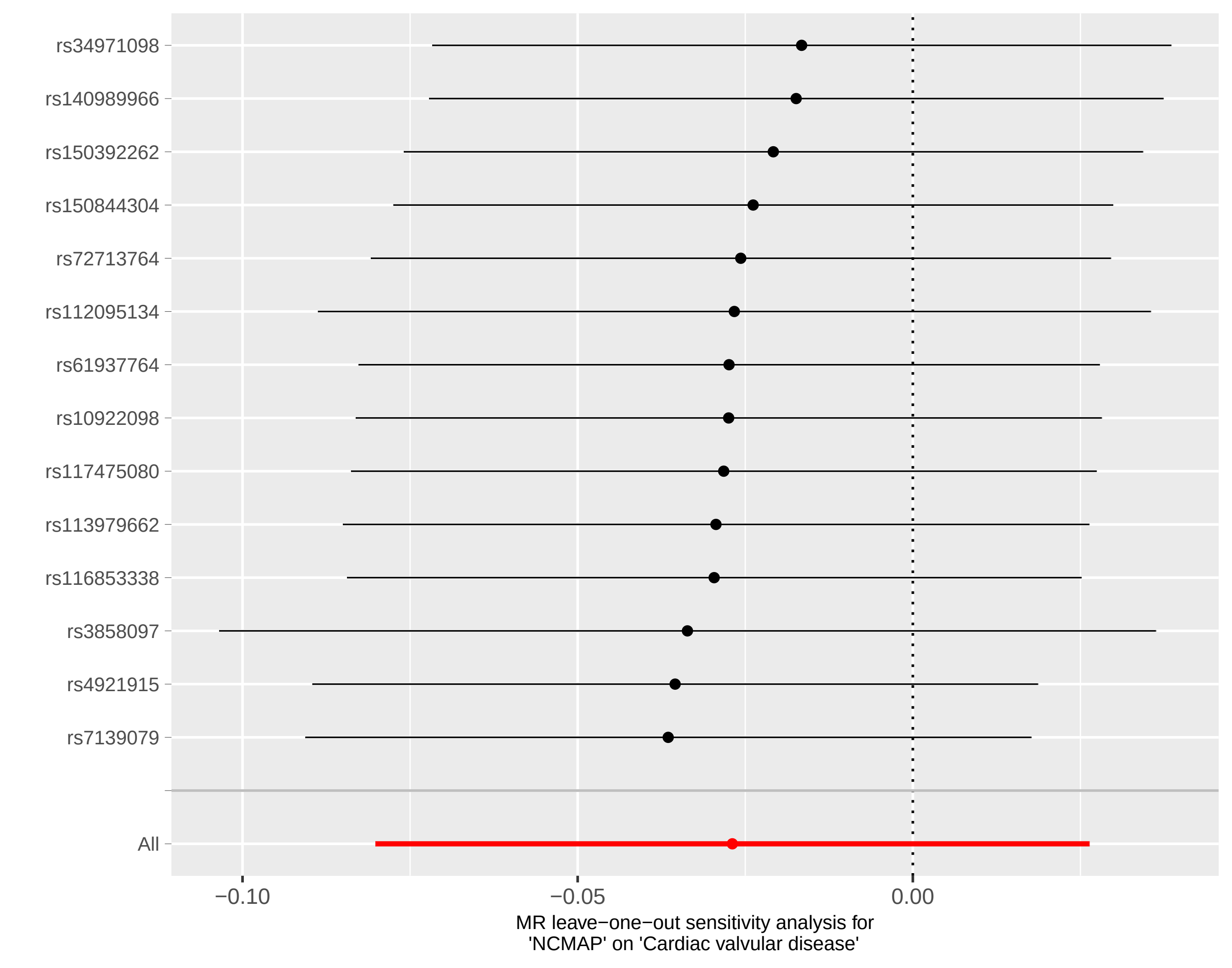
rs34971098
rs140989966
rs150392262
rs150844304
rs72713764
rs112095134
rs61937764
rs10922098
rs117475080
rs113979662
rs116853338
rs3858097
rs4921915
rs7139079
All
−0.10
−0.05
0.00
MR leave−one−out sensitivity analysis for
'NCMAP' on 'Cardiac valvular disease'

Insufficient number of SNPs

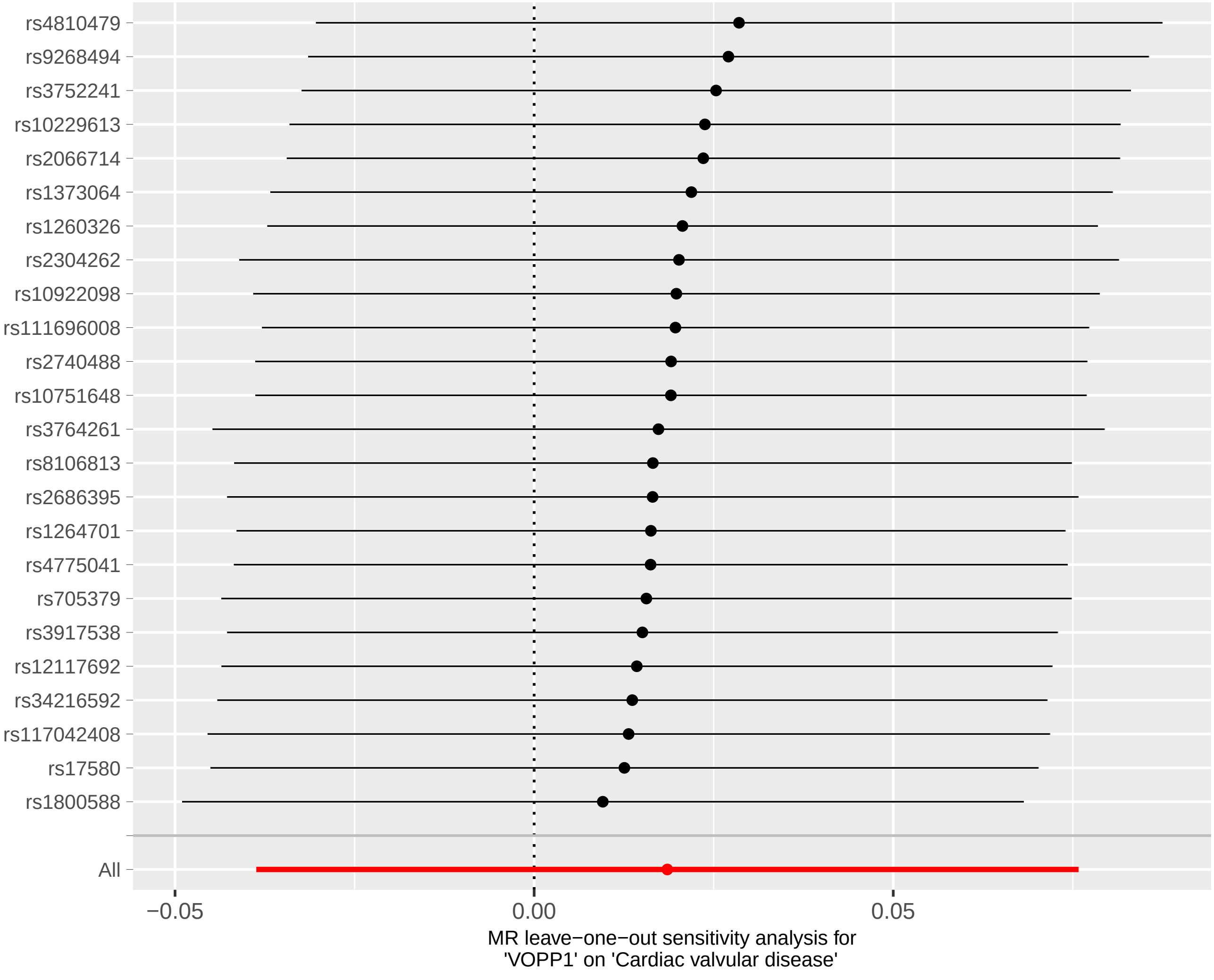
rs4810479
rs9268494
rs3752241
rs10229613
rs2066714
rs1373064
rs1260326
rs2304262
rs10922098
rs111696008
rs2740488
rs10751648
rs3764261
rs8106813
rs2686395
rs1264701
rs4775041
rs705379
rs3917538
rs12117692
rs34216592
rs117042408
rs17580
rs1800588
All
−0.05
0.00
0.05
MR leave−one−out sensitivity analysis for
'VOPP1' on 'Cardiac valvular disease'

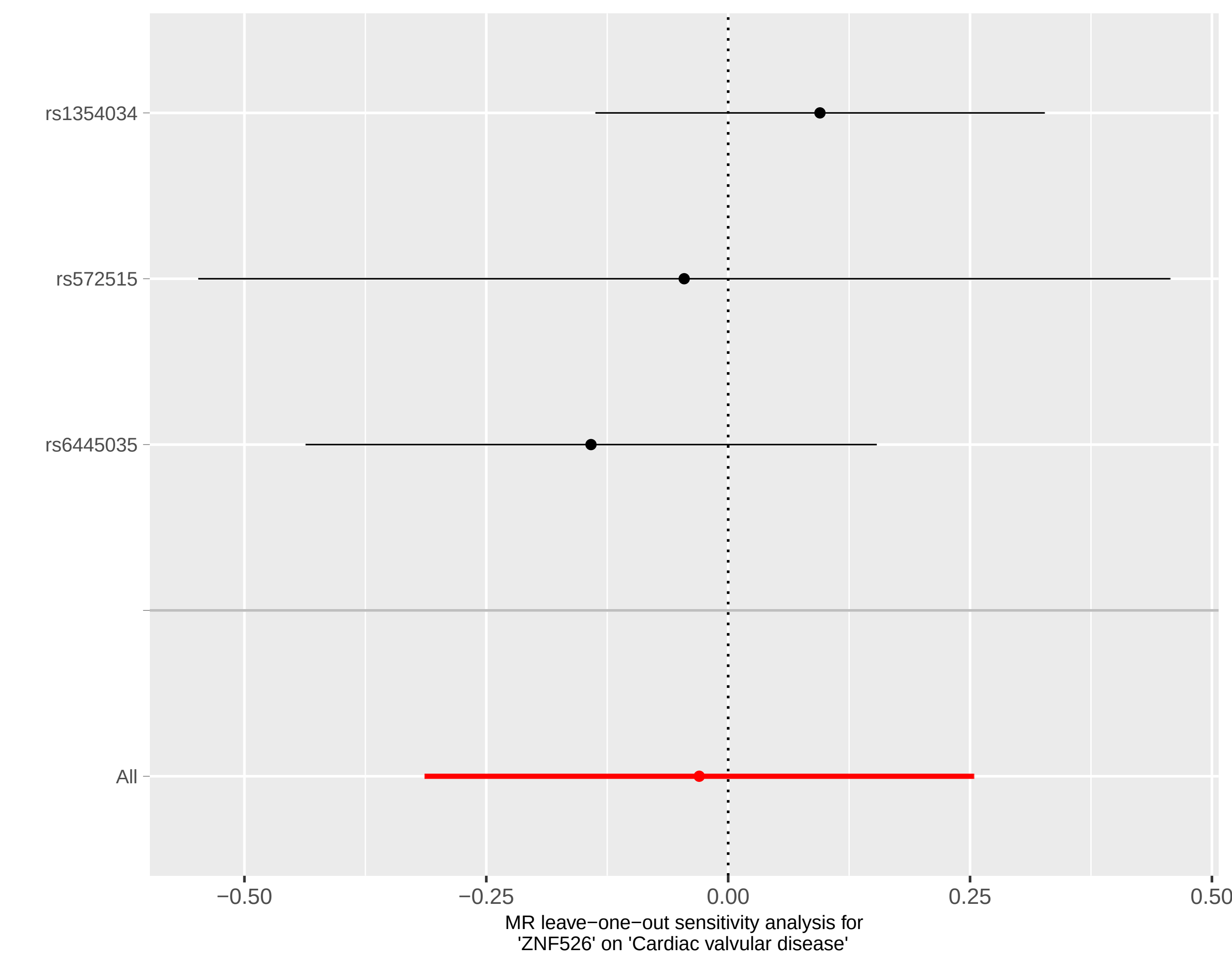
rs1354034
rs572515
rs6445035
All
−0.50
−0.25
0.00
0.25
0.50
MR leave−one−out sensitivity analysis for
'ZNF526' on 'Cardiac valvular disease'

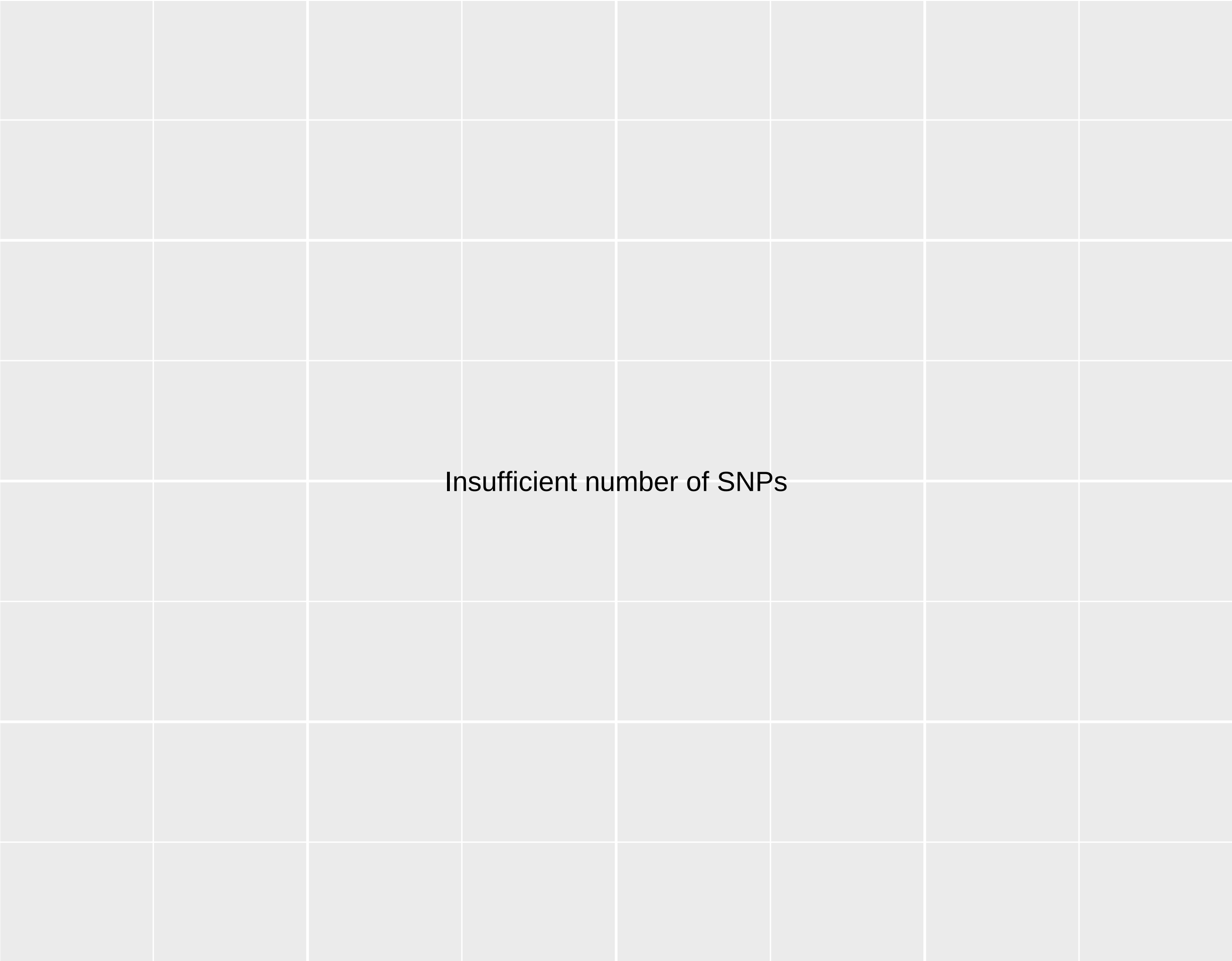
Insufficient number of SNPs

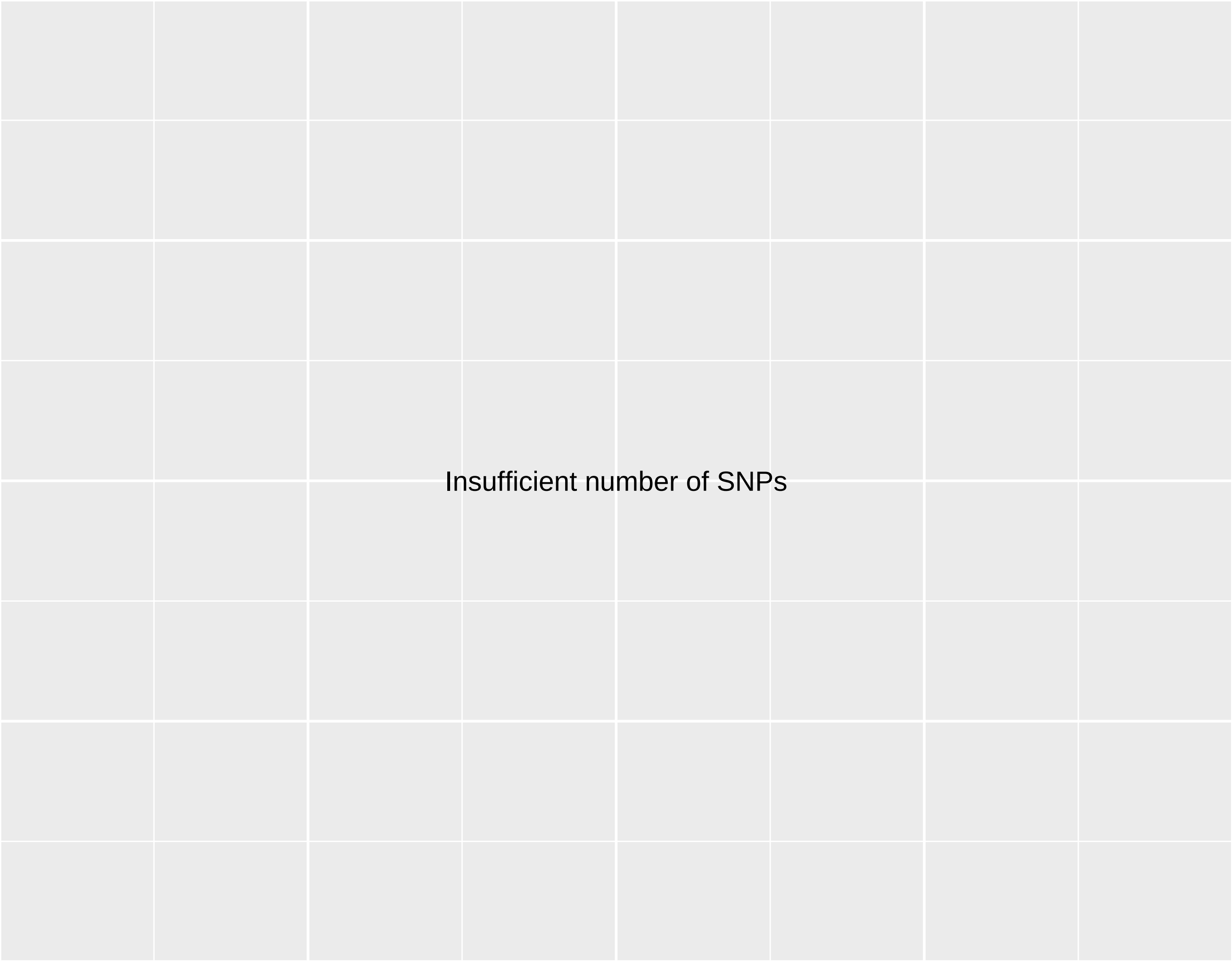
Insufficient number of SNPs

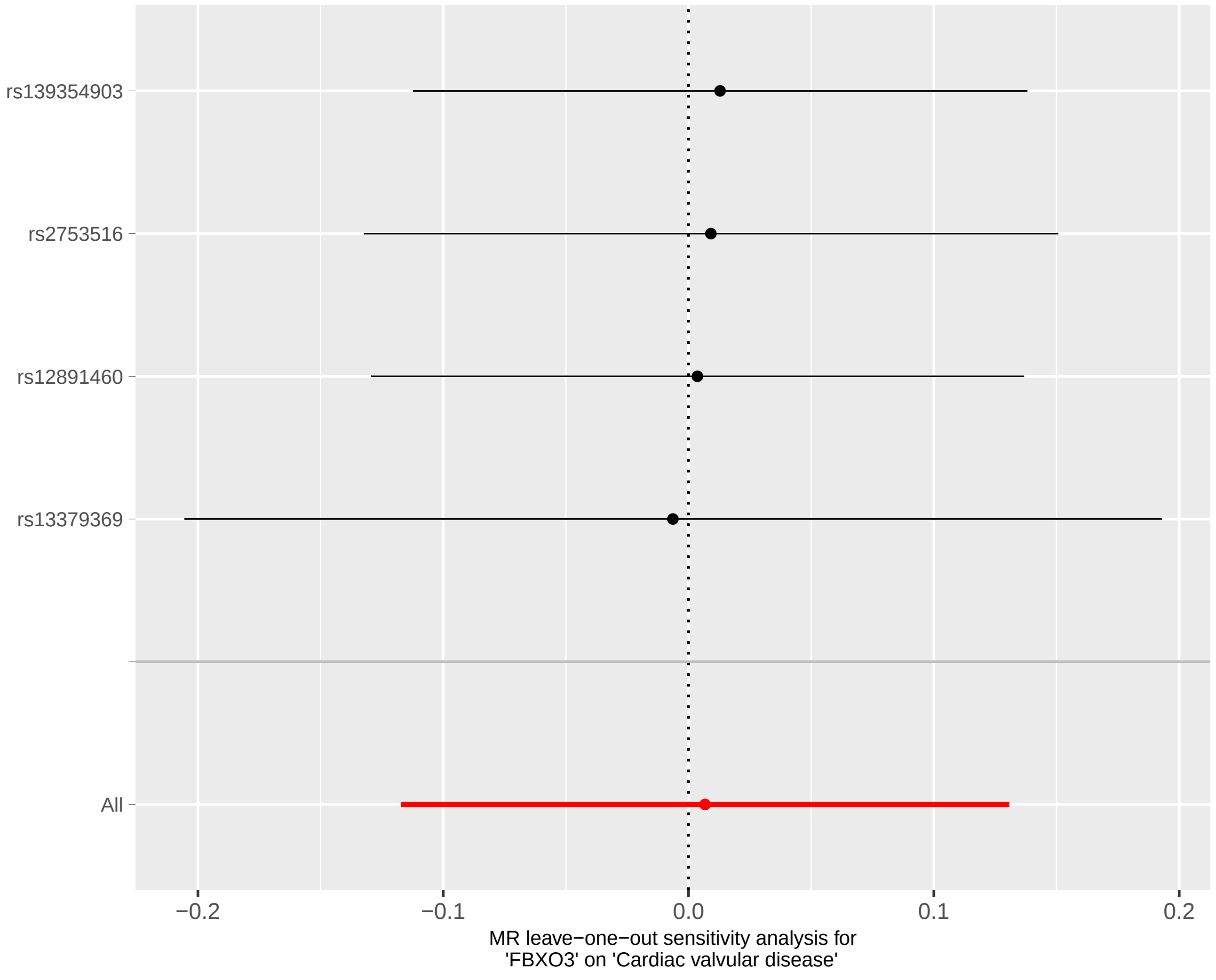

rs139354903
rs2753516
rs12891460
rs13379369
All
−0.2
−0.1
0.0
0.1
0.2
MR leave−one−out sensitivity analysis for
'FBXO3' on 'Cardiac valvular disease'

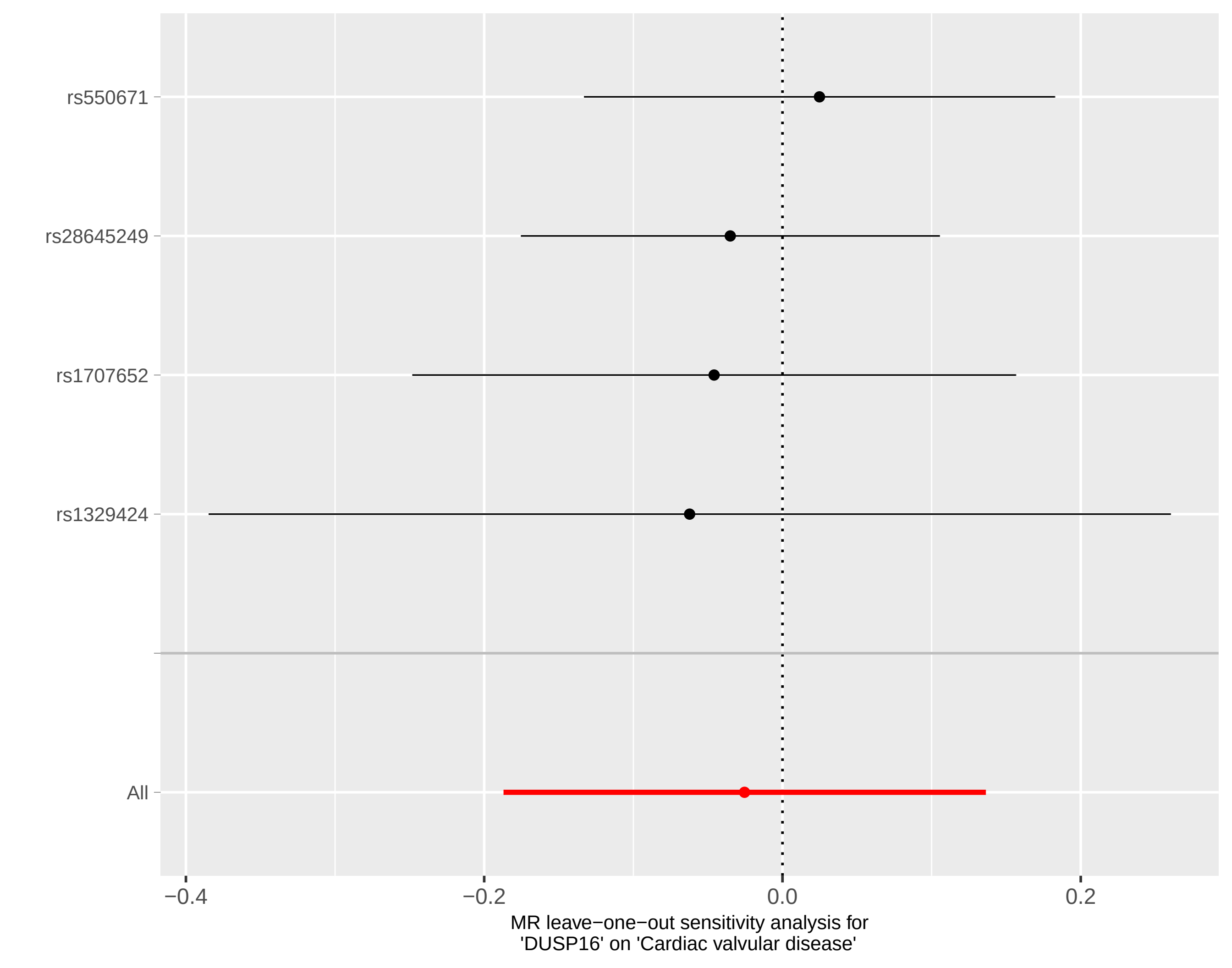
rs550671
rs28645249
rs1707652
rs1329424
All
−0.4
−0.2
0.0
0.2
MR leave−one−out sensitivity analysis for
'DUSP16' on 'Cardiac valvular disease'

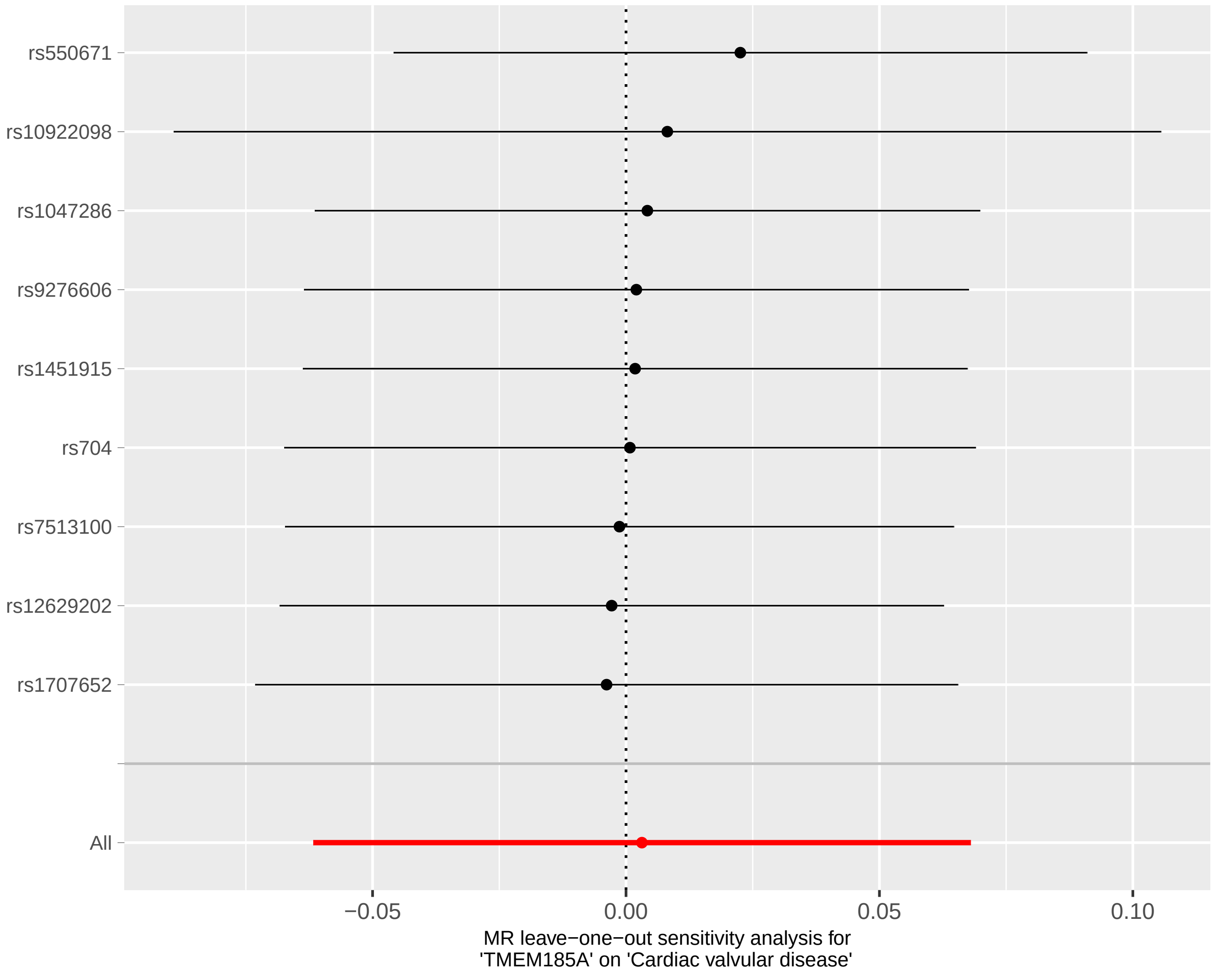

rs550671
rs10922098
rs1047286
rs9276606
rs1451915
rs704
rs7513100
rs12629202
rs1707652
All
−0.05
0.00
0.05
0.10
MR leave−one−out sensitivity analysis for
'TMEM185A' on 'Cardiac valvular disease'

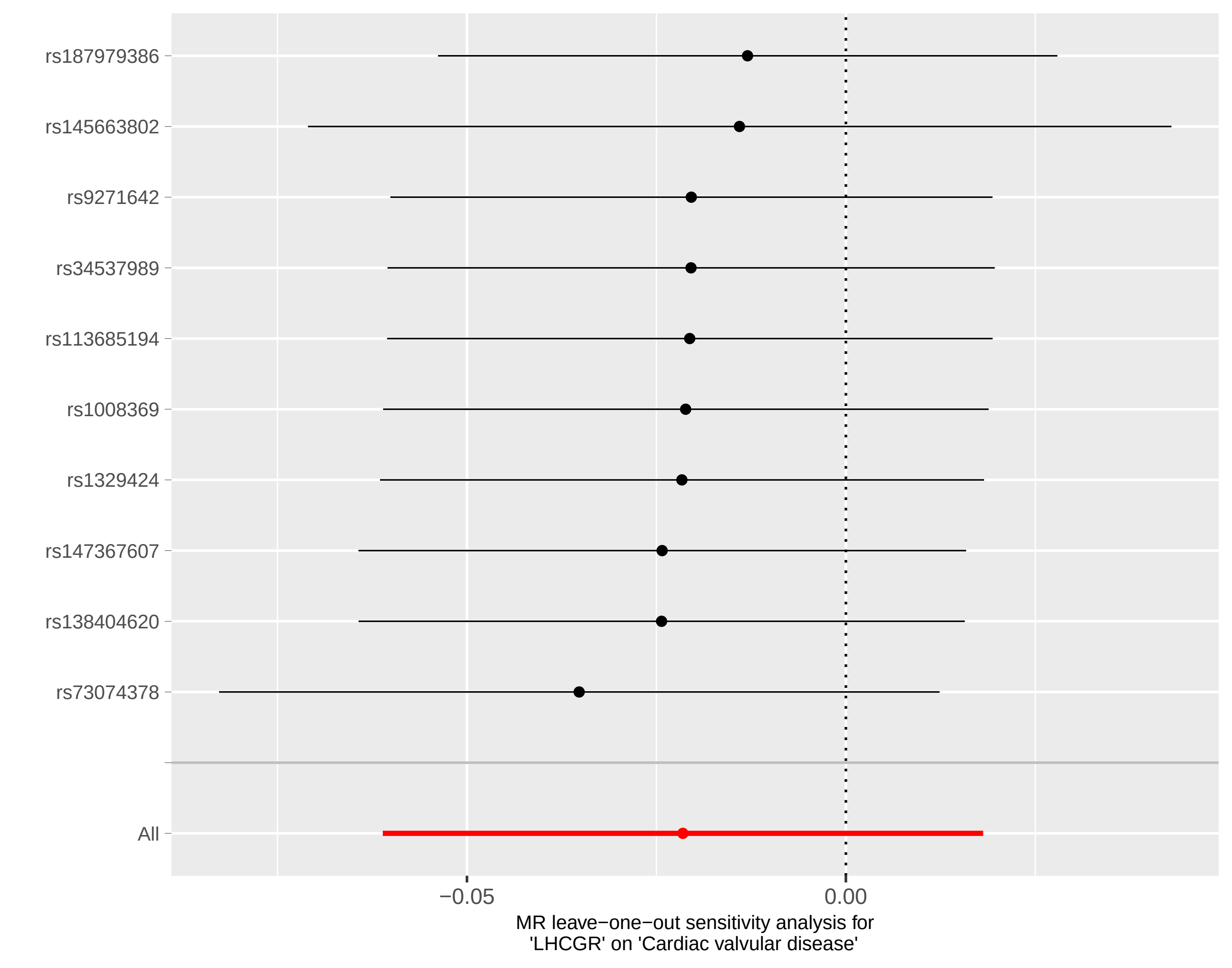
rs187979386
rs145663802
rs9271642
rs34537989
rs113685194
rs1008369
rs1329424
rs147367607
rs138404620
rs73074378
All
−0.05
0.00
MR leave−one−out sensitivity analysis for
'LHCGR' on 'Cardiac valvular disease'

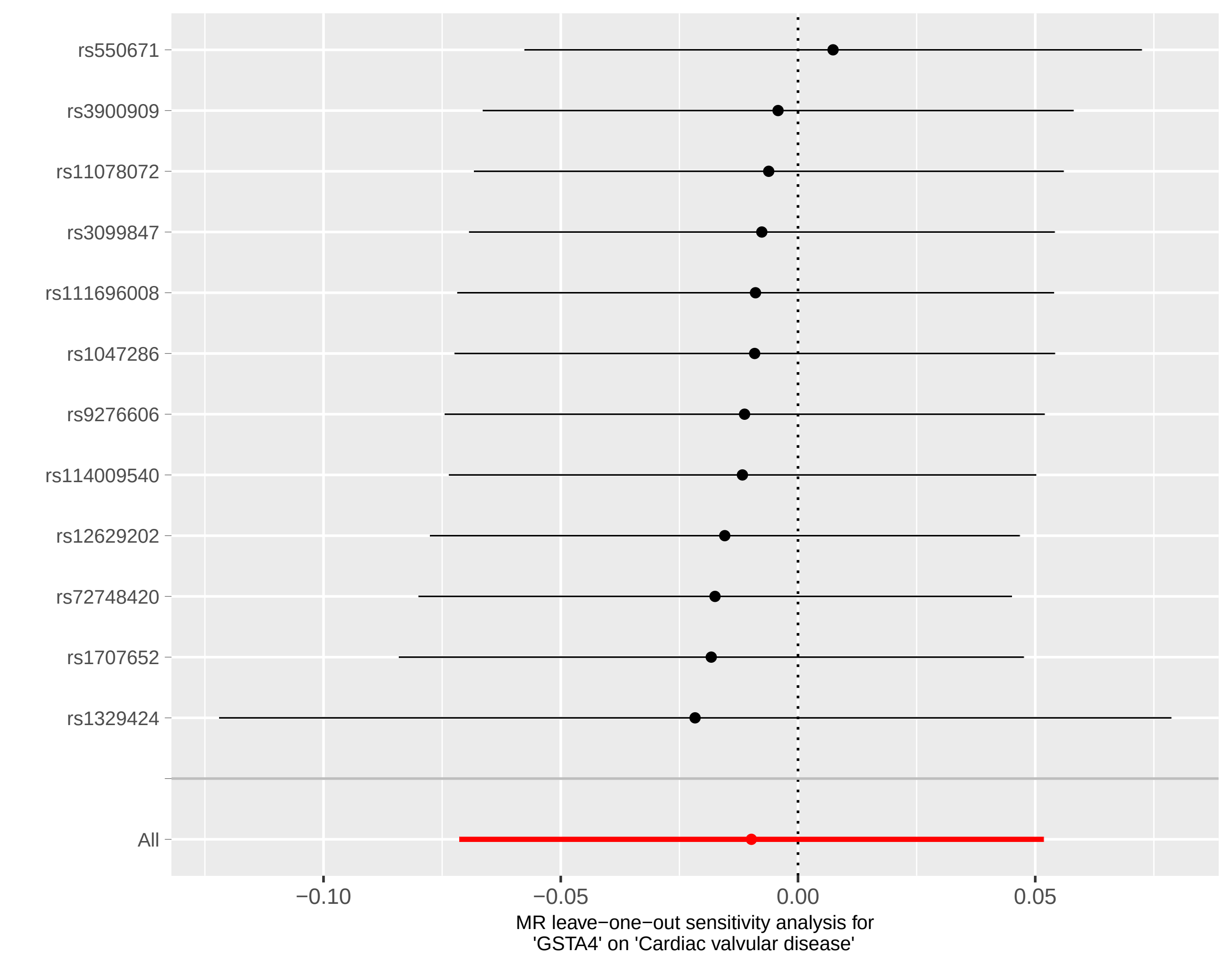

rs550671
rs3900909
rs11078072
rs3099847
rs111696008
rs1047286
rs9276606
rs114009540
rs12629202
rs72748420
rs1707652
rs1329424
All
−0.10
−0.05
0.00
0.05
MR leave−one−out sensitivity analysis for
'GSTA4' on 'Cardiac valvular disease'

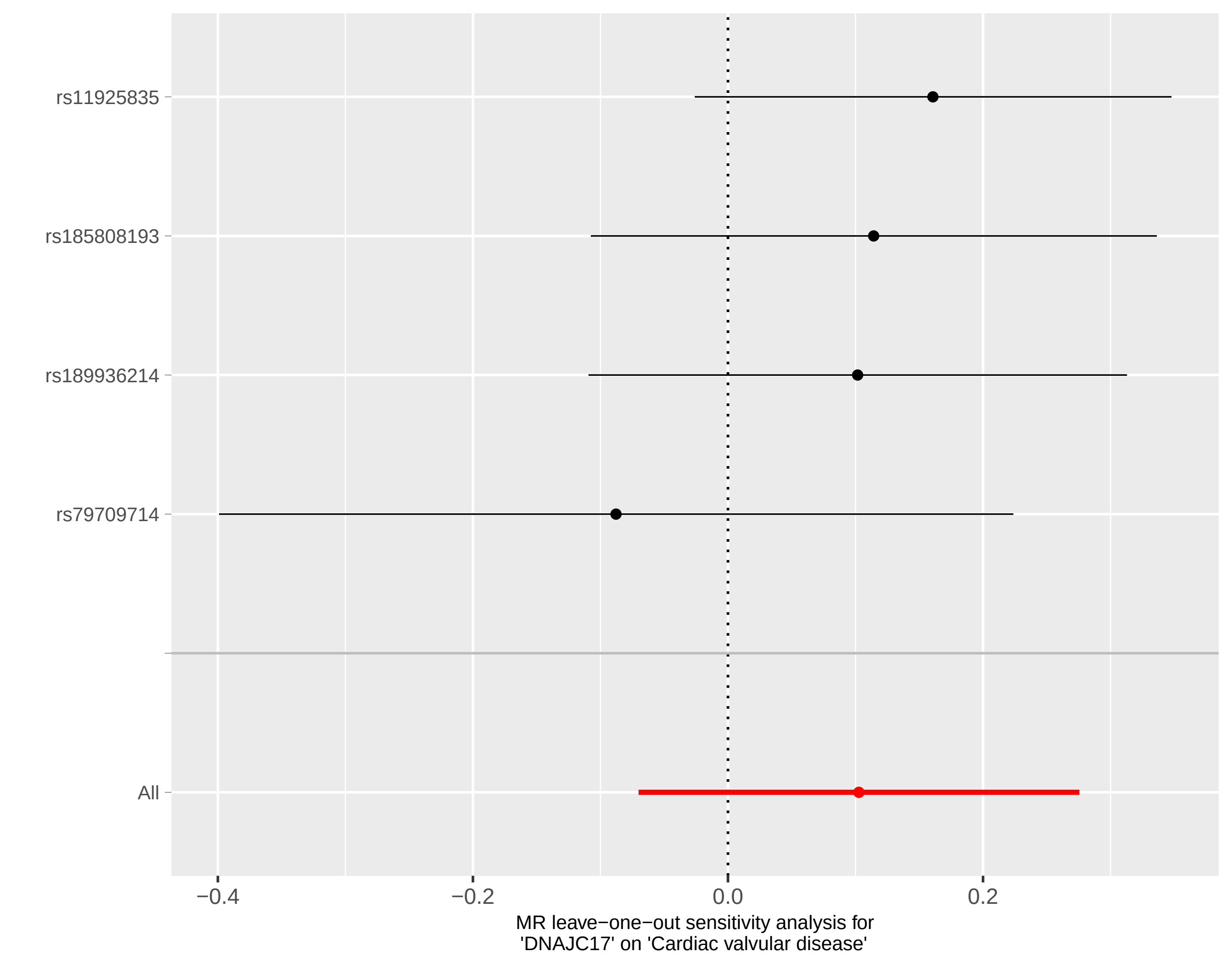
rs11925835
rs185808193
rs189936214
rs79709714
All
−0.4
−0.2
0.0
0.2
MR leave−one−out sensitivity analysis for
'DNAJC17' on 'Cardiac valvular disease'

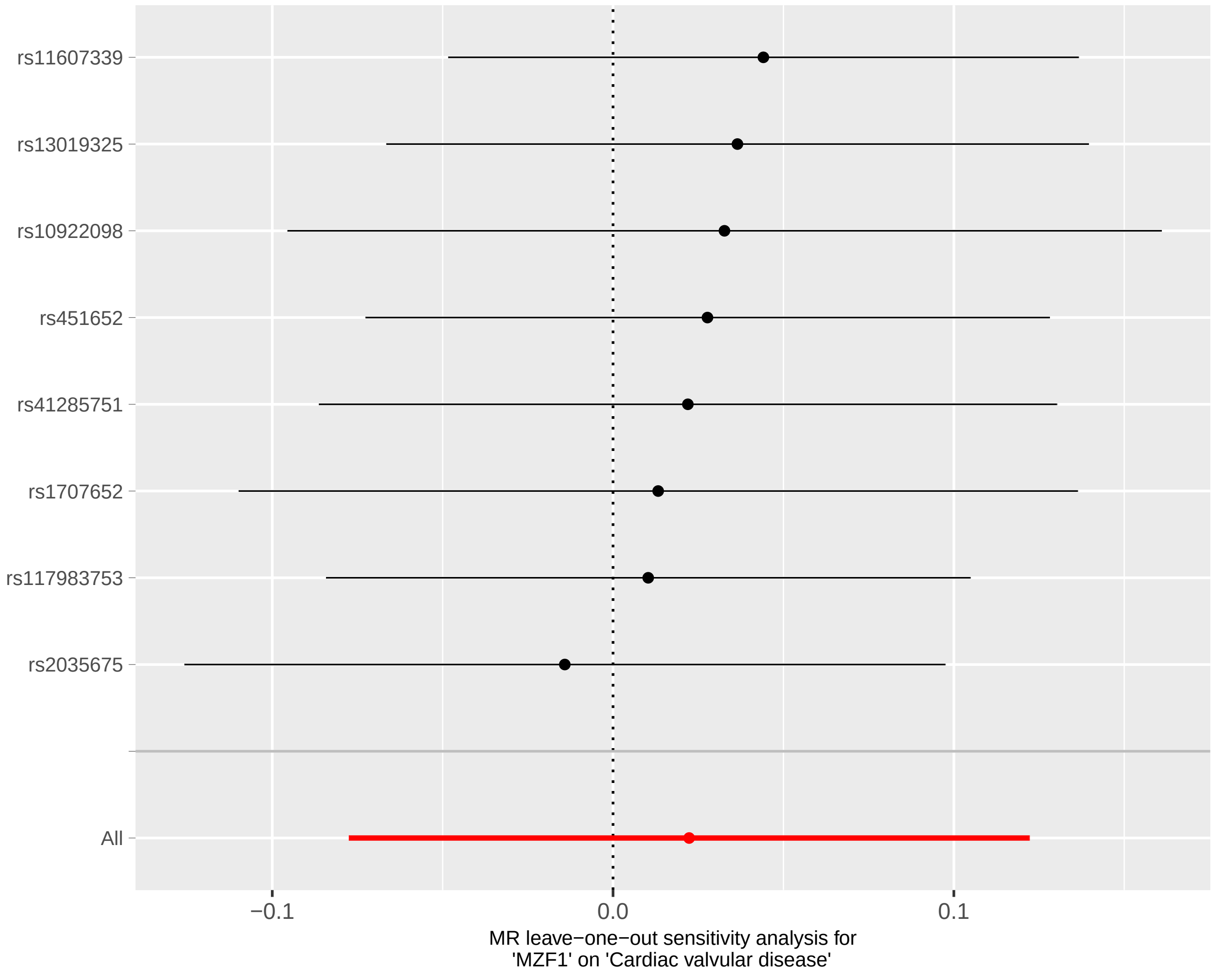
rs11607339
rs13019325
rs10922098
rs451652
rs41285751
rs1707652
rs117983753
rs2035675
All
−0.1
0.0
0.1
MR leave−one−out sensitivity analysis for
'MZF1' on 'Cardiac valvular disease'

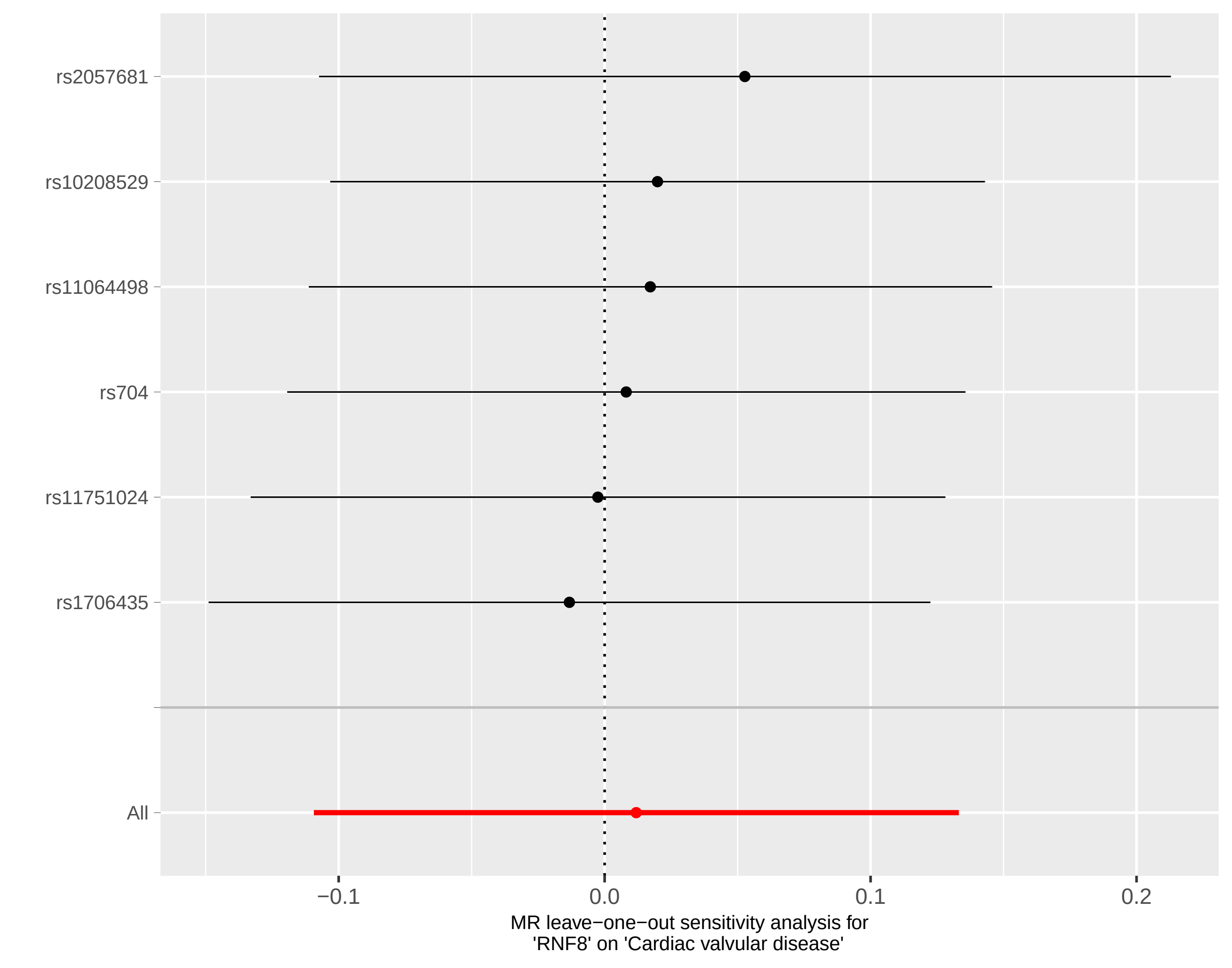
rs2057681
rs10208529
rs11064498
rs704
rs11751024
rs1706435
All
−0.1
0.0
0.1
0.2
MR leave−one−out sensitivity analysis for
'RNF8' on 'Cardiac valvular disease'

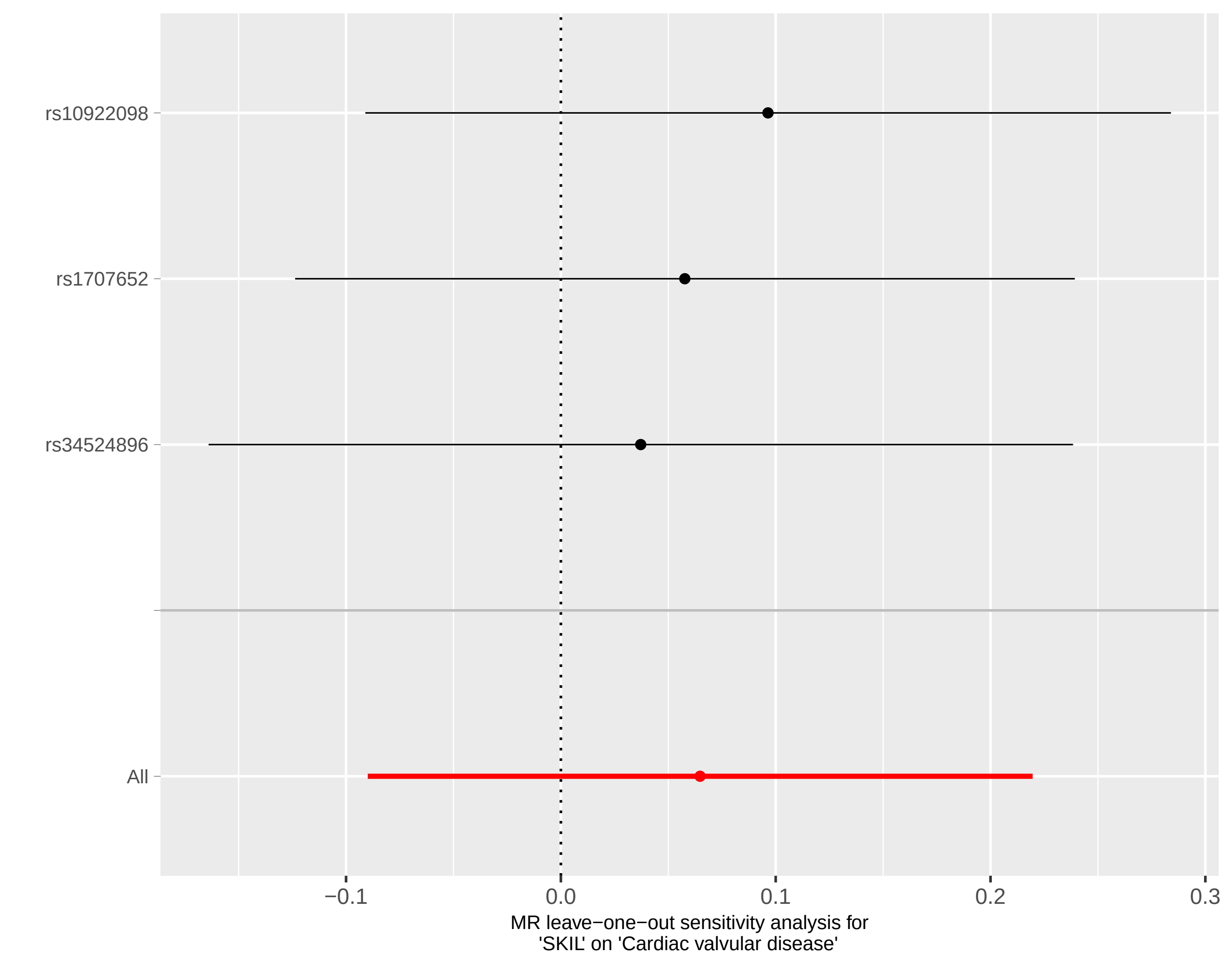

rs10922098
rs1707652
rs34524896
All
−0.1
0.0
0.1
0.2
0.3
MR leave−one−out sensitivity analysis for
'SKIL' on 'Cardiac valvular disease'

Insufficient number of SNPs

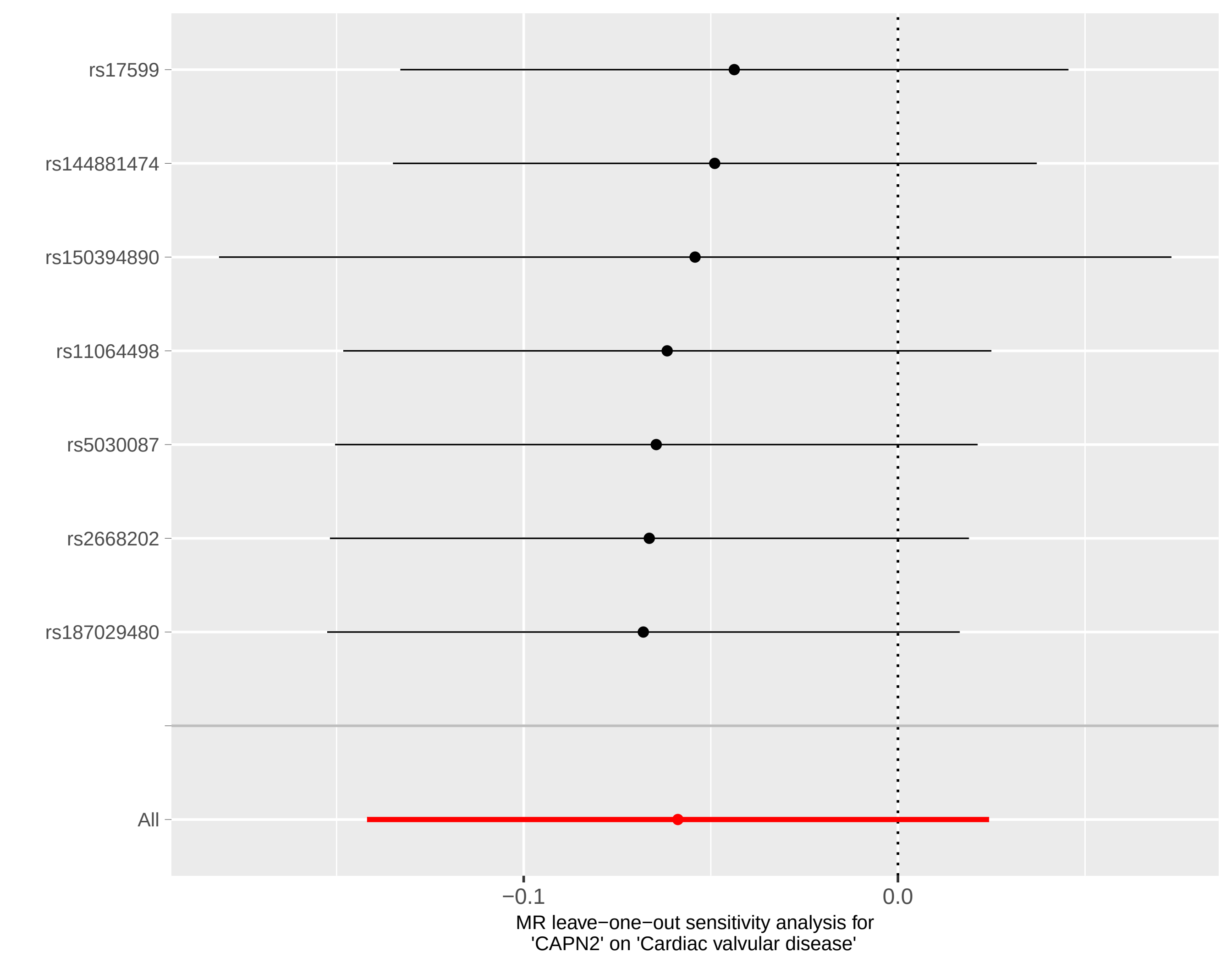
rs17599
rs144881474
rs150394890
rs11064498
rs5030087
rs2668202
rs187029480
All
−0.1
0.0
MR leave−one−out sensitivity analysis for
'CAPN2' on 'Cardiac valvular disease'

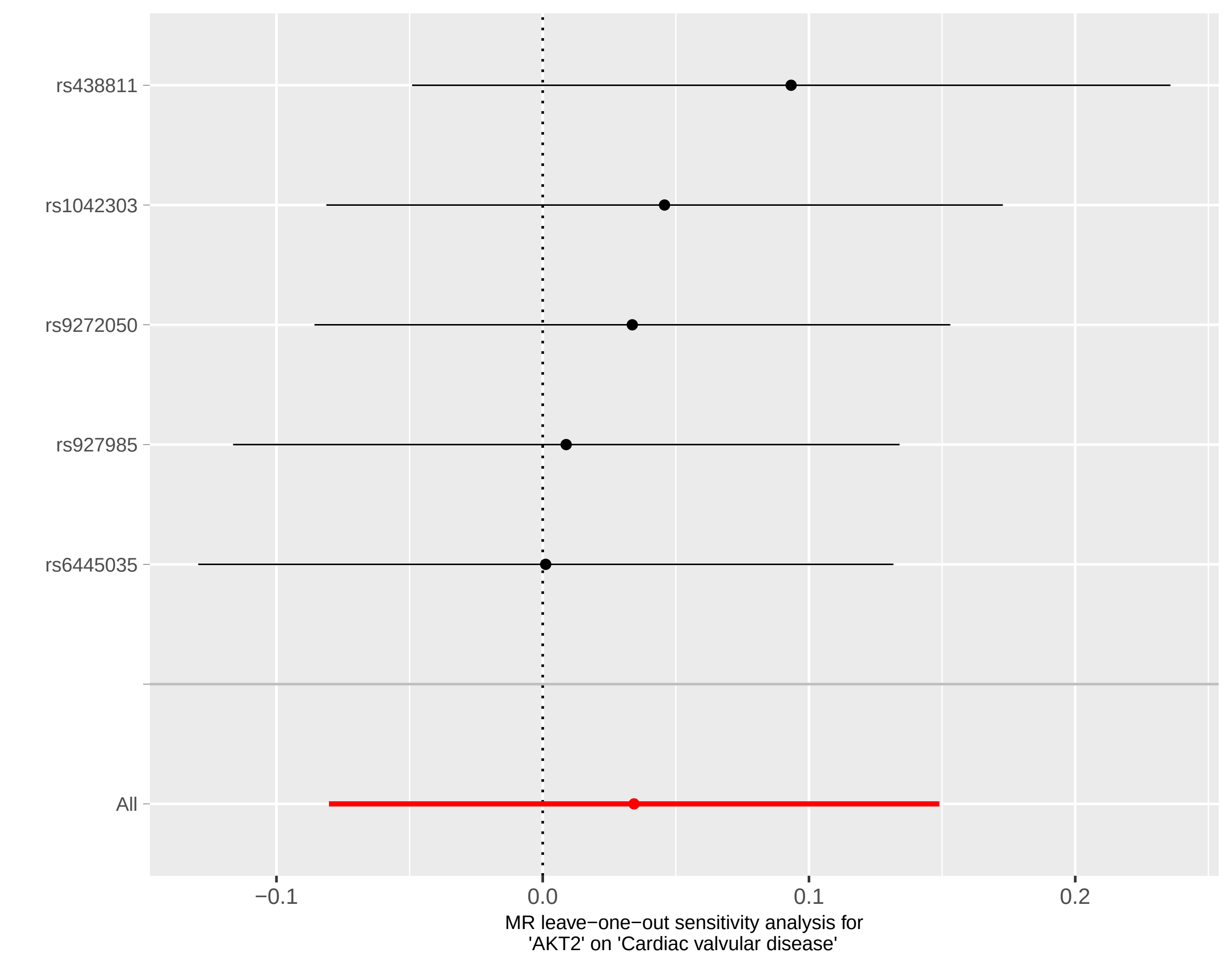
rs438811
rs1042303
rs9272050
rs927985
rs6445035
All
−0.1
0.0
0.1
0.2
MR leave−one−out sensitivity analysis for
'AKT2' on 'Cardiac valvular disease'

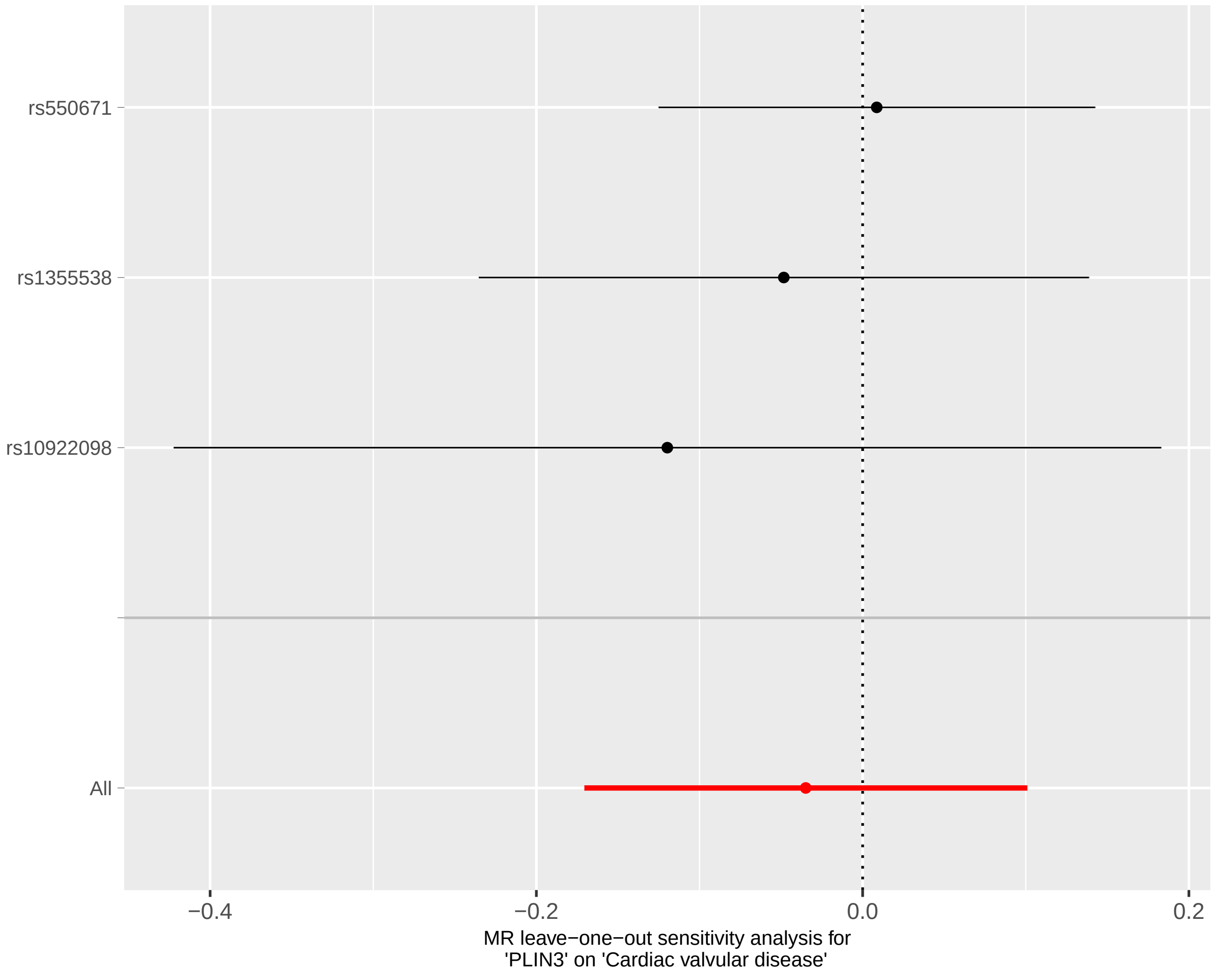
rs550671
rs1355538
rs10922098
All
−0.4
−0.2
0.0
0.2
MR leave−one−out sensitivity analysis for
'PLIN3' on 'Cardiac valvular disease'

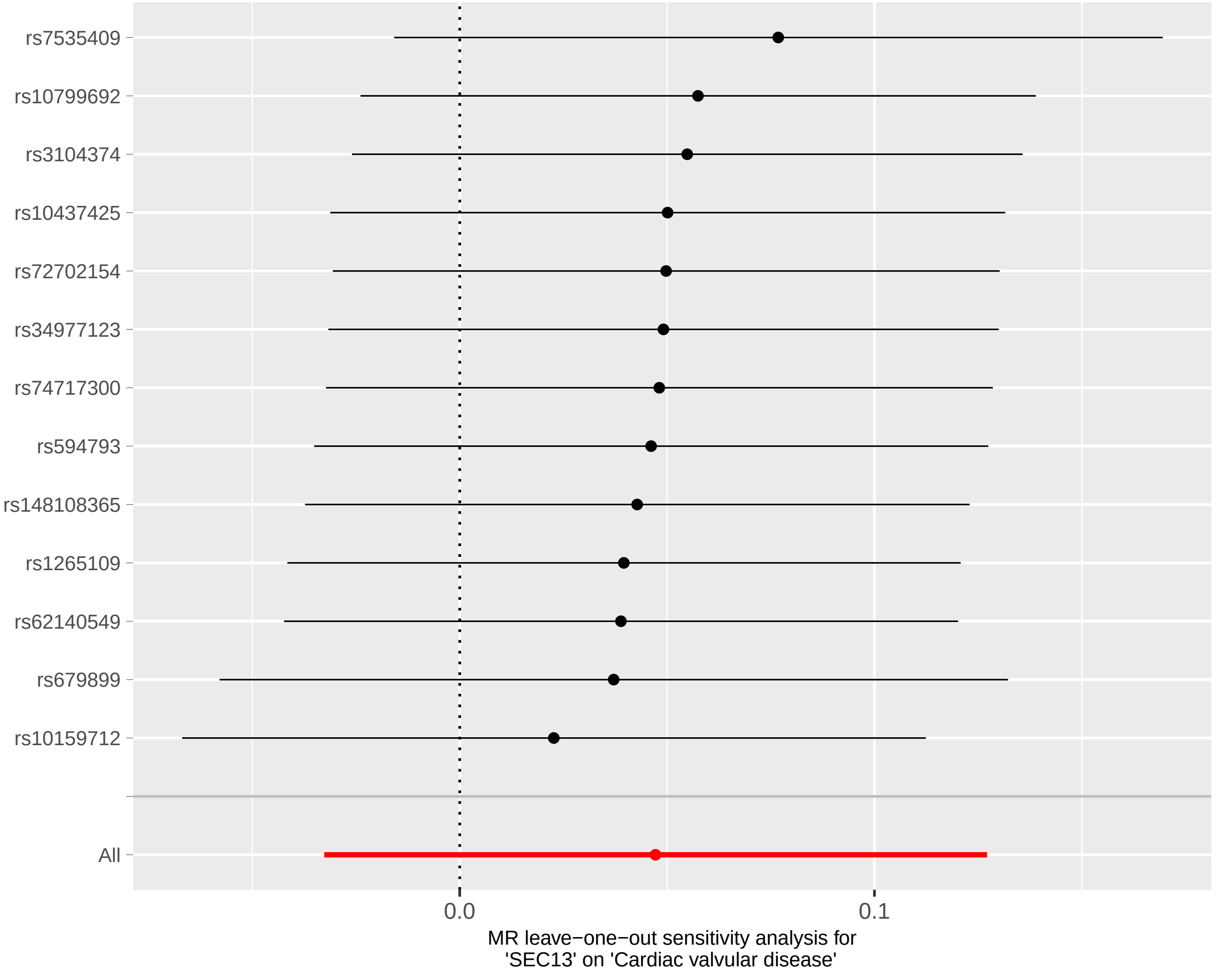
rs7535409
rs10799692
rs3104374
rs10437425
rs72702154
rs34977123
rs74717300
rs594793
rs148108365
rs1265109
rs62140549
rs679899
rs10159712
All
0.0
0.1
MR leave−one−out sensitivity analysis for
'SEC13' on 'Cardiac valvular disease'

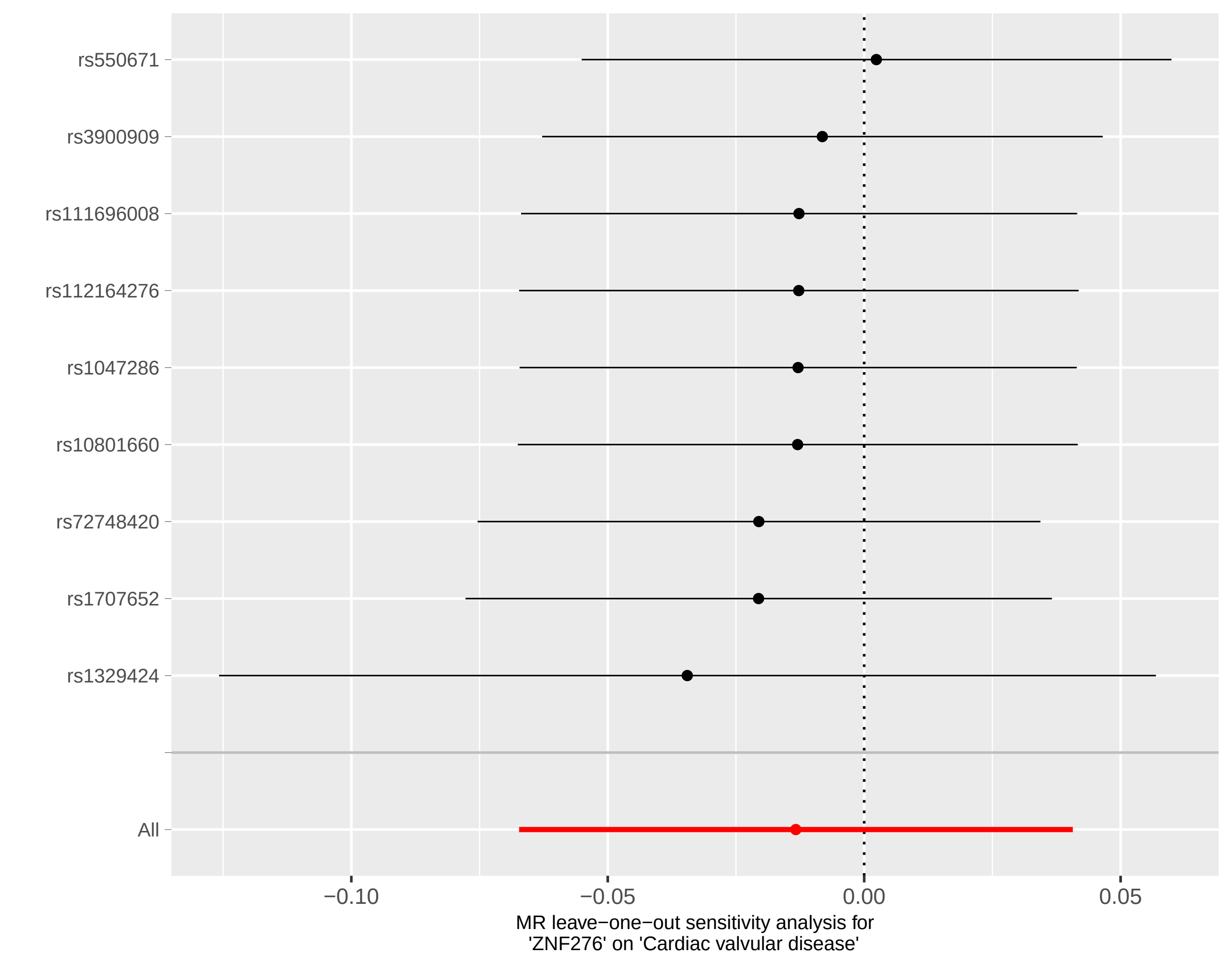

rs550671
rs3900909
rs111696008
rs112164276
rs1047286
rs10801660
rs72748420
rs1707652
rs1329424
All
−0.10
−0.05
0.00
0.05
MR leave−one−out sensitivity analysis for
'ZNF276' on 'Cardiac valvular disease'

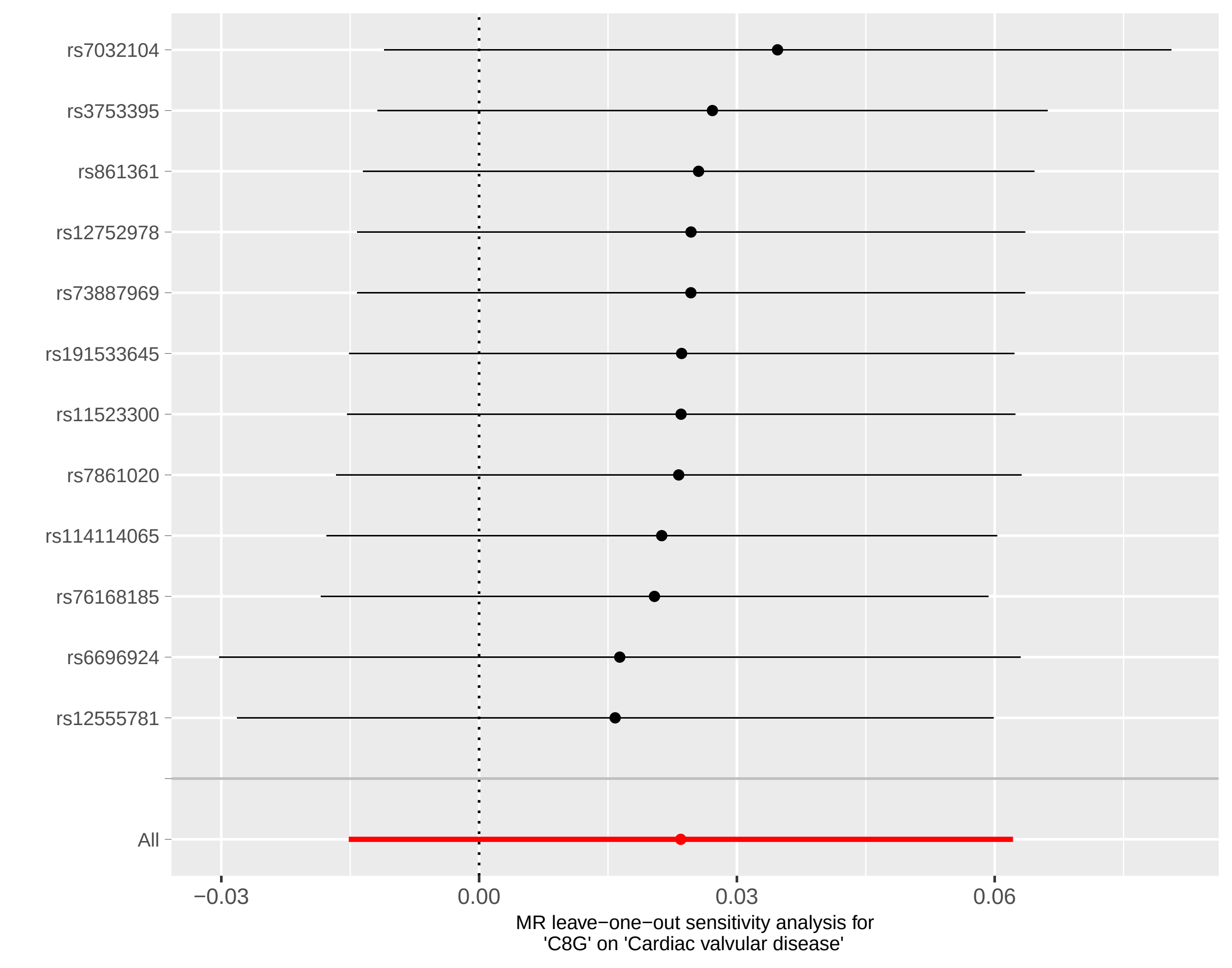
rs7032104
rs3753395
rs861361
rs12752978
rs73887969
rs191533645
rs11523300
rs7861020
rs114114065
rs76168185
rs6696924
rs12555781
All
−0.03
0.00
0.03
0.06
MR leave−one−out sensitivity analysis for
'C8G' on 'Cardiac valvular disease'

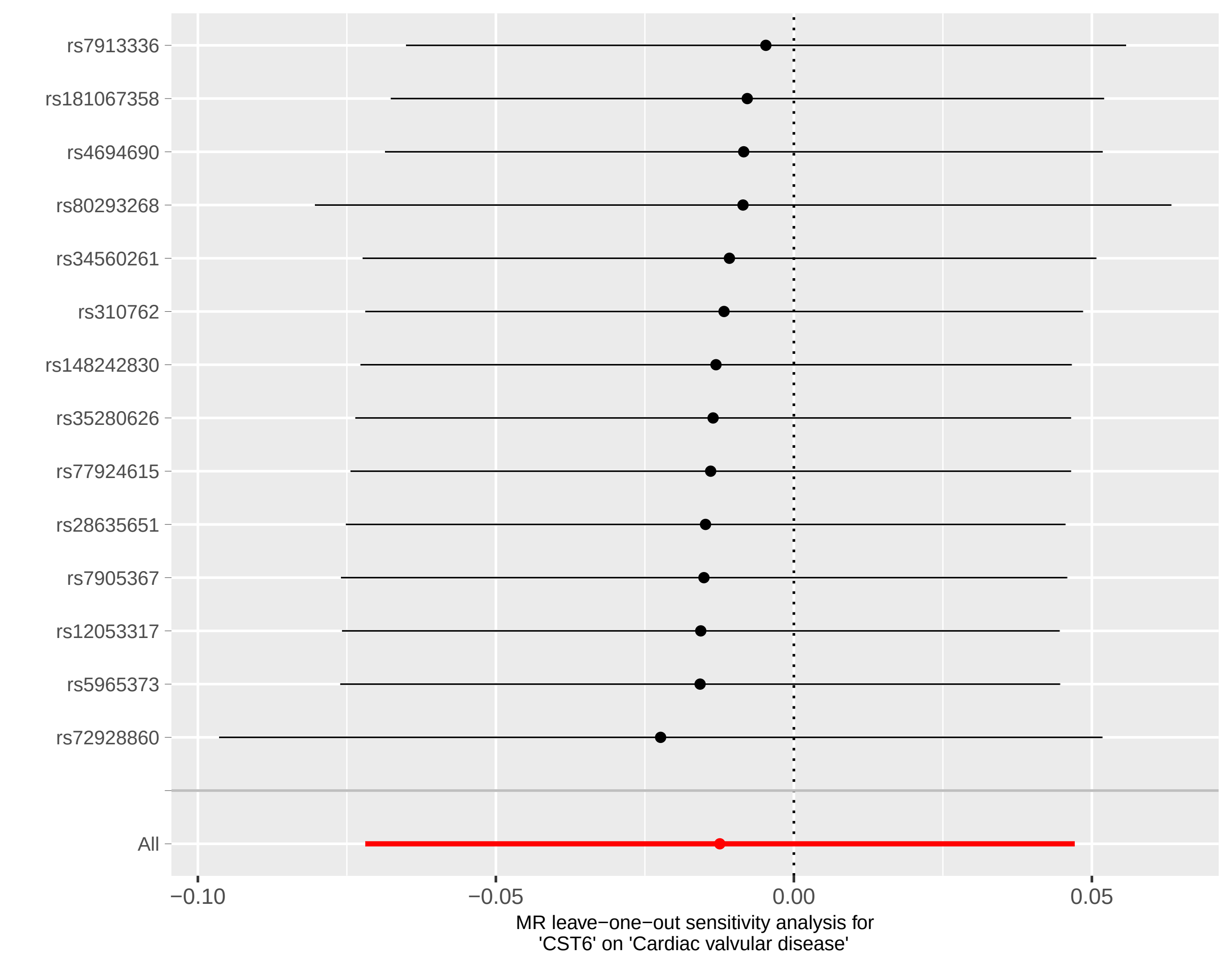
rs7913336
rs181067358
rs4694690
rs80293268
rs34560261
rs310762
rs148242830
rs35280626
rs77924615
rs28635651
rs7905367
rs12053317
rs5965373
rs72928860
All
−0.10
−0.05
0.00
0.05
MR leave−one−out sensitivity analysis for
'CST6' on 'Cardiac valvular disease'

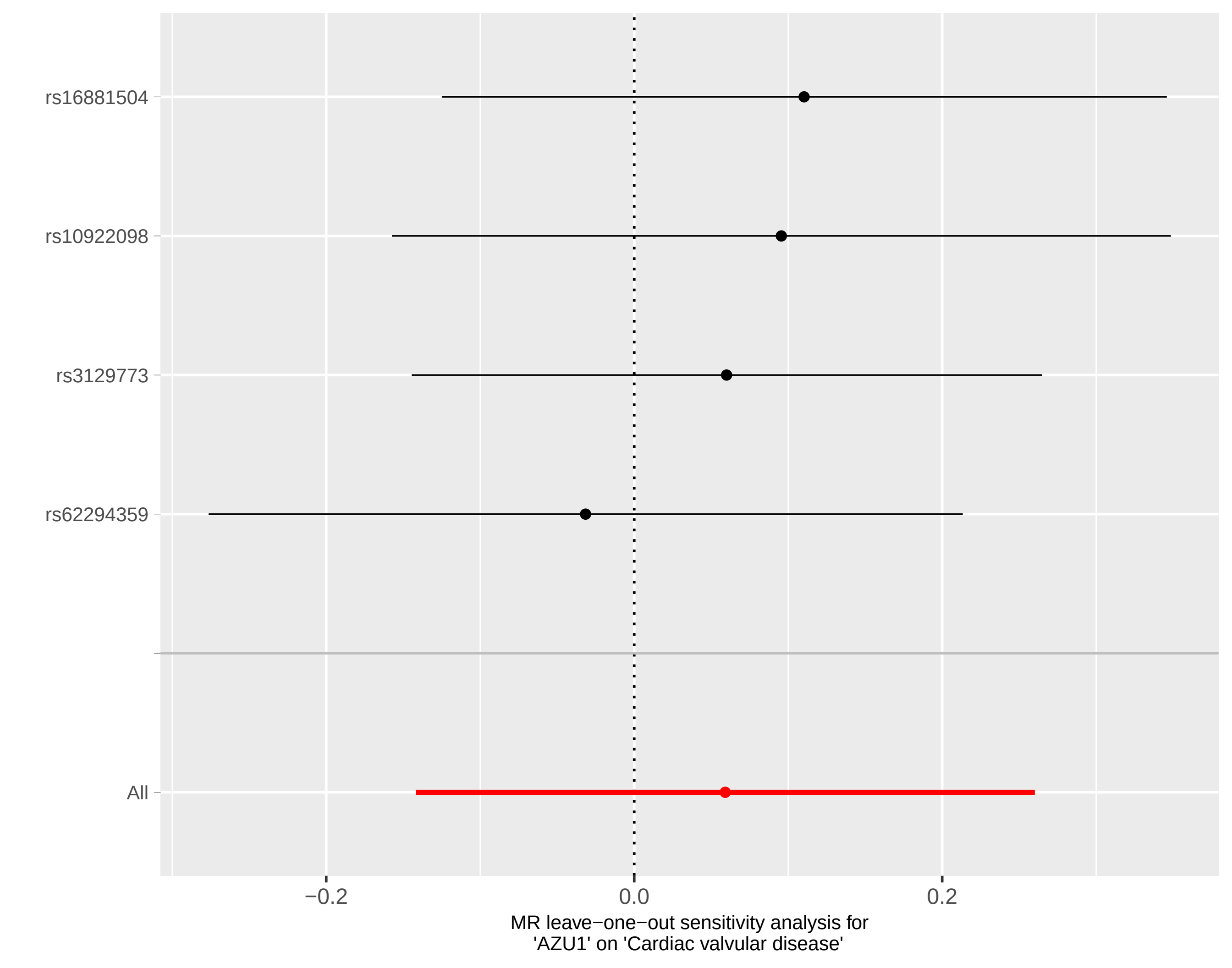
rs16881504
rs10922098
rs3129773
rs62294359
All
−0.2
0.0
0.2
MR leave−one−out sensitivity analysis for
'AZU1' on 'Cardiac valvular disease'

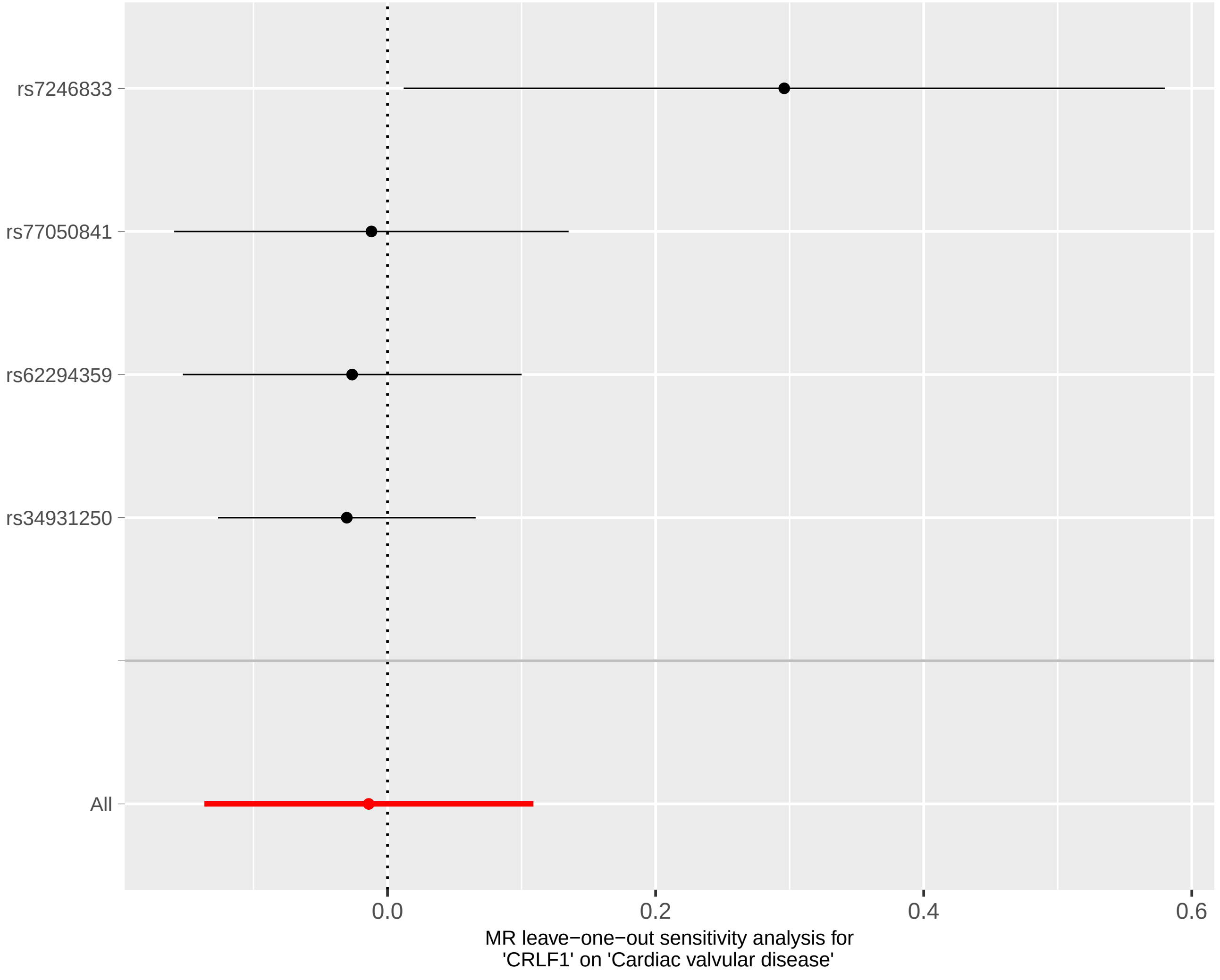
rs7246833
rs77050841
rs62294359
rs34931250
All
0.0
0.2
0.4
0.6
MR leave−one−out sensitivity analysis for
'CRLF1' on 'Cardiac valvular disease'

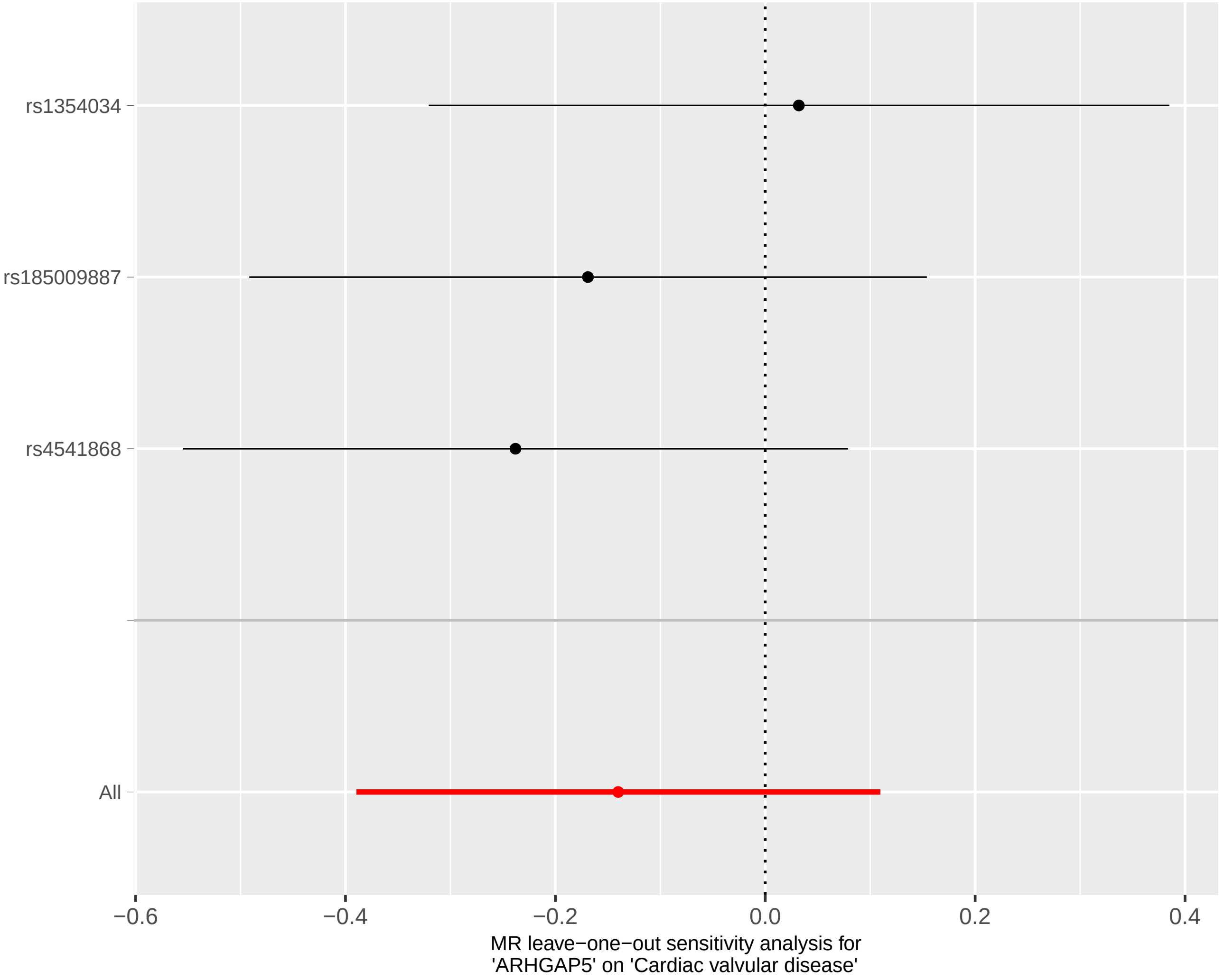

rs1354034
rs185009887
rs4541868
All
−0.6
−0.4
−0.2
0.0
0.2
0.4
MR leave−one−out sensitivity analysis for
'ARHGAP5' on 'Cardiac valvular disease'

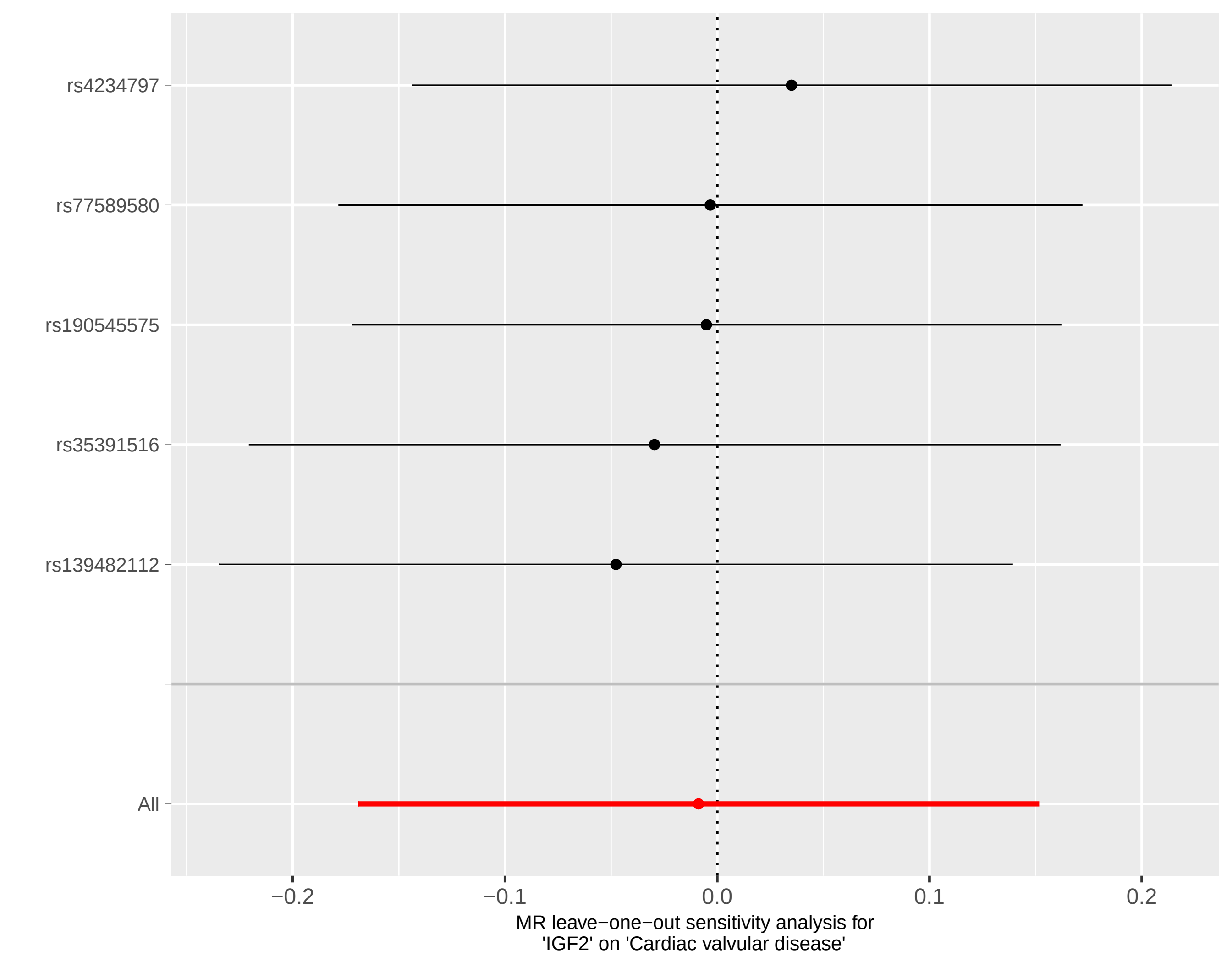
rs4234797
rs77589580
rs190545575
rs35391516
rs139482112
All
−0.2
−0.1
0.0
0.1
0.2
MR leave−one−out sensitivity analysis for
'IGF2' on 'Cardiac valvular disease'

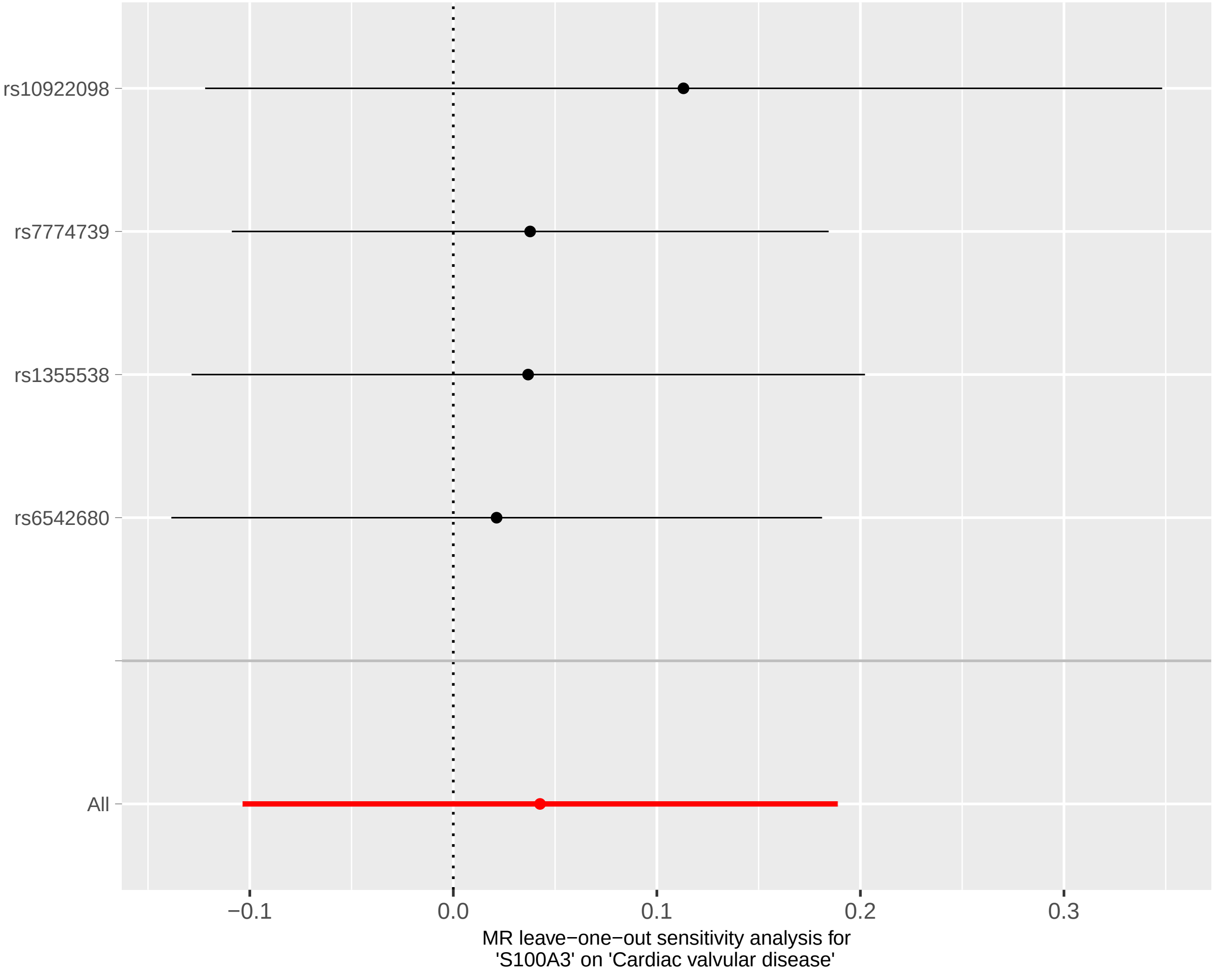
rs10922098
rs7774739
rs1355538
rs6542680
All
−0.1
0.0
0.1
0.2
0.3
MR leave−one−out sensitivity analysis for
'S100A3' on 'Cardiac valvular disease'

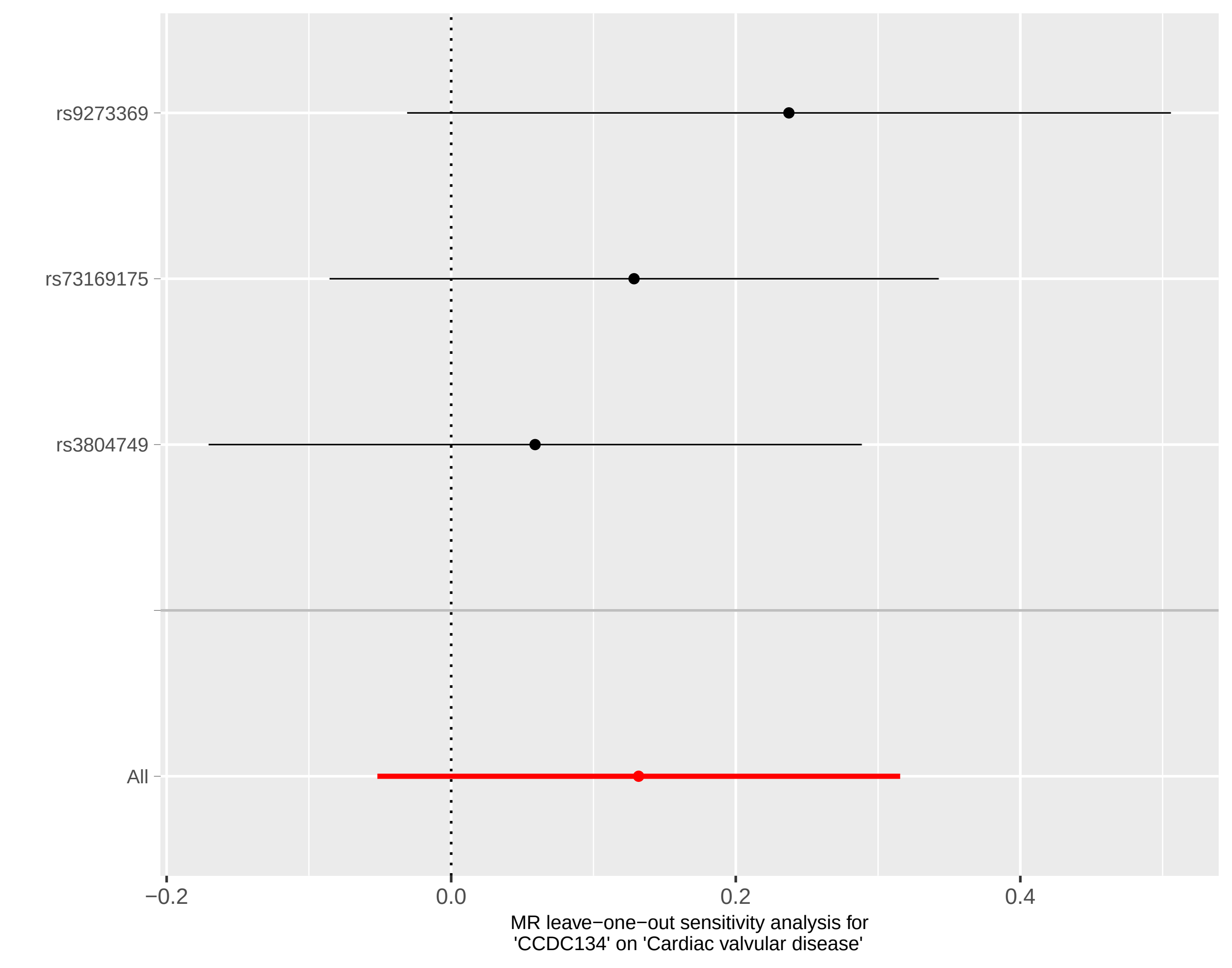

rs9273369
rs73169175
rs3804749
All
−0.2
0.0
0.2
0.4
MR leave−one−out sensitivity analysis for
'CCDC134' on 'Cardiac valvular disease'

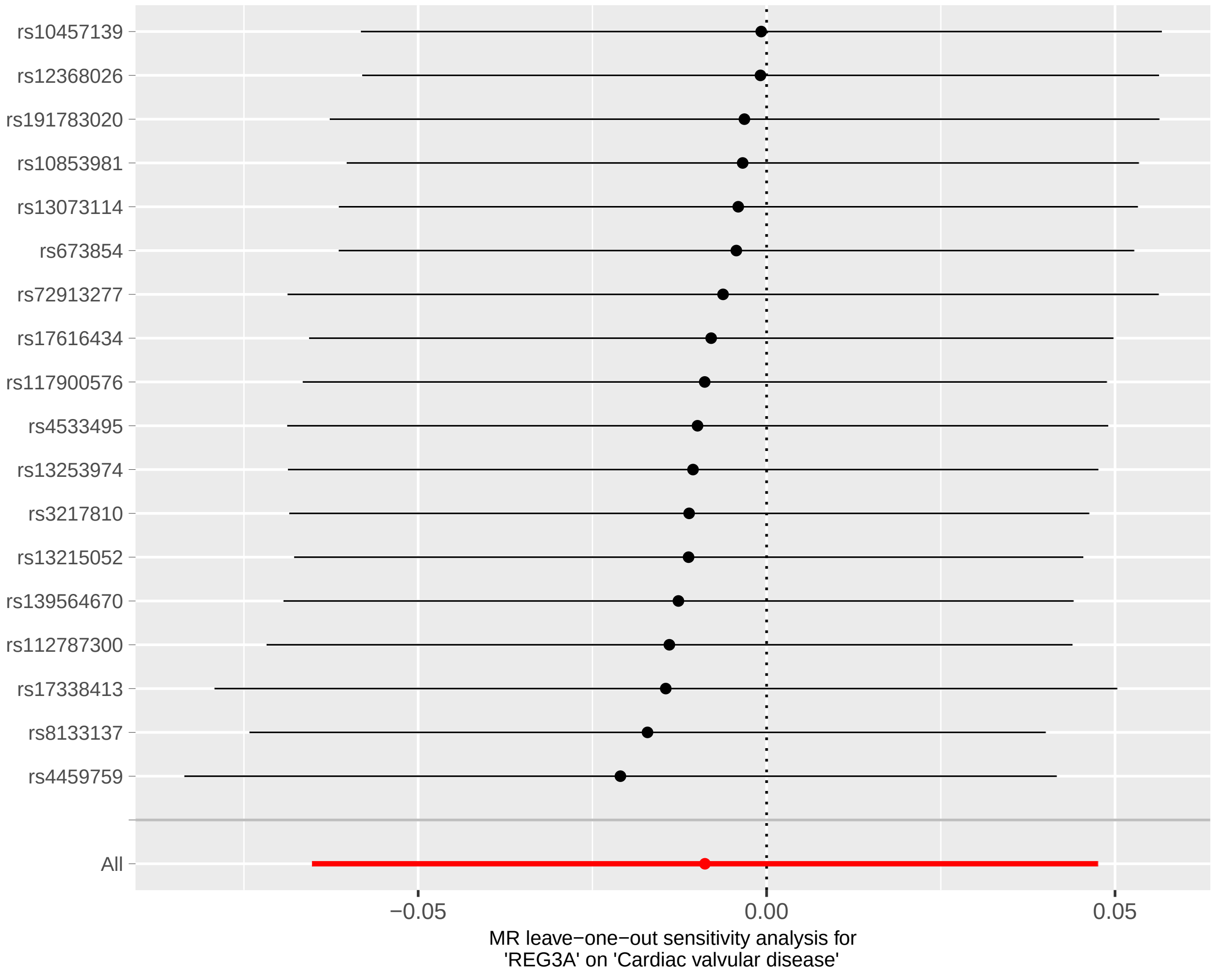

rs10457139
rs12368026
rs191783020
rs10853981
rs13073114
rs673854
rs72913277
rs17616434
rs117900576
rs4533495
rs13253974
rs3217810
rs13215052
rs139564670
rs112787300
rs17338413
rs8133137
rs4459759
All
−0.05
0.00
0.05
MR leave−one−out sensitivity analysis for
'REG3A' on 'Cardiac valvular disease'

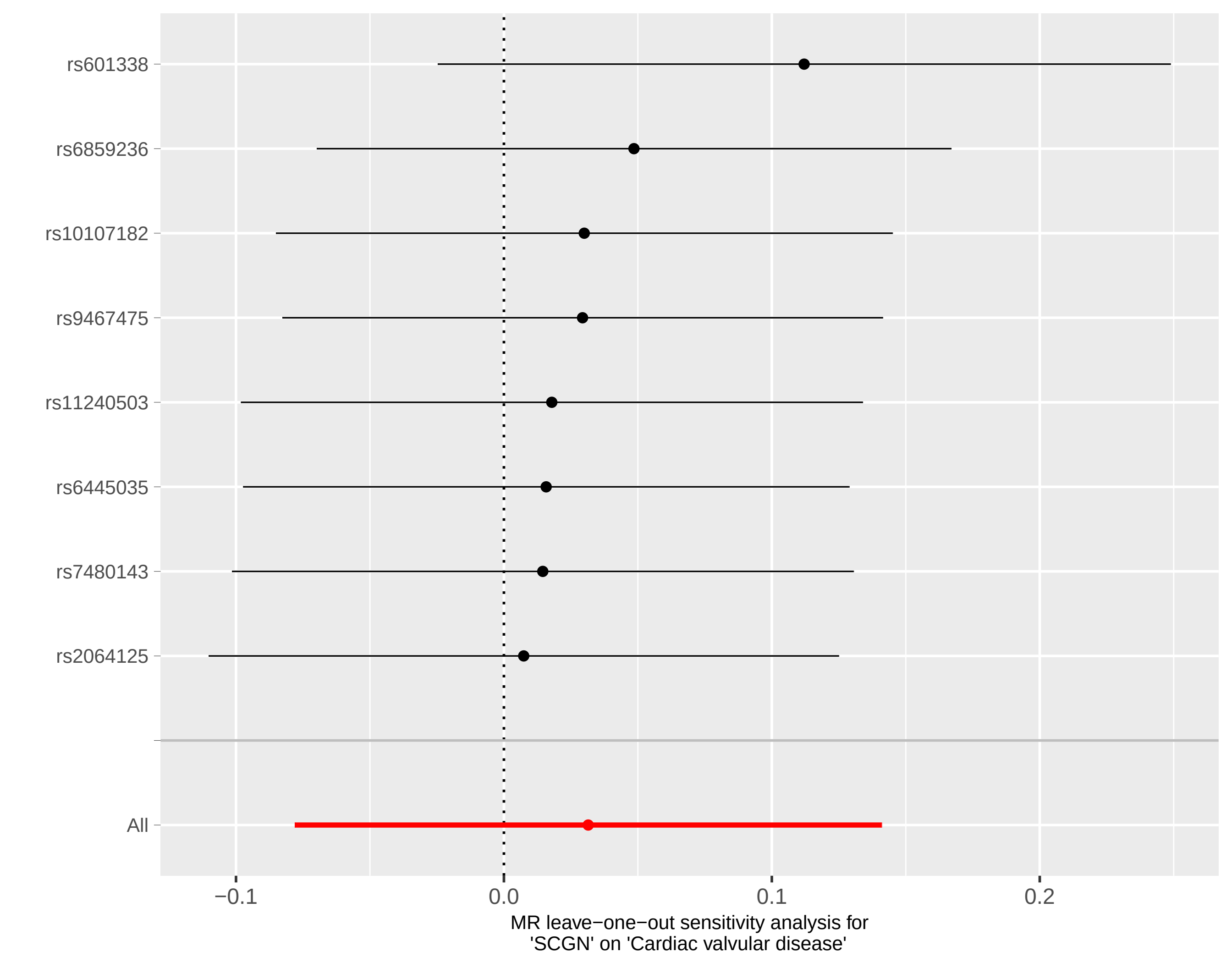
rs601338
rs6859236
rs10107182
rs9467475
rs11240503
rs6445035
rs7480143
rs2064125
All
−0.1
0.0
0.1
0.2
MR leave−one−out sensitivity analysis for
'SCGN' on 'Cardiac valvular disease'

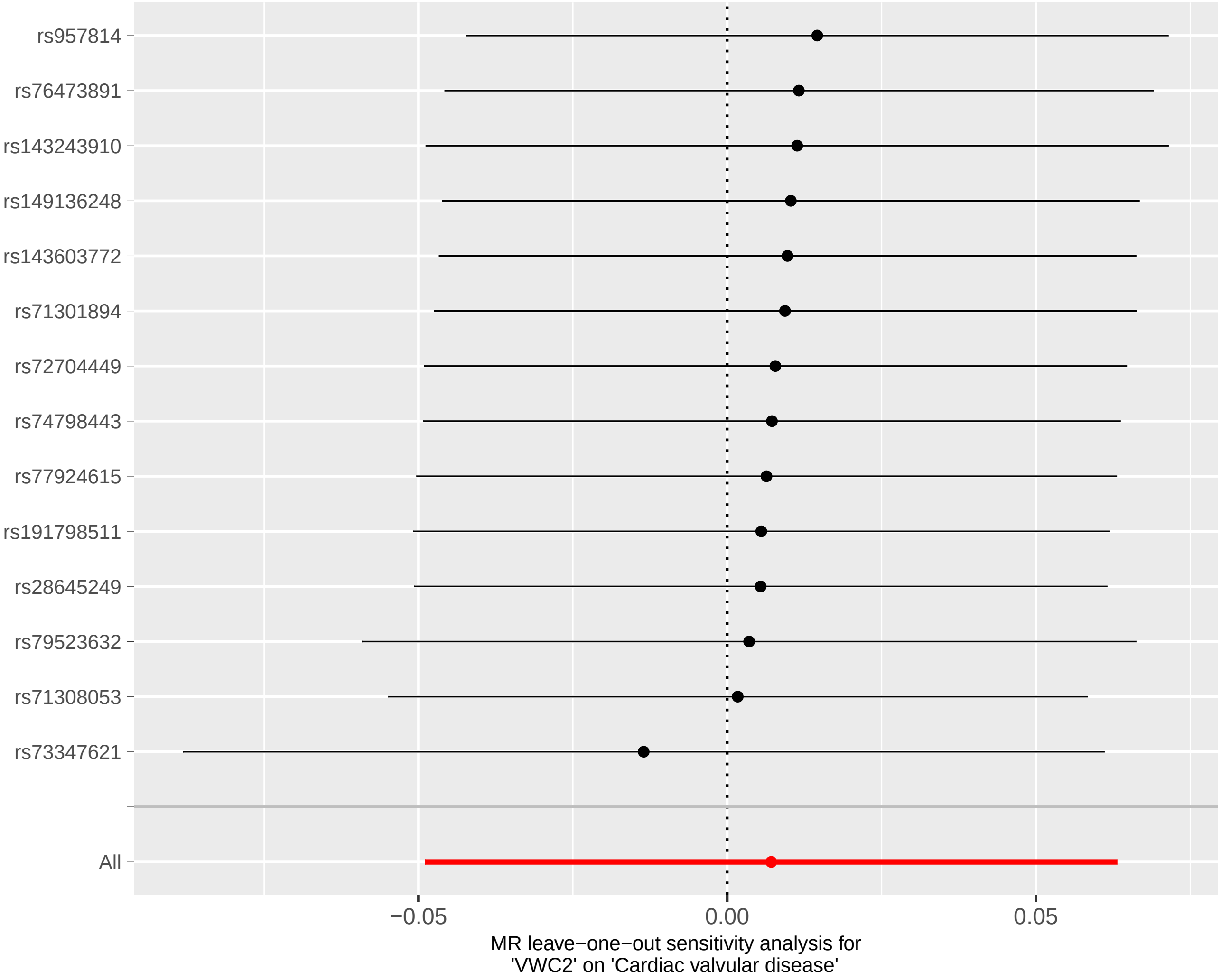
rs957814
rs76473891
rs143243910
rs149136248
rs143603772
rs71301894
rs72704449
rs74798443
rs77924615
rs191798511
rs28645249
rs79523632
rs71308053
rs73347621
All
−0.05
0.00
0.05
MR leave−one−out sensitivity analysis for
'VWC2' on 'Cardiac valvular disease'

Insufficient number of SNPs

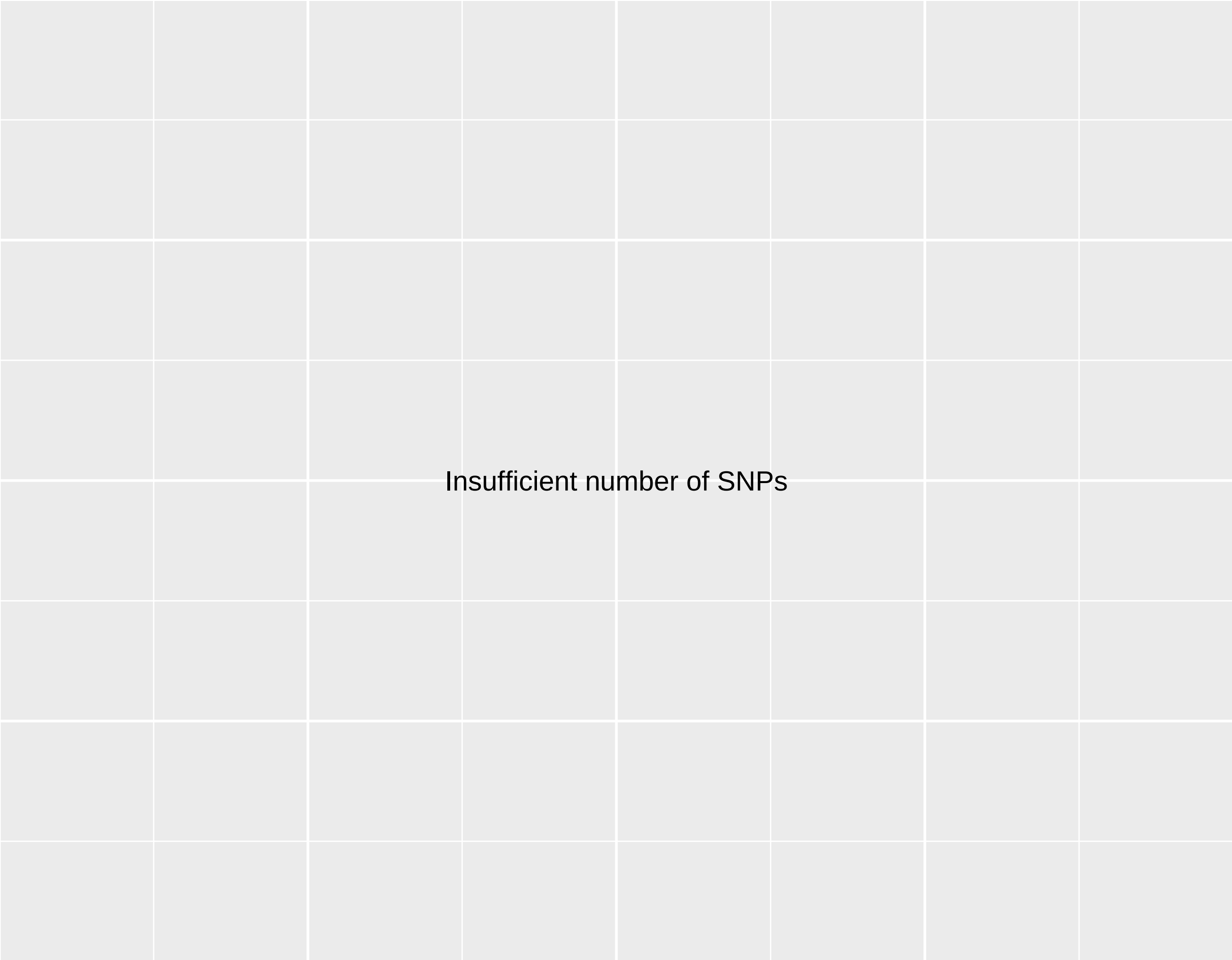
Insufficient number of SNPs

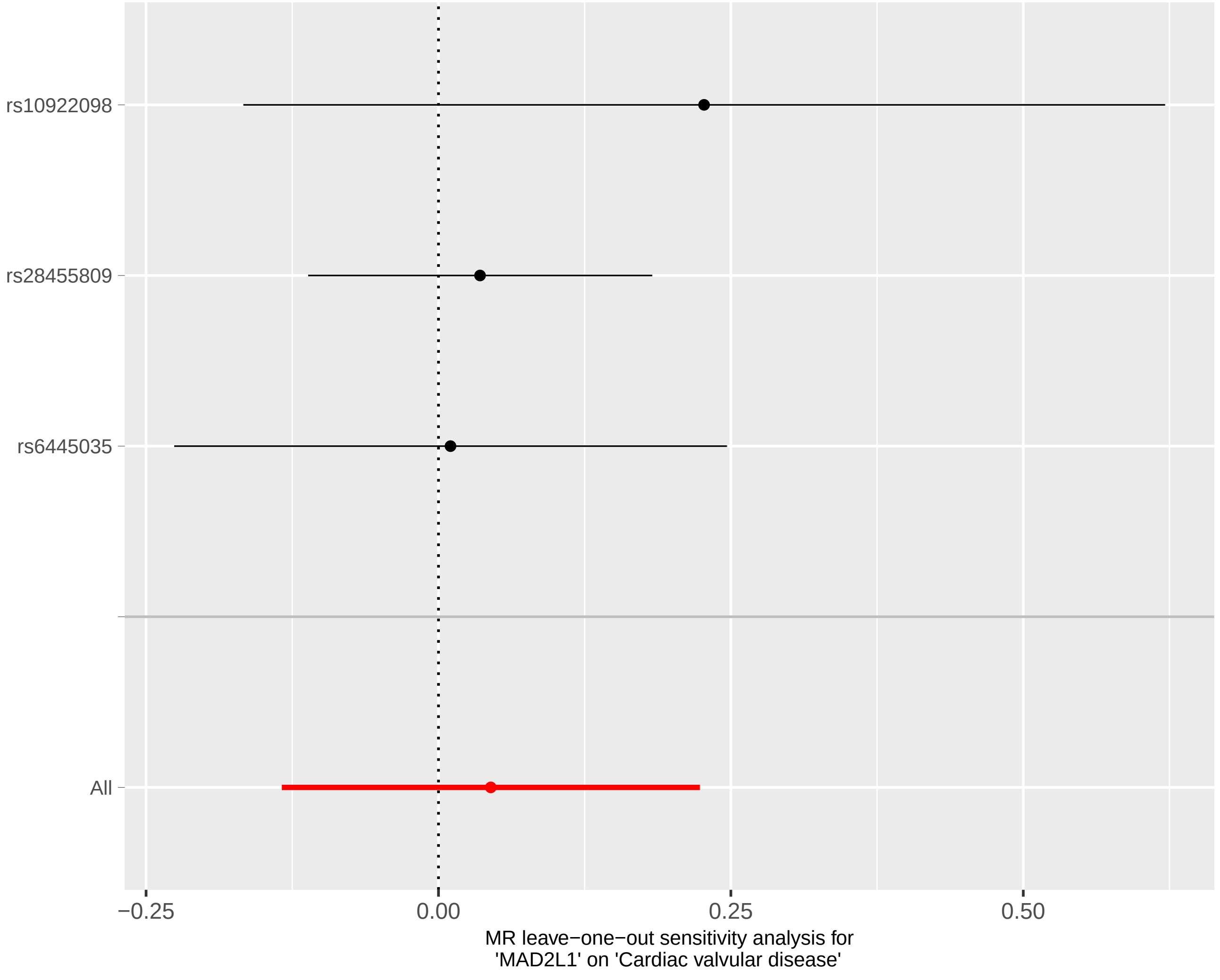
rs10922098
rs28455809
rs6445035
All
−0.25
0.00
0.25
0.50
MR leave−one−out sensitivity analysis for
'MAD2L1' on 'Cardiac valvular disease'

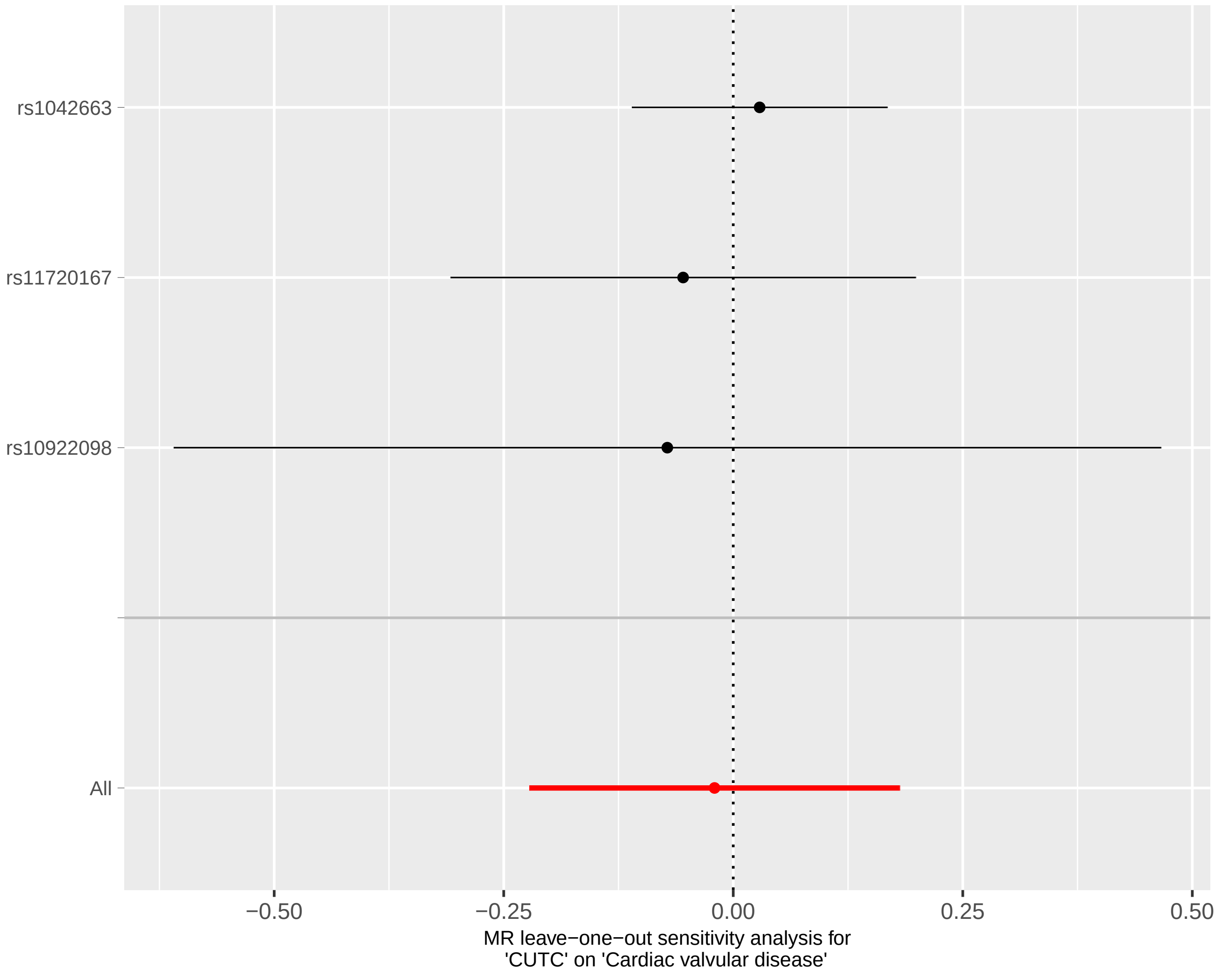

rs1042663
rs11720167
rs10922098
All
−0.50
−0.25
0.00
0.25
0.50
MR leave−one−out sensitivity analysis for
'CUTC' on 'Cardiac valvular disease'

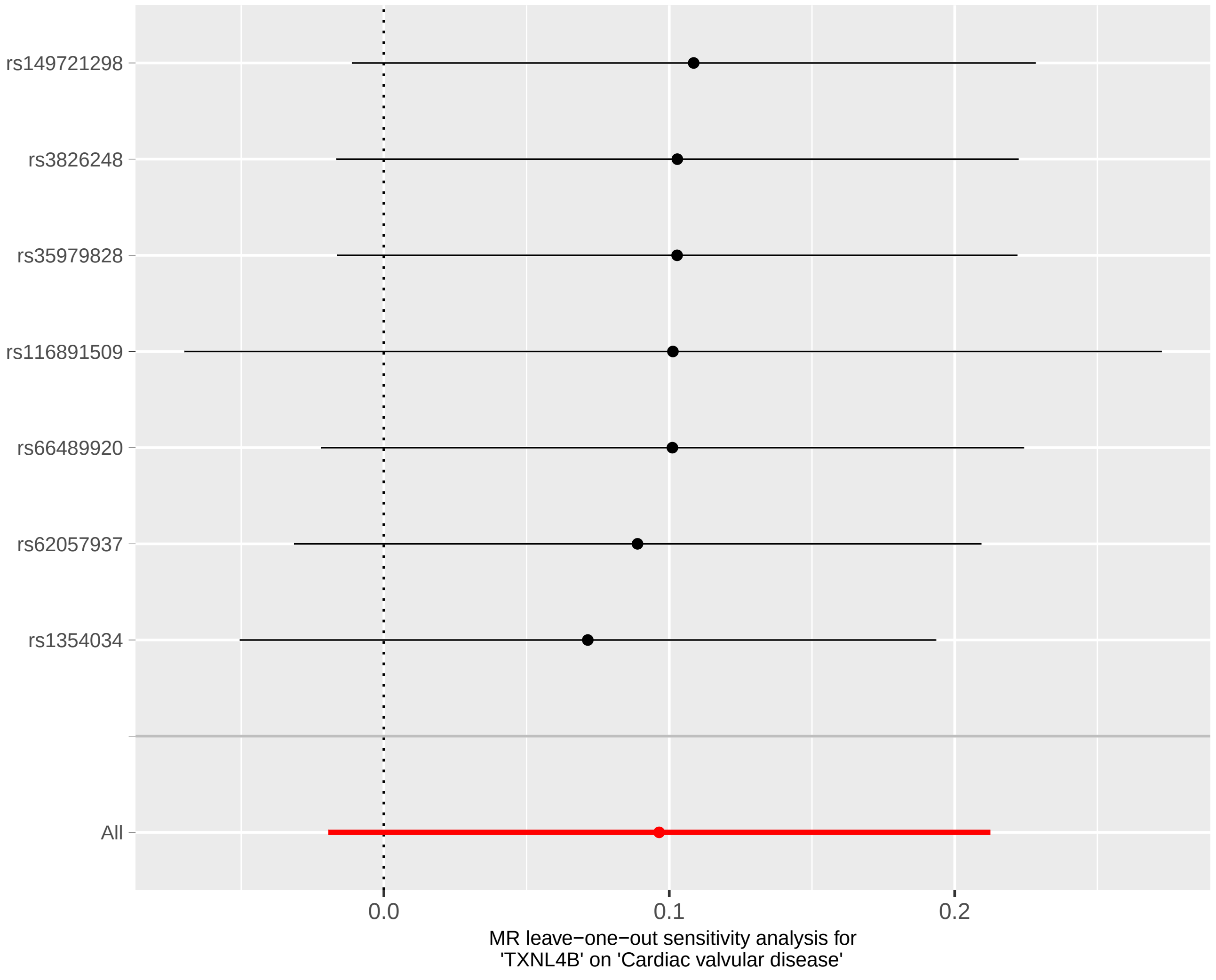
rs149721298
rs3826248
rs35979828
rs116891509
rs66489920
rs62057937
rs1354034
All
0.0
0.1
0.2
MR leave−one−out sensitivity analysis for
'TXNL4B' on 'Cardiac valvular disease'

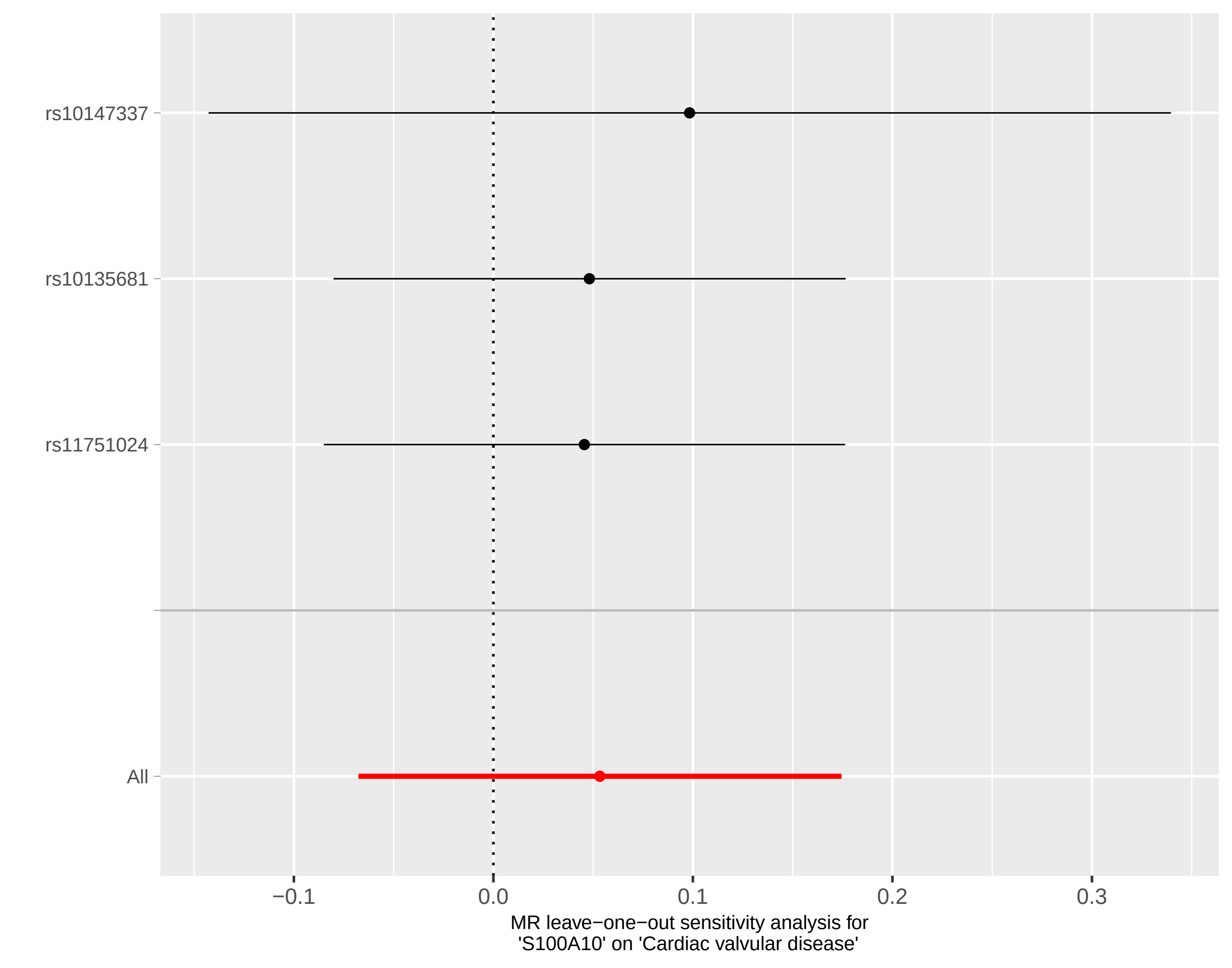

rs10147337
rs10135681
rs11751024
All
−0.1
0.0
0.1
0.2
0.3
MR leave−one−out sensitivity analysis for
'S100A10' on 'Cardiac valvular disease'

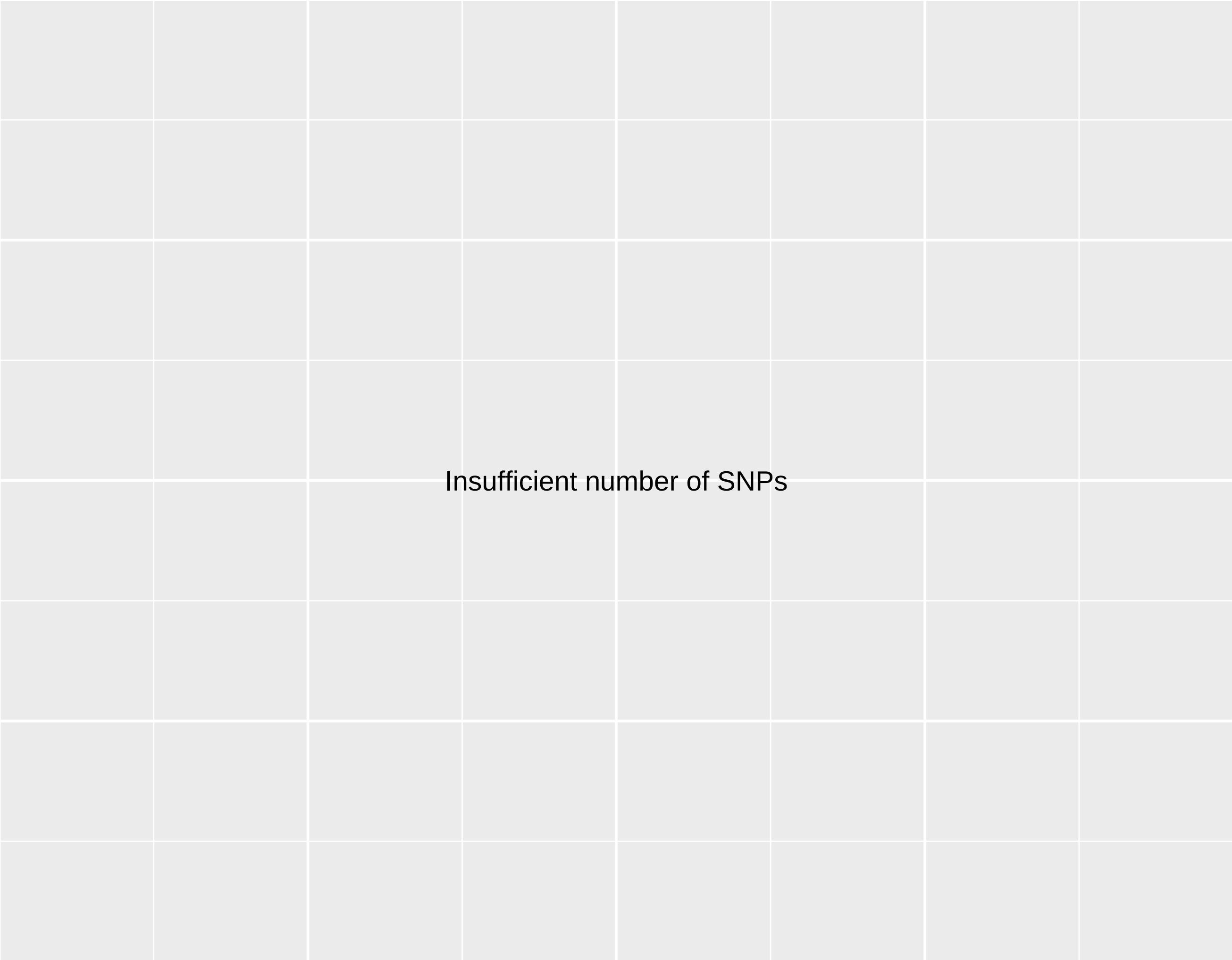
Insufficient number of SNPs

Insufficient number of SNPs

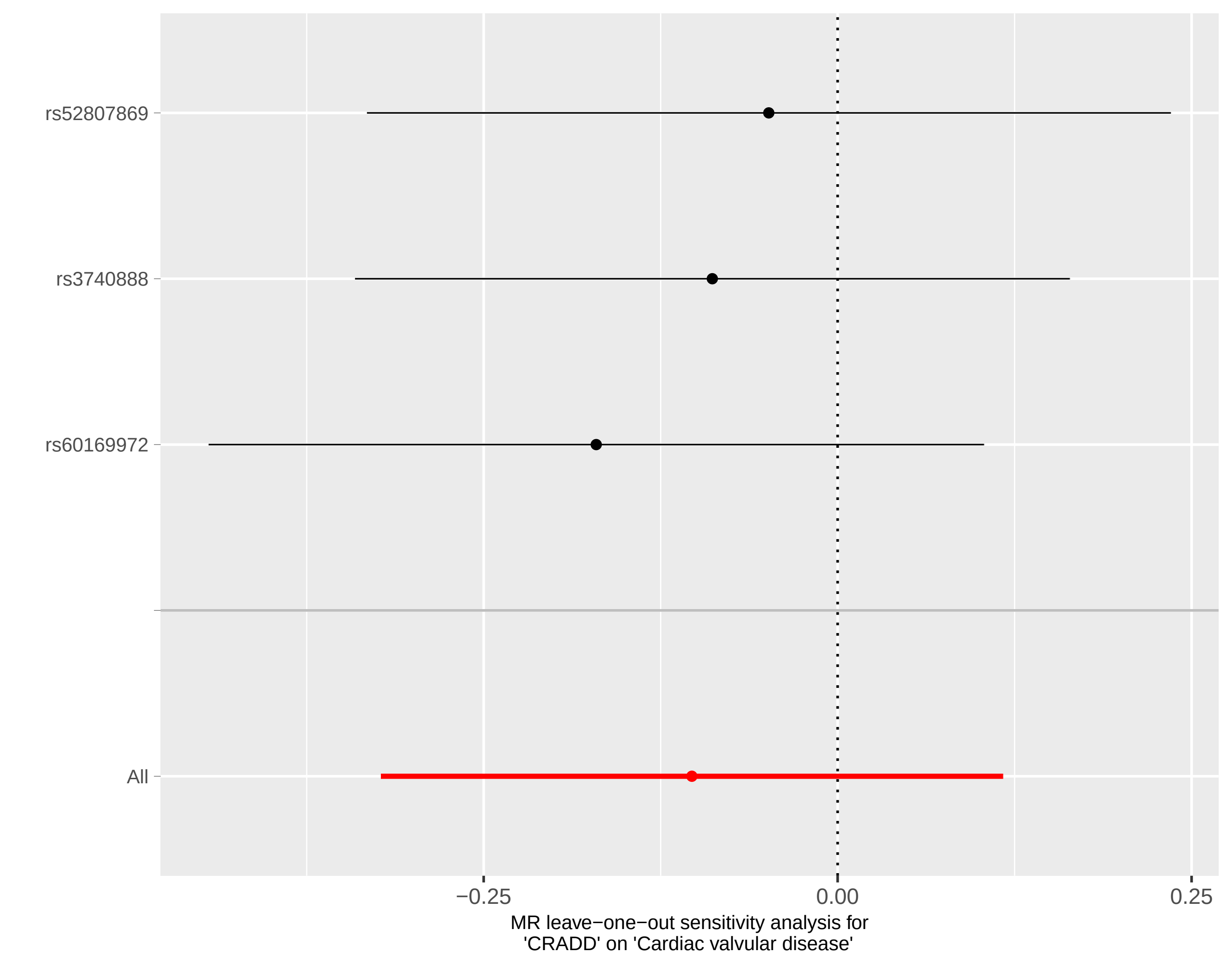
rs52807869
rs3740888
rs60169972
All
−0.25
0.00
0.25
MR leave−one−out sensitivity analysis for
'CRADD' on 'Cardiac valvular disease'

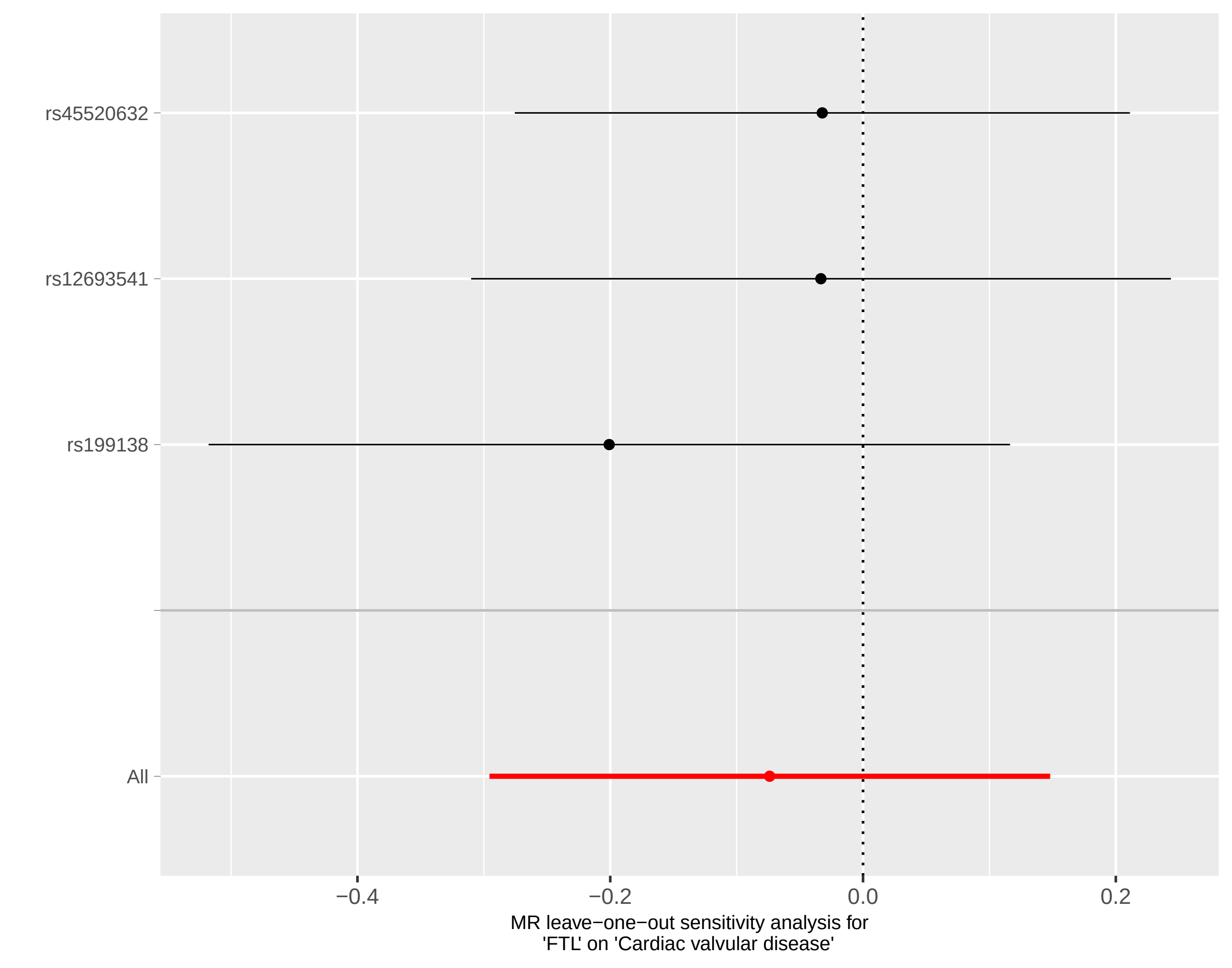
rs45520632
rs12693541
rs199138
All
−0.4
−0.2
0.0
0.2
MR leave−one−out sensitivity analysis for
'FTL' on 'Cardiac valvular disease'

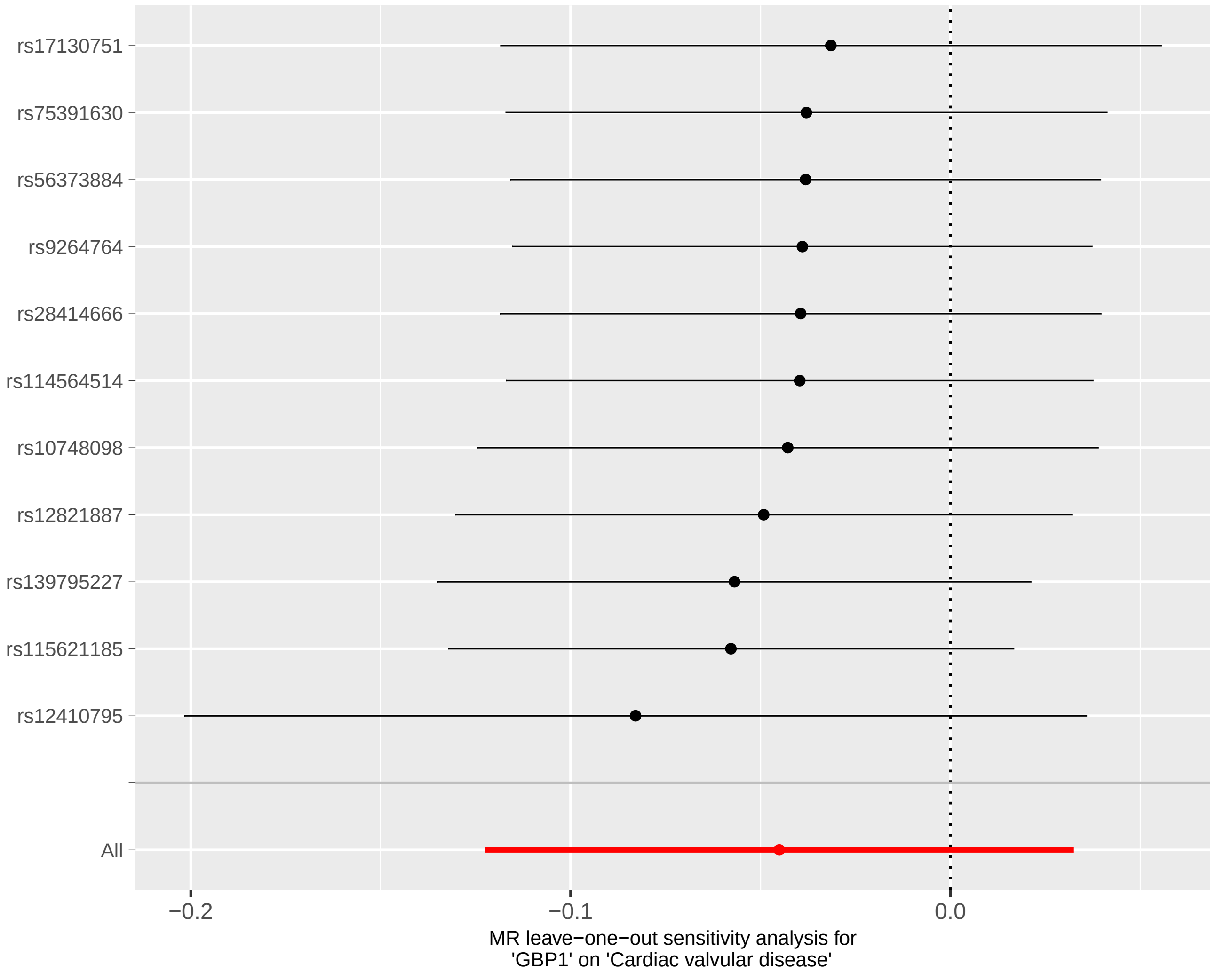
rs17130751
rs75391630
rs56373884
rs9264764
rs28414666
rs114564514
rs10748098
rs12821887
rs139795227
rs115621185
rs12410795
All
−0.2
−0.1
0.0
MR leave−one−out sensitivity analysis for
'GBP1' on 'Cardiac valvular disease'

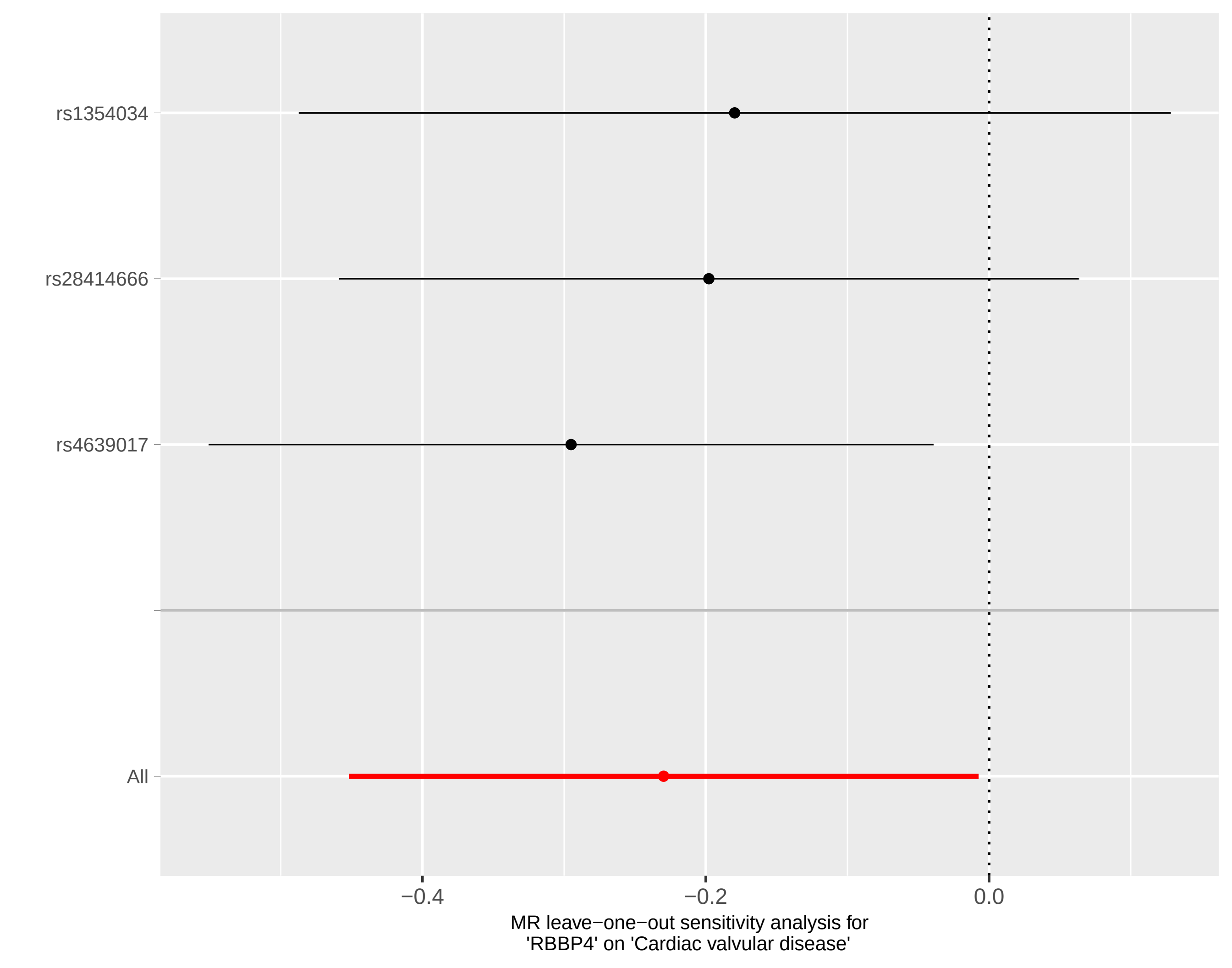

rs1354034
rs28414666
rs4639017
All
−0.4
−0.2
0.0
MR leave−one−out sensitivity analysis for
'RBBP4' on 'Cardiac valvular disease'

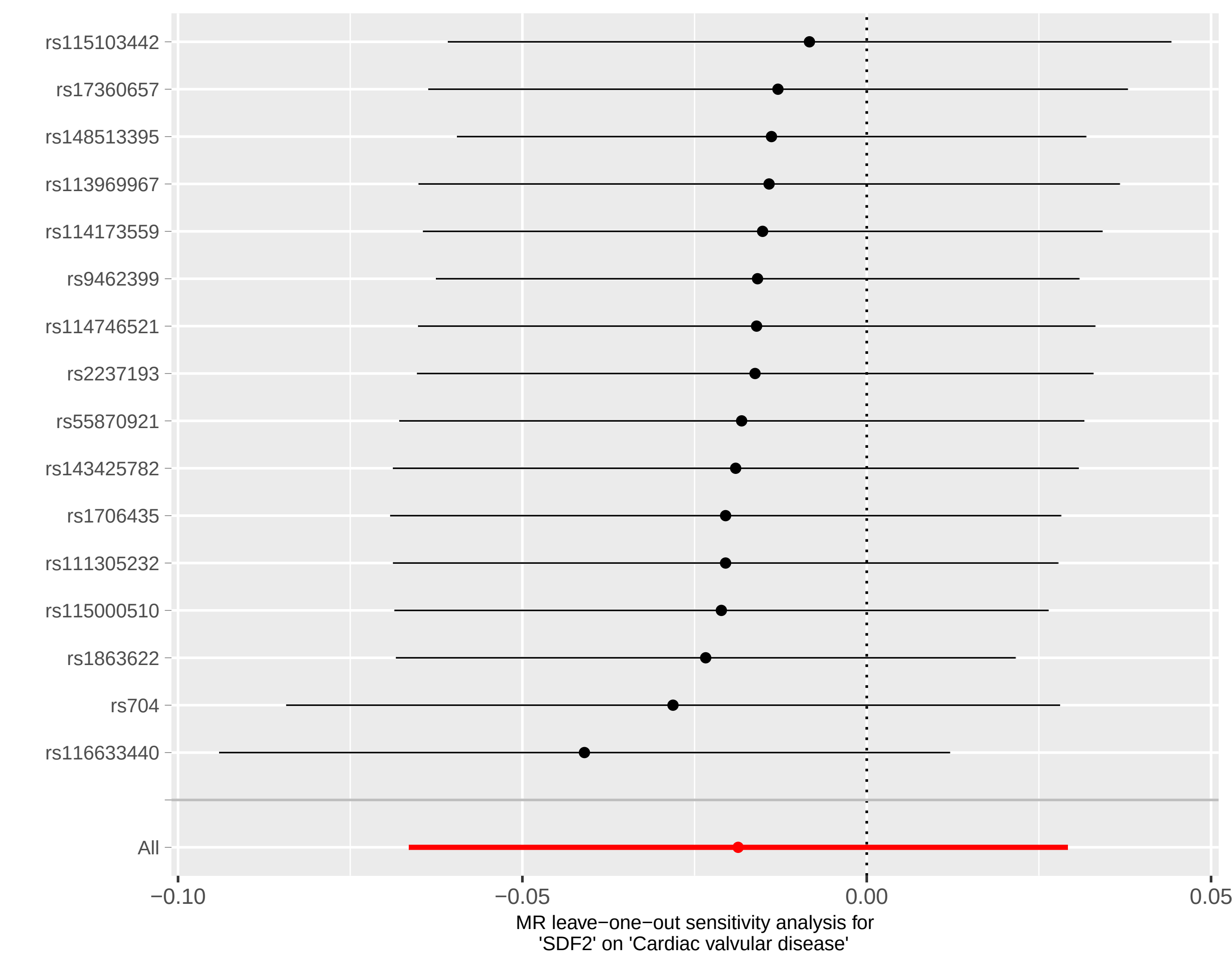
rs115103442
rs17360657
rs148513395
rs113969967
rs114173559
rs9462399
rs114746521
rs2237193
rs55870921
rs143425782
rs1706435
rs111305232
rs115000510
rs1863622
rs704
rs116633440
All
−0.10
−0.05
0.00
0.05
MR leave−one−out sensitivity analysis for
'SDF2' on 'Cardiac valvular disease'

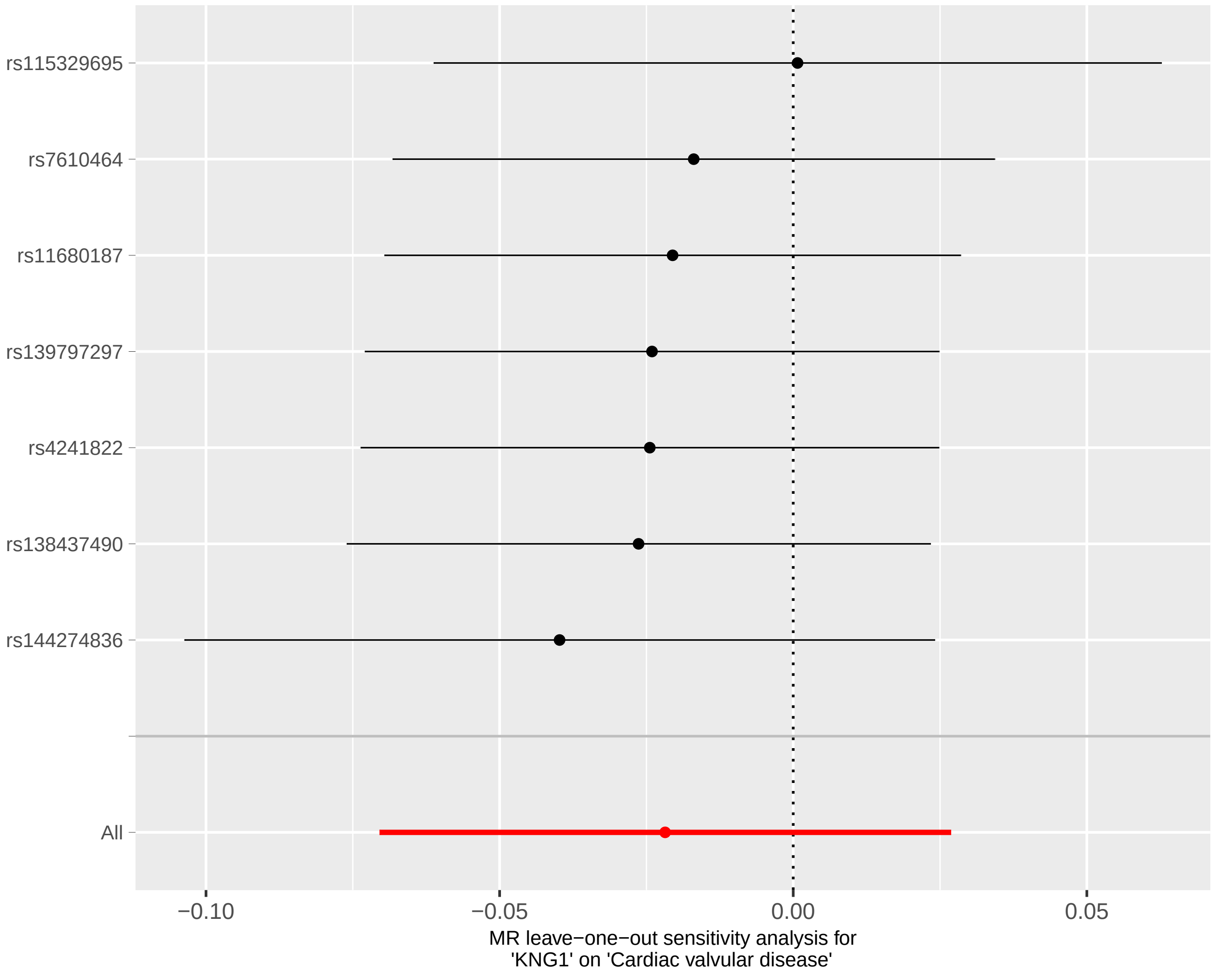
rs115329695
rs7610464
rs11680187
rs139797297
rs4241822
rs138437490
rs144274836
All
−0.10
−0.05
0.00
0.05
MR leave−one−out sensitivity analysis for
'KNG1' on 'Cardiac valvular disease'

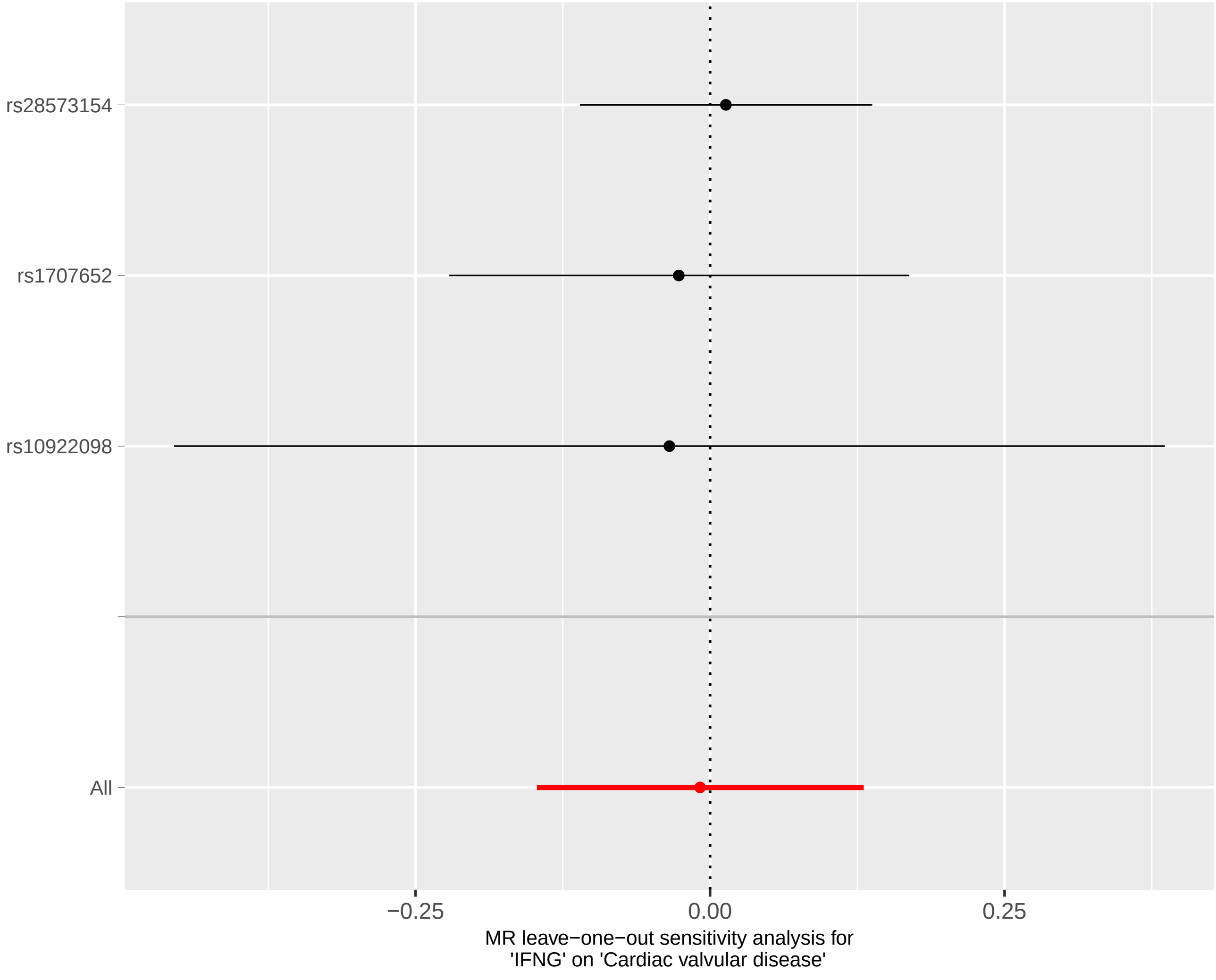
rs28573154
rs1707652
rs10922098
All
−0.25
0.00
0.25
MR leave−one−out sensitivity analysis for
'IFNG' on 'Cardiac valvular disease'

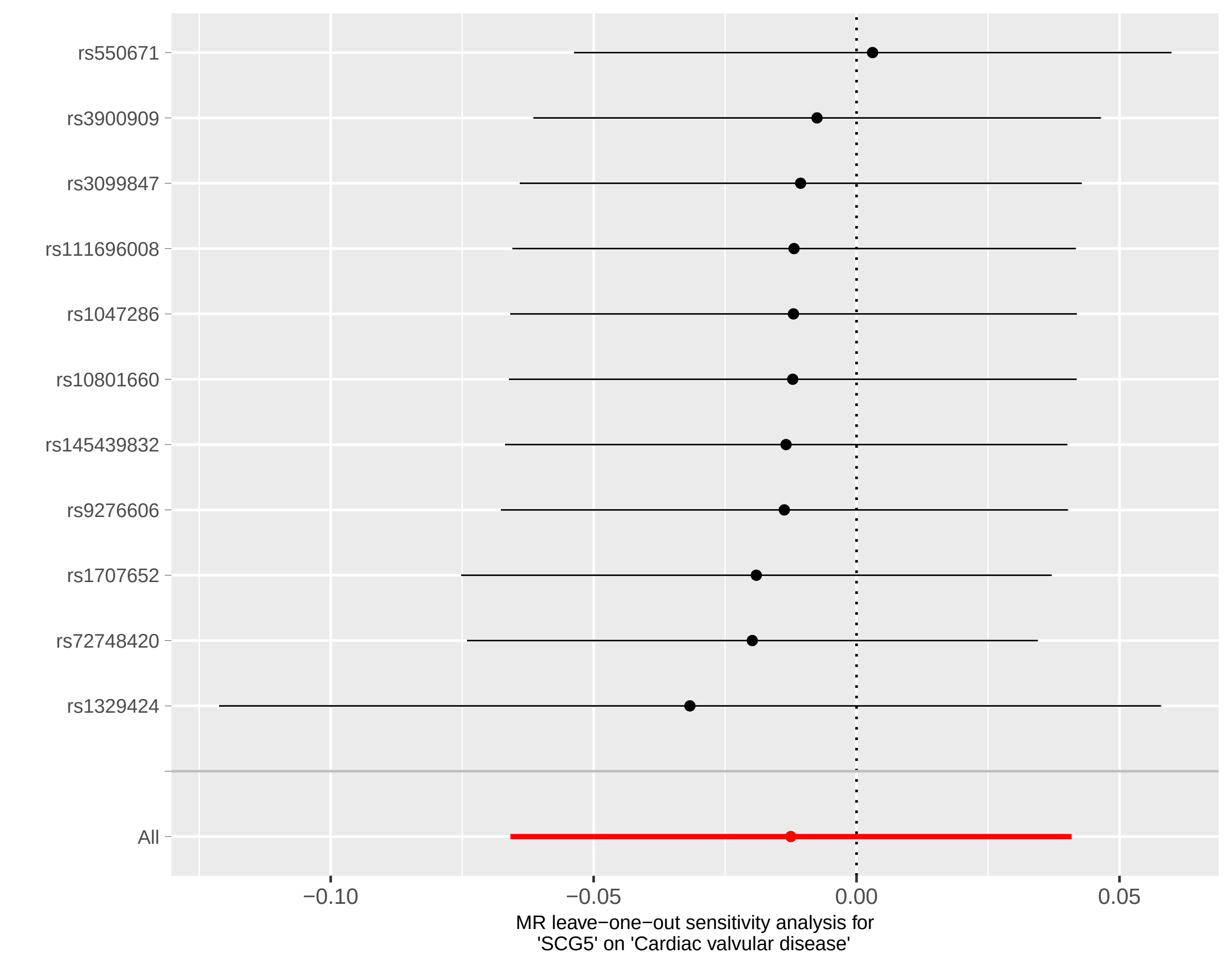

rs550671
rs3900909
rs3099847
rs111696008
rs1047286
rs10801660
rs145439832
rs9276606
rs1707652
rs72748420
rs1329424
All
−0.10
−0.05
0.00
0.05
MR leave−one−out sensitivity analysis for
'SCG5' on 'Cardiac valvular disease'

Insufficient number of SNPs

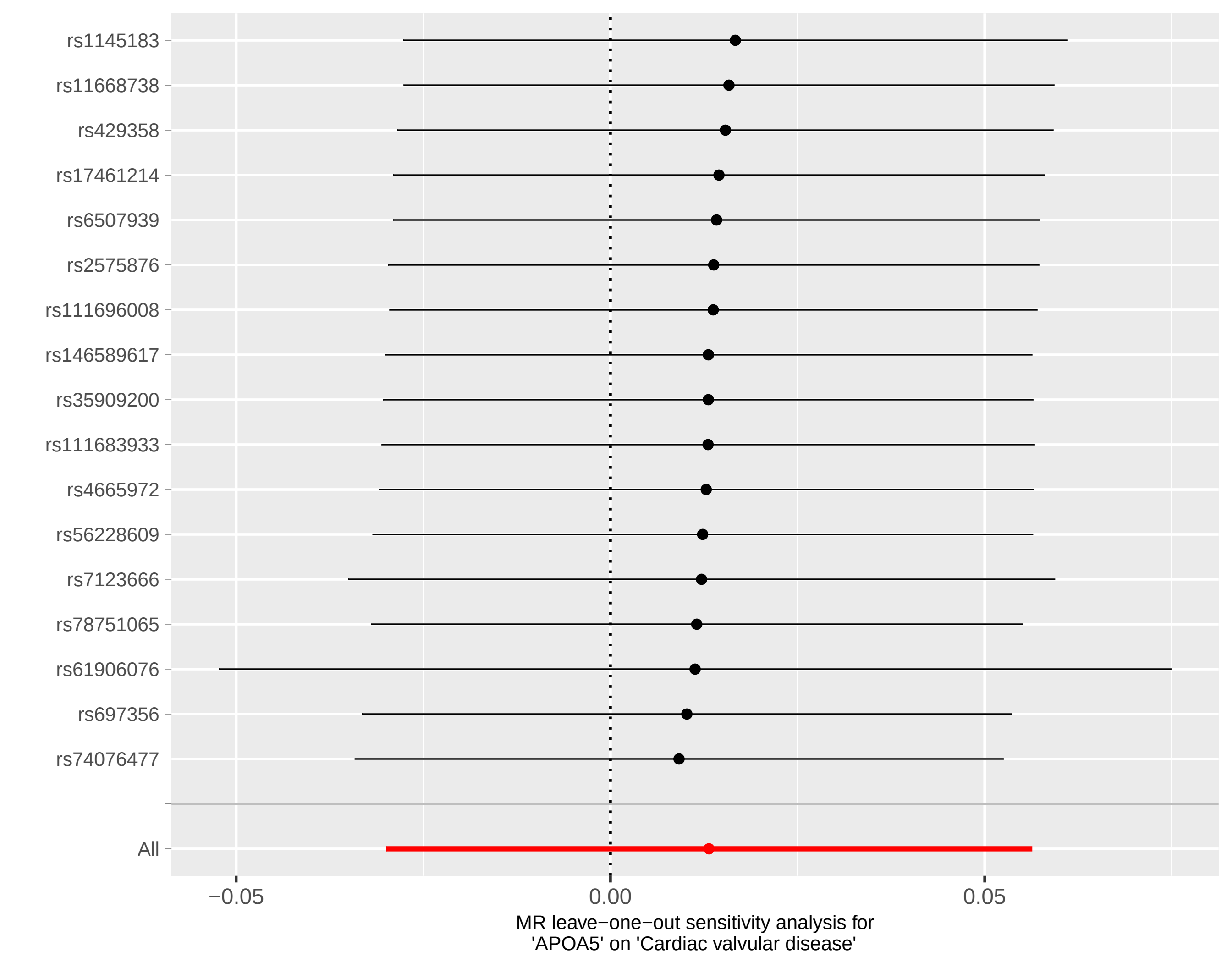
rs1145183
rs11668738
rs429358
rs17461214
rs6507939
rs2575876
rs111696008
rs146589617
rs35909200
rs111683933
rs4665972
rs56228609
rs7123666
rs78751065
rs61906076
rs697356
rs74076477
All
−0.05
0.00
0.05
MR leave−one−out sensitivity analysis for
'APOA5' on 'Cardiac valvular disease'

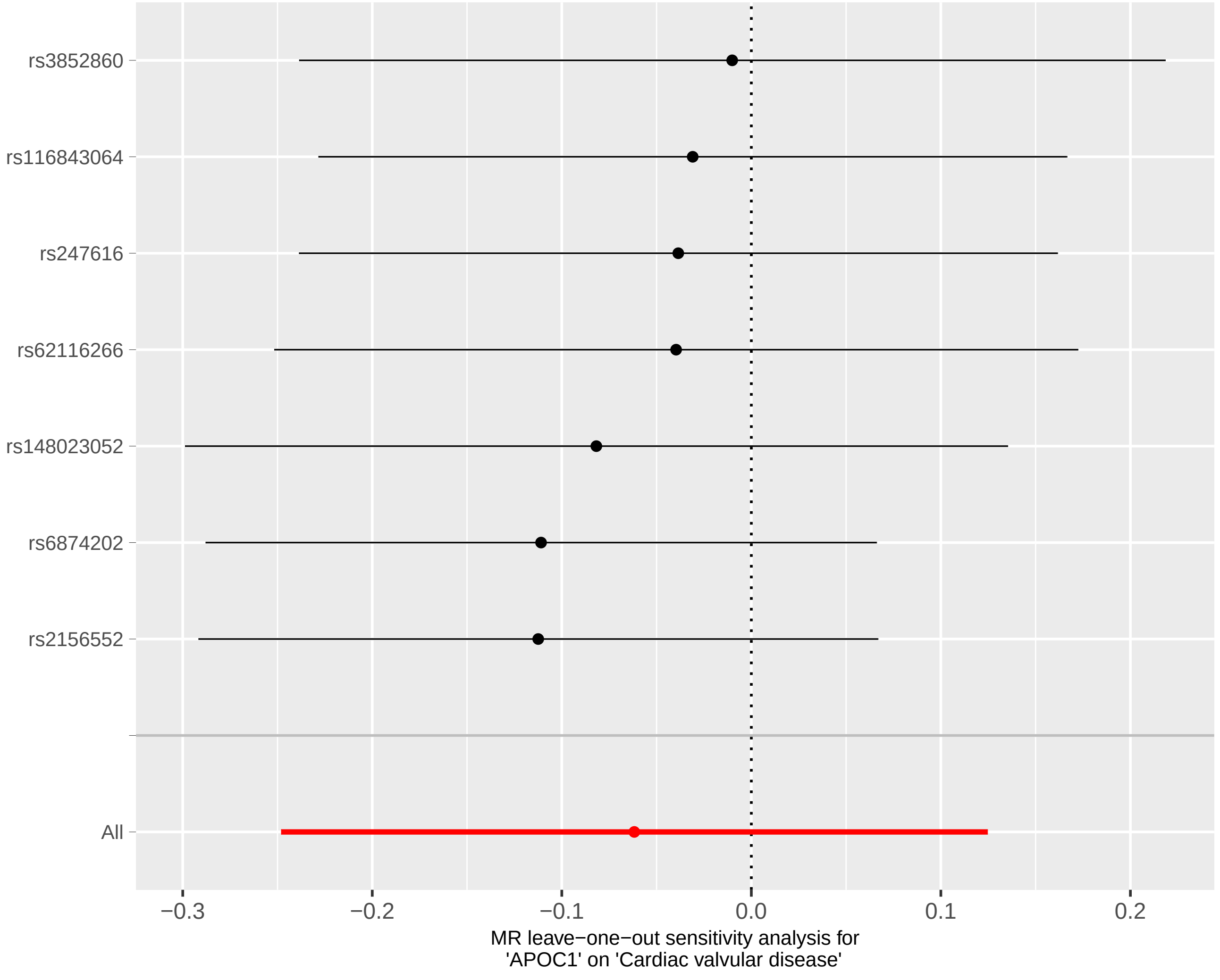
rs3852860
rs116843064
rs247616
rs62116266
rs148023052
rs6874202
rs2156552
All
−0.3
−0.2
−0.1
0.0
0.1
0.2
MR leave−one−out sensitivity analysis for
'APOC1' on 'Cardiac valvular disease'

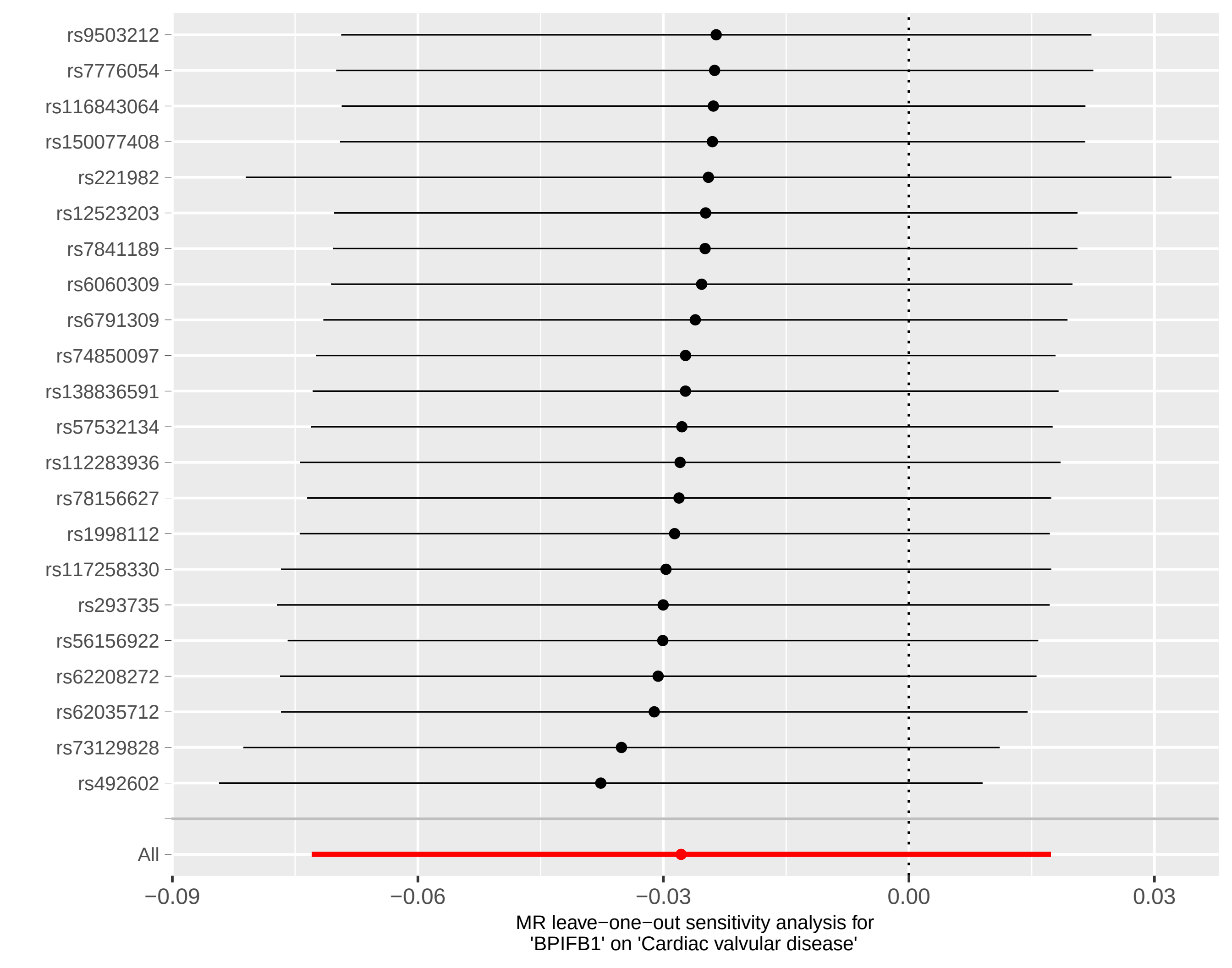
rs9503212
rs7776054
rs116843064
rs150077408
rs221982
rs12523203
rs7841189
rs6060309
rs6791309
rs74850097
rs138836591
rs57532134
rs112283936
rs78156627
rs1998112
rs117258330
rs293735
rs56156922
rs62208272
rs62035712
rs73129828
rs492602
All
−0.09
−0.06
−0.03
0.00
0.03
MR leave−one−out sensitivity analysis for
'BPIFB1' on 'Cardiac valvular disease'

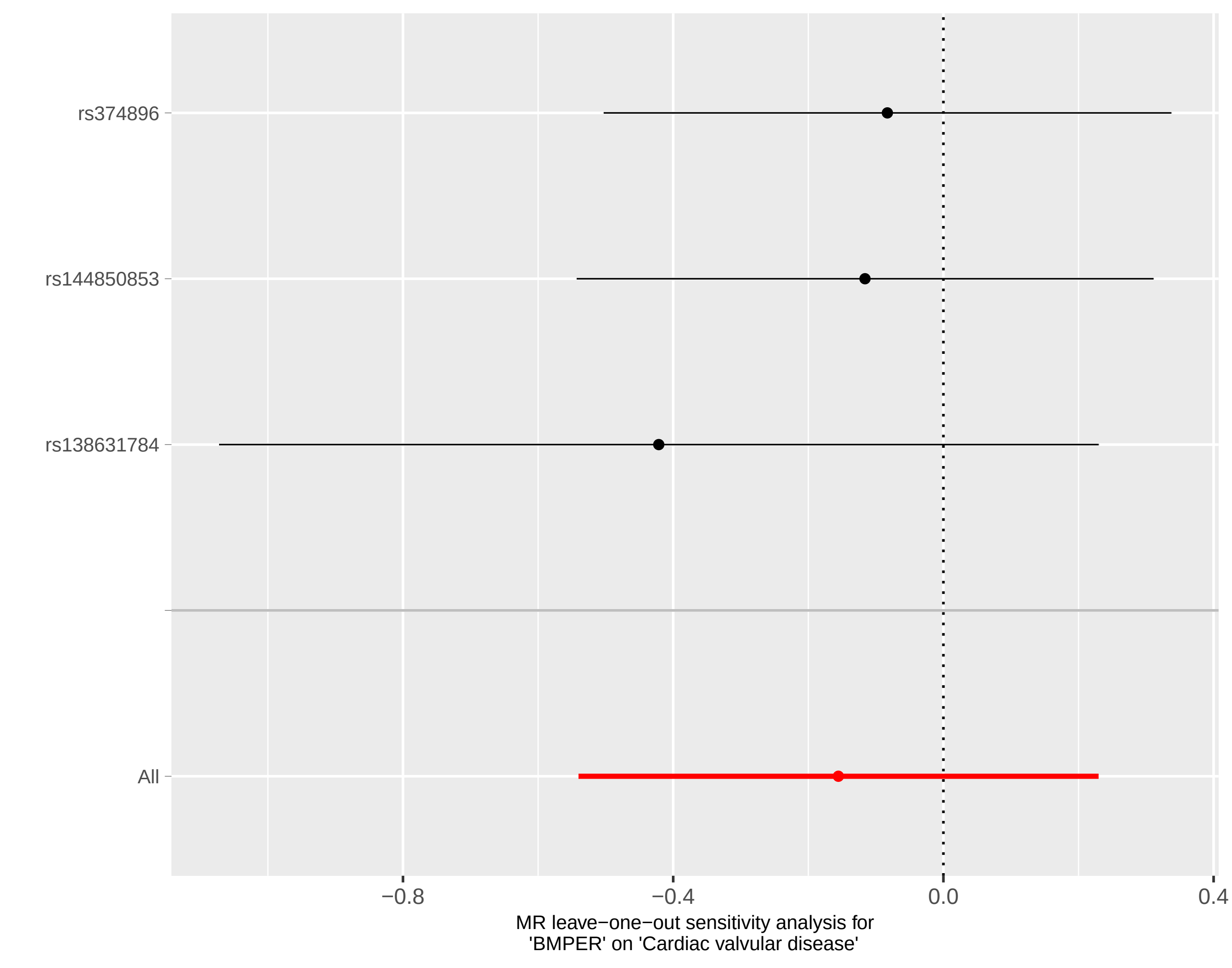
rs374896
rs144850853
rs138631784
All
−0.8
−0.4
0.0
0.4
MR leave−one−out sensitivity analysis for
'BMPER' on 'Cardiac valvular disease'

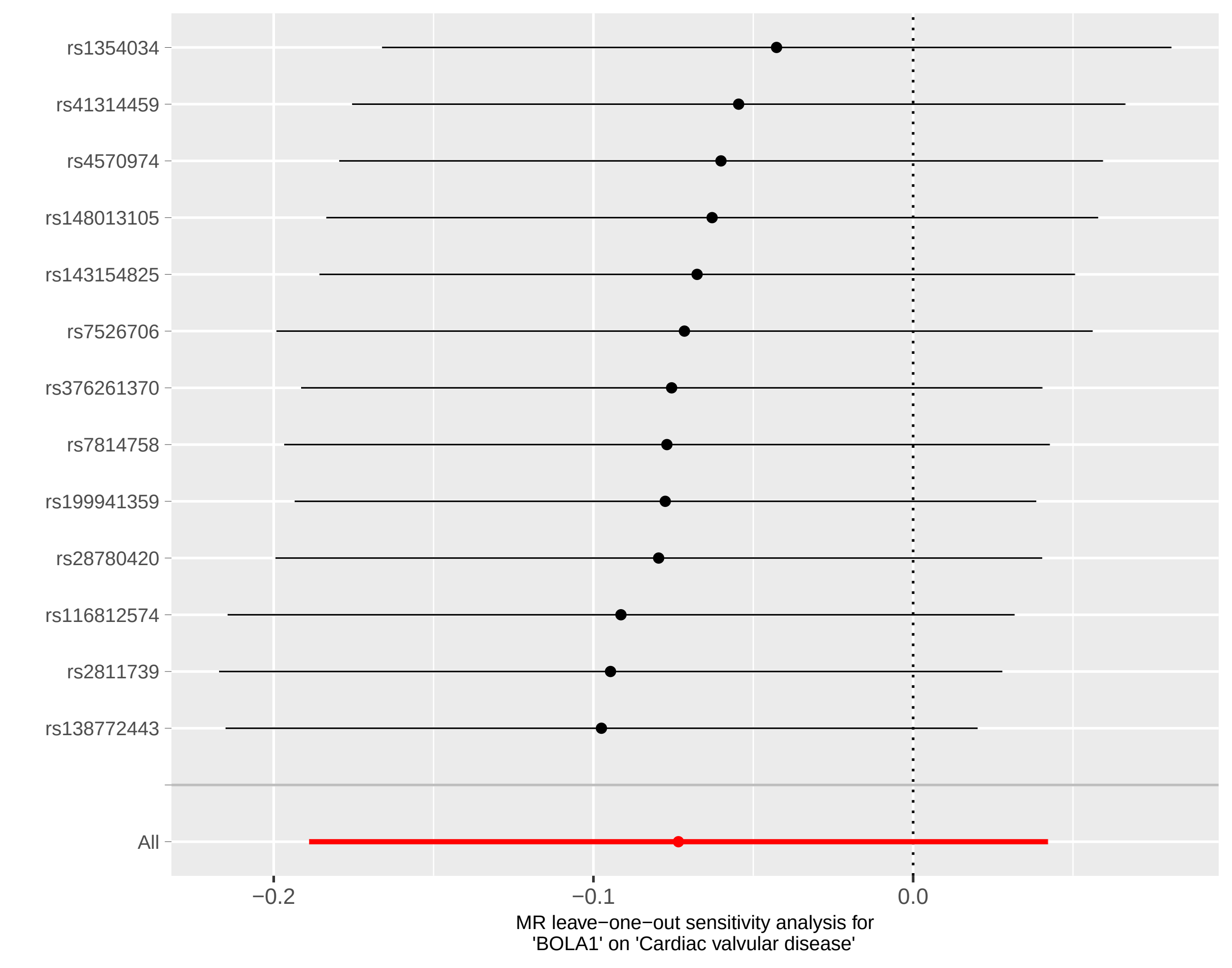

rs1354034
rs41314459
rs4570974
rs148013105
rs143154825
rs7526706
rs376261370
rs7814758
rs199941359
rs28780420
rs116812574
rs2811739
rs138772443
All
−0.2
−0.1
0.0
MR leave−one−out sensitivity analysis for
'BOLA1' on 'Cardiac valvular disease'

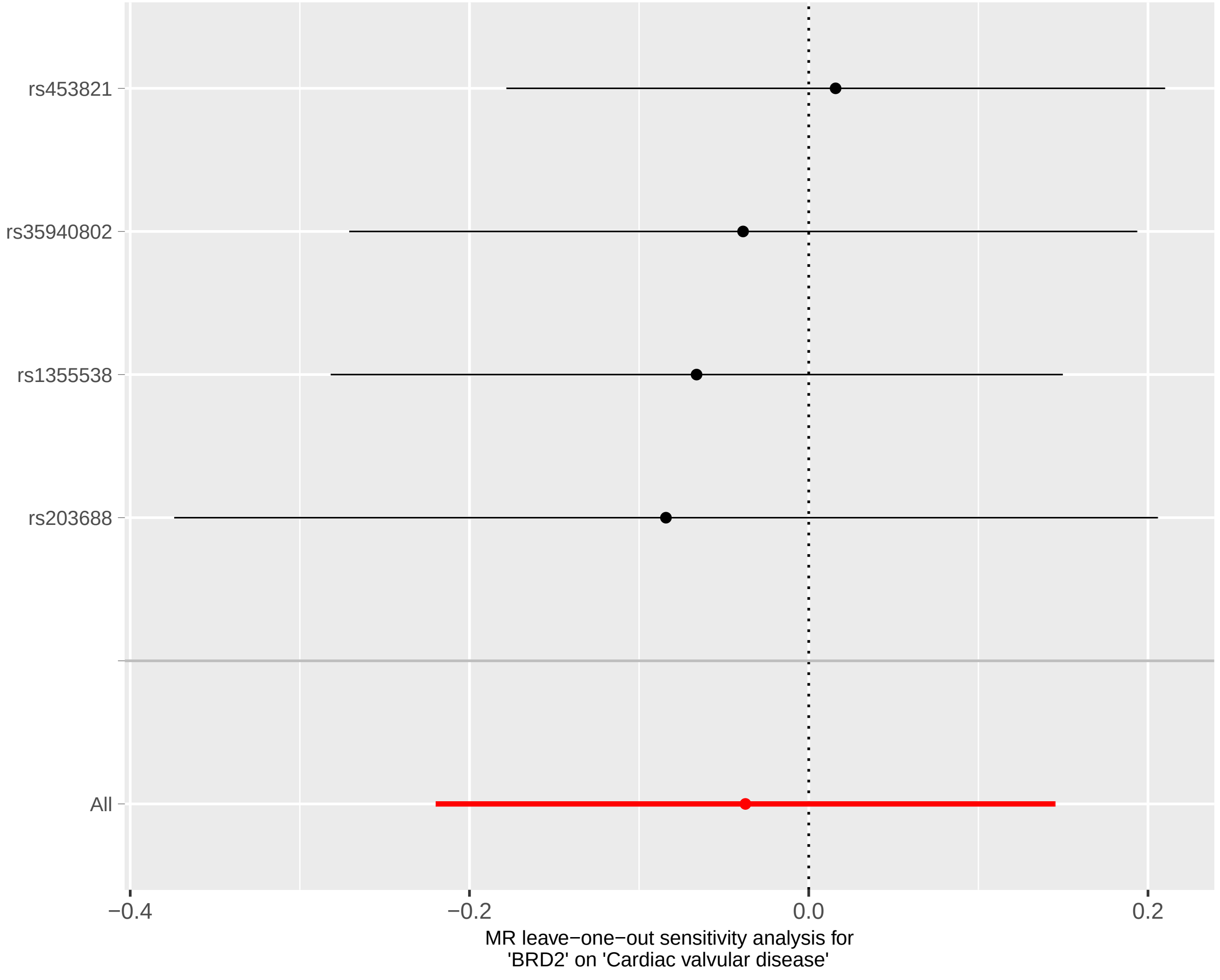
rs453821
rs35940802
rs1355538
rs203688
All
−0.4
−0.2
0.0
0.2
MR leave−one−out sensitivity analysis for
'BRD2' on 'Cardiac valvular disease'

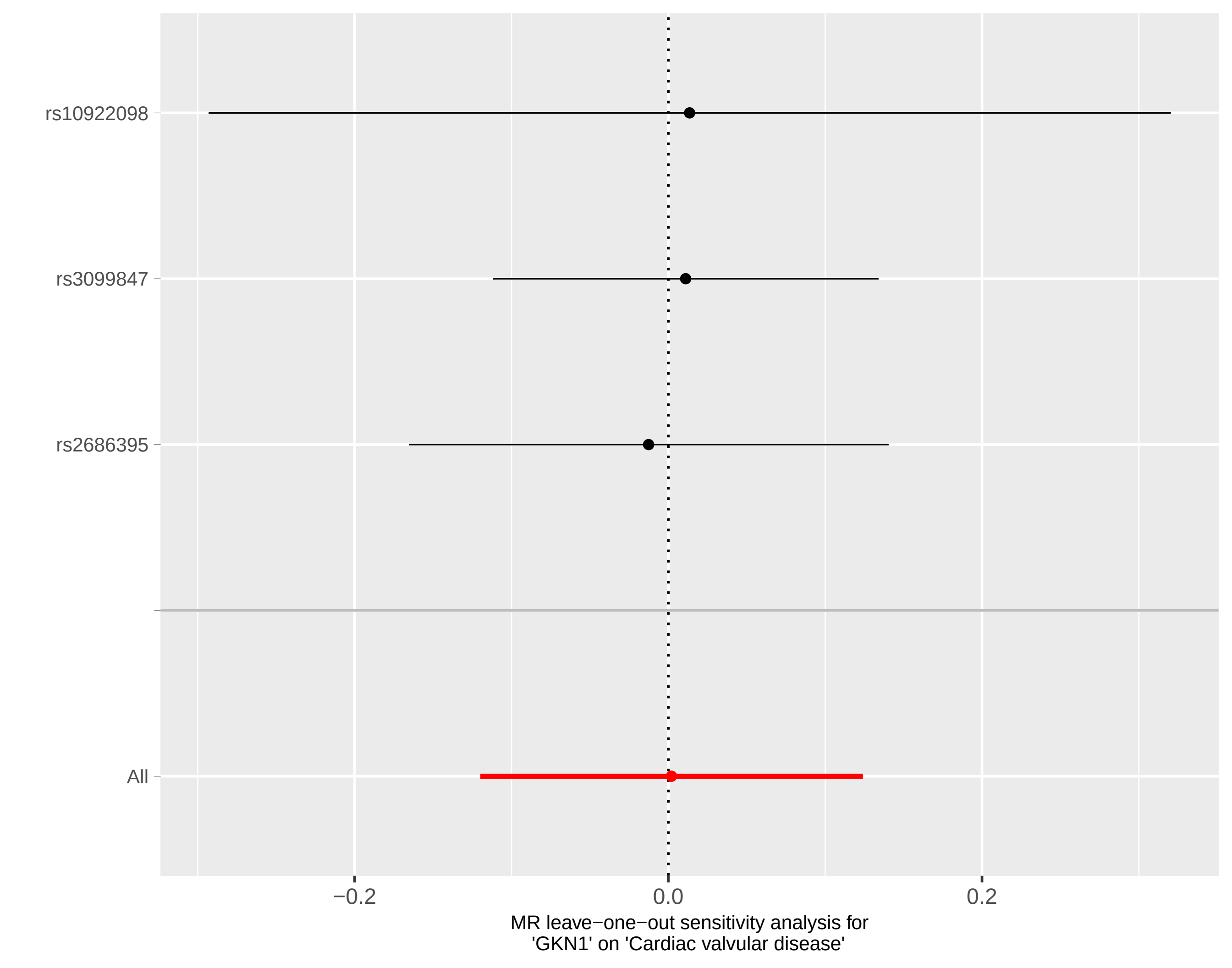

rs10922098
rs3099847
rs2686395
All
−0.2
0.0
0.2
MR leave−one−out sensitivity analysis for
'GKN1' on 'Cardiac valvular disease'

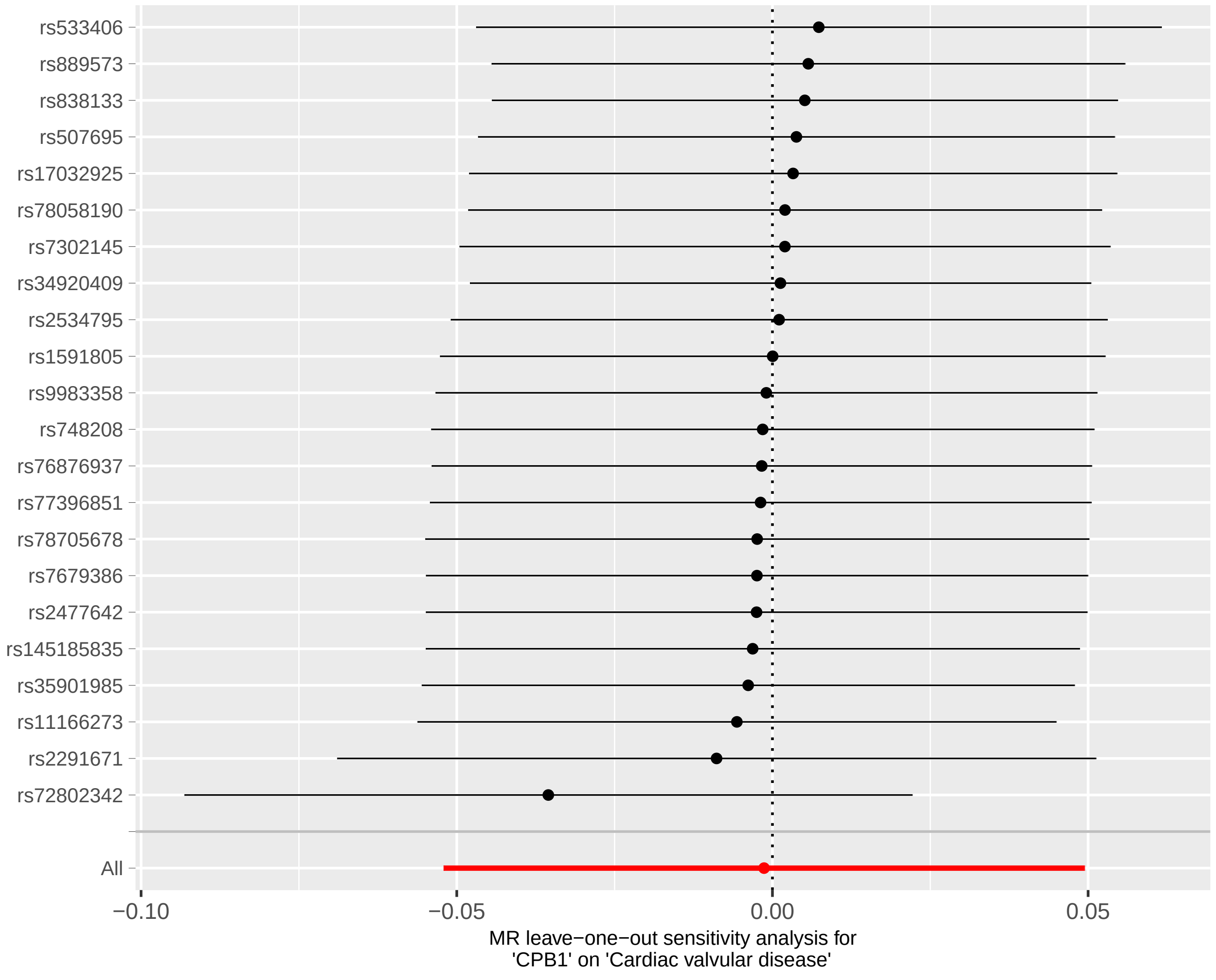
rs533406
rs889573
rs838133
rs507695
rs17032925
rs78058190
rs7302145
rs34920409
rs2534795
rs1591805
rs9983358
rs748208
rs76876937
rs77396851
rs78705678
rs7679386
rs2477642
rs145185835
rs35901985
rs11166273
rs2291671
rs72802342
All
−0.10
−0.05
0.00
0.05
MR leave−one−out sensitivity analysis for
'CPB1' on 'Cardiac valvular disease'

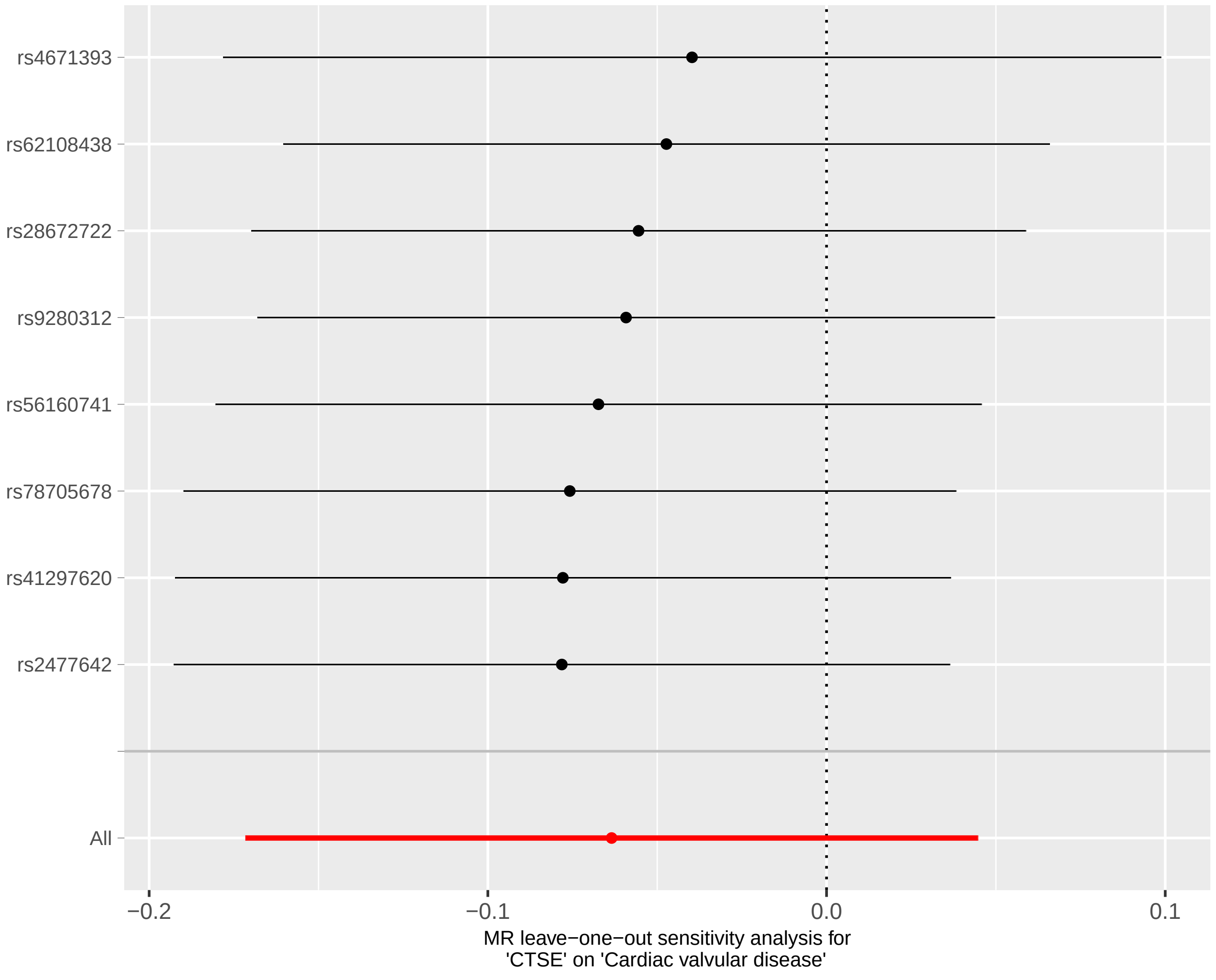
rs4671393
rs62108438
rs28672722
rs9280312
rs56160741
rs78705678
rs41297620
rs2477642
All
−0.2
−0.1
0.0
0.1
MR leave−one−out sensitivity analysis for
'CTSE' on 'Cardiac valvular disease'

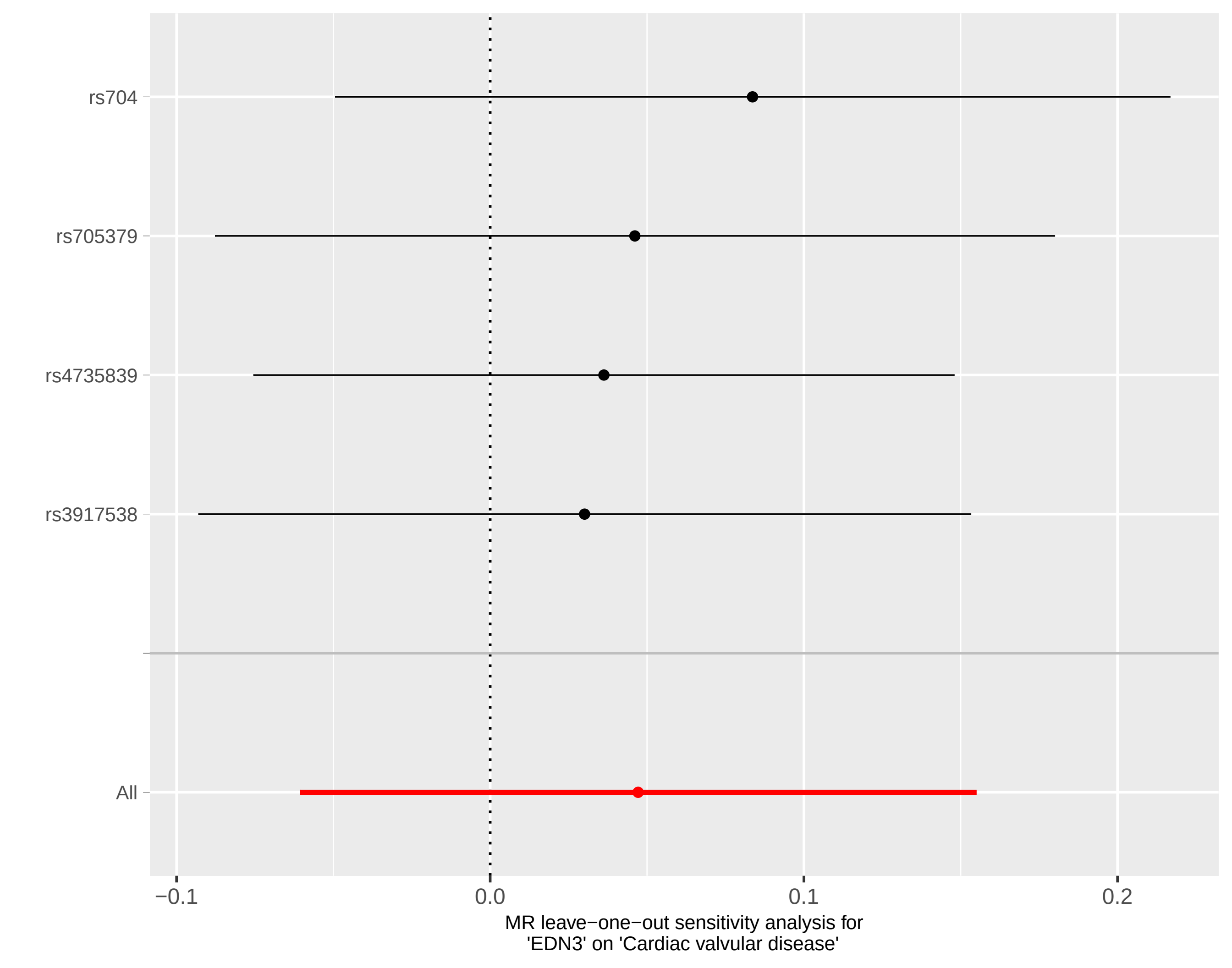

rs704
rs705379
rs4735839
rs3917538
All
−0.1
0.0
0.1
0.2
MR leave−one−out sensitivity analysis for
'EDN3' on 'Cardiac valvular disease'

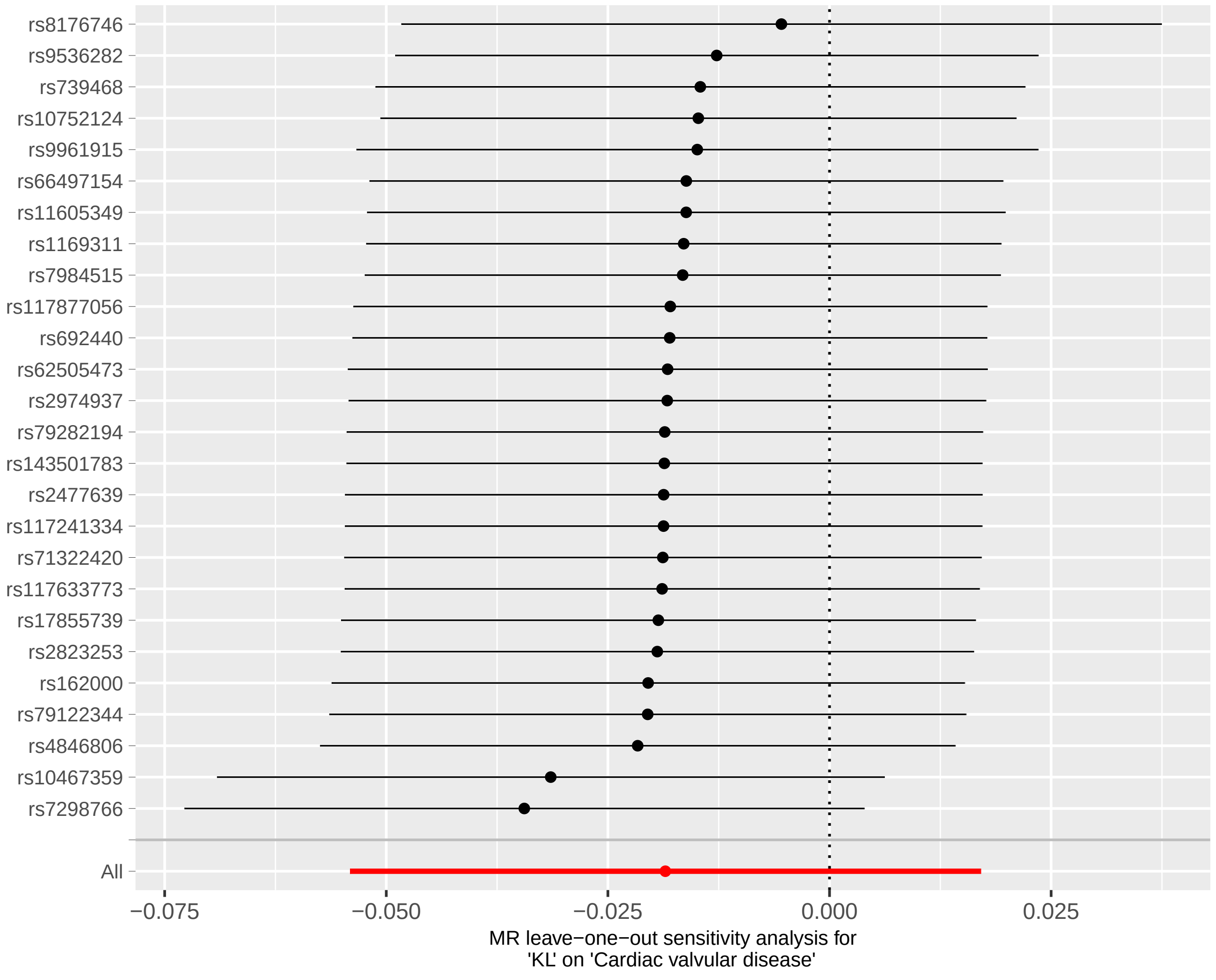

rs8176746
rs9536282
rs739468
rs10752124
rs9961915
rs66497154
rs11605349
rs1169311
rs7984515
rs117877056
rs692440
rs62505473
rs2974937
rs79282194
rs143501783
rs2477639
rs117241334
rs71322420
rs117633773
rs17855739
rs2823253
rs162000
rs79122344
rs4846806
rs10467359
rs7298766
All
−0.075
−0.050
−0.025
0.000
0.025
MR leave−one−out sensitivity analysis for
'KL' on 'Cardiac valvular disease'

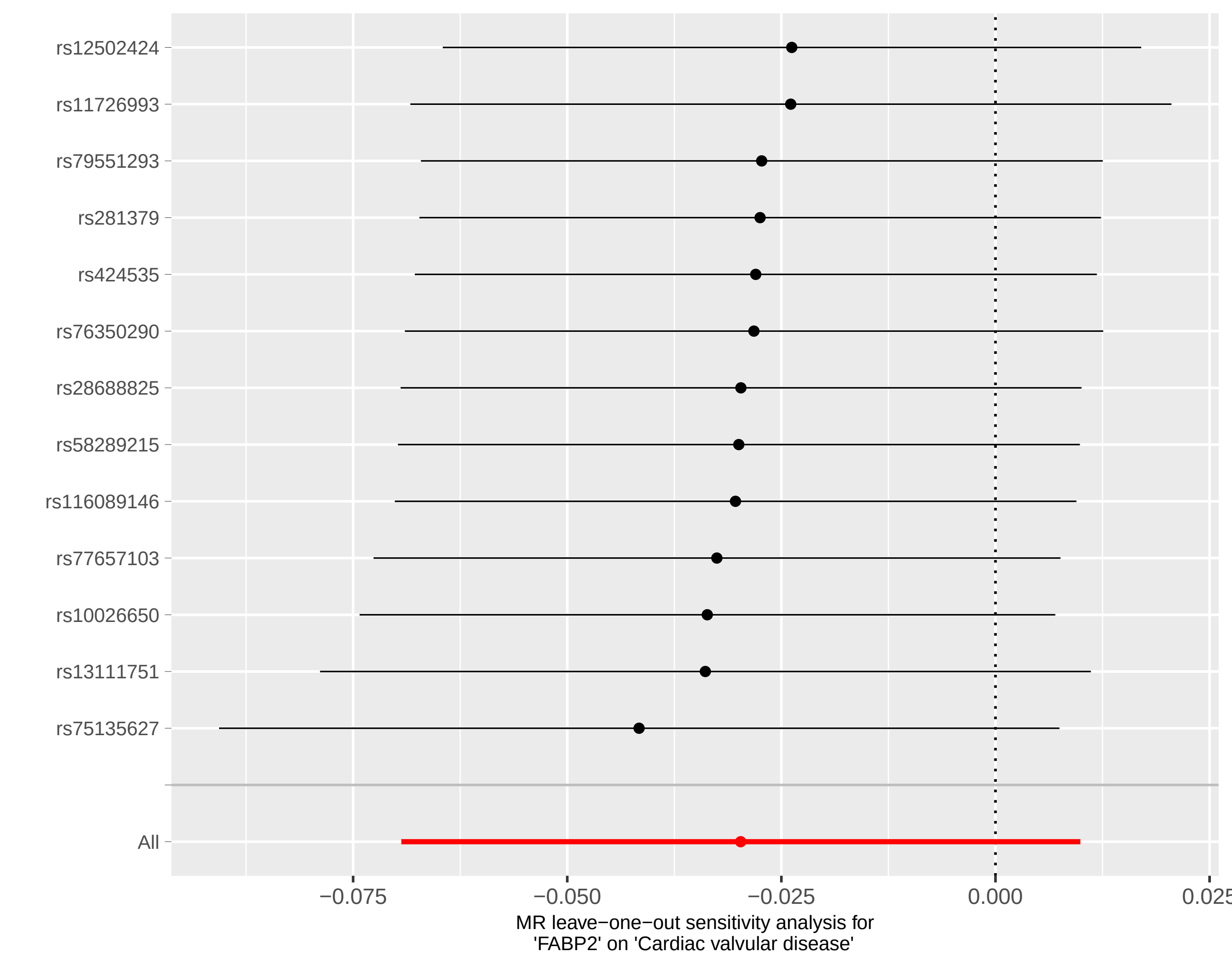
rs12502424
rs11726993
rs79551293
rs281379
rs424535
rs76350290
rs28688825
rs58289215
rs116089146
rs77657103
rs10026650
rs13111751
rs75135627
All
−0.075
−0.050
−0.025
0.000
0.025
MR leave−one−out sensitivity analysis for
'FABP2' on 'Cardiac valvular disease'

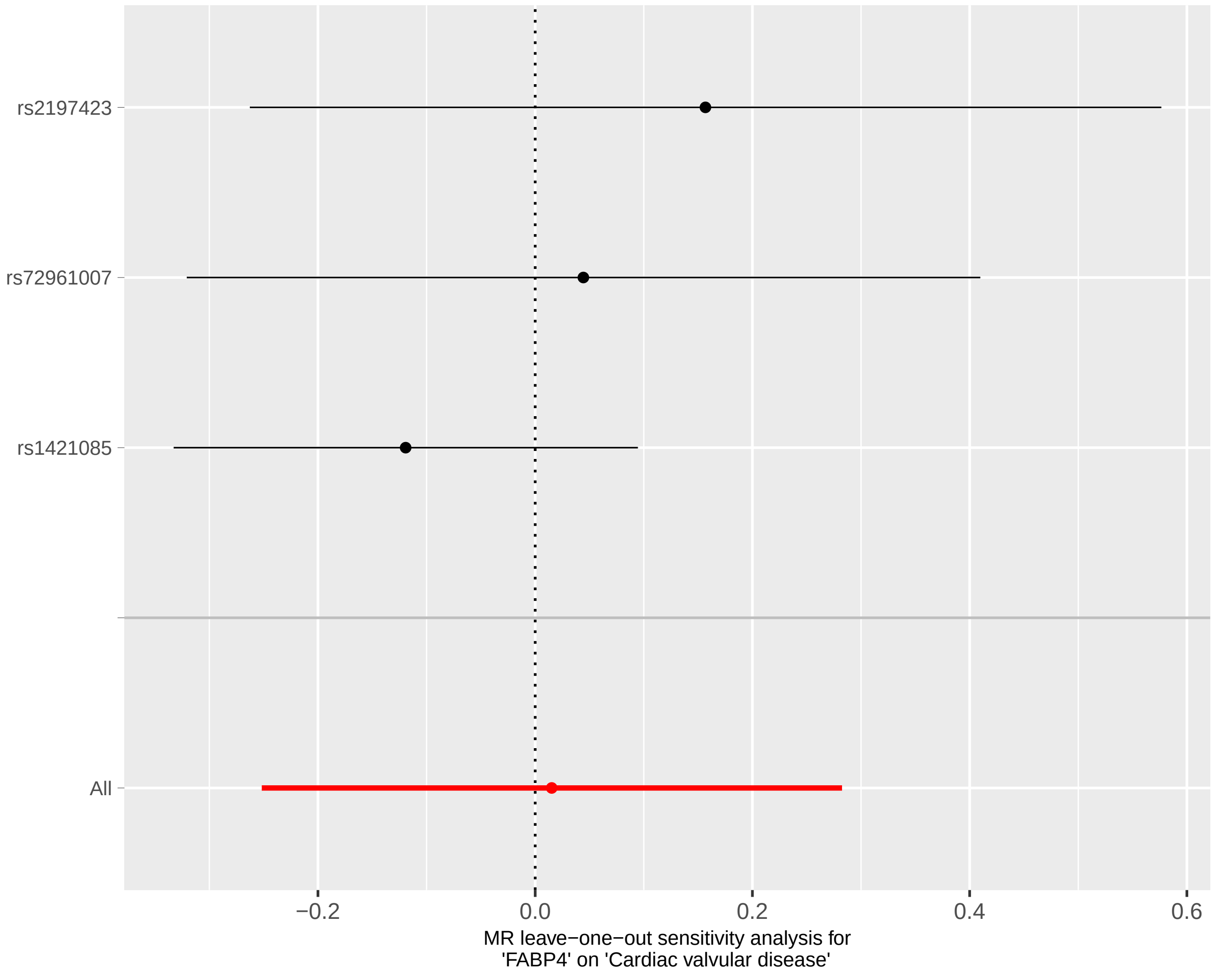
rs2197423
rs72961007
rs1421085
All
−0.2
0.0
0.2
0.4
0.6
MR leave−one−out sensitivity analysis for
'FABP4' on 'Cardiac valvular disease'

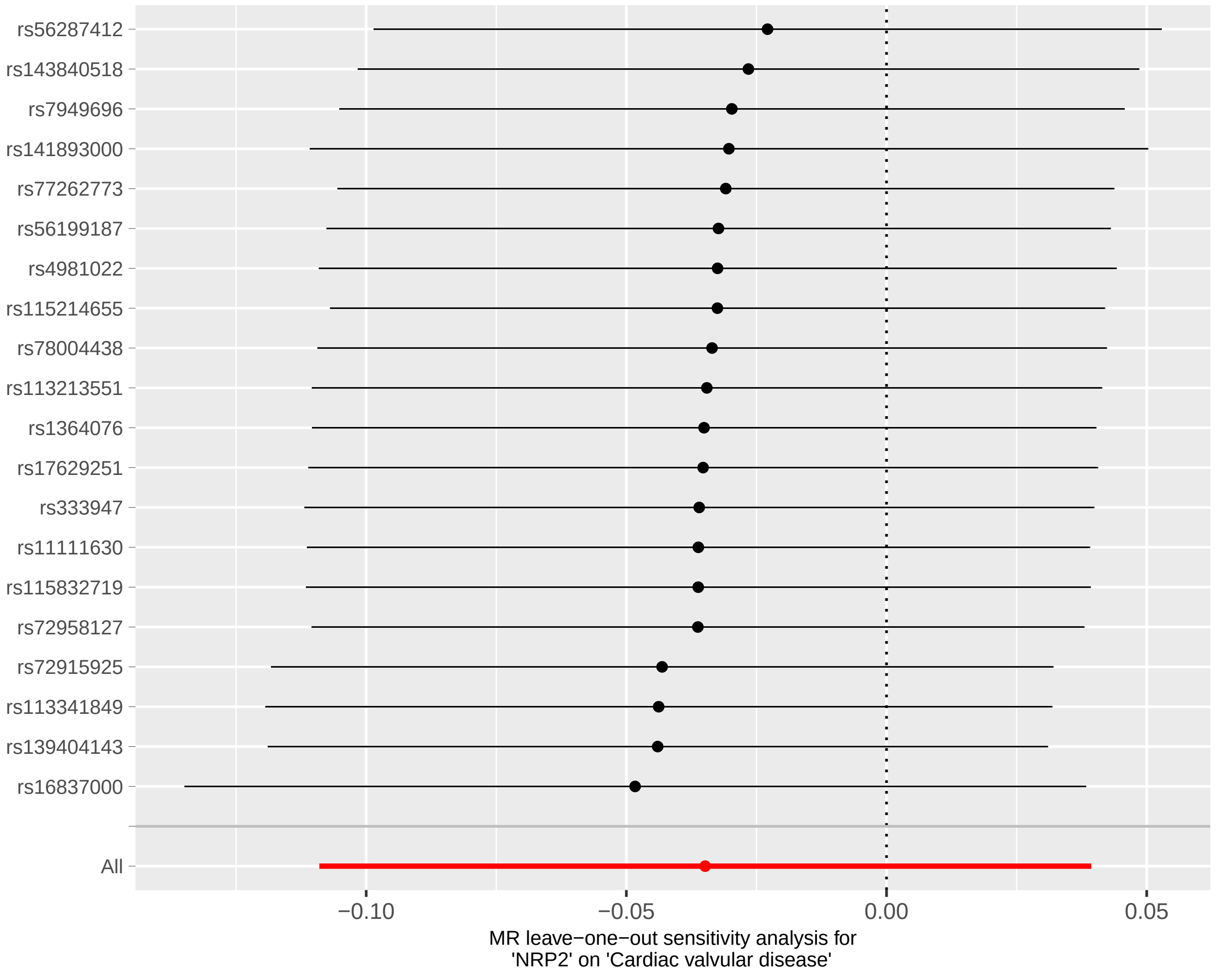
rs56287412
rs143840518
rs7949696
rs141893000
rs77262773
rs56199187
rs4981022
rs115214655
rs78004438
rs113213551
rs1364076
rs17629251
rs333947
rs11111630
rs115832719
rs72958127
rs72915925
rs113341849
rs139404143
rs16837000
All
−0.10
−0.05
0.00
0.05
MR leave−one−out sensitivity analysis for
'NRP2' on 'Cardiac valvular disease'

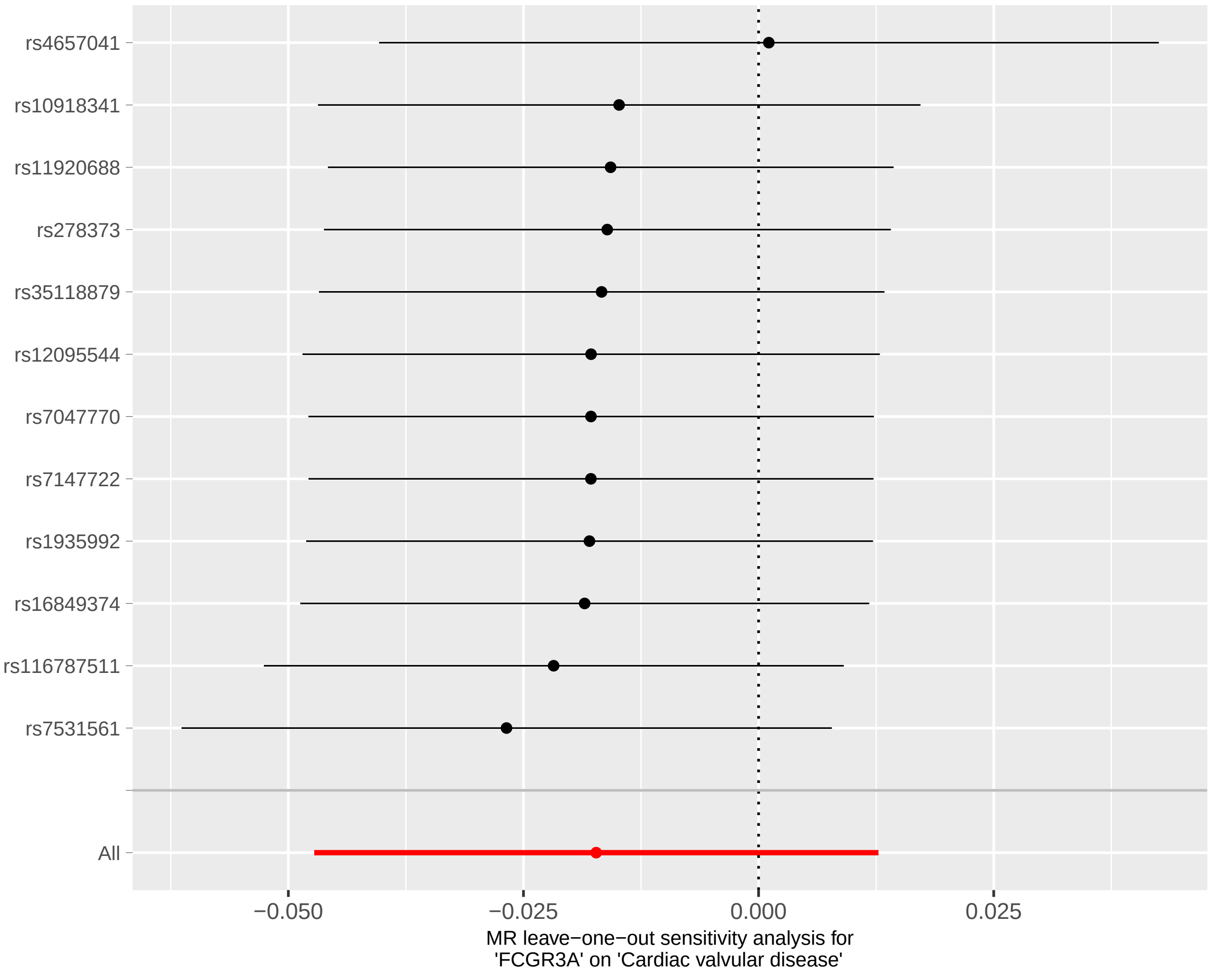

rs4657041
rs10918341
rs11920688
rs278373
rs35118879
rs12095544
rs7047770
rs7147722
rs1935992
rs16849374
rs116787511
rs7531561
All
−0.050
−0.025
0.000
0.025
MR leave−one−out sensitivity analysis for
'FCGR3A' on 'Cardiac valvular disease'

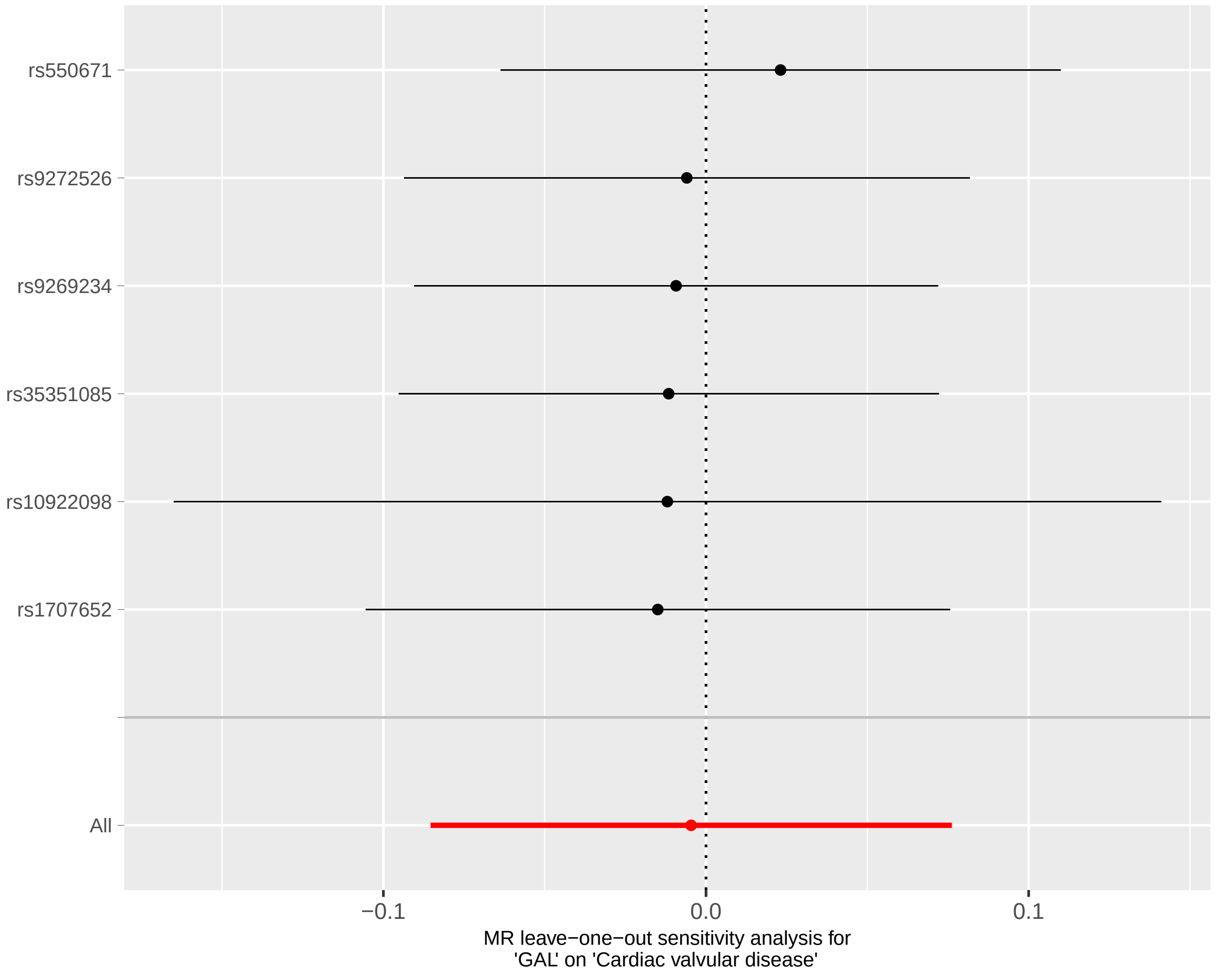
rs550671
rs9272526
rs9269234
rs35351085
rs10922098
rs1707652
All
−0.1
0.0
0.1
MR leave−one−out sensitivity analysis for
'GAL' on 'Cardiac valvular disease'

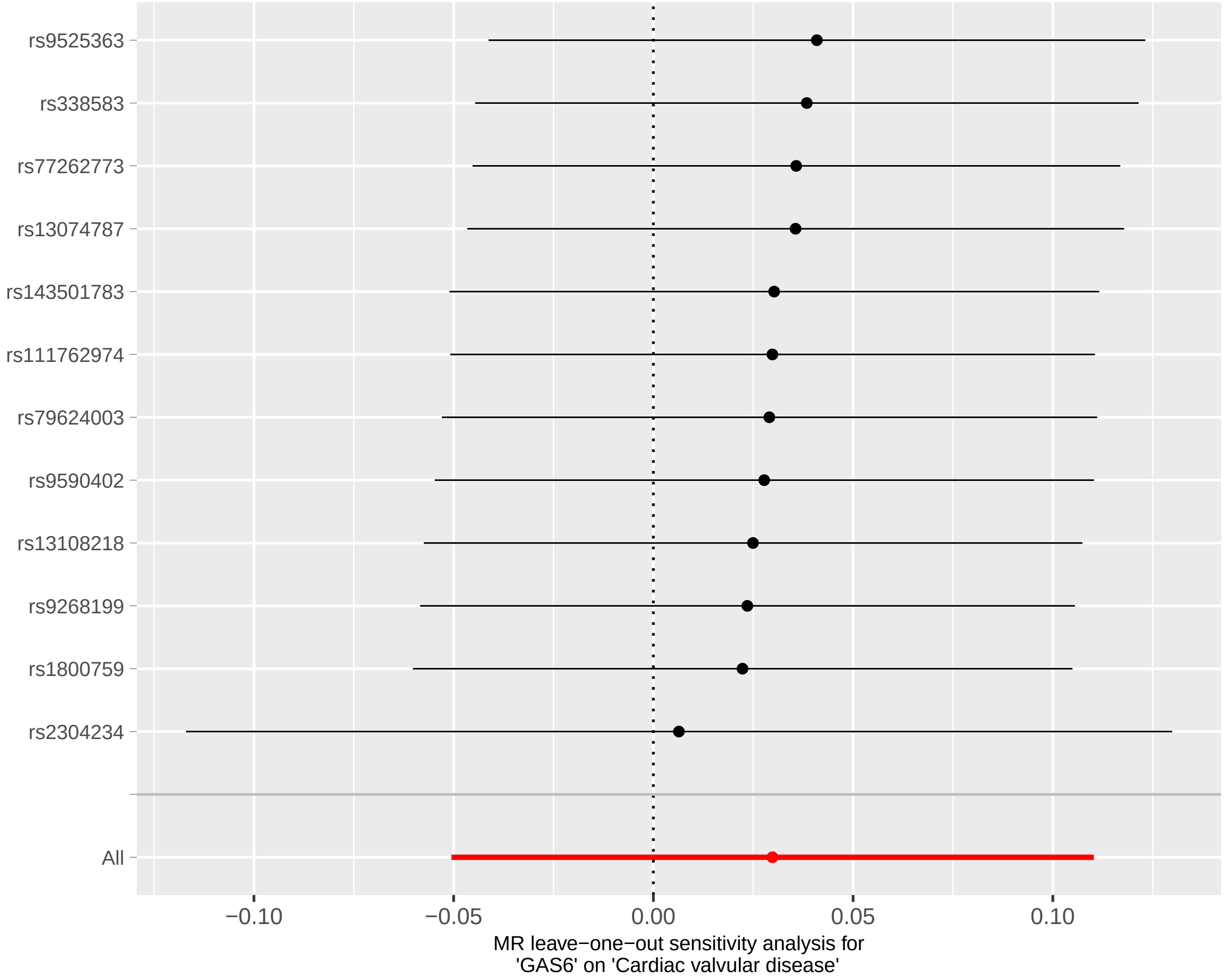

rs9525363
rs338583
rs77262773
rs13074787
rs143501783
rs111762974
rs79624003
rs9590402
rs13108218
rs9268199
rs1800759
rs2304234
All
−0.10
−0.05
0.00
0.05
0.10
MR leave−one−out sensitivity analysis for
'GAS6' on 'Cardiac valvular disease'

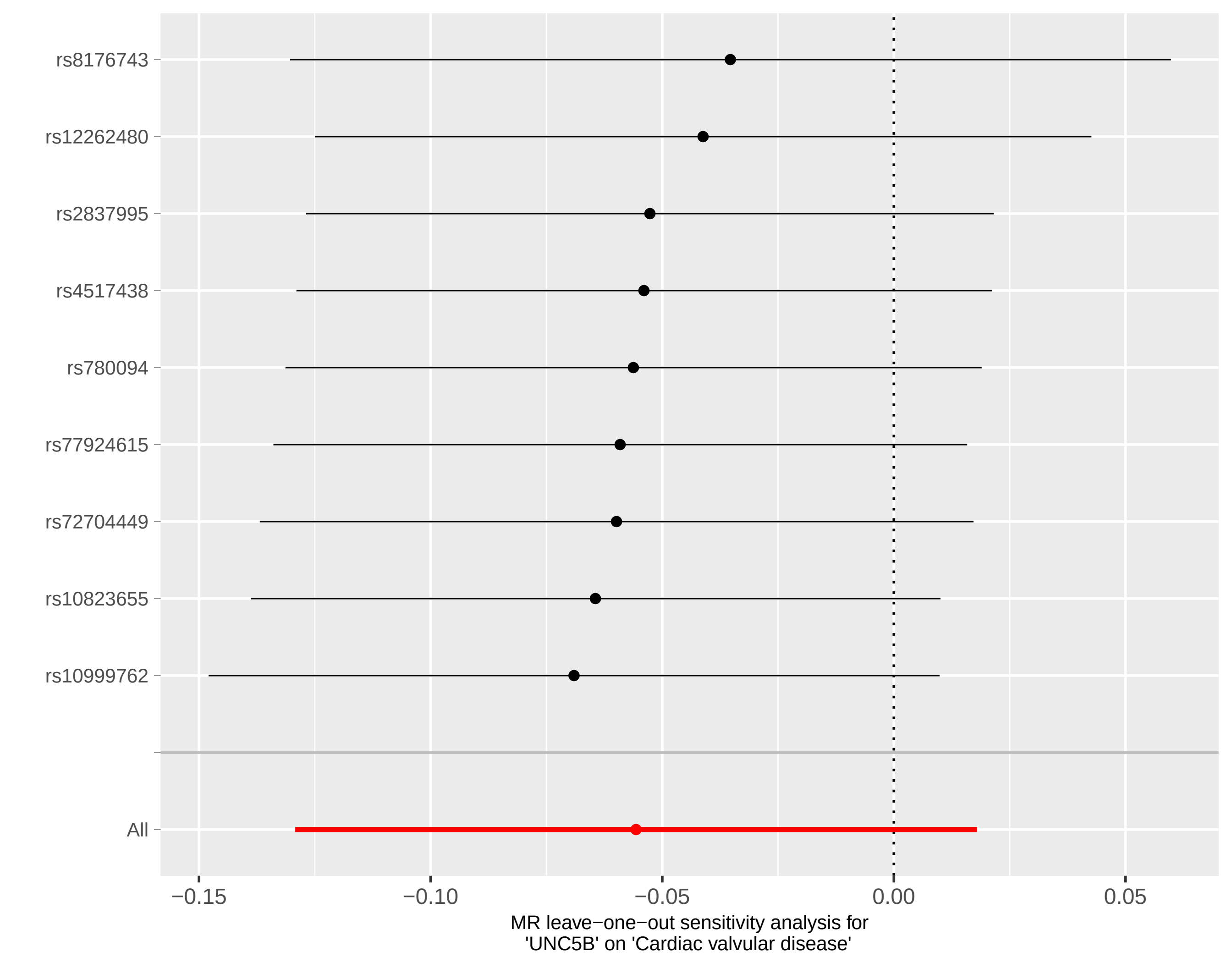
rs8176743
rs12262480
rs2837995
rs4517438
rs780094
rs77924615
rs72704449
rs10823655
rs10999762
All
−0.15
−0.10
−0.05
0.00
0.05
MR leave−one−out sensitivity analysis for
'UNC5B' on 'Cardiac valvular disease'

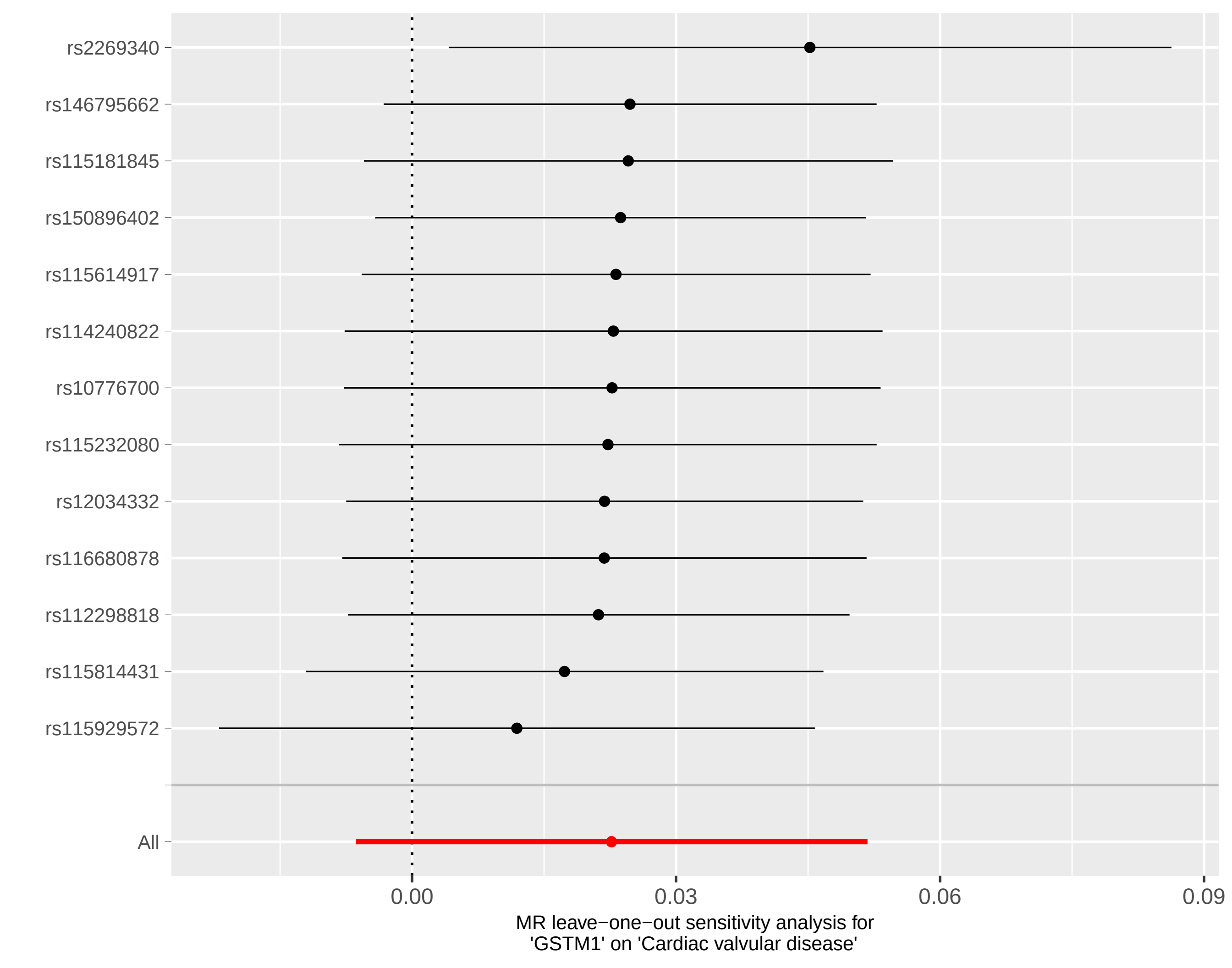

rs2269340
rs146795662
rs115181845
rs150896402
rs115614917
rs114240822
rs10776700
rs115232080
rs12034332
rs116680878
rs112298818
rs115814431
rs115929572
All
0.00
0.03
0.06
0.09
MR leave−one−out sensitivity analysis for
'GSTM1' on 'Cardiac valvular disease'

Insufficient number of SNPs

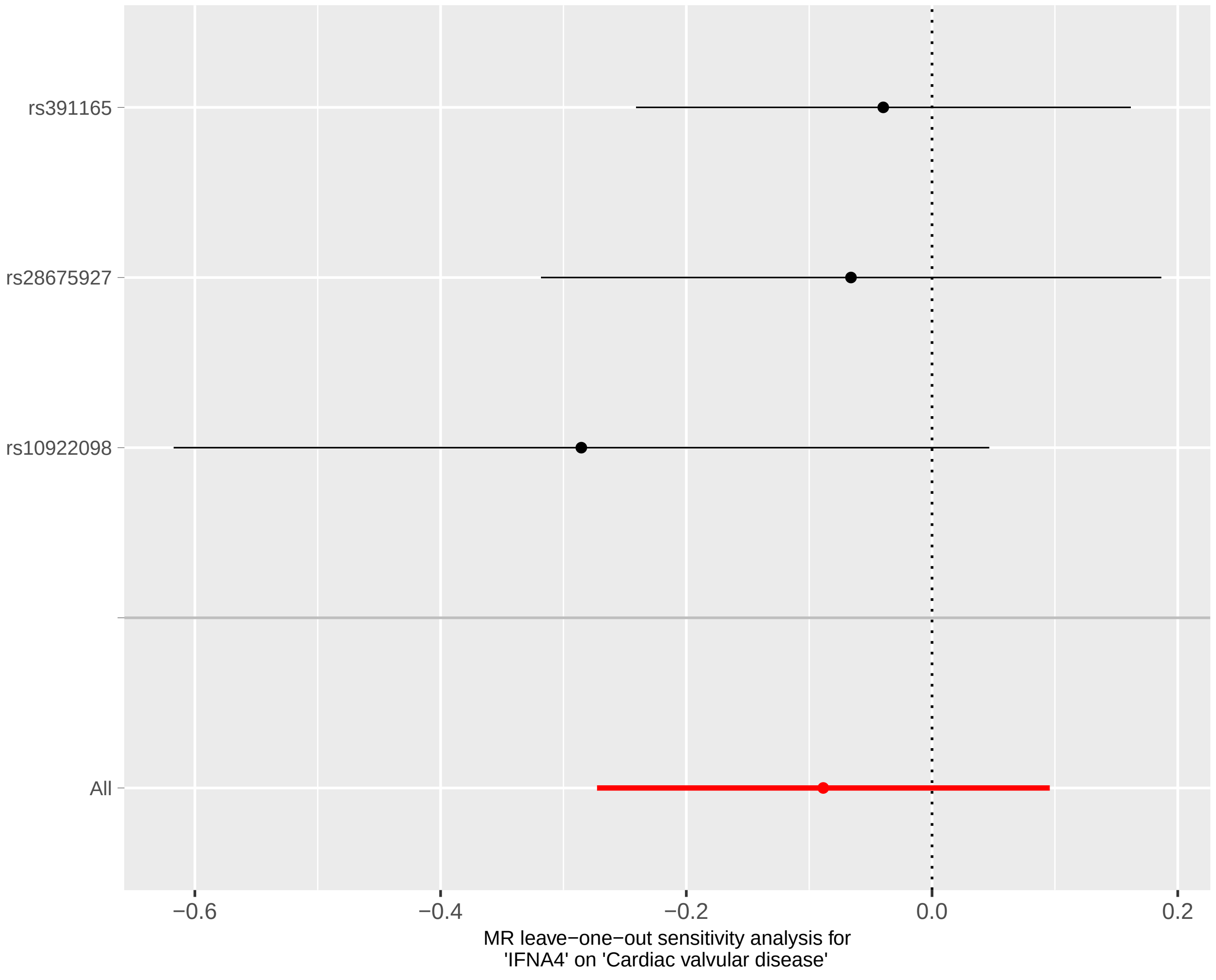
rs391165
rs28675927
rs10922098
All
−0.6
−0.4
−0.2
0.0
0.2
MR leave−one−out sensitivity analysis for
'IFNA4' on 'Cardiac valvular disease'

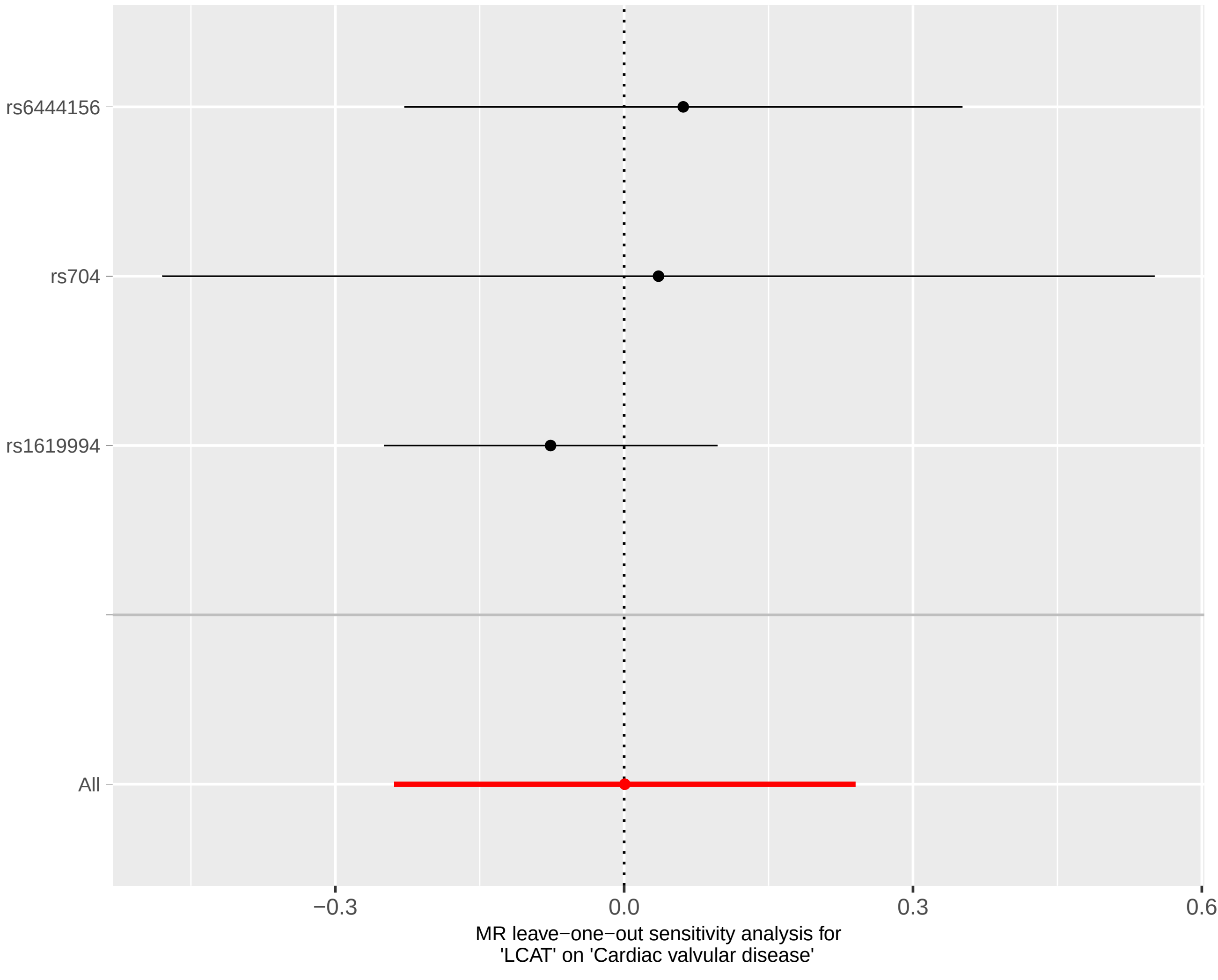
rs6444156
rs704
rs1619994
All
−0.3
0.0
0.3
0.6
MR leave−one−out sensitivity analysis for
'LCAT' on 'Cardiac valvular disease'

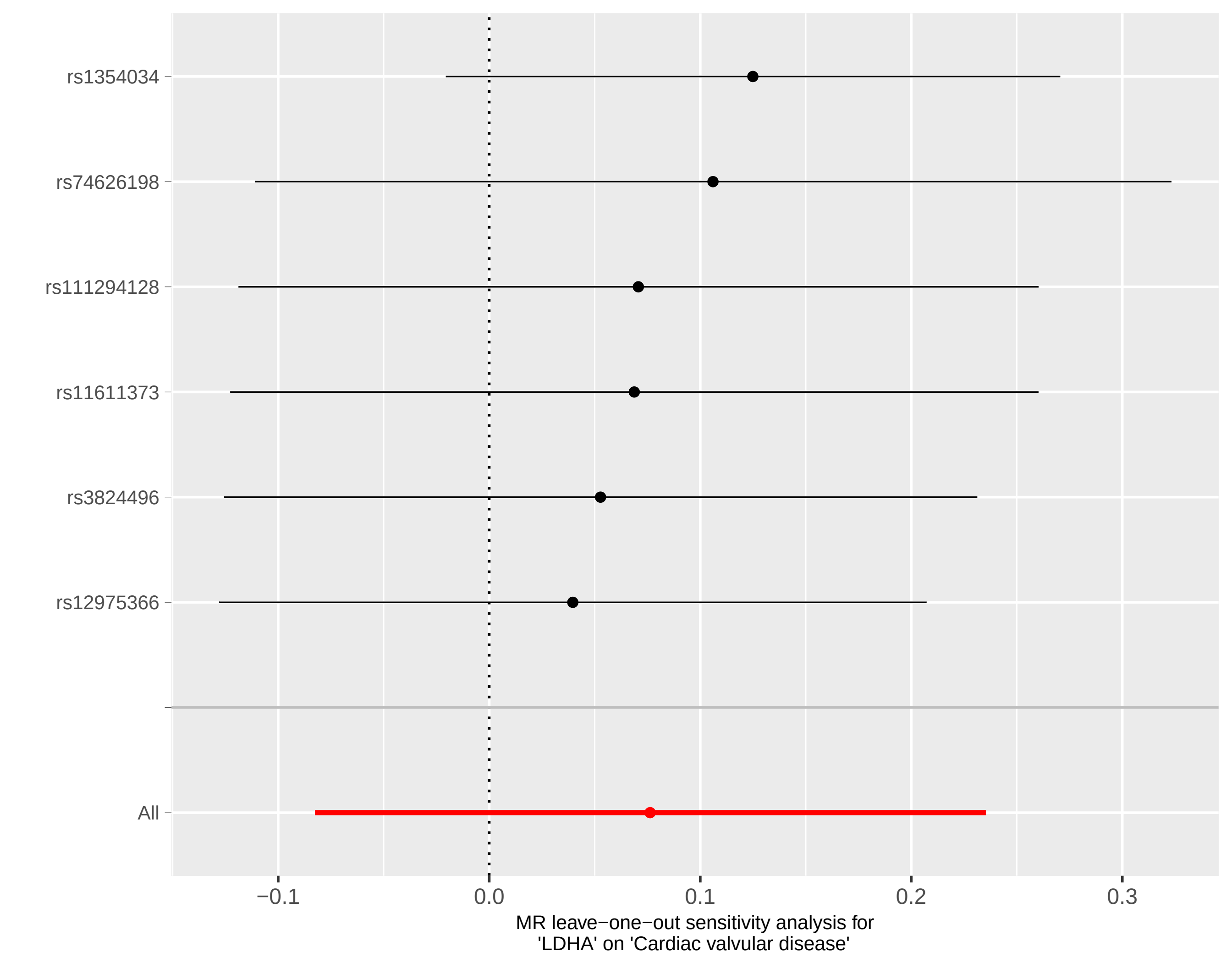
rs1354034
rs74626198
rs111294128
rs11611373
rs3824496
rs12975366
All
−0.1
0.0
0.1
0.2
0.3
MR leave−one−out sensitivity analysis for
'LDHA' on 'Cardiac valvular disease'

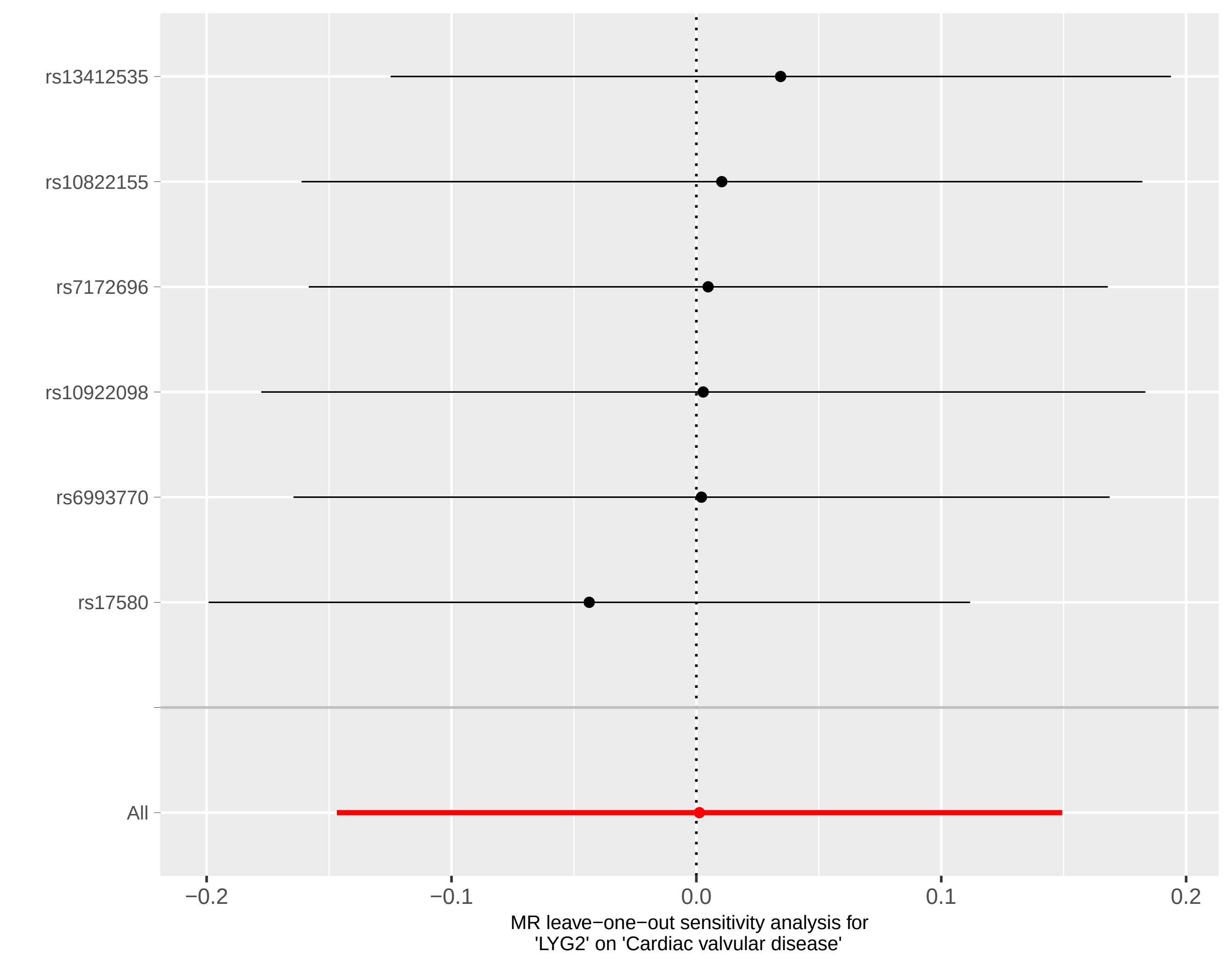
rs13412535
rs10822155
rs7172696
rs10922098
rs6993770
rs17580
All
−0.2
−0.1
0.0
0.1
0.2
MR leave−one−out sensitivity analysis for
'LYG2' on 'Cardiac valvular disease'

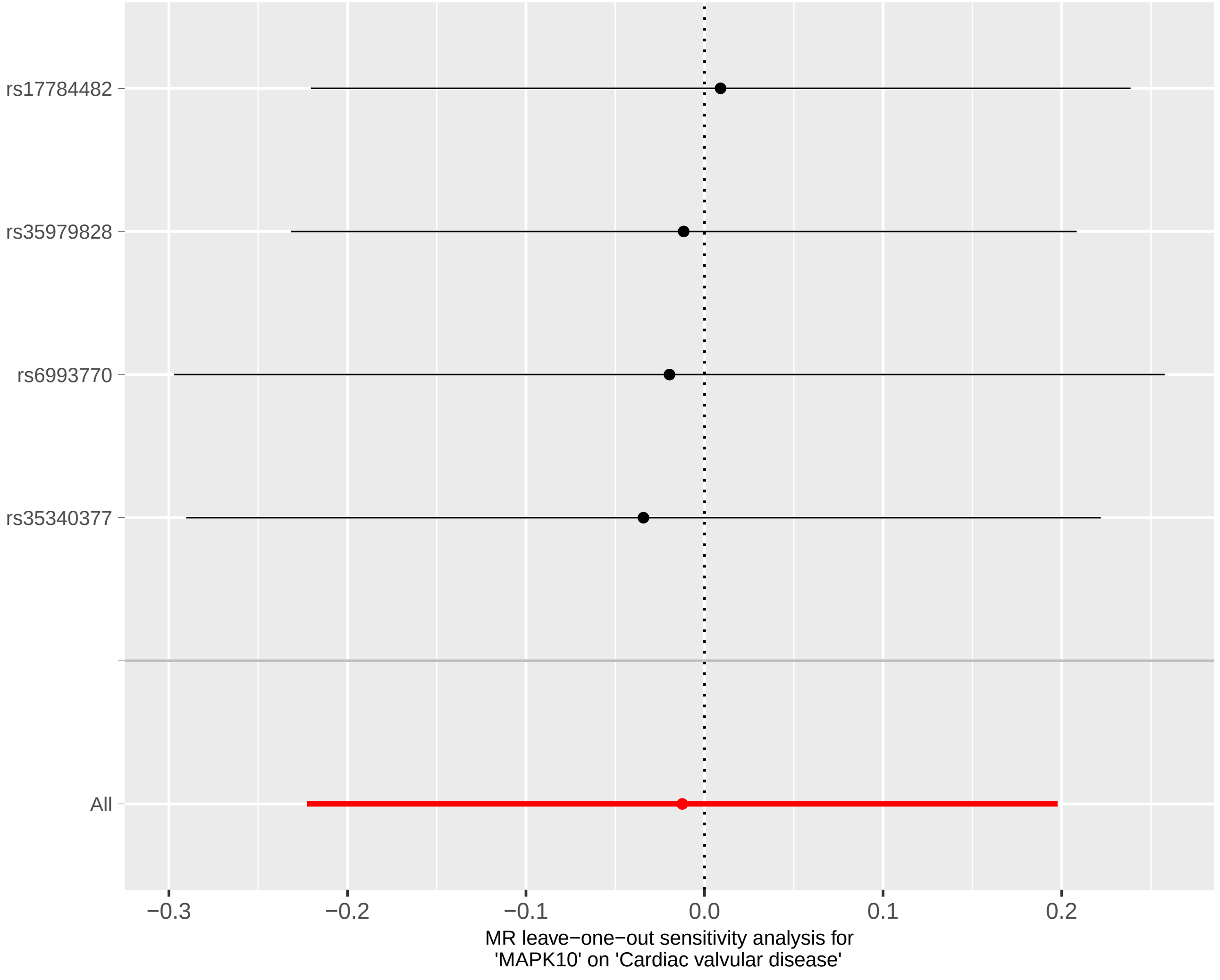

rs17784482
rs35979828
rs6993770
rs35340377
All
−0.3
−0.2
−0.1
0.0
0.1
0.2
MR leave−one−out sensitivity analysis for
'MAPK10' on 'Cardiac valvular disease'

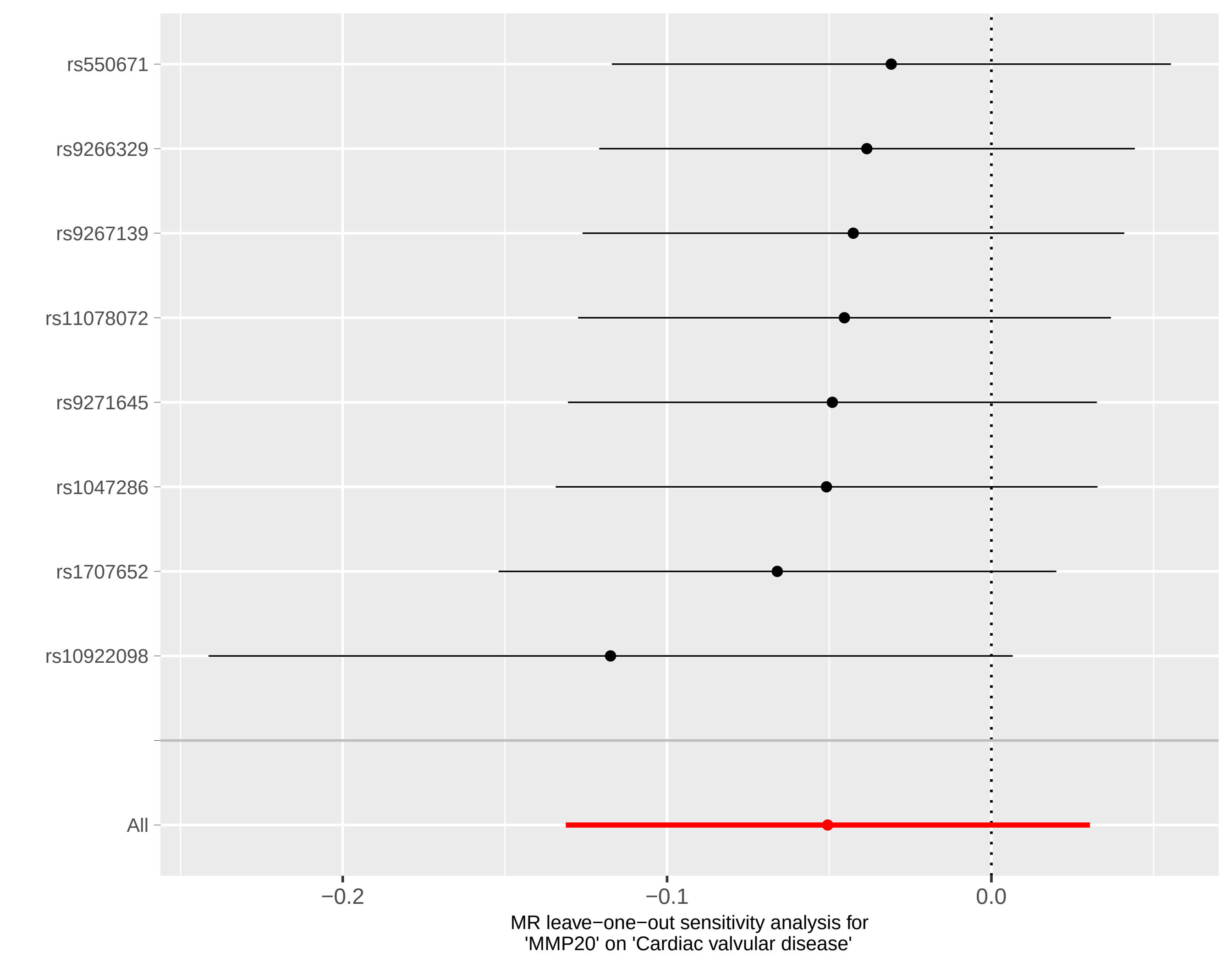
rs550671
rs9266329
rs9267139
rs11078072
rs9271645
rs1047286
rs1707652
rs10922098
All
−0.2
−0.1
0.0
MR leave−one−out sensitivity analysis for
'MMP20' on 'Cardiac valvular disease'

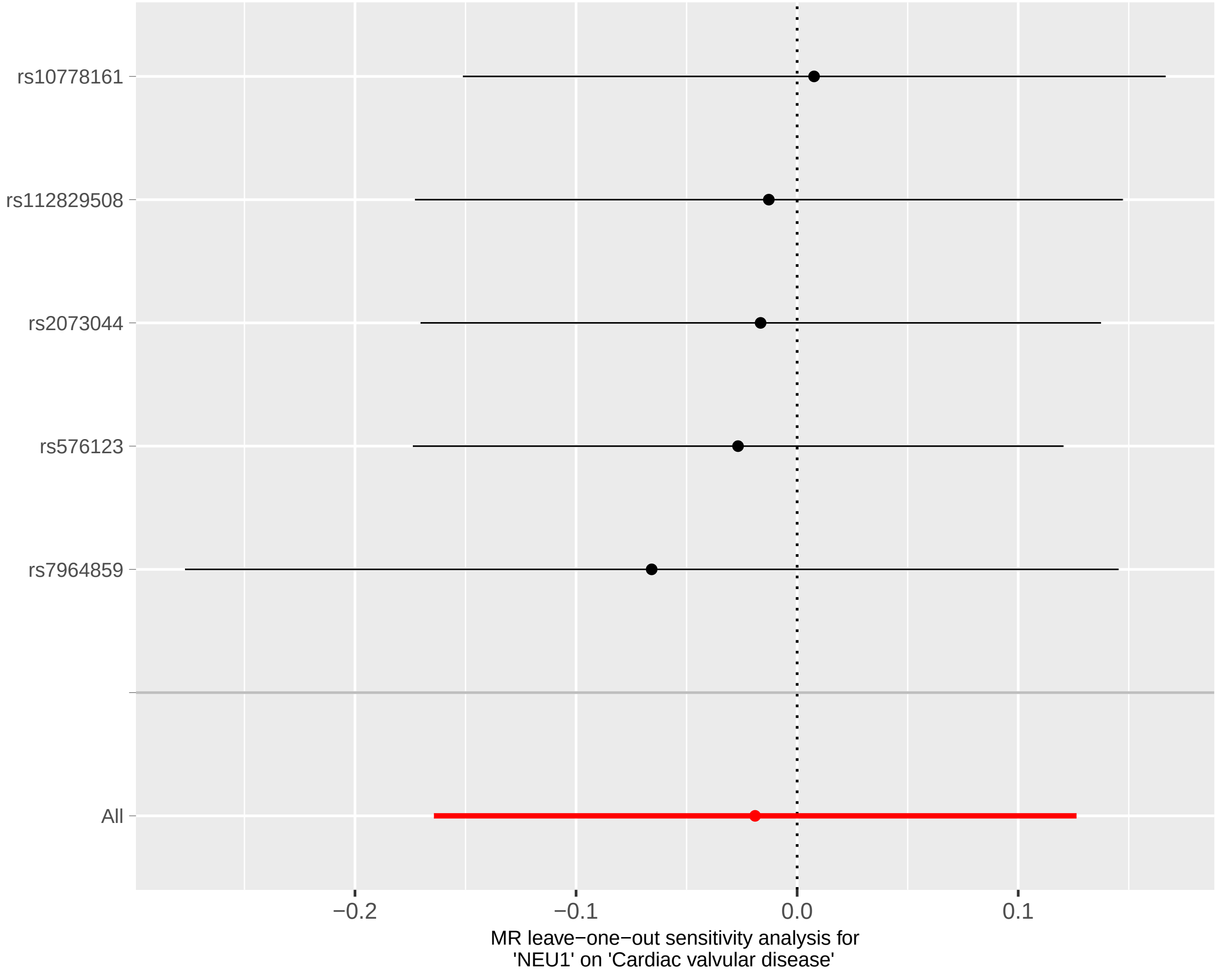
rs10778161
rs112829508
rs2073044
rs576123
rs7964859
All
−0.2
−0.1
0.0
0.1
MR leave−one−out sensitivity analysis for
'NEU1' on 'Cardiac valvular disease'

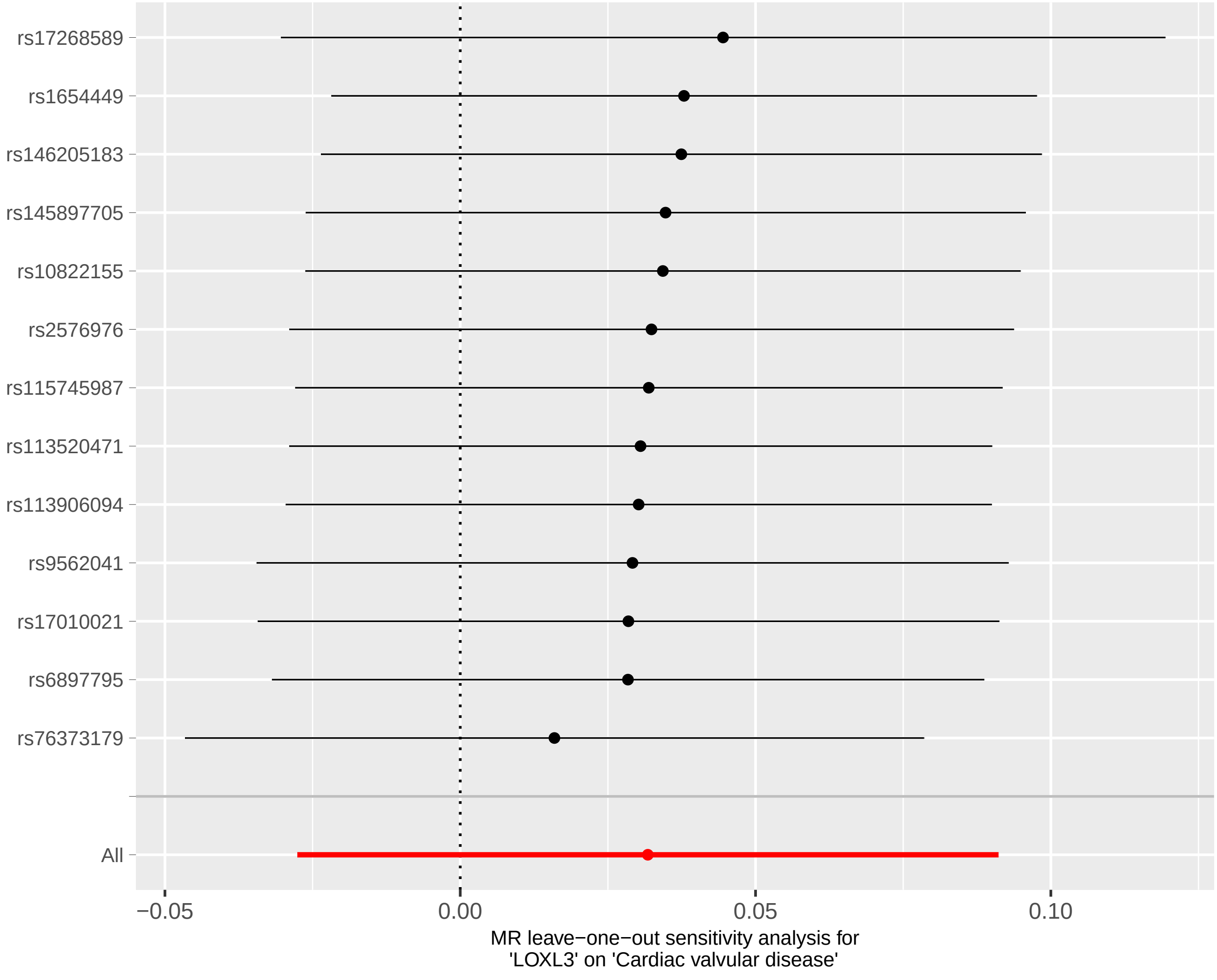
rs17268589
rs1654449
rs146205183
rs145897705
rs10822155
rs2576976
rs115745987
rs113520471
rs113906094
rs9562041
rs17010021
rs6897795
rs76373179
All
−0.05
0.00
0.05
0.10
MR leave−one−out sensitivity analysis for
'LOXL3' on 'Cardiac valvular disease'

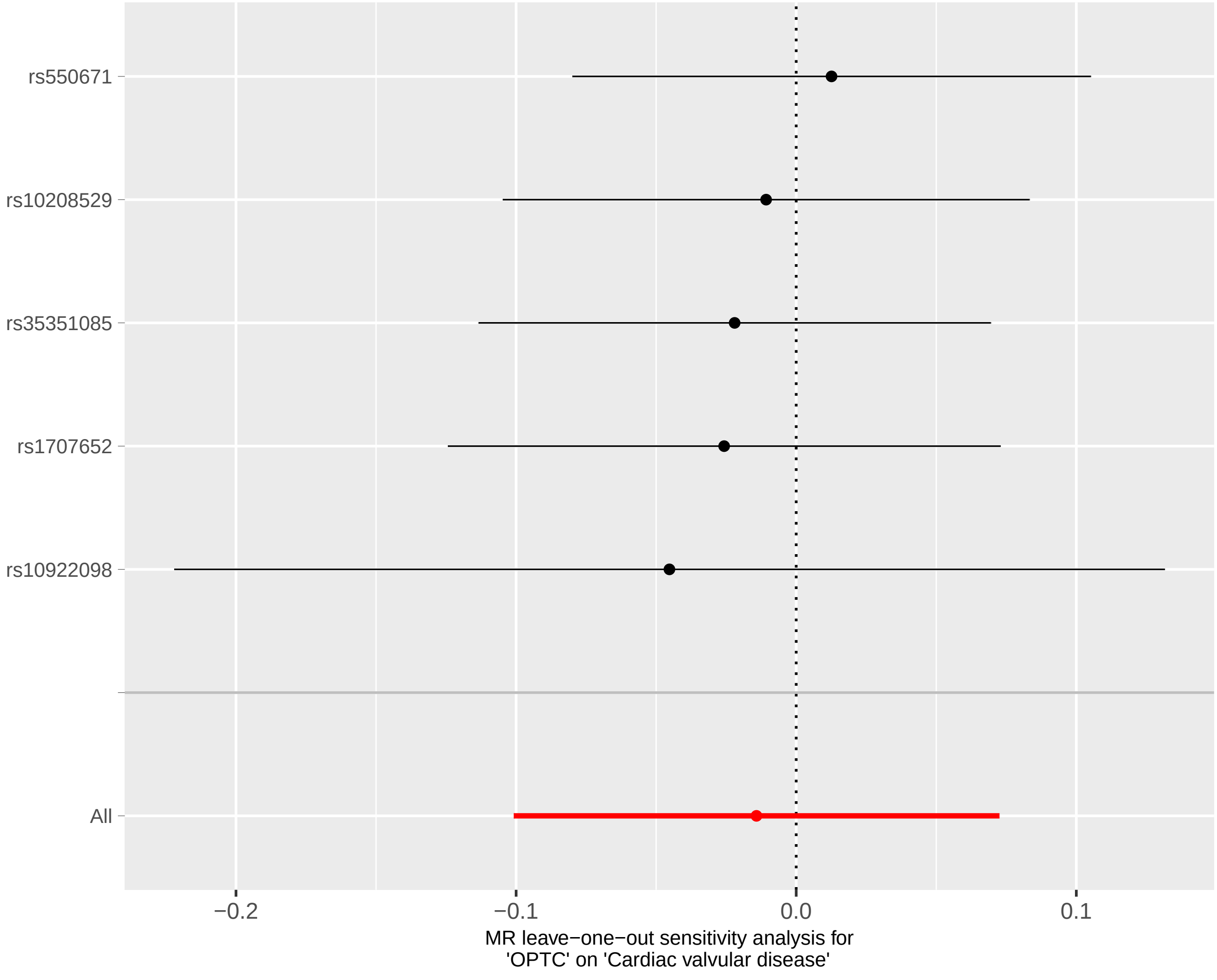
rs550671
rs10208529
rs35351085
rs1707652
rs10922098
All
−0.2
−0.1
0.0
0.1
MR leave−one−out sensitivity analysis for
'OPTC' on 'Cardiac valvular disease'

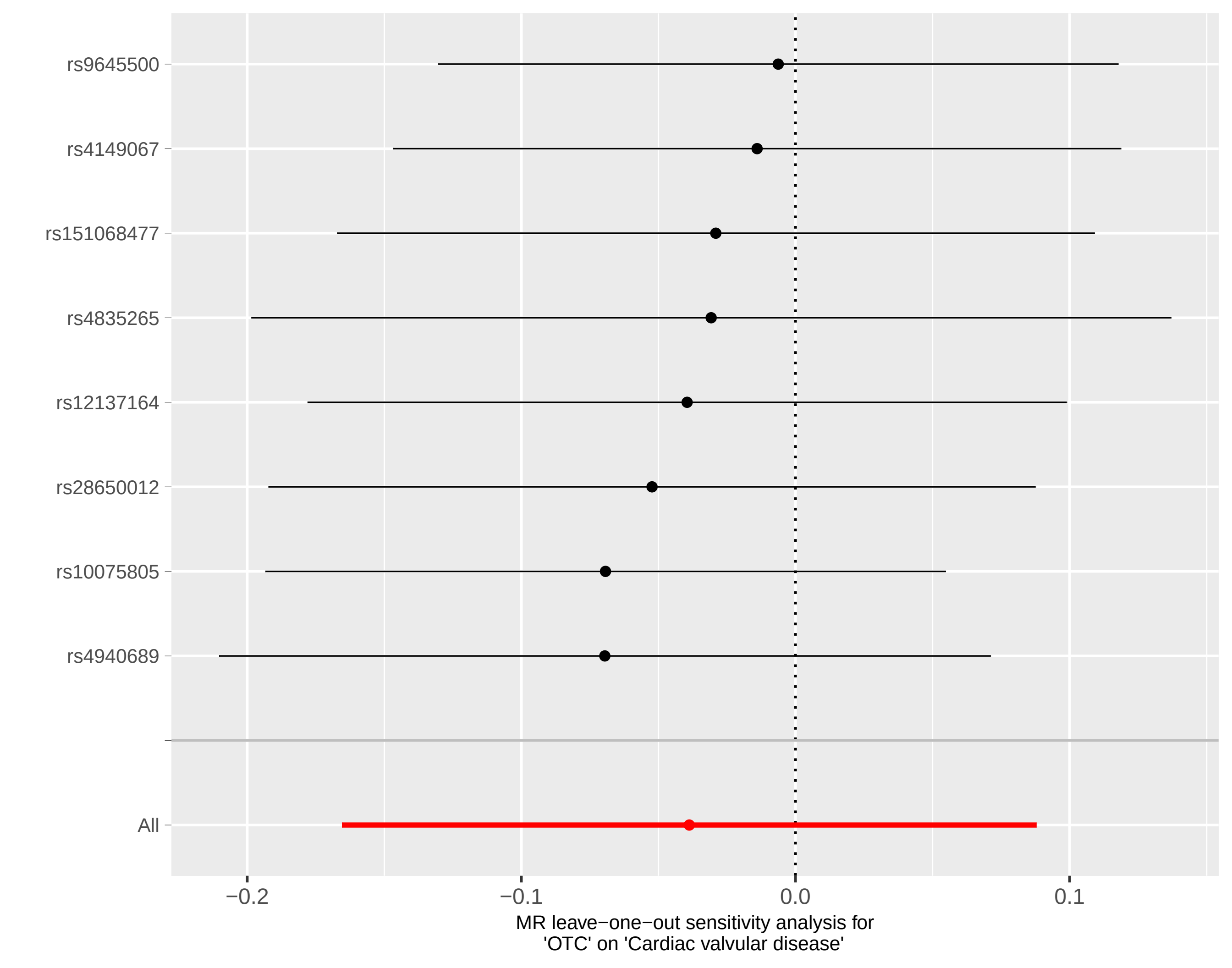
rs9645500
rs4149067
rs151068477
rs4835265
rs12137164
rs28650012
rs10075805
rs4940689
All
−0.2
−0.1
0.0
0.1
MR leave−one−out sensitivity analysis for
'OTC' on 'Cardiac valvular disease'

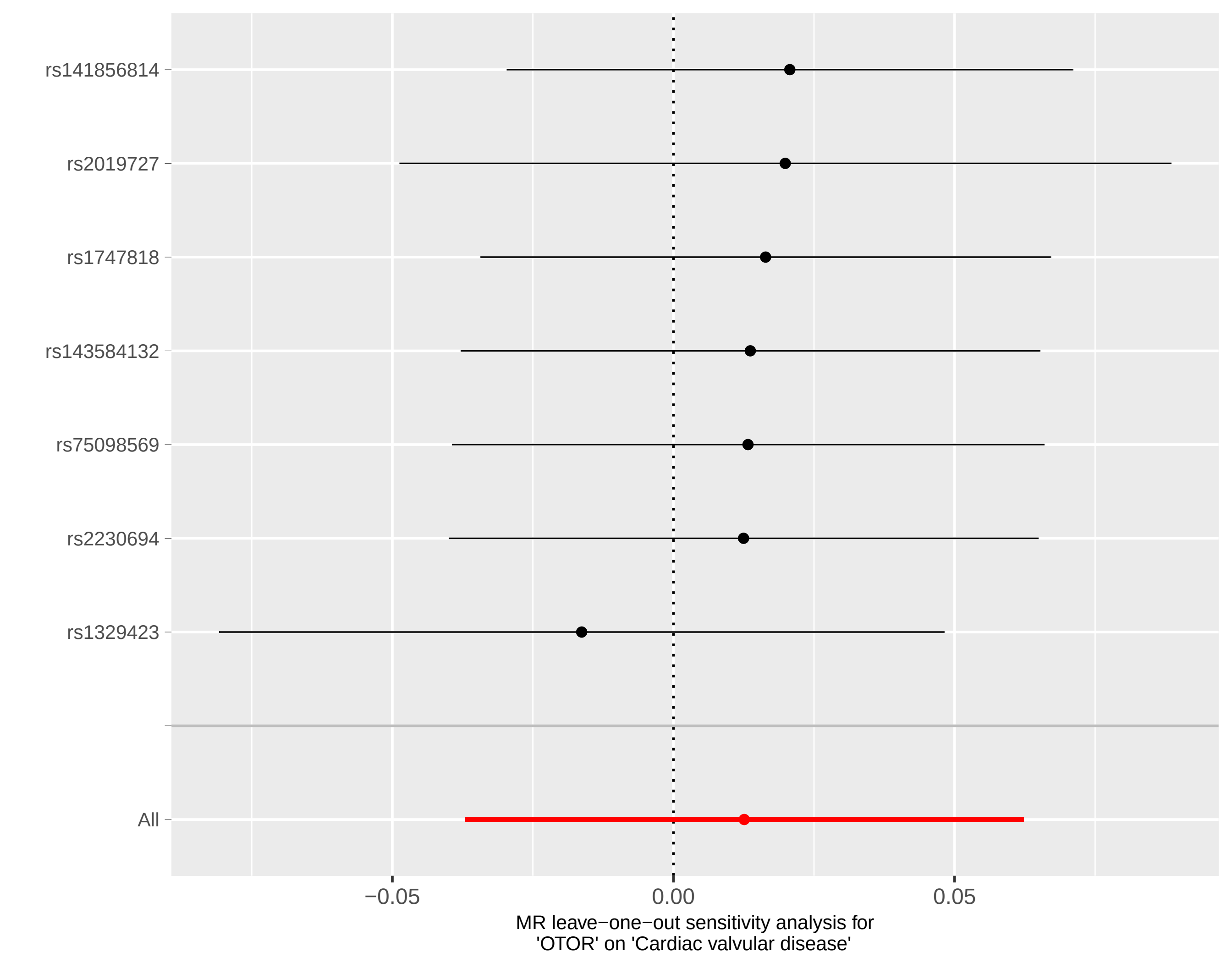
rs141856814
rs2019727
rs1747818
rs143584132
rs75098569
rs2230694
rs1329423
All
−0.05
0.00
0.05
MR leave−one−out sensitivity analysis for
'OTOR' on 'Cardiac valvular disease'

Insufficient number of SNPs

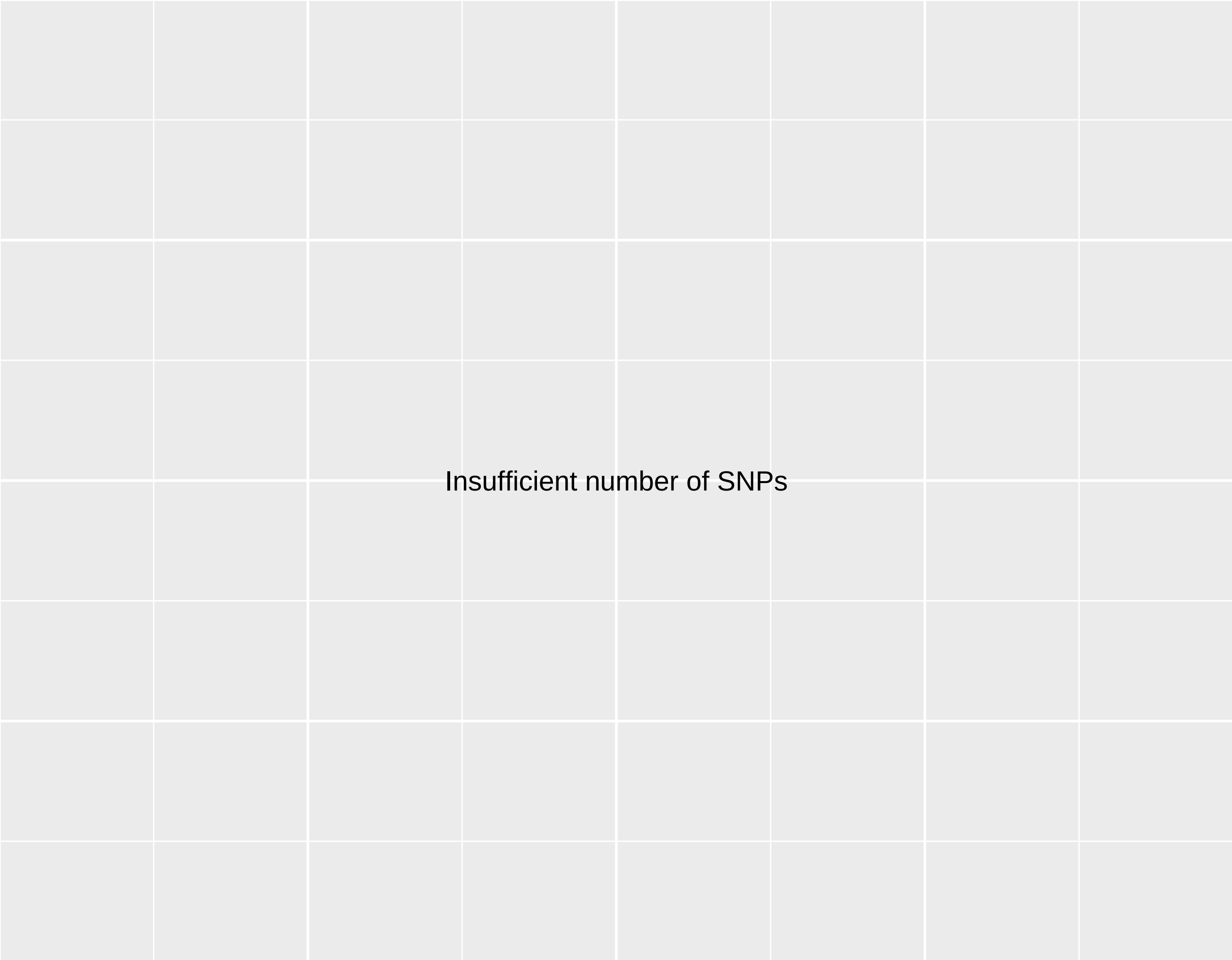
Insufficient number of SNPs

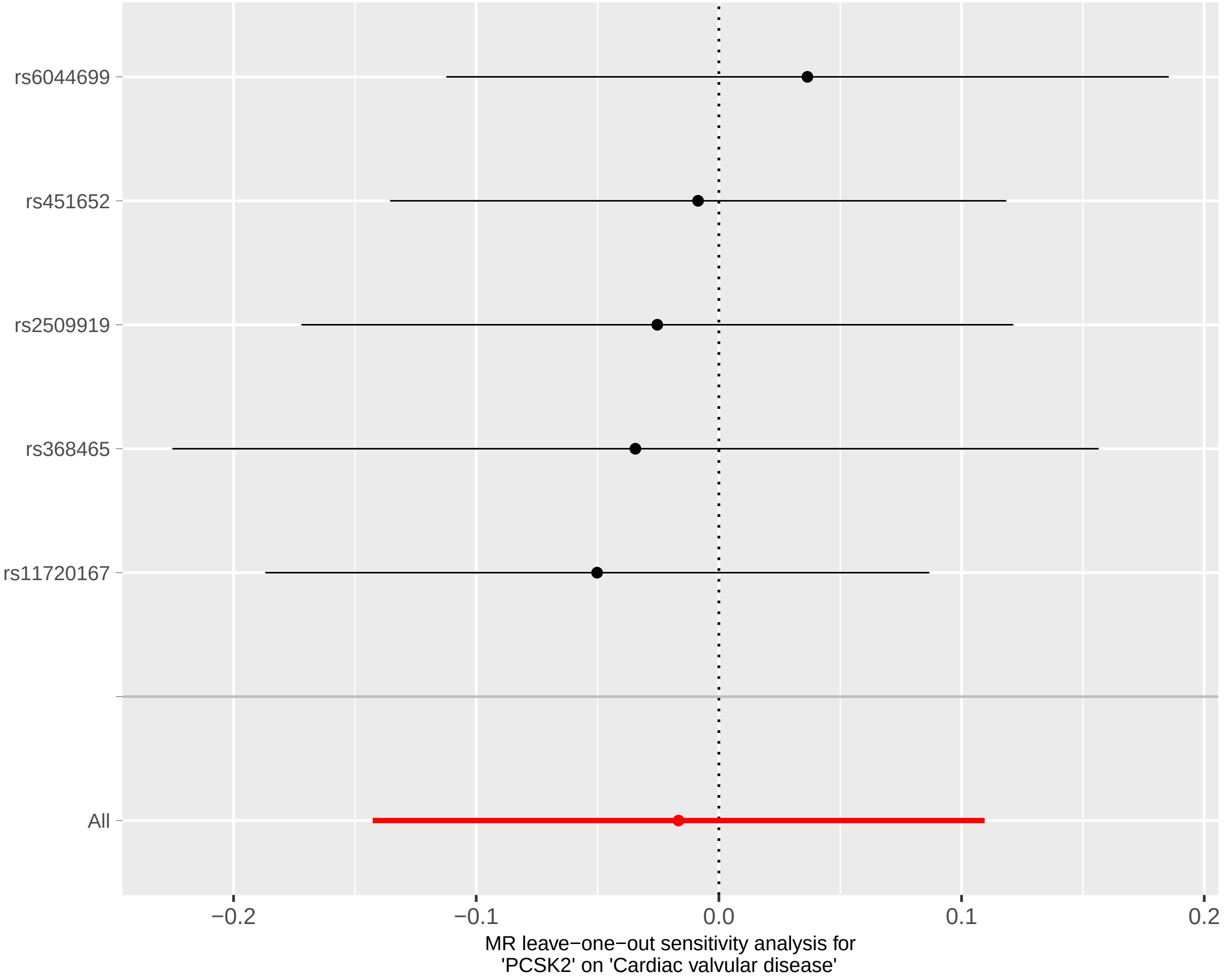
rs6044699
rs451652
rs2509919
rs368465
rs11720167
All
−0.2
−0.1
0.0
0.1
0.2
MR leave−one−out sensitivity analysis for
'PCSK2' on 'Cardiac valvular disease'

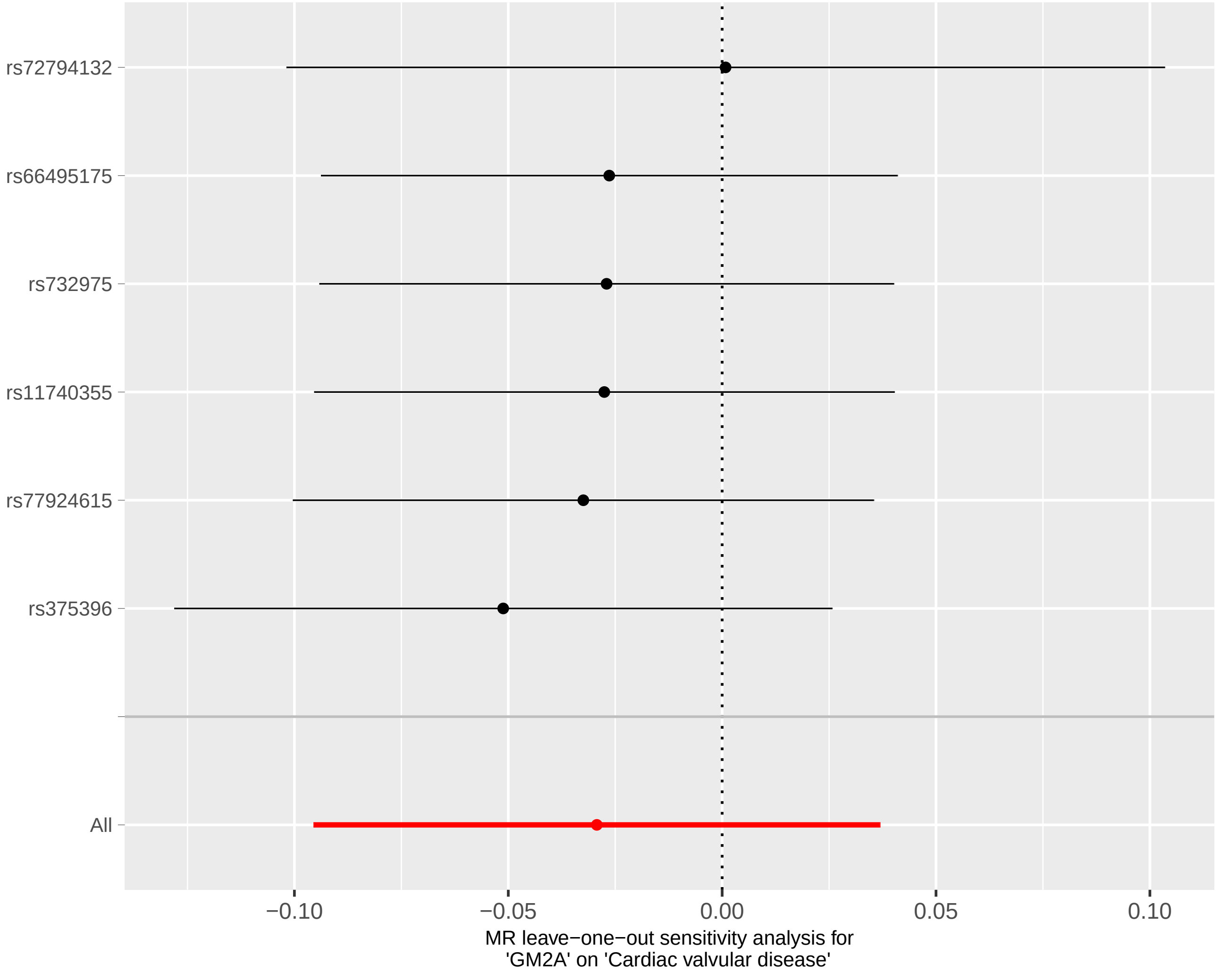

rs72794132
rs66495175
rs732975
rs11740355
rs77924615
rs375396
All
−0.10
−0.05
0.00
0.05
0.10
MR leave−one−out sensitivity analysis for
'GM2A' on 'Cardiac valvular disease'

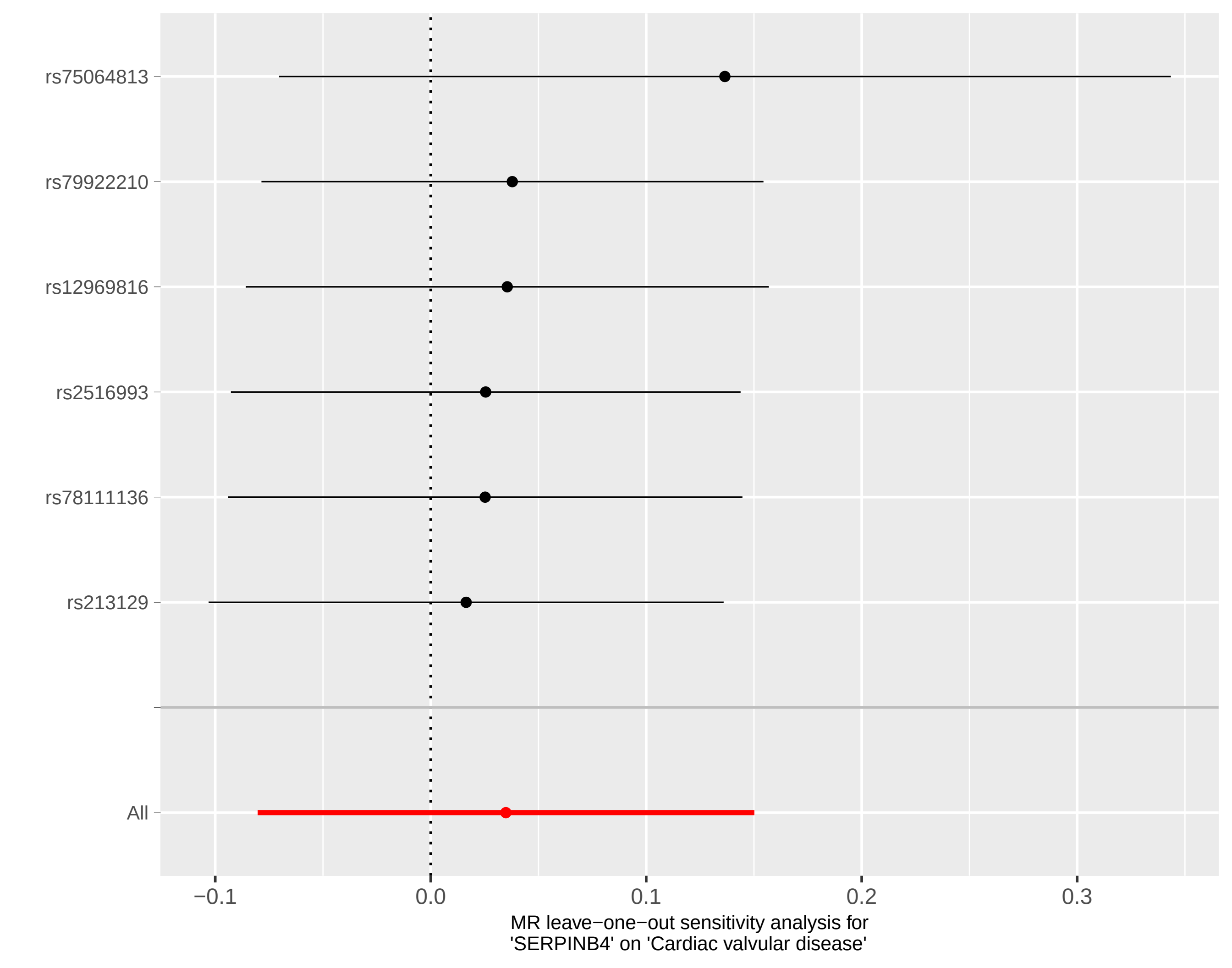
rs75064813
rs79922210
rs12969816
rs2516993
rs78111136
rs213129
All
−0.1
0.0
0.1
0.2
0.3
MR leave−one−out sensitivity analysis for
'SERPINB4' on 'Cardiac valvular disease'

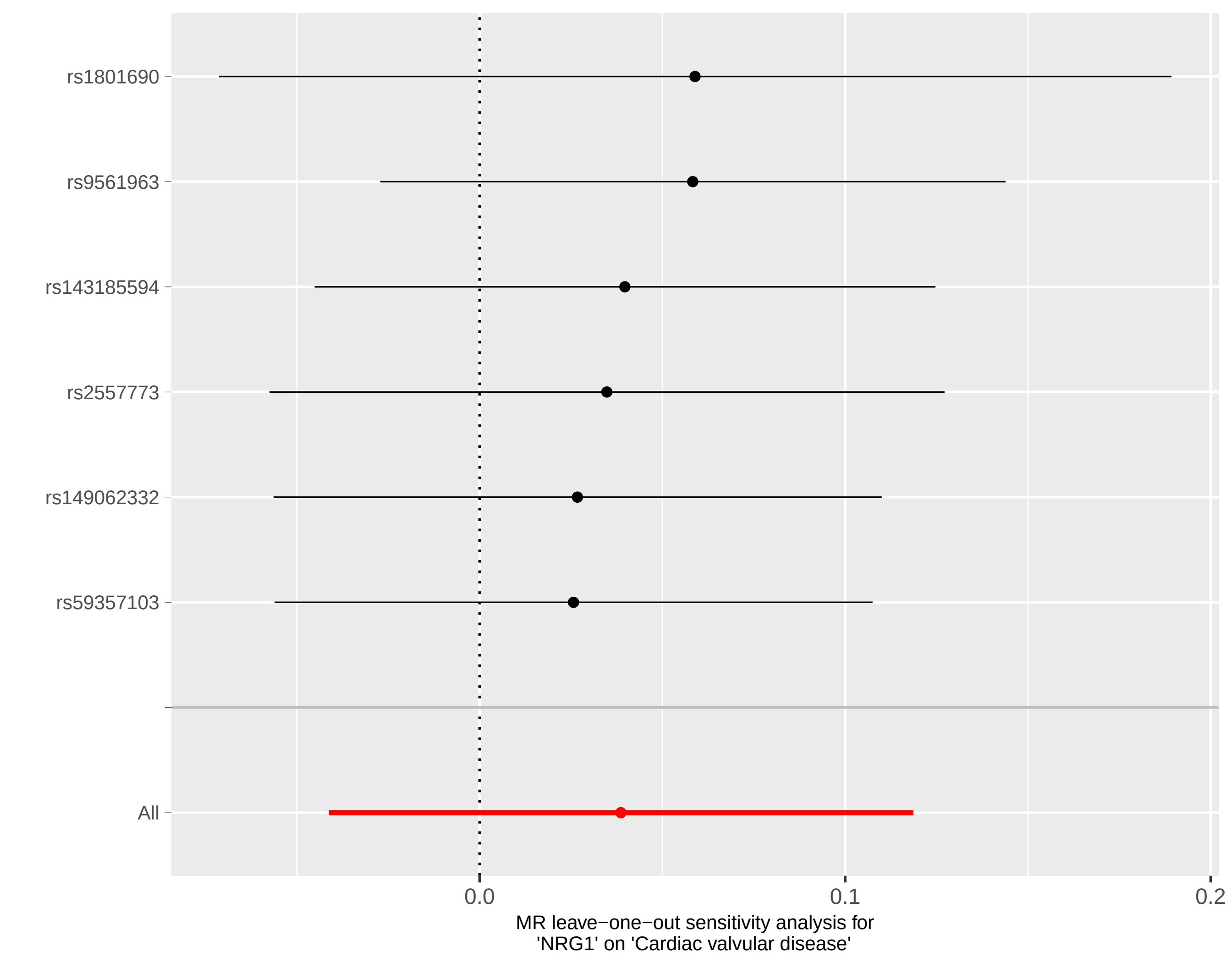

rs1801690
rs9561963
rs143185594
rs2557773
rs149062332
rs59357103
All
0.0
0.1
0.2
MR leave−one−out sensitivity analysis for
'NRG1' on 'Cardiac valvular disease'

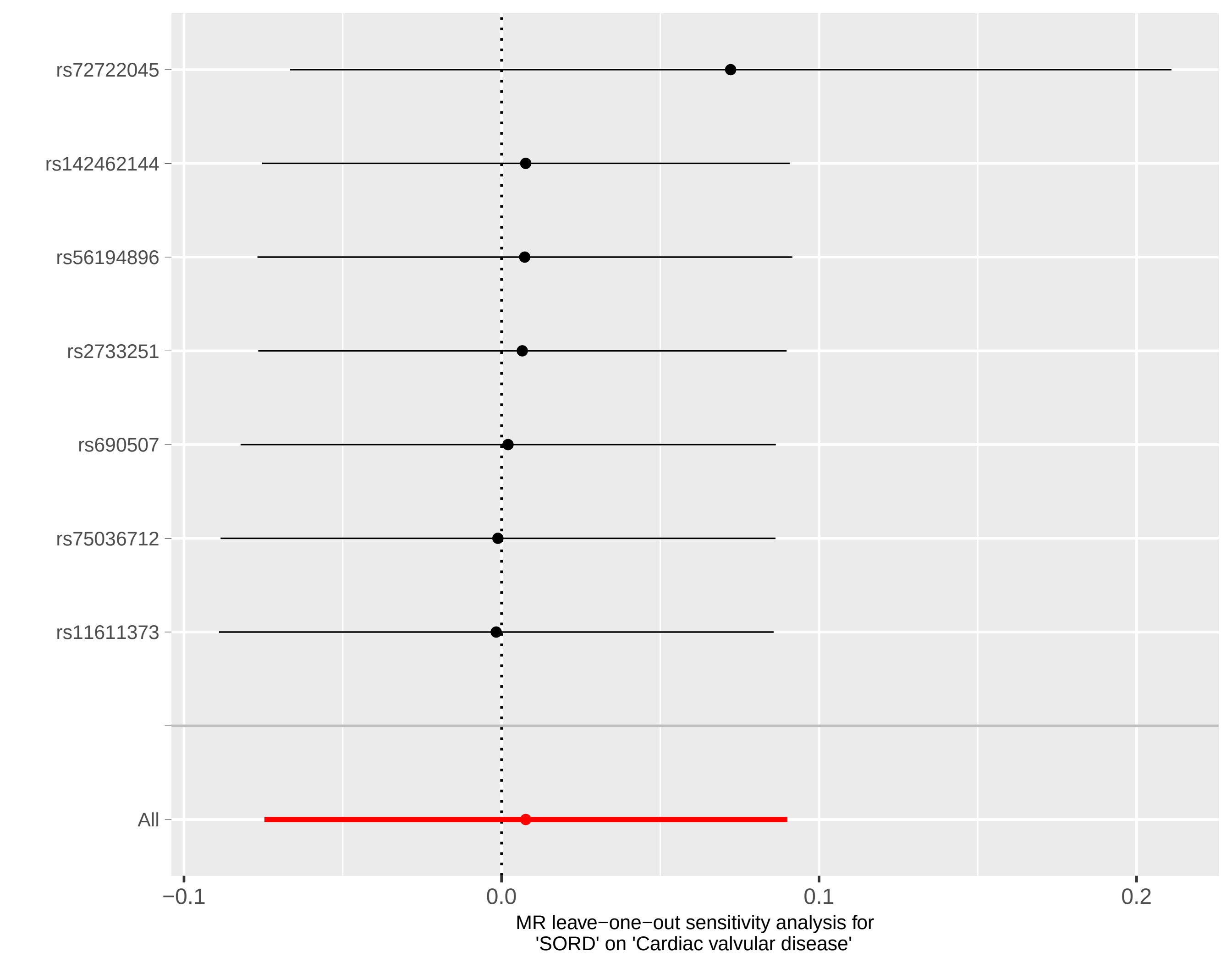

rs72722045
rs142462144
rs56194896
rs2733251
rs690507
rs75036712
rs11611373
All
−0.1
0.0
0.1
0.2
MR leave−one−out sensitivity analysis for
'SORD' on 'Cardiac valvular disease'

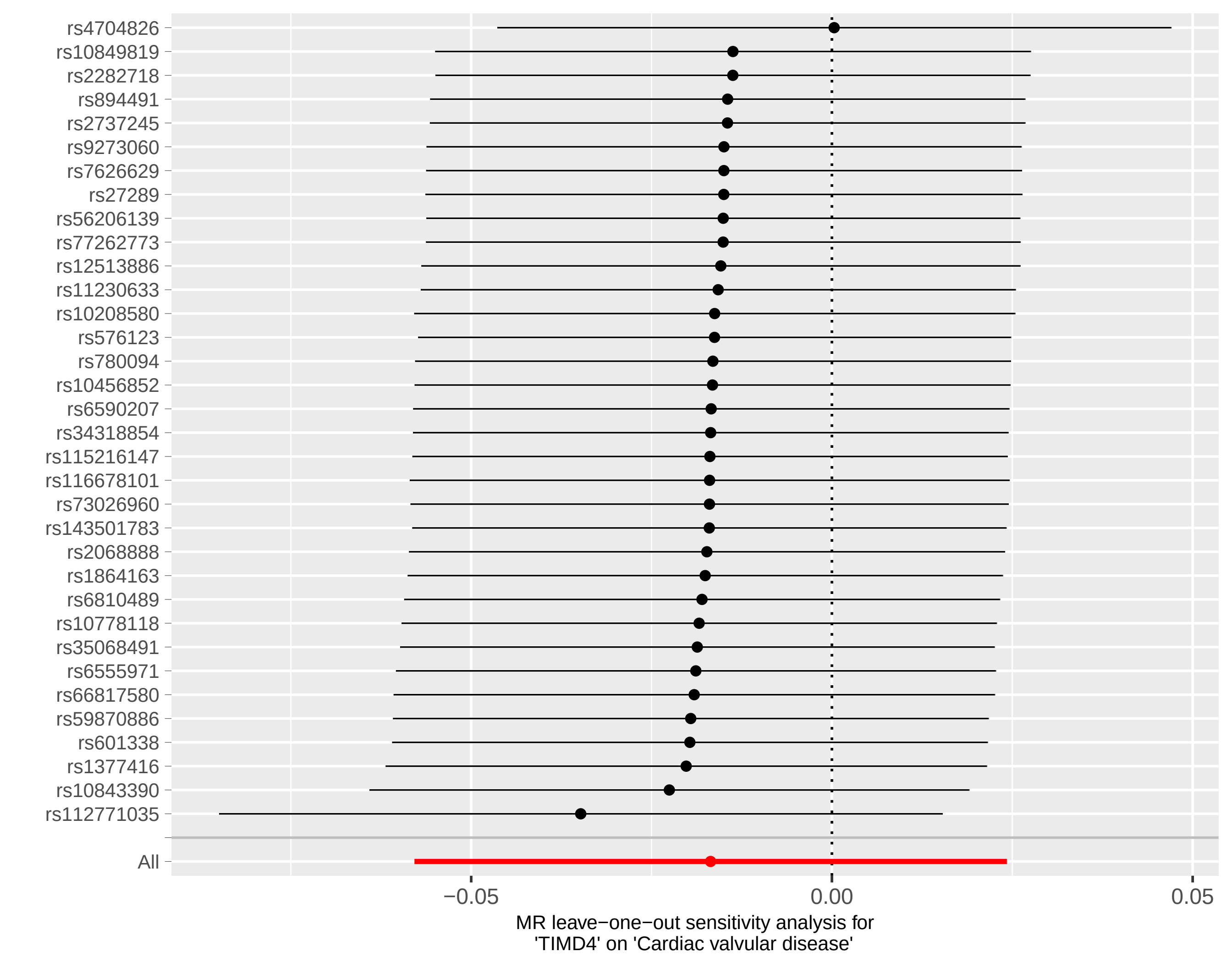
rs4704826
rs10849819
rs2282718
rs894491
rs2737245
rs9273060
rs7626629
rs27289
rs56206139
rs77262773
rs12513886
rs11230633
rs10208580
rs576123
rs780094
rs10456852
rs6590207
rs34318854
rs115216147
rs116678101
rs73026960
rs143501783
rs2068888
rs1864163
rs6810489
rs10778118
rs35068491
rs6555971
rs66817580
rs59870886
rs601338
rs1377416
rs10843390
rs112771035
All
−0.05
0.00
0.05
MR leave−one−out sensitivity analysis for
'TIMD4' on 'Cardiac valvular disease'

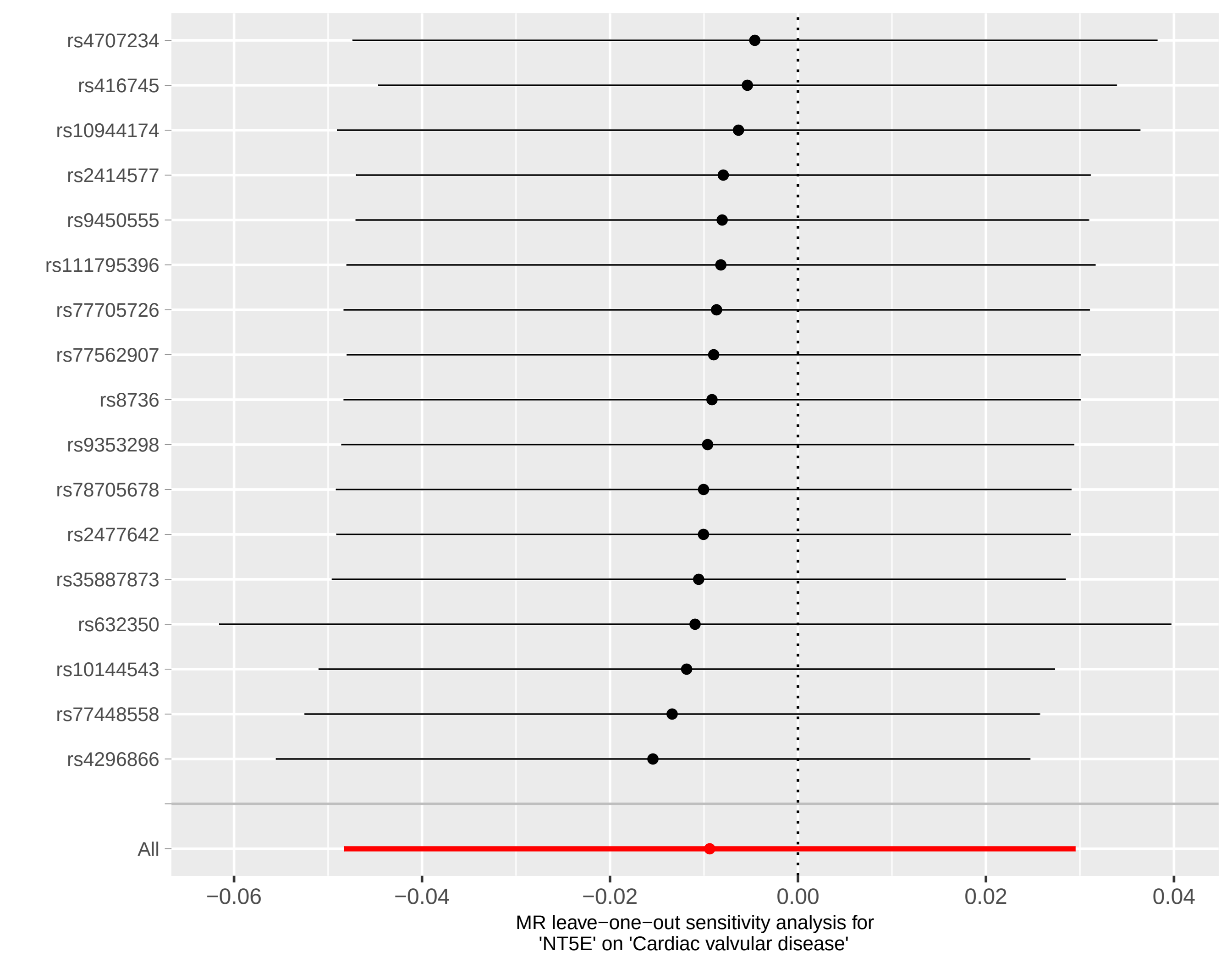
rs4707234
rs416745
rs10944174
rs2414577
rs9450555
rs111795396
rs77705726
rs77562907
rs8736
rs9353298
rs78705678
rs2477642
rs35887873
rs632350
rs10144543
rs77448558
rs4296866
All
−0.06
−0.04
−0.02
0.00
0.02
0.04
MR leave−one−out sensitivity analysis for
'NT5E' on 'Cardiac valvular disease'

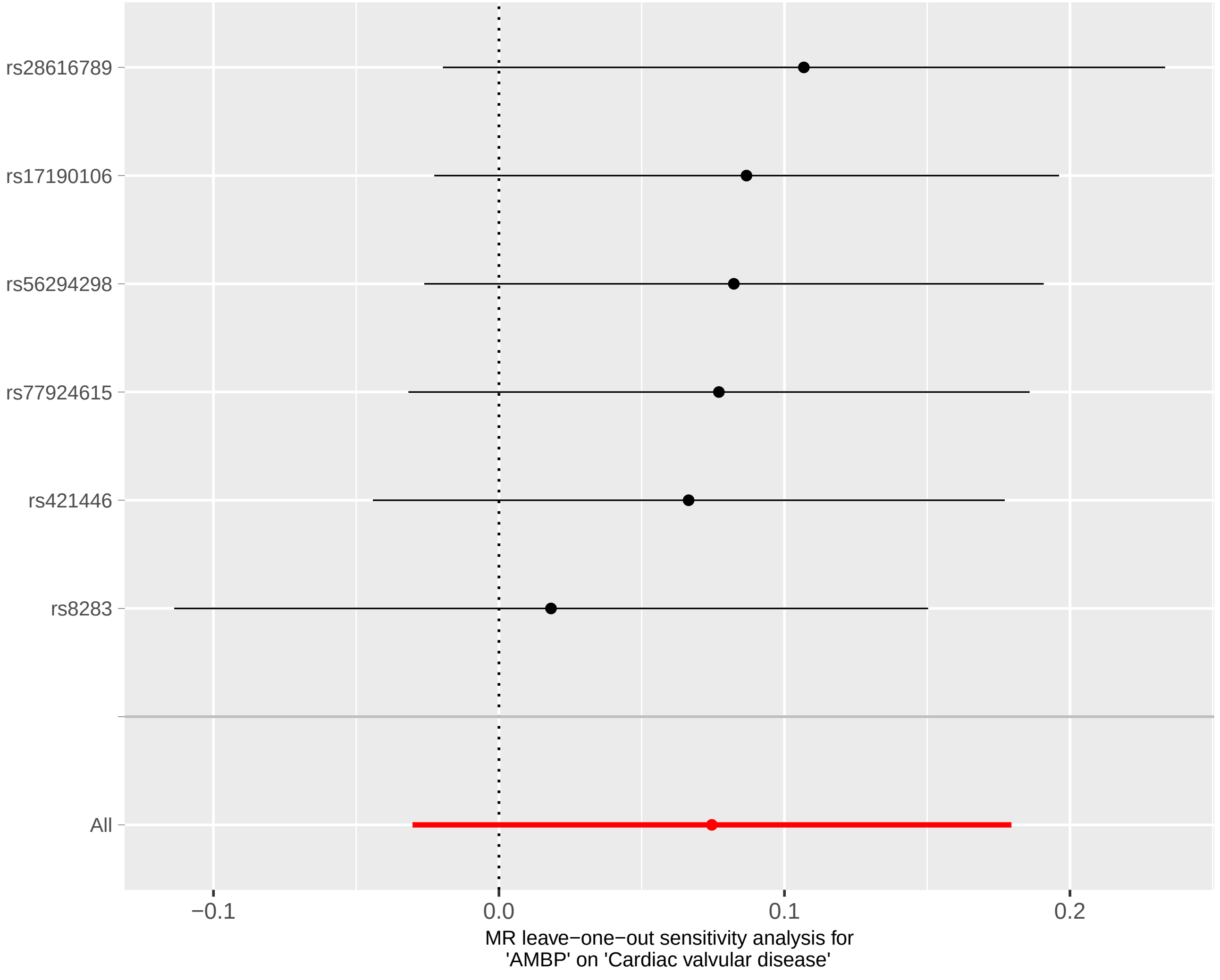
rs28616789
rs17190106
rs56294298
rs77924615
rs421446
rs8283
All
−0.1
0.0
0.1
0.2
MR leave−one−out sensitivity analysis for
'AMBP' on 'Cardiac valvular disease'

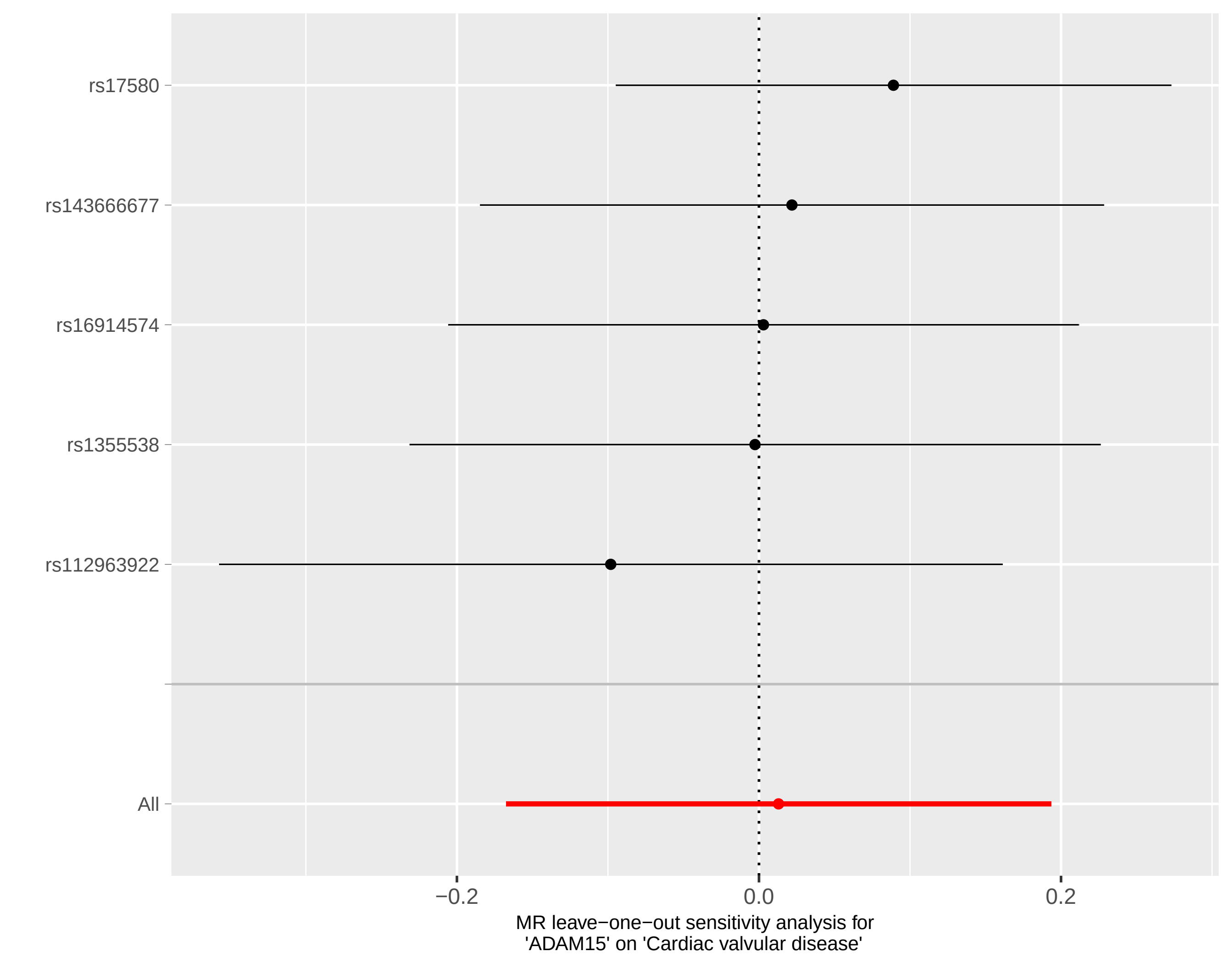
rs17580
rs143666677
rs16914574
rs1355538
rs112963922
All
−0.2
0.0
0.2
MR leave−one−out sensitivity analysis for
'ADAM15' on 'Cardiac valvular disease'

Insufficient number of SNPs

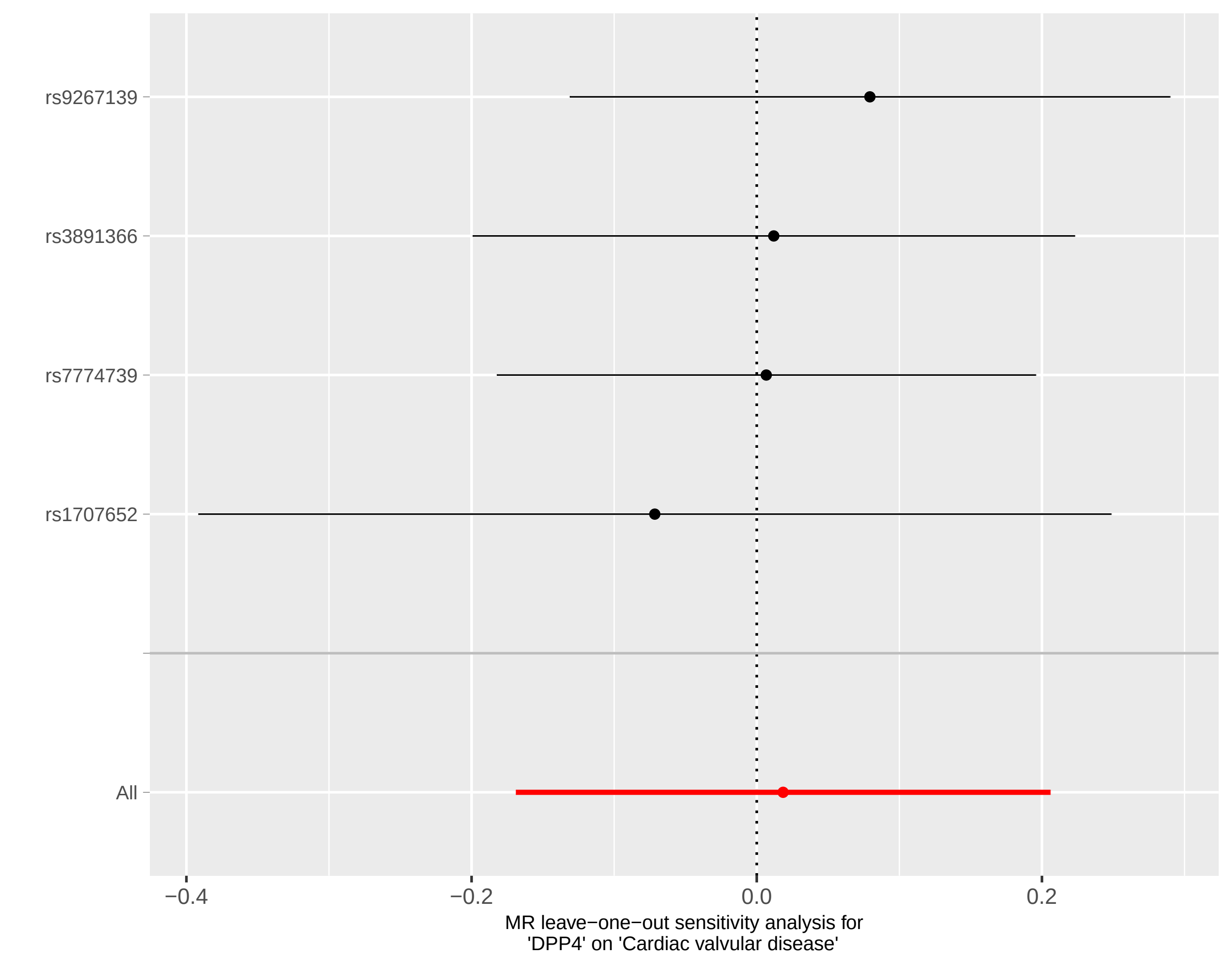
rs9267139
rs3891366
rs7774739
rs1707652
All
−0.4
−0.2
0.0
0.2
MR leave−one−out sensitivity analysis for
'DPP4' on 'Cardiac valvular disease'

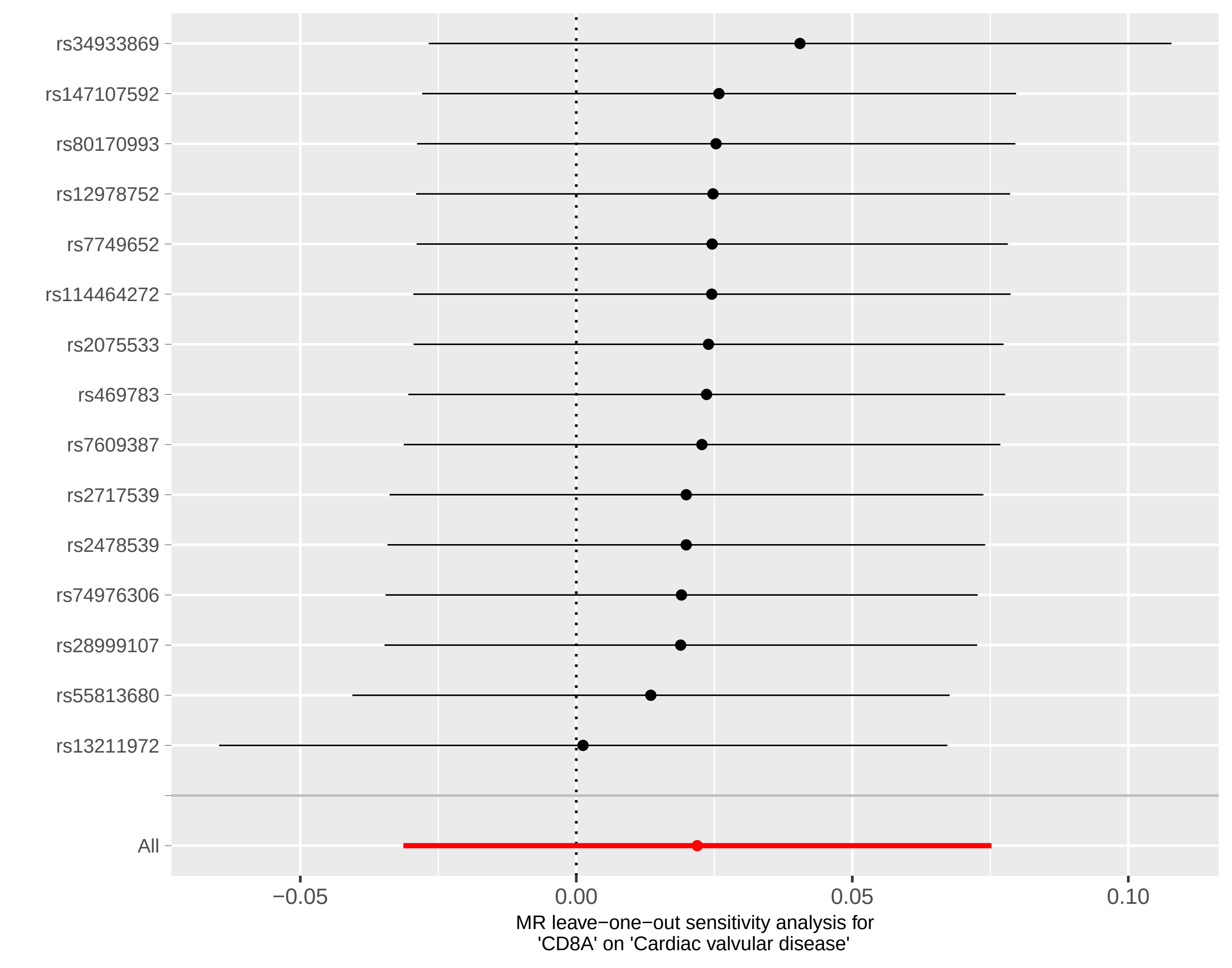
rs34933869
rs147107592
rs80170993
rs12978752
rs7749652
rs114464272
rs2075533
rs469783
rs7609387
rs2717539
rs2478539
rs74976306
rs28999107
rs55813680
rs13211972
All
−0.05
0.00
0.05
0.10
MR leave−one−out sensitivity analysis for
'CD8A' on 'Cardiac valvular disease'

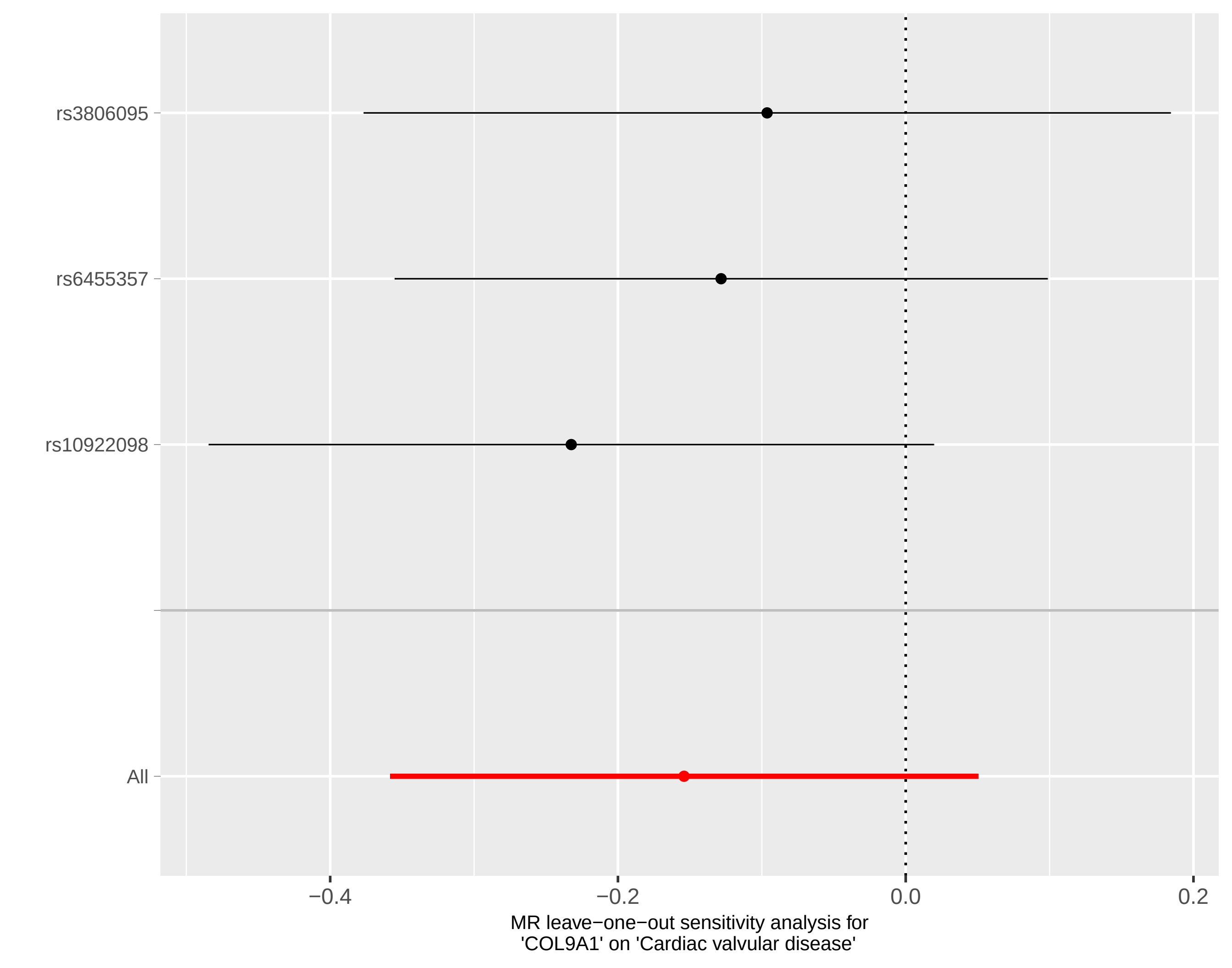

rs3806095
rs6455357
rs10922098
All
−0.4
−0.2
0.0
0.2
MR leave−one−out sensitivity analysis for
'COL9A1' on 'Cardiac valvular disease'

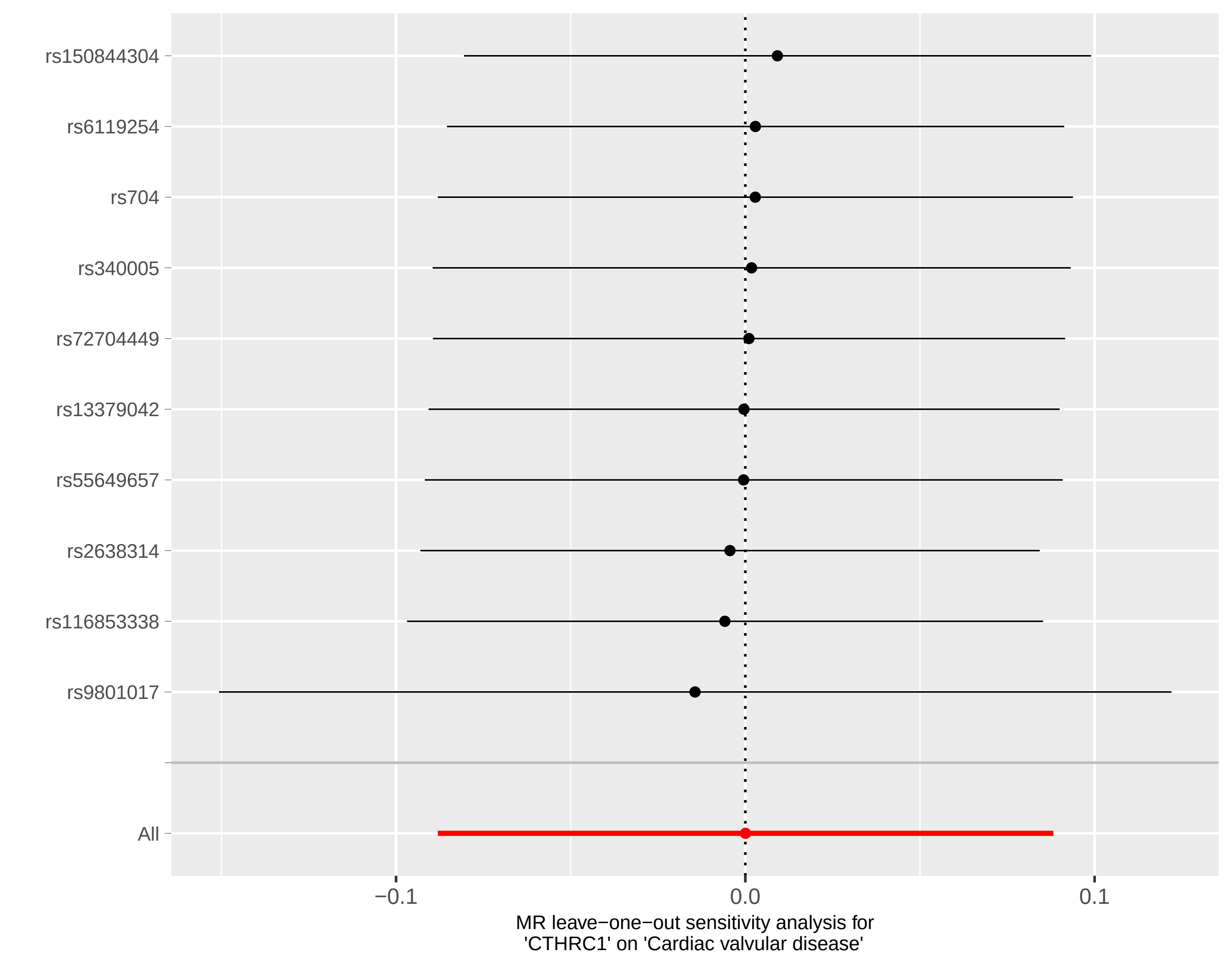

rs150844304
rs6119254
rs704
rs340005
rs72704449
rs13379042
rs55649657
rs2638314
rs116853338
rs9801017
All
−0.1
0.0
0.1
MR leave−one−out sensitivity analysis for
'CTHRC1' on 'Cardiac valvular disease'

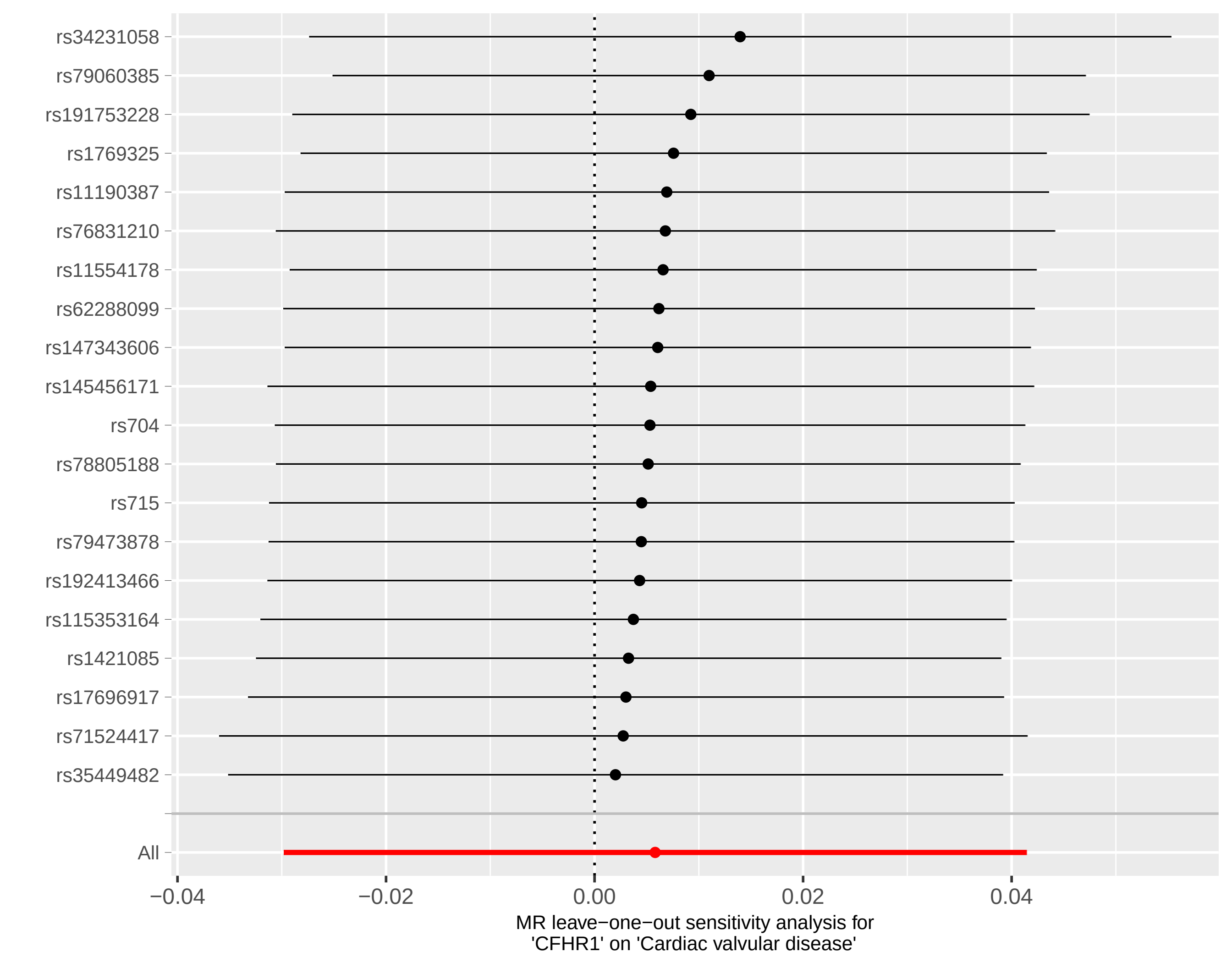
rs34231058
rs79060385
rs191753228
rs1769325
rs11190387
rs76831210
rs11554178
rs62288099
rs147343606
rs145456171
rs704
rs78805188
rs715
rs79473878
rs192413466
rs115353164
rs1421085
rs17696917
rs71524417
rs35449482
All
−0.04
−0.02
0.00
0.02
0.04
MR leave−one−out sensitivity analysis for
'CFHR1' on 'Cardiac valvular disease'

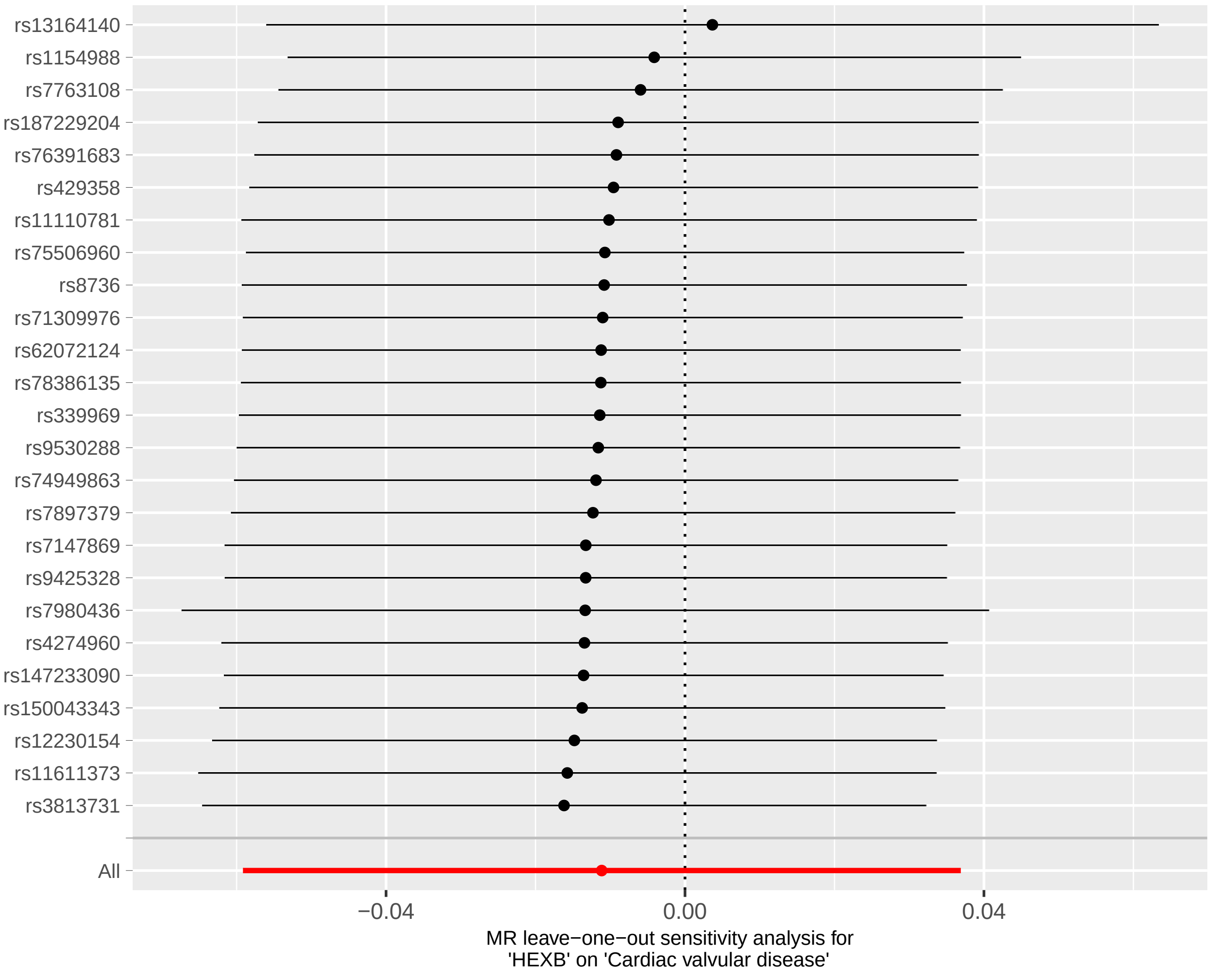
rs13164140
rs1154988
rs7763108
rs187229204
rs76391683
rs429358
rs11110781
rs75506960
rs8736
rs71309976
rs62072124
rs78386135
rs339969
rs9530288
rs74949863
rs7897379
rs7147869
rs9425328
rs7980436
rs4274960
rs147233090
rs150043343
rs12230154
rs11611373
rs3813731
All
−0.04
0.00
0.04
MR leave−one−out sensitivity analysis for
'HEXB' on 'Cardiac valvular disease'

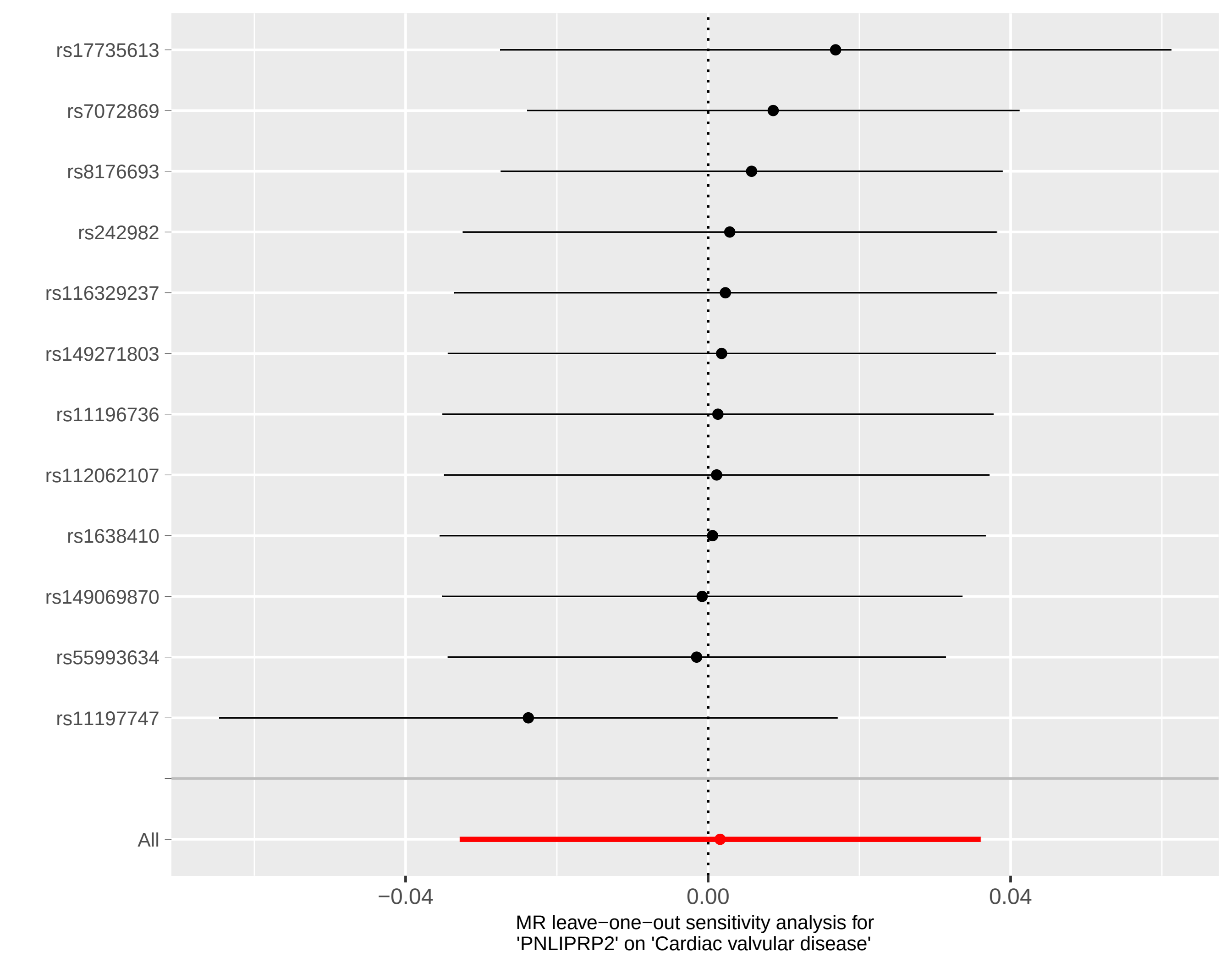
rs17735613
rs7072869
rs8176693
rs242982
rs116329237
rs149271803
rs11196736
rs112062107
rs1638410
rs149069870
rs55993634
rs11197747
All
−0.04
0.00
0.04
MR leave−one−out sensitivity analysis for
'PNLIPRP2' on 'Cardiac valvular disease'

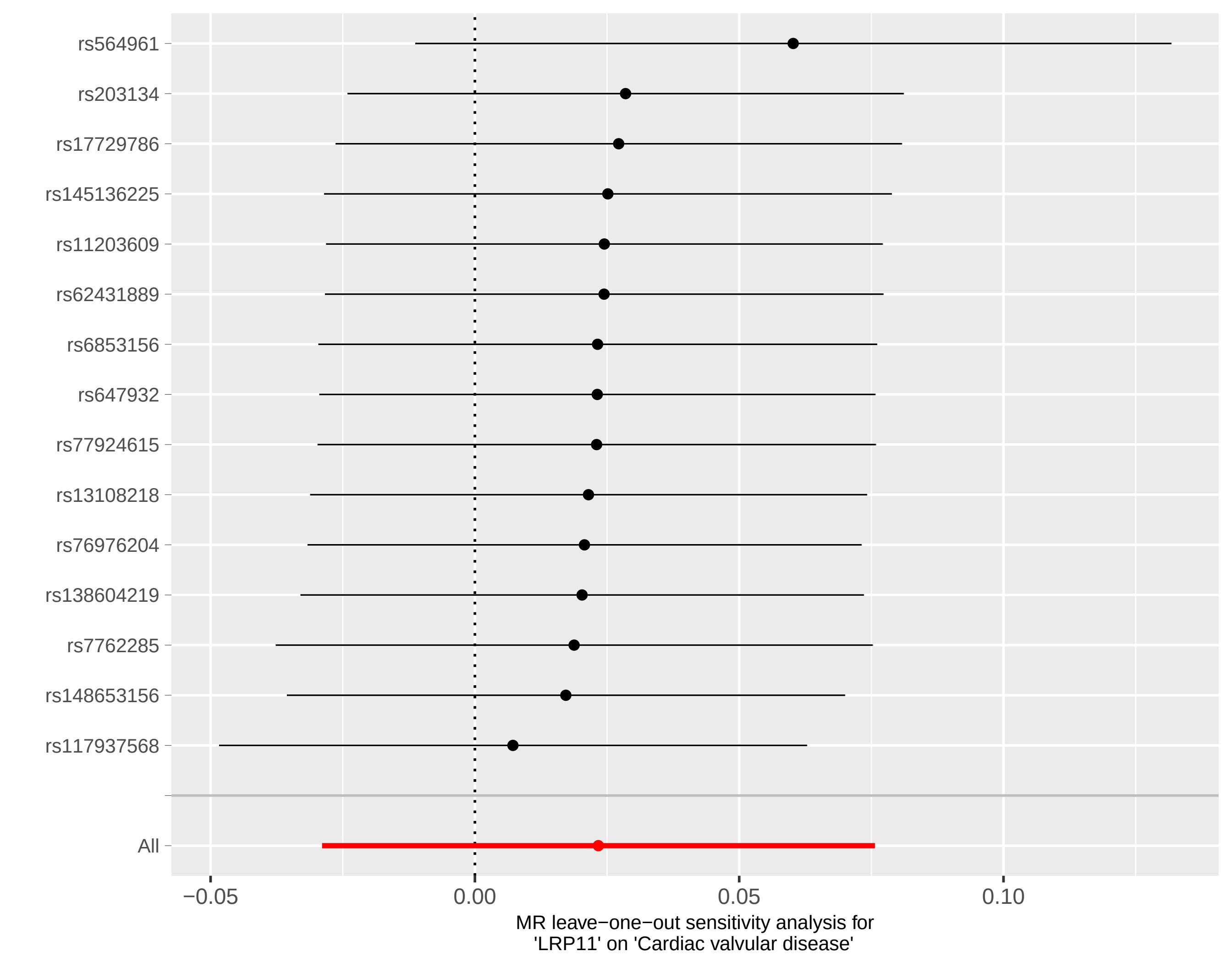
rs564961
rs203134
rs17729786
rs145136225
rs11203609
rs62431889
rs6853156
rs647932
rs77924615
rs13108218
rs76976204
rs138604219
rs7762285
rs148653156
rs117937568
All
−0.05
0.00
0.05
0.10
MR leave−one−out sensitivity analysis for
'LRP11' on 'Cardiac valvular disease'

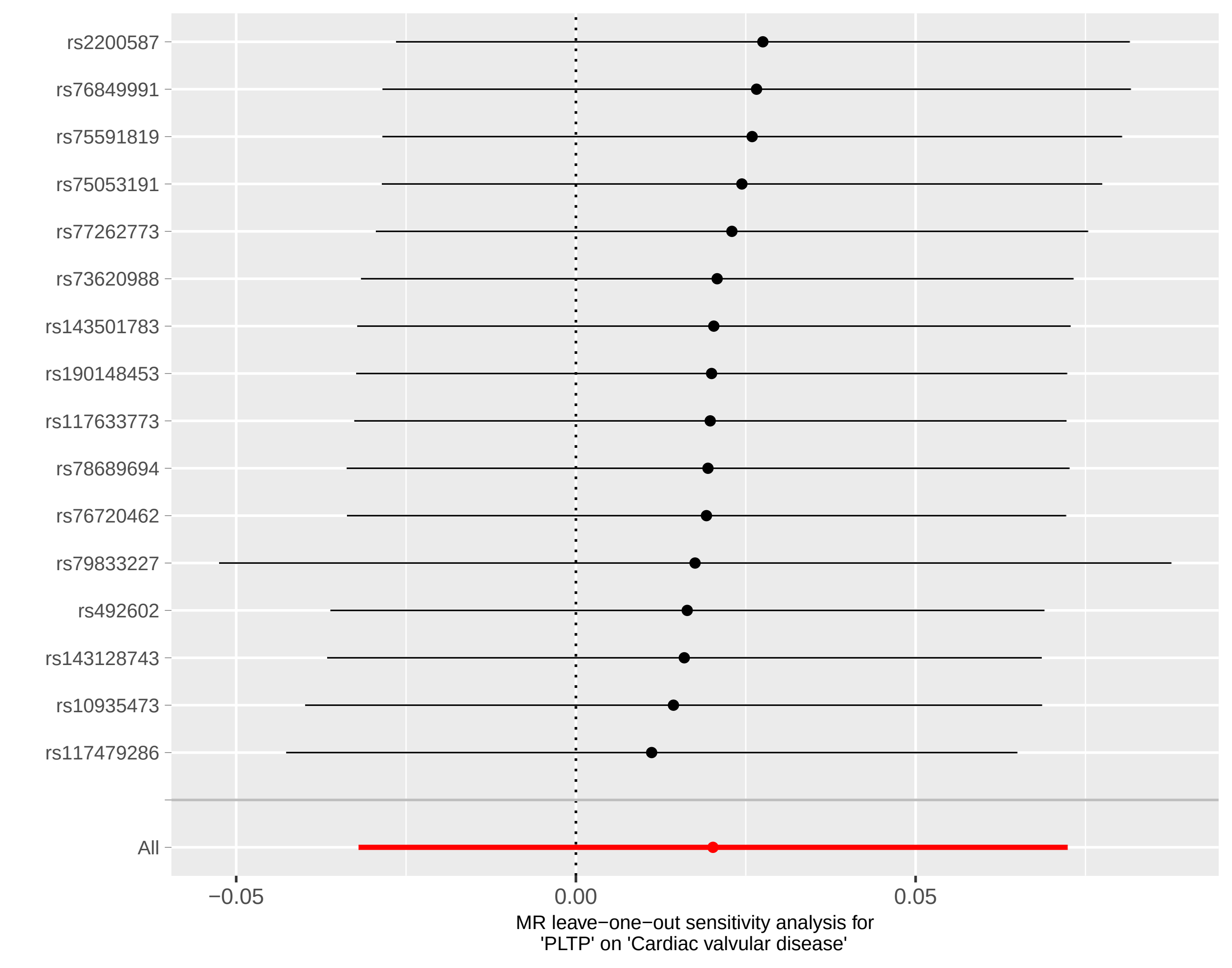
rs2200587
rs76849991
rs75591819
rs75053191
rs77262773
rs73620988
rs143501783
rs190148453
rs117633773
rs78689694
rs76720462
rs79833227
rs492602
rs143128743
rs10935473
rs117479286
All
−0.05
0.00
0.05
MR leave−one−out sensitivity analysis for
'PLTP' on 'Cardiac valvular disease'

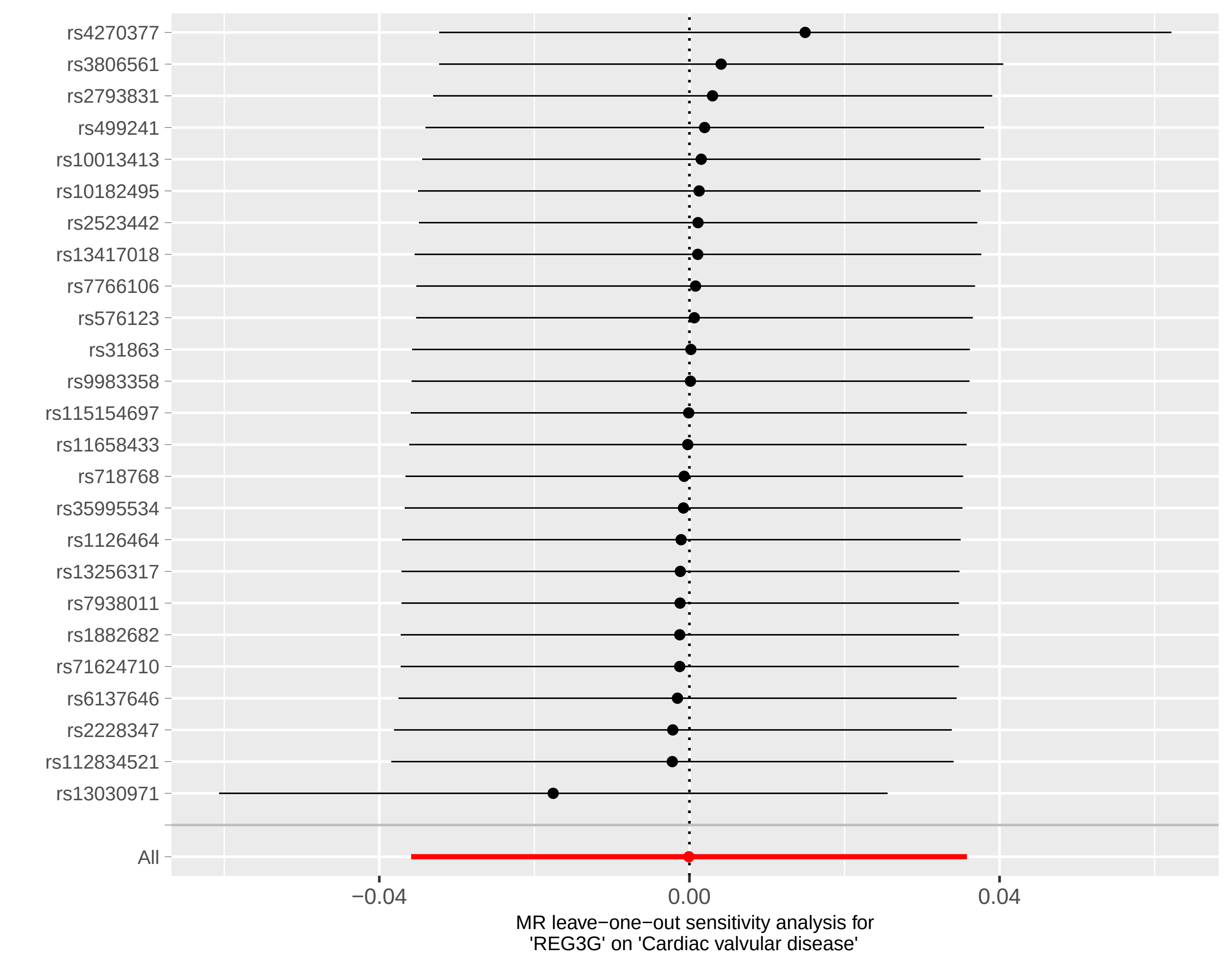
rs4270377
rs3806561
rs2793831
rs499241
rs10013413
rs10182495
rs2523442
rs13417018
rs7766106
rs576123
rs31863
rs9983358
rs115154697
rs11658433
rs718768
rs35995534
rs1126464
rs13256317
rs7938011
rs1882682
rs71624710
rs6137646
rs2228347
rs112834521
rs13030971
All
−0.04
0.00
0.04
MR leave−one−out sensitivity analysis for
'REG3G' on 'Cardiac valvular disease'

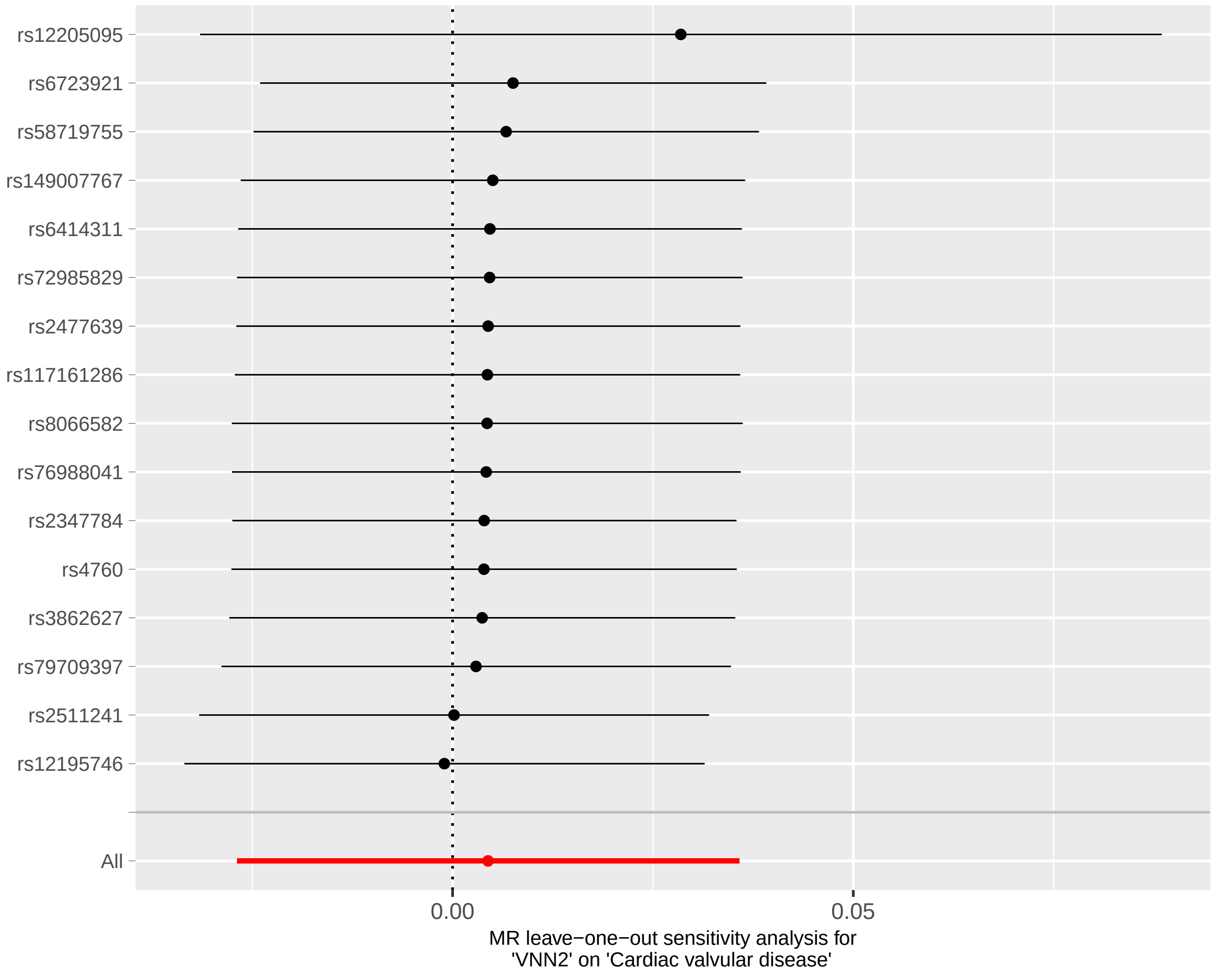

rs12205095
rs6723921
rs58719755
rs149007767
rs6414311
rs72985829
rs2477639
rs117161286
rs8066582
rs76988041
rs2347784
rs4760
rs3862627
rs79709397
rs2511241
rs12195746
All
0.00
0.05
MR leave−one−out sensitivity analysis for
'VNN2' on 'Cardiac valvular disease'

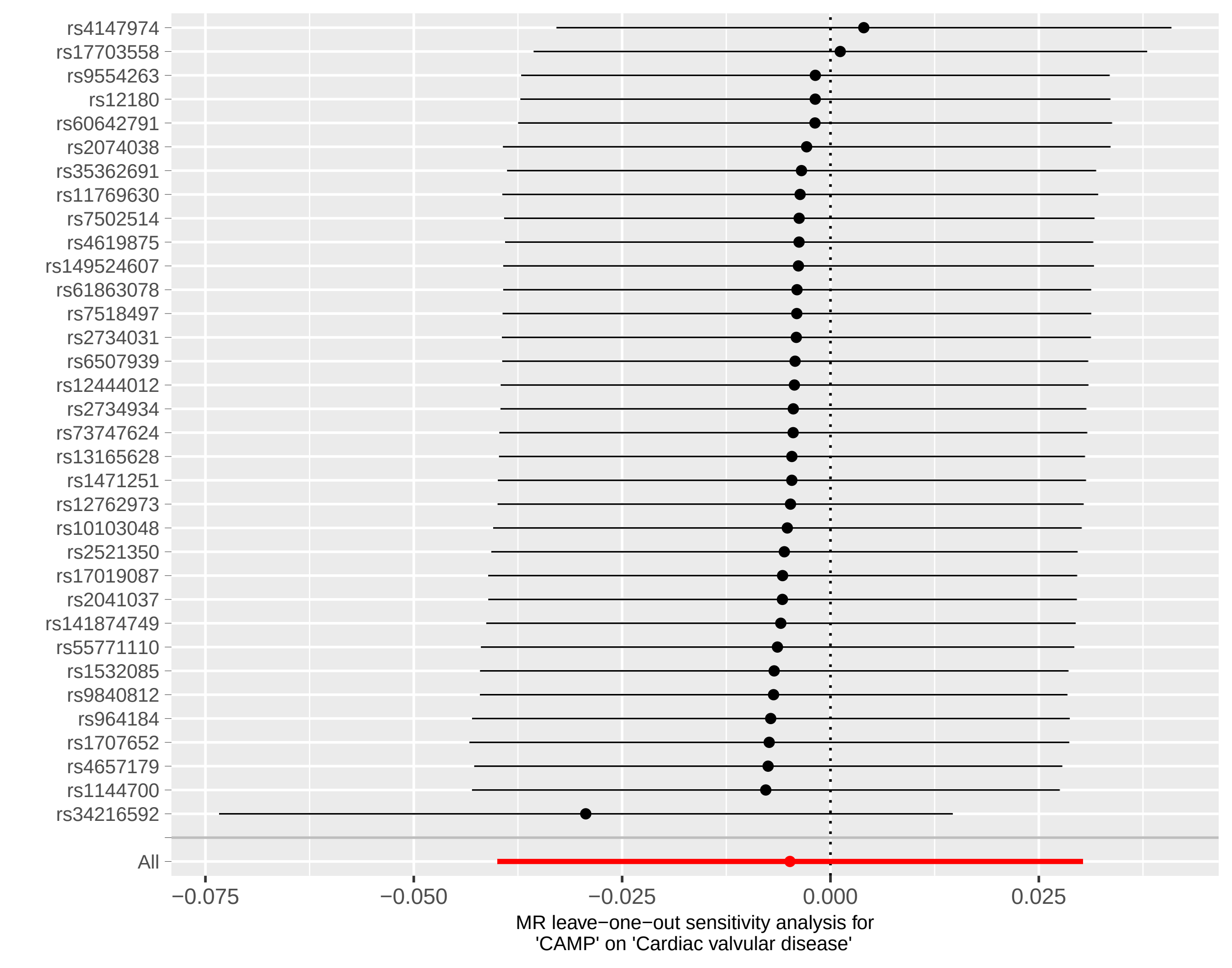

rs4147974
rs17703558
rs9554263
rs12180
rs60642791
rs2074038
rs35362691
rs11769630
rs7502514
rs4619875
rs149524607
rs61863078
rs7518497
rs2734031
rs6507939
rs12444012
rs2734934
rs73747624
rs13165628
rs1471251
rs12762973
rs10103048
rs2521350
rs17019087
rs2041037
rs141874749
rs55771110
rs1532085
rs9840812
rs964184
rs1707652
rs4657179
rs1144700
rs34216592
All
−0.075
−0.050
−0.025
0.000
0.025
MR leave−one−out sensitivity analysis for
'CAMP' on 'Cardiac valvular disease'

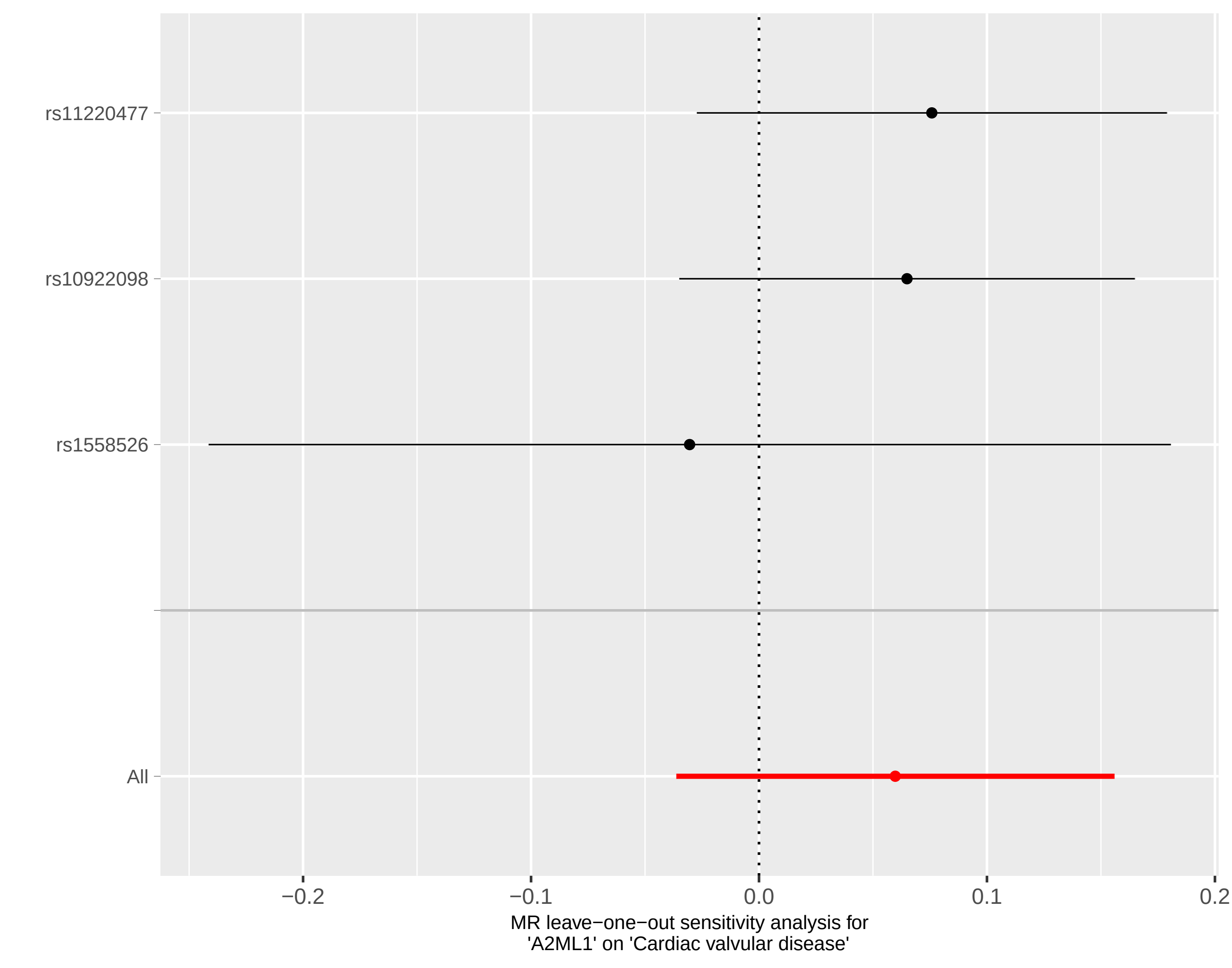

rs11220477
rs10922098
rs1558526
All
−0.2
−0.1
0.0
0.1
0.2
MR leave−one−out sensitivity analysis for
'A2ML1' on 'Cardiac valvular disease'

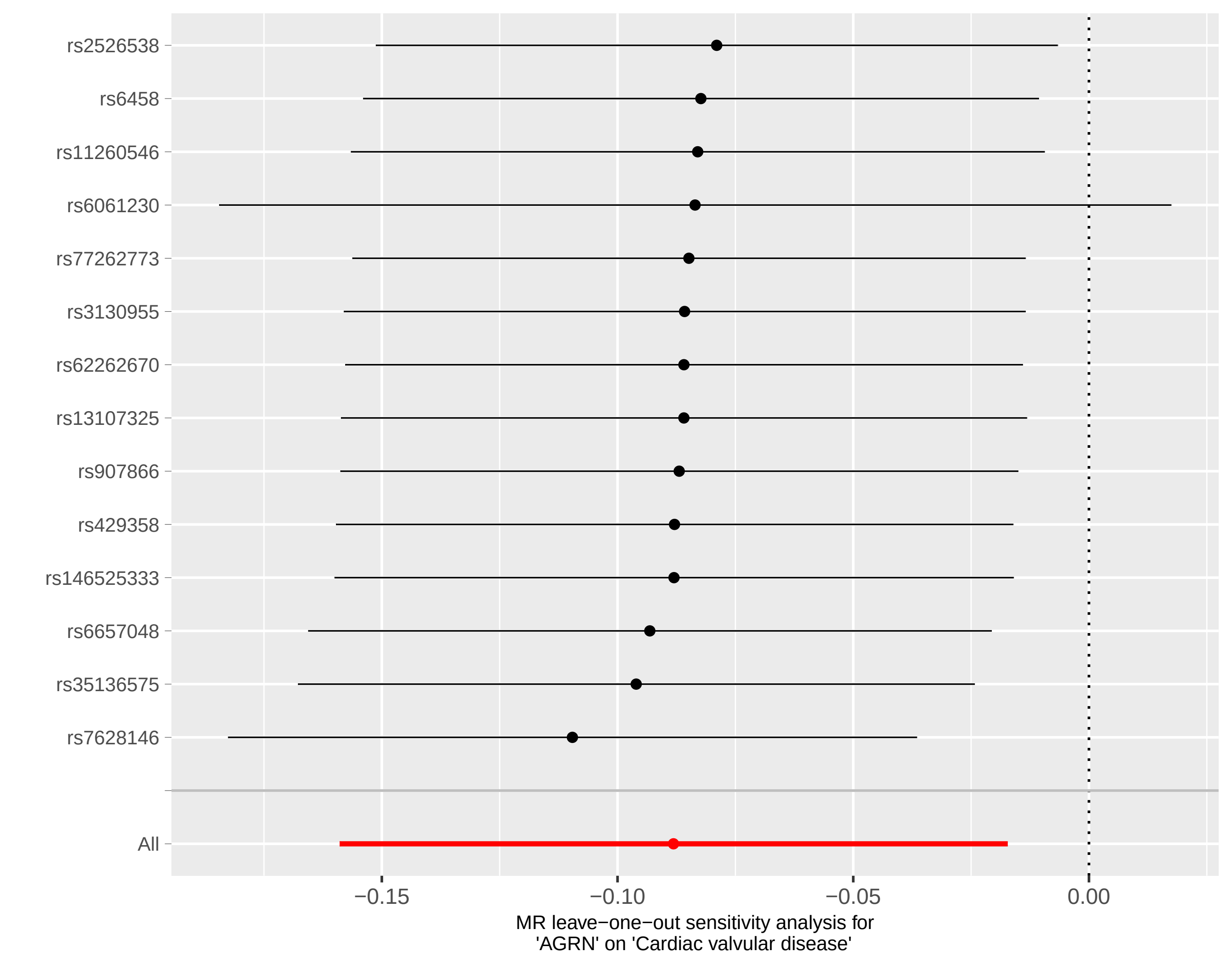
rs2526538
rs6458
rs11260546
rs6061230
rs77262773
rs3130955
rs62262670
rs13107325
rs907866
rs429358
rs146525333
rs6657048
rs35136575
rs7628146
All
−0.15
−0.10
−0.05
0.00
MR leave−one−out sensitivity analysis for
'AGRN' on 'Cardiac valvular disease'

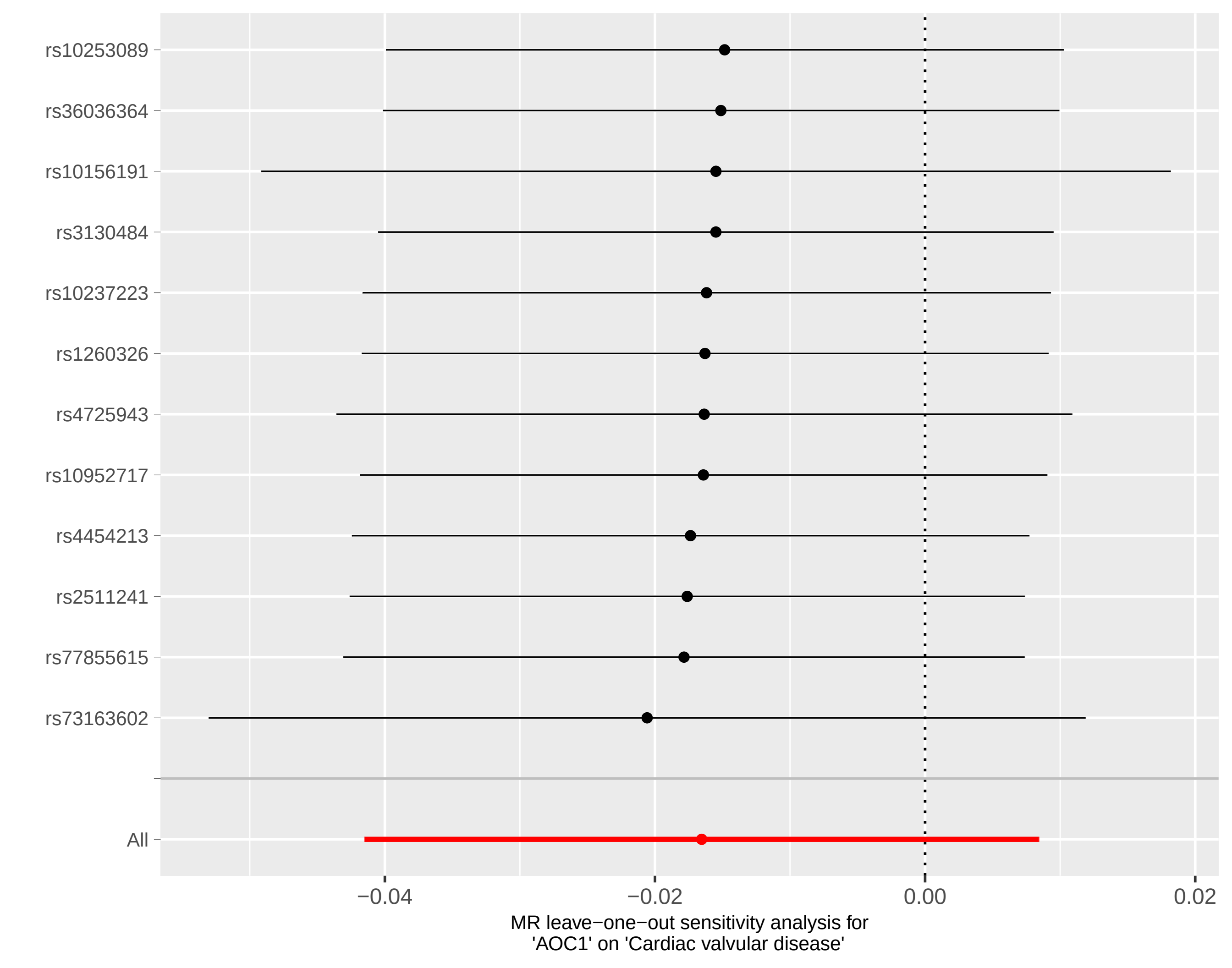
rs10253089
rs36036364
rs10156191
rs3130484
rs10237223
rs1260326
rs4725943
rs10952717
rs4454213
rs2511241
rs77855615
rs73163602
All
−0.04
−0.02
0.00
0.02
MR leave−one−out sensitivity analysis for
'AOC1' on 'Cardiac valvular disease'

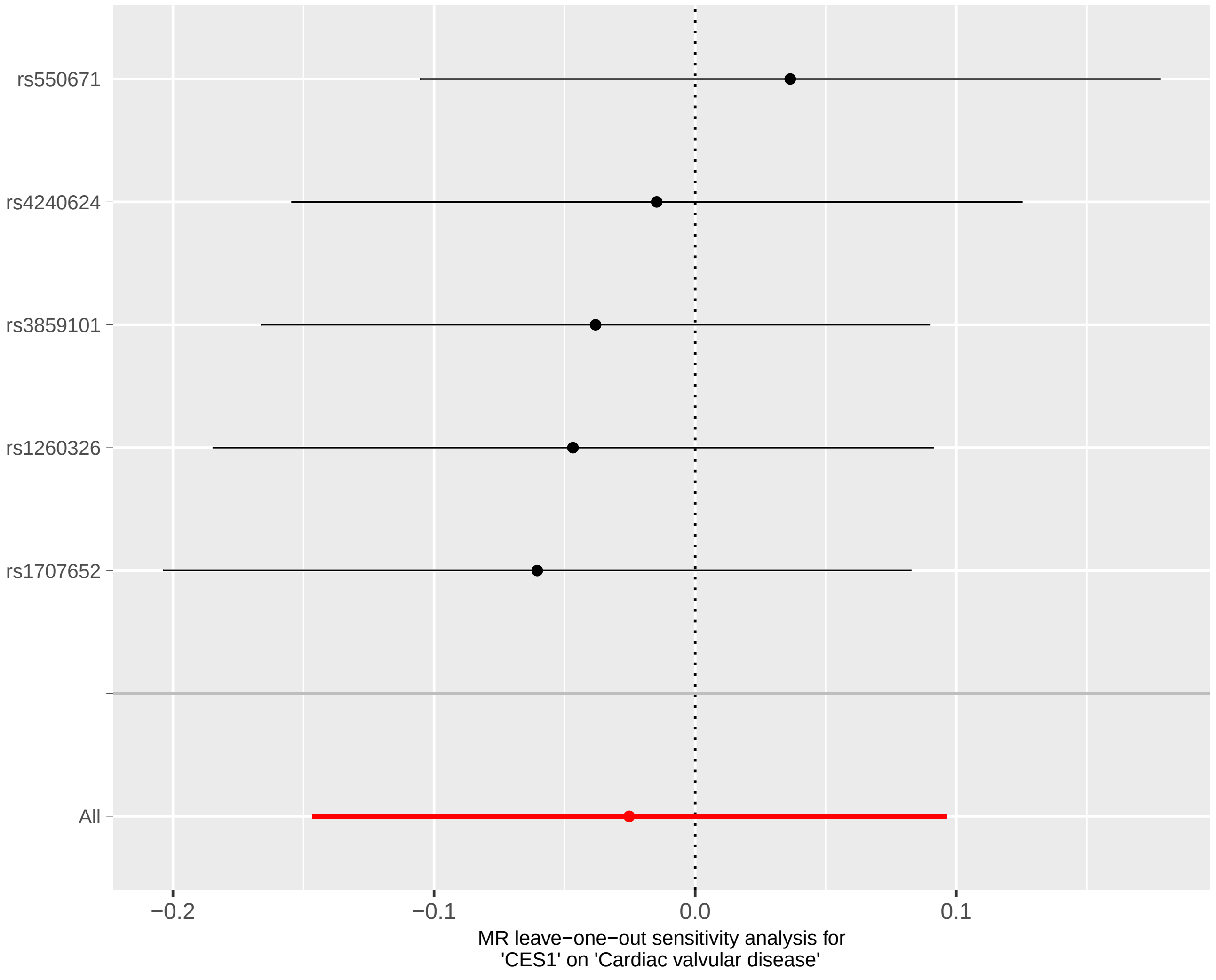
rs550671
rs4240624
rs3859101
rs1260326
rs1707652
All
−0.2
−0.1
0.0
0.1
MR leave−one−out sensitivity analysis for
'CES1' on 'Cardiac valvular disease'

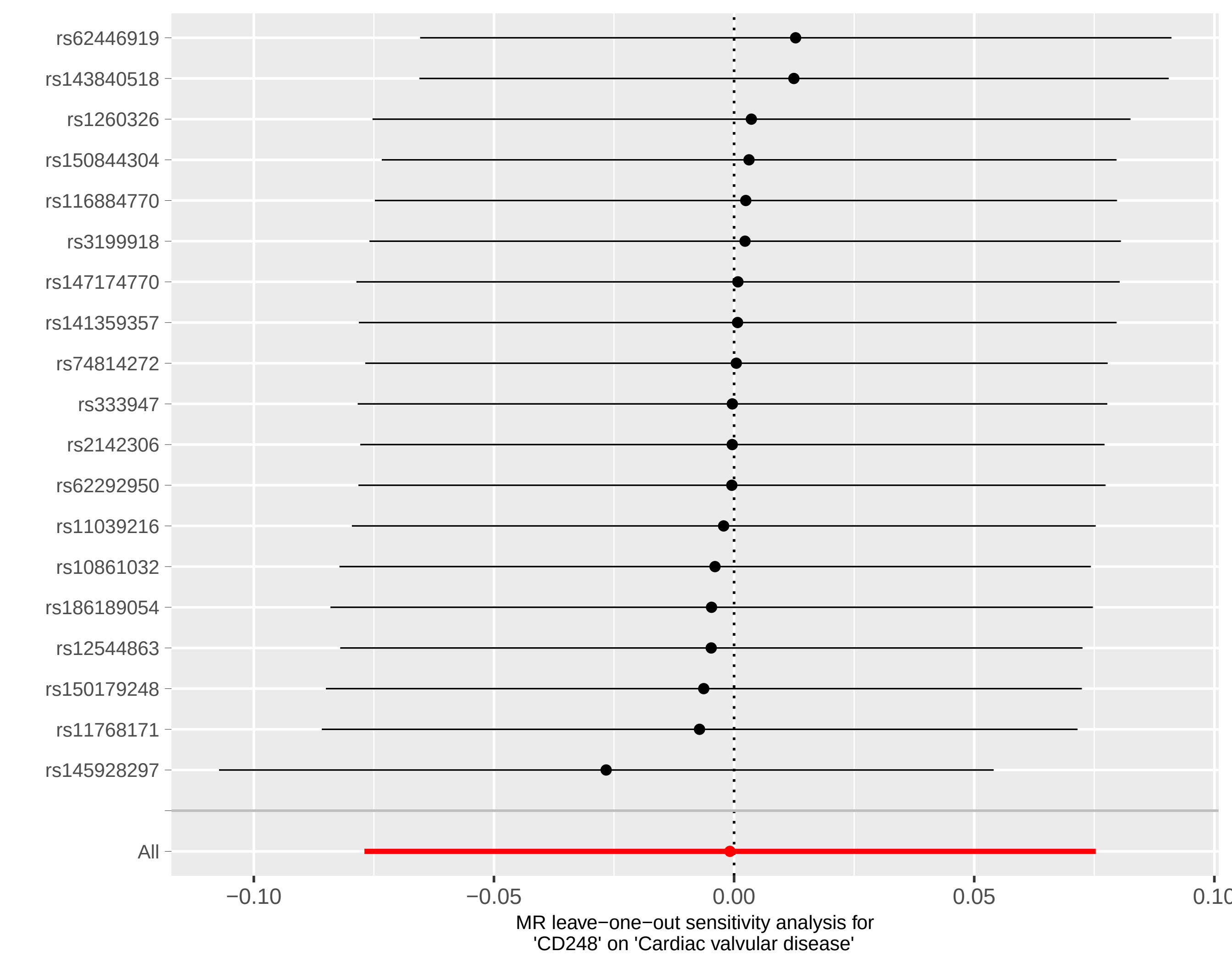
rs62446919
rs143840518
rs1260326
rs150844304
rs116884770
rs3199918
rs147174770
rs141359357
rs74814272
rs333947
rs2142306
rs62292950
rs11039216
rs10861032
rs186189054
rs12544863
rs150179248
rs11768171
rs145928297
All
−0.10
−0.05
0.00
0.05
0.10
MR leave−one−out sensitivity analysis for
'CD248' on 'Cardiac valvular disease'

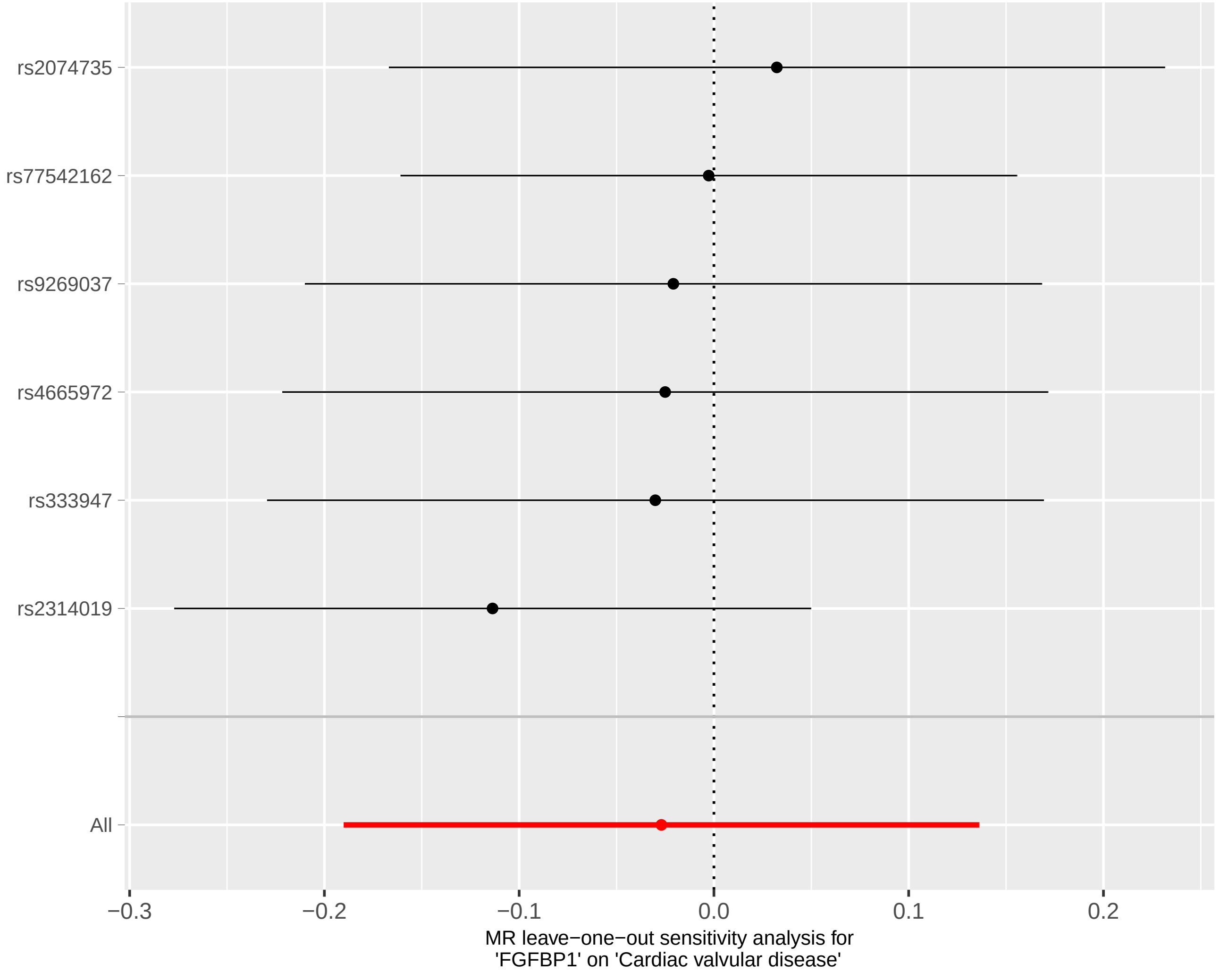
rs2074735
rs77542162
rs9269037
rs4665972
rs333947
rs2314019
All
−0.3
−0.2
−0.1
0.0
0.1
0.2
MR leave−one−out sensitivity analysis for
'FGFBP1' on 'Cardiac valvular disease'

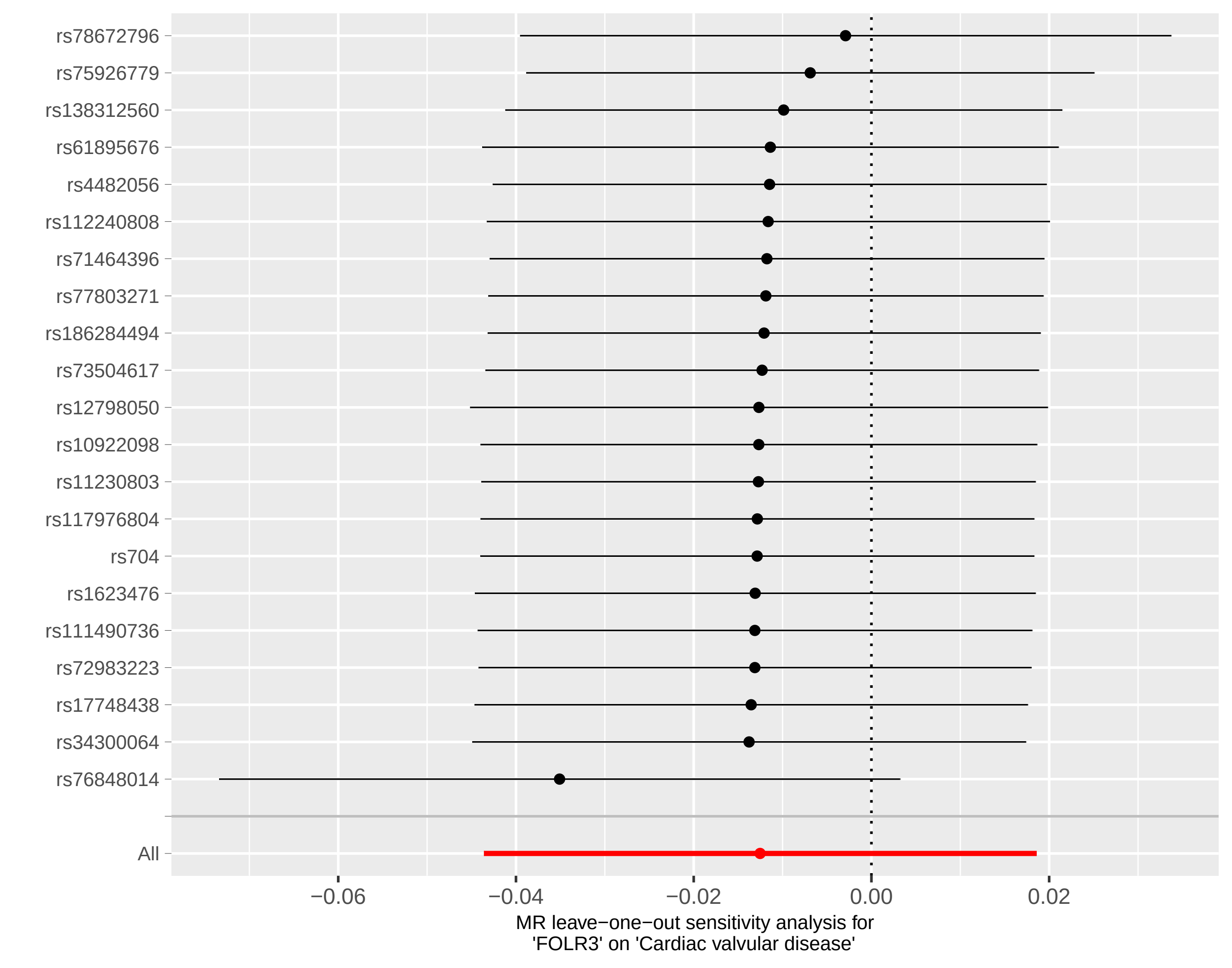
rs78672796
rs75926779
rs138312560
rs61895676
rs4482056
rs112240808
rs71464396
rs77803271
rs186284494
rs73504617
rs12798050
rs10922098
rs11230803
rs117976804
rs704
rs1623476
rs111490736
rs72983223
rs17748438
rs34300064
rs76848014
All
−0.06
−0.04
−0.02
0.00
0.02
MR leave−one−out sensitivity analysis for
'FOLR3' on 'Cardiac valvular disease'

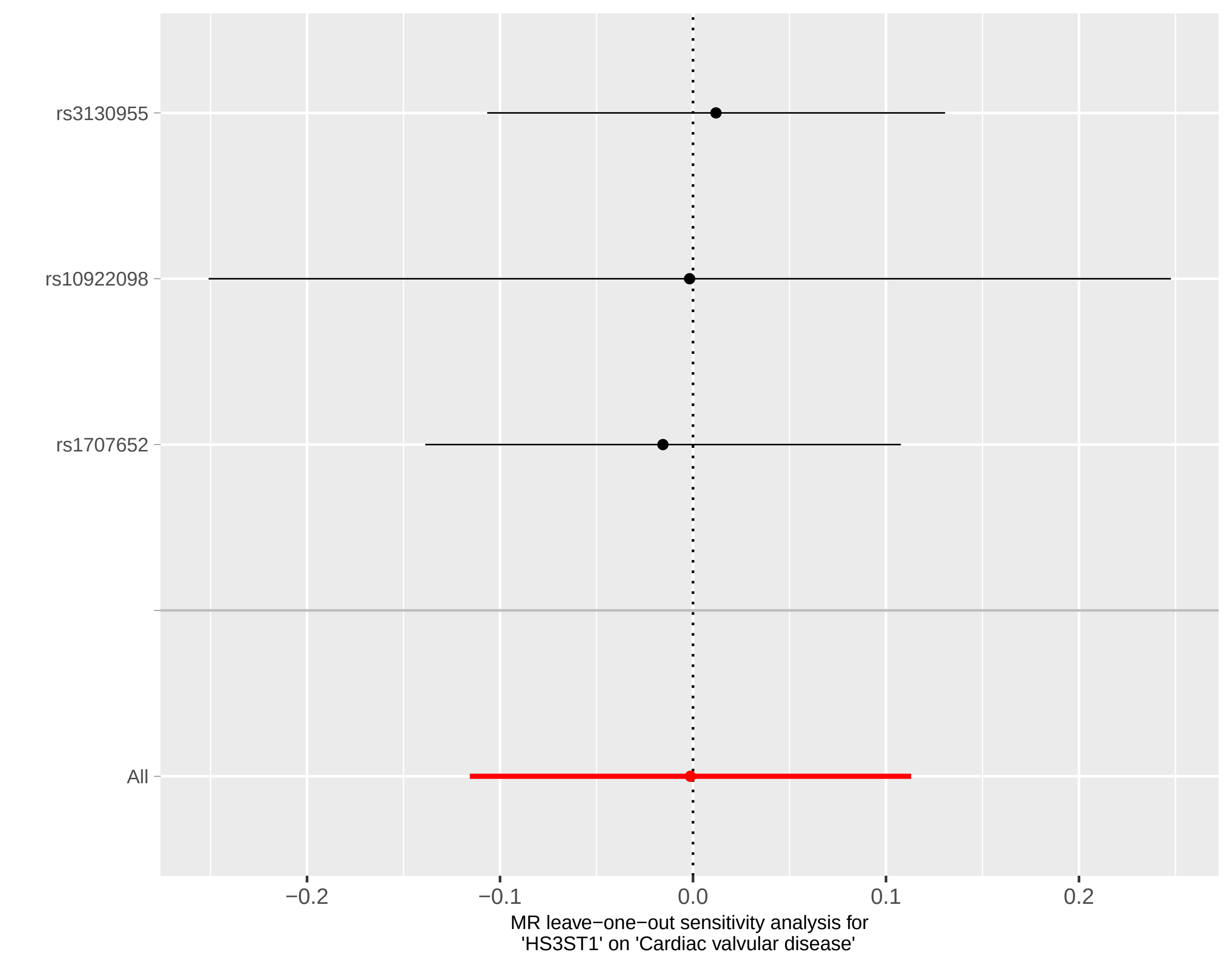
rs3130955
rs10922098
rs1707652
All
−0.2
−0.1
0.0
0.1
0.2
MR leave−one−out sensitivity analysis for
'HS3ST1' on 'Cardiac valvular disease'

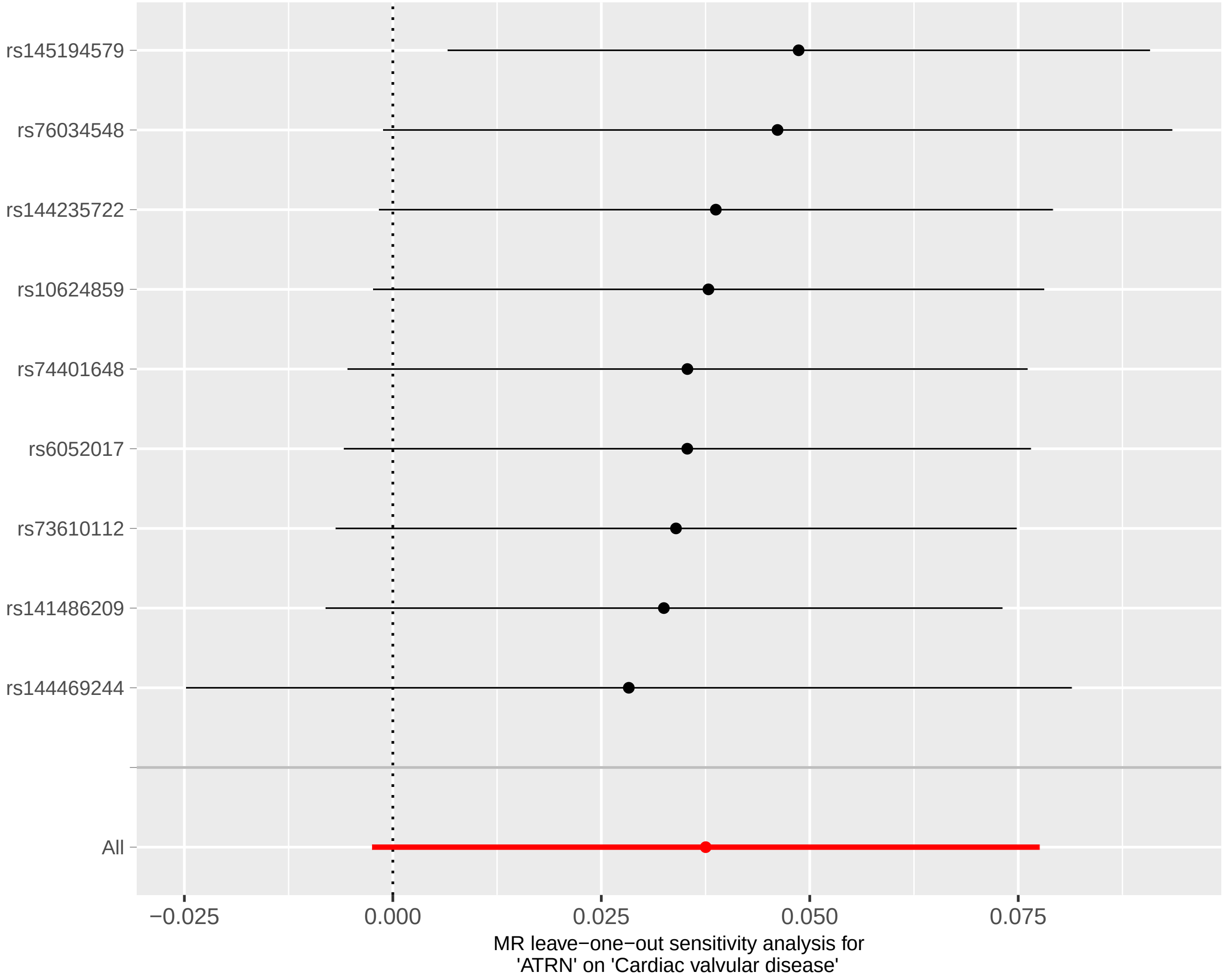
rs145194579
rs76034548
rs144235722
rs10624859
rs74401648
rs6052017
rs73610112
rs141486209
rs144469244
All
−0.025
0.000
0.025
0.050
0.075
MR leave−one−out sensitivity analysis for
'ATRN' on 'Cardiac valvular disease'

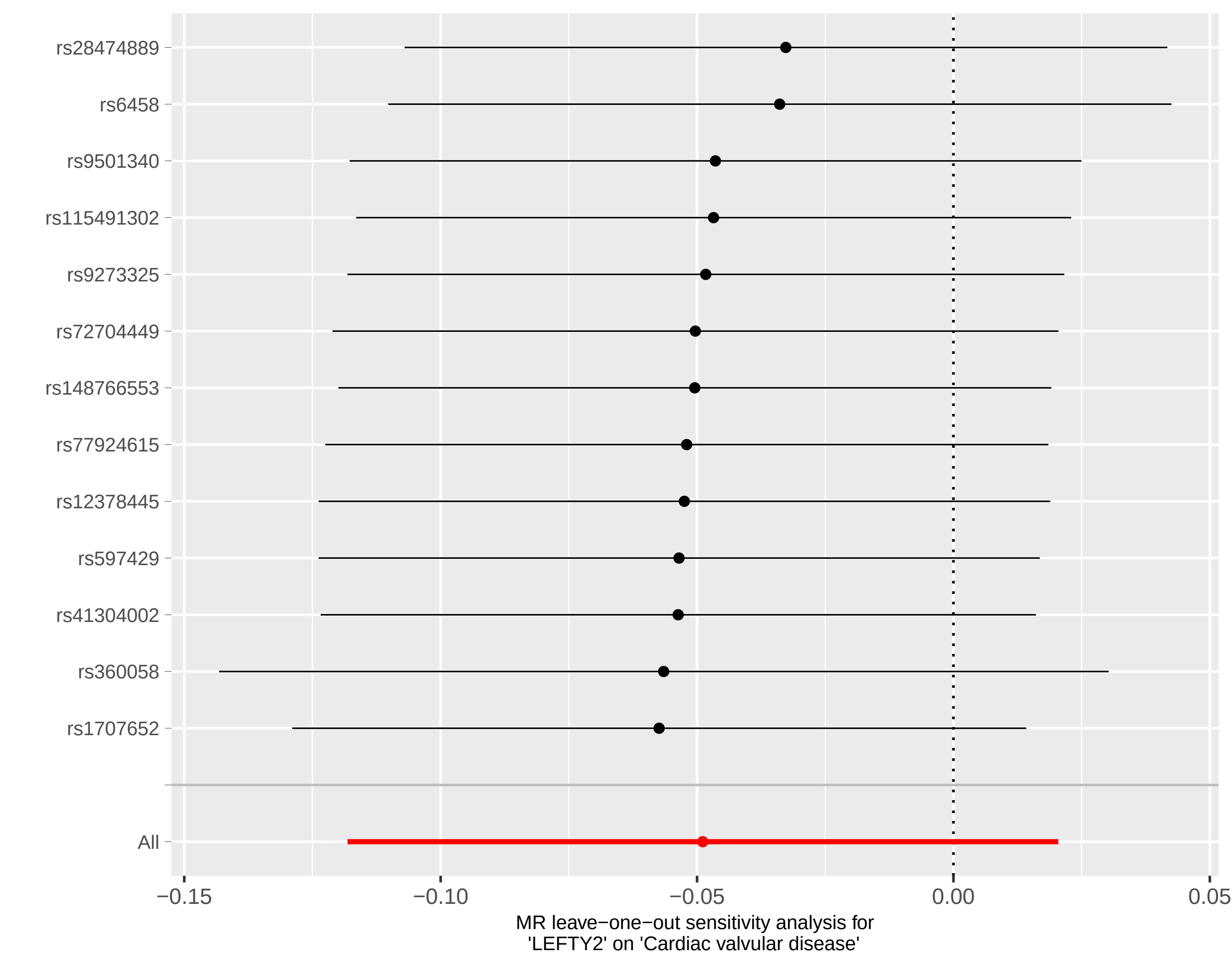
rs28474889
rs6458
rs9501340
rs115491302
rs9273325
rs72704449
rs148766553
rs77924615
rs12378445
rs597429
rs41304002
rs360058
rs1707652
All
−0.15
−0.10
−0.05
0.00
0.05
MR leave−one−out sensitivity analysis for
'LEFTY2' on 'Cardiac valvular disease'

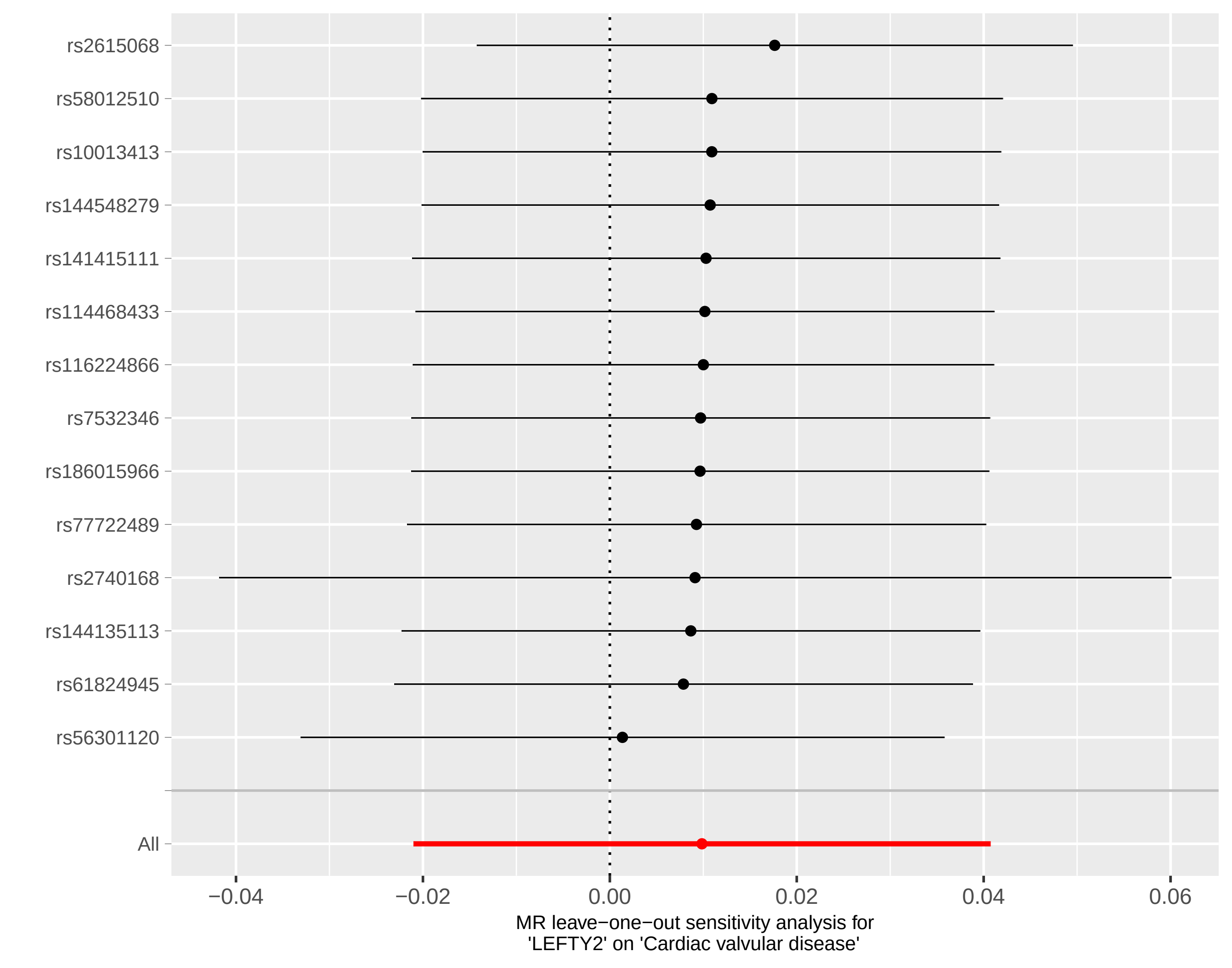
rs2615068
rs58012510
rs10013413
rs144548279
rs141415111
rs114468433
rs116224866
rs7532346
rs186015966
rs77722489
rs2740168
rs144135113
rs61824945
rs56301120
All
−0.04
−0.02
0.00
0.02
0.04
0.06
MR leave−one−out sensitivity analysis for
'LEFTY2' on 'Cardiac valvular disease'

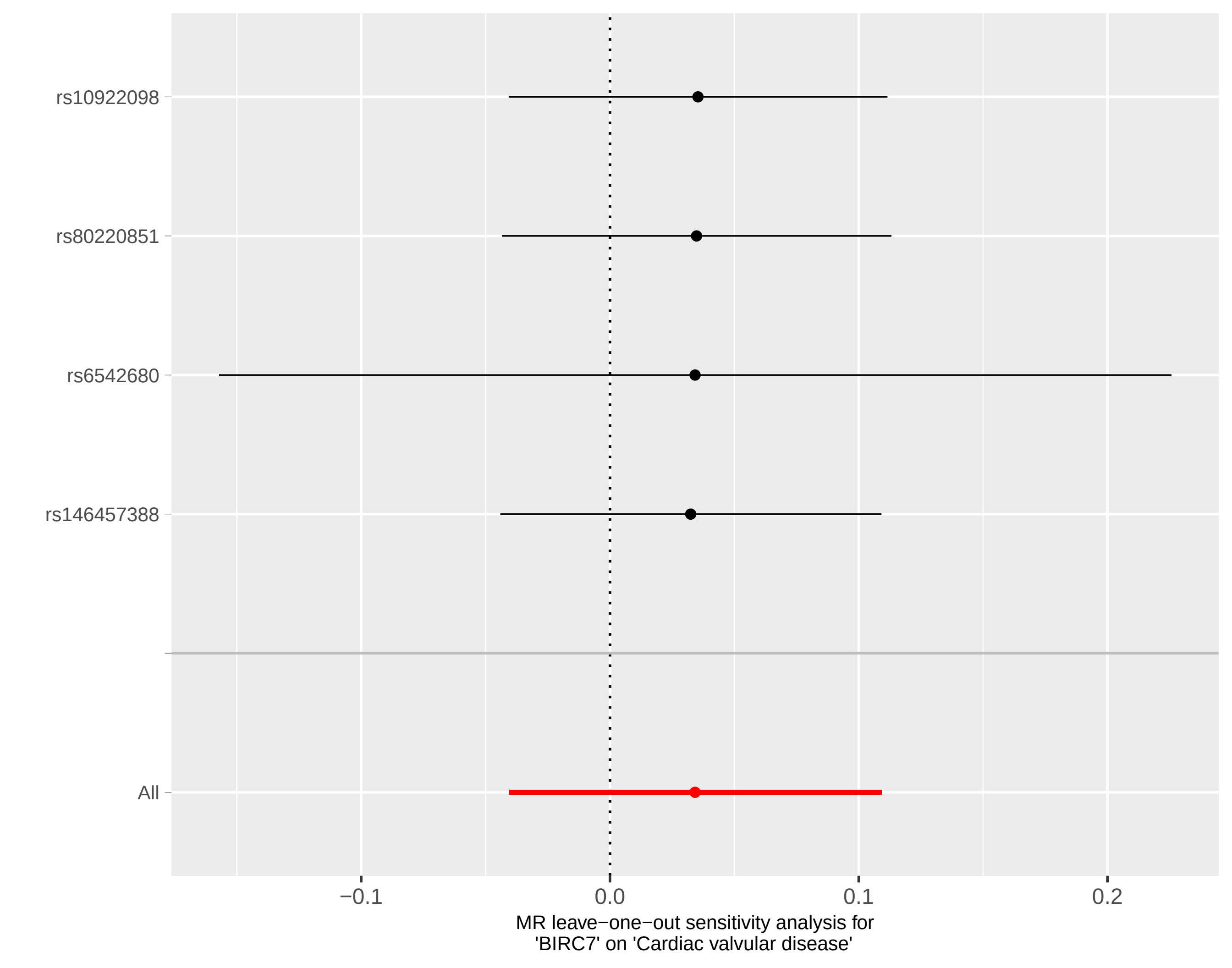
rs10922098
rs80220851
rs6542680
rs146457388
All
−0.1
0.0
0.1
0.2
MR leave−one−out sensitivity analysis for
'BIRC7' on 'Cardiac valvular disease'

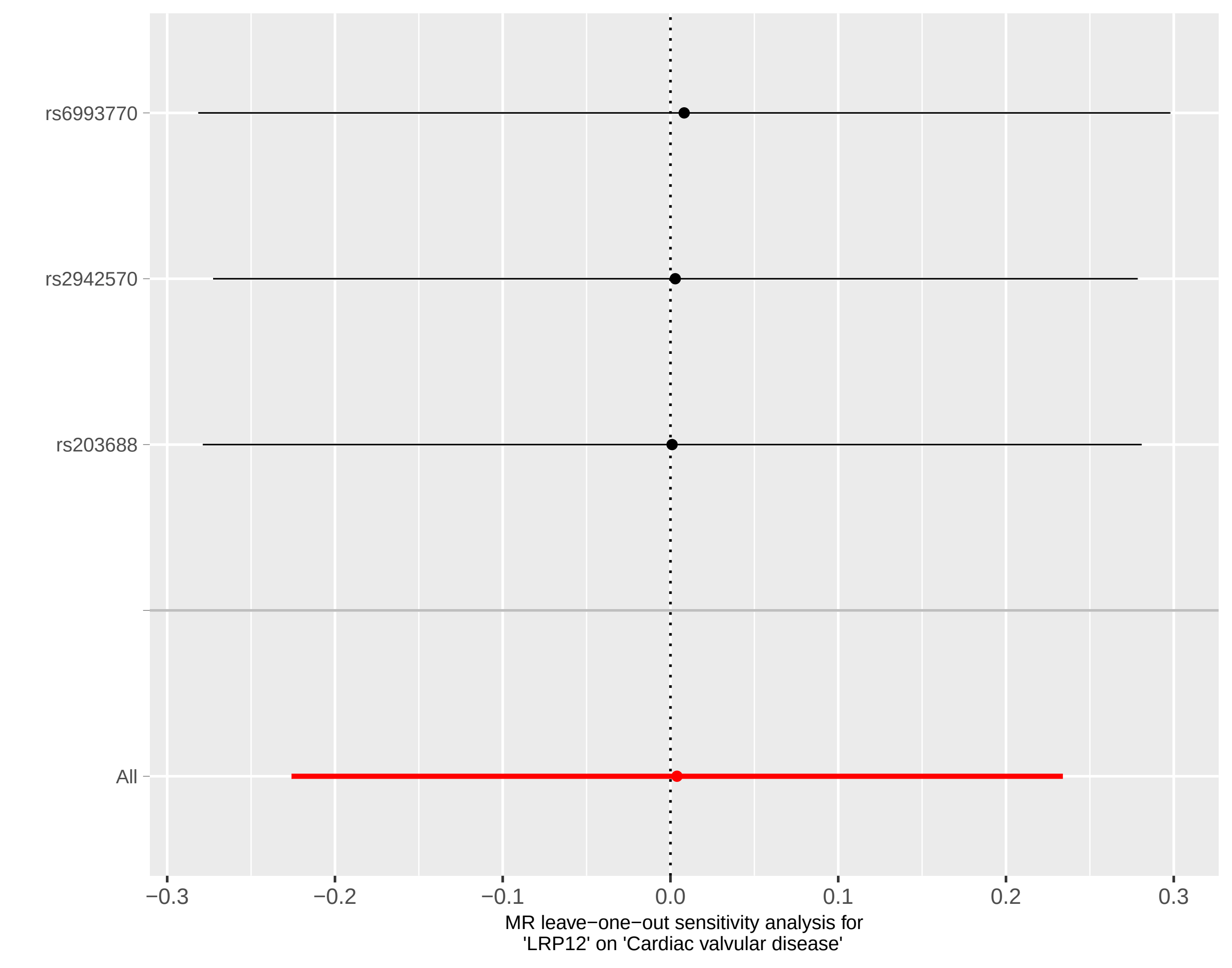
rs6993770
rs2942570
rs203688
All
−0.3
−0.2
−0.1
0.0
0.1
0.2
0.3
MR leave−one−out sensitivity analysis for
'LRP12' on 'Cardiac valvular disease'

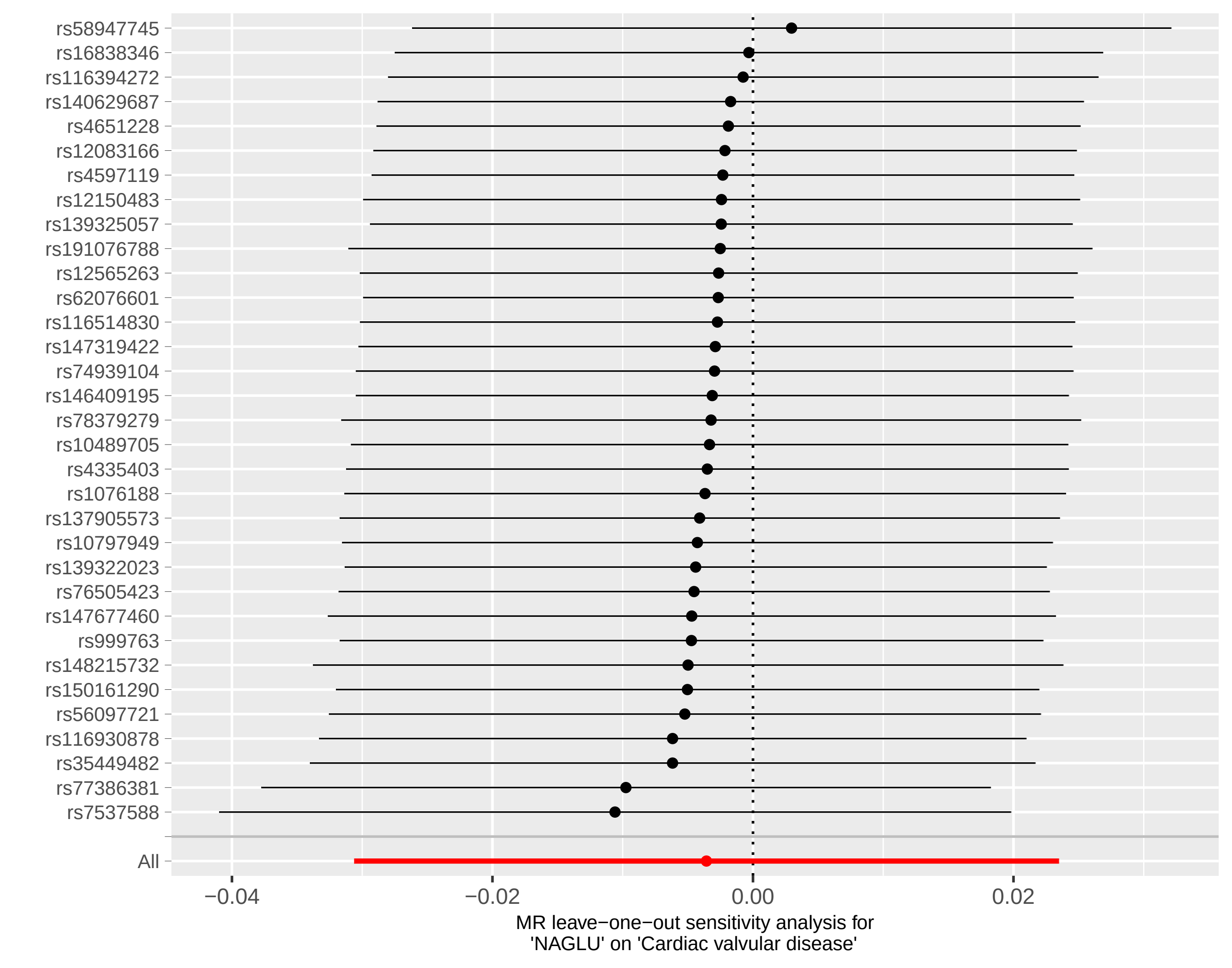
rs58947745
rs16838346
rs116394272
rs140629687
rs4651228
rs12083166
rs4597119
rs12150483
rs139325057
rs191076788
rs12565263
rs62076601
rs116514830
rs147319422
rs74939104
rs146409195
rs78379279
rs10489705
rs4335403
rs1076188
rs137905573
rs10797949
rs139322023
rs76505423
rs147677460
rs999763
rs148215732
rs150161290
rs56097721
rs116930878
rs35449482
rs77386381
rs7537588
All
−0.04
−0.02
0.00
0.02
MR leave−one−out sensitivity analysis for
'NAGLU' on 'Cardiac valvular disease'

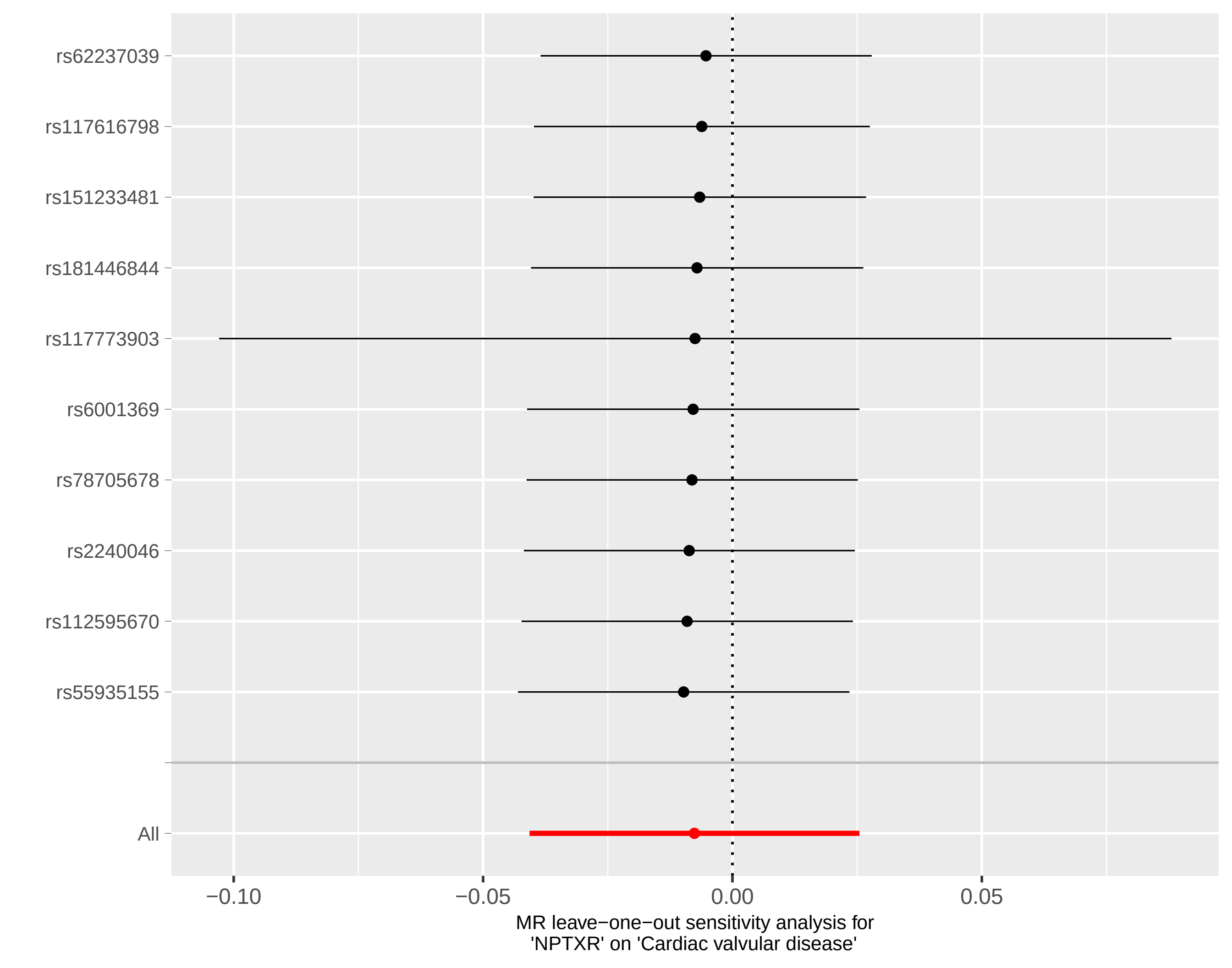
rs62237039
rs117616798
rs151233481
rs181446844
rs117773903
rs6001369
rs78705678
rs2240046
rs112595670
rs55935155
All
−0.10
−0.05
0.00
0.05
MR leave−one−out sensitivity analysis for
'NPTXR' on 'Cardiac valvular disease'

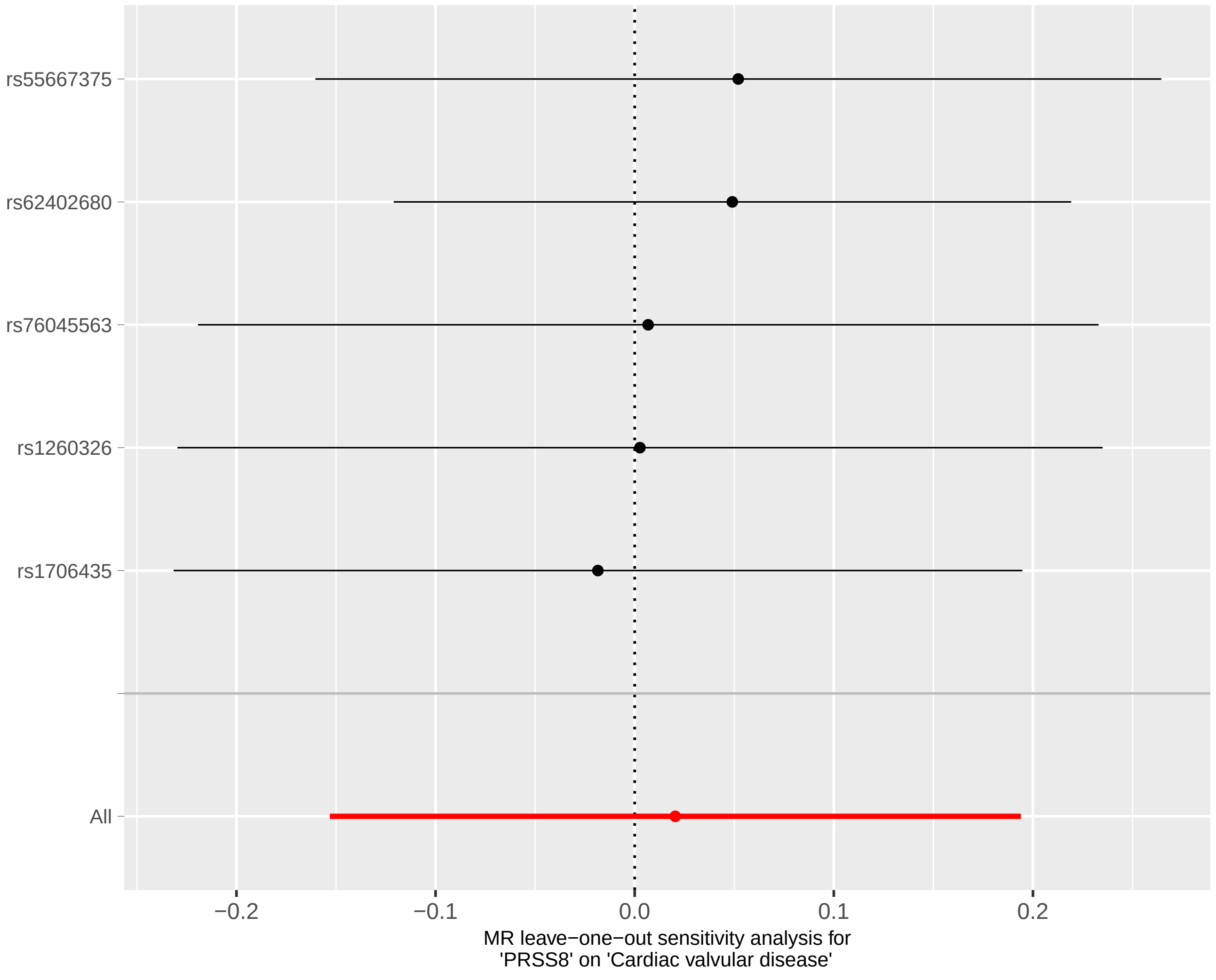

rs55667375
rs62402680
rs76045563
rs1260326
rs1706435
All
−0.2
−0.1
0.0
0.1
0.2
MR leave−one−out sensitivity analysis for
'PRSS8' on 'Cardiac valvular disease'

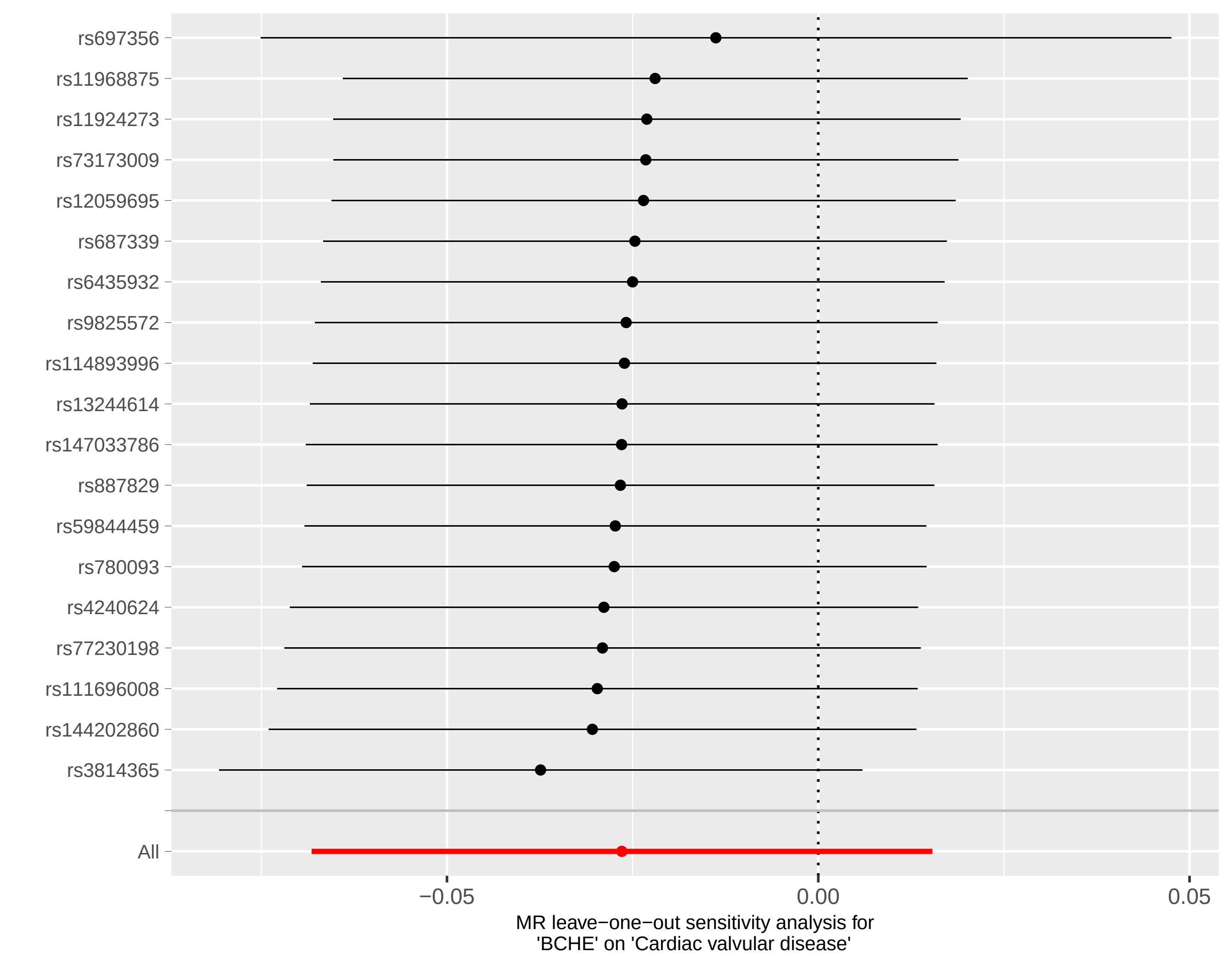
rs697356
rs11968875
rs11924273
rs73173009
rs12059695
rs687339
rs6435932
rs9825572
rs114893996
rs13244614
rs147033786
rs887829
rs59844459
rs780093
rs4240624
rs77230198
rs111696008
rs144202860
rs3814365
All
−0.05
0.00
0.05
MR leave−one−out sensitivity analysis for
'BCHE' on 'Cardiac valvular disease'

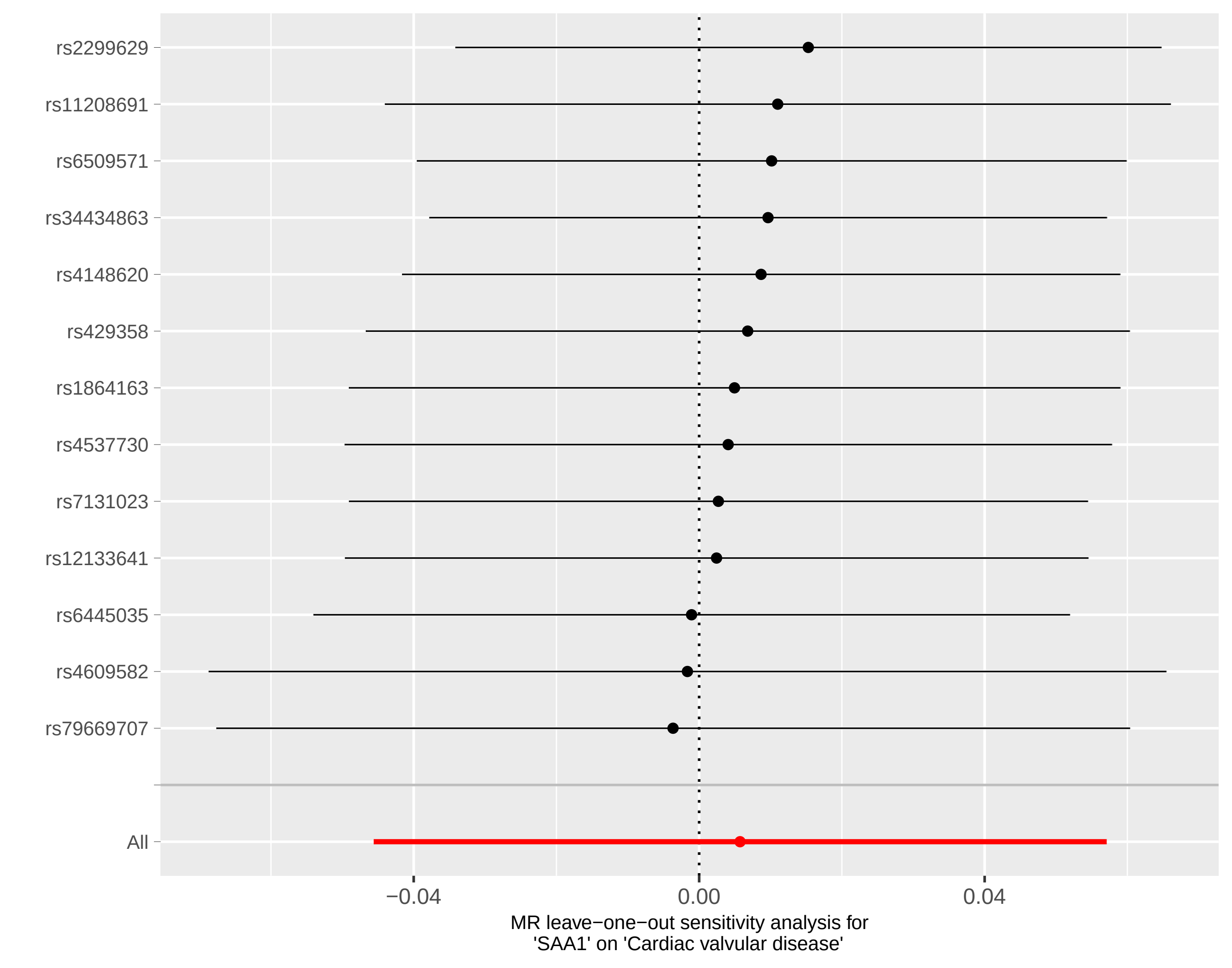
rs2299629
rs11208691
rs6509571
rs34434863
rs4148620
rs429358
rs1864163
rs4537730
rs7131023
rs12133641
rs6445035
rs4609582
rs79669707
All
−0.04
0.00
0.04
MR leave−one−out sensitivity analysis for
'SAA1' on 'Cardiac valvular disease'

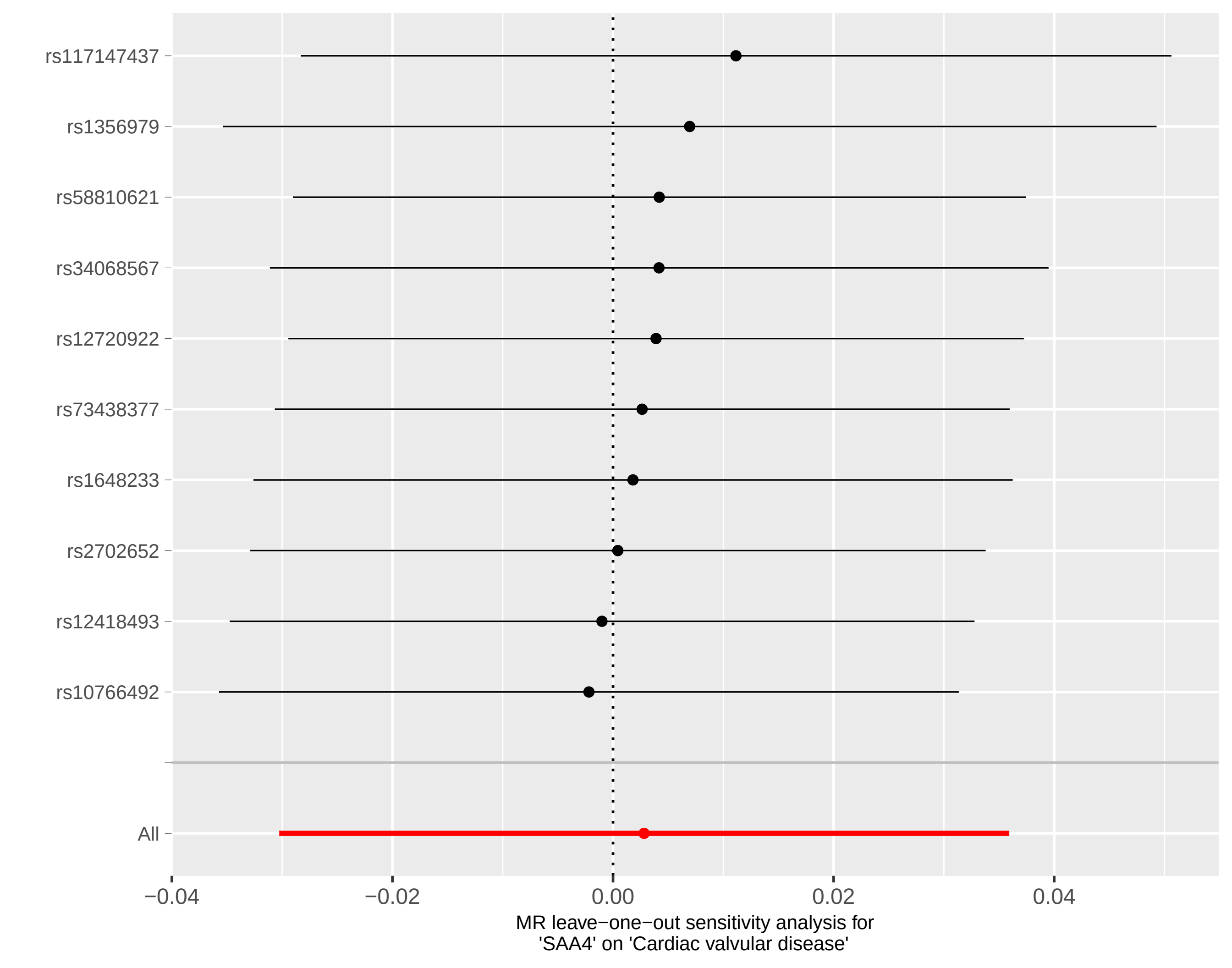

rs117147437
rs1356979
rs58810621
rs34068567
rs12720922
rs73438377
rs1648233
rs2702652
rs12418493
rs10766492
All
−0.04
−0.02
0.00
0.02
0.04
MR leave−one−out sensitivity analysis for
'SAA4' on 'Cardiac valvular disease'

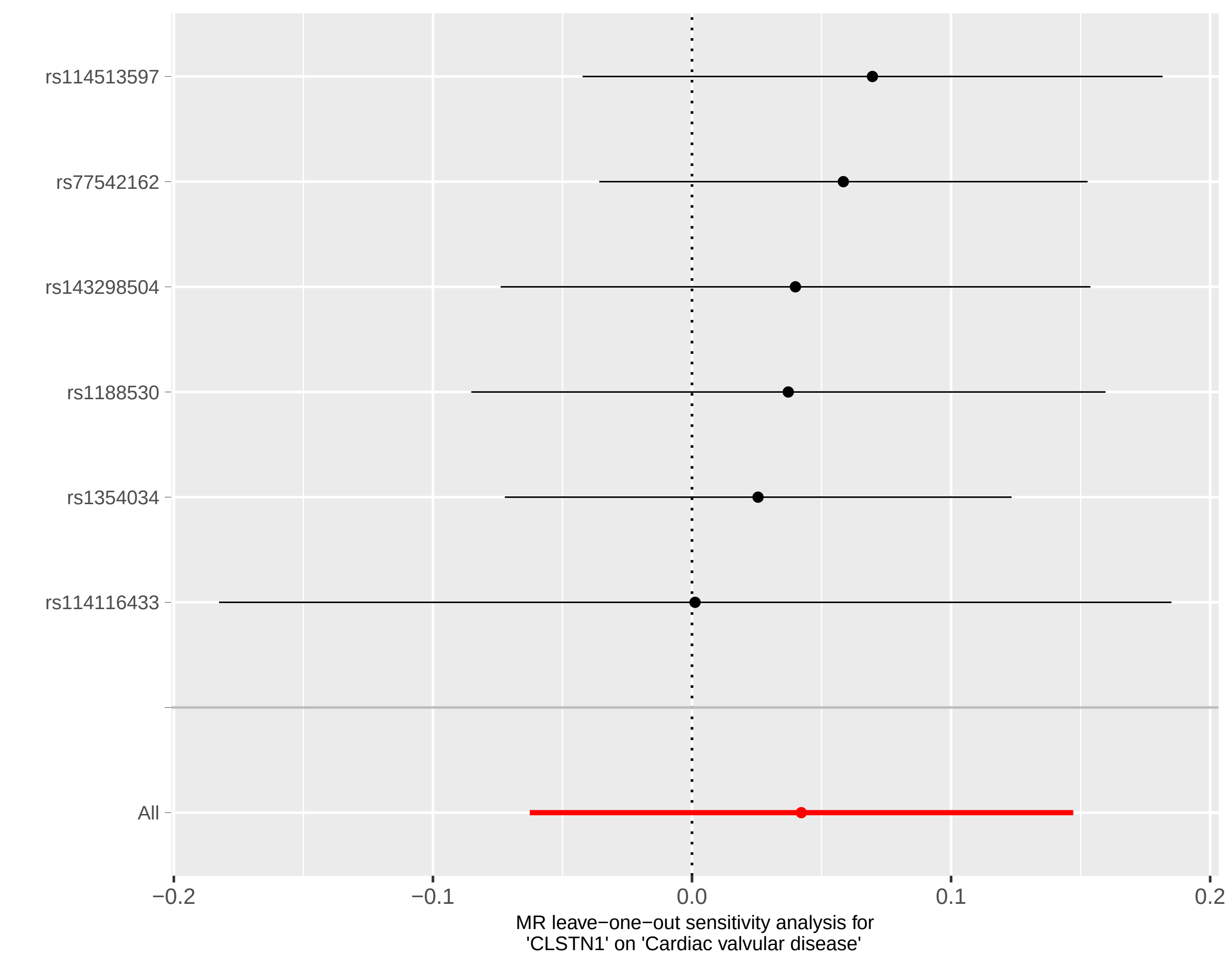
rs114513597
rs77542162
rs143298504
rs1188530
rs1354034
rs114116433
All
−0.2
−0.1
0.0
0.1
0.2
MR leave−one−out sensitivity analysis for
'CLSTN1' on 'Cardiac valvular disease'

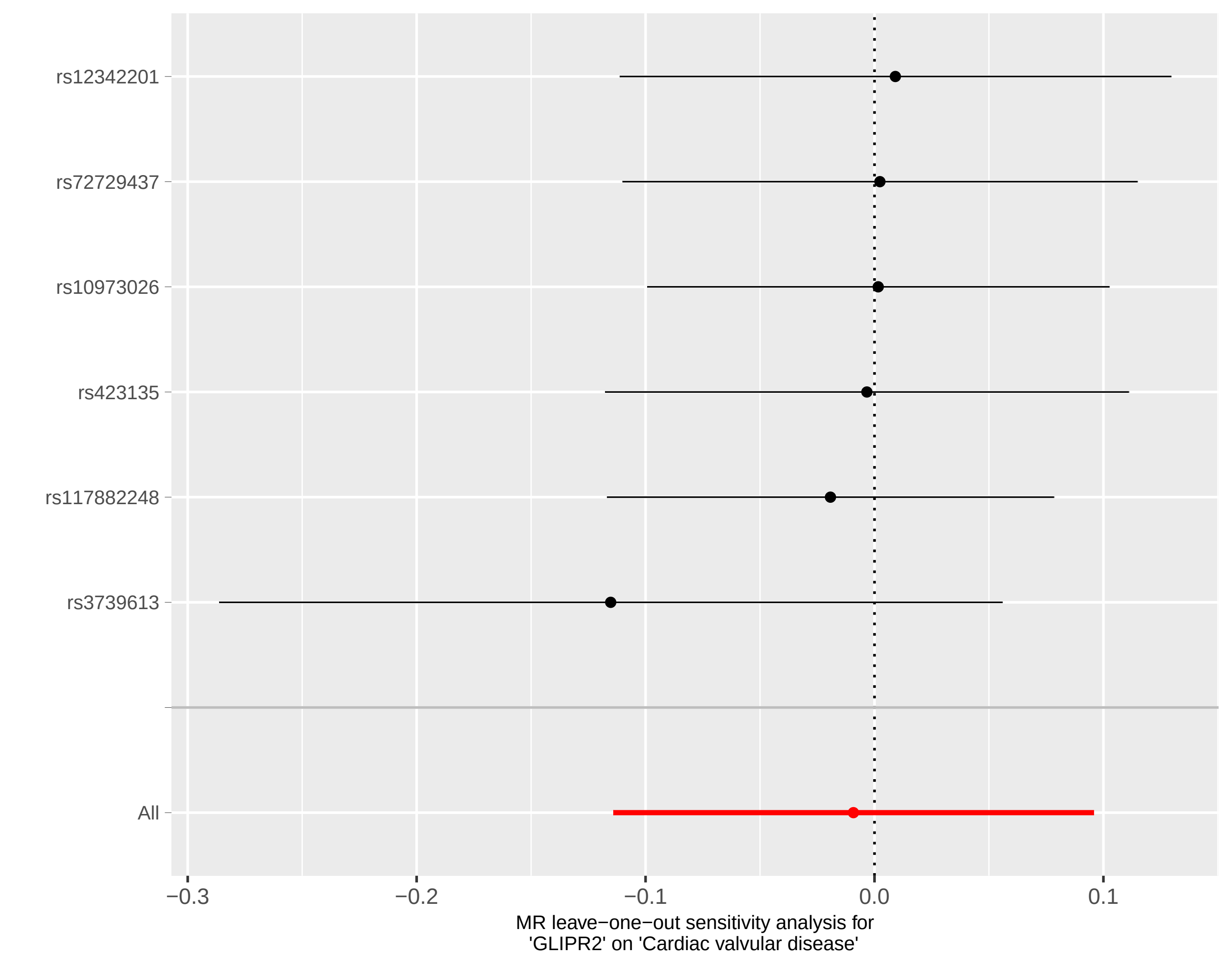
rs12342201
rs72729437
rs10973026
rs423135
rs117882248
rs3739613
All
−0.3
−0.2
−0.1
0.0
0.1
MR leave−one−out sensitivity analysis for
'GLIPR2' on 'Cardiac valvular disease'

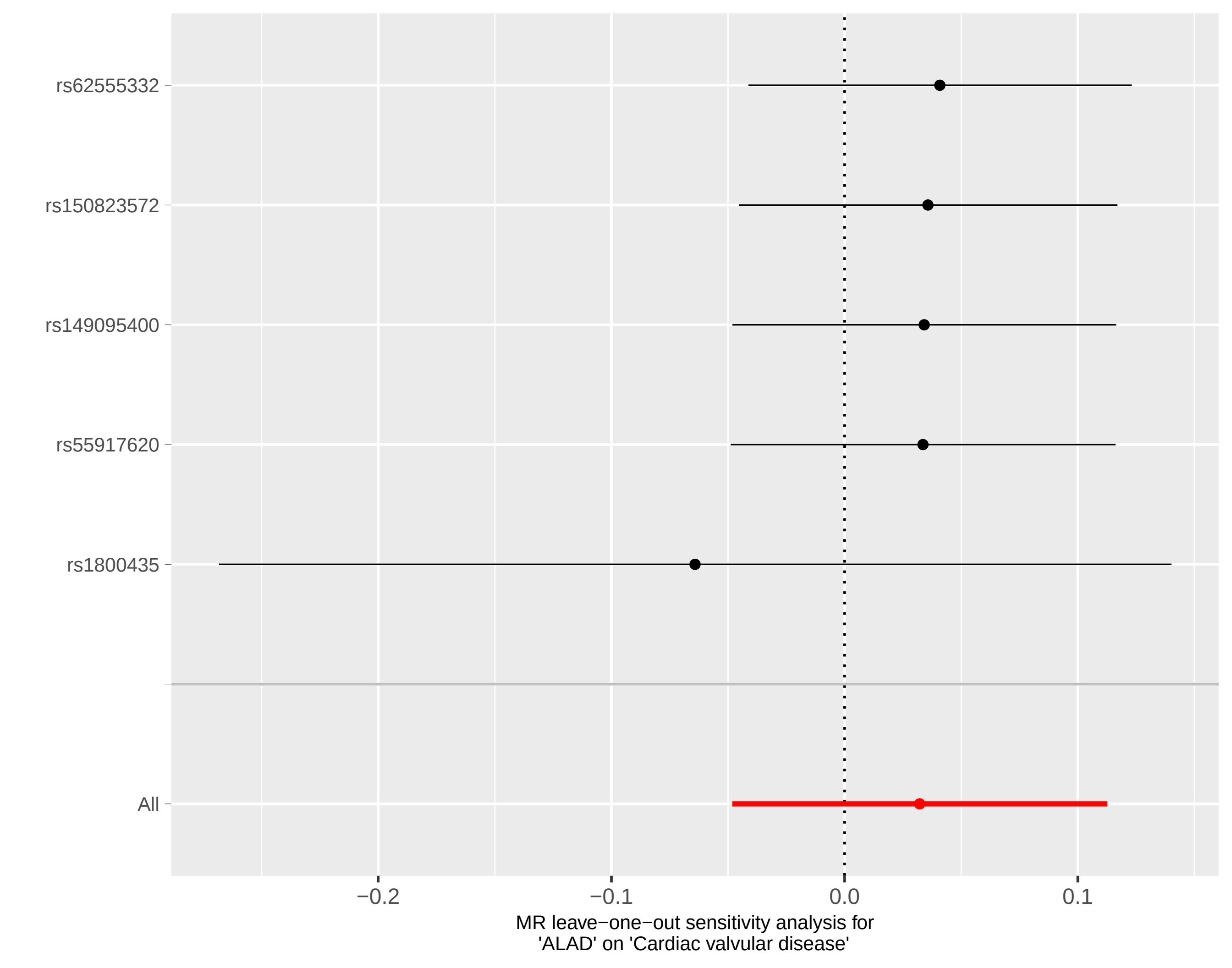
rs62555332
rs150823572
rs149095400
rs55917620
rs1800435
All
−0.2
−0.1
0.0
0.1
MR leave−one−out sensitivity analysis for
'ALAD' on 'Cardiac valvular disease'

Insufficient number of SNPs

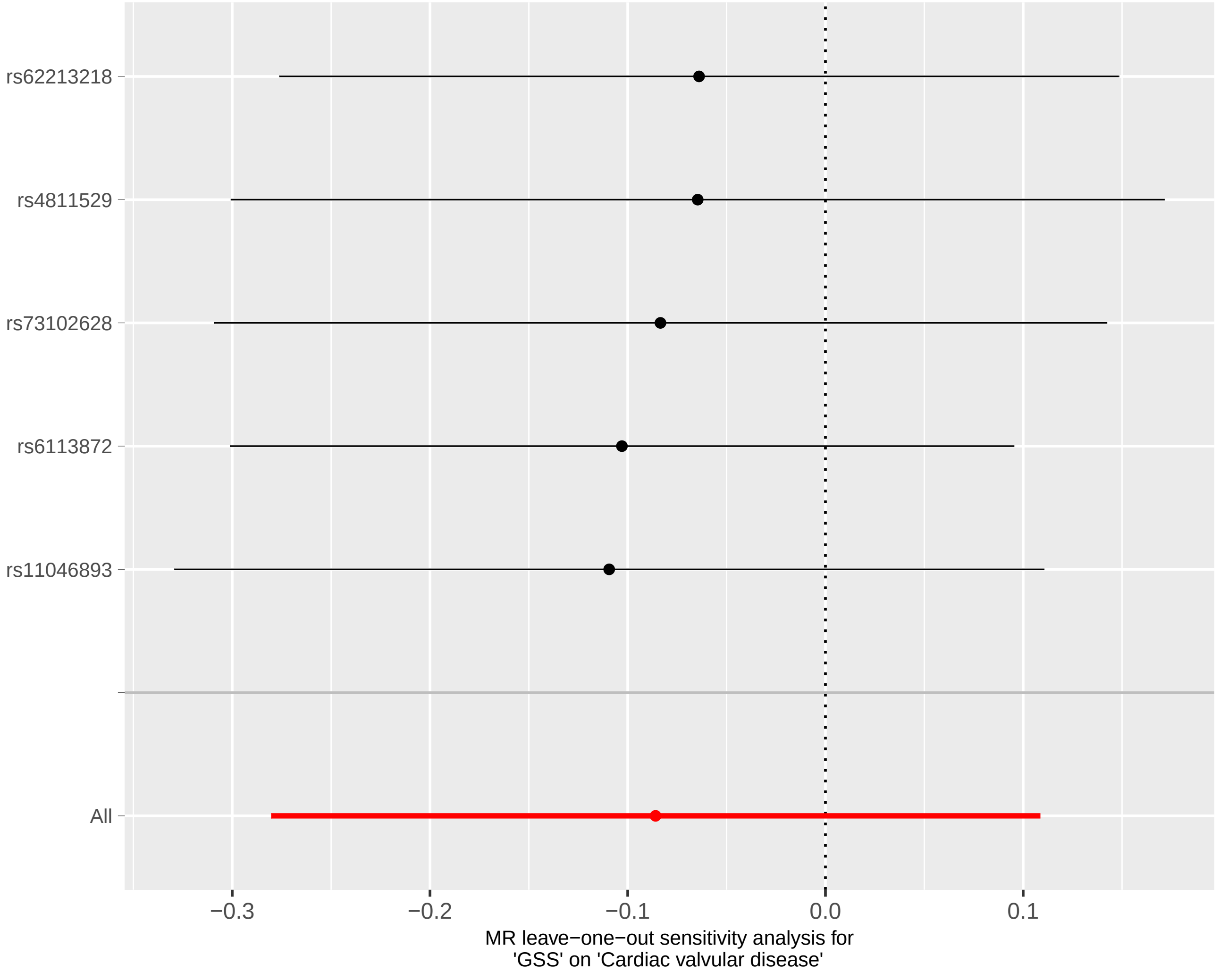

rs62213218
rs4811529
rs73102628
rs6113872
rs11046893
All
−0.3
−0.2
−0.1
0.0
0.1
MR leave−one−out sensitivity analysis for
'GSS' on 'Cardiac valvular disease'

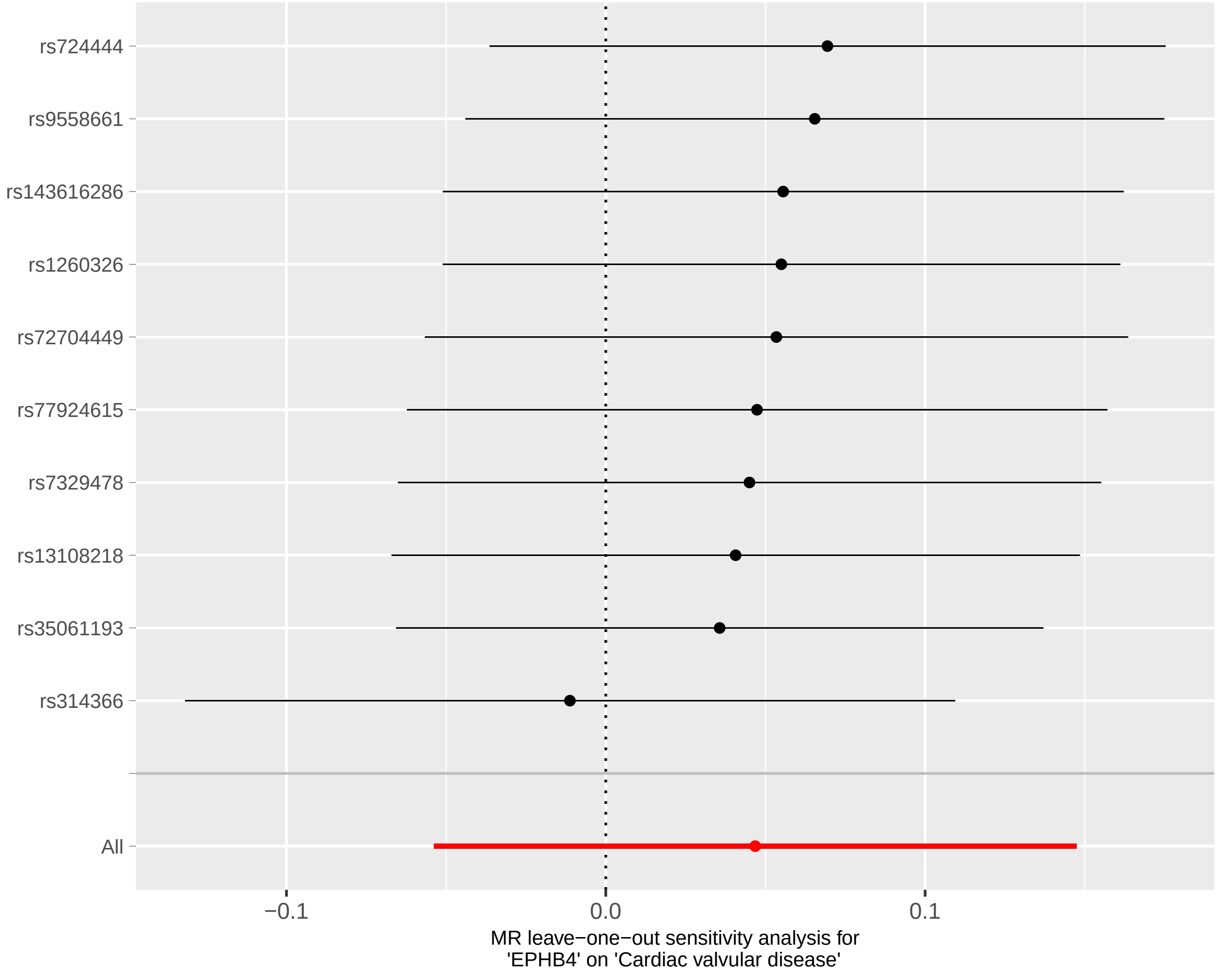
rs724444
rs9558661
rs143616286
rs1260326
rs72704449
rs77924615
rs7329478
rs13108218
rs35061193
rs314366
All
−0.1
0.0
0.1
MR leave−one−out sensitivity analysis for
'EPHB4' on 'Cardiac valvular disease'

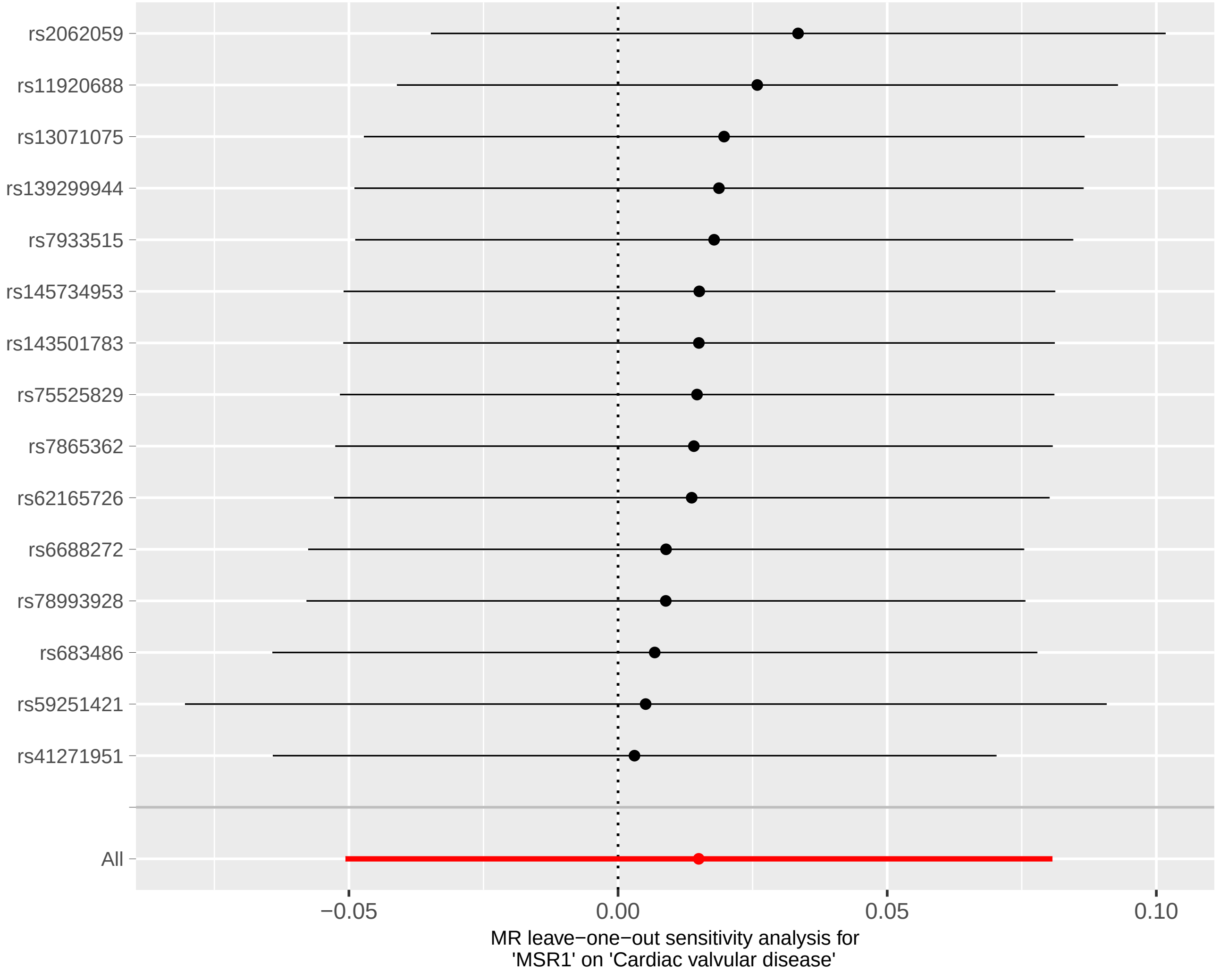
rs2062059
rs11920688
rs13071075
rs139299944
rs7933515
rs145734953
rs143501783
rs75525829
rs7865362
rs62165726
rs6688272
rs78993928
rs683486
rs59251421
rs41271951
All
−0.05
0.00
0.05
0.10
MR leave−one−out sensitivity analysis for
'MSR1' on 'Cardiac valvular disease'

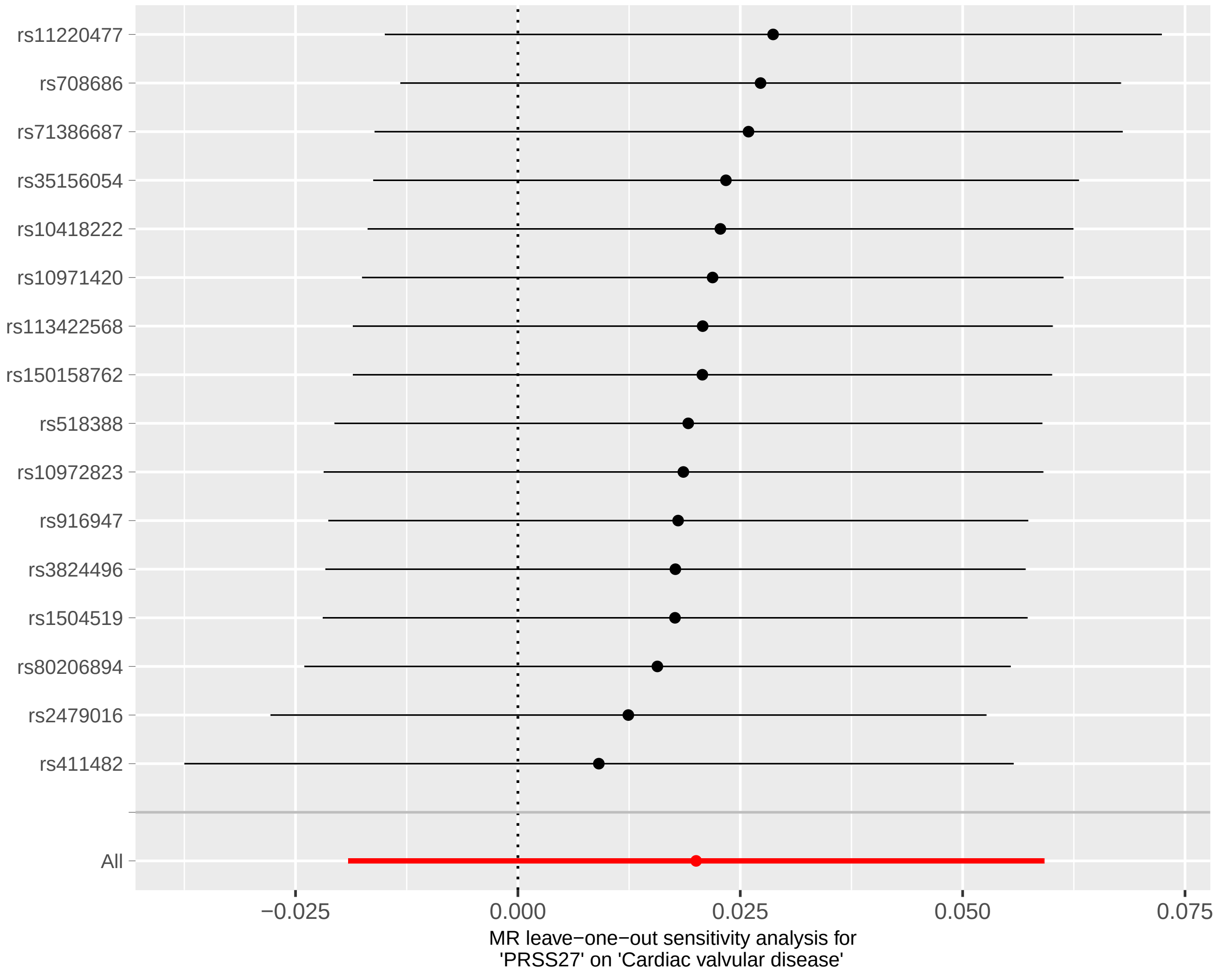

rs11220477
rs708686
rs71386687
rs35156054
rs10418222
rs10971420
rs113422568
rs150158762
rs518388
rs10972823
rs916947
rs3824496
rs1504519
rs80206894
rs2479016
rs411482
All
−0.025
0.000
0.025
0.050
0.075
MR leave−one−out sensitivity analysis for
'PRSS27' on 'Cardiac valvular disease'

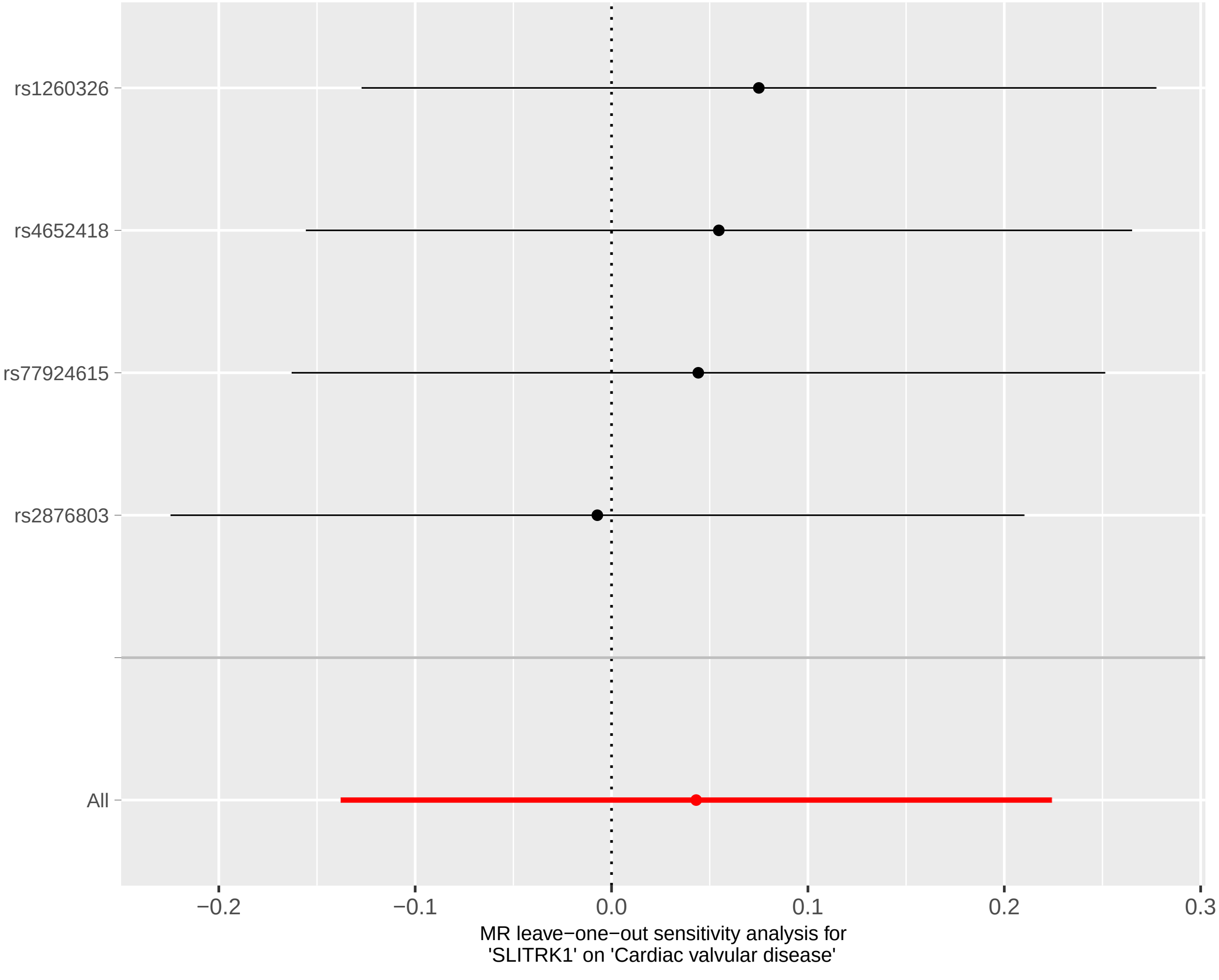
rs1260326
rs4652418
rs77924615
rs2876803
All
−0.2
−0.1
0.0
0.1
0.2
0.3
MR leave−one−out sensitivity analysis for
'SLITRK1' on 'Cardiac valvular disease'

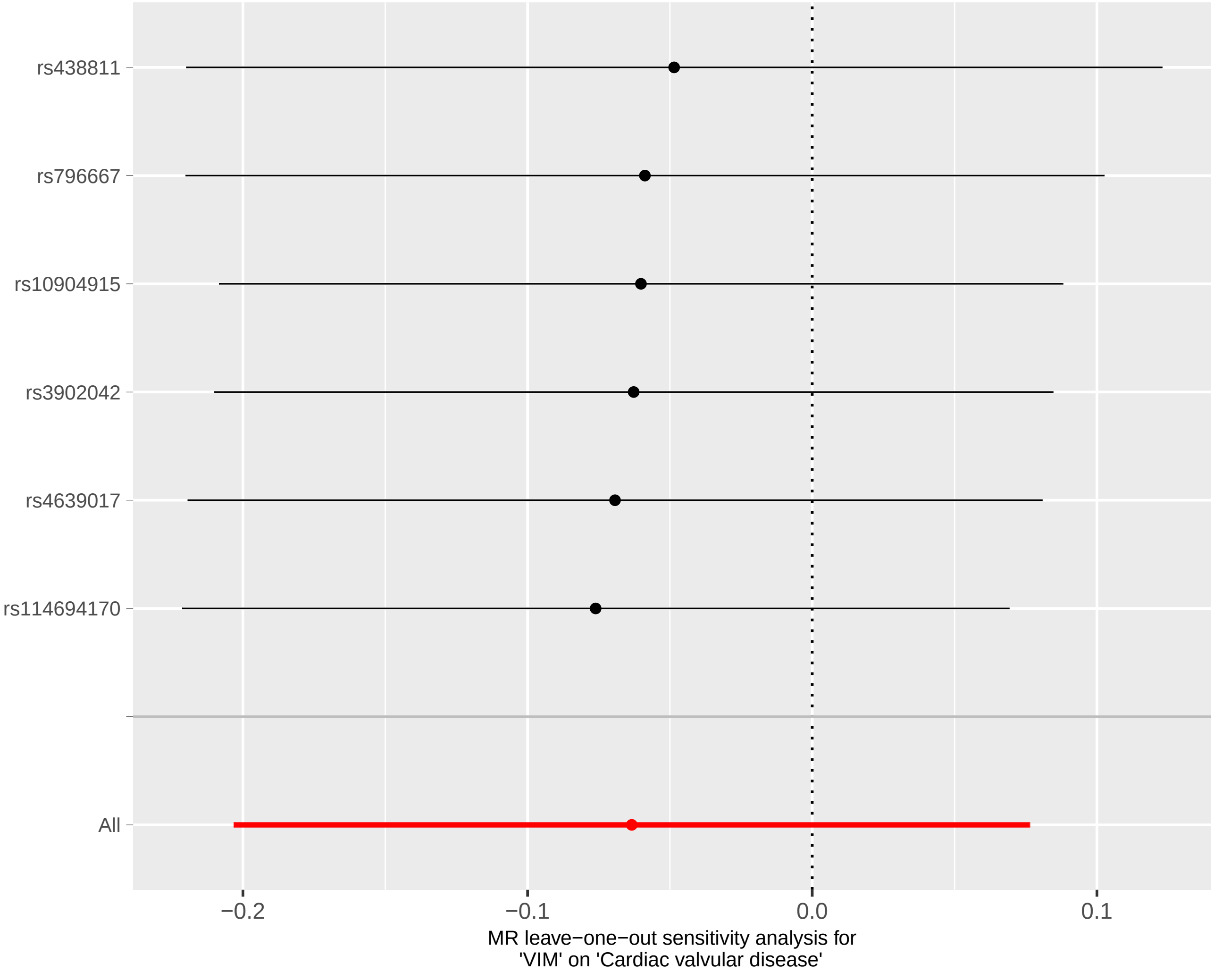
rs438811
rs796667
rs10904915
rs3902042
rs4639017
rs114694170
All
−0.2
−0.1
0.0
0.1
MR leave−one−out sensitivity analysis for
'VIM' on 'Cardiac valvular disease'

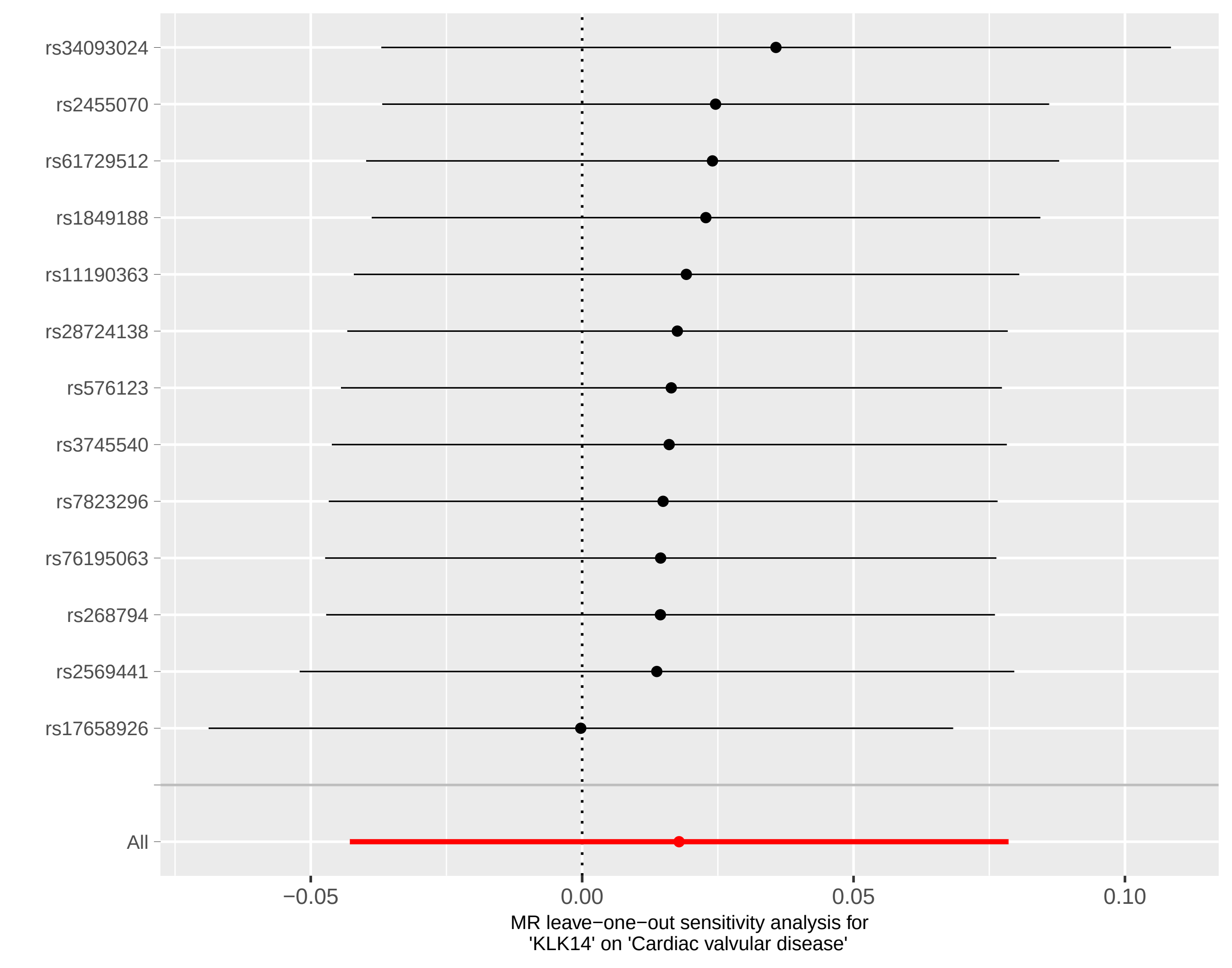
rs34093024
rs2455070
rs61729512
rs1849188
rs11190363
rs28724138
rs576123
rs3745540
rs7823296
rs76195063
rs268794
rs2569441
rs17658926
All
−0.05
0.00
0.05
0.10
MR leave−one−out sensitivity analysis for
'KLK14' on 'Cardiac valvular disease'

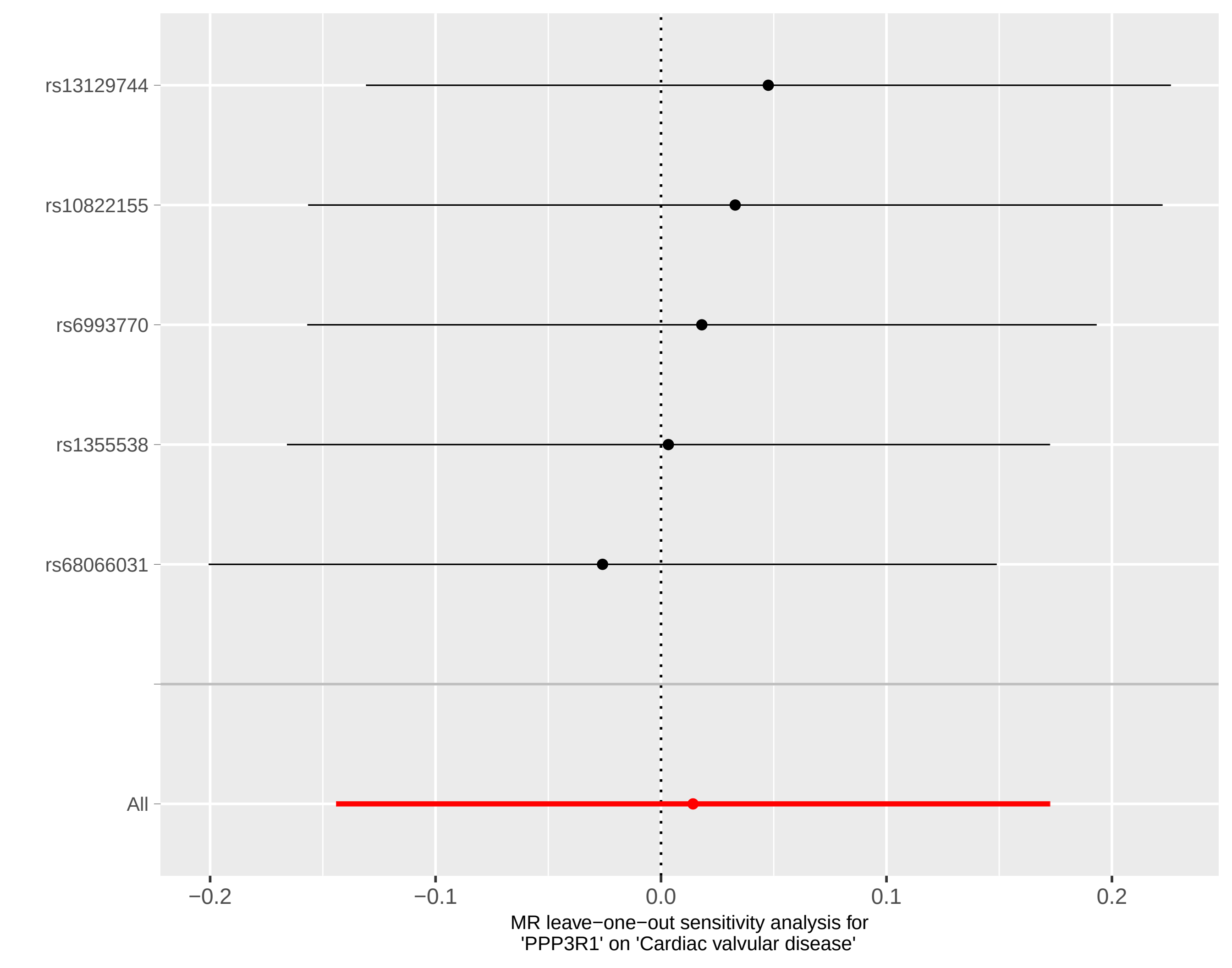
rs13129744
rs10822155
rs6993770
rs1355538
rs68066031
All
−0.2
−0.1
0.0
0.1
0.2
MR leave−one−out sensitivity analysis for
'PPP3R1' on 'Cardiac valvular disease'

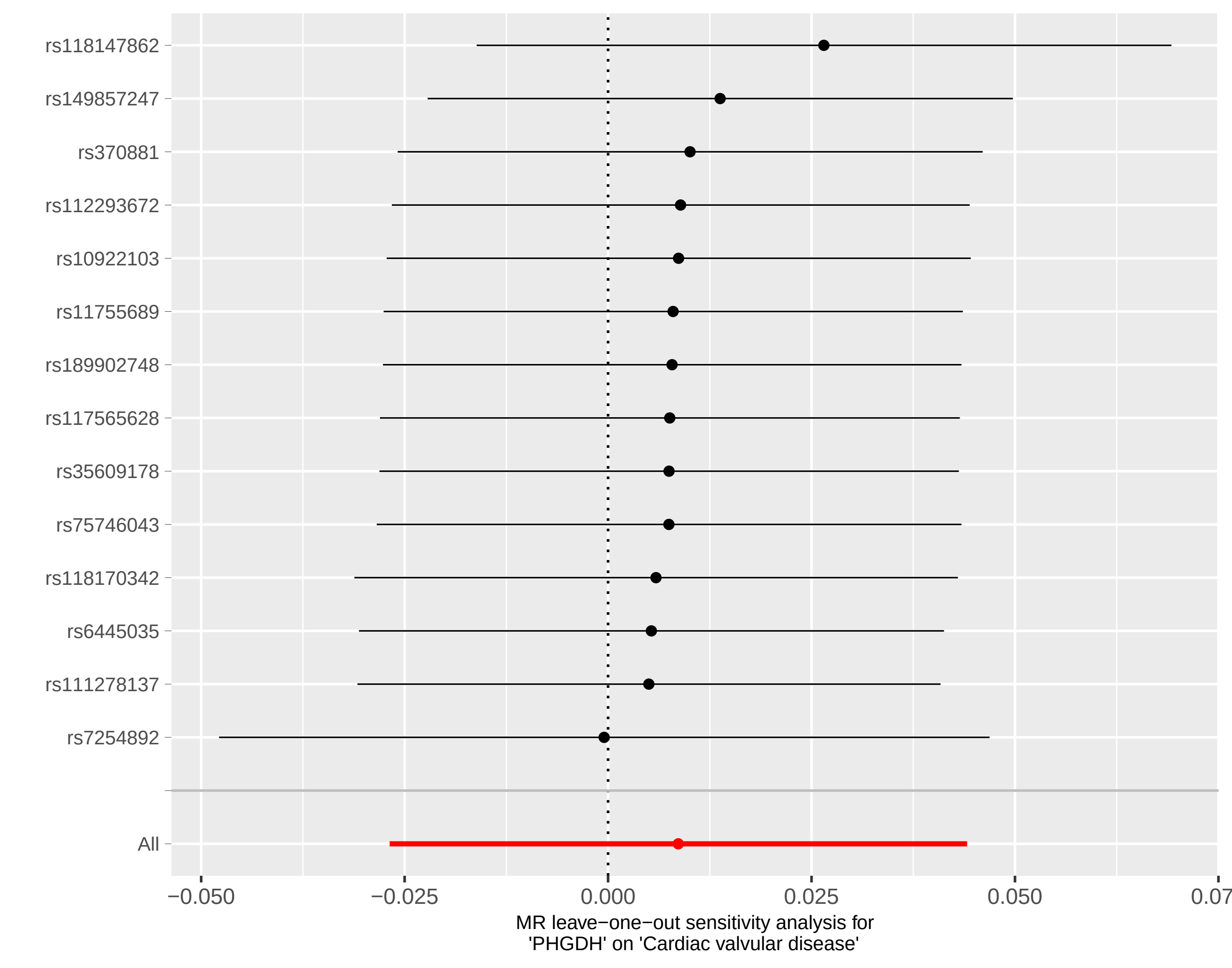
rs118147862
rs149857247
rs370881
rs112293672
rs10922103
rs11755689
rs189902748
rs117565628
rs35609178
rs75746043
rs118170342
rs6445035
rs111278137
rs7254892
All
−0.050
−0.025
0.000
0.025
0.050
MR leave−one−out sensitivity analysis for
'PHGDH' on 'Cardiac valvular disease'

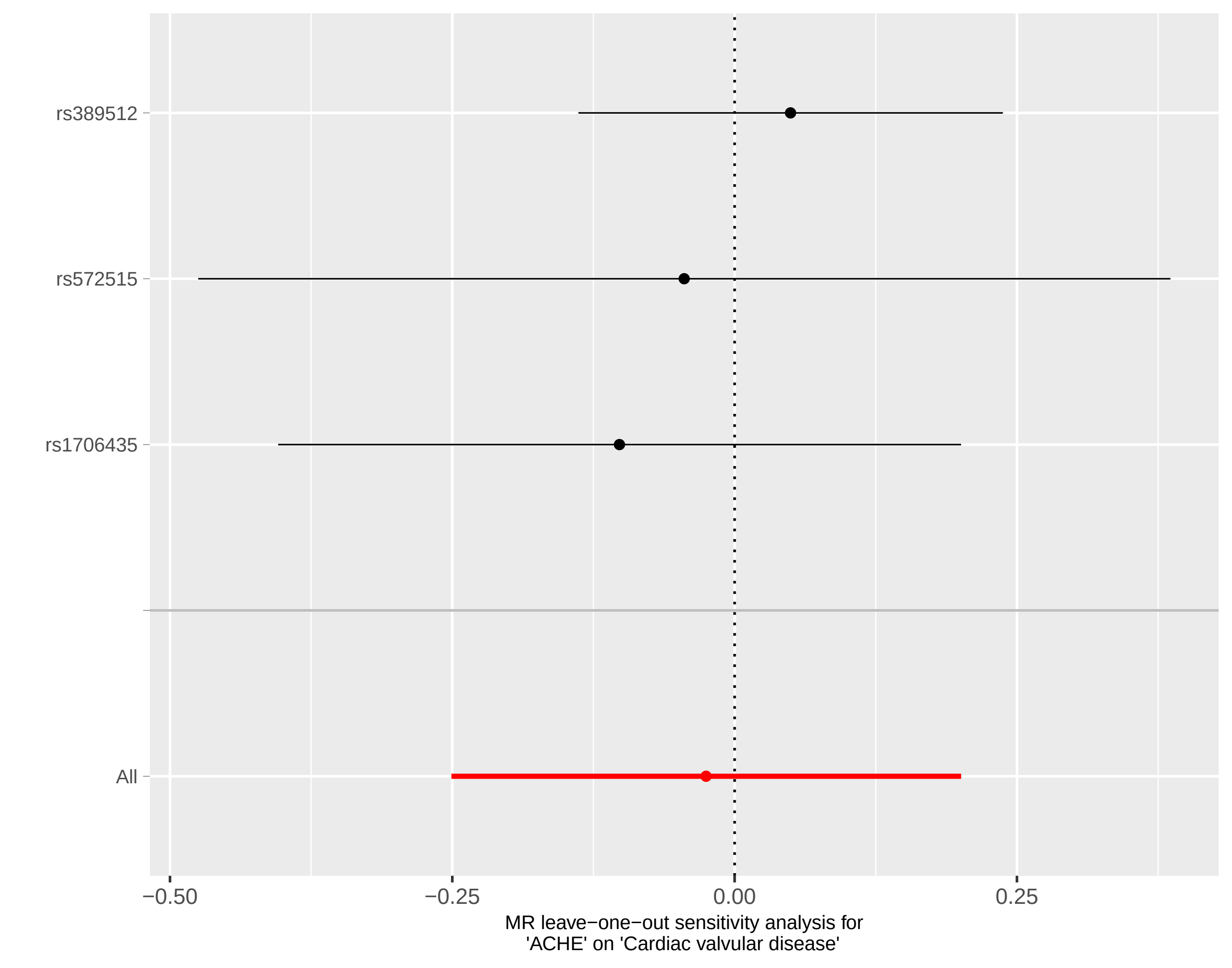
rs389512
rs572515
rs1706435
All
−0.50
−0.25
0.00
0.25
MR leave−one−out sensitivity analysis for
'ACHE' on 'Cardiac valvular disease'

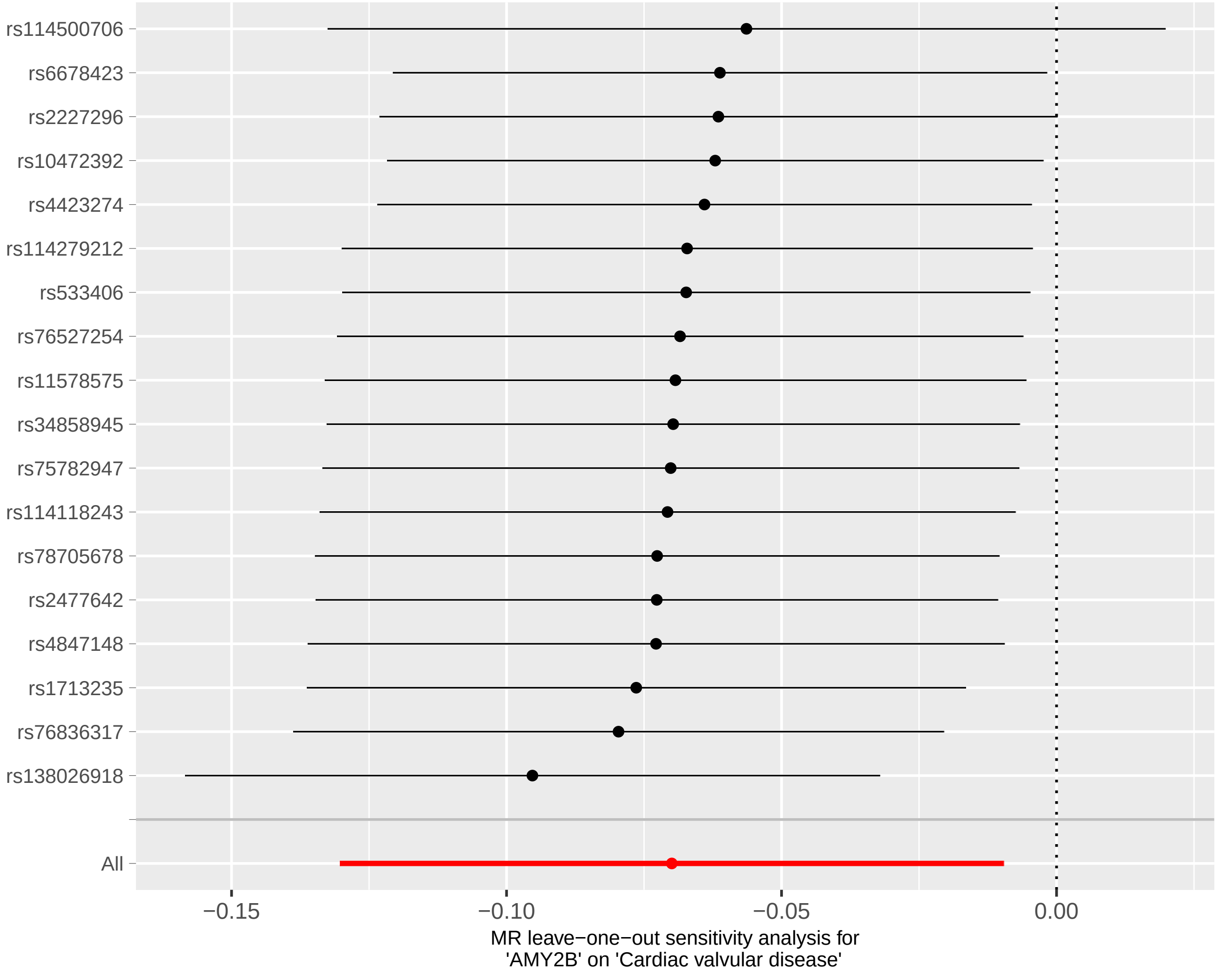

rs114500706
rs6678423
rs2227296
rs10472392
rs4423274
rs114279212
rs533406
rs76527254
rs11578575
rs34858945
rs75782947
rs114118243
rs78705678
rs2477642
rs4847148
rs1713235
rs76836317
rs138026918
All
−0.15
−0.10
−0.05
0.00
MR leave−one−out sensitivity analysis for
'AMY2B' on 'Cardiac valvular disease'

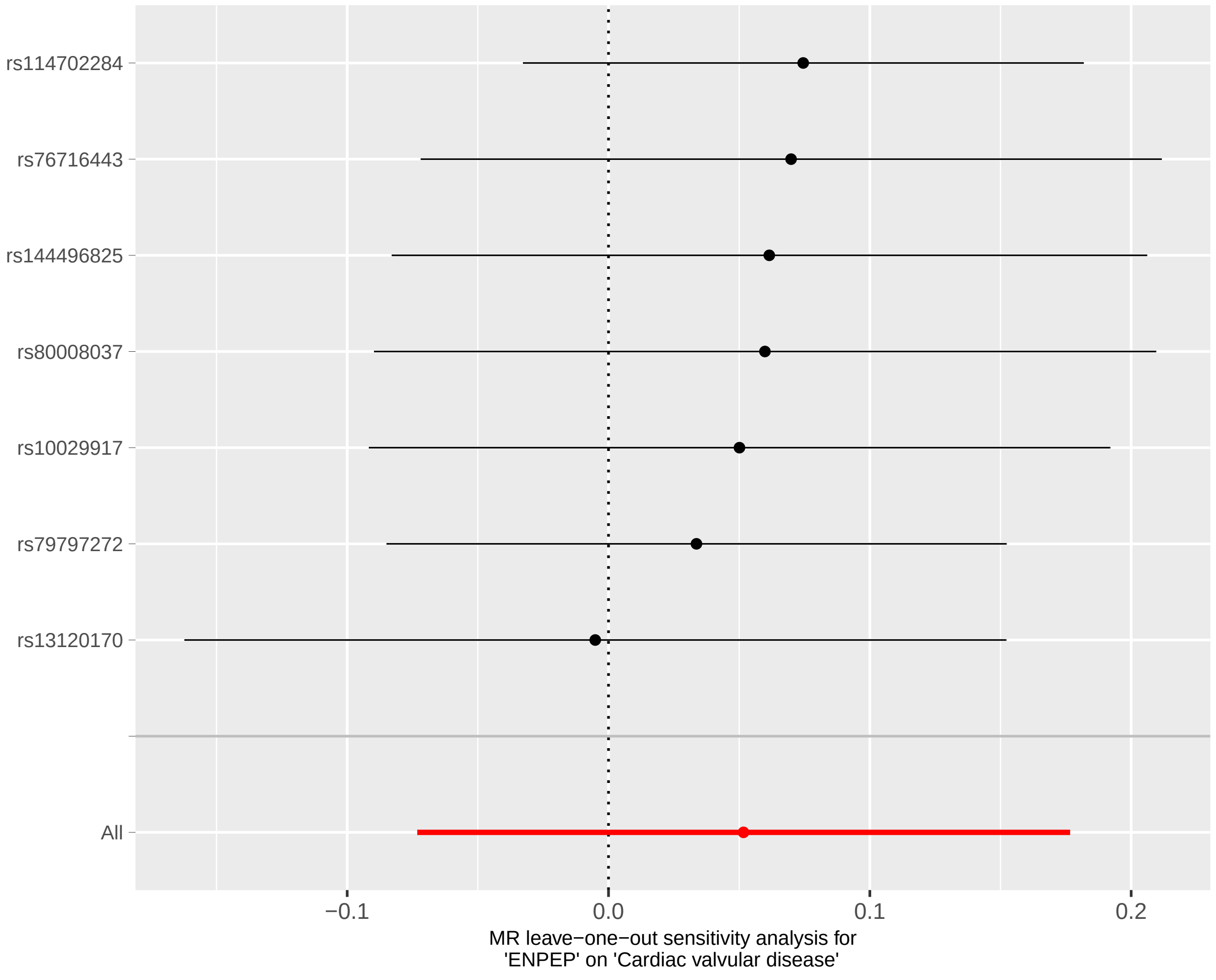
rs114702284
rs76716443
rs144496825
rs80008037
rs10029917
rs79797272
rs13120170
All
−0.1
0.0
0.1
0.2
MR leave−one−out sensitivity analysis for
'ENPEP' on 'Cardiac valvular disease'

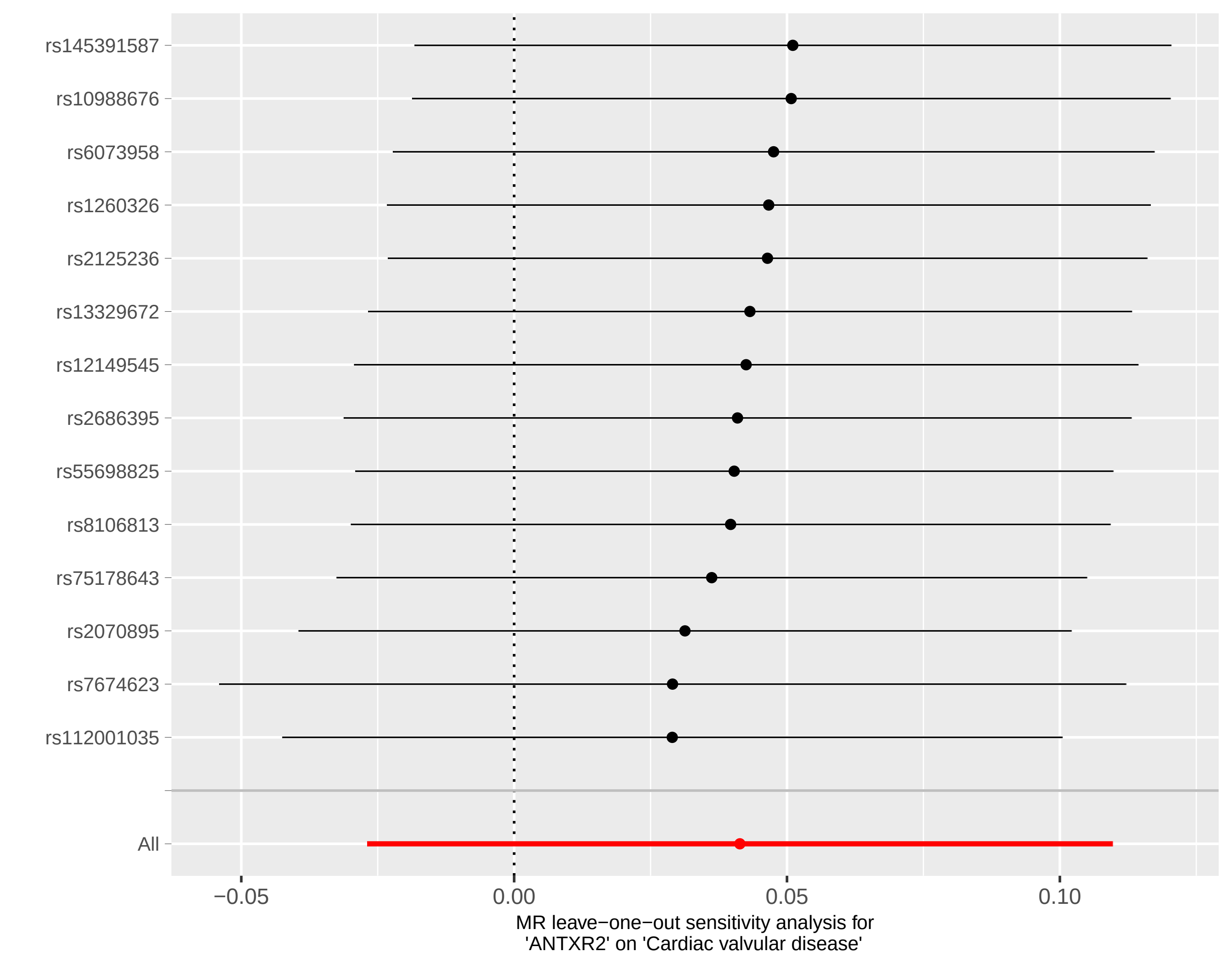
rs145391587
rs10988676
rs6073958
rs1260326
rs2125236
rs13329672
rs12149545
rs2686395
rs55698825
rs8106813
rs75178643
rs2070895
rs7674623
rs112001035
All
−0.05
0.00
0.05
0.10
MR leave−one−out sensitivity analysis for
'ANTXR2' on 'Cardiac valvular disease'

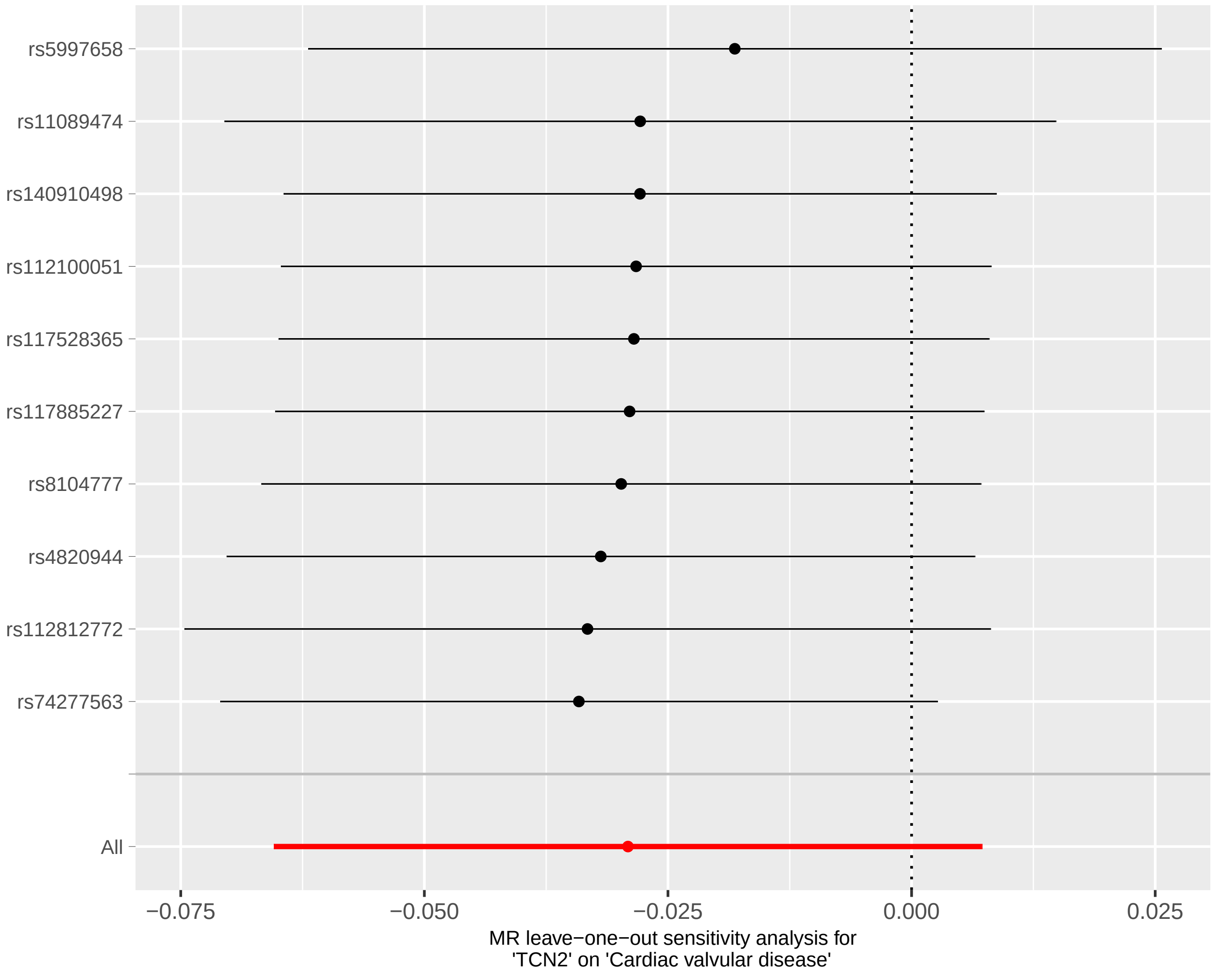
rs5997658
rs11089474
rs140910498
rs112100051
rs117528365
rs117885227
rs8104777
rs4820944
rs112812772
rs74277563
All
−0.075
−0.050
−0.025
0.000
0.025
MR leave−one−out sensitivity analysis for
'TCN2' on 'Cardiac valvular disease'

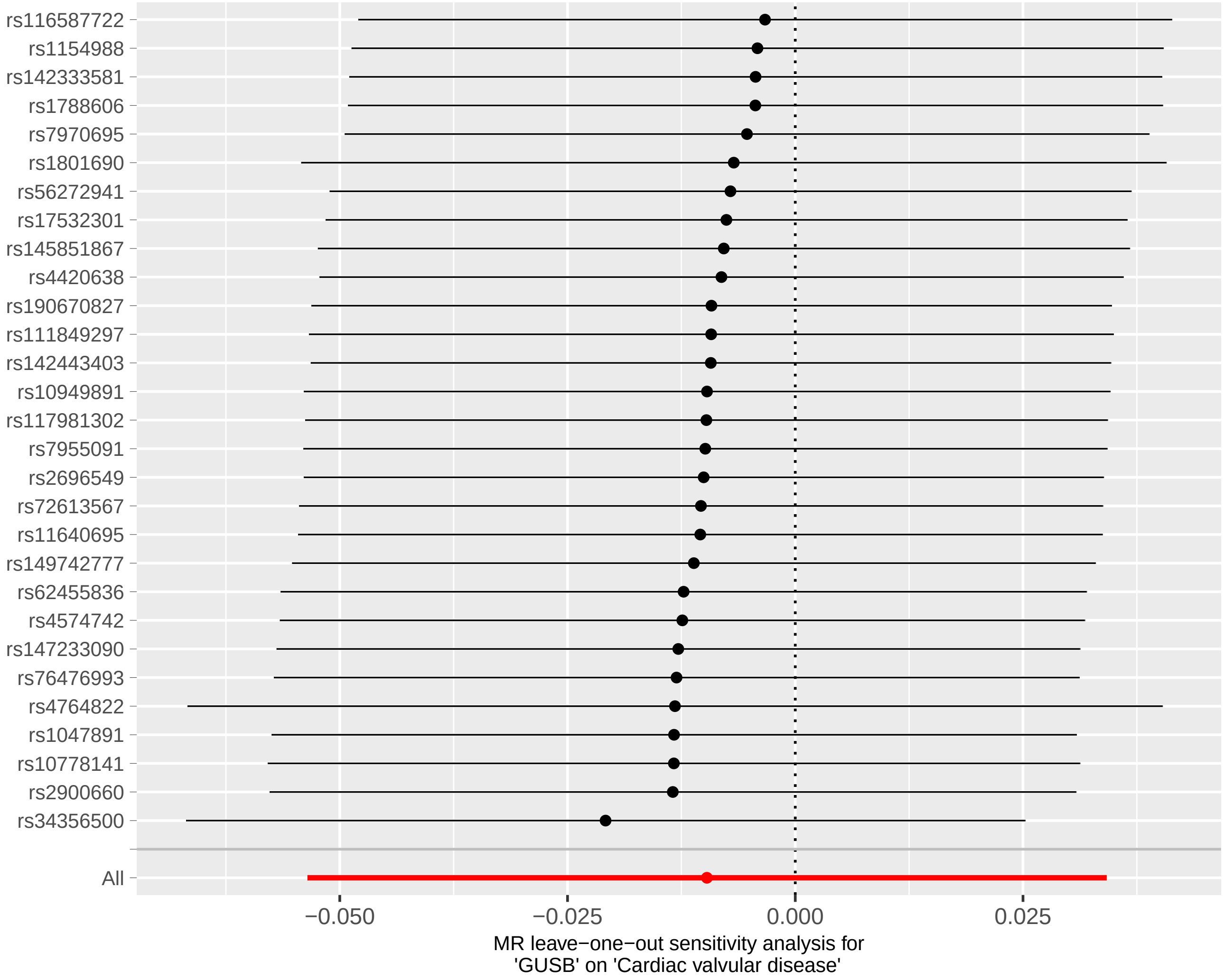
rs116587722
rs1154988
rs142333581
rs1788606
rs7970695
rs1801690
rs56272941
rs17532301
rs145851867
rs4420638
rs190670827
rs111849297
rs142443403
rs10949891
rs117981302
rs7955091
rs2696549
rs72613567
rs11640695
rs149742777
rs62455836
rs4574742
rs147233090
rs76476993
rs4764822
rs1047891
rs10778141
rs2900660
rs34356500
All
−0.050
−0.025
0.000
0.025
MR leave−one−out sensitivity analysis for
'GUSB' on 'Cardiac valvular disease'

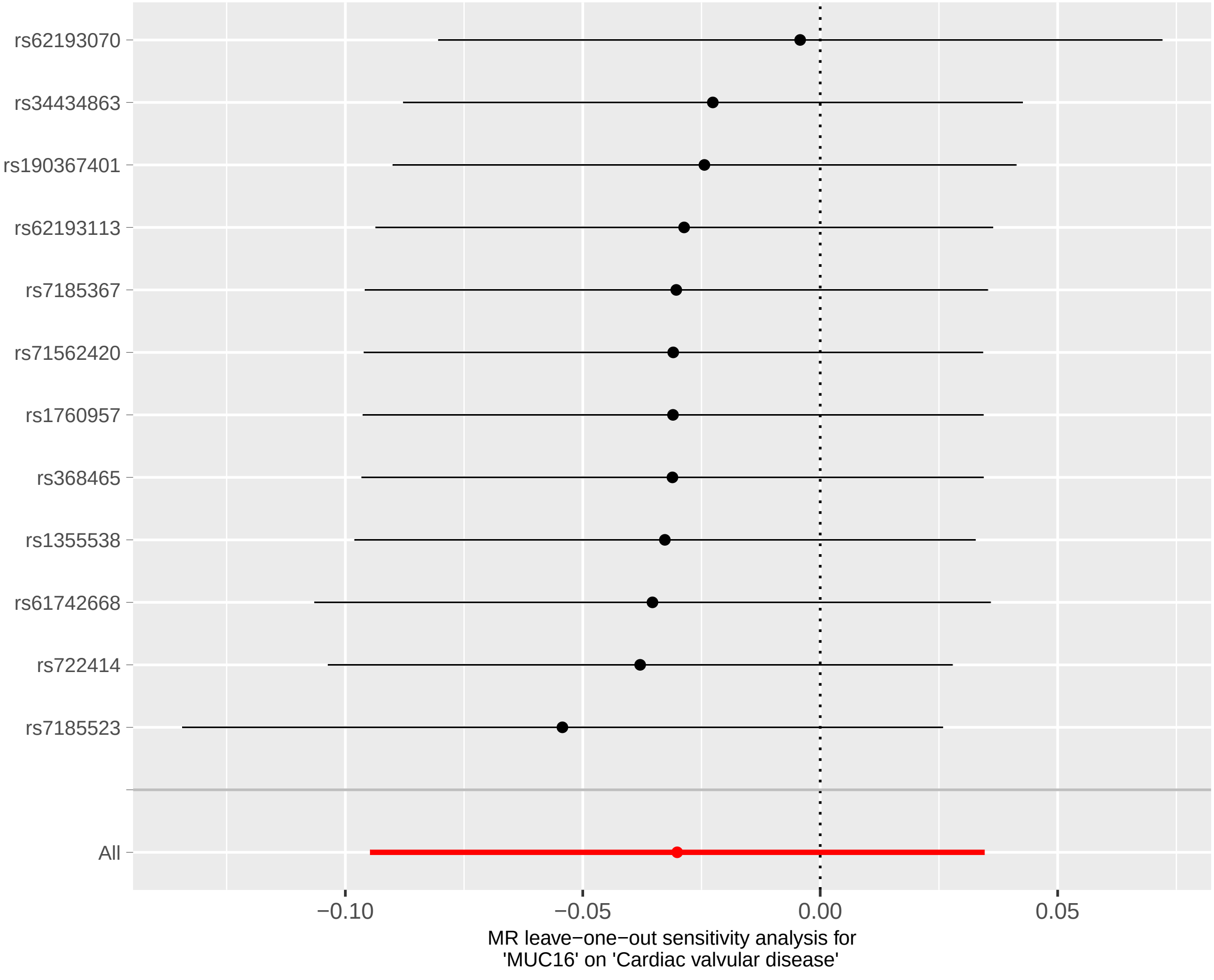
rs62193070
rs34434863
rs190367401
rs62193113
rs7185367
rs71562420
rs1760957
rs368465
rs1355538
rs61742668
rs722414
rs7185523
All
−0.10
−0.05
0.00
0.05
MR leave−one−out sensitivity analysis for
'MUC16' on 'Cardiac valvular disease'

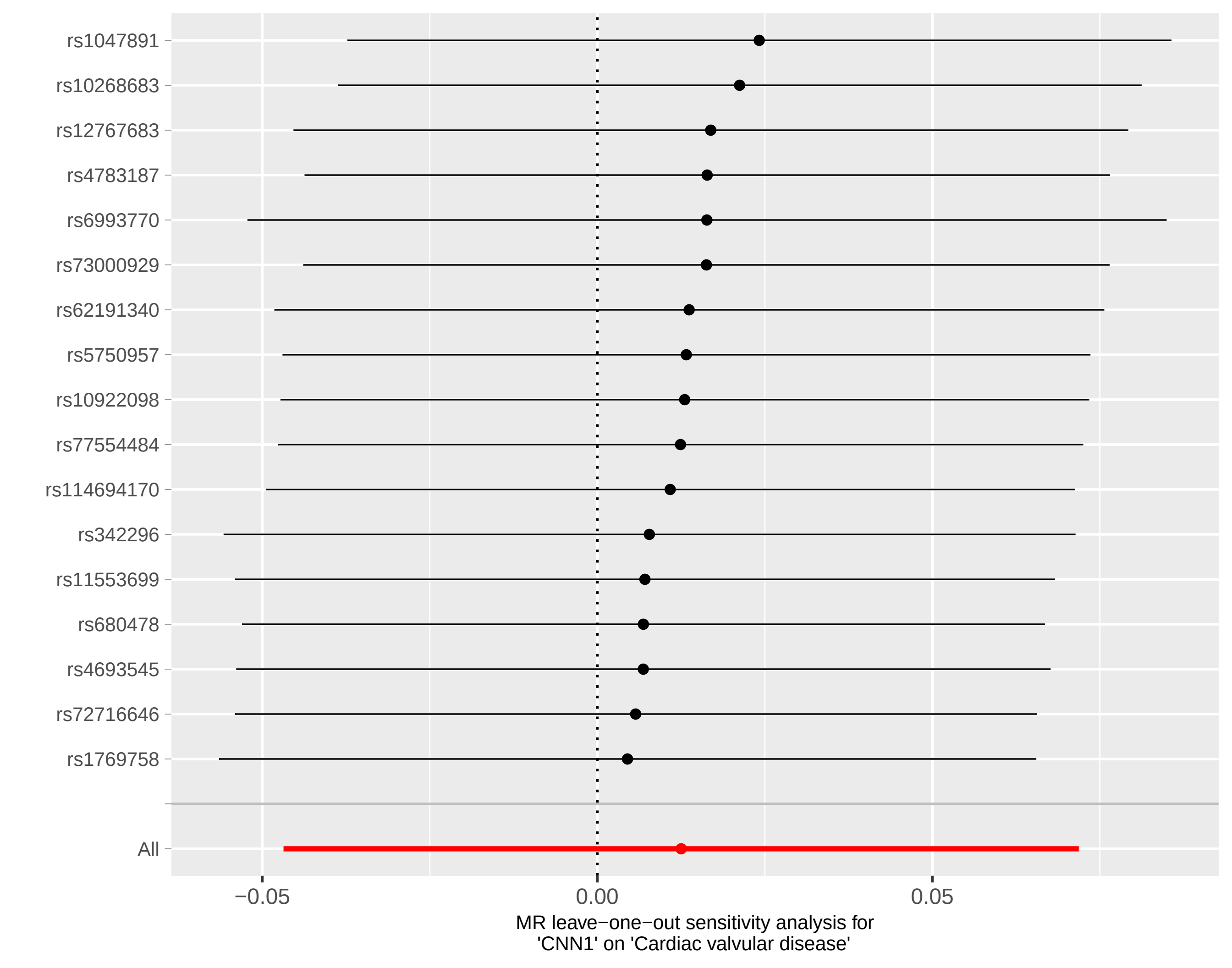
rs1047891
rs10268683
rs12767683
rs4783187
rs6993770
rs73000929
rs62191340
rs5750957
rs10922098
rs77554484
rs114694170
rs342296
rs11553699
rs680478
rs4693545
rs72716646
rs1769758
All
−0.05
0.00
0.05
MR leave−one−out sensitivity analysis for
'CNN1' on 'Cardiac valvular disease'

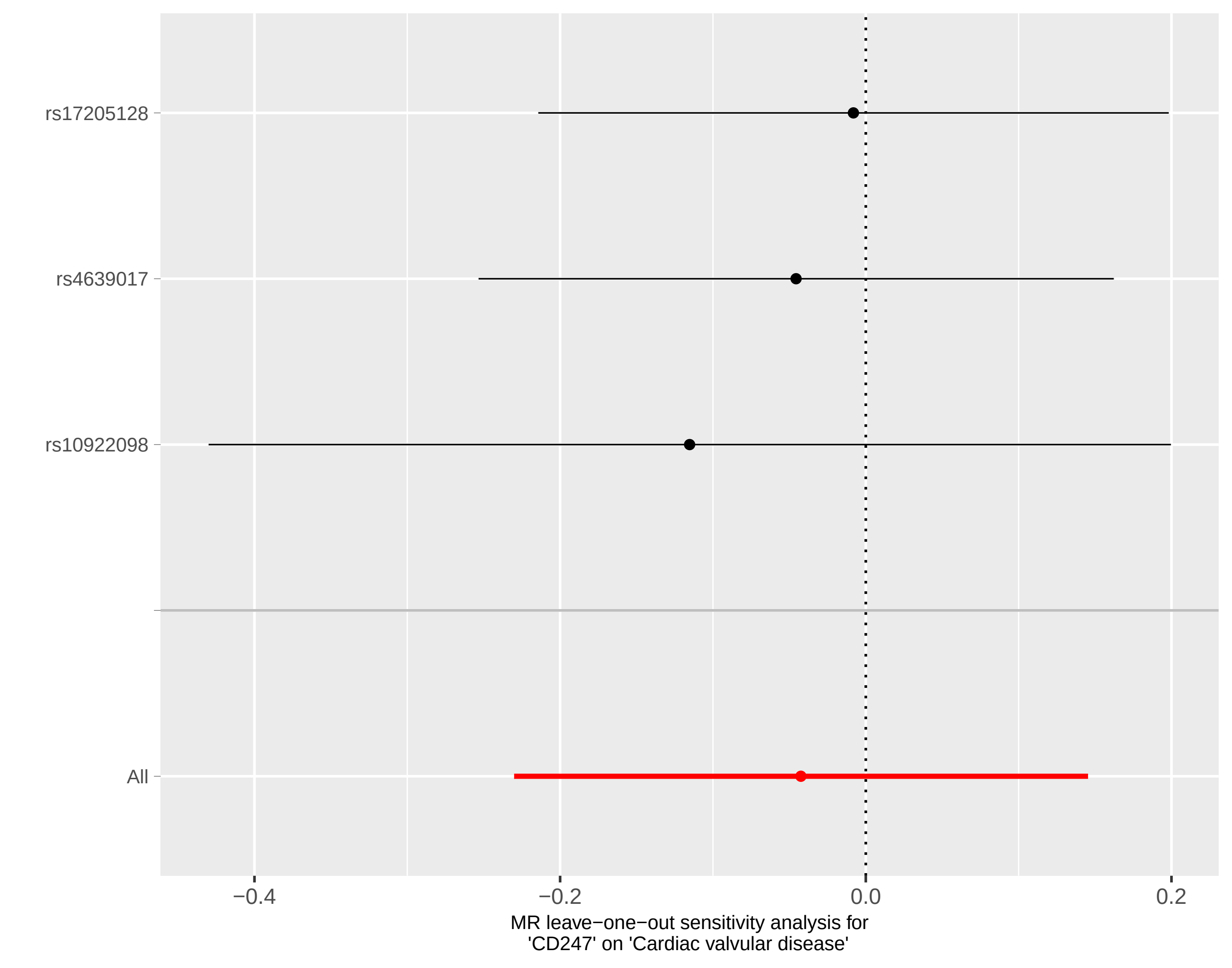

rs17205128
rs4639017
rs10922098
All
−0.4
−0.2
0.0
0.2
MR leave−one−out sensitivity analysis for
'CD247' on 'Cardiac valvular disease'

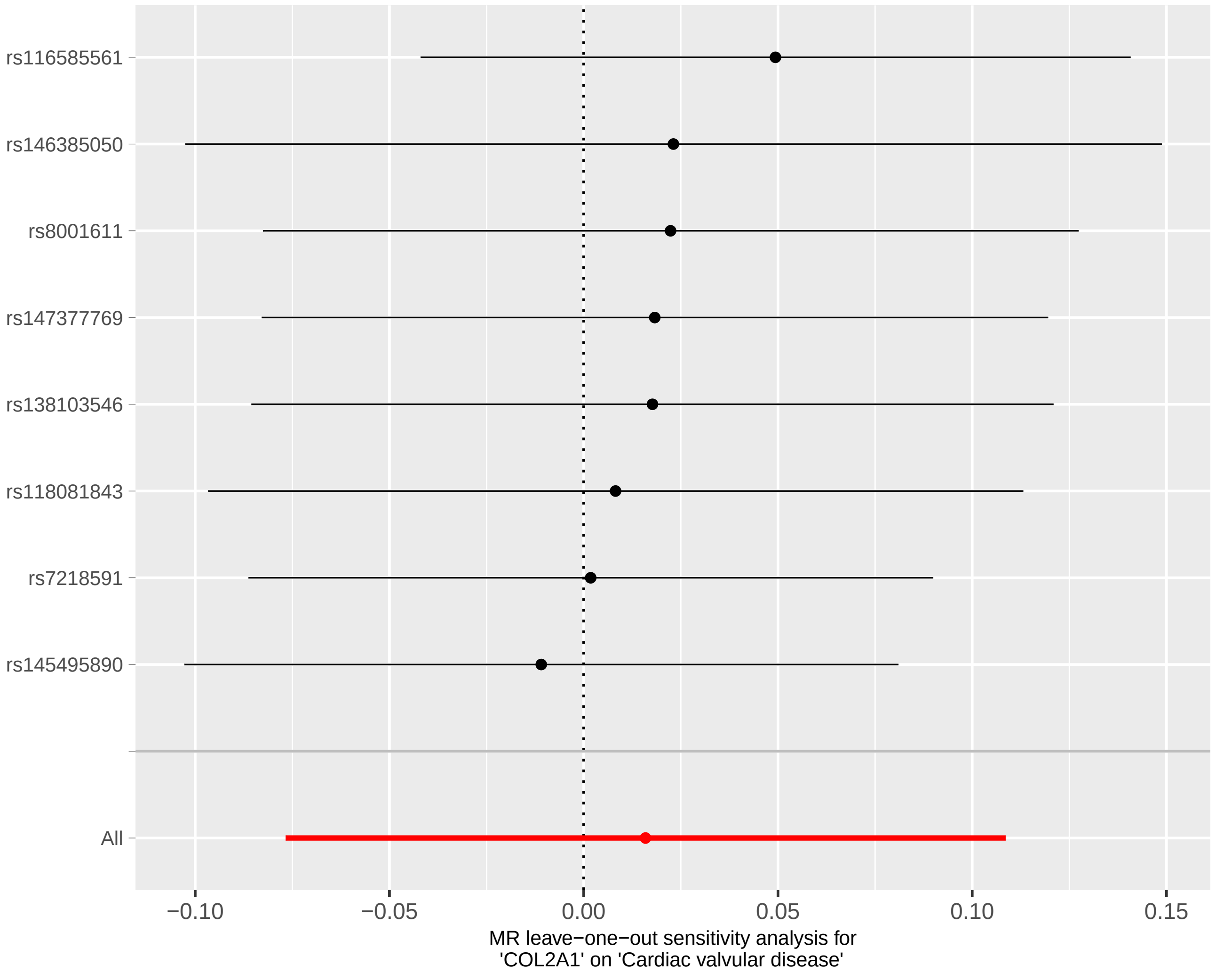

rs116585561
rs146385050
rs8001611
rs147377769
rs138103546
rs118081843
rs7218591
rs145495890
All
−0.10
−0.05
0.00
0.05
0.10
0.15
MR leave−one−out sensitivity analysis for
'COL2A1' on 'Cardiac valvular disease'

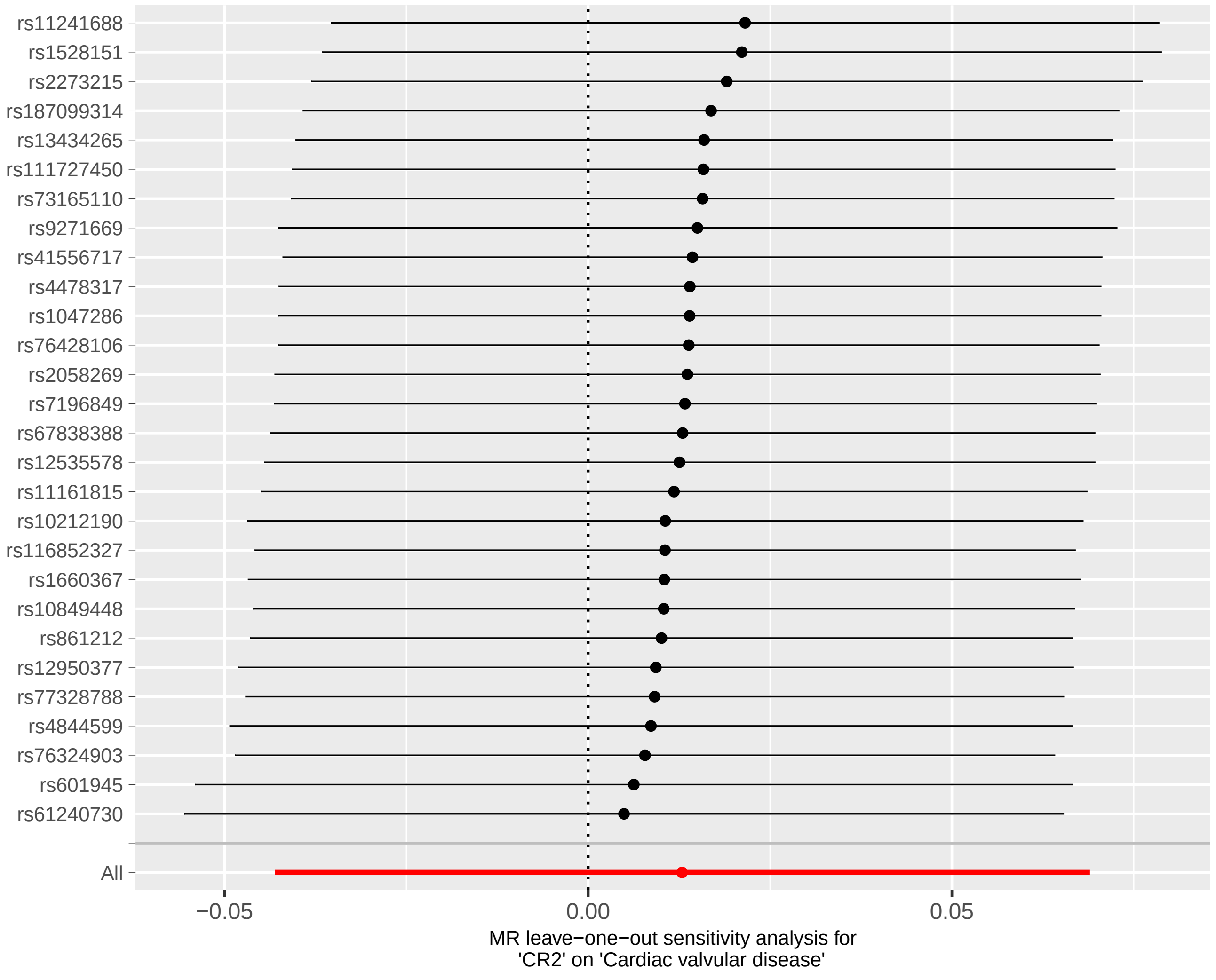
rs11241688
rs1528151
rs2273215
rs187099314
rs13434265
rs111727450
rs73165110
rs9271669
rs41556717
rs4478317
rs1047286
rs76428106
rs2058269
rs7196849
rs67838388
rs12535578
rs11161815
rs10212190
rs116852327
rs1660367
rs10849448
rs861212
rs12950377
rs77328788
rs4844599
rs76324903
rs601945
rs61240730
All
−0.05
0.00
0.05
MR leave−one−out sensitivity analysis for
'CR2' on 'Cardiac valvular disease'

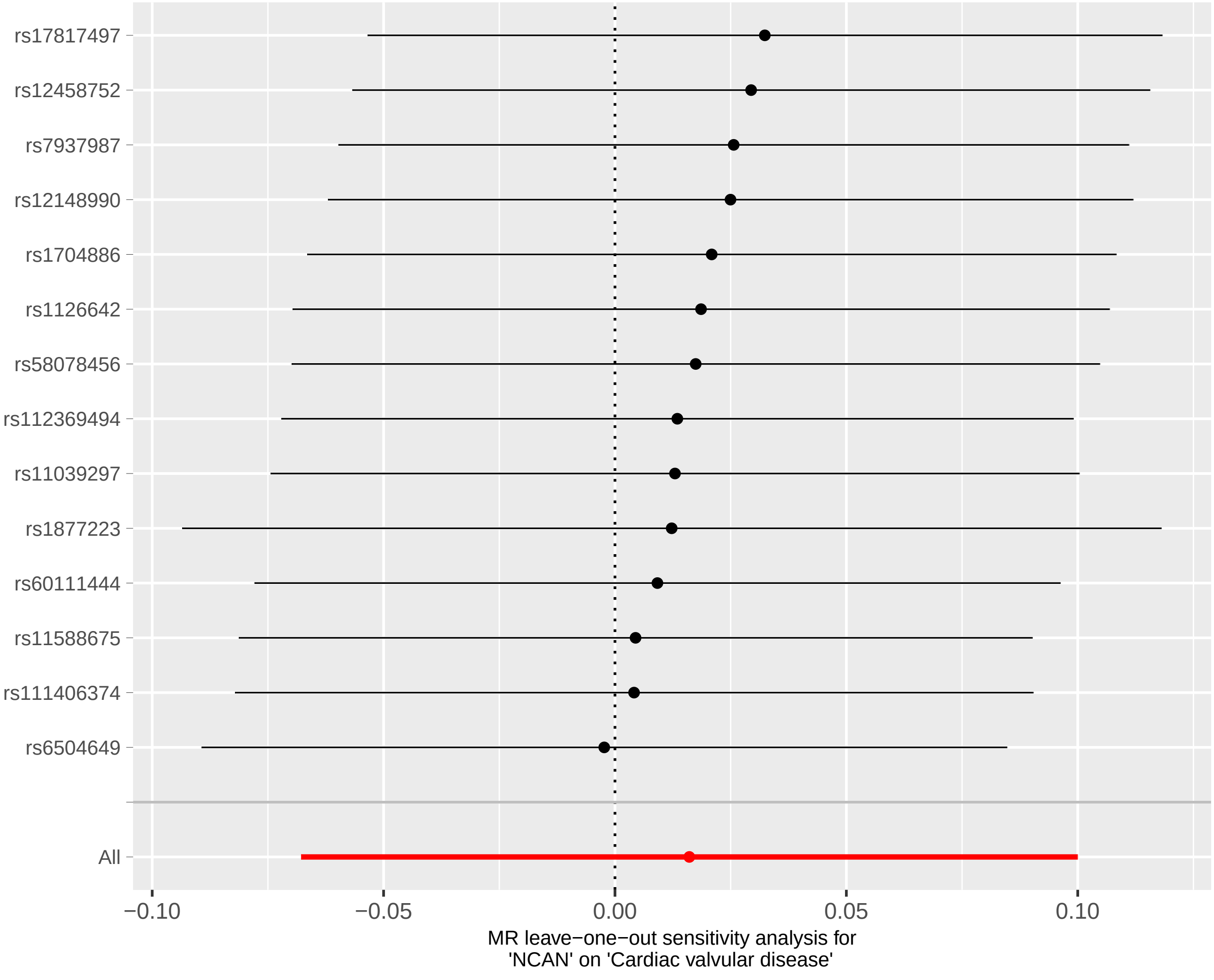
rs17817497
rs12458752
rs7937987
rs12148990
rs1704886
rs1126642
rs58078456
rs112369494
rs11039297
rs1877223
rs60111444
rs11588675
rs111406374
rs6504649
All
−0.10
−0.05
0.00
0.05
0.10
MR leave−one−out sensitivity analysis for
'NCAN' on 'Cardiac valvular disease'

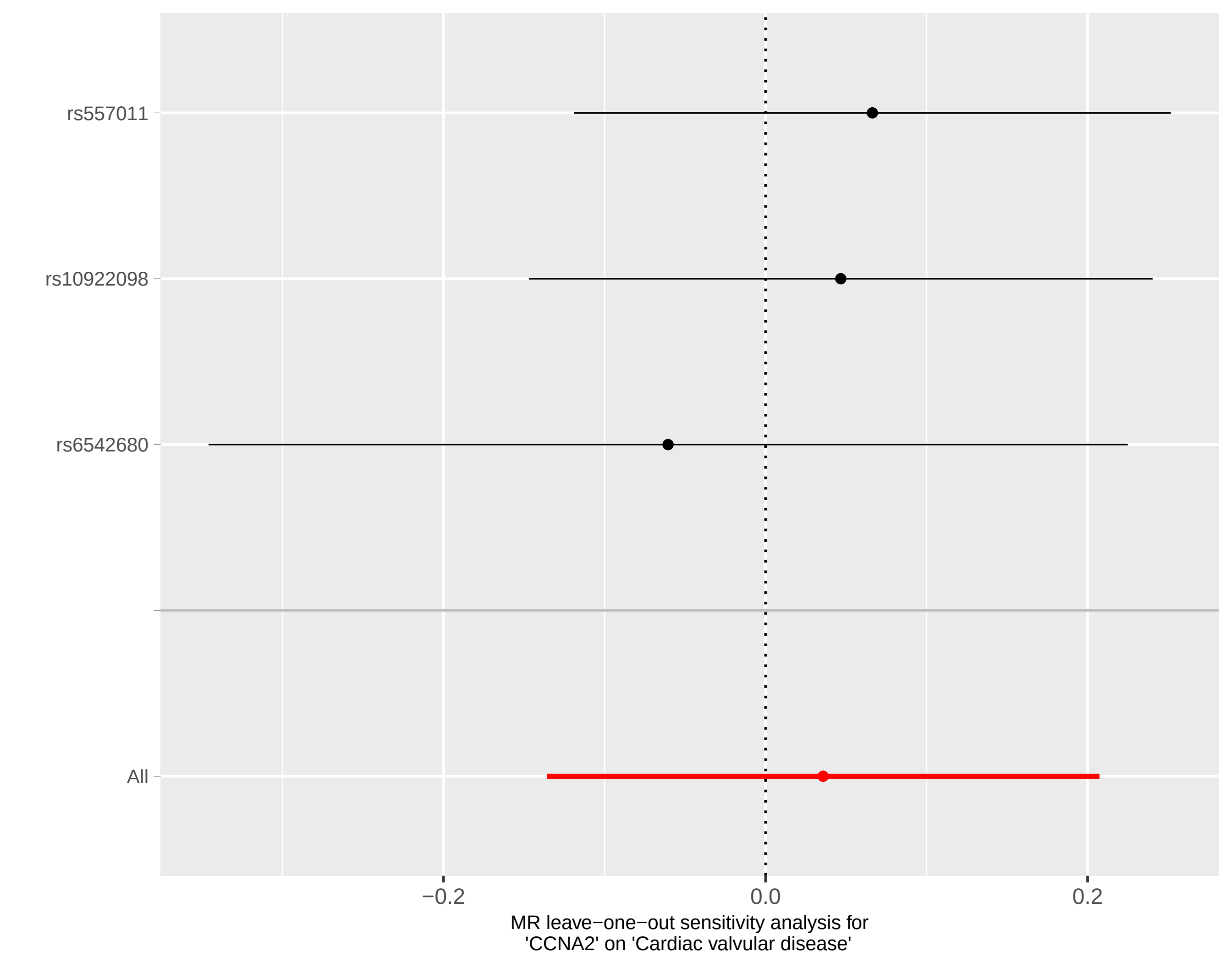
rs557011
rs10922098
rs6542680
All
−0.2
0.0
0.2
MR leave−one−out sensitivity analysis for
'CCNA2' on 'Cardiac valvular disease'

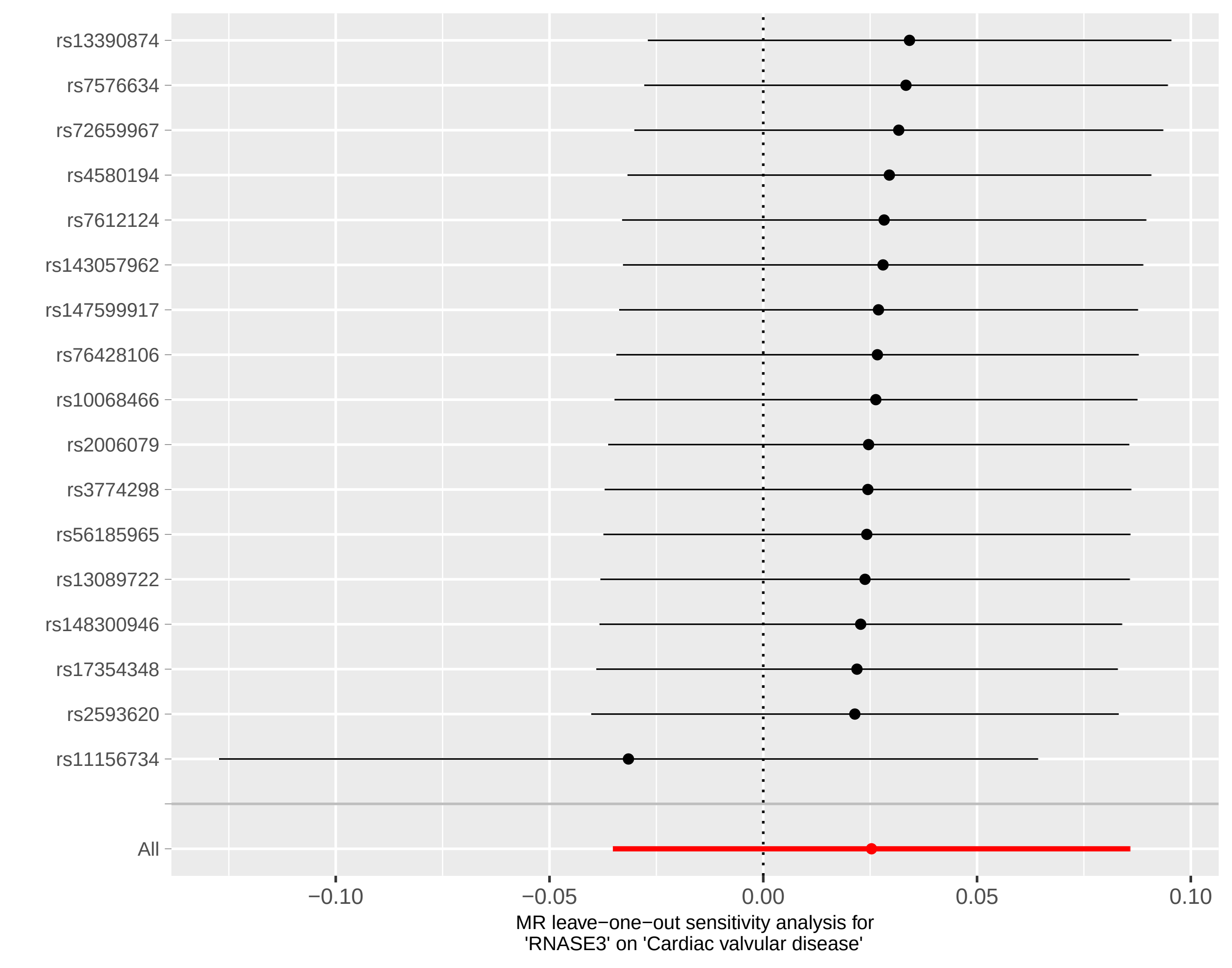
rs13390874
rs7576634
rs72659967
rs4580194
rs7612124
rs143057962
rs147599917
rs76428106
rs10068466
rs2006079
rs3774298
rs56185965
rs13089722
rs148300946
rs17354348
rs2593620
rs11156734
All
−0.10
−0.05
0.00
0.05
0.10
MR leave−one−out sensitivity analysis for
'RNASE3' on 'Cardiac valvular disease'

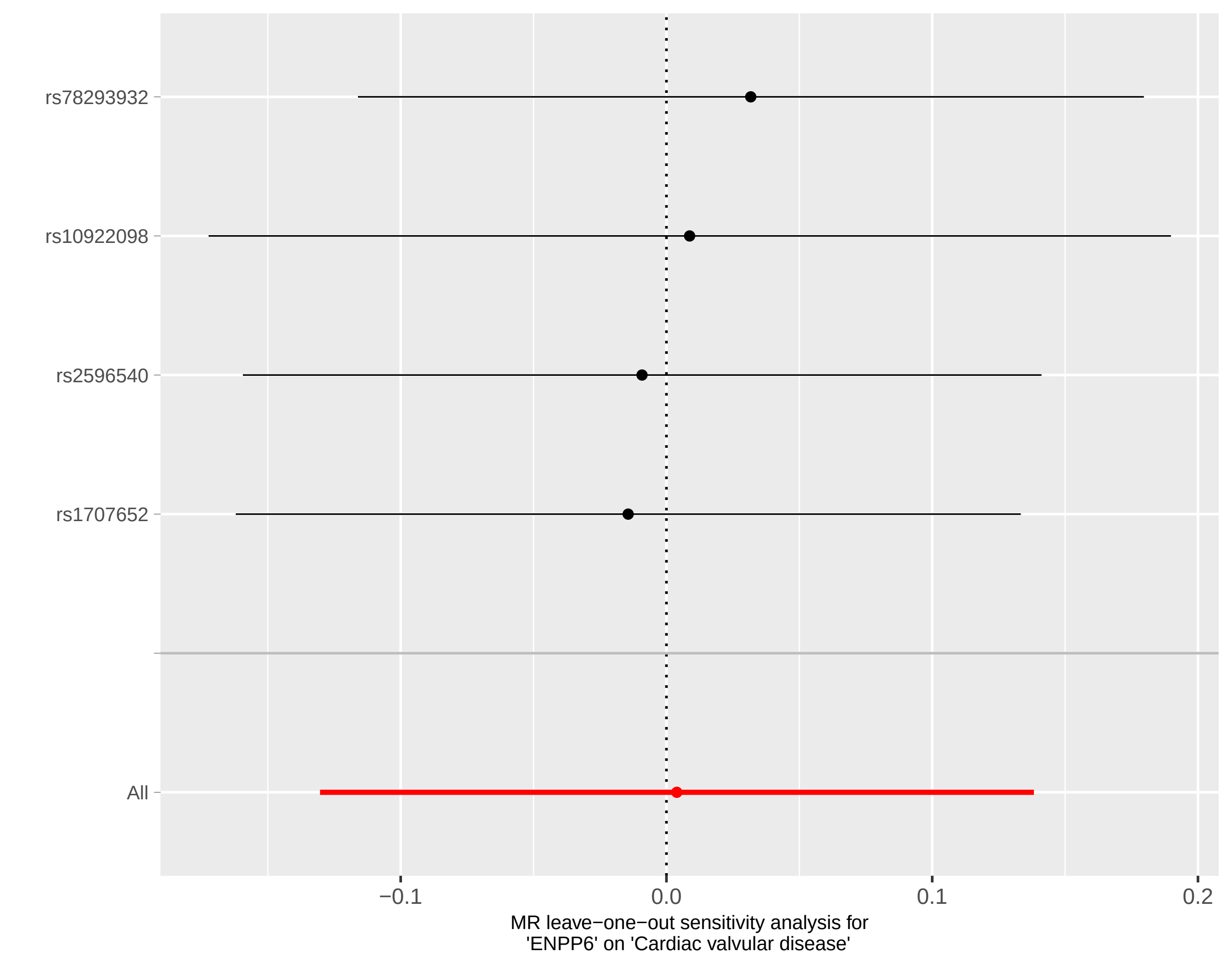
rs78293932
rs10922098
rs2596540
rs1707652
All
−0.1
0.0
0.1
0.2
MR leave−one−out sensitivity analysis for
'ENPP6' on 'Cardiac valvular disease'

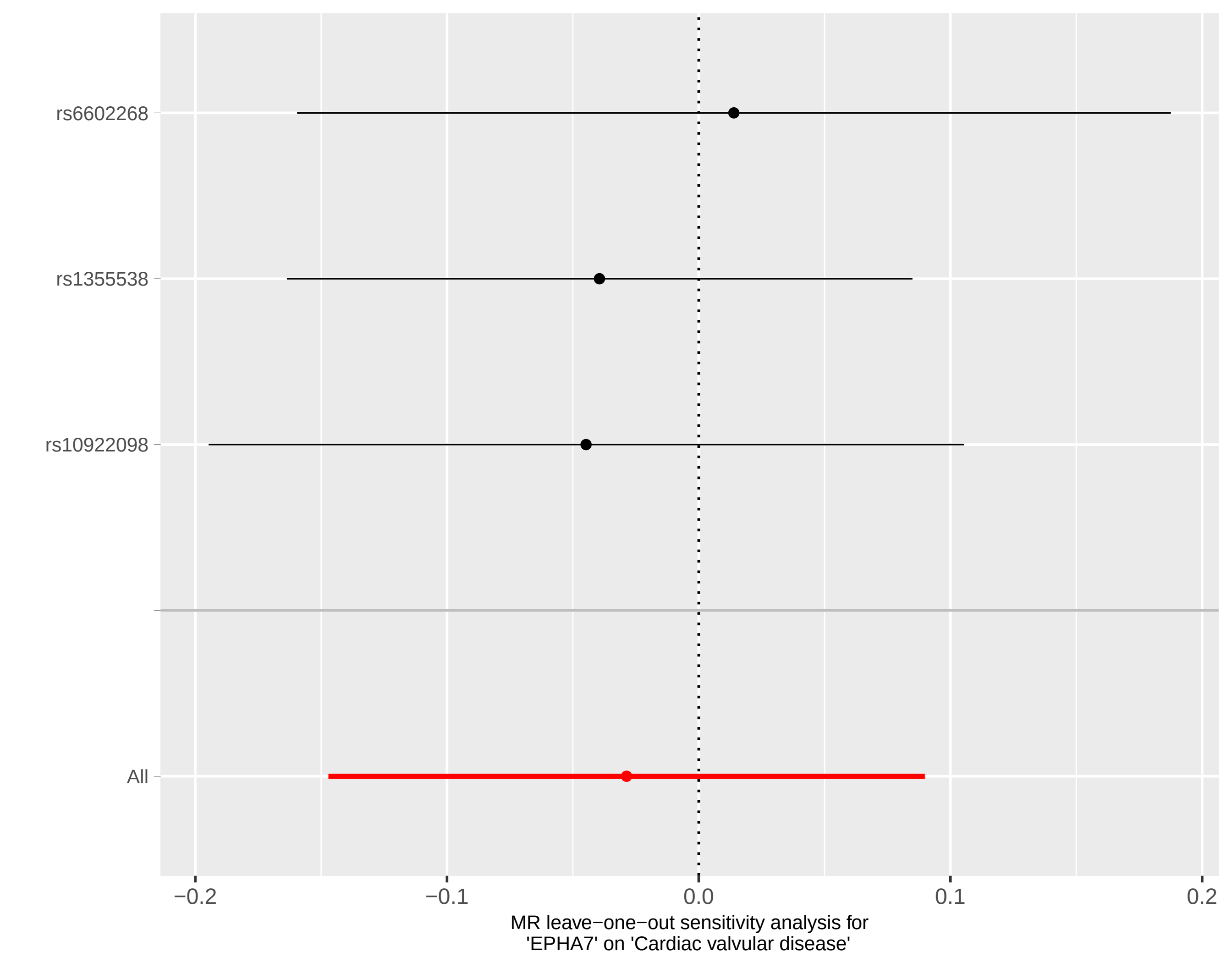
rs6602268
rs1355538
rs10922098
All
−0.2
−0.1
0.0
0.1
0.2
MR leave−one−out sensitivity analysis for
'EPHA7' on 'Cardiac valvular disease'

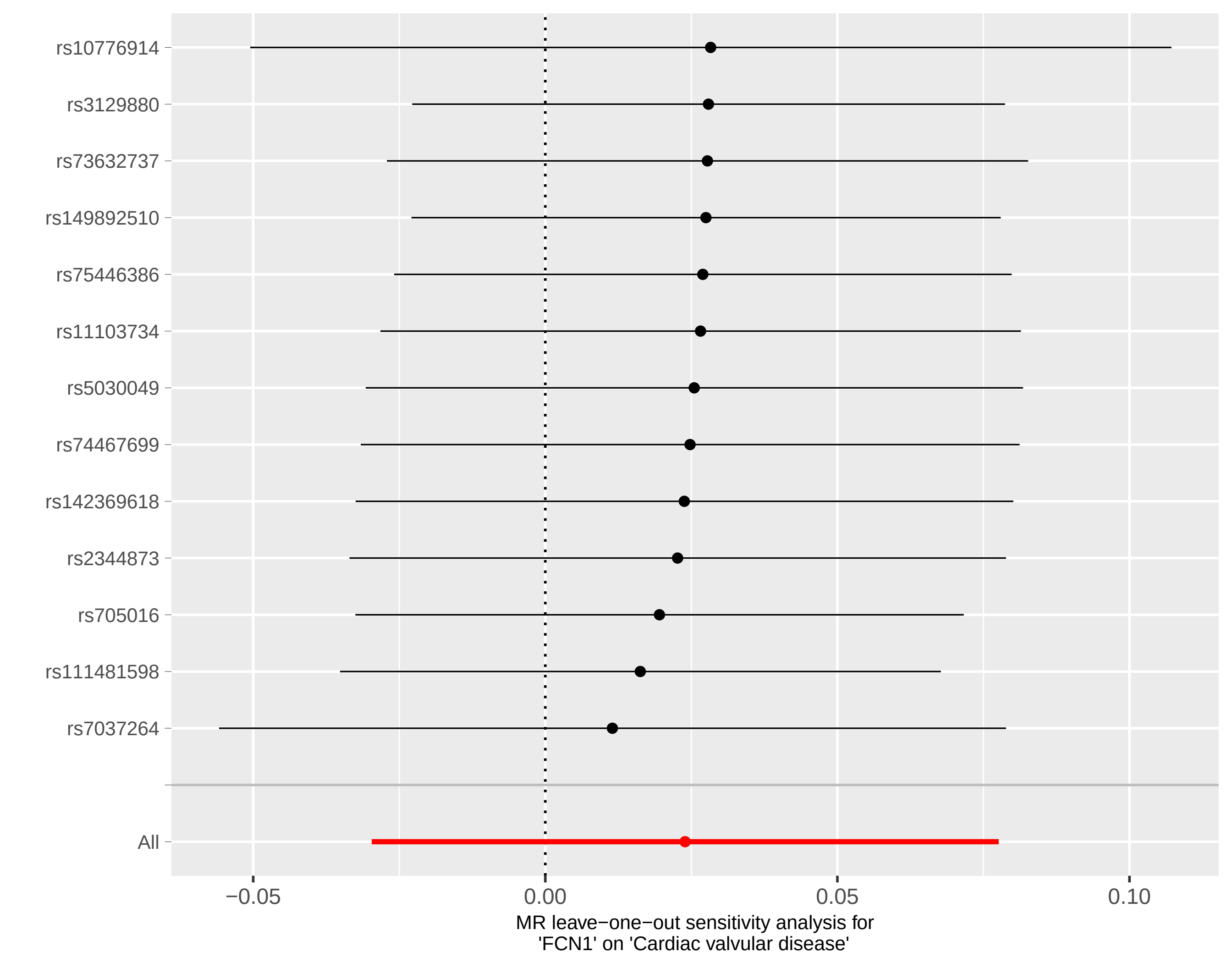

rs10776914
rs3129880
rs73632737
rs149892510
rs75446386
rs11103734
rs5030049
rs74467699
rs142369618
rs2344873
rs705016
rs111481598
rs7037264
All
−0.05
0.00
0.05
0.10
MR leave−one−out sensitivity analysis for
'FCN1' on 'Cardiac valvular disease'

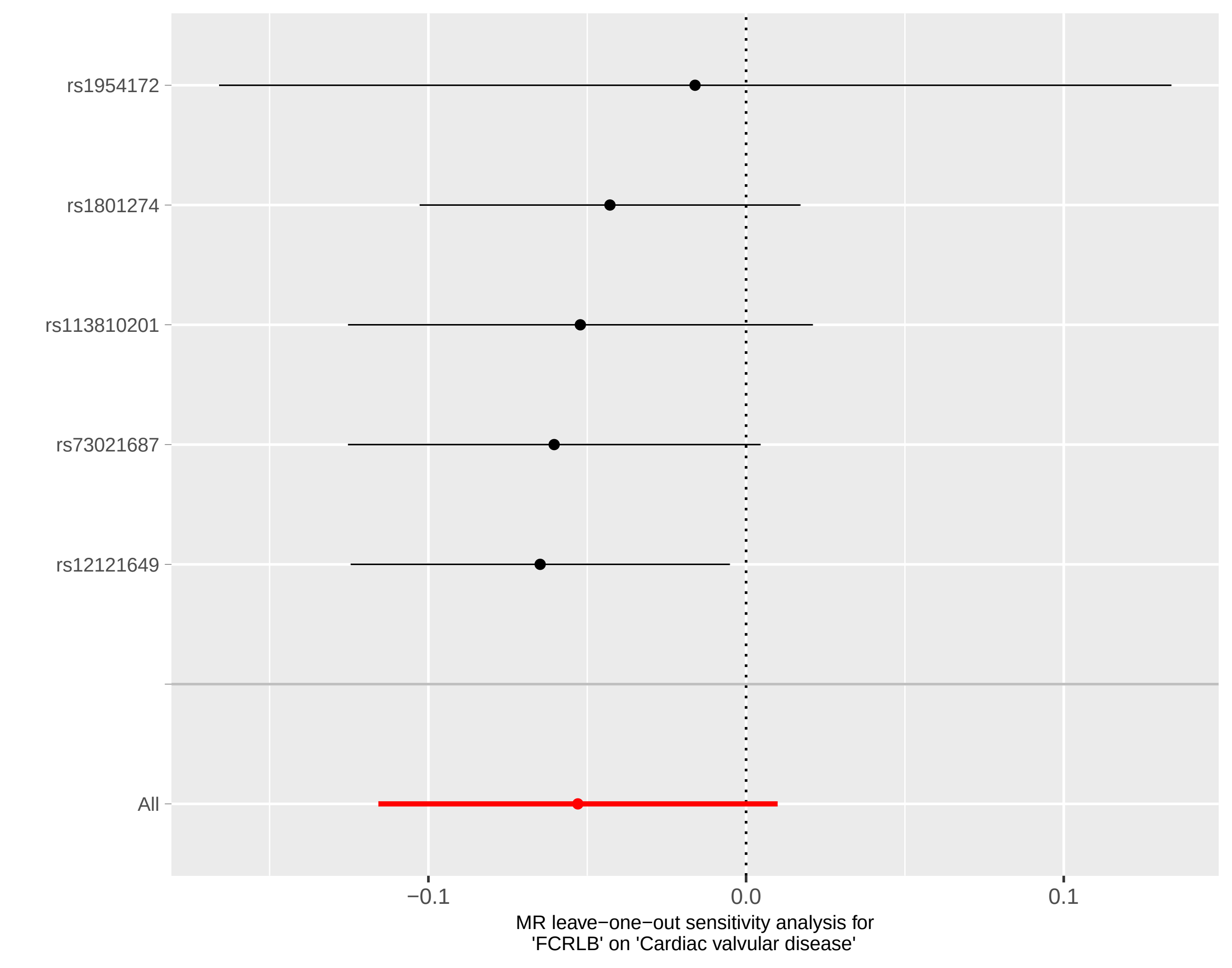
rs1954172
rs1801274
rs113810201
rs73021687
rs12121649
All
−0.1
0.0
0.1
MR leave−one−out sensitivity analysis for
'FCRLB' on 'Cardiac valvular disease'

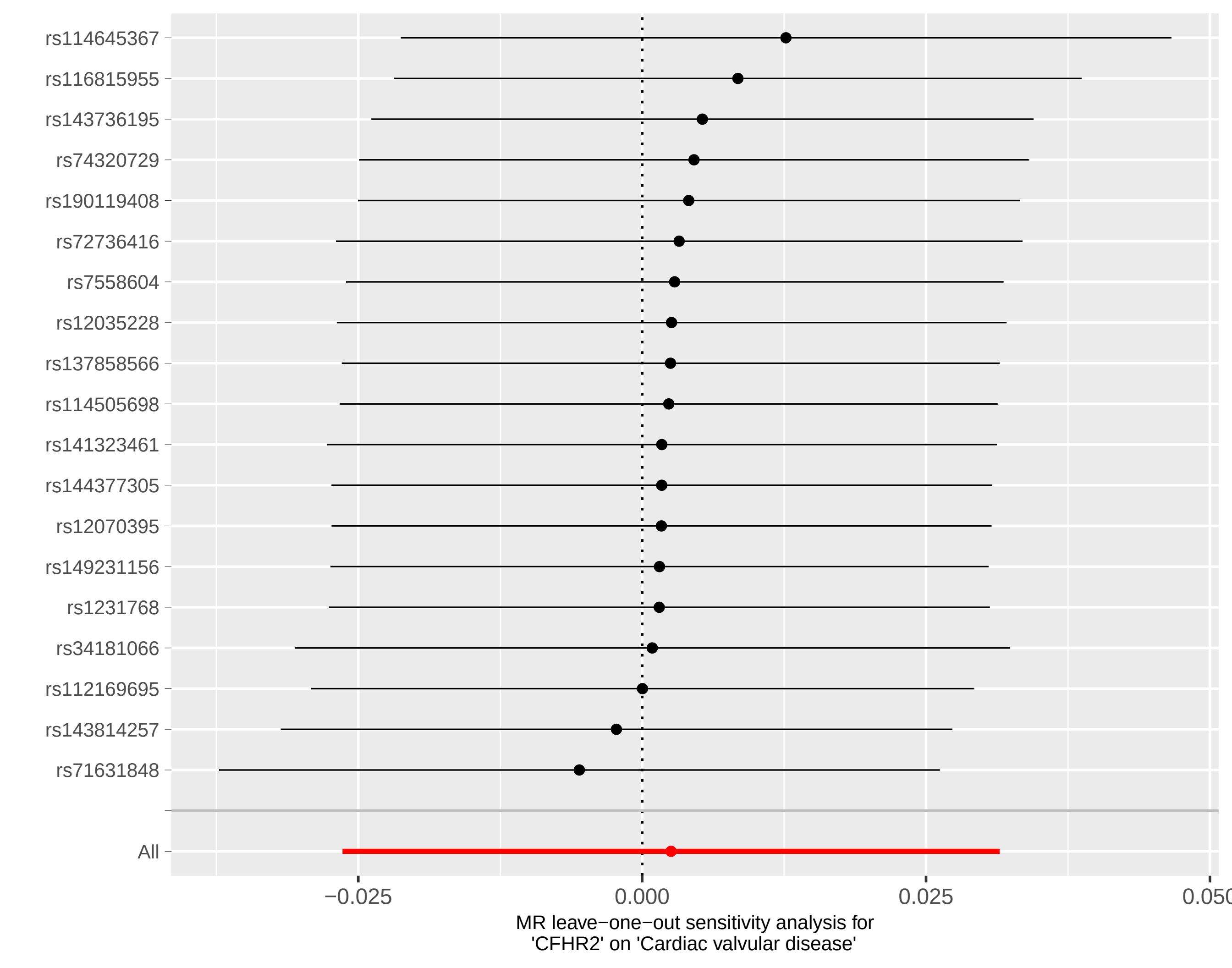

rs114645367
rs116815955
rs143736195
rs74320729
rs190119408
rs72736416
rs7558604
rs12035228
rs137858566
rs114505698
rs141323461
rs144377305
rs12070395
rs149231156
rs1231768
rs34181066
rs112169695
rs143814257
rs71631848
All
−0.025
0.000
0.025
0.050
MR leave−one−out sensitivity analysis for
'CFHR2' on 'Cardiac valvular disease'

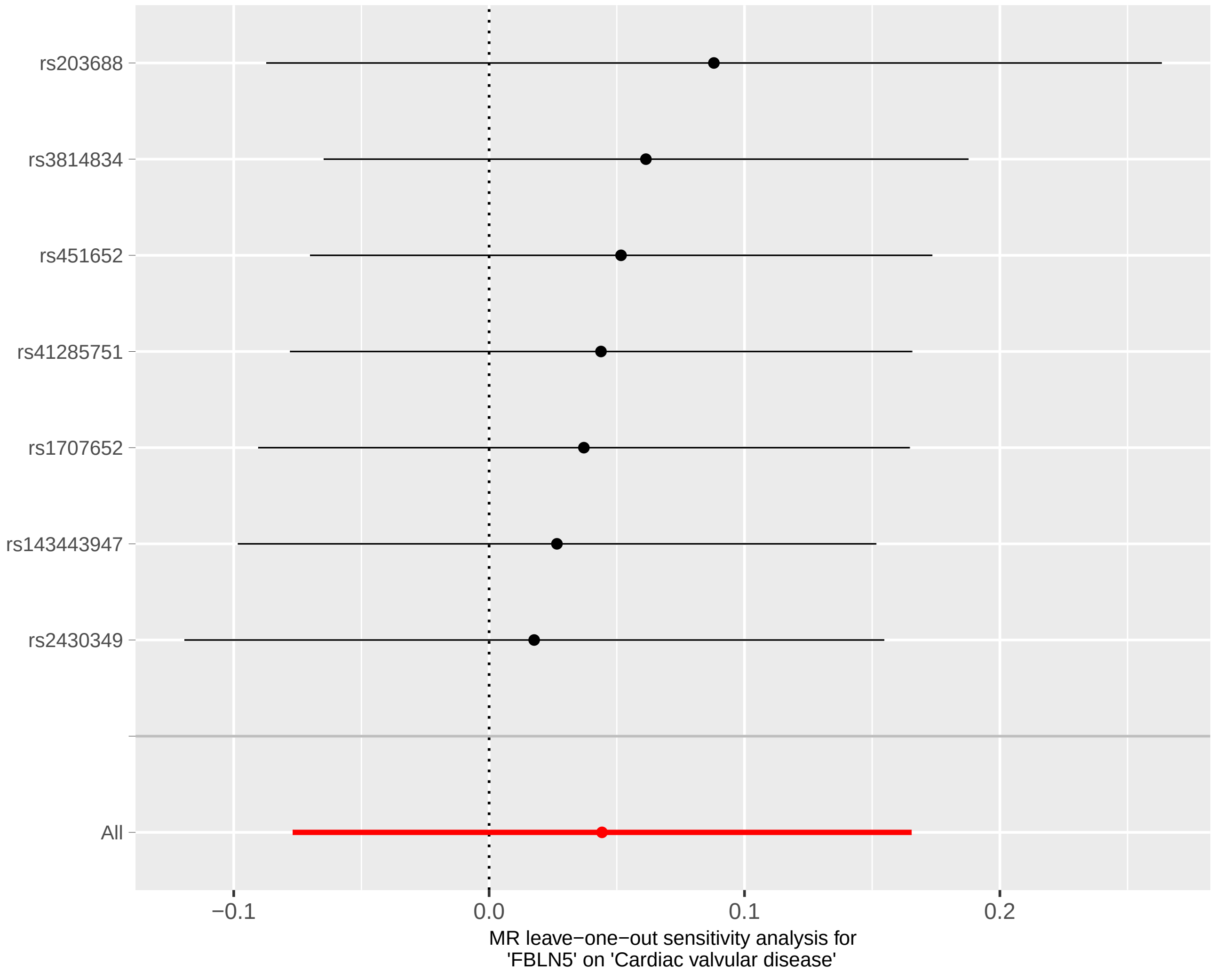
rs203688
rs3814834
rs451652
rs41285751
rs1707652
rs143443947
rs2430349
All
−0.1
0.0
0.1
0.2
MR leave−one−out sensitivity analysis for
'FBLN5' on 'Cardiac valvular disease'

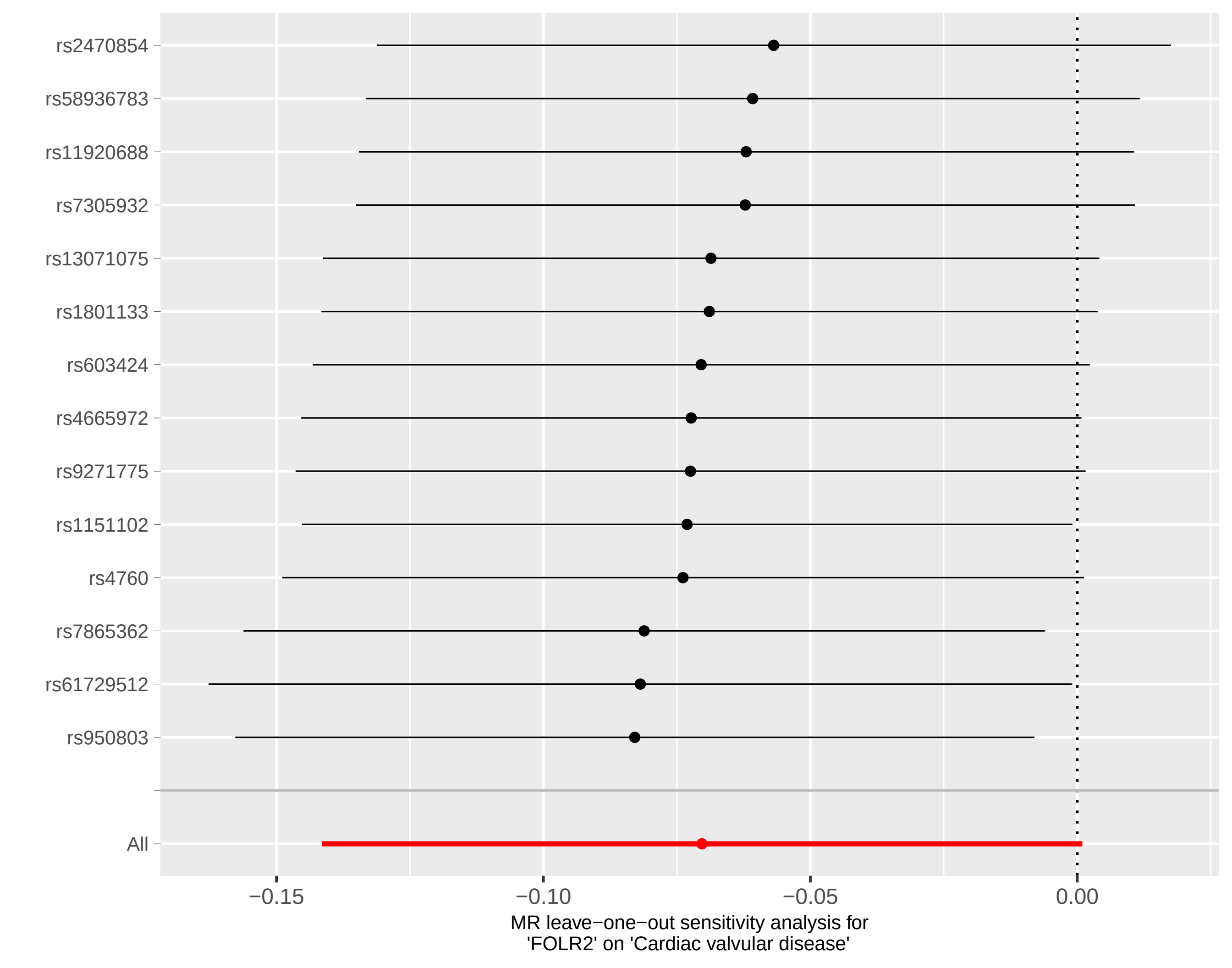
rs2470854
rs58936783
rs11920688
rs7305932
rs13071075
rs1801133
rs603424
rs4665972
rs9271775
rs1151102
rs4760
rs7865362
rs61729512
rs950803
All
−0.15
−0.10
−0.05
0.00
MR leave−one−out sensitivity analysis for
'FOLR2' on 'Cardiac valvular disease'

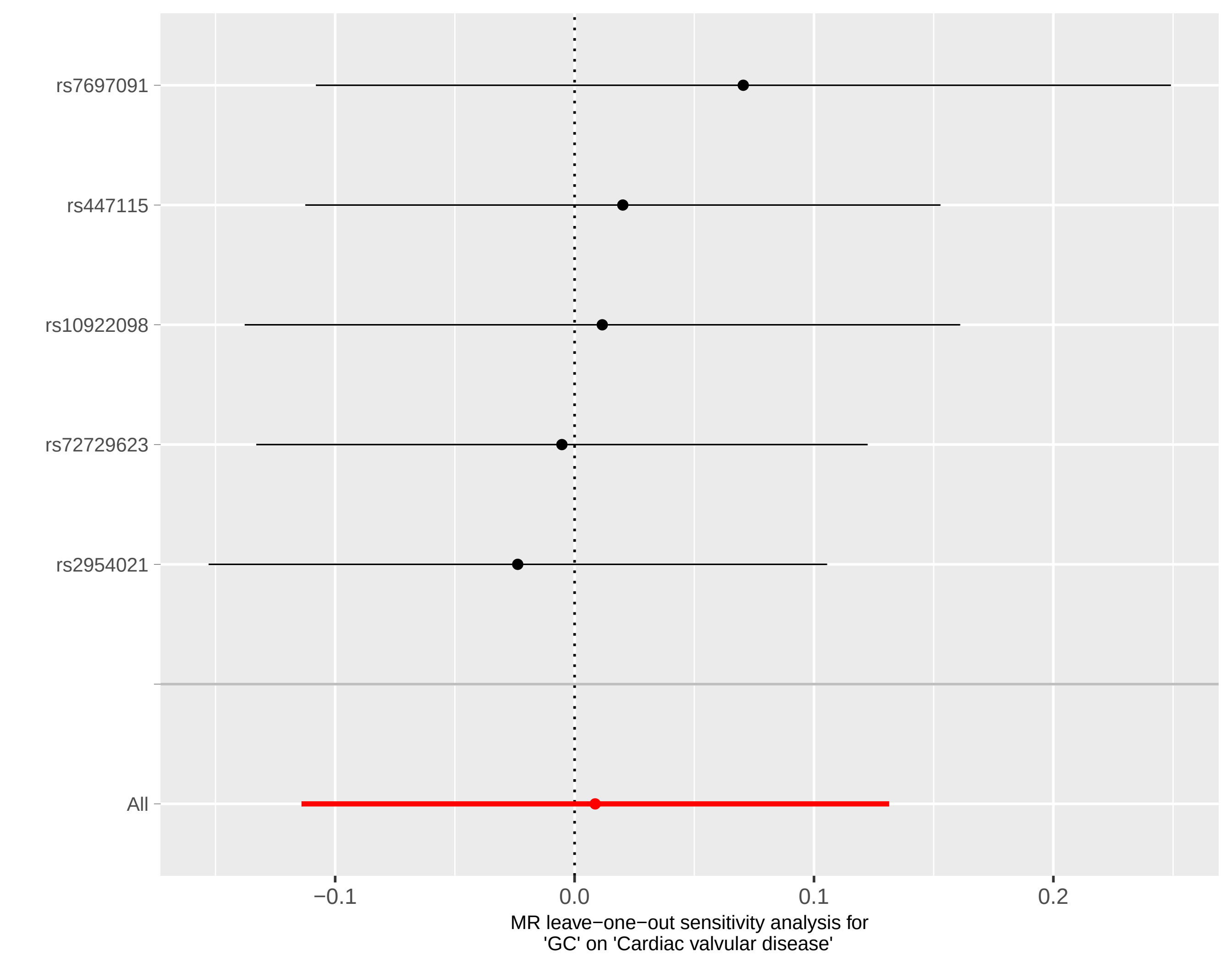

rs7697091
rs447115
rs10922098
rs72729623
rs2954021
All
−0.1
0.0
0.1
0.2
MR leave−one−out sensitivity analysis for
'GC' on 'Cardiac valvular disease'

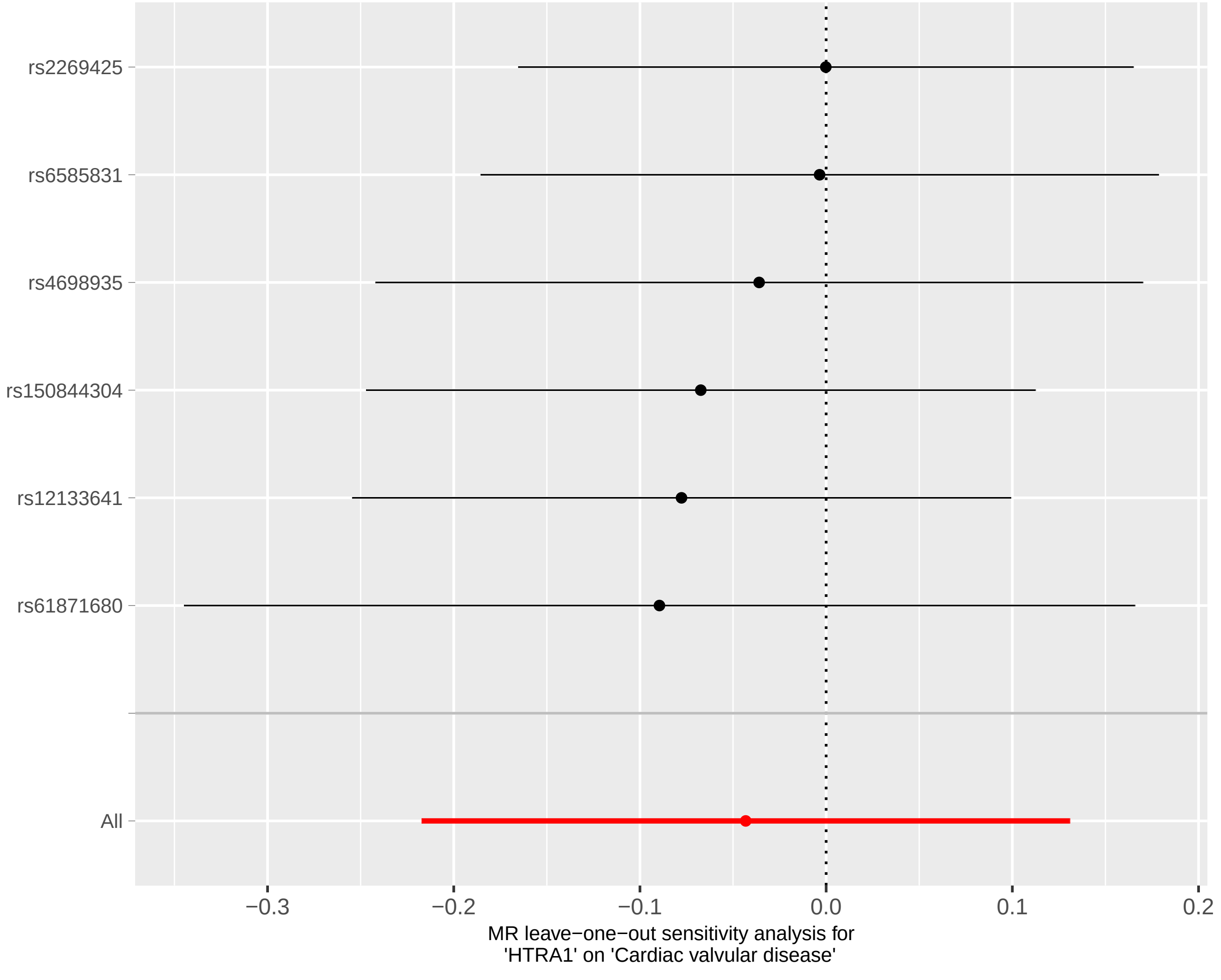

rs2269425
rs6585831
rs4698935
rs150844304
rs12133641
rs61871680
All
−0.3
−0.2
−0.1
0.0
0.1
0.2
MR leave−one−out sensitivity analysis for
'HTRA1' on 'Cardiac valvular disease'

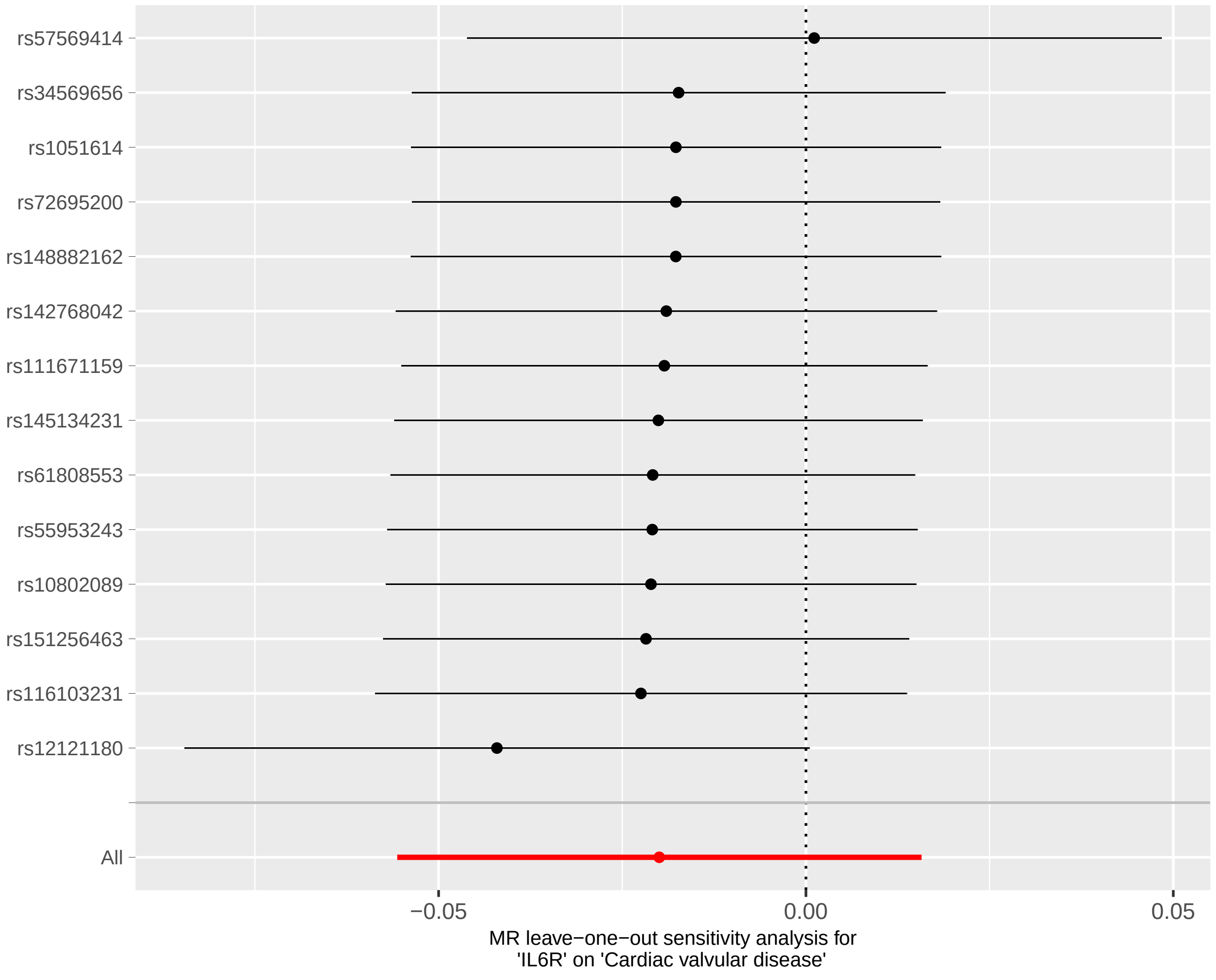
rs57569414
rs34569656
rs1051614
rs72695200
rs148882162
rs142768042
rs111671159
rs145134231
rs61808553
rs55953243
rs10802089
rs151256463
rs116103231
rs12121180
All
−0.05
0.00
0.05
MR leave−one−out sensitivity analysis for
'IL6R' on 'Cardiac valvular disease'

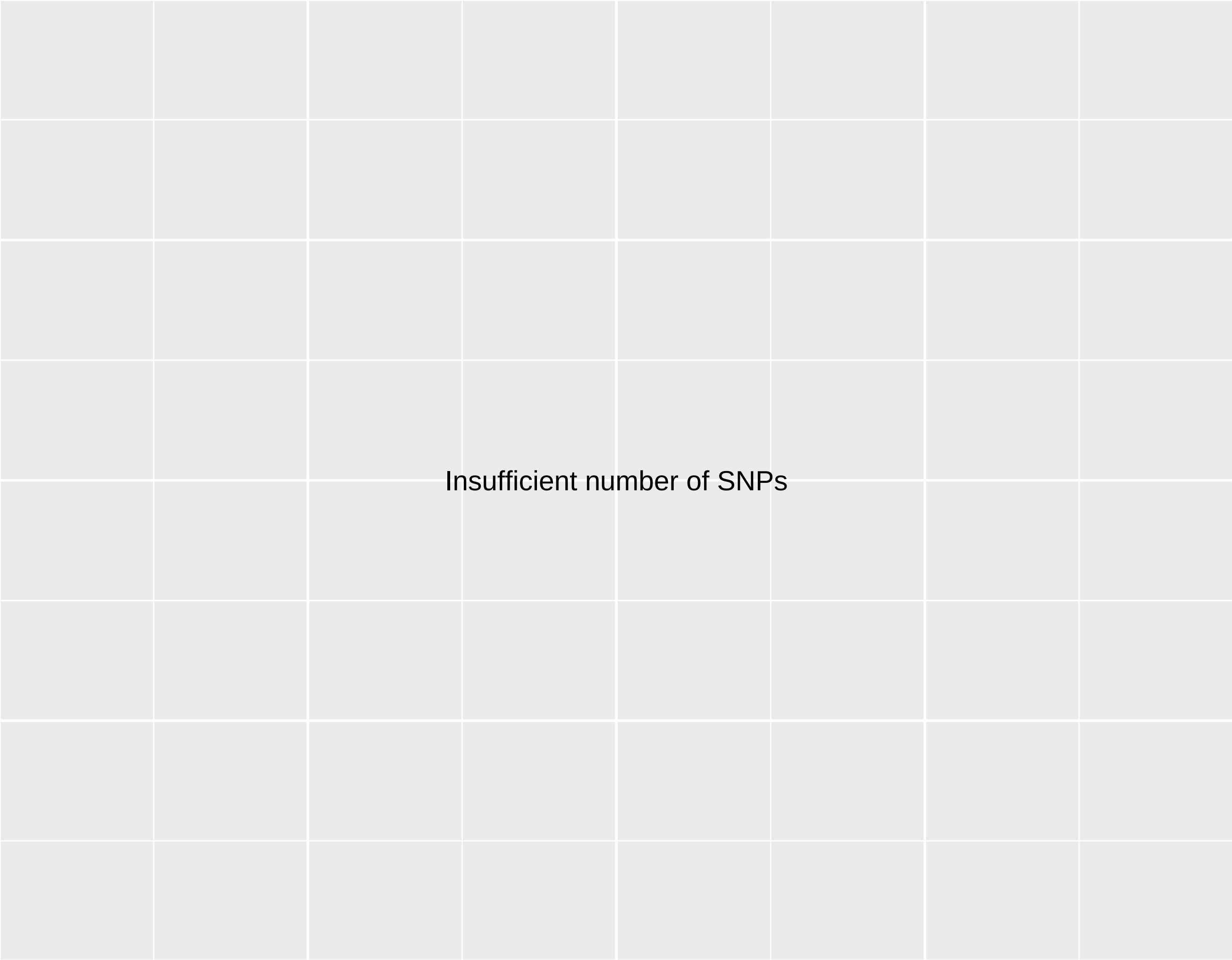
Insufficient number of SNPs

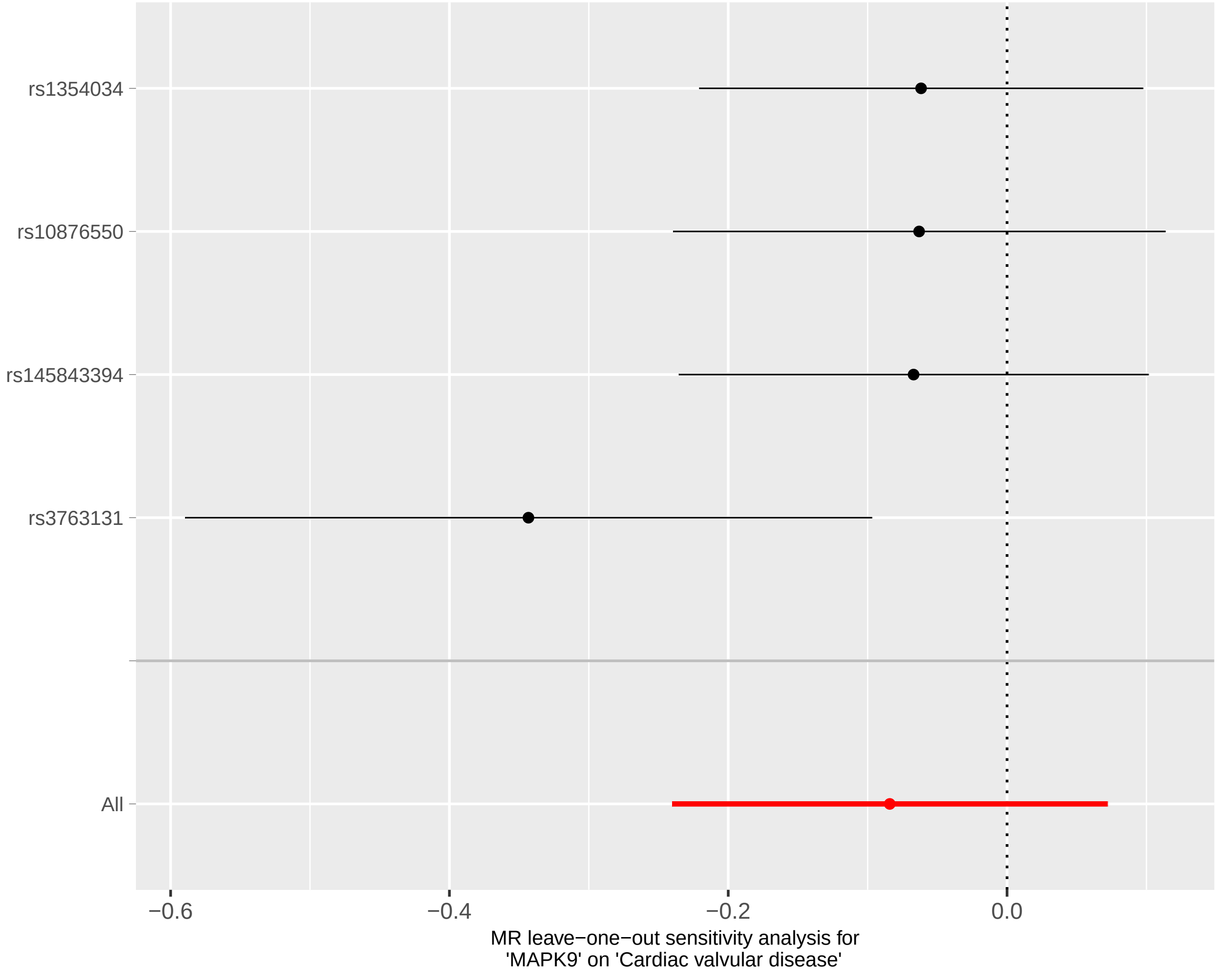

rs1354034
rs10876550
rs145843394
rs3763131
All
−0.6
−0.4
−0.2
0.0
MR leave−one−out sensitivity analysis for
'MAPK9' on 'Cardiac valvular disease'

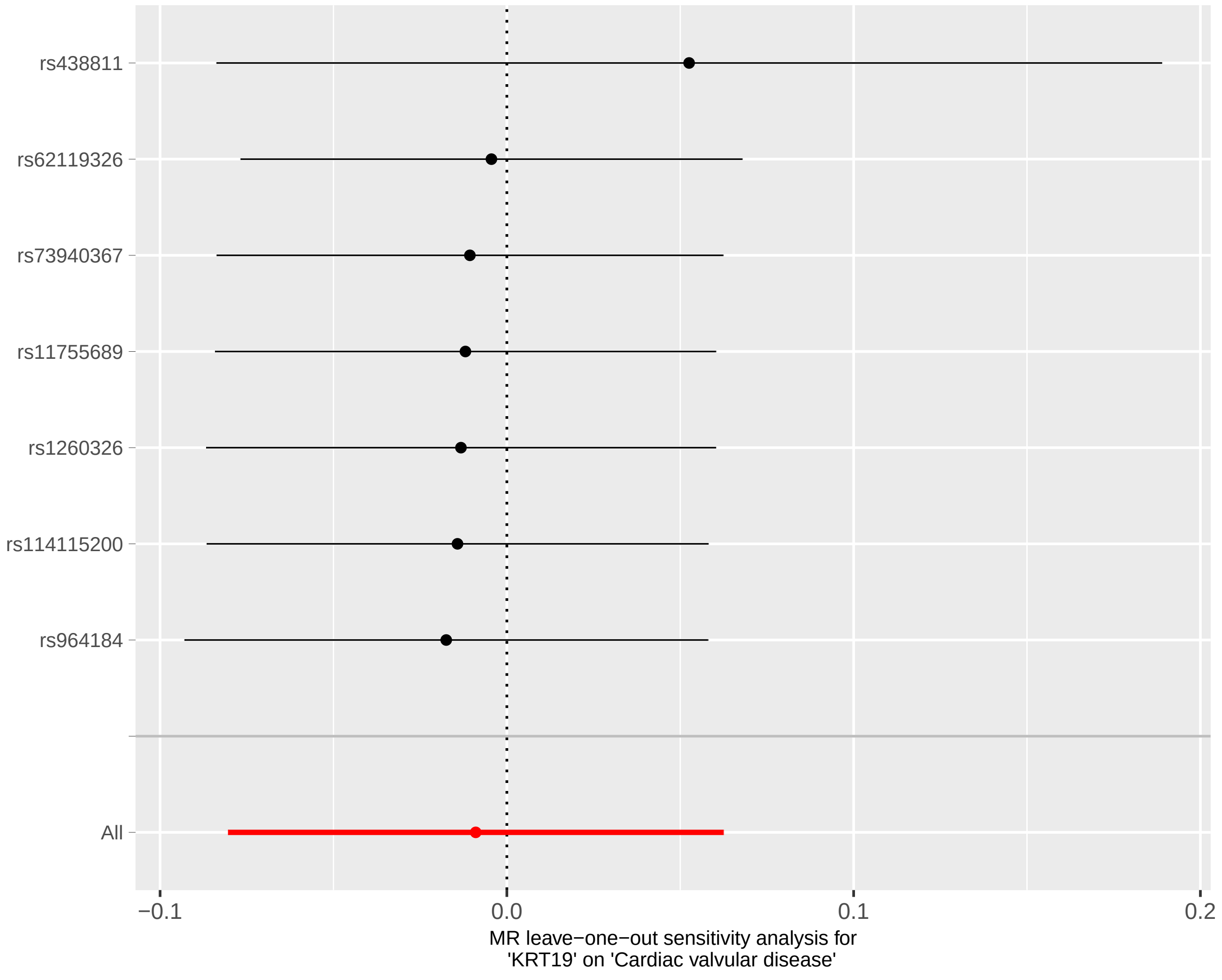
rs438811
rs62119326
rs73940367
rs11755689
rs1260326
rs114115200
rs964184
All
−0.1
0.0
0.1
0.2
MR leave−one−out sensitivity analysis for
'KRT19' on 'Cardiac valvular disease'

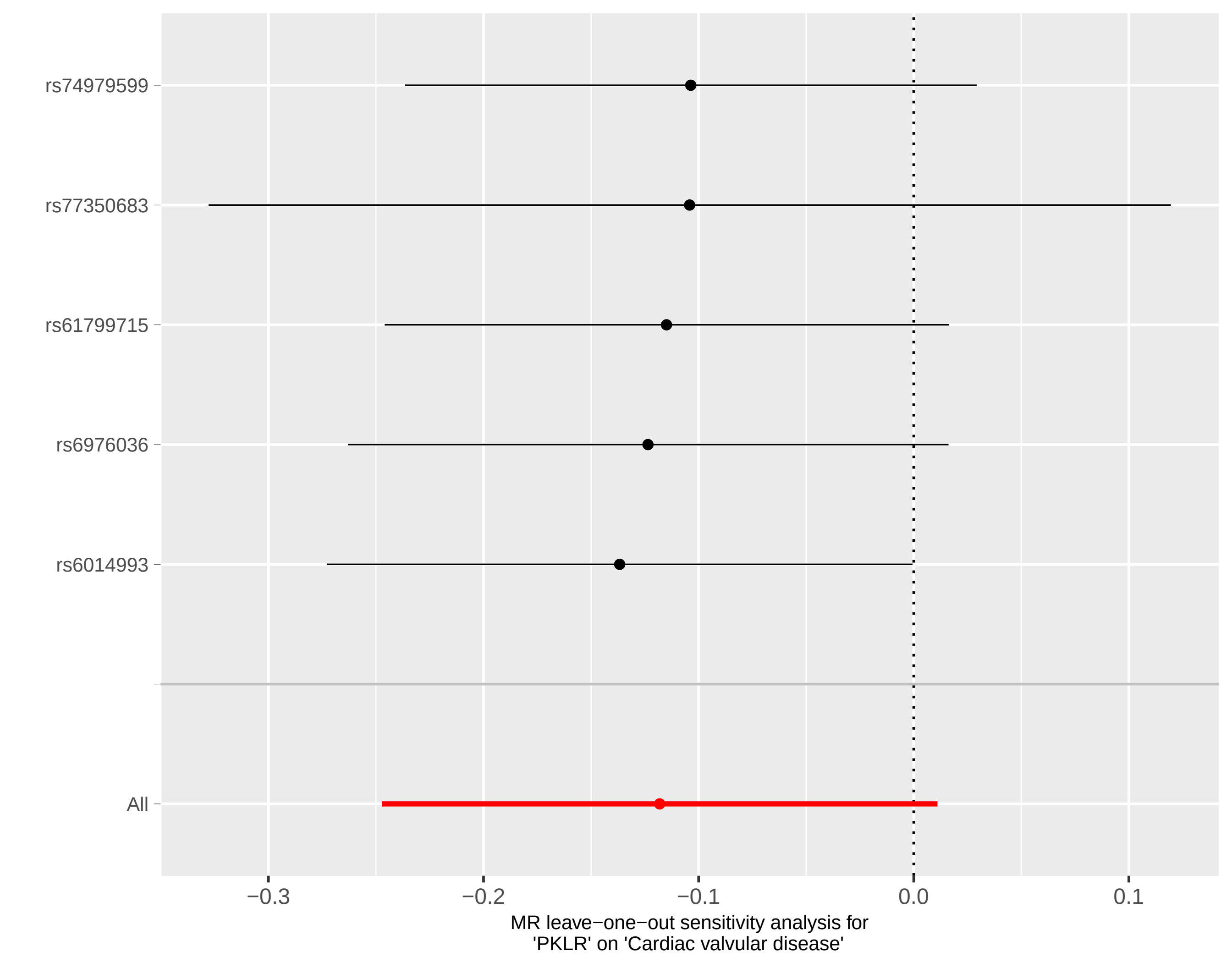
rs74979599
rs77350683
rs61799715
rs6976036
rs6014993
All
−0.3
−0.2
−0.1
0.0
0.1
MR leave−one−out sensitivity analysis for
'PKLR' on 'Cardiac valvular disease'

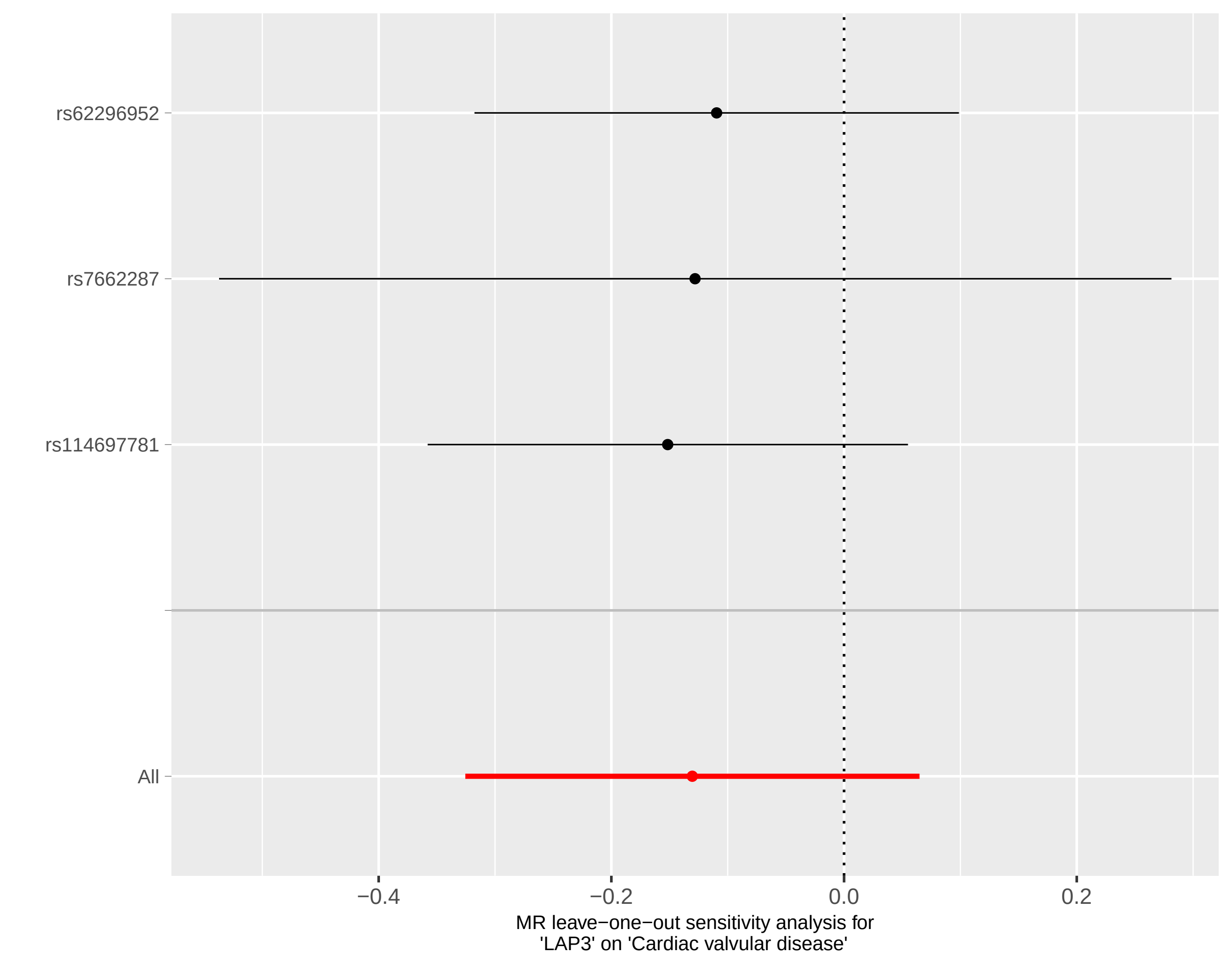

rs62296952
rs7662287
rs114697781
All
−0.4
−0.2
0.0
0.2
MR leave−one−out sensitivity analysis for
'LAP3' on 'Cardiac valvular disease'

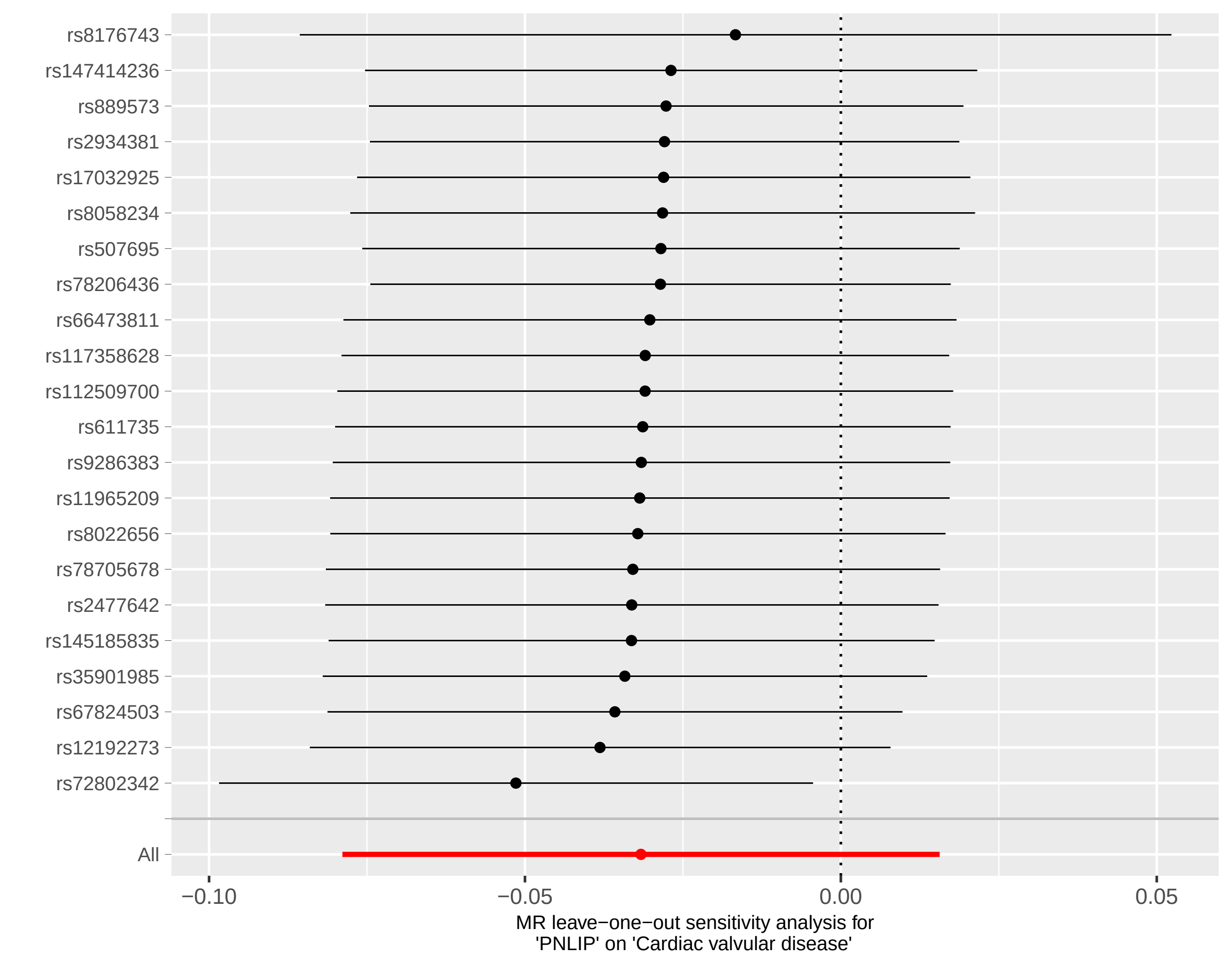
rs8176743
rs147414236
rs889573
rs2934381
rs17032925
rs8058234
rs507695
rs78206436
rs66473811
rs117358628
rs112509700
rs611735
rs9286383
rs11965209
rs8022656
rs78705678
rs2477642
rs145185835
rs35901985
rs67824503
rs12192273
rs72802342
All
−0.10
−0.05
0.00
0.05
MR leave−one−out sensitivity analysis for
'PNLIP' on 'Cardiac valvular disease'

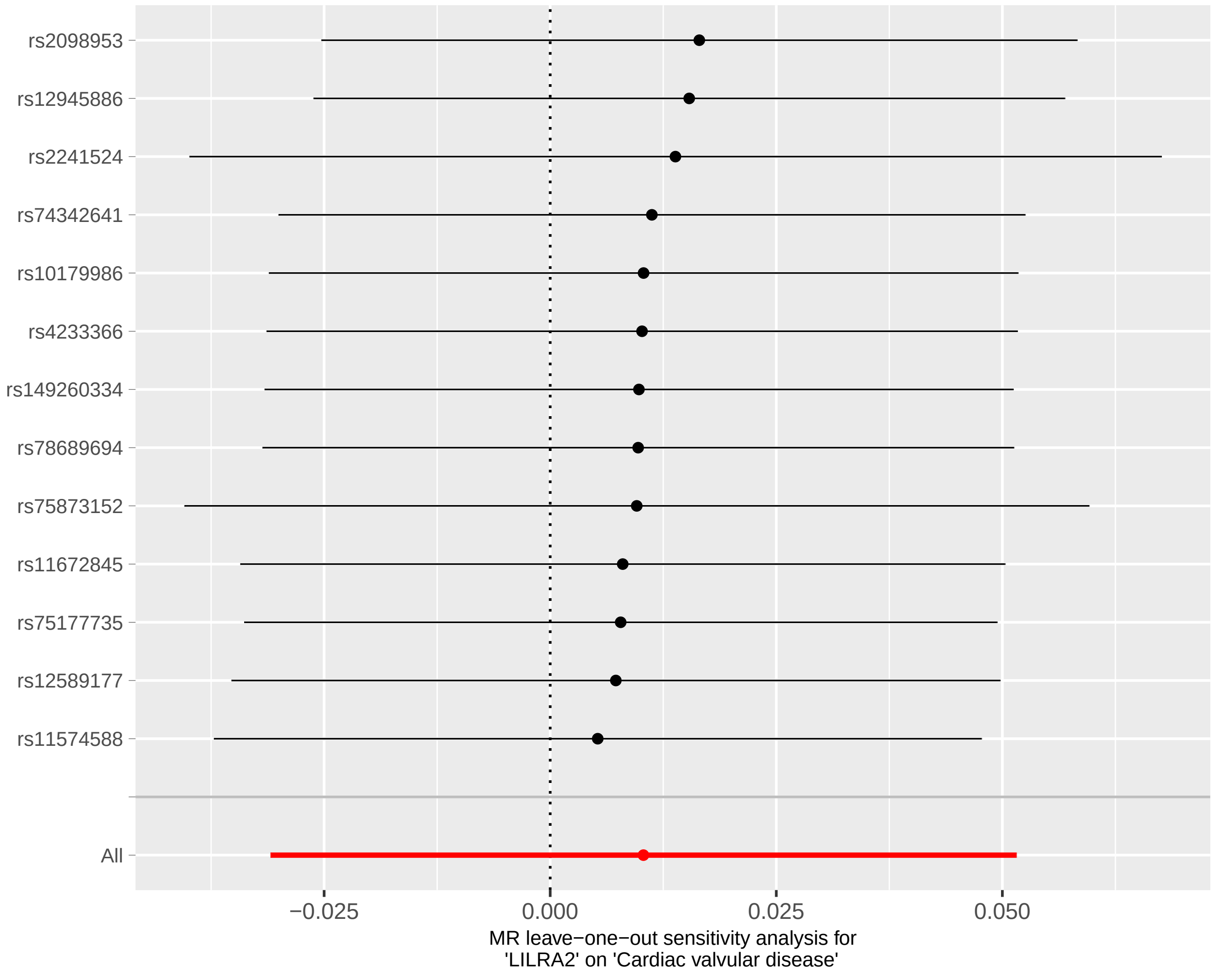
rs2098953
rs12945886
rs2241524
rs74342641
rs10179986
rs4233366
rs149260334
rs78689694
rs75873152
rs11672845
rs75177735
rs12589177
rs11574588
All
−0.025
0.000
0.025
0.050
MR leave−one−out sensitivity analysis for
'LILRA2' on 'Cardiac valvular disease'

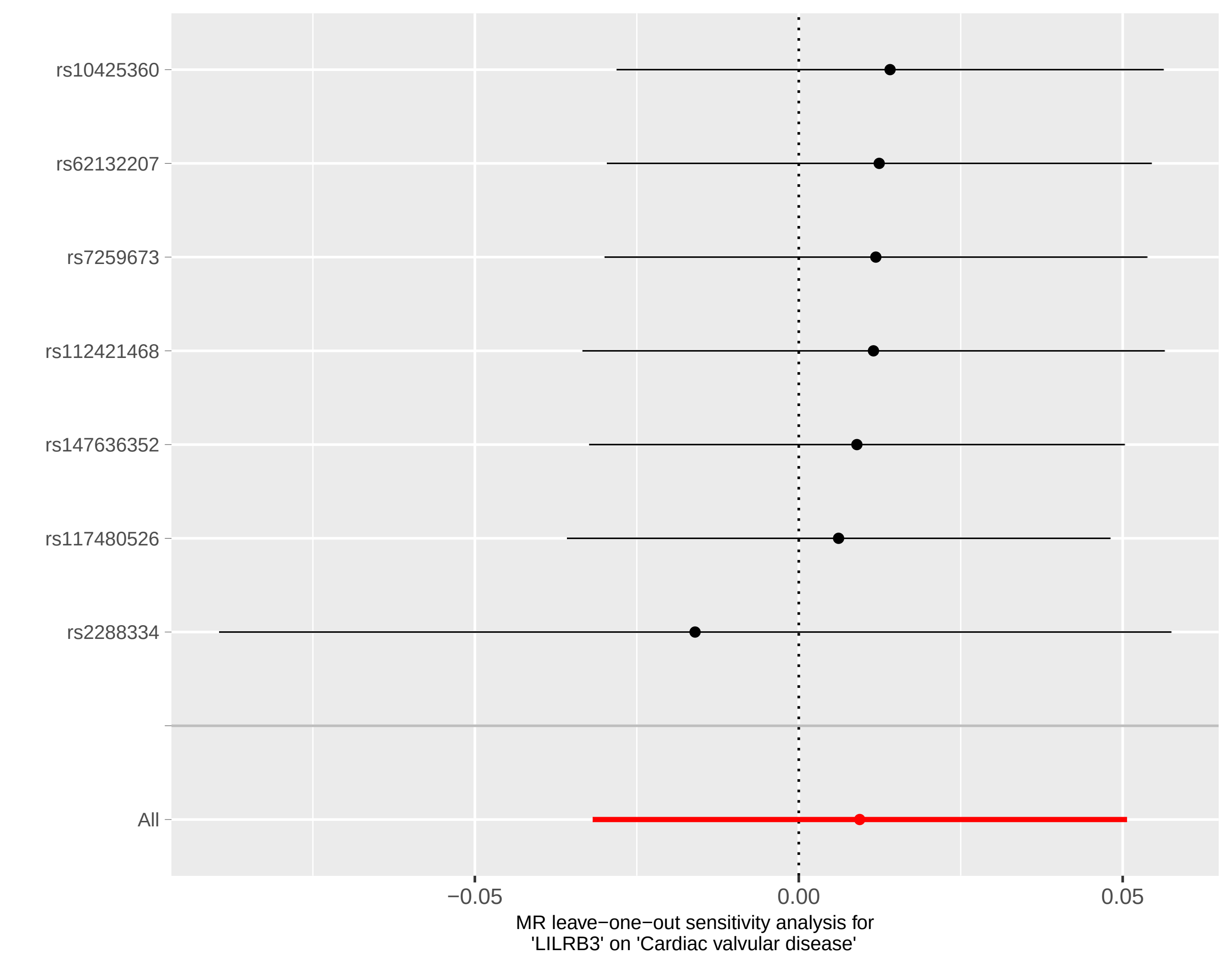
rs10425360
rs62132207
rs7259673
rs112421468
rs147636352
rs117480526
rs2288334
All
−0.05
0.00
0.05
MR leave−one−out sensitivity analysis for
'LILRB3' on 'Cardiac valvular disease'

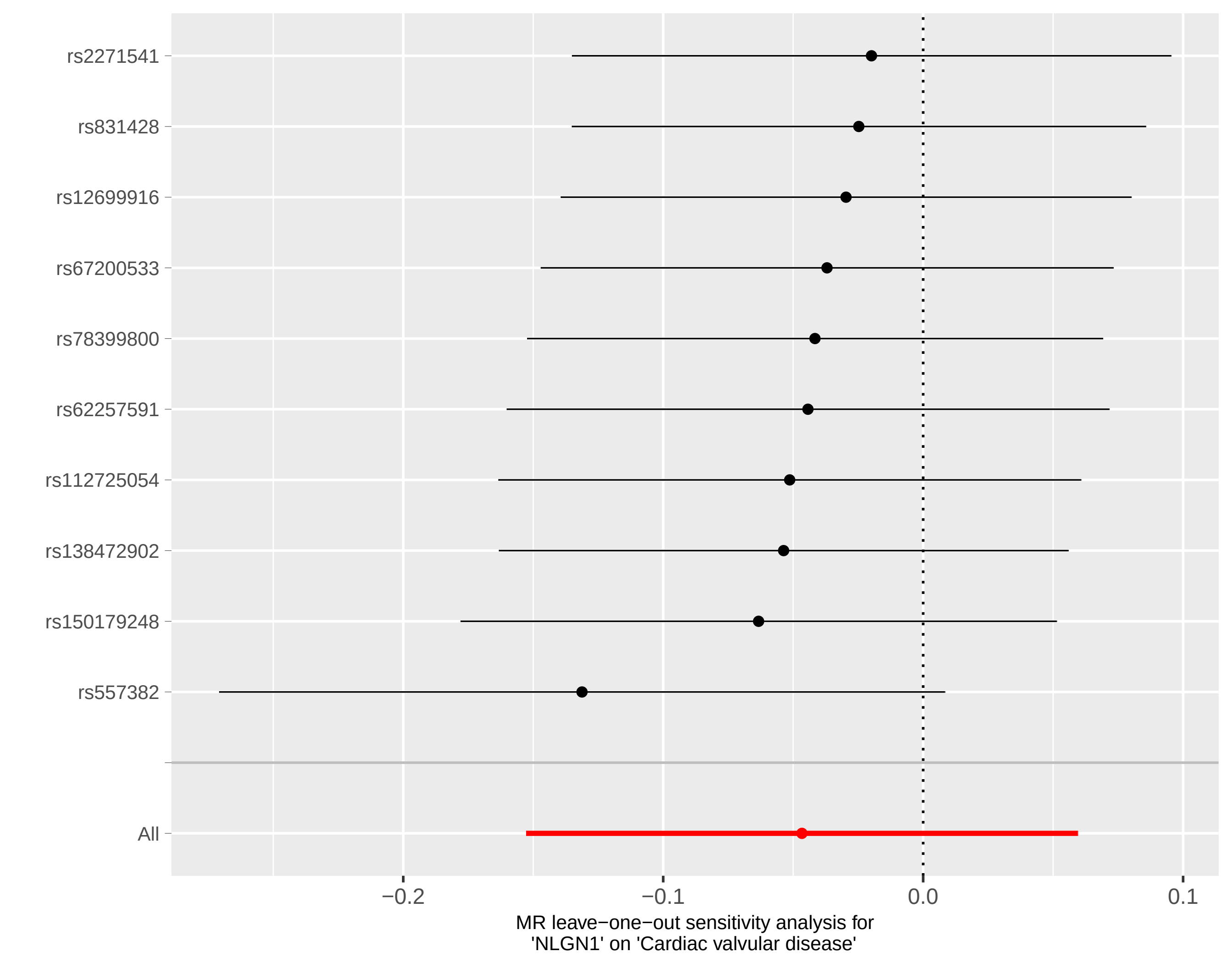

rs2271541
rs831428
rs12699916
rs67200533
rs78399800
rs62257591
rs112725054
rs138472902
rs150179248
rs557382
All
−0.2
−0.1
0.0
0.1
MR leave−one−out sensitivity analysis for
'NLGN1' on 'Cardiac valvular disease'

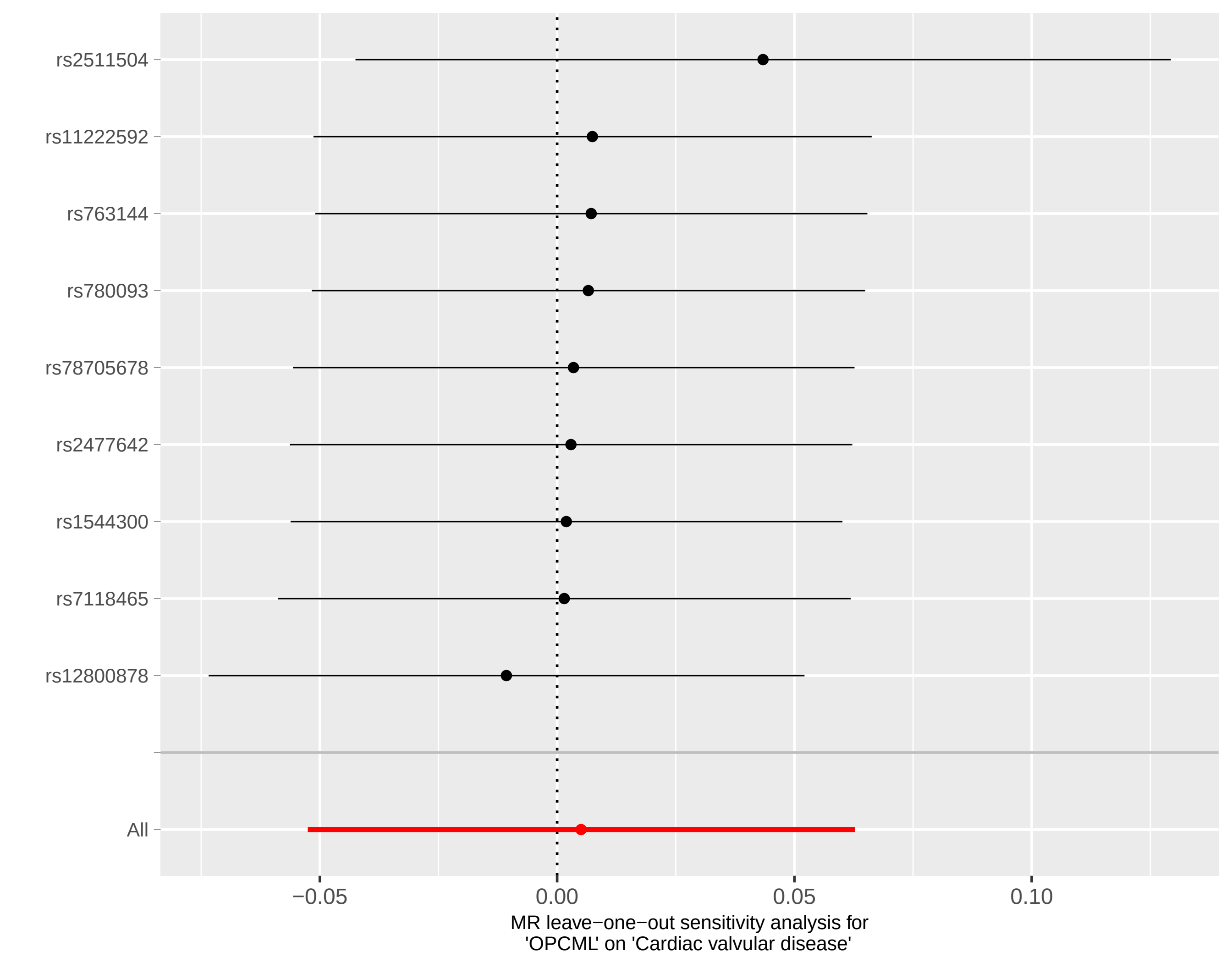
rs2511504
rs11222592
rs763144
rs780093
rs78705678
rs2477642
rs1544300
rs7118465
rs12800878
All
−0.05
0.00
0.05
0.10
MR leave−one−out sensitivity analysis for
'OPCML' on 'Cardiac valvular disease'

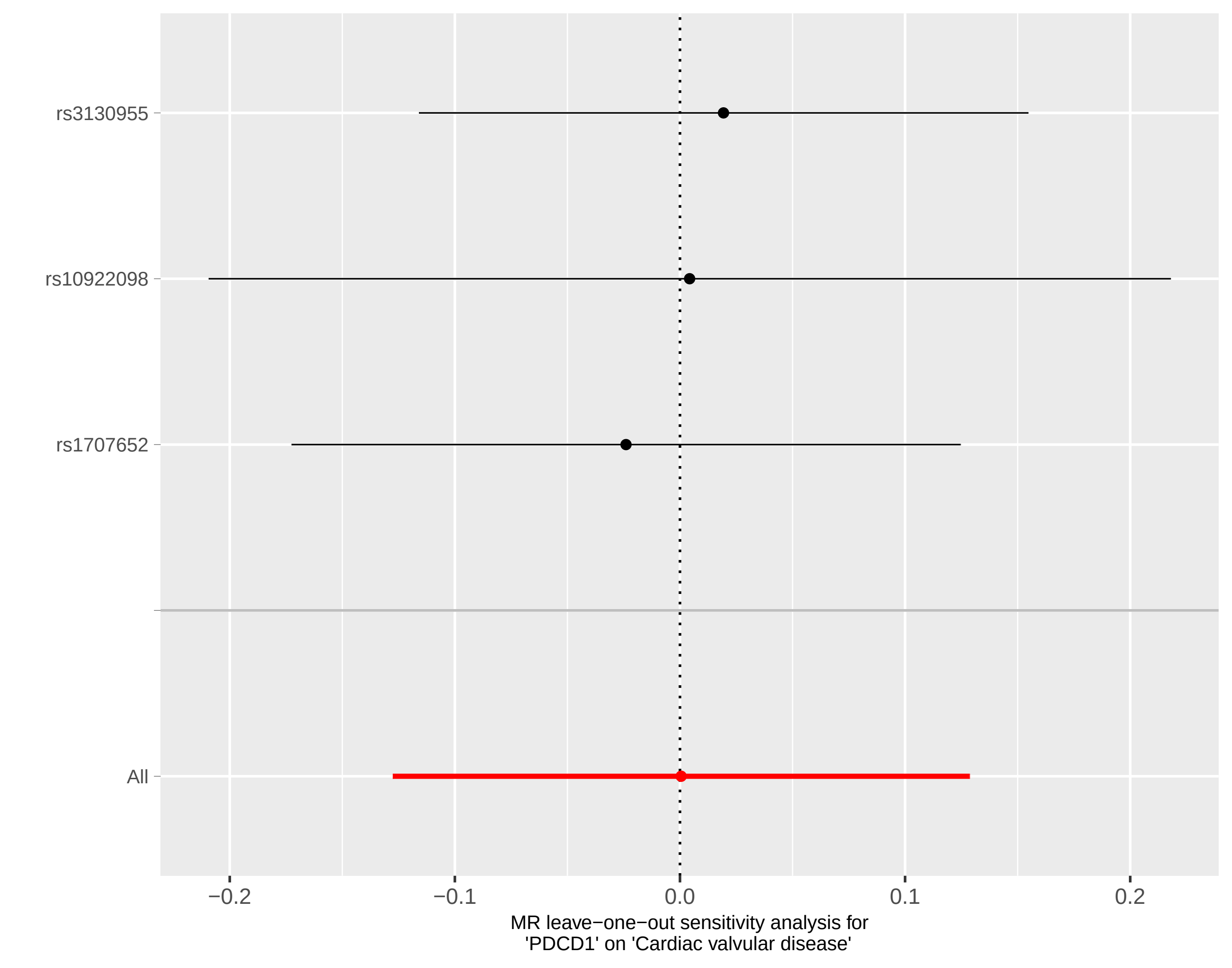

rs3130955
rs10922098
rs1707652
All
−0.2
−0.1
0.0
0.1
0.2
MR leave−one−out sensitivity analysis for
'PDCD1' on 'Cardiac valvular disease'

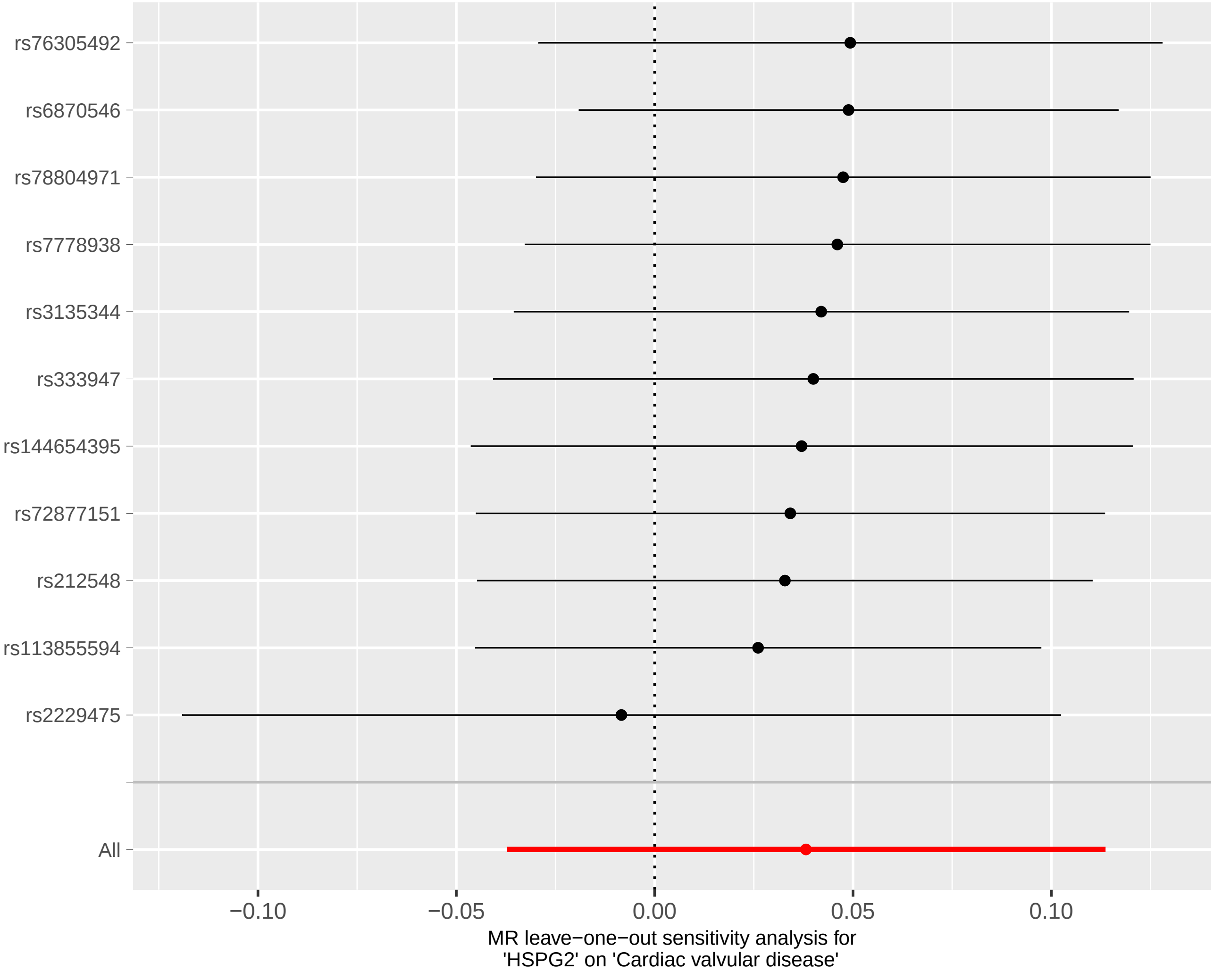
rs76305492
rs6870546
rs78804971
rs7778938
rs3135344
rs333947
rs144654395
rs72877151
rs212548
rs113855594
rs2229475
All
−0.10
−0.05
0.00
0.05
0.10
MR leave−one−out sensitivity analysis for
'HSPG2' on 'Cardiac valvular disease'

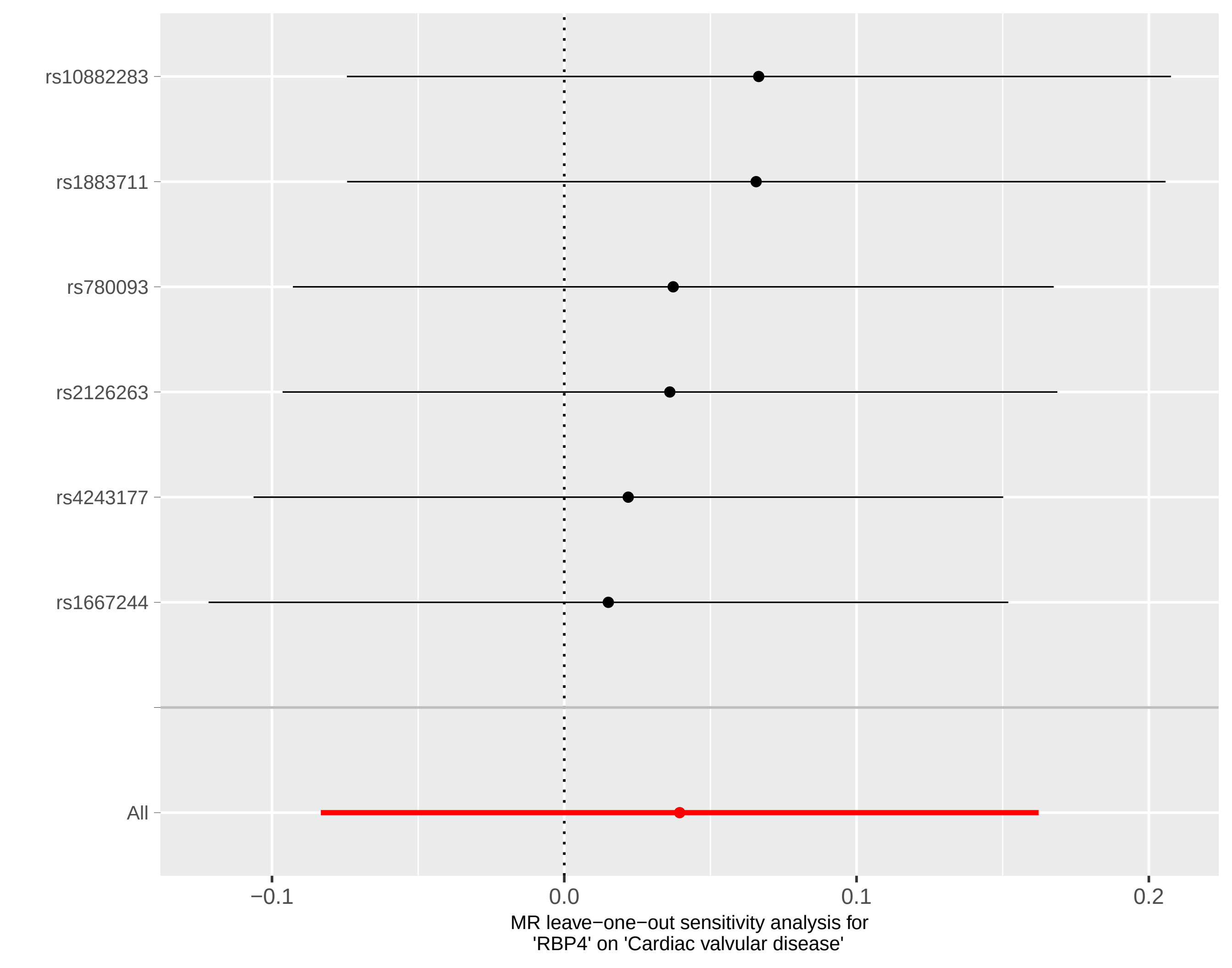
rs10882283
rs1883711
rs780093
rs2126263
rs4243177
rs1667244
All
−0.1
0.0
0.1
0.2
MR leave−one−out sensitivity analysis for
'RBP4' on 'Cardiac valvular disease'

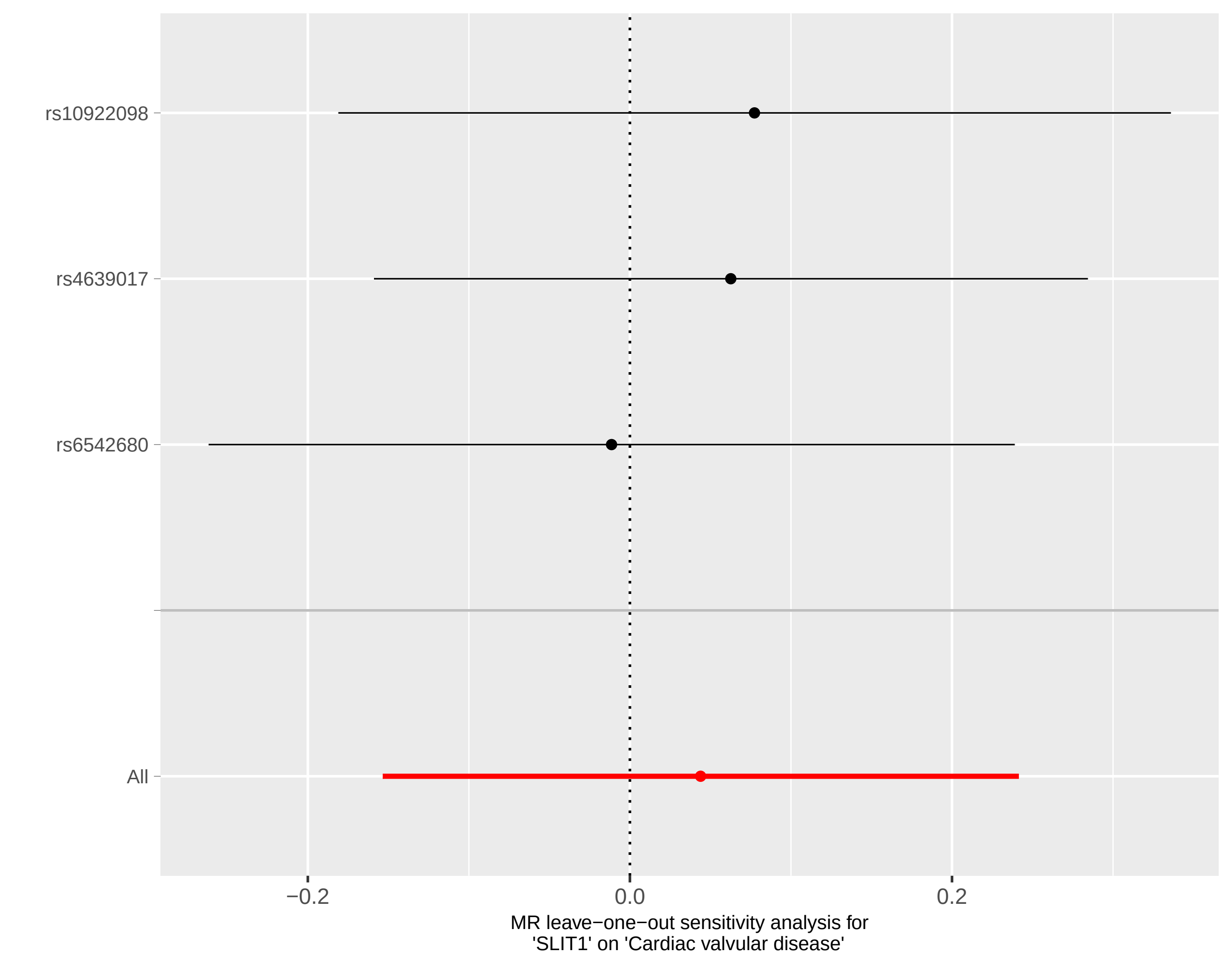
rs10922098
rs4639017
rs6542680
All
−0.2
0.0
0.2
MR leave−one−out sensitivity analysis for
'SLIT1' on 'Cardiac valvular disease'

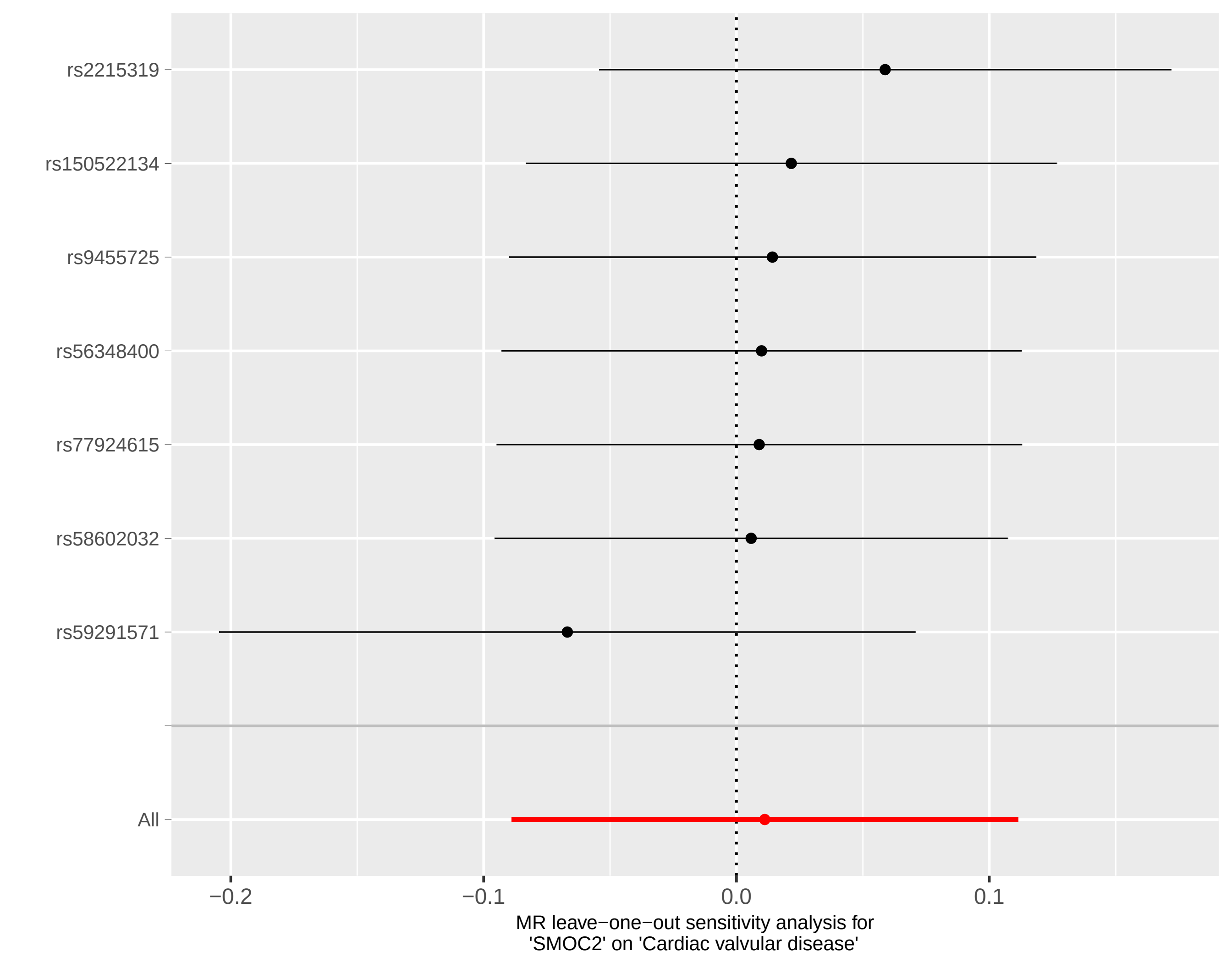
rs2215319
rs150522134
rs9455725
rs56348400
rs77924615
rs58602032
rs59291571
All
−0.2
−0.1
0.0
0.1
MR leave−one−out sensitivity analysis for
'SMOC2' on 'Cardiac valvular disease'

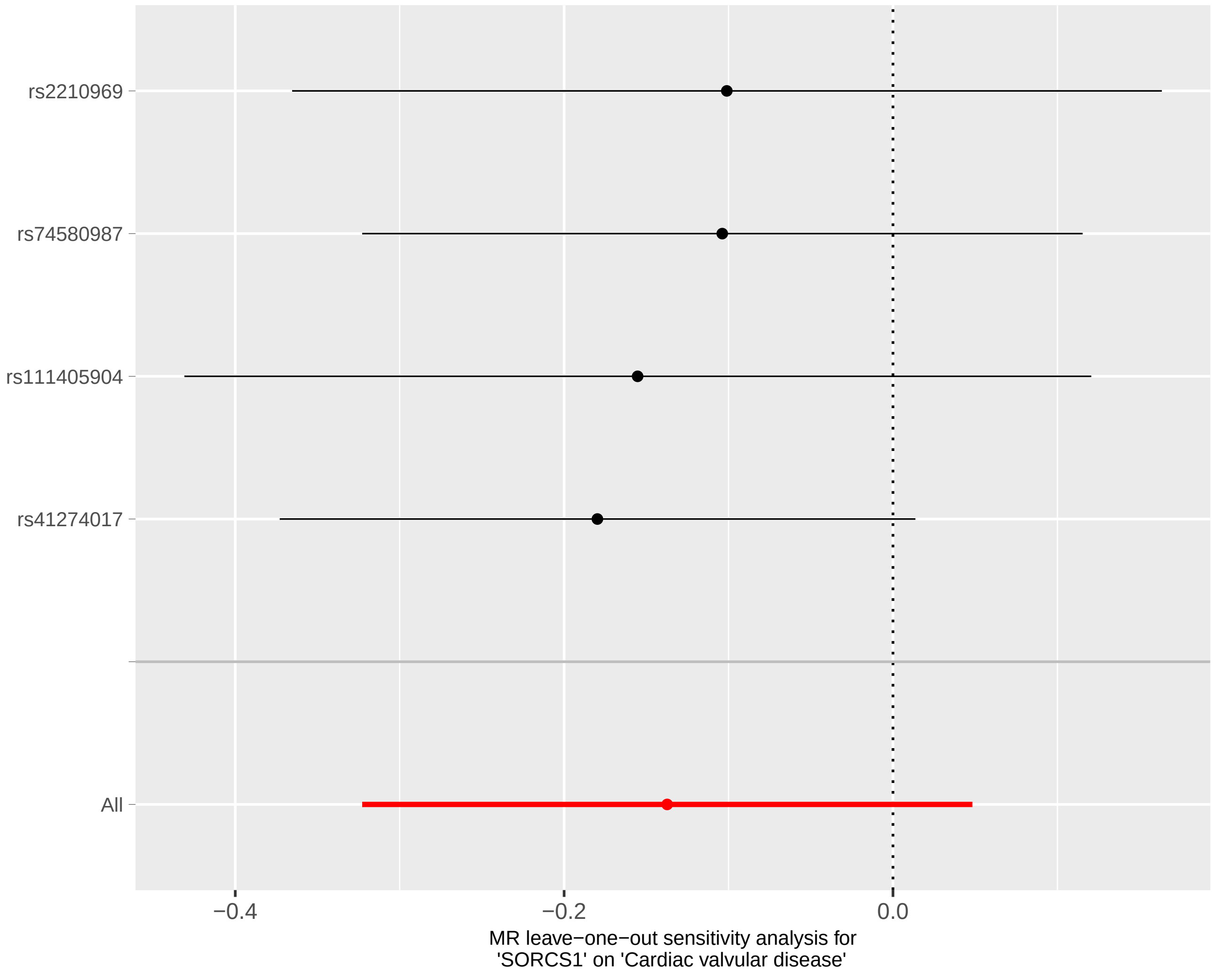
rs2210969
rs74580987
rs111405904
rs41274017
All
−0.4
−0.2
0.0
MR leave−one−out sensitivity analysis for
'SORCS1' on 'Cardiac valvular disease'

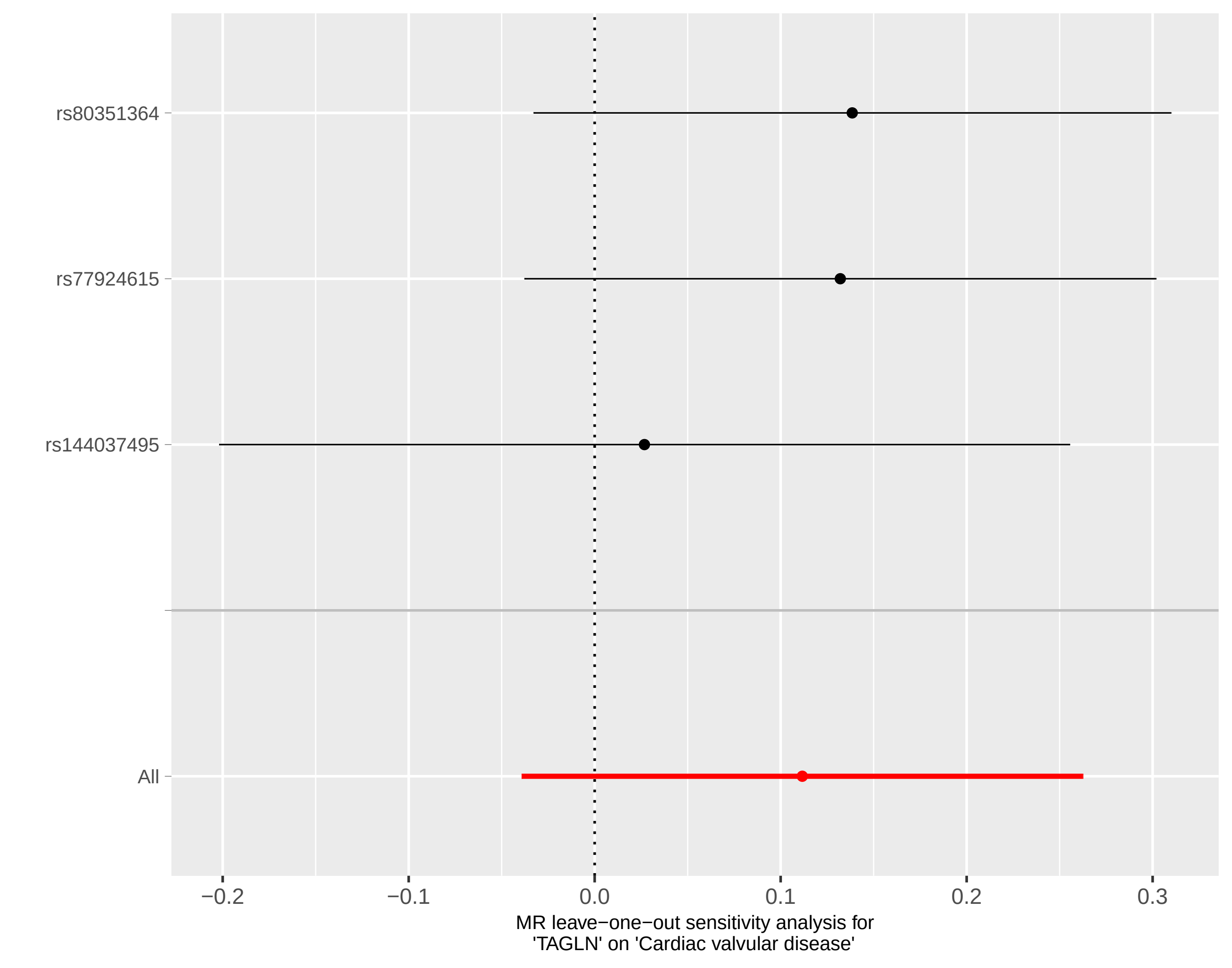
rs80351364
rs77924615
rs144037495
All
−0.2
−0.1
0.0
0.1
0.2
0.3
MR leave−one−out sensitivity analysis for
'TAGLN' on 'Cardiac valvular disease'

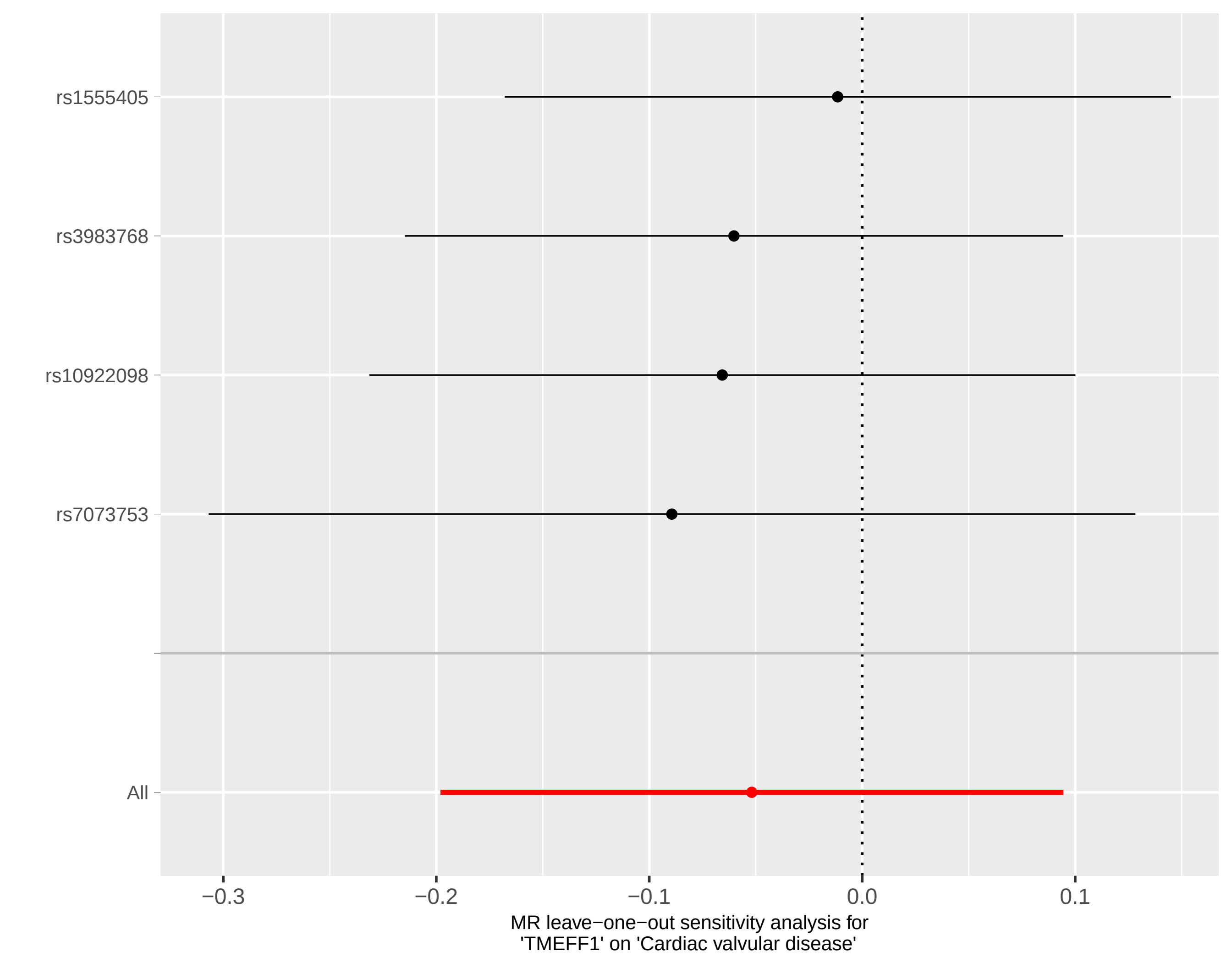

rs1555405
rs3983768
rs10922098
rs7073753
All
−0.3
−0.2
−0.1
0.0
0.1
MR leave−one−out sensitivity analysis for
'TMEFF1' on 'Cardiac valvular disease'

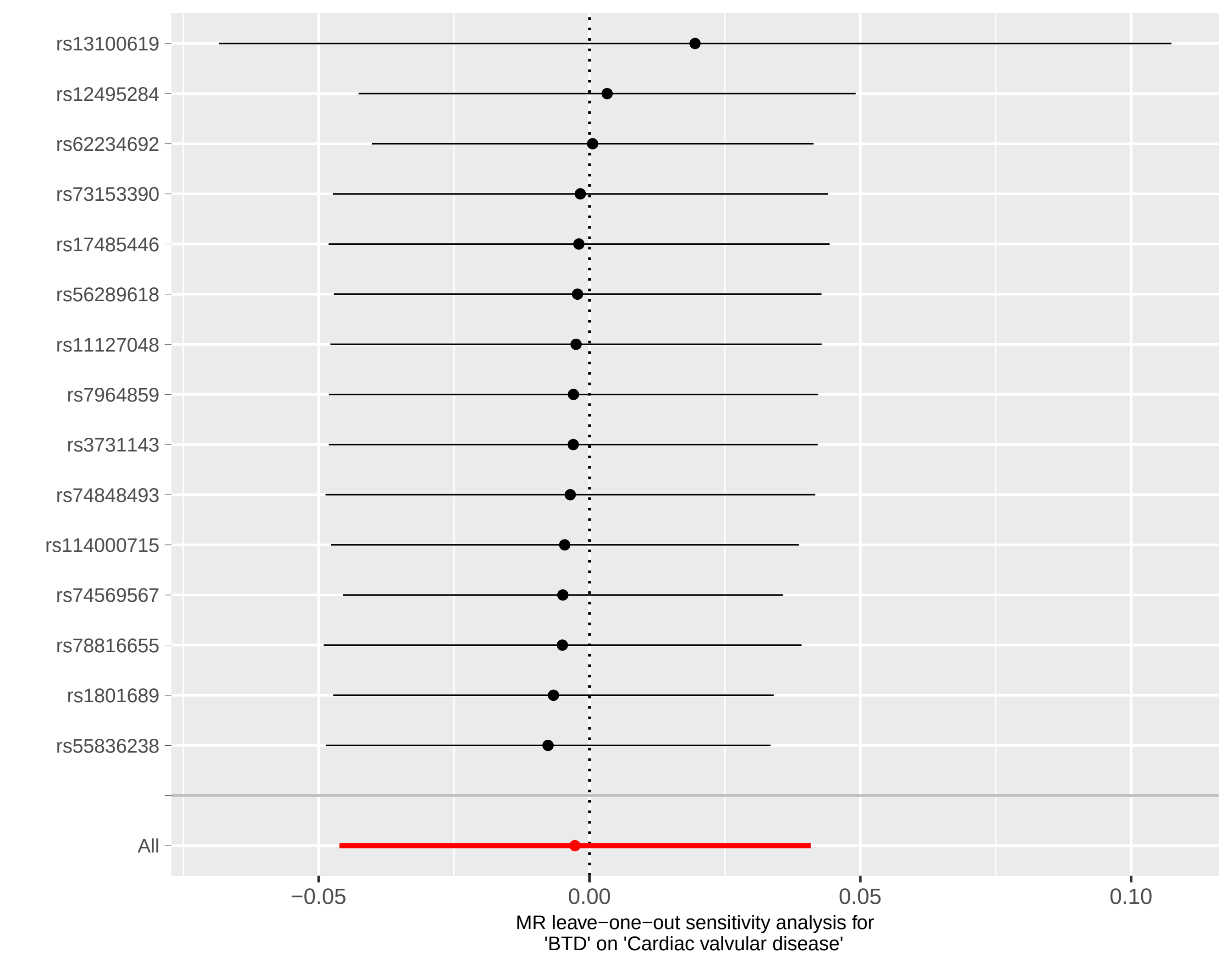
rs13100619
rs12495284
rs62234692
rs73153390
rs17485446
rs56289618
rs11127048
rs7964859
rs3731143
rs74848493
rs114000715
rs74569567
rs78816655
rs1801689
rs55836238
All
−0.05
0.00
0.05
0.10
MR leave−one−out sensitivity analysis for
'BTD' on 'Cardiac valvular disease'

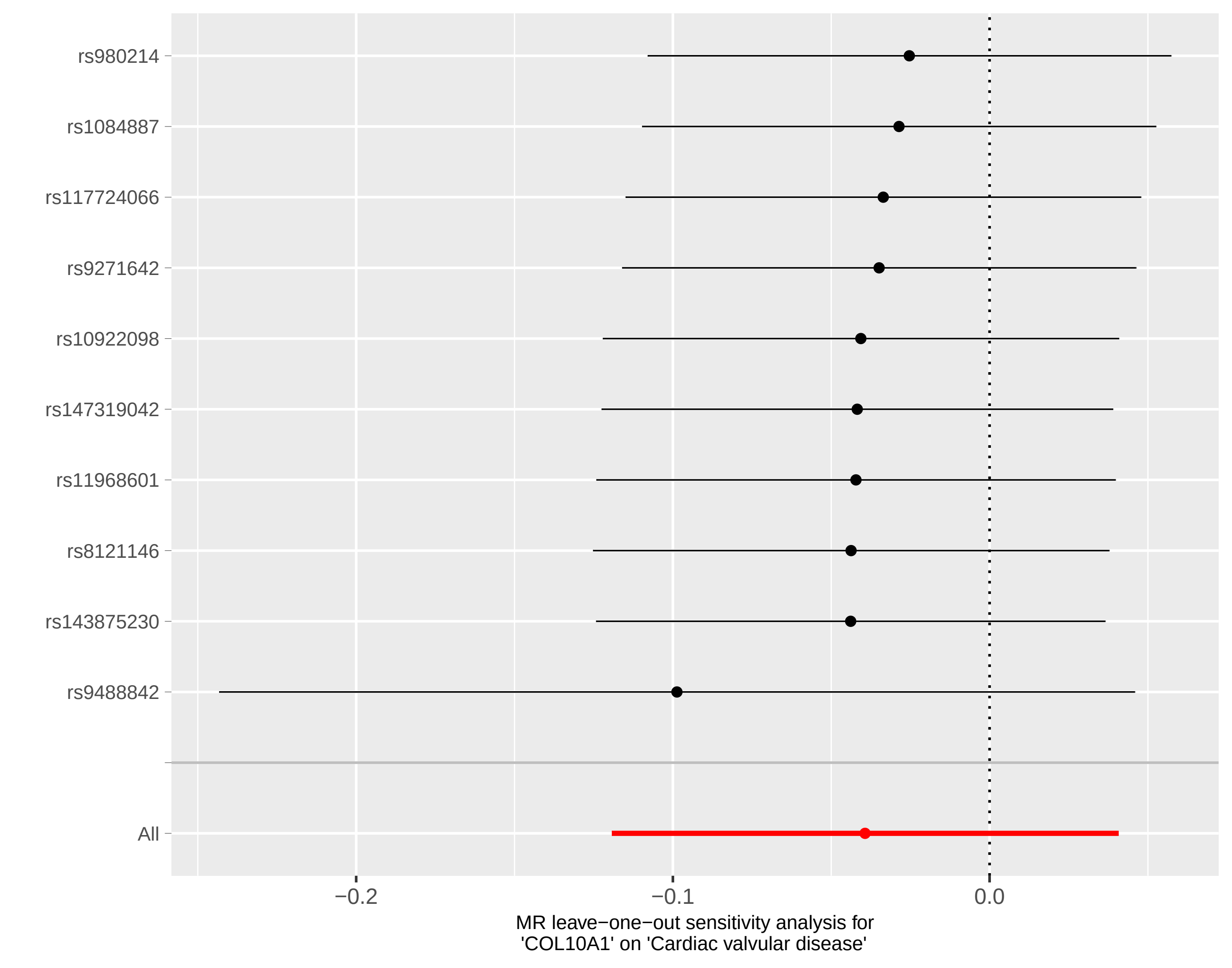
rs980214
rs1084887
rs117724066
rs9271642
rs10922098
rs147319042
rs11968601
rs8121146
rs143875230
rs9488842
All
−0.2
−0.1
0.0
MR leave−one−out sensitivity analysis for
'COL10A1' on 'Cardiac valvular disease'

Insufficient number of SNPs

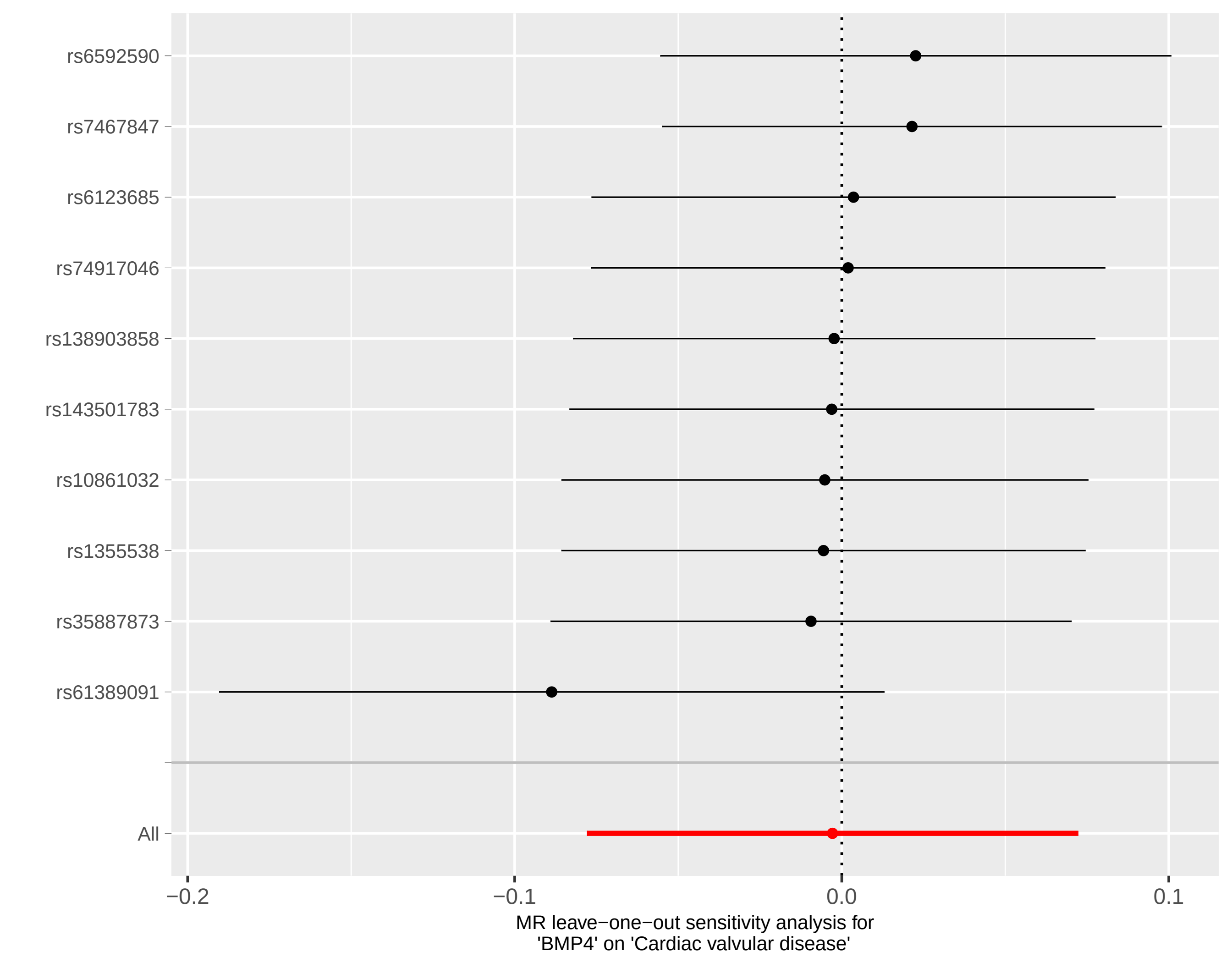

rs6592590
rs7467847
rs6123685
rs74917046
rs138903858
rs143501783
rs10861032
rs1355538
rs35887873
rs61389091
All
−0.2
−0.1
0.0
0.1
MR leave−one−out sensitivity analysis for
'BMP4' on 'Cardiac valvular disease'

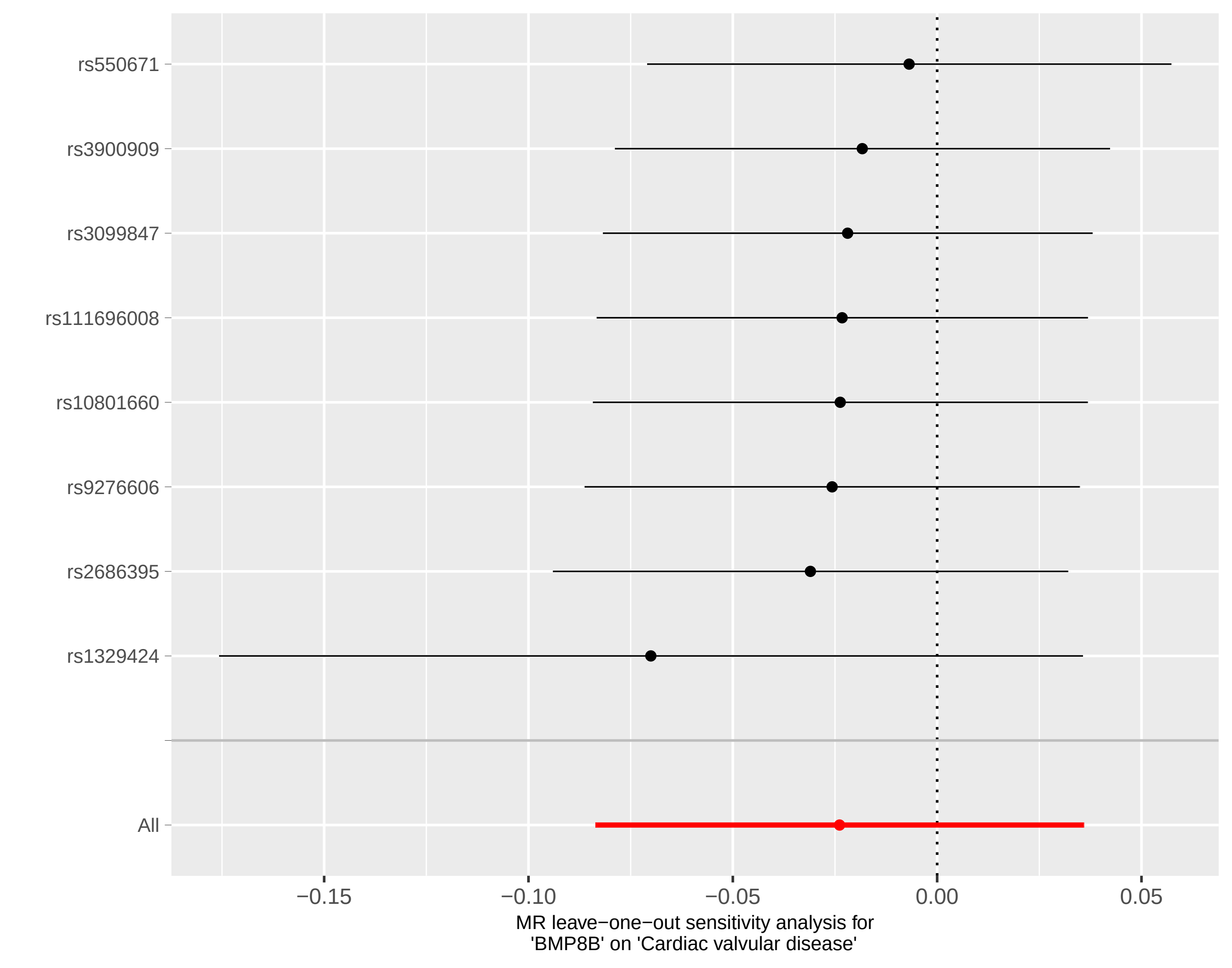
rs550671
rs3900909
rs3099847
rs111696008
rs10801660
rs9276606
rs2686395
rs1329424
All
−0.15
−0.10
−0.05
0.00
0.05
MR leave−one−out sensitivity analysis for
'BMP8B' on 'Cardiac valvular disease'

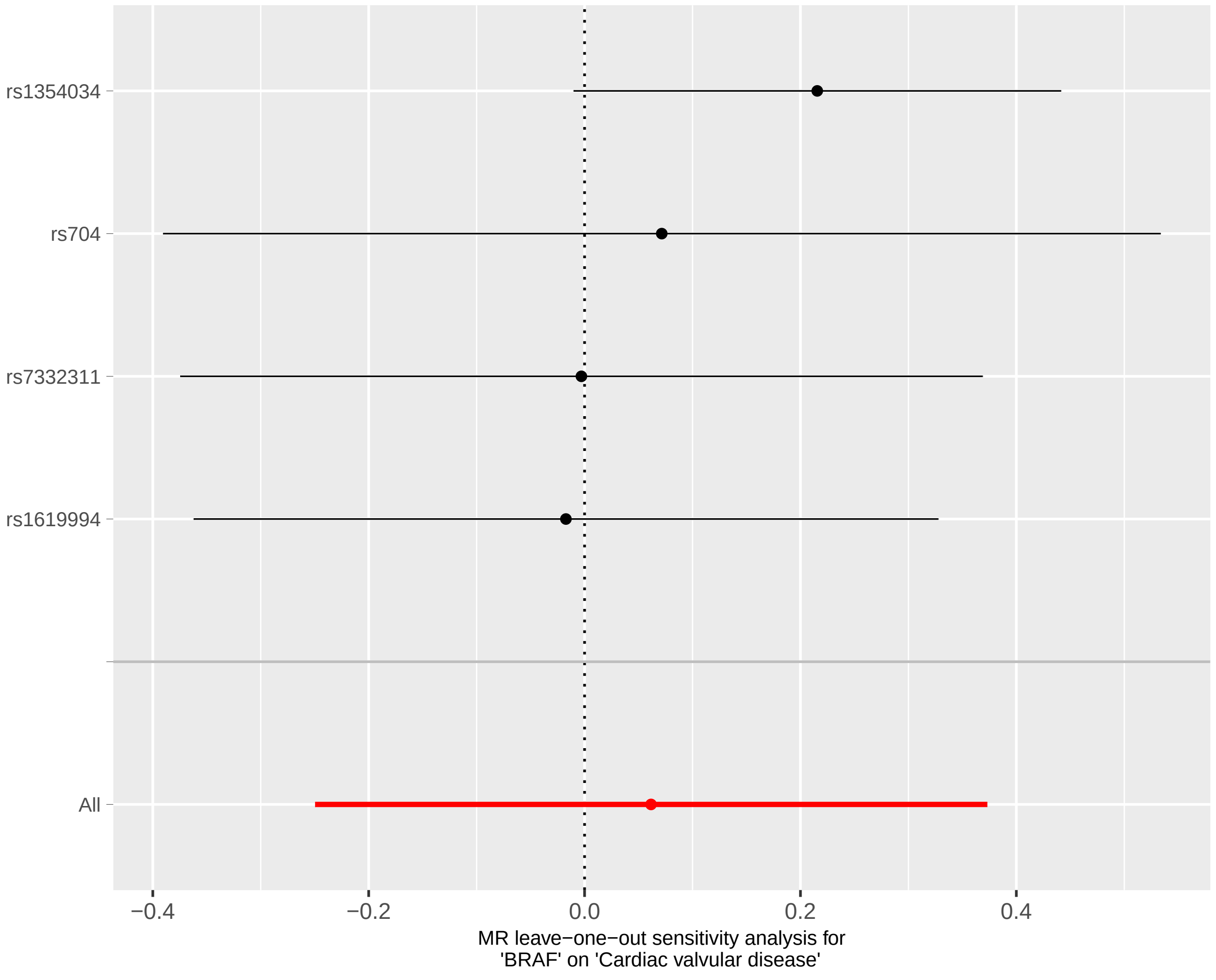

rs1354034
rs704
rs7332311
rs1619994
All
−0.4
−0.2
0.0
0.2
0.4
MR leave−one−out sensitivity analysis for
'BRAF' on 'Cardiac valvular disease'

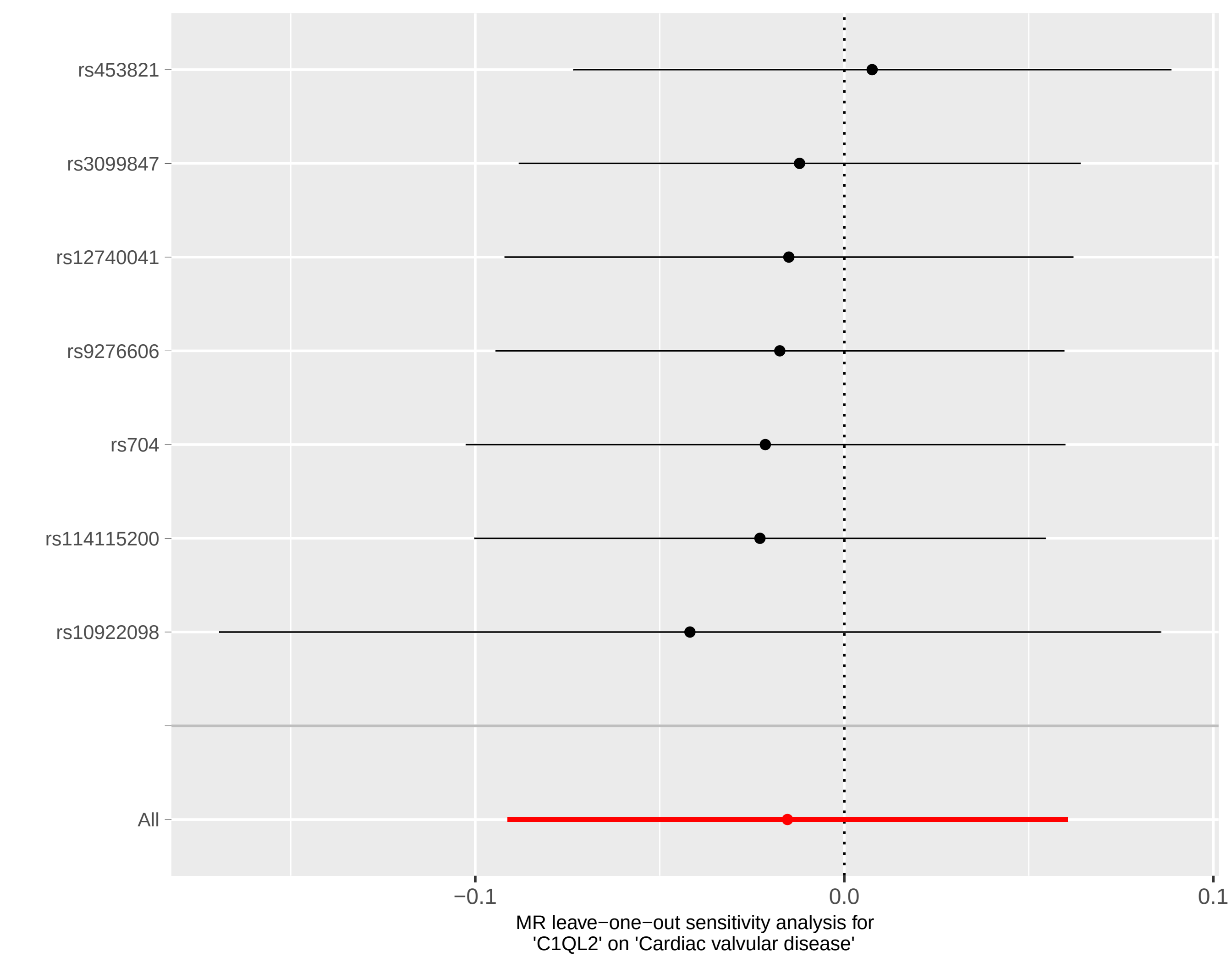

rs453821
rs3099847
rs12740041
rs9276606
rs704
rs114115200
rs10922098
All
−0.1
0.0
0.1
MR leave−one−out sensitivity analysis for
'C1QL2' on 'Cardiac valvular disease'

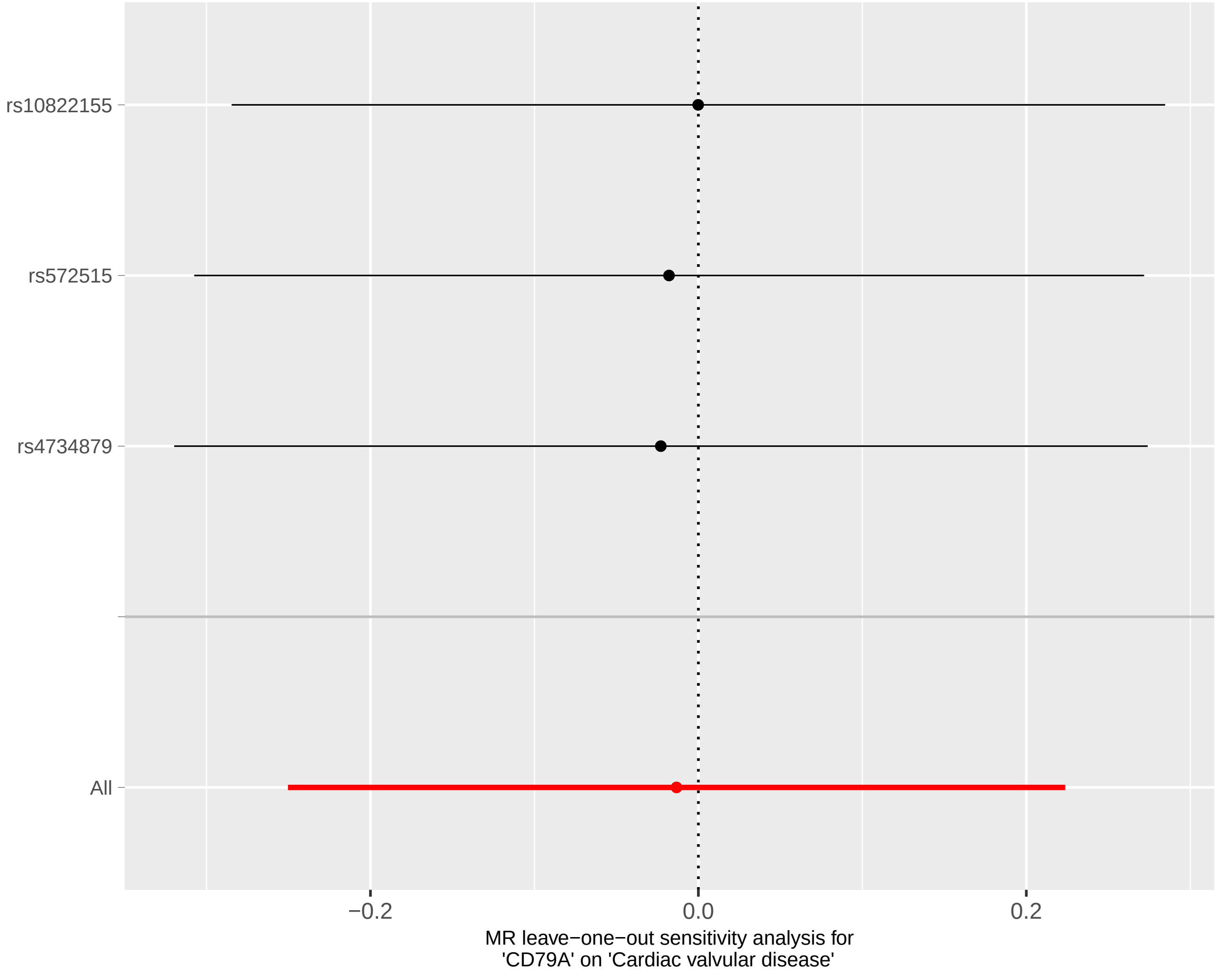
rs10822155
rs572515
rs4734879
All
−0.2
0.0
0.2
MR leave−one−out sensitivity analysis for
'CD79A' on 'Cardiac valvular disease'

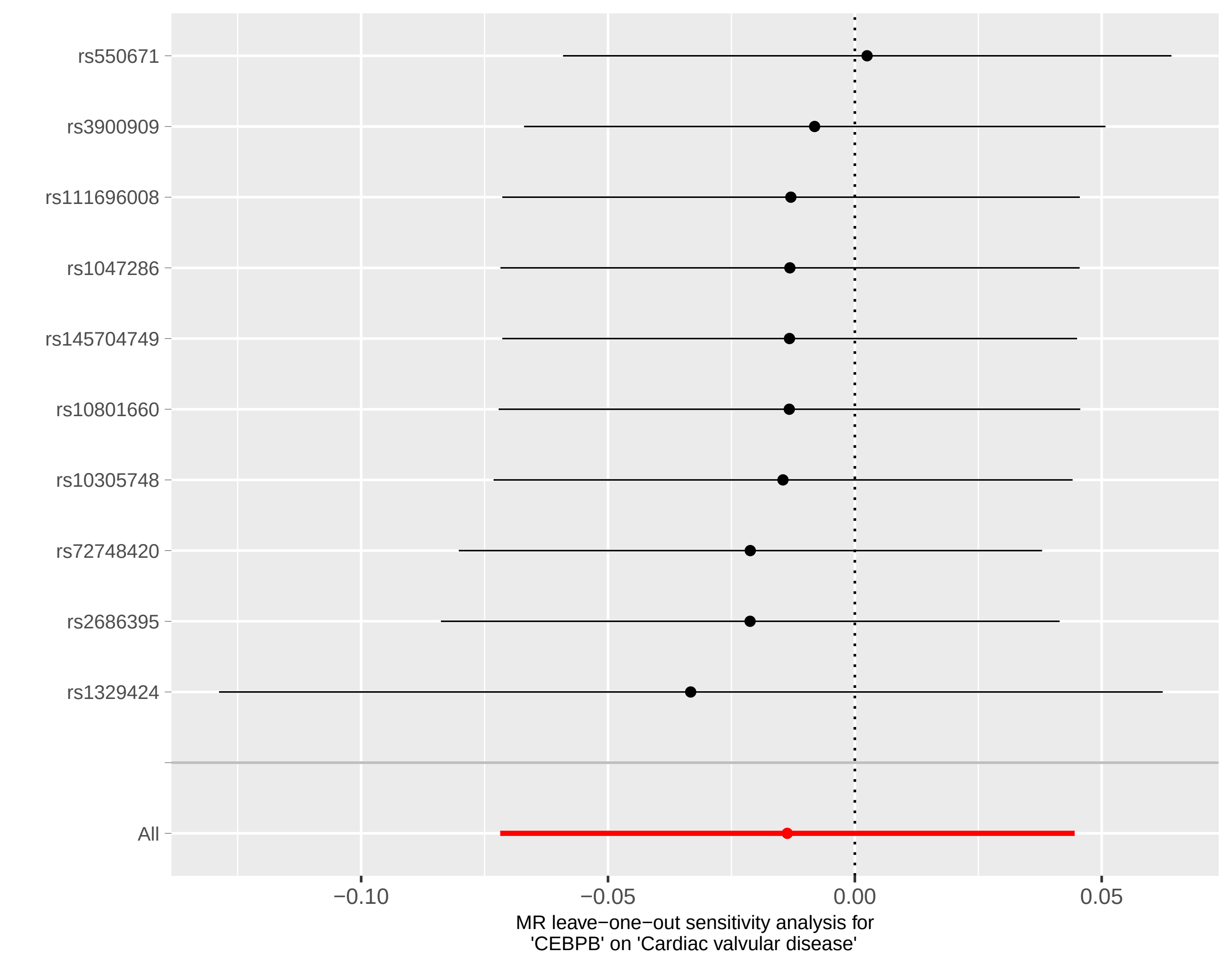

rs550671
rs3900909
rs111696008
rs1047286
rs145704749
rs10801660
rs10305748
rs72748420
rs2686395
rs1329424
All
−0.10
−0.05
0.00
0.05
MR leave−one−out sensitivity analysis for
'CEBPB' on 'Cardiac valvular disease'

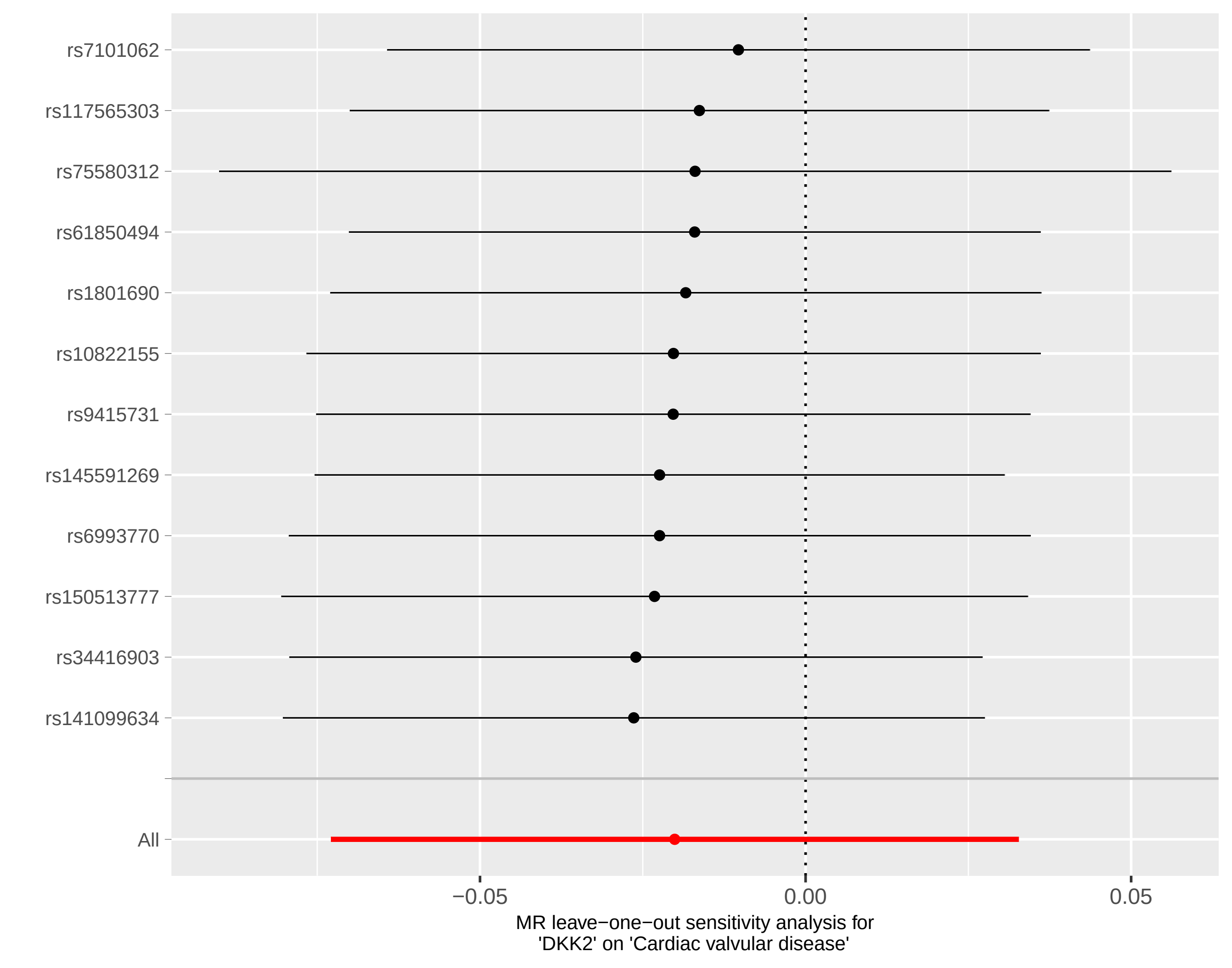
rs7101062
rs117565303
rs75580312
rs61850494
rs1801690
rs10822155
rs9415731
rs145591269
rs6993770
rs150513777
rs34416903
rs141099634
All
−0.05
0.00
0.05
MR leave−one−out sensitivity analysis for
'DKK2' on 'Cardiac valvular disease'

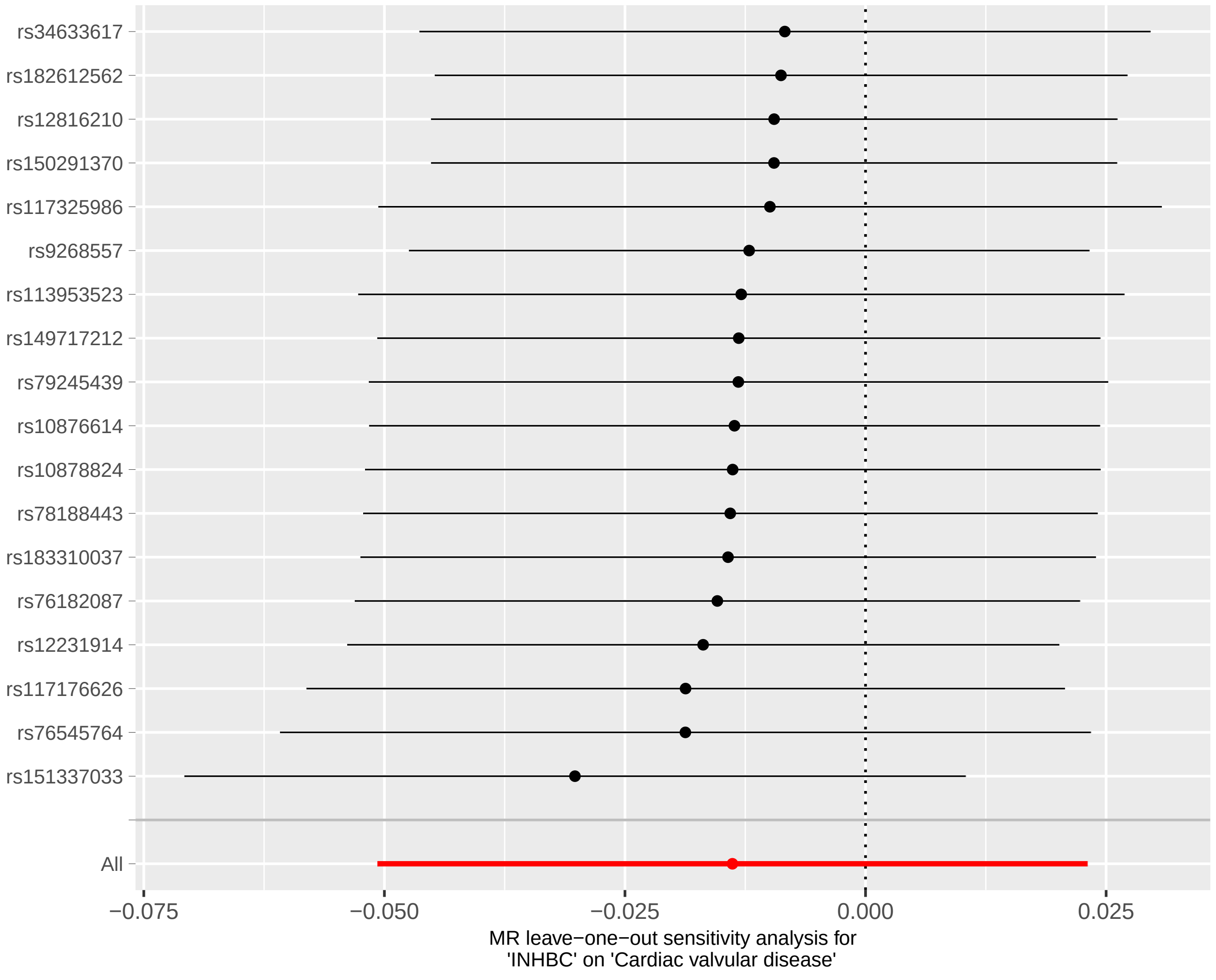
rs34633617
rs182612562
rs12816210
rs150291370
rs117325986
rs9268557
rs113953523
rs149717212
rs79245439
rs10876614
rs10878824
rs78188443
rs183310037
rs76182087
rs12231914
rs117176626
rs76545764
rs151337033
All
−0.075
−0.050
−0.025
0.000
0.025
MR leave−one−out sensitivity analysis for
'INHBC' on 'Cardiac valvular disease'

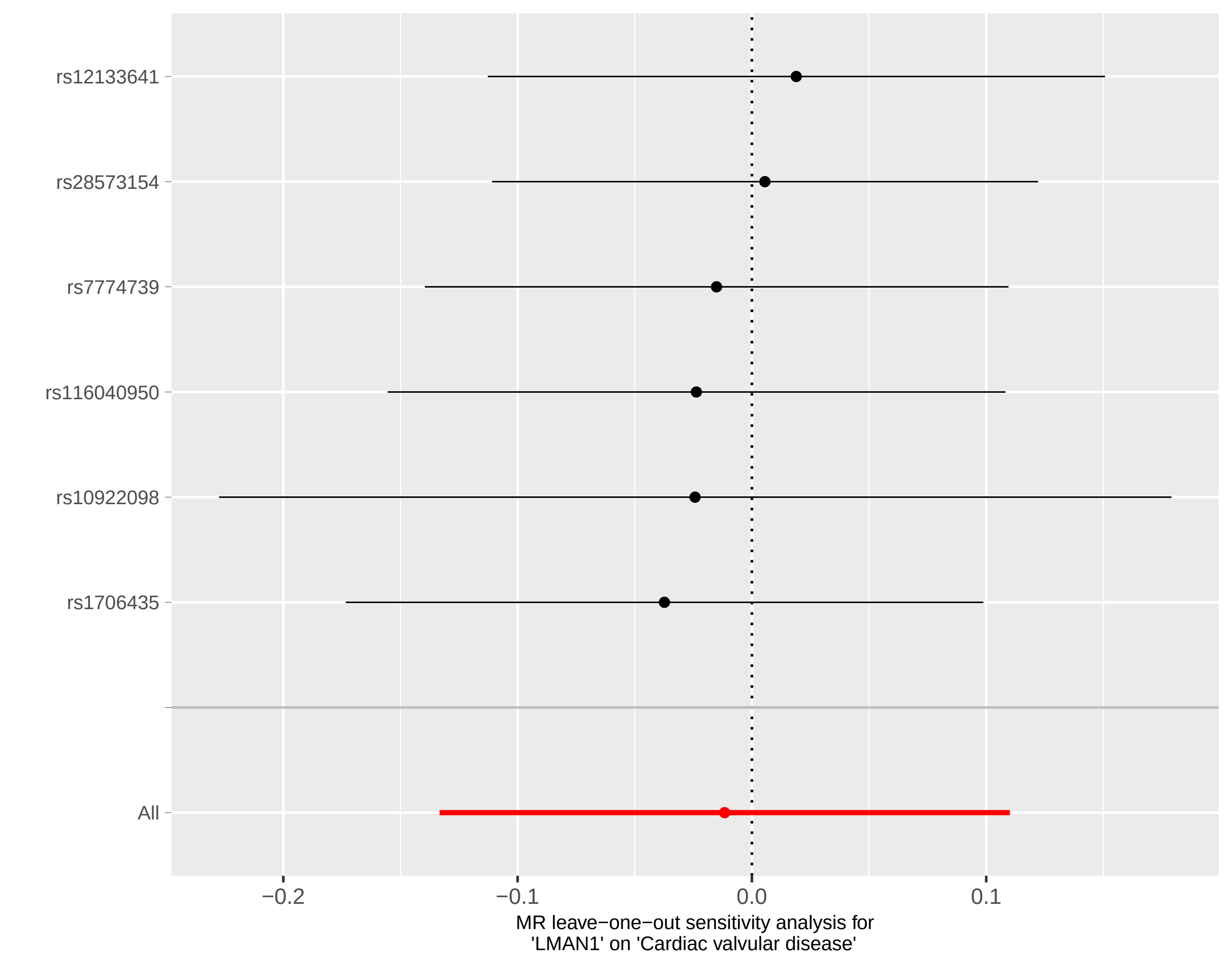
rs12133641
rs28573154
rs7774739
rs116040950
rs10922098
rs1706435
All
−0.2
−0.1
0.0
0.1
MR leave−one−out sensitivity analysis for
'LMAN1' on 'Cardiac valvular disease'

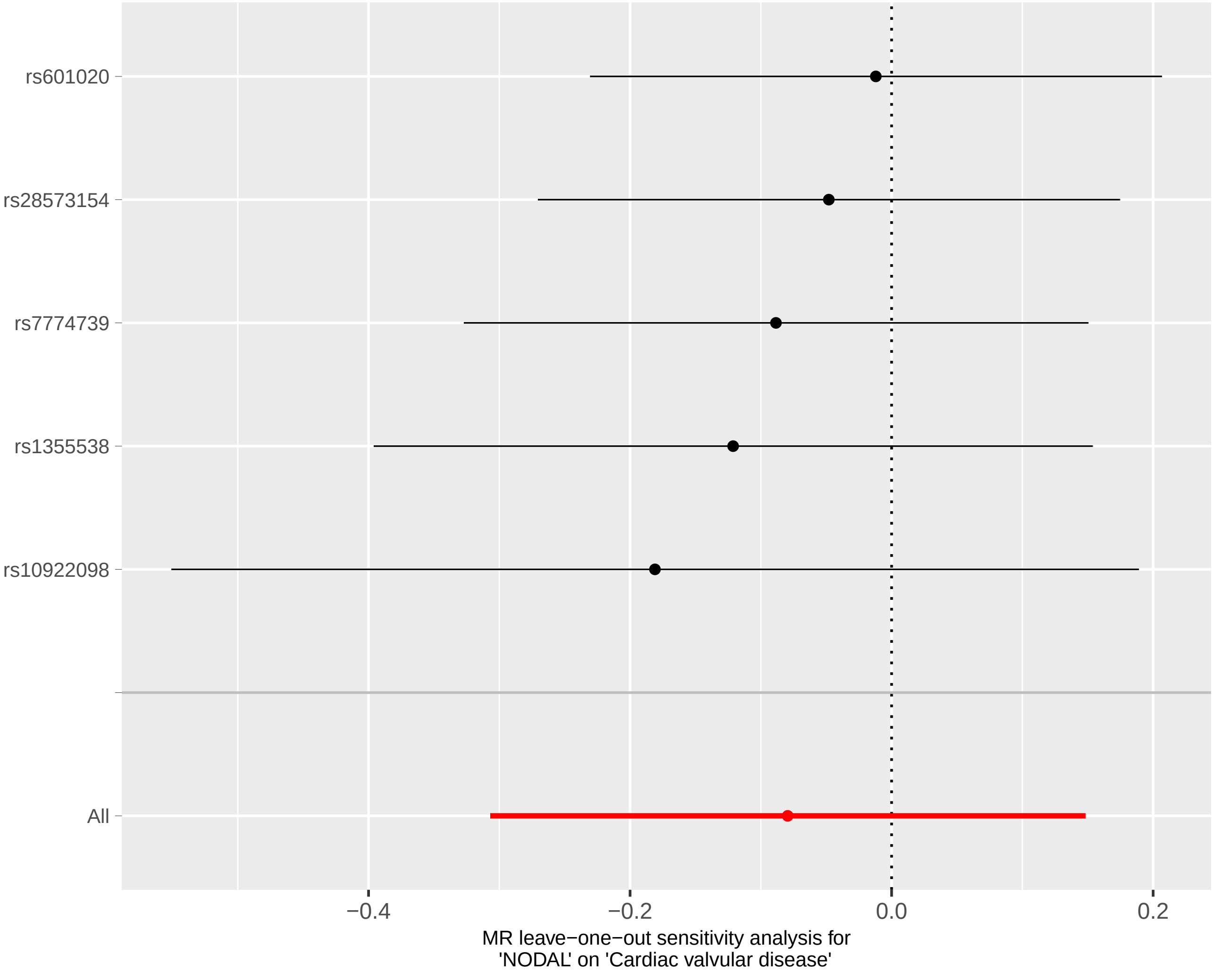

rs601020
rs28573154
rs7774739
rs1355538
rs10922098
All
−0.4
−0.2
0.0
0.2
MR leave−one−out sensitivity analysis for
'NODAL' on 'Cardiac valvular disease'

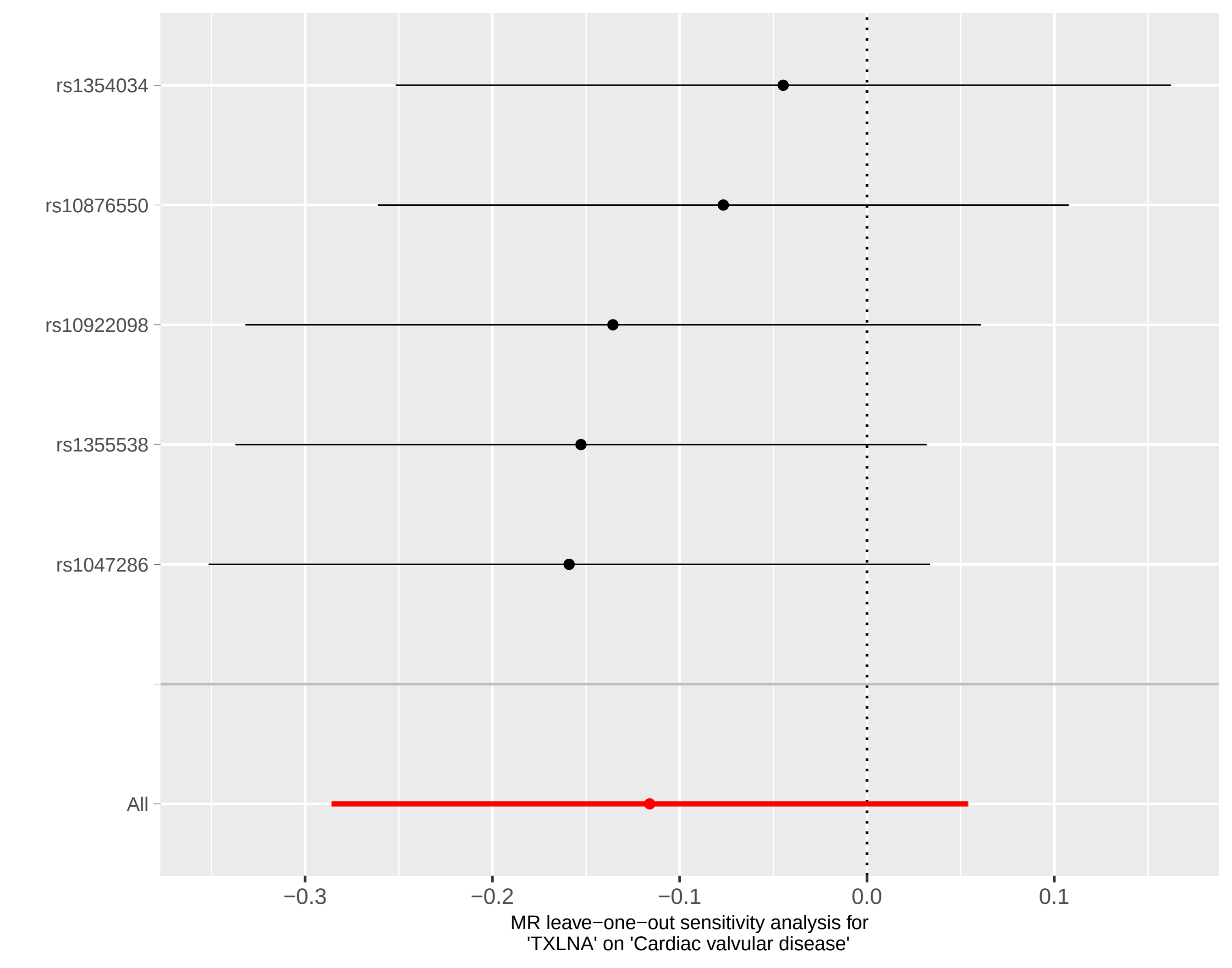

rs1354034
rs10876550
rs10922098
rs1355538
rs1047286
All
−0.3
−0.2
−0.1
0.0
0.1
MR leave−one−out sensitivity analysis for
'TXLNA' on 'Cardiac valvular disease'

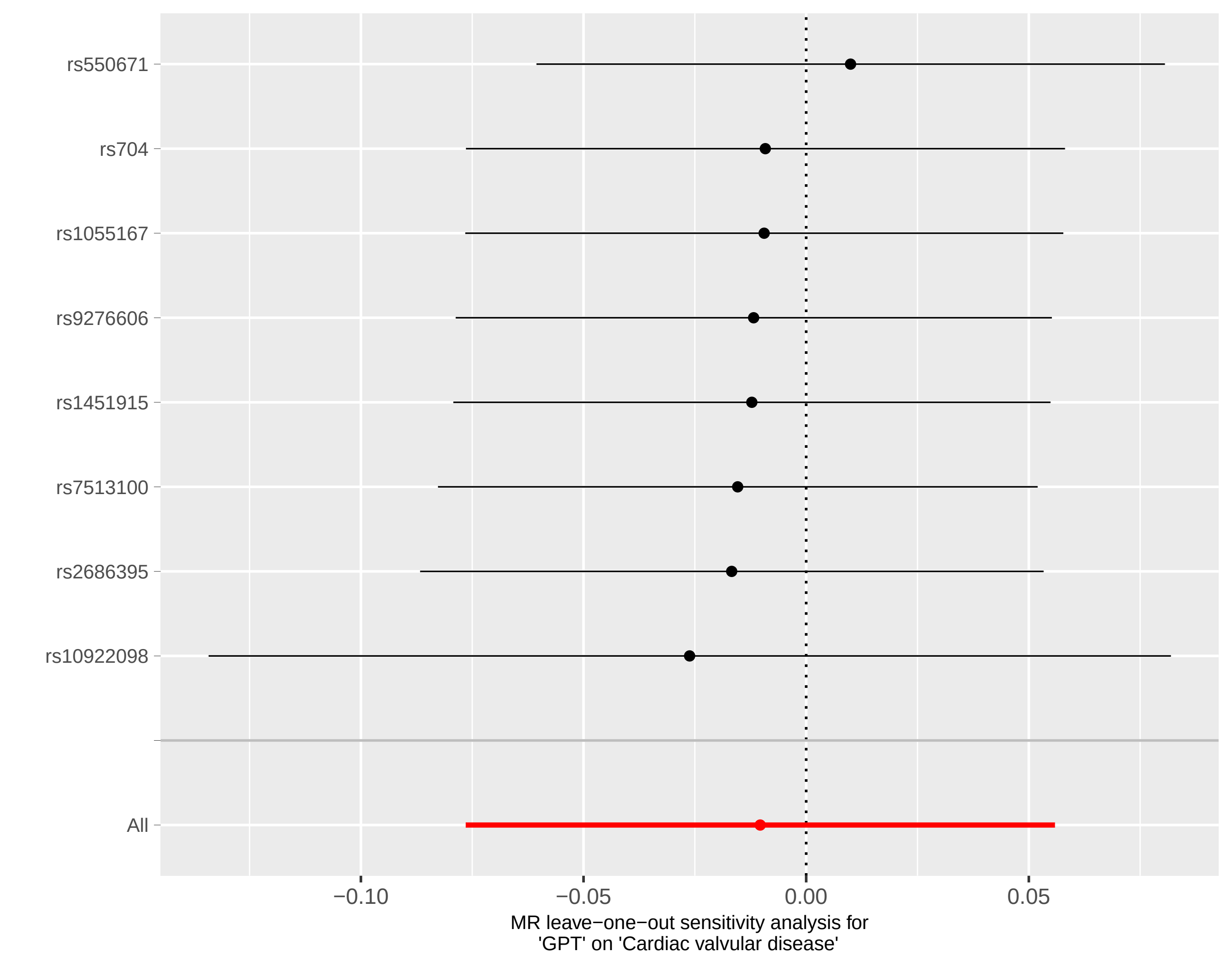

rs550671
rs704
rs1055167
rs9276606
rs1451915
rs7513100
rs2686395
rs10922098
All
−0.10
−0.05
0.00
0.05
MR leave−one−out sensitivity analysis for
'GPT' on 'Cardiac valvular disease'

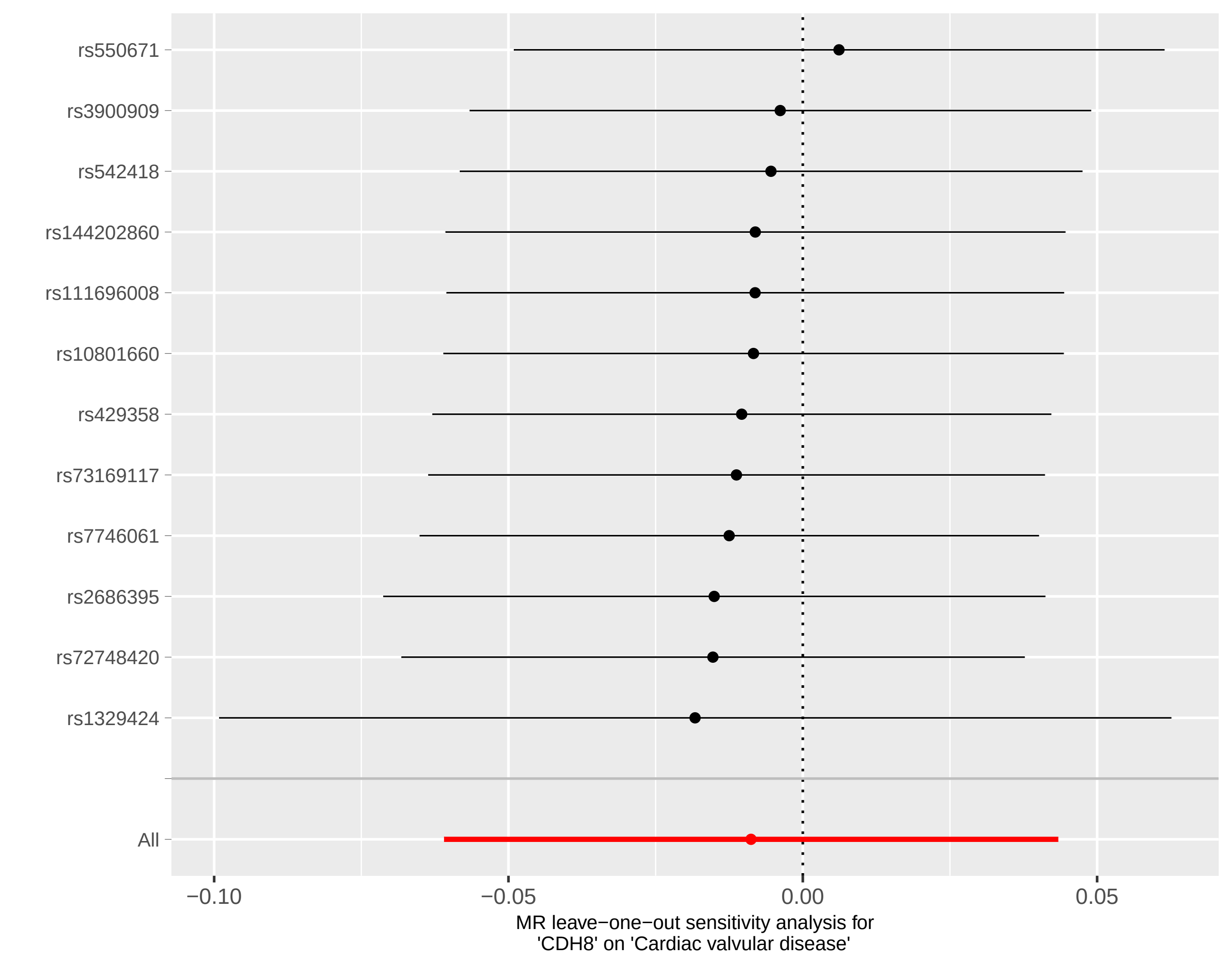
rs550671
rs3900909
rs542418
rs144202860
rs111696008
rs10801660
rs429358
rs73169117
rs7746061
rs2686395
rs72748420
rs1329424
All
−0.10
−0.05
0.00
0.05
MR leave−one−out sensitivity analysis for
'CDH8' on 'Cardiac valvular disease'

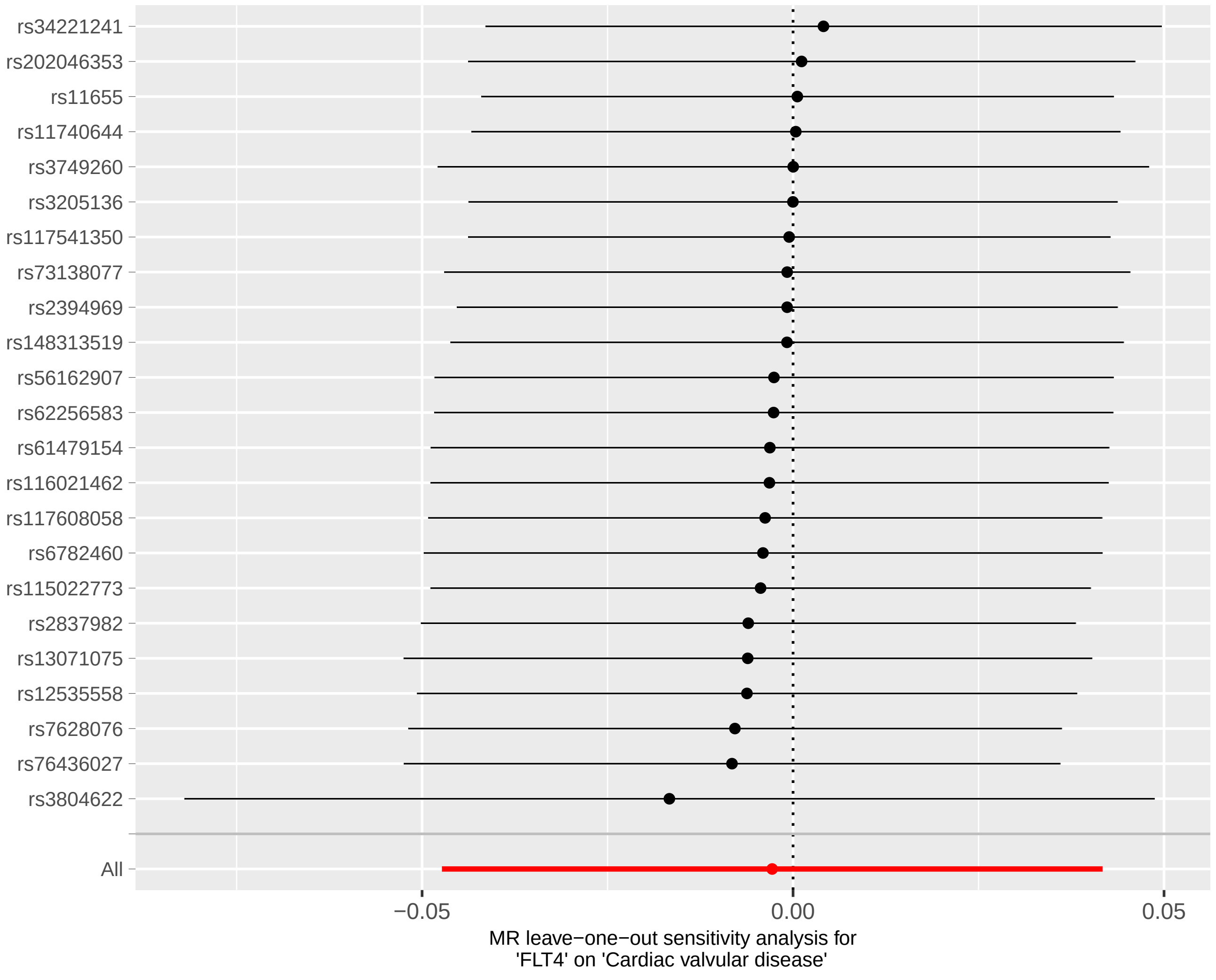
rs34221241
rs202046353
rs11655
rs11740644
rs3749260
rs3205136
rs117541350
rs73138077
rs2394969
rs148313519
rs56162907
rs62256583
rs61479154
rs116021462
rs117608058
rs6782460
rs115022773
rs2837982
rs13071075
rs12535558
rs7628076
rs76436027
rs3804622
All
−0.05
0.00
0.05
MR leave−one−out sensitivity analysis for
'FLT4' on 'Cardiac valvular disease'

Insufficient number of SNPs

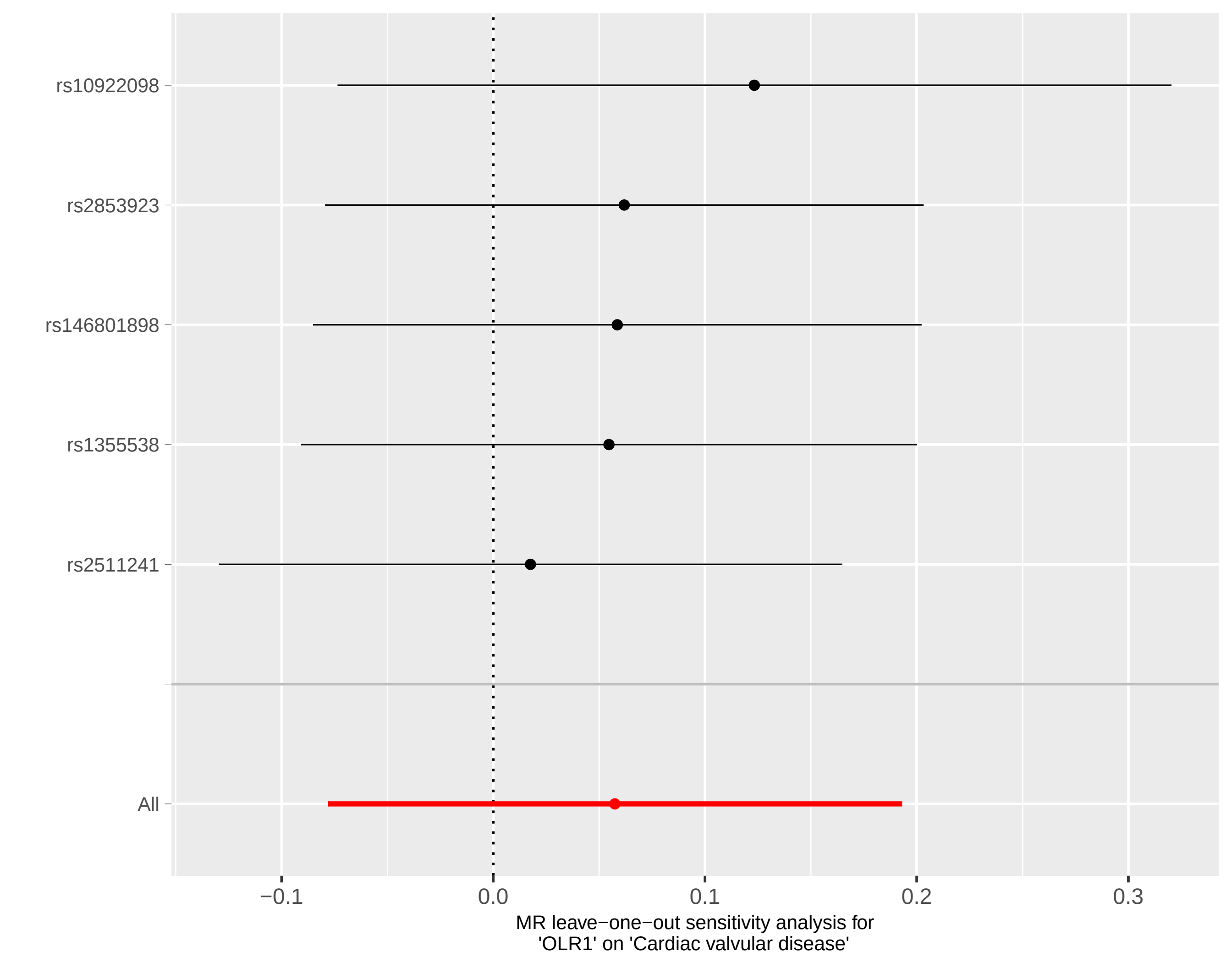

rs10922098
rs2853923
rs146801898
rs1355538
rs2511241
All
−0.1
0.0
0.1
0.2
0.3
MR leave−one−out sensitivity analysis for
'OLR1' on 'Cardiac valvular disease'

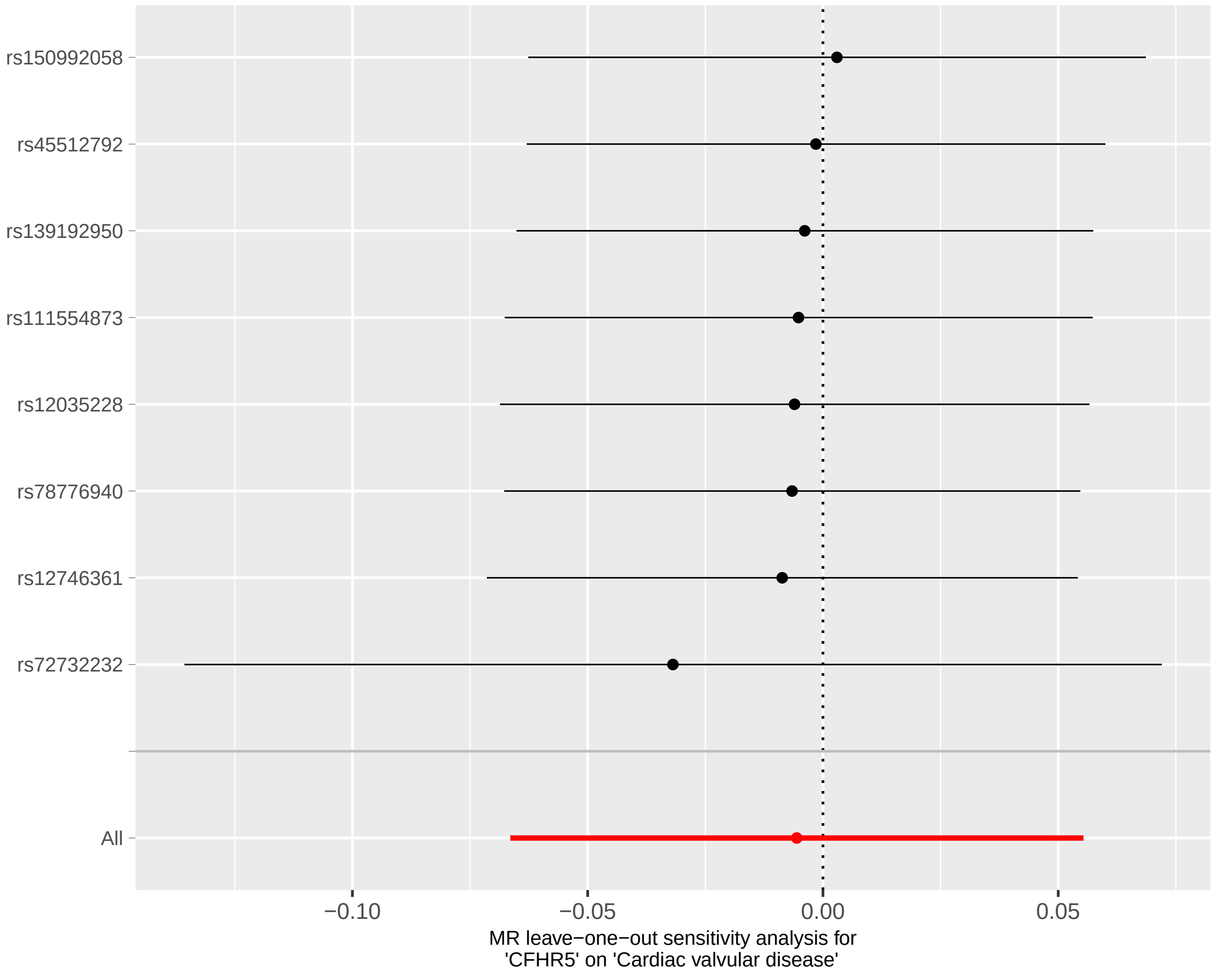
rs150992058
rs45512792
rs139192950
rs111554873
rs12035228
rs78776940
rs12746361
rs72732232
All
−0.10
−0.05
0.00
0.05
MR leave−one−out sensitivity analysis for
'CFHR5' on 'Cardiac valvular disease'

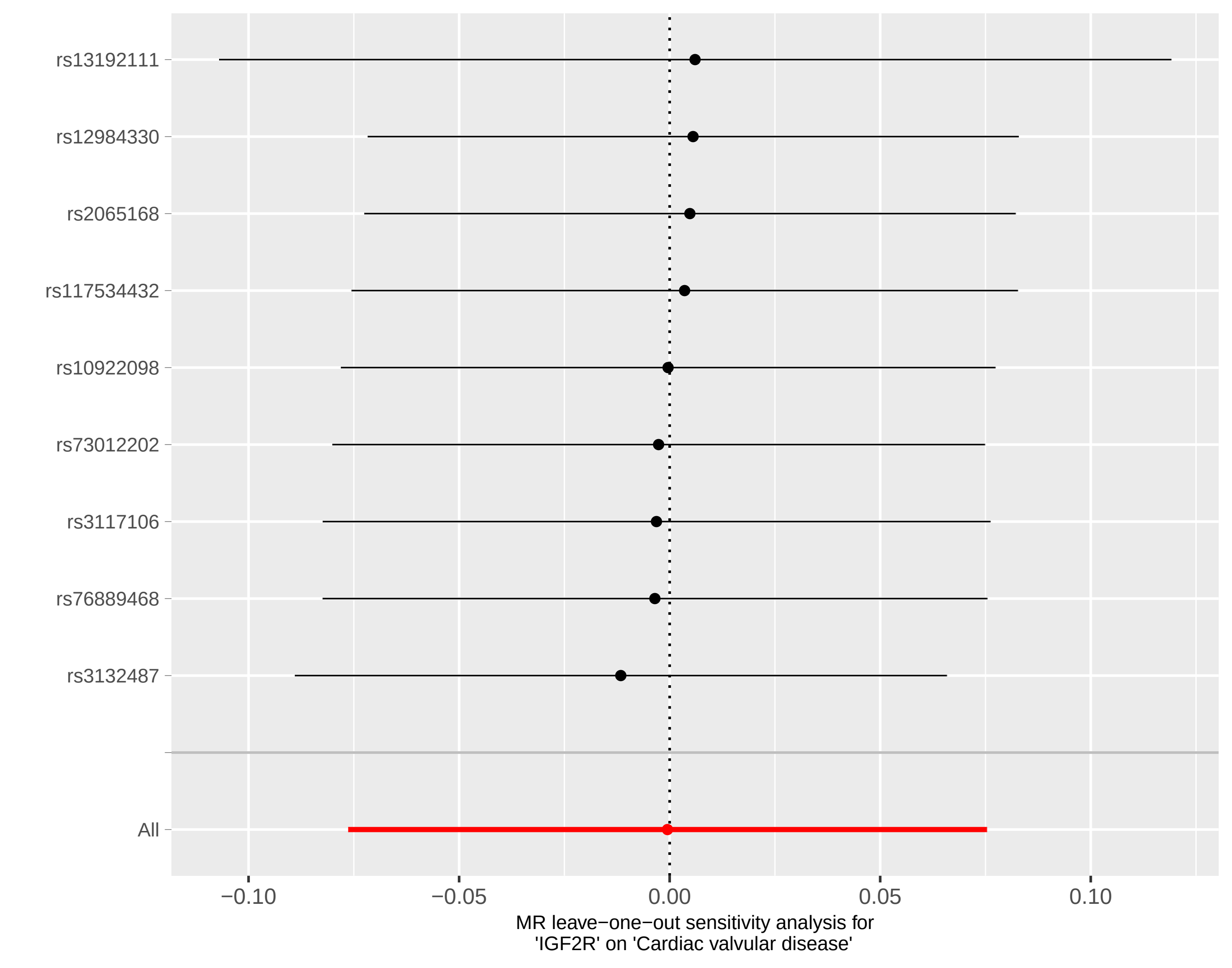
rs13192111
rs12984330
rs2065168
rs117534432
rs10922098
rs73012202
rs3117106
rs76889468
rs3132487
All
−0.10
−0.05
0.00
0.05
0.10
MR leave−one−out sensitivity analysis for
'IGF2R' on 'Cardiac valvular disease'

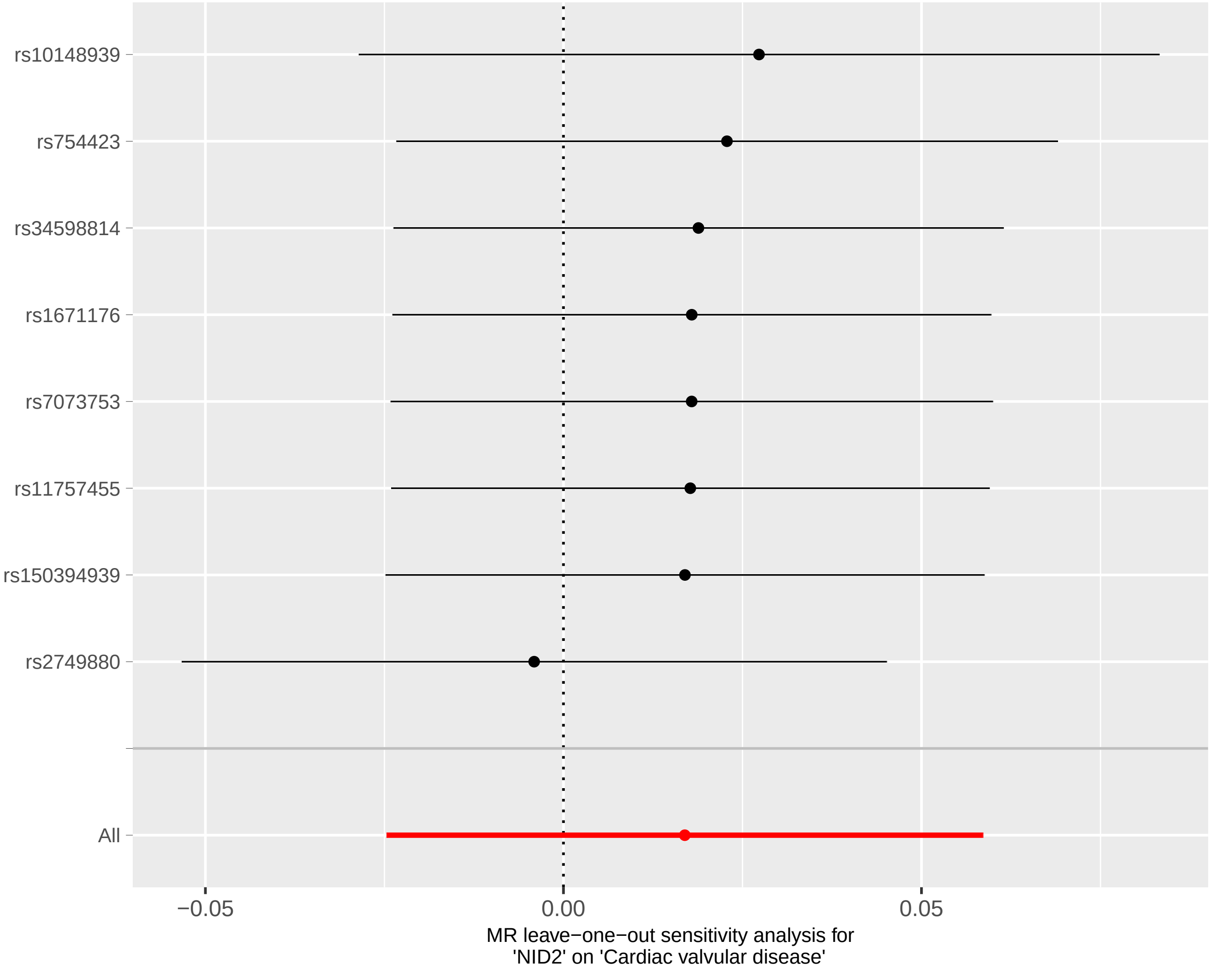

rs10148939
rs754423
rs34598814
rs1671176
rs7073753
rs11757455
rs150394939
rs2749880
All
−0.05
0.00
0.05
MR leave−one−out sensitivity analysis for
'NID2' on 'Cardiac valvular disease'

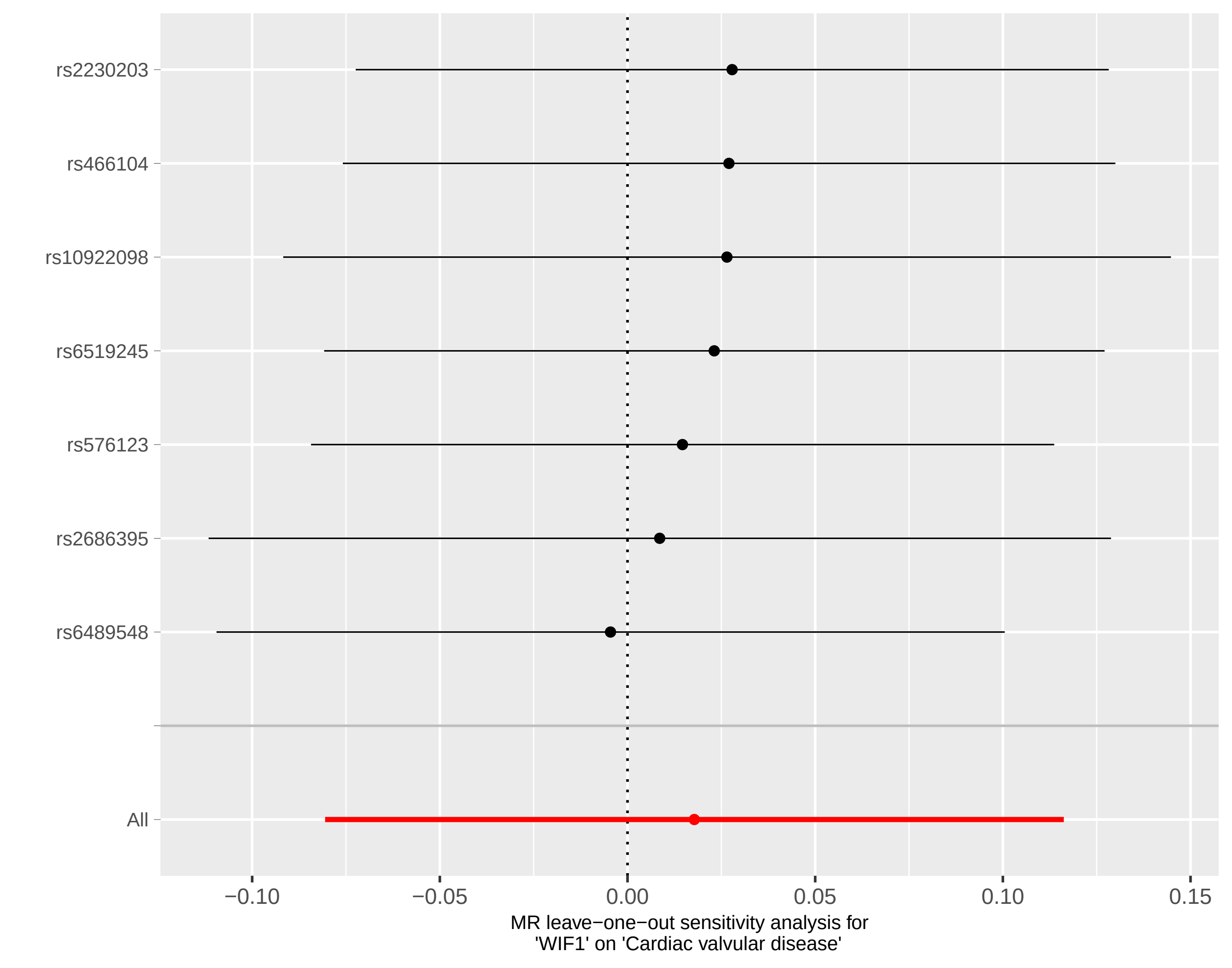
rs2230203
rs466104
rs10922098
rs6519245
rs576123
rs2686395
rs6489548
All
−0.10
−0.05
0.00
0.05
0.10
0.15
MR leave−one−out sensitivity analysis for
'WIF1' on 'Cardiac valvular disease'

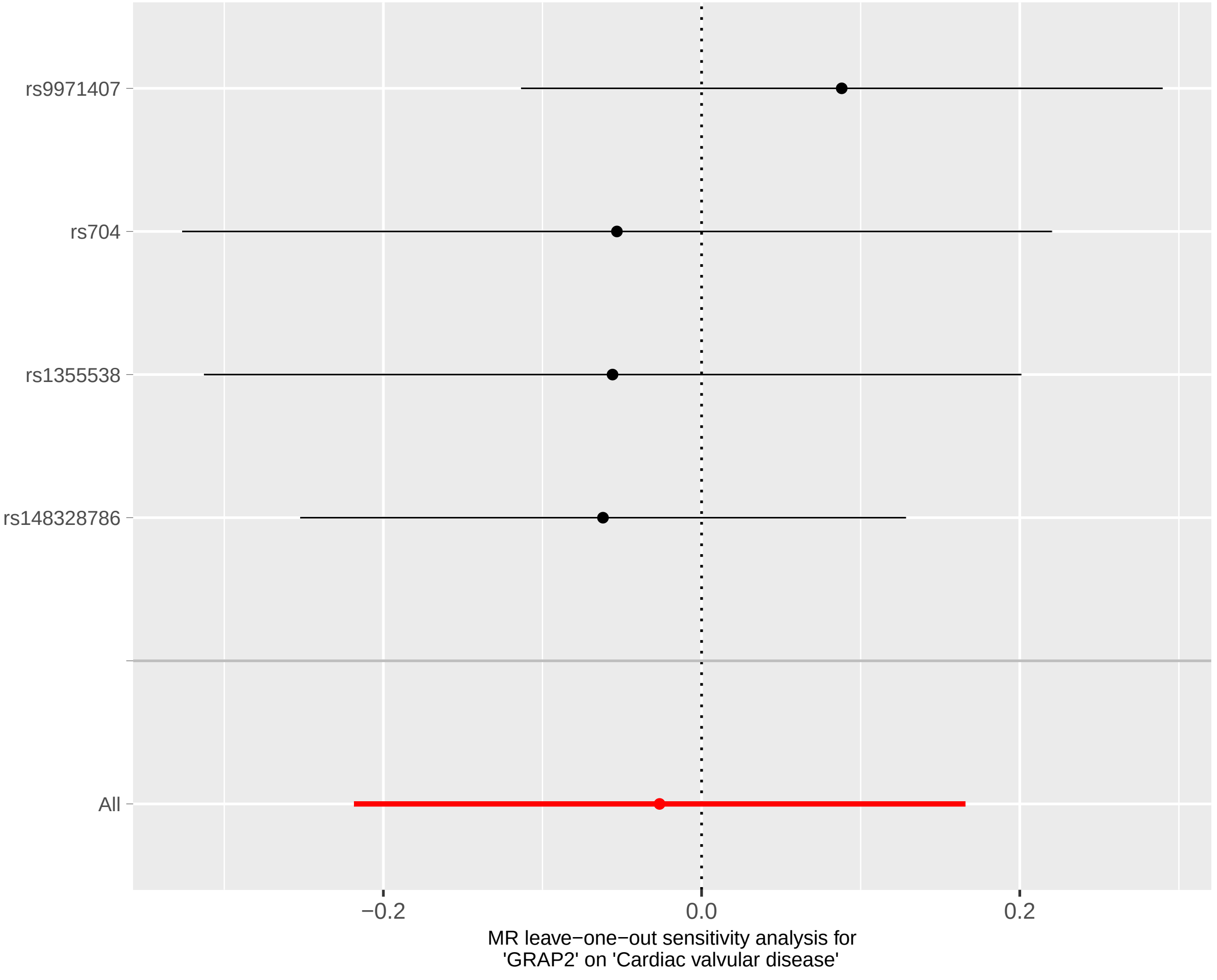
rs9971407
rs704
rs1355538
rs148328786
All
−0.2
0.0
0.2
MR leave−one−out sensitivity analysis for
'GRAP2' on 'Cardiac valvular disease'

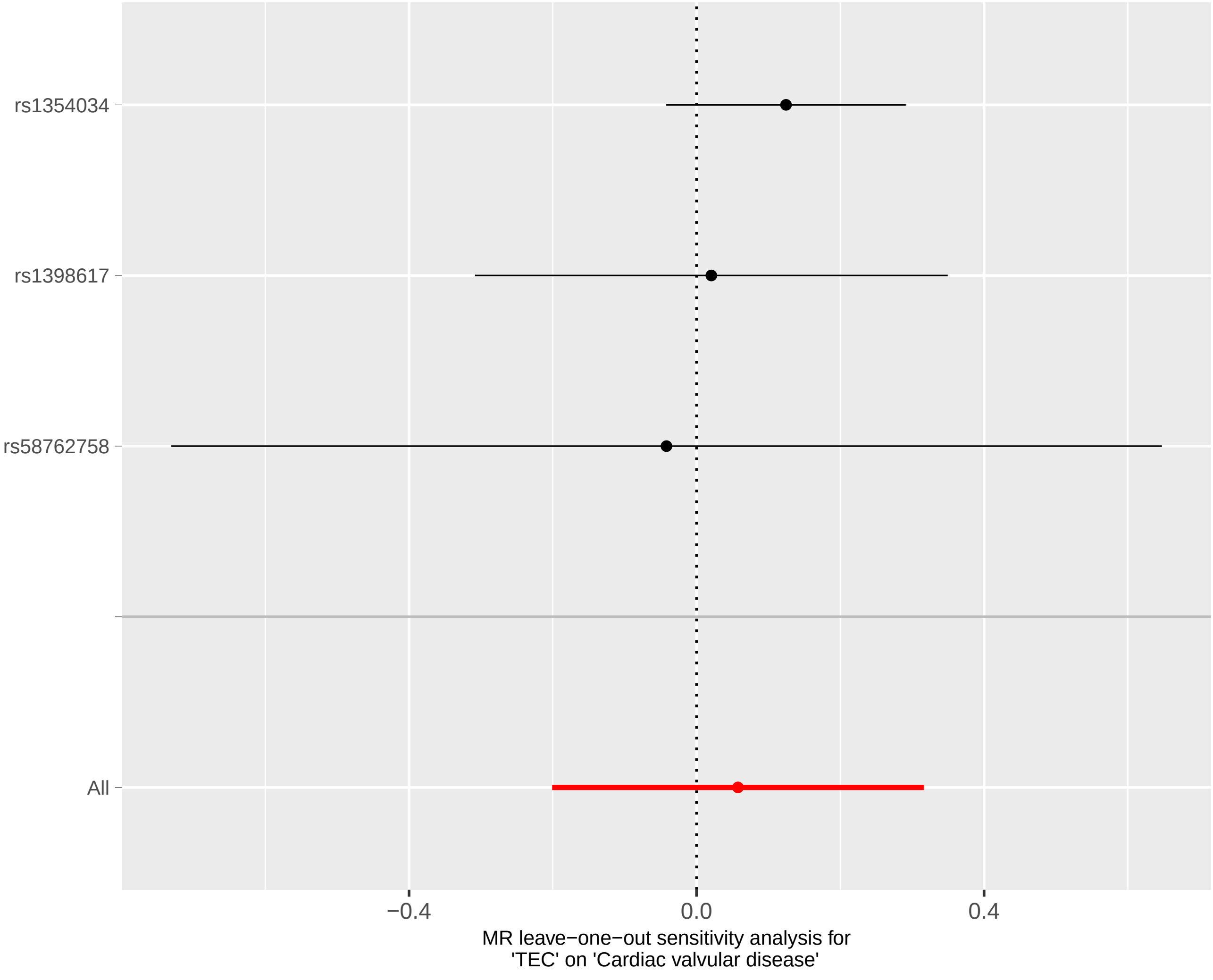
rs1354034
rs1398617
rs58762758
All
−0.4
0.0
0.4
MR leave−one−out sensitivity analysis for
'TEC' on 'Cardiac valvular disease'

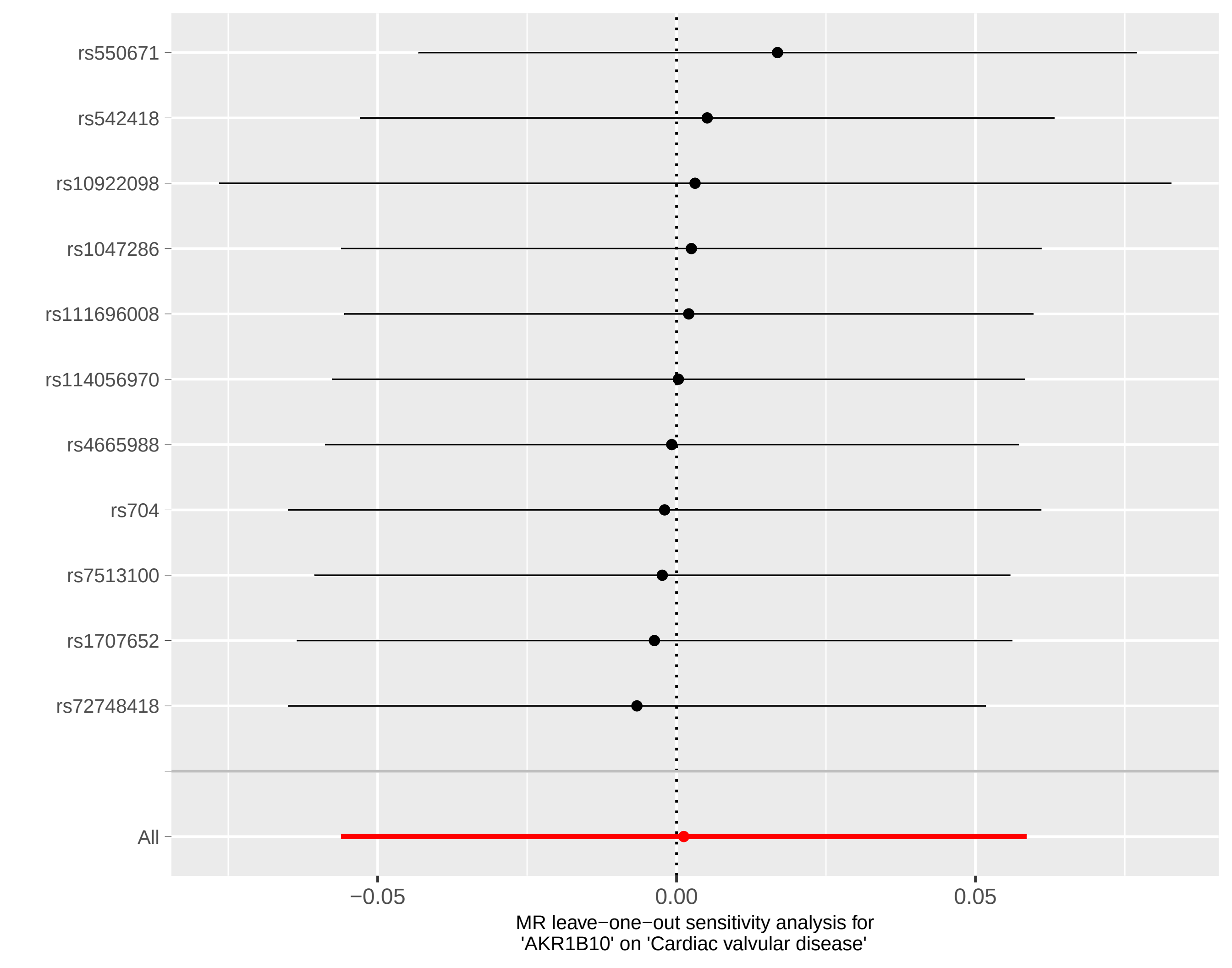
rs550671
rs542418
rs10922098
rs1047286
rs111696008
rs114056970
rs4665988
rs704
rs7513100
rs1707652
rs72748418
All
−0.05
0.00
0.05
MR leave−one−out sensitivity analysis for
'AKR1B10' on 'Cardiac valvular disease'

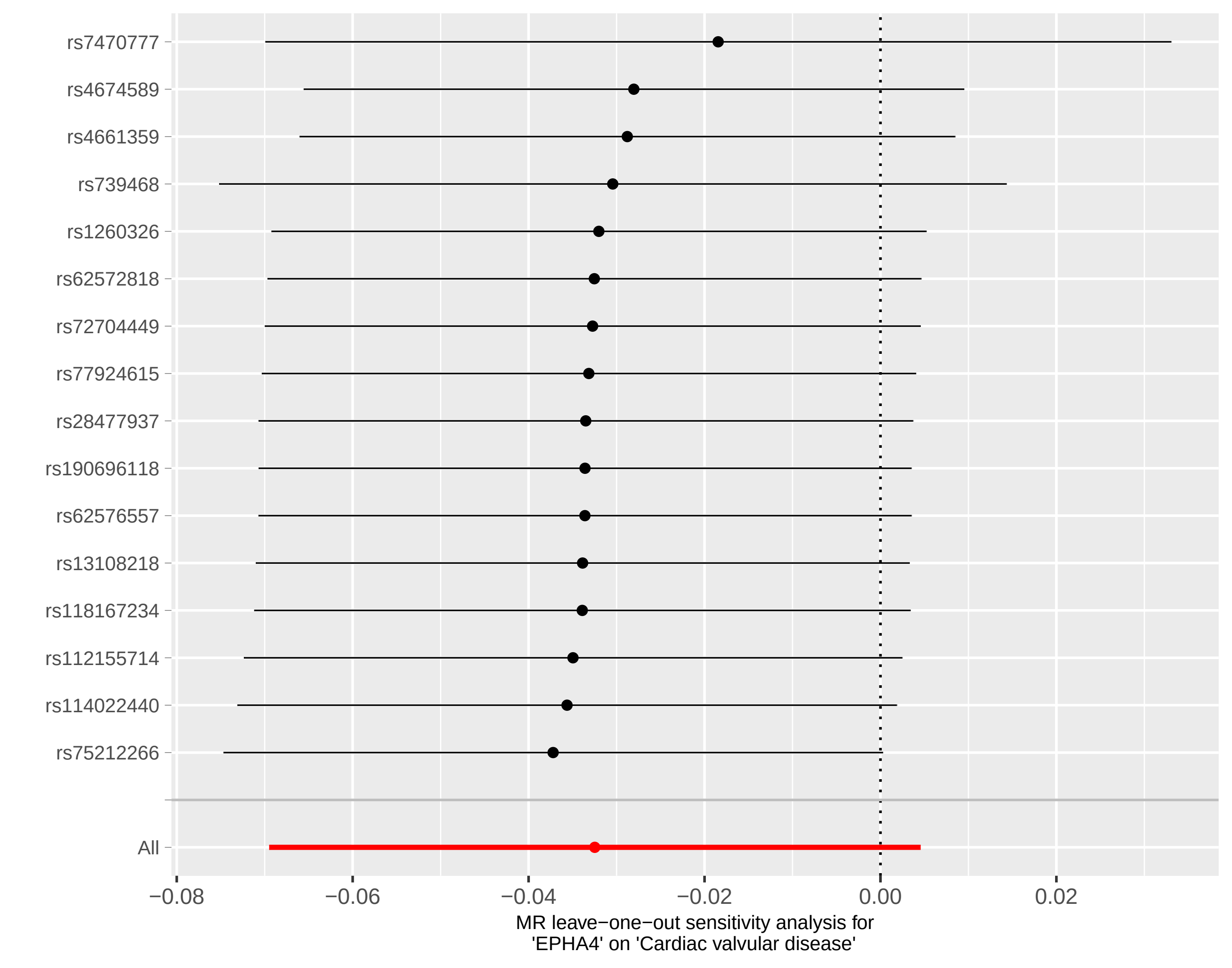
rs7470777
rs4674589
rs4661359
rs739468
rs1260326
rs62572818
rs72704449
rs77924615
rs28477937
rs190696118
rs62576557
rs13108218
rs118167234
rs112155714
rs114022440
rs75212266
All
−0.08
−0.06
−0.04
−0.02
0.00
0.02
MR leave−one−out sensitivity analysis for
'EPHA4' on 'Cardiac valvular disease'

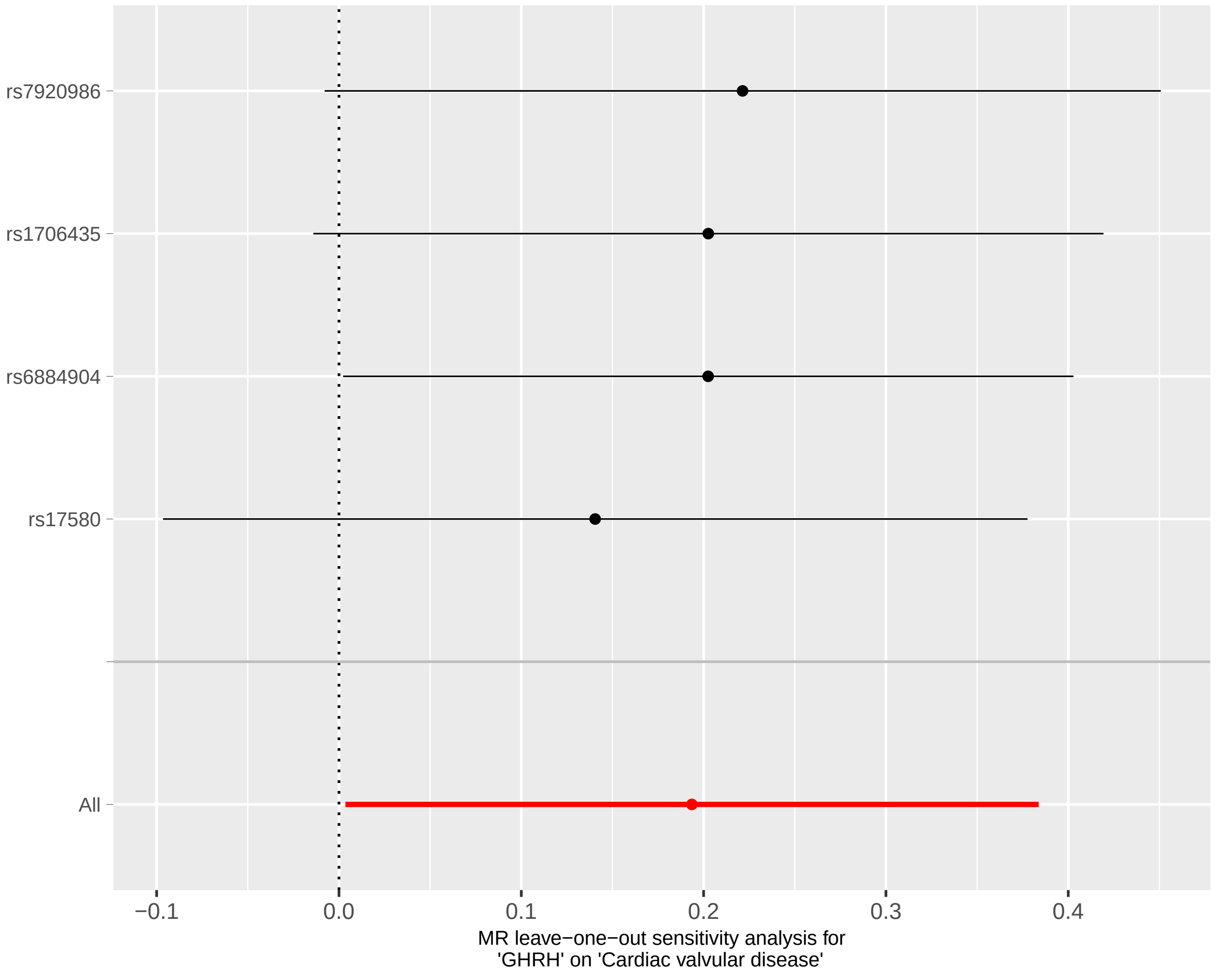

rs7920986
rs1706435
rs6884904
rs17580
All
−0.1
0.0
0.1
0.2
0.3
0.4
MR leave−one−out sensitivity analysis for
'GHRH' on 'Cardiac valvular disease'

Insufficient number of SNPs

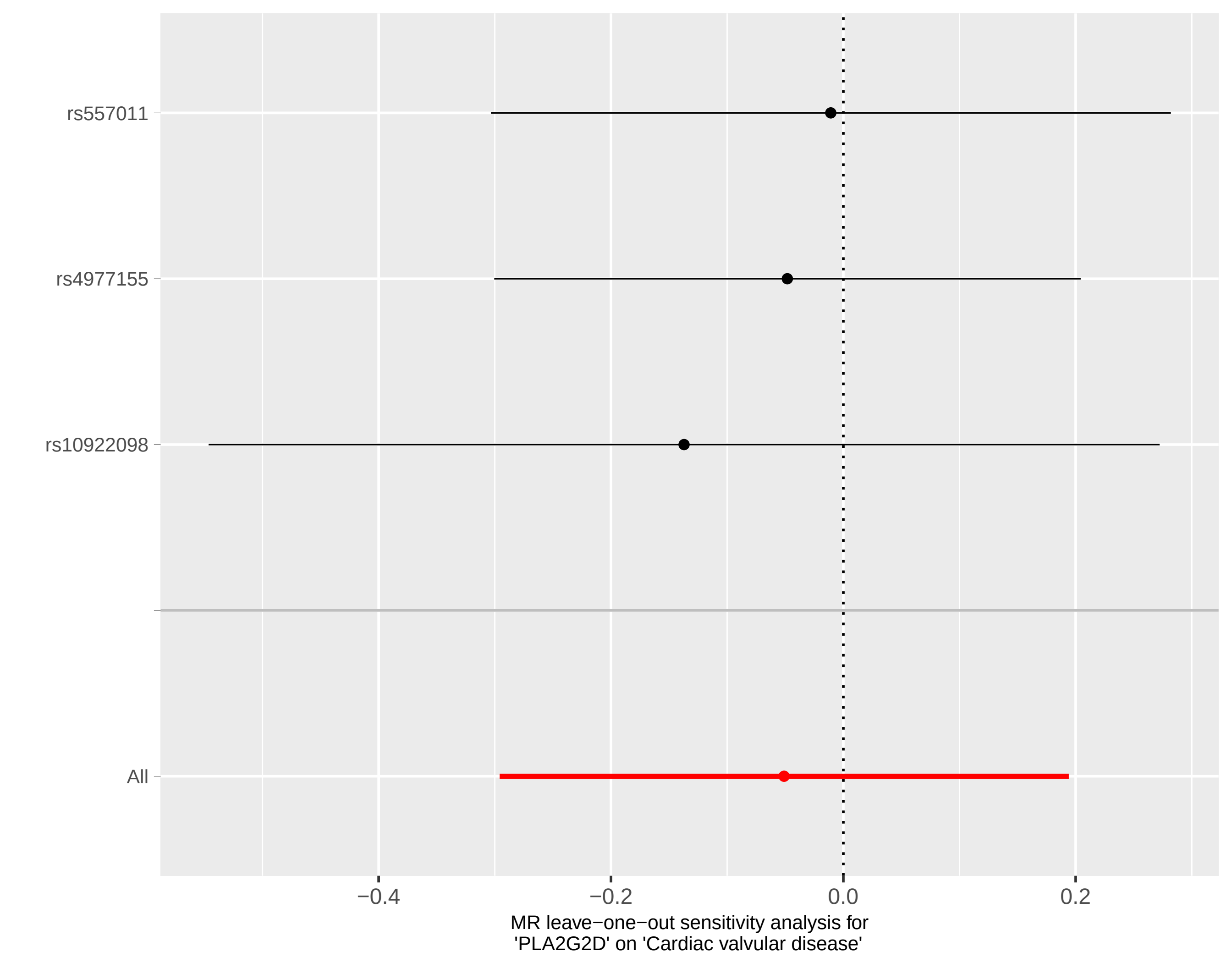
rs557011
rs4977155
rs10922098
All
−0.4
−0.2
0.0
0.2
MR leave−one−out sensitivity analysis for
'PLA2G2D' on 'Cardiac valvular disease'

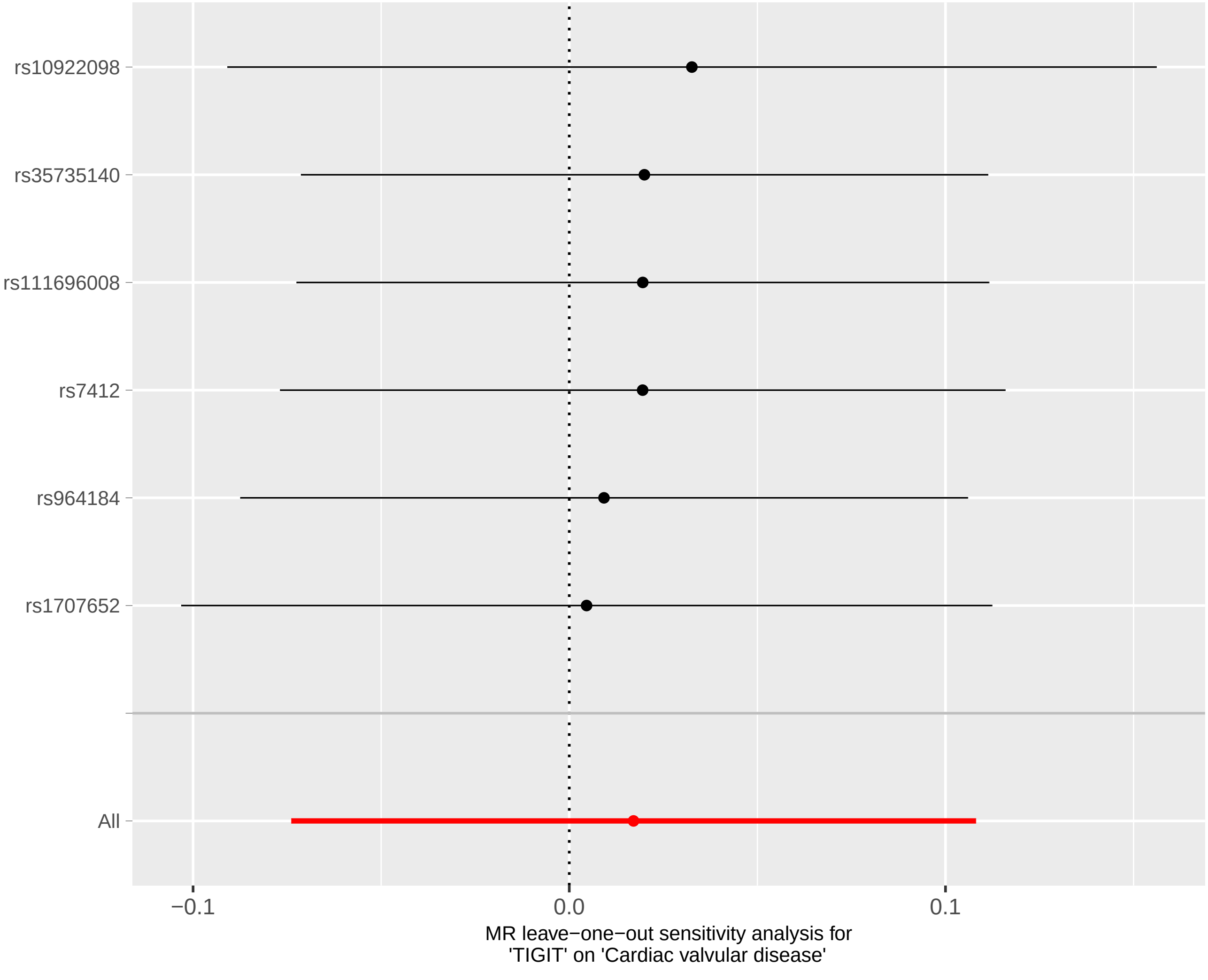
rs10922098
rs35735140
rs111696008
rs7412
rs964184
rs1707652
All
−0.1
0.0
0.1
MR leave−one−out sensitivity analysis for
'TIGIT' on 'Cardiac valvular disease'

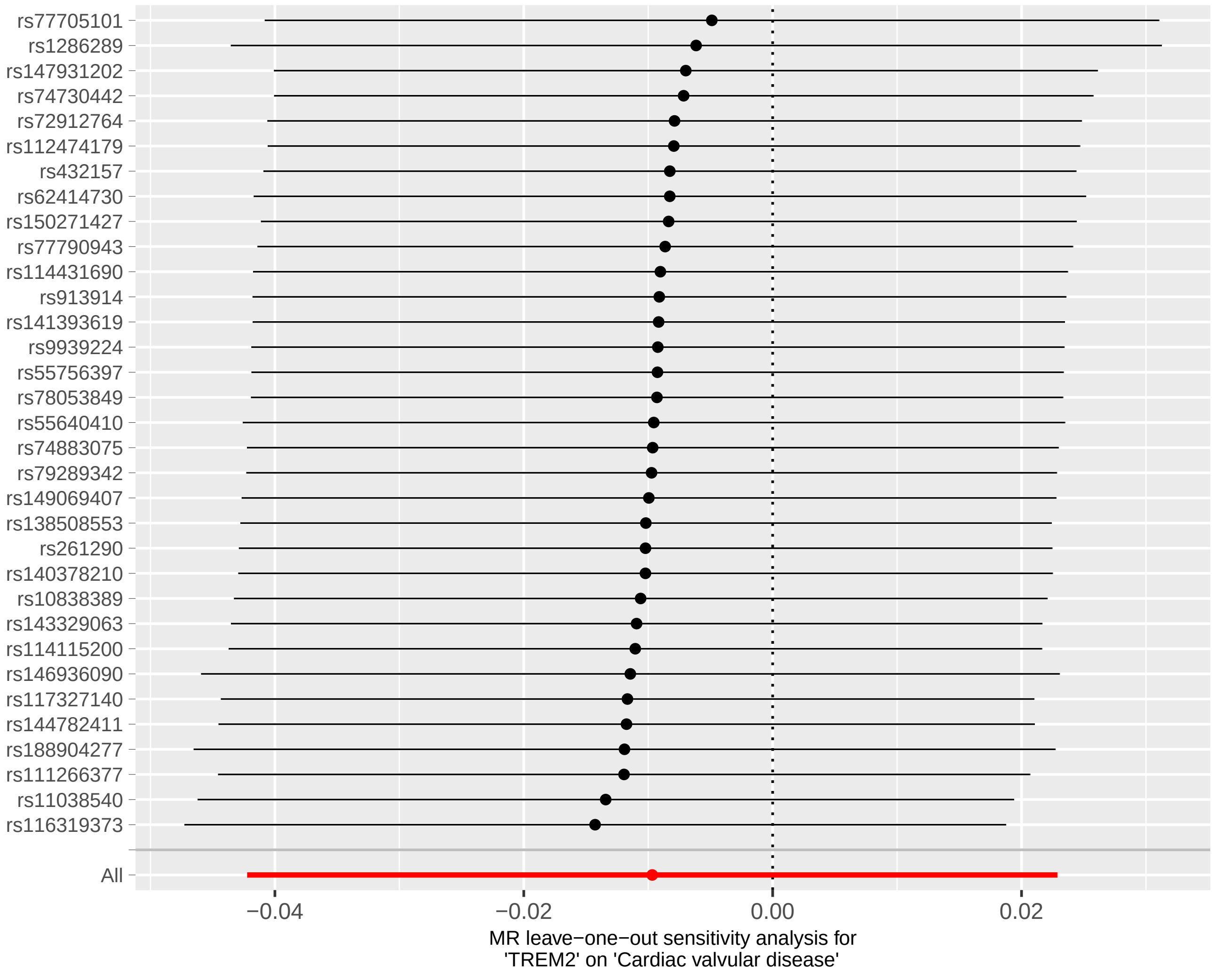

rs77705101
rs1286289
rs147931202
rs74730442
rs72912764
rs112474179
rs432157
rs62414730
rs150271427
rs77790943
rs114431690
rs913914
rs141393619
rs9939224
rs55756397
rs78053849
rs55640410
rs74883075
rs79289342
rs149069407
rs138508553
rs261290
rs140378210
rs10838389
rs143329063
rs114115200
rs146936090
rs117327140
rs144782411
rs188904277
rs111266377
rs11038540
rs116319373
All
−0.04
−0.02
0.00
0.02
MR leave−one−out sensitivity analysis for
'TREM2' on 'Cardiac valvular disease'

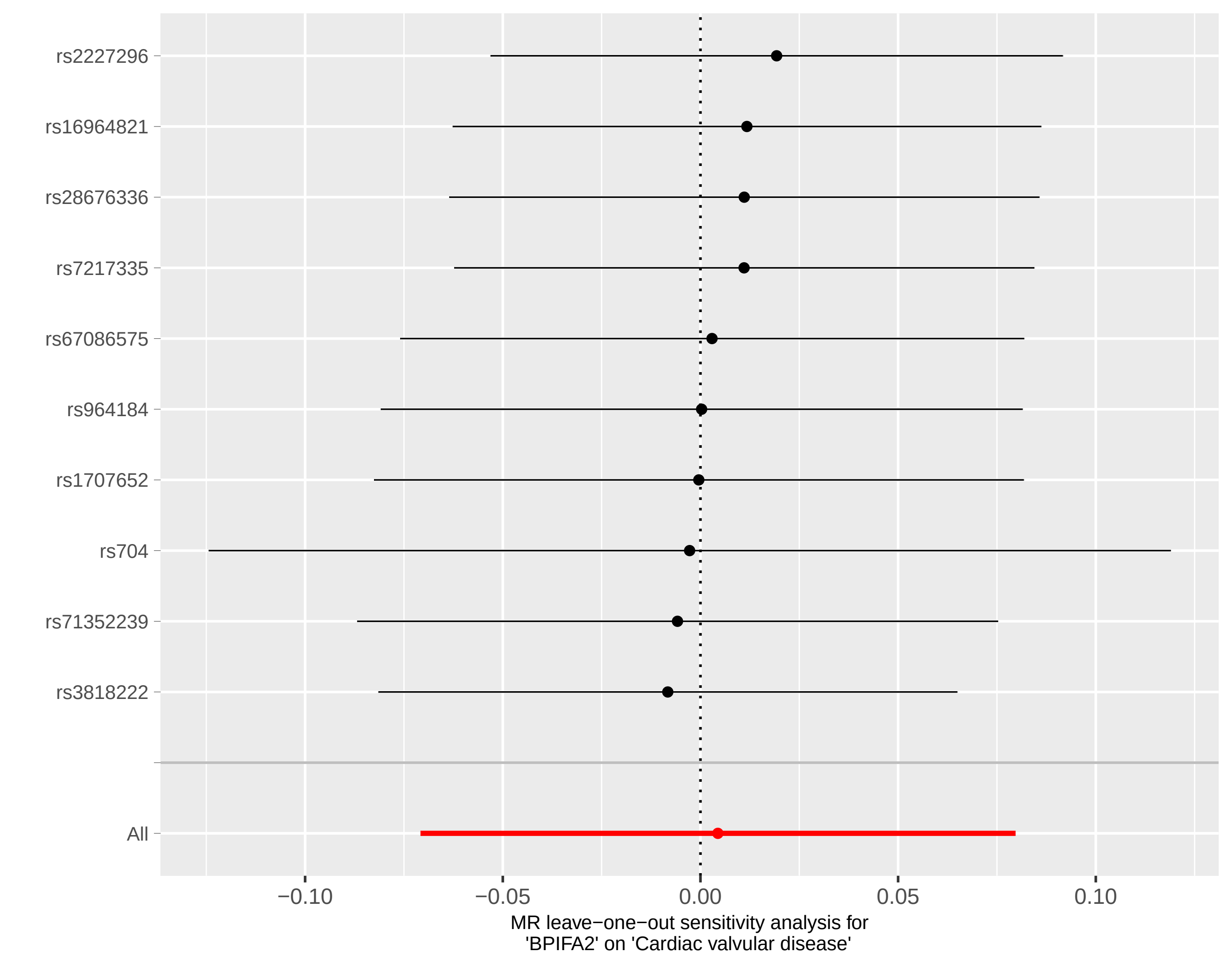
rs2227296
rs16964821
rs28676336
rs7217335
rs67086575
rs964184
rs1707652
rs704
rs71352239
rs3818222
All
−0.10
−0.05
0.00
0.05
0.10
MR leave−one−out sensitivity analysis for
'BPIFA2' on 'Cardiac valvular disease'

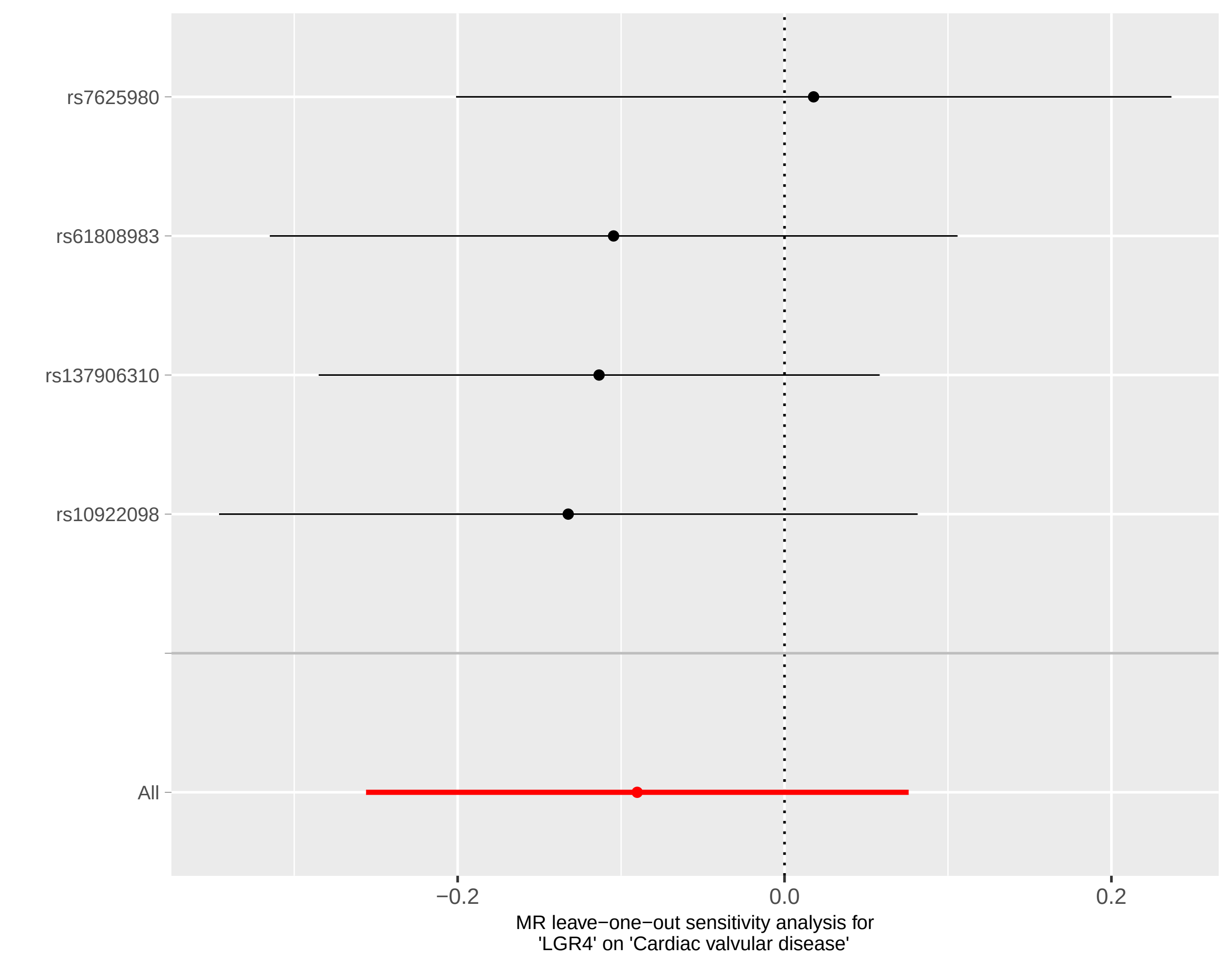

rs7625980
rs61808983
rs137906310
rs10922098
All
−0.2
0.0
0.2
MR leave−one−out sensitivity analysis for
'LGR4' on 'Cardiac valvular disease'

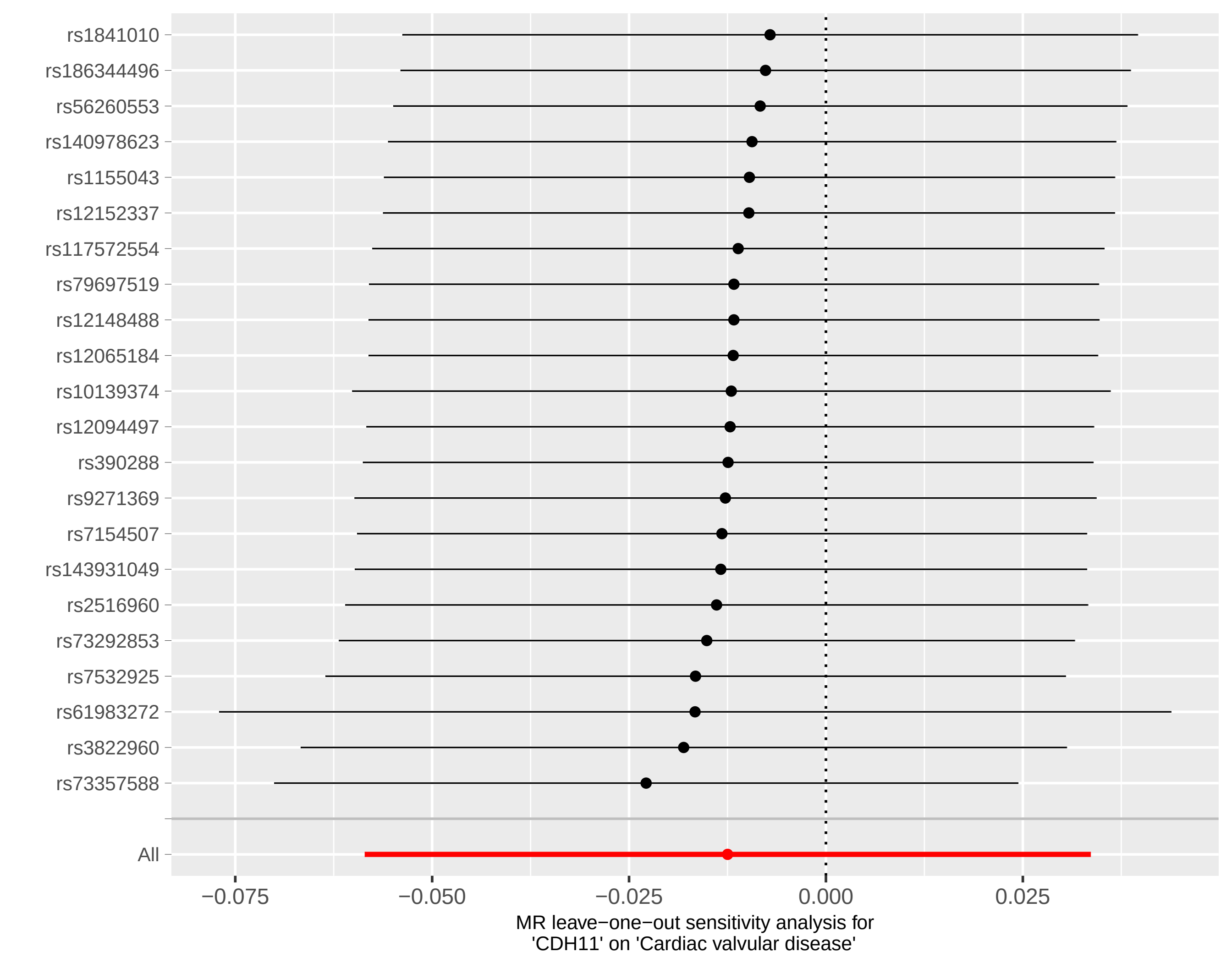

rs1841010
rs186344496
rs56260553
rs140978623
rs1155043
rs12152337
rs117572554
rs79697519
rs12148488
rs12065184
rs10139374
rs12094497
rs390288
rs9271369
rs7154507
rs143931049
rs2516960
rs73292853
rs7532925
rs61983272
rs3822960
rs73357588
All
−0.075
−0.050
−0.025
0.000
0.025
MR leave−one−out sensitivity analysis for
'CDH11' on 'Cardiac valvular disease'

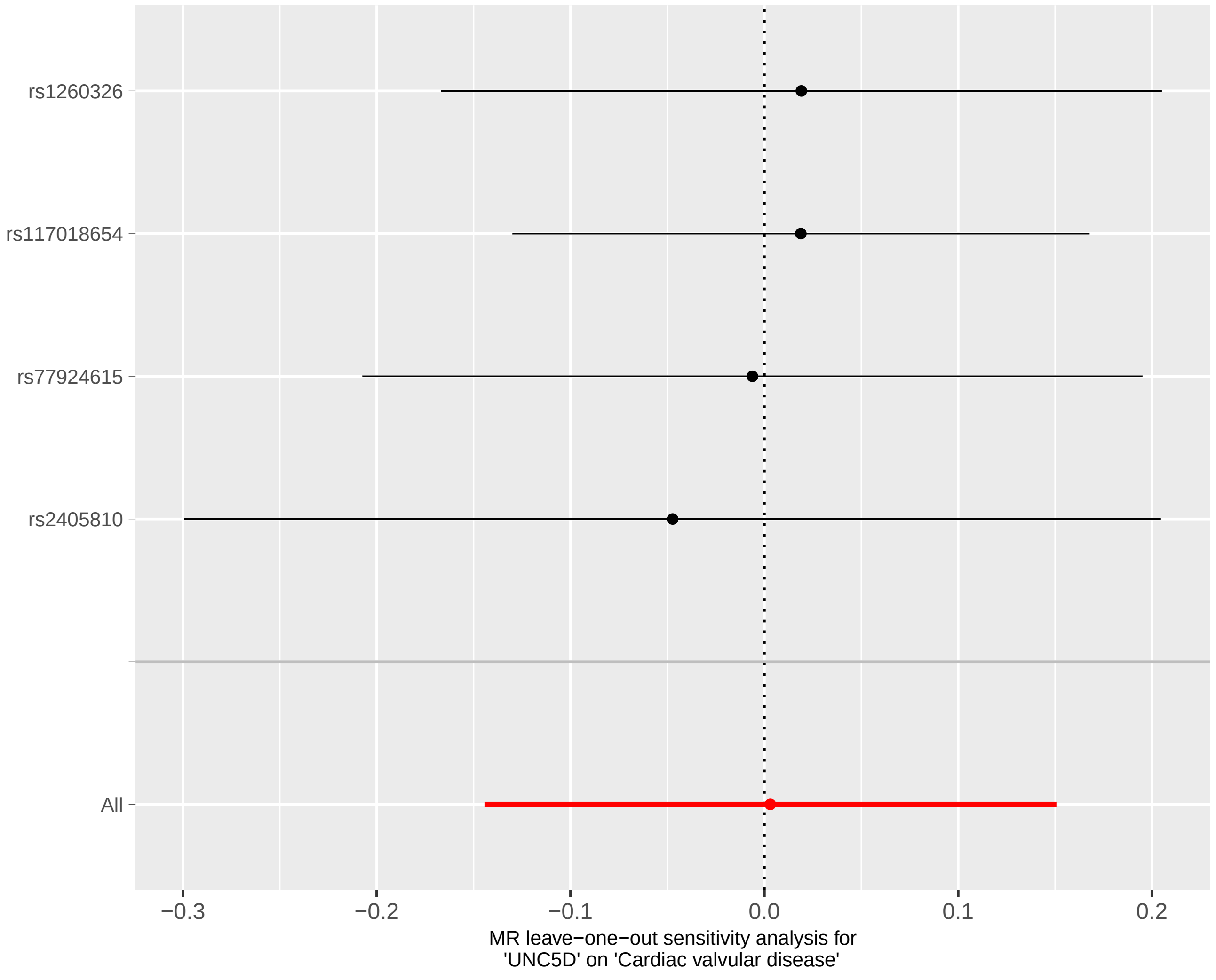
rs1260326
rs117018654
rs77924615
rs2405810
All
−0.3
−0.2
−0.1
0.0
0.1
0.2
MR leave−one−out sensitivity analysis for
'UNC5D' on 'Cardiac valvular disease'

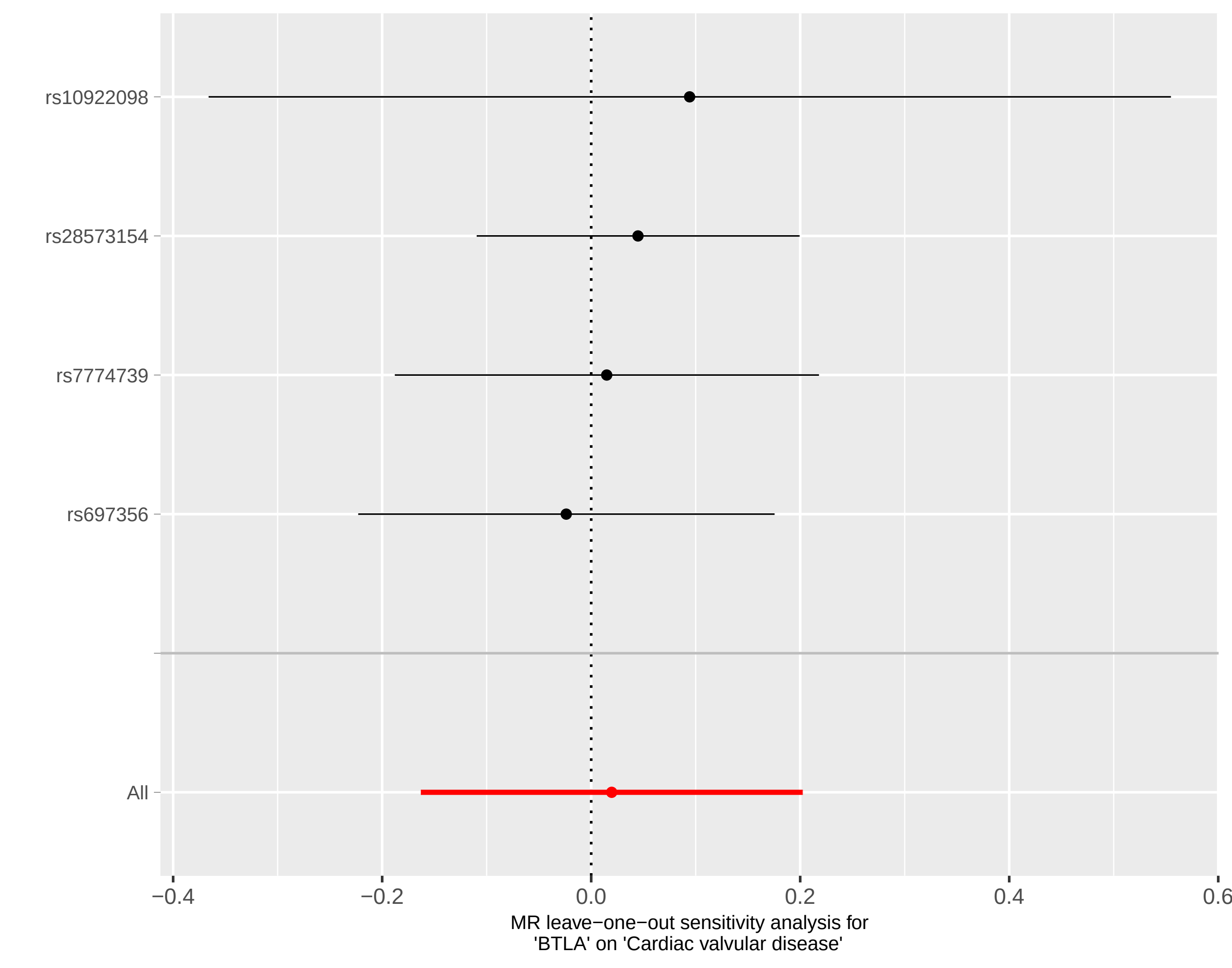
rs10922098
rs28573154
rs7774739
rs697356
All
−0.4
−0.2
0.0
0.2
0.4
0.6
MR leave−one−out sensitivity analysis for
'BTLA' on 'Cardiac valvular disease'

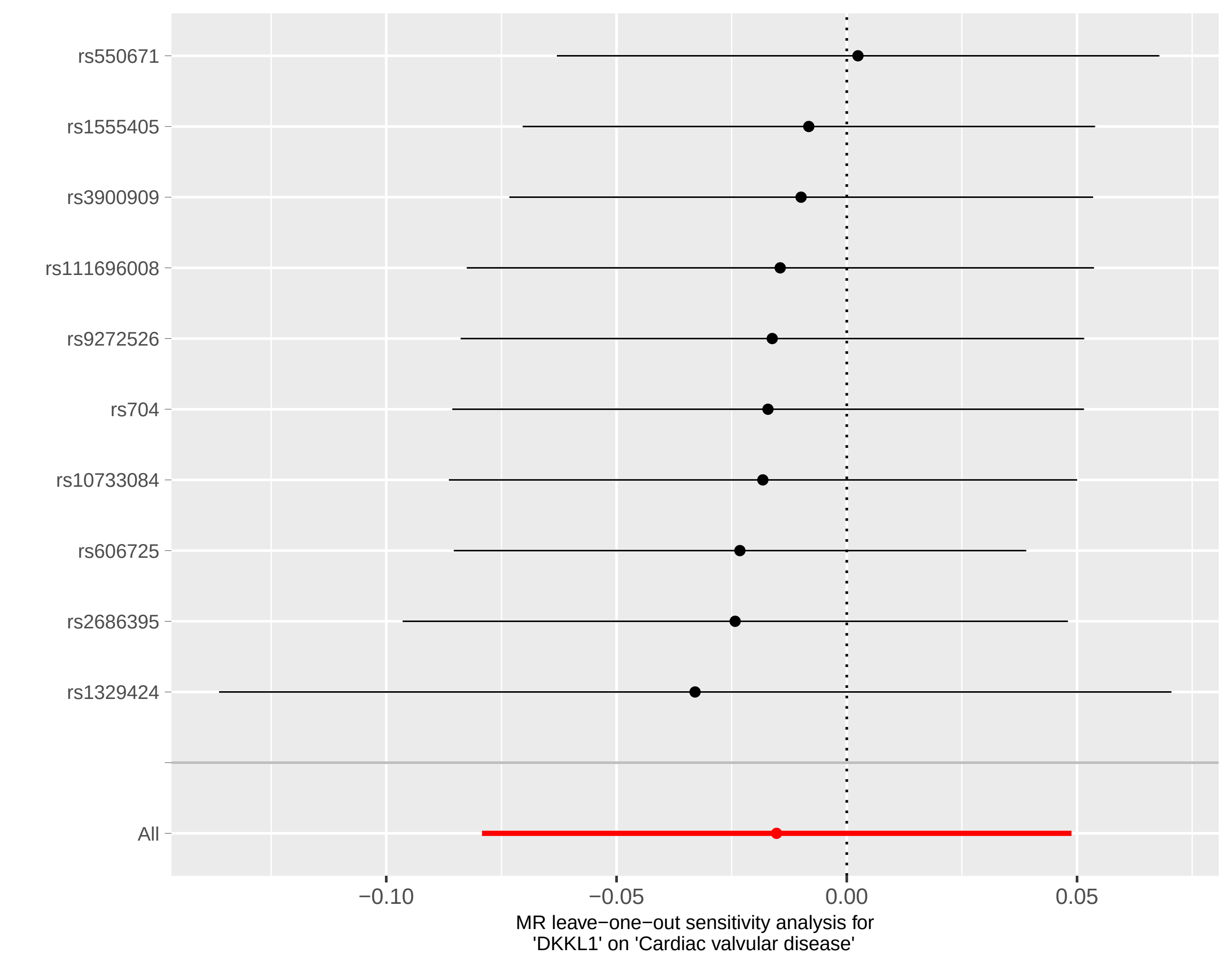

rs550671
rs1555405
rs3900909
rs111696008
rs9272526
rs704
rs10733084
rs606725
rs2686395
rs1329424
All
−0.10
−0.05
0.00
0.05
MR leave−one−out sensitivity analysis for
'DKKL1' on 'Cardiac valvular disease'

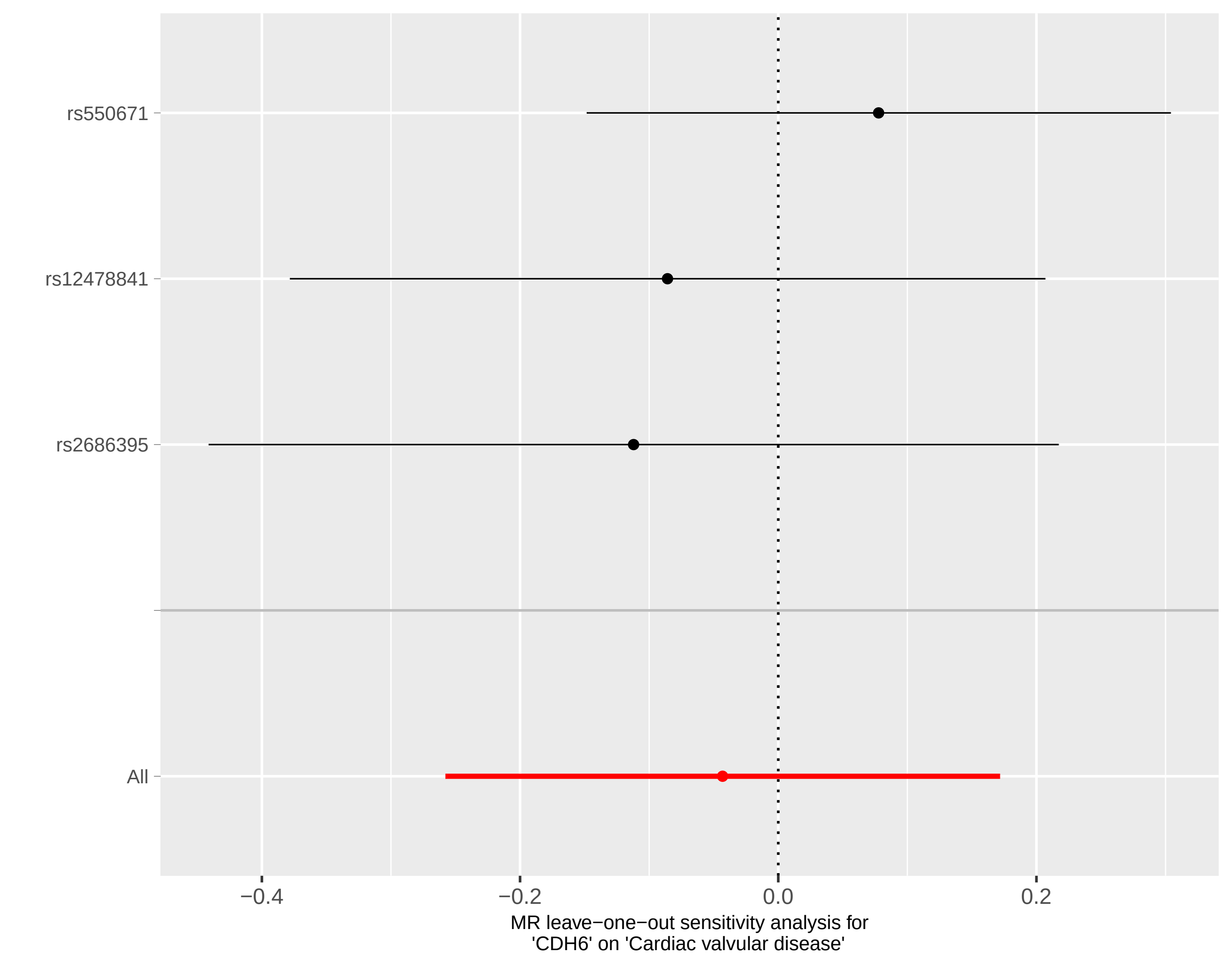
rs550671
rs12478841
rs2686395
All
−0.4
−0.2
0.0
0.2
MR leave−one−out sensitivity analysis for
'CDH6' on 'Cardiac valvular disease'

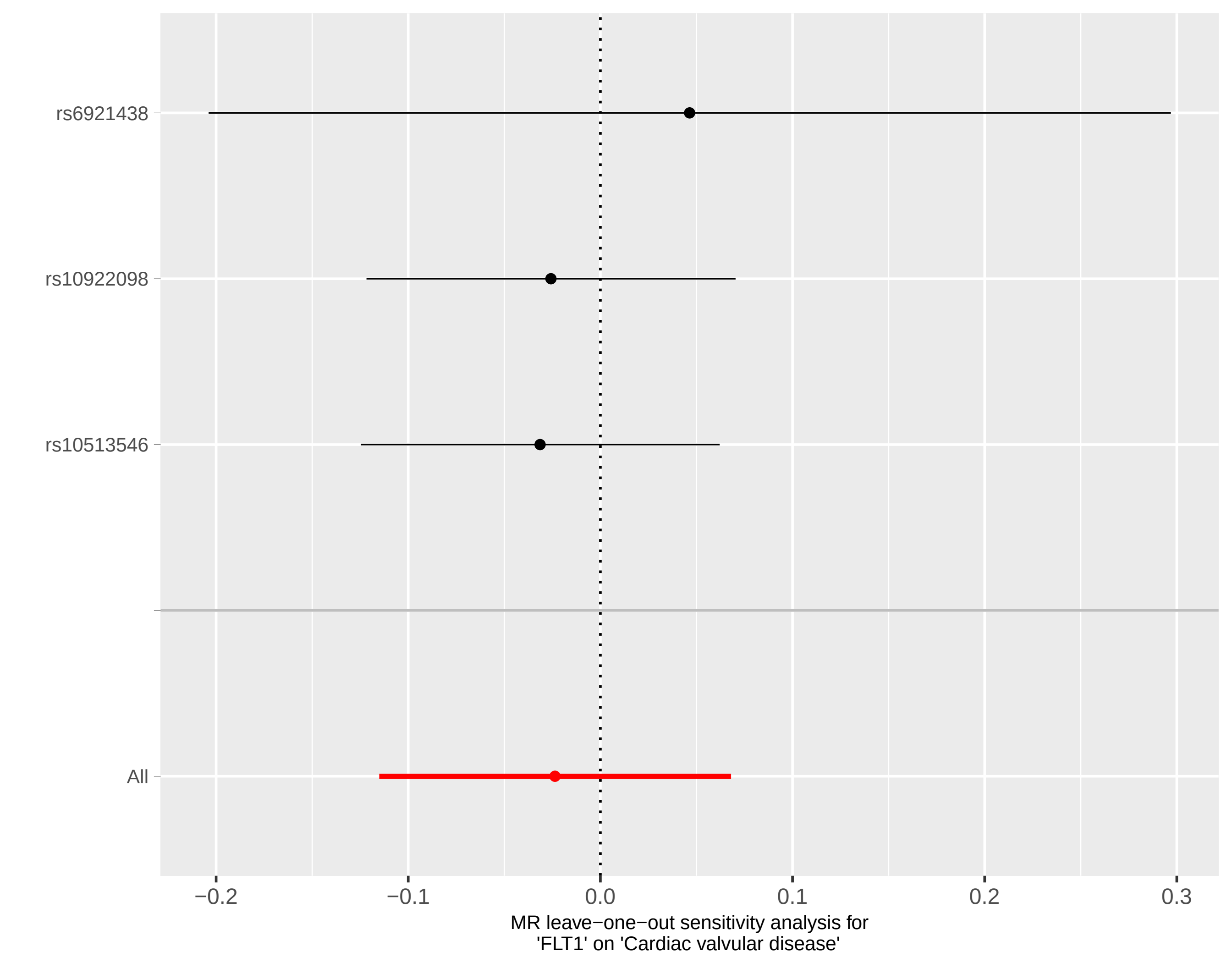
rs6921438
rs10922098
rs10513546
All
−0.2
−0.1
0.0
0.1
0.2
0.3
MR leave−one−out sensitivity analysis for
'FLT1' on 'Cardiac valvular disease'

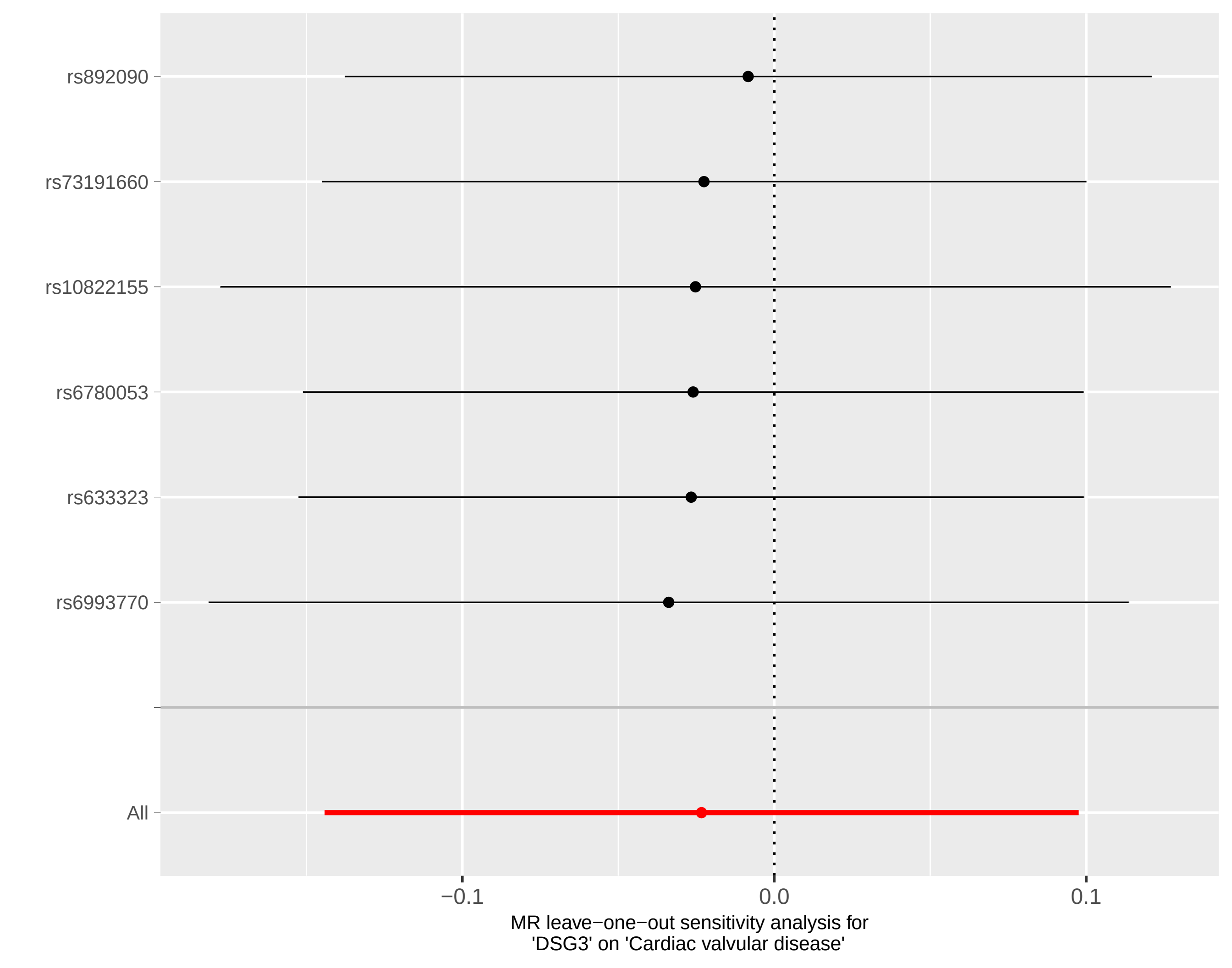

rs892090
rs73191660
rs10822155
rs6780053
rs633323
rs6993770
All
−0.1
0.0
0.1
MR leave−one−out sensitivity analysis for
'DSG3' on 'Cardiac valvular disease'

Insufficient number of SNPs

Insufficient number of SNPs

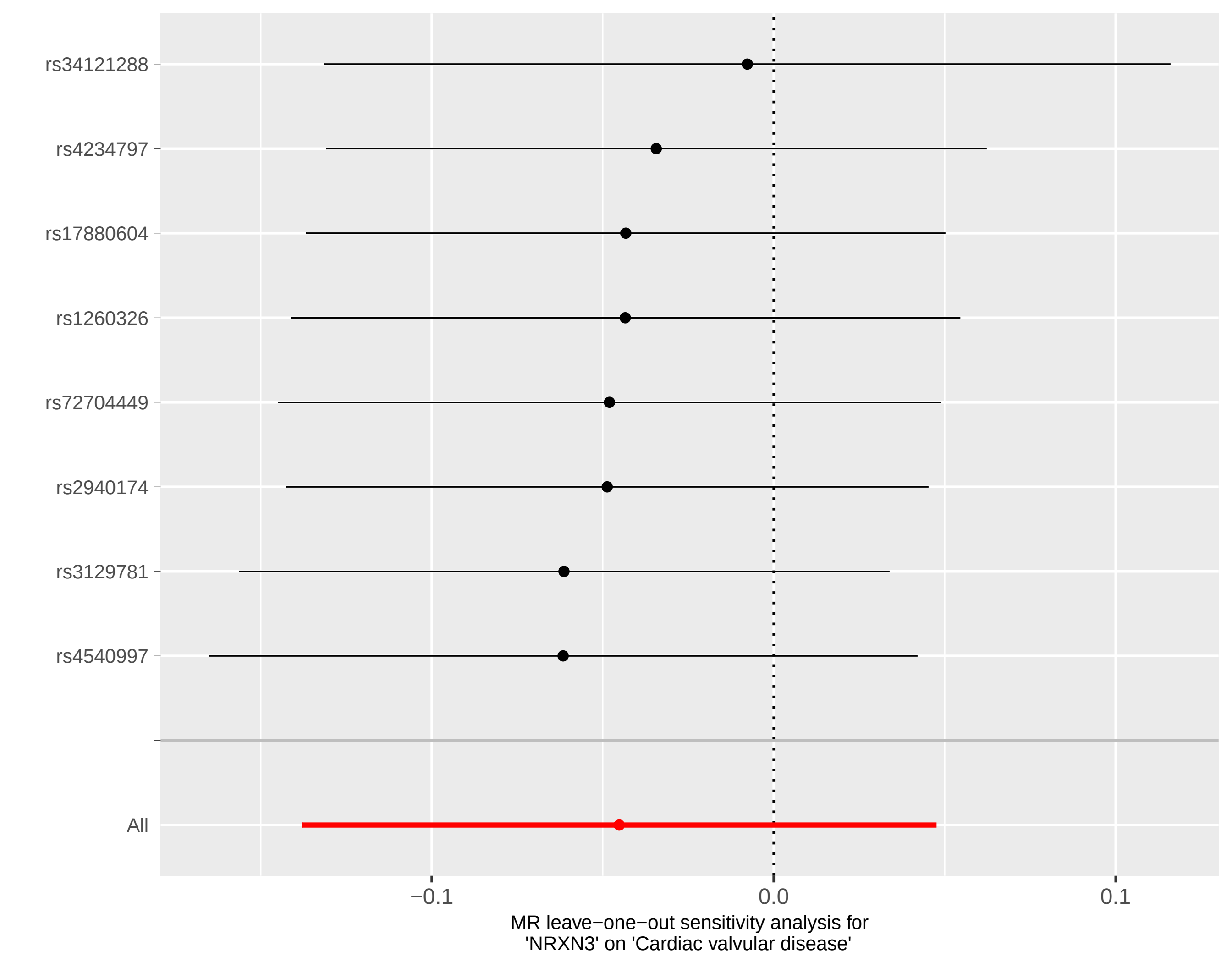
rs34121288
rs4234797
rs17880604
rs1260326
rs72704449
rs2940174
rs3129781
rs4540997
All
−0.1
0.0
0.1
MR leave−one−out sensitivity analysis for
'NRXN3' on 'Cardiac valvular disease'

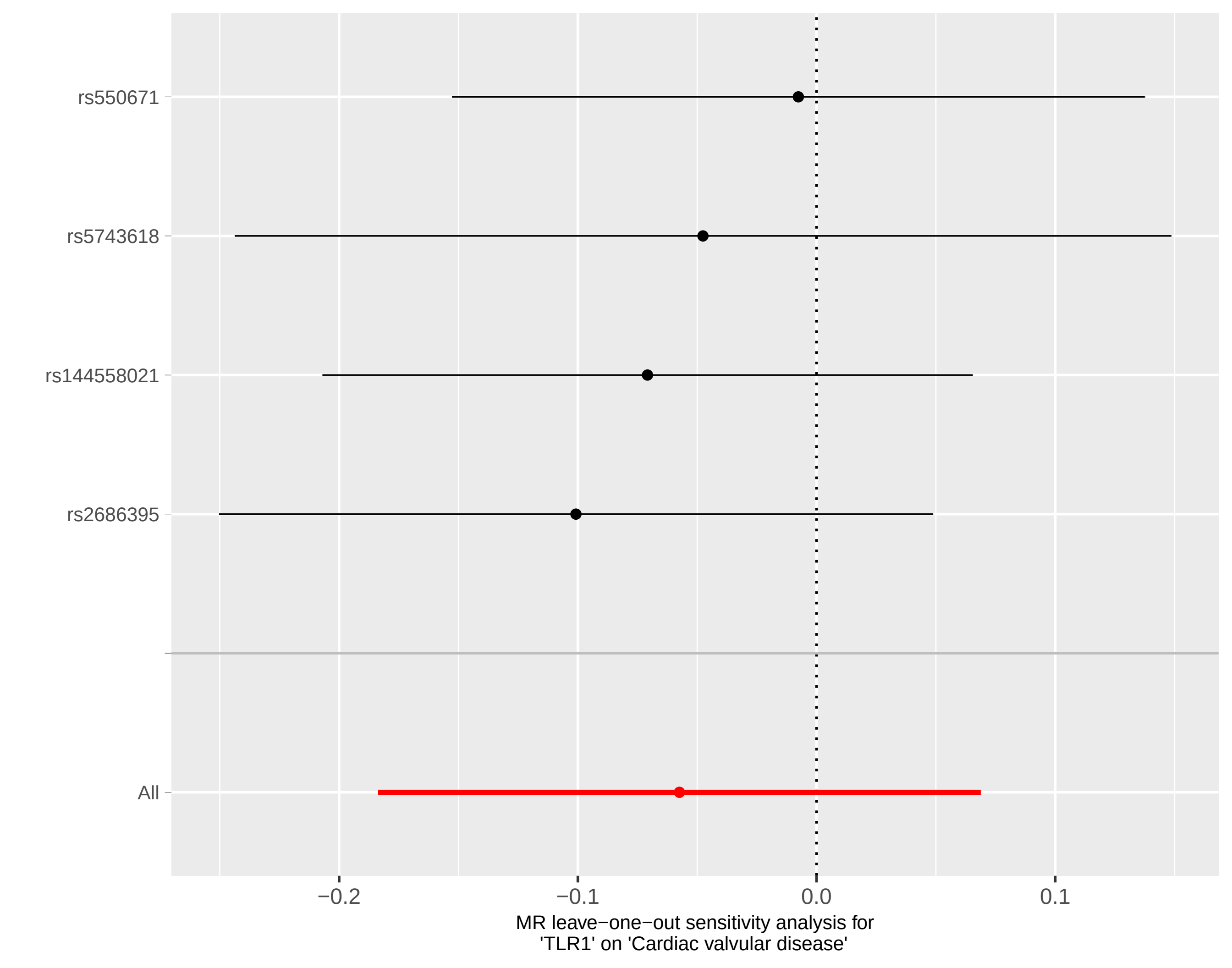
rs550671
rs5743618
rs144558021
rs2686395
All
−0.2
−0.1
0.0
0.1
MR leave−one−out sensitivity analysis for
'TLR1' on 'Cardiac valvular disease'

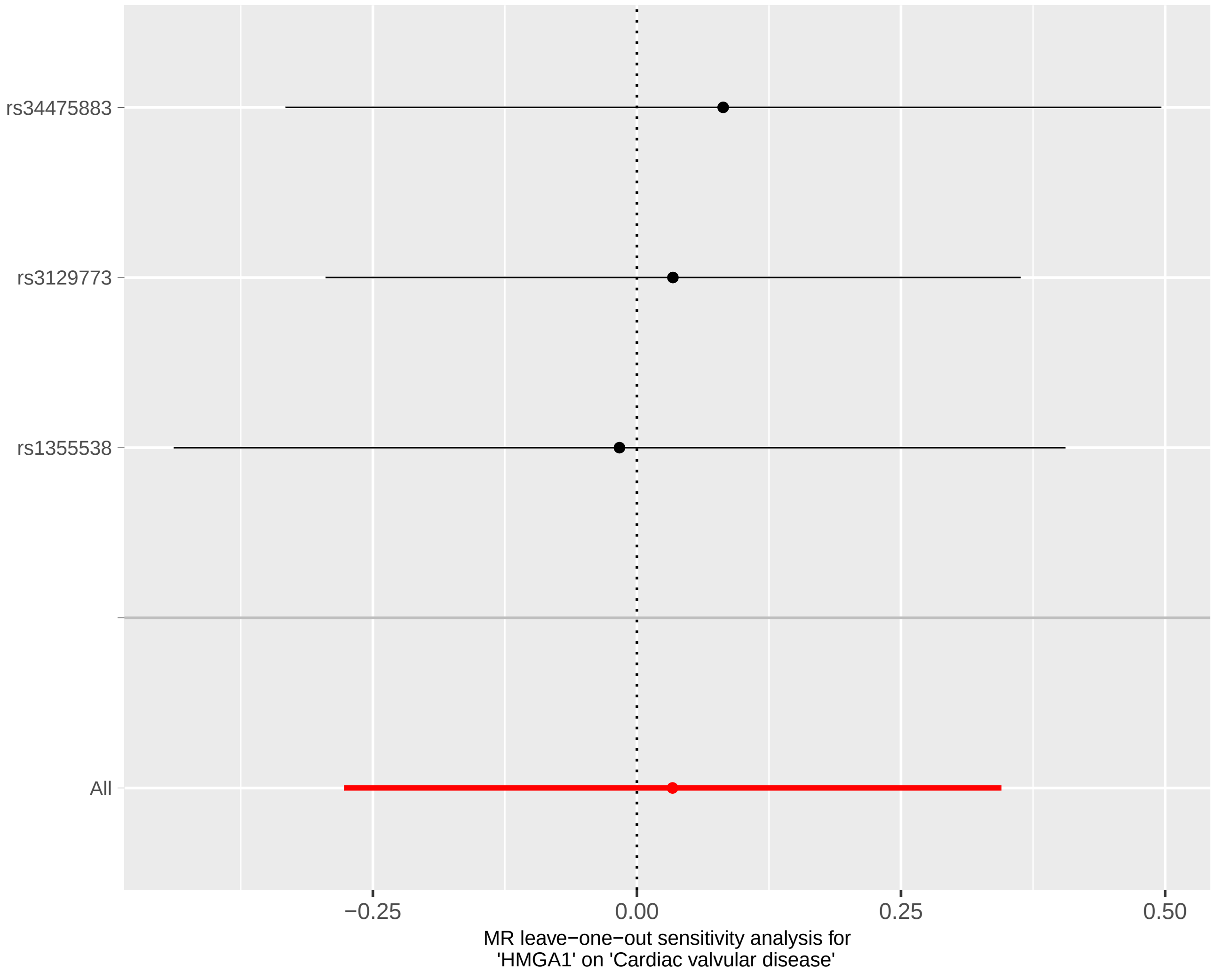

rs34475883
rs3129773
rs1355538
All
−0.25
0.00
0.25
0.50
MR leave−one−out sensitivity analysis for
'HMGA1' on 'Cardiac valvular disease'

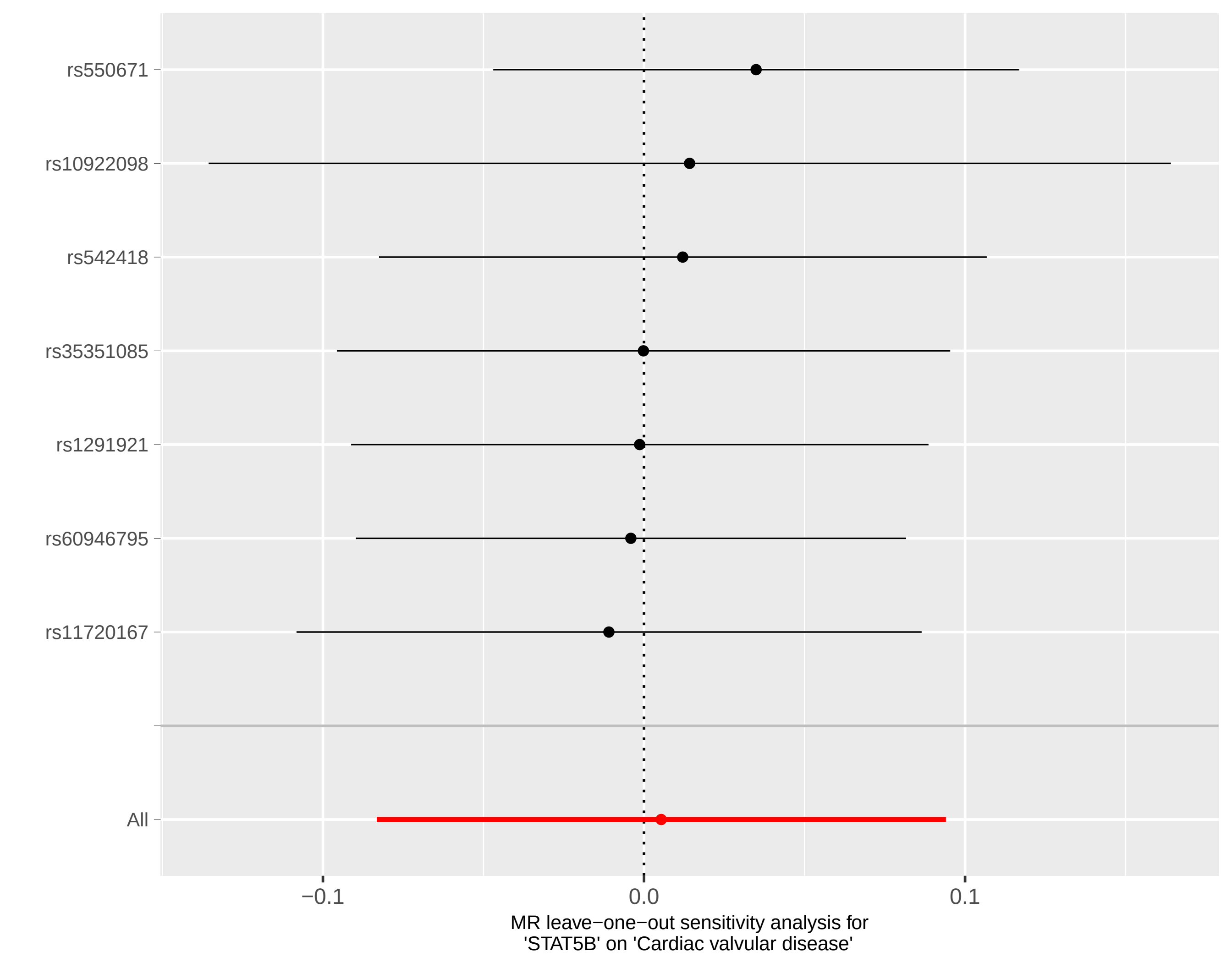
rs550671
rs10922098
rs542418
rs35351085
rs1291921
rs60946795
rs11720167
All
−0.1
0.0
0.1
MR leave−one−out sensitivity analysis for
'STAT5B' on 'Cardiac valvular disease'

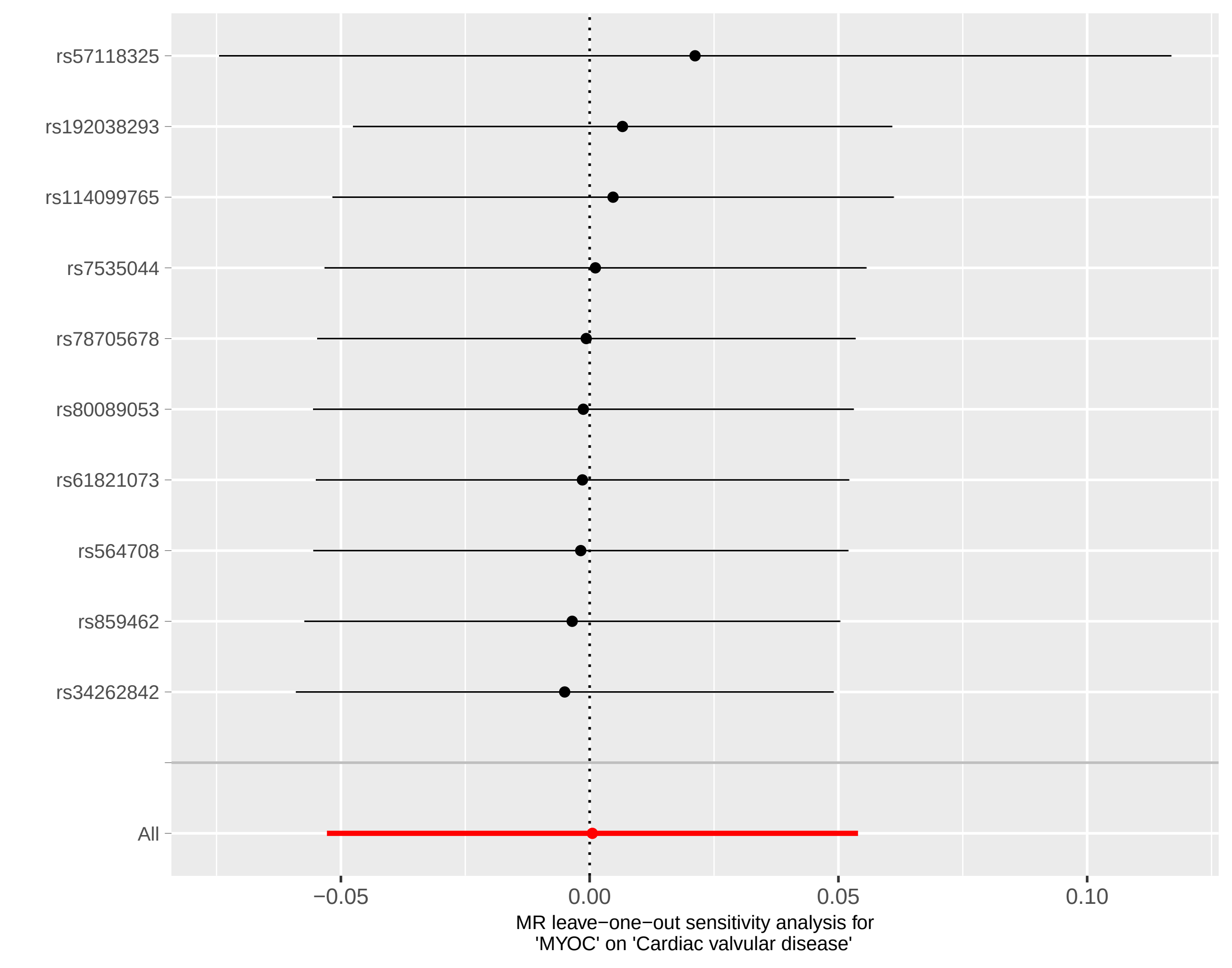

rs57118325
rs192038293
rs114099765
rs7535044
rs78705678
rs80089053
rs61821073
rs564708
rs859462
rs34262842
All
−0.05
0.00
0.05
0.10
MR leave−one−out sensitivity analysis for
'MYOC' on 'Cardiac valvular disease'

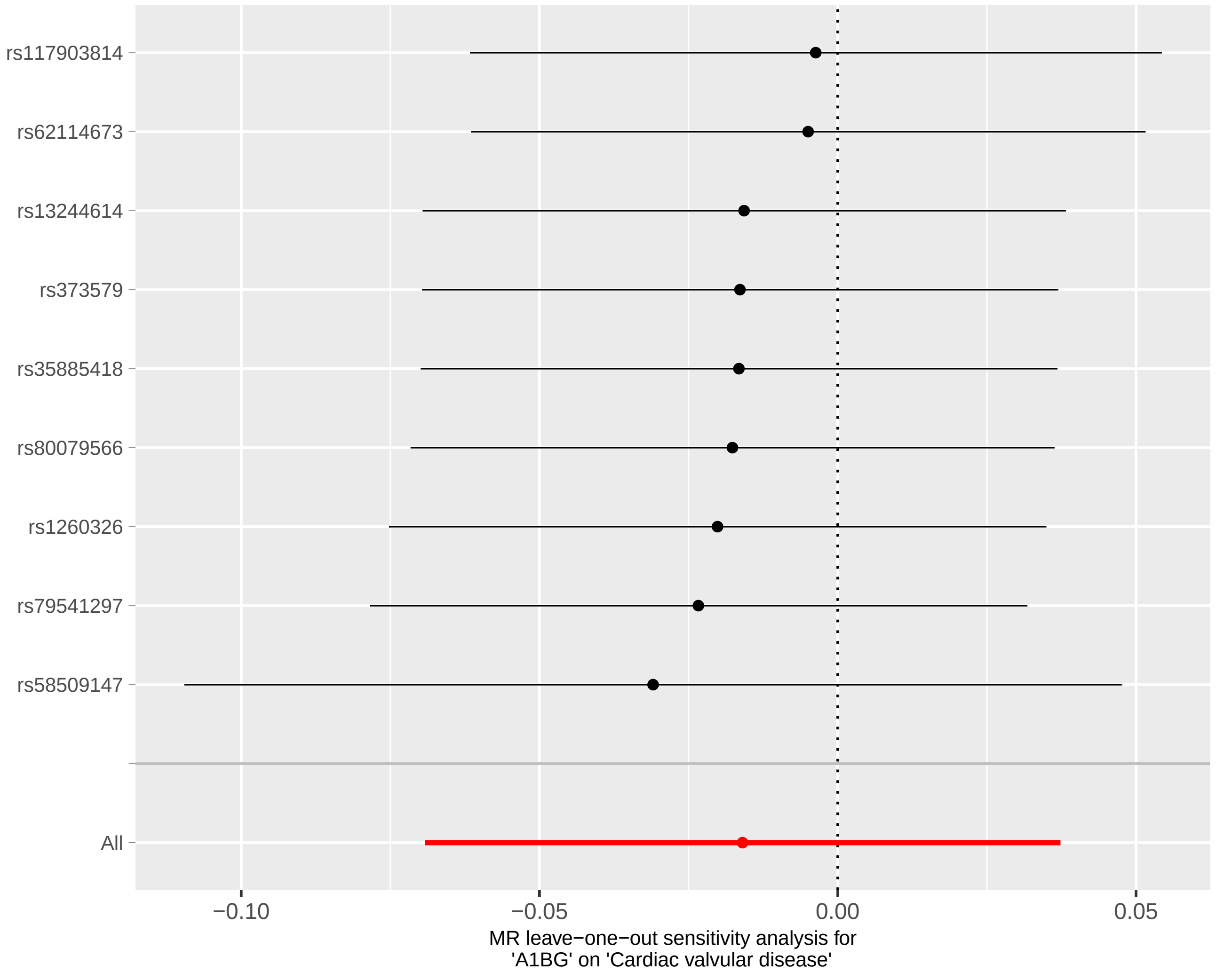
rs117903814
rs62114673
rs13244614
rs373579
rs35885418
rs80079566
rs1260326
rs79541297
rs58509147
All
−0.10
−0.05
0.00
0.05
MR leave−one−out sensitivity analysis for
'A1BG' on 'Cardiac valvular disease'

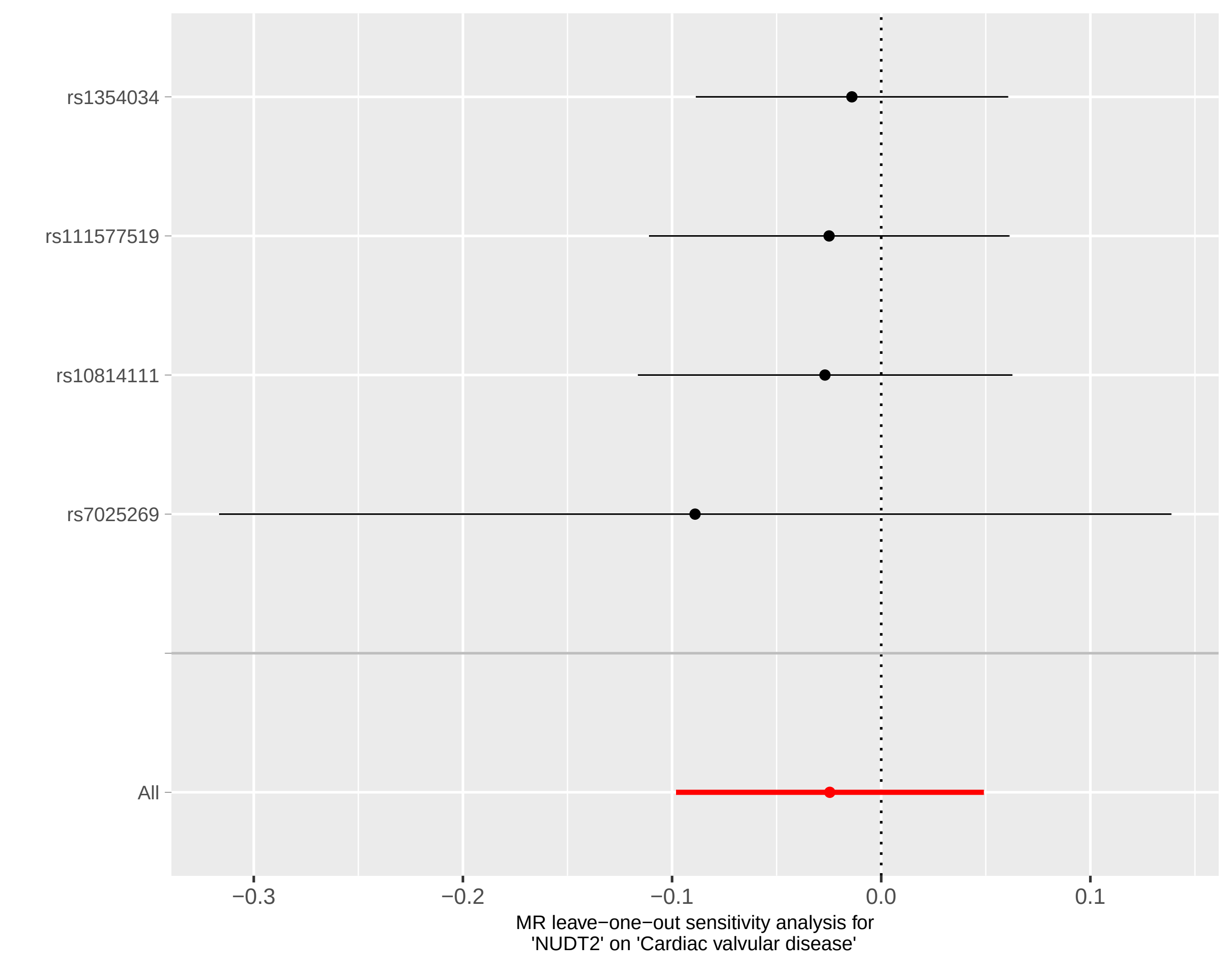
rs1354034
rs111577519
rs10814111
rs7025269
All
−0.3
−0.2
−0.1
0.0
0.1
MR leave−one−out sensitivity analysis for
'NUDT2' on 'Cardiac valvular disease'

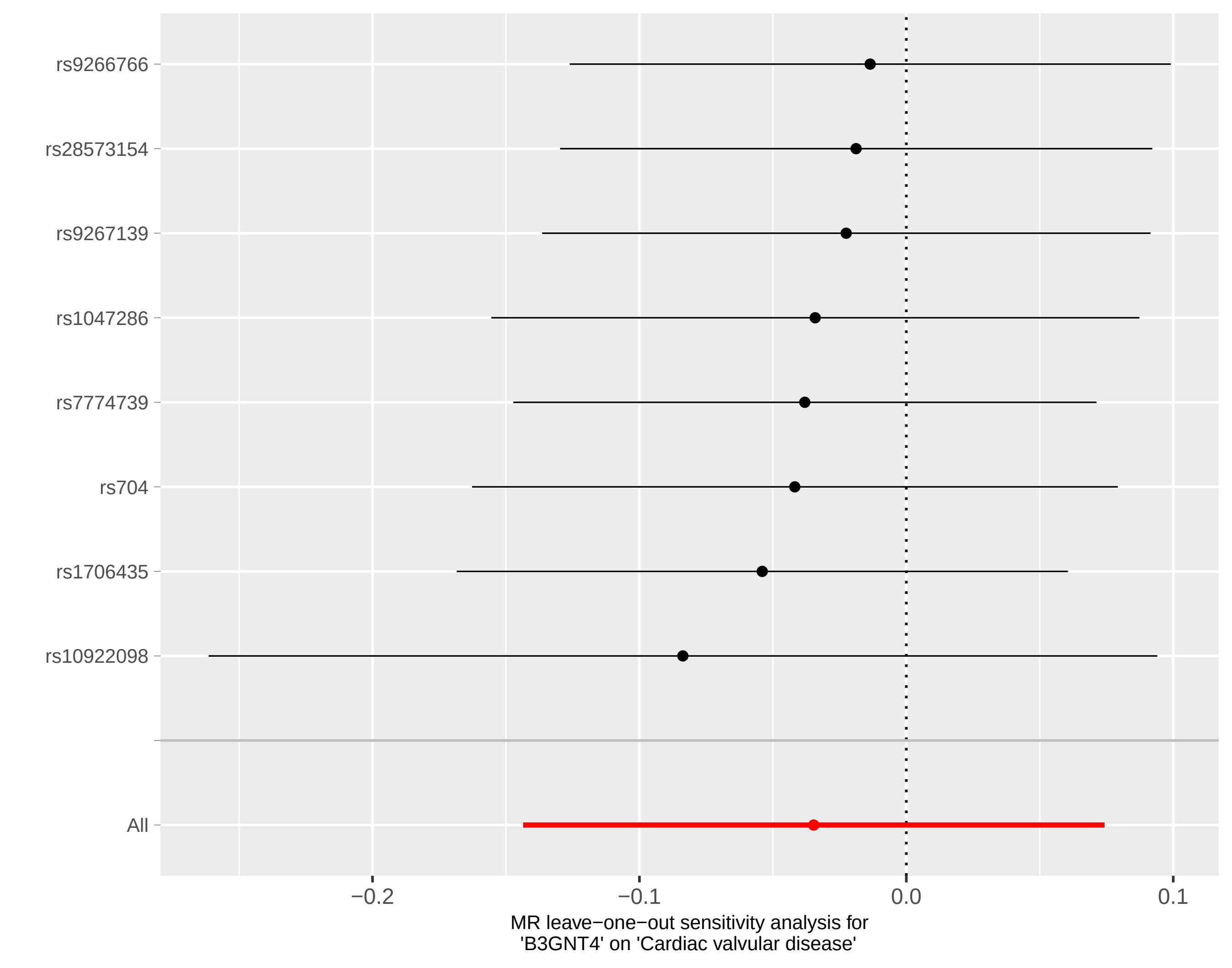

rs9266766
rs28573154
rs9267139
rs1047286
rs7774739
rs704
rs1706435
rs10922098
All
−0.2
−0.1
0.0
0.1
MR leave−one−out sensitivity analysis for
'B3GNT4' on 'Cardiac valvular disease'

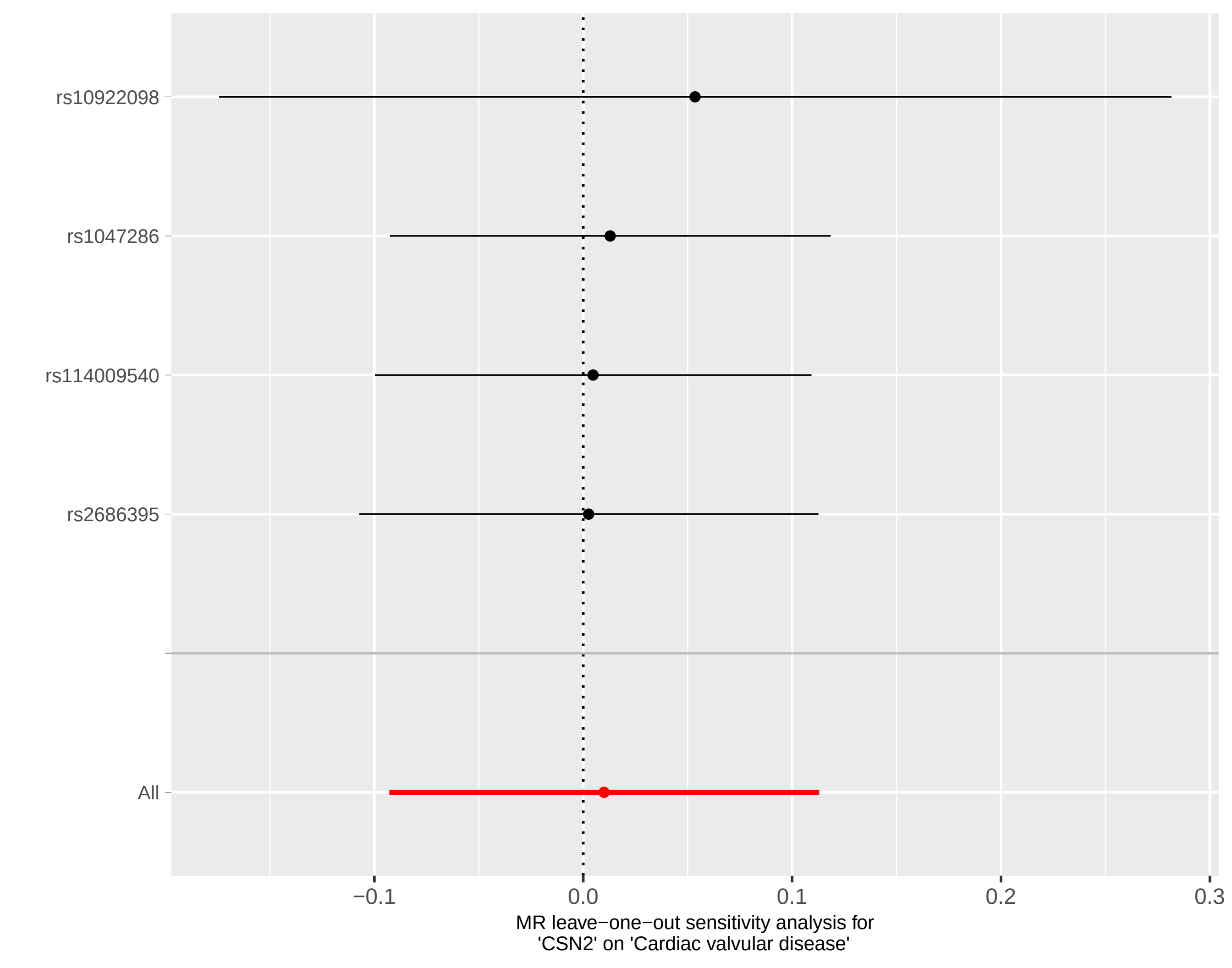

rs10922098
rs1047286
rs114009540
rs2686395
All
−0.1
0.0
0.1
0.2
0.3
MR leave−one−out sensitivity analysis for
'CSN2' on 'Cardiac valvular disease'

Insufficient number of SNPs

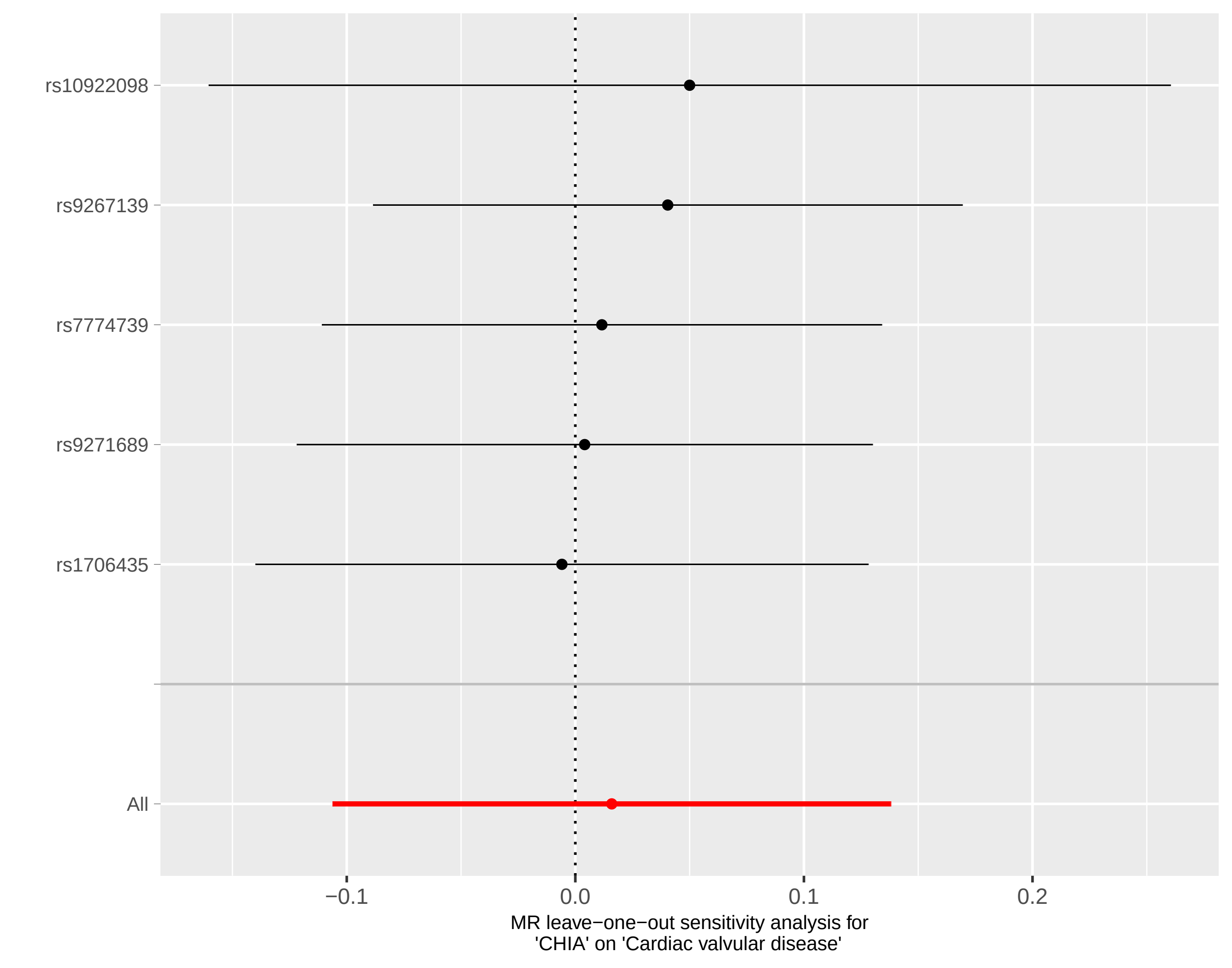
rs10922098
rs9267139
rs7774739
rs9271689
rs1706435
All
−0.1
0.0
0.1
0.2
MR leave−one−out sensitivity analysis for
'CHIA' on 'Cardiac valvular disease'

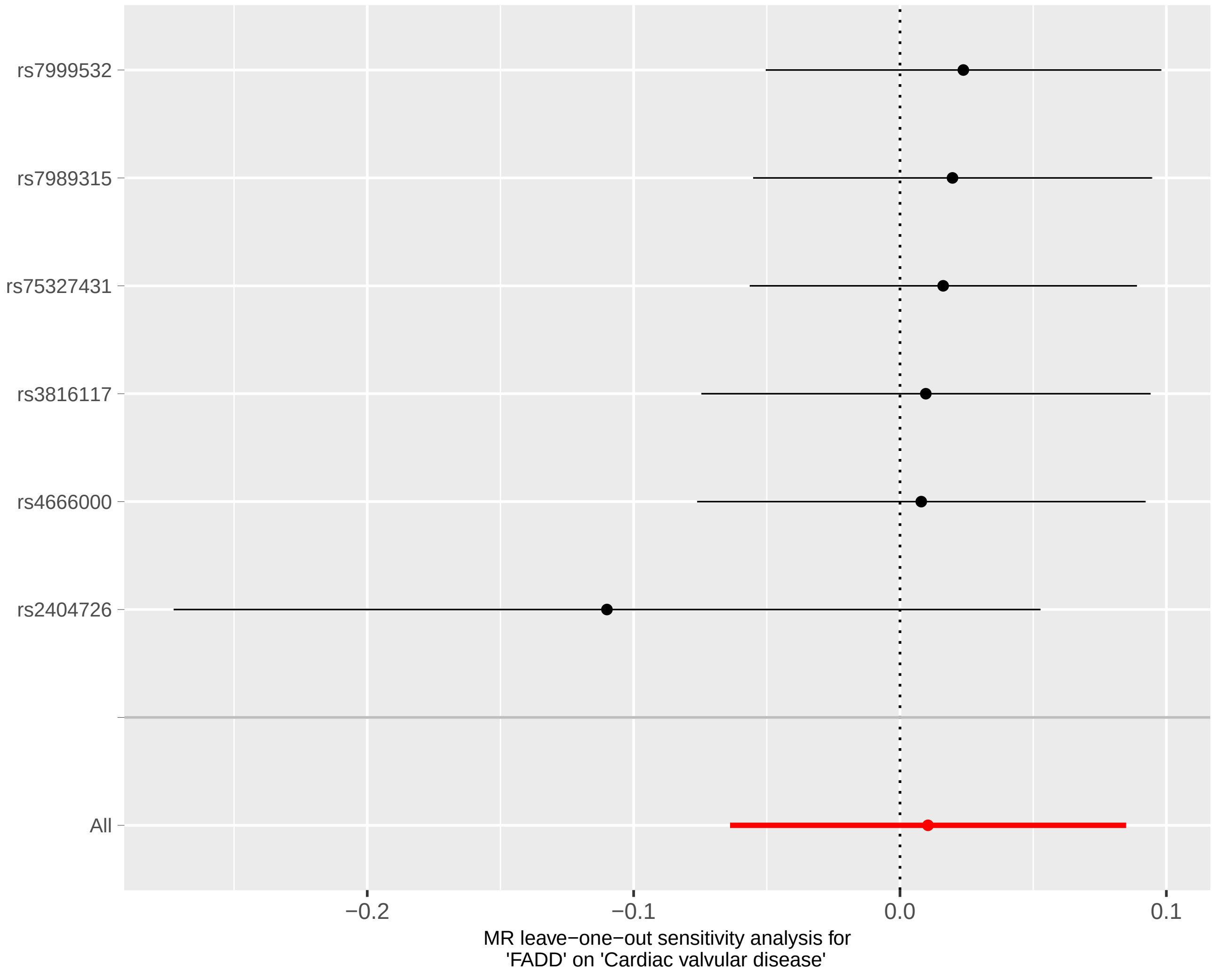

rs7999532
rs7989315
rs75327431
rs3816117
rs4666000
rs2404726
All
−0.2
−0.1
0.0
0.1
MR leave−one−out sensitivity analysis for
'FADD' on 'Cardiac valvular disease'

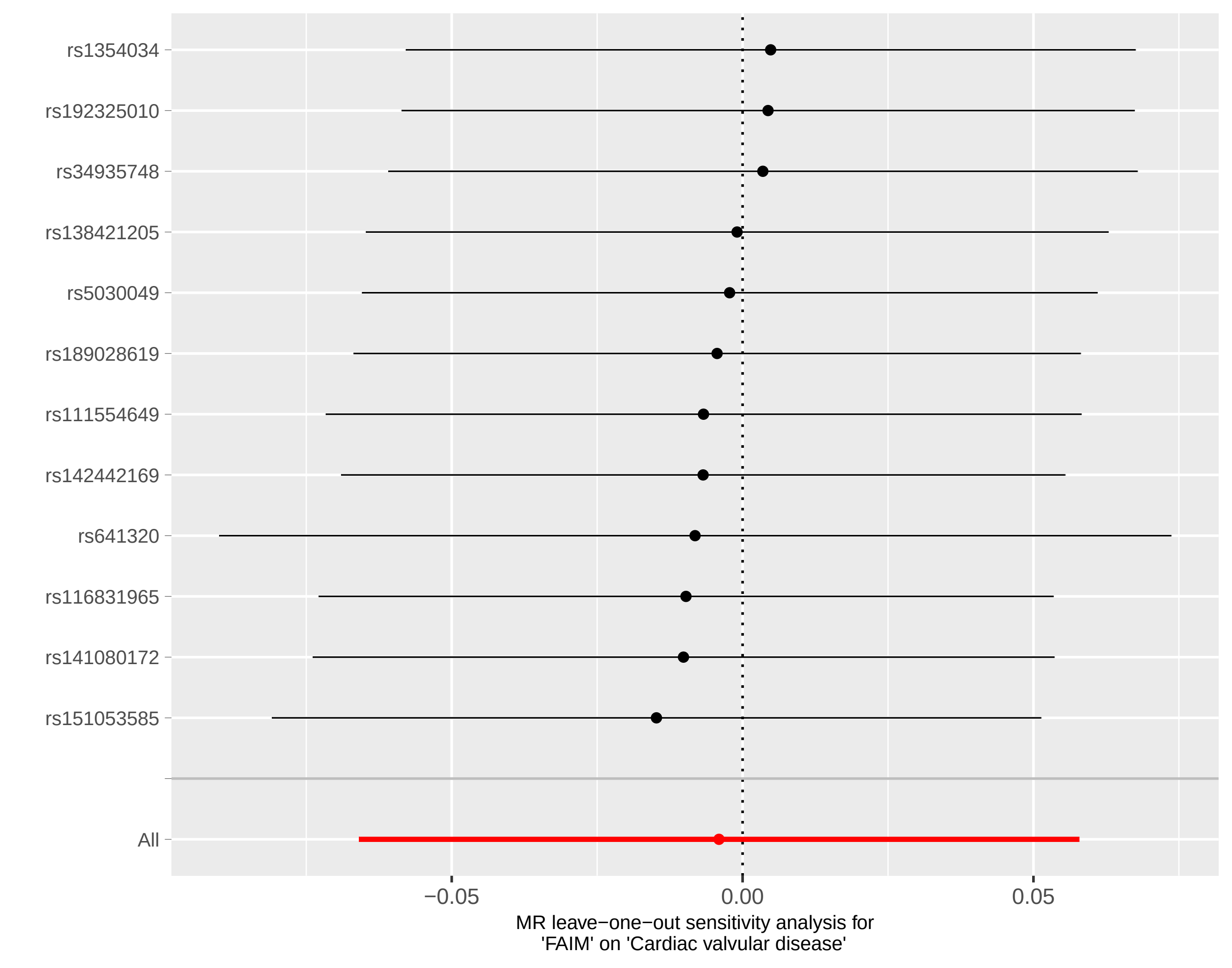

rs1354034
rs192325010
rs34935748
rs138421205
rs5030049
rs189028619
rs111554649
rs142442169
rs641320
rs116831965
rs141080172
rs151053585
All
−0.05
0.00
0.05
MR leave−one−out sensitivity analysis for
'FAIM' on 'Cardiac valvular disease'

Insufficient number of SNPs

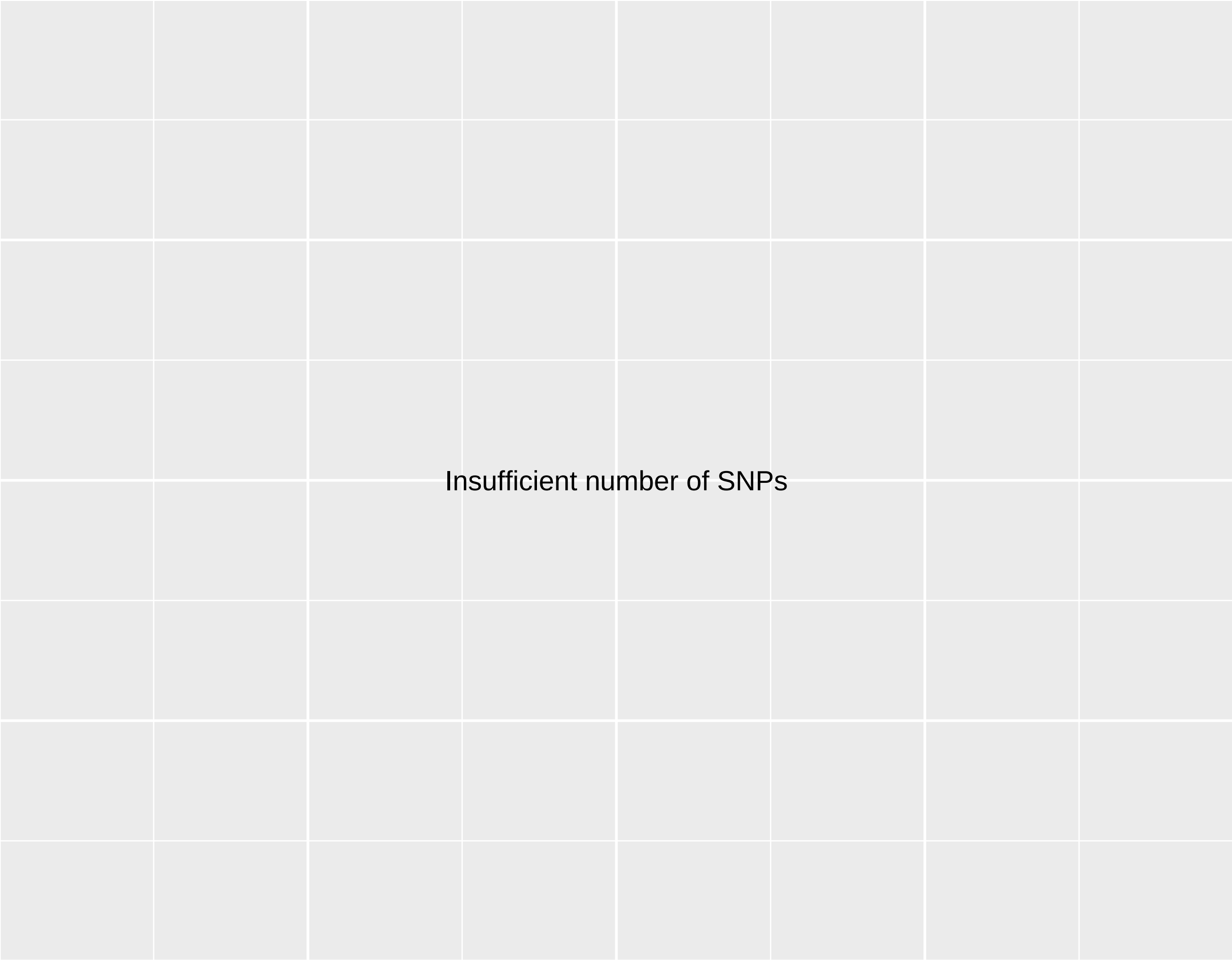
Insufficient number of SNPs

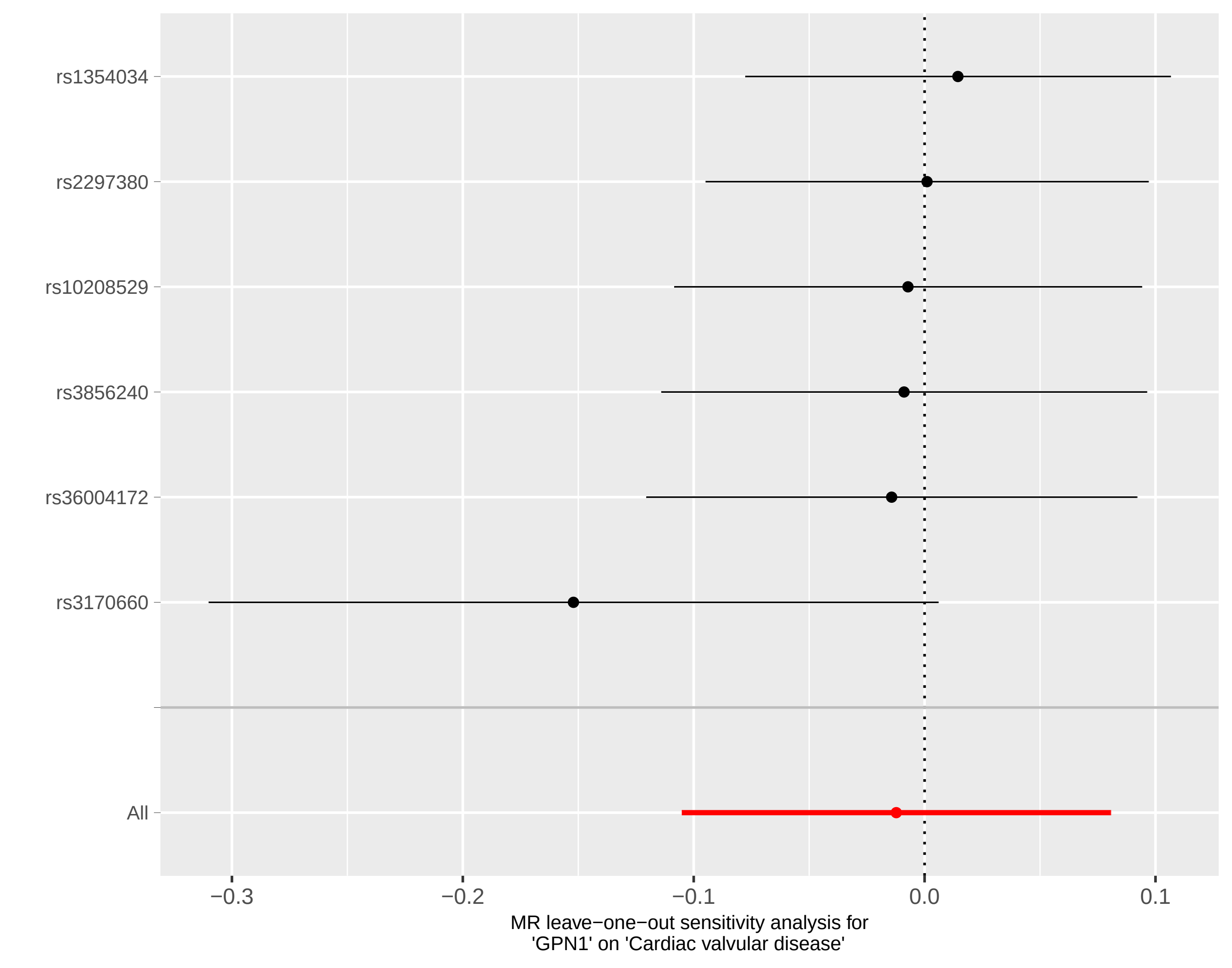

rs1354034
rs2297380
rs10208529
rs3856240
rs36004172
rs3170660
All
−0.3
−0.2
−0.1
0.0
0.1
MR leave−one−out sensitivity analysis for
'GPN1' on 'Cardiac valvular disease'

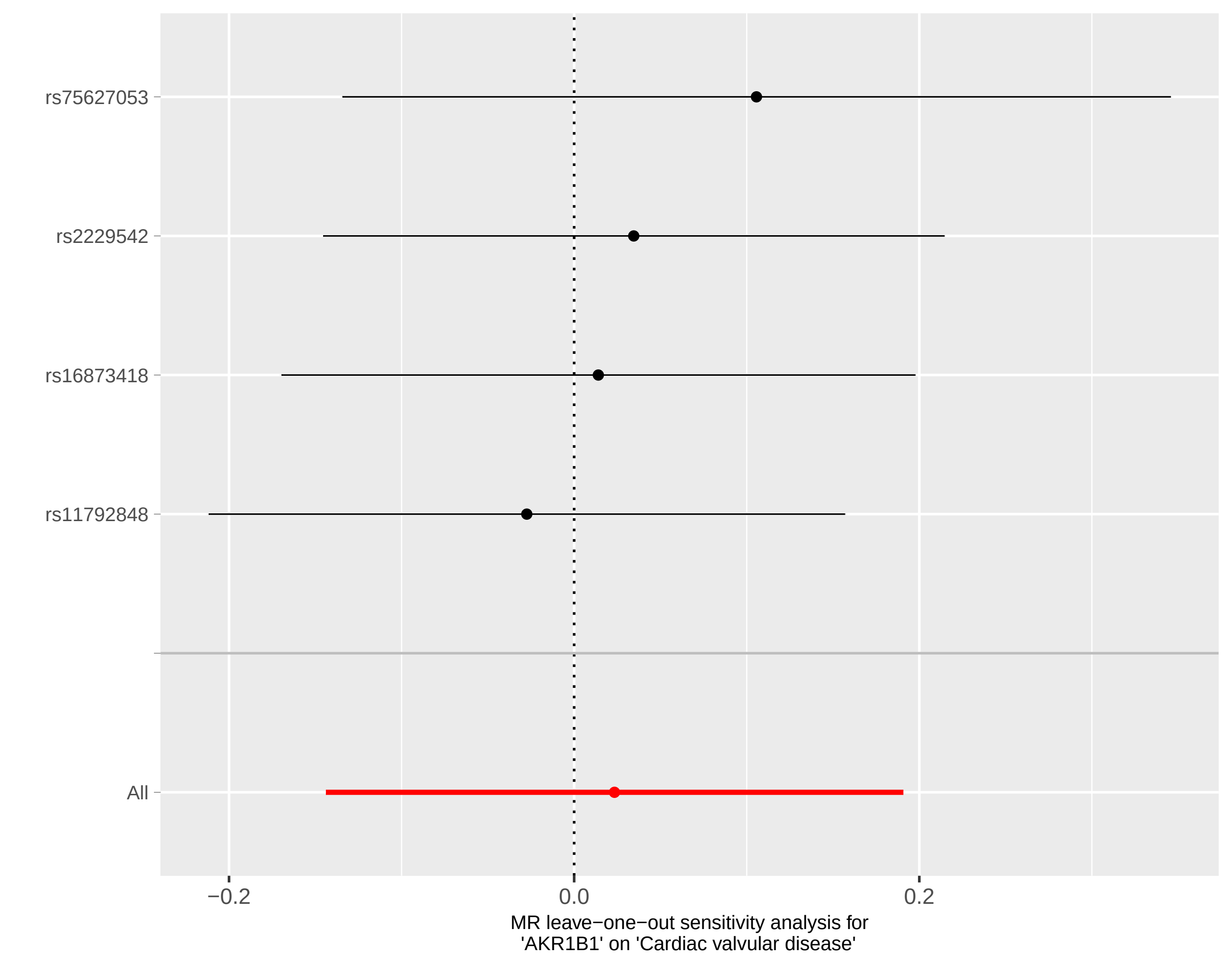
rs75627053
rs2229542
rs16873418
rs11792848
All
−0.2
0.0
0.2
MR leave−one−out sensitivity analysis for
'AKR1B1' on 'Cardiac valvular disease'

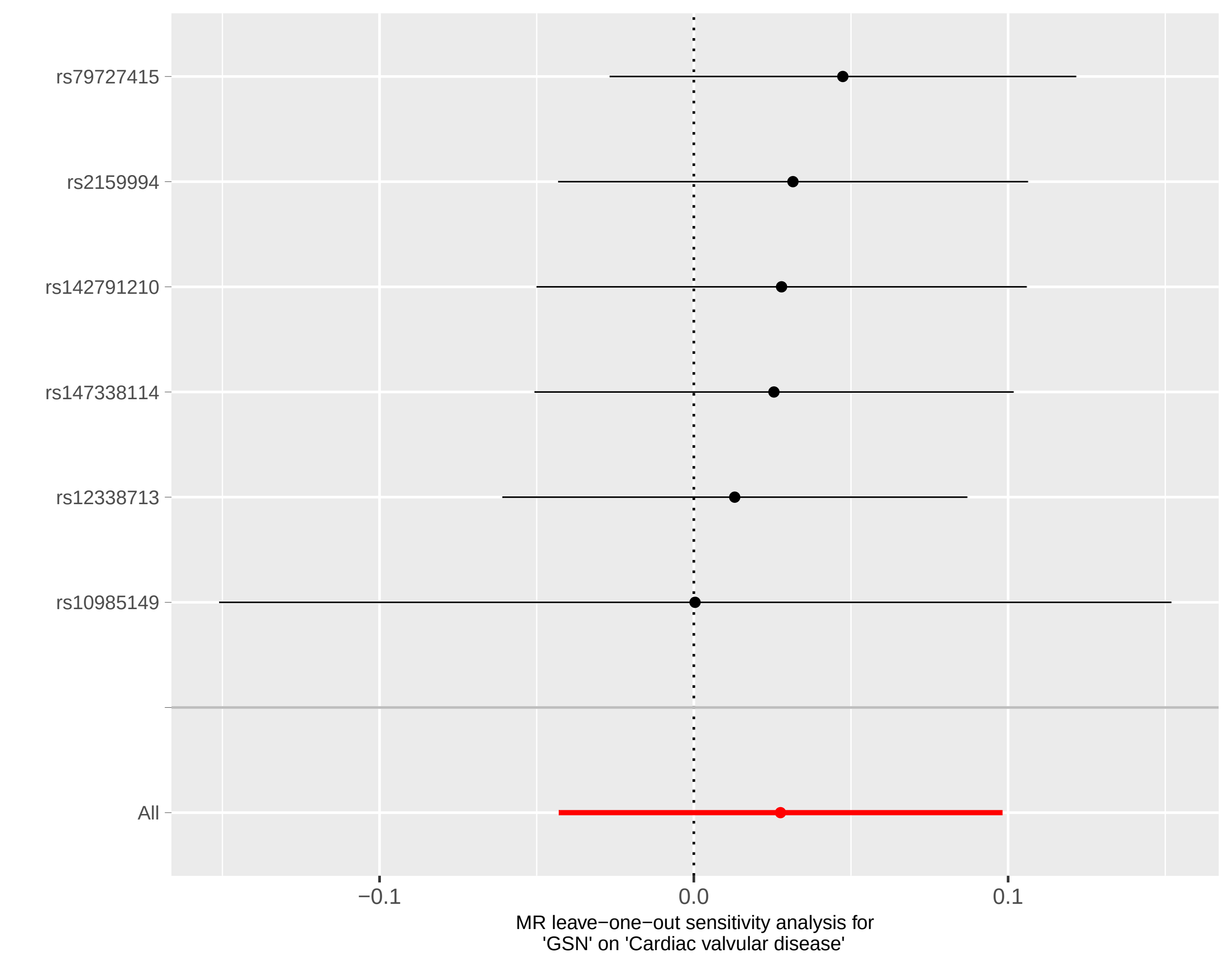

rs79727415
rs2159994
rs142791210
rs147338114
rs12338713
rs10985149
All
−0.1
0.0
0.1
MR leave−one−out sensitivity analysis for
'GSN' on 'Cardiac valvular disease'

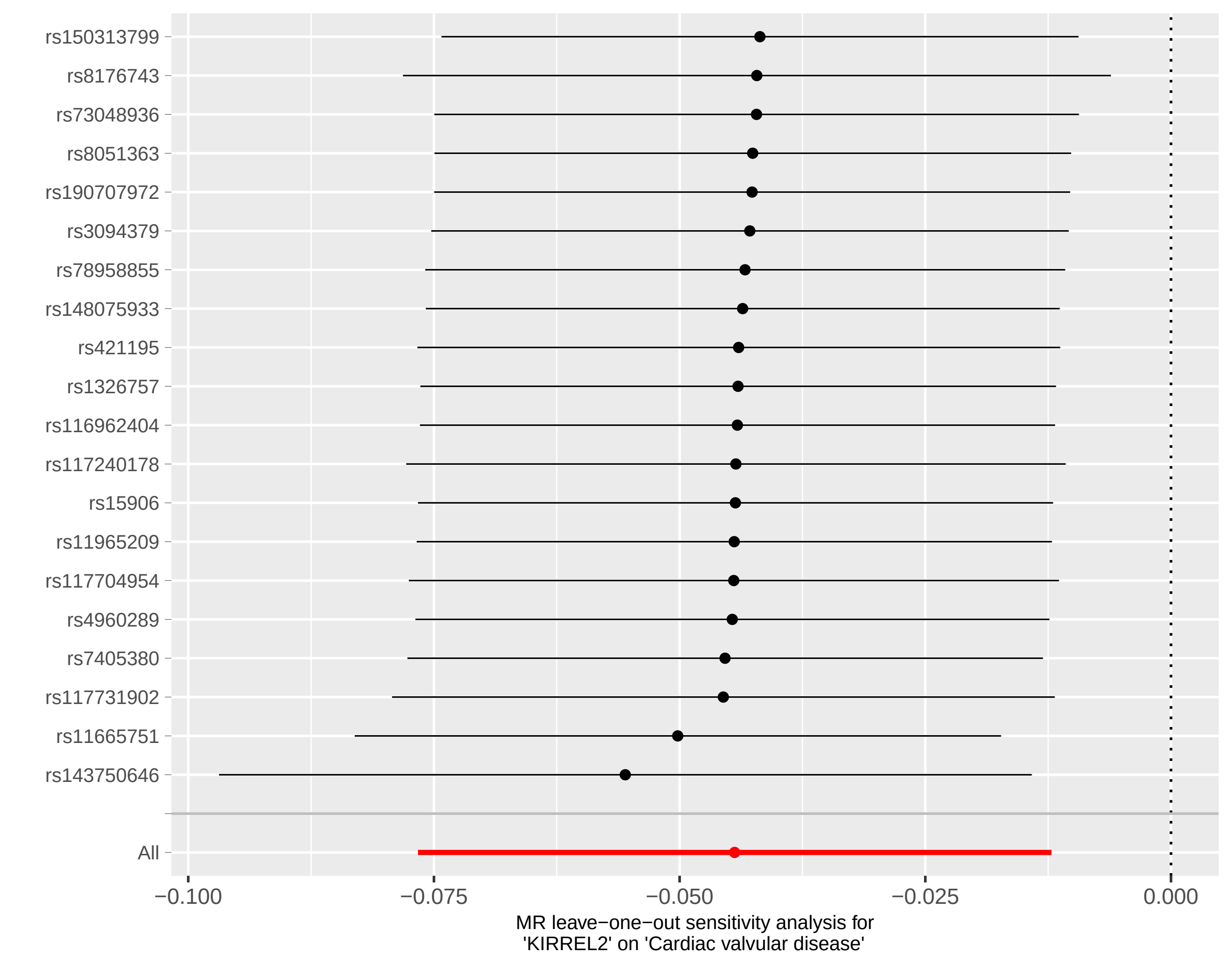
rs150313799
rs8176743
rs73048936
rs8051363
rs190707972
rs3094379
rs78958855
rs148075933
rs421195
rs1326757
rs116962404
rs117240178
rs15906
rs11965209
rs117704954
rs4960289
rs7405380
rs117731902
rs11665751
rs143750646
All
−0.100
−0.075
−0.050
−0.025
0.000
MR leave−one−out sensitivity analysis for
'KIRREL2' on 'Cardiac valvular disease'

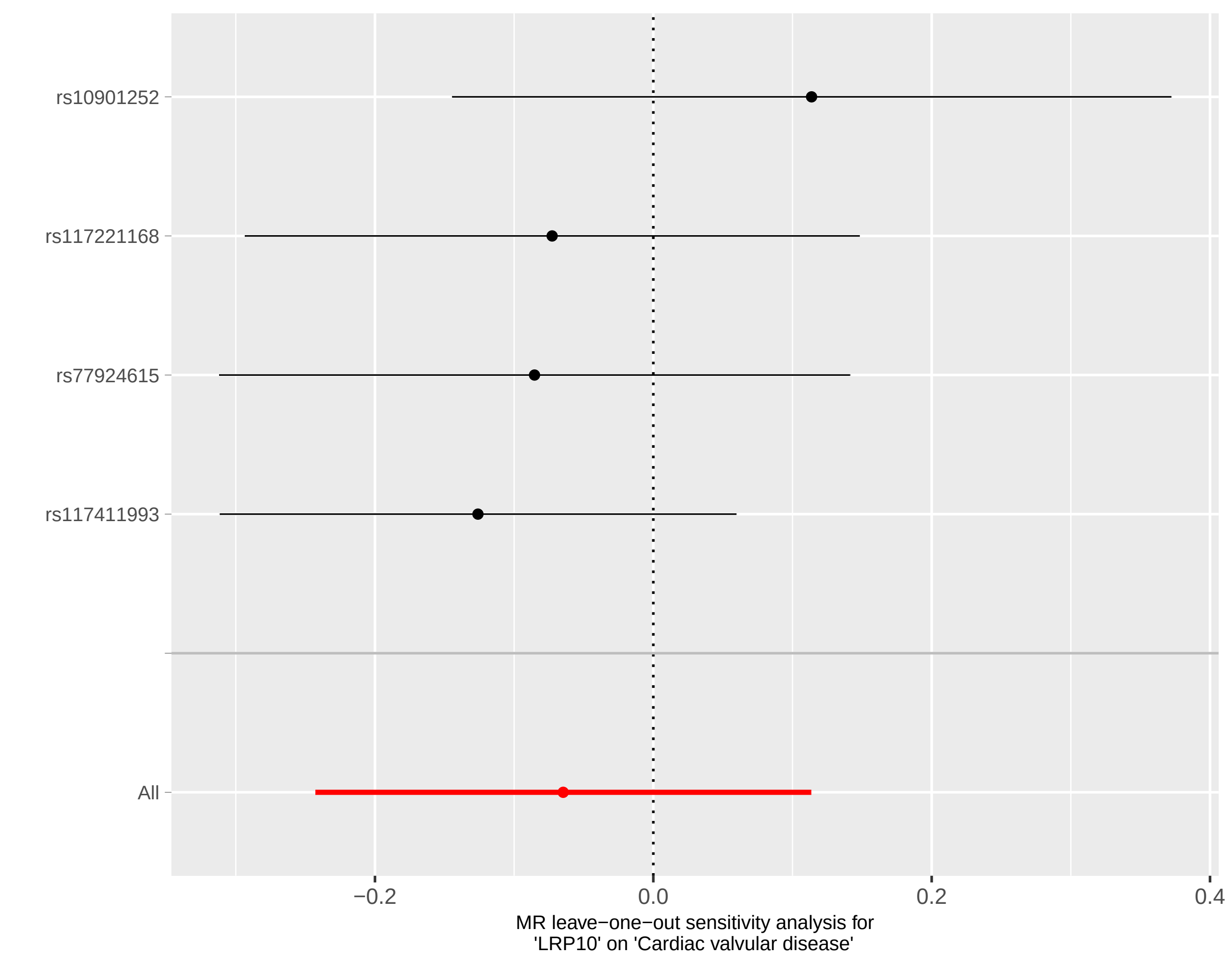

rs10901252
rs117221168
rs77924615
rs117411993
All
−0.2
0.0
0.2
0.4
MR leave−one−out sensitivity analysis for
'LRP10' on 'Cardiac valvular disease'

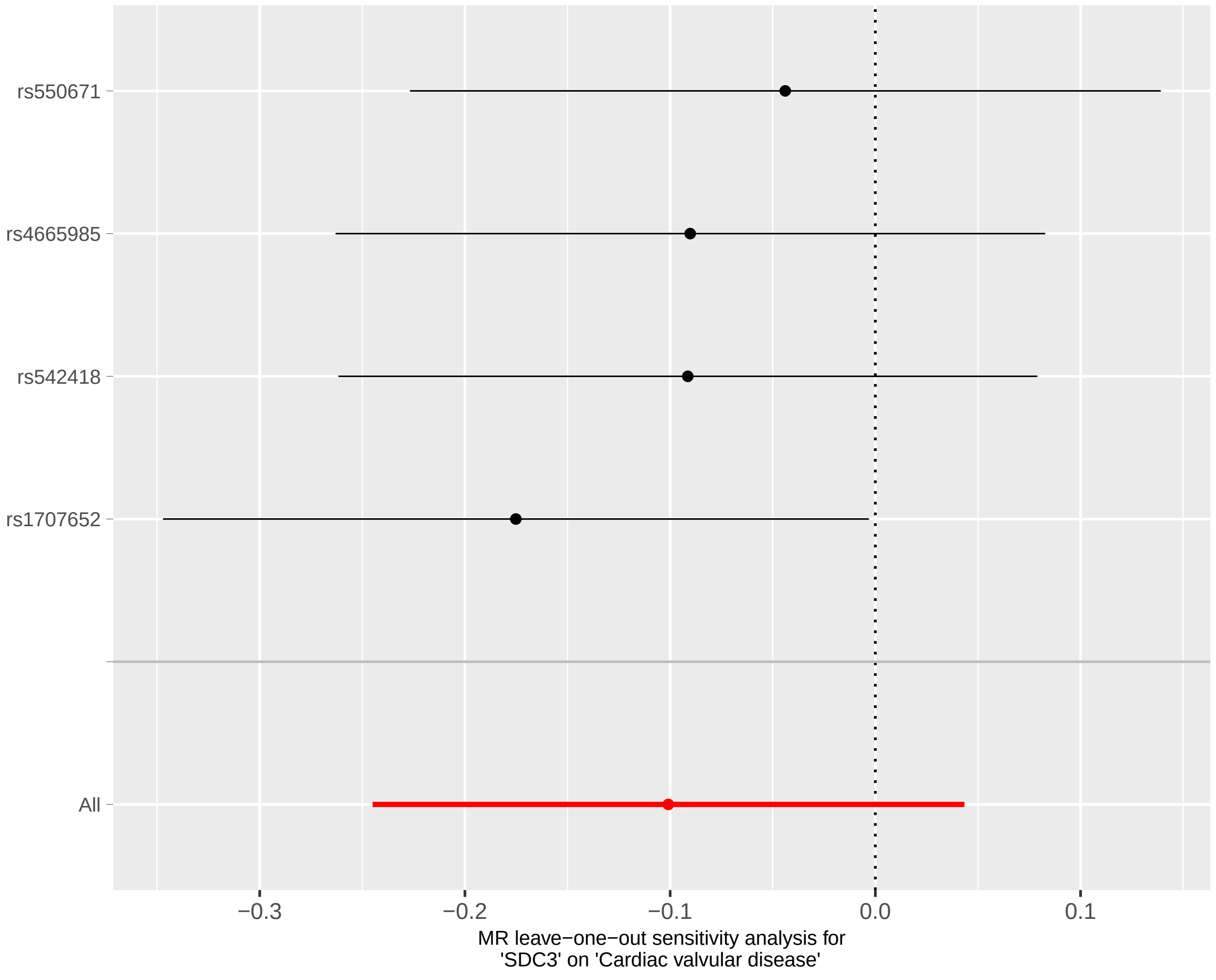
rs550671
rs4665985
rs542418
rs1707652
All
−0.3
−0.2
−0.1
0.0
0.1
MR leave−one−out sensitivity analysis for
'SDC3' on 'Cardiac valvular disease'

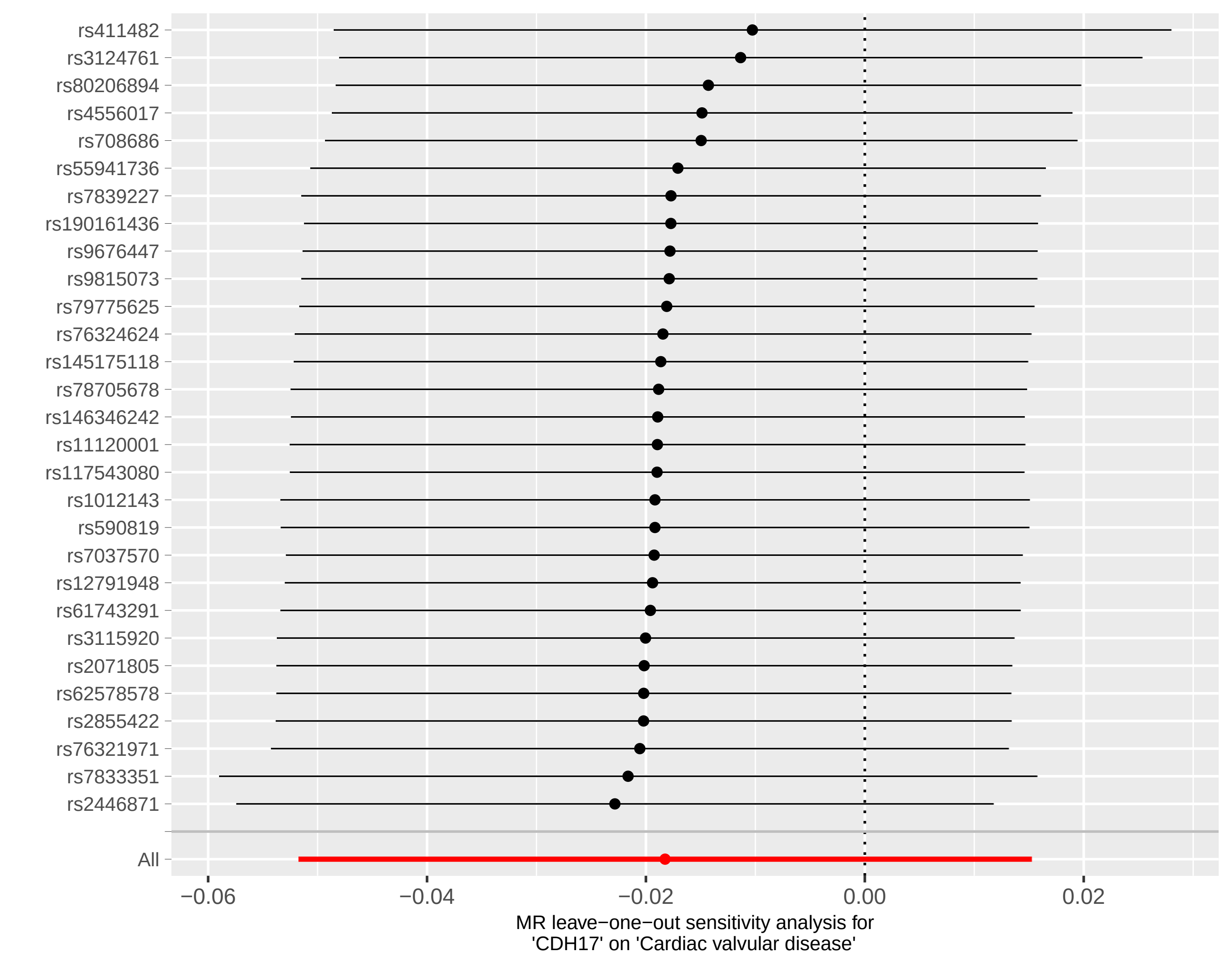
rs411482
rs3124761
rs80206894
rs4556017
rs708686
rs55941736
rs7839227
rs190161436
rs9676447
rs9815073
rs79775625
rs76324624
rs145175118
rs78705678
rs146346242
rs11120001
rs117543080
rs1012143
rs590819
rs7037570
rs12791948
rs61743291
rs3115920
rs2071805
rs62578578
rs2855422
rs76321971
rs7833351
rs2446871
All
−0.06
−0.04
−0.02
0.00
0.02
MR leave−one−out sensitivity analysis for
'CDH17' on 'Cardiac valvular disease'

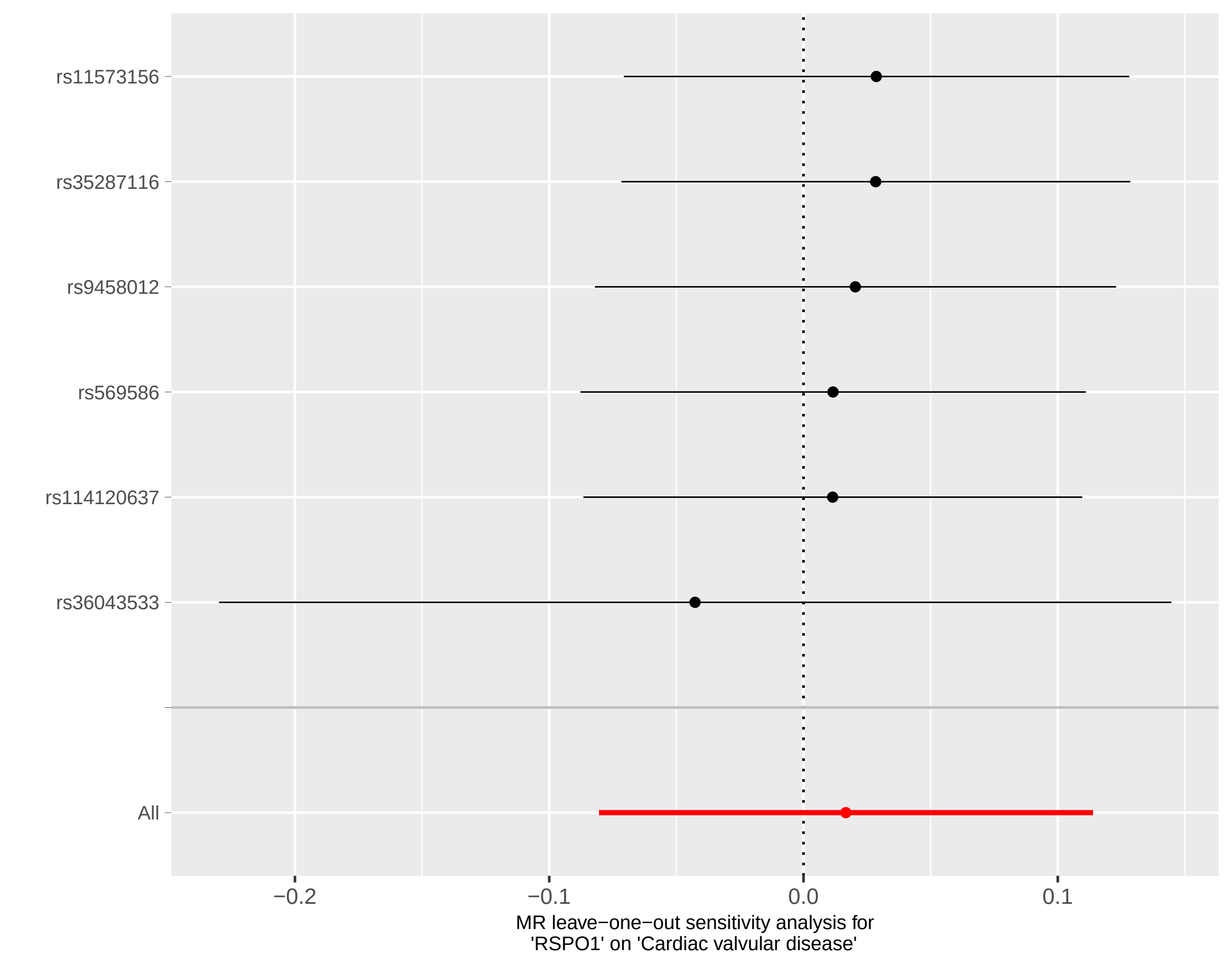

rs11573156
rs35287116
rs9458012
rs569586
rs114120637
rs36043533
All
−0.2
−0.1
0.0
0.1
MR leave−one−out sensitivity analysis for
'RSPO1' on 'Cardiac valvular disease'

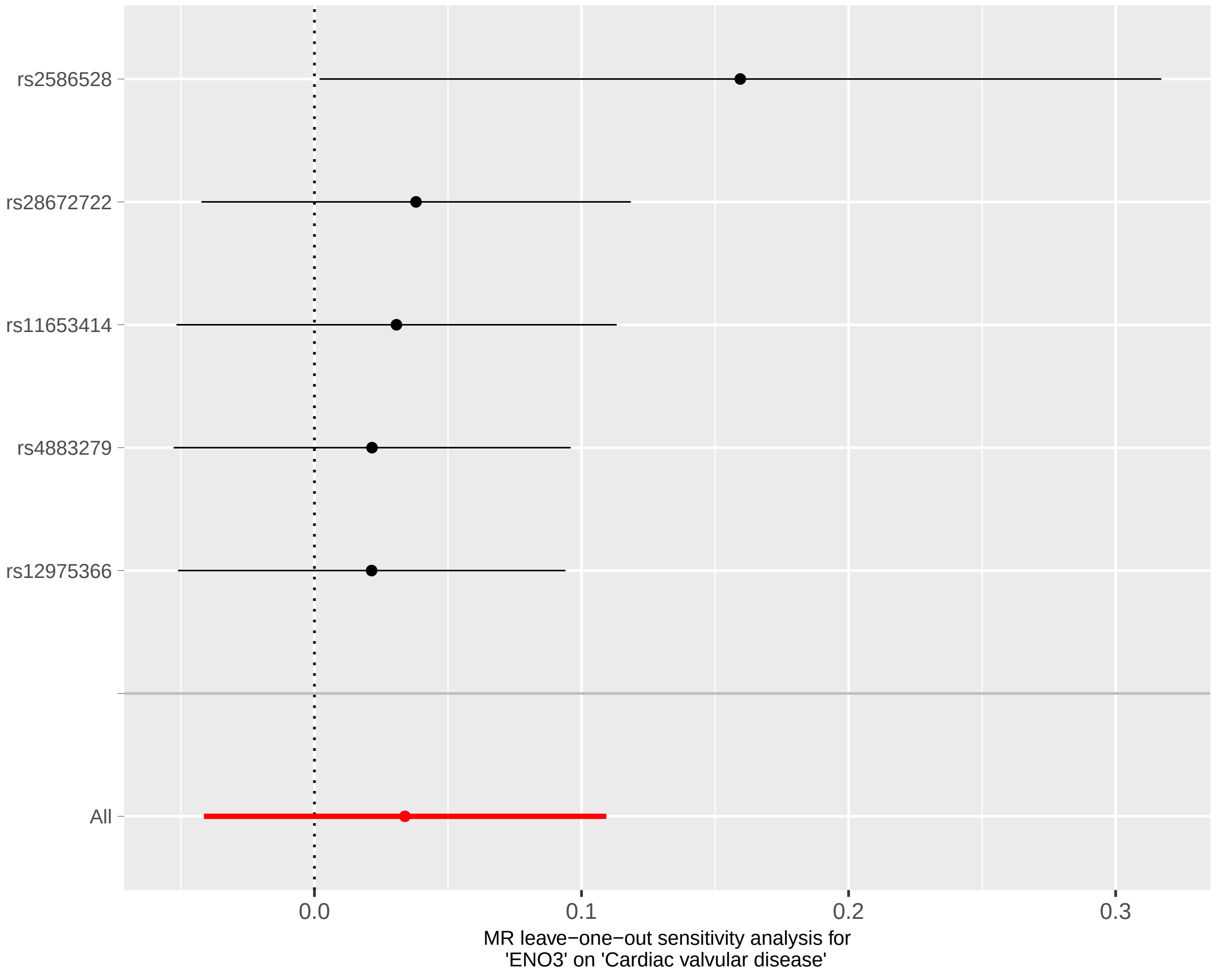
rs2586528
rs28672722
rs11653414
rs4883279
rs12975366
All
0.0
0.1
0.2
0.3
MR leave−one−out sensitivity analysis for
'ENO3' on 'Cardiac valvular disease'

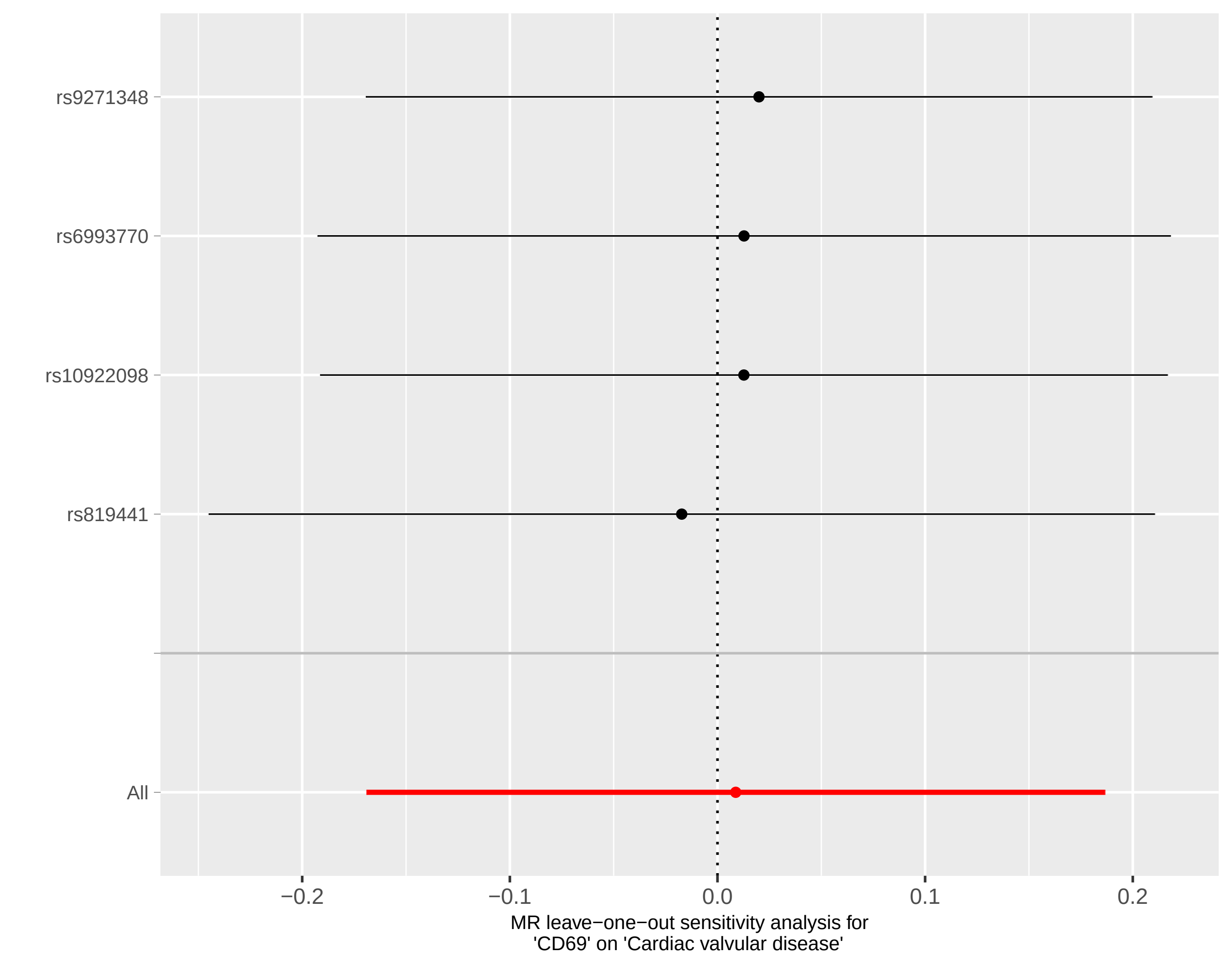
rs9271348
rs6993770
rs10922098
rs819441
All
−0.2
−0.1
0.0
0.1
0.2
MR leave−one−out sensitivity analysis for
'CD69' on 'Cardiac valvular disease'

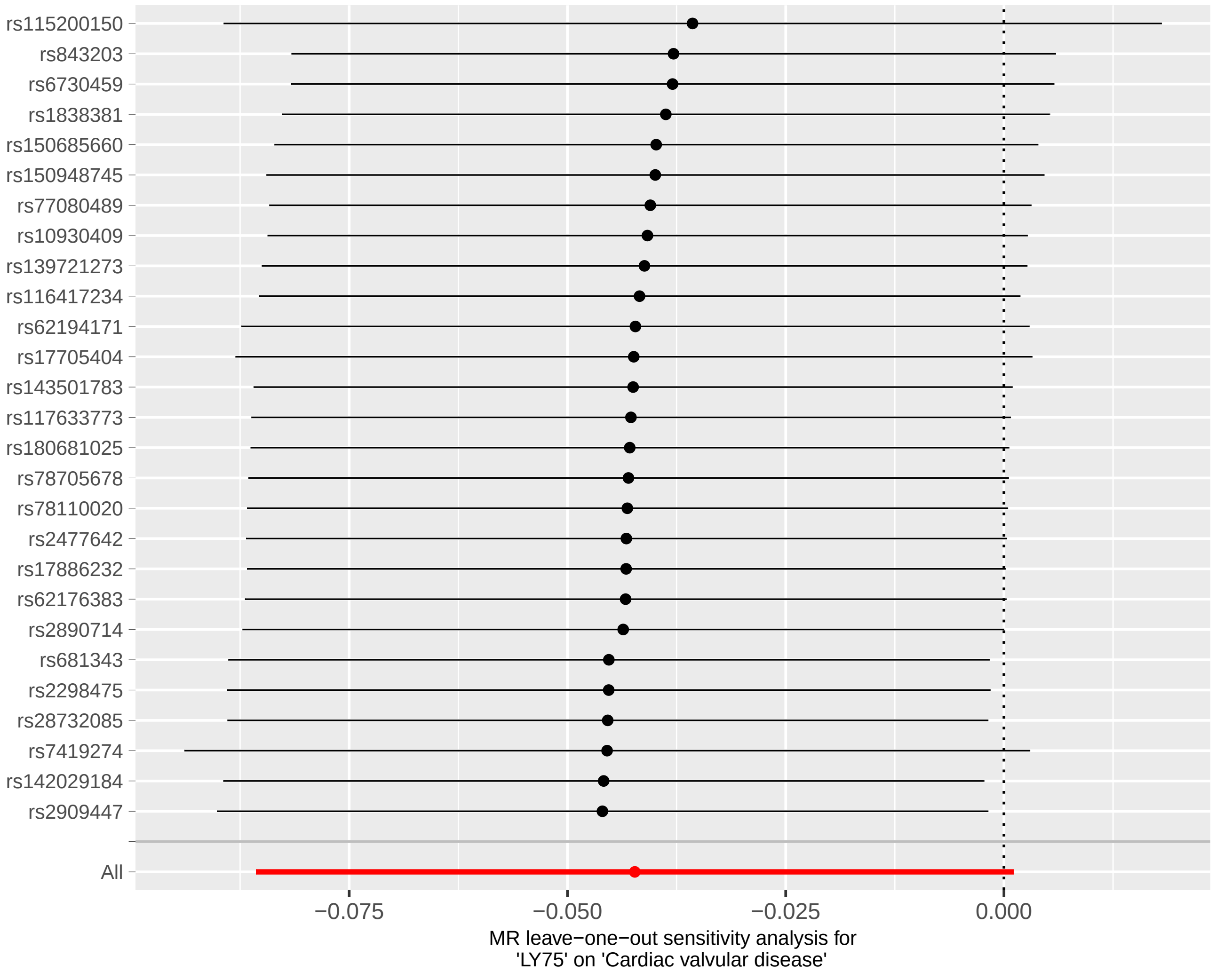
rs115200150
rs843203
rs6730459
rs1838381
rs150685660
rs150948745
rs77080489
rs10930409
rs139721273
rs116417234
rs62194171
rs17705404
rs143501783
rs117633773
rs180681025
rs78705678
rs78110020
rs2477642
rs17886232
rs62176383
rs2890714
rs681343
rs2298475
rs28732085
rs7419274
rs142029184
rs2909447
All
−0.075
−0.050
−0.025
0.000
MR leave−one−out sensitivity analysis for
'LY75' on 'Cardiac valvular disease'

Insufficient number of SNPs

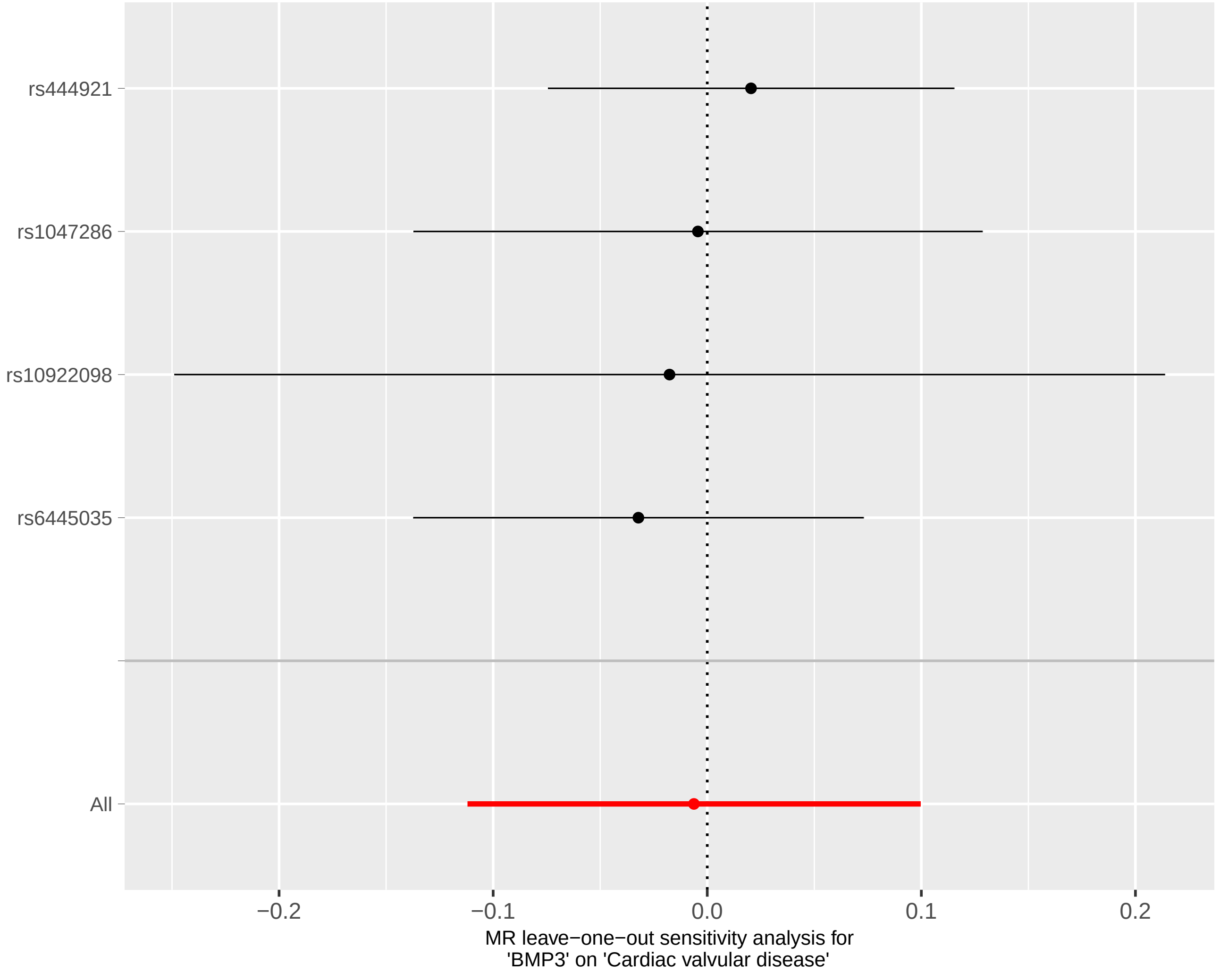
rs444921
rs1047286
rs10922098
rs6445035
All
−0.2
−0.1
0.0
0.1
0.2
MR leave−one−out sensitivity analysis for
'BMP3' on 'Cardiac valvular disease'

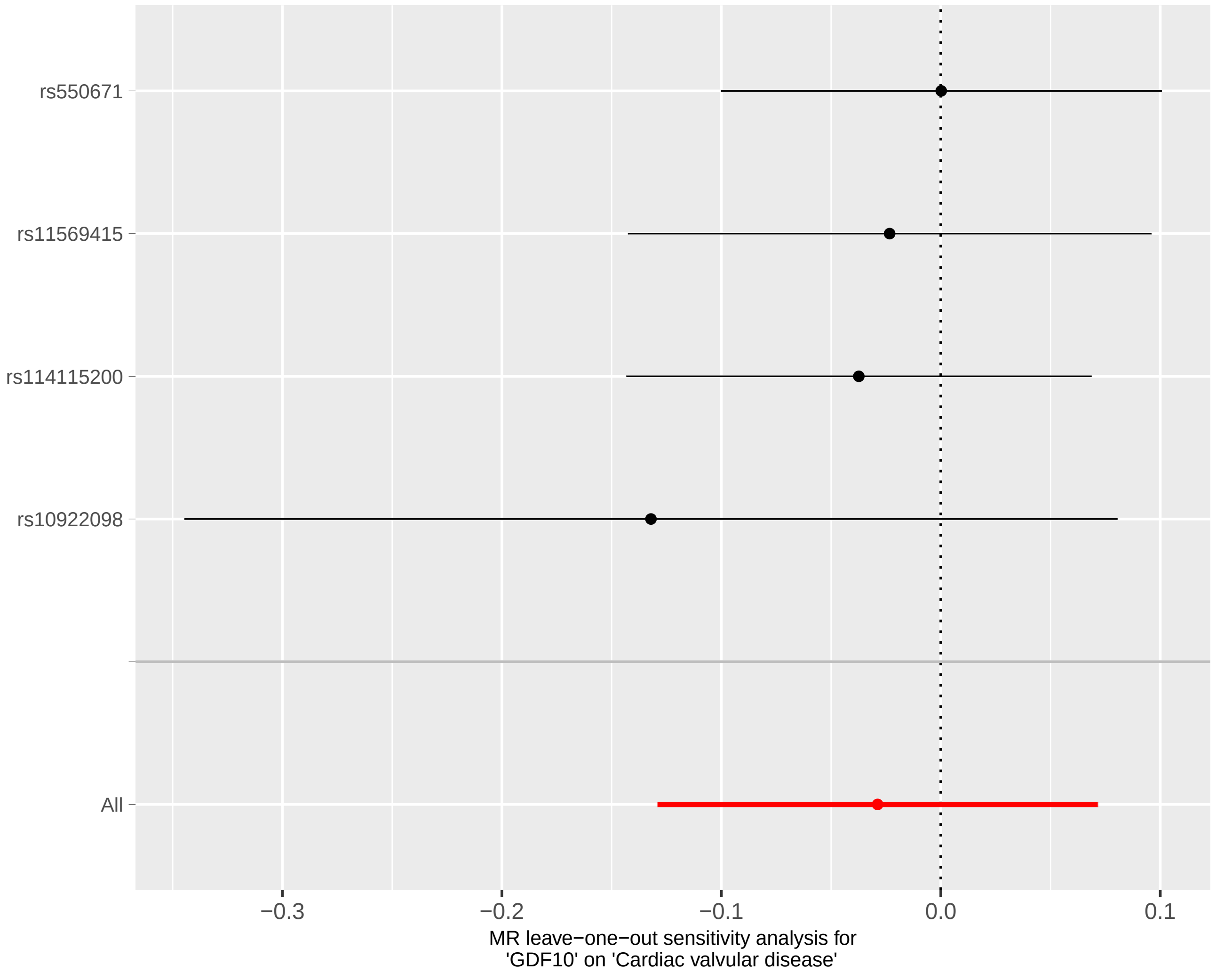

rs550671
rs11569415
rs114115200
rs10922098
All
−0.3
−0.2
−0.1
0.0
0.1
MR leave−one−out sensitivity analysis for
'GDF10' on 'Cardiac valvular disease'

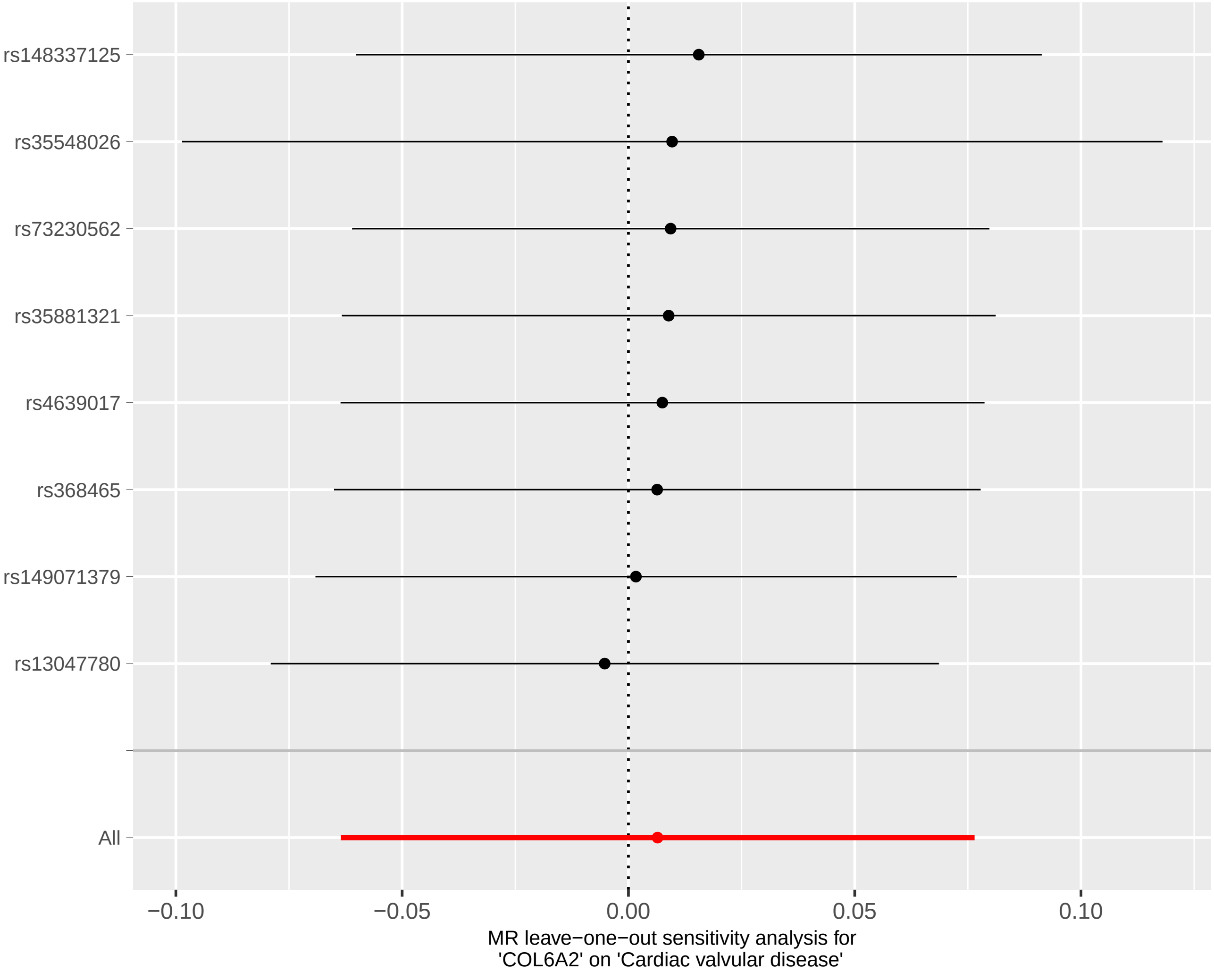

rs148337125
rs35548026
rs73230562
rs35881321
rs4639017
rs368465
rs149071379
rs13047780
All
−0.10
−0.05
0.00
0.05
0.10
MR leave−one−out sensitivity analysis for
'COL6A2' on 'Cardiac valvular disease'

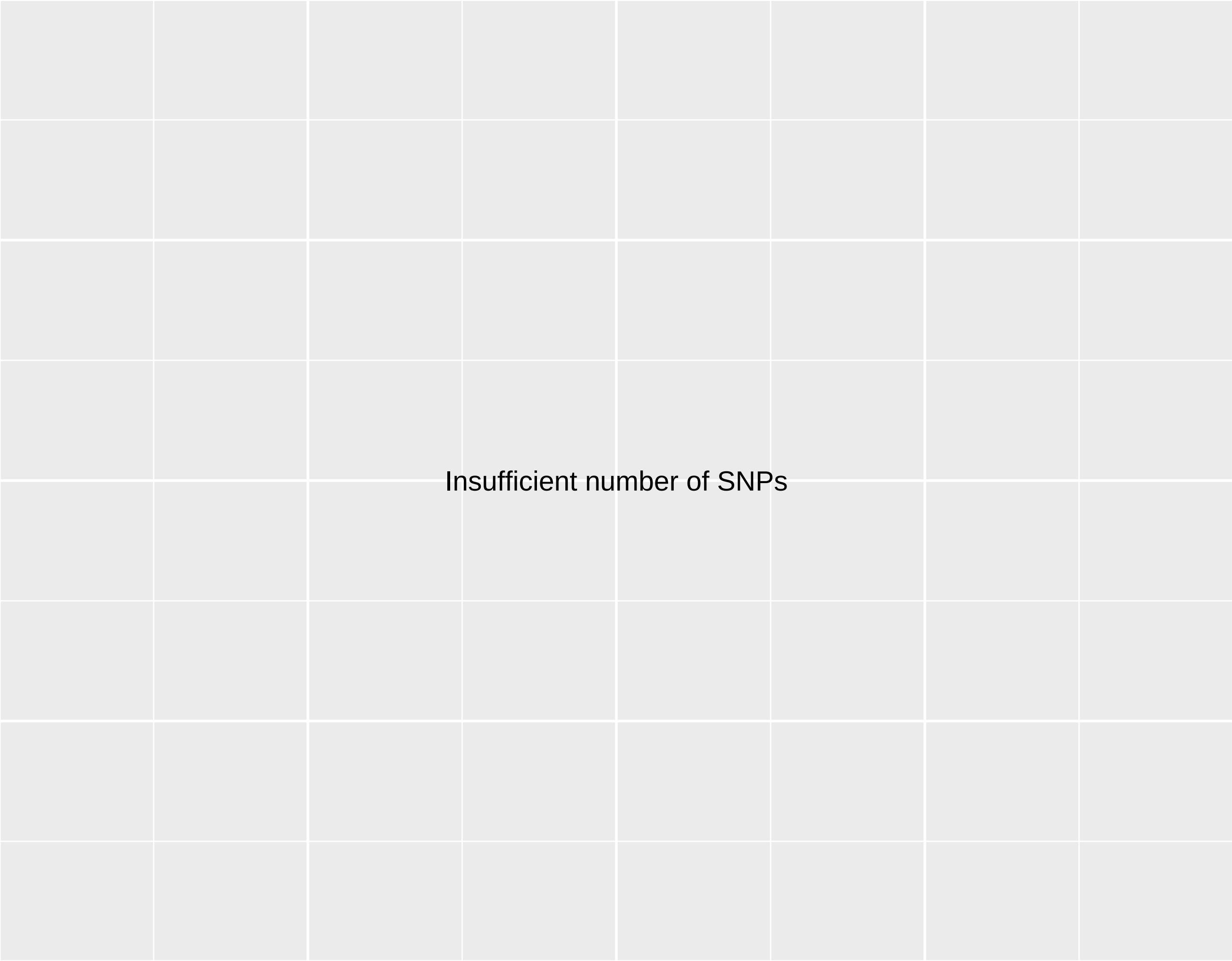
Insufficient number of SNPs

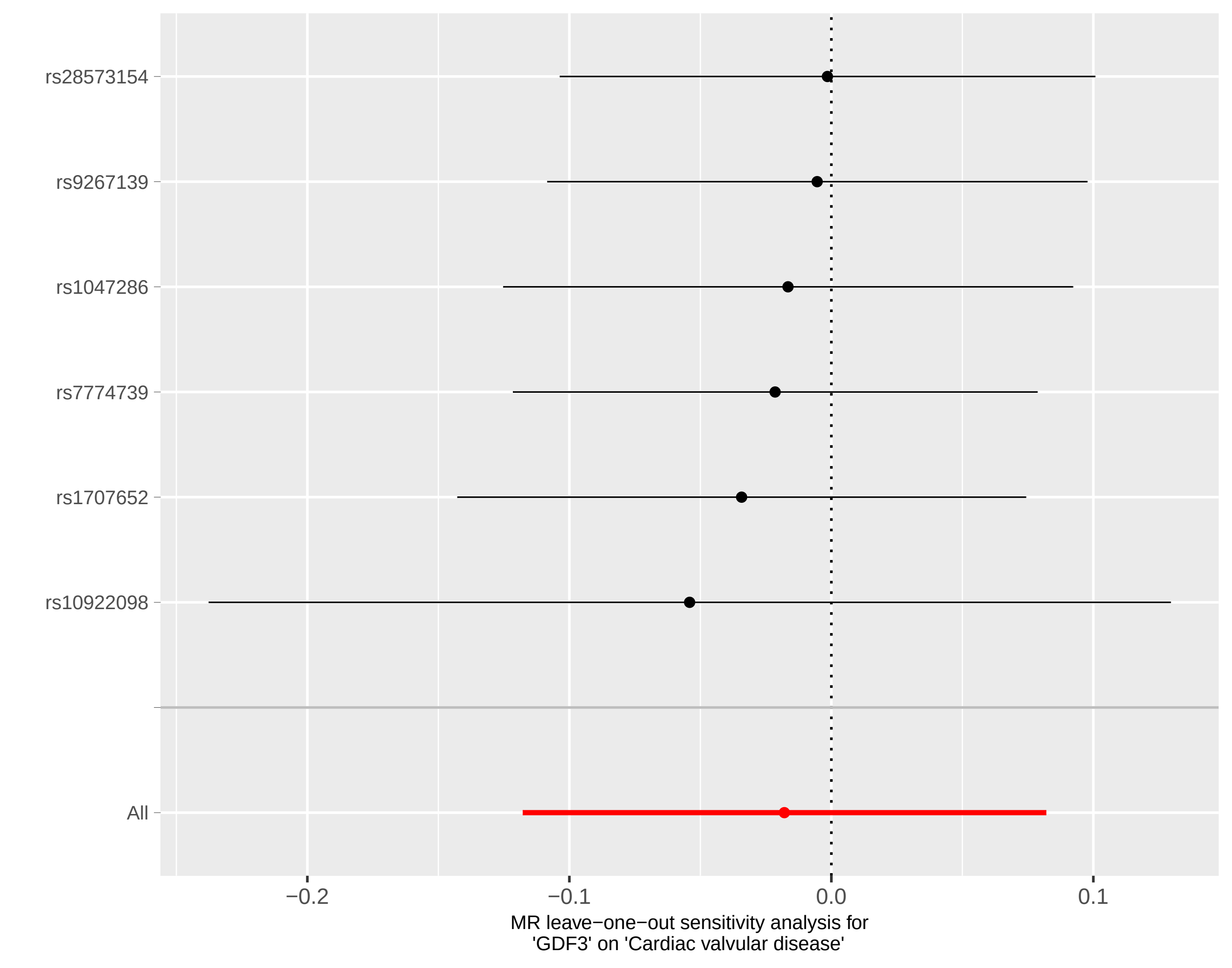
rs28573154
rs9267139
rs1047286
rs7774739
rs1707652
rs10922098
All
−0.2
−0.1
0.0
0.1
MR leave−one−out sensitivity analysis for
'GDF3' on 'Cardiac valvular disease'

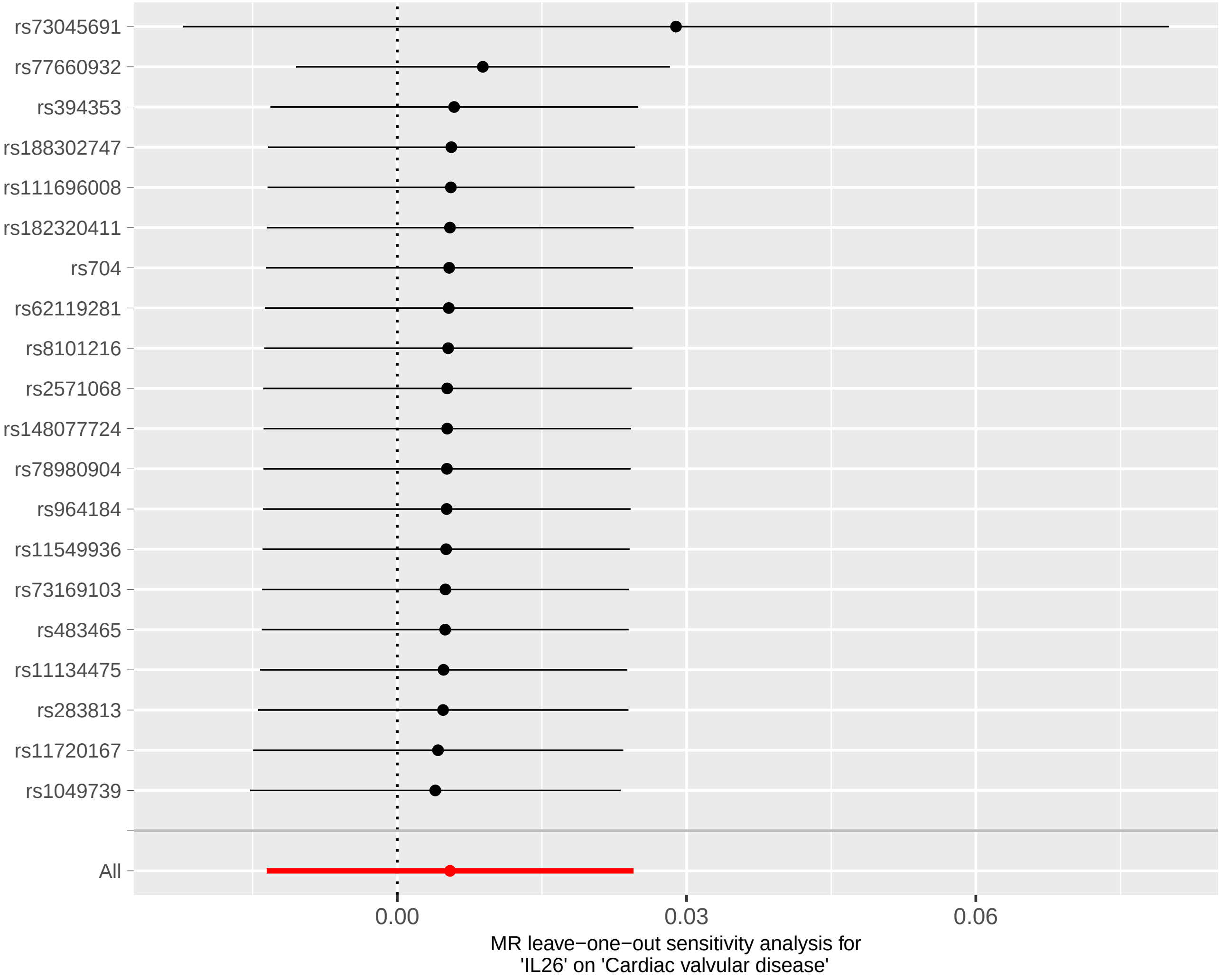
rs73045691
rs77660932
rs394353
rs188302747
rs111696008
rs182320411
rs704
rs62119281
rs8101216
rs2571068
rs148077724
rs78980904
rs964184
rs11549936
rs73169103
rs483465
rs11134475
rs283813
rs11720167
rs1049739
All
0.00
0.03
0.06
MR leave−one−out sensitivity analysis for
'IL26' on 'Cardiac valvular disease'

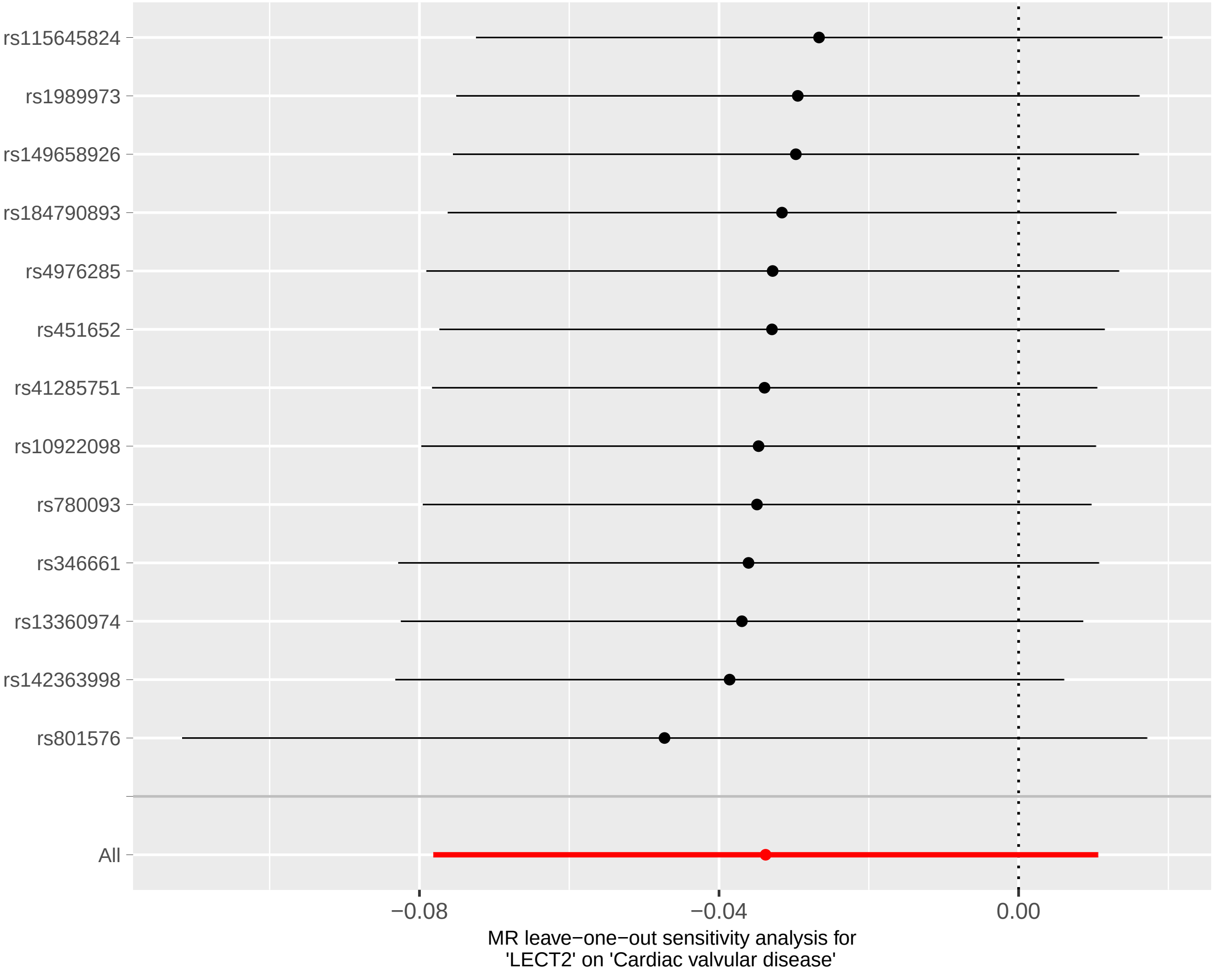
rs115645824
rs1989973
rs149658926
rs184790893
rs4976285
rs451652
rs41285751
rs10922098
rs780093
rs346661
rs13360974
rs142363998
rs801576
All
−0.08
−0.04
0.00
MR leave−one−out sensitivity analysis for
'LECT2' on 'Cardiac valvular disease'

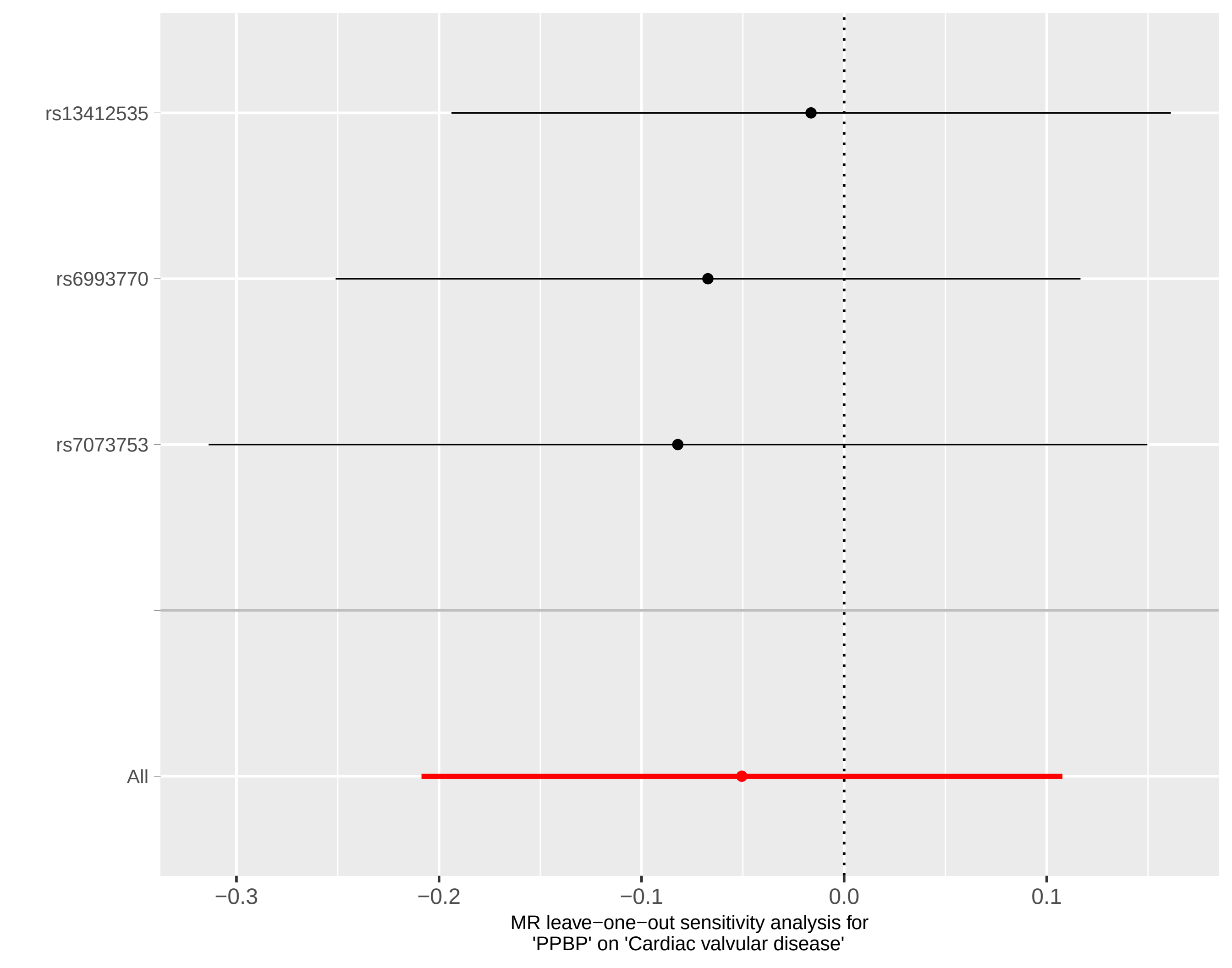
rs13412535
rs6993770
rs7073753
All
−0.3
−0.2
−0.1
0.0
0.1
MR leave−one−out sensitivity analysis for
'PPBP' on 'Cardiac valvular disease'

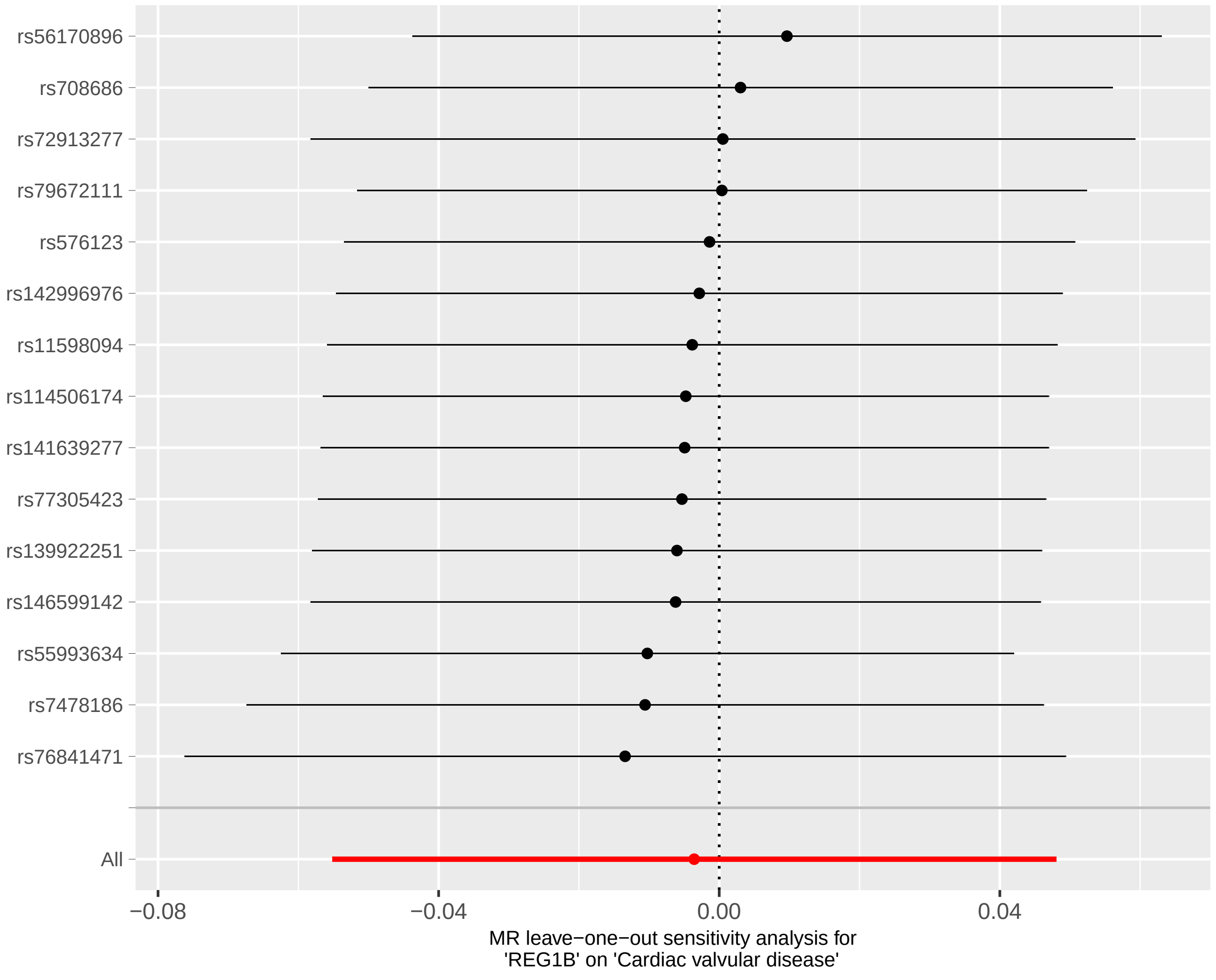
rs56170896
rs708686
rs72913277
rs79672111
rs576123
rs142996976
rs11598094
rs114506174
rs141639277
rs77305423
rs139922251
rs146599142
rs55993634
rs7478186
rs76841471
All
−0.08
−0.04
0.00
0.04
MR leave−one−out sensitivity analysis for
'REG1B' on 'Cardiac valvular disease'

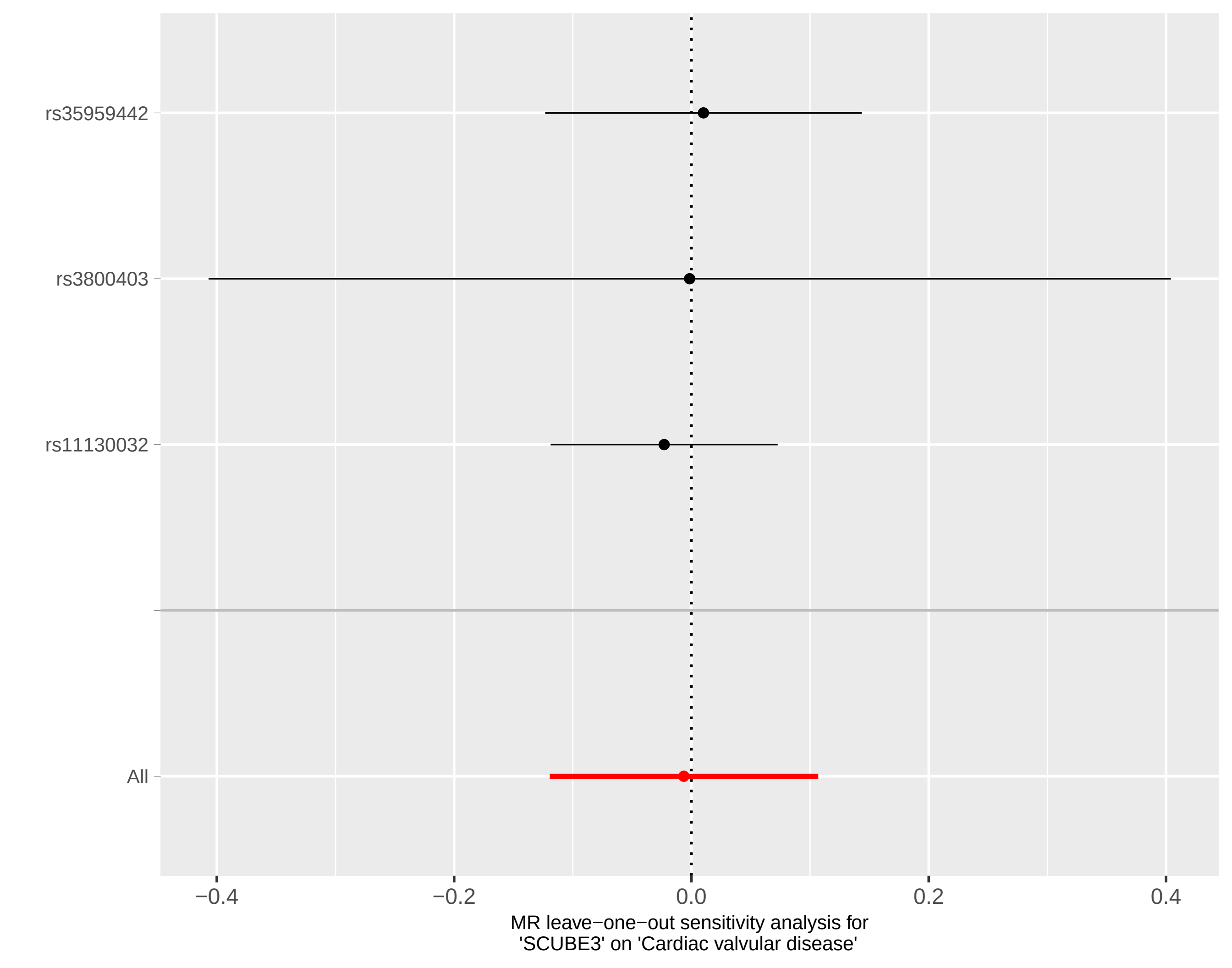
rs35959442
rs3800403
rs11130032
All
−0.4
−0.2
0.0
0.2
0.4
MR leave−one−out sensitivity analysis for
'SCUBE3' on 'Cardiac valvular disease'

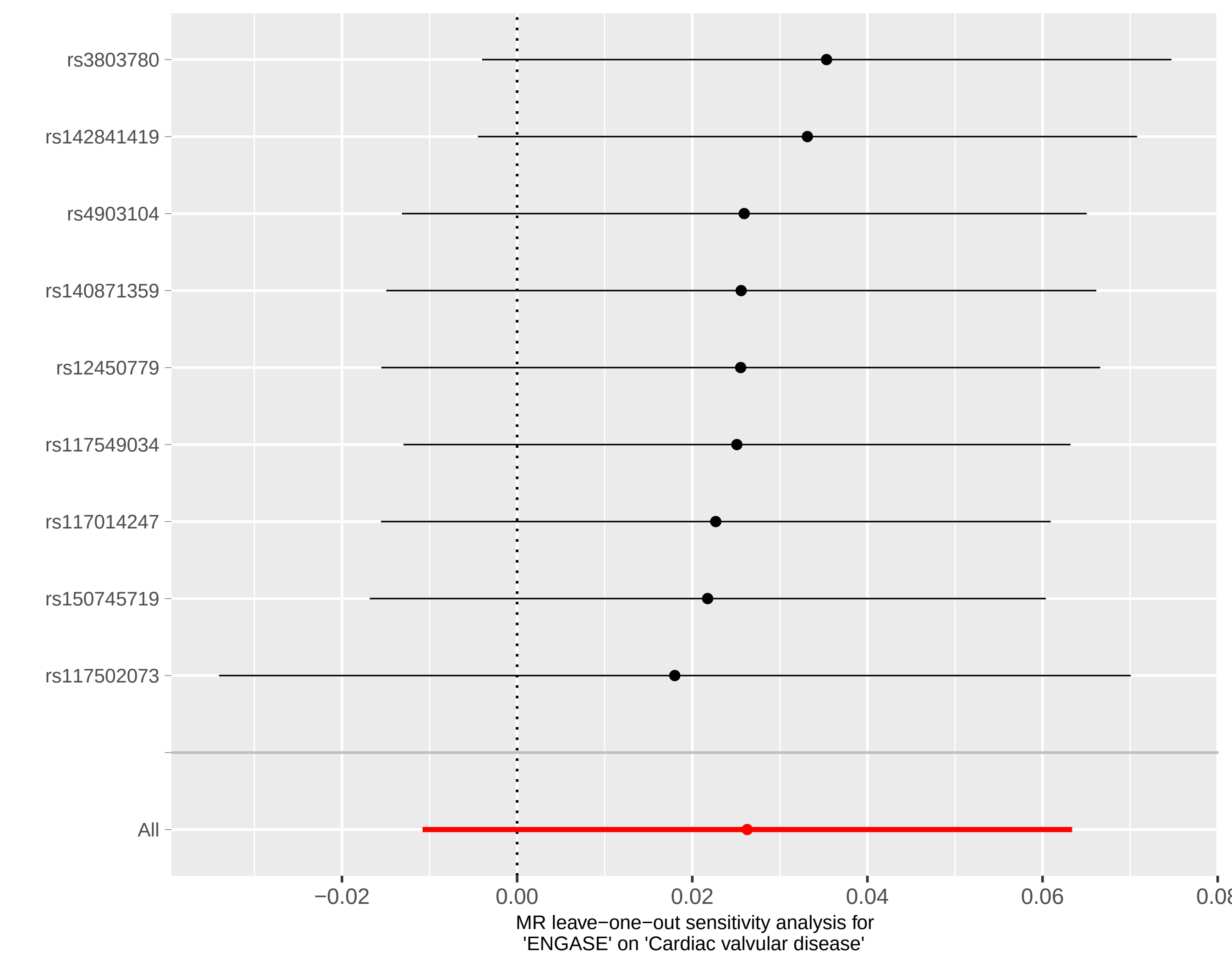
rs3803780
rs142841419
rs4903104
rs140871359
rs12450779
rs117549034
rs117014247
rs150745719
rs117502073
All
−0.02
0.00
0.02
0.04
0.06
MR leave−one−out sensitivity analysis for
'ENGASE' on 'Cardiac valvular disease'

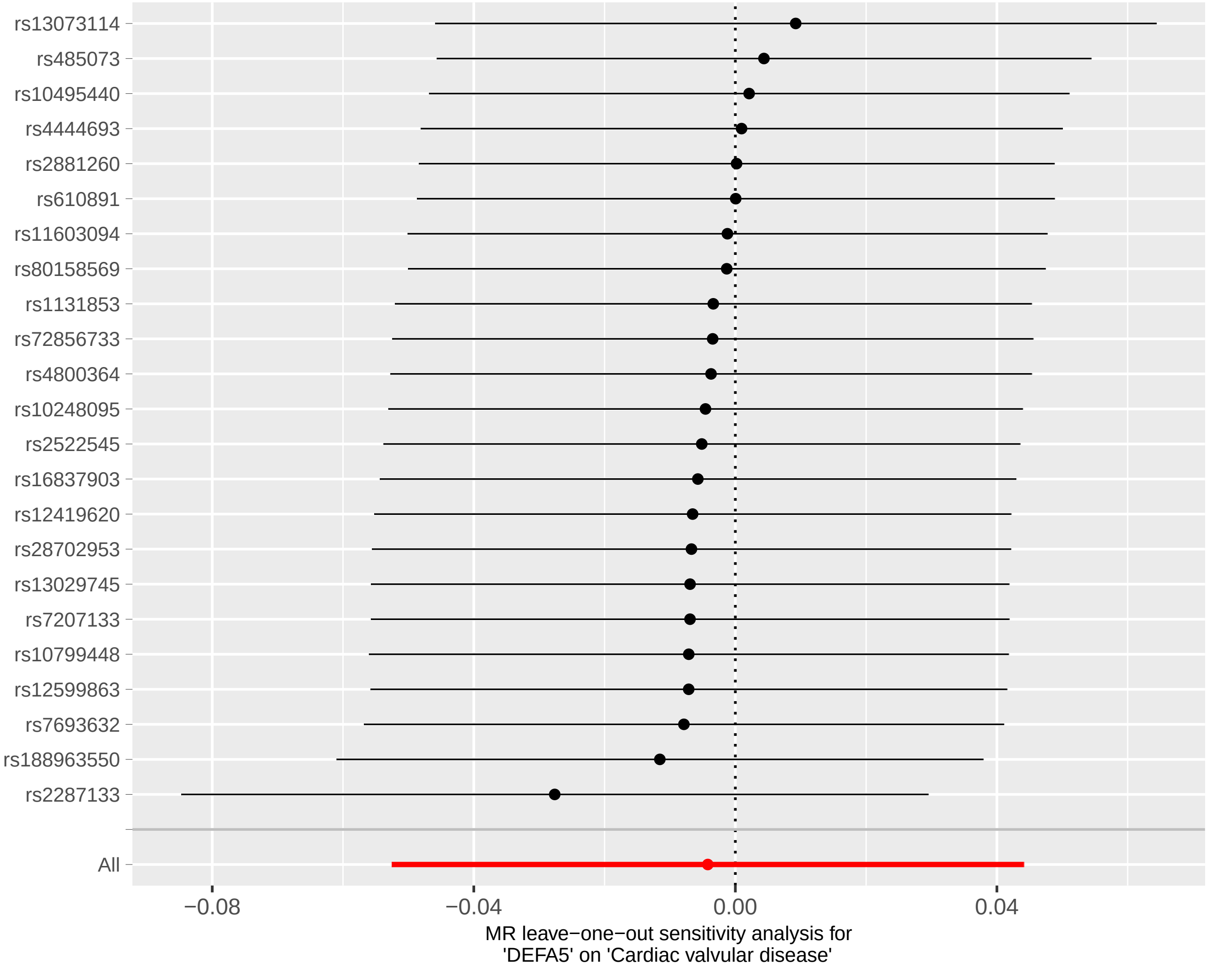
rs13073114
rs485073
rs10495440
rs4444693
rs2881260
rs610891
rs11603094
rs80158569
rs1131853
rs72856733
rs4800364
rs10248095
rs2522545
rs16837903
rs12419620
rs28702953
rs13029745
rs7207133
rs10799448
rs12599863
rs7693632
rs188963550
rs2287133
All
−0.08
−0.04
0.00
0.04
MR leave−one−out sensitivity analysis for
'DEFA5' on 'Cardiac valvular disease'

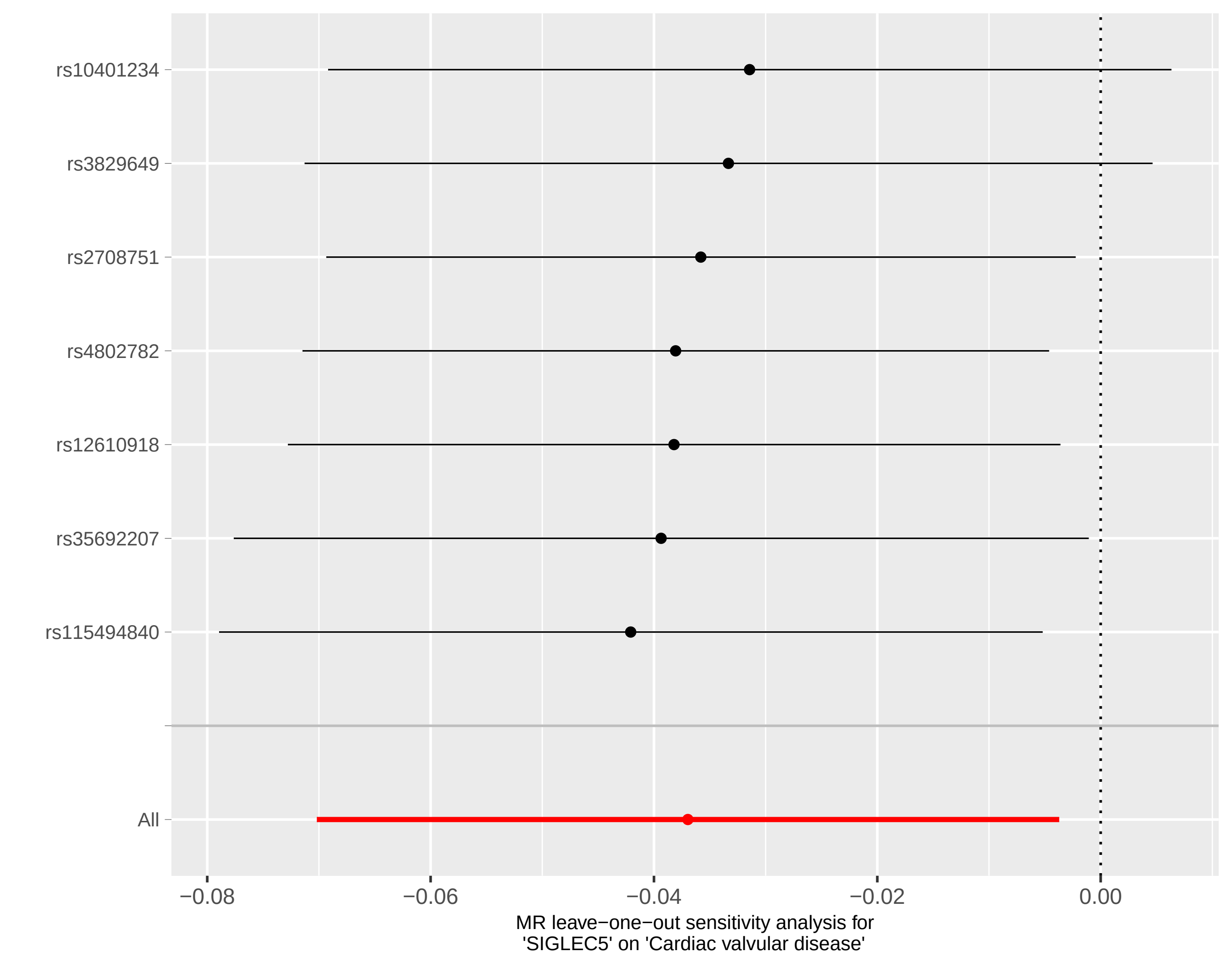

rs10401234
rs3829649
rs2708751
rs4802782
rs12610918
rs35692207
rs115494840
All
−0.08
−0.06
−0.04
−0.02
0.00
MR leave−one−out sensitivity analysis for
'SIGLEC5' on 'Cardiac valvular disease'

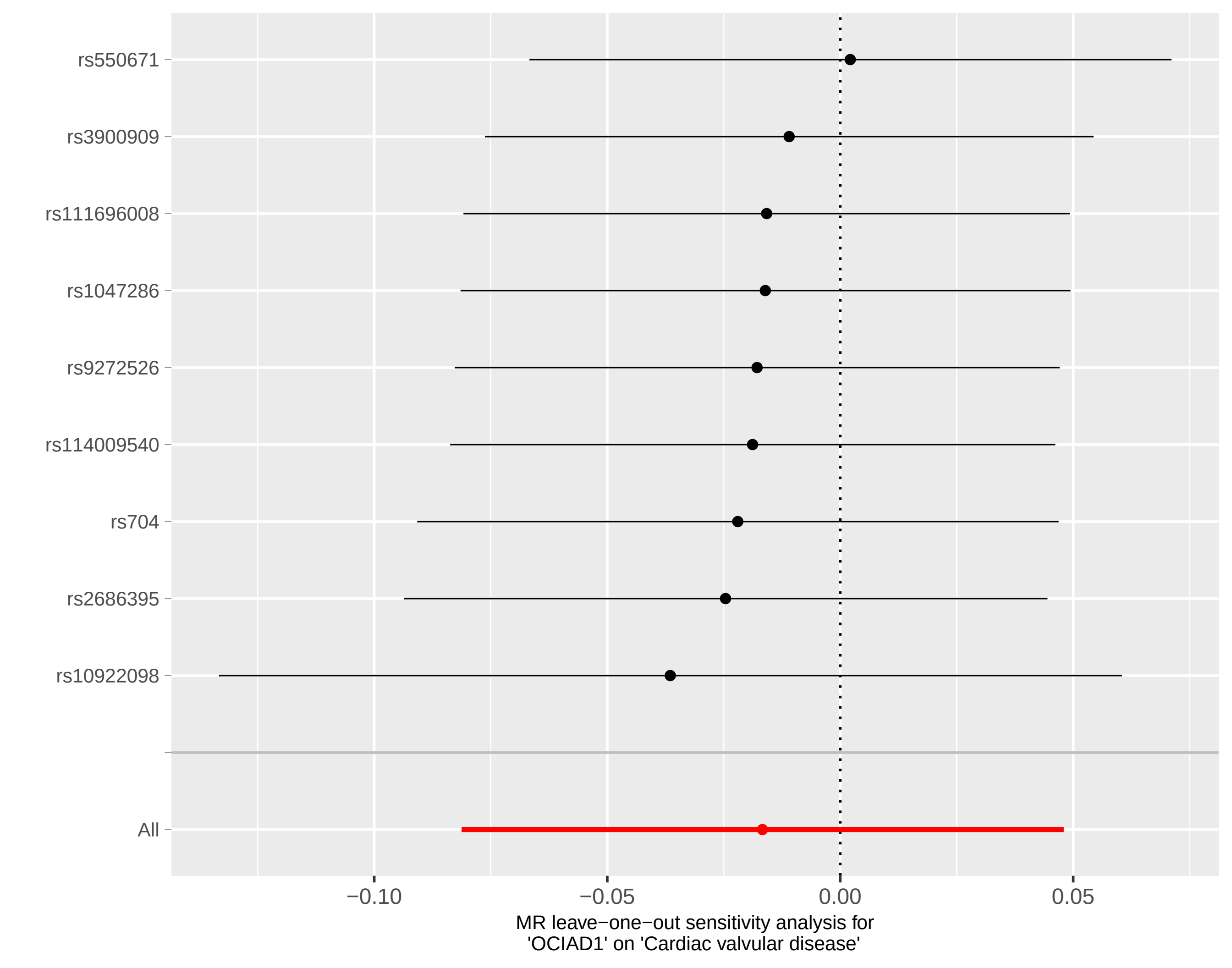

rs550671
rs3900909
rs111696008
rs1047286
rs9272526
rs114009540
rs704
rs2686395
rs10922098
All
−0.10
−0.05
0.00
0.05
MR leave−one−out sensitivity analysis for
'OCIAD1' on 'Cardiac valvular disease'

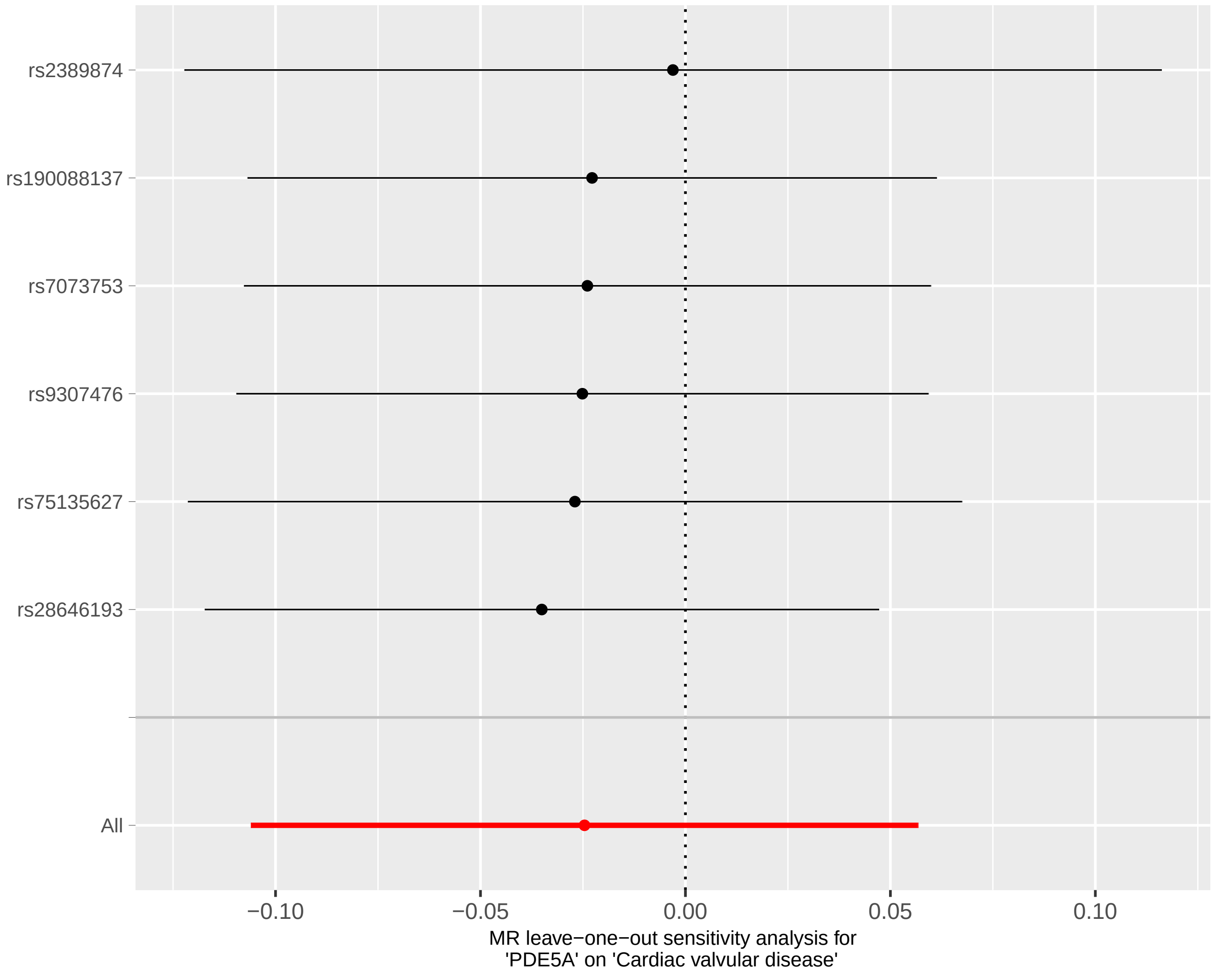
rs2389874
rs190088137
rs7073753
rs9307476
rs75135627
rs28646193
All
−0.10
−0.05
0.00
0.05
0.10
MR leave−one−out sensitivity analysis for
'PDE5A' on 'Cardiac valvular disease'

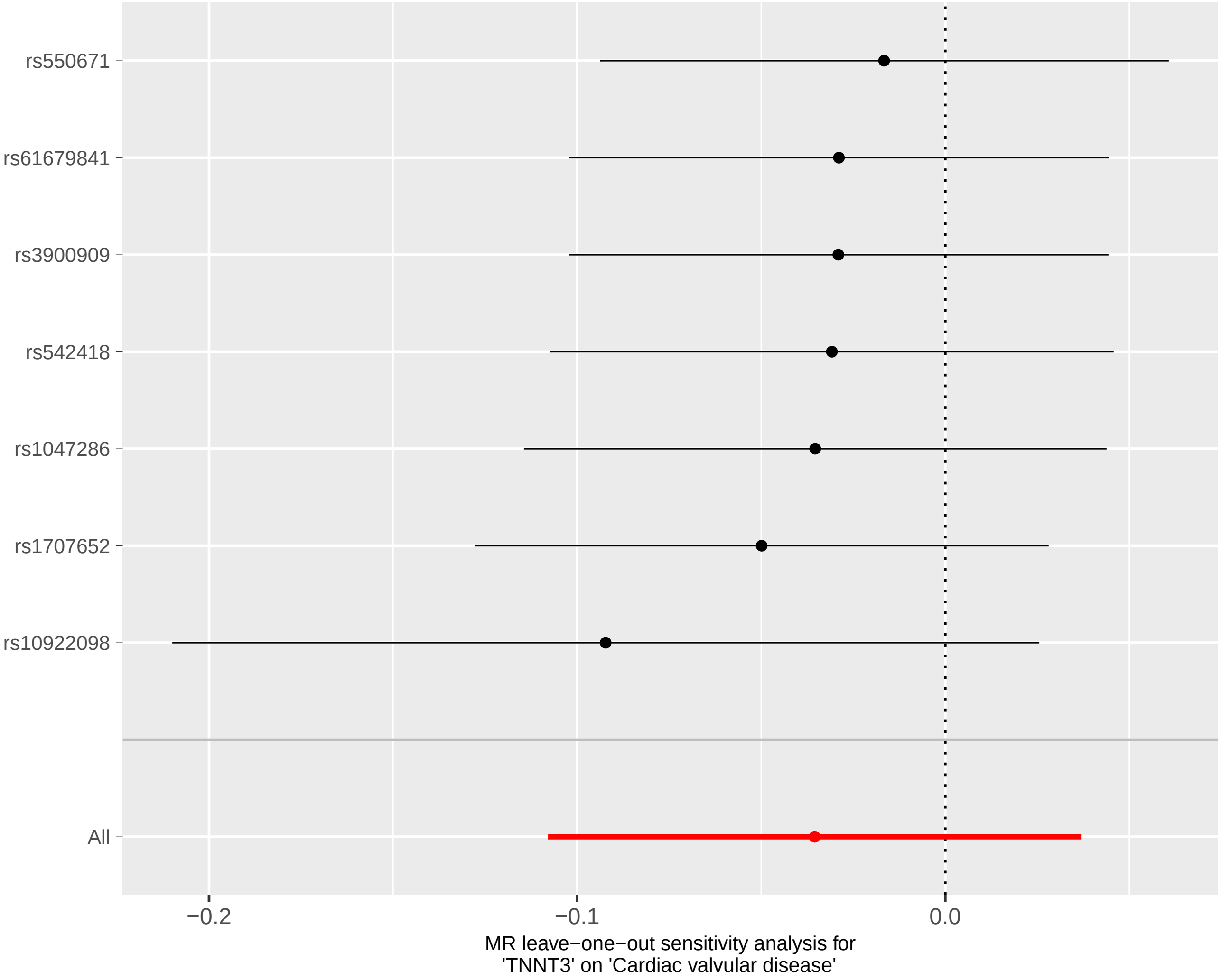

rs550671
rs61679841
rs3900909
rs542418
rs1047286
rs1707652
rs10922098
All
−0.2
−0.1
0.0
MR leave−one−out sensitivity analysis for
'TNNT3' on 'Cardiac valvular disease'

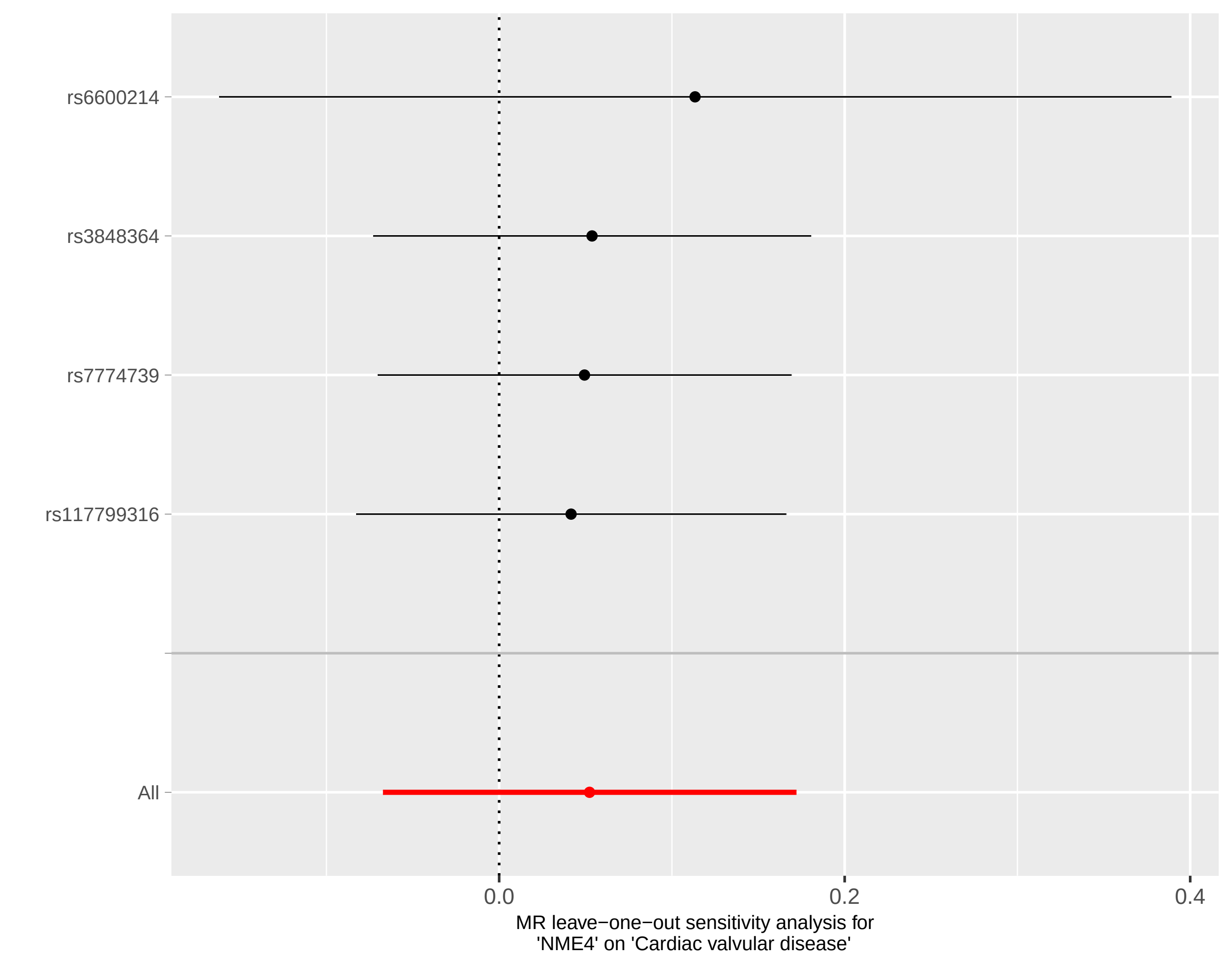
rs6600214
rs3848364
rs7774739
rs117799316
All
0.0
0.2
0.4
MR leave−one−out sensitivity analysis for
'NME4' on 'Cardiac valvular disease'

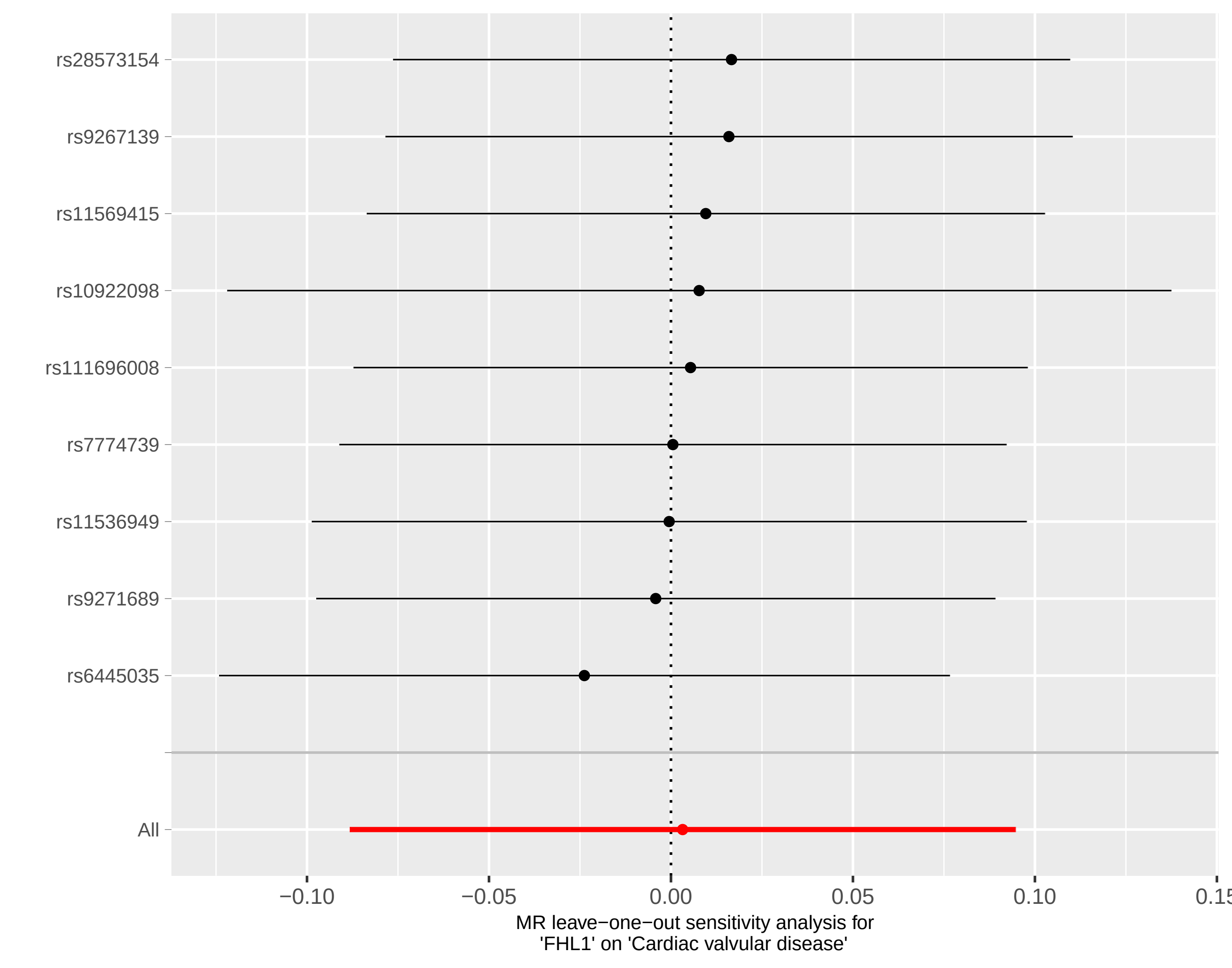
rs28573154
rs9267139
rs11569415
rs10922098
rs111696008
rs7774739
rs11536949
rs9271689
rs6445035
All
−0.10
−0.05
0.00
0.05
0.10
0.15
MR leave−one−out sensitivity analysis for
'FHL1' on 'Cardiac valvular disease'

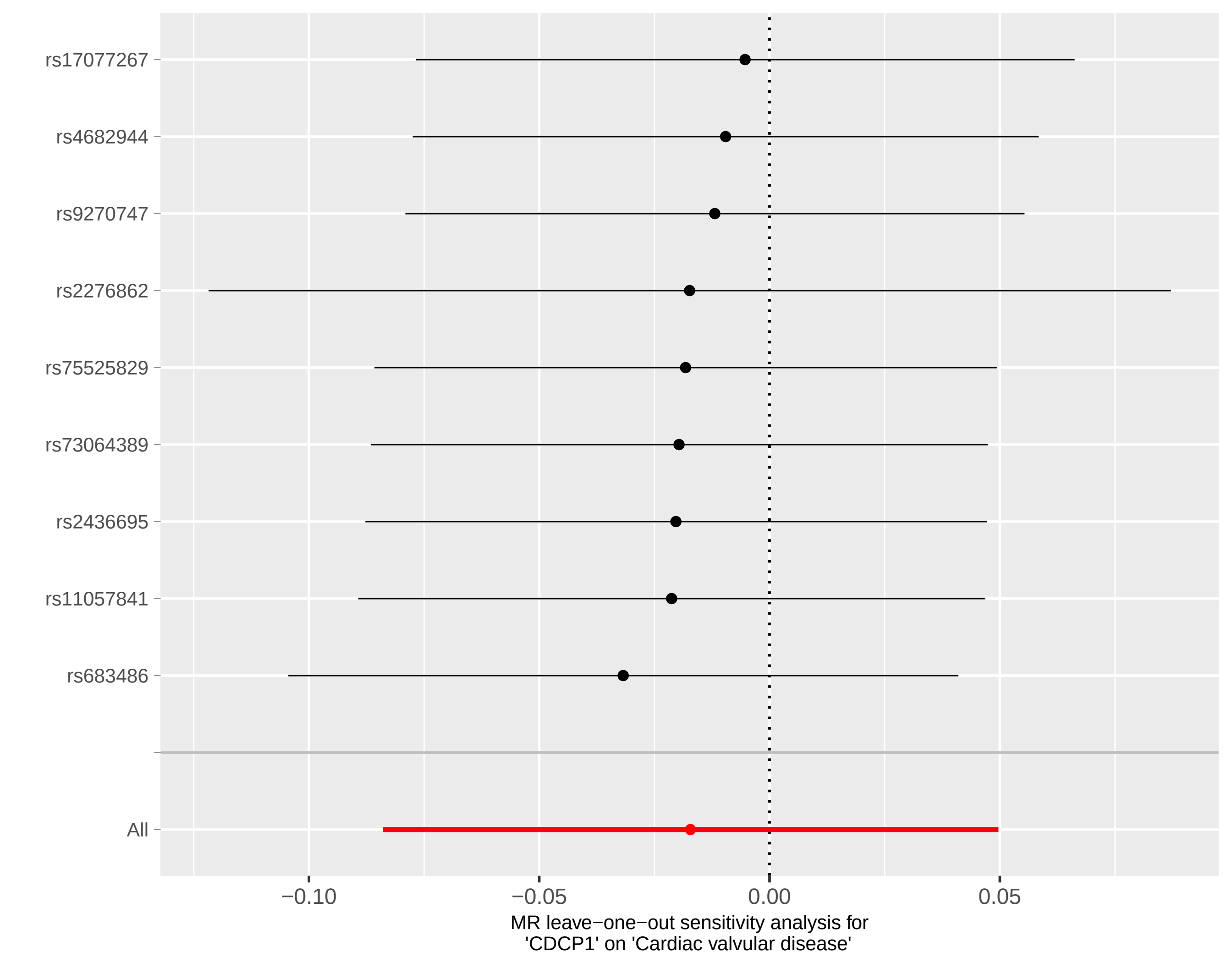

rs17077267
rs4682944
rs9270747
rs2276862
rs75525829
rs73064389
rs2436695
rs11057841
rs683486
All
−0.10
−0.05
0.00
0.05
MR leave−one−out sensitivity analysis for
'CDCP1' on 'Cardiac valvular disease'

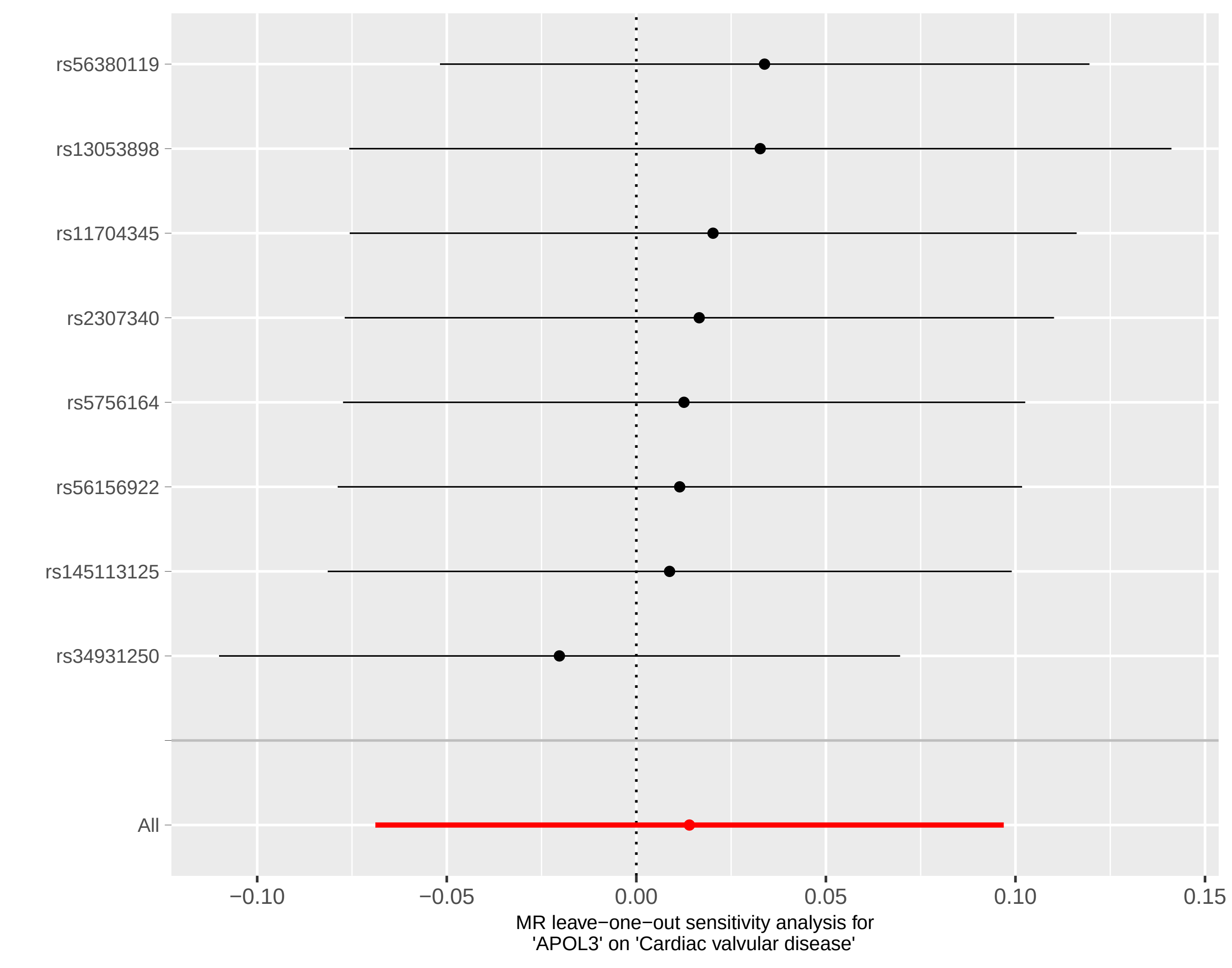
rs56380119
rs13053898
rs11704345
rs2307340
rs5756164
rs56156922
rs145113125
rs34931250
All
−0.10
−0.05
0.00
0.05
0.10
0.15
MR leave−one−out sensitivity analysis for
'APOL3' on 'Cardiac valvular disease'

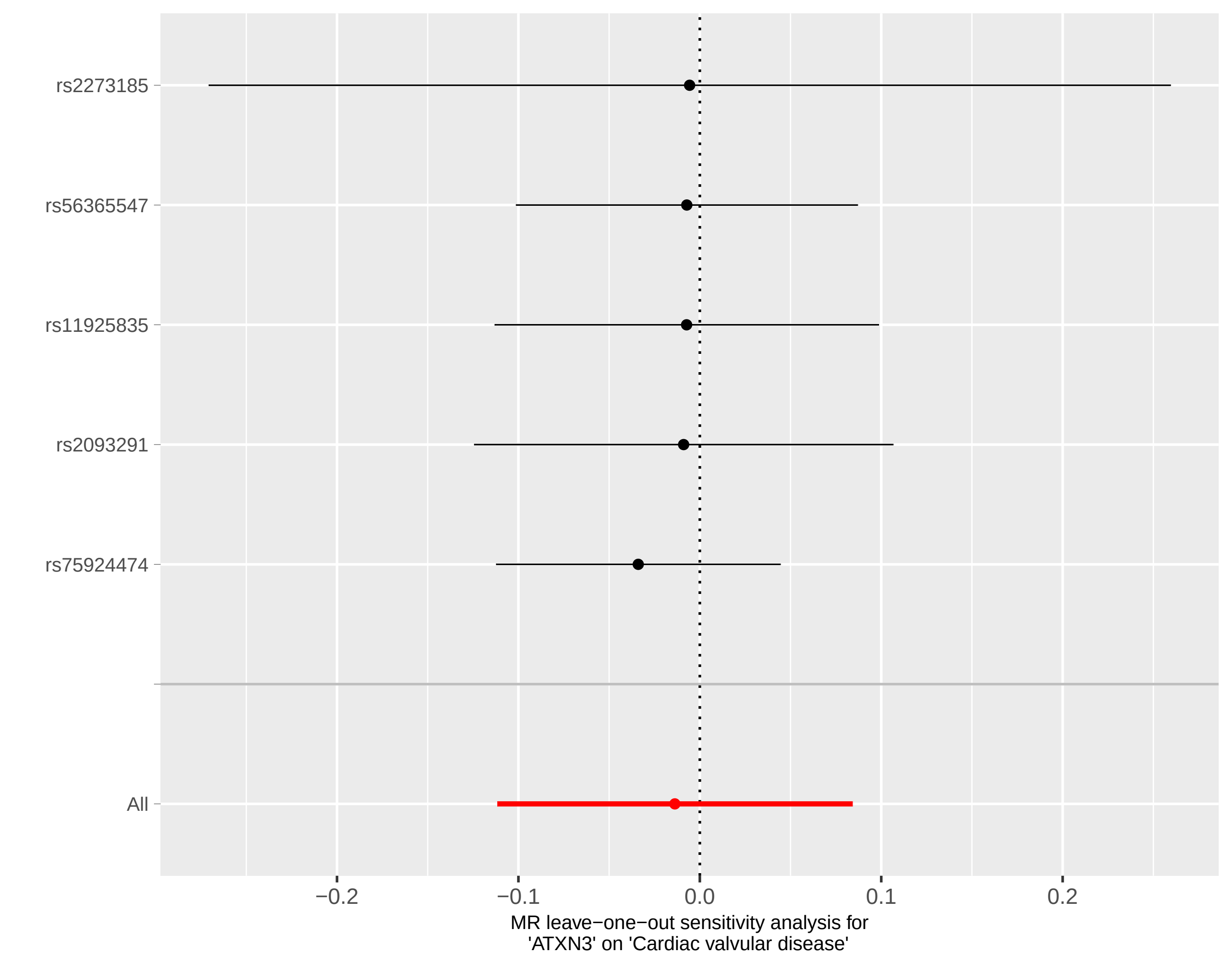
rs2273185
rs56365547
rs11925835
rs2093291
rs75924474
All
−0.2
−0.1
0.0
0.1
0.2
MR leave−one−out sensitivity analysis for
'ATXN3' on 'Cardiac valvular disease'

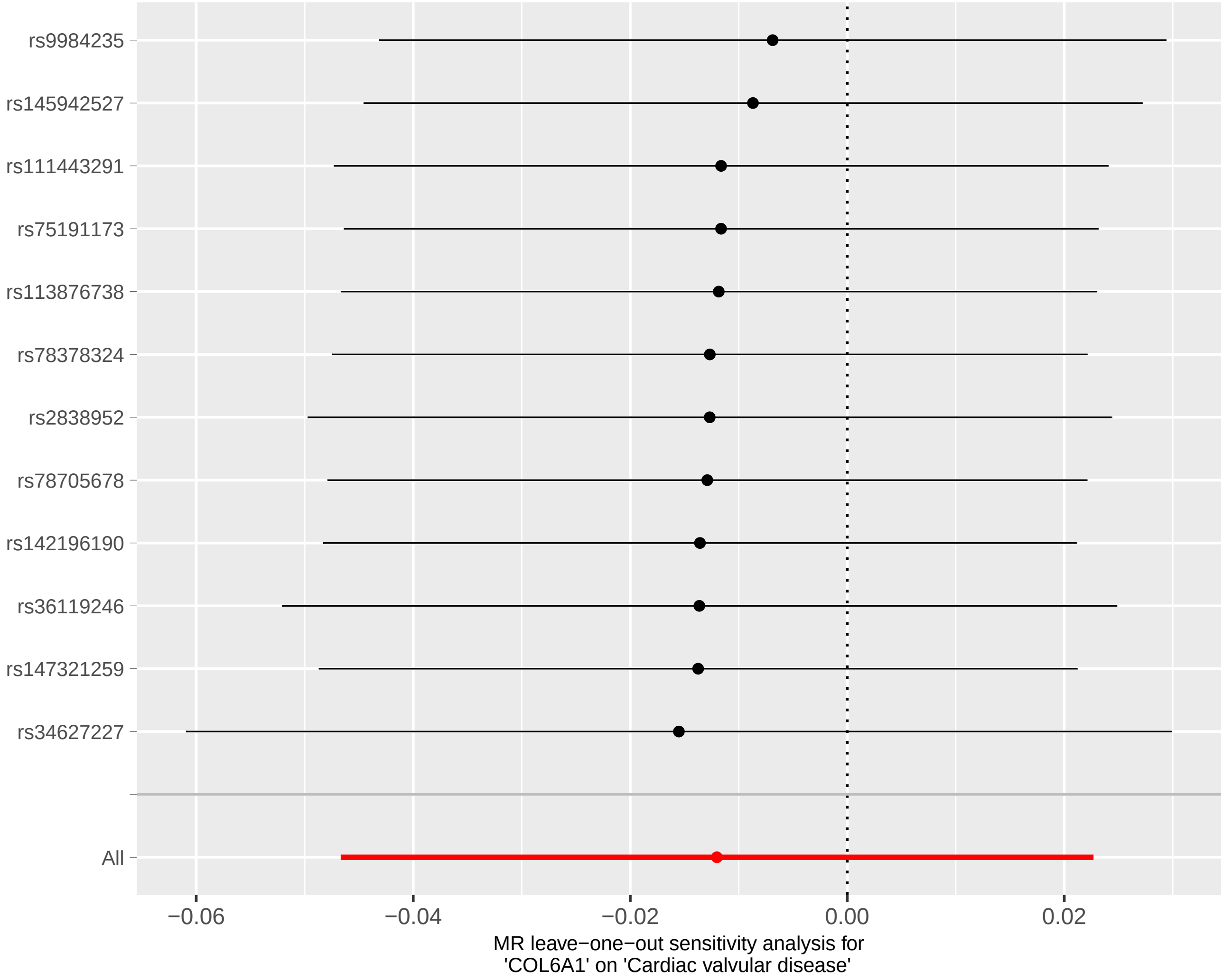
rs9984235
rs145942527
rs111443291
rs75191173
rs113876738
rs78378324
rs2838952
rs78705678
rs142196190
rs36119246
rs147321259
rs34627227
All
−0.06
−0.04
−0.02
0.00
0.02
MR leave−one−out sensitivity analysis for
'COL6A1' on 'Cardiac valvular disease'

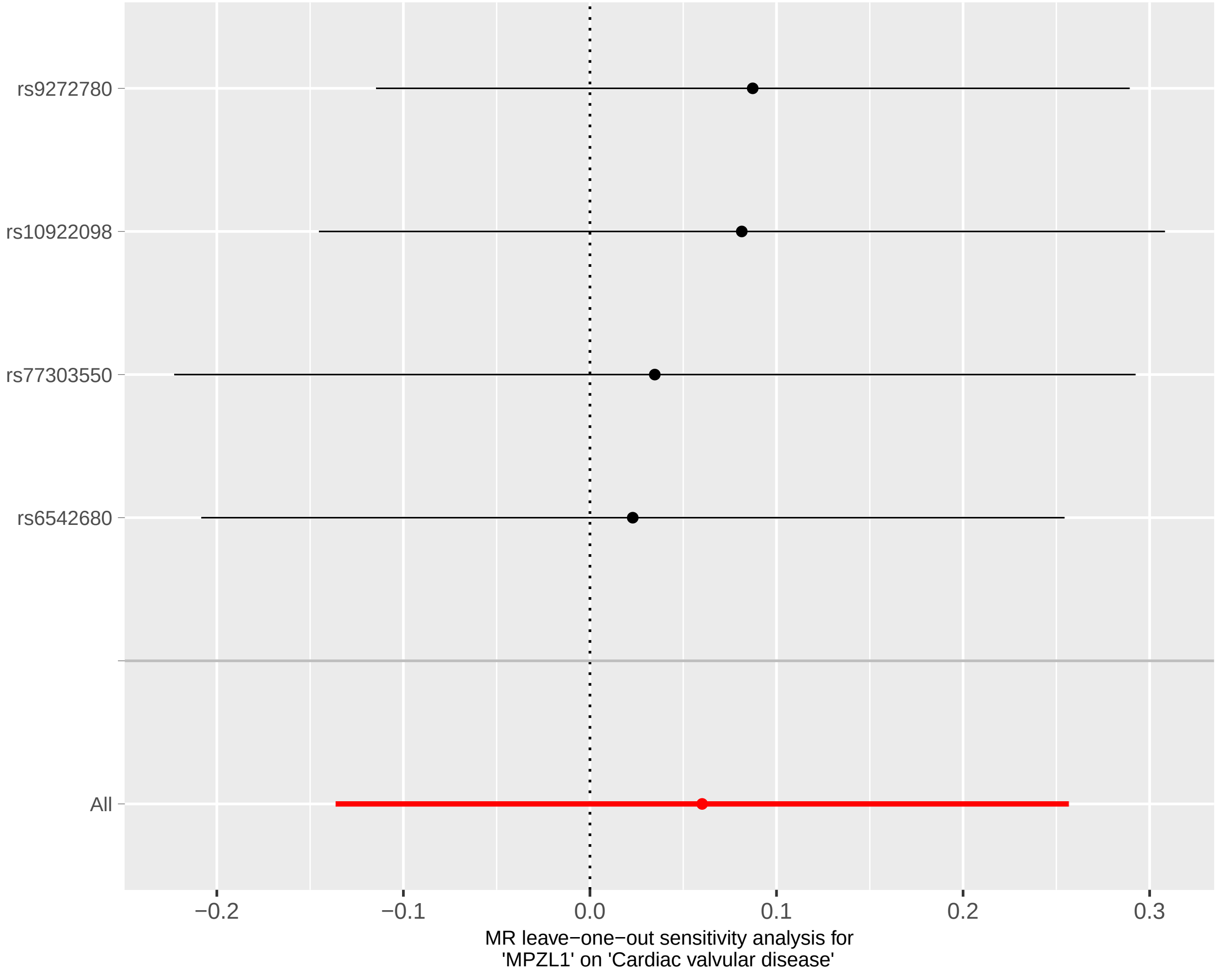
rs9272780
rs10922098
rs77303550
rs6542680
All
−0.2
−0.1
0.0
0.1
0.2
0.3
MR leave−one−out sensitivity analysis for
'MPZL1' on 'Cardiac valvular disease'

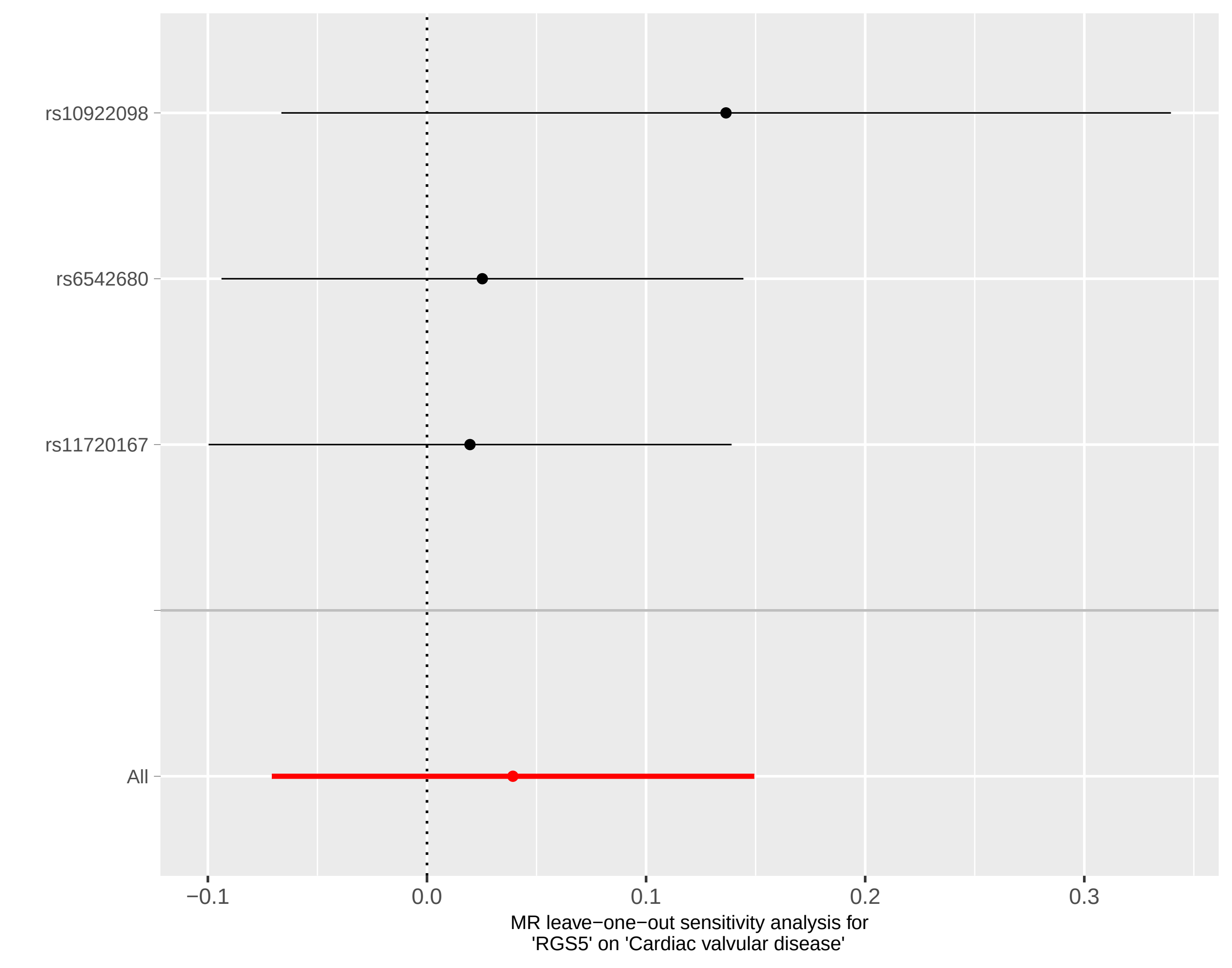
rs10922098
rs6542680
rs11720167
All
−0.1
0.0
0.1
0.2
0.3
MR leave−one−out sensitivity analysis for
'RGS5' on 'Cardiac valvular disease'

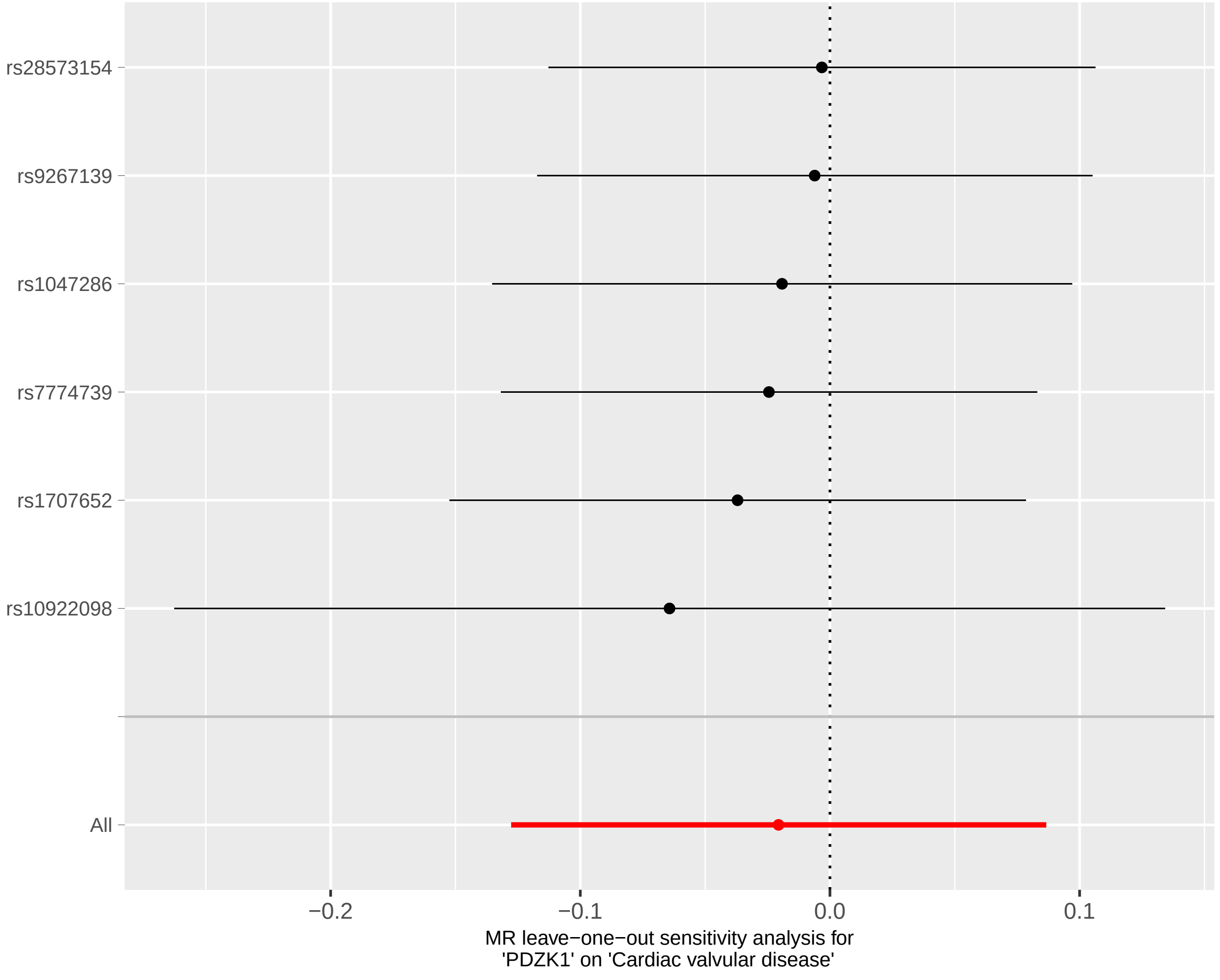
rs28573154
rs9267139
rs1047286
rs7774739
rs1707652
rs10922098
All
−0.2
−0.1
0.0
0.1
MR leave−one−out sensitivity analysis for
'PDZK1' on 'Cardiac valvular disease'

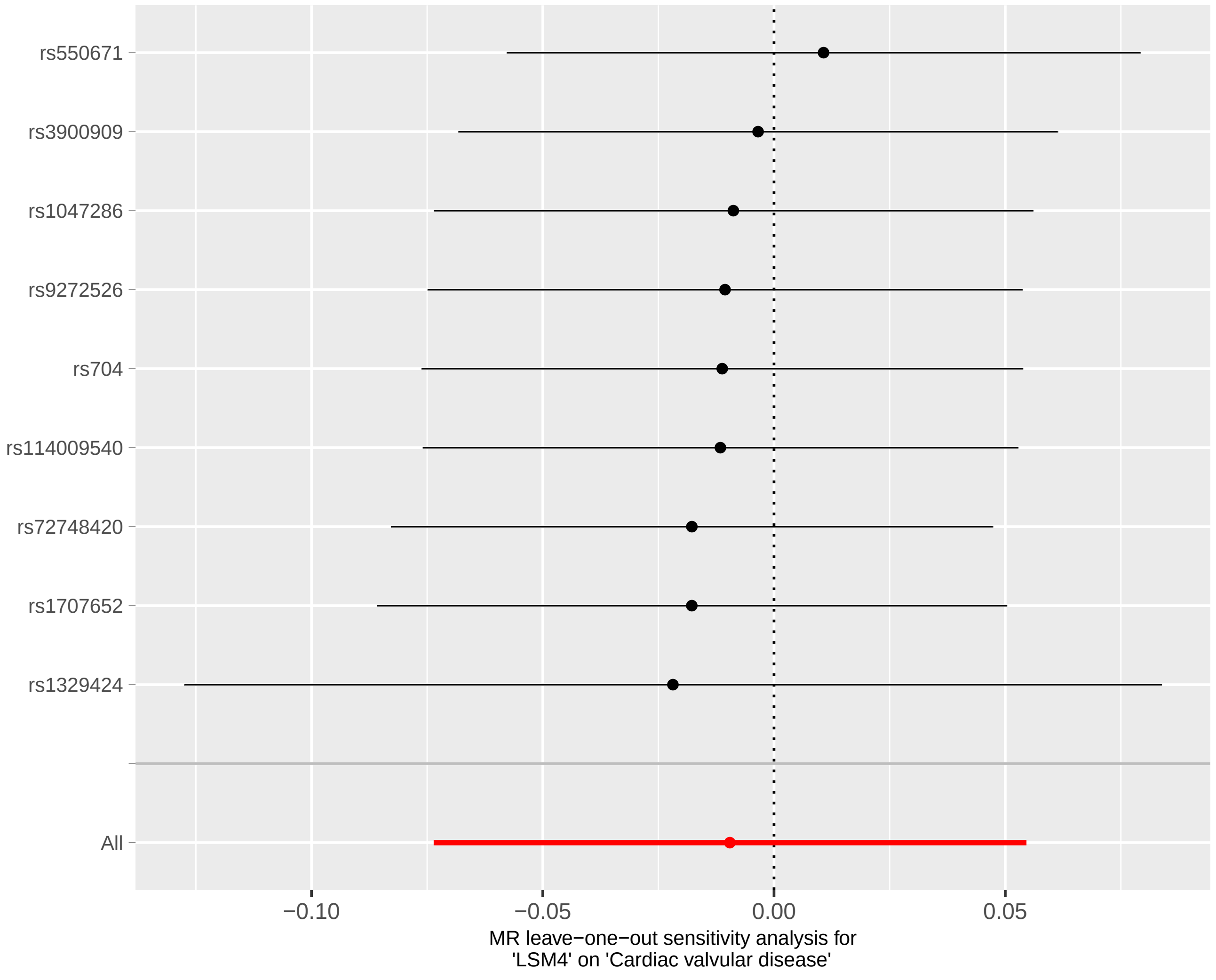
rs550671
rs3900909
rs1047286
rs9272526
rs704
rs114009540
rs72748420
rs1707652
rs1329424
All
−0.10
−0.05
0.00
0.05
MR leave−one−out sensitivity analysis for
'LSM4' on 'Cardiac valvular disease'

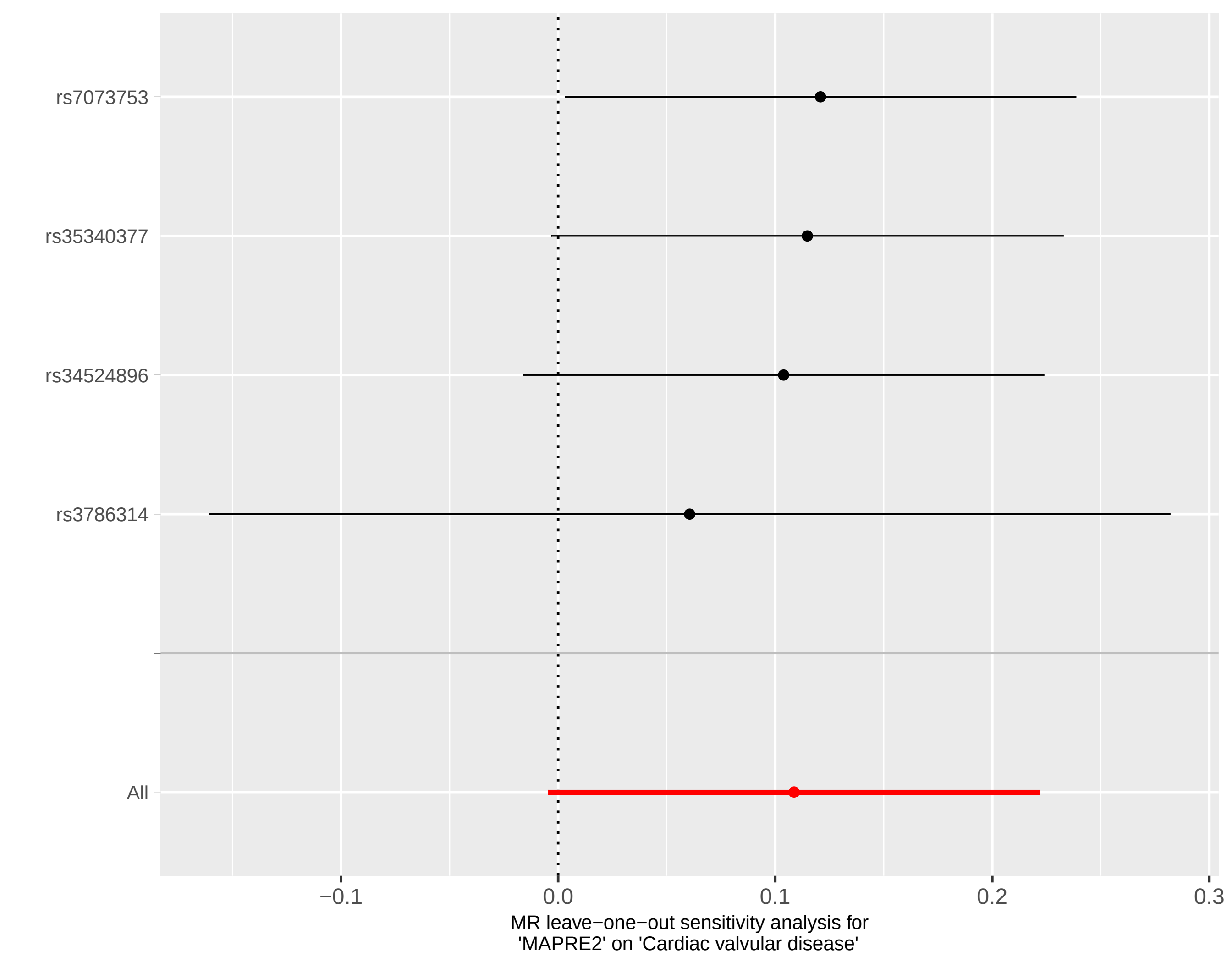
rs7073753
rs35340377
rs34524896
rs3786314
All
−0.1
0.0
0.1
0.2
0.3
MR leave−one−out sensitivity analysis for
'MAPRE2' on 'Cardiac valvular disease'

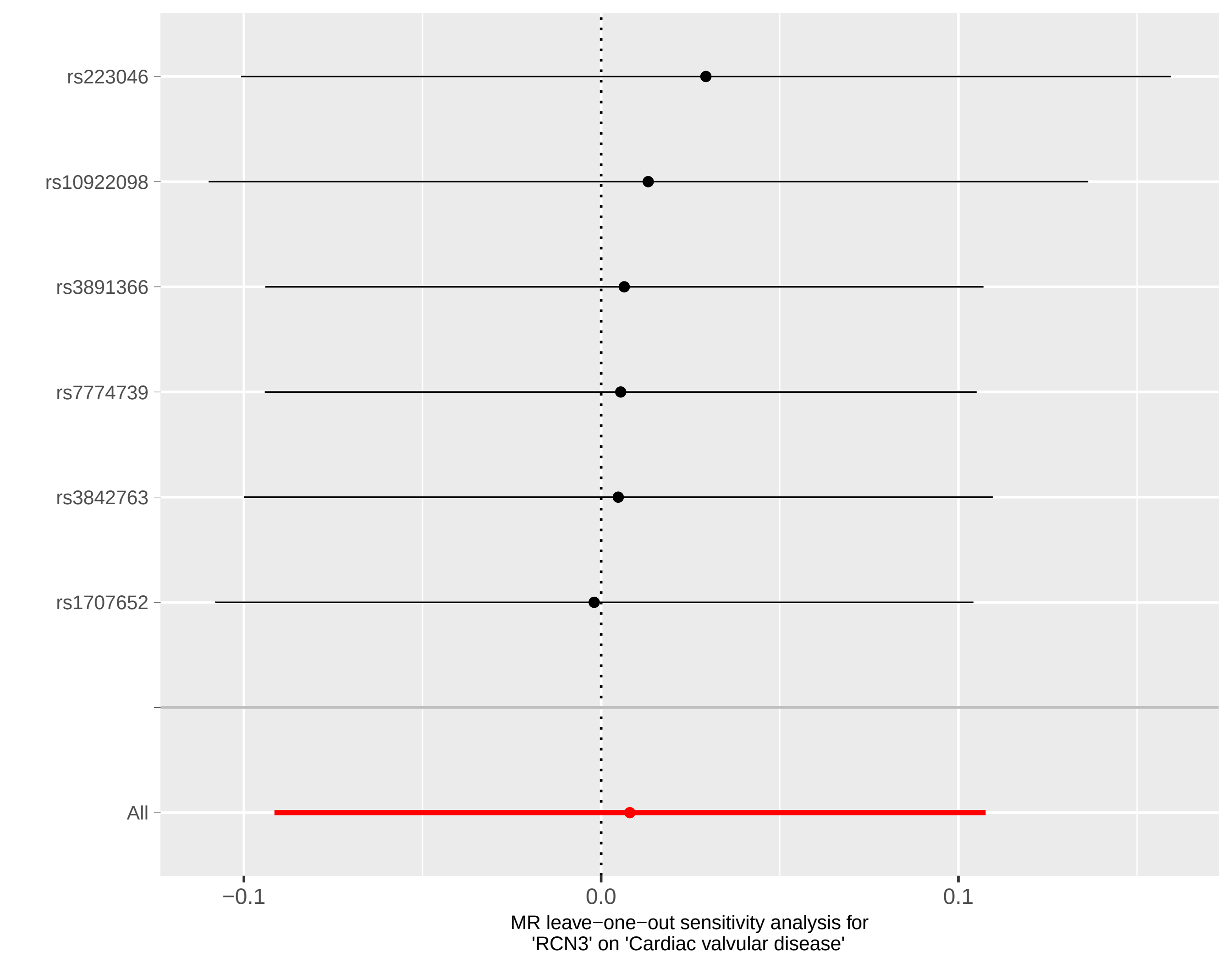
rs223046
rs10922098
rs3891366
rs7774739
rs3842763
rs1707652
All
−0.1
0.0
0.1
MR leave−one−out sensitivity analysis for
'RCN3' on 'Cardiac valvular disease'

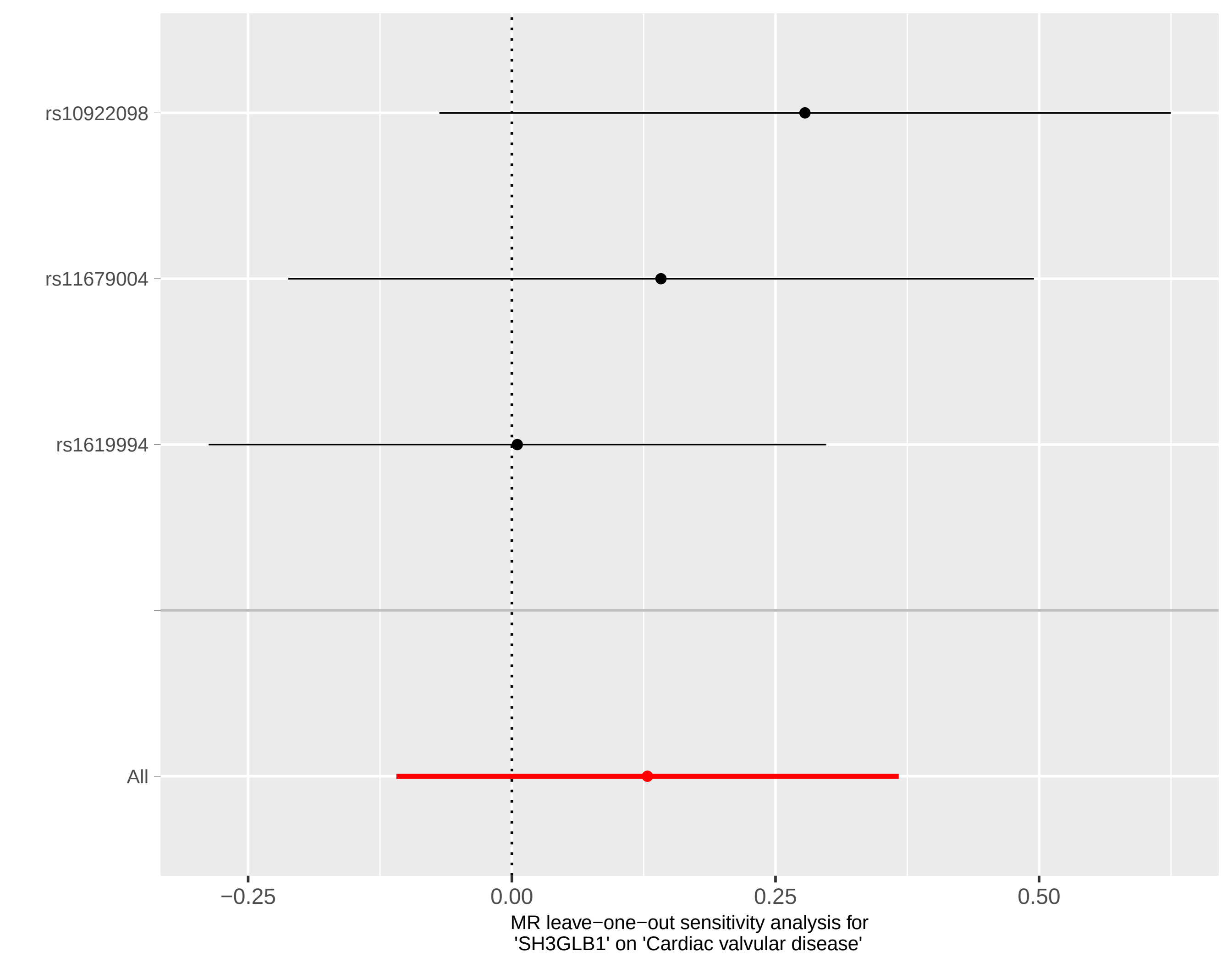

rs10922098
rs11679004
rs1619994
All
−0.25
0.00
0.25
0.50
MR leave−one−out sensitivity analysis for
'SH3GLB1' on 'Cardiac valvular disease'

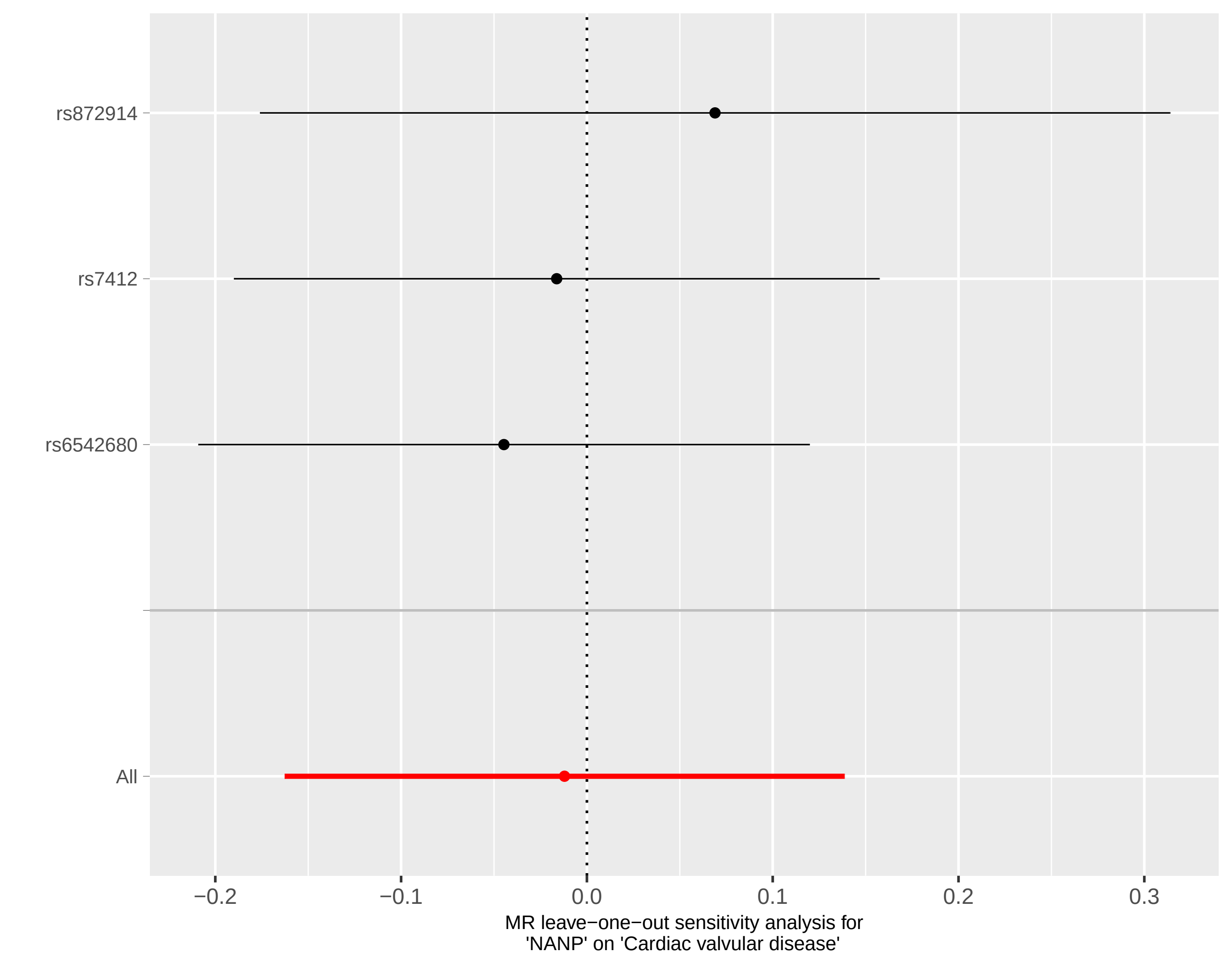
rs872914
rs7412
rs6542680
All
−0.2
−0.1
0.0
0.1
0.2
0.3
MR leave−one−out sensitivity analysis for
'NANP' on 'Cardiac valvular disease'

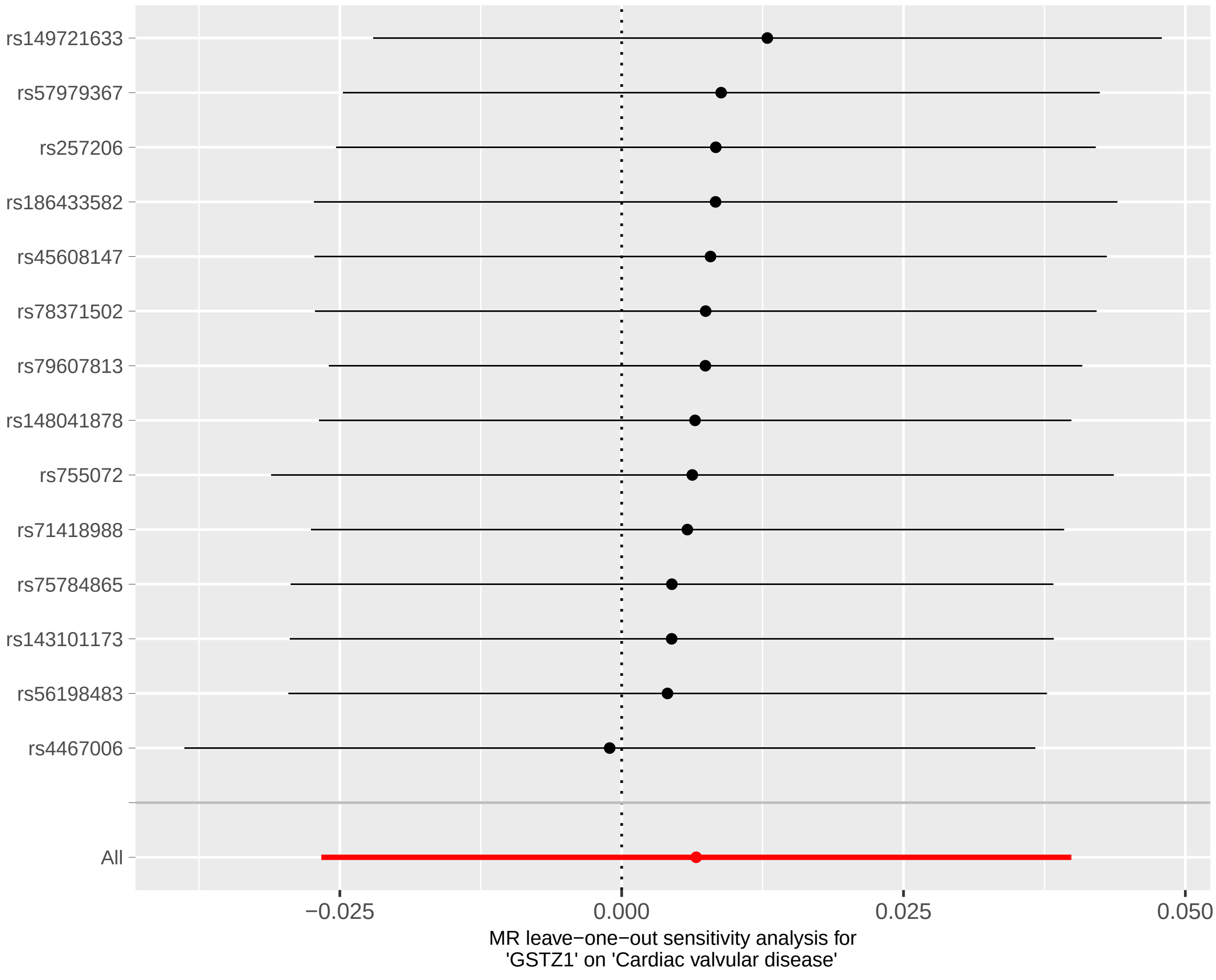
rs149721633
rs57979367
rs257206
rs186433582
rs45608147
rs78371502
rs79607813
rs148041878
rs755072
rs71418988
rs75784865
rs143101173
rs56198483
rs4467006
All
−0.025
0.000
0.025
0.050
MR leave−one−out sensitivity analysis for
'GSTZ1' on 'Cardiac valvular disease'

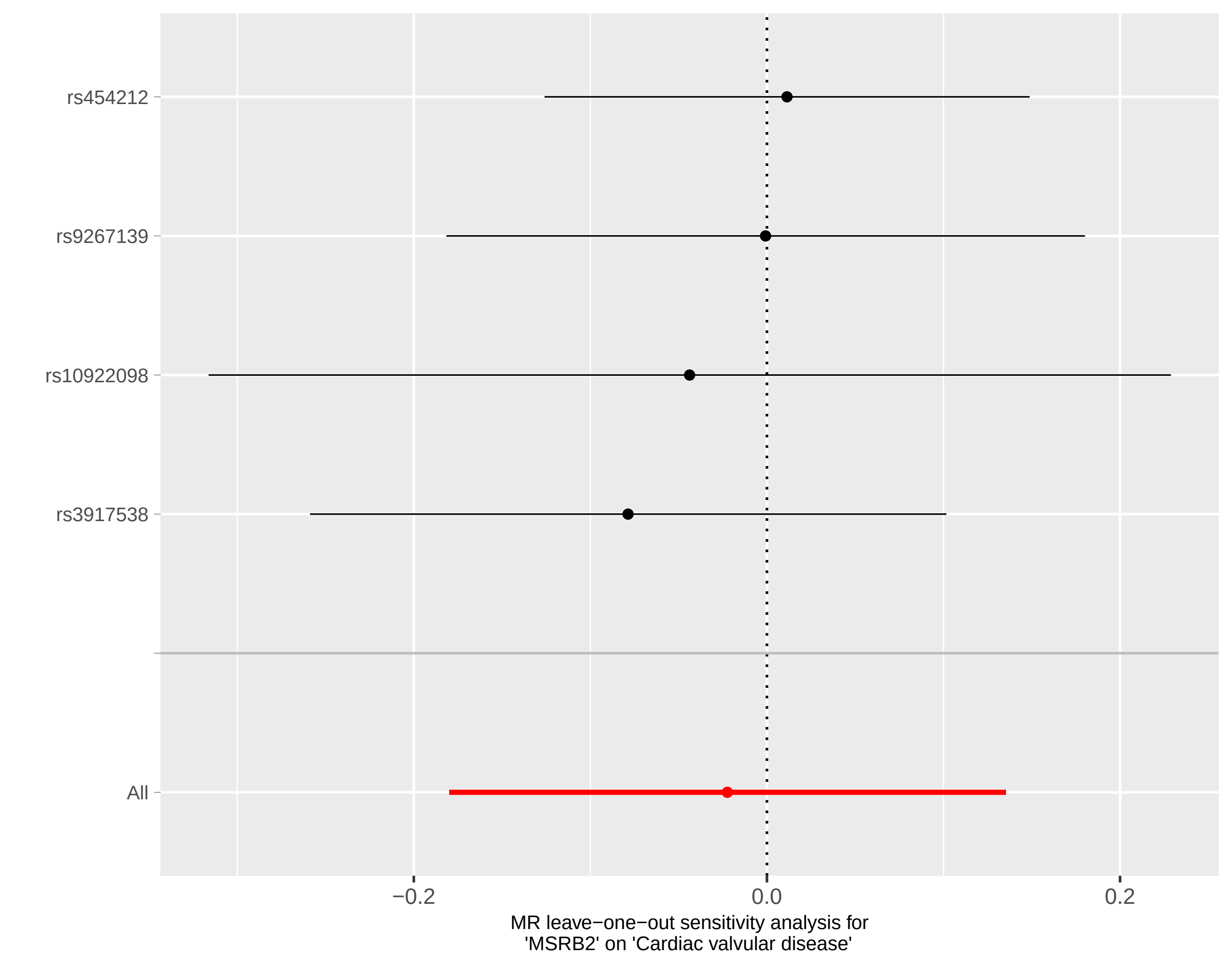
rs454212
rs9267139
rs10922098
rs3917538
All
−0.2
0.0
0.2
MR leave−one−out sensitivity analysis for
'MSRB2' on 'Cardiac valvular disease'

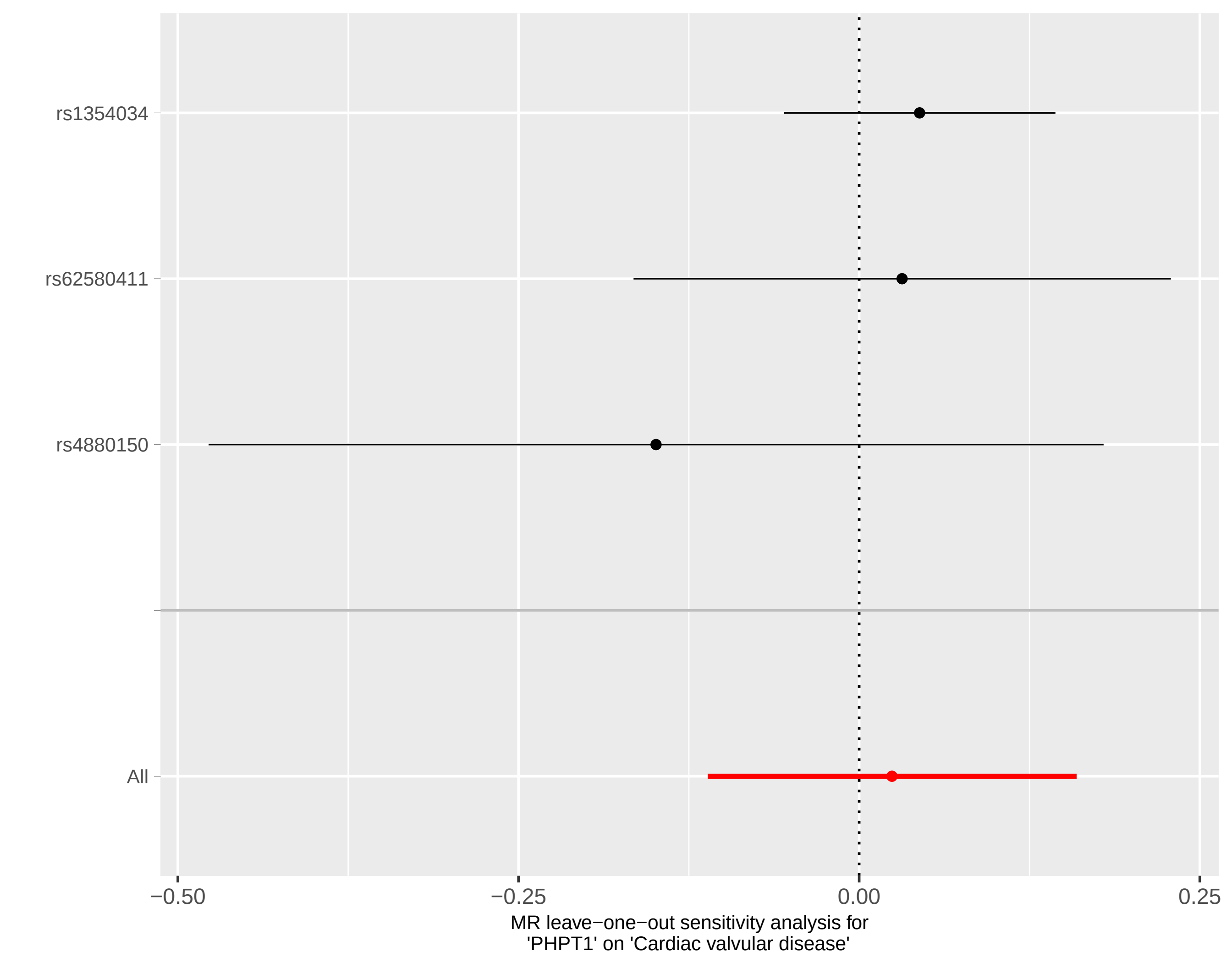
rs1354034
rs62580411
rs4880150
All
−0.50
−0.25
0.00
0.25
MR leave−one−out sensitivity analysis for
'PHPT1' on 'Cardiac valvular disease'

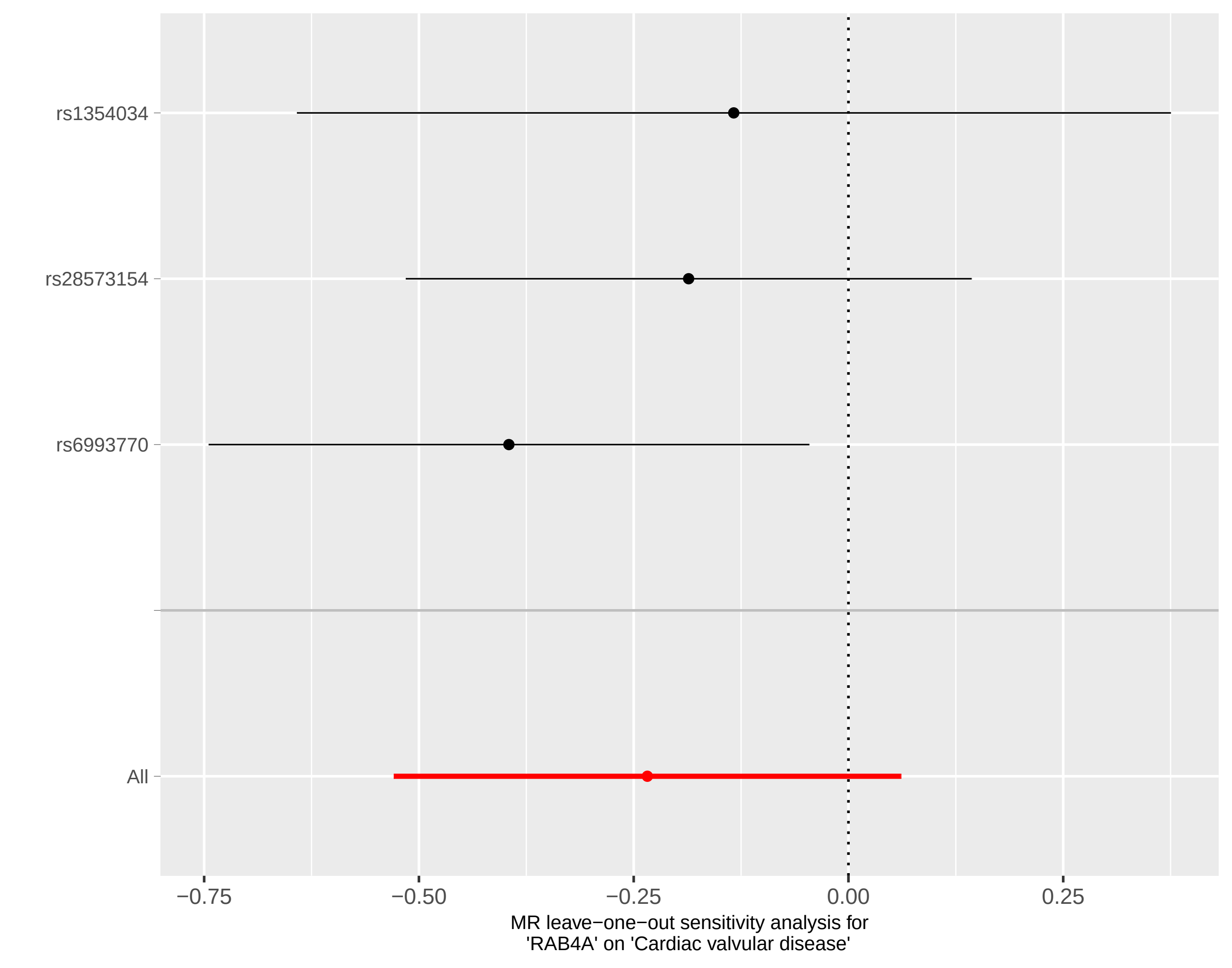

rs1354034
rs28573154
rs6993770
All
−0.75
−0.50
−0.25
0.00
0.25
MR leave−one−out sensitivity analysis for
'RAB4A' on 'Cardiac valvular disease'

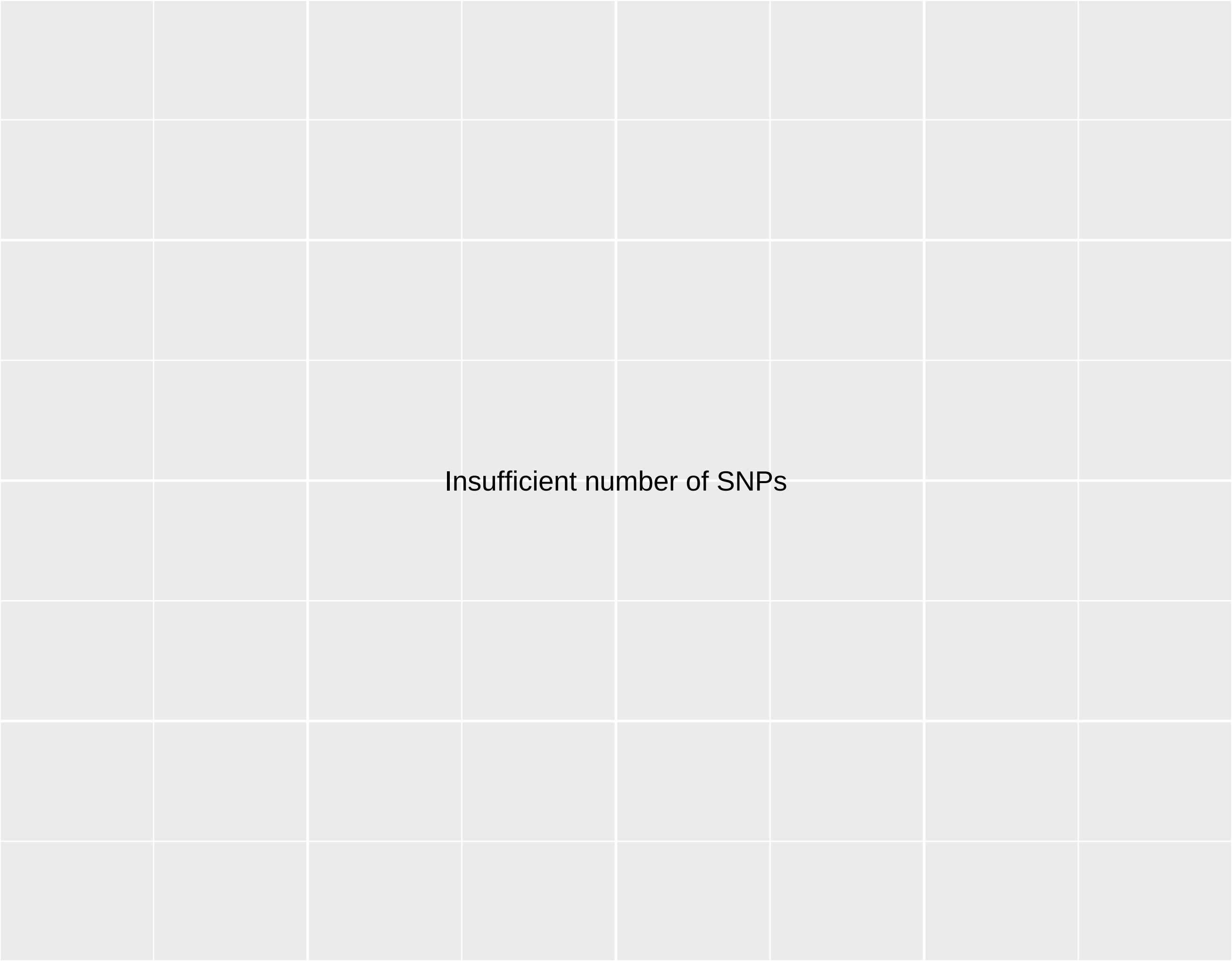
Insufficient number of SNPs

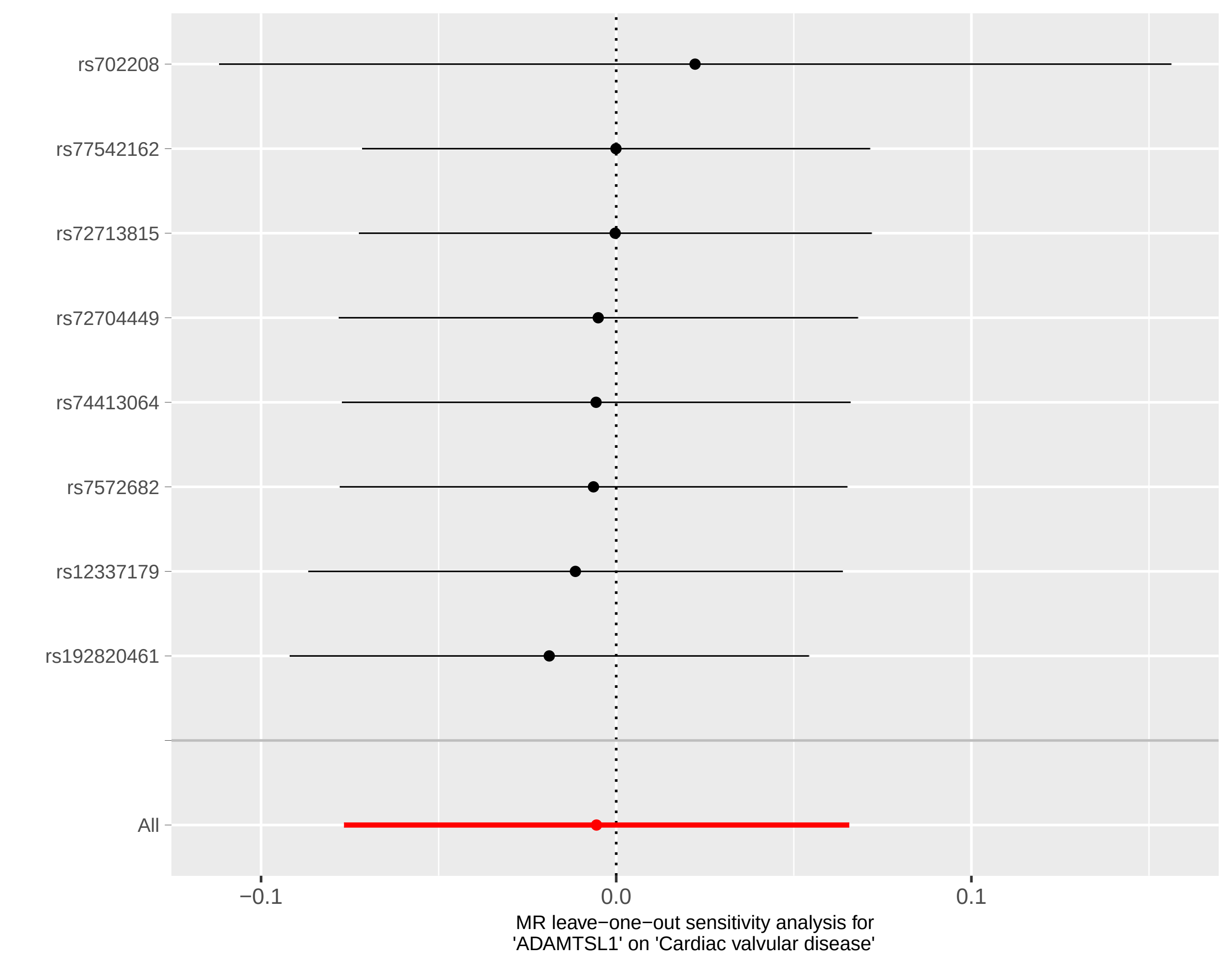
rs702208
rs77542162
rs72713815
rs72704449
rs74413064
rs7572682
rs12337179
rs192820461
All
−0.1
0.0
0.1
MR leave−one−out sensitivity analysis for
'ADAMTSL1' on 'Cardiac valvular disease'

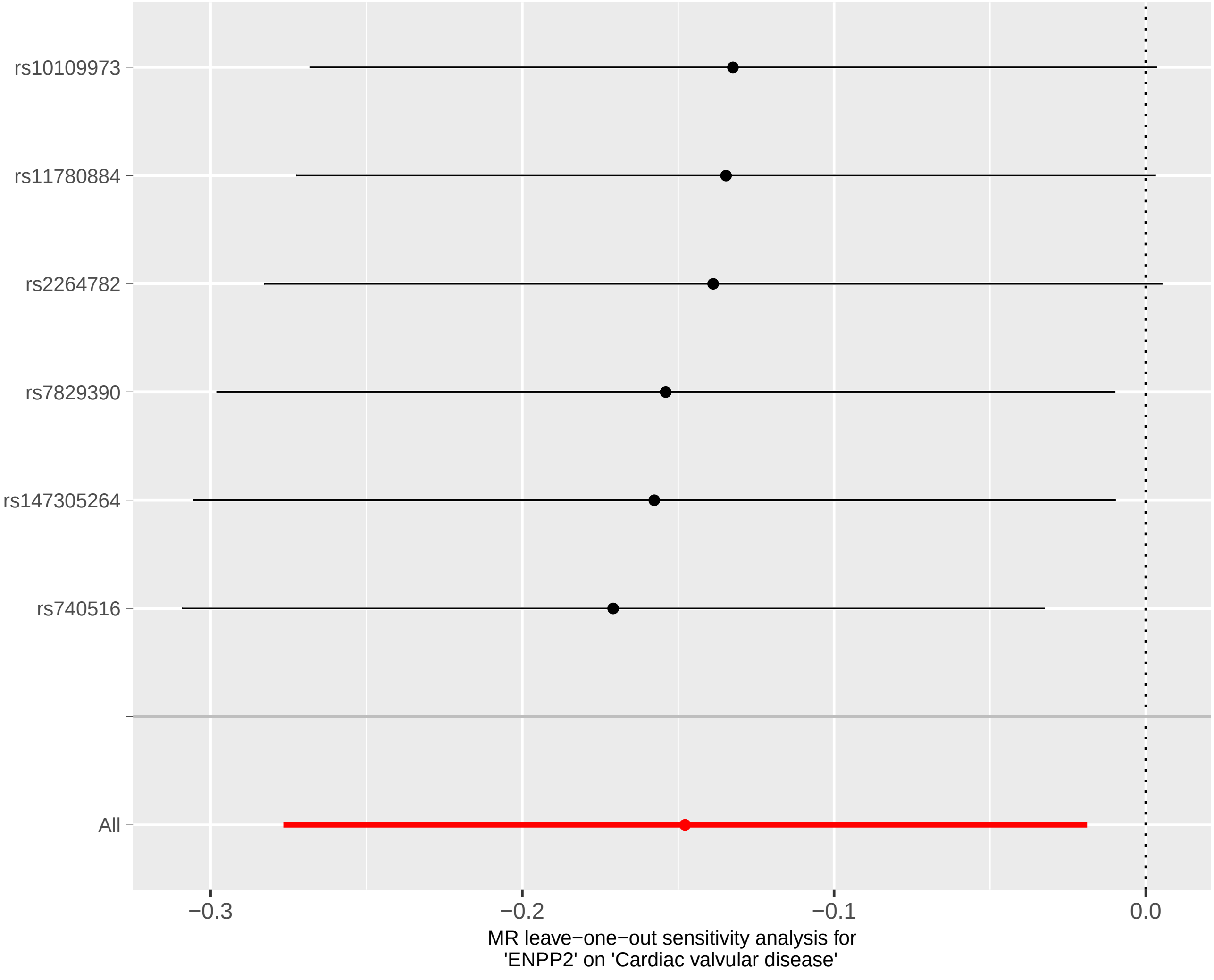
rs10109973
rs11780884
rs2264782
rs7829390
rs147305264
rs740516
All
−0.3
−0.2
−0.1
0.0
MR leave−one−out sensitivity analysis for
'ENPP2' on 'Cardiac valvular disease'

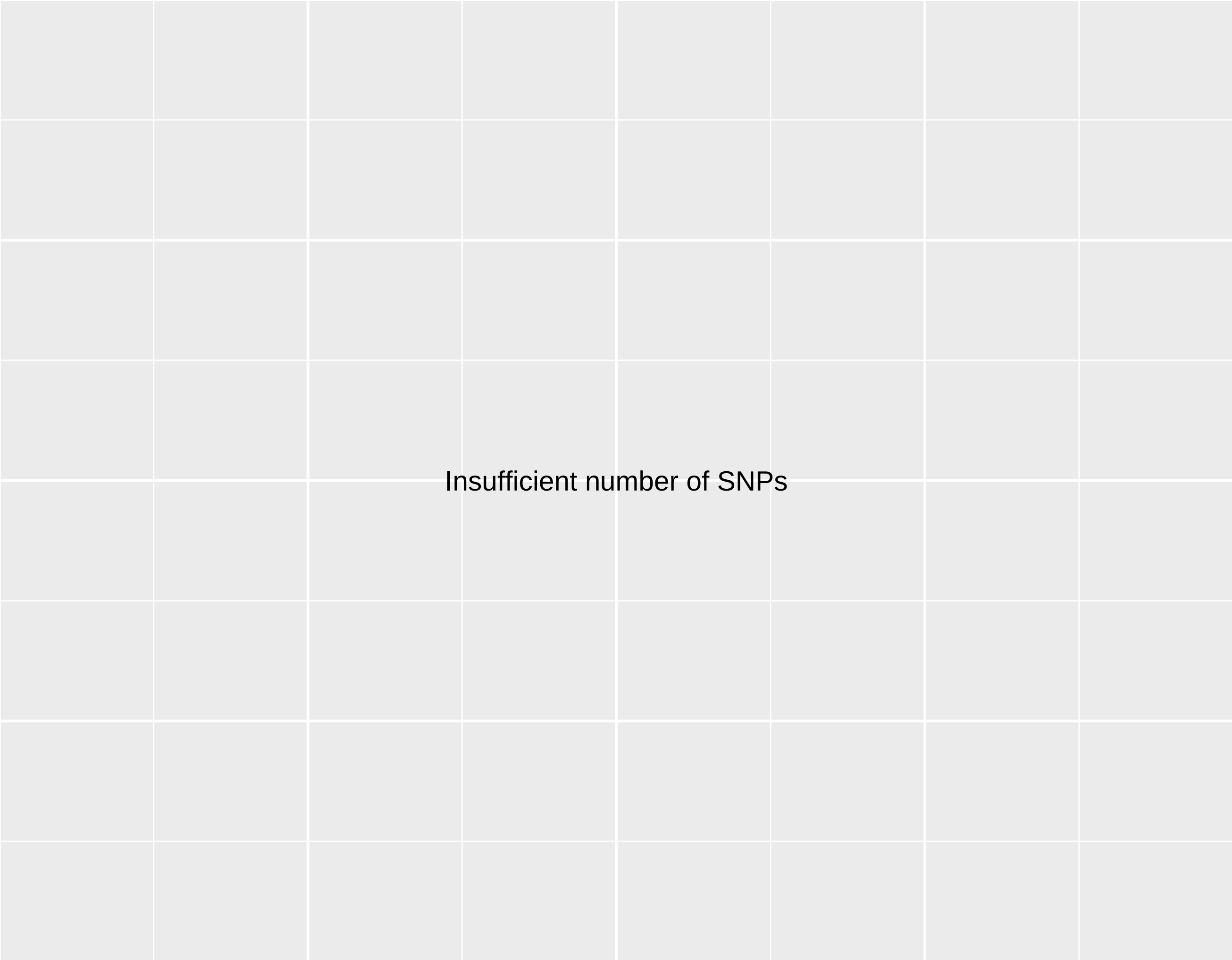
Insufficient number of SNPs

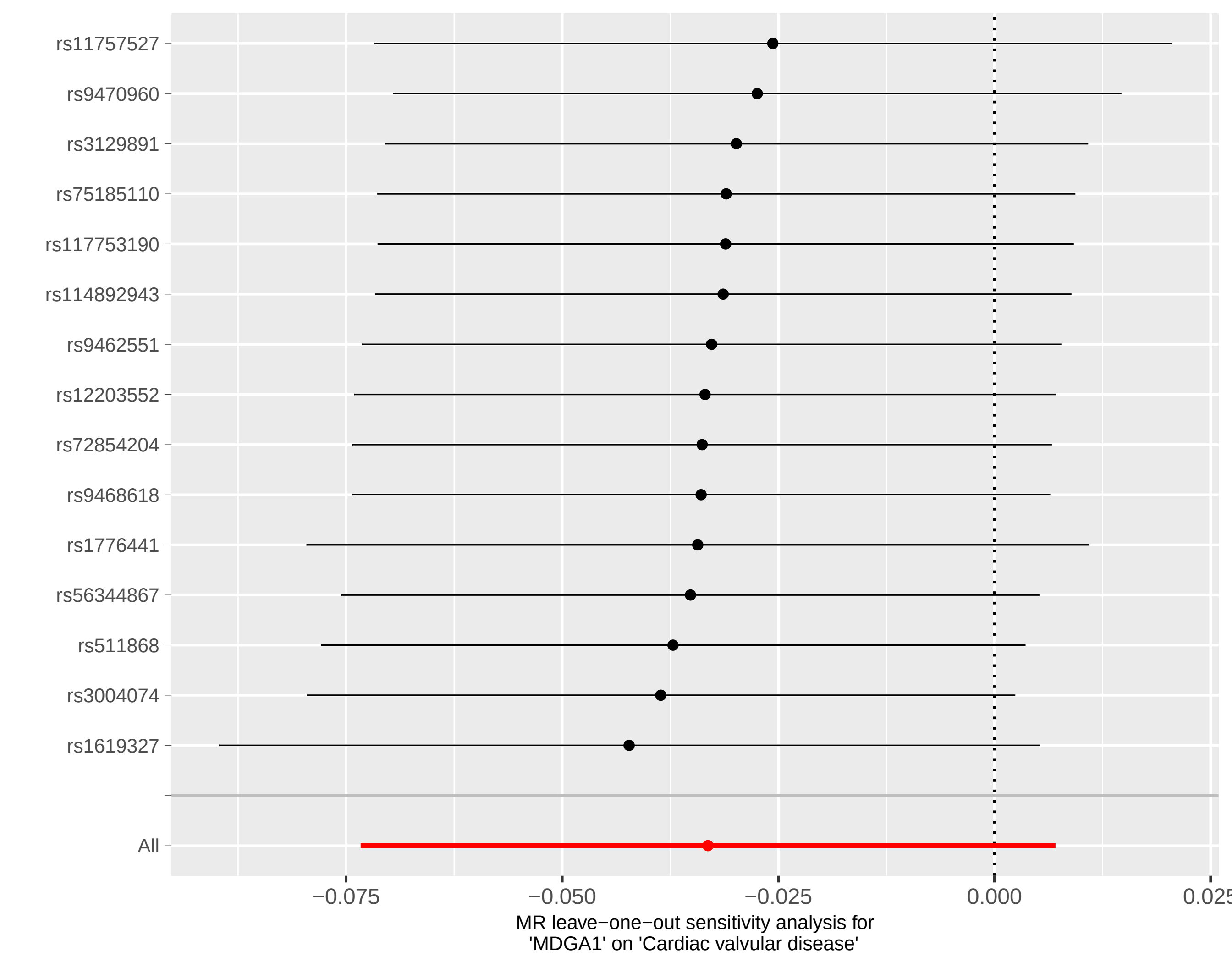
rs11757527
rs9470960
rs3129891
rs75185110
rs117753190
rs114892943
rs9462551
rs12203552
rs72854204
rs9468618
rs1776441
rs56344867
rs511868
rs3004074
rs1619327
All
−0.075
−0.050
−0.025
0.000
0.025
MR leave−one−out sensitivity analysis for
'MDGA1' on 'Cardiac valvular disease'

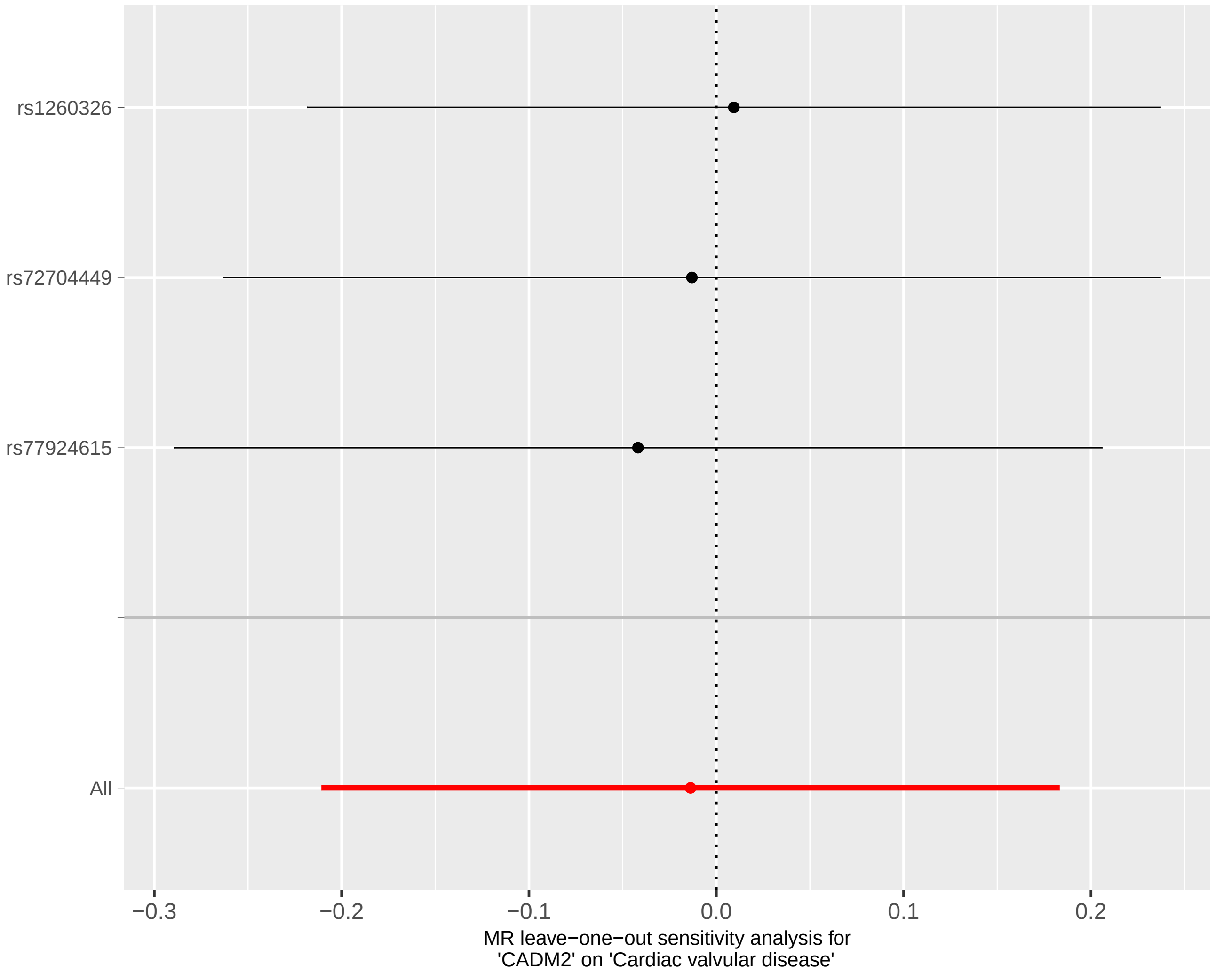
rs1260326
rs72704449
rs77924615
All
−0.3
−0.2
−0.1
0.0
0.1
0.2
MR leave−one−out sensitivity analysis for
'CADM2' on 'Cardiac valvular disease'

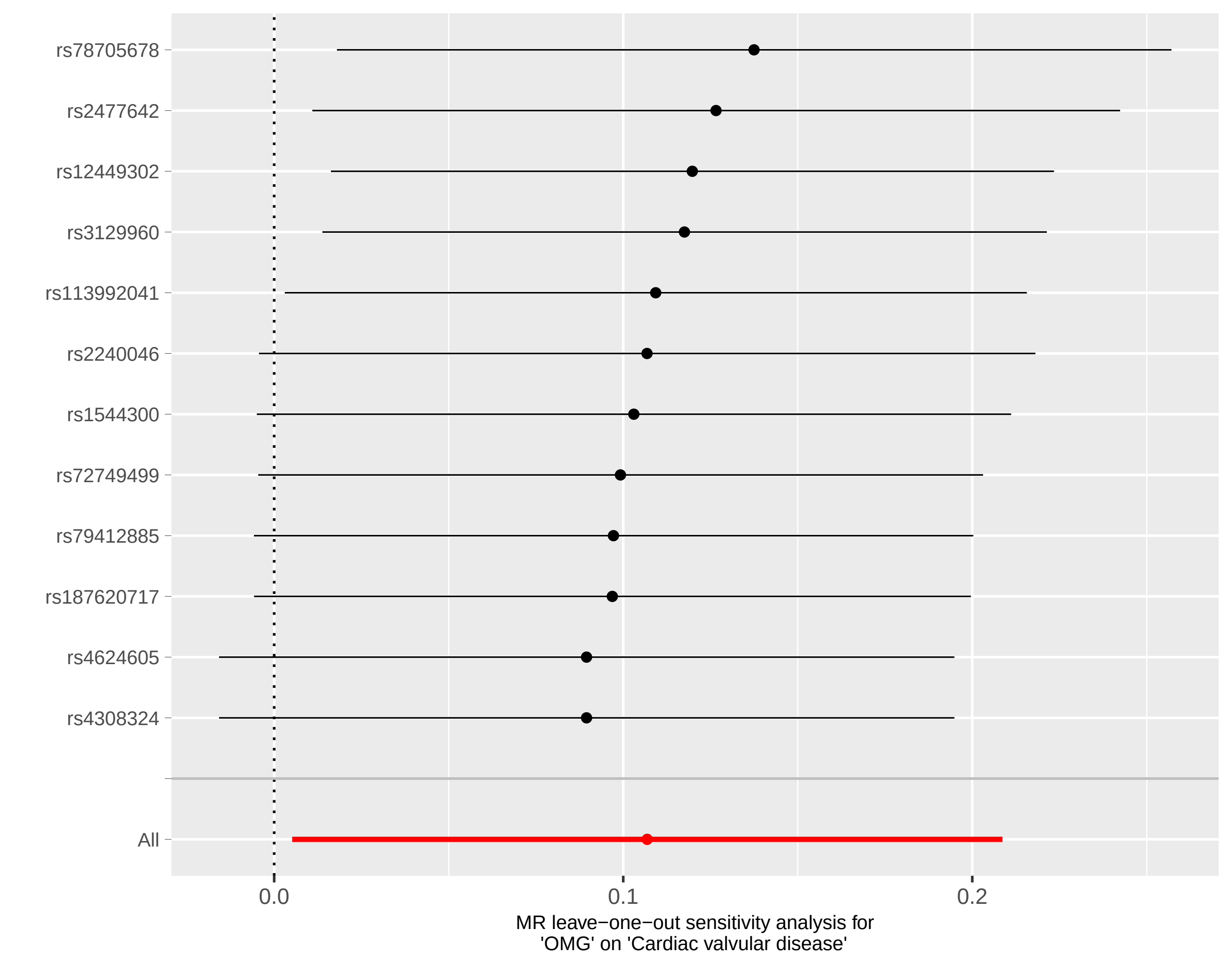
rs78705678
rs2477642
rs12449302
rs3129960
rs113992041
rs2240046
rs1544300
rs72749499
rs79412885
rs187620717
rs4624605
rs4308324
All
0.0
0.1
0.2
MR leave−one−out sensitivity analysis for
'OMG' on 'Cardiac valvular disease'

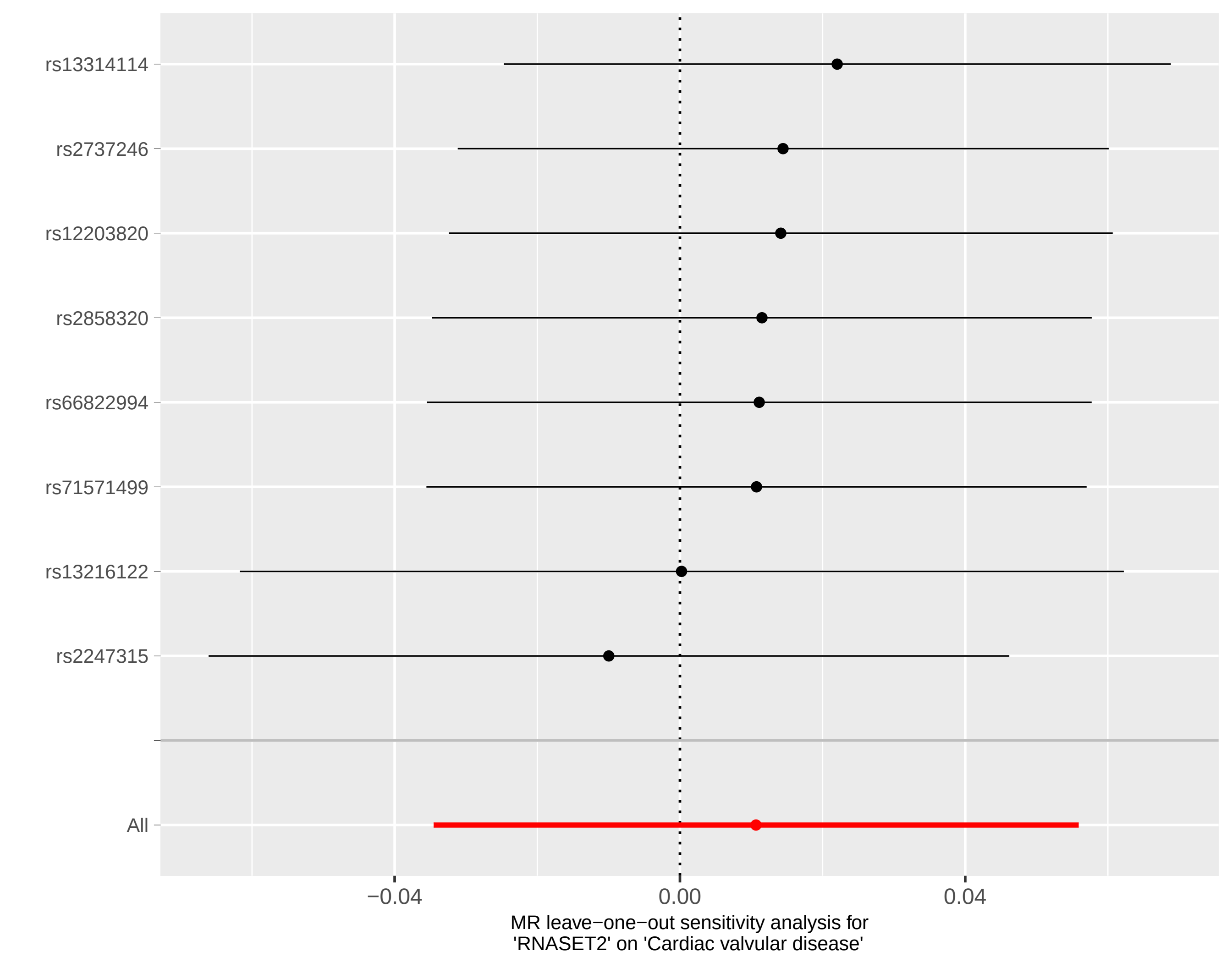

rs13314114
rs2737246
rs12203820
rs2858320
rs66822994
rs71571499
rs13216122
rs2247315
All
−0.04
0.00
0.04
MR leave−one−out sensitivity analysis for
'RNASET2' on 'Cardiac valvular disease'

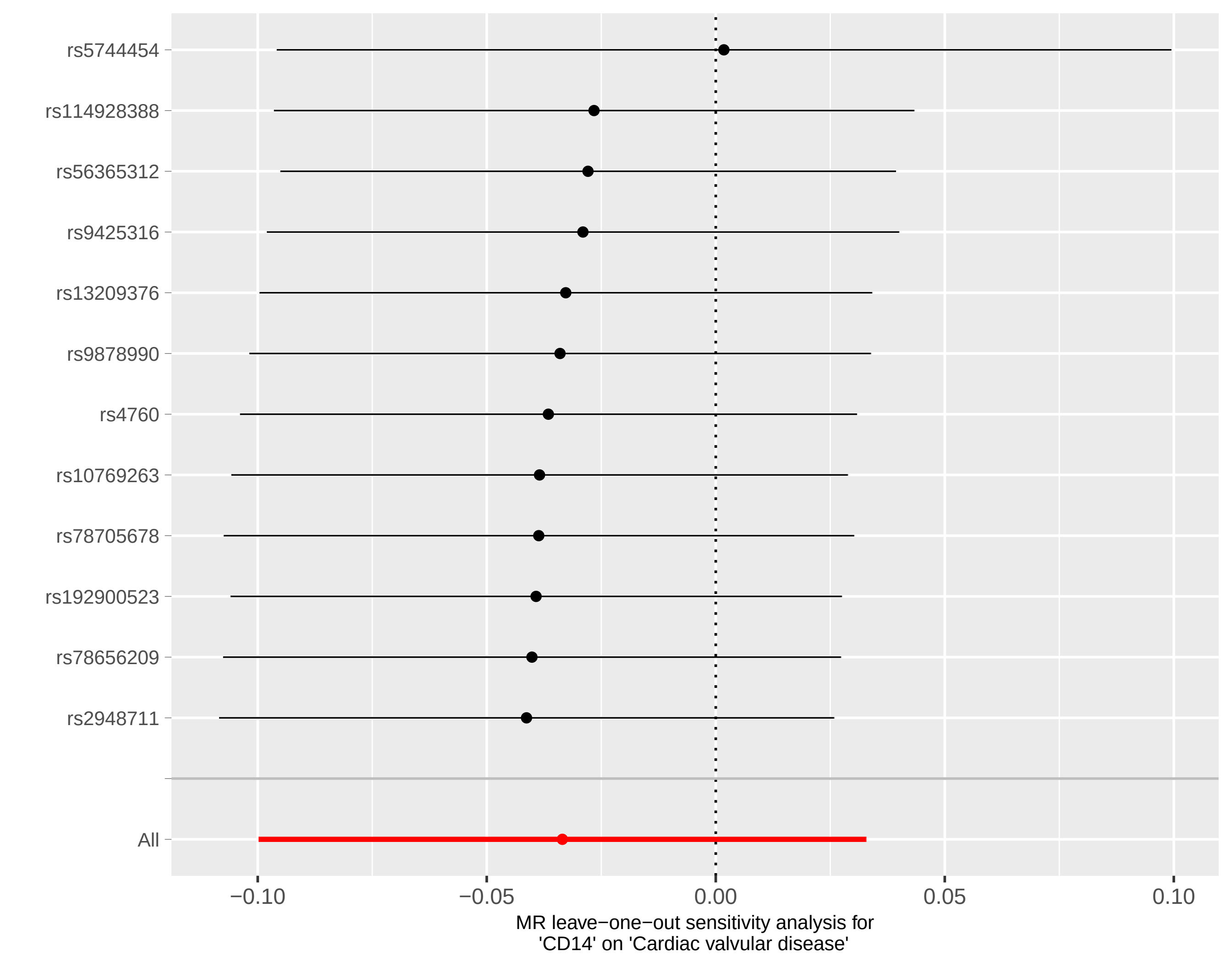
rs5744454
rs114928388
rs56365312
rs9425316
rs13209376
rs9878990
rs4760
rs10769263
rs78705678
rs192900523
rs78656209
rs2948711
All
−0.10
−0.05
0.00
0.05
0.10
MR leave−one−out sensitivity analysis for
'CD14' on 'Cardiac valvular disease'

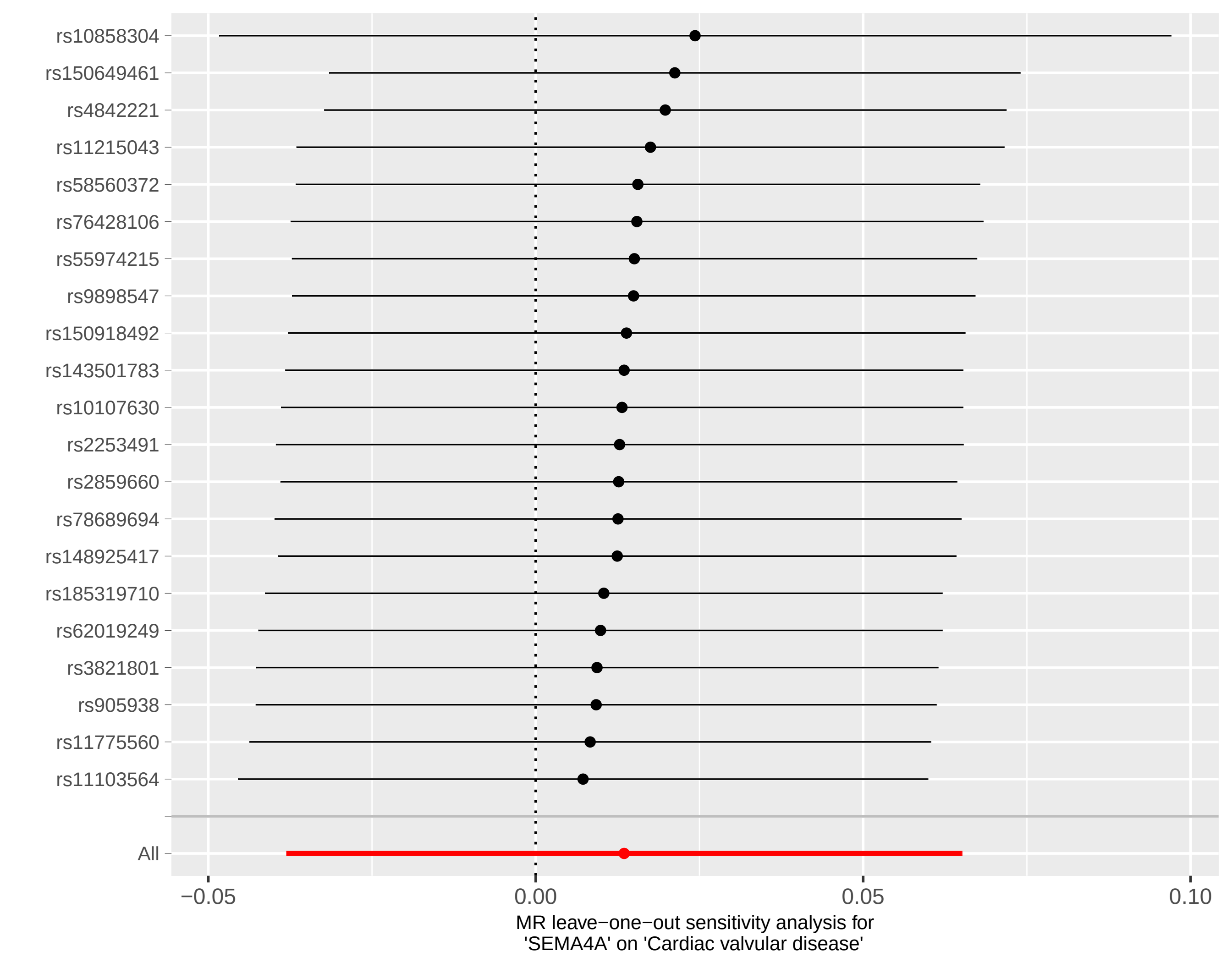
rs10858304
rs150649461
rs4842221
rs11215043
rs58560372
rs76428106
rs55974215
rs9898547
rs150918492
rs143501783
rs10107630
rs2253491
rs2859660
rs78689694
rs148925417
rs185319710
rs62019249
rs3821801
rs905938
rs11775560
rs11103564
All
−0.05
0.00
0.05
0.10
MR leave−one−out sensitivity analysis for
'SEMA4A' on 'Cardiac valvular disease'

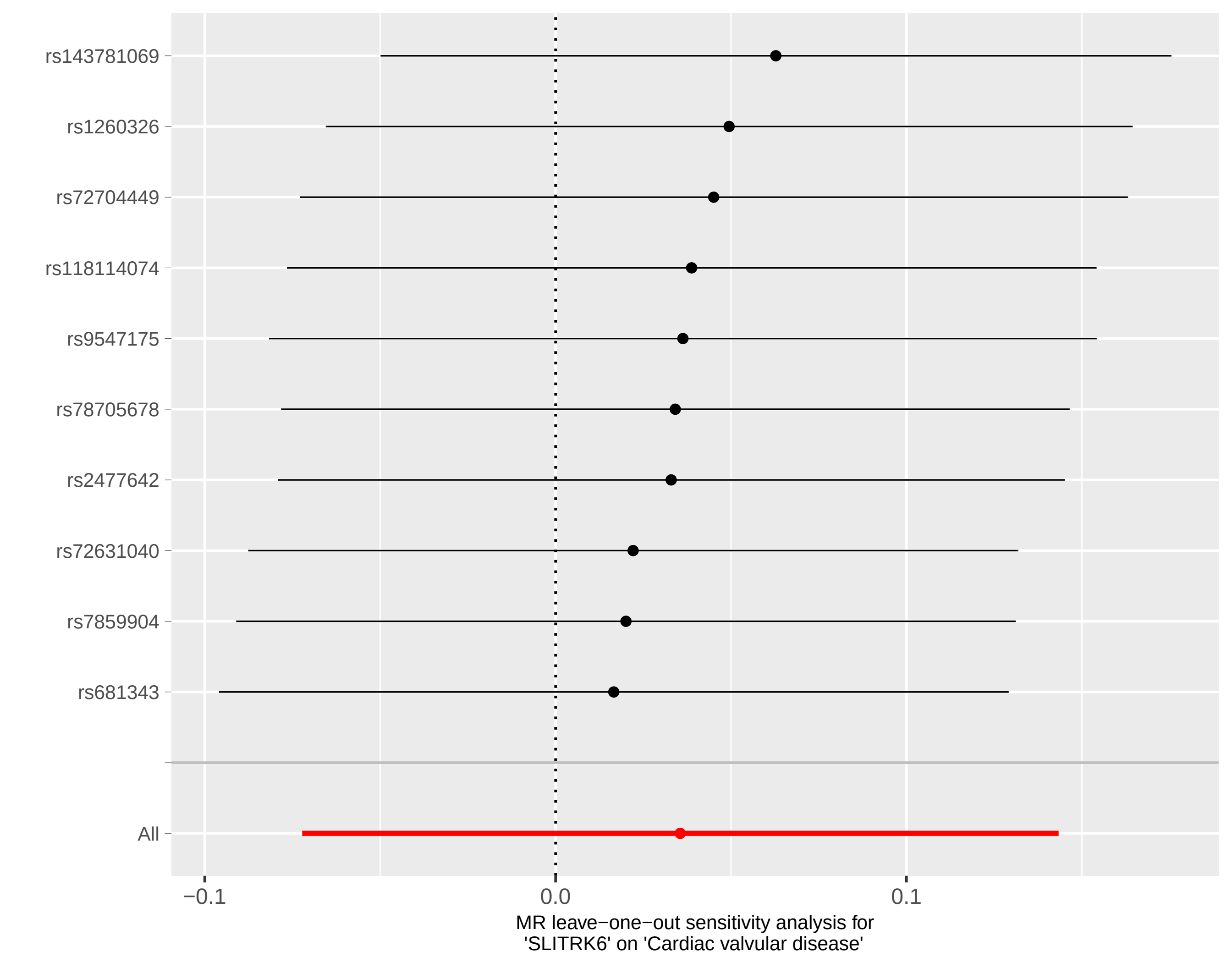

rs143781069
rs1260326
rs72704449
rs118114074
rs9547175
rs78705678
rs2477642
rs72631040
rs7859904
rs681343
All
−0.1
0.0
0.1
MR leave−one−out sensitivity analysis for
'SLITRK6' on 'Cardiac valvular disease'

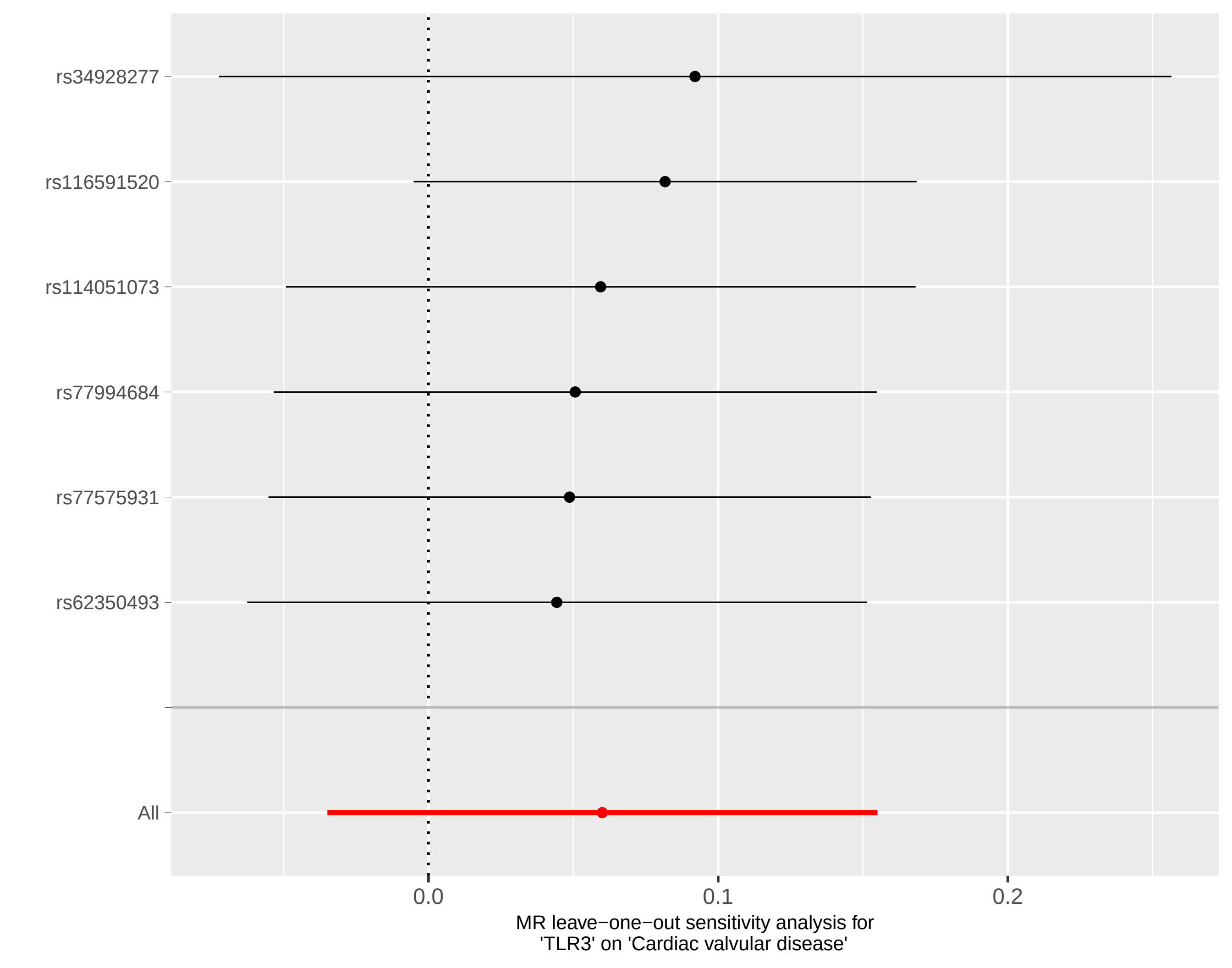
rs34928277
rs116591520
rs114051073
rs77994684
rs77575931
rs62350493
All
0.0
0.1
0.2
MR leave−one−out sensitivity analysis for
'TLR3' on 'Cardiac valvular disease'

Insufficient number of SNPs

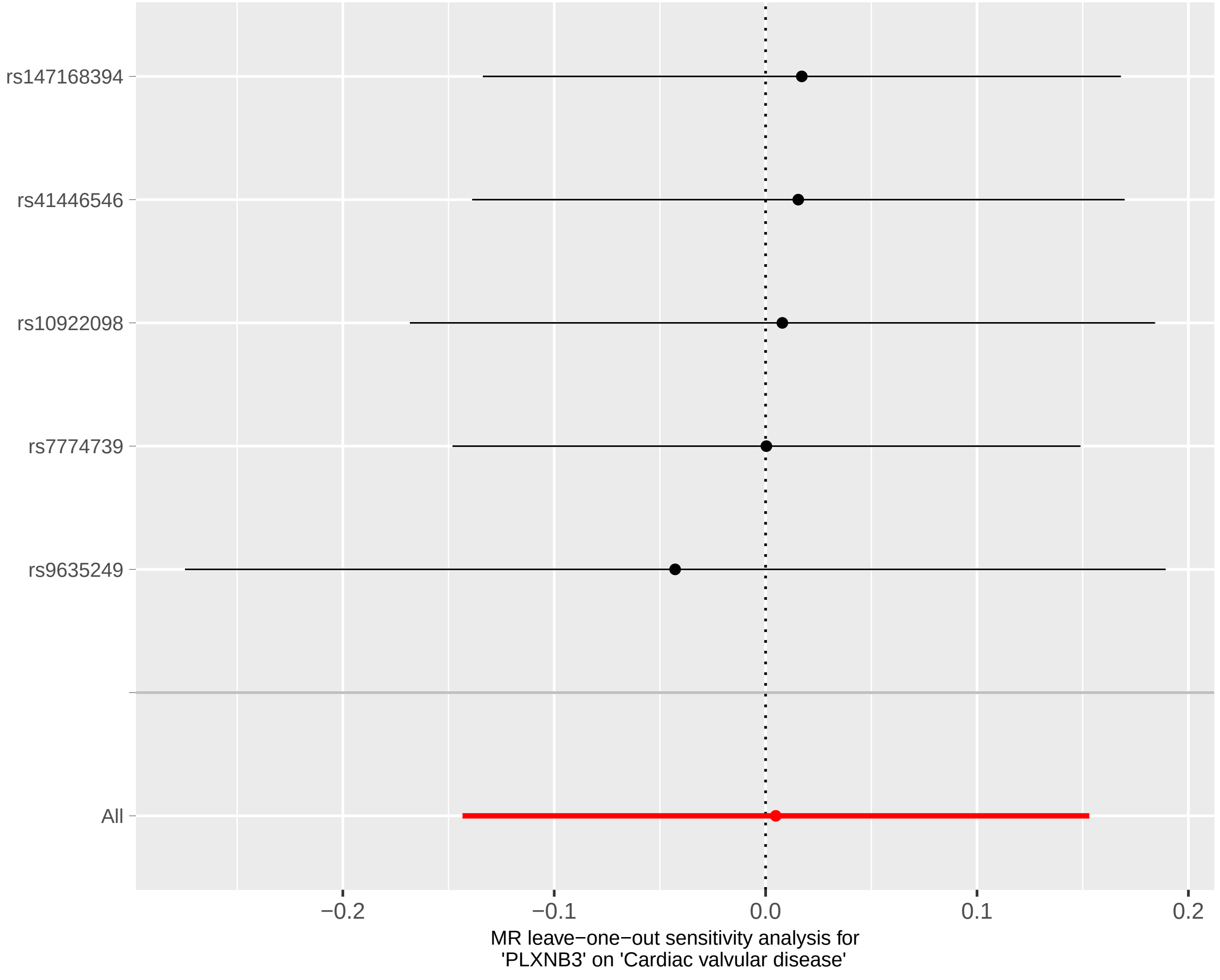
rs147168394
rs41446546
rs10922098
rs7774739
rs9635249
All
−0.2
−0.1
0.0
0.1
0.2
MR leave−one−out sensitivity analysis for
'PLXNB3' on 'Cardiac valvular disease'

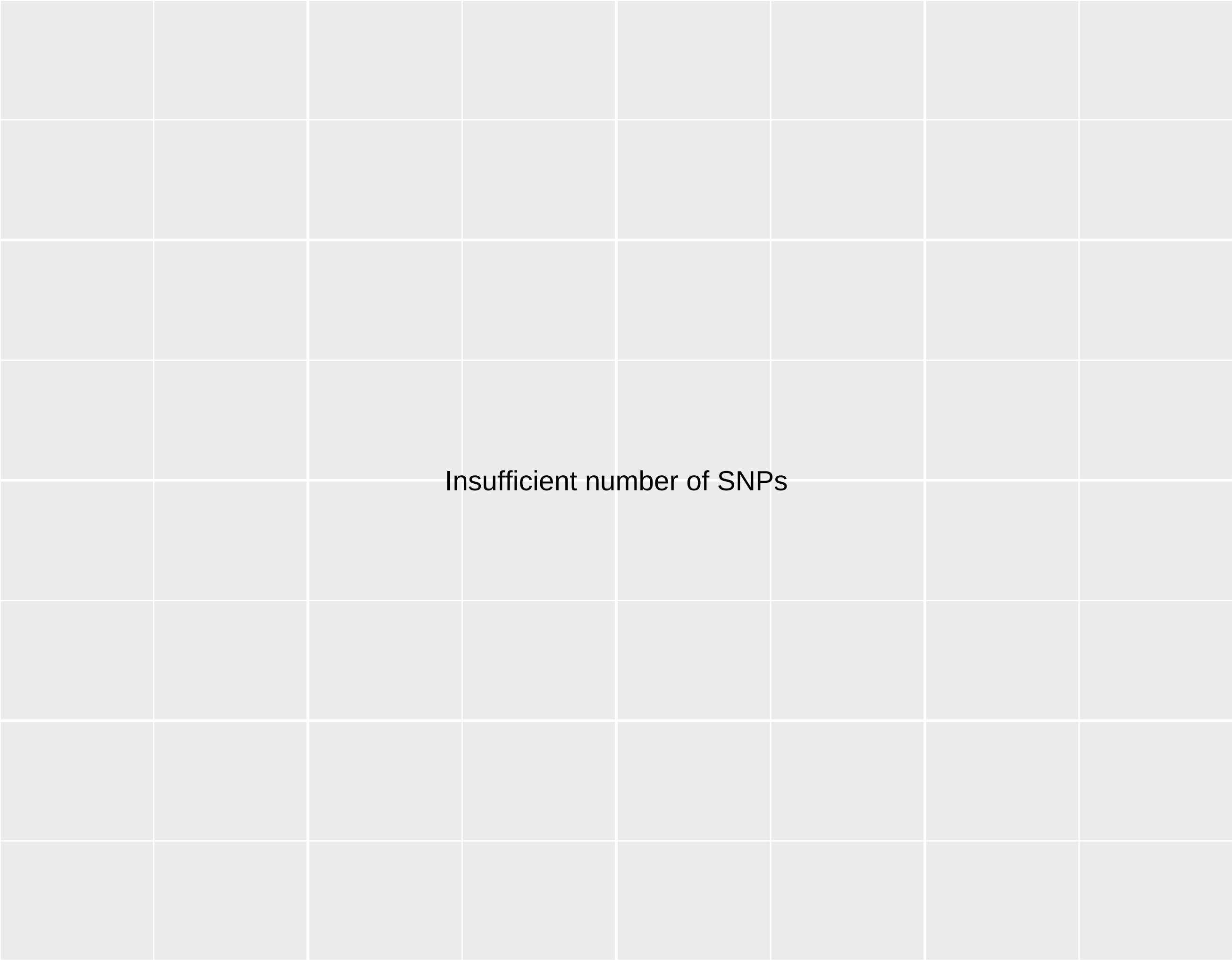
Insufficient number of SNPs

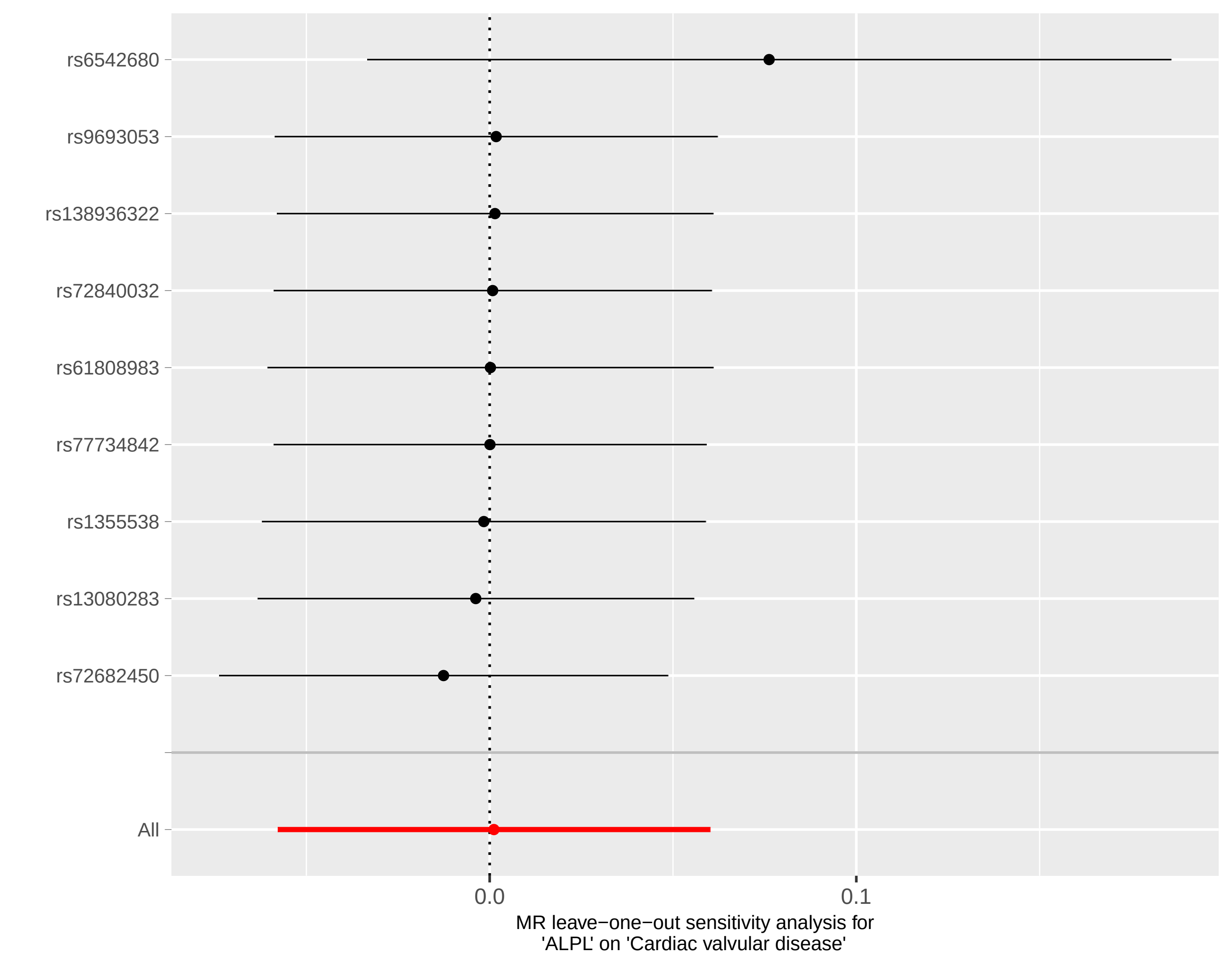

rs6542680
rs9693053
rs138936322
rs72840032
rs61808983
rs77734842
rs1355538
rs13080283
rs72682450
All
0.0
0.1
MR leave−one−out sensitivity analysis for
'ALPL' on 'Cardiac valvular disease'

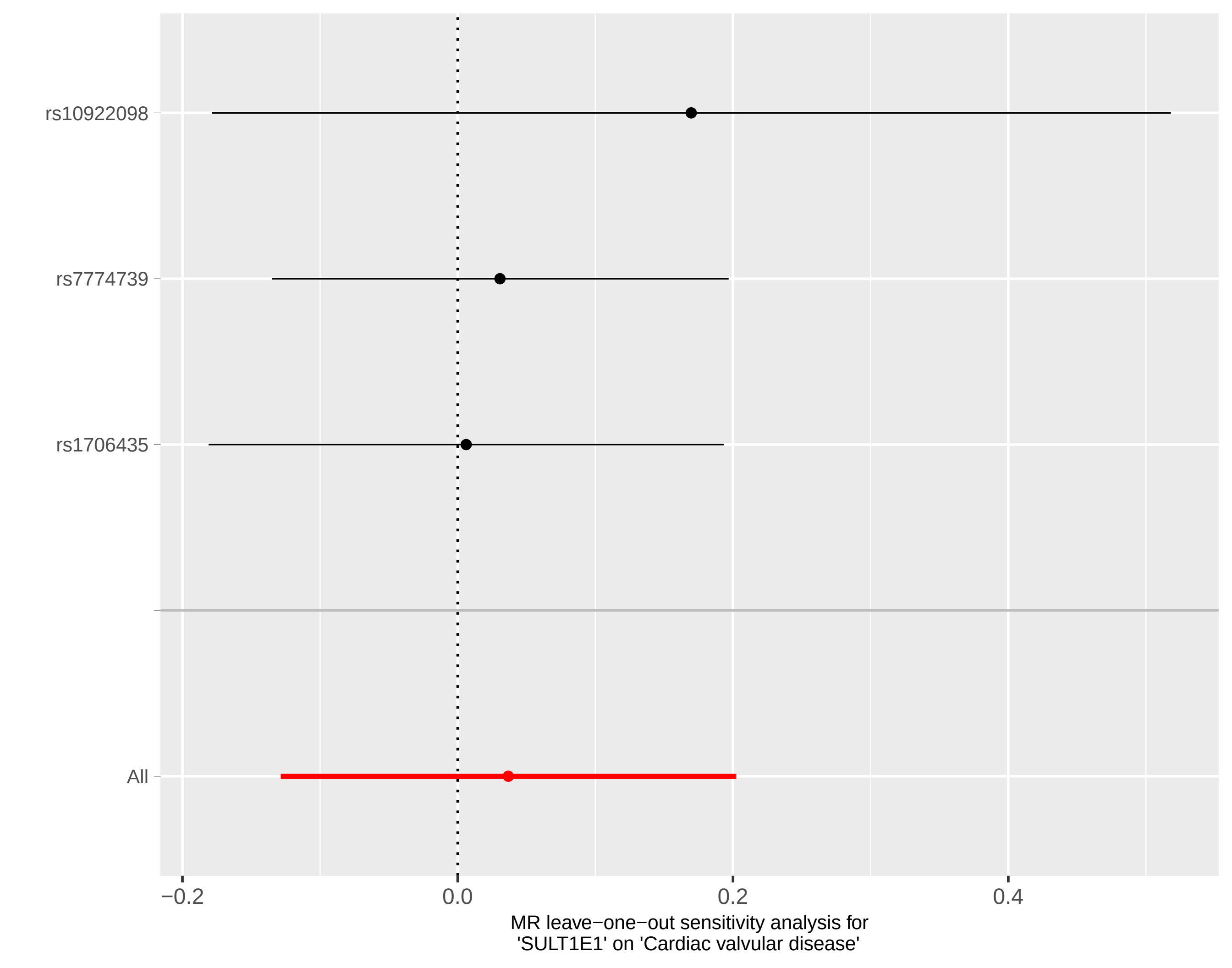
rs10922098
rs7774739
rs1706435
All
−0.2
0.0
0.2
0.4
MR leave−one−out sensitivity analysis for
'SULT1E1' on 'Cardiac valvular disease'

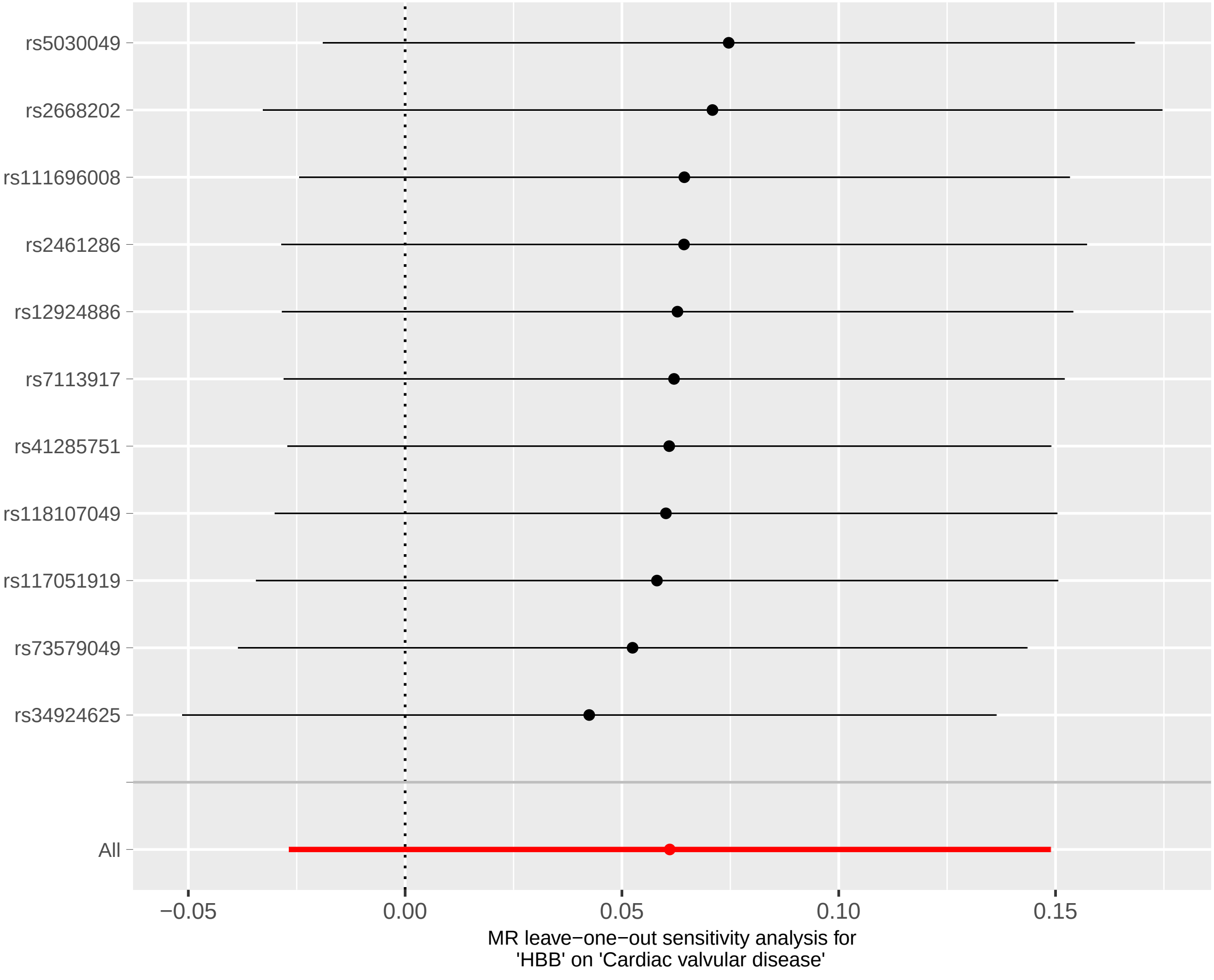
rs5030049
rs2668202
rs111696008
rs2461286
rs12924886
rs7113917
rs41285751
rs118107049
rs117051919
rs73579049
rs34924625
All
−0.05
0.00
0.05
0.10
0.15
MR leave−one−out sensitivity analysis for
'HBB' on 'Cardiac valvular disease'

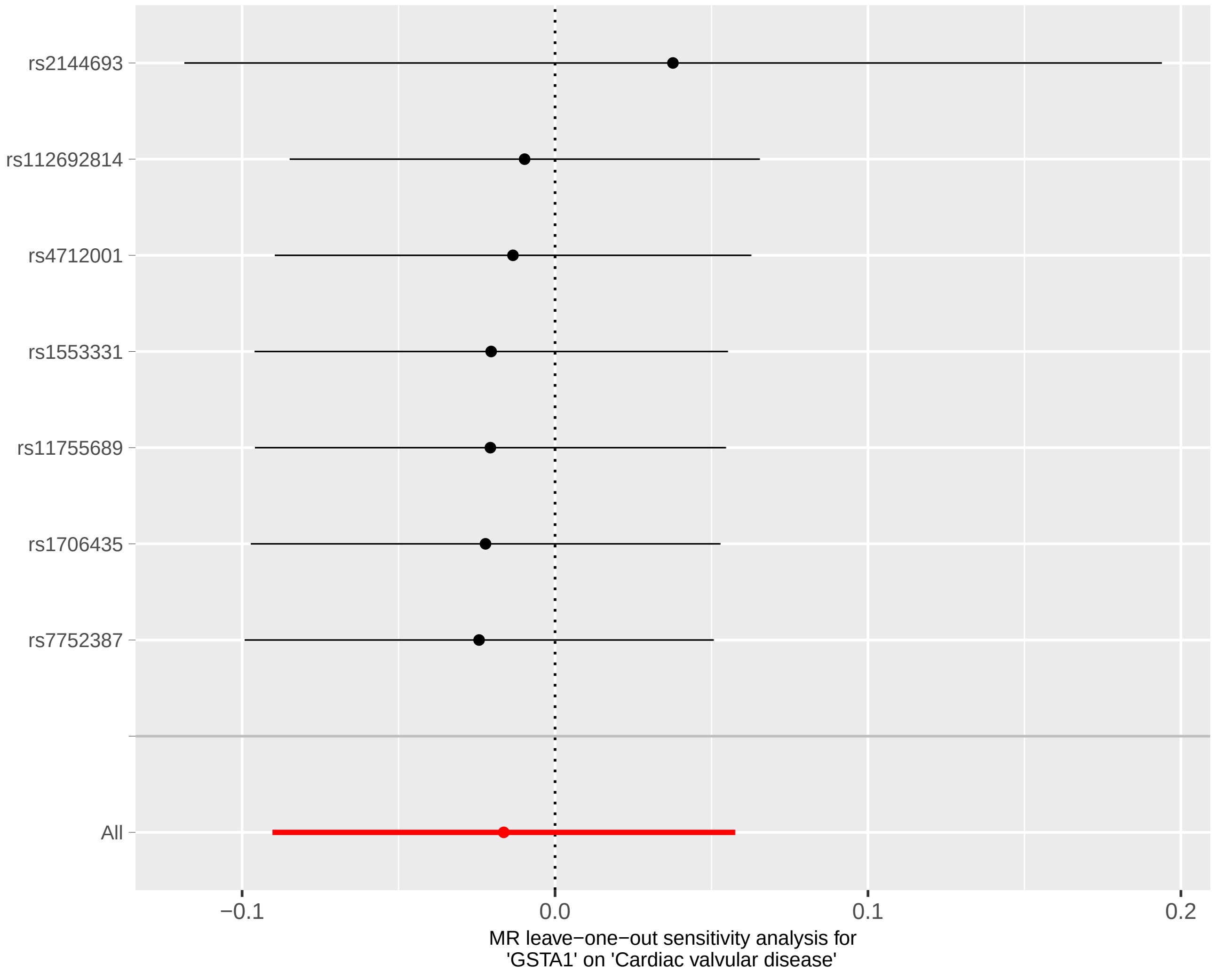
rs2144693
rs112692814
rs4712001
rs1553331
rs11755689
rs1706435
rs7752387
All
−0.1
0.0
0.1
0.2
MR leave−one−out sensitivity analysis for
'GSTA1' on 'Cardiac valvular disease'

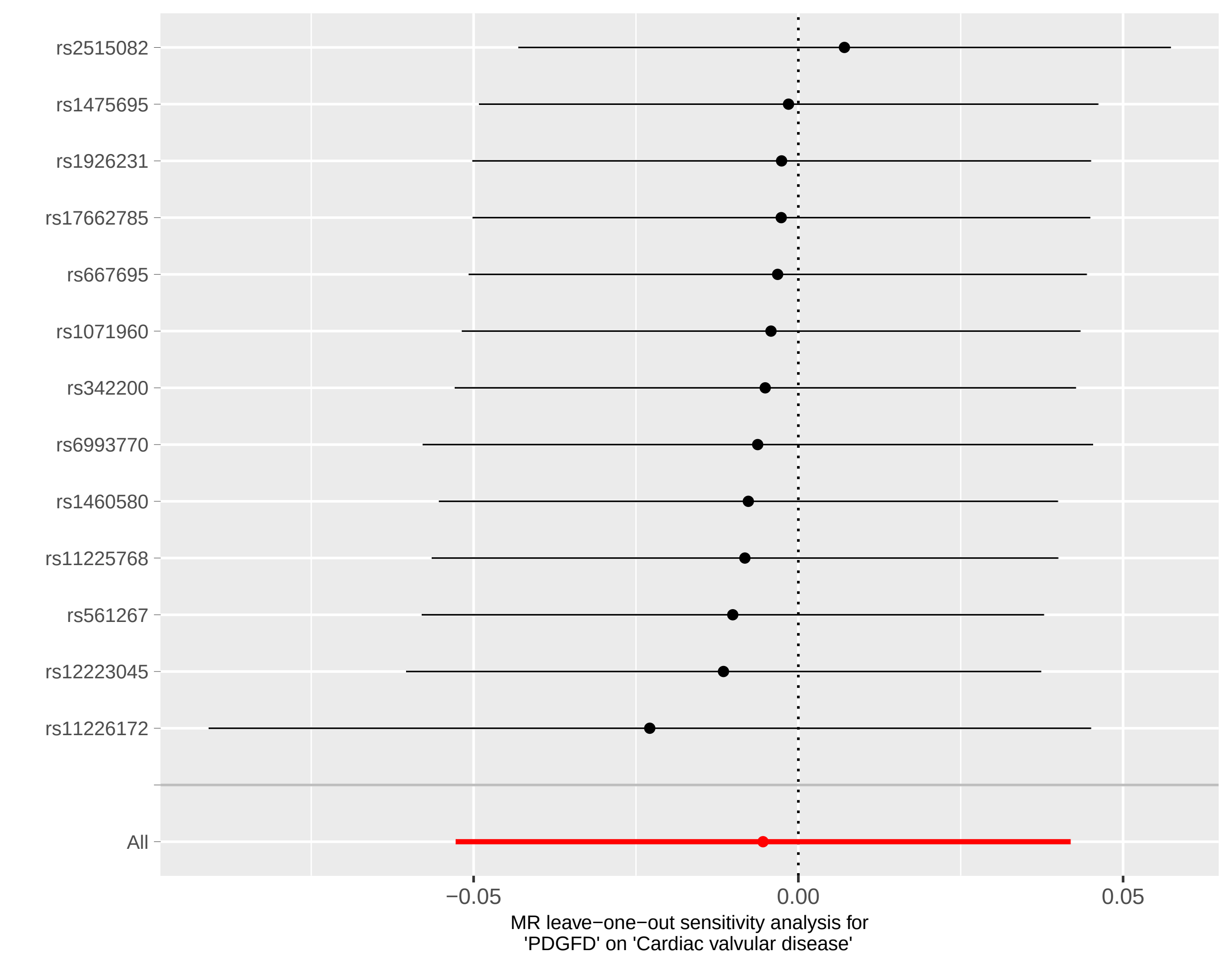
rs2515082
rs1475695
rs1926231
rs17662785
rs667695
rs1071960
rs342200
rs6993770
rs1460580
rs11225768
rs561267
rs12223045
rs11226172
All
−0.05
0.00
0.05
MR leave−one−out sensitivity analysis for
'PDGFD' on 'Cardiac valvular disease'

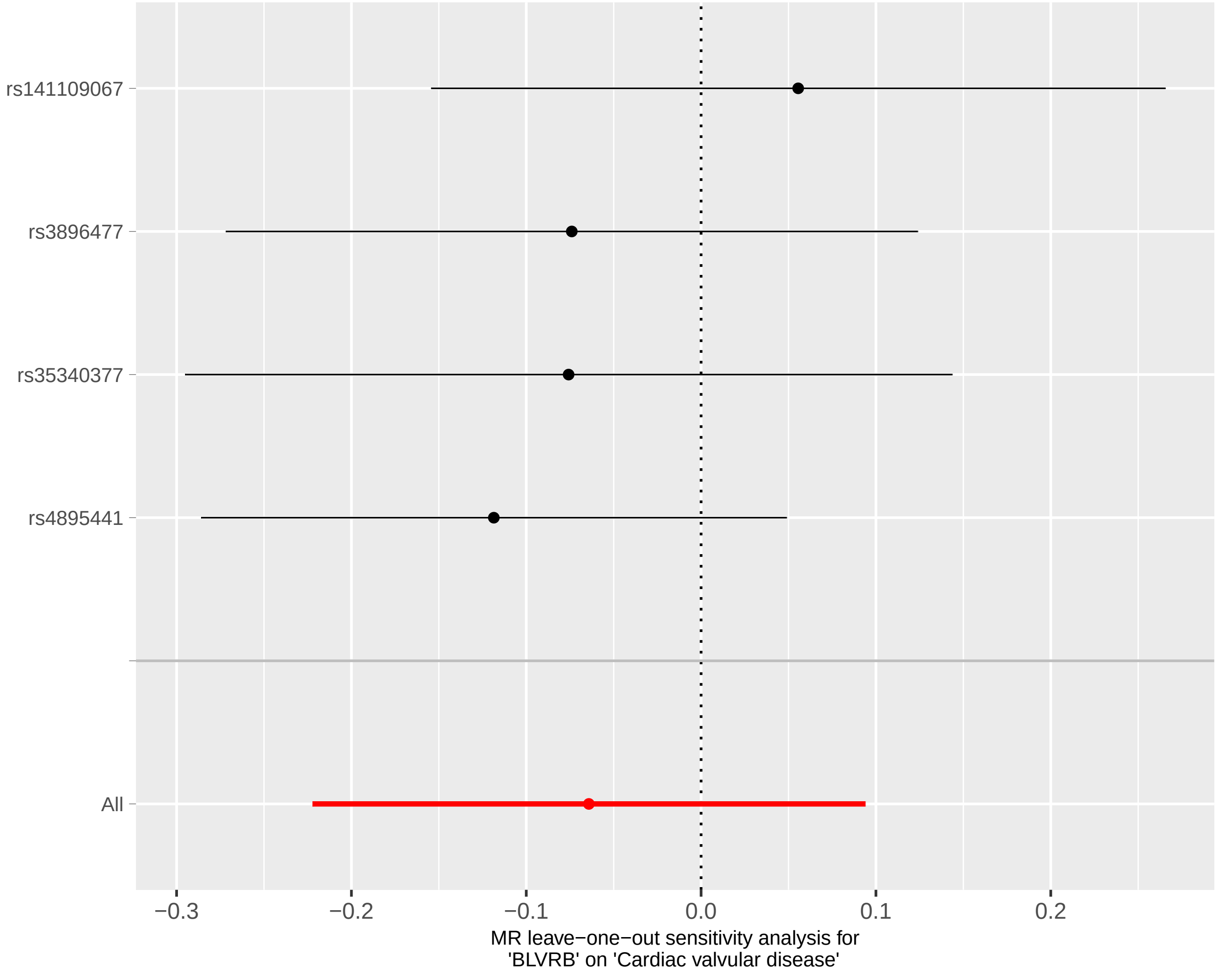

rs141109067
rs3896477
rs35340377
rs4895441
All
−0.3
−0.2
−0.1
0.0
0.1
0.2
MR leave−one−out sensitivity analysis for
'BLVRB' on 'Cardiac valvular disease'

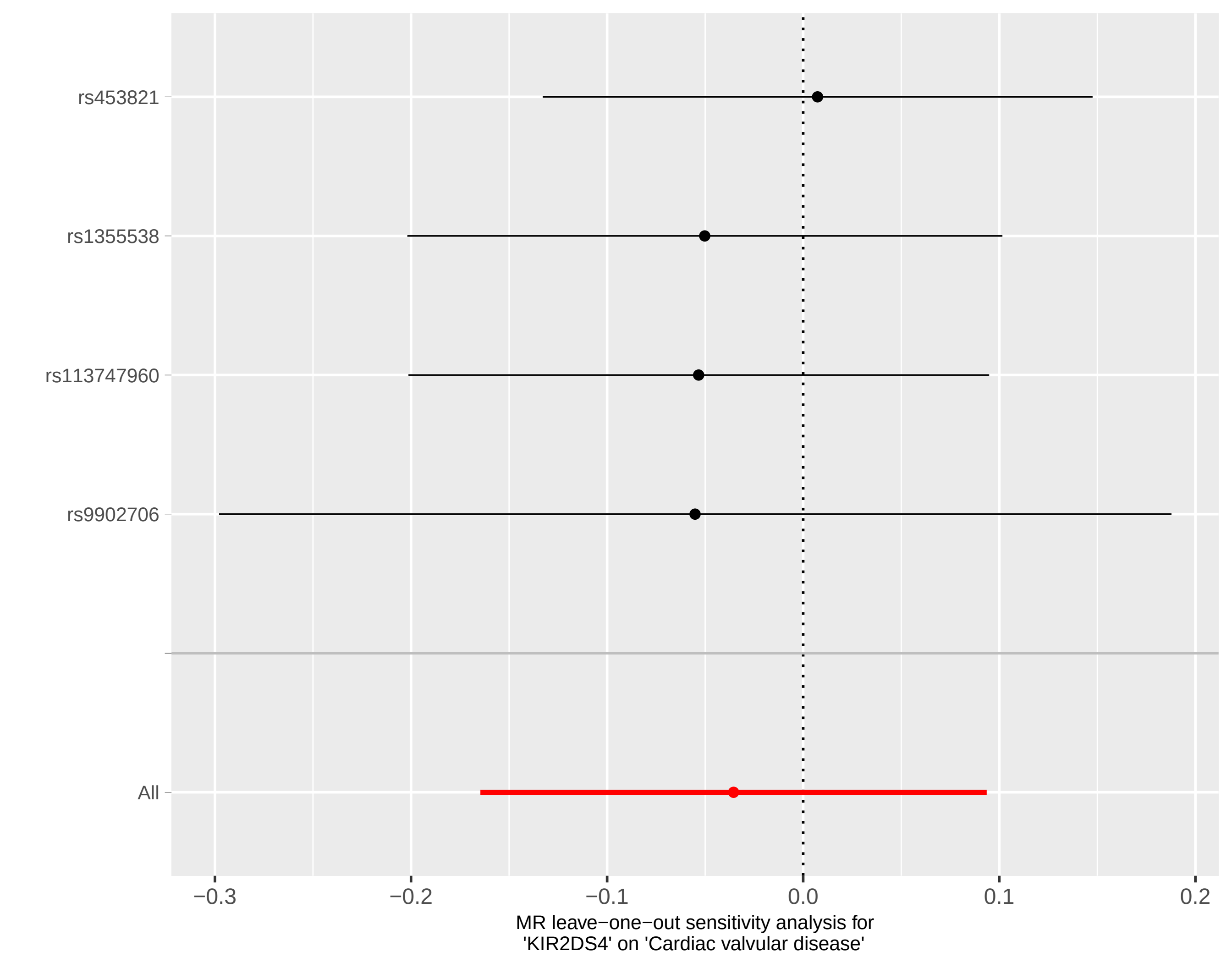

rs453821
rs1355538
rs113747960
rs9902706
All
−0.3
−0.2
−0.1
0.0
0.1
0.2
MR leave−one−out sensitivity analysis for
'KIR2DS4' on 'Cardiac valvular disease'

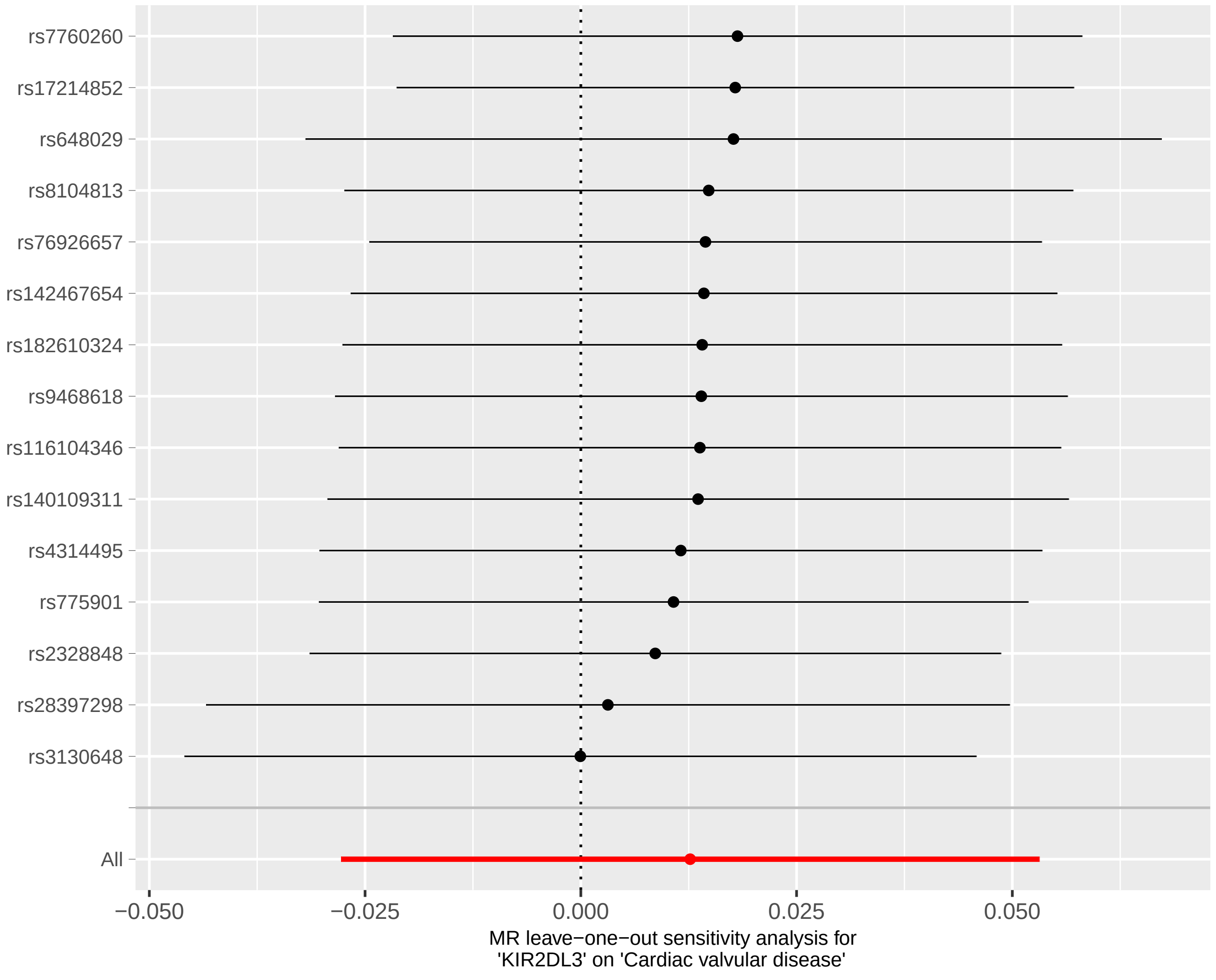
rs7760260
rs17214852
rs648029
rs8104813
rs76926657
rs142467654
rs182610324
rs9468618
rs116104346
rs140109311
rs4314495
rs775901
rs2328848
rs28397298
rs3130648
All
−0.050
−0.025
0.000
0.025
0.050
MR leave−one−out sensitivity analysis for
'KIR2DL3' on 'Cardiac valvular disease'

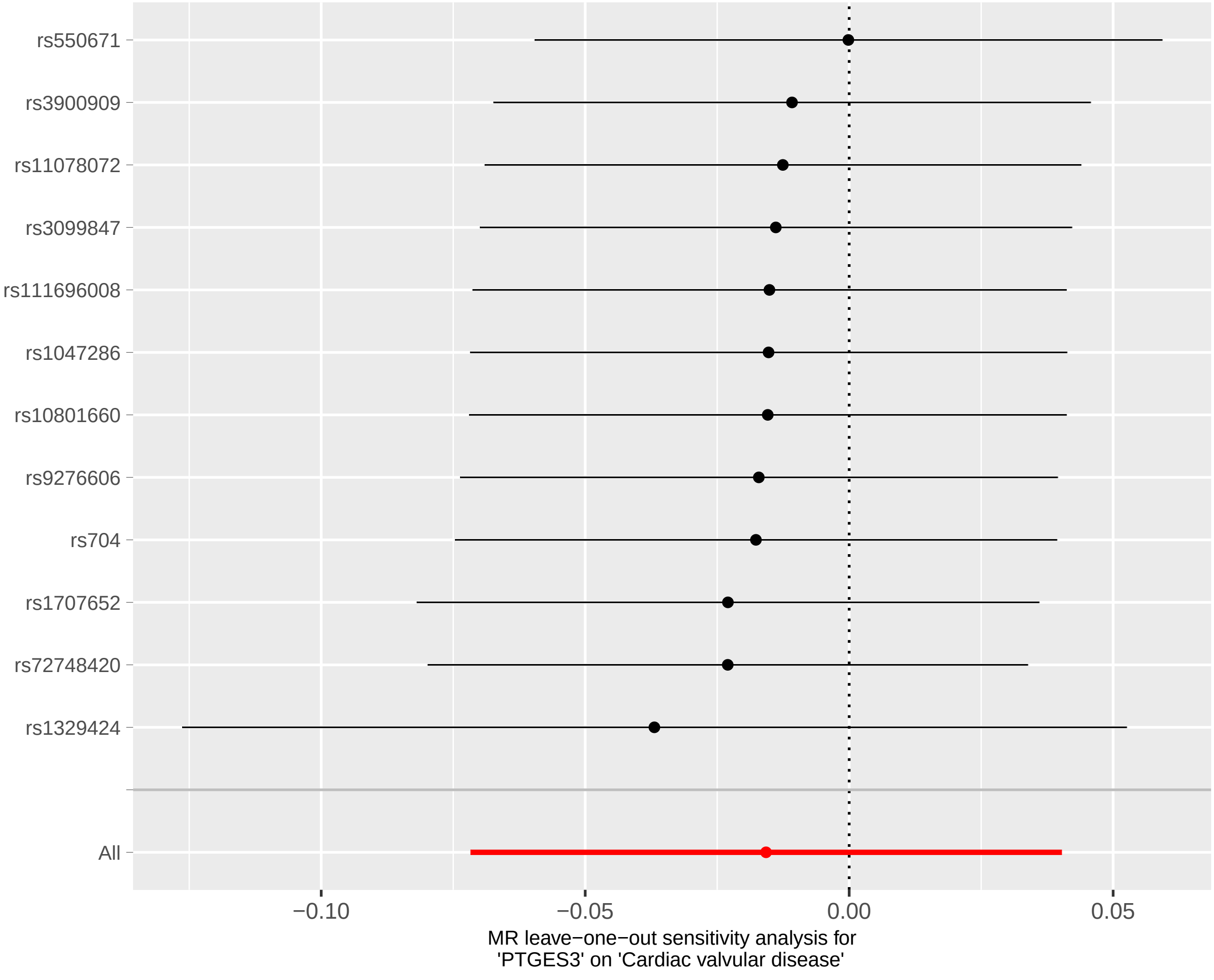

rs550671
rs3900909
rs11078072
rs3099847
rs111696008
rs1047286
rs10801660
rs9276606
rs704
rs1707652
rs72748420
rs1329424
All
−0.10
−0.05
0.00
0.05
MR leave−one−out sensitivity analysis for
'PTGES3' on 'Cardiac valvular disease'

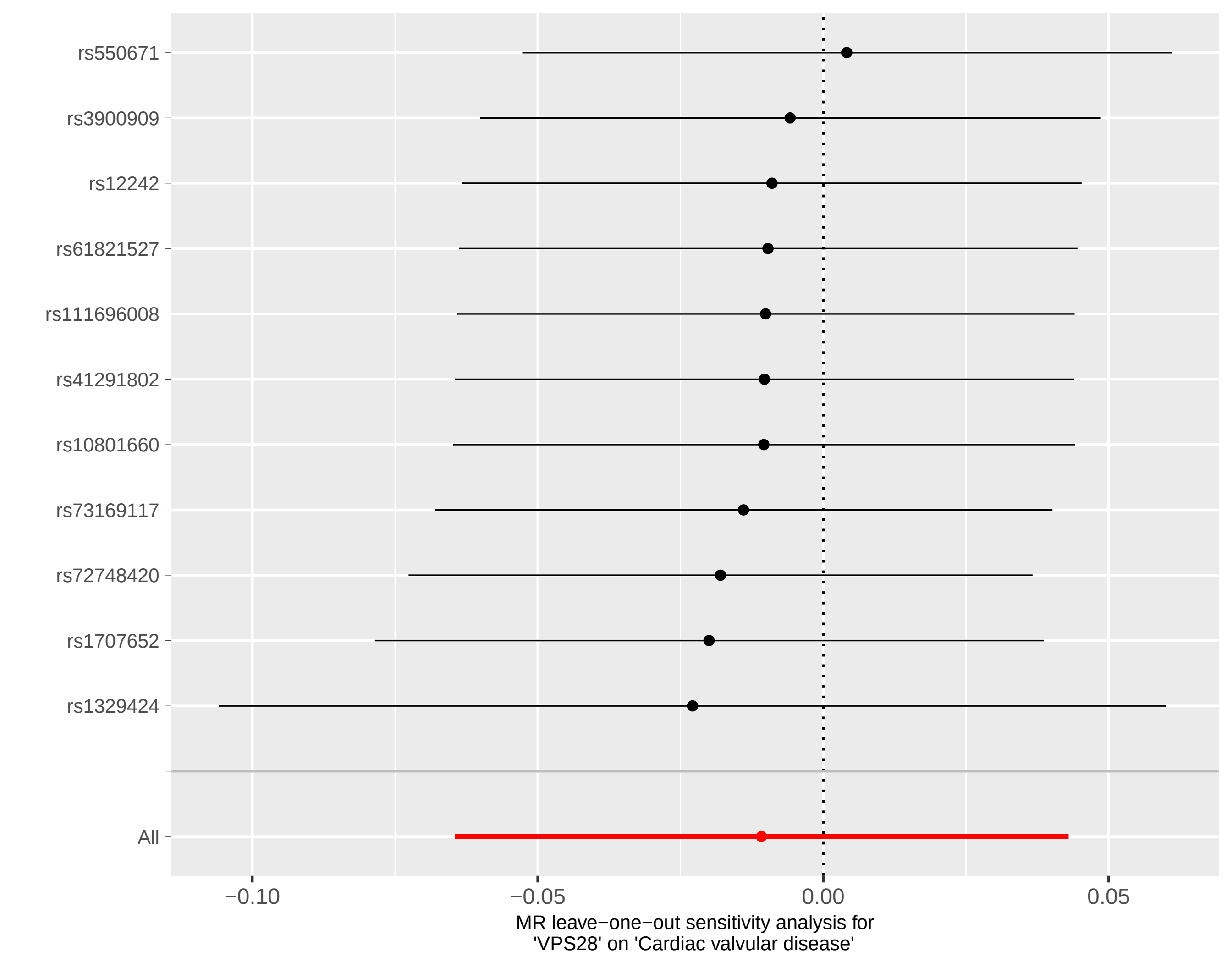
rs550671
rs3900909
rs12242
rs61821527
rs111696008
rs41291802
rs10801660
rs73169117
rs72748420
rs1707652
rs1329424
All
−0.10
−0.05
0.00
0.05
MR leave−one−out sensitivity analysis for
'VPS28' on 'Cardiac valvular disease'

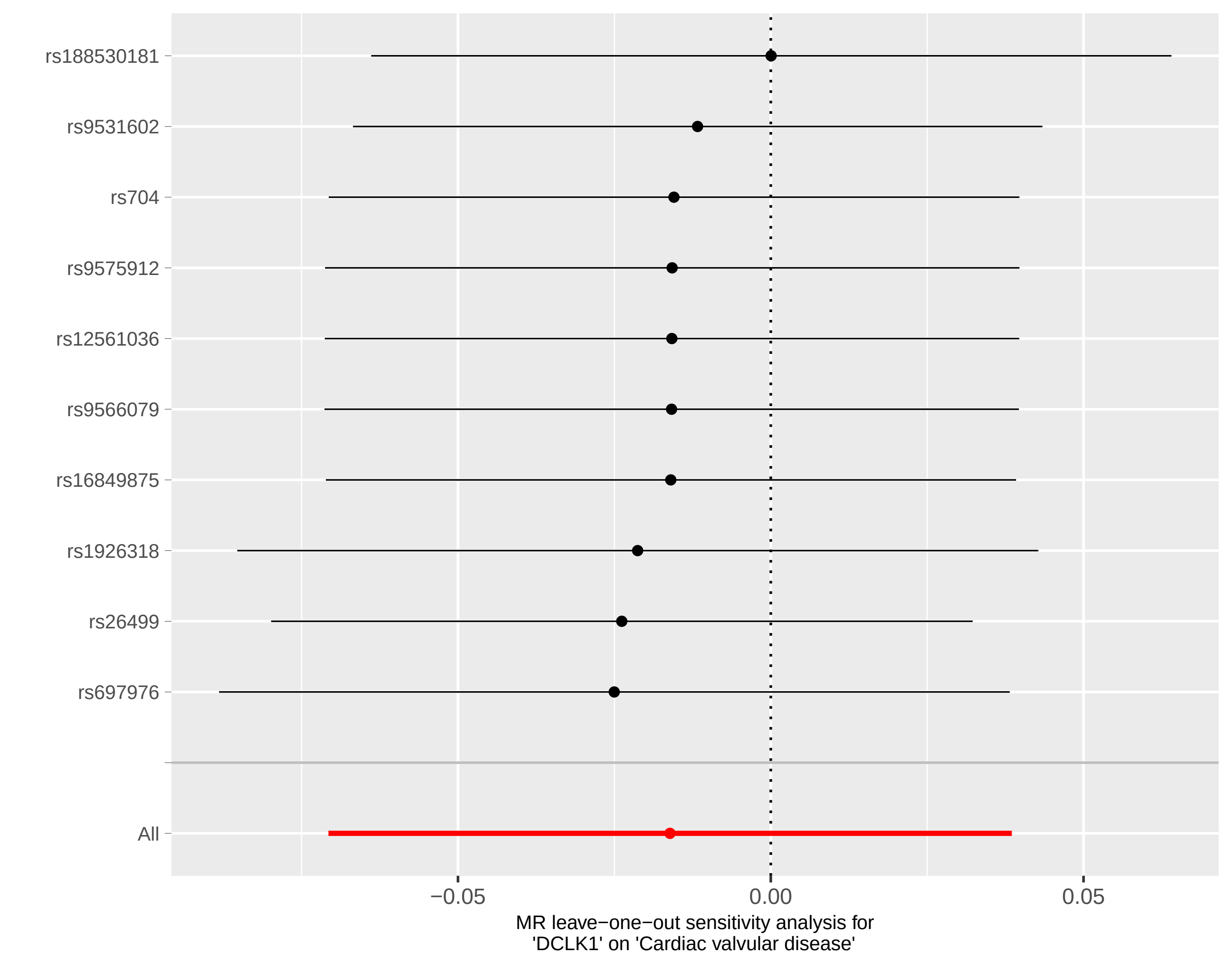

rs188530181
rs9531602
rs704
rs9575912
rs12561036
rs9566079
rs16849875
rs1926318
rs26499
rs697976
All
−0.05
0.00
0.05
MR leave−one−out sensitivity analysis for
'DCLK1' on 'Cardiac valvular disease'

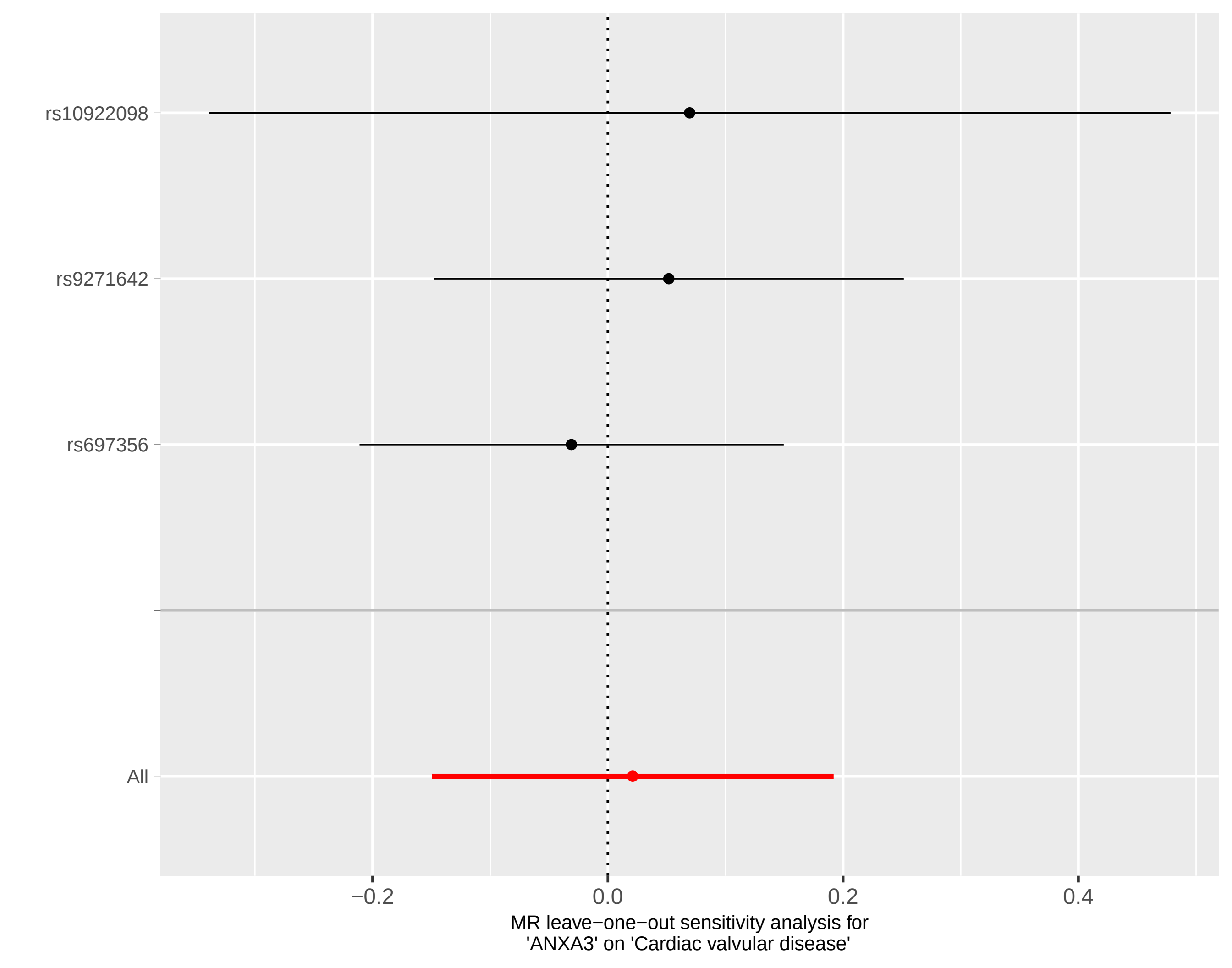

rs10922098
rs9271642
rs697356
All
−0.2
0.0
0.2
0.4
MR leave−one−out sensitivity analysis for
'ANXA3' on 'Cardiac valvular disease'

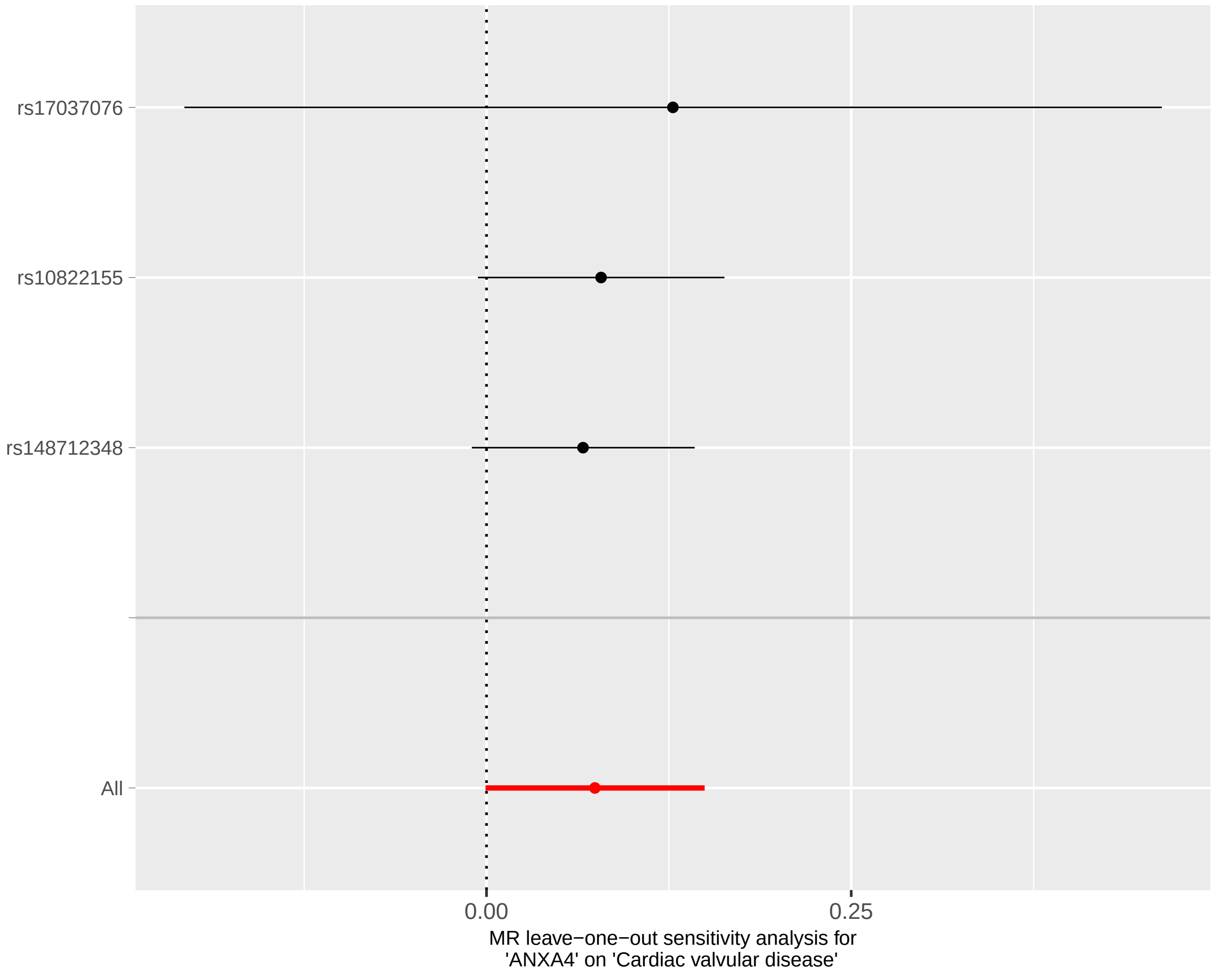
rs17037076
rs10822155
rs148712348
All
0.00
0.25
MR leave−one−out sensitivity analysis for
'ANXA4' on 'Cardiac valvular disease'

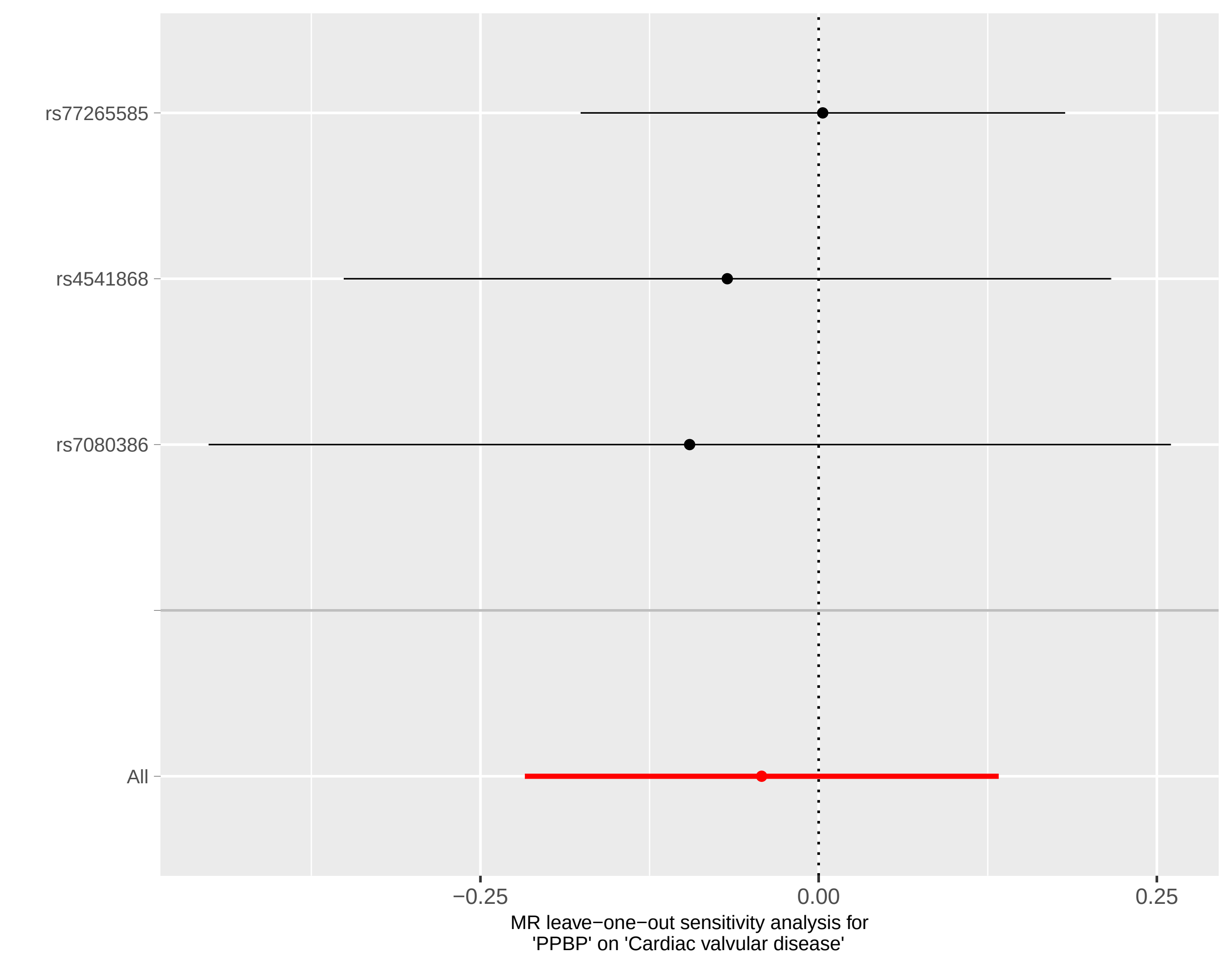
rs77265585
rs4541868
rs7080386
All
−0.25
0.00
0.25
MR leave−one−out sensitivity analysis for
'PPBP' on 'Cardiac valvular disease'

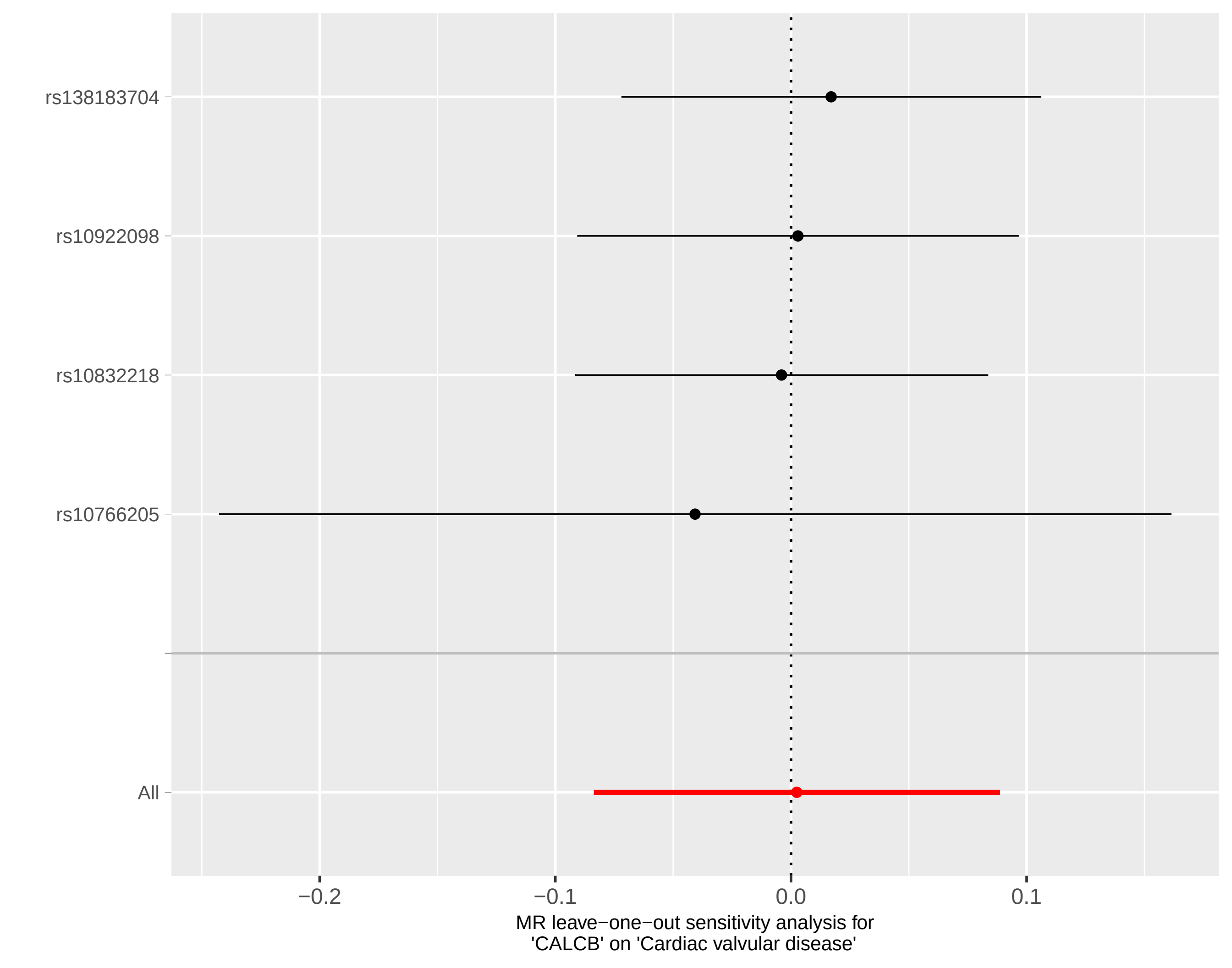
rs138183704
rs10922098
rs10832218
rs10766205
All
−0.2
−0.1
0.0
0.1
MR leave−one−out sensitivity analysis for
'CALCB' on 'Cardiac valvular disease'

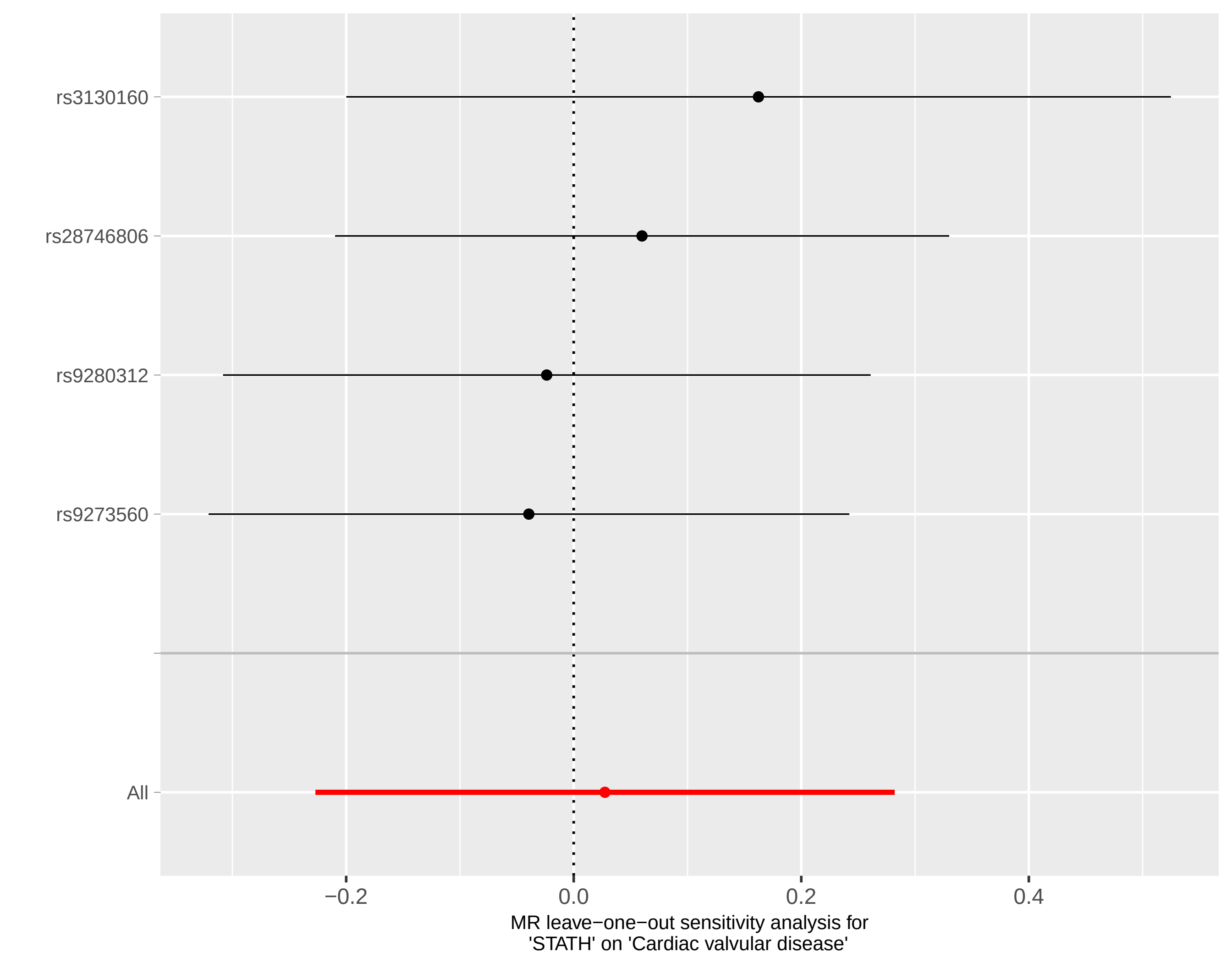

rs3130160
rs28746806
rs9280312
rs9273560
All
−0.2
0.0
0.2
0.4
MR leave−one−out sensitivity analysis for
'STATH' on 'Cardiac valvular disease'

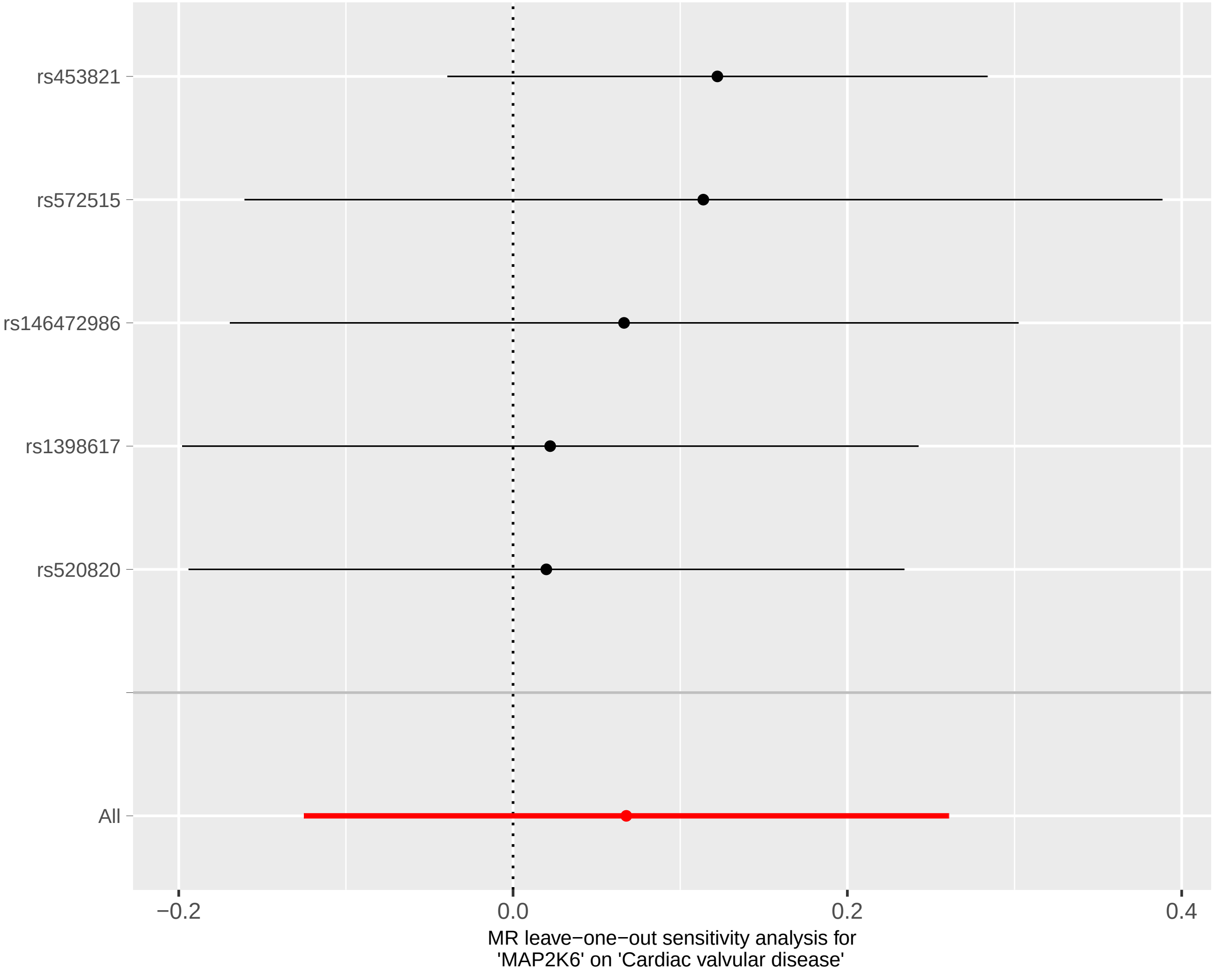
rs453821
rs572515
rs146472986
rs1398617
rs520820
All
−0.2
0.0
0.2
0.4
MR leave−one−out sensitivity analysis for
'MAP2K6' on 'Cardiac valvular disease'

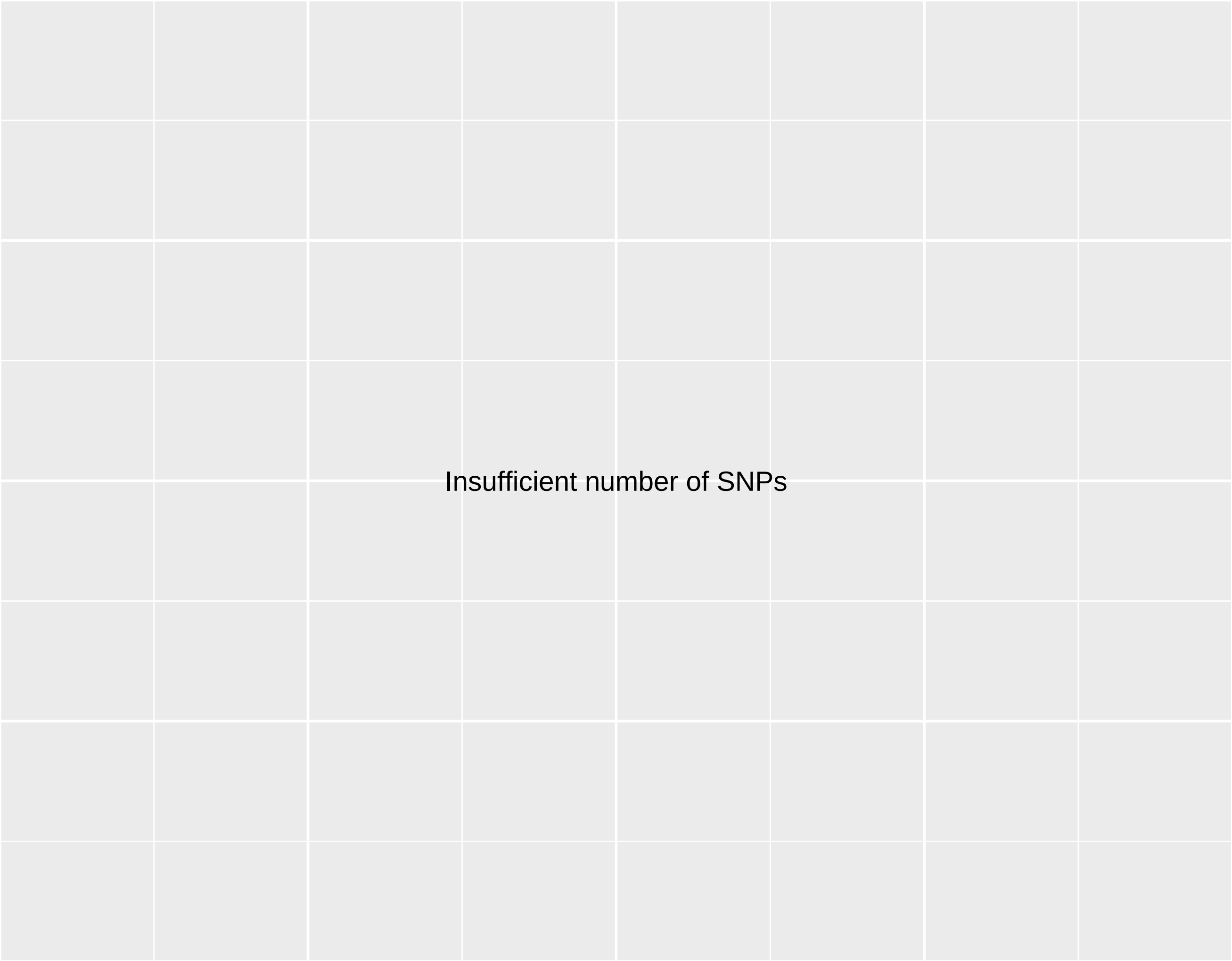
Insufficient number of SNPs

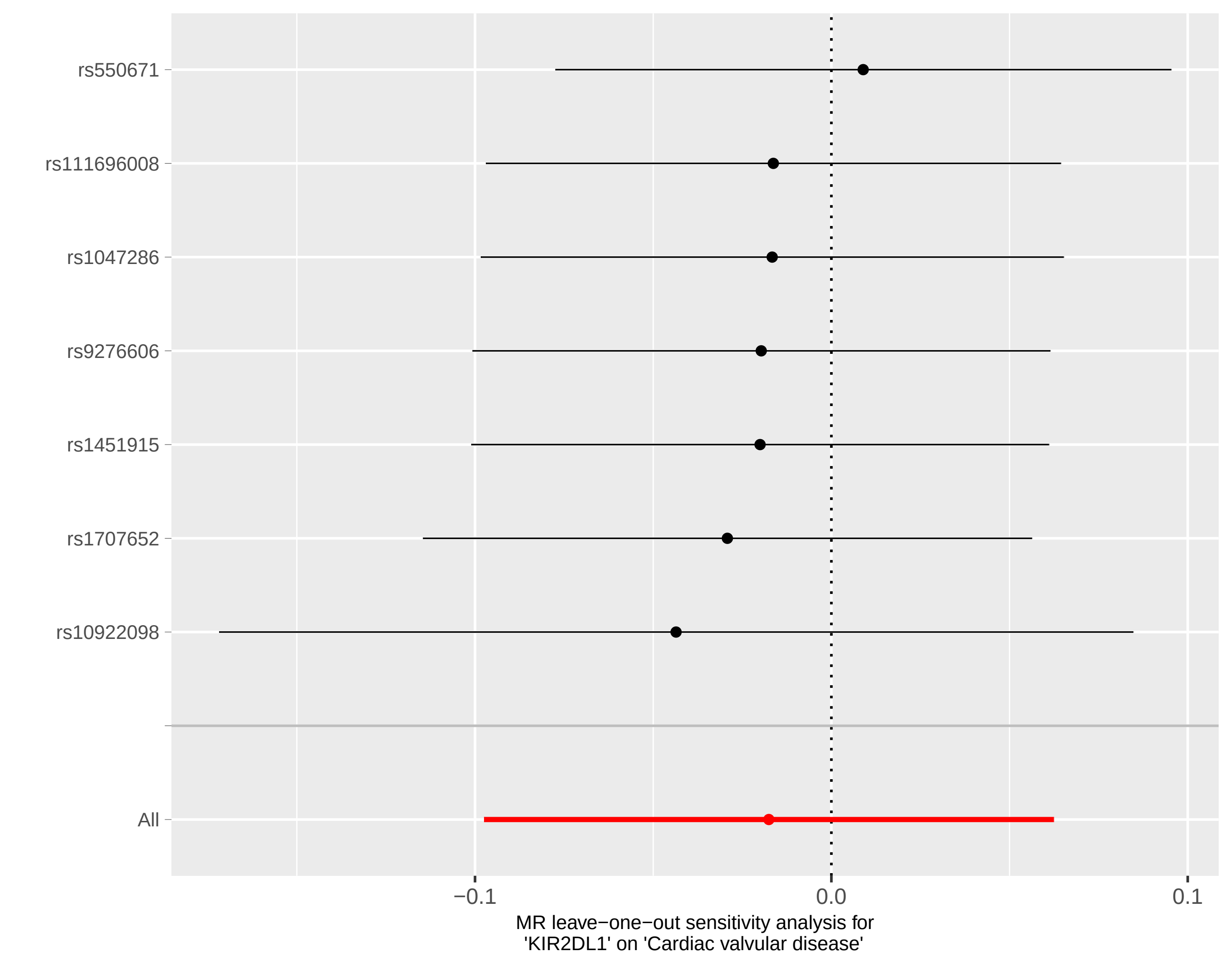
rs550671
rs111696008
rs1047286
rs9276606
rs1451915
rs1707652
rs10922098
All
−0.1
0.0
0.1
MR leave−one−out sensitivity analysis for
'KIR2DL1' on 'Cardiac valvular disease'

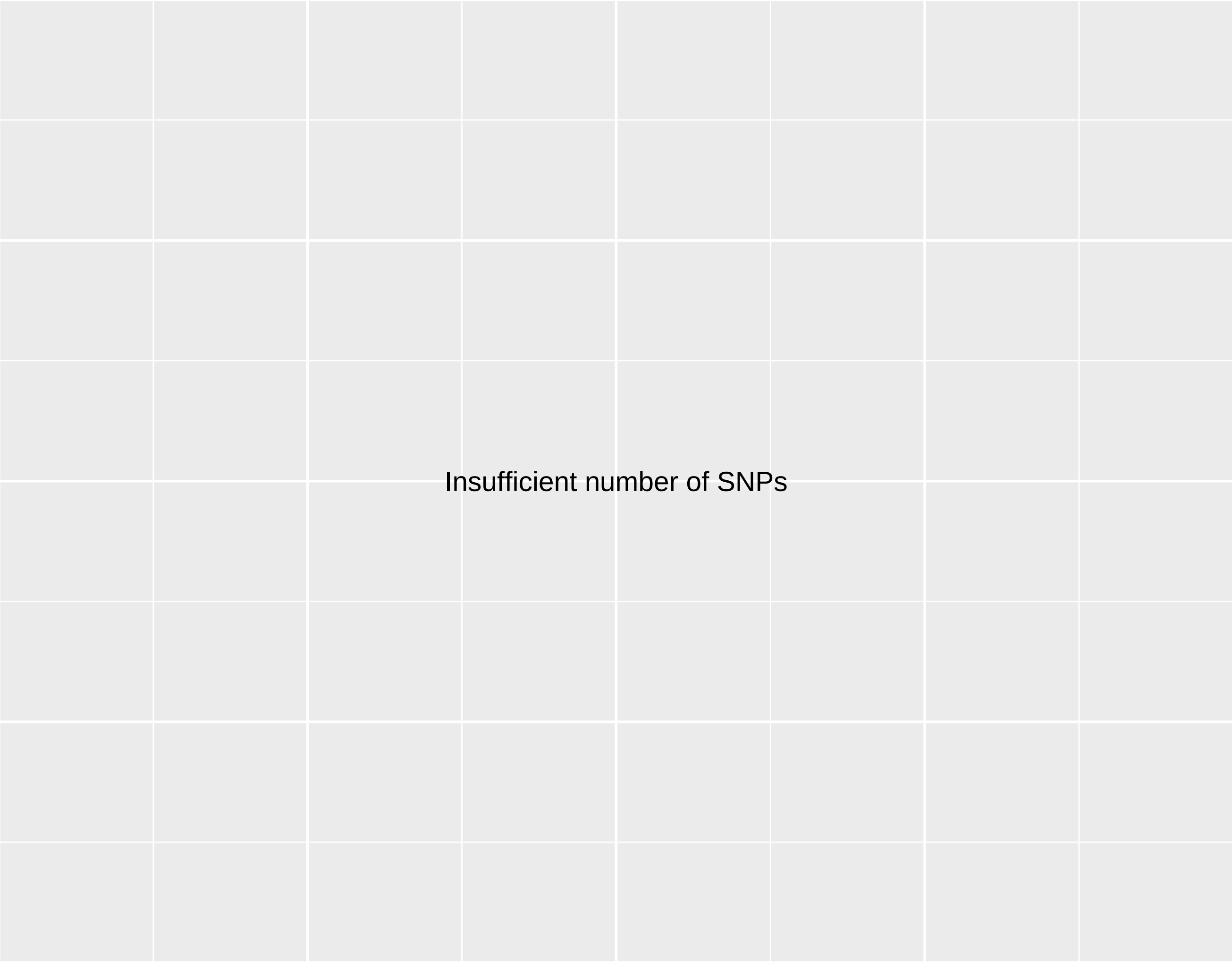
Insufficient number of SNPs

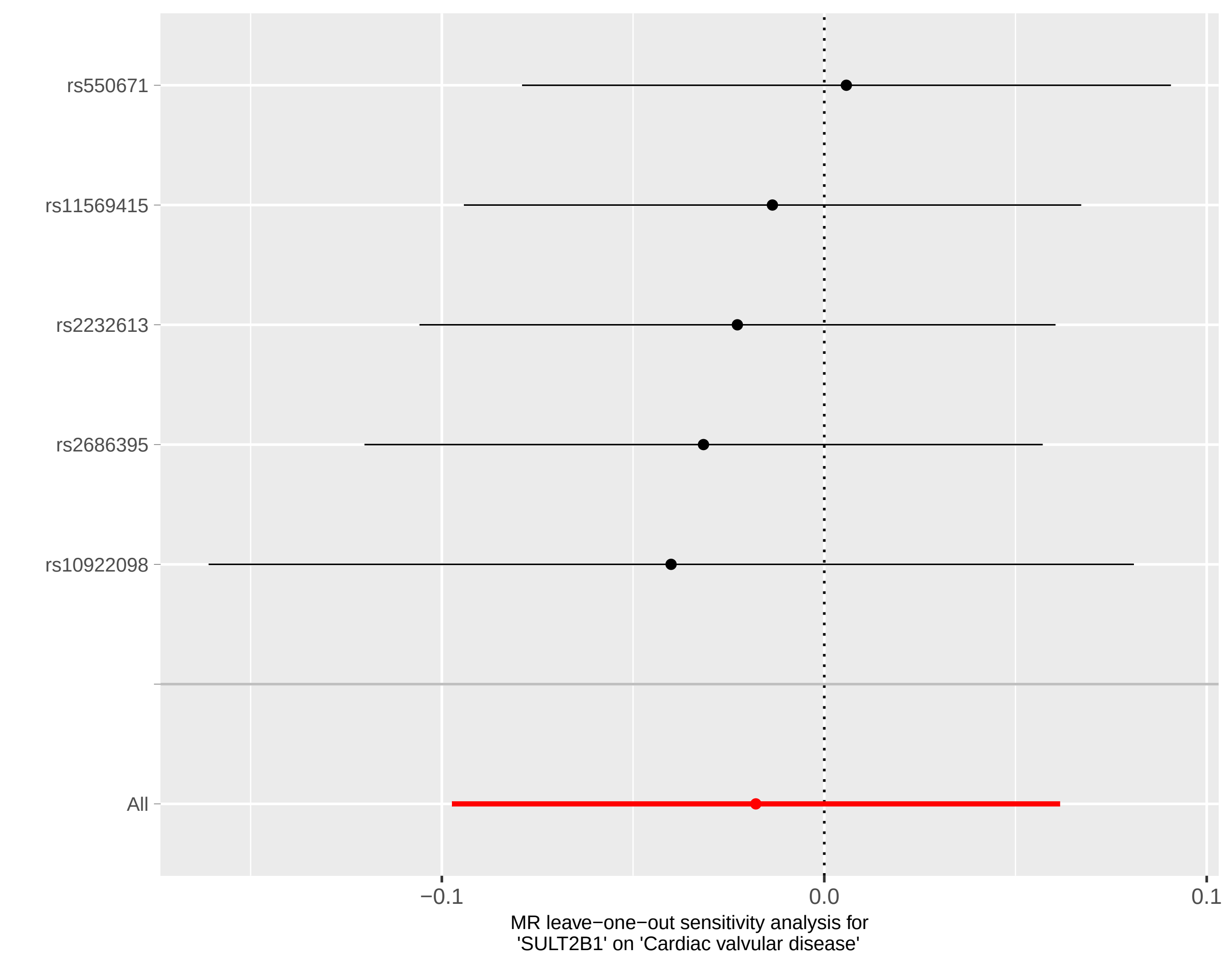

rs550671
rs11569415
rs2232613
rs2686395
rs10922098
All
−0.1
0.0
0.1
MR leave−one−out sensitivity analysis for
'SULT2B1' on 'Cardiac valvular disease'

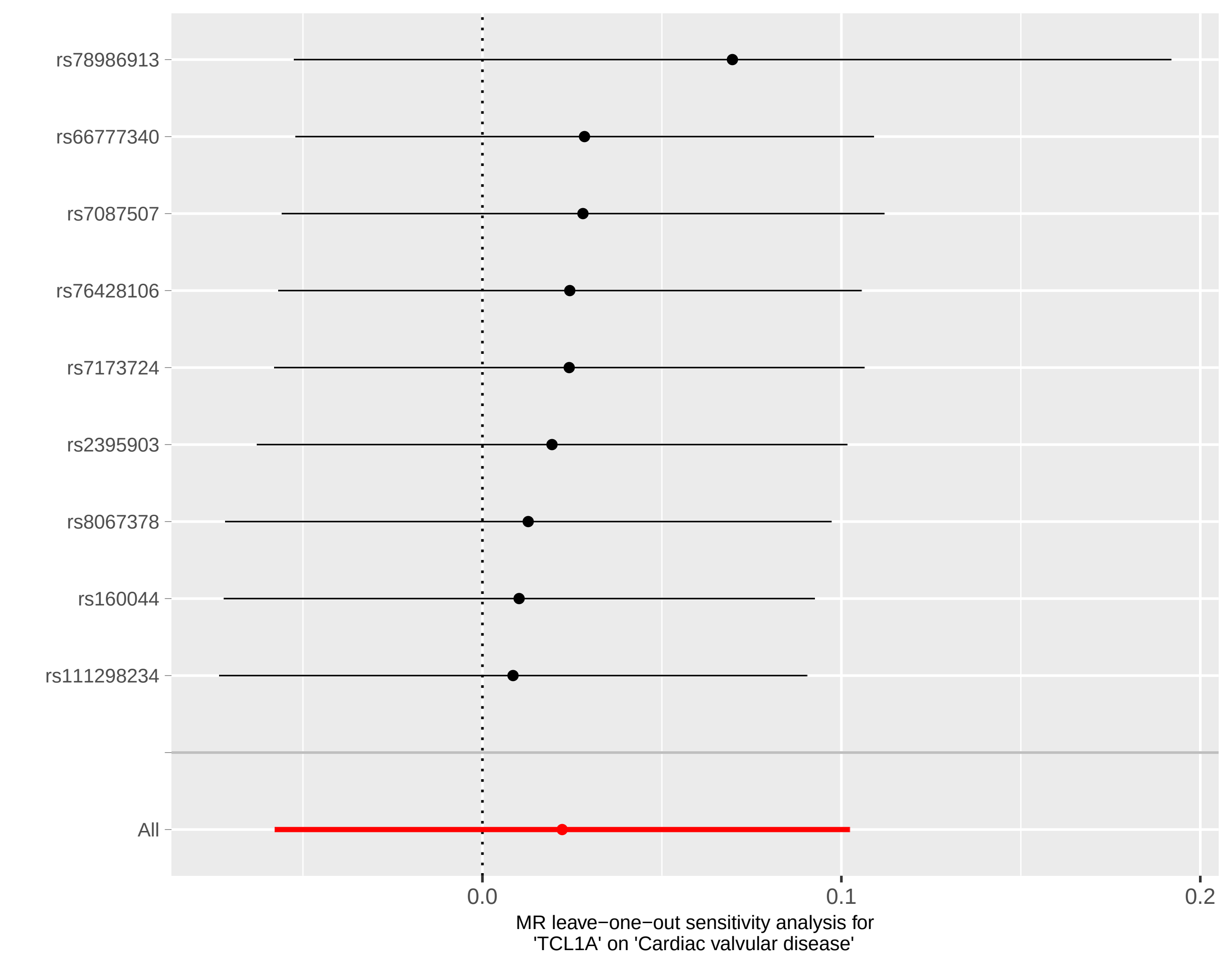

rs78986913
rs66777340
rs7087507
rs76428106
rs7173724
rs2395903
rs8067378
rs160044
rs111298234
All
0.0
0.1
0.2
MR leave−one−out sensitivity analysis for
'TCL1A' on 'Cardiac valvular disease'

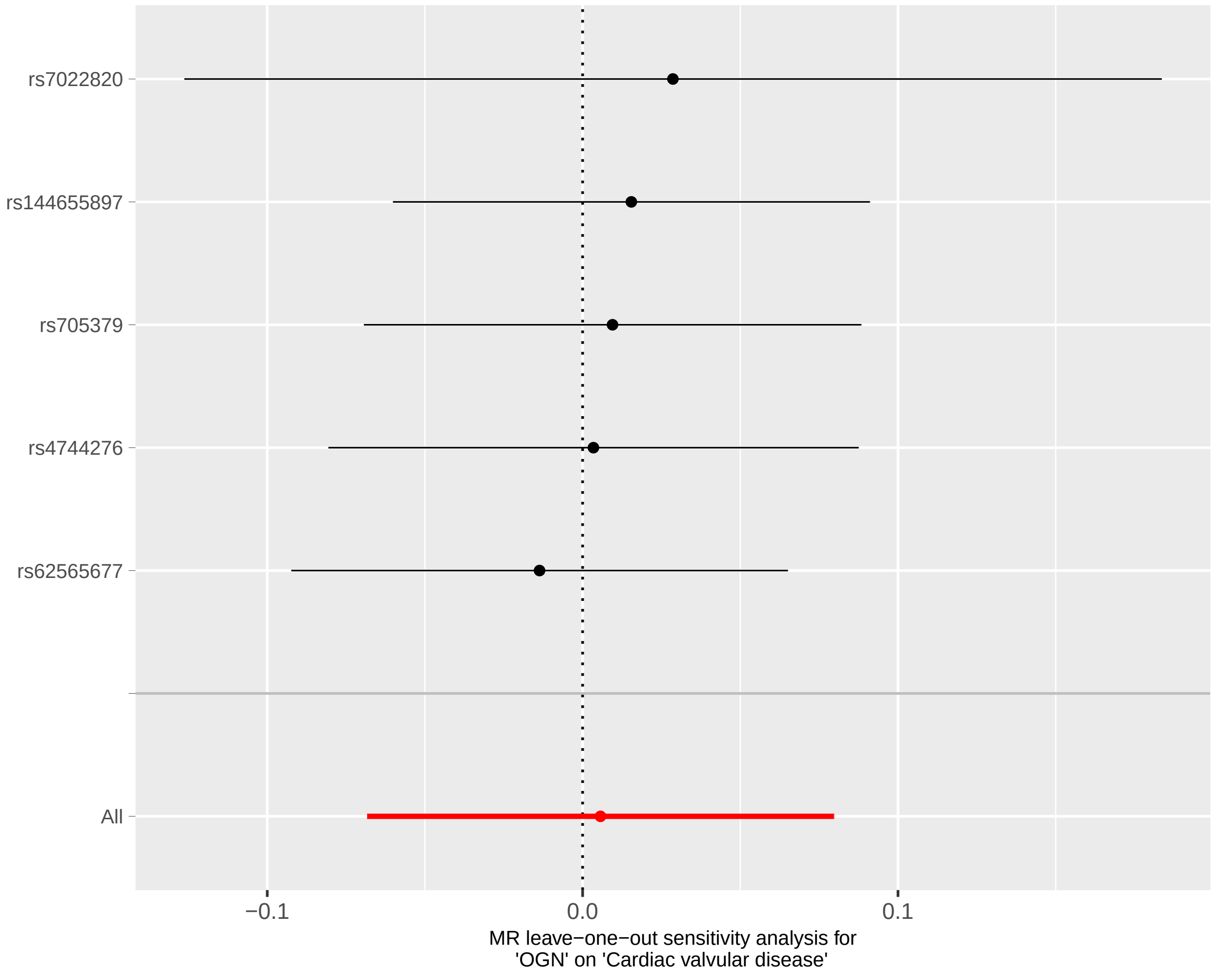

rs7022820
rs144655897
rs705379
rs4744276
rs62565677
All
−0.1
0.0
0.1
MR leave−one−out sensitivity analysis for
'OGN' on 'Cardiac valvular disease'

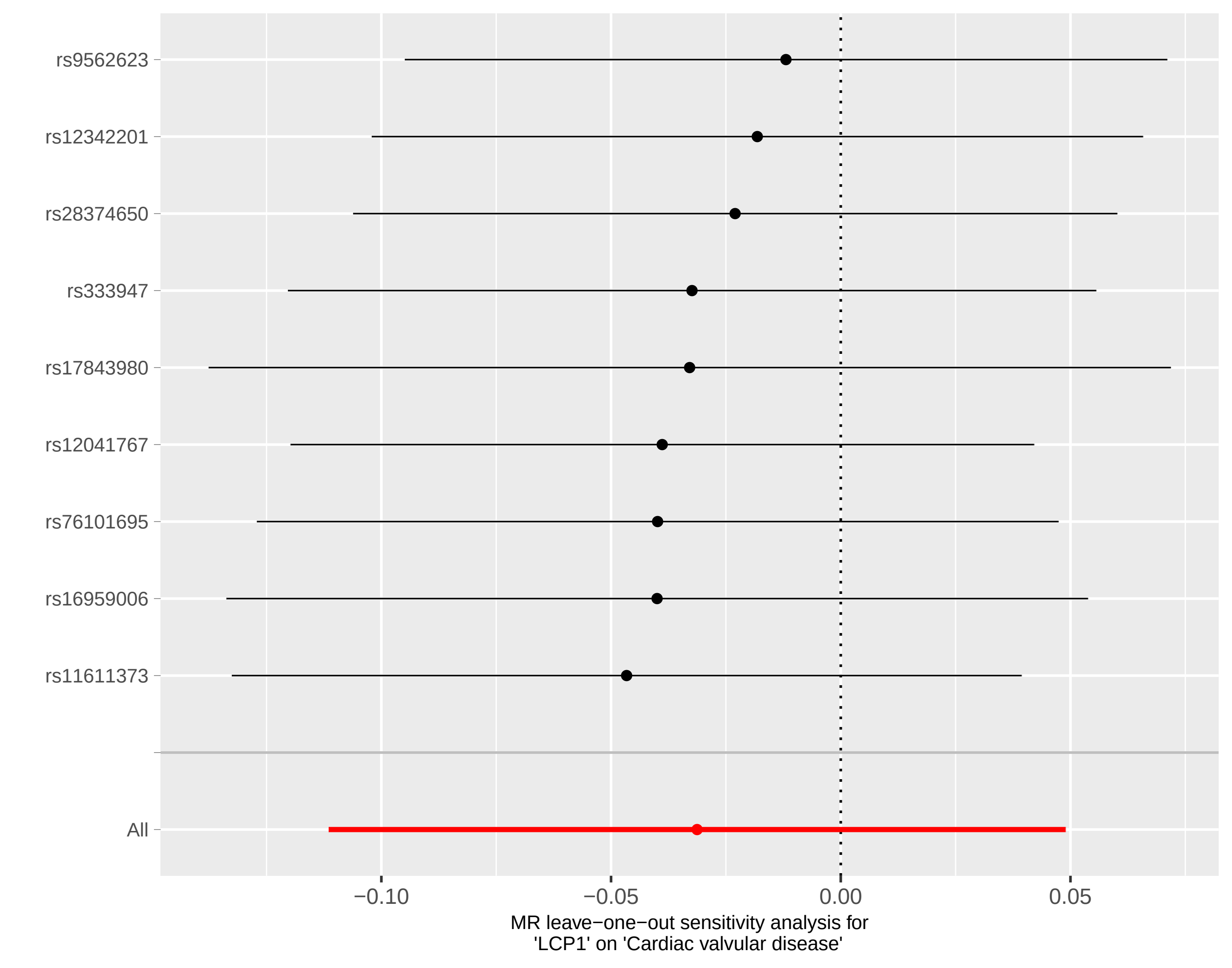

rs9562623
rs12342201
rs28374650
rs333947
rs17843980
rs12041767
rs76101695
rs16959006
rs11611373
All
−0.10
−0.05
0.00
0.05
MR leave−one−out sensitivity analysis for
'LCP1' on 'Cardiac valvular disease'

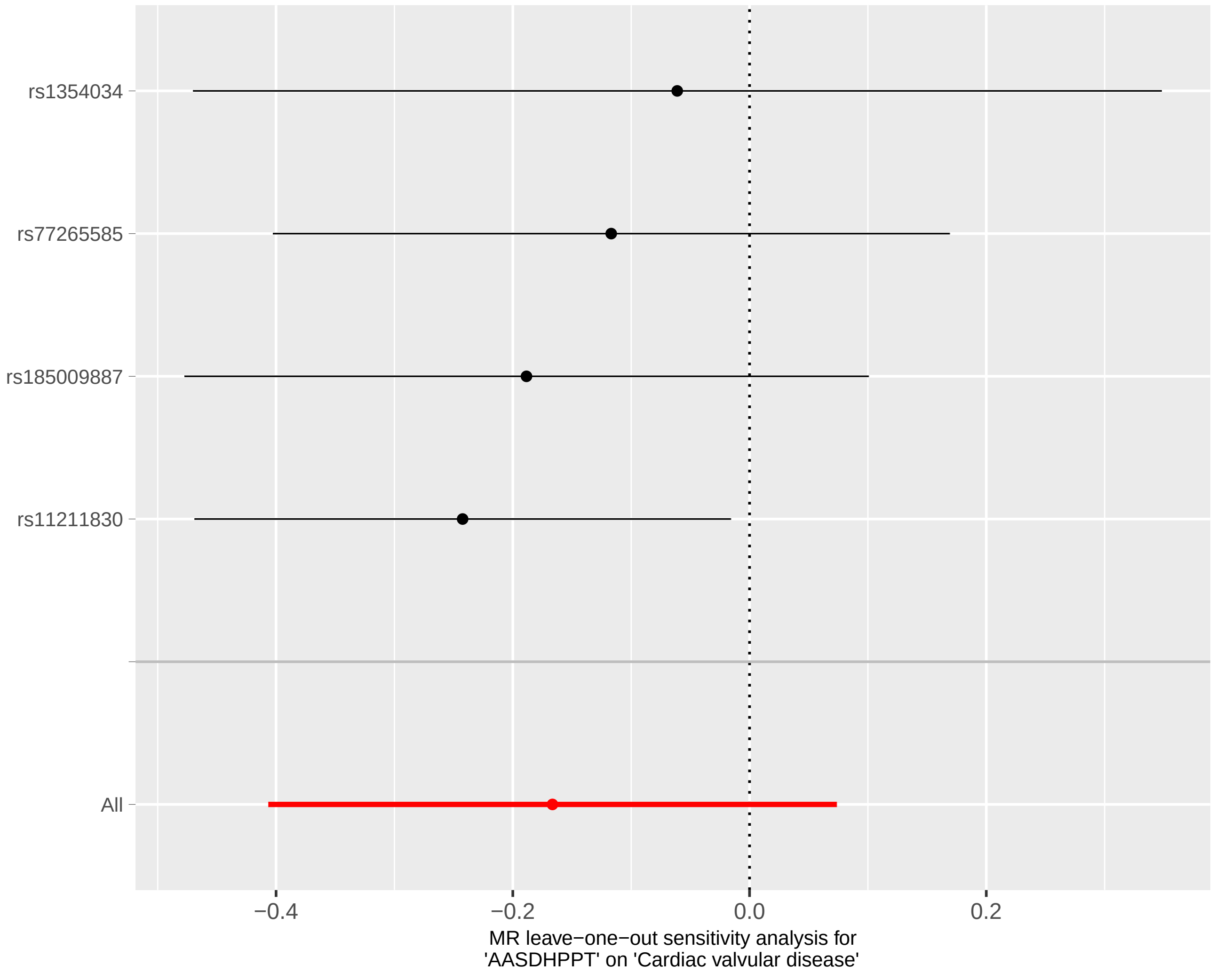

rs1354034
rs77265585
rs185009887
rs11211830
All
−0.4
−0.2
0.0
0.2
MR leave−one−out sensitivity analysis for
'AASDHPPT' on 'Cardiac valvular disease'

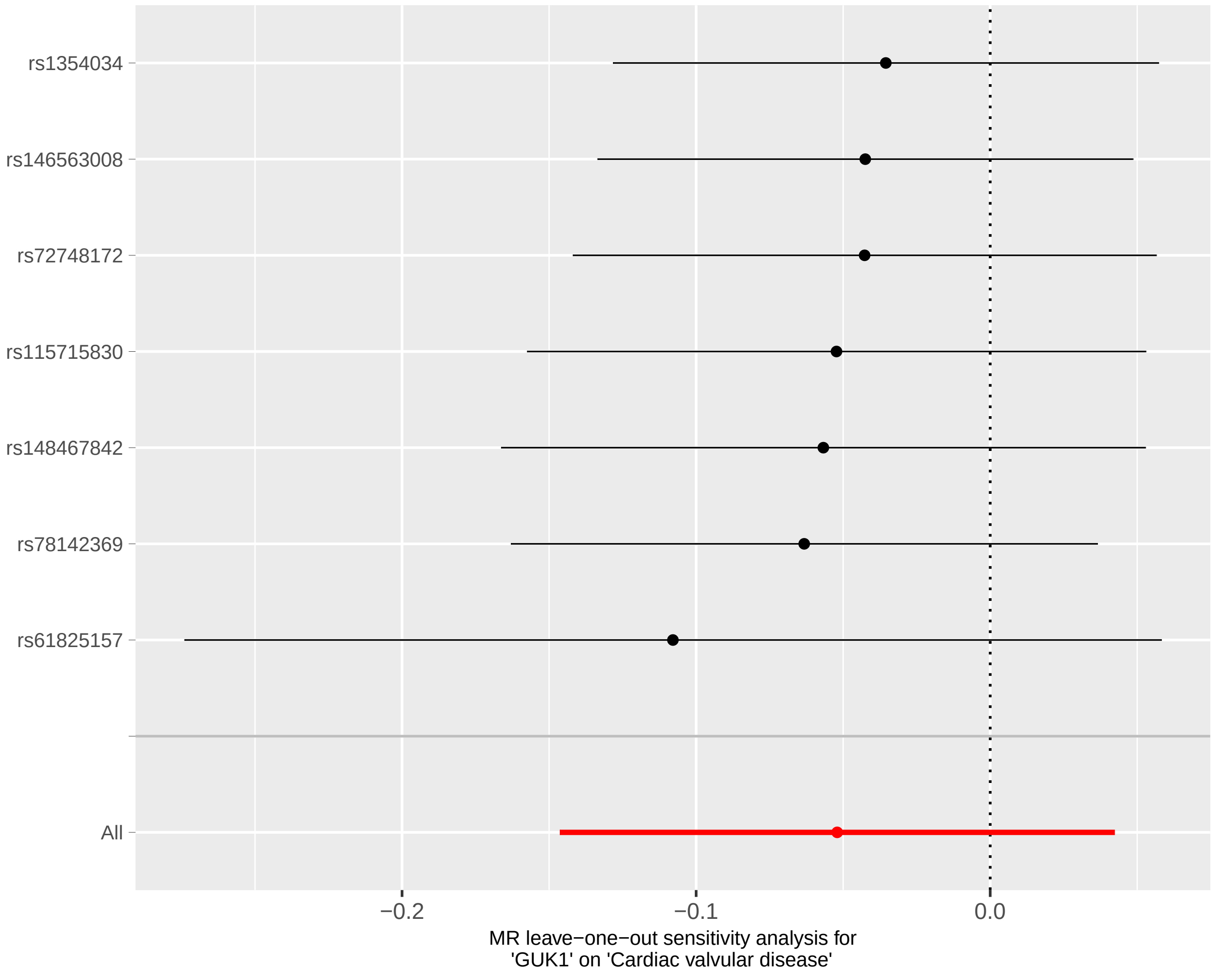

rs1354034
rs146563008
rs72748172
rs115715830
rs148467842
rs78142369
rs61825157
All
−0.2
−0.1
0.0
MR leave−one−out sensitivity analysis for
'GUK1' on 'Cardiac valvular disease'

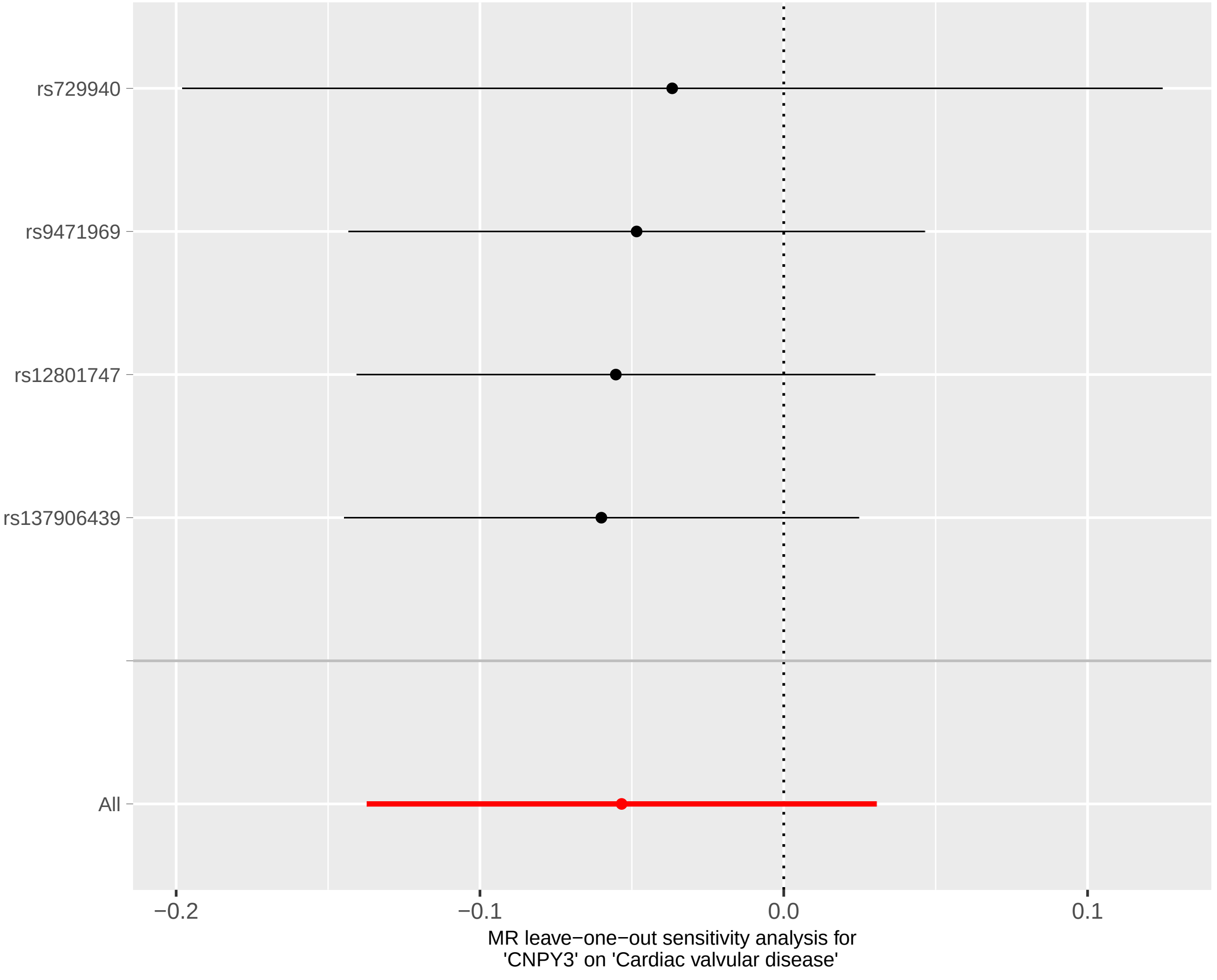

rs729940
rs9471969
rs12801747
rs137906439
All
−0.2
−0.1
0.0
0.1
MR leave−one−out sensitivity analysis for
'CNPY3' on 'Cardiac valvular disease'

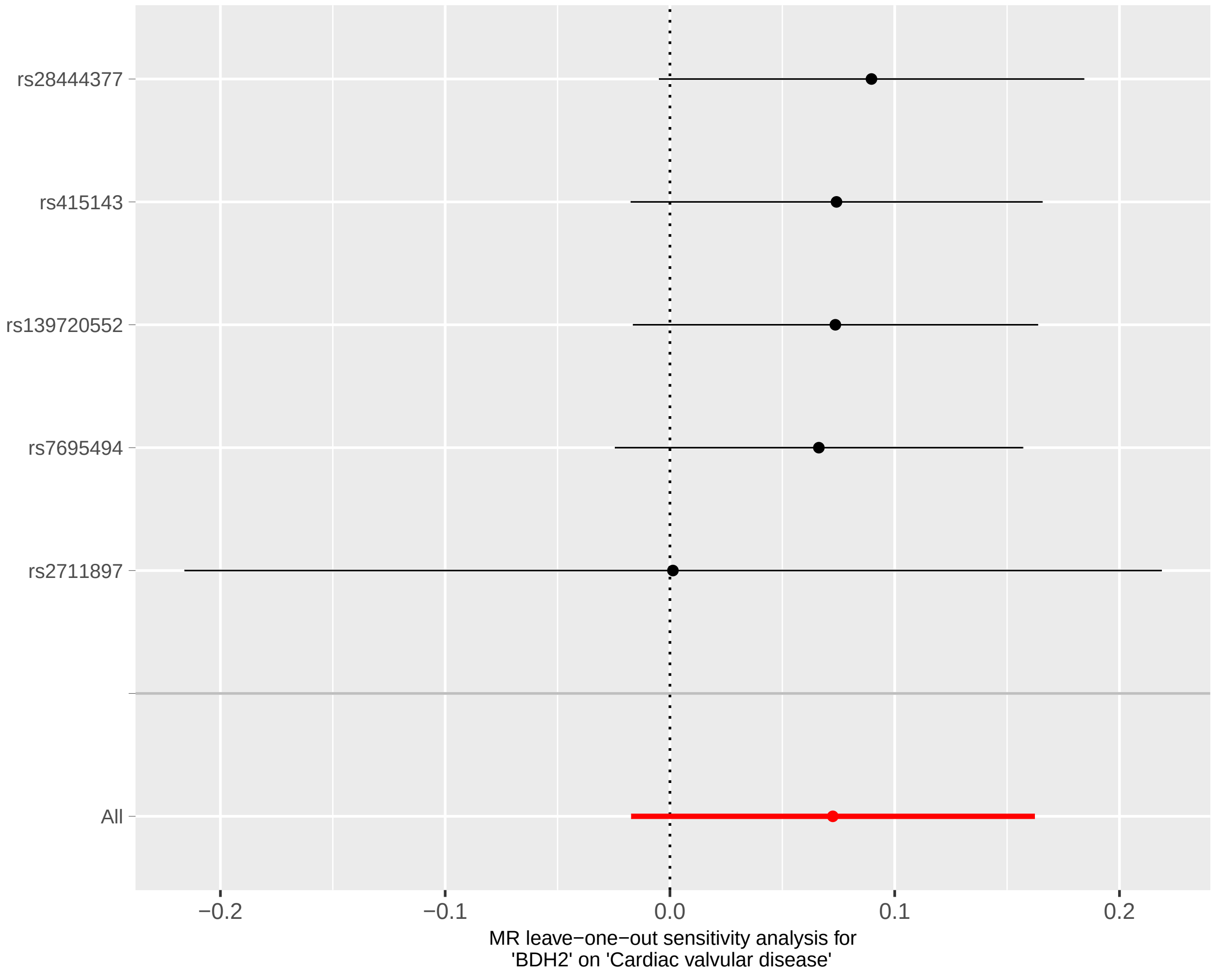
rs28444377
rs415143
rs139720552
rs7695494
rs2711897
All
−0.2
−0.1
0.0
0.1
0.2
MR leave−one−out sensitivity analysis for
'BDH2' on 'Cardiac valvular disease'

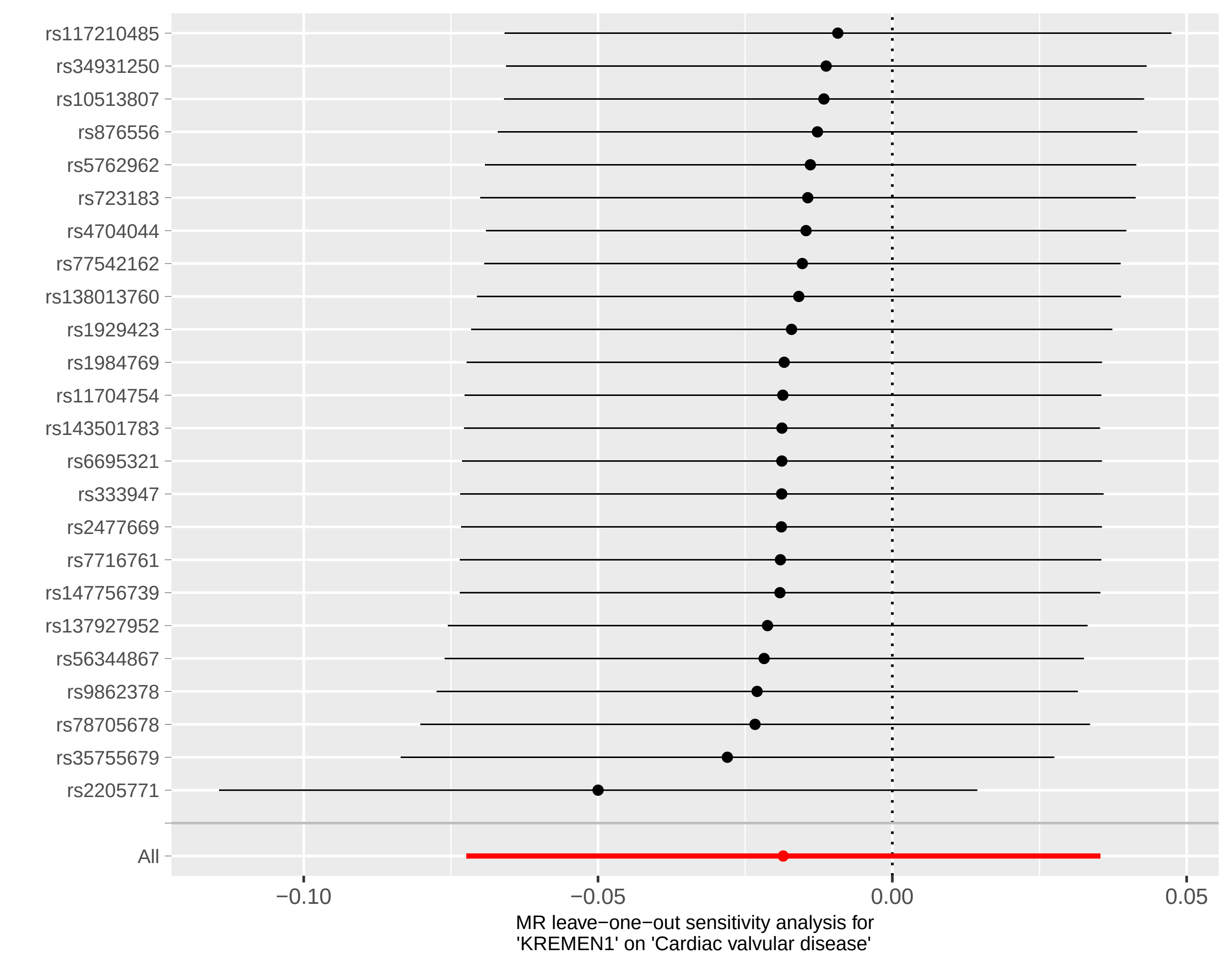
rs117210485
rs34931250
rs10513807
rs876556
rs5762962
rs723183
rs4704044
rs77542162
rs138013760
rs1929423
rs1984769
rs11704754
rs143501783
rs6695321
rs333947
rs2477669
rs7716761
rs147756739
rs137927952
rs56344867
rs9862378
rs78705678
rs35755679
rs2205771
All
−0.10
−0.05
0.00
0.05
MR leave−one−out sensitivity analysis for
'KREMEN1' on 'Cardiac valvular disease'

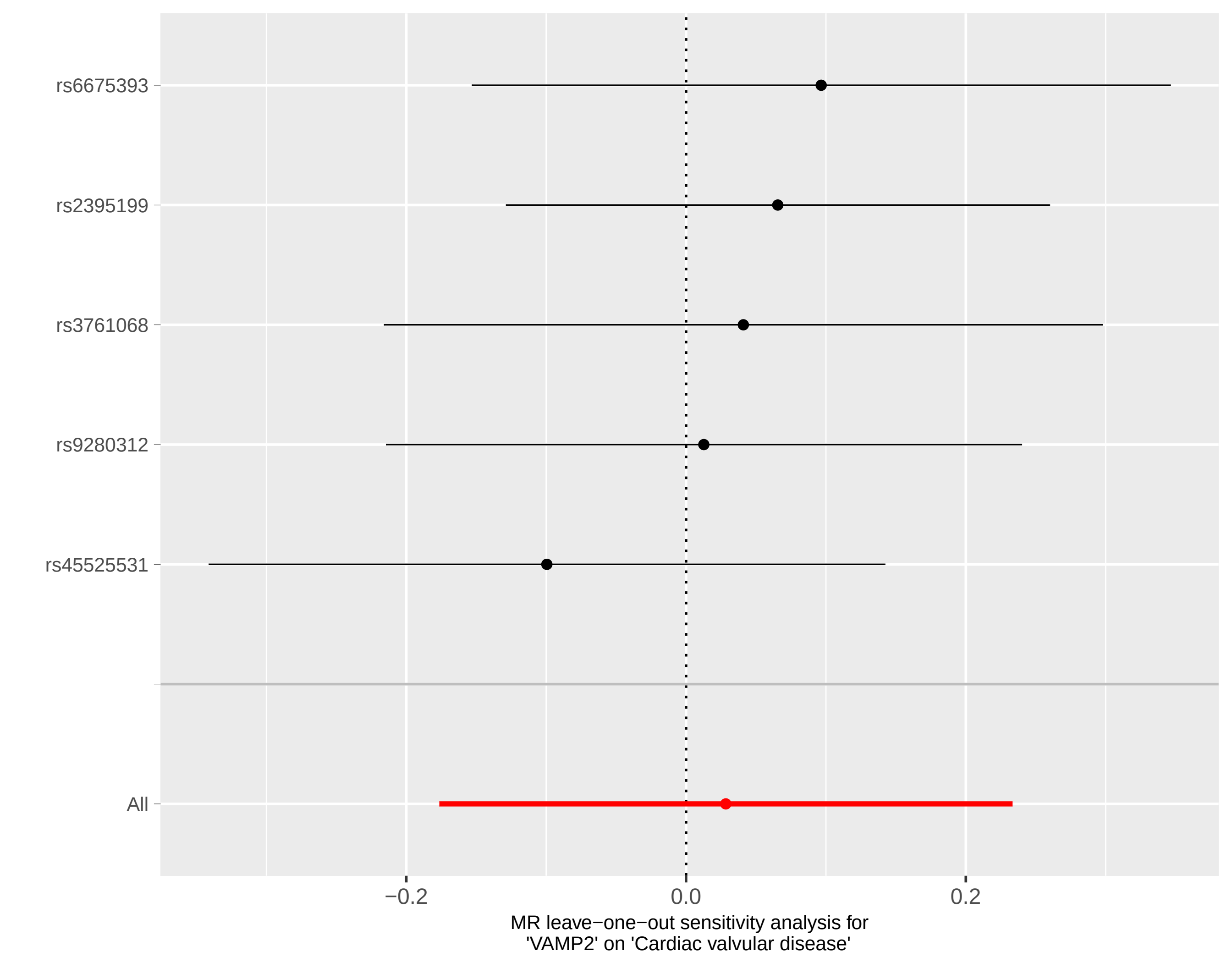
rs6675393
rs2395199
rs3761068
rs9280312
rs45525531
All
−0.2
0.0
0.2
MR leave−one−out sensitivity analysis for
'VAMP2' on 'Cardiac valvular disease'

Insufficient number of SNPs

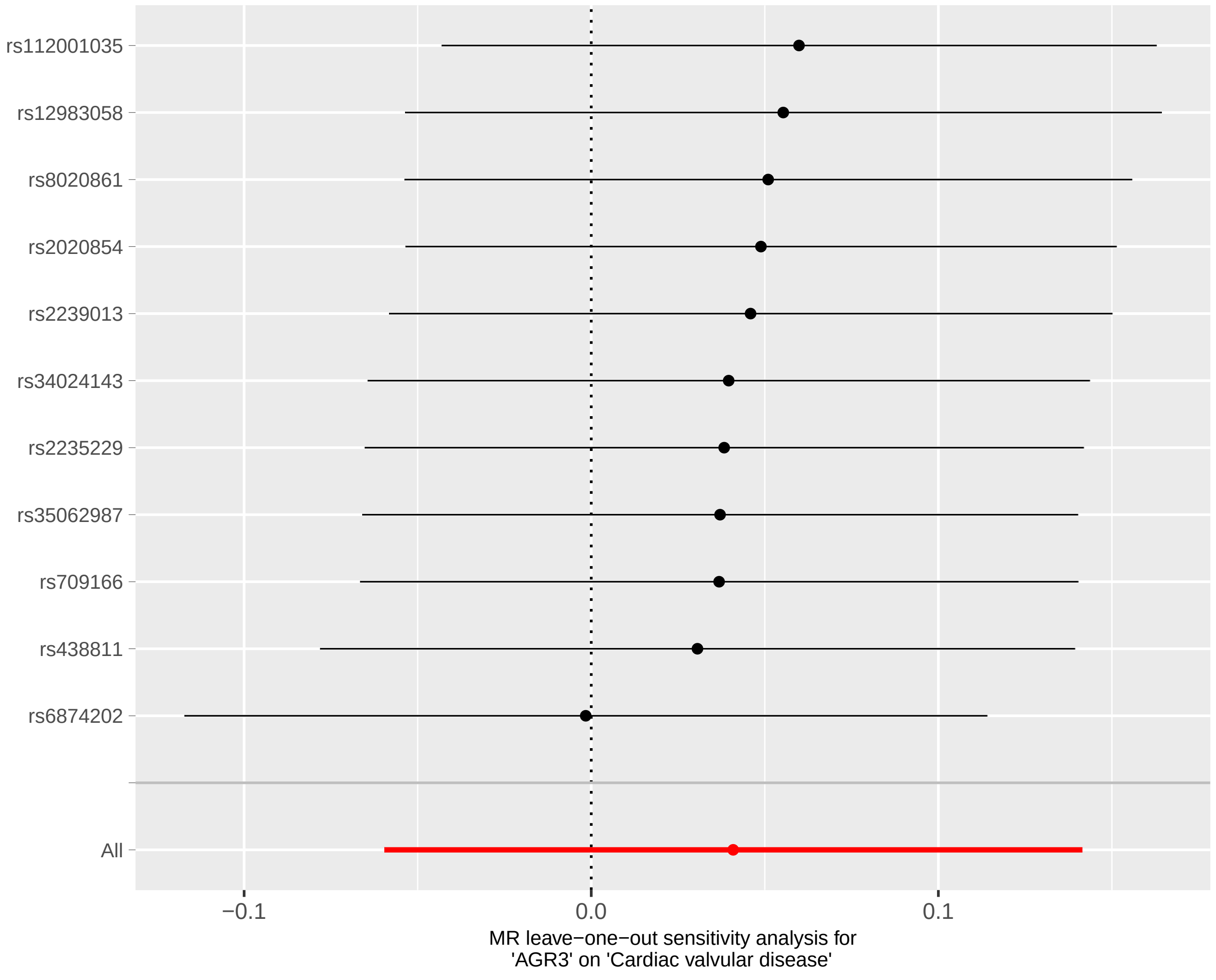
rs112001035
rs12983058
rs8020861
rs2020854
rs2239013
rs34024143
rs2235229
rs35062987
rs709166
rs438811
rs6874202
All
−0.1
0.0
0.1
MR leave−one−out sensitivity analysis for
'AGR3' on 'Cardiac valvular disease'

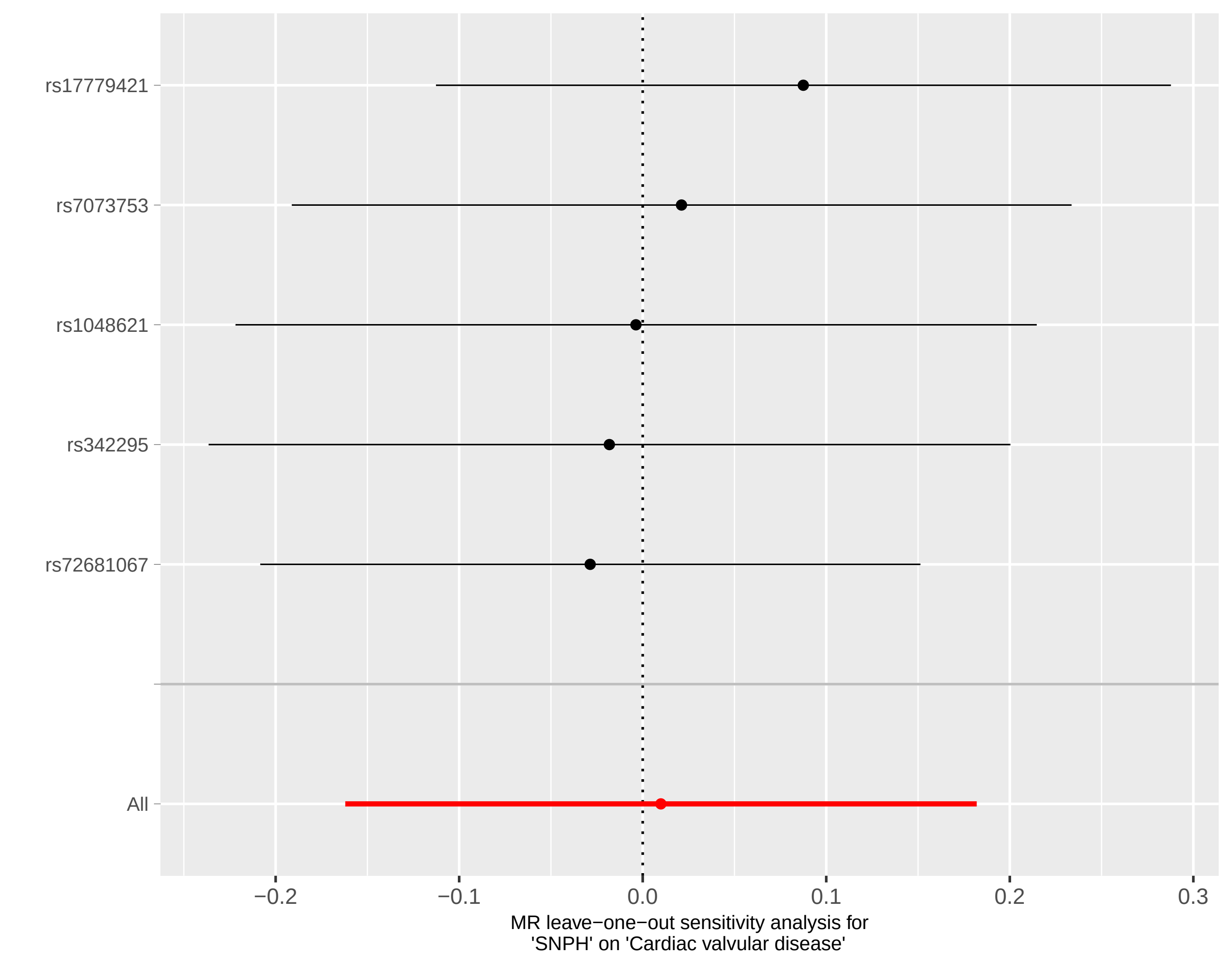
rs17779421
rs7073753
rs1048621
rs342295
rs72681067
All
−0.2
−0.1
0.0
0.1
0.2
0.3
MR leave−one−out sensitivity analysis for
'SNPH' on 'Cardiac valvular disease'

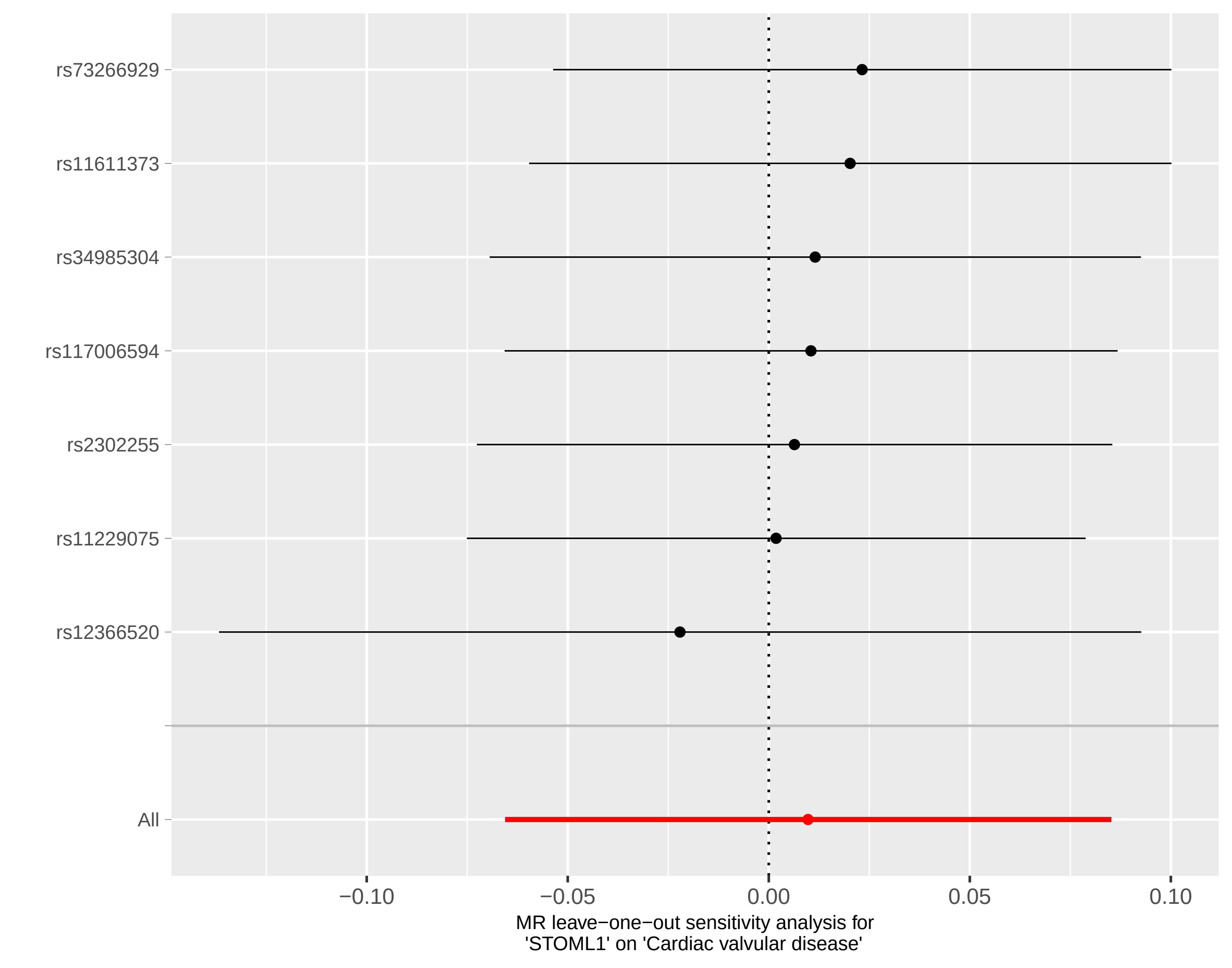
rs73266929
rs11611373
rs34985304
rs117006594
rs2302255
rs11229075
rs12366520
All
−0.10
−0.05
0.00
0.05
0.10
MR leave−one−out sensitivity analysis for
'STOML1' on 'Cardiac valvular disease'

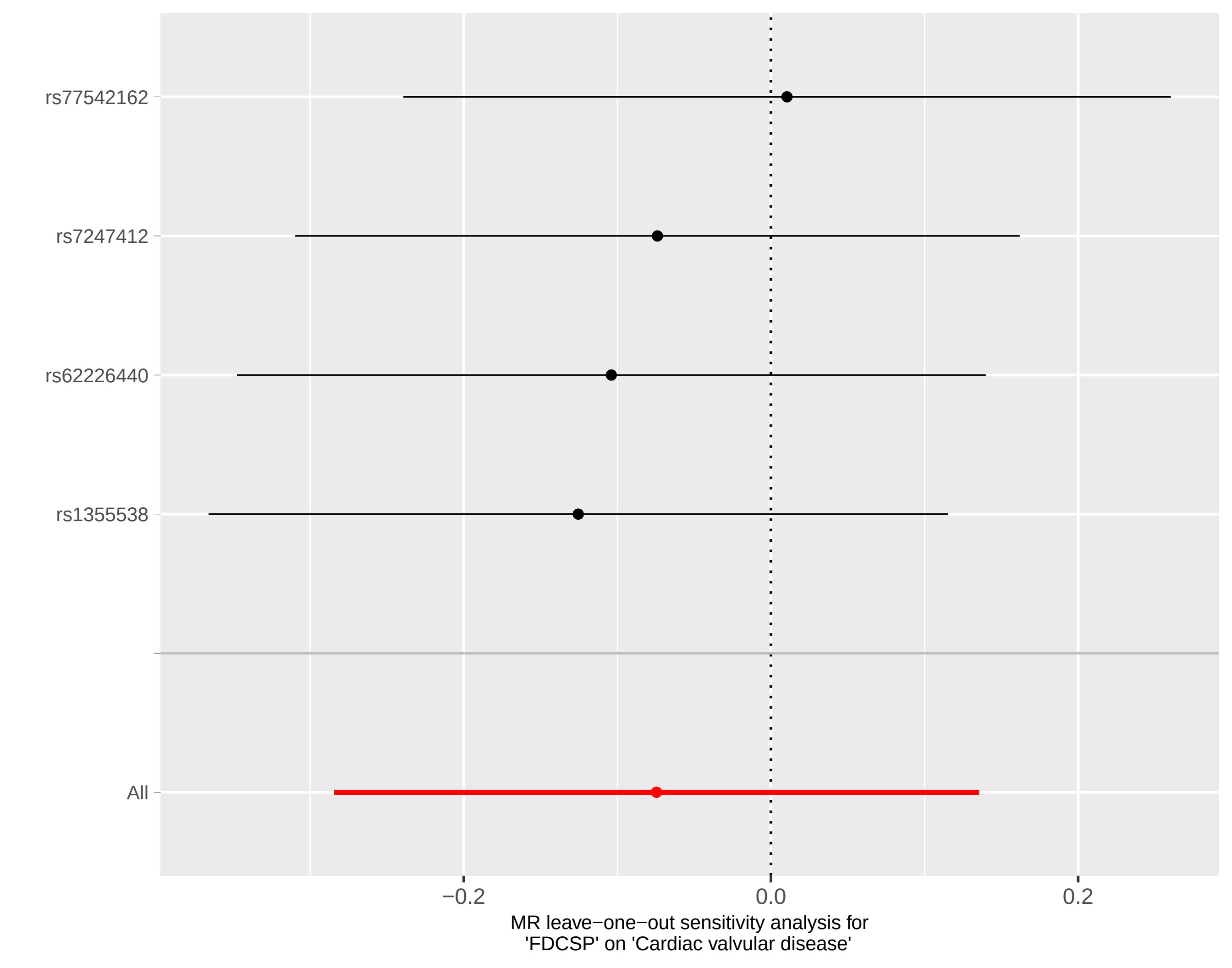

rs77542162
rs7247412
rs62226440
rs1355538
All
−0.2
0.0
0.2
MR leave−one−out sensitivity analysis for
'FDCSP' on 'Cardiac valvular disease'

Insufficient number of SNPs

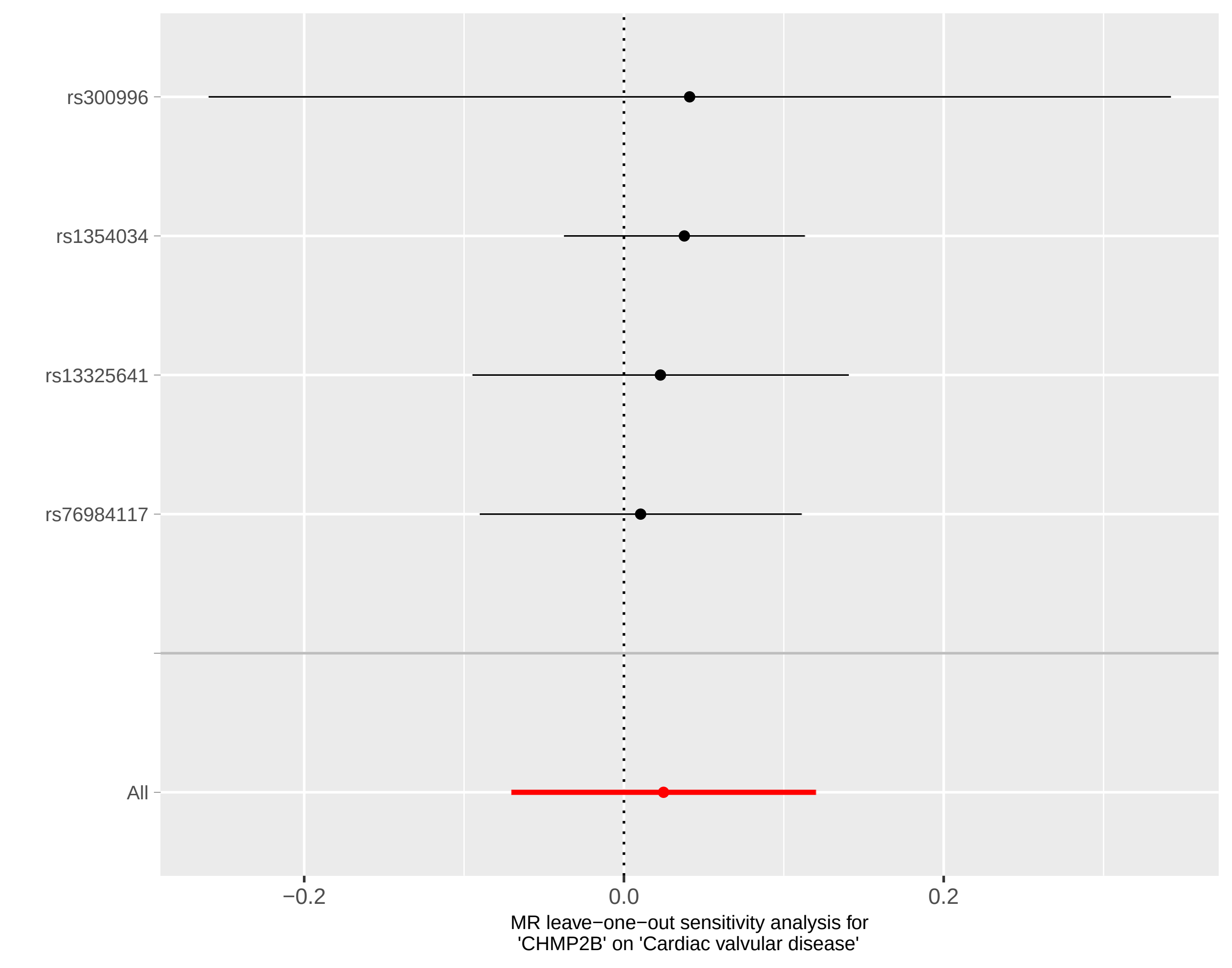
rs300996
rs1354034
rs13325641
rs76984117
All
−0.2
0.0
0.2
MR leave−one−out sensitivity analysis for
'CHMP2B' on 'Cardiac valvular disease'

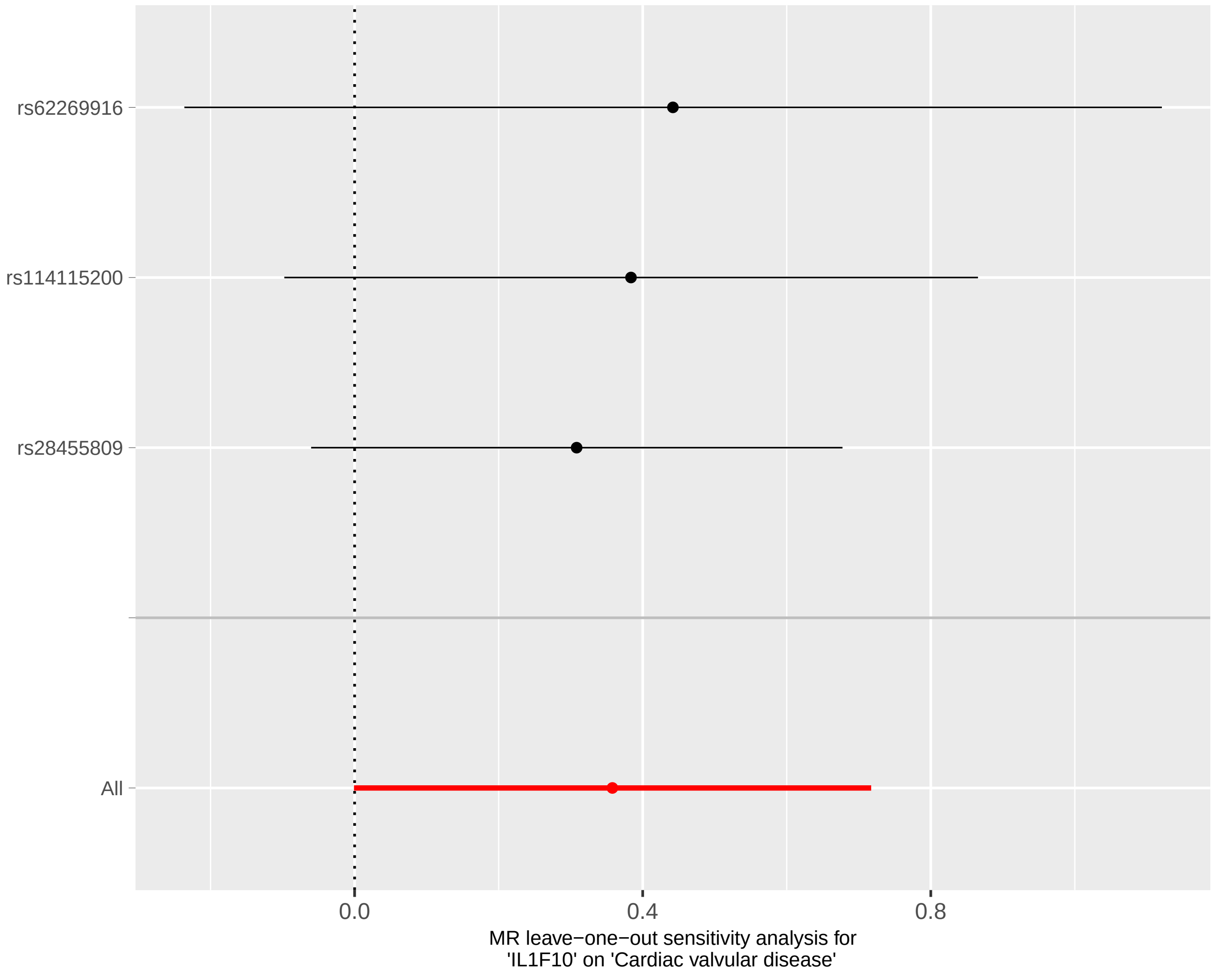
rs62269916
rs114115200
rs28455809
All
0.0
0.4
0.8
MR leave−one−out sensitivity analysis for
'IL1F10' on 'Cardiac valvular disease'

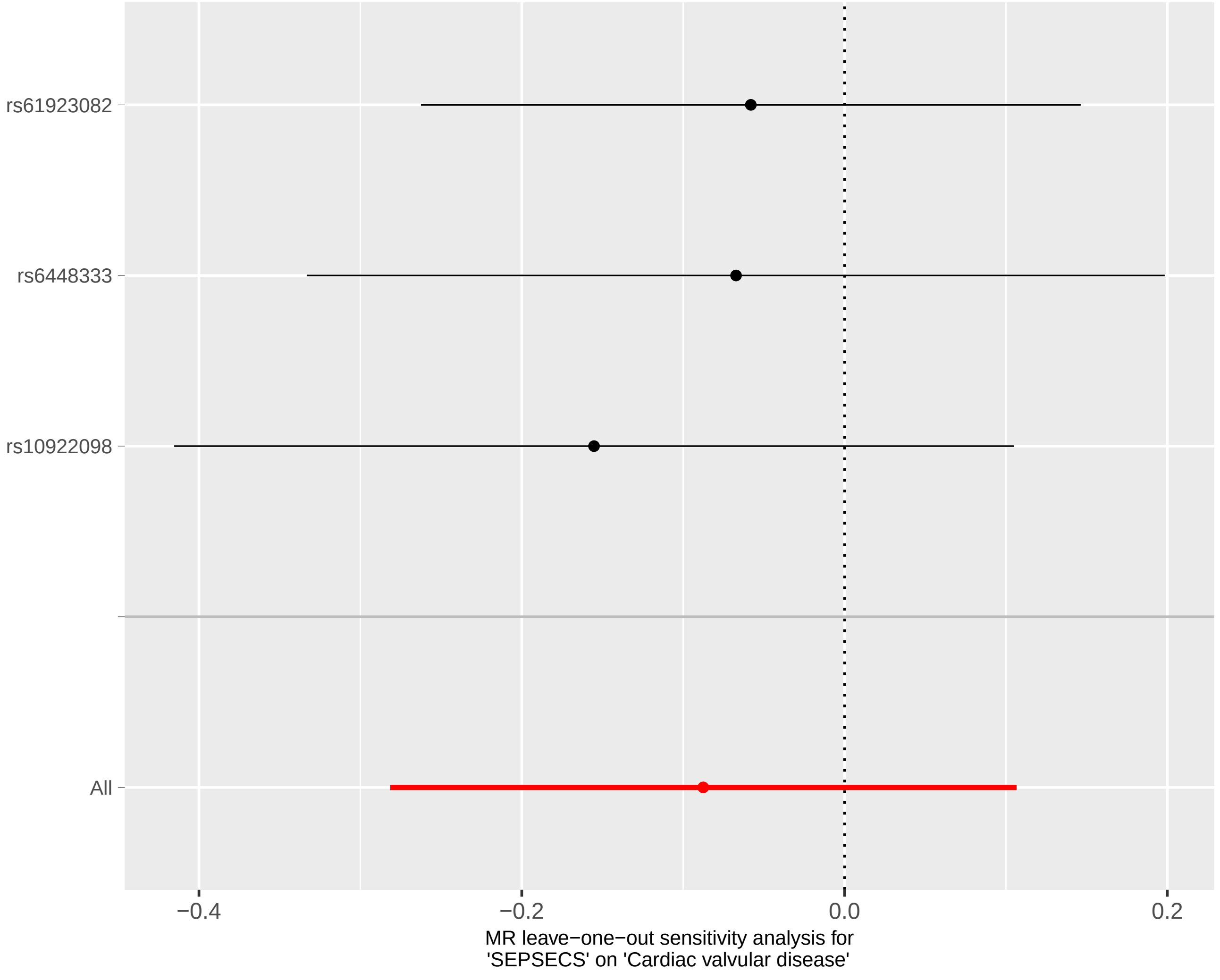
rs61923082
rs6448333
rs10922098
All
−0.4
−0.2
0.0
0.2
MR leave−one−out sensitivity analysis for
'SEPSECS' on 'Cardiac valvular disease'

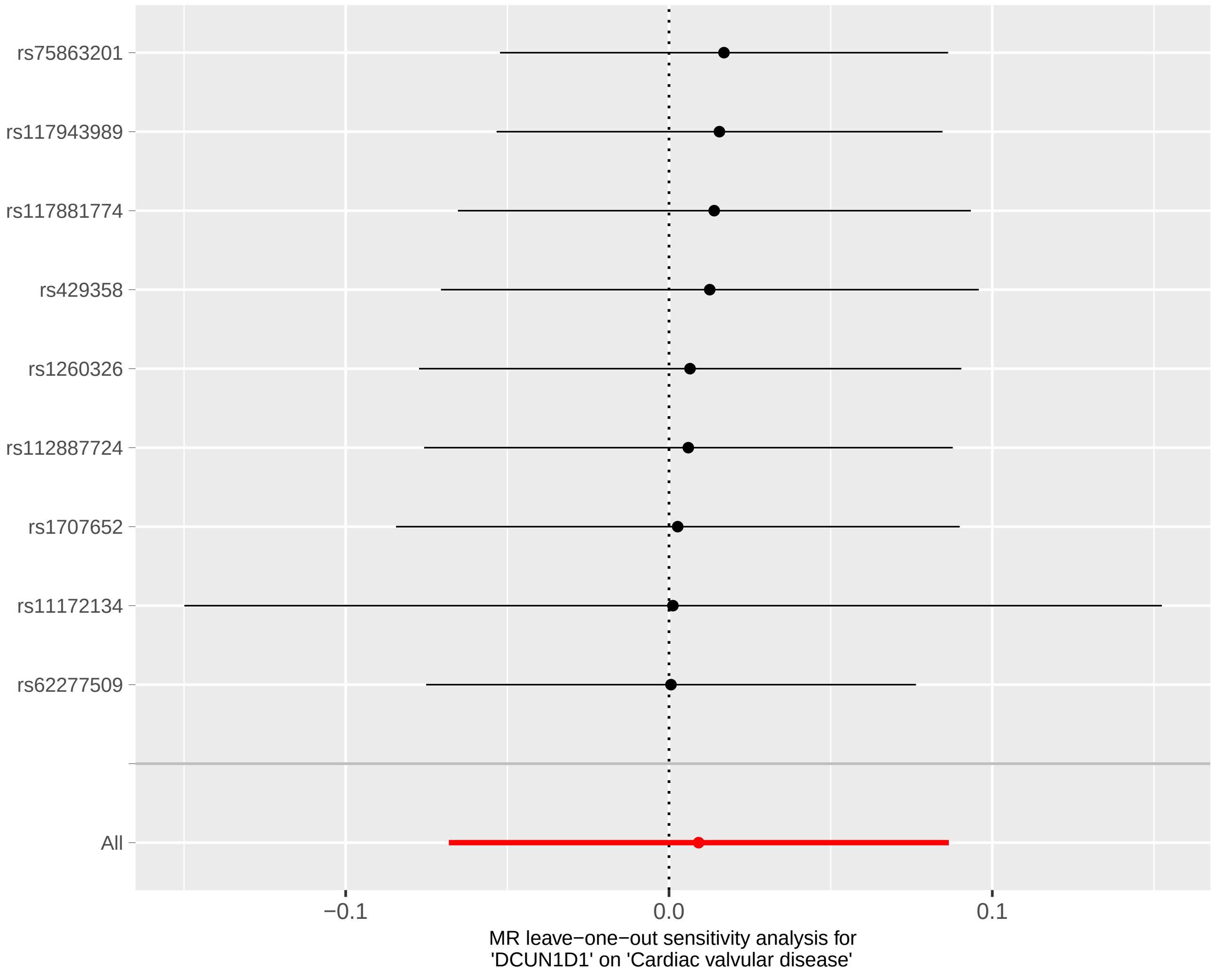

rs75863201
rs117943989
rs117881774
rs429358
rs1260326
rs112887724
rs1707652
rs11172134
rs62277509
All
−0.1
0.0
0.1
MR leave−one−out sensitivity analysis for
'DCUN1D1' on 'Cardiac valvular disease'

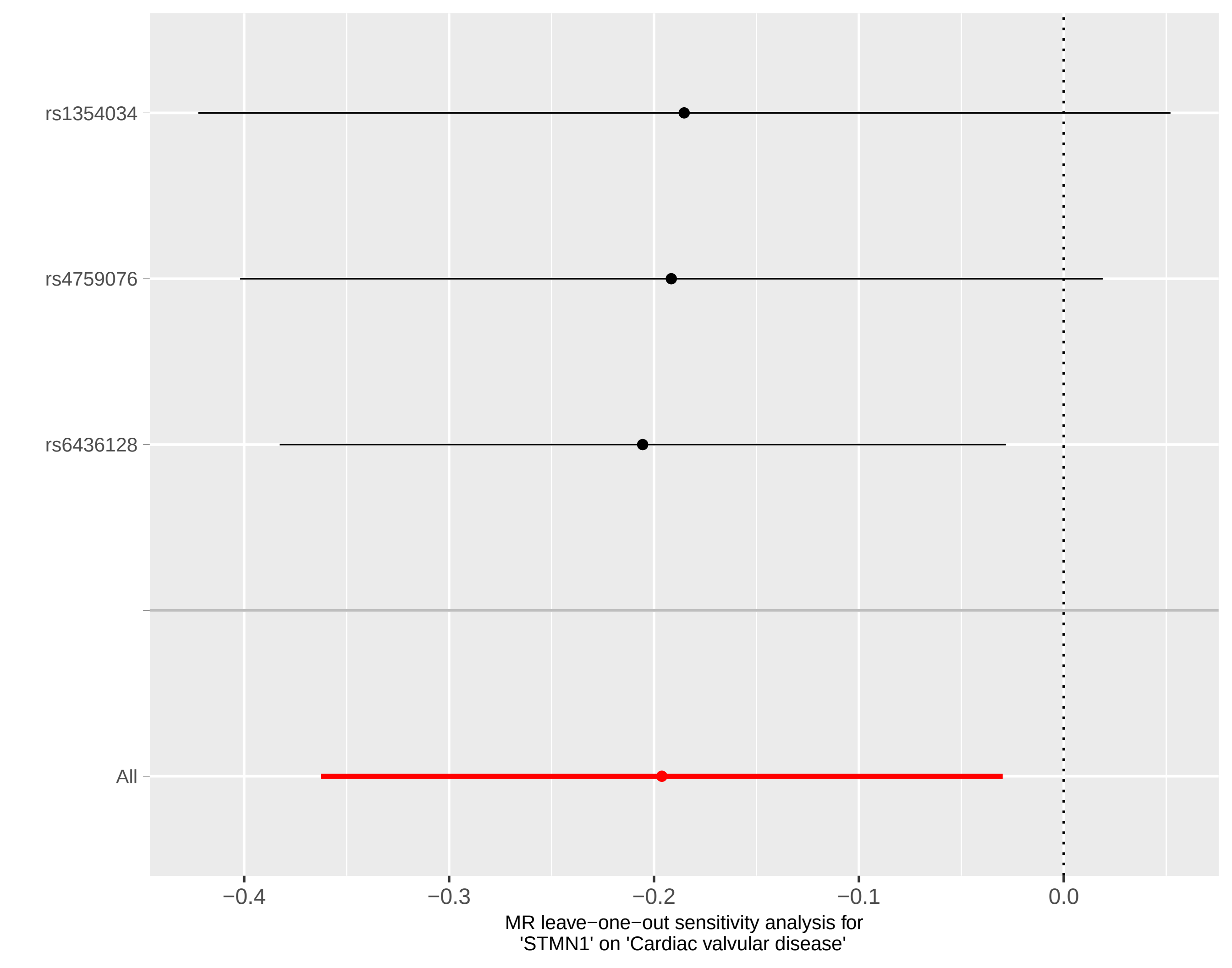
rs1354034
rs4759076
rs6436128
All
−0.4
−0.3
−0.2
−0.1
0.0
MR leave−one−out sensitivity analysis for
'STMN1' on 'Cardiac valvular disease'

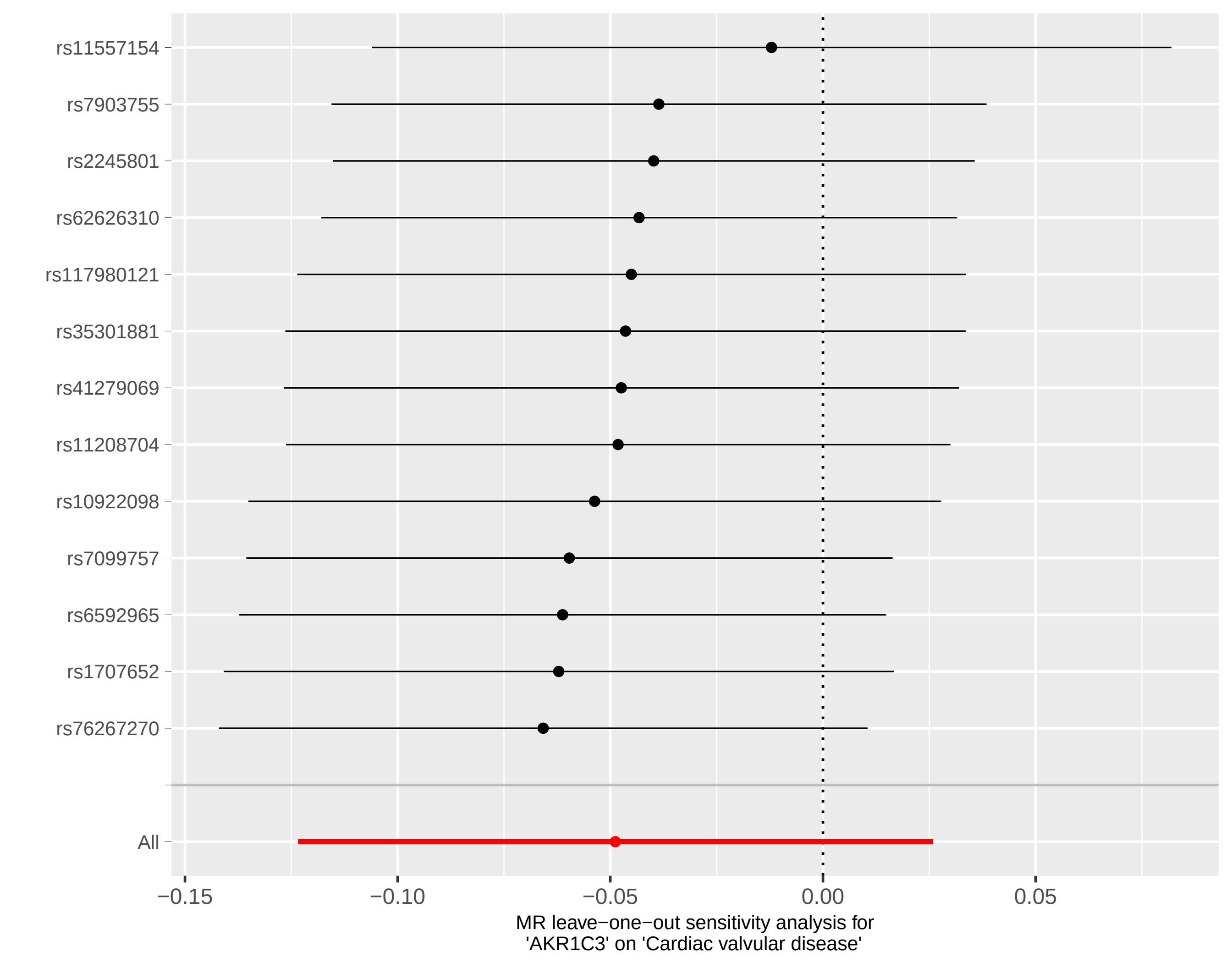

rs11557154
rs7903755
rs2245801
rs62626310
rs117980121
rs35301881
rs41279069
rs11208704
rs10922098
rs7099757
rs6592965
rs1707652
rs76267270
All
−0.15
−0.10
−0.05
0.00
0.05
MR leave−one−out sensitivity analysis for
'AKR1C3' on 'Cardiac valvular disease'

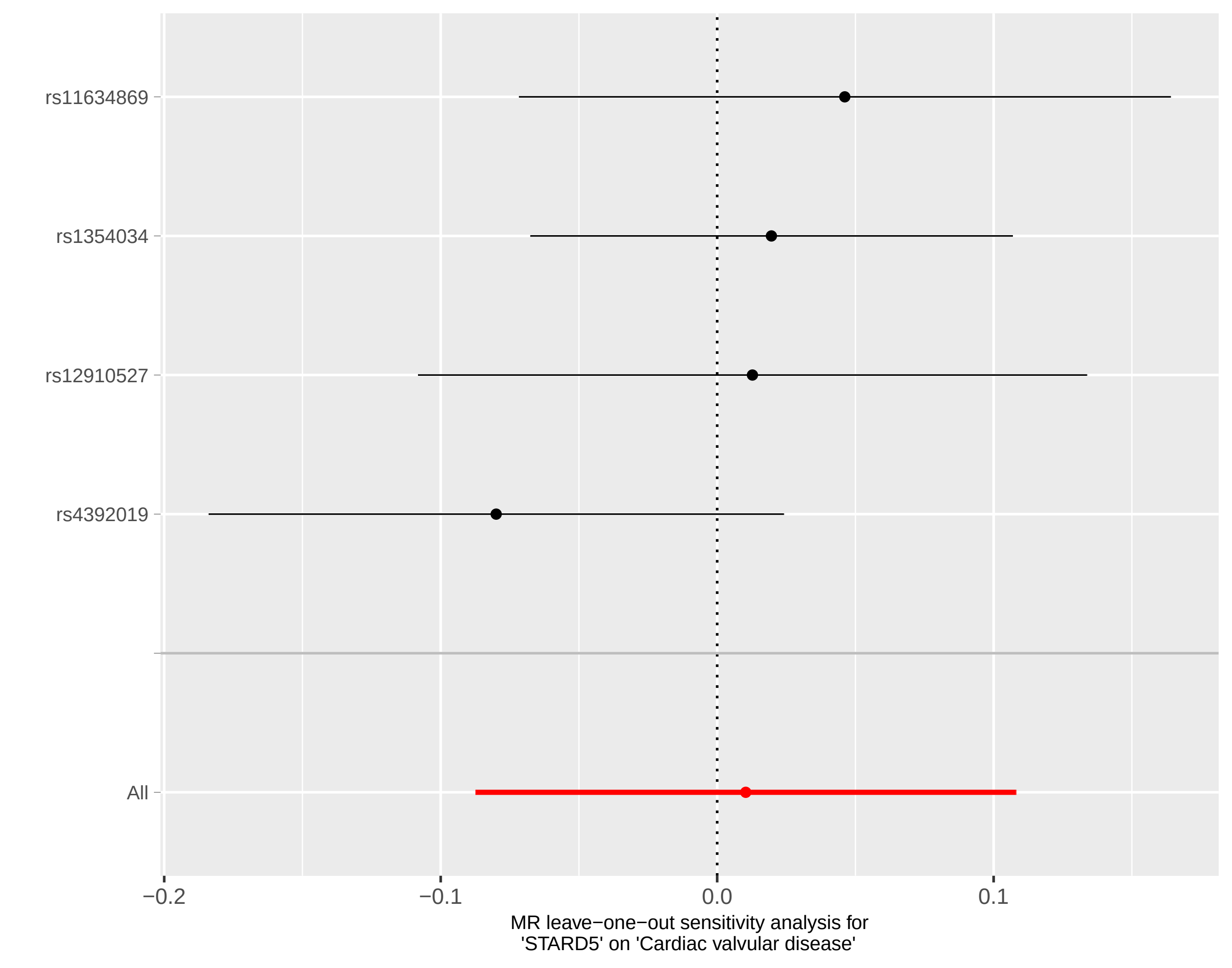
rs11634869
rs1354034
rs12910527
rs4392019
All
−0.2
−0.1
0.0
0.1
MR leave−one−out sensitivity analysis for
'STARD5' on 'Cardiac valvular disease'

Insufficient number of SNPs

Insufficient number of SNPs

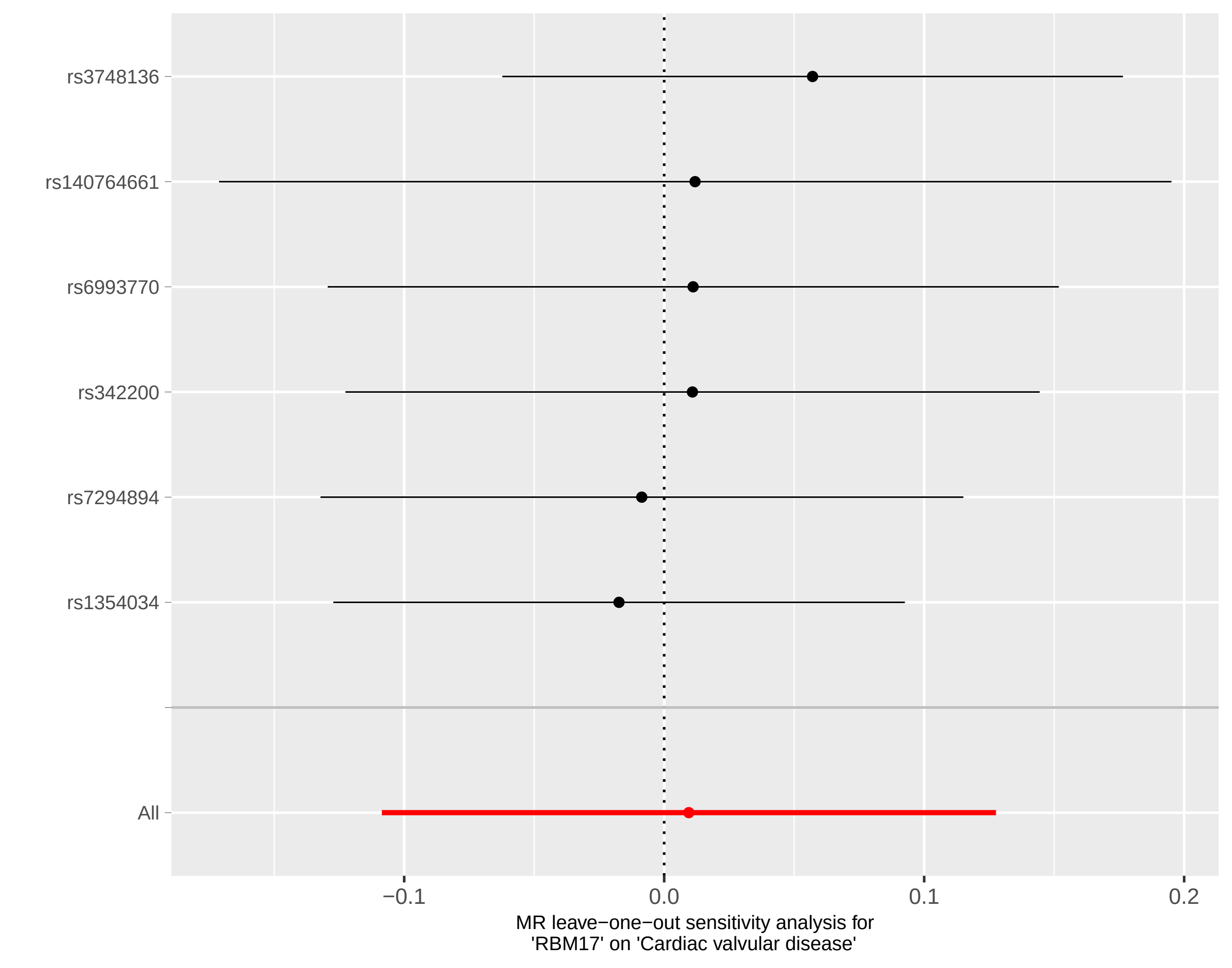
rs3748136
rs140764661
rs6993770
rs342200
rs7294894
rs1354034
All
−0.1
0.0
0.1
0.2
MR leave−one−out sensitivity analysis for
'RBM17' on 'Cardiac valvular disease'

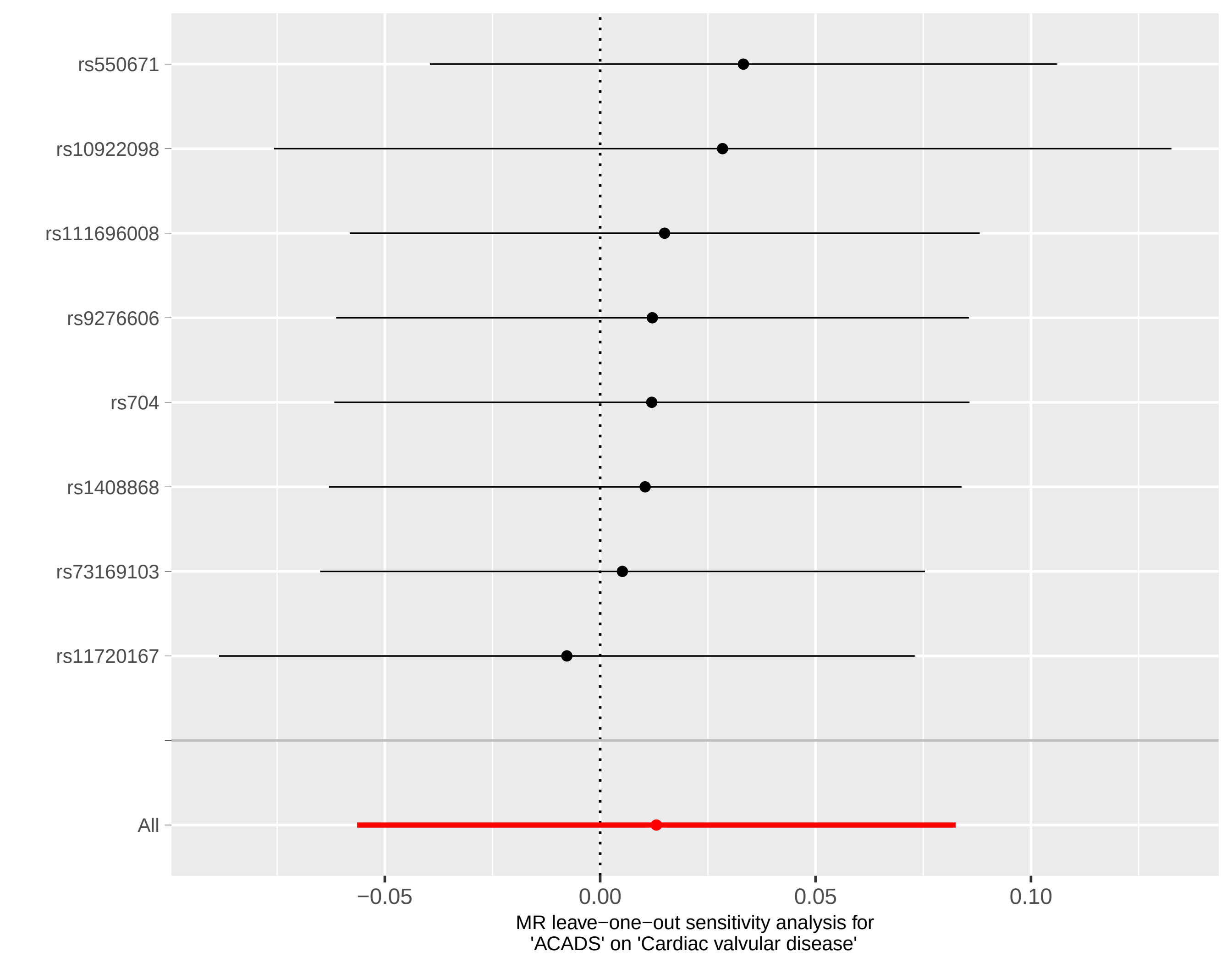

rs550671
rs10922098
rs111696008
rs9276606
rs704
rs1408868
rs73169103
rs11720167
All
−0.05
0.00
0.05
0.10
MR leave−one−out sensitivity analysis for
'ACADS' on 'Cardiac valvular disease'

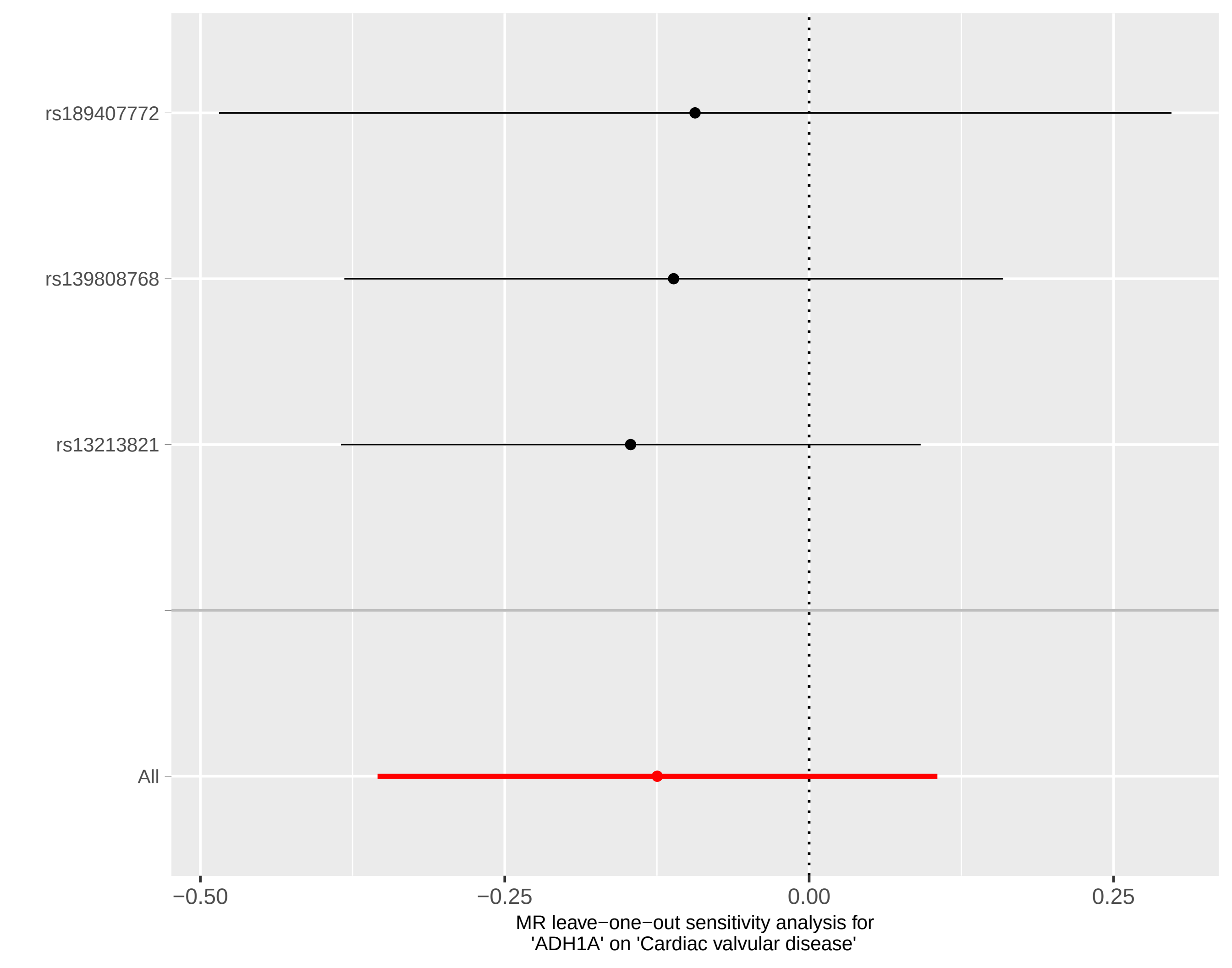
rs189407772
rs139808768
rs13213821
All
−0.50
−0.25
0.00
0.25
MR leave−one−out sensitivity analysis for
'ADH1A' on 'Cardiac valvular disease'

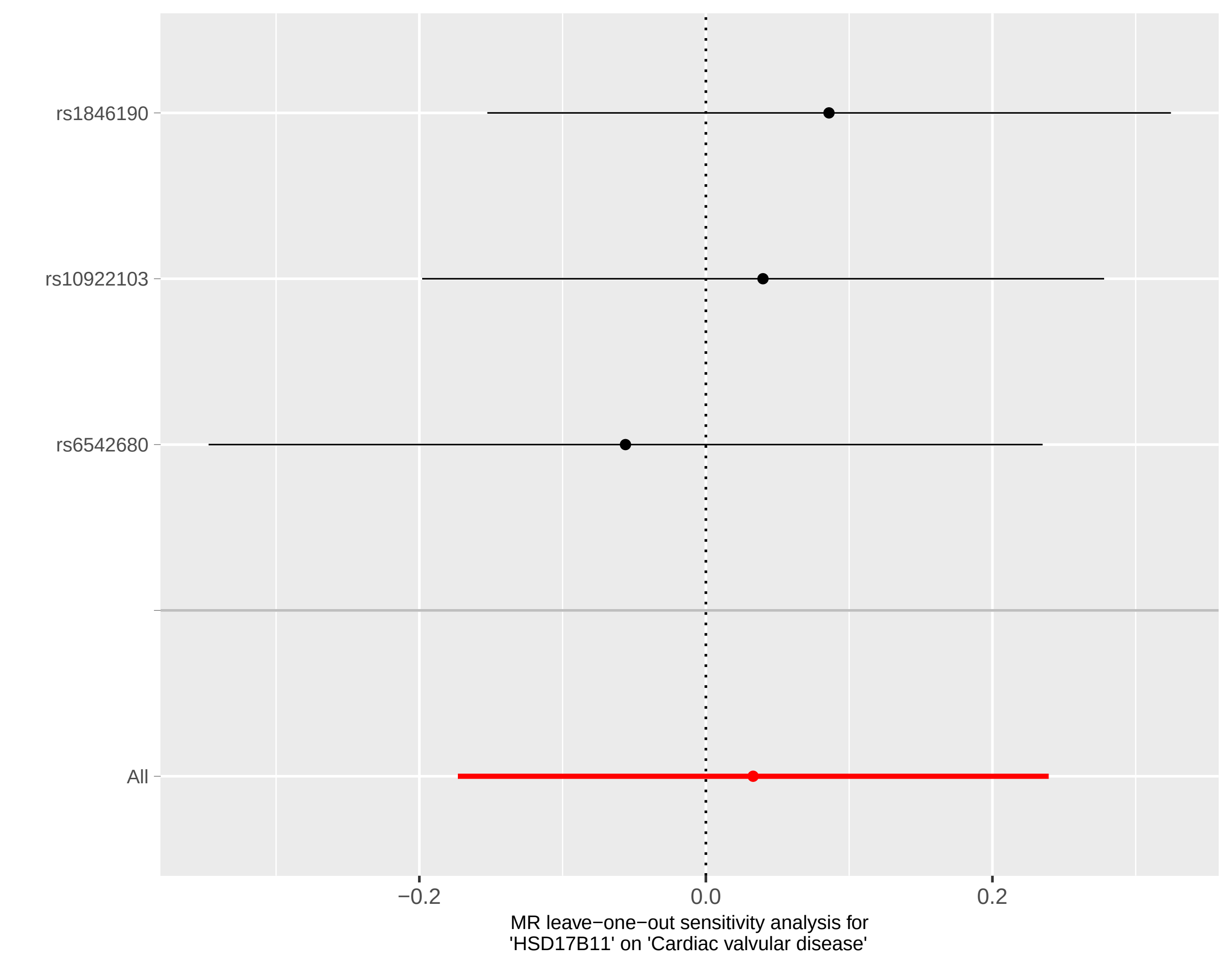

rs1846190
rs10922103
rs6542680
All
−0.2
0.0
0.2
MR leave−one−out sensitivity analysis for
'HSD17B11' on 'Cardiac valvular disease'

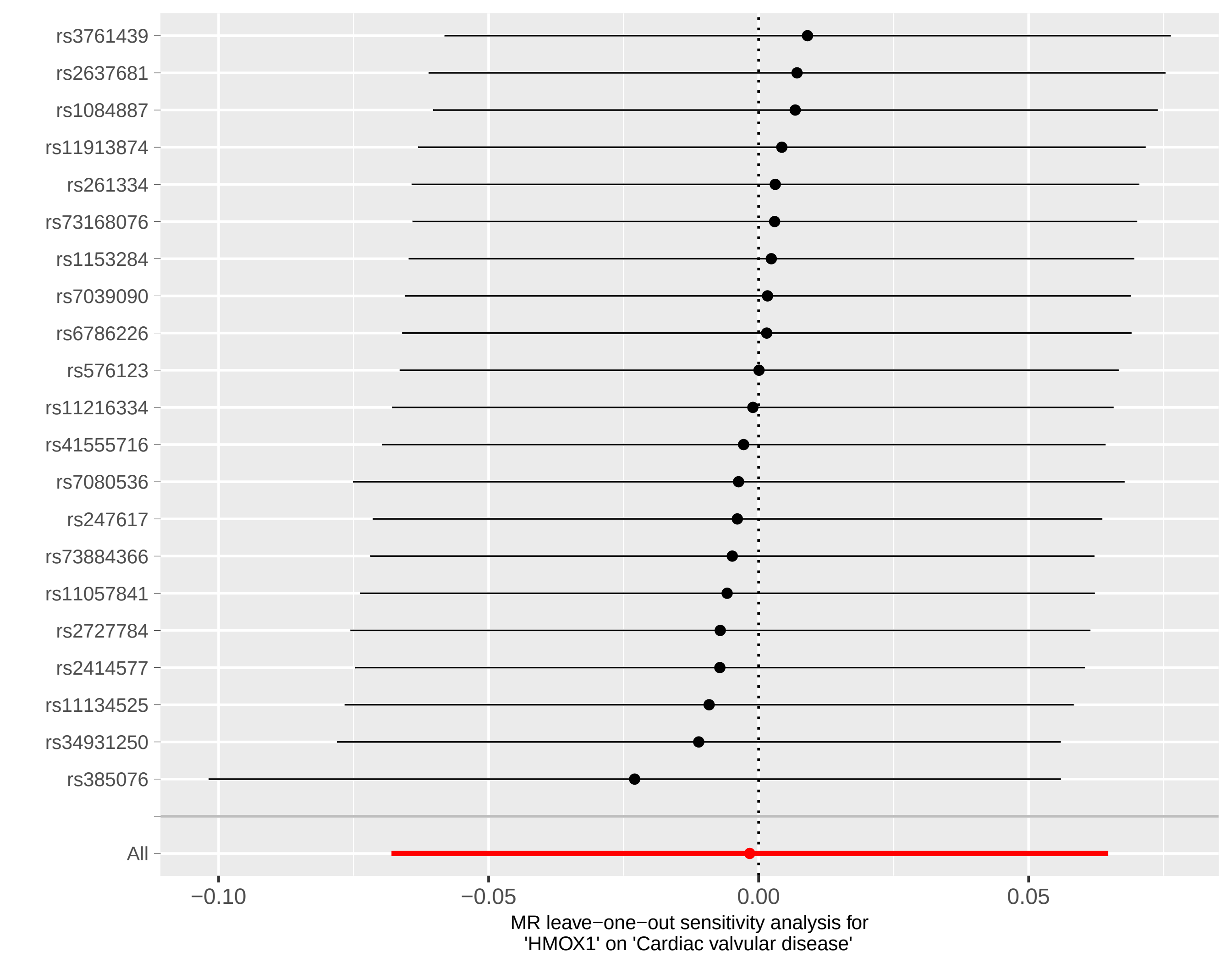
rs3761439
rs2637681
rs1084887
rs11913874
rs261334
rs73168076
rs1153284
rs7039090
rs6786226
rs576123
rs11216334
rs41555716
rs7080536
rs247617
rs73884366
rs11057841
rs2727784
rs2414577
rs11134525
rs34931250
rs385076
All
−0.10
−0.05
0.00
0.05
MR leave−one−out sensitivity analysis for
'HMOX1' on 'Cardiac valvular disease'

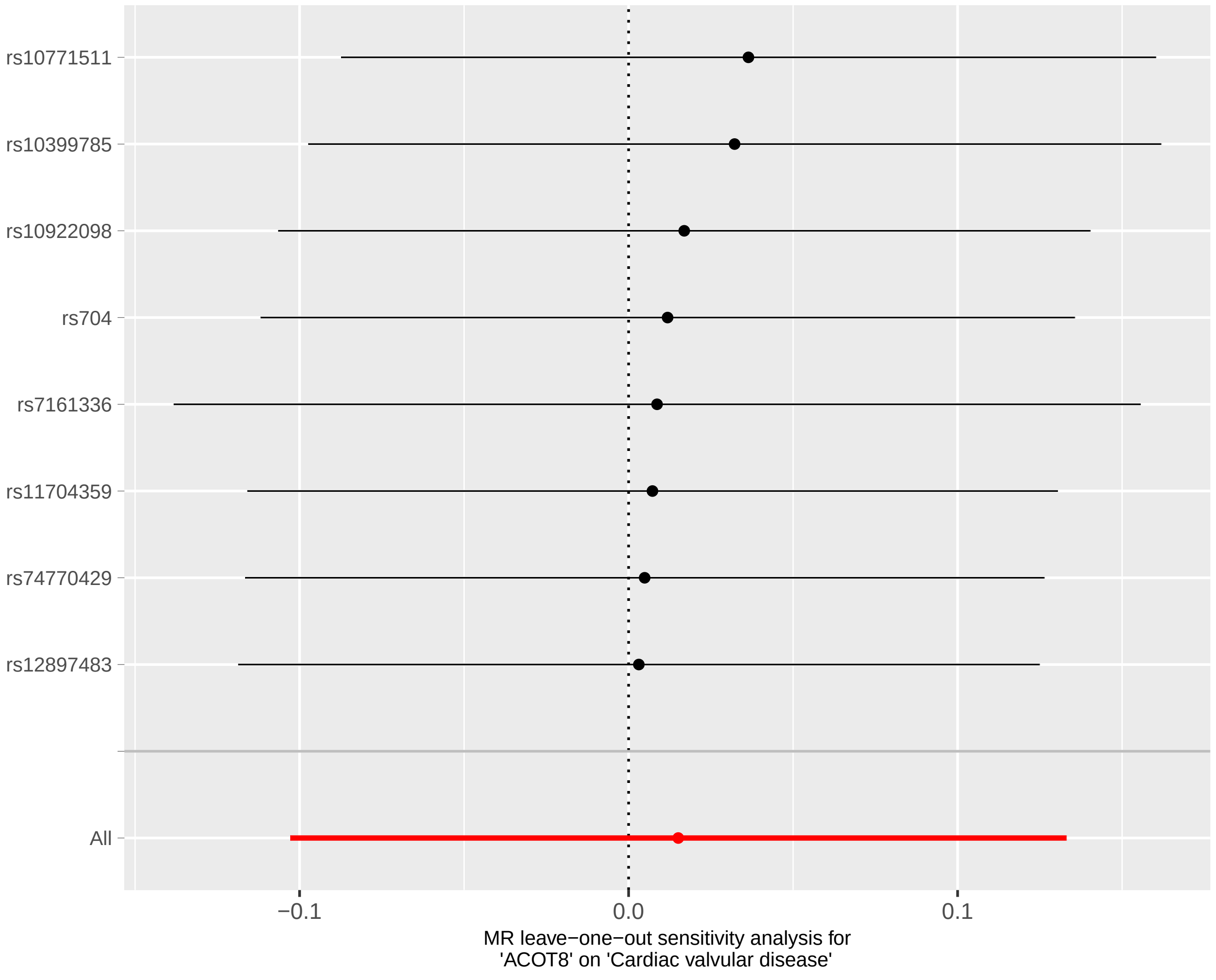
rs10771511
rs10399785
rs10922098
rs704
rs7161336
rs11704359
rs74770429
rs12897483
All
−0.1
0.0
0.1
MR leave−one−out sensitivity analysis for
'ACOT8' on 'Cardiac valvular disease'

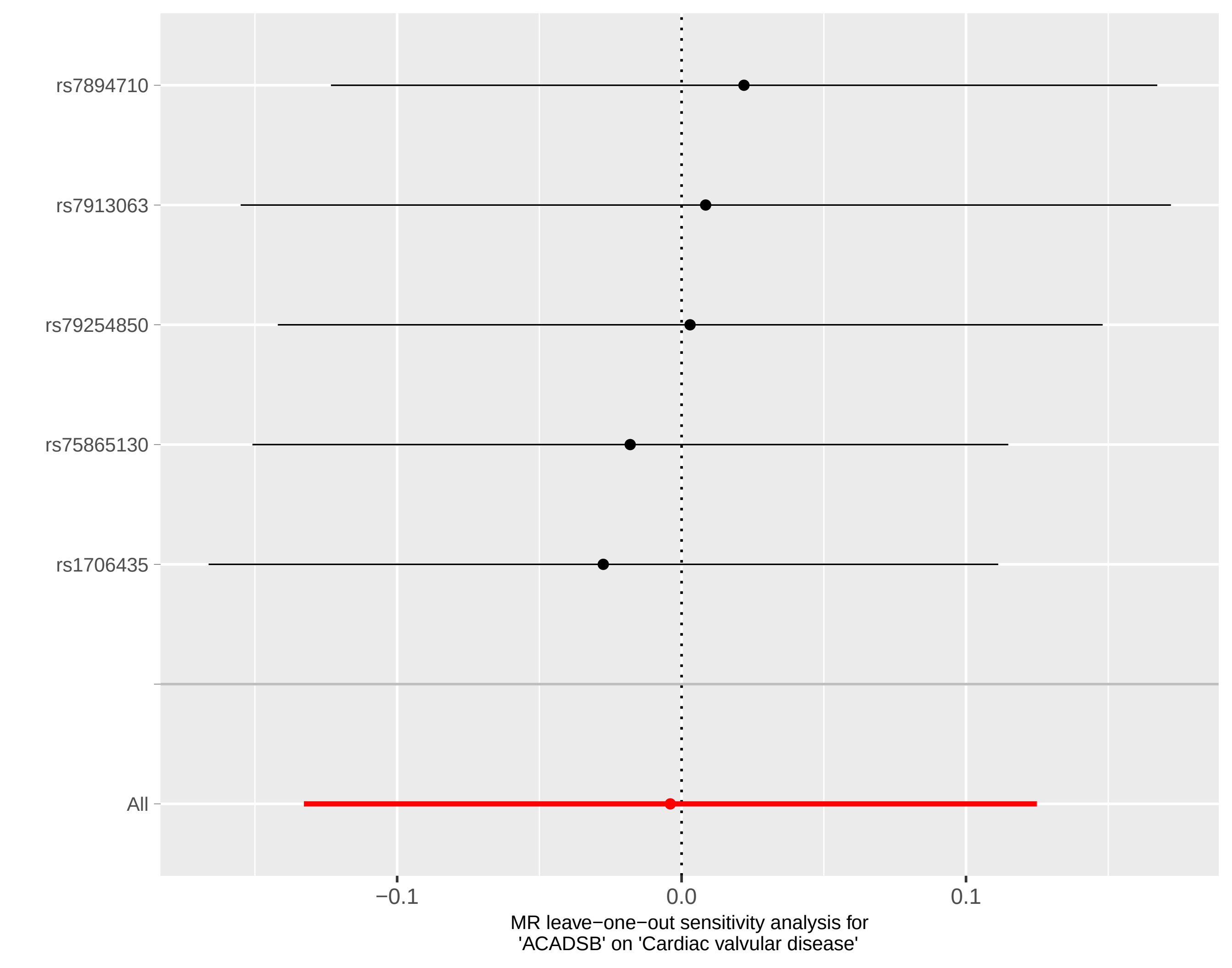
rs7894710
rs7913063
rs79254850
rs75865130
rs1706435
All
−0.1
0.0
0.1
MR leave−one−out sensitivity analysis for
'ACADSB' on 'Cardiac valvular disease'

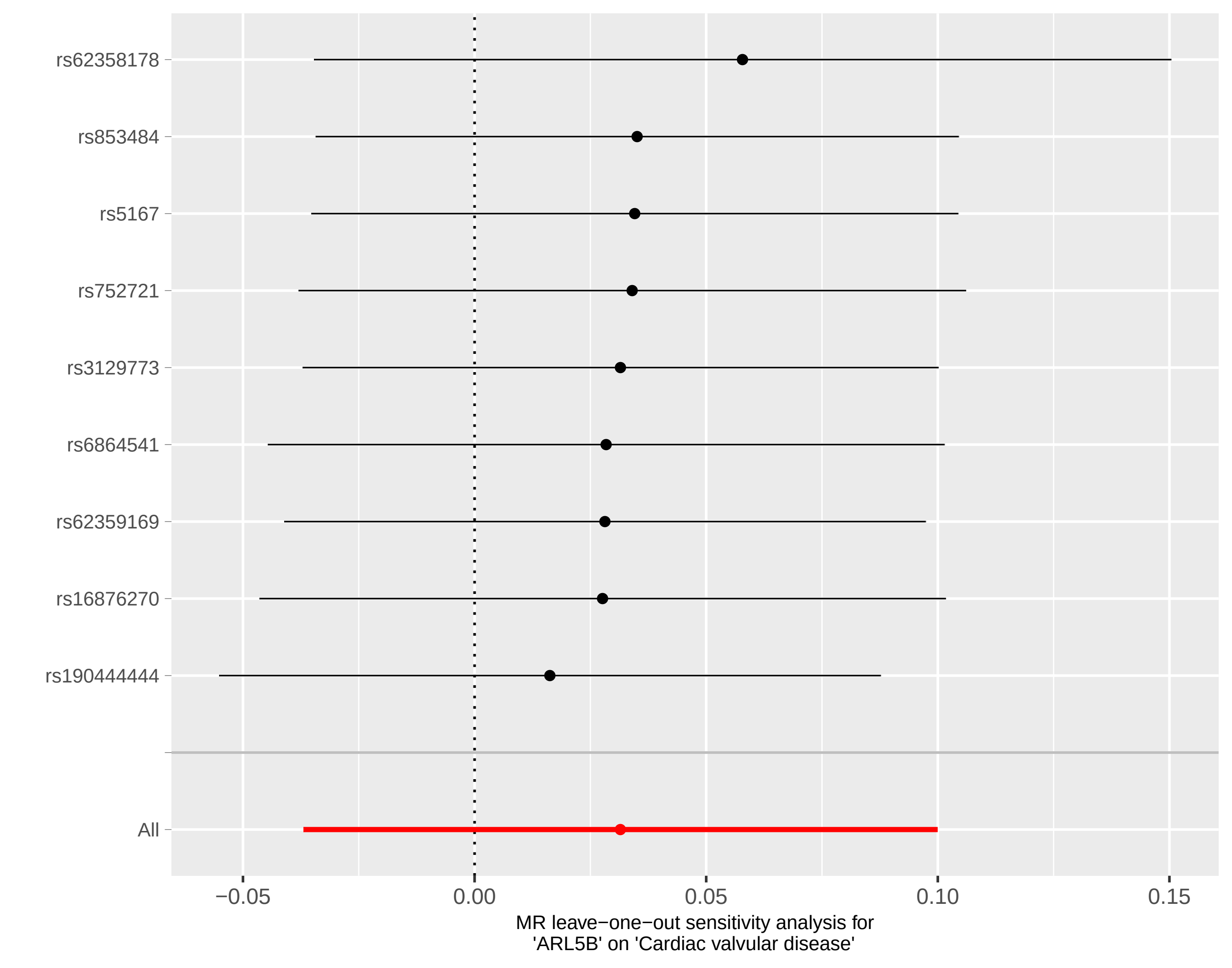

rs62358178
rs853484
rs5167
rs752721
rs3129773
rs6864541
rs62359169
rs16876270
rs190444444
All
−0.05
0.00
0.05
0.10
0.15
MR leave−one−out sensitivity analysis for
'ARL5B' on 'Cardiac valvular disease'

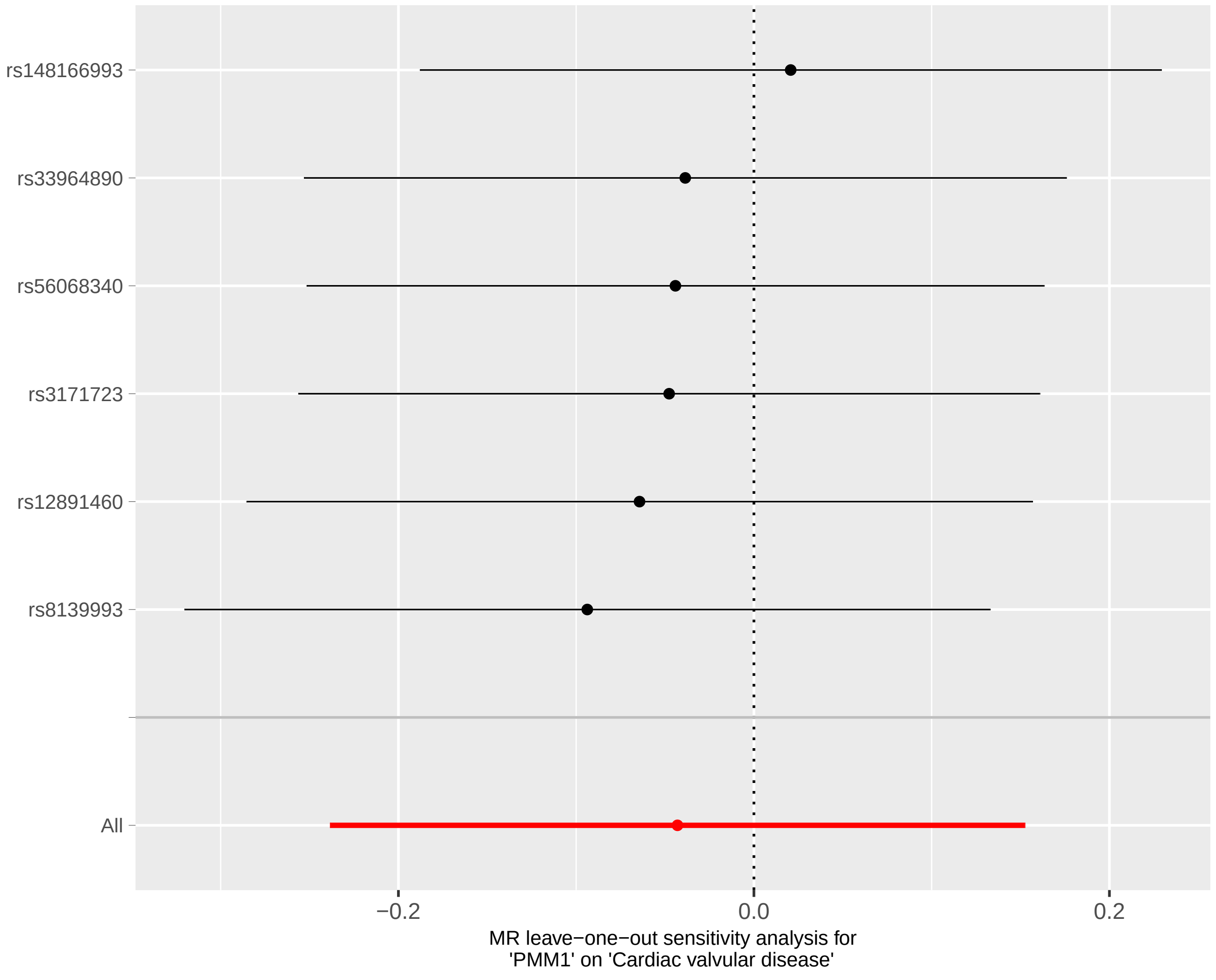
rs148166993
rs33964890
rs56068340
rs3171723
rs12891460
rs8139993
All
−0.2
0.0
0.2
MR leave−one−out sensitivity analysis for
'PMM1' on 'Cardiac valvular disease'

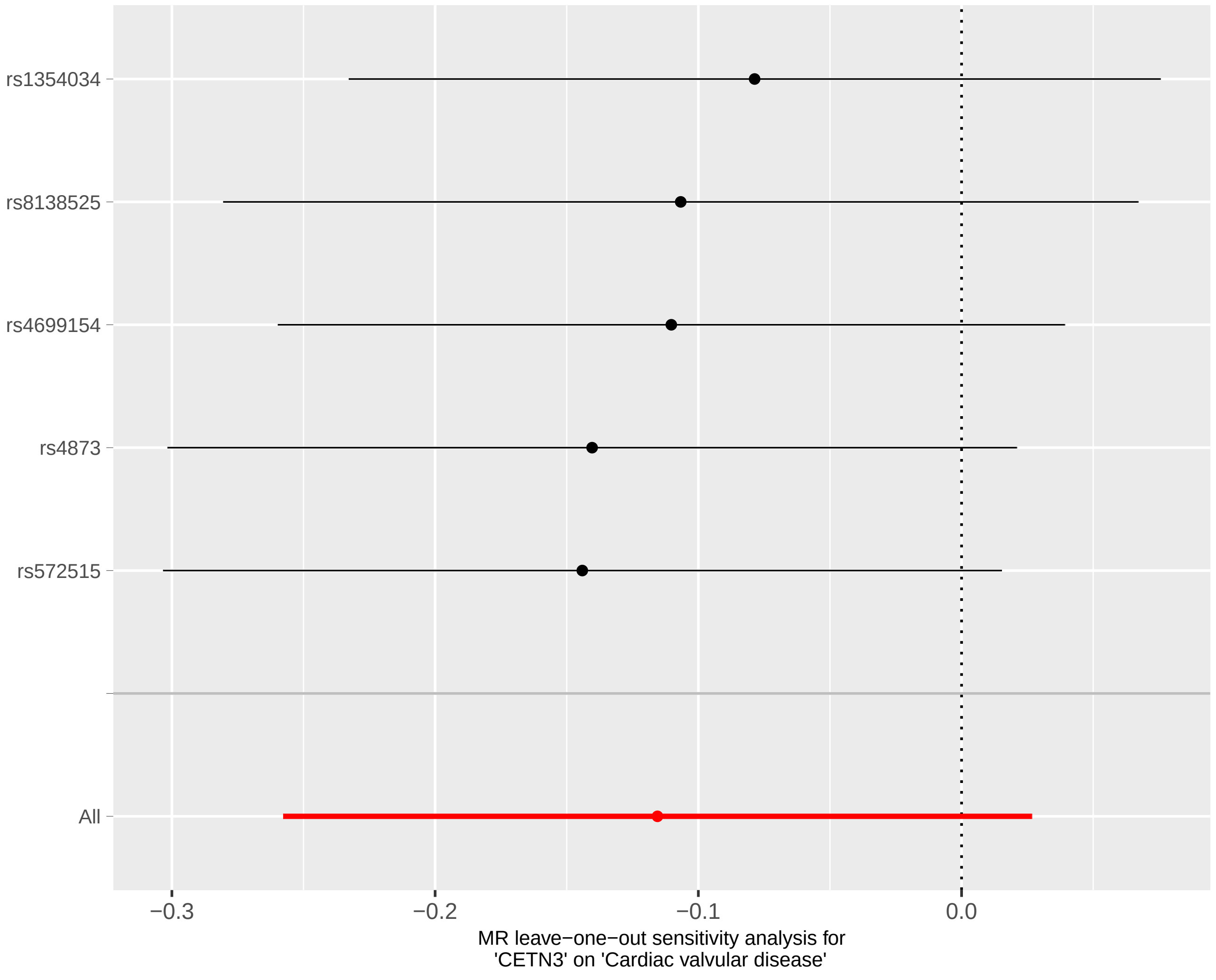
rs1354034
rs8138525
rs4699154
rs4873
rs572515
All
−0.3
−0.2
−0.1
0.0
MR leave−one−out sensitivity analysis for
'CETN3' on 'Cardiac valvular disease'

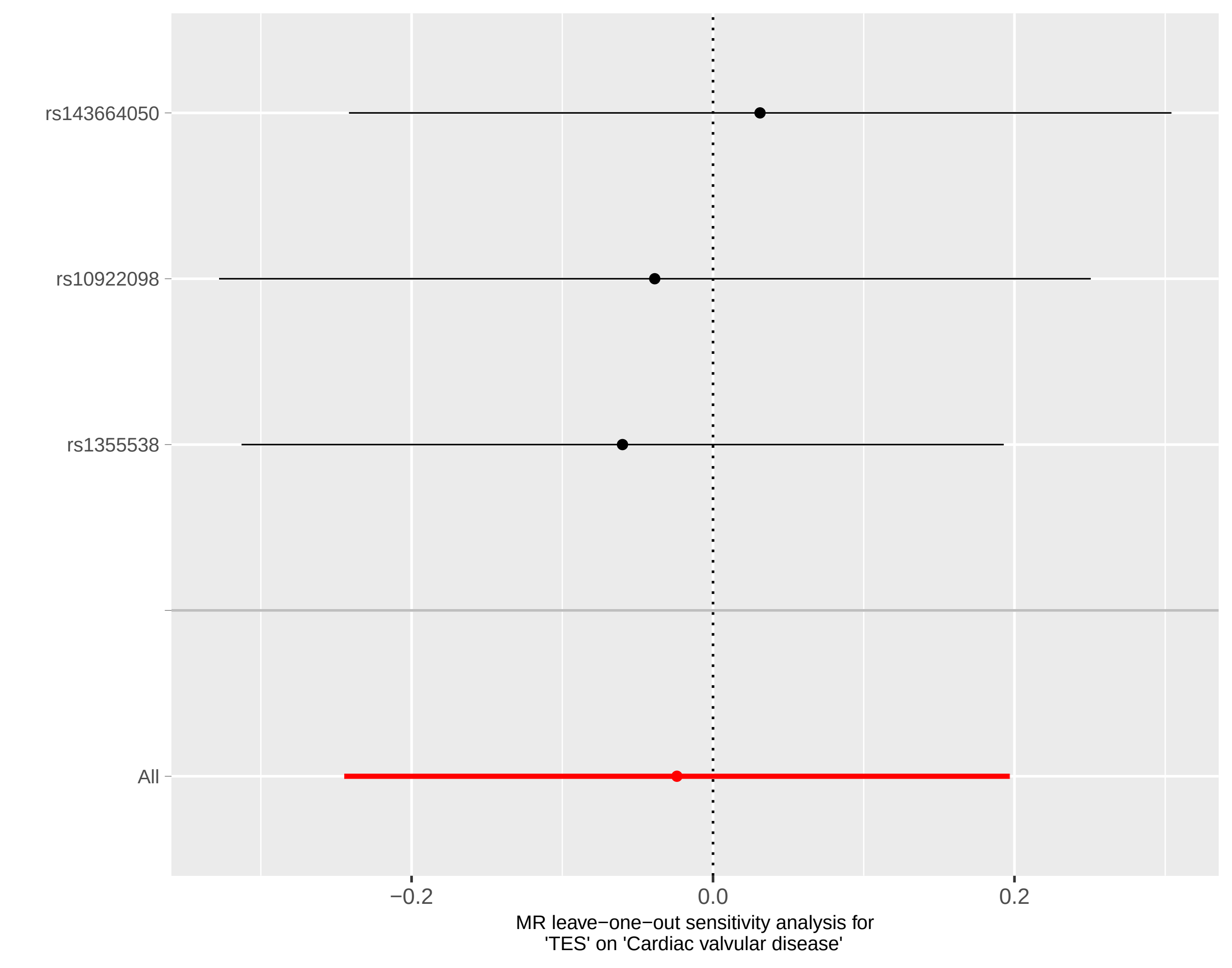
rs143664050
rs10922098
rs1355538
All
−0.2
0.0
0.2
MR leave−one−out sensitivity analysis for
'TES' on 'Cardiac valvular disease'

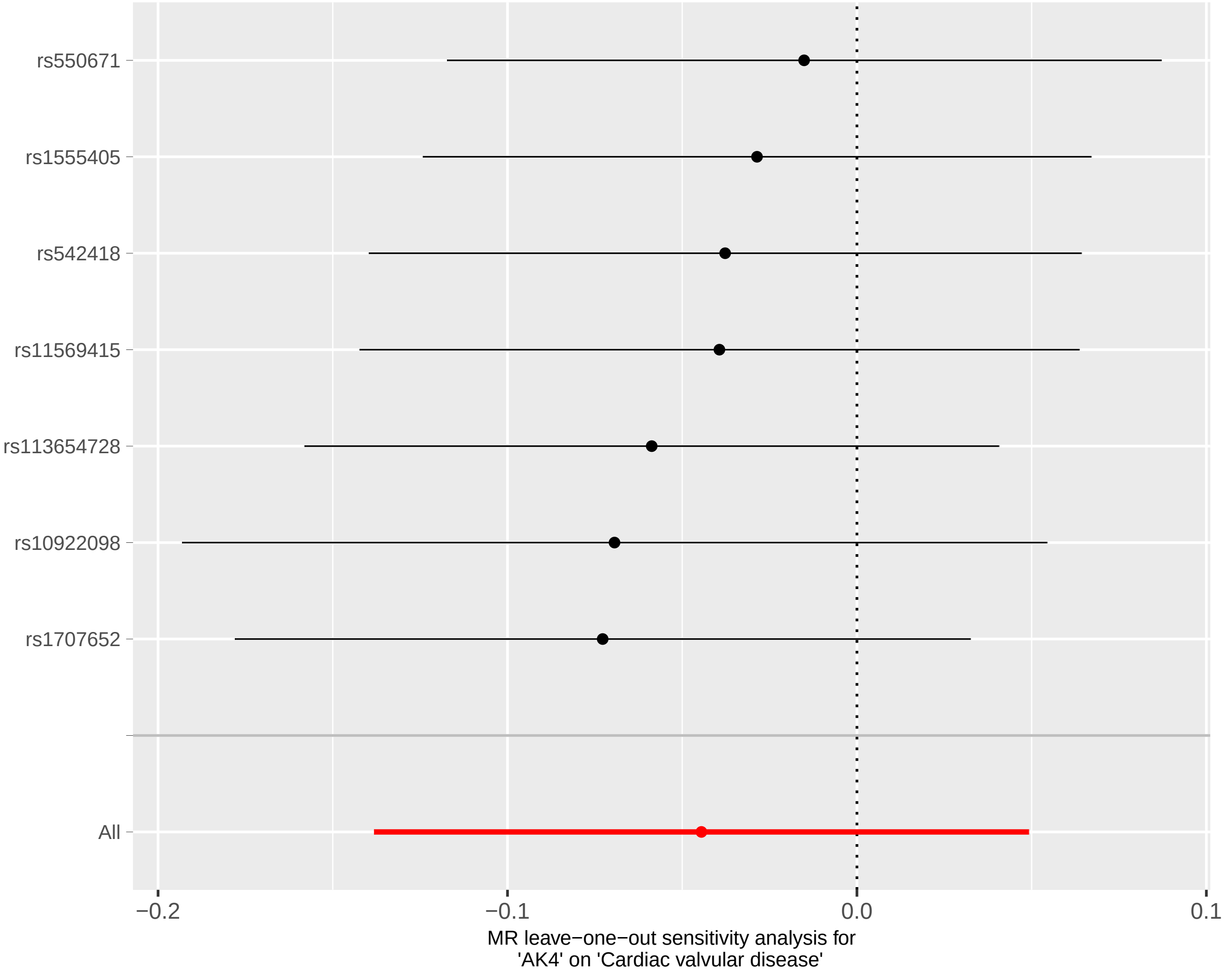

rs550671
rs1555405
rs542418
rs11569415
rs113654728
rs10922098
rs1707652
All
−0.2
−0.1
0.0
0.1
MR leave−one−out sensitivity analysis for
'AK4' on 'Cardiac valvular disease'

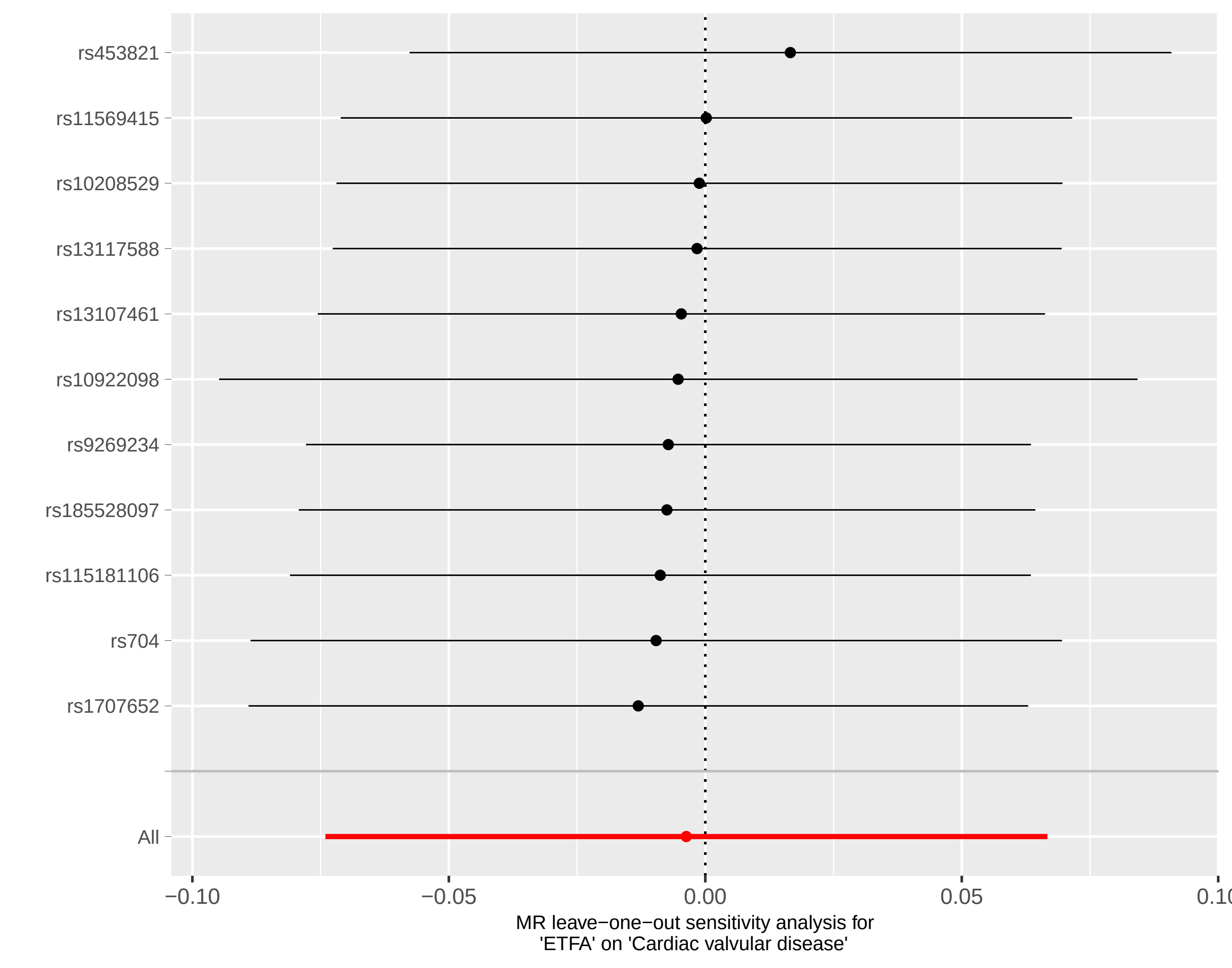
rs453821
rs11569415
rs10208529
rs13117588
rs13107461
rs10922098
rs9269234
rs185528097
rs115181106
rs704
rs1707652
All
−0.10
−0.05
0.00
0.05
MR leave−one−out sensitivity analysis for
'ETFA' on 'Cardiac valvular disease'

Insufficient number of SNPs

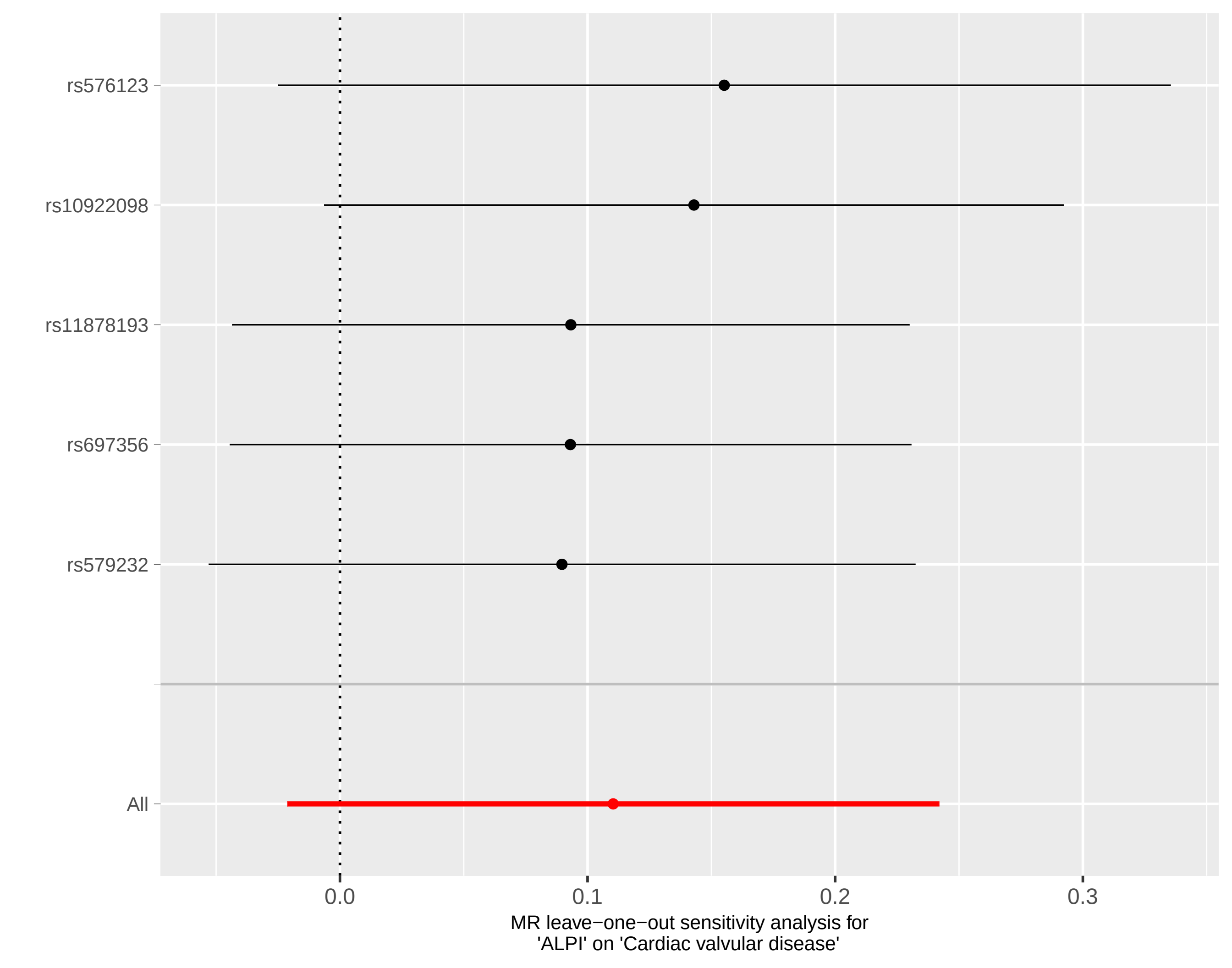
rs576123
rs10922098
rs11878193
rs697356
rs579232
All
0.0
0.1
0.2
0.3
MR leave−one−out sensitivity analysis for
'ALPI' on 'Cardiac valvular disease'

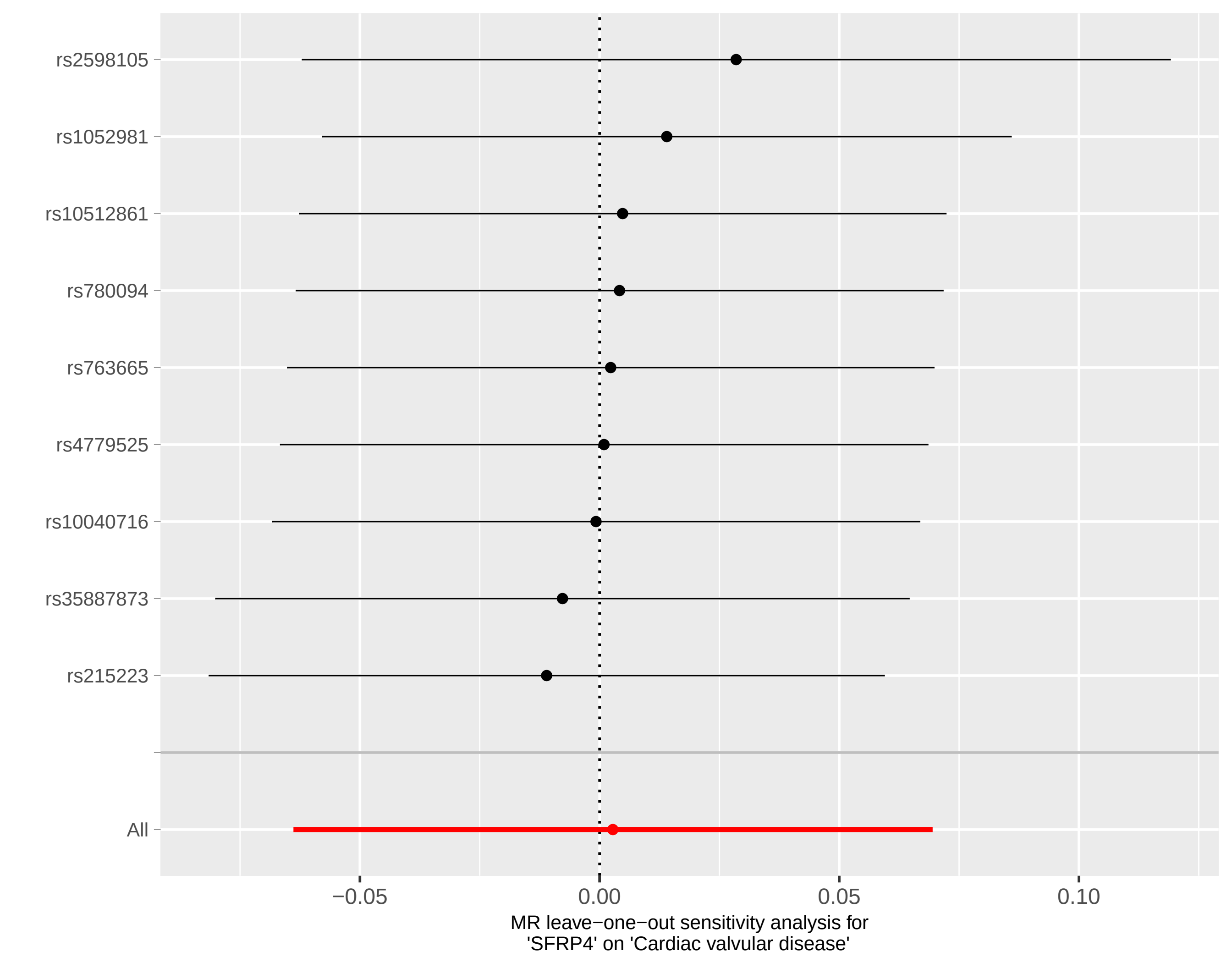
rs2598105
rs1052981
rs10512861
rs780094
rs763665
rs4779525
rs10040716
rs35887873
rs215223
All
−0.05
0.00
0.05
0.10
MR leave−one−out sensitivity analysis for
'SFRP4' on 'Cardiac valvular disease'

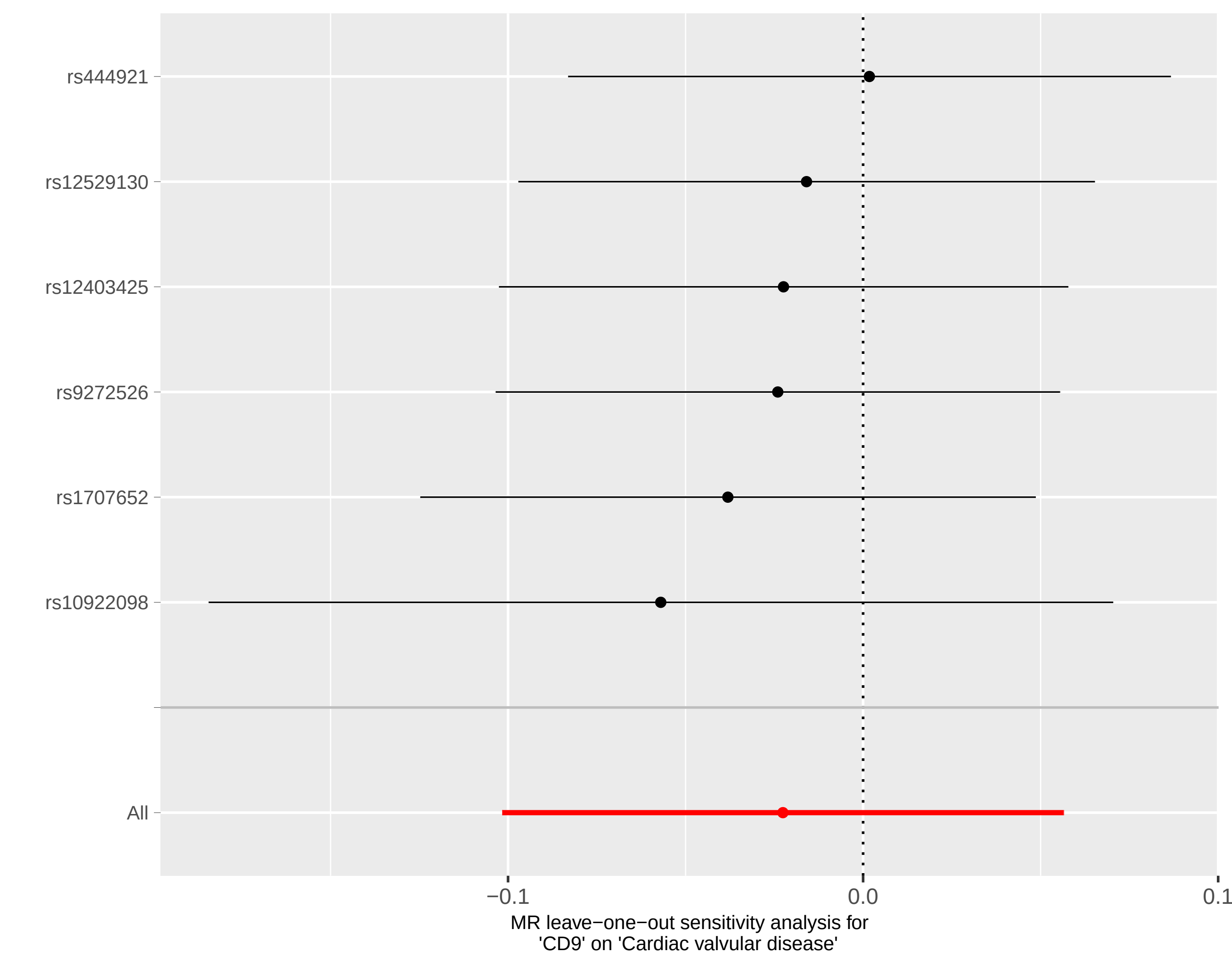
rs444921
rs12529130
rs12403425
rs9272526
rs1707652
rs10922098
All
−0.1
0.0
0.1
MR leave−one−out sensitivity analysis for
'CD9' on 'Cardiac valvular disease'

Insufficient number of SNPs

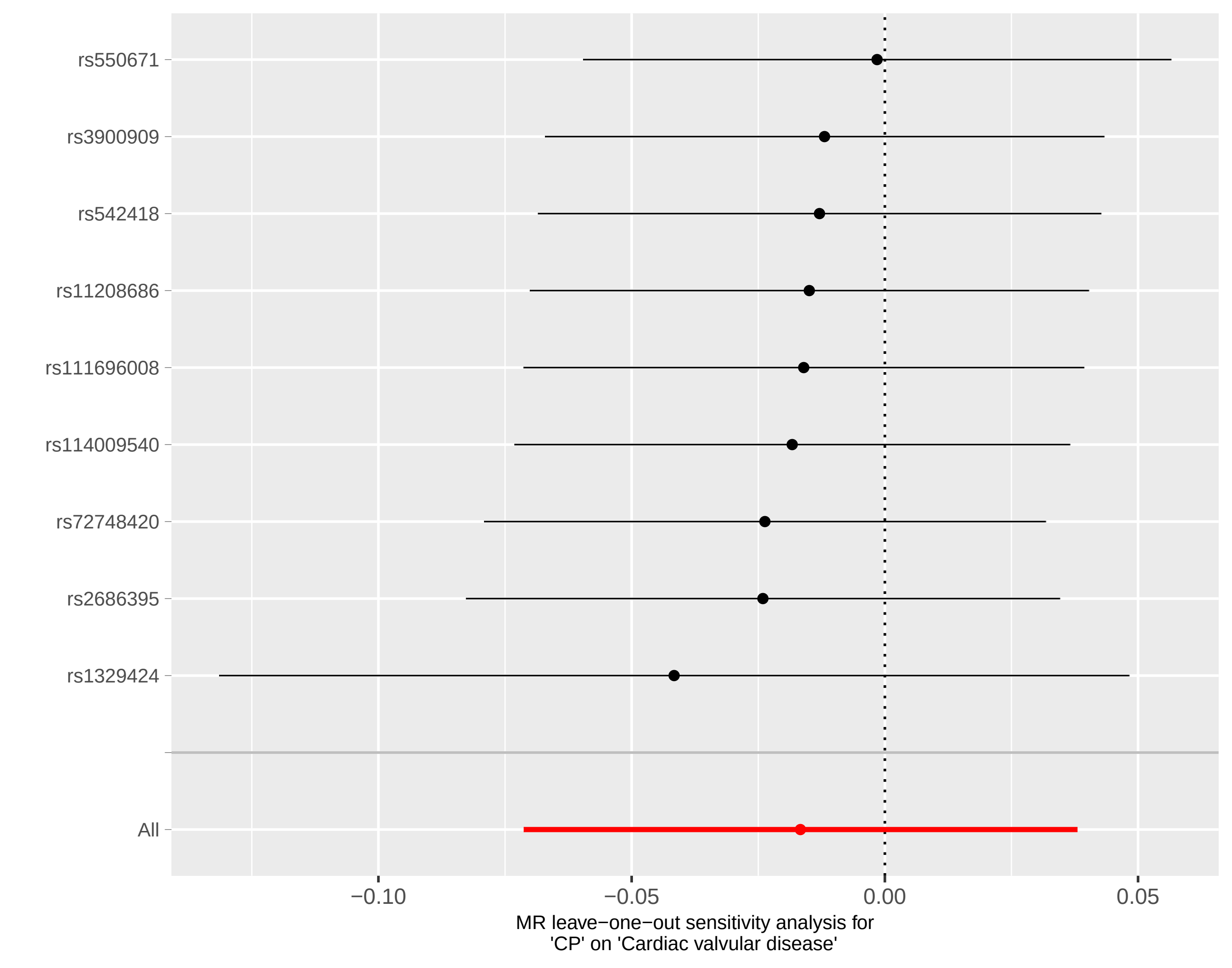
rs550671
rs3900909
rs542418
rs11208686
rs111696008
rs114009540
rs72748420
rs2686395
rs1329424
All
−0.10
−0.05
0.00
0.05
MR leave−one−out sensitivity analysis for
'CP' on 'Cardiac valvular disease'

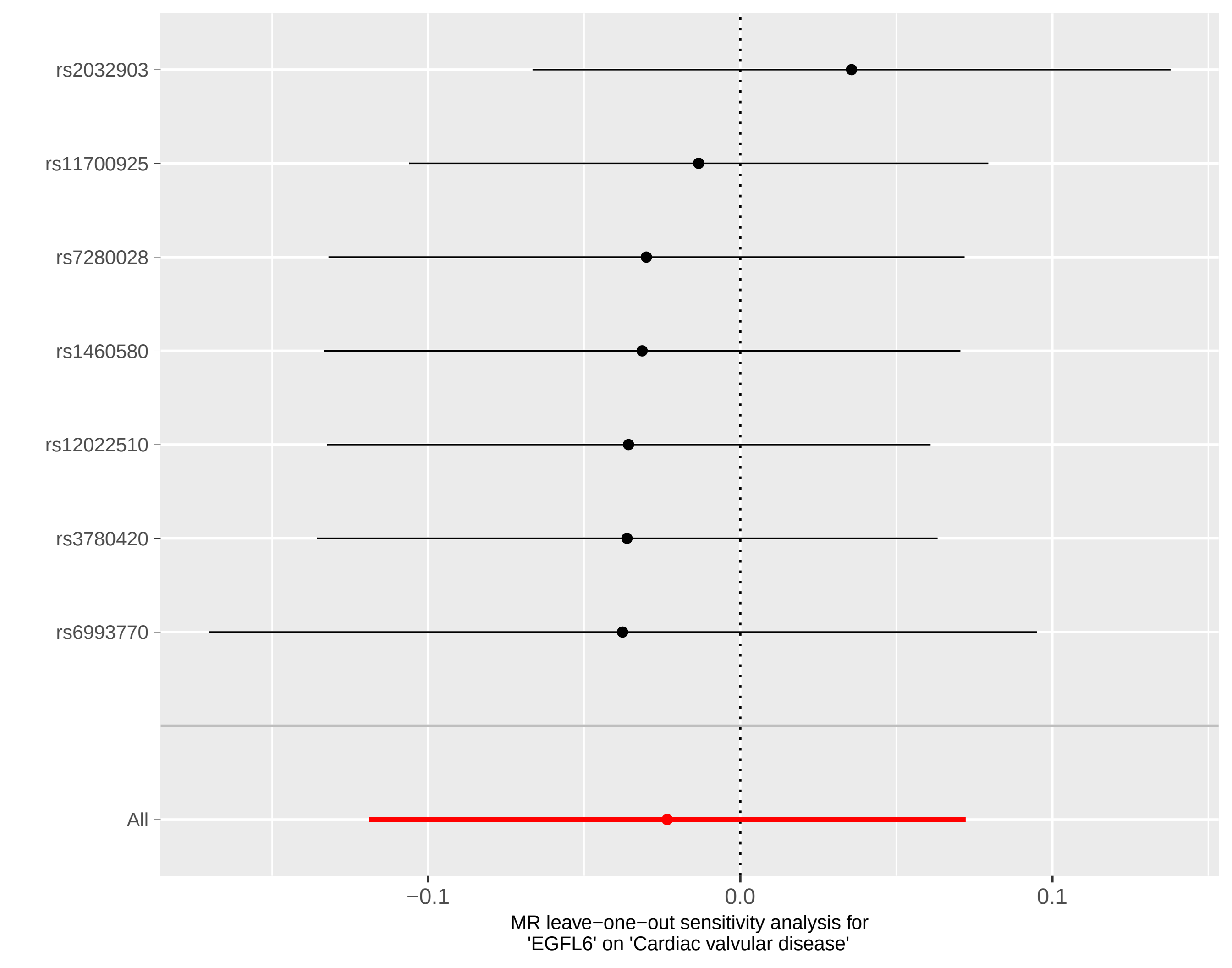
rs2032903
rs11700925
rs7280028
rs1460580
rs12022510
rs3780420
rs6993770
All
−0.1
0.0
0.1
MR leave−one−out sensitivity analysis for
'EGFL6' on 'Cardiac valvular disease'

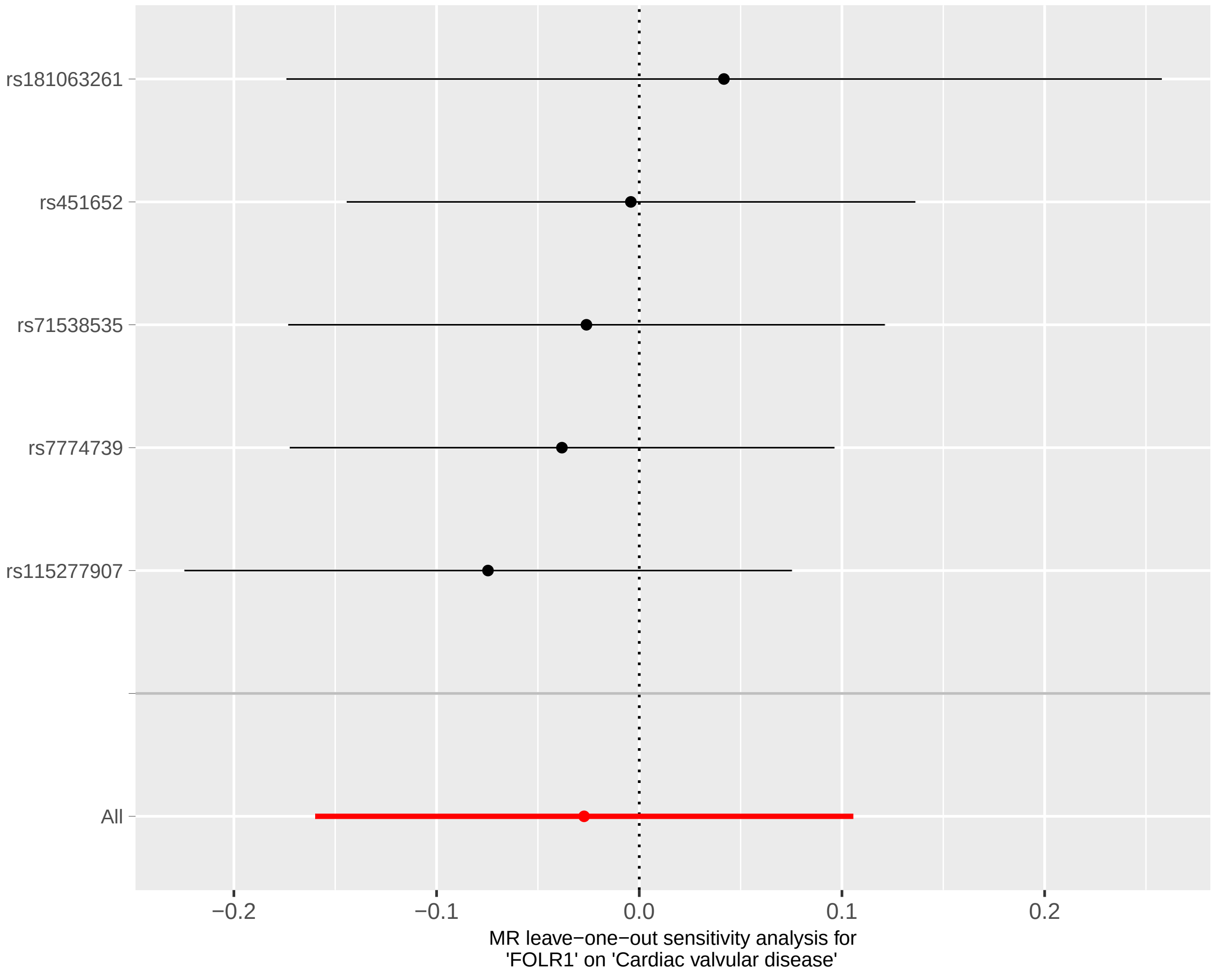

rs181063261
rs451652
rs71538535
rs7774739
rs115277907
All
−0.2
−0.1
0.0
0.1
0.2
MR leave−one−out sensitivity analysis for
'FOLR1' on 'Cardiac valvular disease'

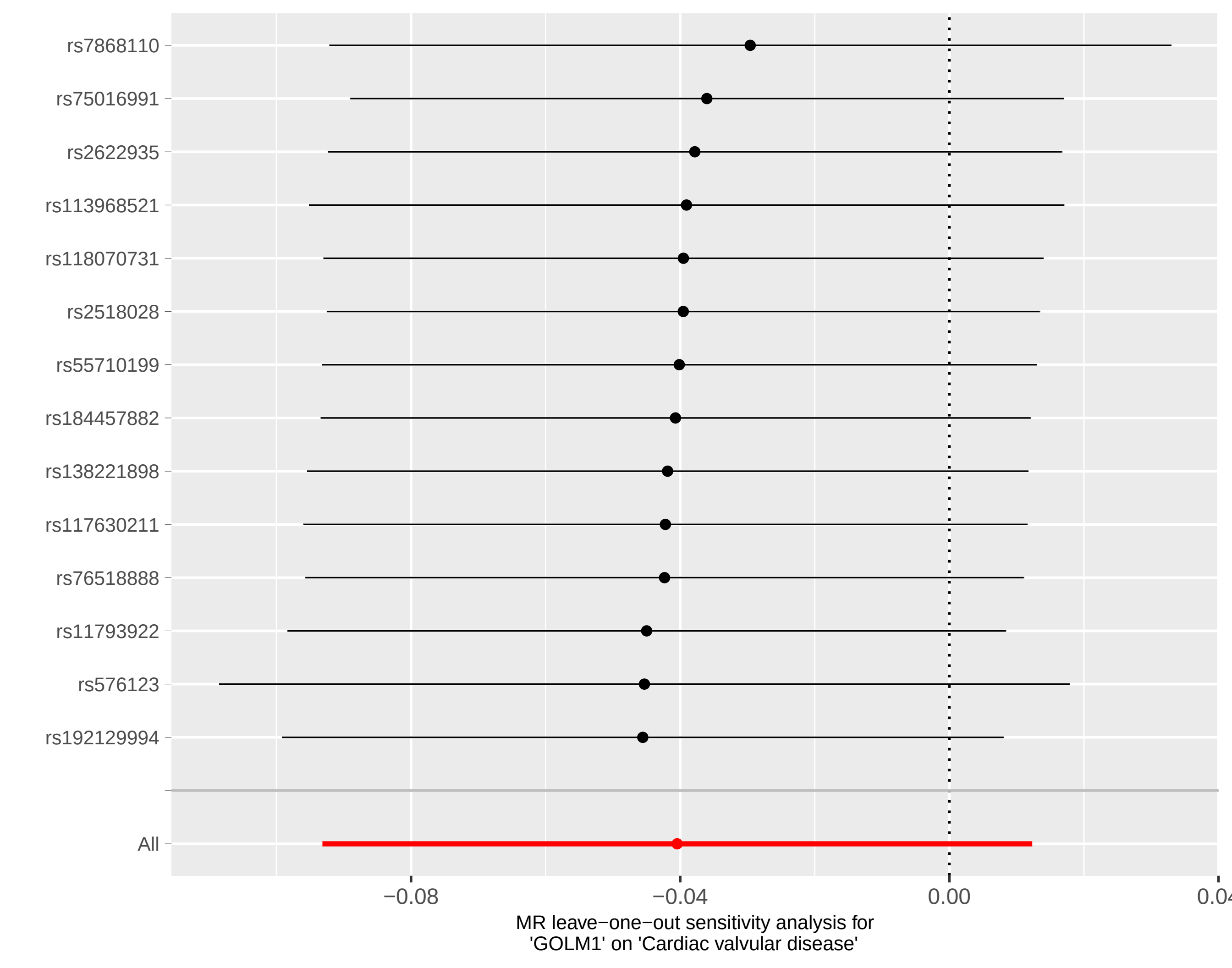
rs7868110
rs75016991
rs2622935
rs113968521
rs118070731
rs2518028
rs55710199
rs184457882
rs138221898
rs117630211
rs76518888
rs11793922
rs576123
rs192129994
All
−0.08
−0.04
0.00
0.04
MR leave−one−out sensitivity analysis for
'GOLM1' on 'Cardiac valvular disease'

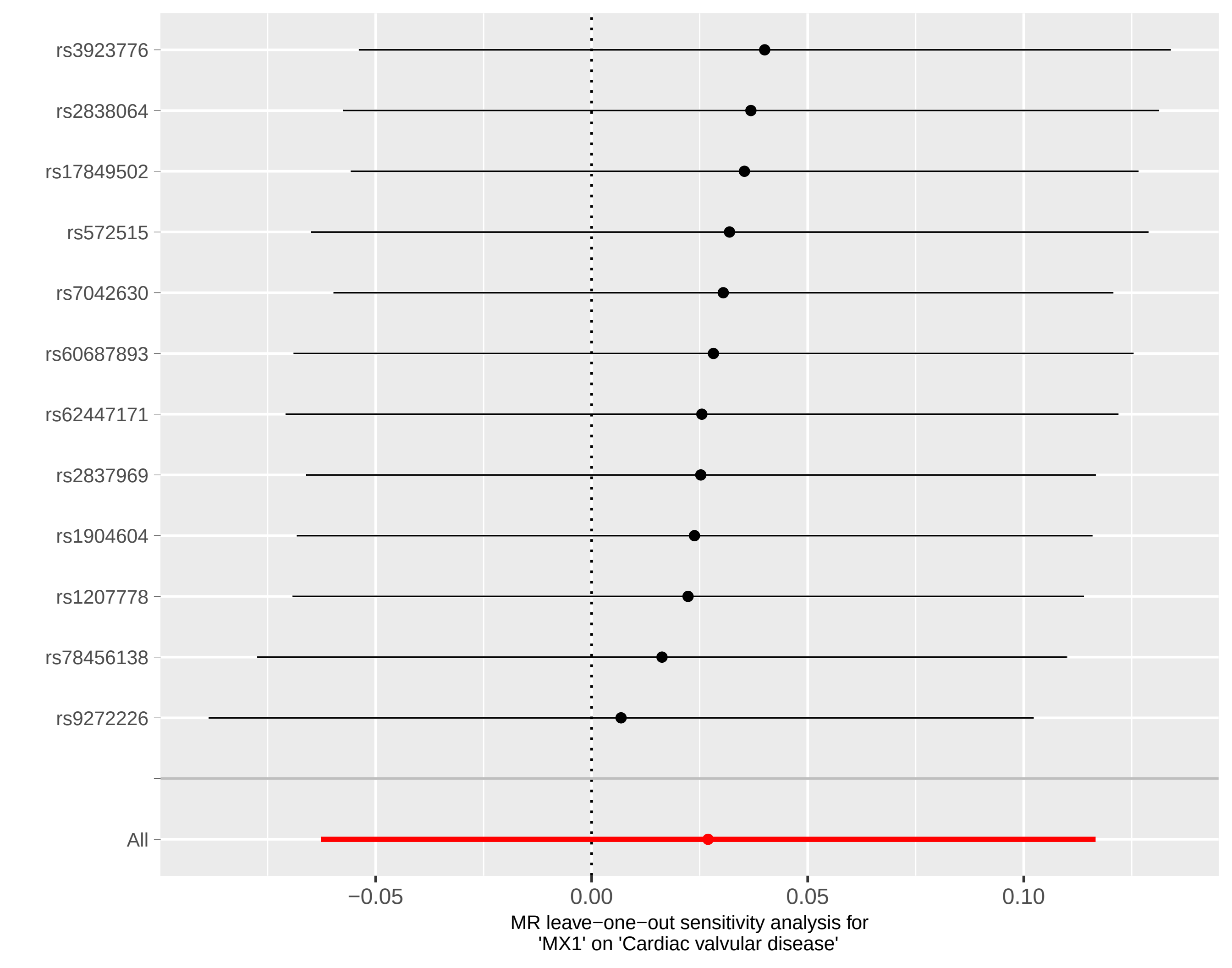

rs3923776
rs2838064
rs17849502
rs572515
rs7042630
rs60687893
rs62447171
rs2837969
rs1904604
rs1207778
rs78456138
rs9272226
All
−0.05
0.00
0.05
0.10
MR leave−one−out sensitivity analysis for
'MX1' on 'Cardiac valvular disease'

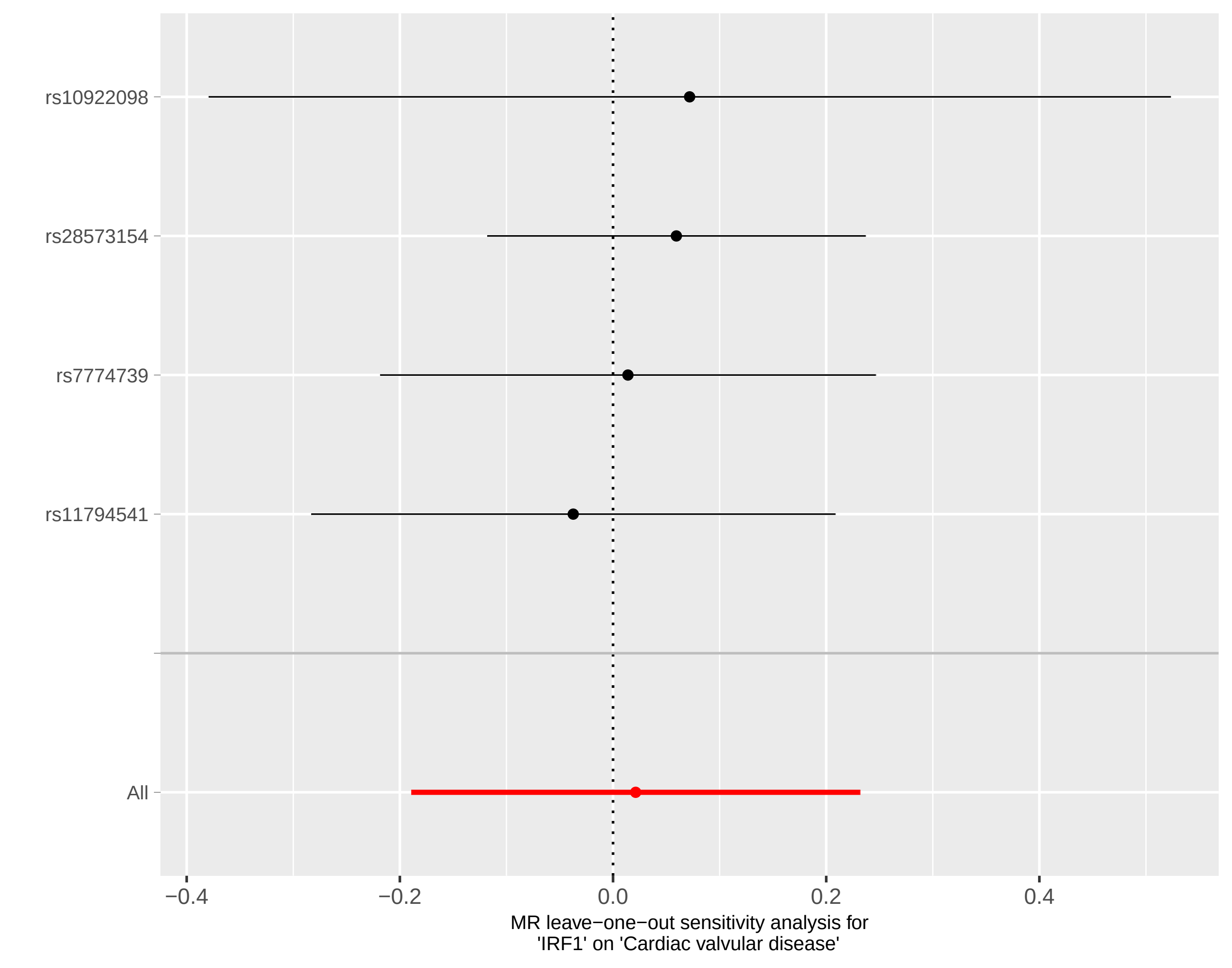
rs10922098
rs28573154
rs7774739
rs11794541
All
−0.4
−0.2
0.0
0.2
0.4
MR leave−one−out sensitivity analysis for
'IRF1' on 'Cardiac valvular disease'

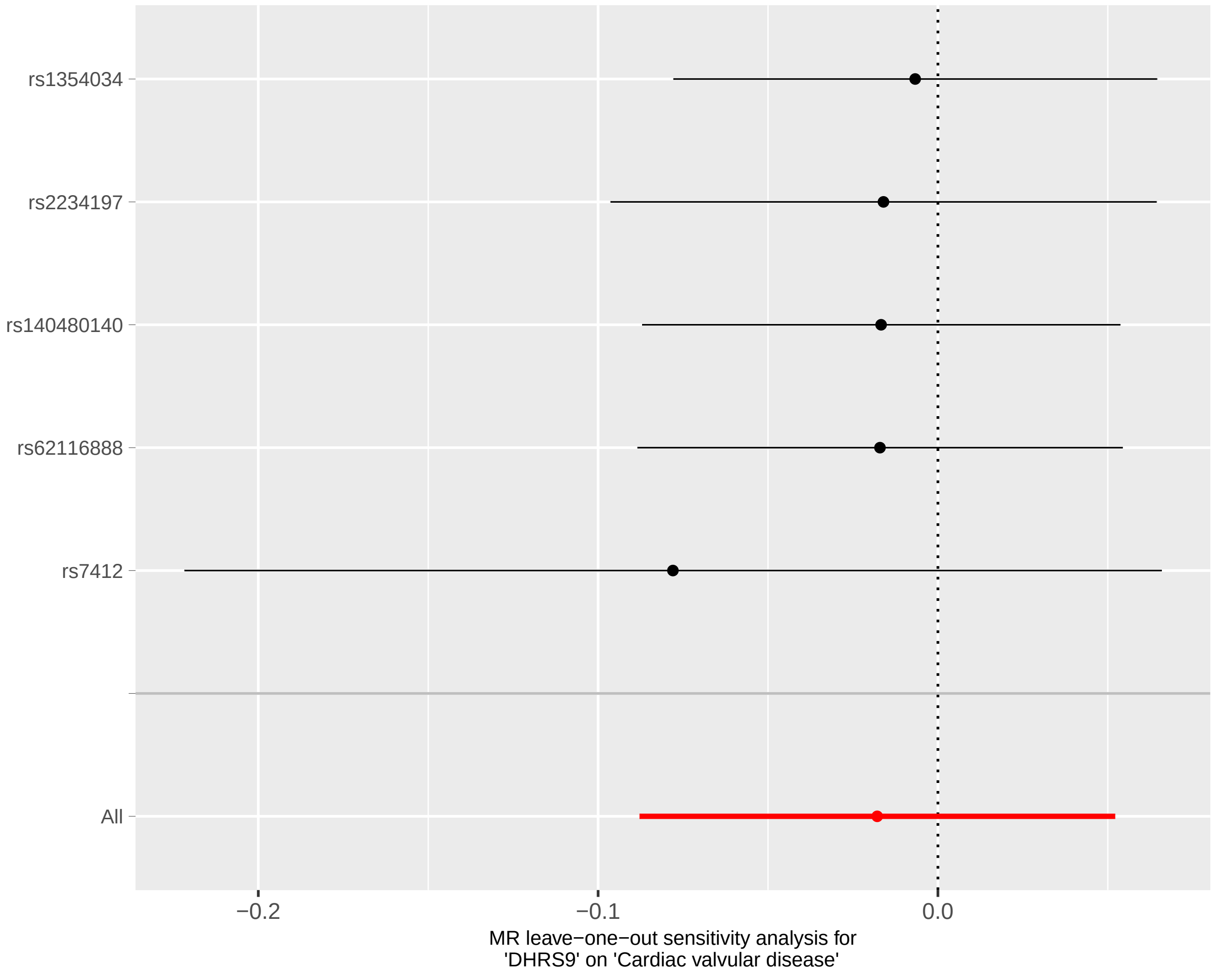
rs1354034
rs2234197
rs140480140
rs62116888
rs7412
All
−0.2
−0.1
0.0
MR leave−one−out sensitivity analysis for
'DHRS9' on 'Cardiac valvular disease'

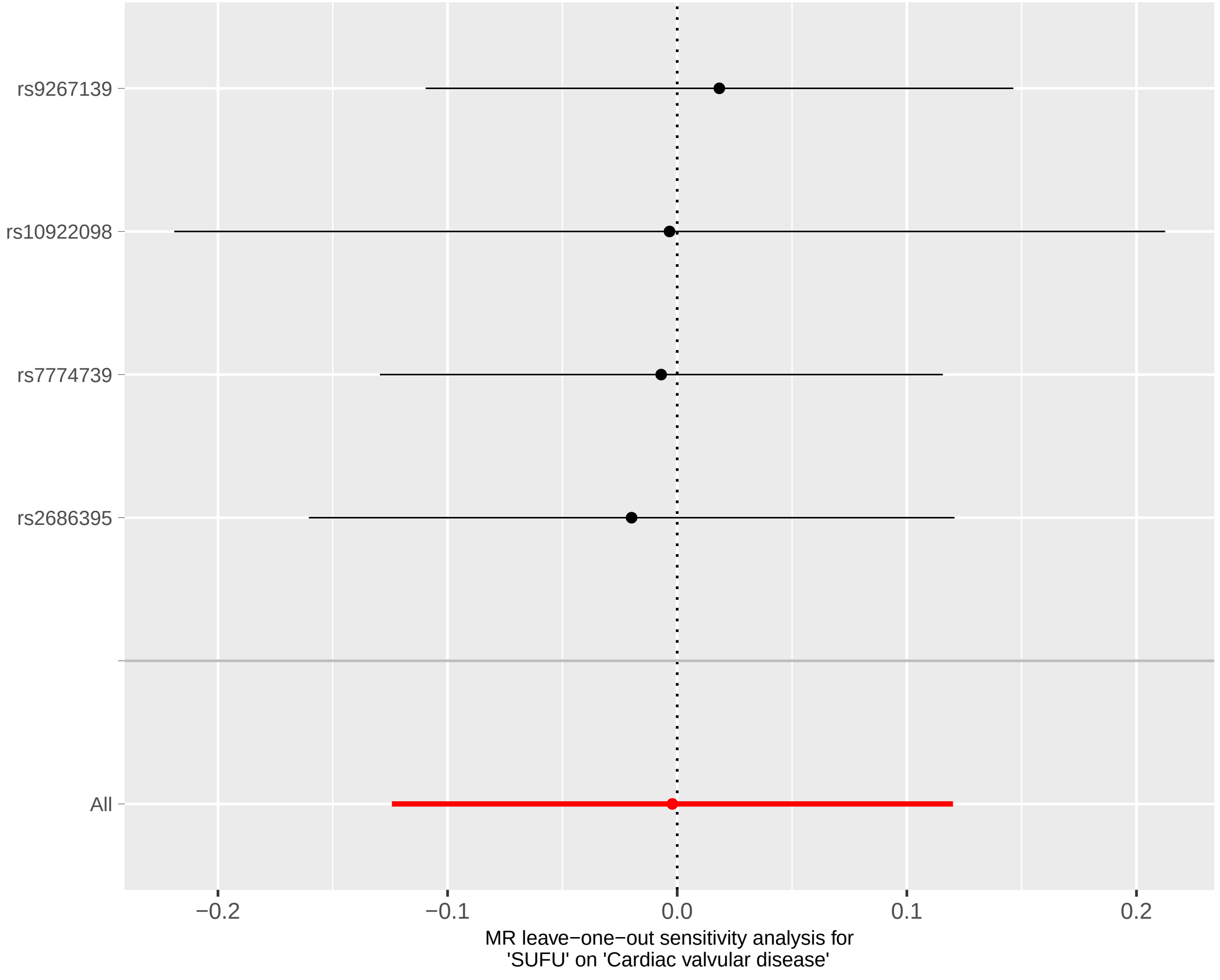
rs9267139
rs10922098
rs7774739
rs2686395
All
−0.2
−0.1
0.0
0.1
0.2
MR leave−one−out sensitivity analysis for
'SUFU' on 'Cardiac valvular disease'

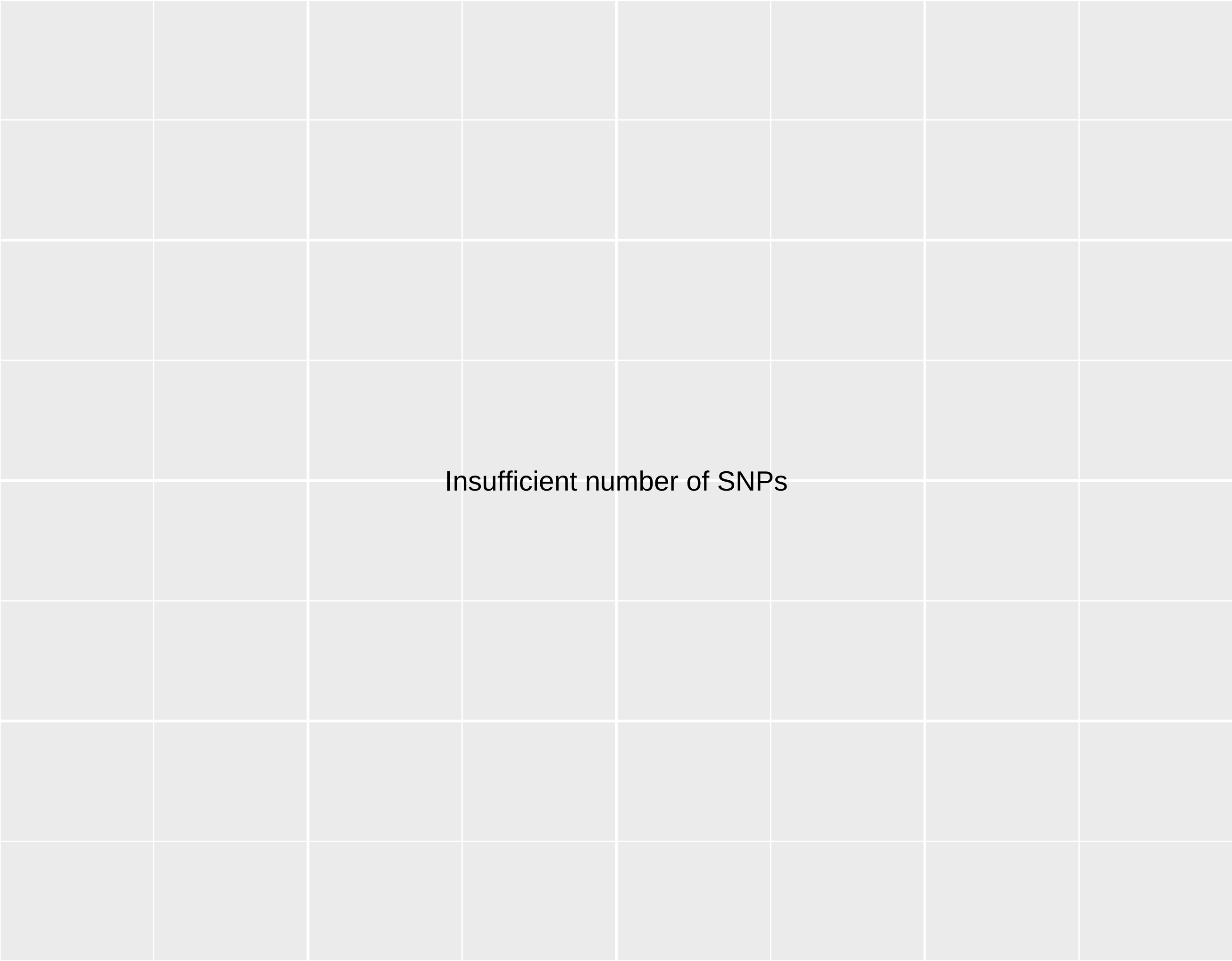
Insufficient number of SNPs

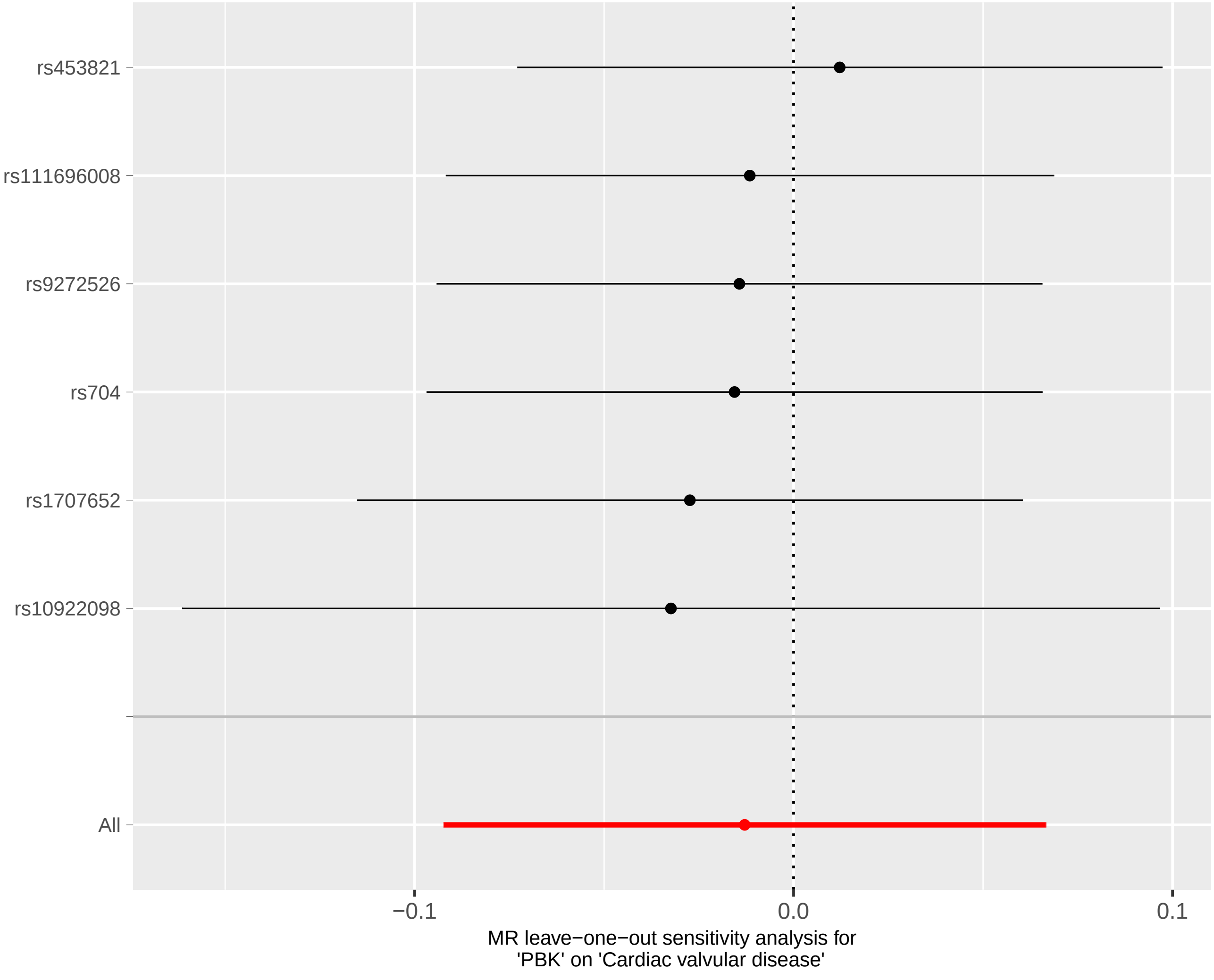
rs453821
rs111696008
rs9272526
rs704
rs1707652
rs10922098
All
−0.1
0.0
0.1
MR leave−one−out sensitivity analysis for
'PBK' on 'Cardiac valvular disease'

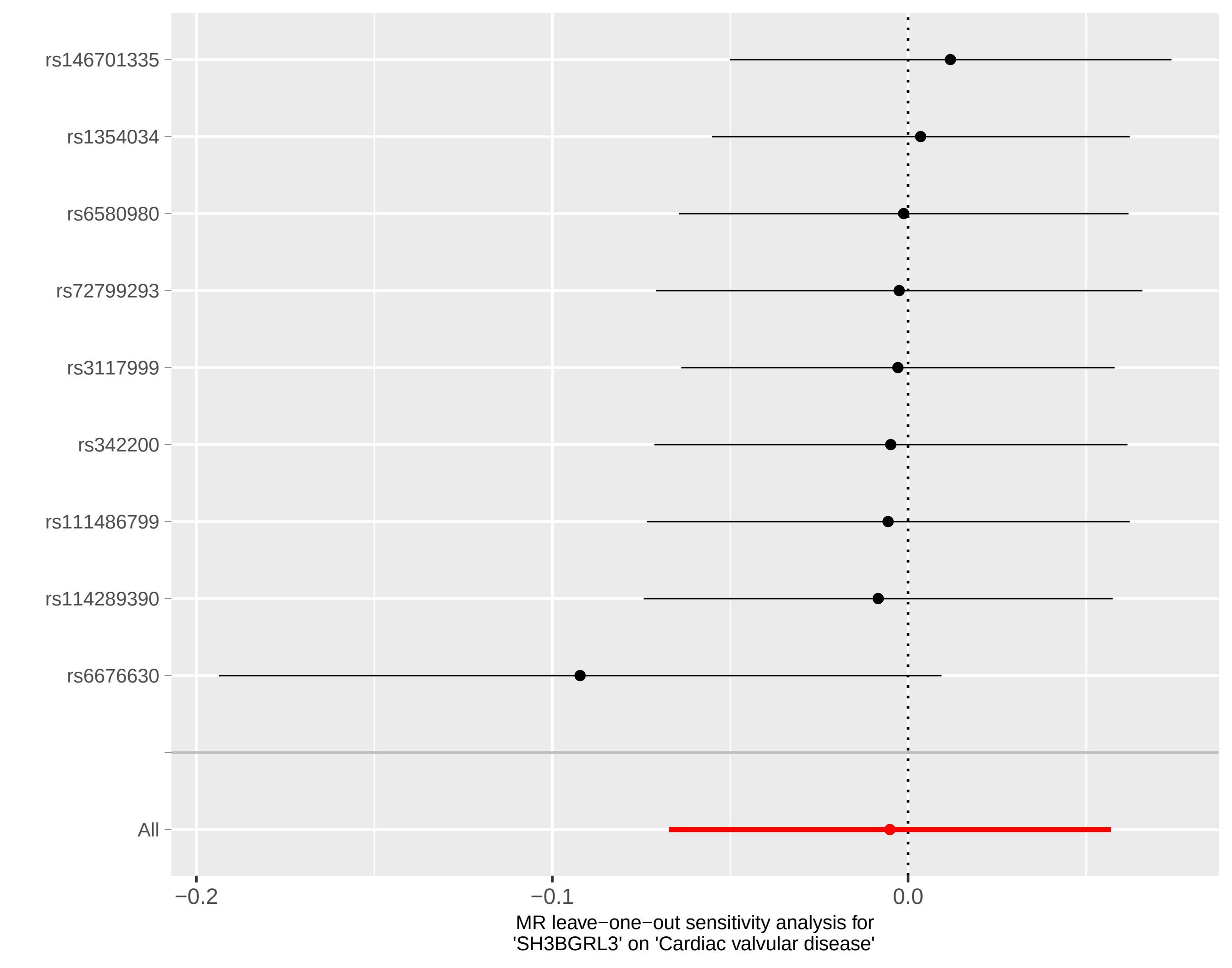
rs146701335
rs1354034
rs6580980
rs72799293
rs3117999
rs342200
rs111486799
rs114289390
rs6676630
All
−0.2
−0.1
0.0
MR leave−one−out sensitivity analysis for
'SH3BGRL3' on 'Cardiac valvular disease'

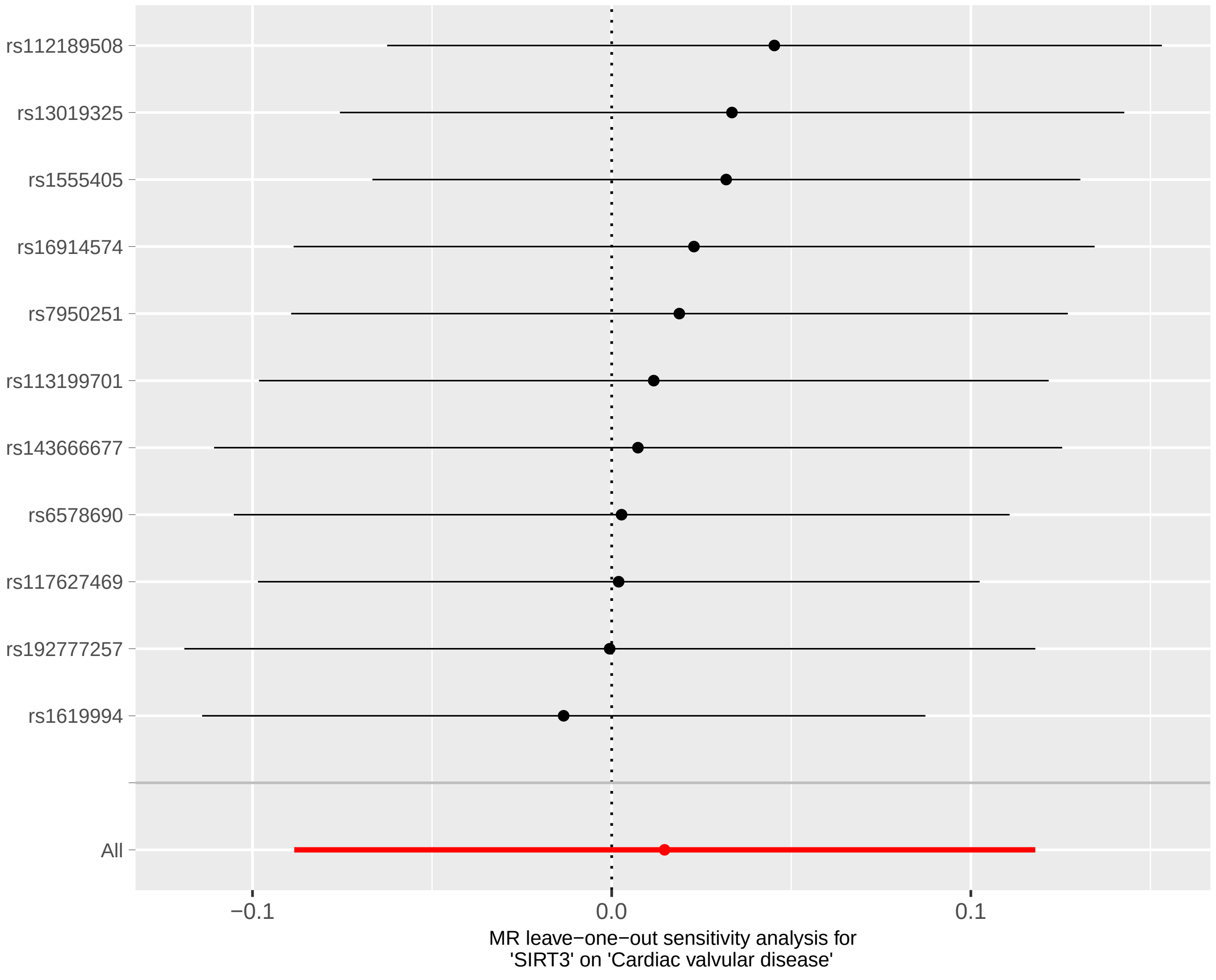
rs112189508
rs13019325
rs1555405
rs16914574
rs7950251
rs113199701
rs143666677
rs6578690
rs117627469
rs192777257
rs1619994
All
−0.1
0.0
0.1
MR leave−one−out sensitivity analysis for
'SIRT3' on 'Cardiac valvular disease'

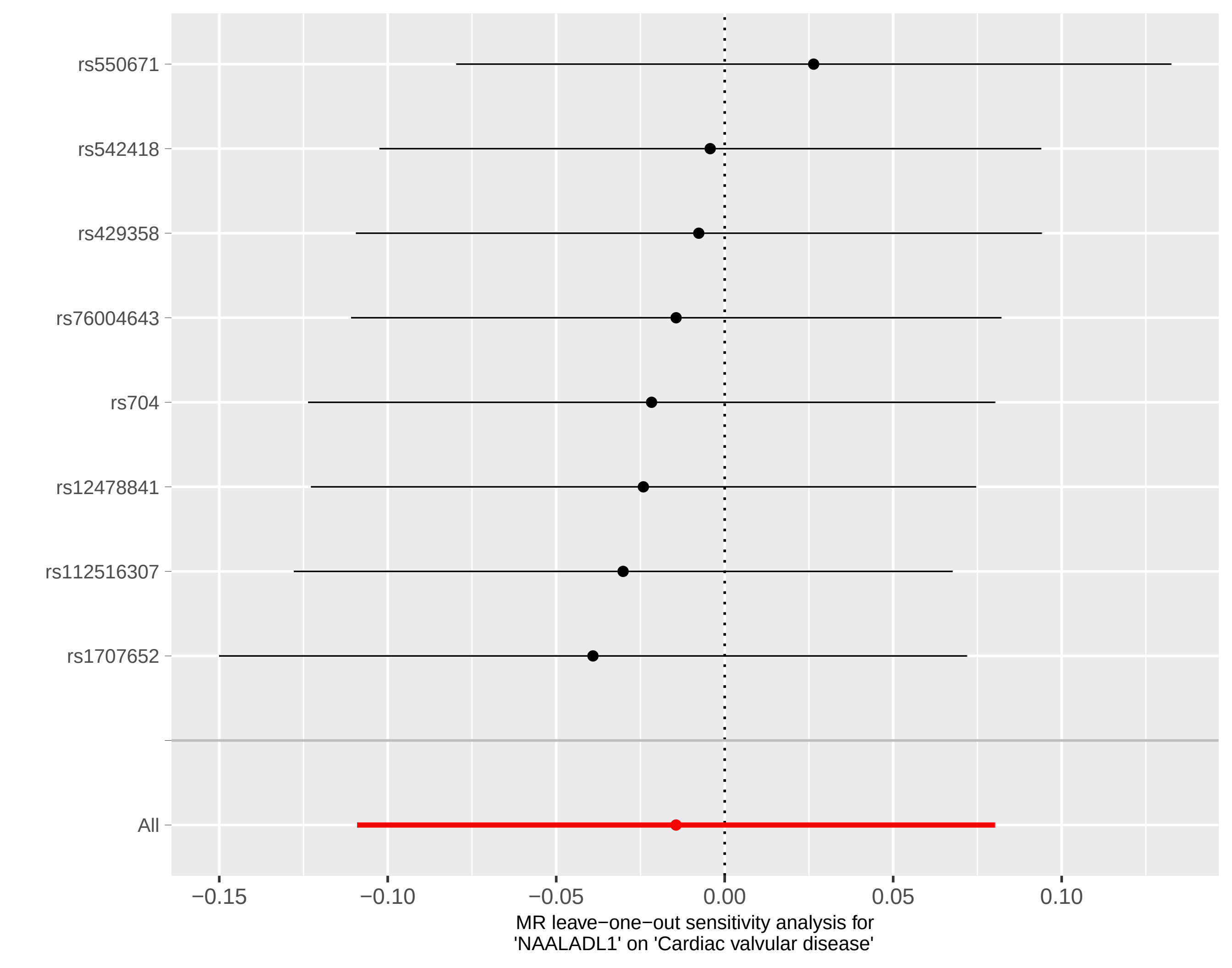

rs550671
rs542418
rs429358
rs76004643
rs704
rs12478841
rs112516307
rs1707652
All
−0.15
−0.10
−0.05
0.00
0.05
0.10
MR leave−one−out sensitivity analysis for
'NAALADL1' on 'Cardiac valvular disease'

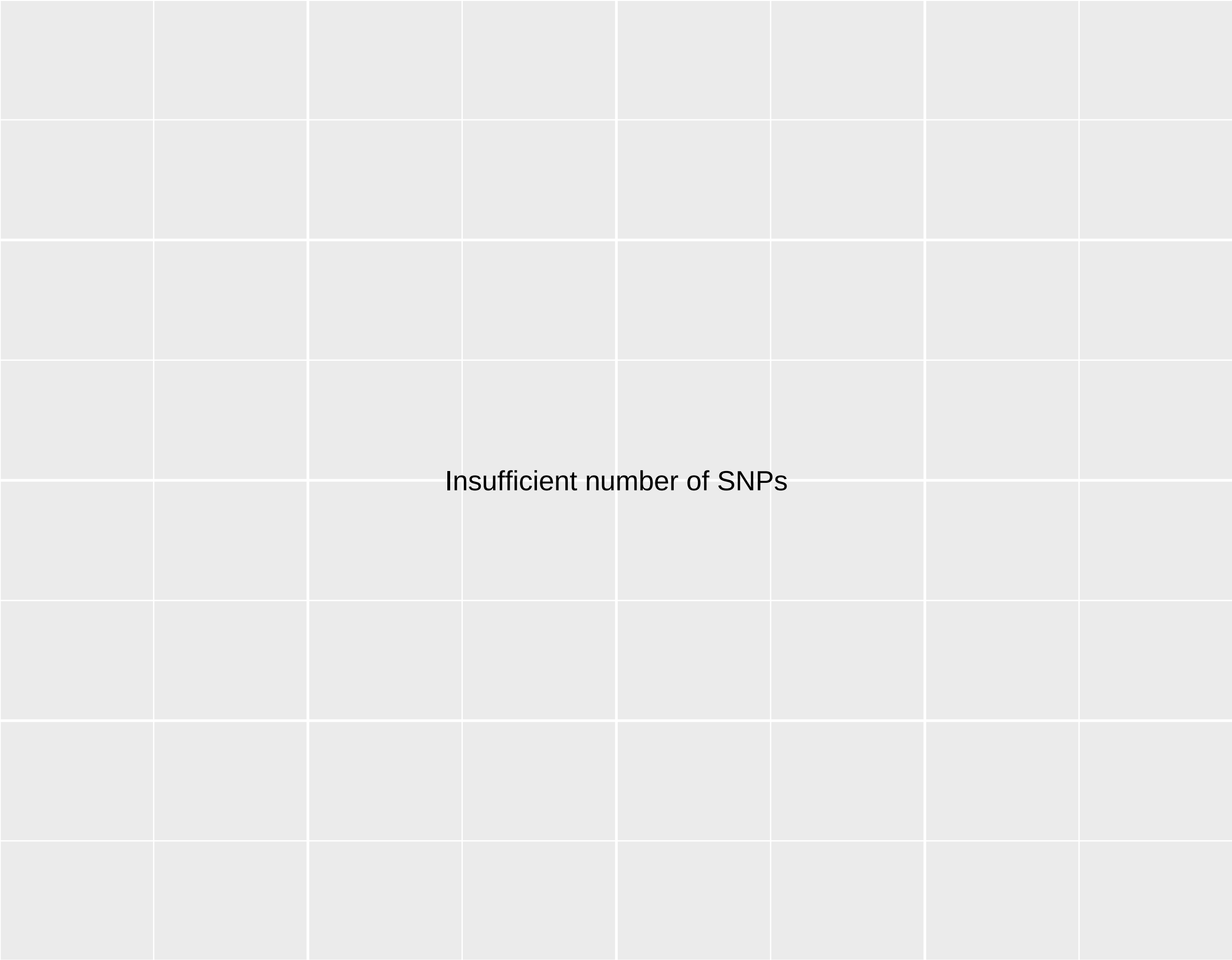
Insufficient number of SNPs

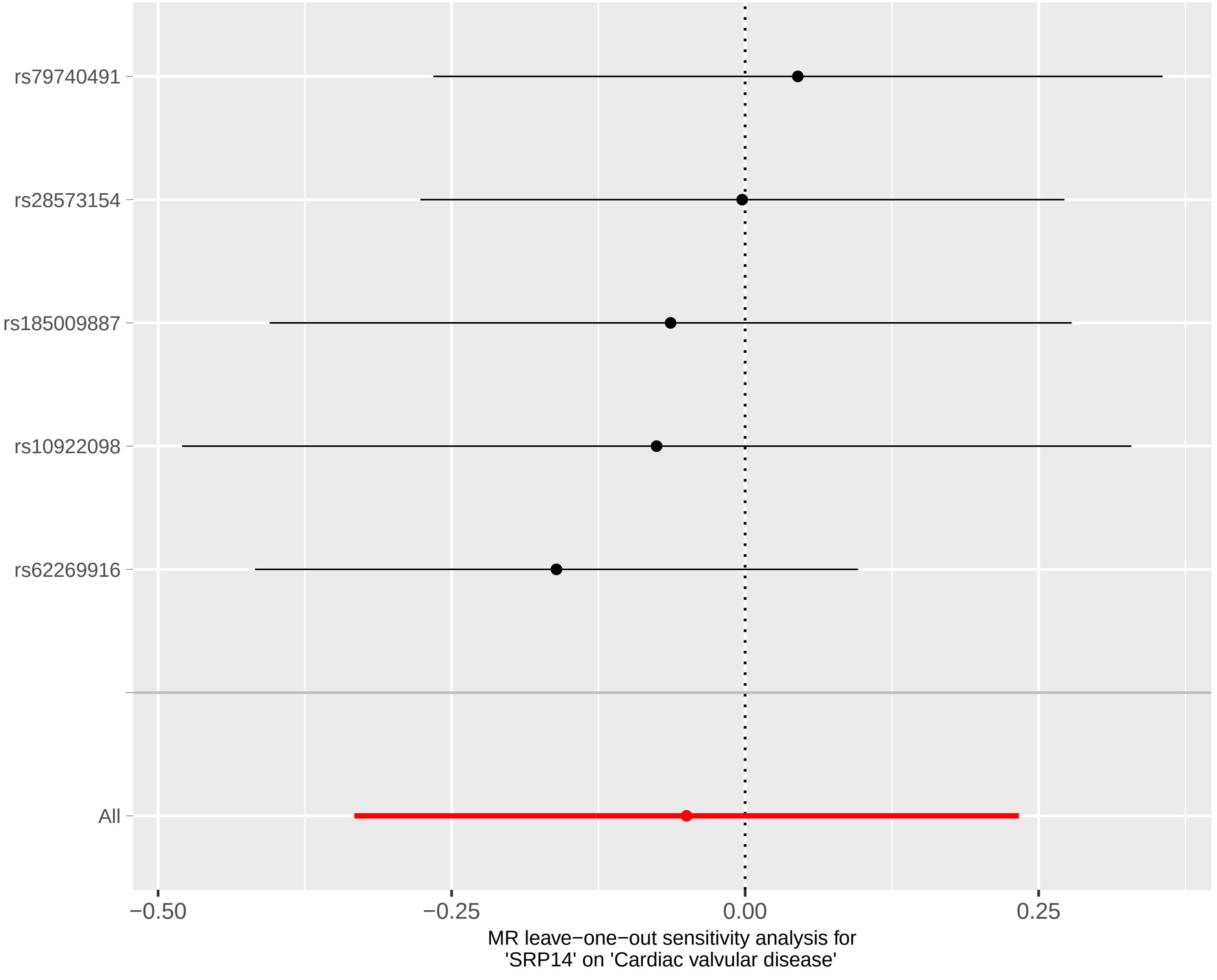

rs79740491
rs28573154
rs185009887
rs10922098
rs62269916
All
−0.50
−0.25
0.00
0.25
MR leave−one−out sensitivity analysis for
'SRP14' on 'Cardiac valvular disease'

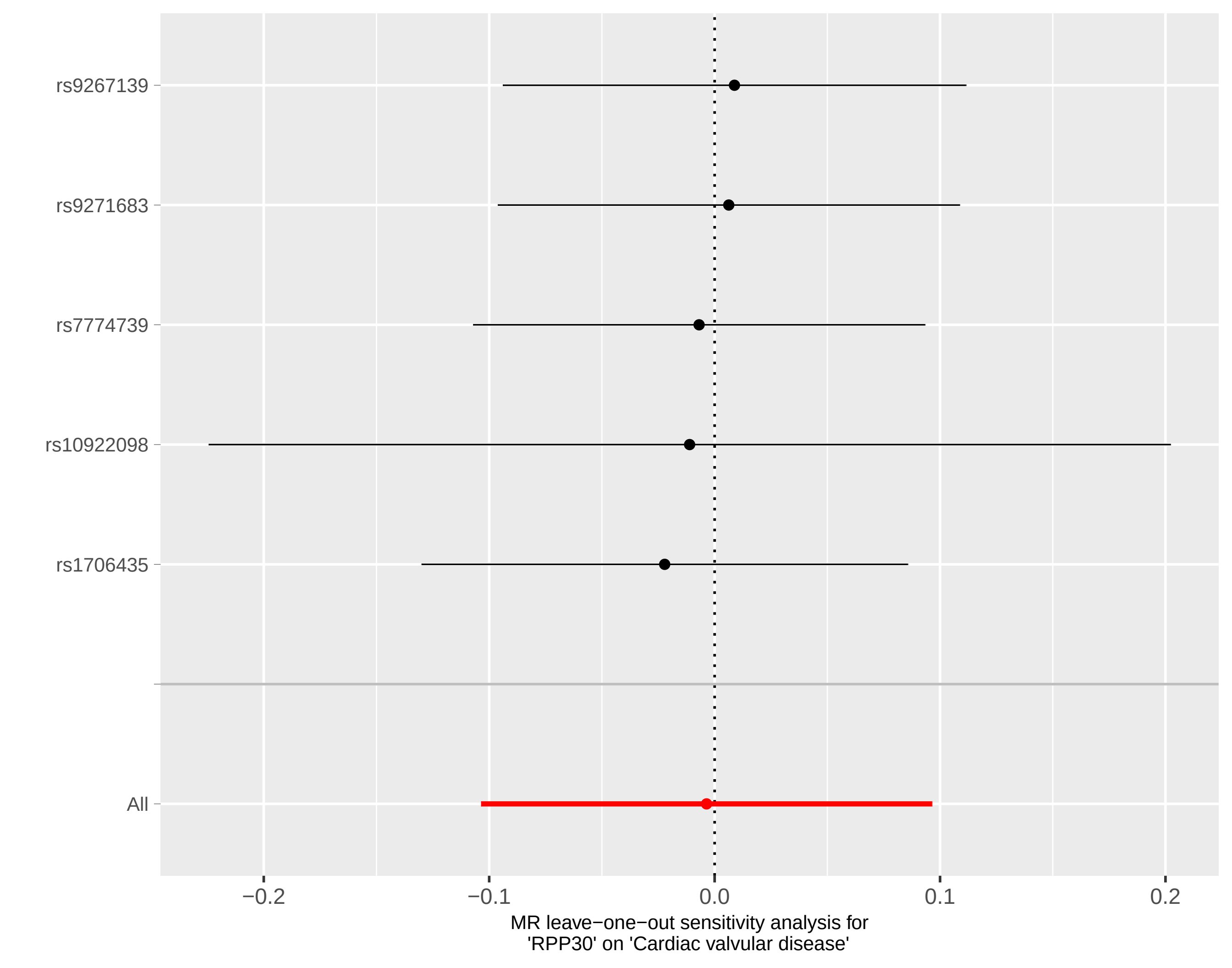
rs9267139
rs9271683
rs7774739
rs10922098
rs1706435
All
−0.2
−0.1
0.0
0.1
0.2
MR leave−one−out sensitivity analysis for
'RPP30' on 'Cardiac valvular disease'

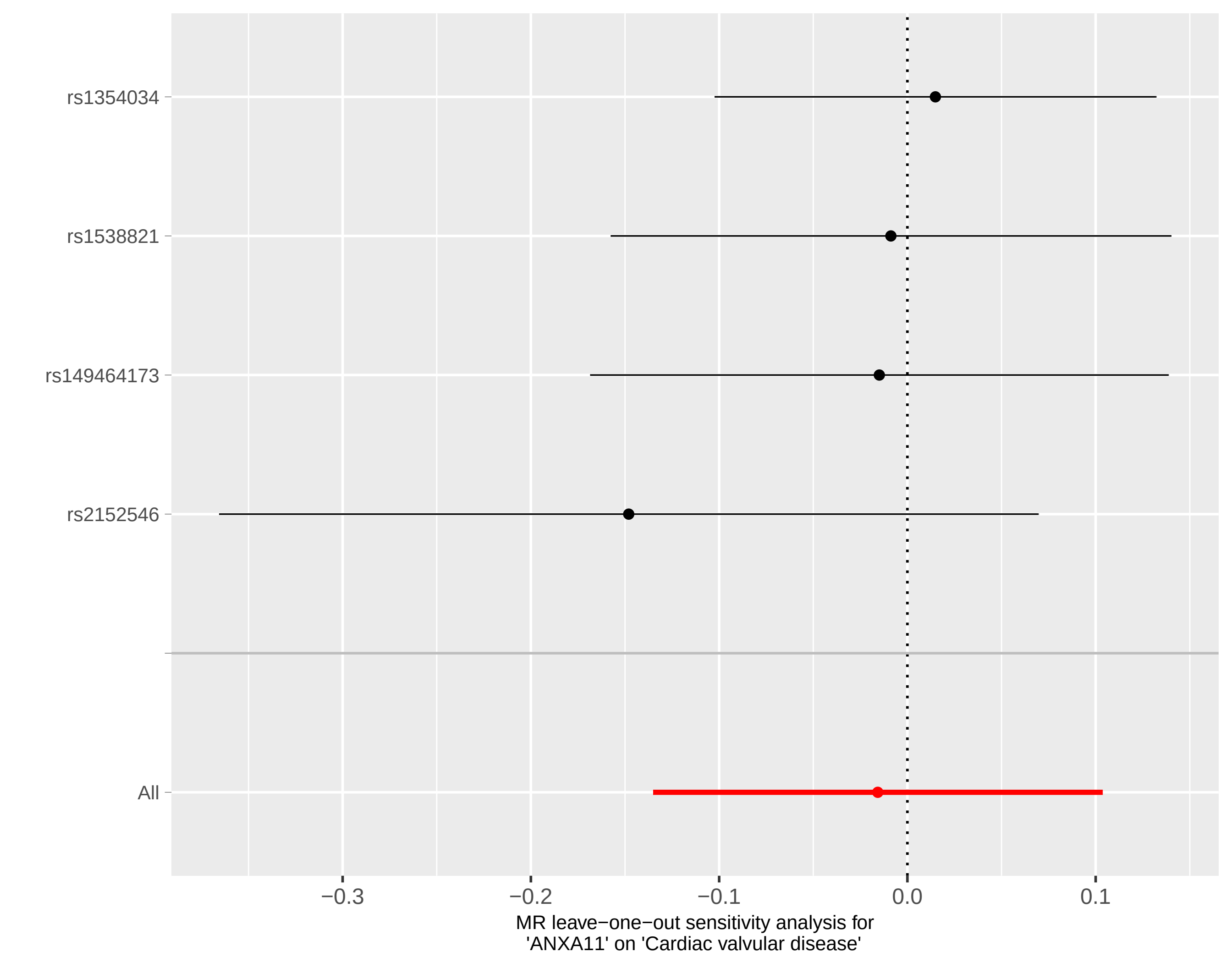
rs1354034
rs1538821
rs149464173
rs2152546
All
−0.3
−0.2
−0.1
0.0
0.1
MR leave−one−out sensitivity analysis for
'ANXA11' on 'Cardiac valvular disease'

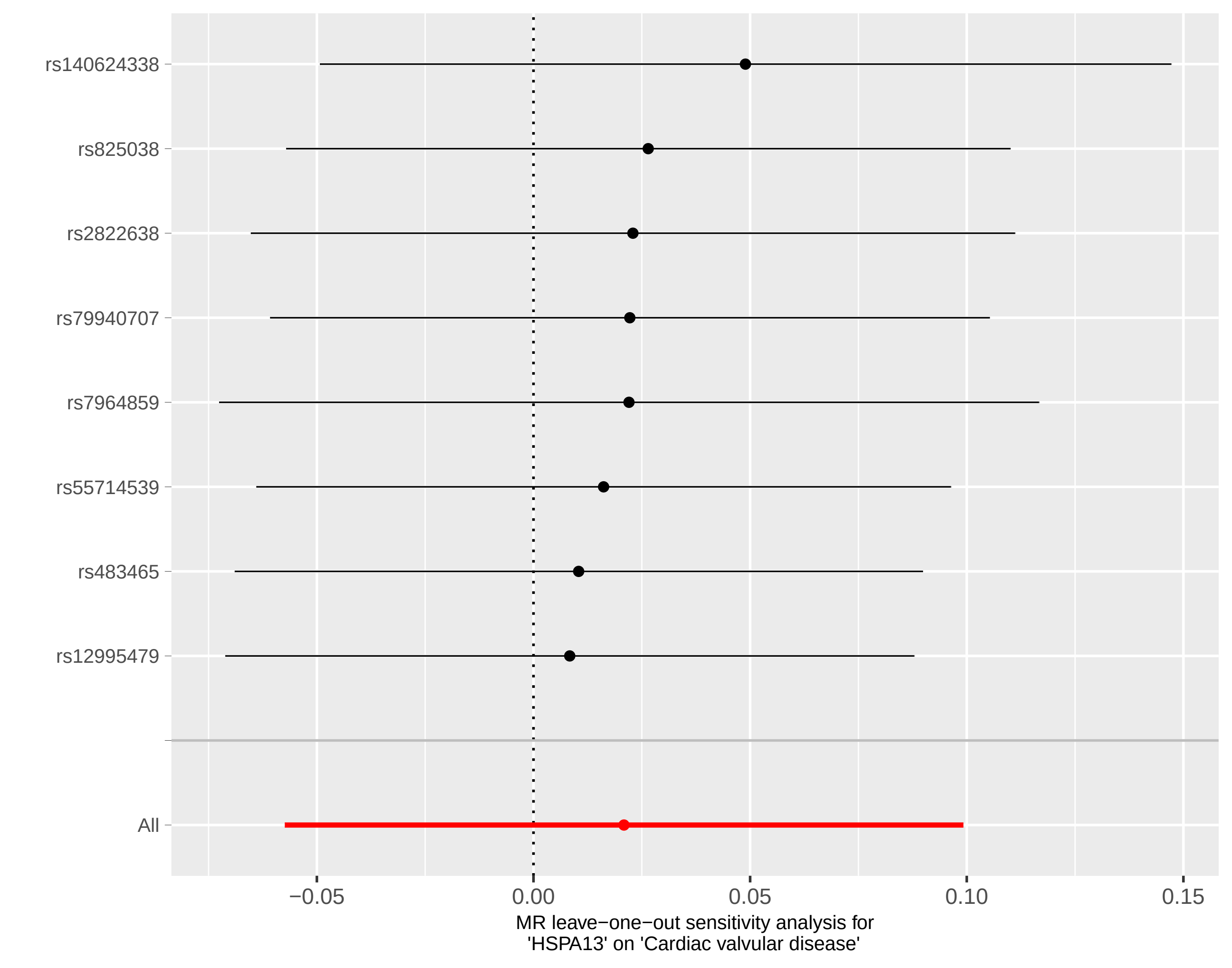
rs140624338
rs825038
rs2822638
rs79940707
rs7964859
rs55714539
rs483465
rs12995479
All
−0.05
0.00
0.05
0.10
0.15
MR leave−one−out sensitivity analysis for
'HSPA13' on 'Cardiac valvular disease'

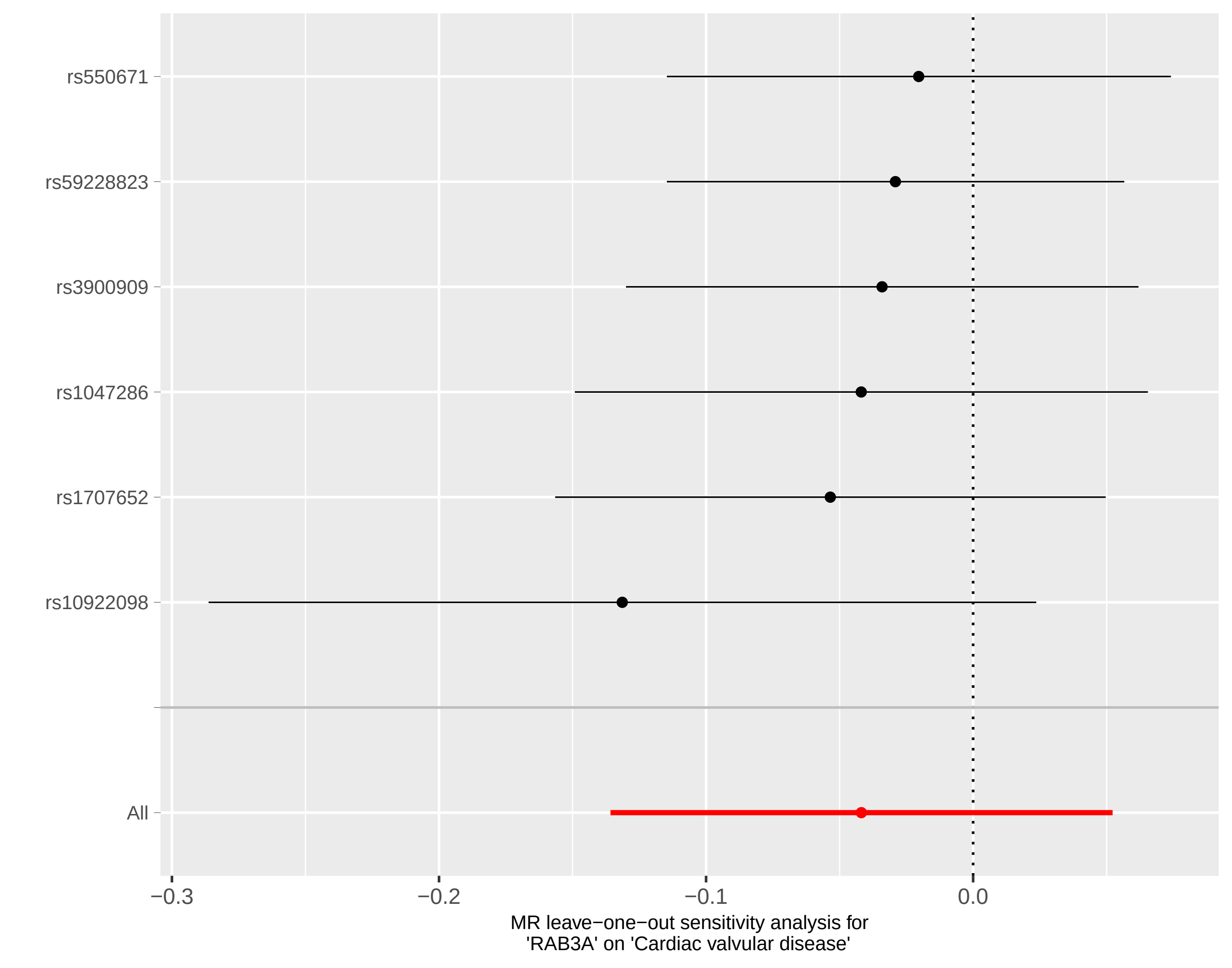
rs550671
rs59228823
rs3900909
rs1047286
rs1707652
rs10922098
All
−0.3
−0.2
−0.1
0.0
MR leave−one−out sensitivity analysis for
'RAB3A' on 'Cardiac valvular disease'

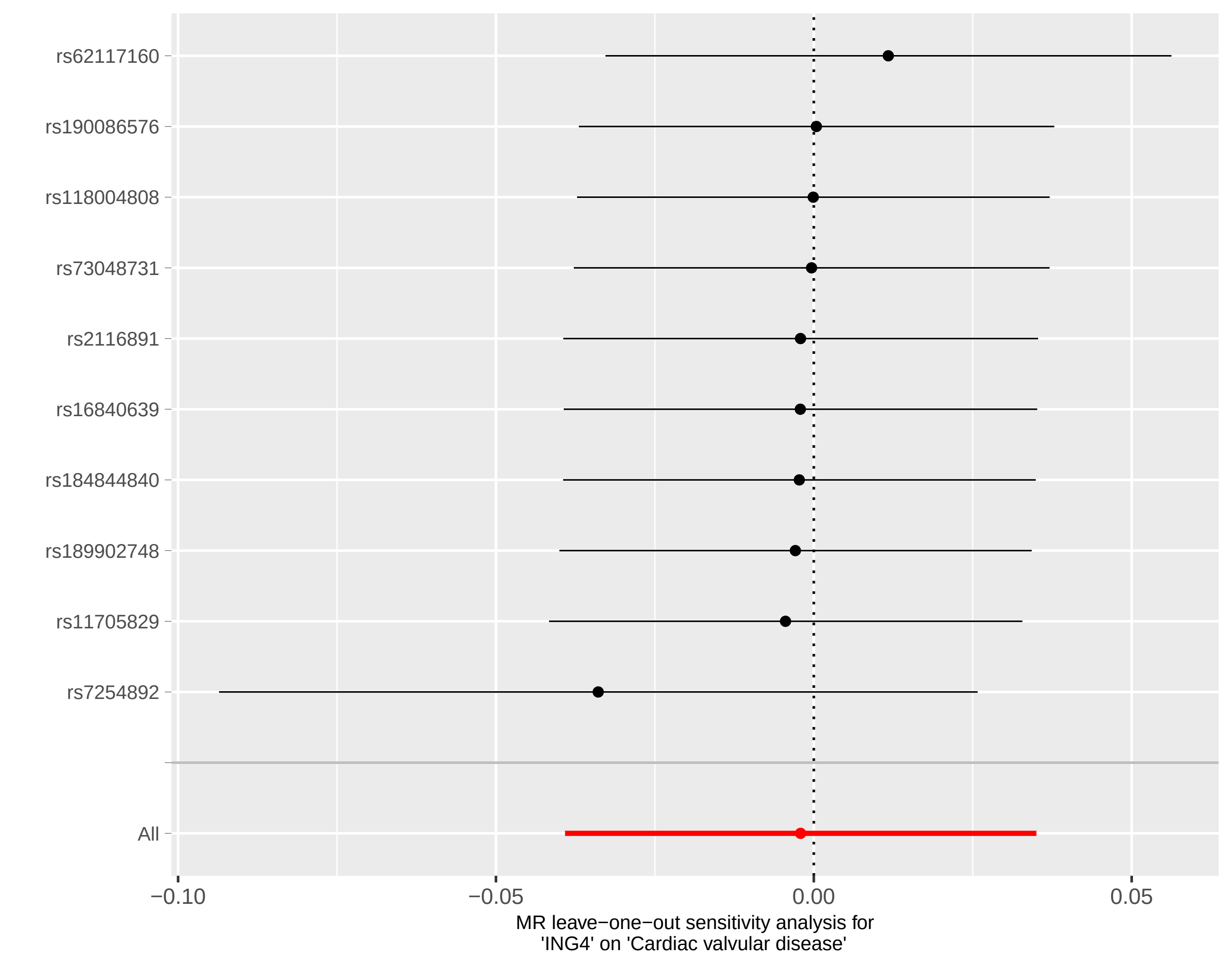
rs62117160
rs190086576
rs118004808
rs73048731
rs2116891
rs16840639
rs184844840
rs189902748
rs11705829
rs7254892
All
−0.10
−0.05
0.00
0.05
MR leave−one−out sensitivity analysis for
'ING4' on 'Cardiac valvular disease'

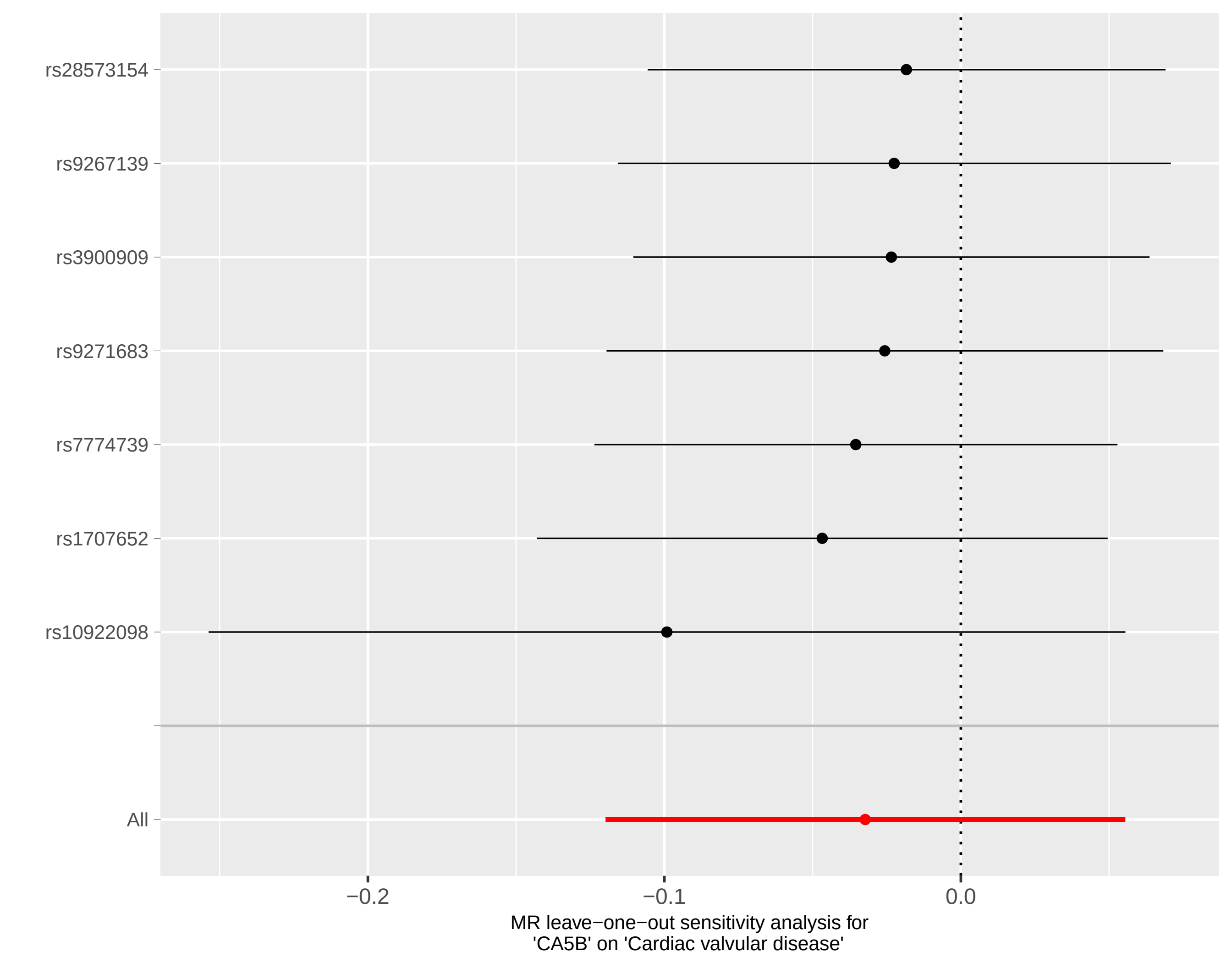
rs28573154
rs9267139
rs3900909
rs9271683
rs7774739
rs1707652
rs10922098
All
−0.2
−0.1
0.0
MR leave−one−out sensitivity analysis for
'CA5B' on 'Cardiac valvular disease'

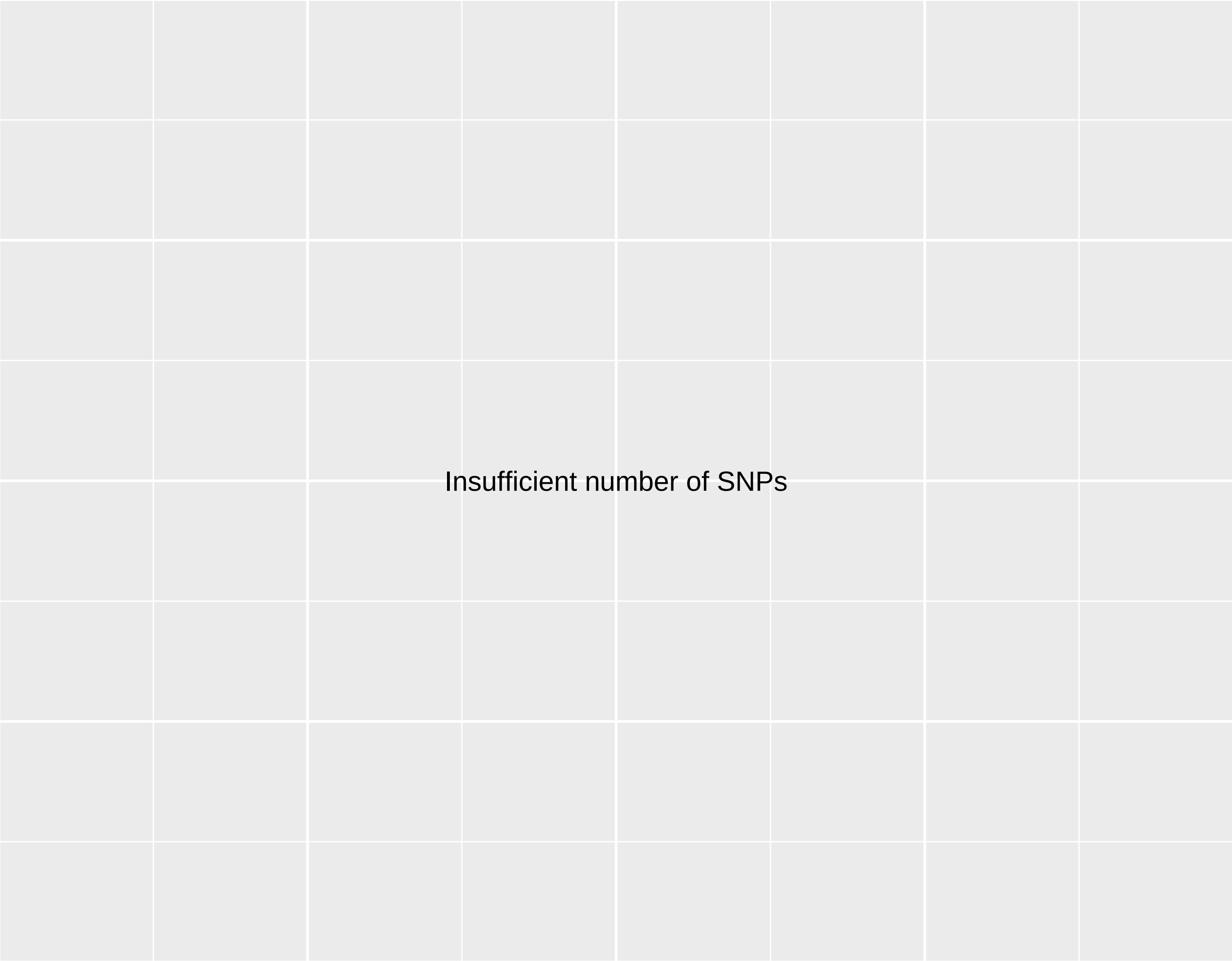
Insufficient number of SNPs

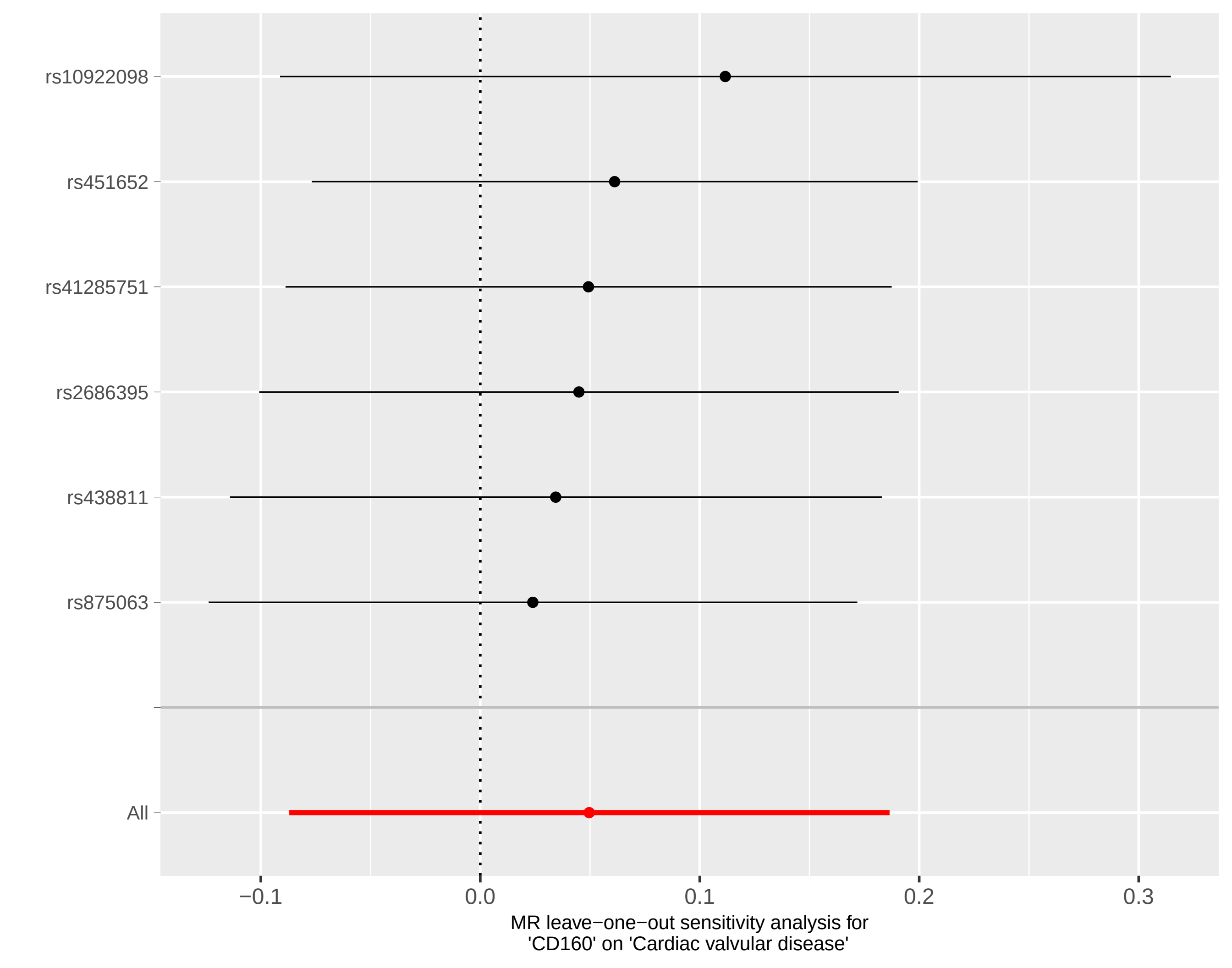
rs10922098
rs451652
rs41285751
rs2686395
rs438811
rs875063
All
−0.1
0.0
0.1
0.2
0.3
MR leave−one−out sensitivity analysis for
'CD160' on 'Cardiac valvular disease'

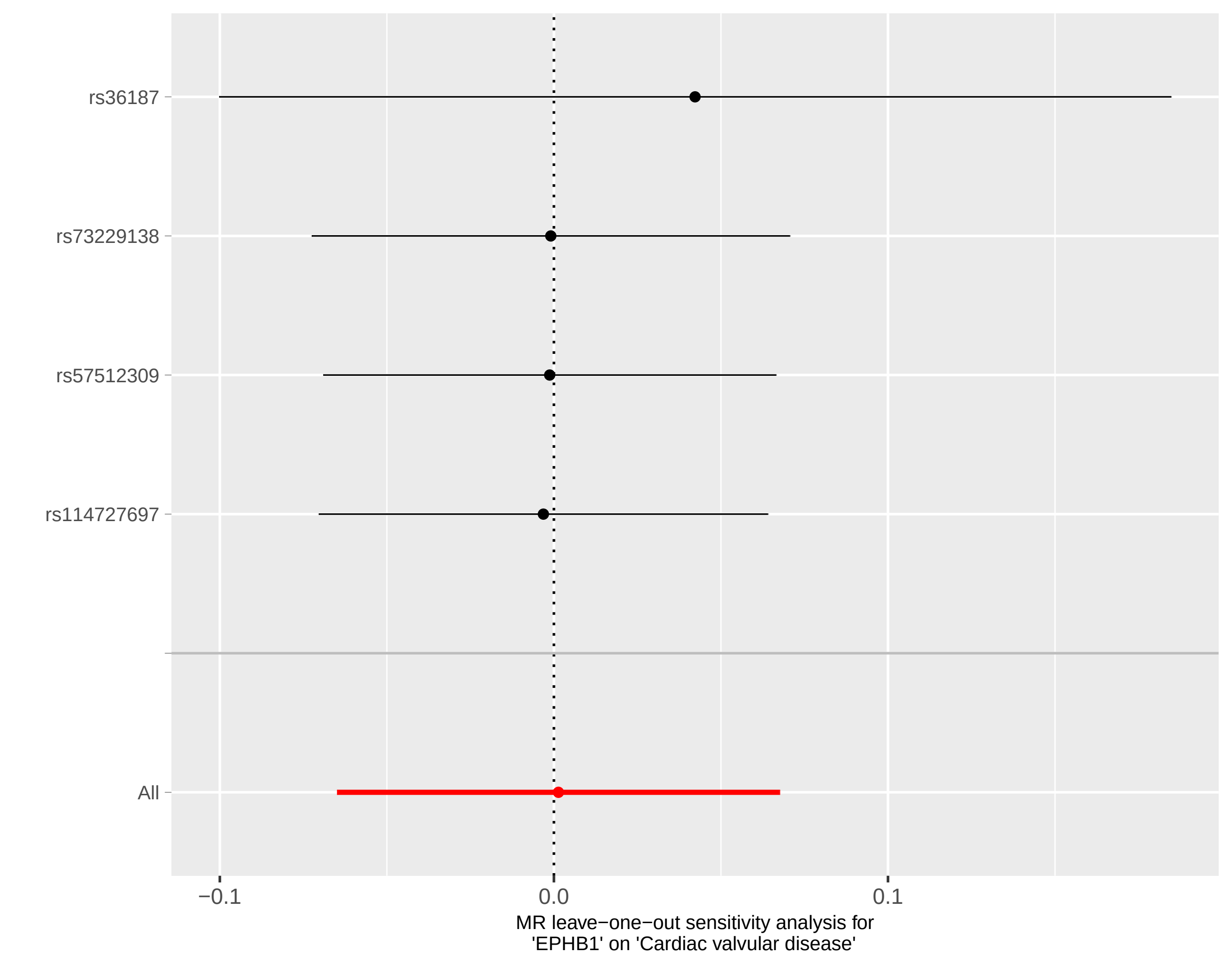

rs36187
rs73229138
rs57512309
rs114727697
All
−0.1
0.0
0.1
MR leave−one−out sensitivity analysis for
'EPHB1' on 'Cardiac valvular disease'

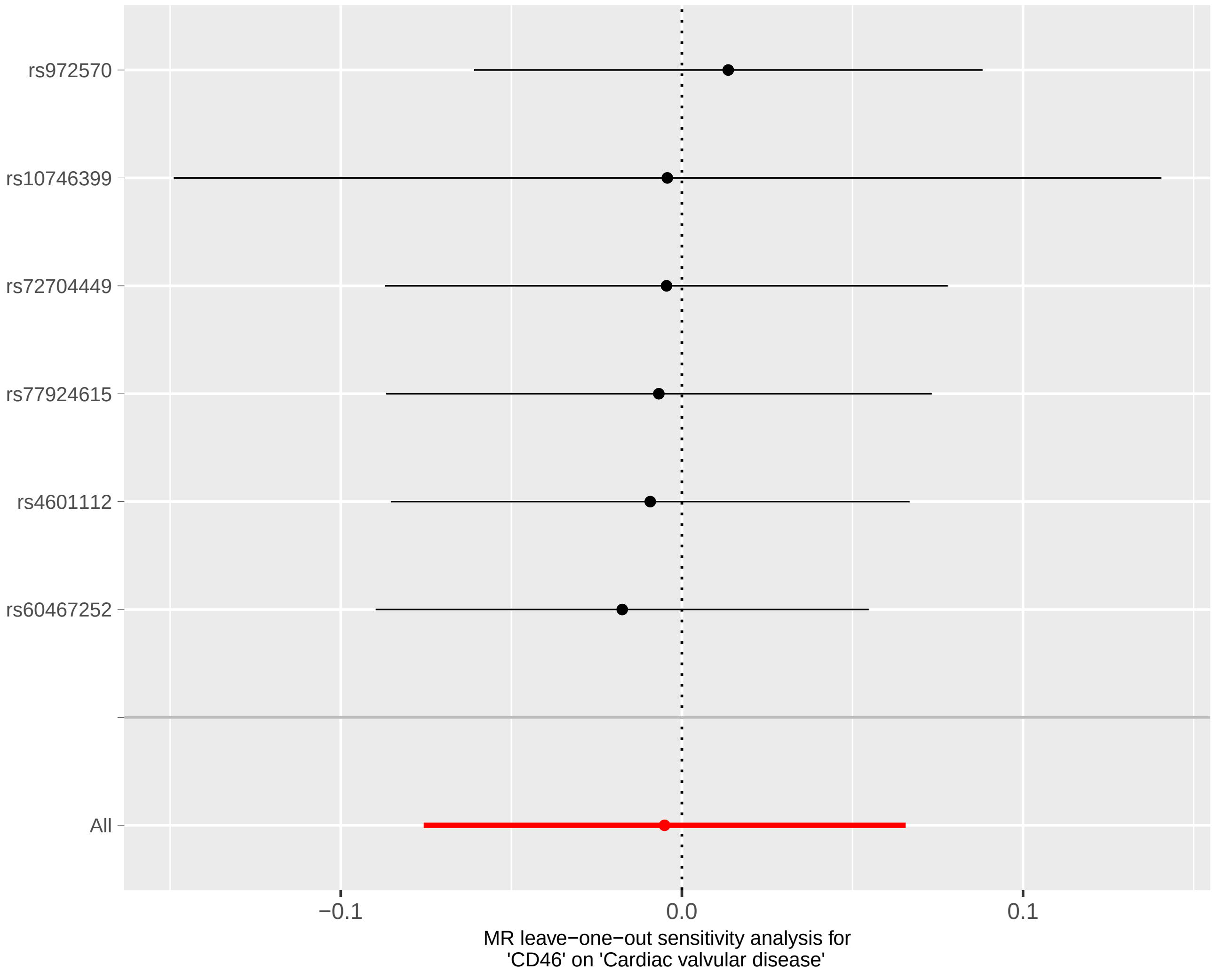
rs972570
rs10746399
rs72704449
rs77924615
rs4601112
rs60467252
All
−0.1
0.0
0.1
MR leave−one−out sensitivity analysis for
'CD46' on 'Cardiac valvular disease'

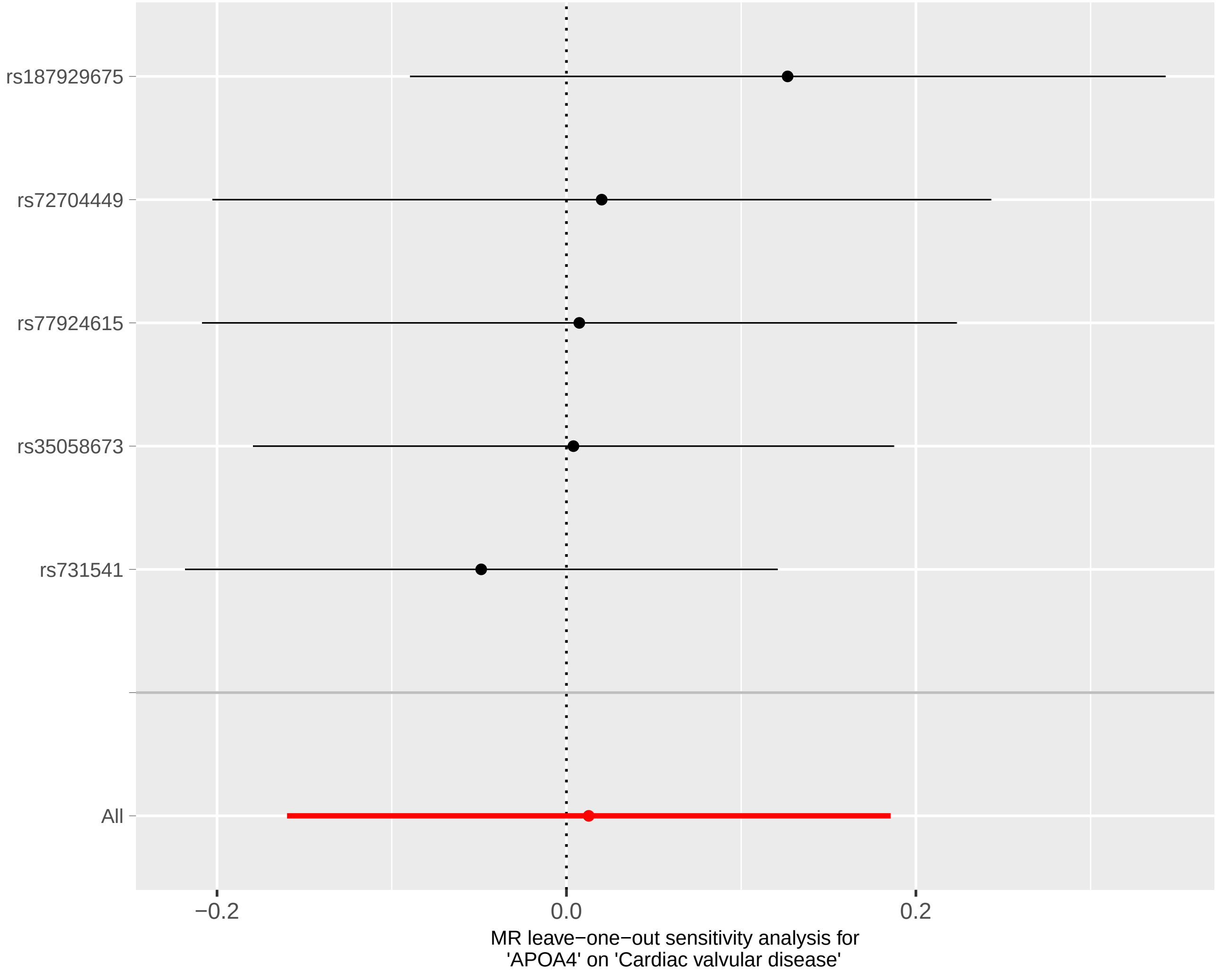
rs187929675
rs72704449
rs77924615
rs35058673
rs731541
All
−0.2
0.0
0.2
MR leave−one−out sensitivity analysis for
'APOA4' on 'Cardiac valvular disease'

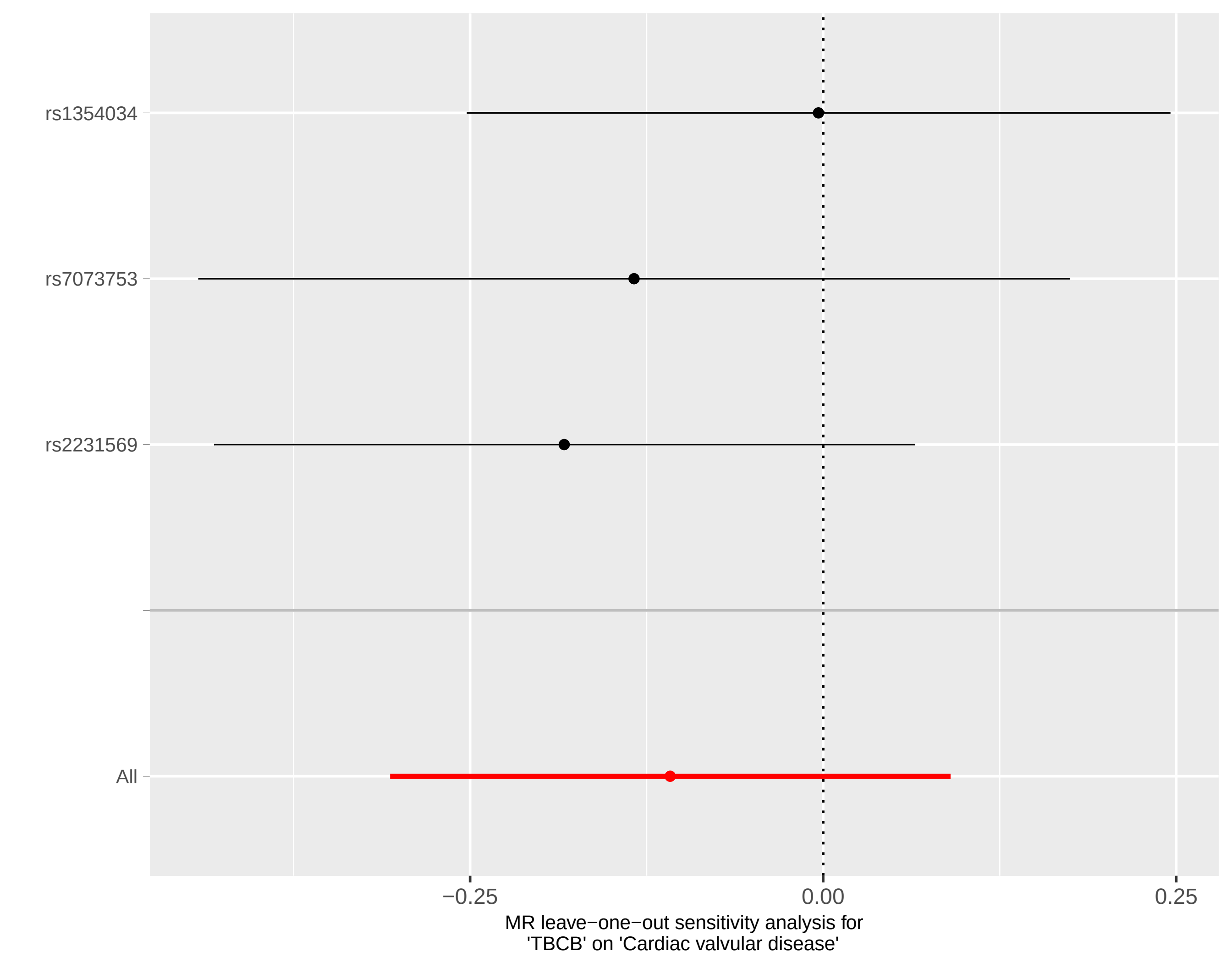
rs1354034
rs7073753
rs2231569
All
−0.25
0.00
0.25
MR leave−one−out sensitivity analysis for
'TBCB' on 'Cardiac valvular disease'

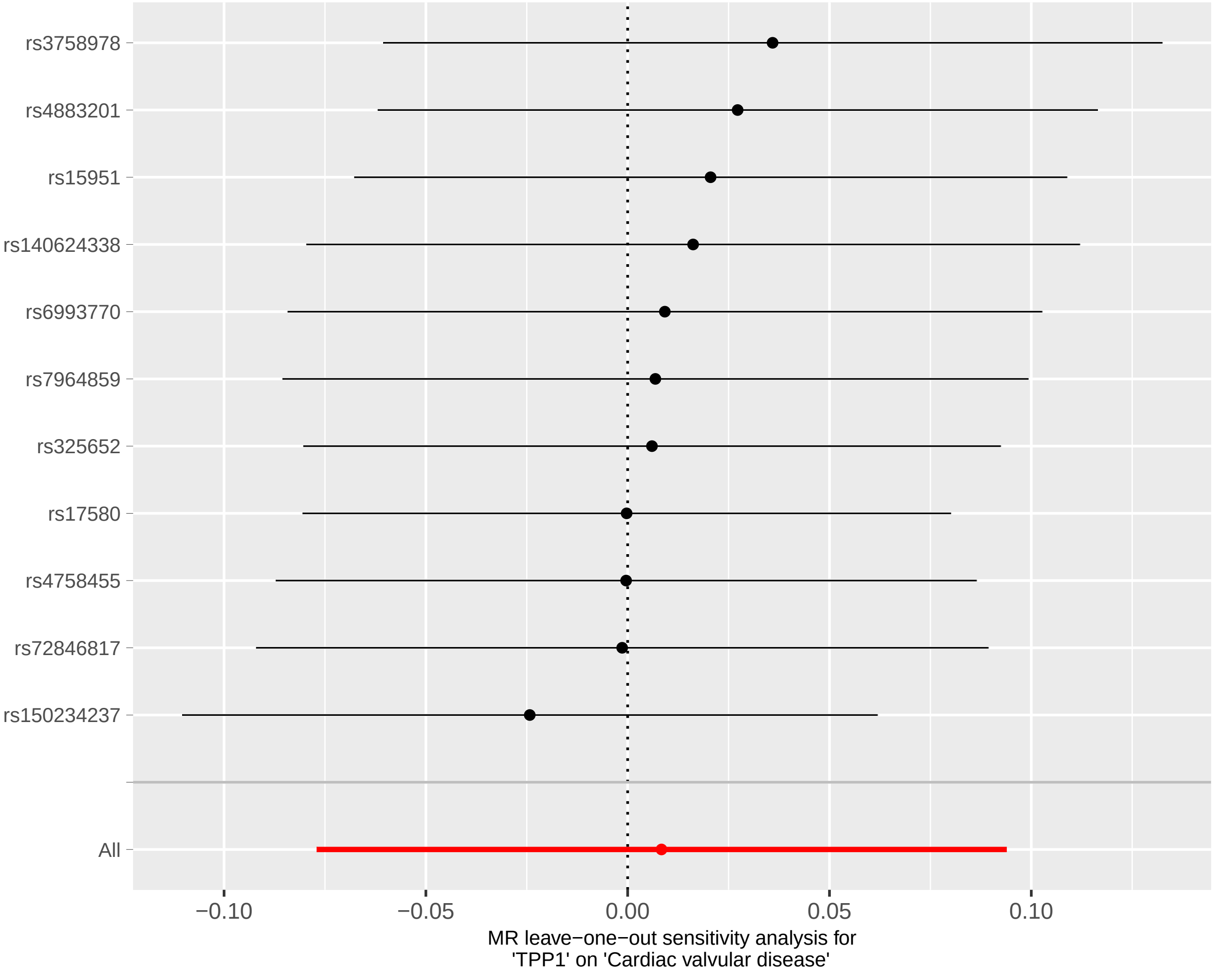

rs3758978
rs4883201
rs15951
rs140624338
rs6993770
rs7964859
rs325652
rs17580
rs4758455
rs72846817
rs150234237
All
−0.10
−0.05
0.00
0.05
0.10
MR leave−one−out sensitivity analysis for
'TPP1' on 'Cardiac valvular disease'

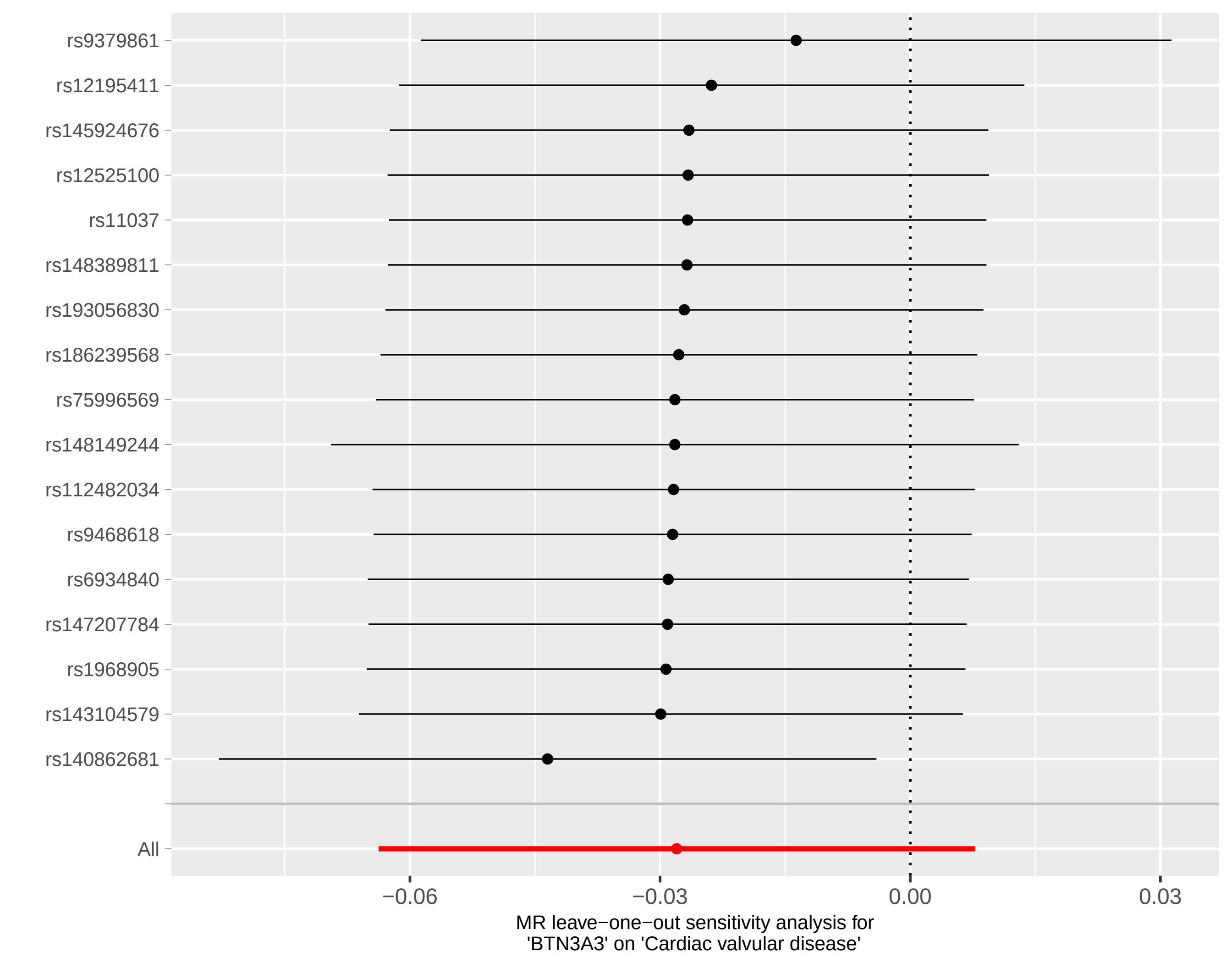

rs9379861
rs12195411
rs145924676
rs12525100
rs11037
rs148389811
rs193056830
rs186239568
rs75996569
rs148149244
rs112482034
rs9468618
rs6934840
rs147207784
rs1968905
rs143104579
rs140862681
All
−0.06
−0.03
0.00
0.03
MR leave−one−out sensitivity analysis for
'BTN3A3' on 'Cardiac valvular disease'

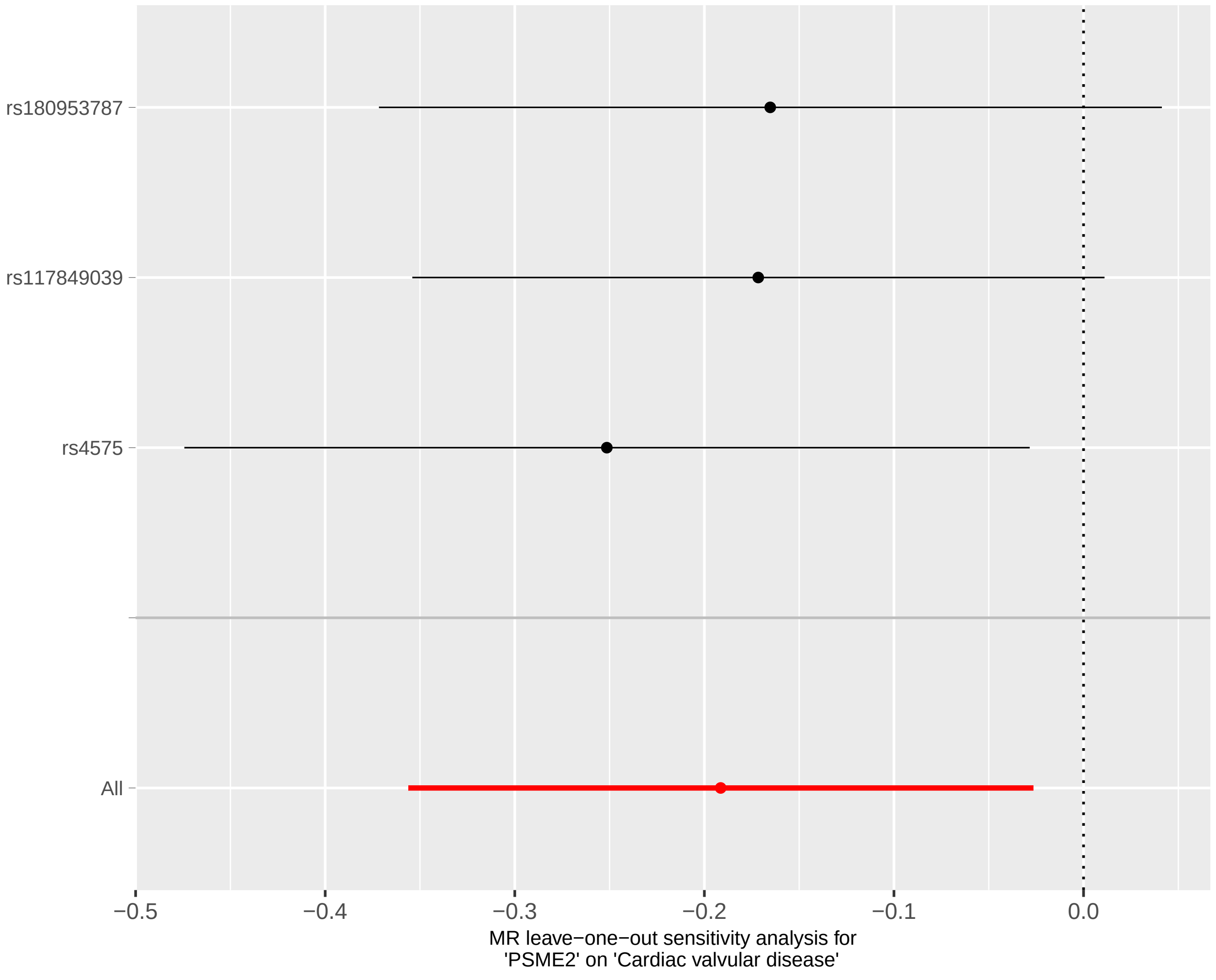
rs180953787
rs117849039
rs4575
All
−0.5
−0.4
−0.3
−0.2
−0.1
0.0
MR leave−one−out sensitivity analysis for
'PSME2' on 'Cardiac valvular disease'

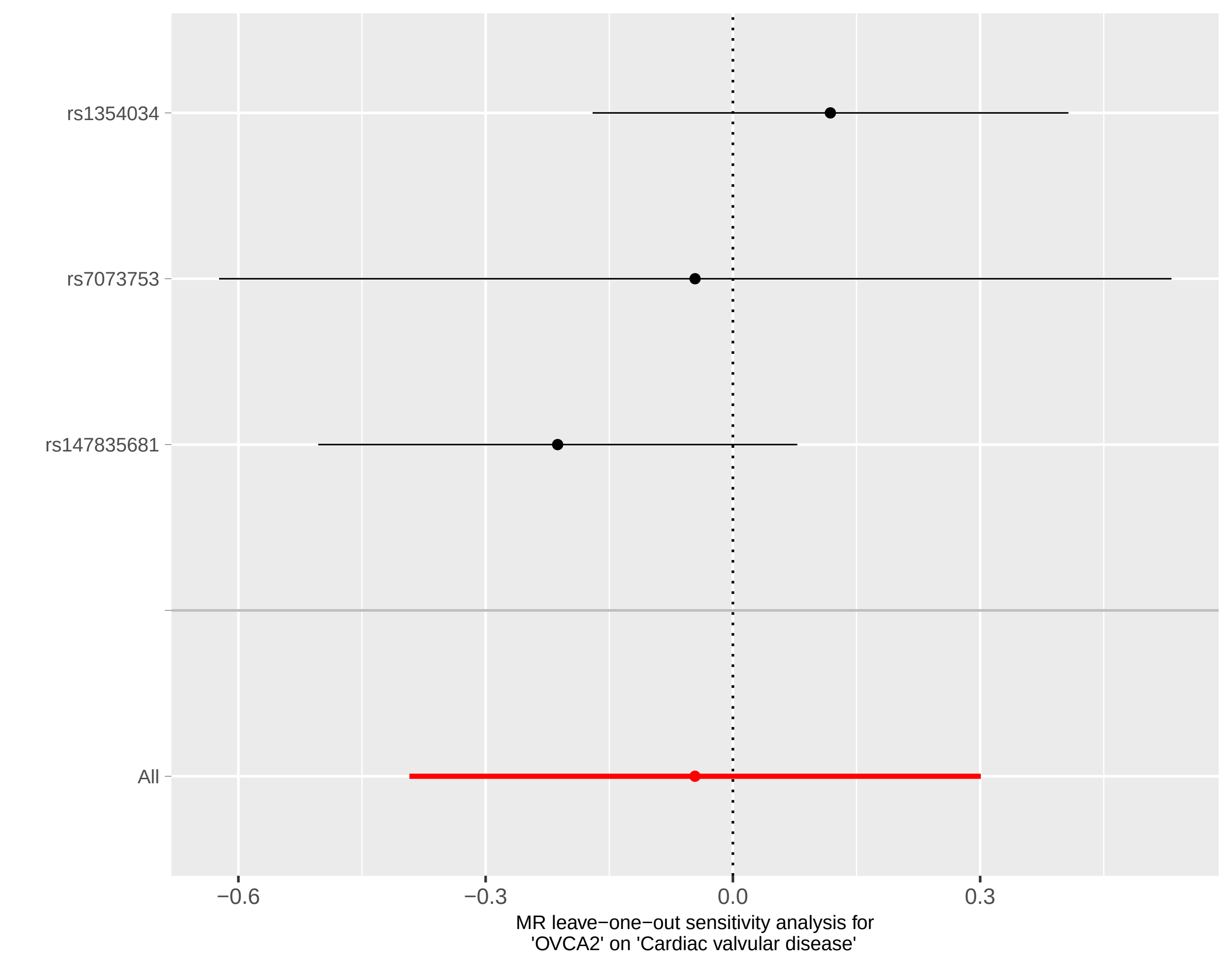
rs1354034
rs7073753
rs147835681
All
−0.6
−0.3
0.0
0.3
MR leave−one−out sensitivity analysis for
'OVCA2' on 'Cardiac valvular disease'

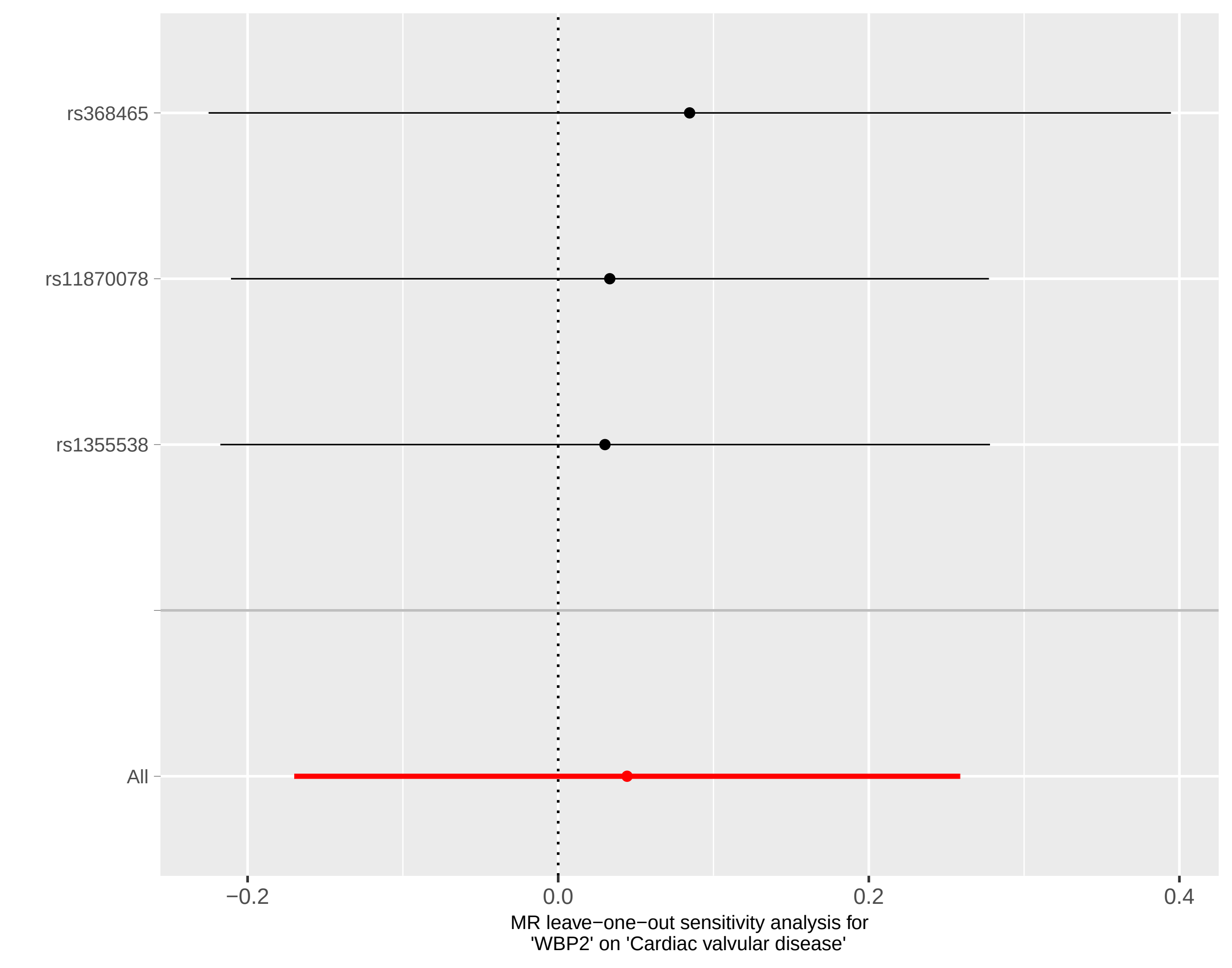
rs368465
rs11870078
rs1355538
All
−0.2
0.0
0.2
0.4
MR leave−one−out sensitivity analysis for
'WBP2' on 'Cardiac valvular disease'

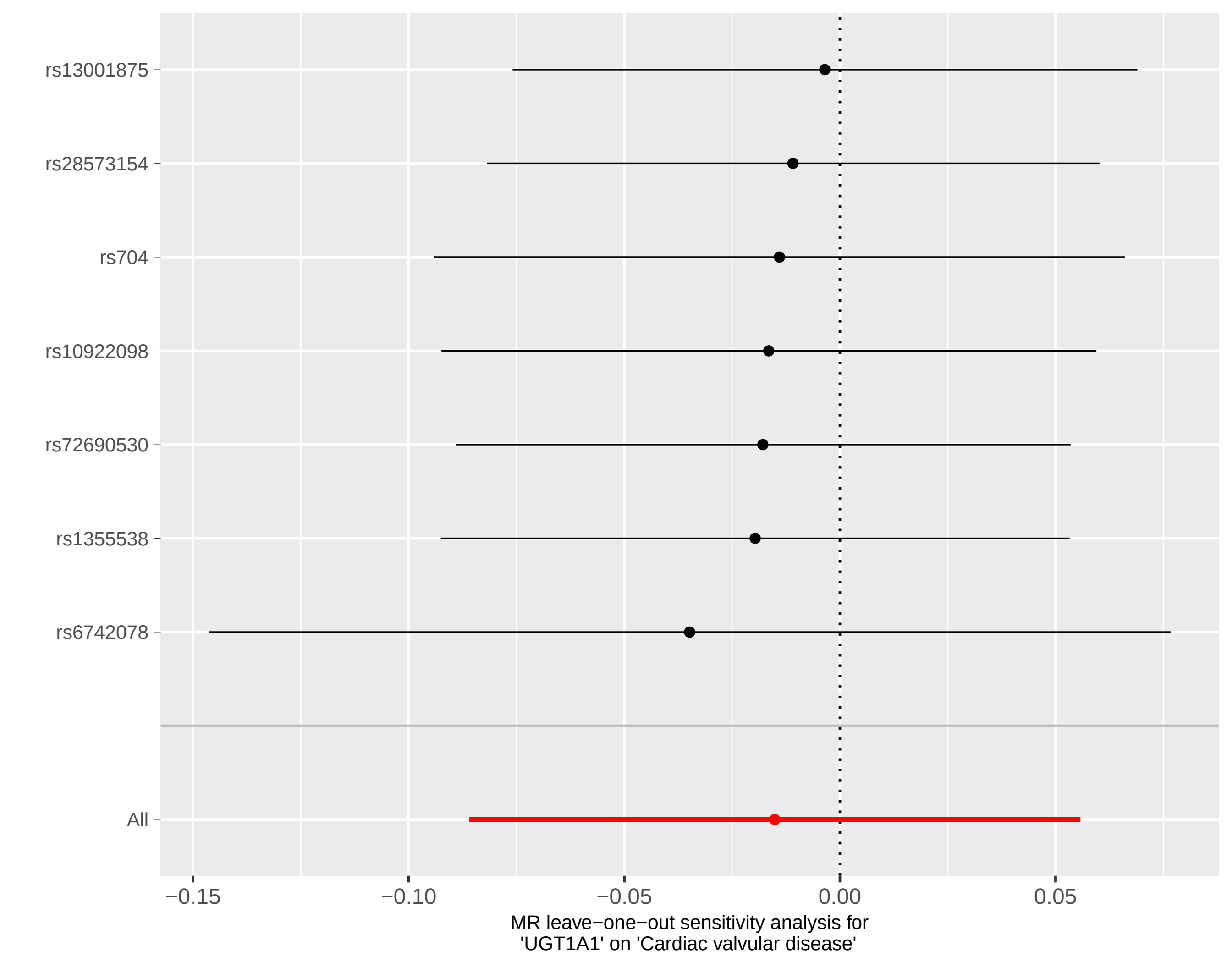
rs13001875
rs28573154
rs704
rs10922098
rs72690530
rs1355538
rs6742078
All
−0.15
−0.10
−0.05
0.00
0.05
MR leave−one−out sensitivity analysis for
'UGT1A1' on 'Cardiac valvular disease'

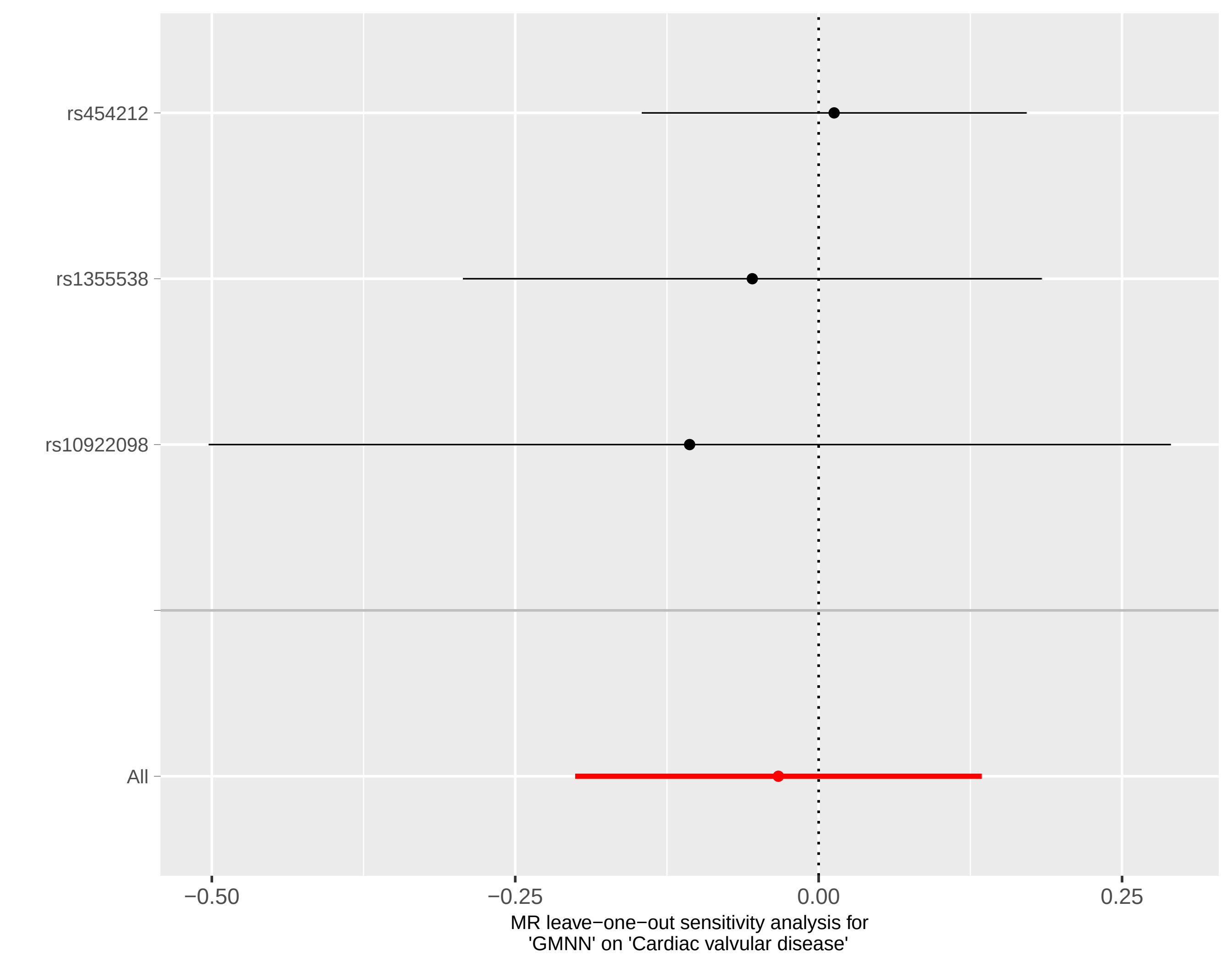
rs454212
rs1355538
rs10922098
All
−0.50
−0.25
0.00
0.25
MR leave−one−out sensitivity analysis for
'GMNN' on 'Cardiac valvular disease'

Insufficient number of SNPs

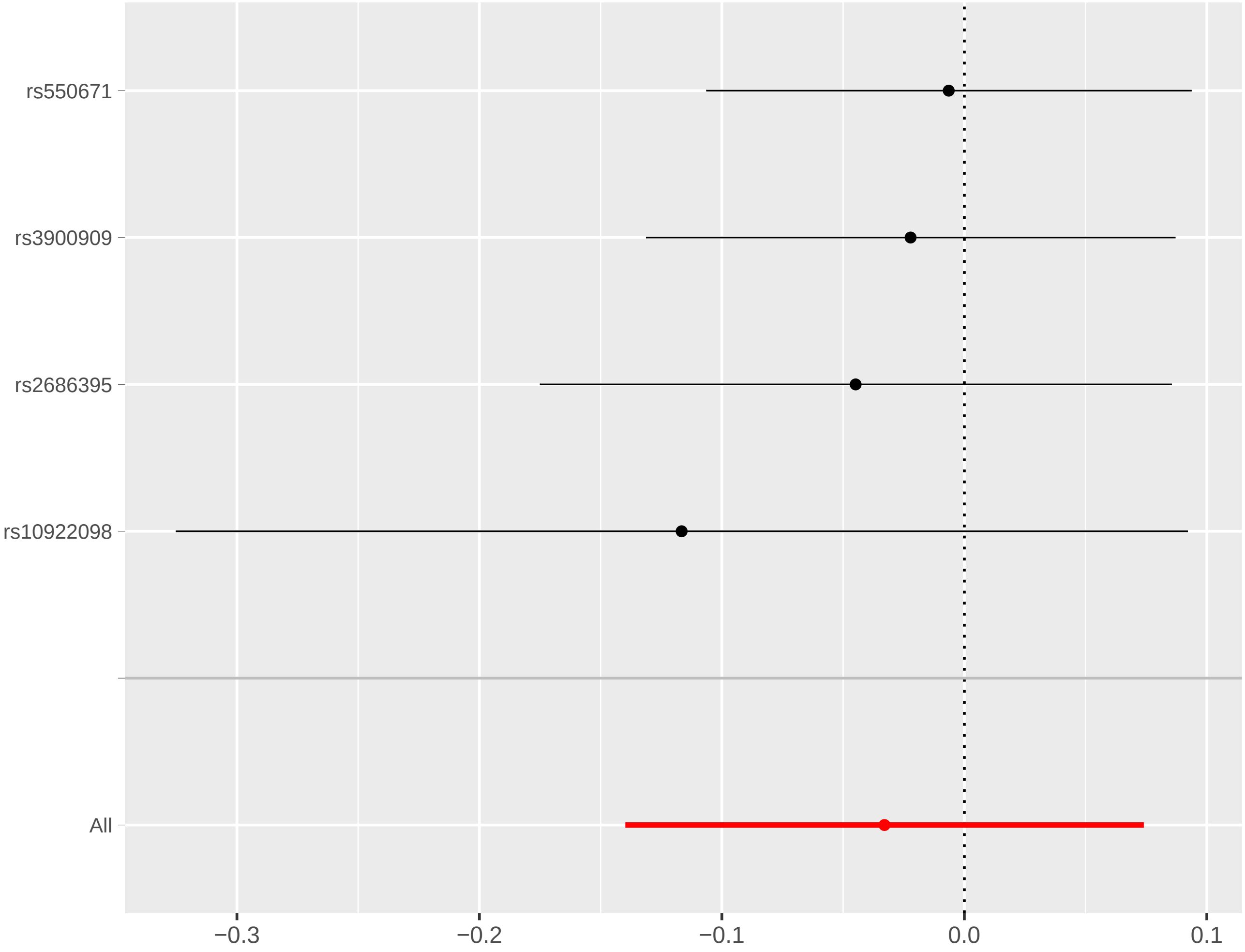

MR leave−one−out sensitivity analysis for
'TPD52L1' on 'Cardiac valvular disease'

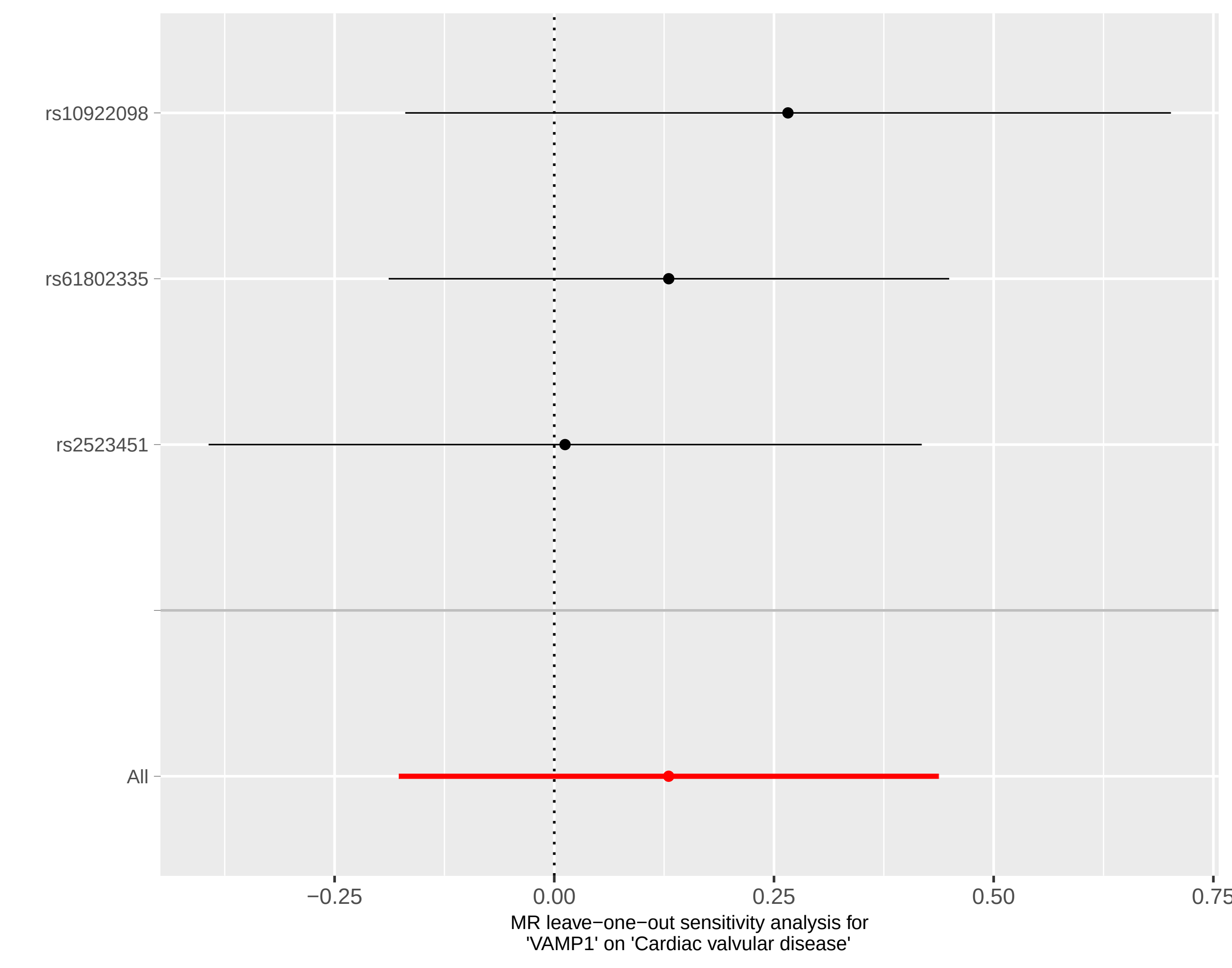
rs10922098
rs61802335
rs2523451
All
−0.25
0.00
0.25
0.50
0.75
MR leave−one−out sensitivity analysis for
'VAMP1' on 'Cardiac valvular disease'

Insufficient number of SNPs

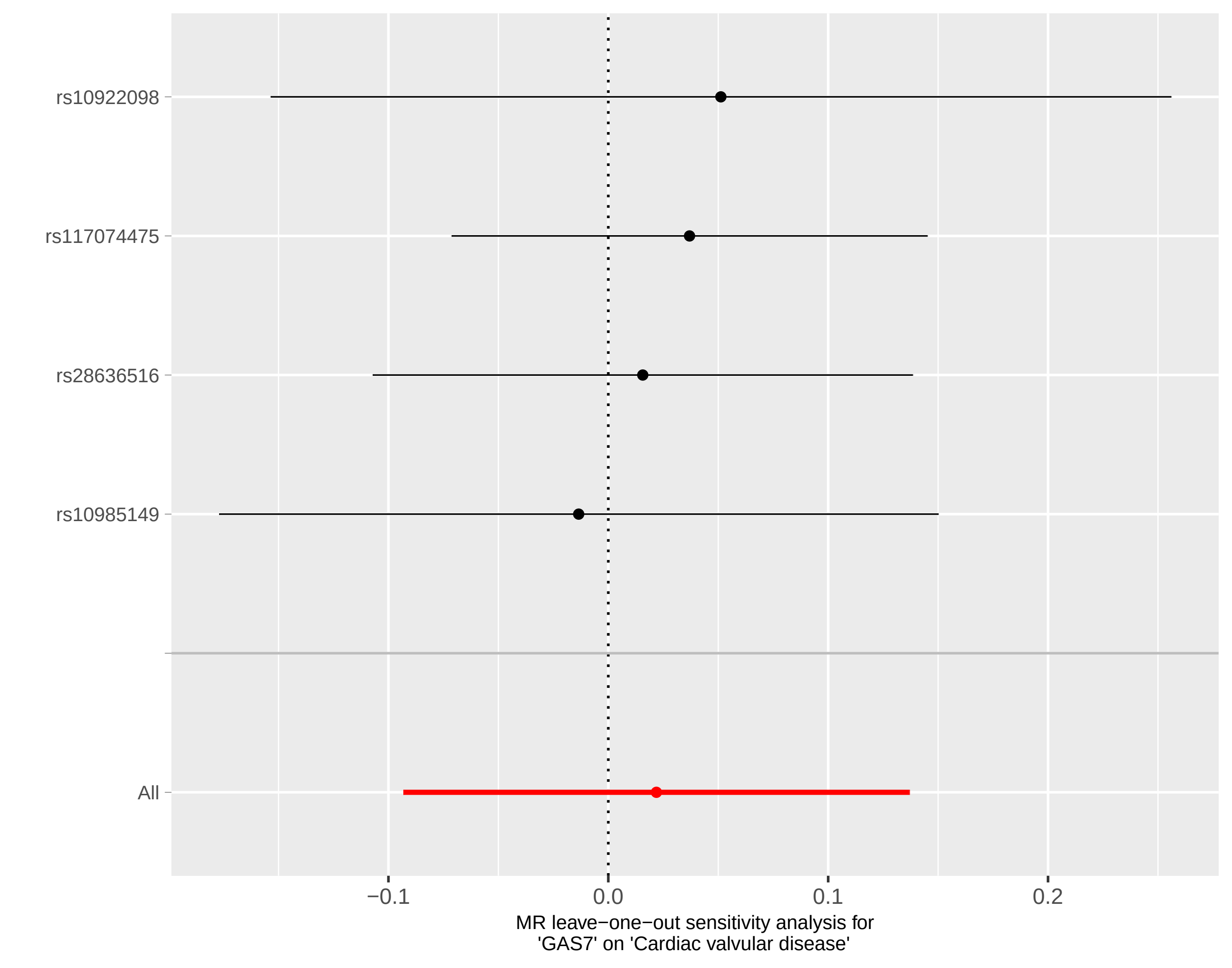
rs10922098
rs117074475
rs28636516
rs10985149
All
−0.1
0.0
0.1
0.2
MR leave−one−out sensitivity analysis for
'GAS7' on 'Cardiac valvular disease'

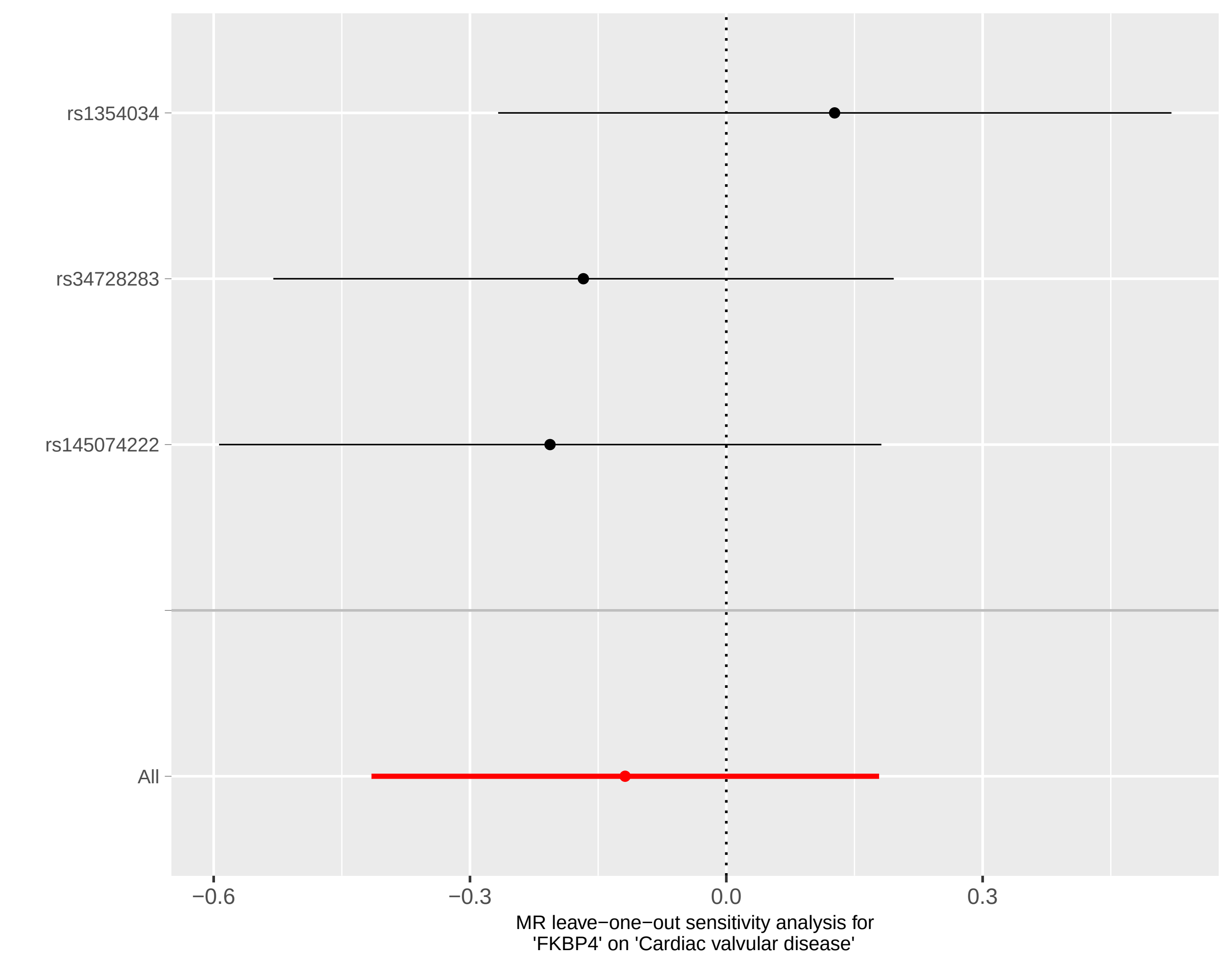

rs1354034
rs34728283
rs145074222
All
−0.6
−0.3
0.0
0.3
MR leave−one−out sensitivity analysis for
'FKBP4' on 'Cardiac valvular disease'

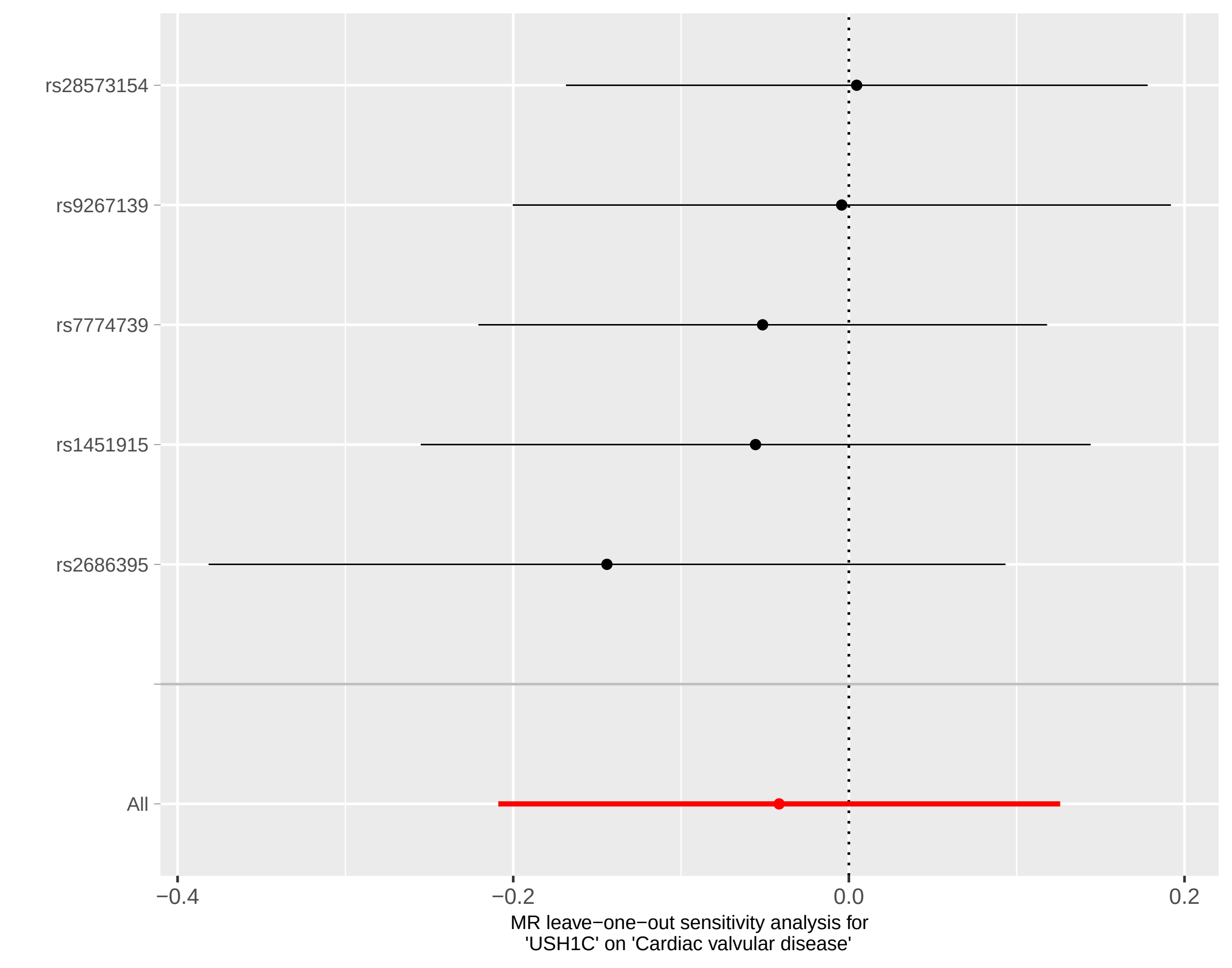
rs28573154
rs9267139
rs7774739
rs1451915
rs2686395
All
−0.4
−0.2
0.0
0.2
MR leave−one−out sensitivity analysis for
'USH1C' on 'Cardiac valvular disease'

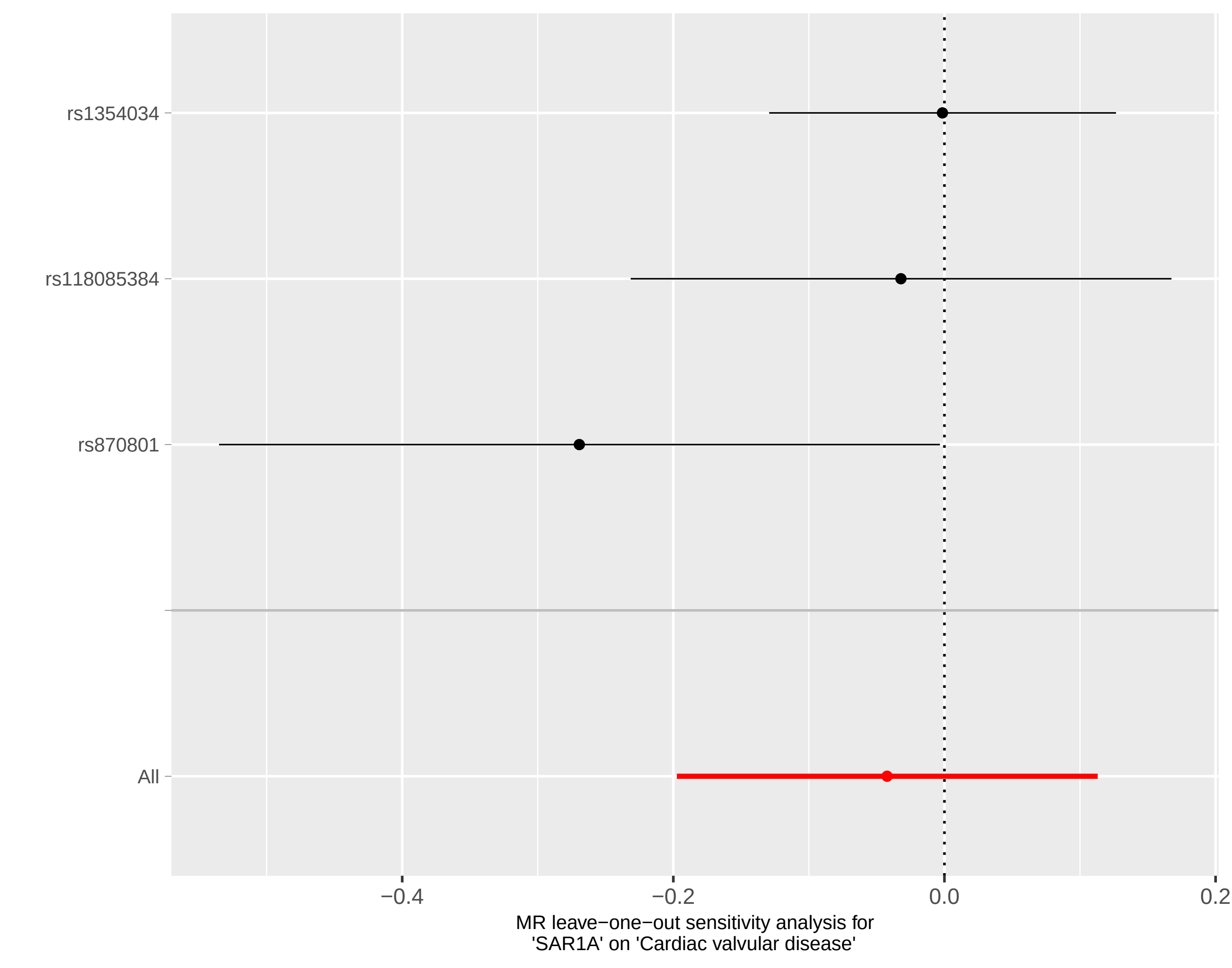

rs1354034
rs118085384
rs870801
All
−0.4
−0.2
0.0
0.2
MR leave−one−out sensitivity analysis for
'SAR1A' on 'Cardiac valvular disease'

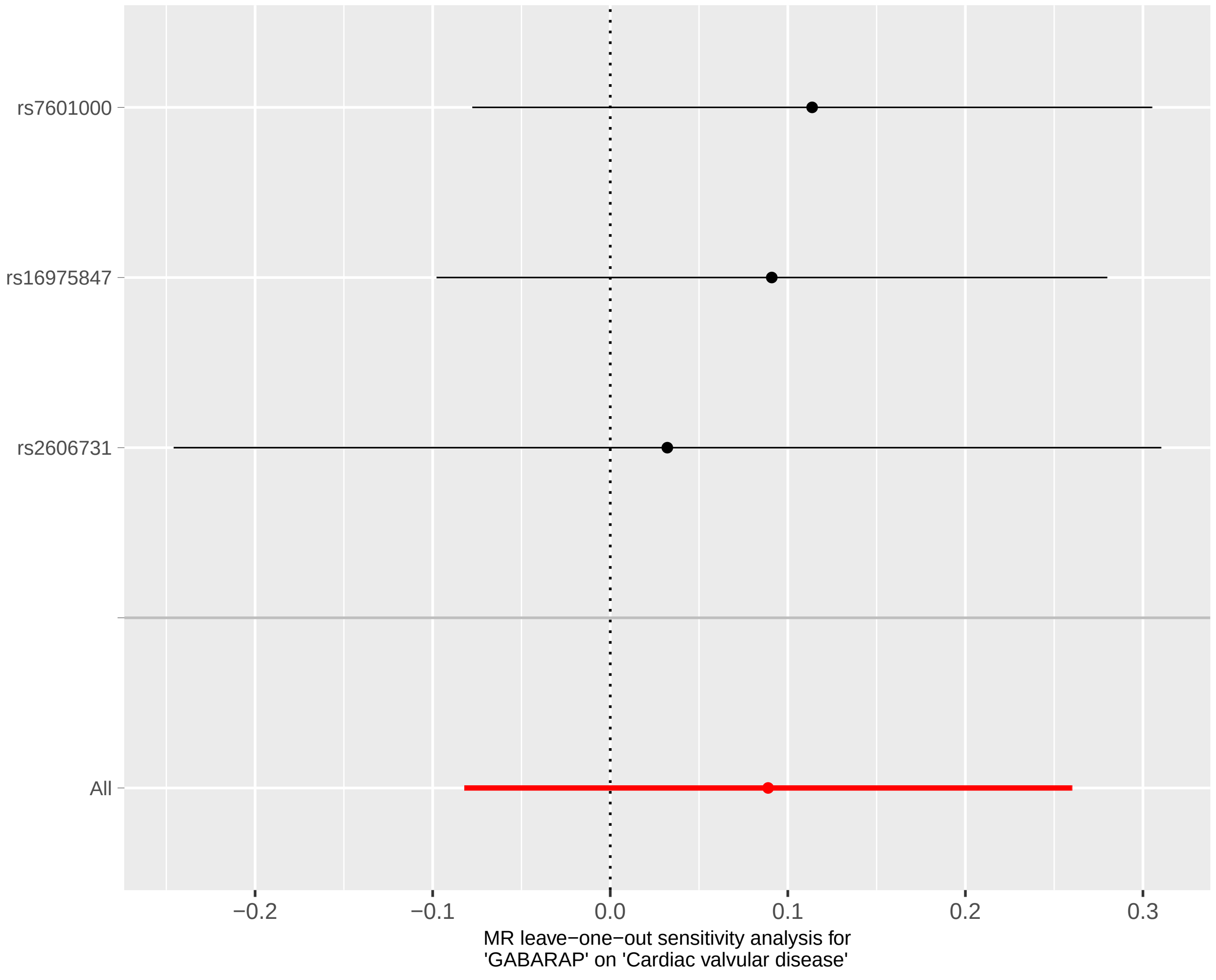
rs7601000
rs16975847
rs2606731
All
−0.2
−0.1
0.0
0.1
0.2
0.3
MR leave−one−out sensitivity analysis for
'GABARAP' on 'Cardiac valvular disease'

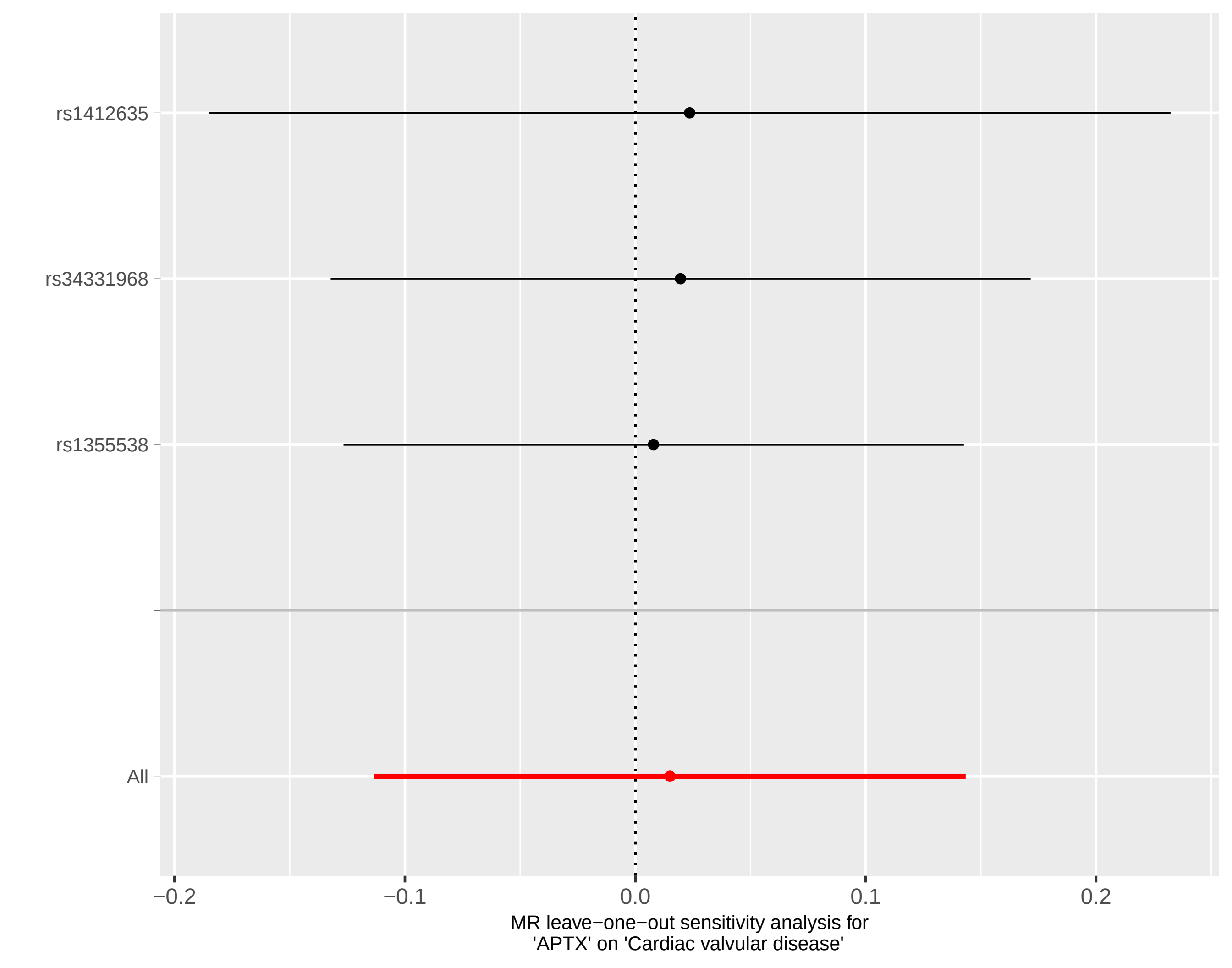
rs1412635
rs34331968
rs1355538
All
−0.2
−0.1
0.0
0.1
0.2
MR leave−one−out sensitivity analysis for
'APTX' on 'Cardiac valvular disease'

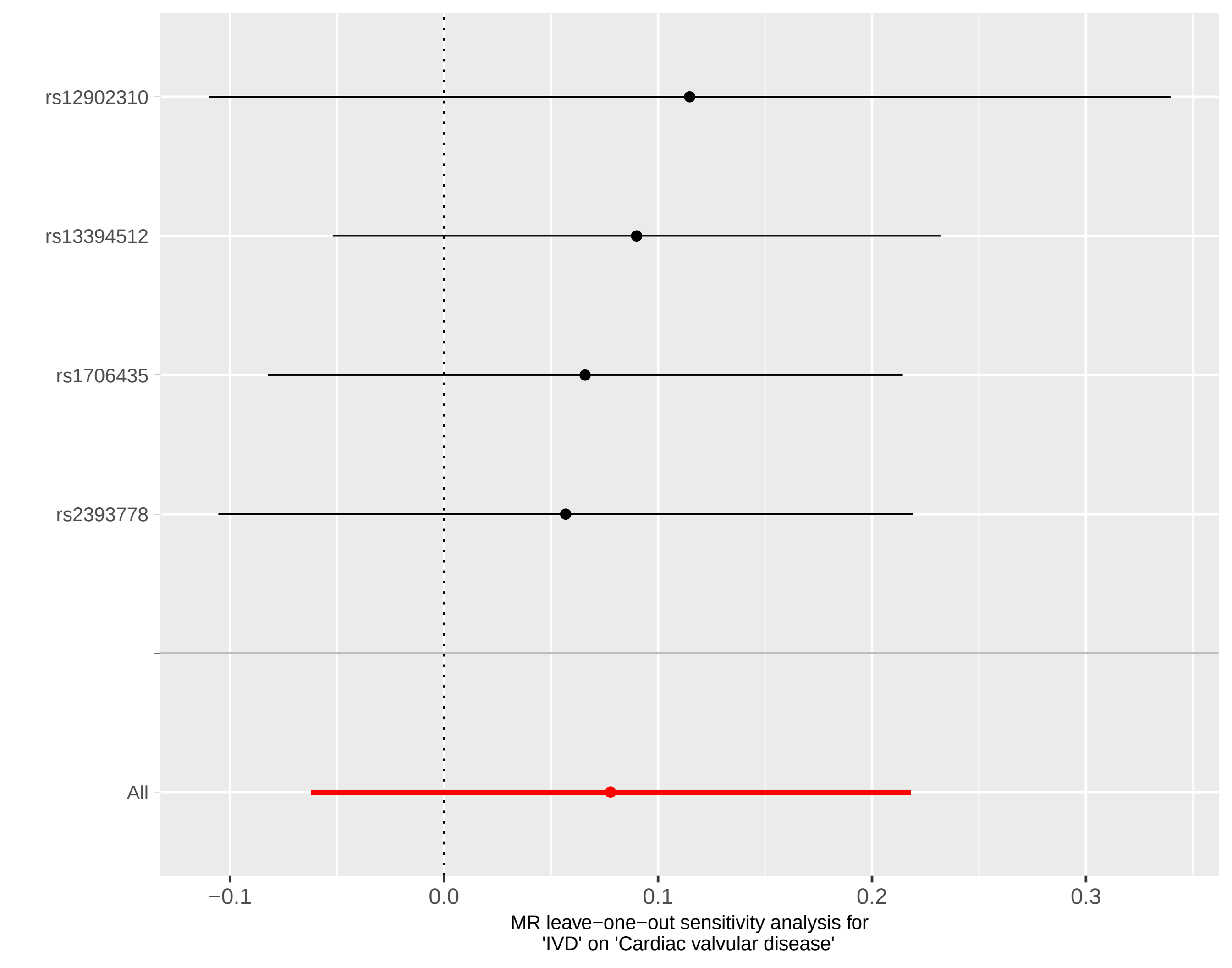

rs12902310
rs13394512
rs1706435
rs2393778
All
−0.1
0.0
0.1
0.2
0.3
MR leave−one−out sensitivity analysis for
'IVD' on 'Cardiac valvular disease'

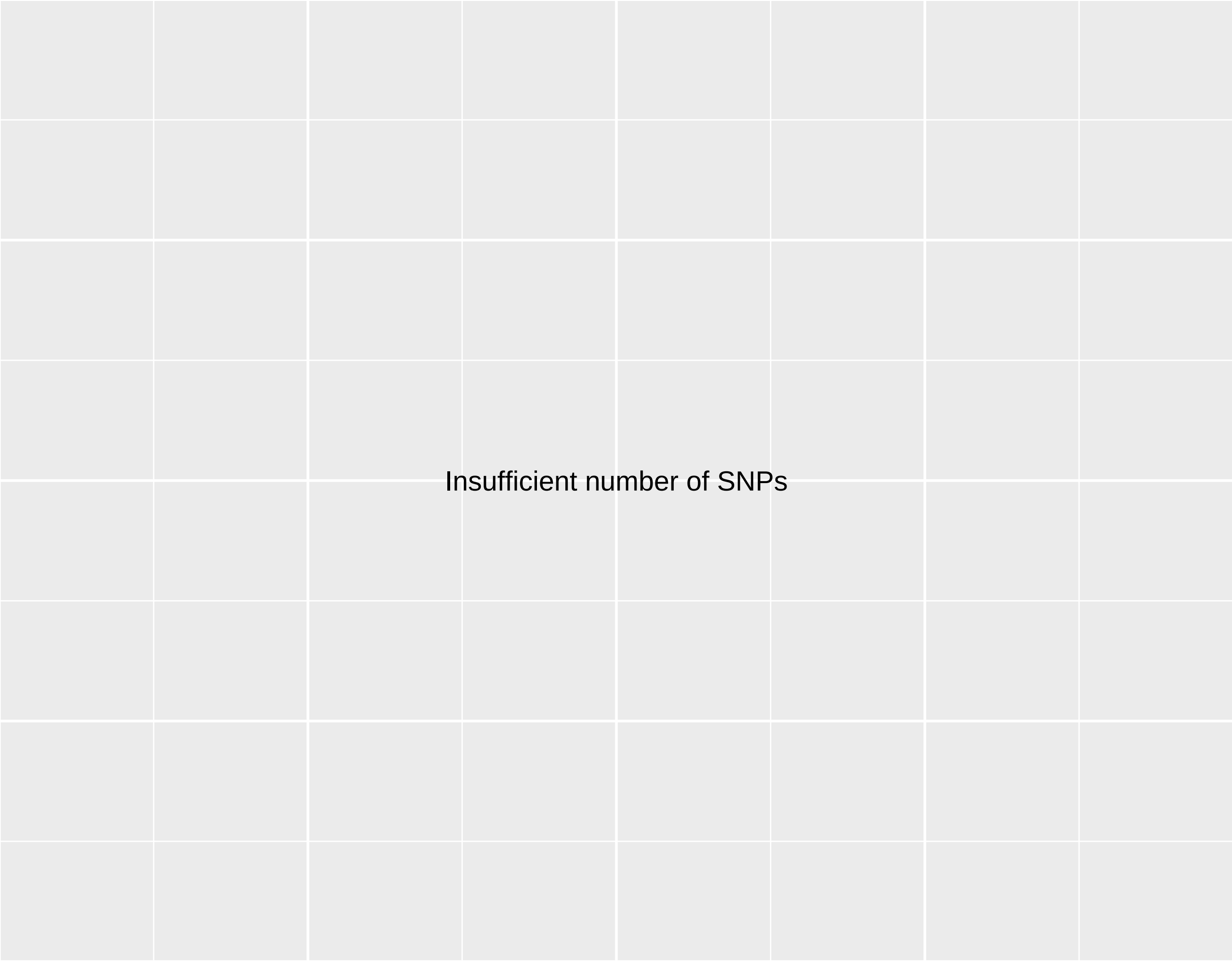
Insufficient number of SNPs

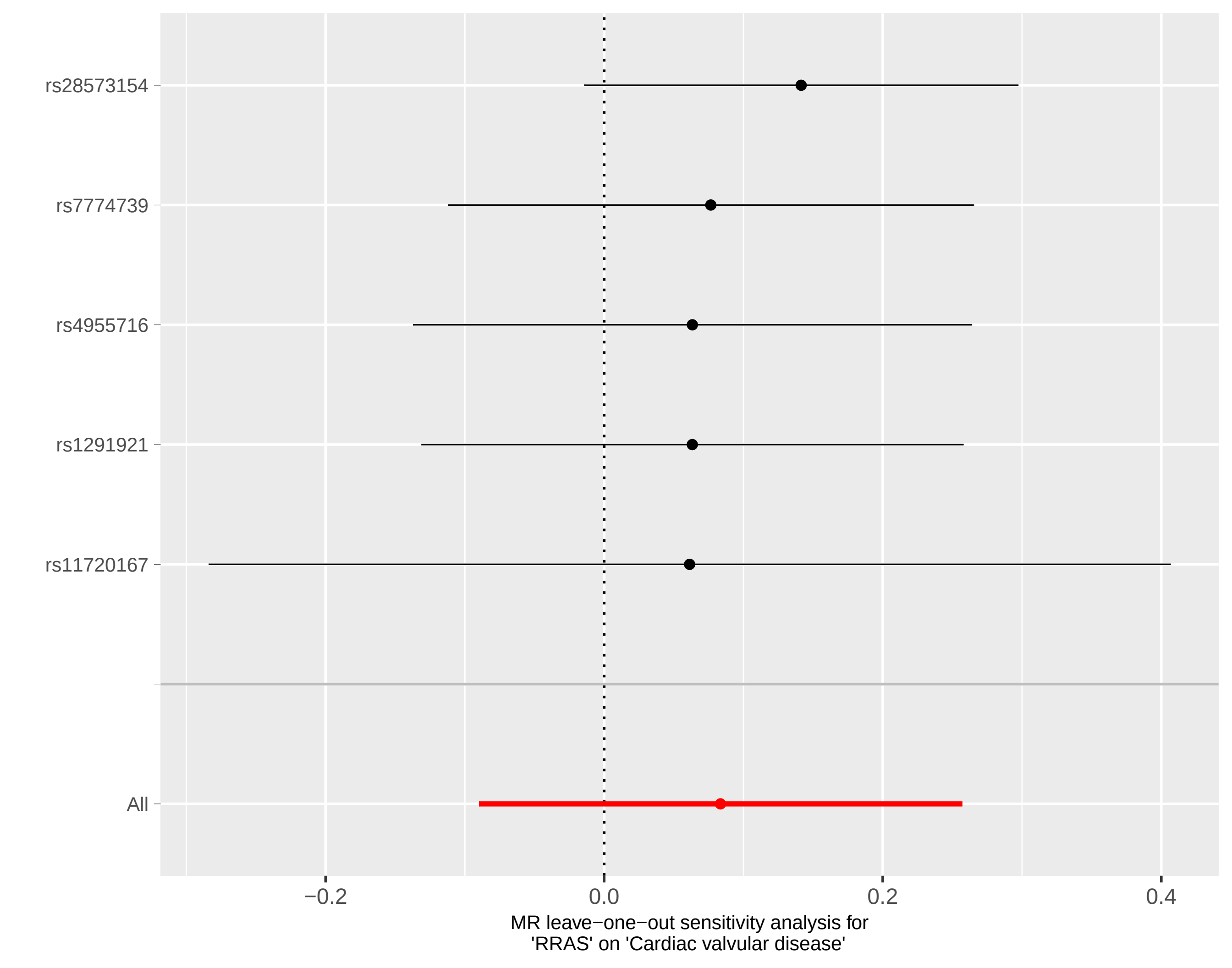

rs28573154
rs7774739
rs4955716
rs1291921
rs11720167
All
−0.2
0.0
0.2
0.4
MR leave−one−out sensitivity analysis for
'RRAS' on 'Cardiac valvular disease'

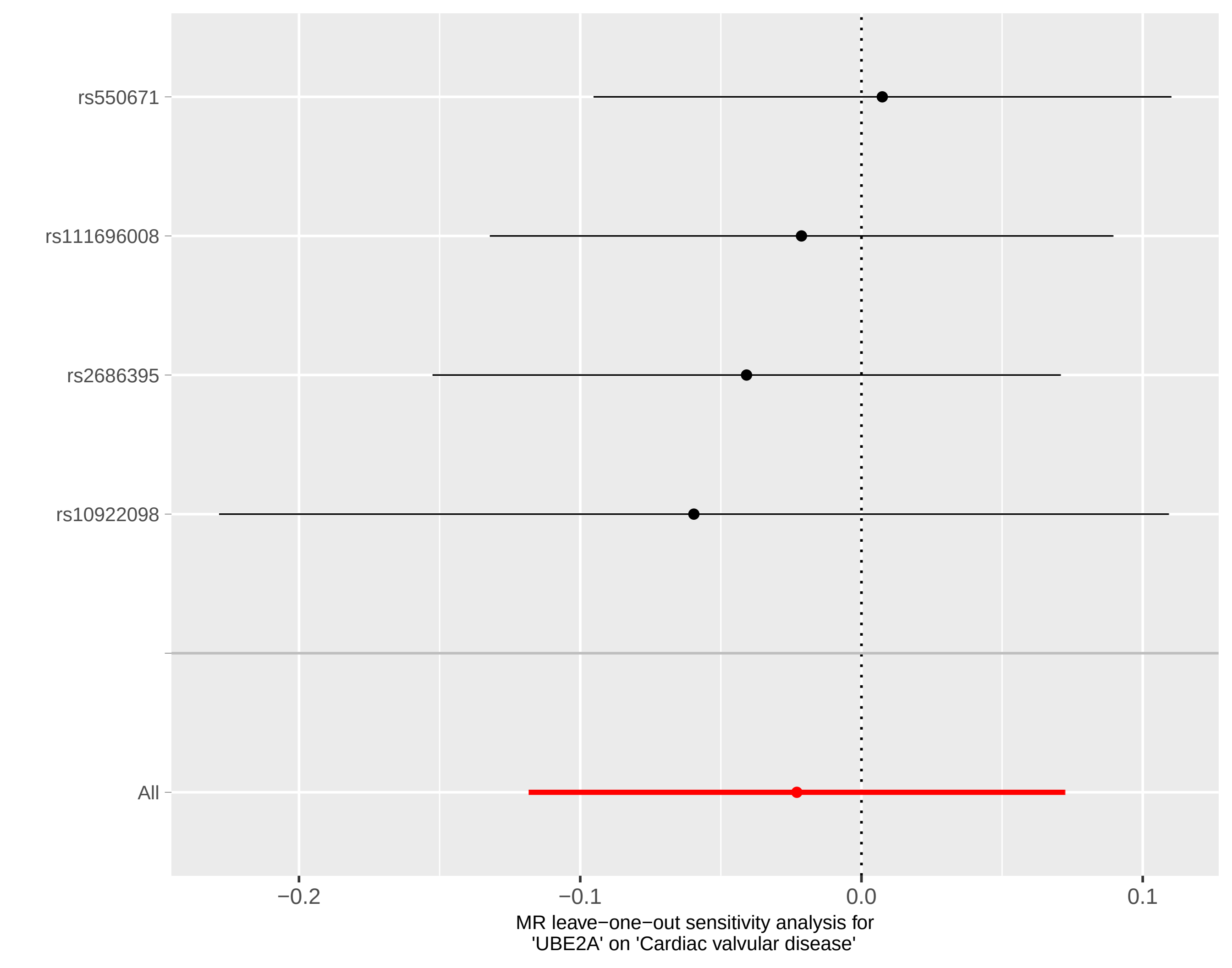
rs550671
rs111696008
rs2686395
rs10922098
All
−0.2
−0.1
0.0
0.1
MR leave−one−out sensitivity analysis for
'UBE2A' on 'Cardiac valvular disease'

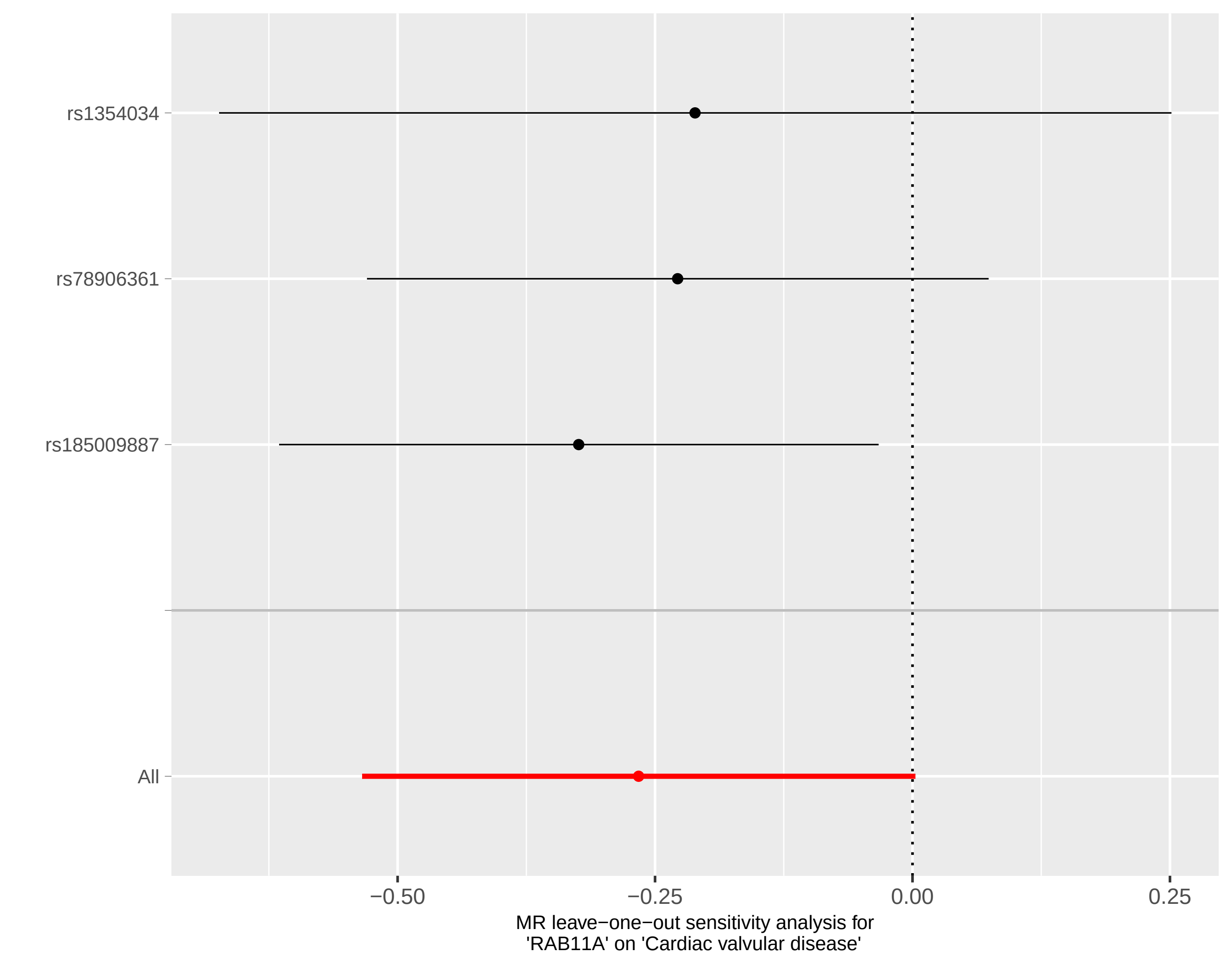
rs1354034
rs78906361
rs185009887
All
−0.50
−0.25
0.00
0.25
MR leave−one−out sensitivity analysis for
'RAB11A' on 'Cardiac valvular disease'

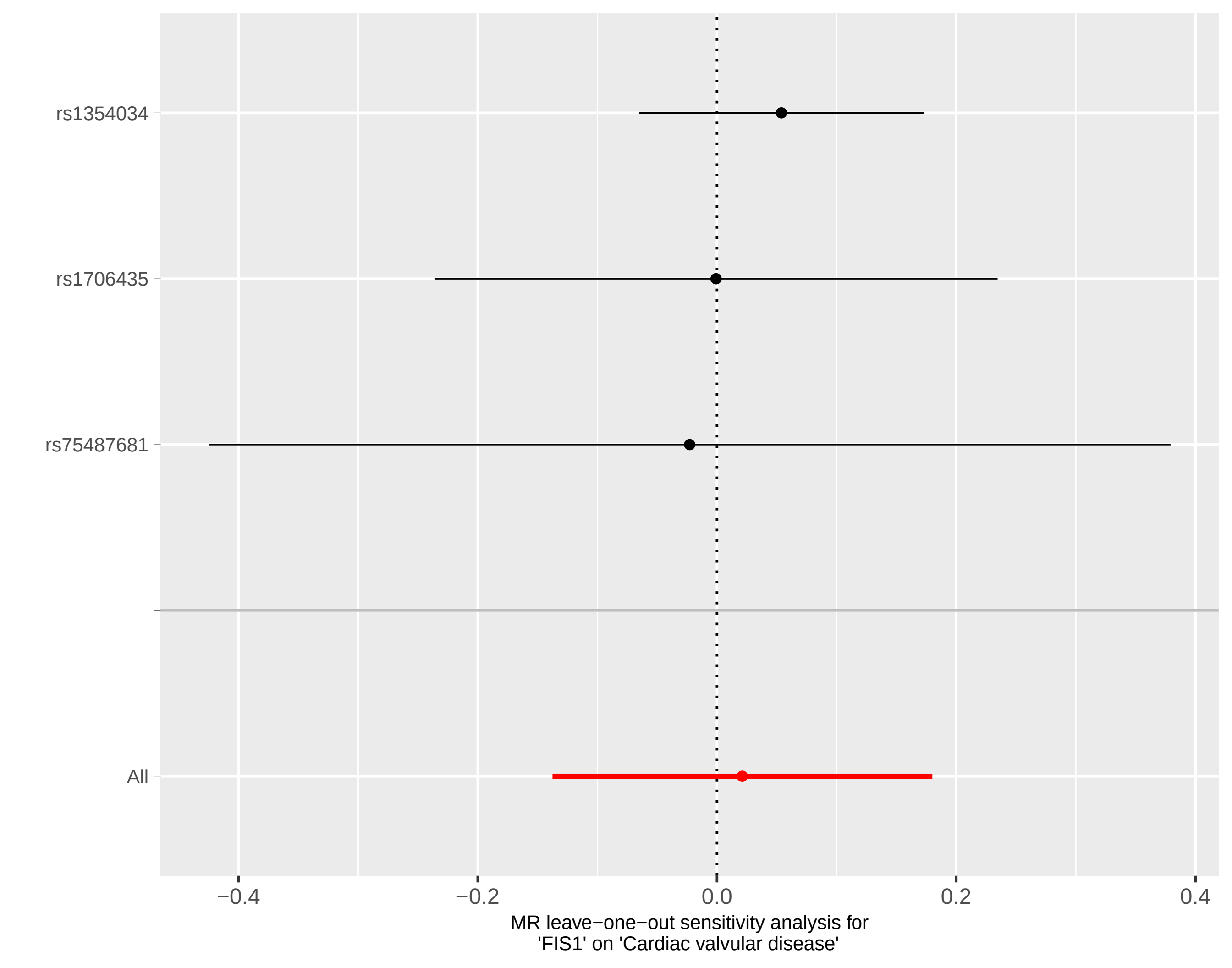
rs1354034
rs1706435
rs75487681
All
−0.4
−0.2
0.0
0.2
0.4
MR leave−one−out sensitivity analysis for
'FIS1' on 'Cardiac valvular disease'

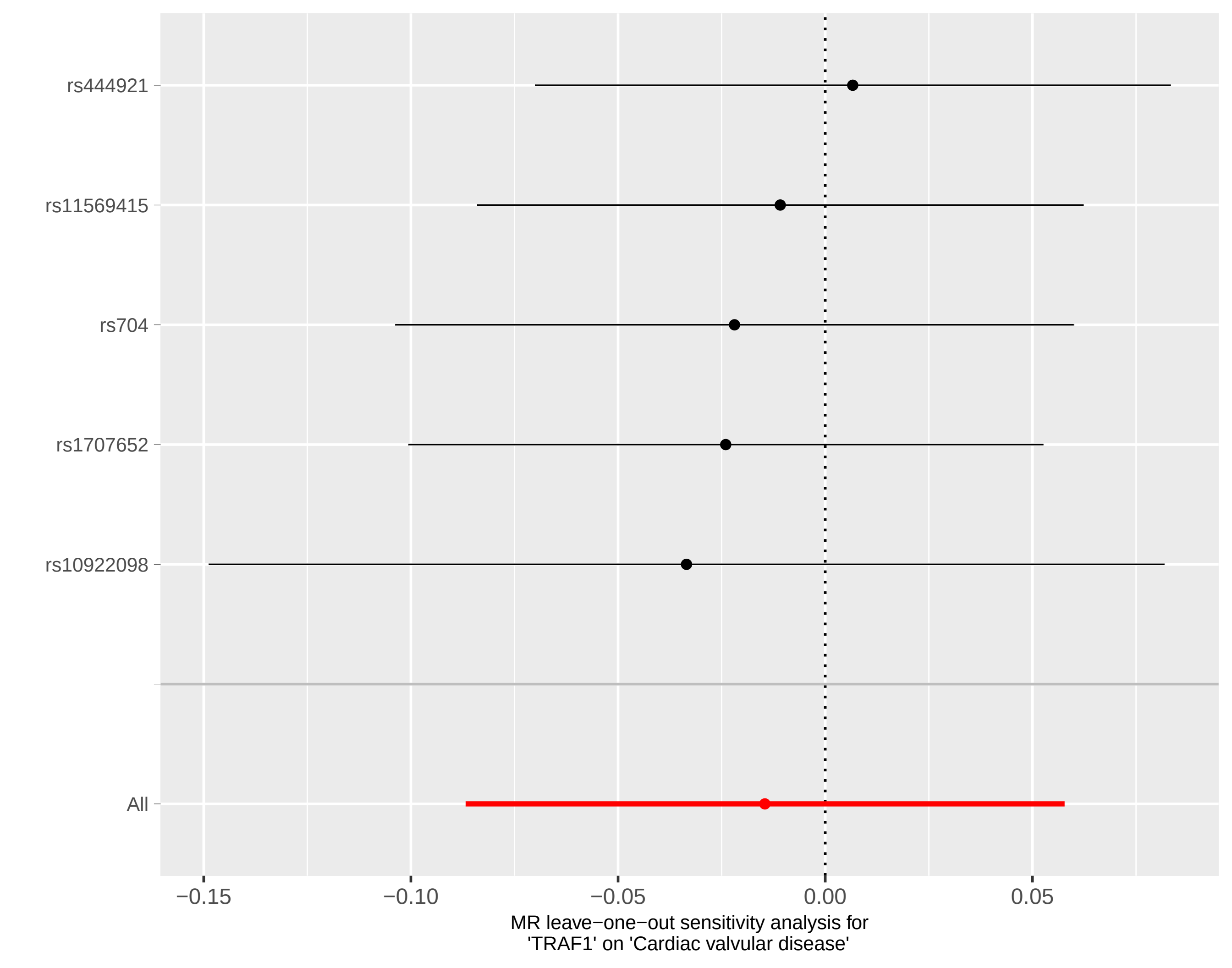
rs444921
rs11569415
rs704
rs1707652
rs10922098
All
−0.15
−0.10
−0.05
0.00
0.05
MR leave−one−out sensitivity analysis for
'TRAF1' on 'Cardiac valvular disease'

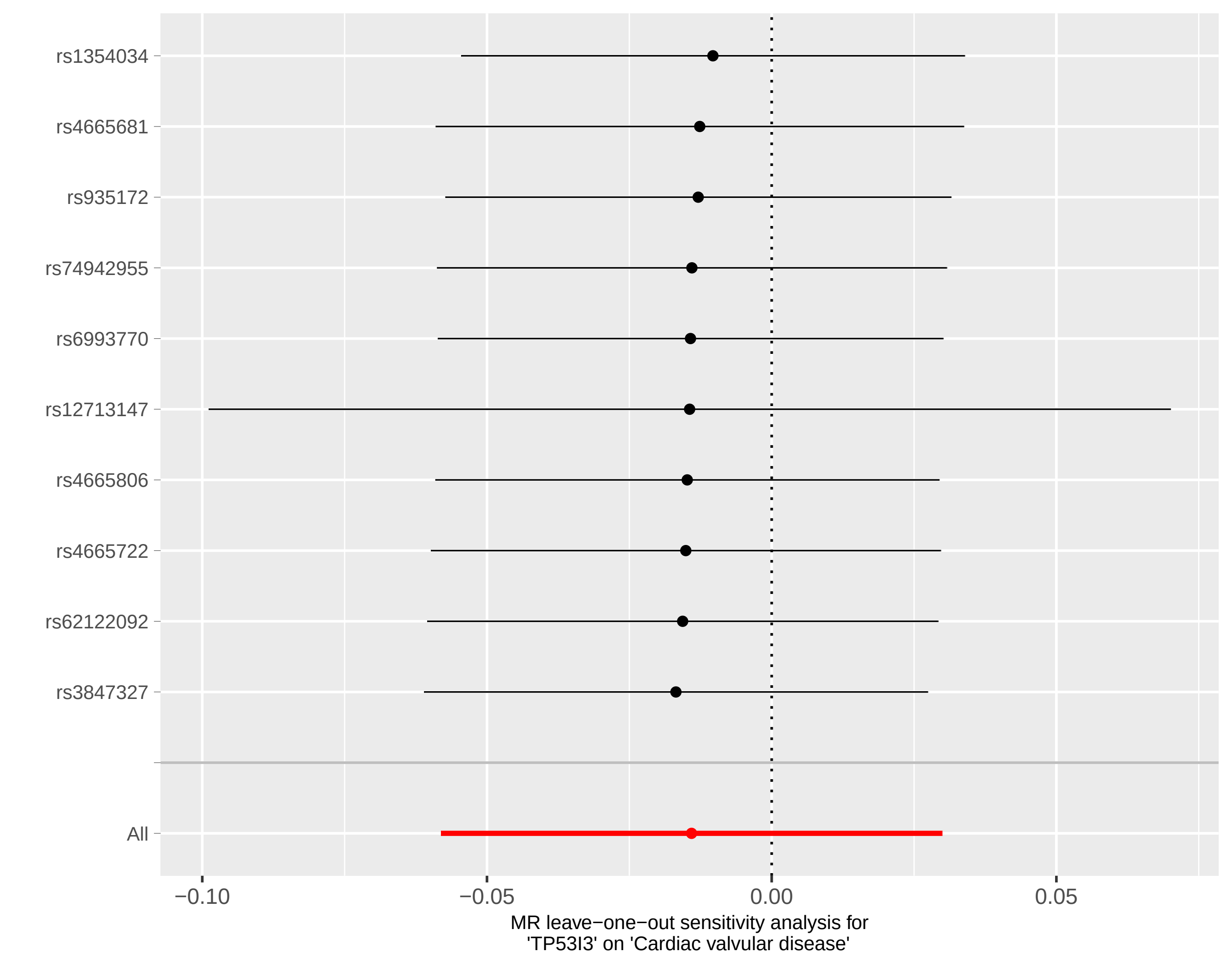
rs1354034
rs4665681
rs935172
rs74942955
rs6993770
rs12713147
rs4665806
rs4665722
rs62122092
rs3847327
All
−0.10
−0.05
0.00
0.05
MR leave−one−out sensitivity analysis for
'TP53I3' on 'Cardiac valvular disease'

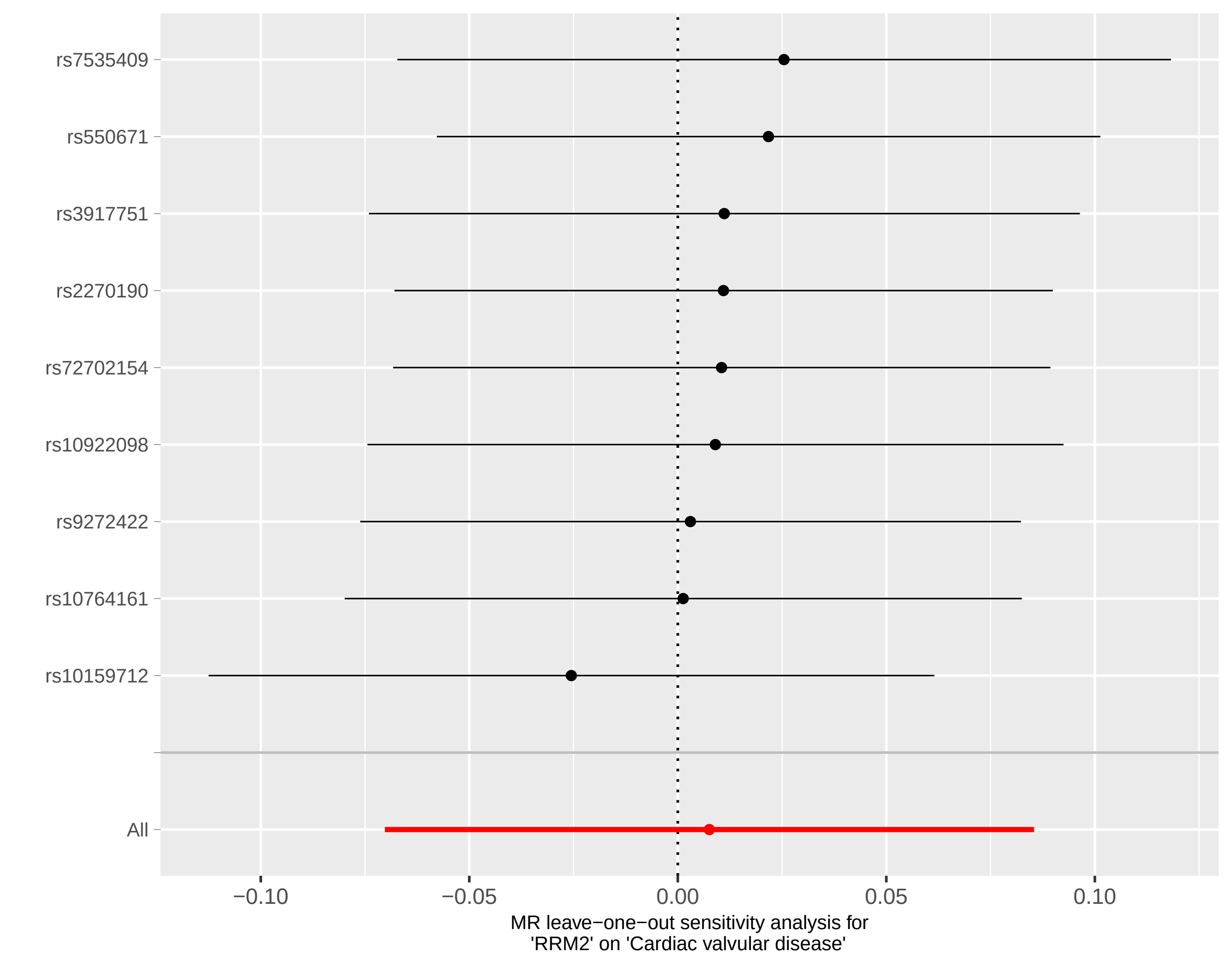

rs7535409
rs550671
rs3917751
rs2270190
rs72702154
rs10922098
rs9272422
rs10764161
rs10159712
All
−0.10
−0.05
0.00
0.05
0.10
MR leave−one−out sensitivity analysis for
'RRM2' on 'Cardiac valvular disease'

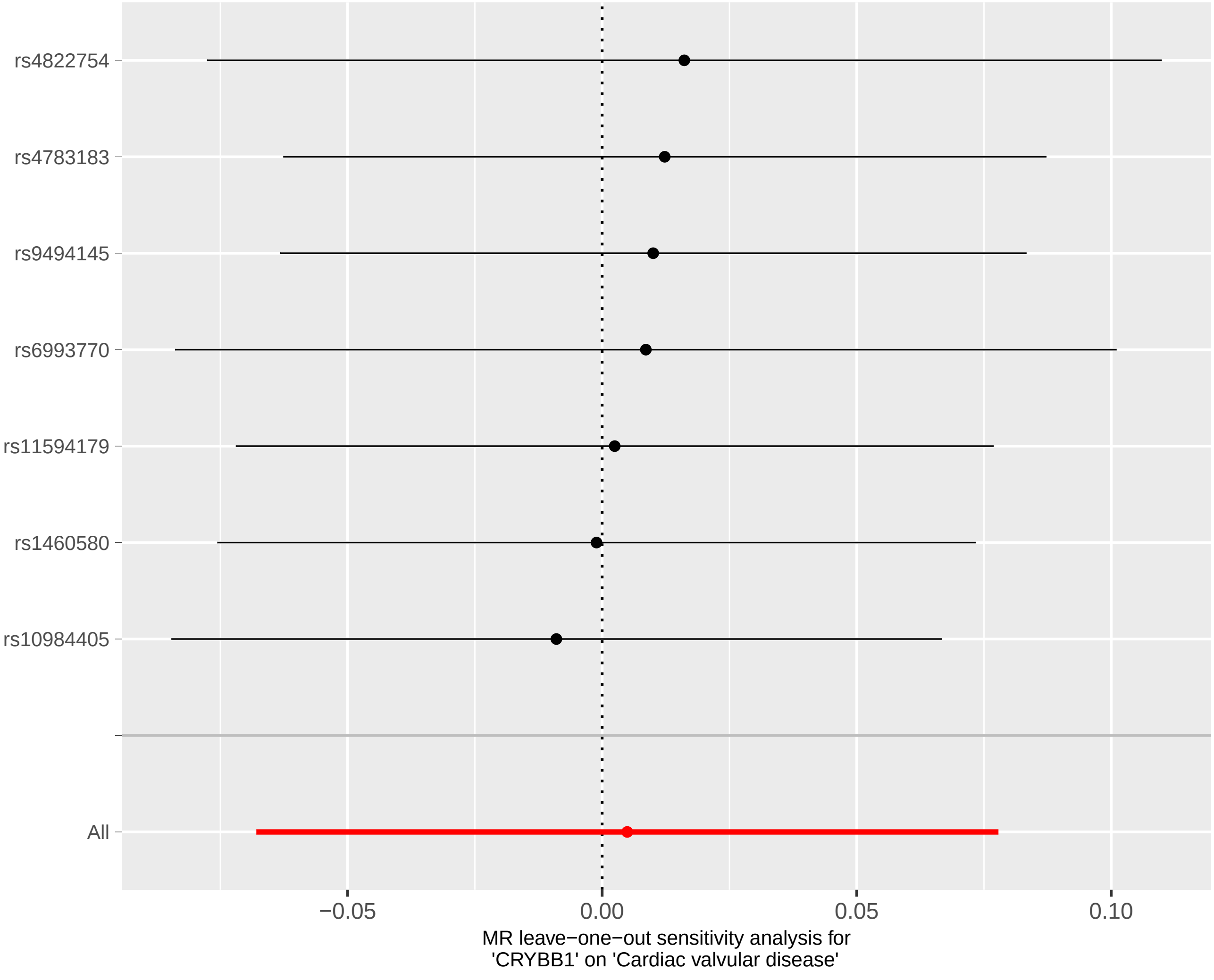

rs4822754
rs4783183
rs9494145
rs6993770
rs11594179
rs1460580
rs10984405
All
−0.05
0.00
0.05
0.10
MR leave−one−out sensitivity analysis for
'CRYBB1' on 'Cardiac valvular disease'

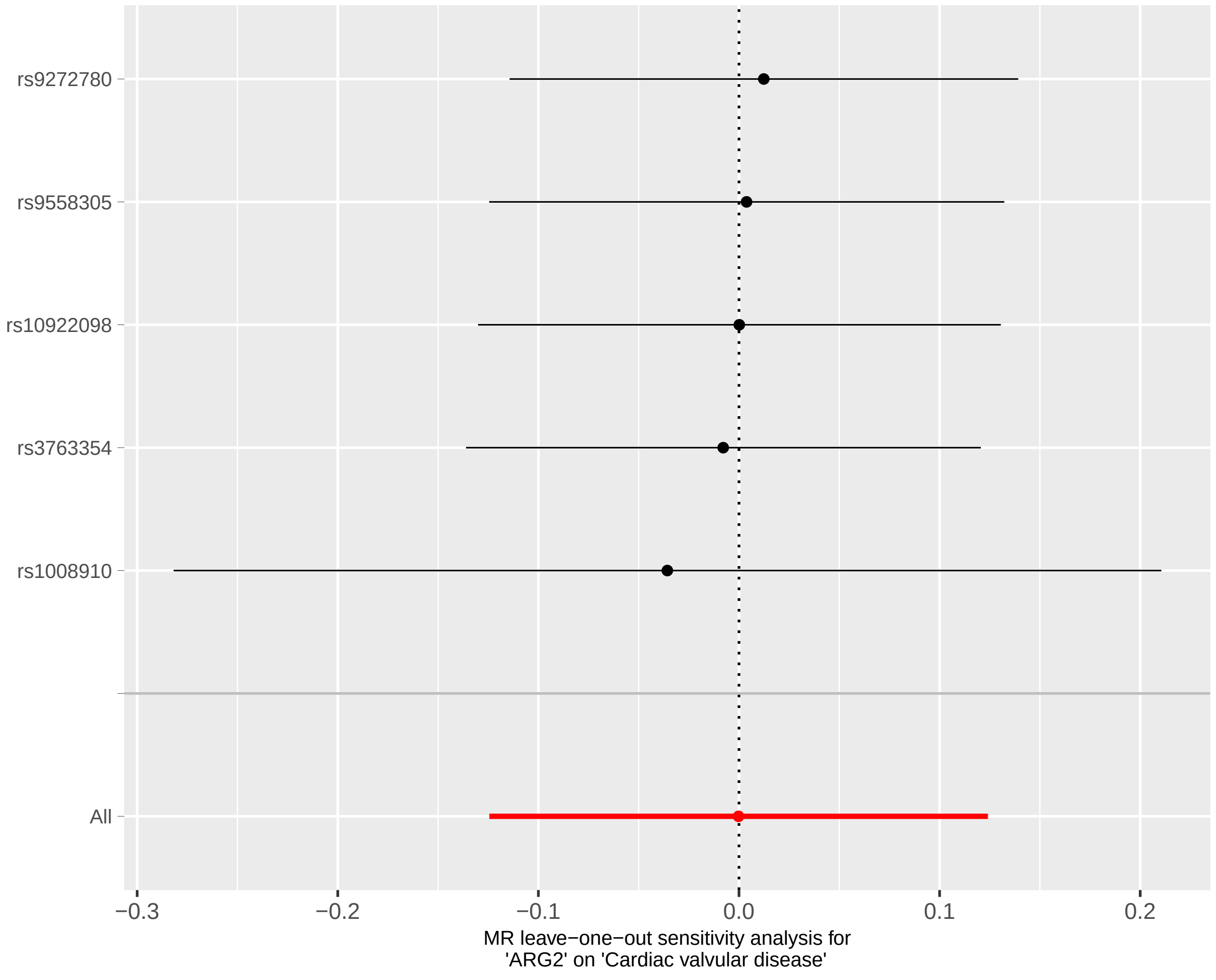

rs9272780
rs9558305
rs10922098
rs3763354
rs1008910
All
−0.3
−0.2
−0.1
0.0
0.1
0.2
MR leave−one−out sensitivity analysis for
'ARG2' on 'Cardiac valvular disease'

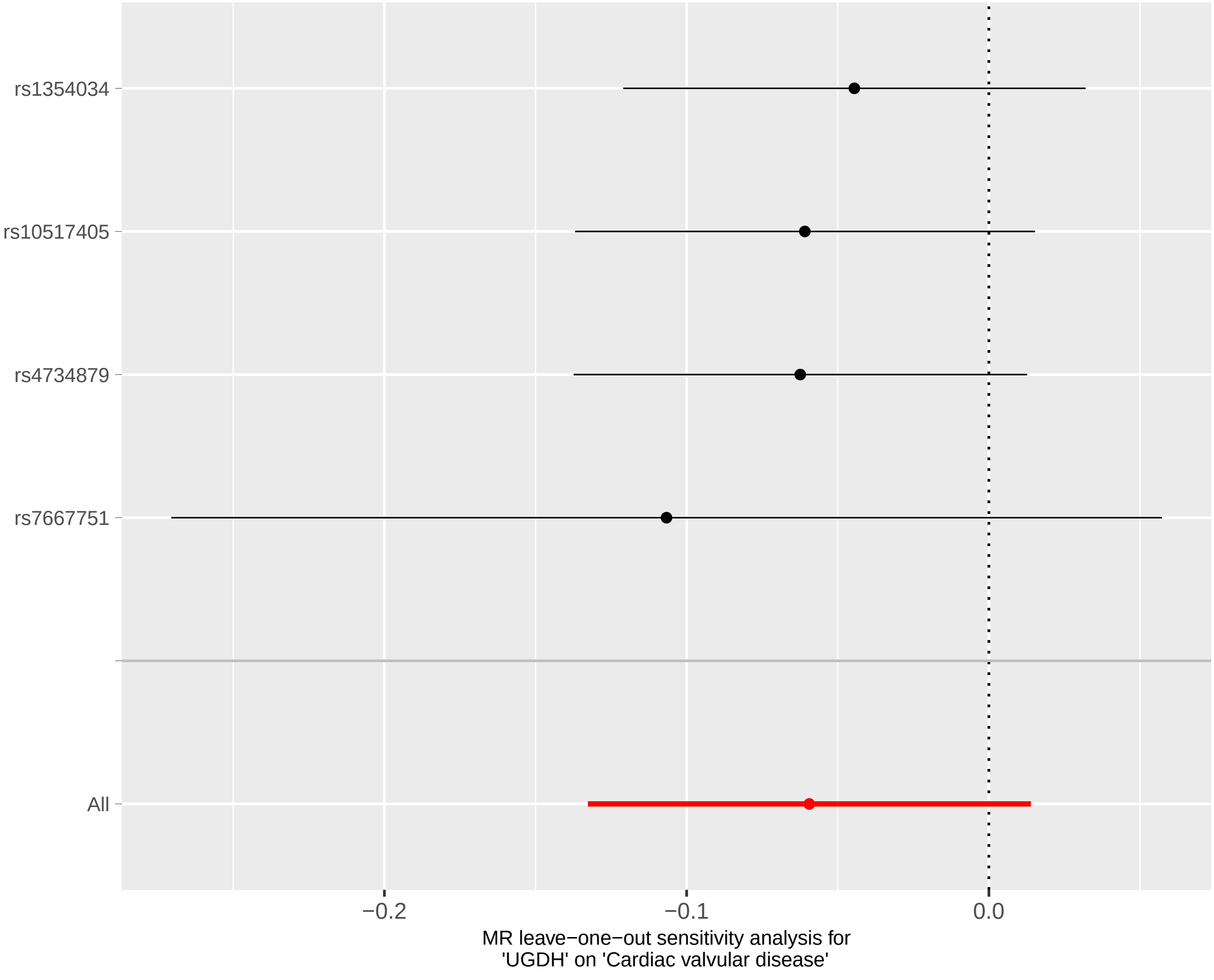

rs1354034
rs10517405
rs4734879
rs7667751
All
−0.2
−0.1
0.0
MR leave−one−out sensitivity analysis for
'UGDH' on 'Cardiac valvular disease'

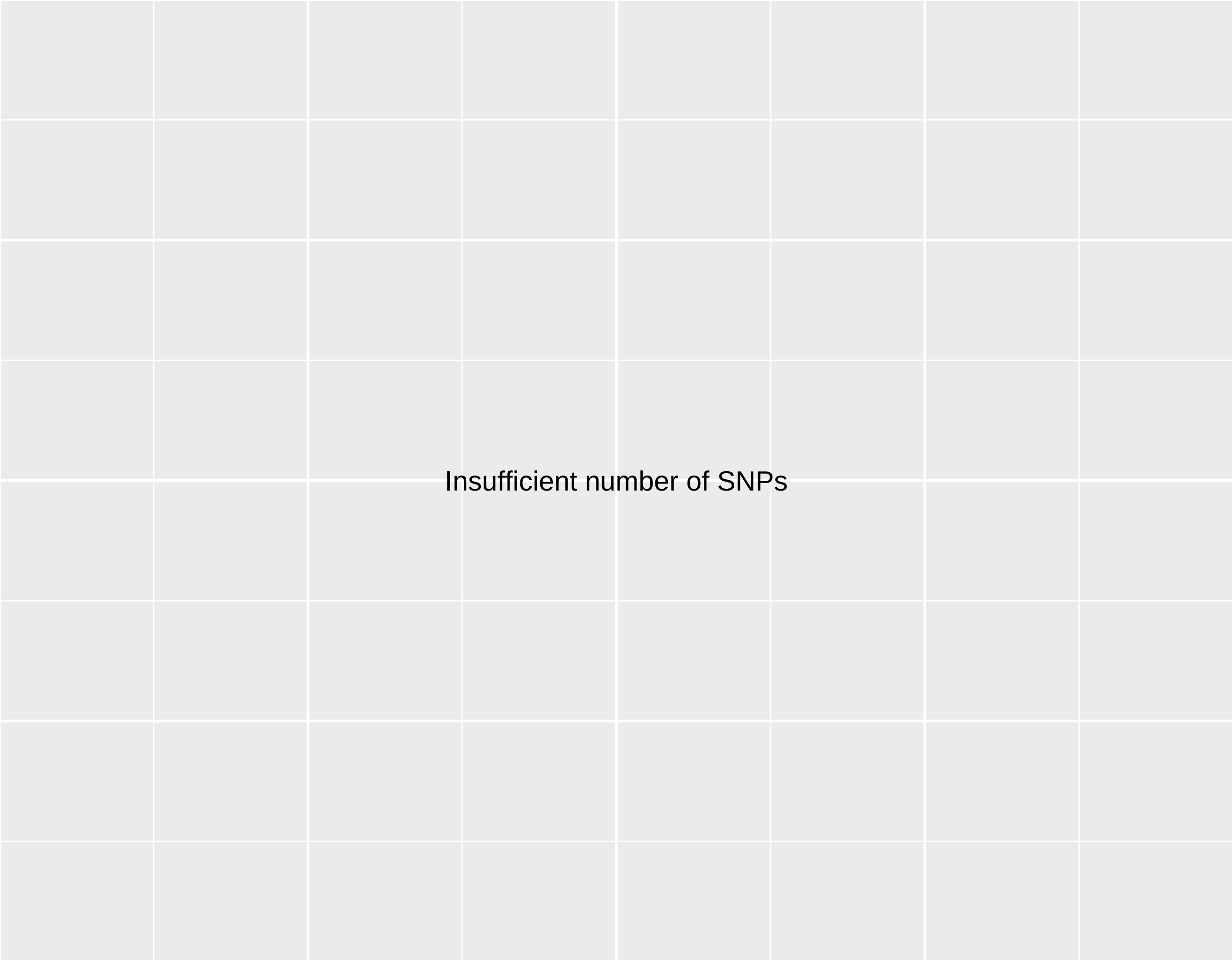
Insufficient number of SNPs

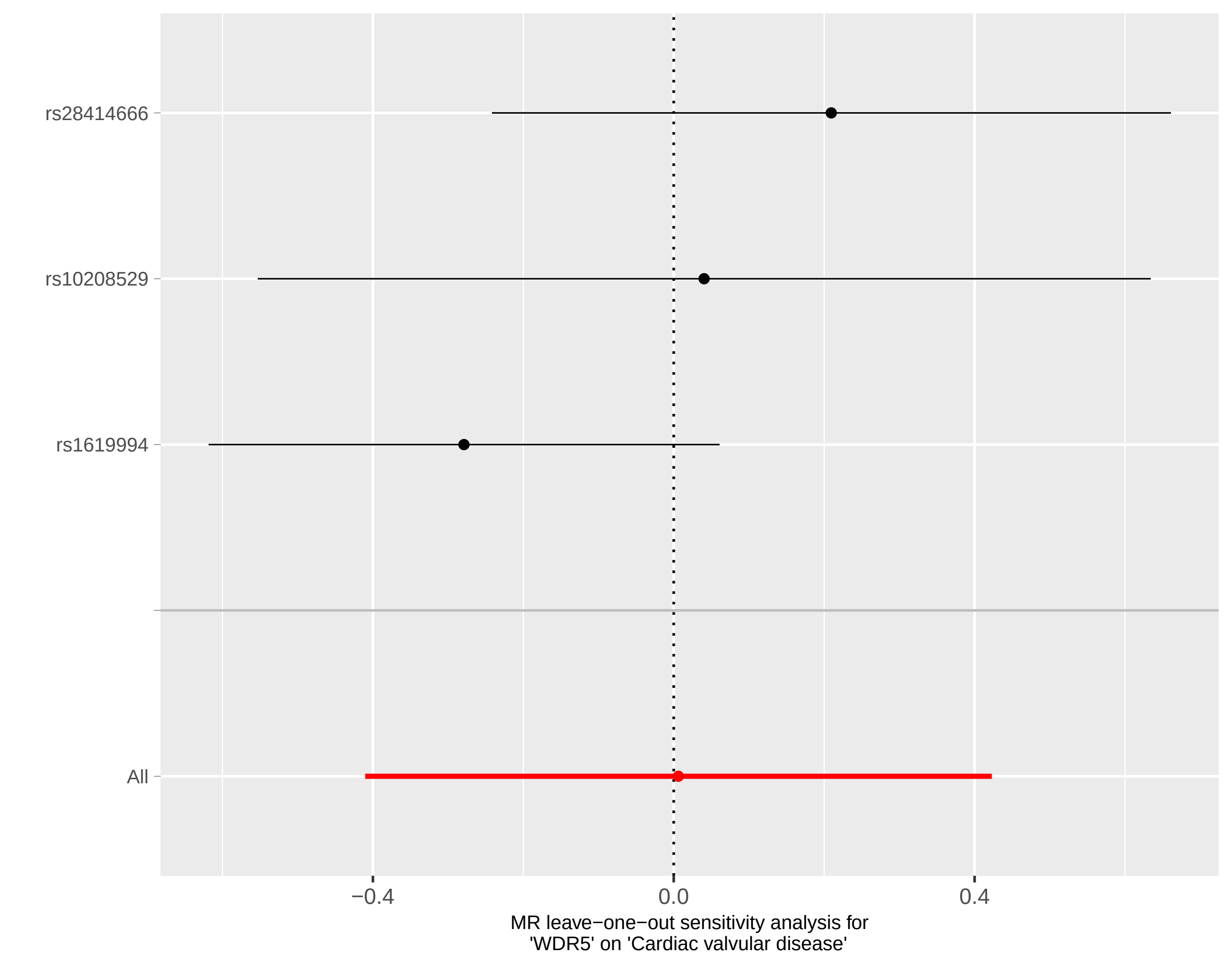
rs28414666
rs10208529
rs1619994
All
−0.4
0.0
0.4
MR leave−one−out sensitivity analysis for
'WDR5' on 'Cardiac valvular disease'

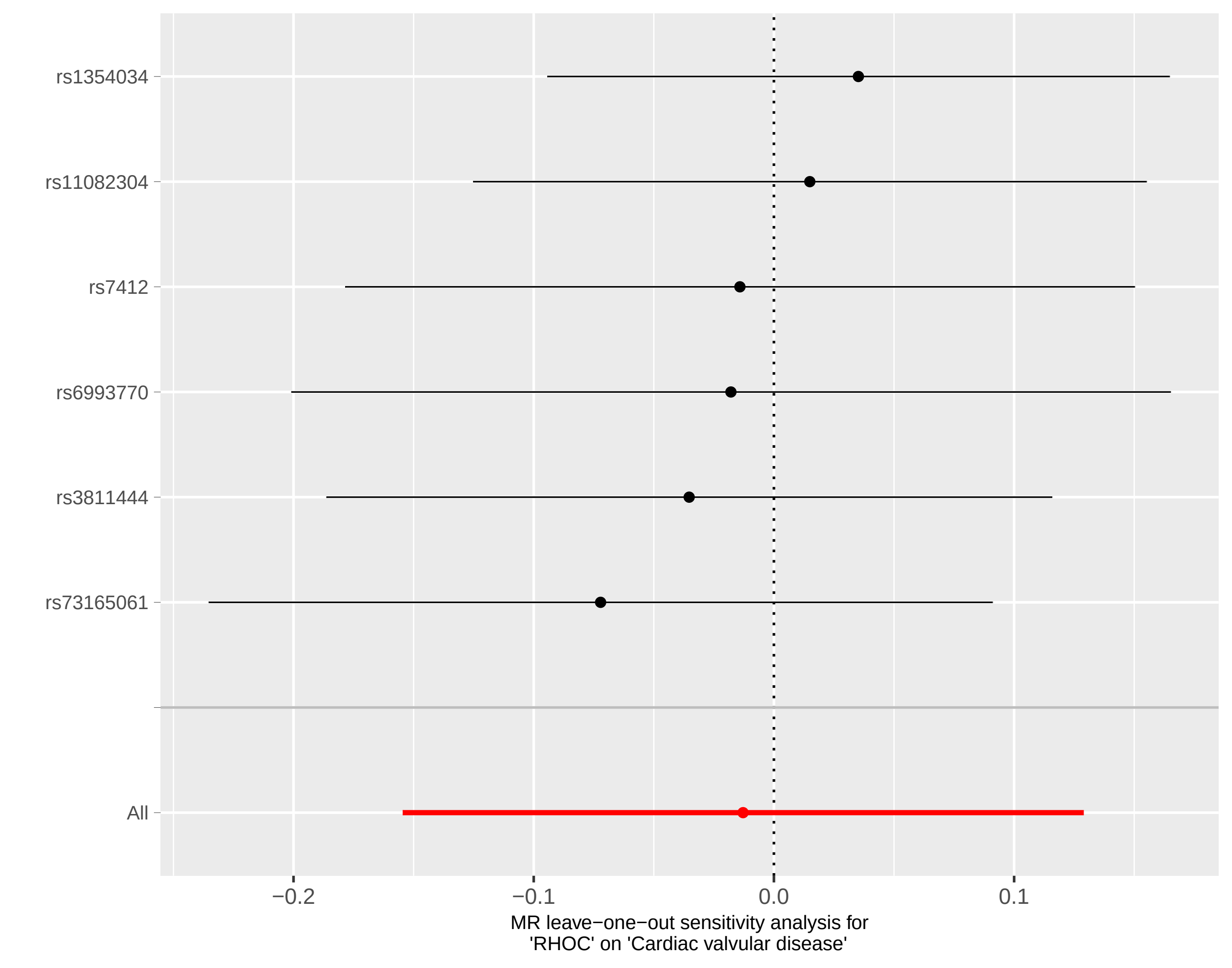
rs1354034
rs11082304
rs7412
rs6993770
rs3811444
rs73165061
All
−0.2
−0.1
0.0
0.1
MR leave−one−out sensitivity analysis for
'RHOC' on 'Cardiac valvular disease'

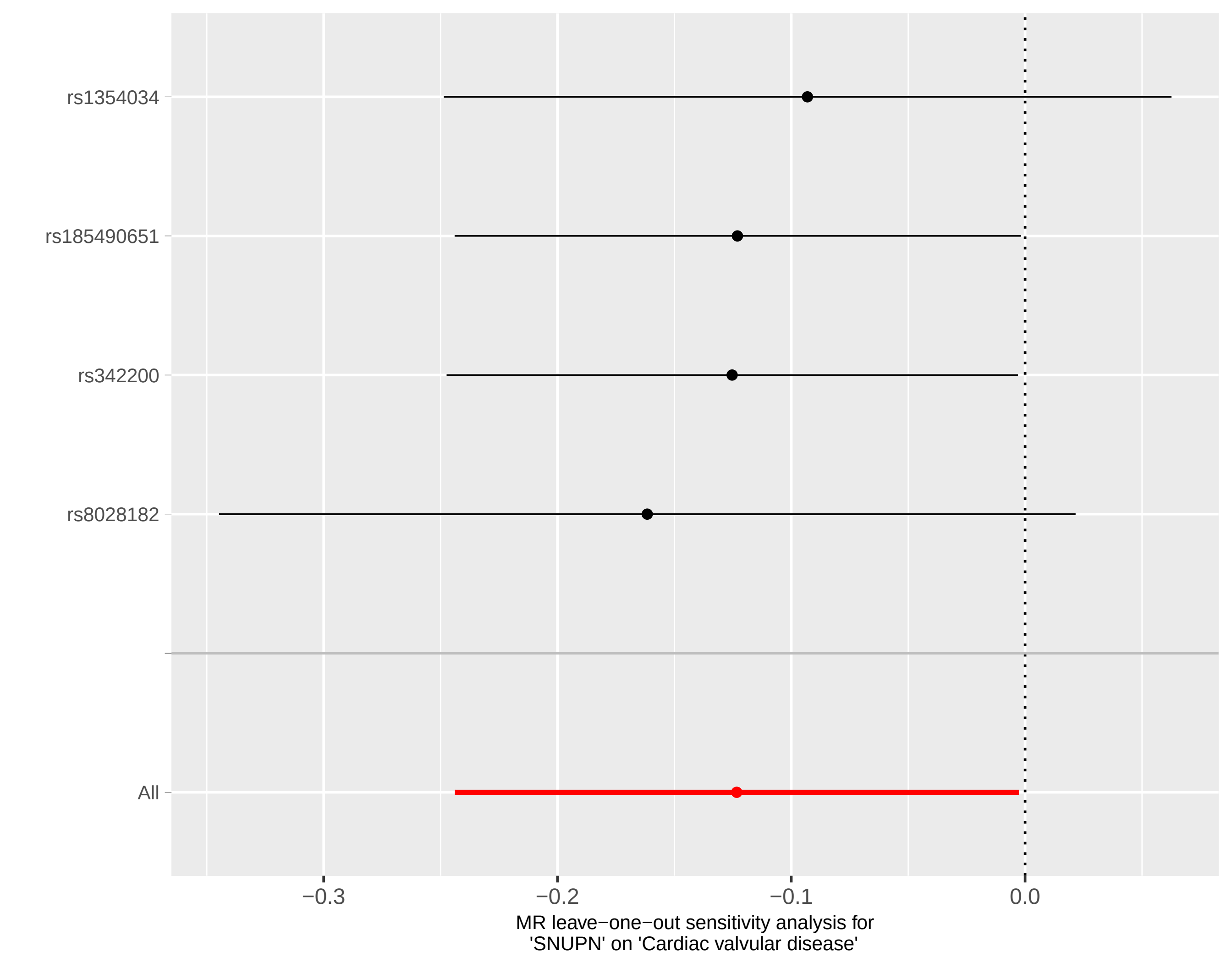
rs1354034
rs185490651
rs342200
rs8028182
All
−0.3
−0.2
−0.1
0.0
MR leave−one−out sensitivity analysis for
'SNUPN' on 'Cardiac valvular disease'

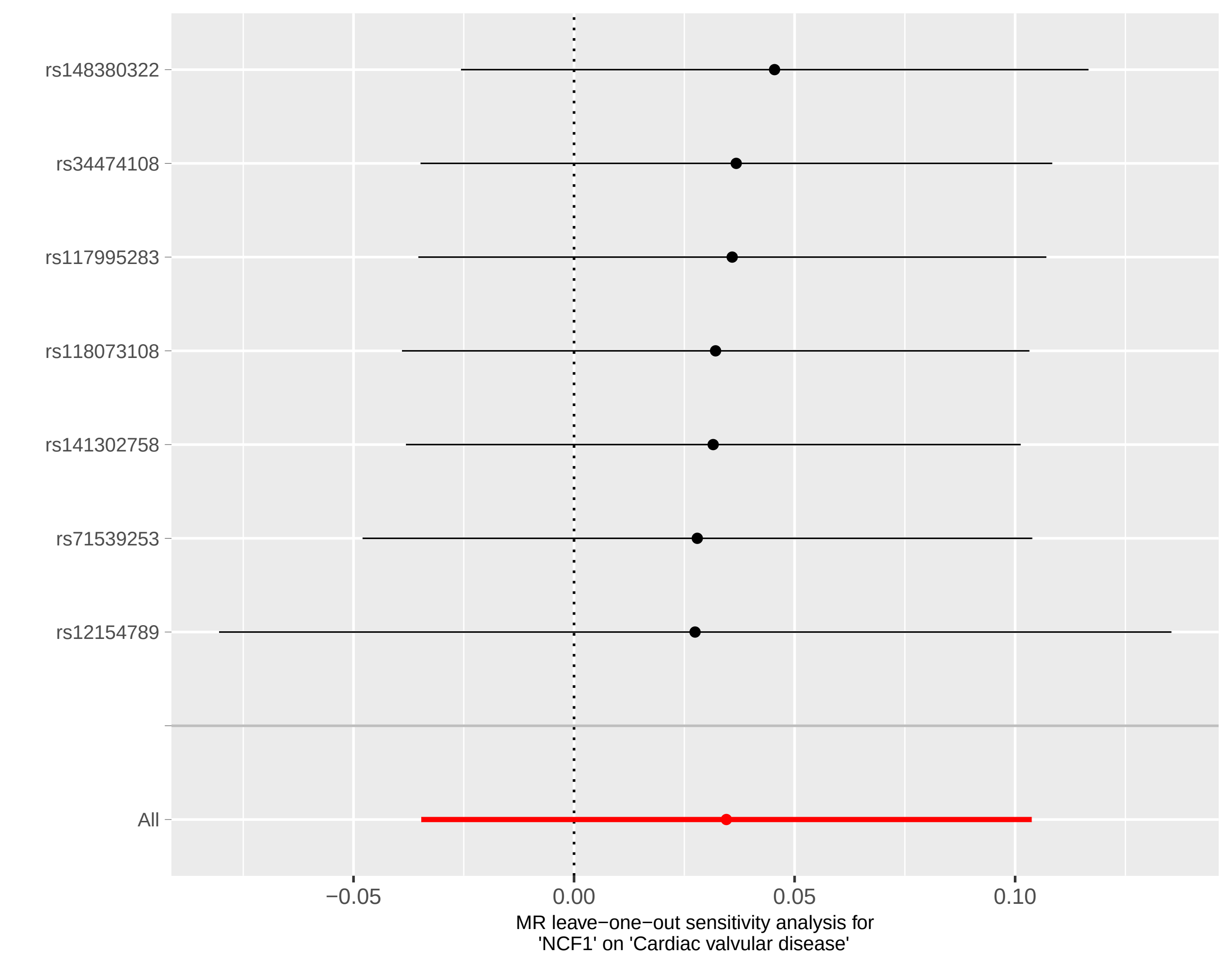
rs148380322
rs34474108
rs117995283
rs118073108
rs141302758
rs71539253
rs12154789
All
−0.05
0.00
0.05
0.10
MR leave−one−out sensitivity analysis for
'NCF1' on 'Cardiac valvular disease'

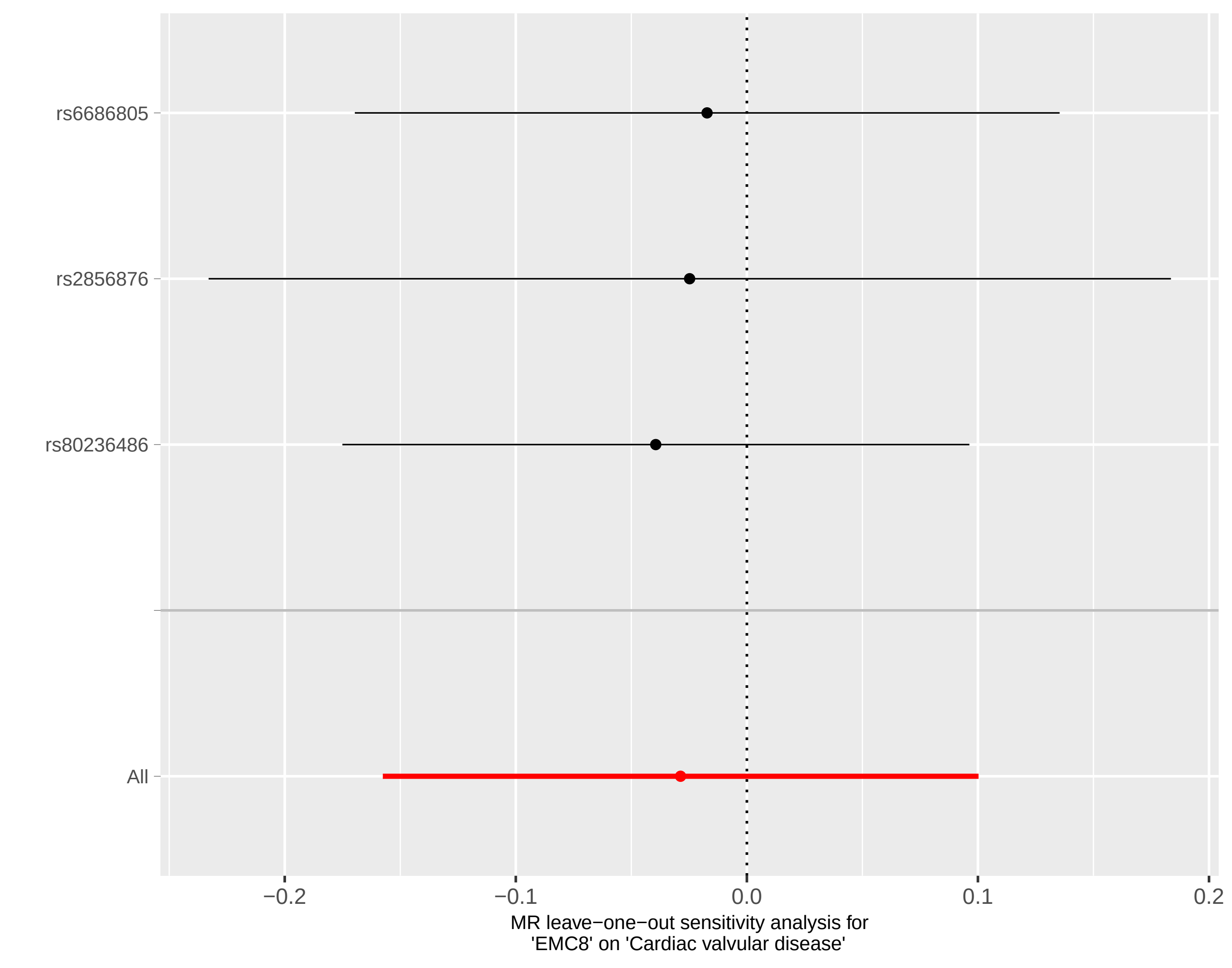
rs6686805
rs2856876
rs80236486
All
−0.2
−0.1
0.0
0.1
0.2
MR leave−one−out sensitivity analysis for
'EMC8' on 'Cardiac valvular disease'

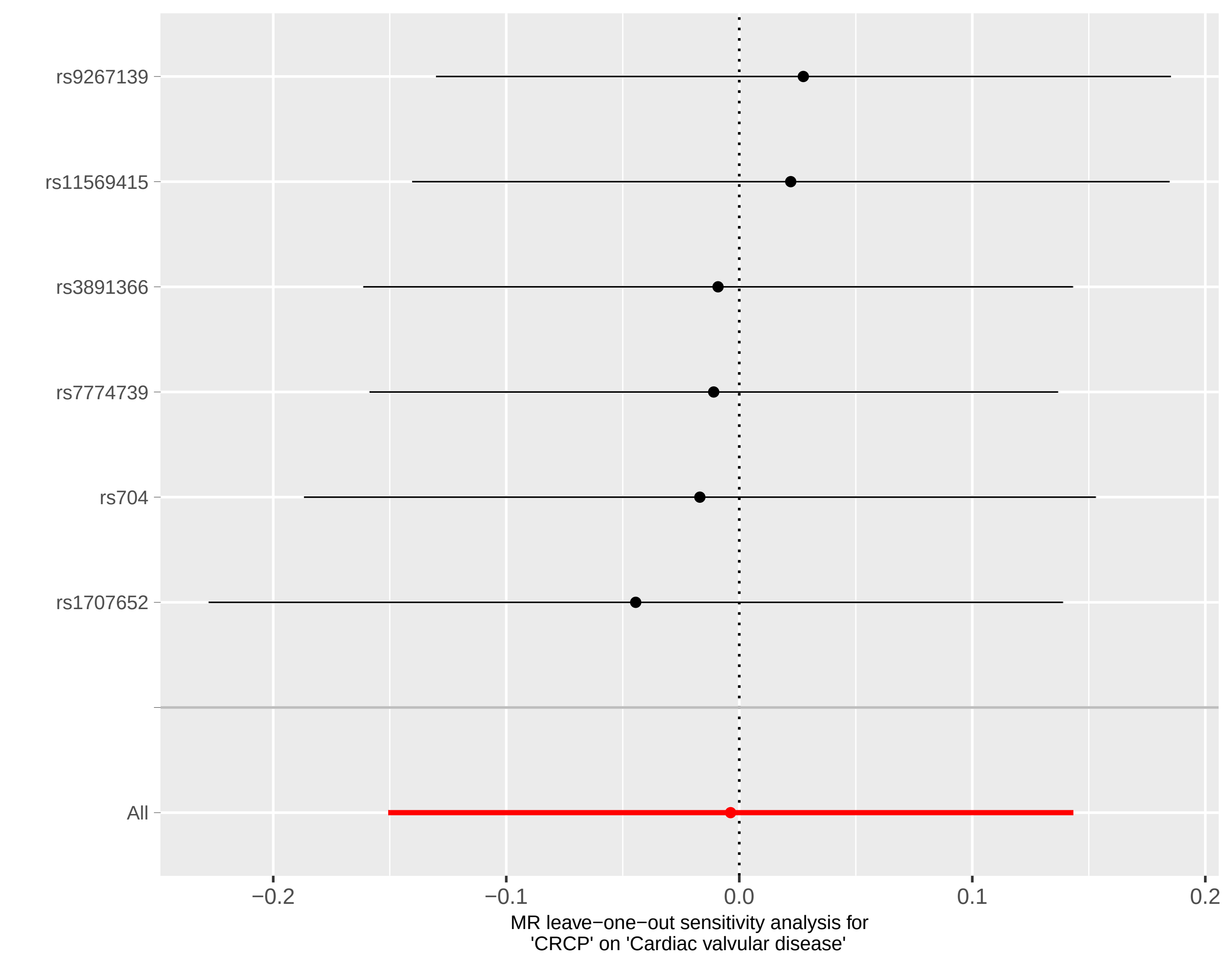
rs9267139
rs11569415
rs3891366
rs7774739
rs704
rs1707652
All
−0.2
−0.1
0.0
0.1
0.2
MR leave−one−out sensitivity analysis for
'CRCP' on 'Cardiac valvular disease'

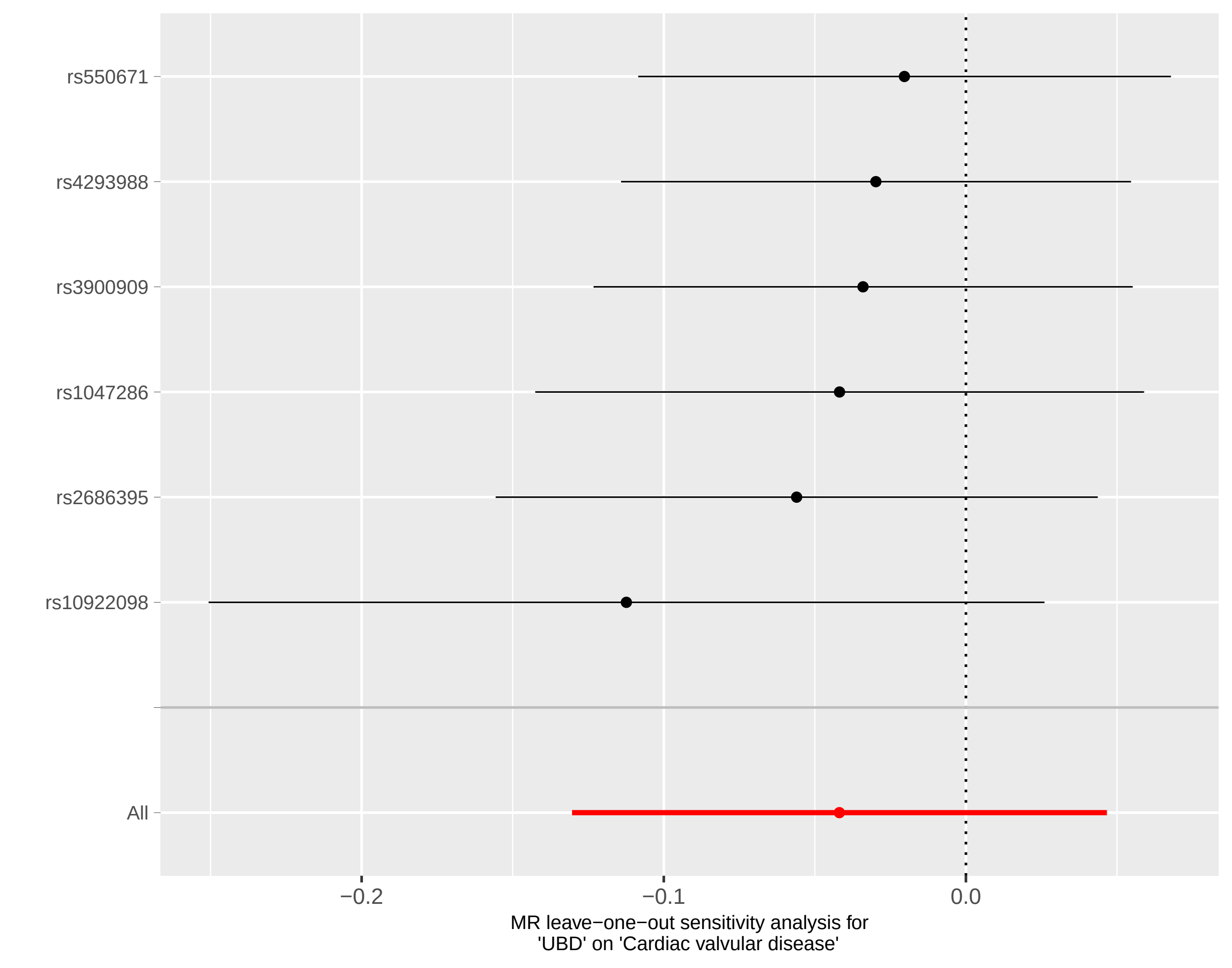
rs550671
rs4293988
rs3900909
rs1047286
rs2686395
rs10922098
All
−0.2
−0.1
0.0
MR leave−one−out sensitivity analysis for
'UBD' on 'Cardiac valvular disease'

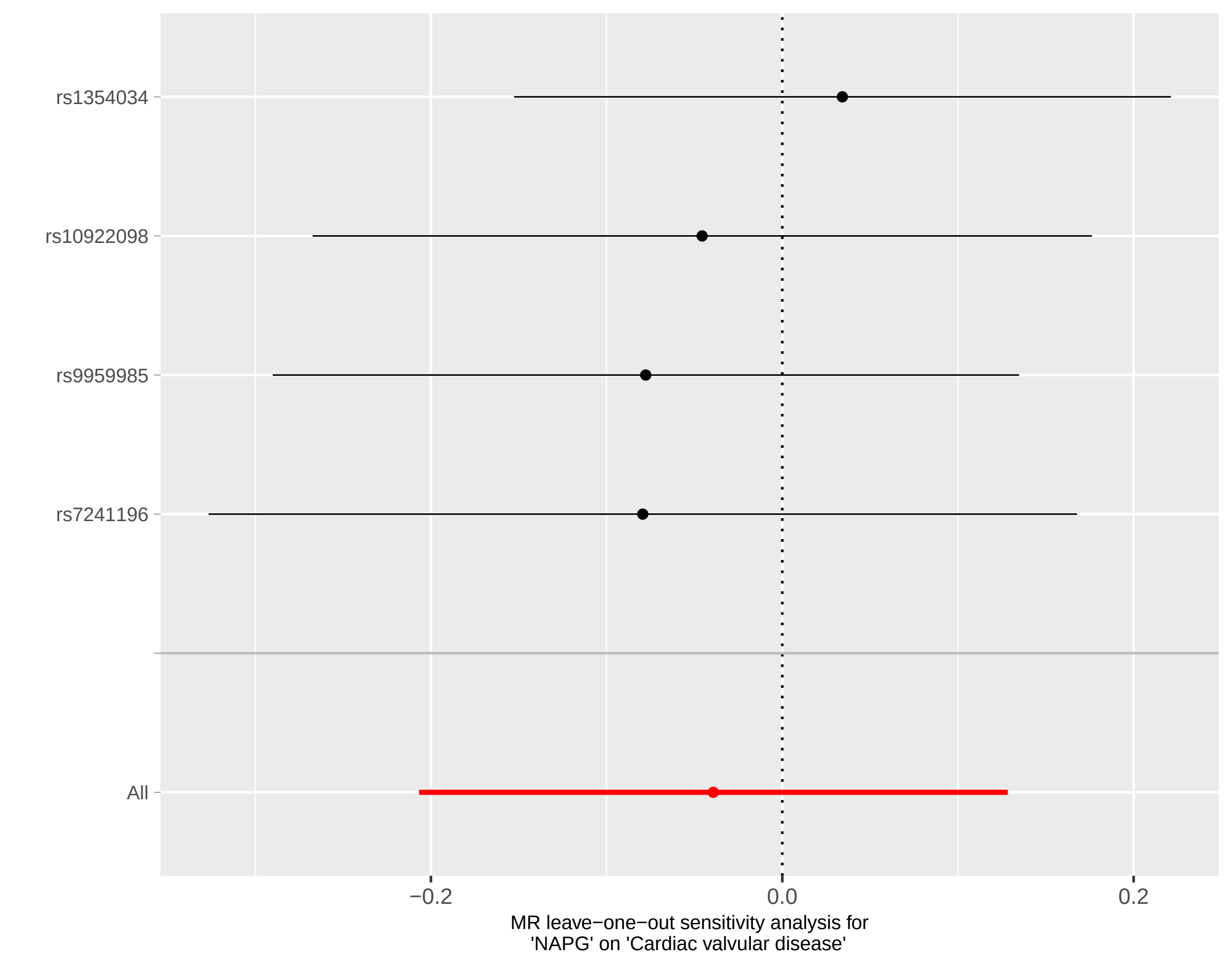
rs1354034
rs10922098
rs9959985
rs7241196
All
−0.2
0.0
0.2
MR leave−one−out sensitivity analysis for
'NAPG' on 'Cardiac valvular disease'

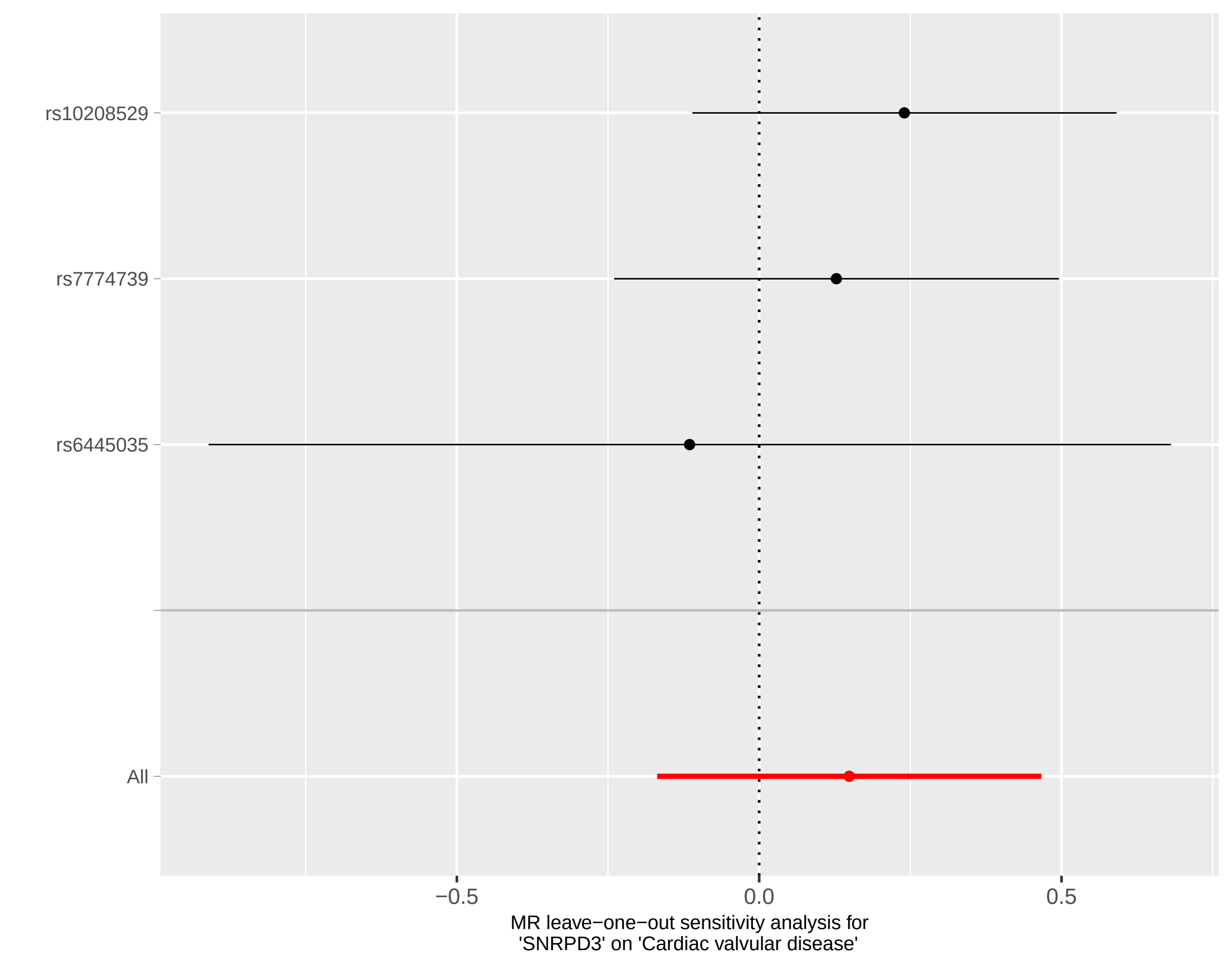
rs10208529
rs7774739
rs6445035
All
−0.5
0.0
0.5
MR leave−one−out sensitivity analysis for
'SNRPD3' on 'Cardiac valvular disease'

Insufficient number of SNPs

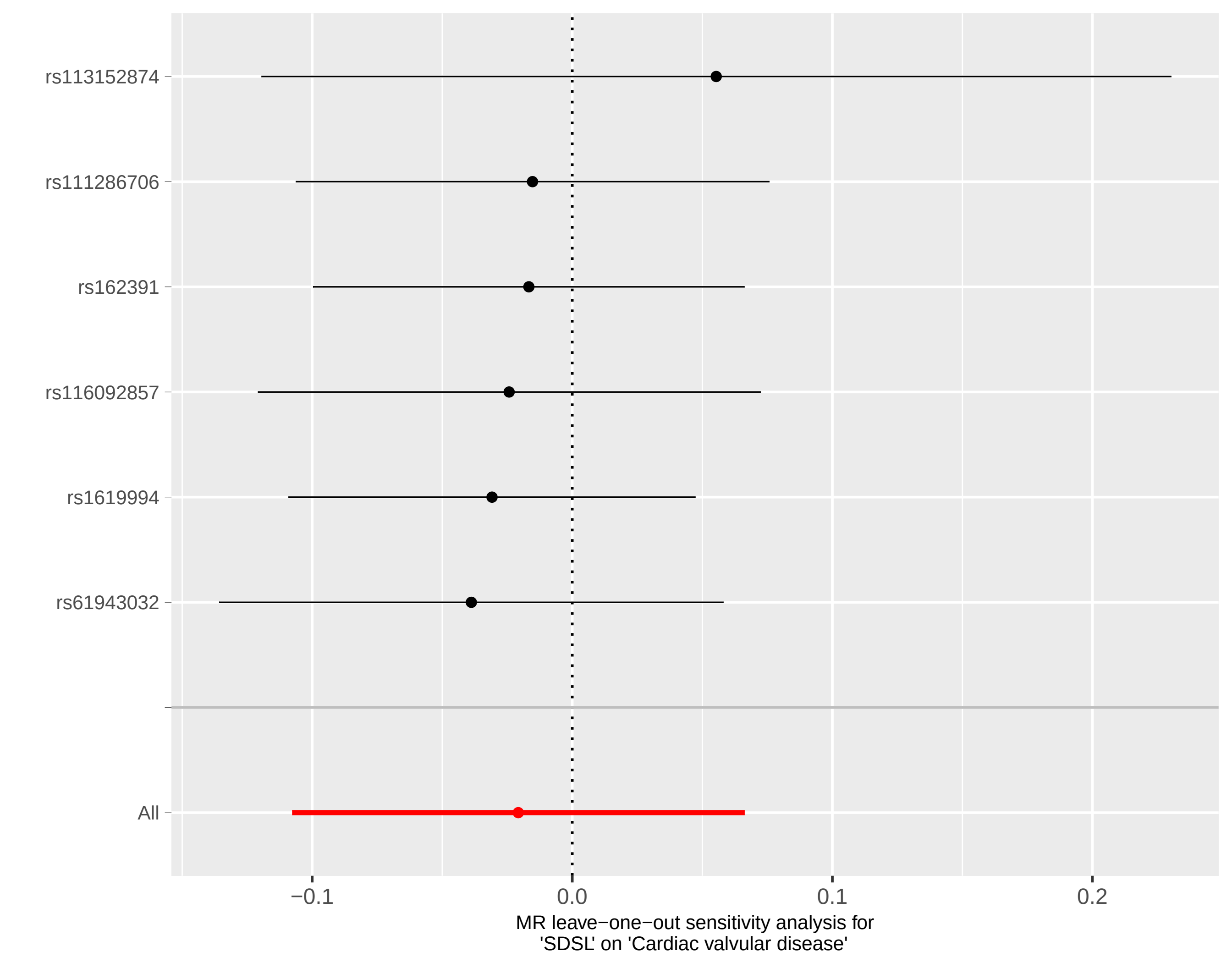

rs113152874
rs111286706
rs162391
rs116092857
rs1619994
rs61943032
All
−0.1
0.0
0.1
0.2
MR leave−one−out sensitivity analysis for
'SDSL' on 'Cardiac valvular disease'

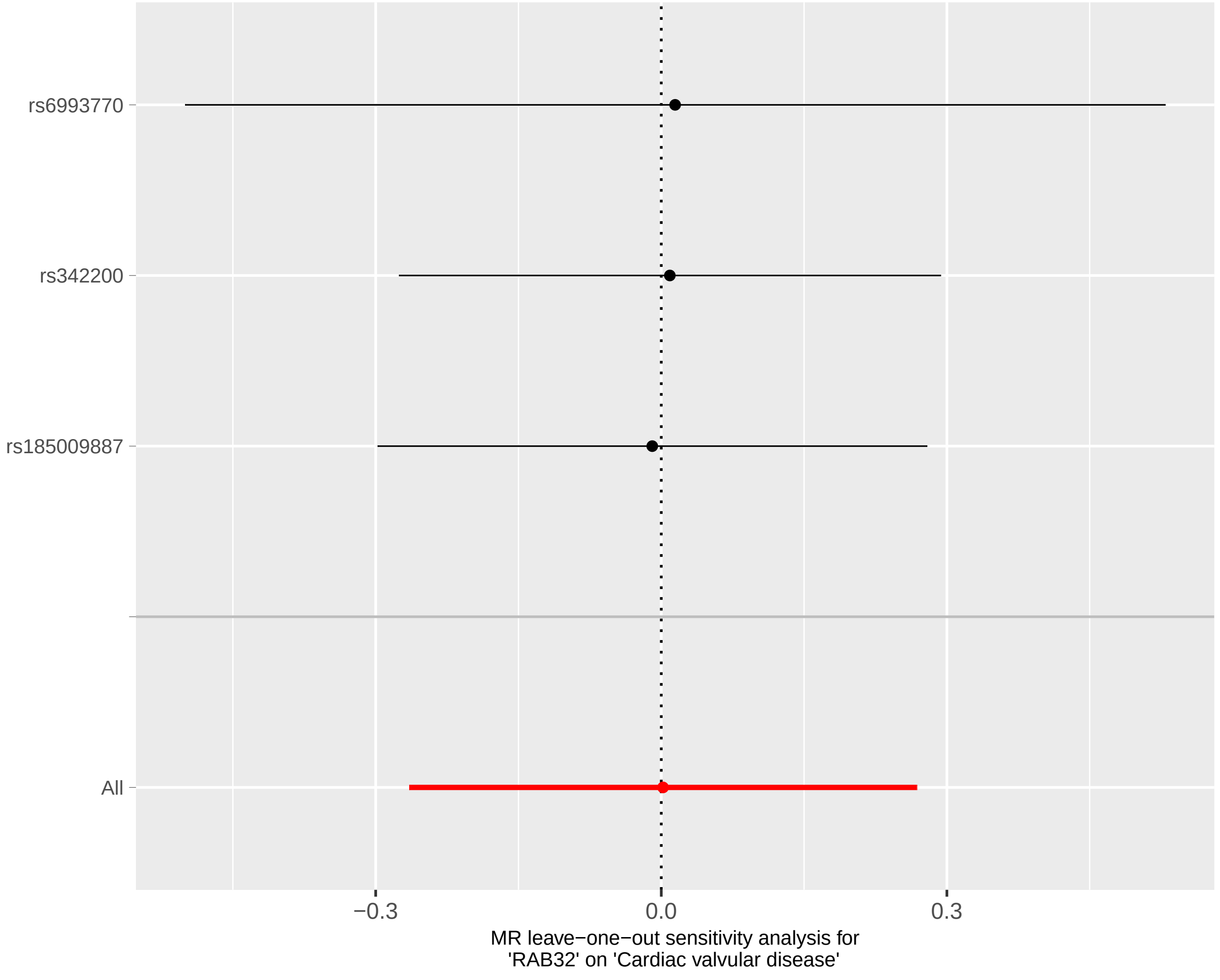
rs6993770
rs342200
rs185009887
All
−0.3
0.0
0.3
MR leave−one−out sensitivity analysis for
'RAB32' on 'Cardiac valvular disease'

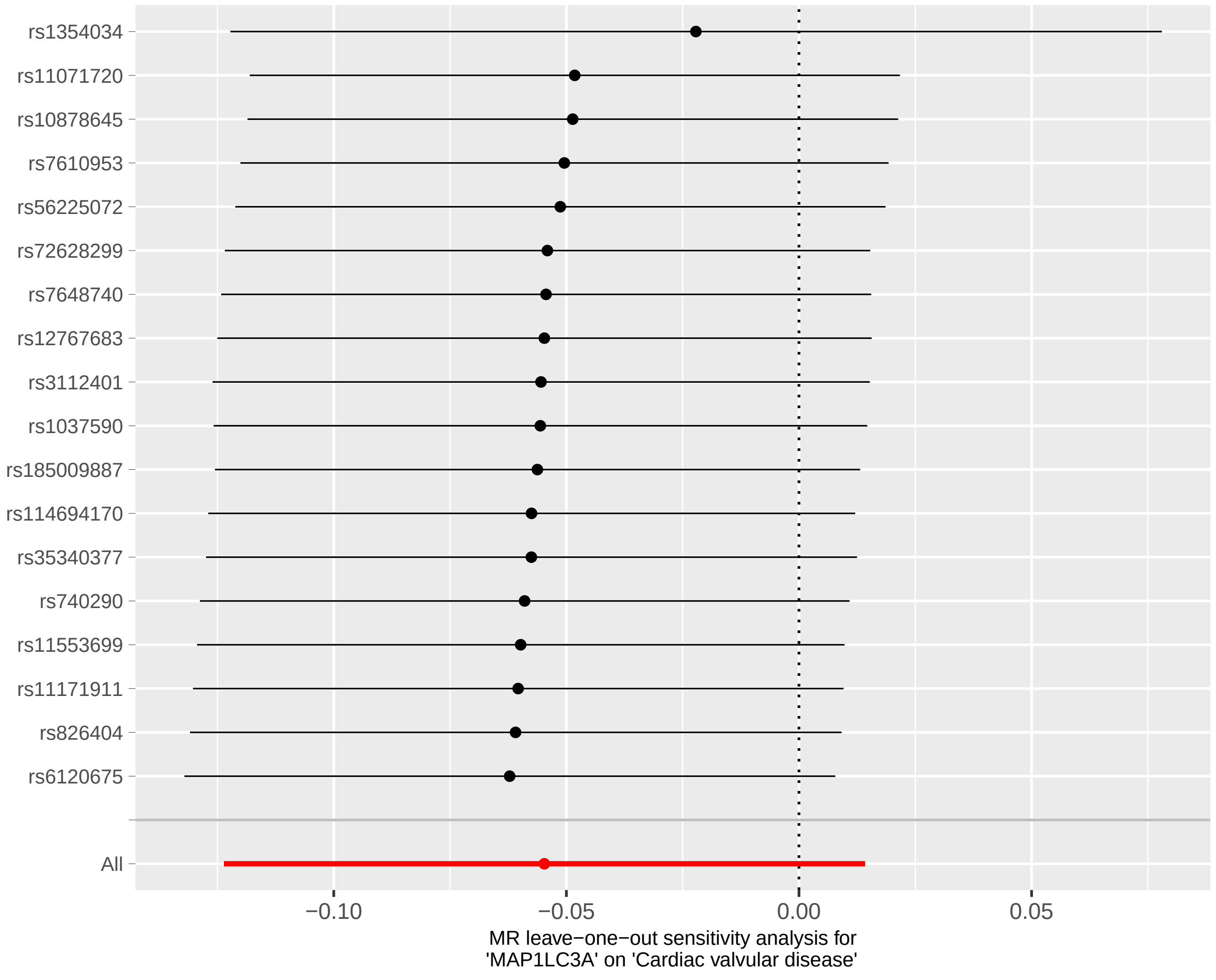
rs1354034
rs11071720
rs10878645
rs7610953
rs56225072
rs72628299
rs7648740
rs12767683
rs3112401
rs1037590
rs185009887
rs114694170
rs35340377
rs740290
rs11553699
rs11171911
rs826404
rs6120675
All
−0.10
−0.05
0.00
0.05
MR leave−one−out sensitivity analysis for
'MAP1LC3A' on 'Cardiac valvular disease'

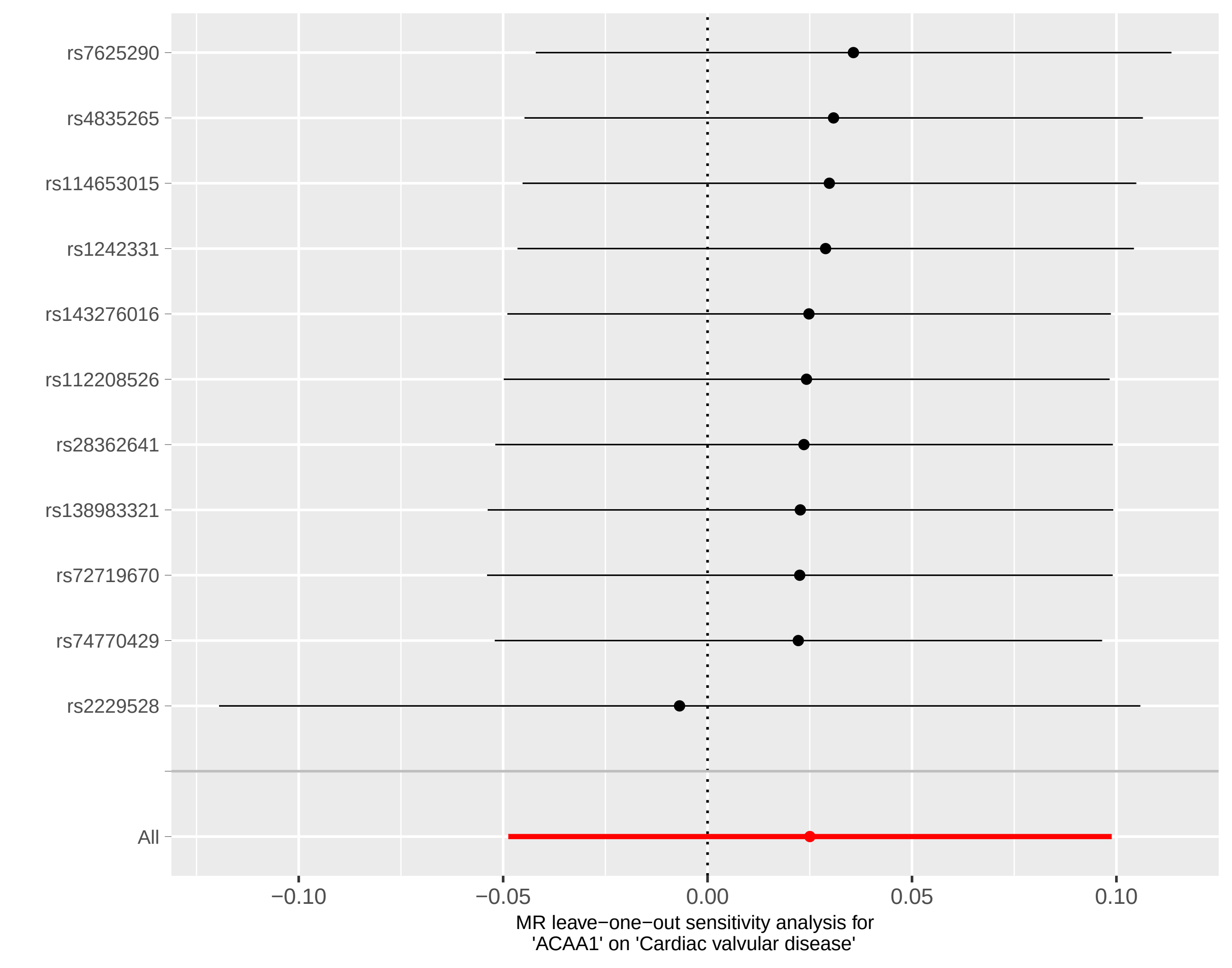
rs7625290
rs4835265
rs114653015
rs1242331
rs143276016
rs112208526
rs28362641
rs138983321
rs72719670
rs74770429
rs2229528
All
−0.10
−0.05
0.00
0.05
0.10
MR leave−one−out sensitivity analysis for
'ACAA1' on 'Cardiac valvular disease'

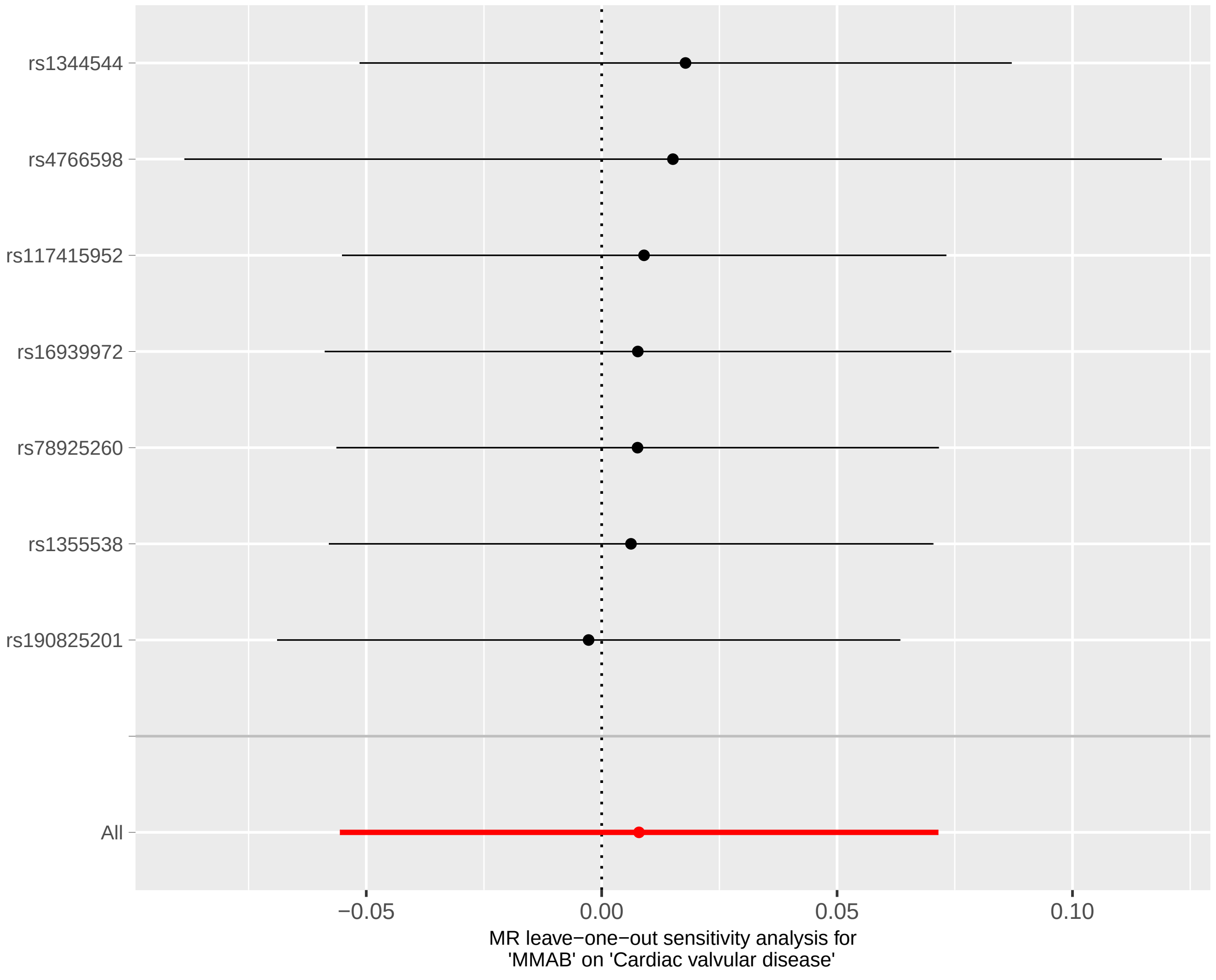
rs1344544
rs4766598
rs117415952
rs16939972
rs78925260
rs1355538
rs190825201
All
−0.05
0.00
0.05
0.10
MR leave−one−out sensitivity analysis for
'MMAB' on 'Cardiac valvular disease'

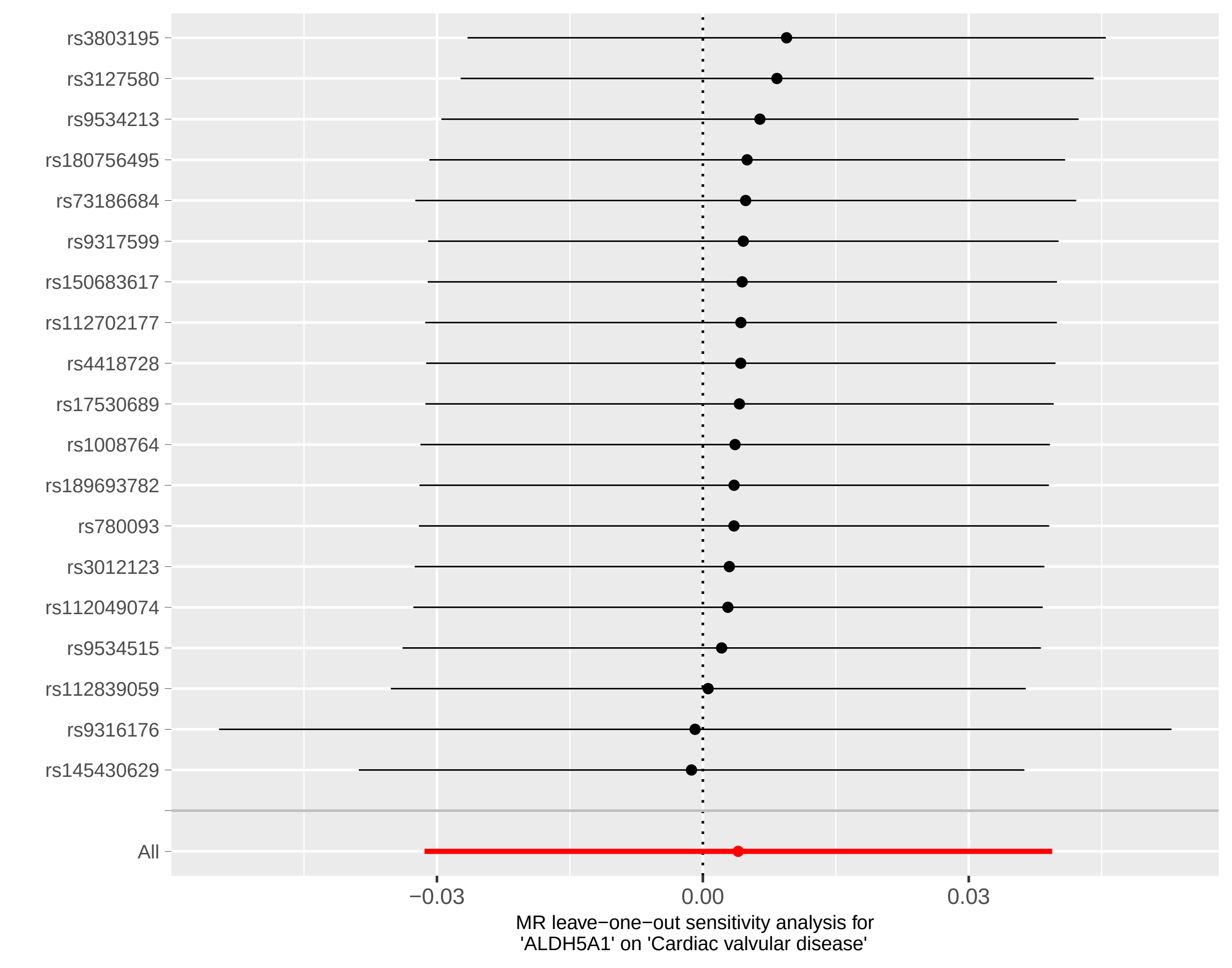

rs3803195
rs3127580
rs9534213
rs180756495
rs73186684
rs9317599
rs150683617
rs112702177
rs4418728
rs17530689
rs1008764
rs189693782
rs780093
rs3012123
rs112049074
rs9534515
rs112839059
rs9316176
rs145430629
All
−0.03
0.00
0.03
MR leave−one−out sensitivity analysis for
'ALDH5A1' on 'Cardiac valvular disease'

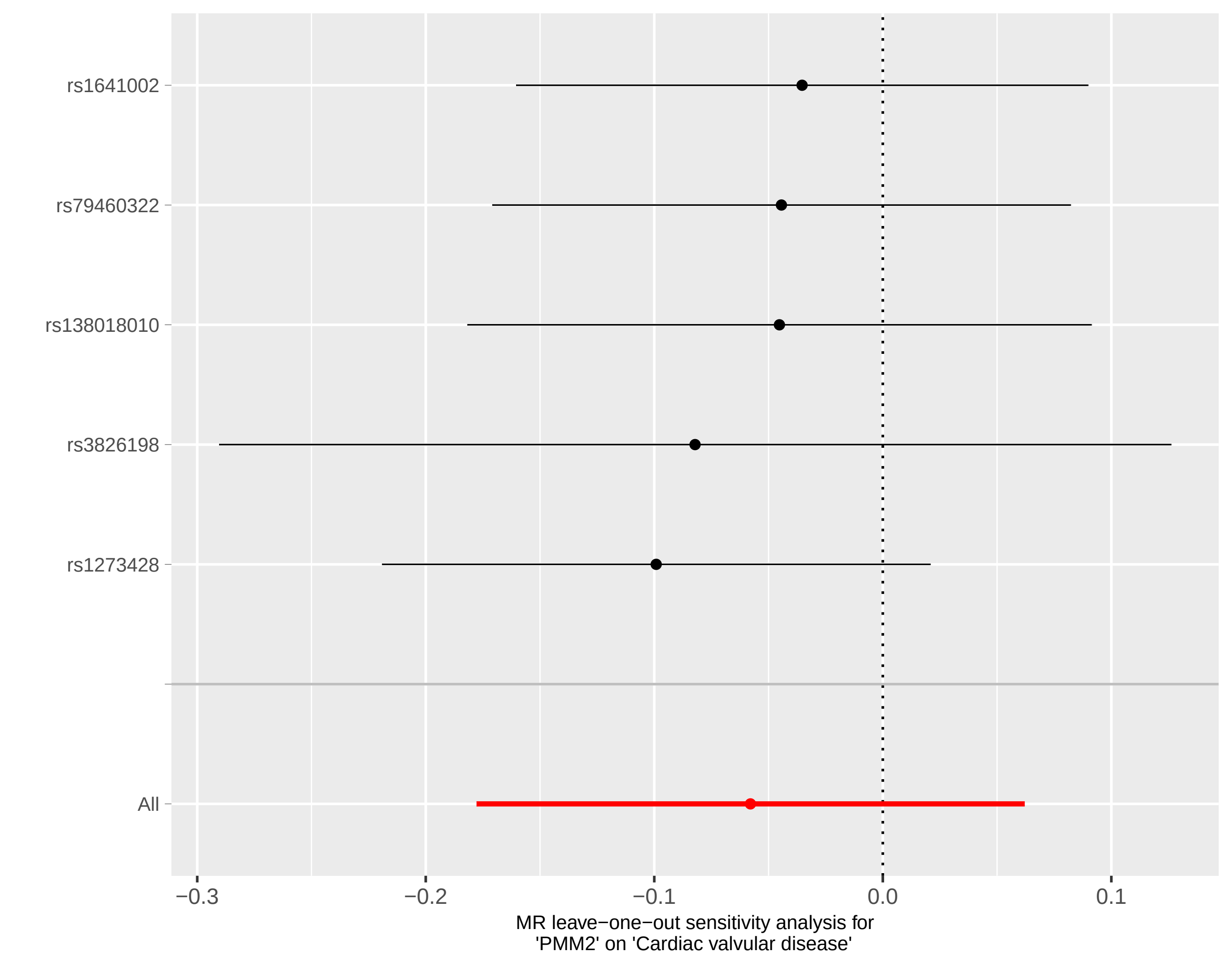

rs1641002
rs79460322
rs138018010
rs3826198
rs1273428
All
−0.3
−0.2
−0.1
0.0
0.1
MR leave−one−out sensitivity analysis for
'PMM2' on 'Cardiac valvular disease'

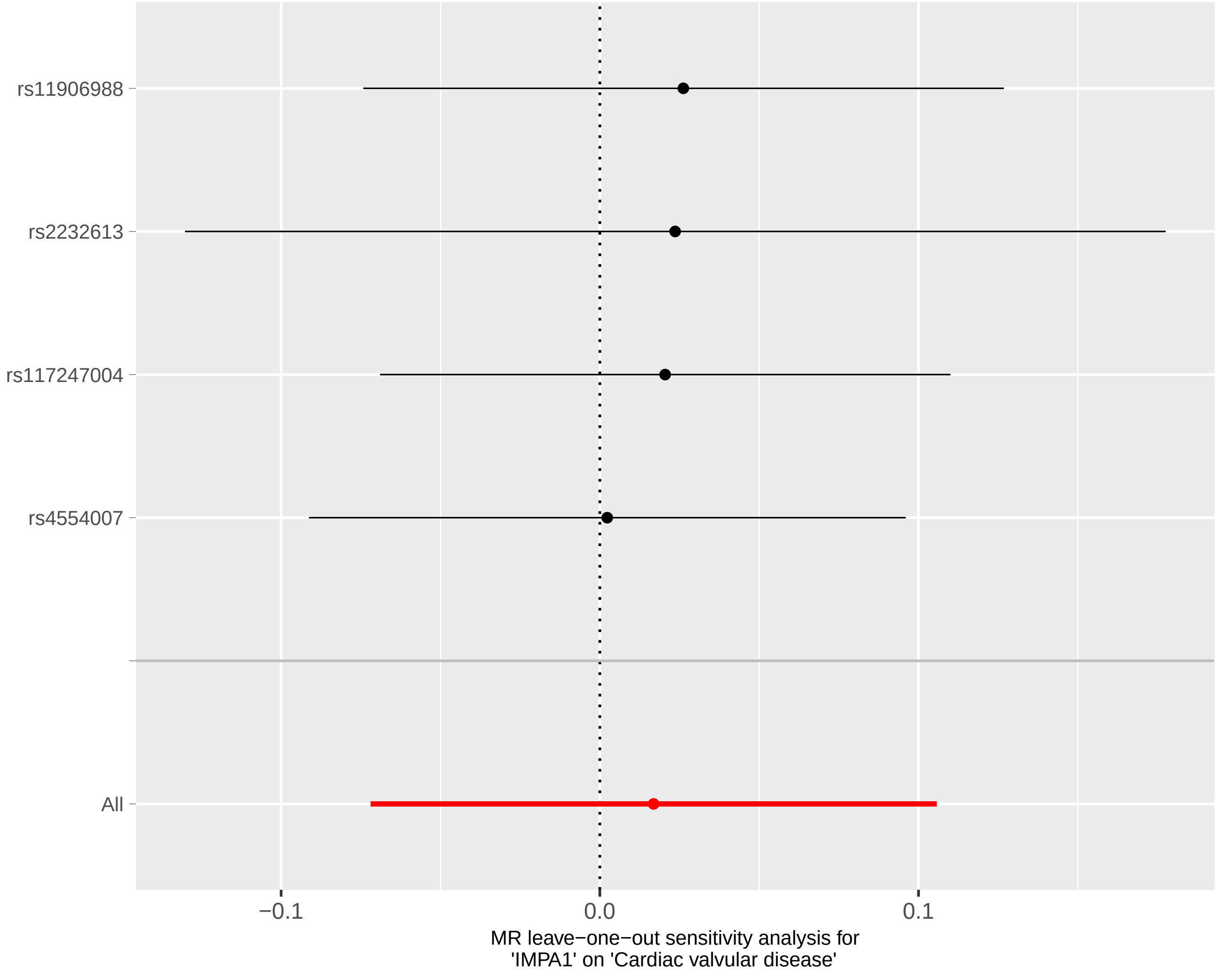

rs11906988
rs2232613
rs117247004
rs4554007
All
−0.1
0.0
0.1
MR leave−one−out sensitivity analysis for
'IMPA1' on 'Cardiac valvular disease'

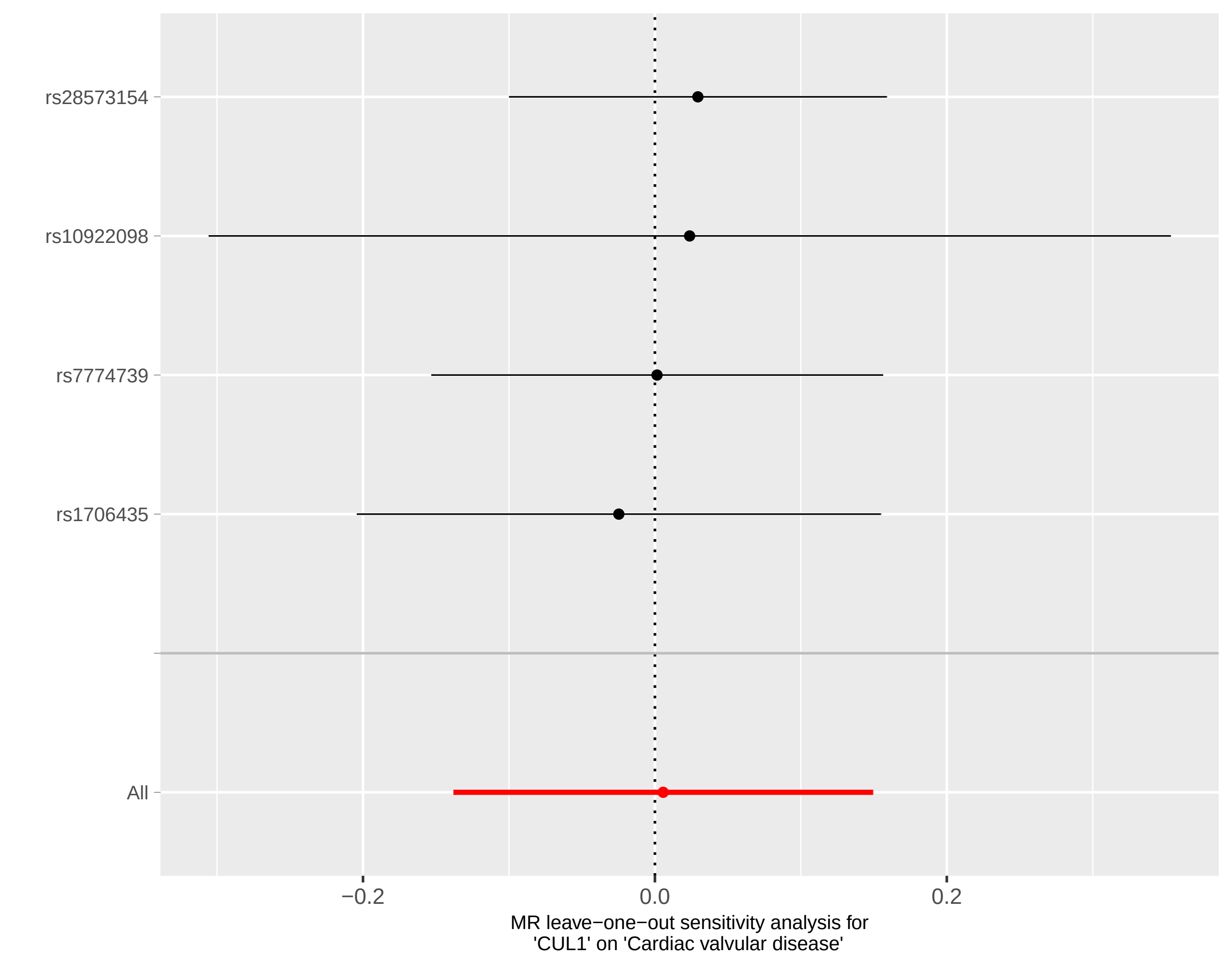
rs28573154
rs10922098
rs7774739
rs1706435
All
−0.2
0.0
0.2
MR leave−one−out sensitivity analysis for
'CUL1' on 'Cardiac valvular disease'

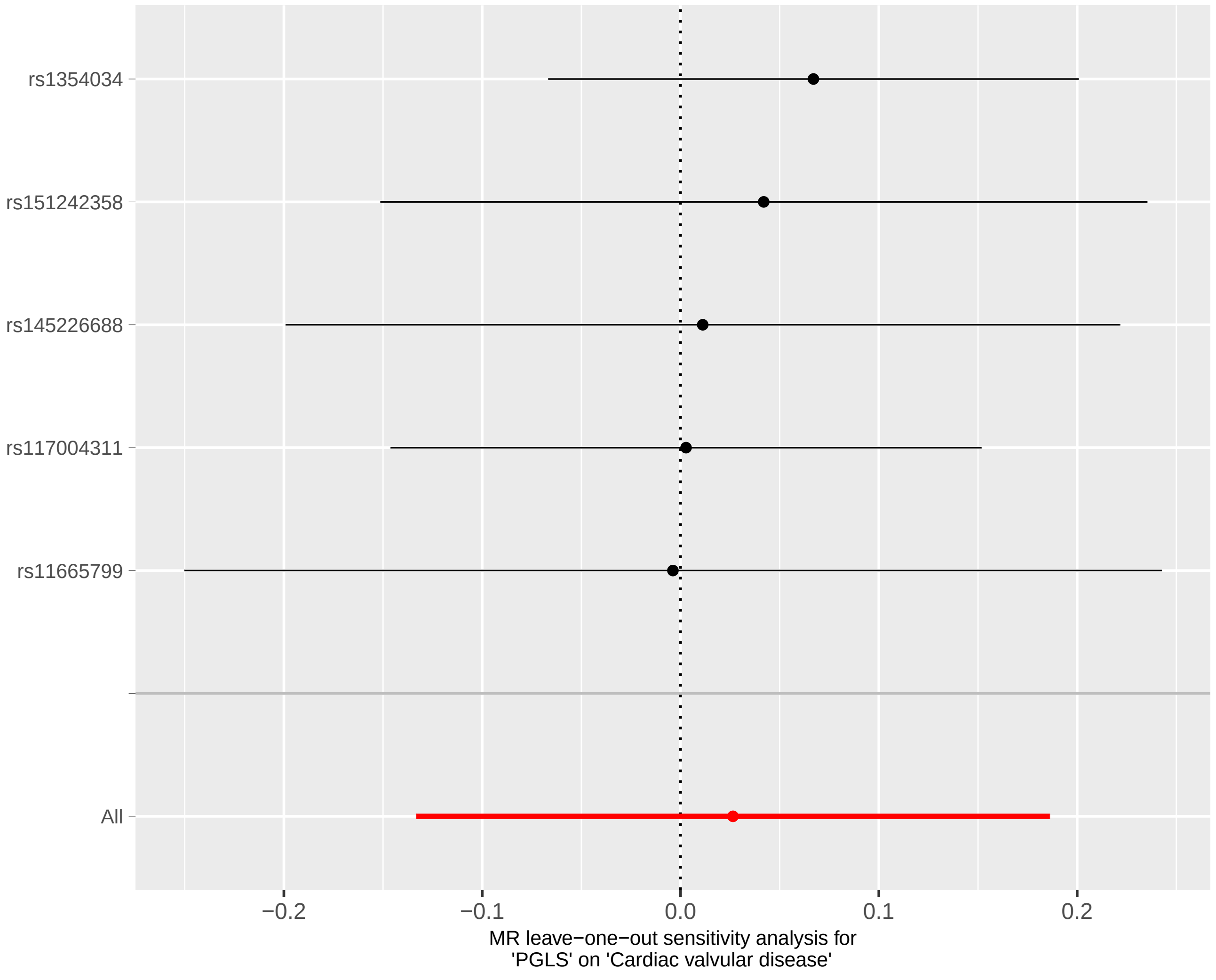
rs1354034
rs151242358
rs145226688
rs117004311
rs11665799
All
−0.2
−0.1
0.0
0.1
0.2
MR leave−one−out sensitivity analysis for
'PGLS' on 'Cardiac valvular disease'

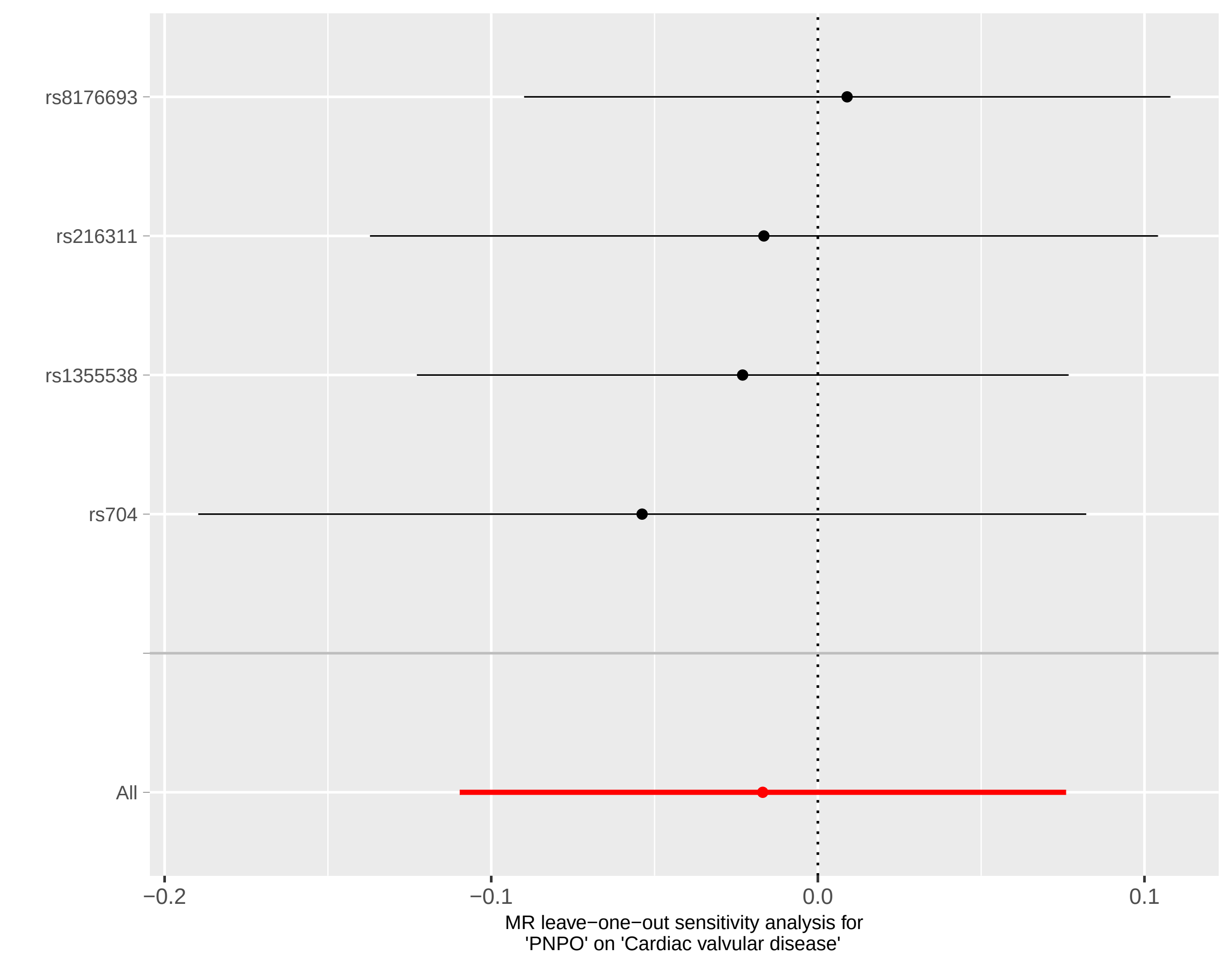
rs8176693
rs216311
rs1355538
rs704
All
−0.2
−0.1
0.0
0.1
MR leave−one−out sensitivity analysis for
'PNPO' on 'Cardiac valvular disease'

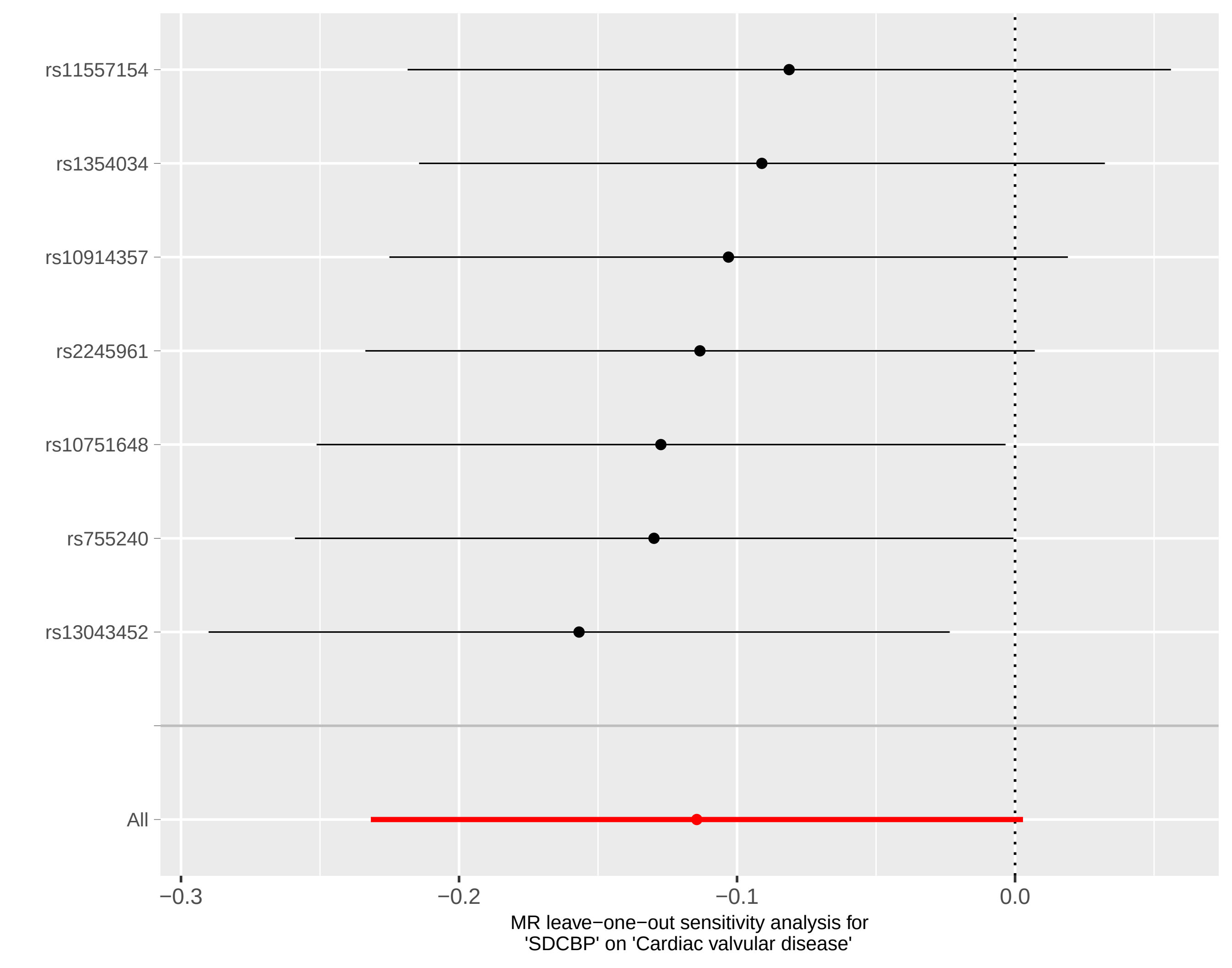
rs11557154
rs1354034
rs10914357
rs2245961
rs10751648
rs755240
rs13043452
All
−0.3
−0.2
−0.1
0.0
MR leave−one−out sensitivity analysis for
'SDCBP' on 'Cardiac valvular disease'

Insufficient number of SNPs

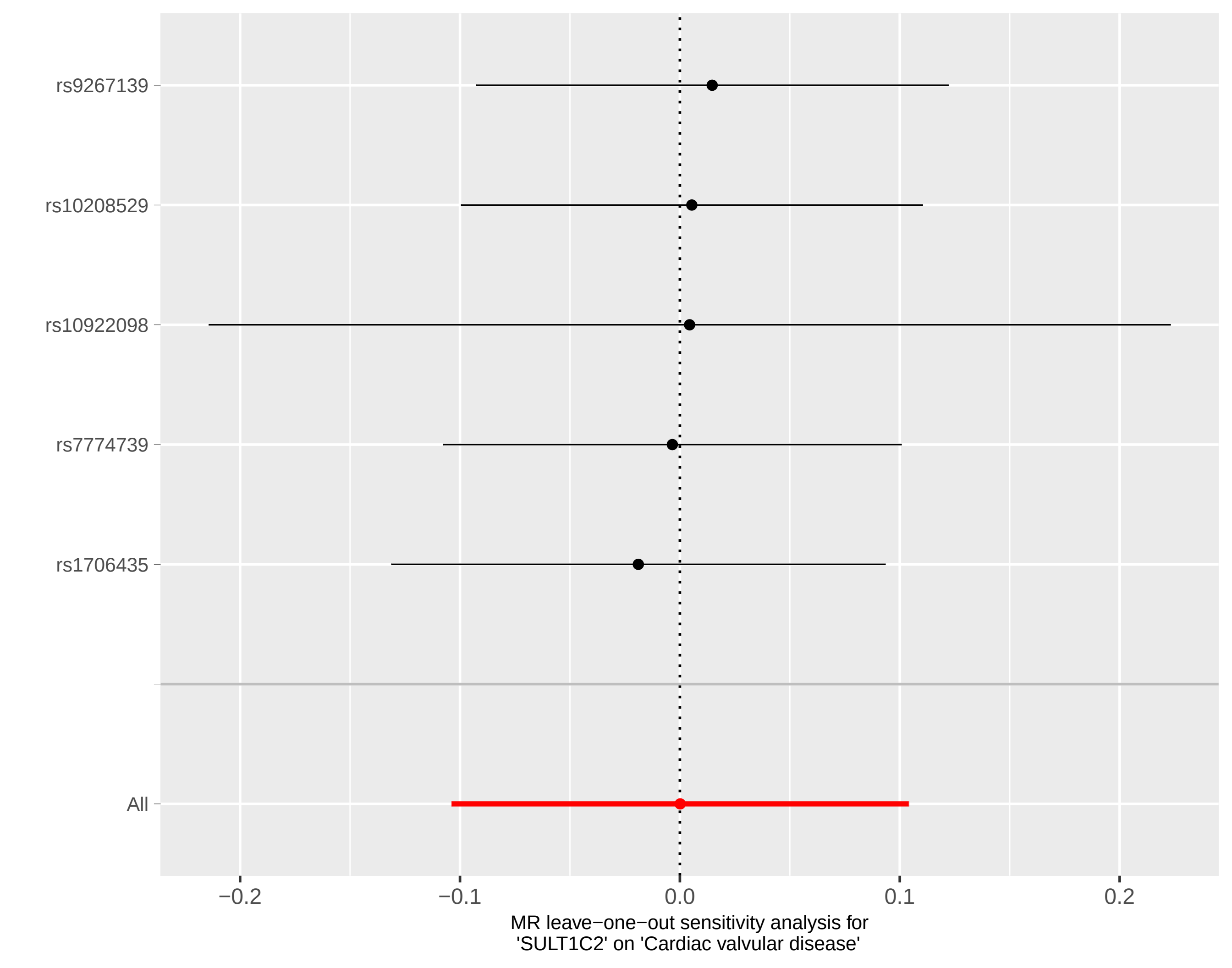
rs9267139
rs10208529
rs10922098
rs7774739
rs1706435
All
−0.2
−0.1
0.0
0.1
0.2
MR leave−one−out sensitivity analysis for
'SULT1C2' on 'Cardiac valvular disease'

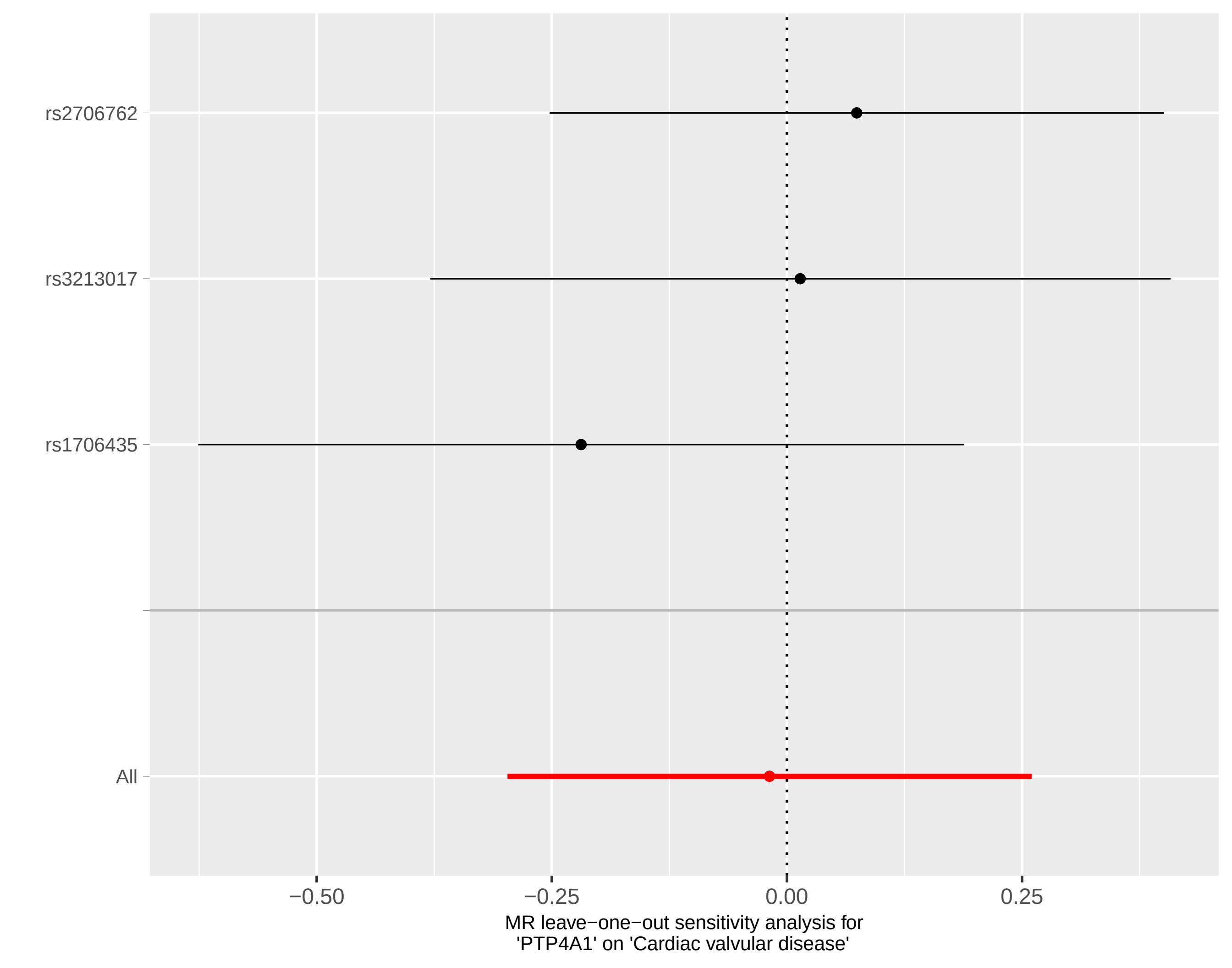
rs2706762
rs3213017
rs1706435
All
−0.50
−0.25
0.00
0.25
MR leave−one−out sensitivity analysis for
'PTP4A1' on 'Cardiac valvular disease'

Insufficient number of SNPs

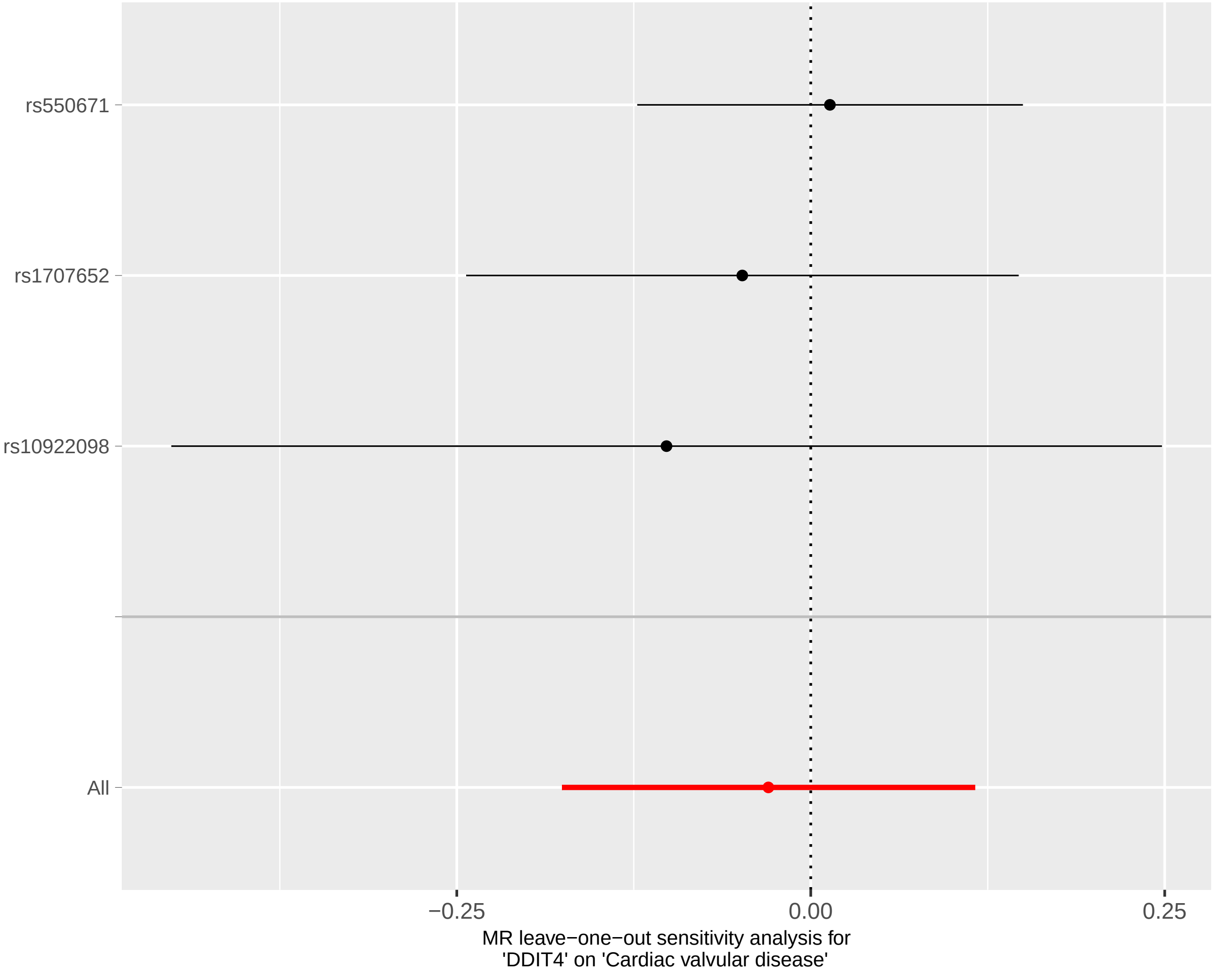
rs550671
rs1707652
rs10922098
All
−0.25
0.00
0.25
MR leave−one−out sensitivity analysis for
'DDIT4' on 'Cardiac valvular disease'

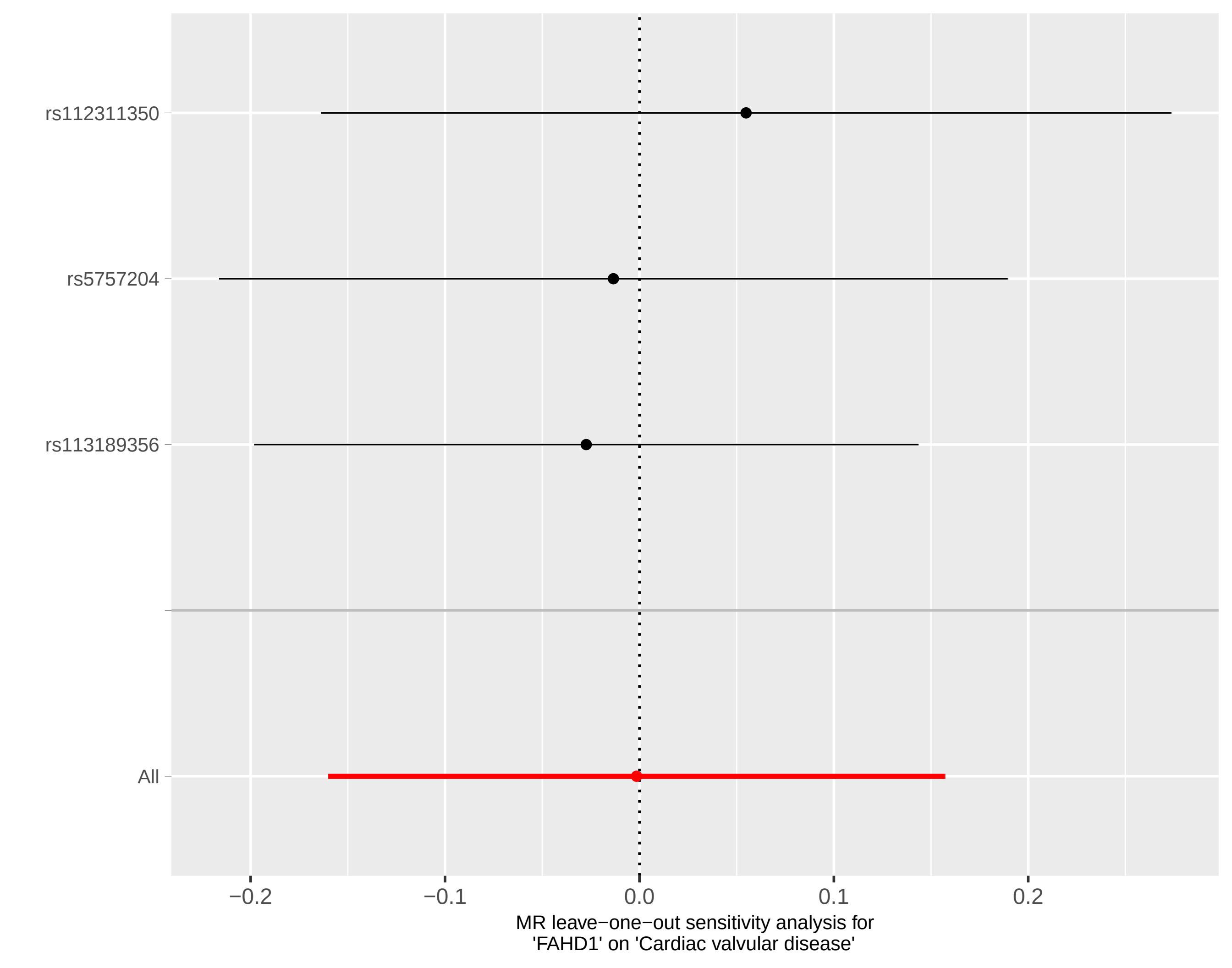

rs112311350
rs5757204
rs113189356
All
−0.2
−0.1
0.0
0.1
0.2
MR leave−one−out sensitivity analysis for
'FAHD1' on 'Cardiac valvular disease'

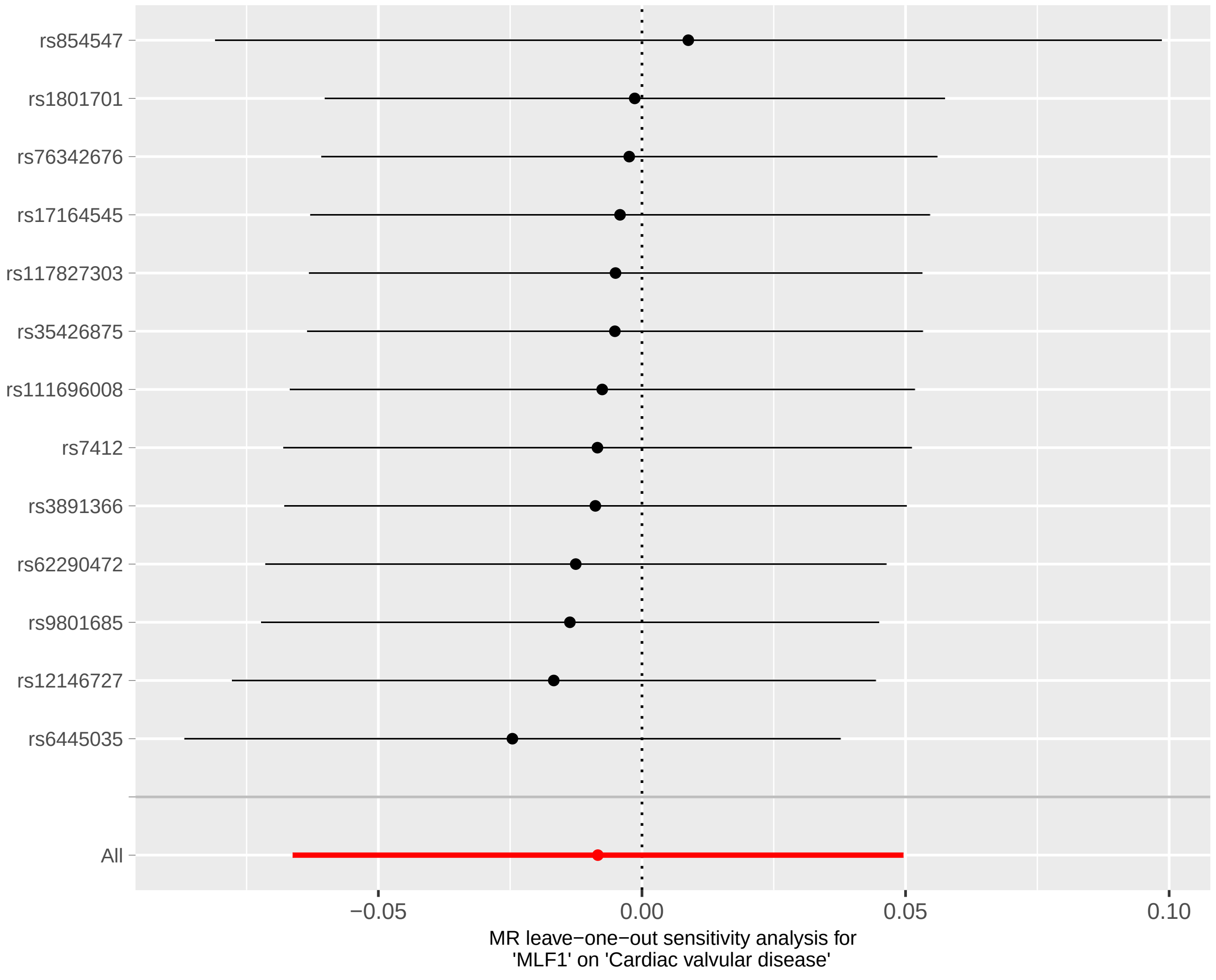
rs854547
rs1801701
rs76342676
rs17164545
rs117827303
rs35426875
rs111696008
rs7412
rs3891366
rs62290472
rs9801685
rs12146727
rs6445035
All
−0.05
0.00
0.05
0.10
MR leave−one−out sensitivity analysis for
'MLF1' on 'Cardiac valvular disease'

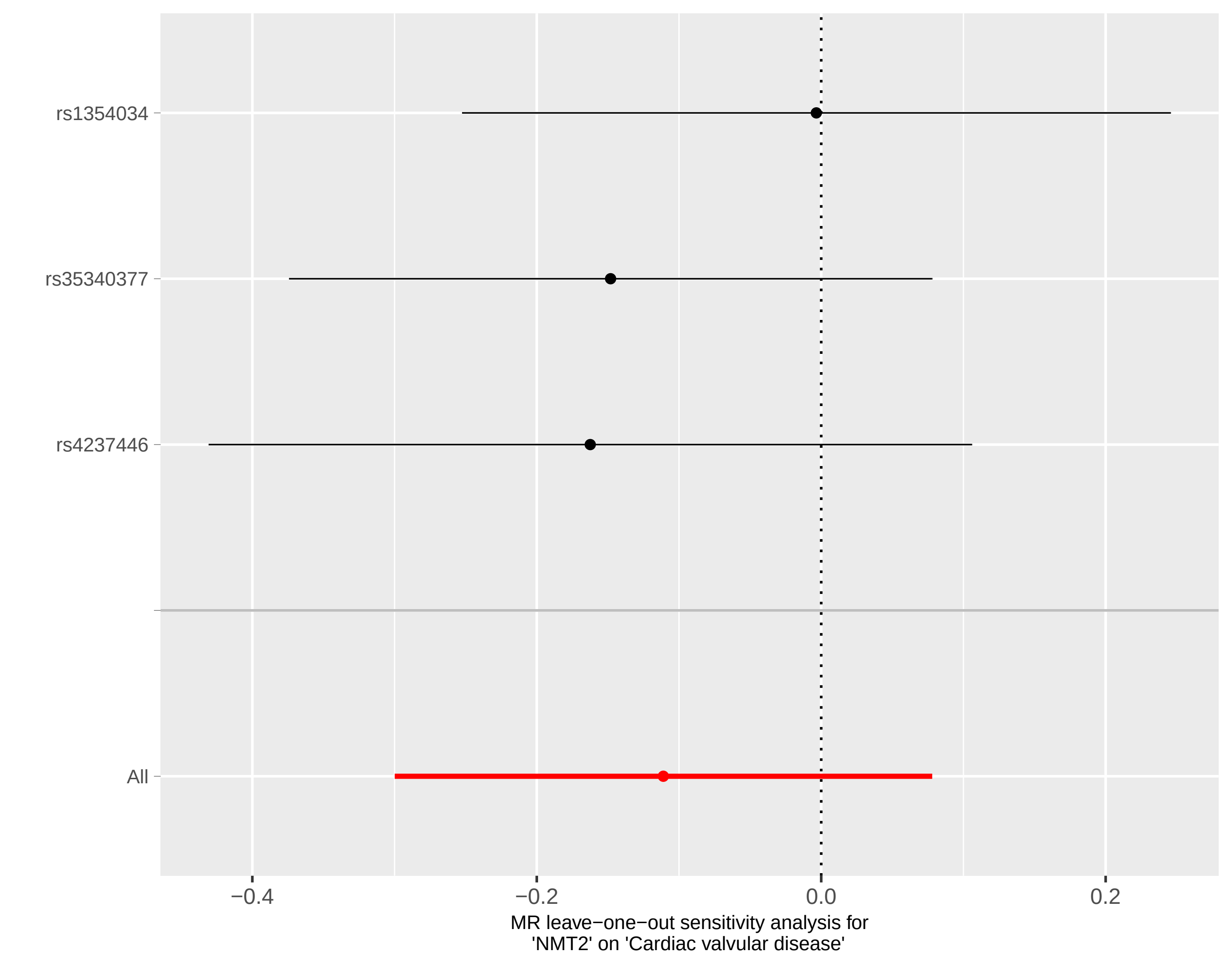

rs1354034
rs35340377
rs4237446
All
−0.4
−0.2
0.0
0.2
MR leave−one−out sensitivity analysis for
'NMT2' on 'Cardiac valvular disease'

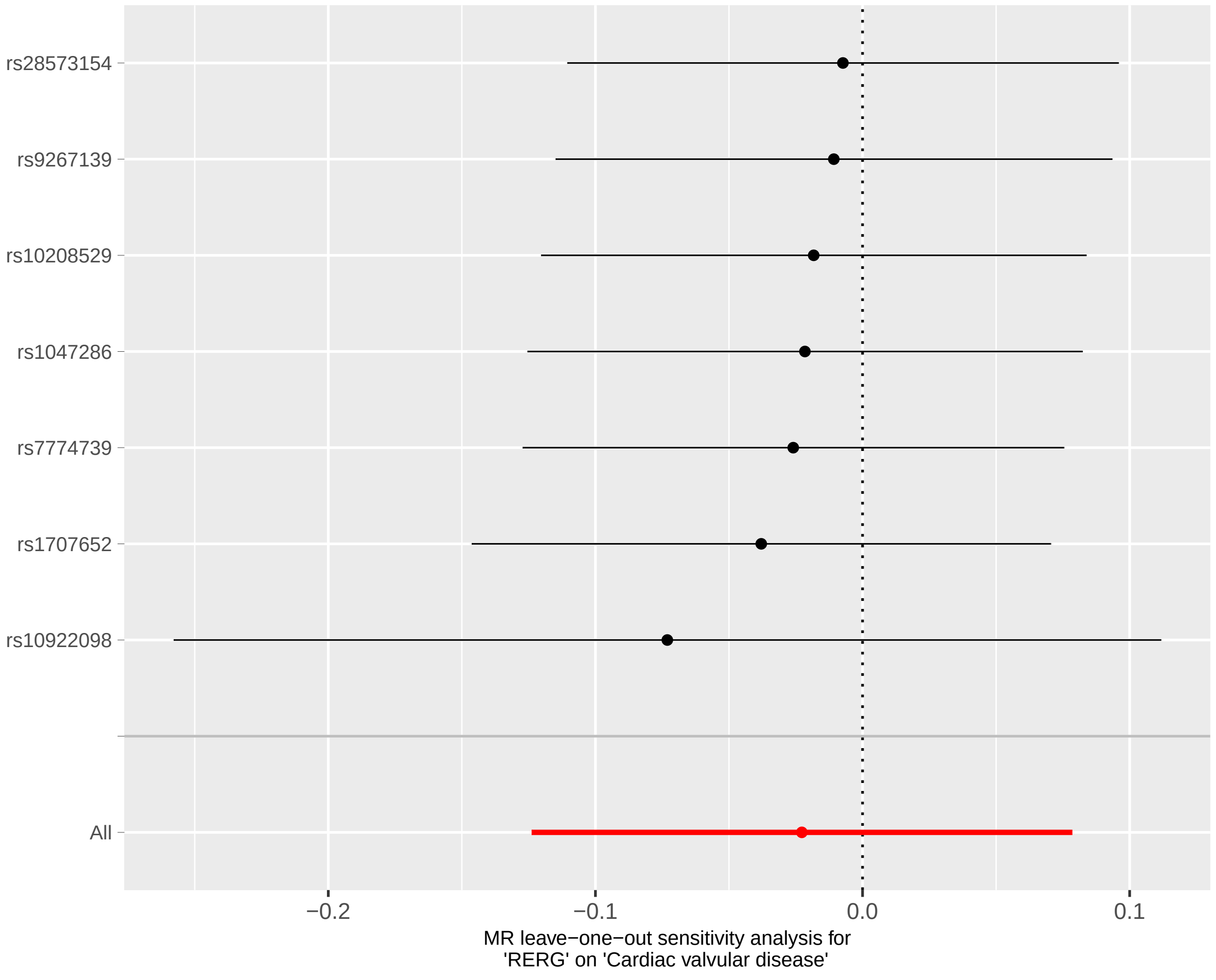
rs28573154
rs9267139
rs10208529
rs1047286
rs7774739
rs1707652
rs10922098
All
−0.2
−0.1
0.0
0.1
MR leave−one−out sensitivity analysis for
'RERG' on 'Cardiac valvular disease'

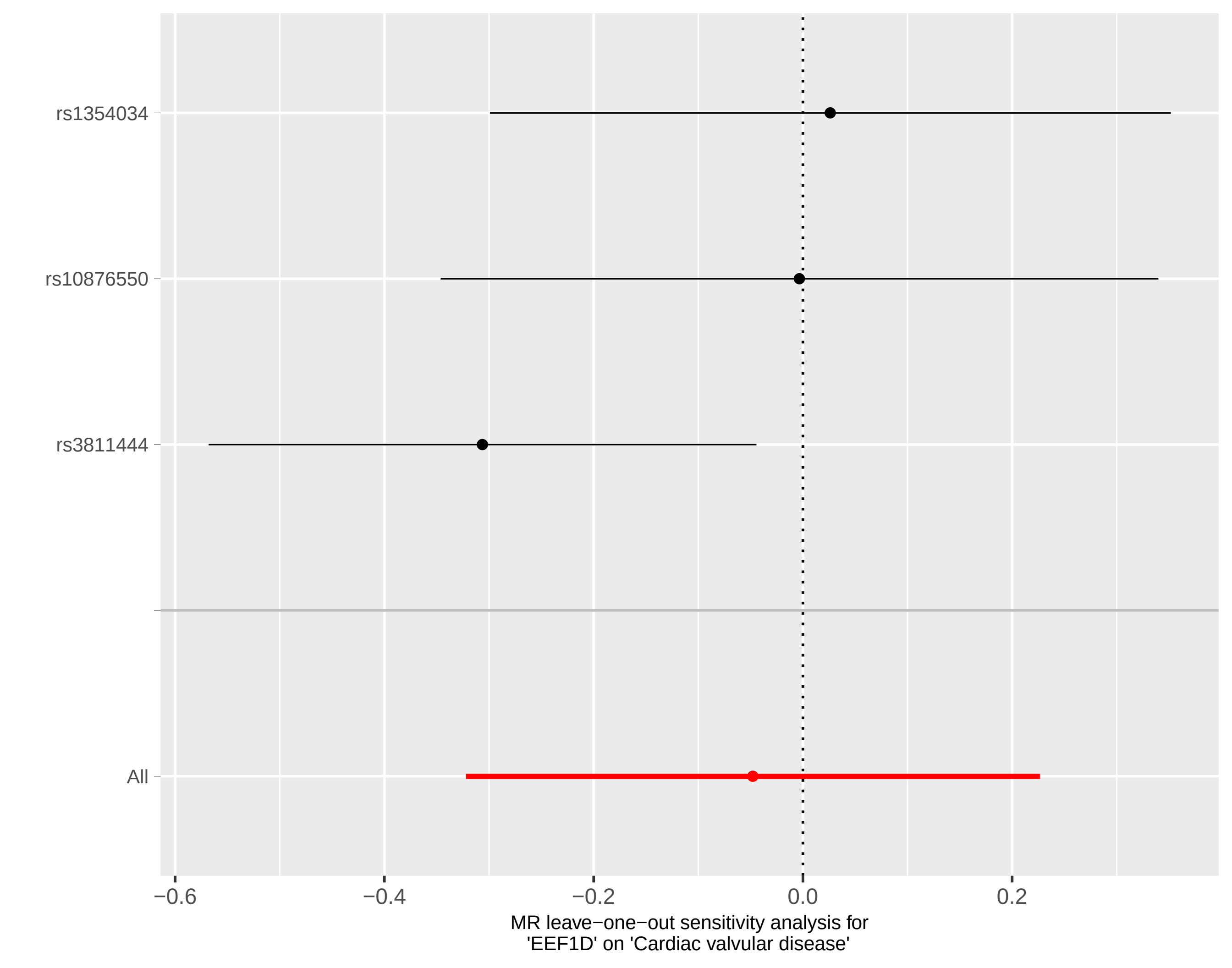
rs1354034
rs10876550
rs3811444
All
−0.6
−0.4
−0.2
0.0
0.2
MR leave−one−out sensitivity analysis for
'EEF1D' on 'Cardiac valvular disease'

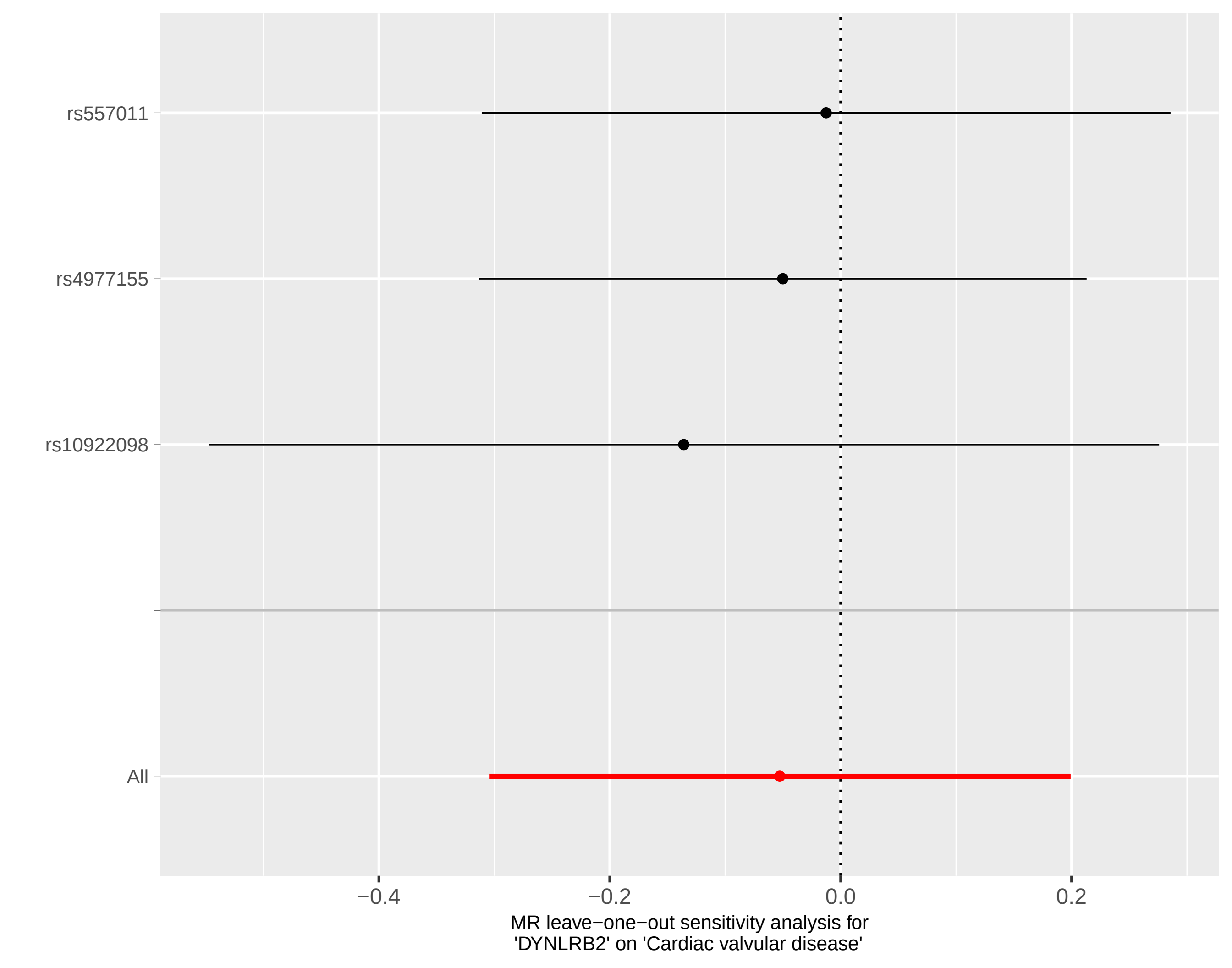
rs557011
rs4977155
rs10922098
All
−0.4
−0.2
0.0
0.2
MR leave−one−out sensitivity analysis for
'DYNLRB2' on 'Cardiac valvular disease'

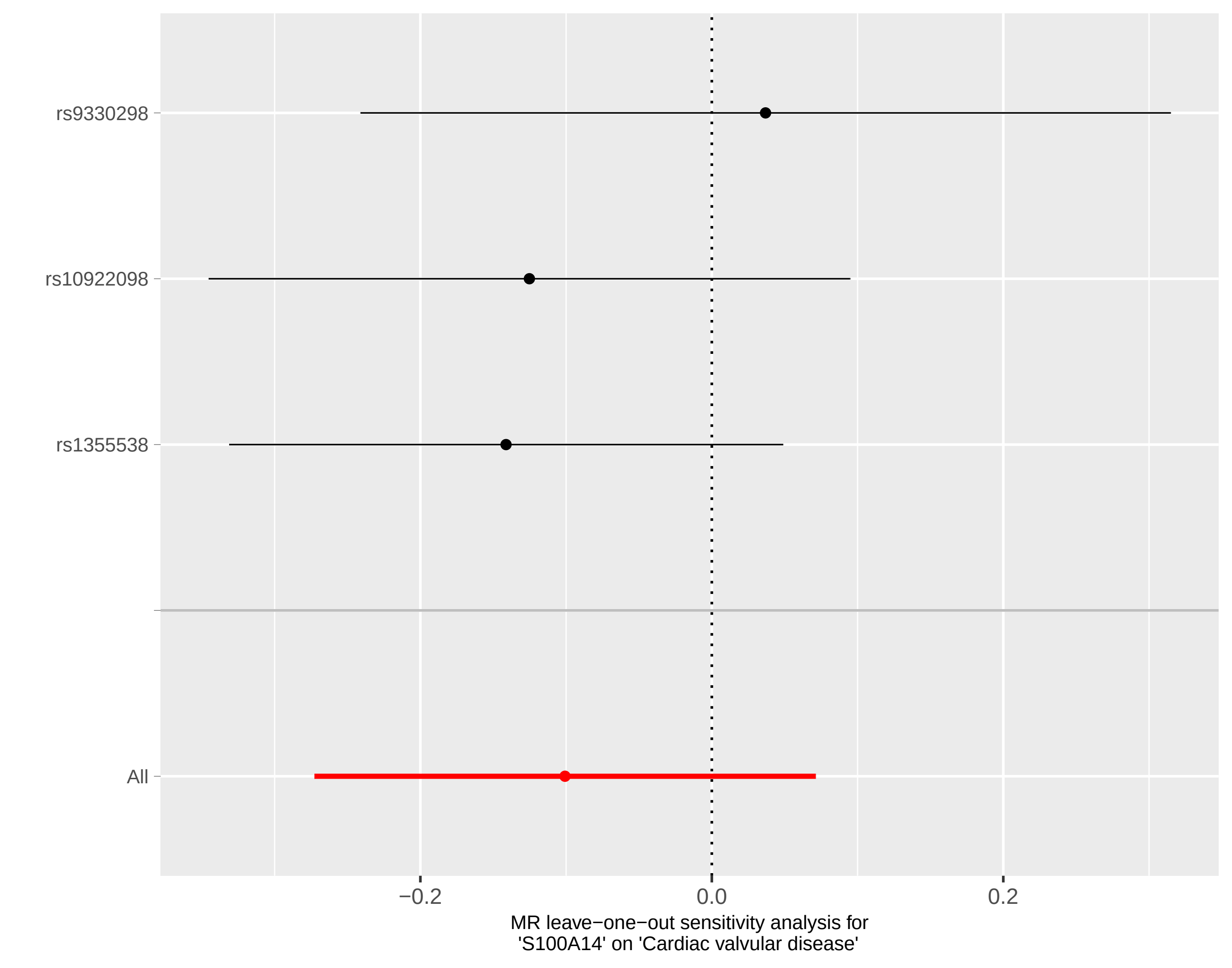

rs9330298
rs10922098
rs1355538
All
−0.2
0.0
0.2
MR leave−one−out sensitivity analysis for
'S100A14' on 'Cardiac valvular disease'

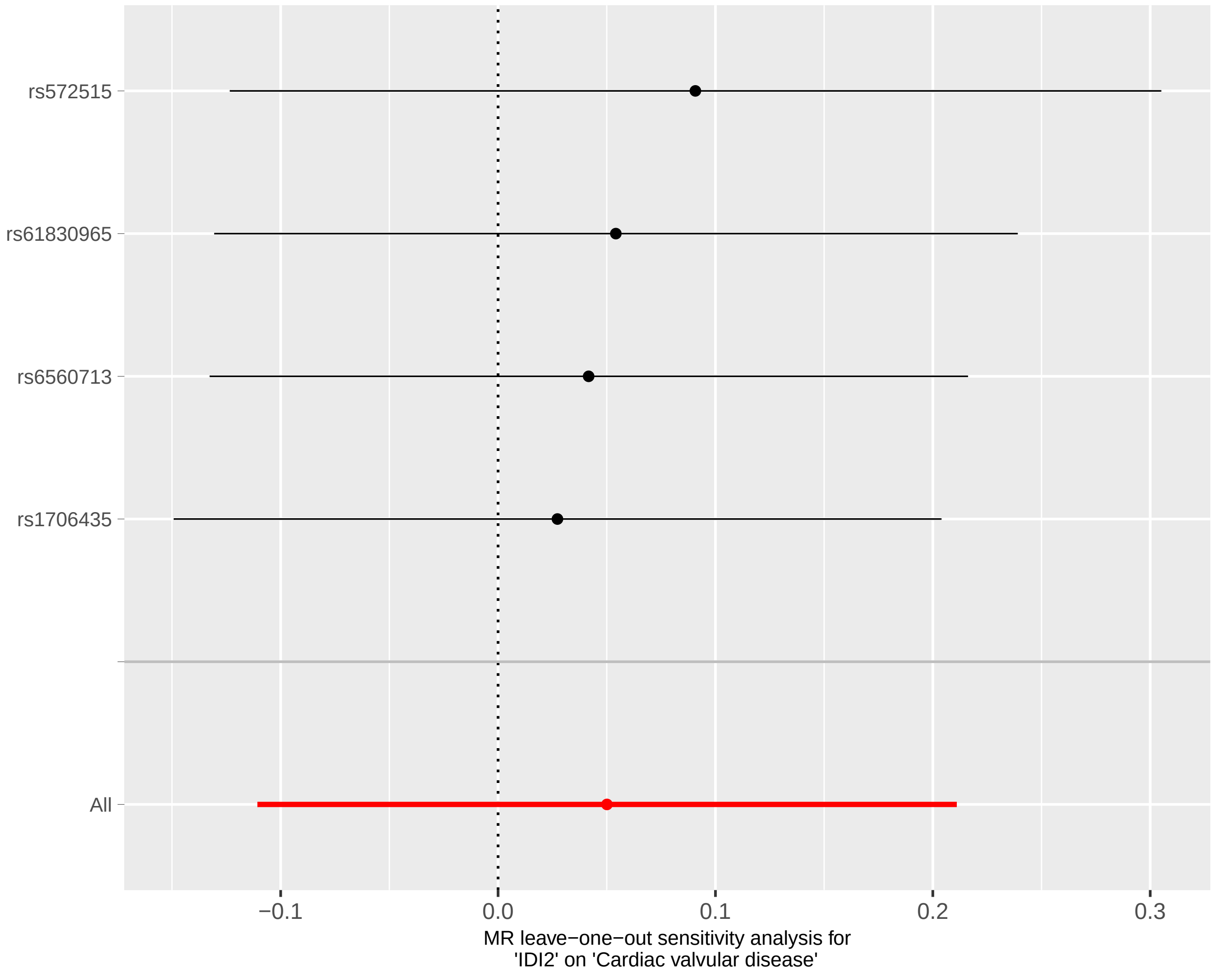
rs572515
rs61830965
rs6560713
rs1706435
All
−0.1
0.0
0.1
0.2
0.3
MR leave−one−out sensitivity analysis for
'IDI2' on 'Cardiac valvular disease'

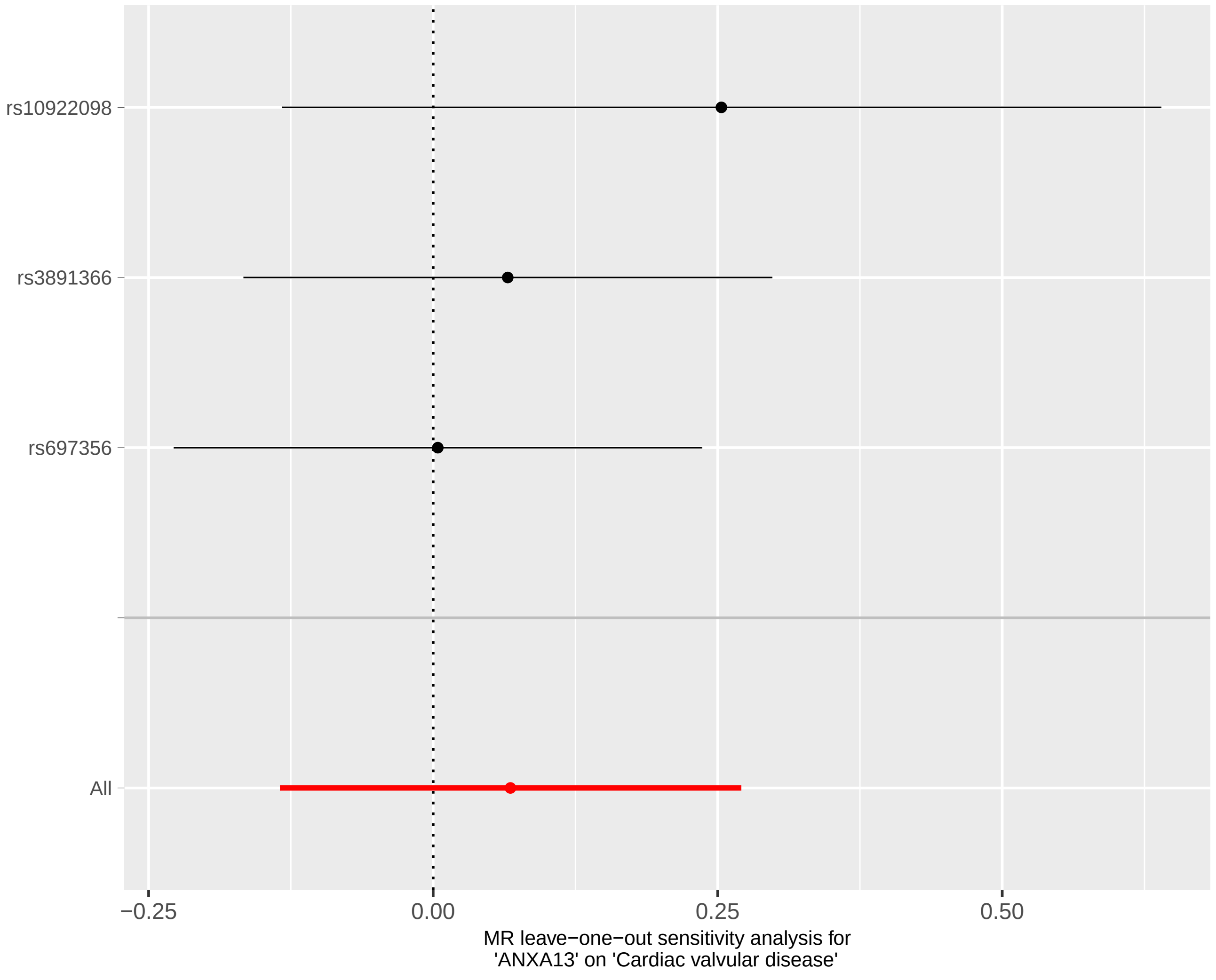
rs10922098
rs3891366
rs697356
All
−0.25
0.00
0.25
0.50
MR leave−one−out sensitivity analysis for
'ANXA13' on 'Cardiac valvular disease'

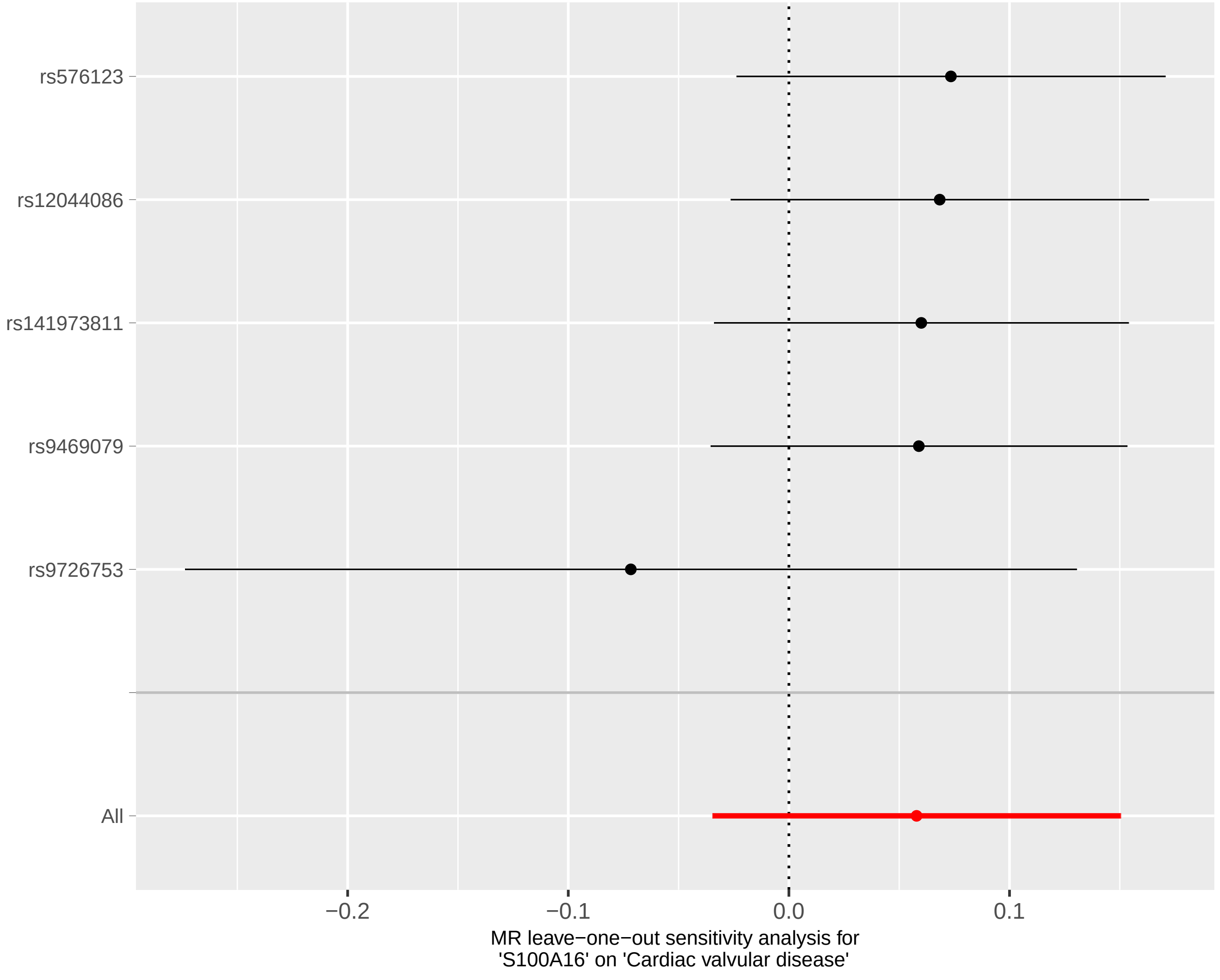

rs576123
rs12044086
rs141973811
rs9469079
rs9726753
All
−0.2
−0.1
0.0
0.1
MR leave−one−out sensitivity analysis for
'S100A16' on 'Cardiac valvular disease'

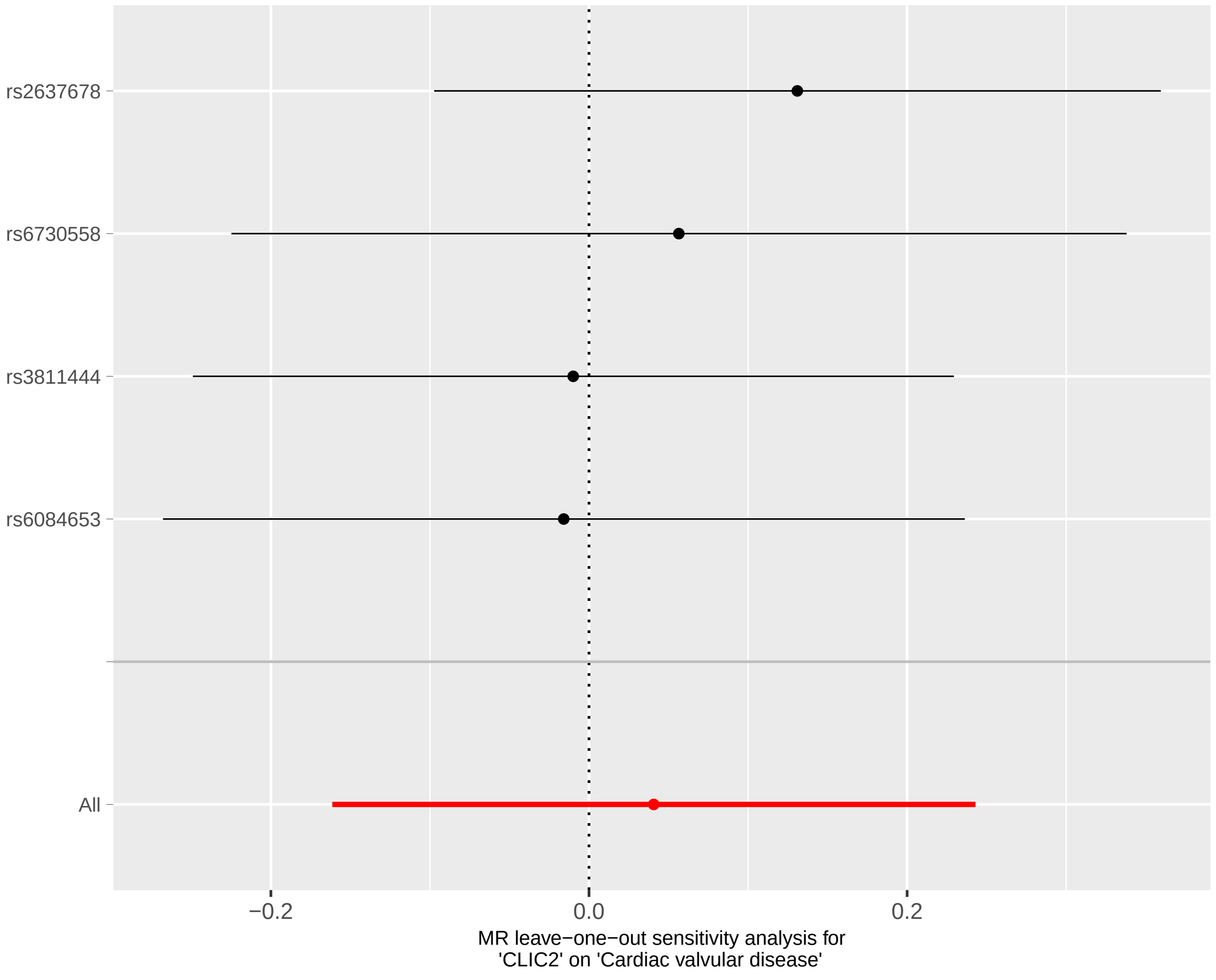

rs2637678
rs6730558
rs3811444
rs6084653
All
−0.2
0.0
0.2
MR leave−one−out sensitivity analysis for
'CLIC2' on 'Cardiac valvular disease'

Insufficient number of SNPs

Insufficient number of SNPs

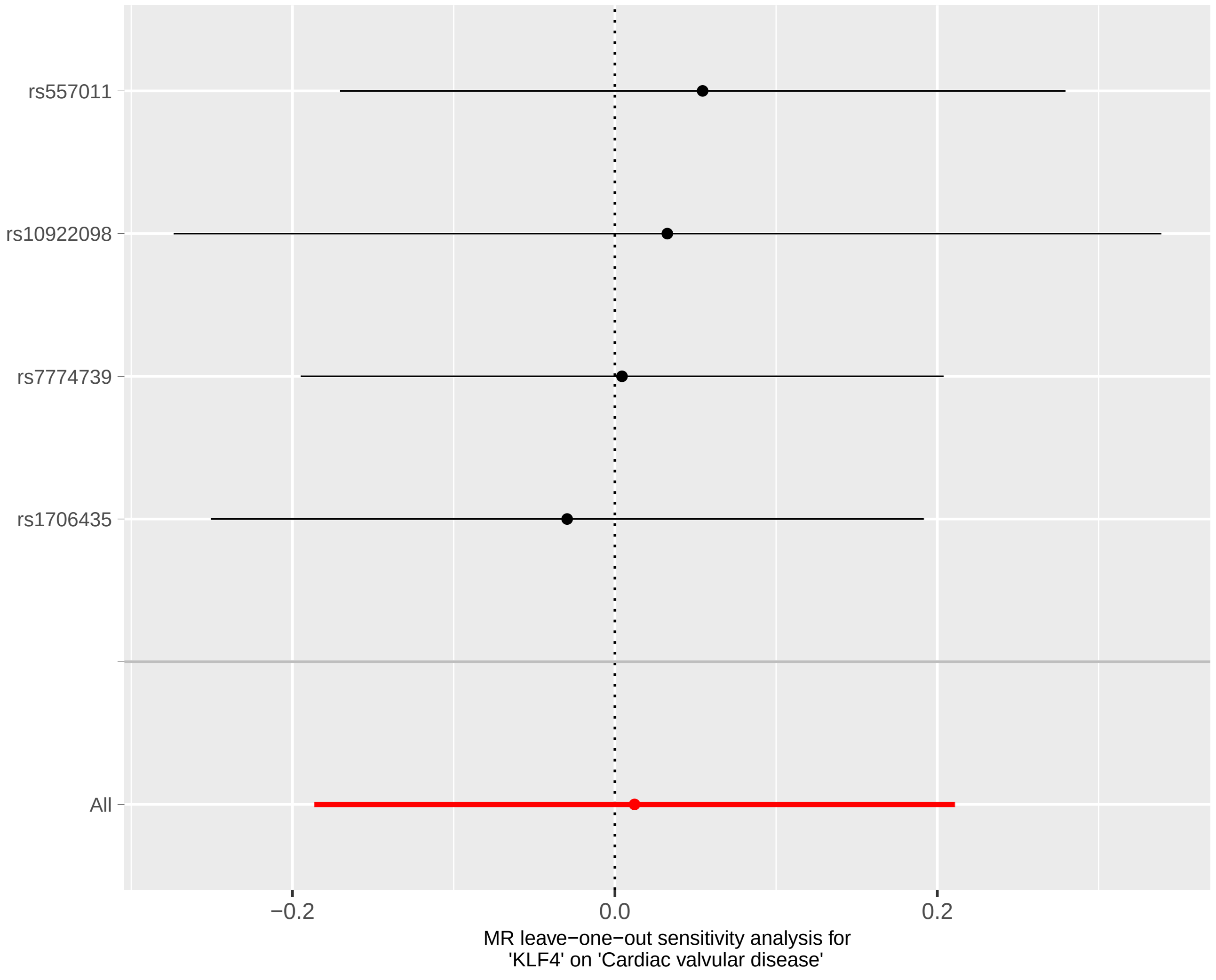

rs557011
rs10922098
rs7774739
rs1706435
All
−0.2
0.0
0.2
MR leave−one−out sensitivity analysis for
'KLF4' on 'Cardiac valvular disease'

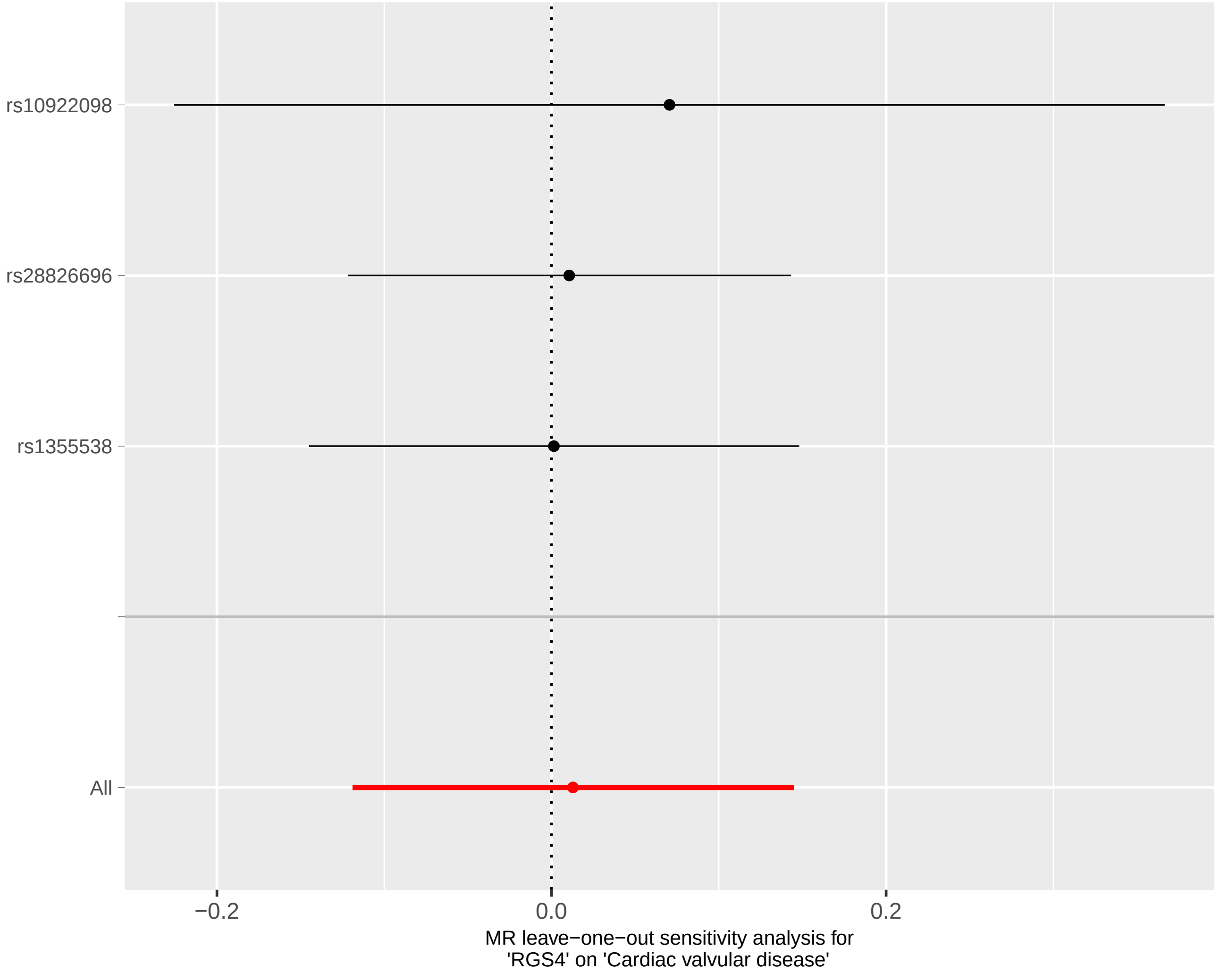

rs10922098
rs28826696
rs1355538
All
−0.2
0.0
0.2
MR leave−one−out sensitivity analysis for
'RGS4' on 'Cardiac valvular disease'

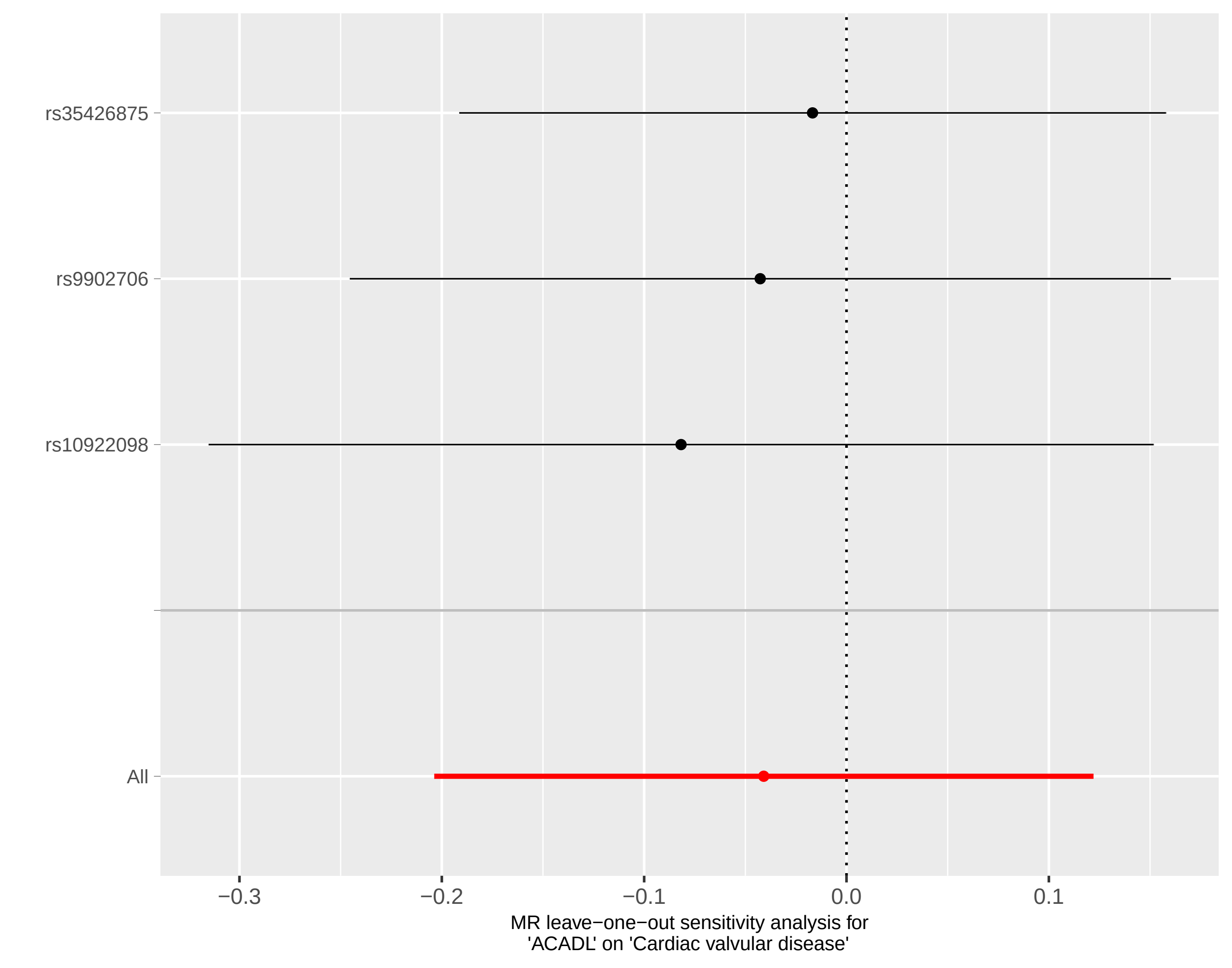

rs35426875
rs9902706
rs10922098
All
−0.3
−0.2
−0.1
0.0
0.1
MR leave−one−out sensitivity analysis for
'ACADL' on 'Cardiac valvular disease'

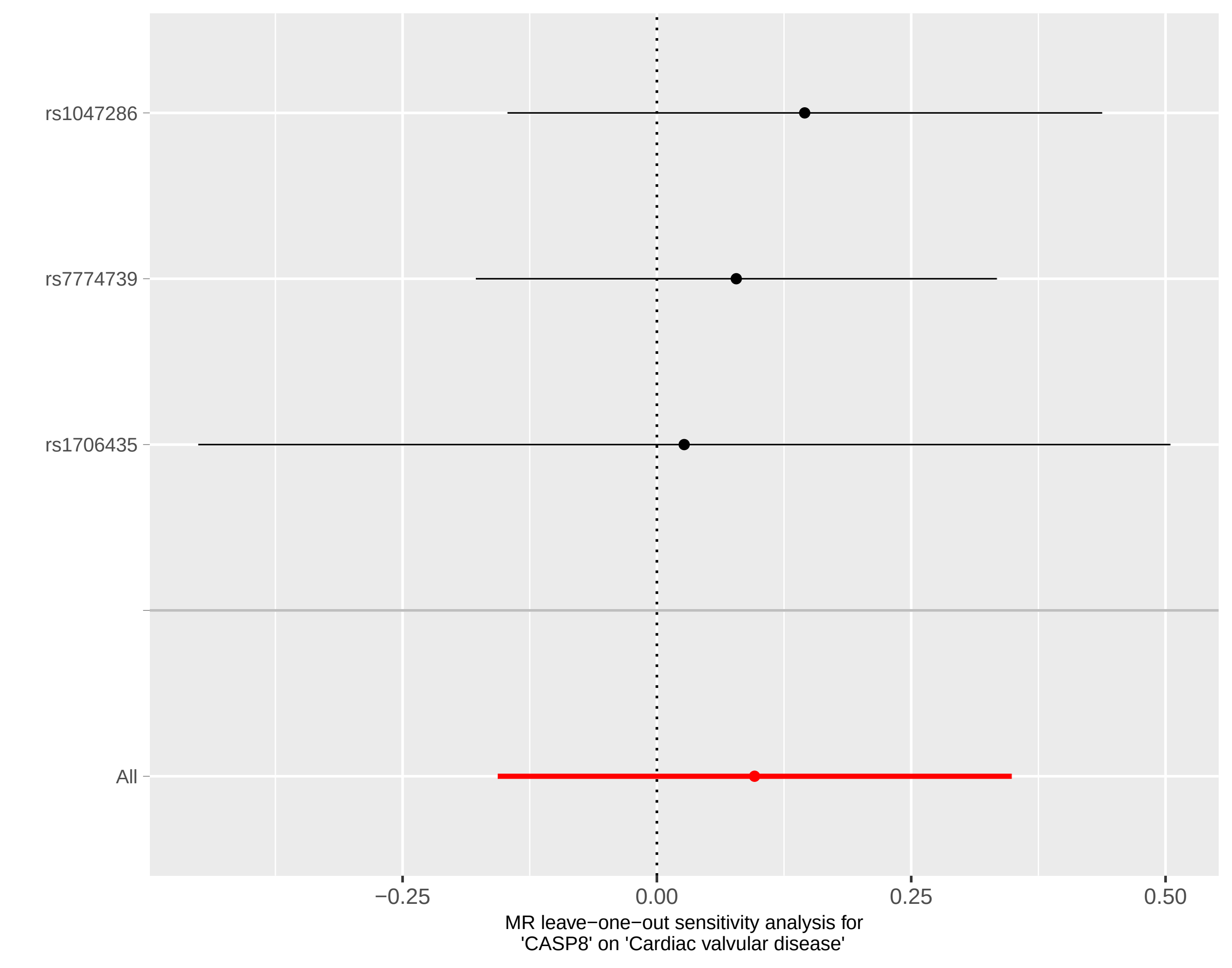
rs1047286
rs7774739
rs1706435
All
−0.25
0.00
0.25
0.50
MR leave−one−out sensitivity analysis for
'CASP8' on 'Cardiac valvular disease'

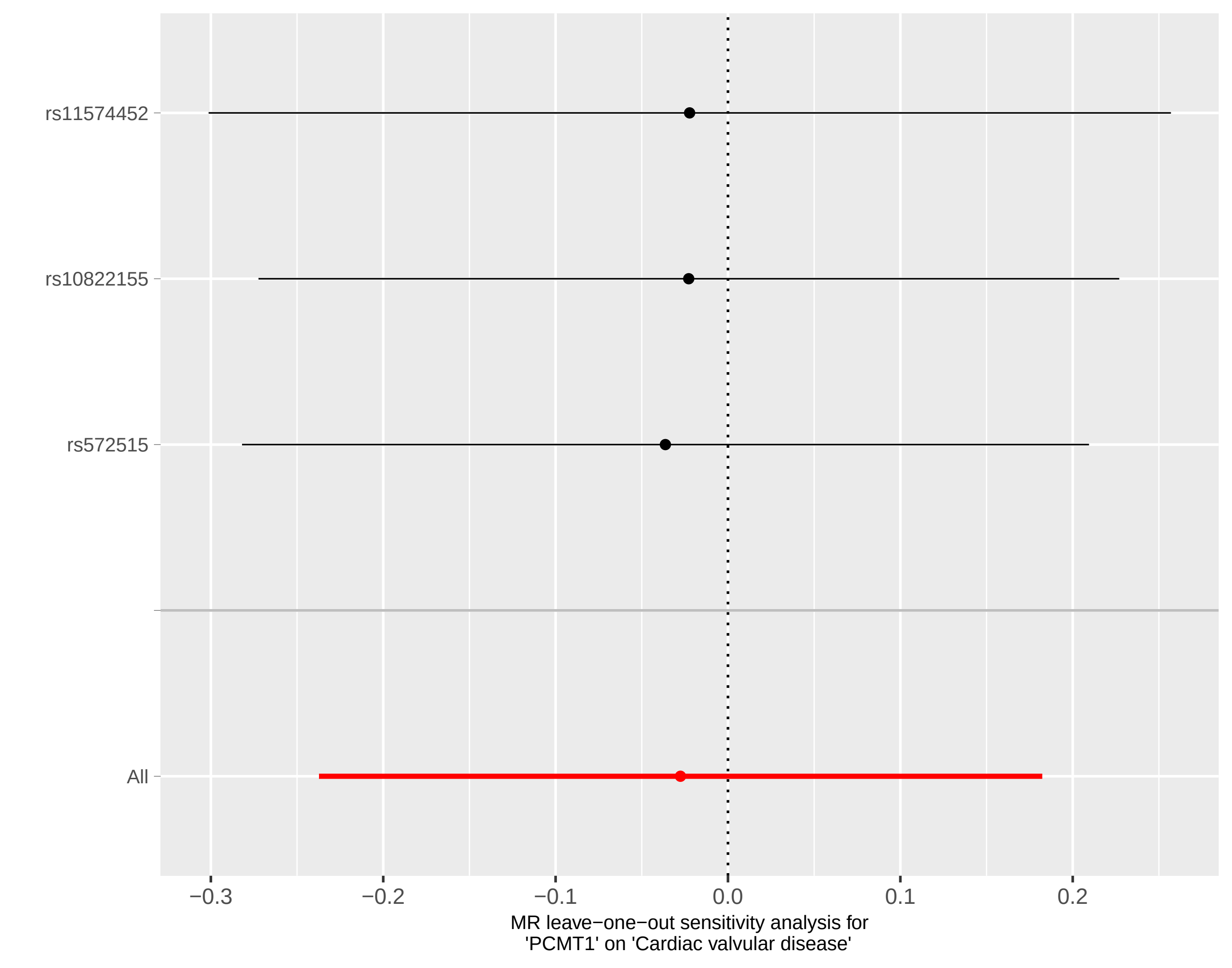
rs11574452
rs10822155
rs572515
All
−0.3
−0.2
−0.1
0.0
0.1
0.2
MR leave−one−out sensitivity analysis for
'PCMT1' on 'Cardiac valvular disease'

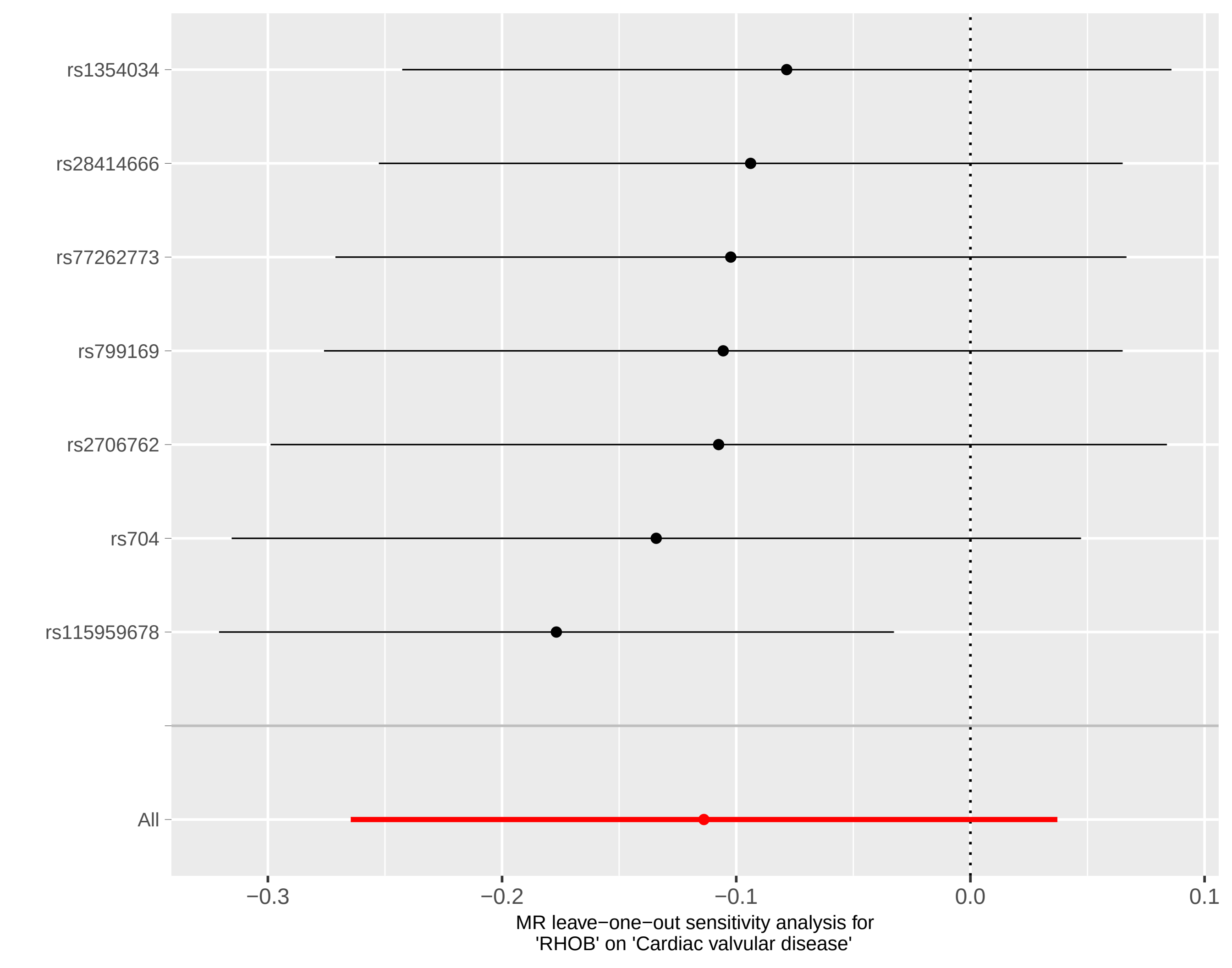
rs1354034
rs28414666
rs77262773
rs799169
rs2706762
rs704
rs115959678
All
−0.3
−0.2
−0.1
0.0
0.1
MR leave−one−out sensitivity analysis for
'RHOB' on 'Cardiac valvular disease'

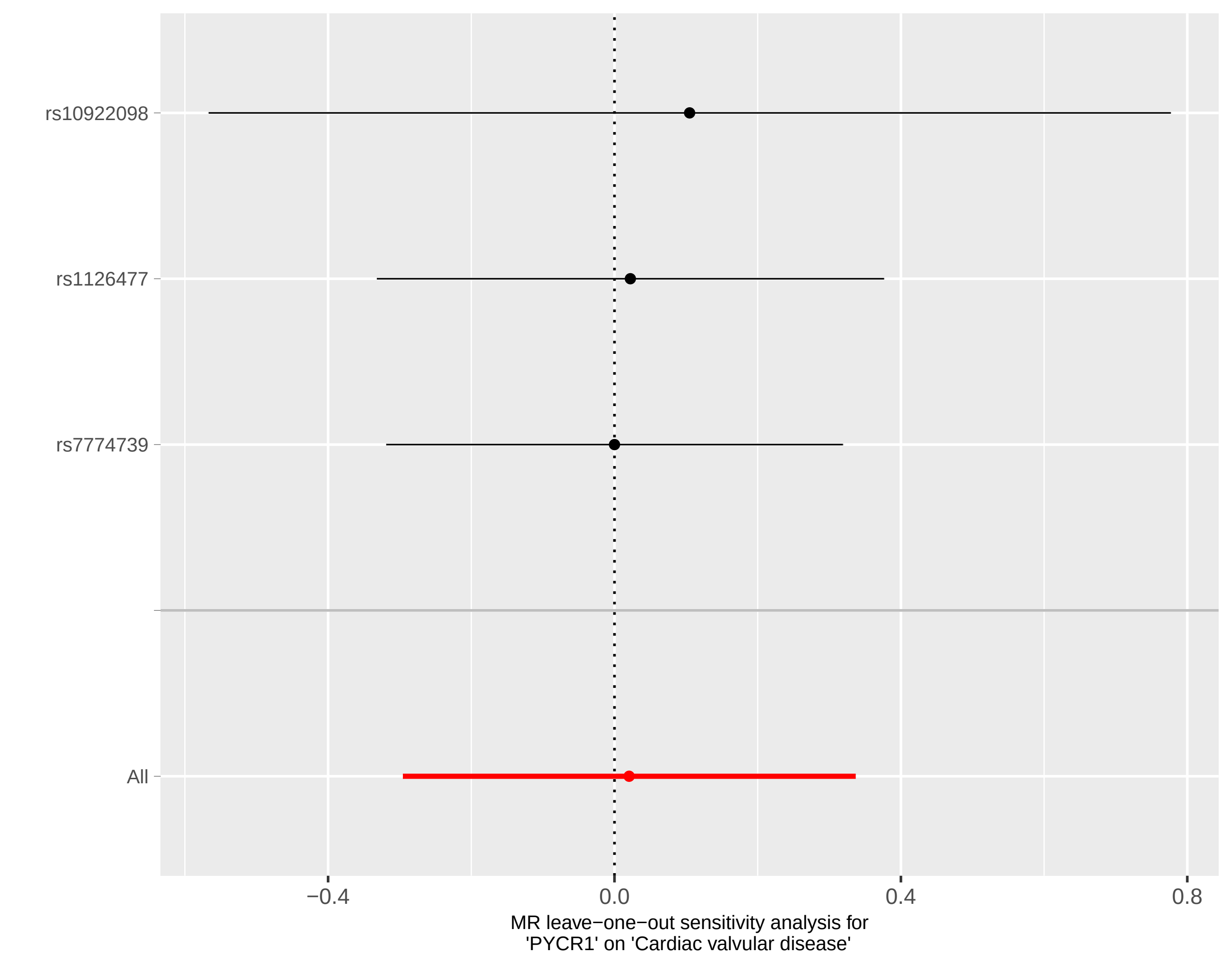
rs10922098
rs1126477
rs7774739
All
−0.4
0.0
0.4
0.8
MR leave−one−out sensitivity analysis for
'PYCR1' on 'Cardiac valvular disease'

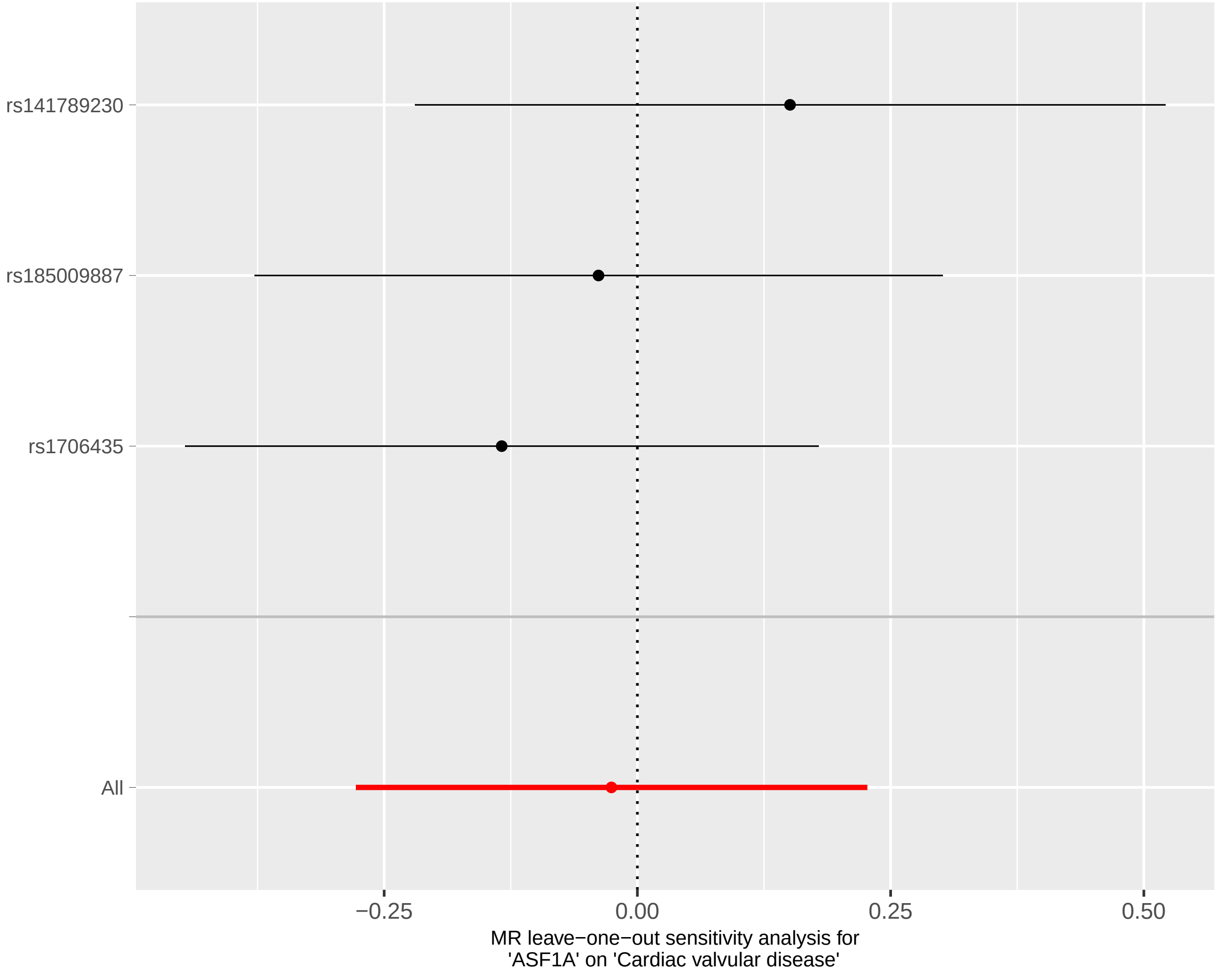
rs141789230
rs185009887
rs1706435
All
−0.25
0.00
0.25
0.50
MR leave−one−out sensitivity analysis for
'ASF1A' on 'Cardiac valvular disease'

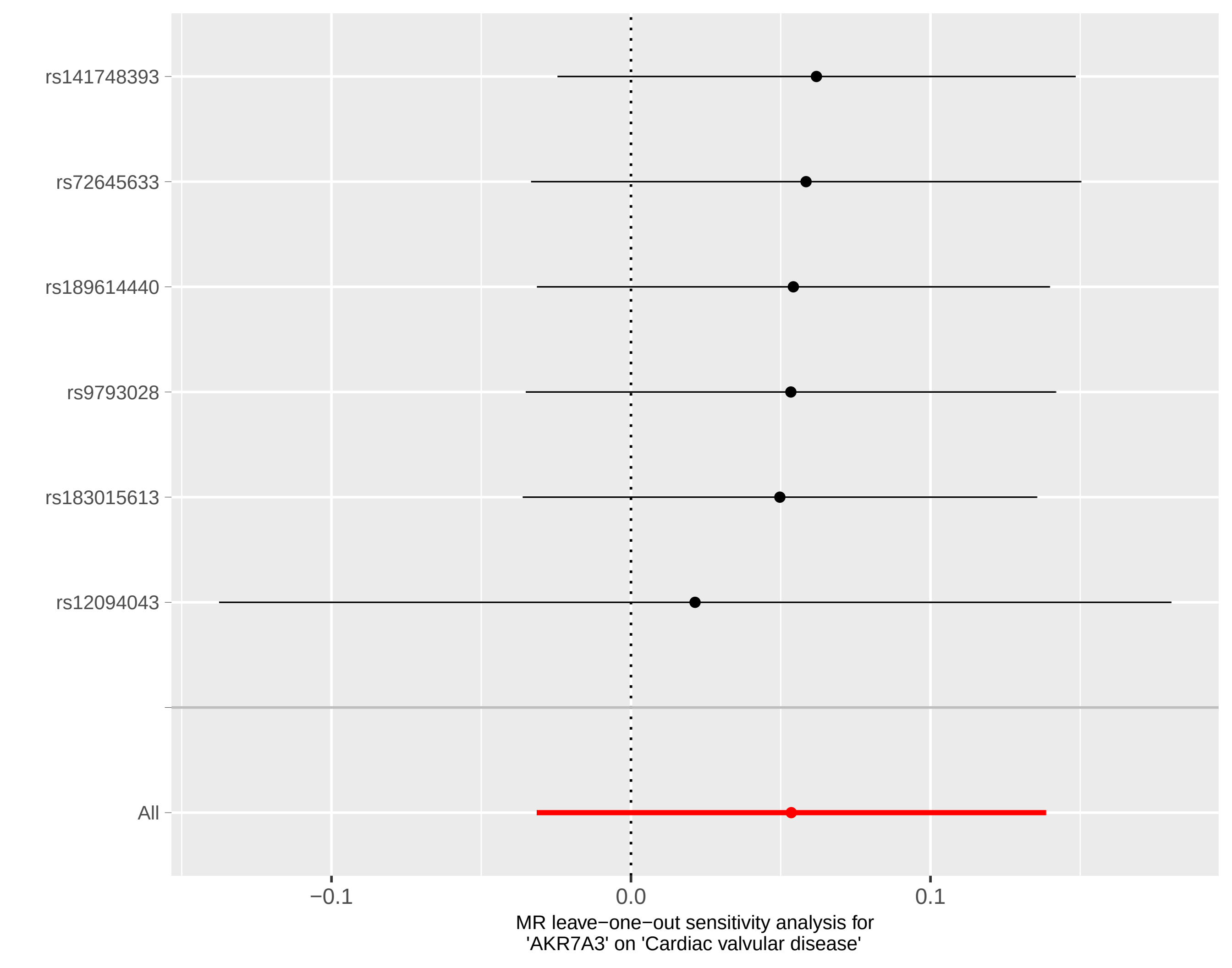

rs141748393
rs72645633
rs189614440
rs9793028
rs183015613
rs12094043
All
−0.1
0.0
0.1
MR leave−one−out sensitivity analysis for
'AKR7A3' on 'Cardiac valvular disease'

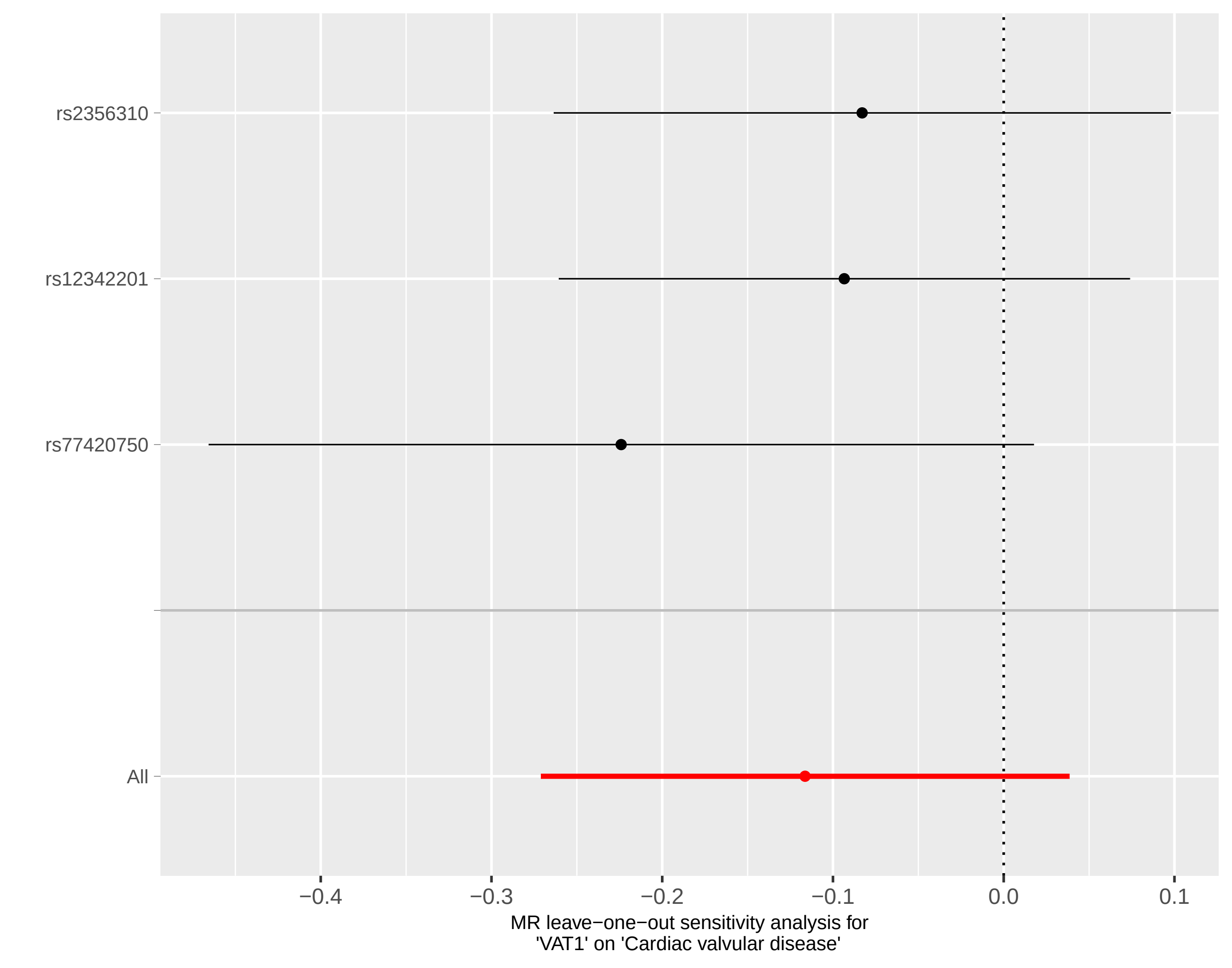
rs2356310
rs12342201
rs77420750
All
−0.4
−0.3
−0.2
−0.1
0.0
0.1
MR leave−one−out sensitivity analysis for
'VAT1' on 'Cardiac valvular disease'

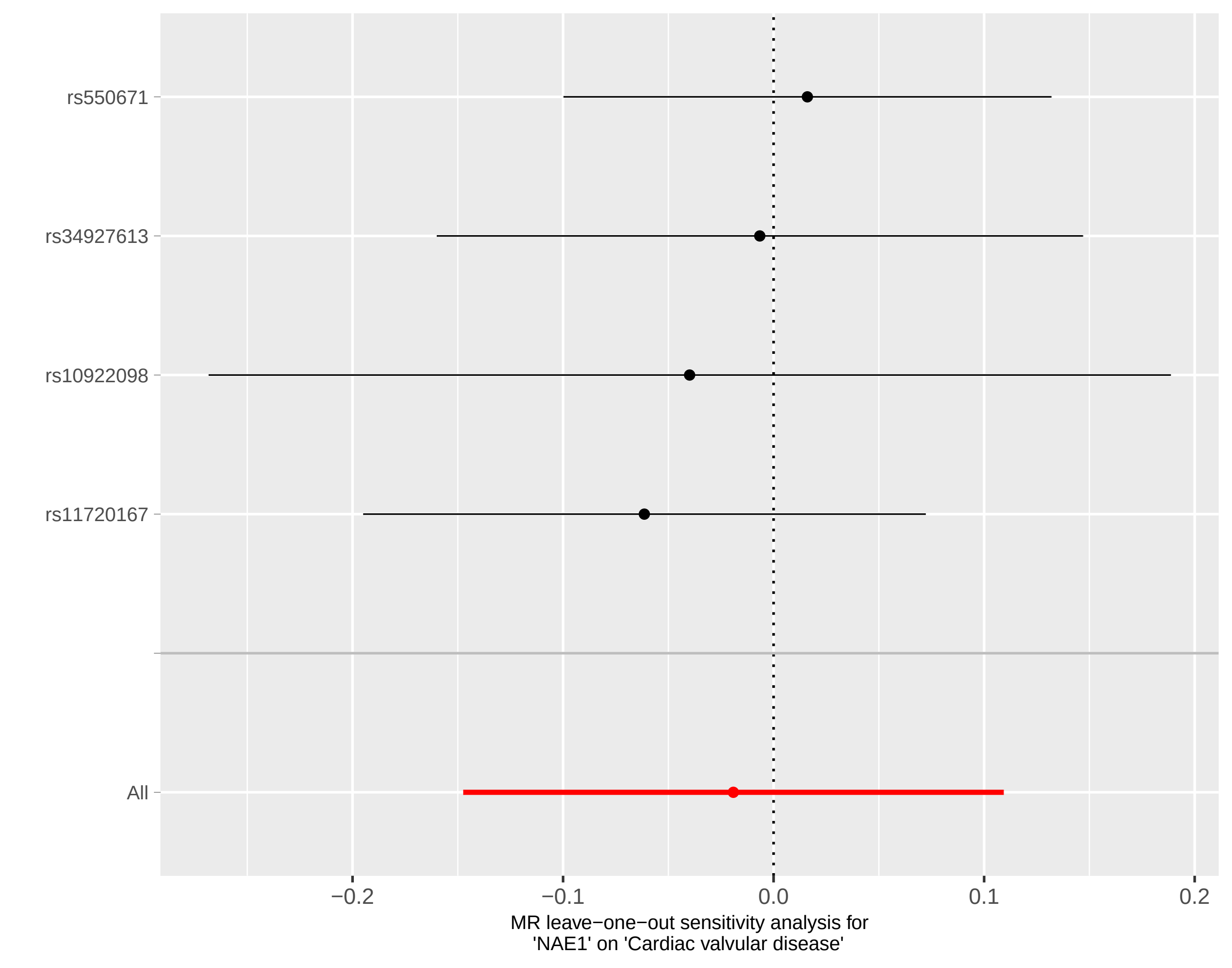

rs550671
rs34927613
rs10922098
rs11720167
All
−0.2
−0.1
0.0
0.1
0.2
MR leave−one−out sensitivity analysis for
'NAE1' on 'Cardiac valvular disease'

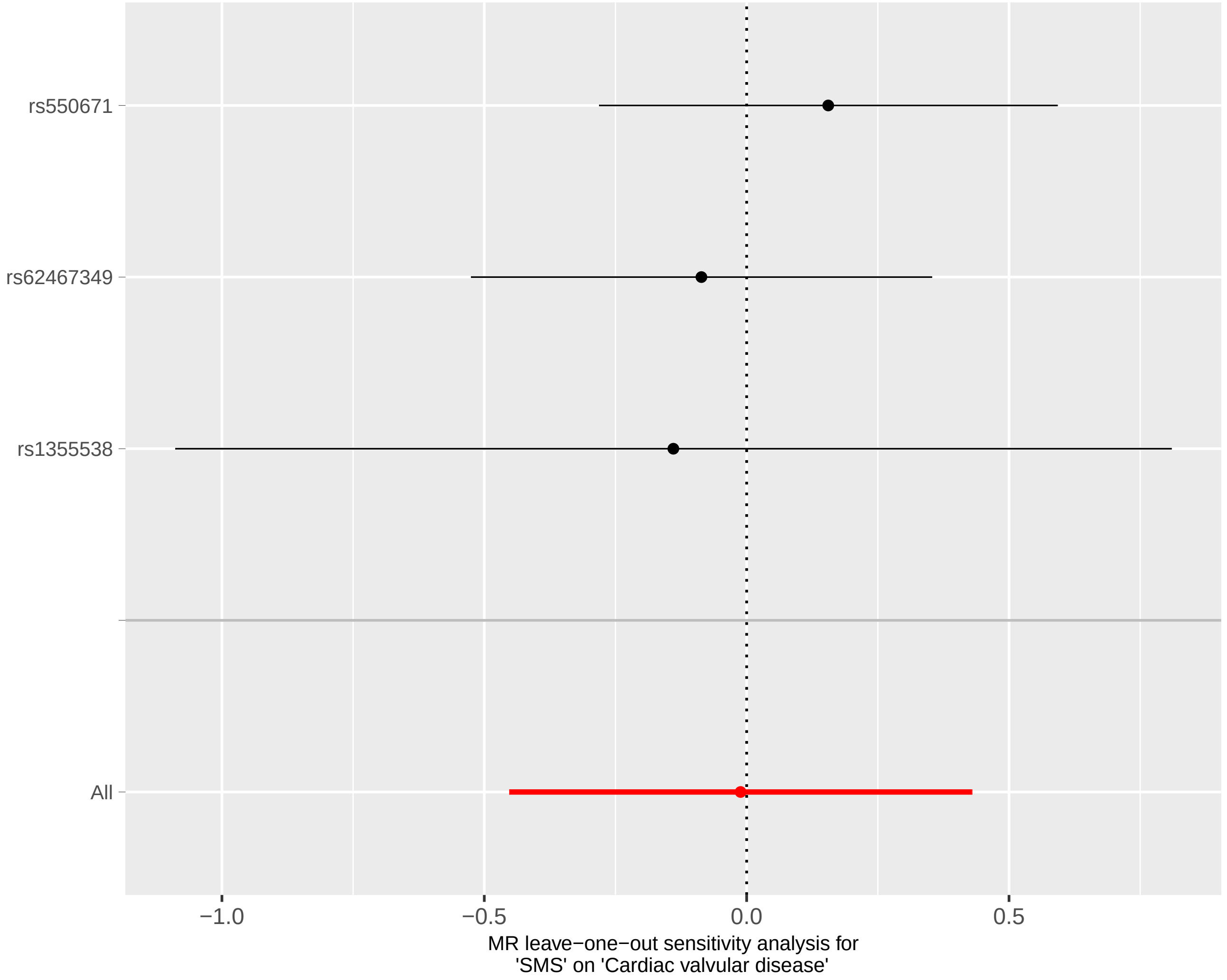
rs550671
rs62467349
rs1355538
All
−1.0
−0.5
0.0
0.5
MR leave−one−out sensitivity analysis for
'SMS' on 'Cardiac valvular disease'

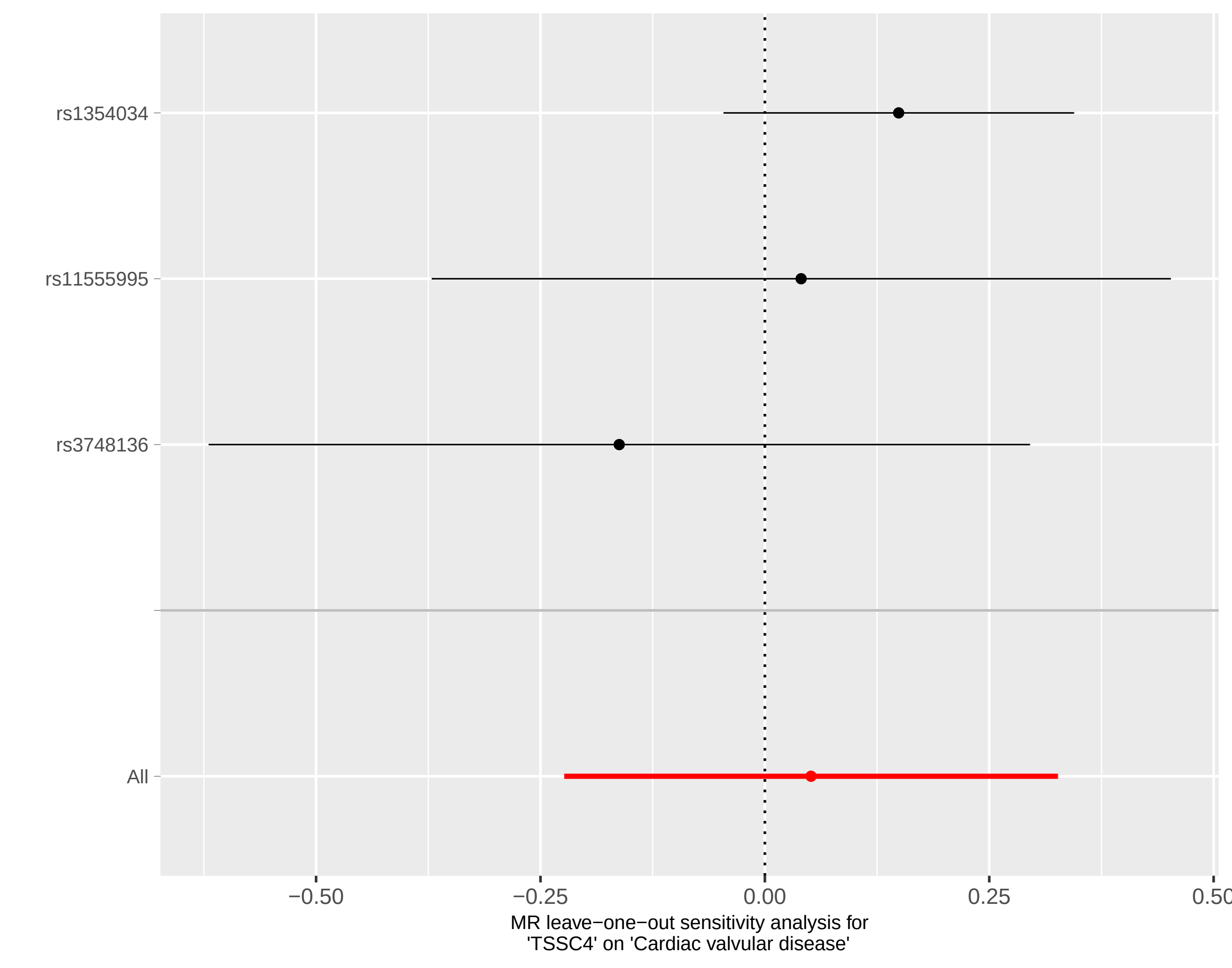
rs1354034
rs11555995
rs3748136
All
−0.50
−0.25
0.00
0.25
0.50
MR leave−one−out sensitivity analysis for
'TSSC4' on 'Cardiac valvular disease'

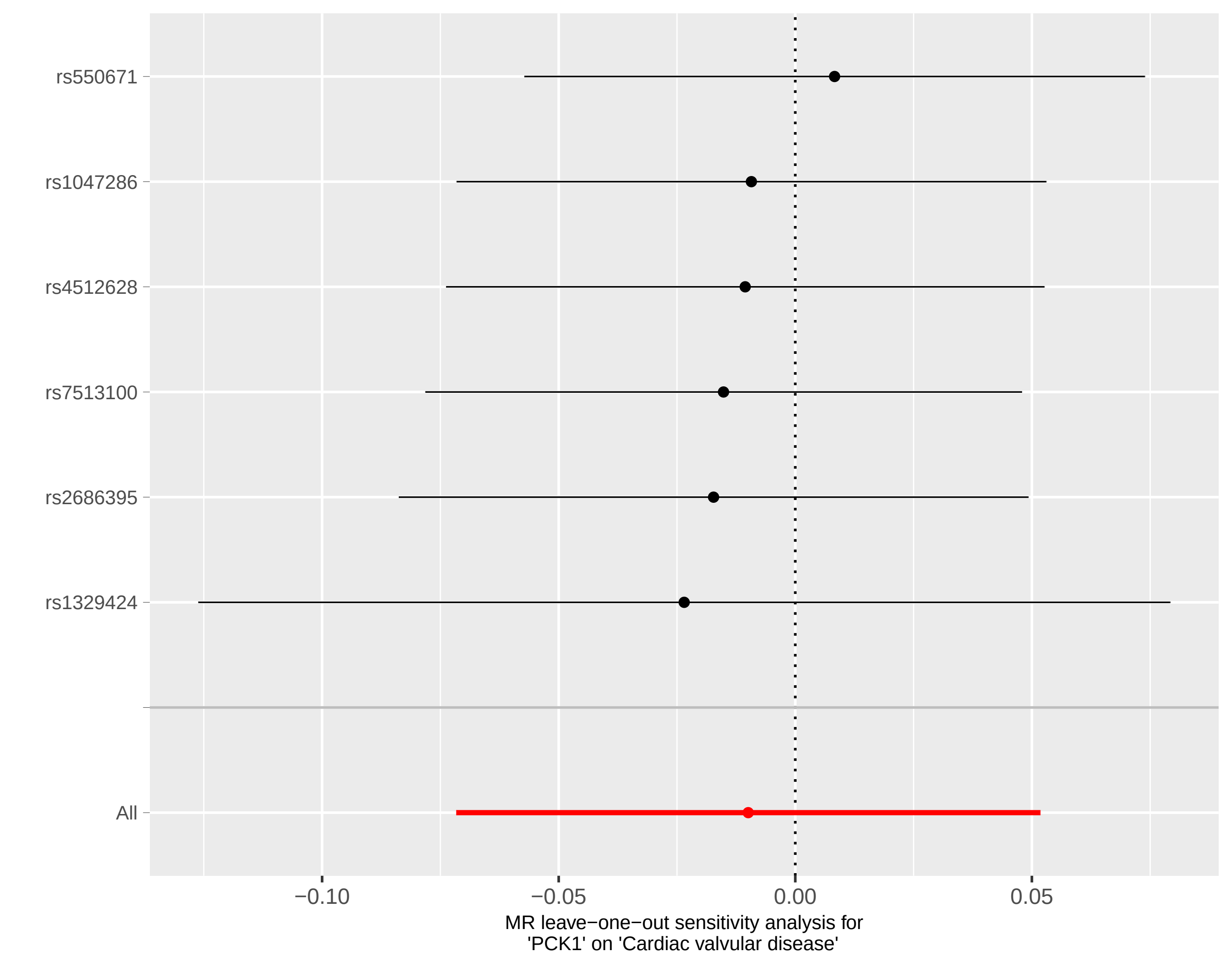

rs550671
rs1047286
rs4512628
rs7513100
rs2686395
rs1329424
All
−0.10
−0.05
0.00
0.05
MR leave−one−out sensitivity analysis for
'PCK1' on 'Cardiac valvular disease'

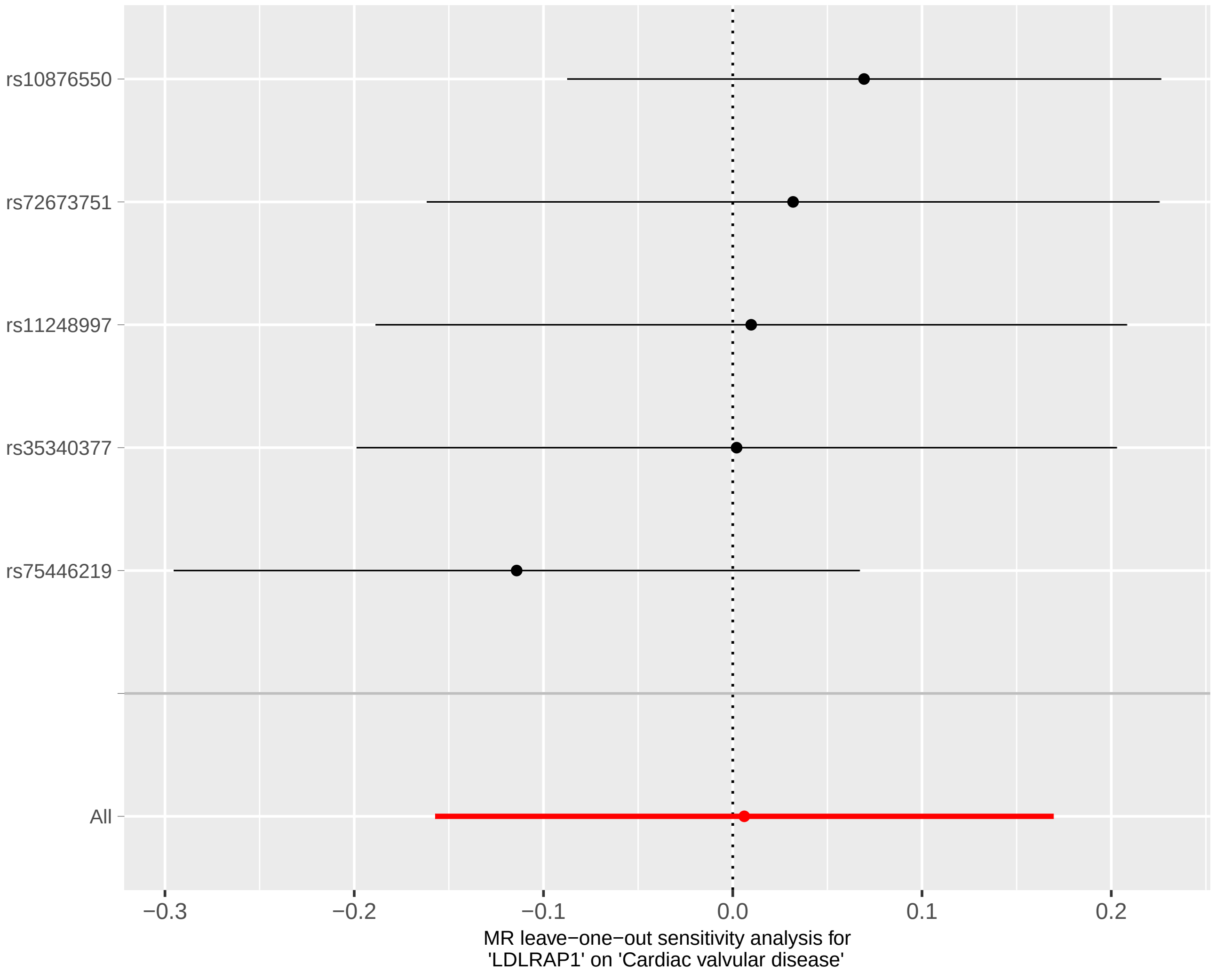

rs10876550
rs72673751
rs11248997
rs35340377
rs75446219
All
−0.3
−0.2
−0.1
0.0
0.1
0.2
MR leave−one−out sensitivity analysis for
'LDLRAP1' on 'Cardiac valvular disease'

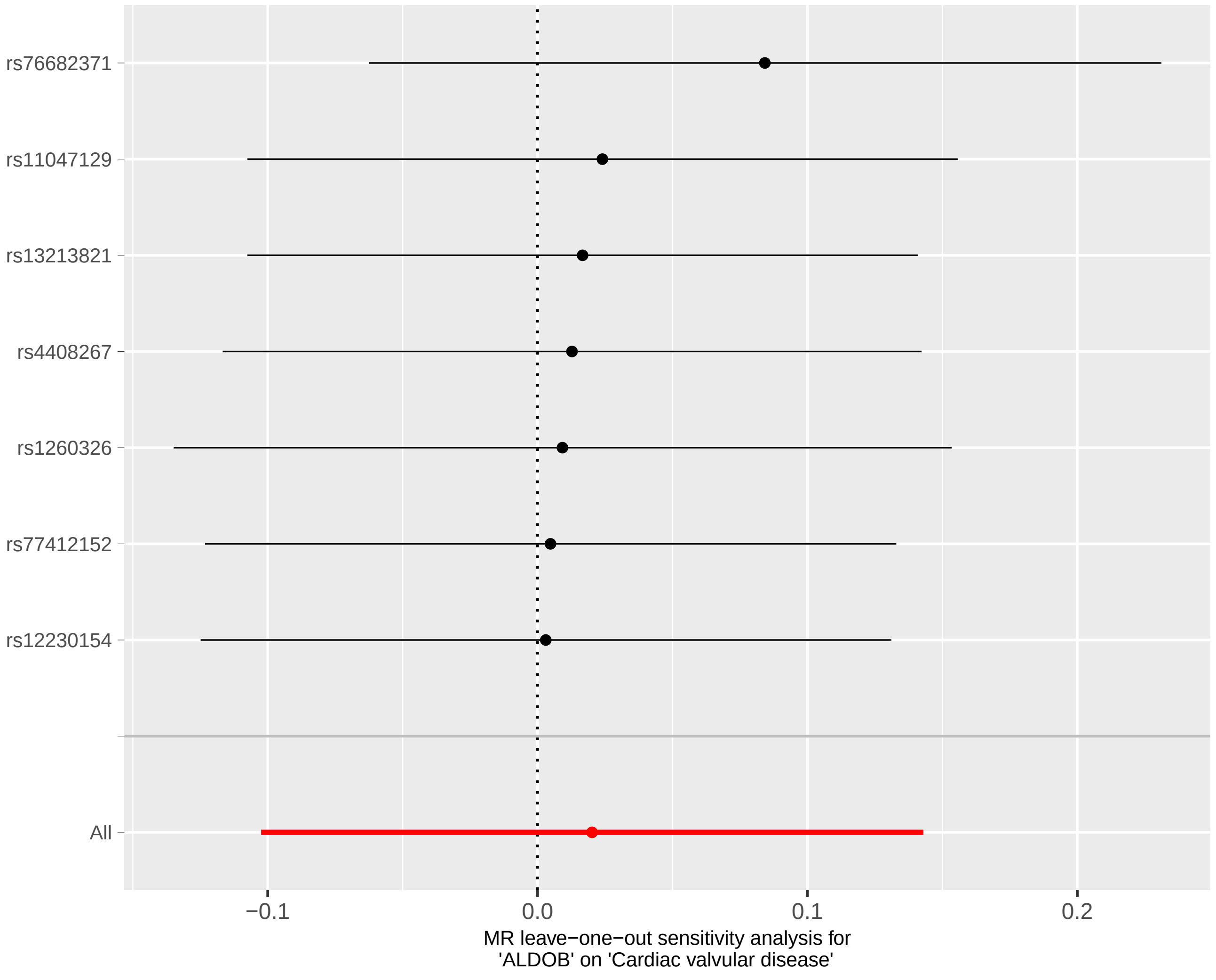
rs76682371
rs11047129
rs13213821
rs4408267
rs1260326
rs77412152
rs12230154
All
−0.1
0.0
0.1
0.2
MR leave−one−out sensitivity analysis for
'ALDOB' on 'Cardiac valvular disease'

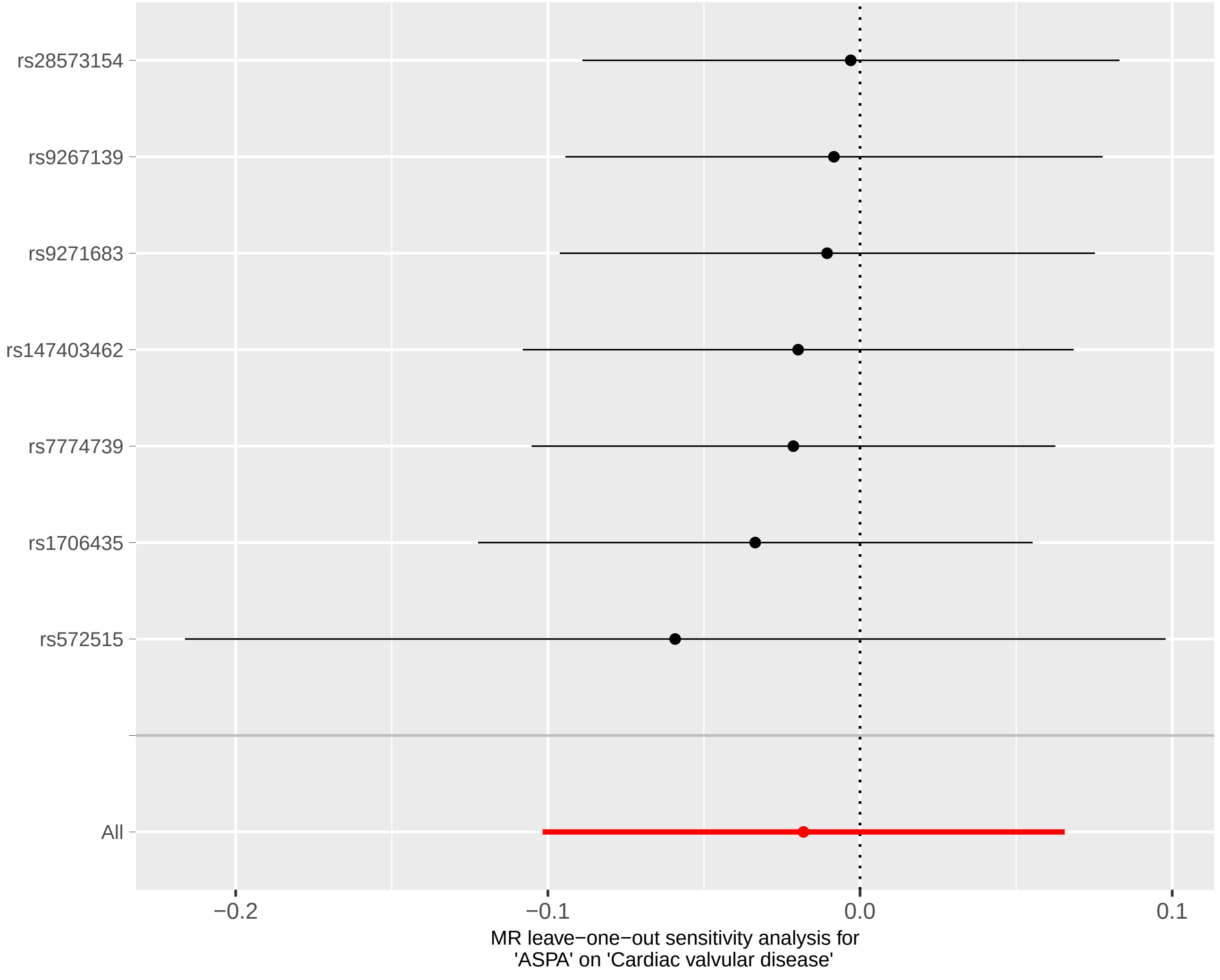
rs28573154
rs9267139
rs9271683
rs147403462
rs7774739
rs1706435
rs572515
All
−0.2
−0.1
0.0
0.1
MR leave−one−out sensitivity analysis for
'ASPA' on 'Cardiac valvular disease'

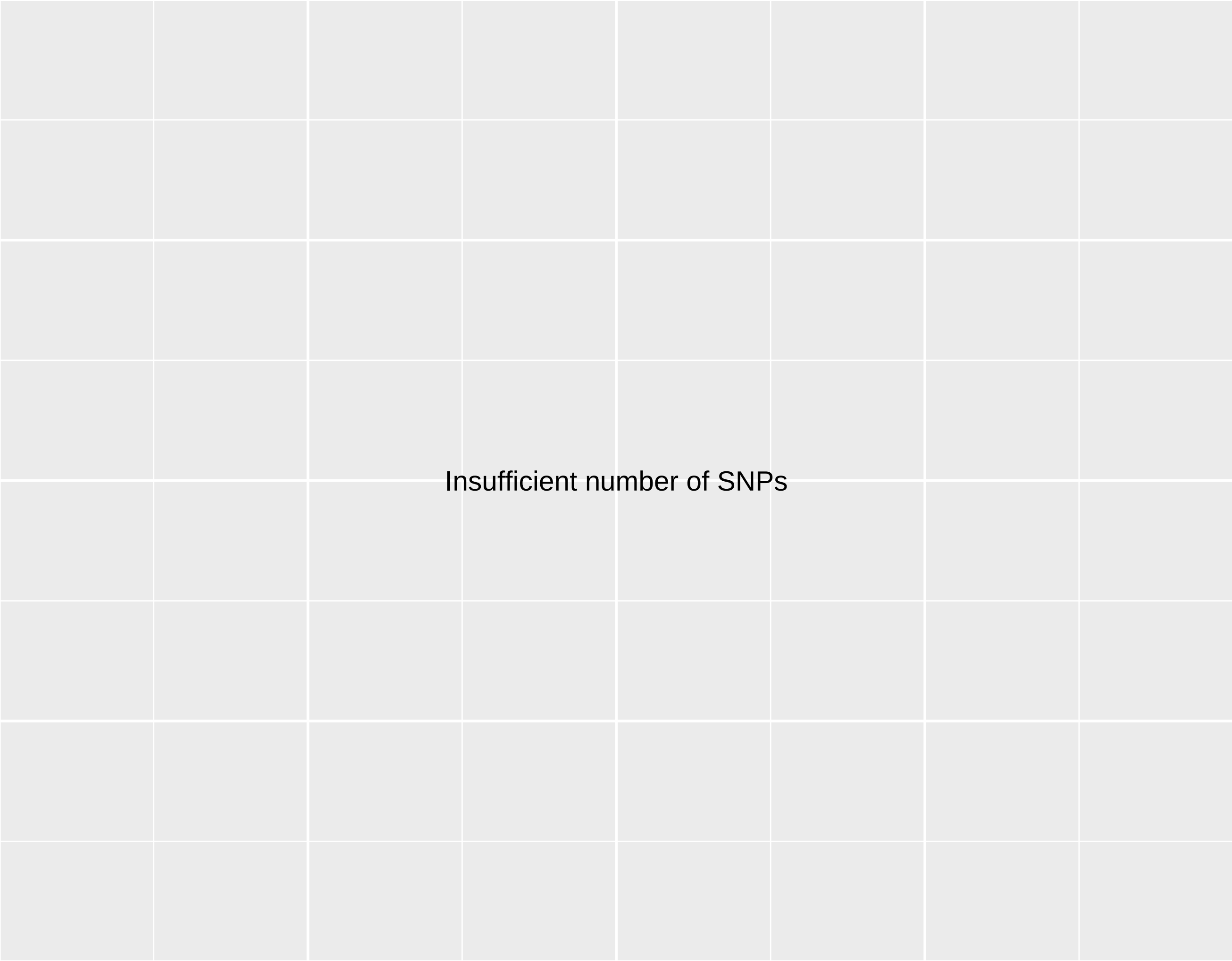
Insufficient number of SNPs

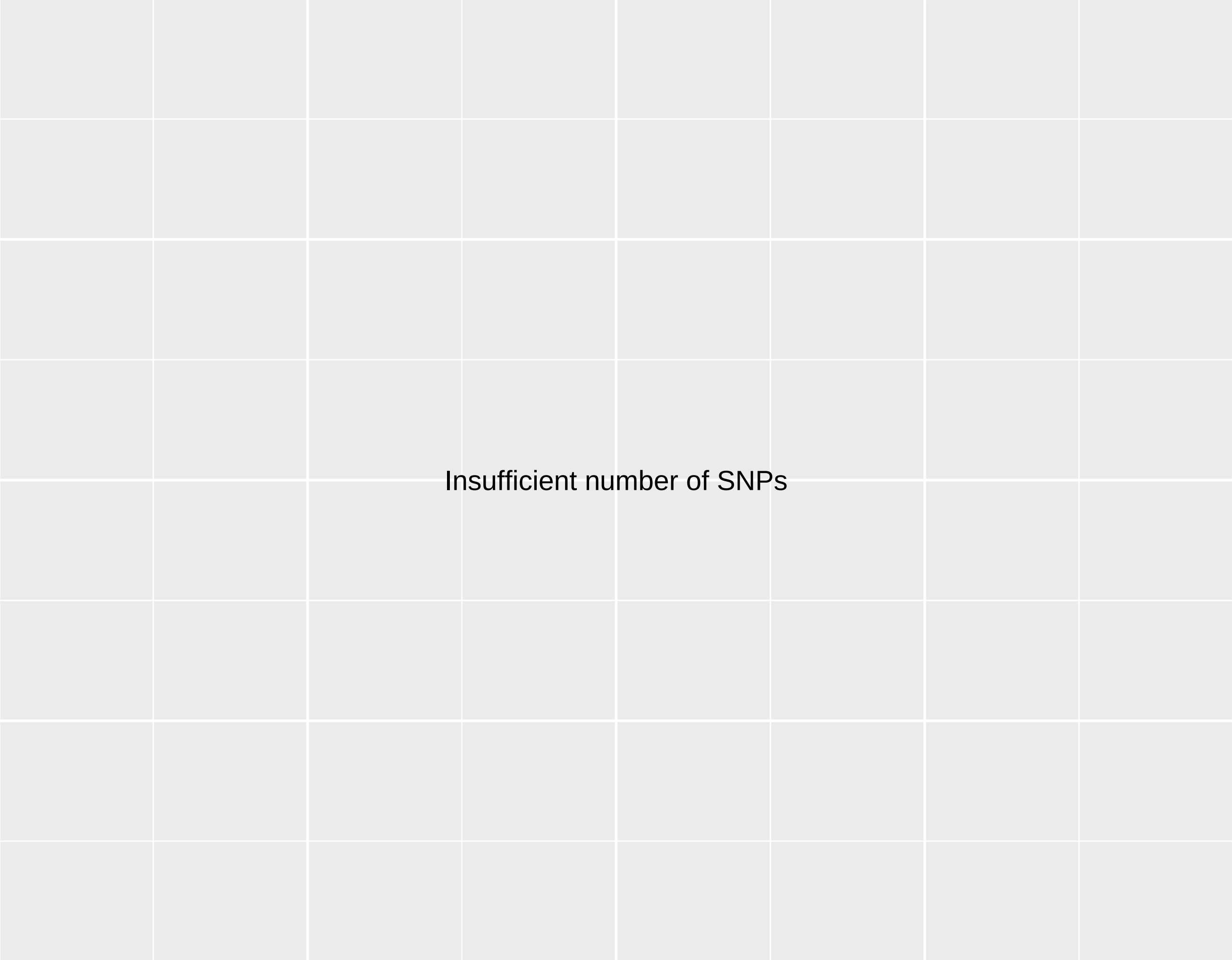
Insufficient number of SNPs

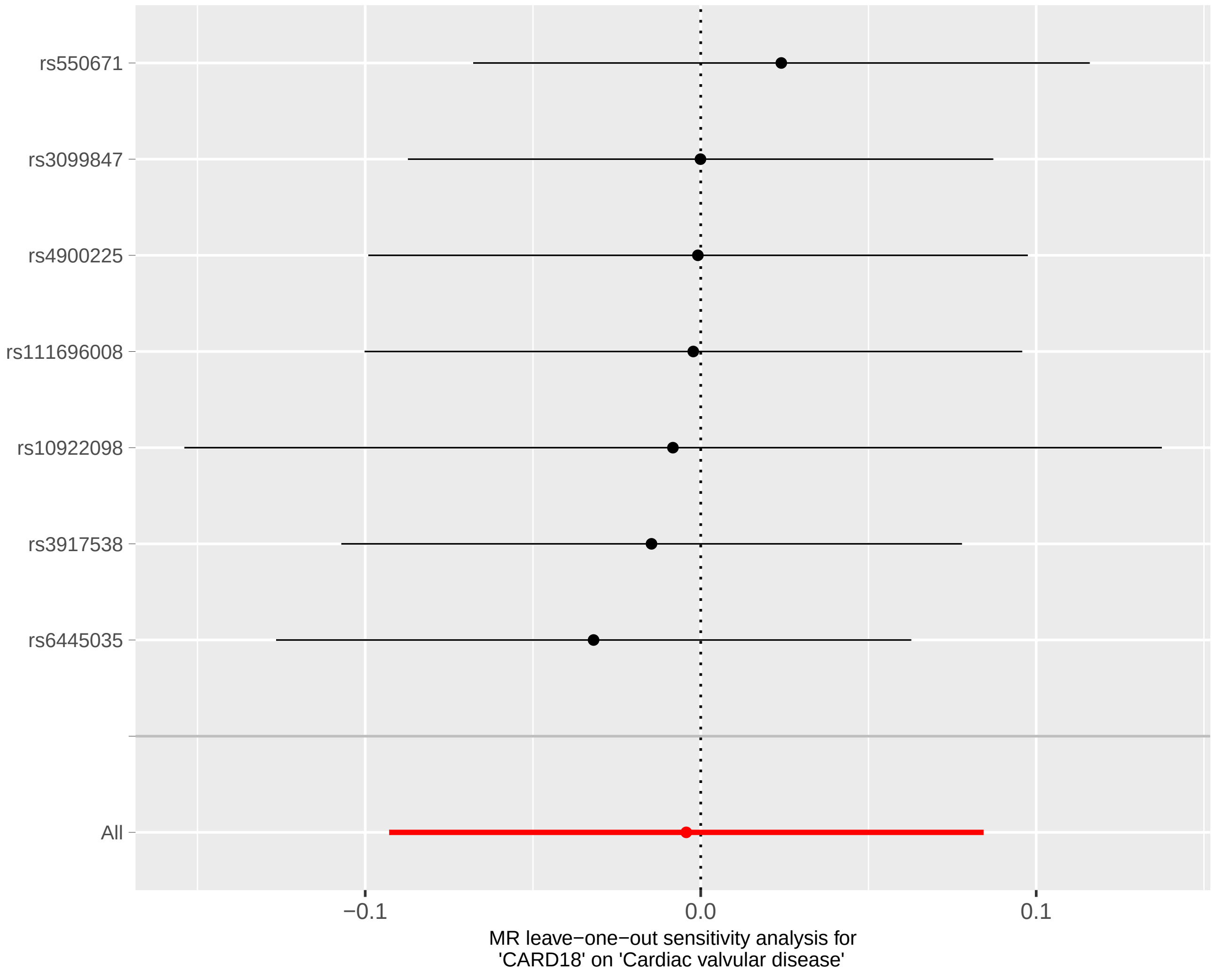
rs550671
rs3099847
rs4900225
rs111696008
rs10922098
rs3917538
rs6445035
All
−0.1
0.0
0.1
MR leave−one−out sensitivity analysis for
'CARD18' on 'Cardiac valvular disease'

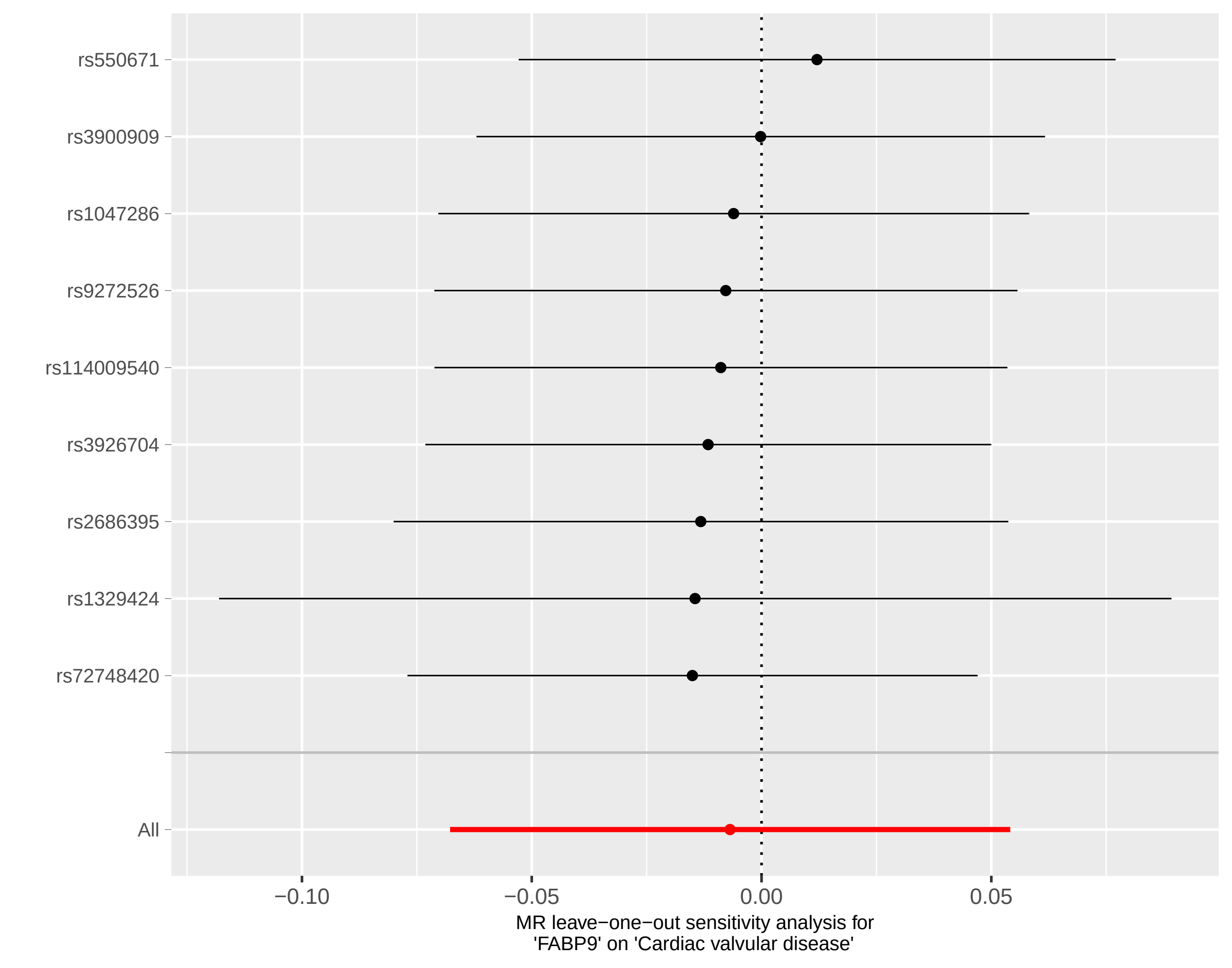
rs550671
rs3900909
rs1047286
rs9272526
rs114009540
rs3926704
rs2686395
rs1329424
rs72748420
All
−0.10
−0.05
0.00
0.05
MR leave−one−out sensitivity analysis for
'FABP9' on 'Cardiac valvular disease'

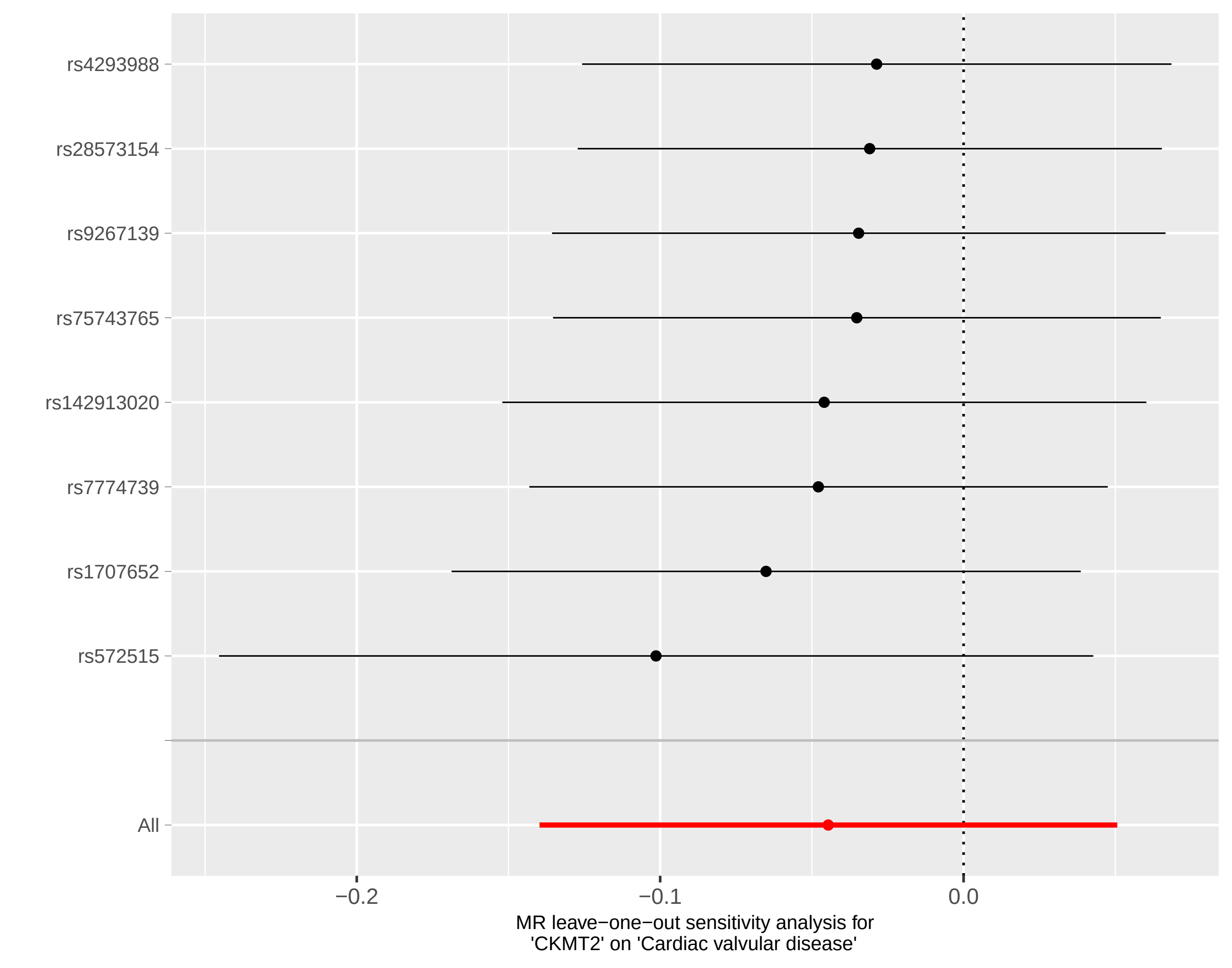
rs4293988
rs28573154
rs9267139
rs75743765
rs142913020
rs7774739
rs1707652
rs572515
All
−0.2
−0.1
0.0
MR leave−one−out sensitivity analysis for
'CKMT2' on 'Cardiac valvular disease'

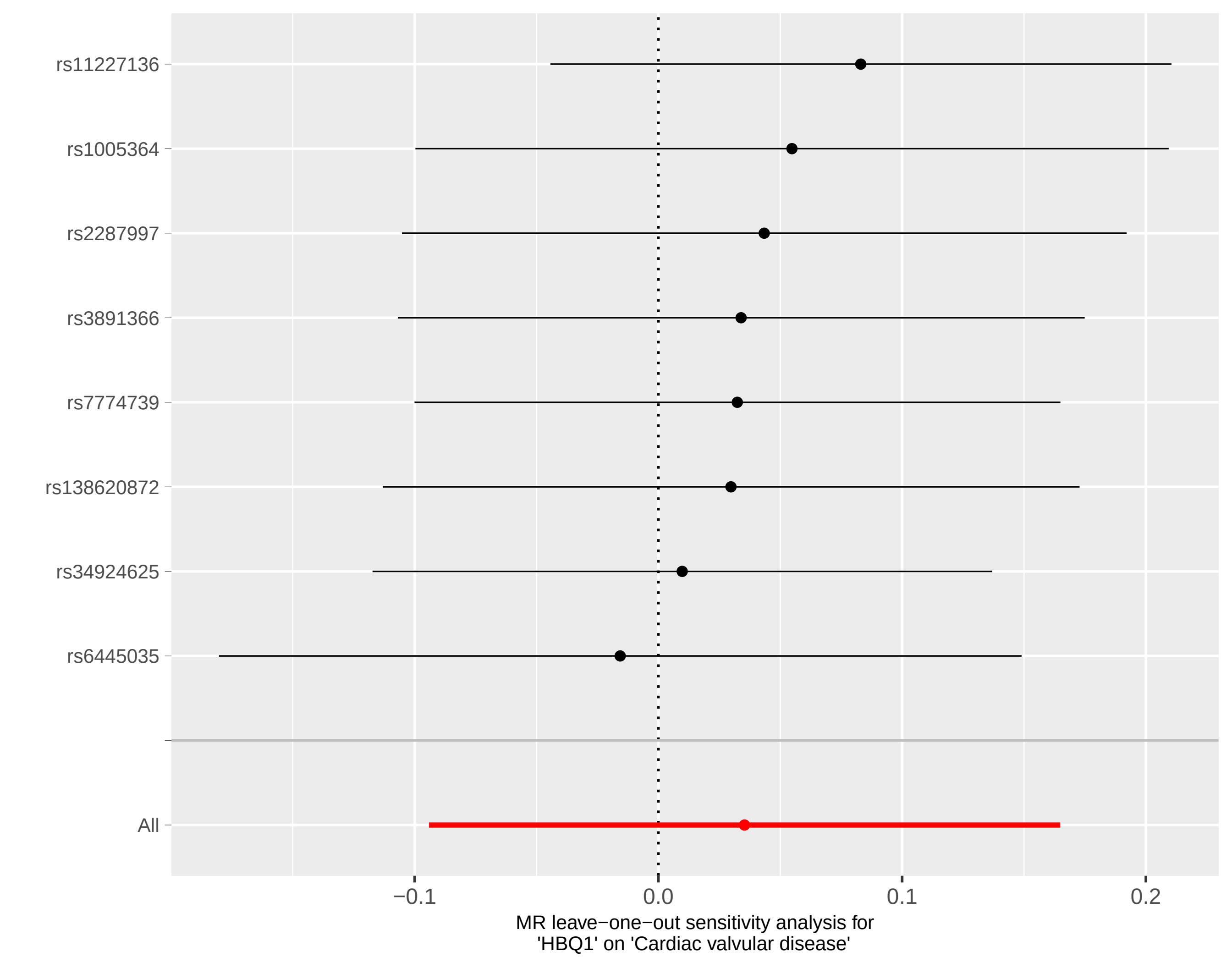
rs11227136
rs1005364
rs2287997
rs3891366
rs7774739
rs138620872
rs34924625
rs6445035
All
−0.1
0.0
0.1
0.2
MR leave−one−out sensitivity analysis for
'HBQ1' on 'Cardiac valvular disease'

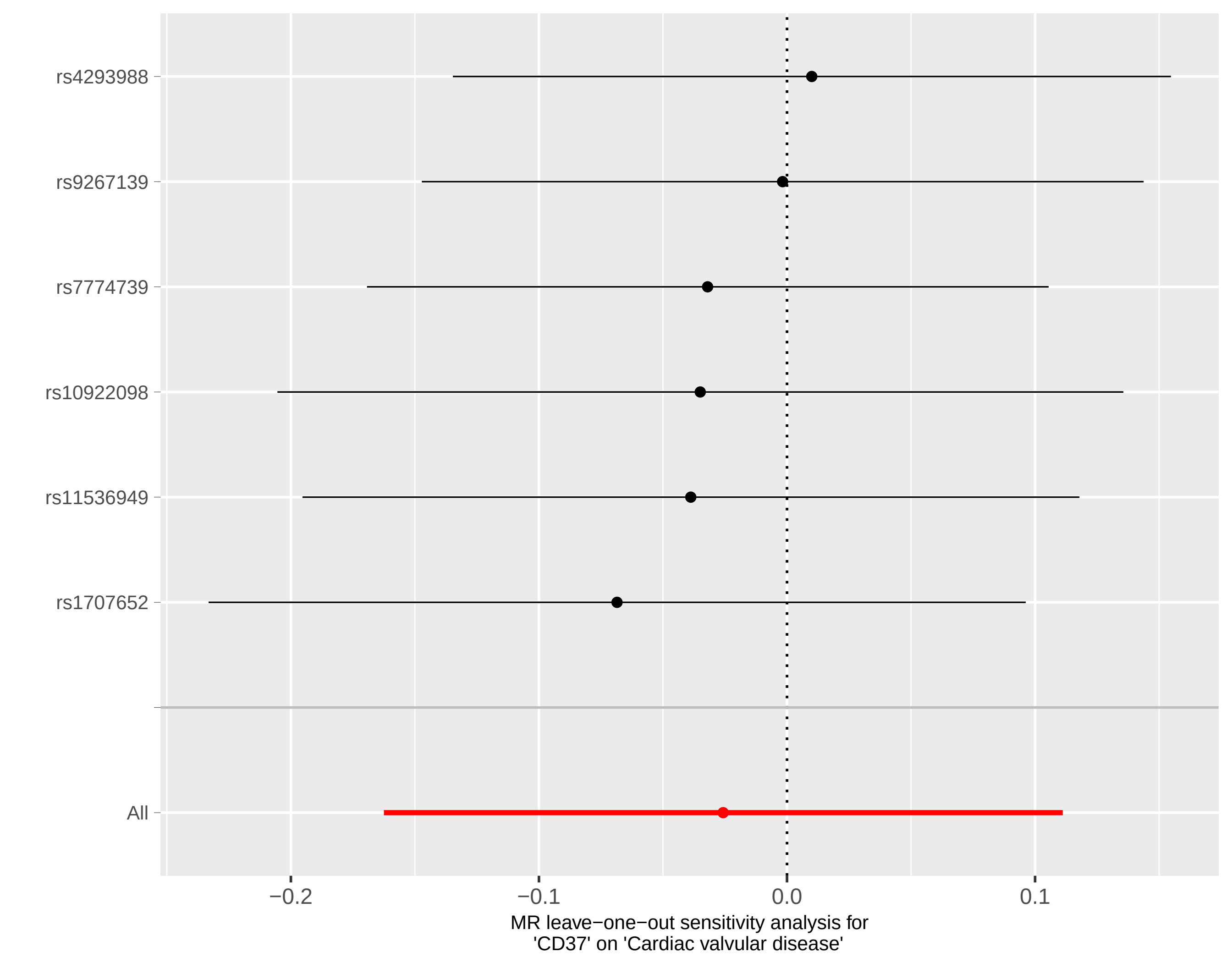

rs4293988
rs9267139
rs7774739
rs10922098
rs11536949
rs1707652
All
−0.2
−0.1
0.0
0.1
MR leave−one−out sensitivity analysis for
'CD37' on 'Cardiac valvular disease'

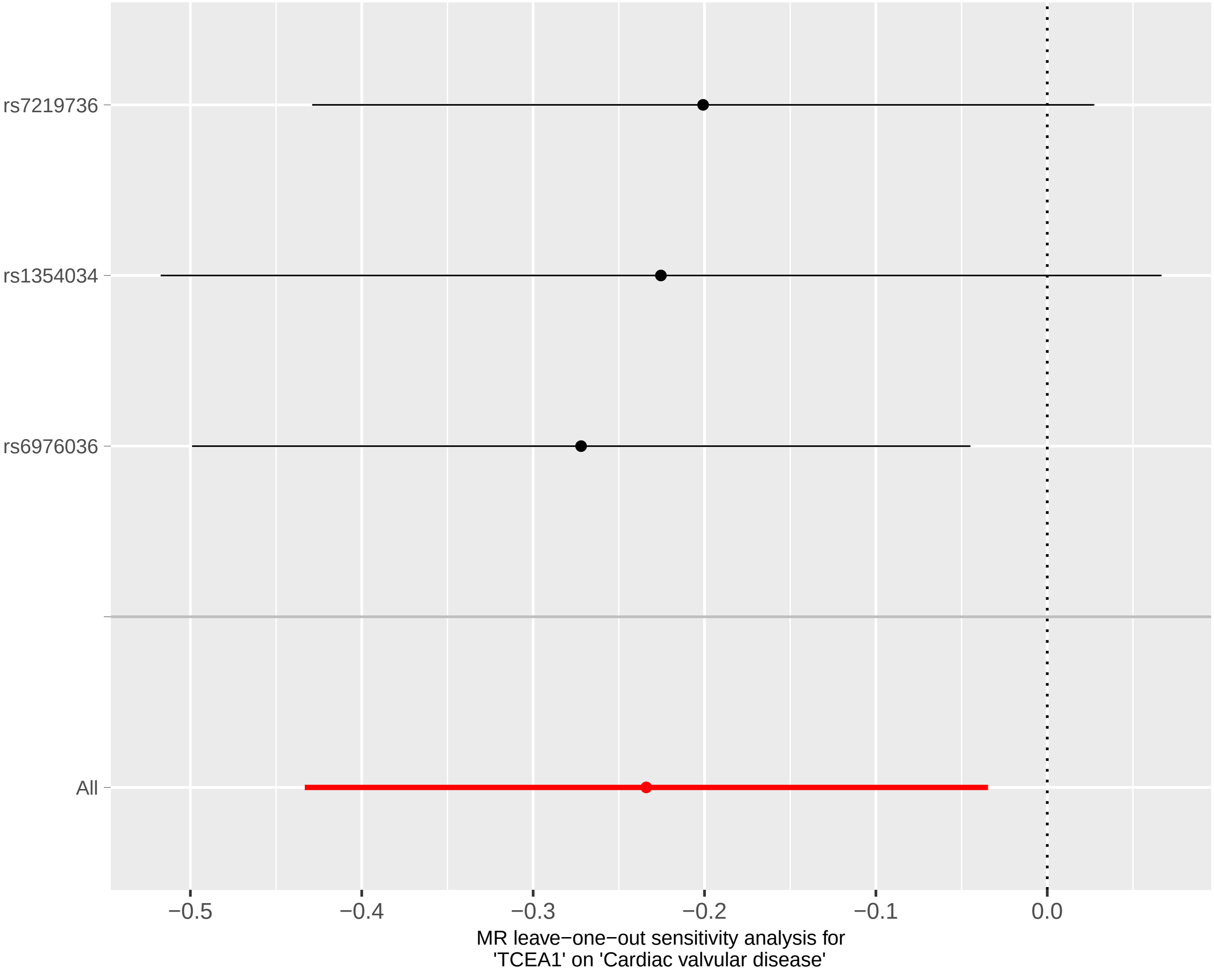

rs7219736
rs1354034
rs6976036
All
−0.5
−0.4
−0.3
−0.2
−0.1
0.0
MR leave−one−out sensitivity analysis for
'TCEA1' on 'Cardiac valvular disease'

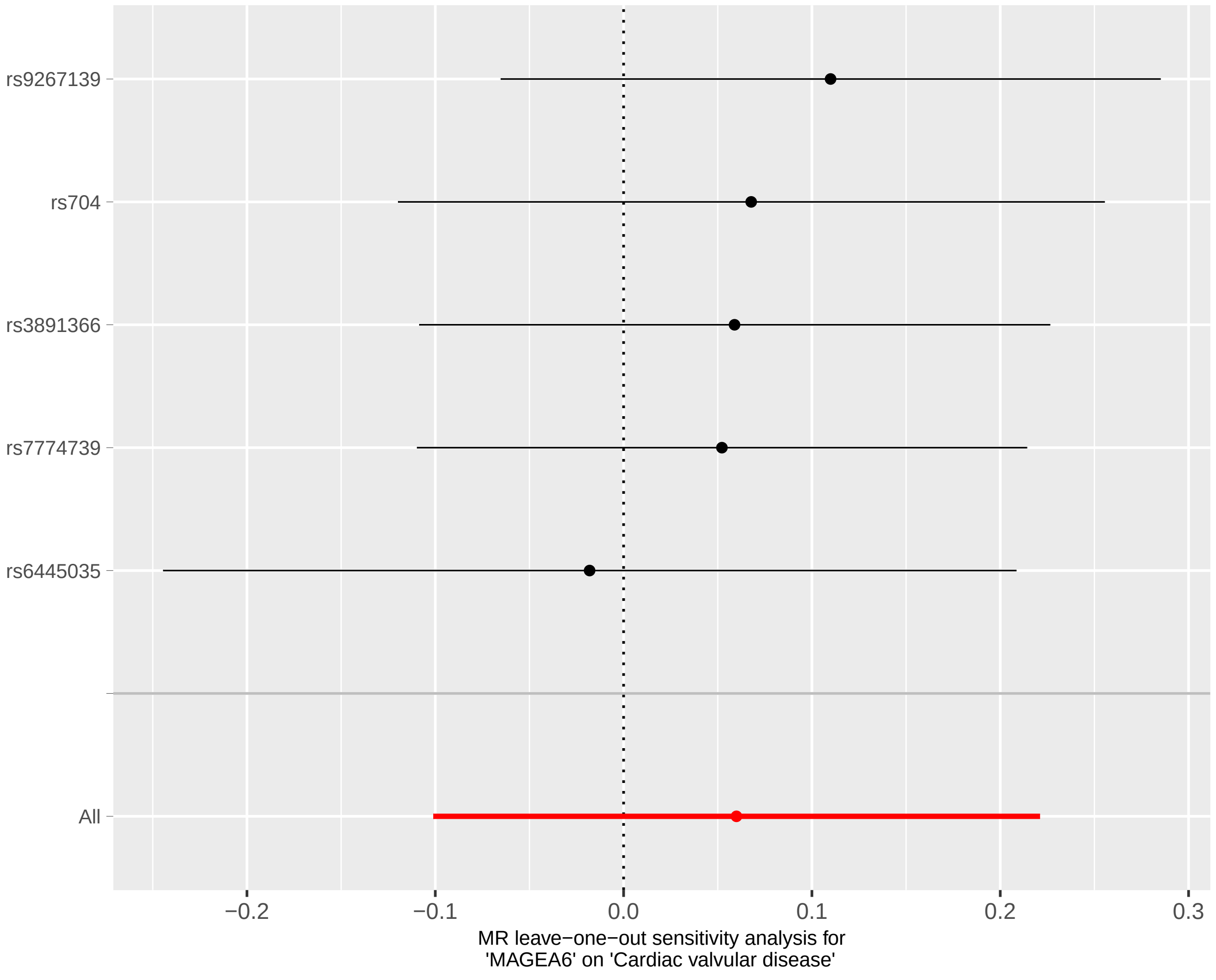
rs9267139
rs704
rs3891366
rs7774739
rs6445035
All
−0.2
−0.1
0.0
0.1
0.2
0.3
MR leave−one−out sensitivity analysis for
'MAGEA6' on 'Cardiac valvular disease'

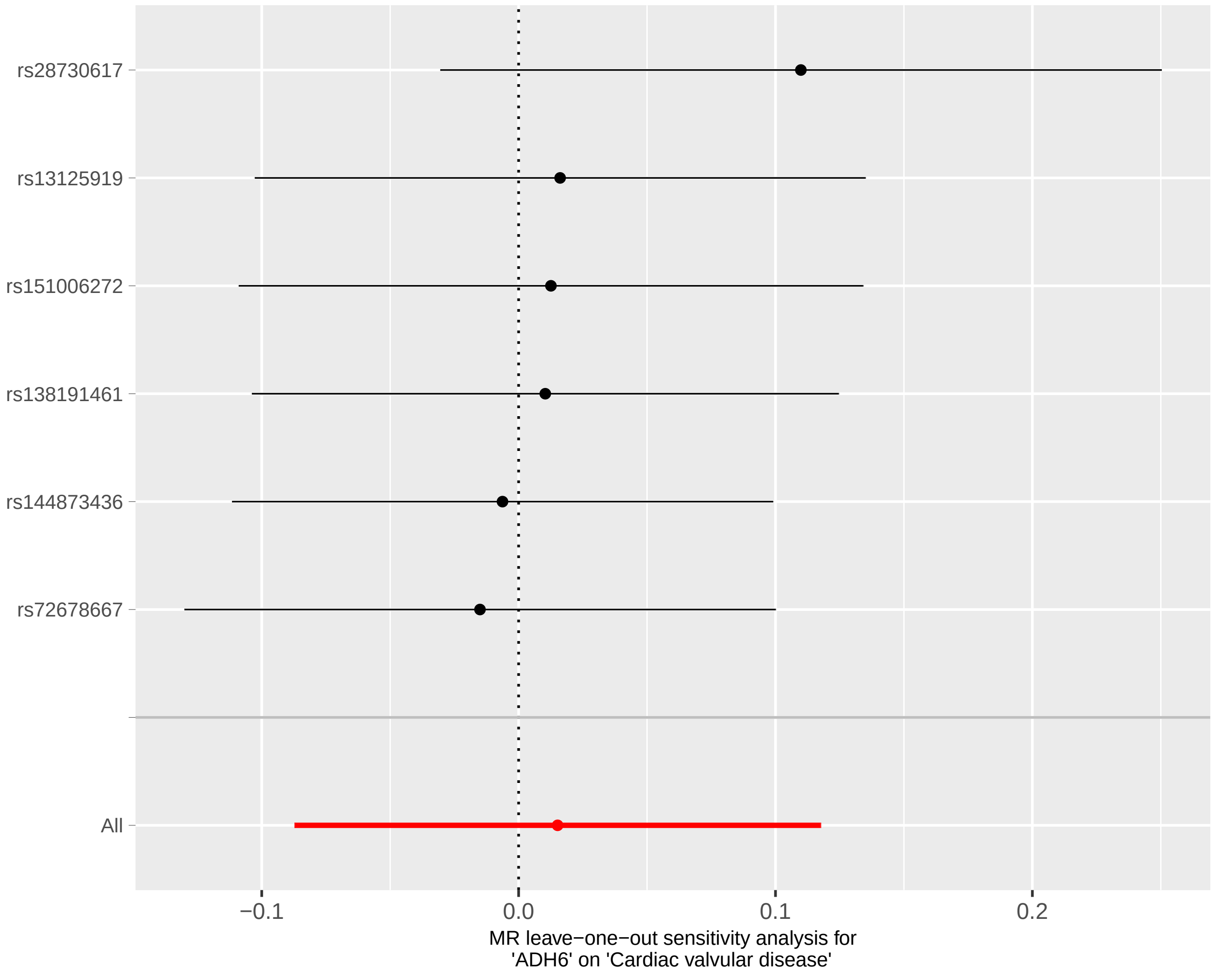
rs28730617
rs13125919
rs151006272
rs138191461
rs144873436
rs72678667
All
−0.1
0.0
0.1
0.2
MR leave−one−out sensitivity analysis for
'ADH6' on 'Cardiac valvular disease'

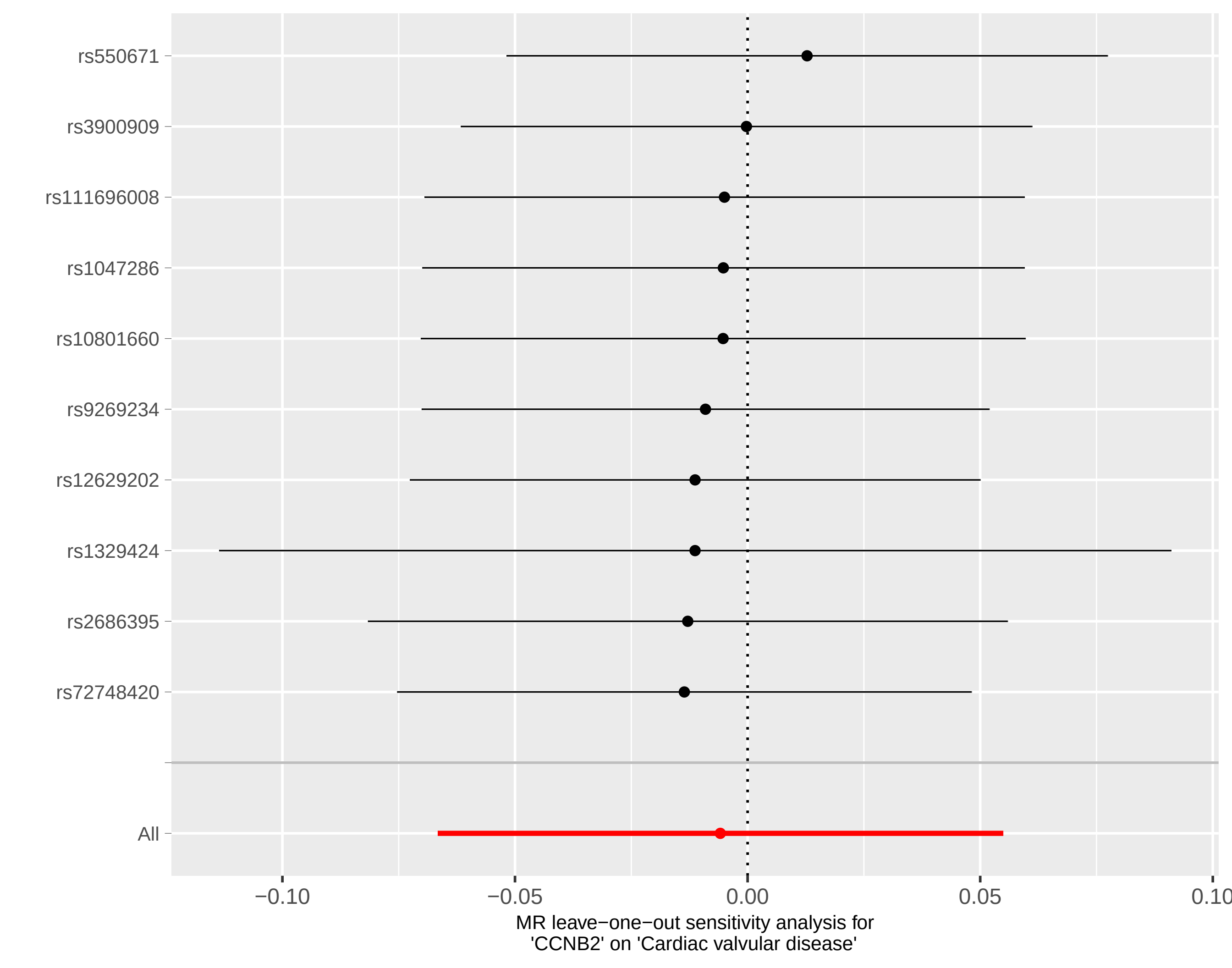
rs550671
rs3900909
rs111696008
rs1047286
rs10801660
rs9269234
rs12629202
rs1329424
rs2686395
rs72748420
All
−0.10
−0.05
0.00
0.05
0.10
MR leave−one−out sensitivity analysis for
'CCNB2' on 'Cardiac valvular disease'

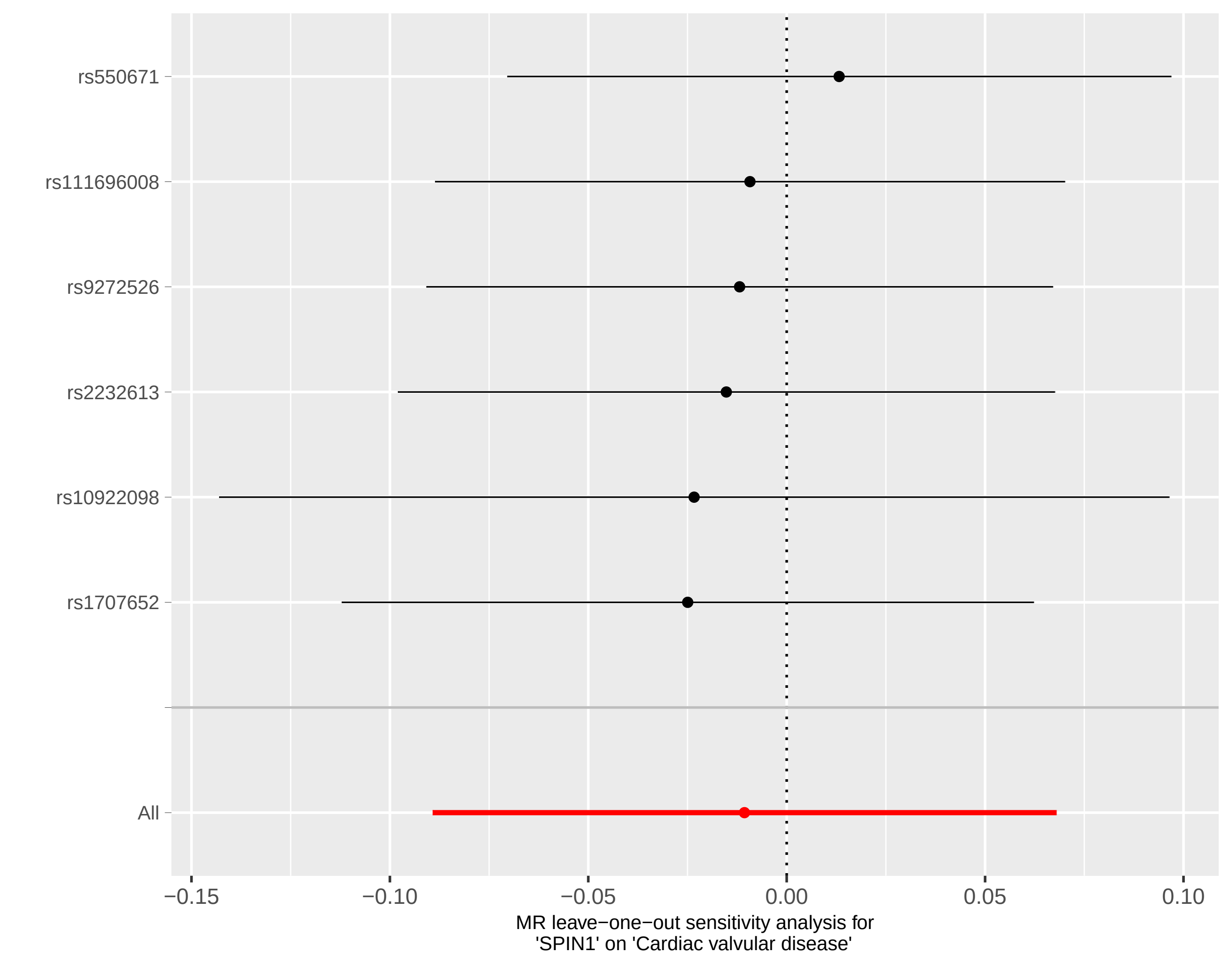

rs550671
rs111696008
rs9272526
rs2232613
rs10922098
rs1707652
All
−0.15
−0.10
−0.05
0.00
0.05
0.10
MR leave−one−out sensitivity analysis for
'SPIN1' on 'Cardiac valvular disease'

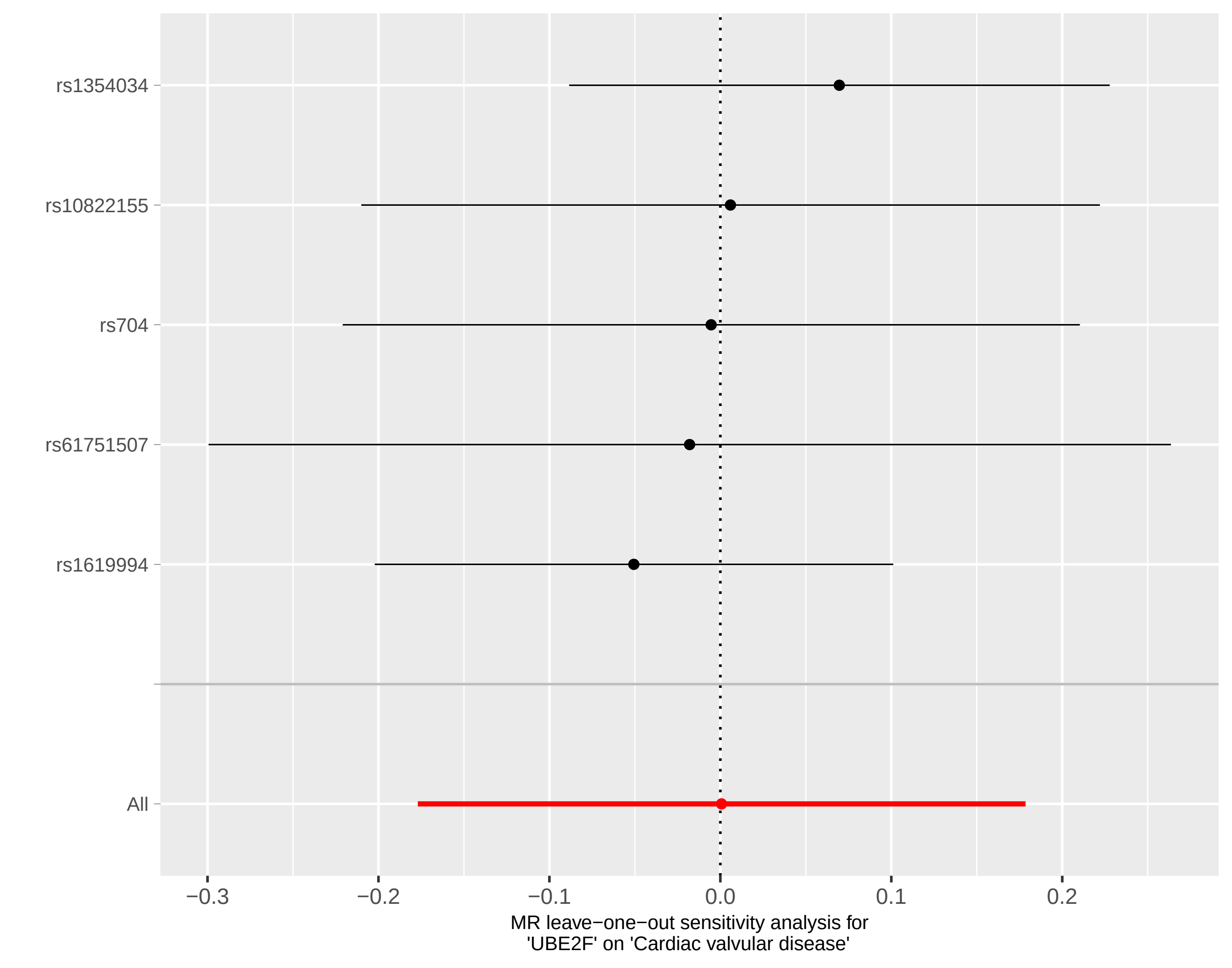
rs1354034
rs10822155
rs704
rs61751507
rs1619994
All
−0.3
−0.2
−0.1
0.0
0.1
0.2
MR leave−one−out sensitivity analysis for
'UBE2F' on 'Cardiac valvular disease'

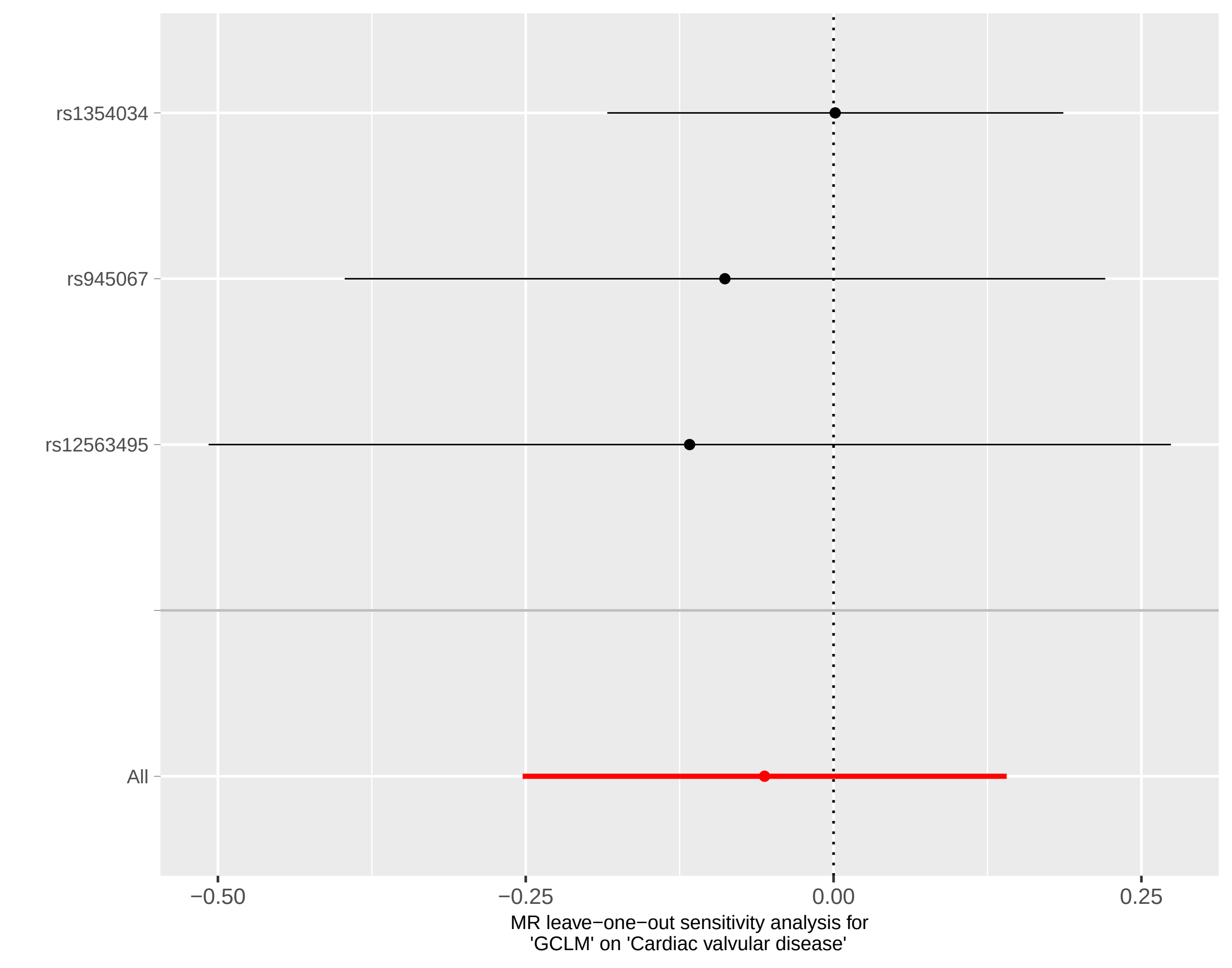
rs1354034
rs945067
rs12563495
All
−0.50
−0.25
0.00
0.25
MR leave−one−out sensitivity analysis for
'GCLM' on 'Cardiac valvular disease'

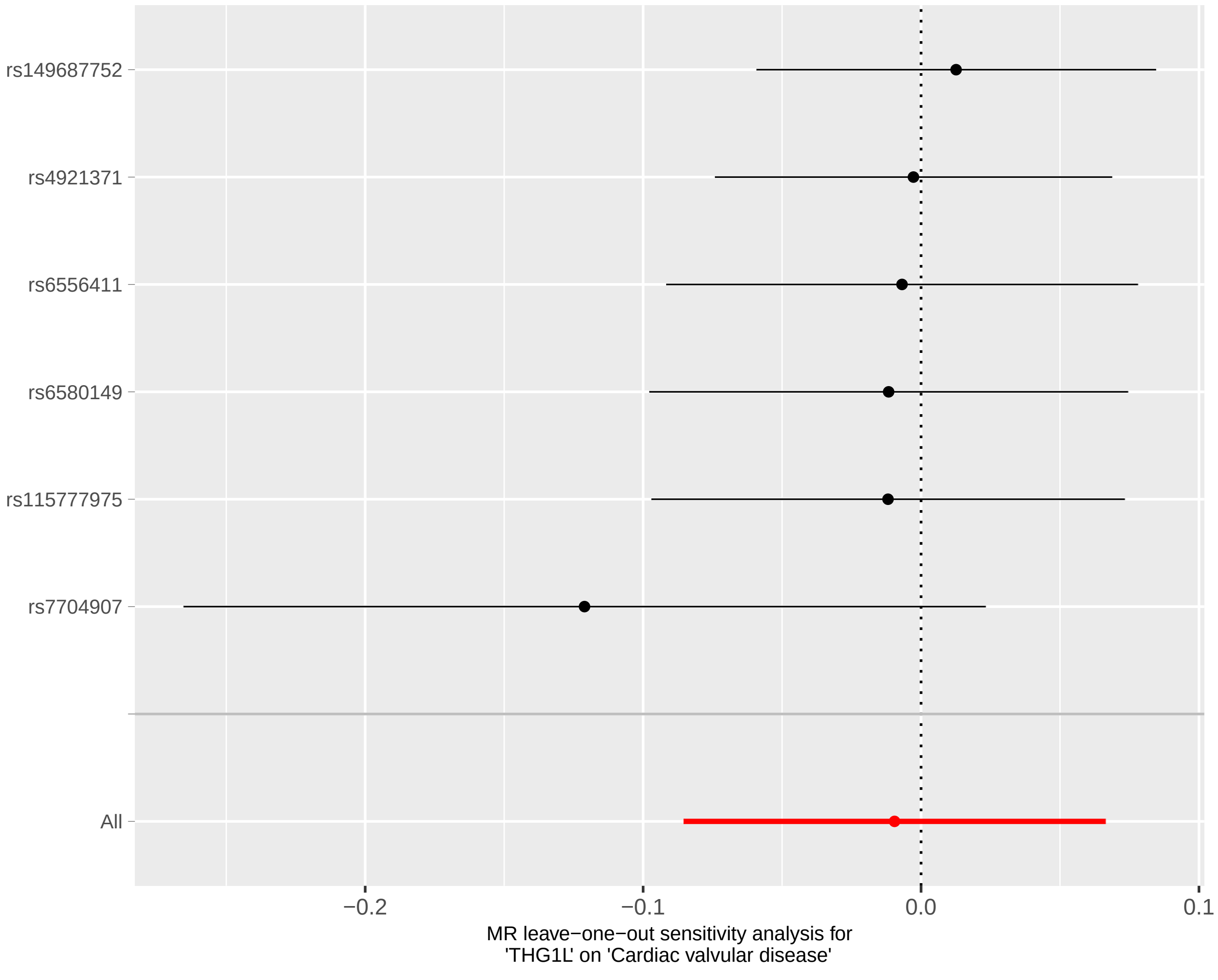
rs149687752
rs4921371
rs6556411
rs6580149
rs115777975
rs7704907
All
−0.2
−0.1
0.0
0.1
MR leave−one−out sensitivity analysis for
'THG1L' on 'Cardiac valvular disease'

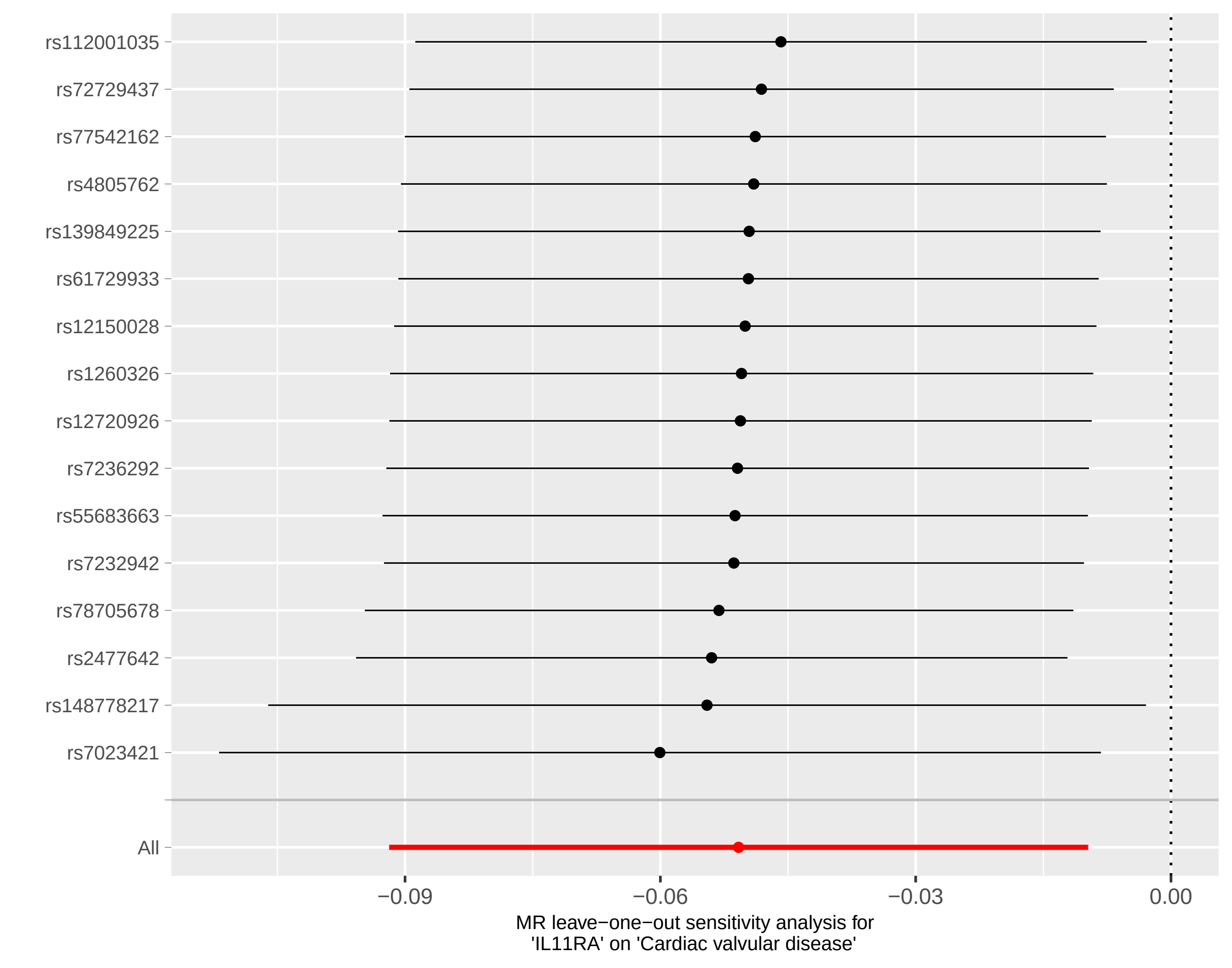

rs112001035
rs72729437
rs77542162
rs4805762
rs139849225
rs61729933
rs12150028
rs1260326
rs12720926
rs7236292
rs55683663
rs7232942
rs78705678
rs2477642
rs148778217
rs7023421
All
−0.09
−0.06
−0.03
0.00
MR leave−one−out sensitivity analysis for
'IL11RA' on 'Cardiac valvular disease'

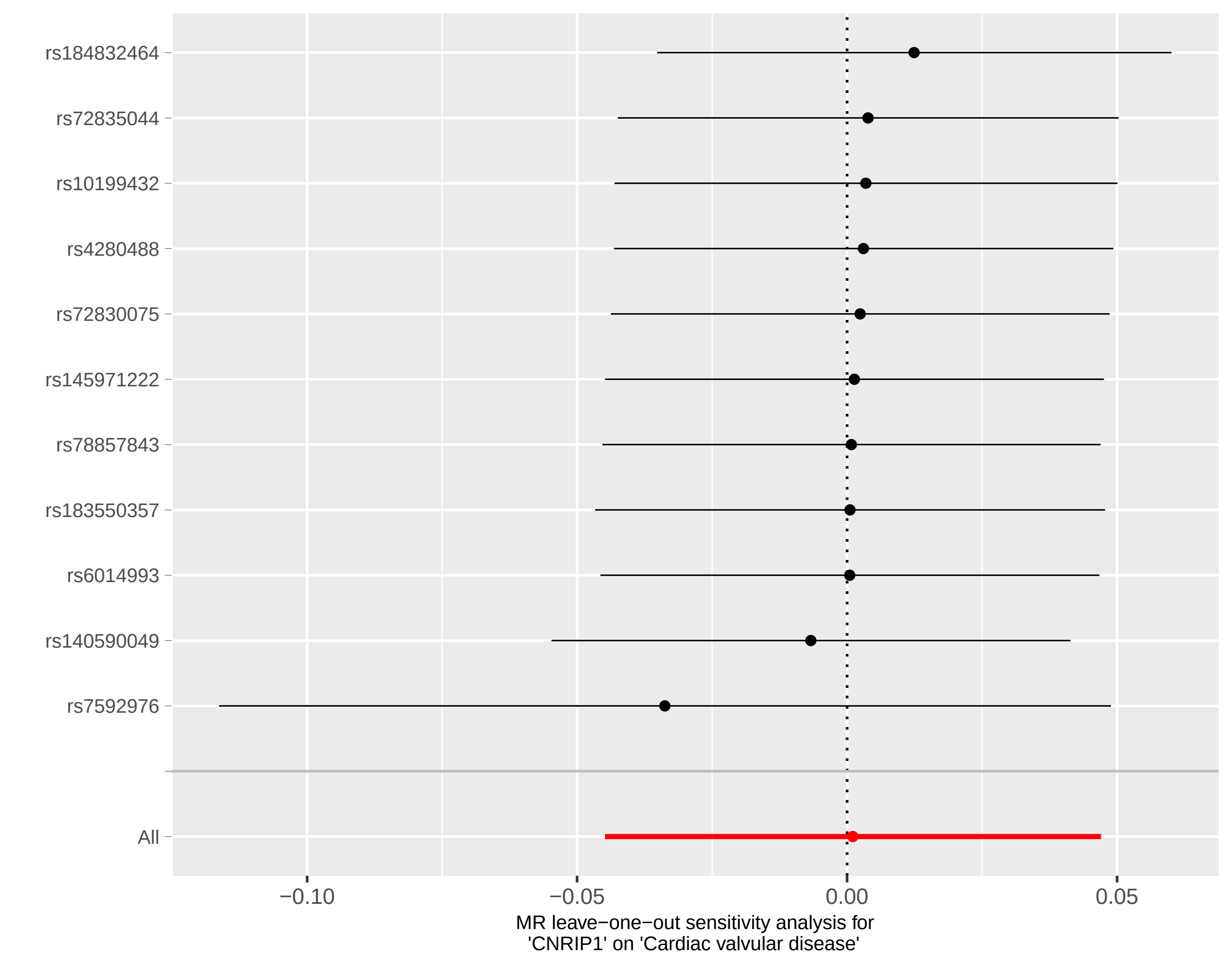
rs184832464
rs72835044
rs10199432
rs4280488
rs72830075
rs145971222
rs78857843
rs183550357
rs6014993
rs140590049
rs7592976
All
−0.10
−0.05
0.00
0.05
MR leave−one−out sensitivity analysis for
'CNRIP1' on 'Cardiac valvular disease'

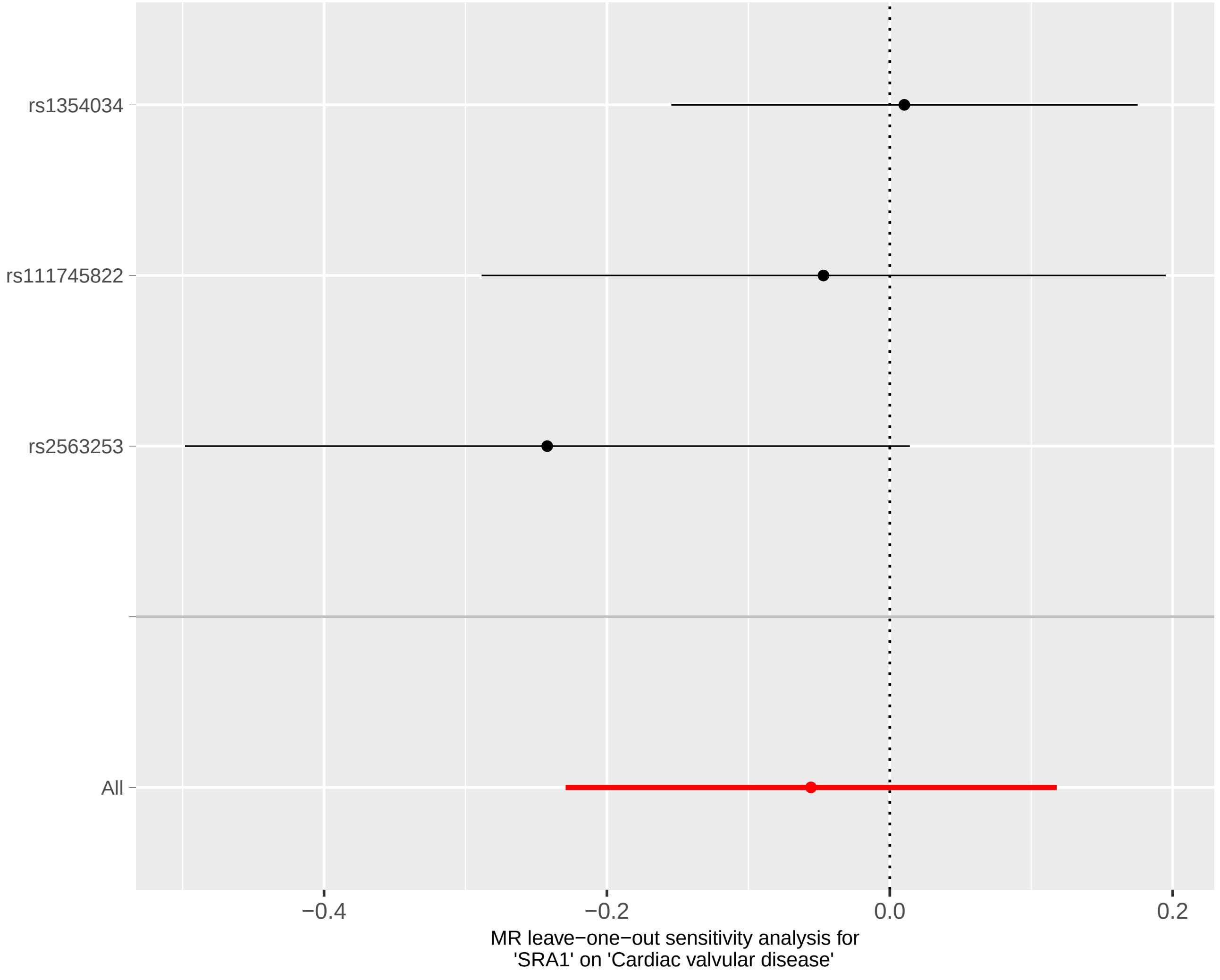
rs1354034
rs111745822
rs2563253
All
−0.4
−0.2
0.0
0.2
MR leave−one−out sensitivity analysis for
'SRA1' on 'Cardiac valvular disease'

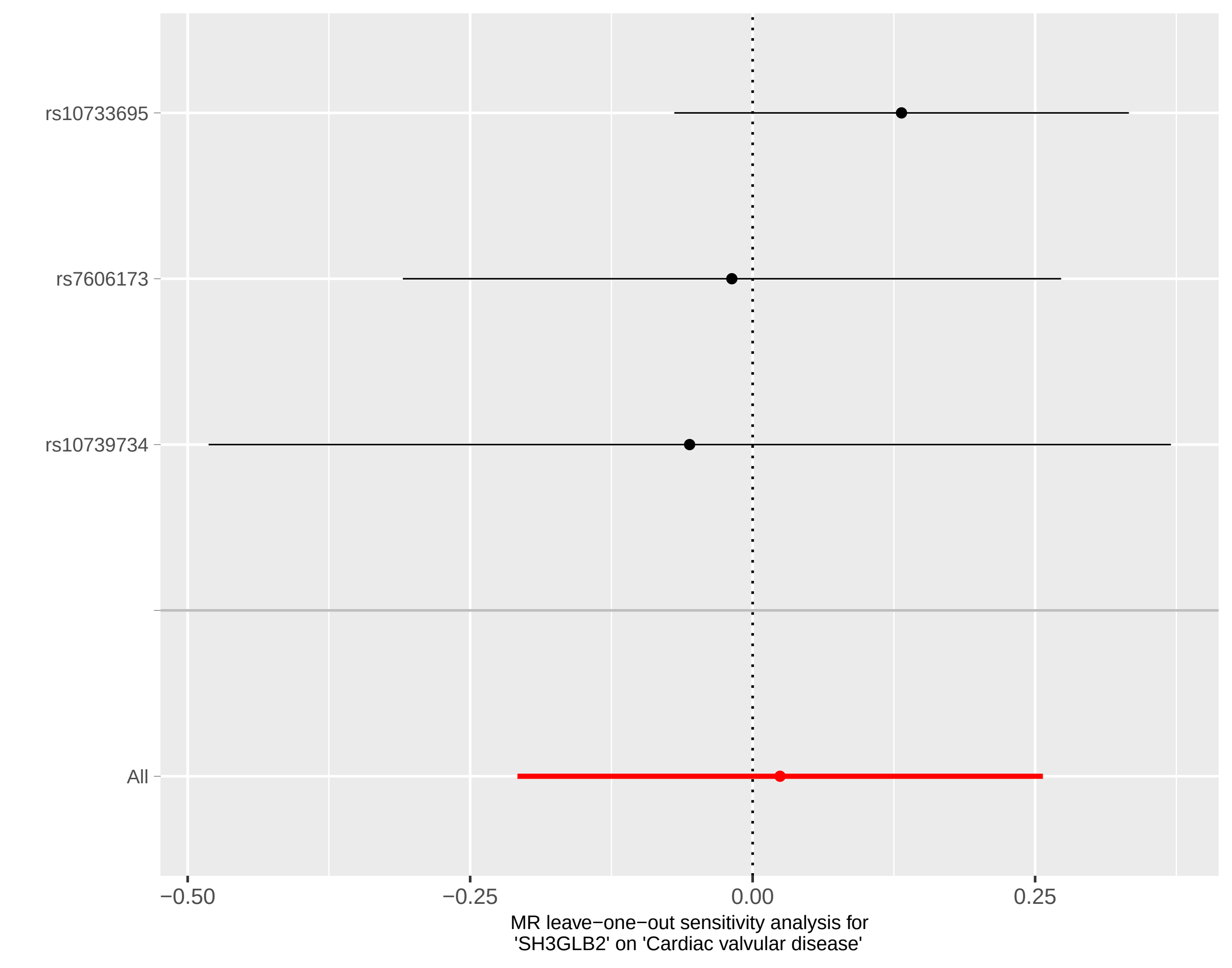
rs10733695
rs7606173
rs10739734
All
−0.50
−0.25
0.00
0.25
MR leave−one−out sensitivity analysis for
'SH3GLB2' on 'Cardiac valvular disease'

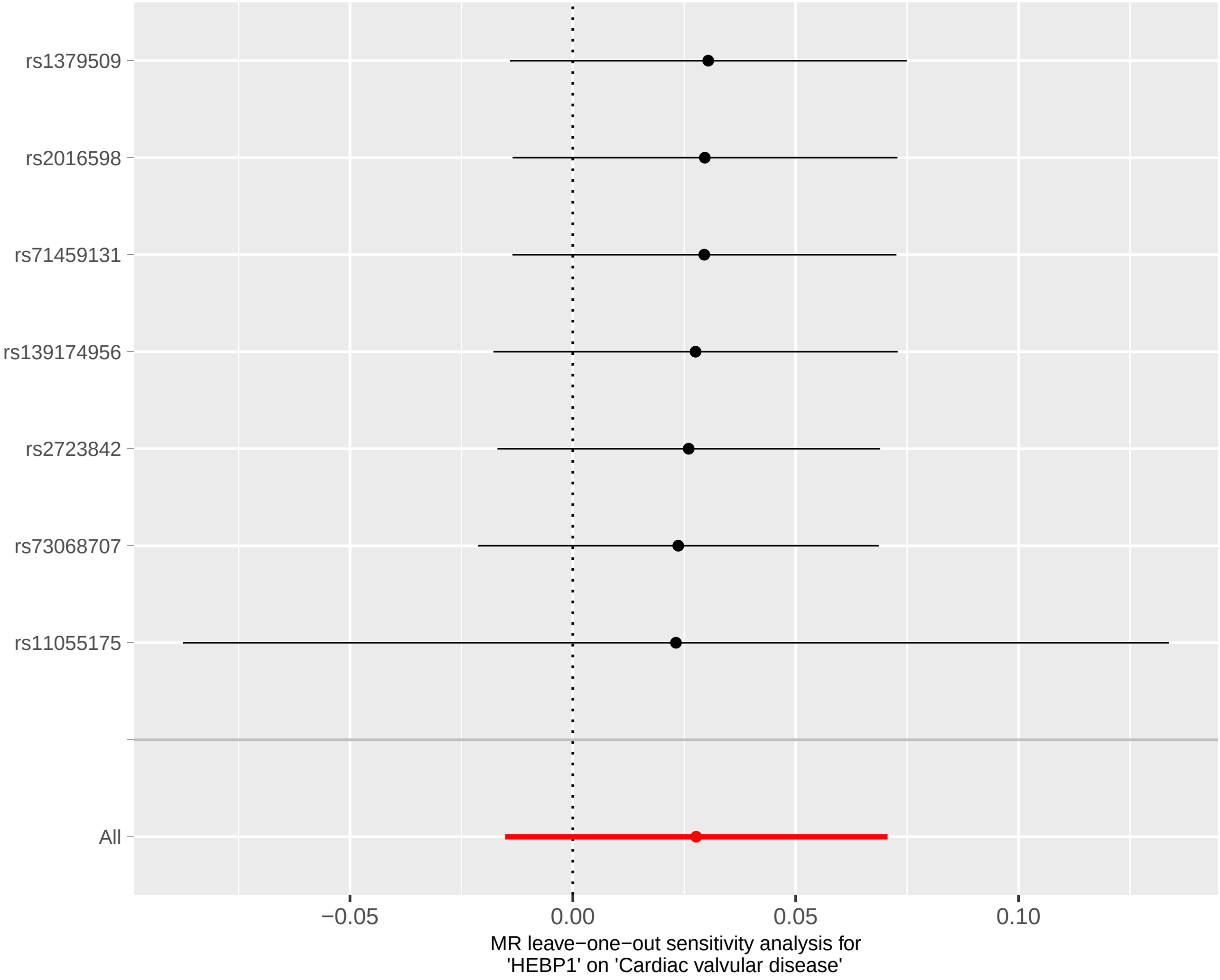
rs1379509
rs2016598
rs71459131
rs139174956
rs2723842
rs73068707
rs11055175
All
−0.05
0.00
0.05
0.10
MR leave−one−out sensitivity analysis for
'HEBP1' on 'Cardiac valvular disease'

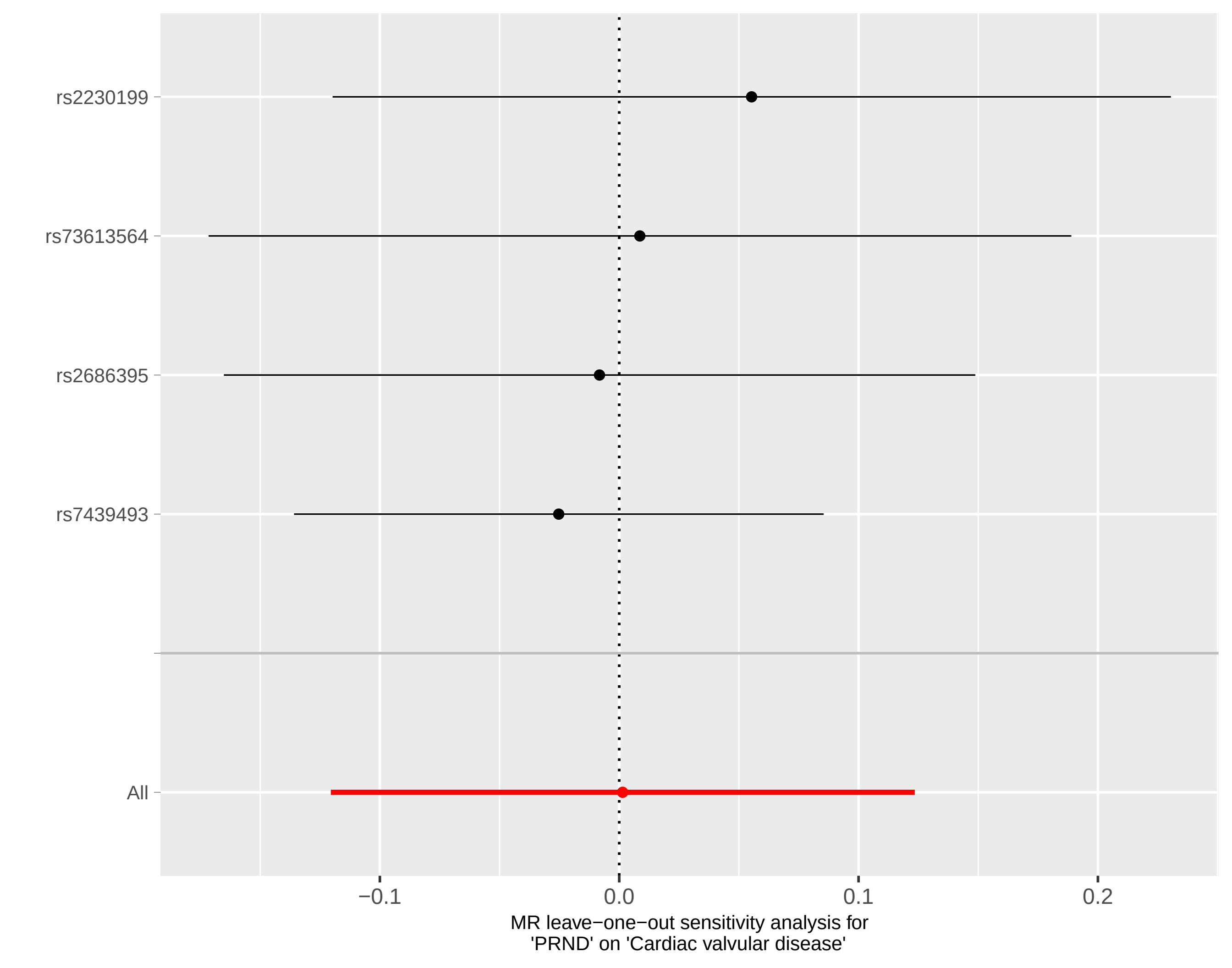

rs2230199
rs73613564
rs2686395
rs7439493
All
−0.1
0.0
0.1
0.2
MR leave−one−out sensitivity analysis for
'PRND' on 'Cardiac valvular disease'

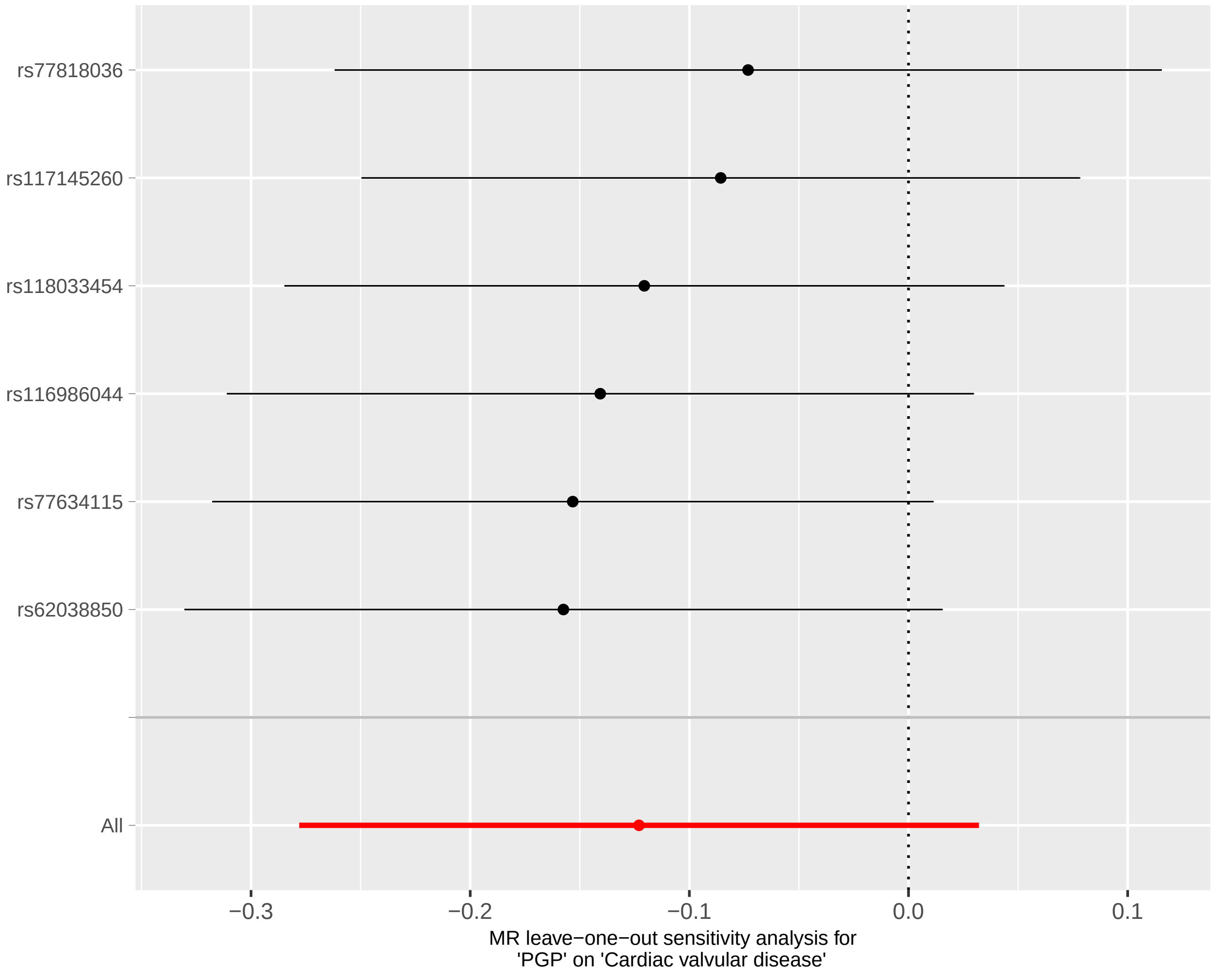
rs77818036
rs117145260
rs118033454
rs116986044
rs77634115
rs62038850
All
−0.3
−0.2
−0.1
0.0
0.1
MR leave−one−out sensitivity analysis for
'PGP' on 'Cardiac valvular disease'

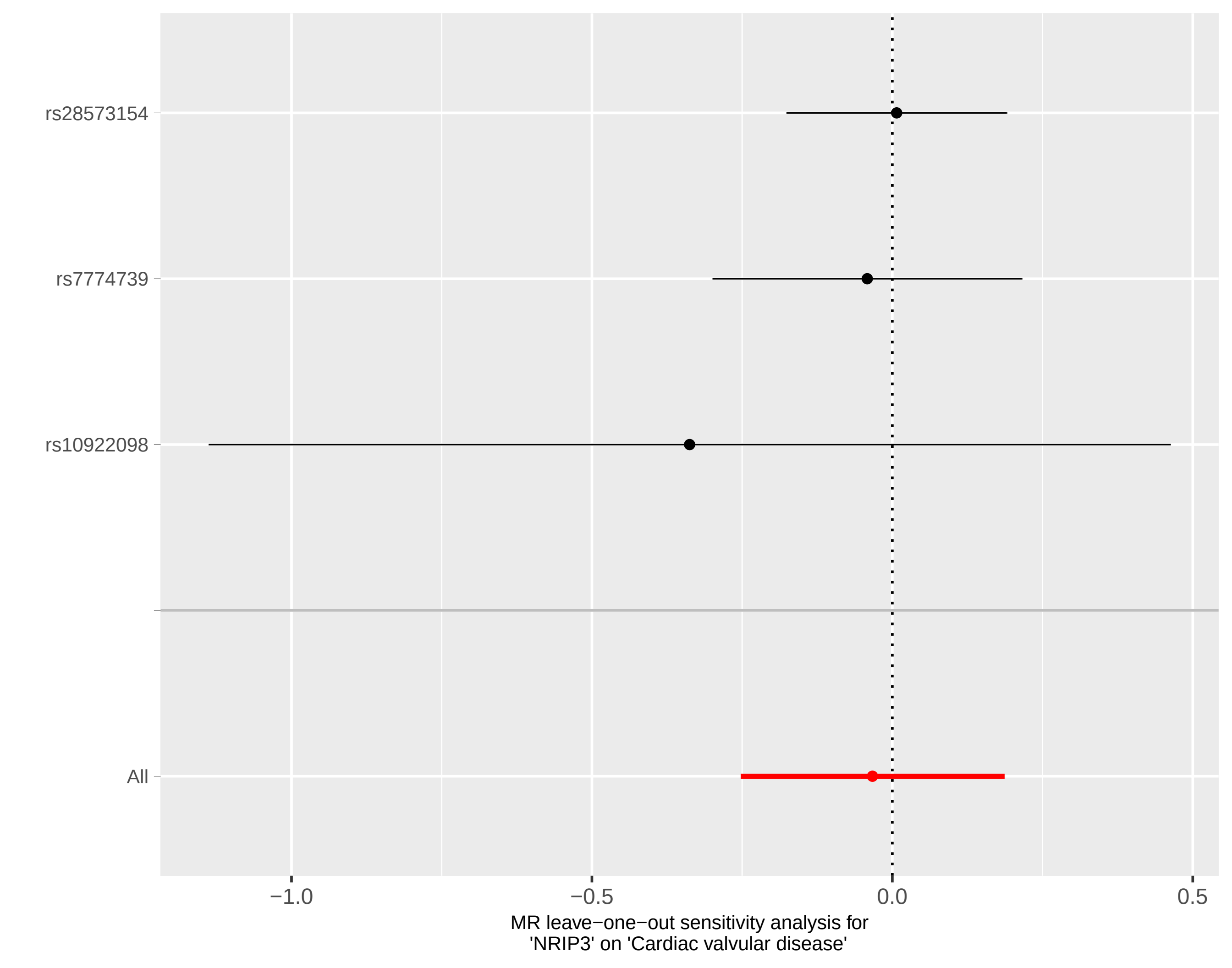

rs28573154
rs7774739
rs10922098
All
−1.0
−0.5
0.0
0.5
MR leave−one−out sensitivity analysis for
'NRIP3' on 'Cardiac valvular disease'

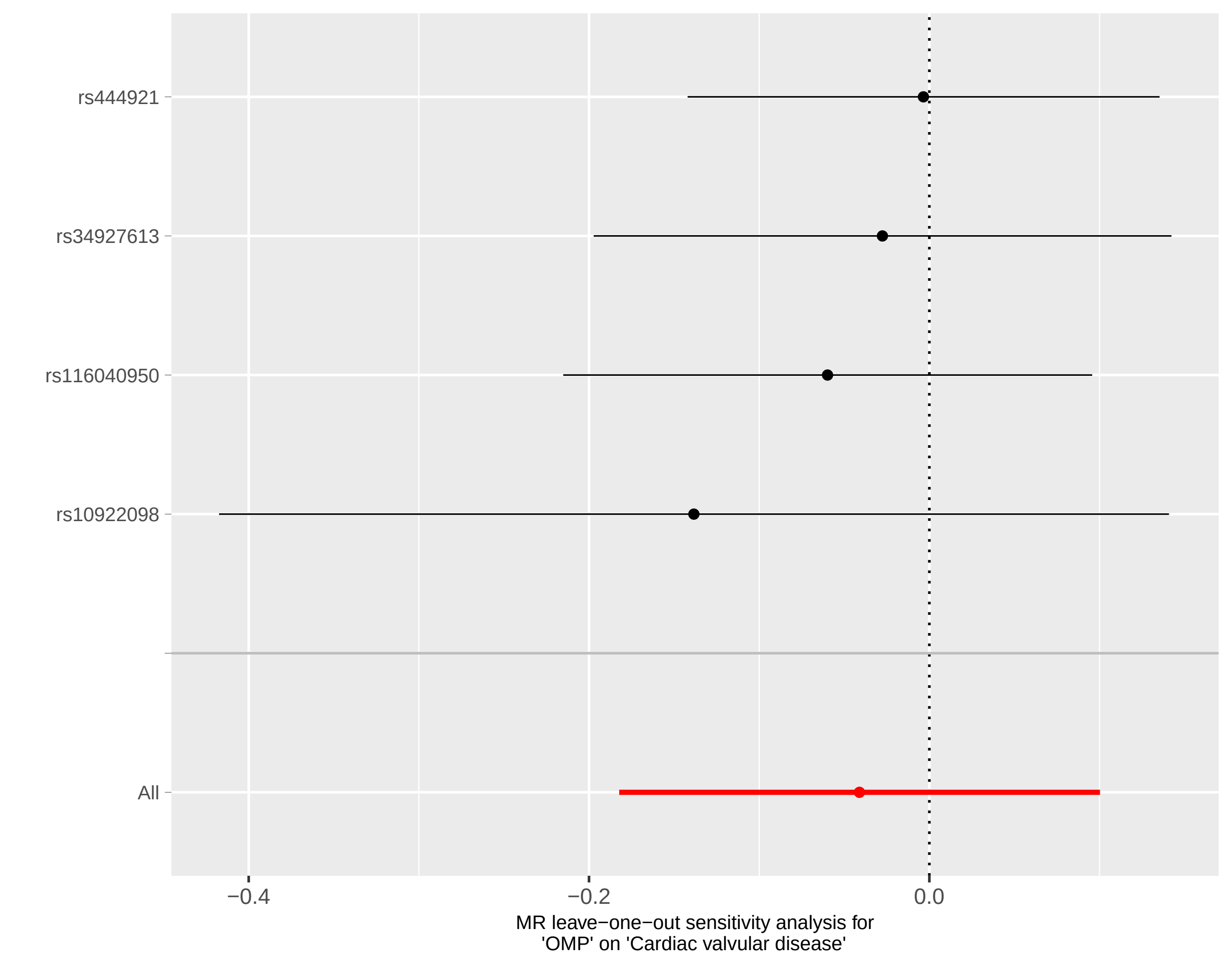
rs444921
rs34927613
rs116040950
rs10922098
All
−0.4
−0.2
0.0
MR leave−one−out sensitivity analysis for
'OMP' on 'Cardiac valvular disease'

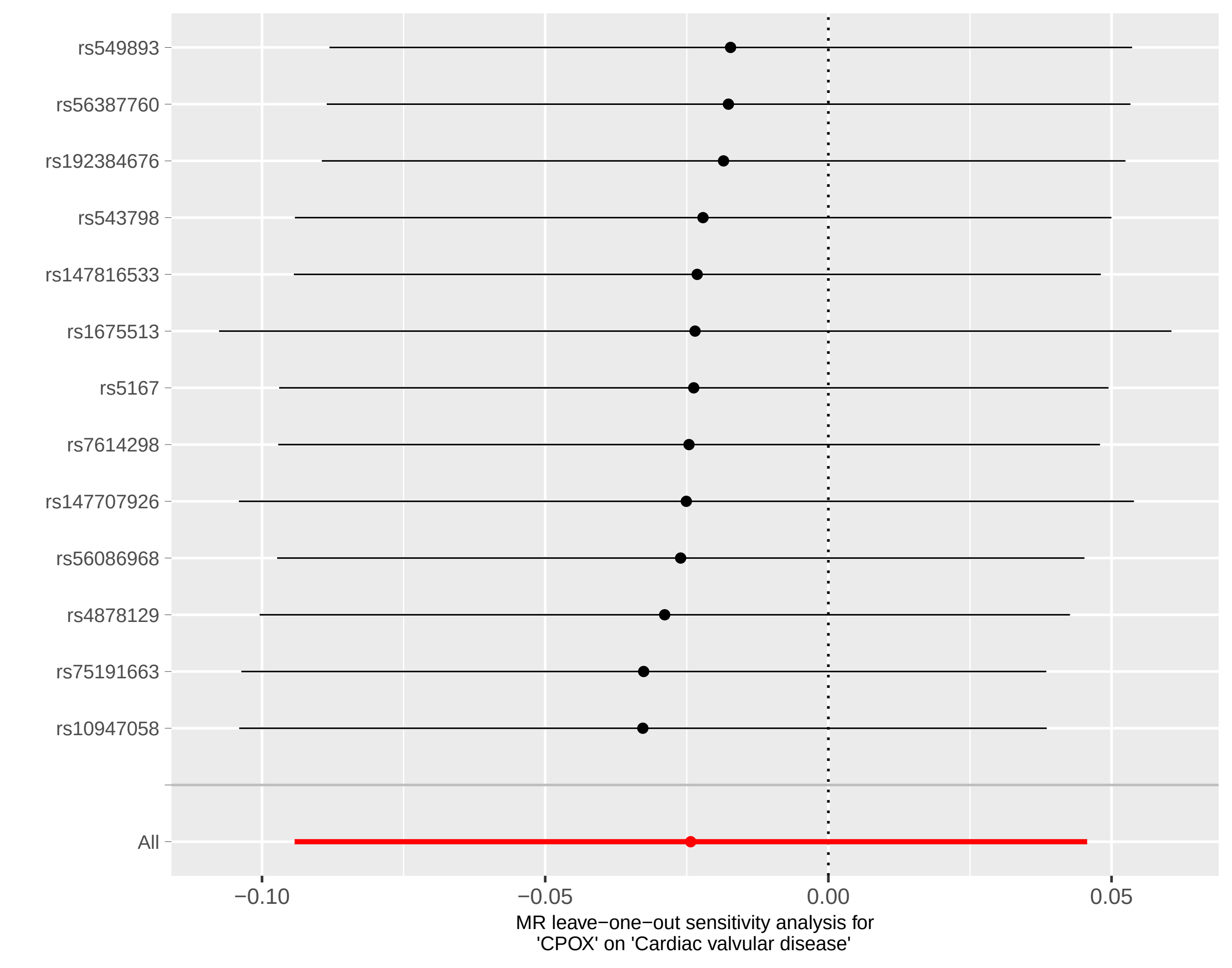
rs549893
rs56387760
rs192384676
rs543798
rs147816533
rs1675513
rs5167
rs7614298
rs147707926
rs56086968
rs4878129
rs75191663
rs10947058
All
−0.10
−0.05
0.00
0.05
MR leave−one−out sensitivity analysis for
'CPOX' on 'Cardiac valvular disease'

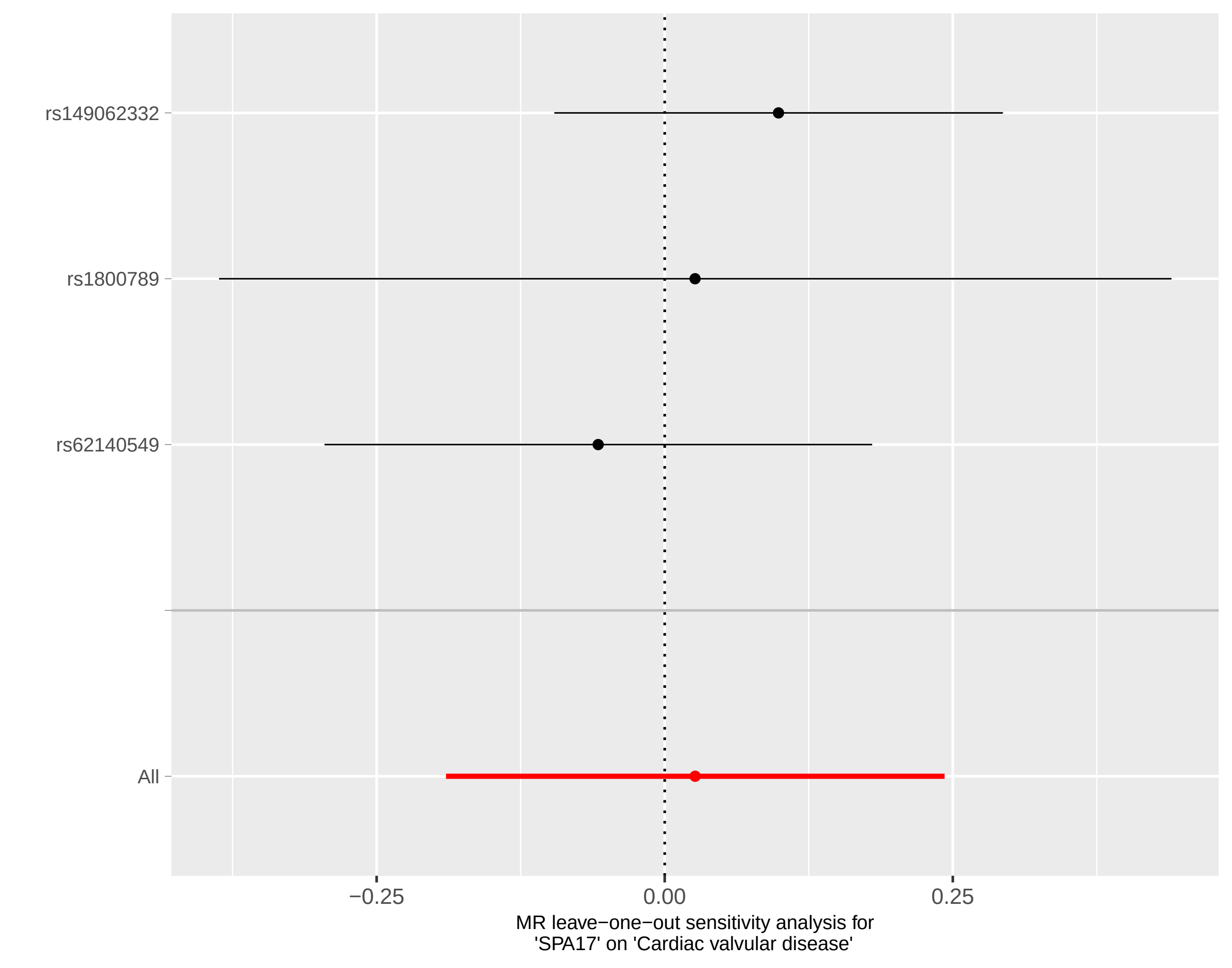
rs149062332
rs1800789
rs62140549
All
−0.25
0.00
0.25
MR leave−one−out sensitivity analysis for
'SPA17' on 'Cardiac valvular disease'

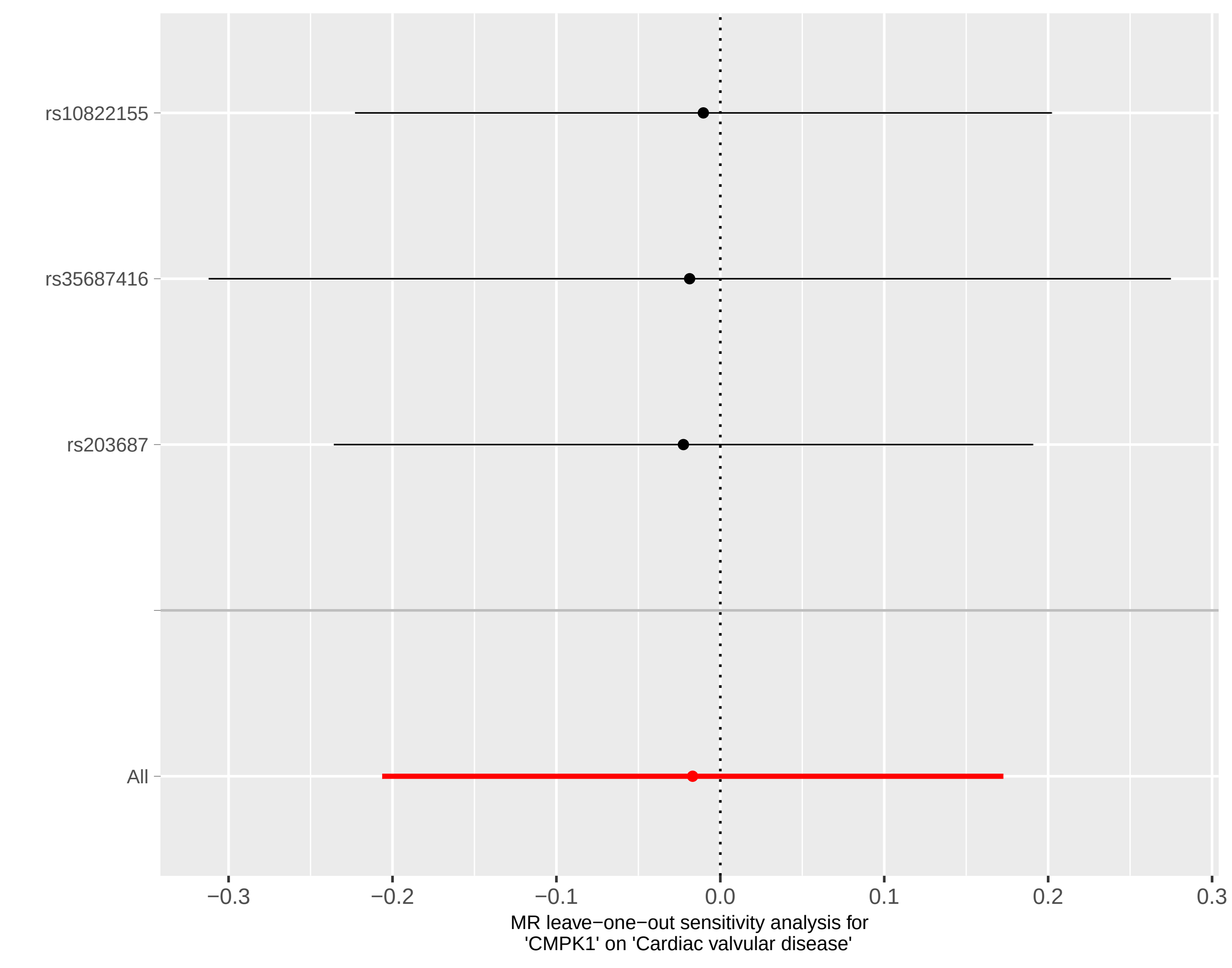
rs10822155
rs35687416
rs203687
All
−0.3
−0.2
−0.1
0.0
0.1
0.2
0.3
MR leave−one−out sensitivity analysis for
'CMPK1' on 'Cardiac valvular disease'

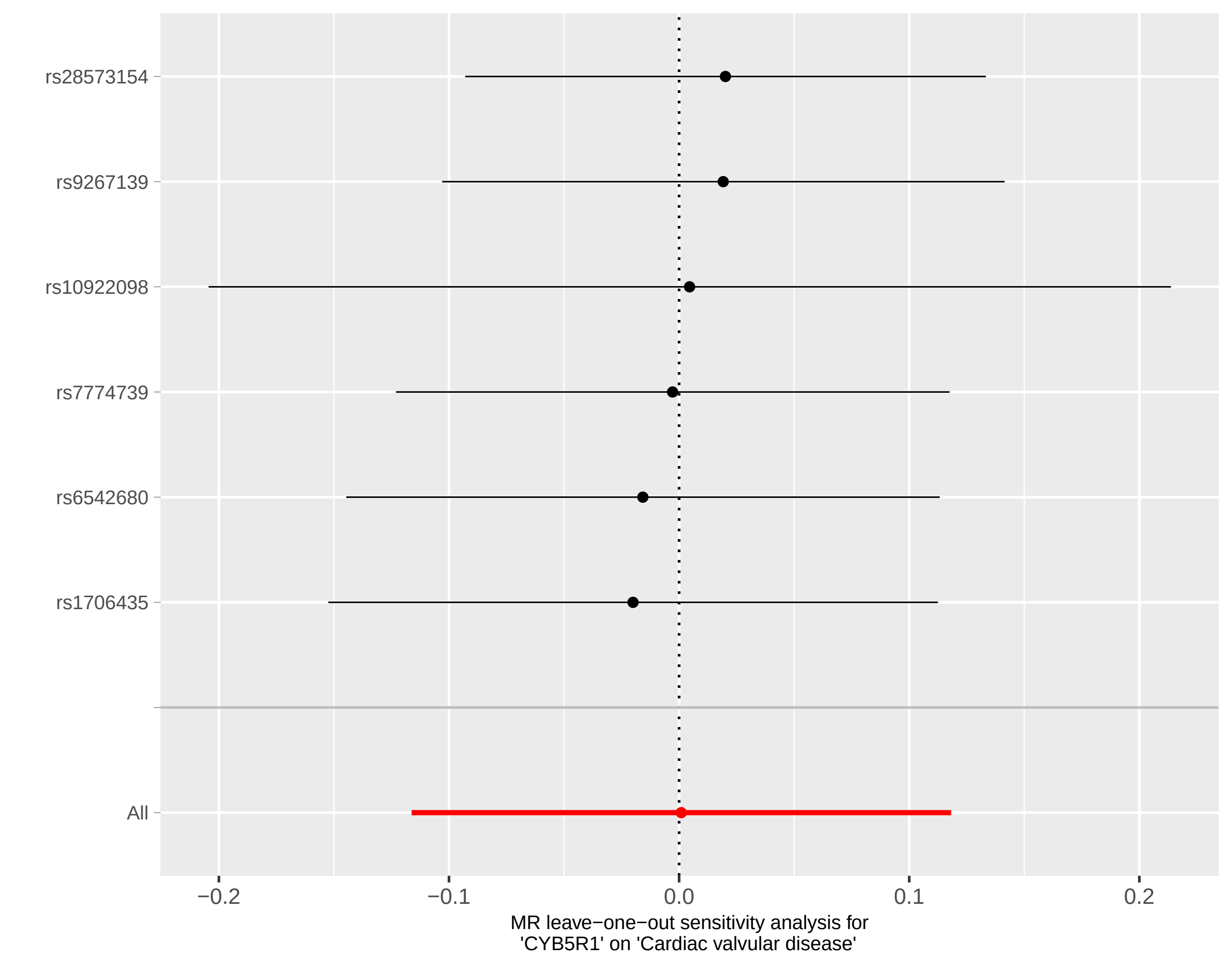
rs28573154
rs9267139
rs10922098
rs7774739
rs6542680
rs1706435
All
−0.2
−0.1
0.0
0.1
0.2
MR leave−one−out sensitivity analysis for
'CYB5R1' on 'Cardiac valvular disease'

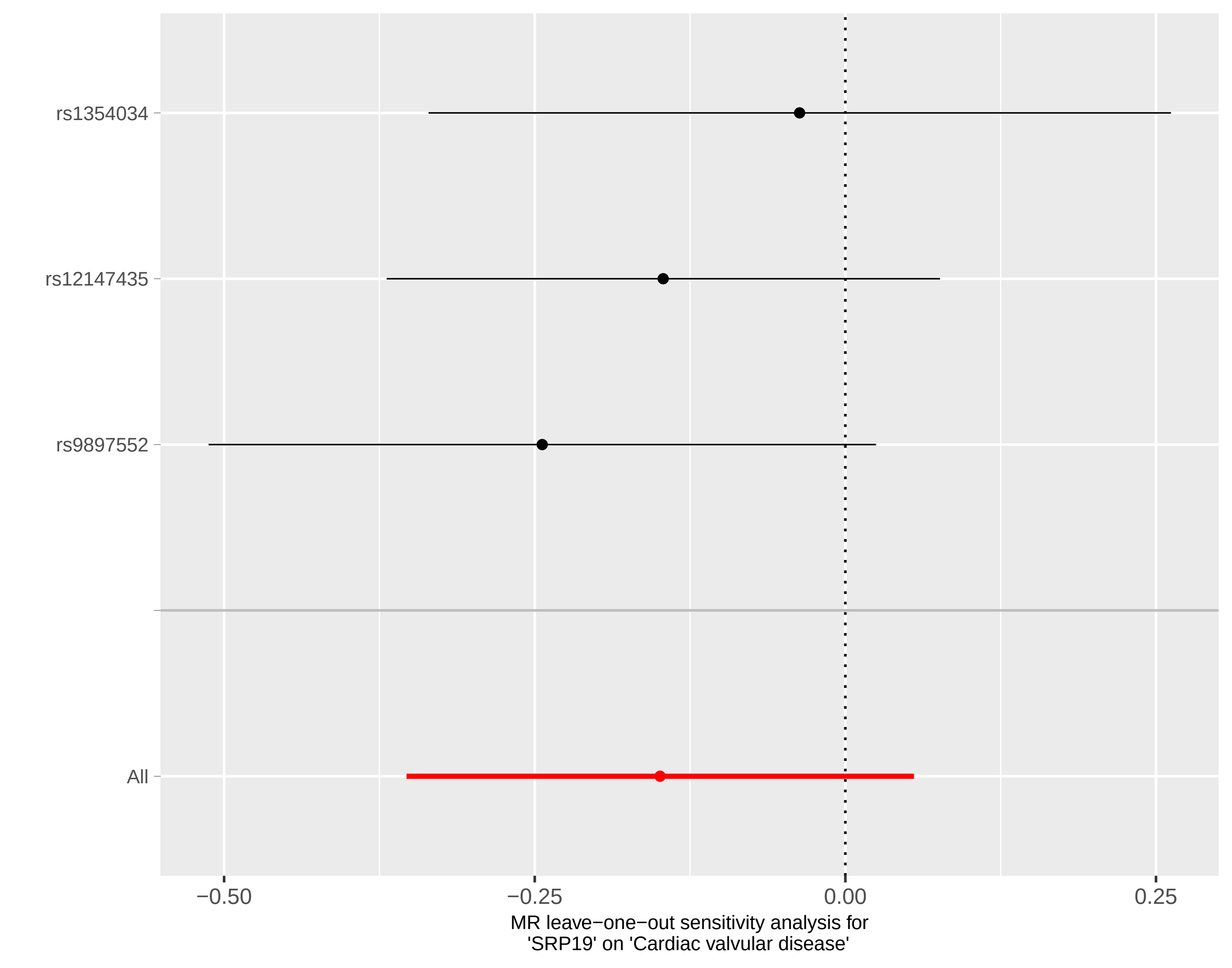

rs1354034
rs12147435
rs9897552
All
−0.50
−0.25
0.00
0.25
MR leave−one−out sensitivity analysis for
'SRP19' on 'Cardiac valvular disease'

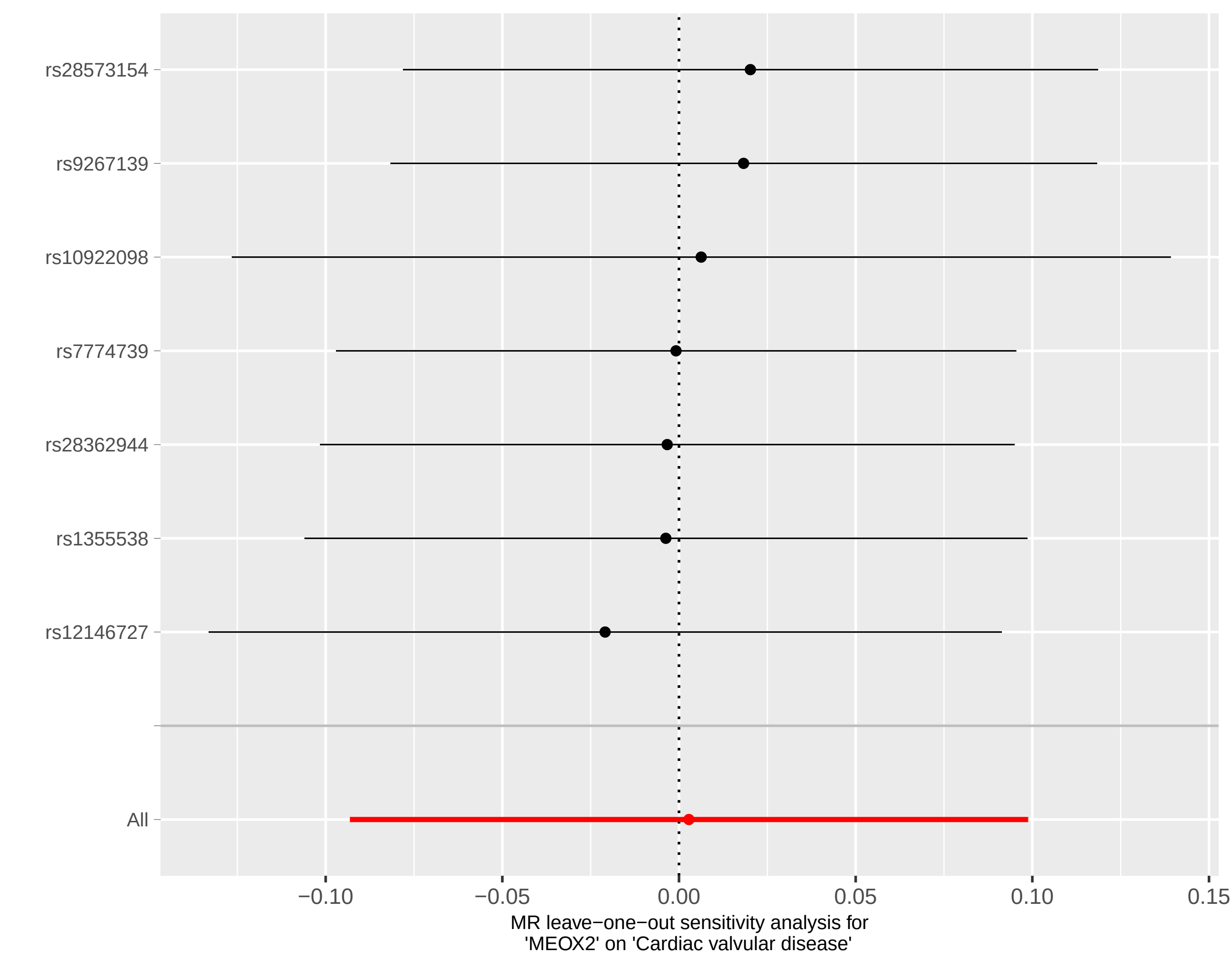
rs28573154
rs9267139
rs10922098
rs7774739
rs28362944
rs1355538
rs12146727
All
−0.10
−0.05
0.00
0.05
0.10
0.15
MR leave−one−out sensitivity analysis for
'MEOX2' on 'Cardiac valvular disease'

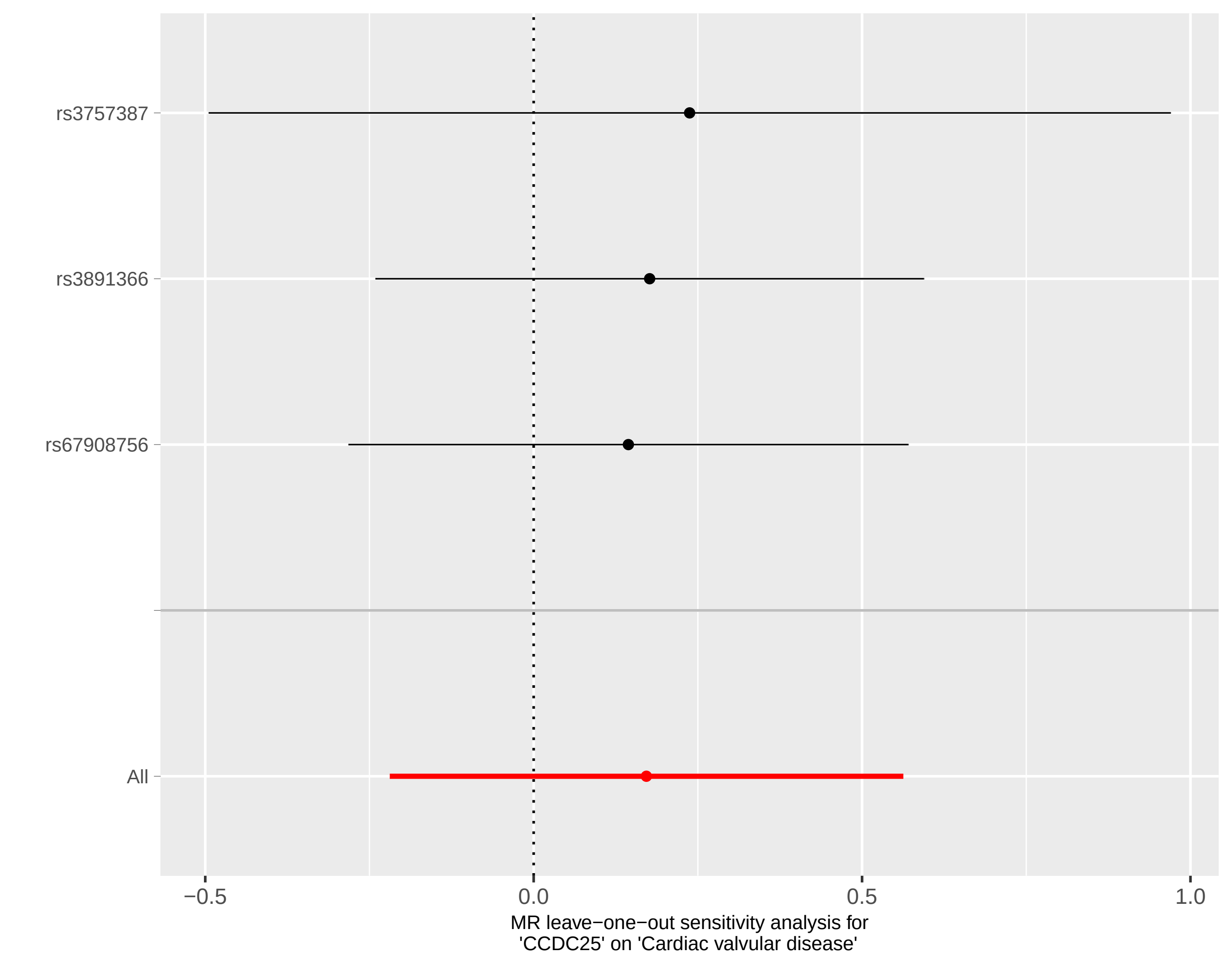

rs3757387
rs3891366
rs67908756
All
−0.5
0.0
0.5
1.0
MR leave−one−out sensitivity analysis for
'CCDC25' on 'Cardiac valvular disease'

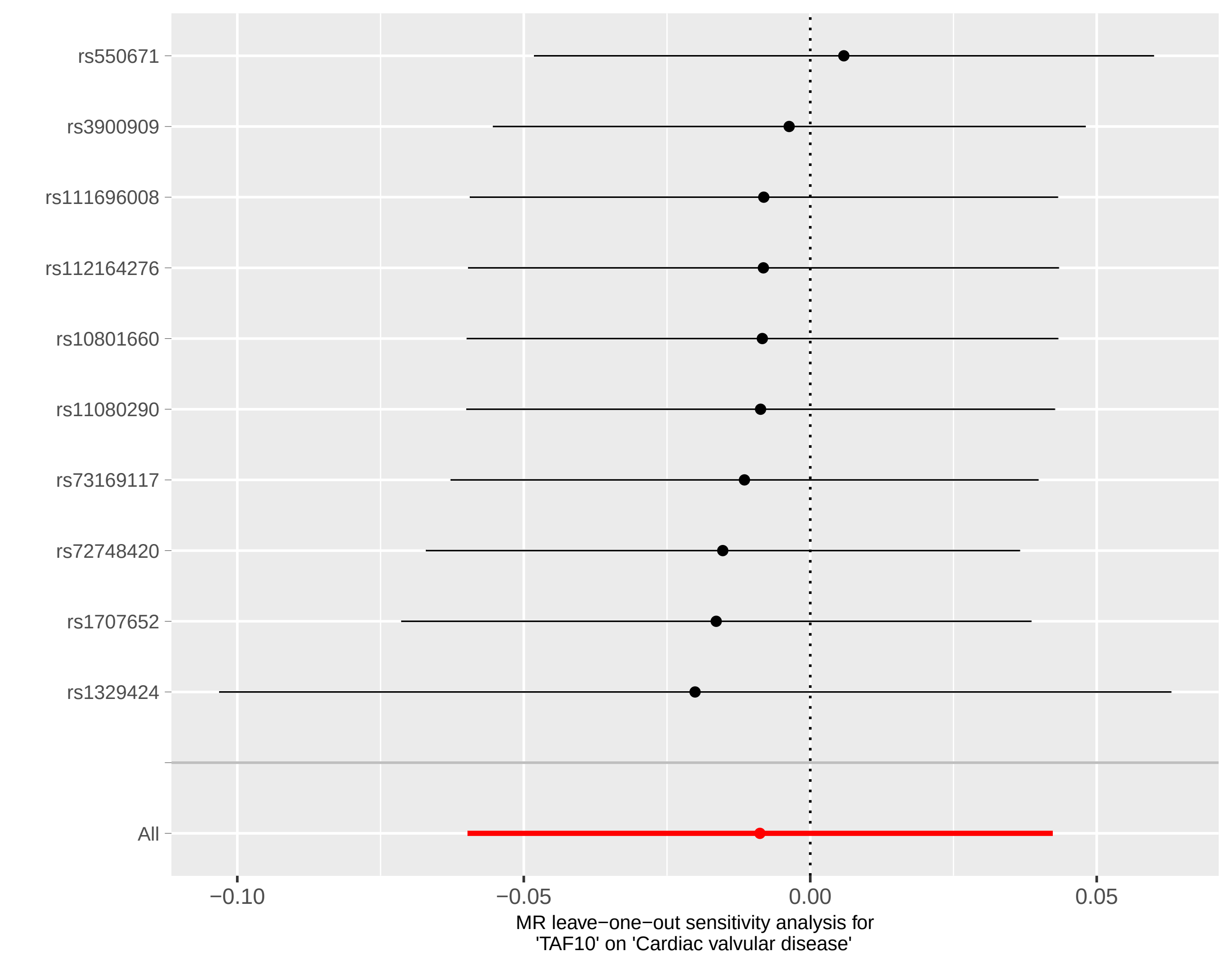
rs550671
rs3900909
rs111696008
rs112164276
rs10801660
rs11080290
rs73169117
rs72748420
rs1707652
rs1329424
All
−0.10
−0.05
0.00
0.05
MR leave−one−out sensitivity analysis for
'TAF10' on 'Cardiac valvular disease'

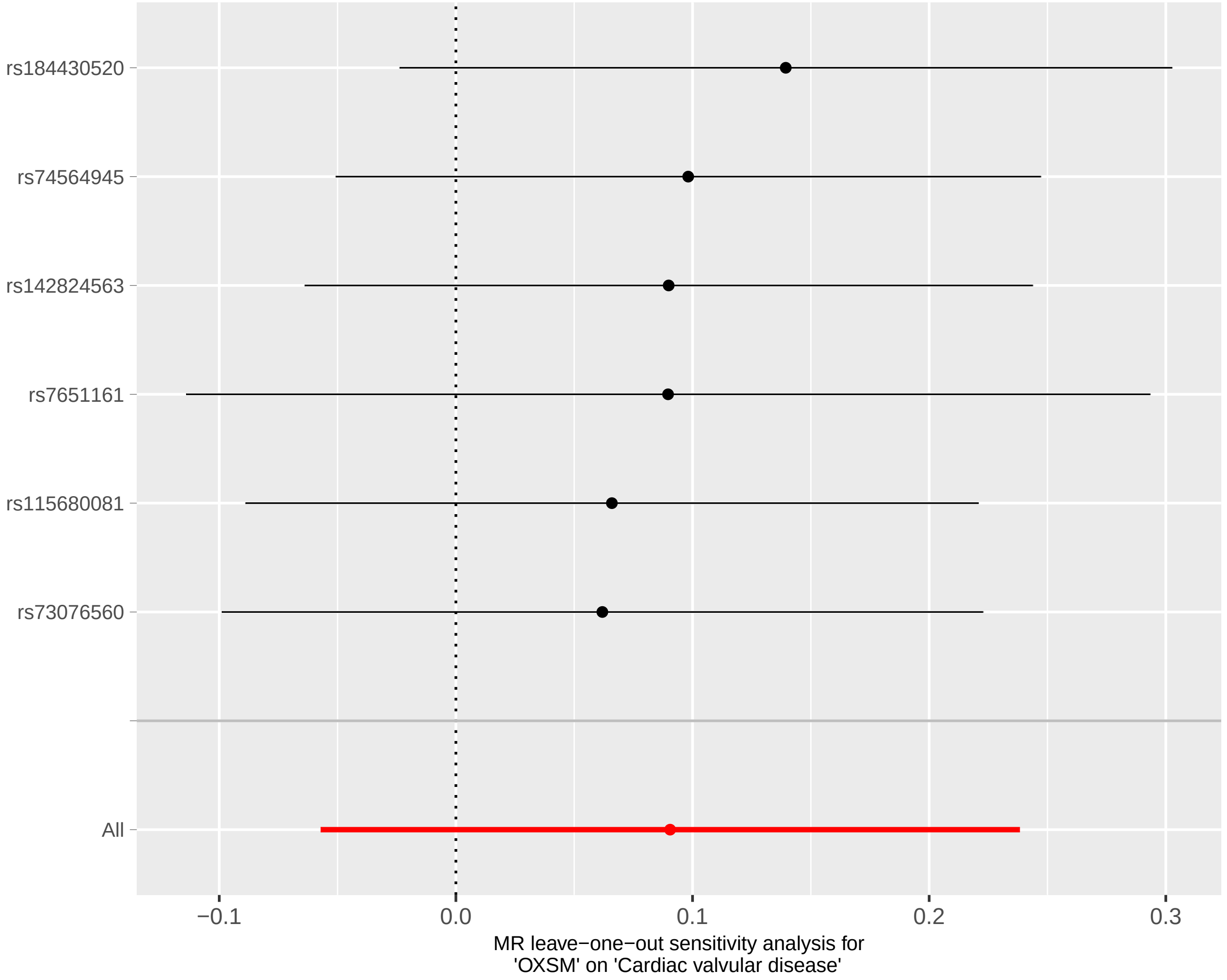

rs184430520
rs74564945
rs142824563
rs7651161
rs115680081
rs73076560
All
−0.1
0.0
0.1
0.2
0.3
MR leave−one−out sensitivity analysis for
'OXSM' on 'Cardiac valvular disease'

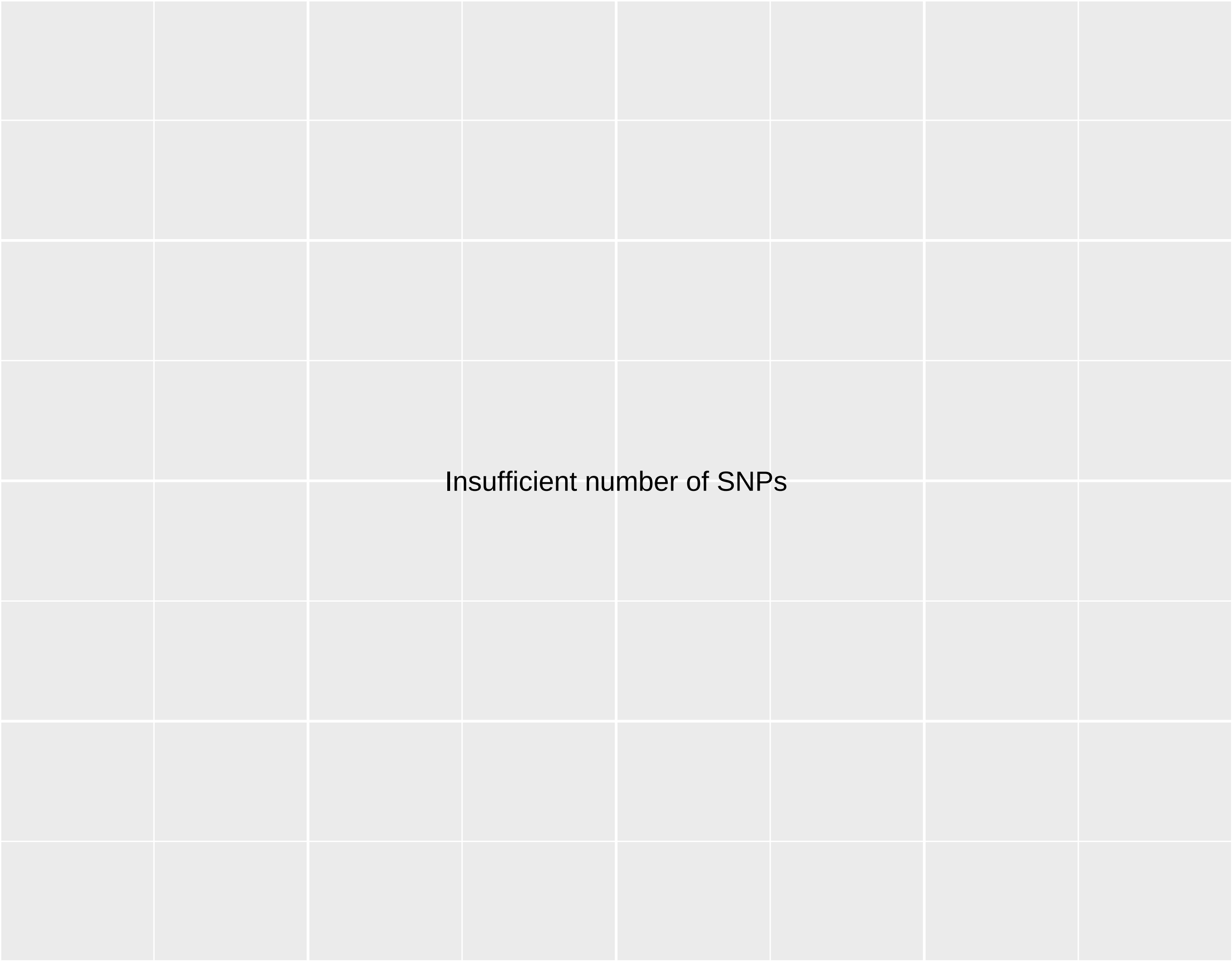
Insufficient number of SNPs

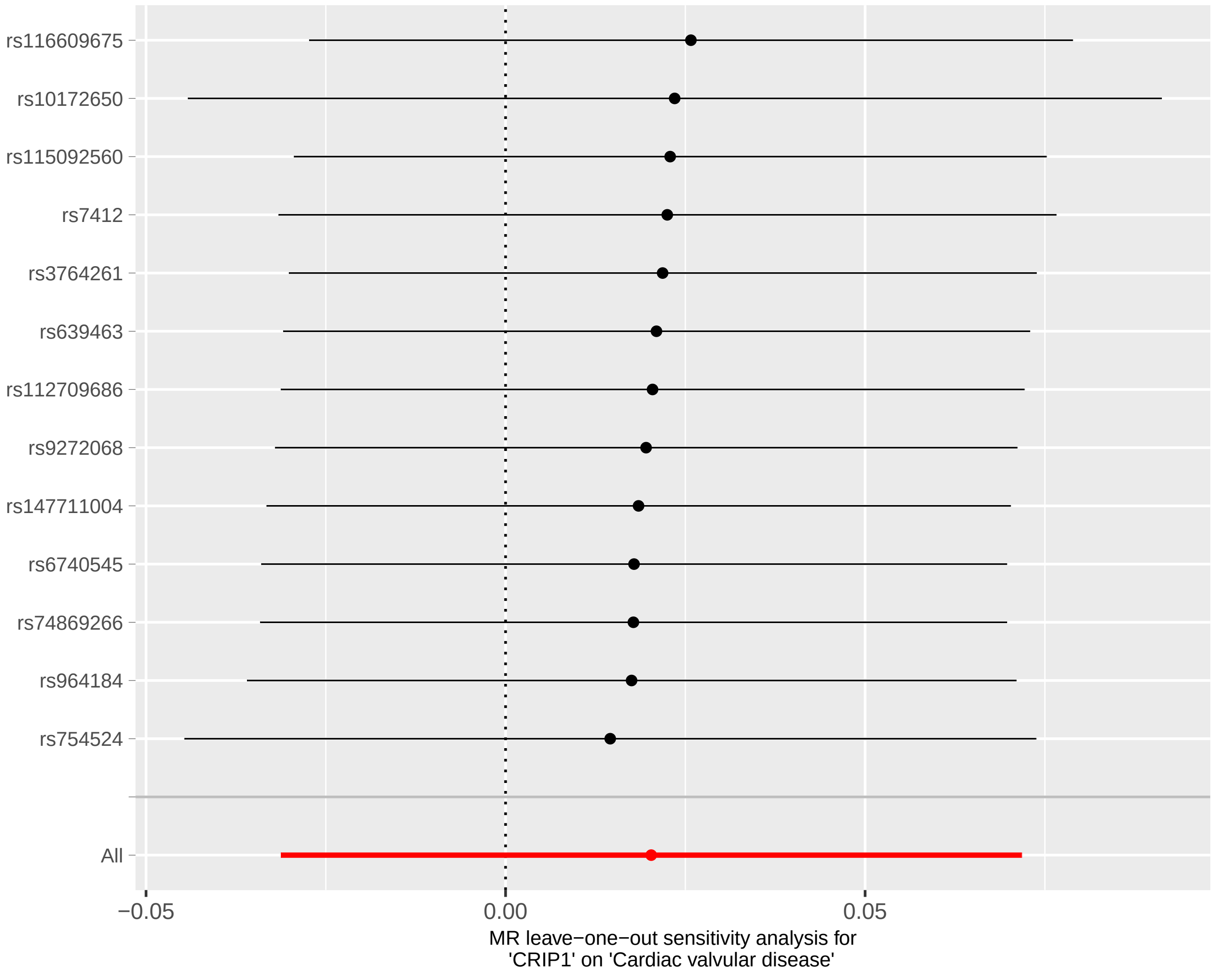
rs116609675
rs10172650
rs115092560
rs7412
rs3764261
rs639463
rs112709686
rs9272068
rs147711004
rs6740545
rs74869266
rs964184
rs754524
All
−0.05
0.00
0.05
MR leave−one−out sensitivity analysis for
'CRIP1' on 'Cardiac valvular disease'

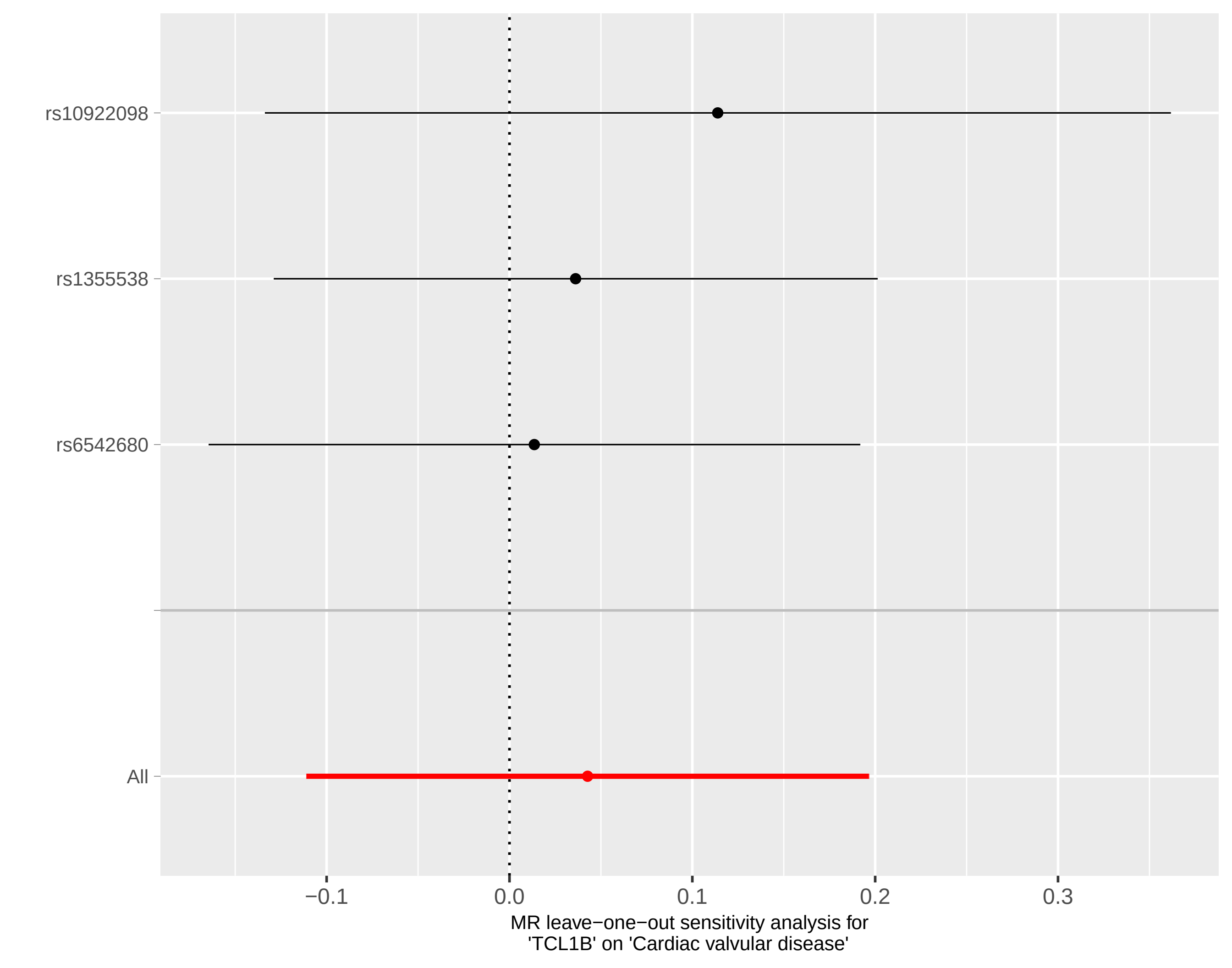
rs10922098
rs1355538
rs6542680
All
−0.1
0.0
0.1
0.2
0.3
MR leave−one−out sensitivity analysis for
'TCL1B' on 'Cardiac valvular disease'

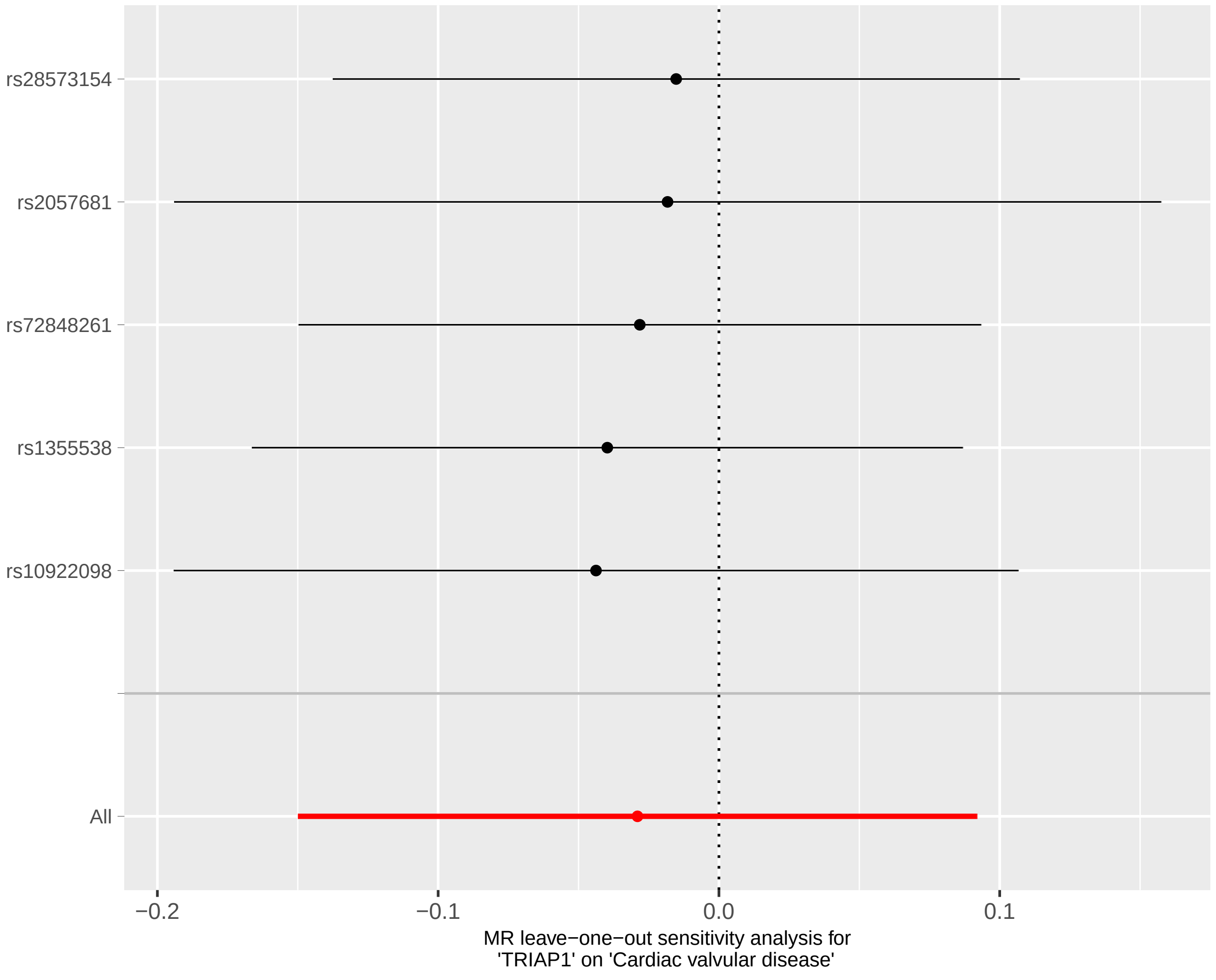

rs28573154
rs2057681
rs72848261
rs1355538
rs10922098
All
−0.2
−0.1
0.0
0.1
MR leave−one−out sensitivity analysis for
'TRIAP1' on 'Cardiac valvular disease'

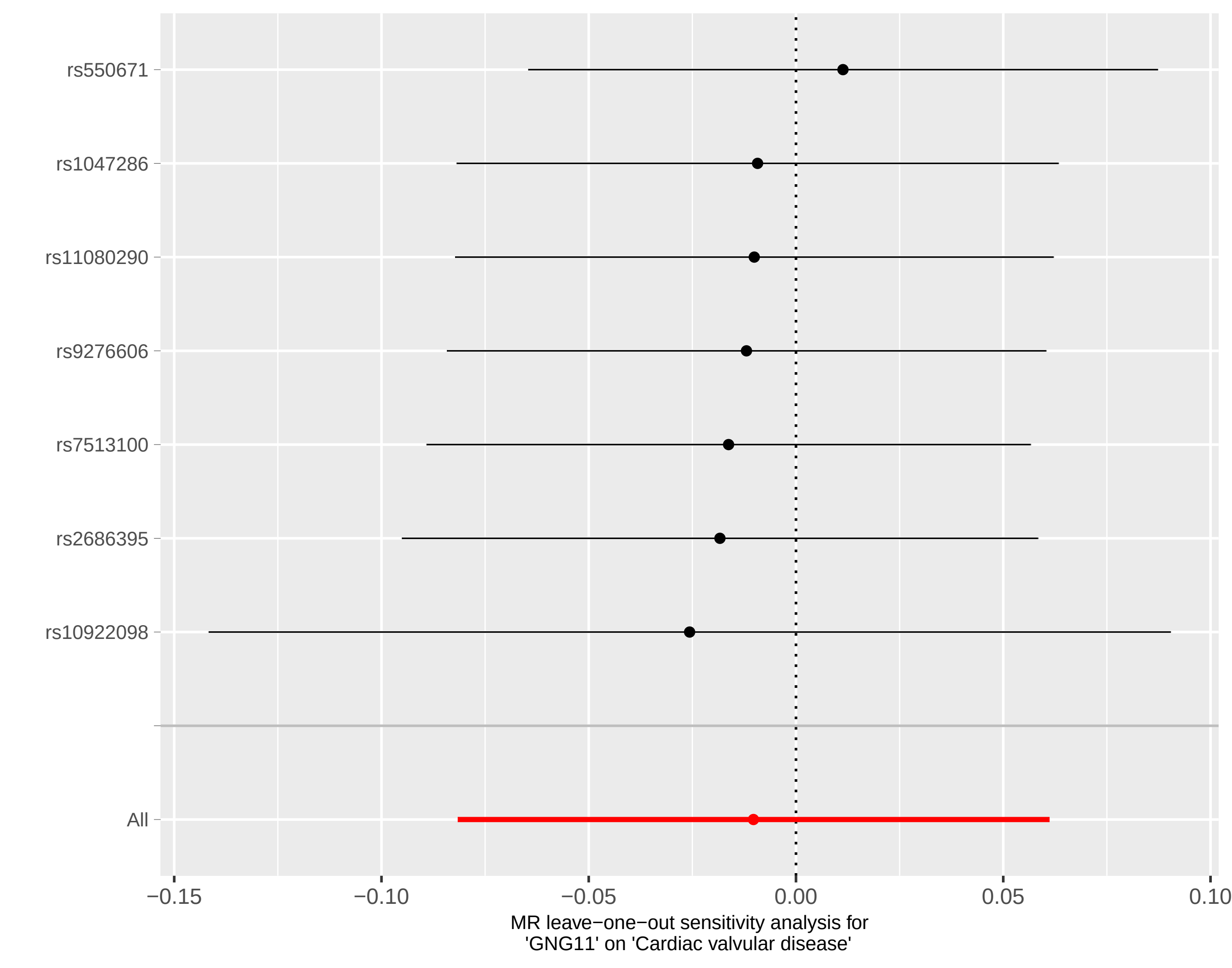

rs550671
rs1047286
rs11080290
rs9276606
rs7513100
rs2686395
rs10922098
All
−0.15
−0.10
−0.05
0.00
0.05
0.10
MR leave−one−out sensitivity analysis for
'GNG11' on 'Cardiac valvular disease'

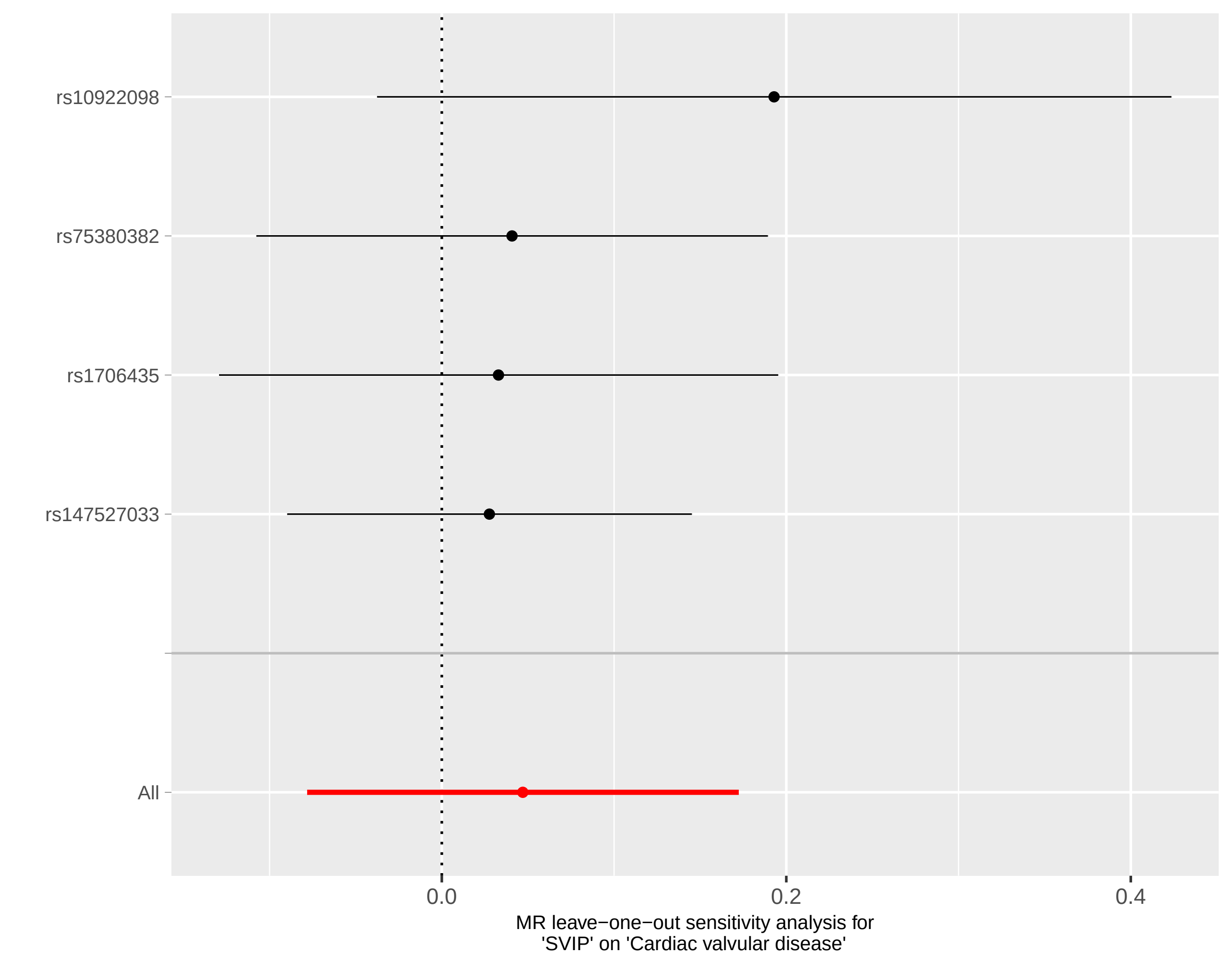
rs10922098
rs75380382
rs1706435
rs147527033
All
0.0
0.2
0.4
MR leave−one−out sensitivity analysis for
'SVIP' on 'Cardiac valvular disease'

Insufficient number of SNPs

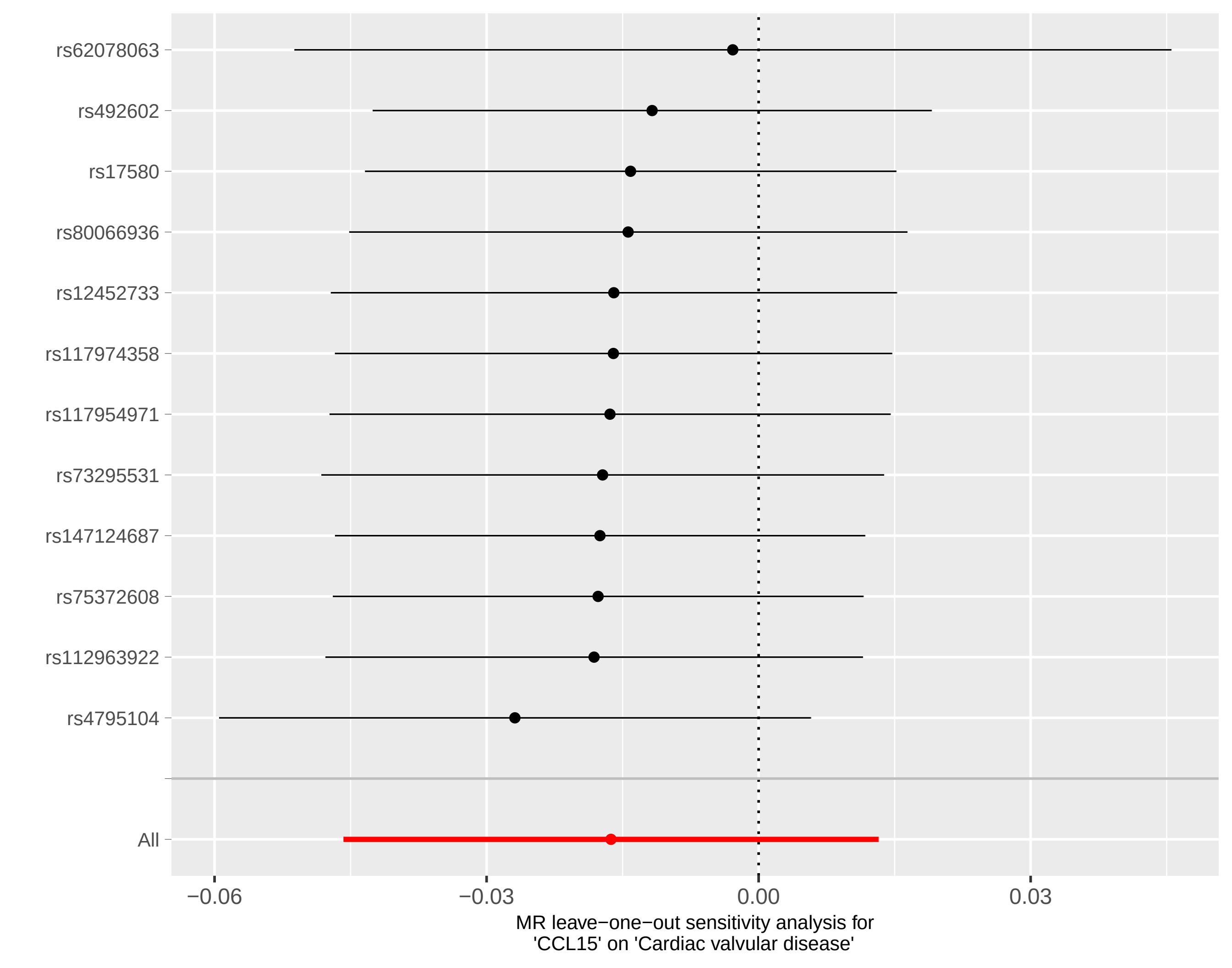
rs62078063
rs492602
rs17580
rs80066936
rs12452733
rs117974358
rs117954971
rs73295531
rs147124687
rs75372608
rs112963922
rs4795104
All
−0.06
−0.03
0.00
0.03
MR leave−one−out sensitivity analysis for
'CCL15' on 'Cardiac valvular disease'

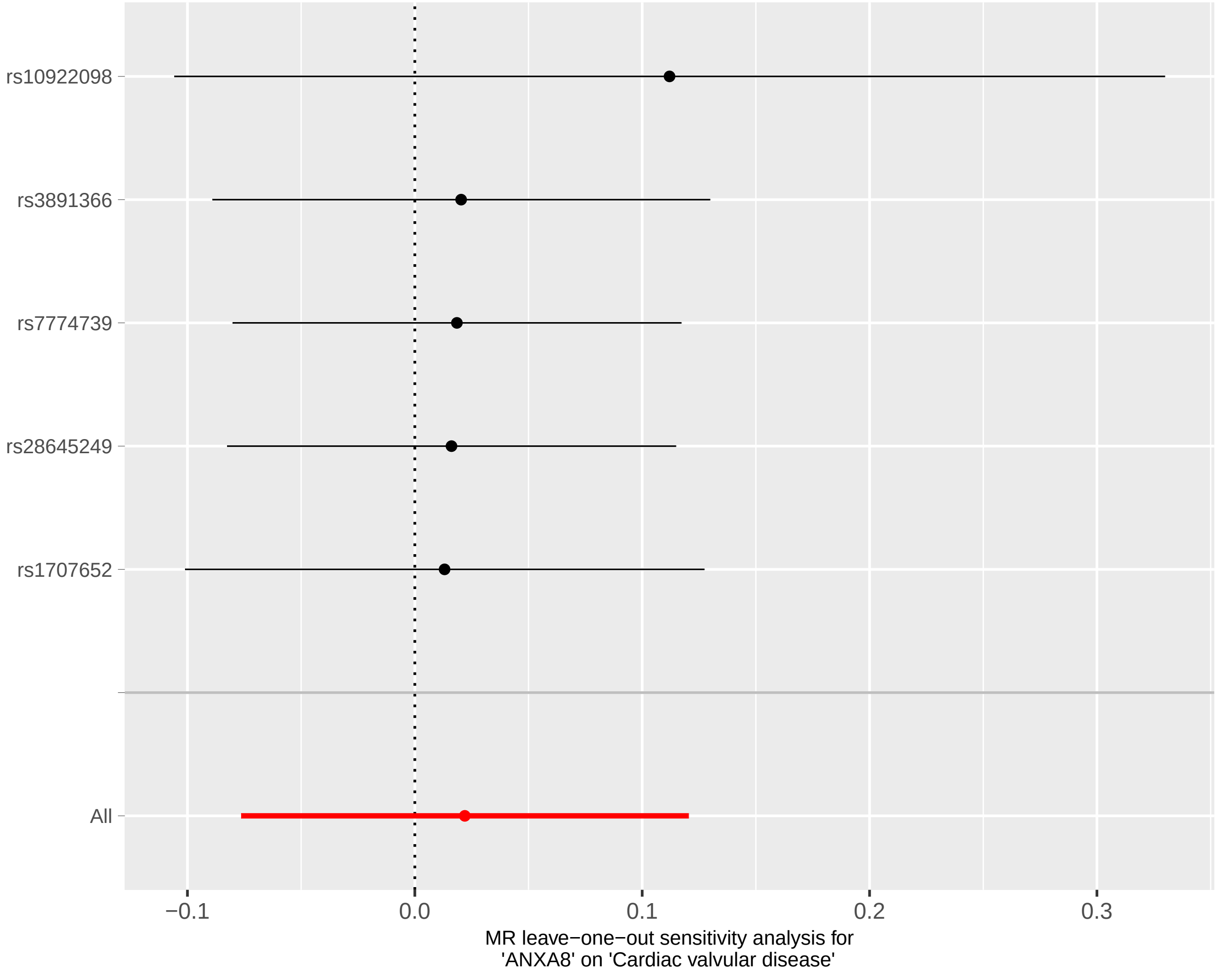

rs10922098
rs3891366
rs7774739
rs28645249
rs1707652
All
−0.1
0.0
0.1
0.2
0.3
MR leave−one−out sensitivity analysis for
'ANXA8' on 'Cardiac valvular disease'

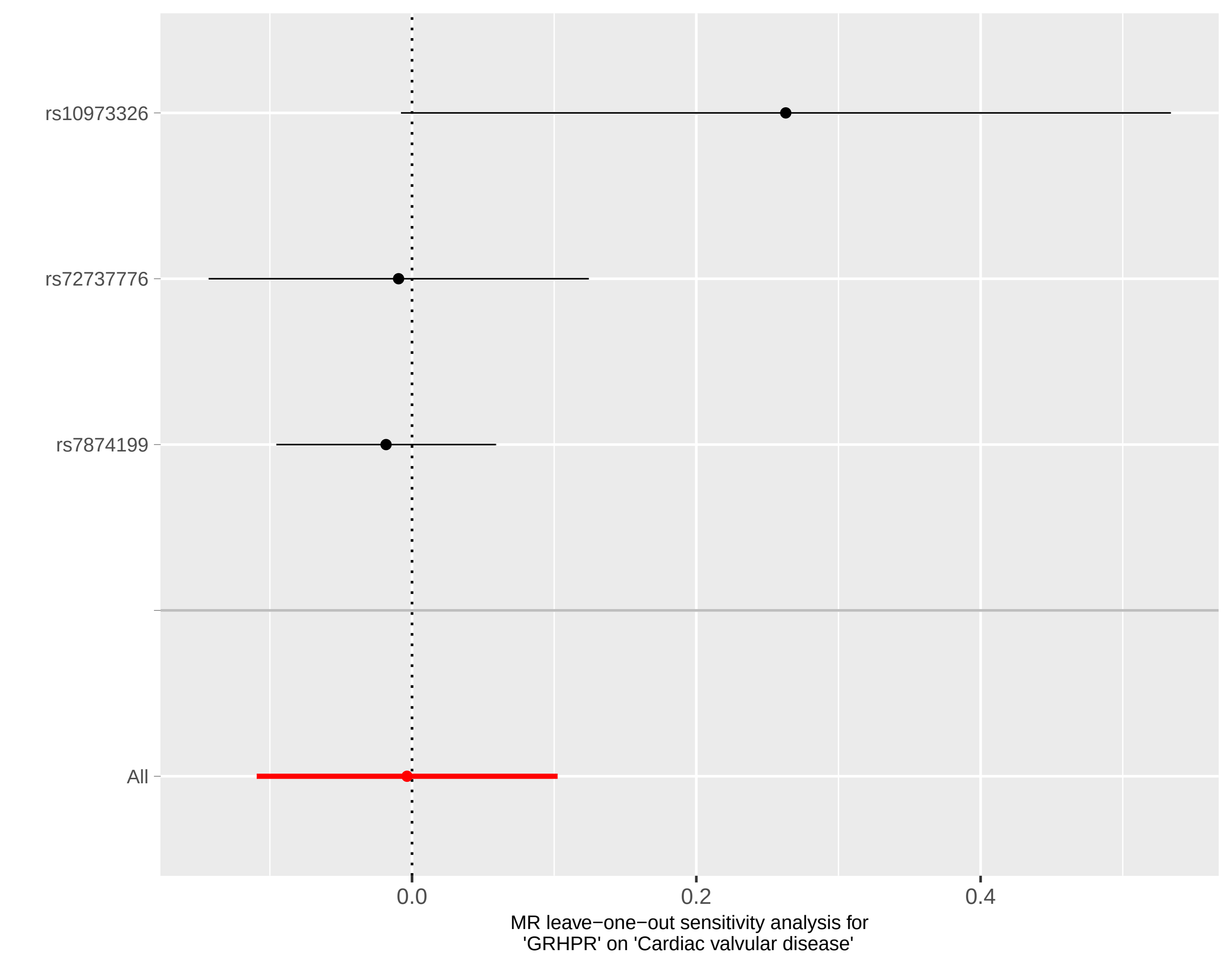
rs10973326
rs72737776
rs7874199
All
0.0
0.2
0.4
MR leave−one−out sensitivity analysis for
'GRHPR' on 'Cardiac valvular disease'

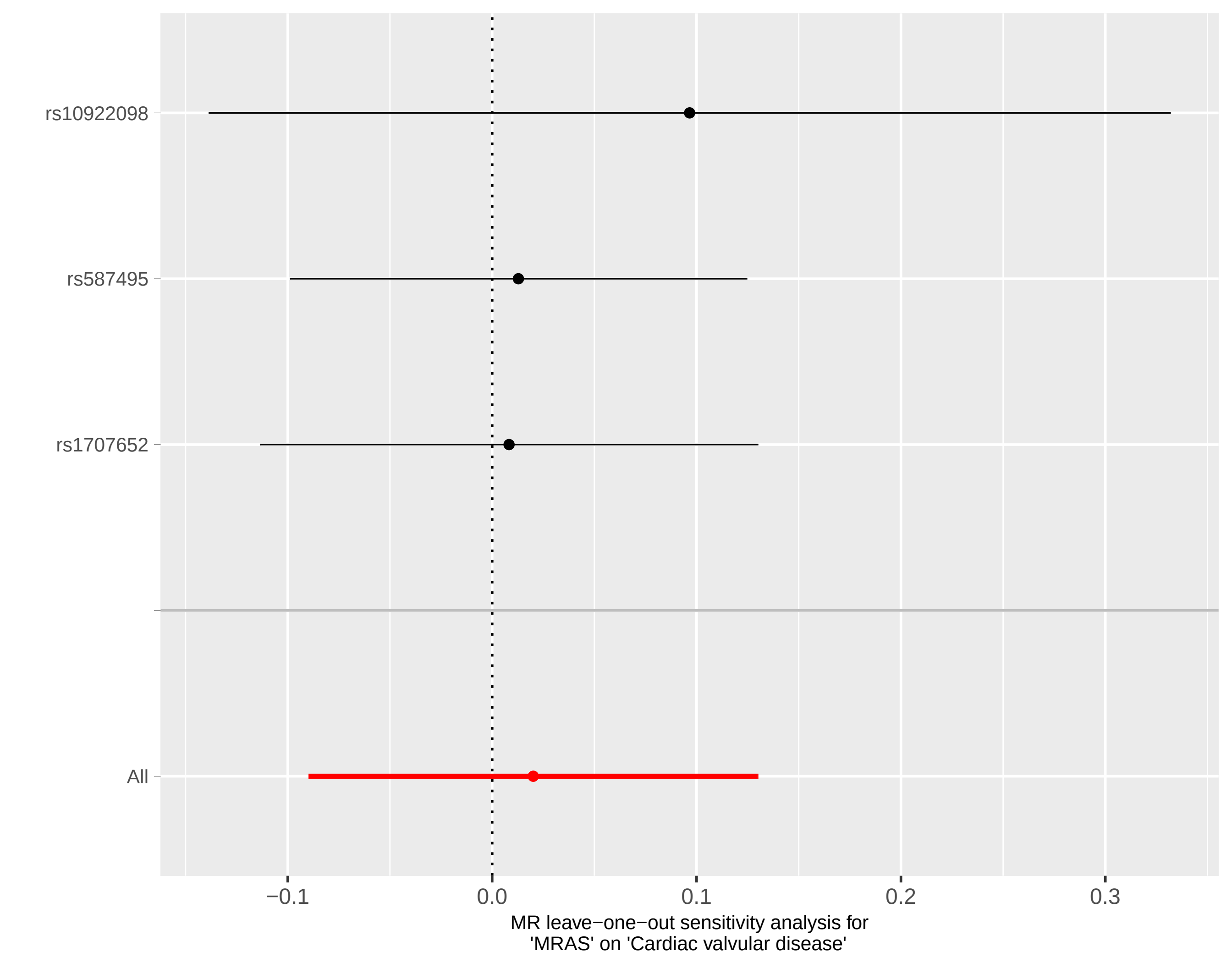

rs10922098
rs587495
rs1707652
All
−0.1
0.0
0.1
0.2
0.3
MR leave−one−out sensitivity analysis for
'MRAS' on 'Cardiac valvular disease'

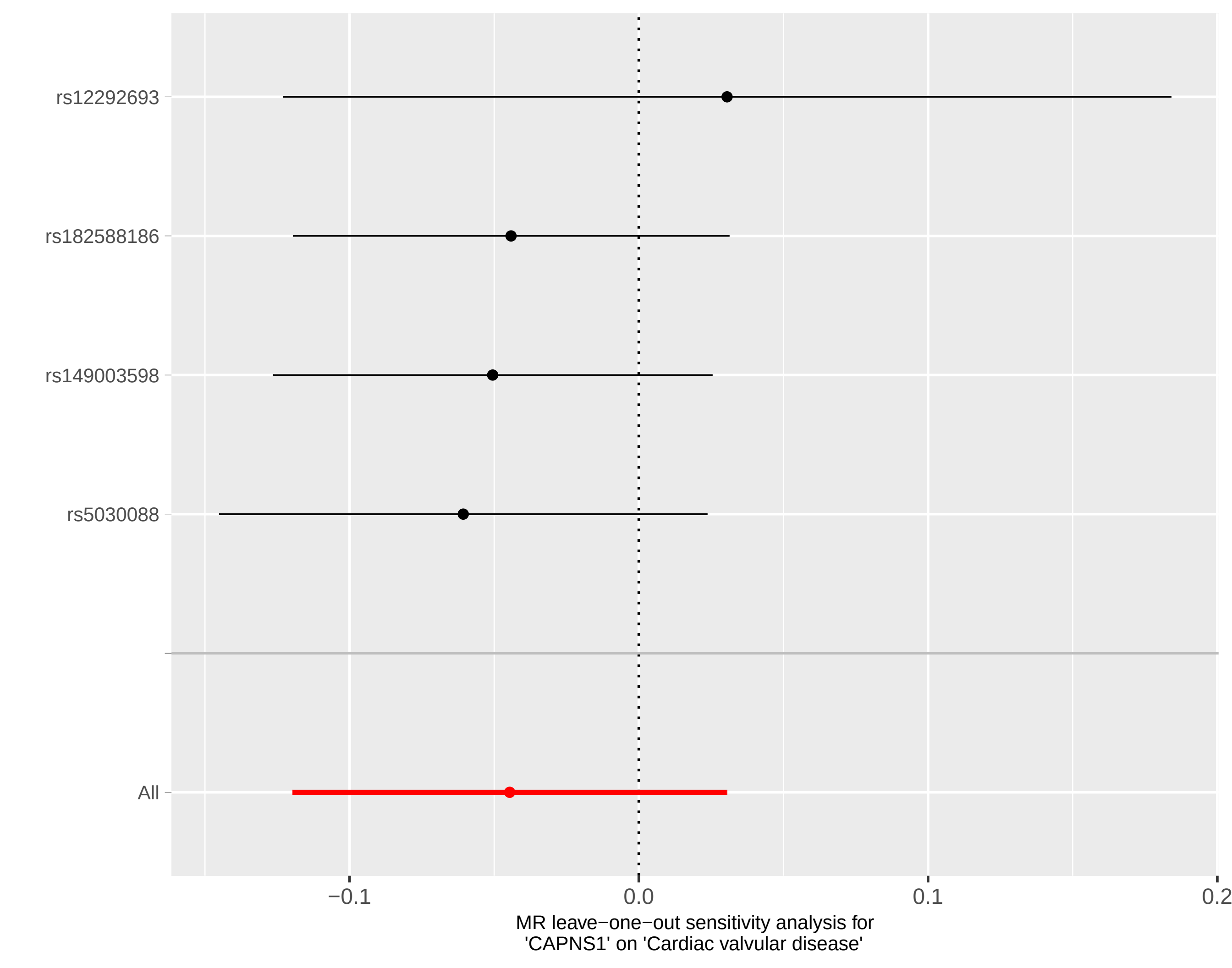
rs12292693
rs182588186
rs149003598
rs5030088
All
−0.1
0.0
0.1
0.2
MR leave−one−out sensitivity analysis for
'CAPNS1' on 'Cardiac valvular disease'

Insufficient number of SNPs

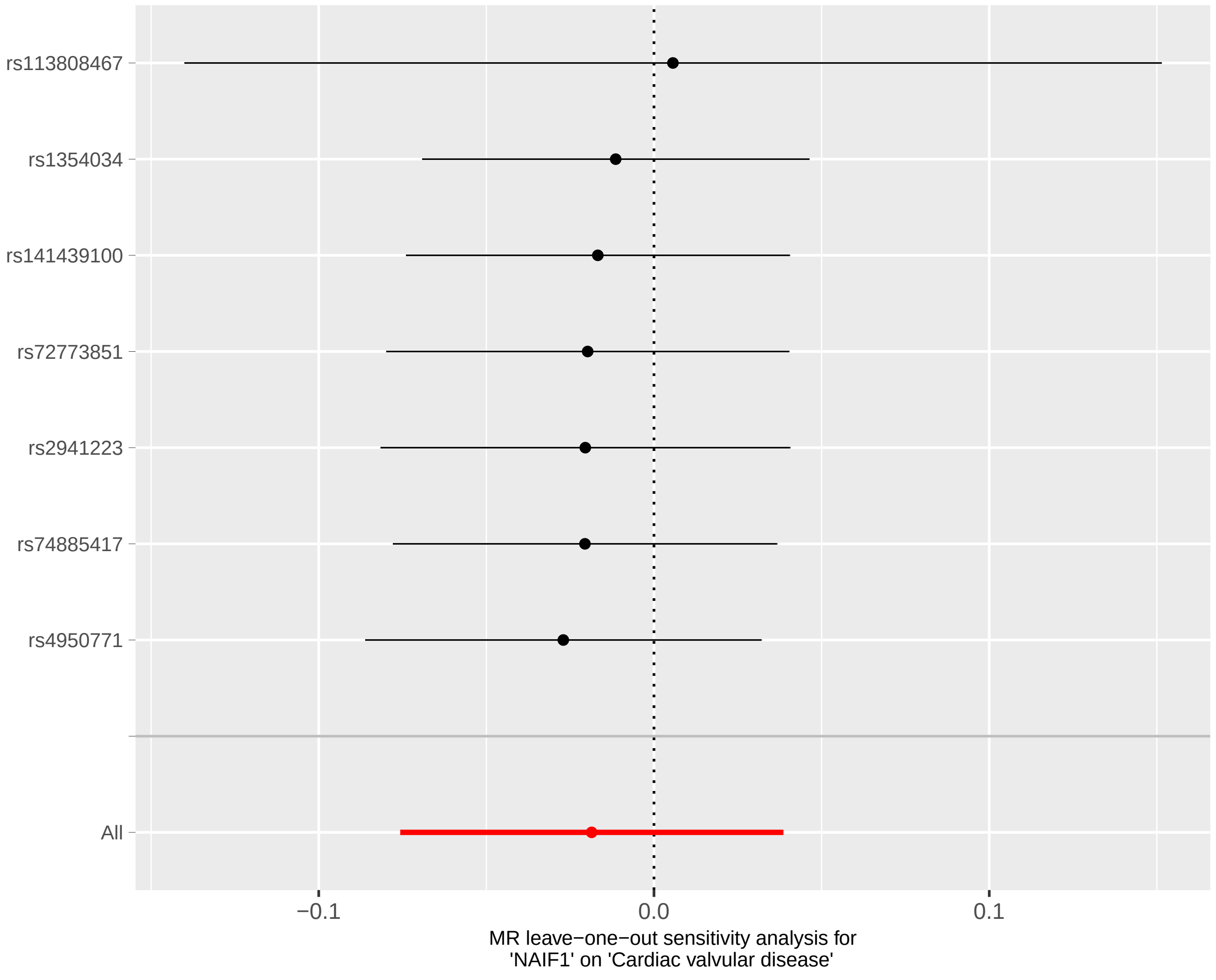

rs113808467
rs1354034
rs141439100
rs72773851
rs2941223
rs74885417
rs4950771
All
−0.1
0.0
0.1
MR leave−one−out sensitivity analysis for
'NAIF1' on 'Cardiac valvular disease'

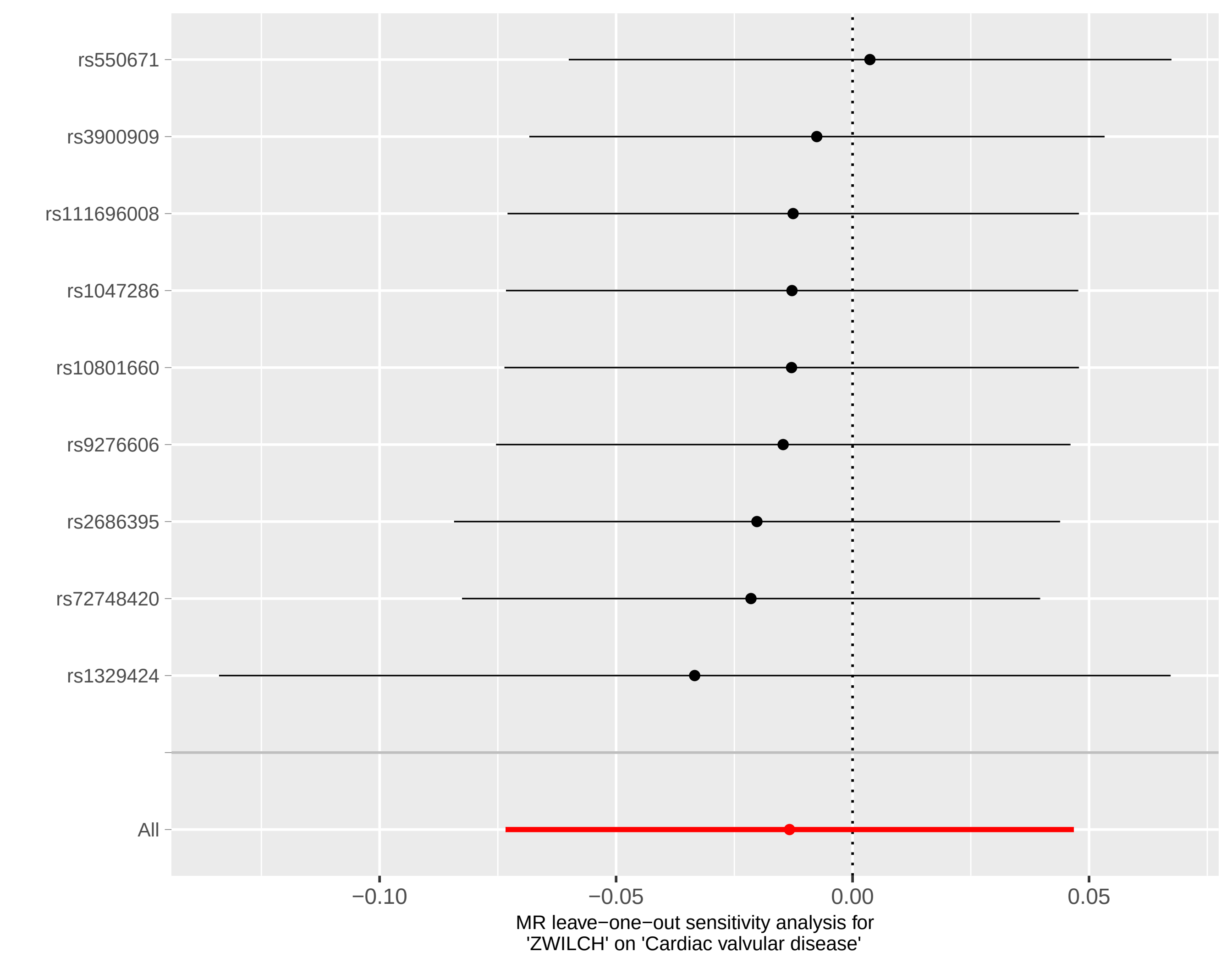

rs550671
rs3900909
rs111696008
rs1047286
rs10801660
rs9276606
rs2686395
rs72748420
rs1329424
All
−0.10
−0.05
0.00
0.05
MR leave−one−out sensitivity analysis for
'ZWILCH' on 'Cardiac valvular disease'

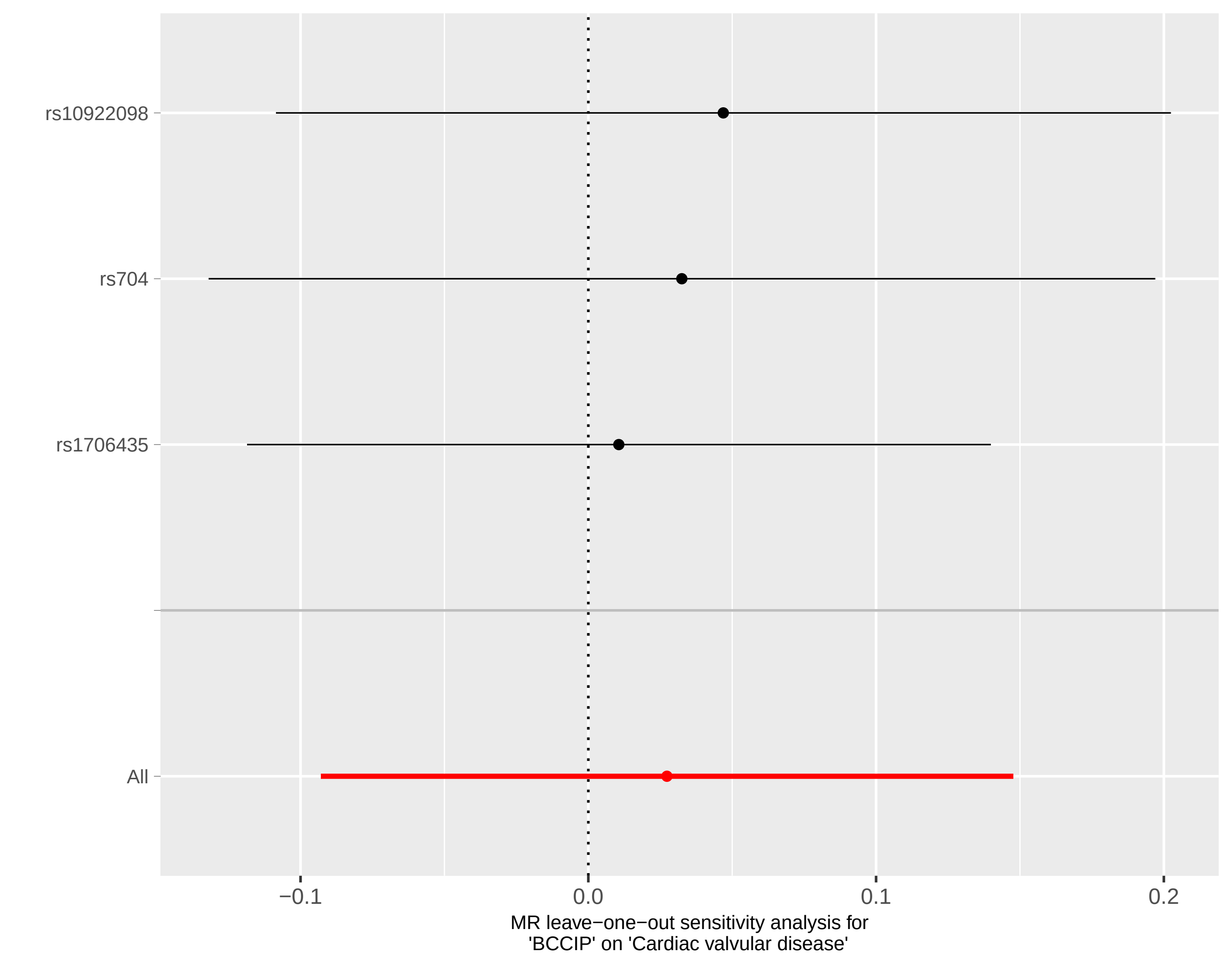

rs10922098
rs704
rs1706435
All
−0.1
0.0
0.1
0.2
MR leave−one−out sensitivity analysis for
'BCCIP' on 'Cardiac valvular disease'

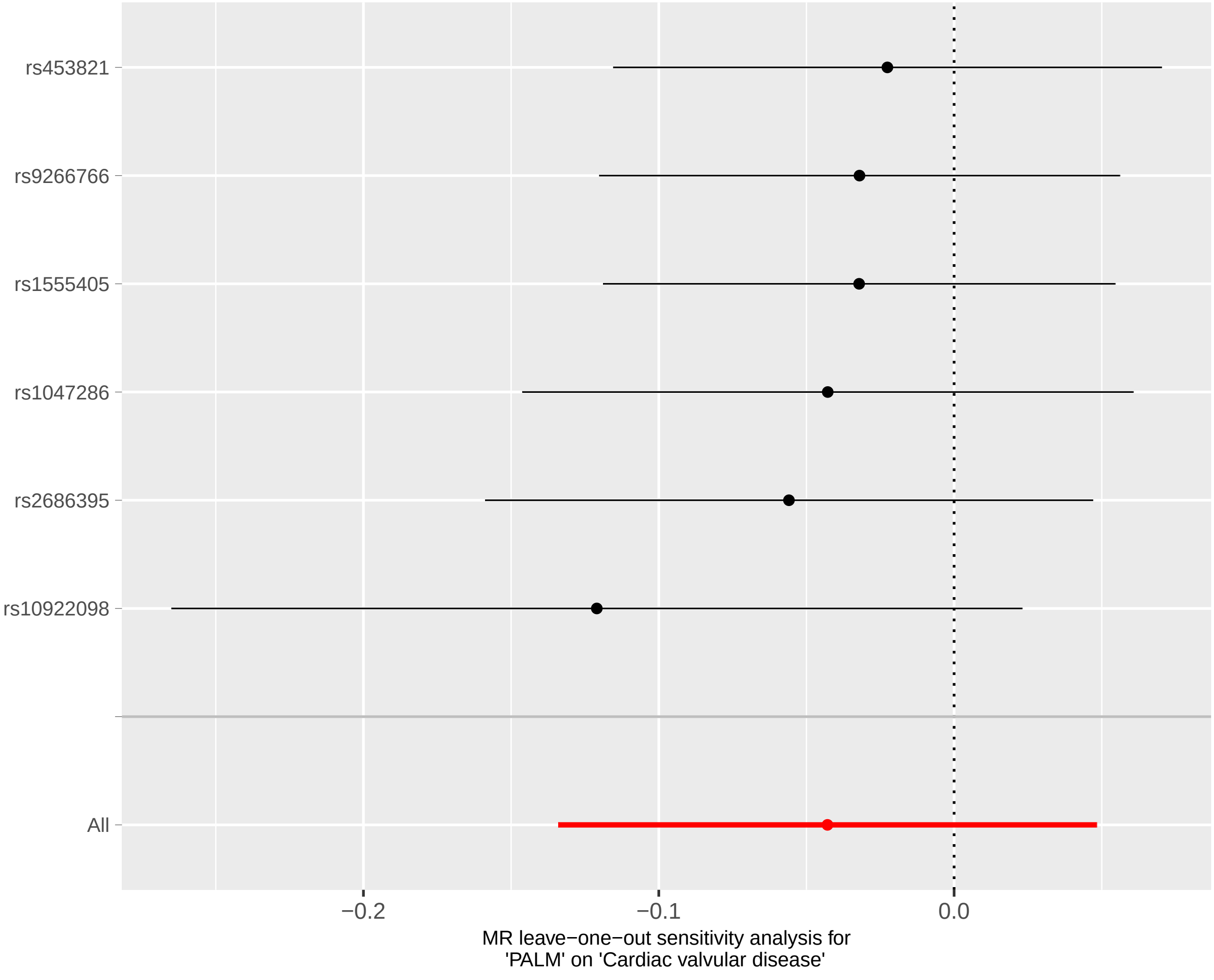
rs453821
rs9266766
rs1555405
rs1047286
rs2686395
rs10922098
All
−0.2
−0.1
0.0
MR leave−one−out sensitivity analysis for
'PALM' on 'Cardiac valvular disease'

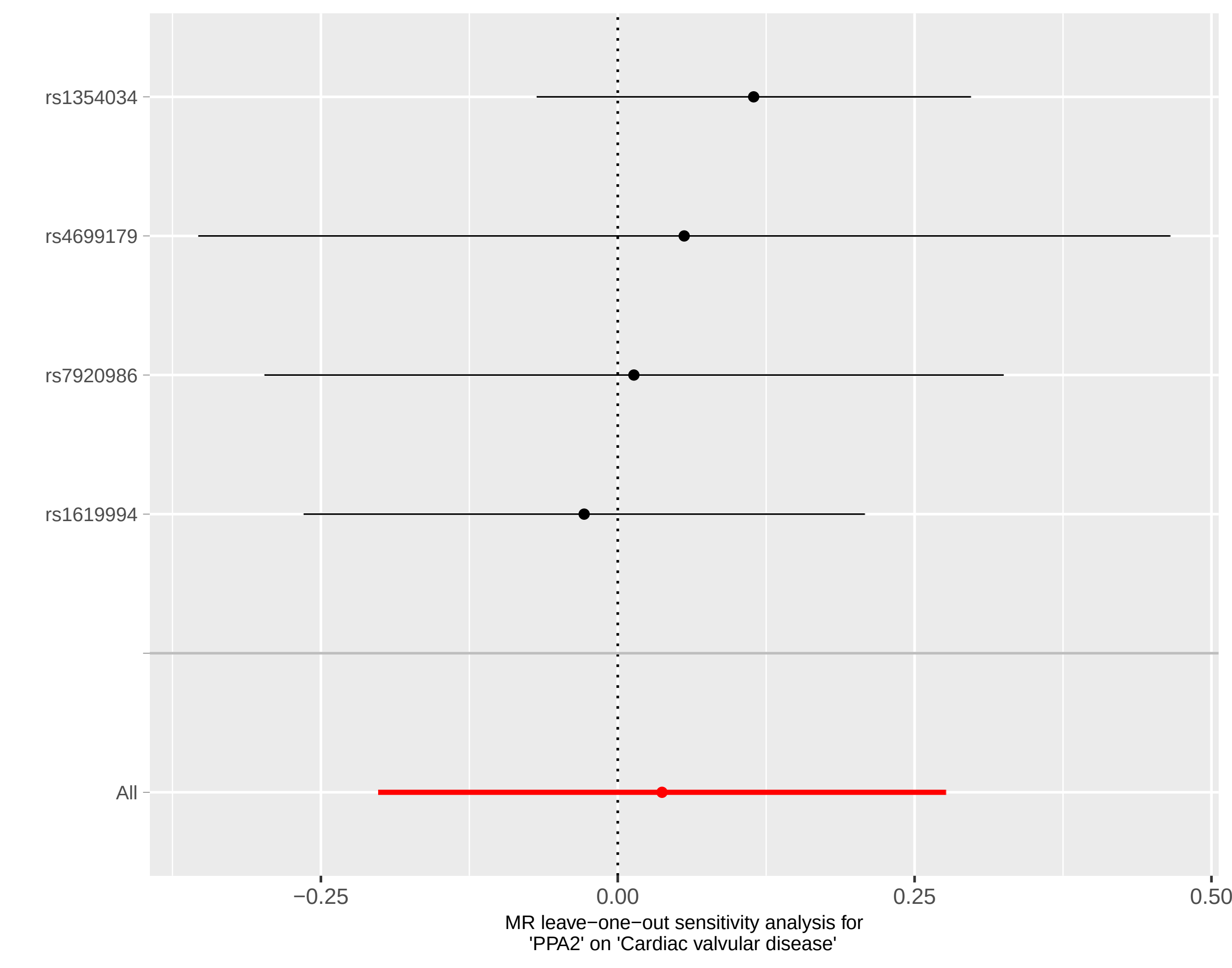
rs1354034
rs4699179
rs7920986
rs1619994
All
−0.25
0.00
0.25
0.50
MR leave−one−out sensitivity analysis for
'PPA2' on 'Cardiac valvular disease'

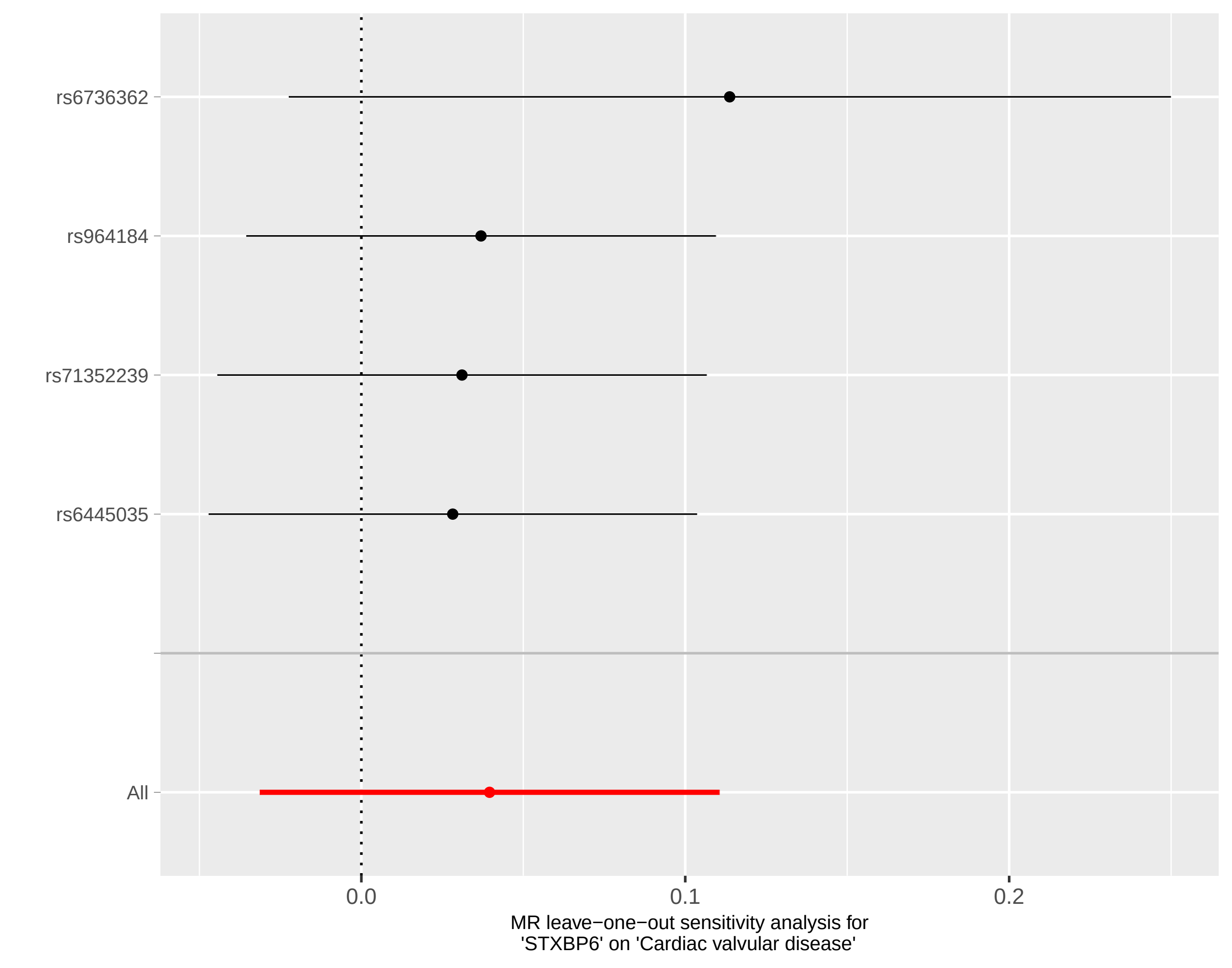

rs6736362
rs964184
rs71352239
rs6445035
All
0.0
0.1
0.2
MR leave−one−out sensitivity analysis for
'STXBP6' on 'Cardiac valvular disease'

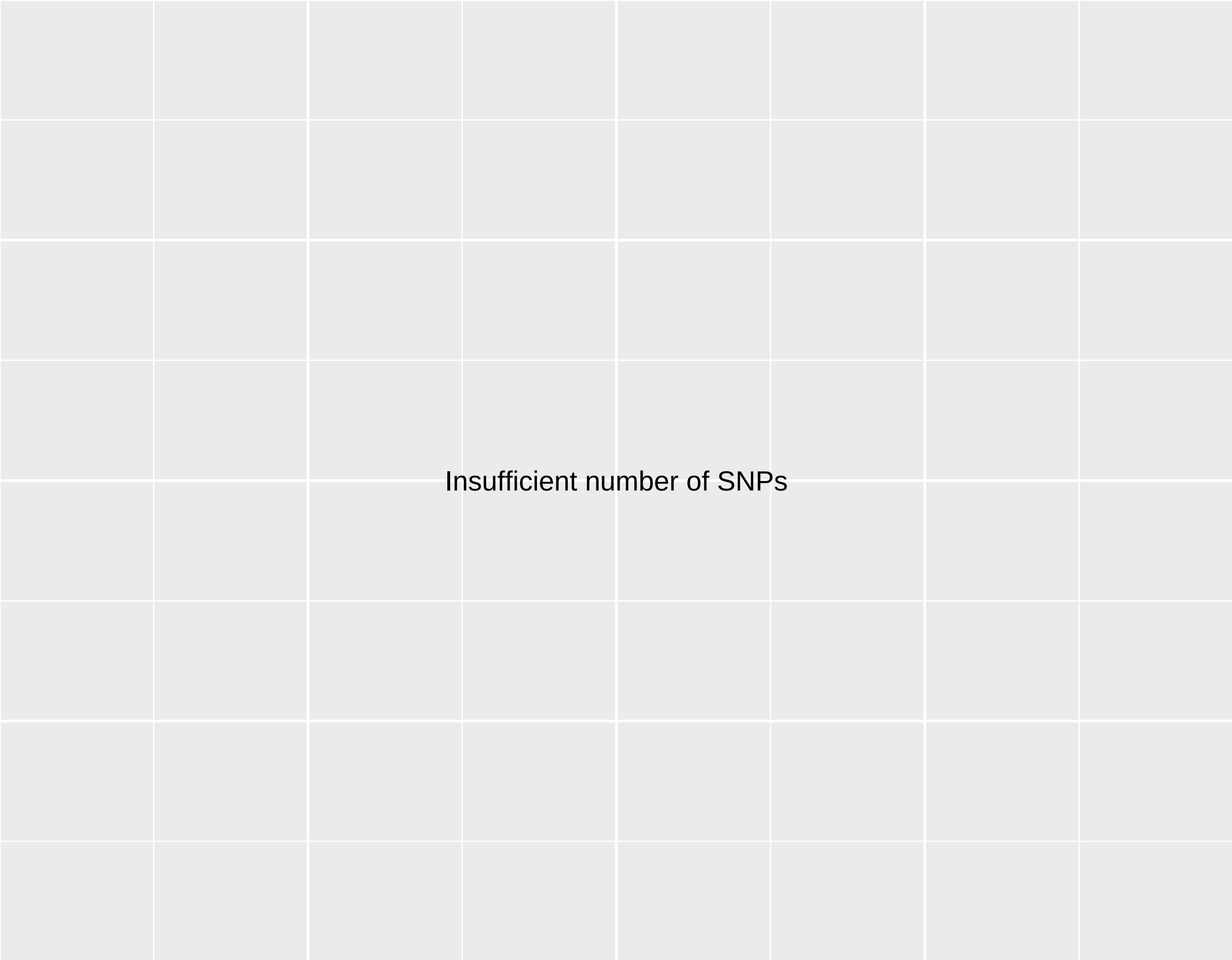
Insufficient number of SNPs

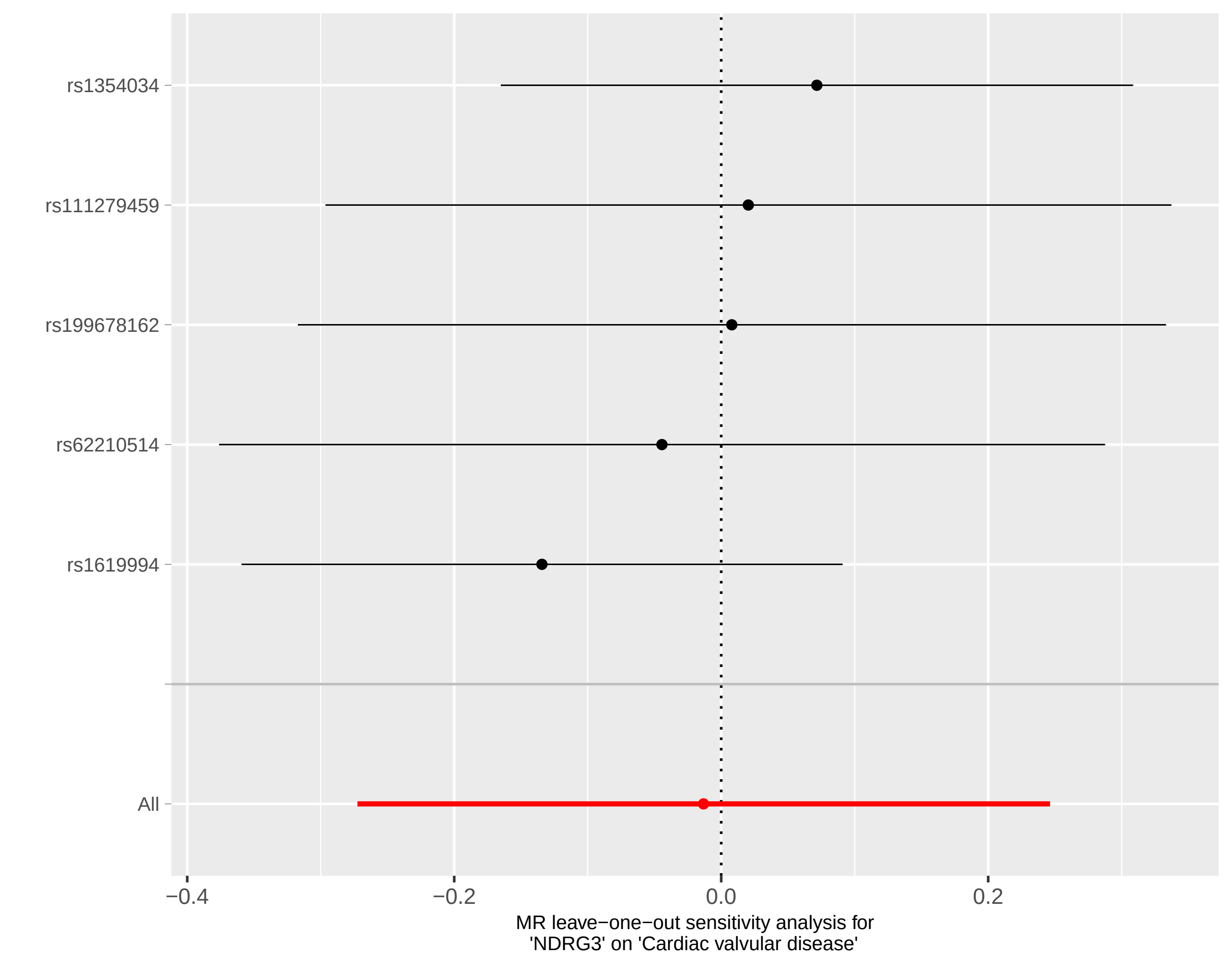
rs1354034
rs111279459
rs199678162
rs62210514
rs1619994
All
−0.4
−0.2
0.0
0.2
MR leave−one−out sensitivity analysis for
'NDRG3' on 'Cardiac valvular disease'

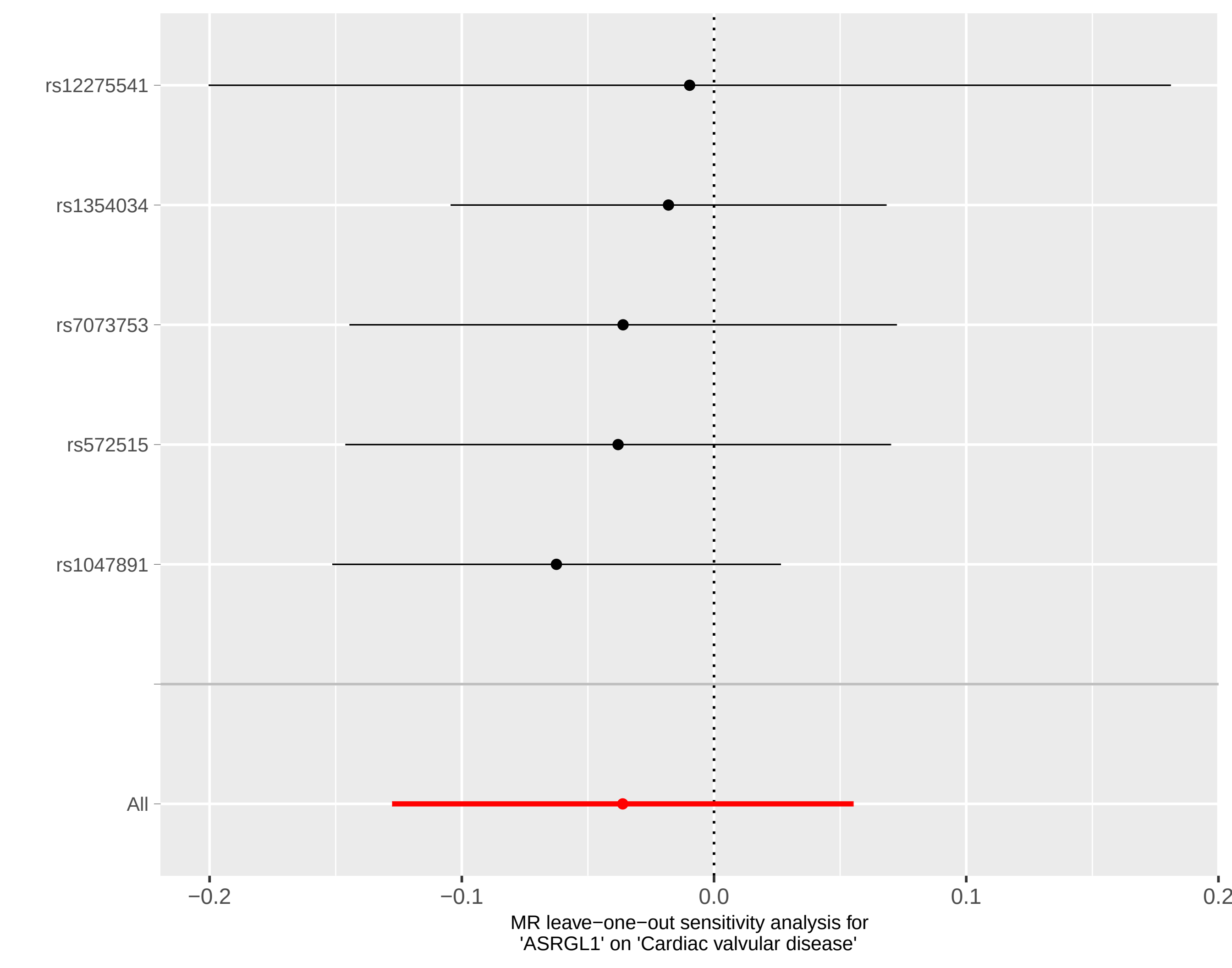

rs12275541
rs1354034
rs7073753
rs572515
rs1047891
All
−0.2
−0.1
0.0
0.1
0.2
MR leave−one−out sensitivity analysis for
'ASRGL1' on 'Cardiac valvular disease'

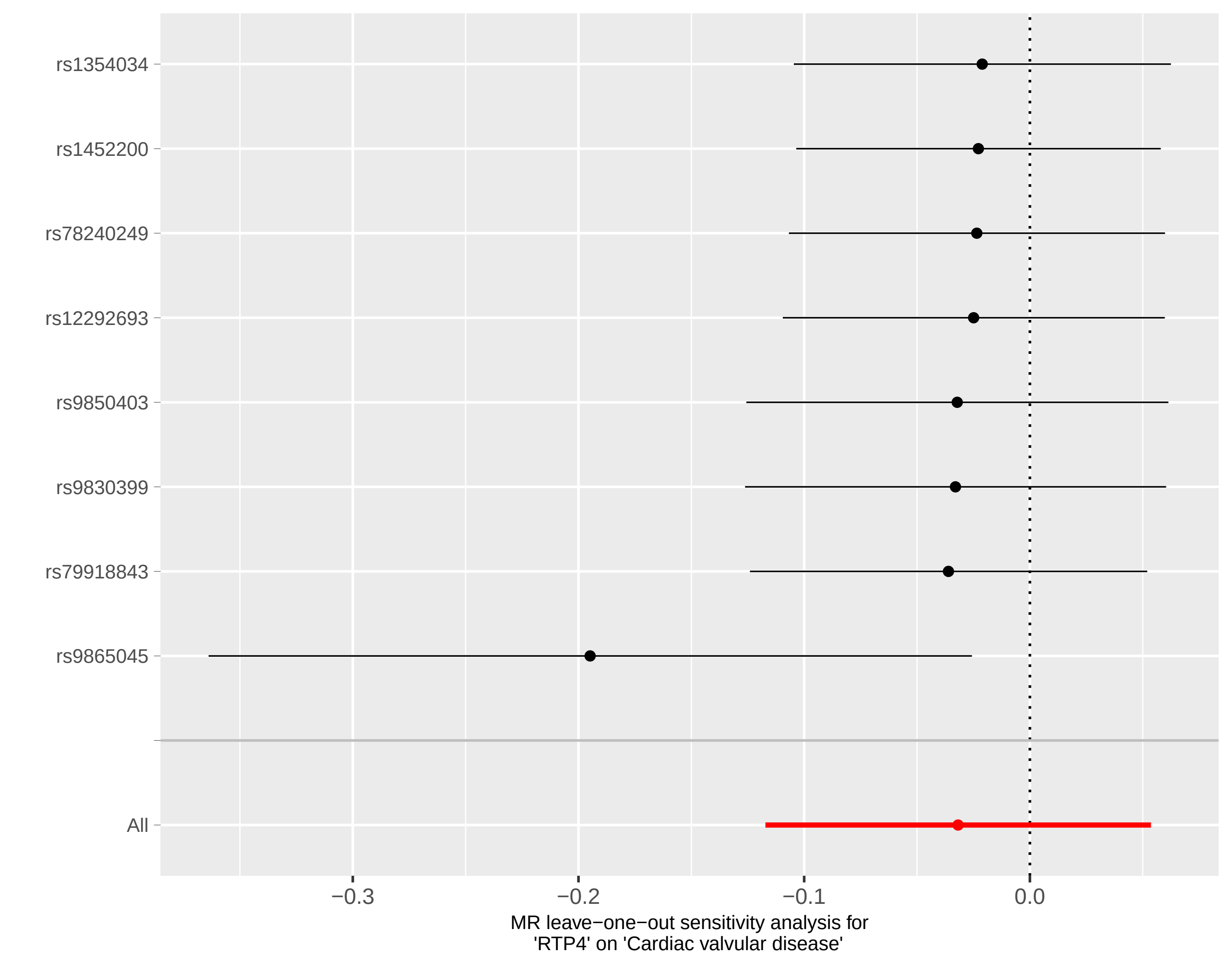

rs1354034
rs1452200
rs78240249
rs12292693
rs9850403
rs9830399
rs79918843
rs9865045
All
−0.3
−0.2
−0.1
0.0
MR leave−one−out sensitivity analysis for
'RTP4' on 'Cardiac valvular disease'

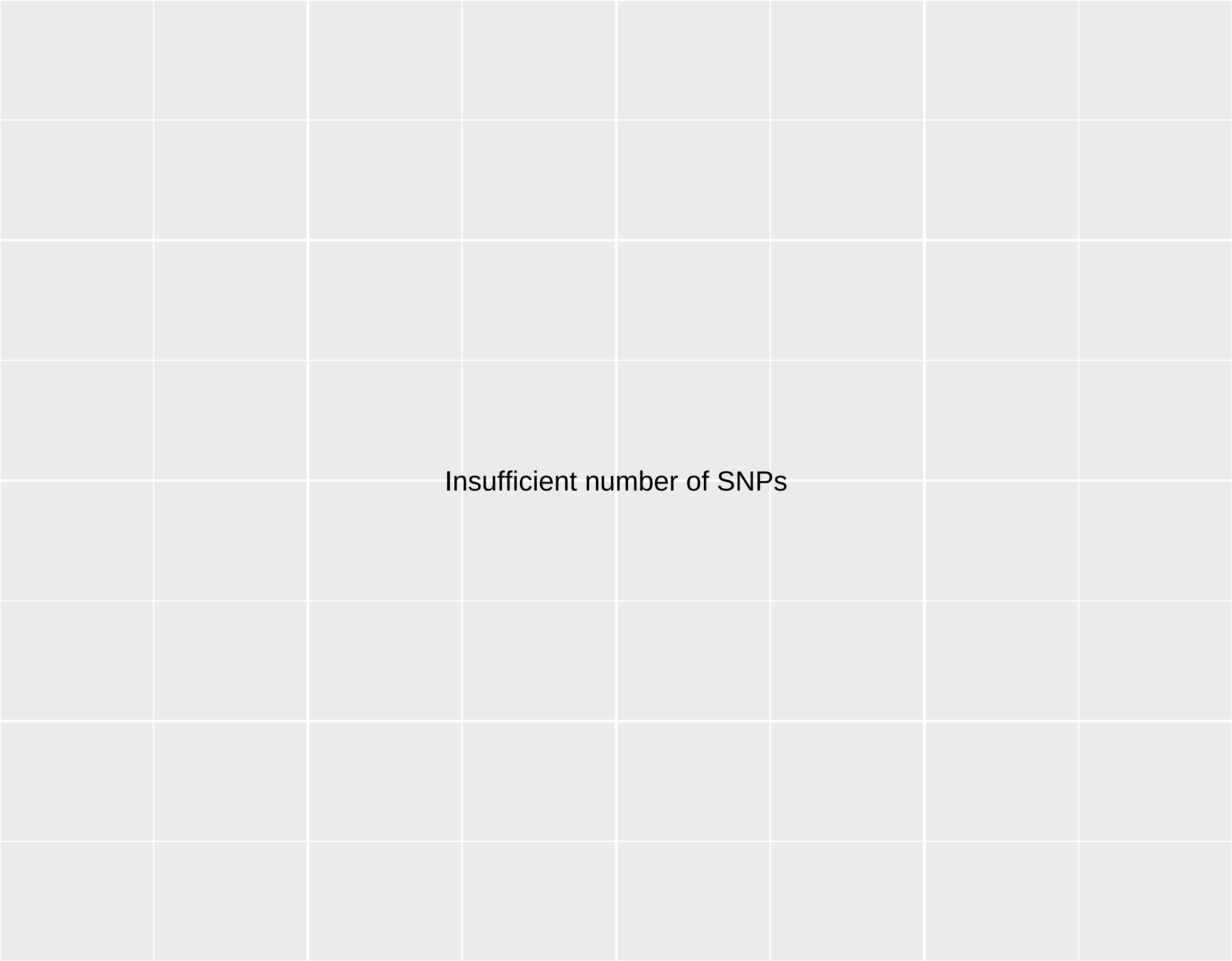
Insufficient number of SNPs

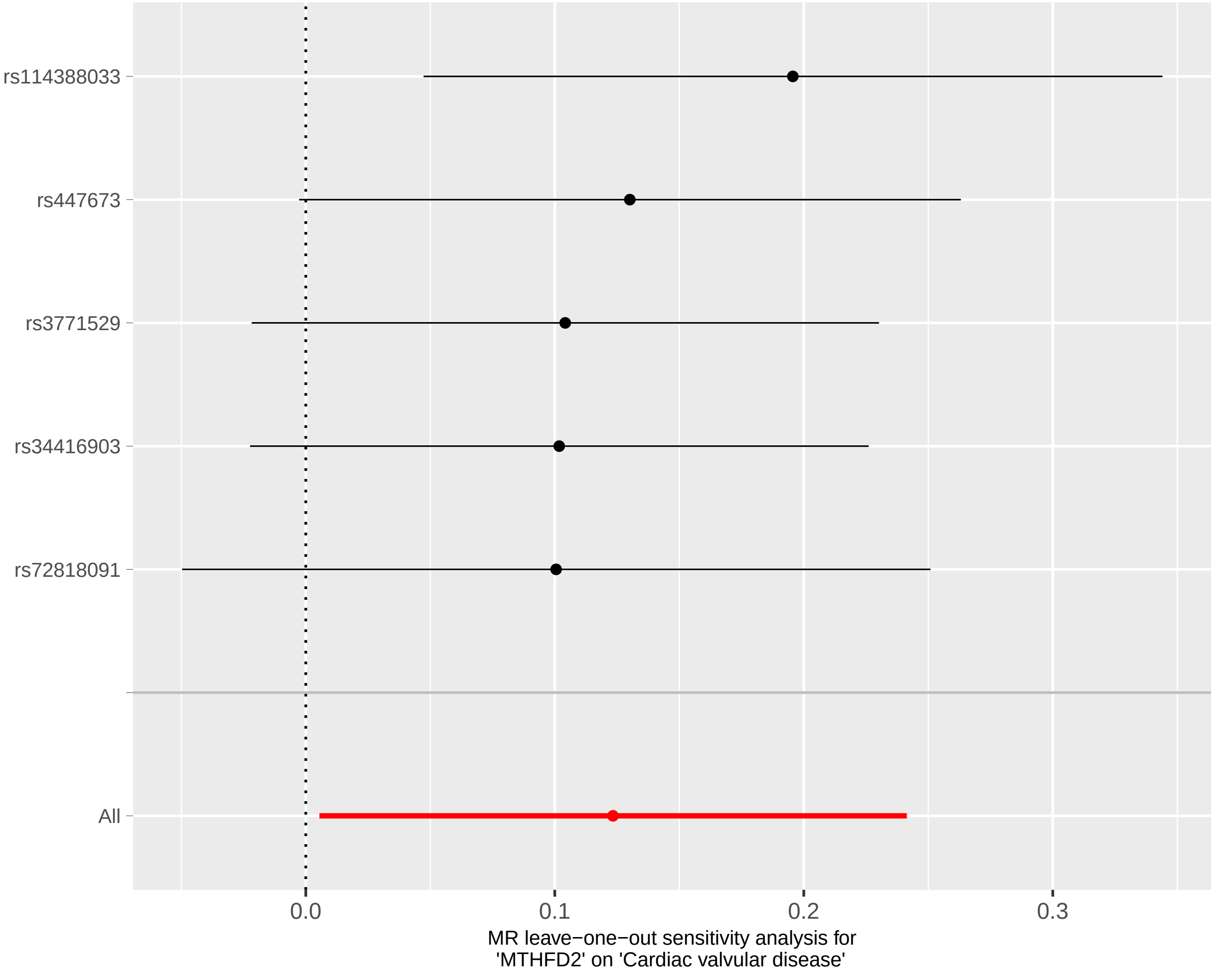
rs114388033
rs447673
rs3771529
rs34416903
rs72818091
All
0.0
0.1
0.2
0.3
MR leave−one−out sensitivity analysis for
'MTHFD2' on 'Cardiac valvular disease'

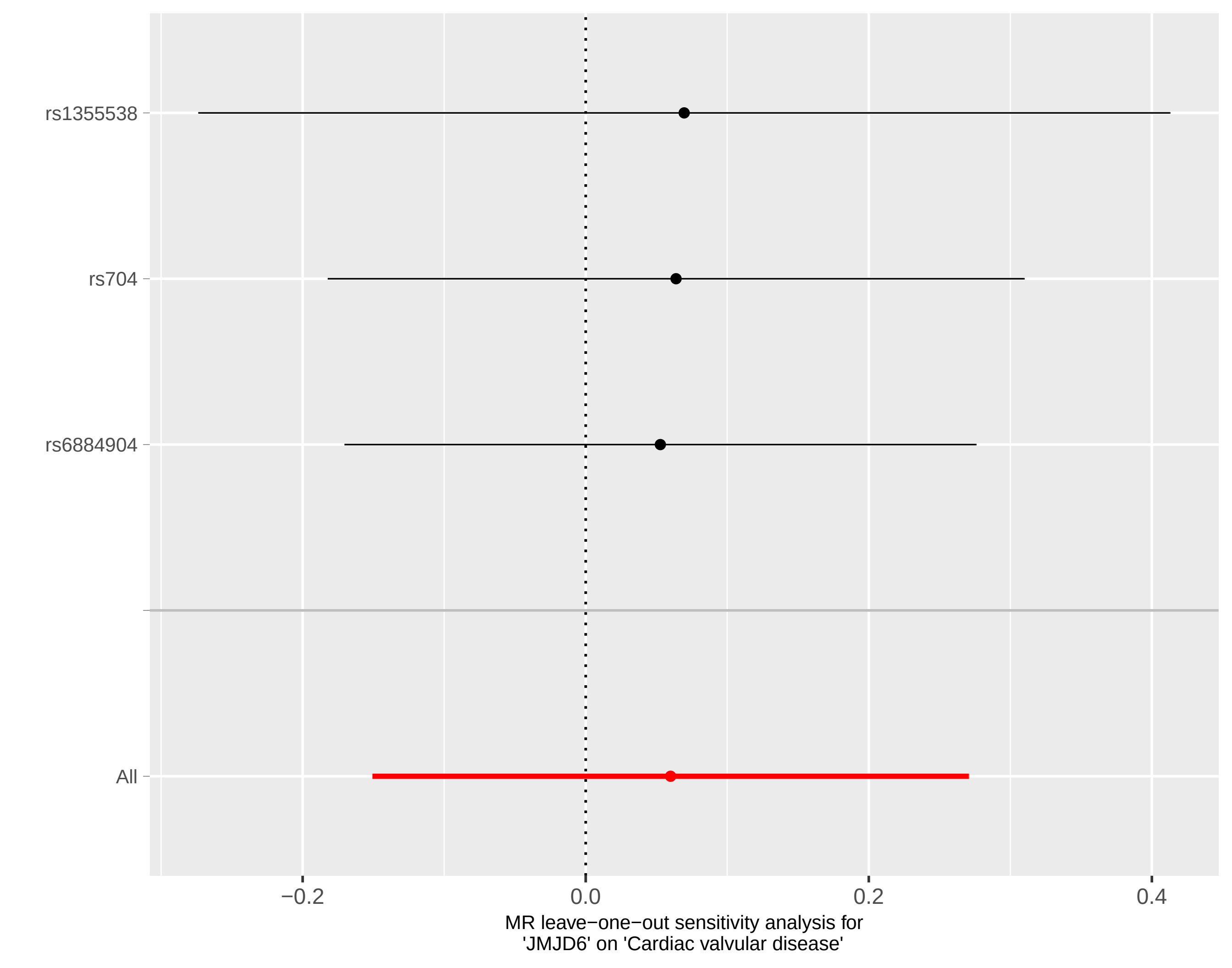
rs1355538
rs704
rs6884904
All
−0.2
0.0
0.2
0.4
MR leave−one−out sensitivity analysis for
'JMJD6' on 'Cardiac valvular disease'

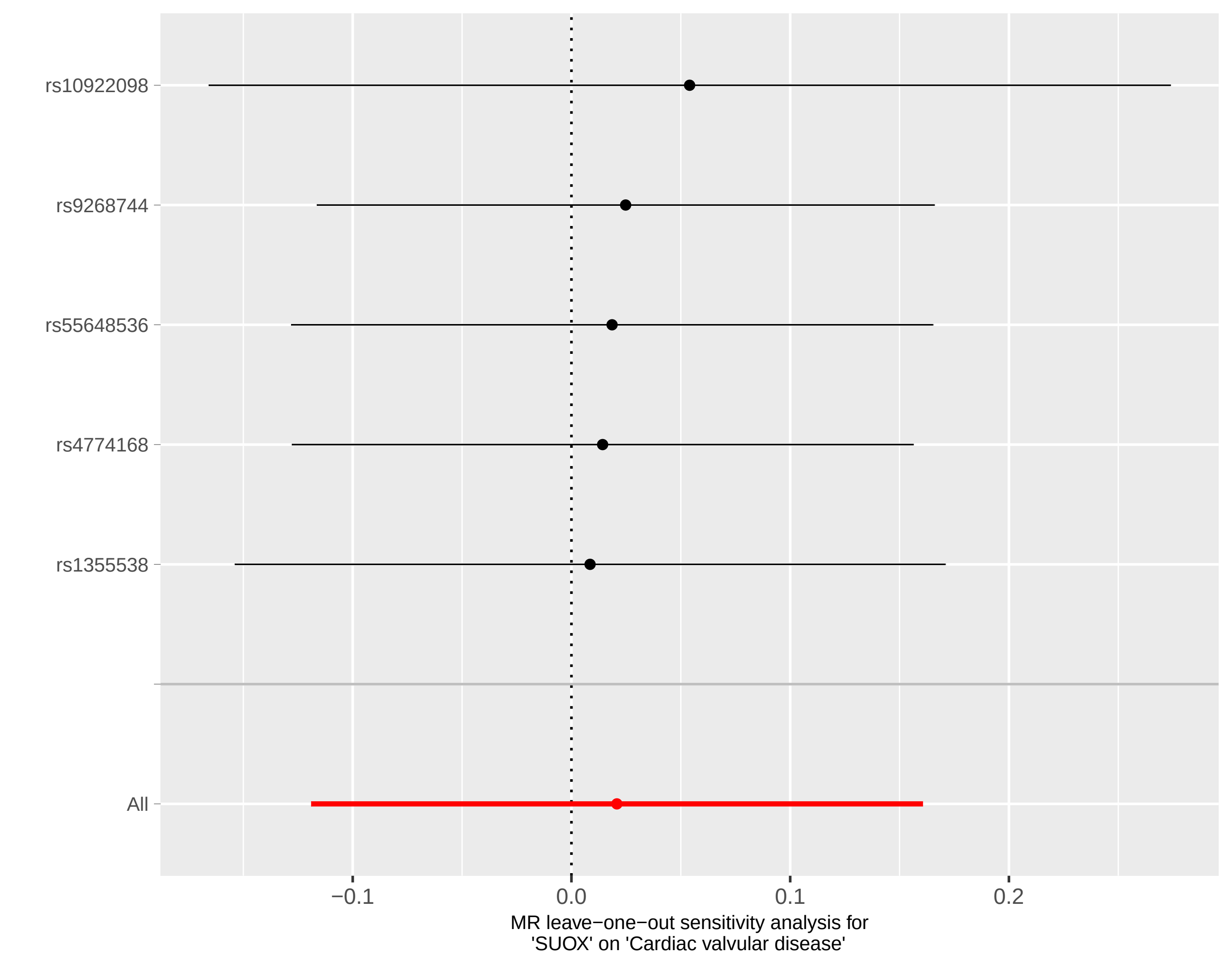
rs10922098
rs9268744
rs55648536
rs4774168
rs1355538
All
−0.1
0.0
0.1
0.2
MR leave−one−out sensitivity analysis for
'SUOX' on 'Cardiac valvular disease'

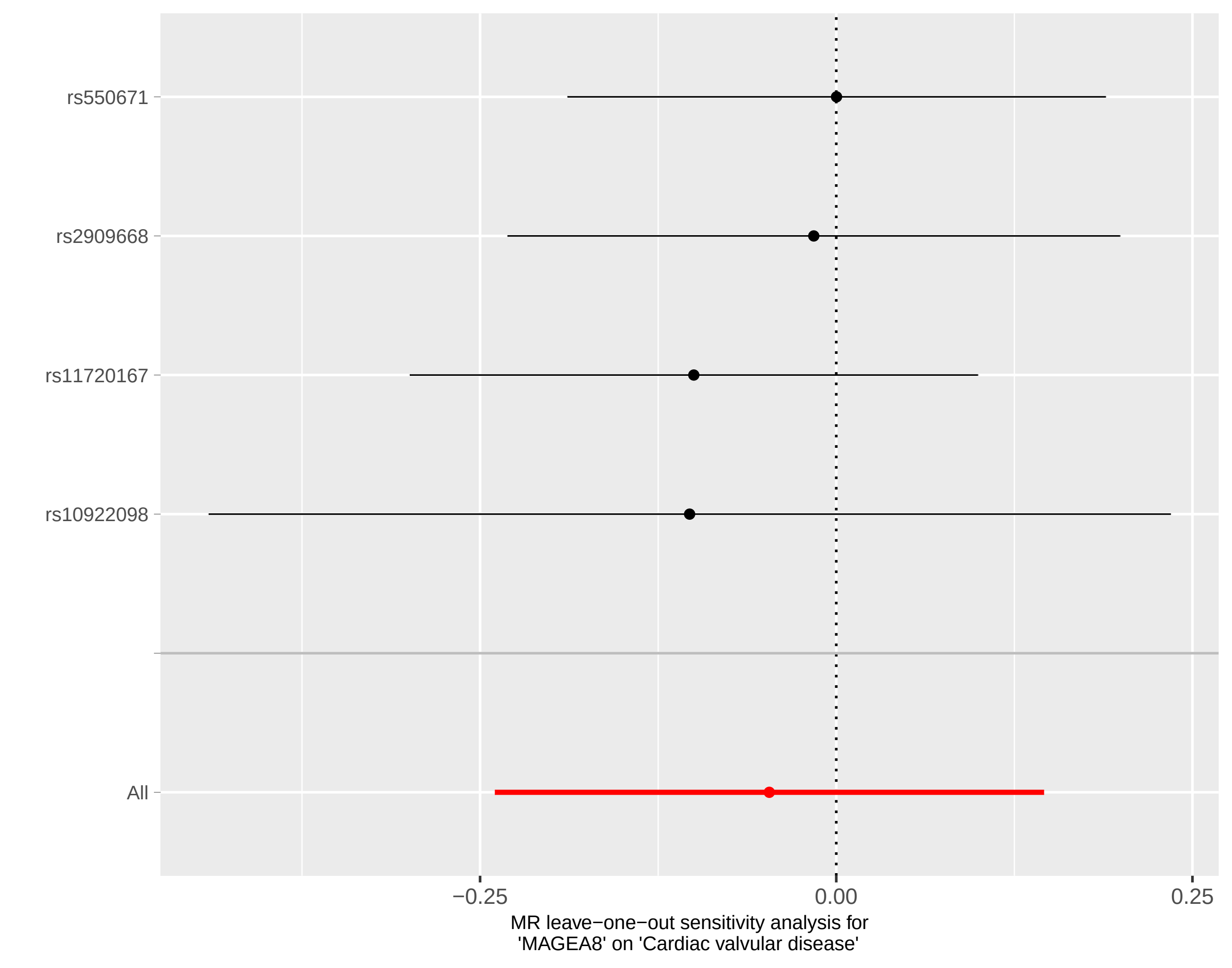
rs550671
rs2909668
rs11720167
rs10922098
All
−0.25
0.00
0.25
MR leave−one−out sensitivity analysis for
'MAGEA8' on 'Cardiac valvular disease'

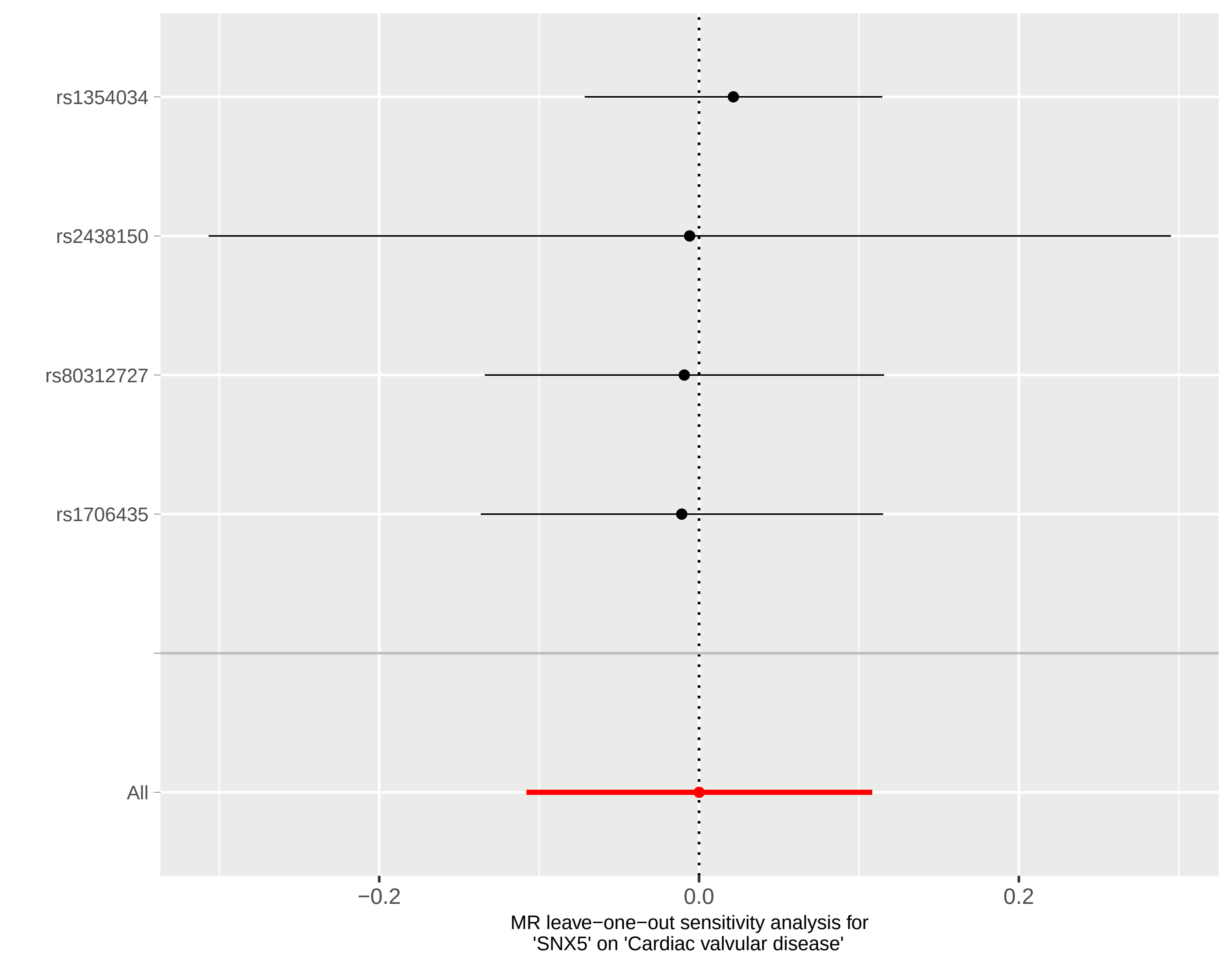

rs1354034
rs2438150
rs80312727
rs1706435
All
−0.2
0.0
0.2
MR leave−one−out sensitivity analysis for
'SNX5' on 'Cardiac valvular disease'

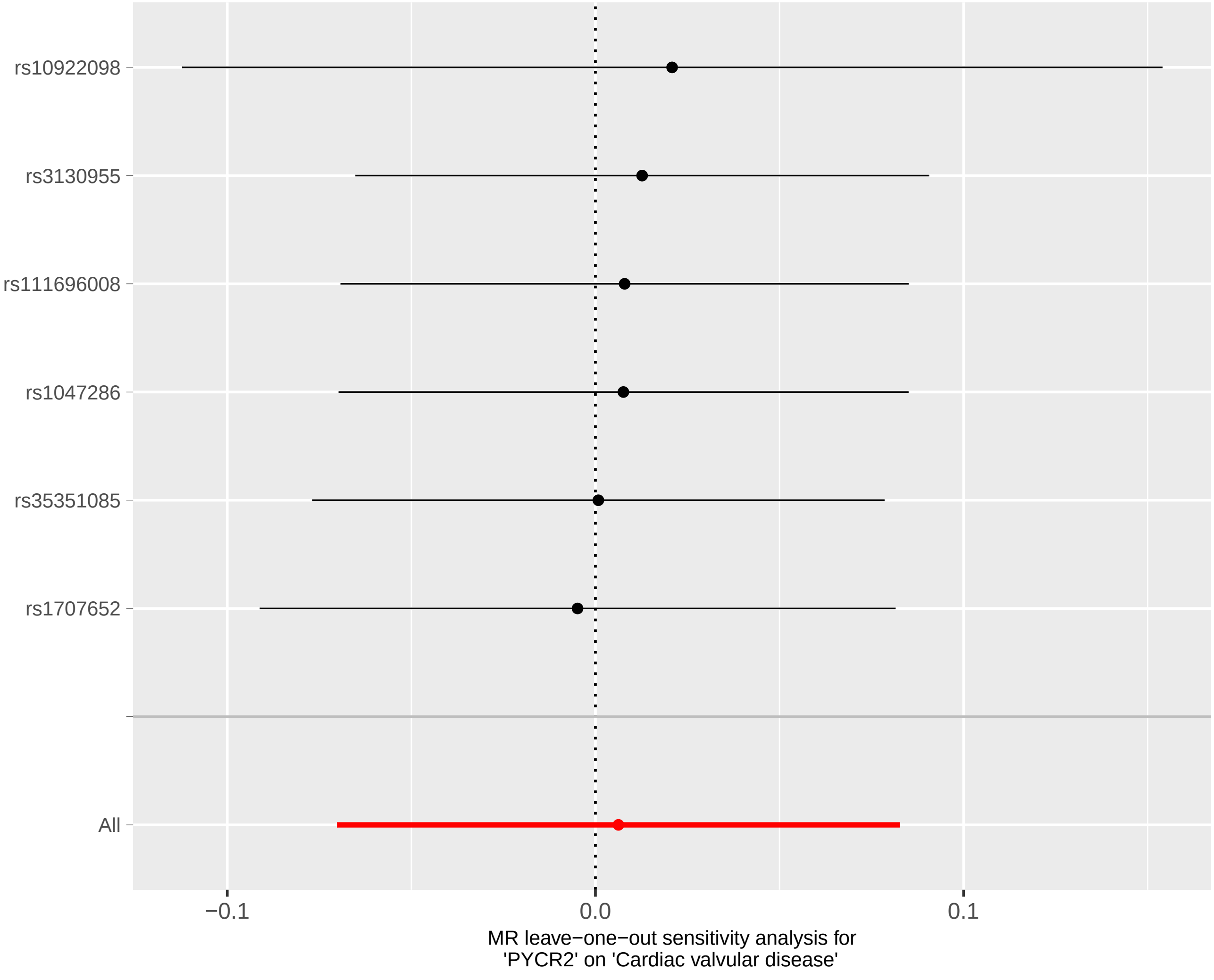

rs10922098
rs3130955
rs111696008
rs1047286
rs35351085
rs1707652
All
−0.1
0.0
0.1
MR leave−one−out sensitivity analysis for
'PYCR2' on 'Cardiac valvular disease'

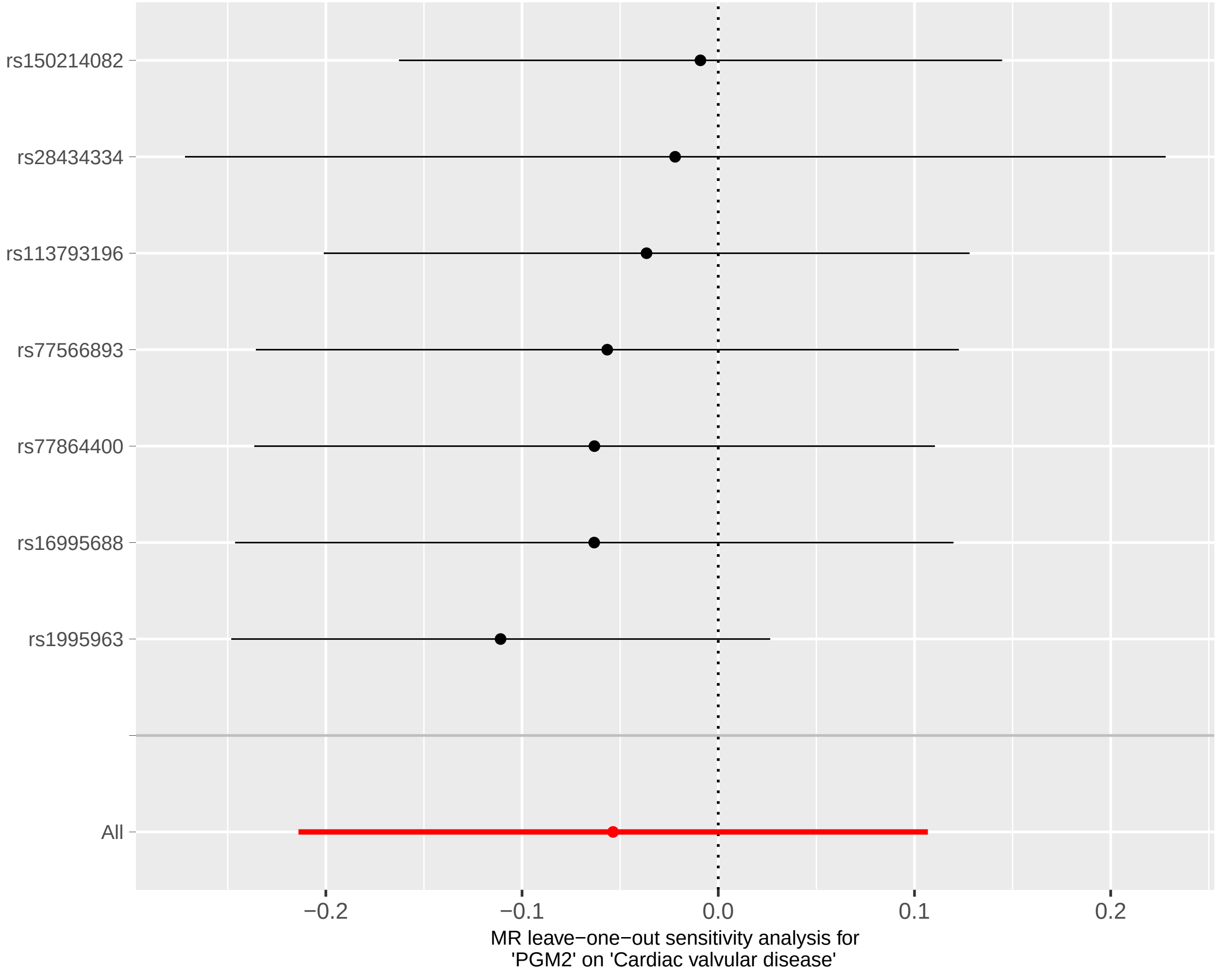
rs150214082
rs28434334
rs113793196
rs77566893
rs77864400
rs16995688
rs1995963
All
−0.2
−0.1
0.0
0.1
0.2
MR leave−one−out sensitivity analysis for
'PGM2' on 'Cardiac valvular disease'

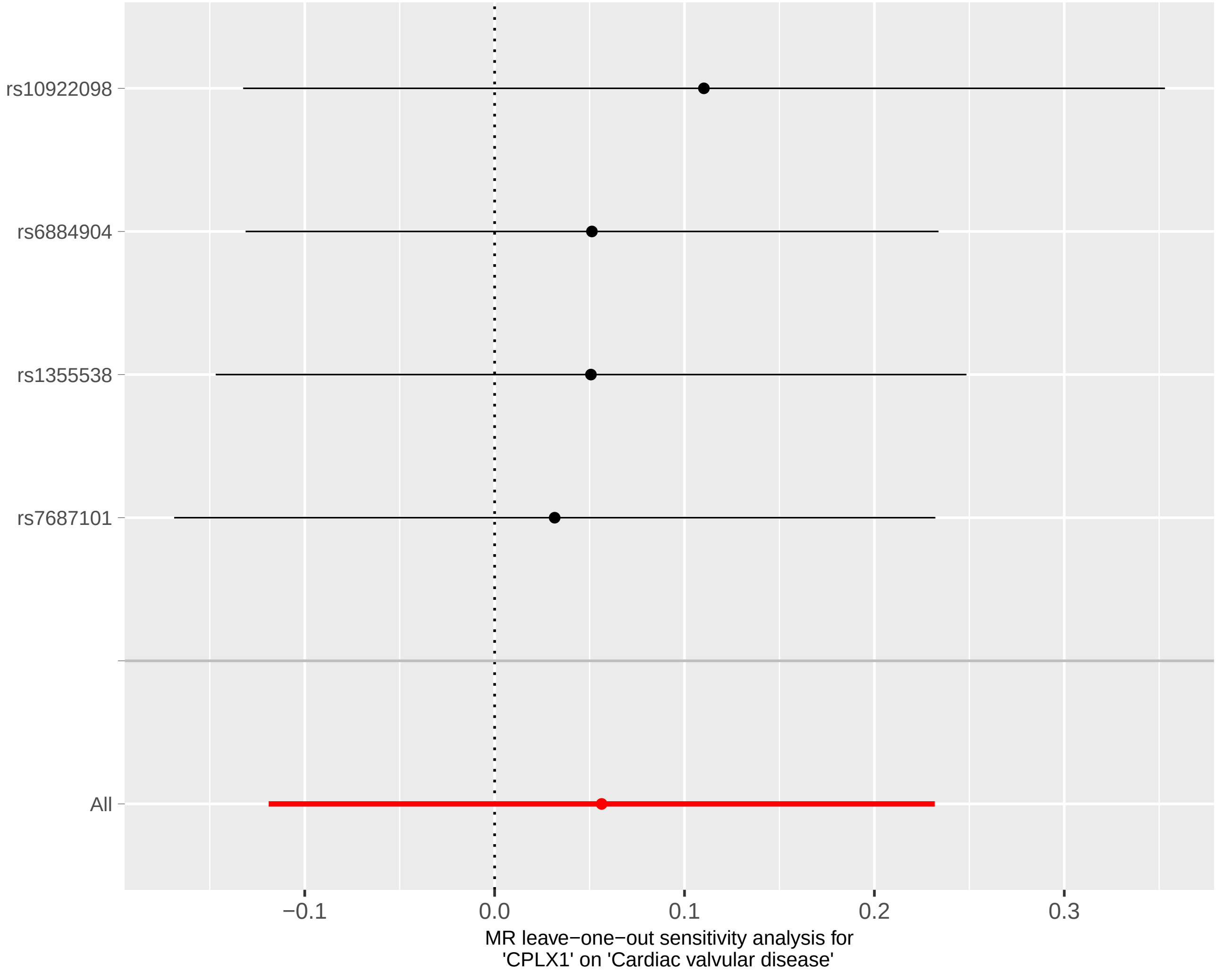
rs10922098
rs6884904
rs1355538
rs7687101
All
−0.1
0.0
0.1
0.2
0.3
MR leave−one−out sensitivity analysis for
'CPLX1' on 'Cardiac valvular disease'

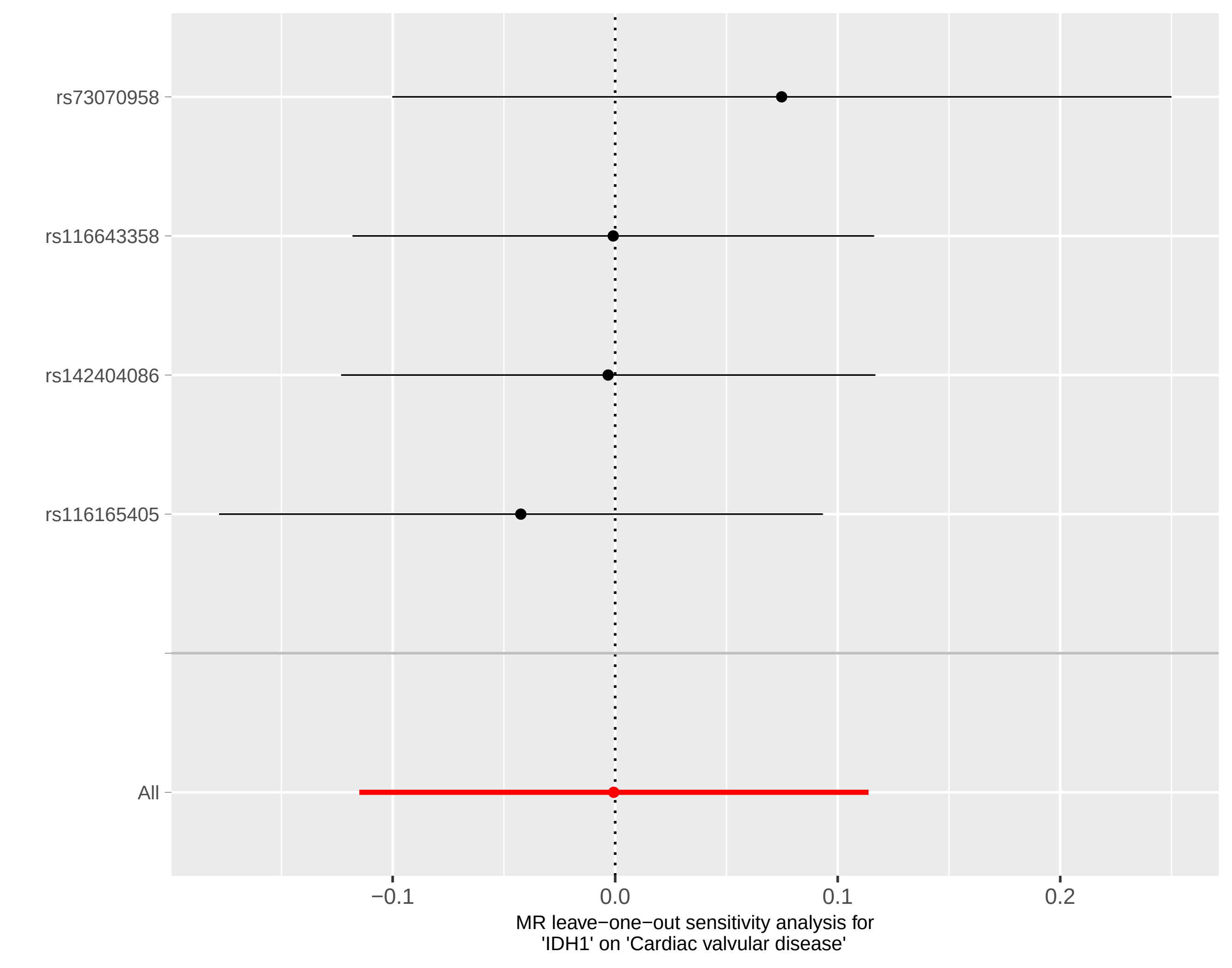
rs73070958
rs116643358
rs142404086
rs116165405
All
−0.1
0.0
0.1
0.2
MR leave−one−out sensitivity analysis for
'IDH1' on 'Cardiac valvular disease'

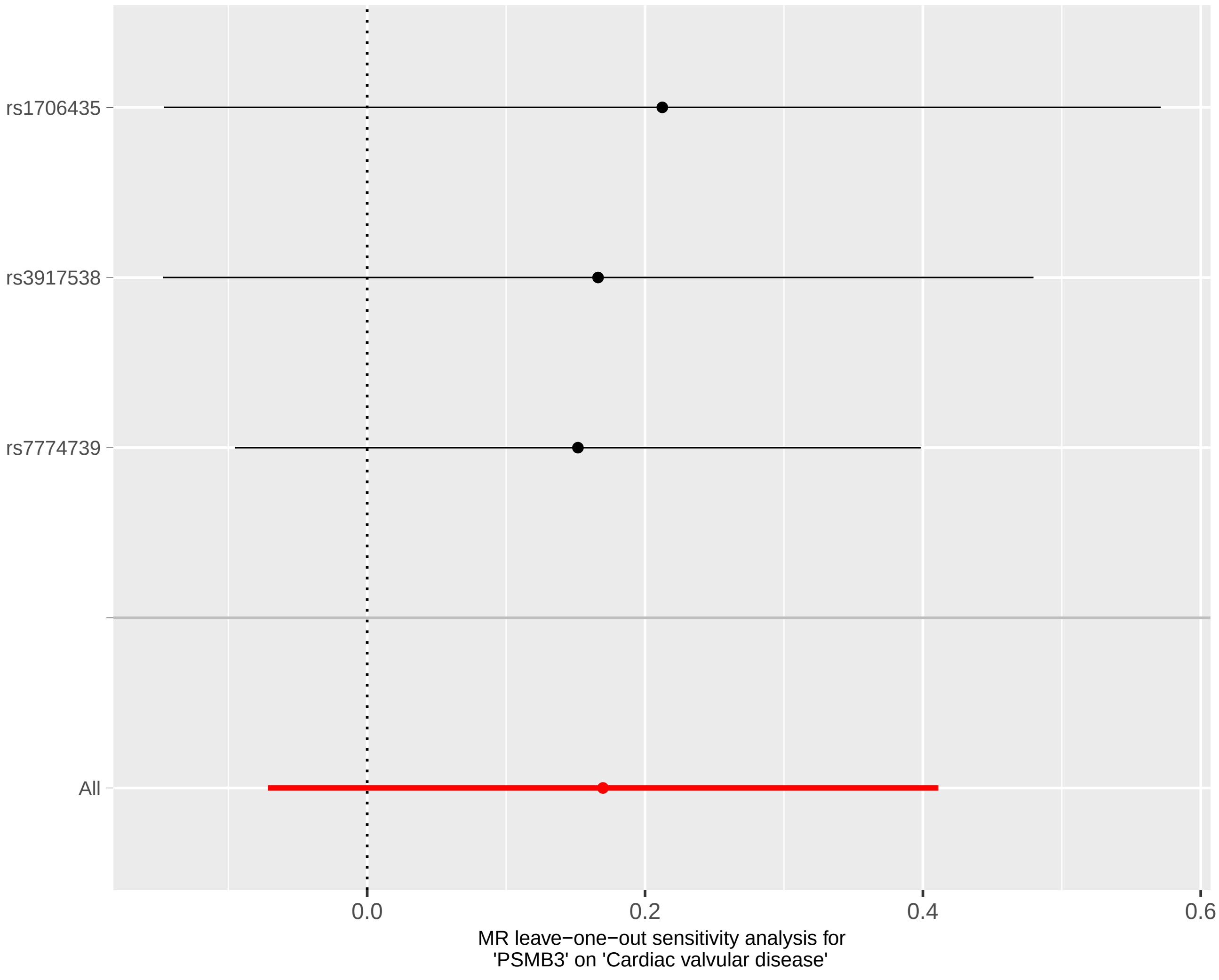

rs1706435
rs3917538
rs7774739
All
0.0
0.2
0.4
0.6
MR leave−one−out sensitivity analysis for
'PSMB3' on 'Cardiac valvular disease'

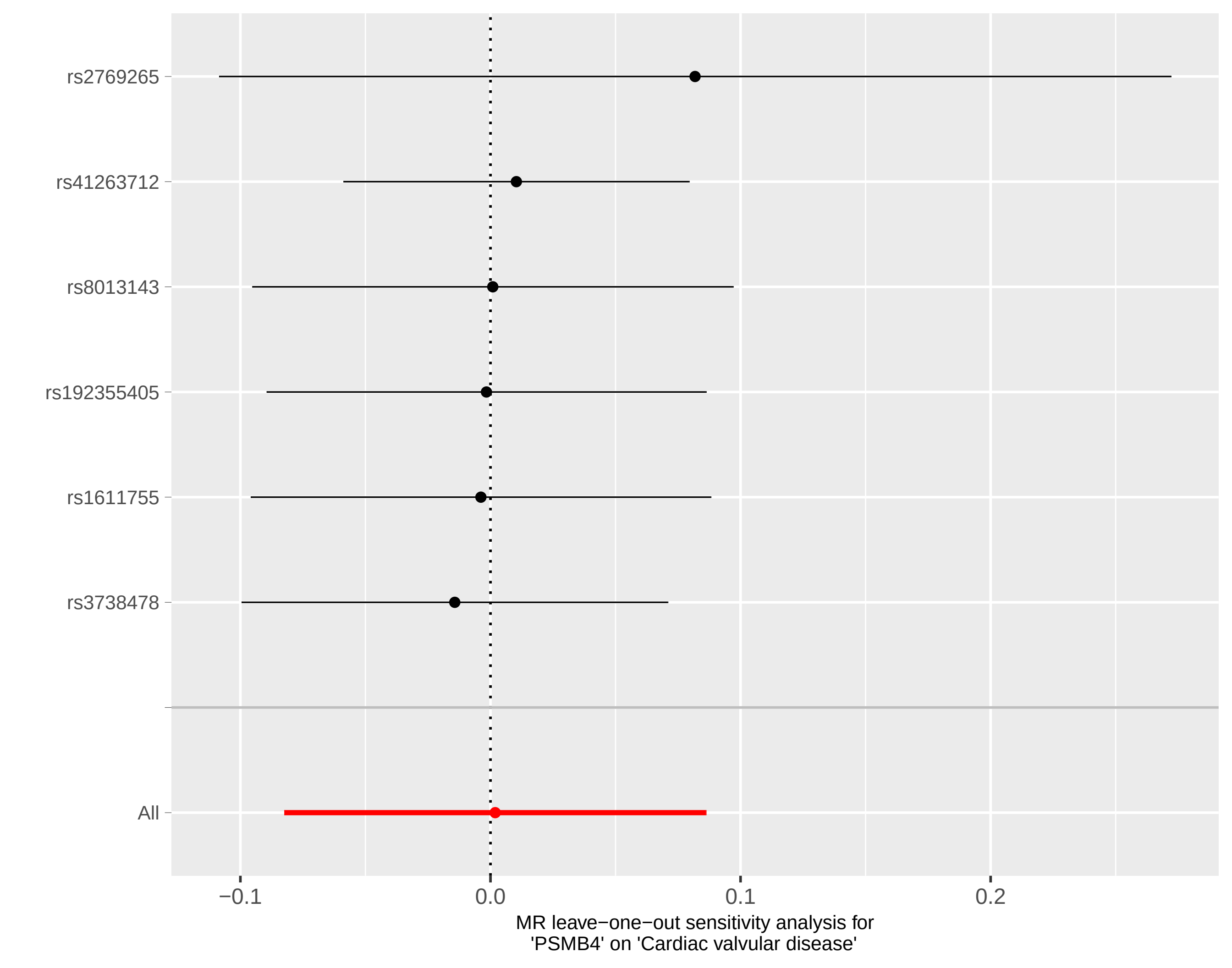

rs2769265
rs41263712
rs8013143
rs192355405
rs1611755
rs3738478
All
−0.1
0.0
0.1
0.2
MR leave−one−out sensitivity analysis for
'PSMB4' on 'Cardiac valvular disease'

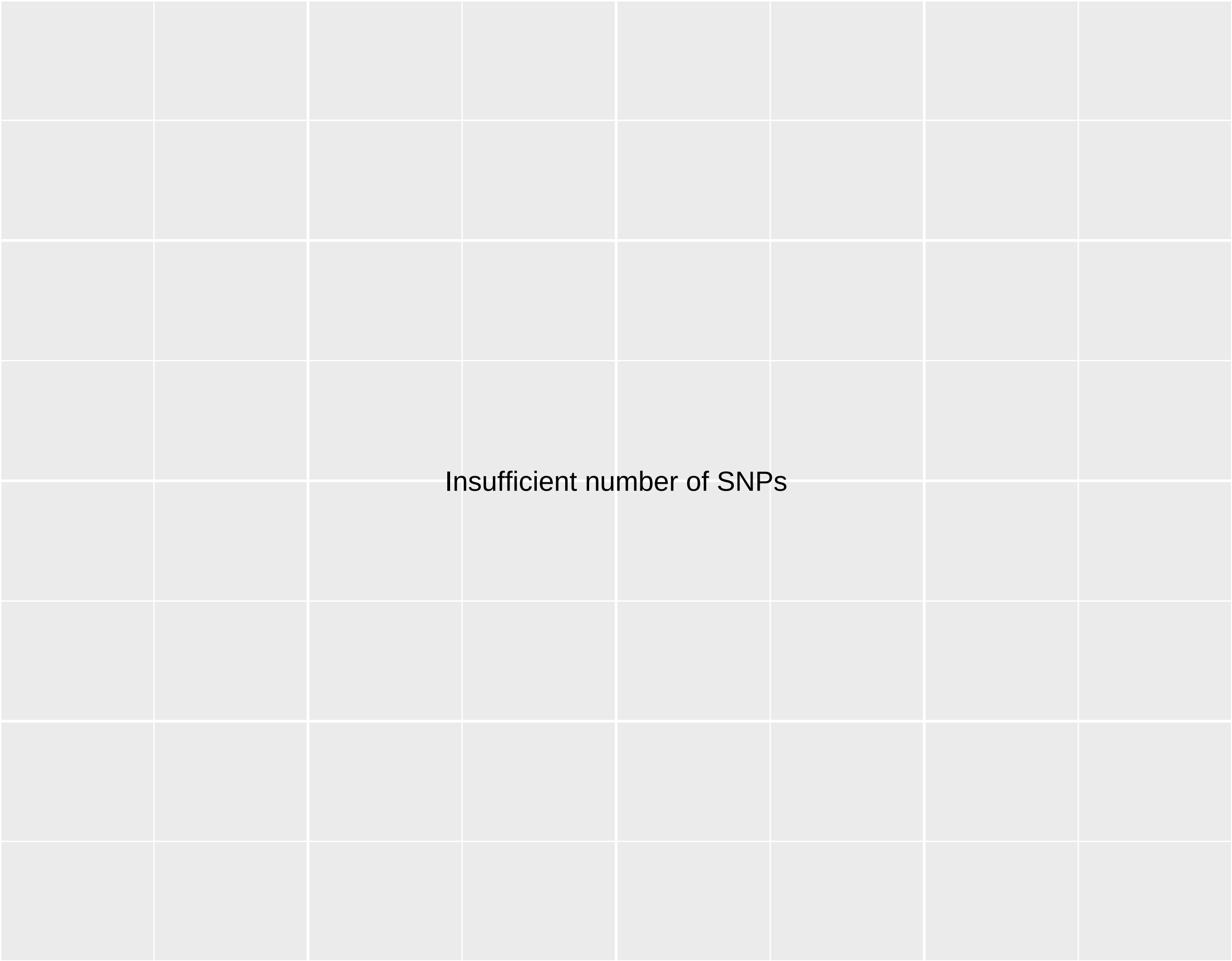
Insufficient number of SNPs

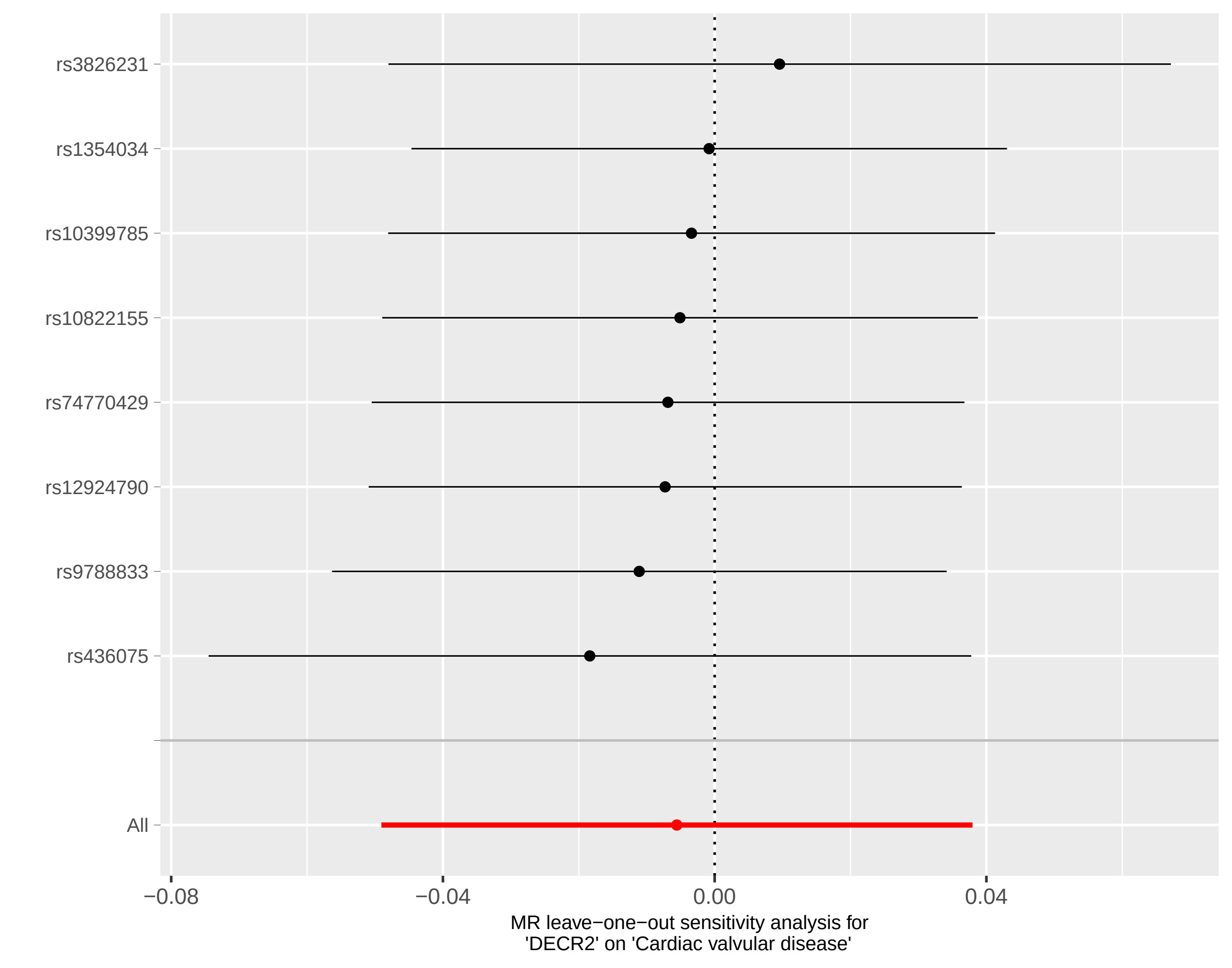
rs3826231
rs1354034
rs10399785
rs10822155
rs74770429
rs12924790
rs9788833
rs436075
All
−0.08
−0.04
0.00
0.04
MR leave−one−out sensitivity analysis for
'DECR2' on 'Cardiac valvular disease'

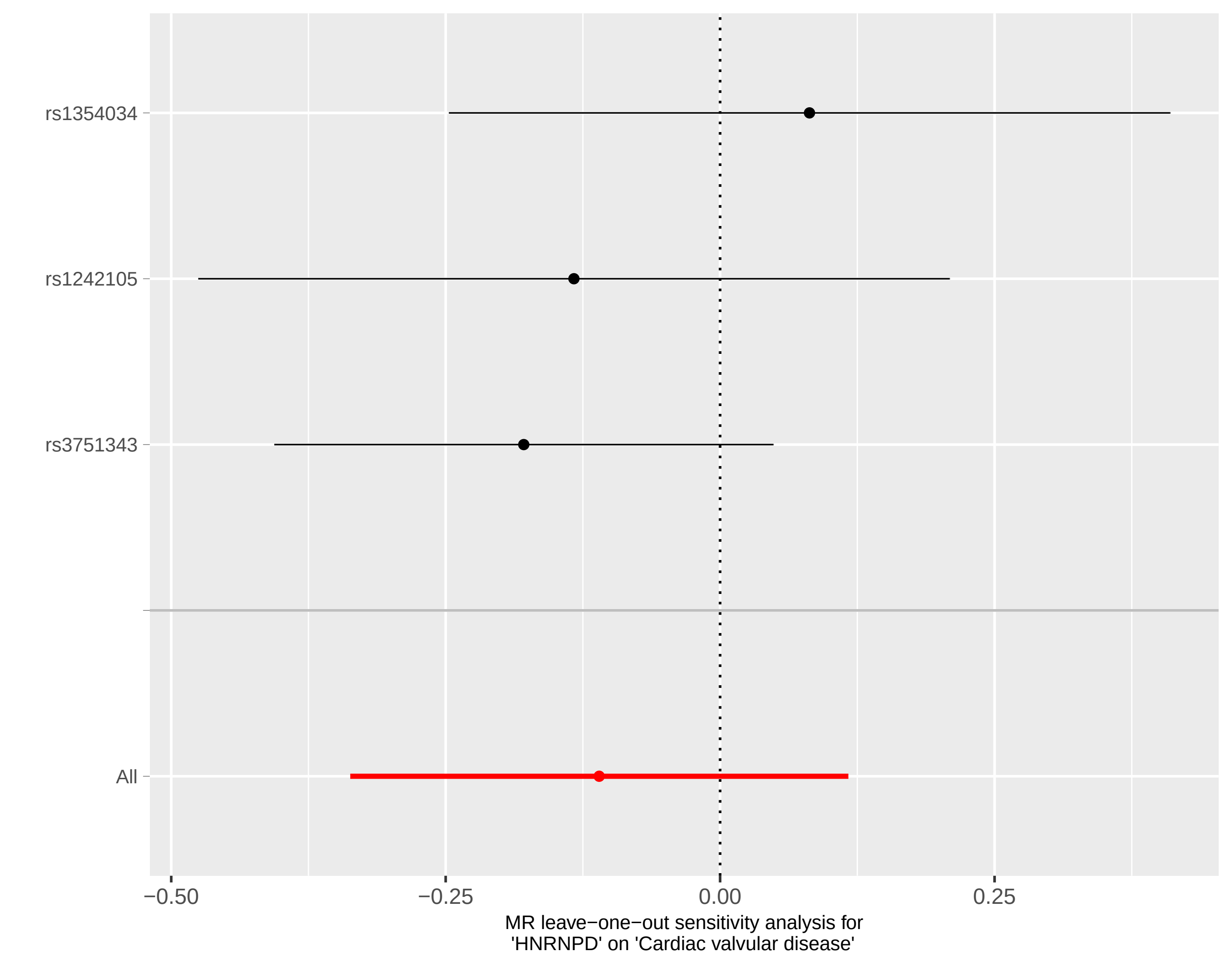
rs1354034
rs1242105
rs3751343
All
−0.50
−0.25
0.00
0.25
MR leave−one−out sensitivity analysis for
'HNRNPD' on 'Cardiac valvular disease'

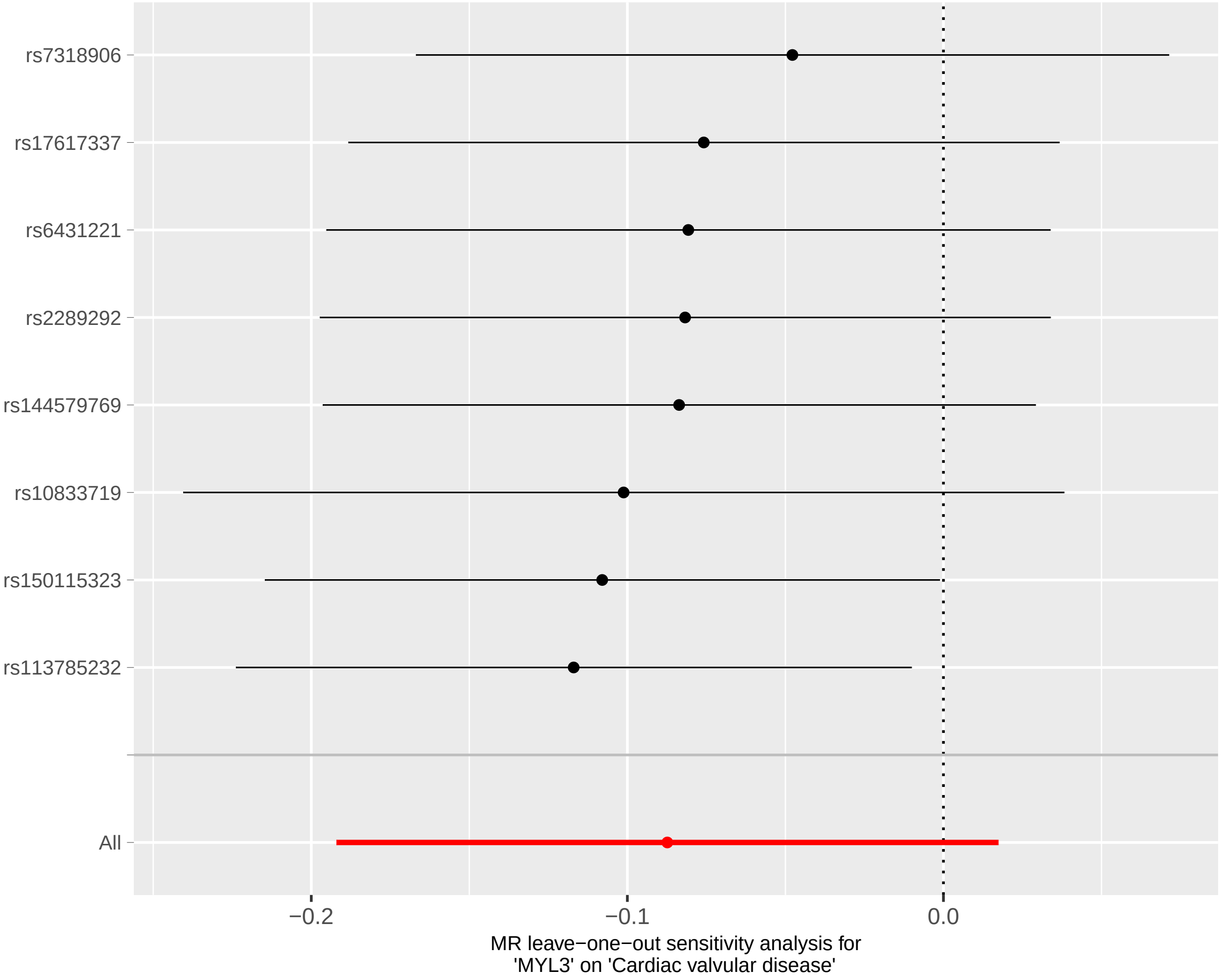

rs7318906
rs17617337
rs6431221
rs2289292
rs144579769
rs10833719
rs150115323
rs113785232
All
−0.2
−0.1
0.0
MR leave−one−out sensitivity analysis for
'MYL3' on 'Cardiac valvular disease'

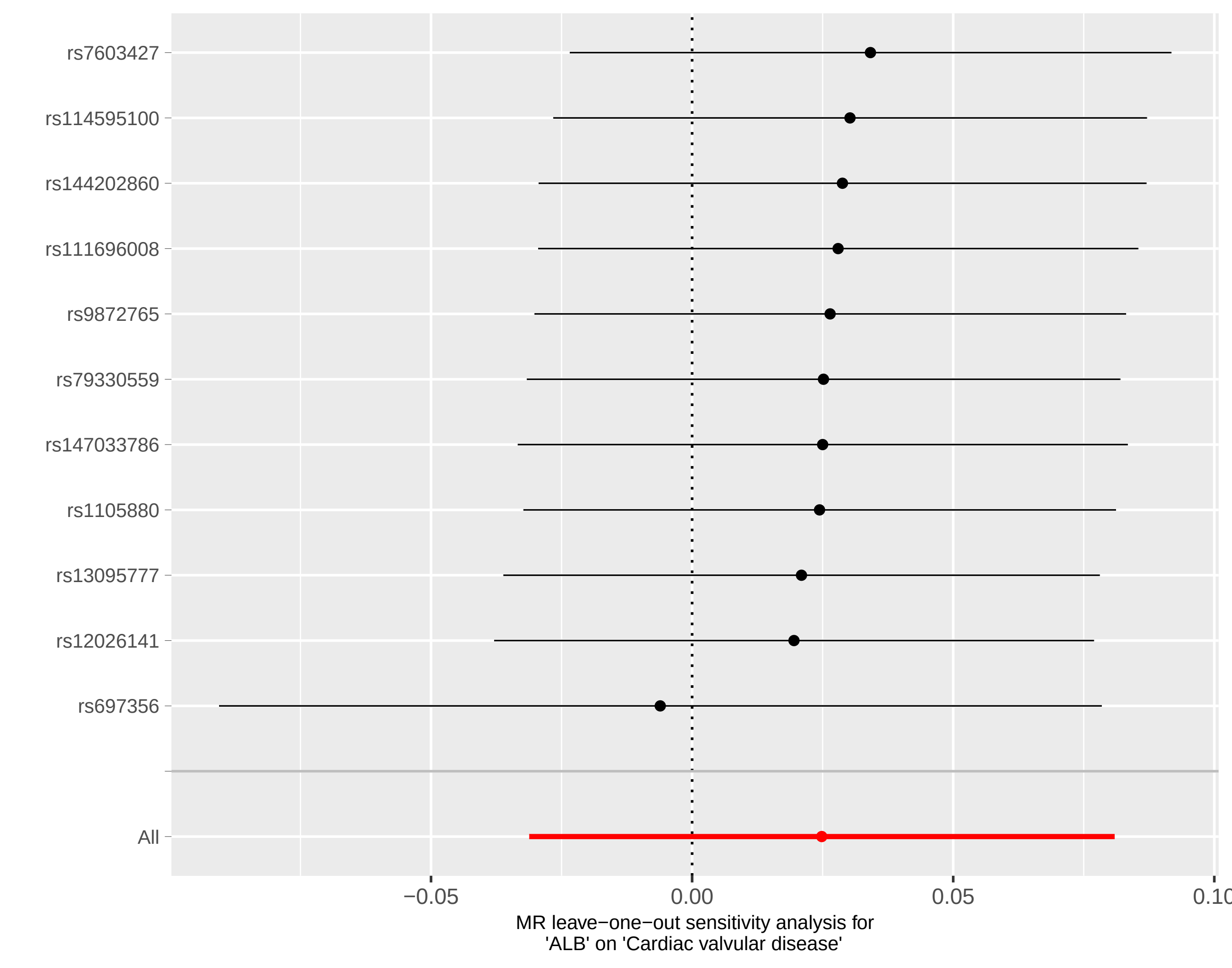
rs7603427
rs114595100
rs144202860
rs111696008
rs9872765
rs79330559
rs147033786
rs1105880
rs13095777
rs12026141
rs697356
All
−0.05
0.00
0.05
0.10
MR leave−one−out sensitivity analysis for
'ALB' on 'Cardiac valvular disease'

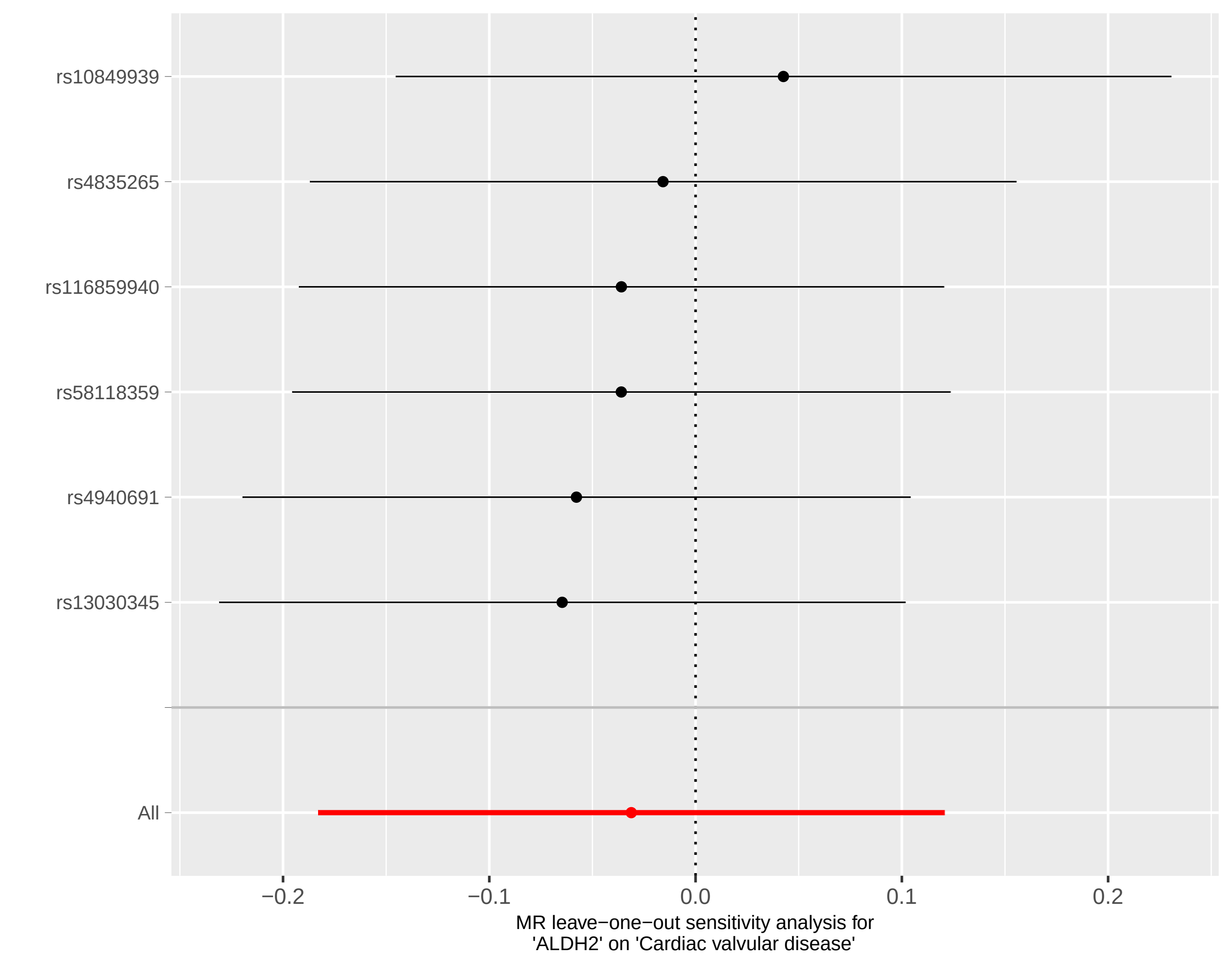
rs10849939
rs4835265
rs116859940
rs58118359
rs4940691
rs13030345
All
−0.2
−0.1
0.0
0.1
0.2
MR leave−one−out sensitivity analysis for
'ALDH2' on 'Cardiac valvular disease'

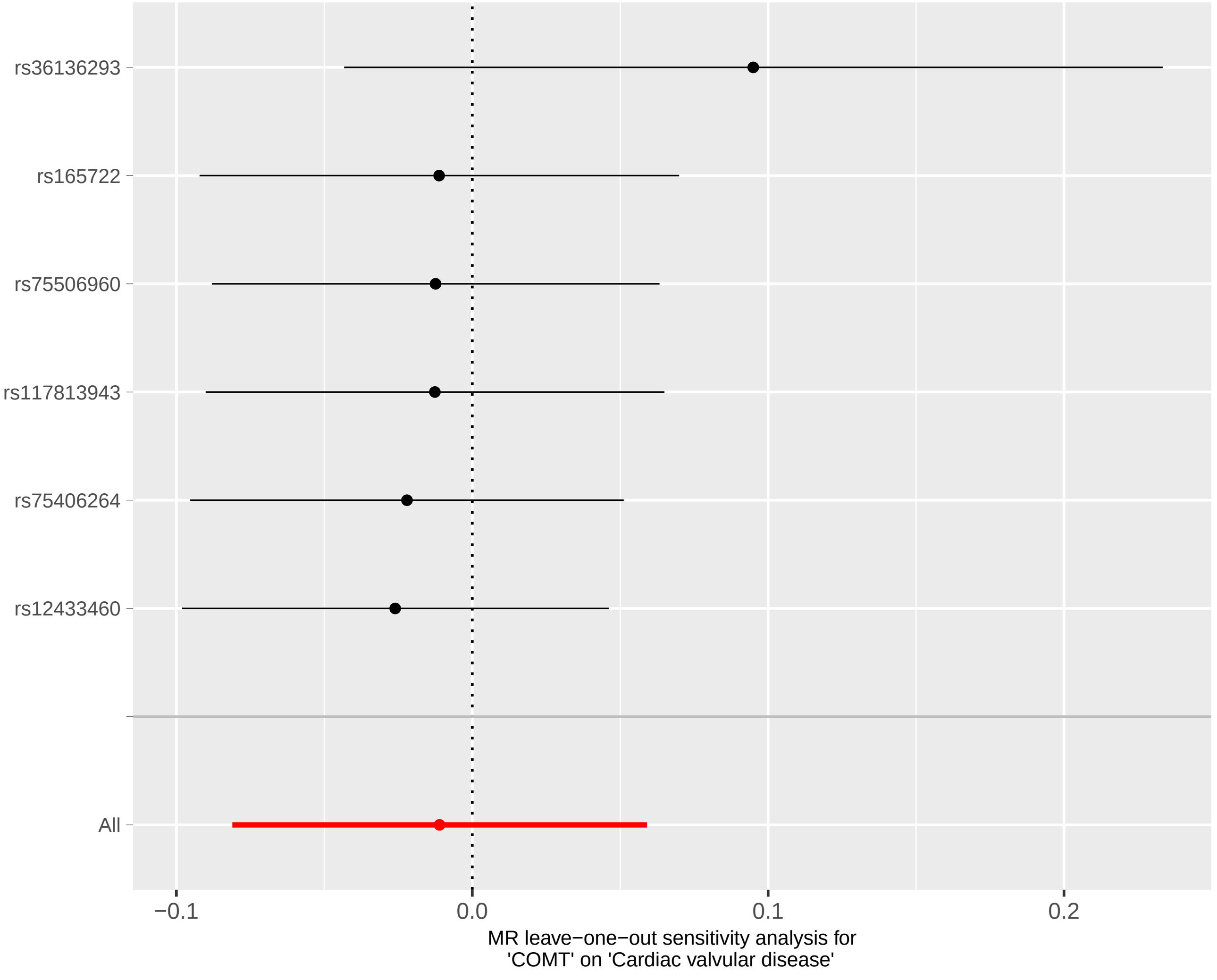

rs36136293
rs165722
rs75506960
rs117813943
rs75406264
rs12433460
All
−0.1
0.0
0.1
0.2
MR leave−one−out sensitivity analysis for
'COMT' on 'Cardiac valvular disease'

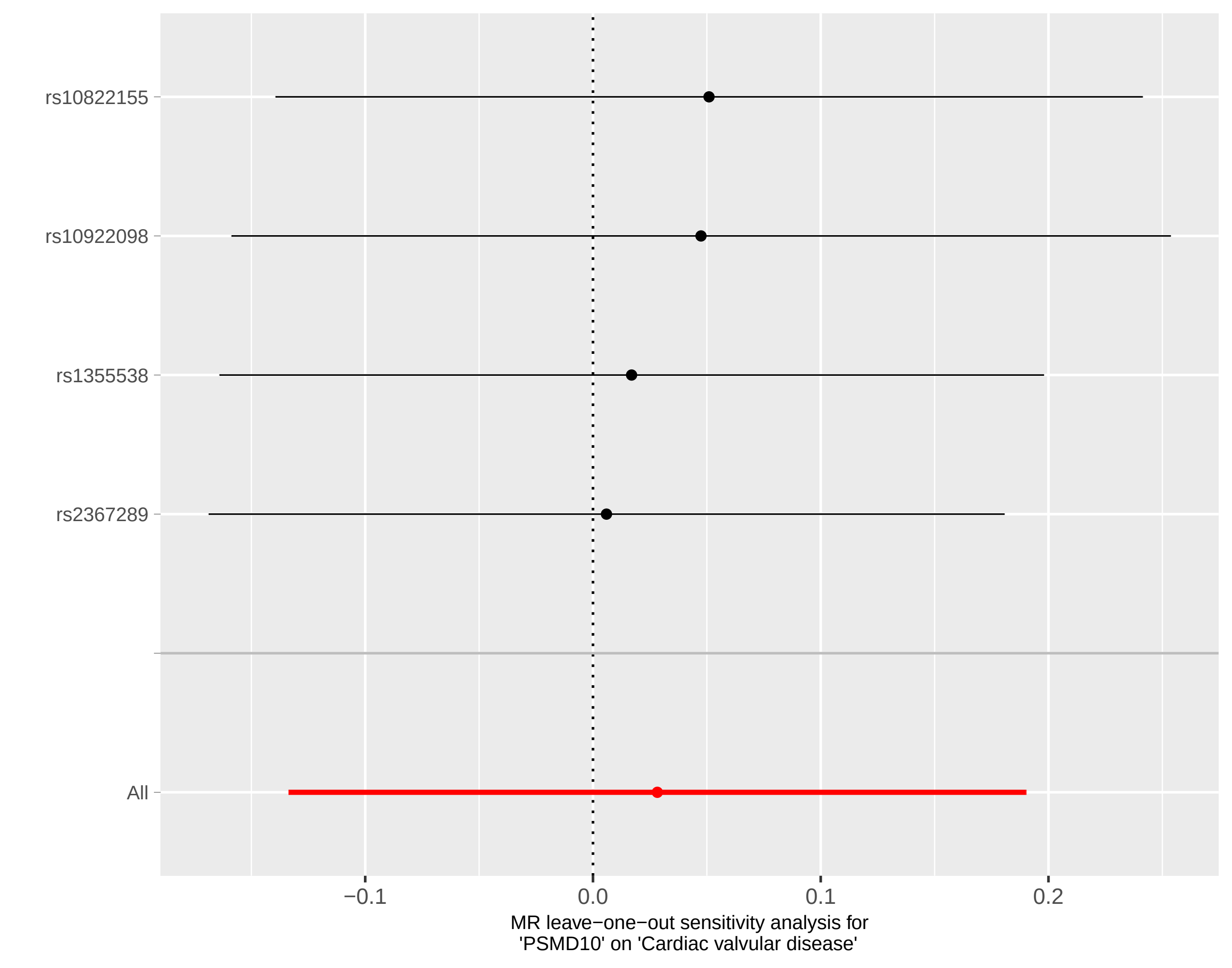
rs10822155
rs10922098
rs1355538
rs2367289
All
−0.1
0.0
0.1
0.2
MR leave−one−out sensitivity analysis for
'PSMD10' on 'Cardiac valvular disease'

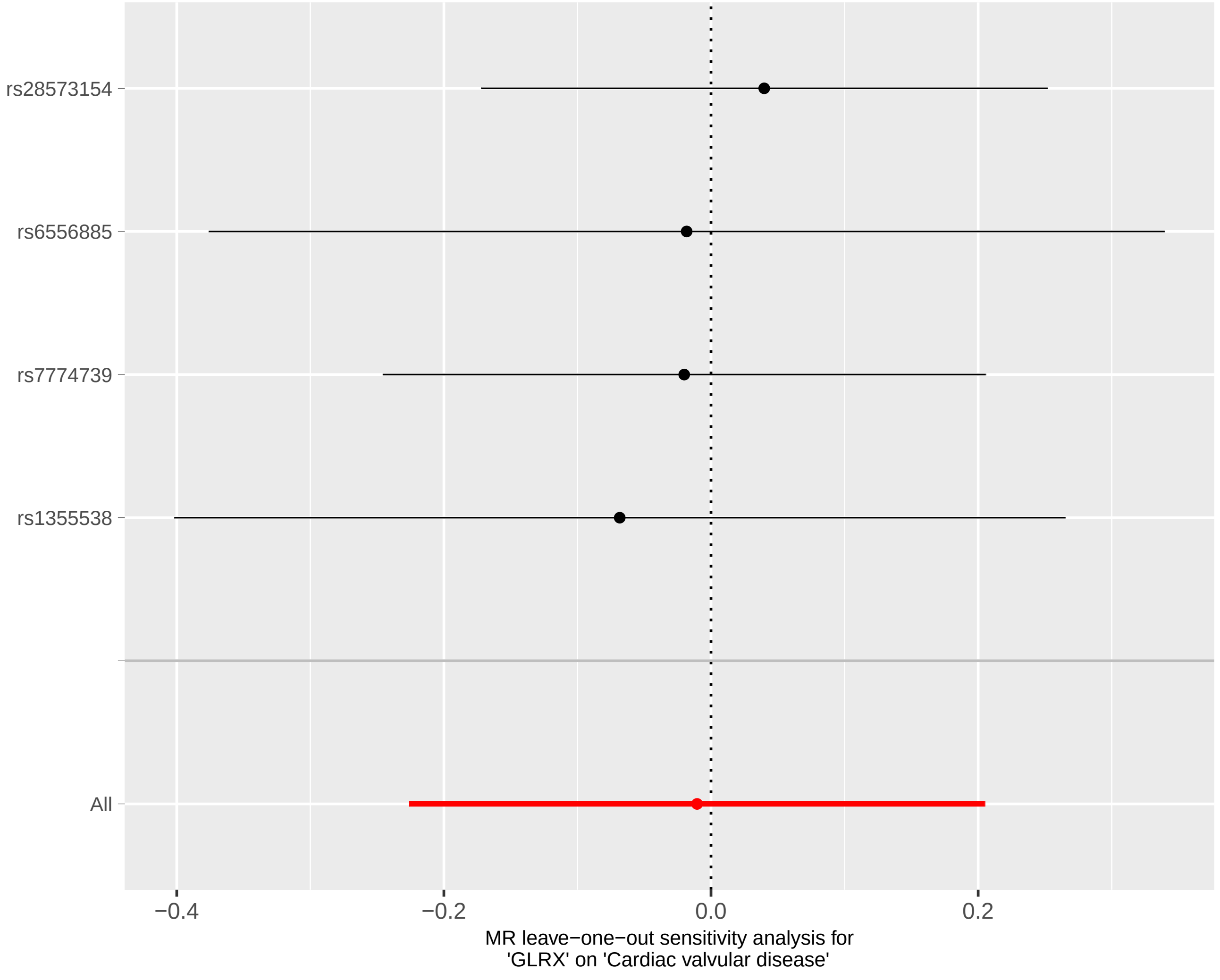
rs28573154
rs6556885
rs7774739
rs1355538
All
−0.4
−0.2
0.0
0.2
MR leave−one−out sensitivity analysis for
'GLRX' on 'Cardiac valvular disease'

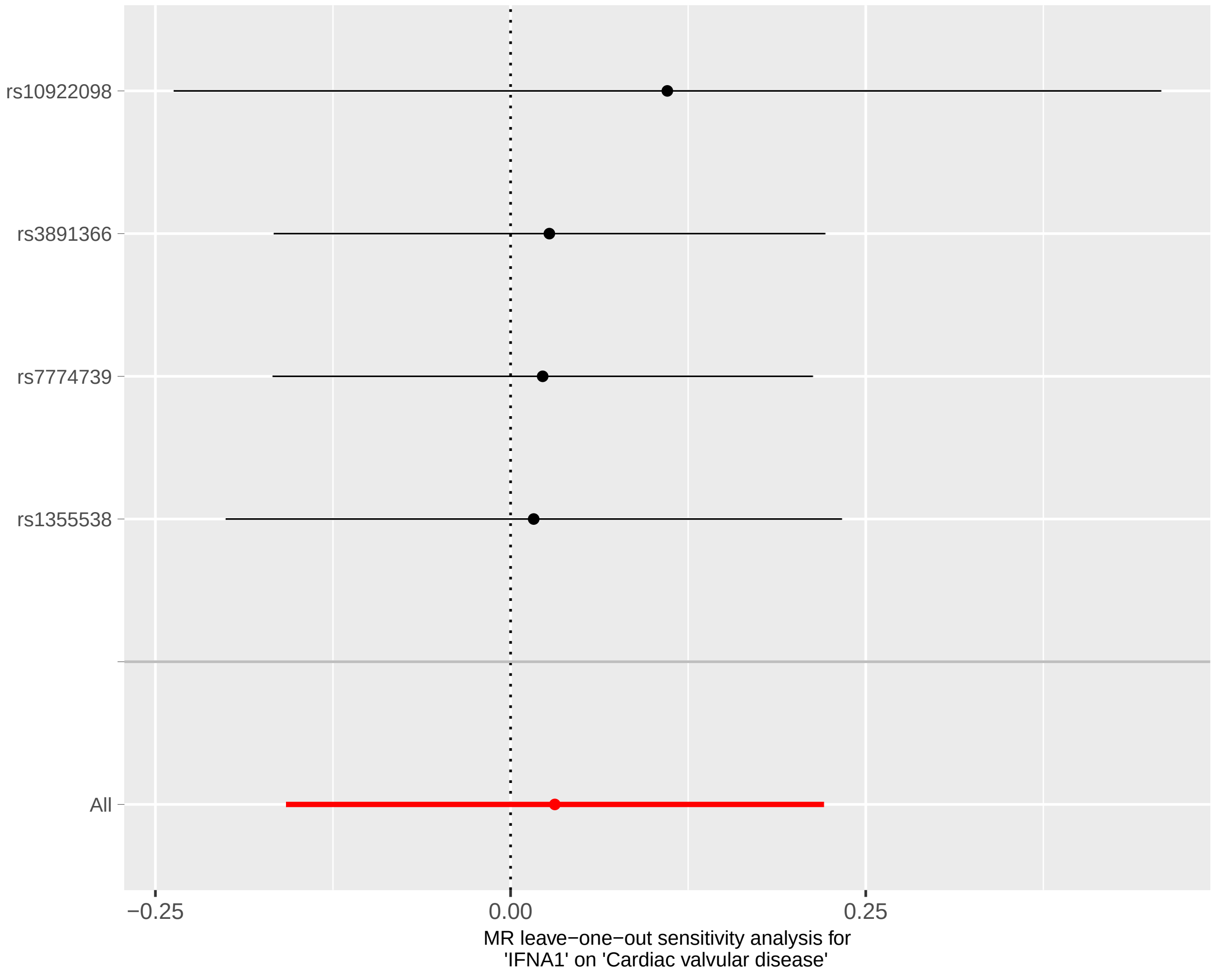
rs10922098
rs3891366
rs7774739
rs1355538
All
−0.25
0.00
0.25
MR leave−one−out sensitivity analysis for
'IFNA1' on 'Cardiac valvular disease'

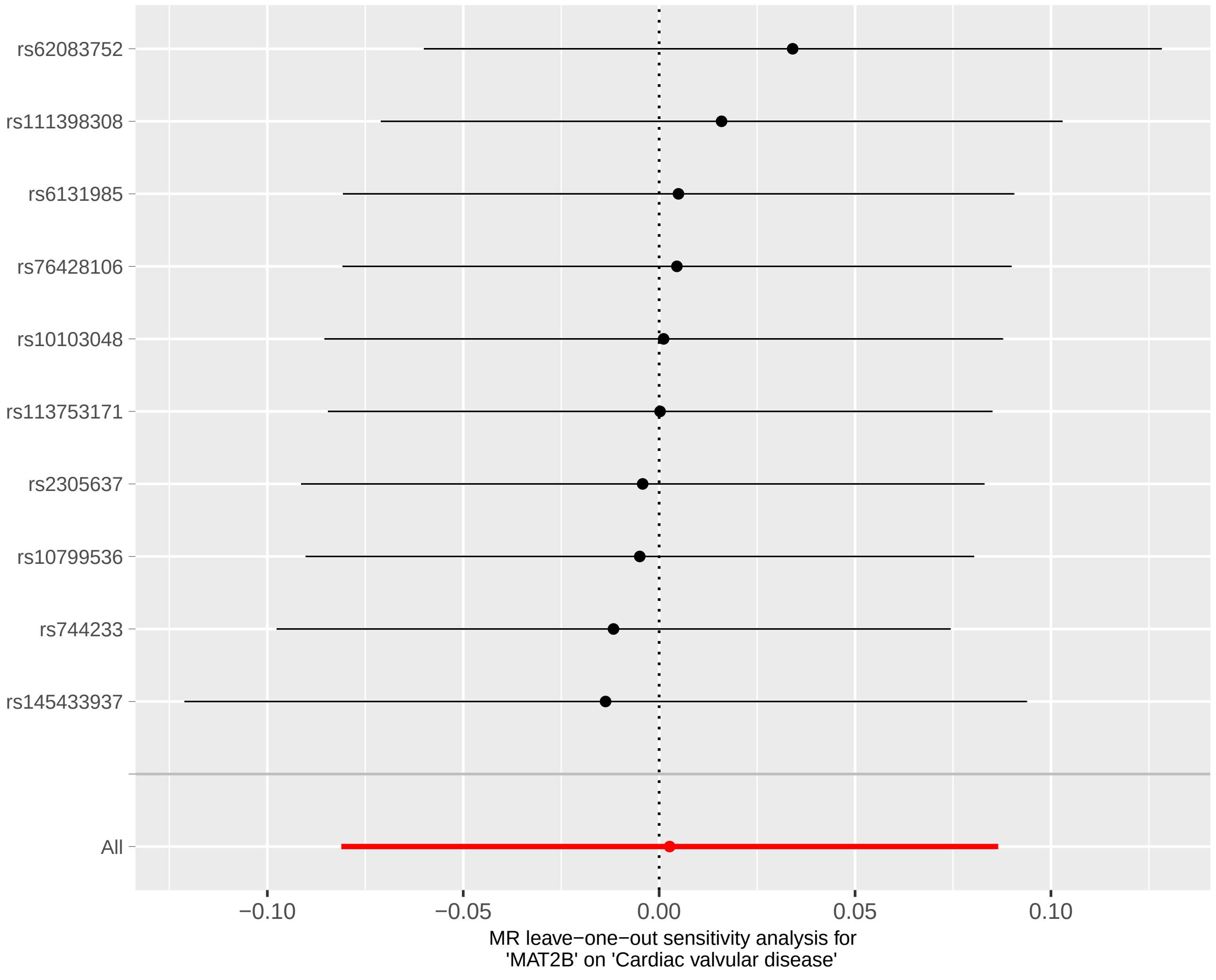

rs62083752
rs111398308
rs6131985
rs76428106
rs10103048
rs113753171
rs2305637
rs10799536
rs744233
rs145433937
All
−0.10
−0.05
0.00
0.05
0.10
MR leave−one−out sensitivity analysis for
'MAT2B' on 'Cardiac valvular disease'

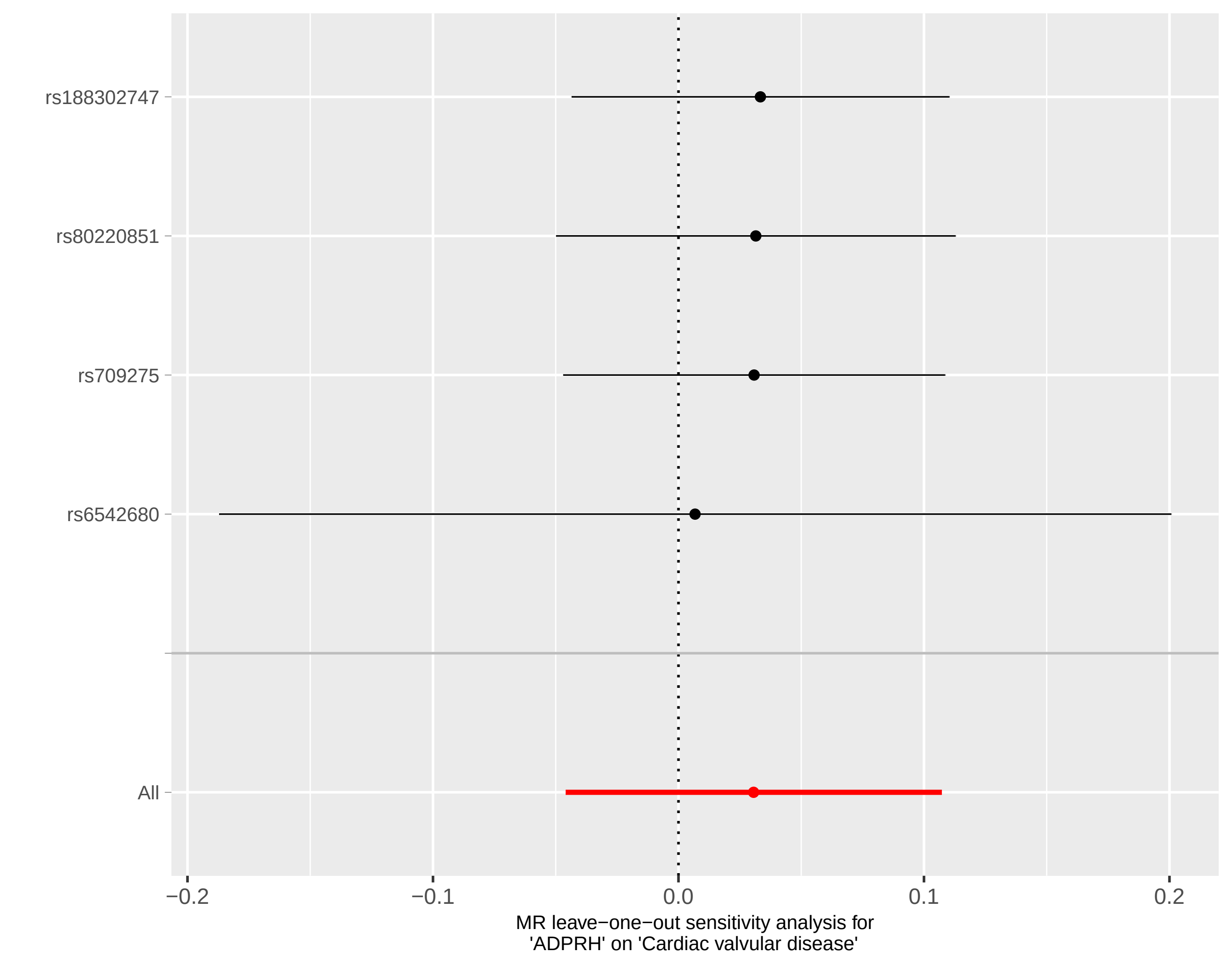
rs188302747
rs80220851
rs709275
rs6542680
All
−0.2
−0.1
0.0
0.1
0.2
MR leave−one−out sensitivity analysis for
'ADPRH' on 'Cardiac valvular disease'

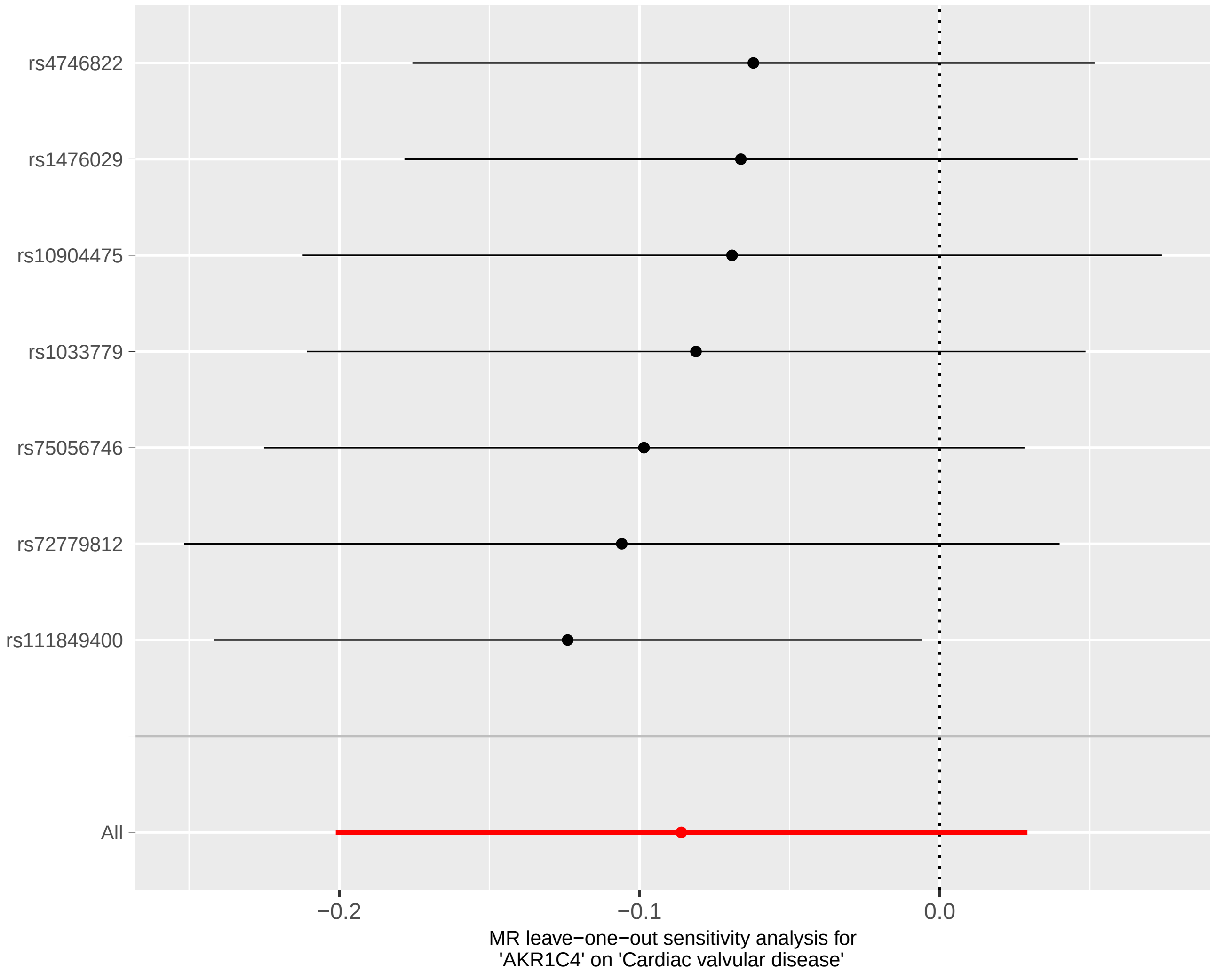
rs4746822
rs1476029
rs10904475
rs1033779
rs75056746
rs72779812
rs111849400
All
−0.2
−0.1
0.0
MR leave−one−out sensitivity analysis for
'AKR1C4' on 'Cardiac valvular disease'

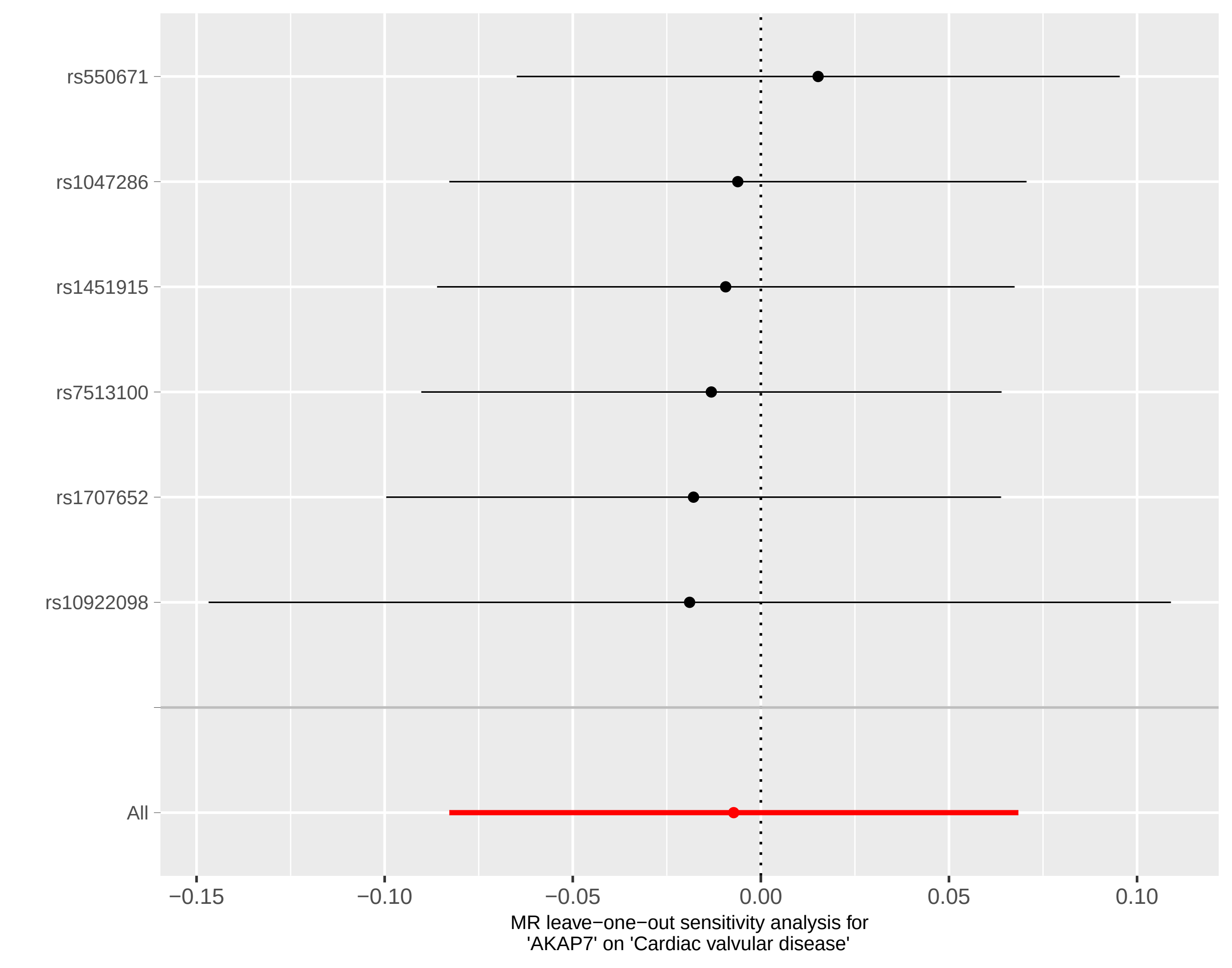

rs550671
rs1047286
rs1451915
rs7513100
rs1707652
rs10922098
All
−0.15
−0.10
−0.05
0.00
0.05
0.10
MR leave−one−out sensitivity analysis for
'AKAP7' on 'Cardiac valvular disease'

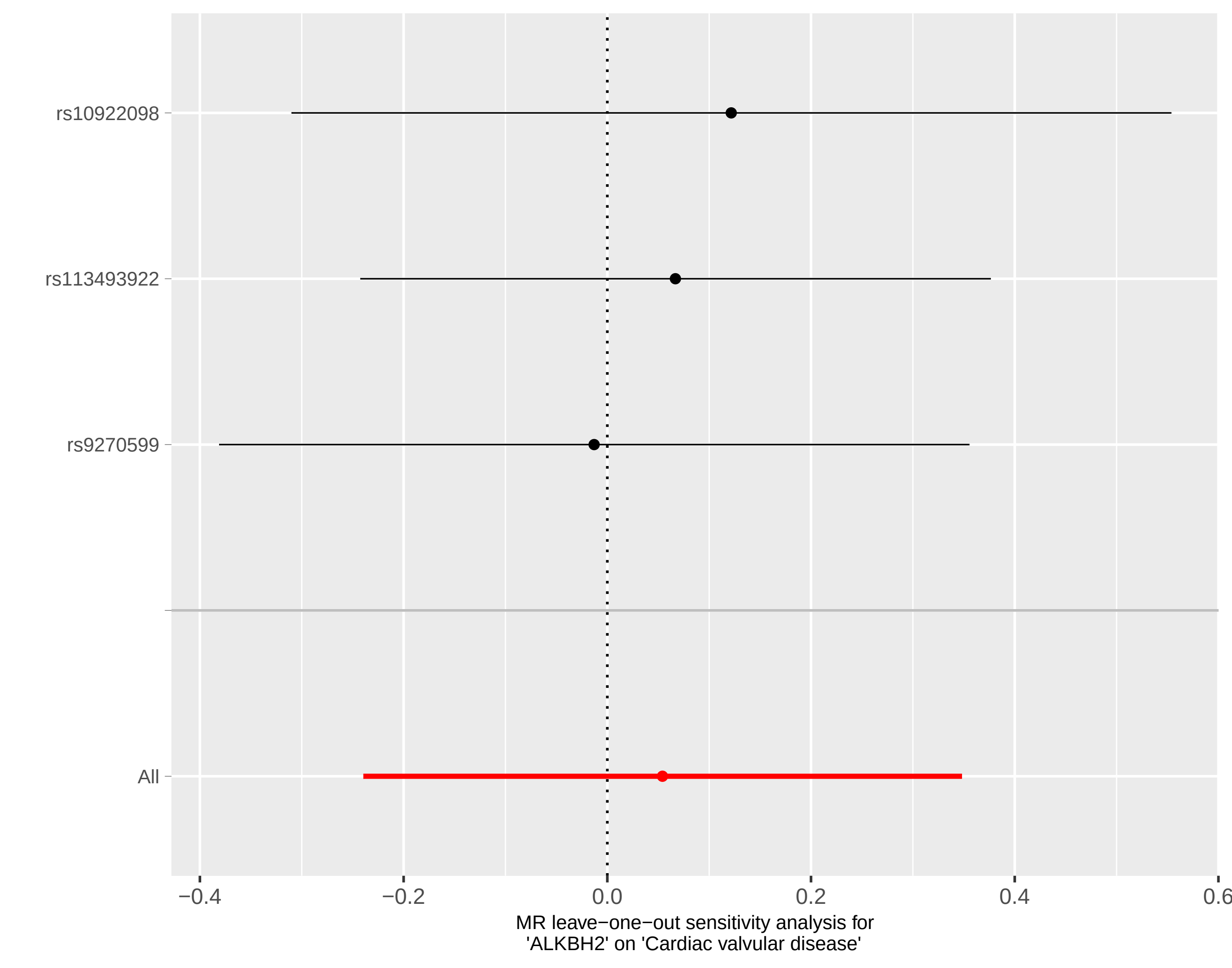

rs10922098
rs113493922
rs9270599
All
−0.4
−0.2
0.0
0.2
0.4
0.6
MR leave−one−out sensitivity analysis for
'ALKBH2' on 'Cardiac valvular disease'

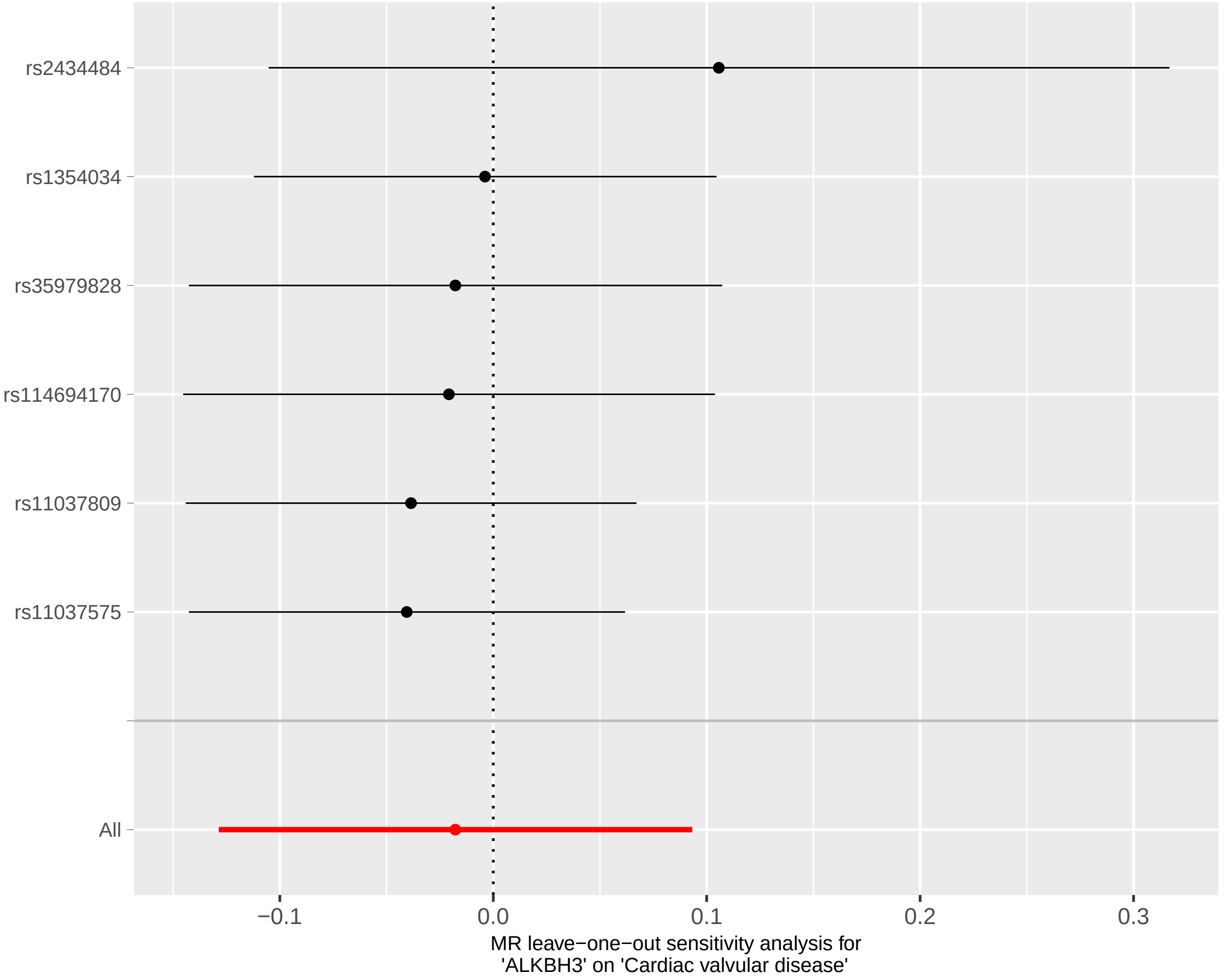
rs2434484
rs1354034
rs35979828
rs114694170
rs11037809
rs11037575
All
−0.1
0.0
0.1
0.2
0.3
MR leave−one−out sensitivity analysis for
'ALKBH3' on 'Cardiac valvular disease'

Insufficient number of SNPs

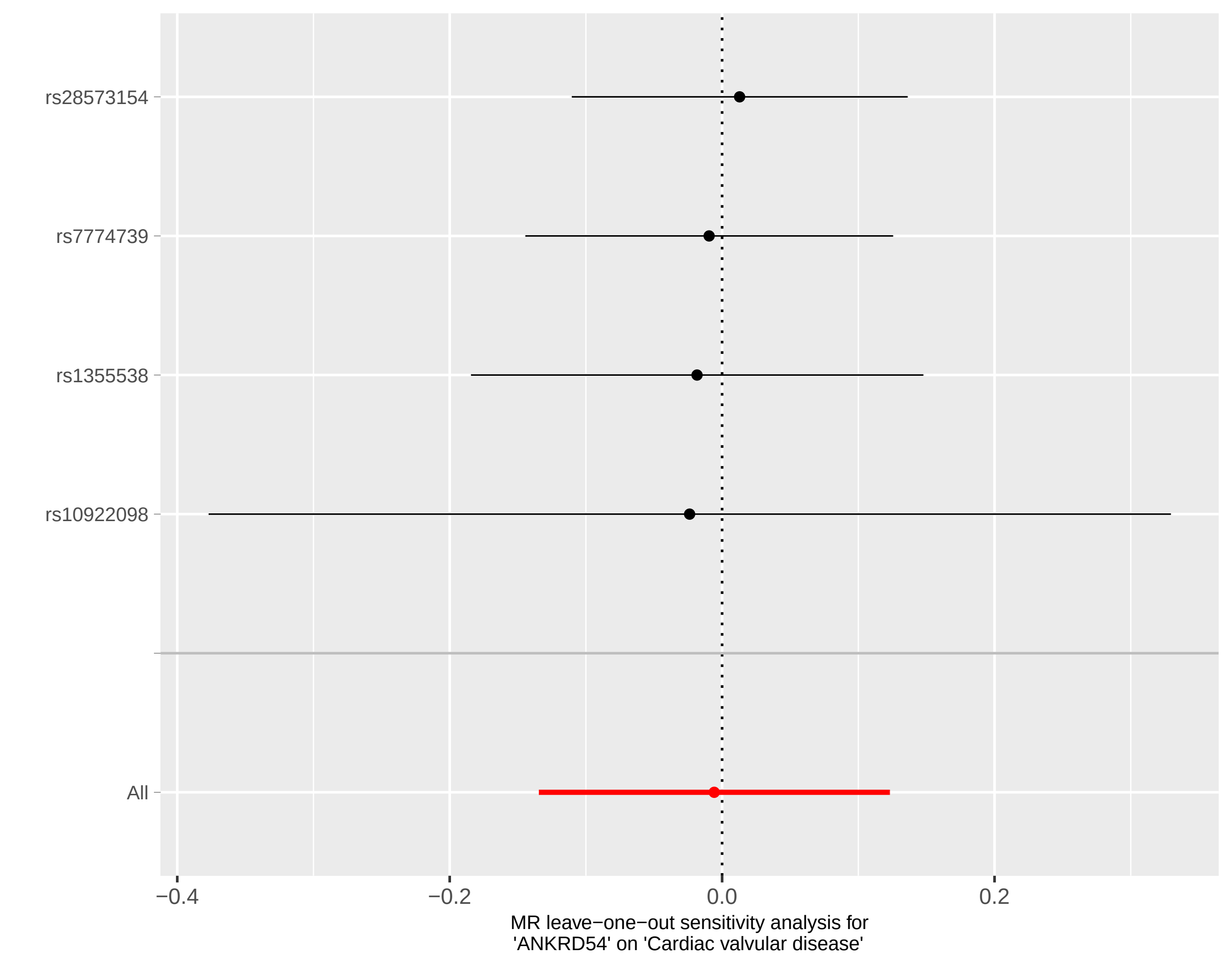
rs28573154
rs7774739
rs1355538
rs10922098
All
−0.4
−0.2
0.0
0.2
MR leave−one−out sensitivity analysis for
'ANKRD54' on 'Cardiac valvular disease'

Insufficient number of SNPs

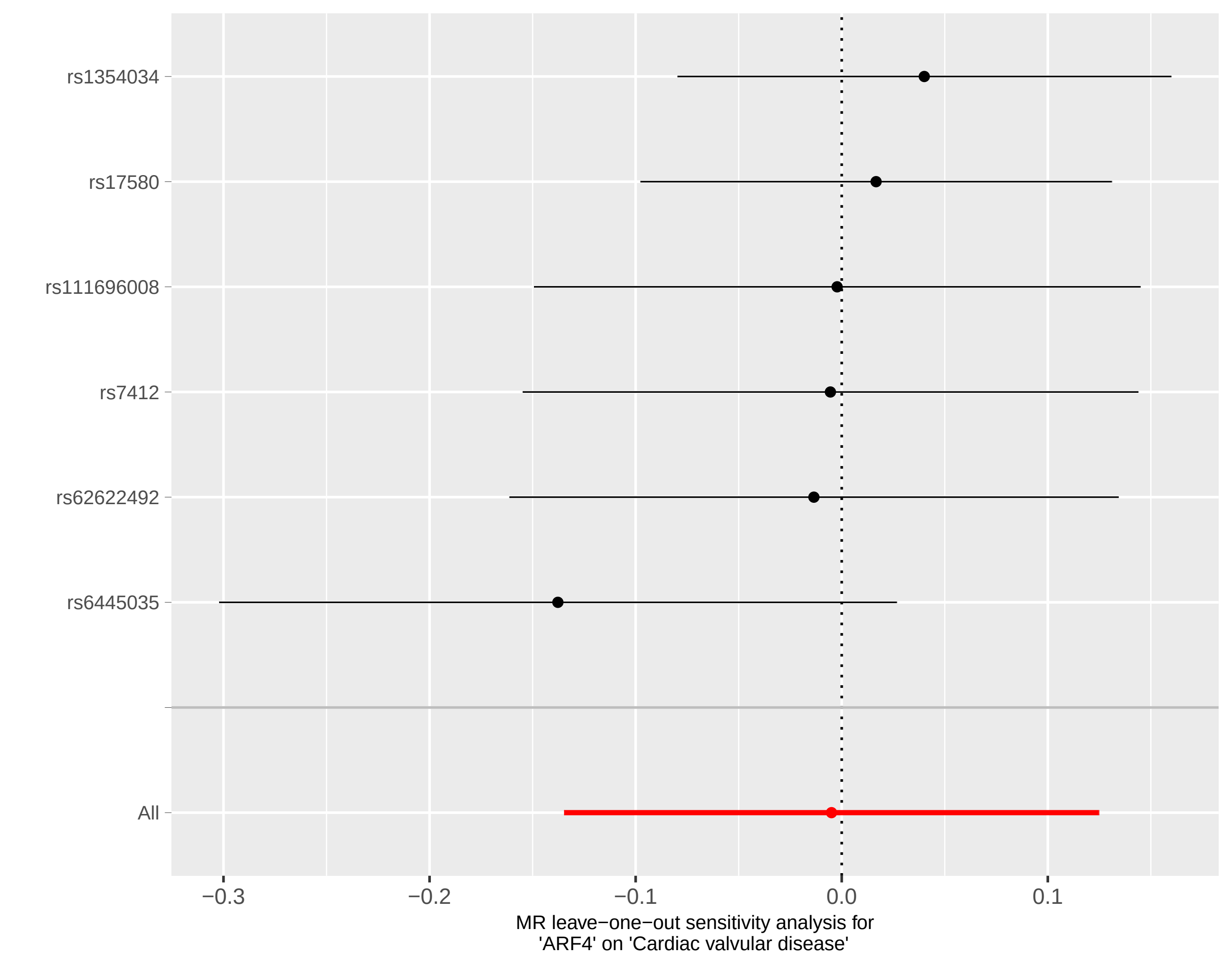

rs1354034
rs17580
rs111696008
rs7412
rs62622492
rs6445035
All
−0.3
−0.2
−0.1
0.0
0.1
MR leave−one−out sensitivity analysis for
'ARF4' on 'Cardiac valvular disease'

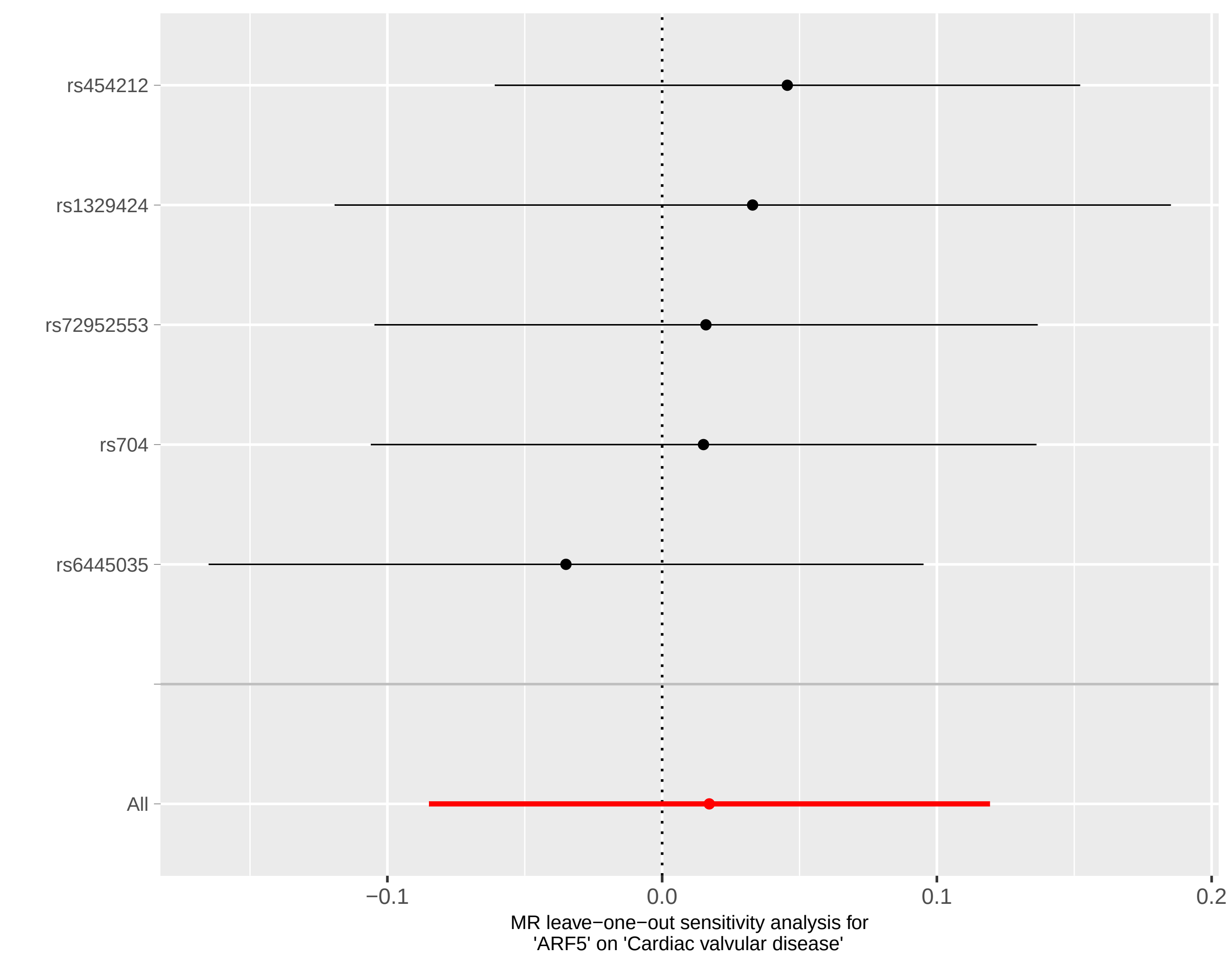

rs454212
rs1329424
rs72952553
rs704
rs6445035
All
−0.1
0.0
0.1
0.2
MR leave−one−out sensitivity analysis for
'ARF5' on 'Cardiac valvular disease'

Insufficient number of SNPs

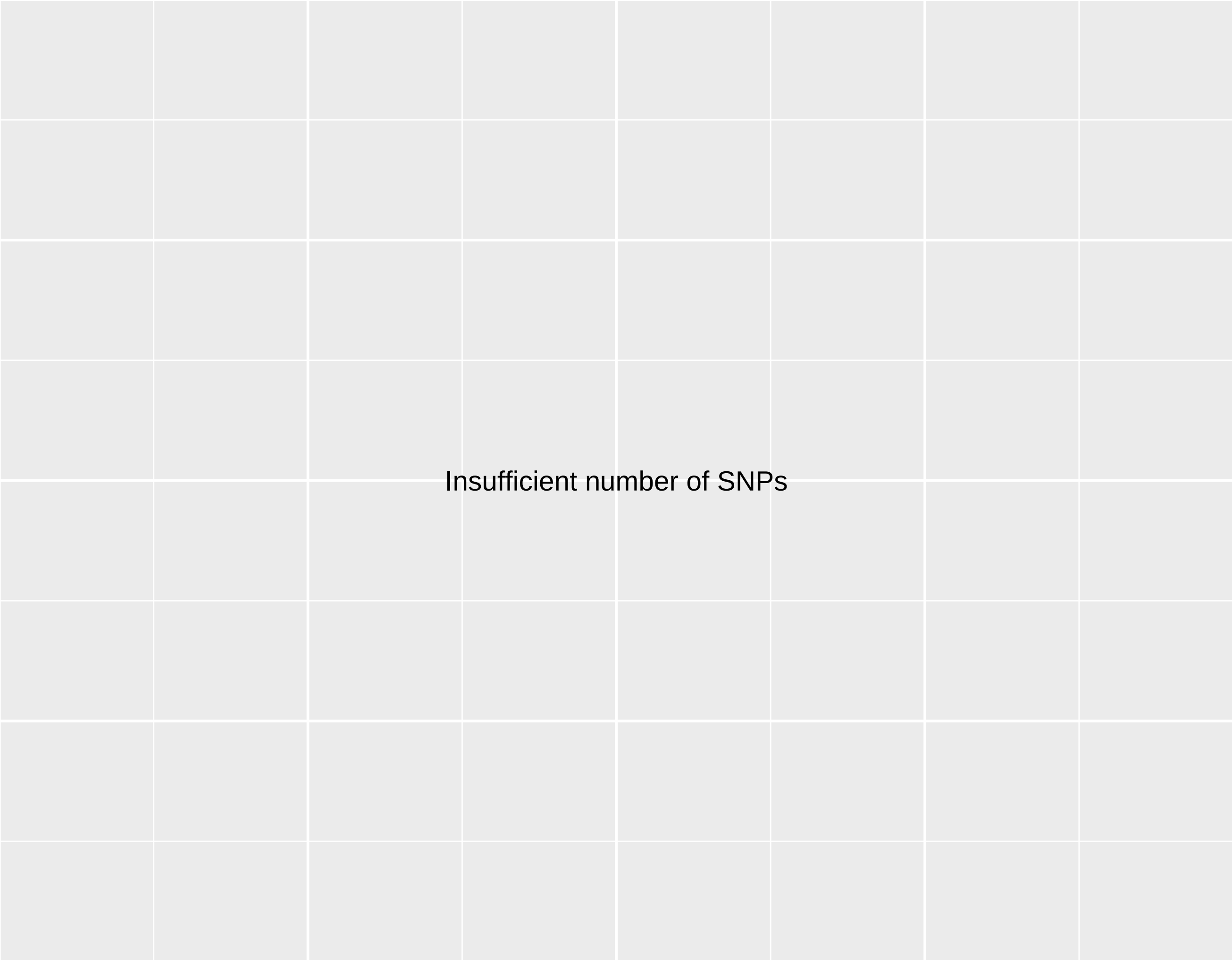
Insufficient number of SNPs

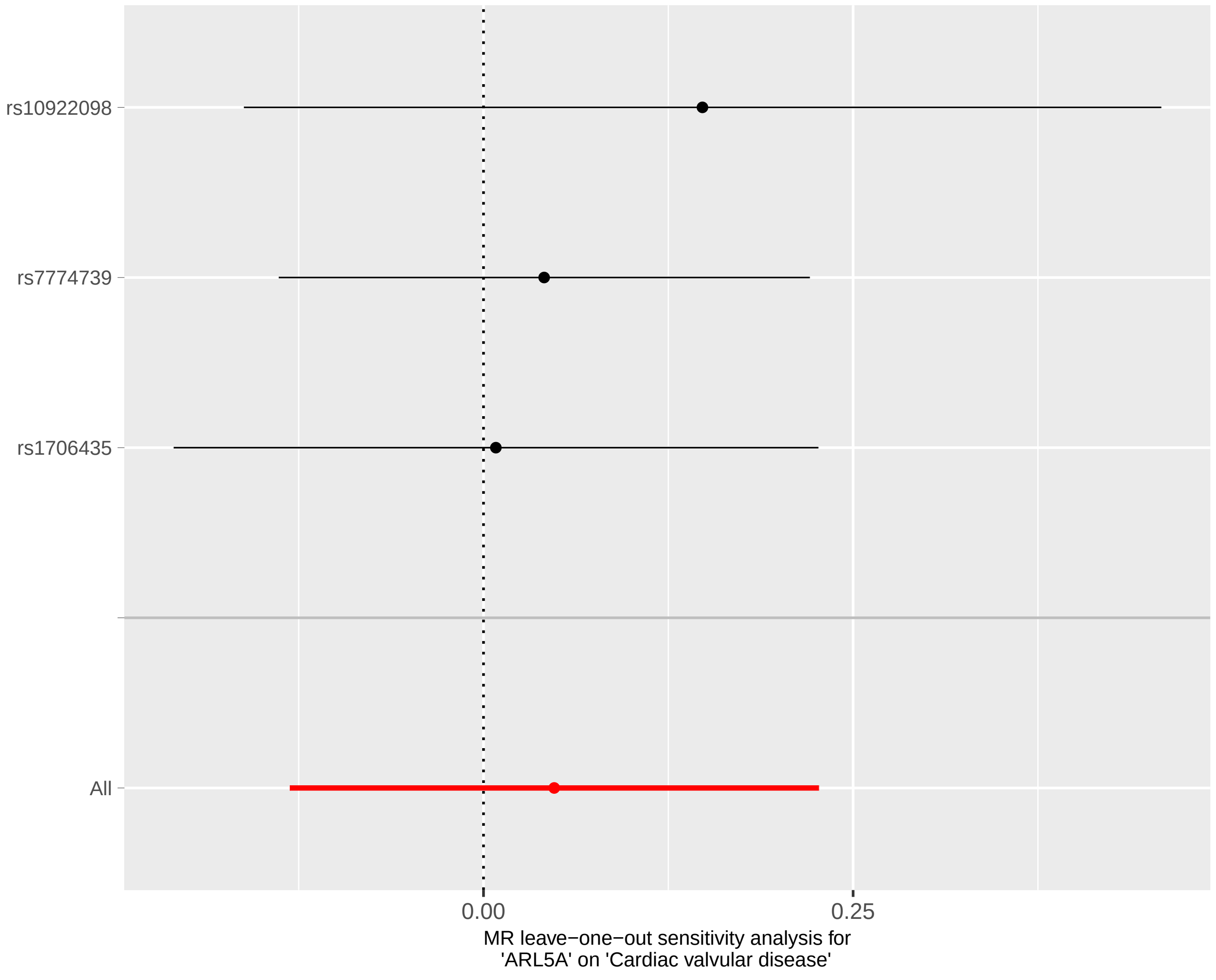
rs10922098
rs7774739
rs1706435
All
0.00
0.25
MR leave−one−out sensitivity analysis for
'ARL5A' on 'Cardiac valvular disease'

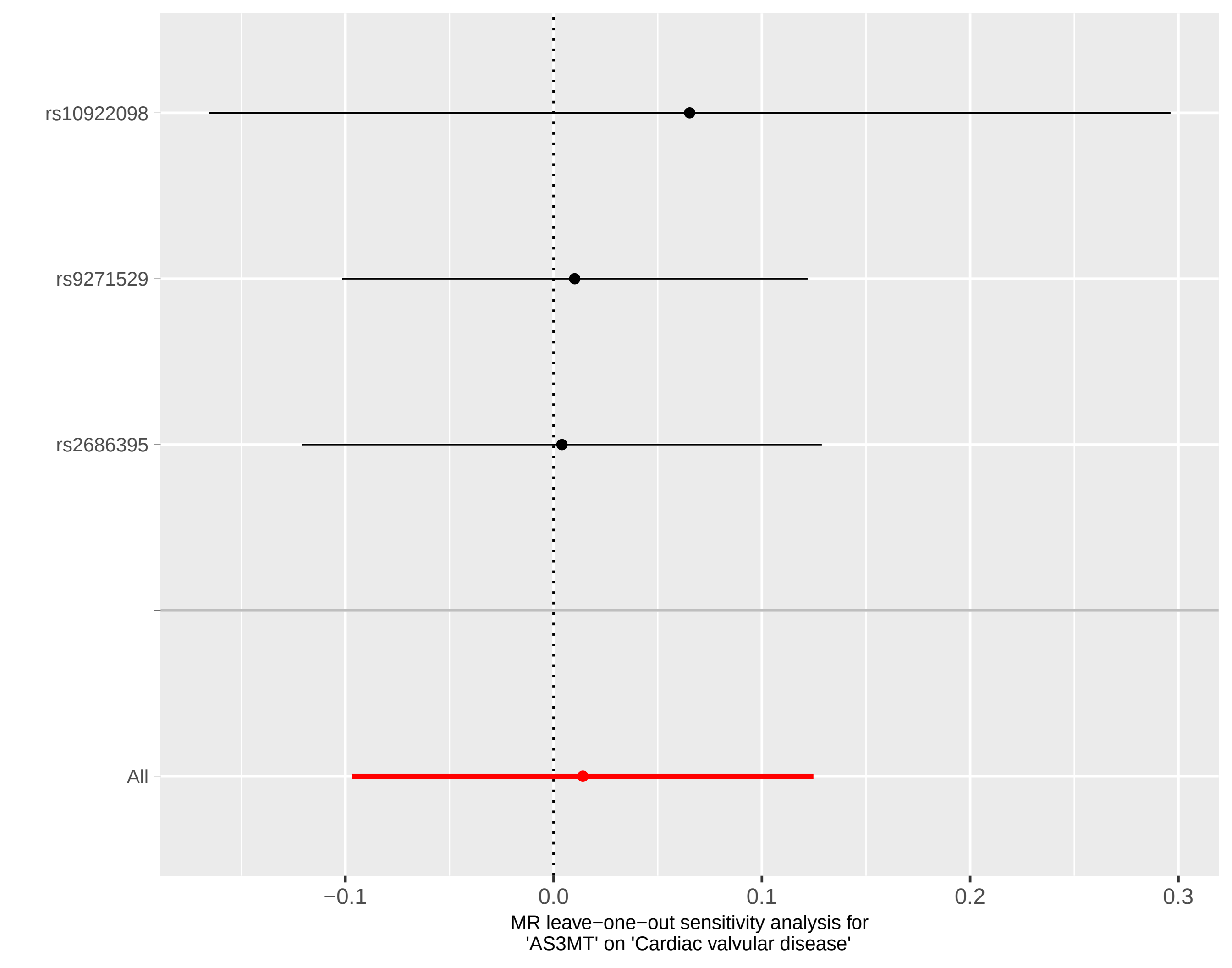

rs10922098
rs9271529
rs2686395
All
−0.1
0.0
0.1
0.2
0.3
MR leave−one−out sensitivity analysis for
'AS3MT' on 'Cardiac valvular disease'

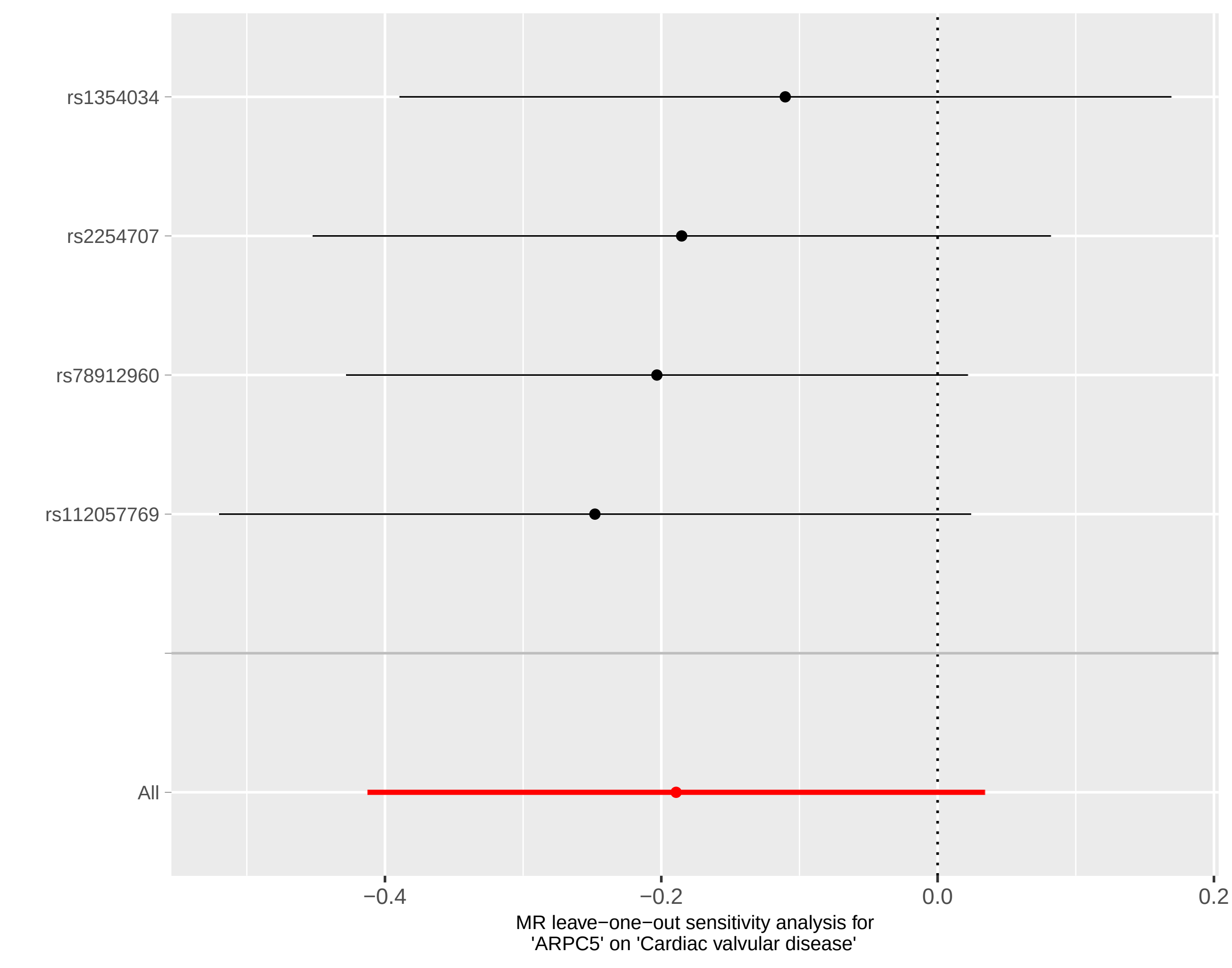

rs1354034
rs2254707
rs78912960
rs112057769
All
−0.4
−0.2
0.0
0.2
MR leave−one−out sensitivity analysis for
'ARPC5' on 'Cardiac valvular disease'

Insufficient number of SNPs

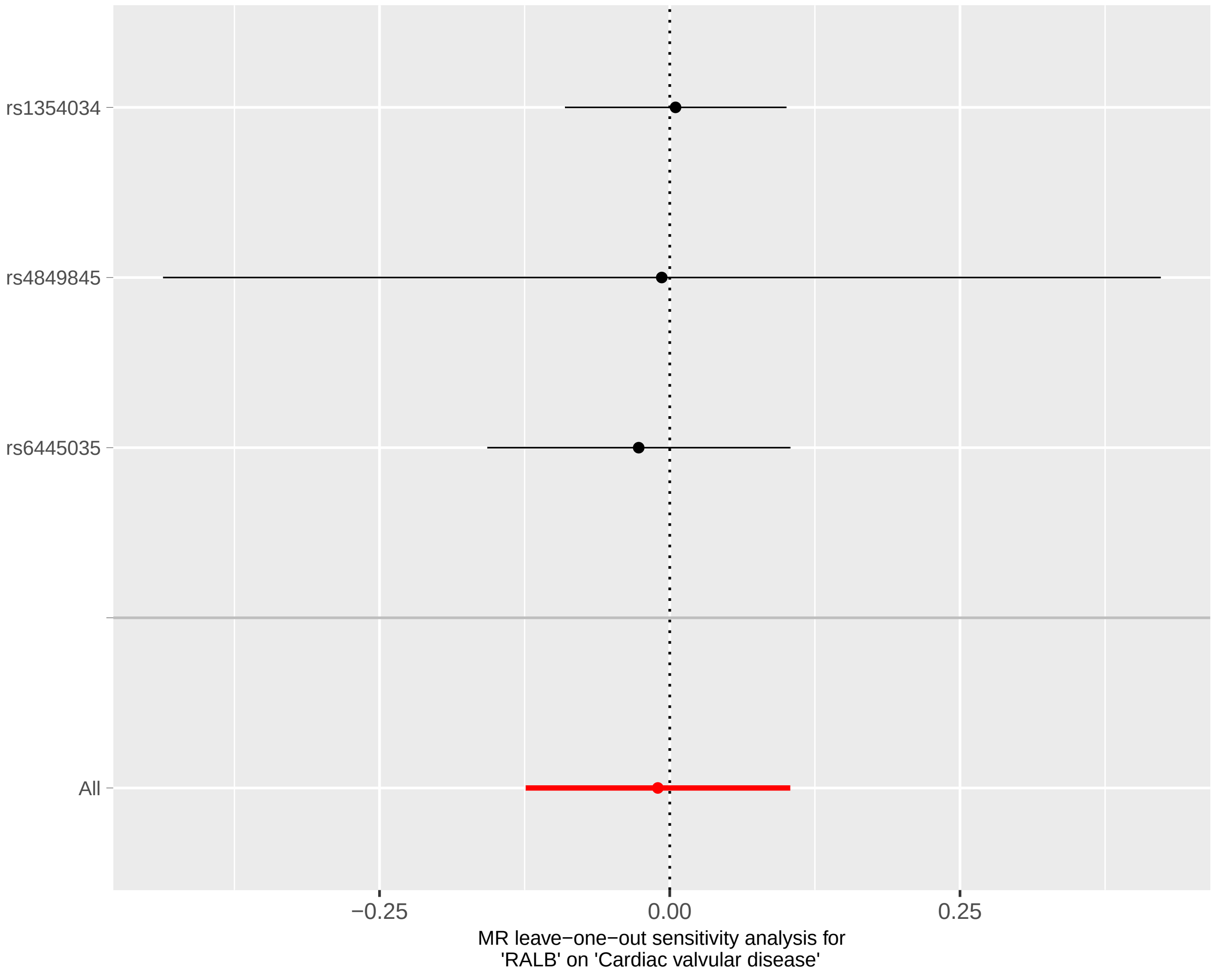
rs1354034
rs4849845
rs6445035
All
−0.25
0.00
0.25
MR leave−one−out sensitivity analysis for
'RALB' on 'Cardiac valvular disease'

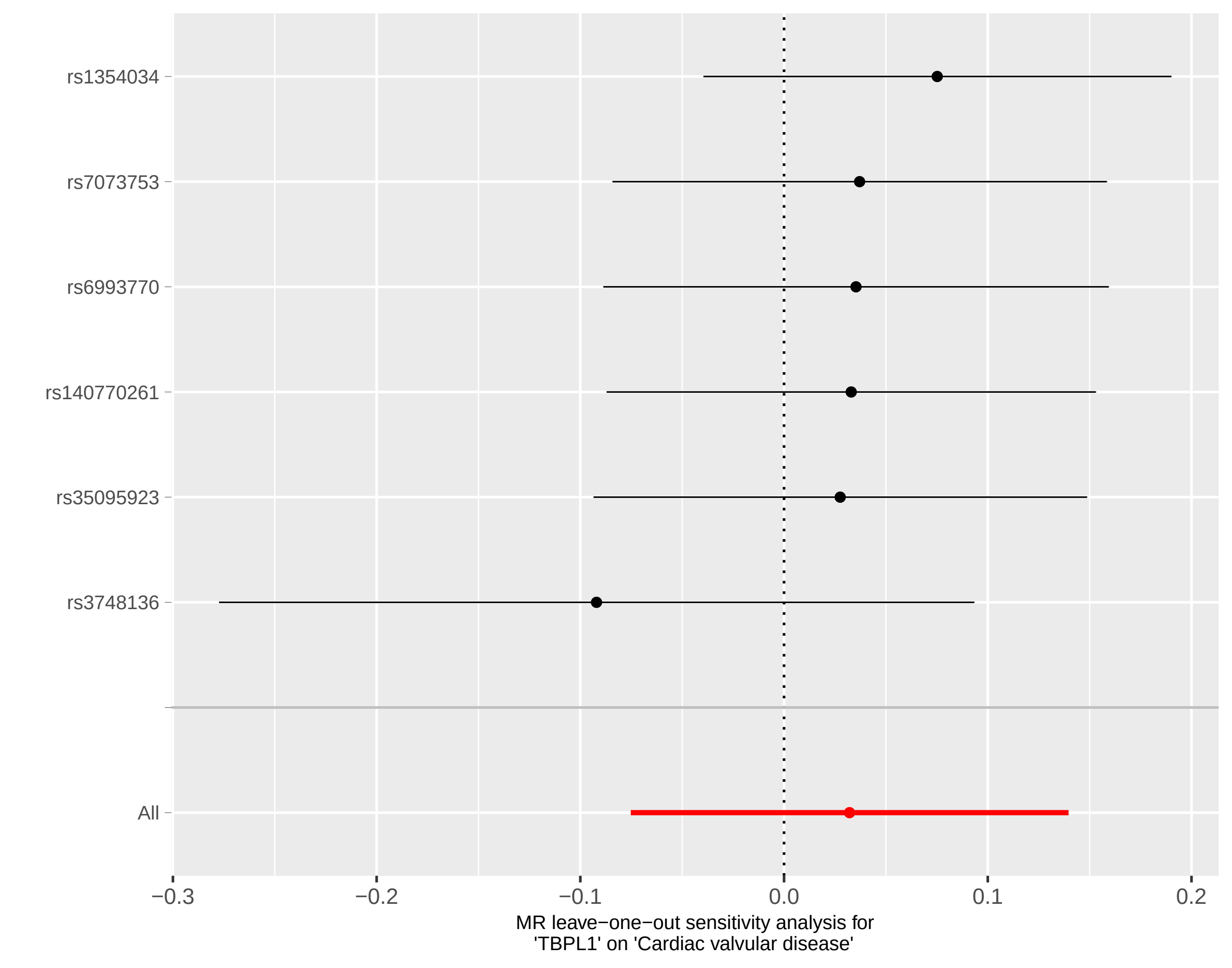

rs1354034
rs7073753
rs6993770
rs140770261
rs35095923
rs3748136
All
−0.3
−0.2
−0.1
0.0
0.1
0.2
MR leave−one−out sensitivity analysis for
'TBPL1' on 'Cardiac valvular disease'

Insufficient number of SNPs

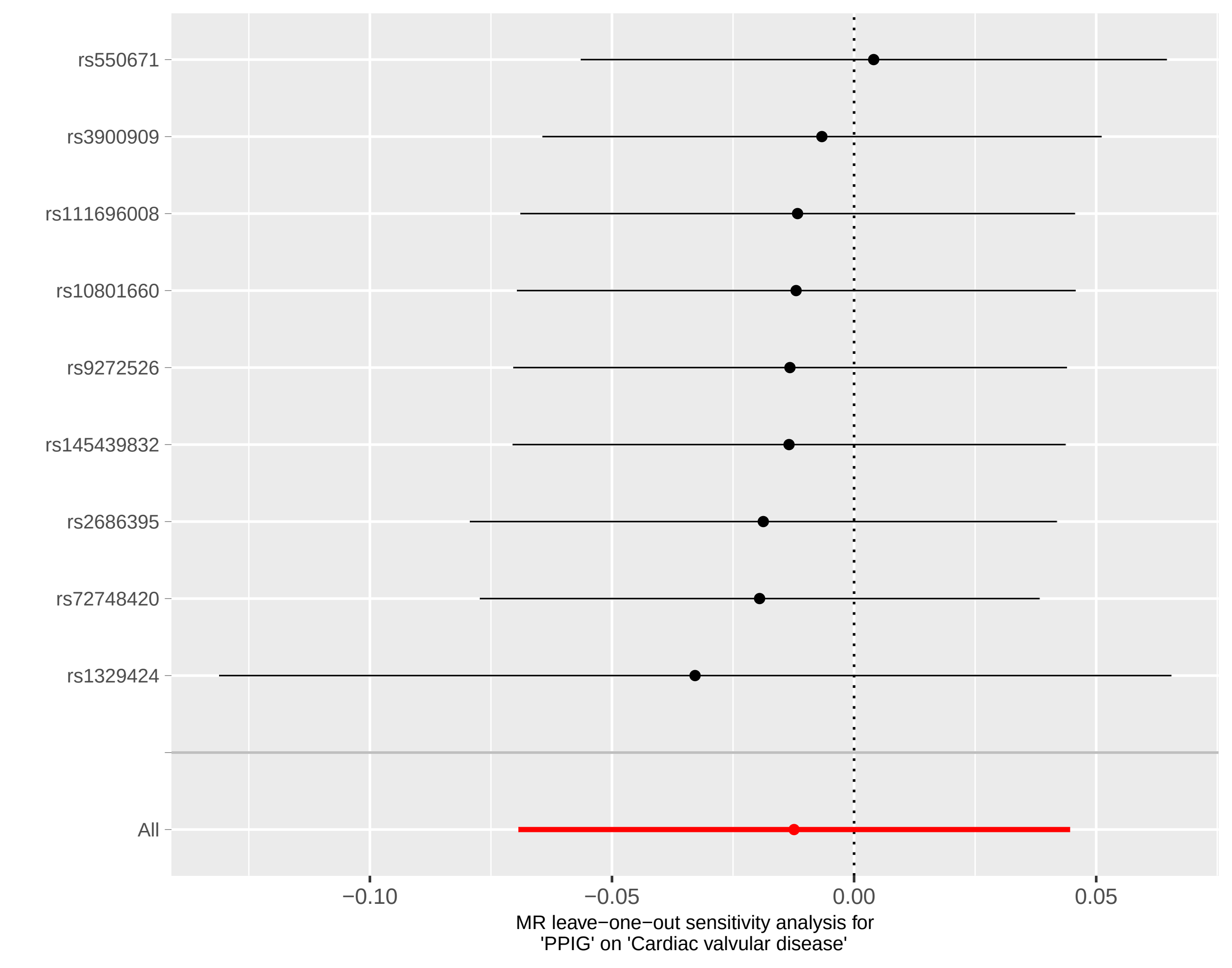
rs550671
rs3900909
rs111696008
rs10801660
rs9272526
rs145439832
rs2686395
rs72748420
rs1329424
All
−0.10
−0.05
0.00
0.05
MR leave−one−out sensitivity analysis for
'PPIG' on 'Cardiac valvular disease'

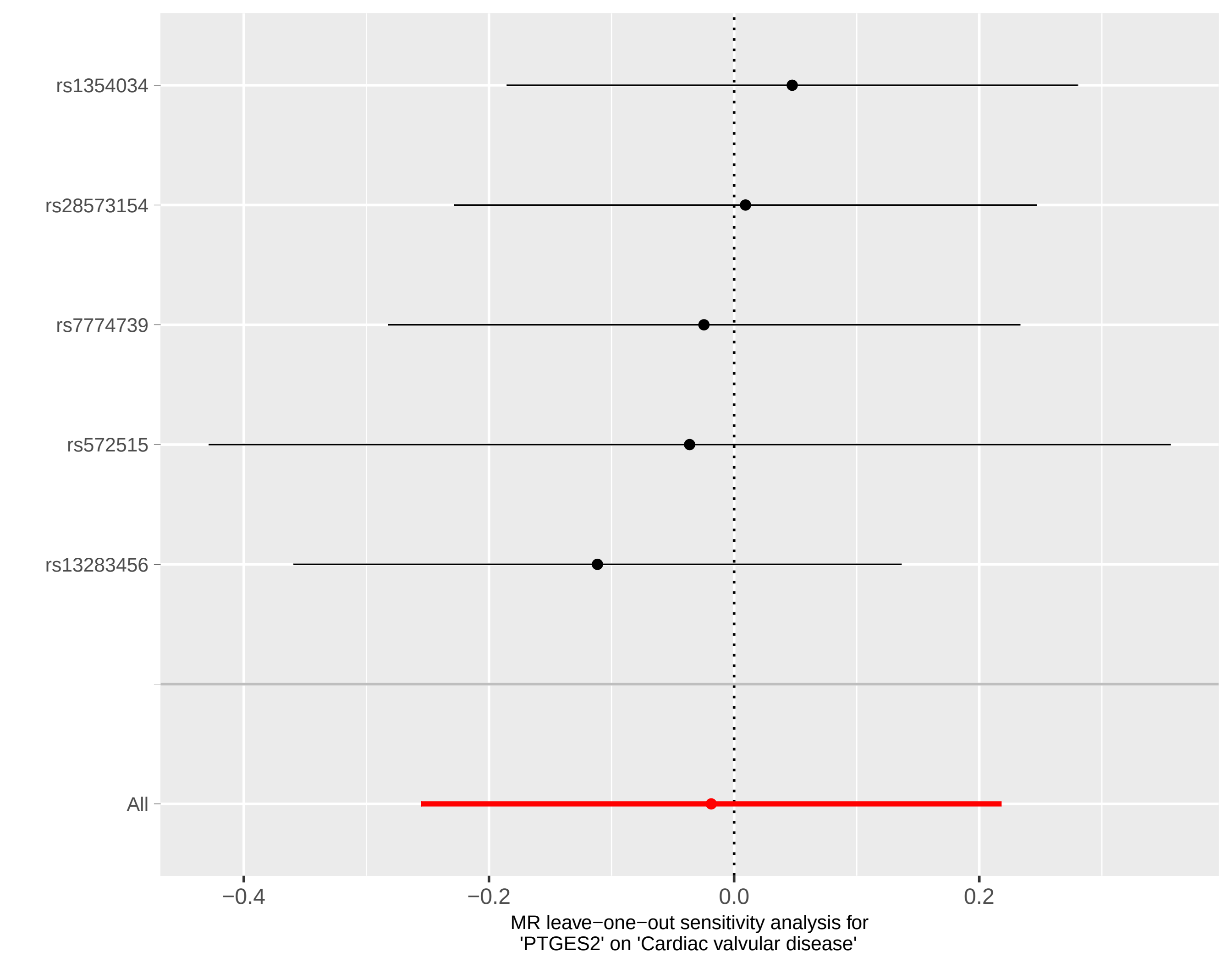
rs1354034
rs28573154
rs7774739
rs572515
rs13283456
All
−0.4
−0.2
0.0
0.2
MR leave−one−out sensitivity analysis for
'PTGES2' on 'Cardiac valvular disease'

Insufficient number of SNPs

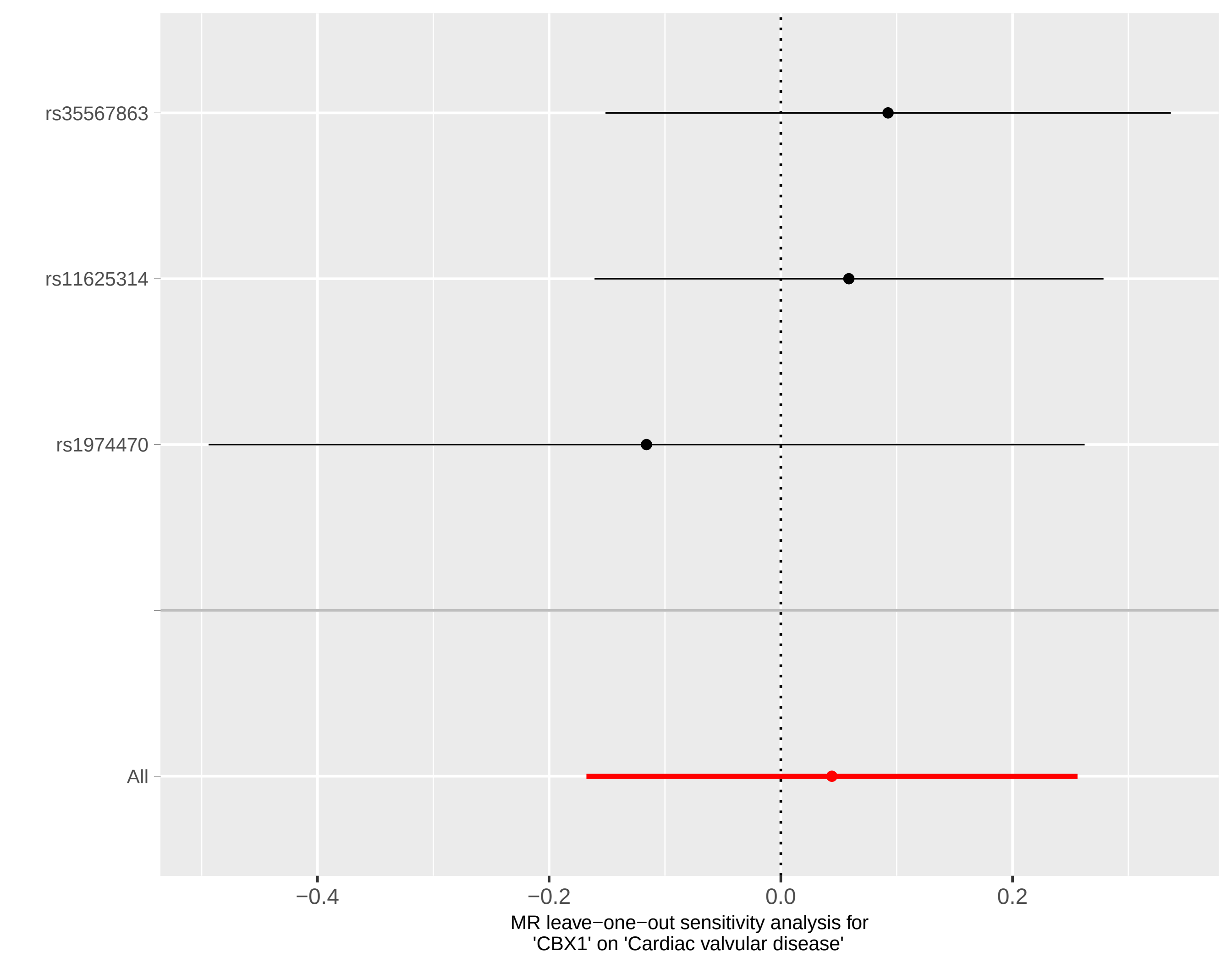
rs35567863
rs11625314
rs1974470
All
−0.4
−0.2
0.0
0.2
MR leave−one−out sensitivity analysis for
'CBX1' on 'Cardiac valvular disease'

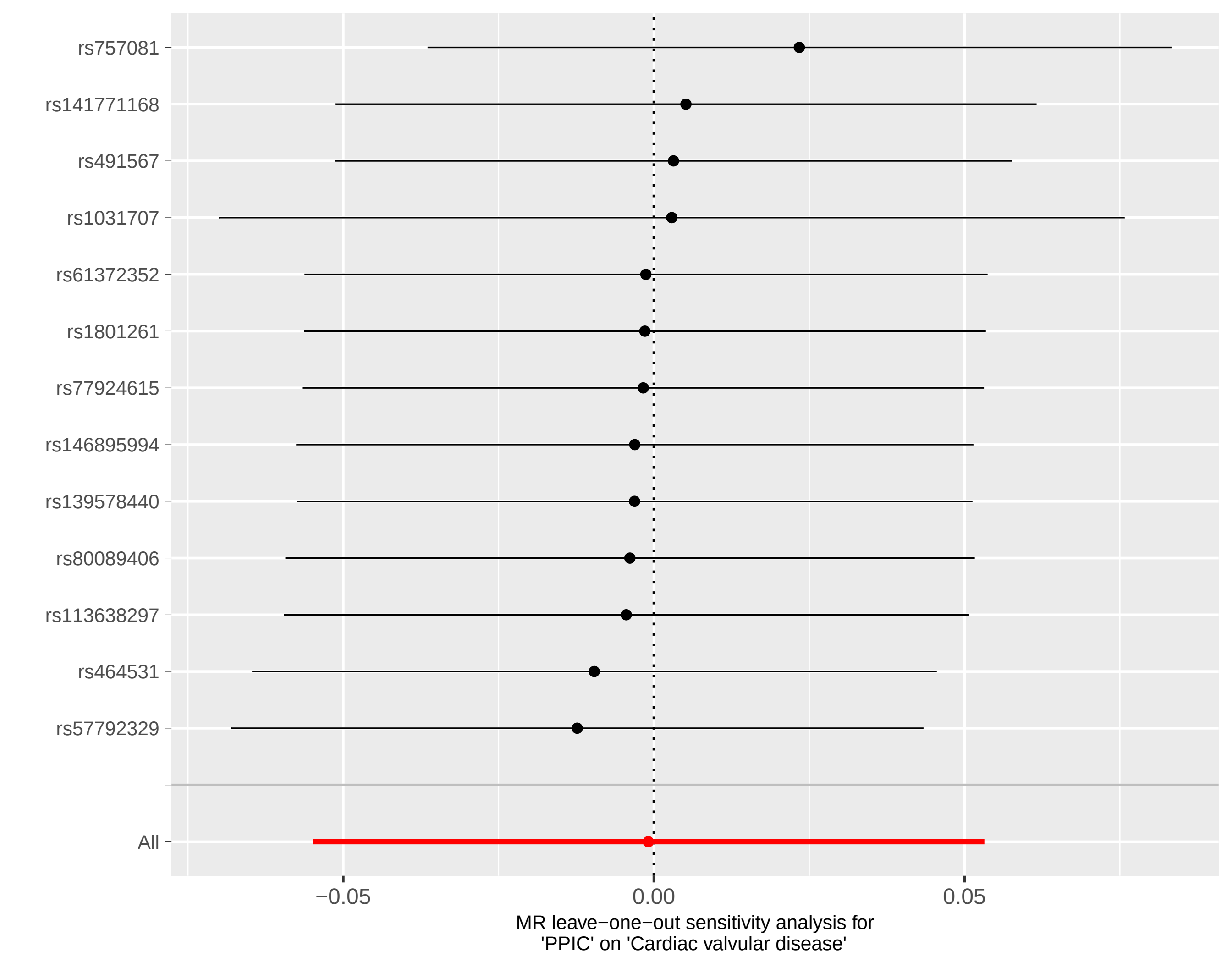
rs757081
rs141771168
rs491567
rs1031707
rs61372352
rs1801261
rs77924615
rs146895994
rs139578440
rs80089406
rs113638297
rs464531
rs57792329
All
−0.05
0.00
0.05
MR leave−one−out sensitivity analysis for
'PPIC' on 'Cardiac valvular disease'

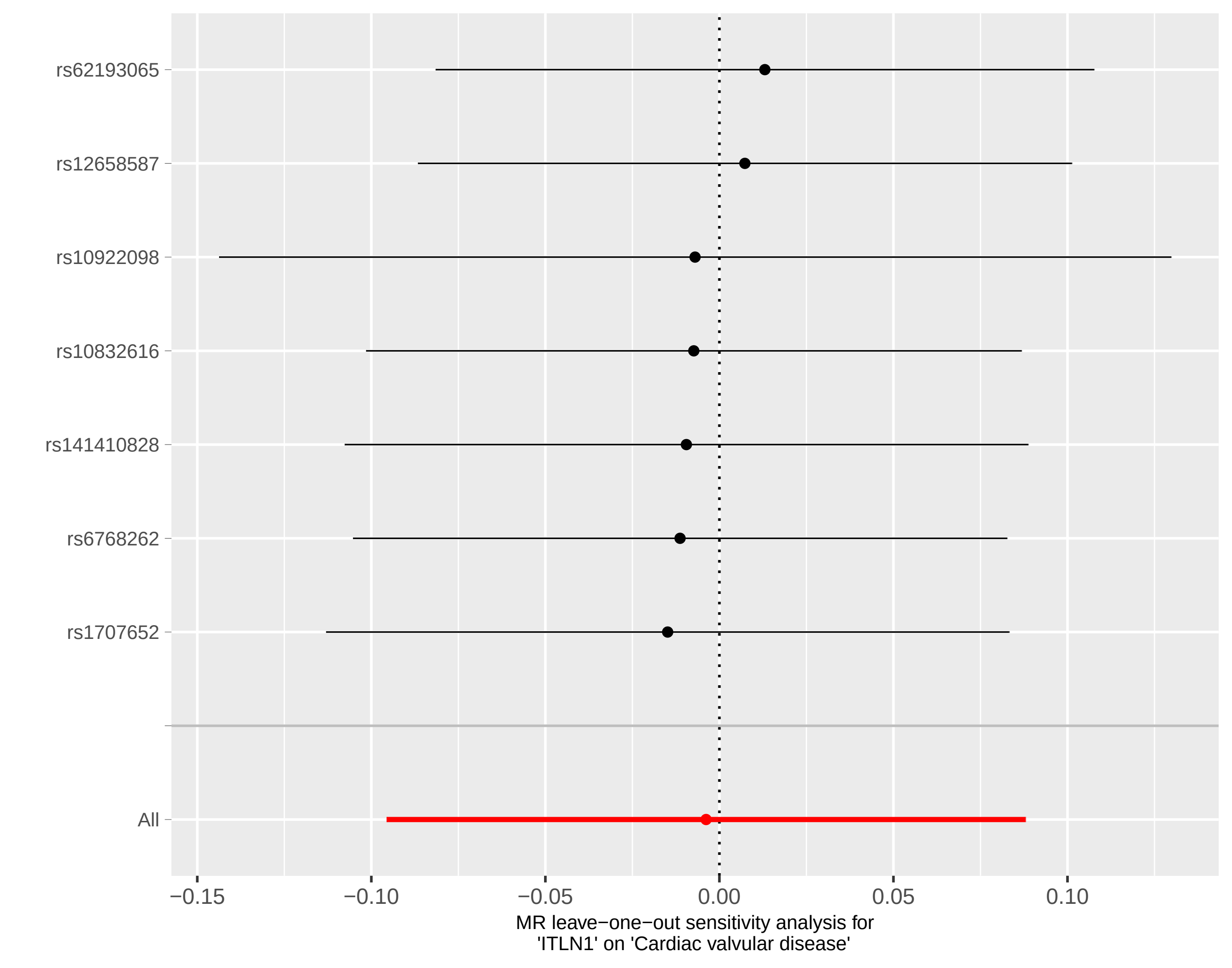

rs62193065
rs12658587
rs10922098
rs10832616
rs141410828
rs6768262
rs1707652
All
−0.15
−0.10
−0.05
0.00
0.05
0.10
MR leave−one−out sensitivity analysis for
'ITLN1' on 'Cardiac valvular disease'

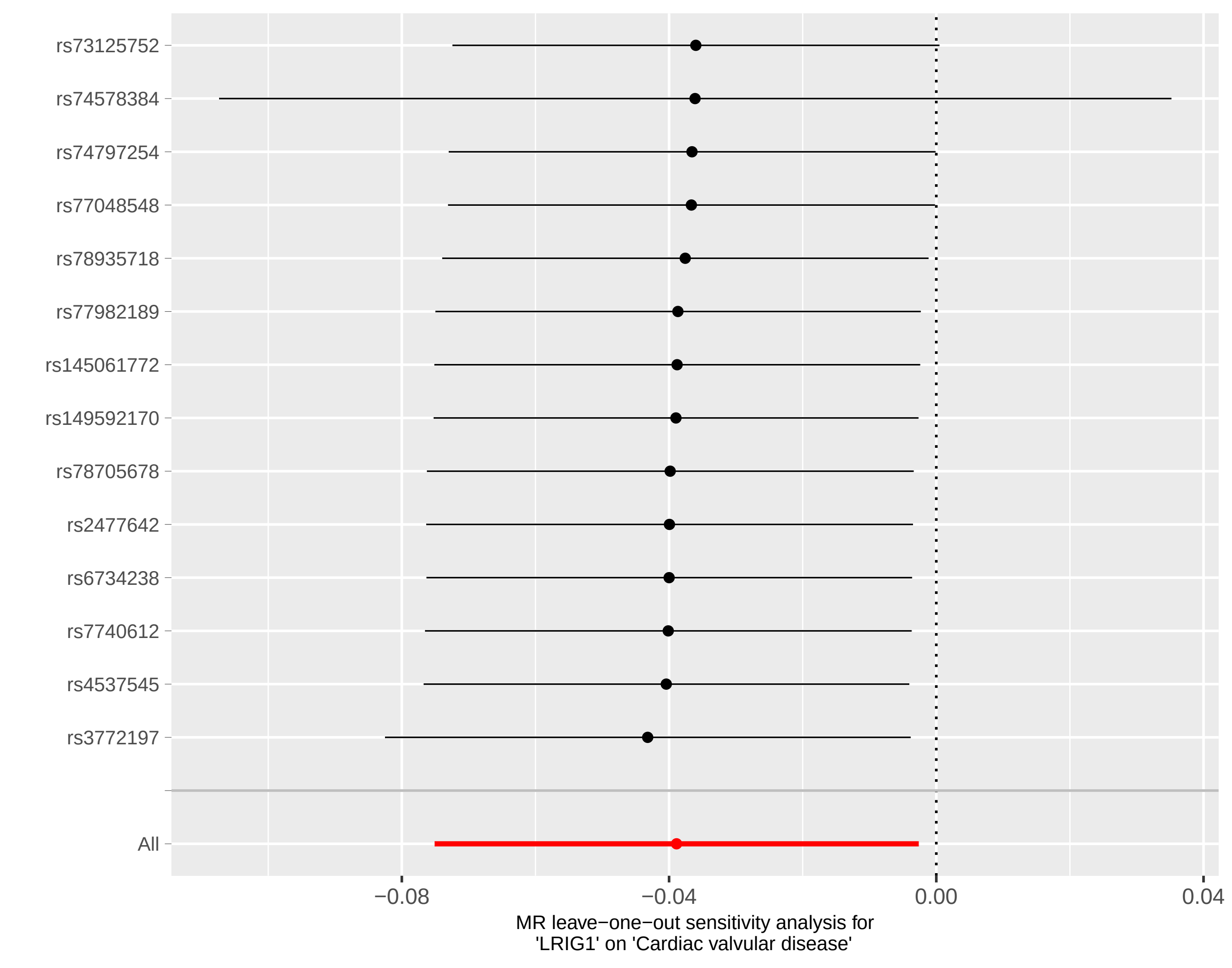
rs73125752
rs74578384
rs74797254
rs77048548
rs78935718
rs77982189
rs145061772
rs149592170
rs78705678
rs2477642
rs6734238
rs7740612
rs4537545
rs3772197
All
−0.08
−0.04
0.00
0.04
MR leave−one−out sensitivity analysis for
'LRIG1' on 'Cardiac valvular disease'

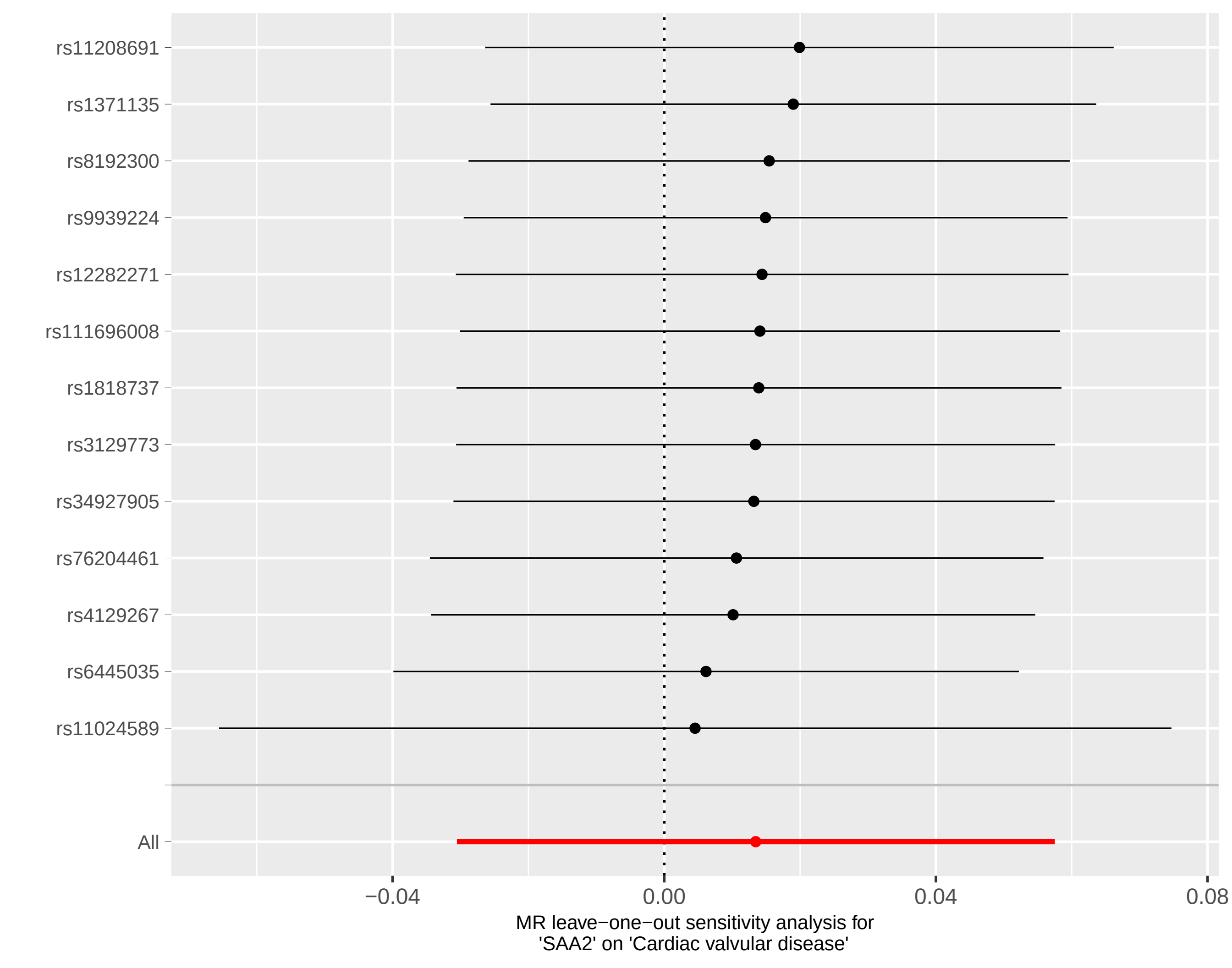
rs11208691
rs1371135
rs8192300
rs9939224
rs12282271
rs111696008
rs1818737
rs3129773
rs34927905
rs76204461
rs4129267
rs6445035
rs11024589
All
−0.04
0.00
0.04
0.08
MR leave−one−out sensitivity analysis for
'SAA2' on 'Cardiac valvular disease'

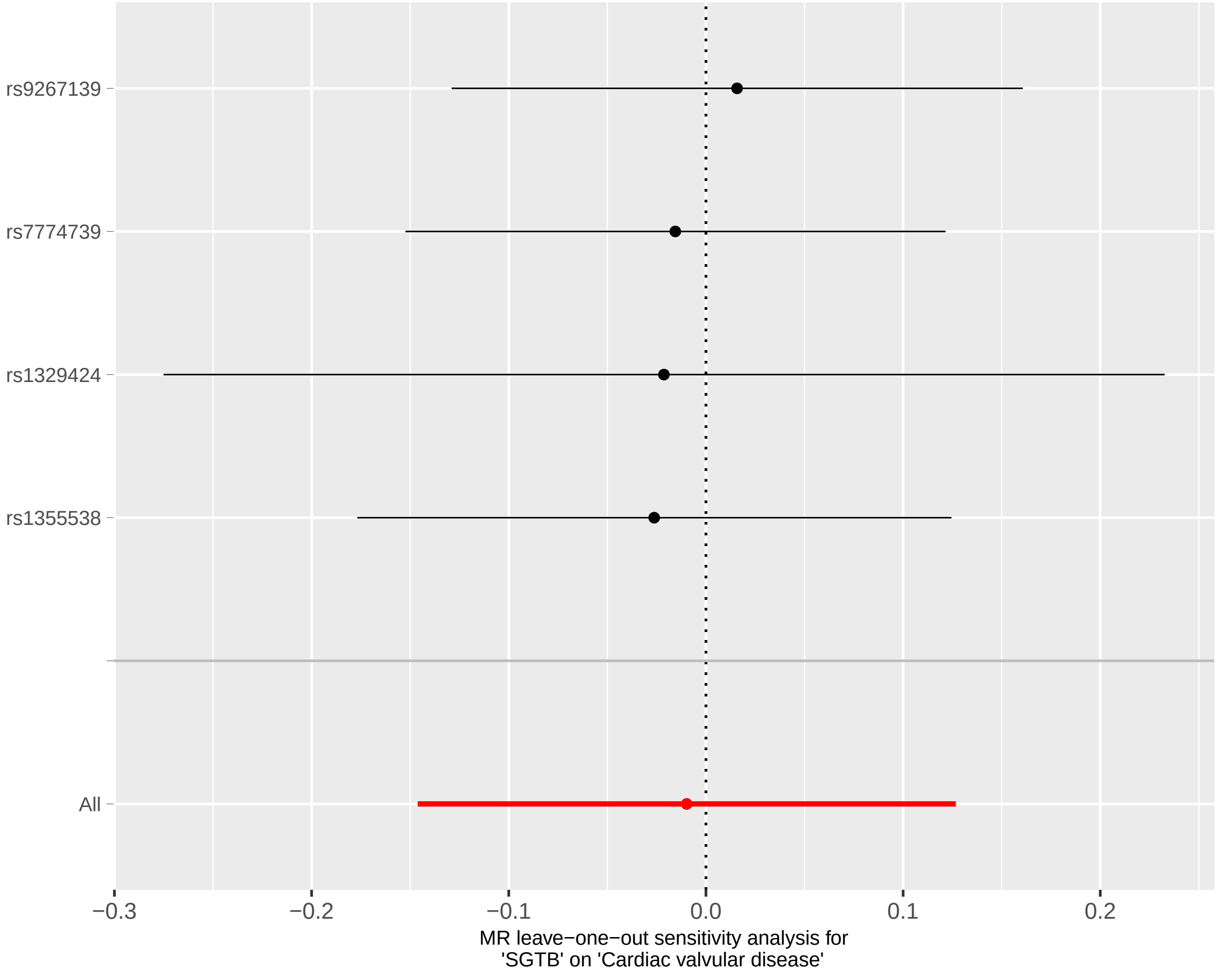
rs9267139
rs7774739
rs1329424
rs1355538
All
−0.3
−0.2
−0.1
0.0
0.1
0.2
MR leave−one−out sensitivity analysis for
'SGTB' on 'Cardiac valvular disease'

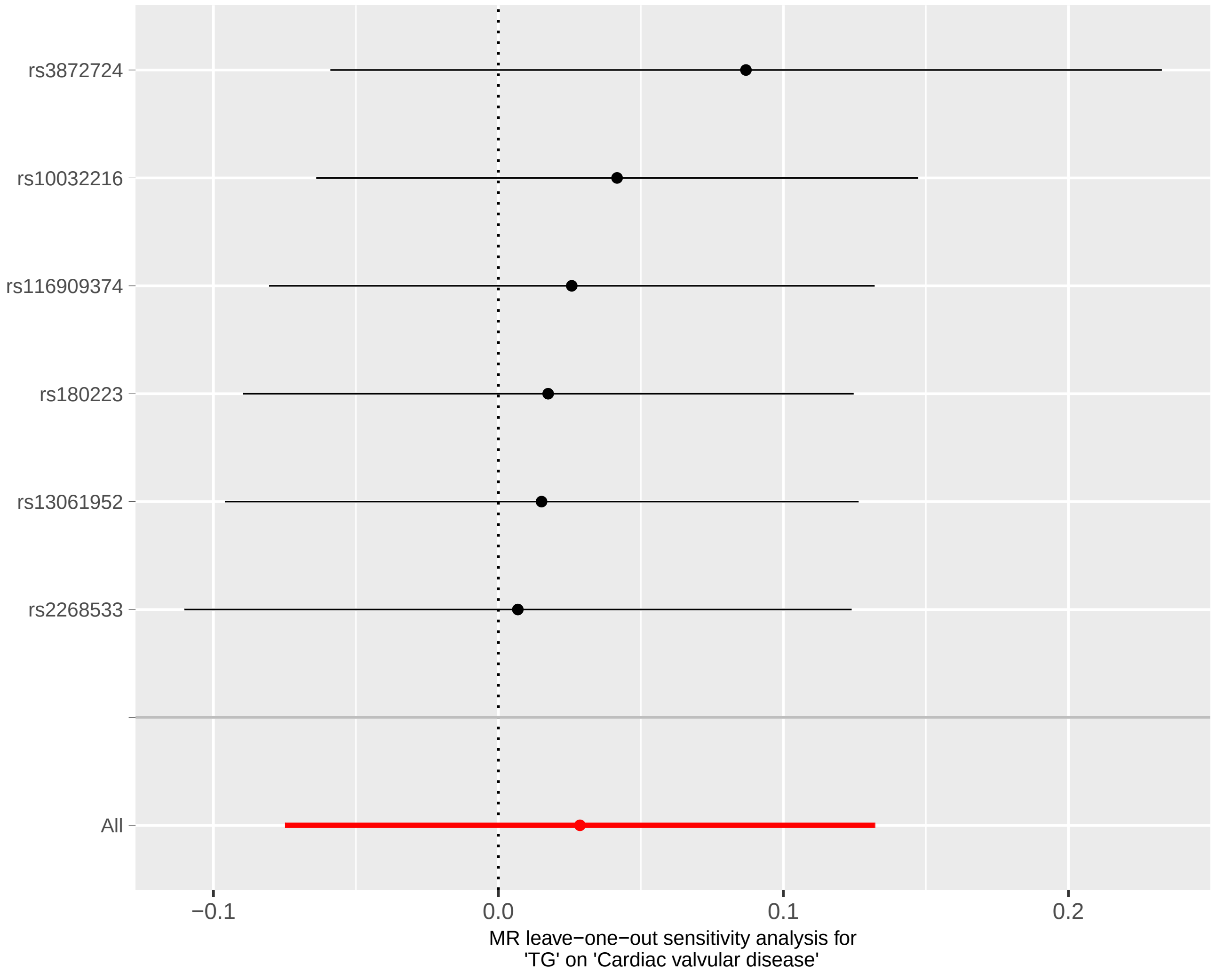
rs3872724
rs10032216
rs116909374
rs180223
rs13061952
rs2268533
All
−0.1
0.0
0.1
0.2
MR leave−one−out sensitivity analysis for
'TG' on 'Cardiac valvular disease'

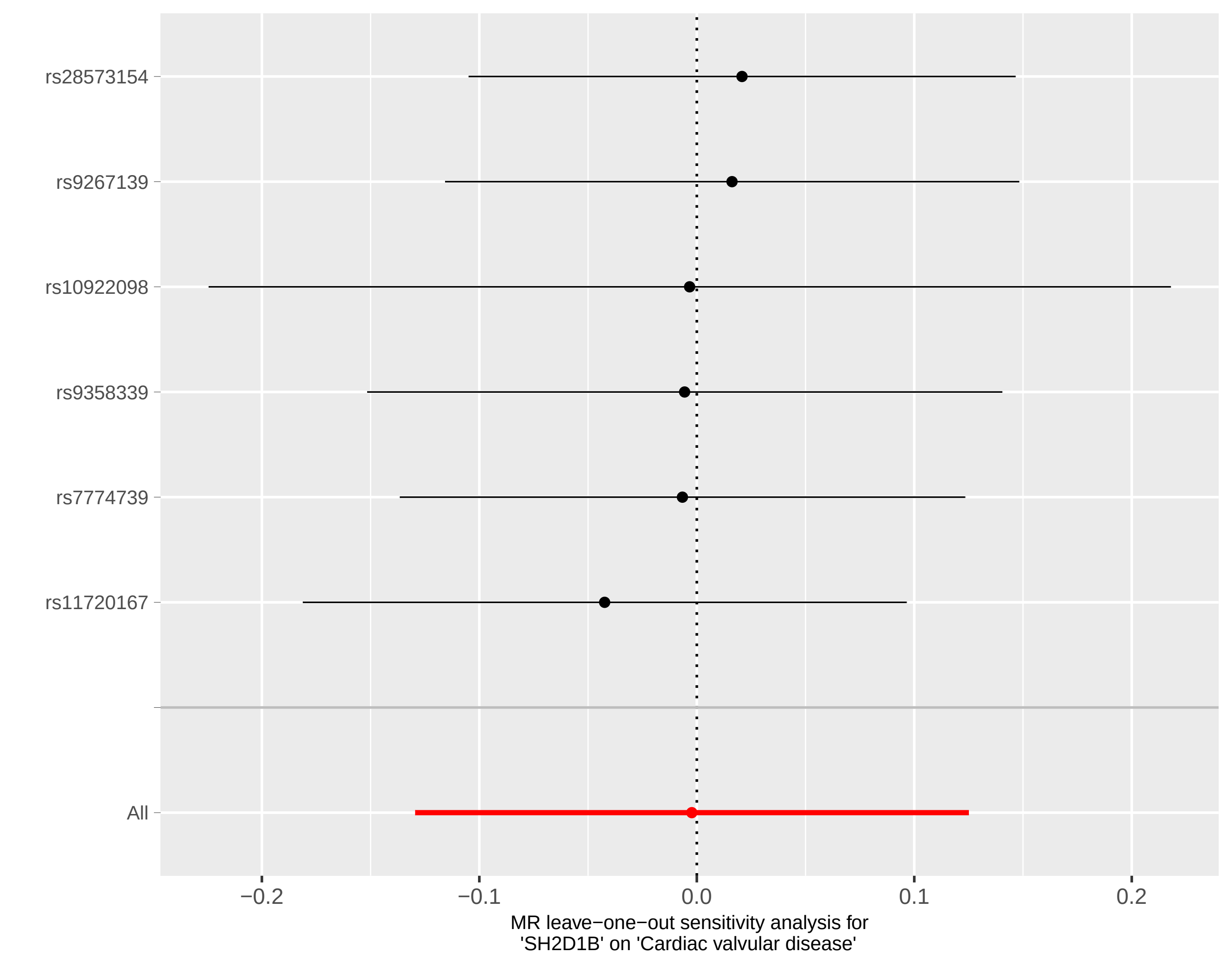

rs28573154
rs9267139
rs10922098
rs9358339
rs7774739
rs11720167
All
−0.2
−0.1
0.0
0.1
0.2
MR leave−one−out sensitivity analysis for
'SH2D1B' on 'Cardiac valvular disease'

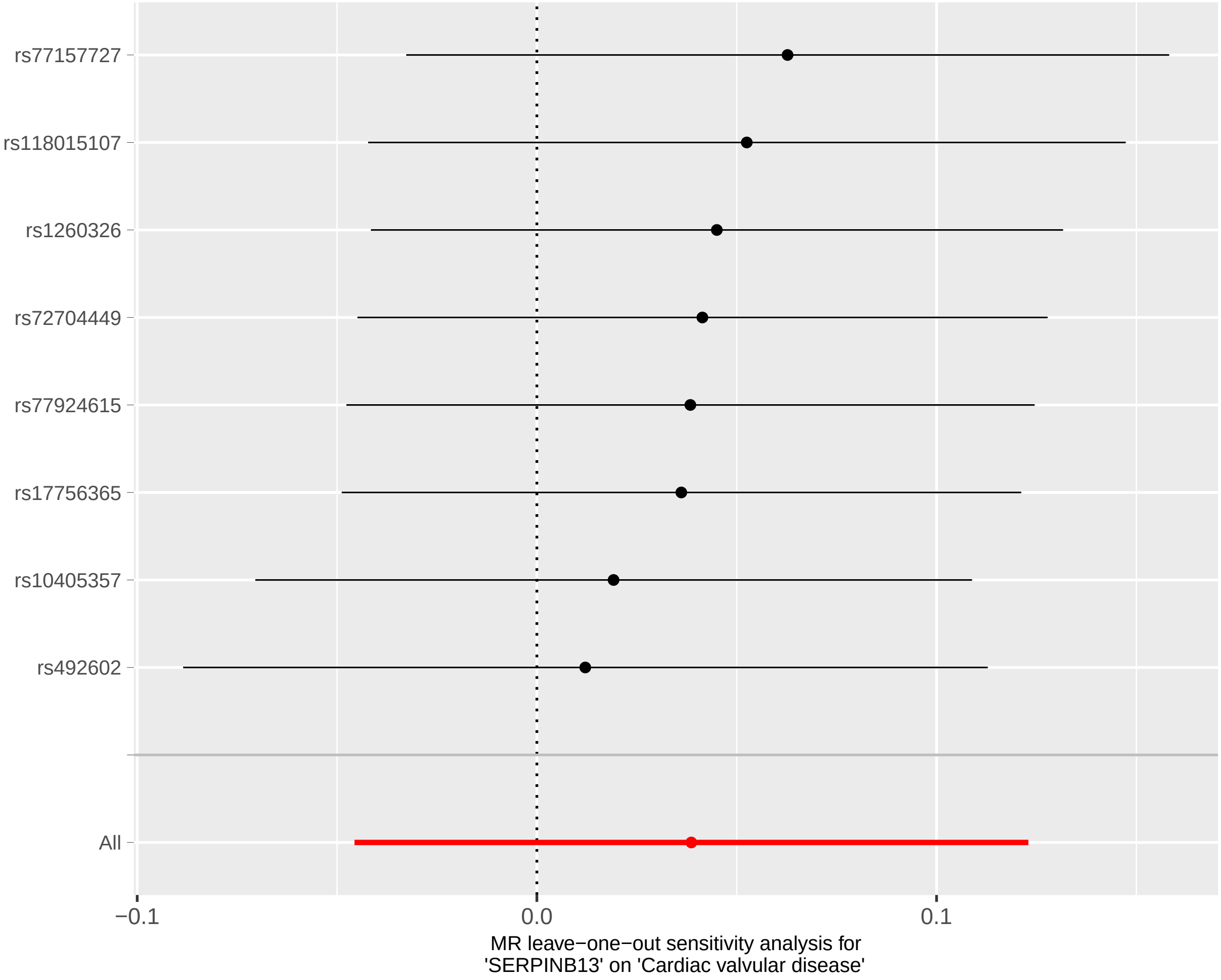

rs77157727
rs118015107
rs1260326
rs72704449
rs77924615
rs17756365
rs10405357
rs492602
All
−0.1
0.0
0.1
MR leave−one−out sensitivity analysis for
'SERPINB13' on 'Cardiac valvular disease'

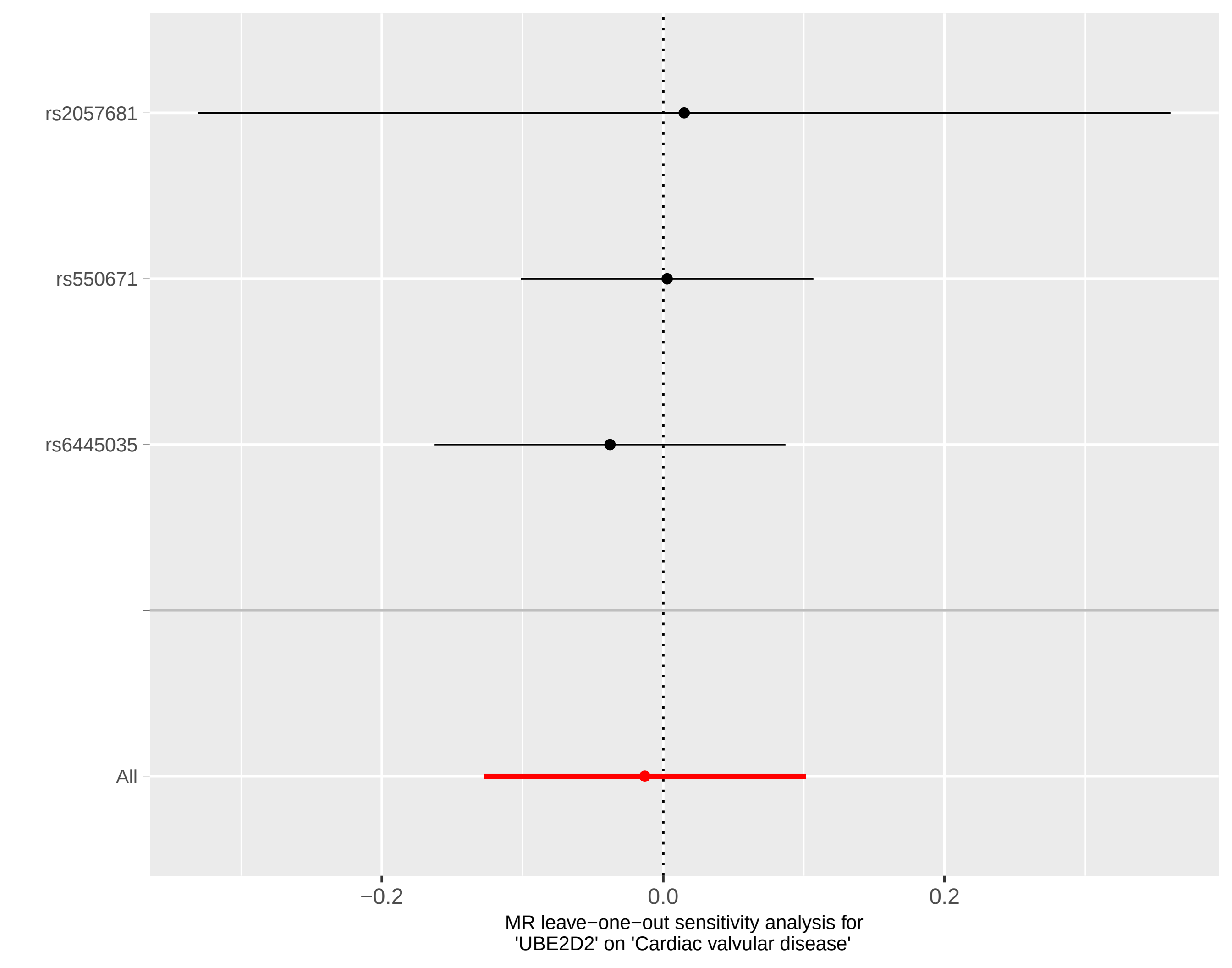
rs2057681
rs550671
rs6445035
All
−0.2
0.0
0.2
MR leave−one−out sensitivity analysis for
'UBE2D2' on 'Cardiac valvular disease'

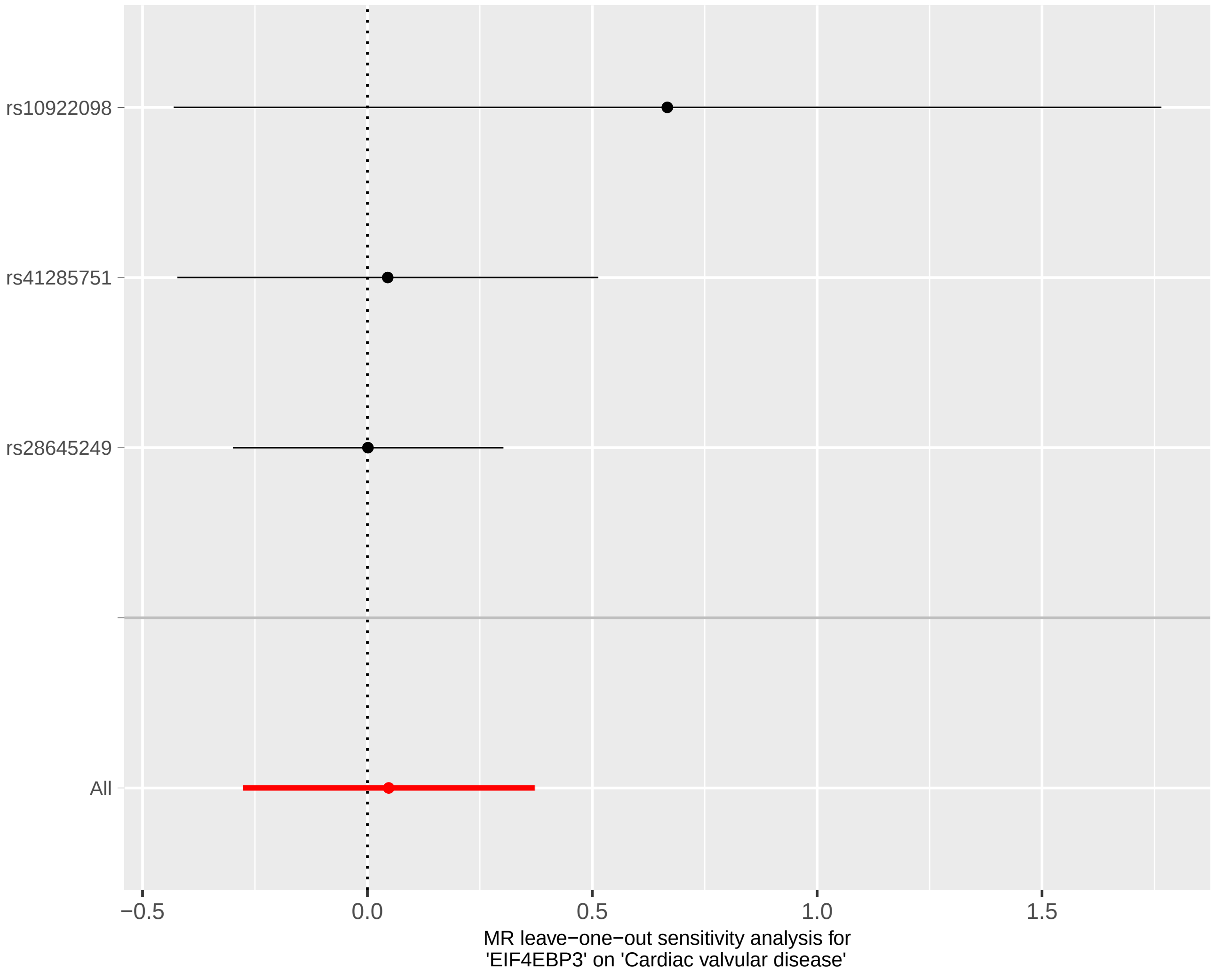
rs10922098
rs41285751
rs28645249
All
−0.5
0.0
0.5
1.0
1.5
MR leave−one−out sensitivity analysis for
'EIF4EBP3' on 'Cardiac valvular disease'

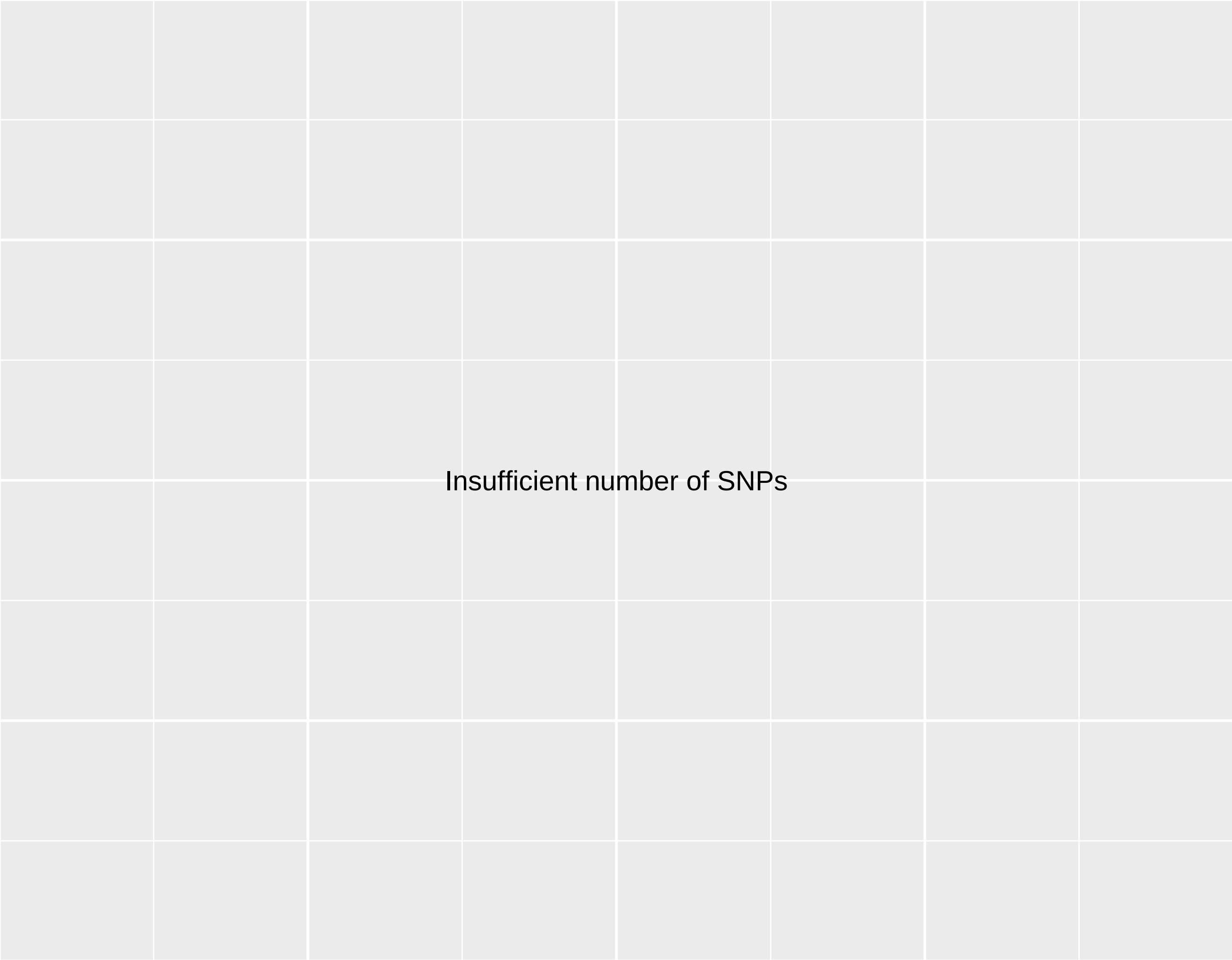
Insufficient number of SNPs

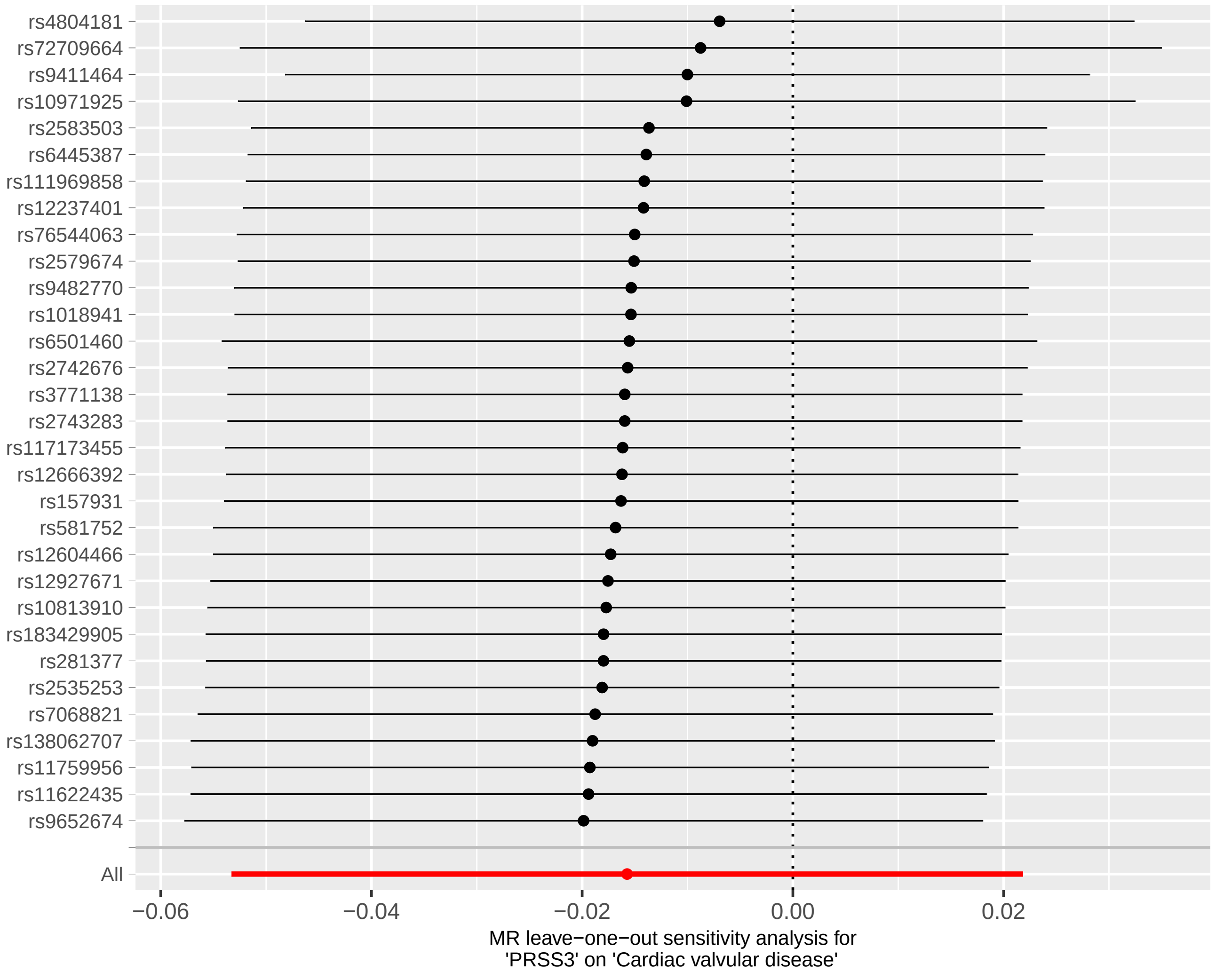
rs4804181
rs72709664
rs9411464
rs10971925
rs2583503
rs6445387
rs111969858
rs12237401
rs76544063
rs2579674
rs9482770
rs1018941
rs6501460
rs2742676
rs3771138
rs2743283
rs117173455
rs12666392
rs157931
rs581752
rs12604466
rs12927671
rs10813910
rs183429905
rs281377
rs2535253
rs7068821
rs138062707
rs11759956
rs11622435
rs9652674
All
−0.06
−0.04
−0.02
0.00
0.02
MR leave−one−out sensitivity analysis for
'PRSS3' on 'Cardiac valvular disease'

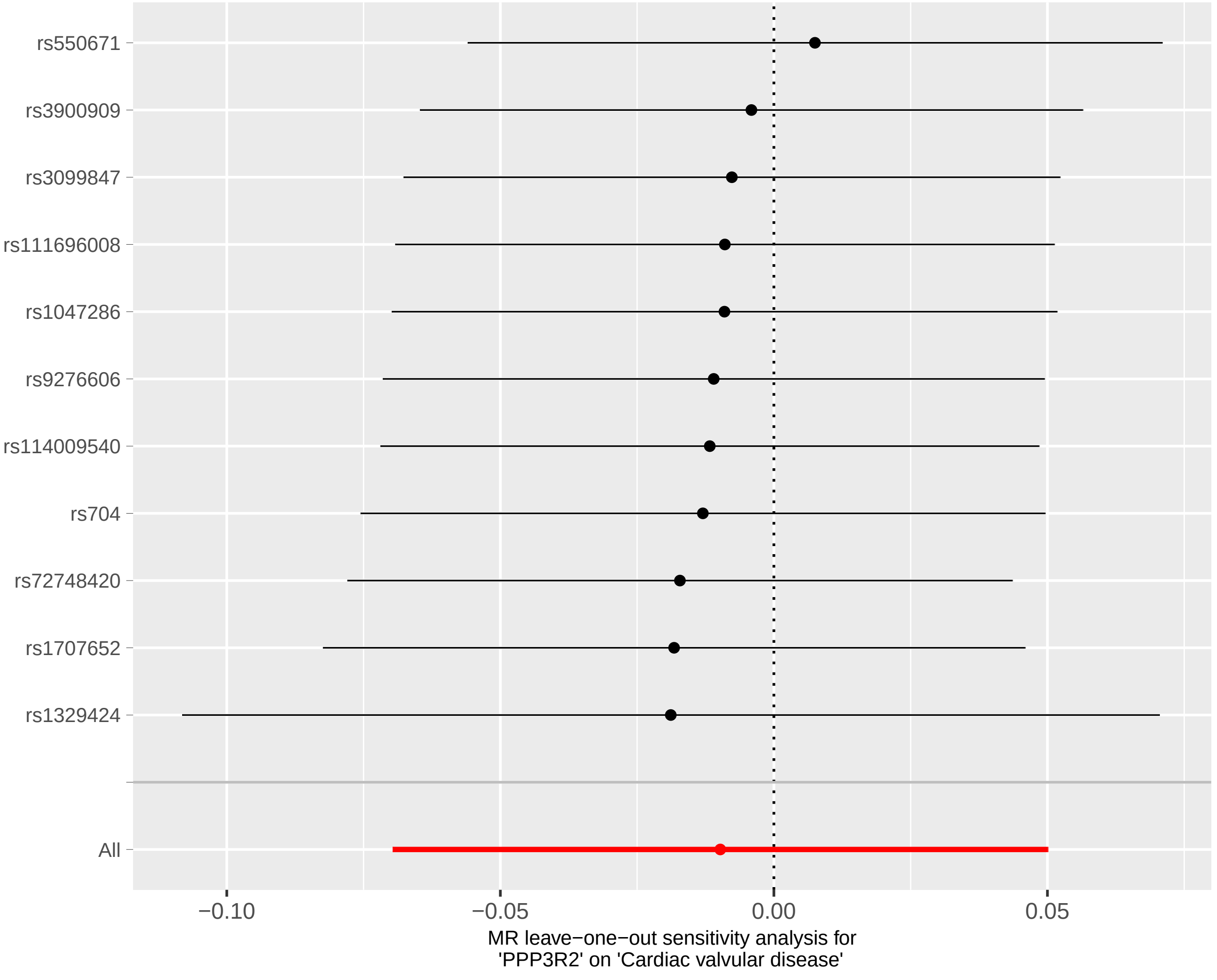

rs550671
rs3900909
rs3099847
rs111696008
rs1047286
rs9276606
rs114009540
rs704
rs72748420
rs1707652
rs1329424
All
−0.10
−0.05
0.00
0.05
MR leave−one−out sensitivity analysis for
'PPP3R2' on 'Cardiac valvular disease'

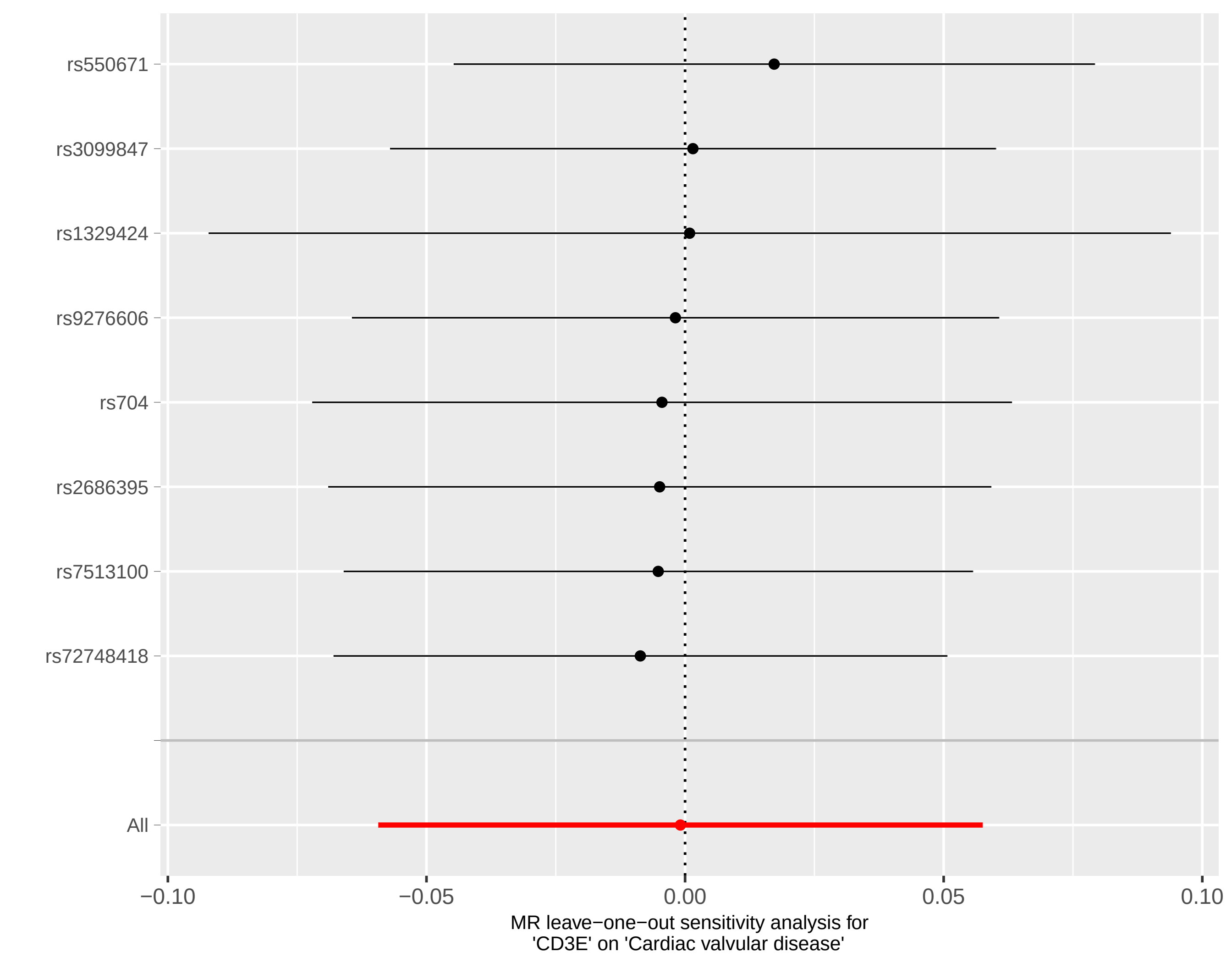
rs550671
rs3099847
rs1329424
rs9276606
rs704
rs2686395
rs7513100
rs72748418
All
−0.10
−0.05
0.00
0.05
0.10
MR leave−one−out sensitivity analysis for
'CD3E' on 'Cardiac valvular disease'

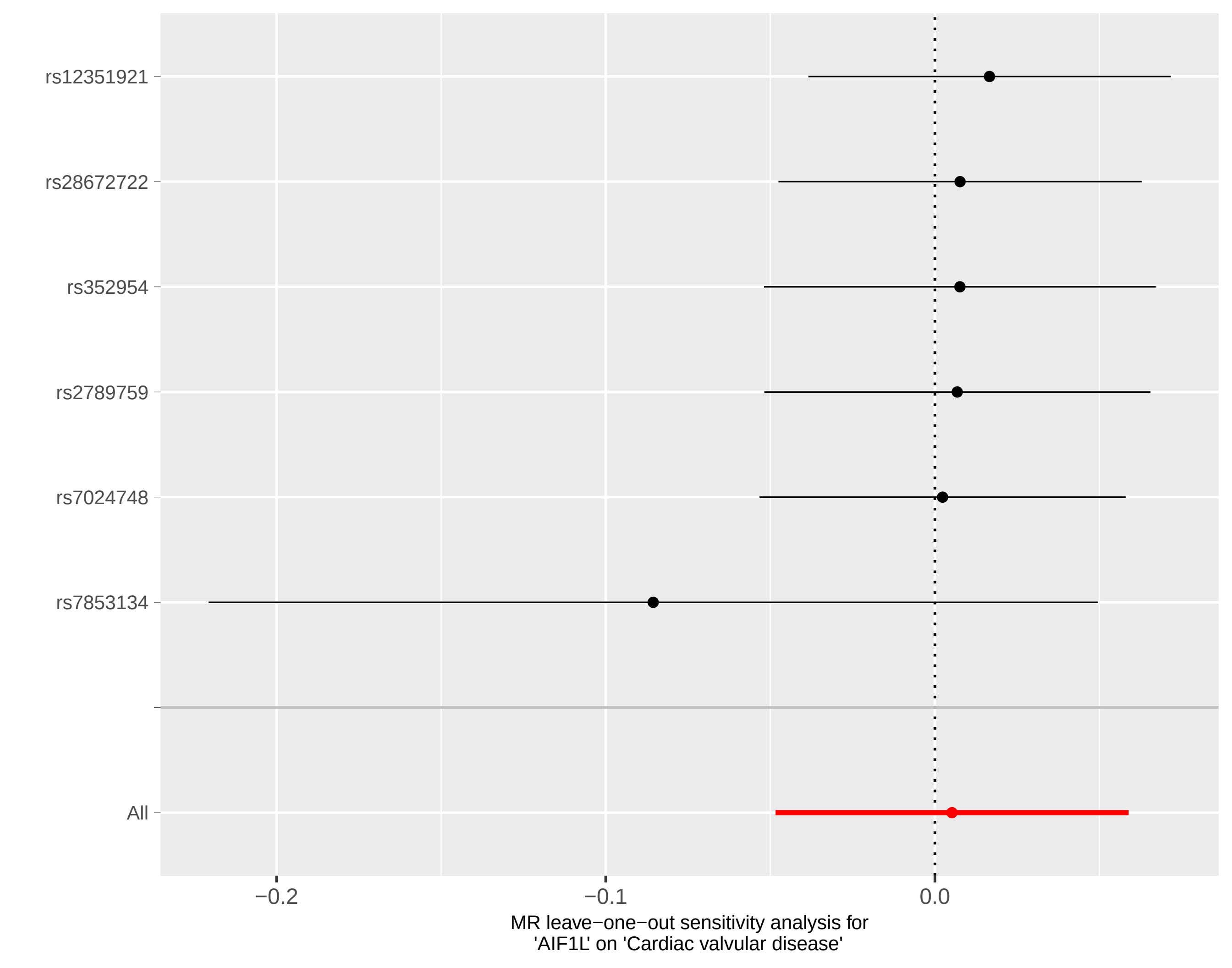
rs12351921
rs28672722
rs352954
rs2789759
rs7024748
rs7853134
All
−0.2
−0.1
0.0
MR leave−one−out sensitivity analysis for
'AIF1L' on 'Cardiac valvular disease'

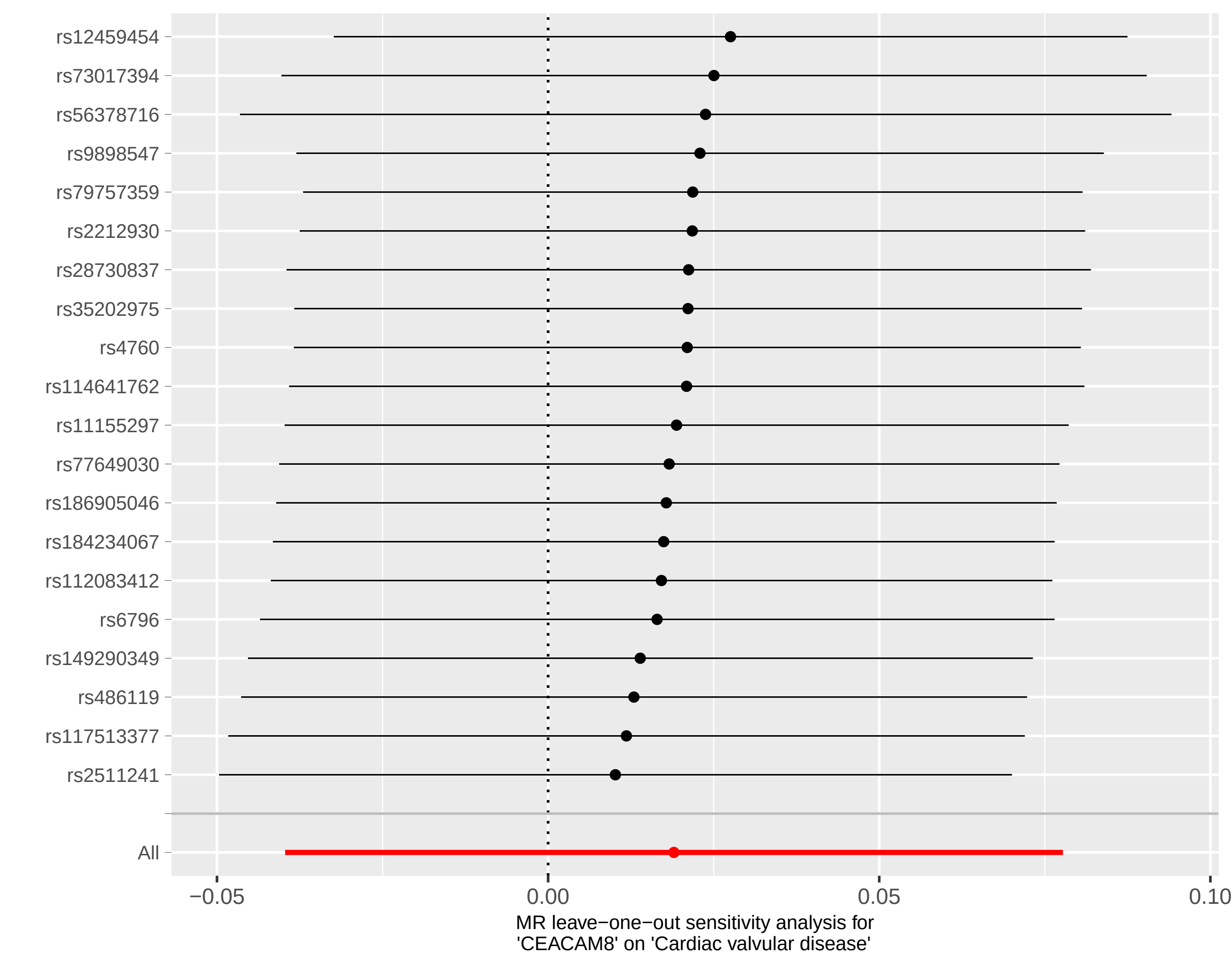

rs12459454
rs73017394
rs56378716
rs9898547
rs79757359
rs2212930
rs28730837
rs35202975
rs4760
rs114641762
rs11155297
rs77649030
rs186905046
rs184234067
rs112083412
rs6796
rs149290349
rs486119
rs117513377
rs2511241
All
−0.05
0.00
0.05
0.10
MR leave−one−out sensitivity analysis for
'CEACAM8' on 'Cardiac valvular disease'

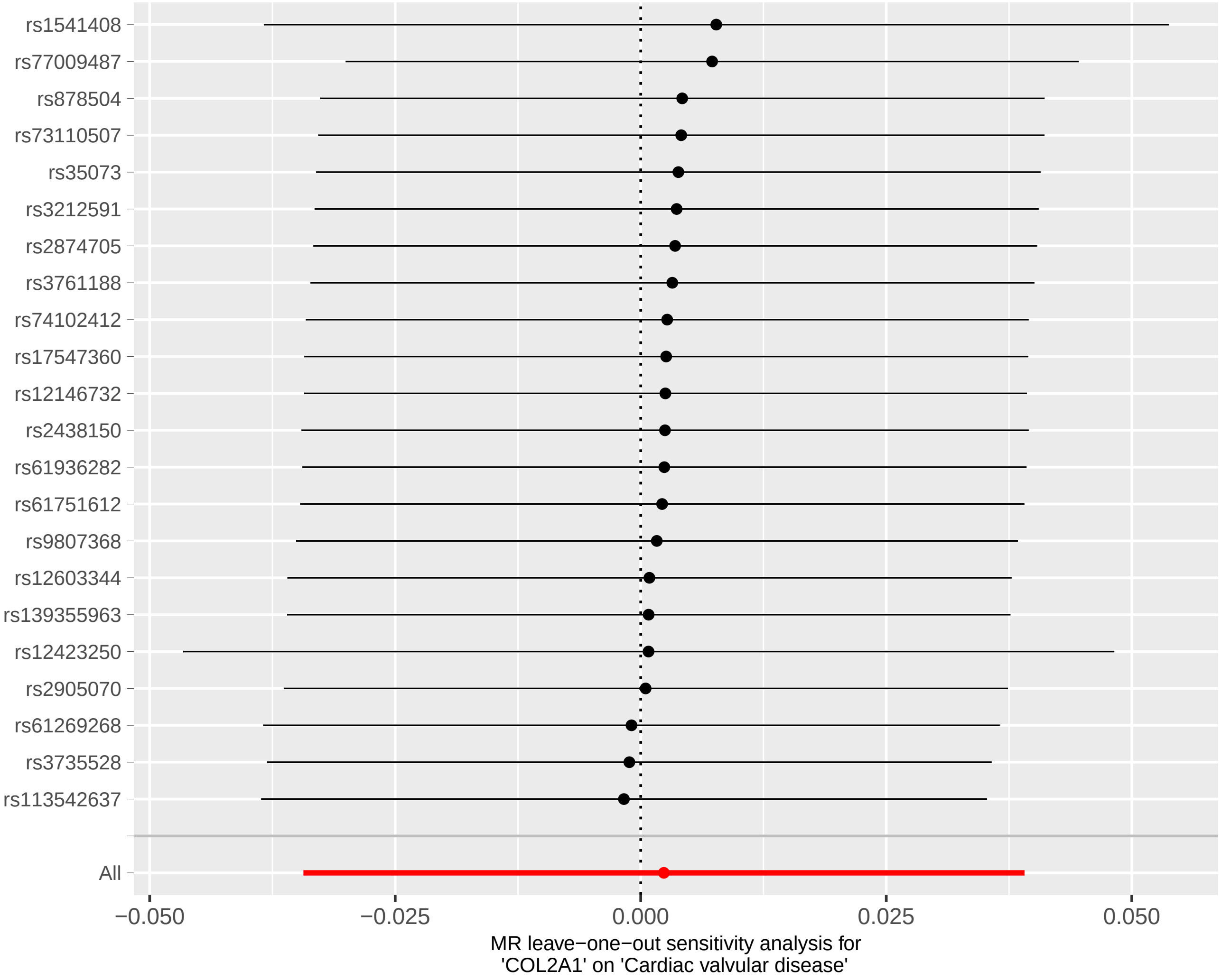
rs1541408
rs77009487
rs878504
rs73110507
rs35073
rs3212591
rs2874705
rs3761188
rs74102412
rs17547360
rs12146732
rs2438150
rs61936282
rs61751612
rs9807368
rs12603344
rs139355963
rs12423250
rs2905070
rs61269268
rs3735528
rs113542637
All
−0.050
−0.025
0.000
0.025
0.050
MR leave−one−out sensitivity analysis for
'COL2A1' on 'Cardiac valvular disease'

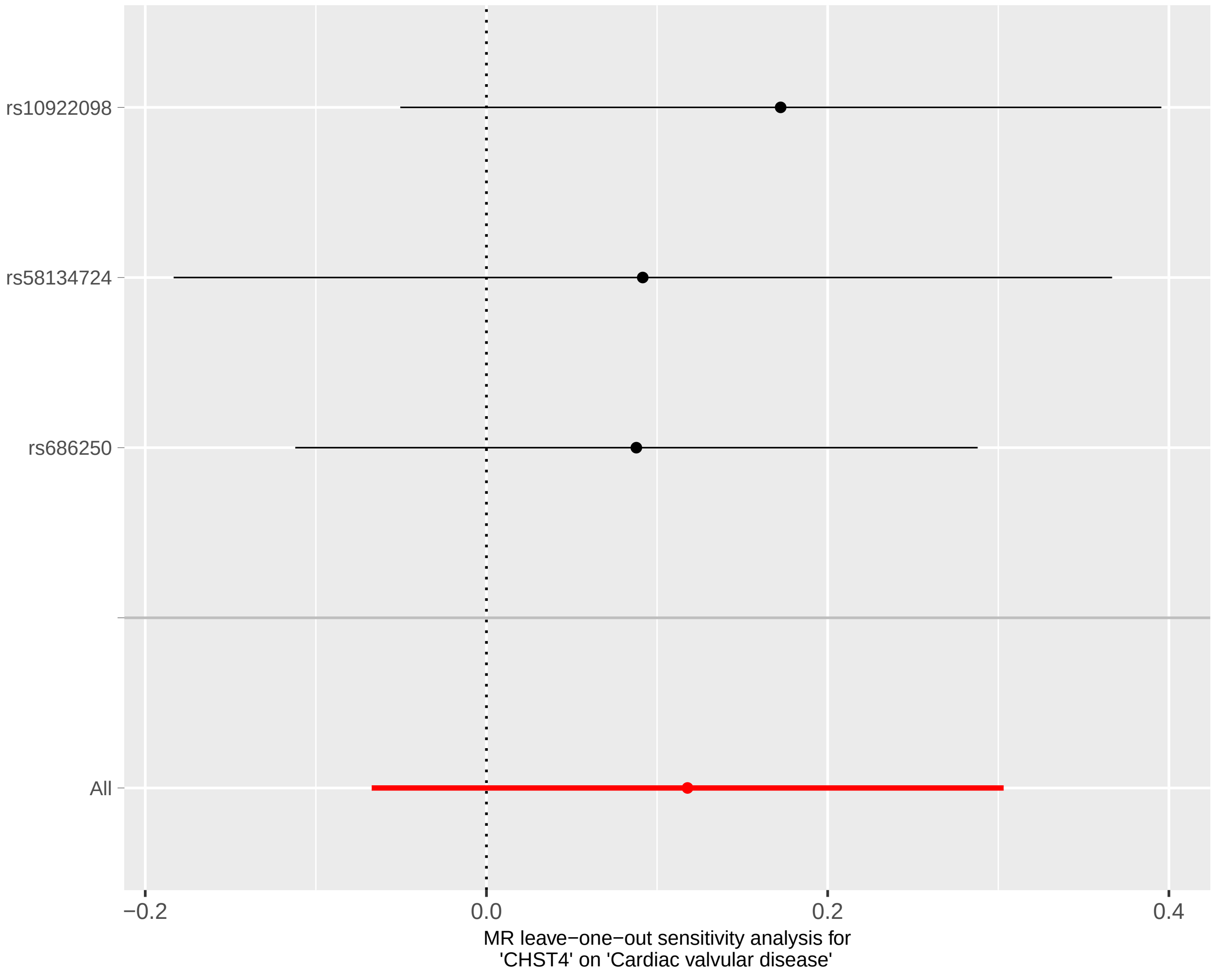
rs10922098
rs58134724
rs686250
All
−0.2
0.0
0.2
0.4
MR leave−one−out sensitivity analysis for
'CHST4' on 'Cardiac valvular disease'

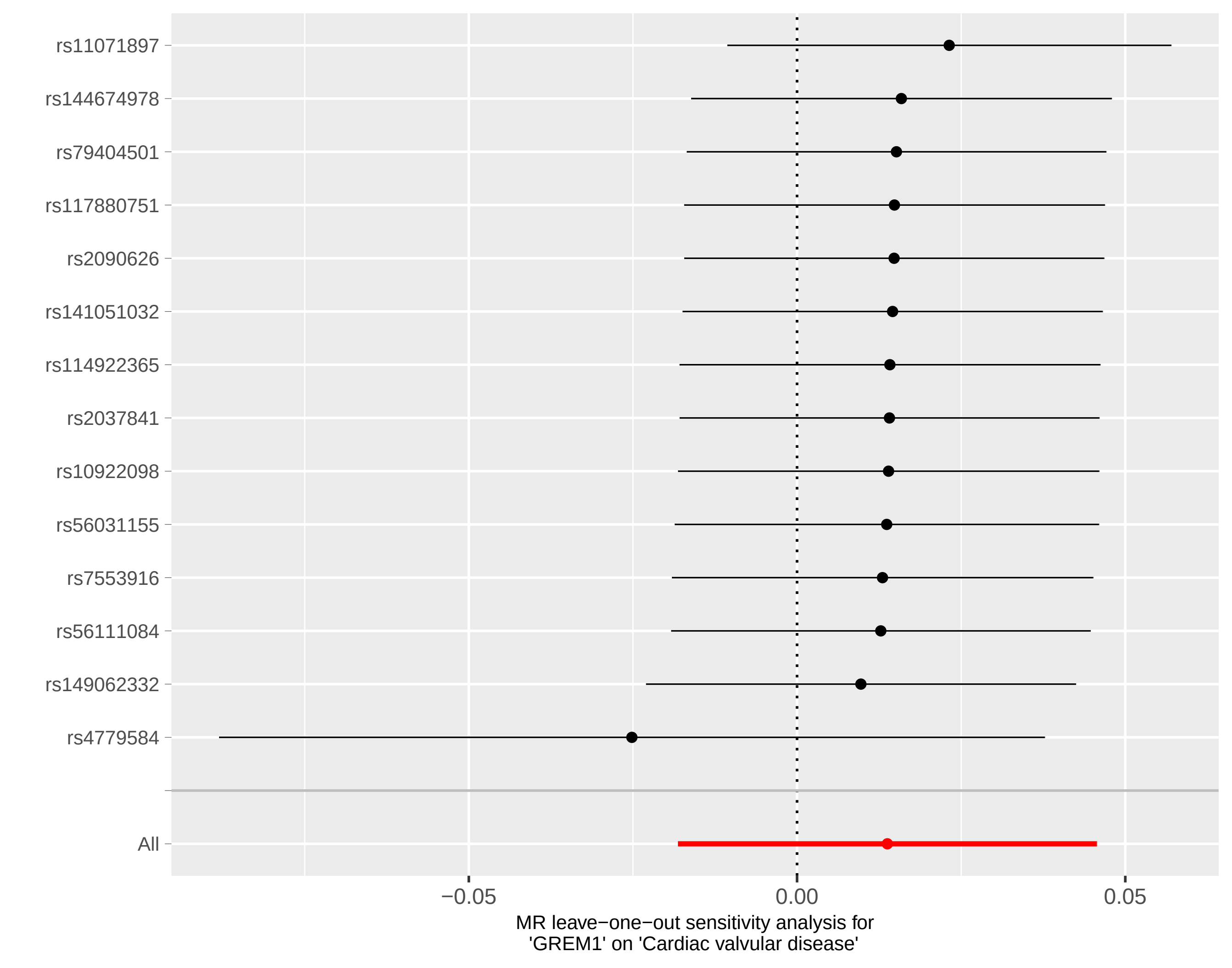

rs11071897
rs144674978
rs79404501
rs117880751
rs2090626
rs141051032
rs114922365
rs2037841
rs10922098
rs56031155
rs7553916
rs56111084
rs149062332
rs4779584
All
−0.05
0.00
0.05
MR leave−one−out sensitivity analysis for
'GREM1' on 'Cardiac valvular disease'

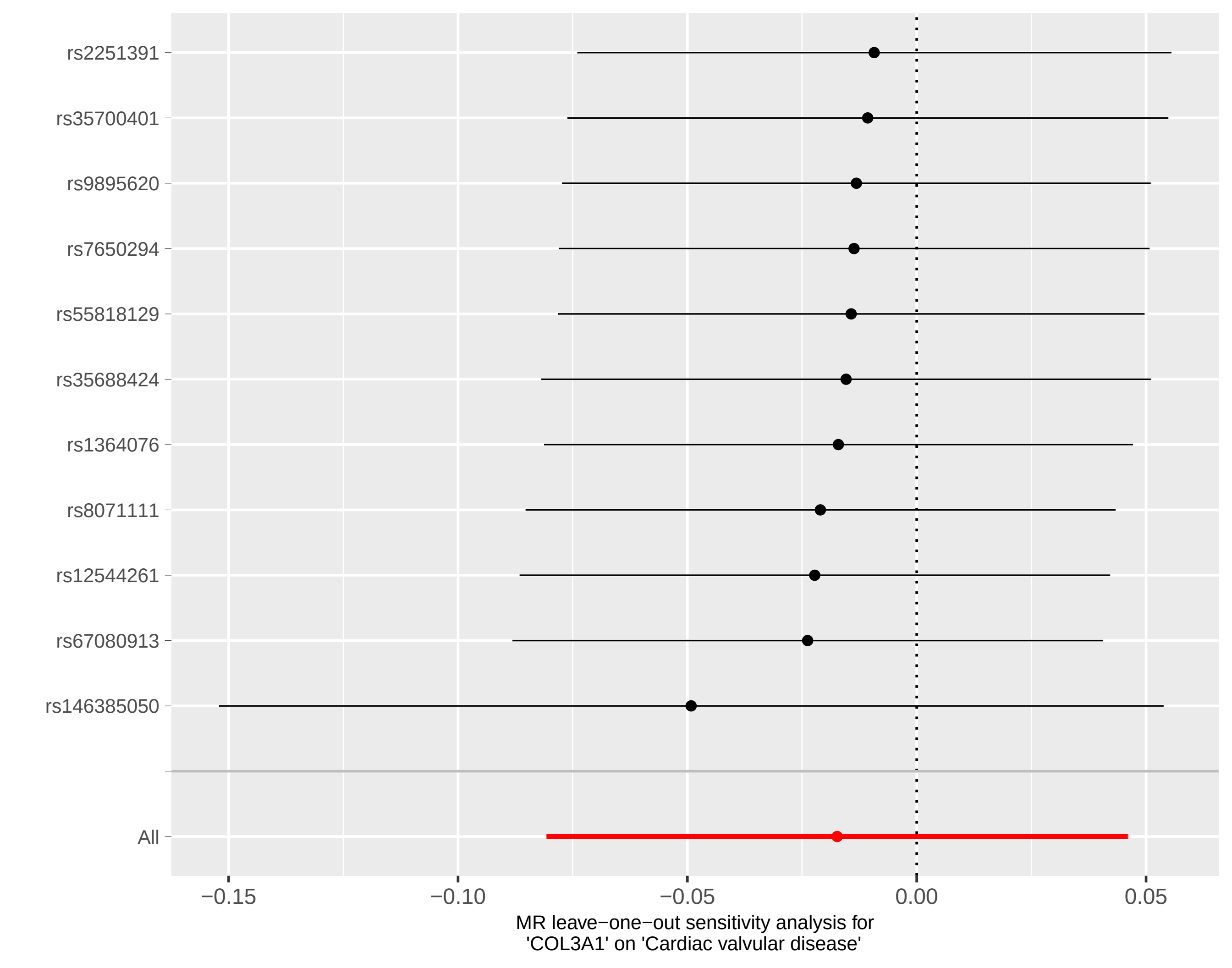
rs2251391
rs35700401
rs9895620
rs7650294
rs55818129
rs35688424
rs1364076
rs8071111
rs12544261
rs67080913
rs146385050
All
−0.15
−0.10
−0.05
0.00
0.05
MR leave−one−out sensitivity analysis for
'COL3A1' on 'Cardiac valvular disease'

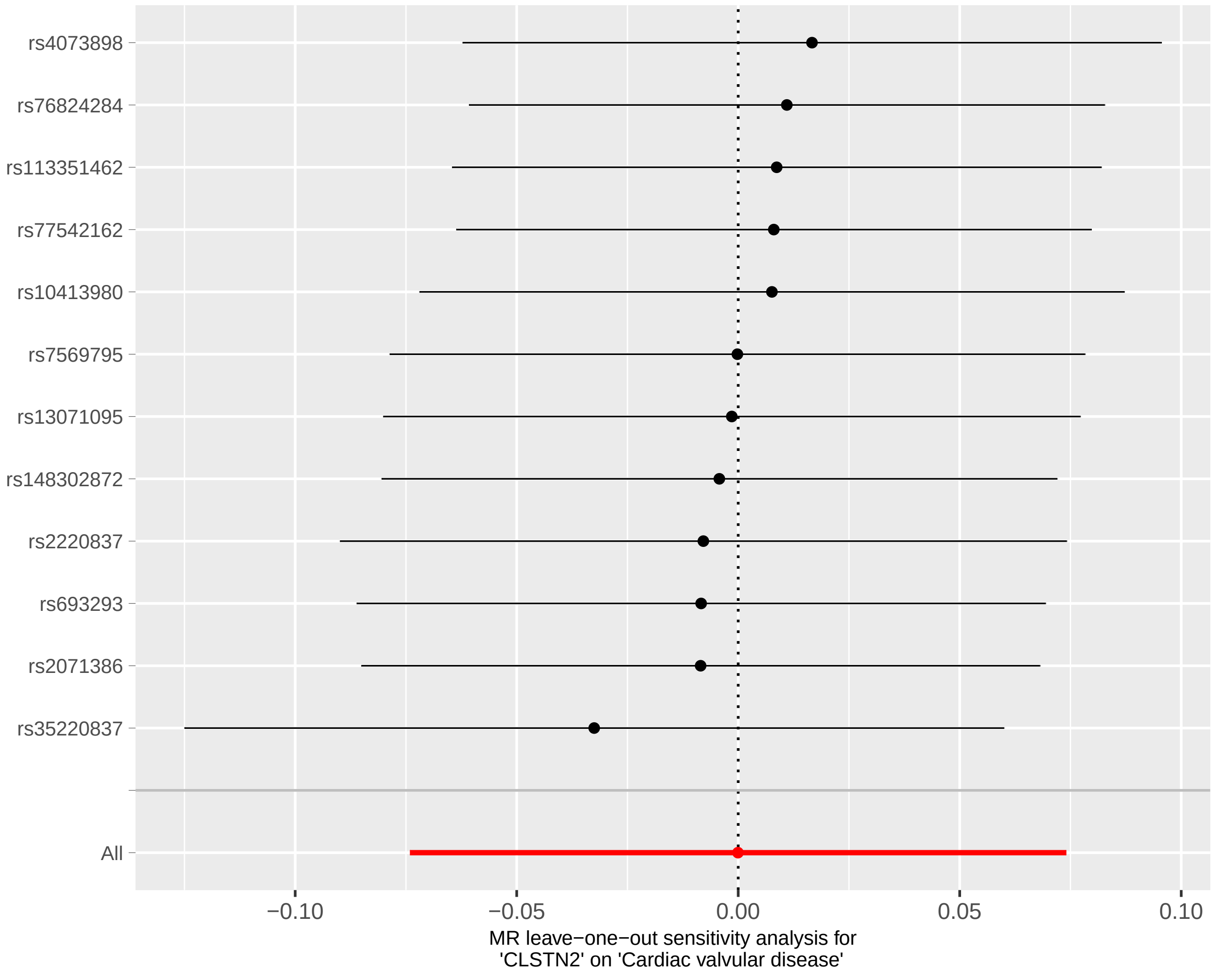
rs4073898
rs76824284
rs113351462
rs77542162
rs10413980
rs7569795
rs13071095
rs148302872
rs2220837
rs693293
rs2071386
rs35220837
All
−0.10
−0.05
0.00
0.05
0.10
MR leave−one−out sensitivity analysis for
'CLSTN2' on 'Cardiac valvular disease'

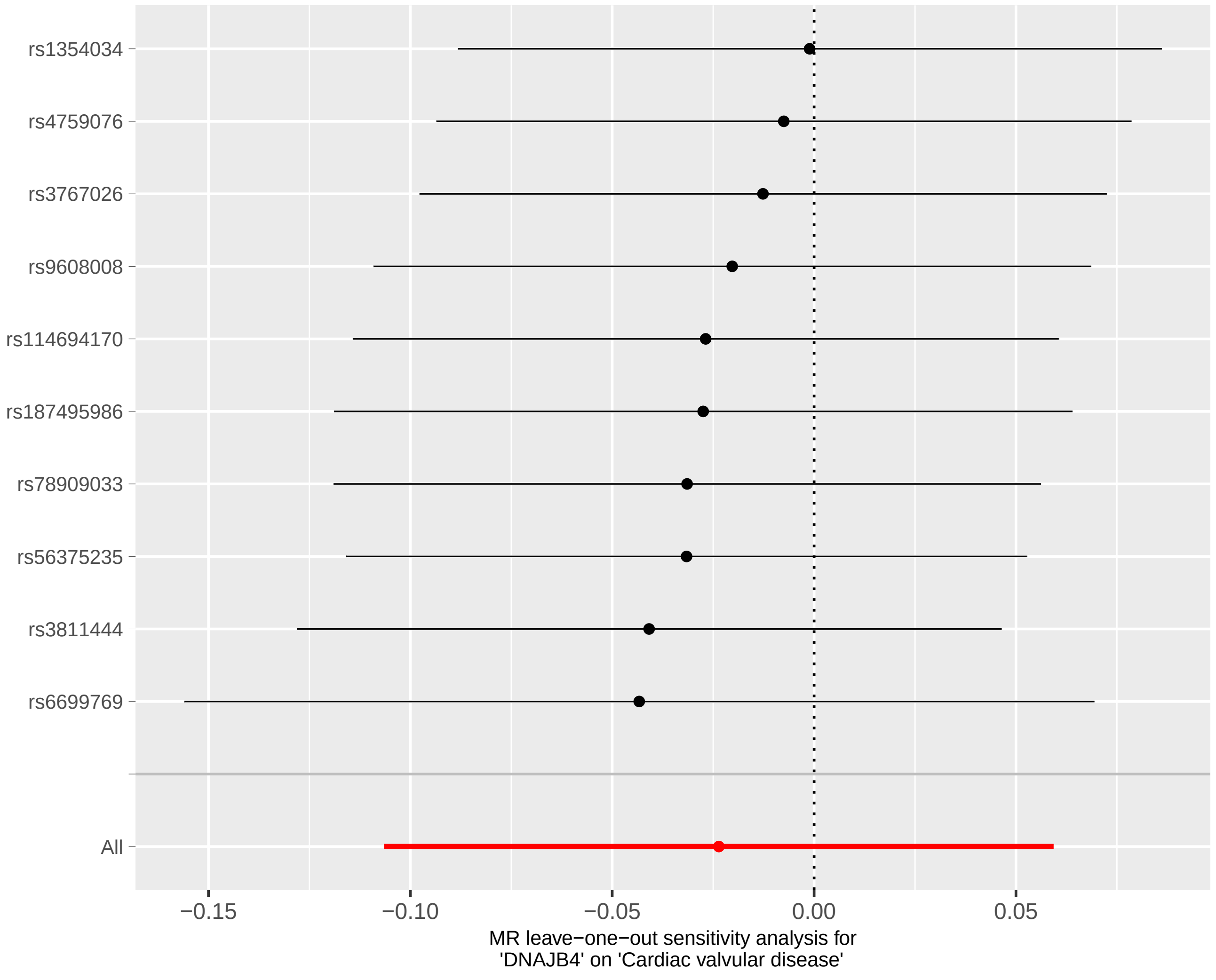

rs1354034
rs4759076
rs3767026
rs9608008
rs114694170
rs187495986
rs78909033
rs56375235
rs3811444
rs6699769
All
−0.15
−0.10
−0.05
0.00
0.05
MR leave−one−out sensitivity analysis for
'DNAJB4' on 'Cardiac valvular disease'

Insufficient number of SNPs

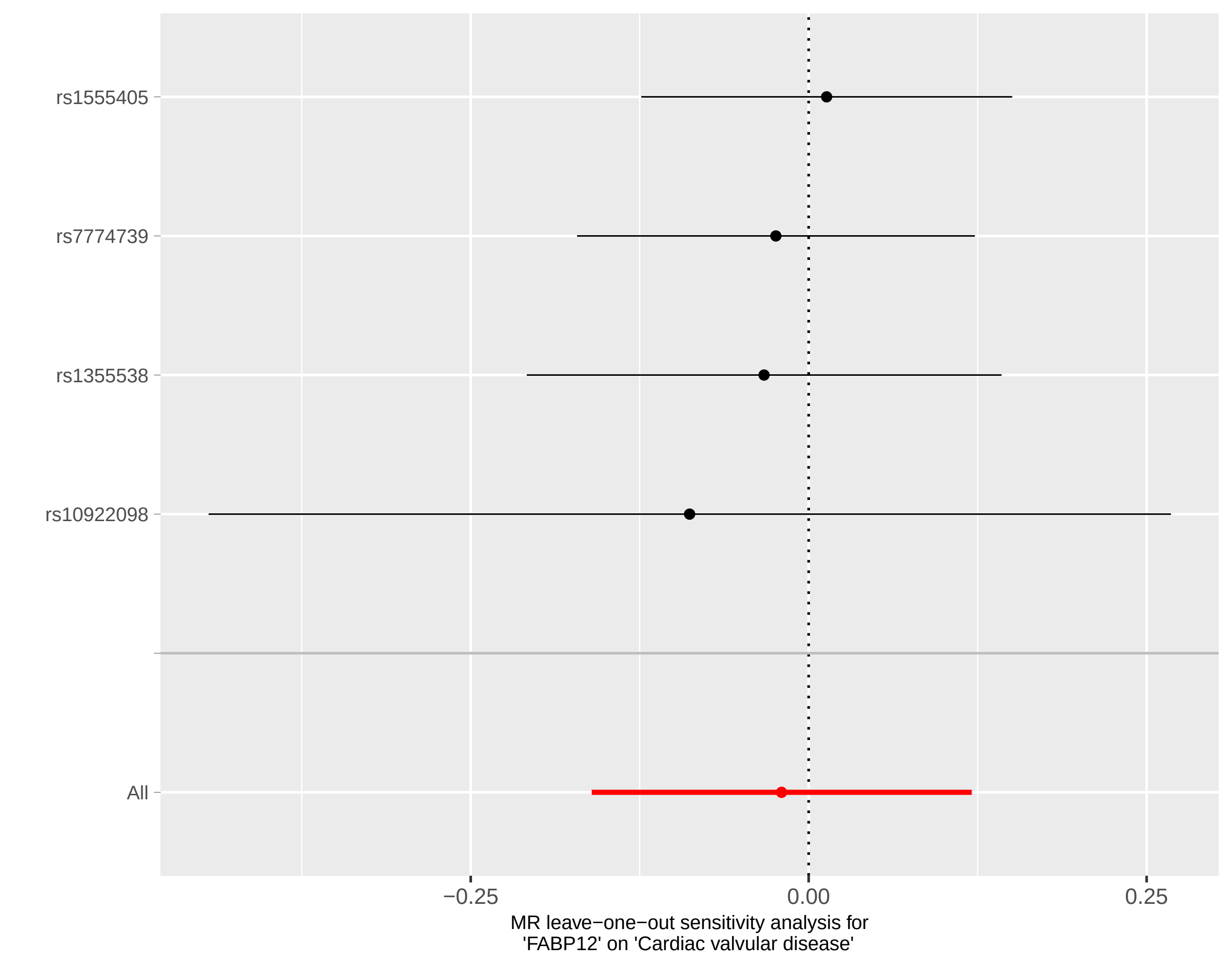
rs1555405
rs7774739
rs1355538
rs10922098
All
−0.25
0.00
0.25
MR leave−one−out sensitivity analysis for
'FABP12' on 'Cardiac valvular disease'

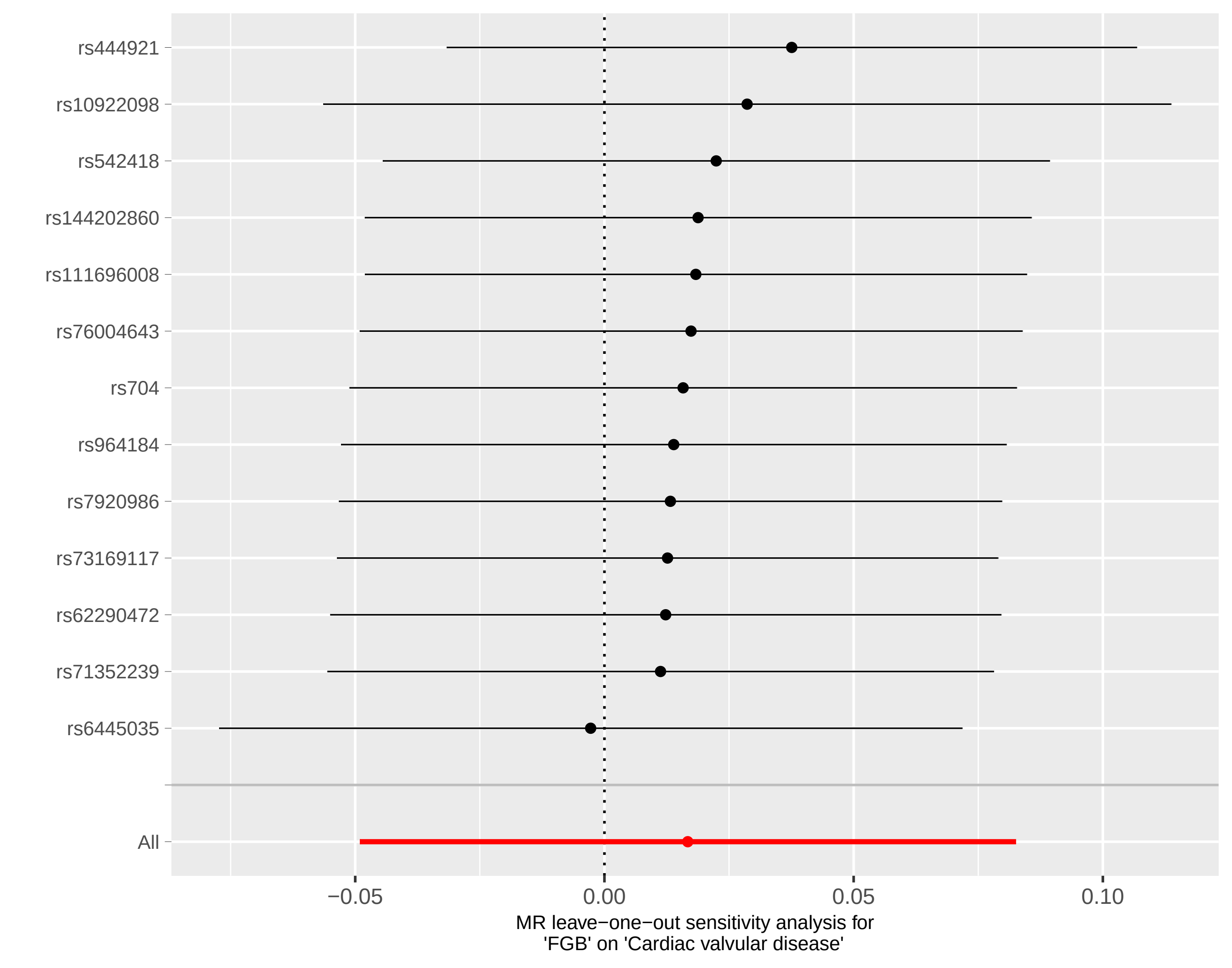

rs444921
rs10922098
rs542418
rs144202860
rs111696008
rs76004643
rs704
rs964184
rs7920986
rs73169117
rs62290472
rs71352239
rs6445035
All
−0.05
0.00
0.05
0.10
MR leave−one−out sensitivity analysis for
'FGB' on 'Cardiac valvular disease'

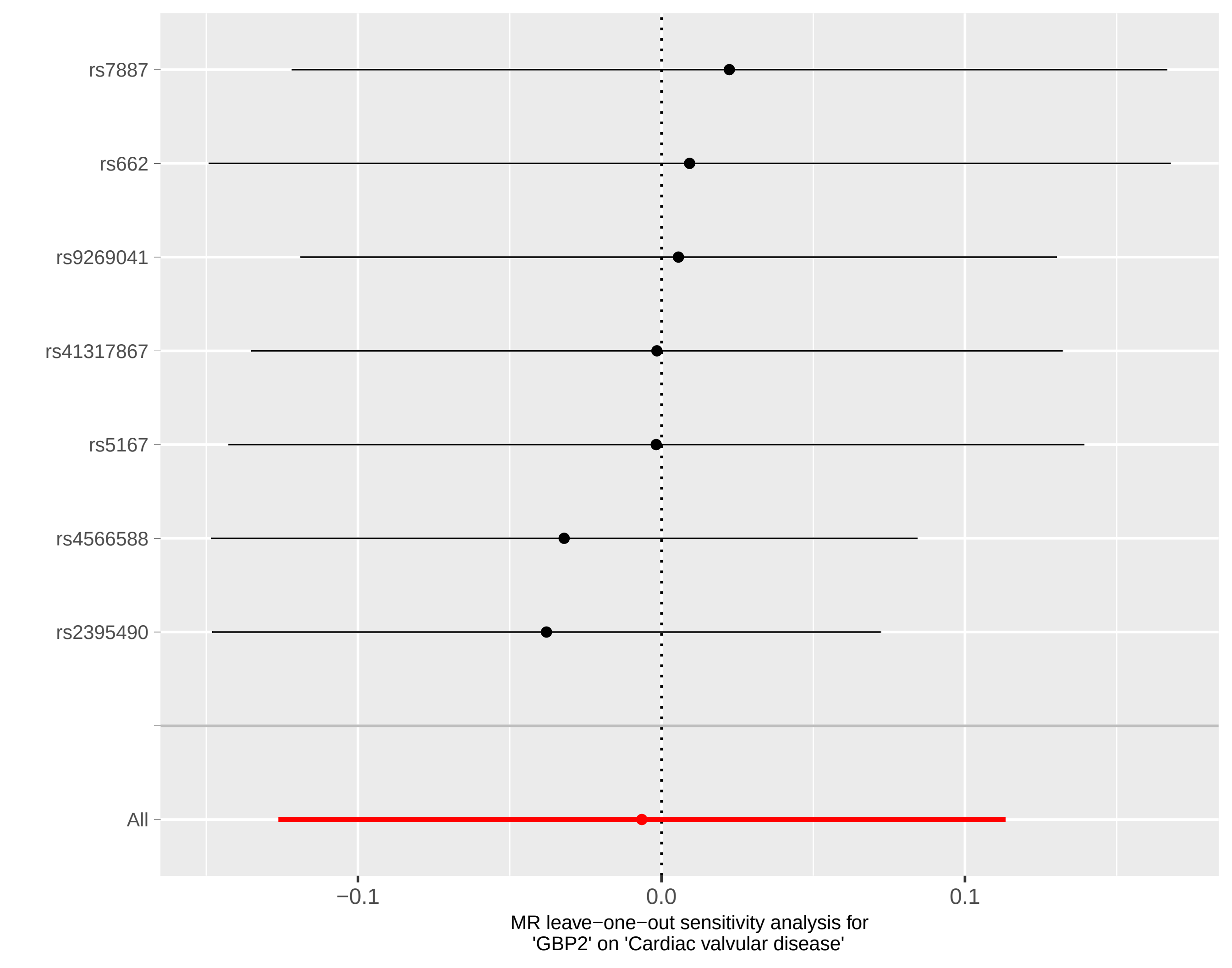

rs7887
rs662
rs9269041
rs41317867
rs5167
rs4566588
rs2395490
All
−0.1
0.0
0.1
MR leave−one−out sensitivity analysis for
'GBP2' on 'Cardiac valvular disease'

Insufficient number of SNPs

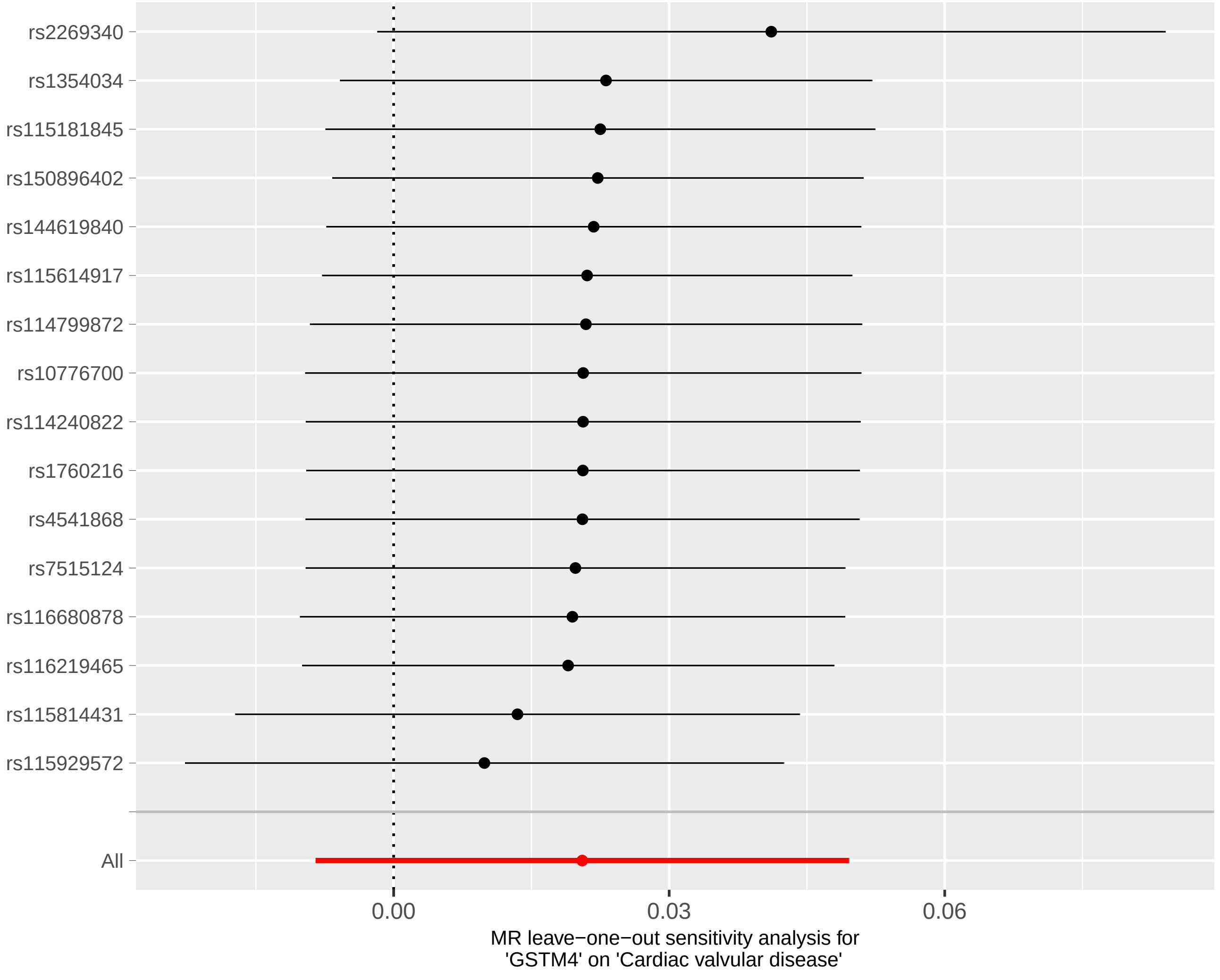

rs2269340
rs1354034
rs115181845
rs150896402
rs144619840
rs115614917
rs114799872
rs10776700
rs114240822
rs1760216
rs4541868
rs7515124
rs116680878
rs116219465
rs115814431
rs115929572
All
0.00
0.03
0.06
MR leave−one−out sensitivity analysis for
'GSTM4' on 'Cardiac valvular disease'

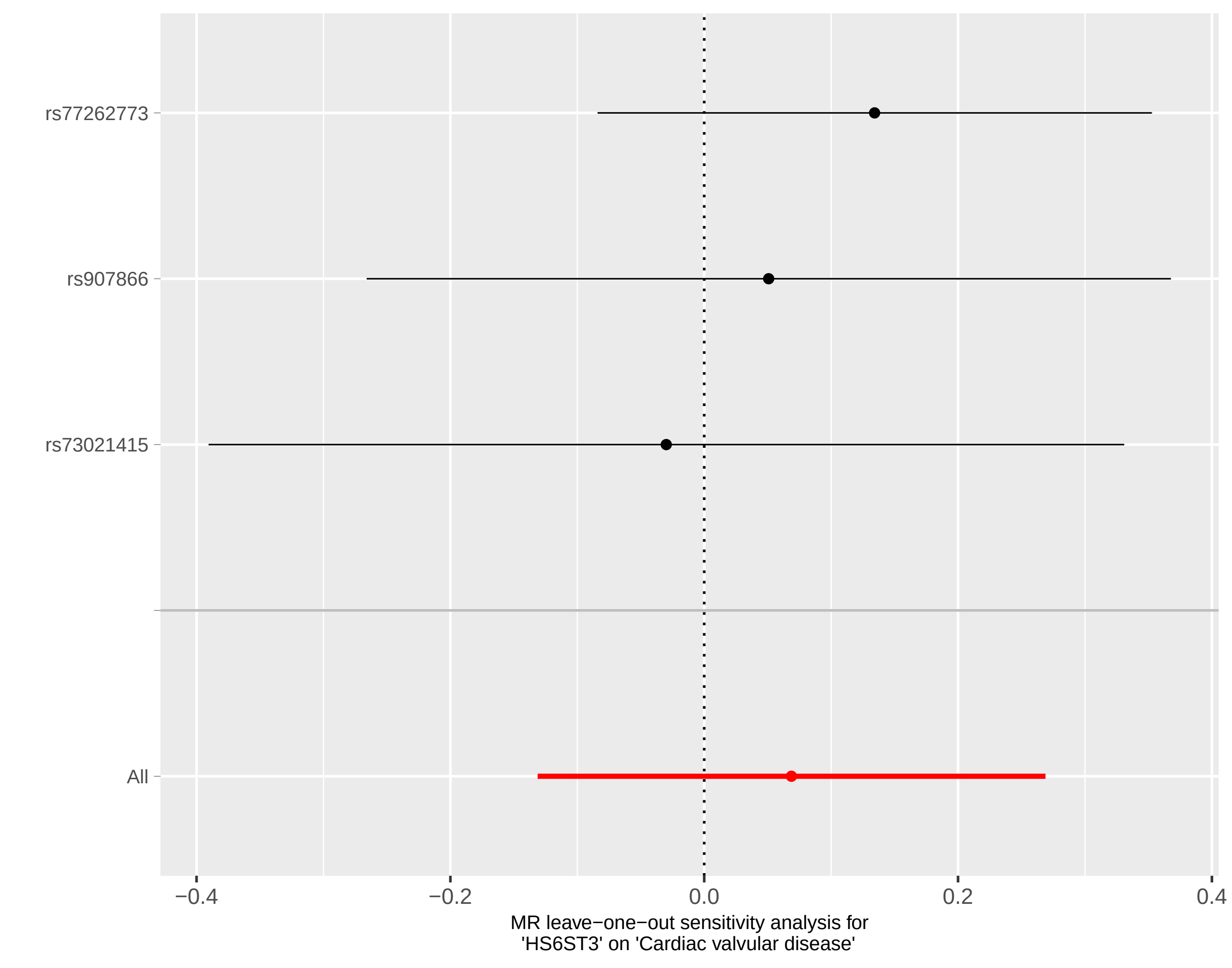
rs77262773
rs907866
rs73021415
All
−0.4
−0.2
0.0
0.2
0.4
MR leave−one−out sensitivity analysis for
'HS6ST3' on 'Cardiac valvular disease'

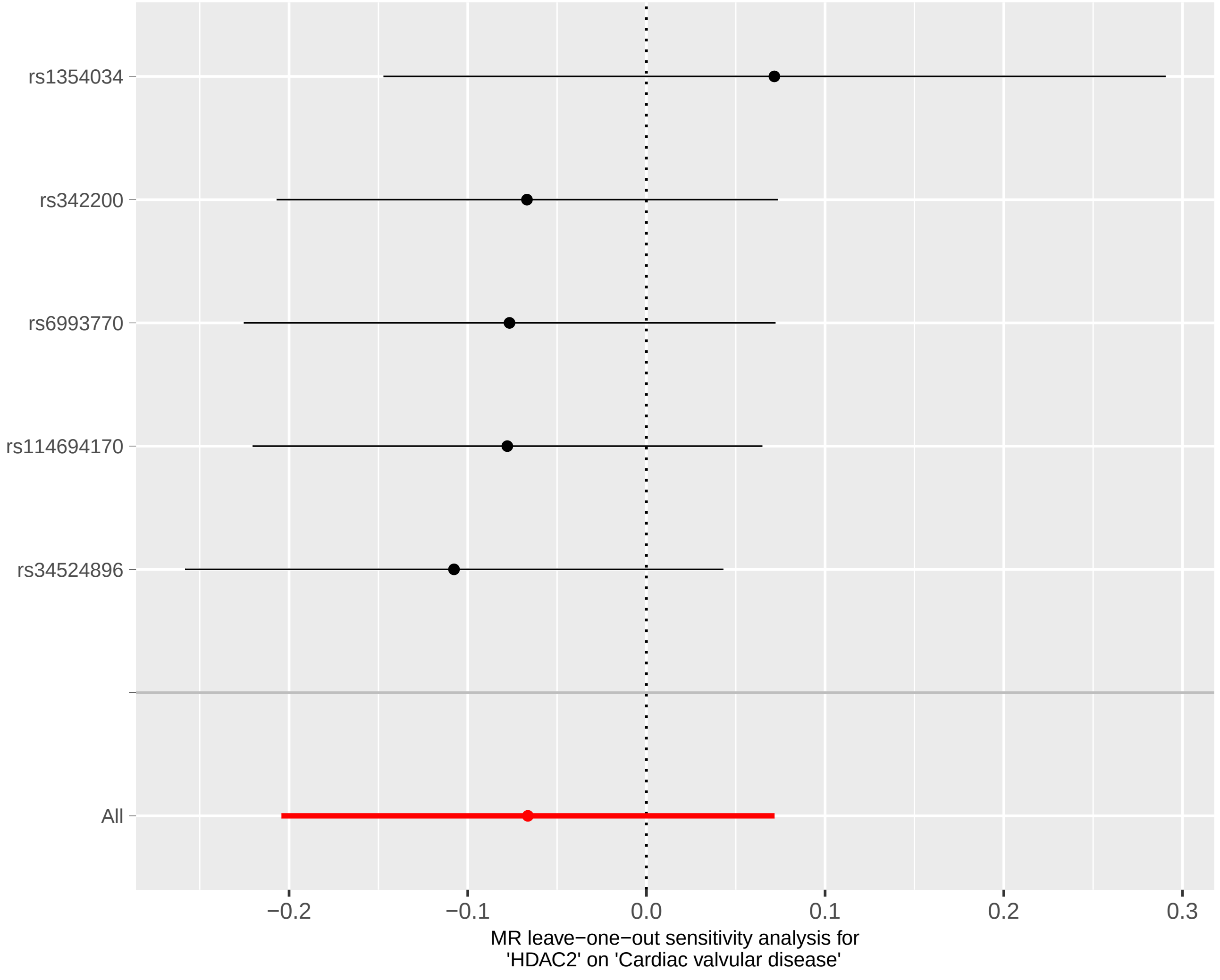
rs1354034
rs342200
rs6993770
rs114694170
rs34524896
All
−0.2
−0.1
0.0
0.1
0.2
0.3
MR leave−one−out sensitivity analysis for
'HDAC2' on 'Cardiac valvular disease'

Insufficient number of SNPs

Insufficient number of SNPs

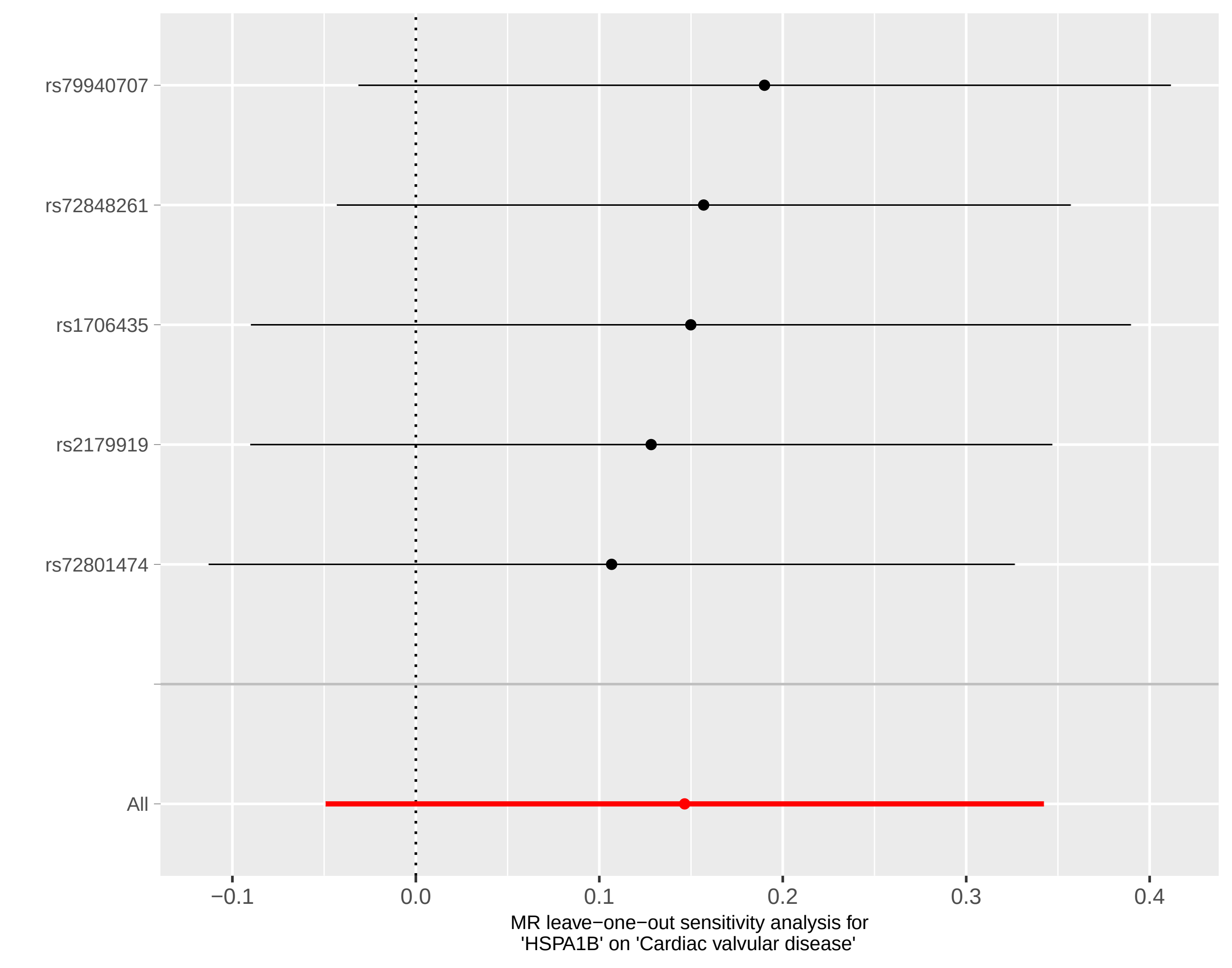
rs79940707
rs72848261
rs1706435
rs2179919
rs72801474
All
−0.1
0.0
0.1
0.2
0.3
0.4
MR leave−one−out sensitivity analysis for
'HSPA1B' on 'Cardiac valvular disease'

Insufficient number of SNPs

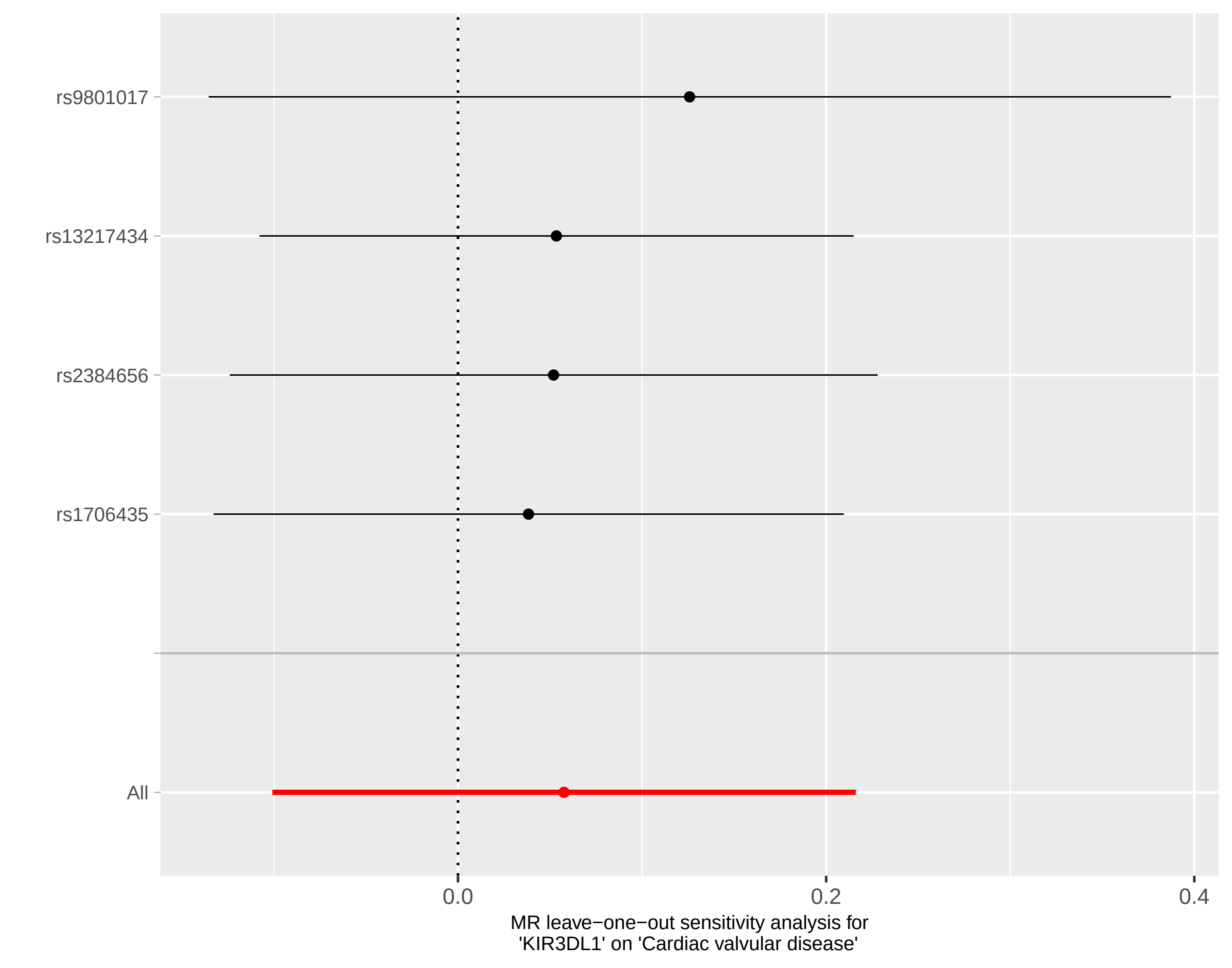

rs9801017
rs13217434
rs2384656
rs1706435
All
0.0
0.2
0.4
MR leave−one−out sensitivity analysis for
'KIR3DL1' on 'Cardiac valvular disease'

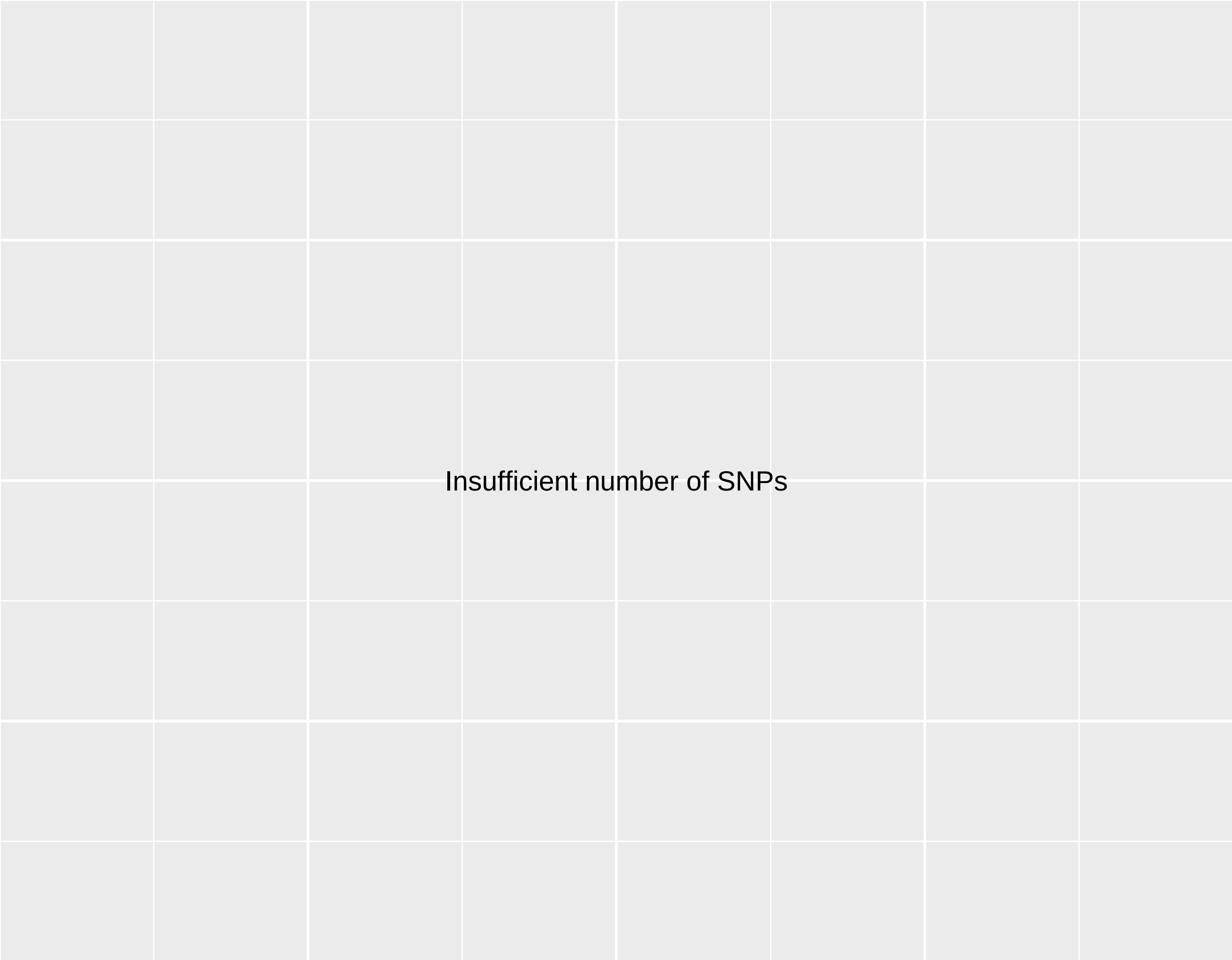
Insufficient number of SNPs

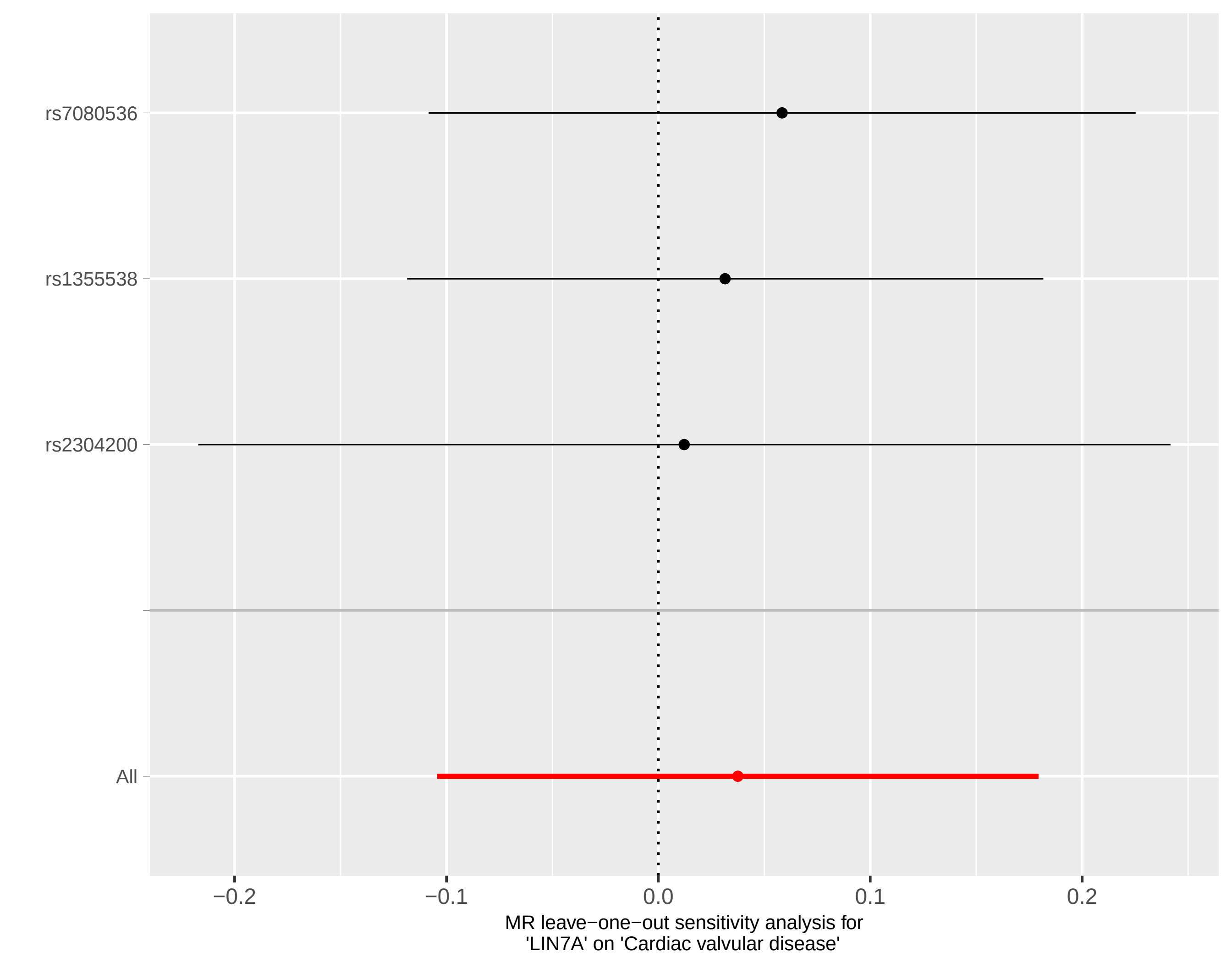

rs7080536
rs1355538
rs2304200
All
−0.2
−0.1
0.0
0.1
0.2
MR leave−one−out sensitivity analysis for
'LIN7A' on 'Cardiac valvular disease'

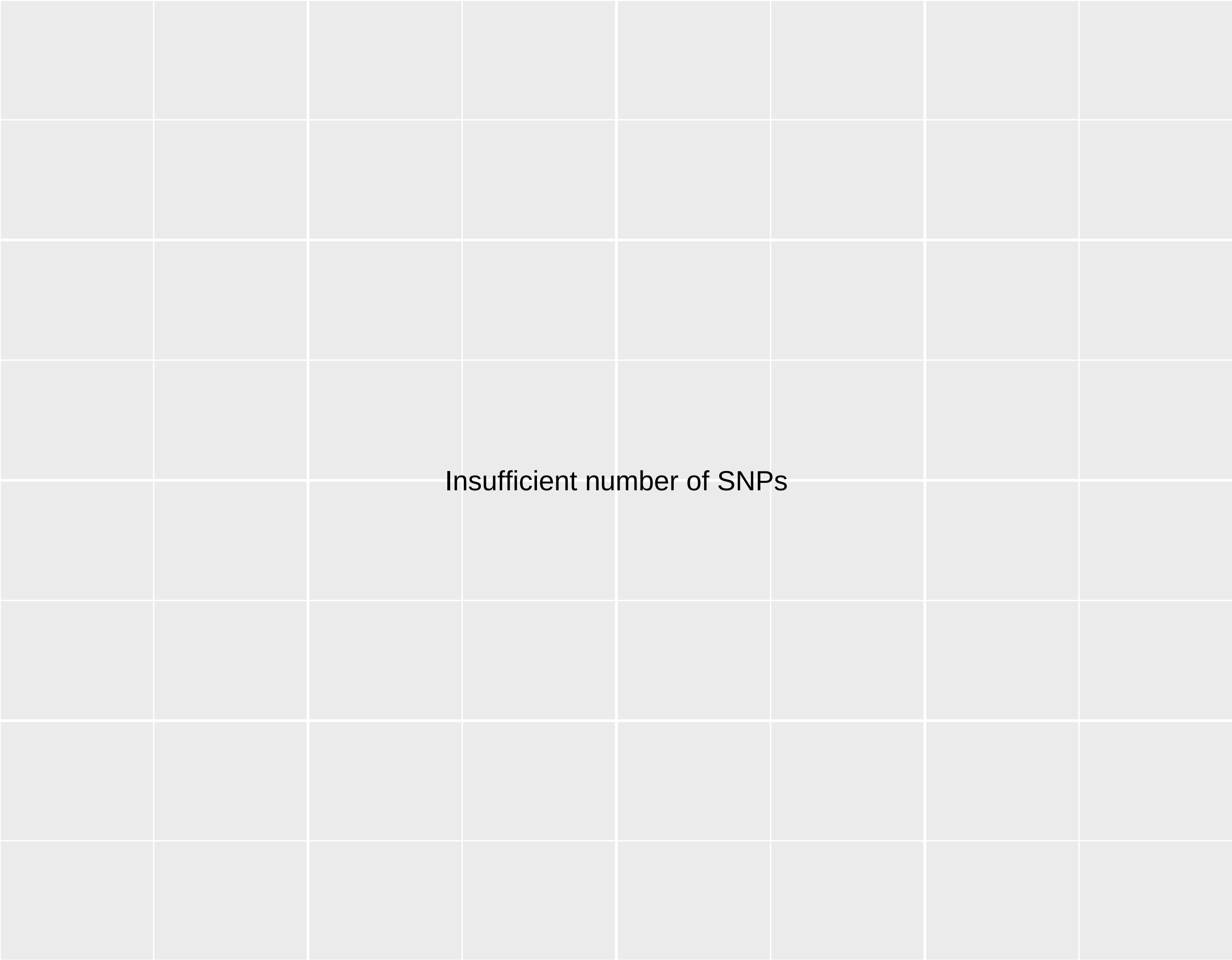
Insufficient number of SNPs

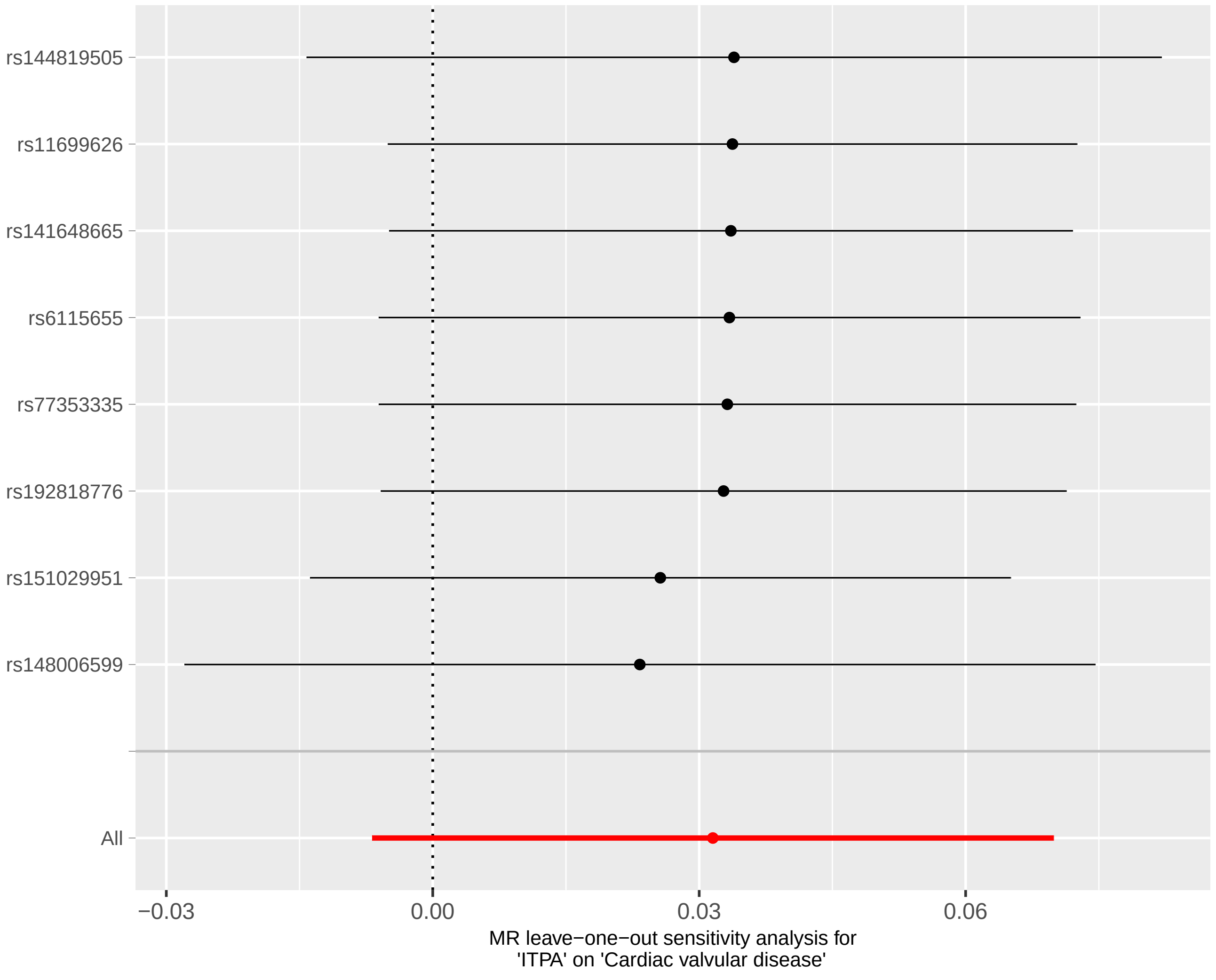

rs144819505
rs11699626
rs141648665
rs6115655
rs77353335
rs192818776
rs151029951
rs148006599
All
−0.03
0.00
0.03
0.06
MR leave−one−out sensitivity analysis for
'ITPA' on 'Cardiac valvular disease'

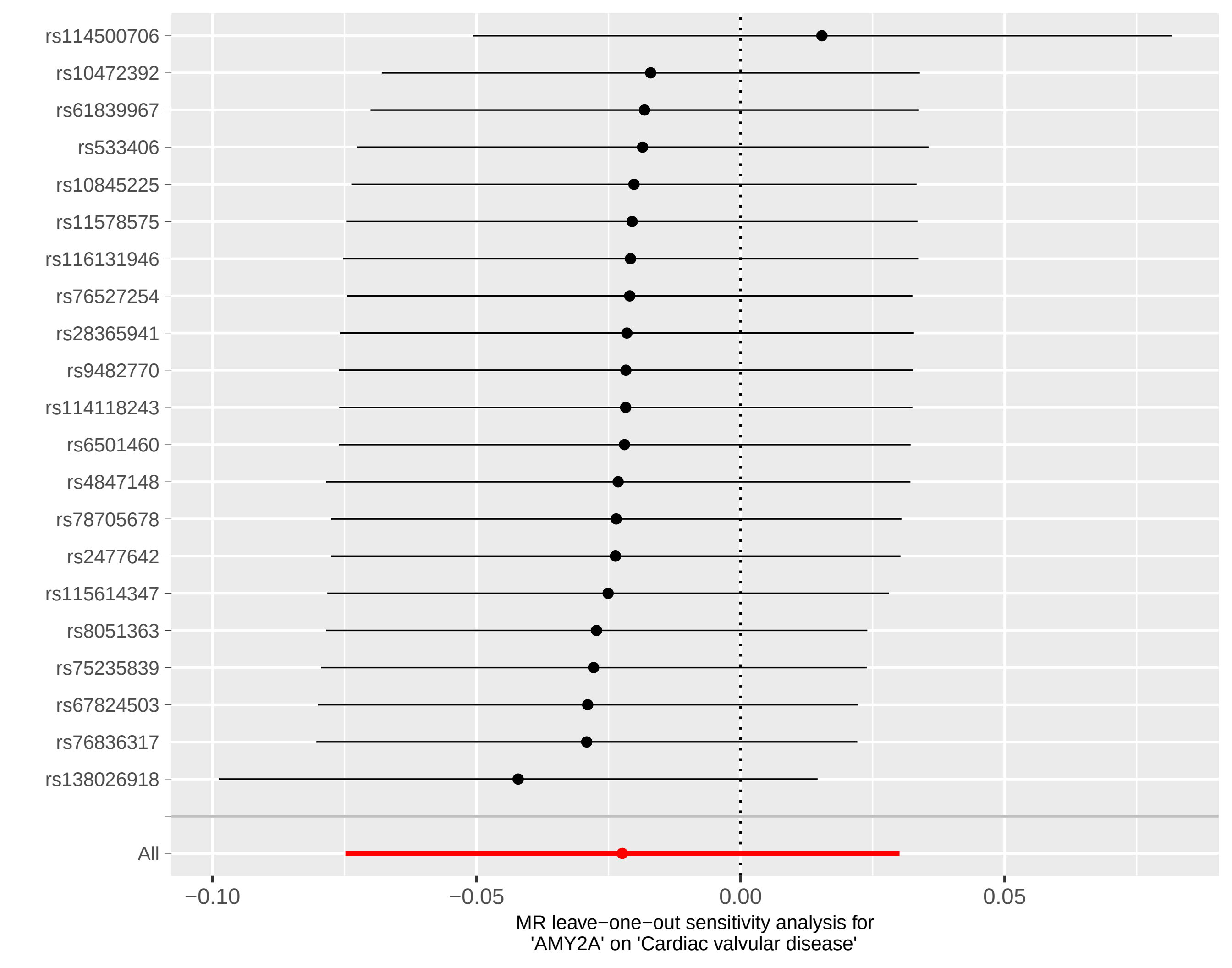
rs114500706
rs10472392
rs61839967
rs533406
rs10845225
rs11578575
rs116131946
rs76527254
rs28365941
rs9482770
rs114118243
rs6501460
rs4847148
rs78705678
rs2477642
rs115614347
rs8051363
rs75235839
rs67824503
rs76836317
rs138026918
All
−0.10
−0.05
0.00
0.05
MR leave−one−out sensitivity analysis for
'AMY2A' on 'Cardiac valvular disease'

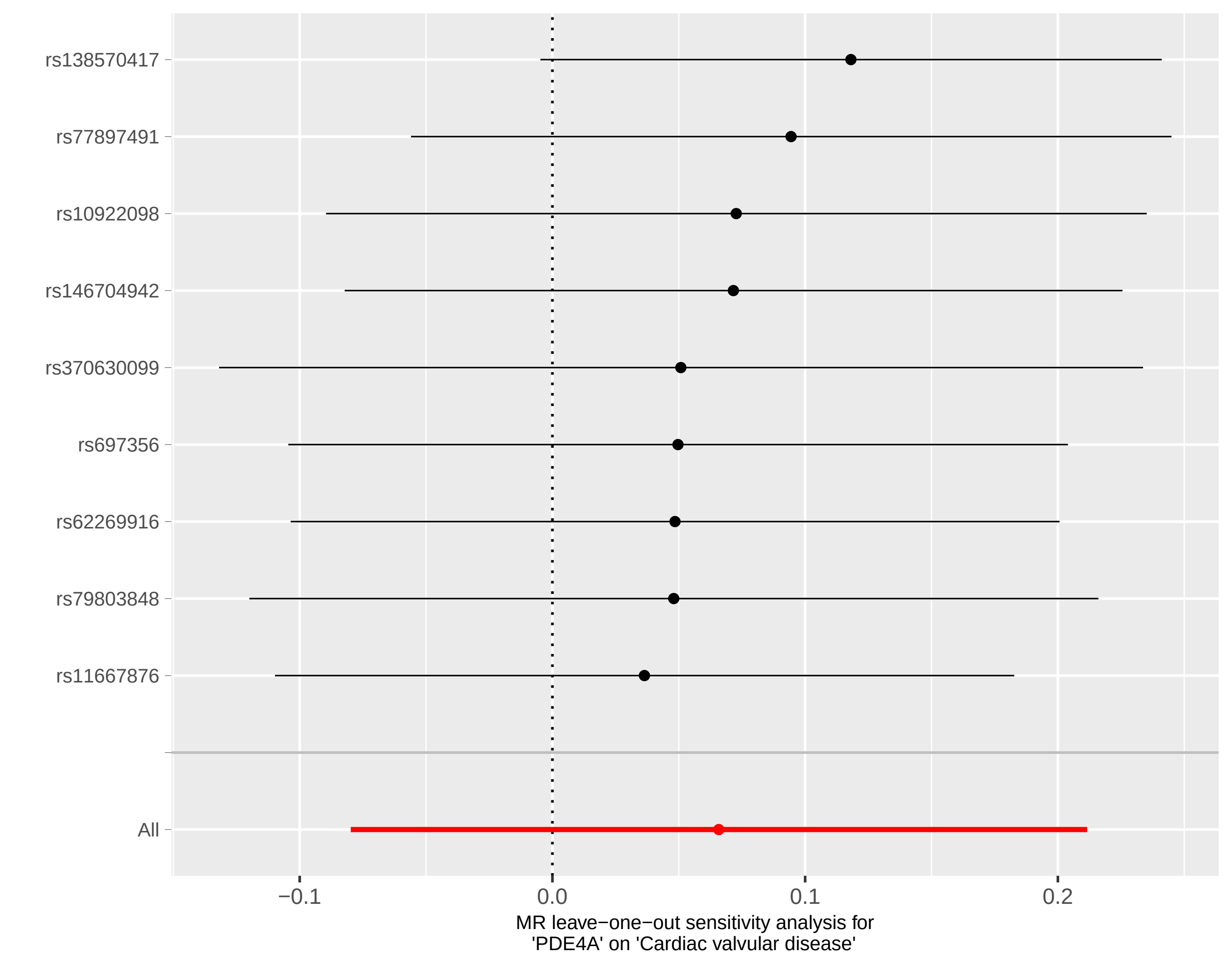
rs138570417
rs77897491
rs10922098
rs146704942
rs370630099
rs697356
rs62269916
rs79803848
rs11667876
All
−0.1
0.0
0.1
0.2
MR leave−one−out sensitivity analysis for
'PDE4A' on 'Cardiac valvular disease'

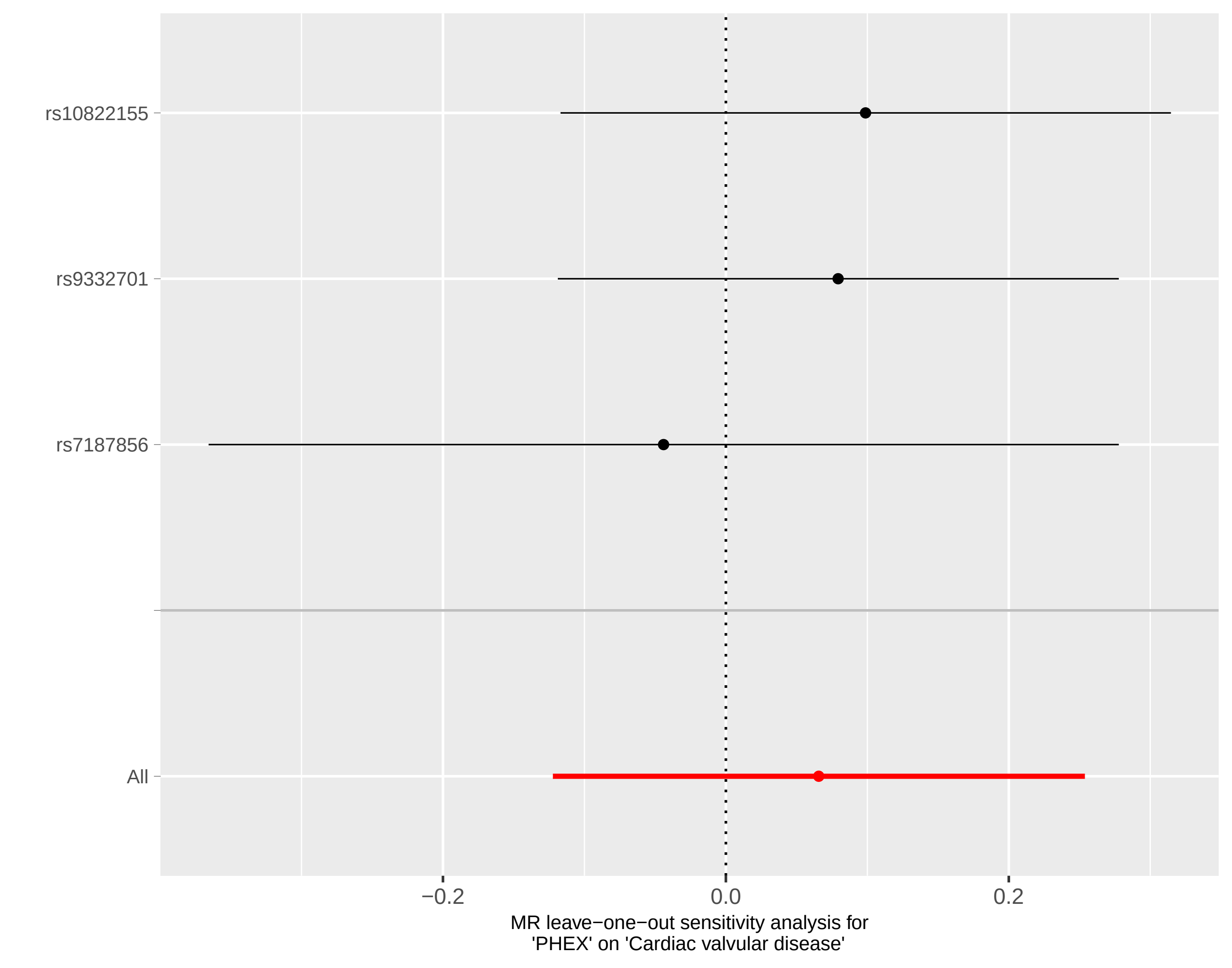
rs10822155
rs9332701
rs7187856
All
−0.2
0.0
0.2
MR leave−one−out sensitivity analysis for
'PHEX' on 'Cardiac valvular disease'

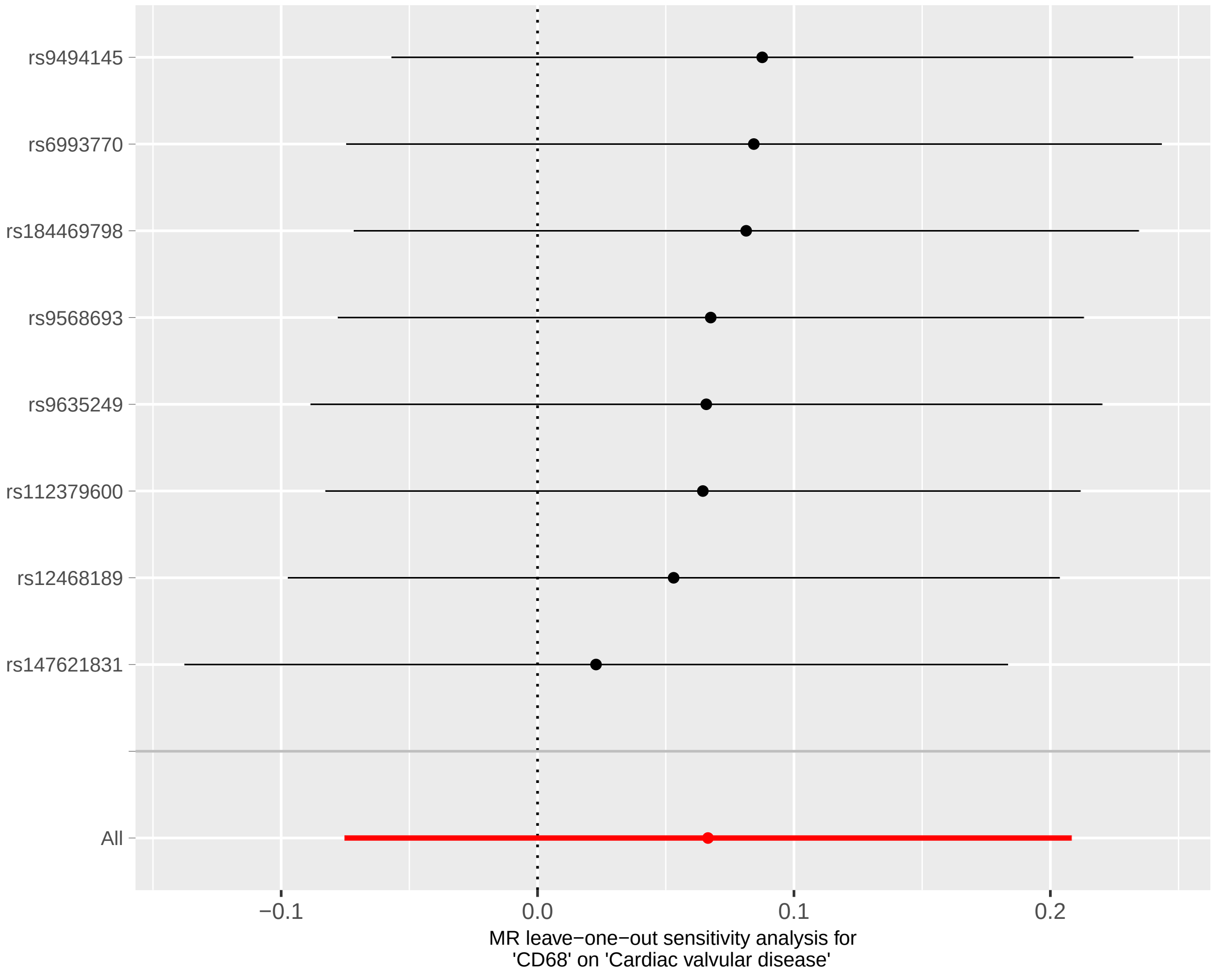

rs9494145
rs6993770
rs184469798
rs9568693
rs9635249
rs112379600
rs12468189
rs147621831
All
−0.1
0.0
0.1
0.2
MR leave−one−out sensitivity analysis for
'CD68' on 'Cardiac valvular disease'

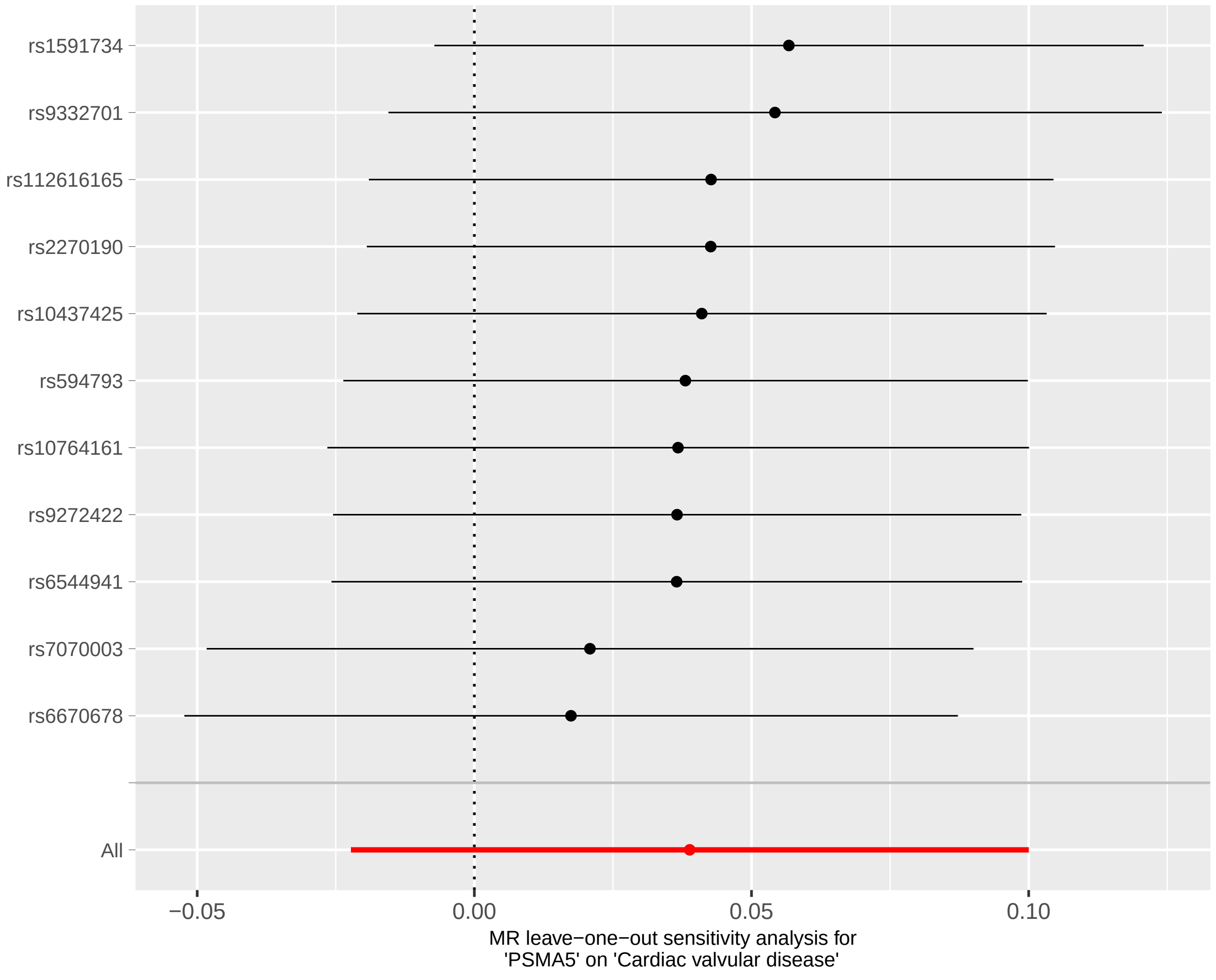
rs1591734
rs9332701
rs112616165
rs2270190
rs10437425
rs594793
rs10764161
rs9272422
rs6544941
rs7070003
rs6670678
All
−0.05
0.00
0.05
0.10
MR leave−one−out sensitivity analysis for
'PSMA5' on 'Cardiac valvular disease'

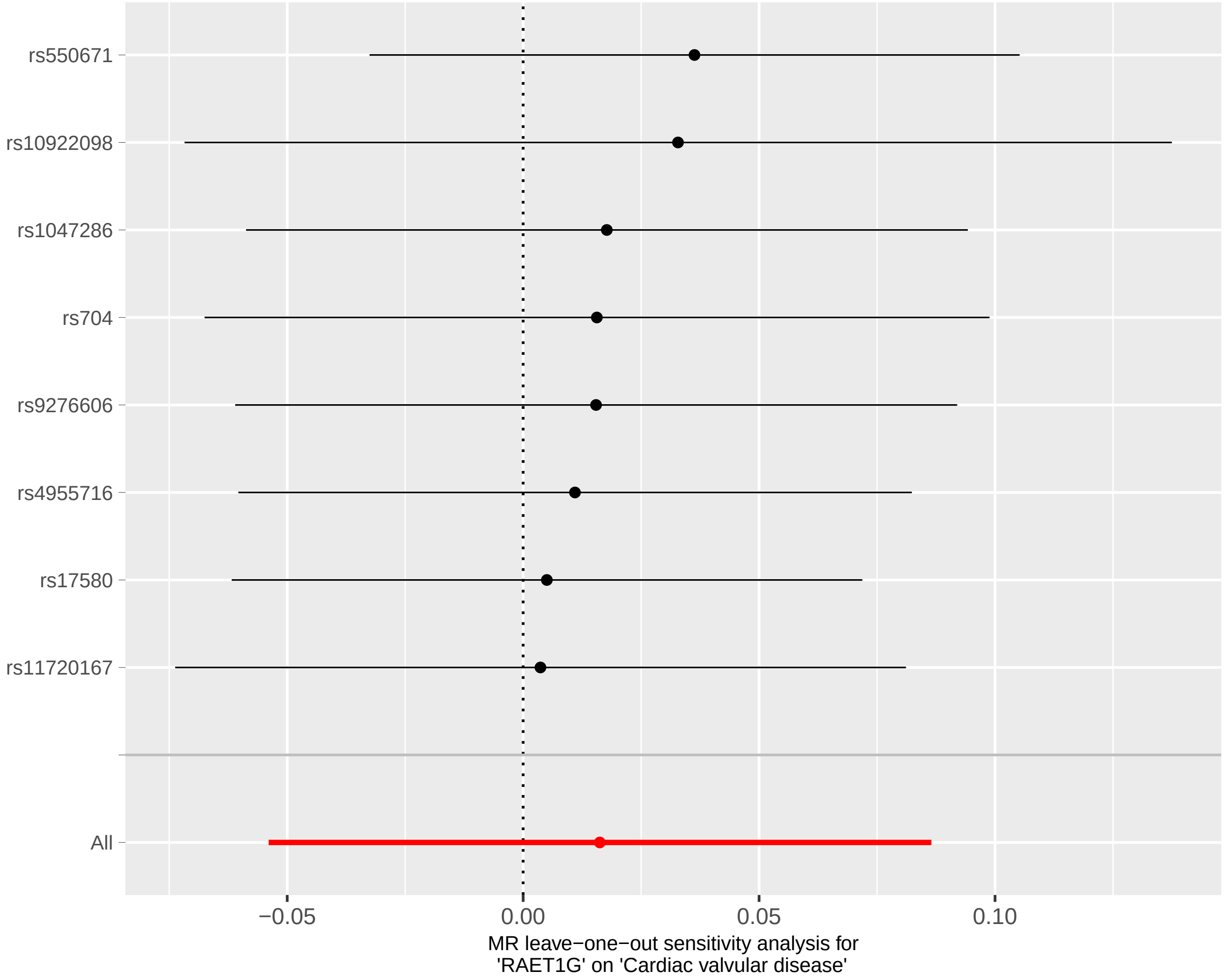

rs550671
rs10922098
rs1047286
rs704
rs9276606
rs4955716
rs17580
rs11720167
All
−0.05
0.00
0.05
0.10
MR leave−one−out sensitivity analysis for
'RAET1G' on 'Cardiac valvular disease'

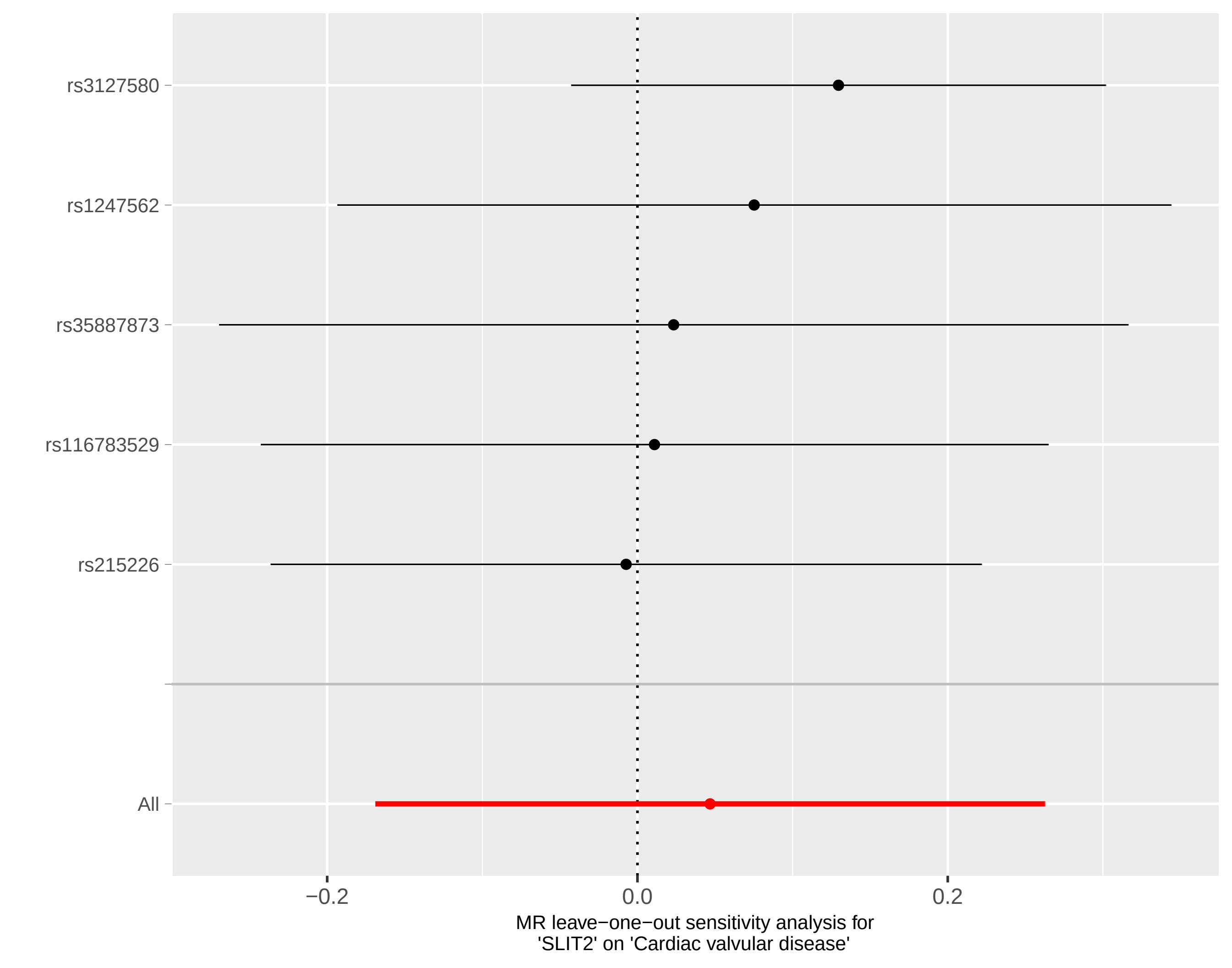
rs3127580
rs1247562
rs35887873
rs116783529
rs215226
All
−0.2
0.0
0.2
MR leave−one−out sensitivity analysis for
'SLIT2' on 'Cardiac valvular disease'

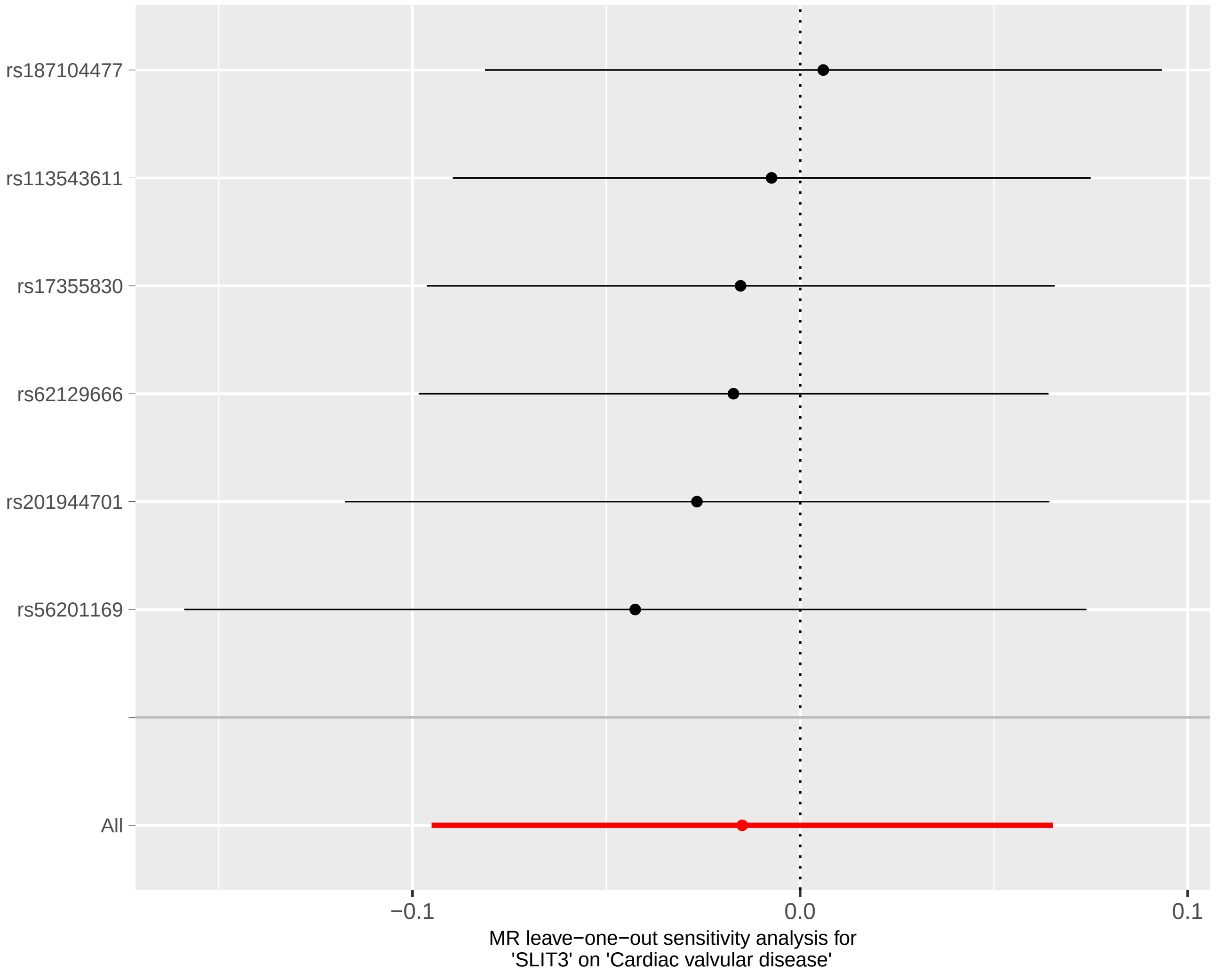
rs187104477
rs113543611
rs17355830
rs62129666
rs201944701
rs56201169
All
−0.1
0.0
0.1
MR leave−one−out sensitivity analysis for
'SLIT3' on 'Cardiac valvular disease'

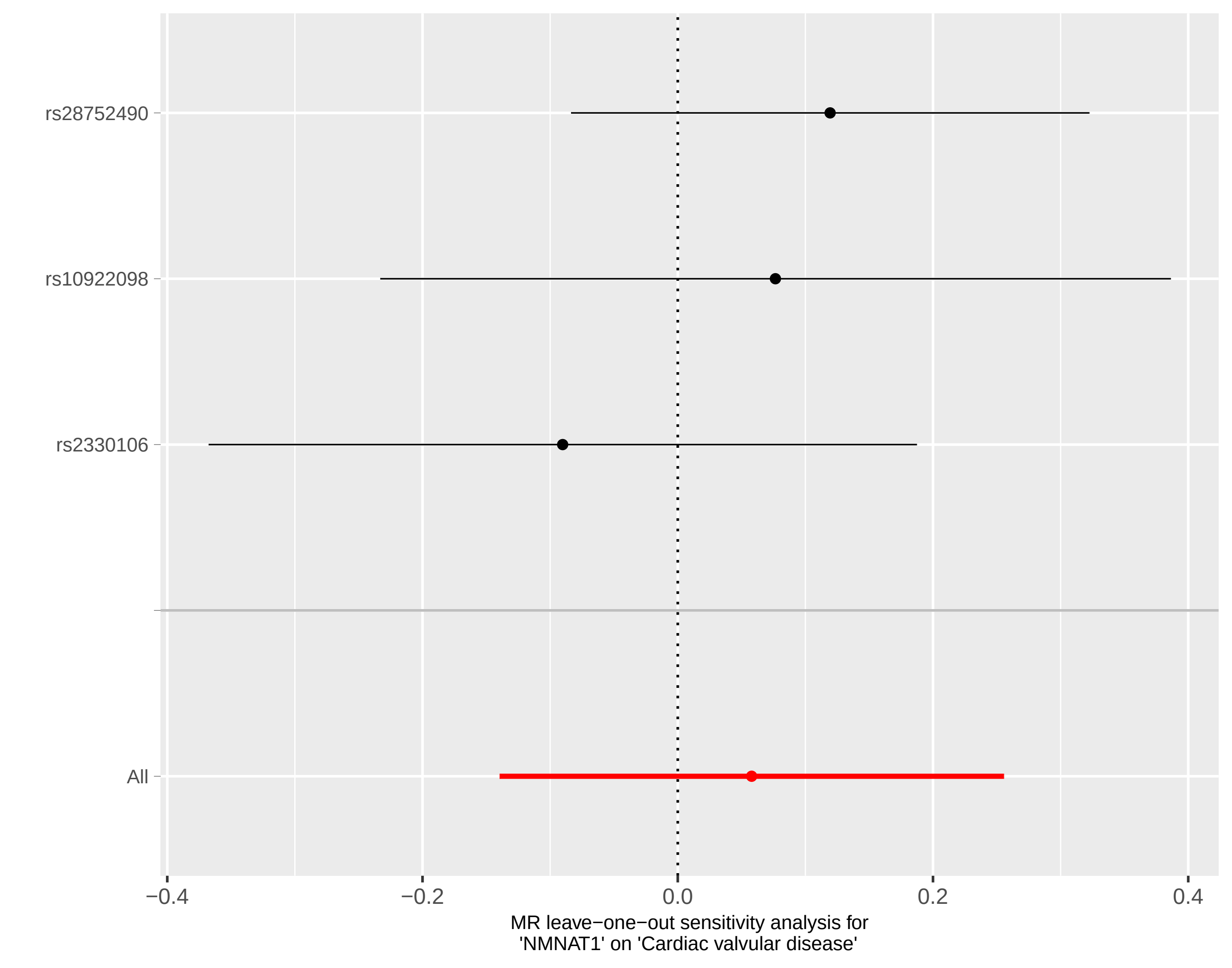

rs28752490
rs10922098
rs2330106
All
−0.4
−0.2
0.0
0.2
0.4
MR leave−one−out sensitivity analysis for
'NMNAT1' on 'Cardiac valvular disease'

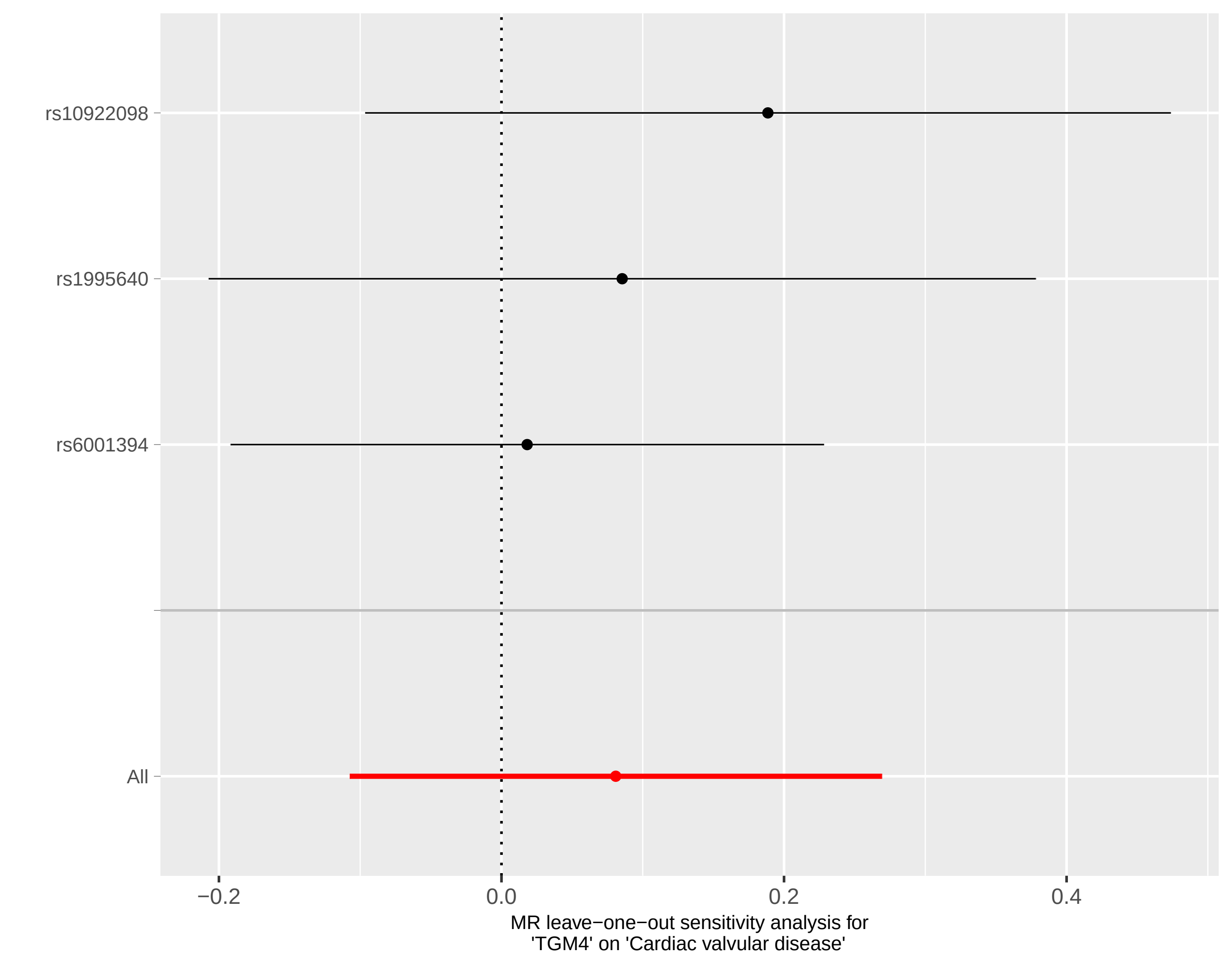

rs10922098
rs1995640
rs6001394
All
−0.2
0.0
0.2
0.4
MR leave−one−out sensitivity analysis for
'TGM4' on 'Cardiac valvular disease'

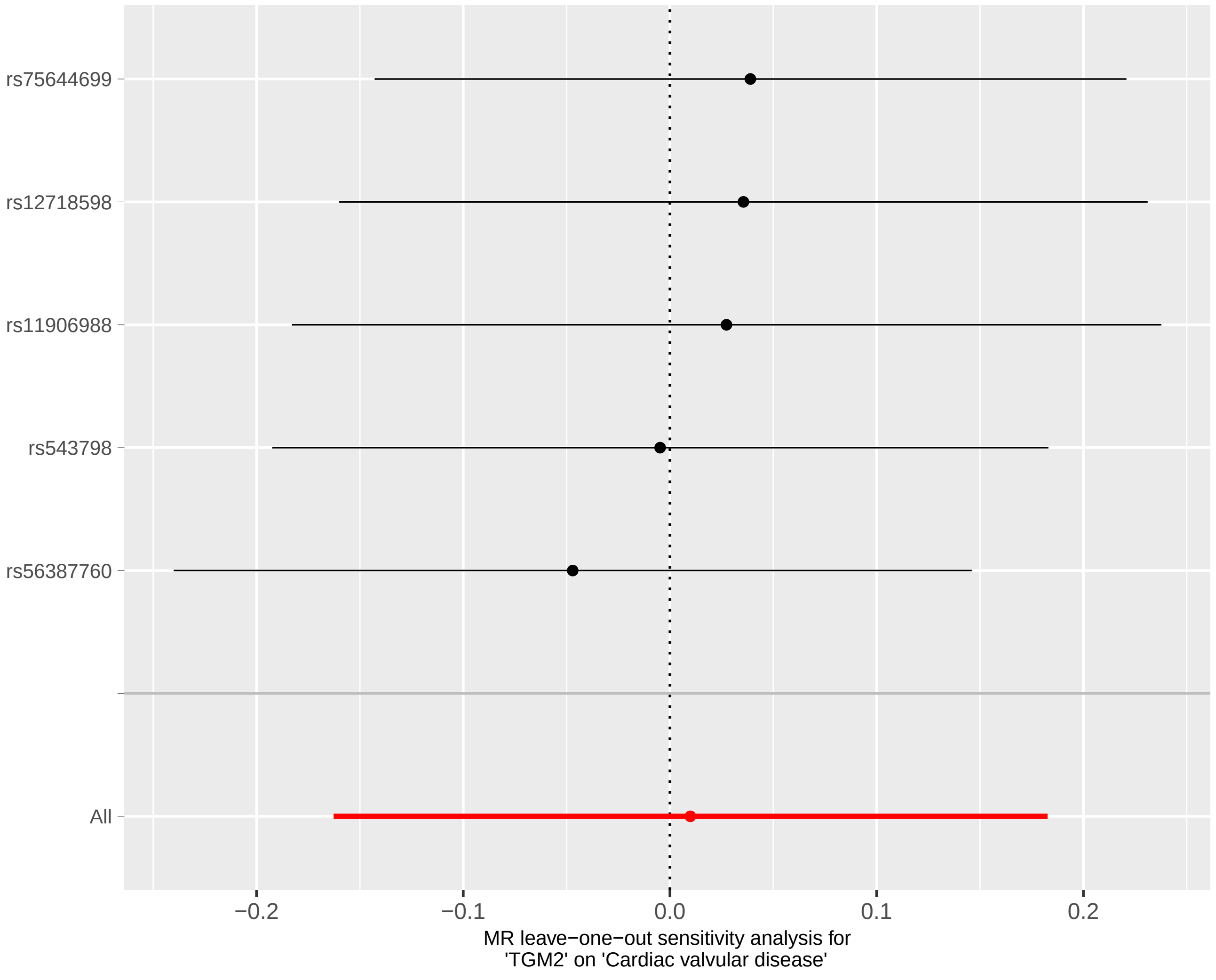
rs75644699
rs12718598
rs11906988
rs543798
rs56387760
All
−0.2
−0.1
0.0
0.1
0.2
MR leave−one−out sensitivity analysis for
'TGM2' on 'Cardiac valvular disease'

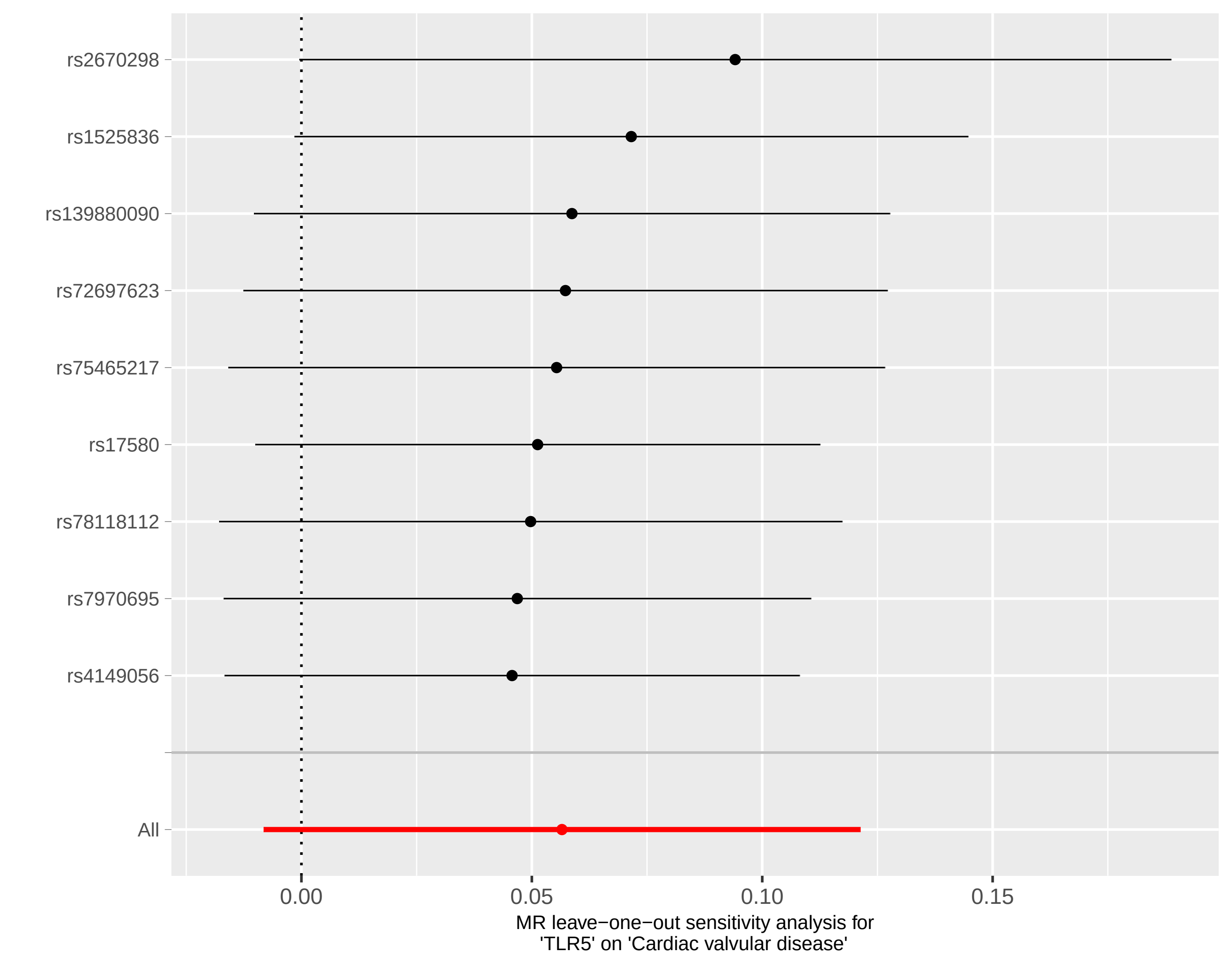

rs2670298
rs1525836
rs139880090
rs72697623
rs75465217
rs17580
rs78118112
rs7970695
rs4149056
All
0.00
0.05
0.10
0.15
MR leave−one−out sensitivity analysis for
'TLR5' on 'Cardiac valvular disease'

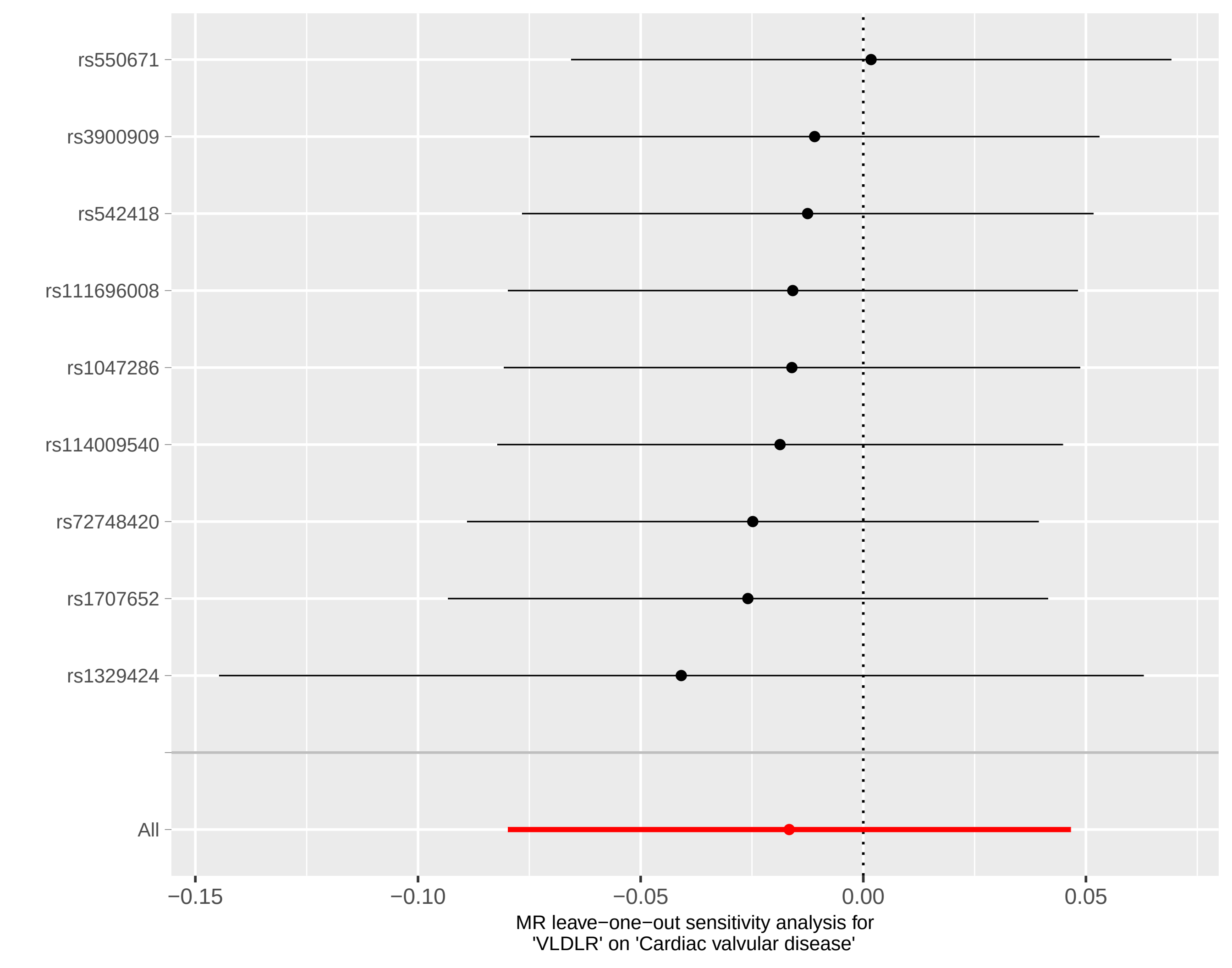
rs550671
rs3900909
rs542418
rs111696008
rs1047286
rs114009540
rs72748420
rs1707652
rs1329424
All
−0.15
−0.10
−0.05
0.00
0.05
MR leave−one−out sensitivity analysis for
'VLDLR' on 'Cardiac valvular disease'

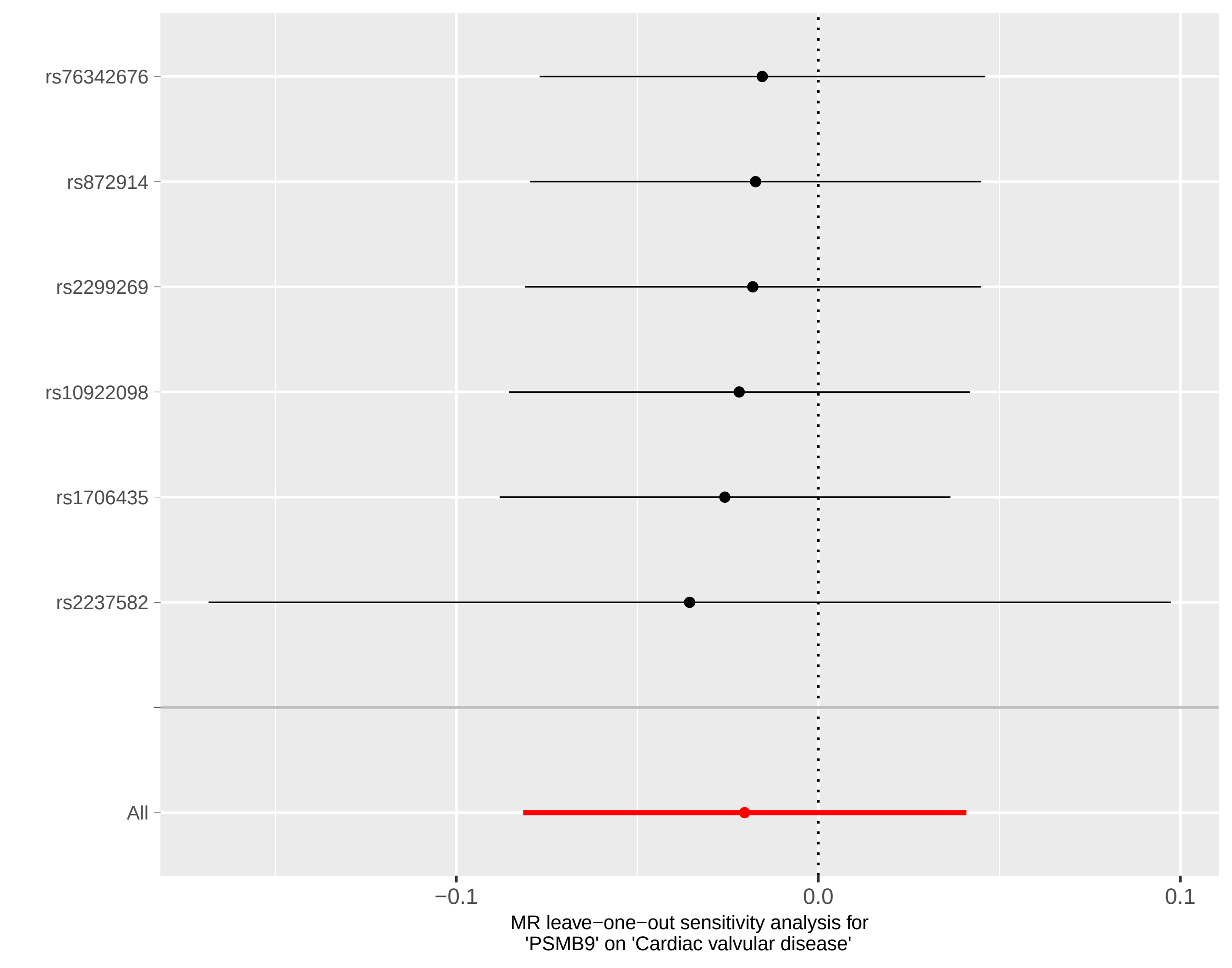
rs76342676
rs872914
rs2299269
rs10922098
rs1706435
rs2237582
All
−0.1
0.0
0.1
MR leave−one−out sensitivity analysis for
'PSMB9' on 'Cardiac valvular disease'

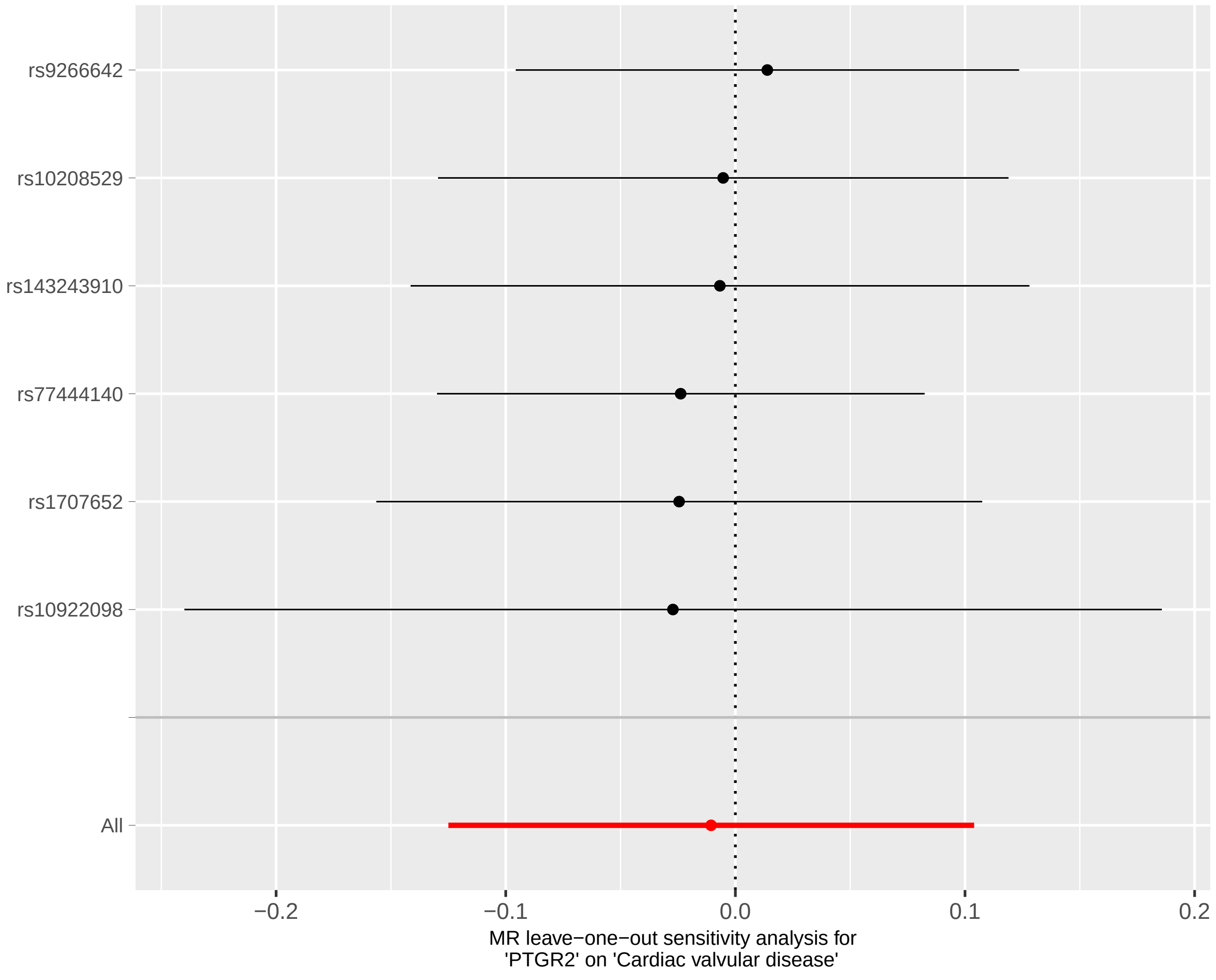
rs9266642
rs10208529
rs143243910
rs77444140
rs1707652
rs10922098
All
−0.2
−0.1
0.0
0.1
0.2
MR leave−one−out sensitivity analysis for
'PTGR2' on 'Cardiac valvular disease'

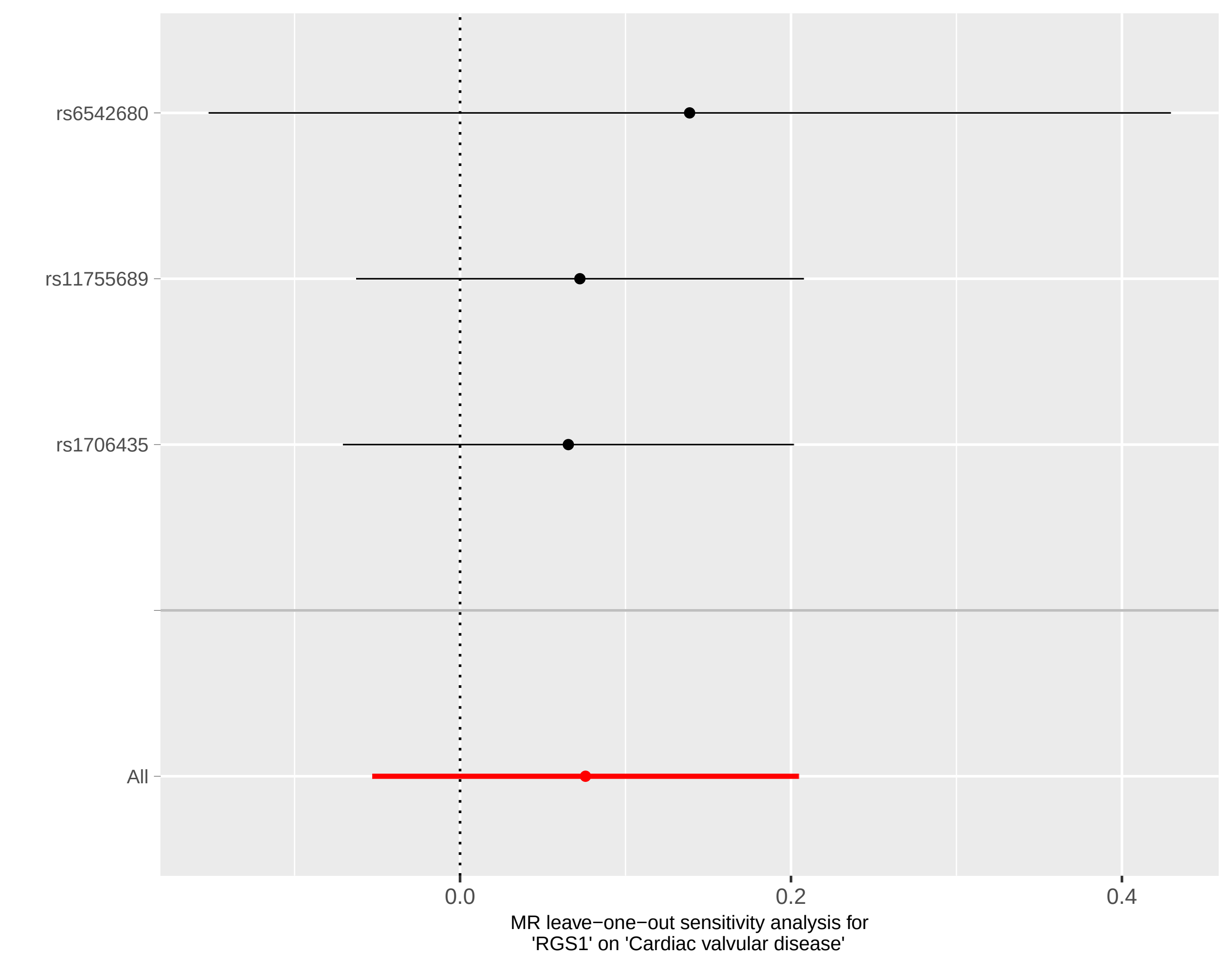

rs6542680
rs11755689
rs1706435
All
0.0
0.2
0.4
MR leave−one−out sensitivity analysis for
'RGS1' on 'Cardiac valvular disease'

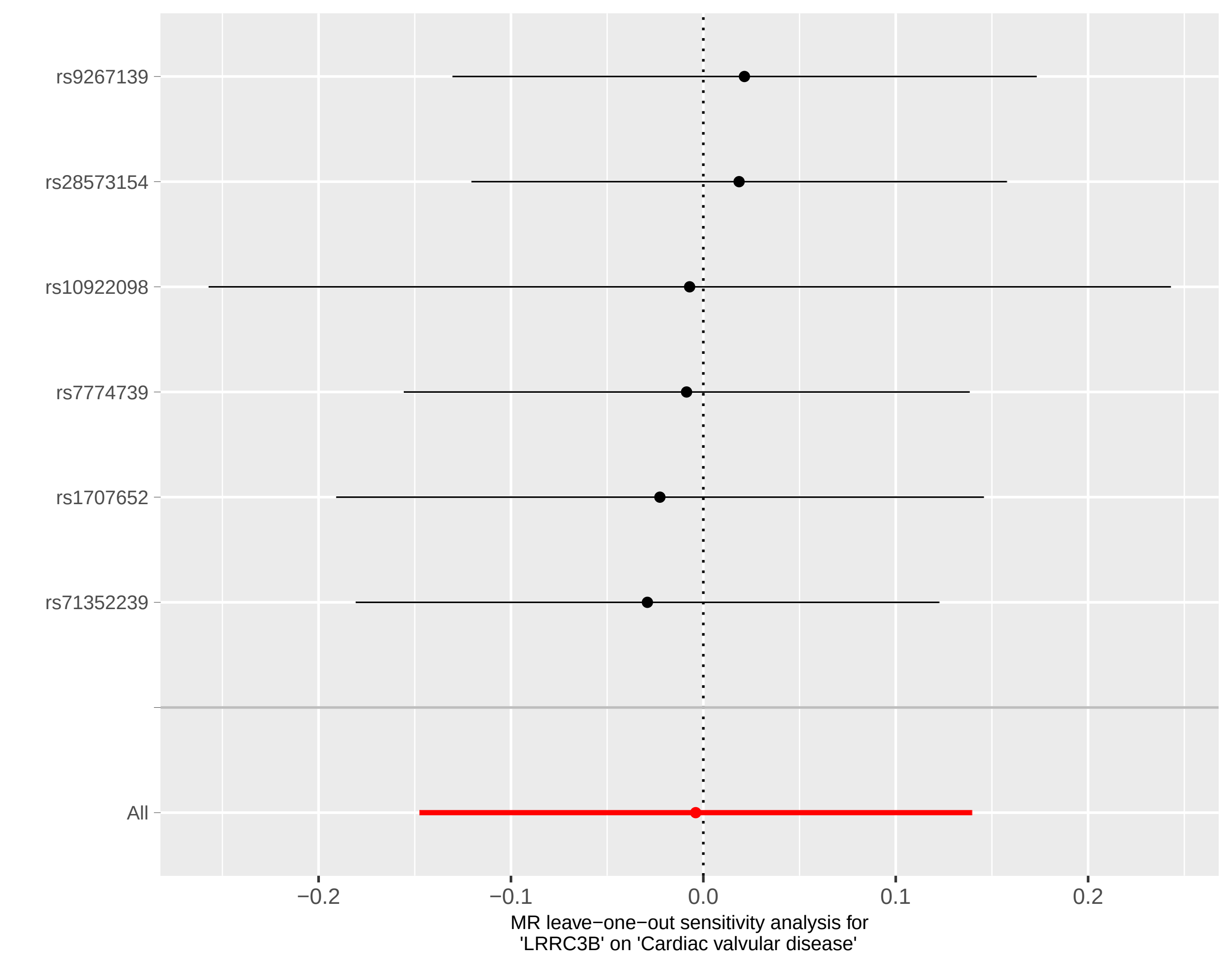

rs9267139
rs28573154
rs10922098
rs7774739
rs1707652
rs71352239
All
−0.2
−0.1
0.0
0.1
0.2
MR leave−one−out sensitivity analysis for
'LRRC3B' on 'Cardiac valvular disease'

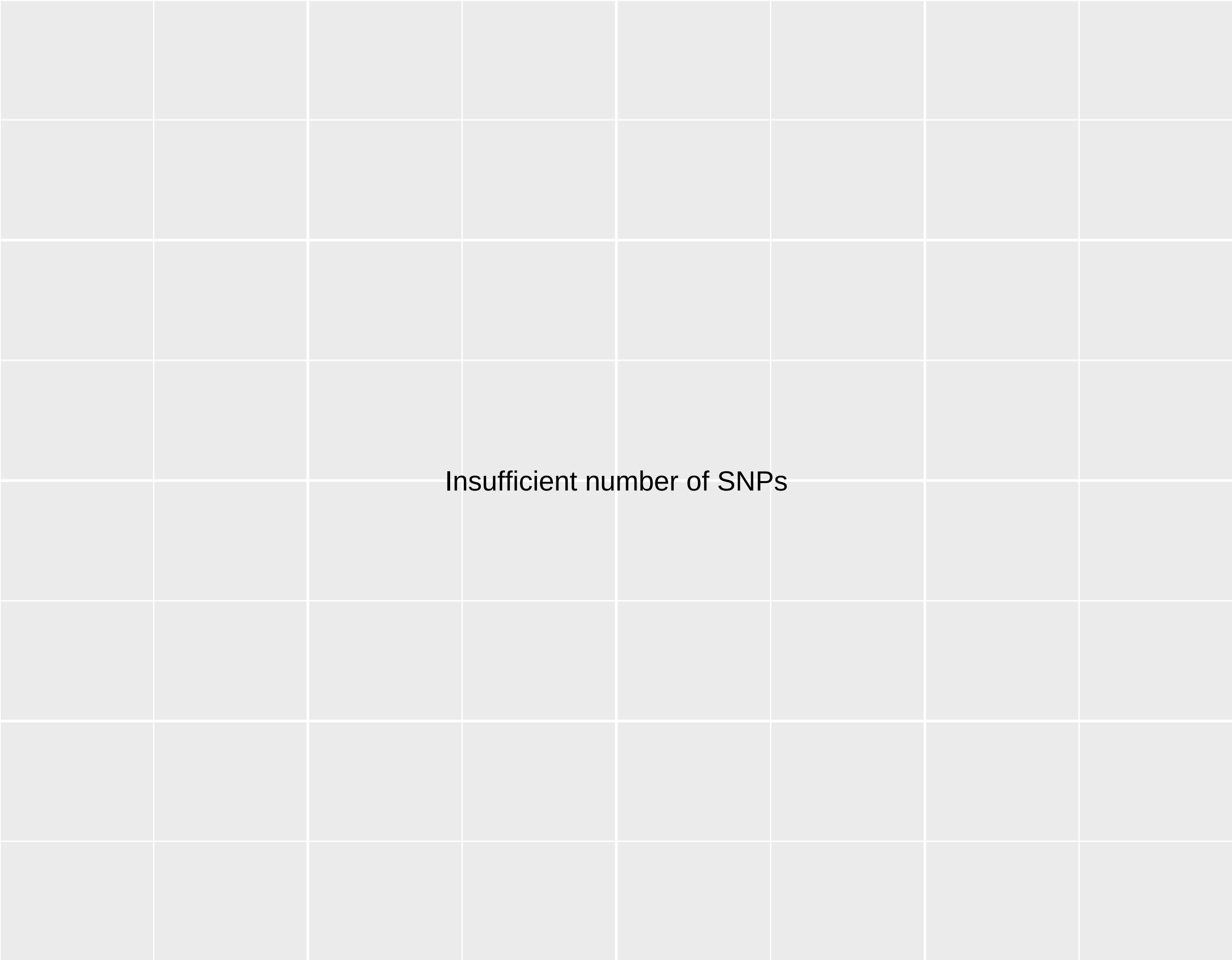
Insufficient number of SNPs

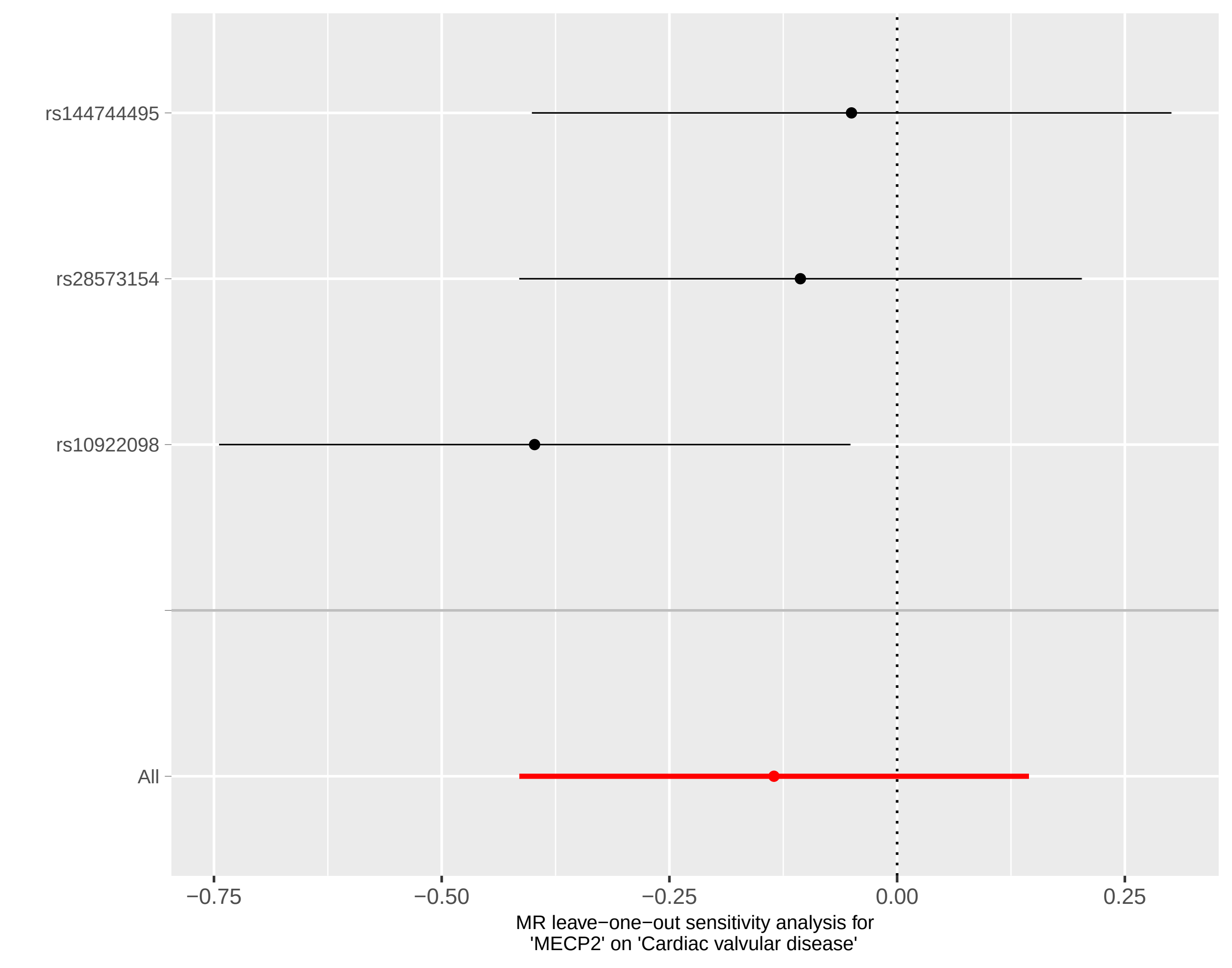
rs144744495
rs28573154
rs10922098
All
−0.75
−0.50
−0.25
0.00
0.25
MR leave−one−out sensitivity analysis for
'MECP2' on 'Cardiac valvular disease'

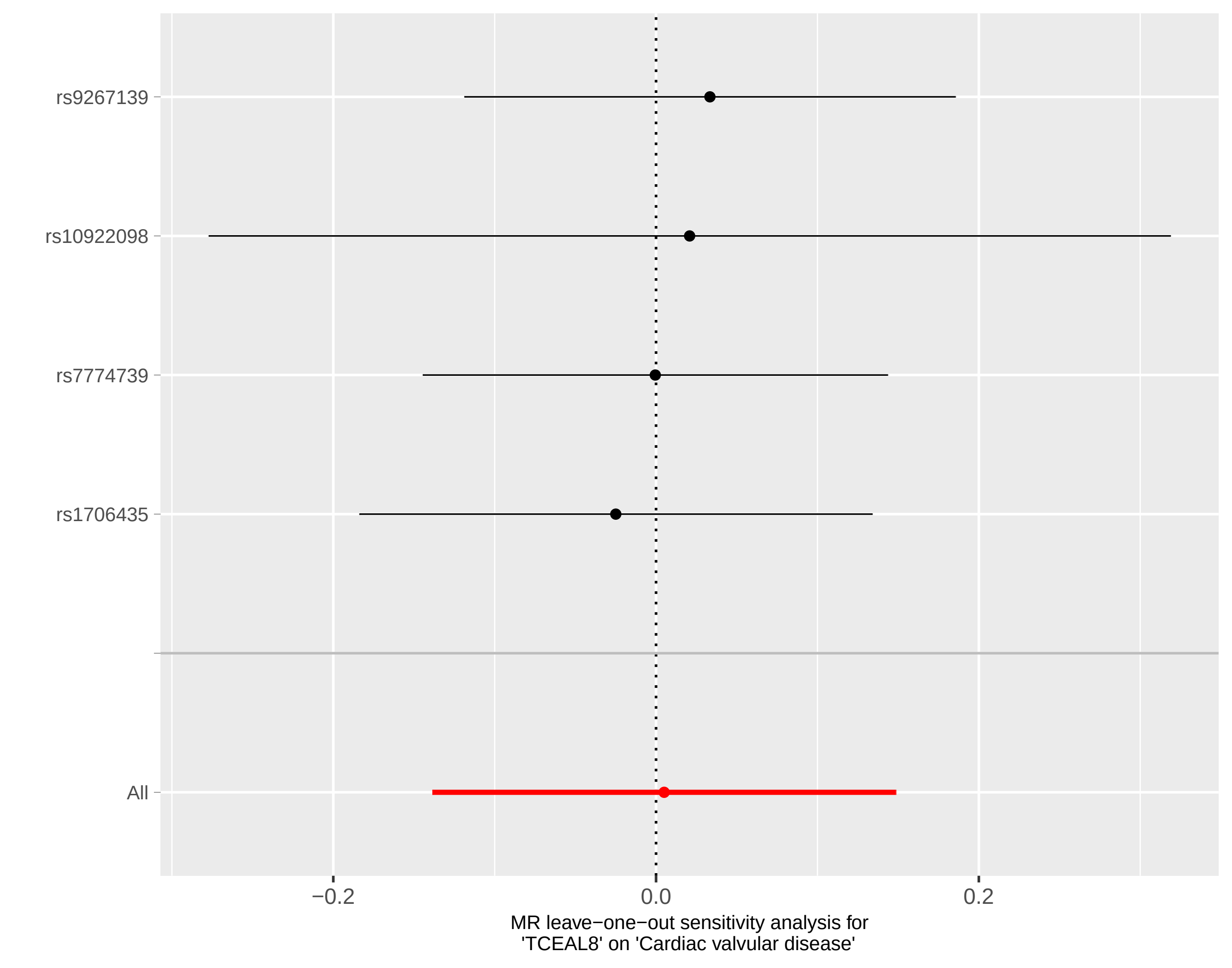
rs9267139
rs10922098
rs7774739
rs1706435
All
−0.2
0.0
0.2
MR leave−one−out sensitivity analysis for
'TCEAL8' on 'Cardiac valvular disease'

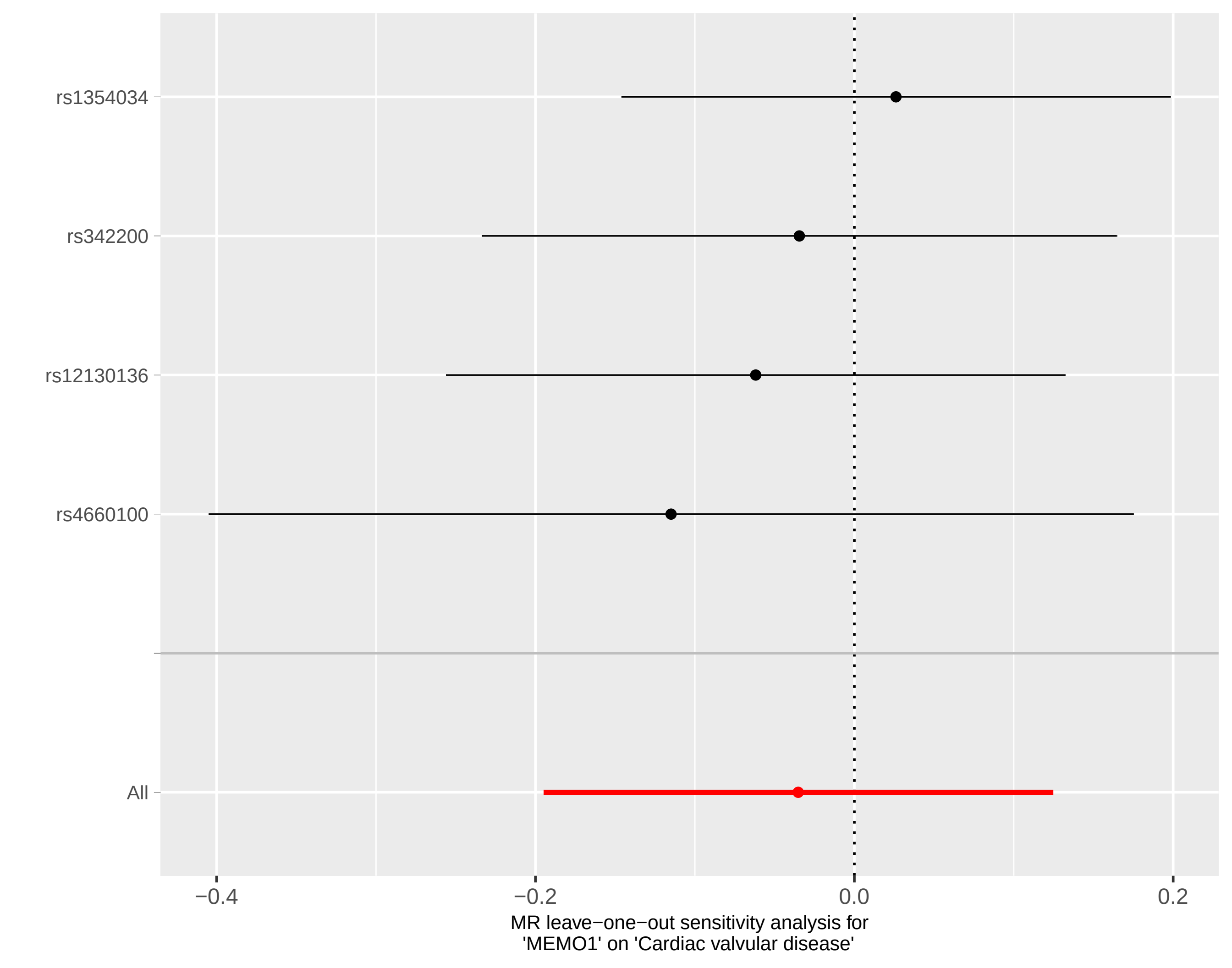
rs1354034
rs342200
rs12130136
rs4660100
All
−0.4
−0.2
0.0
0.2
MR leave−one−out sensitivity analysis for
'MEMO1' on 'Cardiac valvular disease'

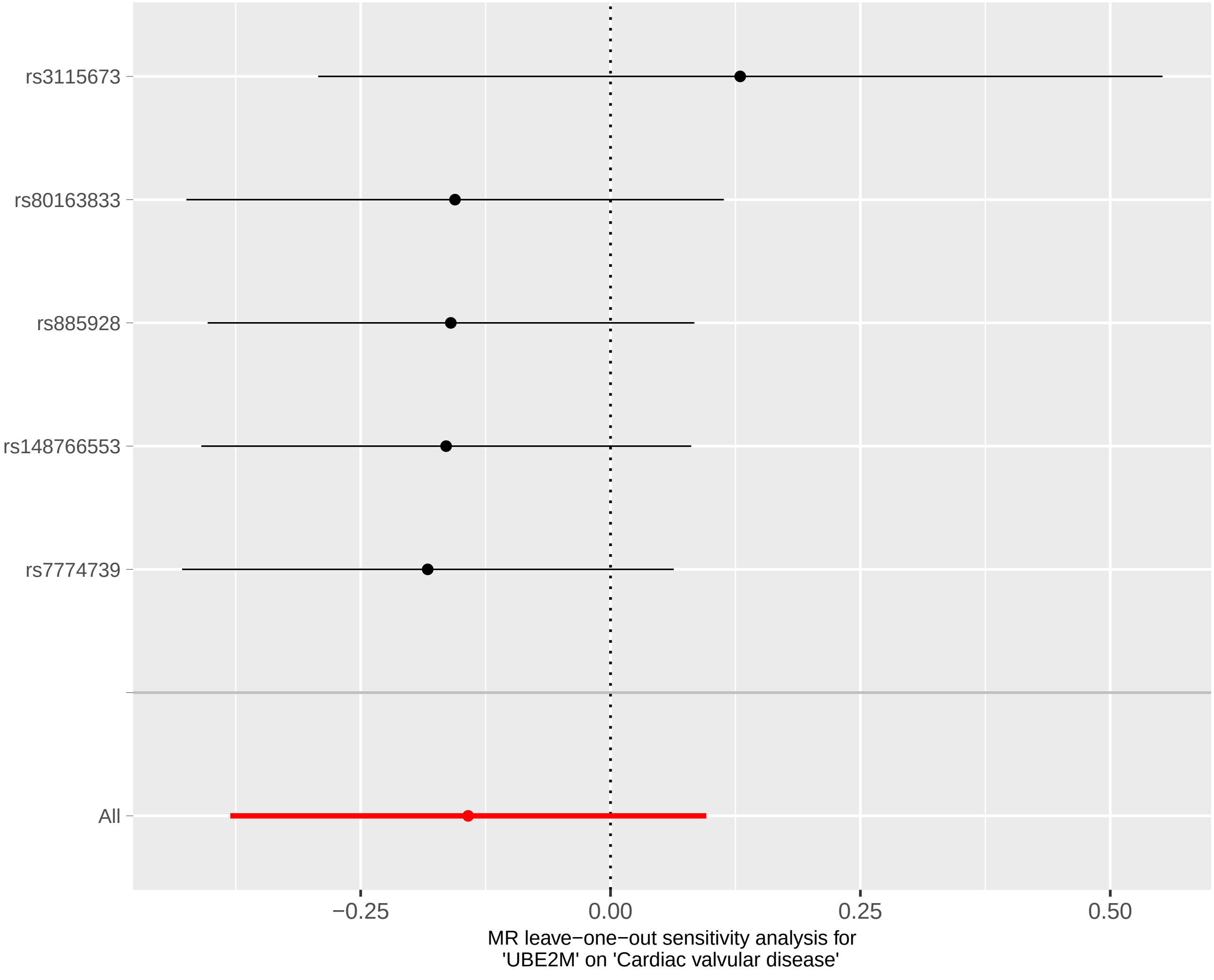
rs3115673
rs80163833
rs885928
rs148766553
rs7774739
All
−0.25
0.00
0.25
0.50
MR leave−one−out sensitivity analysis for
'UBE2M' on 'Cardiac valvular disease'

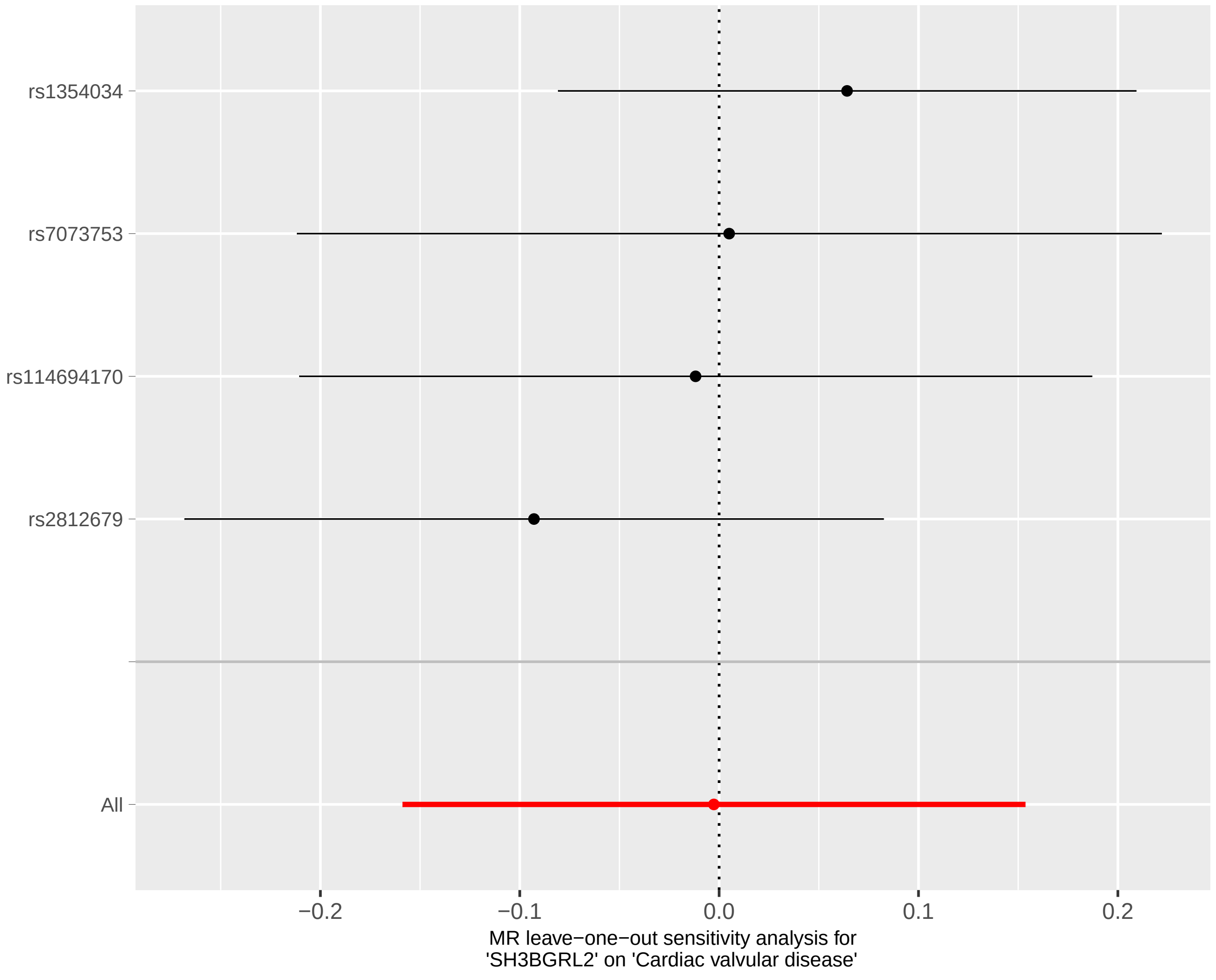
rs1354034
rs7073753
rs114694170
rs2812679
All
−0.2
−0.1
0.0
0.1
0.2
MR leave−one−out sensitivity analysis for
'SH3BGRL2' on 'Cardiac valvular disease'

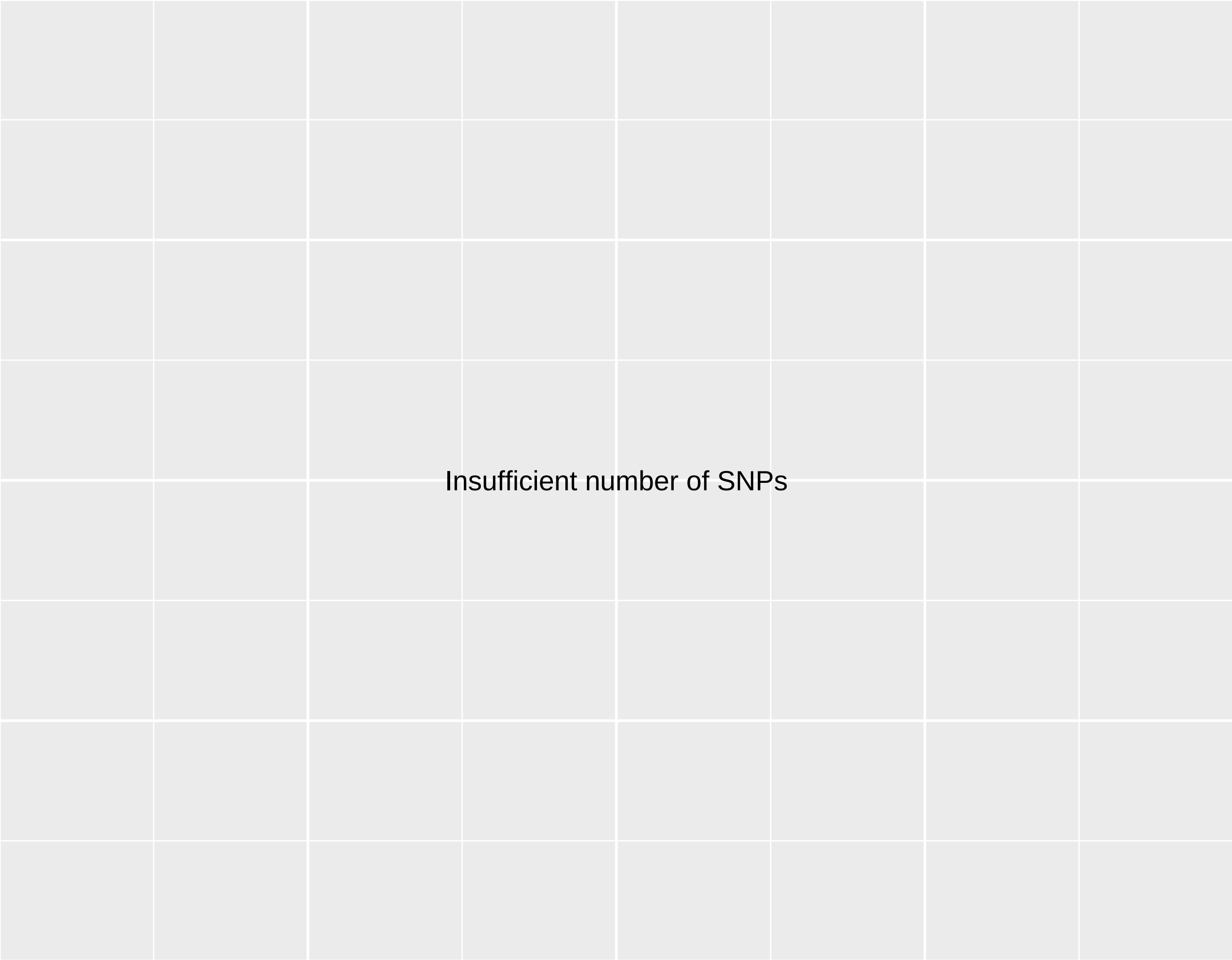
Insufficient number of SNPs

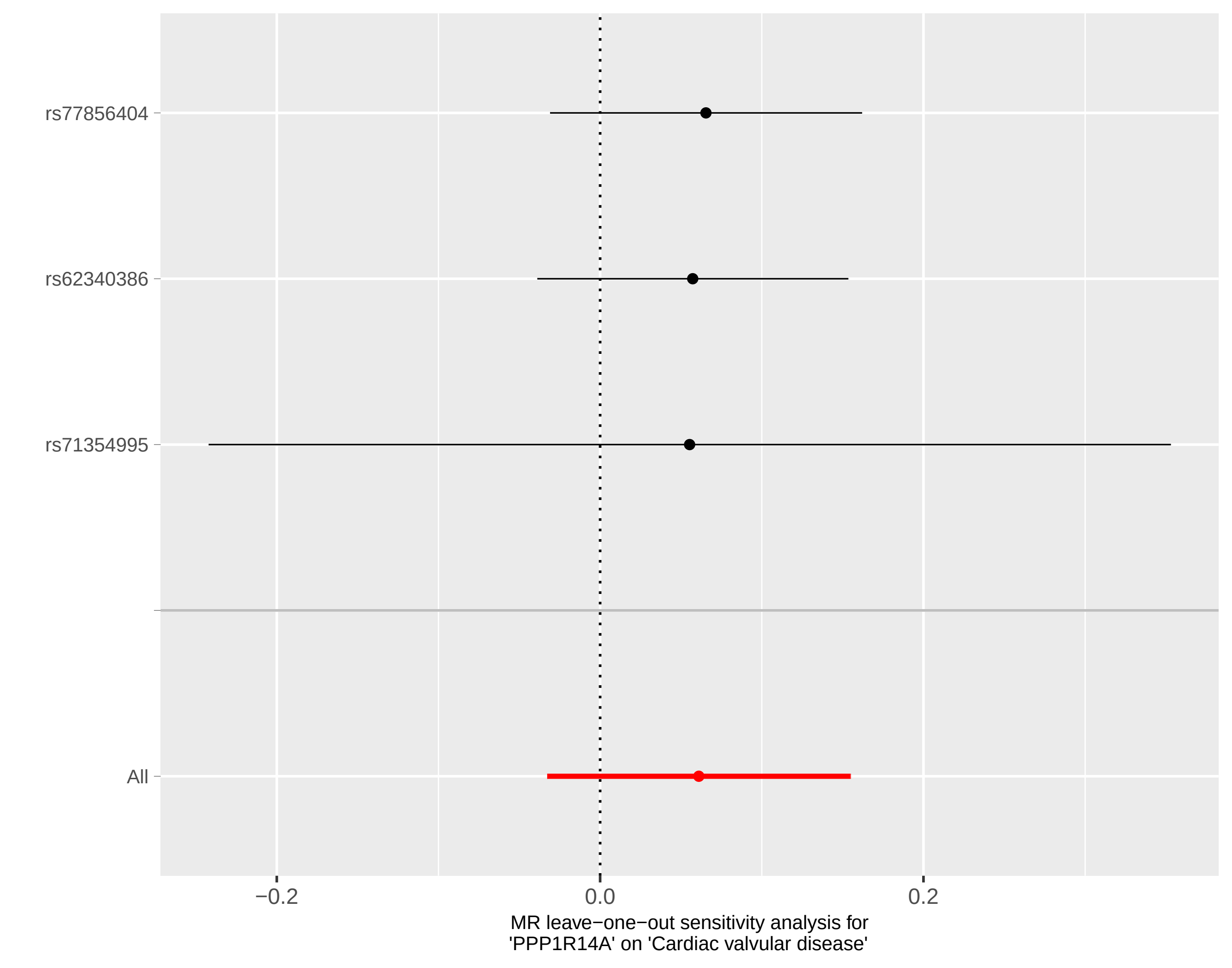

rs77856404
rs62340386
rs71354995
All
−0.2
0.0
0.2
MR leave−one−out sensitivity analysis for
'PPP1R14A' on 'Cardiac valvular disease'

Insufficient number of SNPs

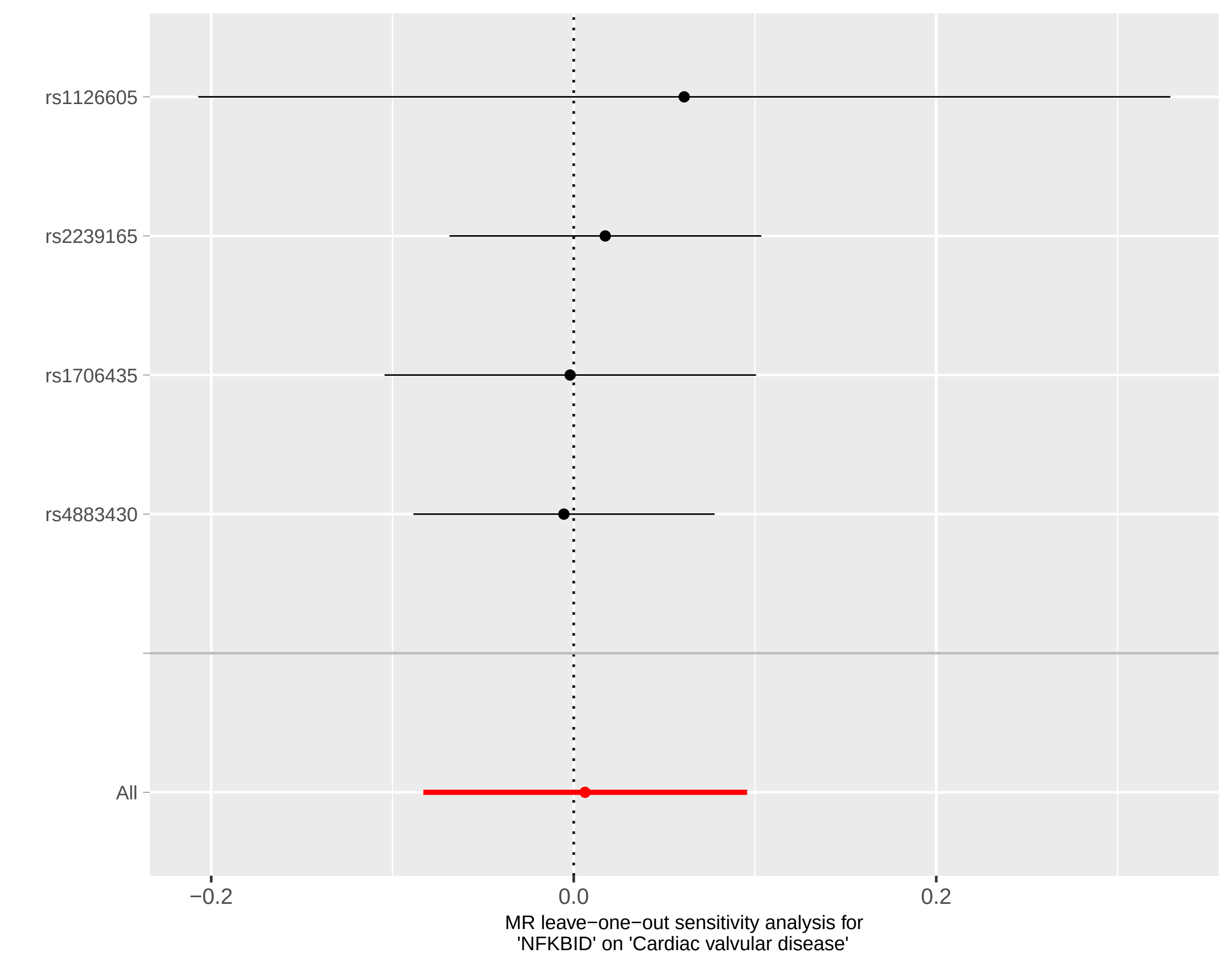

rs1126605
rs2239165
rs1706435
rs4883430
All
−0.2
0.0
0.2
MR leave−one−out sensitivity analysis for
'NFKBID' on 'Cardiac valvular disease'

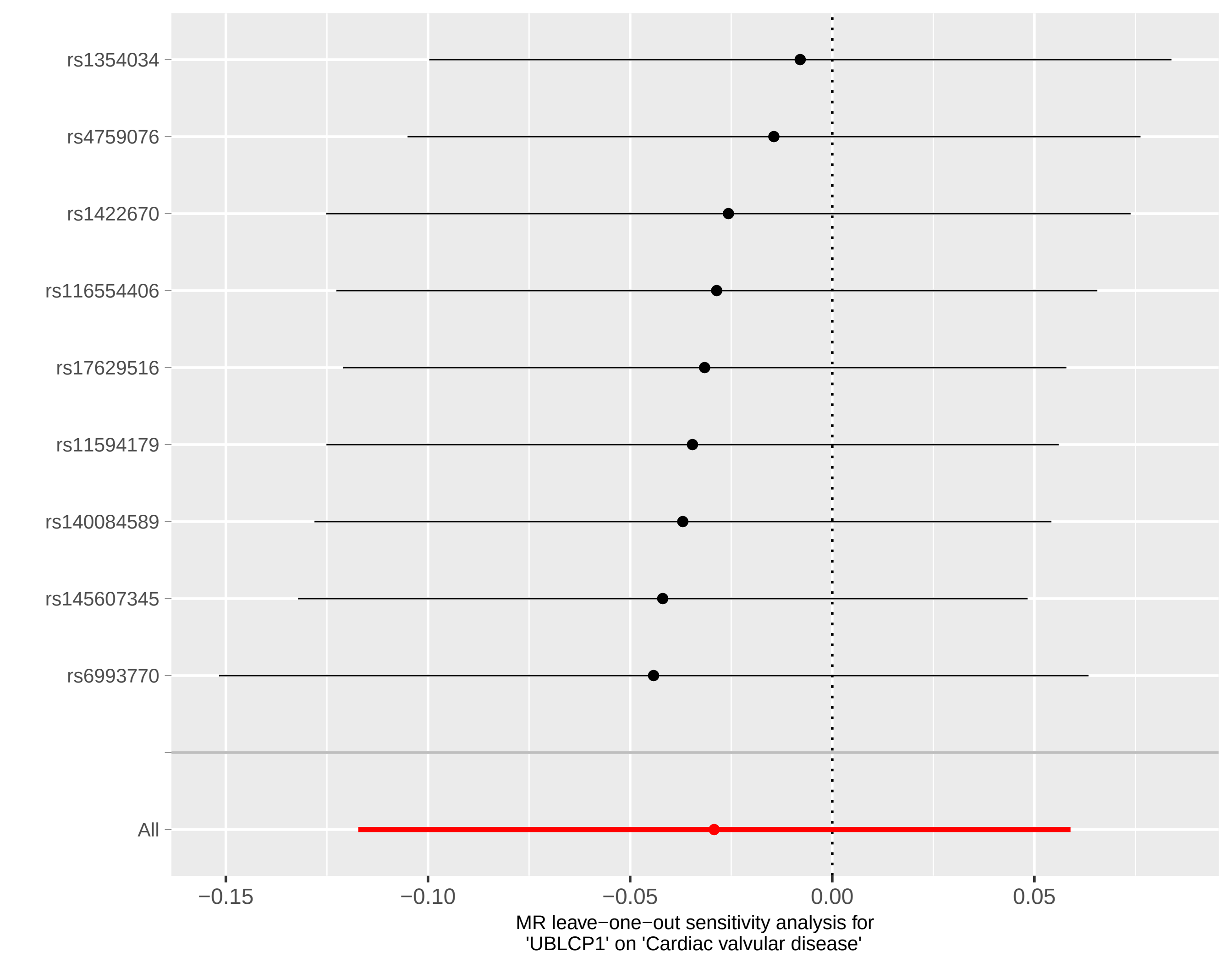

rs1354034
rs4759076
rs1422670
rs116554406
rs17629516
rs11594179
rs140084589
rs145607345
rs6993770
All
−0.15
−0.10
−0.05
0.00
0.05
MR leave−one−out sensitivity analysis for
'UBLCP1' on 'Cardiac valvular disease'

Insufficient number of SNPs

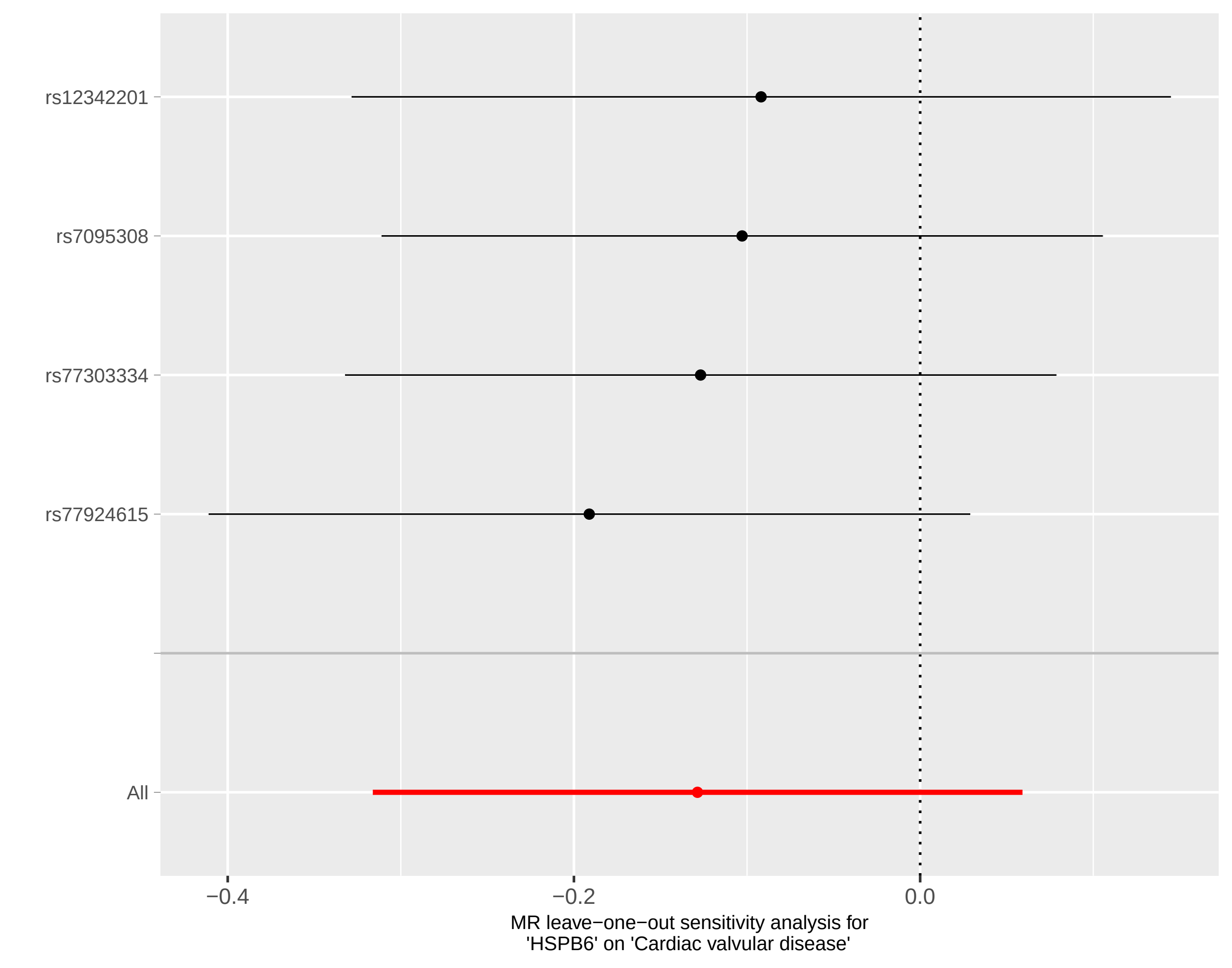

rs12342201
rs7095308
rs77303334
rs77924615
All
−0.4
−0.2
0.0
MR leave−one−out sensitivity analysis for
'HSPB6' on 'Cardiac valvular disease'

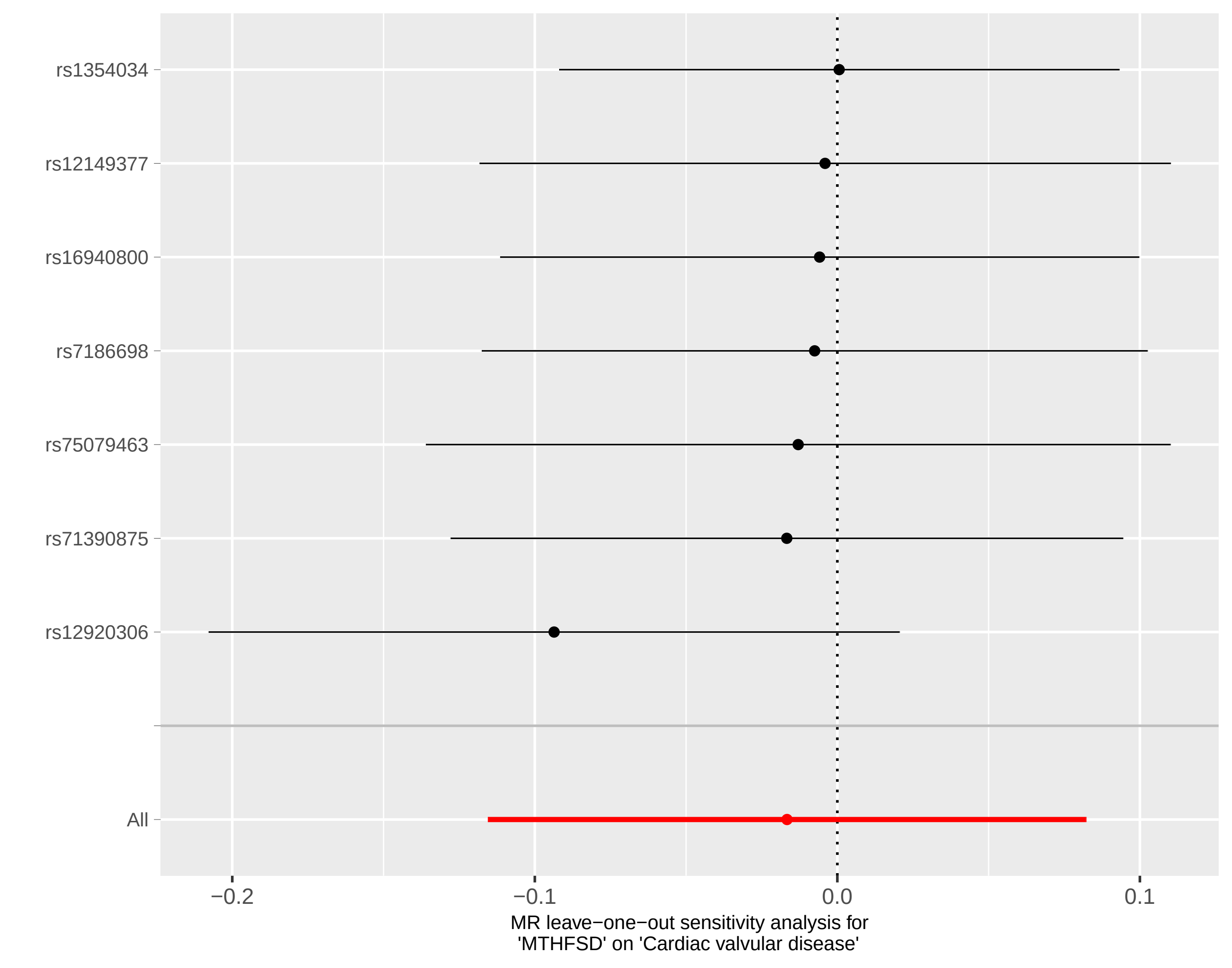

rs1354034
rs12149377
rs16940800
rs7186698
rs75079463
rs71390875
rs12920306
All
−0.2
−0.1
0.0
0.1
MR leave−one−out sensitivity analysis for
'MTHFSD' on 'Cardiac valvular disease'

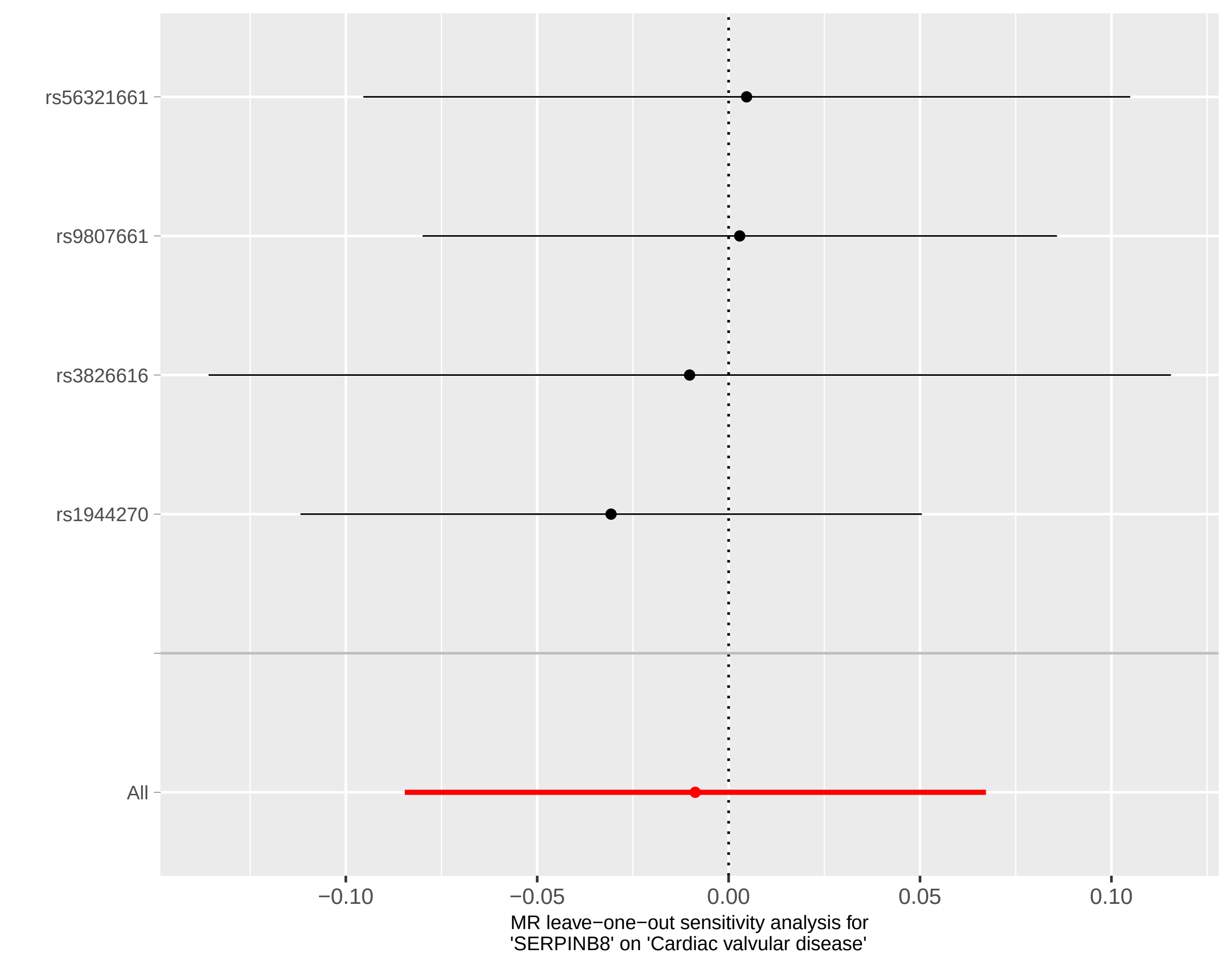
rs56321661
rs9807661
rs3826616
rs1944270
All
−0.10
−0.05
0.00
0.05
0.10
MR leave−one−out sensitivity analysis for
'SERPINB8' on 'Cardiac valvular disease'

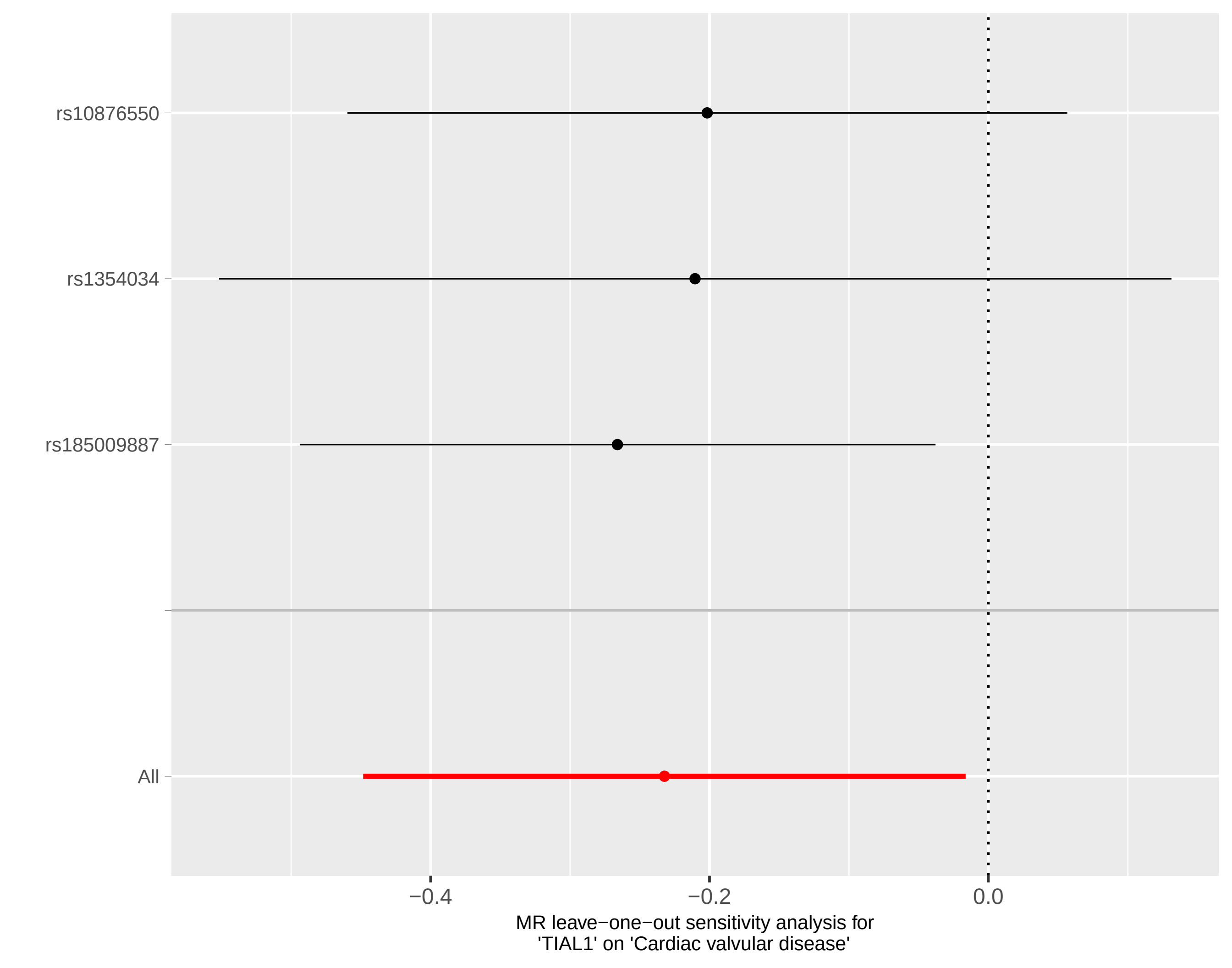

rs10876550
rs1354034
rs185009887
All
−0.4
−0.2
0.0
MR leave−one−out sensitivity analysis for
'TIAL1' on 'Cardiac valvular disease'

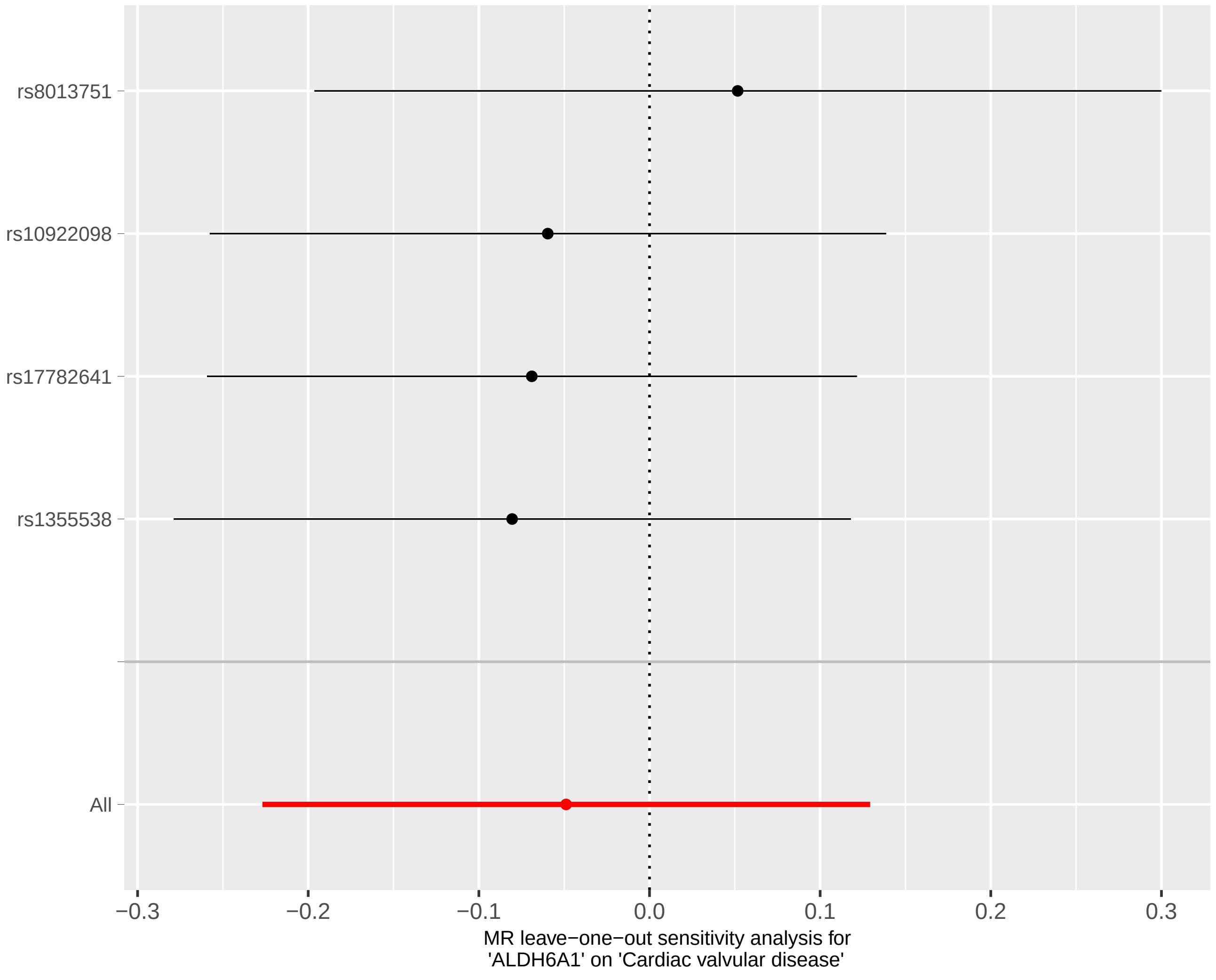
rs8013751
rs10922098
rs17782641
rs1355538
All
−0.3
−0.2
−0.1
0.0
0.1
0.2
0.3
MR leave−one−out sensitivity analysis for
'ALDH6A1' on 'Cardiac valvular disease'

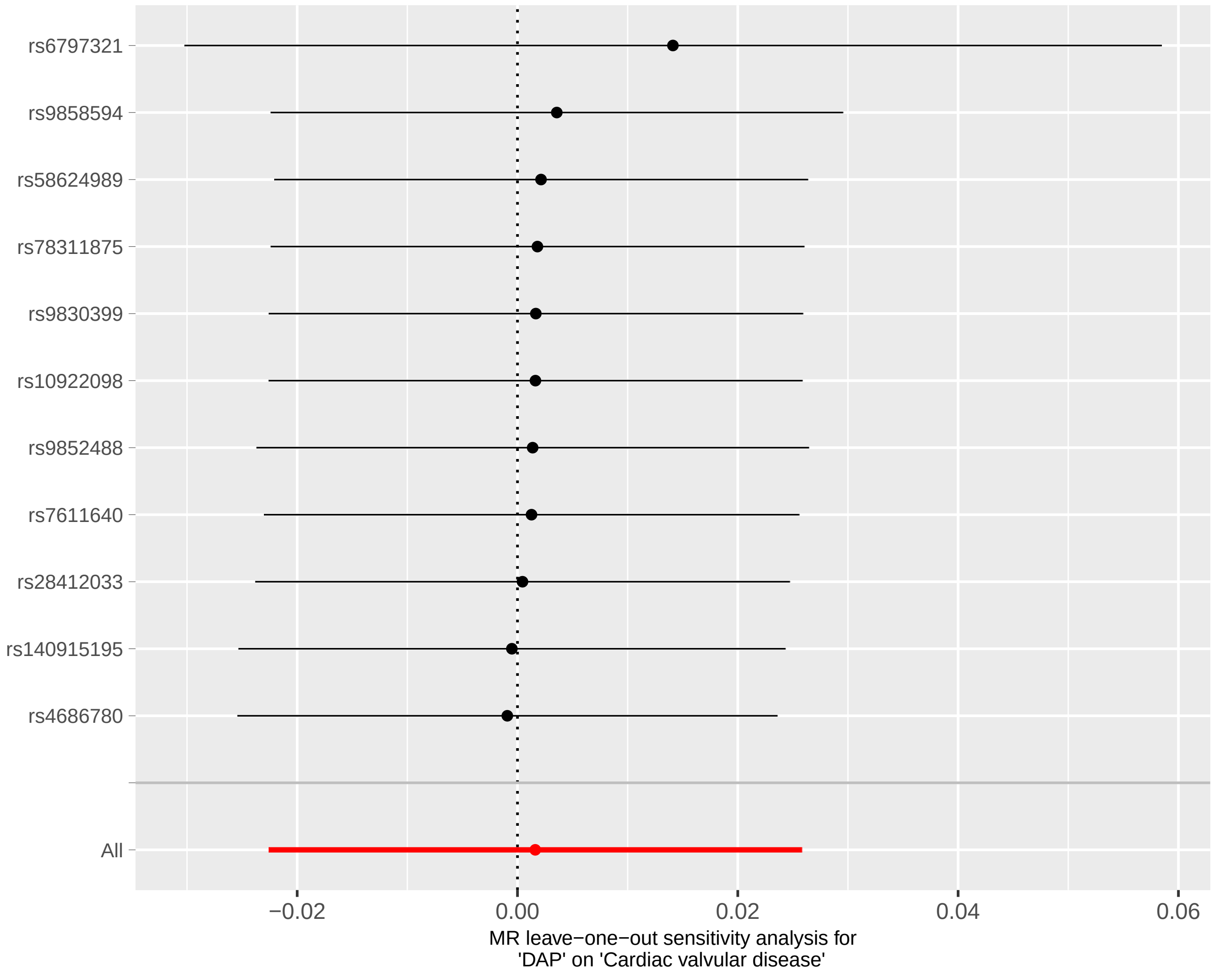

rs6797321
rs9858594
rs58624989
rs78311875
rs9830399
rs10922098
rs9852488
rs7611640
rs28412033
rs140915195
rs4686780
All
−0.02
0.00
0.02
0.04
0.06
MR leave−one−out sensitivity analysis for
'DAP' on 'Cardiac valvular disease'

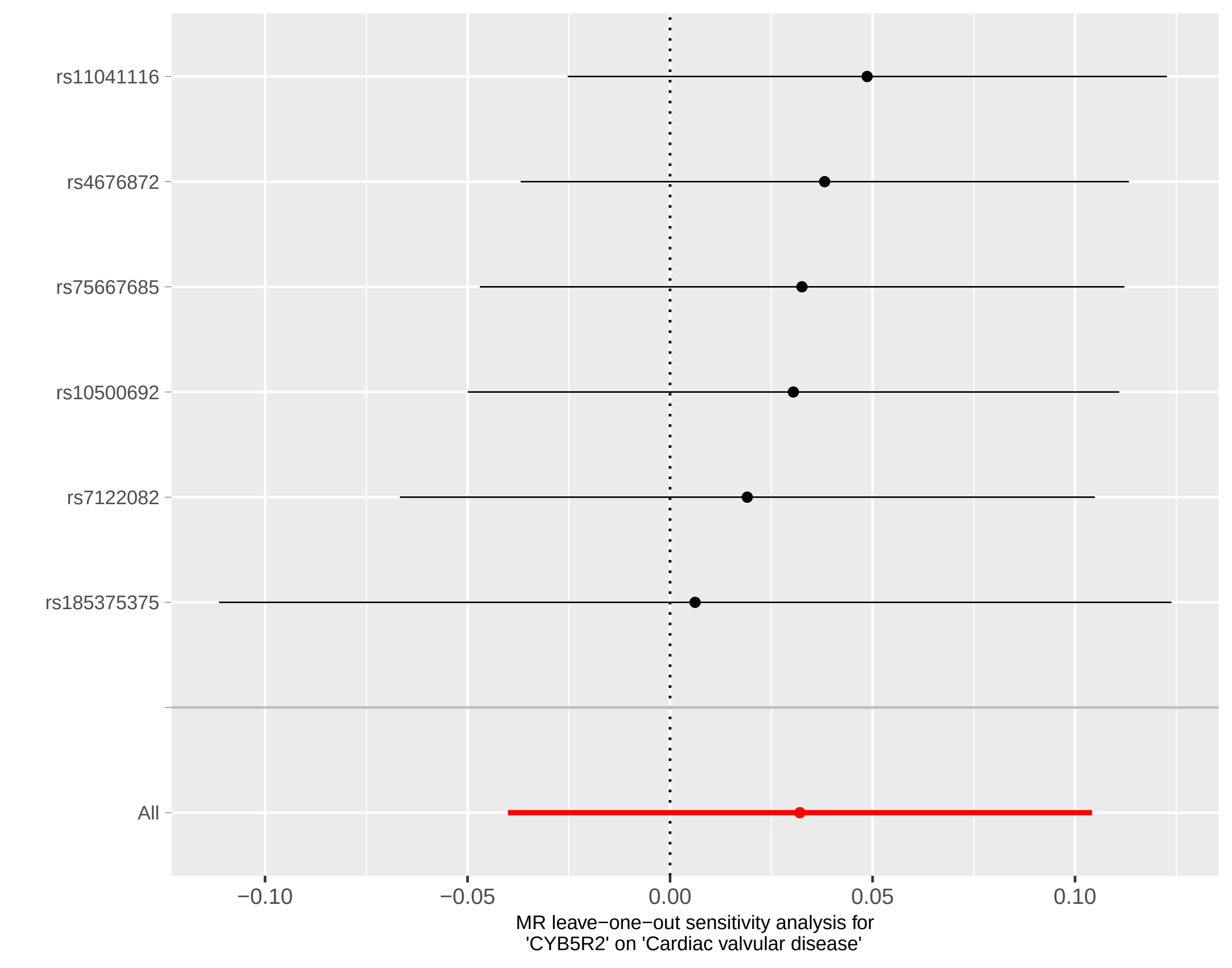

rs11041116
rs4676872
rs75667685
rs10500692
rs7122082
rs185375375
All
−0.10
−0.05
0.00
0.05
0.10
MR leave−one−out sensitivity analysis for
'CYB5R2' on 'Cardiac valvular disease'

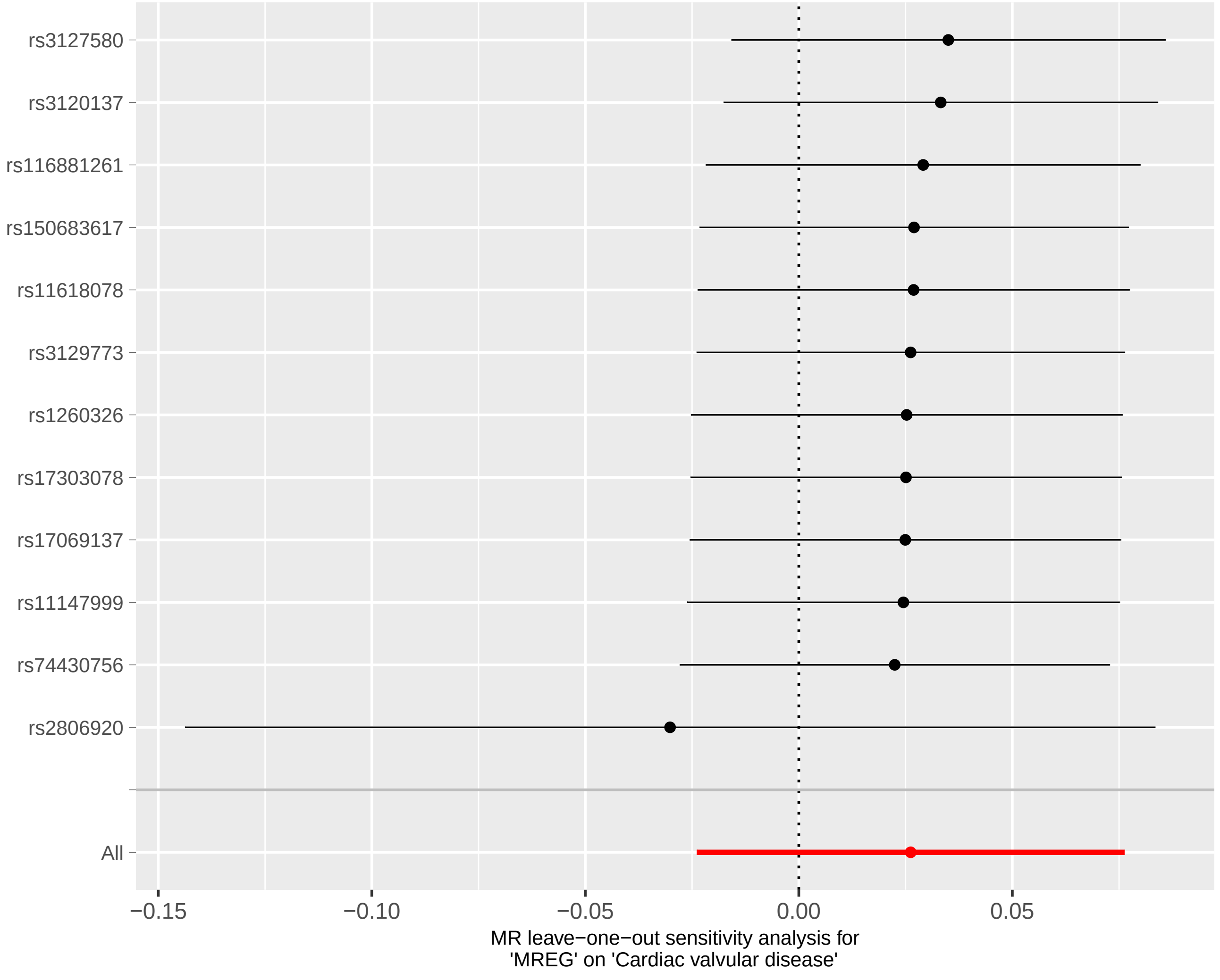

rs3127580
rs3120137
rs116881261
rs150683617
rs11618078
rs3129773
rs1260326
rs17303078
rs17069137
rs11147999
rs74430756
rs2806920
All
−0.15
−0.10
−0.05
0.00
0.05
MR leave−one−out sensitivity analysis for
'MREG' on 'Cardiac valvular disease'

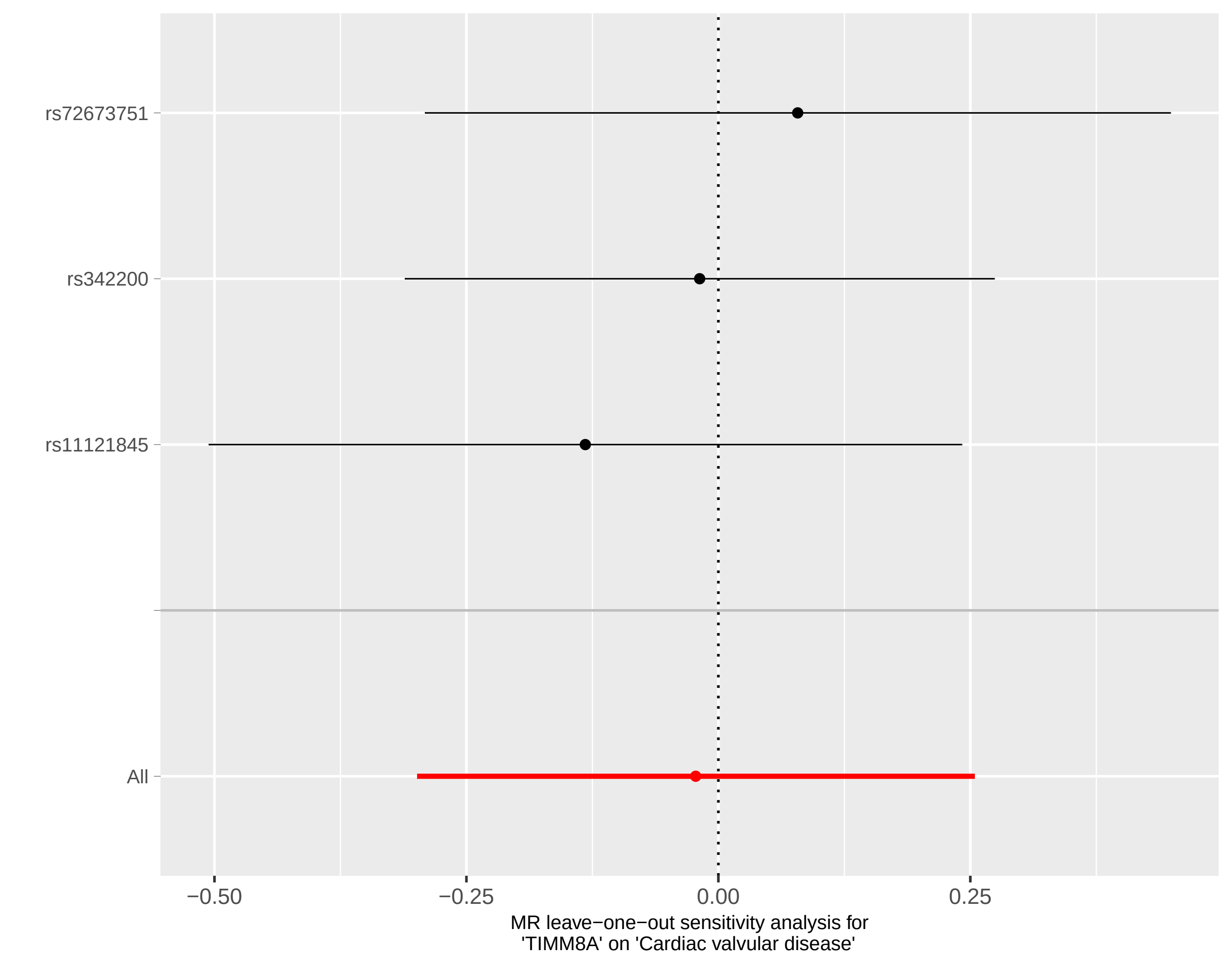
rs72673751
rs342200
rs11121845
All
−0.50
−0.25
0.00
0.25
MR leave−one−out sensitivity analysis for
'TIMM8A' on 'Cardiac valvular disease'

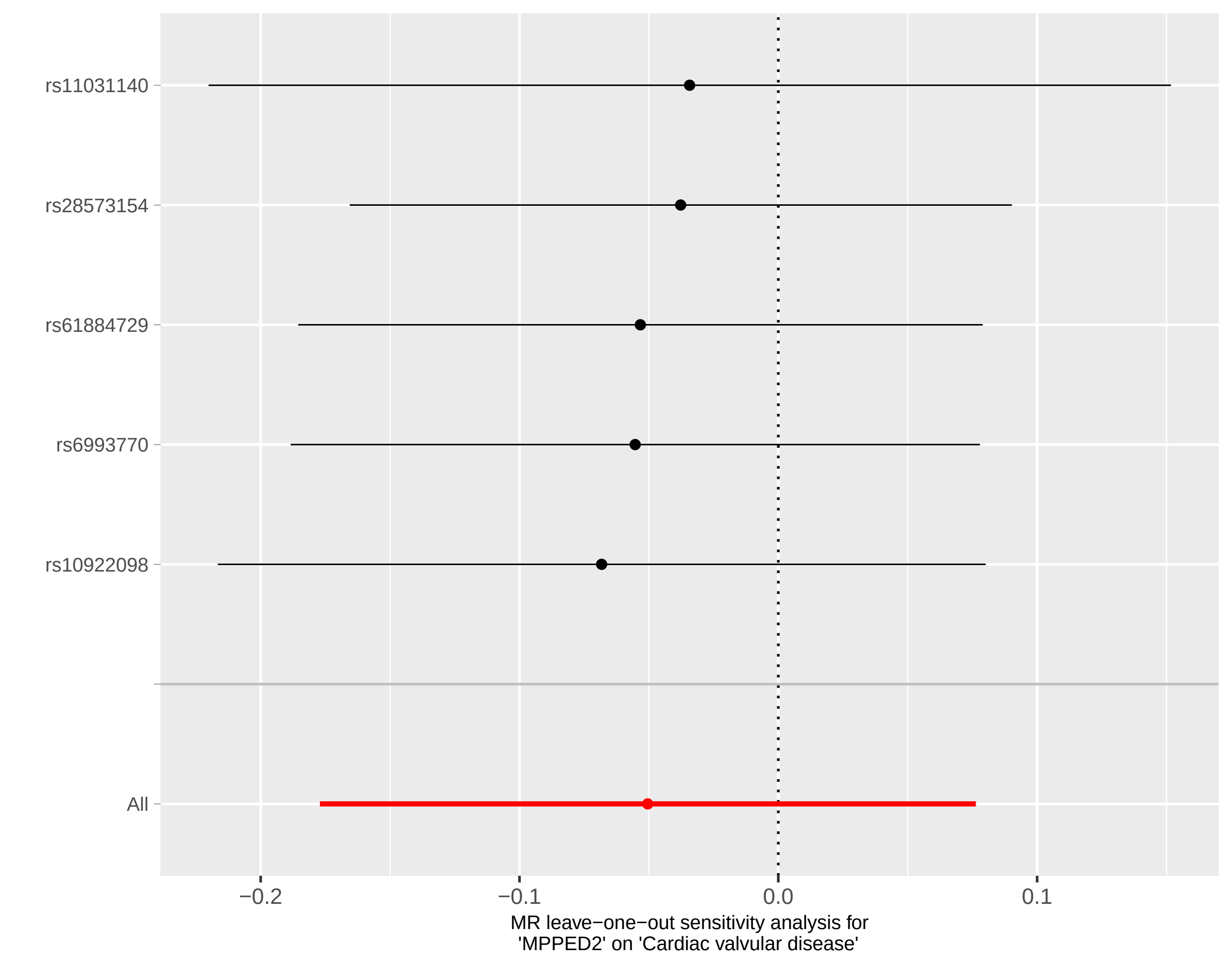
rs11031140
rs28573154
rs61884729
rs6993770
rs10922098
All
−0.2
−0.1
0.0
0.1
MR leave−one−out sensitivity analysis for
'MPPED2' on 'Cardiac valvular disease'

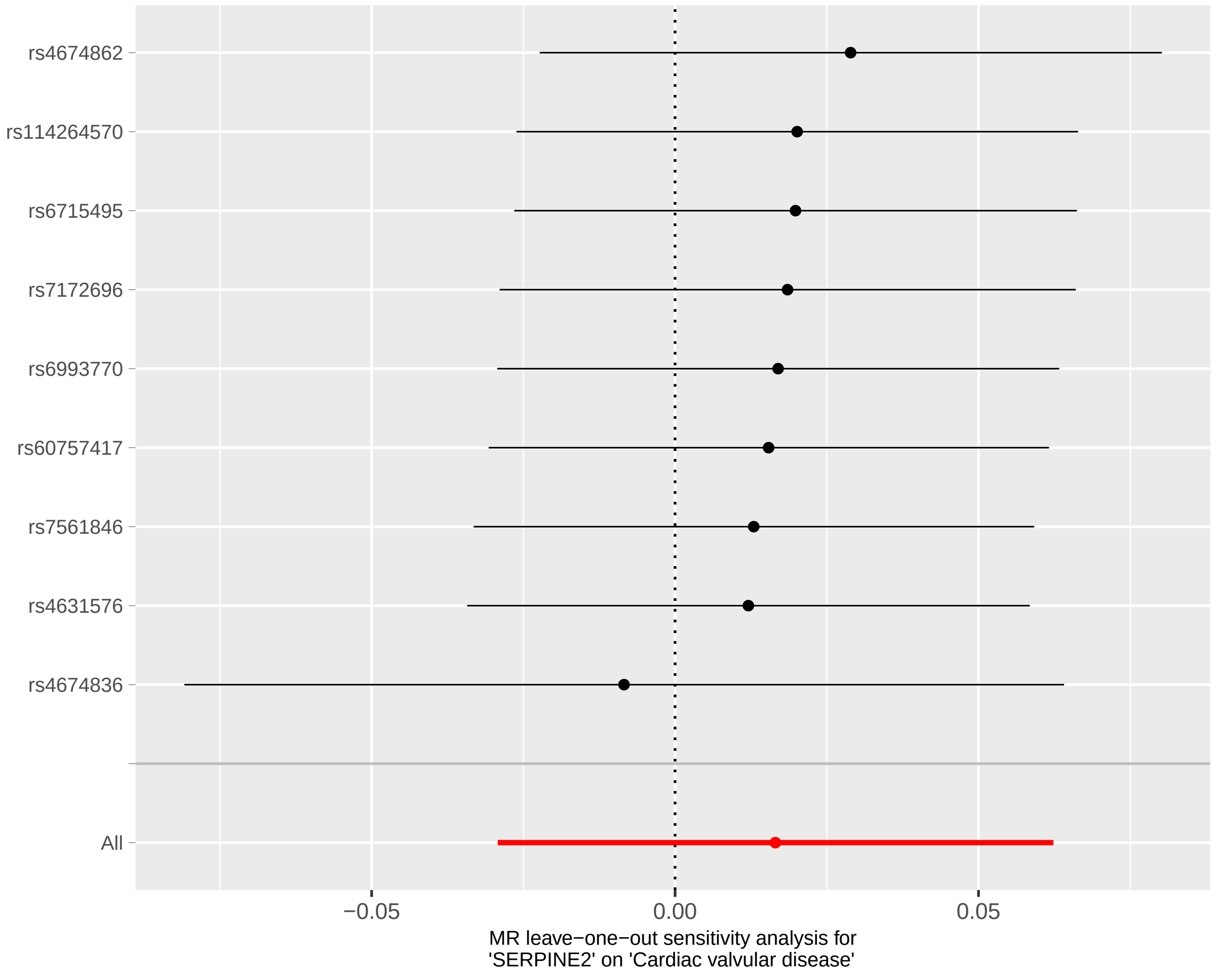

rs4674862
rs114264570
rs6715495
rs7172696
rs6993770
rs60757417
rs7561846
rs4631576
rs4674836
All
−0.05
0.00
0.05
MR leave−one−out sensitivity analysis for
'SERPINE2' on 'Cardiac valvular disease'

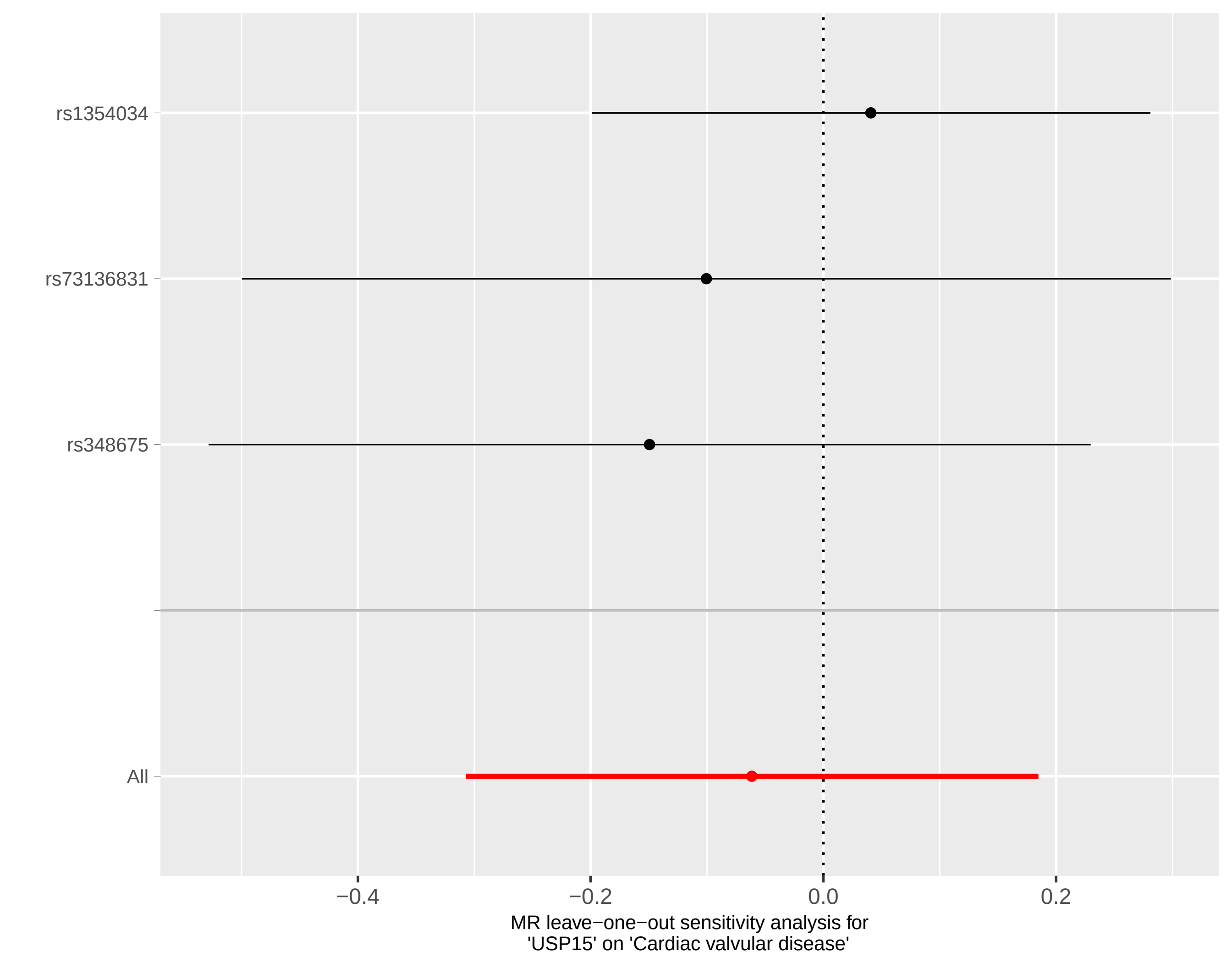

rs1354034
rs73136831
rs348675
All
−0.4
−0.2
0.0
0.2
MR leave−one−out sensitivity analysis for
'USP15' on 'Cardiac valvular disease'

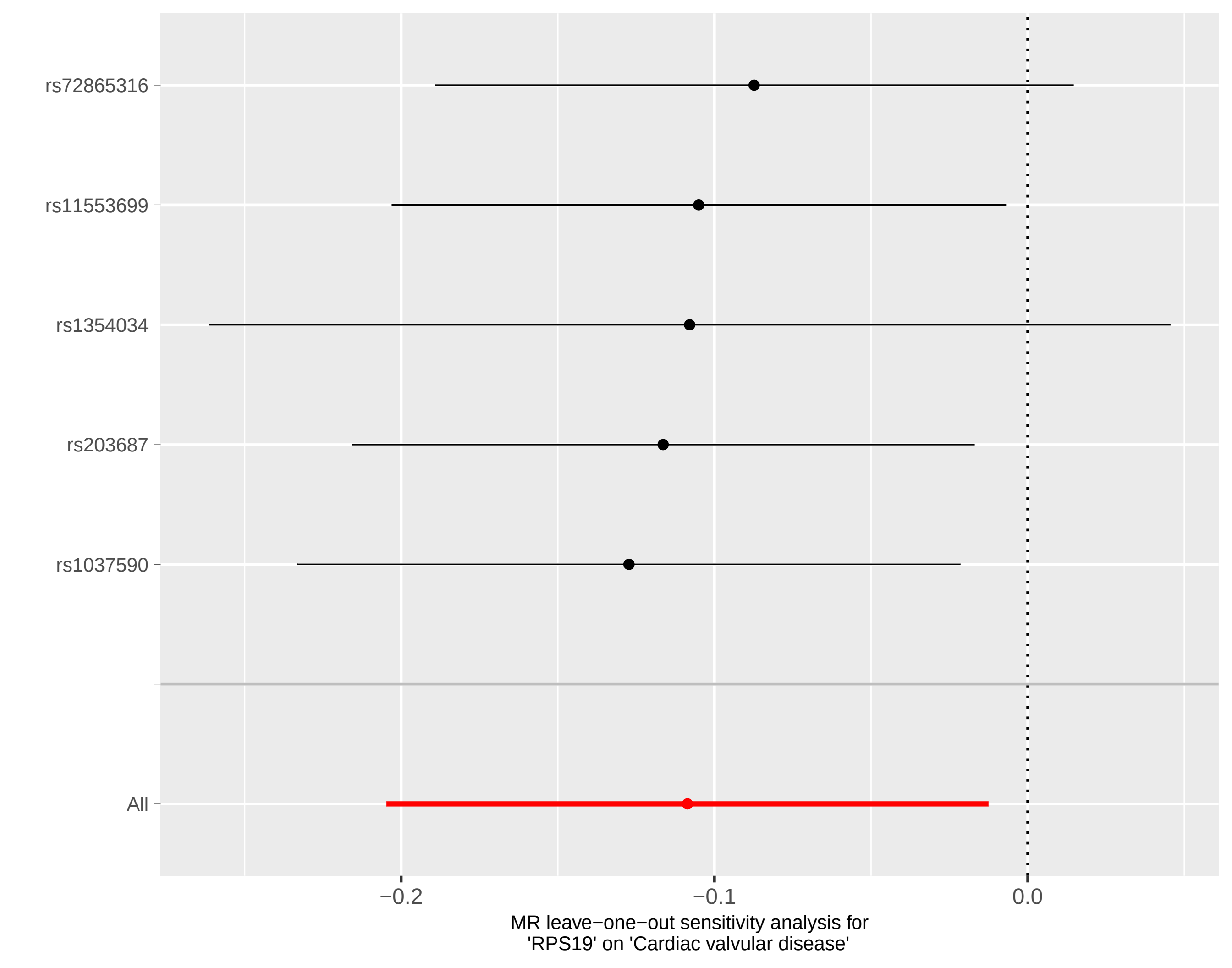
rs72865316
rs11553699
rs1354034
rs203687
rs1037590
All
−0.2
−0.1
0.0
MR leave−one−out sensitivity analysis for
'RPS19' on 'Cardiac valvular disease'

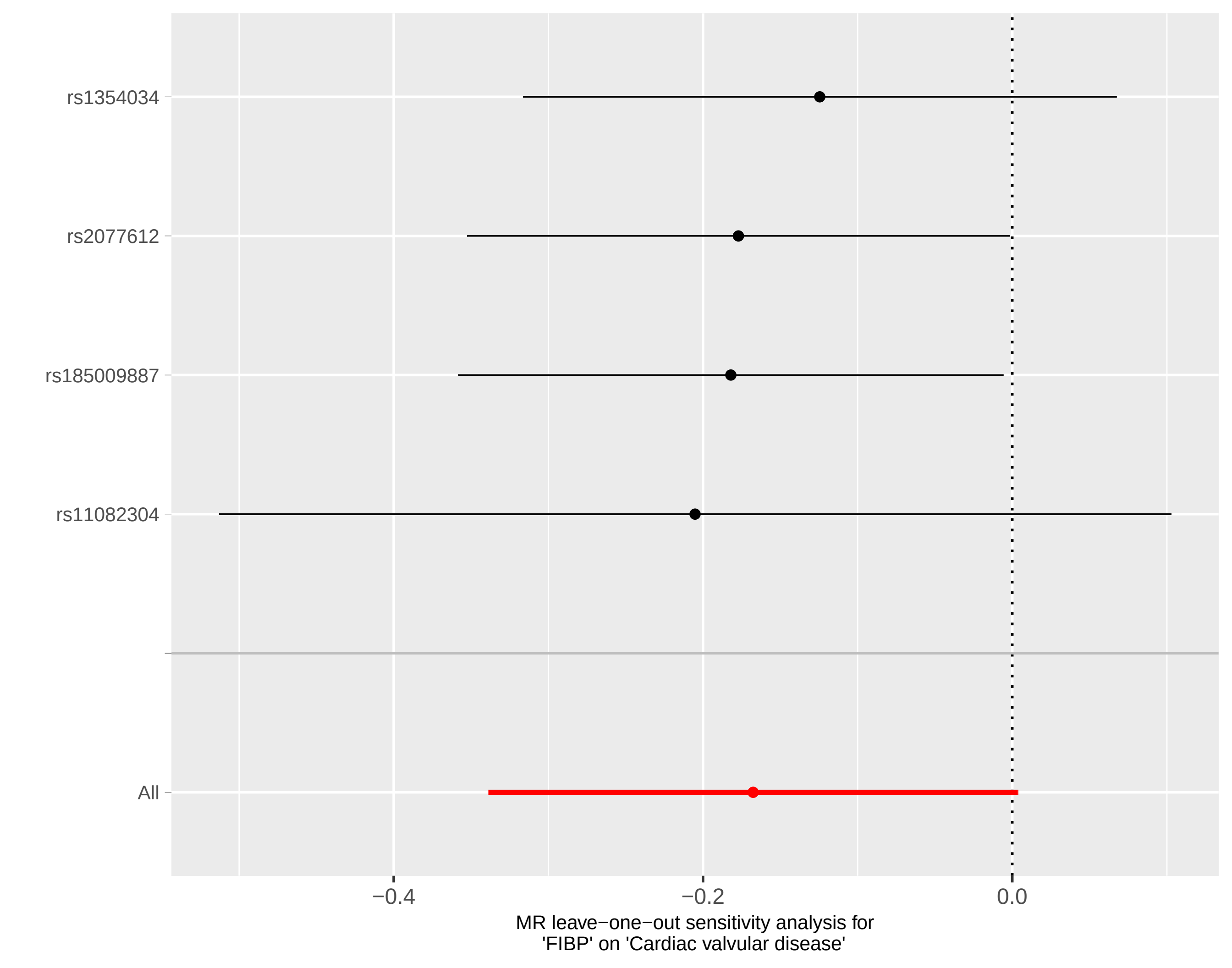
rs1354034
rs2077612
rs185009887
rs11082304
All
−0.4
−0.2
0.0
MR leave−one−out sensitivity analysis for
'FIBP' on 'Cardiac valvular disease'

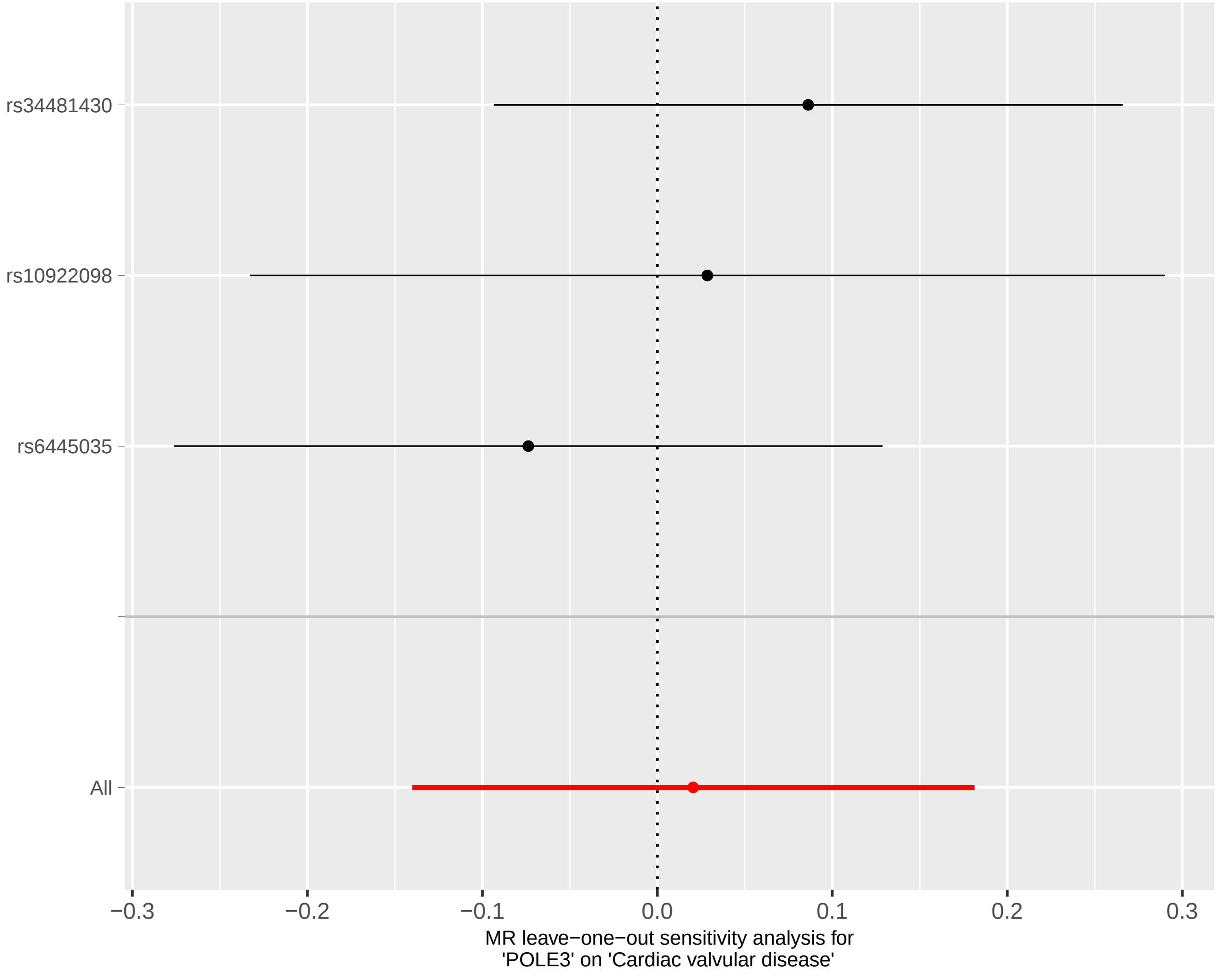
rs34481430
rs10922098
rs6445035
All
−0.3
−0.2
−0.1
0.0
0.1
0.2
0.3
MR leave−one−out sensitivity analysis for
'POLE3' on 'Cardiac valvular disease'

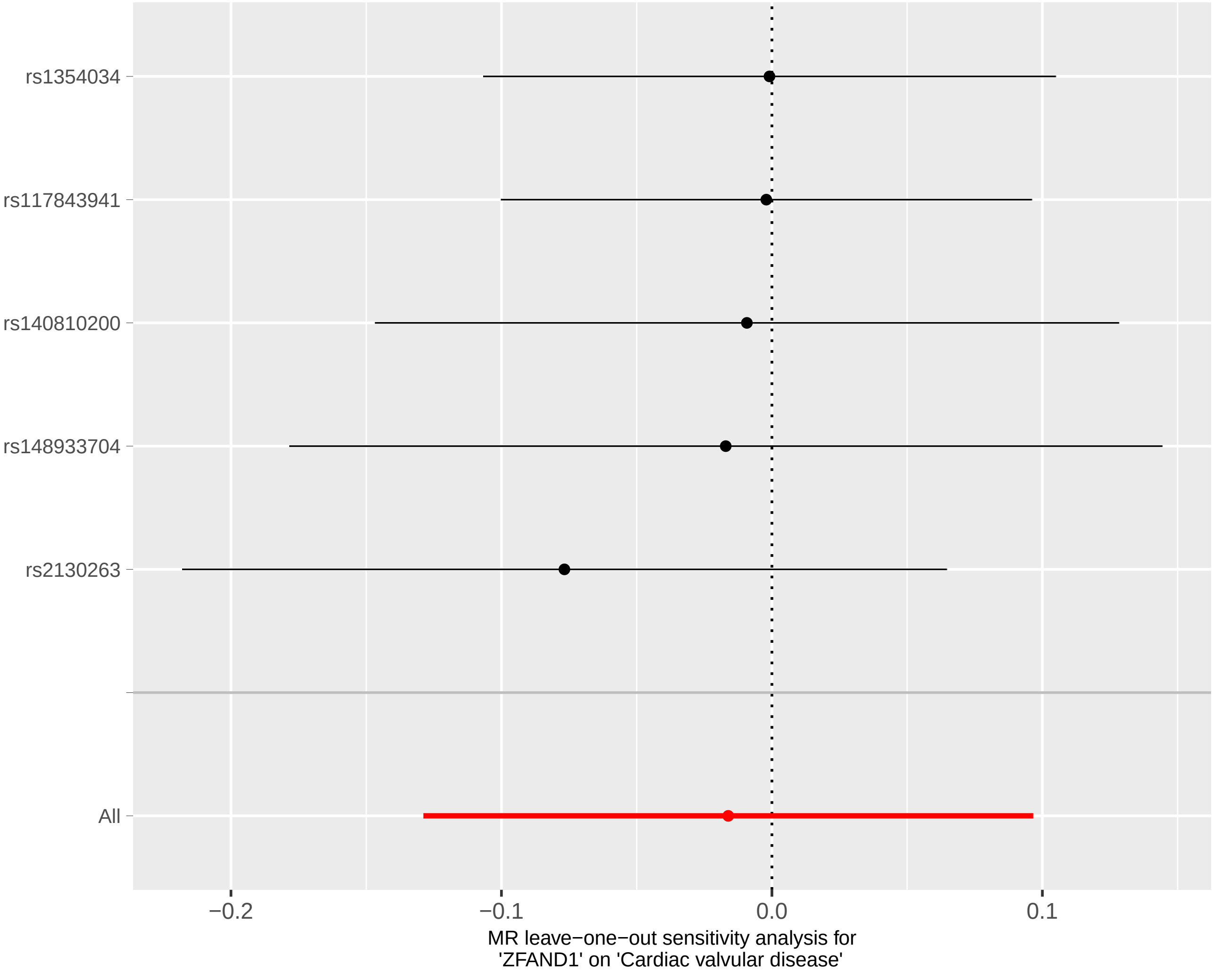
rs1354034
rs117843941
rs140810200
rs148933704
rs2130263
All
−0.2
−0.1
0.0
0.1
MR leave−one−out sensitivity analysis for
'ZFAND1' on 'Cardiac valvular disease'

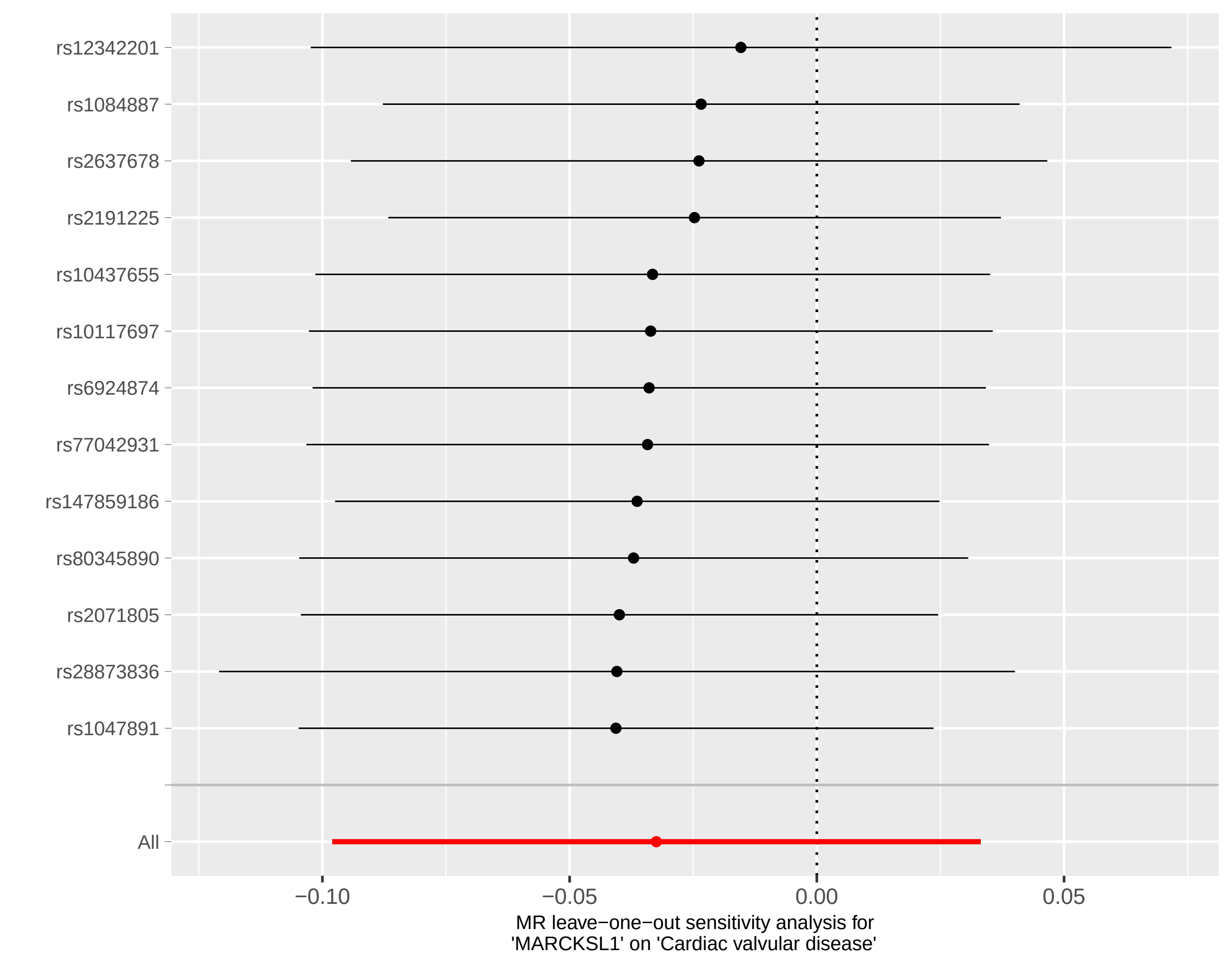
rs12342201
rs1084887
rs2637678
rs2191225
rs10437655
rs10117697
rs6924874
rs77042931
rs147859186
rs80345890
rs2071805
rs28873836
rs1047891
All
−0.10
−0.05
0.00
0.05
MR leave−one−out sensitivity analysis for
'MARCKSL1' on 'Cardiac valvular disease'

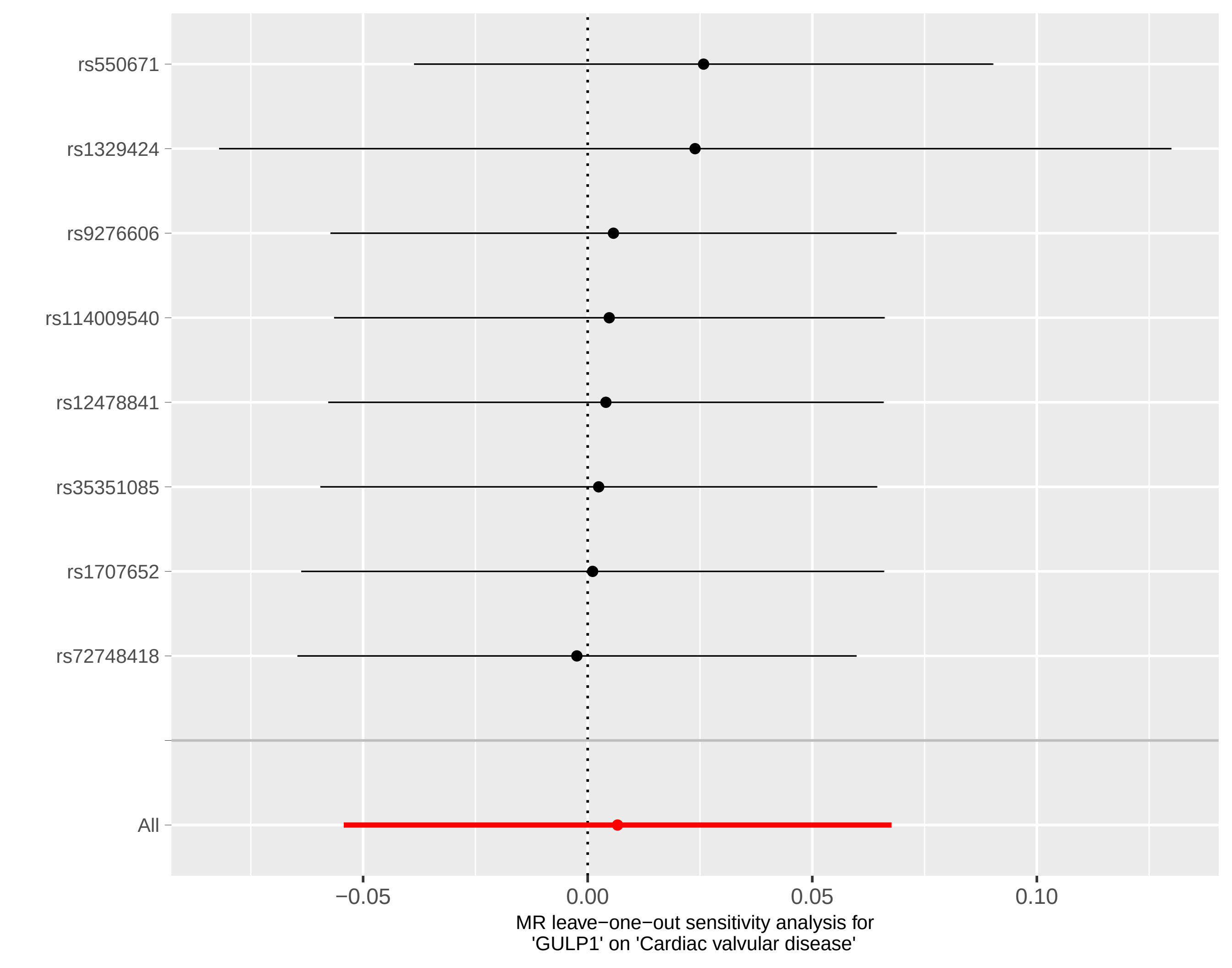

rs550671
rs1329424
rs9276606
rs114009540
rs12478841
rs35351085
rs1707652
rs72748418
All
−0.05
0.00
0.05
0.10
MR leave−one−out sensitivity analysis for
'GULP1' on 'Cardiac valvular disease'

Insufficient number of SNPs

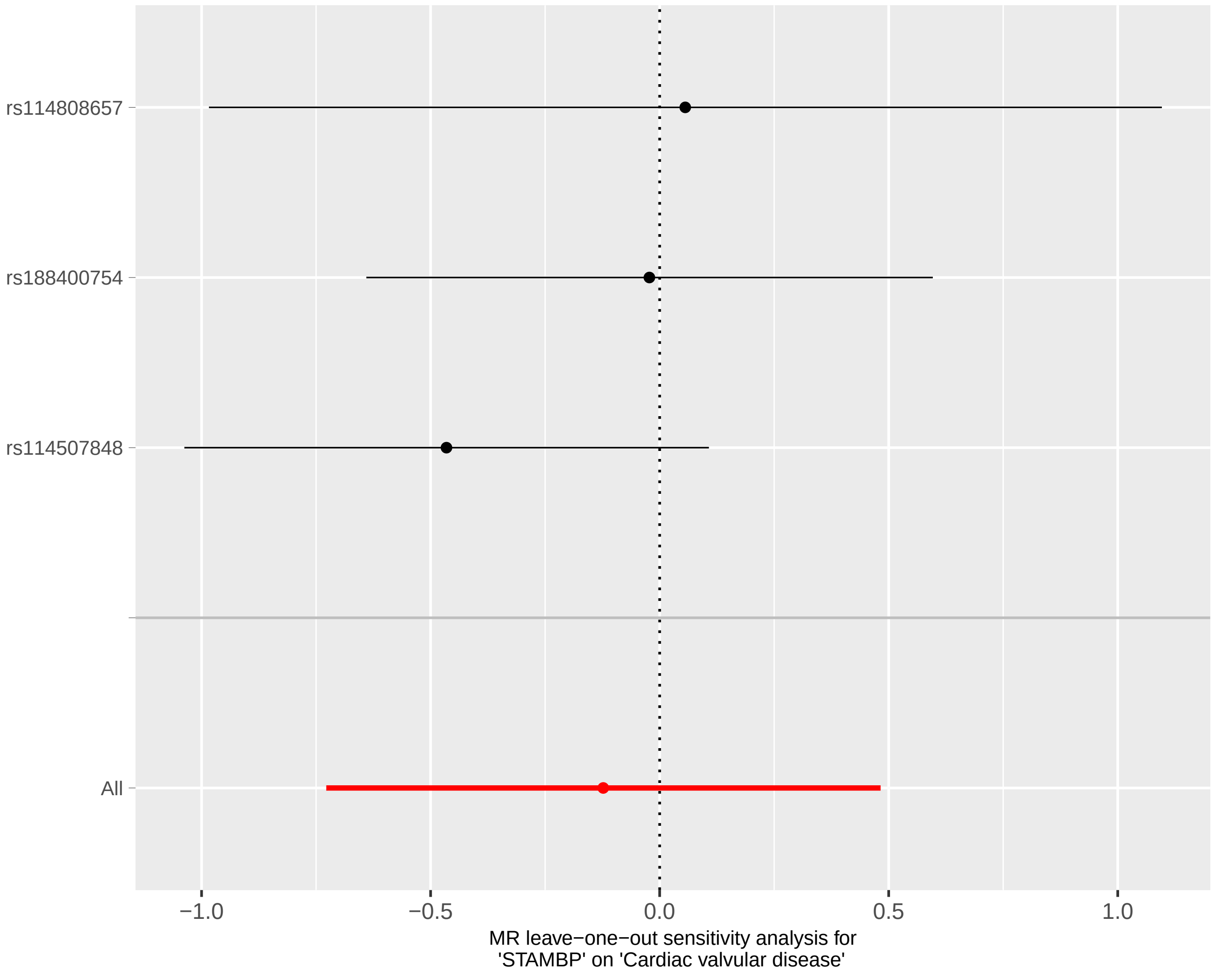

rs114808657
rs188400754
rs114507848
All
−1.0
−0.5
0.0
0.5
1.0
MR leave−one−out sensitivity analysis for
'STAMBP' on 'Cardiac valvular disease'

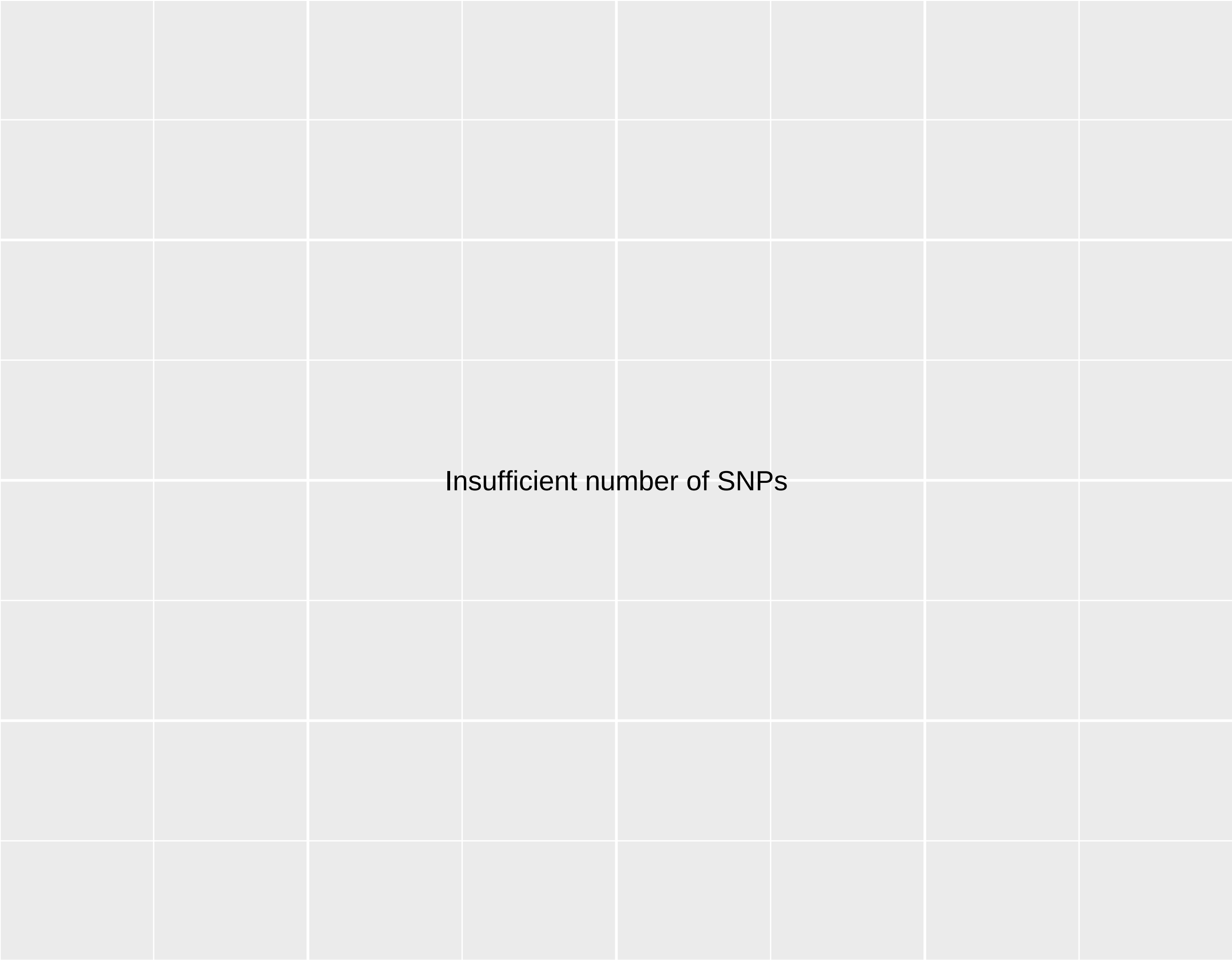
Insufficient number of SNPs

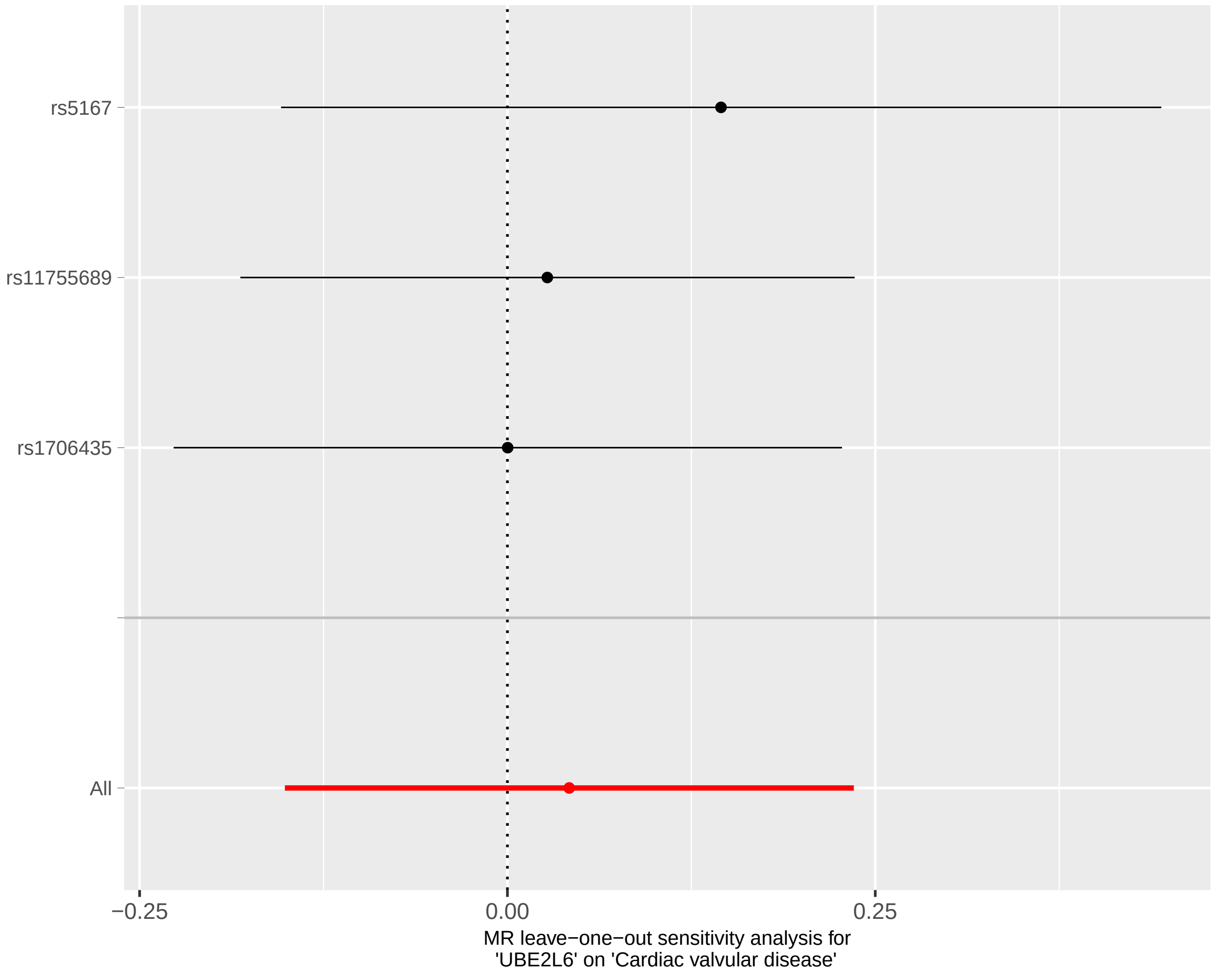

rs5167
rs11755689
rs1706435
All
−0.25
0.00
0.25
MR leave−one−out sensitivity analysis for
'UBE2L6' on 'Cardiac valvular disease'

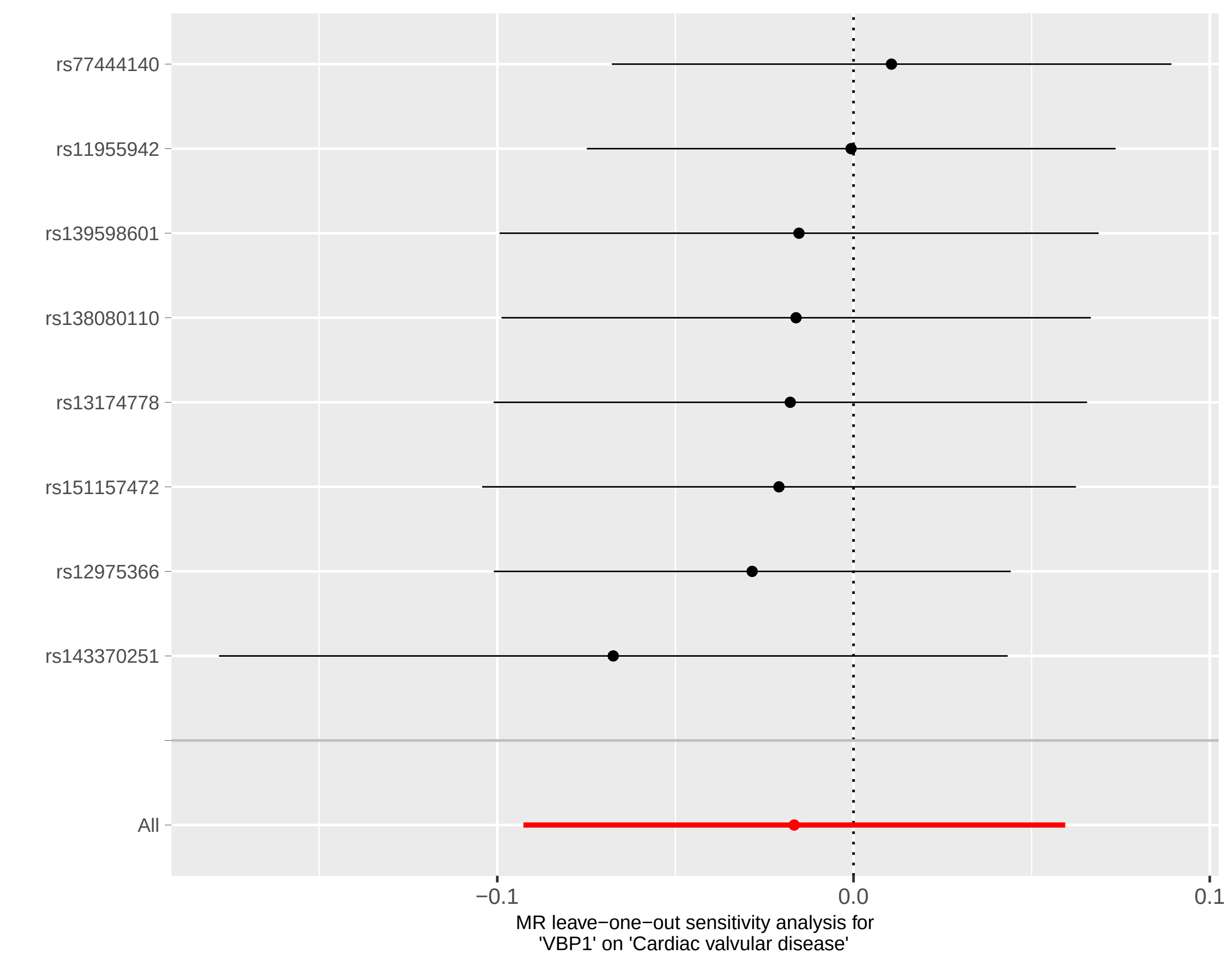
rs77444140
rs11955942
rs139598601
rs138080110
rs13174778
rs151157472
rs12975366
rs143370251
All
−0.1
0.0
0.1
MR leave−one−out sensitivity analysis for
'VBP1' on 'Cardiac valvular disease'

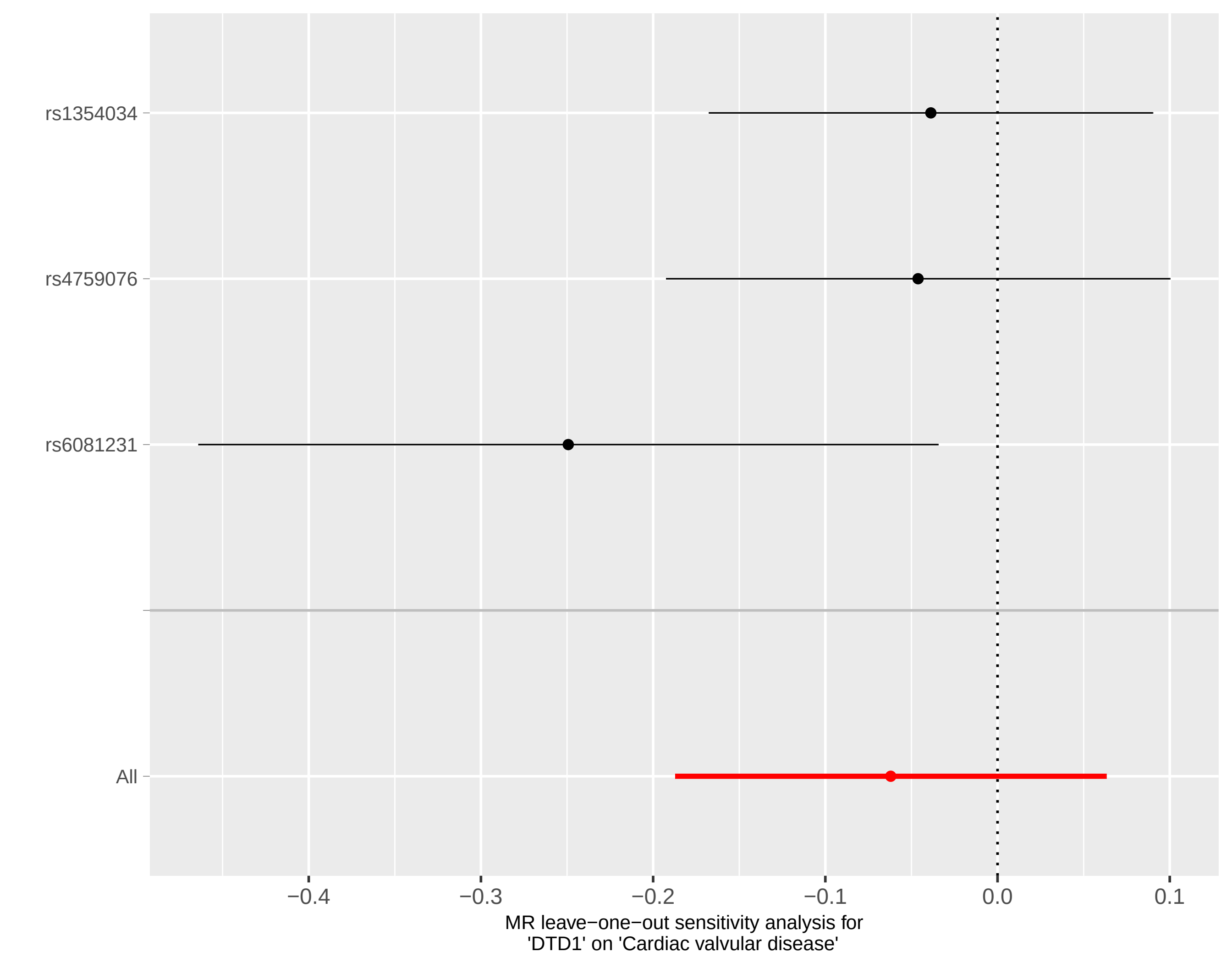
rs1354034
rs4759076
rs6081231
All
−0.4
−0.3
−0.2
−0.1
0.0
0.1
MR leave−one−out sensitivity analysis for
'DTD1' on 'Cardiac valvular disease'

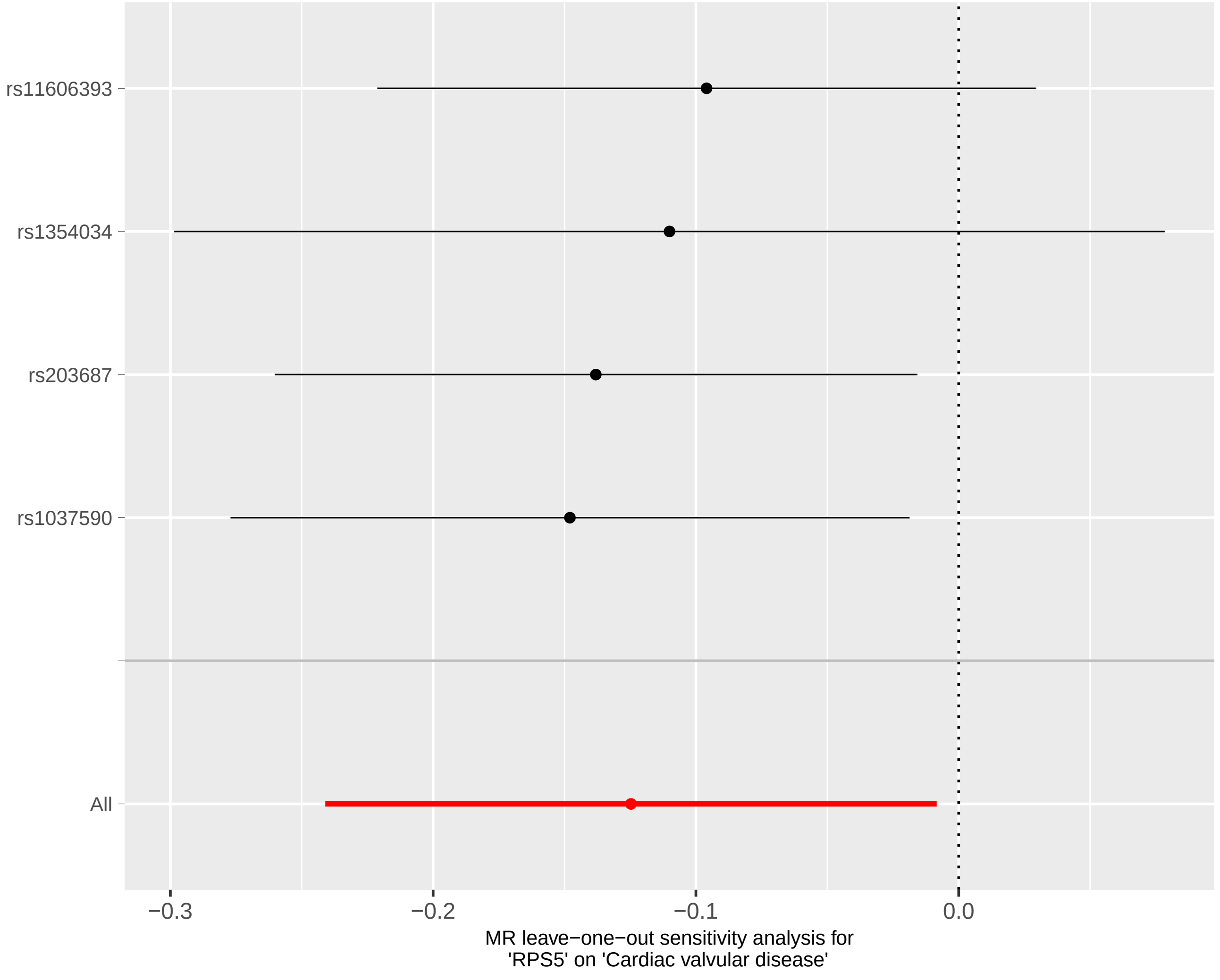
rs11606393
rs1354034
rs203687
rs1037590
All
−0.3
−0.2
−0.1
0.0
MR leave−one−out sensitivity analysis for
'RPS5' on 'Cardiac valvular disease'

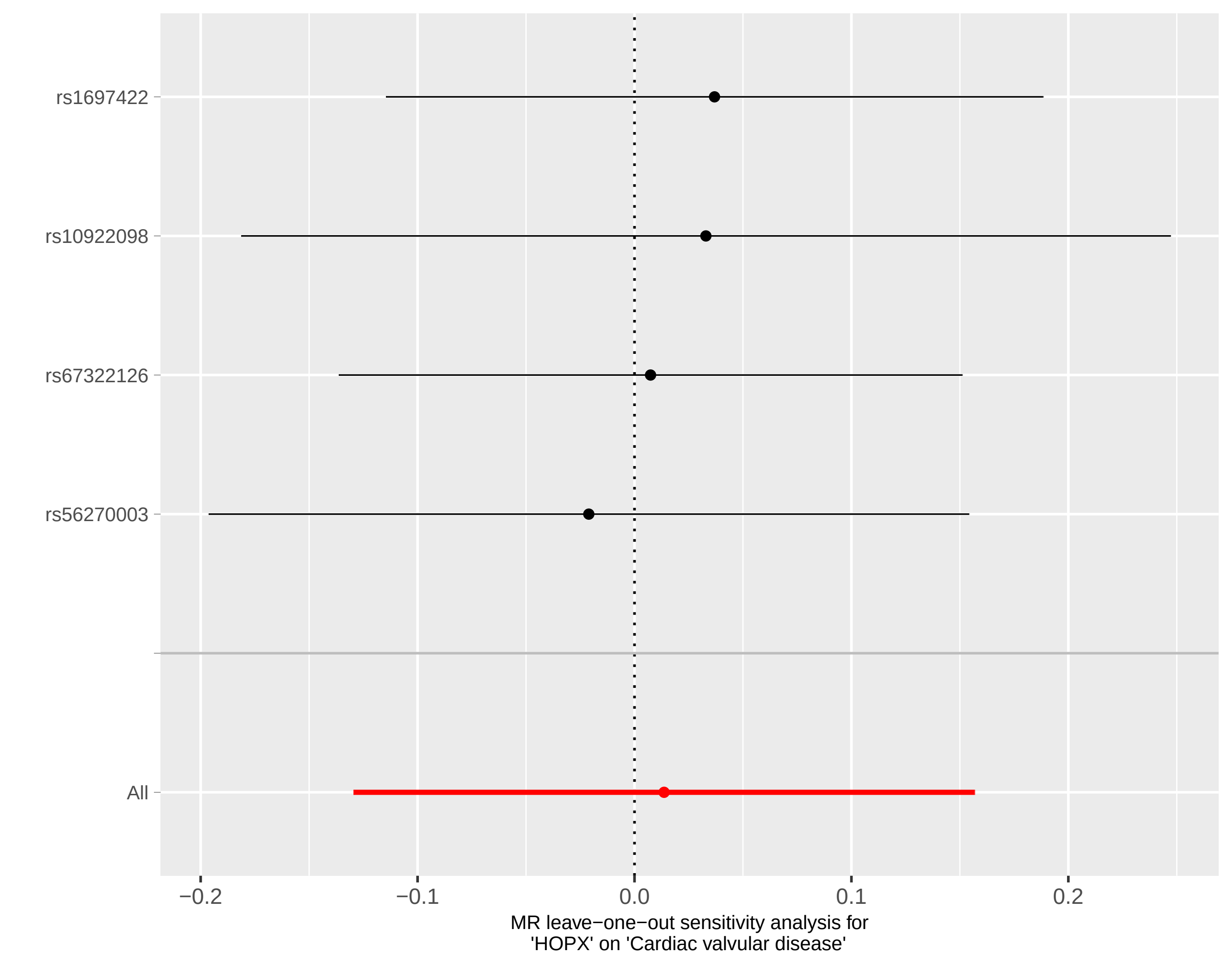
rs1697422
rs10922098
rs67322126
rs56270003
All
−0.2
−0.1
0.0
0.1
0.2
MR leave−one−out sensitivity analysis for
'HOPX' on 'Cardiac valvular disease'

Insufficient number of SNPs

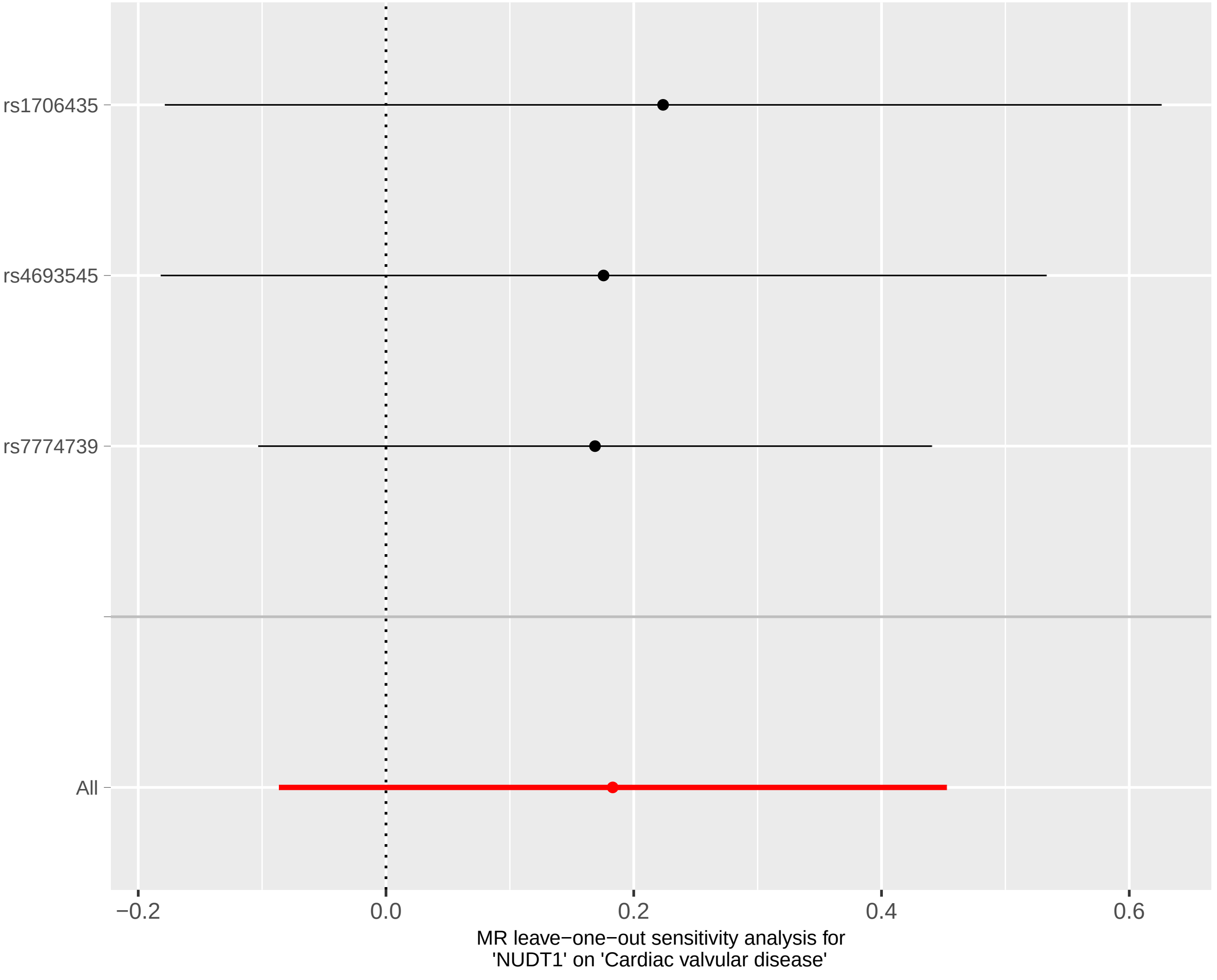
rs1706435
rs4693545
rs7774739
All
−0.2
0.0
0.2
0.4
0.6
MR leave−one−out sensitivity analysis for
'NUDT1' on 'Cardiac valvular disease'

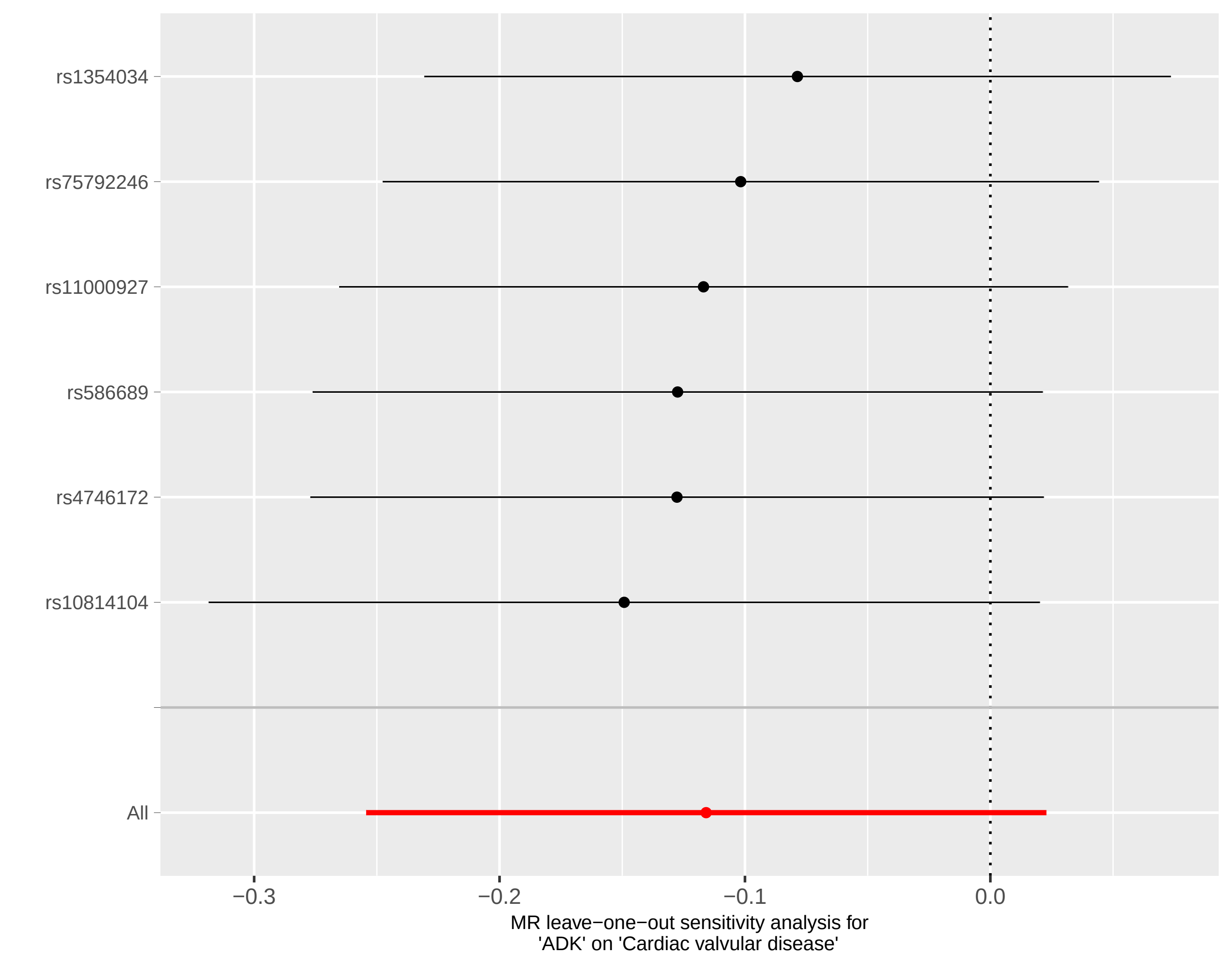

rs1354034
rs75792246
rs11000927
rs586689
rs4746172
rs10814104
All
−0.3
−0.2
−0.1
0.0
MR leave−one−out sensitivity analysis for
'ADK' on 'Cardiac valvular disease'

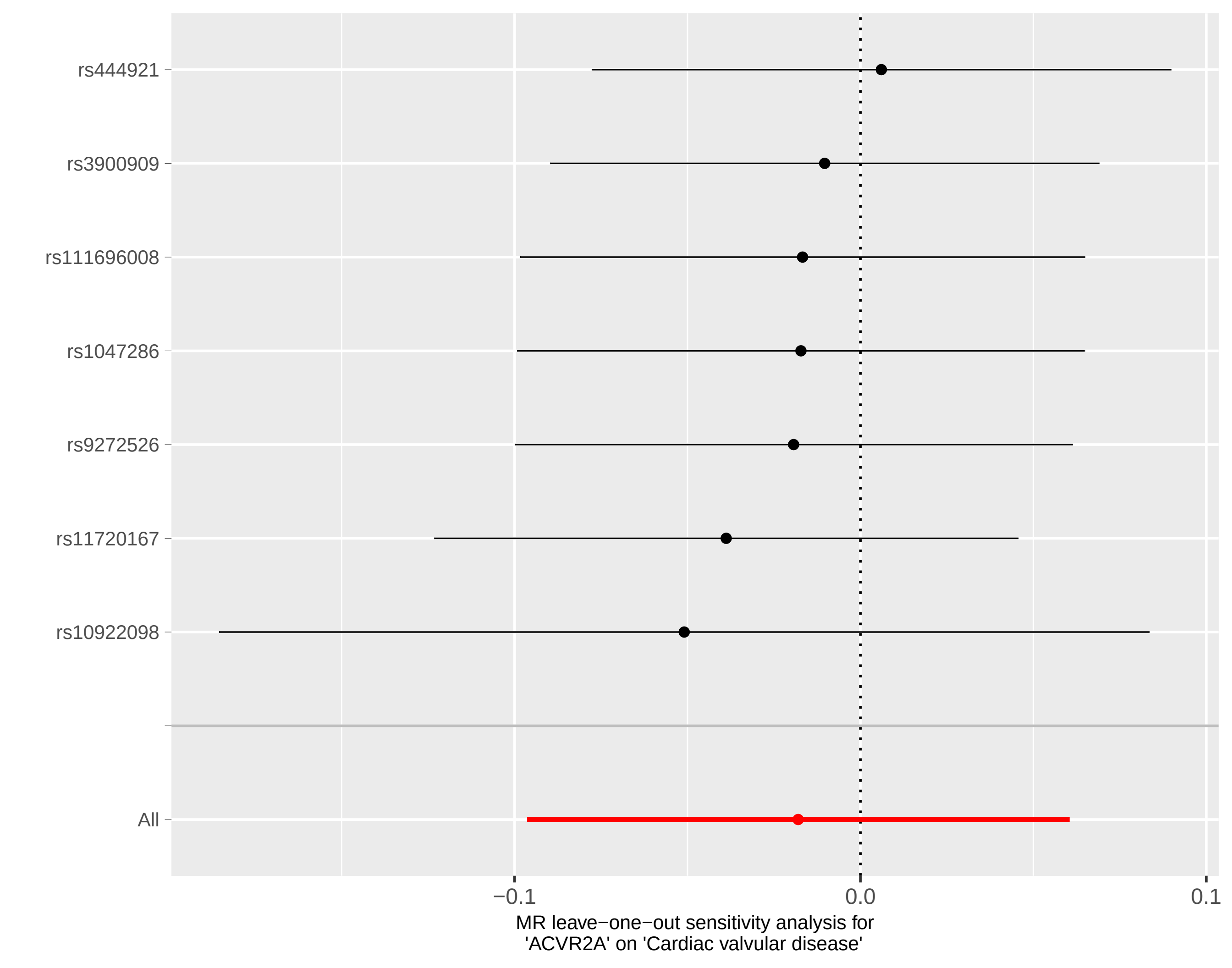
rs444921
rs3900909
rs111696008
rs1047286
rs9272526
rs11720167
rs10922098
All
−0.1
0.0
0.1
MR leave−one−out sensitivity analysis for
'ACVR2A' on 'Cardiac valvular disease'

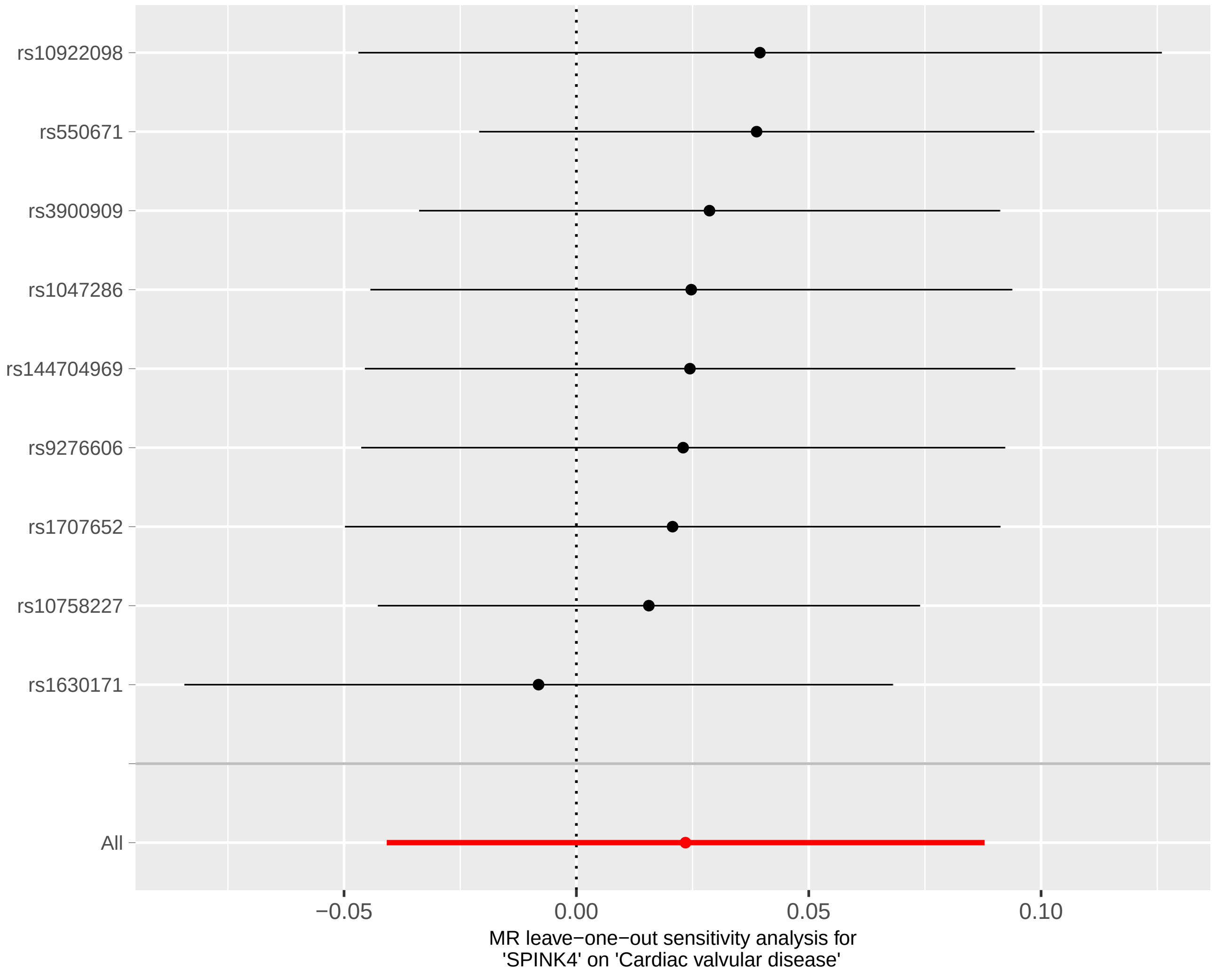
rs10922098
rs550671
rs3900909
rs1047286
rs144704969
rs9276606
rs1707652
rs10758227
rs1630171
All
−0.05
0.00
0.05
0.10
MR leave−one−out sensitivity analysis for
'SPINK4' on 'Cardiac valvular disease'

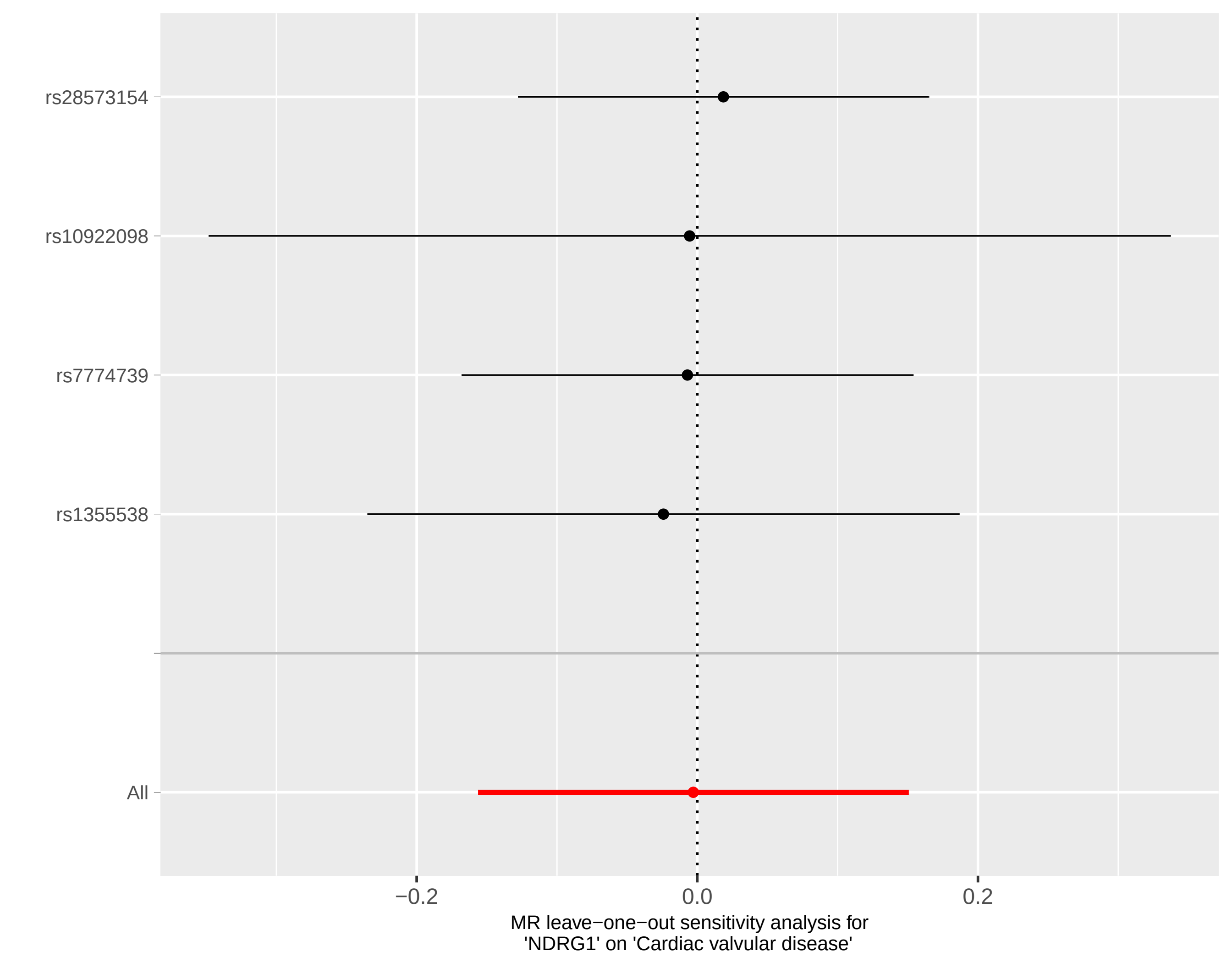
rs28573154
rs10922098
rs7774739
rs1355538
All
−0.2
0.0
0.2
MR leave−one−out sensitivity analysis for
'NDRG1' on 'Cardiac valvular disease'

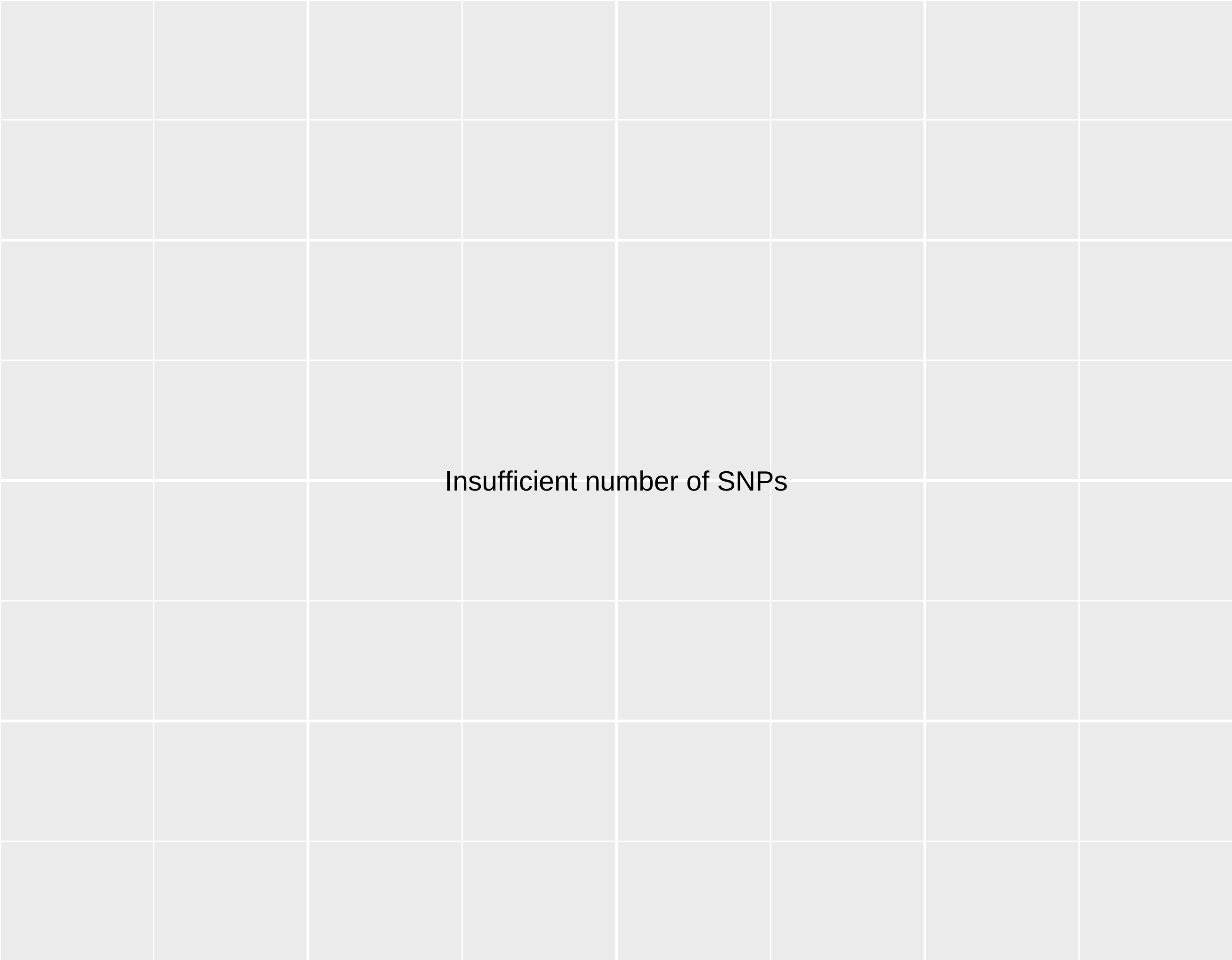
Insufficient number of SNPs

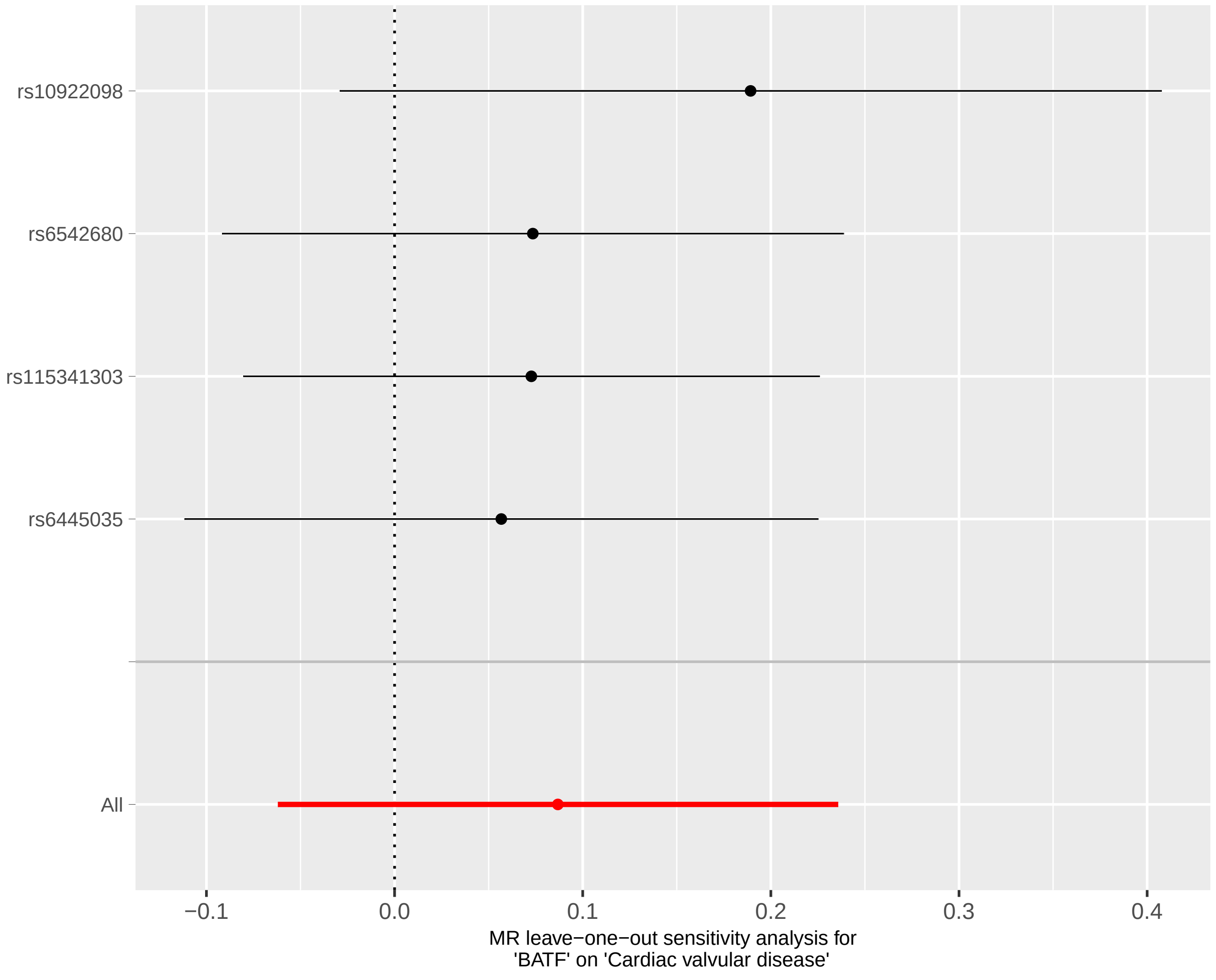

rs10922098
rs6542680
rs115341303
rs6445035
All
−0.1
0.0
0.1
0.2
0.3
0.4
MR leave−one−out sensitivity analysis for
'BATF' on 'Cardiac valvular disease'

Insufficient number of SNPs

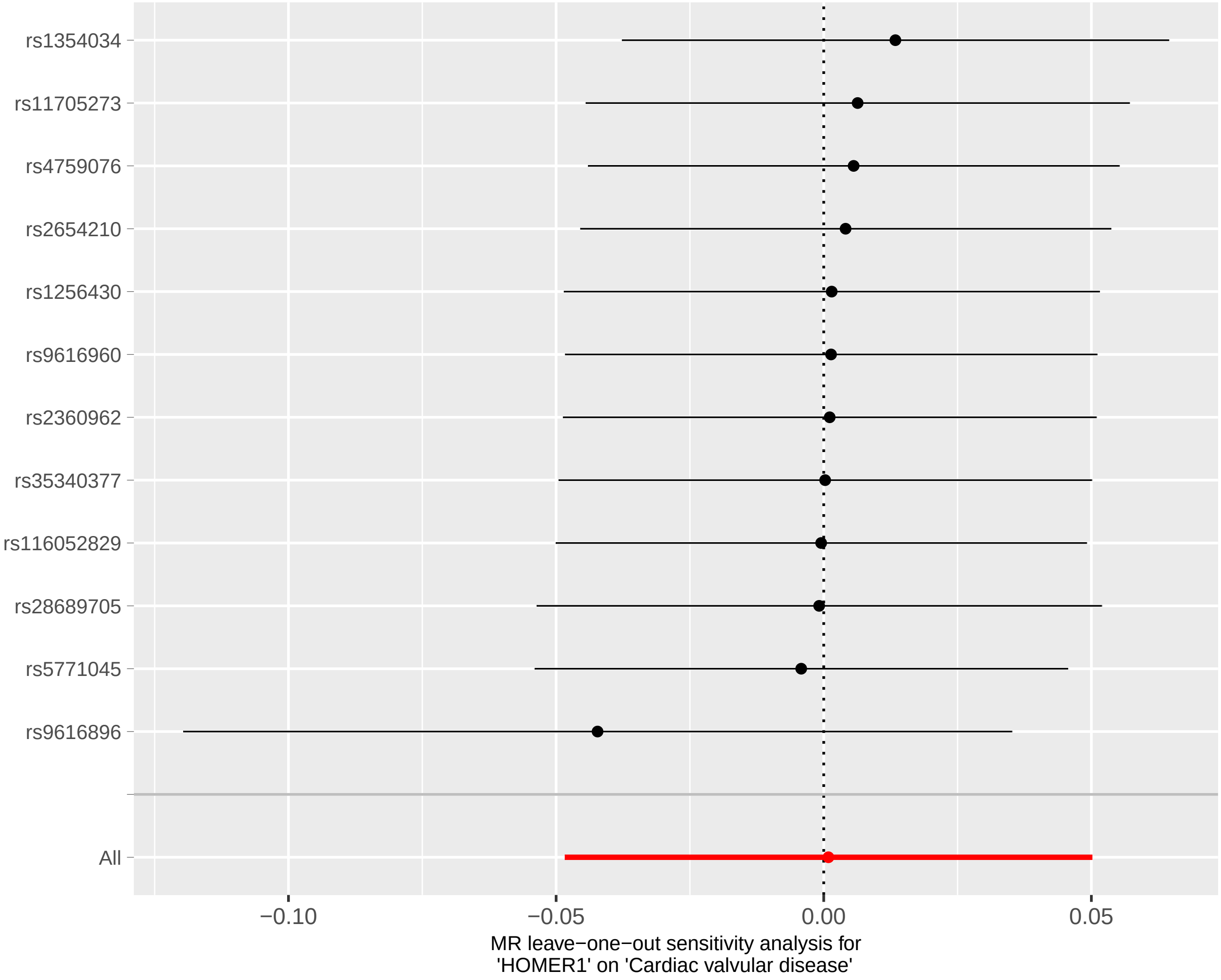

rs1354034
rs11705273
rs4759076
rs2654210
rs1256430
rs9616960
rs2360962
rs35340377
rs116052829
rs28689705
rs5771045
rs9616896
All
−0.10
−0.05
0.00
0.05
MR leave−one−out sensitivity analysis for
'HOMER1' on 'Cardiac valvular disease'

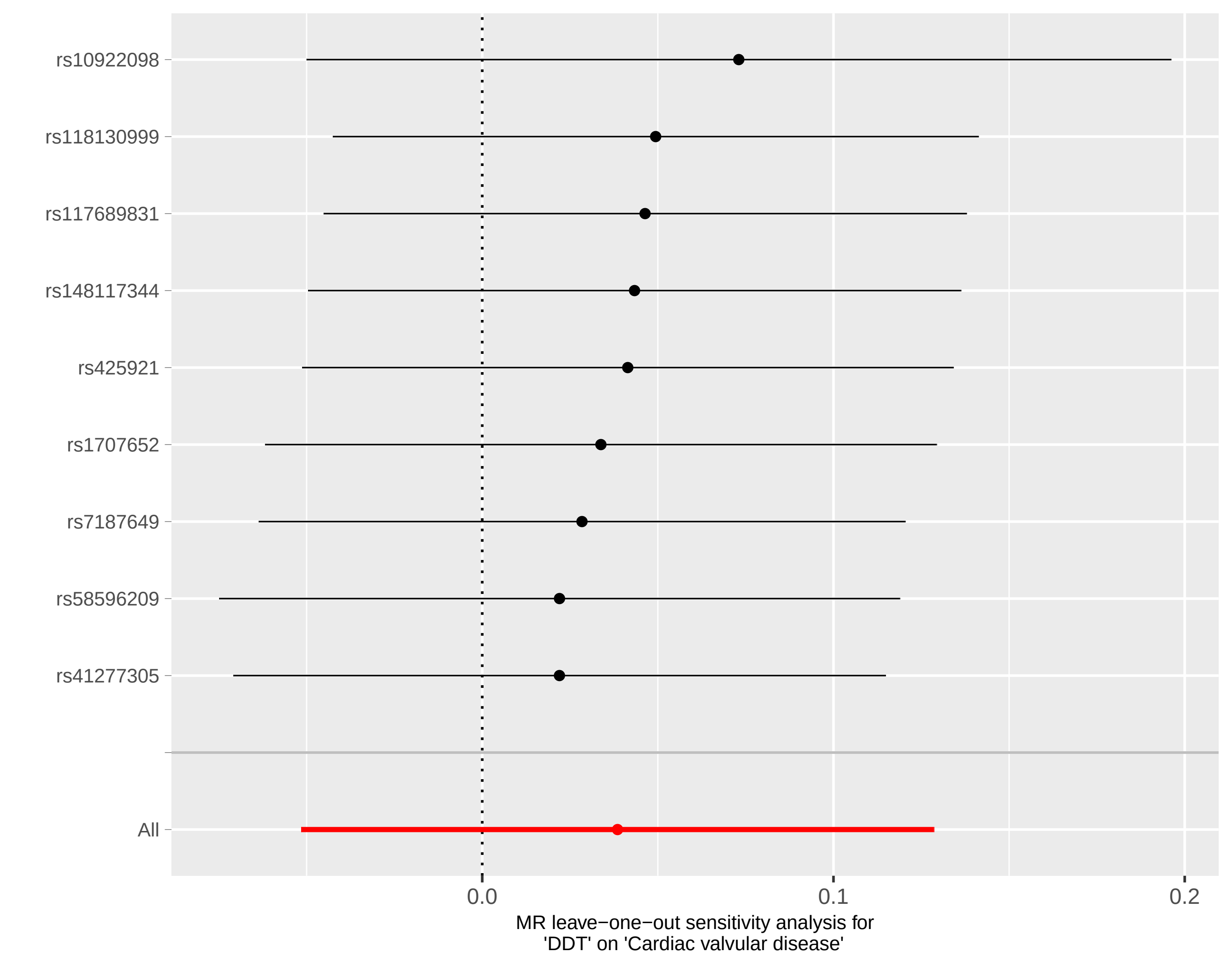

rs10922098
rs118130999
rs117689831
rs148117344
rs425921
rs1707652
rs7187649
rs58596209
rs41277305
All
0.0
0.1
0.2
MR leave−one−out sensitivity analysis for
'DDT' on 'Cardiac valvular disease'

Insufficient number of SNPs

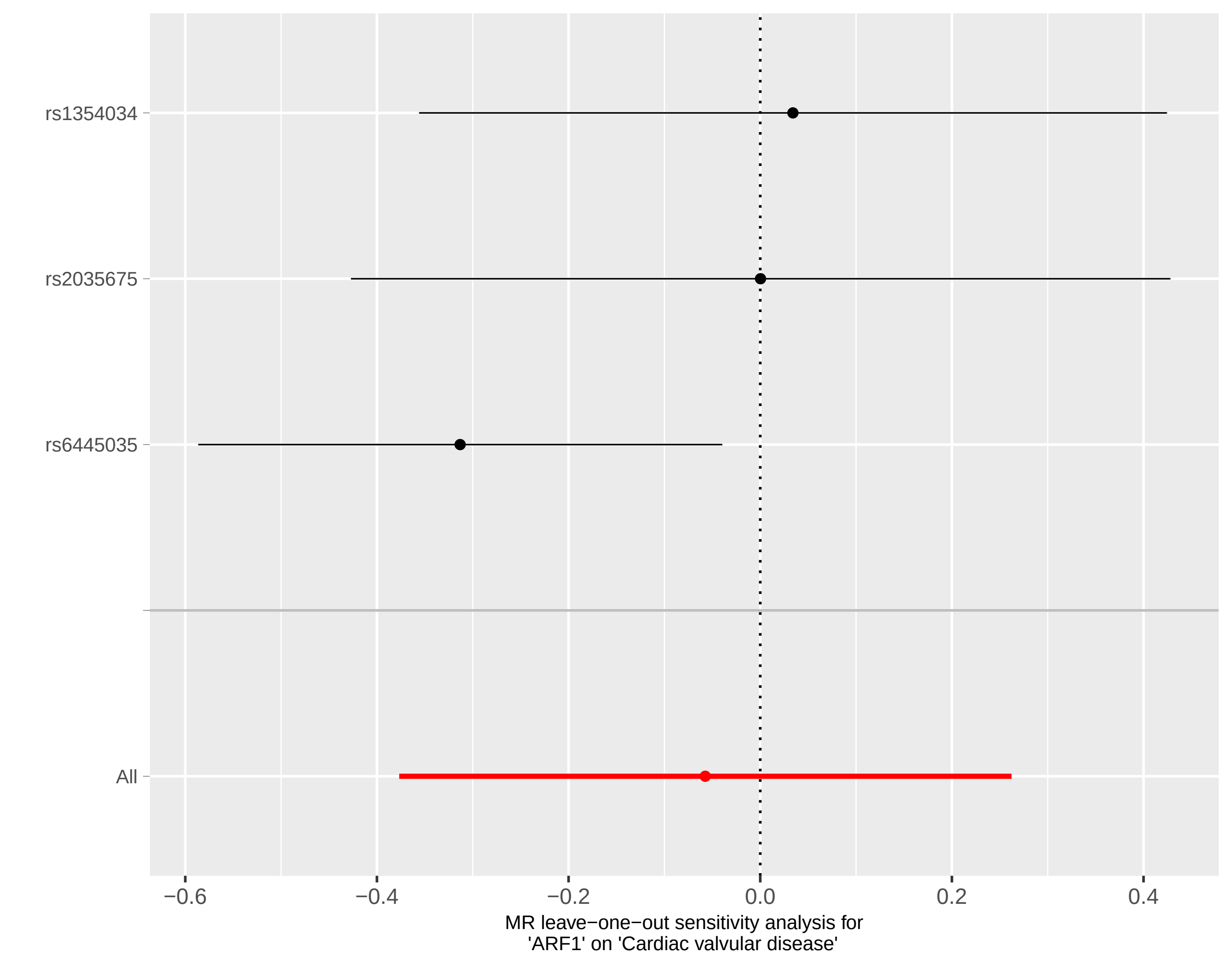
rs1354034
rs2035675
rs6445035
All
−0.6
−0.4
−0.2
0.0
0.2
0.4
MR leave−one−out sensitivity analysis for
'ARF1' on 'Cardiac valvular disease'

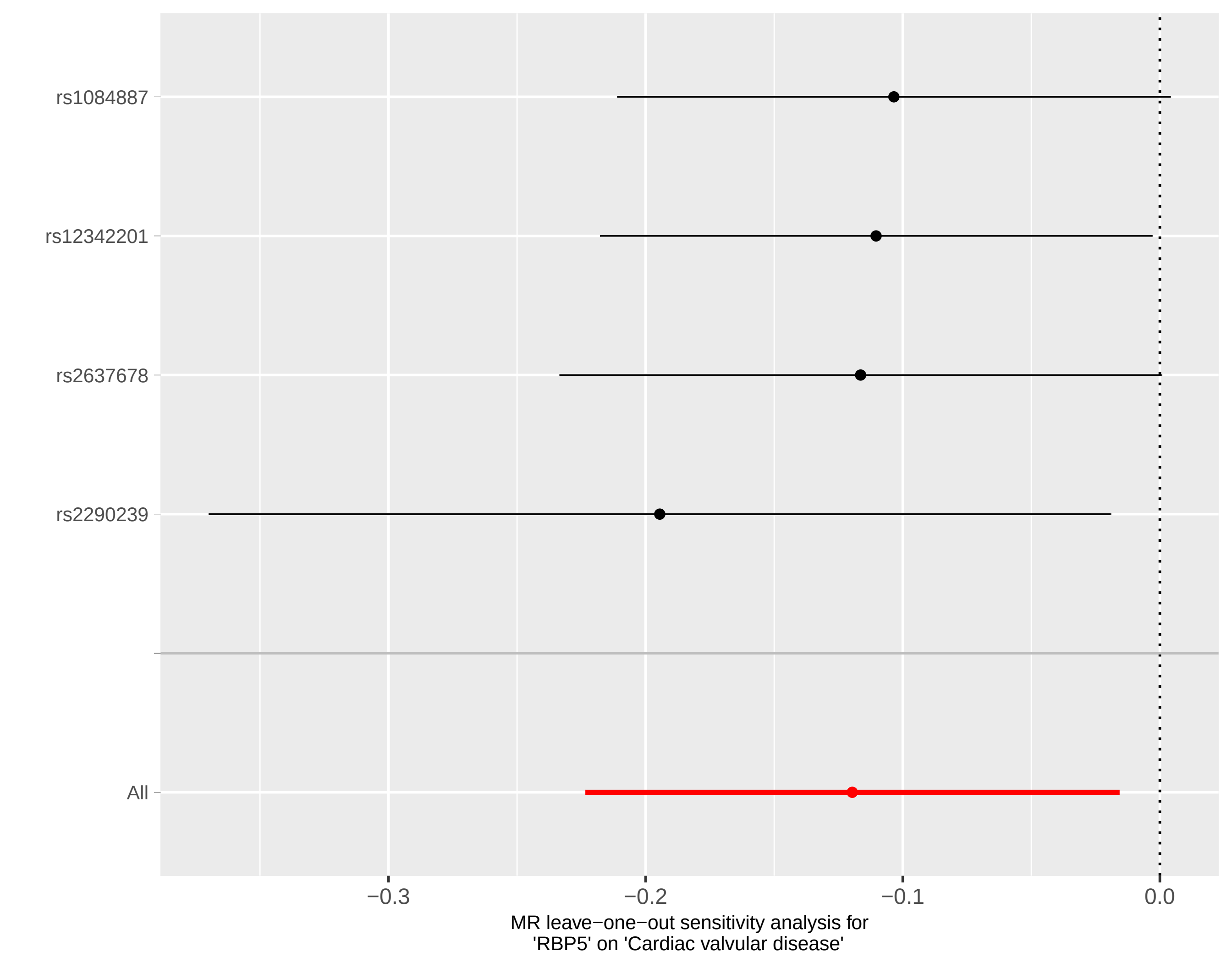
rs1084887
rs12342201
rs2637678
rs2290239
All
−0.3
−0.2
−0.1
0.0
MR leave−one−out sensitivity analysis for
'RBP5' on 'Cardiac valvular disease'

Insufficient number of SNPs

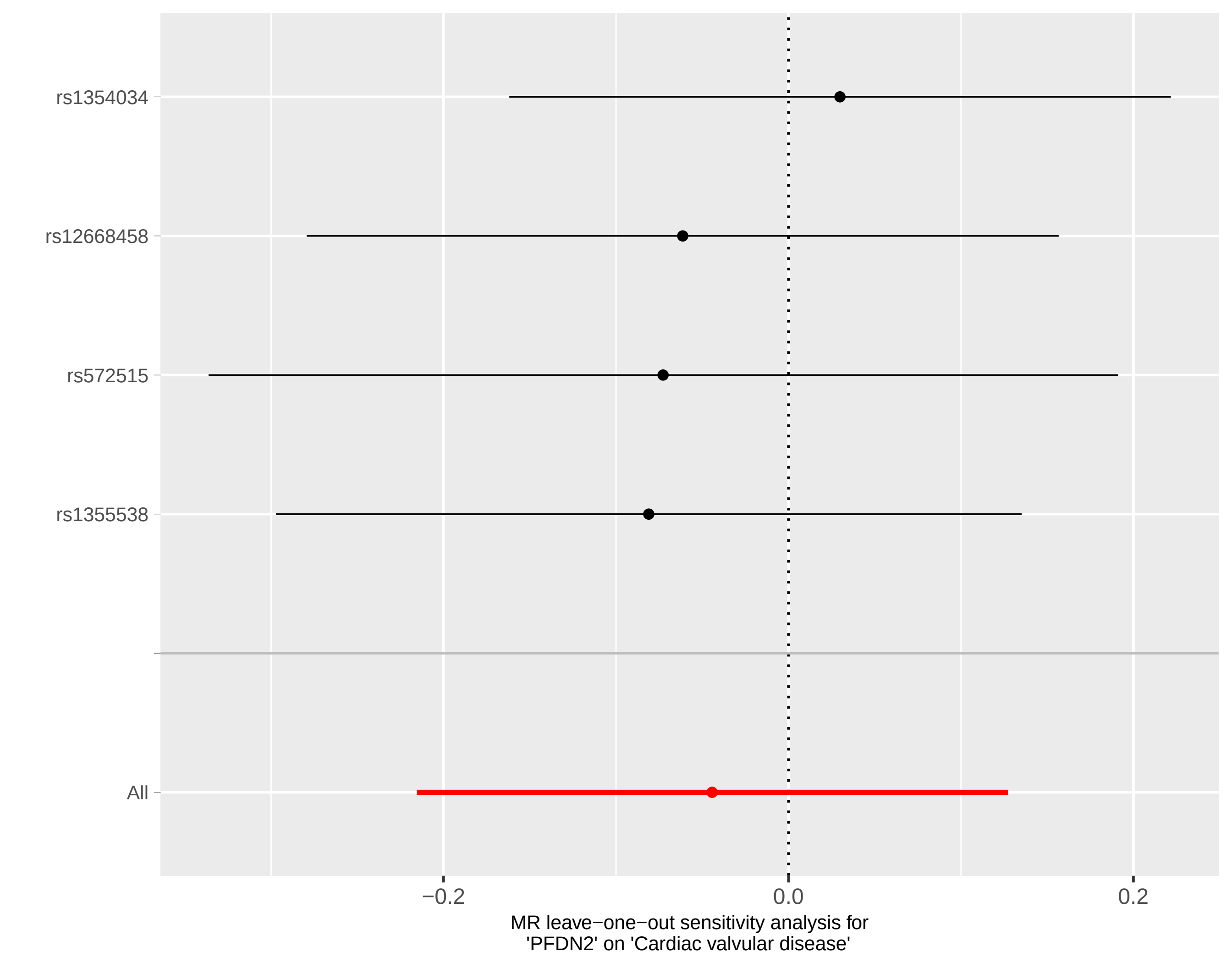
rs1354034
rs12668458
rs572515
rs1355538
All
−0.2
0.0
0.2
MR leave−one−out sensitivity analysis for
'PFDN2' on 'Cardiac valvular disease'

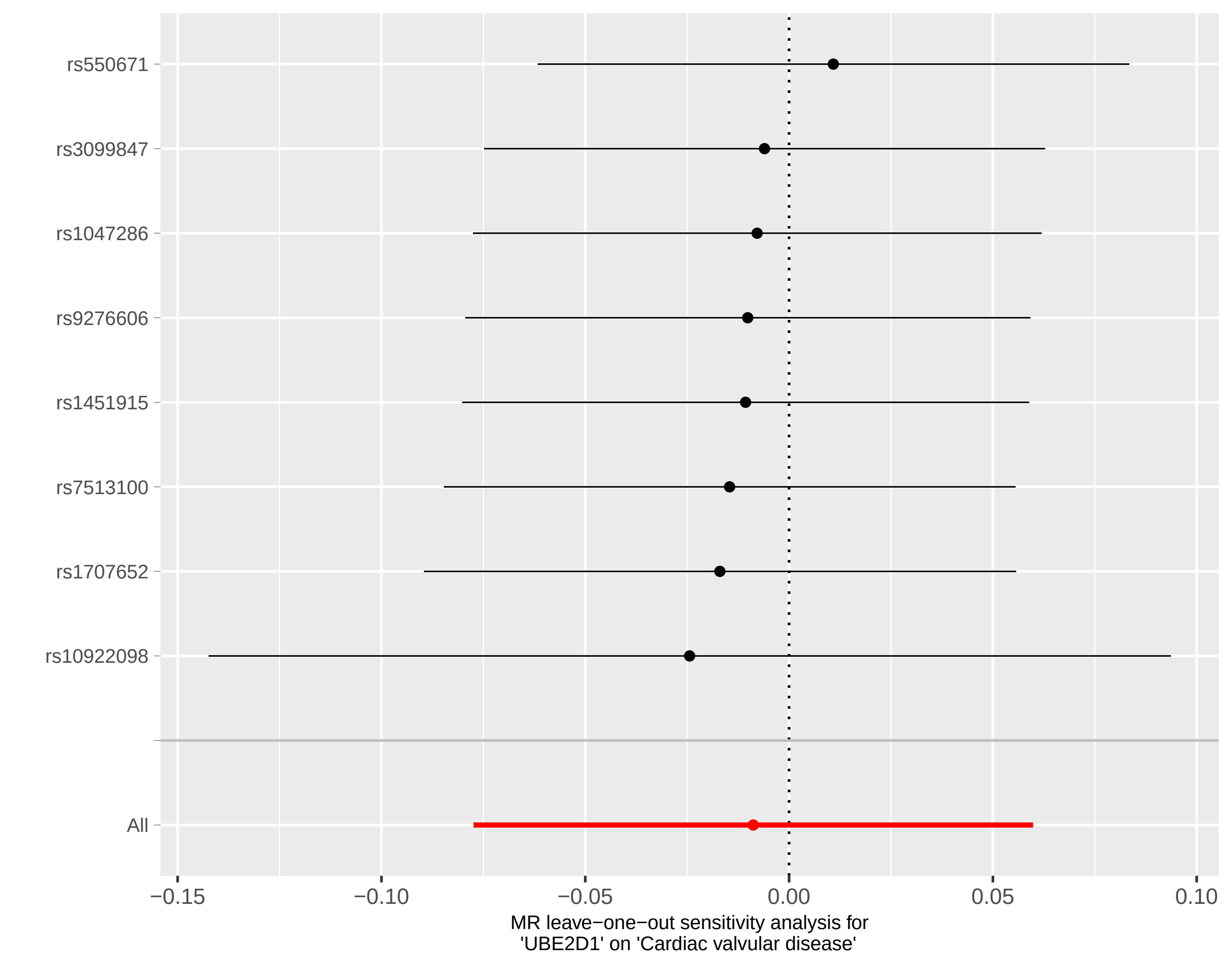
rs550671
rs3099847
rs1047286
rs9276606
rs1451915
rs7513100
rs1707652
rs10922098
All
−0.15
−0.10
−0.05
0.00
0.05
0.10
MR leave−one−out sensitivity analysis for
'UBE2D1' on 'Cardiac valvular disease'

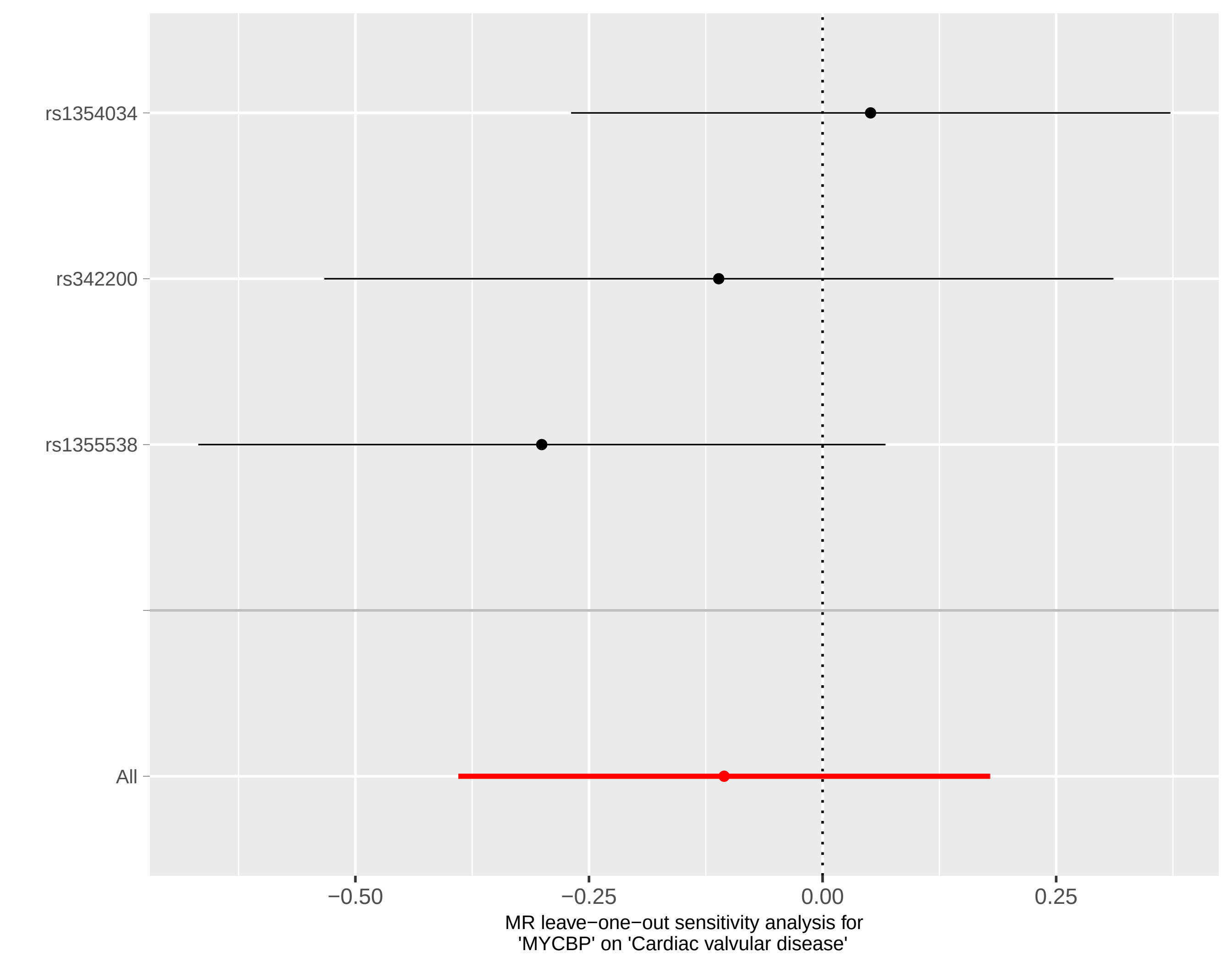
rs1354034
rs342200
rs1355538
All
−0.50
−0.25
0.00
0.25
MR leave−one−out sensitivity analysis for
'MYCBP' on 'Cardiac valvular disease'

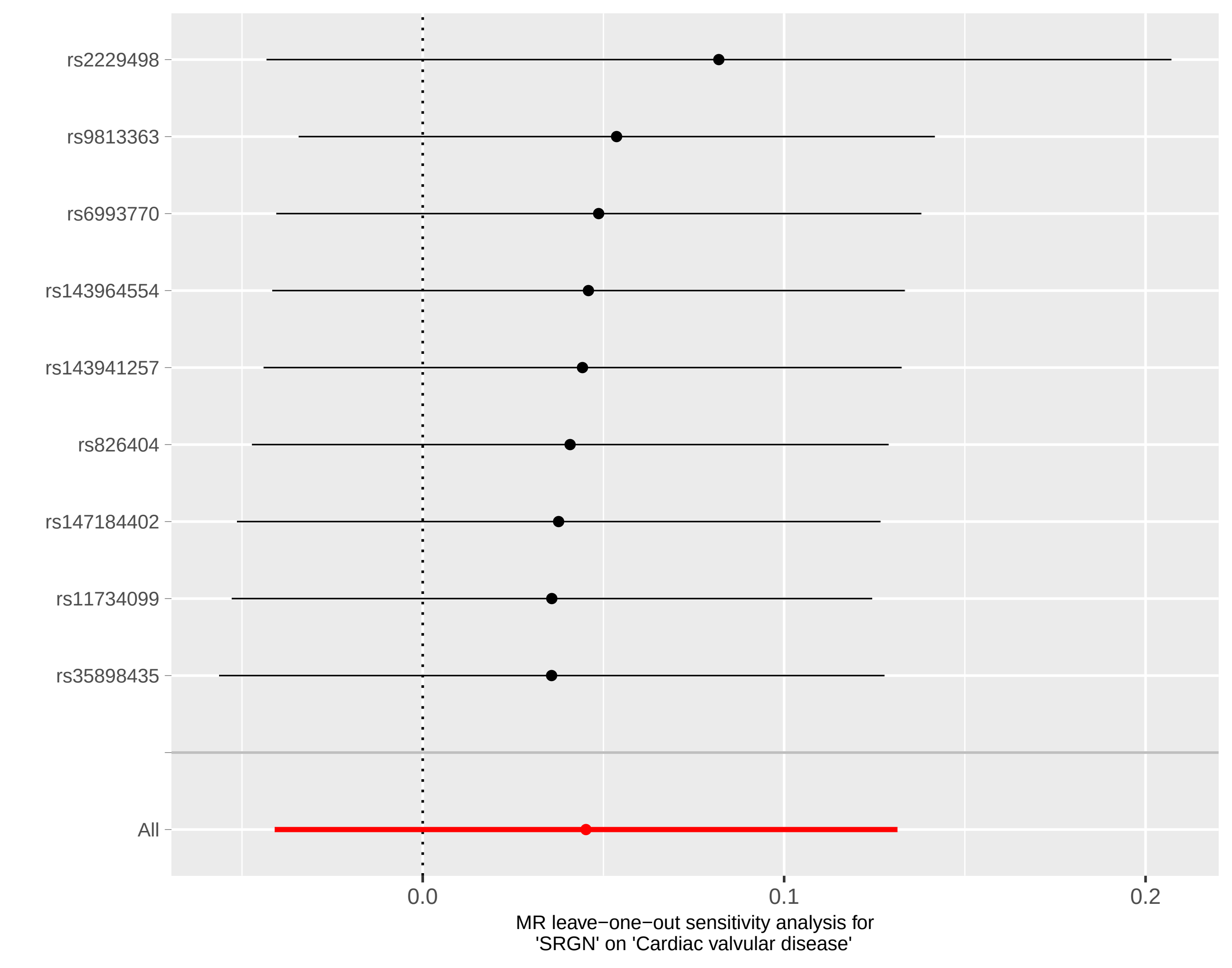
rs2229498
rs9813363
rs6993770
rs143964554
rs143941257
rs826404
rs147184402
rs11734099
rs35898435
All
0.0
0.1
0.2
MR leave−one−out sensitivity analysis for
'SRGN' on 'Cardiac valvular disease'

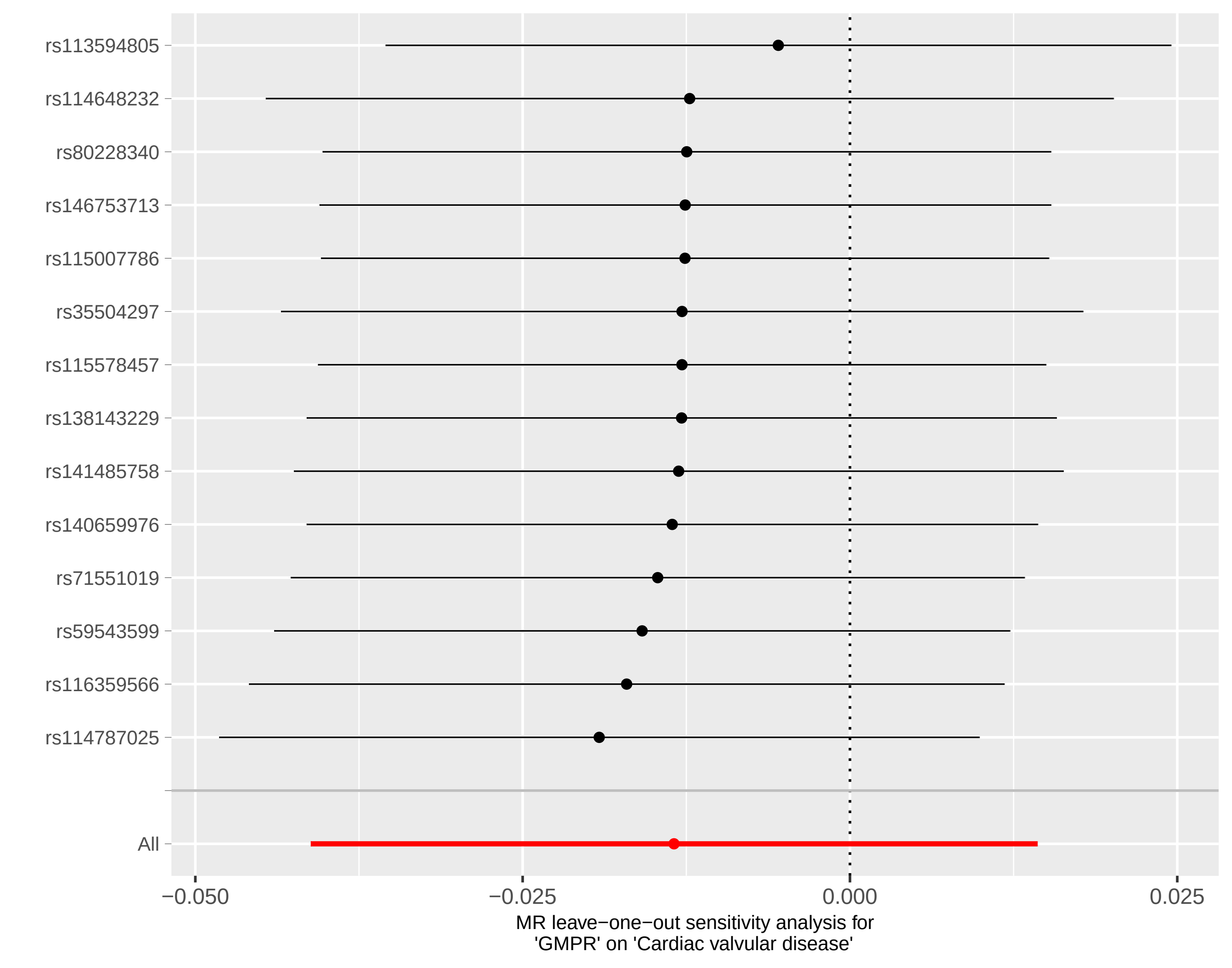

rs113594805
rs114648232
rs80228340
rs146753713
rs115007786
rs35504297
rs115578457
rs138143229
rs141485758
rs140659976
rs71551019
rs59543599
rs116359566
rs114787025
All
−0.050
−0.025
0.000
0.025
MR leave−one−out sensitivity analysis for
'GMPR' on 'Cardiac valvular disease'

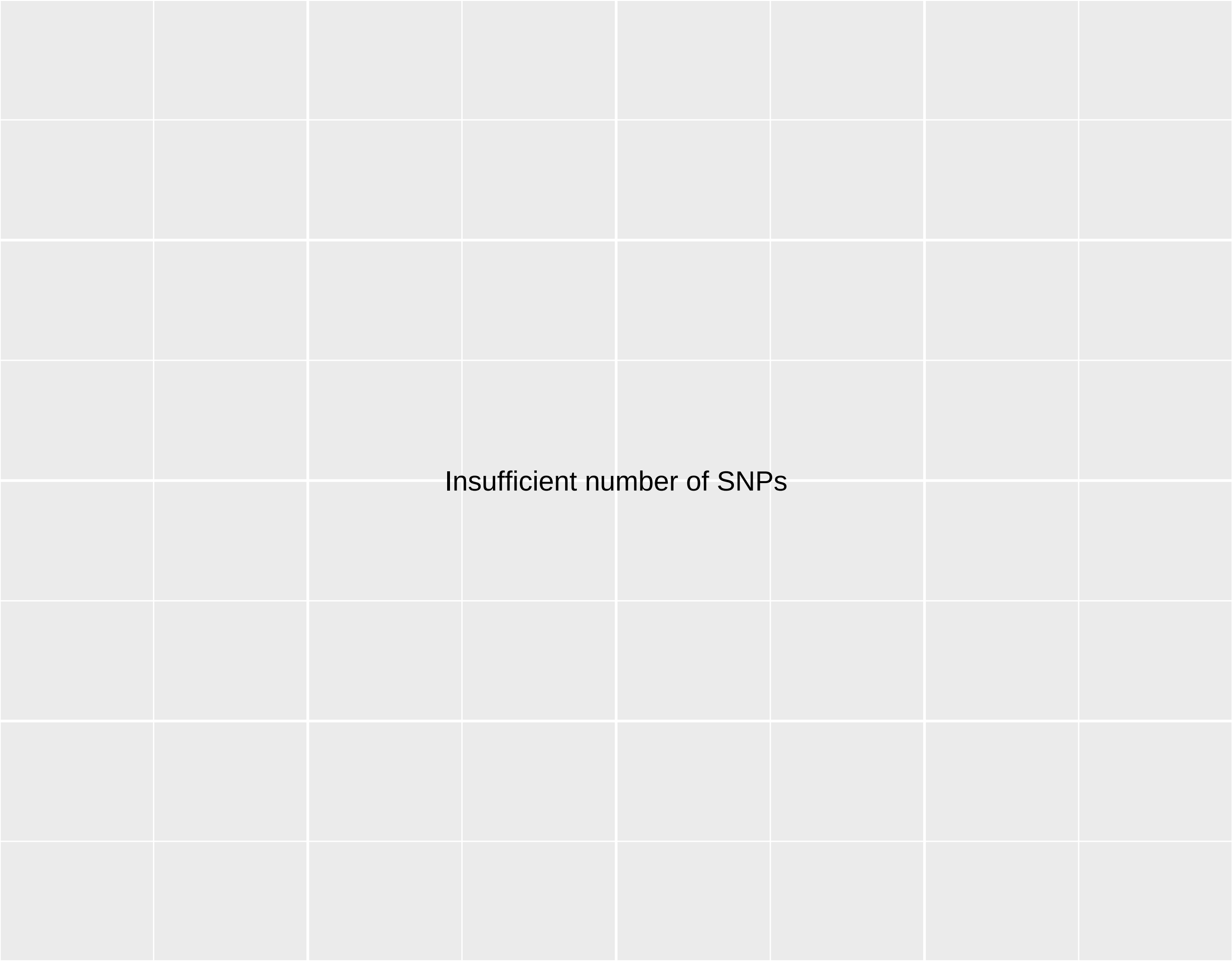
Insufficient number of SNPs

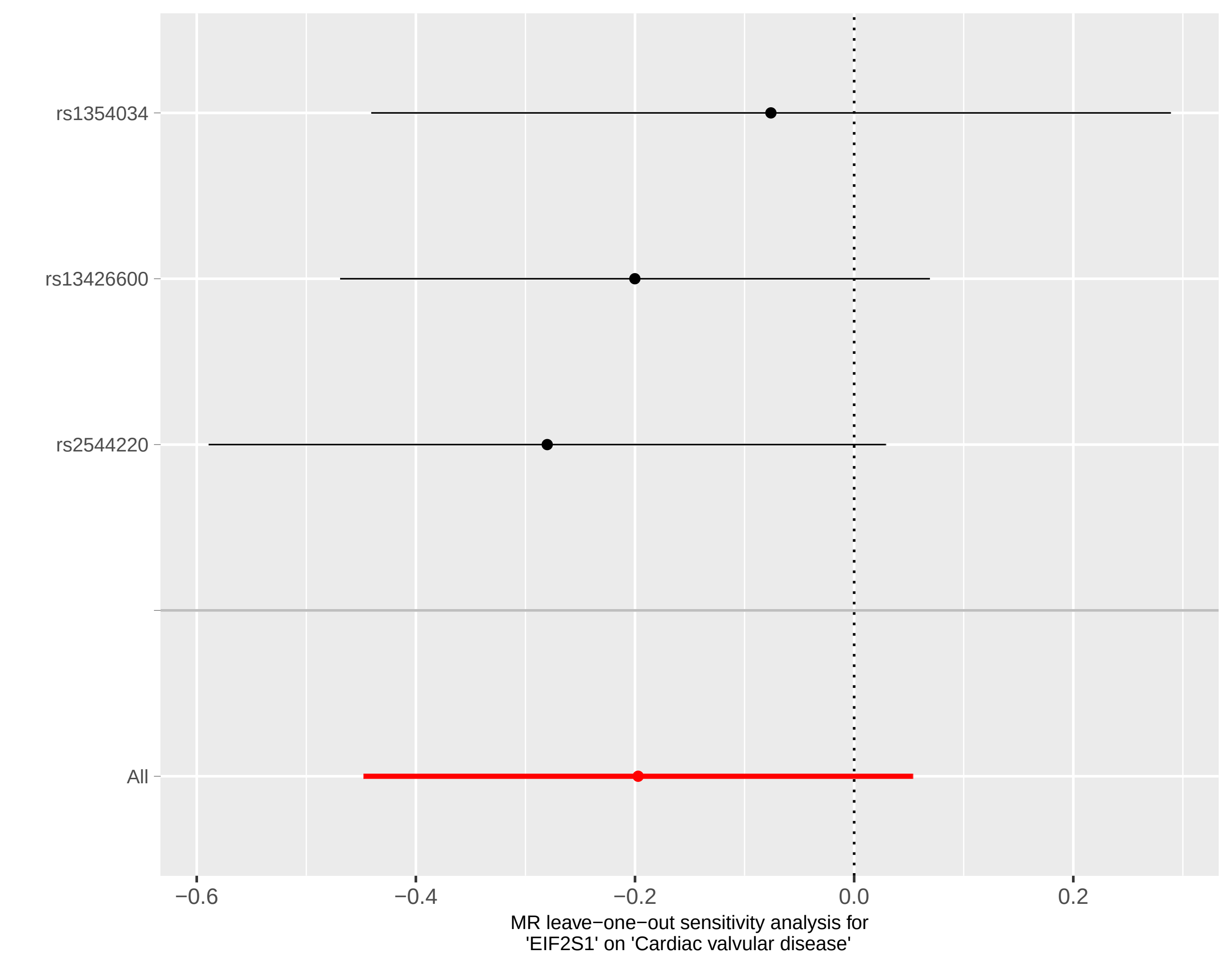
rs1354034
rs13426600
rs2544220
All
−0.6
−0.4
−0.2
0.0
0.2
MR leave−one−out sensitivity analysis for
'EIF2S1' on 'Cardiac valvular disease'

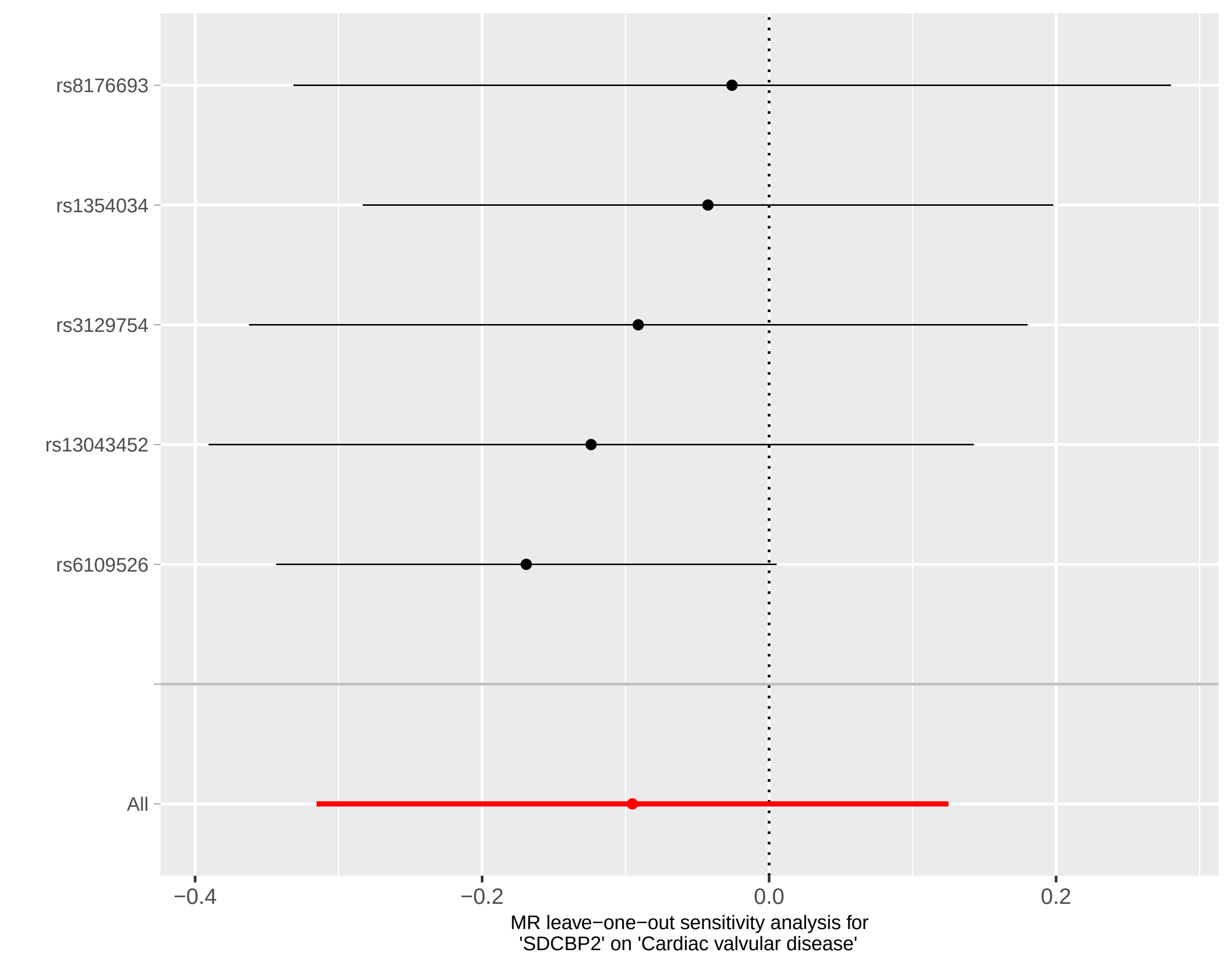
rs8176693
rs1354034
rs3129754
rs13043452
rs6109526
All
−0.4
−0.2
0.0
0.2
MR leave−one−out sensitivity analysis for
'SDCBP2' on 'Cardiac valvular disease'

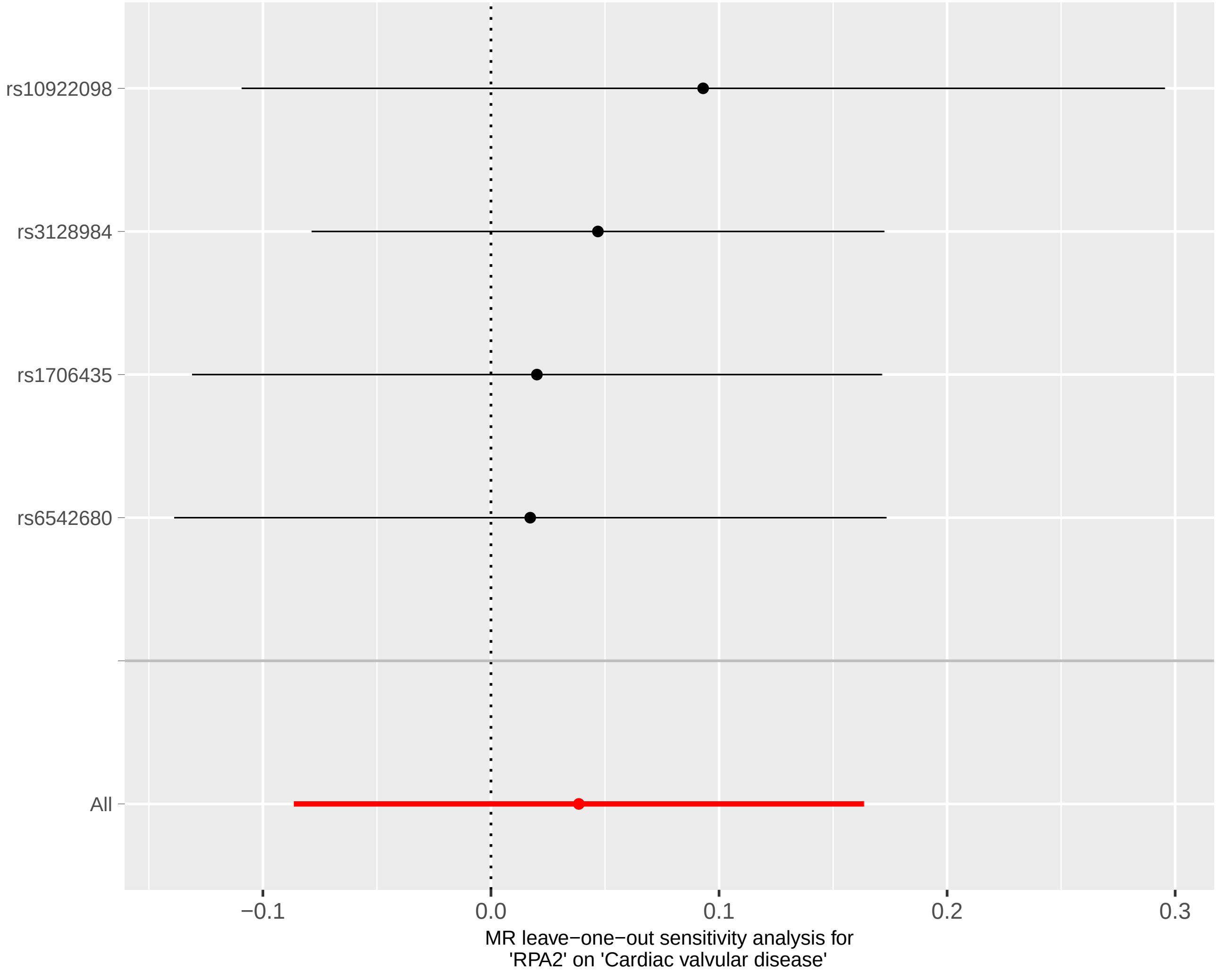

rs10922098
rs3128984
rs1706435
rs6542680
All
−0.1
0.0
0.1
0.2
0.3
MR leave−one−out sensitivity analysis for
'RPA2' on 'Cardiac valvular disease'

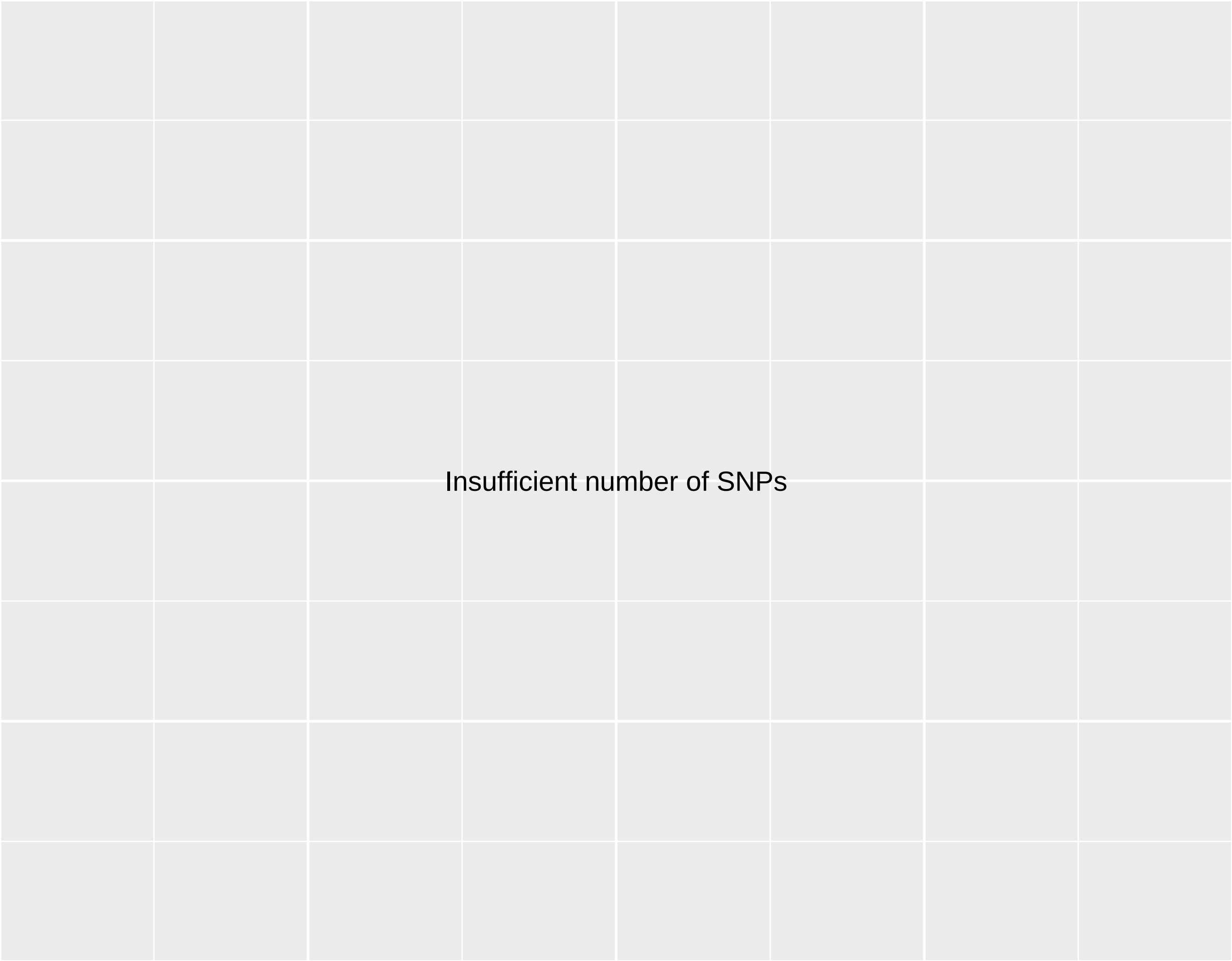
Insufficient number of SNPs

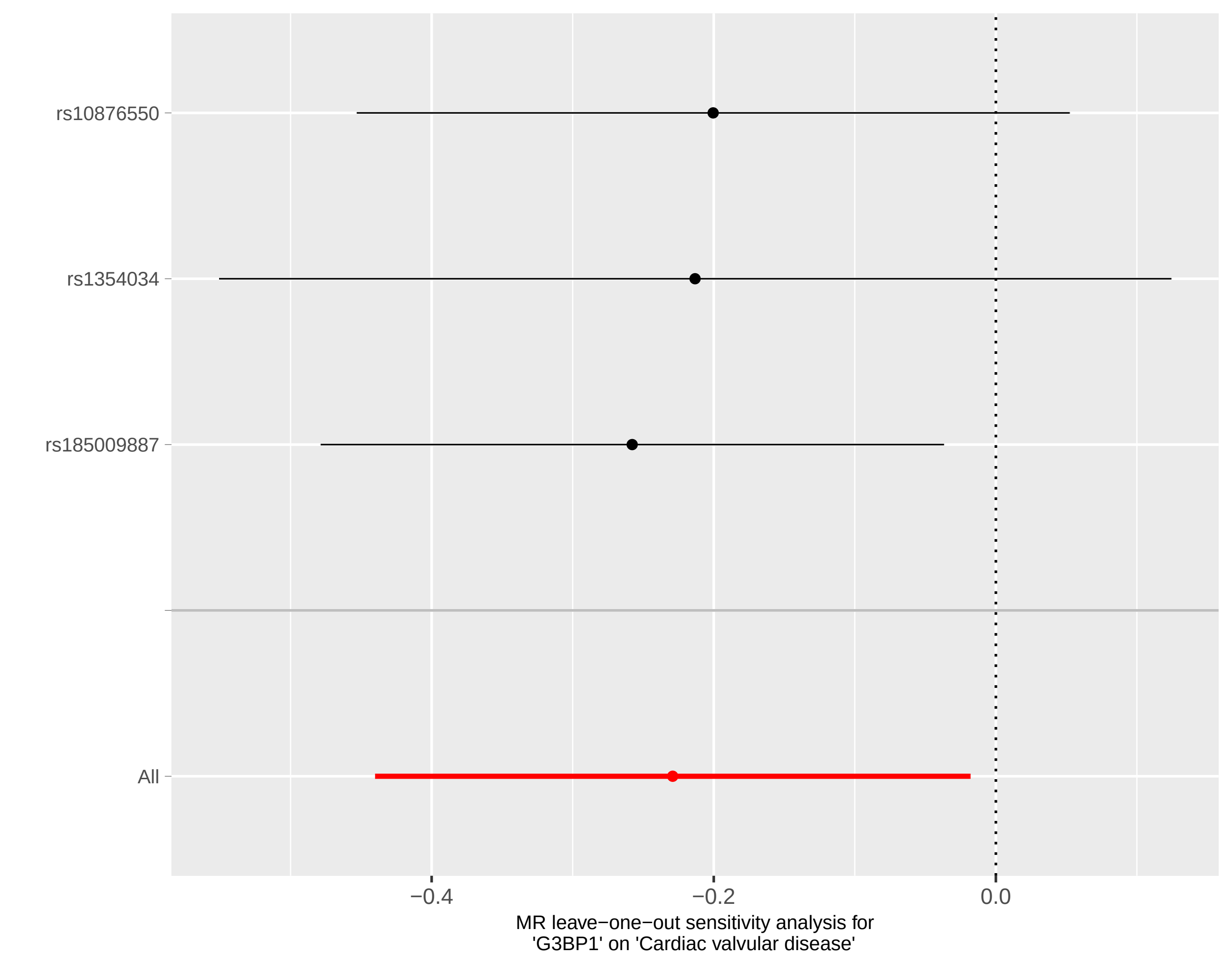

rs10876550
rs1354034
rs185009887
All
−0.4
−0.2
0.0
MR leave−one−out sensitivity analysis for
'G3BP1' on 'Cardiac valvular disease'

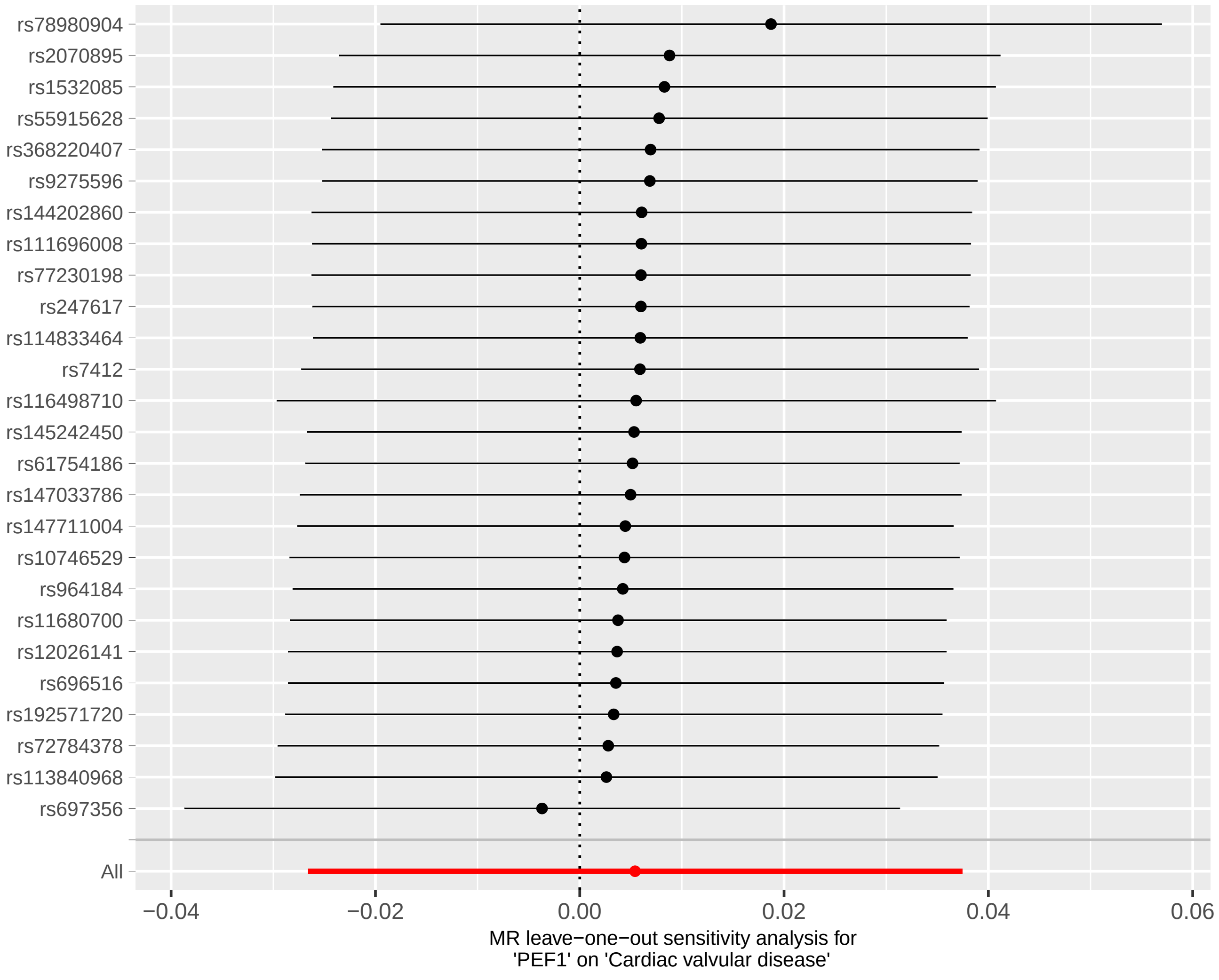

rs78980904
rs2070895
rs1532085
rs55915628
rs368220407
rs9275596
rs144202860
rs111696008
rs77230198
rs247617
rs114833464
rs7412
rs116498710
rs145242450
rs61754186
rs147033786
rs147711004
rs10746529
rs964184
rs11680700
rs12026141
rs696516
rs192571720
rs72784378
rs113840968
rs697356
All
−0.04
−0.02
0.00
0.02
0.04
0.06
MR leave−one−out sensitivity analysis for
'PEF1' on 'Cardiac valvular disease'

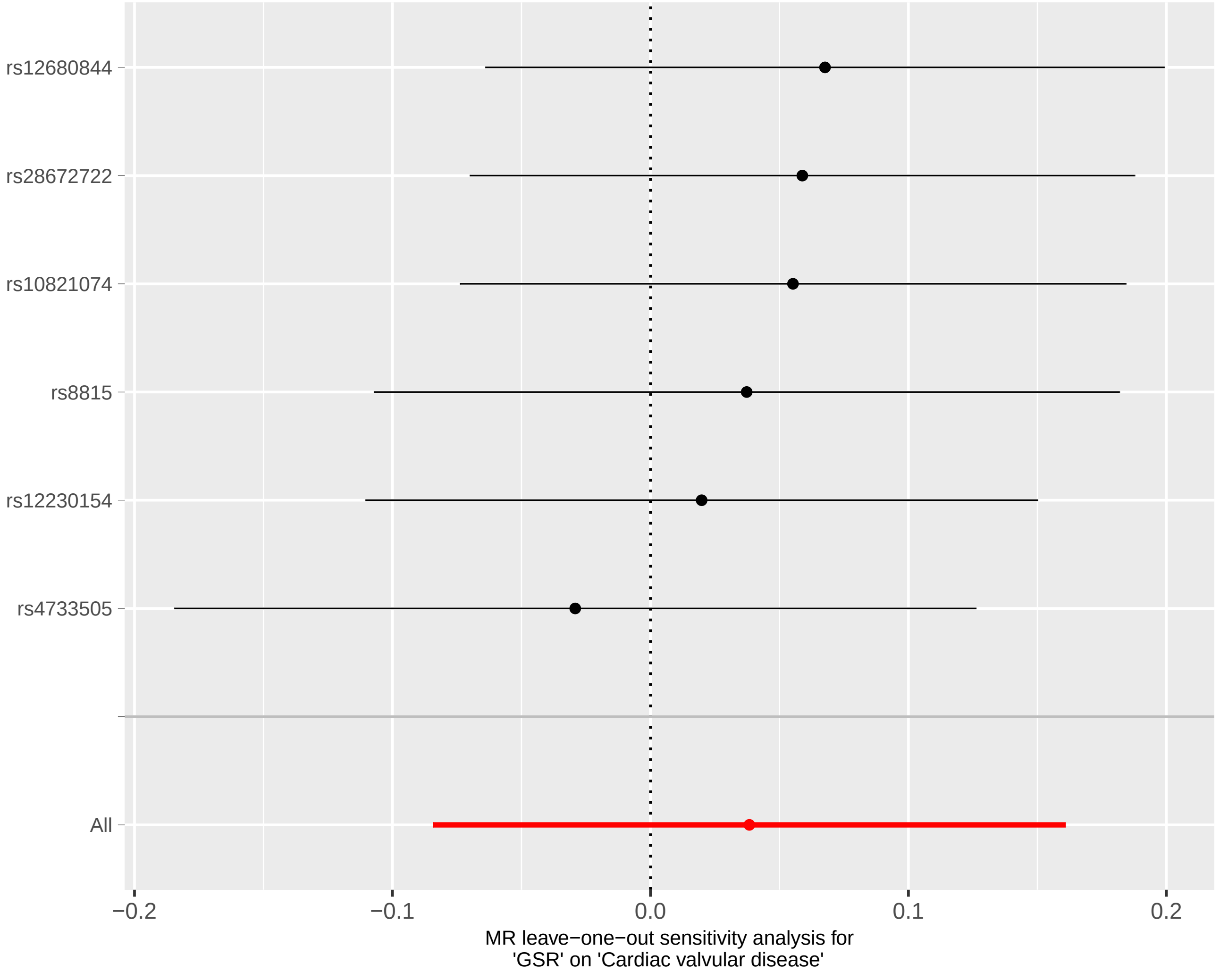

rs12680844
rs28672722
rs10821074
rs8815
rs12230154
rs4733505
All
−0.2
−0.1
0.0
0.1
0.2
MR leave−one−out sensitivity analysis for
'GSR' on 'Cardiac valvular disease'

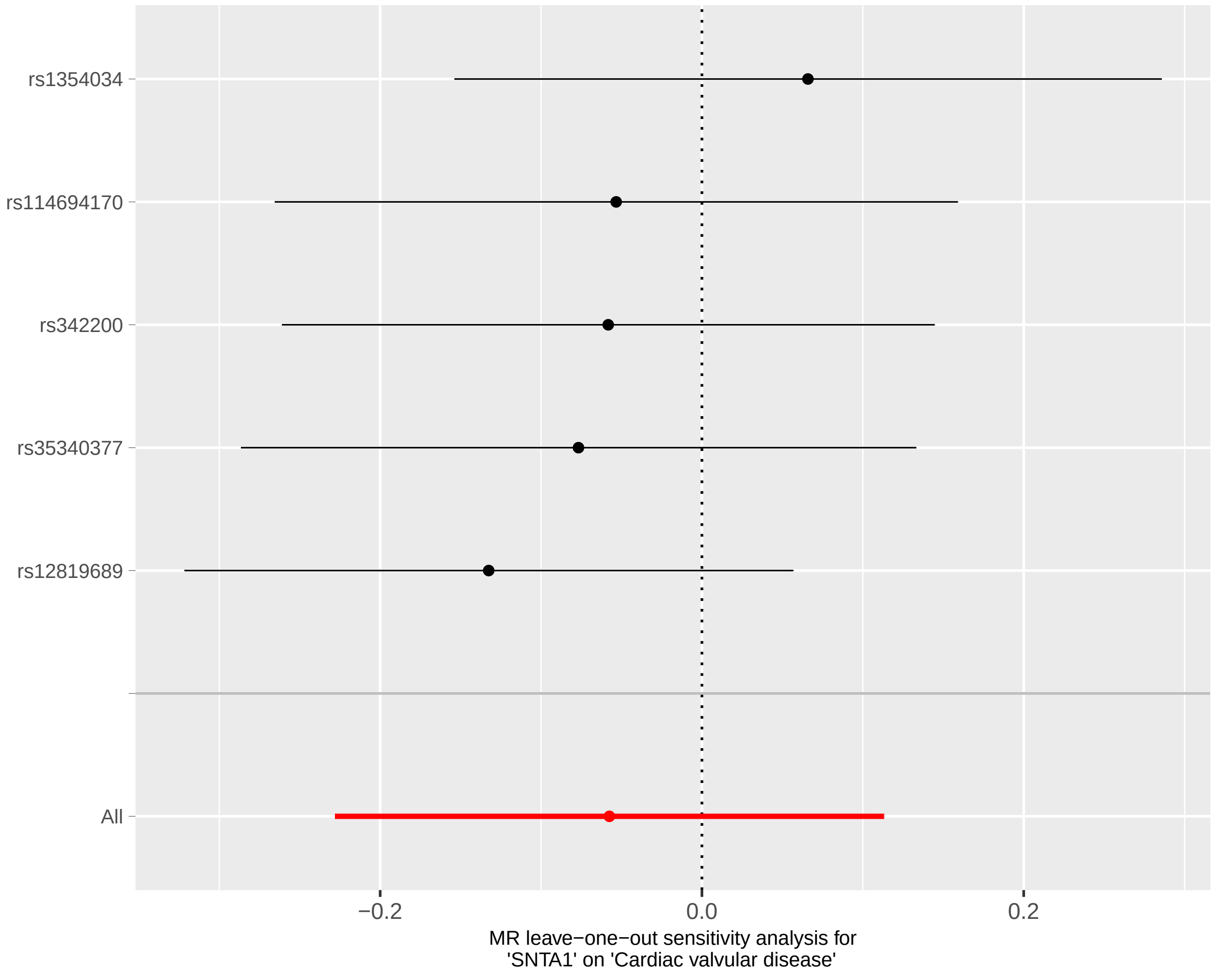
rs1354034
rs114694170
rs342200
rs35340377
rs12819689
All
−0.2
0.0
0.2
MR leave−one−out sensitivity analysis for
'SNTA1' on 'Cardiac valvular disease'

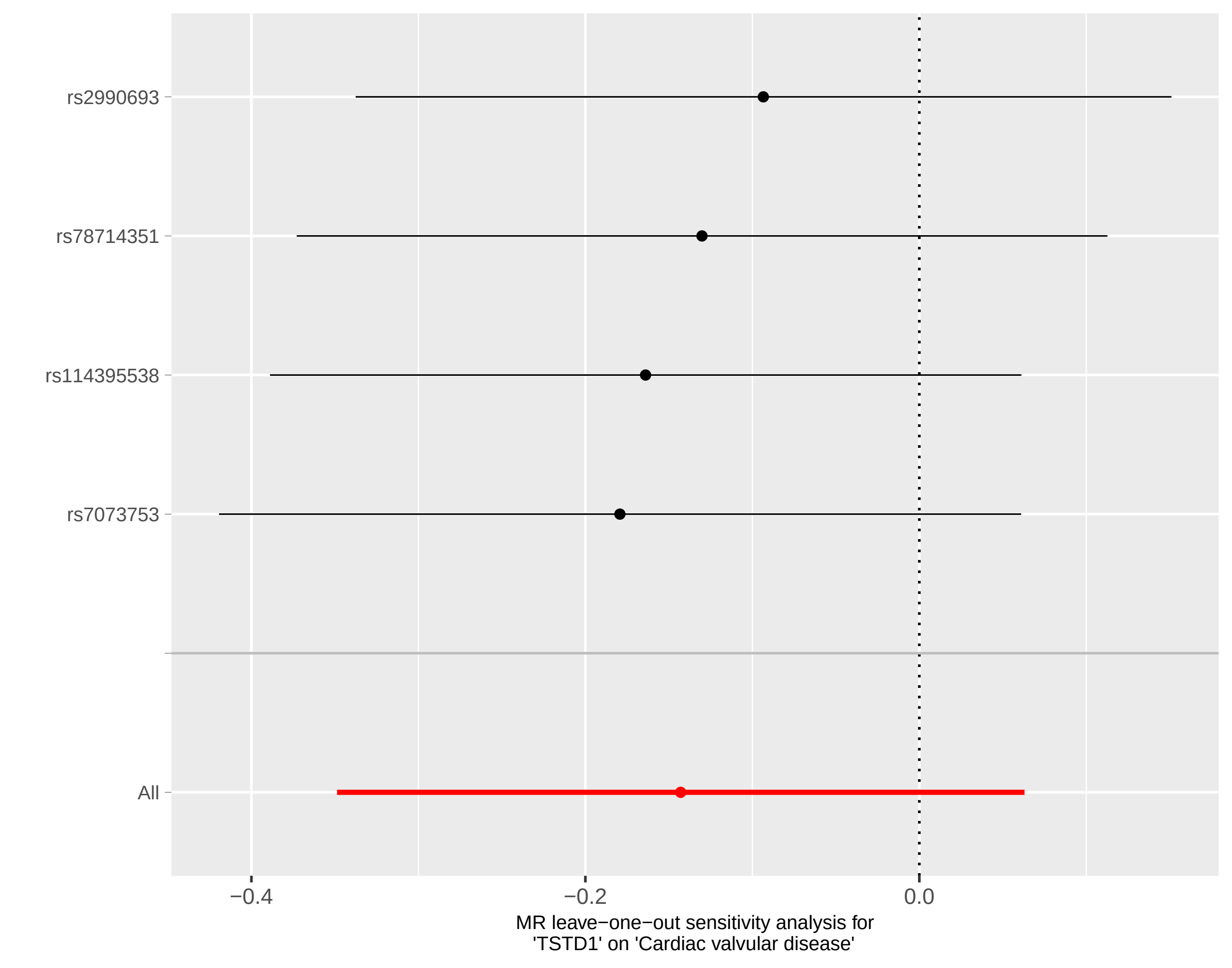
rs2990693
rs78714351
rs114395538
rs7073753
All
−0.4
−0.2
0.0
MR leave−one−out sensitivity analysis for
'TSTD1' on 'Cardiac valvular disease'

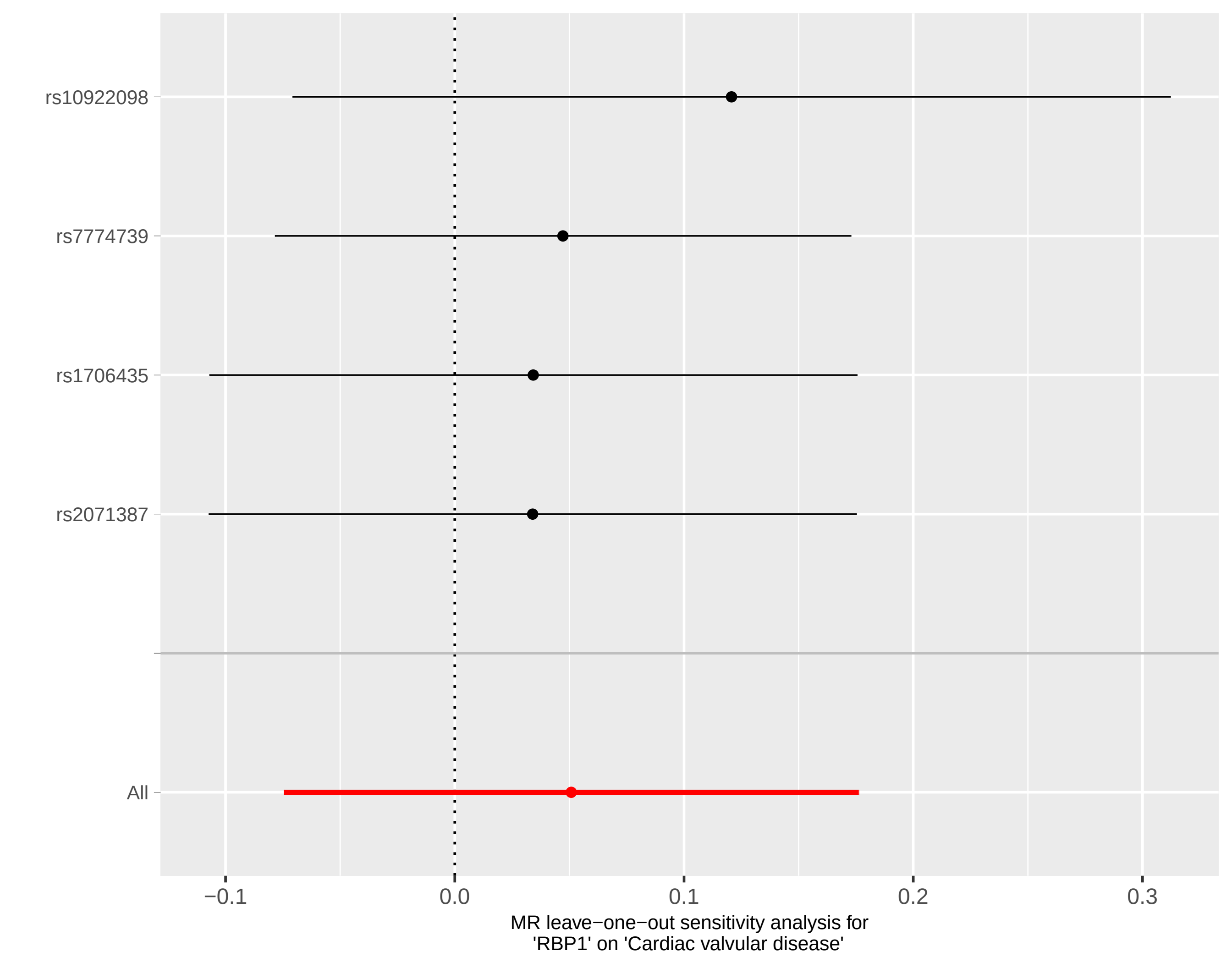
rs10922098
rs7774739
rs1706435
rs2071387
All
−0.1
0.0
0.1
0.2
0.3
MR leave−one−out sensitivity analysis for
'RBP1' on 'Cardiac valvular disease'

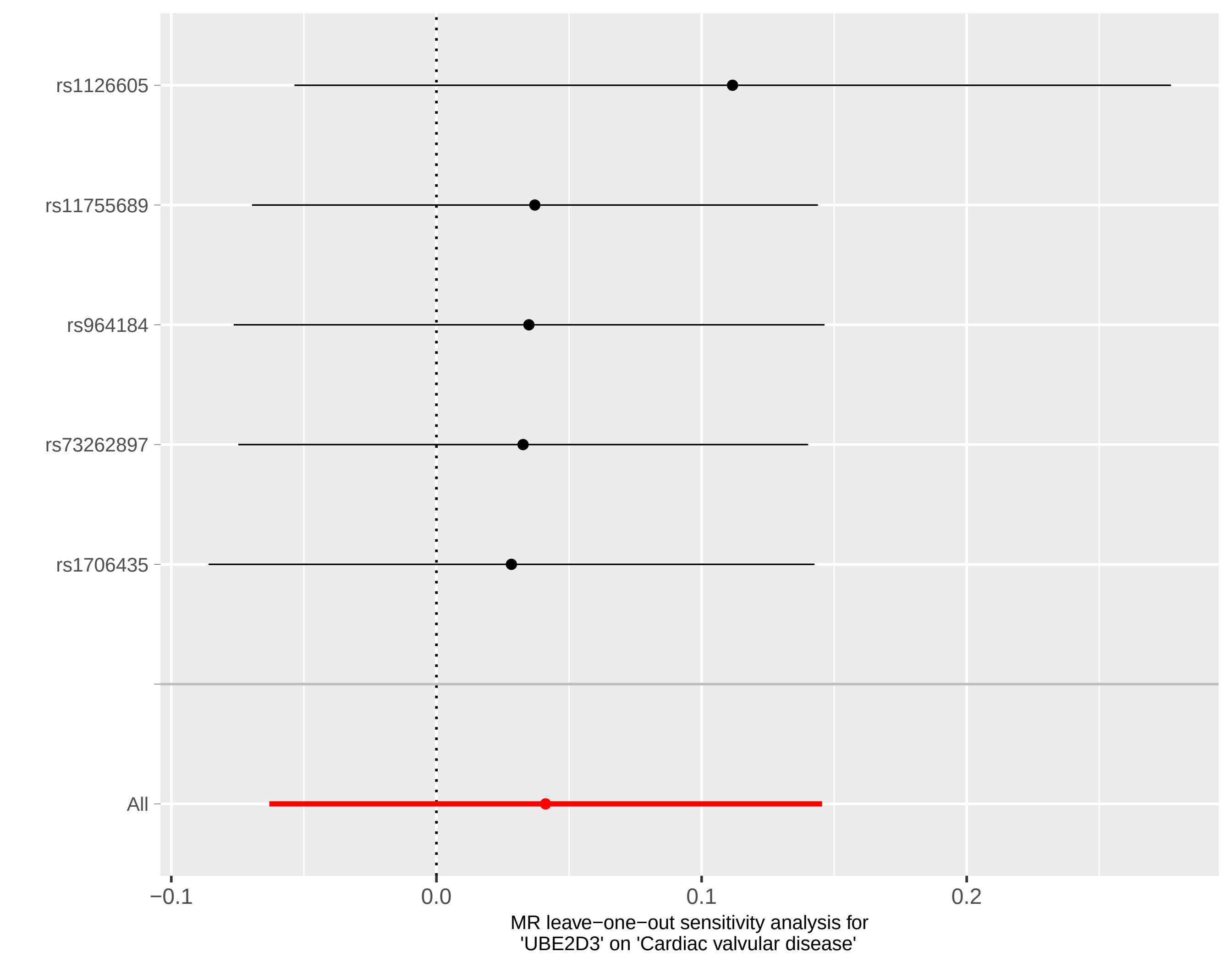
rs1126605
rs11755689
rs964184
rs73262897
rs1706435
All
−0.1
0.0
0.1
0.2
MR leave−one−out sensitivity analysis for
'UBE2D3' on 'Cardiac valvular disease'

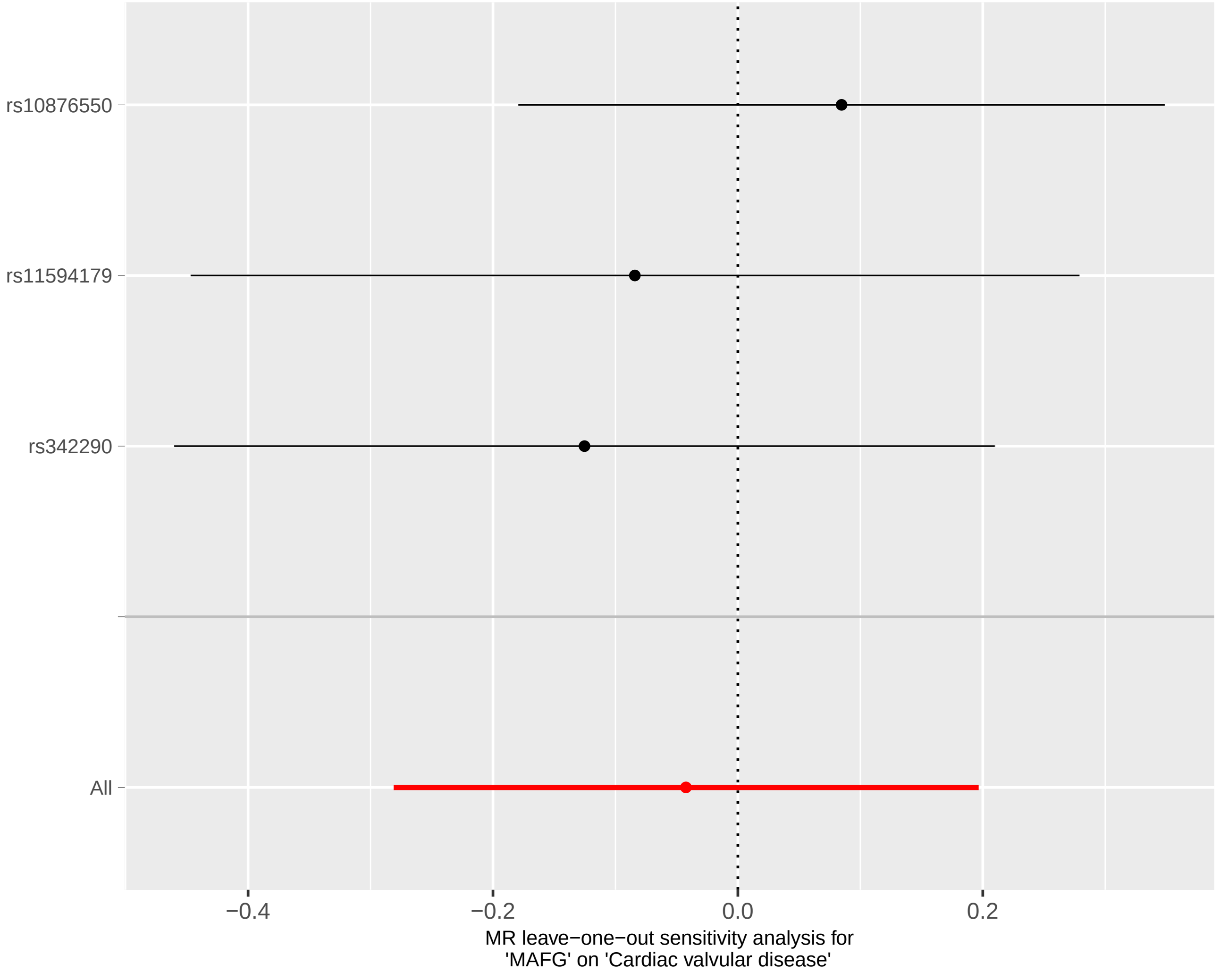
rs10876550
rs11594179
rs342290
All
−0.4
−0.2
0.0
0.2
MR leave−one−out sensitivity analysis for
'MAFG' on 'Cardiac valvular disease'

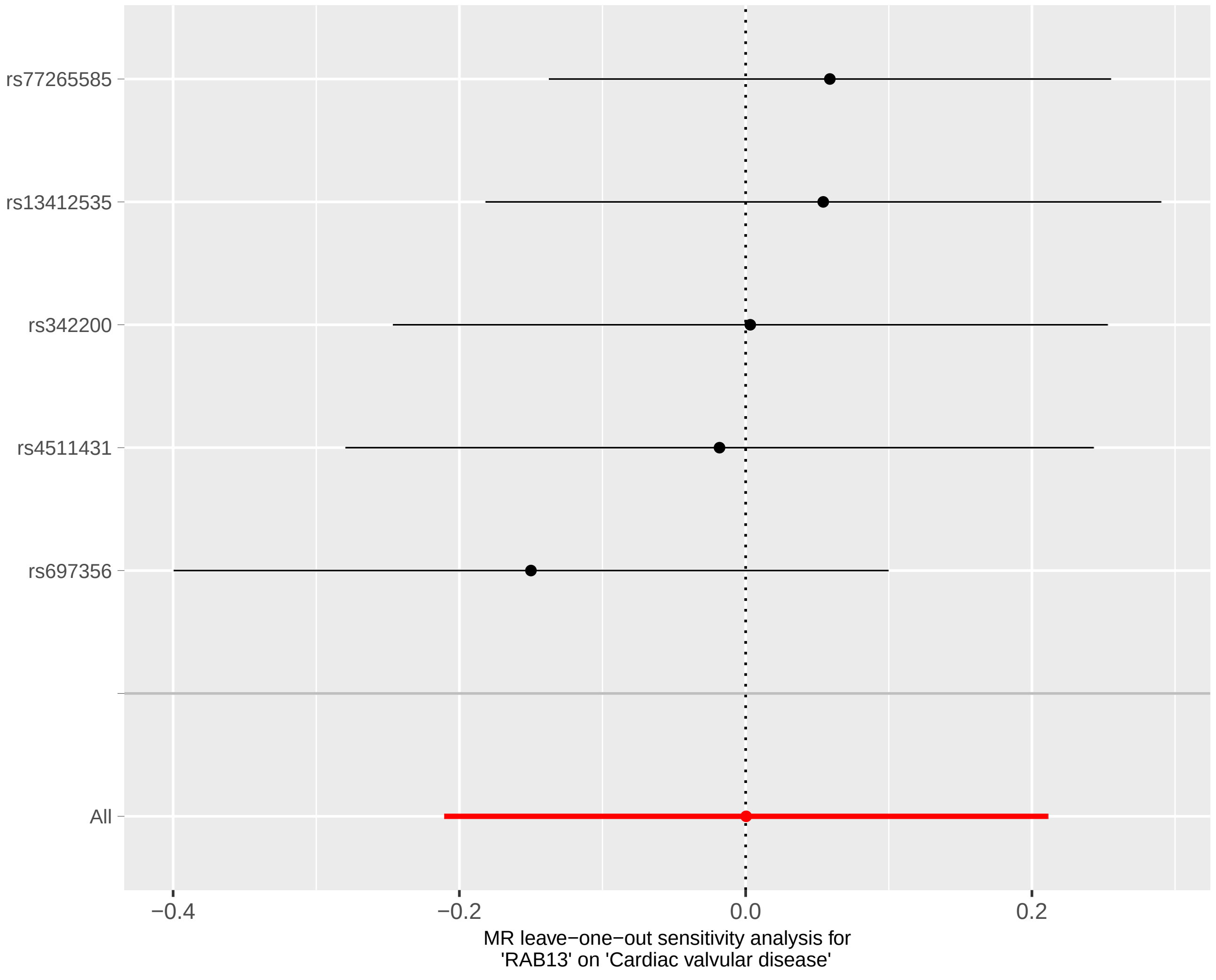
rs77265585
rs13412535
rs342200
rs4511431
rs697356
All
−0.4
−0.2
0.0
0.2
MR leave−one−out sensitivity analysis for
'RAB13' on 'Cardiac valvular disease'

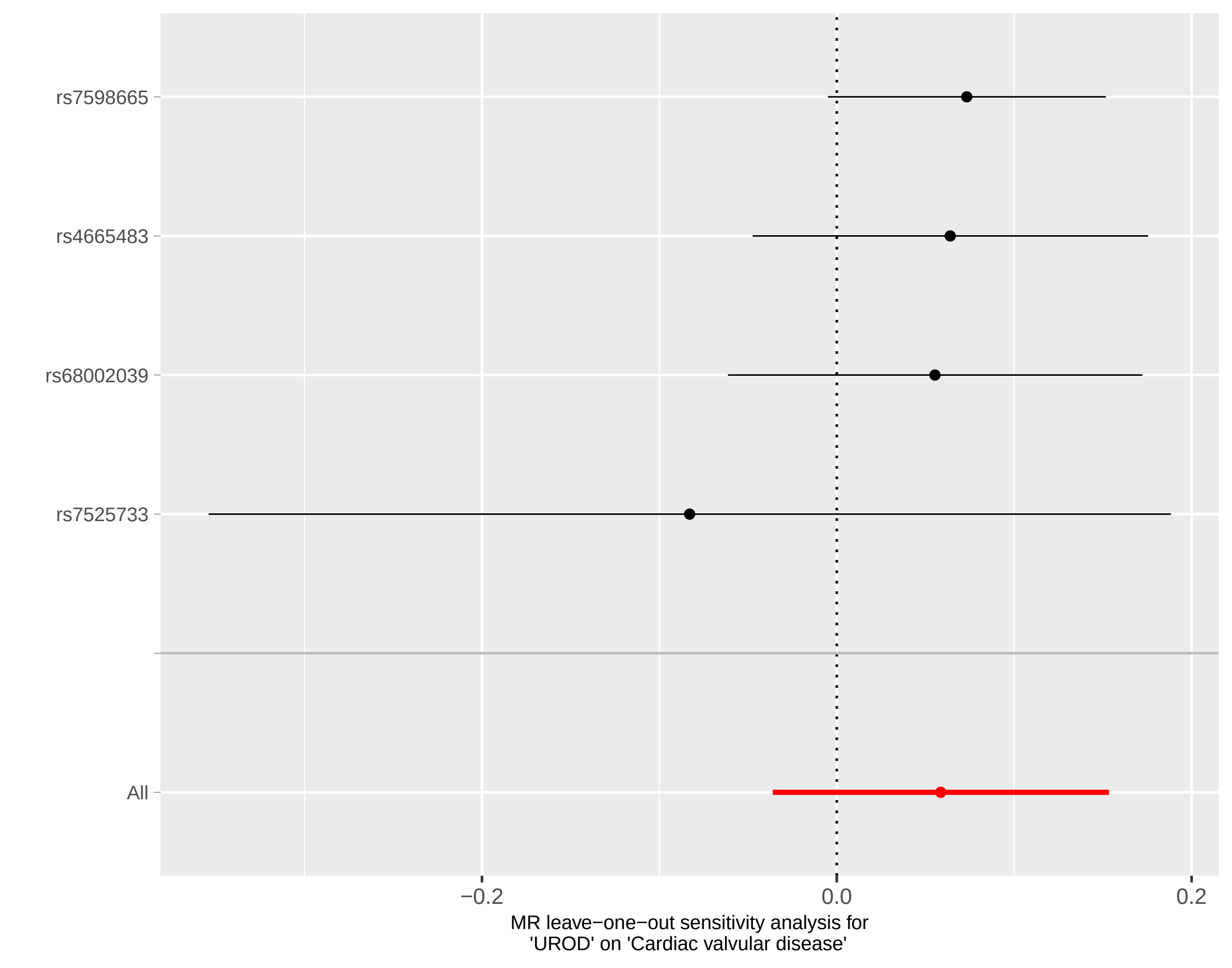

rs7598665
rs4665483
rs68002039
rs7525733
All
−0.2
0.0
0.2
MR leave−one−out sensitivity analysis for
'UROD' on 'Cardiac valvular disease'

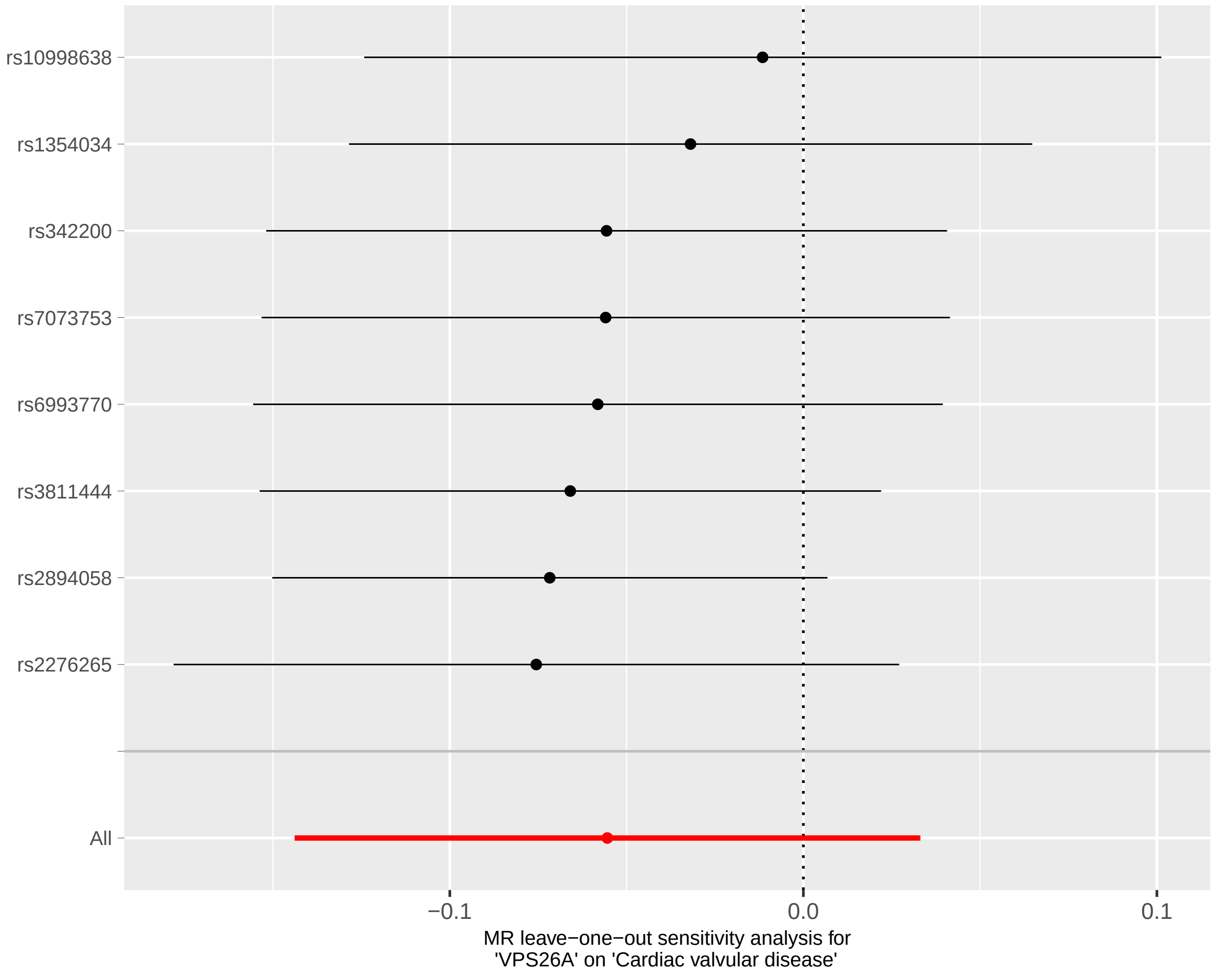

rs10998638
rs1354034
rs342200
rs7073753
rs6993770
rs3811444
rs2894058
rs2276265
All
−0.1
0.0
0.1
MR leave−one−out sensitivity analysis for
'VPS26A' on 'Cardiac valvular disease'

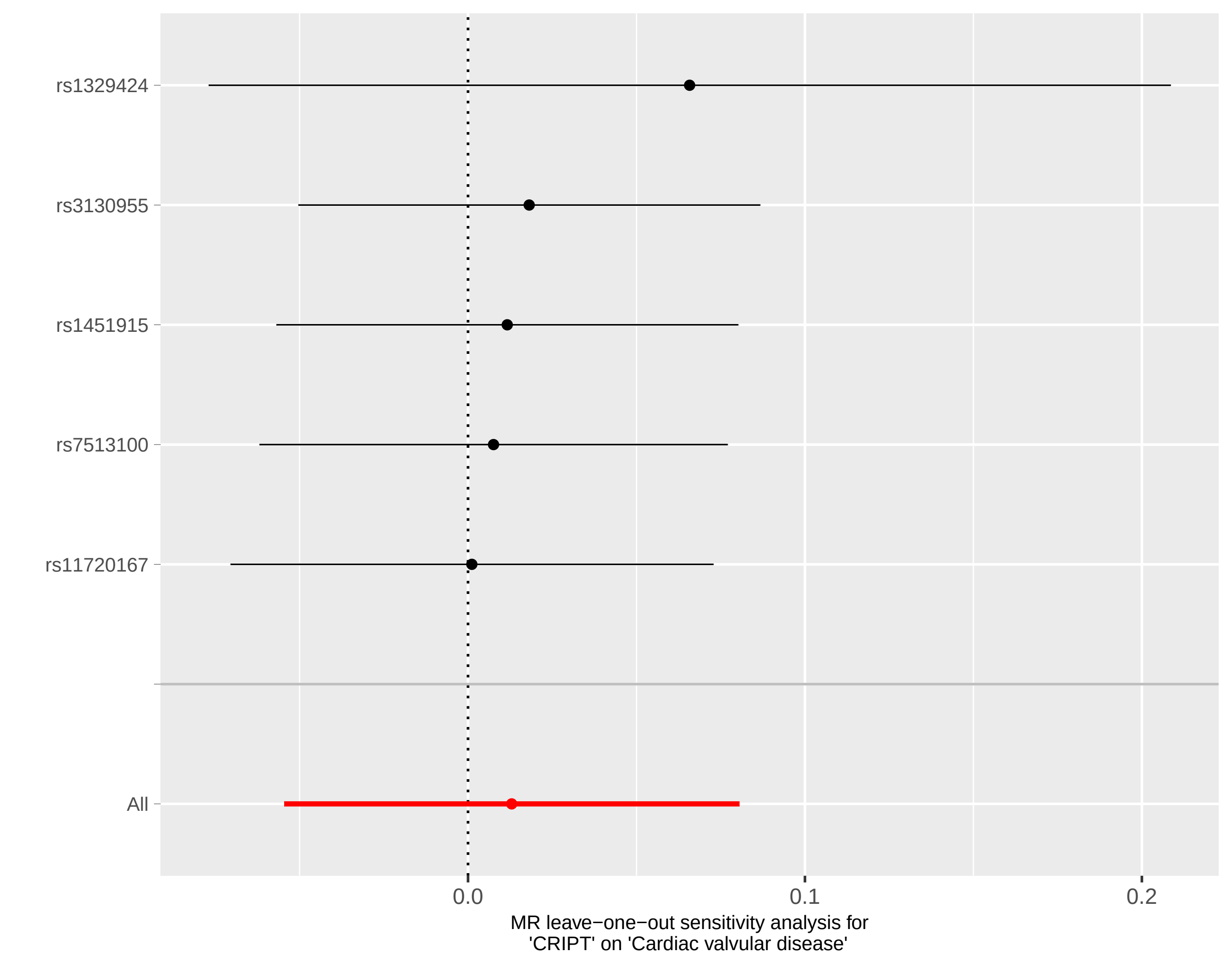
rs1329424
rs3130955
rs1451915
rs7513100
rs11720167
All
0.0
0.1
0.2
MR leave−one−out sensitivity analysis for
'CRIPT' on 'Cardiac valvular disease'

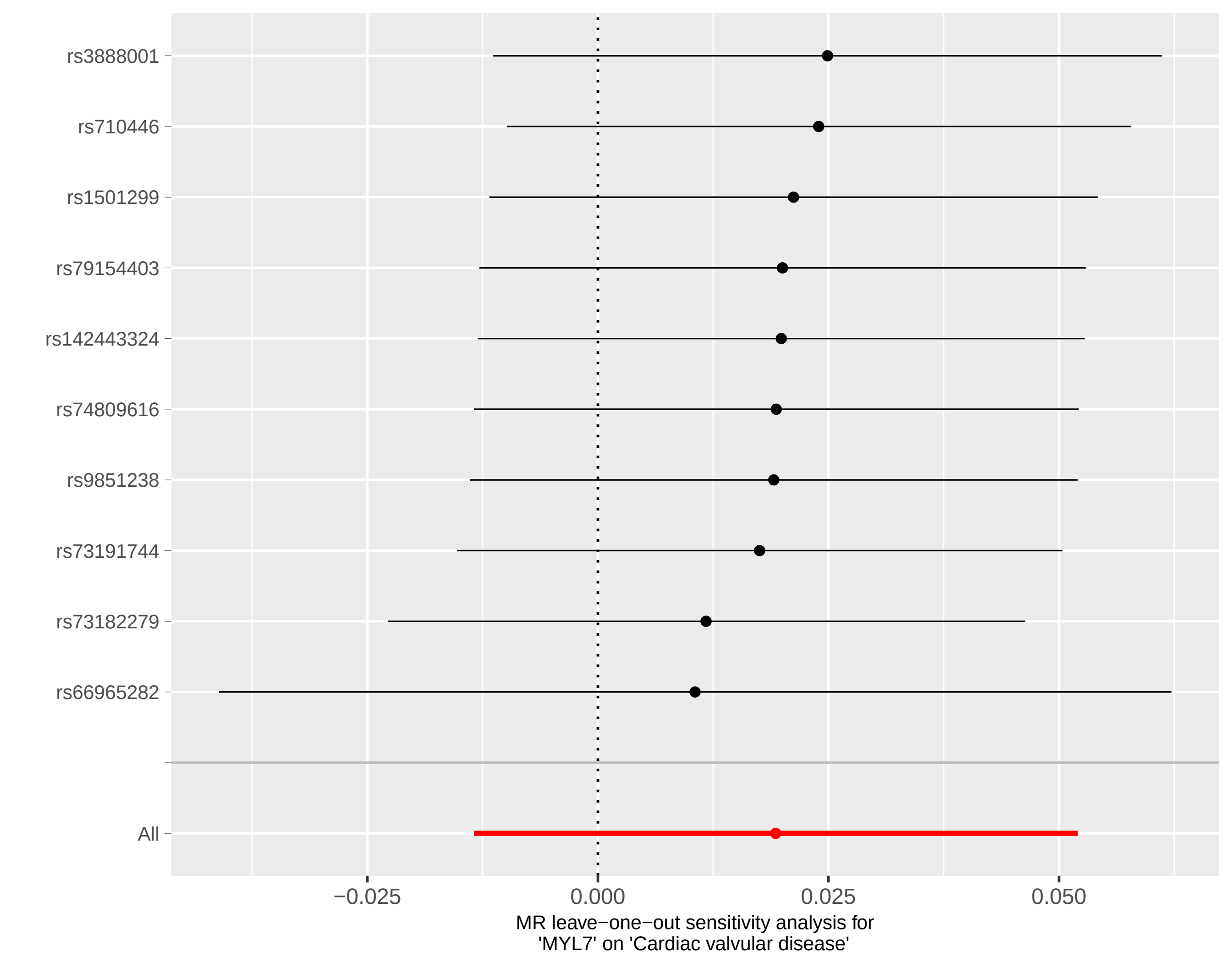

rs3888001
rs710446
rs1501299
rs79154403
rs142443324
rs74809616
rs9851238
rs73191744
rs73182279
rs66965282
All
−0.025
0.000
0.025
0.050
MR leave−one−out sensitivity analysis for
'MYL7' on 'Cardiac valvular disease'

Insufficient number of SNPs

Insufficient number of SNPs

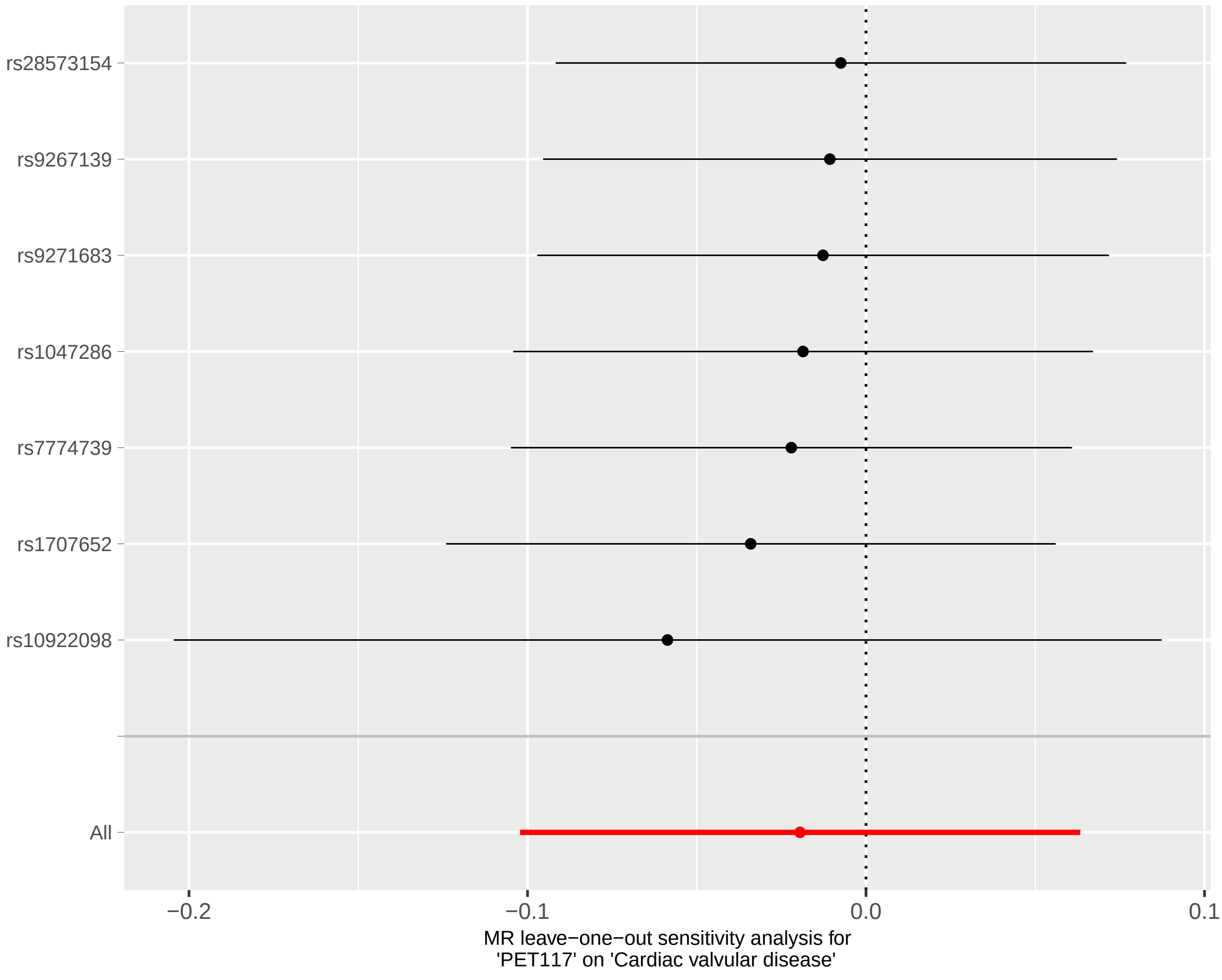
rs28573154
rs9267139
rs9271683
rs1047286
rs7774739
rs1707652
rs10922098
All
−0.2
−0.1
0.0
0.1
MR leave−one−out sensitivity analysis for
'PET117' on 'Cardiac valvular disease'

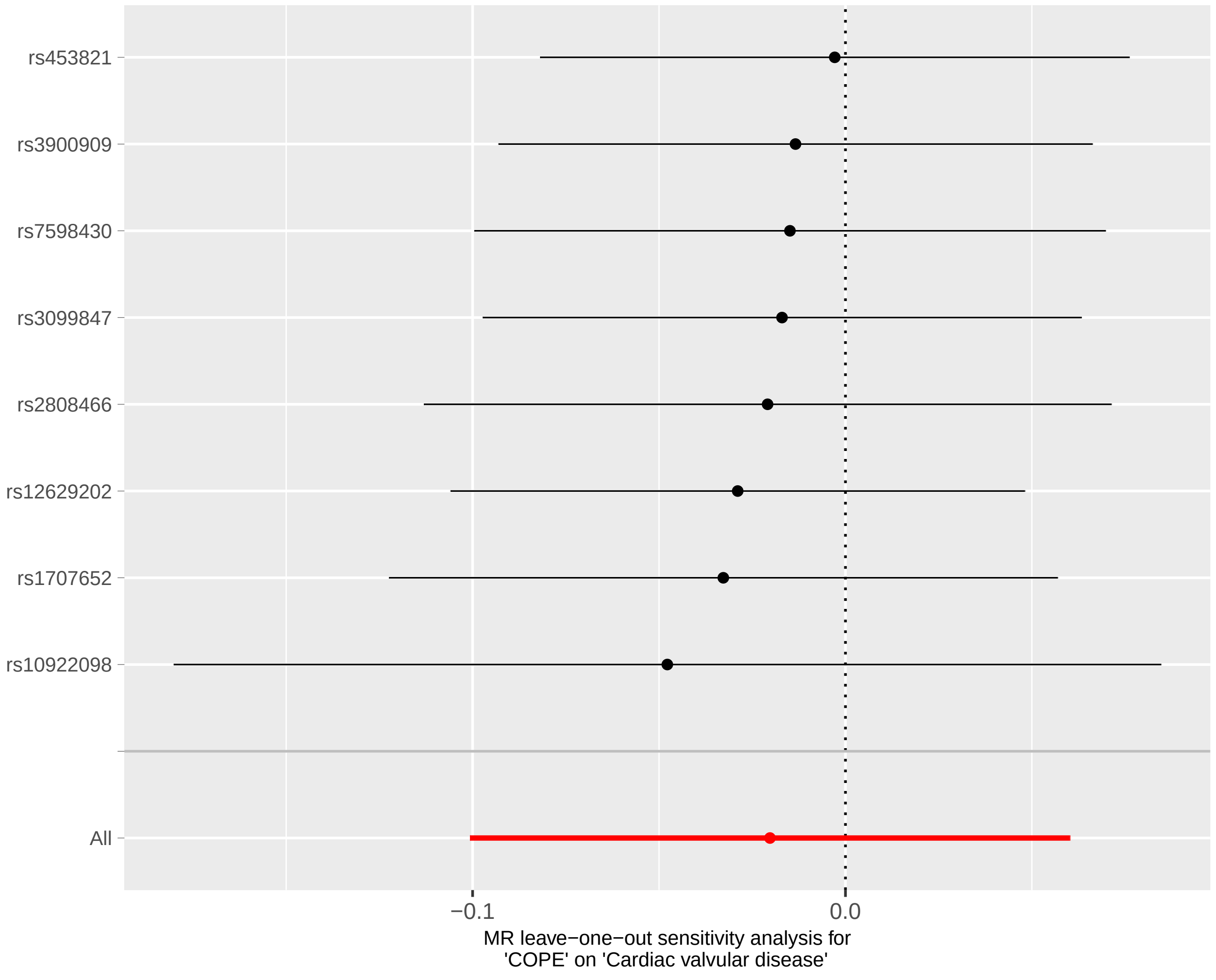
rs453821
rs3900909
rs7598430
rs3099847
rs2808466
rs12629202
rs1707652
rs10922098
All
−0.1
0.0
MR leave−one−out sensitivity analysis for
'COPE' on 'Cardiac valvular disease'

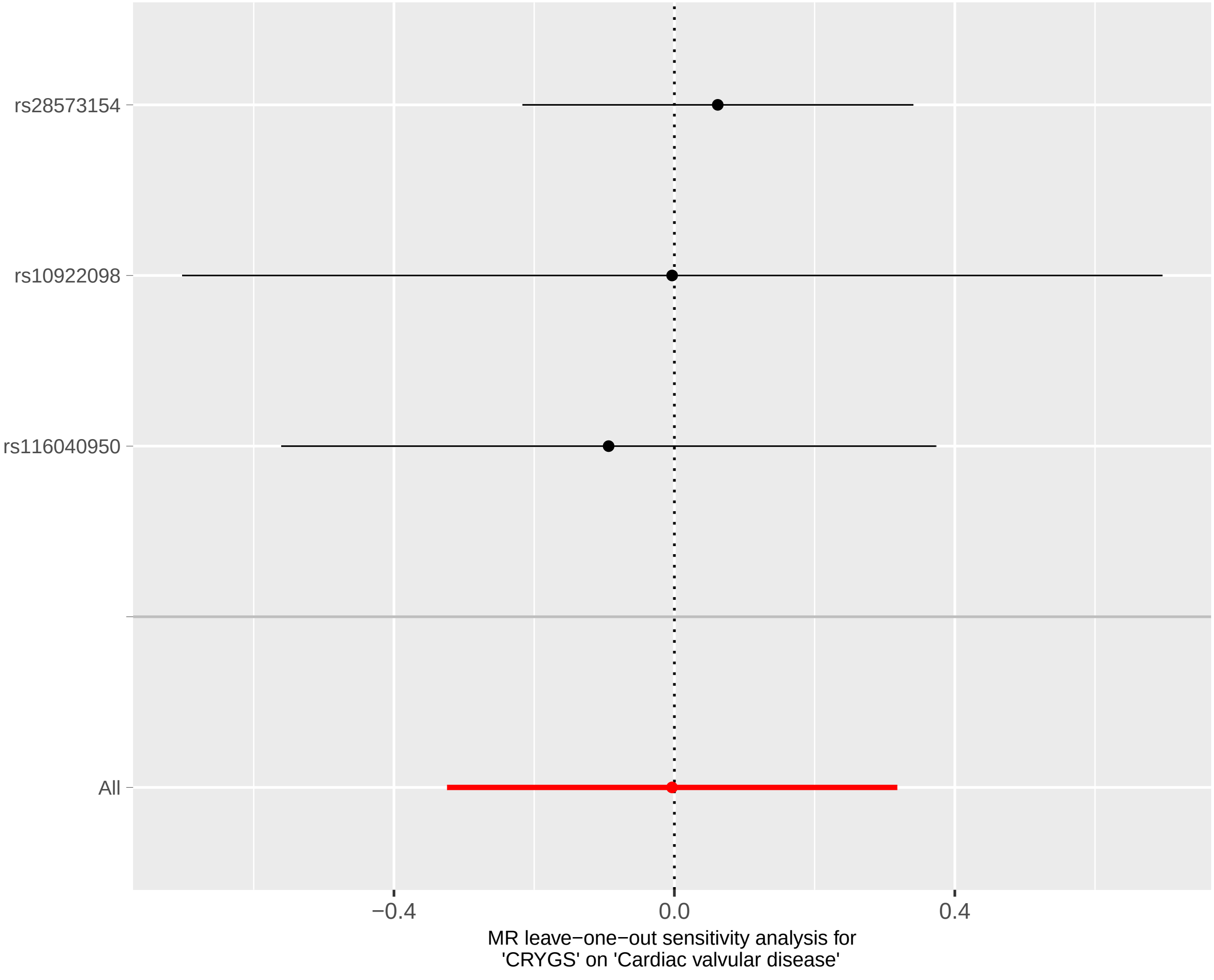
rs28573154
rs10922098
rs116040950
All
−0.4
0.0
0.4
MR leave−one−out sensitivity analysis for
'CRYGS' on 'Cardiac valvular disease'

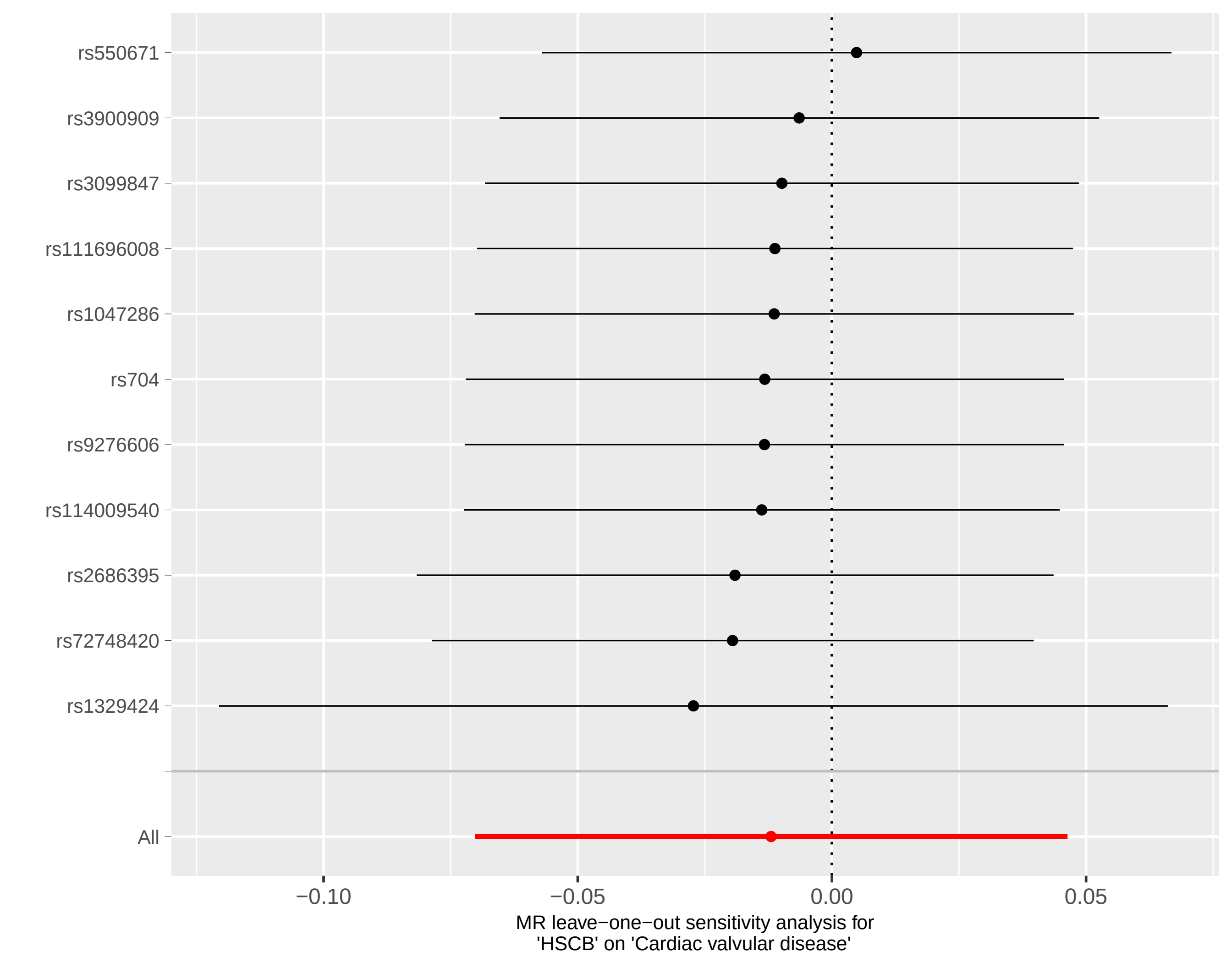
rs550671
rs3900909
rs3099847
rs111696008
rs1047286
rs704
rs9276606
rs114009540
rs2686395
rs72748420
rs1329424
All
−0.10
−0.05
0.00
0.05
MR leave−one−out sensitivity analysis for
'HSCB' on 'Cardiac valvular disease'

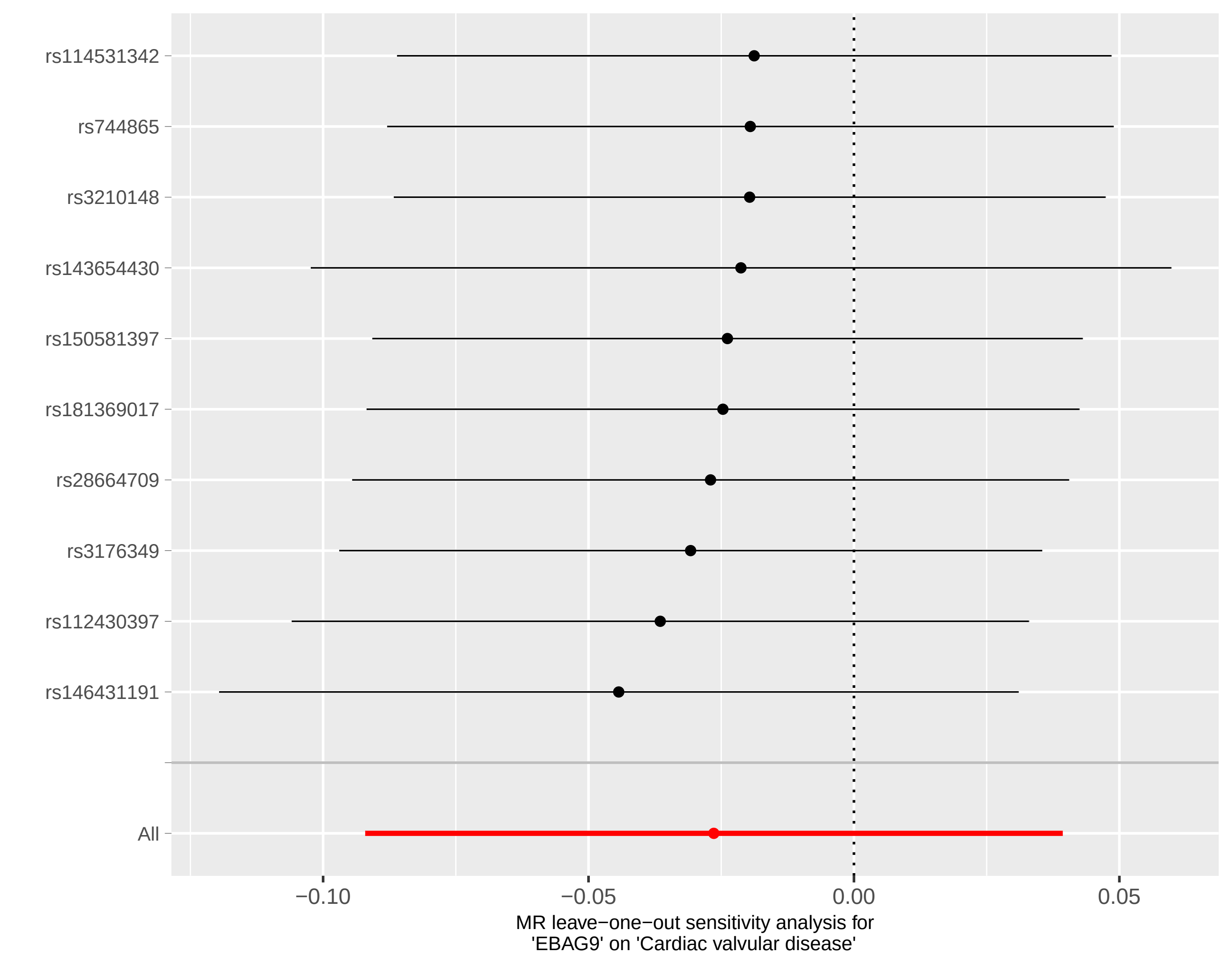
rs114531342
rs744865
rs3210148
rs143654430
rs150581397
rs181369017
rs28664709
rs3176349
rs112430397
rs146431191
All
−0.10
−0.05
0.00
0.05
MR leave−one−out sensitivity analysis for
'EBAG9' on 'Cardiac valvular disease'

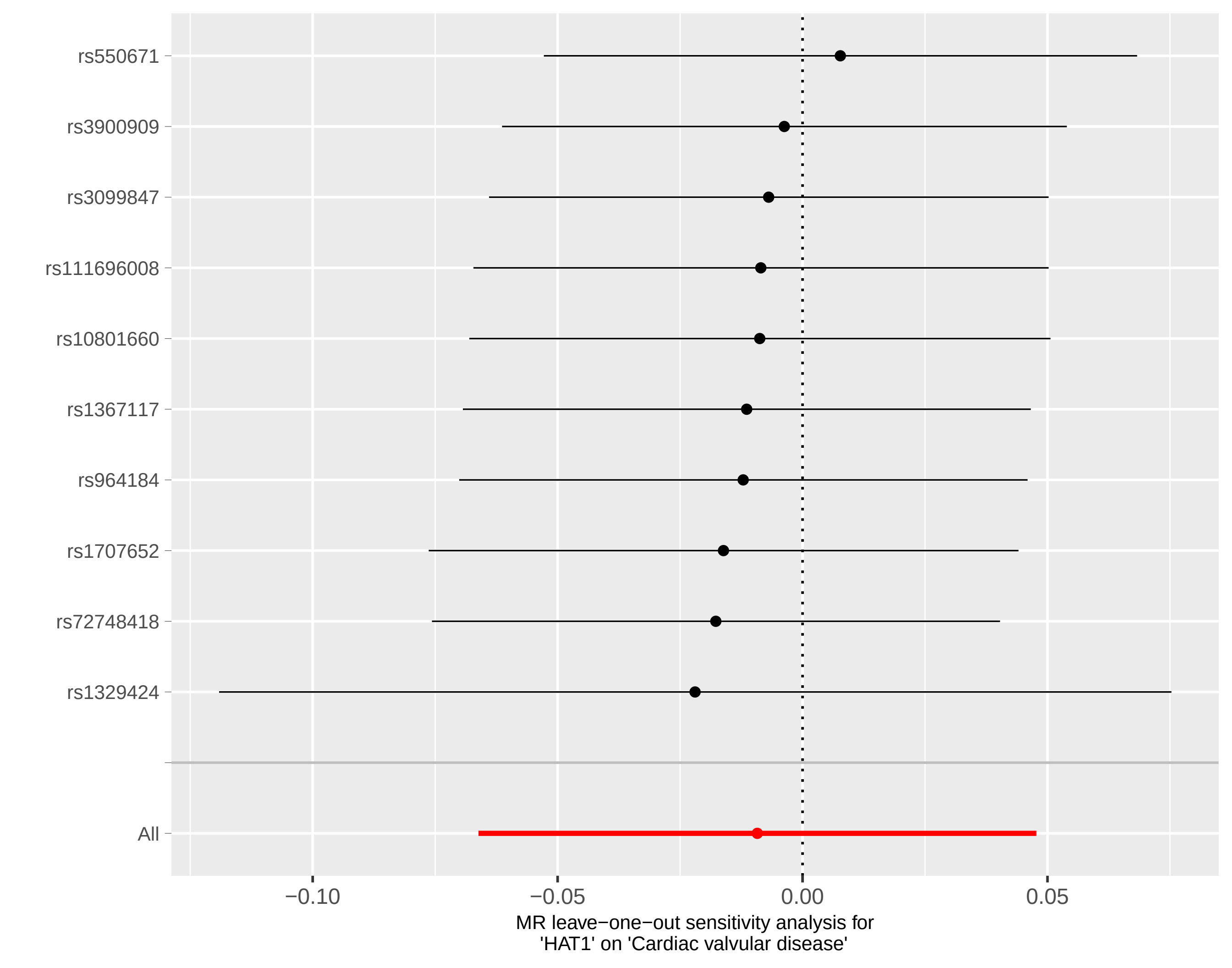

rs550671
rs3900909
rs3099847
rs111696008
rs10801660
rs1367117
rs964184
rs1707652
rs72748418
rs1329424
All
−0.10
−0.05
0.00
0.05
MR leave−one−out sensitivity analysis for
'HAT1' on 'Cardiac valvular disease'

Insufficient number of SNPs

Insufficient number of SNPs

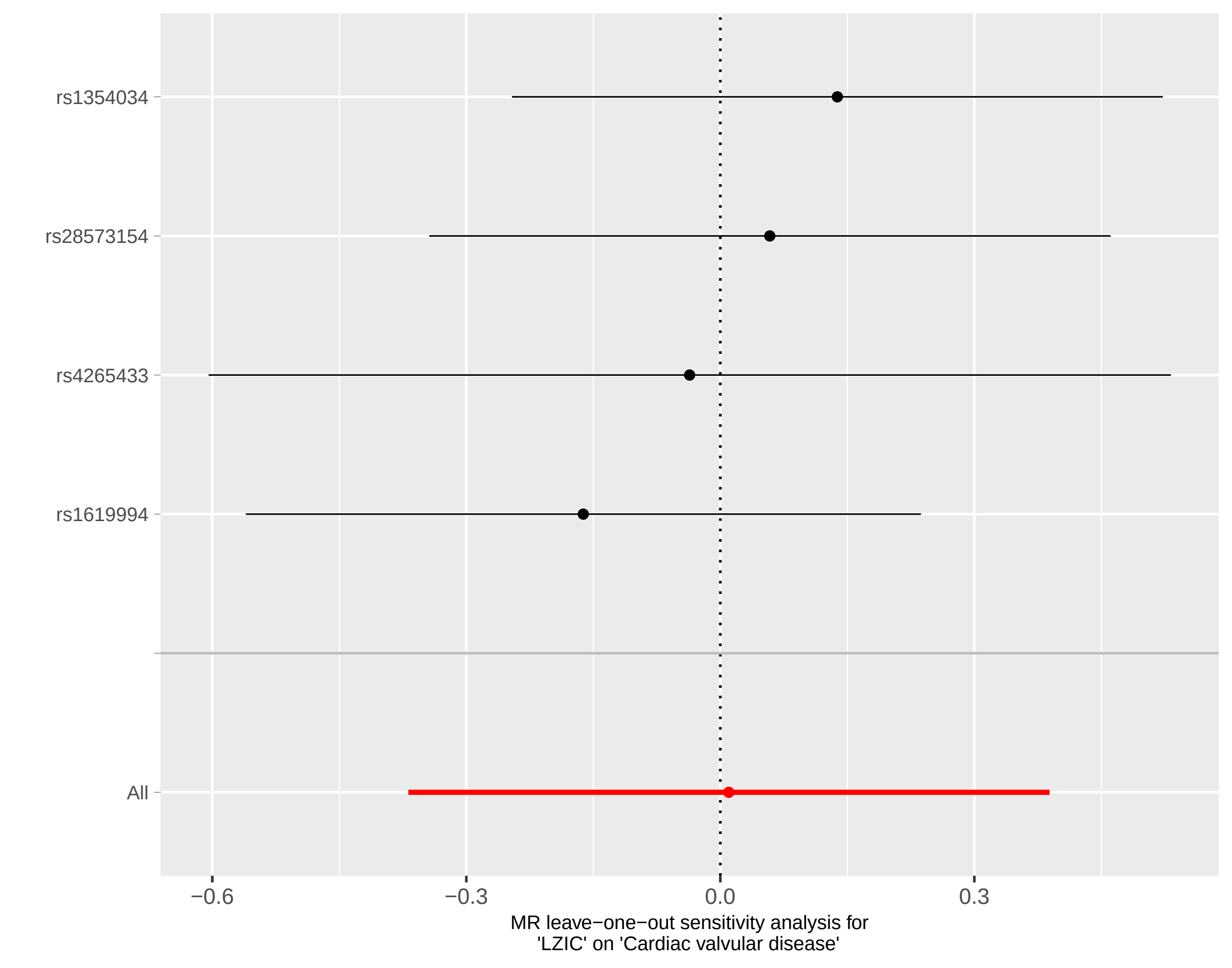
rs1354034
rs28573154
rs4265433
rs1619994
All
−0.6
−0.3
0.0
0.3
MR leave−one−out sensitivity analysis for
'LZIC' on 'Cardiac valvular disease'

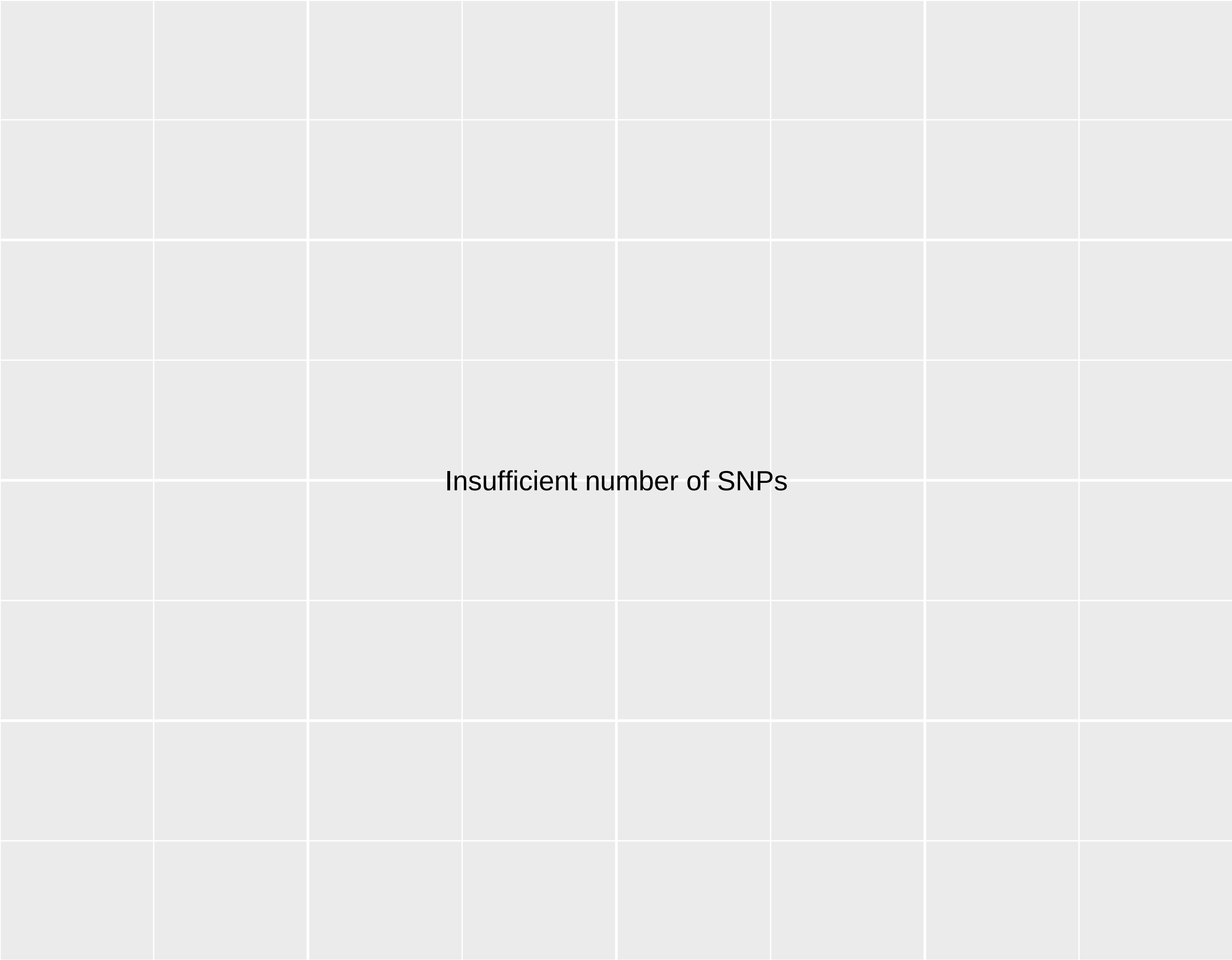
Insufficient number of SNPs

Insufficient number of SNPs

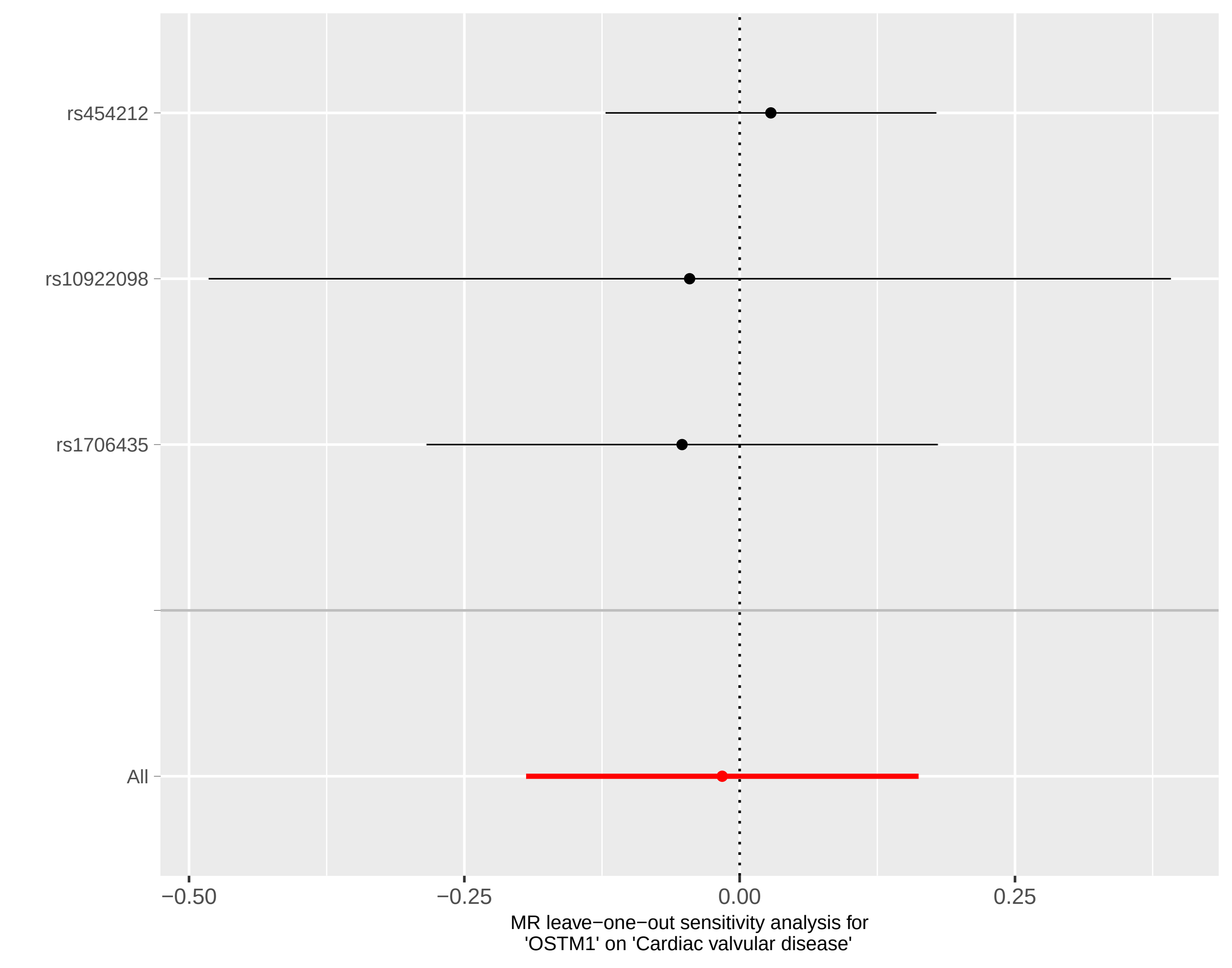
rs454212
rs10922098
rs1706435
All
−0.50
−0.25
0.00
0.25
MR leave−one−out sensitivity analysis for
'OSTM1' on 'Cardiac valvular disease'

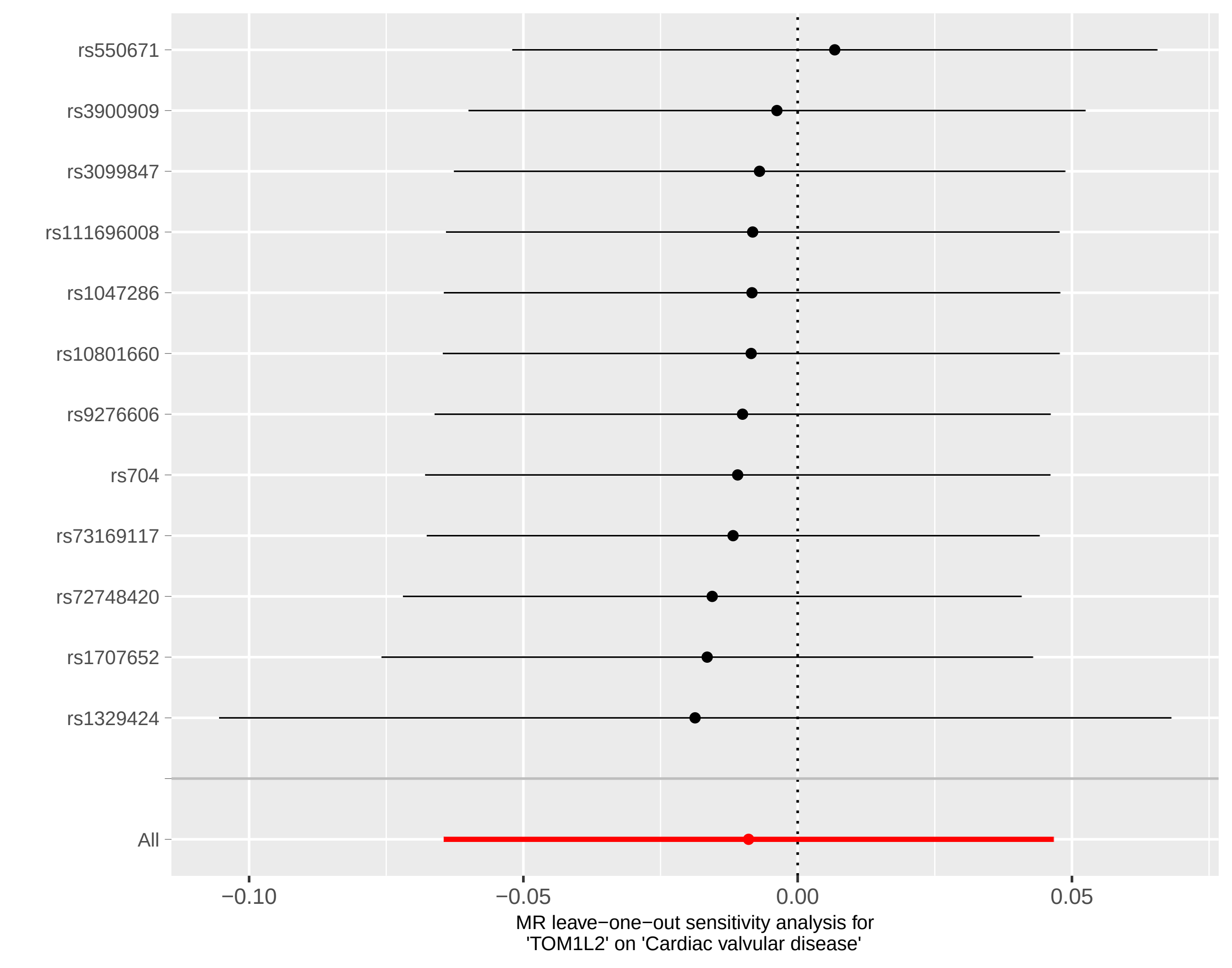

rs550671
rs3900909
rs3099847
rs111696008
rs1047286
rs10801660
rs9276606
rs704
rs73169117
rs72748420
rs1707652
rs1329424
All
−0.10
−0.05
0.00
0.05
MR leave−one−out sensitivity analysis for
'TOM1L2' on 'Cardiac valvular disease'

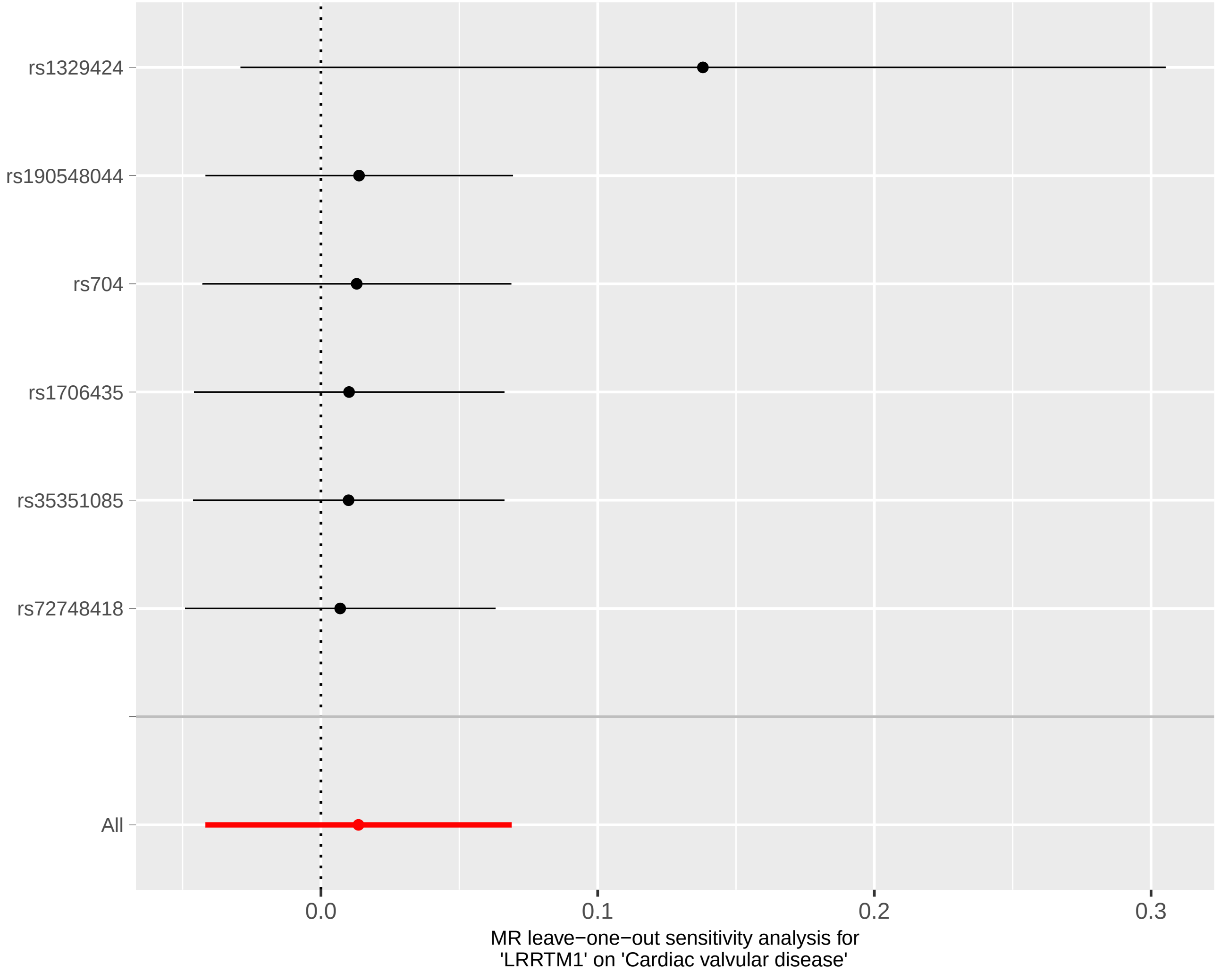

rs1329424
rs190548044
rs704
rs1706435
rs35351085
rs72748418
All
0.0
0.1
0.2
0.3
MR leave−one−out sensitivity analysis for
'LRRTM1' on 'Cardiac valvular disease'

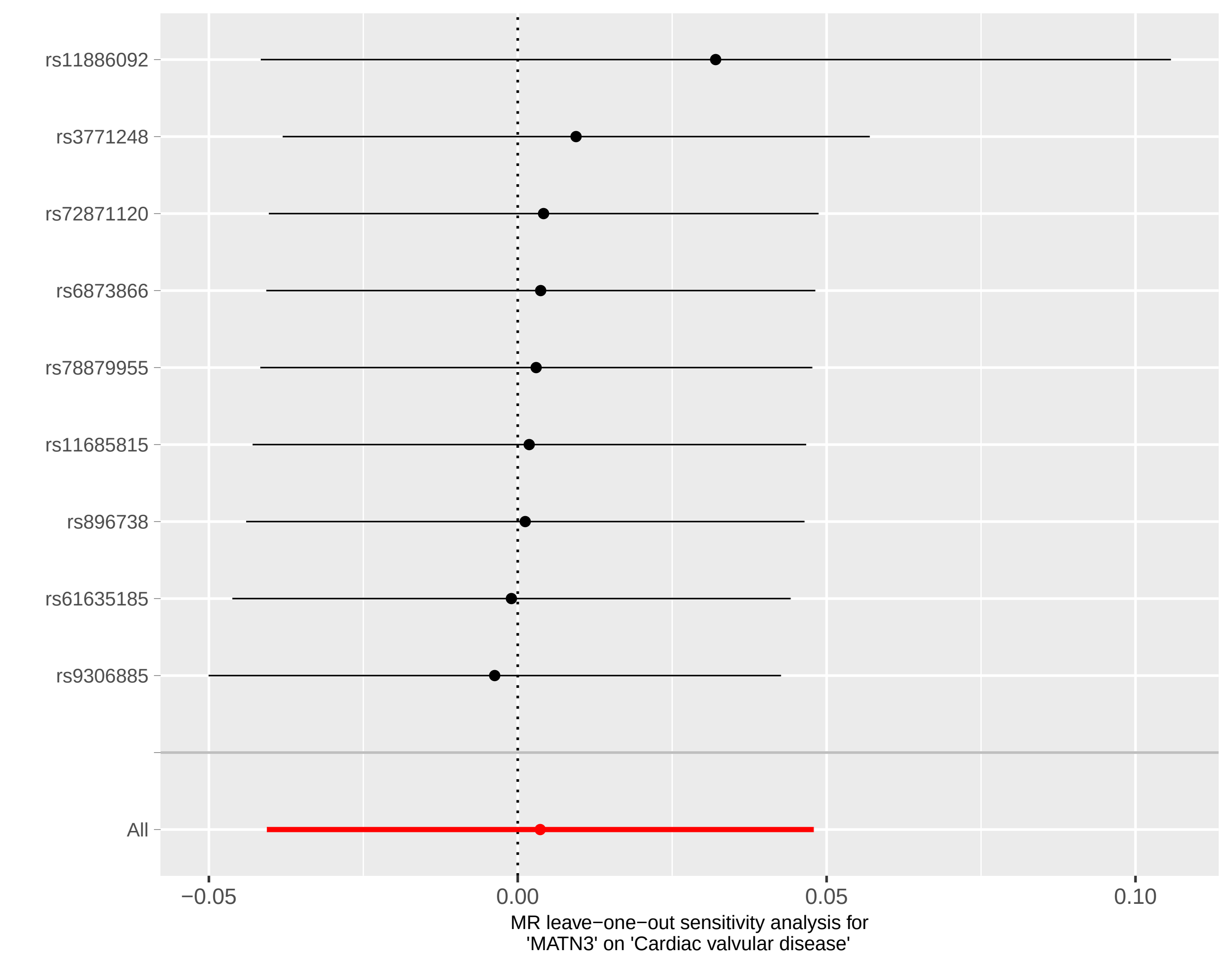
rs11886092
rs3771248
rs72871120
rs6873866
rs78879955
rs11685815
rs896738
rs61635185
rs9306885
All
−0.05
0.00
0.05
0.10
MR leave−one−out sensitivity analysis for
'MATN3' on 'Cardiac valvular disease'

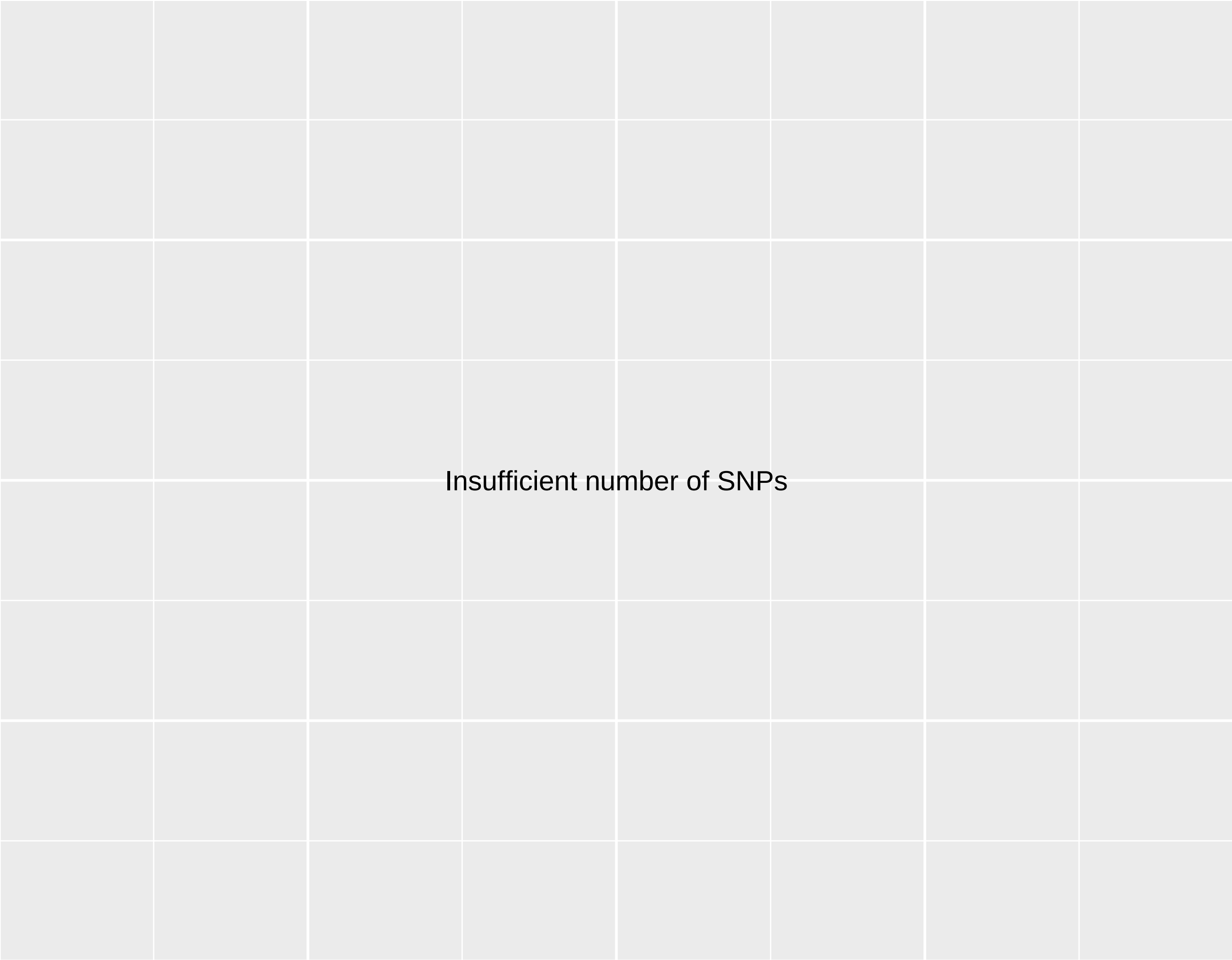
Insufficient number of SNPs

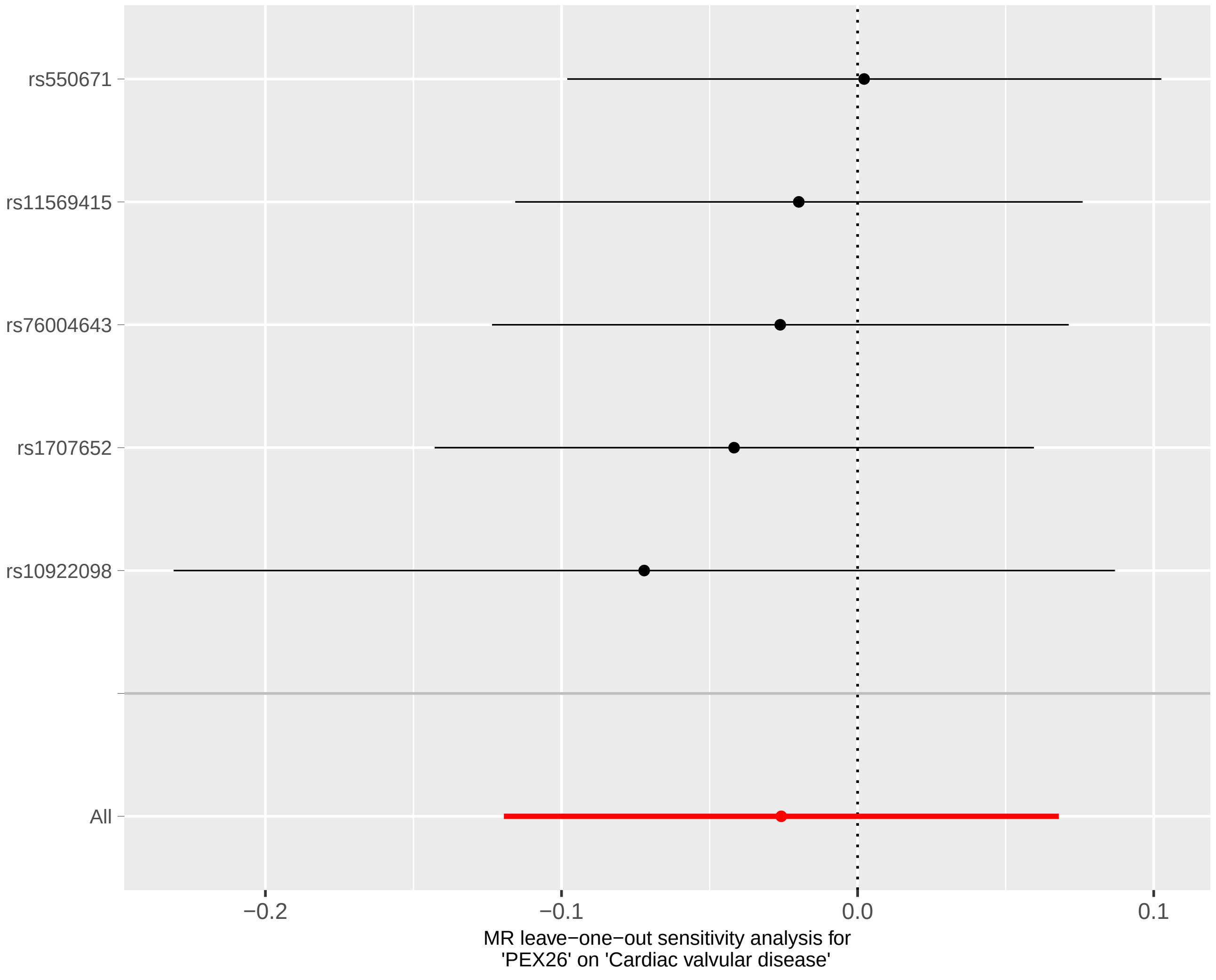

rs550671
rs11569415
rs76004643
rs1707652
rs10922098
All
−0.2
−0.1
0.0
0.1
MR leave−one−out sensitivity analysis for
'PEX26' on 'Cardiac valvular disease'

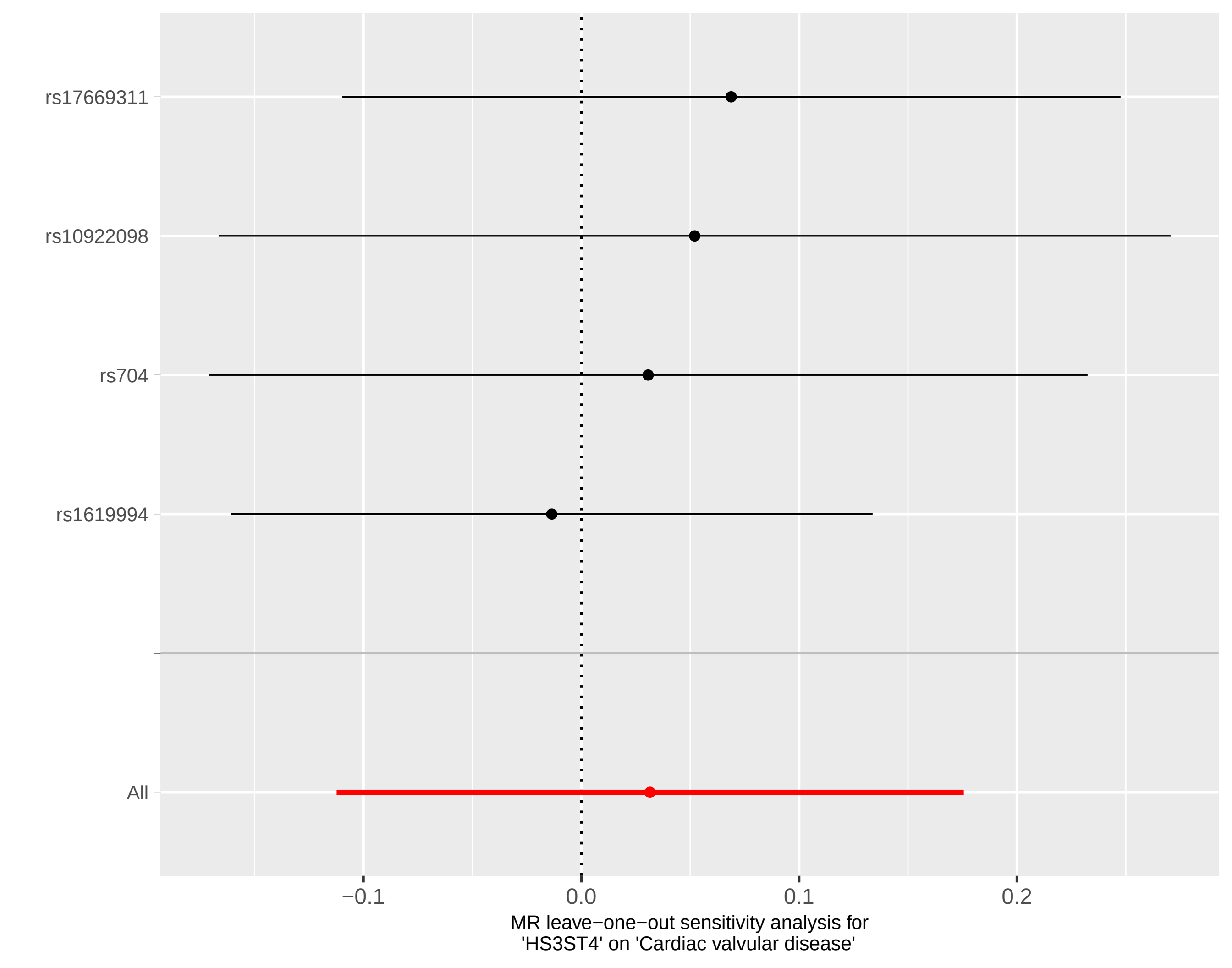
rs17669311
rs10922098
rs704
rs1619994
All
−0.1
0.0
0.1
0.2
MR leave−one−out sensitivity analysis for
'HS3ST4' on 'Cardiac valvular disease'

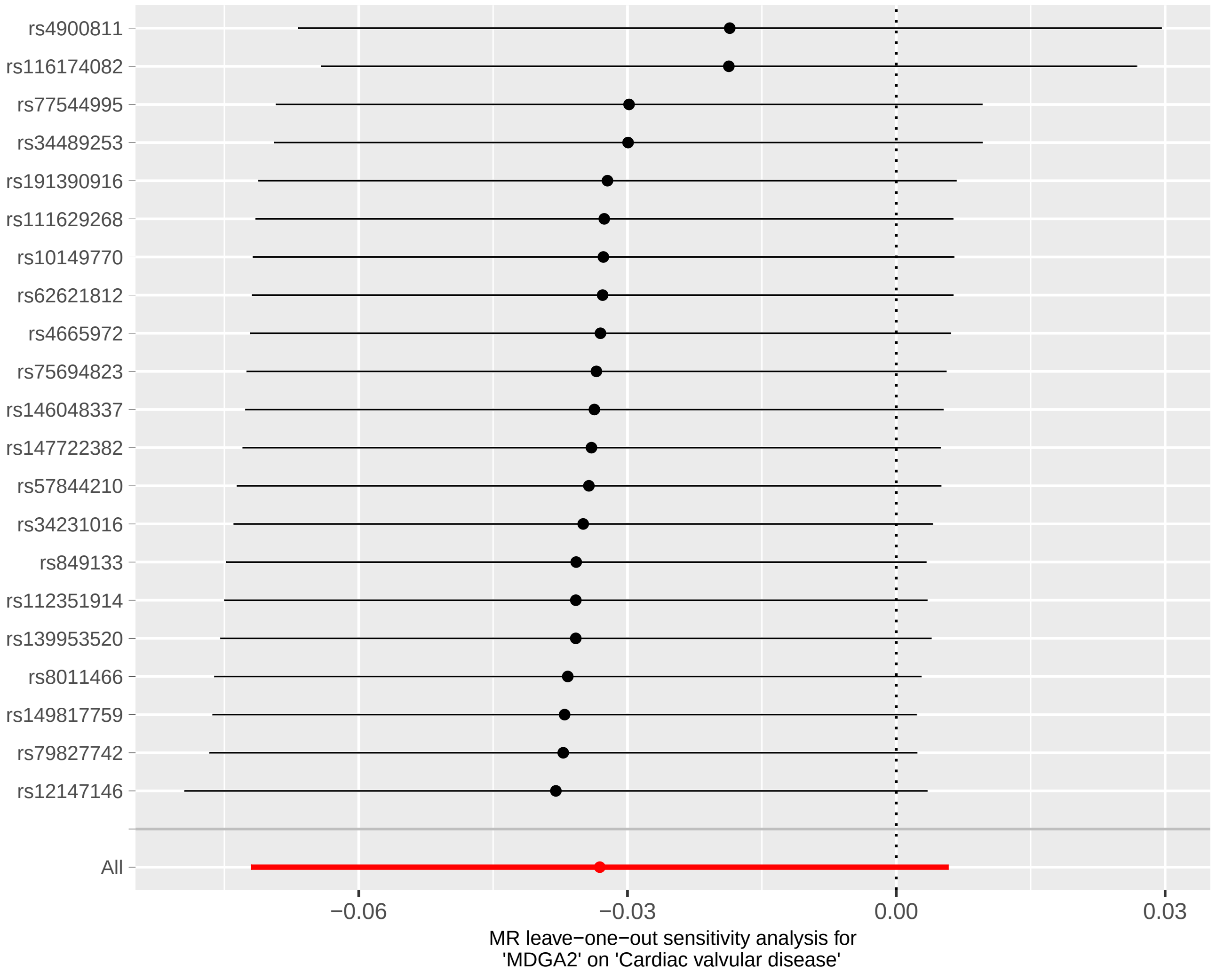
rs4900811
rs116174082
rs77544995
rs34489253
rs191390916
rs111629268
rs10149770
rs62621812
rs4665972
rs75694823
rs146048337
rs147722382
rs57844210
rs34231016
rs849133
rs112351914
rs139953520
rs8011466
rs149817759
rs79827742
rs12147146
All
−0.06
−0.03
0.00
0.03
MR leave−one−out sensitivity analysis for
'MDGA2' on 'Cardiac valvular disease'

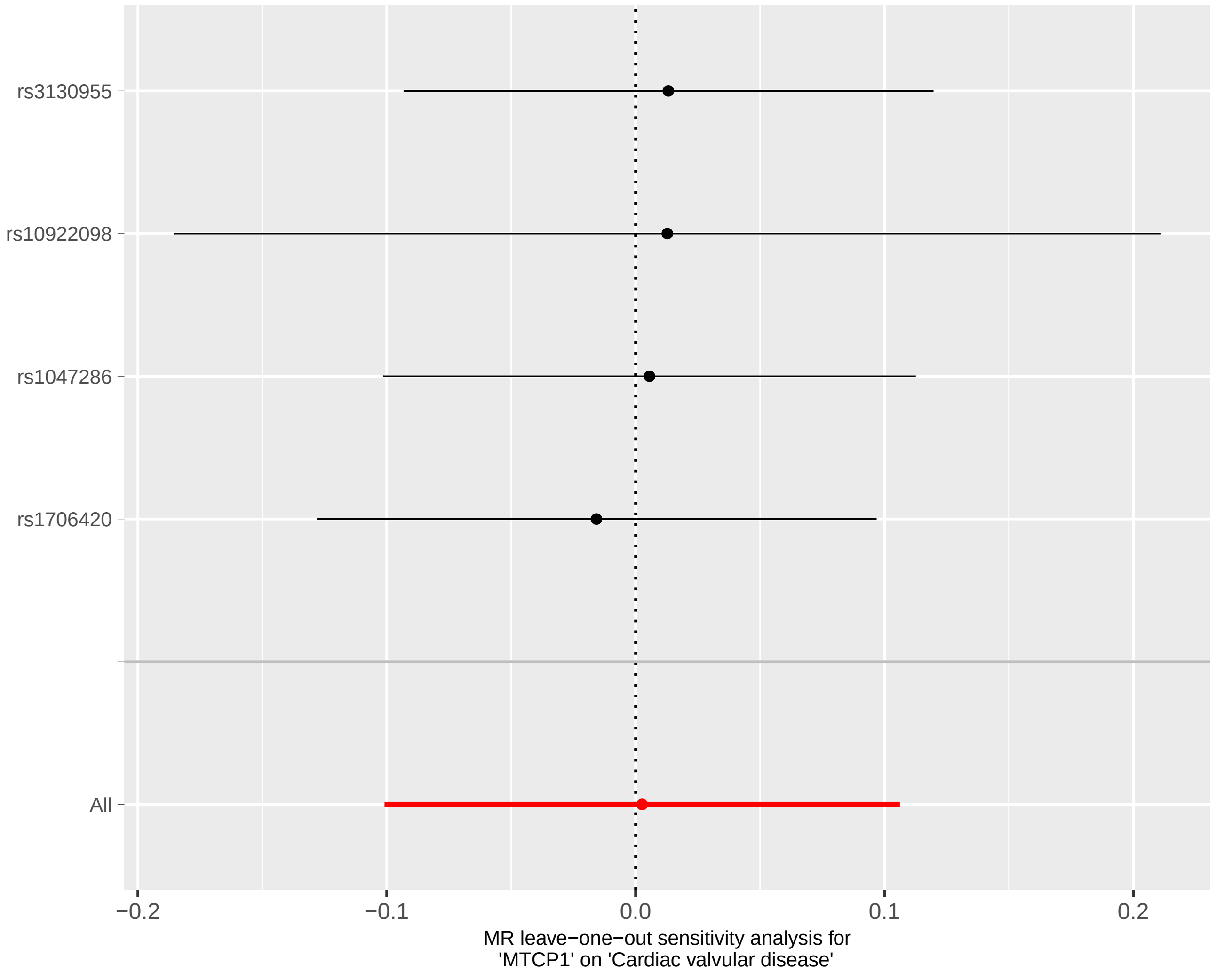
rs3130955
rs10922098
rs1047286
rs1706420
All
−0.2
−0.1
0.0
0.1
0.2
MR leave−one−out sensitivity analysis for
'MTCP1' on 'Cardiac valvular disease'

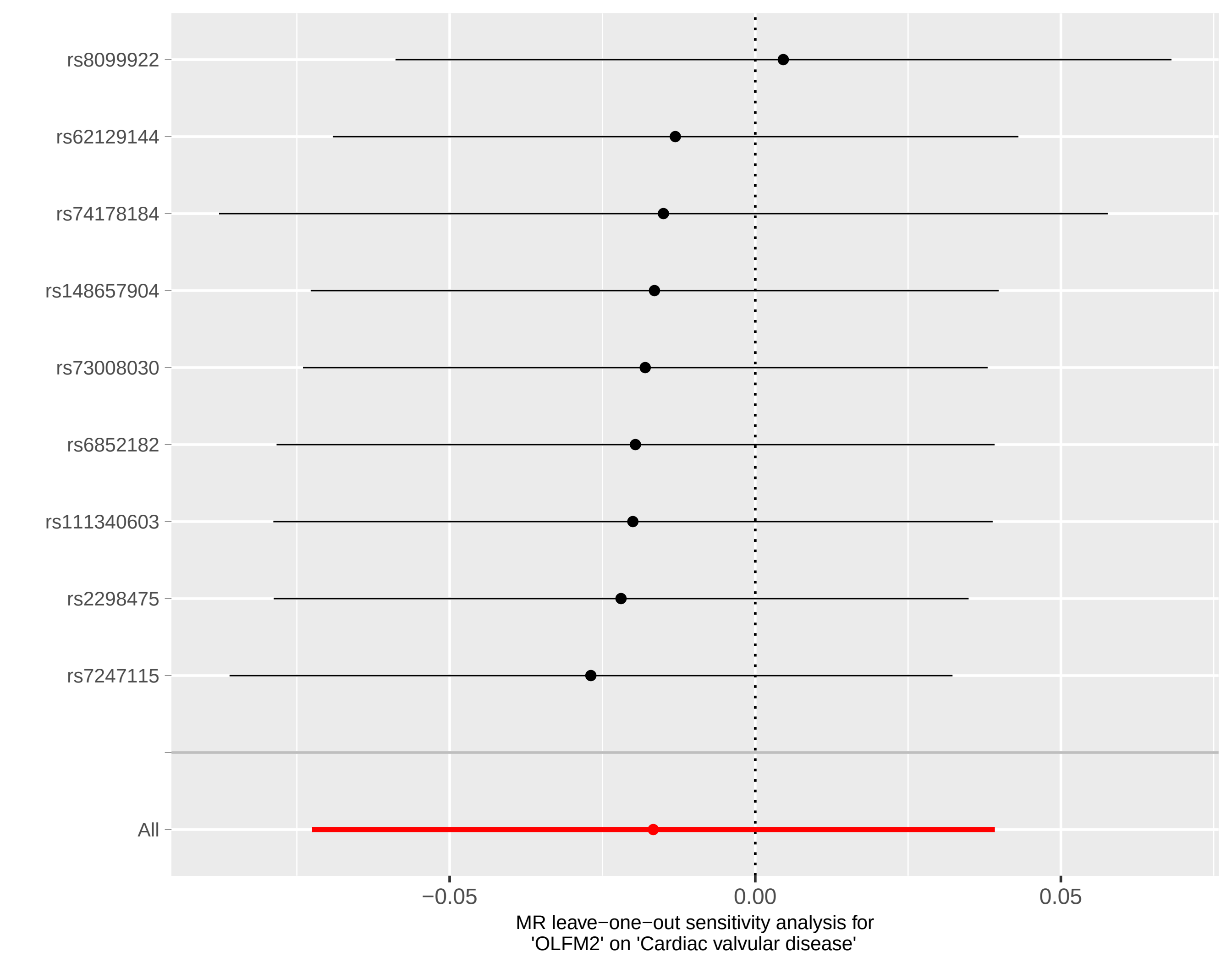
rs8099922
rs62129144
rs74178184
rs148657904
rs73008030
rs6852182
rs111340603
rs2298475
rs7247115
All
−0.05
0.00
0.05
MR leave−one−out sensitivity analysis for
'OLFM2' on 'Cardiac valvular disease'

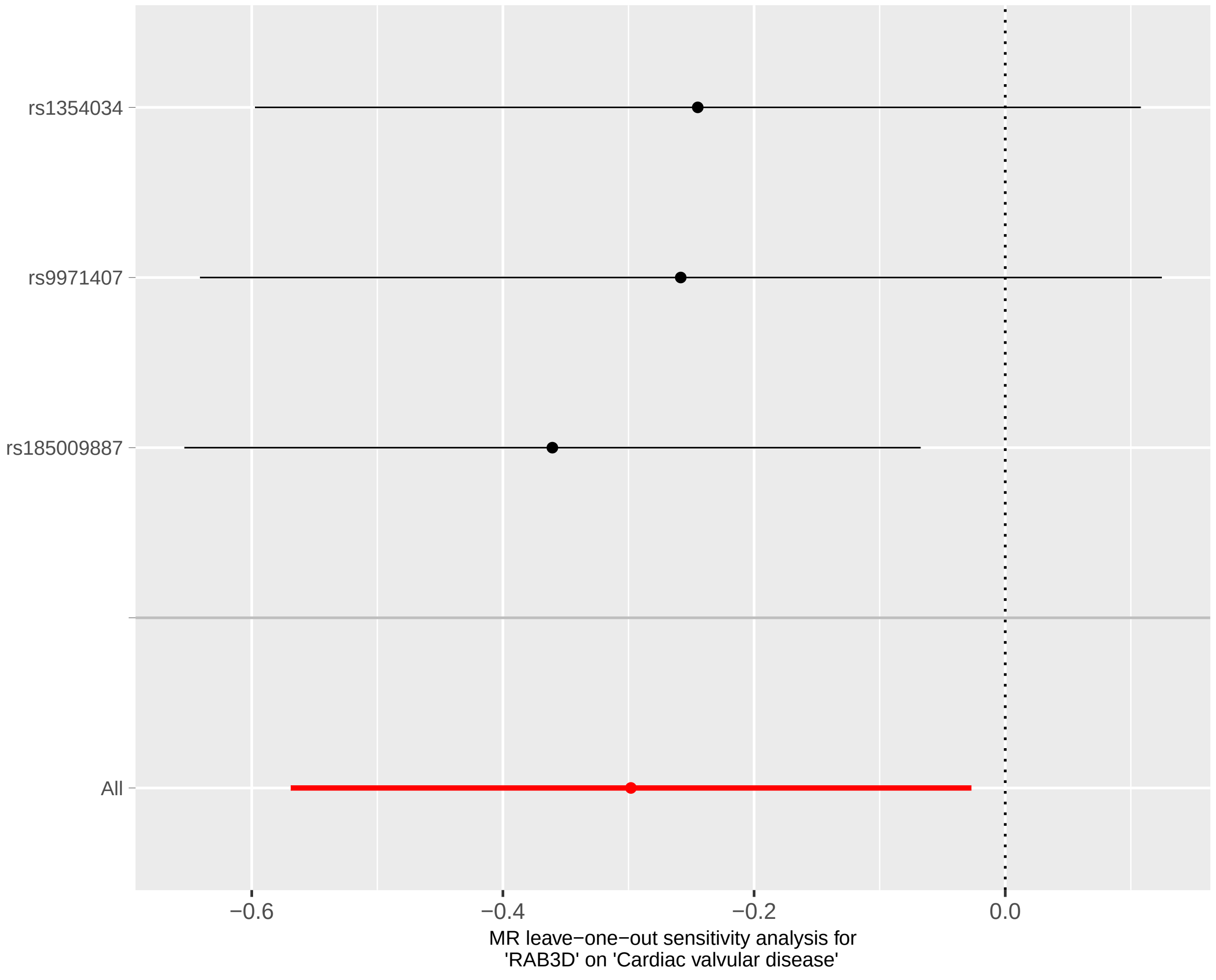
rs1354034
rs9971407
rs185009887
All
−0.6
−0.4
−0.2
0.0
MR leave−one−out sensitivity analysis for
'RAB3D' on 'Cardiac valvular disease'

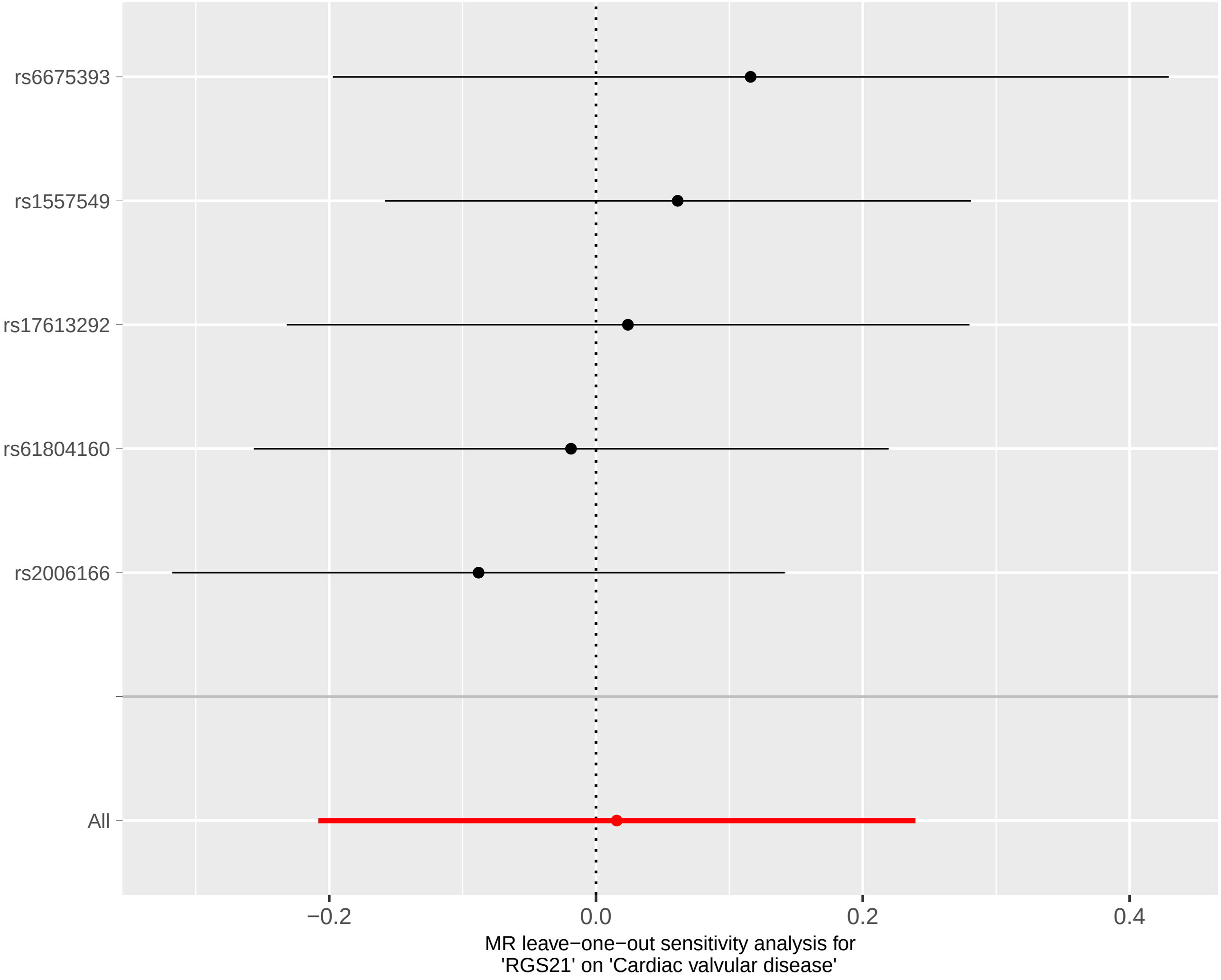

rs6675393
rs1557549
rs17613292
rs61804160
rs2006166
All
−0.2
0.0
0.2
0.4
MR leave−one−out sensitivity analysis for
'RGS21' on 'Cardiac valvular disease'

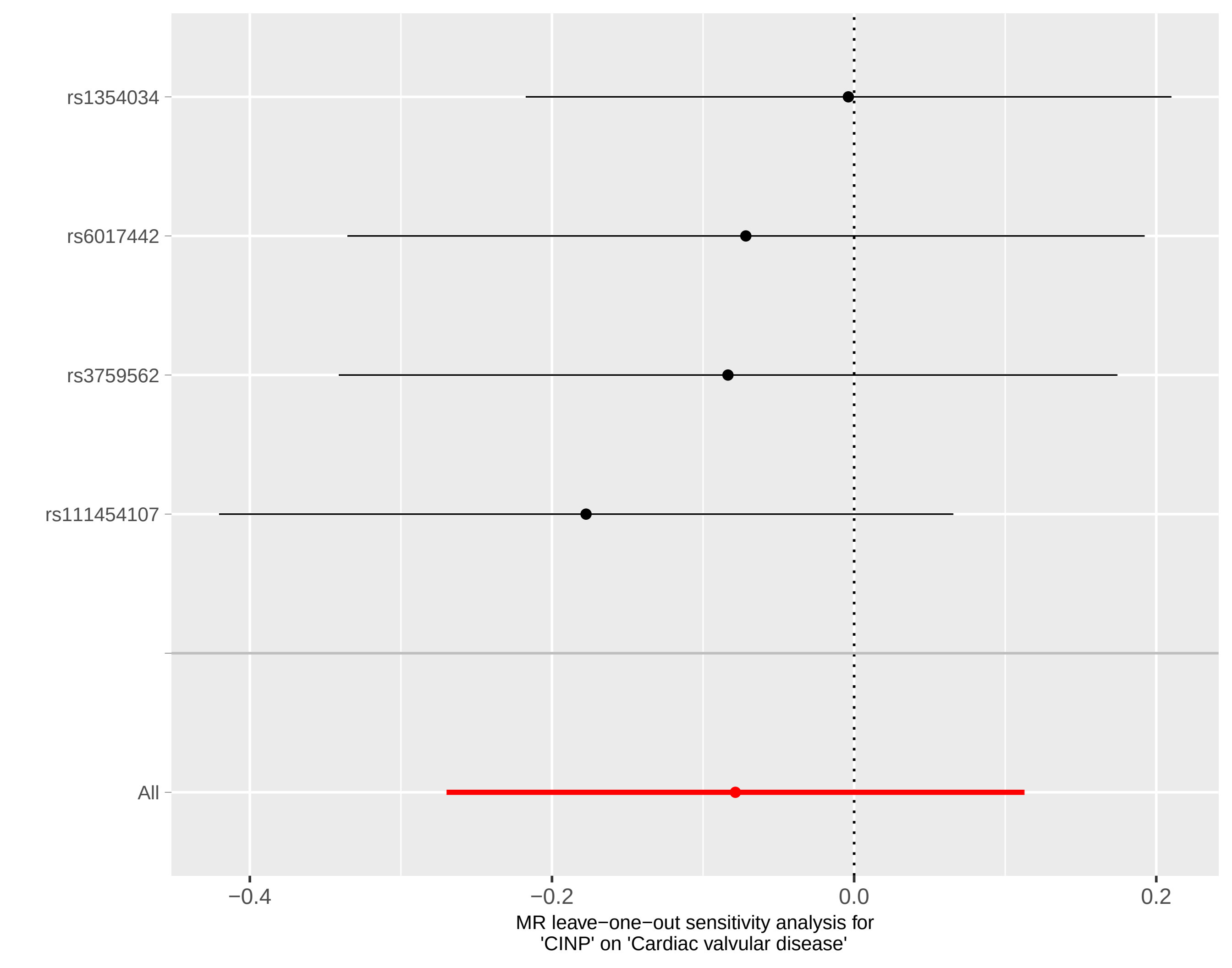
rs1354034
rs6017442
rs3759562
rs111454107
All
−0.4
−0.2
0.0
0.2
MR leave−one−out sensitivity analysis for
'CINP' on 'Cardiac valvular disease'

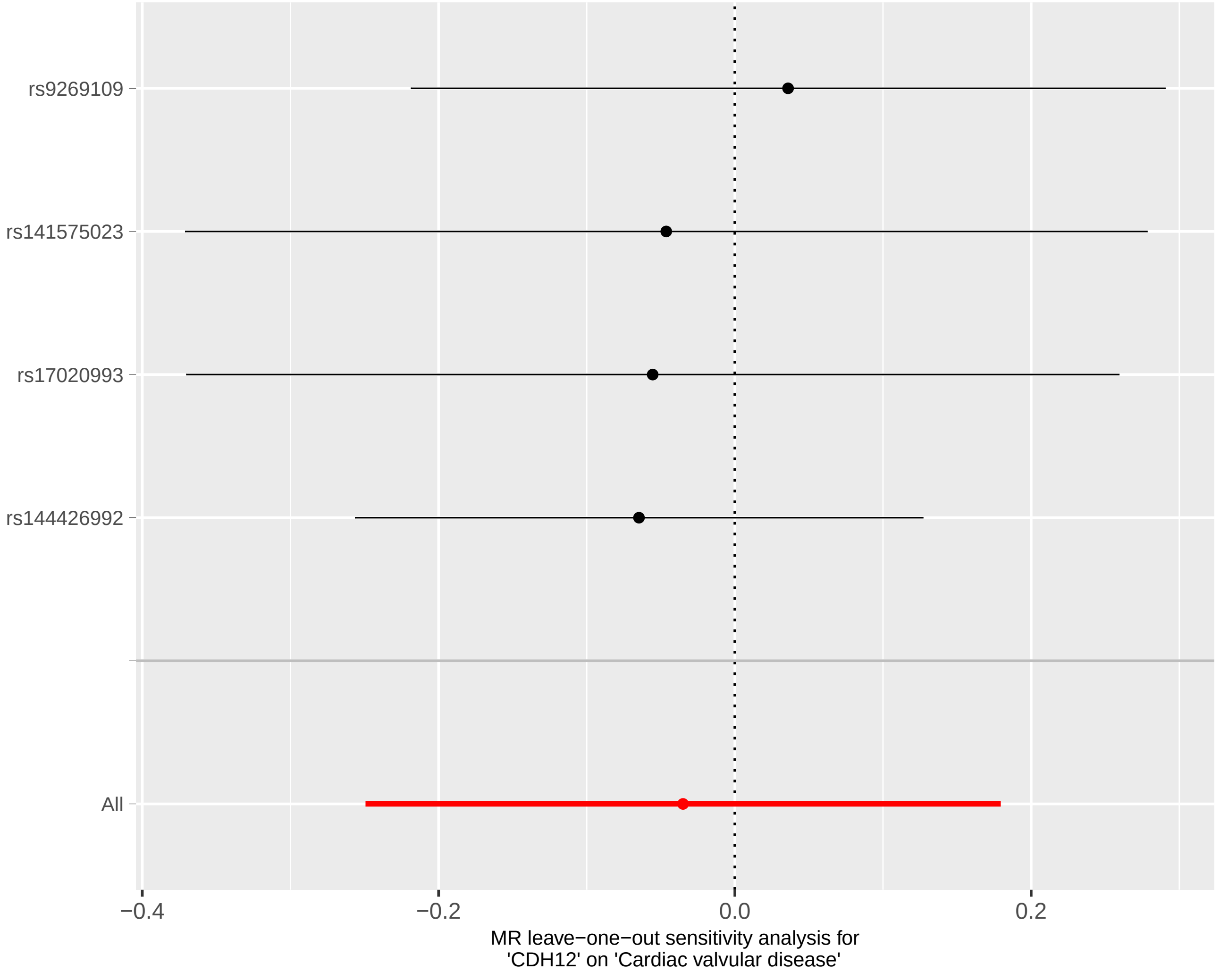
rs9269109
rs141575023
rs17020993
rs144426992
All
−0.4
−0.2
0.0
0.2
MR leave−one−out sensitivity analysis for
'CDH12' on 'Cardiac valvular disease'

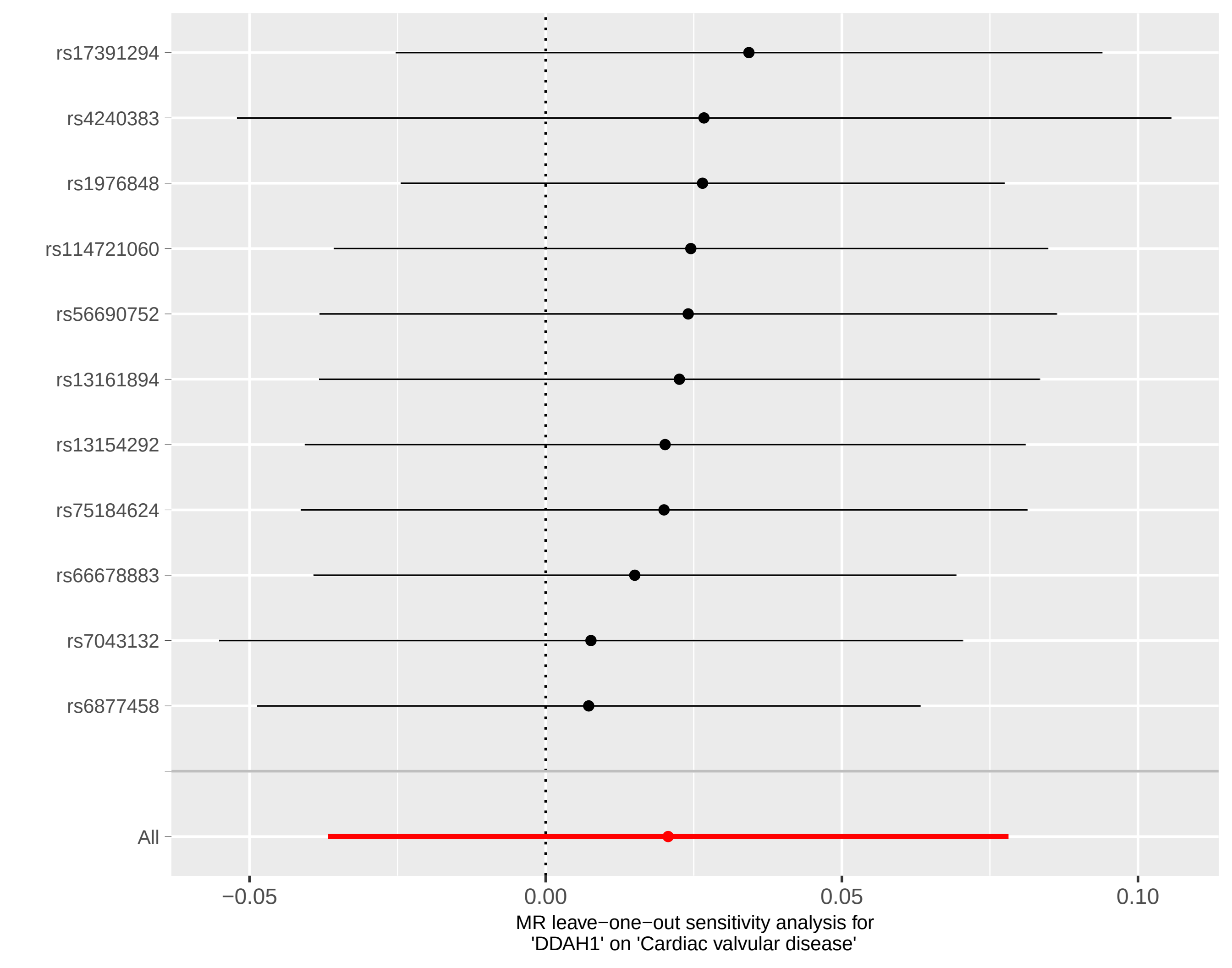
rs17391294
rs4240383
rs1976848
rs114721060
rs56690752
rs13161894
rs13154292
rs75184624
rs66678883
rs7043132
rs6877458
All
−0.05
0.00
0.05
0.10
MR leave−one−out sensitivity analysis for
'DDAH1' on 'Cardiac valvular disease'

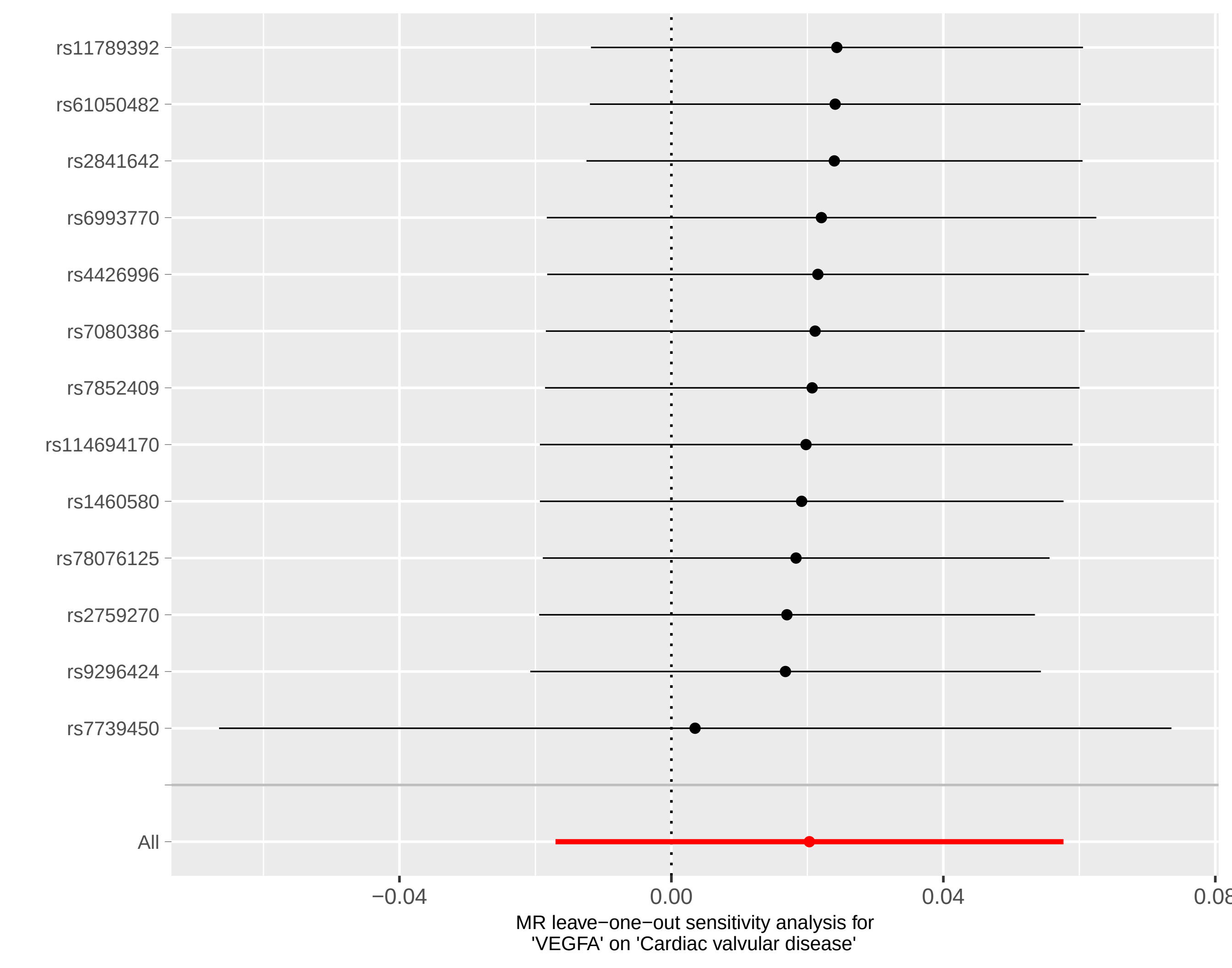

rs11789392
rs61050482
rs2841642
rs6993770
rs4426996
rs7080386
rs7852409
rs114694170
rs1460580
rs78076125
rs2759270
rs9296424
rs7739450
All
−0.04
0.00
0.04
0.08
MR leave−one−out sensitivity analysis for
'VEGFA' on 'Cardiac valvular disease'

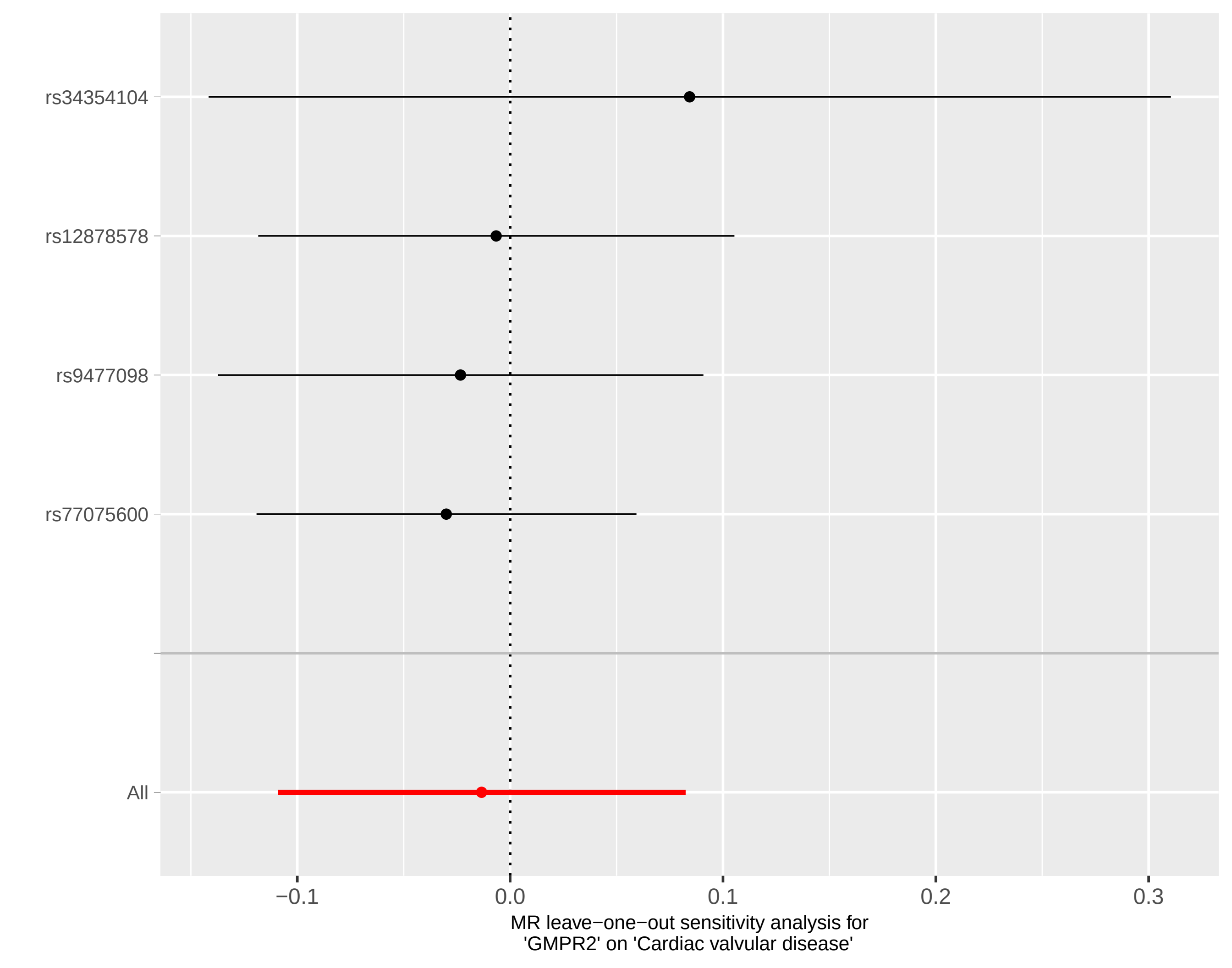
rs34354104
rs12878578
rs9477098
rs77075600
All
−0.1
0.0
0.1
0.2
0.3
MR leave−one−out sensitivity analysis for
'GMPR2' on 'Cardiac valvular disease'

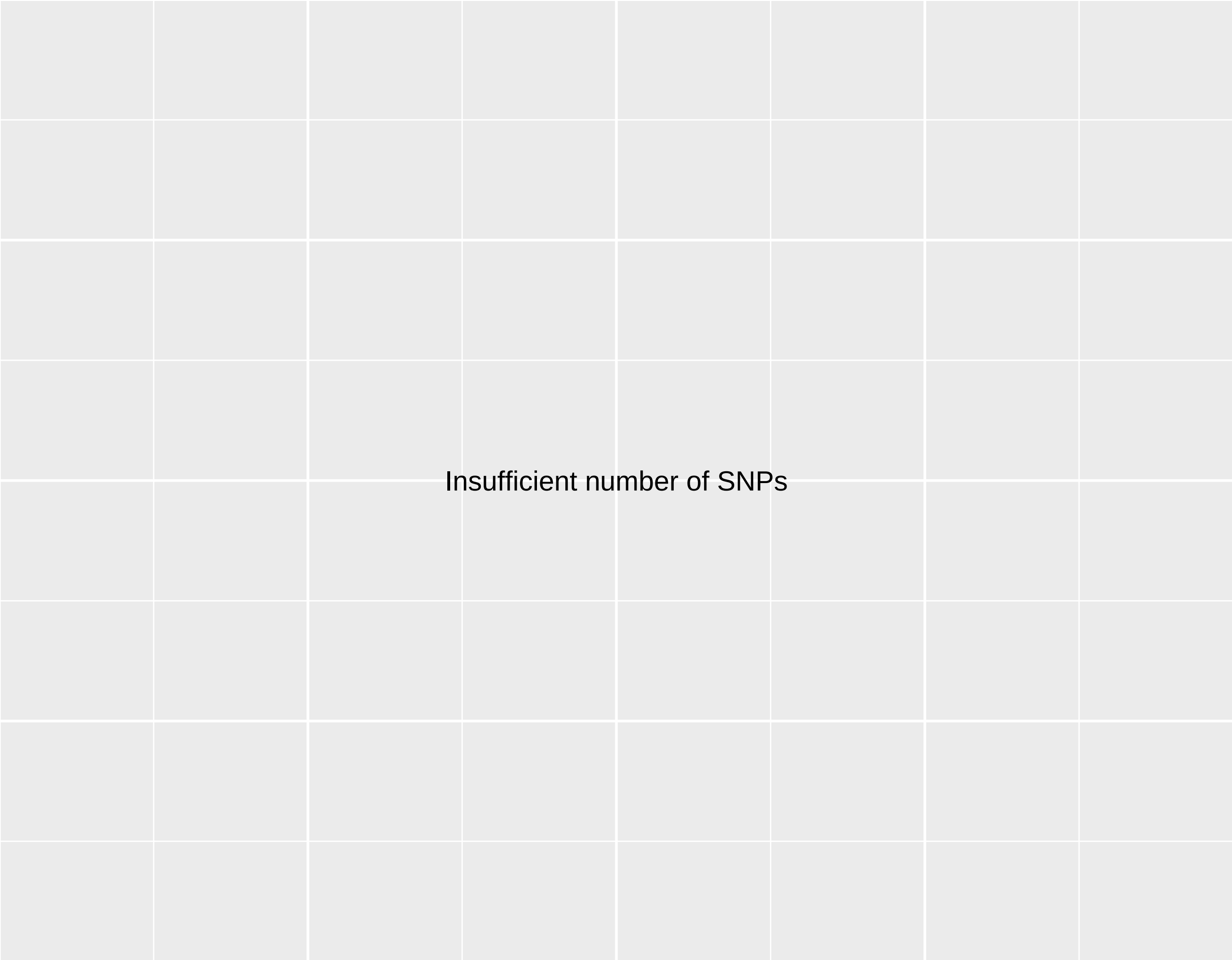
Insufficient number of SNPs

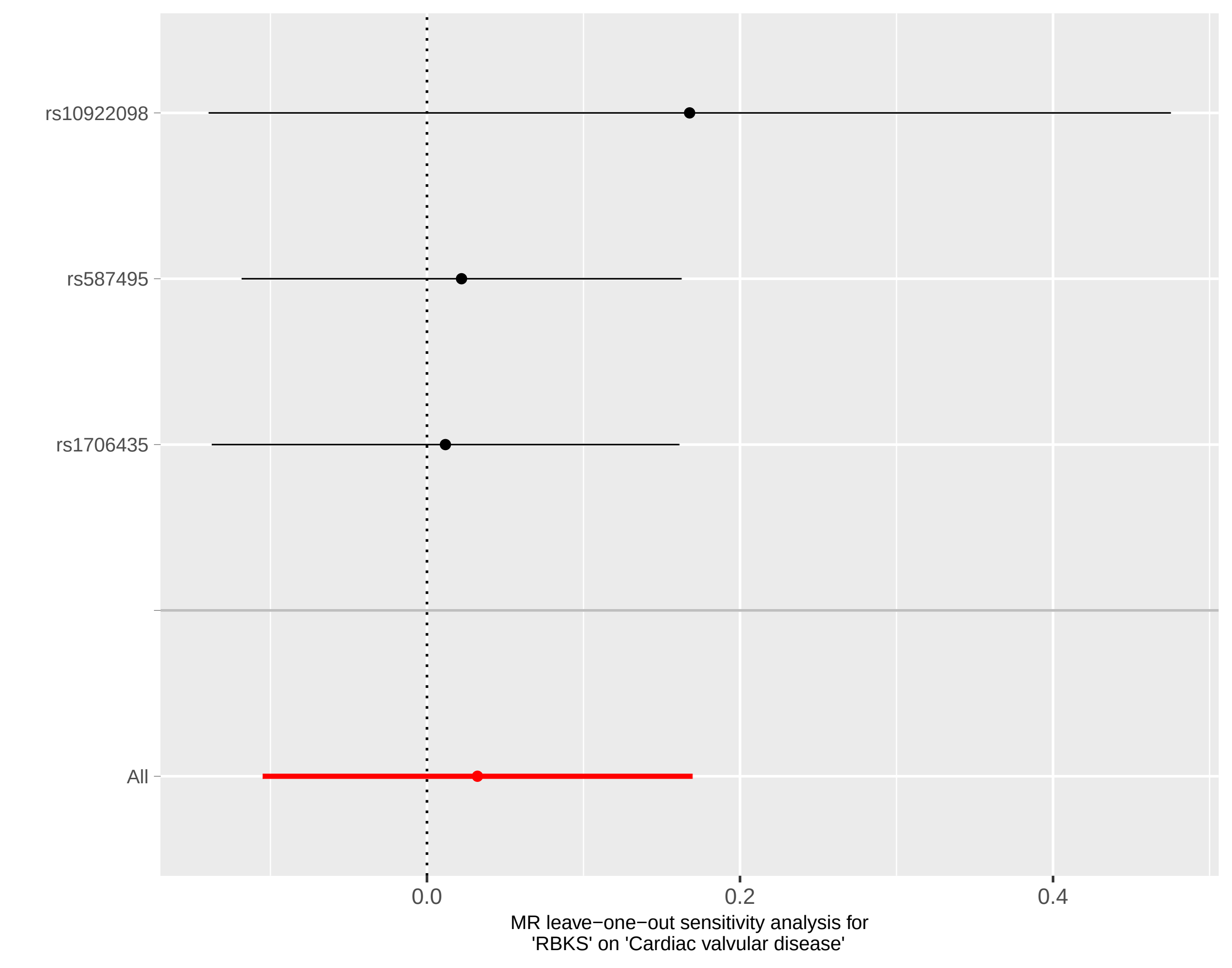
rs10922098
rs587495
rs1706435
All
0.0
0.2
0.4
MR leave−one−out sensitivity analysis for
'RBKS' on 'Cardiac valvular disease'

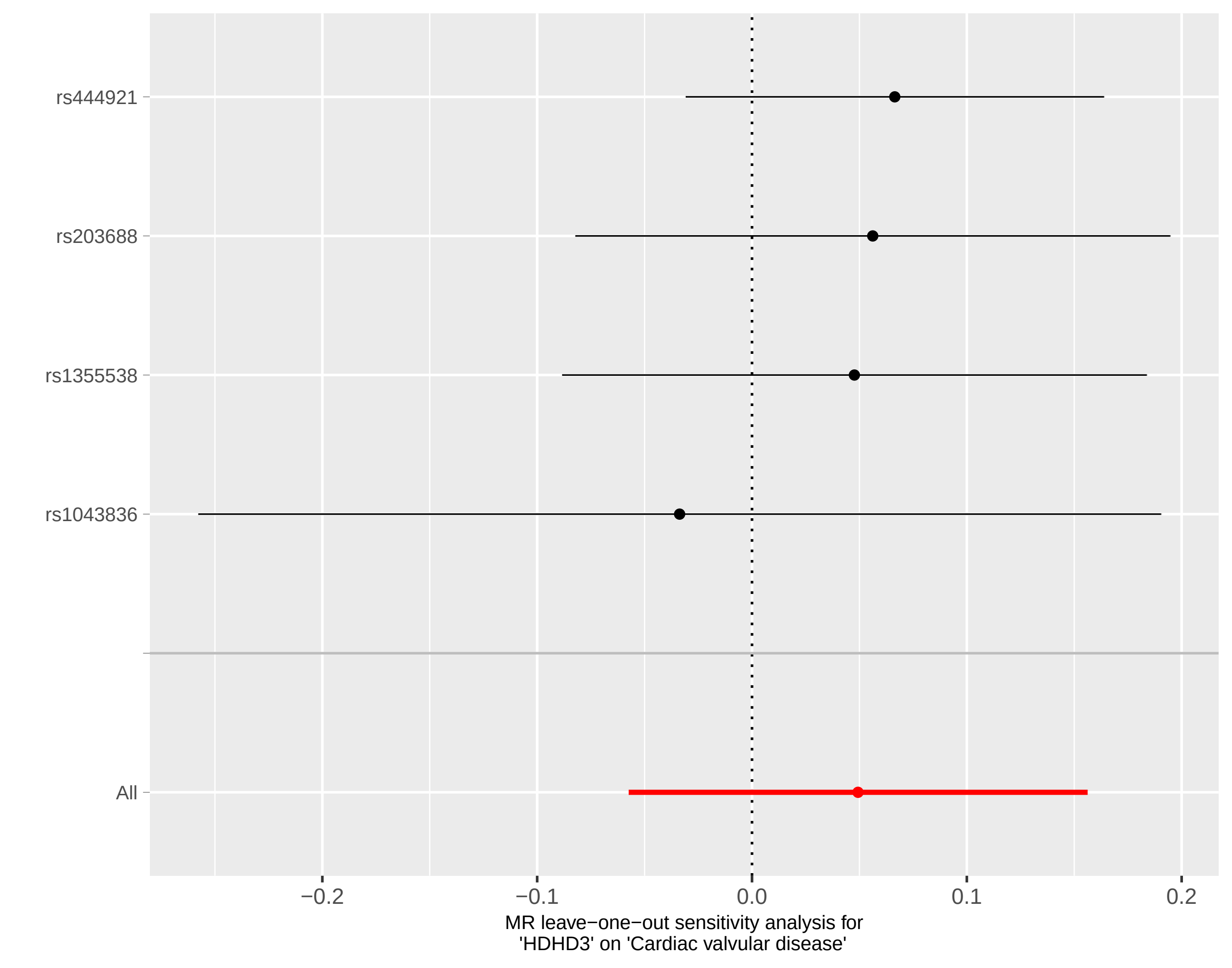
rs444921
rs203688
rs1355538
rs1043836
All
−0.2
−0.1
0.0
0.1
0.2
MR leave−one−out sensitivity analysis for
'HDHD3' on 'Cardiac valvular disease'

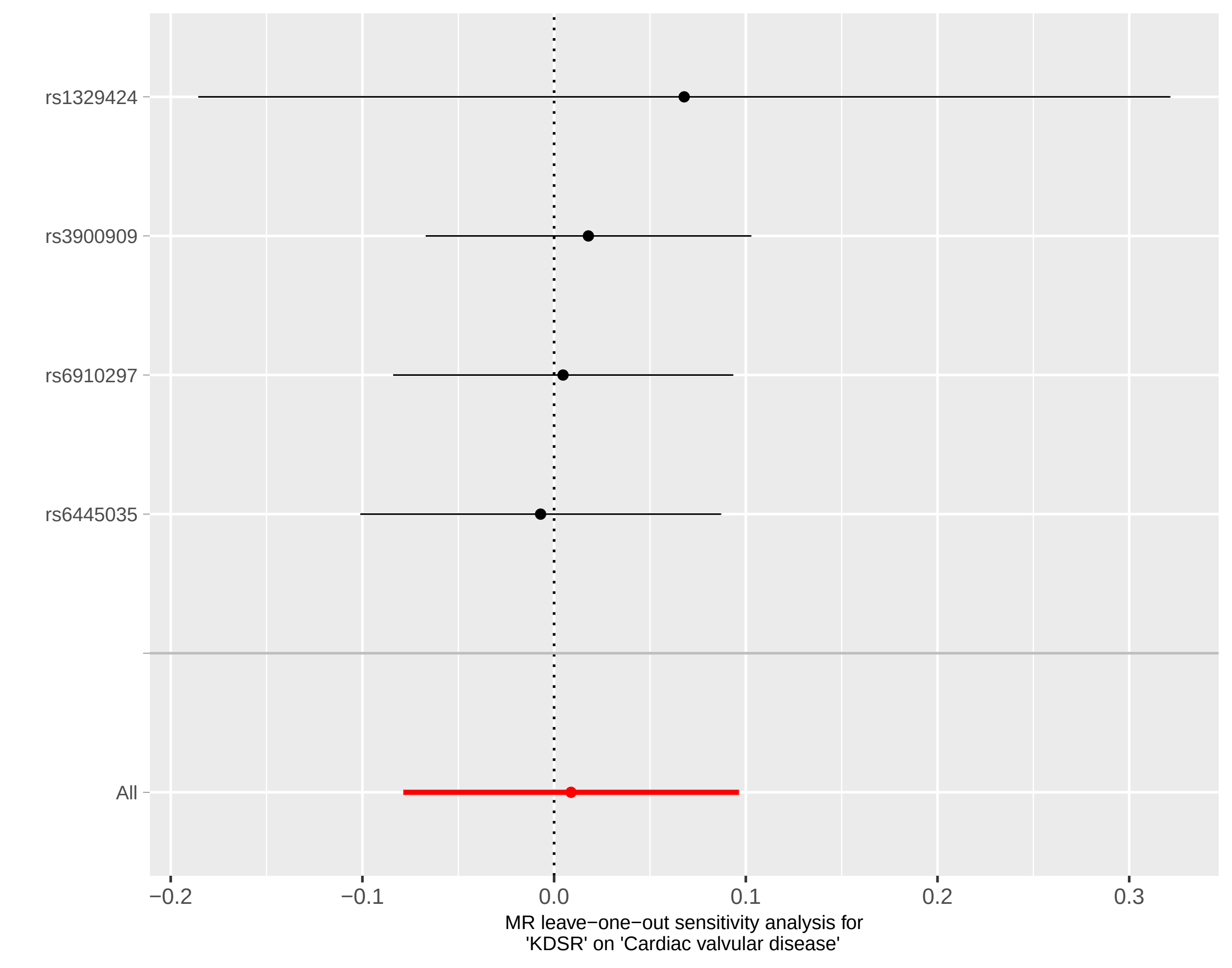
rs1329424
rs3900909
rs6910297
rs6445035
All
−0.2
−0.1
0.0
0.1
0.2
0.3
MR leave−one−out sensitivity analysis for
'KDSR' on 'Cardiac valvular disease'

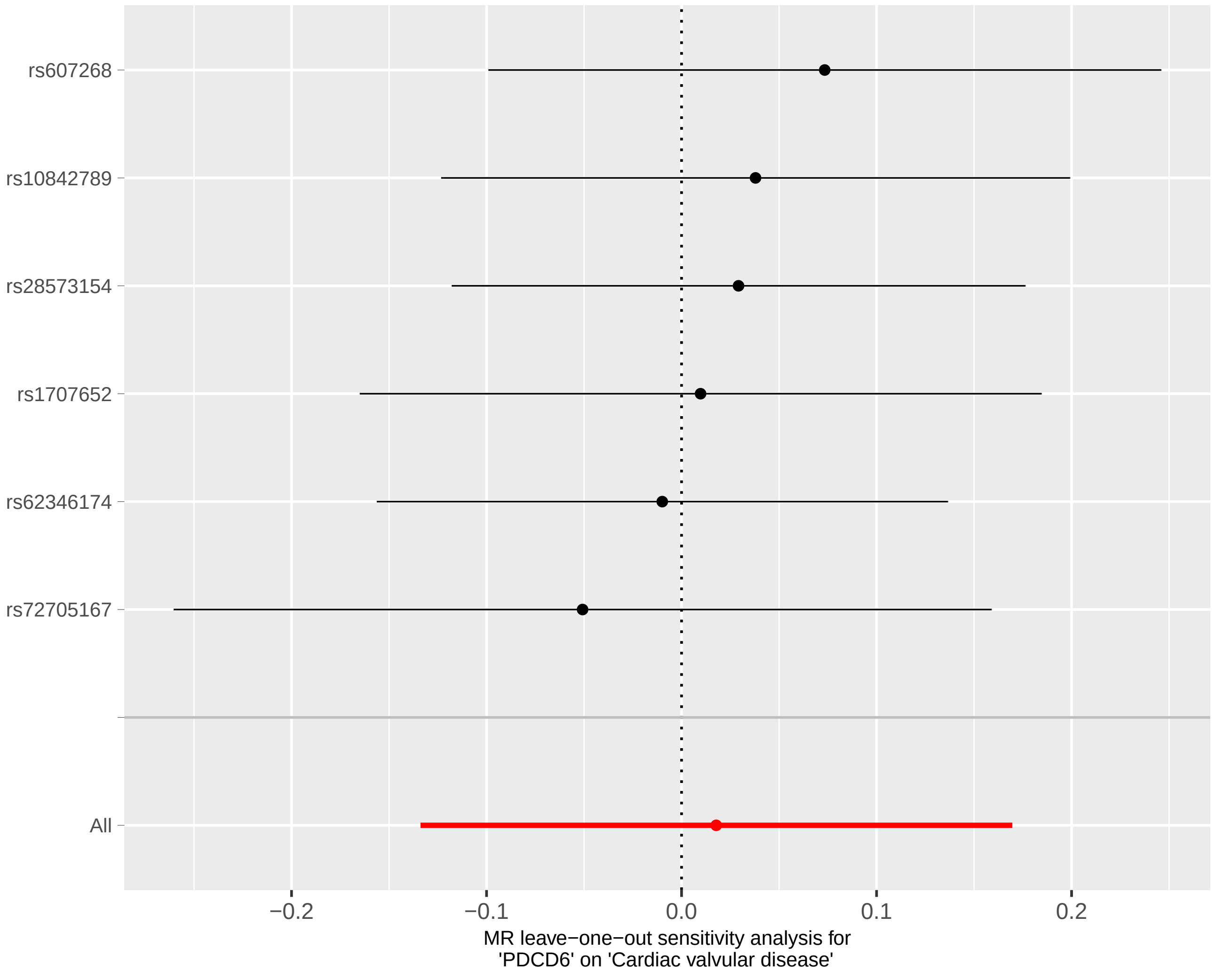
rs607268
rs10842789
rs28573154
rs1707652
rs62346174
rs72705167
All
−0.2
−0.1
0.0
0.1
0.2
MR leave−one−out sensitivity analysis for
'PDCD6' on 'Cardiac valvular disease'

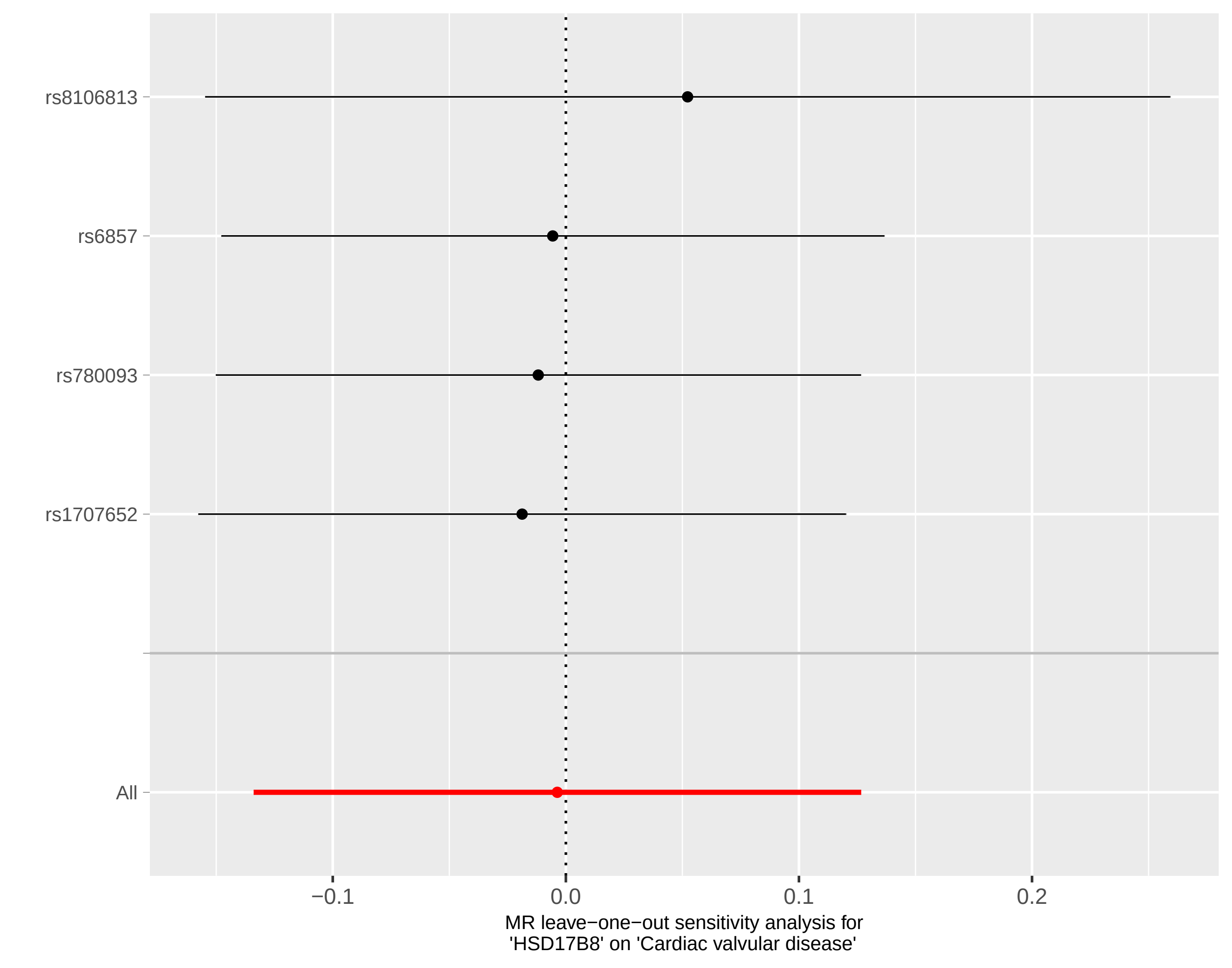
rs8106813
rs6857
rs780093
rs1707652
All
−0.1
0.0
0.1
0.2
MR leave−one−out sensitivity analysis for
'HSD17B8' on 'Cardiac valvular disease'

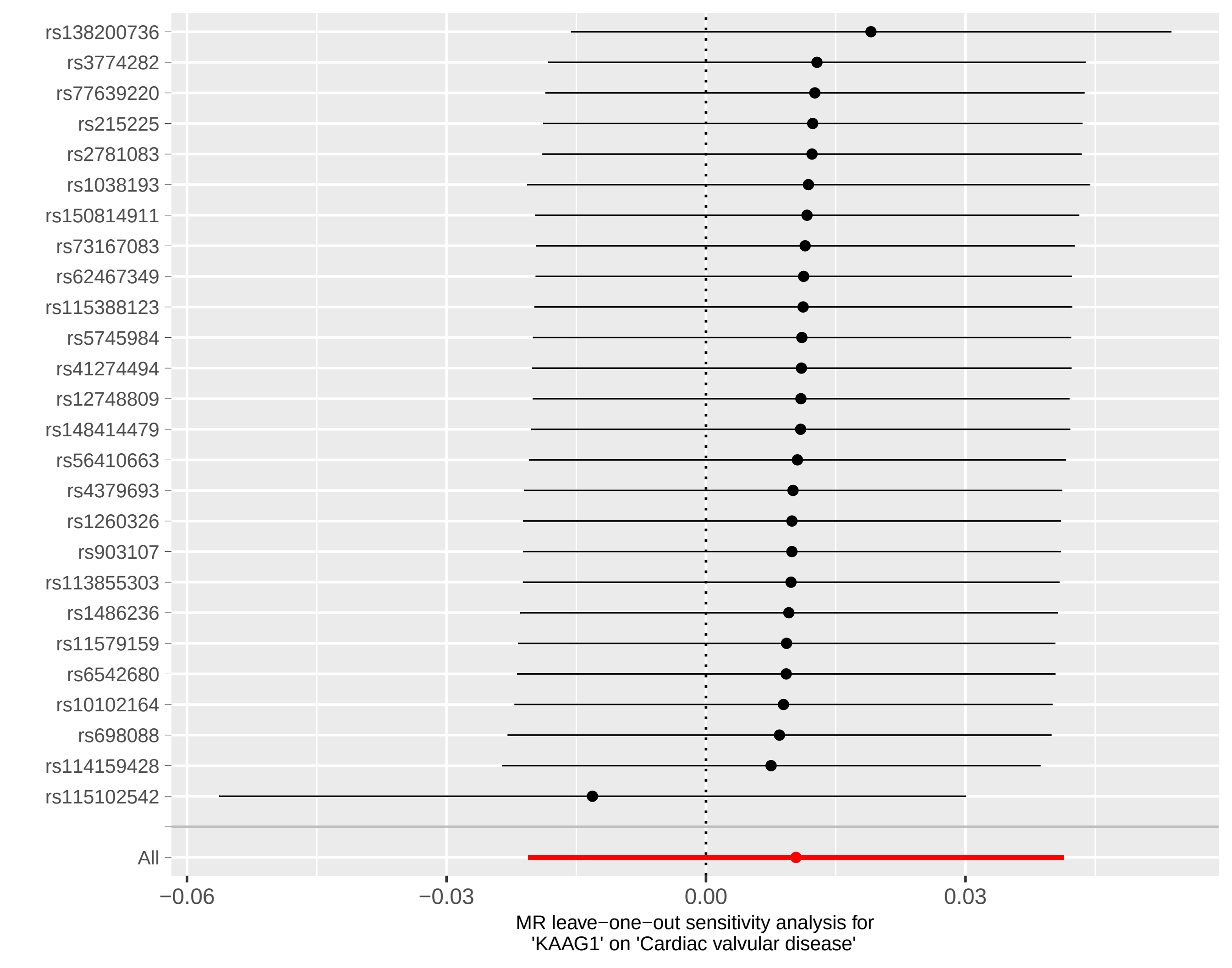
rs138200736
rs3774282
rs77639220
rs215225
rs2781083
rs1038193
rs150814911
rs73167083
rs62467349
rs115388123
rs5745984
rs41274494
rs12748809
rs148414479
rs56410663
rs4379693
rs1260326
rs903107
rs113855303
rs1486236
rs11579159
rs6542680
rs10102164
rs698088
rs114159428
rs115102542
All
−0.06
−0.03
0.00
0.03
MR leave−one−out sensitivity analysis for
'KAAG1' on 'Cardiac valvular disease'

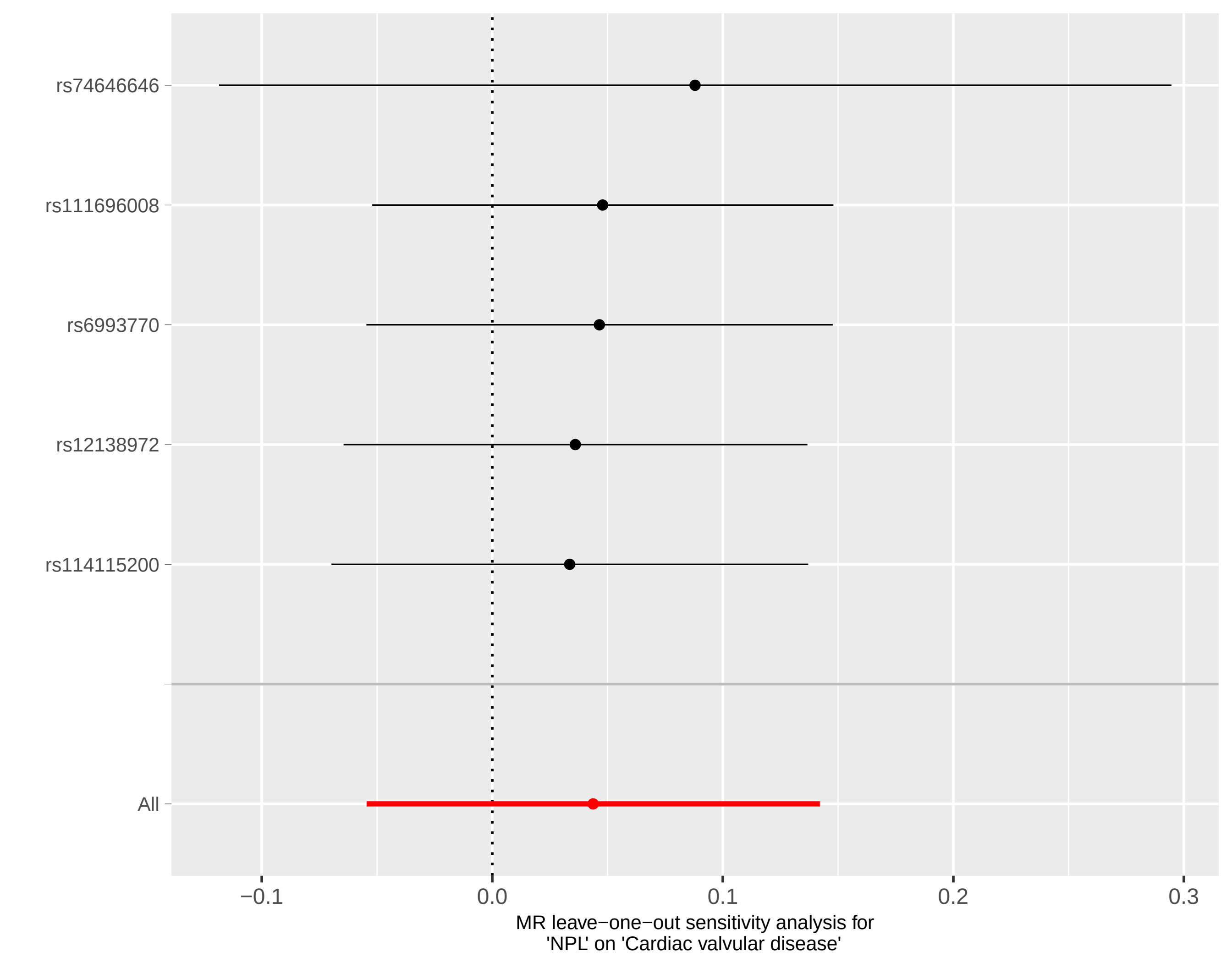

rs74646646
rs111696008
rs6993770
rs12138972
rs114115200
All
−0.1
0.0
0.1
0.2
0.3
MR leave−one−out sensitivity analysis for
'NPL' on 'Cardiac valvular disease'

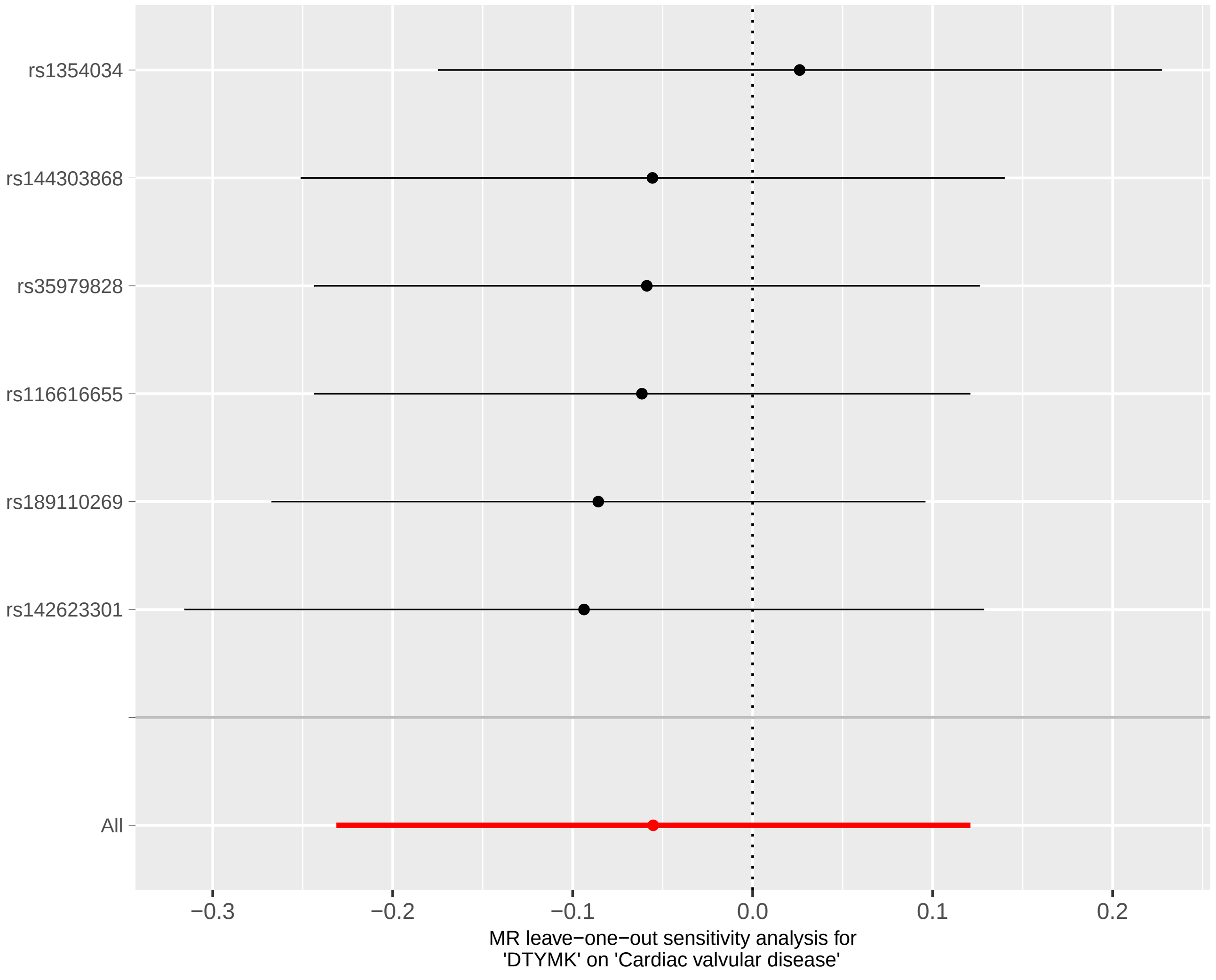
rs1354034
rs144303868
rs35979828
rs116616655
rs189110269
rs142623301
All
−0.3
−0.2
−0.1
0.0
0.1
0.2
MR leave−one−out sensitivity analysis for
'DTYMK' on 'Cardiac valvular disease'

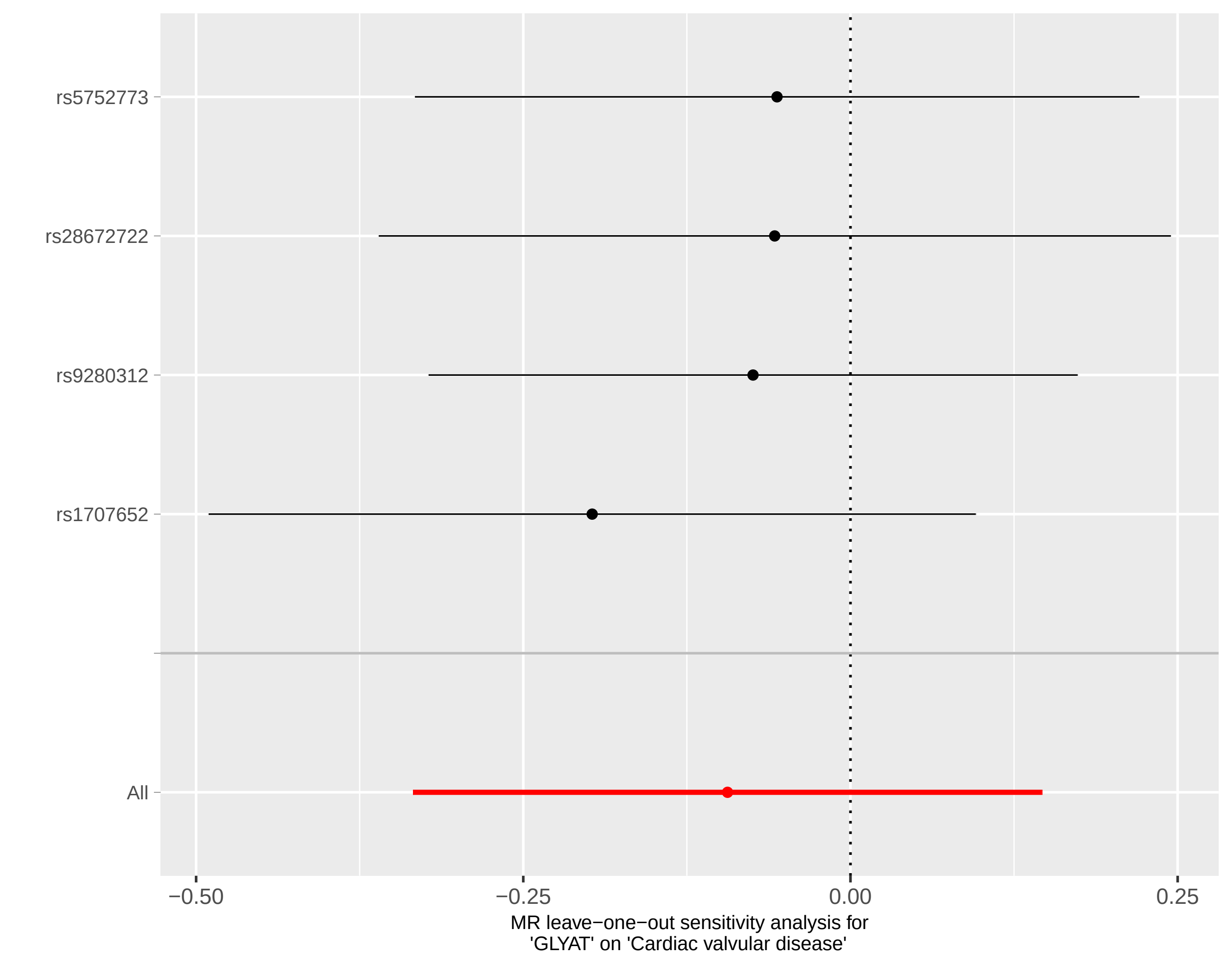
rs5752773
rs28672722
rs9280312
rs1707652
All
−0.50
−0.25
0.00
0.25
MR leave−one−out sensitivity analysis for
'GLYAT' on 'Cardiac valvular disease'

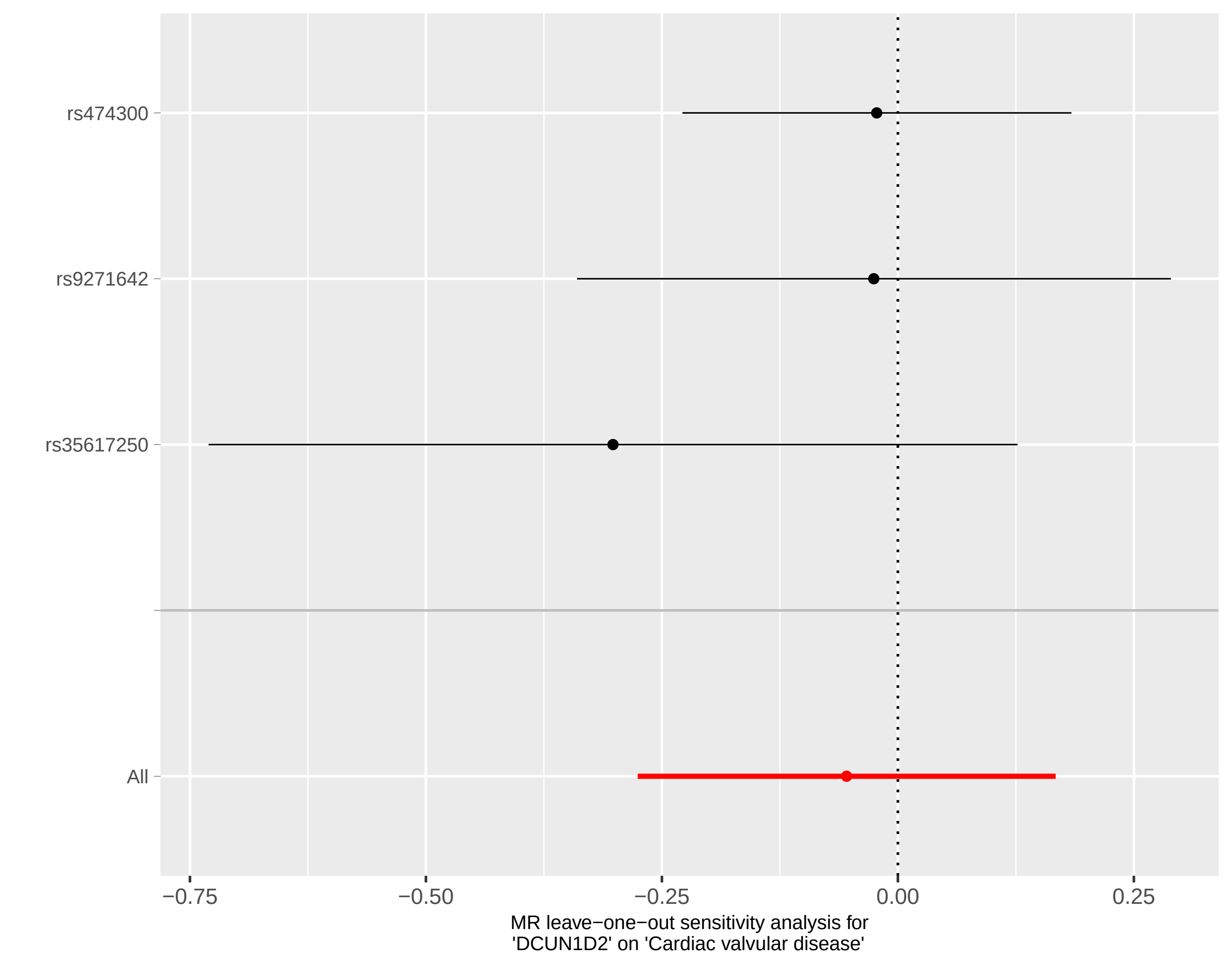

rs474300
rs9271642
rs35617250
All
−0.75
−0.50
−0.25
0.00
0.25
MR leave−one−out sensitivity analysis for
'DCUN1D2' on 'Cardiac valvular disease'

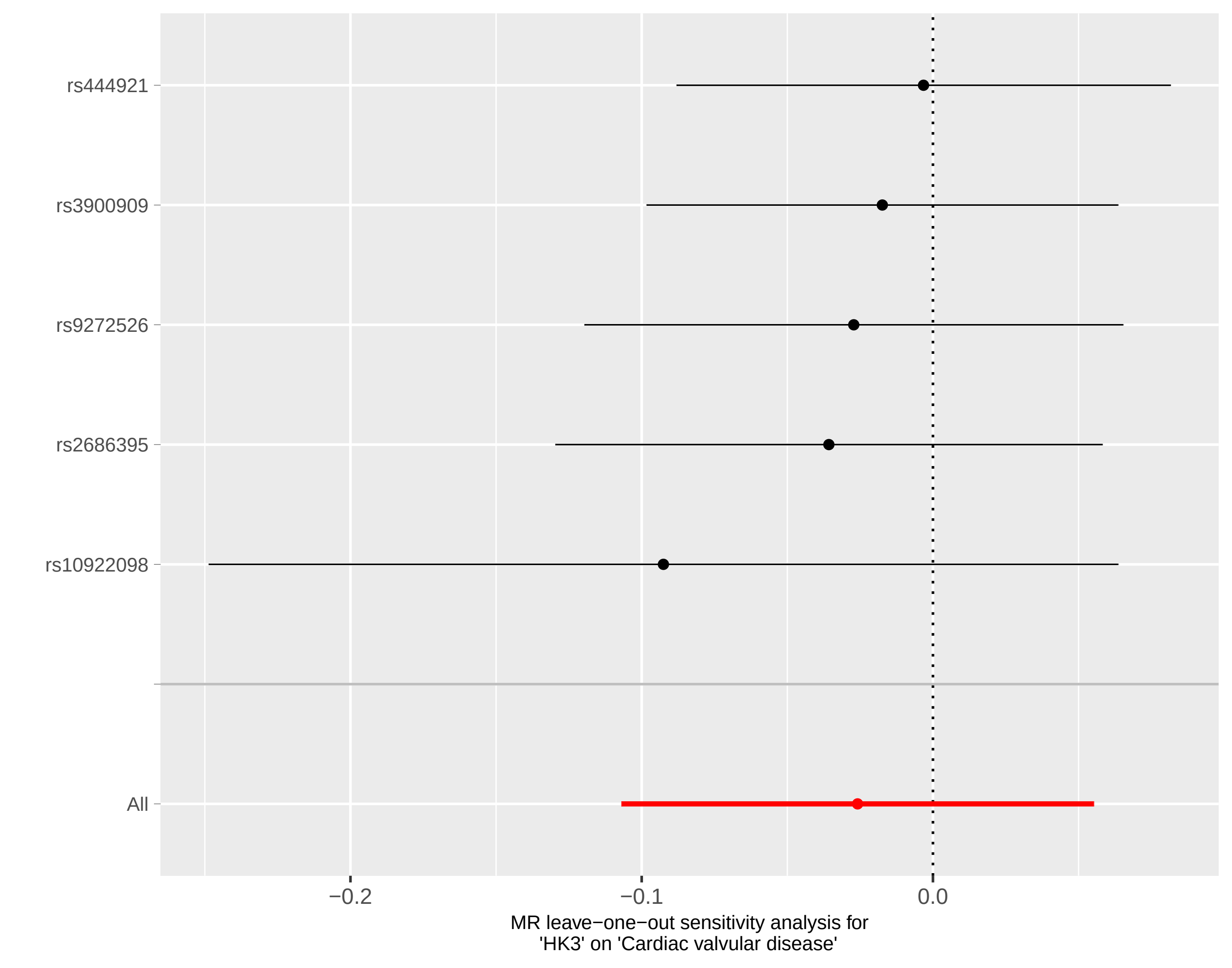
rs444921
rs3900909
rs9272526
rs2686395
rs10922098
All
−0.2
−0.1
0.0
MR leave−one−out sensitivity analysis for
'HK3' on 'Cardiac valvular disease'

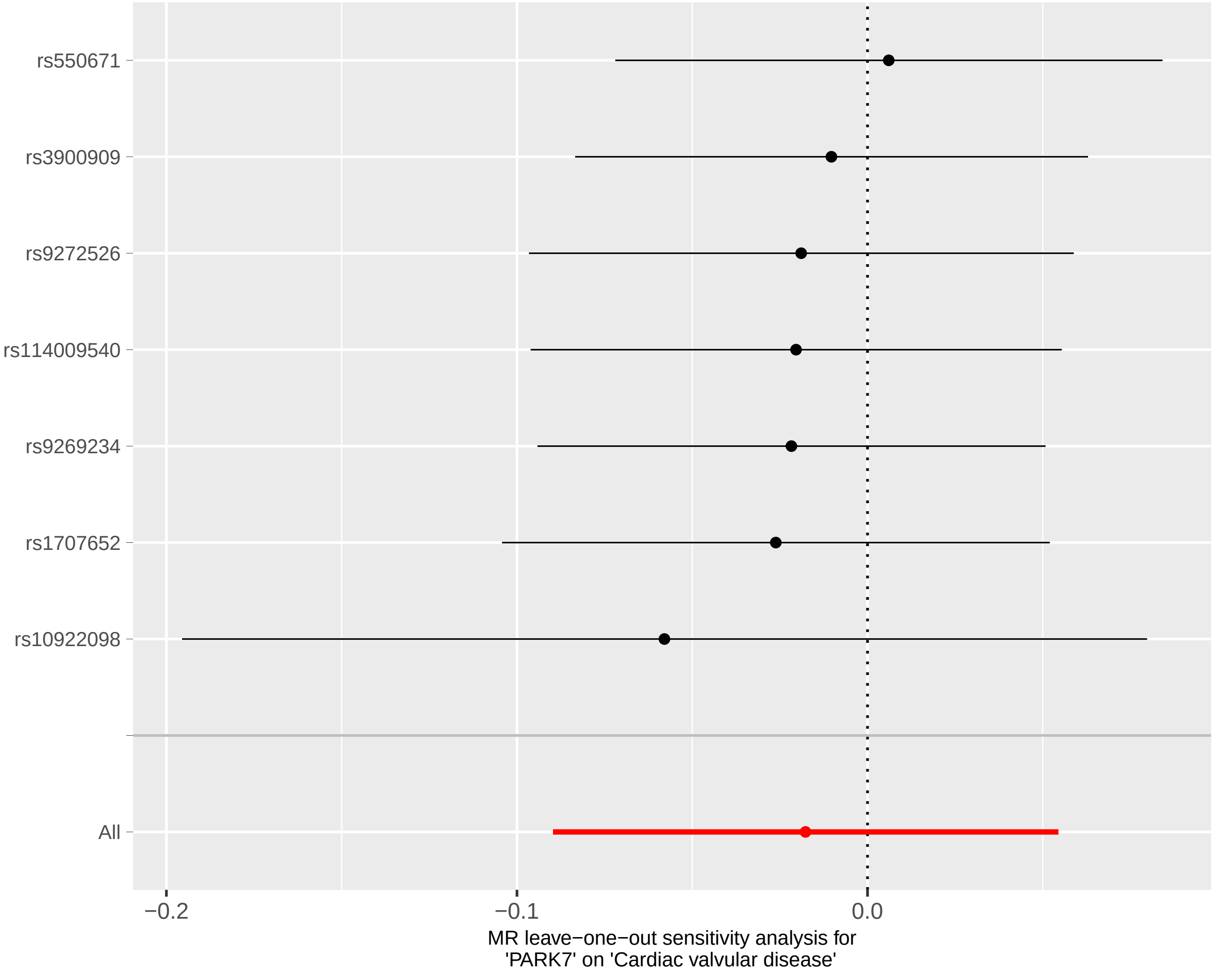

rs550671
rs3900909
rs9272526
rs114009540
rs9269234
rs1707652
rs10922098
All
−0.2
−0.1
0.0
MR leave−one−out sensitivity analysis for
'PARK7' on 'Cardiac valvular disease'

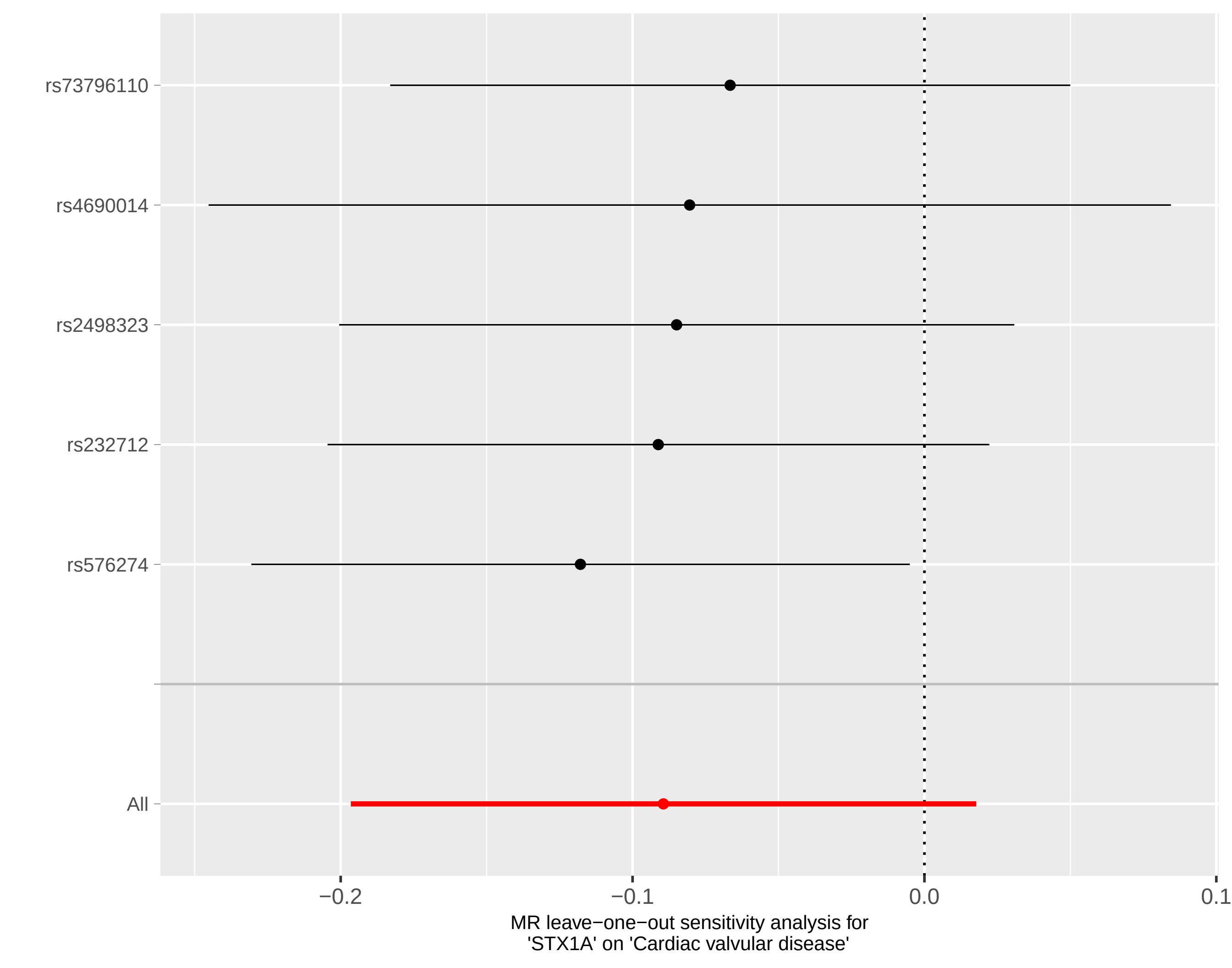
rs73796110
rs4690014
rs2498323
rs232712
rs576274
All
−0.2
−0.1
0.0
0.1
MR leave−one−out sensitivity analysis for
'STX1A' on 'Cardiac valvular disease'

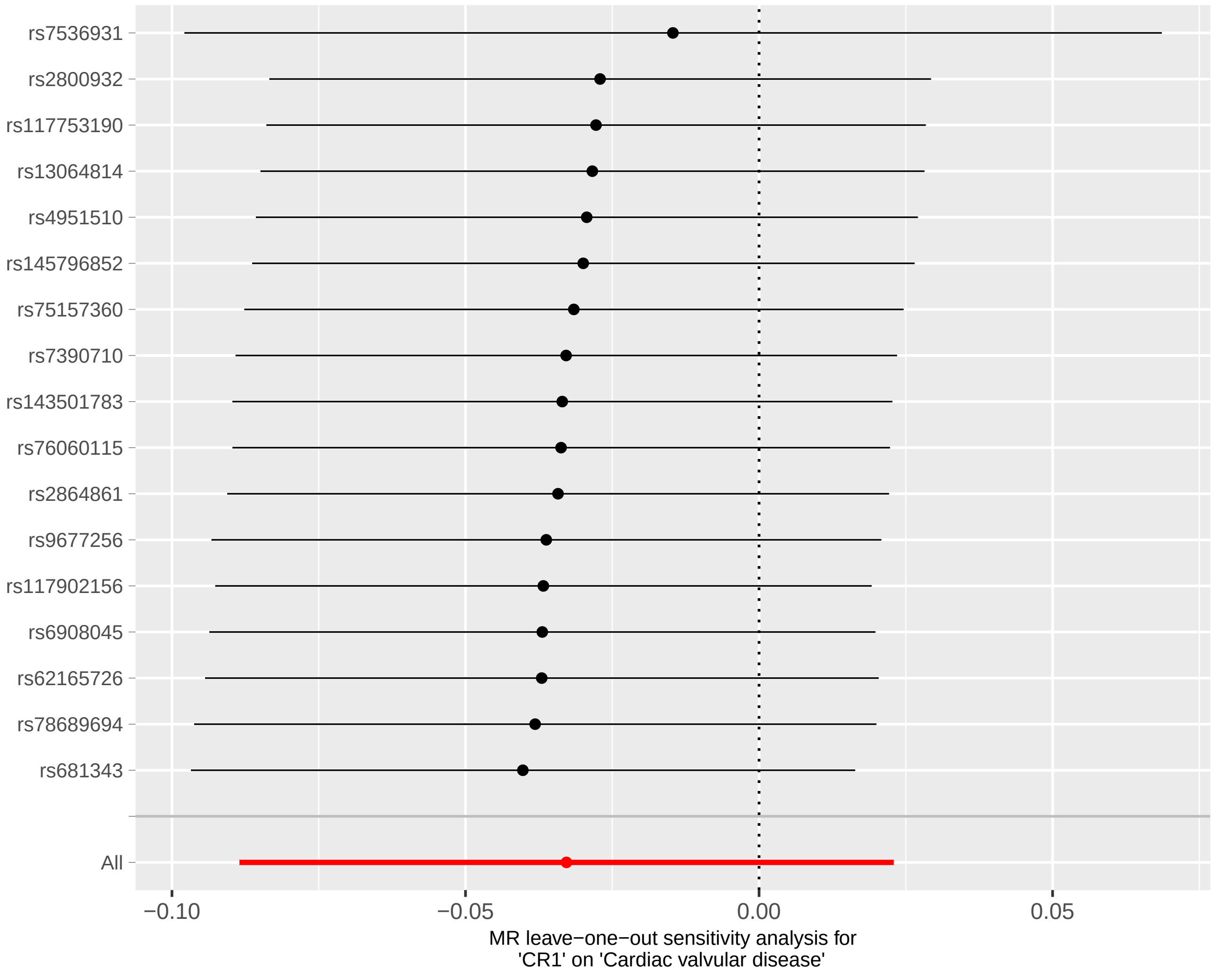

rs7536931
rs2800932
rs117753190
rs13064814
rs4951510
rs145796852
rs75157360
rs7390710
rs143501783
rs76060115
rs2864861
rs9677256
rs117902156
rs6908045
rs62165726
rs78689694
rs681343
All
−0.10
−0.05
0.00
0.05
MR leave−one−out sensitivity analysis for
'CR1' on 'Cardiac valvular disease'

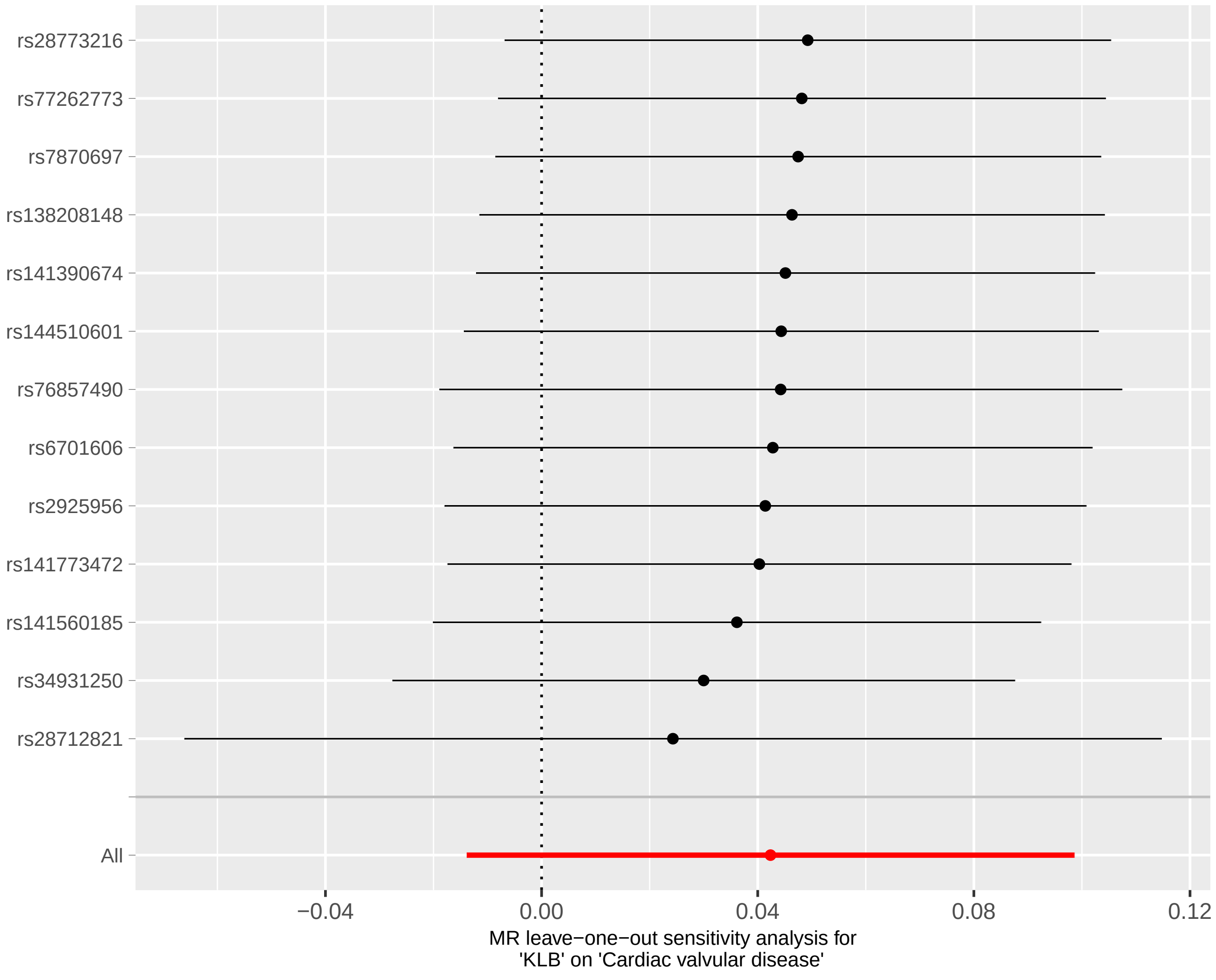

rs28773216
rs77262773
rs7870697
rs138208148
rs141390674
rs144510601
rs76857490
rs6701606
rs2925956
rs141773472
rs141560185
rs34931250
rs28712821
All
−0.04
0.00
0.04
0.08
0.12
MR leave−one−out sensitivity analysis for
'KLB' on 'Cardiac valvular disease'

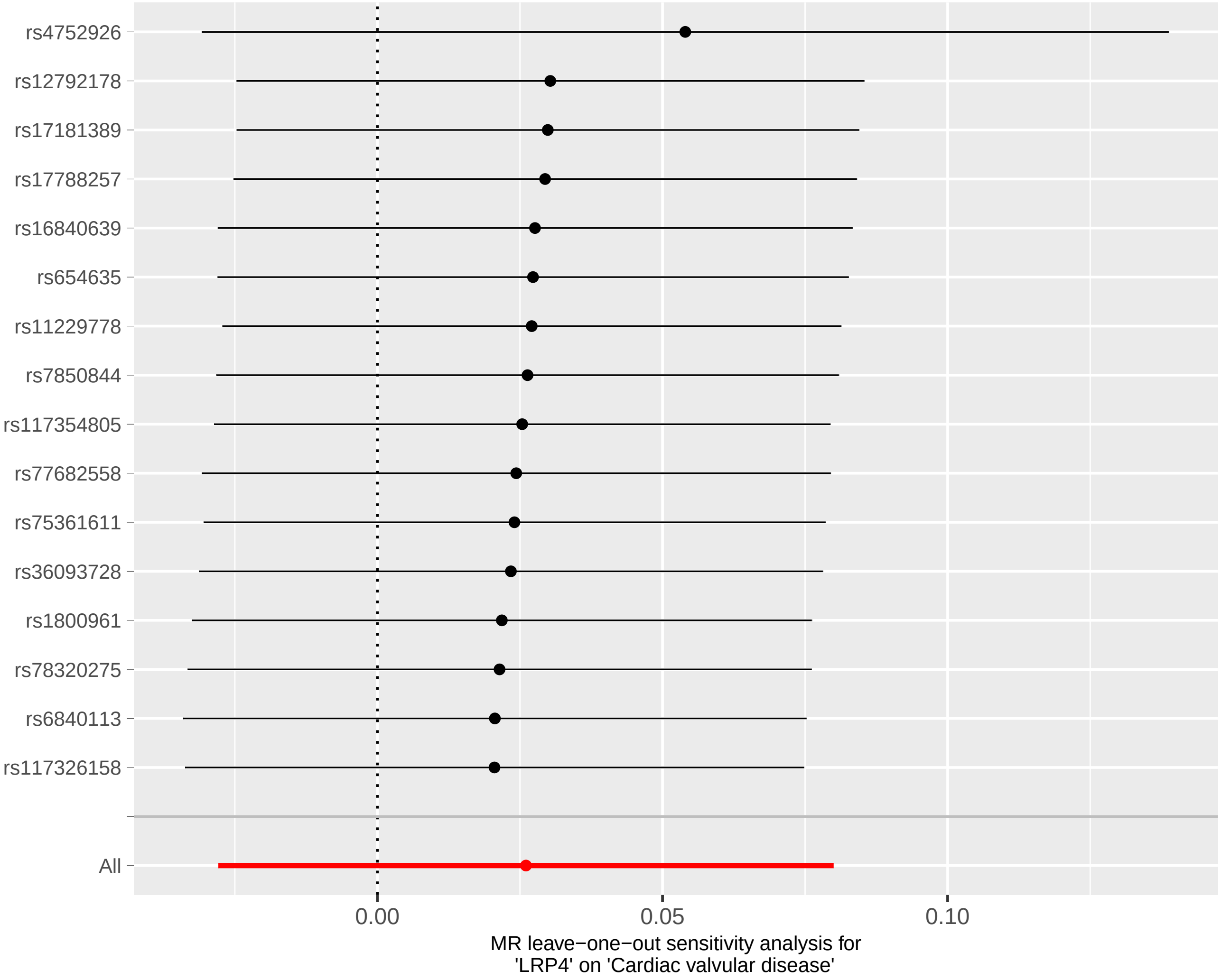
rs4752926
rs12792178
rs17181389
rs17788257
rs16840639
rs654635
rs11229778
rs7850844
rs117354805
rs77682558
rs75361611
rs36093728
rs1800961
rs78320275
rs6840113
rs117326158
All
0.00
0.05
0.10
MR leave−one−out sensitivity analysis for
'LRP4' on 'Cardiac valvular disease'

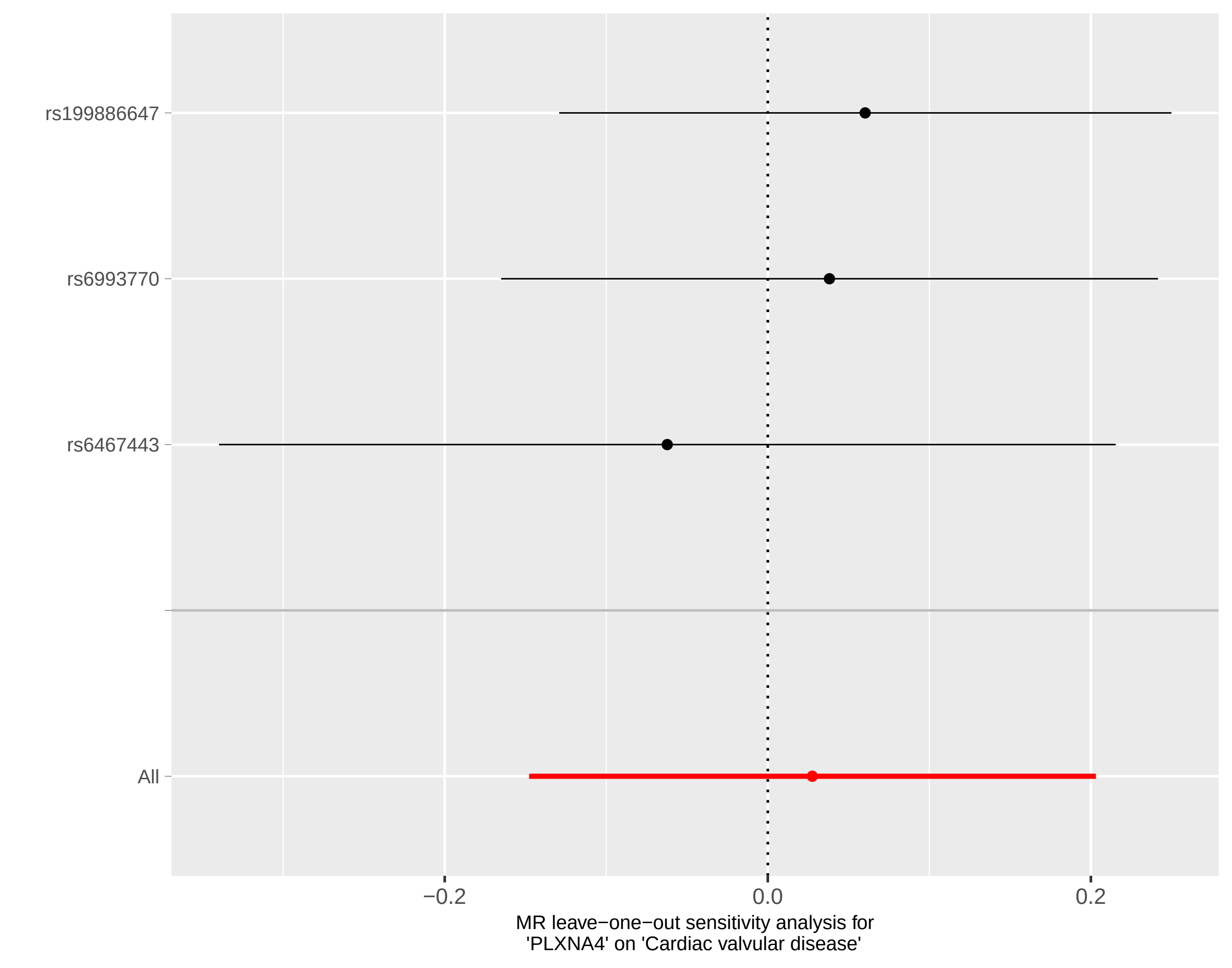
rs199886647
rs6993770
rs6467443
All
−0.2
0.0
0.2
MR leave−one−out sensitivity analysis for
'PLXNA4' on 'Cardiac valvular disease'

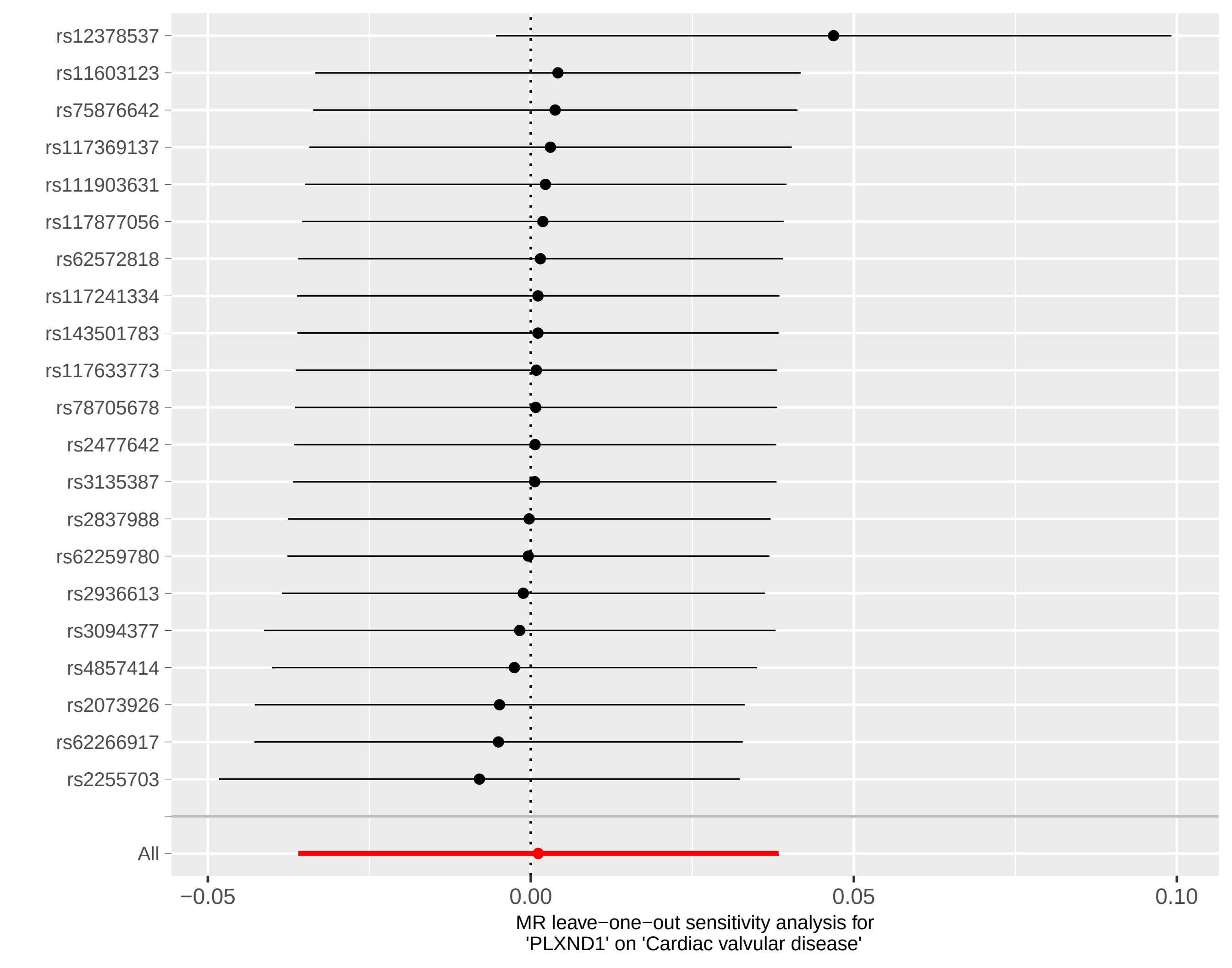

rs12378537
rs11603123
rs75876642
rs117369137
rs111903631
rs117877056
rs62572818
rs117241334
rs143501783
rs117633773
rs78705678
rs2477642
rs3135387
rs2837988
rs62259780
rs2936613
rs3094377
rs4857414
rs2073926
rs62266917
rs2255703
All
−0.05
0.00
0.05
0.10
MR leave−one−out sensitivity analysis for
'PLXND1' on 'Cardiac valvular disease'

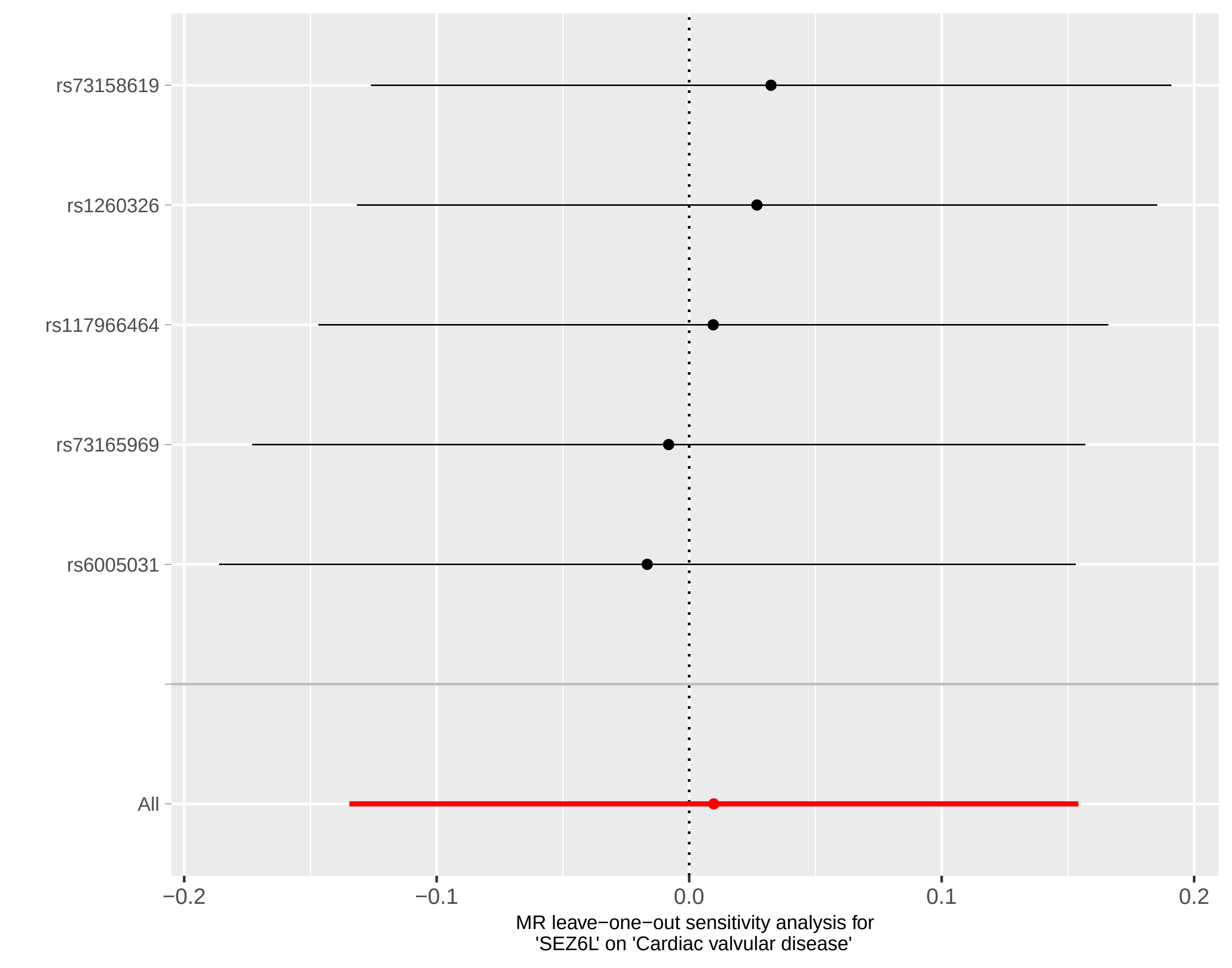
rs73158619
rs1260326
rs117966464
rs73165969
rs6005031
All
−0.2
−0.1
0.0
0.1
0.2
MR leave−one−out sensitivity analysis for
'SEZ6L' on 'Cardiac valvular disease'

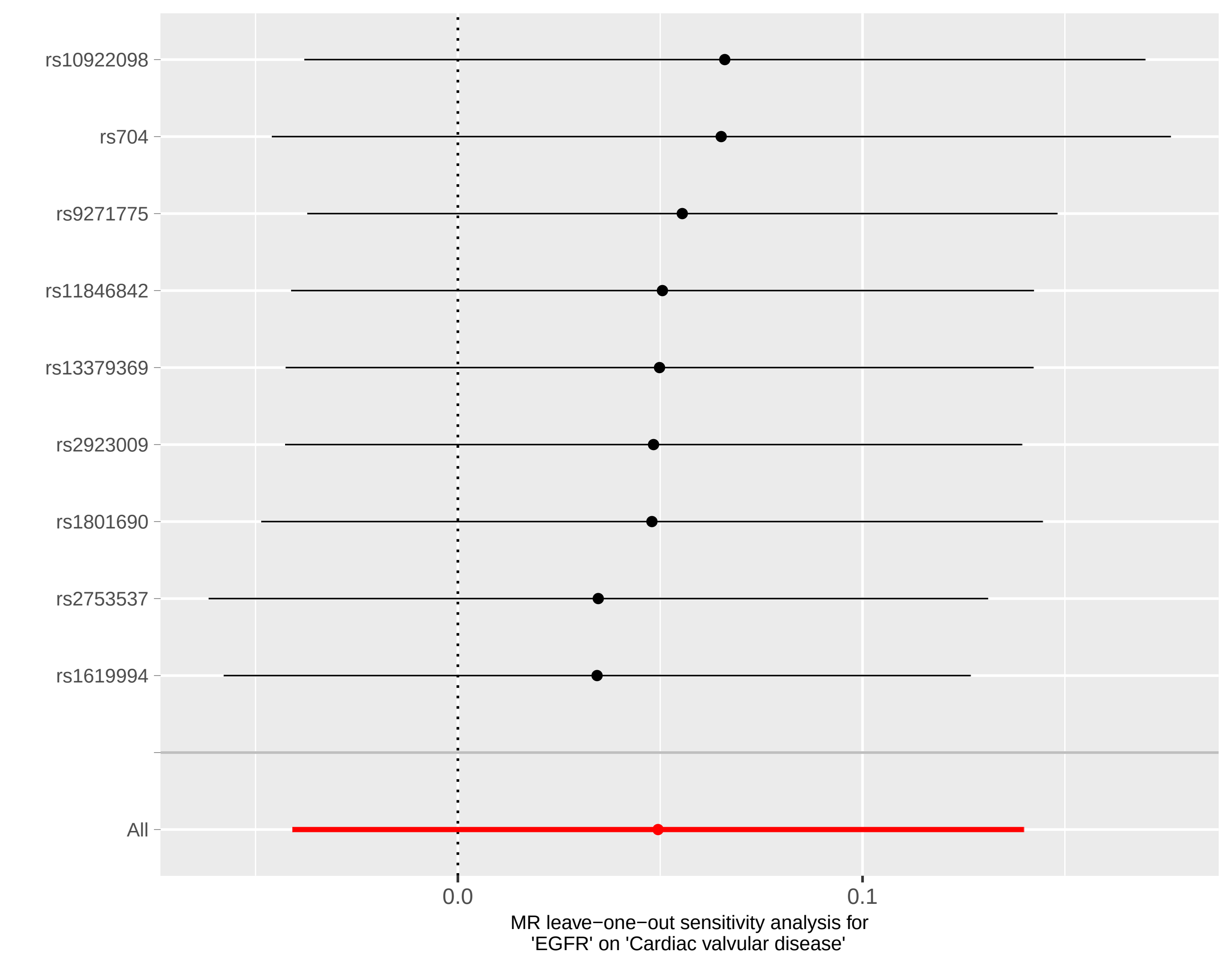

rs10922098
rs704
rs9271775
rs11846842
rs13379369
rs2923009
rs1801690
rs2753537
rs1619994
All
0.0
0.1
MR leave−one−out sensitivity analysis for
'EGFR' on 'Cardiac valvular disease'

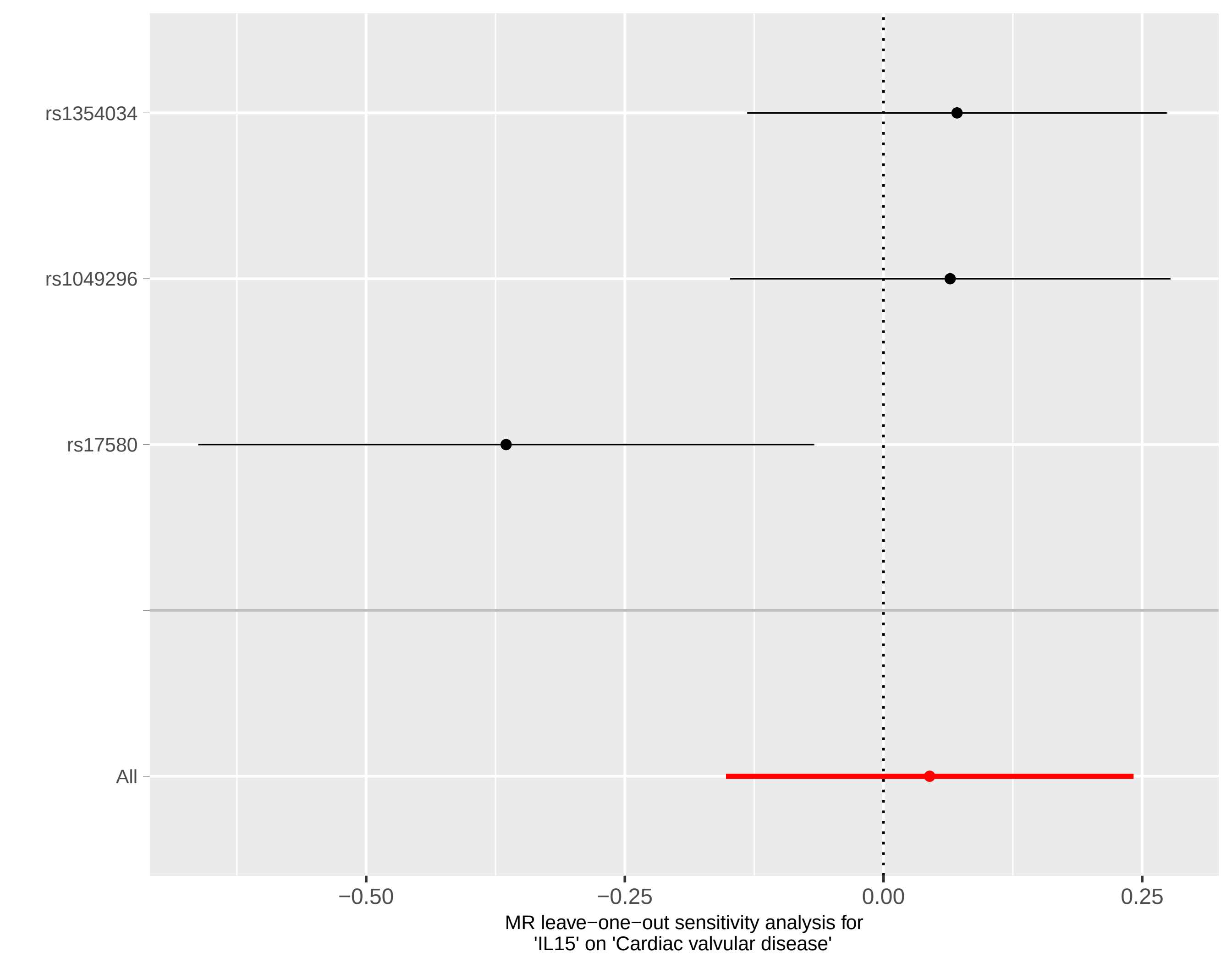
rs1354034
rs1049296
rs17580
All
−0.50
−0.25
0.00
0.25
MR leave−one−out sensitivity analysis for
'IL15' on 'Cardiac valvular disease'

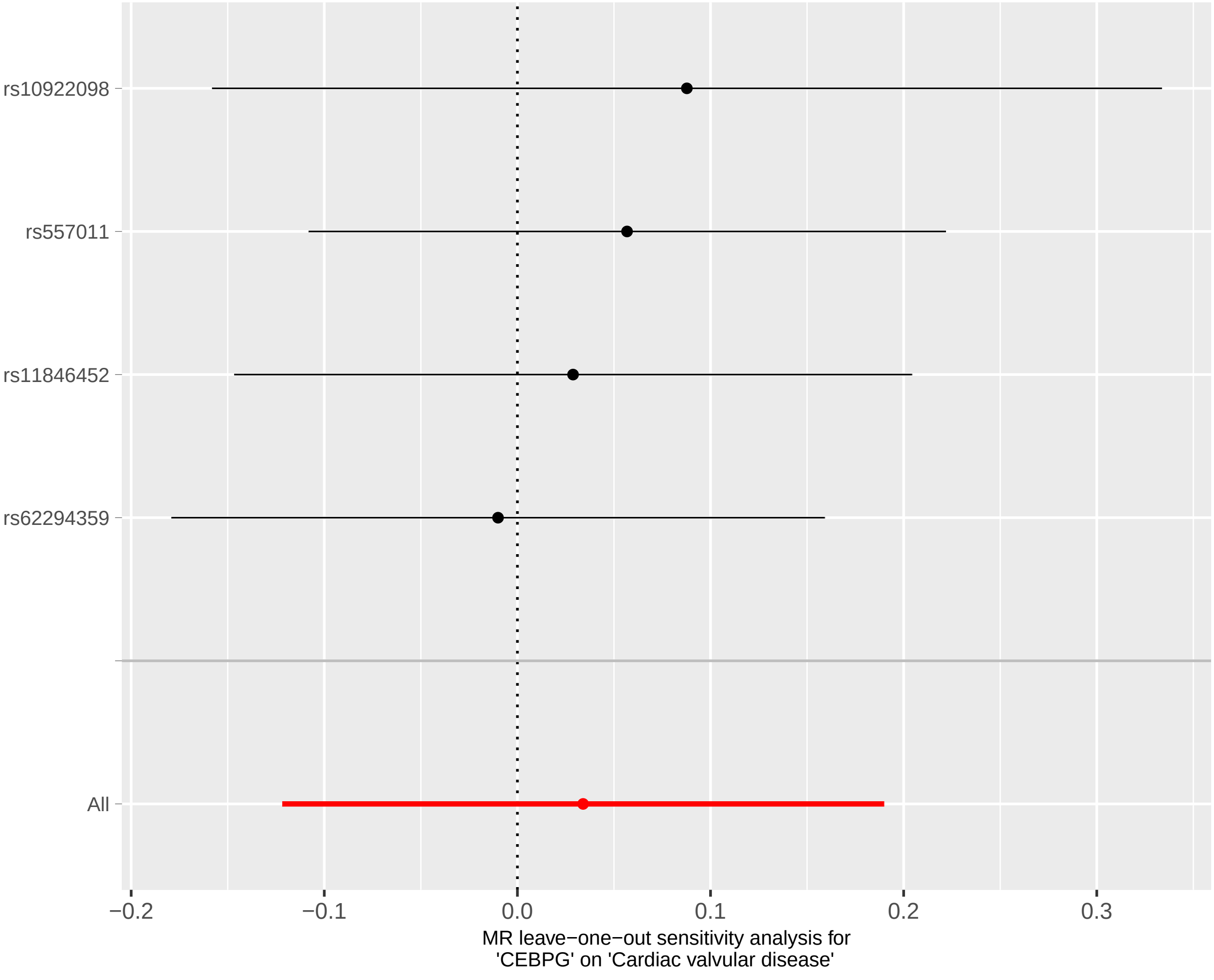

rs10922098
rs557011
rs11846452
rs62294359
All
−0.2
−0.1
0.0
0.1
0.2
0.3
MR leave−one−out sensitivity analysis for
'CEBPG' on 'Cardiac valvular disease'

Insufficient number of SNPs

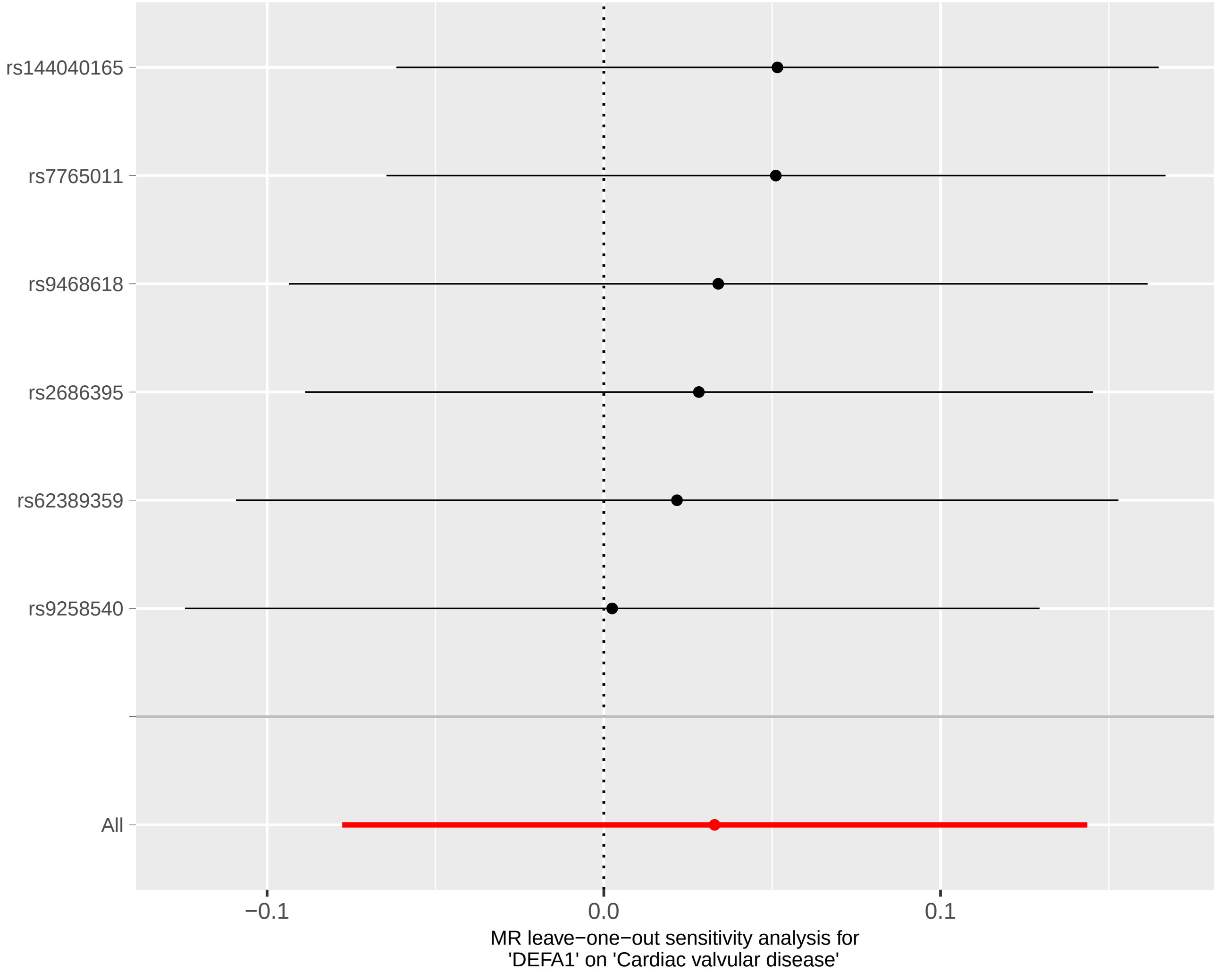
rs144040165
rs7765011
rs9468618
rs2686395
rs62389359
rs9258540
All
−0.1
0.0
0.1
MR leave−one−out sensitivity analysis for
'DEFA1' on 'Cardiac valvular disease'

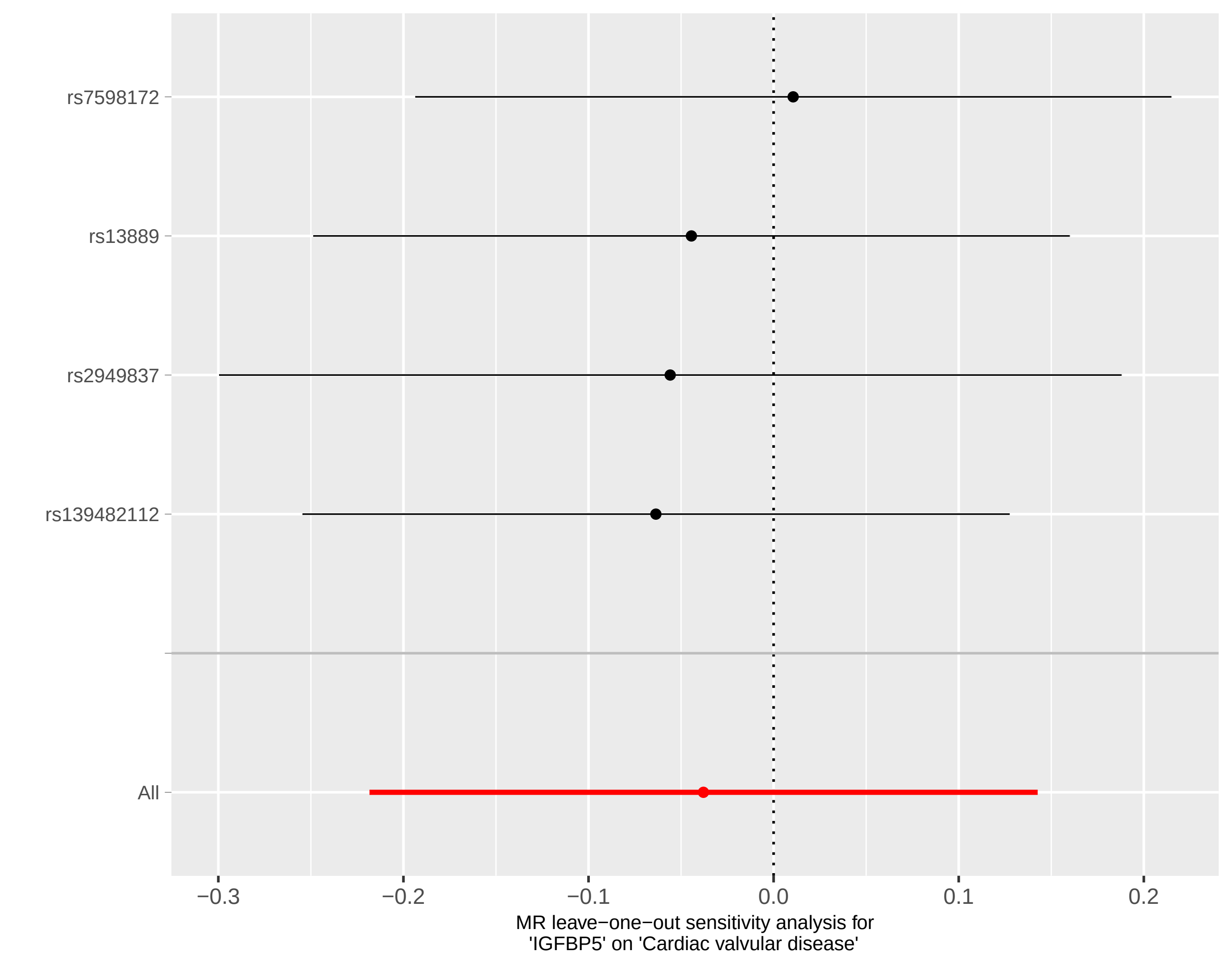
rs7598172
rs13889
rs2949837
rs139482112
All
−0.3
−0.2
−0.1
0.0
0.1
0.2
MR leave−one−out sensitivity analysis for
'IGFBP5' on 'Cardiac valvular disease'

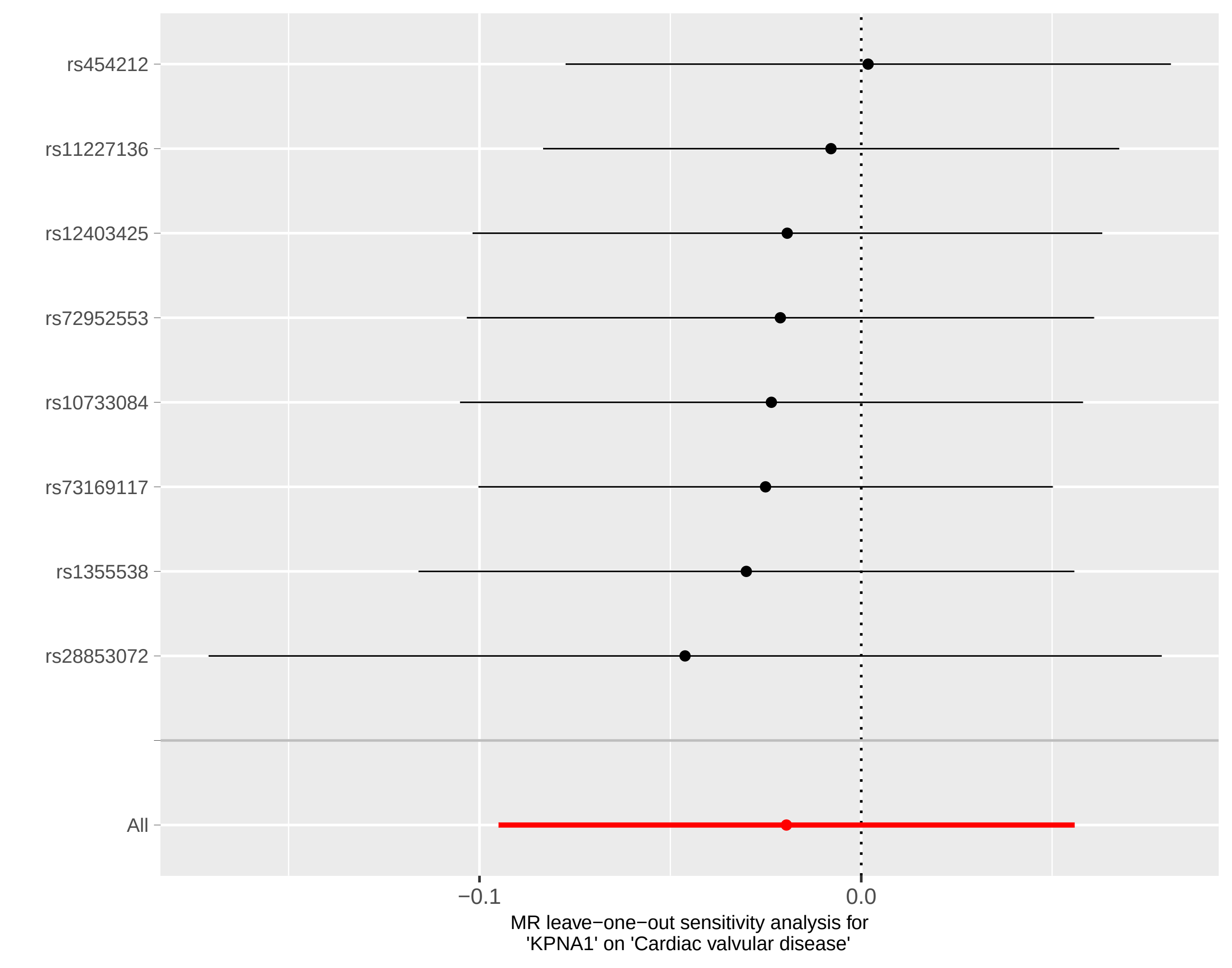
rs454212
rs11227136
rs12403425
rs72952553
rs10733084
rs73169117
rs1355538
rs28853072
All
−0.1
0.0
MR leave−one−out sensitivity analysis for
'KPNA1' on 'Cardiac valvular disease'

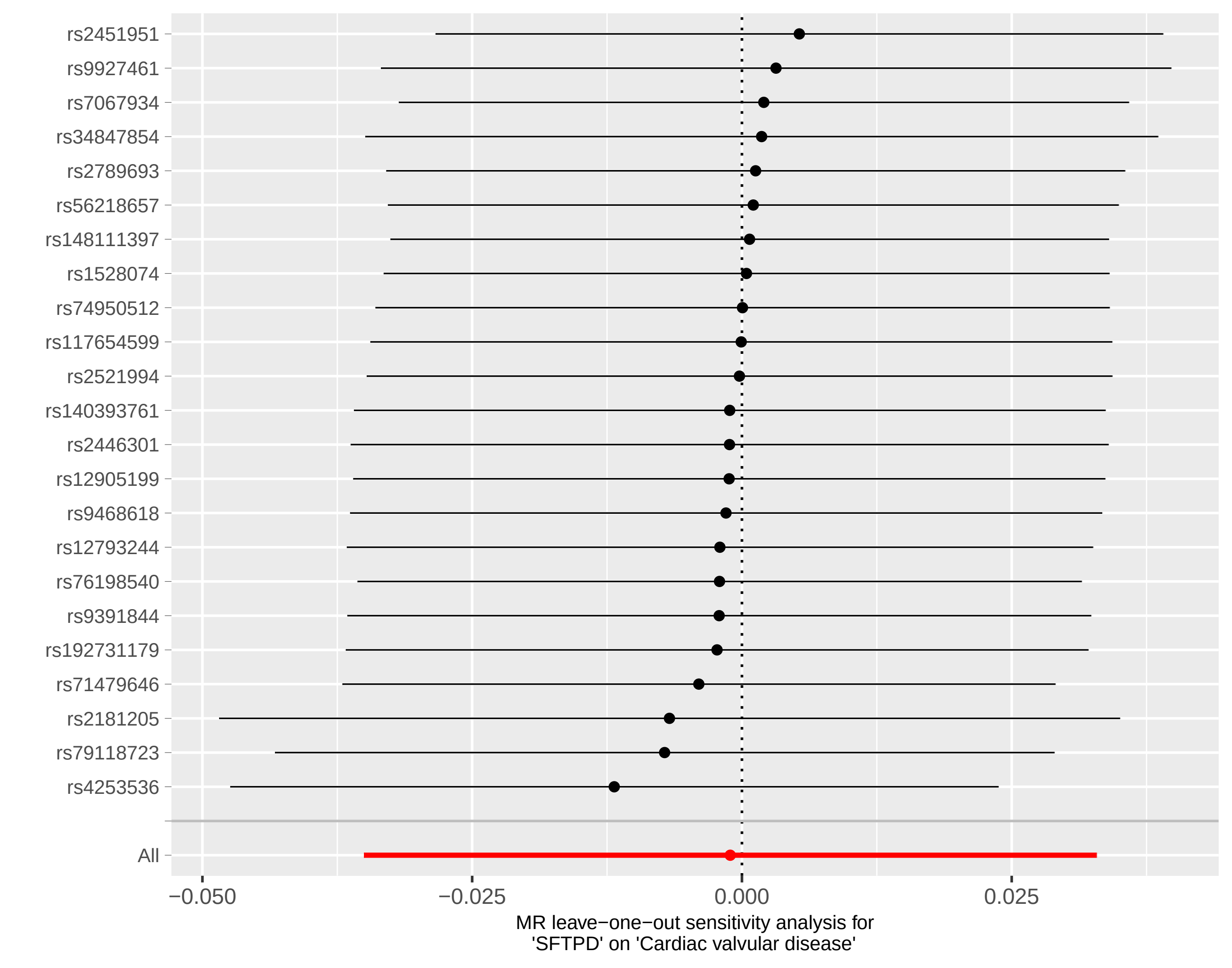
rs2451951
rs9927461
rs7067934
rs34847854
rs2789693
rs56218657
rs148111397
rs1528074
rs74950512
rs117654599
rs2521994
rs140393761
rs2446301
rs12905199
rs9468618
rs12793244
rs76198540
rs9391844
rs192731179
rs71479646
rs2181205
rs79118723
rs4253536
All
−0.050
−0.025
0.000
0.025
MR leave−one−out sensitivity analysis for
'SFTPD' on 'Cardiac valvular disease'

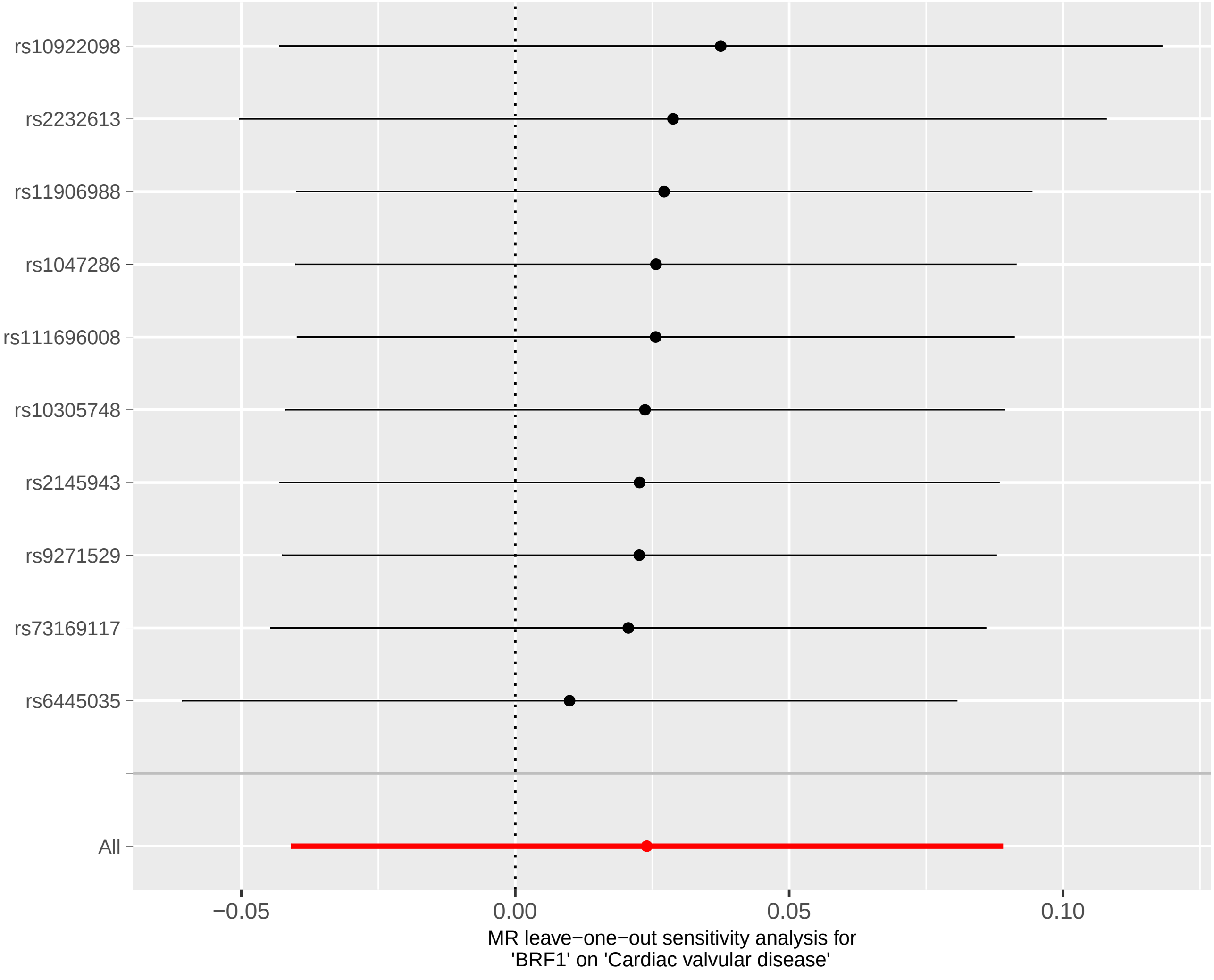

rs10922098
rs2232613
rs11906988
rs1047286
rs111696008
rs10305748
rs2145943
rs9271529
rs73169117
rs6445035
All
−0.05
0.00
0.05
0.10
MR leave−one−out sensitivity analysis for
'BRF1' on 'Cardiac valvular disease'

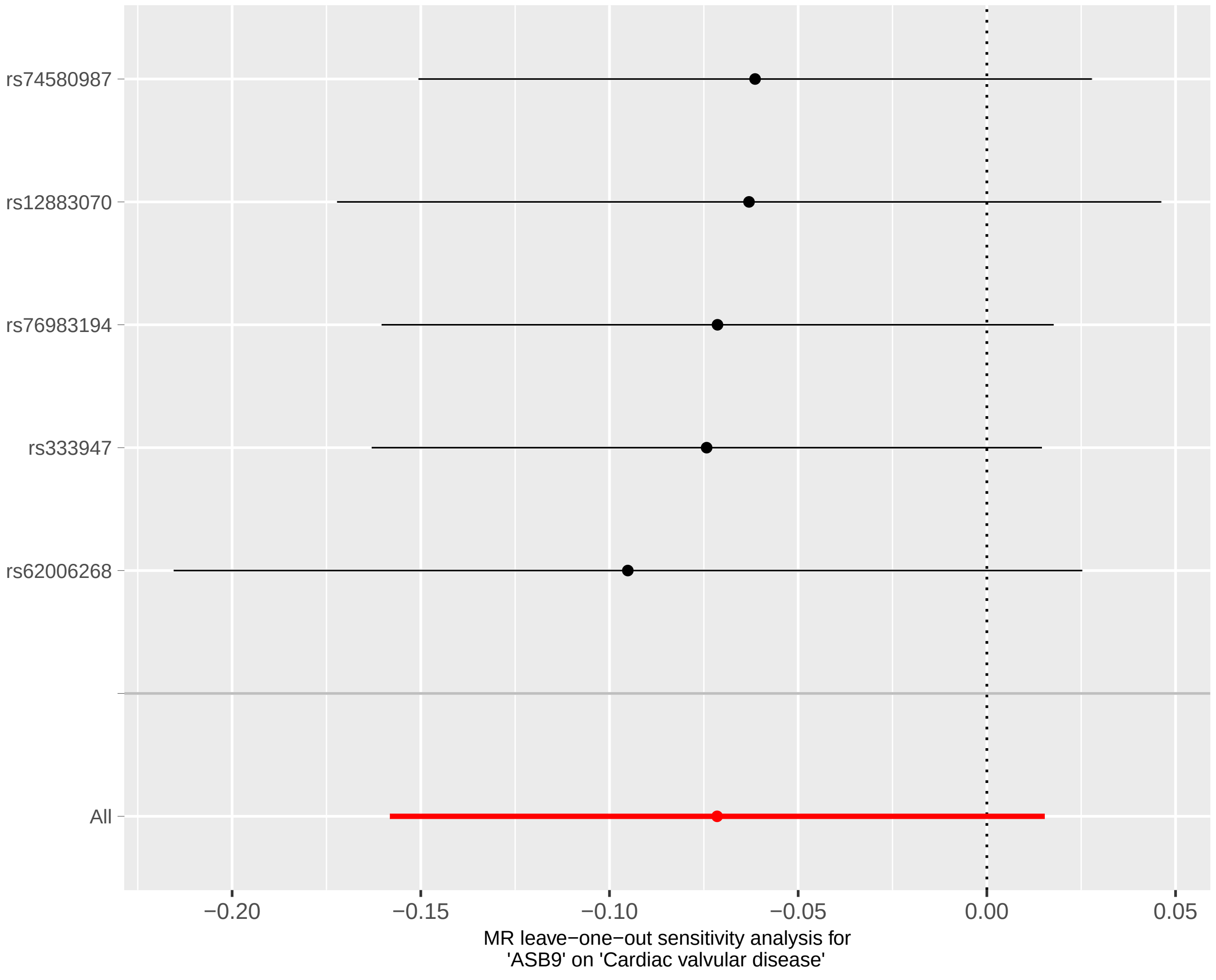
rs74580987
rs12883070
rs76983194
rs333947
rs62006268
All
−0.20
−0.15
−0.10
−0.05
0.00
0.05
MR leave−one−out sensitivity analysis for
'ASB9' on 'Cardiac valvular disease'

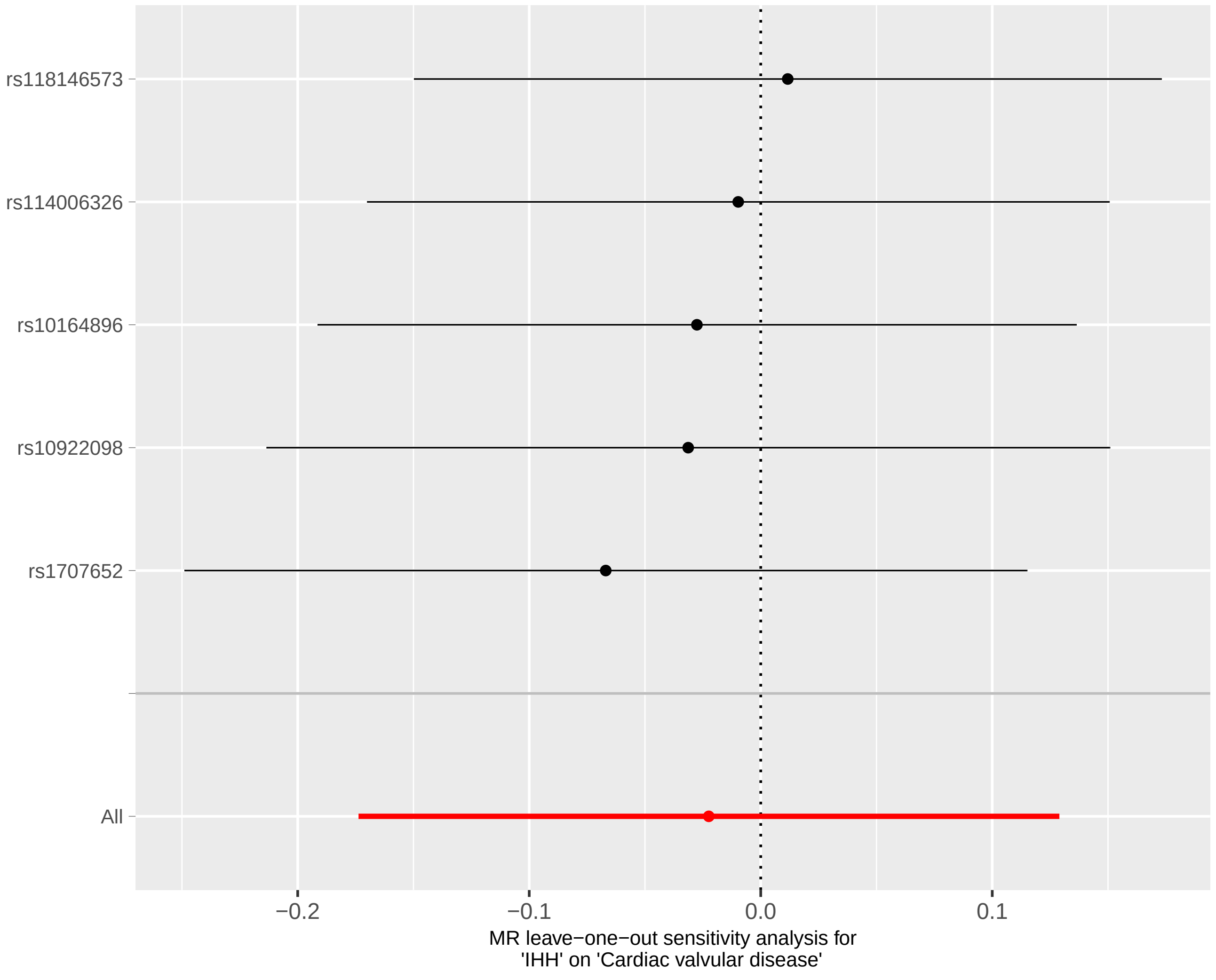

rs118146573
rs114006326
rs10164896
rs10922098
rs1707652
All
−0.2
−0.1
0.0
0.1
MR leave−one−out sensitivity analysis for
'IHH' on 'Cardiac valvular disease'

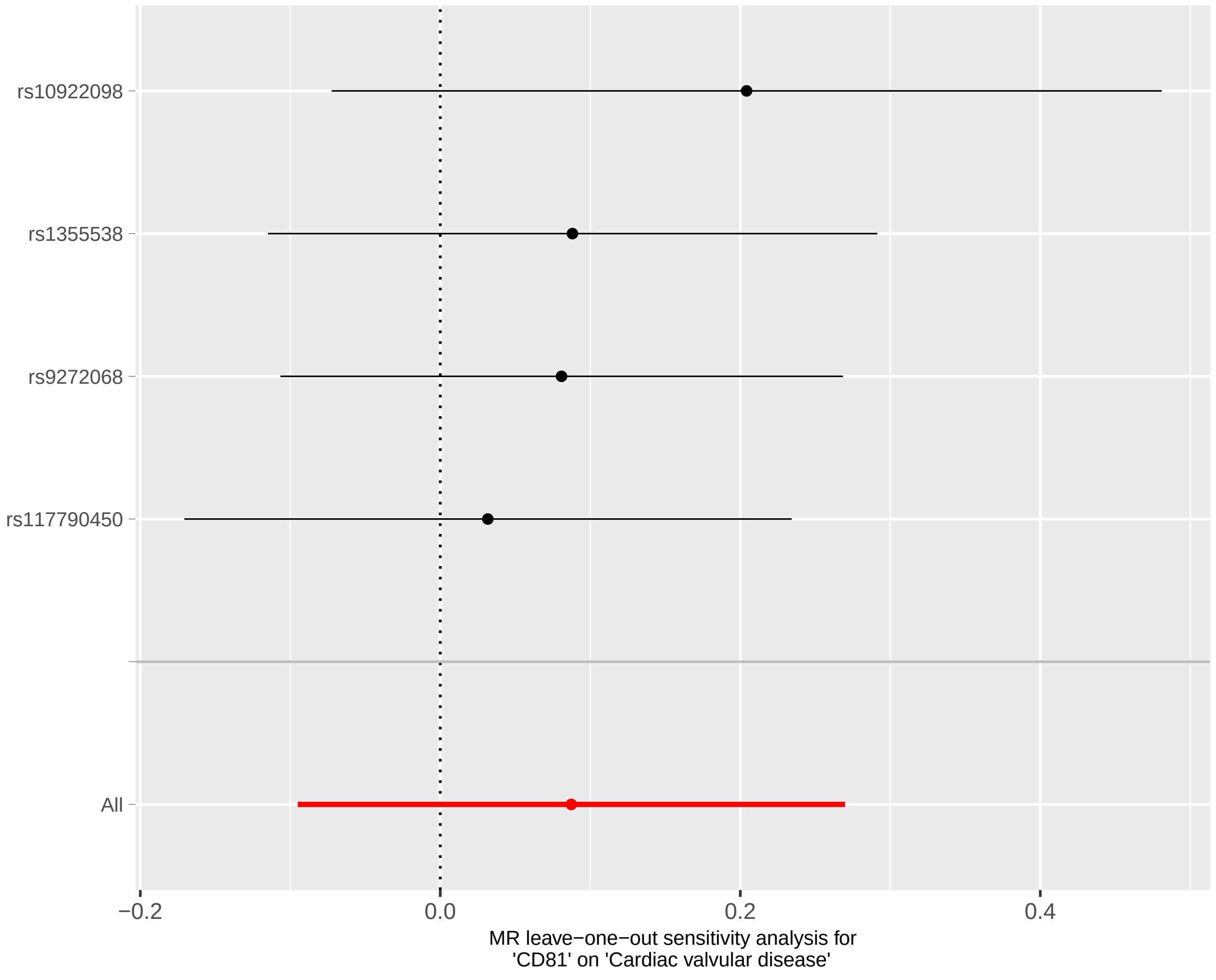
rs10922098
rs1355538
rs9272068
rs117790450
All
−0.2
0.0
0.2
0.4
MR leave−one−out sensitivity analysis for
'CD81' on 'Cardiac valvular disease'

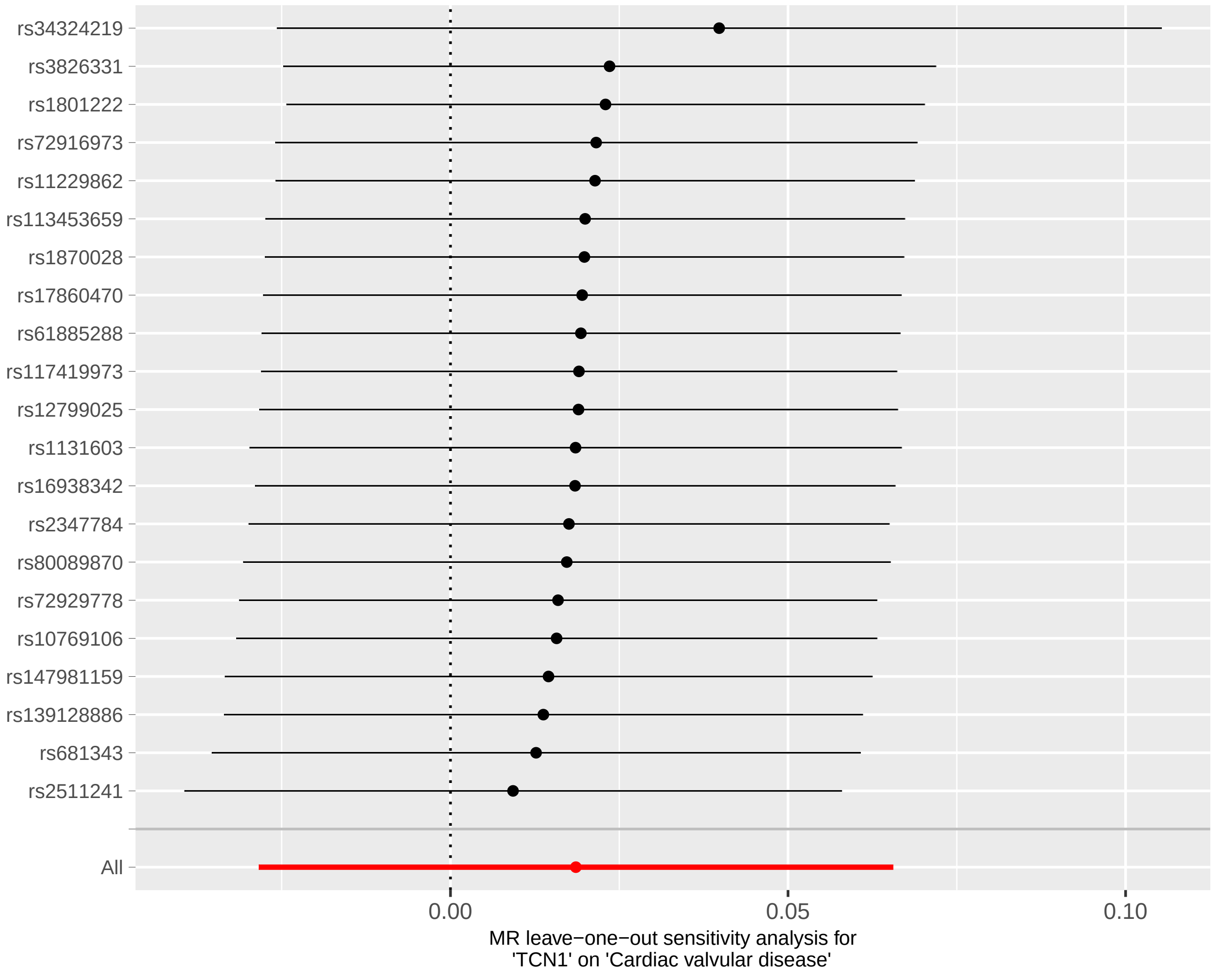
rs34324219
rs3826331
rs1801222
rs72916973
rs11229862
rs113453659
rs1870028
rs17860470
rs61885288
rs117419973
rs12799025
rs1131603
rs16938342
rs2347784
rs80089870
rs72929778
rs10769106
rs147981159
rs139128886
rs681343
rs2511241
All
0.00
0.05
0.10
MR leave−one−out sensitivity analysis for
'TCN1' on 'Cardiac valvular disease'

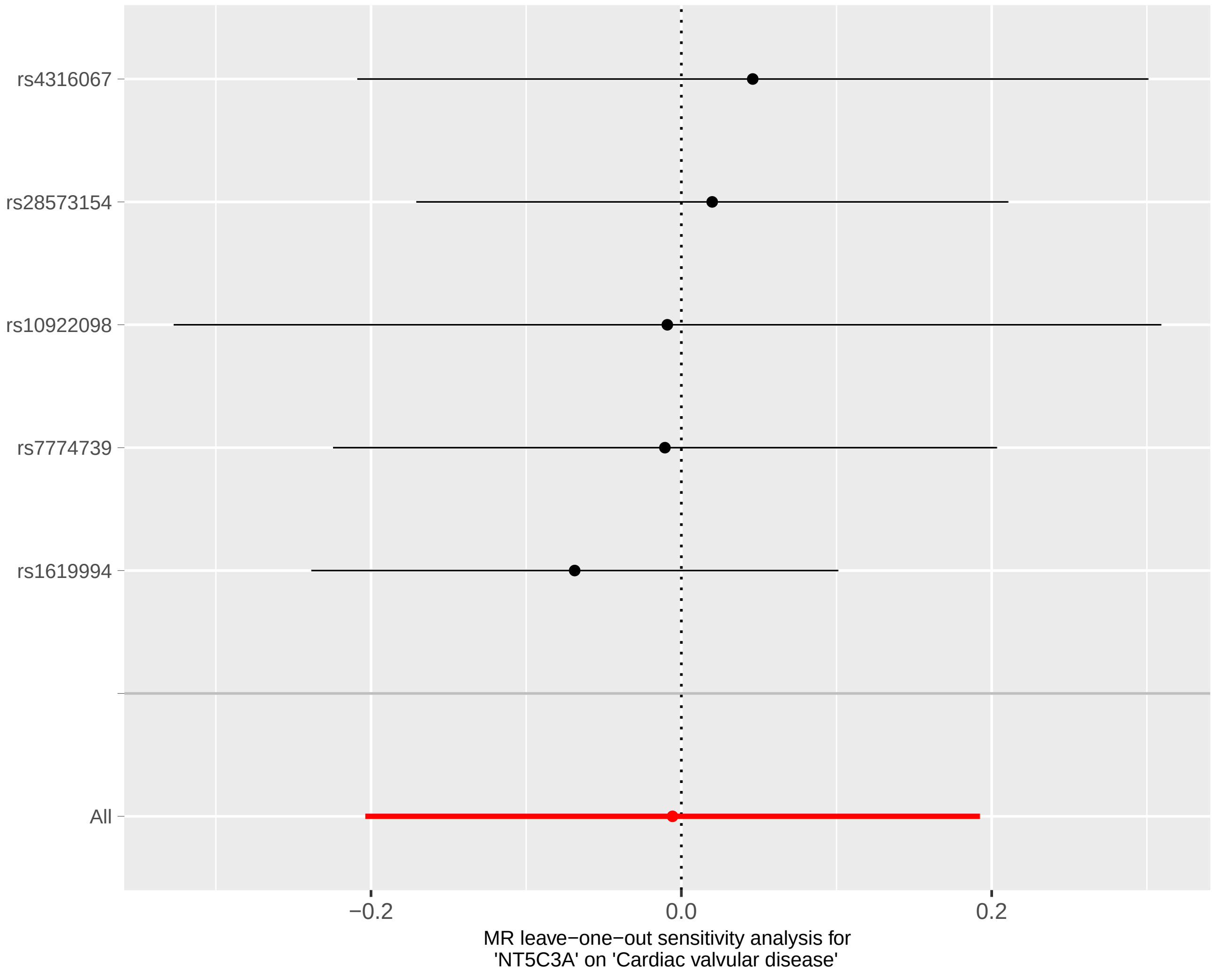

rs4316067
rs28573154
rs10922098
rs7774739
rs1619994
All
−0.2
0.0
0.2
MR leave−one−out sensitivity analysis for
'NT5C3A' on 'Cardiac valvular disease'

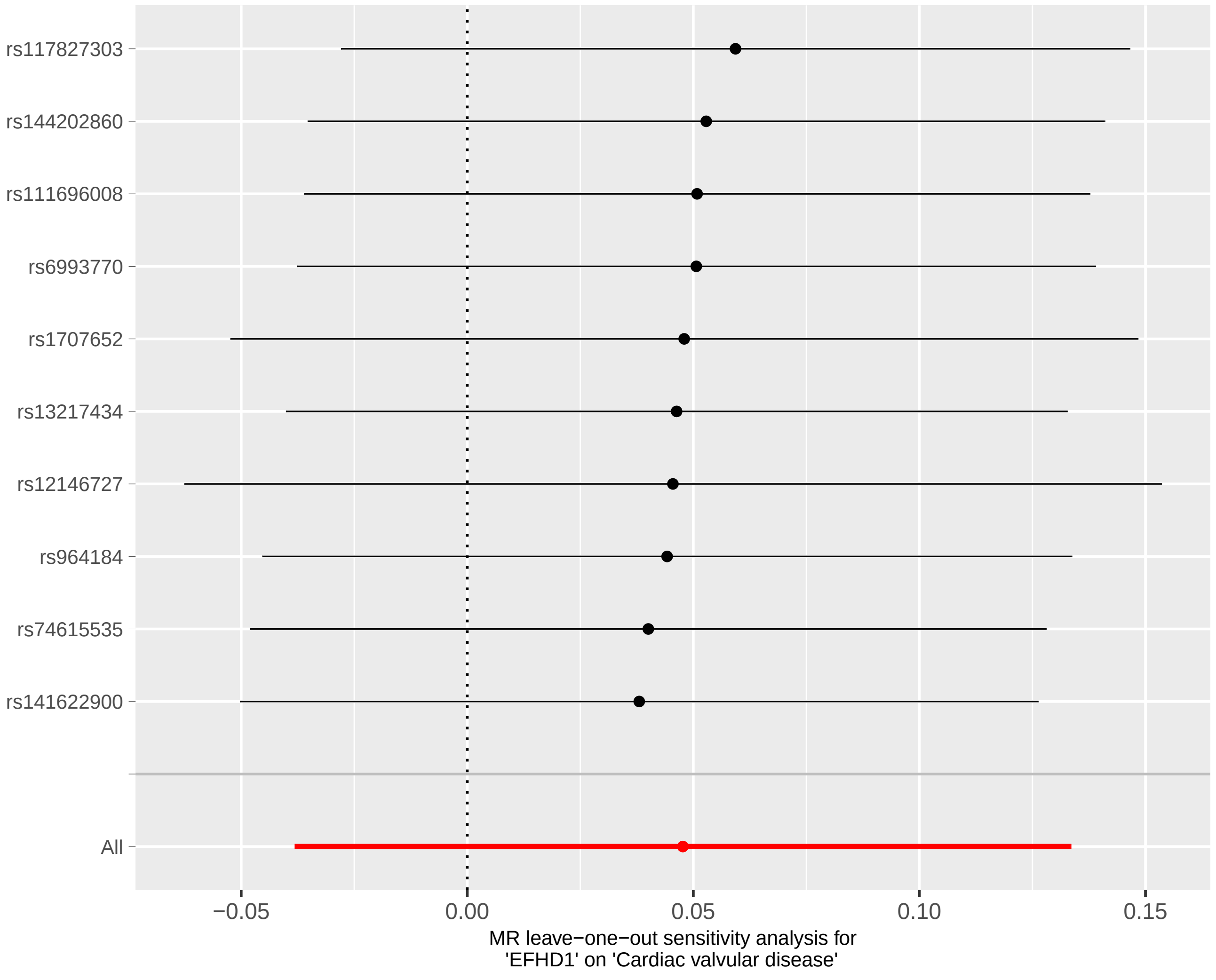
rs117827303
rs144202860
rs111696008
rs6993770
rs1707652
rs13217434
rs12146727
rs964184
rs74615535
rs141622900
All
−0.05
0.00
0.05
0.10
0.15
MR leave−one−out sensitivity analysis for
'EFHD1' on 'Cardiac valvular disease'

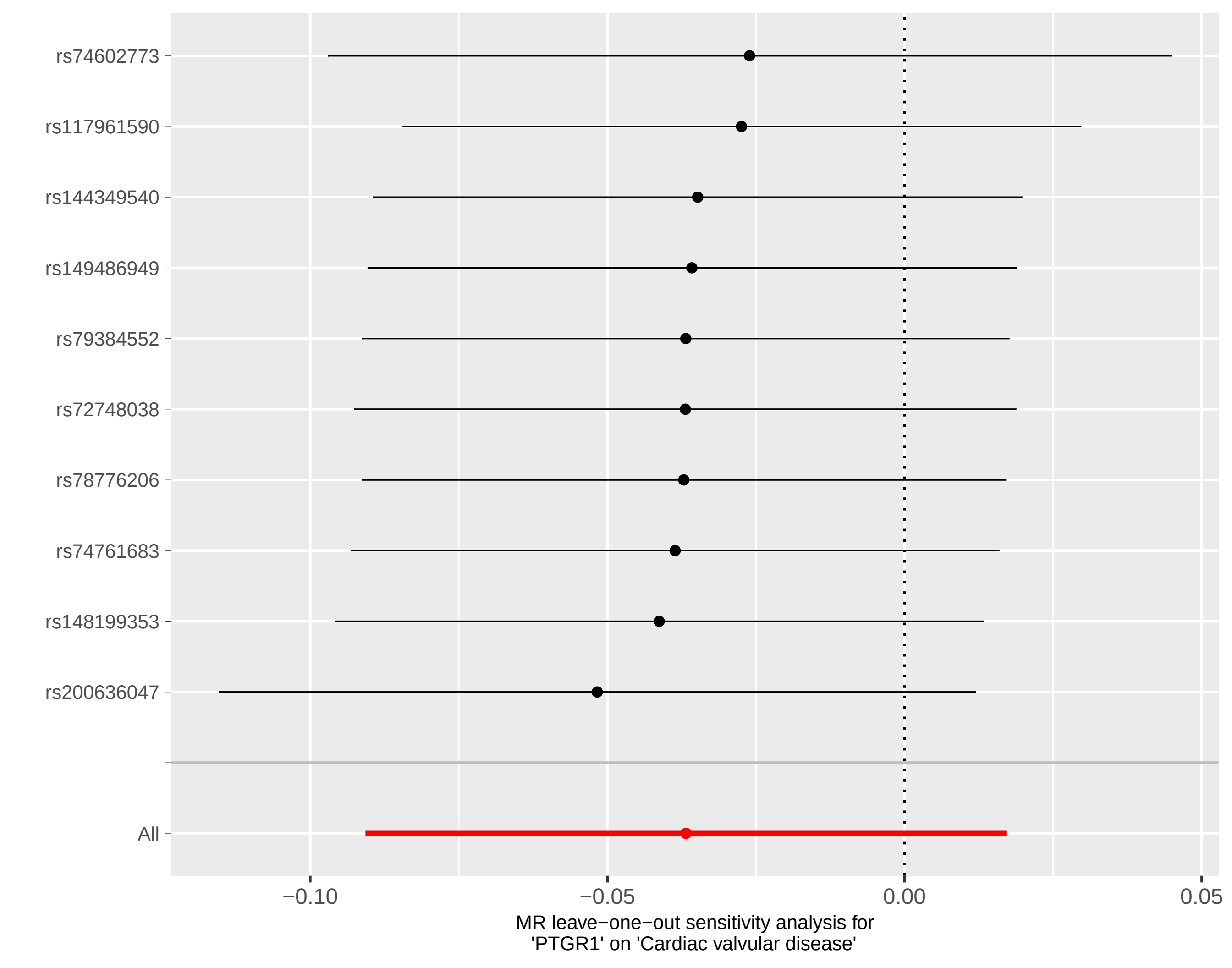

rs74602773
rs117961590
rs144349540
rs149486949
rs79384552
rs72748038
rs78776206
rs74761683
rs148199353
rs200636047
All
−0.10
−0.05
0.00
0.05
MR leave−one−out sensitivity analysis for
'PTGR1' on 'Cardiac valvular disease'

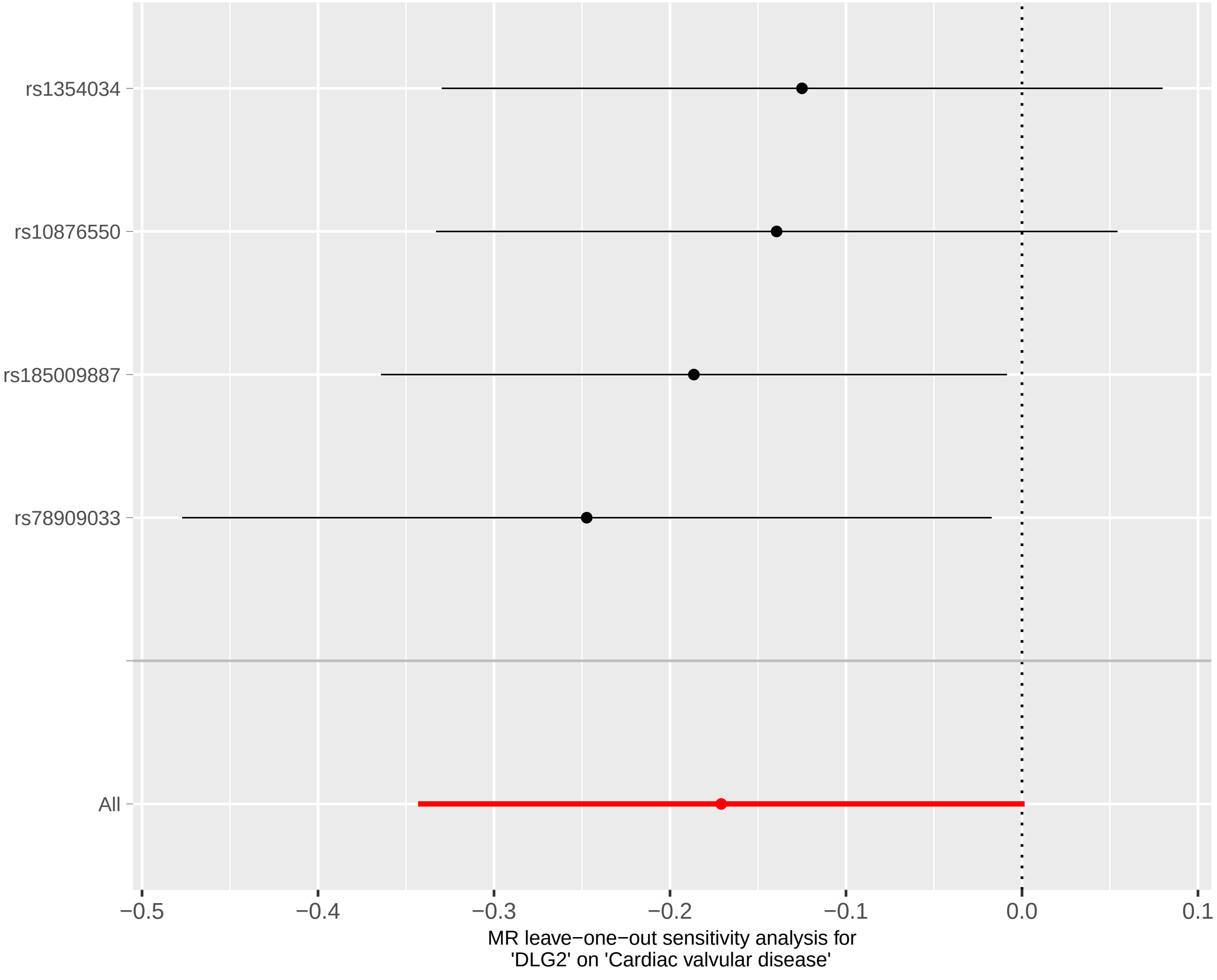

rs1354034
rs10876550
rs185009887
rs78909033
All
−0.5
−0.4
−0.3
−0.2
−0.1
0.0
0.1
MR leave−one−out sensitivity analysis for
'DLG2' on 'Cardiac valvular disease'

Insufficient number of SNPs

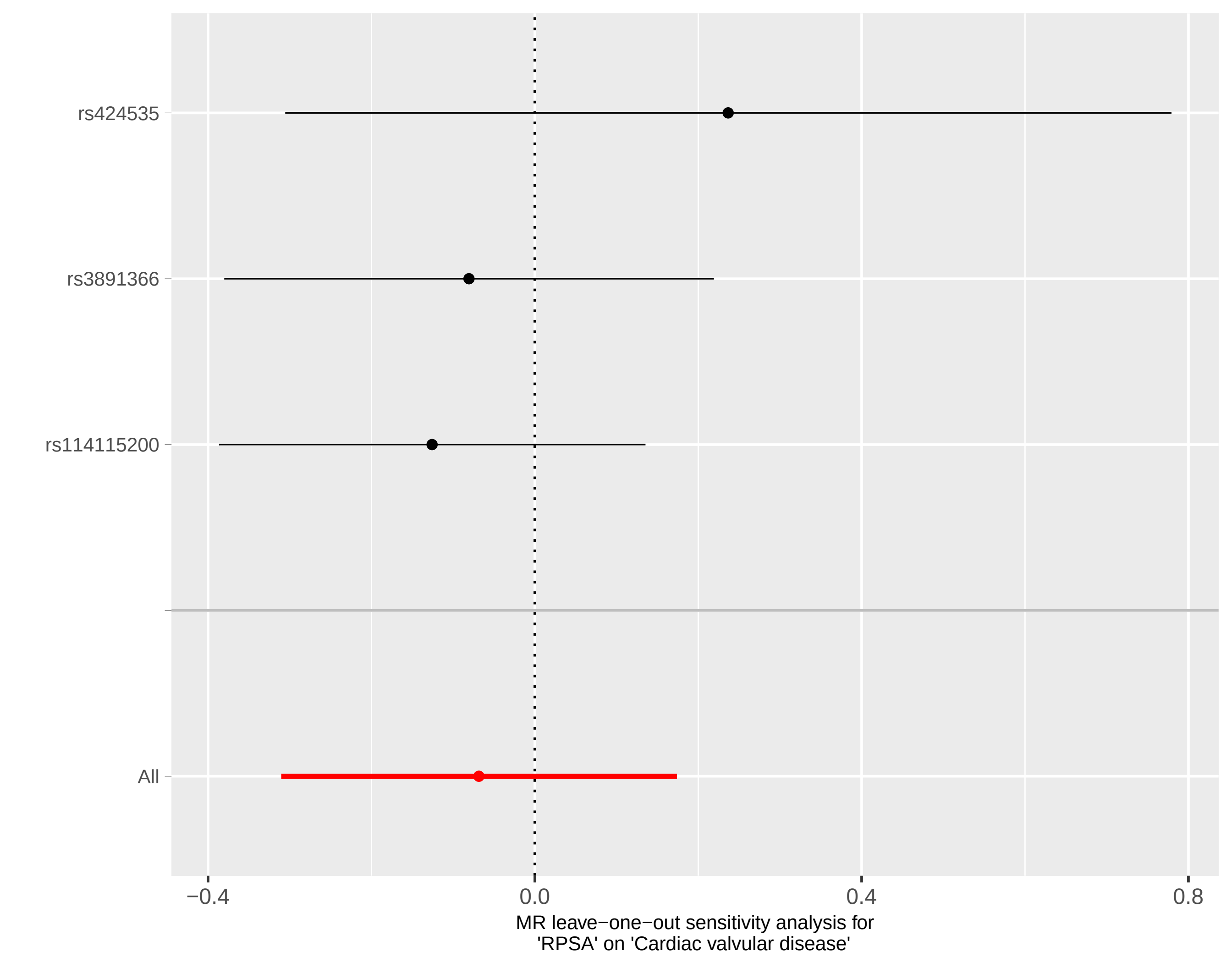

rs424535
rs3891366
rs114115200
All
−0.4
0.0
0.4
0.8
MR leave−one−out sensitivity analysis for
'RPSA' on 'Cardiac valvular disease'

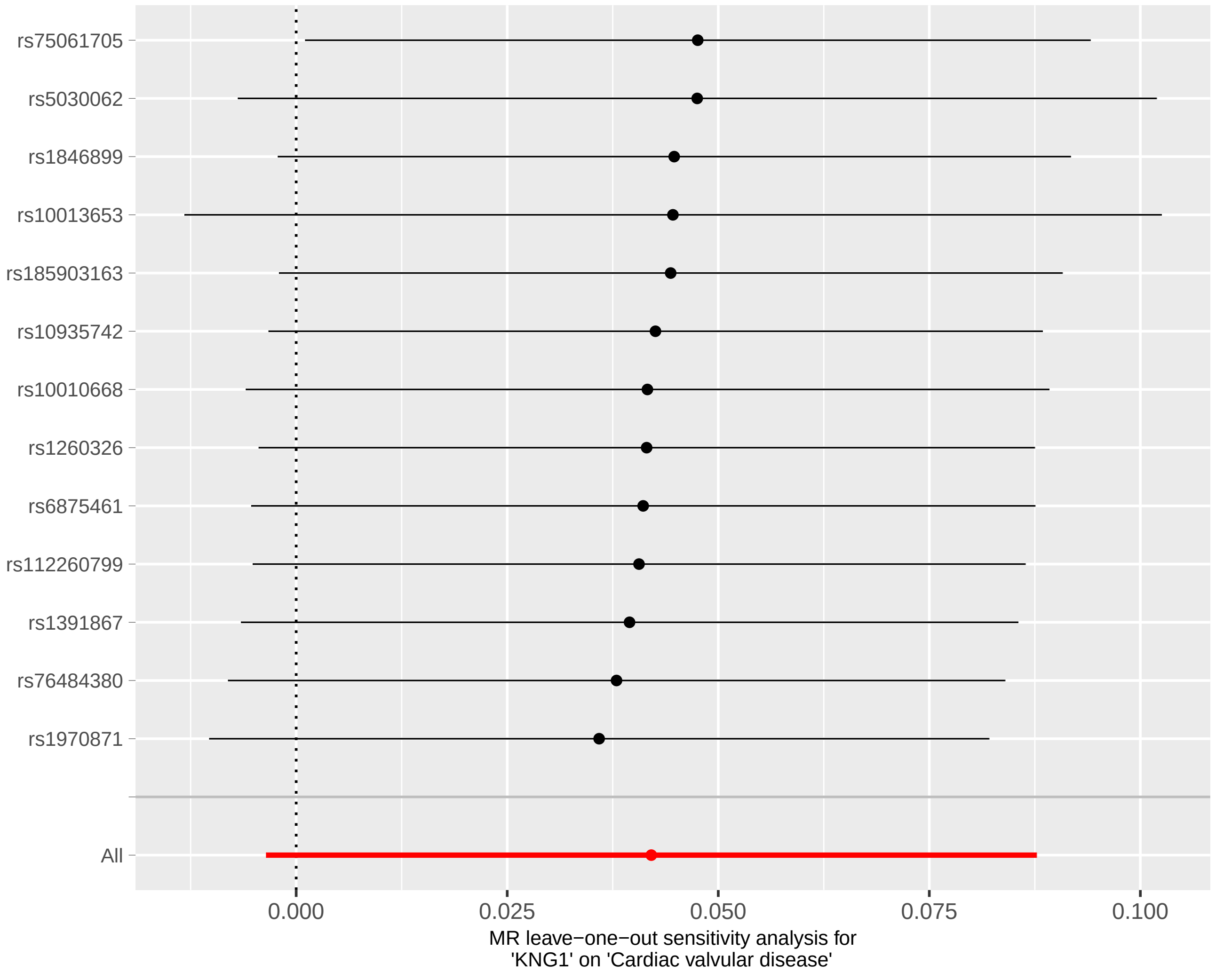
rs75061705
rs5030062
rs1846899
rs10013653
rs185903163
rs10935742
rs10010668
rs1260326
rs6875461
rs112260799
rs1391867
rs76484380
rs1970871
All
0.000
0.025
0.050
0.075
0.100
MR leave−one−out sensitivity analysis for
'KNG1' on 'Cardiac valvular disease'

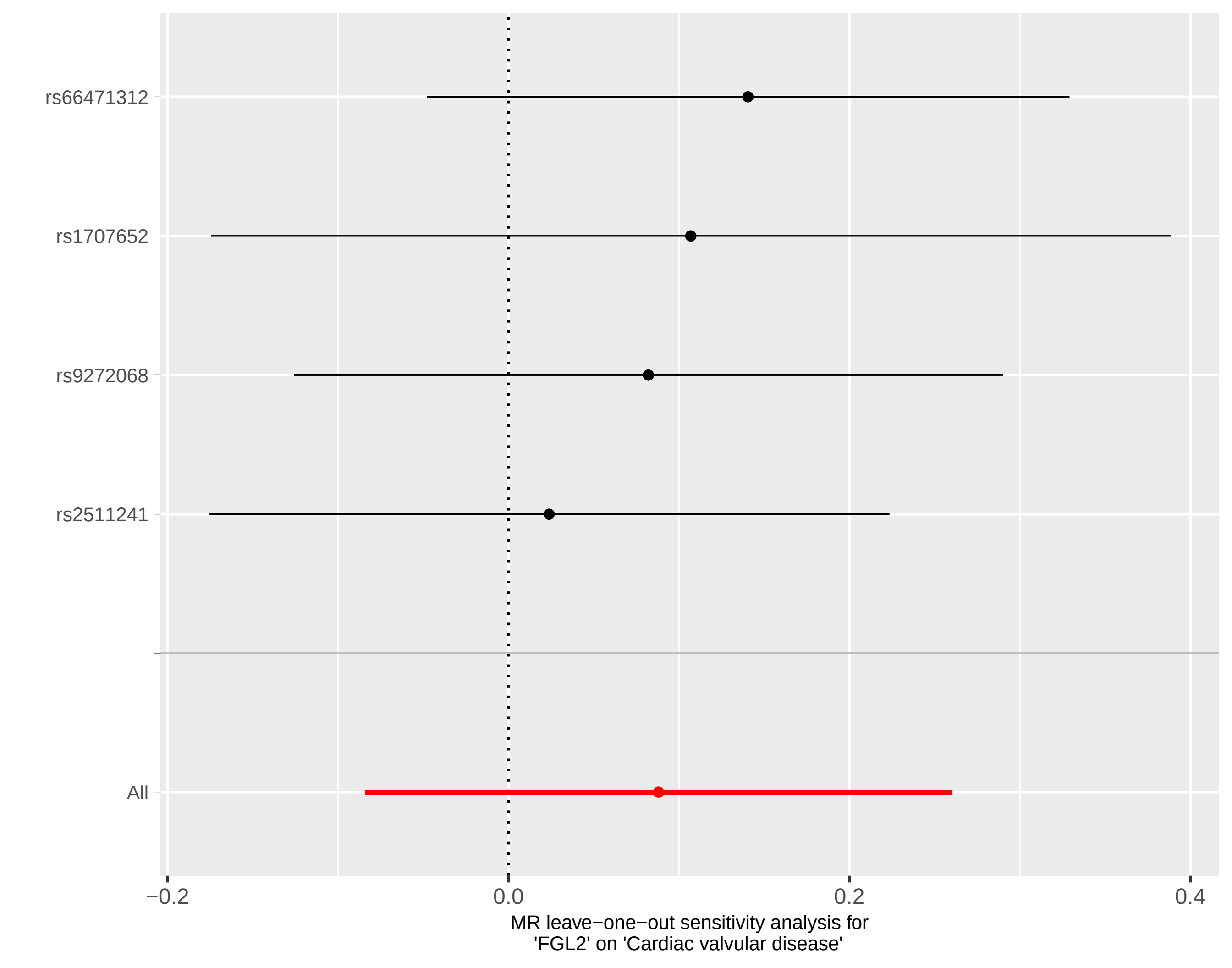
rs66471312
rs1707652
rs9272068
rs2511241
All
−0.2
0.0
0.2
0.4
MR leave−one−out sensitivity analysis for
'FGL2' on 'Cardiac valvular disease'

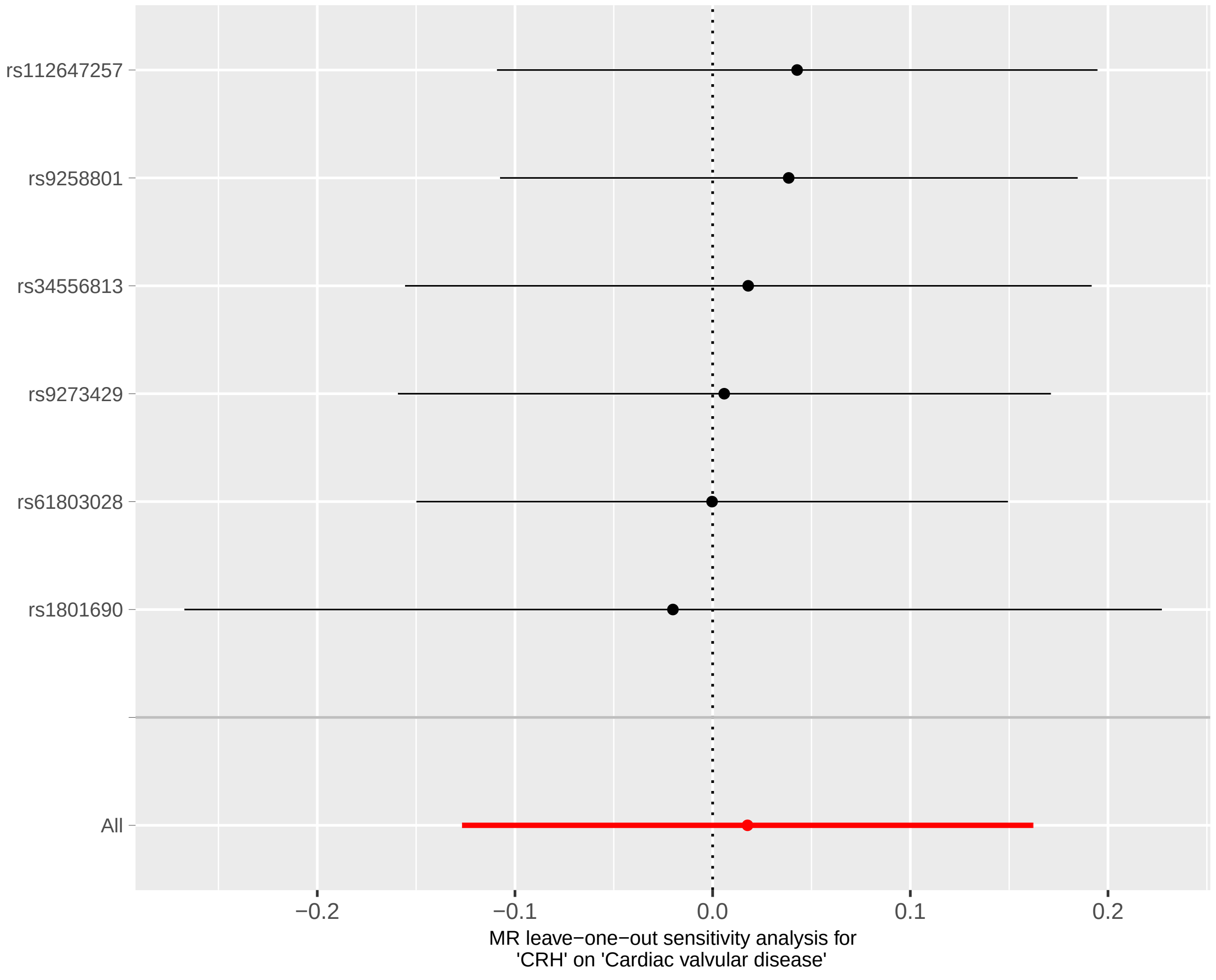

rs112647257
rs9258801
rs34556813
rs9273429
rs61803028
rs1801690
All
−0.2
−0.1
0.0
0.1
0.2
MR leave−one−out sensitivity analysis for
'CRH' on 'Cardiac valvular disease'

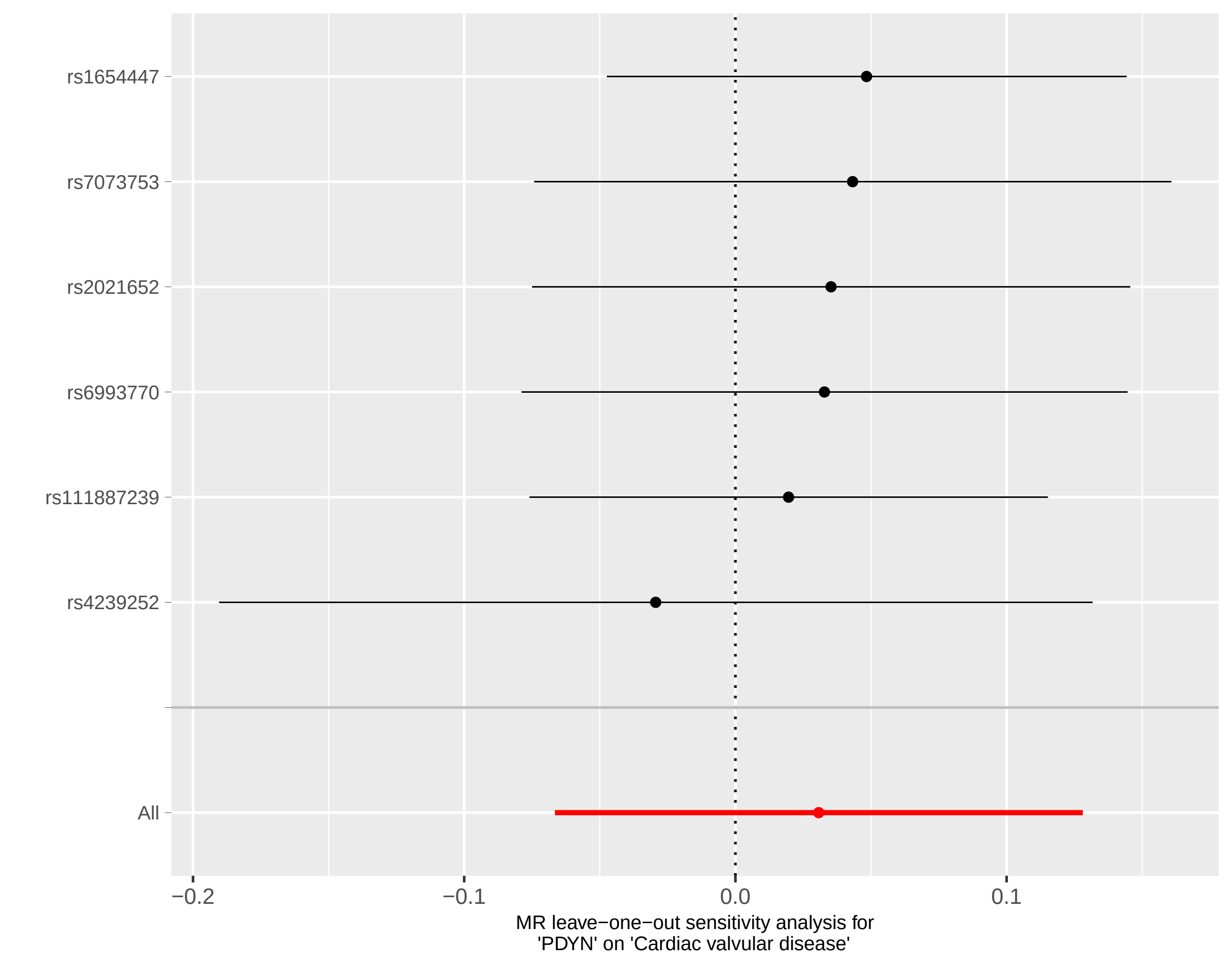
rs1654447
rs7073753
rs2021652
rs6993770
rs111887239
rs4239252
All
−0.2
−0.1
0.0
0.1
MR leave−one−out sensitivity analysis for
'PDYN' on 'Cardiac valvular disease'

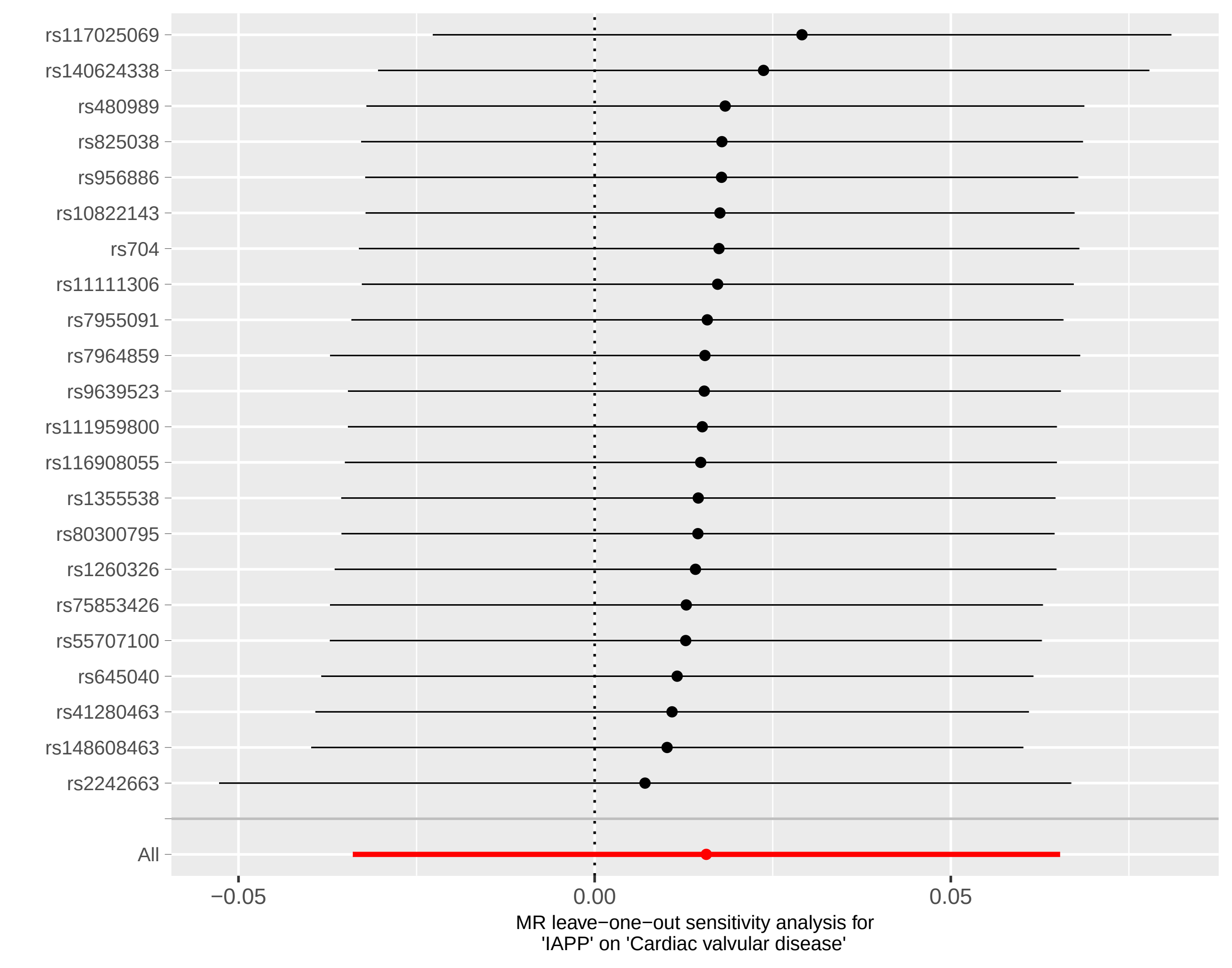
rs117025069
rs140624338
rs480989
rs825038
rs956886
rs10822143
rs704
rs11111306
rs7955091
rs7964859
rs9639523
rs111959800
rs116908055
rs1355538
rs80300795
rs1260326
rs75853426
rs55707100
rs645040
rs41280463
rs148608463
rs2242663
All
−0.05
0.00
0.05
MR leave−one−out sensitivity analysis for
'IAPP' on 'Cardiac valvular disease'

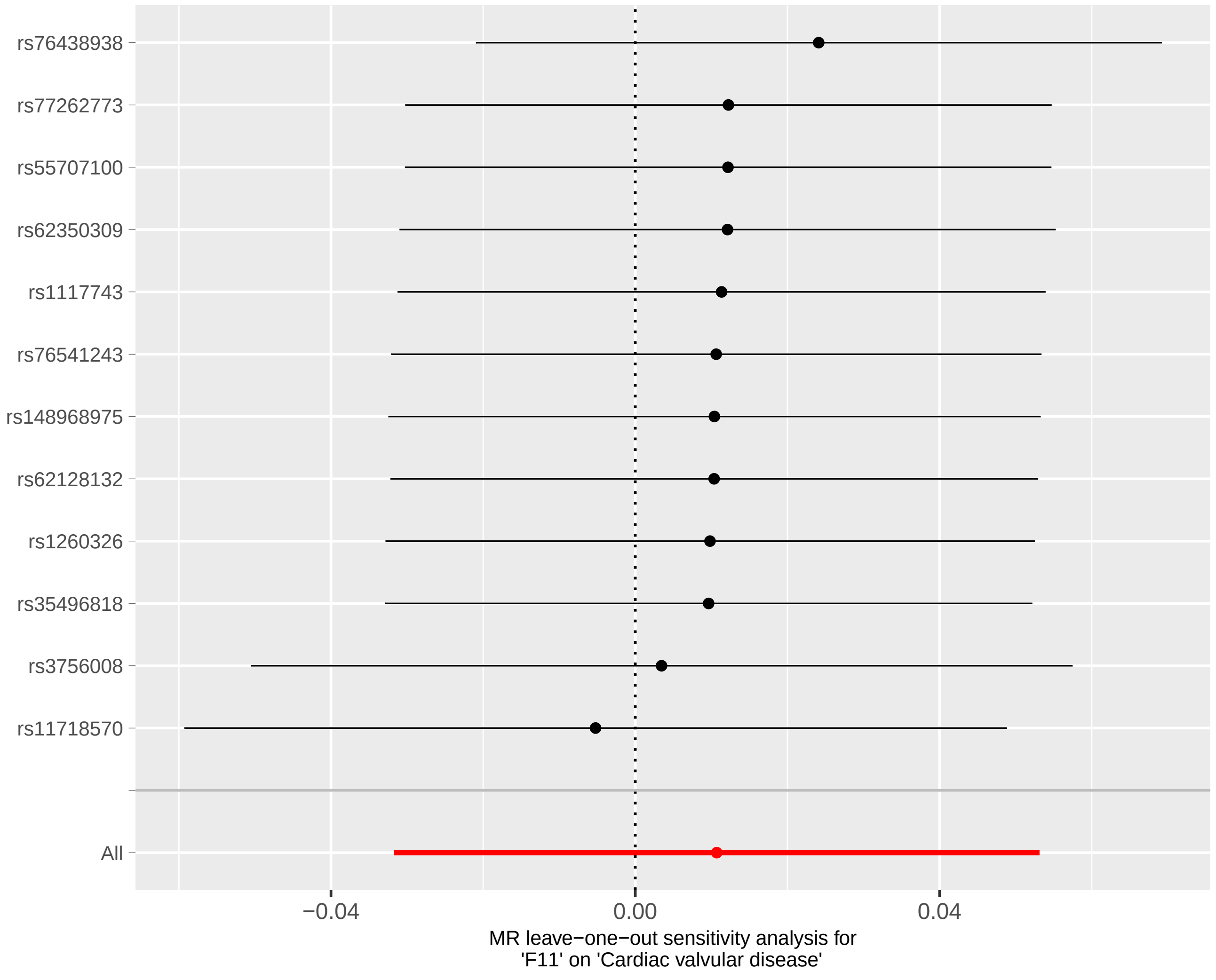
rs76438938
rs77262773
rs55707100
rs62350309
rs1117743
rs76541243
rs148968975
rs62128132
rs1260326
rs35496818
rs3756008
rs11718570
All
−0.04
0.00
0.04
MR leave−one−out sensitivity analysis for
'F11' on 'Cardiac valvular disease'

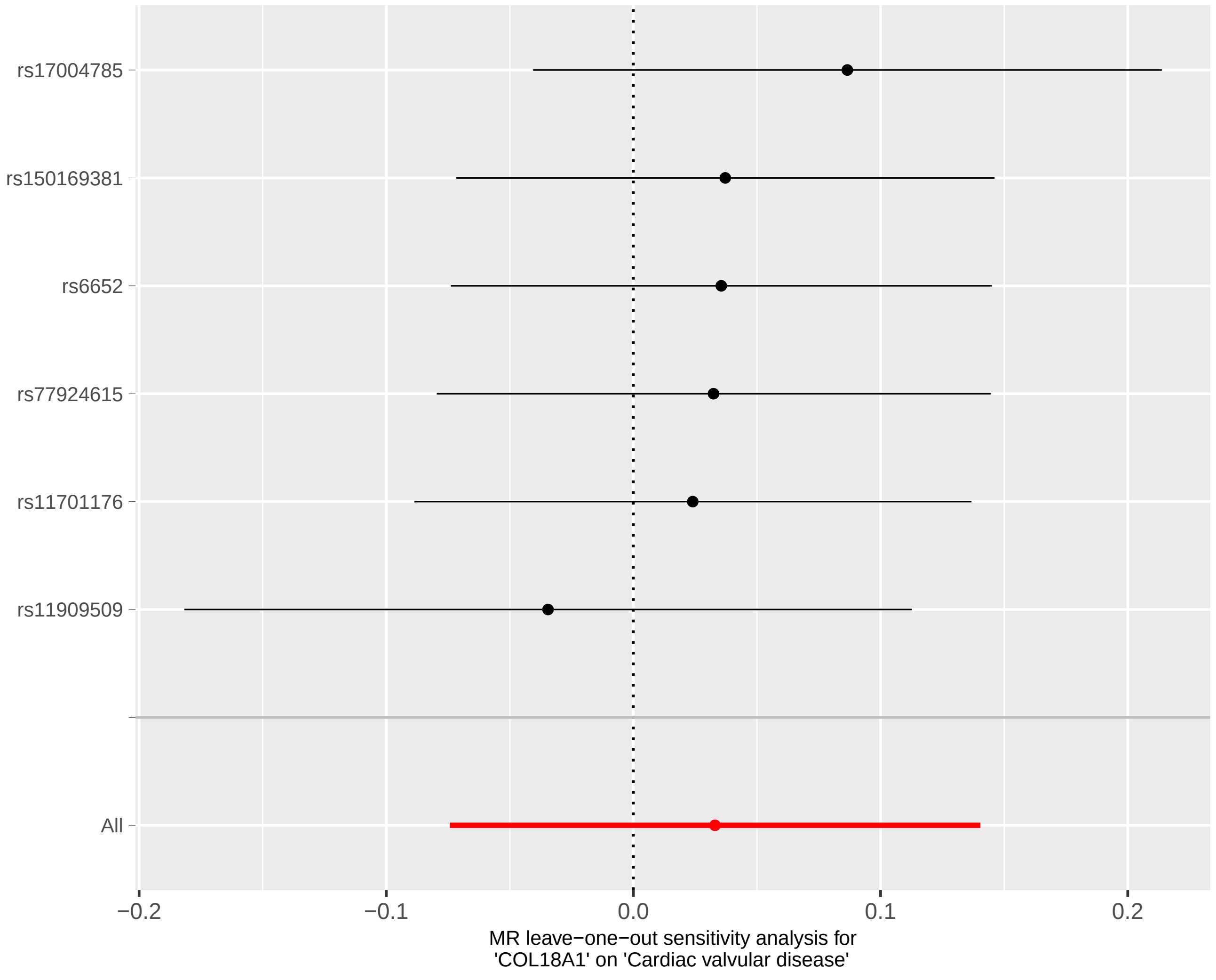

rs17004785
rs150169381
rs6652
rs77924615
rs11701176
rs11909509
All
−0.2
−0.1
0.0
0.1
0.2
MR leave−one−out sensitivity analysis for
'COL18A1' on 'Cardiac valvular disease'

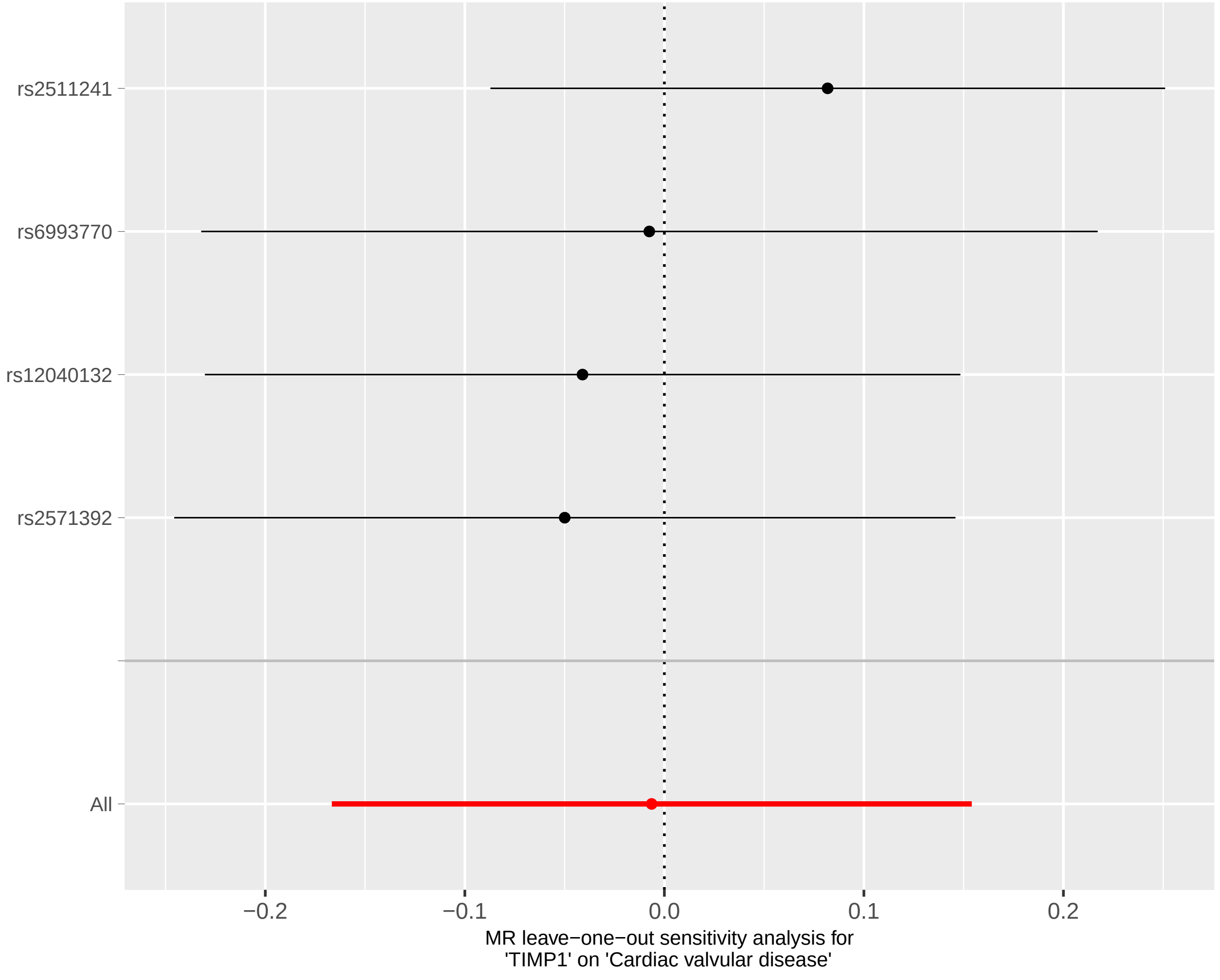
rs2511241
rs6993770
rs12040132
rs2571392
All
−0.2
−0.1
0.0
0.1
0.2
MR leave−one−out sensitivity analysis for
'TIMP1' on 'Cardiac valvular disease'

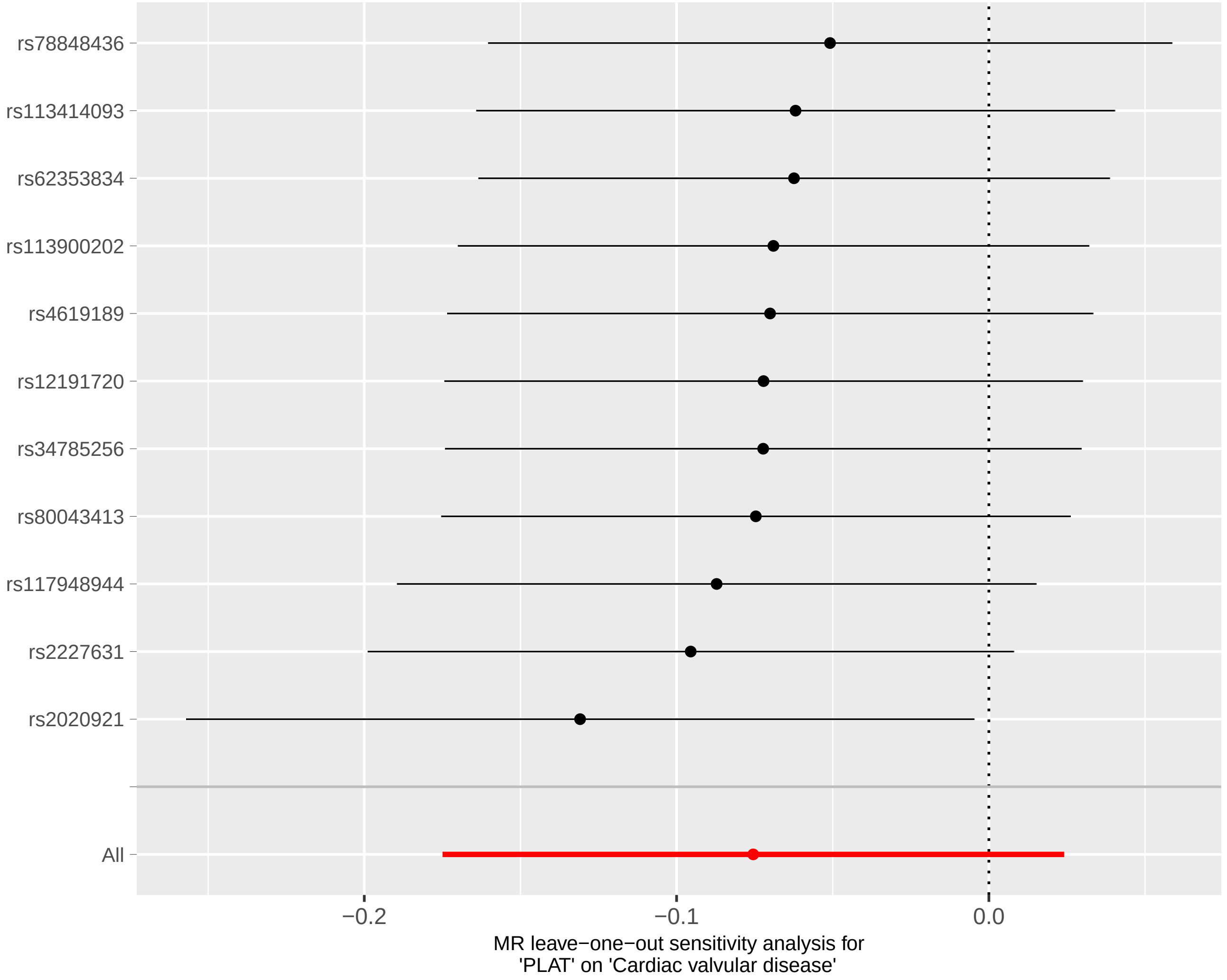
rs78848436
rs113414093
rs62353834
rs113900202
rs4619189
rs12191720
rs34785256
rs80043413
rs117948944
rs2227631
rs2020921
All
−0.2
−0.1
0.0
MR leave−one−out sensitivity analysis for
'PLAT' on 'Cardiac valvular disease'

Insufficient number of SNPs

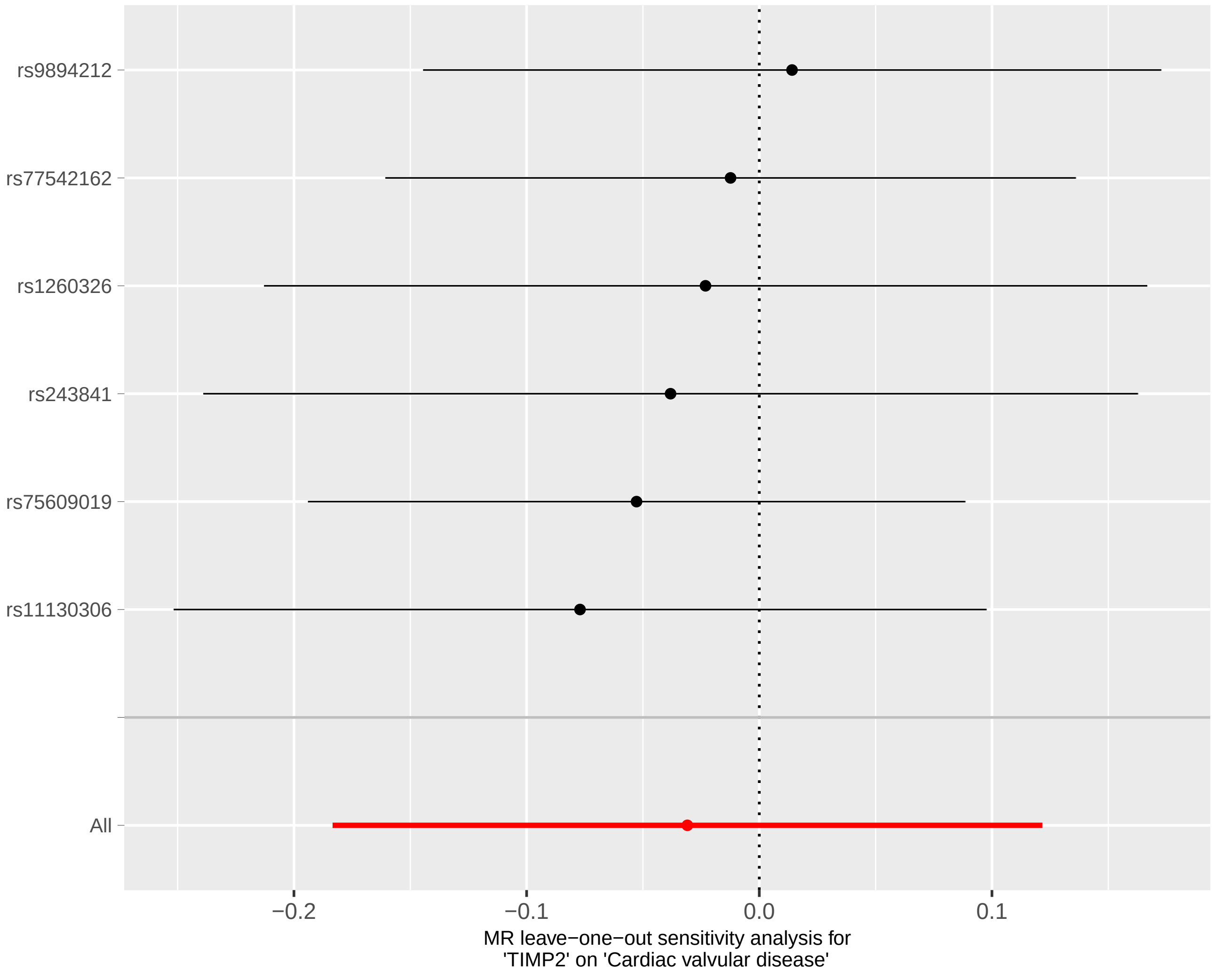
rs9894212
rs77542162
rs1260326
rs243841
rs75609019
rs11130306
All
−0.2
−0.1
0.0
0.1
MR leave−one−out sensitivity analysis for
'TIMP2' on 'Cardiac valvular disease'

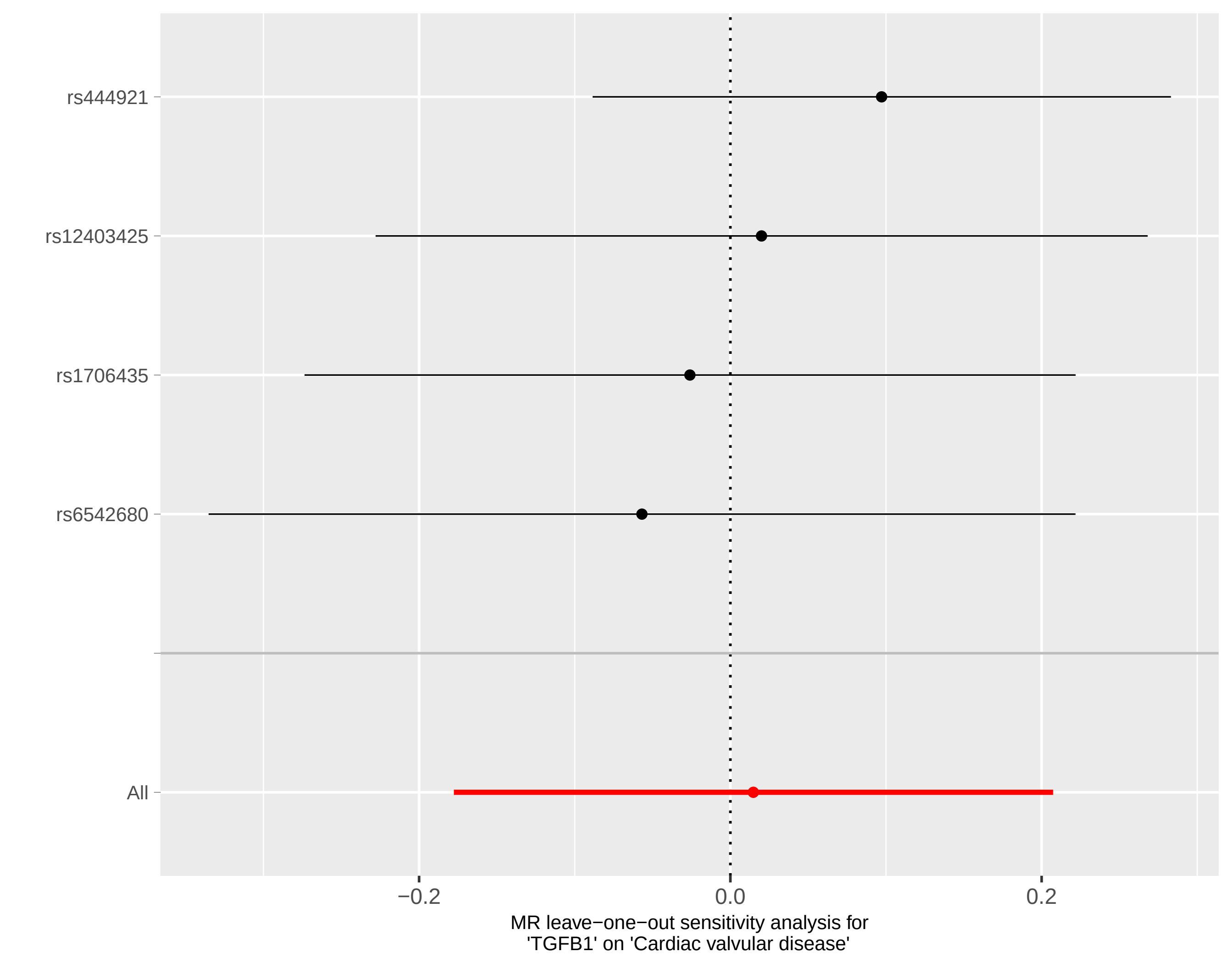
rs444921
rs12403425
rs1706435
rs6542680
All
−0.2
0.0
0.2
MR leave−one−out sensitivity analysis for
'TGFB1' on 'Cardiac valvular disease'

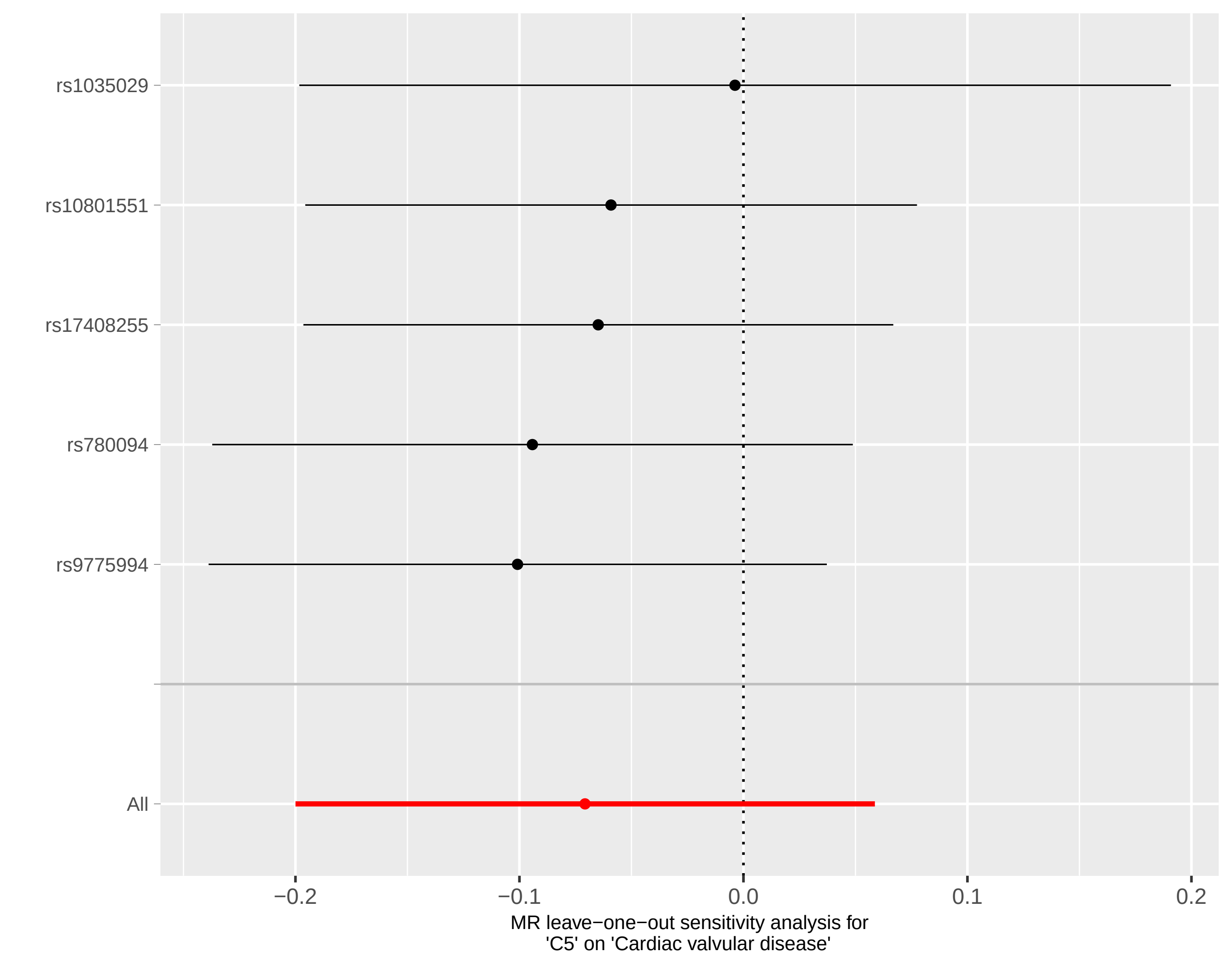
rs1035029
rs10801551
rs17408255
rs780094
rs9775994
All
−0.2
−0.1
0.0
0.1
0.2
MR leave−one−out sensitivity analysis for
'C5' on 'Cardiac valvular disease'

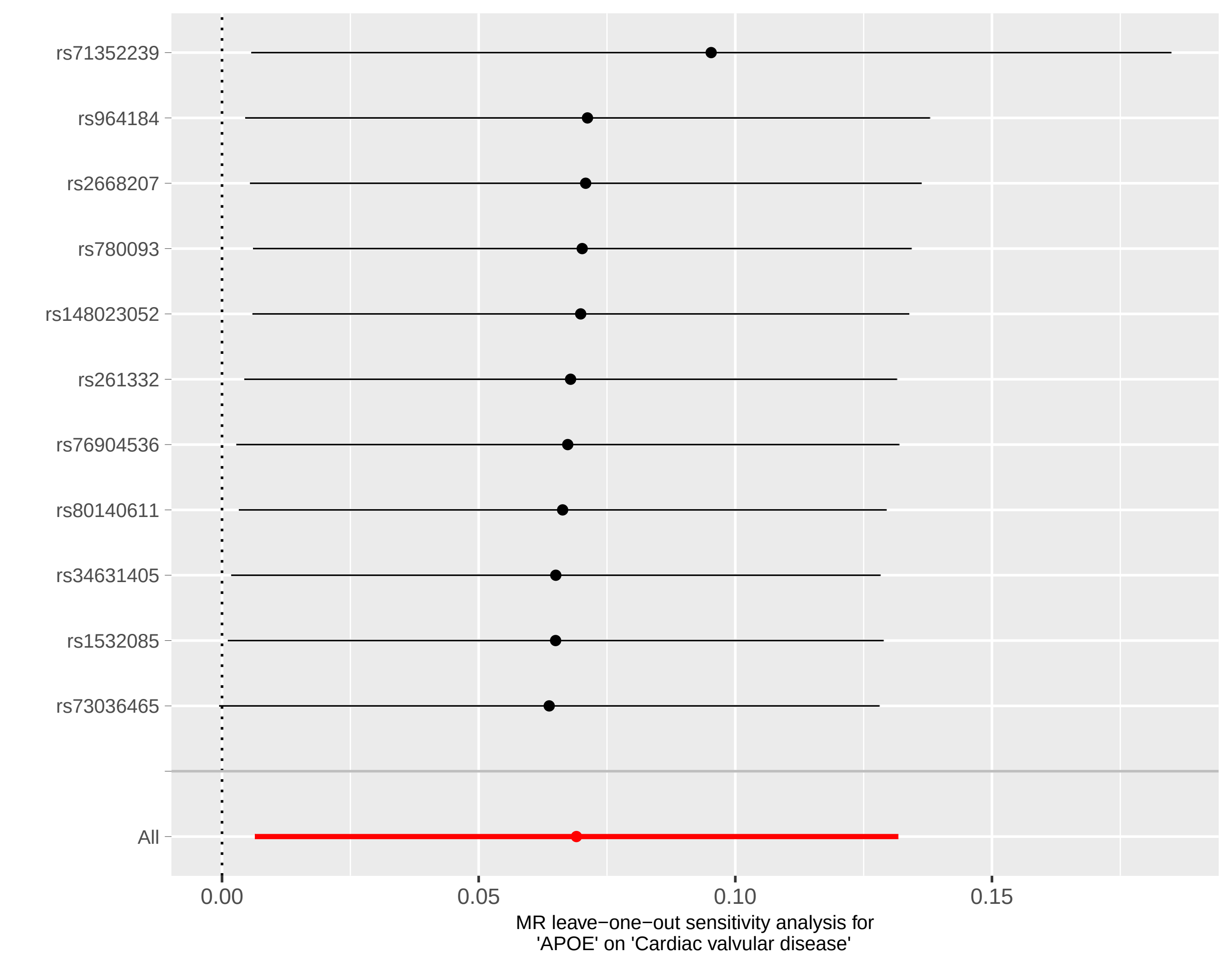

rs71352239
rs964184
rs2668207
rs780093
rs148023052
rs261332
rs76904536
rs80140611
rs34631405
rs1532085
rs73036465
All
0.00
0.05
0.10
0.15
MR leave−one−out sensitivity analysis for
'APOE' on 'Cardiac valvular disease'

Insufficient number of SNPs

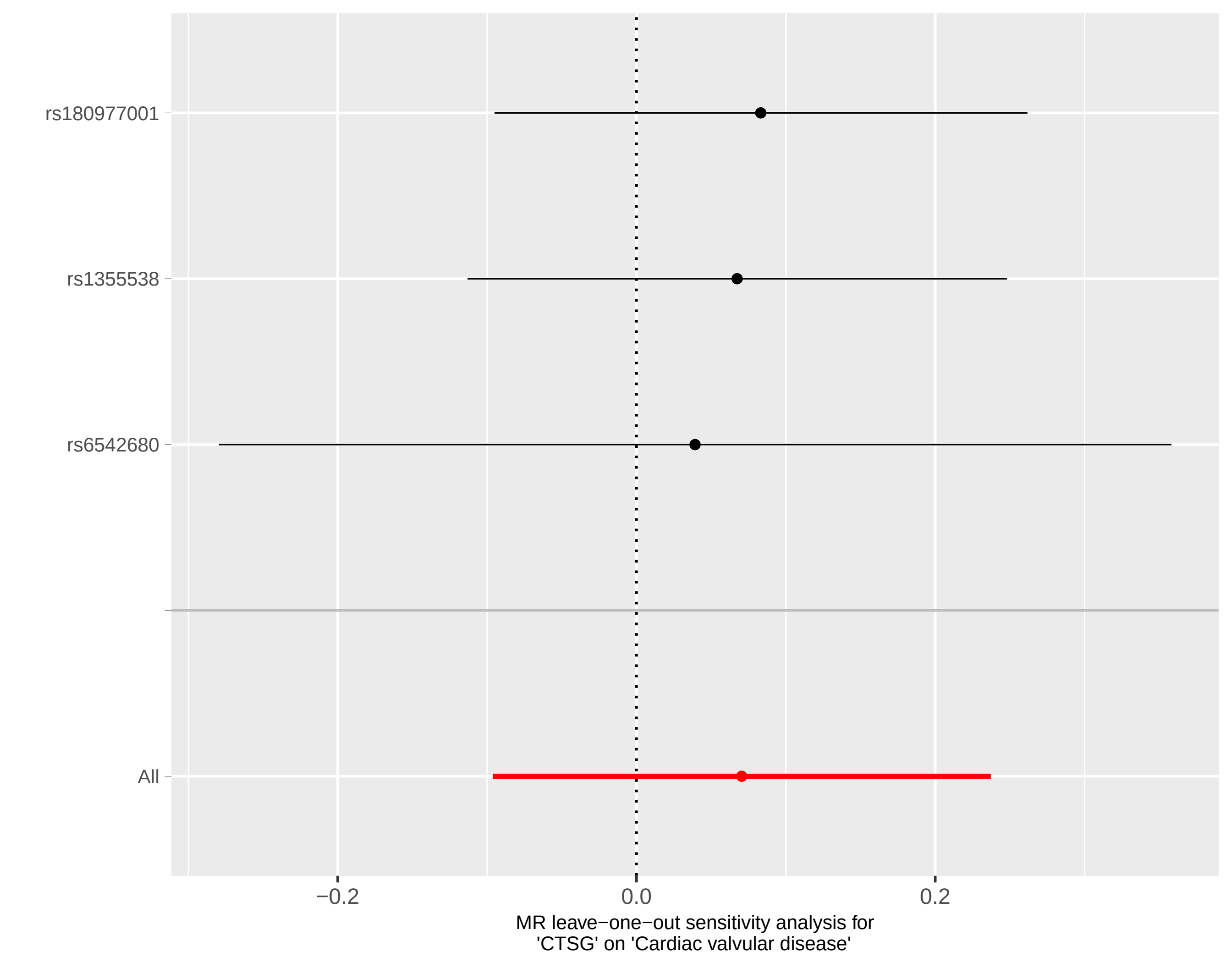

rs180977001
rs1355538
rs6542680
All
−0.2
0.0
0.2
MR leave−one−out sensitivity analysis for
'CTSG' on 'Cardiac valvular disease'

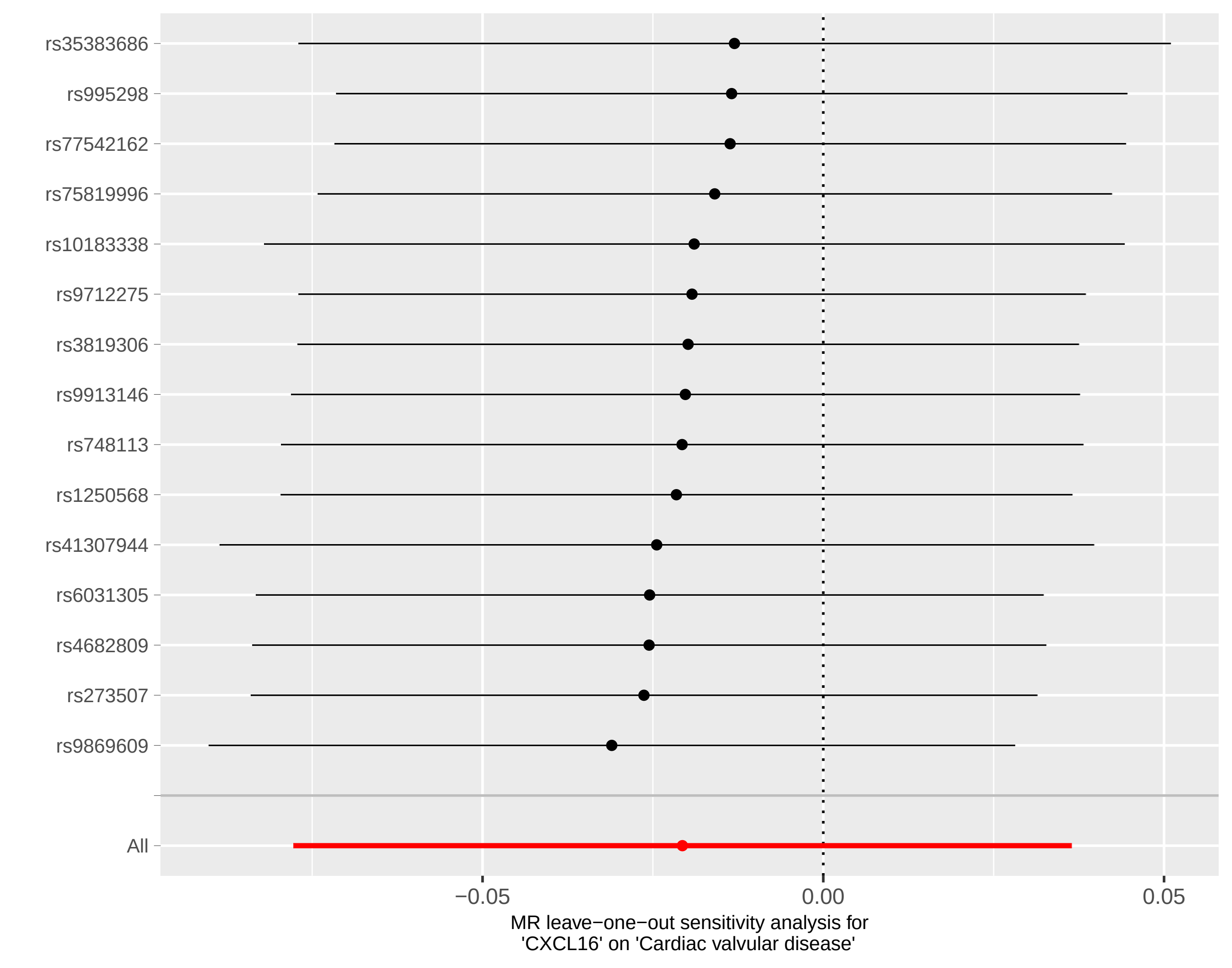
rs35383686
rs995298
rs77542162
rs75819996
rs10183338
rs9712275
rs3819306
rs9913146
rs748113
rs1250568
rs41307944
rs6031305
rs4682809
rs273507
rs9869609
All
−0.05
0.00
0.05
MR leave−one−out sensitivity analysis for
'CXCL16' on 'Cardiac valvular disease'

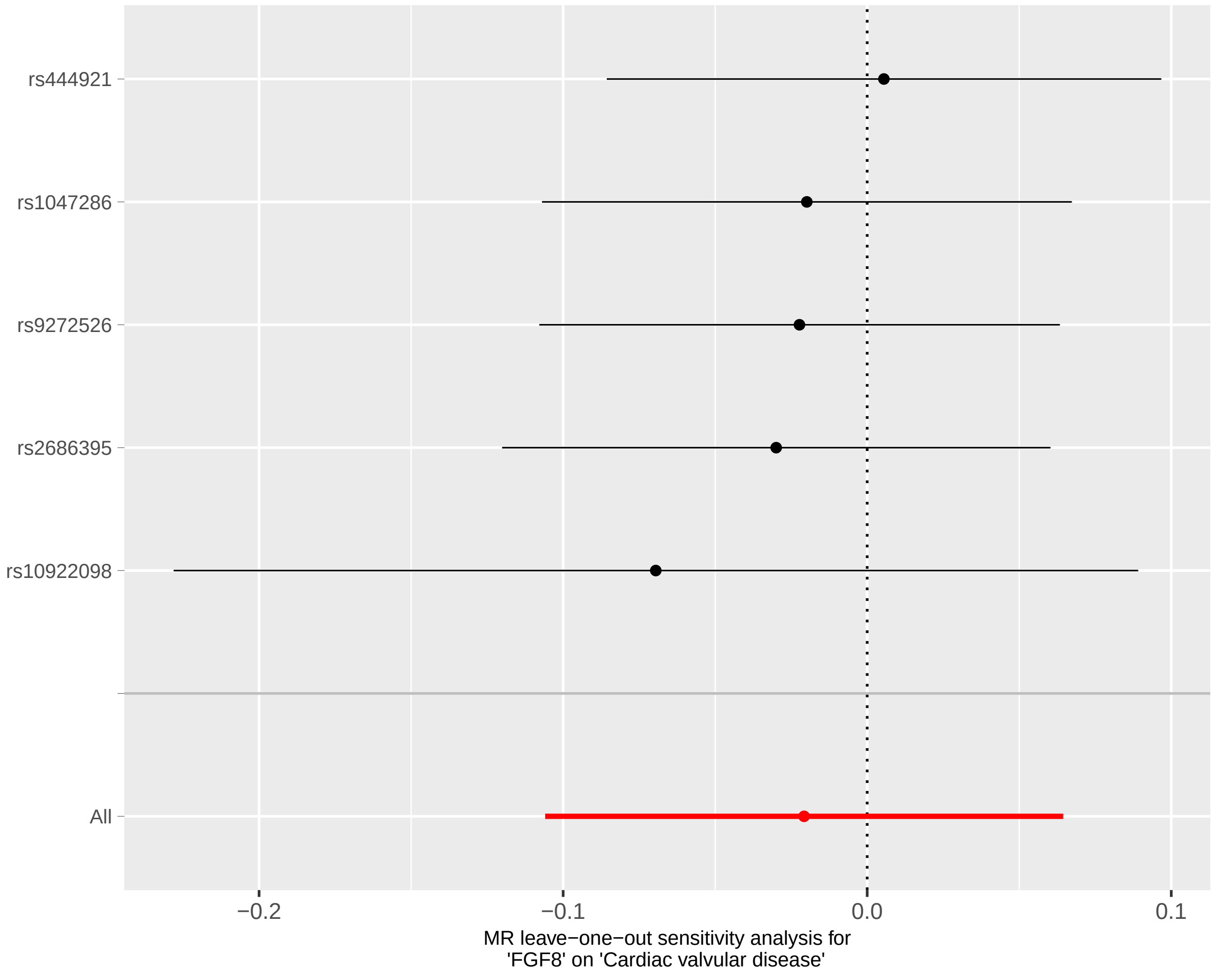
rs444921
rs1047286
rs9272526
rs2686395
rs10922098
All
−0.2
−0.1
0.0
0.1
MR leave−one−out sensitivity analysis for
'FGF8' on 'Cardiac valvular disease'

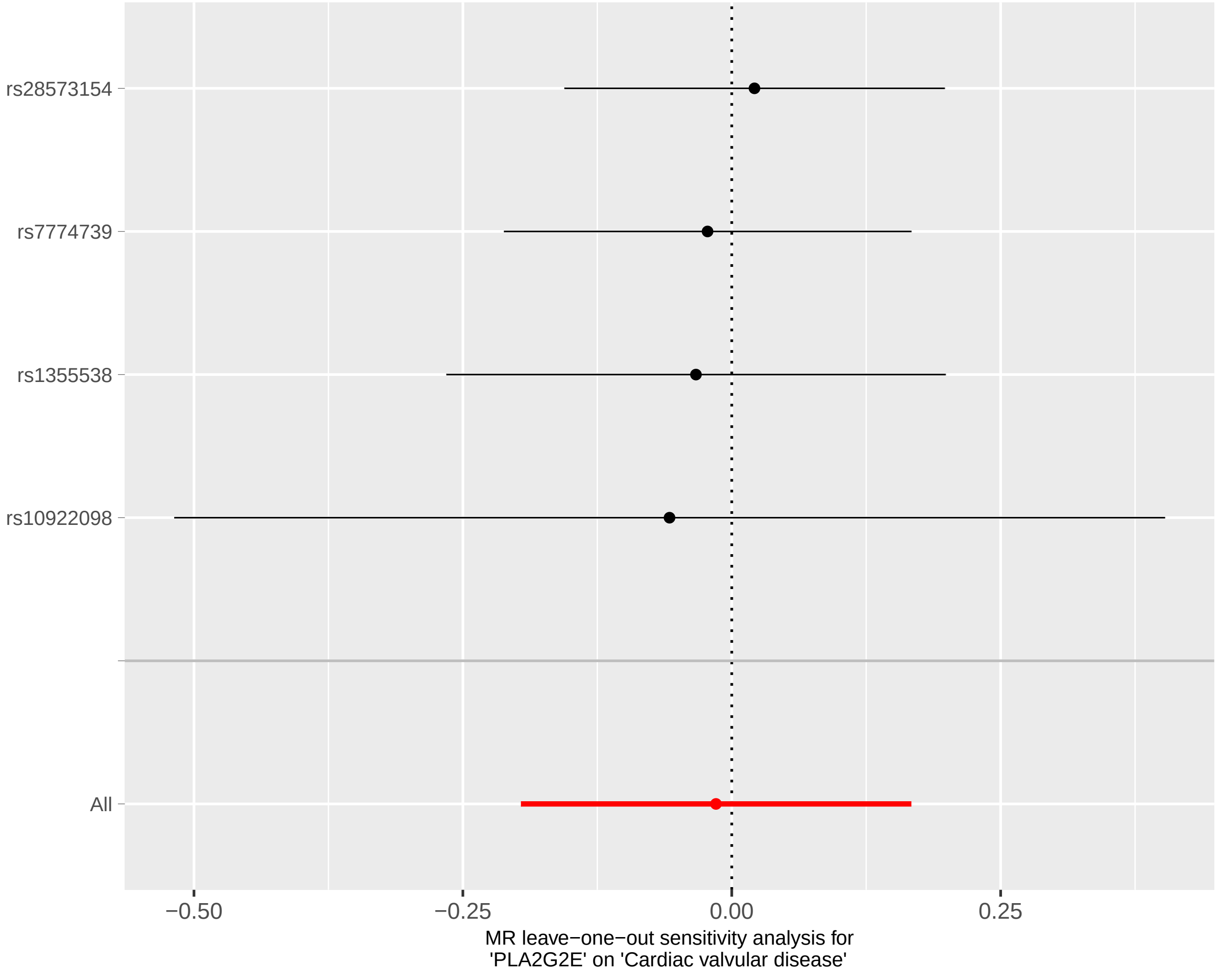

rs28573154
rs7774739
rs1355538
rs10922098
All
−0.50
−0.25
0.00
0.25
MR leave−one−out sensitivity analysis for
'PLA2G2E' on 'Cardiac valvular disease'

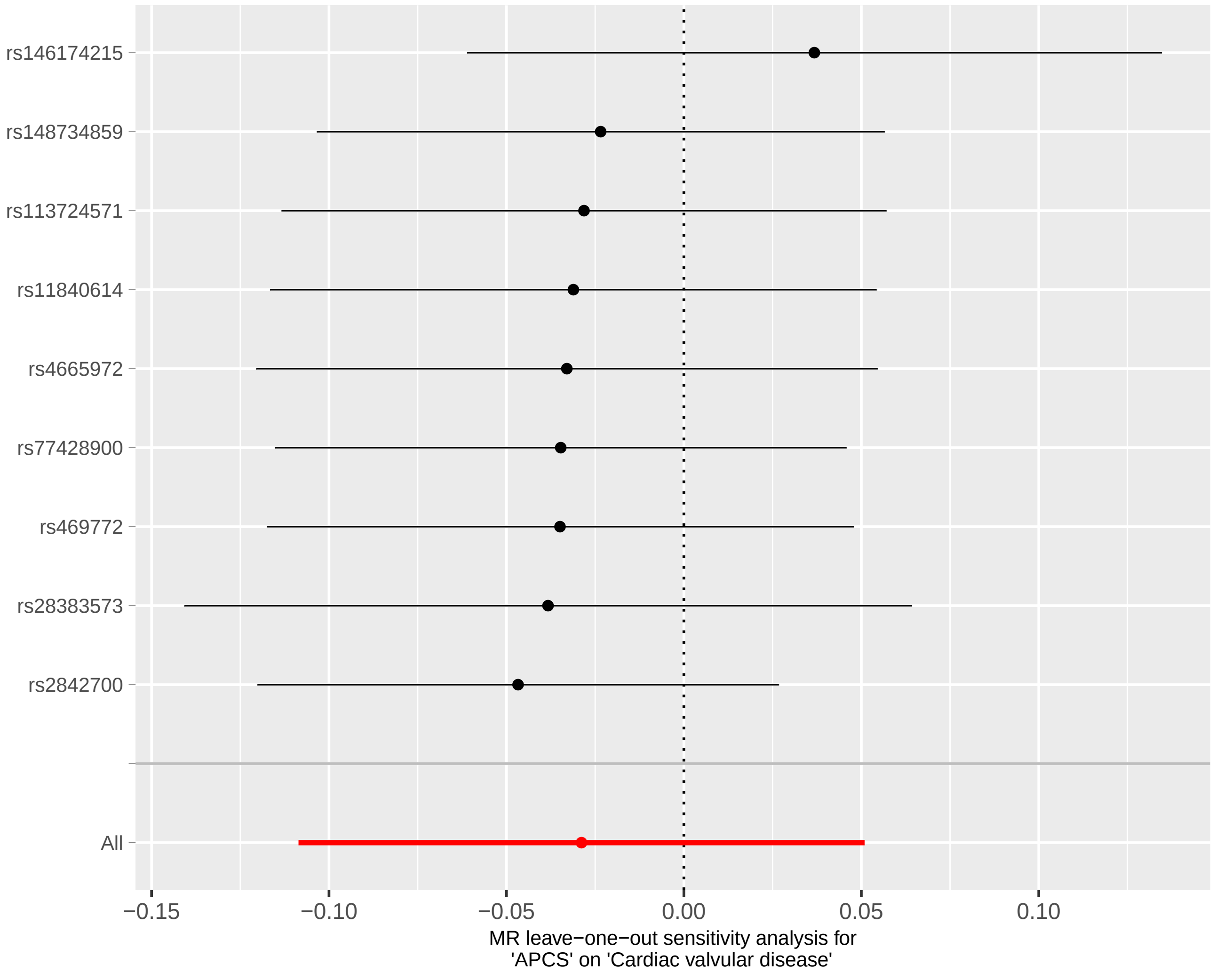
rs146174215
rs148734859
rs113724571
rs11840614
rs4665972
rs77428900
rs469772
rs28383573
rs2842700
All
−0.15
−0.10
−0.05
0.00
0.05
0.10
MR leave−one−out sensitivity analysis for
'APCS' on 'Cardiac valvular disease'

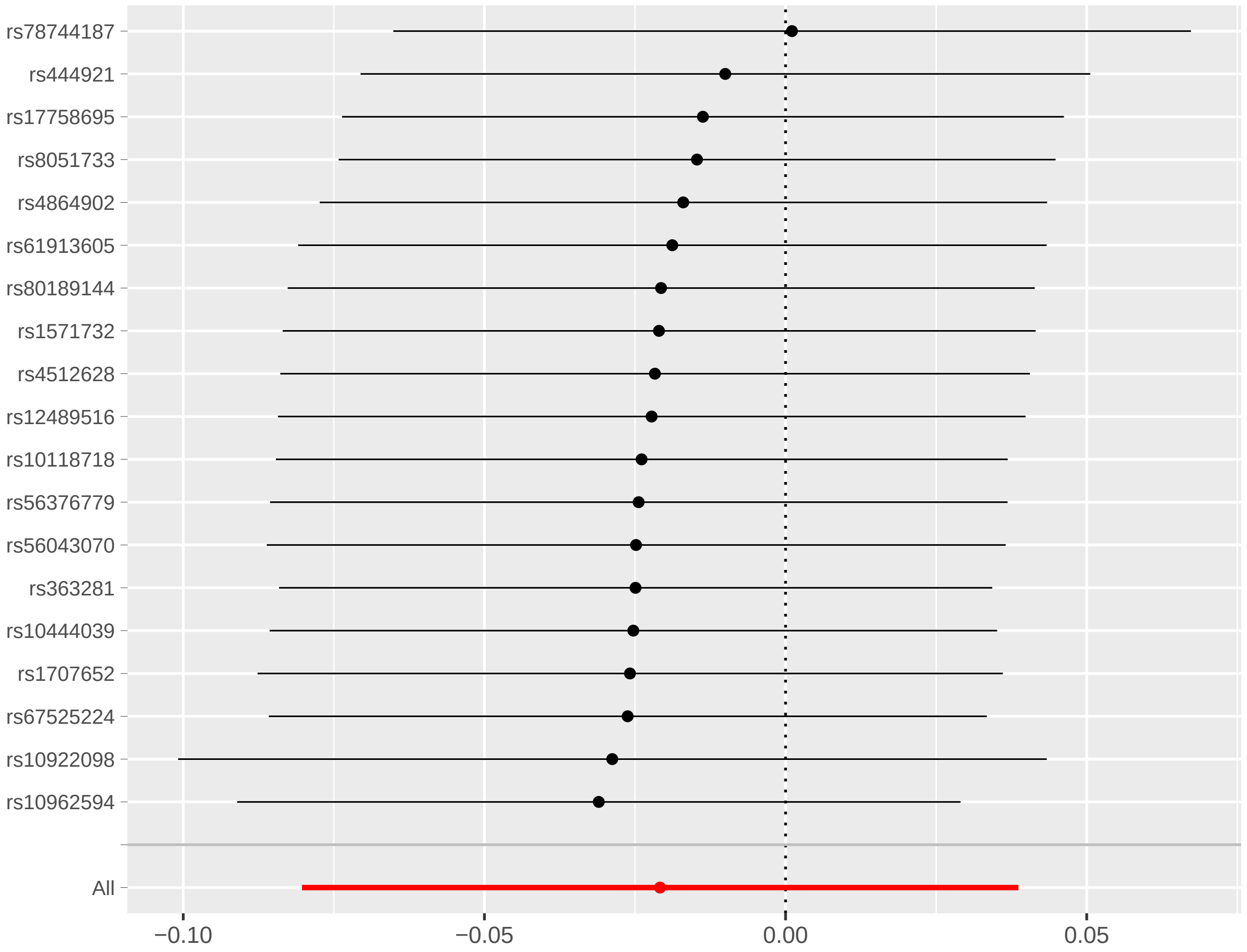

MR leave−one−out sensitivity analysis for
'KIT' on 'Cardiac valvular disease'

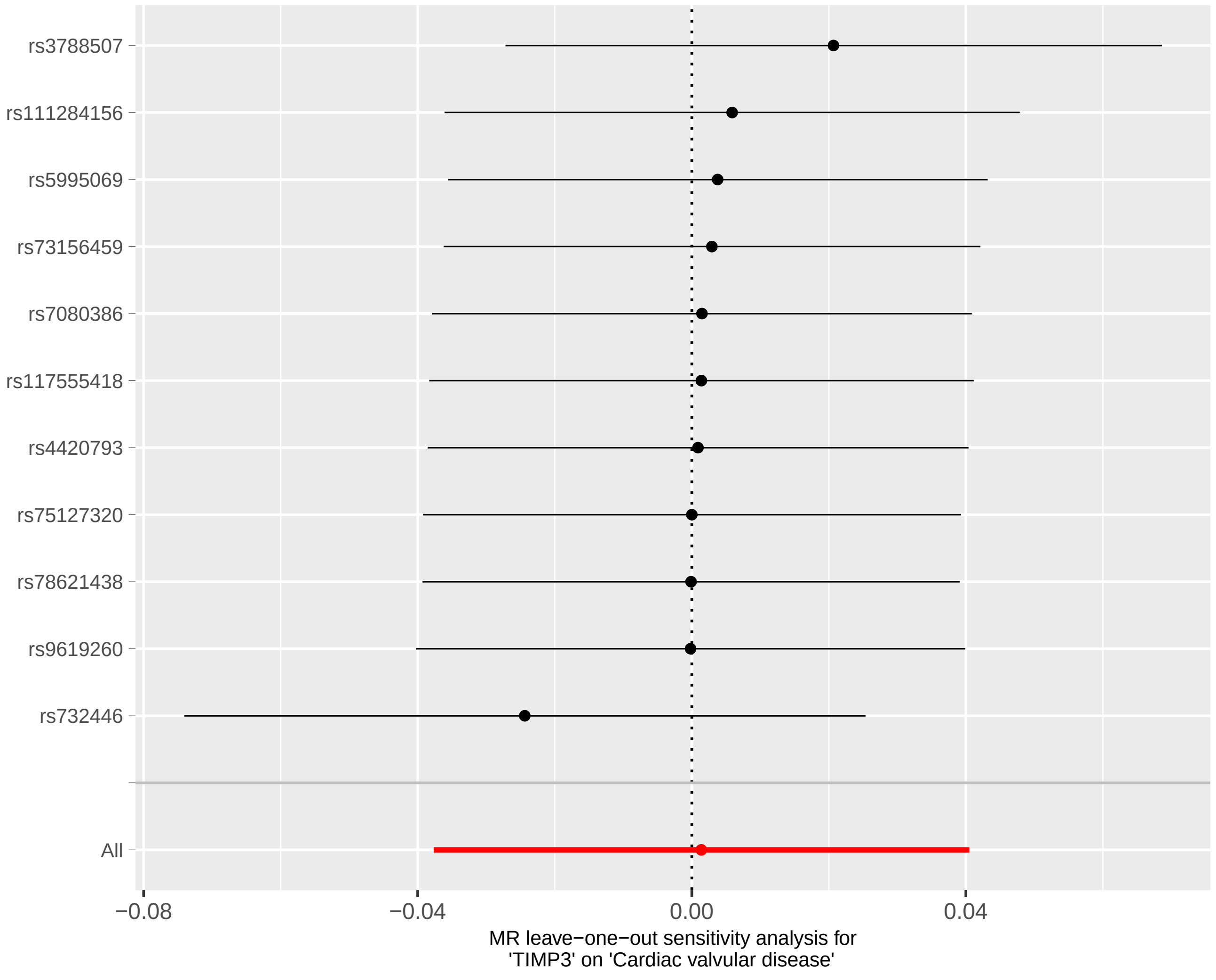

rs3788507
rs111284156
rs5995069
rs73156459
rs7080386
rs117555418
rs4420793
rs75127320
rs78621438
rs9619260
rs732446
All
−0.08
−0.04
0.00
0.04
MR leave−one−out sensitivity analysis for
'TIMP3' on 'Cardiac valvular disease'

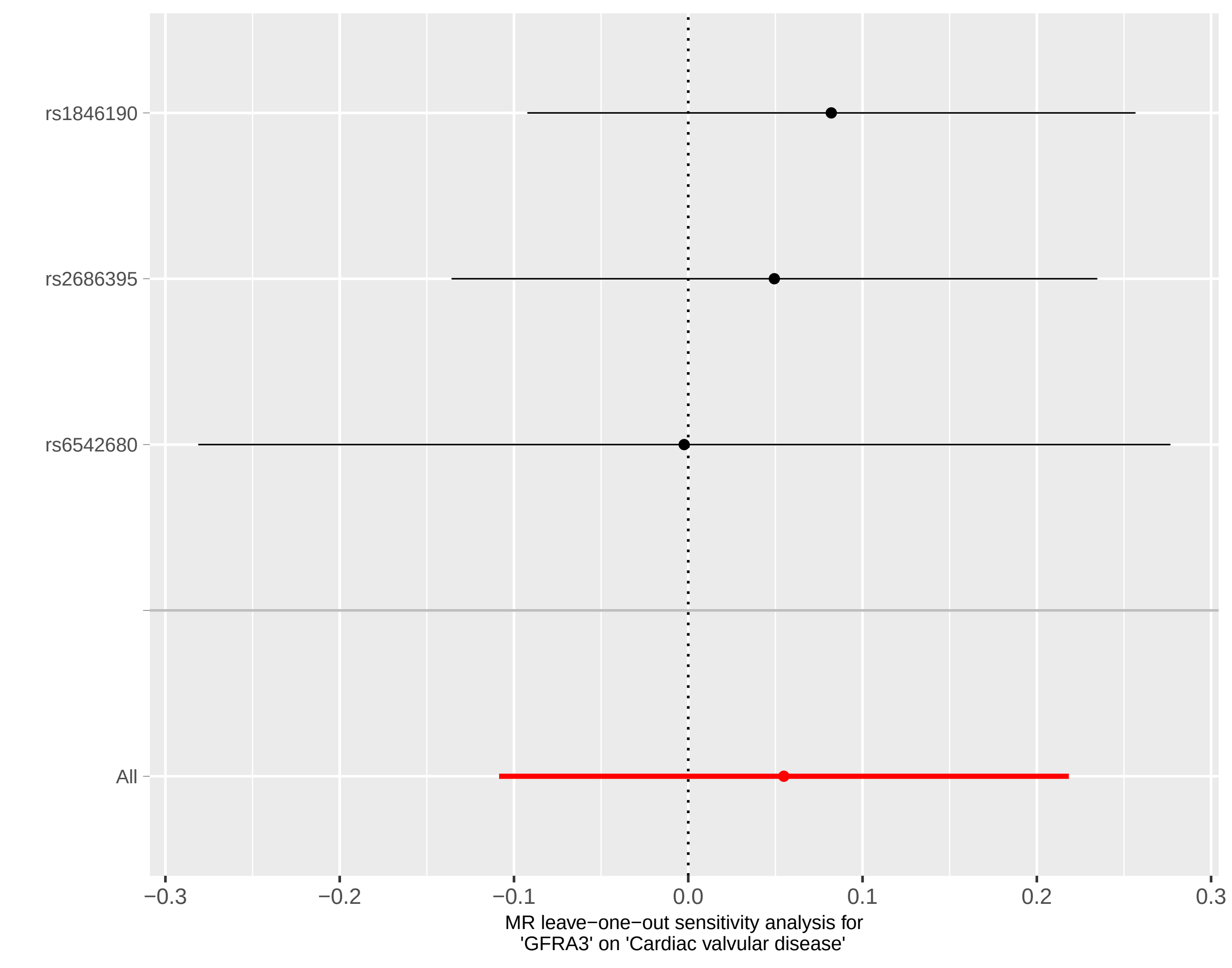
rs1846190
rs2686395
rs6542680
All
−0.3
−0.2
−0.1
0.0
0.1
0.2
0.3
MR leave−one−out sensitivity analysis for
'GFRA3' on 'Cardiac valvular disease'

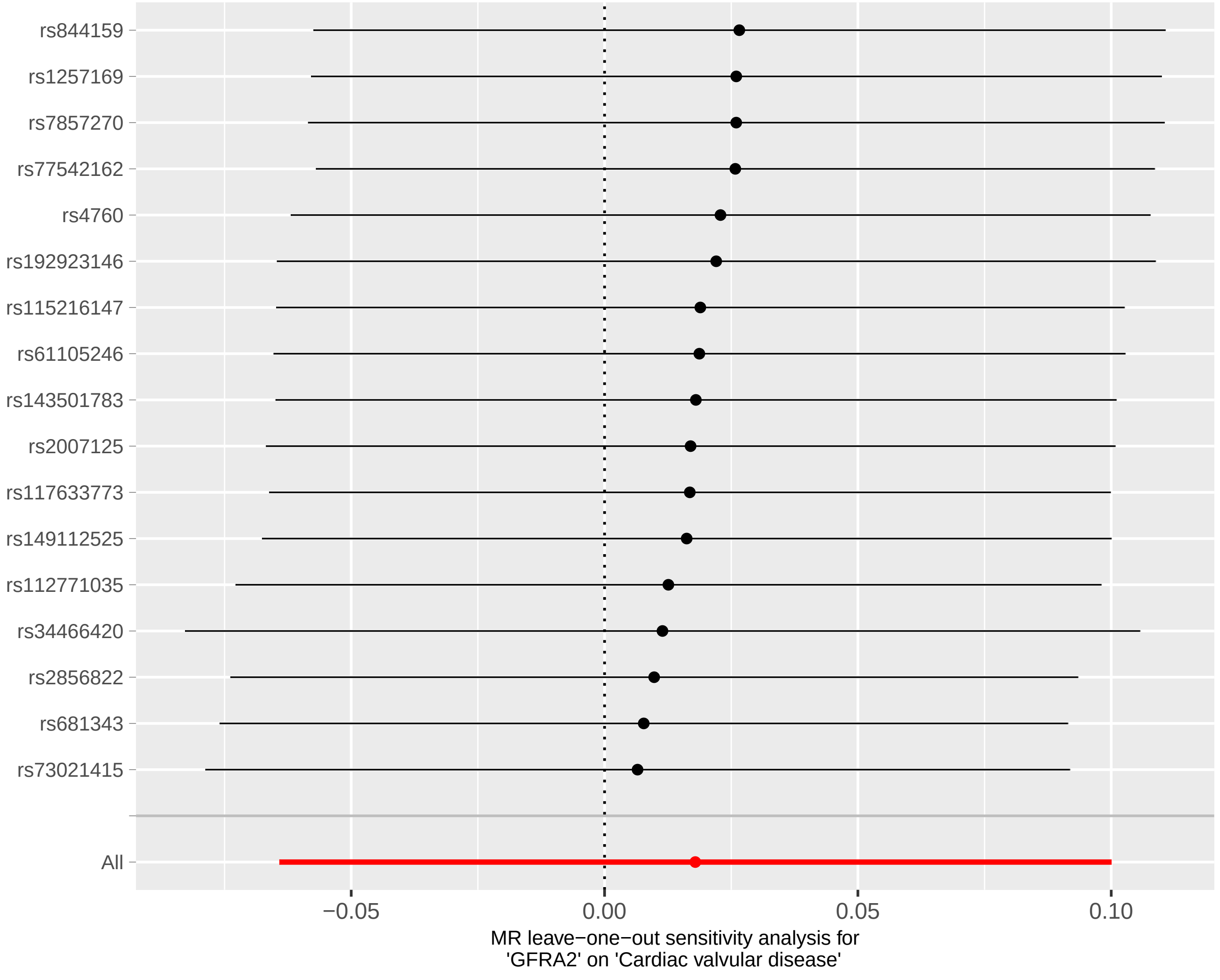
rs844159
rs1257169
rs7857270
rs77542162
rs4760
rs192923146
rs115216147
rs61105246
rs143501783
rs2007125
rs117633773
rs149112525
rs112771035
rs34466420
rs2856822
rs681343
rs73021415
All
−0.05
0.00
0.05
0.10
MR leave−one−out sensitivity analysis for
'GFRA2' on 'Cardiac valvular disease'

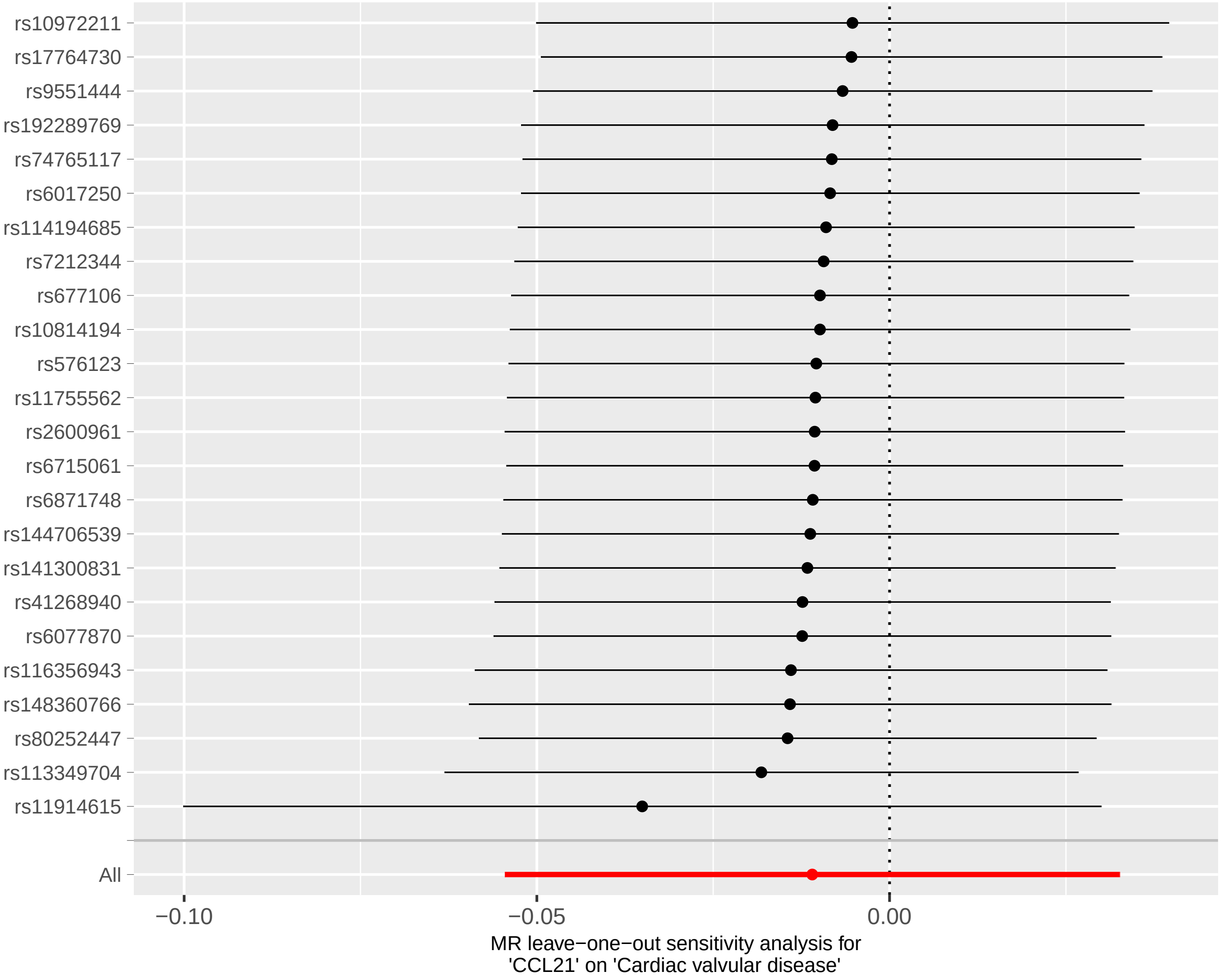
rs10972211
rs17764730
rs9551444
rs192289769
rs74765117
rs6017250
rs114194685
rs7212344
rs677106
rs10814194
rs576123
rs11755562
rs2600961
rs6715061
rs6871748
rs144706539
rs141300831
rs41268940
rs6077870
rs116356943
rs148360766
rs80252447
rs113349704
rs11914615
All
−0.10
−0.05
0.00
MR leave−one−out sensitivity analysis for
'CCL21' on 'Cardiac valvular disease'

Insufficient number of SNPs

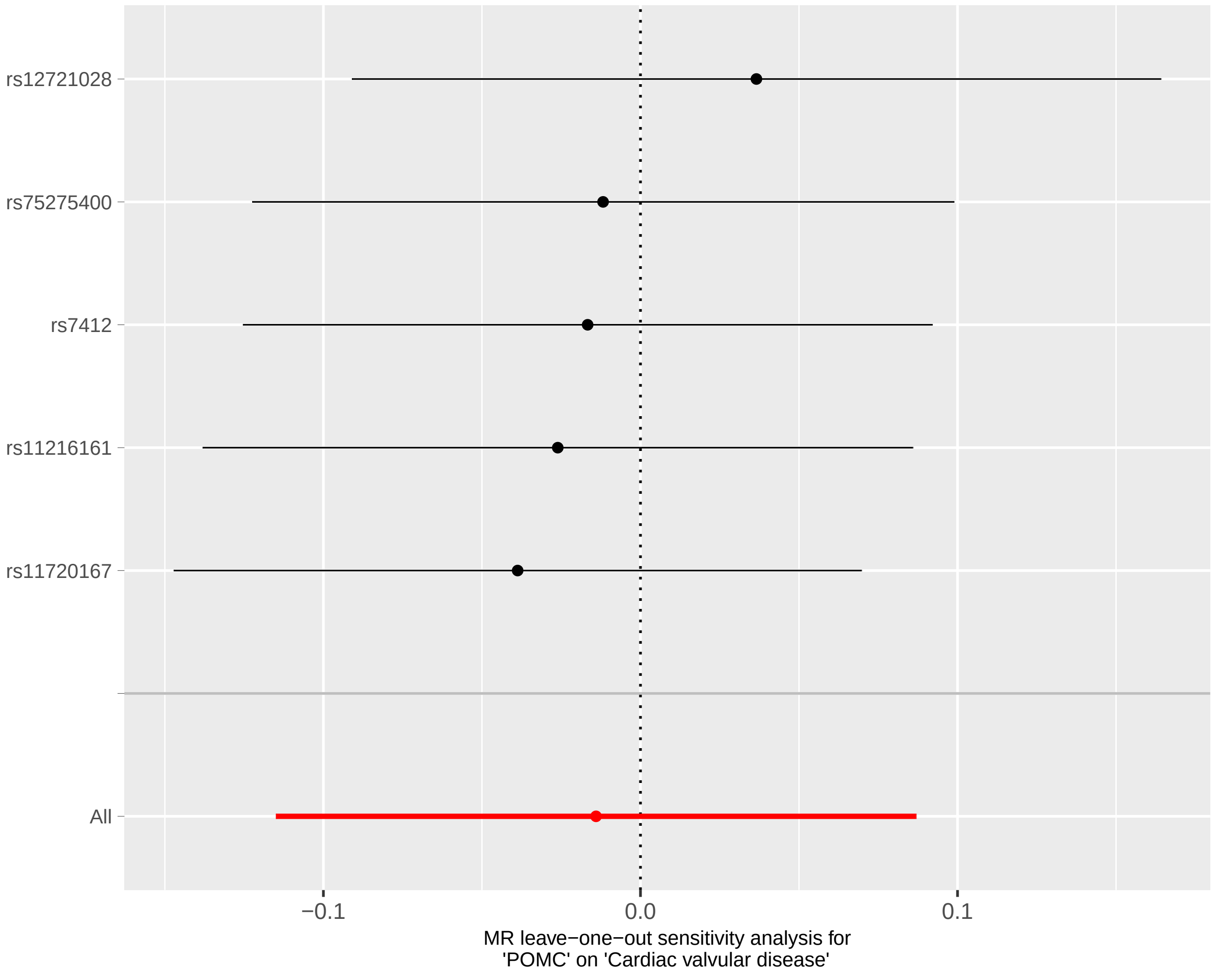
rs12721028
rs75275400
rs7412
rs11216161
rs11720167
All
−0.1
0.0
0.1
MR leave−one−out sensitivity analysis for
'POMC' on 'Cardiac valvular disease'

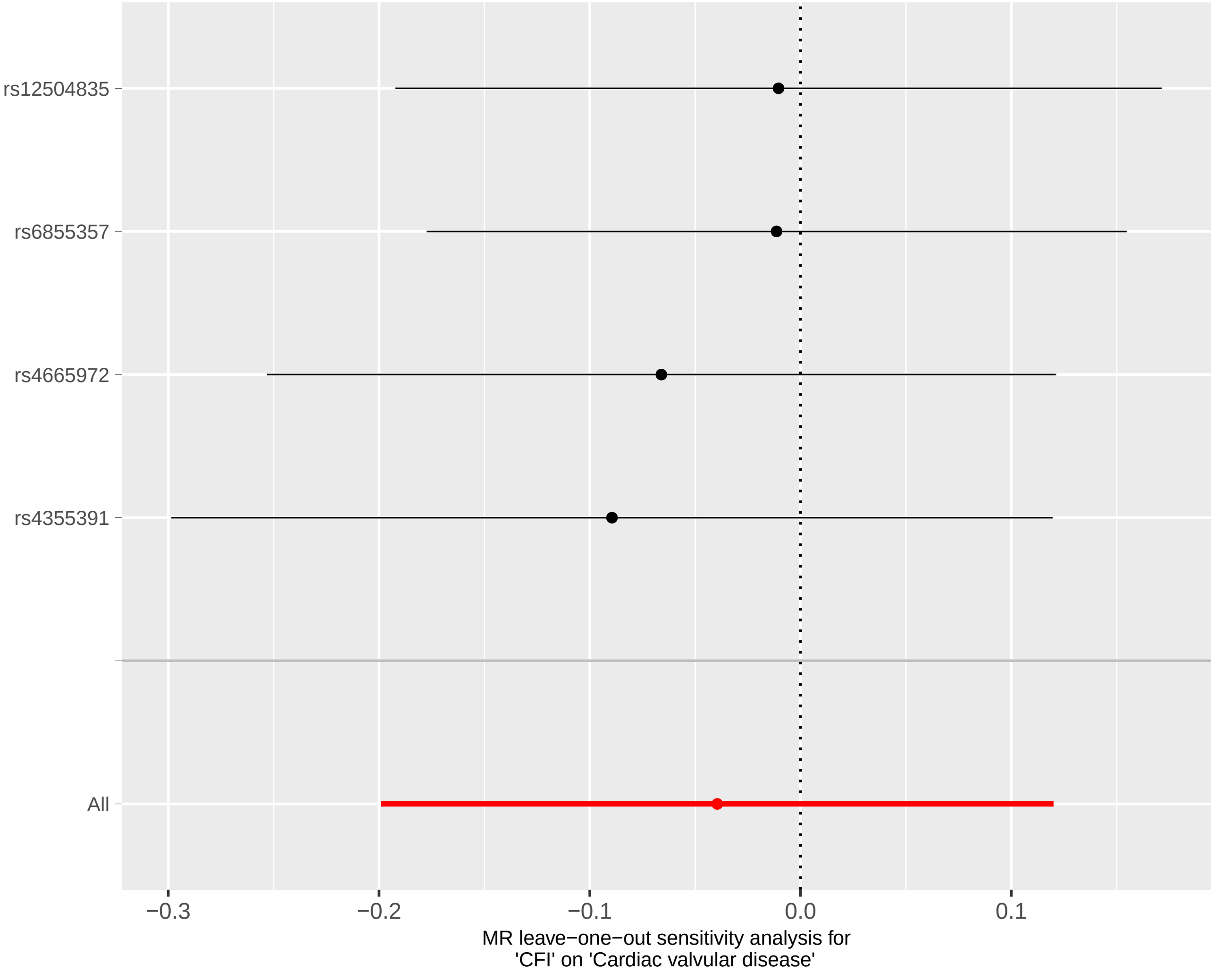
rs12504835
rs6855357
rs4665972
rs4355391
All
−0.3
−0.2
−0.1
0.0
0.1
MR leave−one−out sensitivity analysis for
'CFI' on 'Cardiac valvular disease'

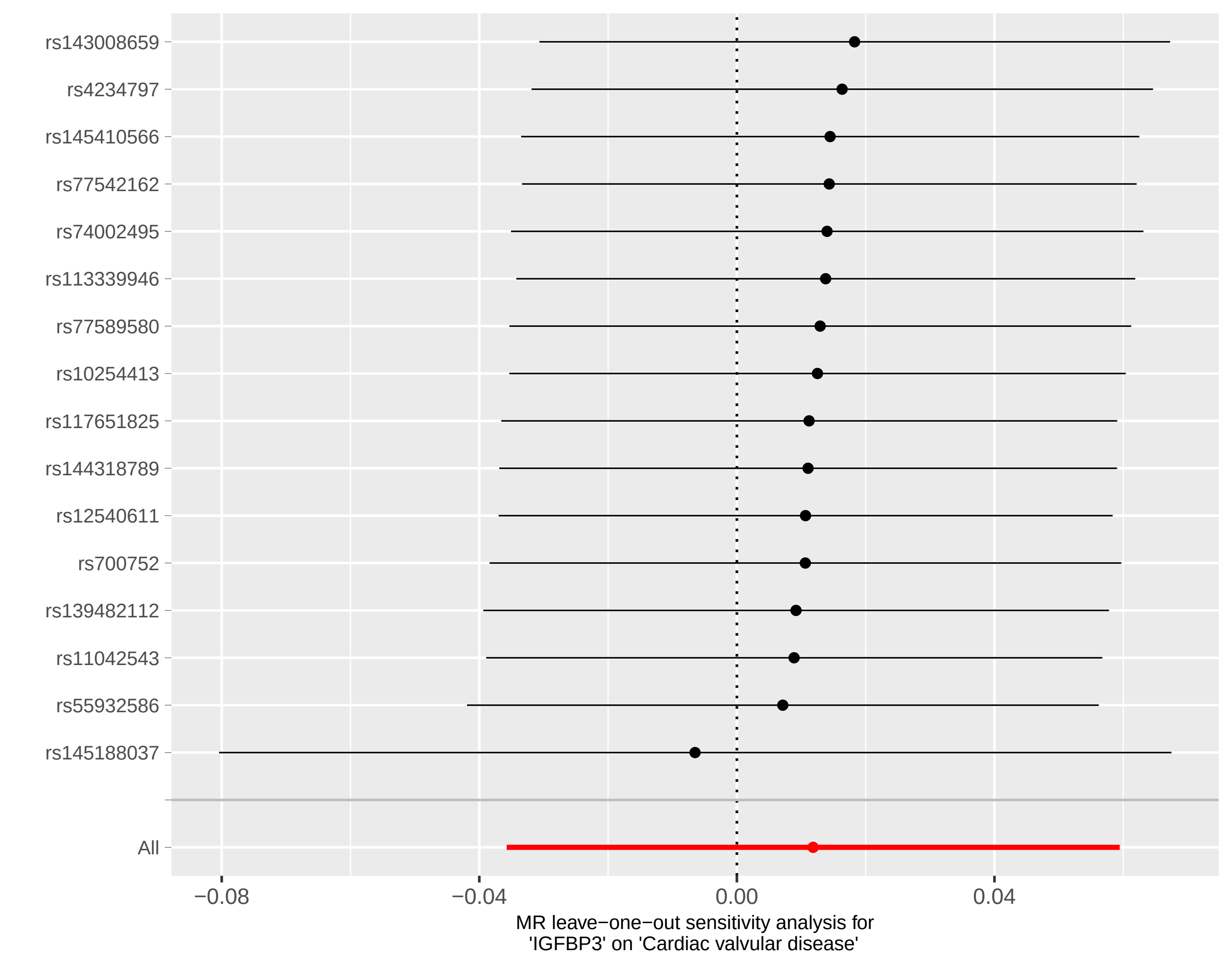
rs143008659
rs4234797
rs145410566
rs77542162
rs74002495
rs113339946
rs77589580
rs10254413
rs117651825
rs144318789
rs12540611
rs700752
rs139482112
rs11042543
rs55932586
rs145188037
All
−0.08
−0.04
0.00
0.04
MR leave−one−out sensitivity analysis for
'IGFBP3' on 'Cardiac valvular disease'

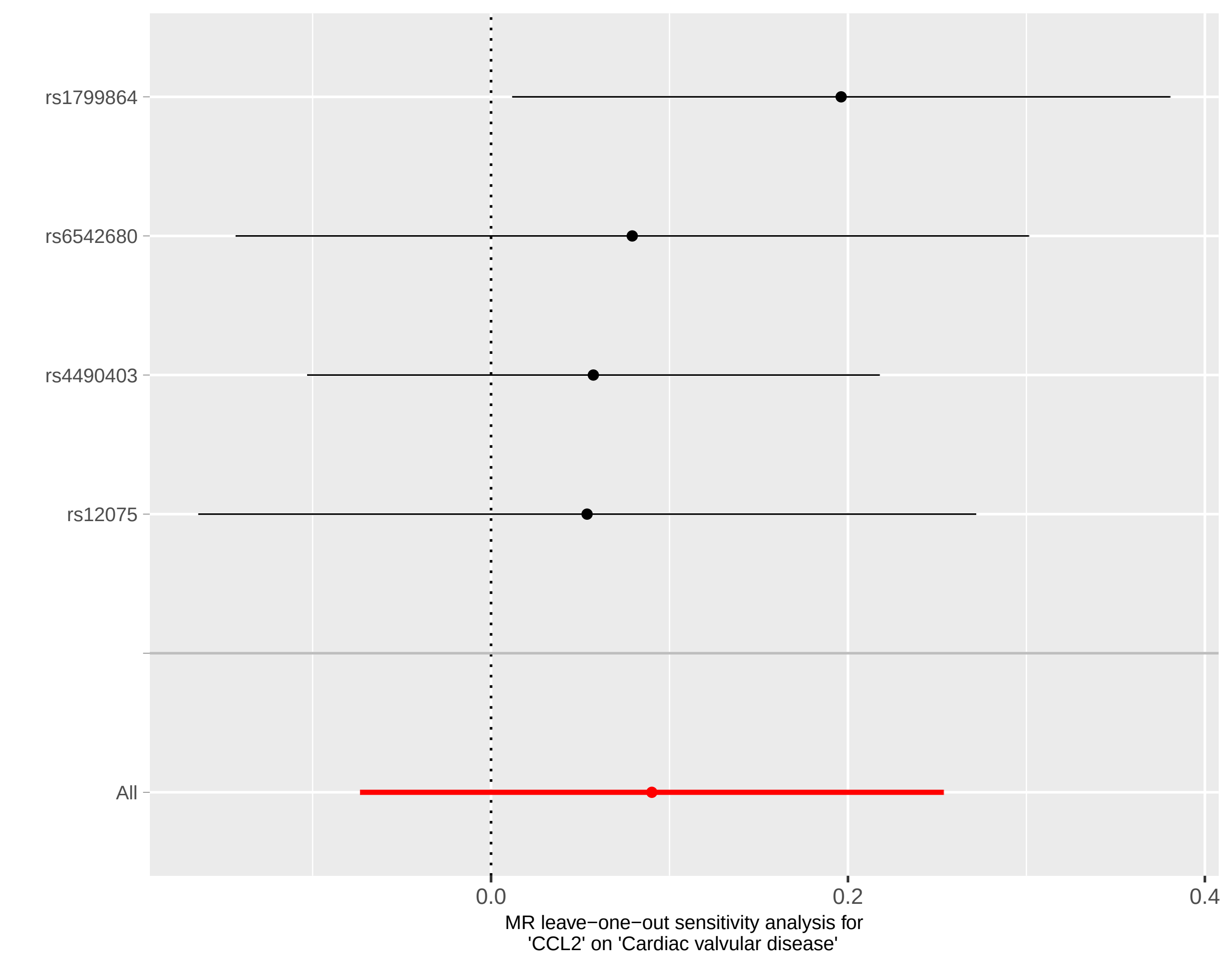

rs1799864
rs6542680
rs4490403
rs12075
All
0.0
0.2
0.4
MR leave−one−out sensitivity analysis for
'CCL2' on 'Cardiac valvular disease'

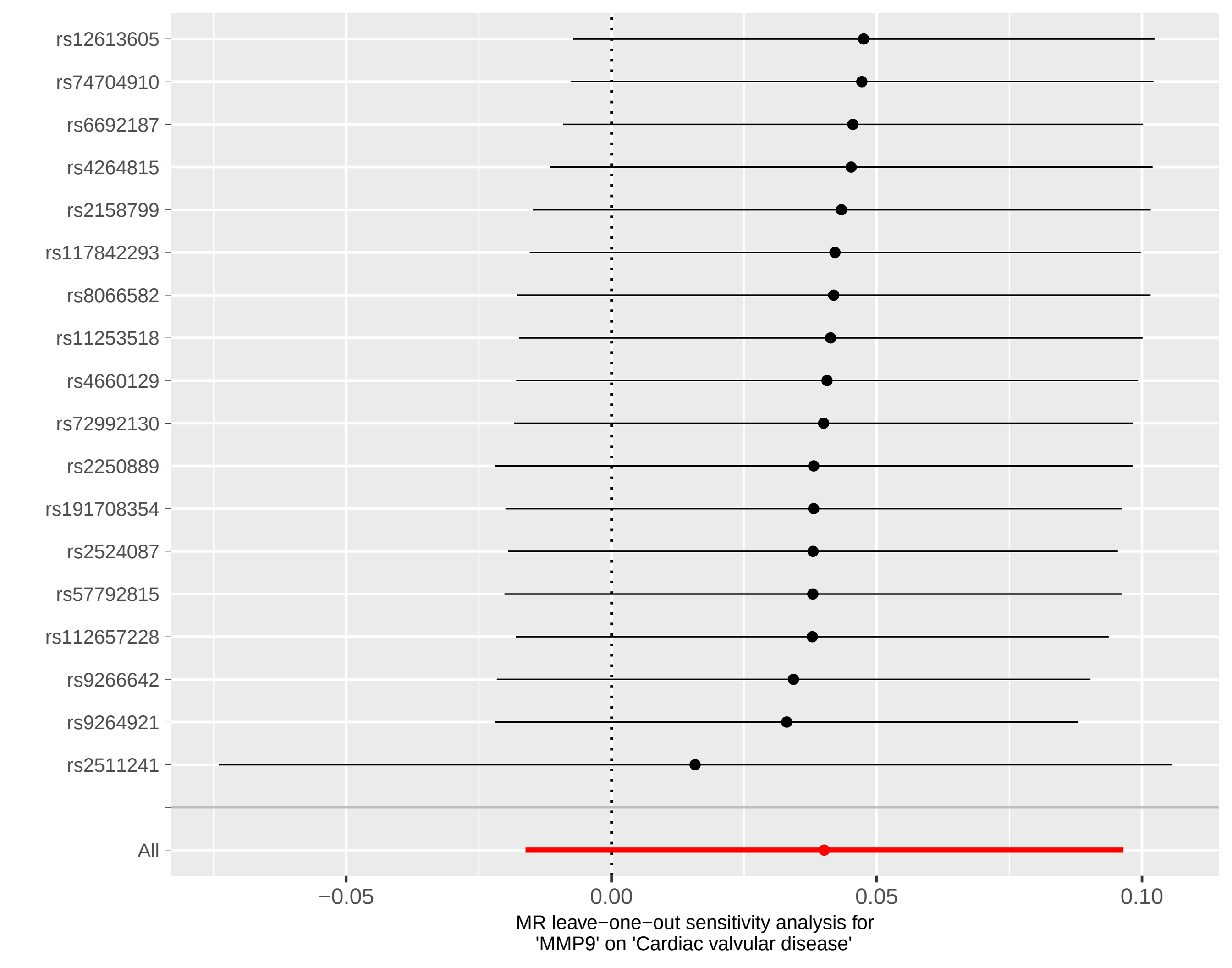
rs12613605
rs74704910
rs6692187
rs4264815
rs2158799
rs117842293
rs8066582
rs11253518
rs4660129
rs72992130
rs2250889
rs191708354
rs2524087
rs57792815
rs112657228
rs9266642
rs9264921
rs2511241
All
−0.05
0.00
0.05
0.10
MR leave−one−out sensitivity analysis for
'MMP9' on 'Cardiac valvular disease'

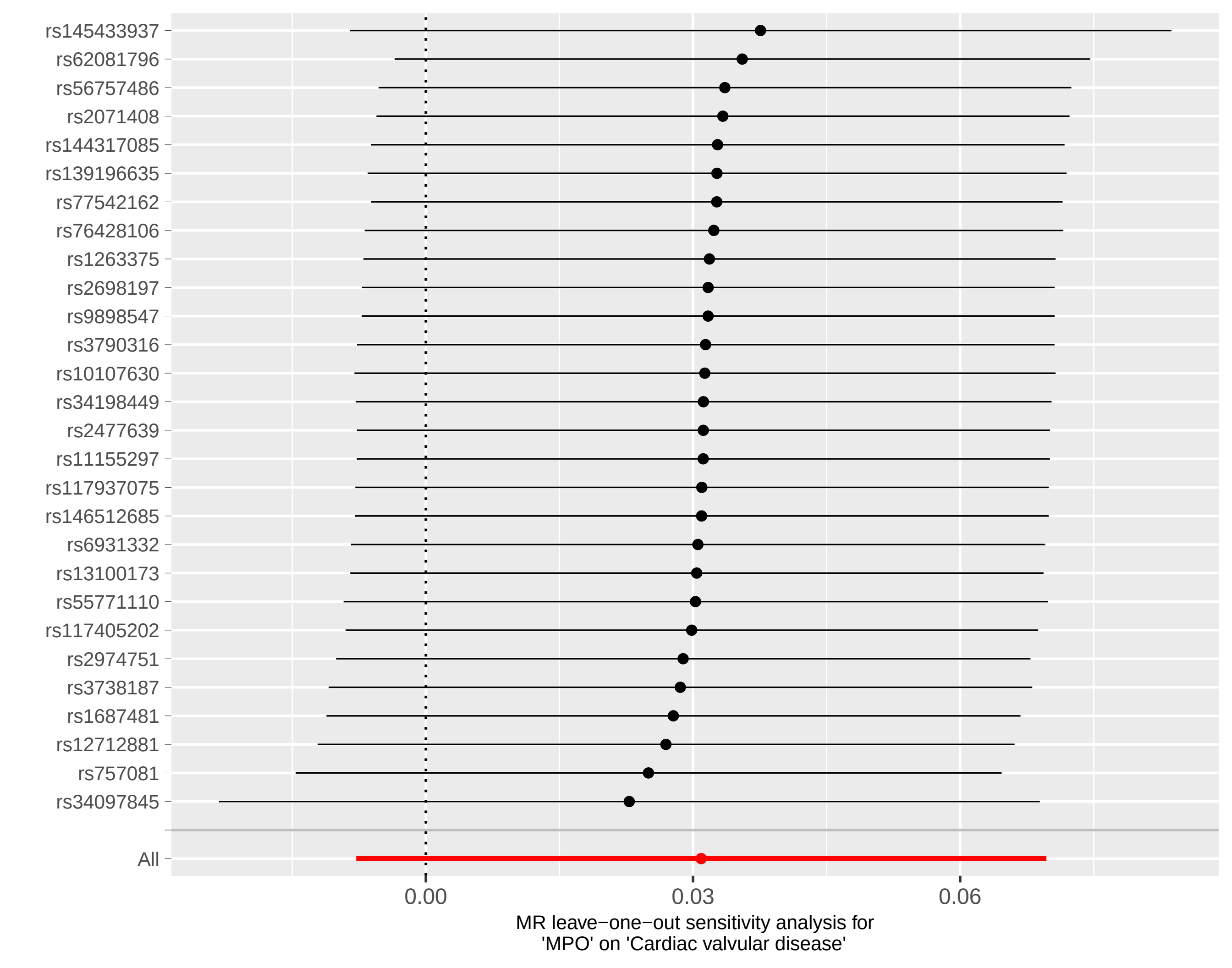
rs145433937
rs62081796
rs56757486
rs2071408
rs144317085
rs139196635
rs77542162
rs76428106
rs1263375
rs2698197
rs9898547
rs3790316
rs10107630
rs34198449
rs2477639
rs11155297
rs117937075
rs146512685
rs6931332
rs13100173
rs55771110
rs117405202
rs2974751
rs3738187
rs1687481
rs12712881
rs757081
rs34097845
All
0.00
0.03
0.06
MR leave−one−out sensitivity analysis for
'MPO' on 'Cardiac valvular disease'

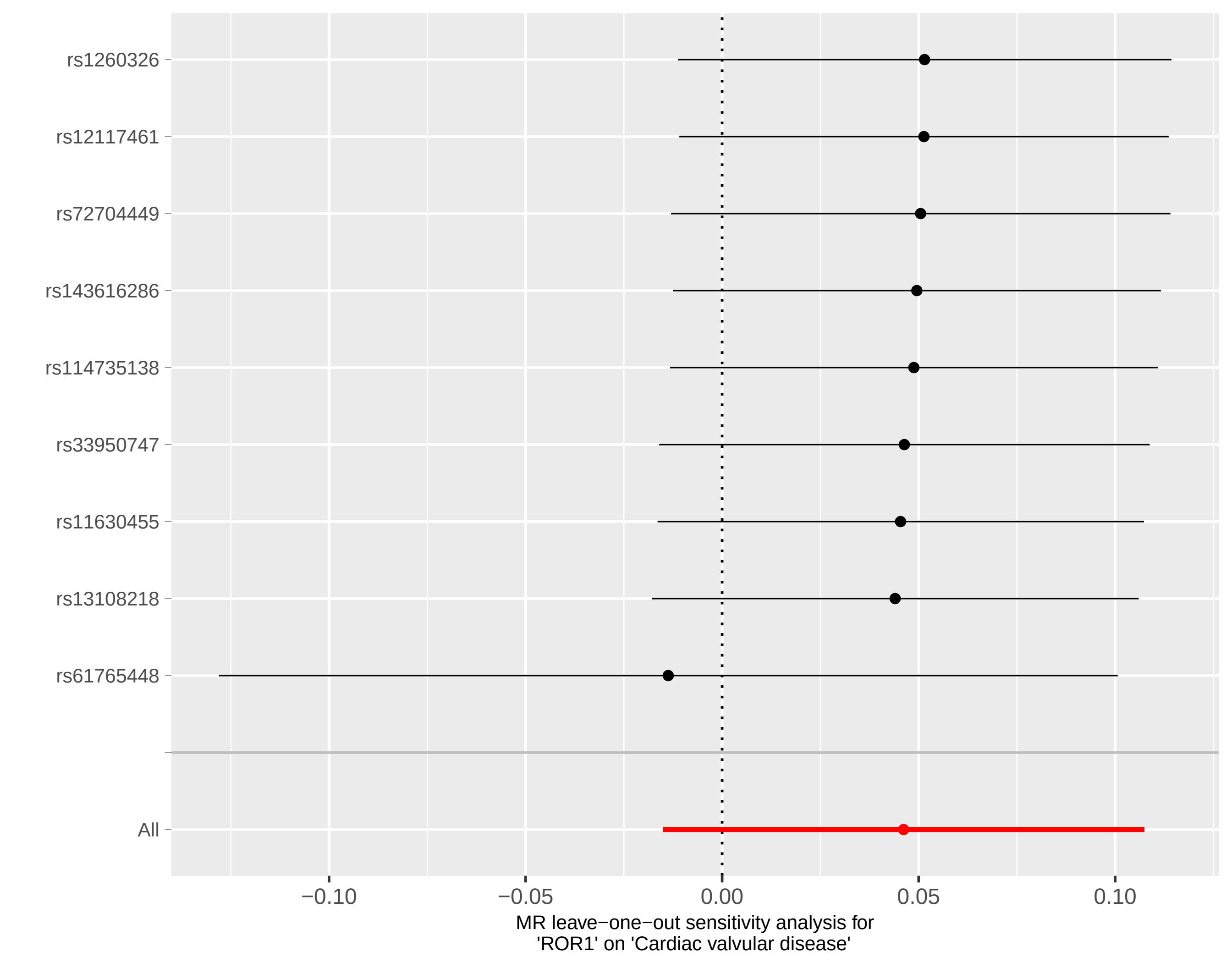
rs1260326
rs12117461
rs72704449
rs143616286
rs114735138
rs33950747
rs11630455
rs13108218
rs61765448
All
−0.10
−0.05
0.00
0.05
0.10
MR leave−one−out sensitivity analysis for
'ROR1' on 'Cardiac valvular disease'

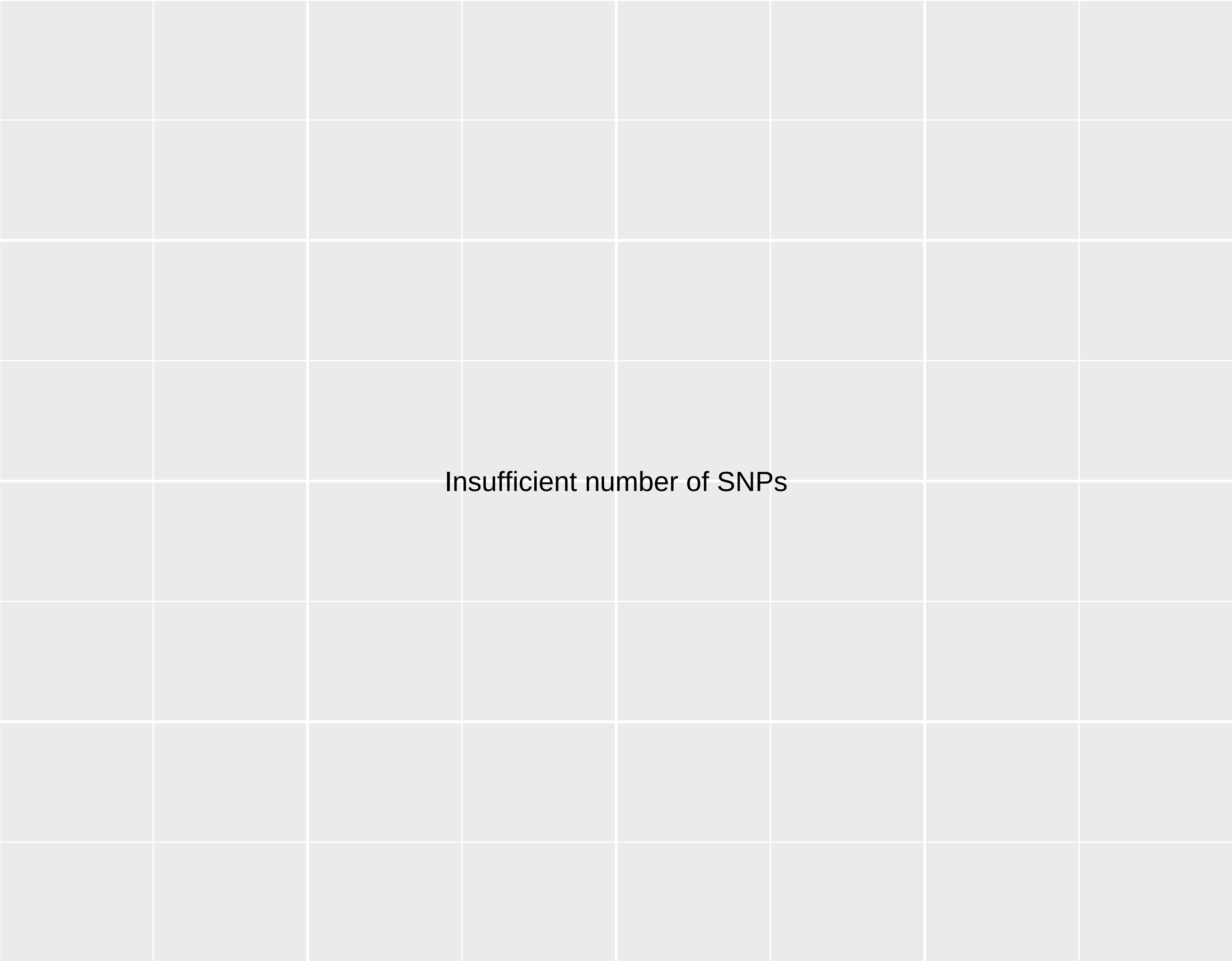
Insufficient number of SNPs

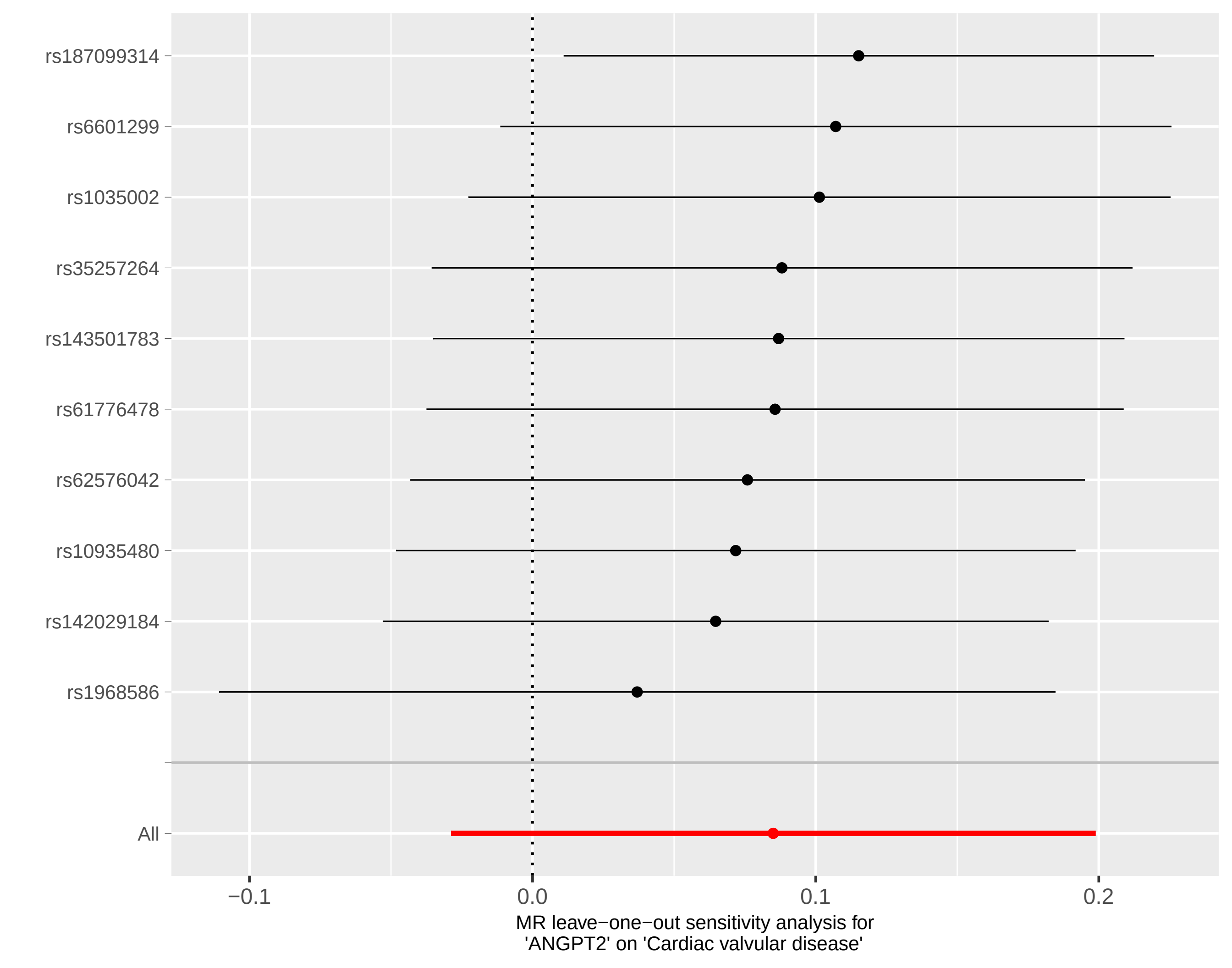

rs187099314
rs6601299
rs1035002
rs35257264
rs143501783
rs61776478
rs62576042
rs10935480
rs142029184
rs1968586
All
−0.1
0.0
0.1
0.2
MR leave−one−out sensitivity analysis for
'ANGPT2' on 'Cardiac valvular disease'

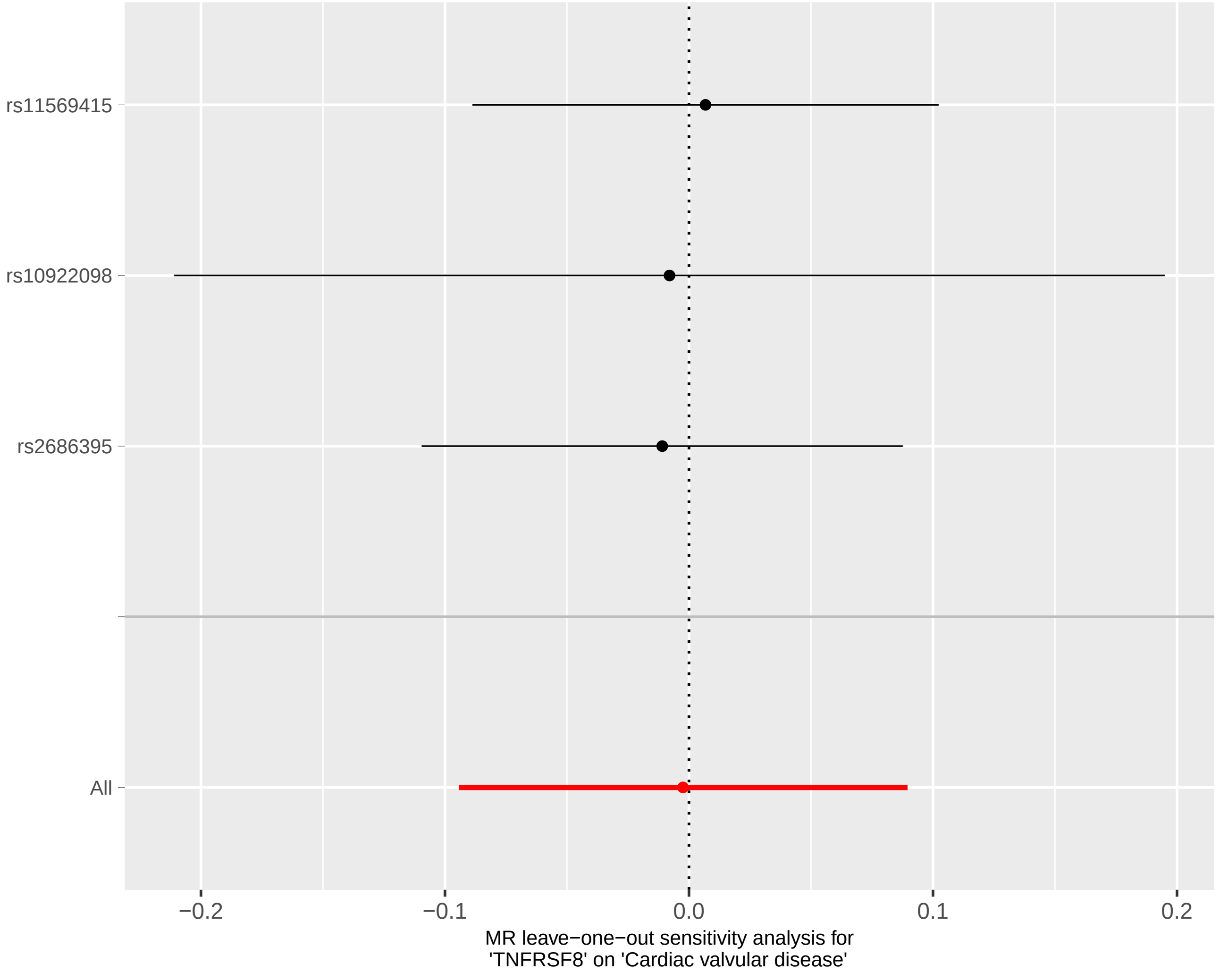
rs11569415
rs10922098
rs2686395
All
−0.2
−0.1
0.0
0.1
0.2
MR leave−one−out sensitivity analysis for
'TNFRSF8' on 'Cardiac valvular disease'

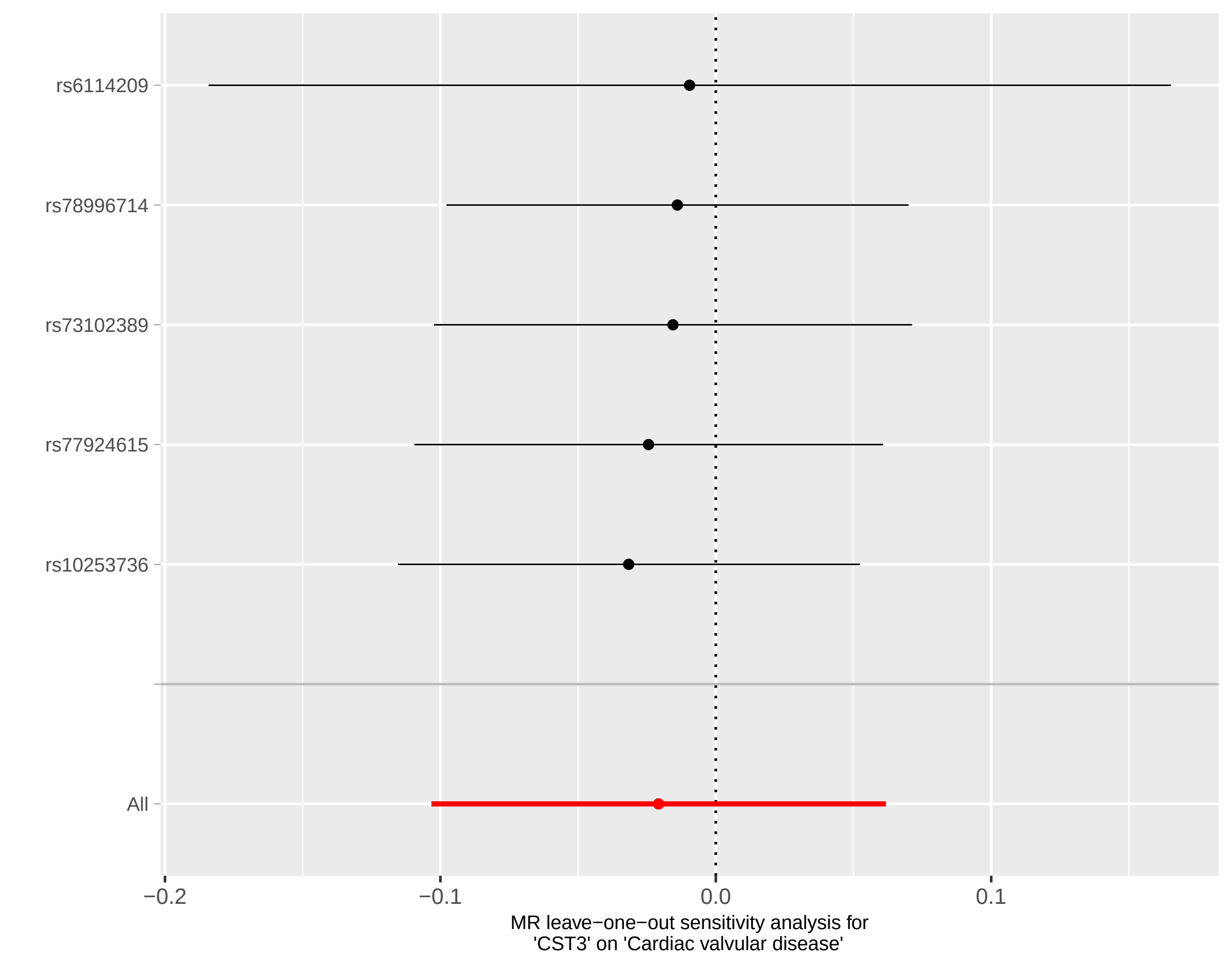

rs6114209
rs78996714
rs73102389
rs77924615
rs10253736
All
−0.2
−0.1
0.0
0.1
MR leave−one−out sensitivity analysis for
'CST3' on 'Cardiac valvular disease'

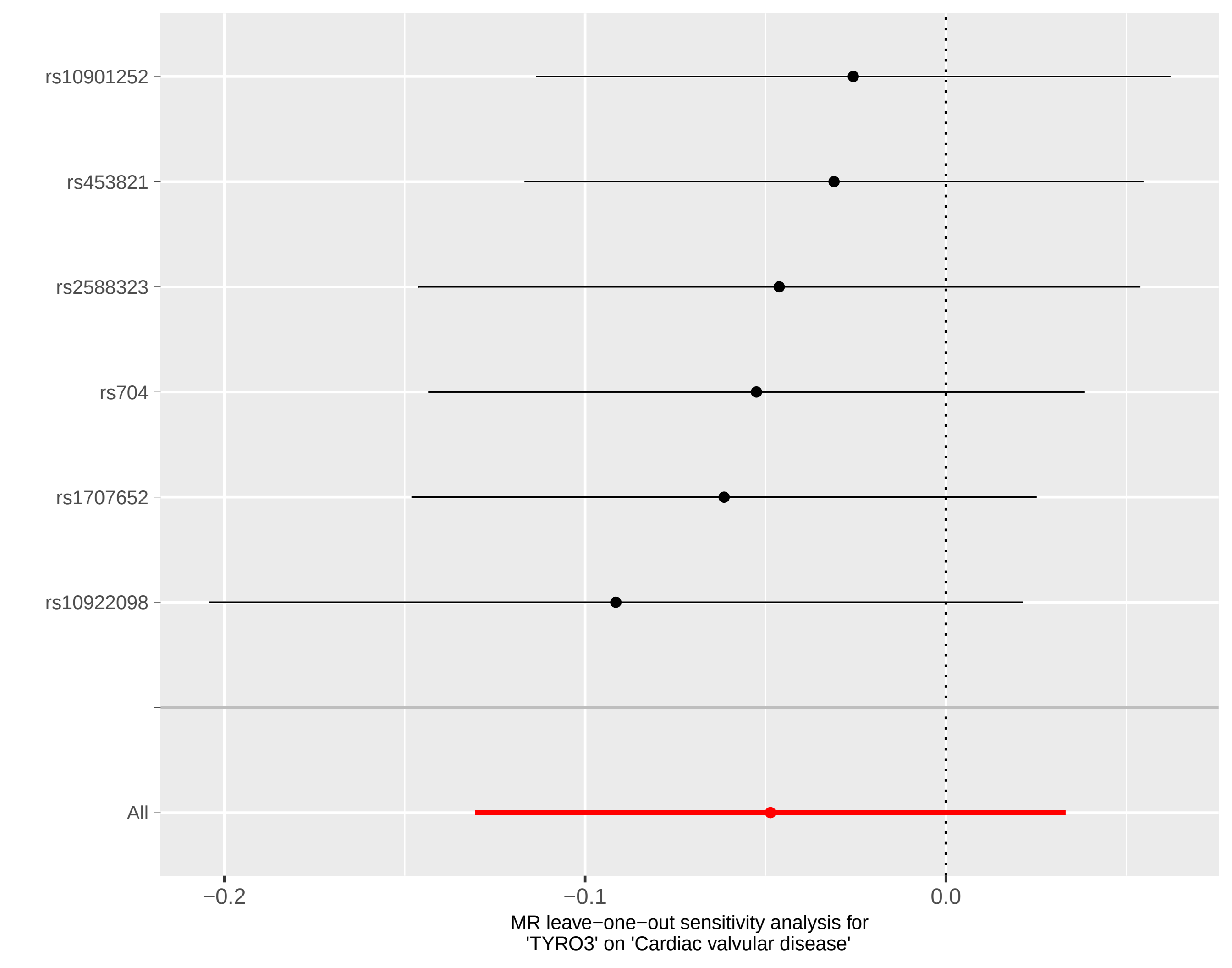
rs10901252
rs453821
rs2588323
rs704
rs1707652
rs10922098
All
−0.2
−0.1
0.0
MR leave−one−out sensitivity analysis for
'TYRO3' on 'Cardiac valvular disease'

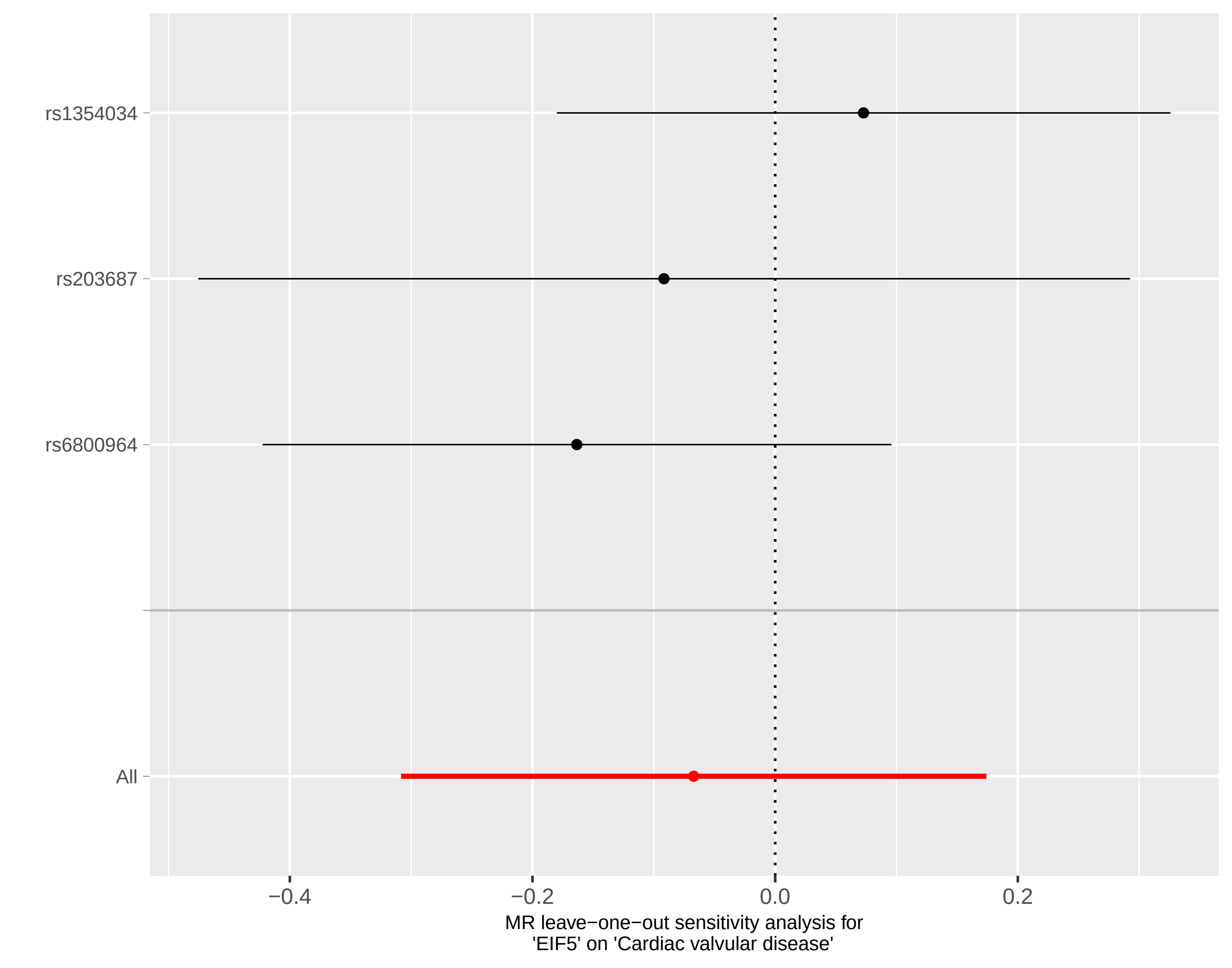

rs1354034
rs203687
rs6800964
All
−0.4
−0.2
0.0
0.2
MR leave−one−out sensitivity analysis for
'EIF5' on 'Cardiac valvular disease'

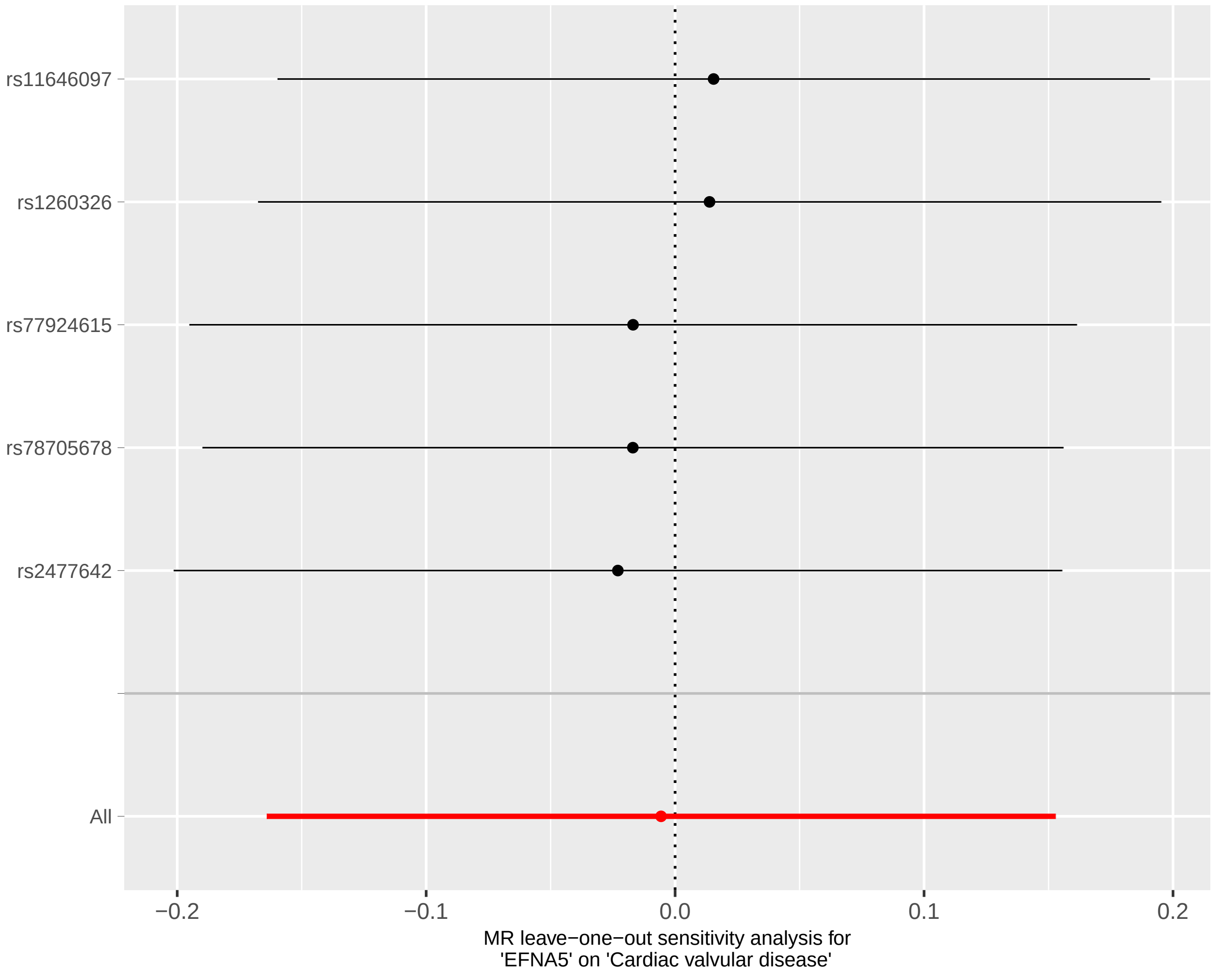
rs11646097
rs1260326
rs77924615
rs78705678
rs2477642
All
−0.2
−0.1
0.0
0.1
0.2
MR leave−one−out sensitivity analysis for
'EFNA5' on 'Cardiac valvular disease'

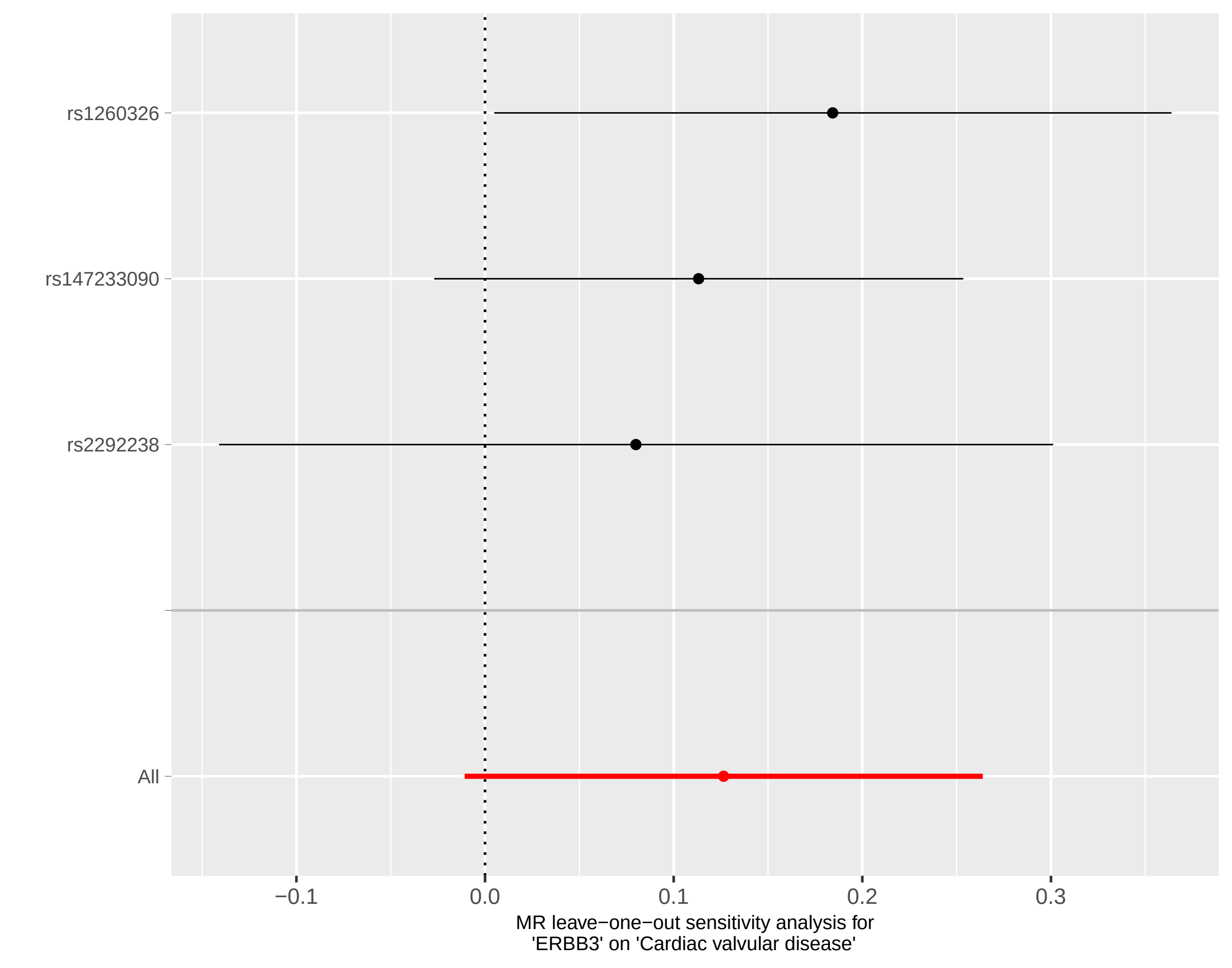
rs1260326
rs147233090
rs2292238
All
−0.1
0.0
0.1
0.2
0.3
MR leave−one−out sensitivity analysis for
'ERBB3' on 'Cardiac valvular disease'

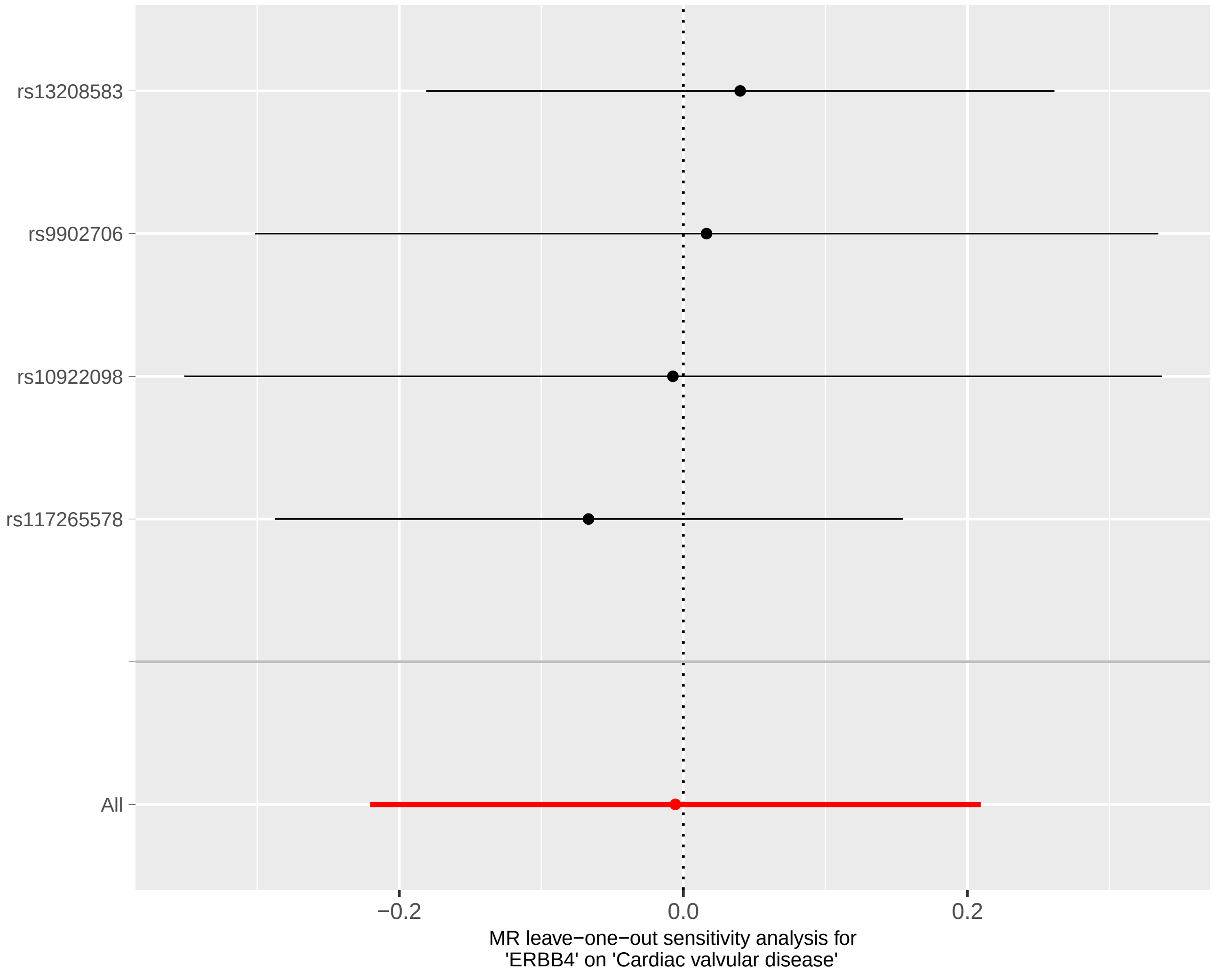

rs13208583
rs9902706
rs10922098
rs117265578
All
−0.2
0.0
0.2
MR leave−one−out sensitivity analysis for
'ERBB4' on 'Cardiac valvular disease'

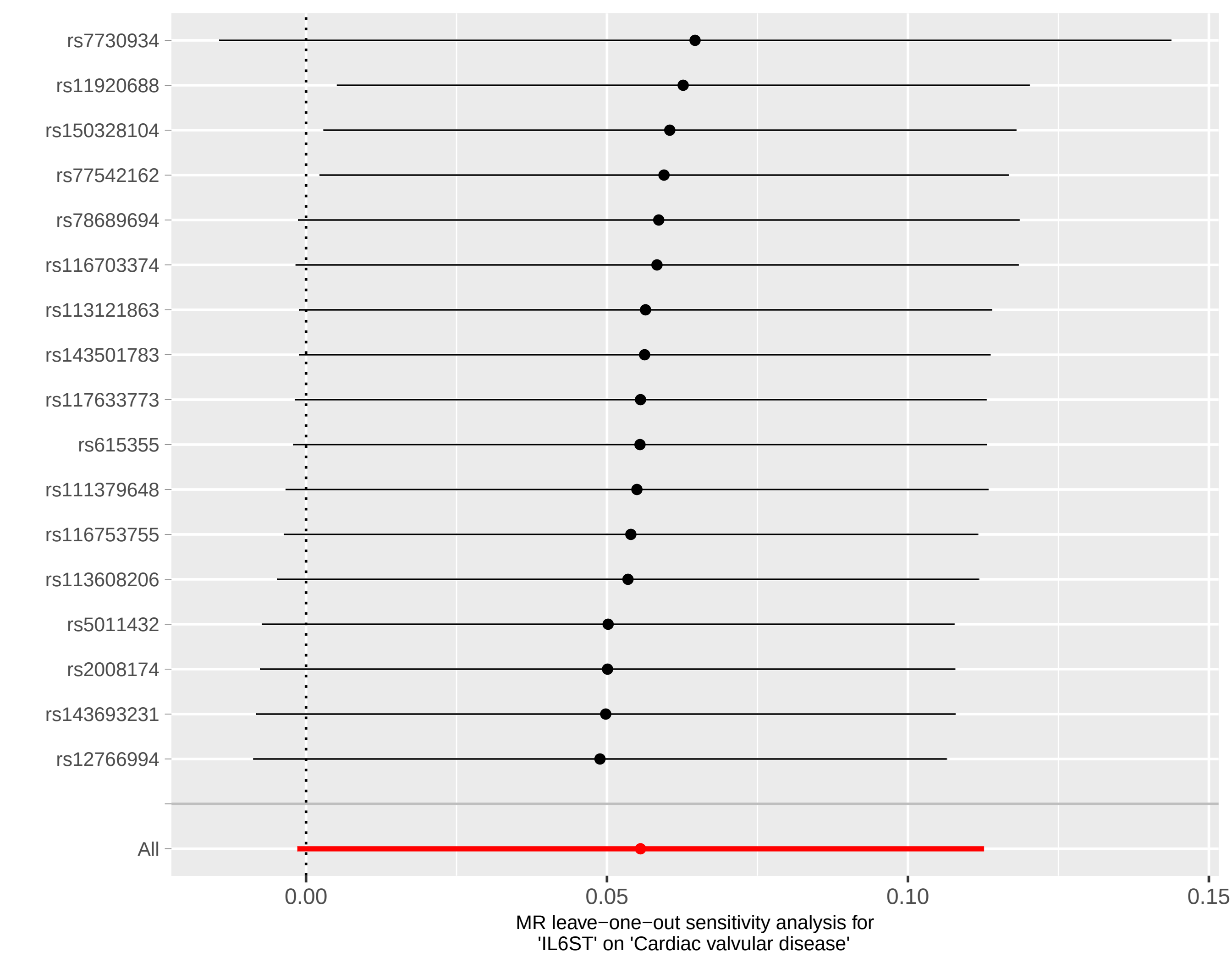
rs7730934
rs11920688
rs150328104
rs77542162
rs78689694
rs116703374
rs113121863
rs143501783
rs117633773
rs615355
rs111379648
rs116753755
rs113608206
rs5011432
rs2008174
rs143693231
rs12766994
All
0.00
0.05
0.10
0.15
MR leave−one−out sensitivity analysis for
'IL6ST' on 'Cardiac valvular disease'

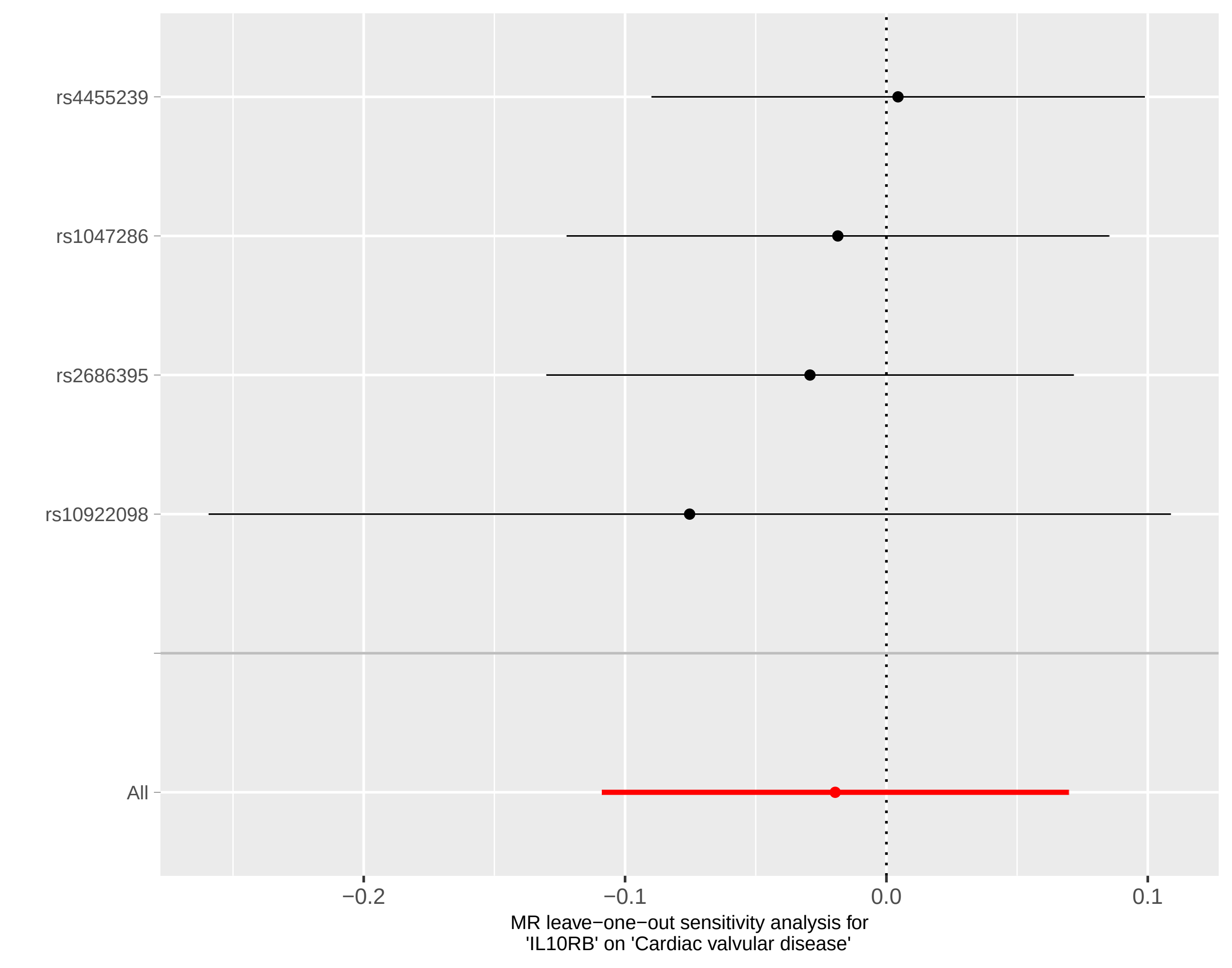
rs4455239
rs1047286
rs2686395
rs10922098
All
−0.2
−0.1
0.0
0.1
MR leave−one−out sensitivity analysis for
'IL10RB' on 'Cardiac valvular disease'

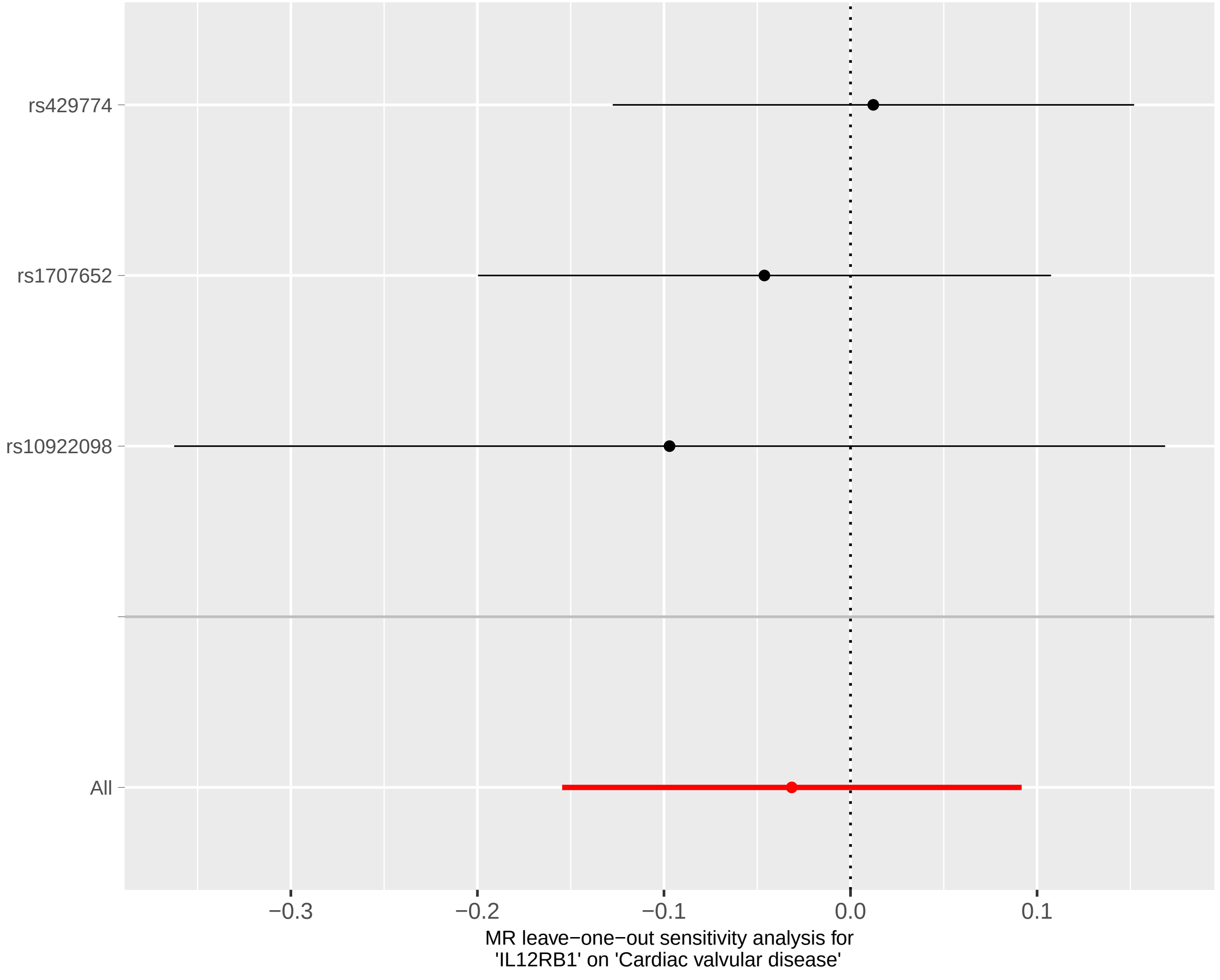
rs429774
rs1707652
rs10922098
All
−0.3
−0.2
−0.1
0.0
0.1
MR leave−one−out sensitivity analysis for
'IL12RB1' on 'Cardiac valvular disease'

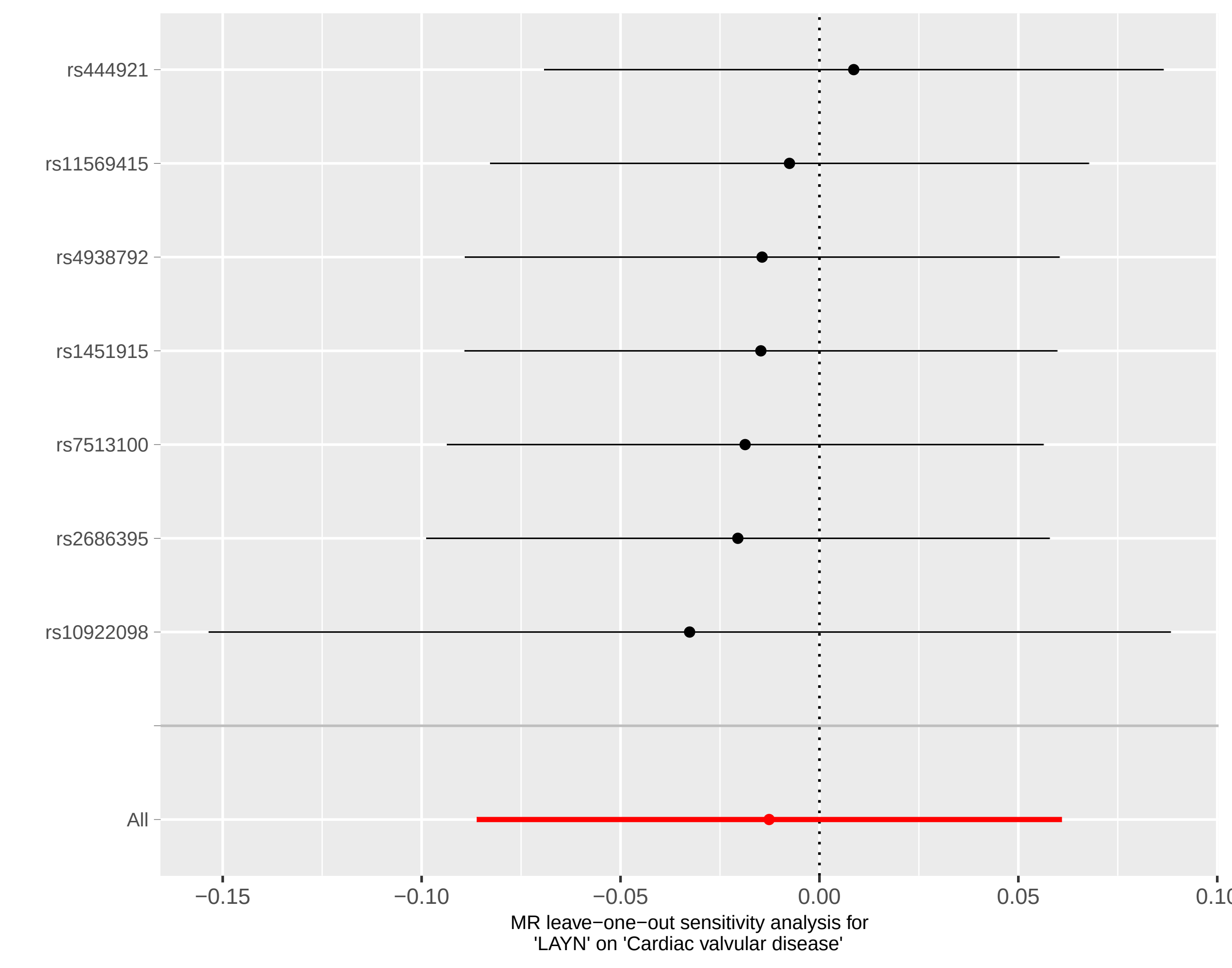

rs444921
rs11569415
rs4938792
rs1451915
rs7513100
rs2686395
rs10922098
All
−0.15
−0.10
−0.05
0.00
0.05
0.10
MR leave−one−out sensitivity analysis for
'LAYN' on 'Cardiac valvular disease'

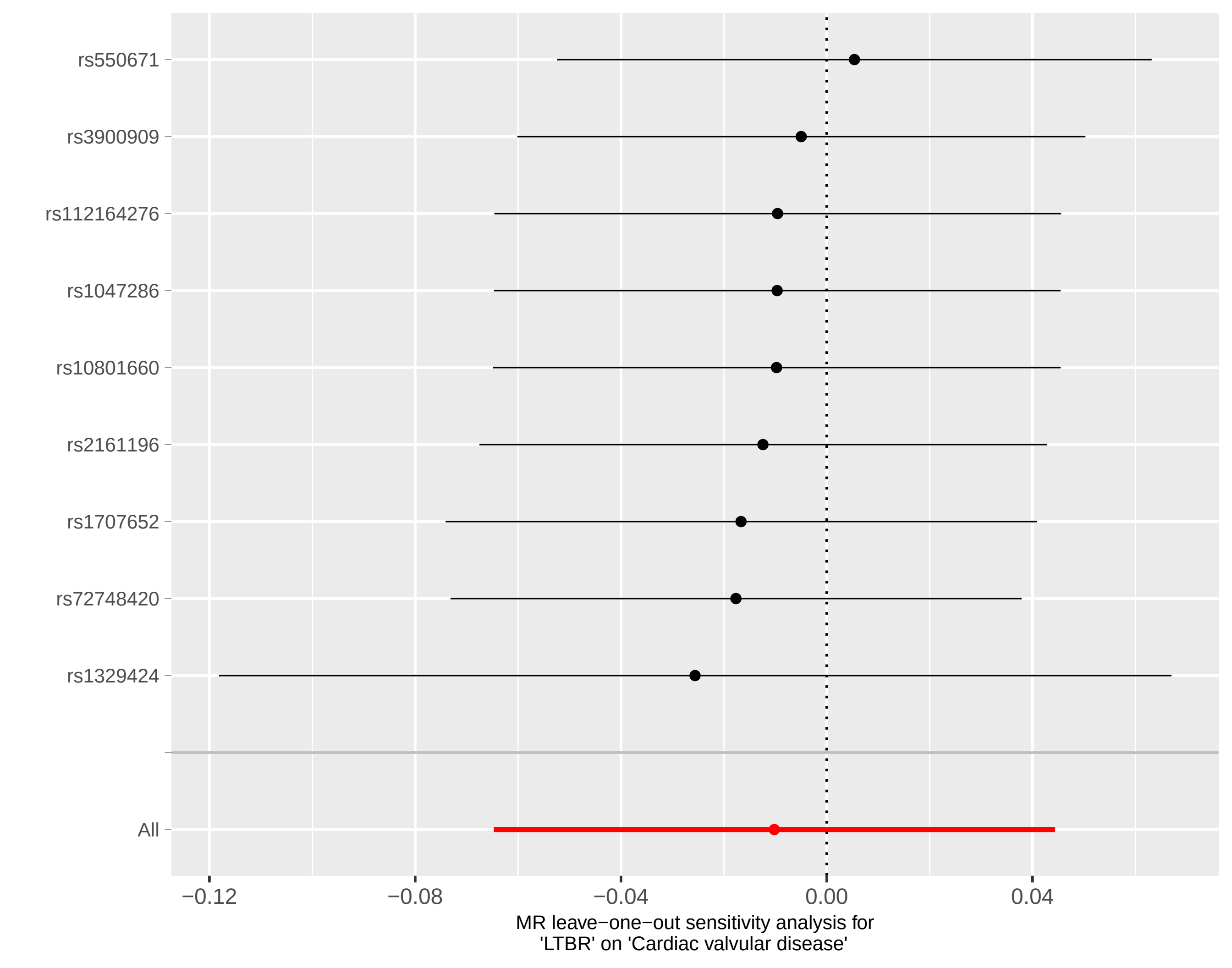
rs550671
rs3900909
rs112164276
rs1047286
rs10801660
rs2161196
rs1707652
rs72748420
rs1329424
All
−0.12
−0.08
−0.04
0.00
0.04
MR leave−one−out sensitivity analysis for
'LTBR' on 'Cardiac valvular disease'

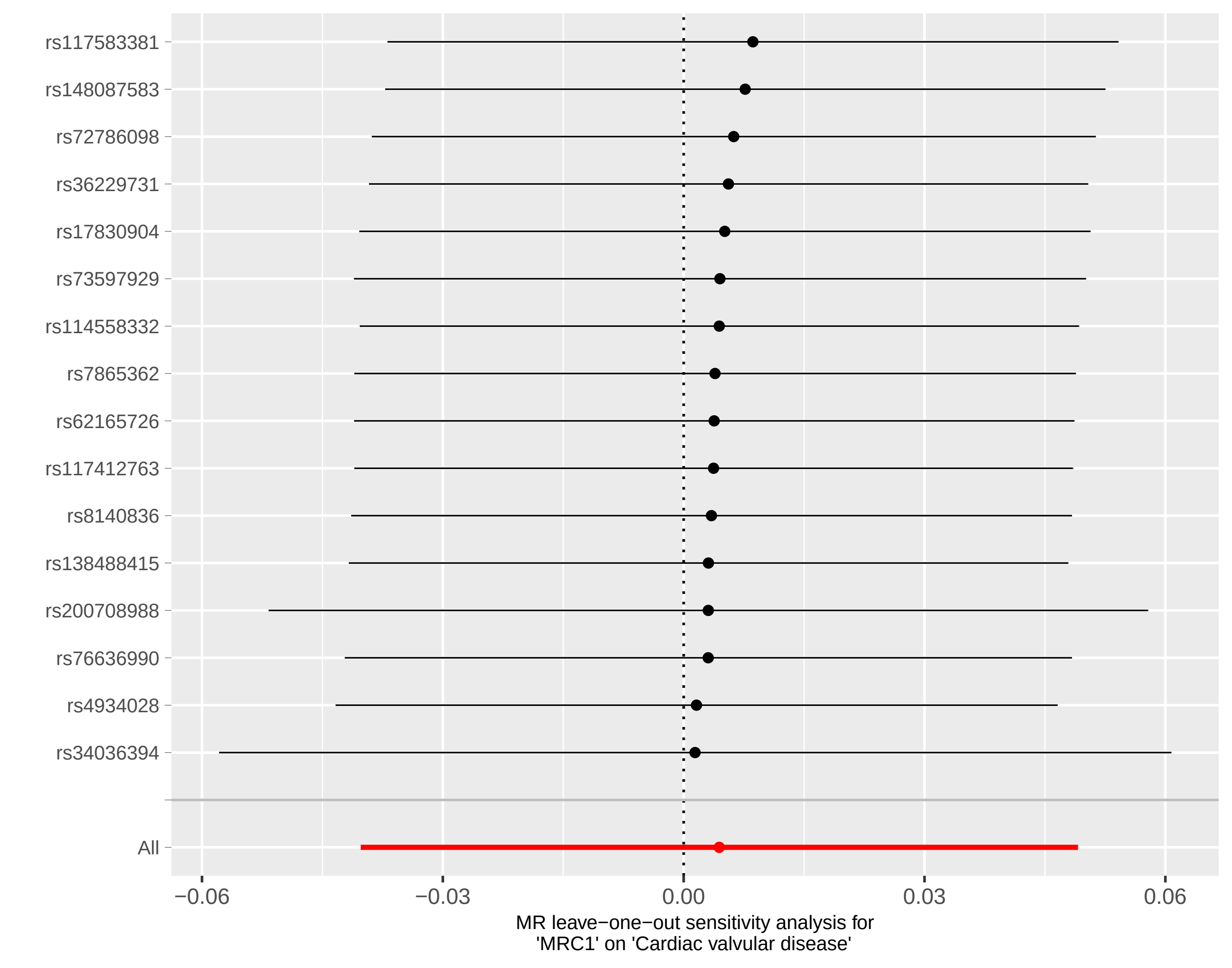
rs117583381
rs148087583
rs72786098
rs36229731
rs17830904
rs73597929
rs114558332
rs7865362
rs62165726
rs117412763
rs8140836
rs138488415
rs200708988
rs76636990
rs4934028
rs34036394
All
−0.06
−0.03
0.00
0.03
0.06
MR leave−one−out sensitivity analysis for
'MRC1' on 'Cardiac valvular disease'

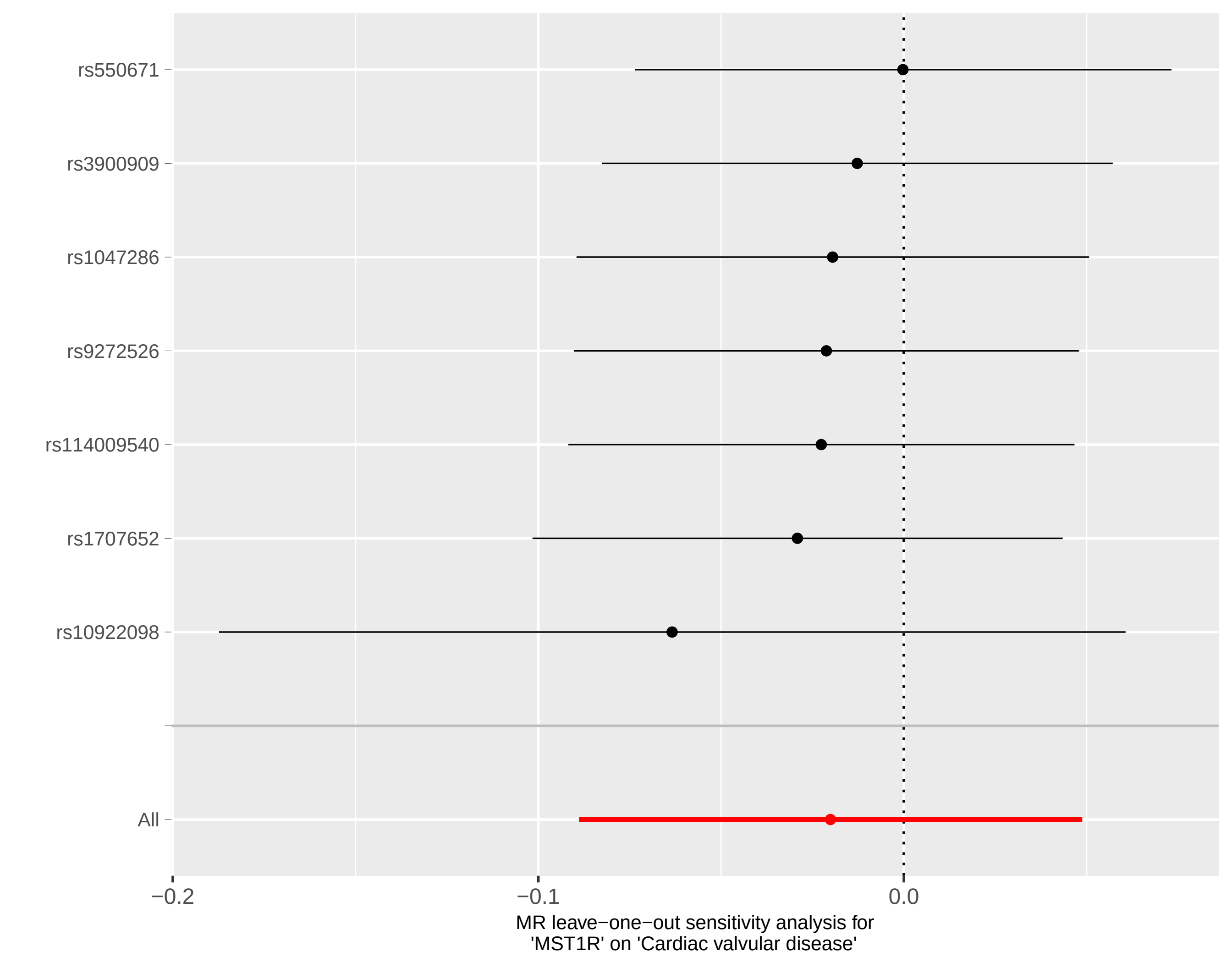
rs550671
rs3900909
rs1047286
rs9272526
rs114009540
rs1707652
rs10922098
All
−0.2
−0.1
0.0
MR leave−one−out sensitivity analysis for
'MST1R' on 'Cardiac valvular disease'

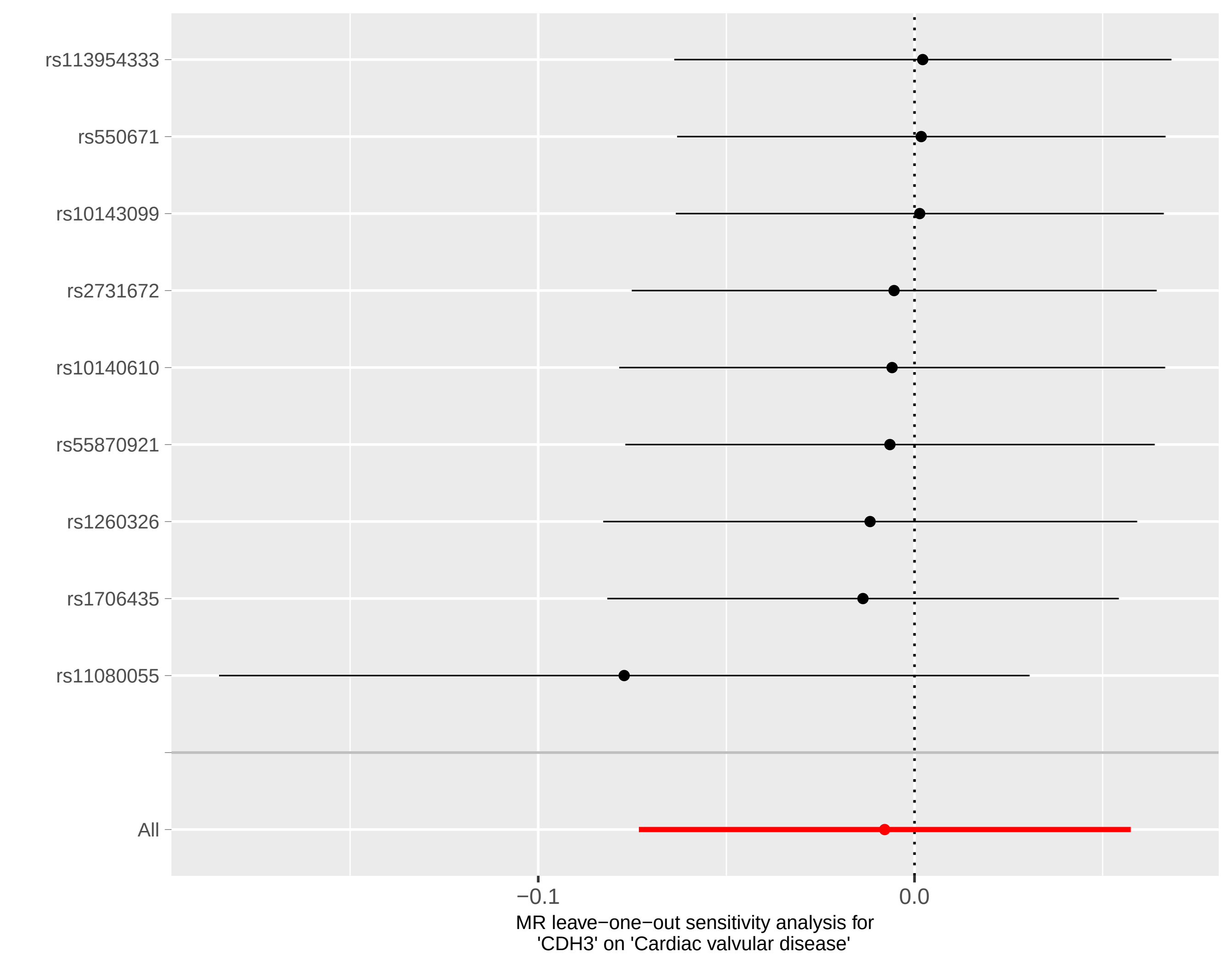
rs113954333
rs550671
rs10143099
rs2731672
rs10140610
rs55870921
rs1260326
rs1706435
rs11080055
All
−0.1
0.0
MR leave−one−out sensitivity analysis for
'CDH3' on 'Cardiac valvular disease'

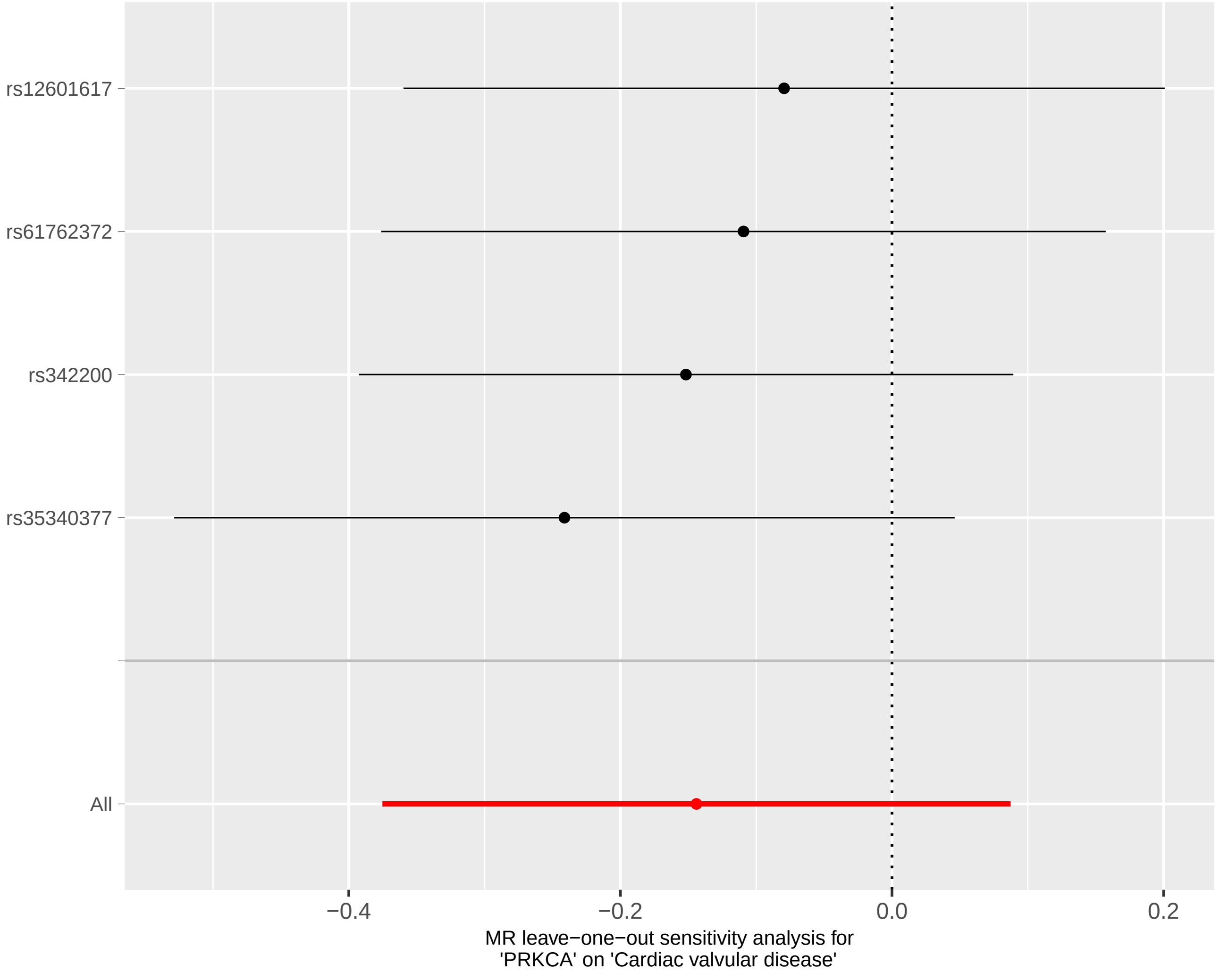
rs12601617
rs61762372
rs342200
rs35340377
All
−0.4
−0.2
0.0
0.2
MR leave−one−out sensitivity analysis for
'PRKCA' on 'Cardiac valvular disease'

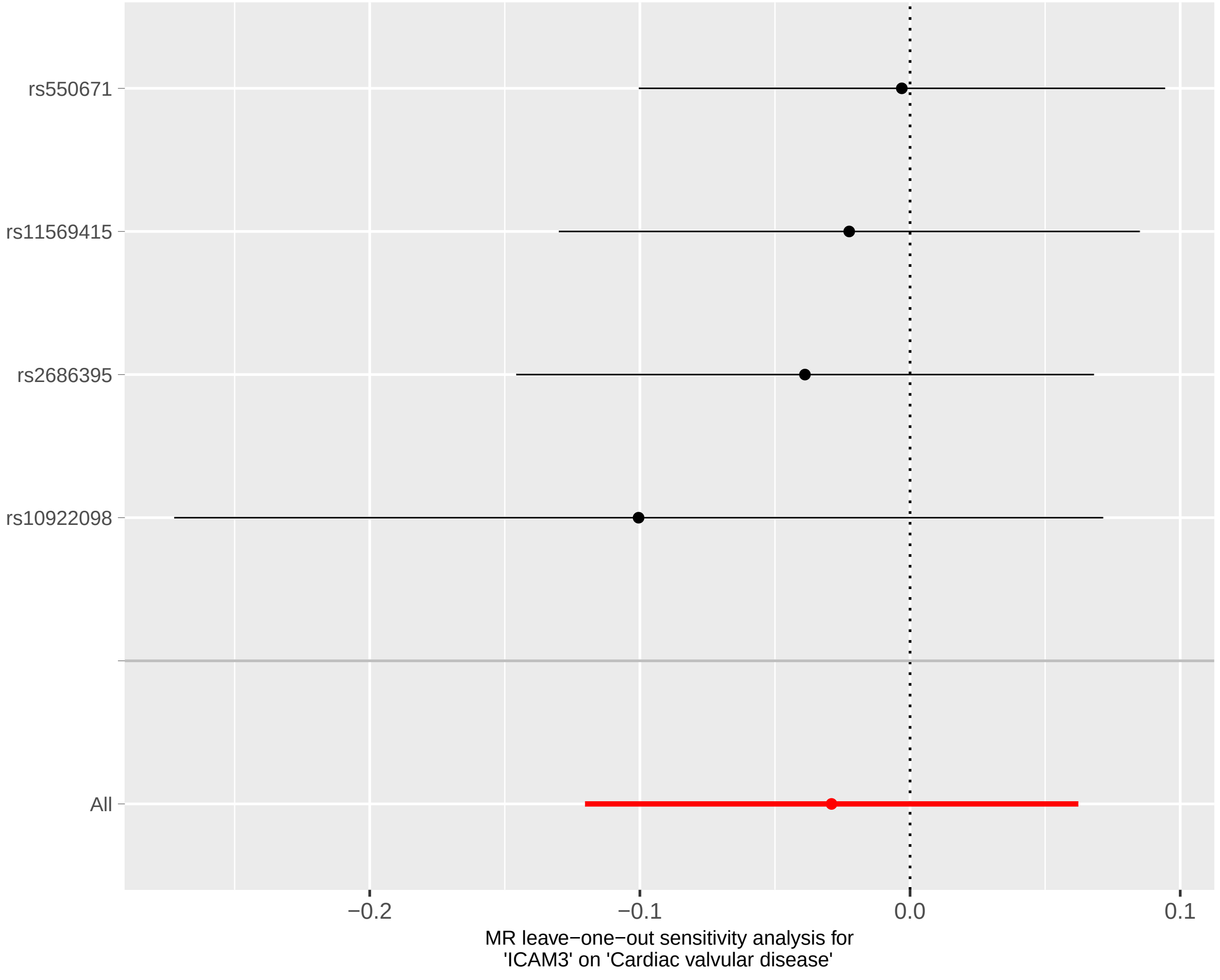
rs550671
rs11569415
rs2686395
rs10922098
All
−0.2
−0.1
0.0
0.1
MR leave−one−out sensitivity analysis for
'ICAM3' on 'Cardiac valvular disease'

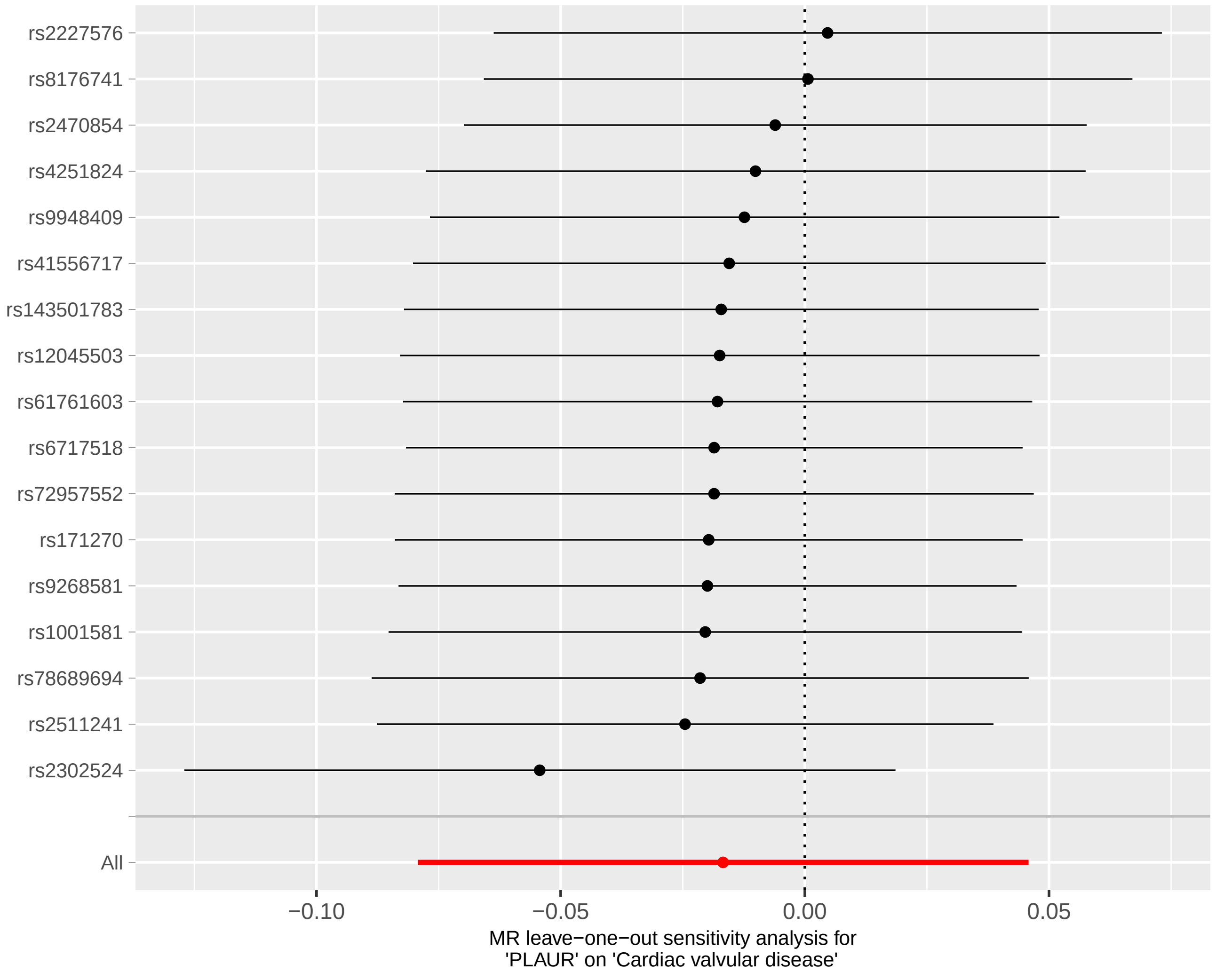
rs2227576
rs8176741
rs2470854
rs4251824
rs9948409
rs41556717
rs143501783
rs12045503
rs61761603
rs6717518
rs72957552
rs171270
rs9268581
rs1001581
rs78689694
rs2511241
rs2302524
All
−0.10
−0.05
0.00
0.05
MR leave−one−out sensitivity analysis for
'PLAUR' on 'Cardiac valvular disease'

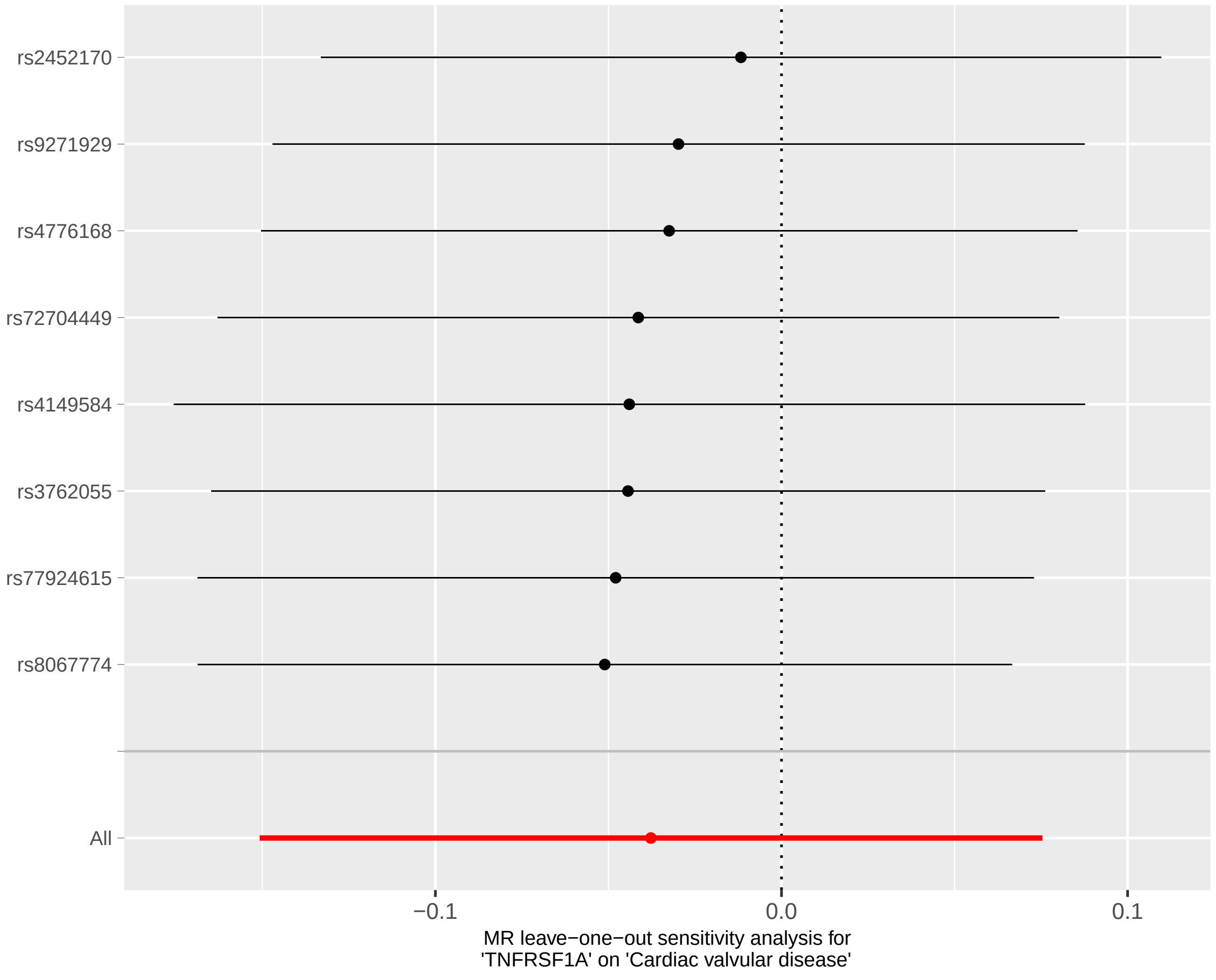

rs2452170
rs9271929
rs4776168
rs72704449
rs4149584
rs3762055
rs77924615
rs8067774
All
−0.1
0.0
0.1
MR leave−one−out sensitivity analysis for
'TNFRSF1A' on 'Cardiac valvular disease'

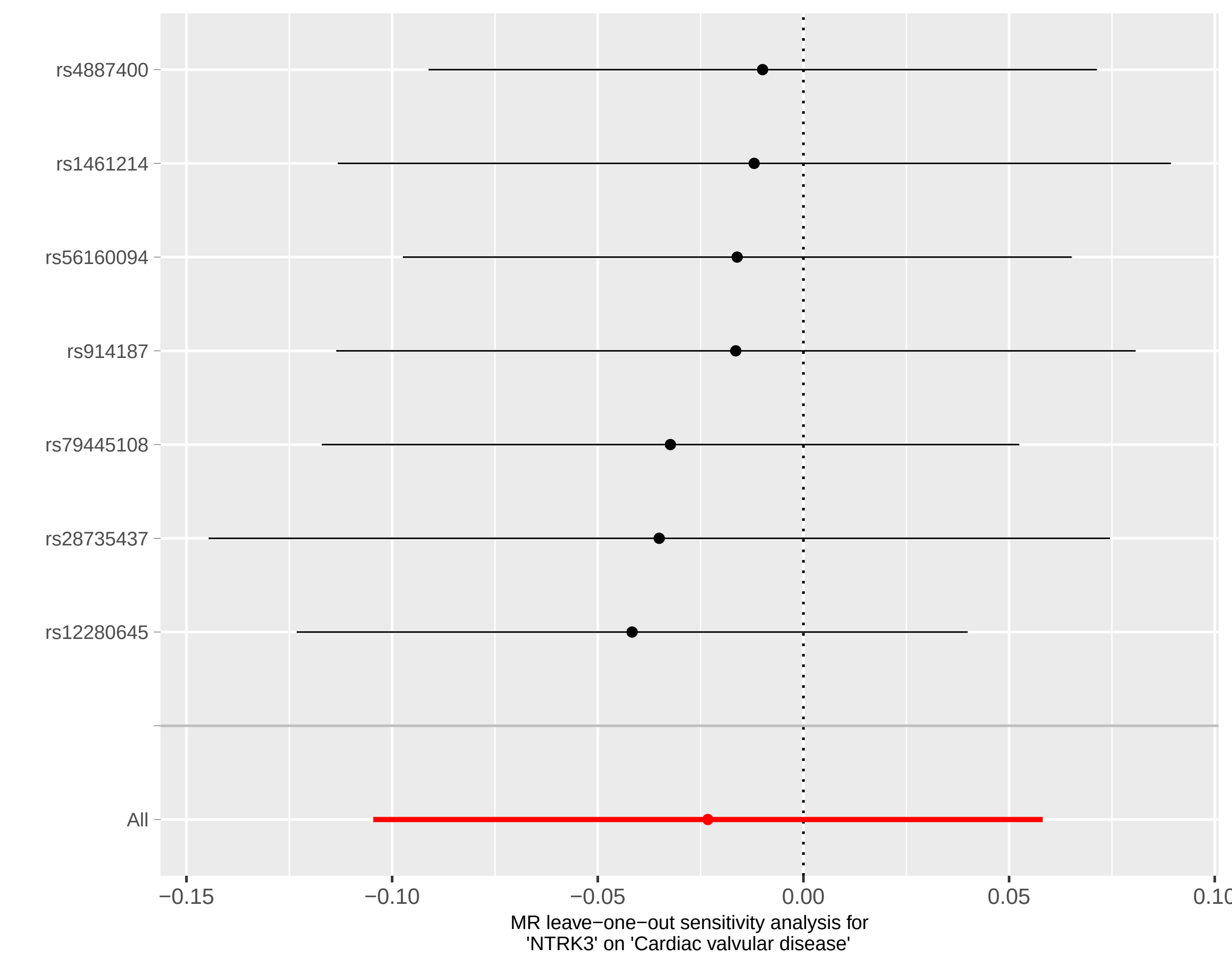
rs4887400
rs1461214
rs56160094
rs914187
rs79445108
rs28735437
rs12280645
All
−0.15
−0.10
−0.05
0.00
0.05
MR leave−one−out sensitivity analysis for
'NTRK3' on 'Cardiac valvular disease'

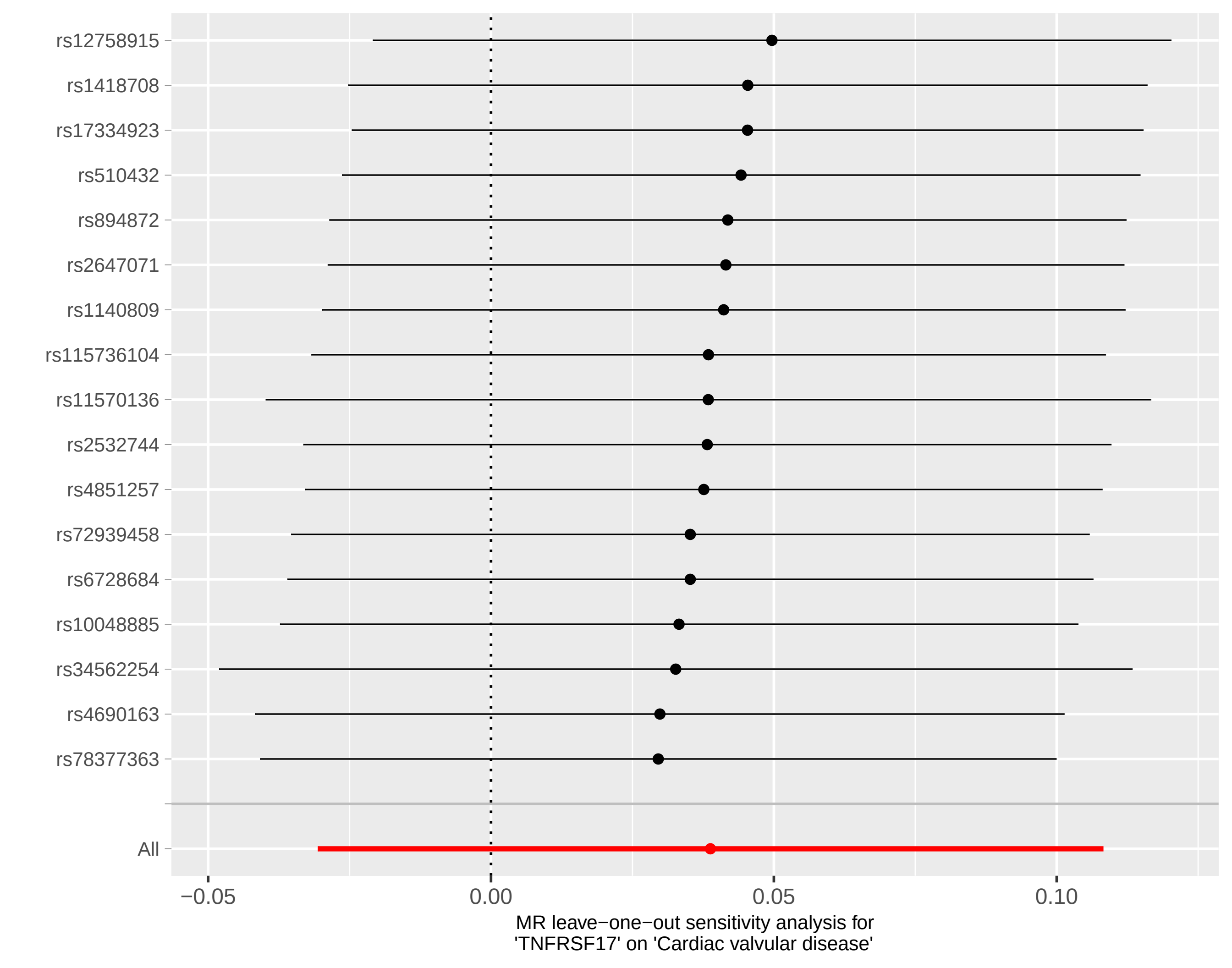
rs12758915
rs1418708
rs17334923
rs510432
rs894872
rs2647071
rs1140809
rs115736104
rs11570136
rs2532744
rs4851257
rs72939458
rs6728684
rs10048885
rs34562254
rs4690163
rs78377363
All
−0.05
0.00
0.05
0.10
MR leave−one−out sensitivity analysis for
'TNFRSF17' on 'Cardiac valvular disease'

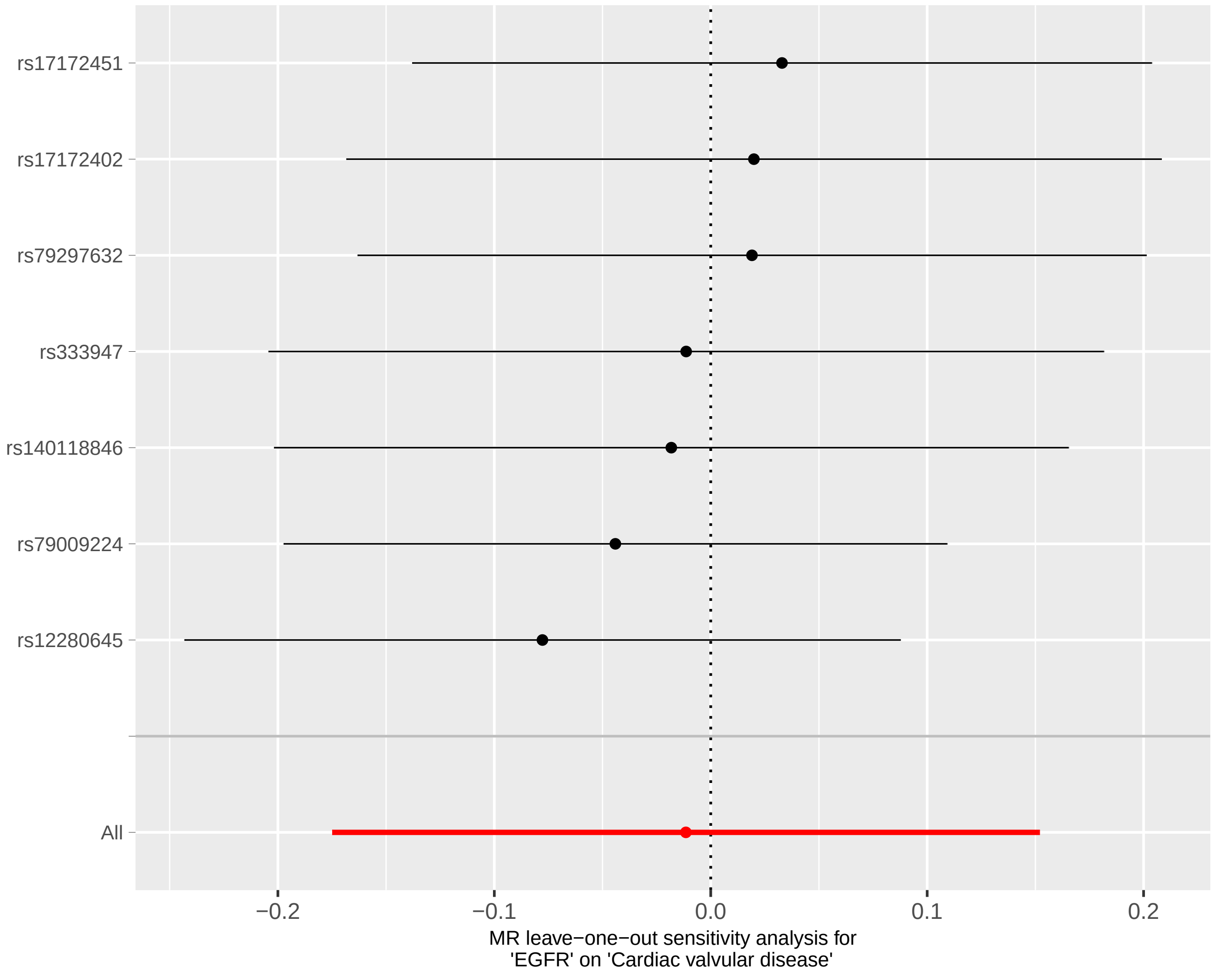

rs17172451
rs17172402
rs79297632
rs333947
rs140118846
rs79009224
rs12280645
All
−0.2
−0.1
0.0
0.1
0.2
MR leave−one−out sensitivity analysis for
'EGFR' on 'Cardiac valvular disease'

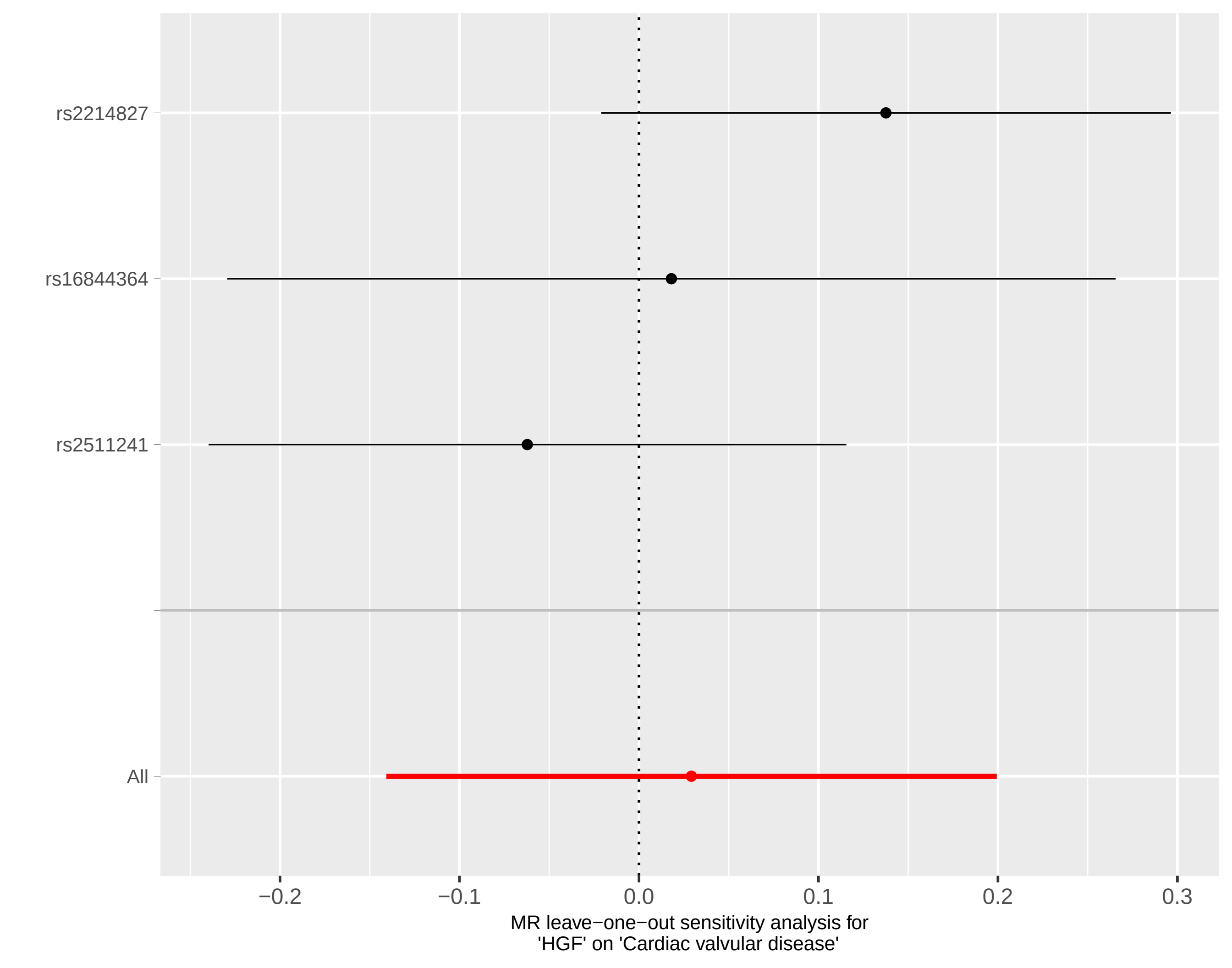
rs2214827
rs16844364
rs2511241
All
−0.2
−0.1
0.0
0.1
0.2
0.3
MR leave−one−out sensitivity analysis for
'HGF' on 'Cardiac valvular disease'

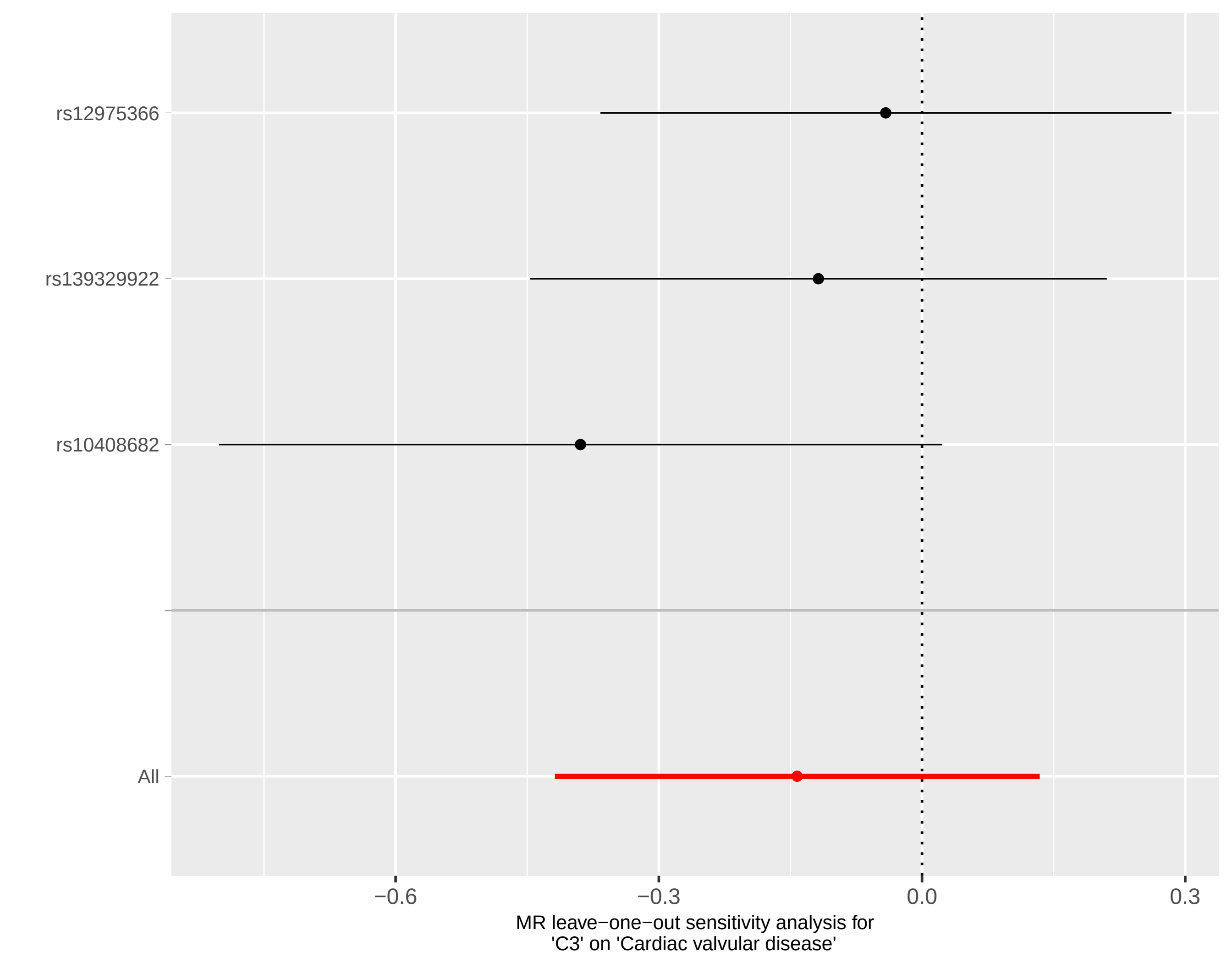

rs12975366
rs139329922
rs10408682
All
−0.6
−0.3
0.0
0.3
MR leave−one−out sensitivity analysis for
'C3' on 'Cardiac valvular disease'

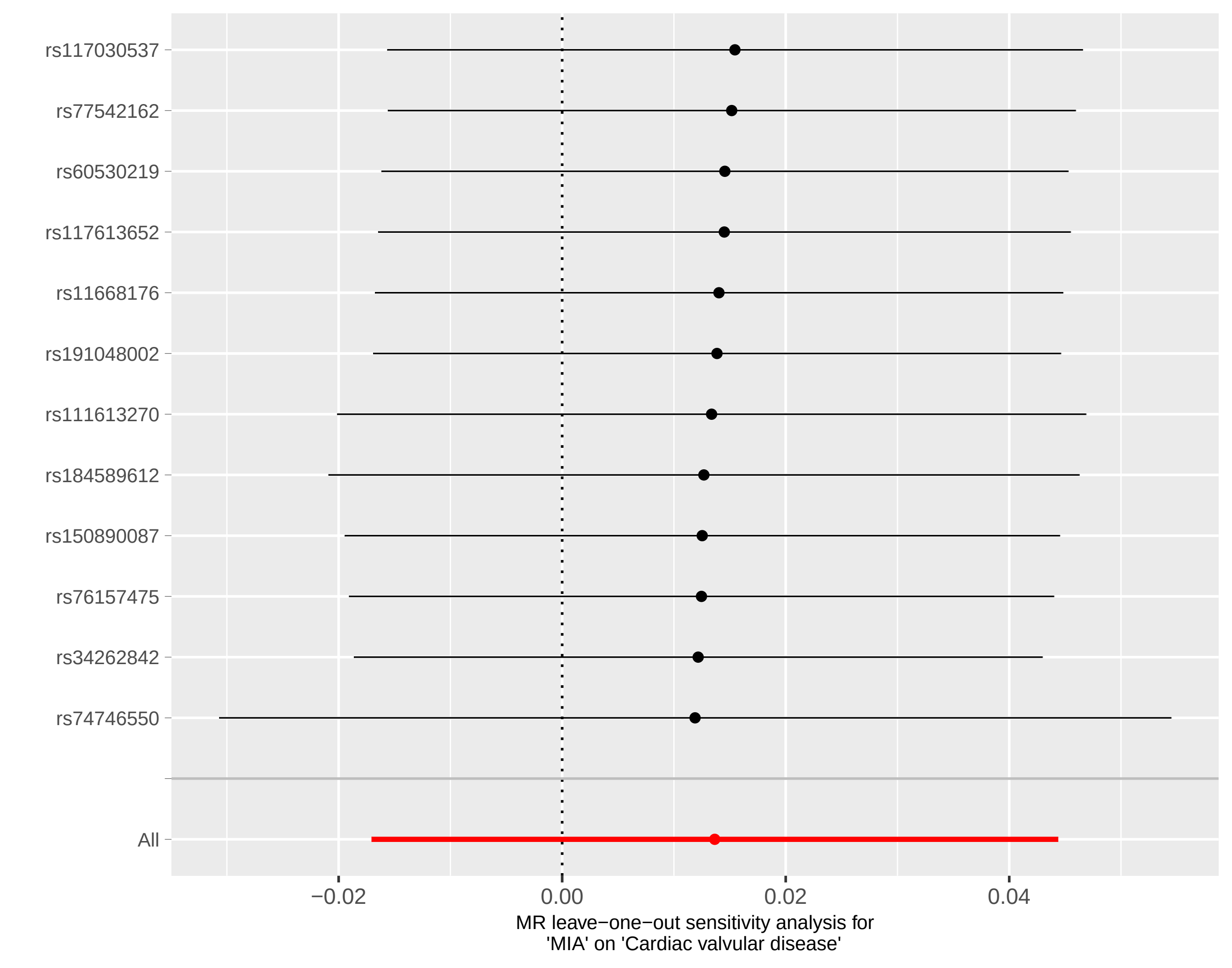
rs117030537
rs77542162
rs60530219
rs117613652
rs11668176
rs191048002
rs111613270
rs184589612
rs150890087
rs76157475
rs34262842
rs74746550
All
−0.02
0.00
0.02
0.04
MR leave−one−out sensitivity analysis for
'MIA' on 'Cardiac valvular disease'

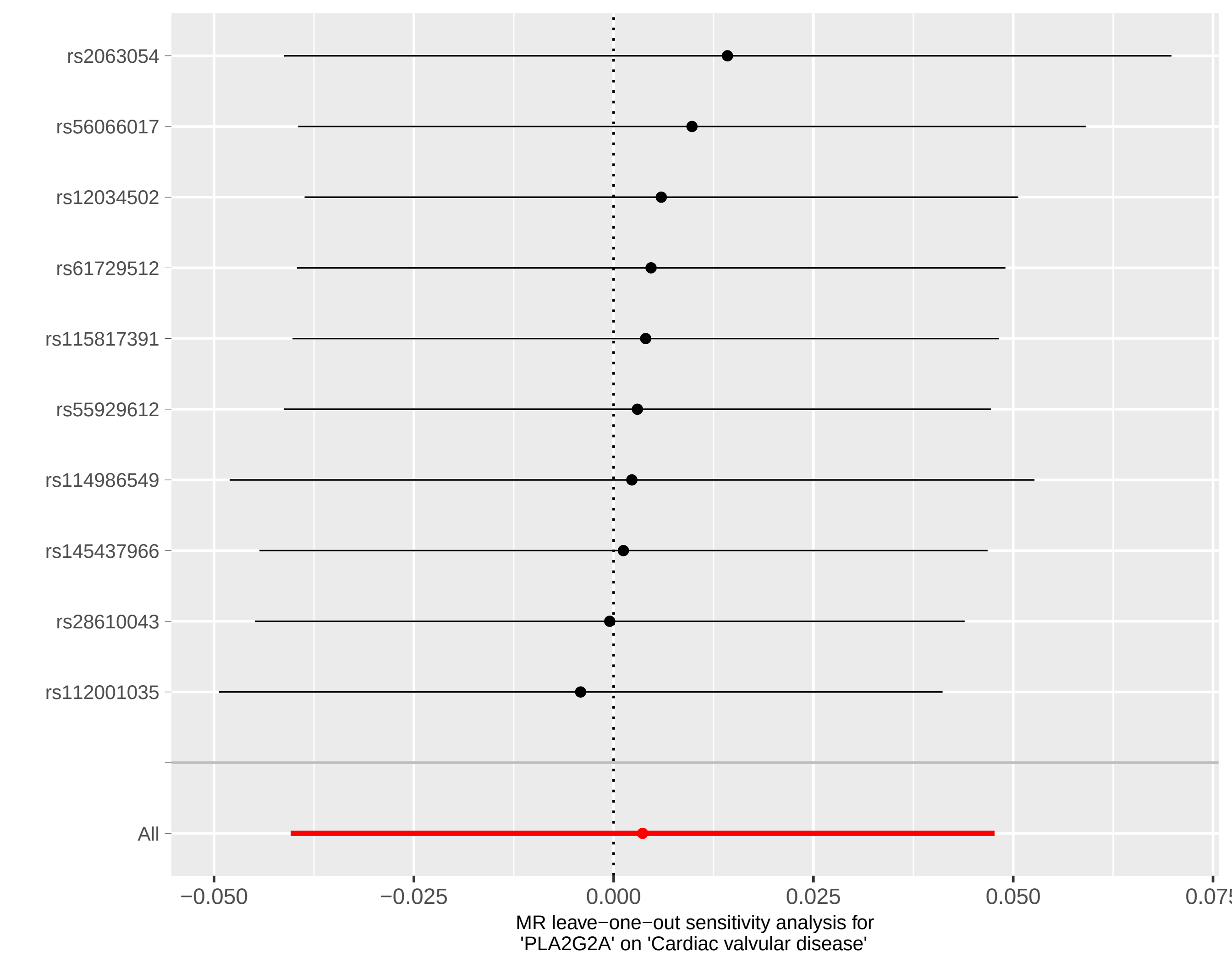
rs2063054
rs56066017
rs12034502
rs61729512
rs115817391
rs55929612
rs114986549
rs145437966
rs28610043
rs112001035
All
−0.050
−0.025
0.000
0.025
0.050
MR leave−one−out sensitivity analysis for
'PLA2G2A' on 'Cardiac valvular disease'

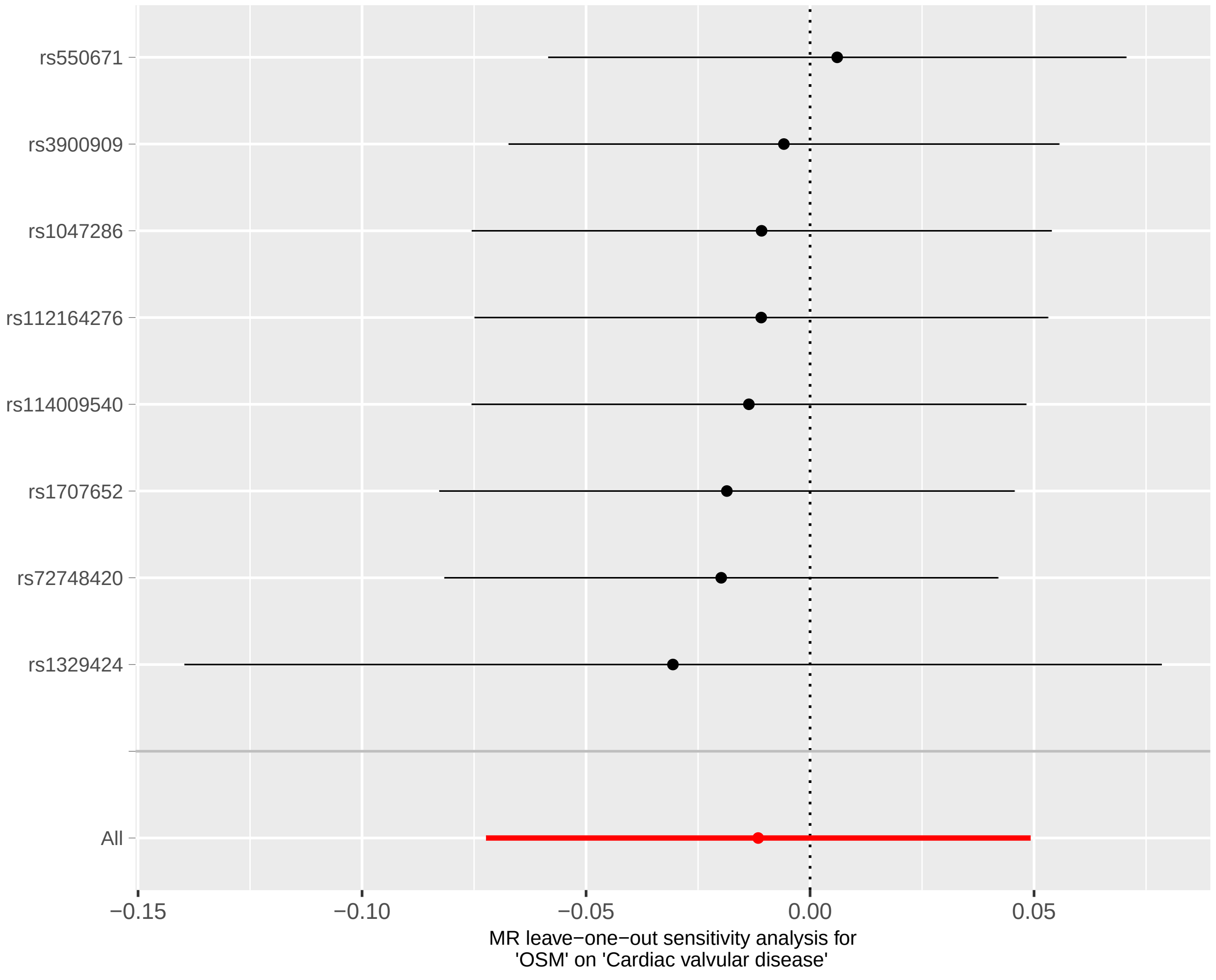

rs550671
rs3900909
rs1047286
rs112164276
rs114009540
rs1707652
rs72748420
rs1329424
All
−0.15
−0.10
−0.05
0.00
0.05
MR leave−one−out sensitivity analysis for
'OSM' on 'Cardiac valvular disease'

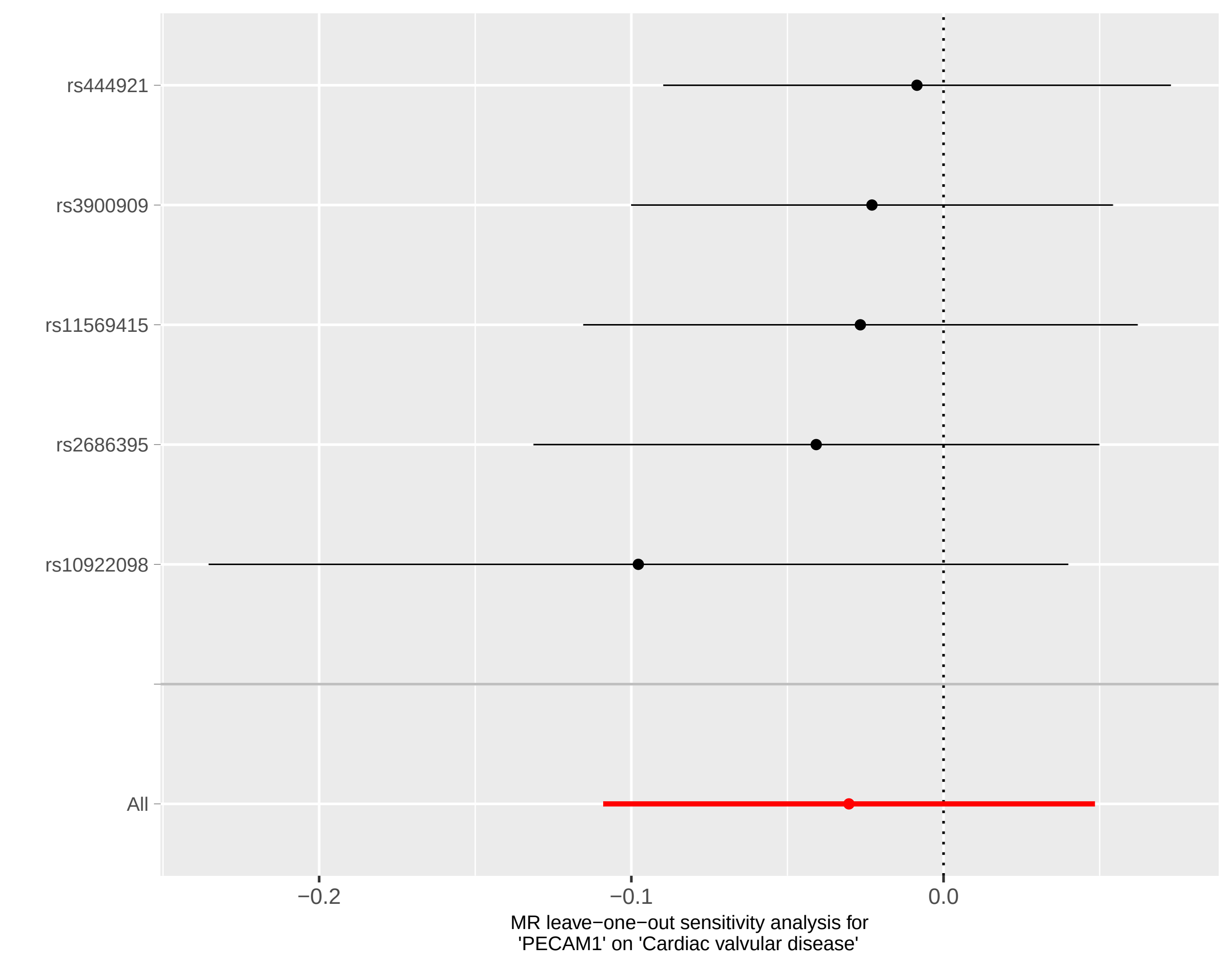
rs444921
rs3900909
rs11569415
rs2686395
rs10922098
All
−0.2
−0.1
0.0
MR leave−one−out sensitivity analysis for
'PECAM1' on 'Cardiac valvular disease'

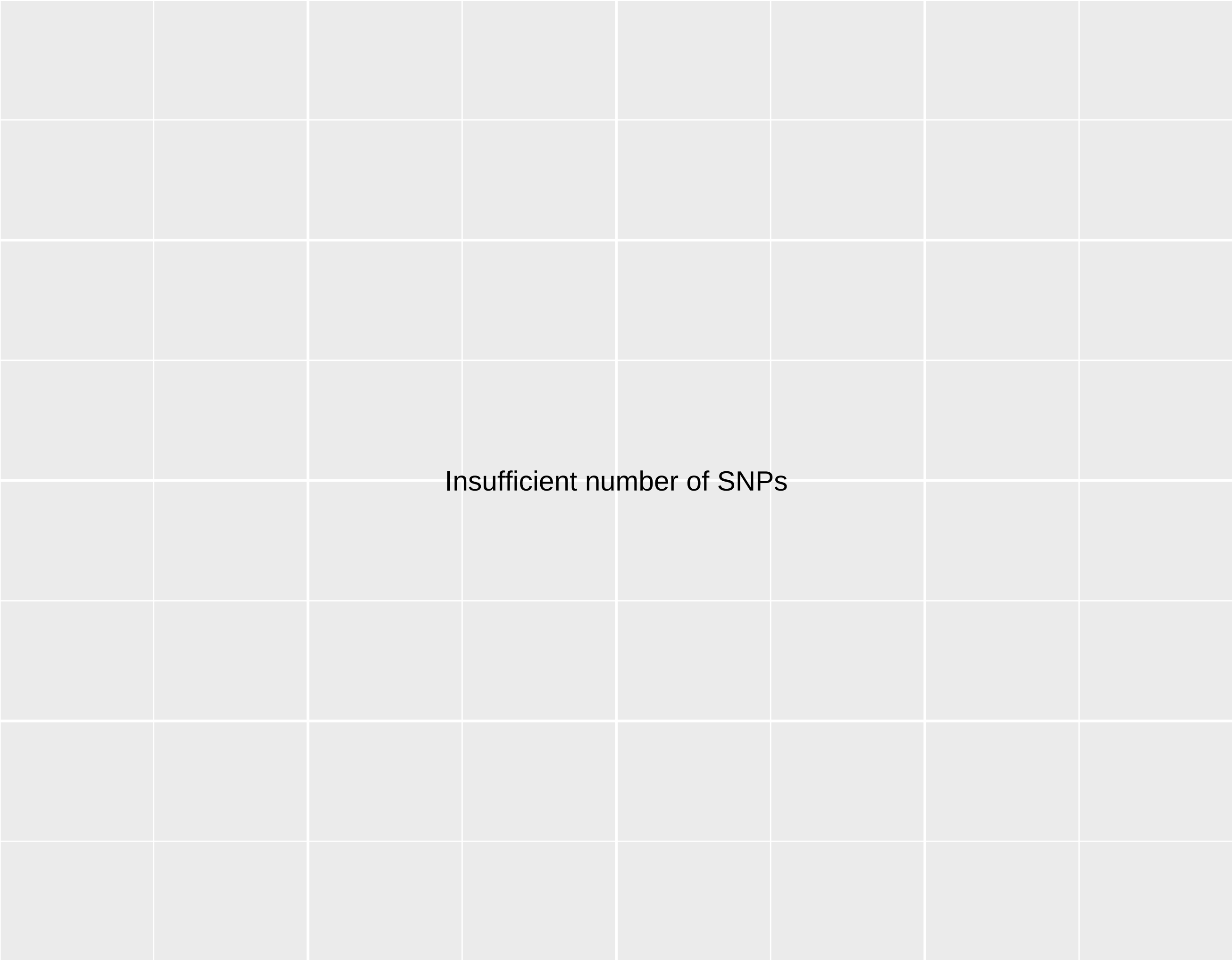
Insufficient number of SNPs

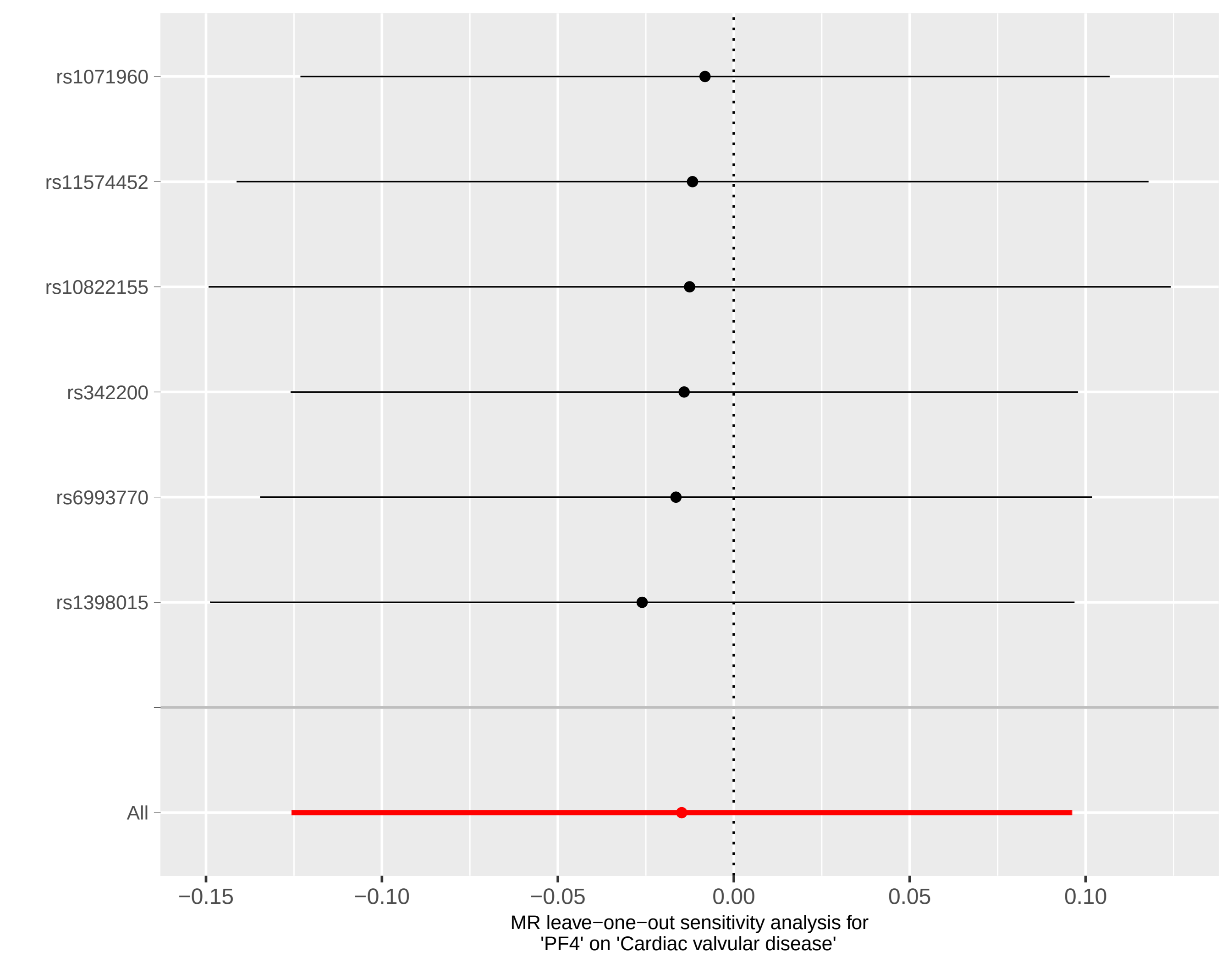
rs1071960
rs11574452
rs10822155
rs342200
rs6993770
rs1398015
All
−0.15
−0.10
−0.05
0.00
0.05
0.10
MR leave−one−out sensitivity analysis for
'PF4' on 'Cardiac valvular disease'

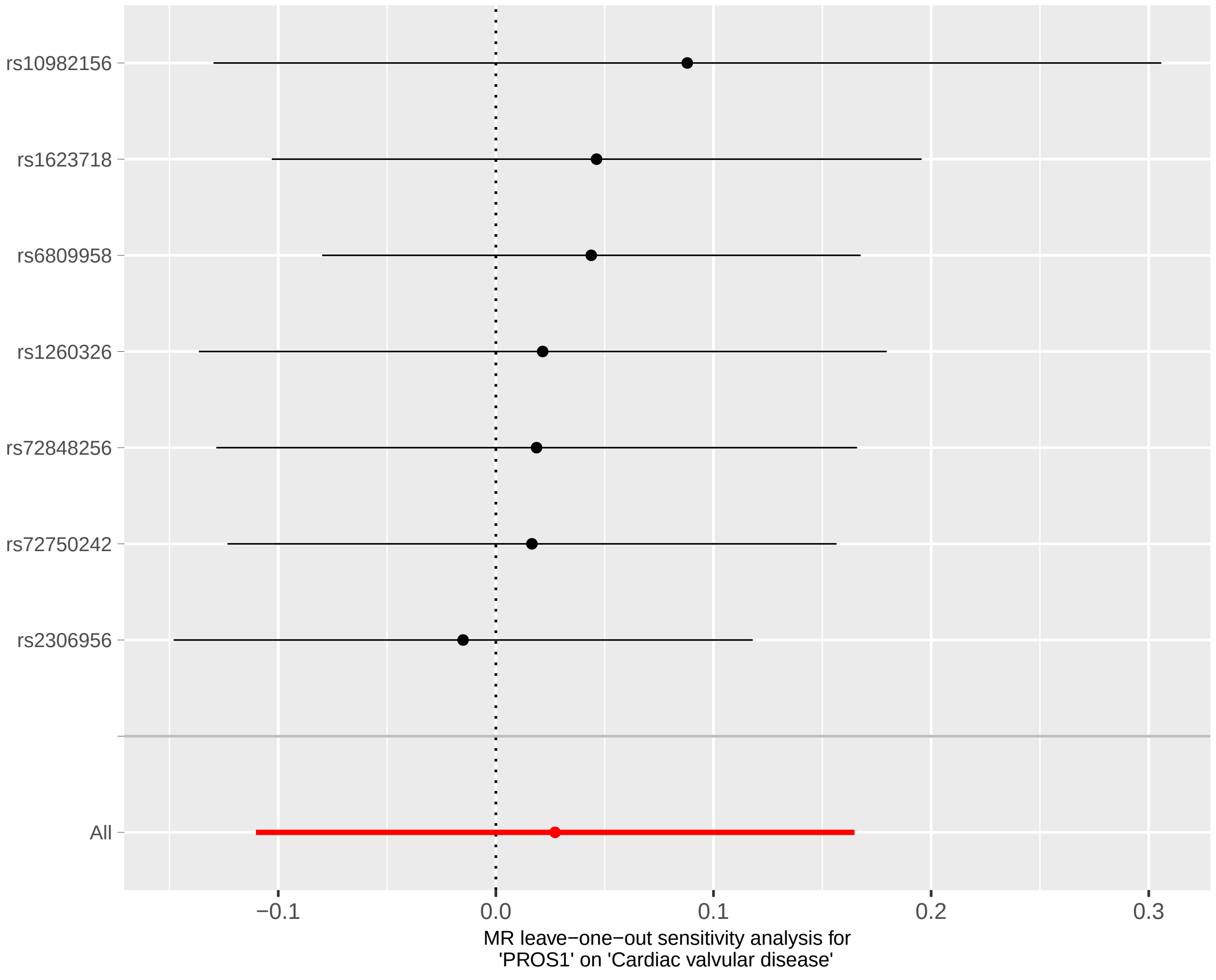

rs10982156
rs1623718
rs6809958
rs1260326
rs72848256
rs72750242
rs2306956
All
−0.1
0.0
0.1
0.2
0.3
MR leave−one−out sensitivity analysis for
'PROS1' on 'Cardiac valvular disease'

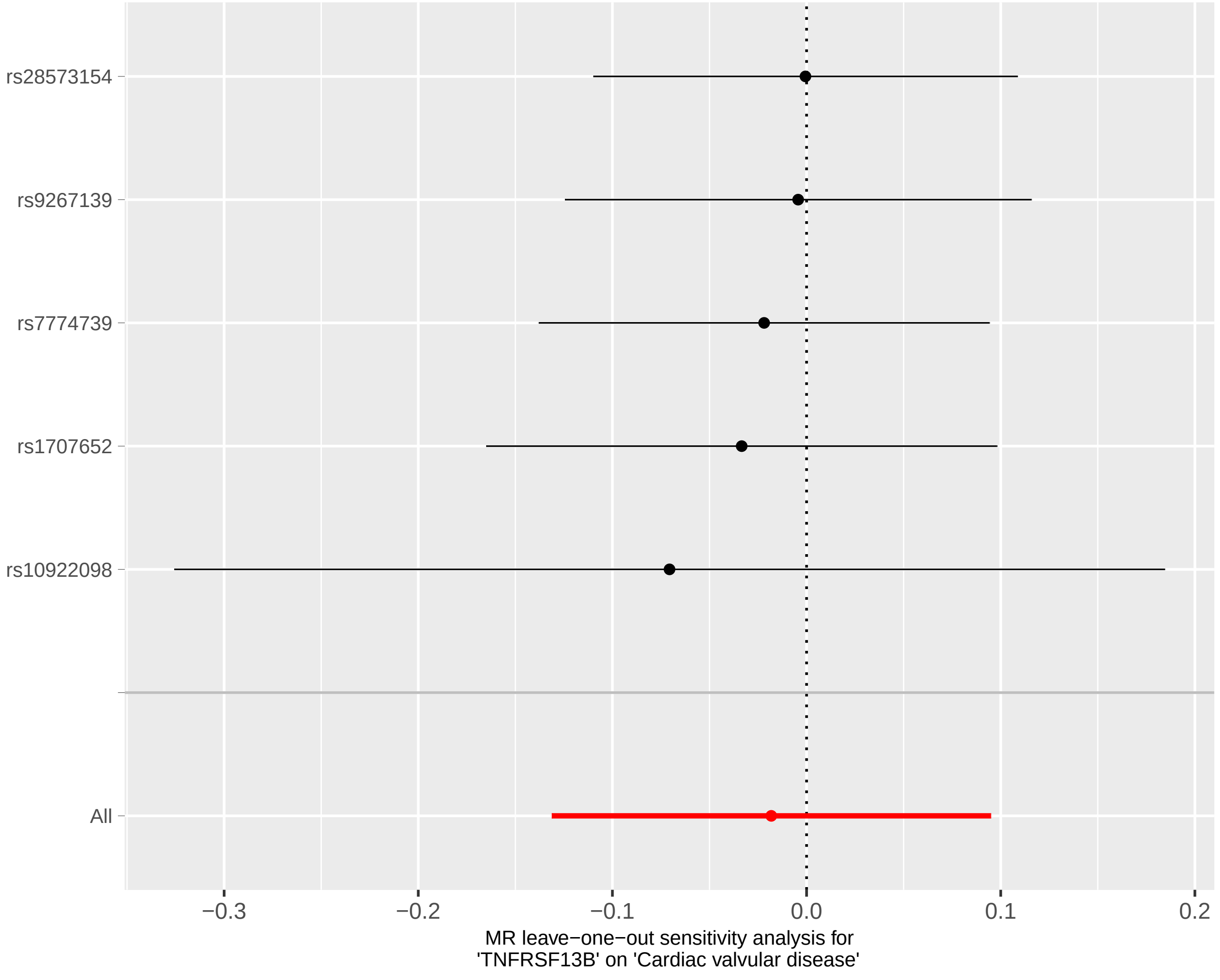

rs28573154
rs9267139
rs7774739
rs1707652
rs10922098
All
−0.3
−0.2
−0.1
0.0
0.1
0.2
MR leave−one−out sensitivity analysis for
'TNFRSF13B' on 'Cardiac valvular disease'

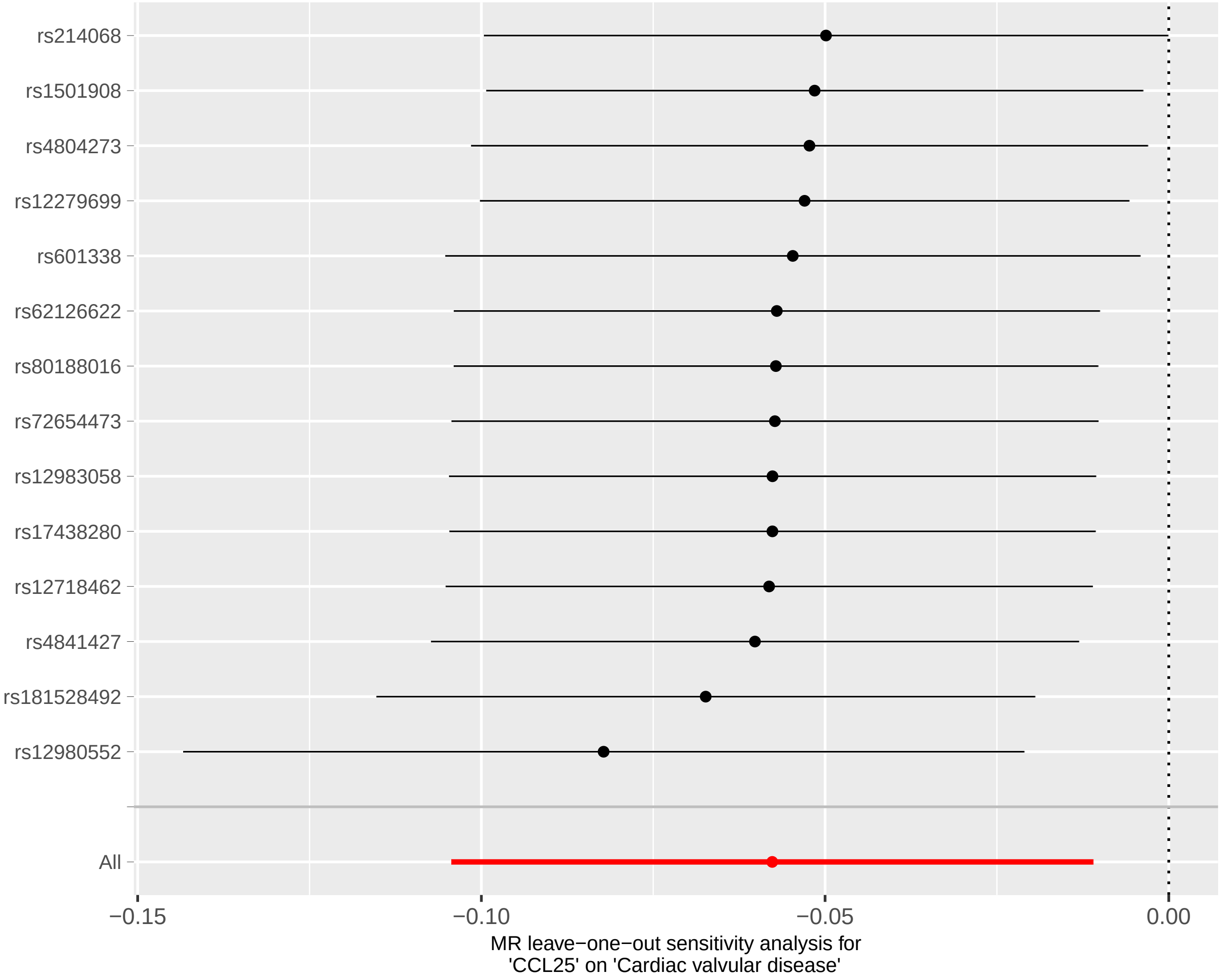
rs214068
rs1501908
rs4804273
rs12279699
rs601338
rs62126622
rs80188016
rs72654473
rs12983058
rs17438280
rs12718462
rs4841427
rs181528492
rs12980552
All
−0.15
−0.10
−0.05
0.00
MR leave−one−out sensitivity analysis for
'CCL25' on 'Cardiac valvular disease'

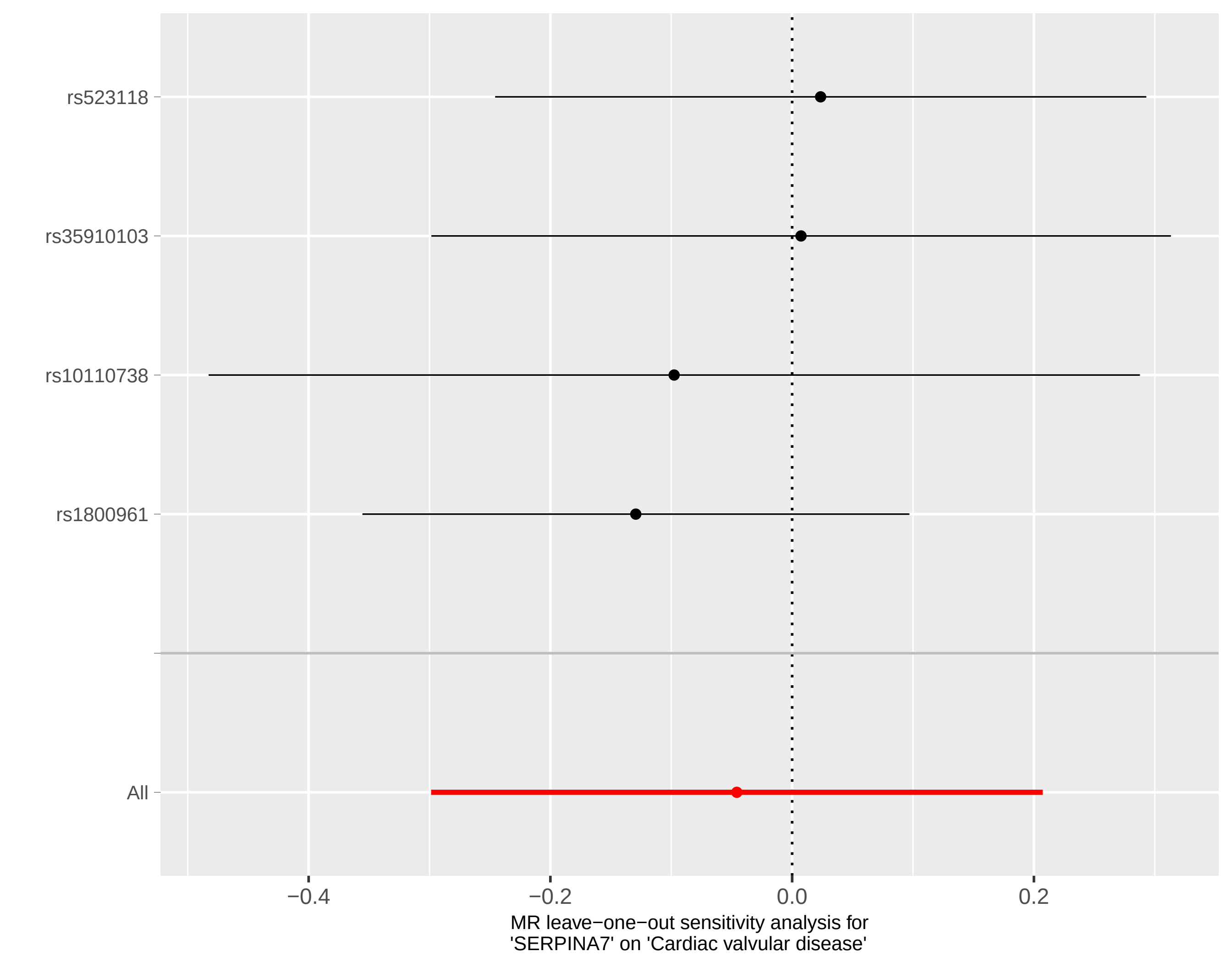

rs523118
rs35910103
rs10110738
rs1800961
All
−0.4
−0.2
0.0
0.2
MR leave−one−out sensitivity analysis for
'SERPINA7' on 'Cardiac valvular disease'

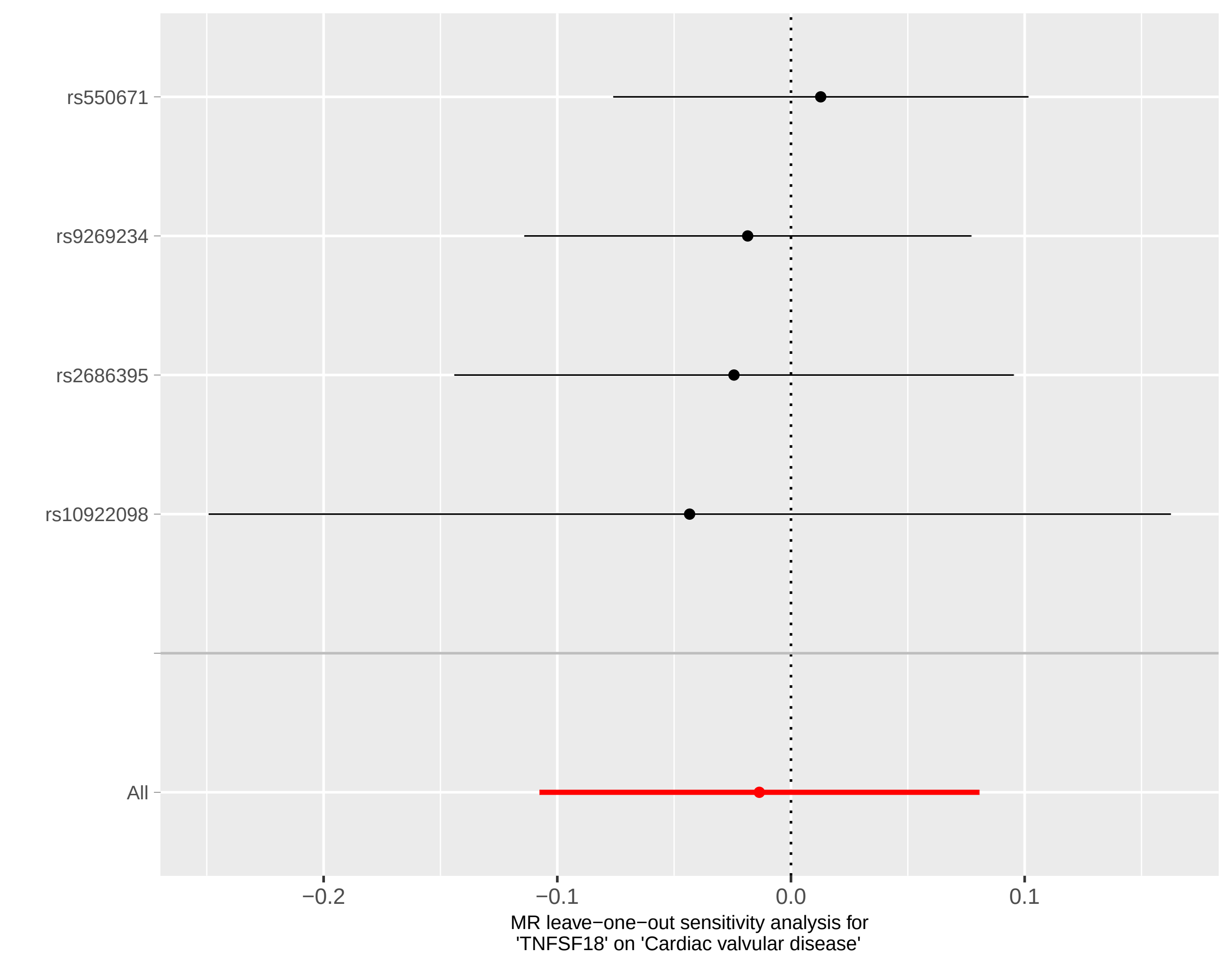

rs550671
rs9269234
rs2686395
rs10922098
All
−0.2
−0.1
0.0
0.1
MR leave−one−out sensitivity analysis for
'TNFSF18' on 'Cardiac valvular disease'

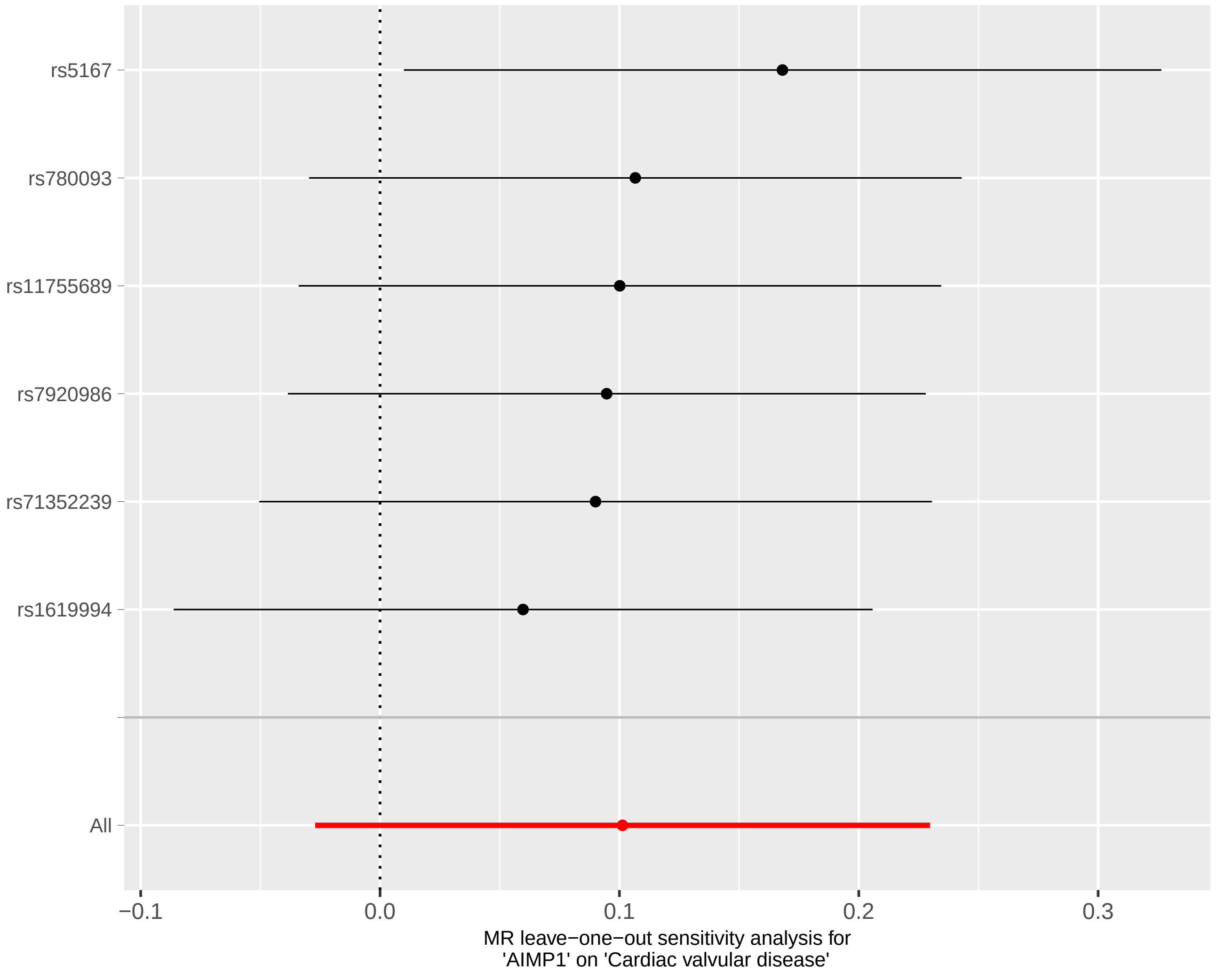

rs5167
rs780093
rs11755689
rs7920986
rs71352239
rs1619994
All
−0.1
0.0
0.1
0.2
0.3
MR leave−one−out sensitivity analysis for
'AIMP1' on 'Cardiac valvular disease'

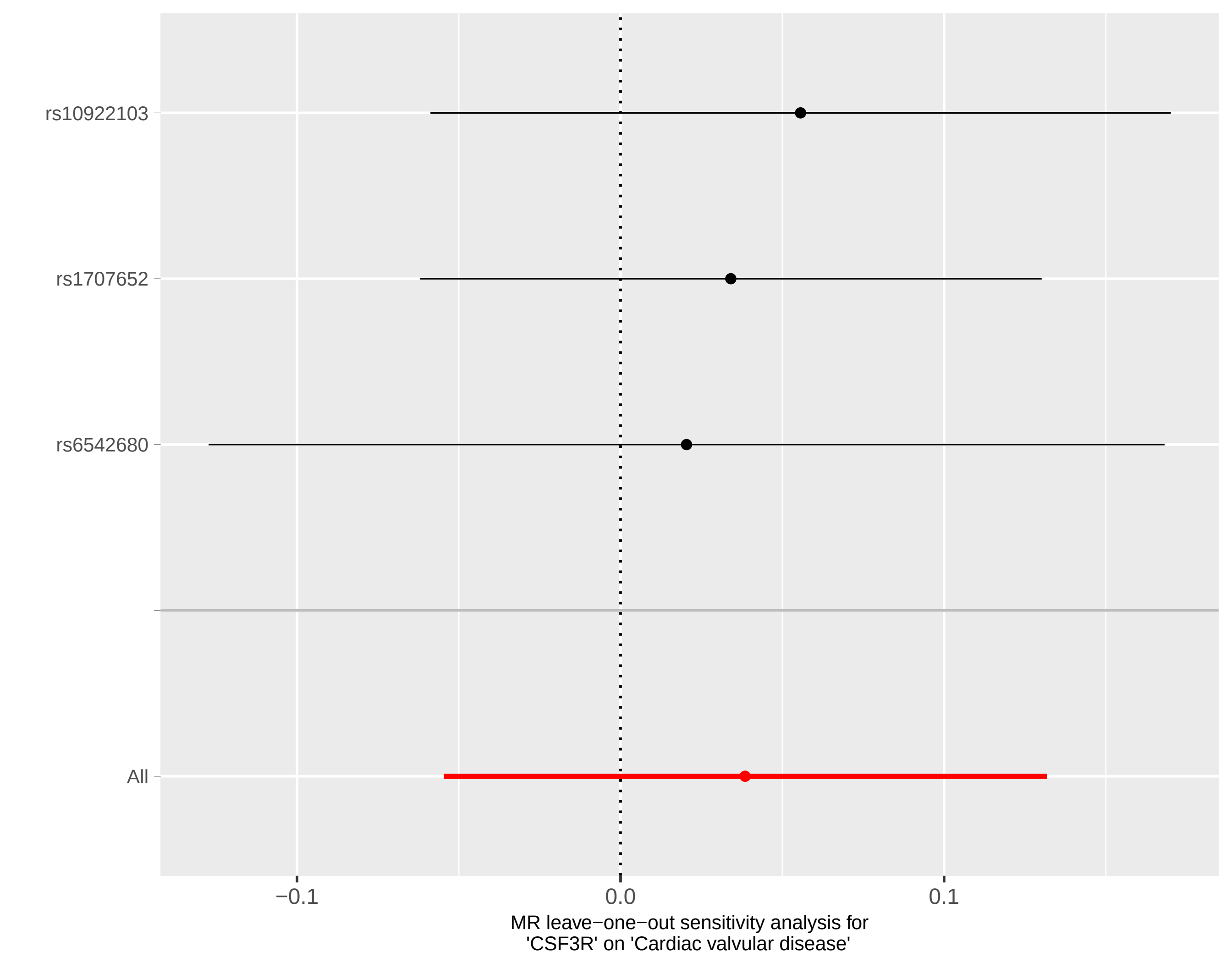

rs10922103
rs1707652
rs6542680
All
−0.1
0.0
0.1
MR leave−one−out sensitivity analysis for
'CSF3R' on 'Cardiac valvular disease'

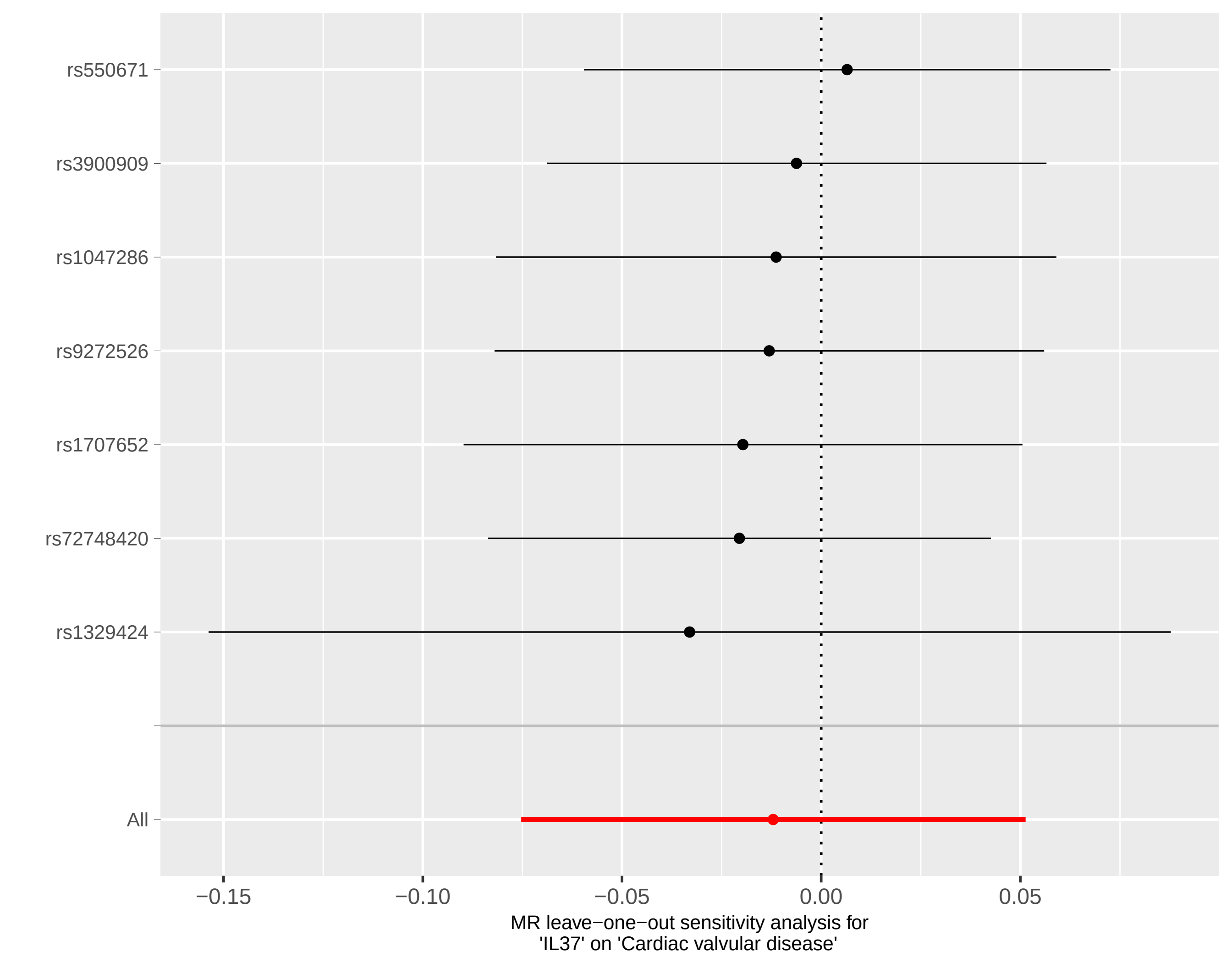
rs550671
rs3900909
rs1047286
rs9272526
rs1707652
rs72748420
rs1329424
All
−0.15
−0.10
−0.05
0.00
0.05
MR leave−one−out sensitivity analysis for
'IL37' on 'Cardiac valvular disease'

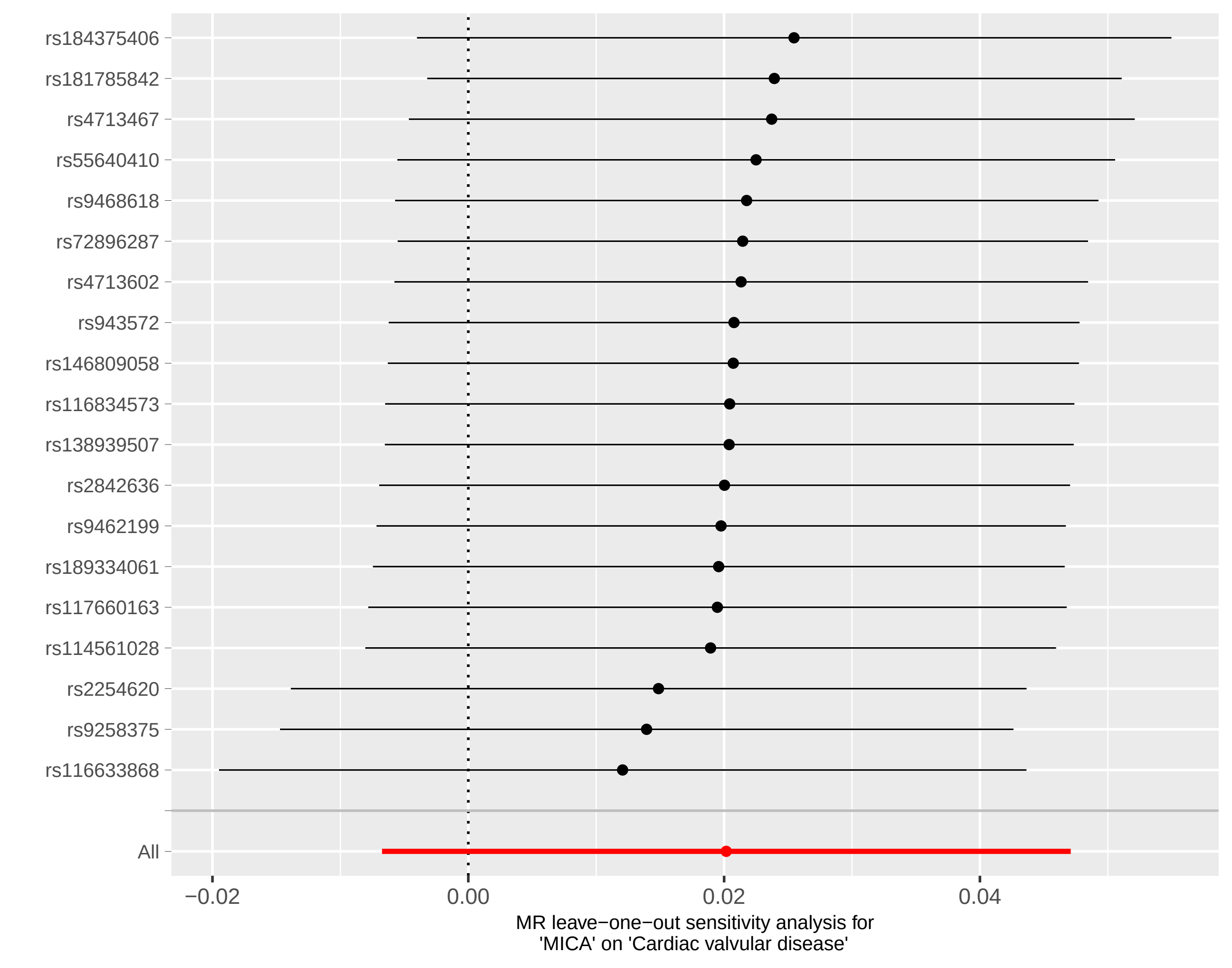

rs184375406
rs181785842
rs4713467
rs55640410
rs9468618
rs72896287
rs4713602
rs943572
rs146809058
rs116834573
rs138939507
rs2842636
rs9462199
rs189334061
rs117660163
rs114561028
rs2254620
rs9258375
rs116633868
All
−0.02
0.00
0.02
0.04
MR leave−one−out sensitivity analysis for
'MICA' on 'Cardiac valvular disease'

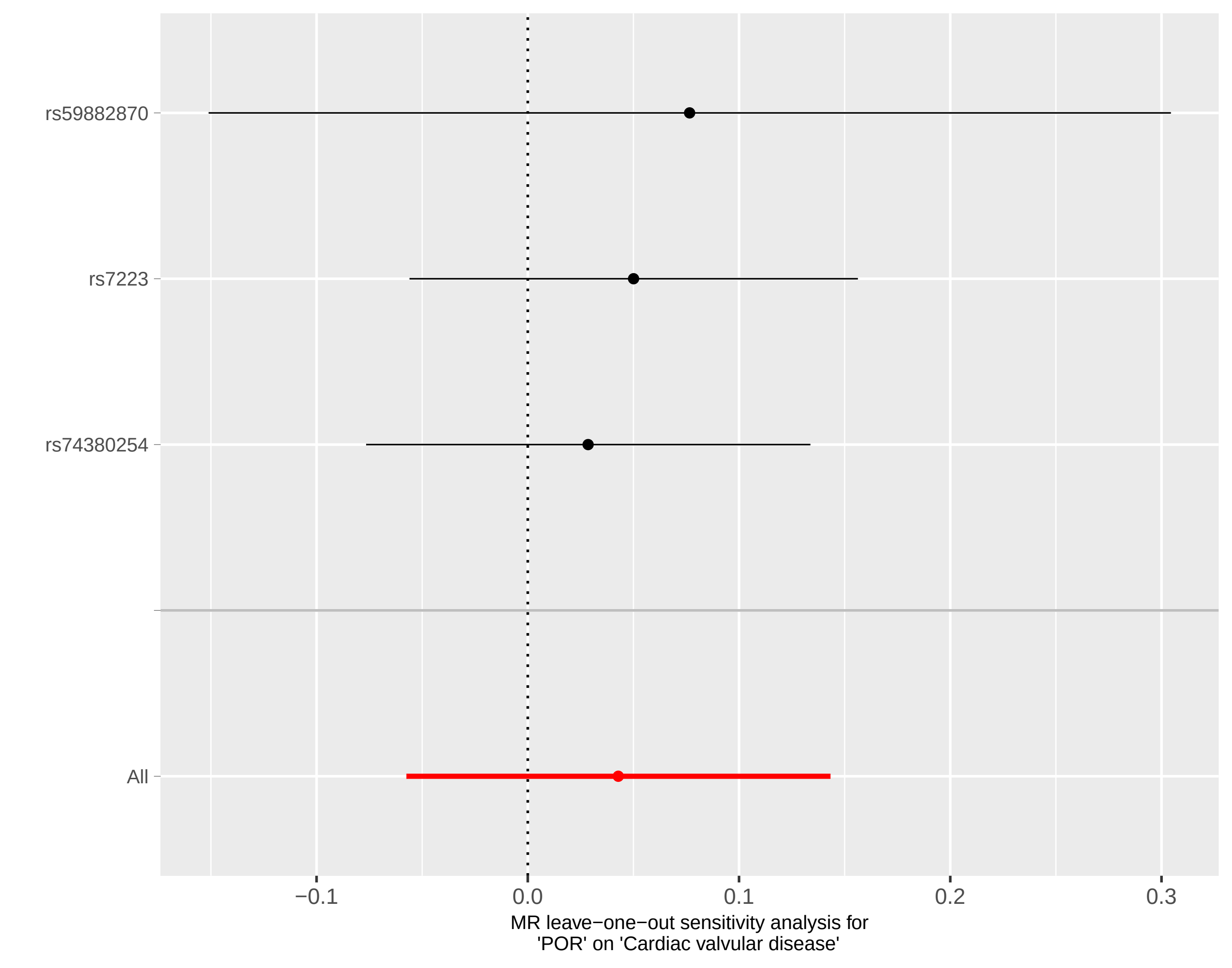
rs59882870
rs7223
rs74380254
All
−0.1
0.0
0.1
0.2
0.3
MR leave−one−out sensitivity analysis for
'POR' on 'Cardiac valvular disease'

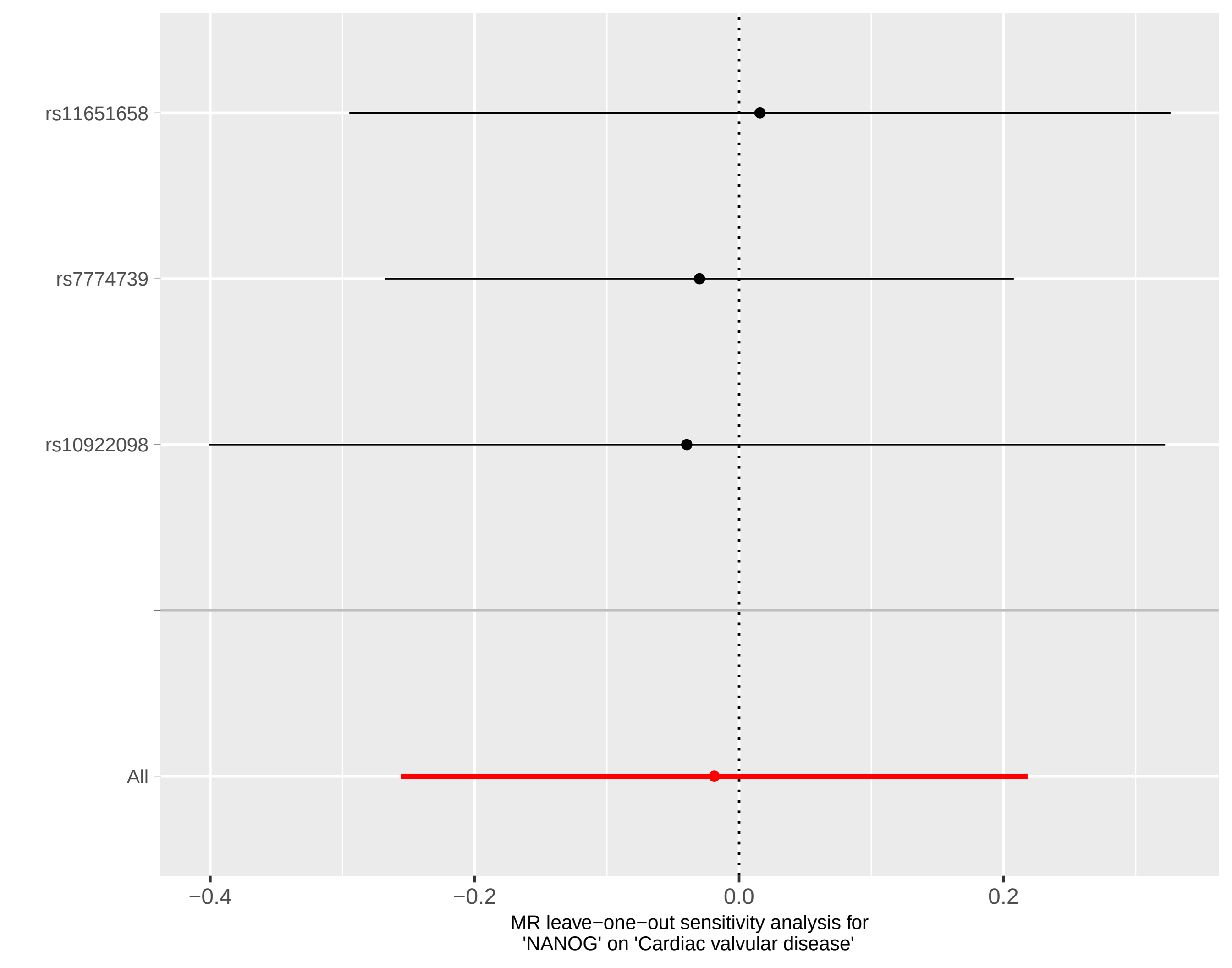
rs11651658
rs7774739
rs10922098
All
−0.4
−0.2
0.0
0.2
MR leave−one−out sensitivity analysis for
'NANOG' on 'Cardiac valvular disease'

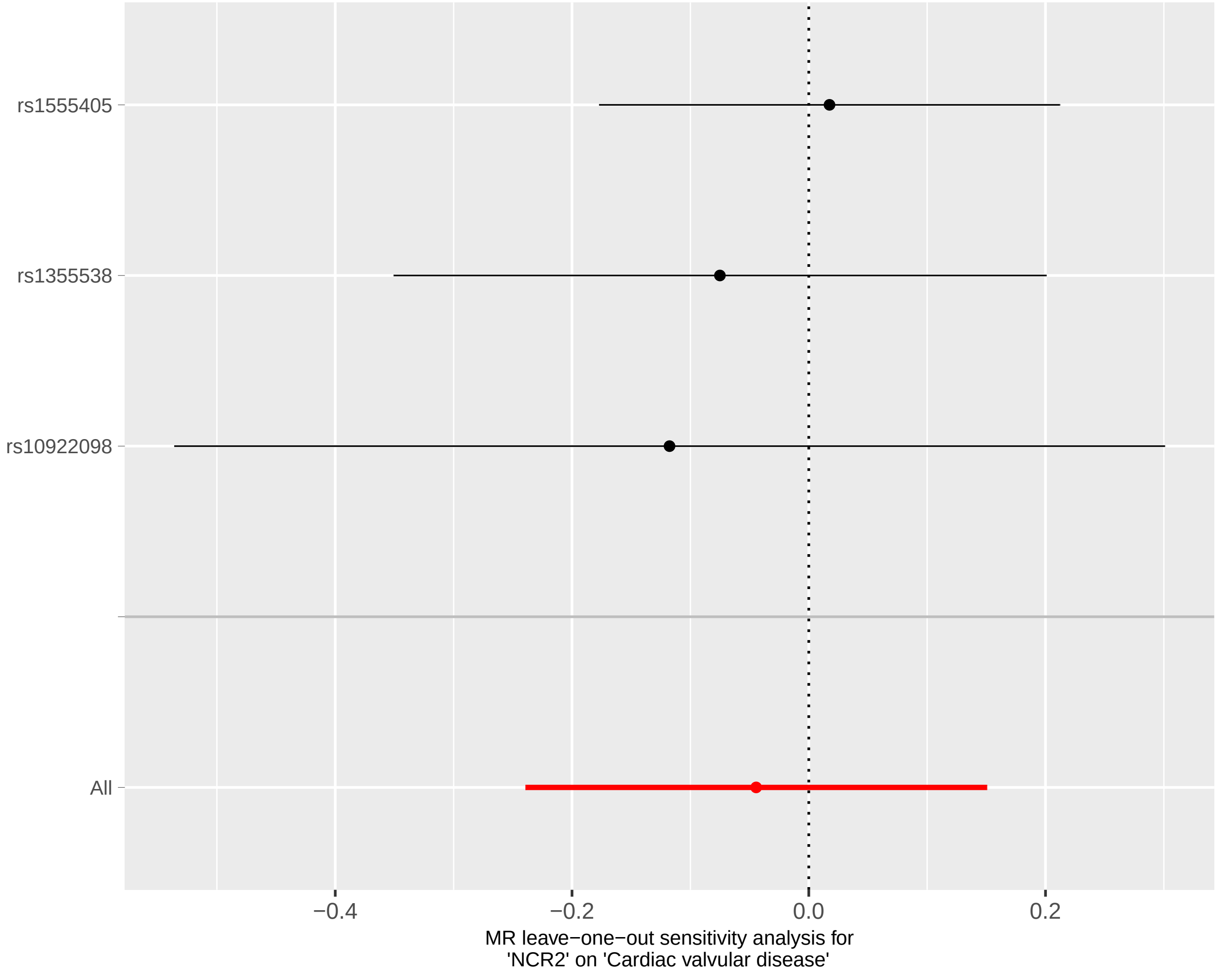

rs1555405
rs1355538
rs10922098
All
−0.4
−0.2
0.0
0.2
MR leave−one−out sensitivity analysis for
'NCR2' on 'Cardiac valvular disease'

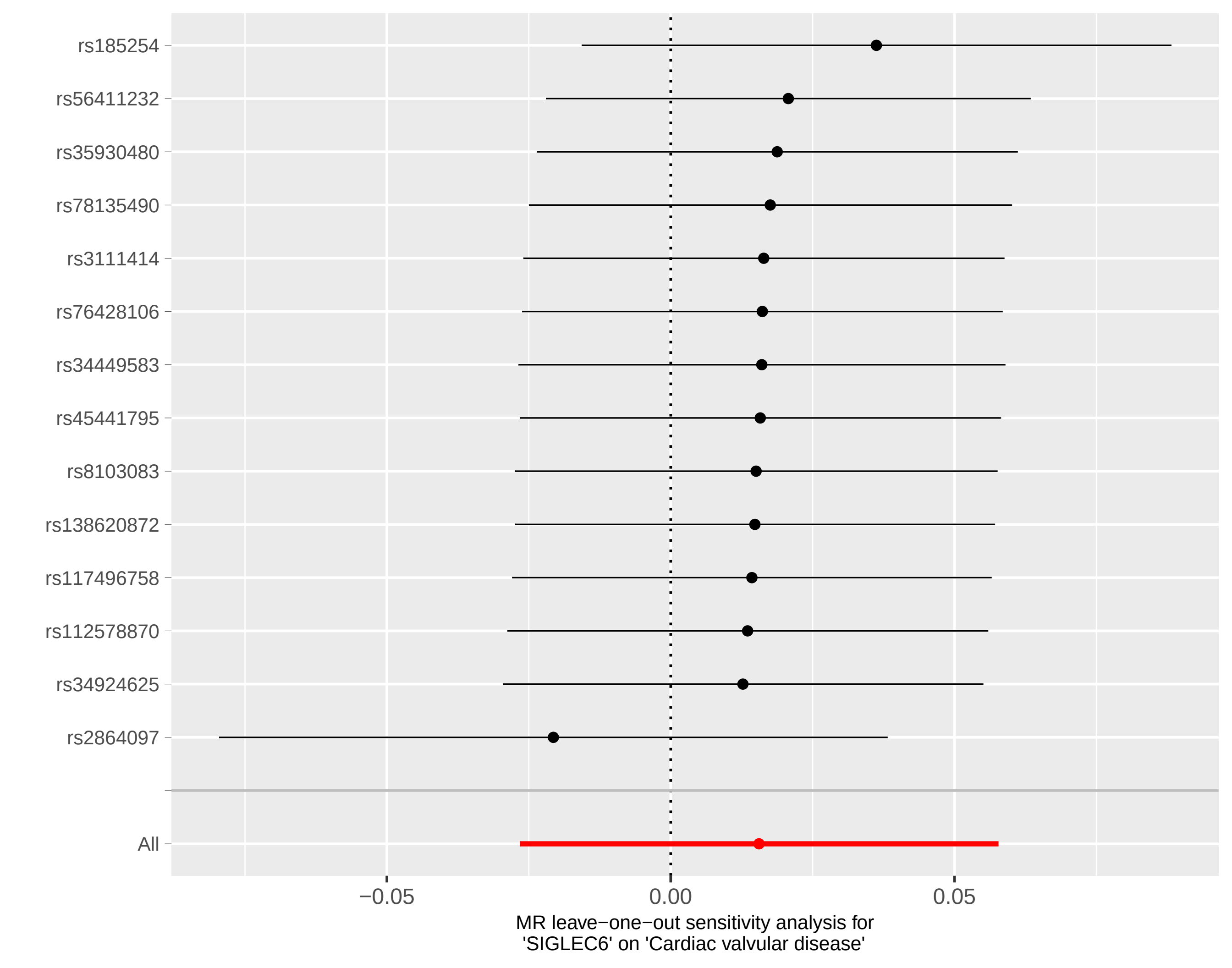
rs185254
rs56411232
rs35930480
rs78135490
rs3111414
rs76428106
rs34449583
rs45441795
rs8103083
rs138620872
rs117496758
rs112578870
rs34924625
rs2864097
All
−0.05
0.00
0.05
MR leave−one−out sensitivity analysis for
'SIGLEC6' on 'Cardiac valvular disease'

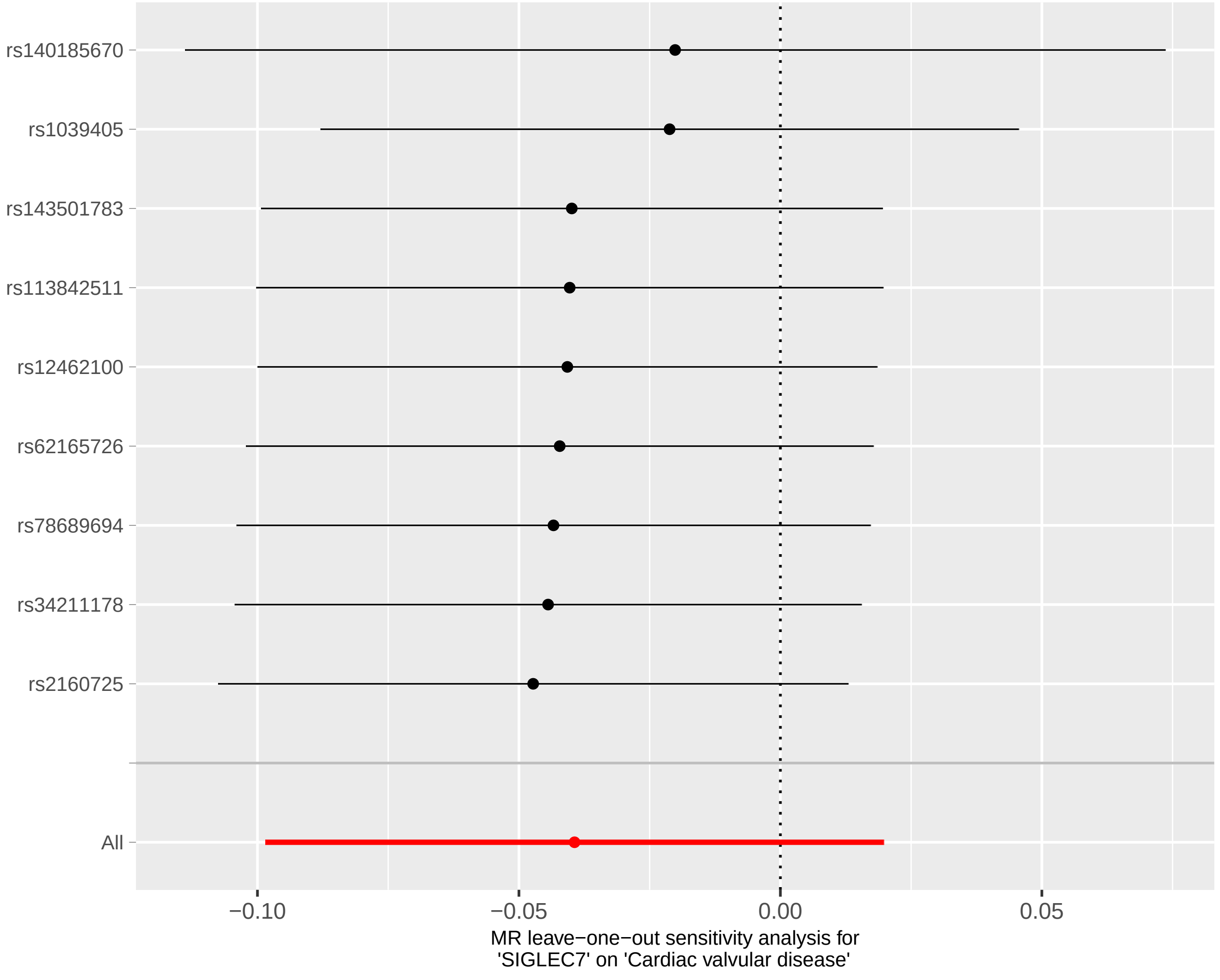

rs140185670
rs1039405
rs143501783
rs113842511
rs12462100
rs62165726
rs78689694
rs34211178
rs2160725
All
−0.10
−0.05
0.00
0.05
MR leave−one−out sensitivity analysis for
'SIGLEC7' on 'Cardiac valvular disease'

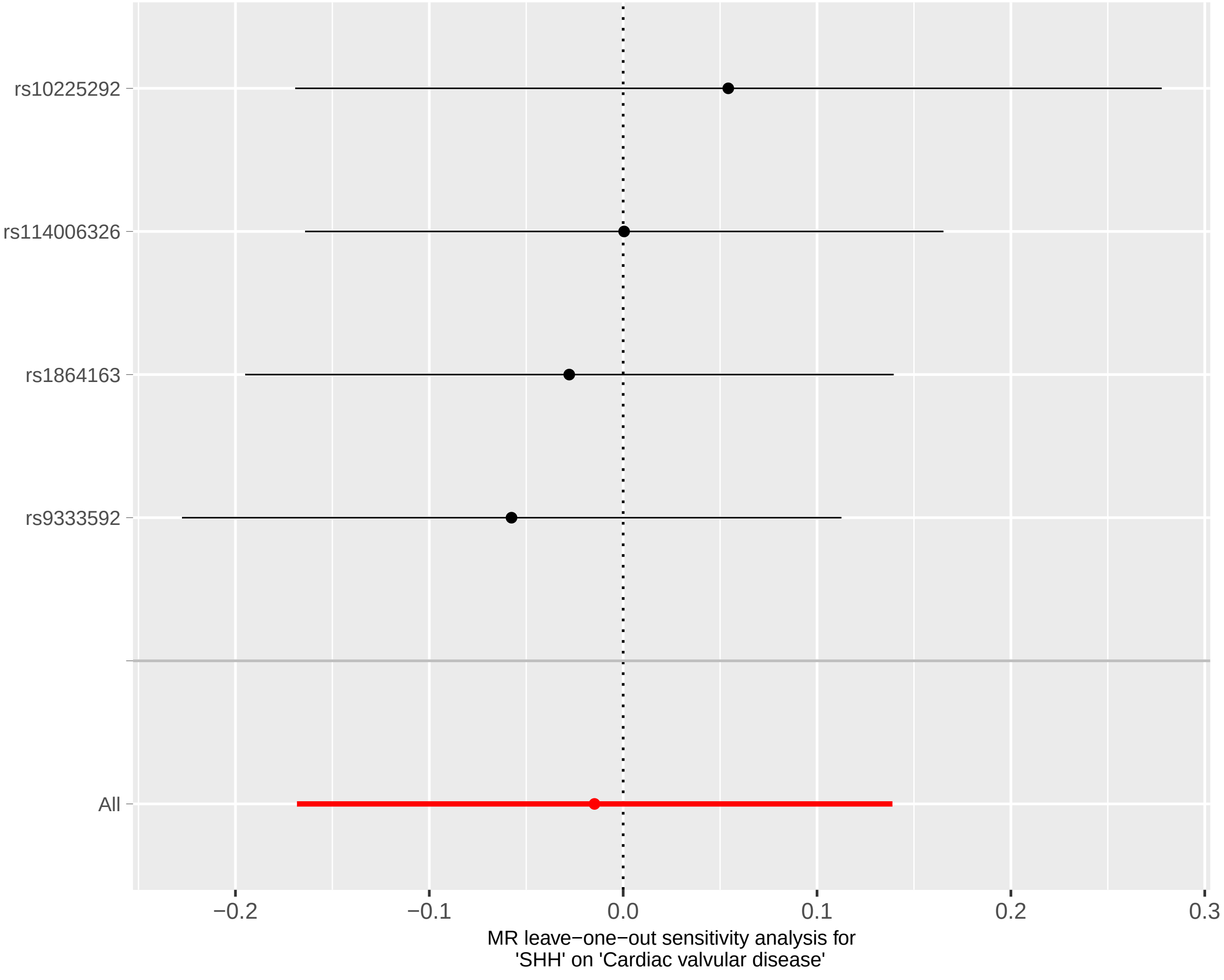
rs10225292
rs114006326
rs1864163
rs9333592
All
−0.2
−0.1
0.0
0.1
0.2
0.3
MR leave−one−out sensitivity analysis for
'SHH' on 'Cardiac valvular disease'

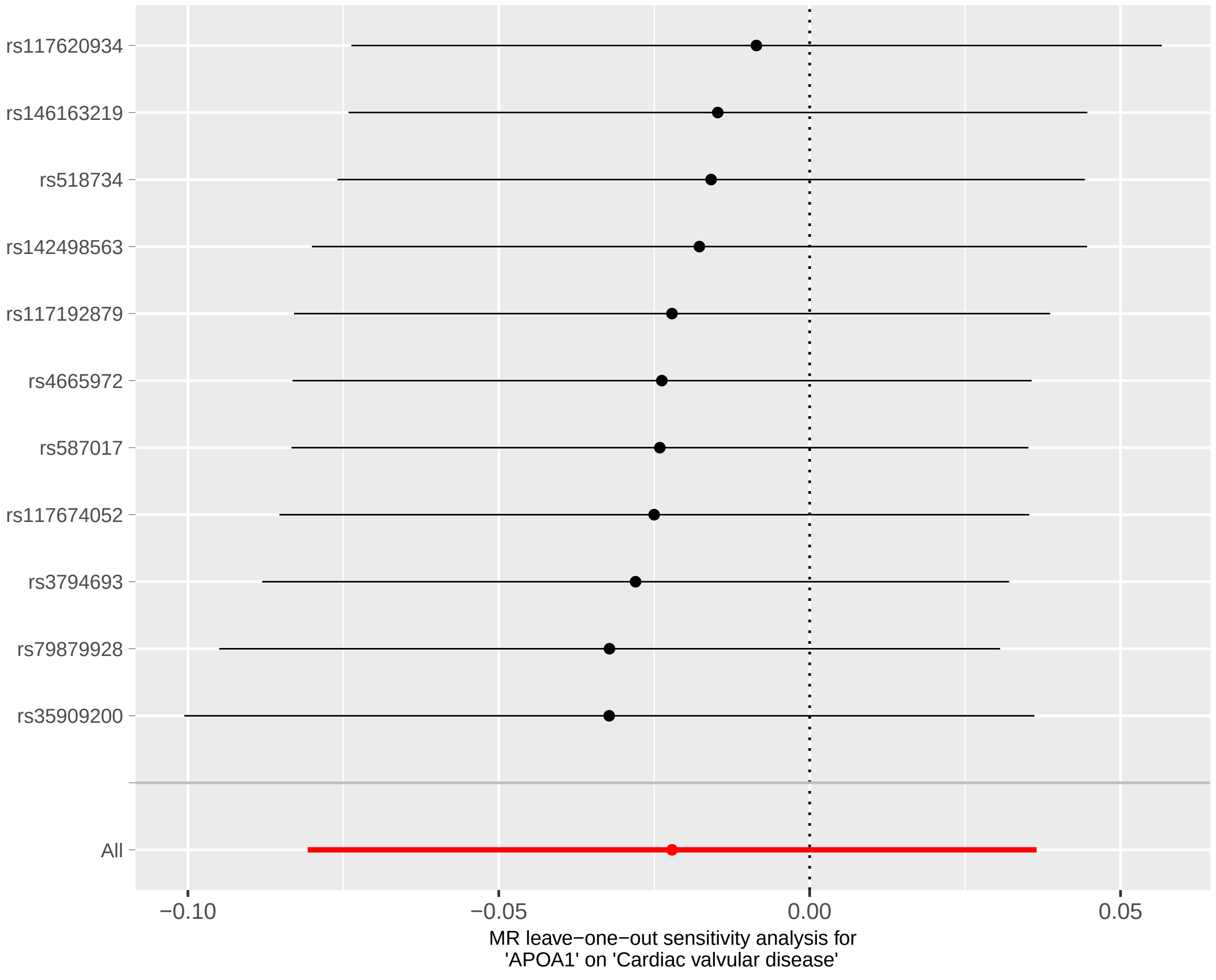

rs117620934
rs146163219
rs518734
rs142498563
rs117192879
rs4665972
rs587017
rs117674052
rs3794693
rs79879928
rs35909200
All
−0.10
−0.05
0.00
0.05
MR leave−one−out sensitivity analysis for
'APOA1' on 'Cardiac valvular disease'

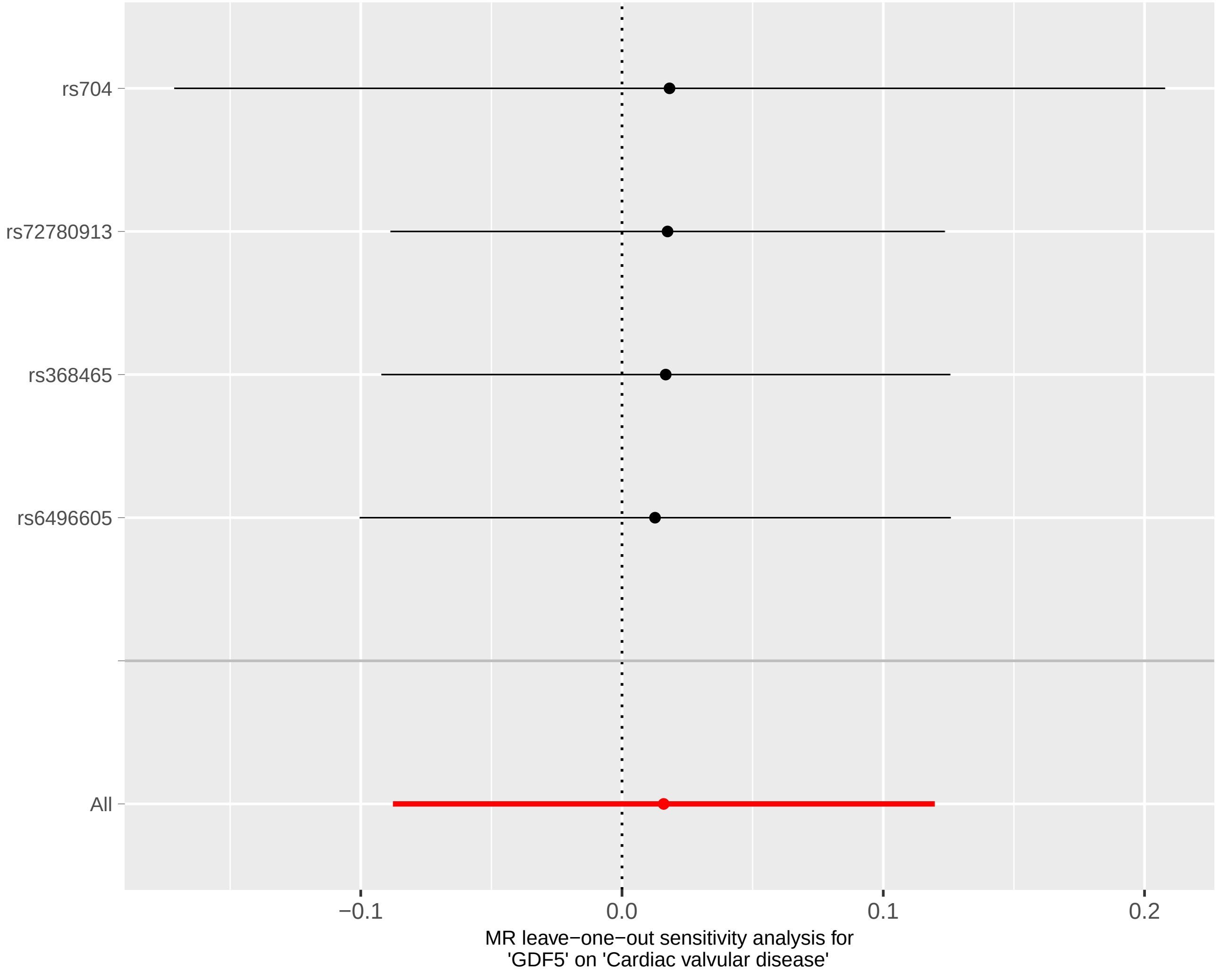
rs704
rs72780913
rs368465
rs6496605
All
−0.1
0.0
0.1
0.2
MR leave−one−out sensitivity analysis for
'GDF5' on 'Cardiac valvular disease'

Insufficient number of SNPs

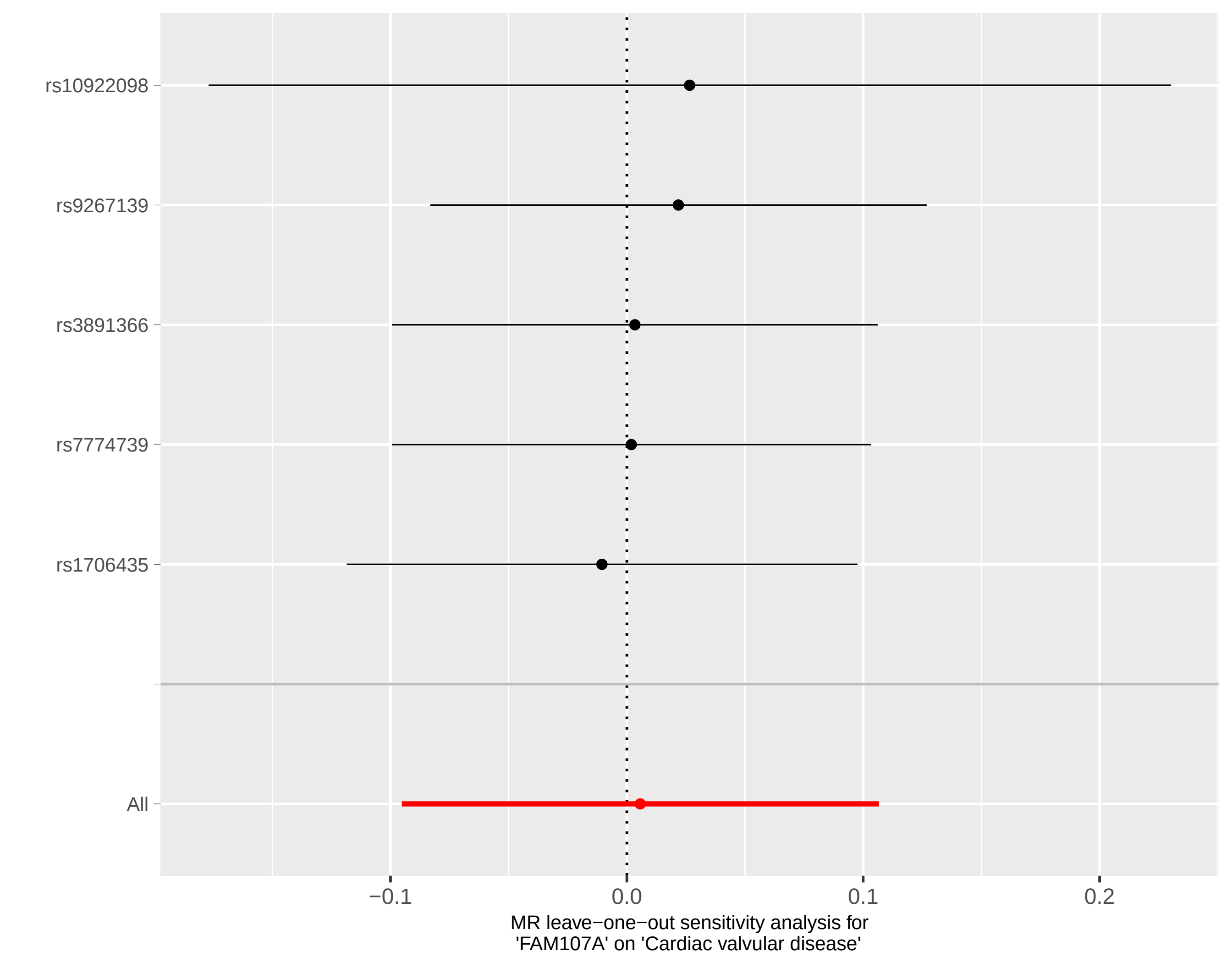
rs10922098
rs9267139
rs3891366
rs7774739
rs1706435
All
−0.1
0.0
0.1
0.2
MR leave−one−out sensitivity analysis for
'FAM107A' on 'Cardiac valvular disease'

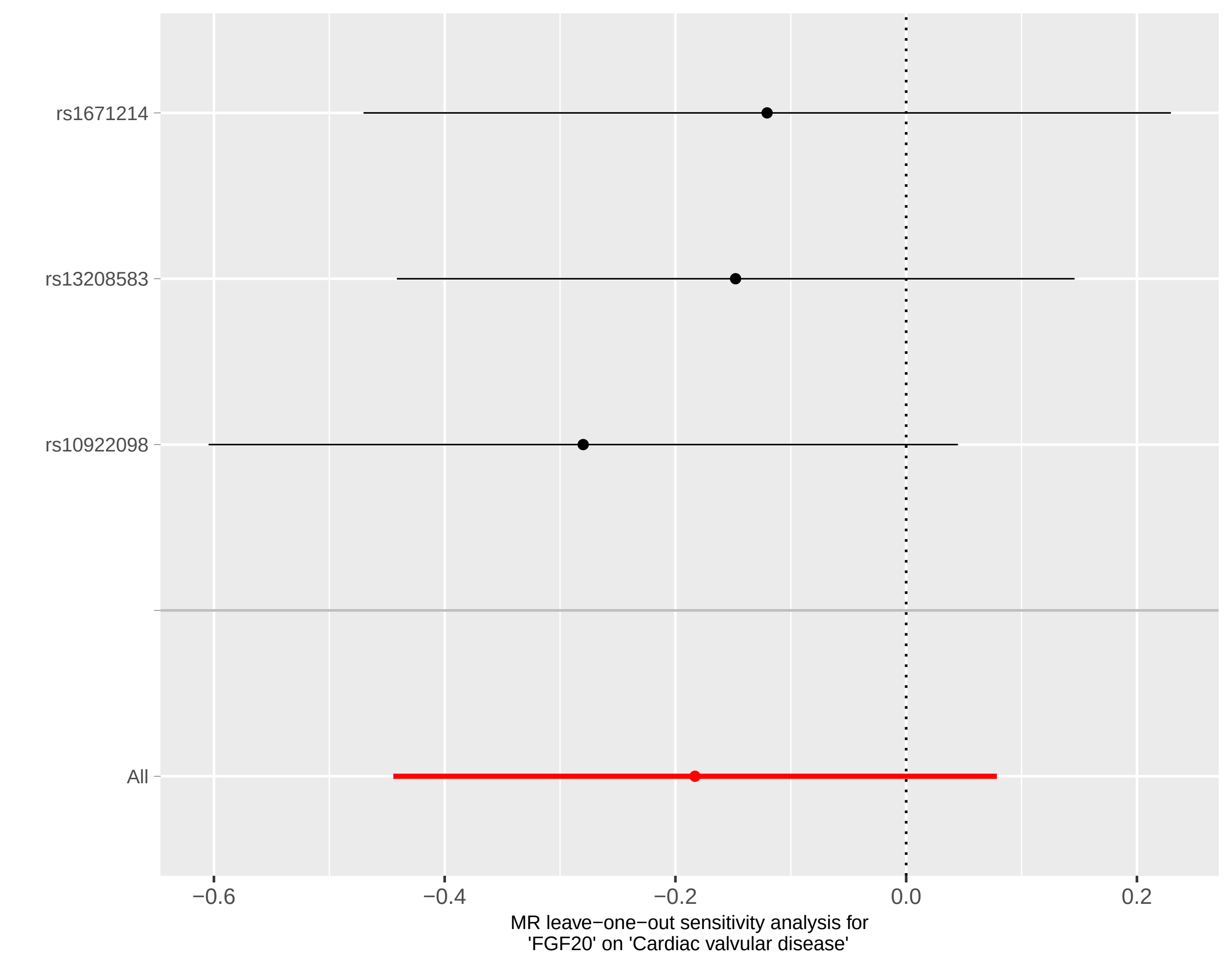

rs1671214
rs13208583
rs10922098
All
−0.6
−0.4
−0.2
0.0
0.2
MR leave−one−out sensitivity analysis for
'FGF20' on 'Cardiac valvular disease'

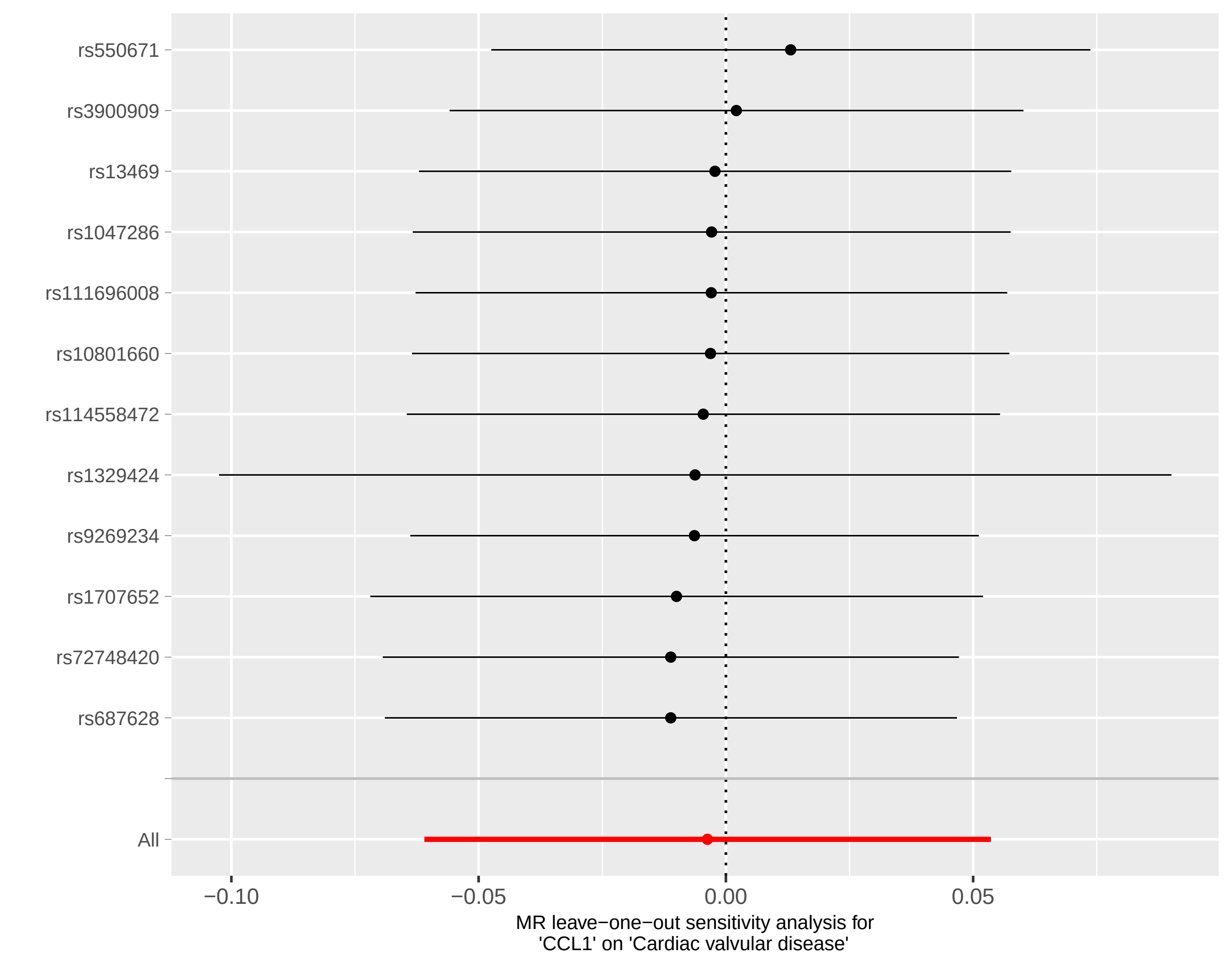

rs550671
rs3900909
rs13469
rs1047286
rs111696008
rs10801660
rs114558472
rs1329424
rs9269234
rs1707652
rs72748420
rs687628
All
−0.10
−0.05
0.00
0.05
MR leave−one−out sensitivity analysis for
'CCL1' on 'Cardiac valvular disease'

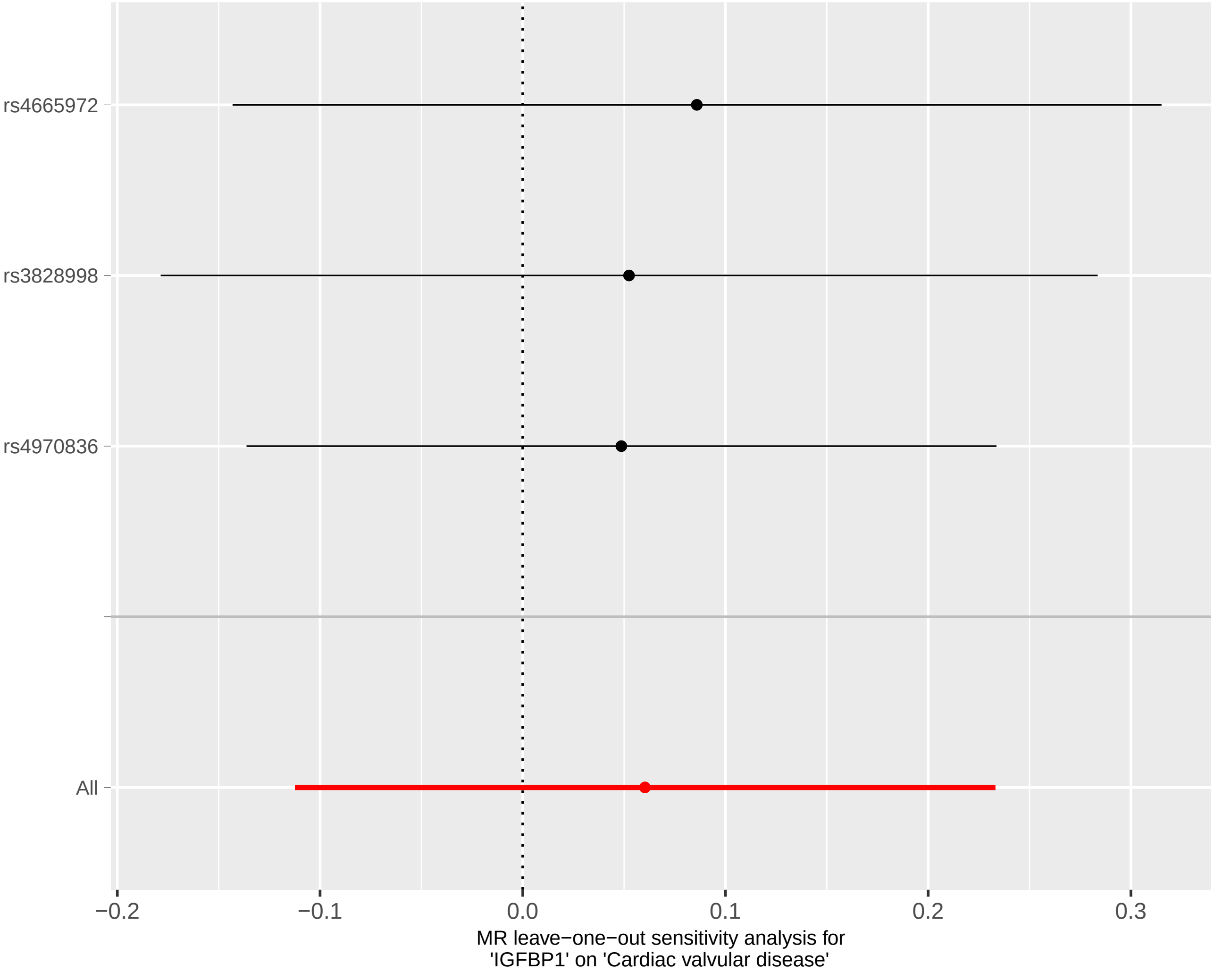
rs4665972
rs3828998
rs4970836
All
−0.2
−0.1
0.0
0.1
0.2
0.3
MR leave−one−out sensitivity analysis for
'IGFBP1' on 'Cardiac valvular disease'

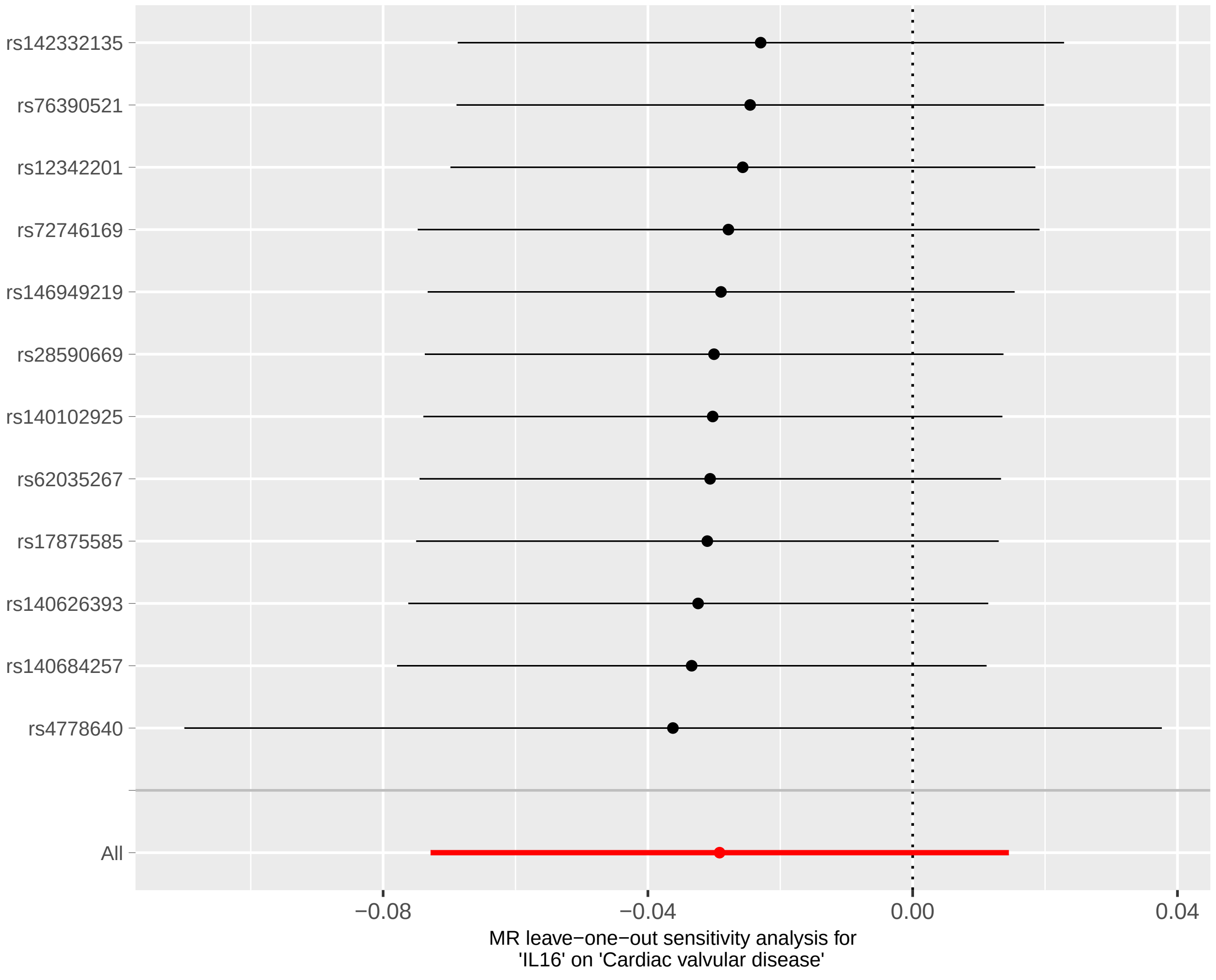
rs142332135
rs76390521
rs12342201
rs72746169
rs146949219
rs28590669
rs140102925
rs62035267
rs17875585
rs140626393
rs140684257
rs4778640
All
−0.08
−0.04
0.00
0.04
MR leave−one−out sensitivity analysis for
'IL16' on 'Cardiac valvular disease'

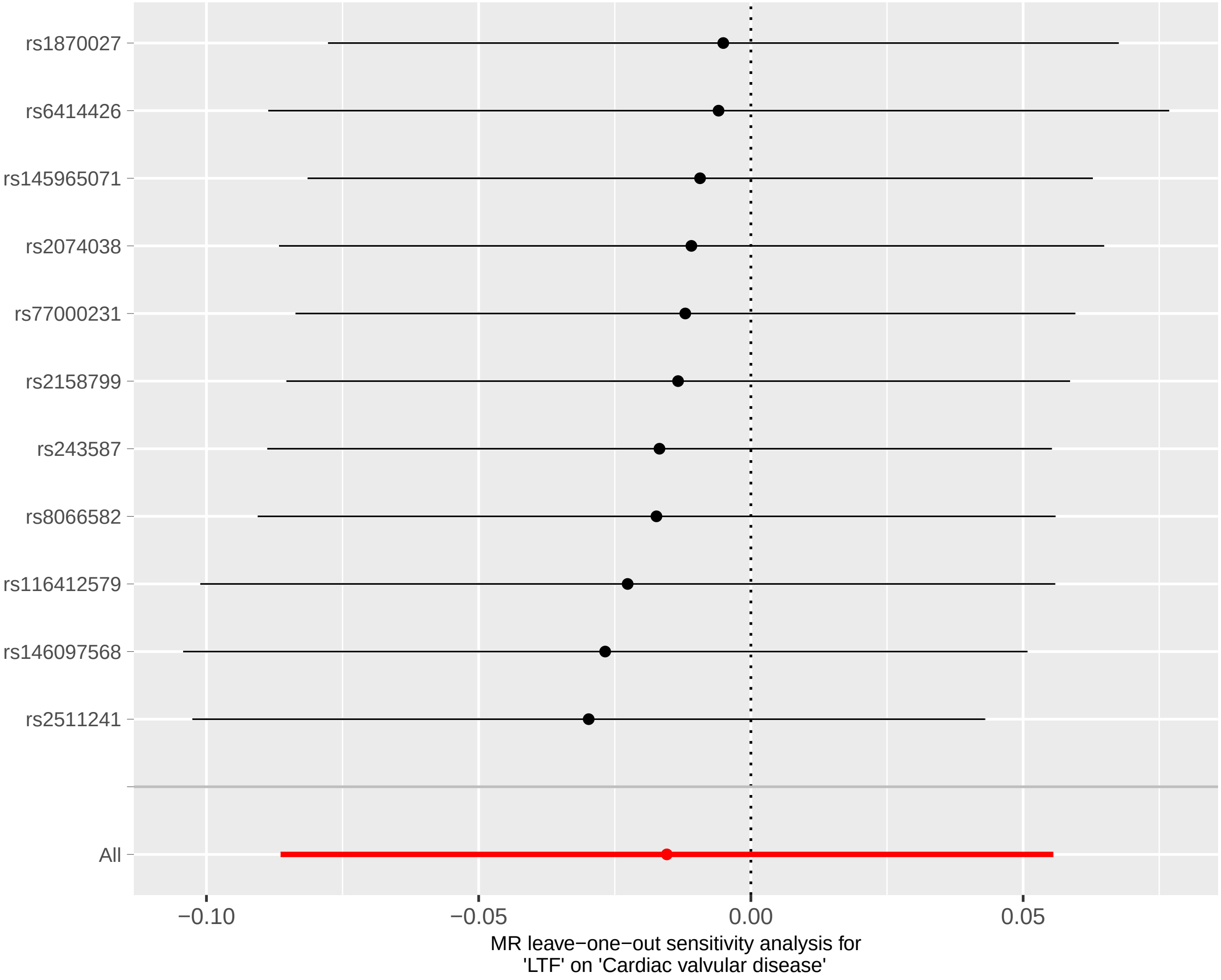
rs1870027
rs6414426
rs145965071
rs2074038
rs77000231
rs2158799
rs243587
rs8066582
rs116412579
rs146097568
rs2511241
All
−0.10
−0.05
0.00
0.05
MR leave−one−out sensitivity analysis for
'LTF' on 'Cardiac valvular disease'

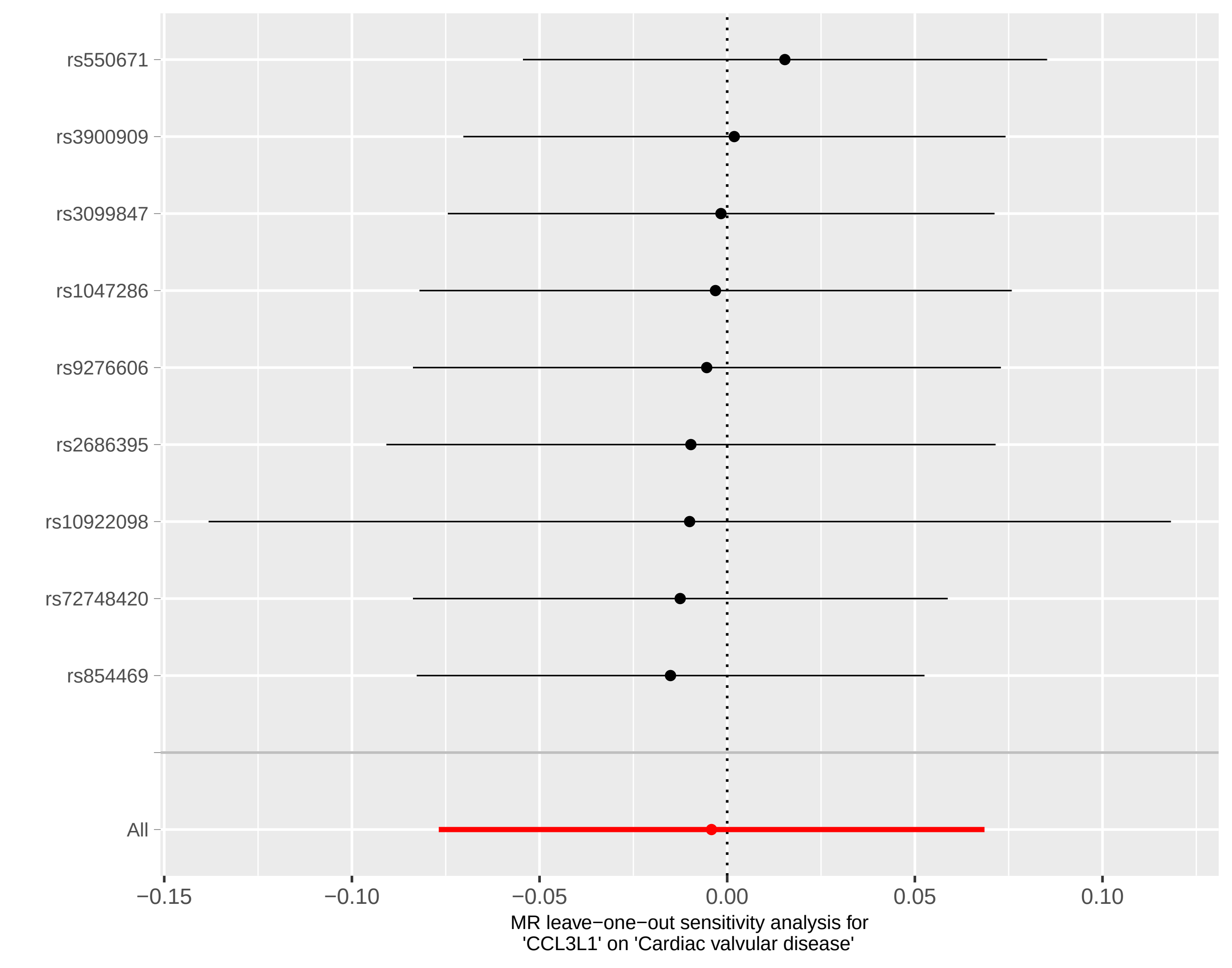
rs550671
rs3900909
rs3099847
rs1047286
rs9276606
rs2686395
rs10922098
rs72748420
rs854469
All
−0.15
−0.10
−0.05
0.00
0.05
0.10
MR leave−one−out sensitivity analysis for
'CCL3L1' on 'Cardiac valvular disease'

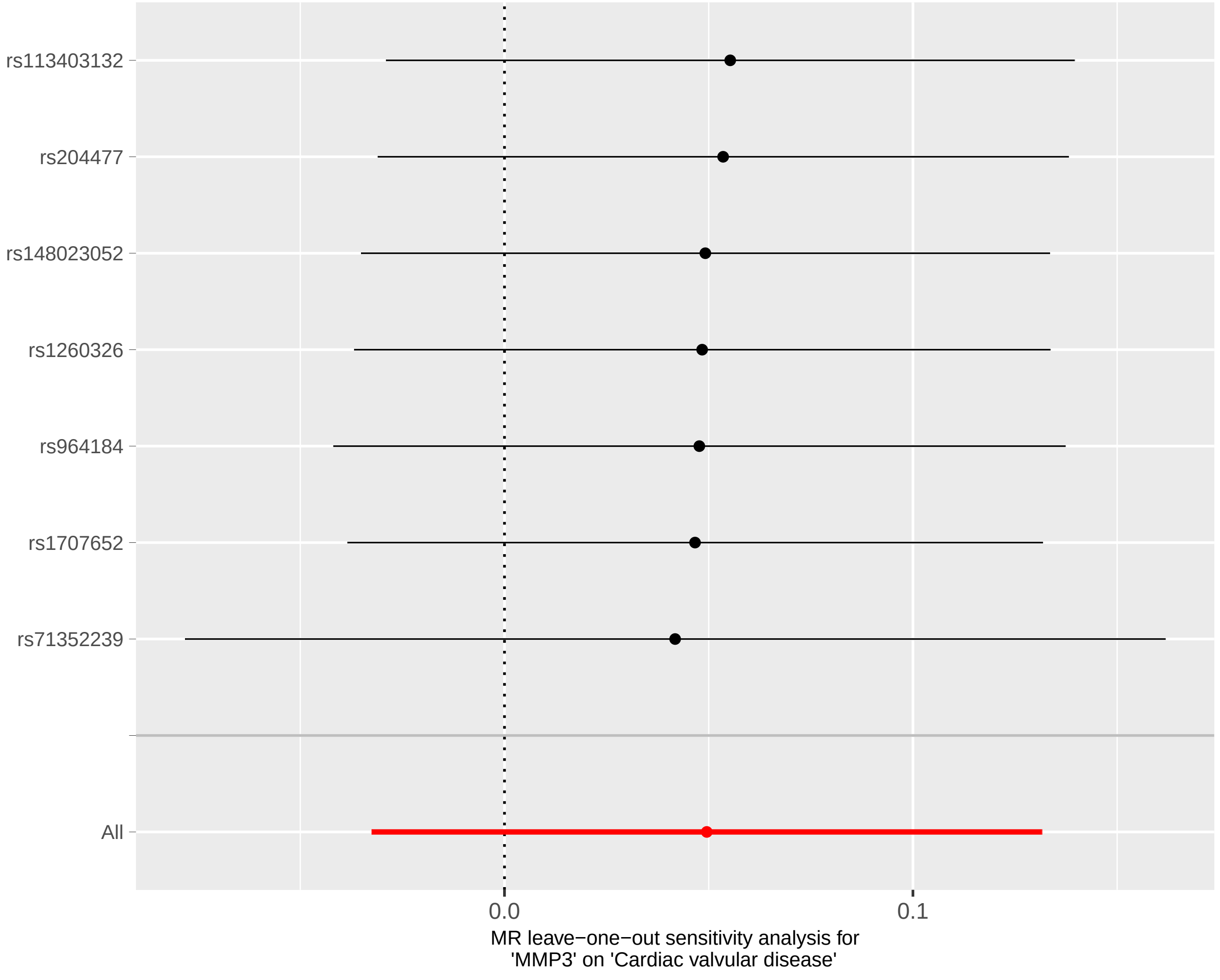
rs113403132
rs204477
rs148023052
rs1260326
rs964184
rs1707652
rs71352239
All
0.0
0.1
MR leave−one−out sensitivity analysis for
'MMP3' on 'Cardiac valvular disease'

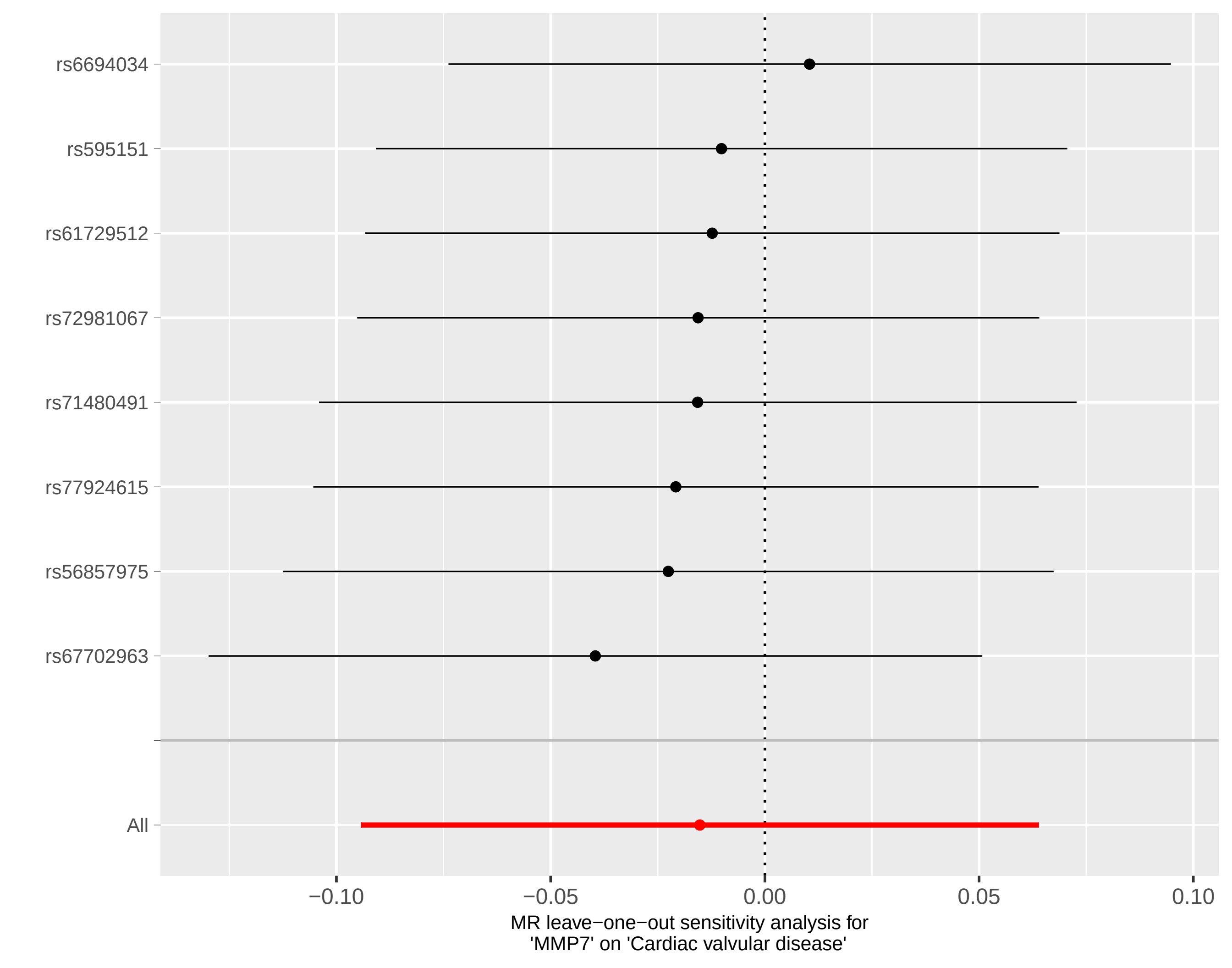
rs6694034
rs595151
rs61729512
rs72981067
rs71480491
rs77924615
rs56857975
rs67702963
All
−0.10
−0.05
0.00
0.05
0.10
MR leave−one−out sensitivity analysis for
'MMP7' on 'Cardiac valvular disease'

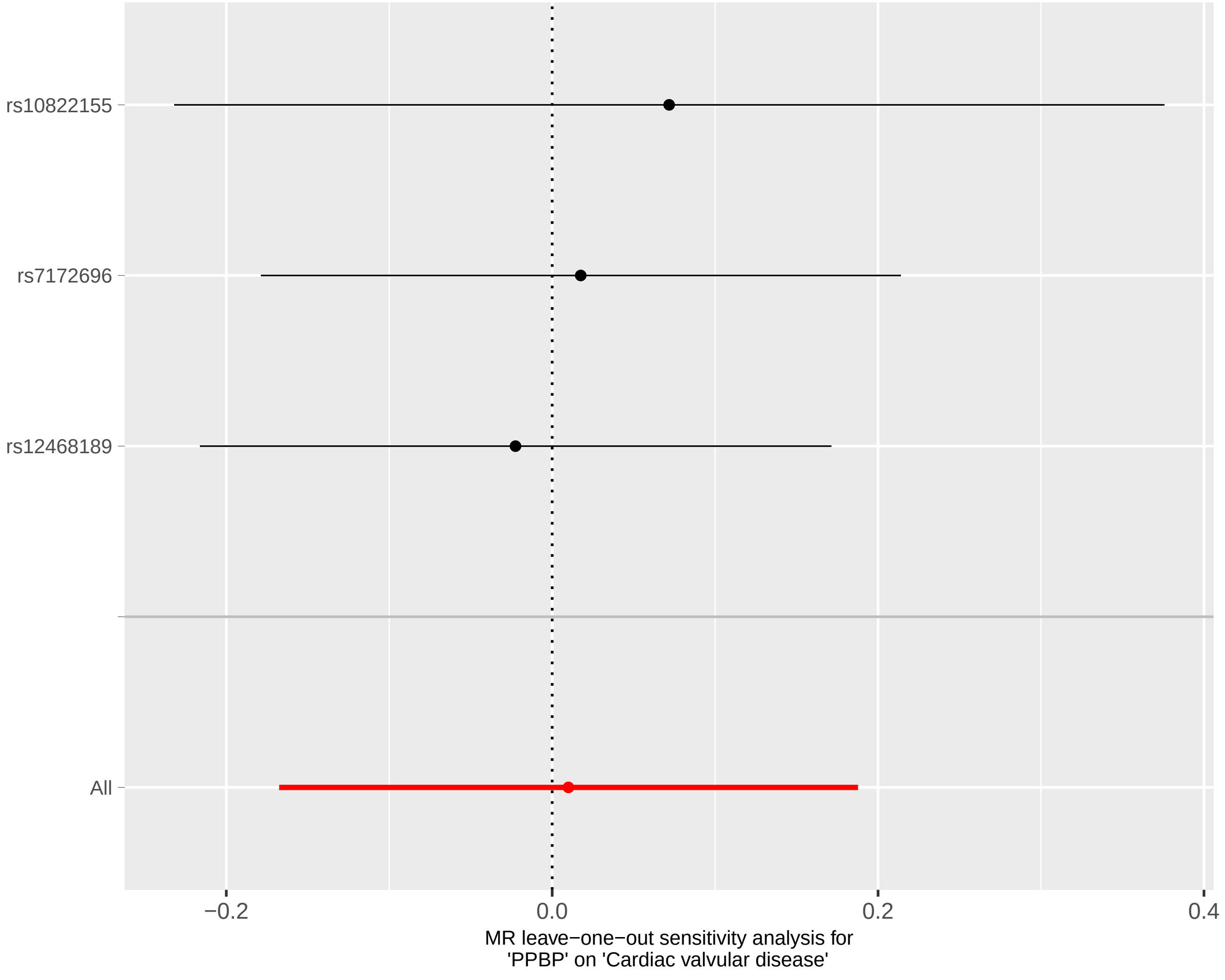

rs10822155
rs7172696
rs12468189
All
−0.2
0.0
0.2
0.4
MR leave−one−out sensitivity analysis for
'PPBP' on 'Cardiac valvular disease'

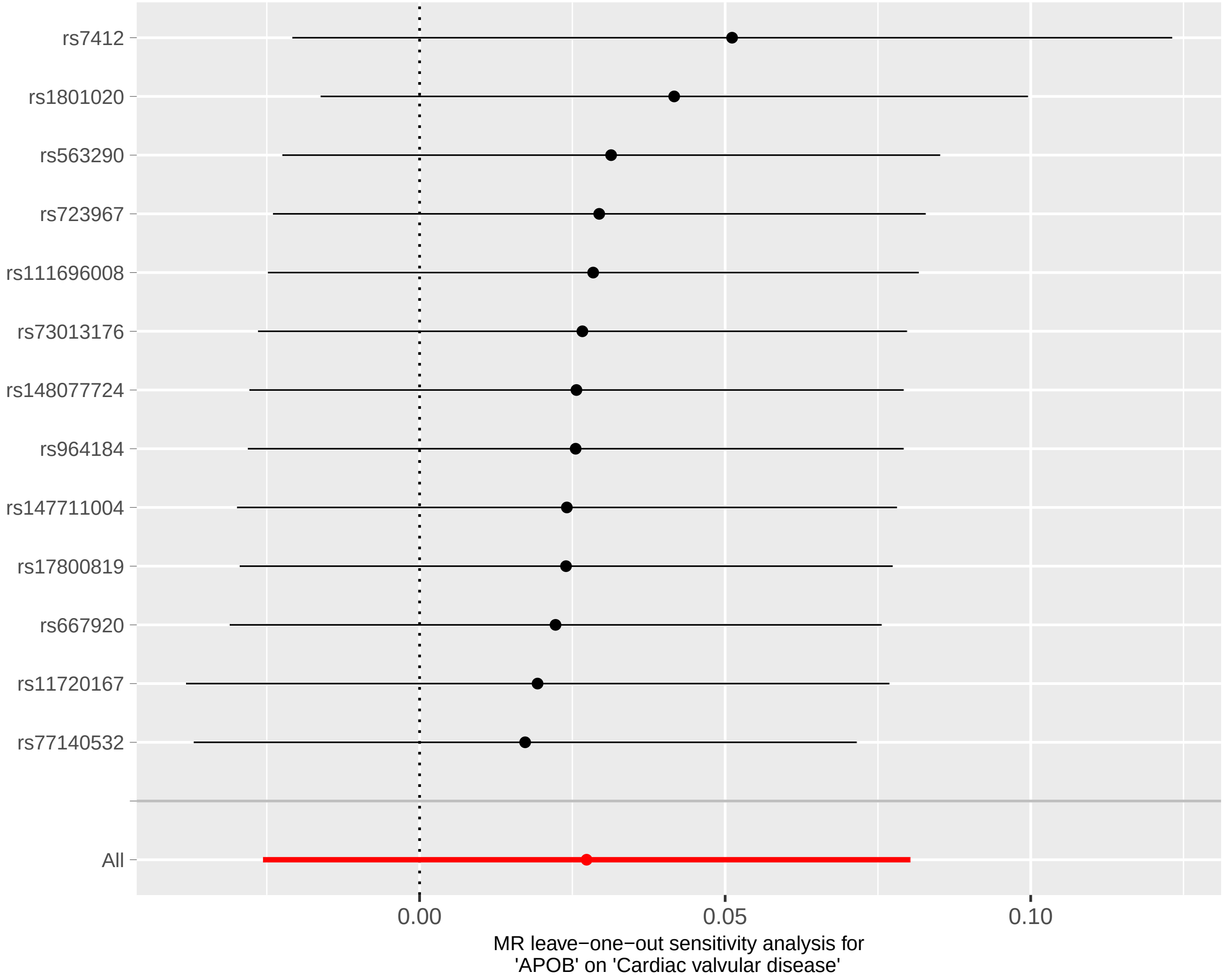

rs7412
rs1801020
rs563290
rs723967
rs111696008
rs73013176
rs148077724
rs964184
rs147711004
rs17800819
rs667920
rs11720167
rs77140532
All
0.00
0.05
0.10
MR leave−one−out sensitivity analysis for
'APOB' on 'Cardiac valvular disease'

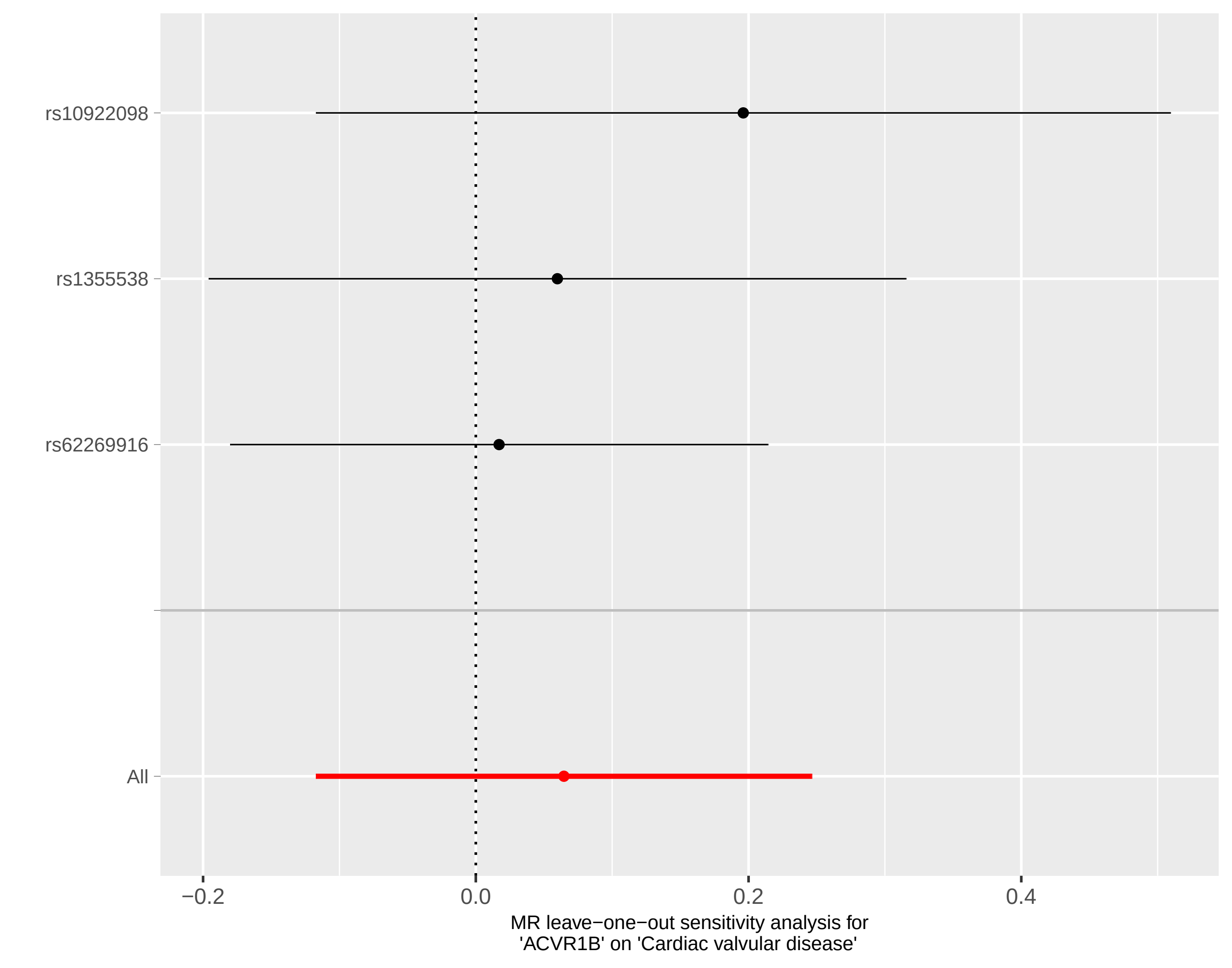
rs10922098
rs1355538
rs62269916
All
−0.2
0.0
0.2
0.4
MR leave−one−out sensitivity analysis for
'ACVR1B' on 'Cardiac valvular disease'

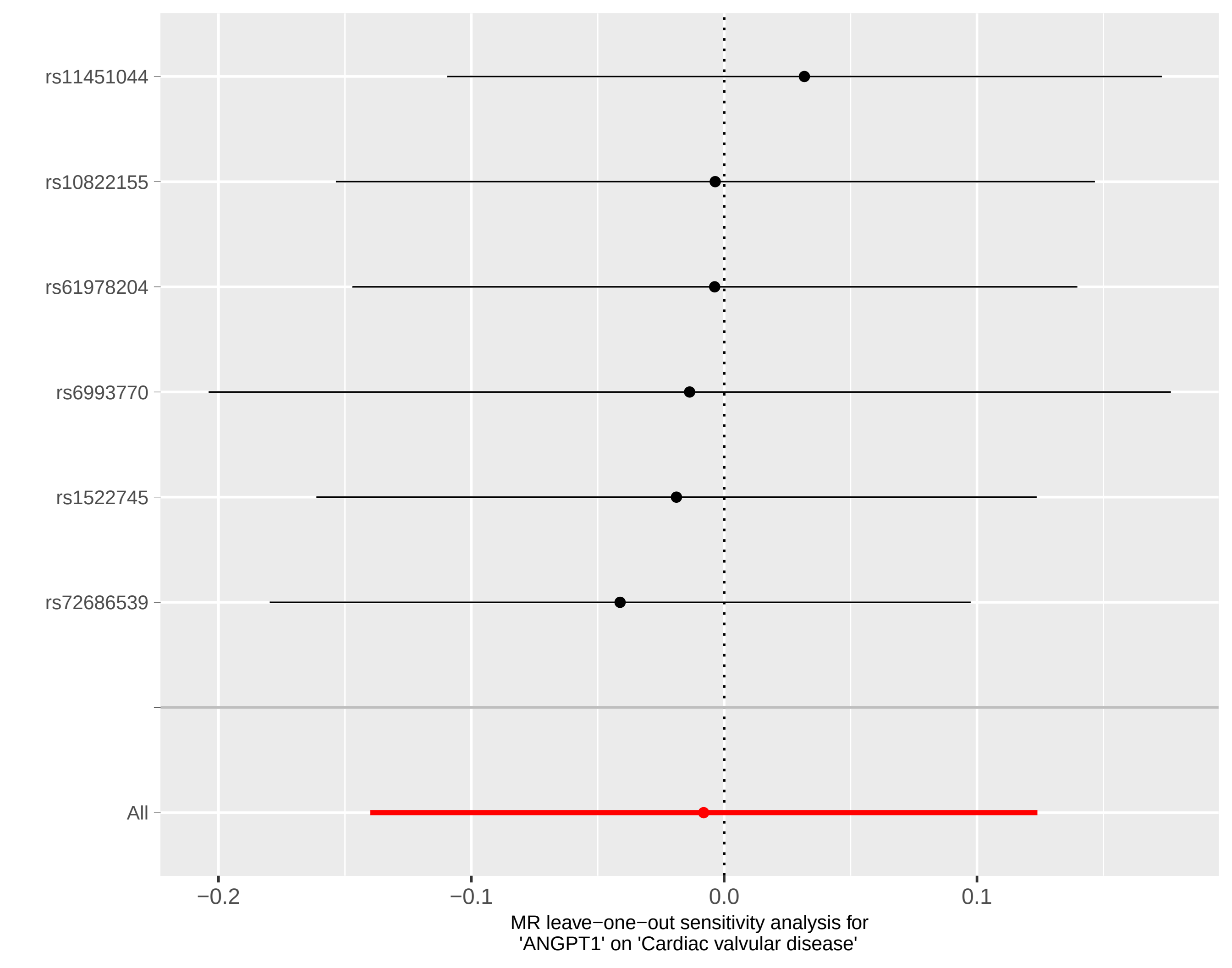

rs11451044
rs10822155
rs61978204
rs6993770
rs1522745
rs72686539
All
−0.2
−0.1
0.0
0.1
MR leave−one−out sensitivity analysis for
'ANGPT1' on 'Cardiac valvular disease'

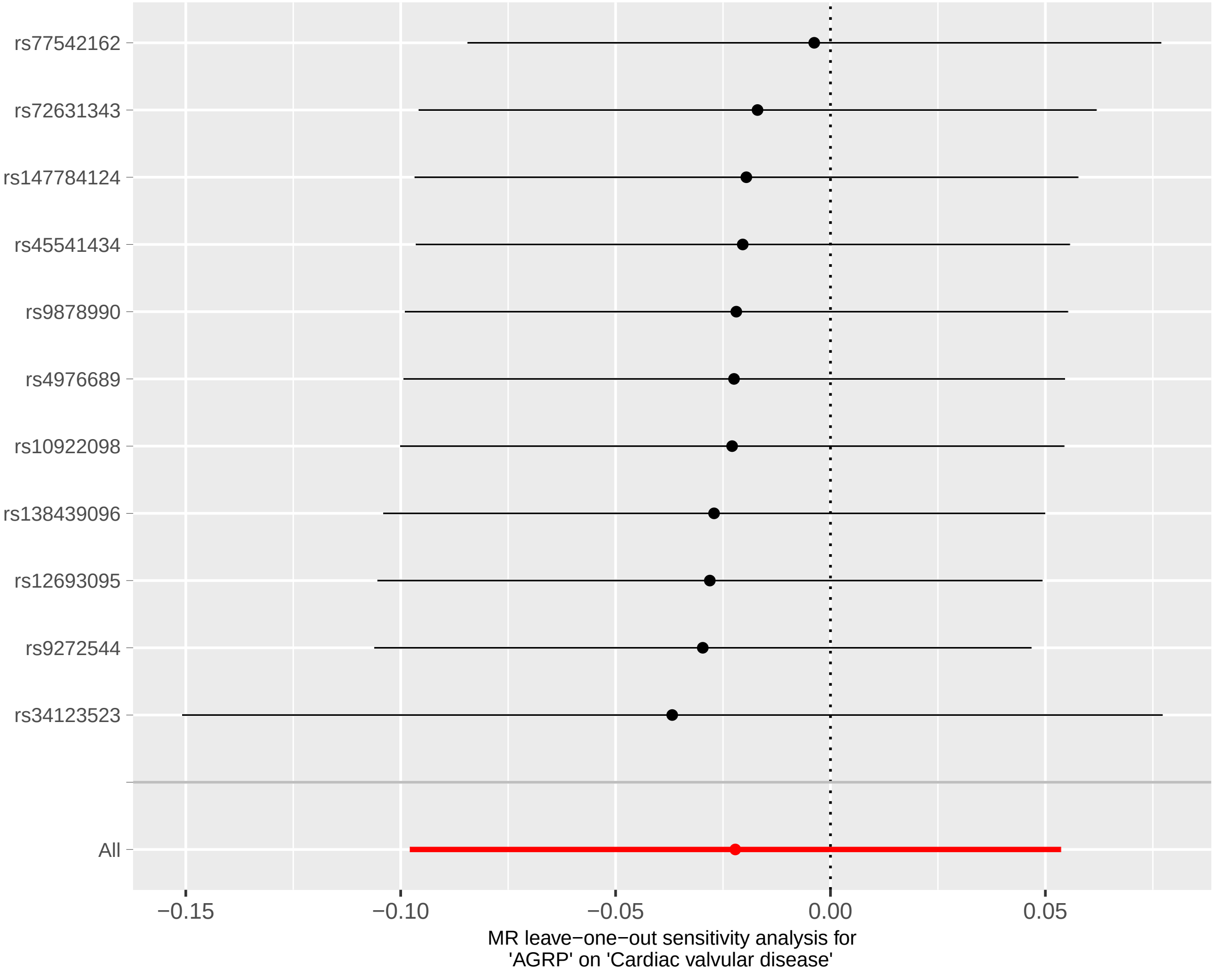

rs77542162
rs72631343
rs147784124
rs45541434
rs9878990
rs4976689
rs10922098
rs138439096
rs12693095
rs9272544
rs34123523
All
−0.15
−0.10
−0.05
0.00
0.05
MR leave−one−out sensitivity analysis for
'AGRP' on 'Cardiac valvular disease'

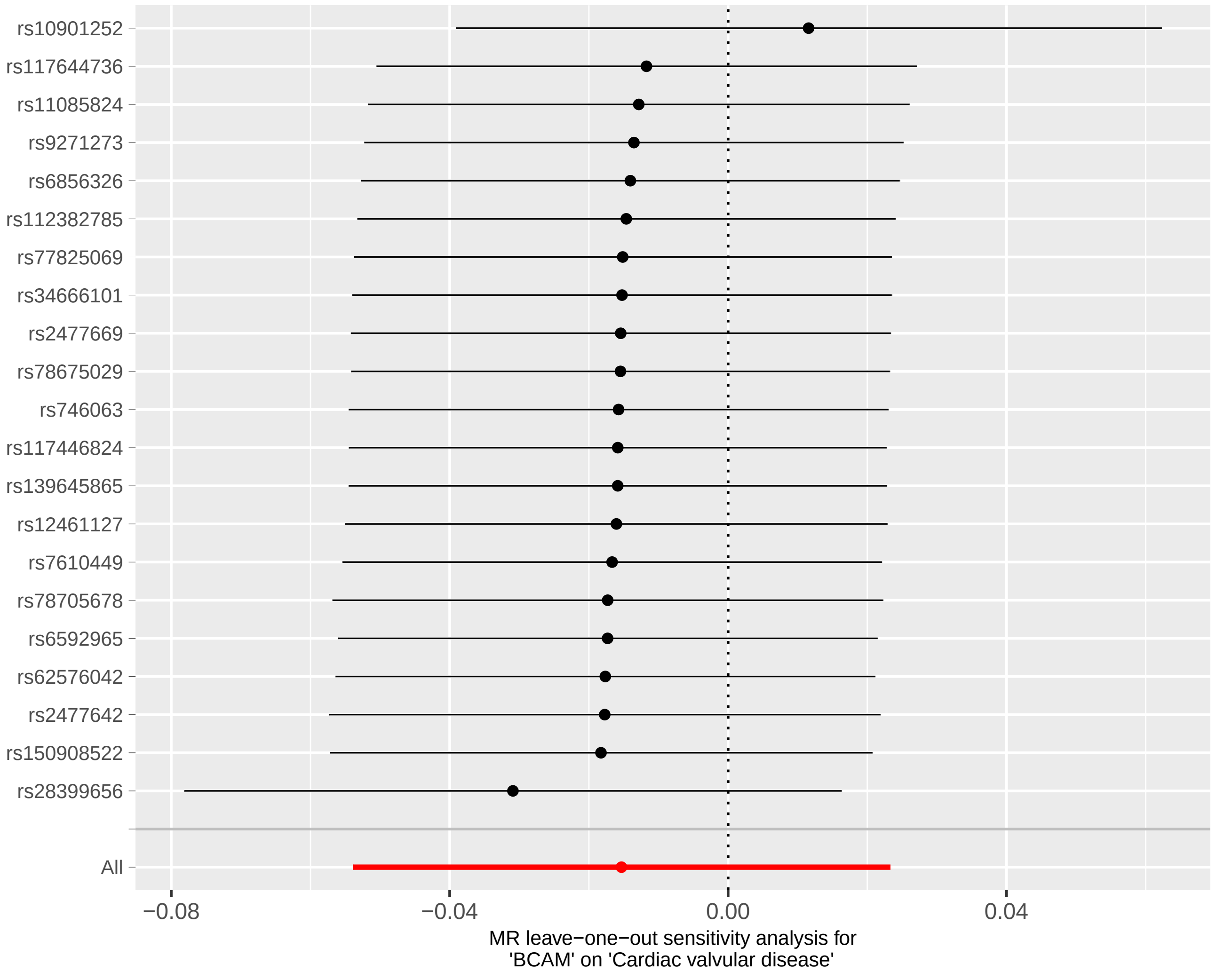
rs10901252
rs117644736
rs11085824
rs9271273
rs6856326
rs112382785
rs77825069
rs34666101
rs2477669
rs78675029
rs746063
rs117446824
rs139645865
rs12461127
rs7610449
rs78705678
rs6592965
rs62576042
rs2477642
rs150908522
rs28399656
All
−0.08
−0.04
0.00
0.04
MR leave−one−out sensitivity analysis for
'BCAM' on 'Cardiac valvular disease'

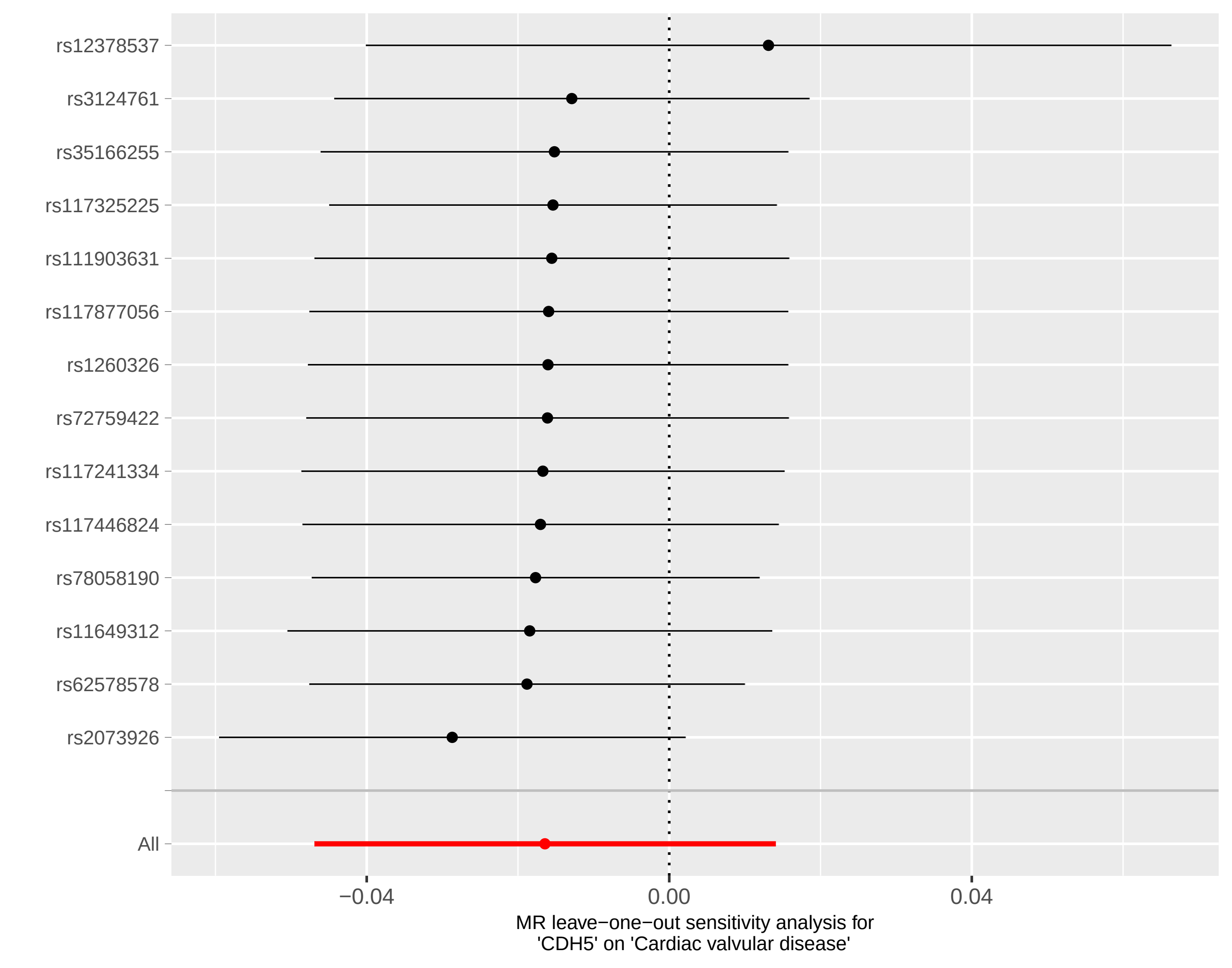

rs12378537
rs3124761
rs35166255
rs117325225
rs111903631
rs117877056
rs1260326
rs72759422
rs117241334
rs117446824
rs78058190
rs11649312
rs62578578
rs2073926
All
−0.04
0.00
0.04
MR leave−one−out sensitivity analysis for
'CDH5' on 'Cardiac valvular disease'

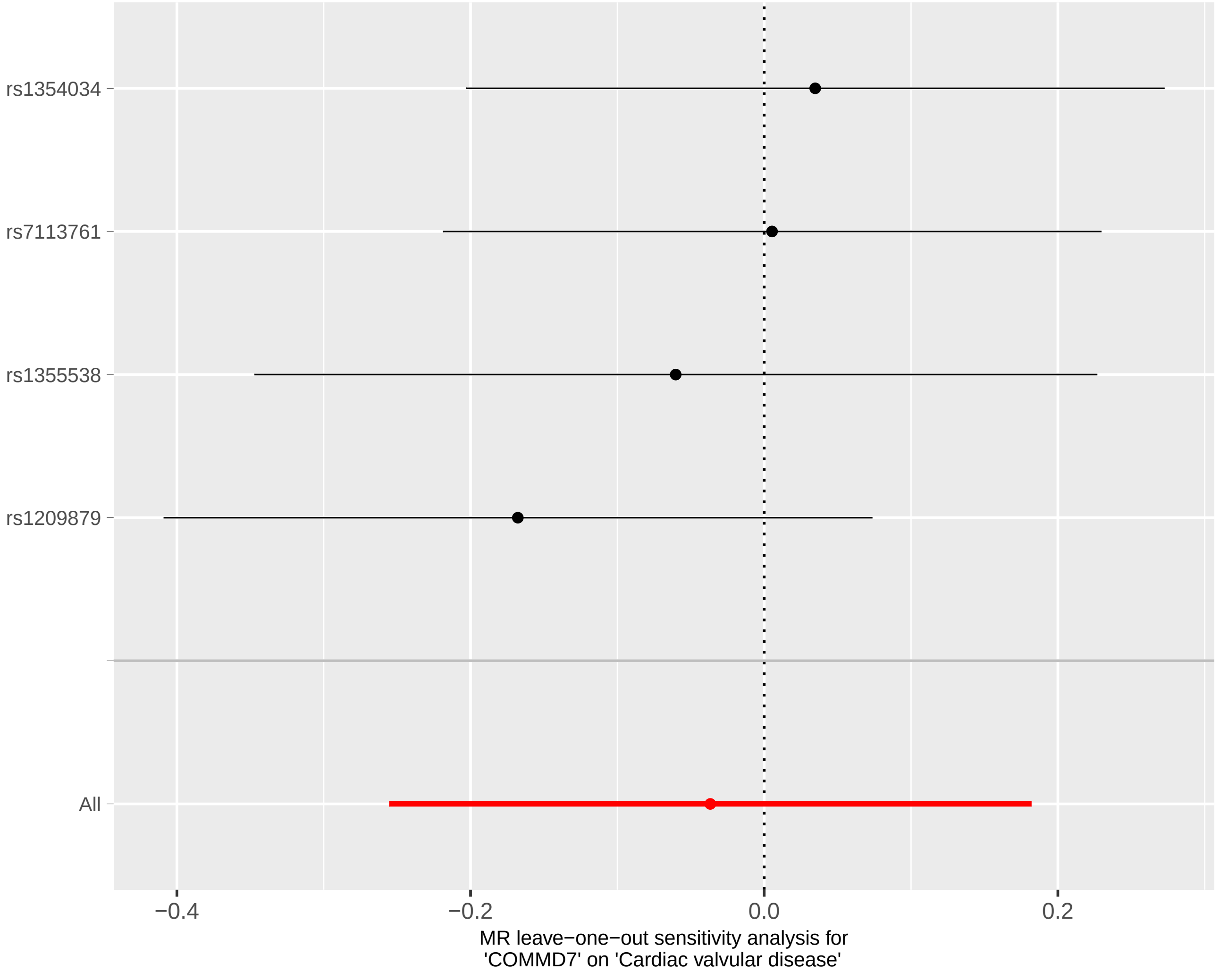
rs1354034
rs7113761
rs1355538
rs1209879
All
−0.4
−0.2
0.0
0.2
MR leave−one−out sensitivity analysis for
'COMMD7' on 'Cardiac valvular disease'

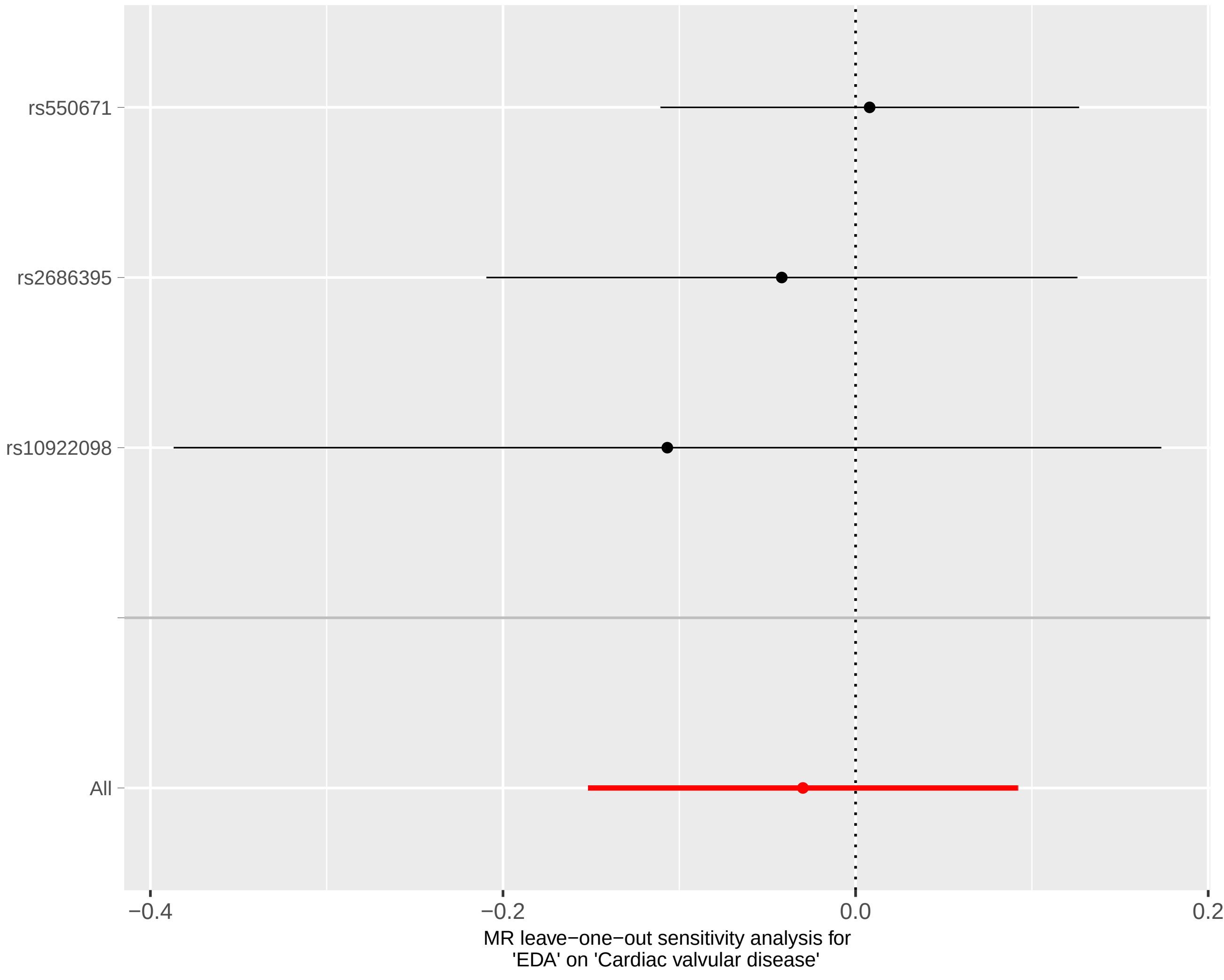
rs550671
rs2686395
rs10922098
All
−0.4
−0.2
0.0
0.2
MR leave−one−out sensitivity analysis for
'EDA' on 'Cardiac valvular disease'

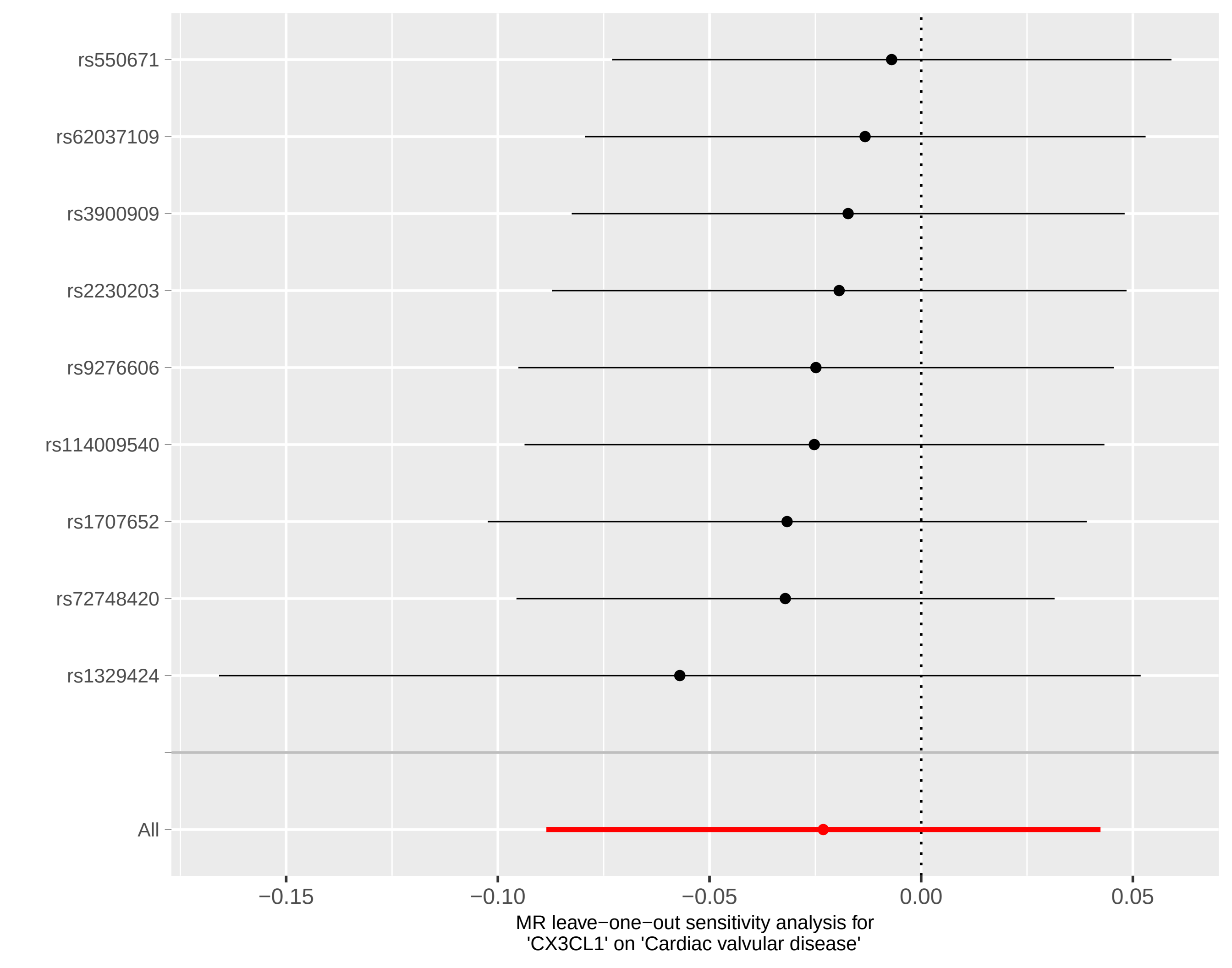

rs550671
rs62037109
rs3900909
rs2230203
rs9276606
rs114009540
rs1707652
rs72748420
rs1329424
All
−0.15
−0.10
−0.05
0.00
0.05
MR leave−one−out sensitivity analysis for
'CX3CL1' on 'Cardiac valvular disease'

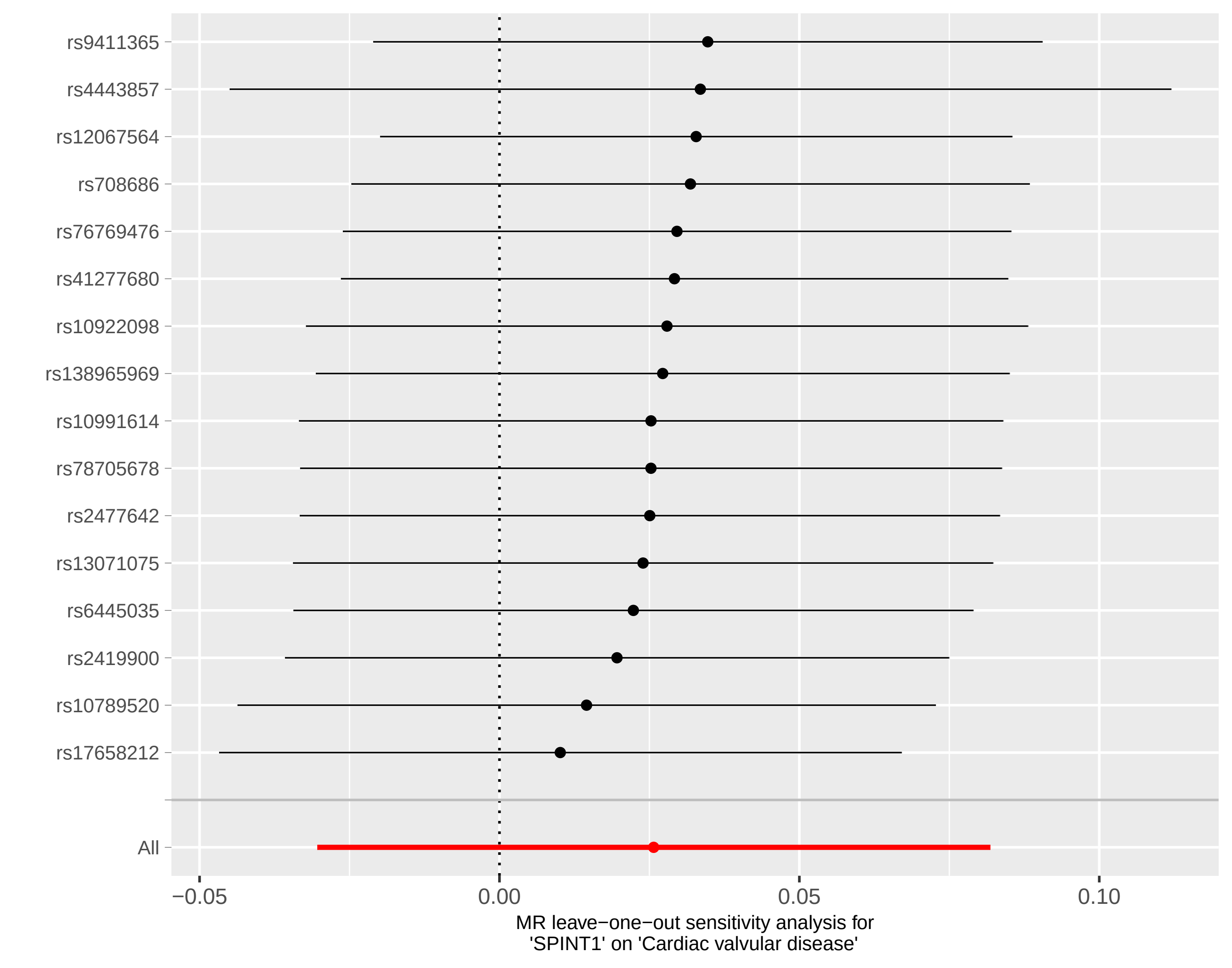

rs9411365
rs4443857
rs12067564
rs708686
rs76769476
rs41277680
rs10922098
rs138965969
rs10991614
rs78705678
rs2477642
rs13071075
rs6445035
rs2419900
rs10789520
rs17658212
All
−0.05
0.00
0.05
0.10
MR leave−one−out sensitivity analysis for
'SPINT1' on 'Cardiac valvular disease'

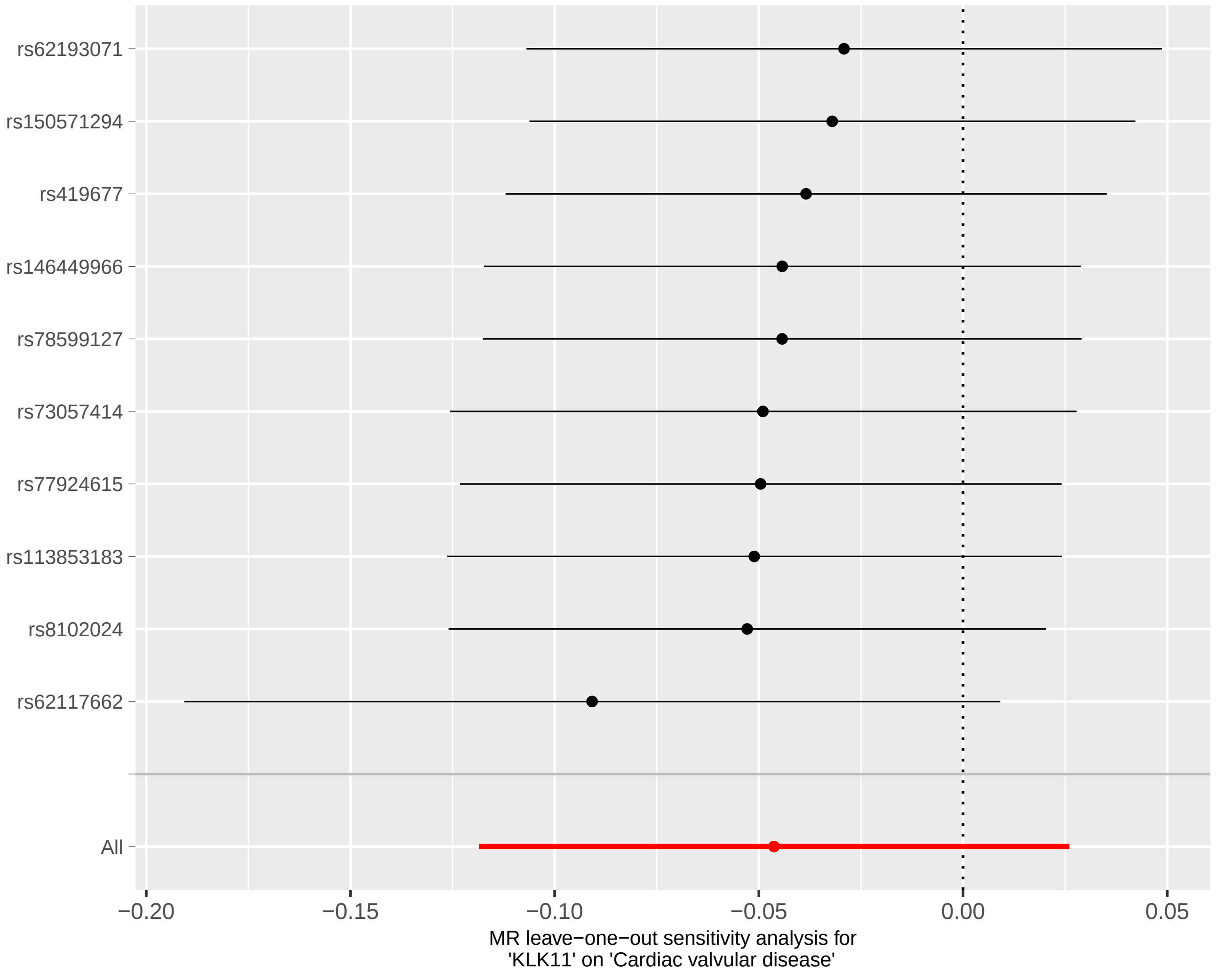

rs62193071
rs150571294
rs419677
rs146449966
rs78599127
rs73057414
rs77924615
rs113853183
rs8102024
rs62117662
All
−0.20
−0.15
−0.10
−0.05
0.00
0.05
MR leave−one−out sensitivity analysis for
'KLK11' on 'Cardiac valvular disease'

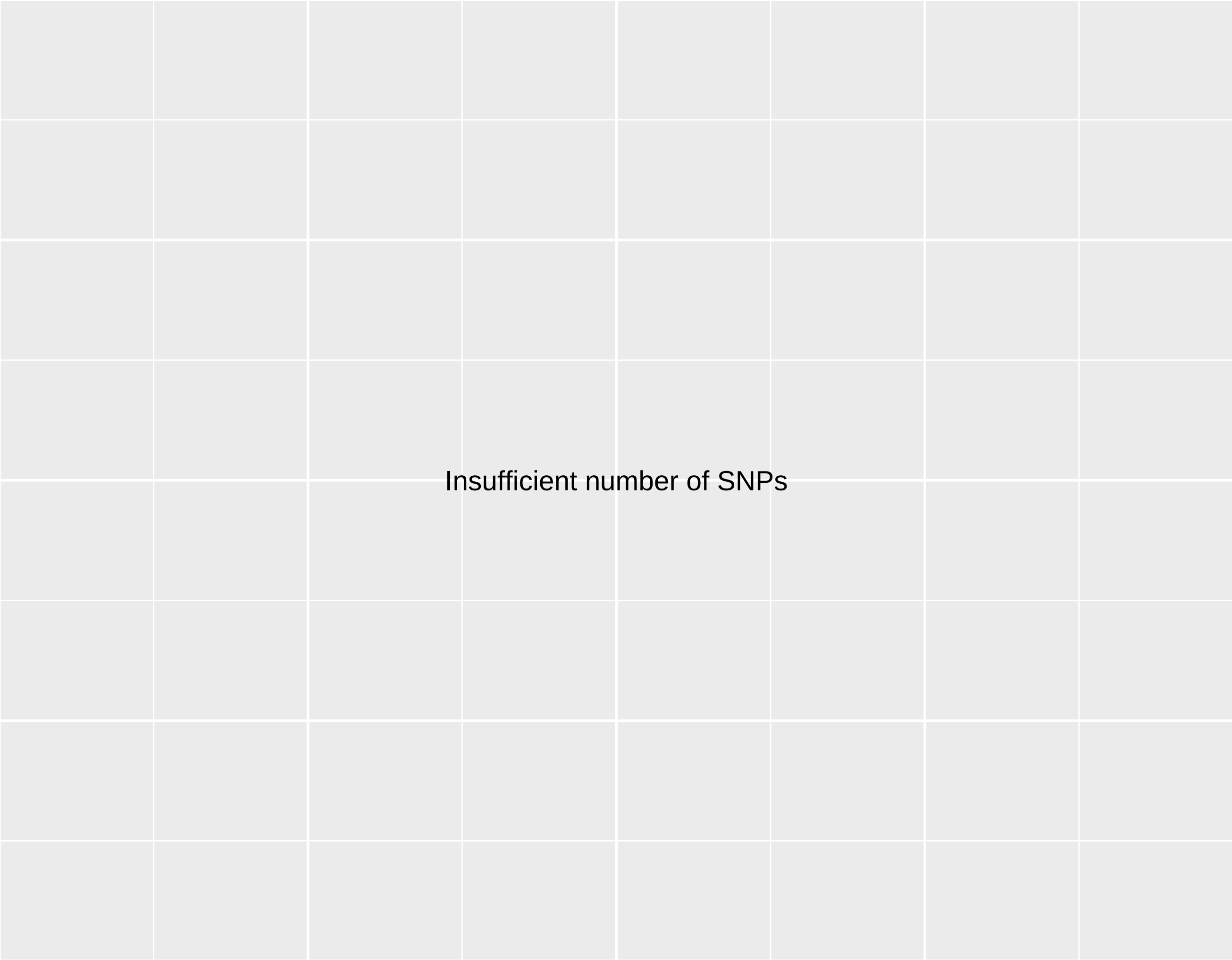
Insufficient number of SNPs

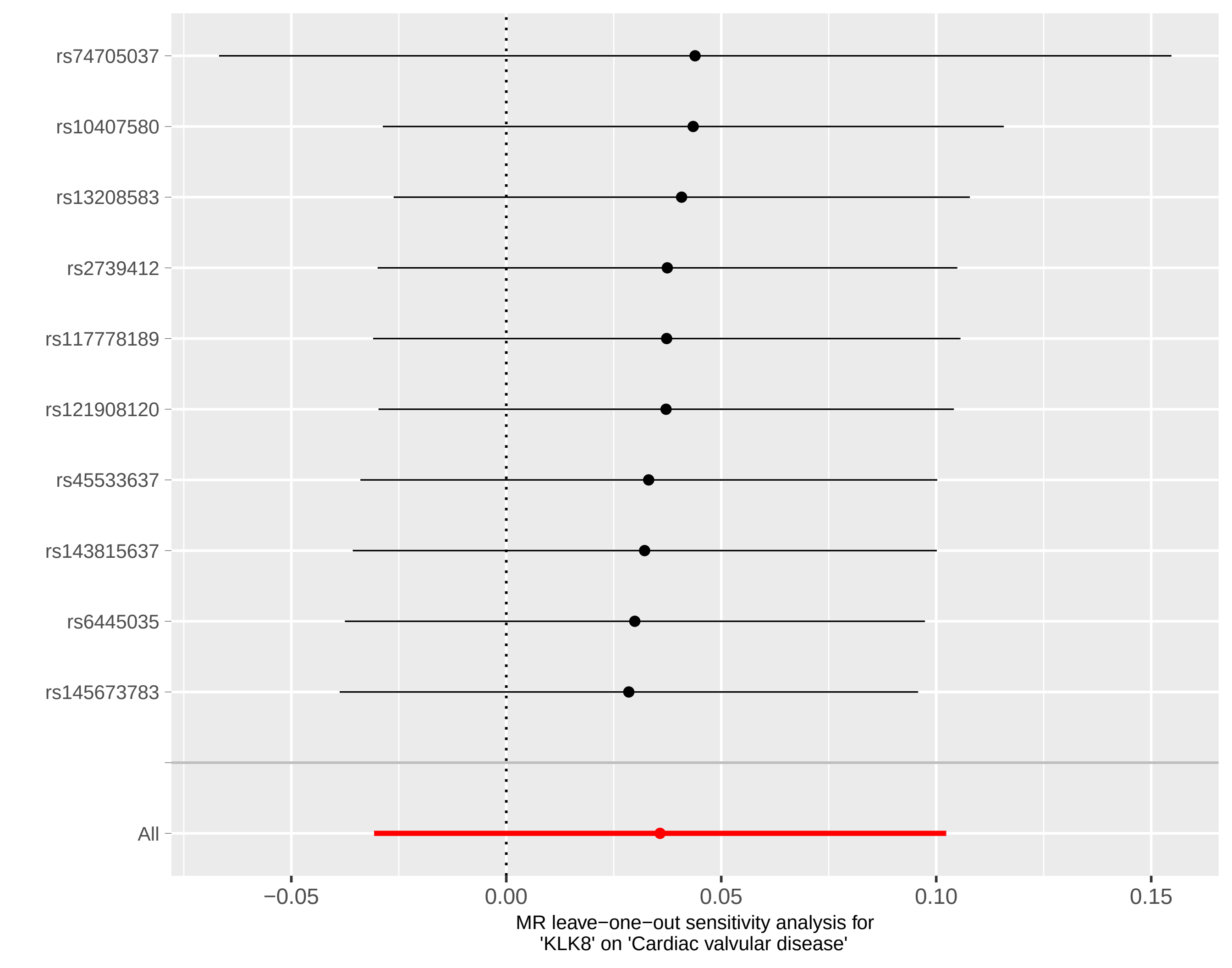
rs74705037
rs10407580
rs13208583
rs2739412
rs117778189
rs121908120
rs45533637
rs143815637
rs6445035
rs145673783
All
−0.05
0.00
0.05
0.10
0.15
MR leave−one−out sensitivity analysis for
'KLK8' on 'Cardiac valvular disease'

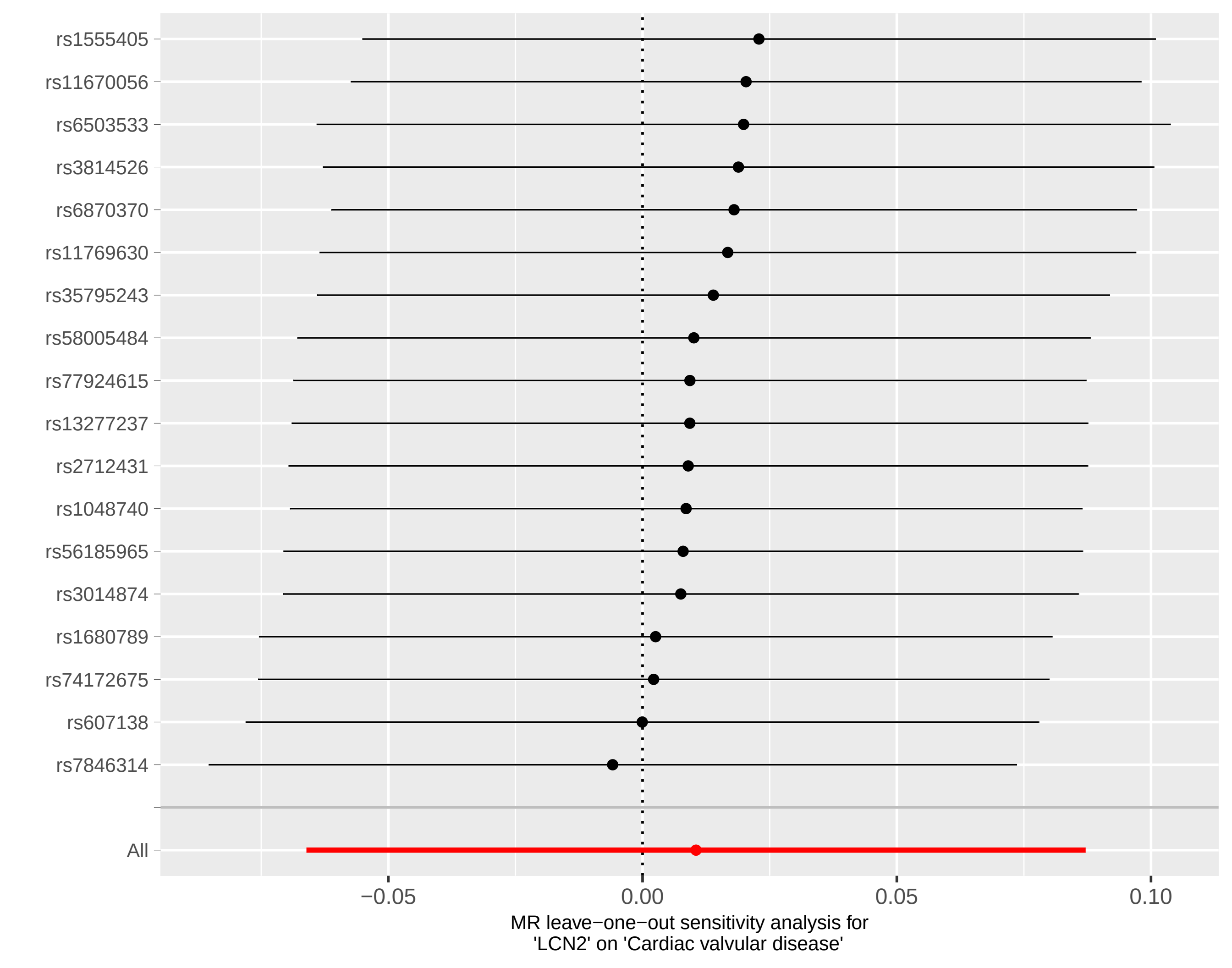
rs1555405
rs11670056
rs6503533
rs3814526
rs6870370
rs11769630
rs35795243
rs58005484
rs77924615
rs13277237
rs2712431
rs1048740
rs56185965
rs3014874
rs1680789
rs74172675
rs607138
rs7846314
All
−0.05
0.00
0.05
0.10
MR leave−one−out sensitivity analysis for
'LCN2' on 'Cardiac valvular disease'

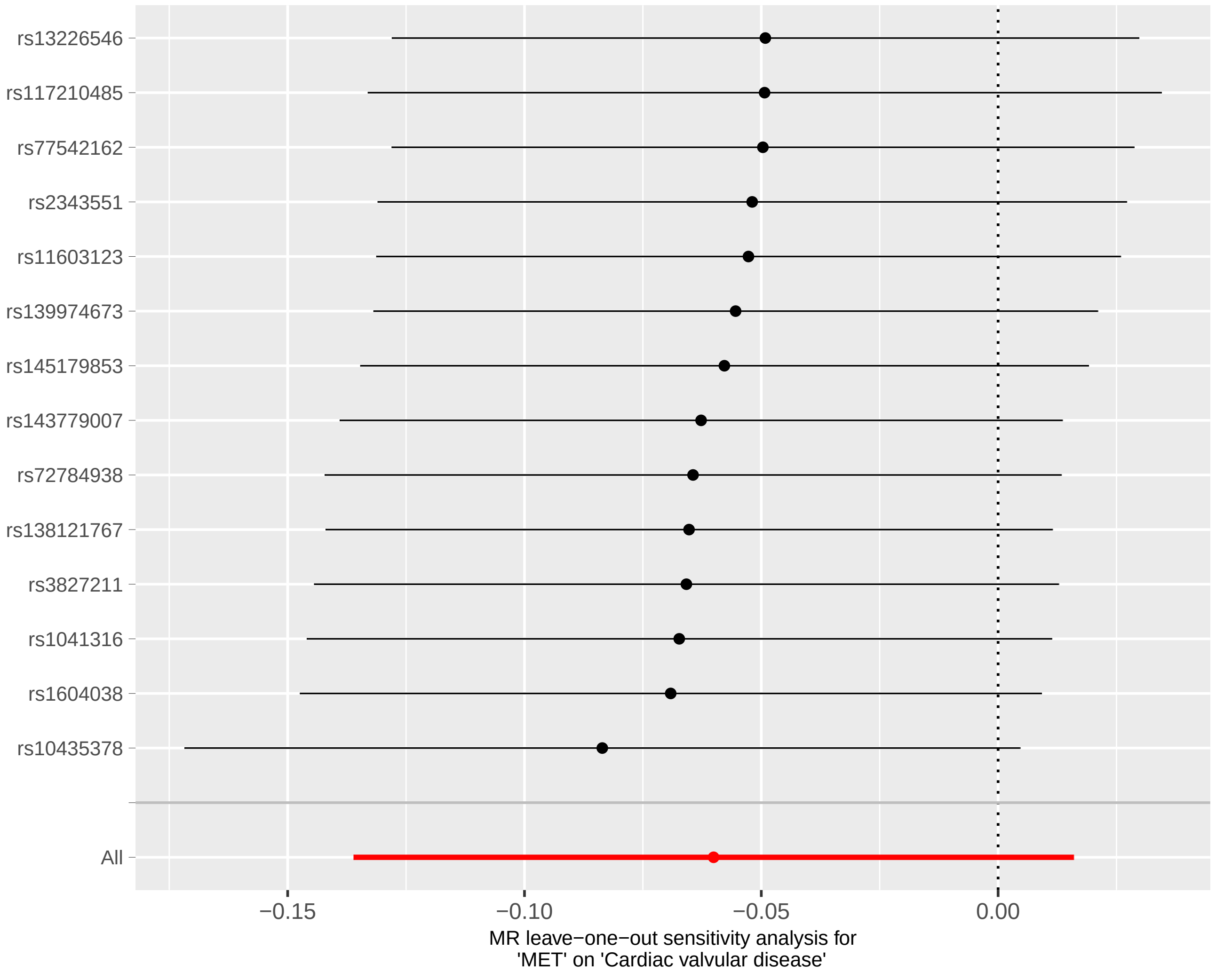

rs13226546
rs117210485
rs77542162
rs2343551
rs11603123
rs139974673
rs145179853
rs143779007
rs72784938
rs138121767
rs3827211
rs1041316
rs1604038
rs10435378
All
−0.15
−0.10
−0.05
0.00
MR leave−one−out sensitivity analysis for
'MET' on 'Cardiac valvular disease'

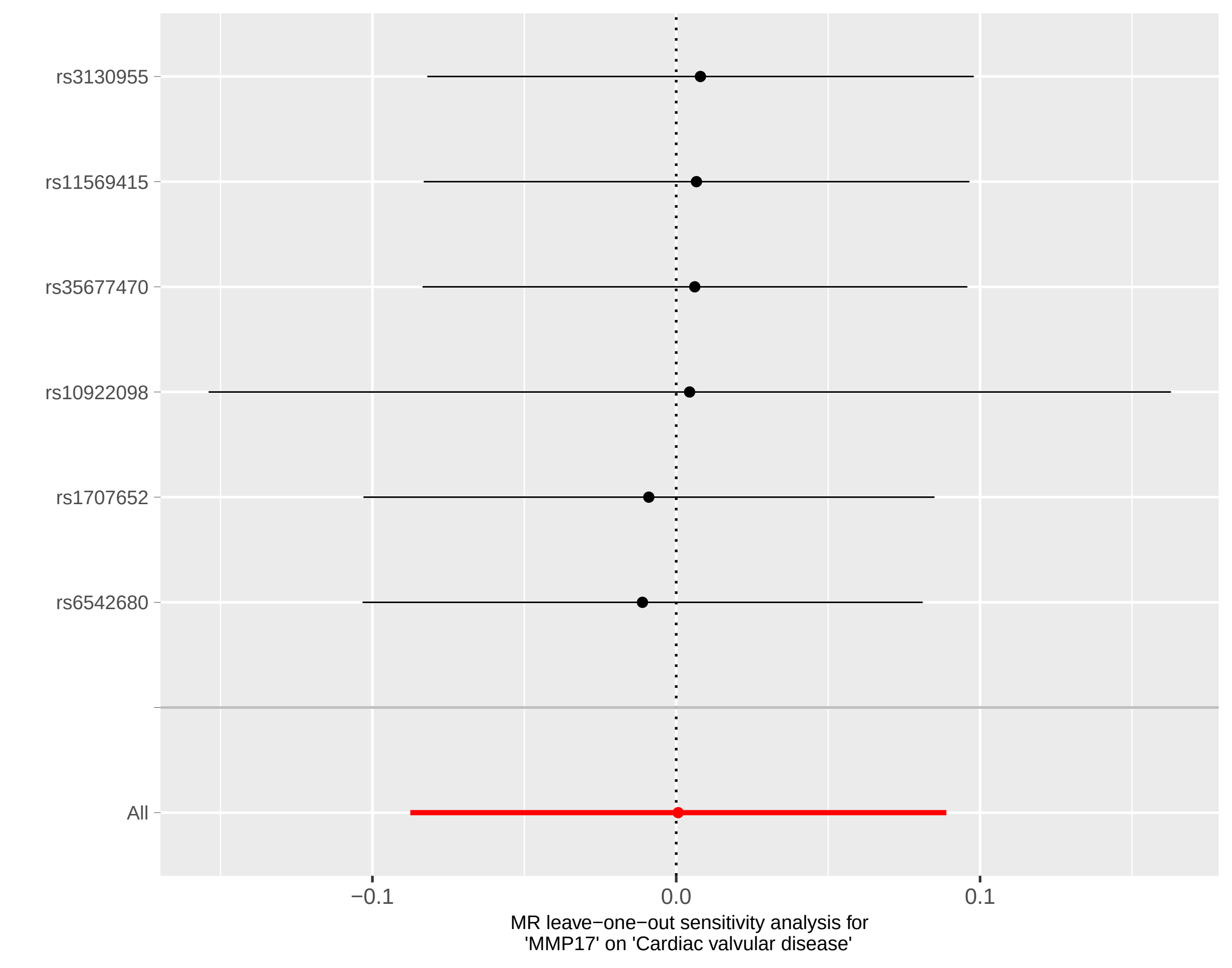

rs3130955
rs11569415
rs35677470
rs10922098
rs1707652
rs6542680
All
−0.1
0.0
0.1
MR leave−one−out sensitivity analysis for
'MMP17' on 'Cardiac valvular disease'

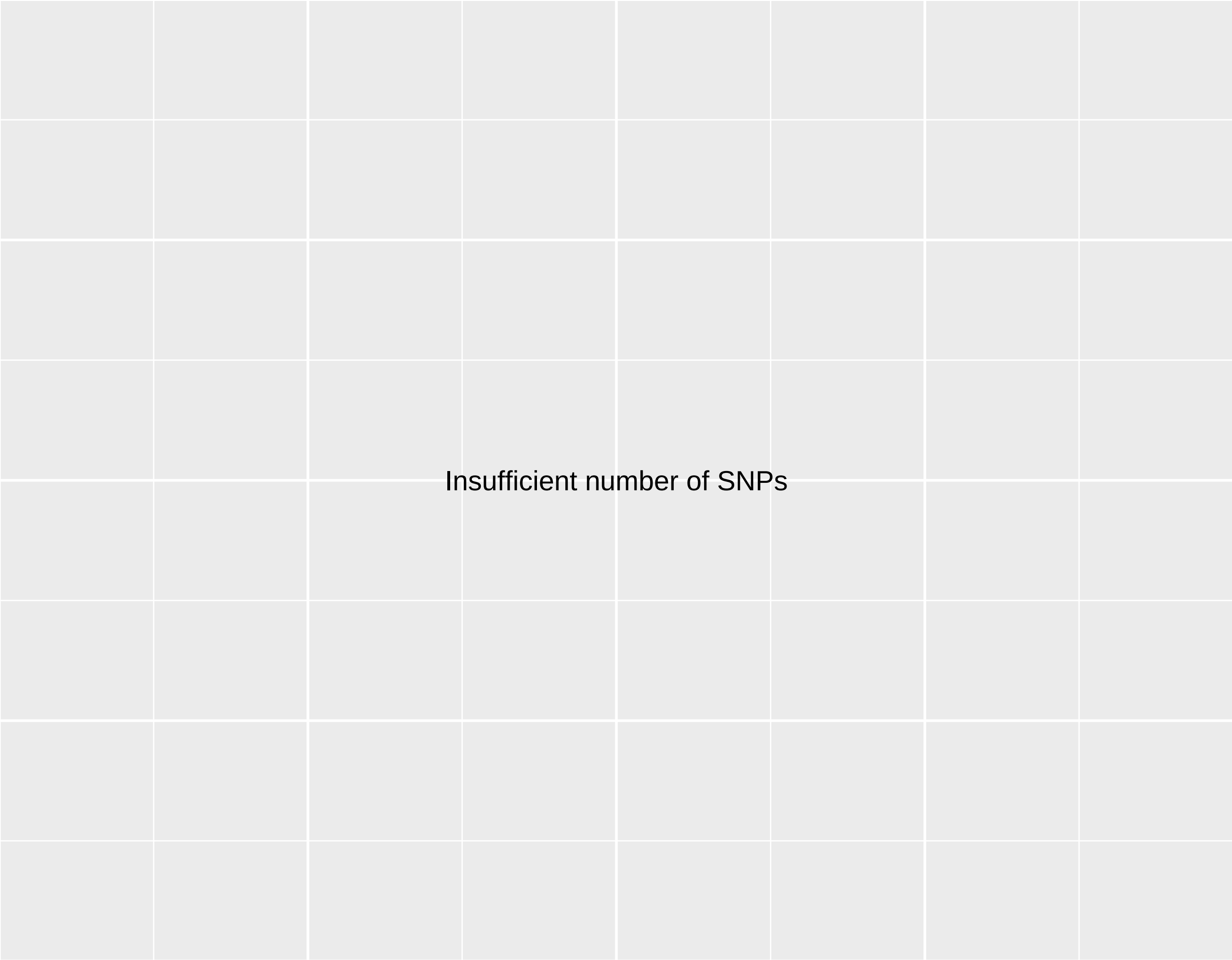
Insufficient number of SNPs

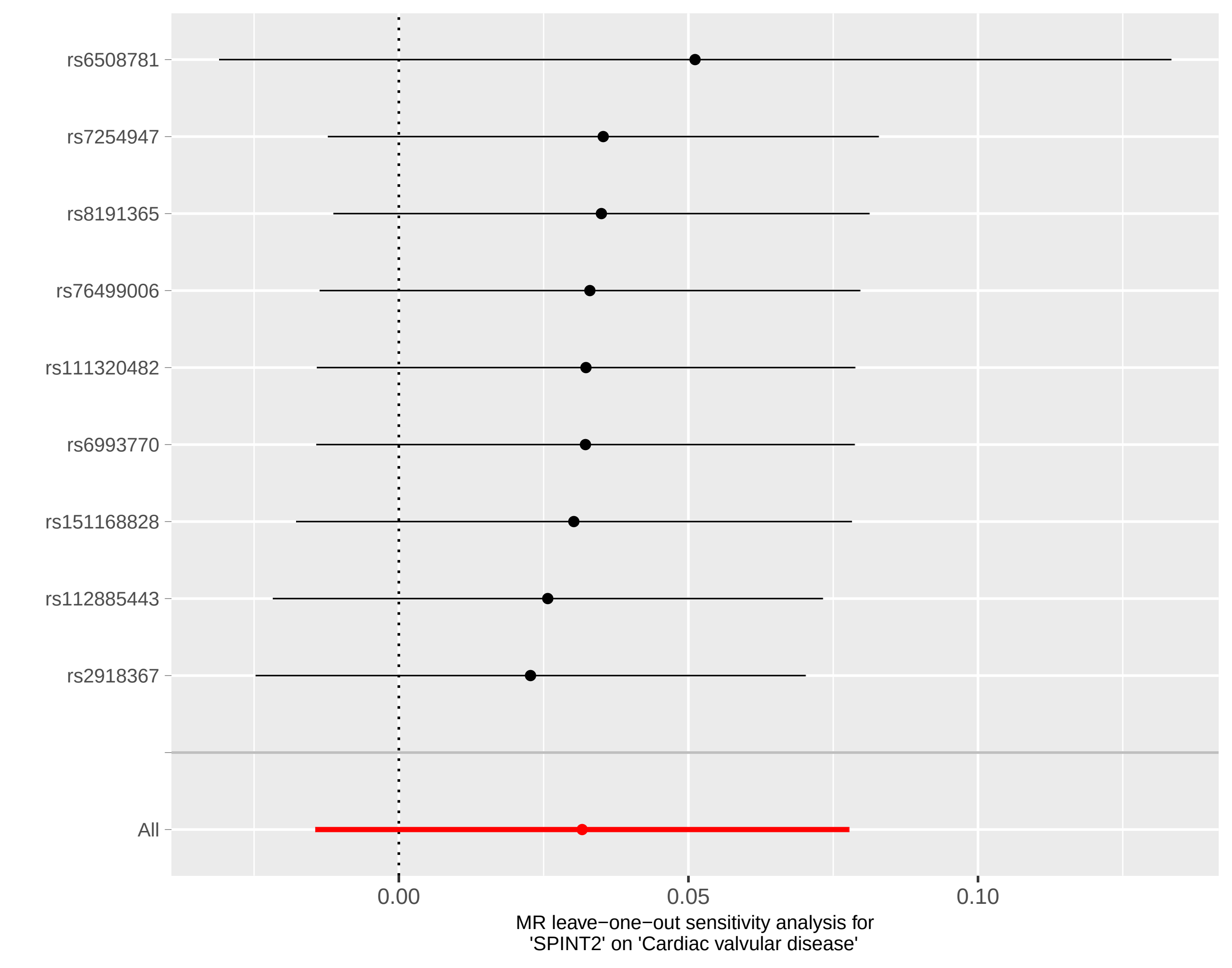
rs6508781
rs7254947
rs8191365
rs76499006
rs111320482
rs6993770
rs151168828
rs112885443
rs2918367
All
0.00
0.05
0.10
MR leave−one−out sensitivity analysis for
'SPINT2' on 'Cardiac valvular disease'

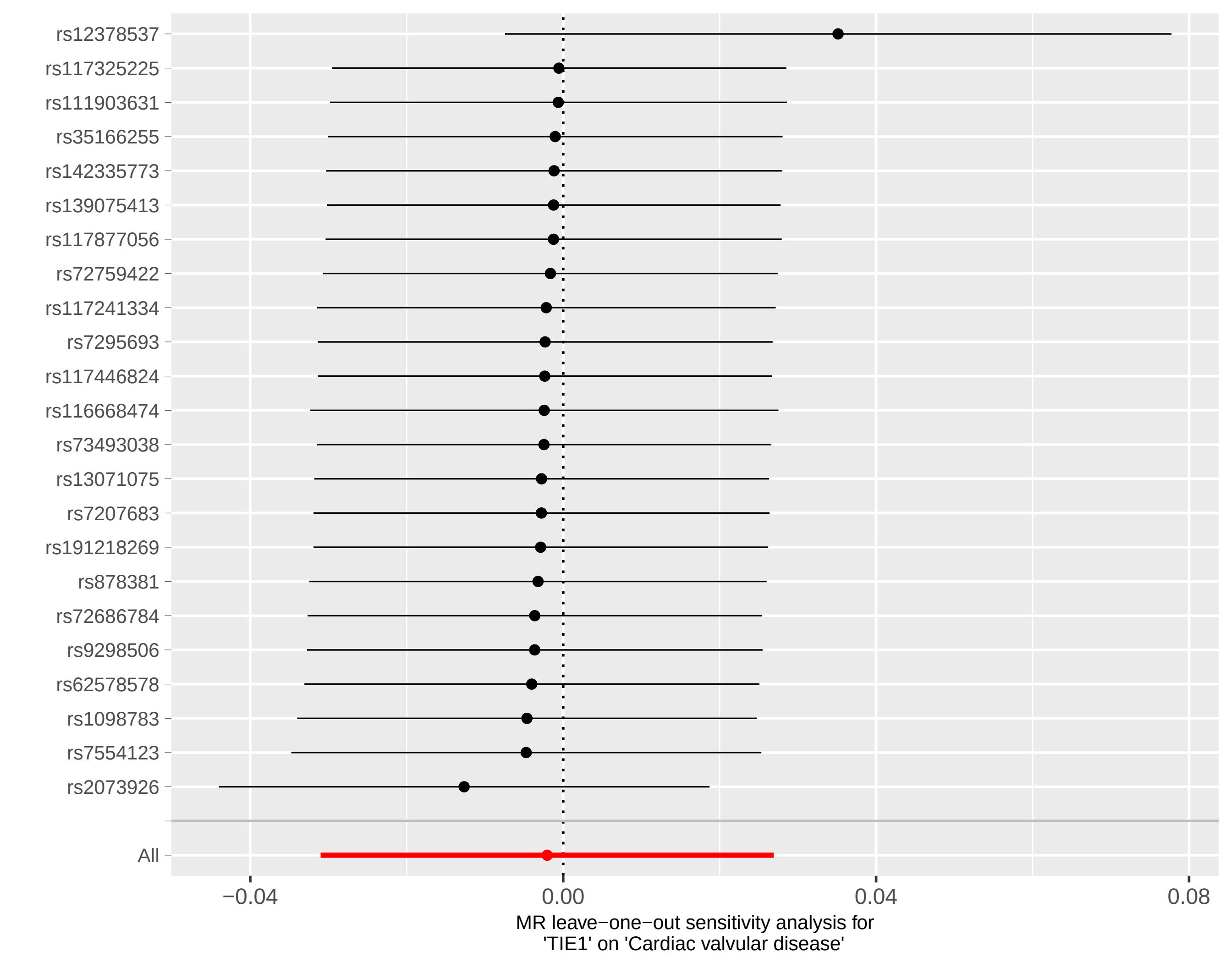
rs12378537
rs117325225
rs111903631
rs35166255
rs142335773
rs139075413
rs117877056
rs72759422
rs117241334
rs7295693
rs117446824
rs116668474
rs73493038
rs13071075
rs7207683
rs191218269
rs878381
rs72686784
rs9298506
rs62578578
rs1098783
rs7554123
rs2073926
All
−0.04
0.00
0.04
0.08
MR leave−one−out sensitivity analysis for
'TIE1' on 'Cardiac valvular disease'

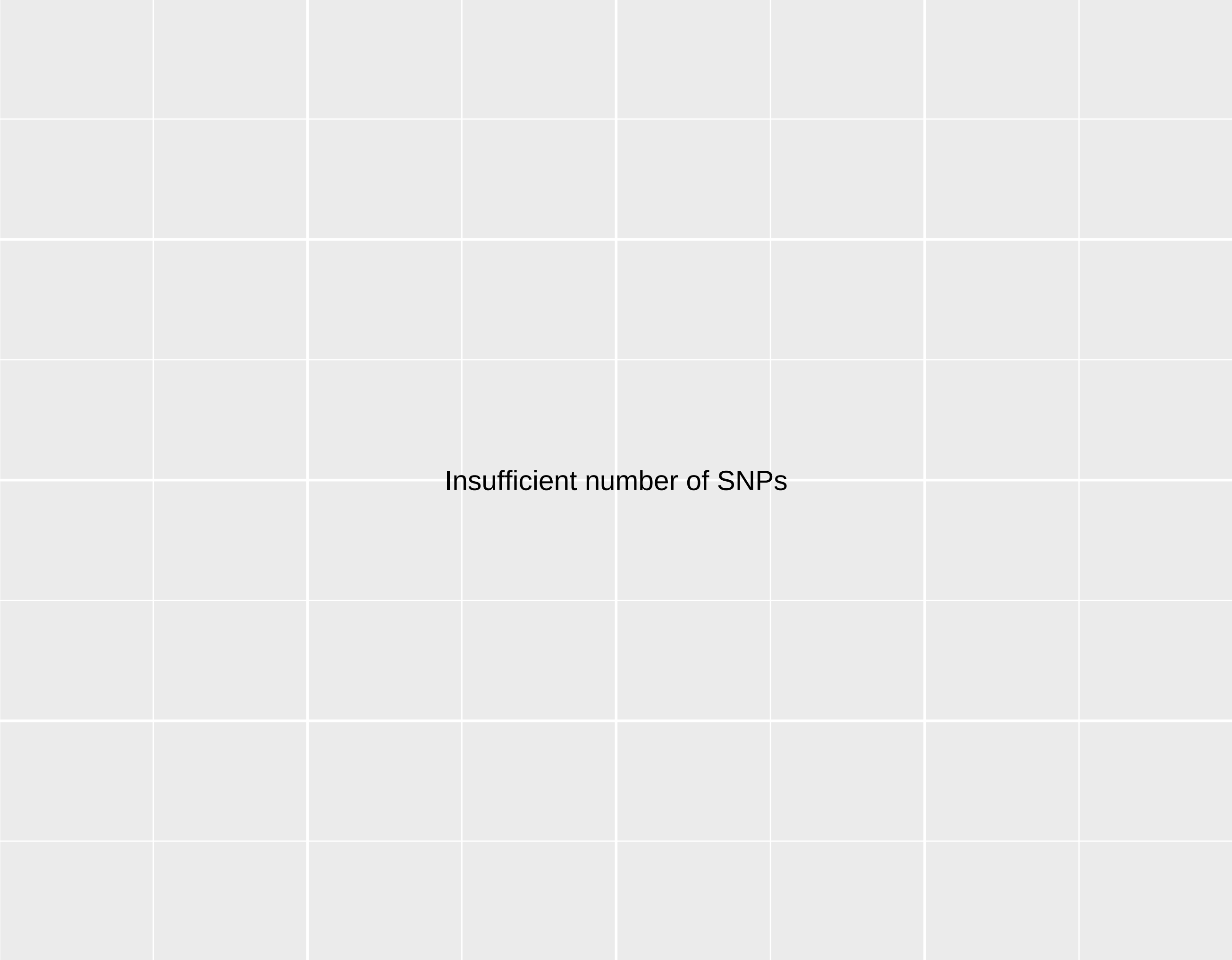
Insufficient number of SNPs

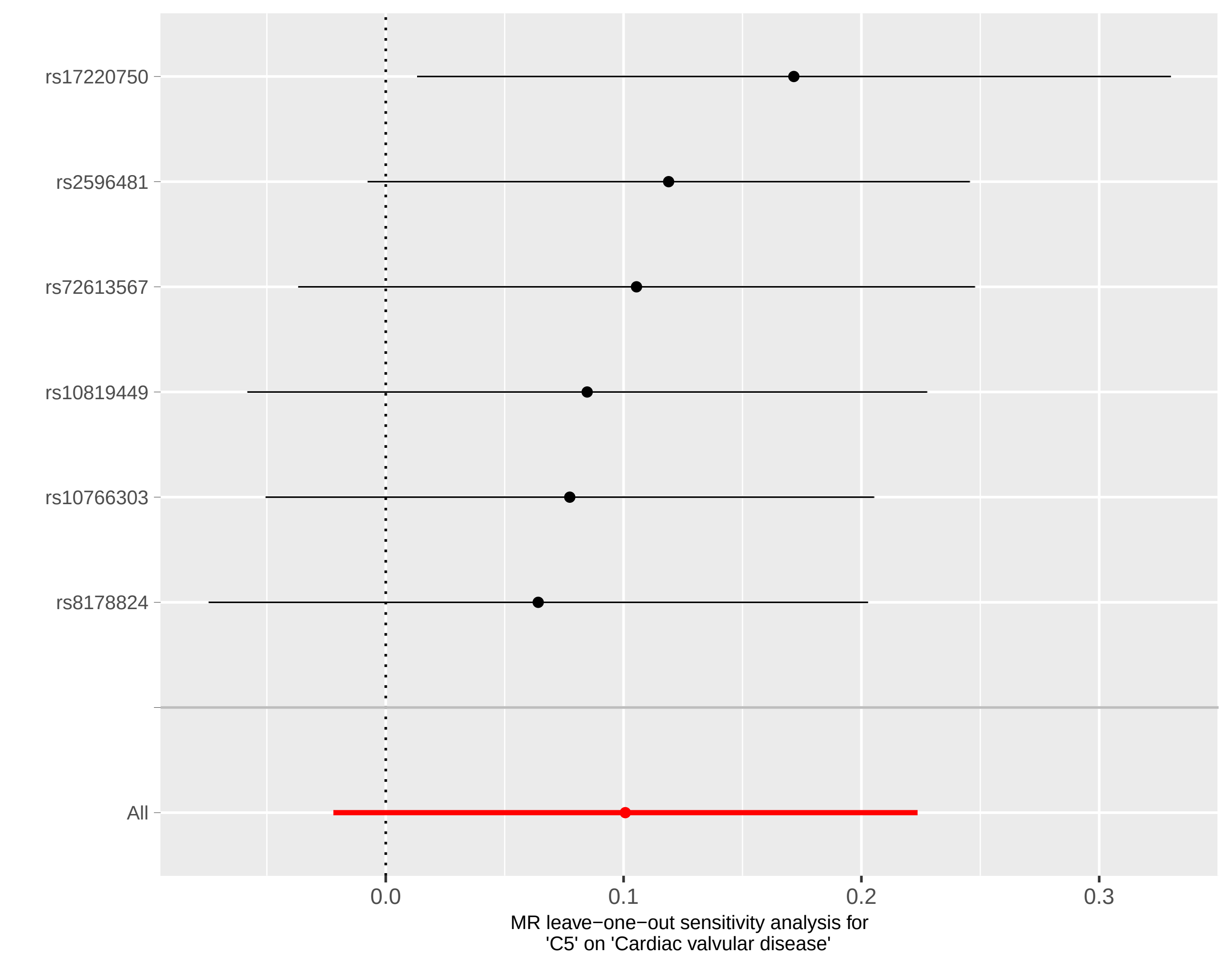
rs17220750
rs2596481
rs72613567
rs10819449
rs10766303
rs8178824
All
0.0
0.1
0.2
0.3
MR leave−one−out sensitivity analysis for
'C5' on 'Cardiac valvular disease'

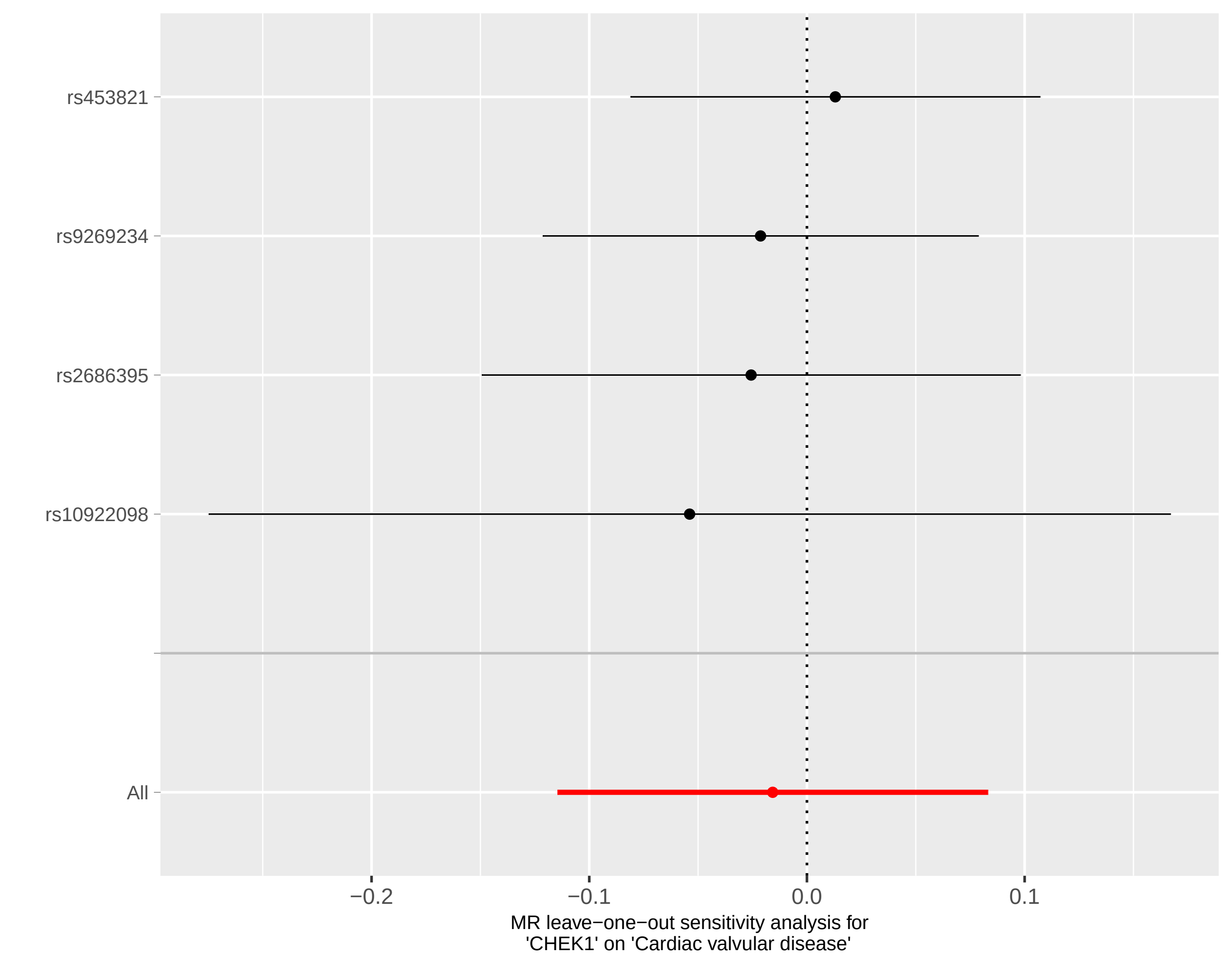
rs453821
rs9269234
rs2686395
rs10922098
All
−0.2
−0.1
0.0
0.1
MR leave−one−out sensitivity analysis for
'CHEK1' on 'Cardiac valvular disease'

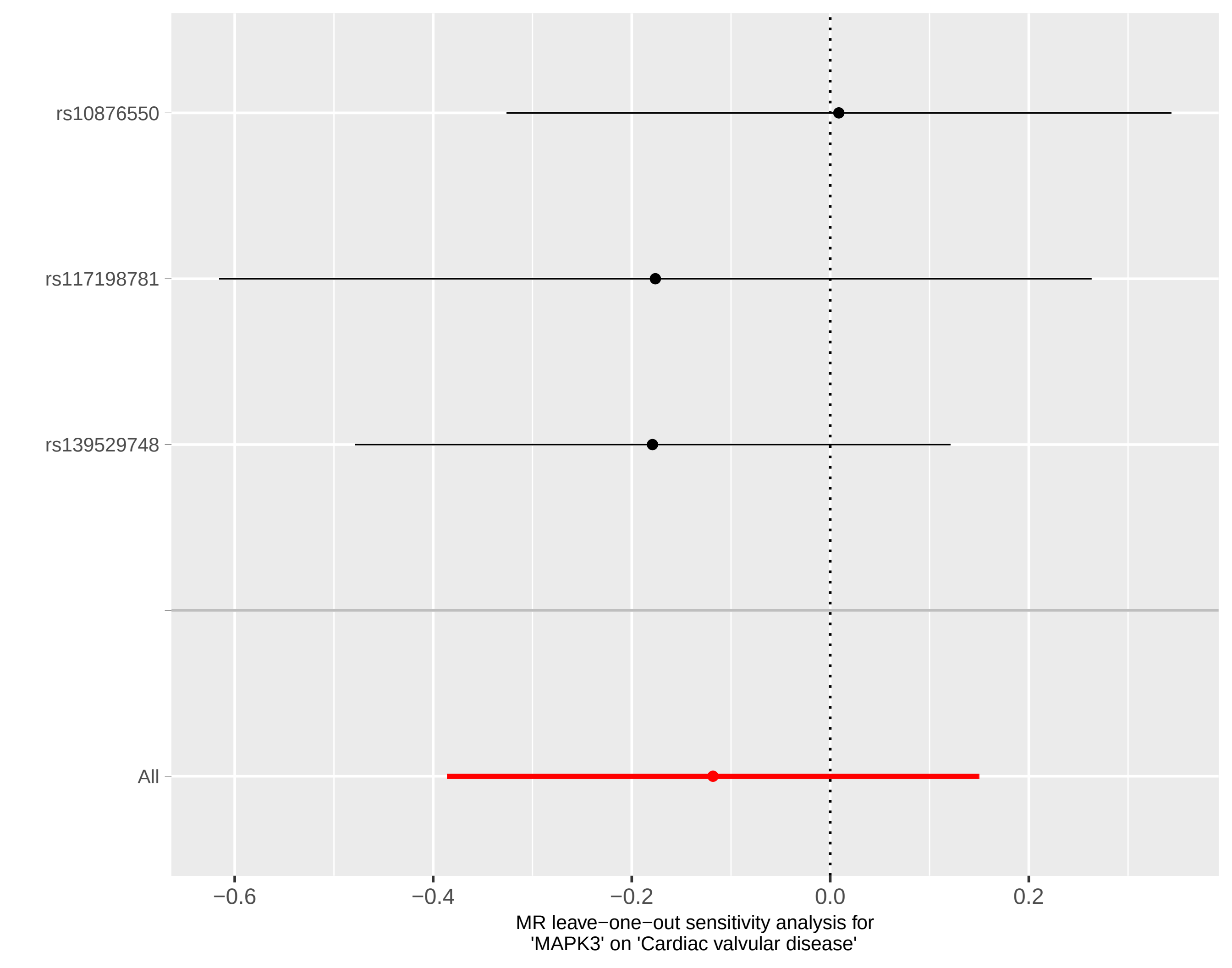
rs10876550
rs117198781
rs139529748
All
−0.6
−0.4
−0.2
0.0
0.2
MR leave−one−out sensitivity analysis for
'MAPK3' on 'Cardiac valvular disease'

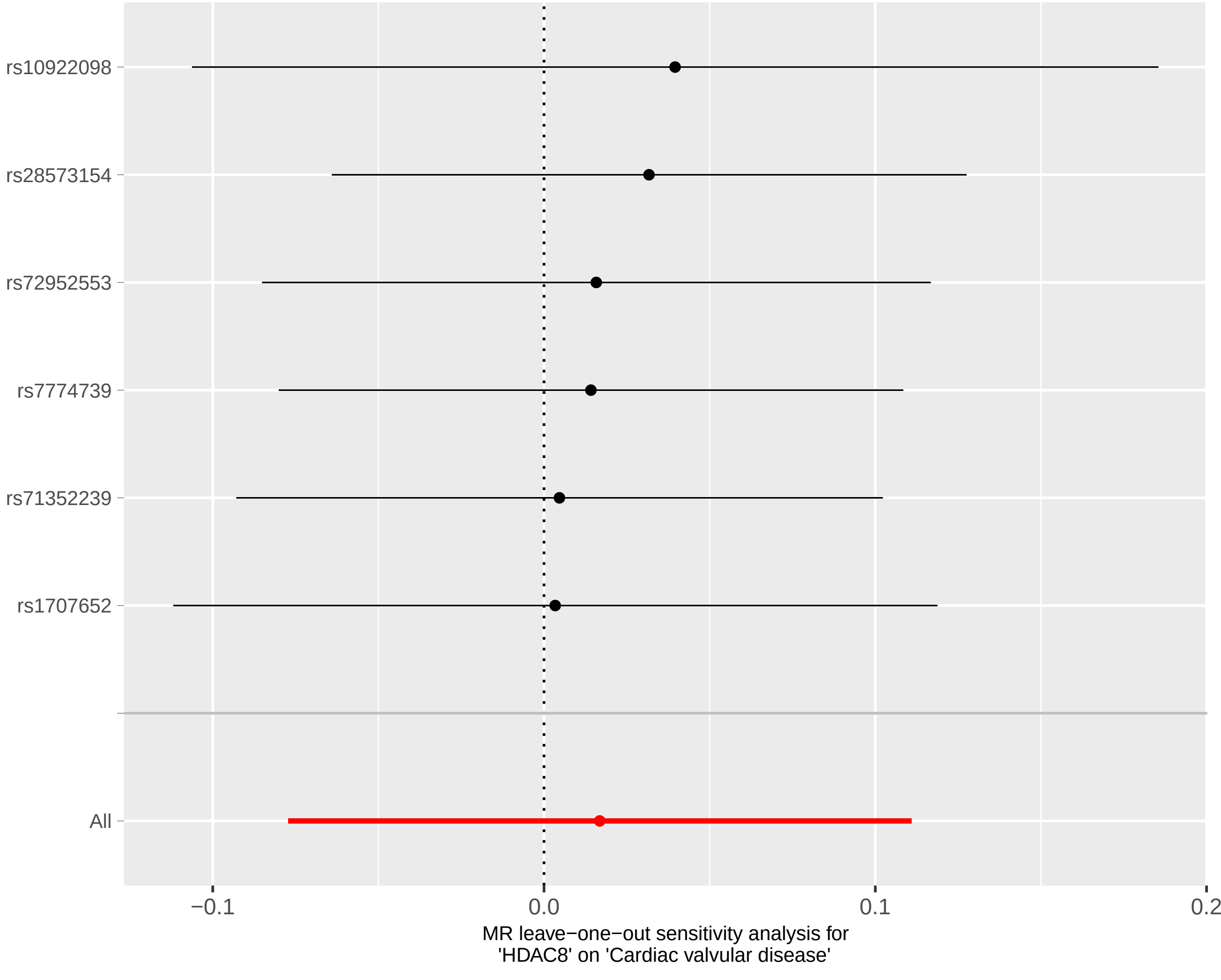
rs10922098
rs28573154
rs72952553
rs7774739
rs71352239
rs1707652
All
−0.1
0.0
0.1
0.2
MR leave−one−out sensitivity analysis for
'HDAC8' on 'Cardiac valvular disease'

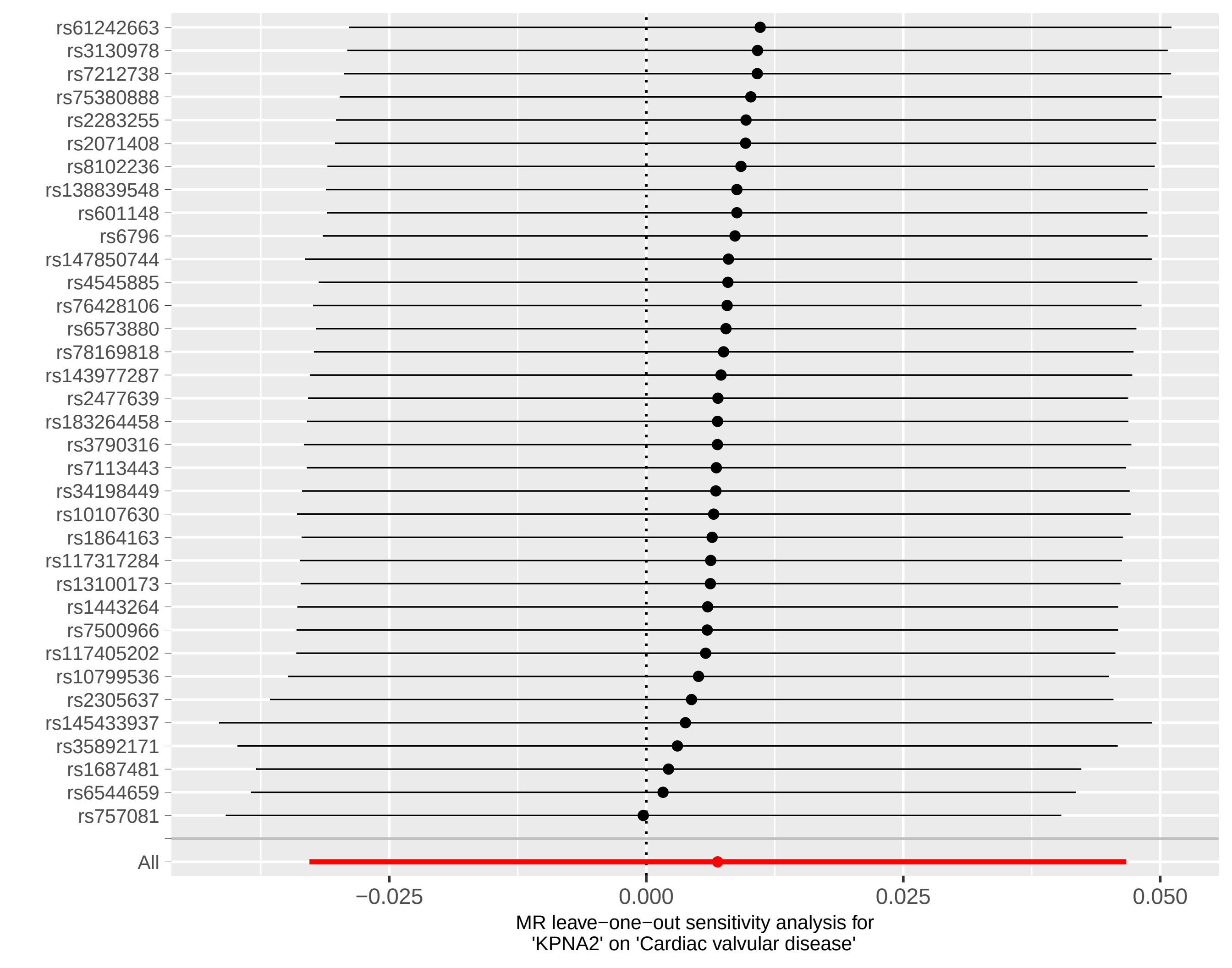
rs61242663
rs3130978
rs7212738
rs75380888
rs2283255
rs2071408
rs8102236
rs138839548
rs601148
rs6796
rs147850744
rs4545885
rs76428106
rs6573880
rs78169818
rs143977287
rs2477639
rs183264458
rs3790316
rs7113443
rs34198449
rs10107630
rs1864163
rs117317284
rs13100173
rs1443264
rs7500966
rs117405202
rs10799536
rs2305637
rs145433937
rs35892171
rs1687481
rs6544659
rs757081
All
−0.025
0.000
0.025
0.050
MR leave−one−out sensitivity analysis for
'KPNA2' on 'Cardiac valvular disease'

Insufficient number of SNPs

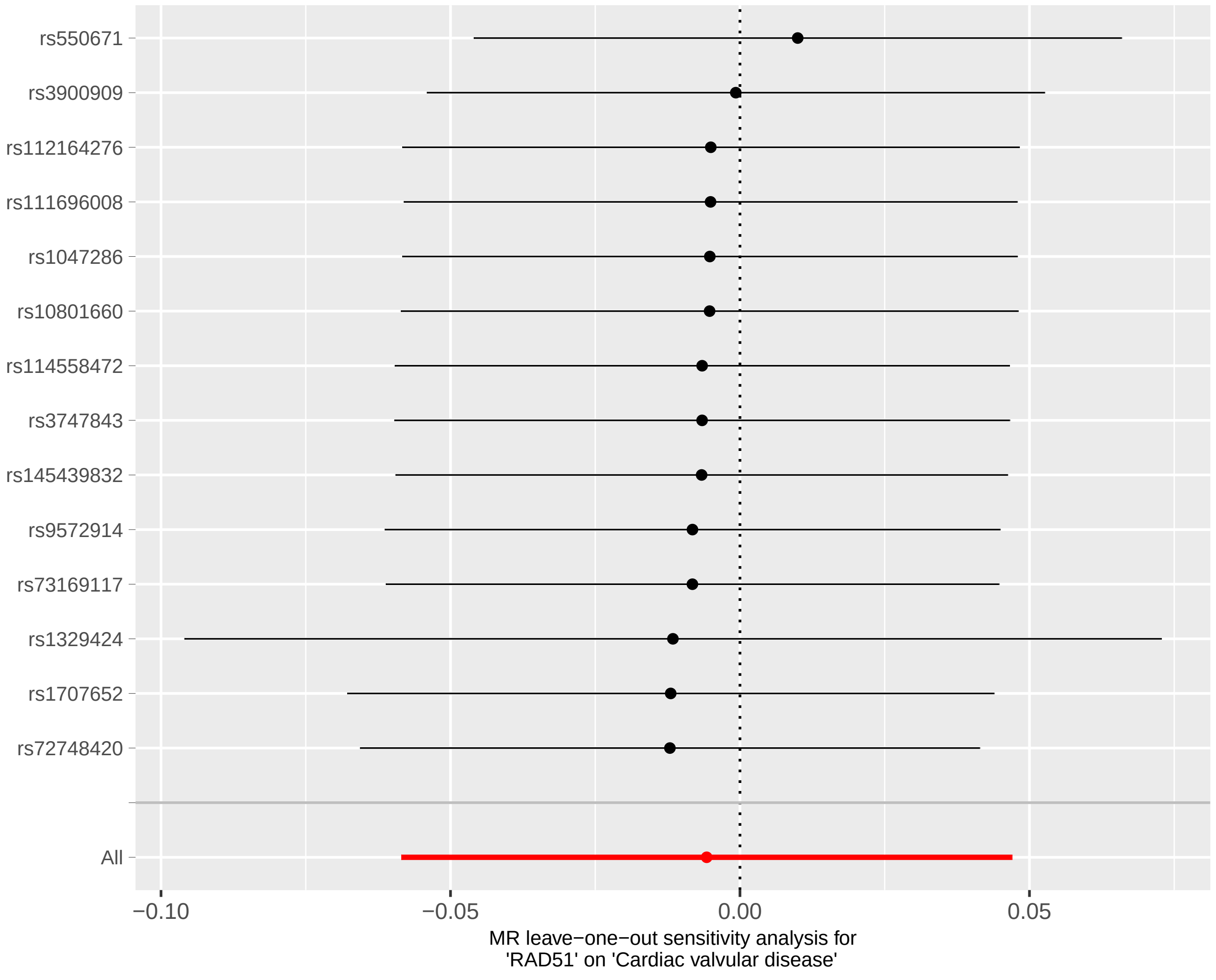
rs550671
rs3900909
rs112164276
rs111696008
rs1047286
rs10801660
rs114558472
rs3747843
rs145439832
rs9572914
rs73169117
rs1329424
rs1707652
rs72748420
All
−0.10
−0.05
0.00
0.05
MR leave−one−out sensitivity analysis for
'RAD51' on 'Cardiac valvular disease'

Insufficient number of SNPs

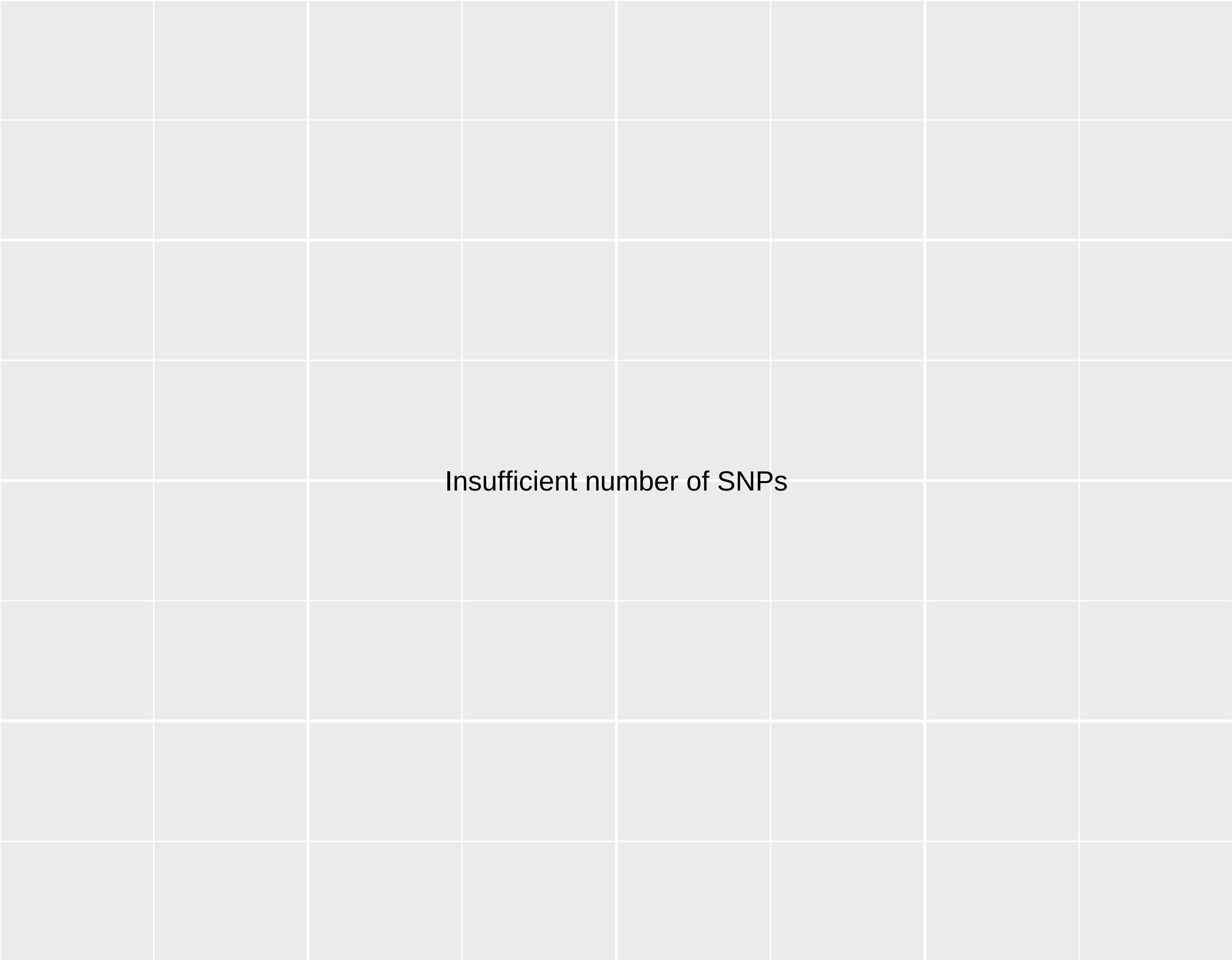
Insufficient number of SNPs

Insufficient number of SNPs

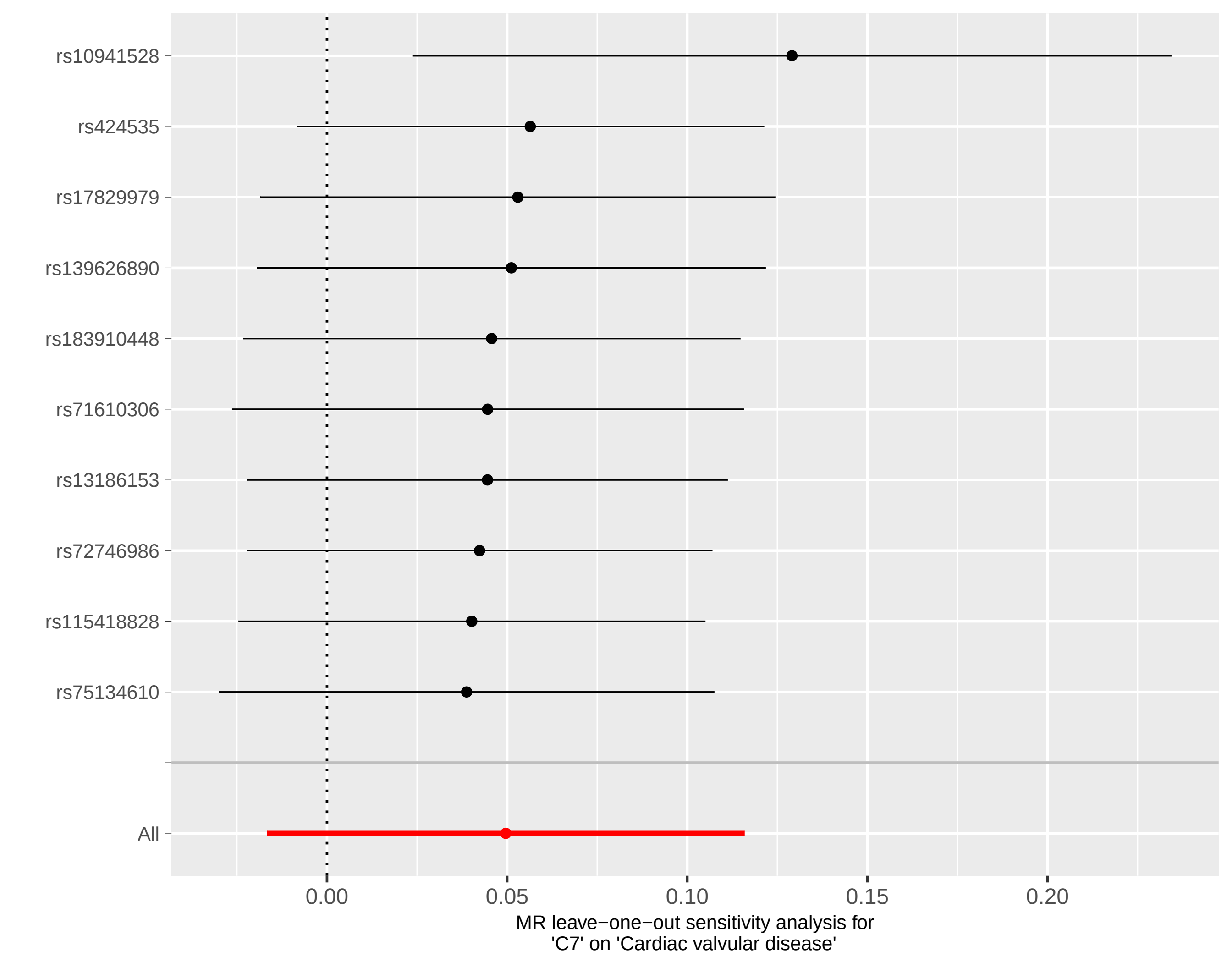

rs10941528
rs424535
rs17829979
rs139626890
rs183910448
rs71610306
rs13186153
rs72746986
rs115418828
rs75134610
All
0.00
0.05
0.10
0.15
0.20
MR leave−one−out sensitivity analysis for
'C7' on 'Cardiac valvular disease'

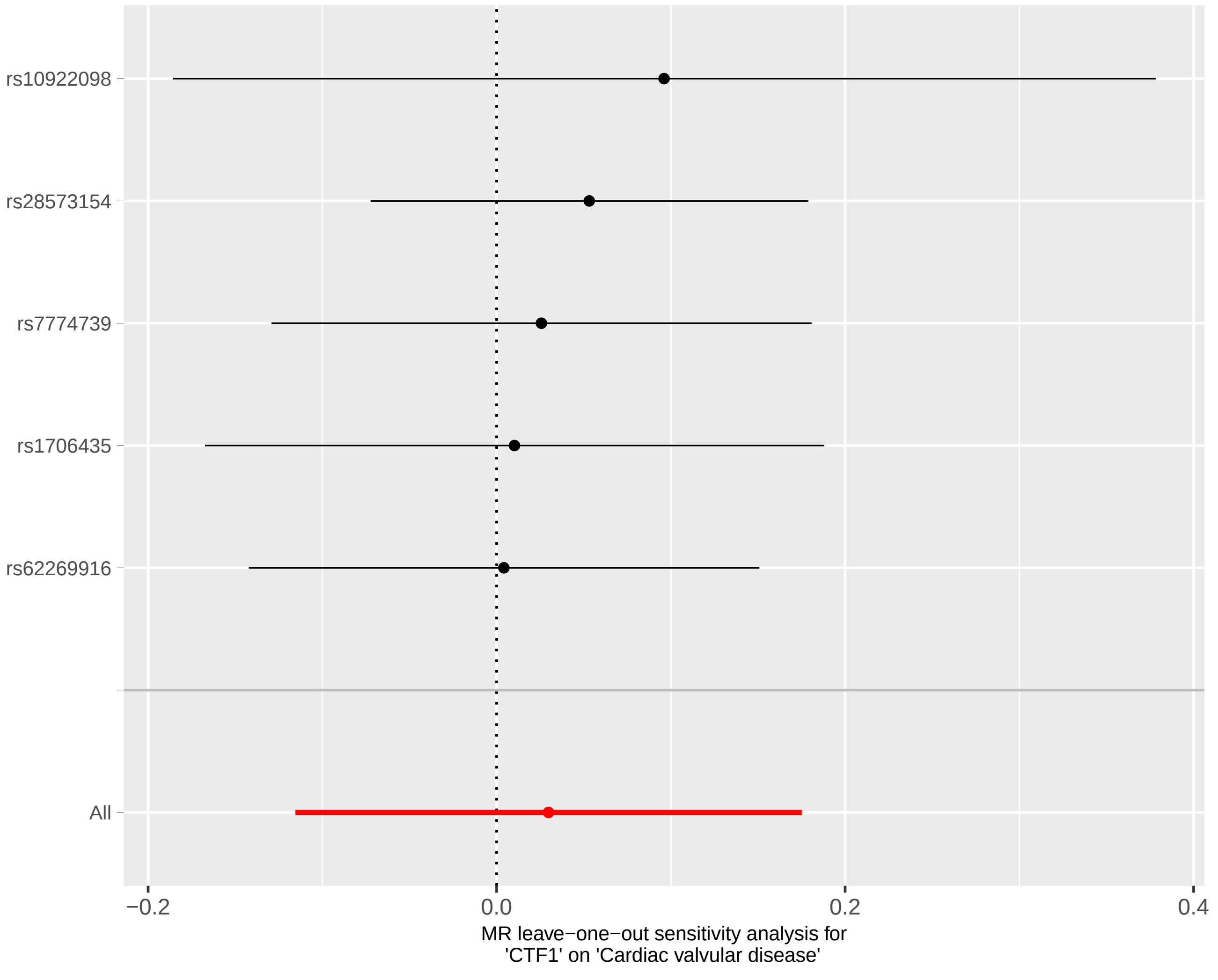
rs10922098
rs28573154
rs7774739
rs1706435
rs62269916
All
−0.2
0.0
0.2
0.4
MR leave−one−out sensitivity analysis for
'CTF1' on 'Cardiac valvular disease'

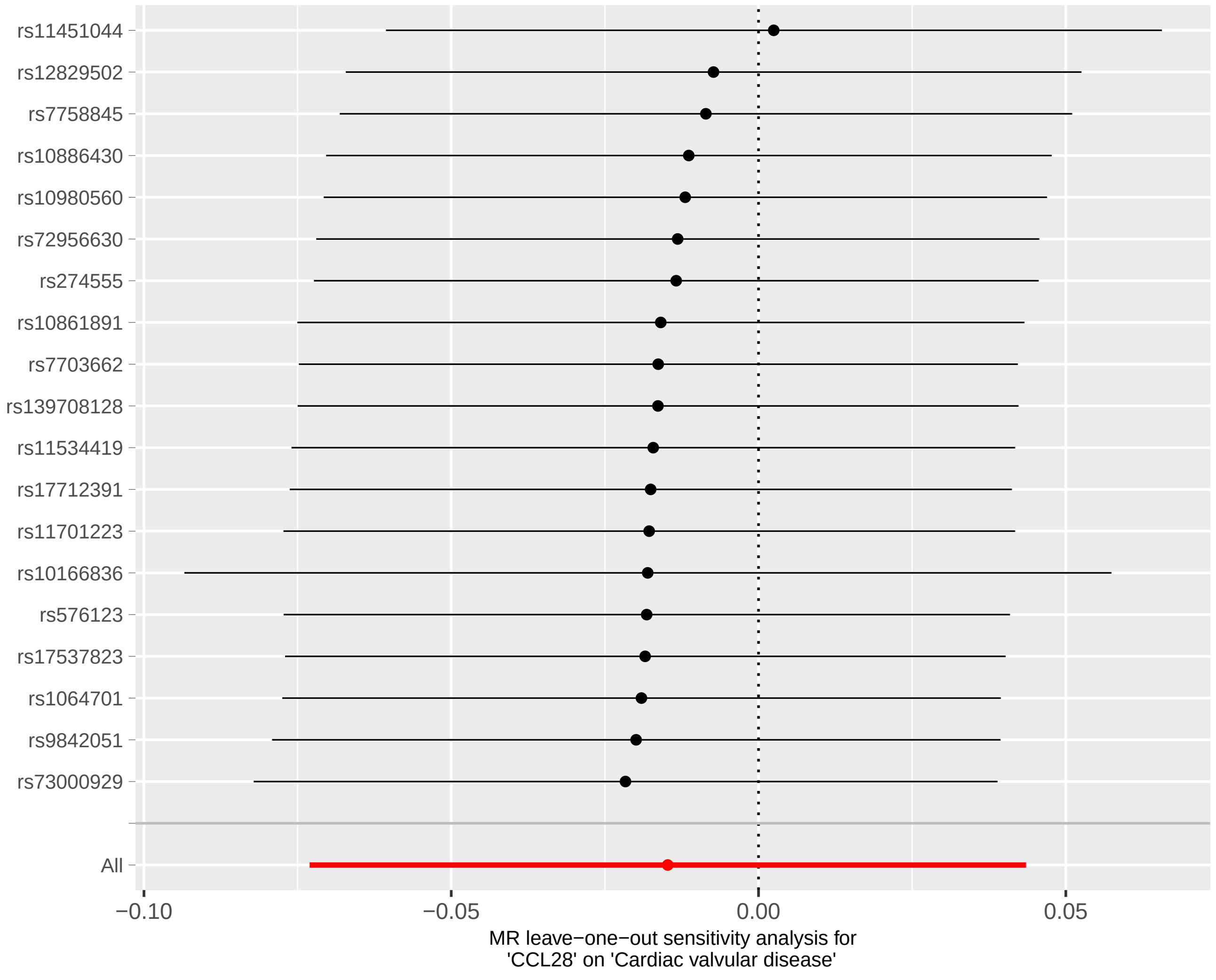

rs11451044
rs12829502
rs7758845
rs10886430
rs10980560
rs72956630
rs274555
rs10861891
rs7703662
rs139708128
rs11534419
rs17712391
rs11701223
rs10166836
rs576123
rs17537823
rs1064701
rs9842051
rs73000929
All
−0.10
−0.05
0.00
0.05
MR leave−one−out sensitivity analysis for
'CCL28' on 'Cardiac valvular disease'

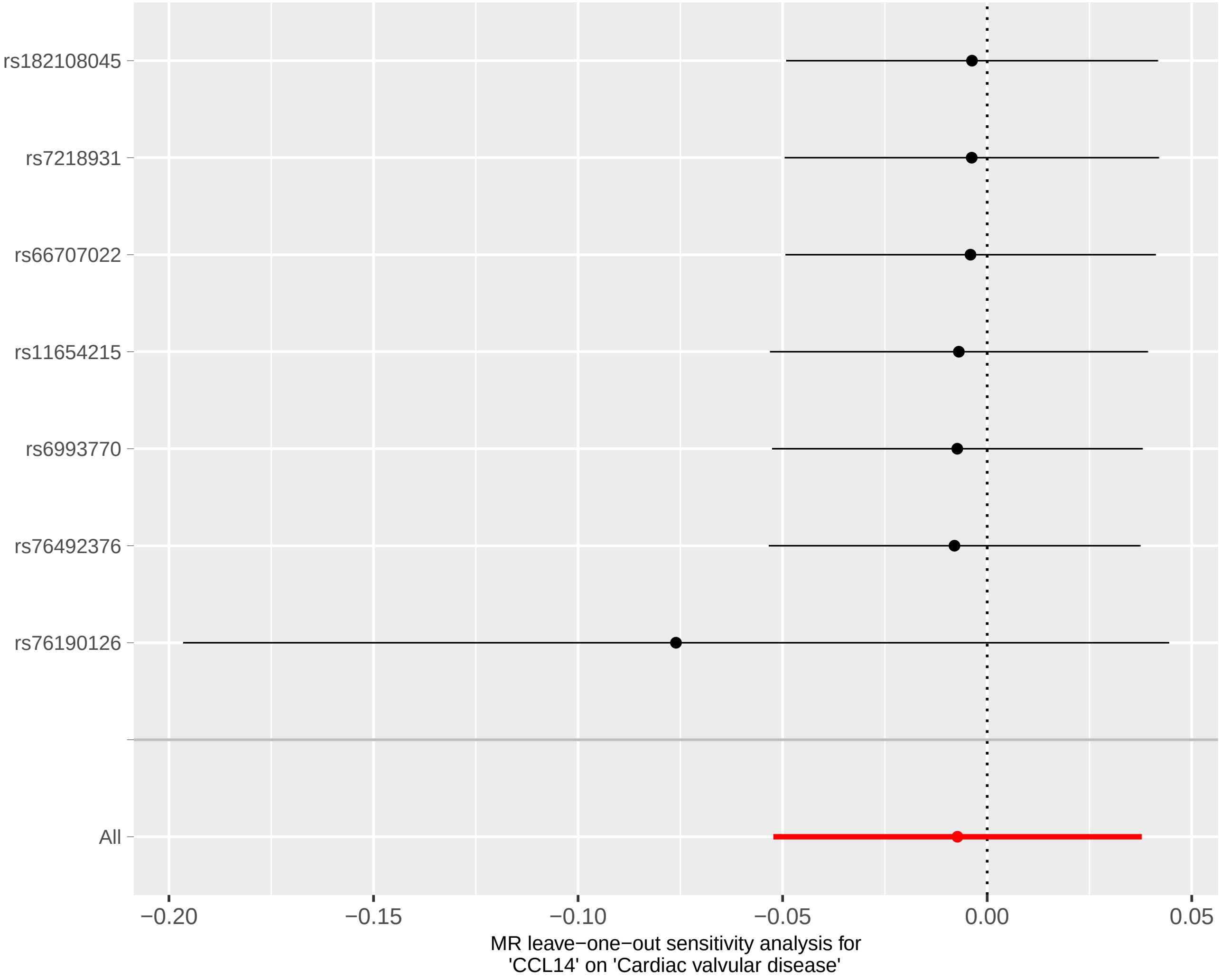
rs182108045
rs7218931
rs66707022
rs11654215
rs6993770
rs76492376
rs76190126
All
−0.20
−0.15
−0.10
−0.05
0.00
0.05
MR leave−one−out sensitivity analysis for
'CCL14' on 'Cardiac valvular disease'

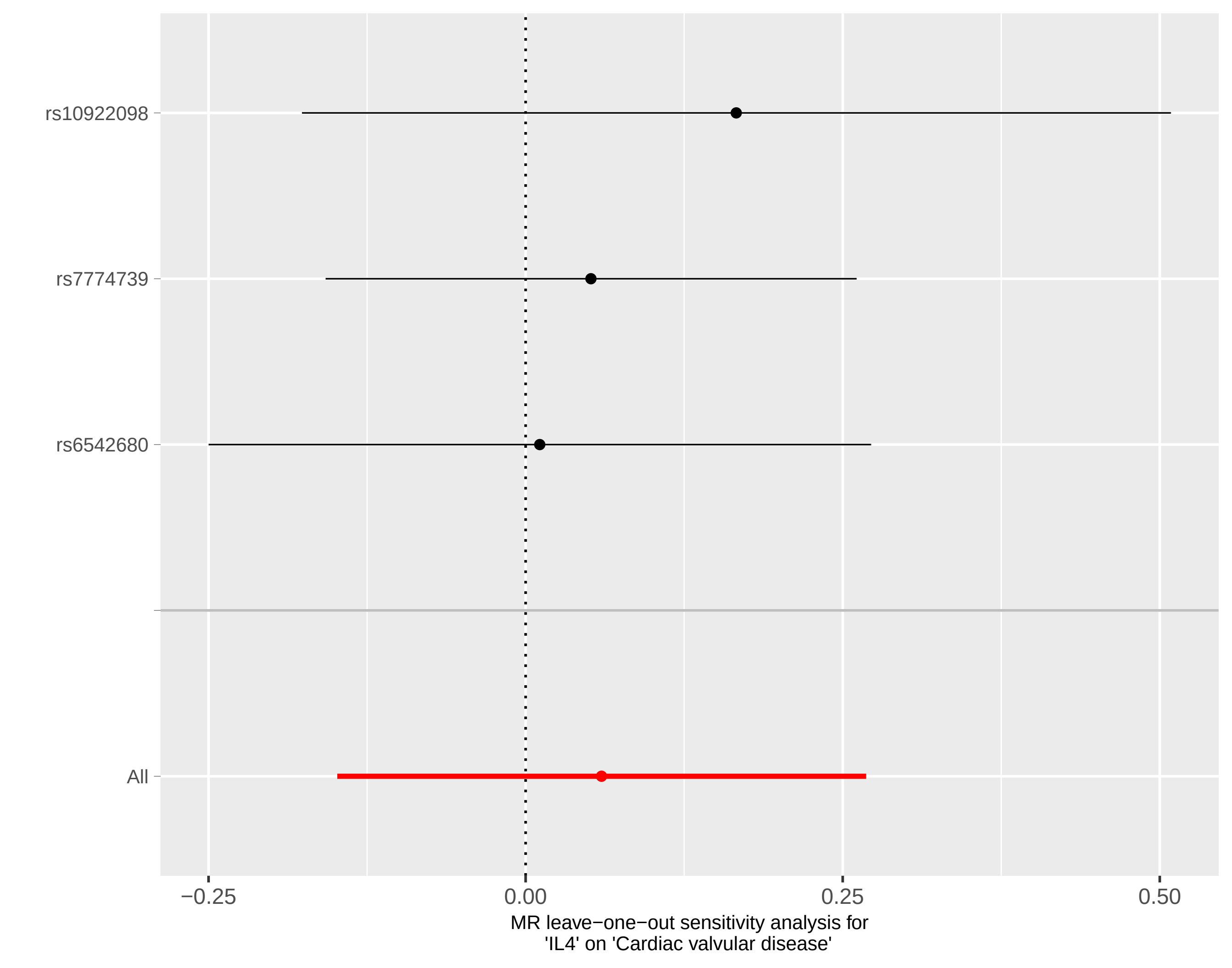
rs10922098
rs7774739
rs6542680
All
−0.25
0.00
0.25
0.50
MR leave−one−out sensitivity analysis for
'IL4' on 'Cardiac valvular disease'

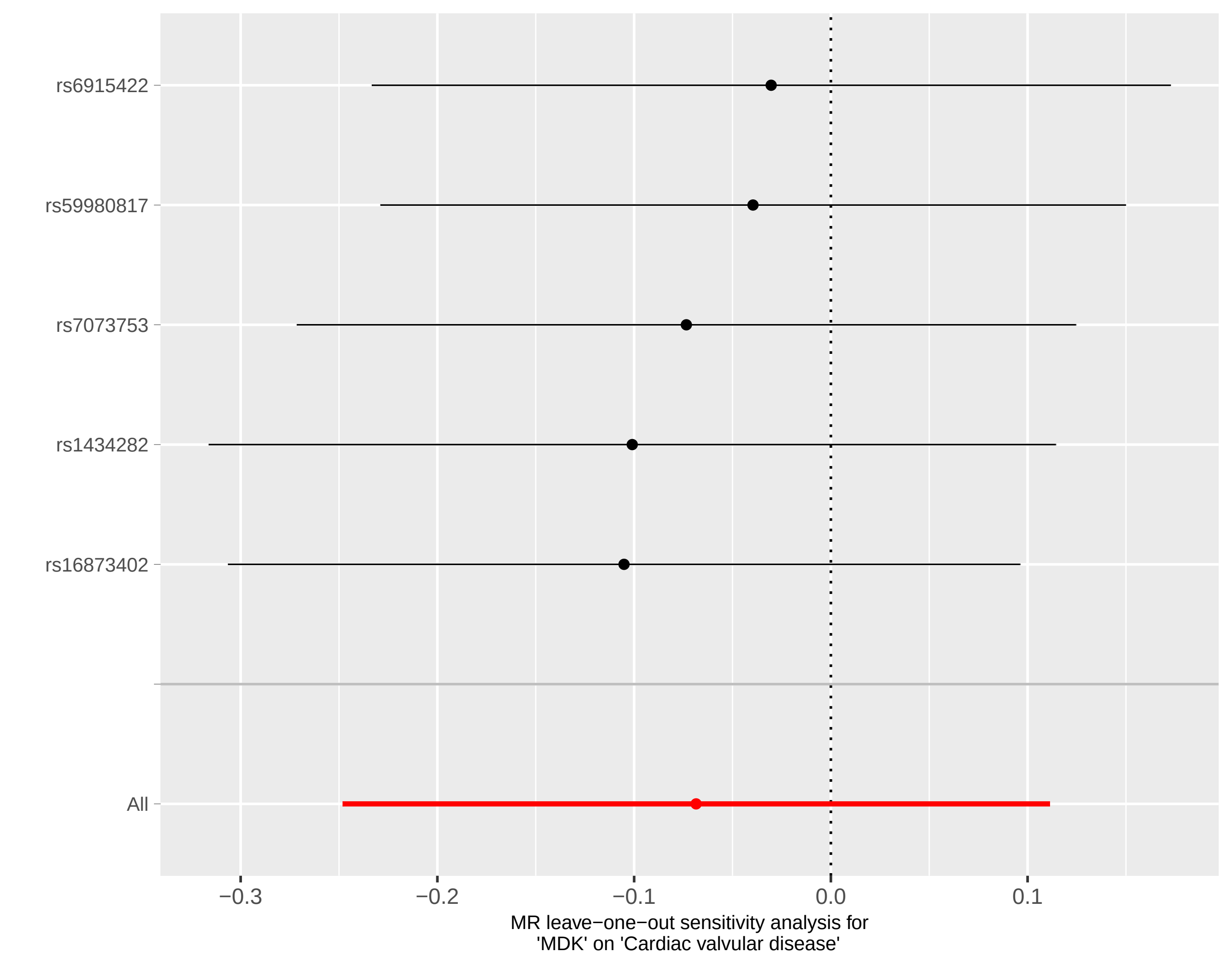
rs6915422
rs59980817
rs7073753
rs1434282
rs16873402
All
−0.3
−0.2
−0.1
0.0
0.1
MR leave−one−out sensitivity analysis for
'MDK' on 'Cardiac valvular disease'

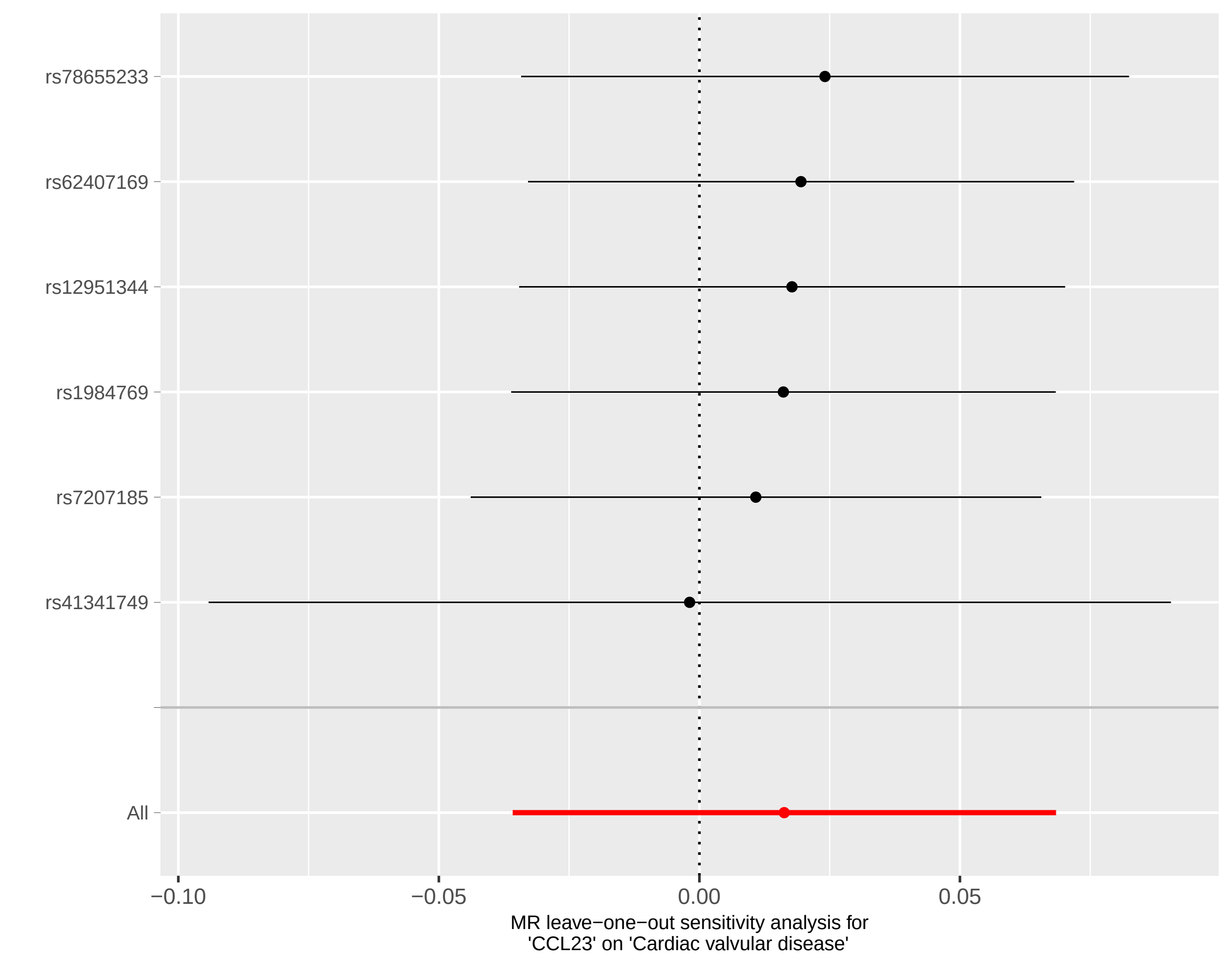

rs78655233
rs62407169
rs12951344
rs1984769
rs7207185
rs41341749
All
−0.10
−0.05
0.00
0.05
MR leave−one−out sensitivity analysis for
'CCL23' on 'Cardiac valvular disease'

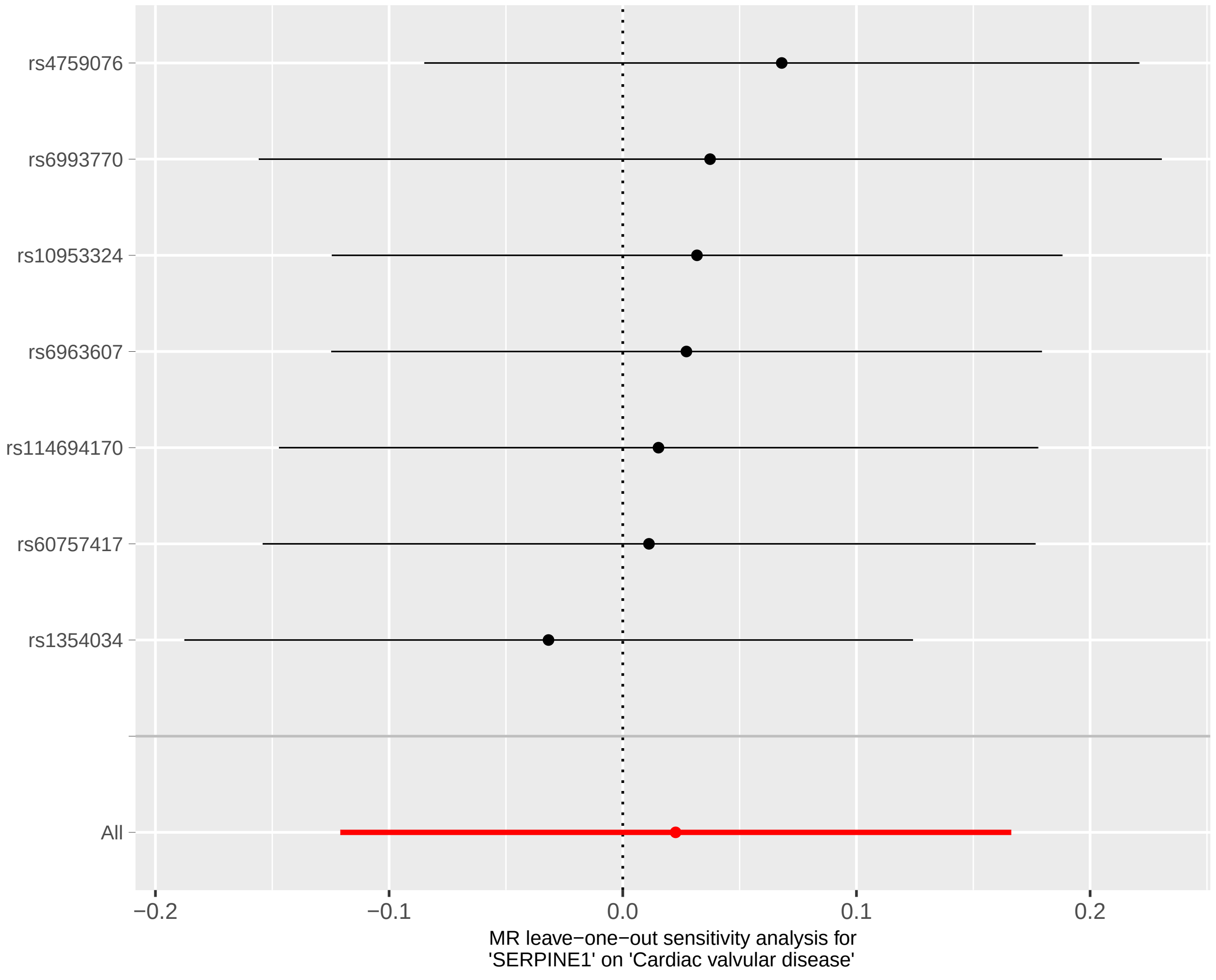

rs4759076
rs6993770
rs10953324
rs6963607
rs114694170
rs60757417
rs1354034
All
−0.2
−0.1
0.0
0.1
0.2
MR leave−one−out sensitivity analysis for
'SERPINE1' on 'Cardiac valvular disease'

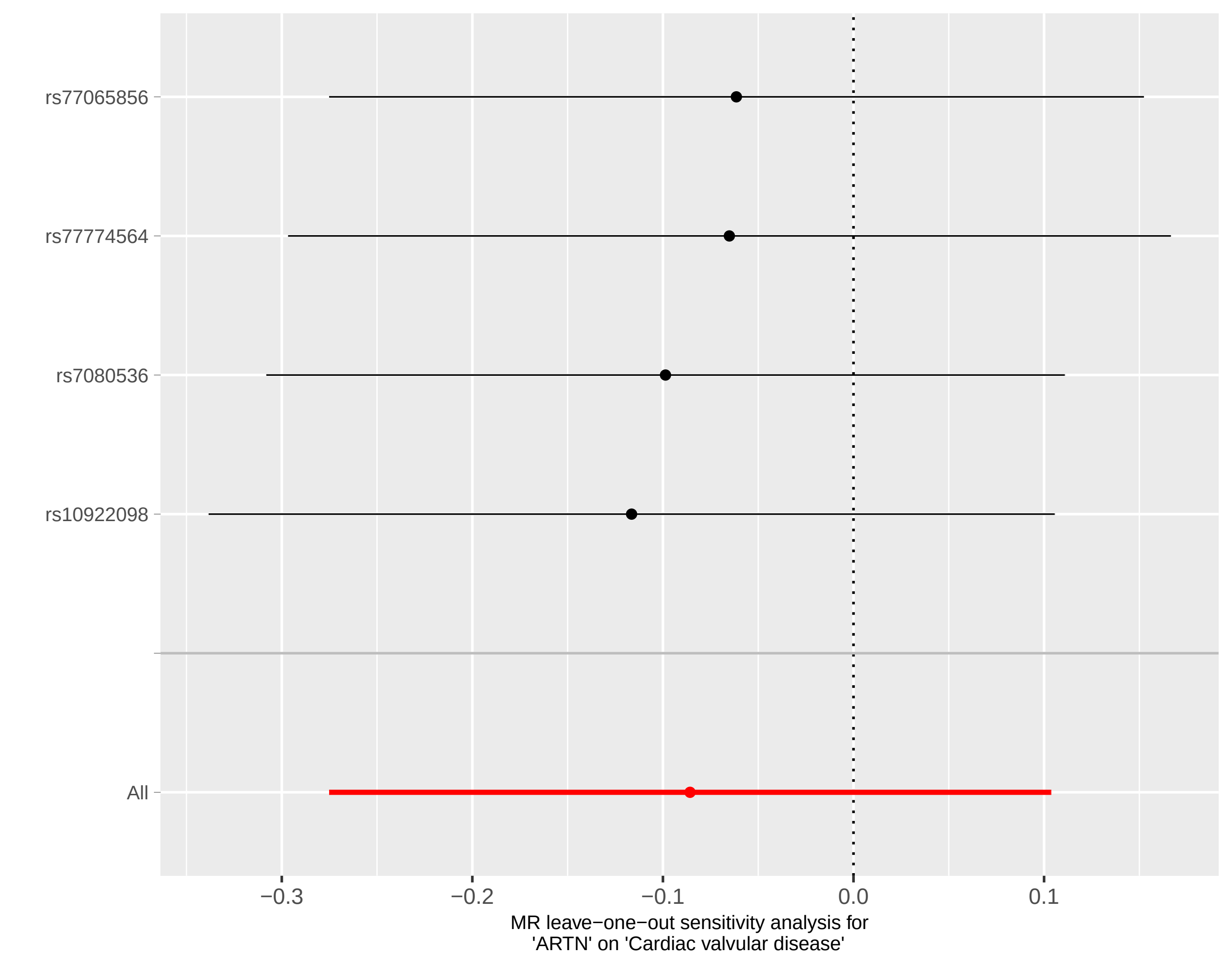

rs77065856
rs77774564
rs7080536
rs10922098
All
−0.3
−0.2
−0.1
0.0
0.1
MR leave−one−out sensitivity analysis for
'ARTN' on 'Cardiac valvular disease'

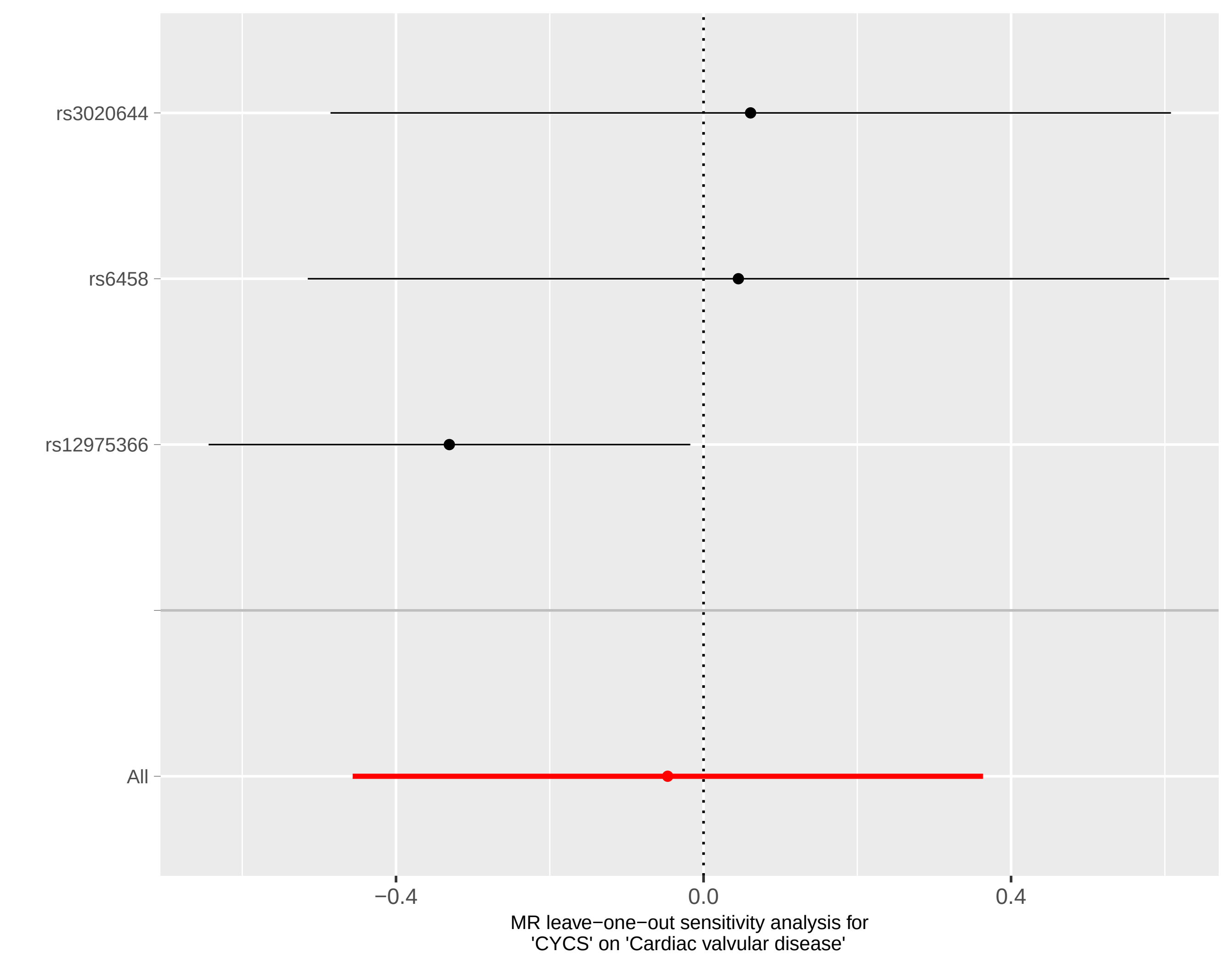
rs3020644
rs6458
rs12975366
All
−0.4
0.0
0.4
MR leave−one−out sensitivity analysis for
'CYCS' on 'Cardiac valvular disease'

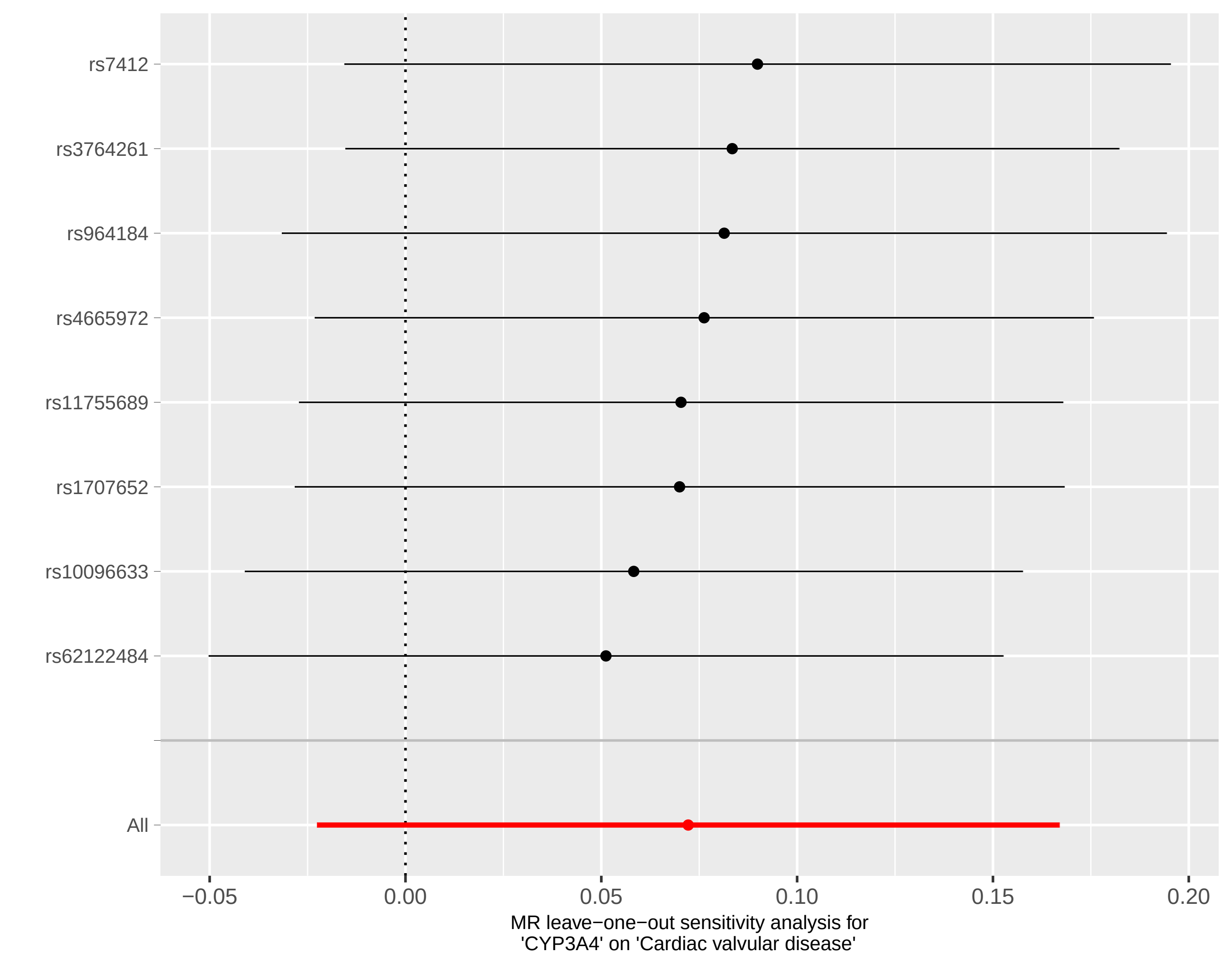
rs7412
rs3764261
rs964184
rs4665972
rs11755689
rs1707652
rs10096633
rs62122484
All
−0.05
0.00
0.05
0.10
0.15
0.20
MR leave−one−out sensitivity analysis for
'CYP3A4' on 'Cardiac valvular disease'

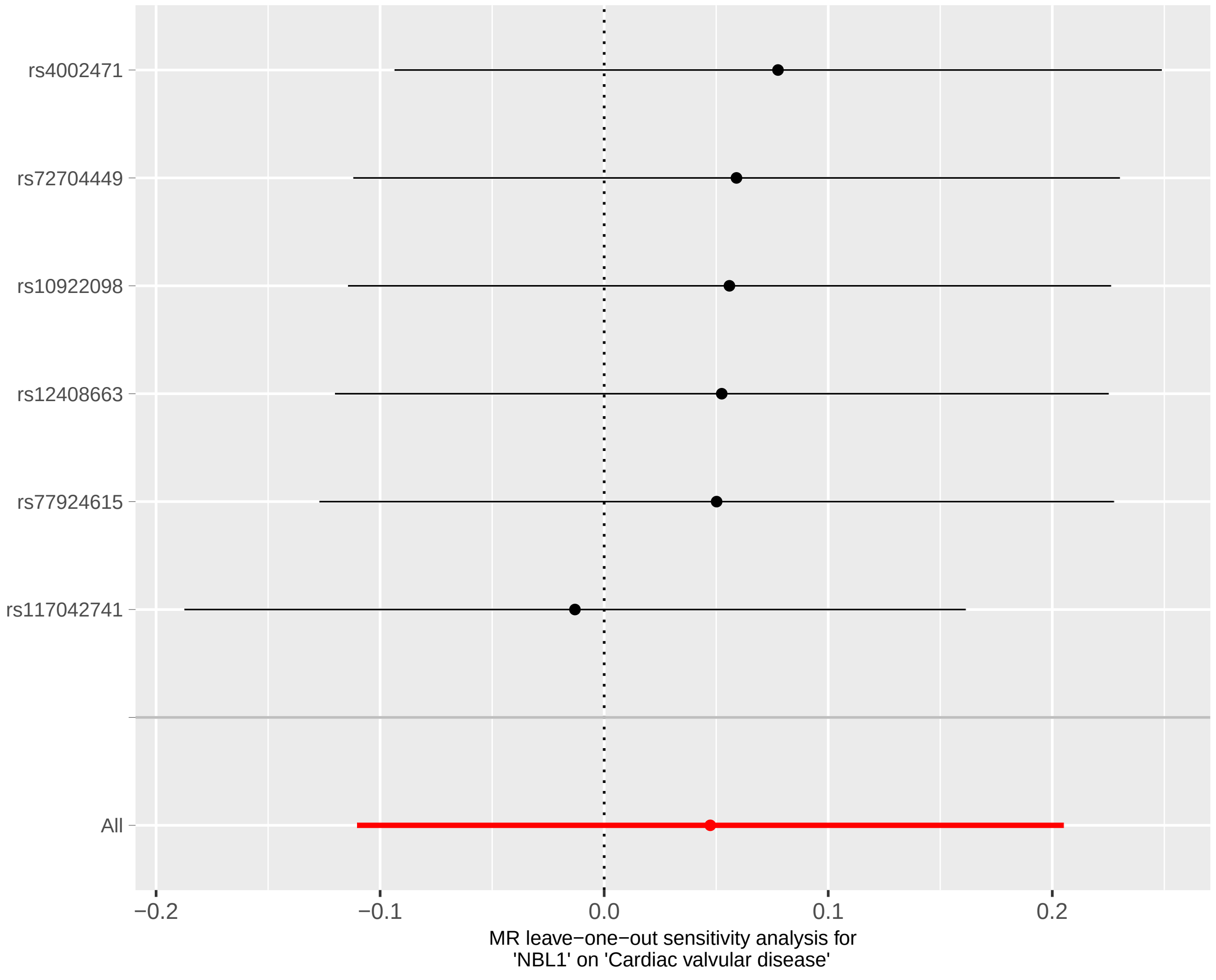

rs4002471
rs72704449
rs10922098
rs12408663
rs77924615
rs117042741
All
−0.2
−0.1
0.0
0.1
0.2
MR leave−one−out sensitivity analysis for
'NBL1' on 'Cardiac valvular disease'

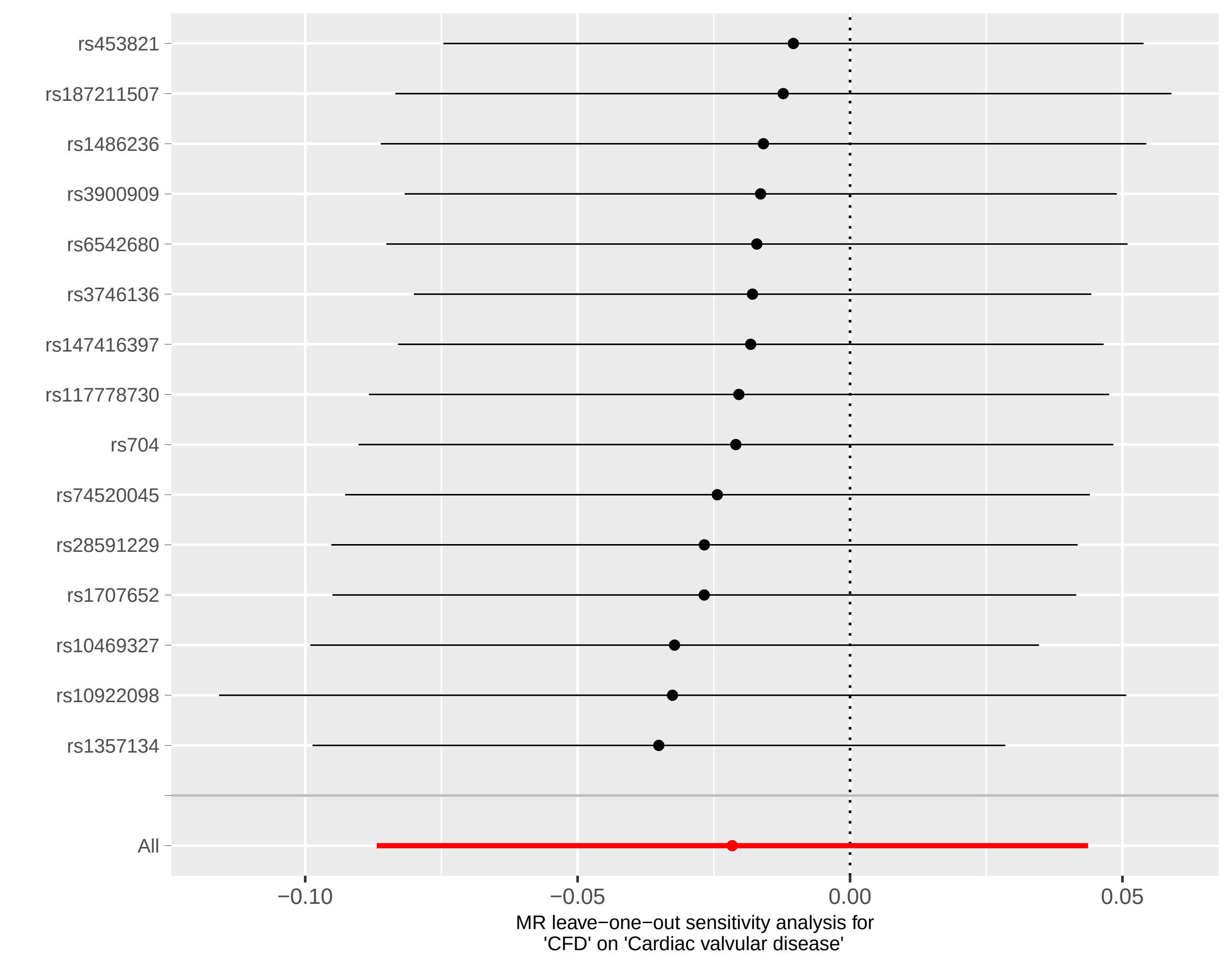

rs453821
rs187211507
rs1486236
rs3900909
rs6542680
rs3746136
rs147416397
rs117778730
rs704
rs74520045
rs28591229
rs1707652
rs10469327
rs10922098
rs1357134
All
−0.10
−0.05
0.00
0.05
MR leave−one−out sensitivity analysis for
'CFD' on 'Cardiac valvular disease'

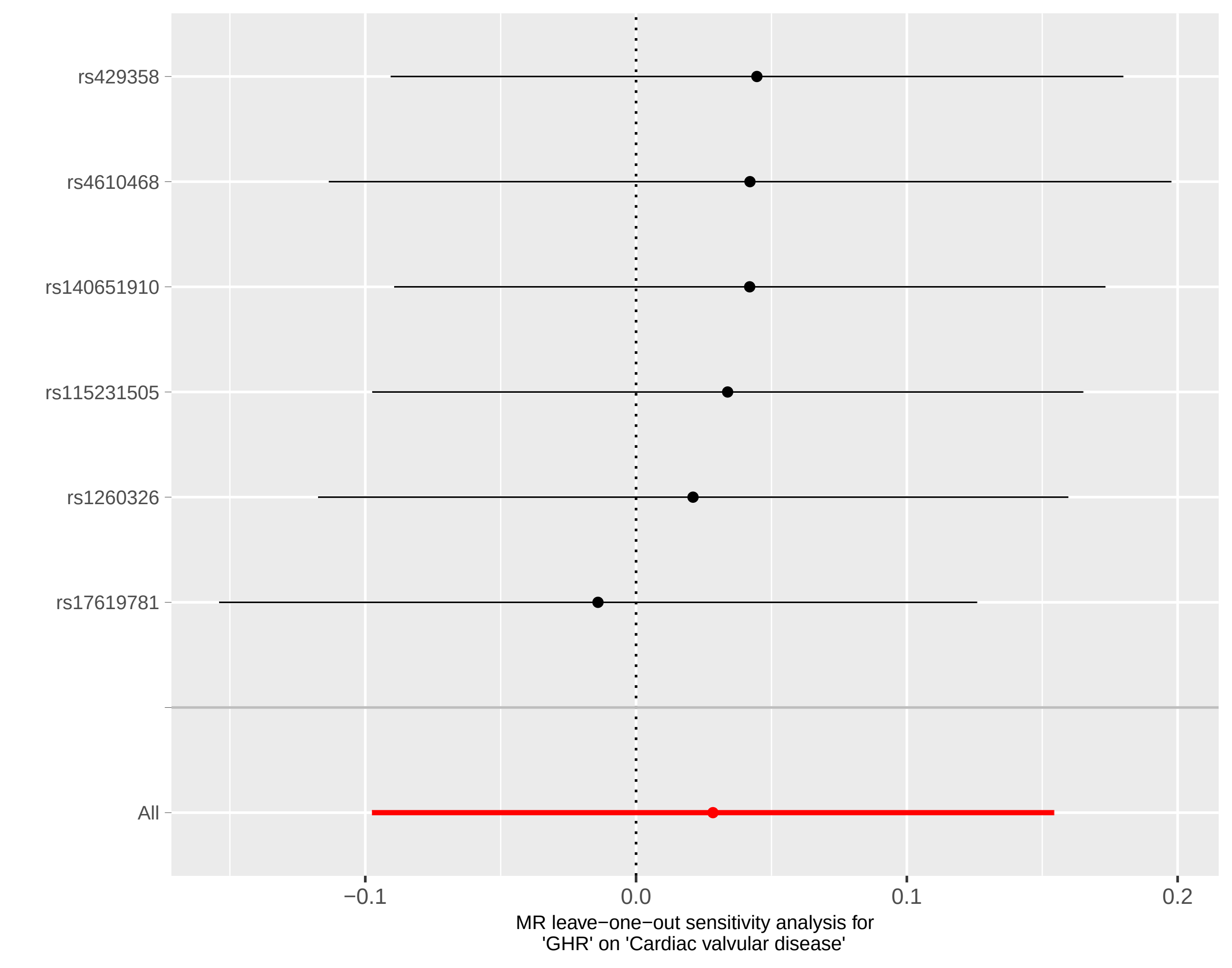
rs429358
rs4610468
rs140651910
rs115231505
rs1260326
rs17619781
All
−0.1
0.0
0.1
0.2
MR leave−one−out sensitivity analysis for
'GHR' on 'Cardiac valvular disease'

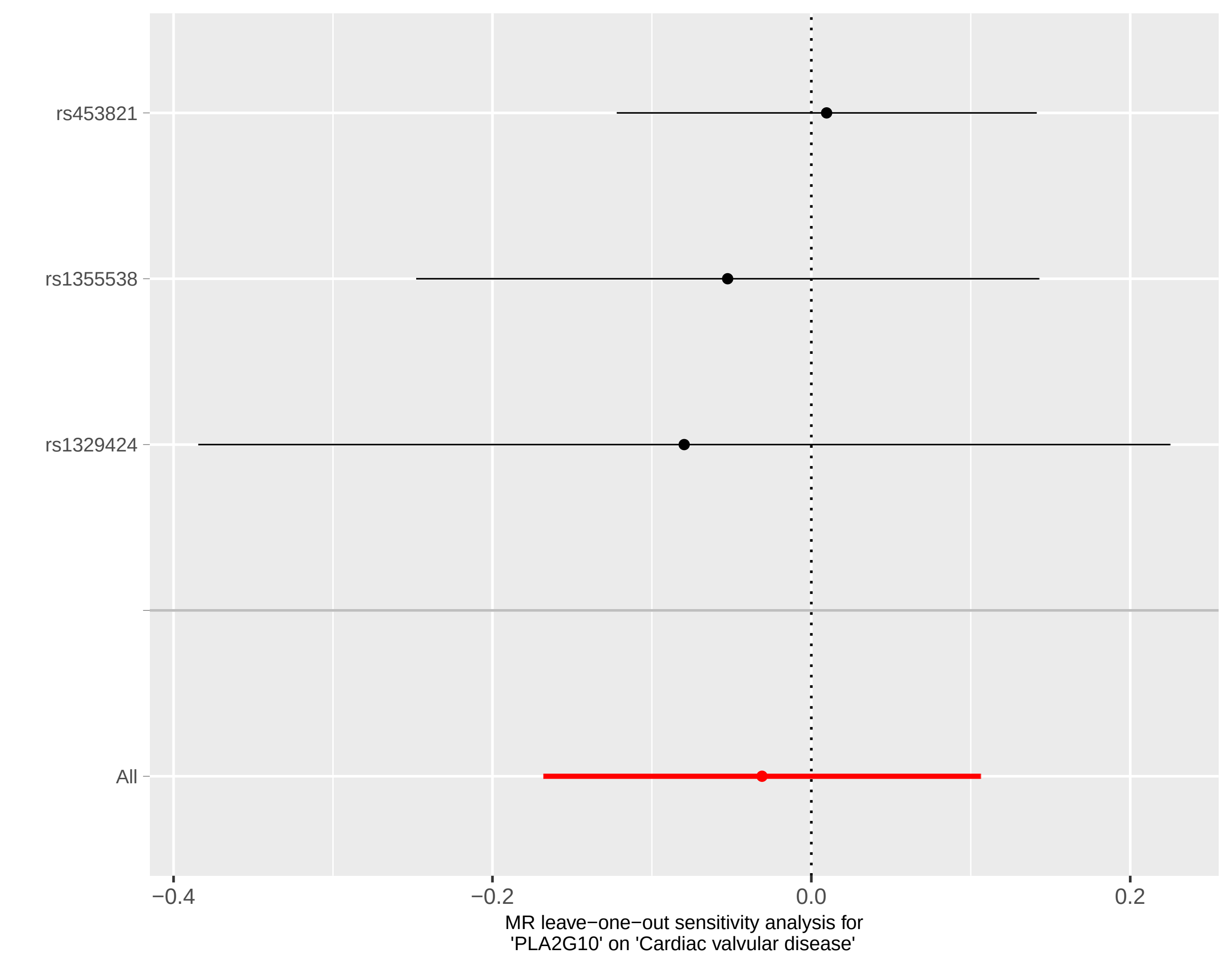
rs453821
rs1355538
rs1329424
All
−0.4
−0.2
0.0
0.2
MR leave−one−out sensitivity analysis for
'PLA2G10' on 'Cardiac valvular disease'

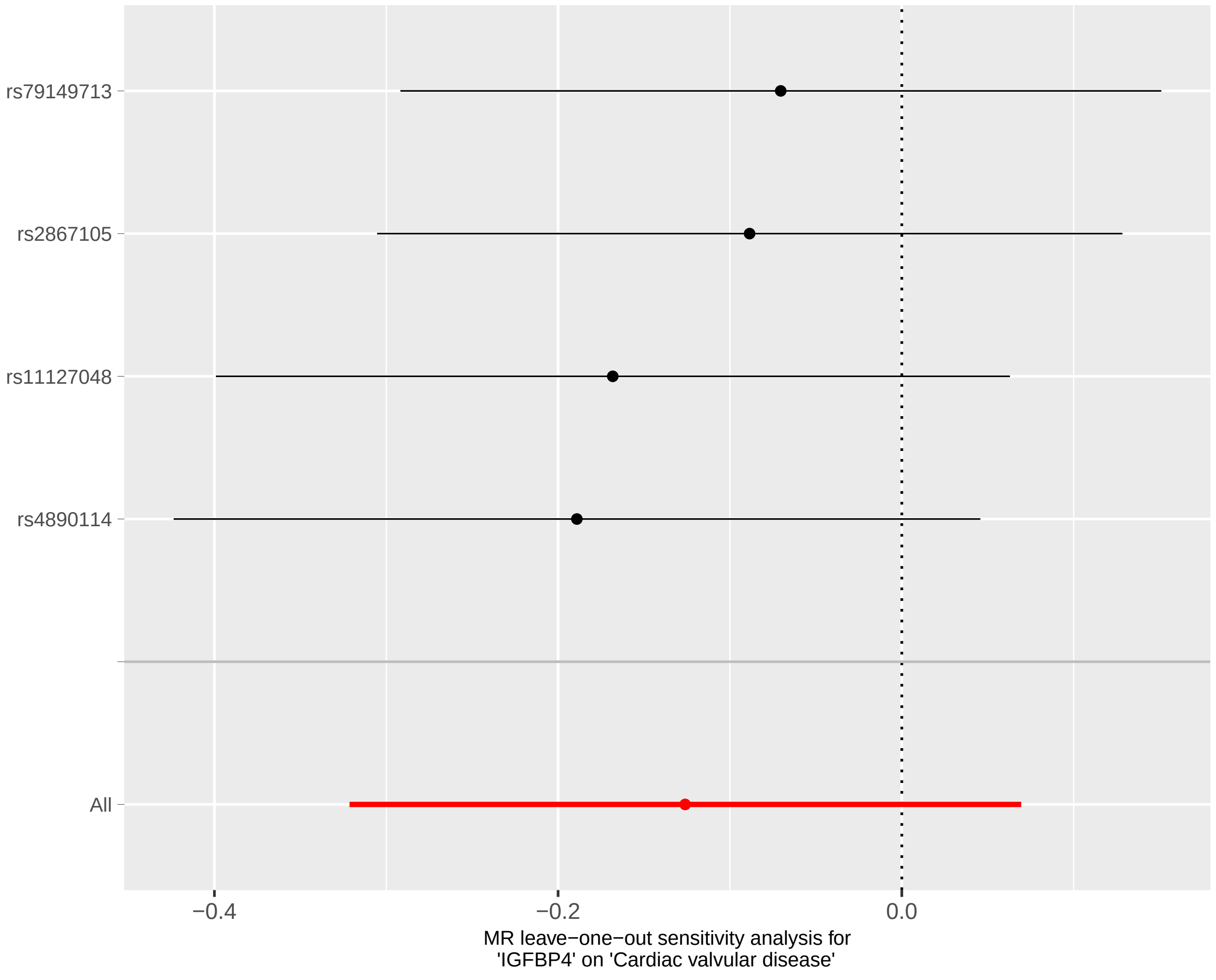
rs79149713
rs2867105
rs11127048
rs4890114
All
−0.4
−0.2
0.0
MR leave−one−out sensitivity analysis for
'IGFBP4' on 'Cardiac valvular disease'

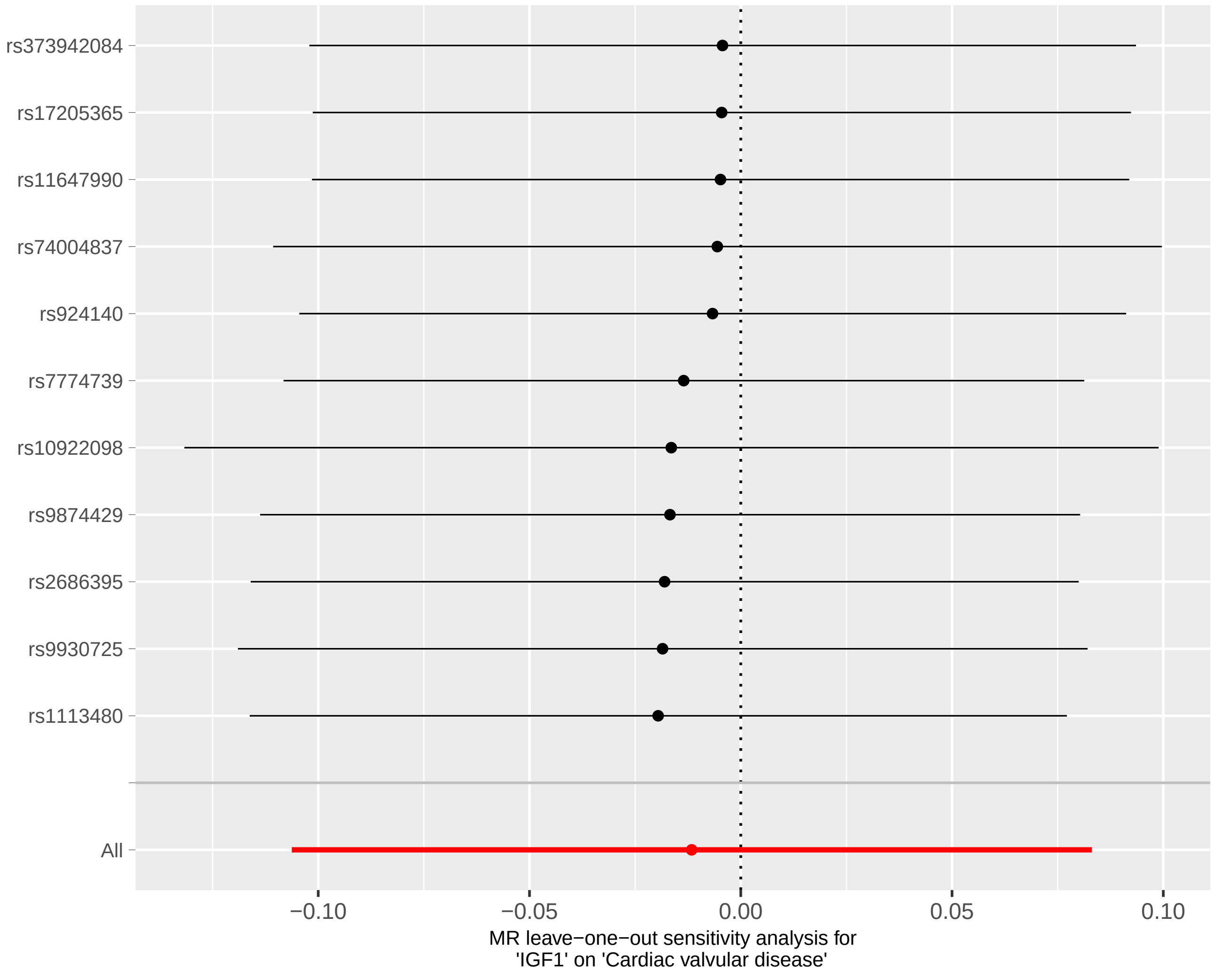
rs373942084
rs17205365
rs11647990
rs74004837
rs924140
rs7774739
rs10922098
rs9874429
rs2686395
rs9930725
rs1113480
All
−0.10
−0.05
0.00
0.05
0.10
MR leave−one−out sensitivity analysis for
'IGF1' on 'Cardiac valvular disease'

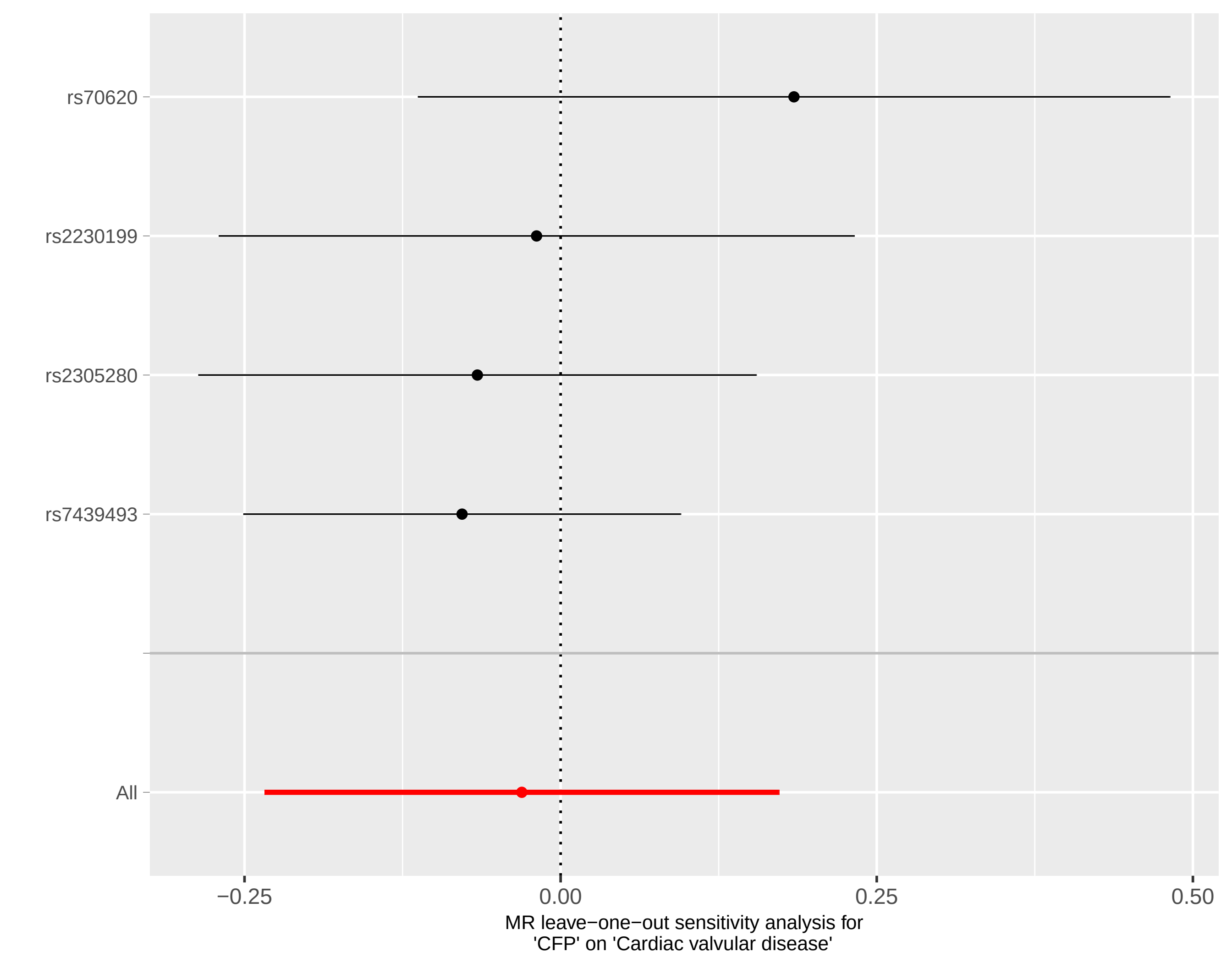

rs70620
rs2230199
rs2305280
rs7439493
All
−0.25
0.00
0.25
0.50
MR leave−one−out sensitivity analysis for
'CFP' on 'Cardiac valvular disease'

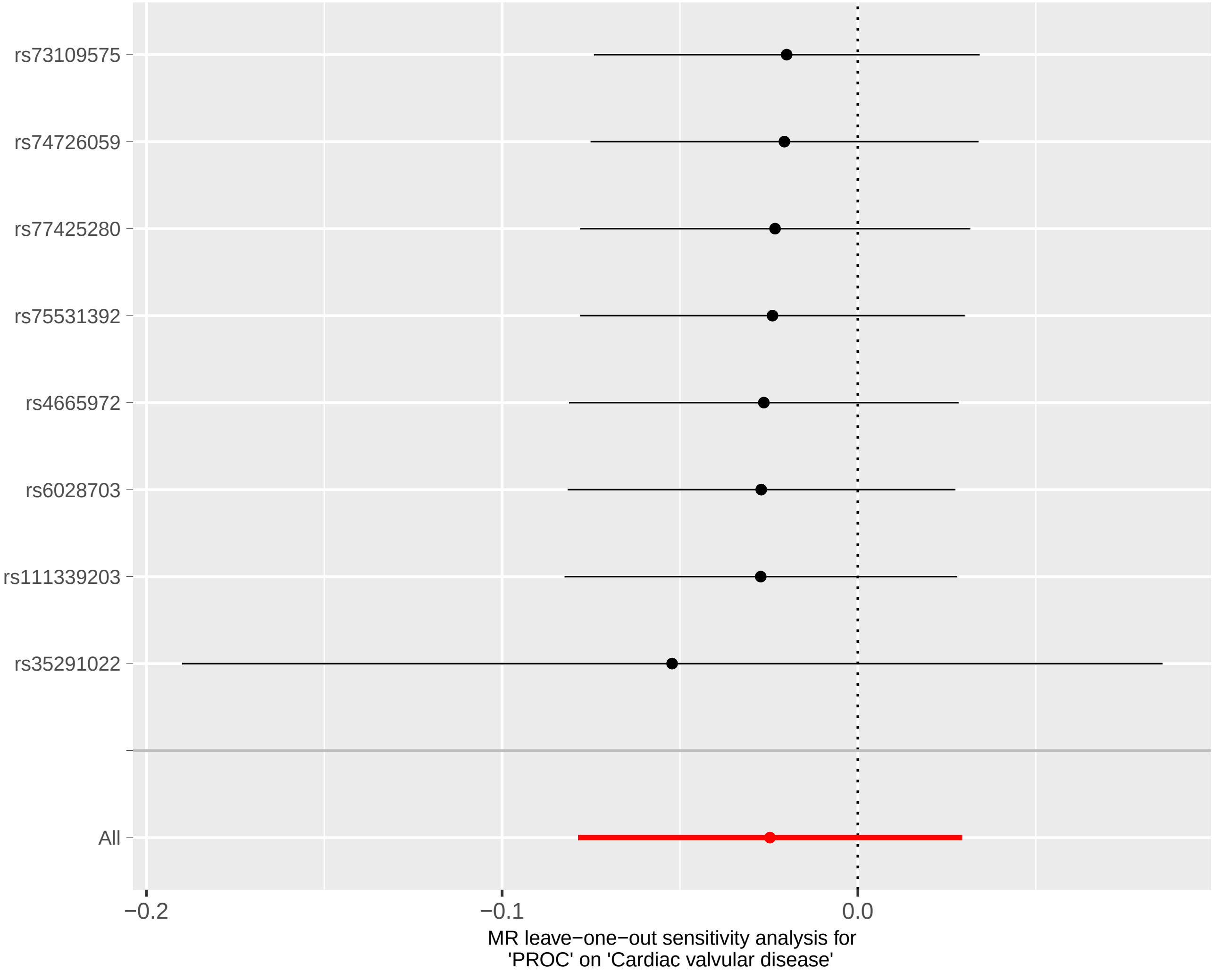
rs73109575
rs74726059
rs77425280
rs75531392
rs4665972
rs6028703
rs111339203
rs35291022
All
−0.2
−0.1
0.0
MR leave−one−out sensitivity analysis for
'PROC' on 'Cardiac valvular disease'

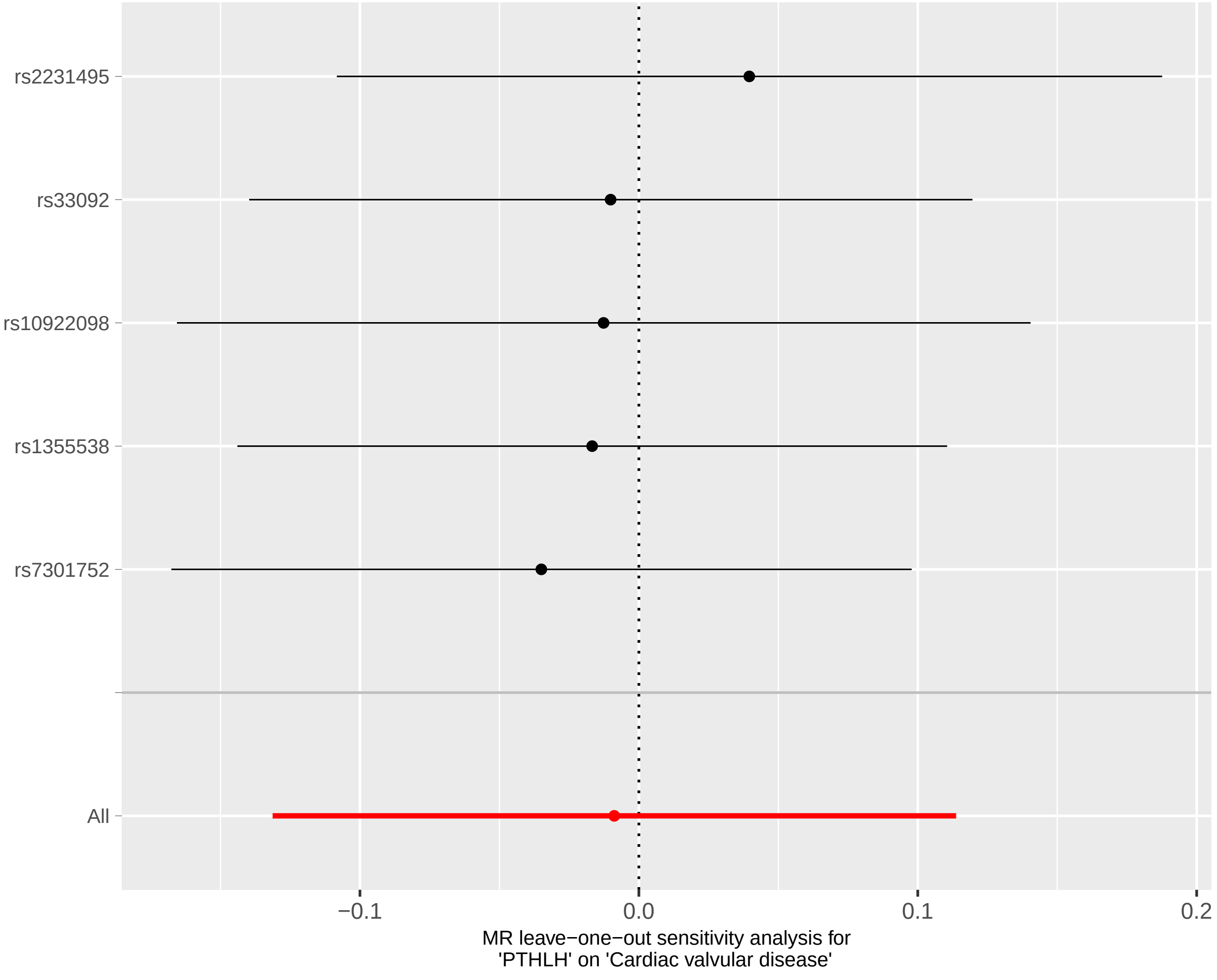

rs2231495
rs33092
rs10922098
rs1355538
rs7301752
All
−0.1
0.0
0.1
0.2
MR leave−one−out sensitivity analysis for
'PTHLH' on 'Cardiac valvular disease'

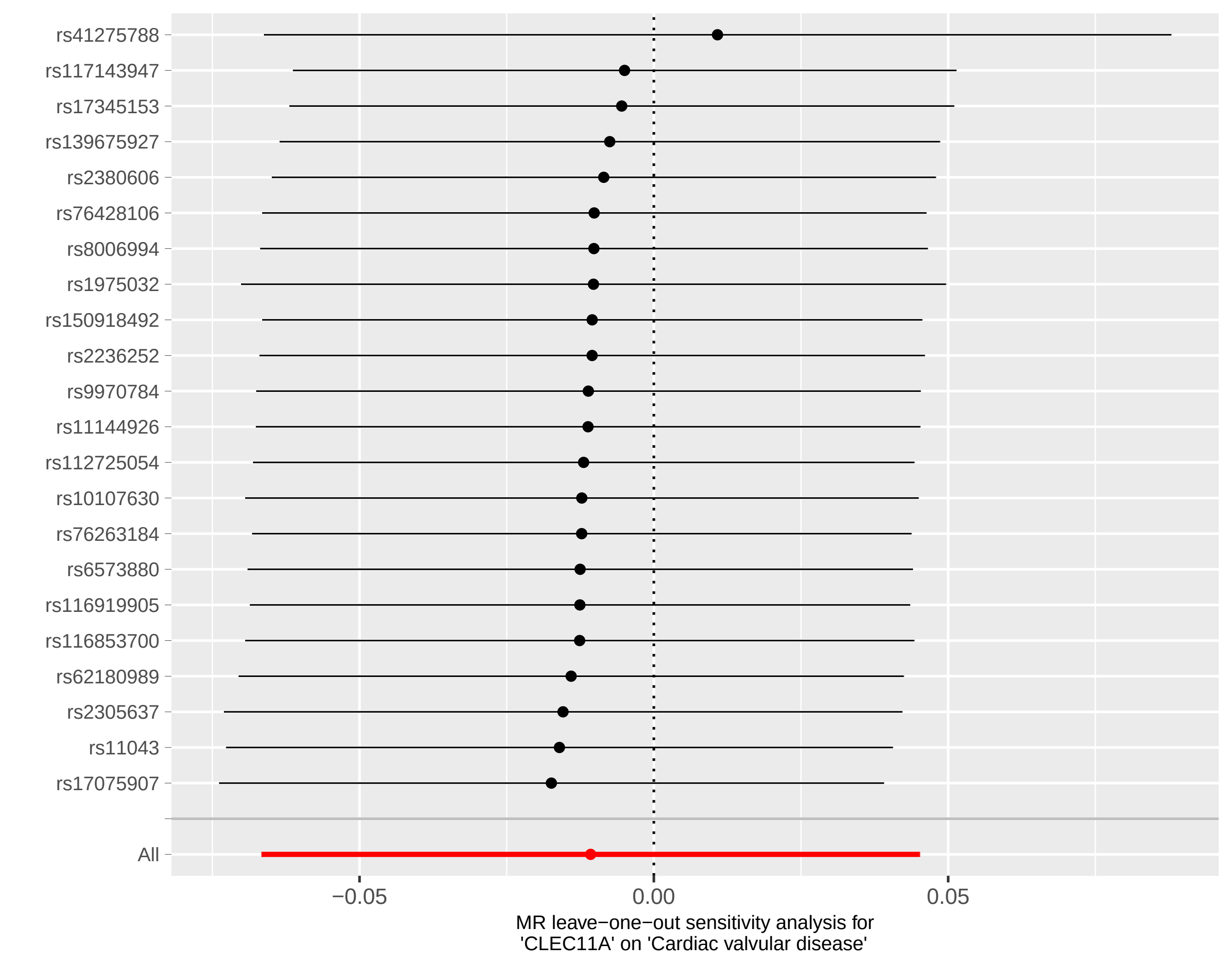
rs41275788
rs117143947
rs17345153
rs139675927
rs2380606
rs76428106
rs8006994
rs1975032
rs150918492
rs2236252
rs9970784
rs11144926
rs112725054
rs10107630
rs76263184
rs6573880
rs116919905
rs116853700
rs62180989
rs2305637
rs11043
rs17075907
All
−0.05
0.00
0.05
MR leave−one−out sensitivity analysis for
'CLEC11A' on 'Cardiac valvular disease'

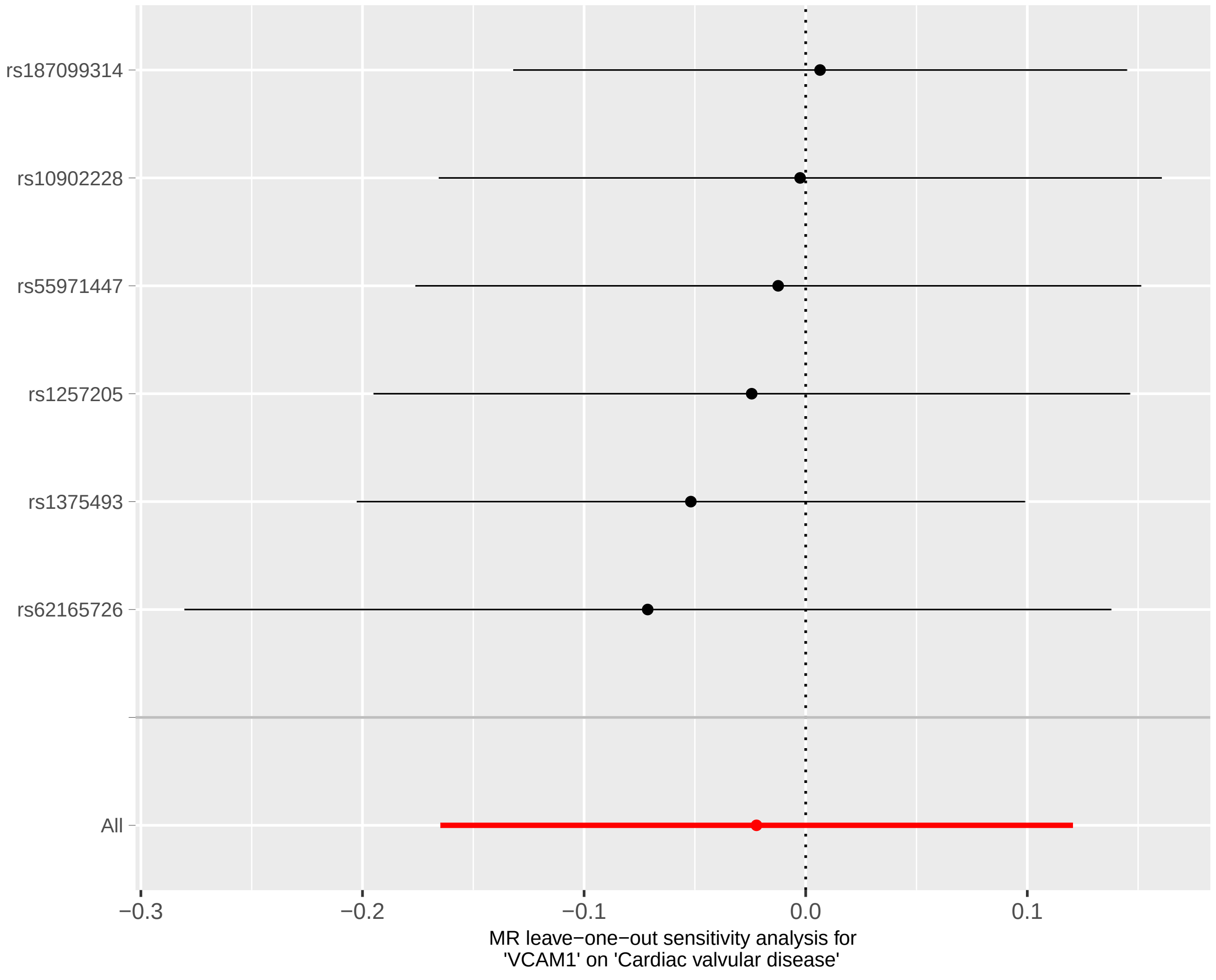
rs187099314
rs10902228
rs55971447
rs1257205
rs1375493
rs62165726
All
−0.3
−0.2
−0.1
0.0
0.1
MR leave−one−out sensitivity analysis for
'VCAM1' on 'Cardiac valvular disease'

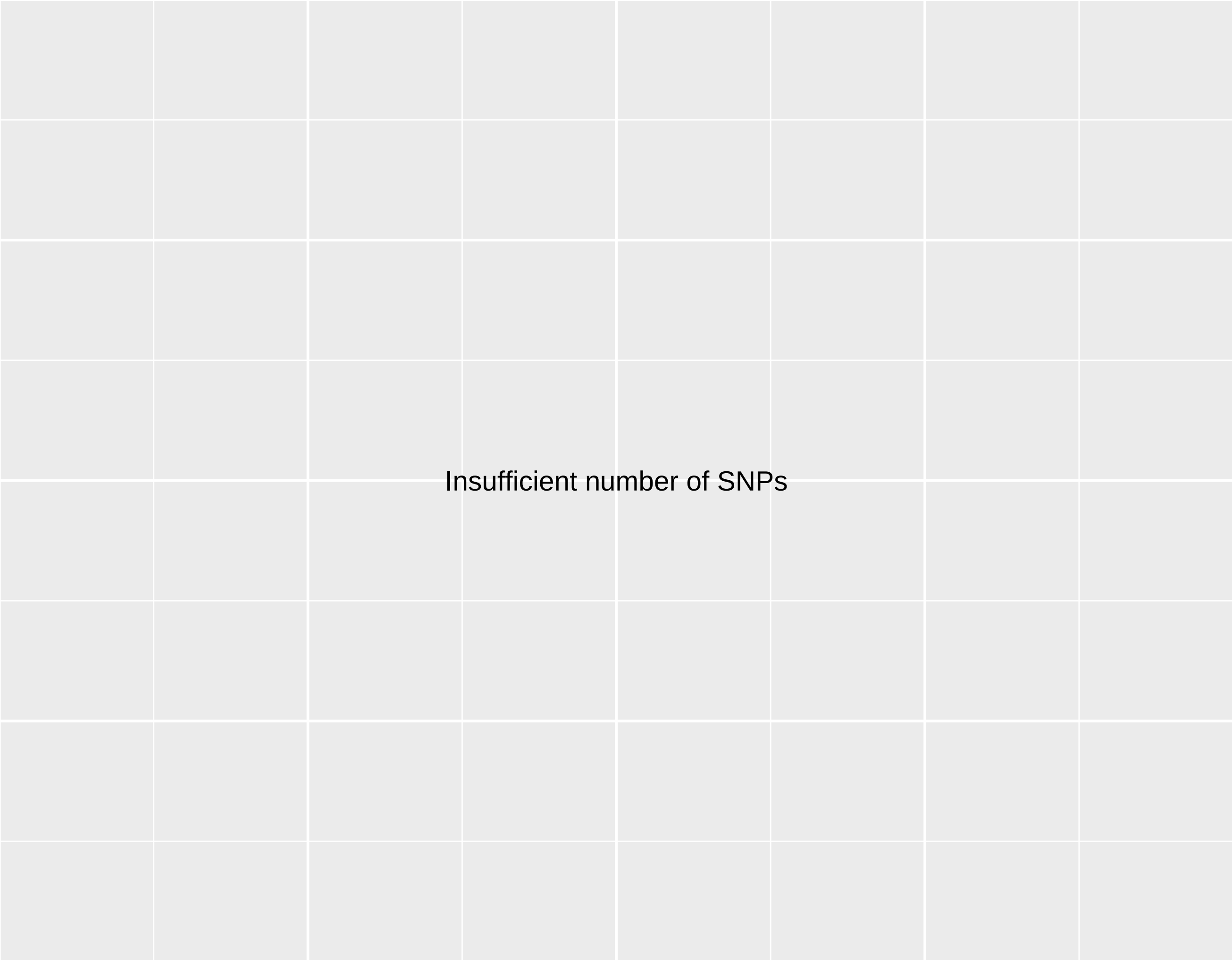
Insufficient number of SNPs

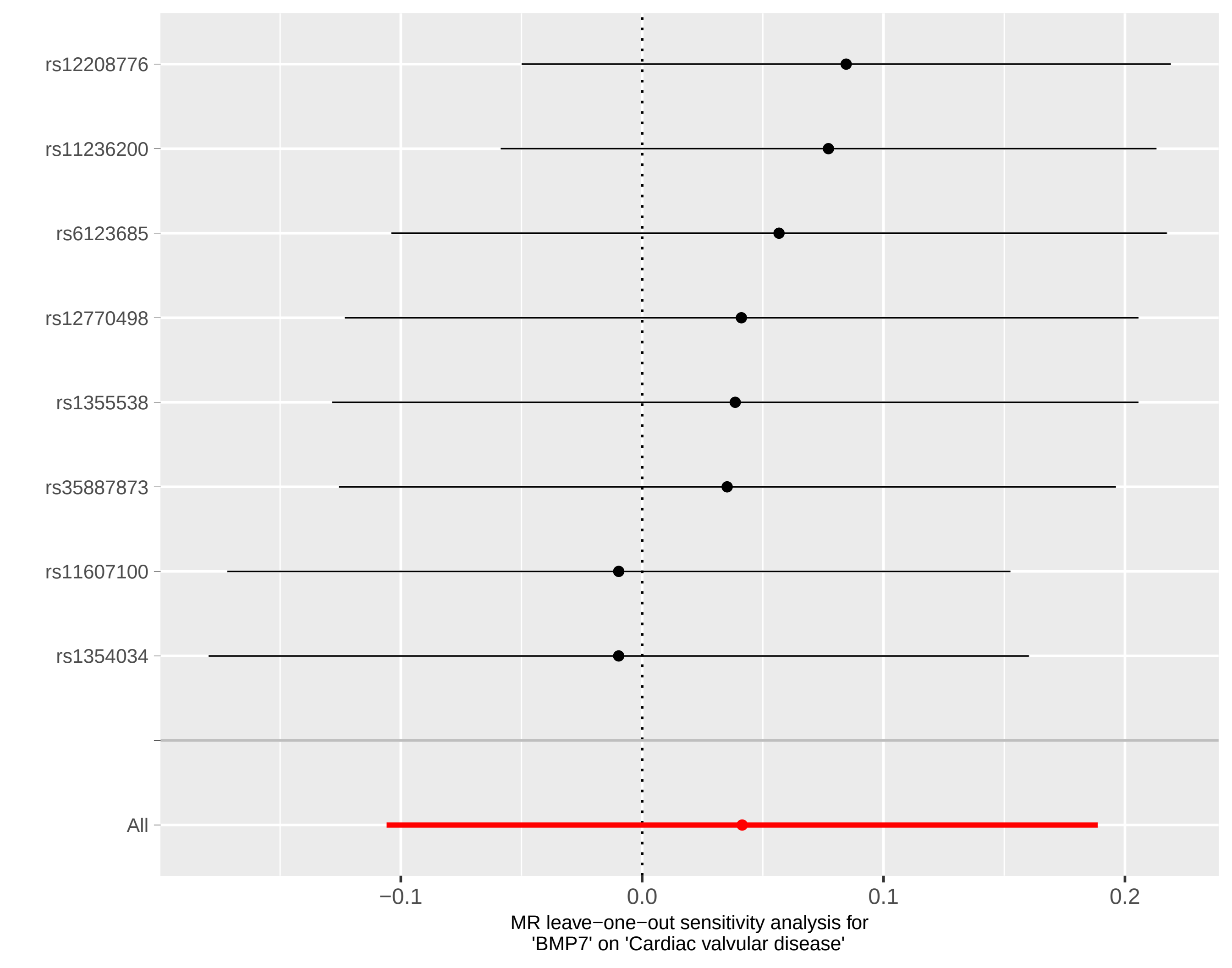
rs12208776
rs11236200
rs6123685
rs12770498
rs1355538
rs35887873
rs11607100
rs1354034
All
−0.1
0.0
0.1
0.2
MR leave−one−out sensitivity analysis for
'BMP7' on 'Cardiac valvular disease'

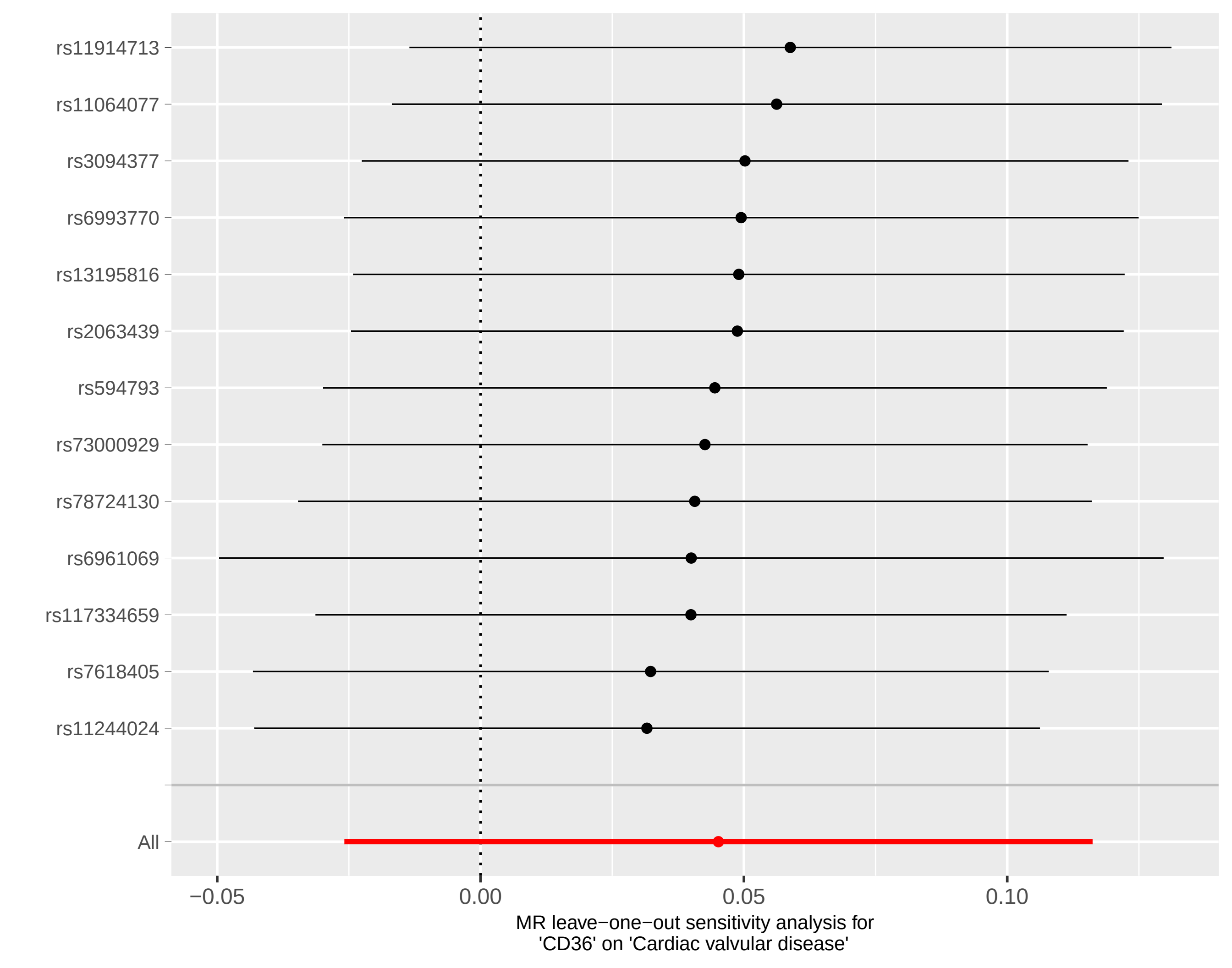
rs11914713
rs11064077
rs3094377
rs6993770
rs13195816
rs2063439
rs594793
rs73000929
rs78724130
rs6961069
rs117334659
rs7618405
rs11244024
All
−0.05
0.00
0.05
0.10
MR leave−one−out sensitivity analysis for
'CD36' on 'Cardiac valvular disease'

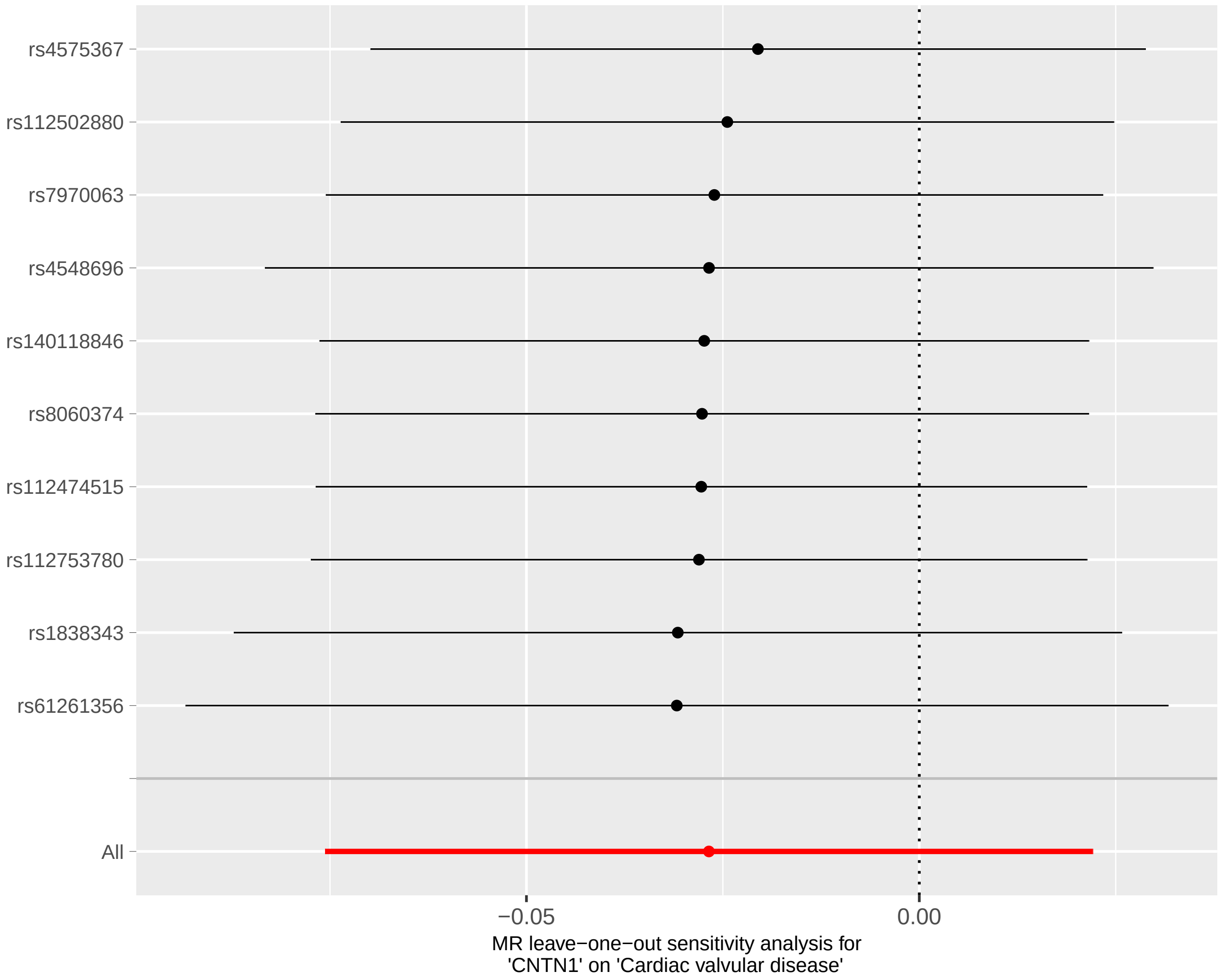
rs4575367
rs112502880
rs7970063
rs4548696
rs140118846
rs8060374
rs112474515
rs112753780
rs1838343
rs61261356
All
−0.05
0.00
MR leave−one−out sensitivity analysis for
'CNTN1' on 'Cardiac valvular disease'

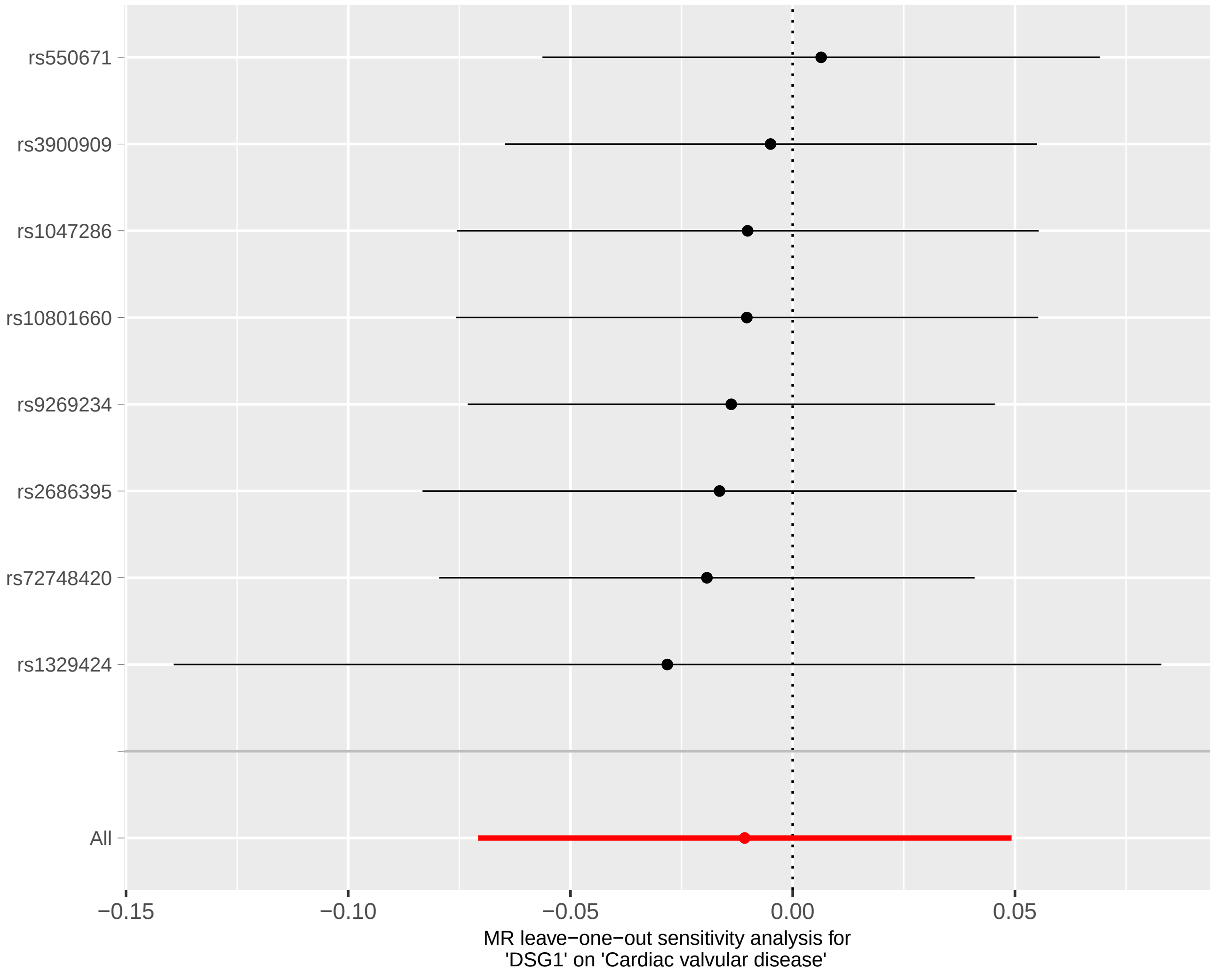
rs550671
rs3900909
rs1047286
rs10801660
rs9269234
rs2686395
rs72748420
rs1329424
All
−0.15
−0.10
−0.05
0.00
0.05
MR leave−one−out sensitivity analysis for
'DSG1' on 'Cardiac valvular disease'

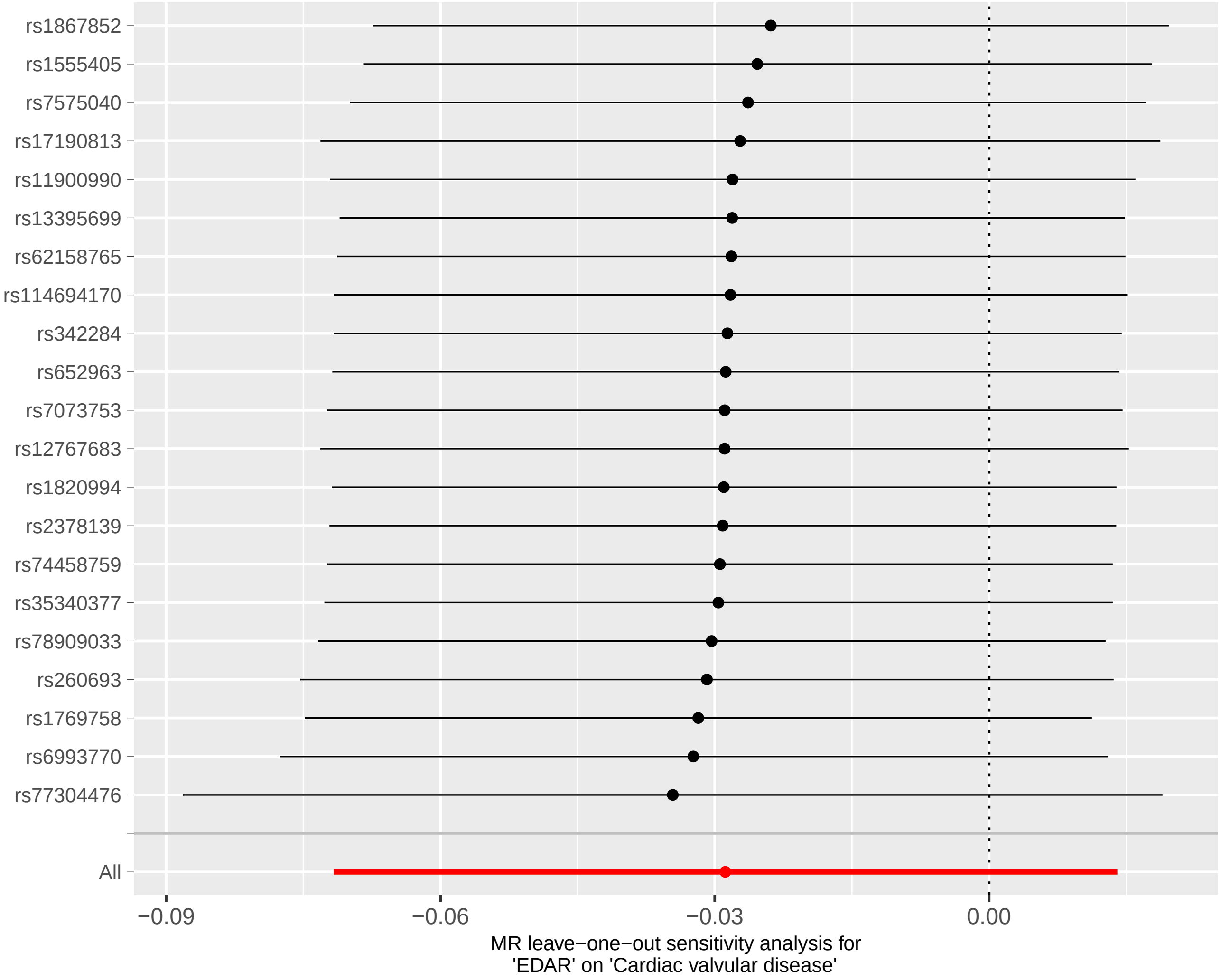

rs1867852
rs1555405
rs7575040
rs17190813
rs11900990
rs13395699
rs62158765
rs114694170
rs342284
rs652963
rs7073753
rs12767683
rs1820994
rs2378139
rs74458759
rs35340377
rs78909033
rs260693
rs1769758
rs6993770
rs77304476
All
−0.09
−0.06
−0.03
0.00
MR leave−one−out sensitivity analysis for
'EDAR' on 'Cardiac valvular disease'

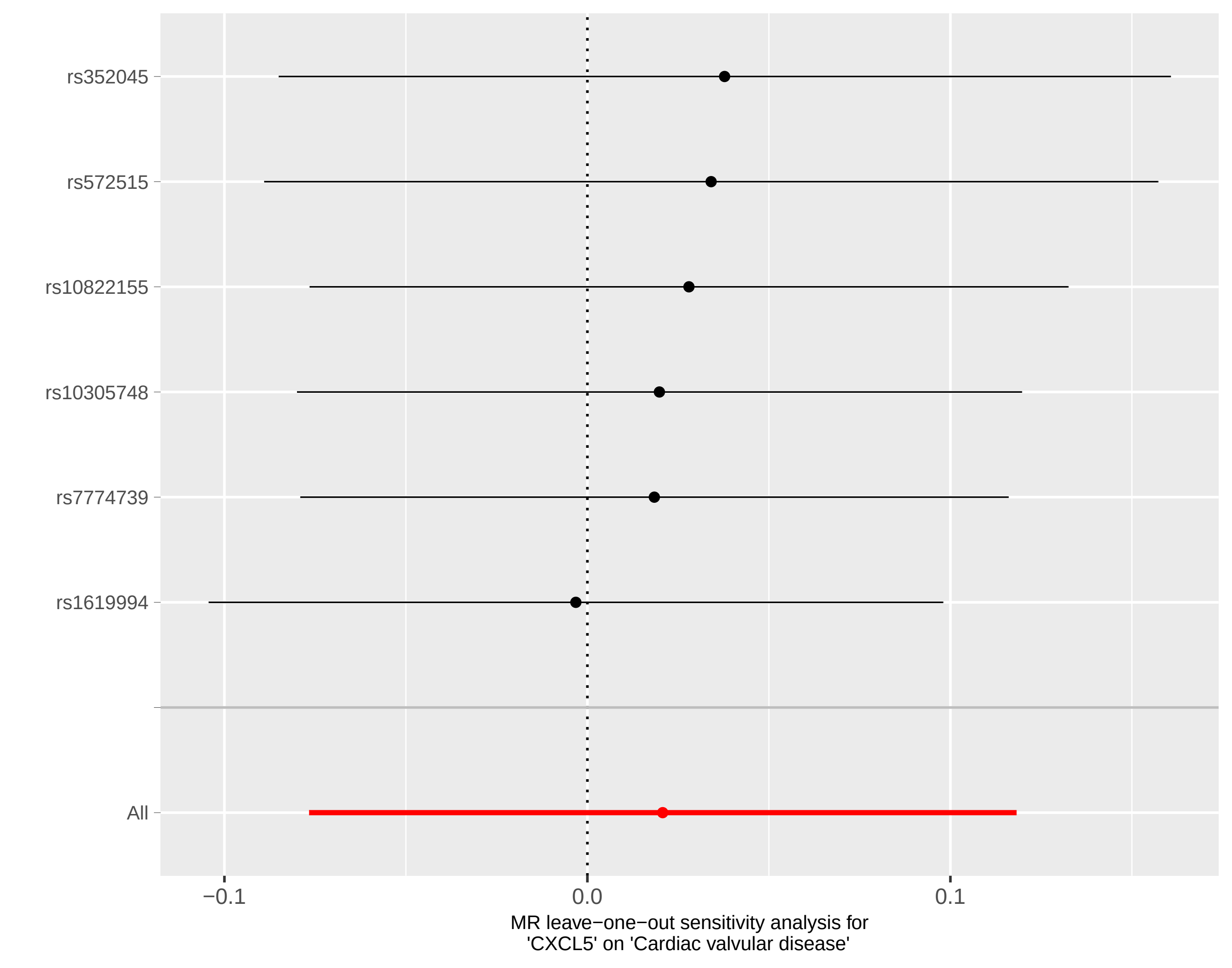
rs352045
rs572515
rs10822155
rs10305748
rs7774739
rs1619994
All
−0.1
0.0
0.1
MR leave−one−out sensitivity analysis for
'CXCL5' on 'Cardiac valvular disease'

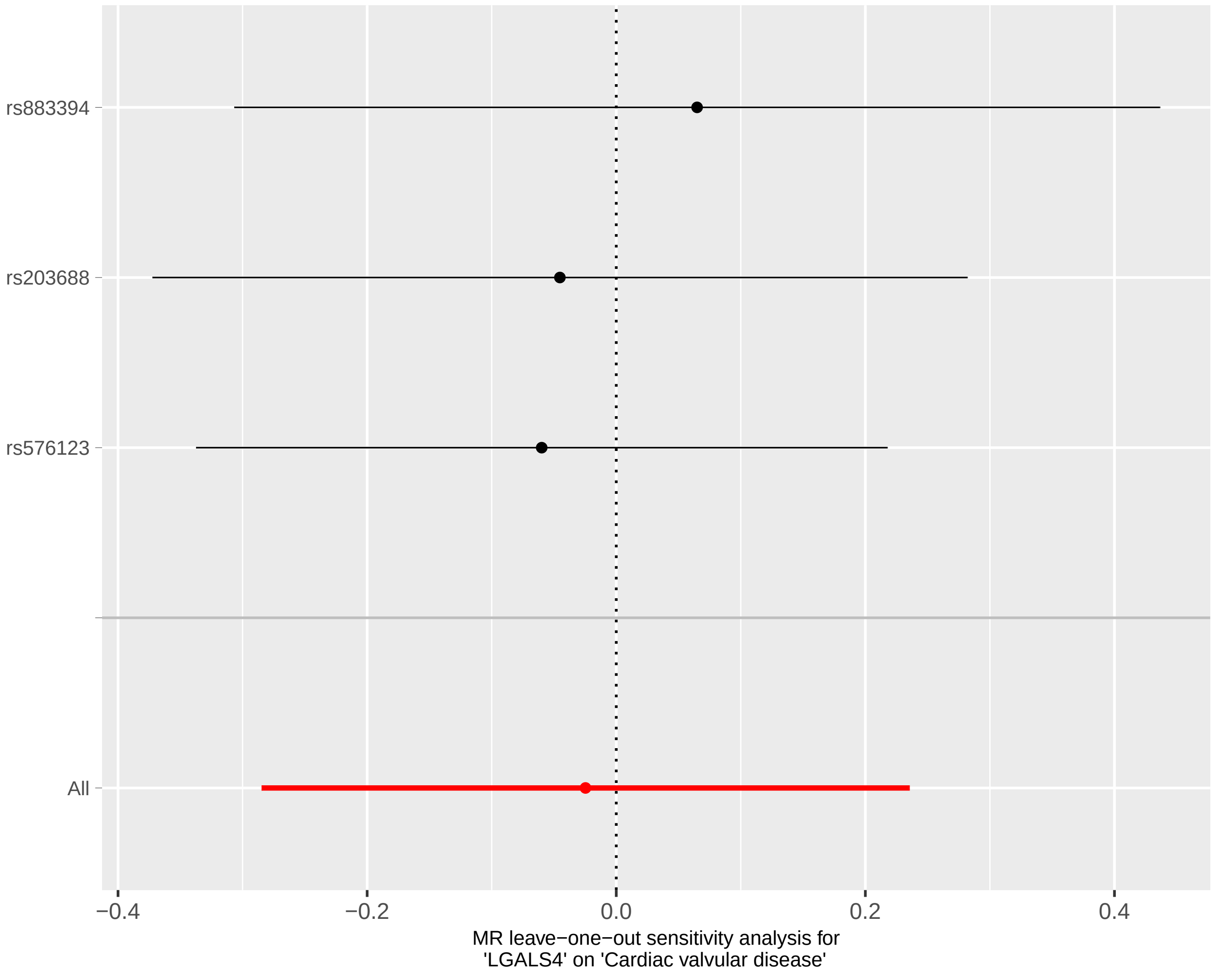
rs883394
rs203688
rs576123
All
−0.4
−0.2
0.0
0.2
0.4
MR leave−one−out sensitivity analysis for
'LGALS4' on 'Cardiac valvular disease'

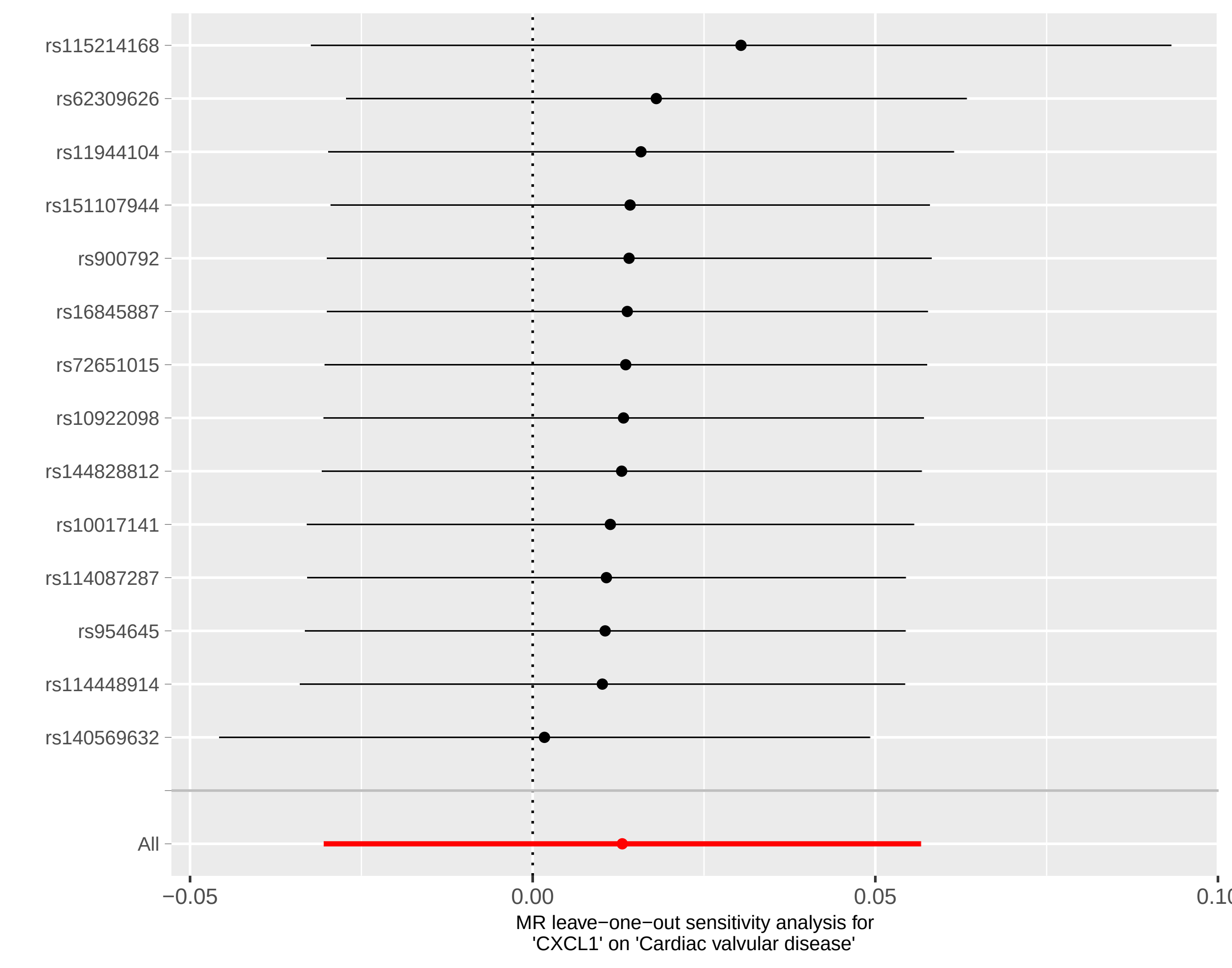
rs115214168
rs62309626
rs11944104
rs151107944
rs900792
rs16845887
rs72651015
rs10922098
rs144828812
rs10017141
rs114087287
rs954645
rs114448914
rs140569632
All
−0.05
0.00
0.05
0.10
MR leave−one−out sensitivity analysis for
'CXCL1' on 'Cardiac valvular disease'

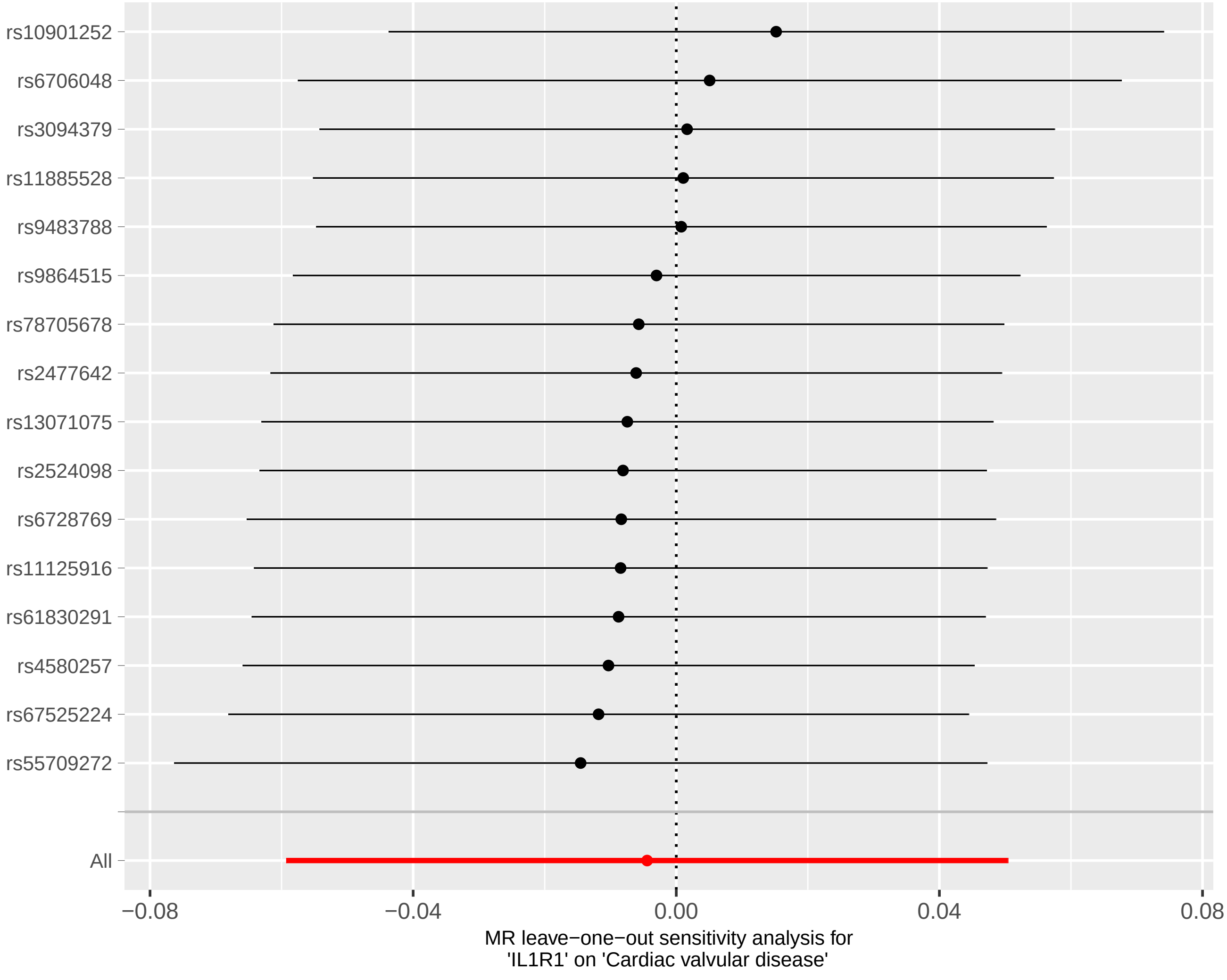

rs10901252
rs6706048
rs3094379
rs11885528
rs9483788
rs9864515
rs78705678
rs2477642
rs13071075
rs2524098
rs6728769
rs11125916
rs61830291
rs4580257
rs67525224
rs55709272
All
−0.08
−0.04
0.00
0.04
0.08
MR leave−one−out sensitivity analysis for
'IL1R1' on 'Cardiac valvular disease'

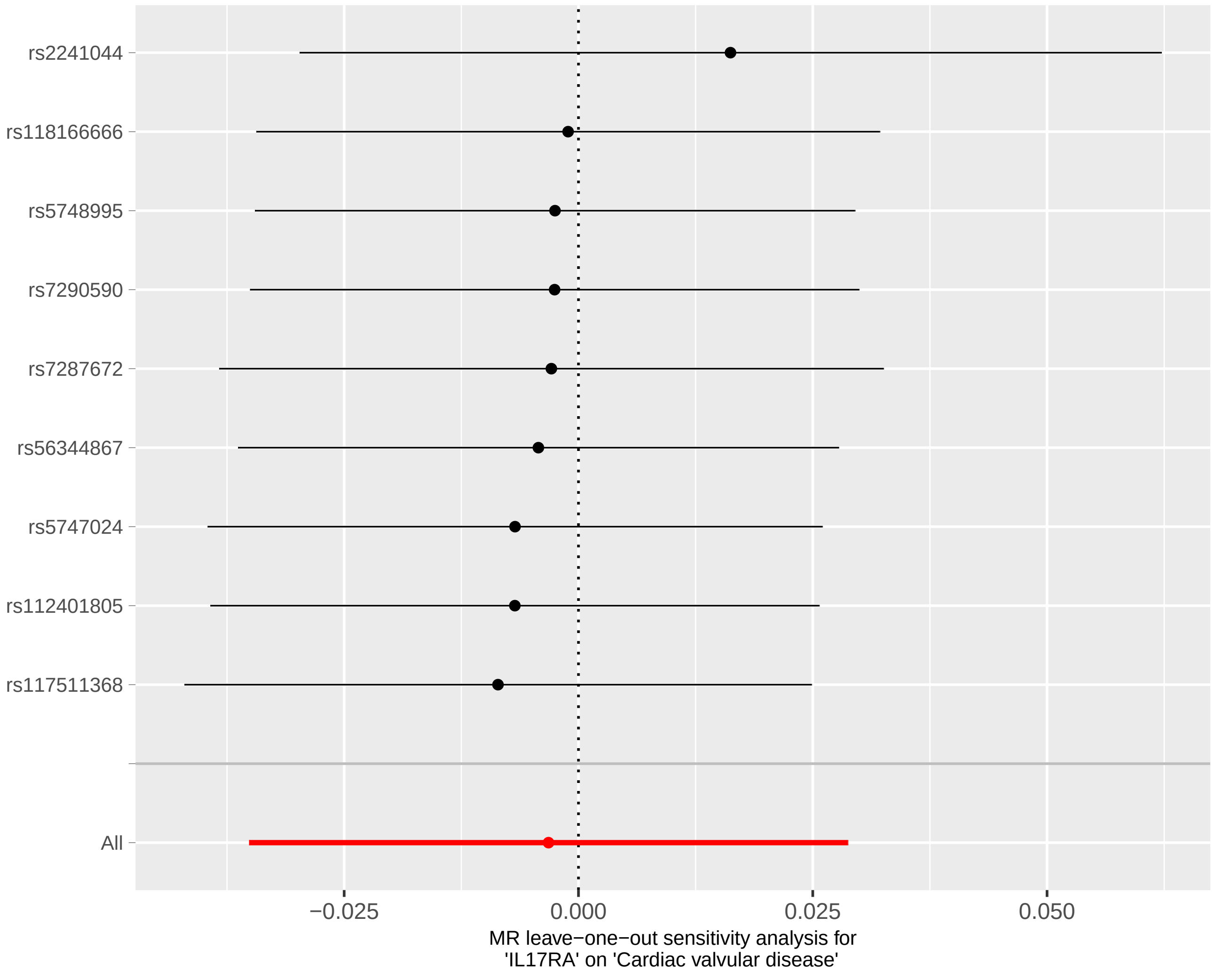

rs2241044
rs118166666
rs5748995
rs7290590
rs7287672
rs56344867
rs5747024
rs112401805
rs117511368
All
−0.025
0.000
0.025
0.050
MR leave−one−out sensitivity analysis for
'IL17RA' on 'Cardiac valvular disease'

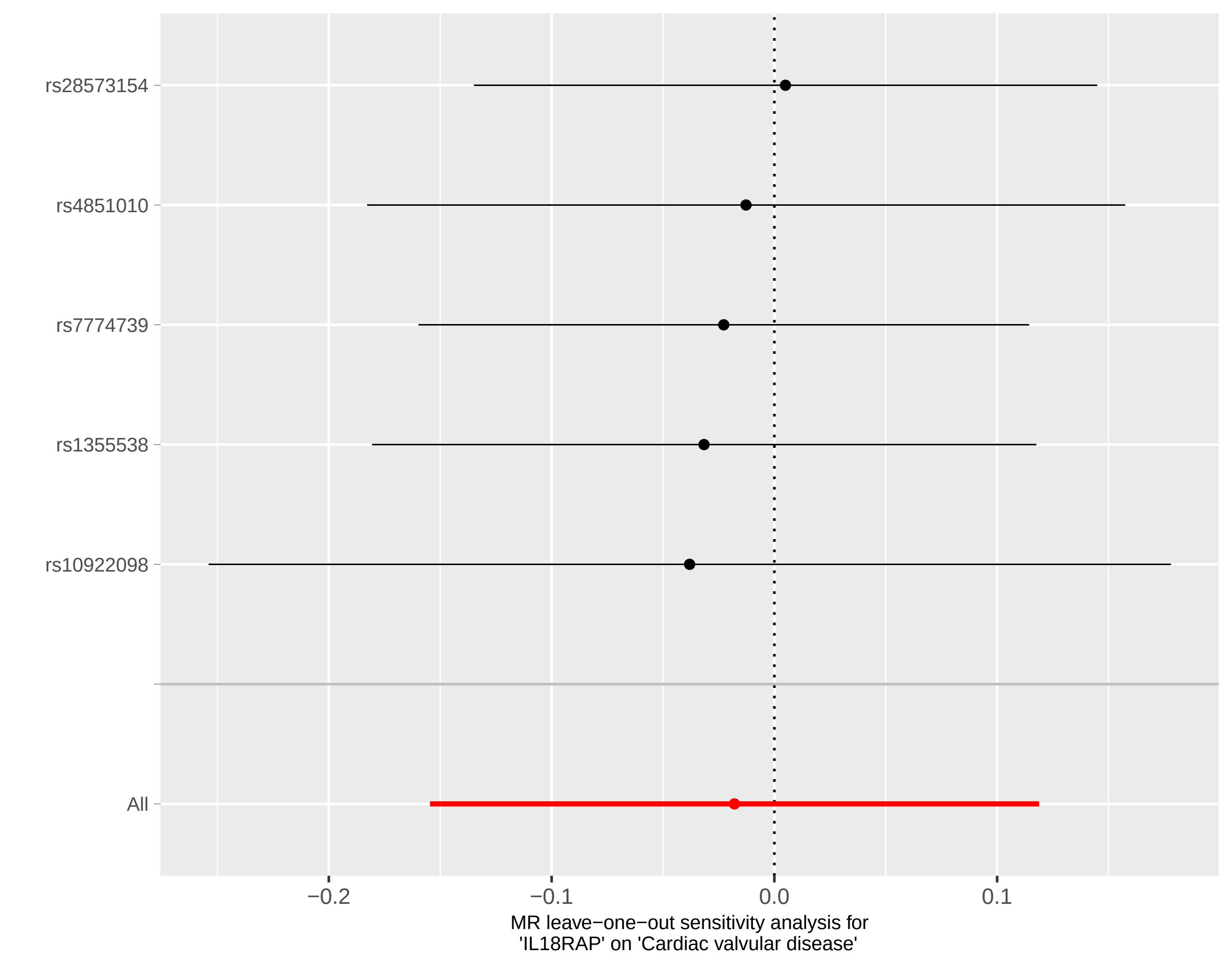

rs28573154
rs4851010
rs7774739
rs1355538
rs10922098
All
−0.2
−0.1
0.0
0.1
MR leave−one−out sensitivity analysis for
'IL18RAP' on 'Cardiac valvular disease'

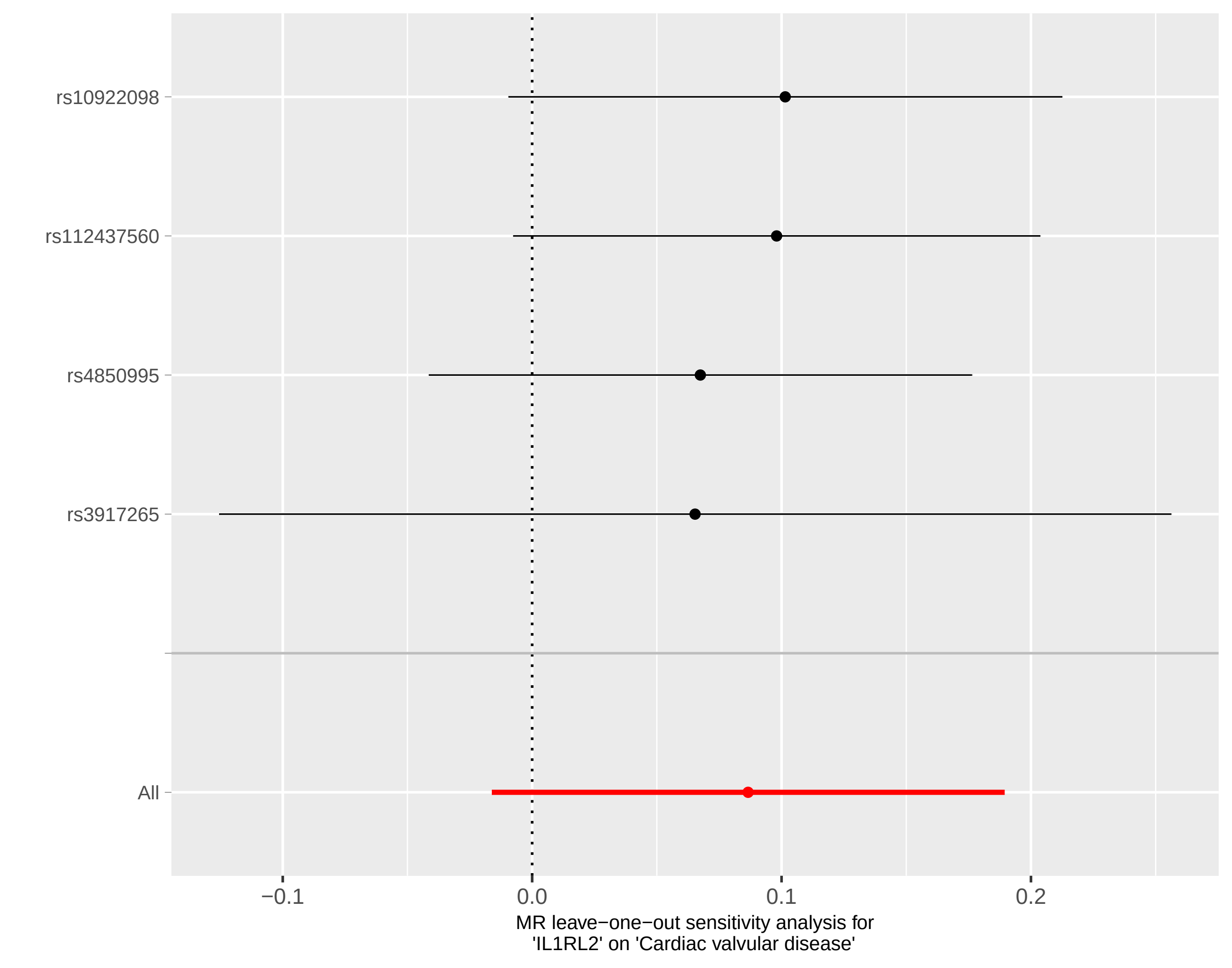
rs10922098
rs112437560
rs4850995
rs3917265
All
−0.1
0.0
0.1
0.2
MR leave−one−out sensitivity analysis for
'IL1RL2' on 'Cardiac valvular disease'

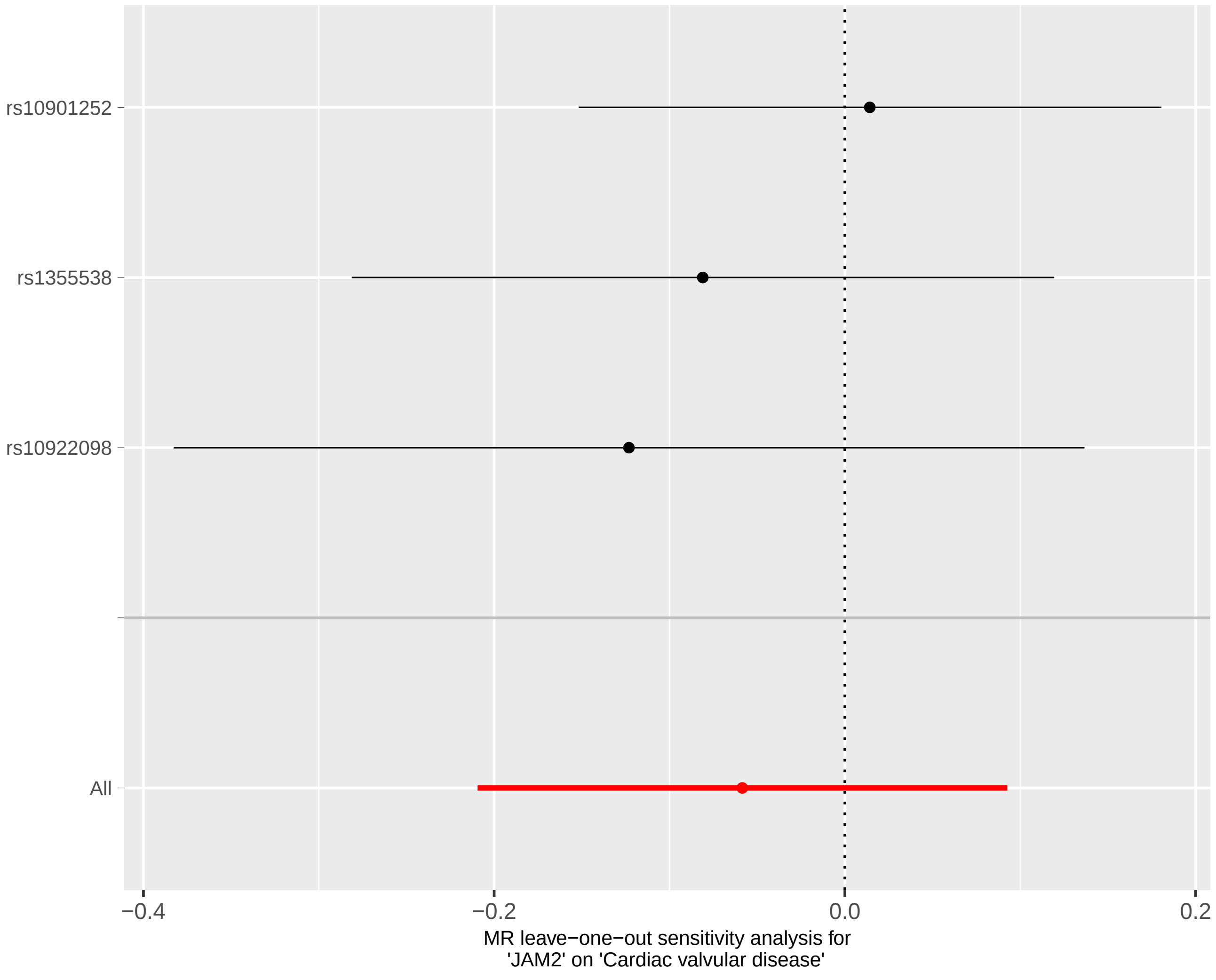

rs10901252
rs1355538
rs10922098
All
−0.4
−0.2
0.0
0.2
MR leave−one−out sensitivity analysis for
'JAM2' on 'Cardiac valvular disease'

Insufficient number of SNPs

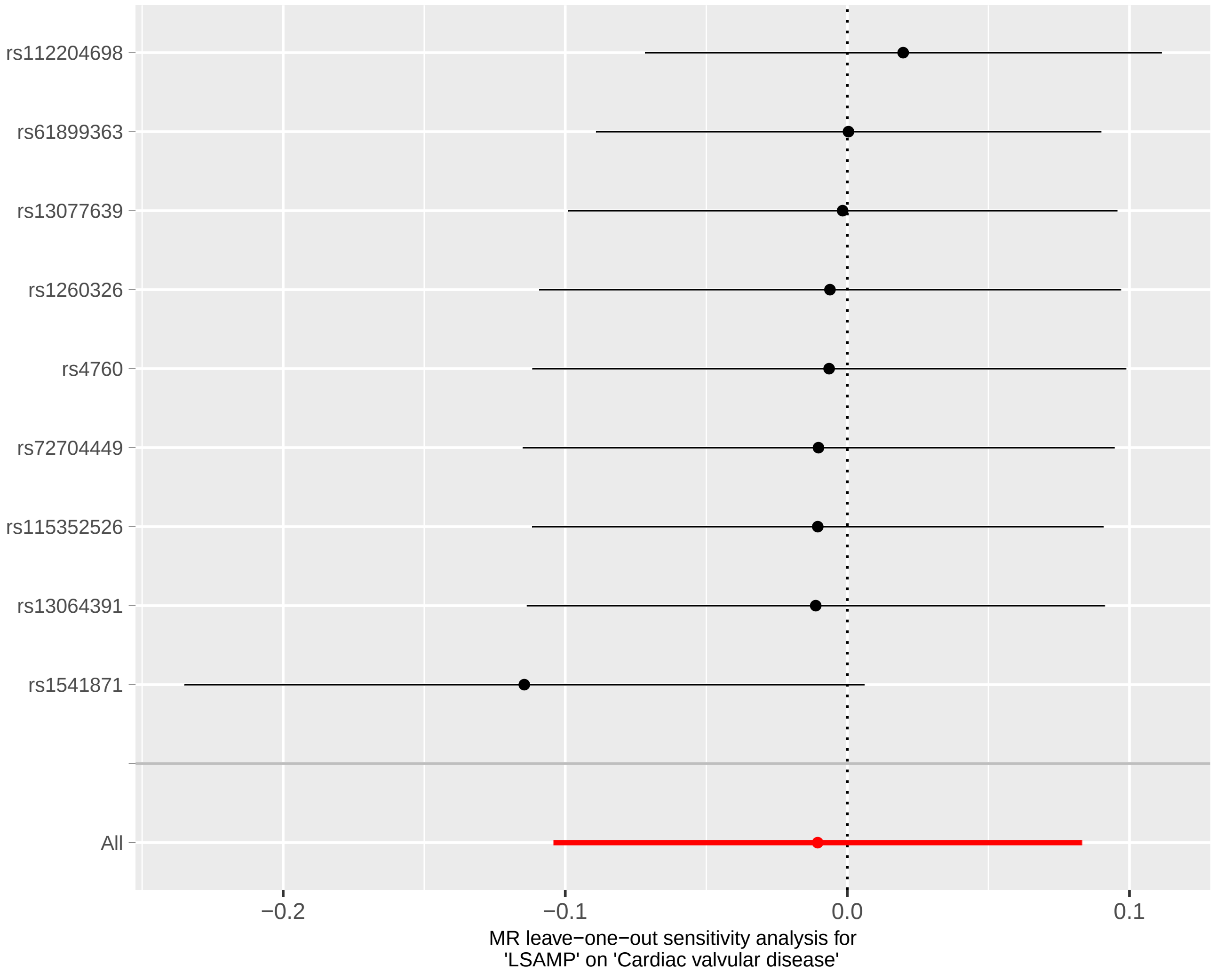
rs112204698
rs61899363
rs13077639
rs1260326
rs4760
rs72704449
rs115352526
rs13064391
rs1541871
All
−0.2
−0.1
0.0
0.1
MR leave−one−out sensitivity analysis for
'LSAMP' on 'Cardiac valvular disease'

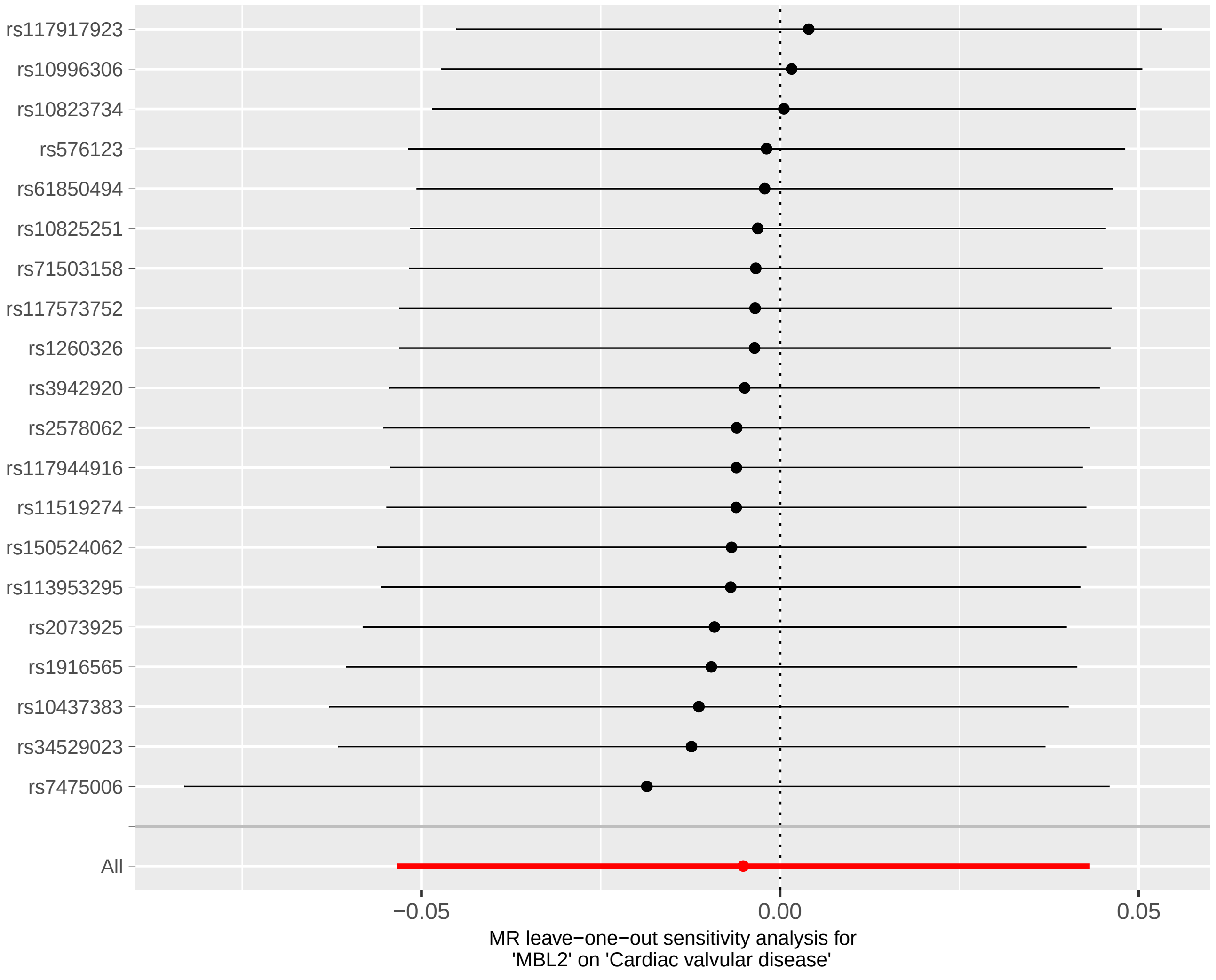
rs117917923
rs10996306
rs10823734
rs576123
rs61850494
rs10825251
rs71503158
rs117573752
rs1260326
rs3942920
rs2578062
rs117944916
rs11519274
rs150524062
rs113953295
rs2073925
rs1916565
rs10437383
rs34529023
rs7475006
All
−0.05
0.00
0.05
MR leave−one−out sensitivity analysis for
'MBL2' on 'Cardiac valvular disease'

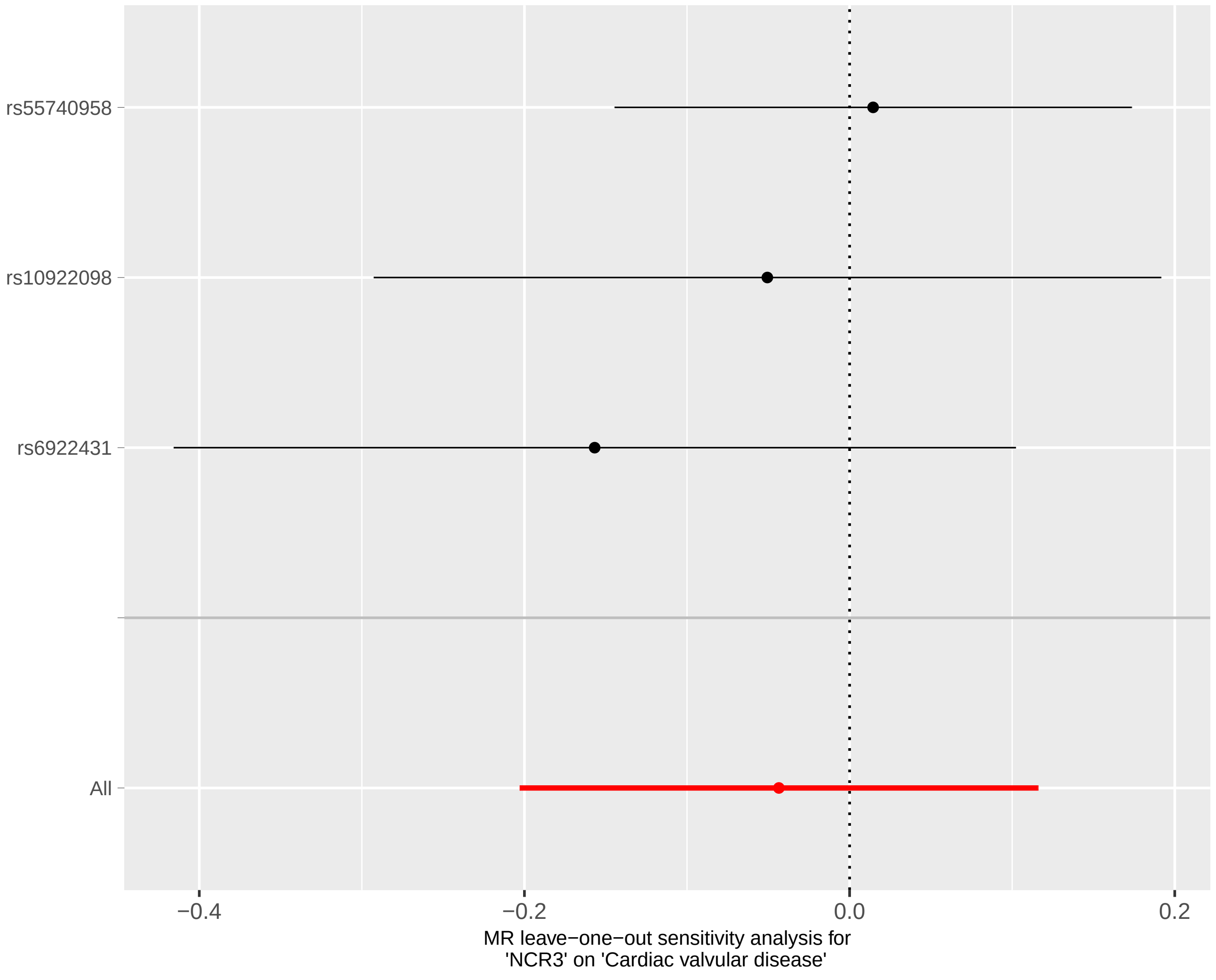

rs55740958
rs10922098
rs6922431
All
−0.4
−0.2
0.0
0.2
MR leave−one−out sensitivity analysis for
'NCR3' on 'Cardiac valvular disease'

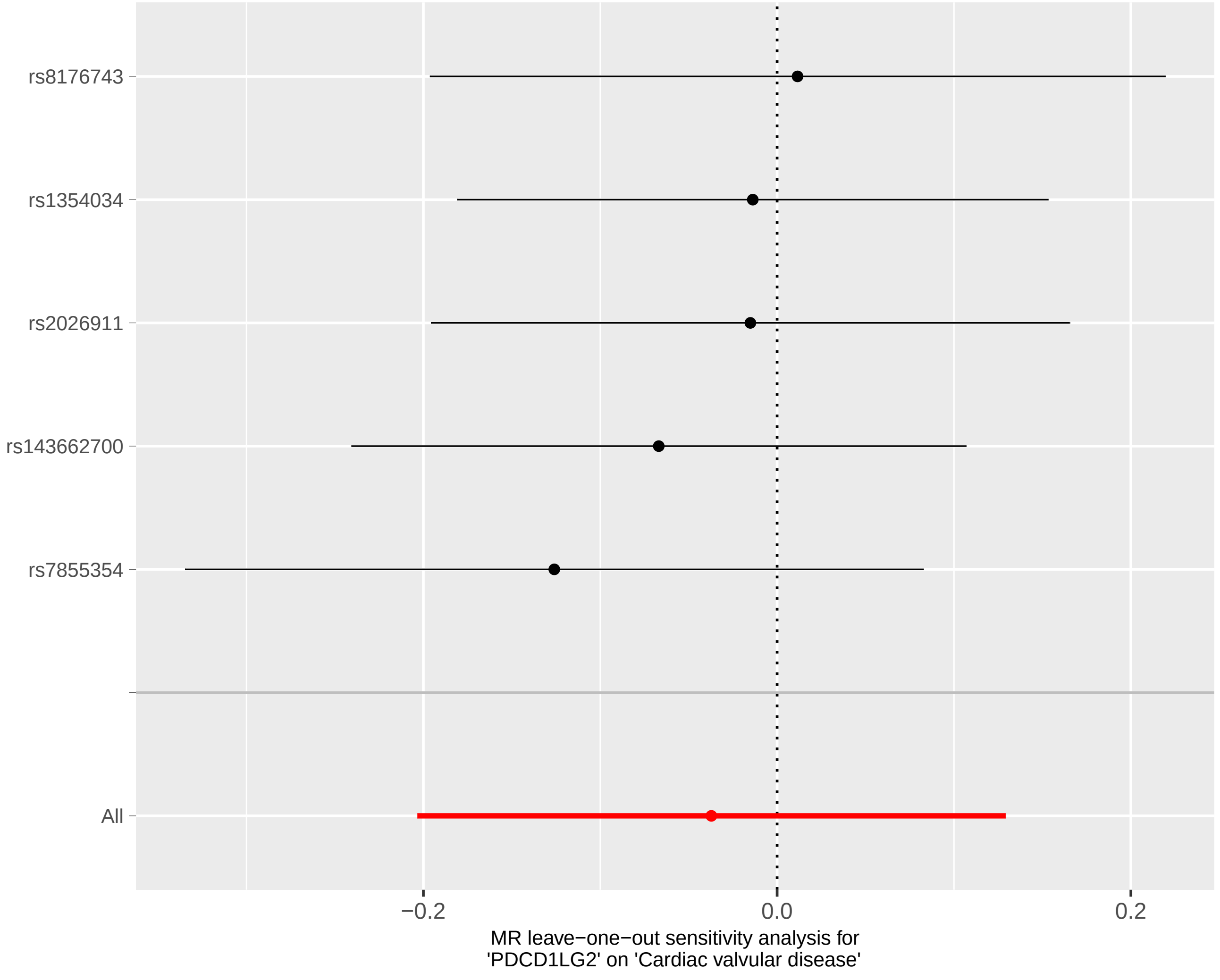
rs8176743
rs1354034
rs2026911
rs143662700
rs7855354
All
−0.2
0.0
0.2
MR leave−one−out sensitivity analysis for
'PDCD1LG2' on 'Cardiac valvular disease'

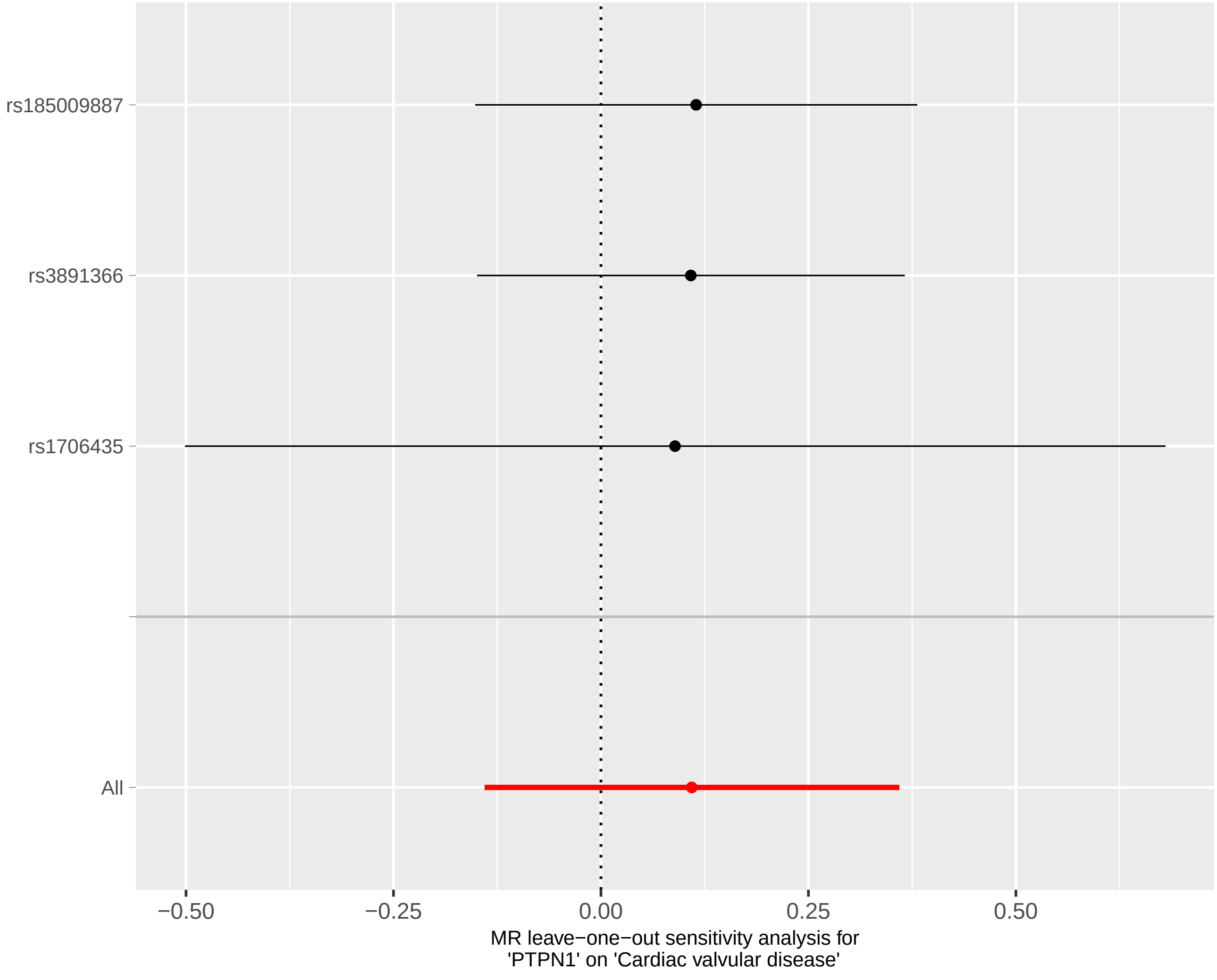

rs185009887
rs3891366
rs1706435
All
−0.50
−0.25
0.00
0.25
0.50
MR leave−one−out sensitivity analysis for
'PTPN1' on 'Cardiac valvular disease'

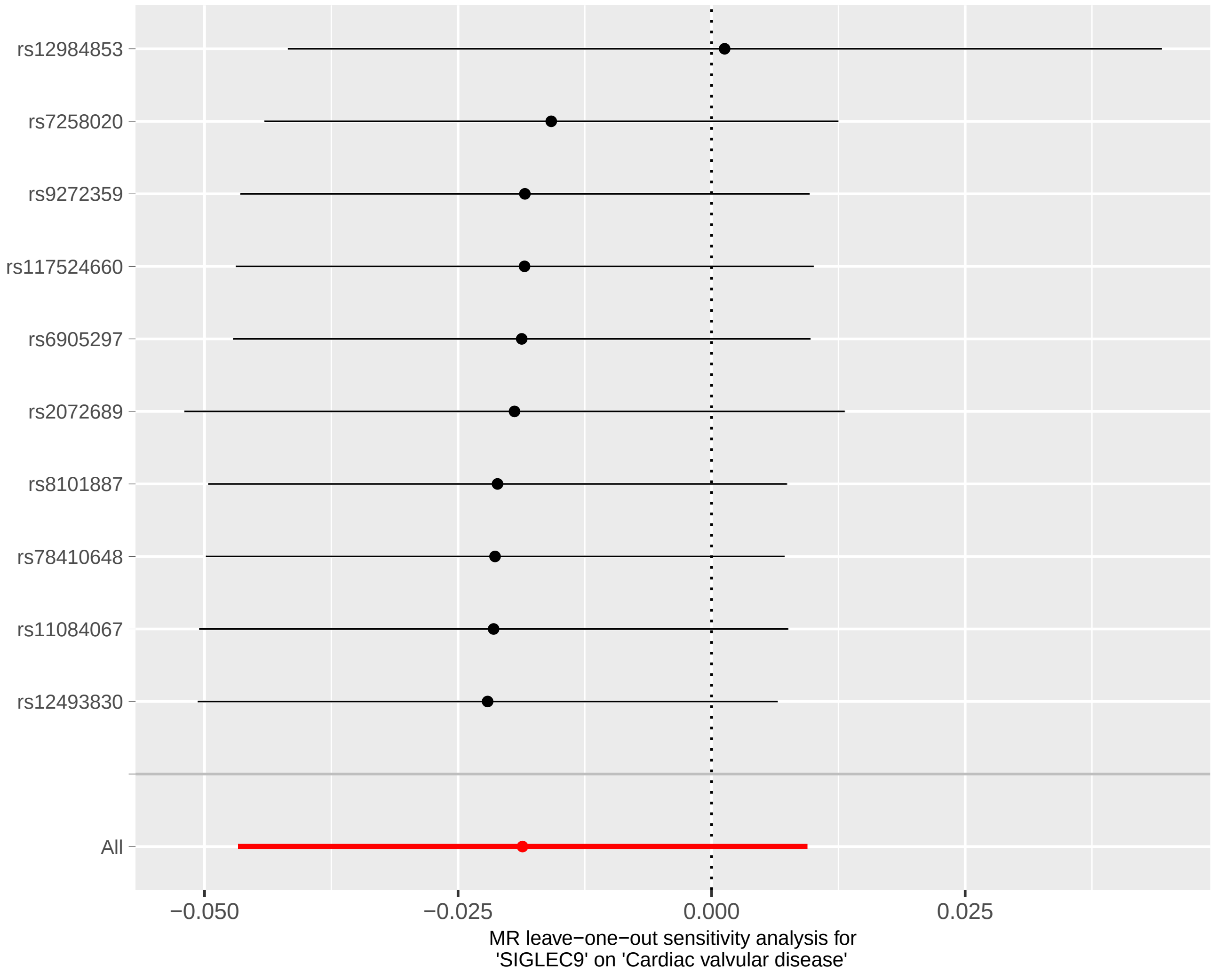

rs12984853
rs7258020
rs9272359
rs117524660
rs6905297
rs2072689
rs8101887
rs78410648
rs11084067
rs12493830
All
−0.050
−0.025
0.000
0.025
MR leave−one−out sensitivity analysis for
'SIGLEC9' on 'Cardiac valvular disease'

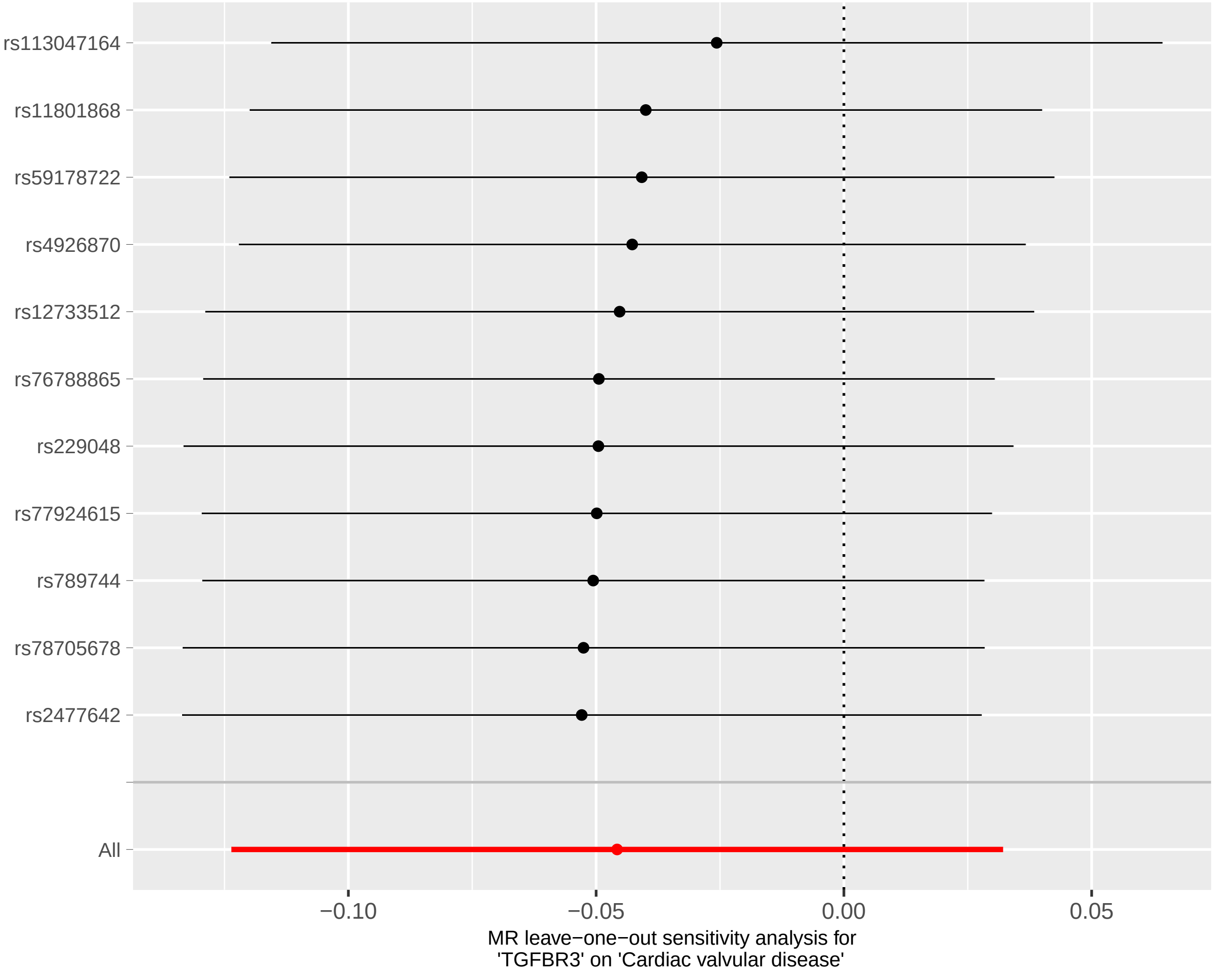
rs113047164
rs11801868
rs59178722
rs4926870
rs12733512
rs76788865
rs229048
rs77924615
rs789744
rs78705678
rs2477642
All
−0.10
−0.05
0.00
0.05
MR leave−one−out sensitivity analysis for
'TGFBR3' on 'Cardiac valvular disease'

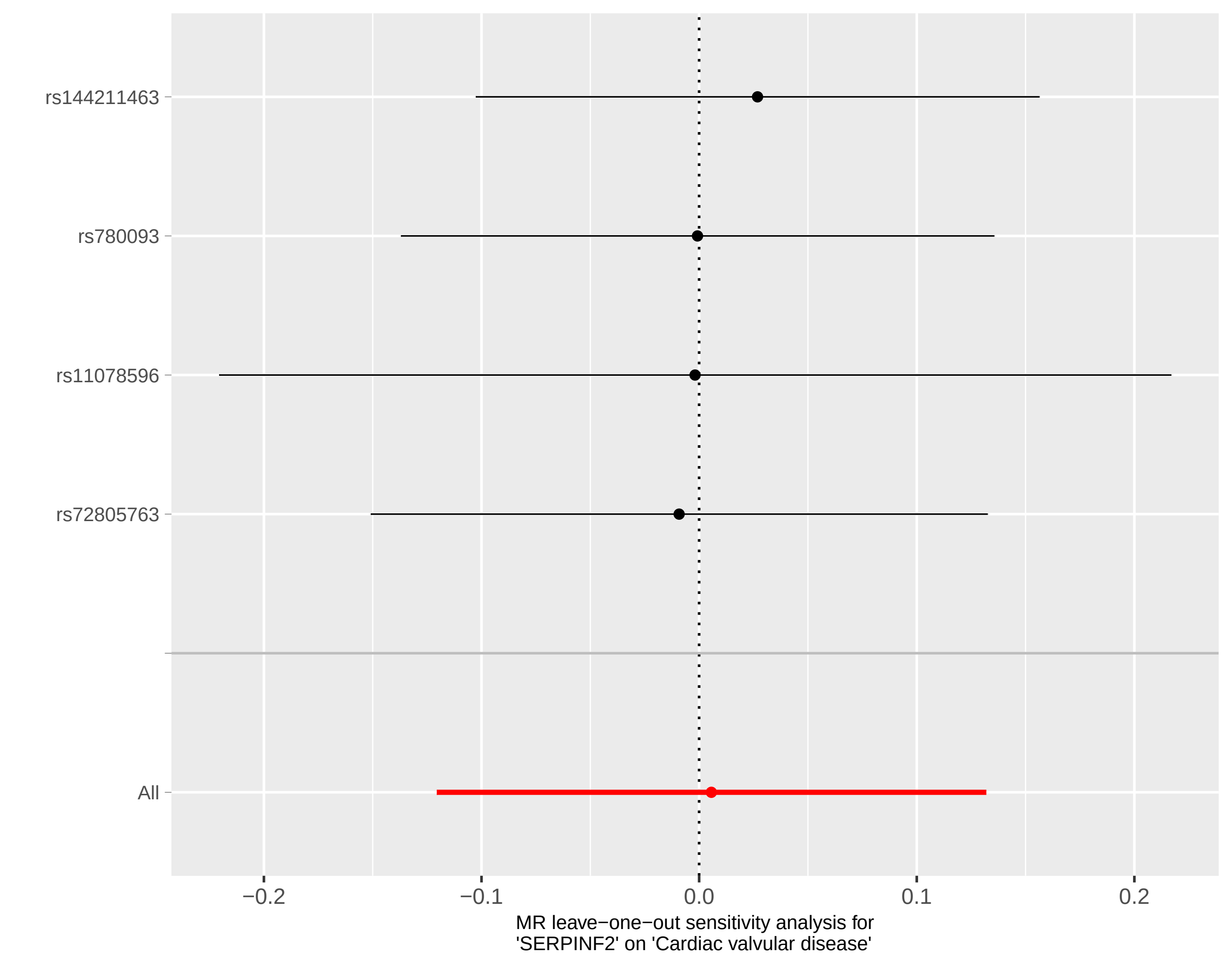

rs144211463
rs780093
rs11078596
rs72805763
All
−0.2
−0.1
0.0
0.1
0.2
MR leave−one−out sensitivity analysis for
'SERPINF2' on 'Cardiac valvular disease'

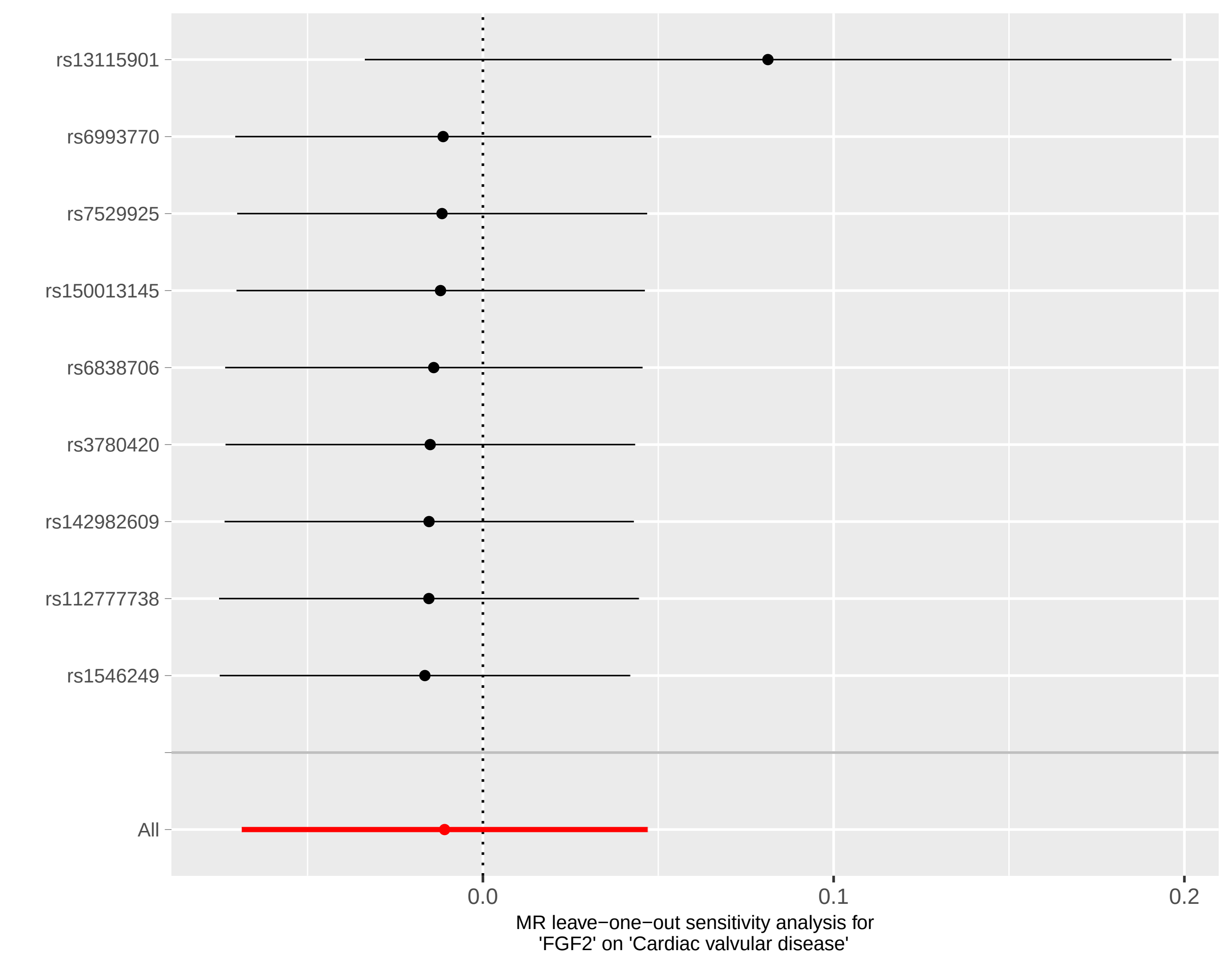

rs13115901
rs6993770
rs7529925
rs150013145
rs6838706
rs3780420
rs142982609
rs112777738
rs1546249
All
0.0
0.1
0.2
MR leave−one−out sensitivity analysis for
'FGF2' on 'Cardiac valvular disease'

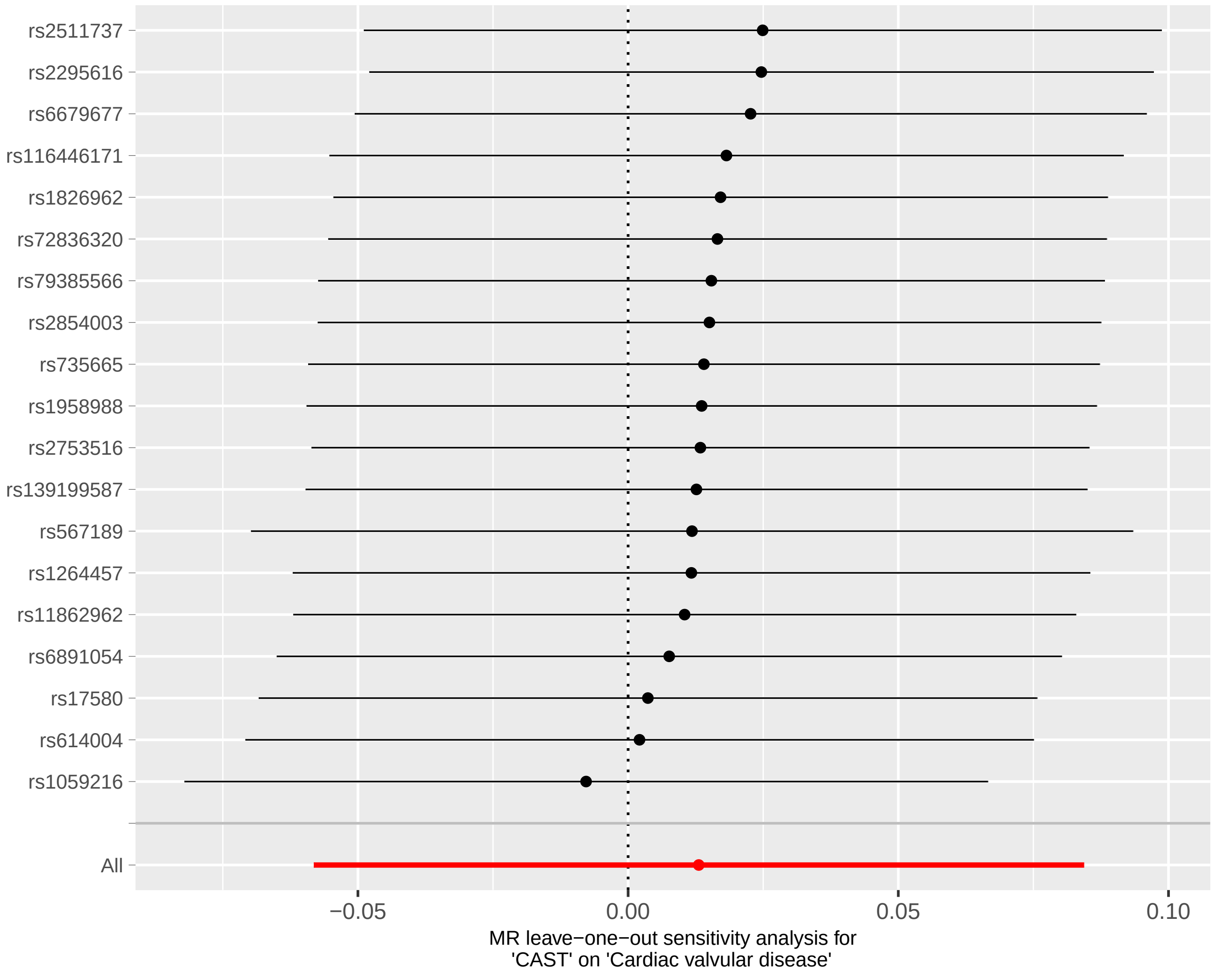
rs2511737
rs2295616
rs6679677
rs116446171
rs1826962
rs72836320
rs79385566
rs2854003
rs735665
rs1958988
rs2753516
rs139199587
rs567189
rs1264457
rs11862962
rs6891054
rs17580
rs614004
rs1059216
All
−0.05
0.00
0.05
0.10
MR leave−one−out sensitivity analysis for
'CAST' on 'Cardiac valvular disease'

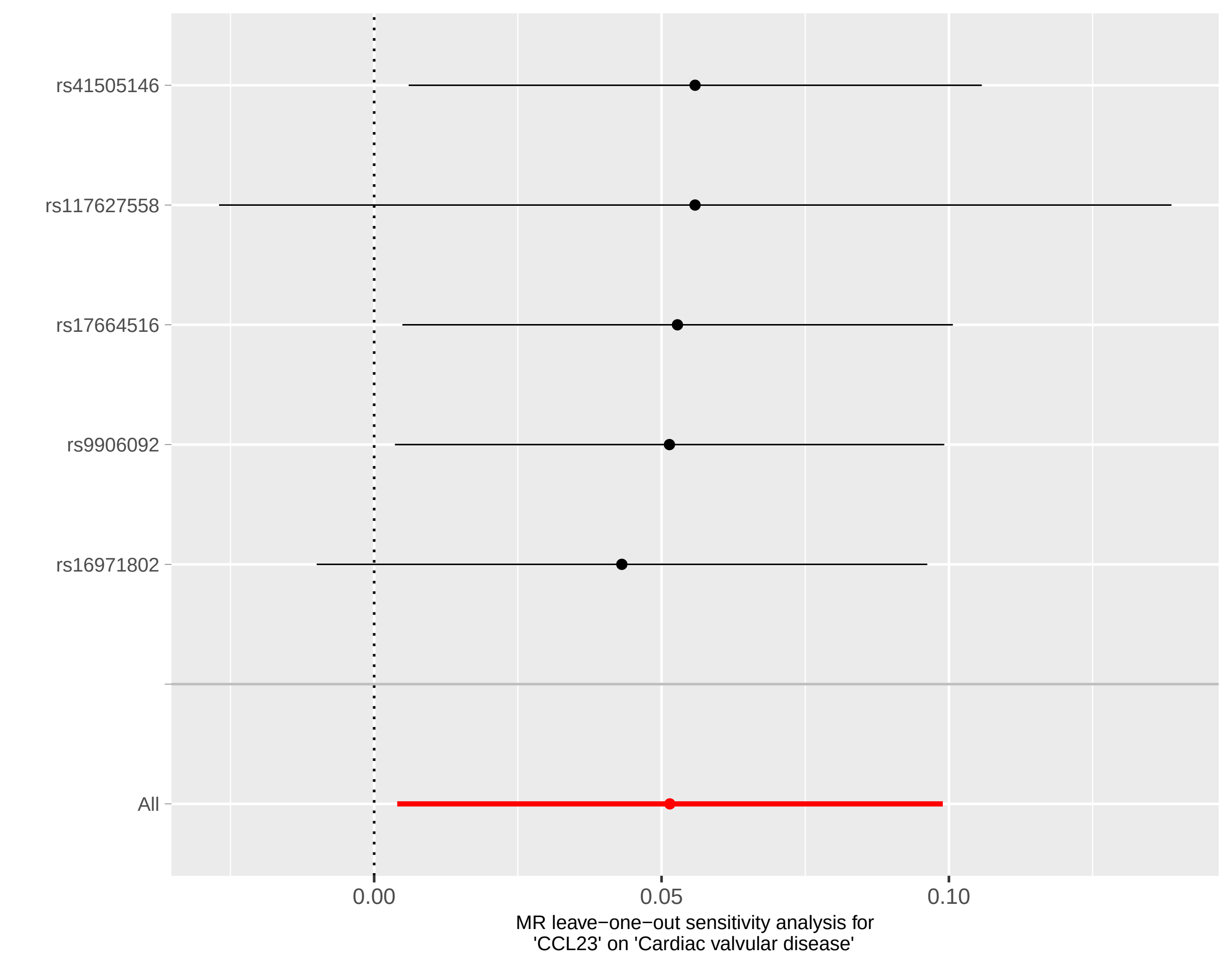
rs41505146
rs117627558
rs17664516
rs9906092
rs16971802
All
0.00
0.05
0.10
MR leave−one−out sensitivity analysis for
'CCL23' on 'Cardiac valvular disease'

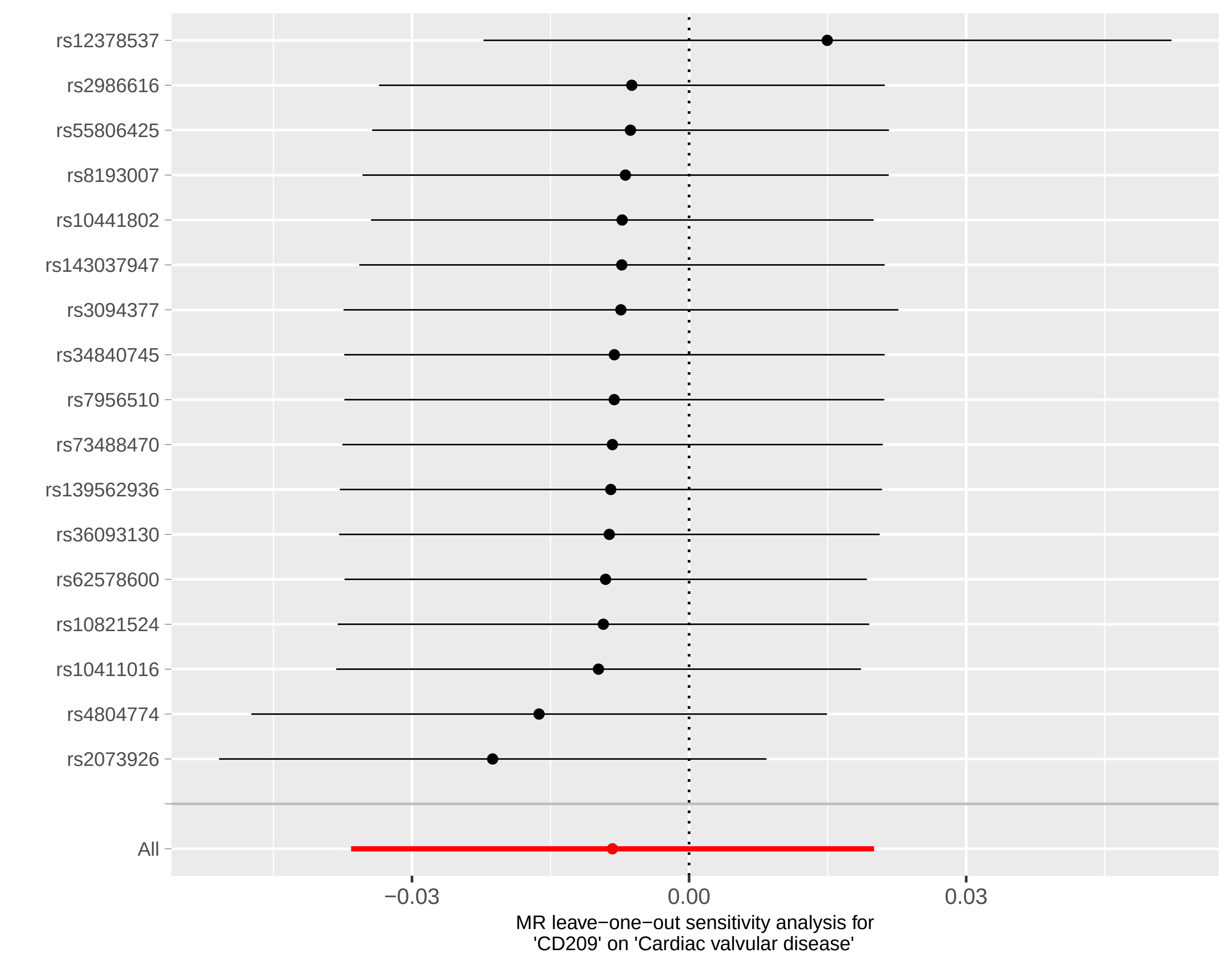
rs12378537
rs2986616
rs55806425
rs8193007
rs10441802
rs143037947
rs3094377
rs34840745
rs7956510
rs73488470
rs139562936
rs36093130
rs62578600
rs10821524
rs10411016
rs4804774
rs2073926
All
−0.03
0.00
0.03
MR leave−one−out sensitivity analysis for
'CD209' on 'Cardiac valvular disease'

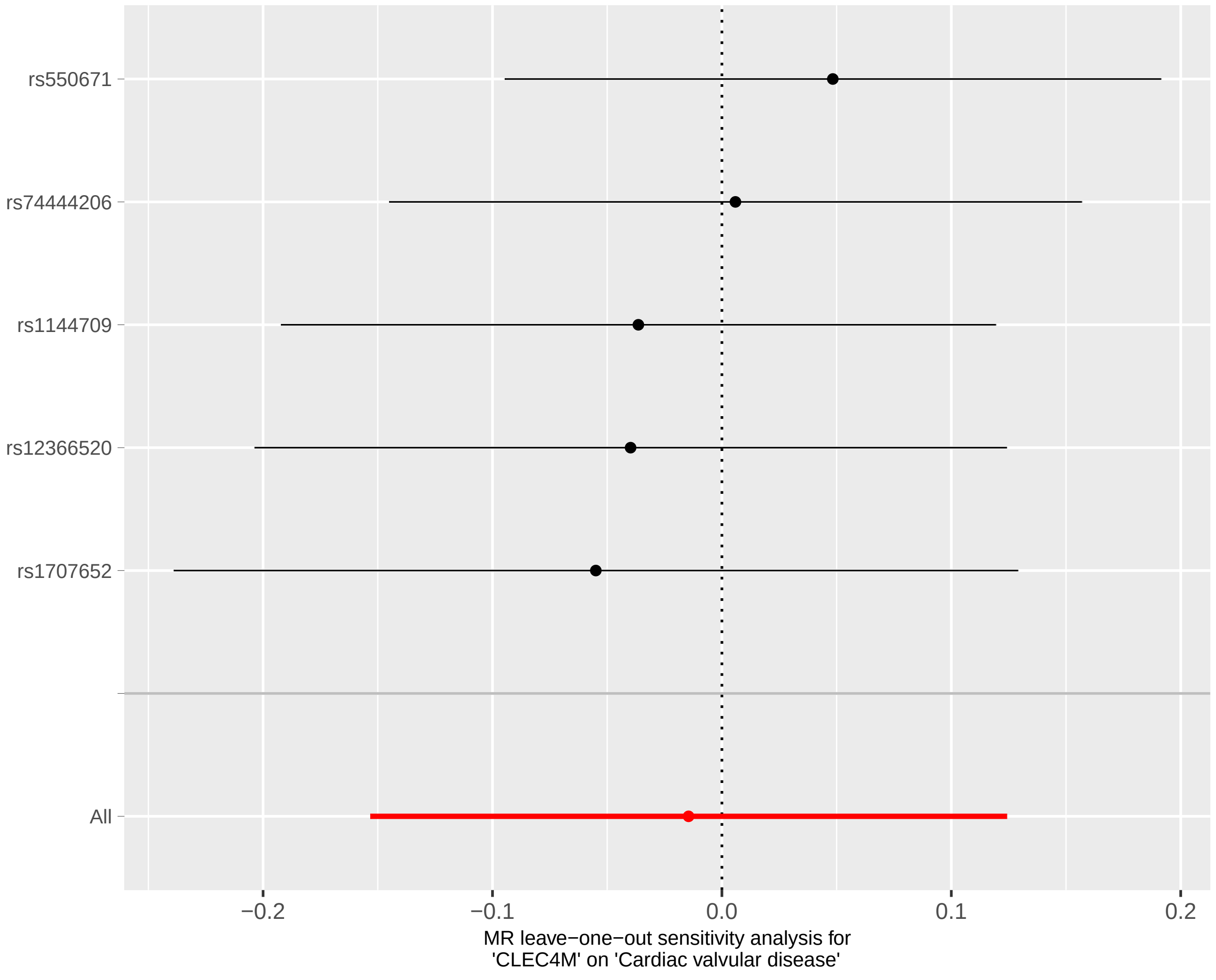
rs550671
rs74444206
rs1144709
rs12366520
rs1707652
All
−0.2
−0.1
0.0
0.1
0.2
MR leave−one−out sensitivity analysis for
'CLEC4M' on 'Cardiac valvular disease'

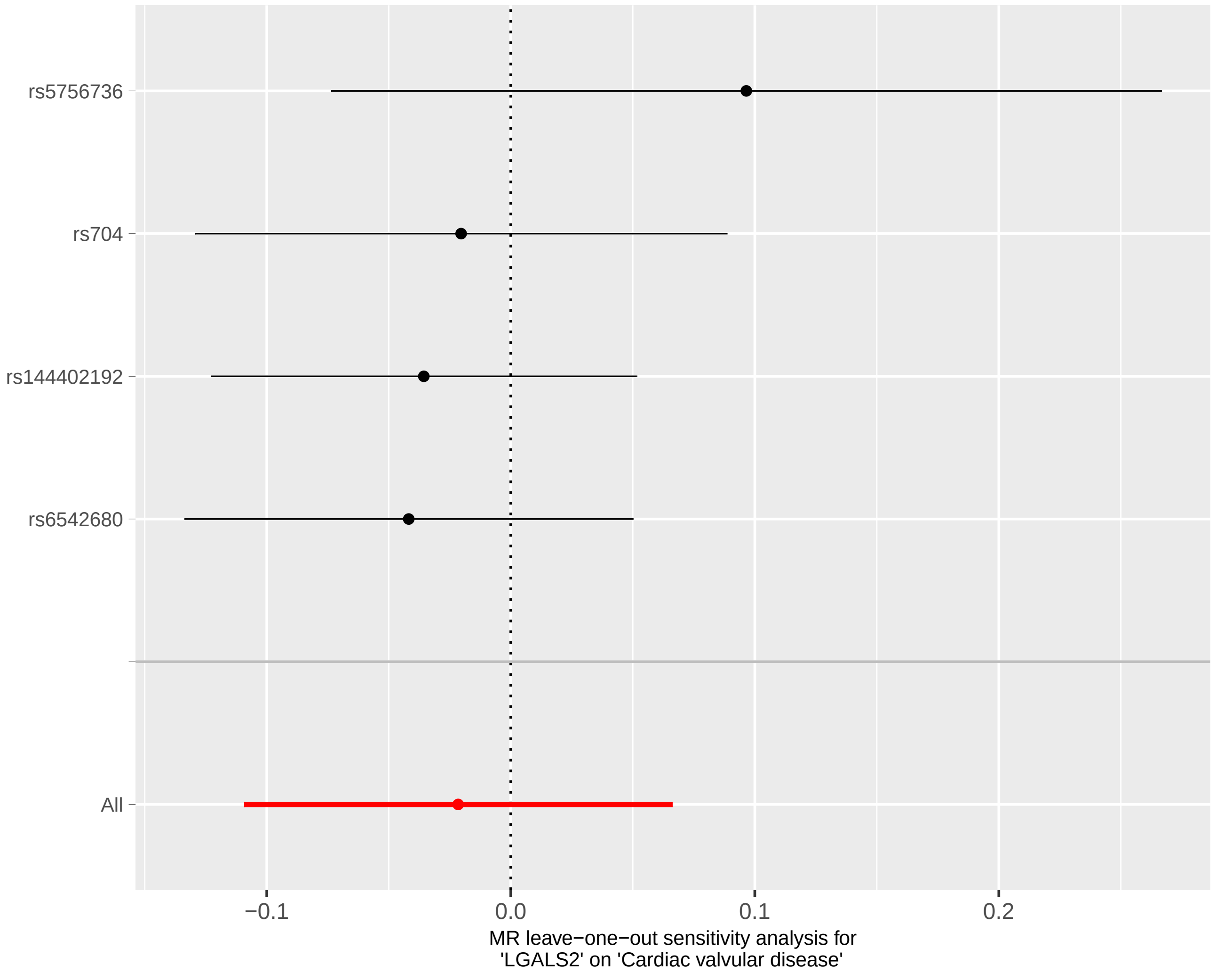
rs5756736
rs704
rs144402192
rs6542680
All
−0.1
0.0
0.1
0.2
MR leave−one−out sensitivity analysis for
'LGALS2' on 'Cardiac valvular disease'

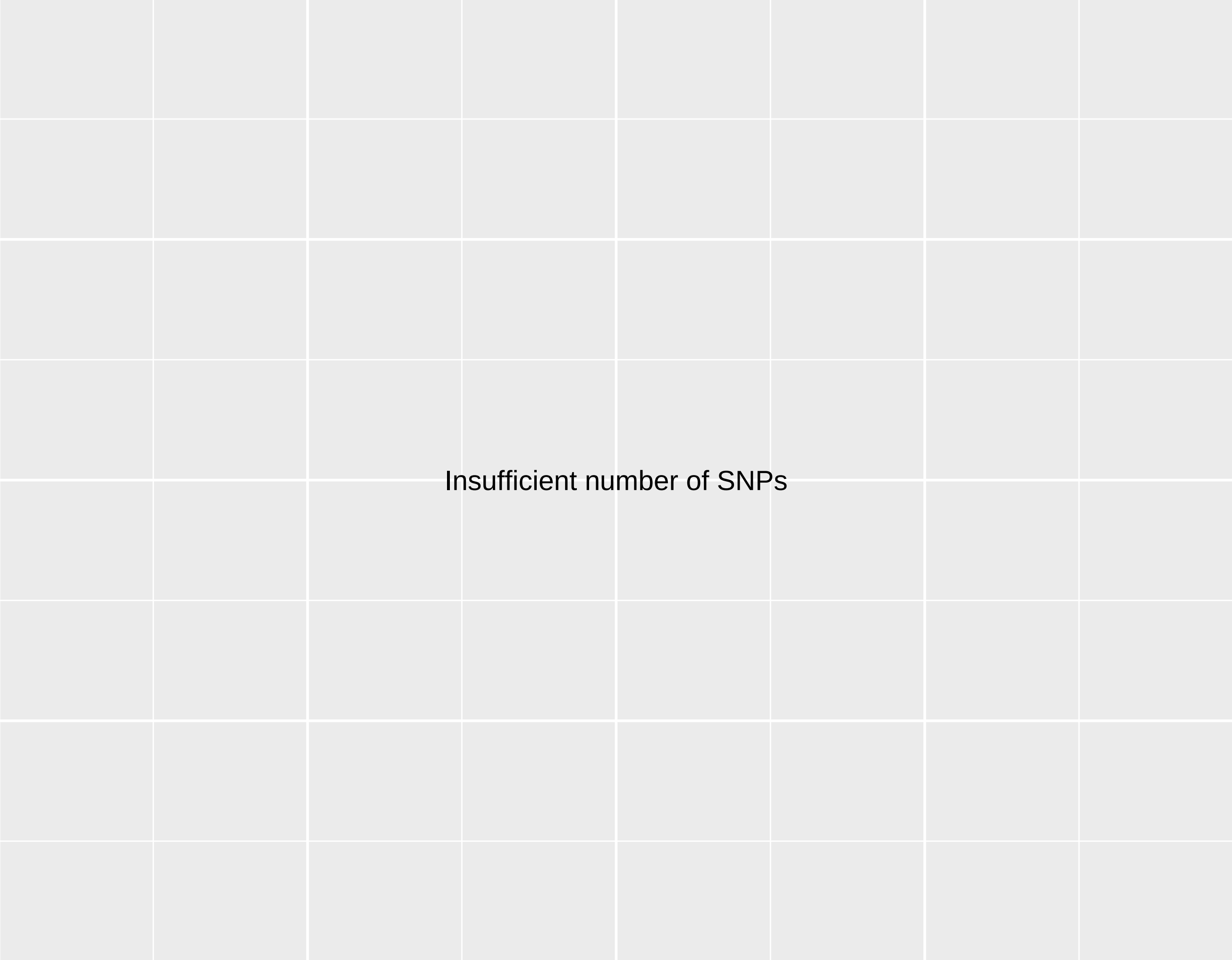
Insufficient number of SNPs

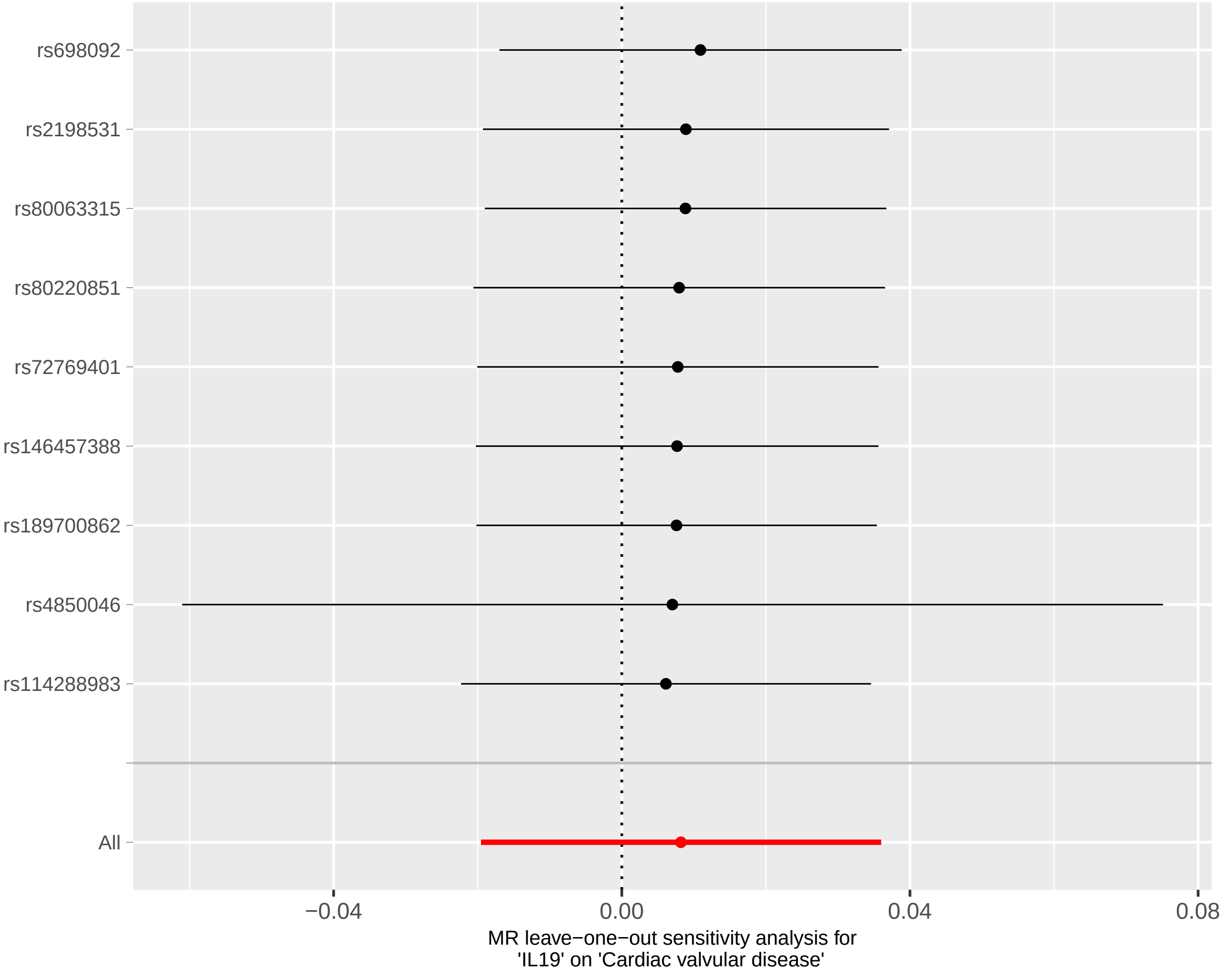

rs698092
rs2198531
rs80063315
rs80220851
rs72769401
rs146457388
rs189700862
rs4850046
rs114288983
All
−0.04
0.00
0.04
0.08
MR leave−one−out sensitivity analysis for
'IL19' on 'Cardiac valvular disease'

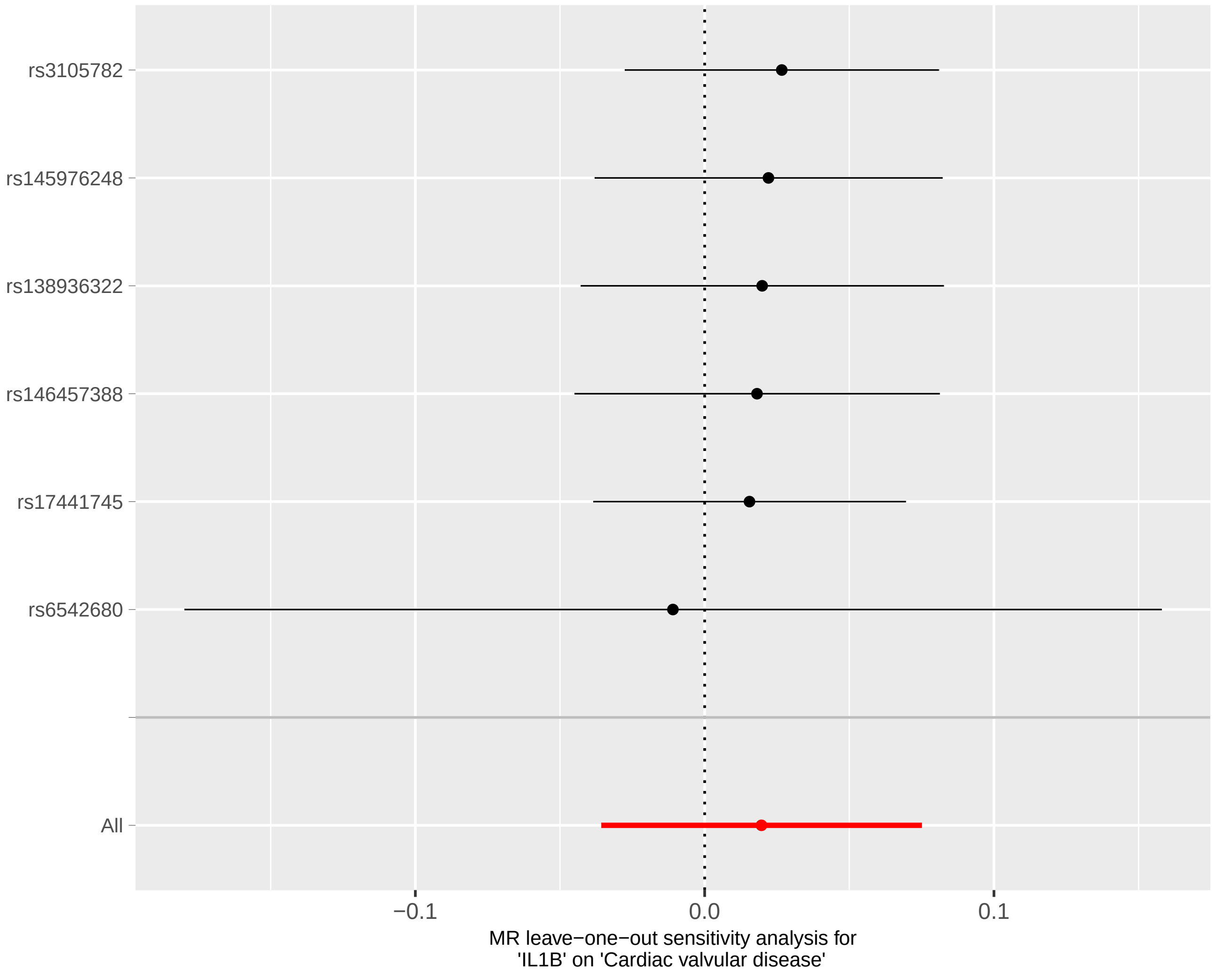
rs3105782
rs145976248
rs138936322
rs146457388
rs17441745
rs6542680
All
−0.1
0.0
0.1
MR leave−one−out sensitivity analysis for
'IL1B' on 'Cardiac valvular disease'

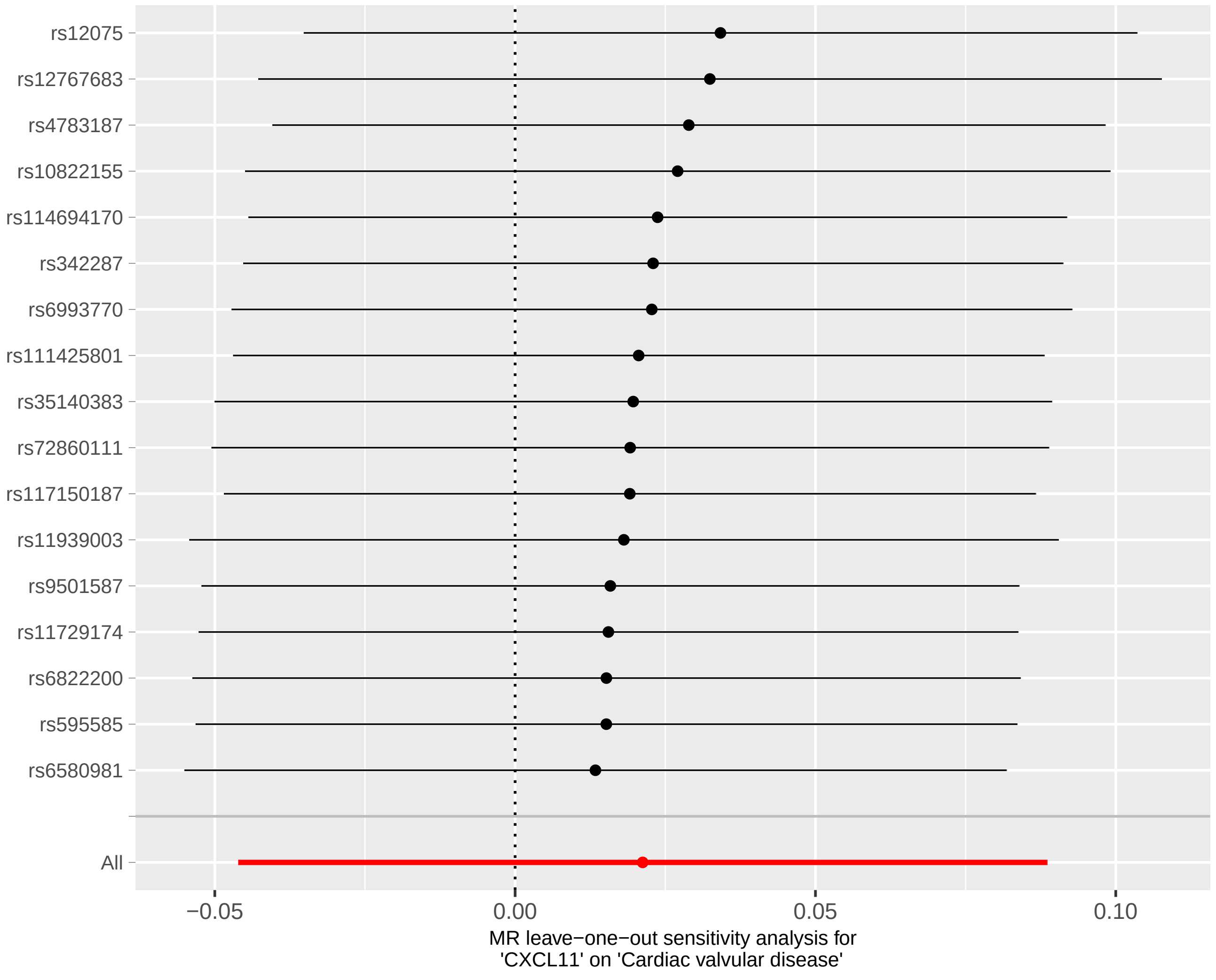
rs12075
rs12767683
rs4783187
rs10822155
rs114694170
rs342287
rs6993770
rs111425801
rs35140383
rs72860111
rs117150187
rs11939003
rs9501587
rs11729174
rs6822200
rs595585
rs6580981
All
−0.05
0.00
0.05
0.10
MR leave−one−out sensitivity analysis for
'CXCL11' on 'Cardiac valvular disease'

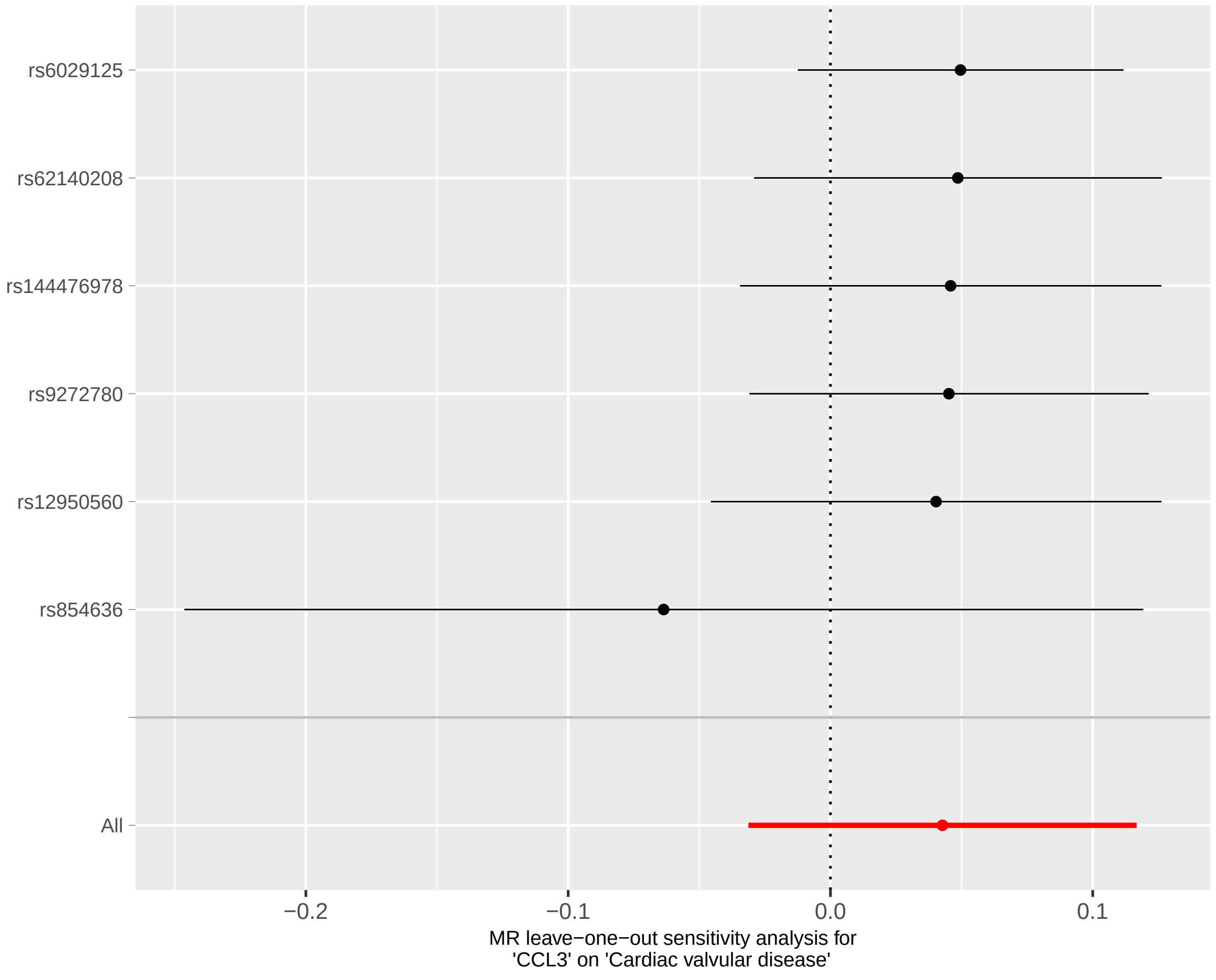
rs6029125
rs62140208
rs144476978
rs9272780
rs12950560
rs854636
All
−0.2
−0.1
0.0
0.1
MR leave−one−out sensitivity analysis for
'CCL3' on 'Cardiac valvular disease'

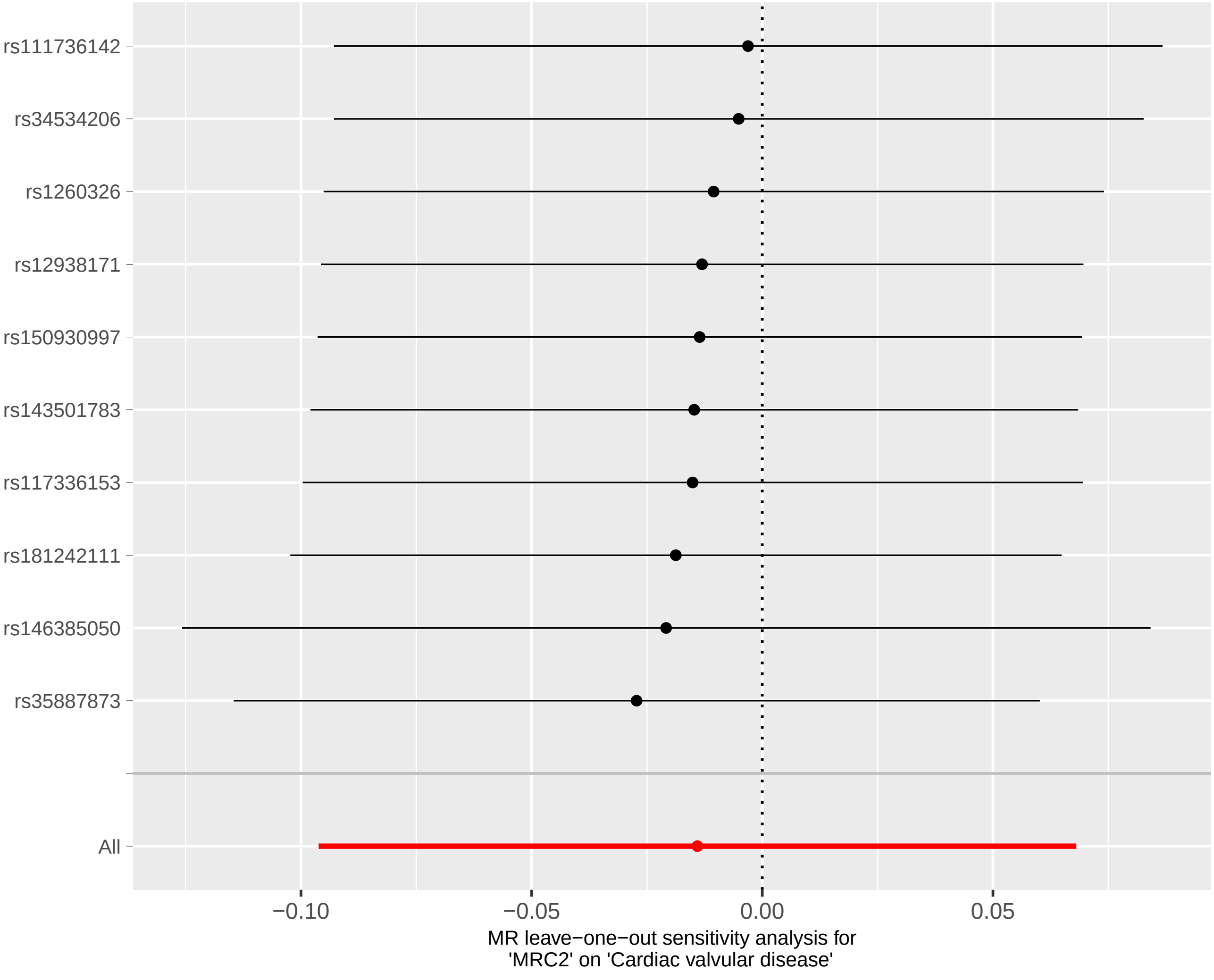

rs111736142
rs34534206
rs1260326
rs12938171
rs150930997
rs143501783
rs117336153
rs181242111
rs146385050
rs35887873
All
−0.10
−0.05
0.00
0.05
MR leave−one−out sensitivity analysis for
'MRC2' on 'Cardiac valvular disease'

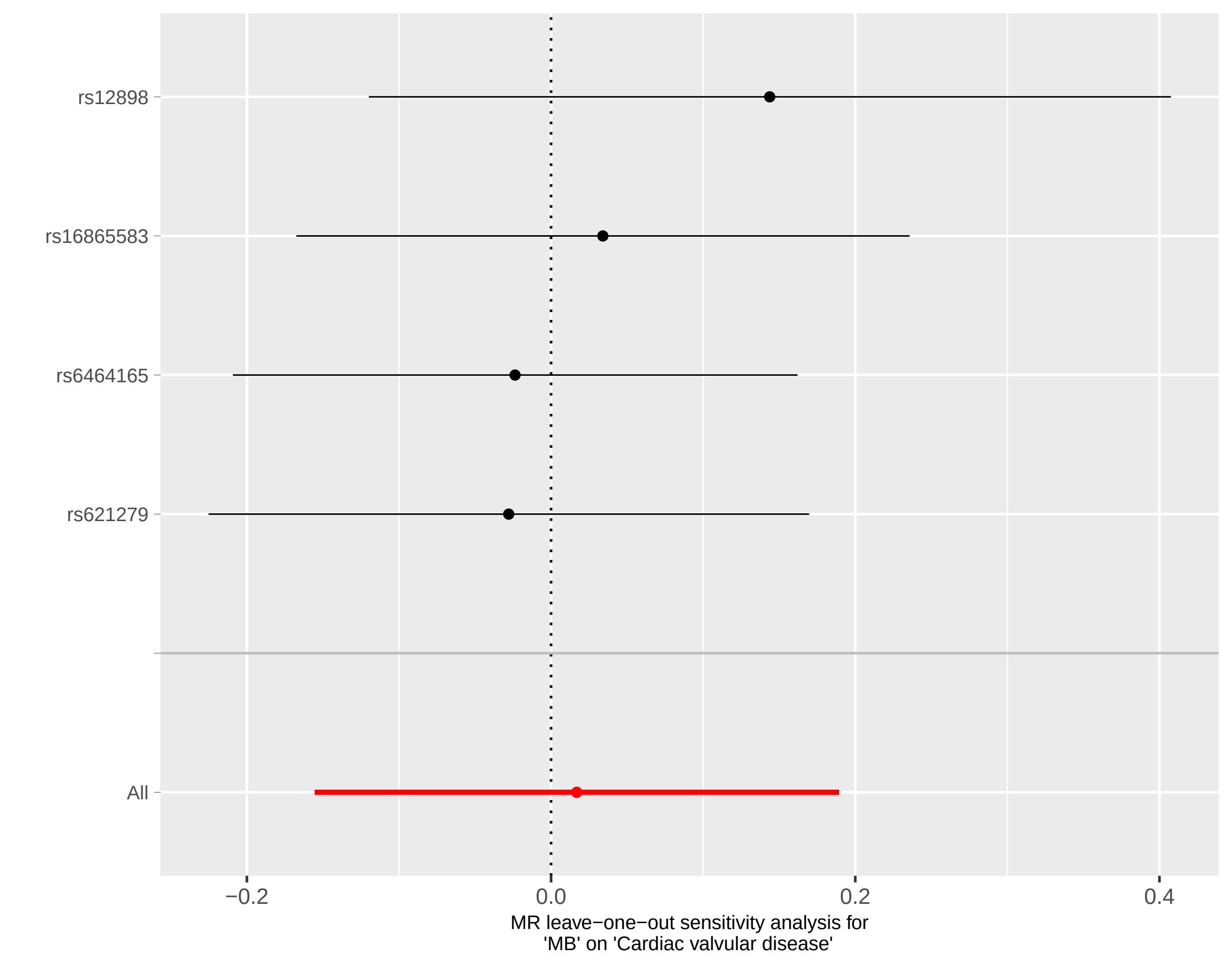

rs12898
rs16865583
rs6464165
rs621279
All
−0.2
0.0
0.2
0.4
MR leave−one−out sensitivity analysis for
'MB' on 'Cardiac valvular disease'

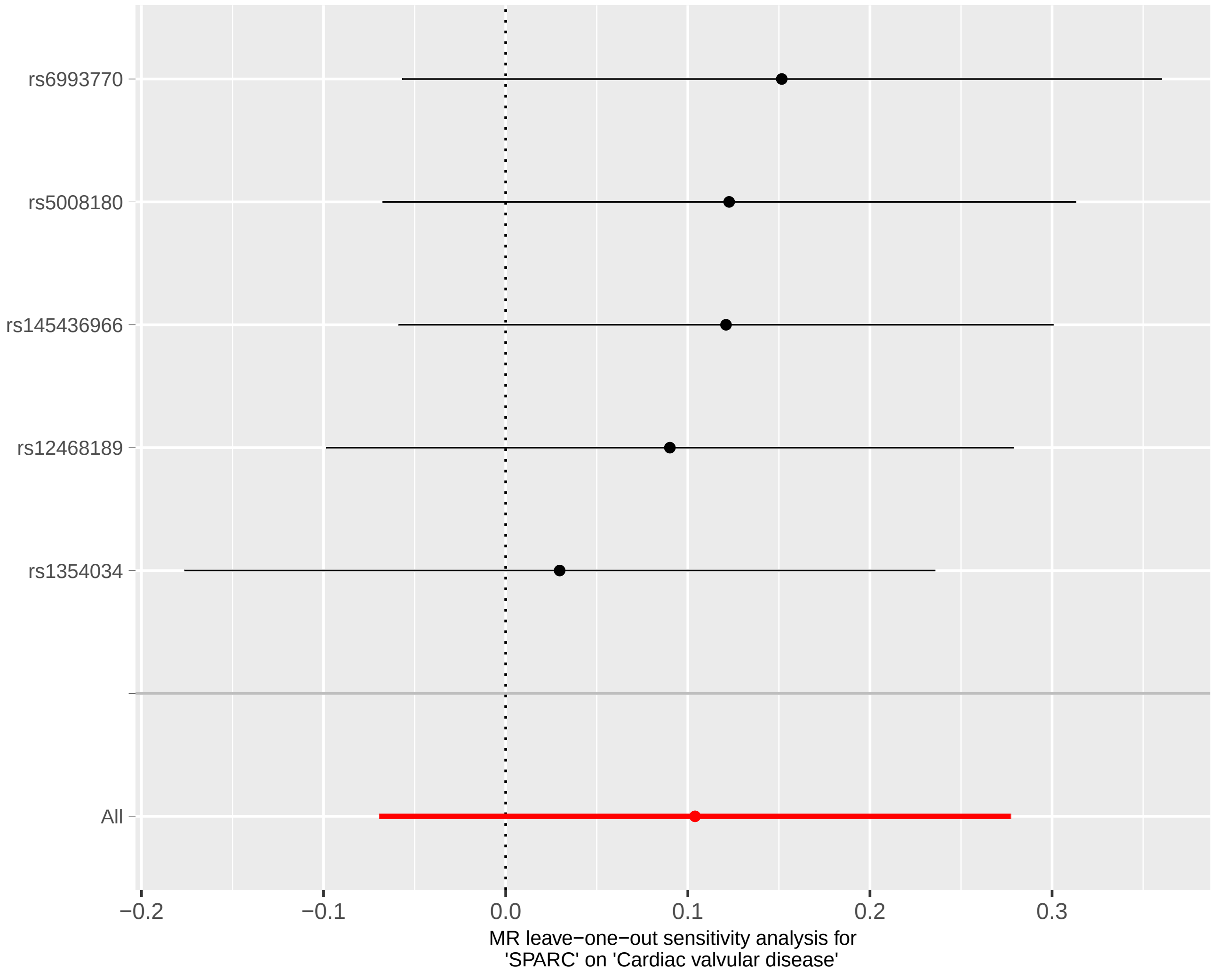
rs6993770
rs5008180
rs145436966
rs12468189
rs1354034
All
−0.2
−0.1
0.0
0.1
0.2
0.3
MR leave−one−out sensitivity analysis for
'SPARC' on 'Cardiac valvular disease'

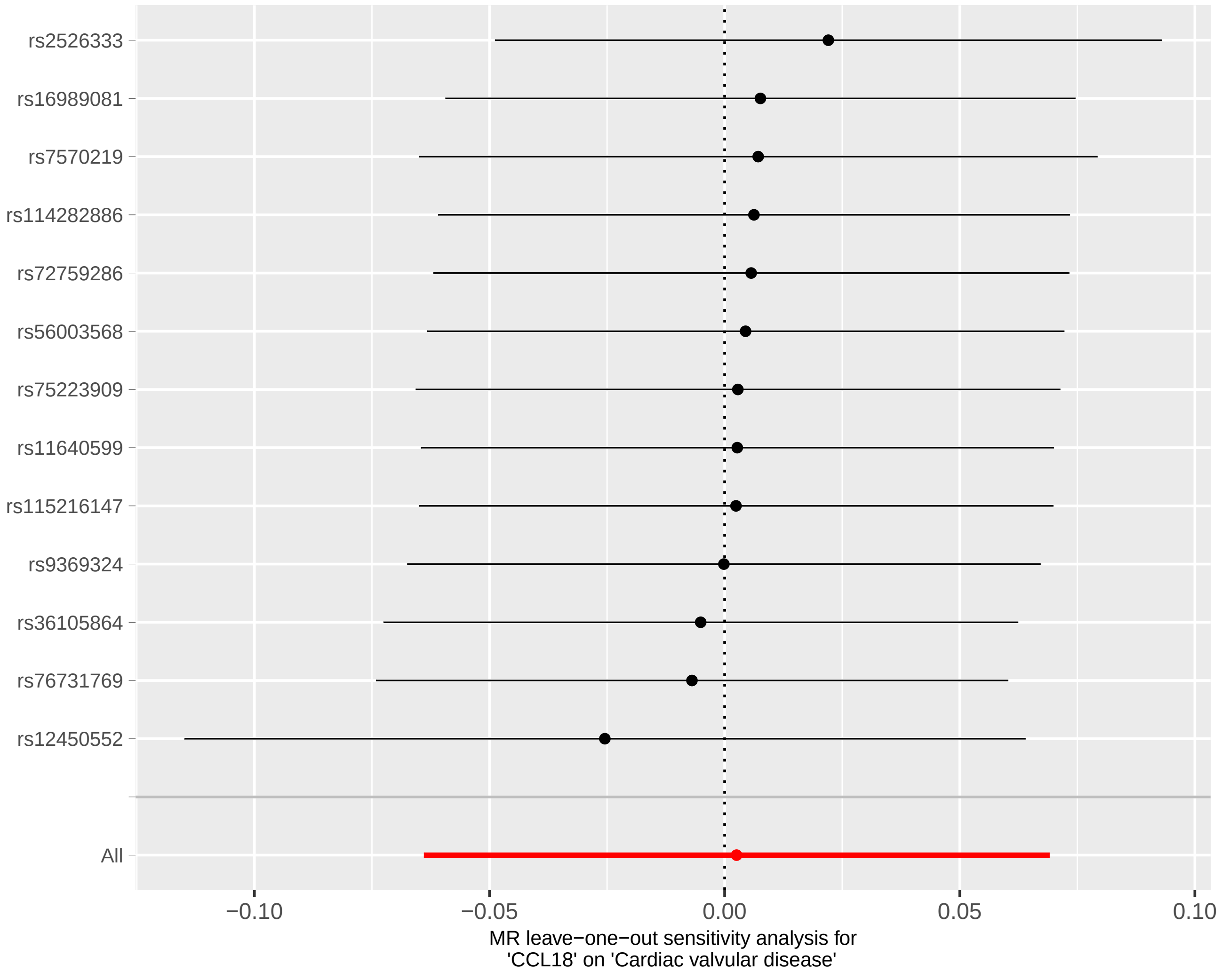
rs2526333
rs16989081
rs7570219
rs114282886
rs72759286
rs56003568
rs75223909
rs11640599
rs115216147
rs9369324
rs36105864
rs76731769
rs12450552
All
−0.10
−0.05
0.00
0.05
0.10
MR leave−one−out sensitivity analysis for
'CCL18' on 'Cardiac valvular disease'

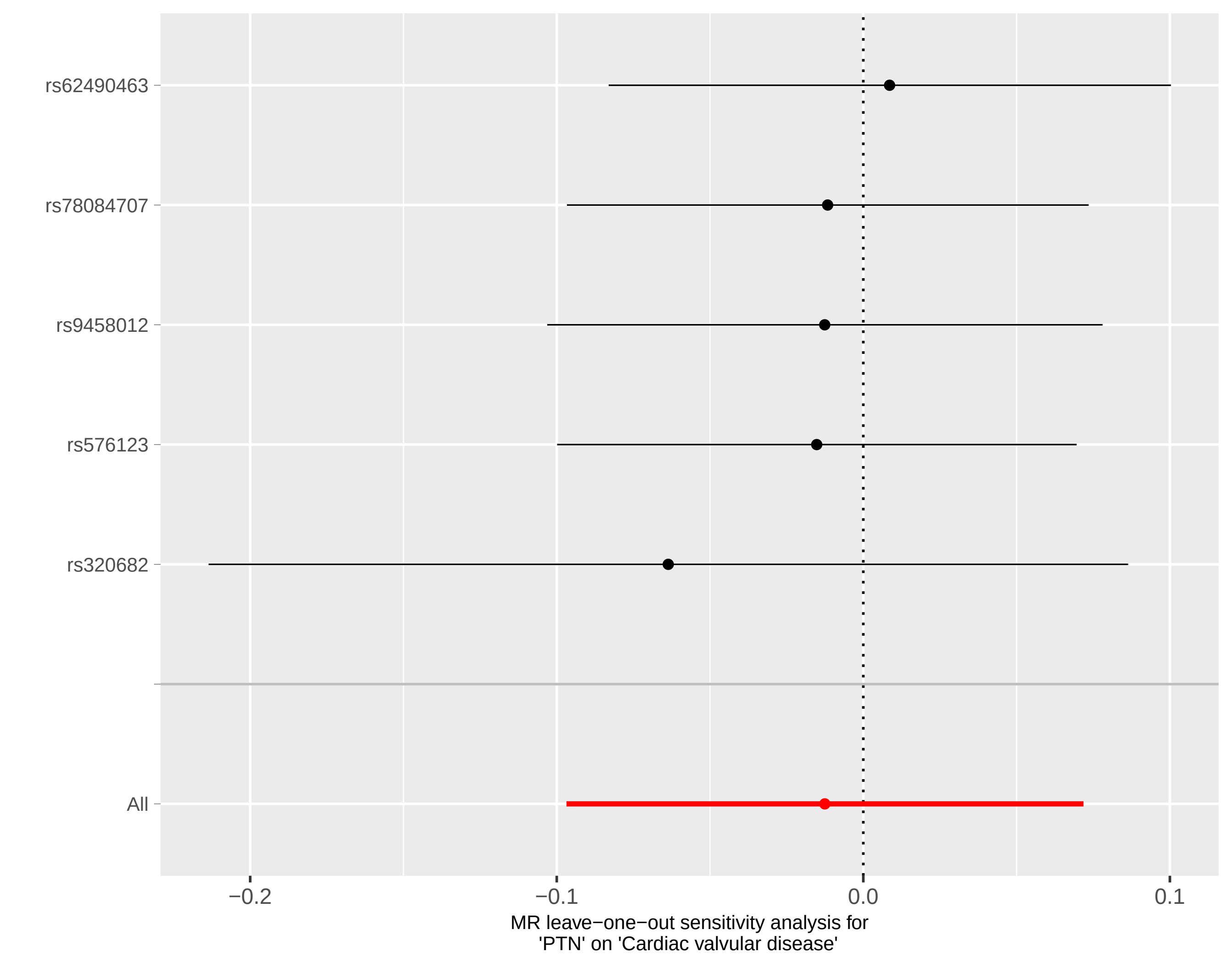

rs62490463
rs78084707
rs9458012
rs576123
rs320682
All
−0.2
−0.1
0.0
0.1
MR leave−one−out sensitivity analysis for
'PTN' on 'Cardiac valvular disease'

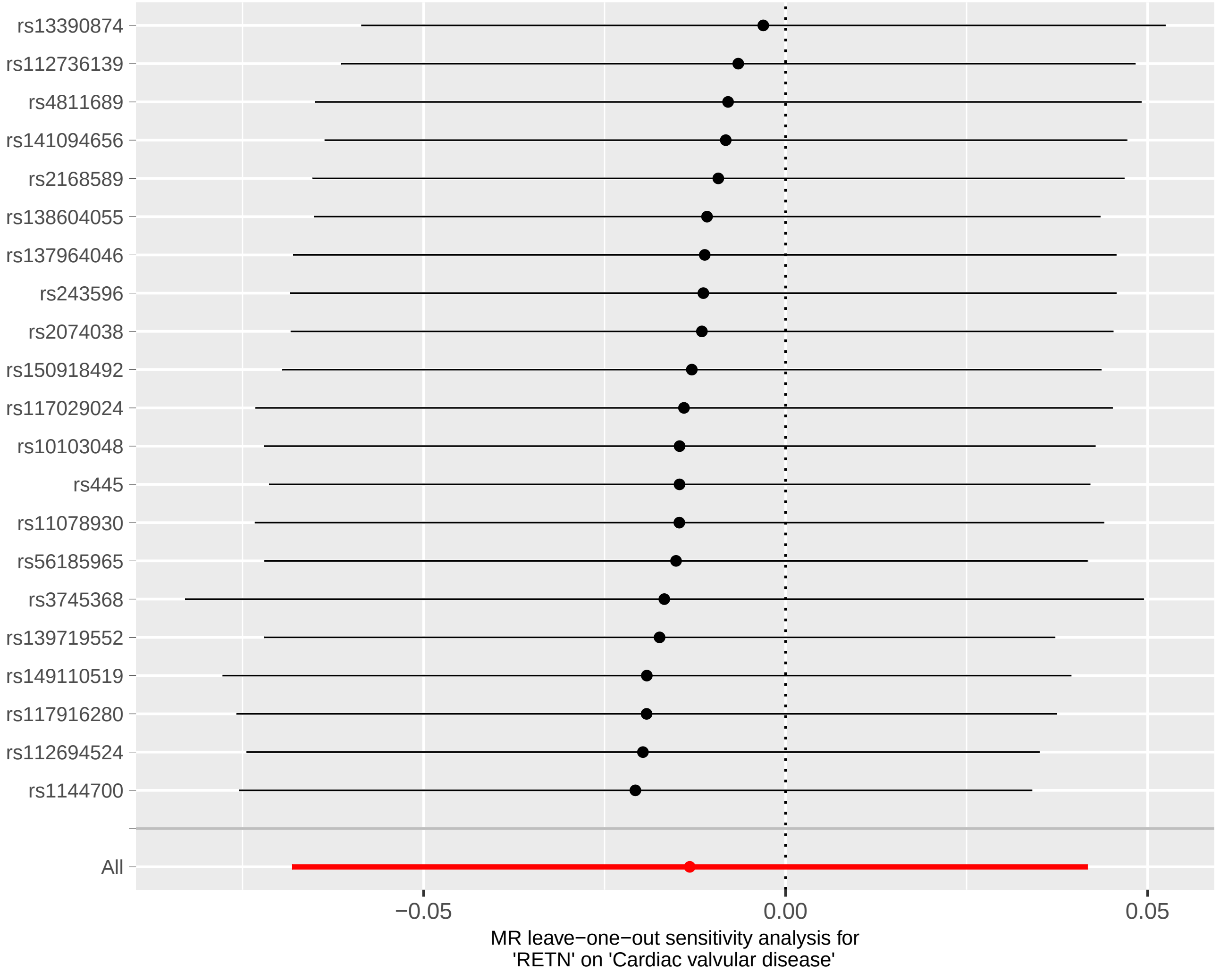
rs13390874
rs112736139
rs4811689
rs141094656
rs2168589
rs138604055
rs137964046
rs243596
rs2074038
rs150918492
rs117029024
rs10103048
rs445
rs11078930
rs56185965
rs3745368
rs139719552
rs149110519
rs117916280
rs112694524
rs1144700
All
−0.05
0.00
0.05
MR leave−one−out sensitivity analysis for
'RETN' on 'Cardiac valvular disease'

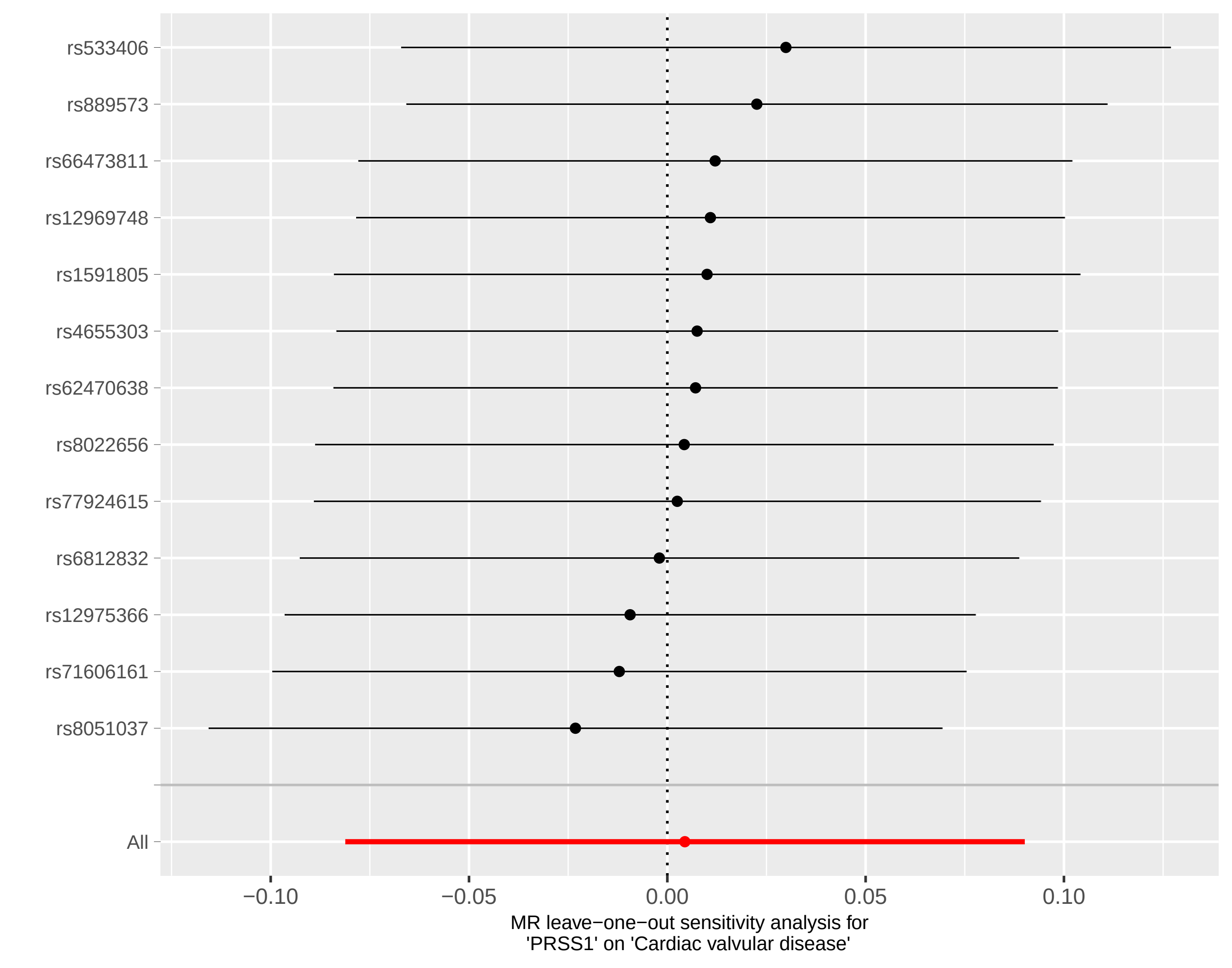
rs533406
rs889573
rs66473811
rs12969748
rs1591805
rs4655303
rs62470638
rs8022656
rs77924615
rs6812832
rs12975366
rs71606161
rs8051037
All
−0.10
−0.05
0.00
0.05
0.10
MR leave−one−out sensitivity analysis for
'PRSS1' on 'Cardiac valvular disease'

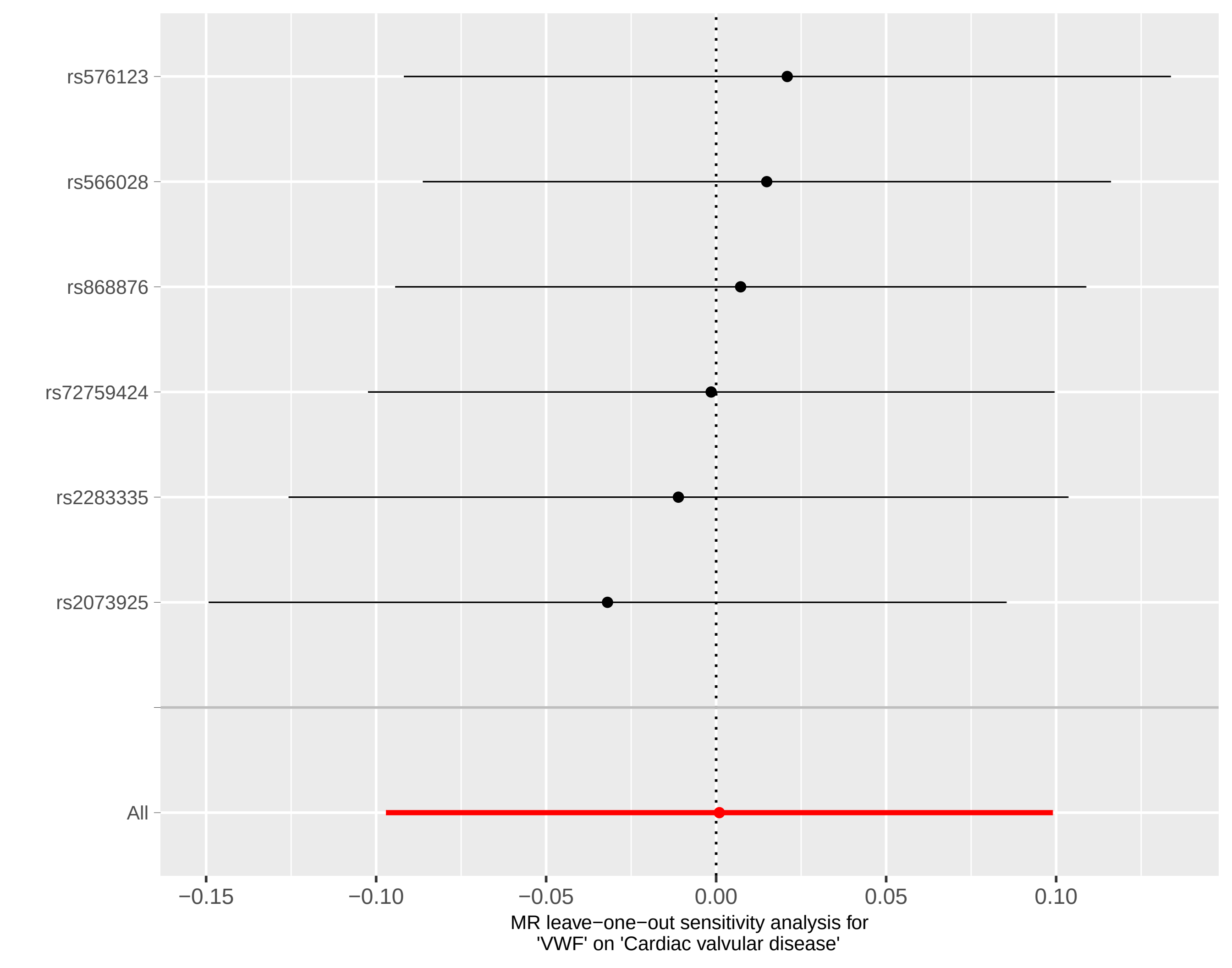
rs576123
rs566028
rs868876
rs72759424
rs2283335
rs2073925
All
−0.15
−0.10
−0.05
0.00
0.05
0.10
MR leave−one−out sensitivity analysis for
'VWF' on 'Cardiac valvular disease'

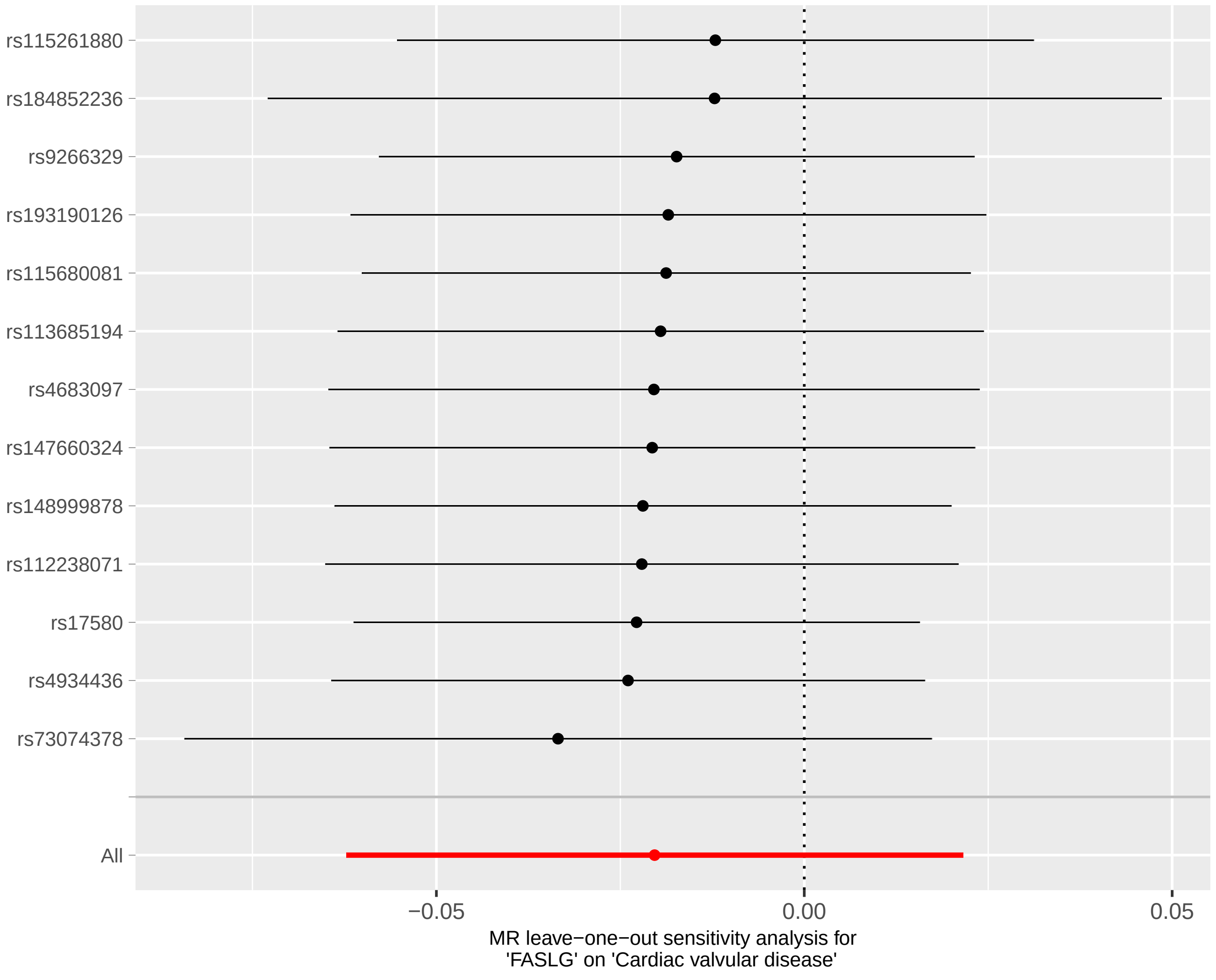

rs115261880
rs184852236
rs9266329
rs193190126
rs115680081
rs113685194
rs4683097
rs147660324
rs148999878
rs112238071
rs17580
rs4934436
rs73074378
All
−0.05
0.00
0.05
MR leave−one−out sensitivity analysis for
'FASLG' on 'Cardiac valvular disease'

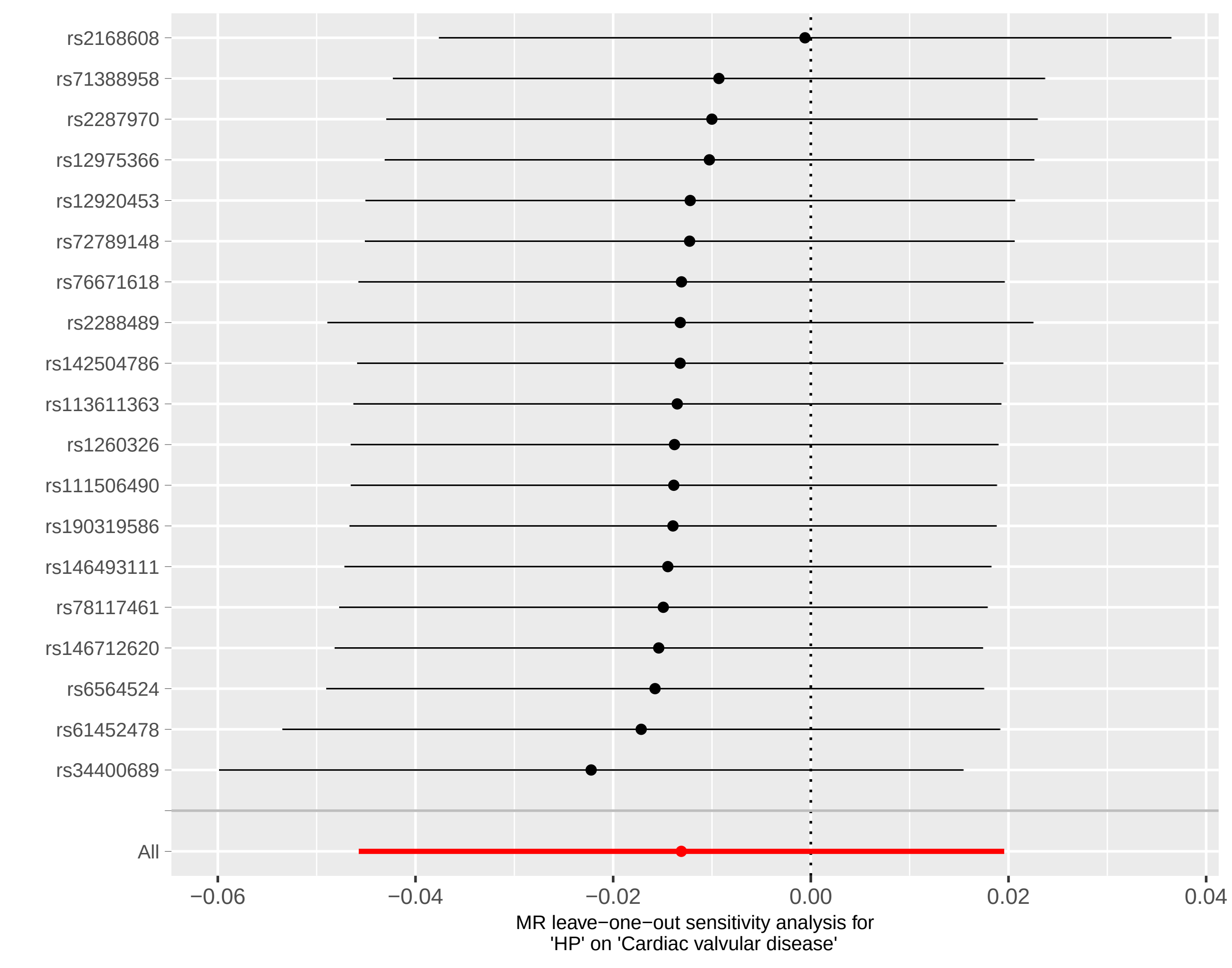

rs2168608
rs71388958
rs2287970
rs12975366
rs12920453
rs72789148
rs76671618
rs2288489
rs142504786
rs113611363
rs1260326
rs111506490
rs190319586
rs146493111
rs78117461
rs146712620
rs6564524
rs61452478
rs34400689
All
−0.06
−0.04
−0.02
0.00
0.02
0.04
MR leave−one−out sensitivity analysis for
'HP' on 'Cardiac valvular disease'

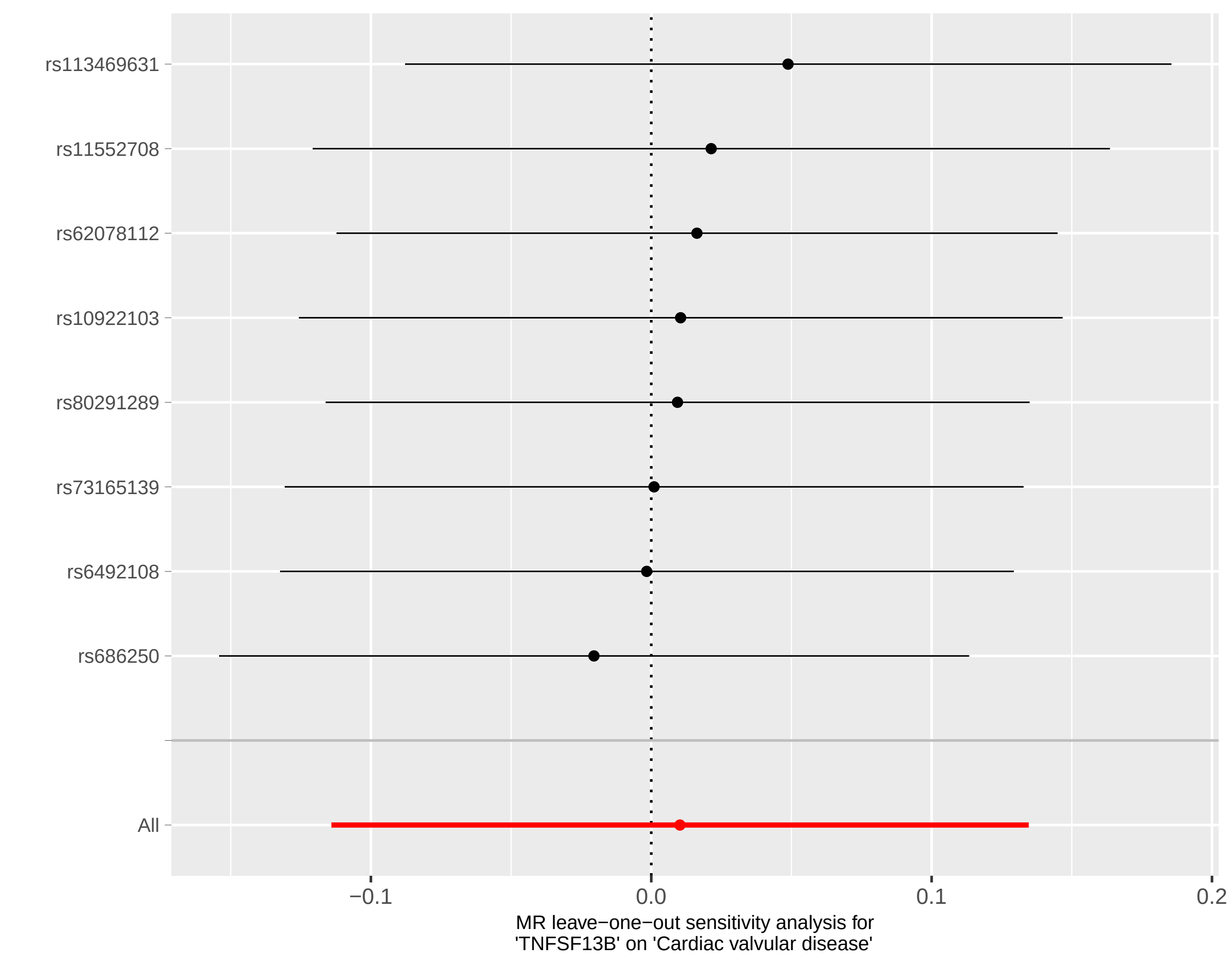
rs113469631
rs11552708
rs62078112
rs10922103
rs80291289
rs73165139
rs6492108
rs686250
All
−0.1
0.0
0.1
0.2
MR leave−one−out sensitivity analysis for
'TNFSF13B' on 'Cardiac valvular disease'

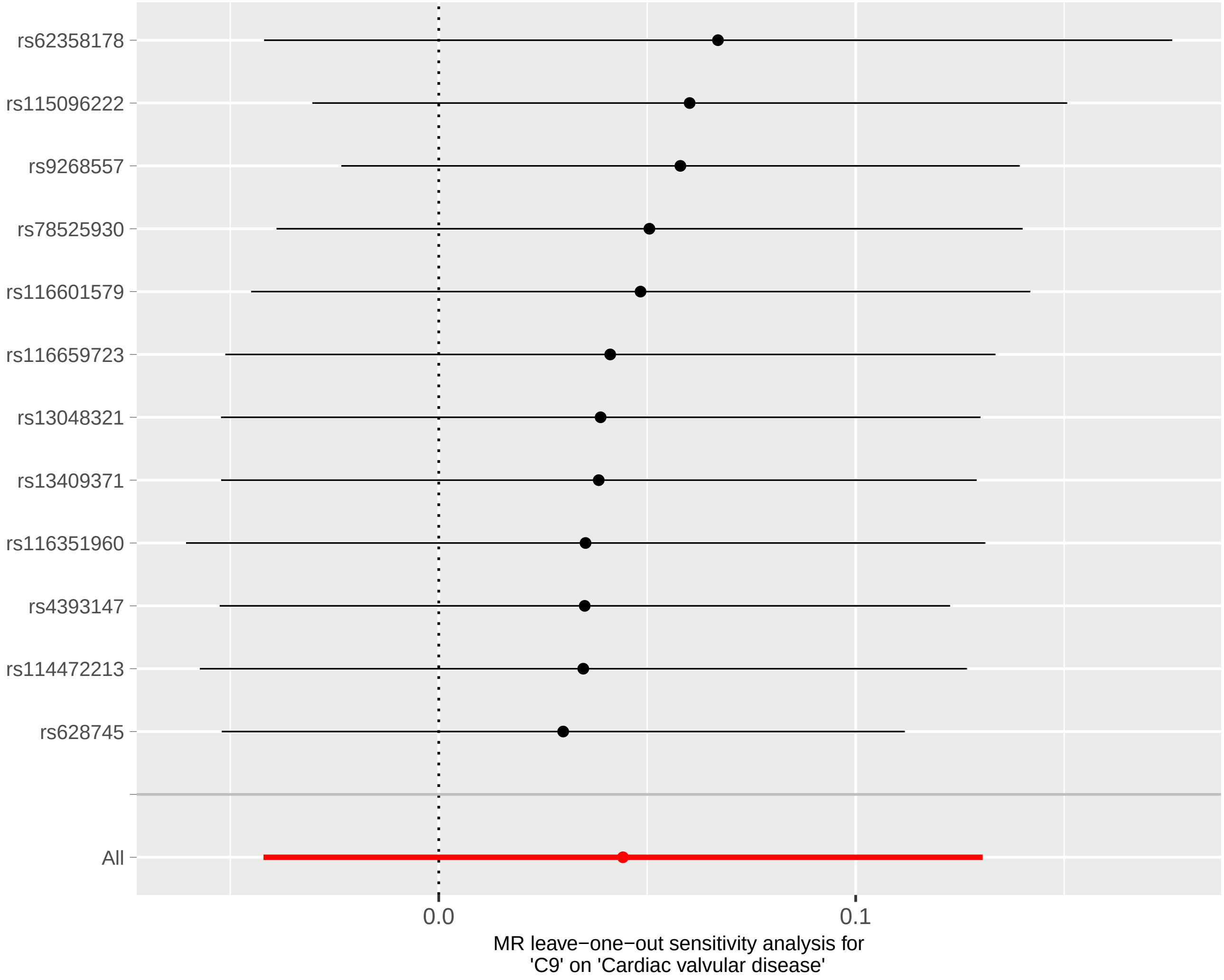
rs62358178
rs115096222
rs9268557
rs78525930
rs116601579
rs116659723
rs13048321
rs13409371
rs116351960
rs4393147
rs114472213
rs628745
All
0.0
0.1
MR leave−one−out sensitivity analysis for
'C9' on 'Cardiac valvular disease'

Insufficient number of SNPs

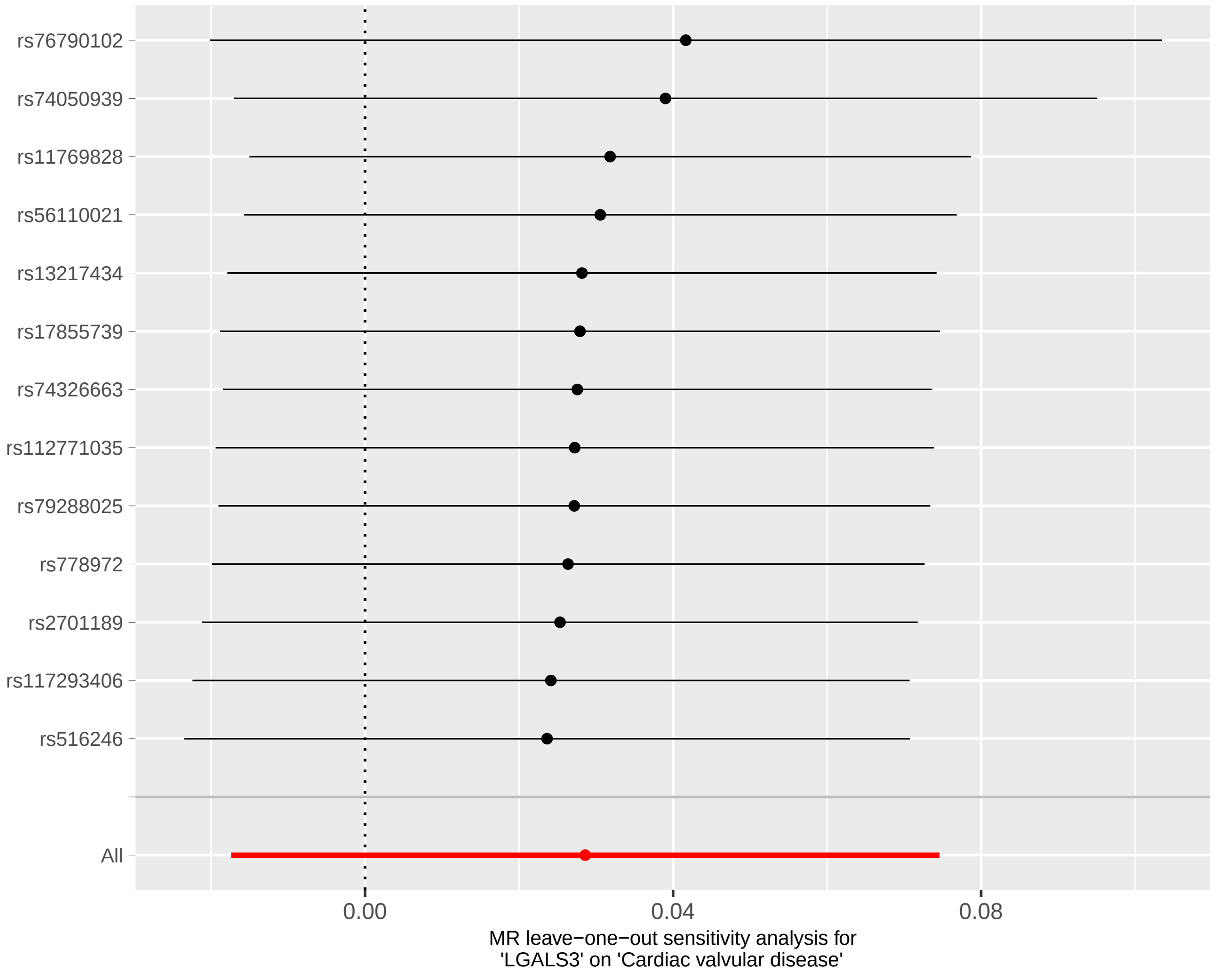
rs76790102
rs74050939
rs11769828
rs56110021
rs13217434
rs17855739
rs74326663
rs112771035
rs79288025
rs778972
rs2701189
rs117293406
rs516246
All
0.00
0.04
0.08
MR leave−one−out sensitivity analysis for
'LGALS3' on 'Cardiac valvular disease'

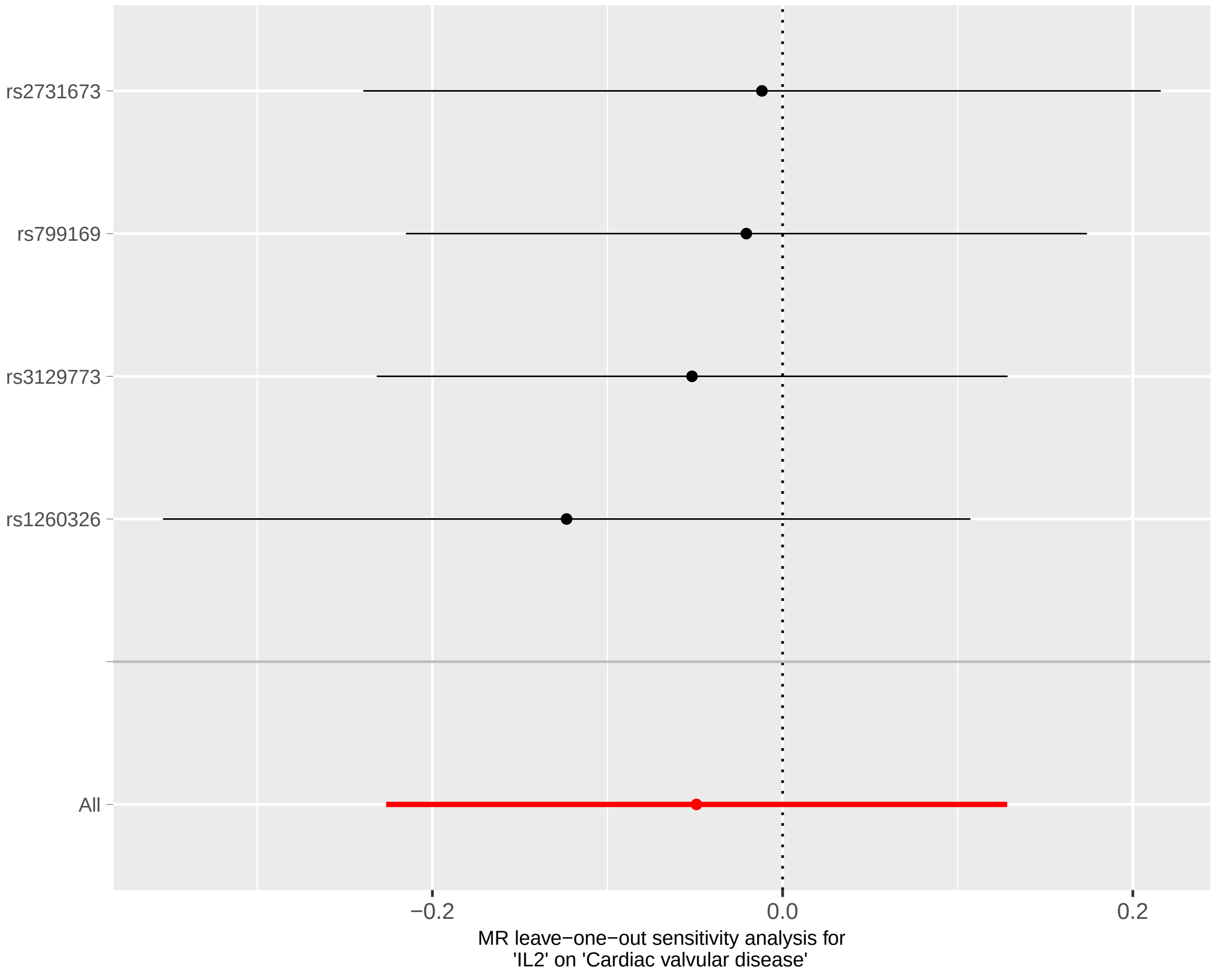
rs2731673
rs799169
rs3129773
rs1260326
All
−0.2
0.0
0.2
MR leave−one−out sensitivity analysis for
'IL2' on 'Cardiac valvular disease'

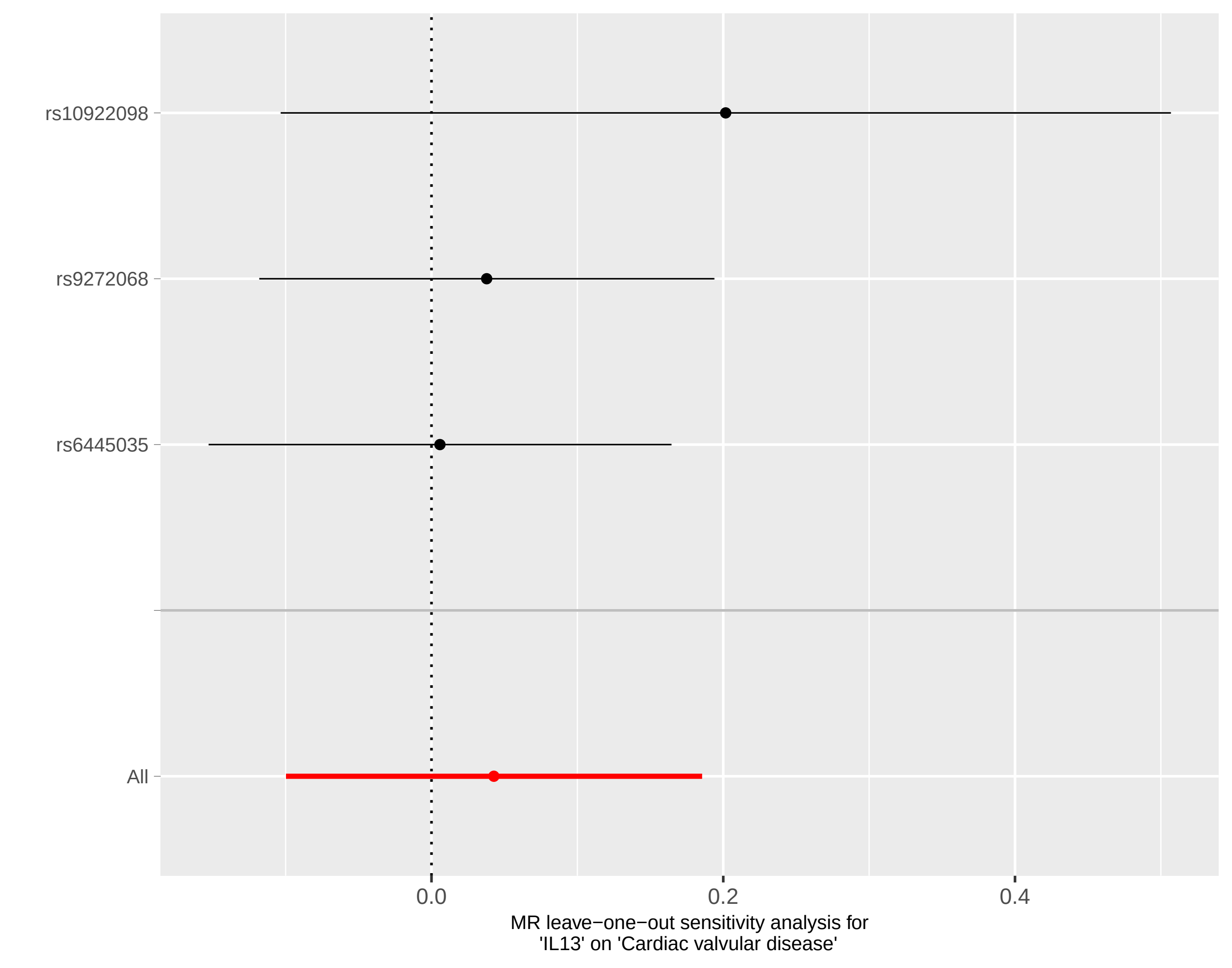
rs10922098
rs9272068
rs6445035
All
0.0
0.2
0.4
MR leave−one−out sensitivity analysis for
'IL13' on 'Cardiac valvular disease'

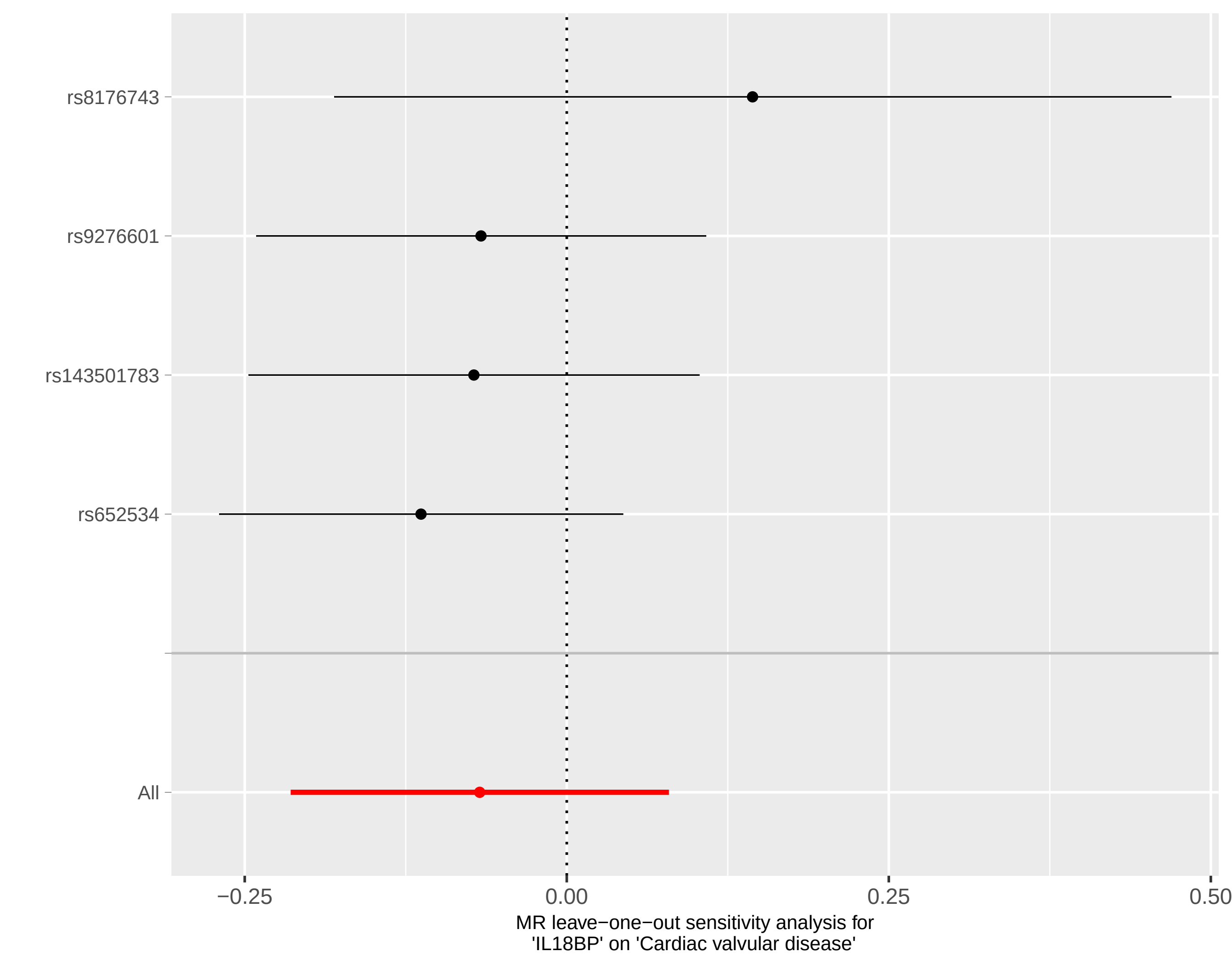
rs8176743
rs9276601
rs143501783
rs652534
All
−0.25
0.00
0.25
0.50
MR leave−one−out sensitivity analysis for
'IL18BP' on 'Cardiac valvular disease'

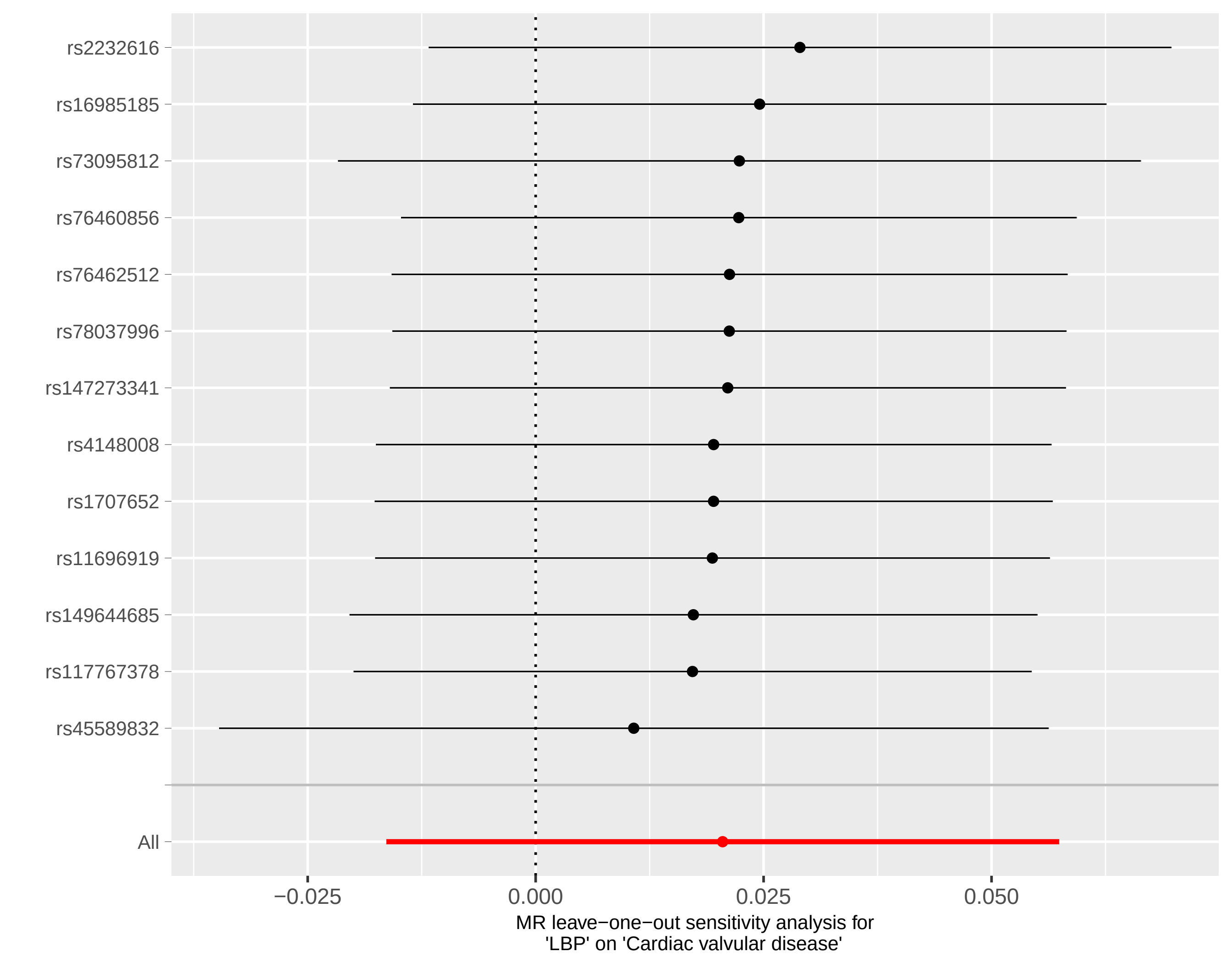
rs2232616
rs16985185
rs73095812
rs76460856
rs76462512
rs78037996
rs147273341
rs4148008
rs1707652
rs11696919
rs149644685
rs117767378
rs45589832
All
−0.025
0.000
0.025
0.050
MR leave−one−out sensitivity analysis for
'LBP' on 'Cardiac valvular disease'

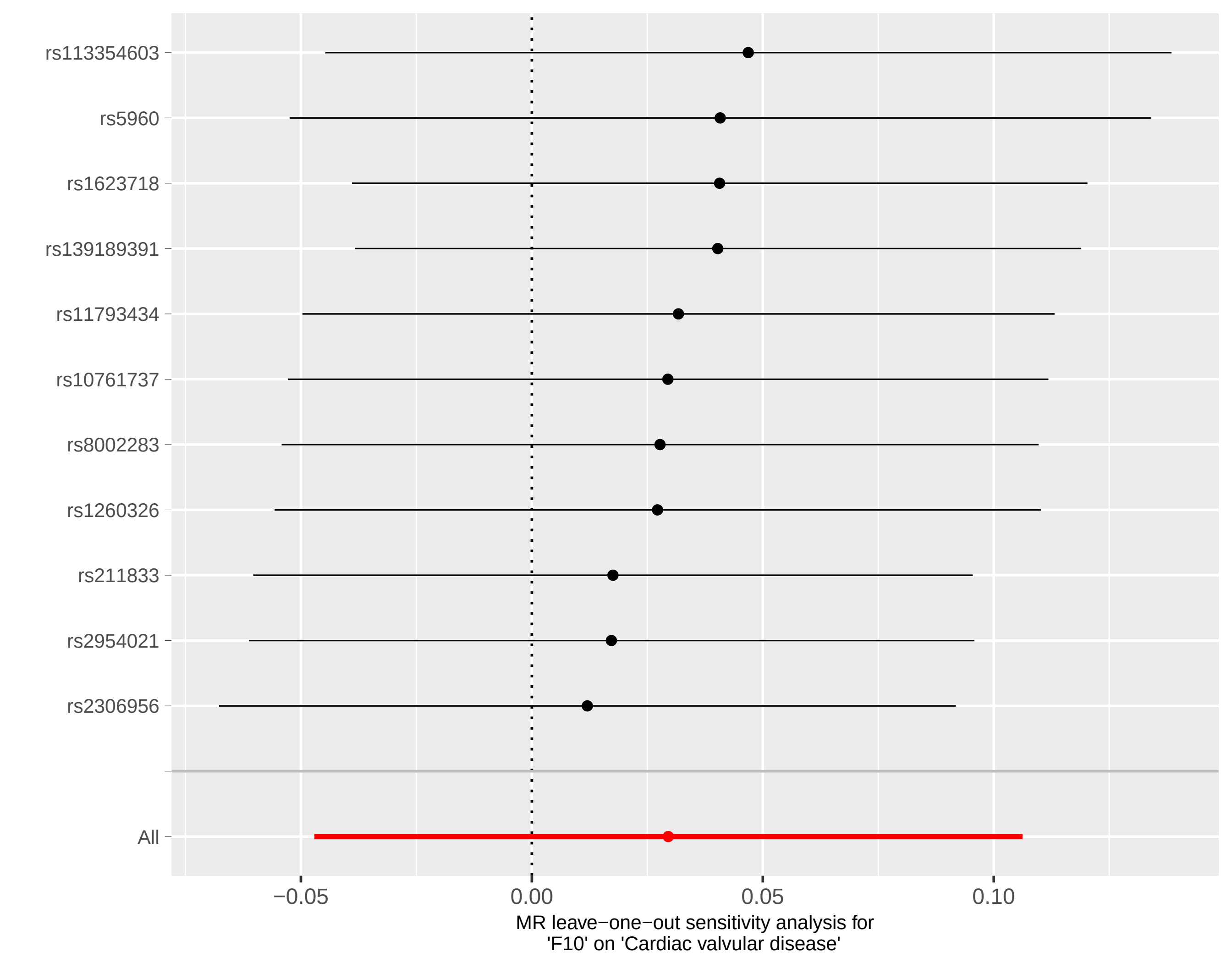

rs113354603
rs5960
rs1623718
rs139189391
rs11793434
rs10761737
rs8002283
rs1260326
rs211833
rs2954021
rs2306956
All
−0.05
0.00
0.05
0.10
MR leave−one−out sensitivity analysis for
'F10' on 'Cardiac valvular disease'

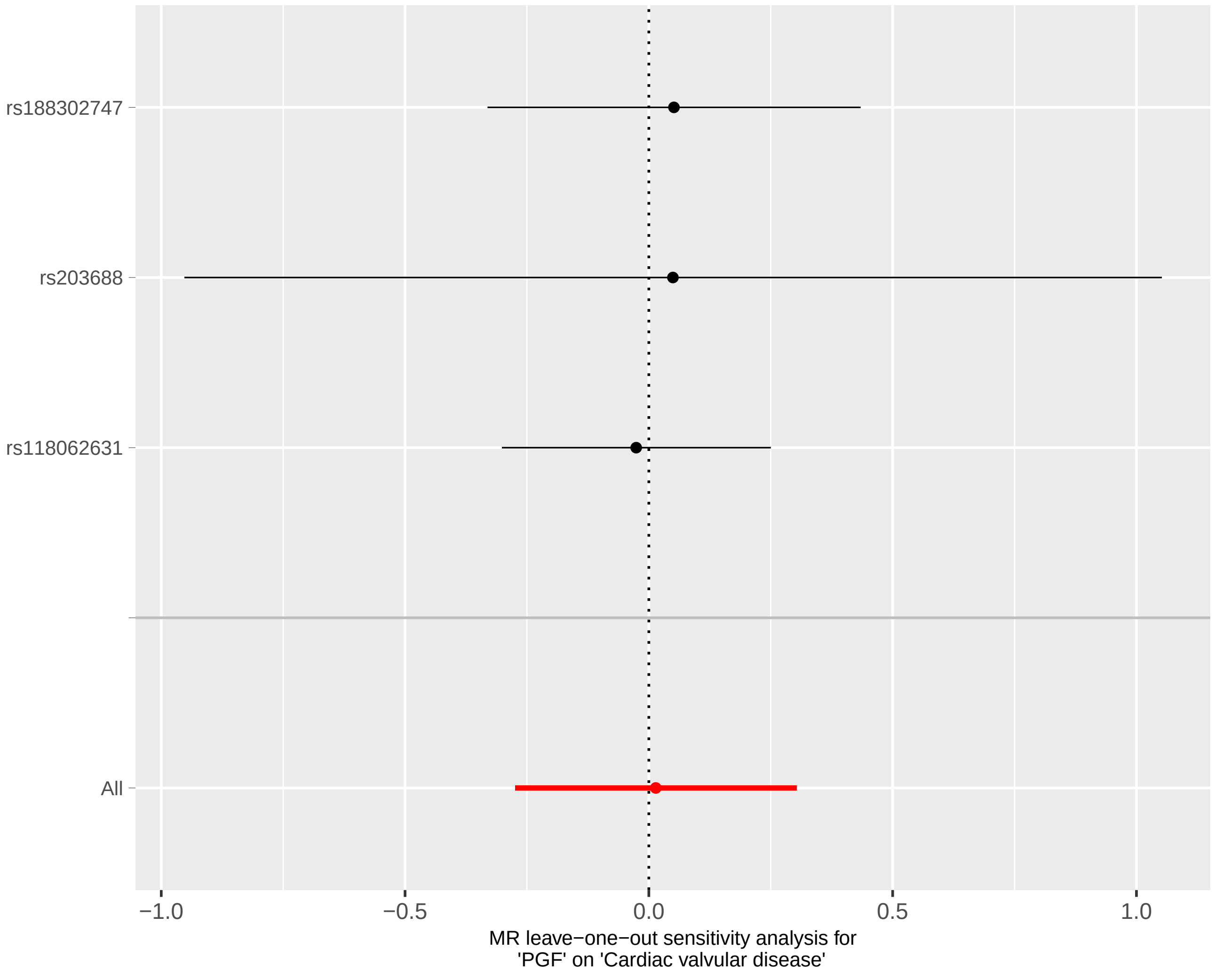

rs188302747
rs203688
rs118062631
All
−1.0
−0.5
0.0
0.5
1.0
MR leave−one−out sensitivity analysis for
'PGF' on 'Cardiac valvular disease'

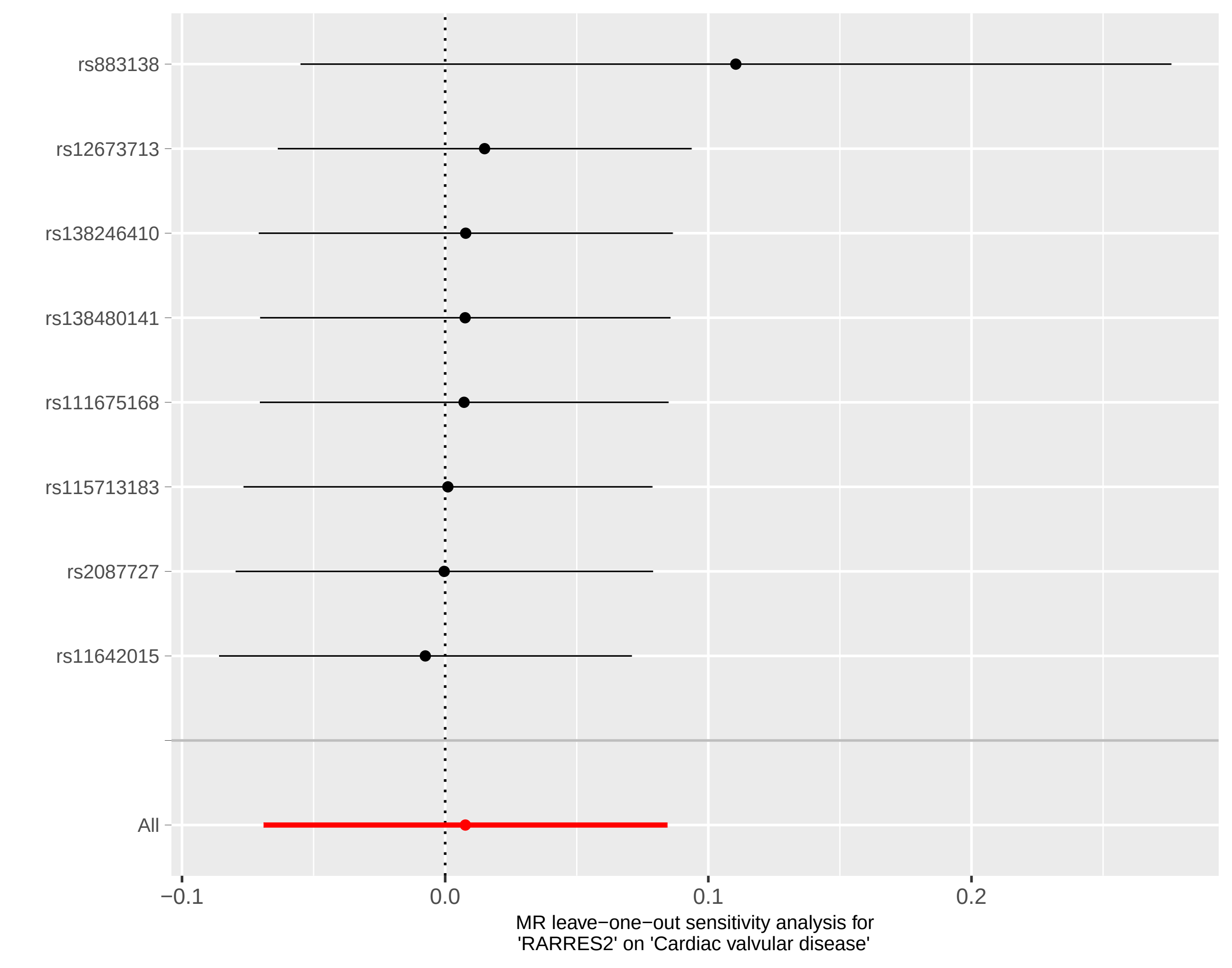
rs883138
rs12673713
rs138246410
rs138480141
rs111675168
rs115713183
rs2087727
rs11642015
All
−0.1
0.0
0.1
0.2
MR leave−one−out sensitivity analysis for
'RARRES2' on 'Cardiac valvular disease'

Insufficient number of SNPs

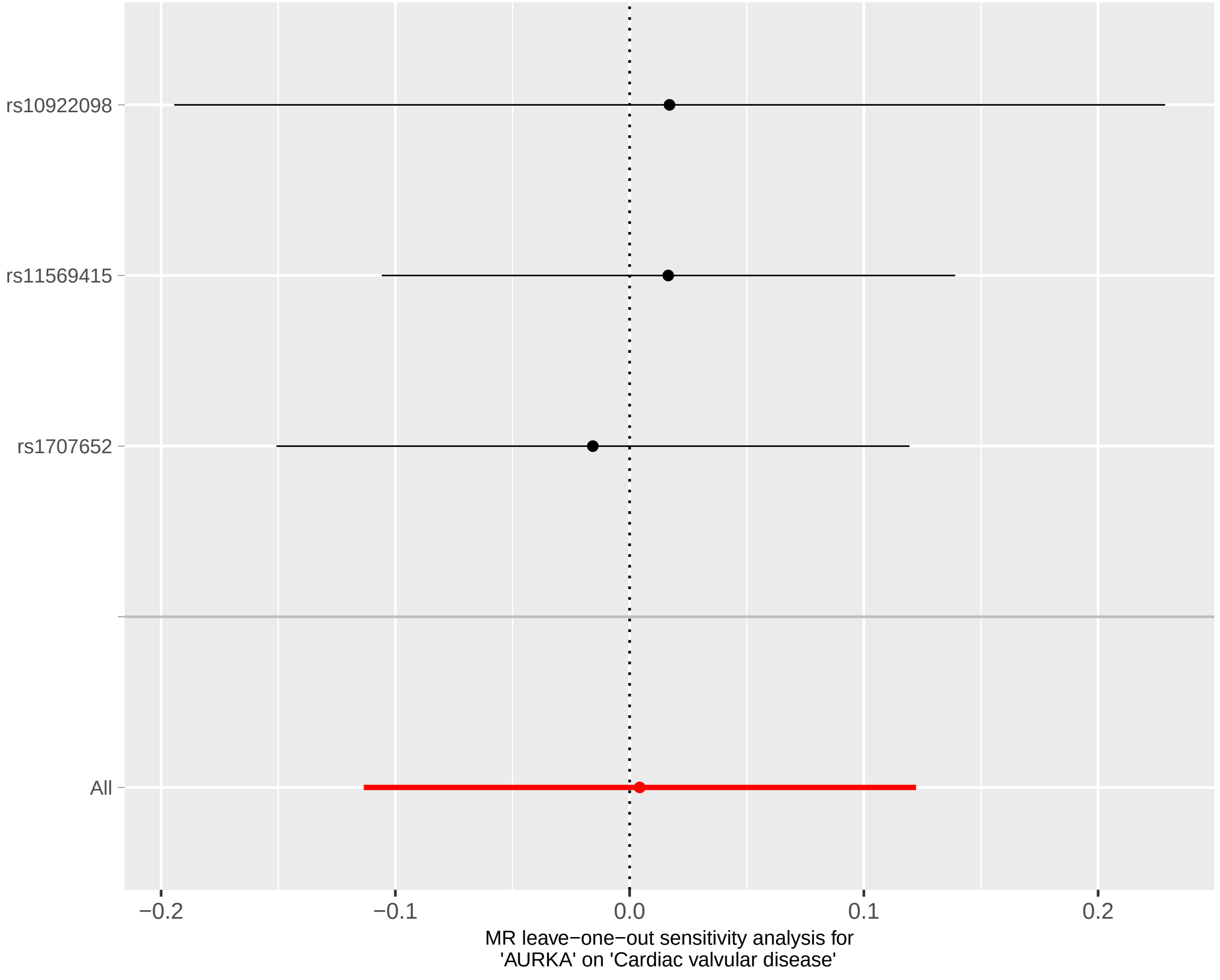
rs10922098
rs11569415
rs1707652
All
−0.2
−0.1
0.0
0.1
0.2
MR leave−one−out sensitivity analysis for
'AURKA' on 'Cardiac valvular disease'

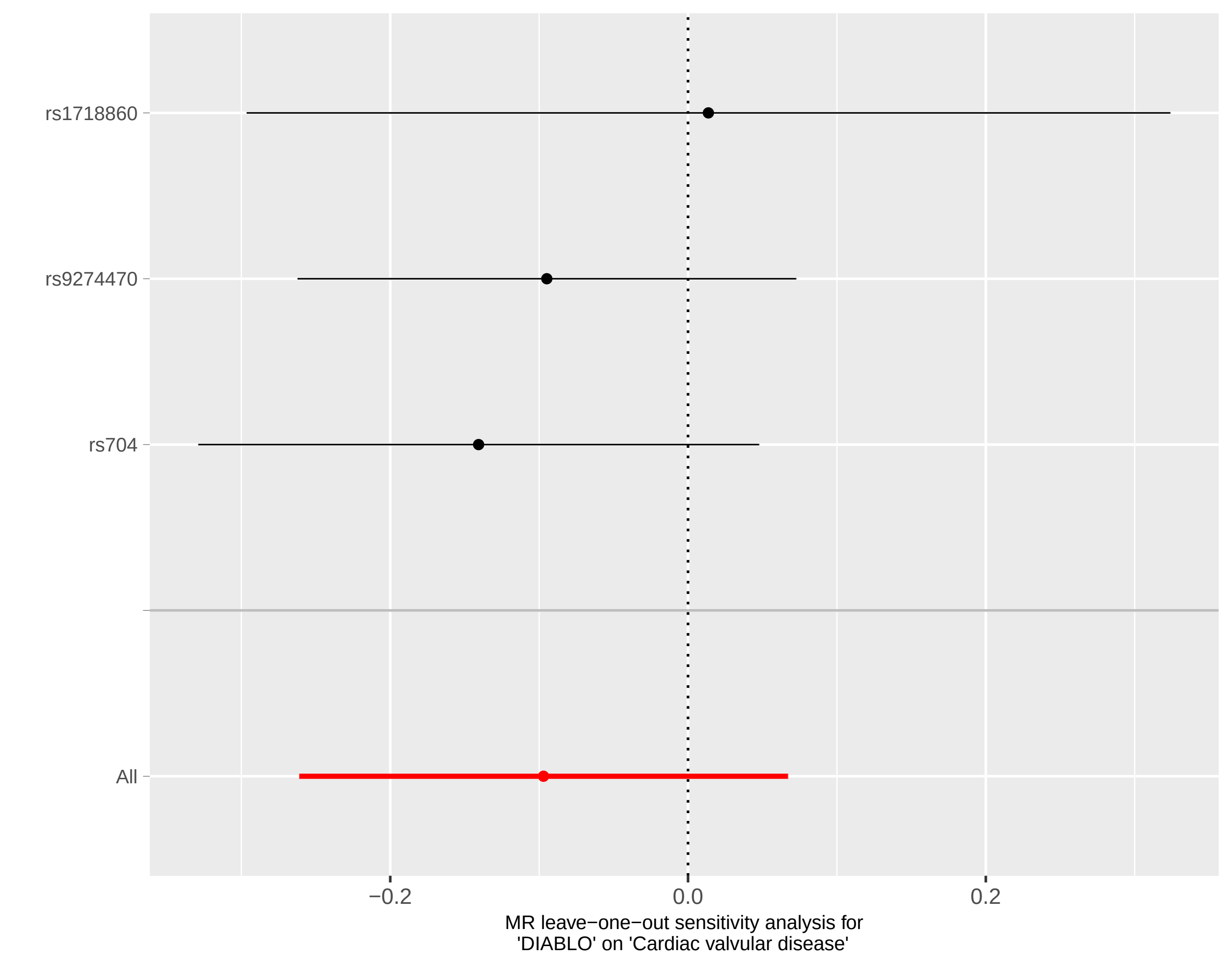
rs1718860
rs9274470
rs704
All
−0.2
0.0
0.2
MR leave−one−out sensitivity analysis for
'DIABLO' on 'Cardiac valvular disease'

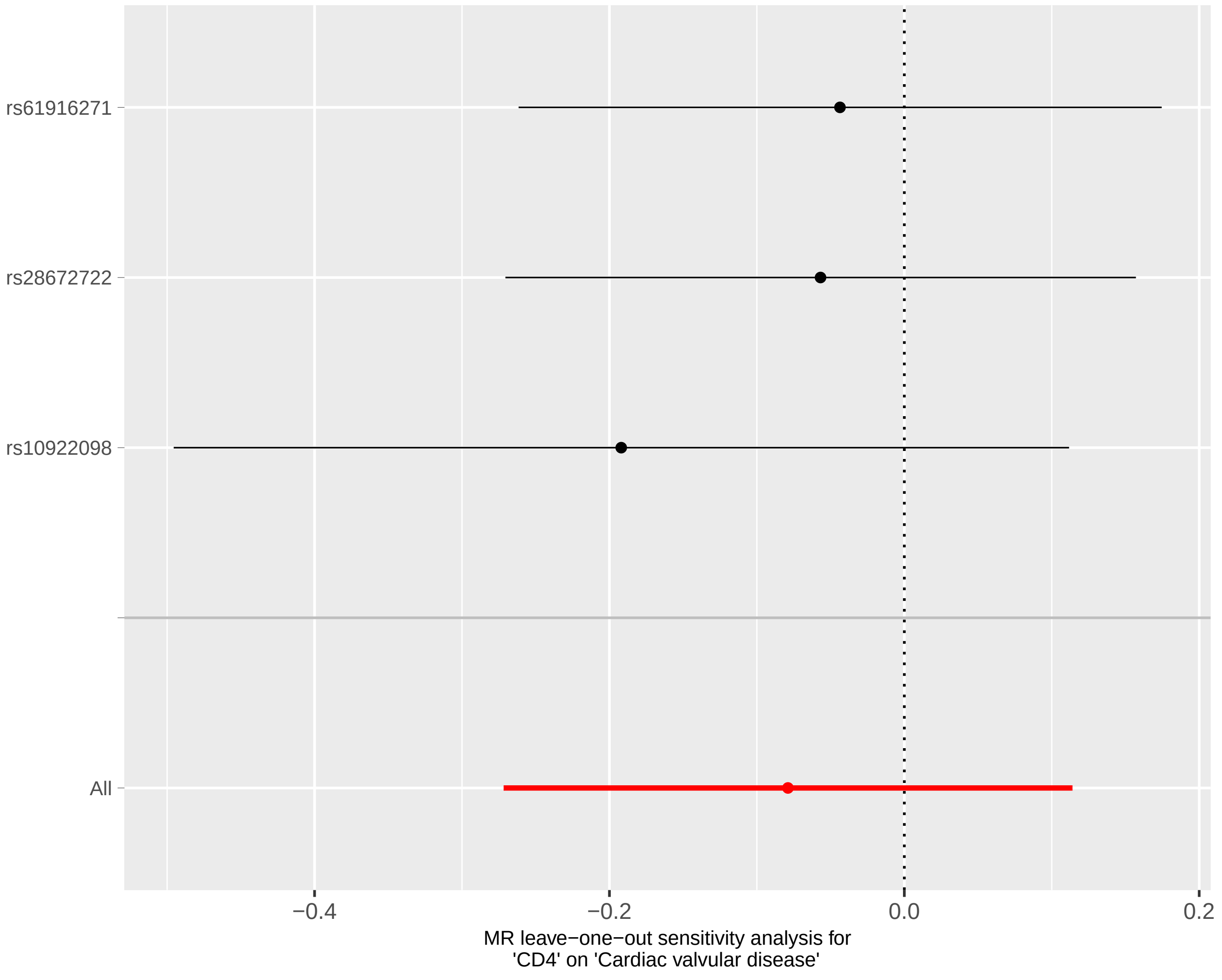
rs61916271
rs28672722
rs10922098
All
−0.4
−0.2
0.0
0.2
MR leave−one−out sensitivity analysis for
'CD4' on 'Cardiac valvular disease'

Insufficient number of SNPs

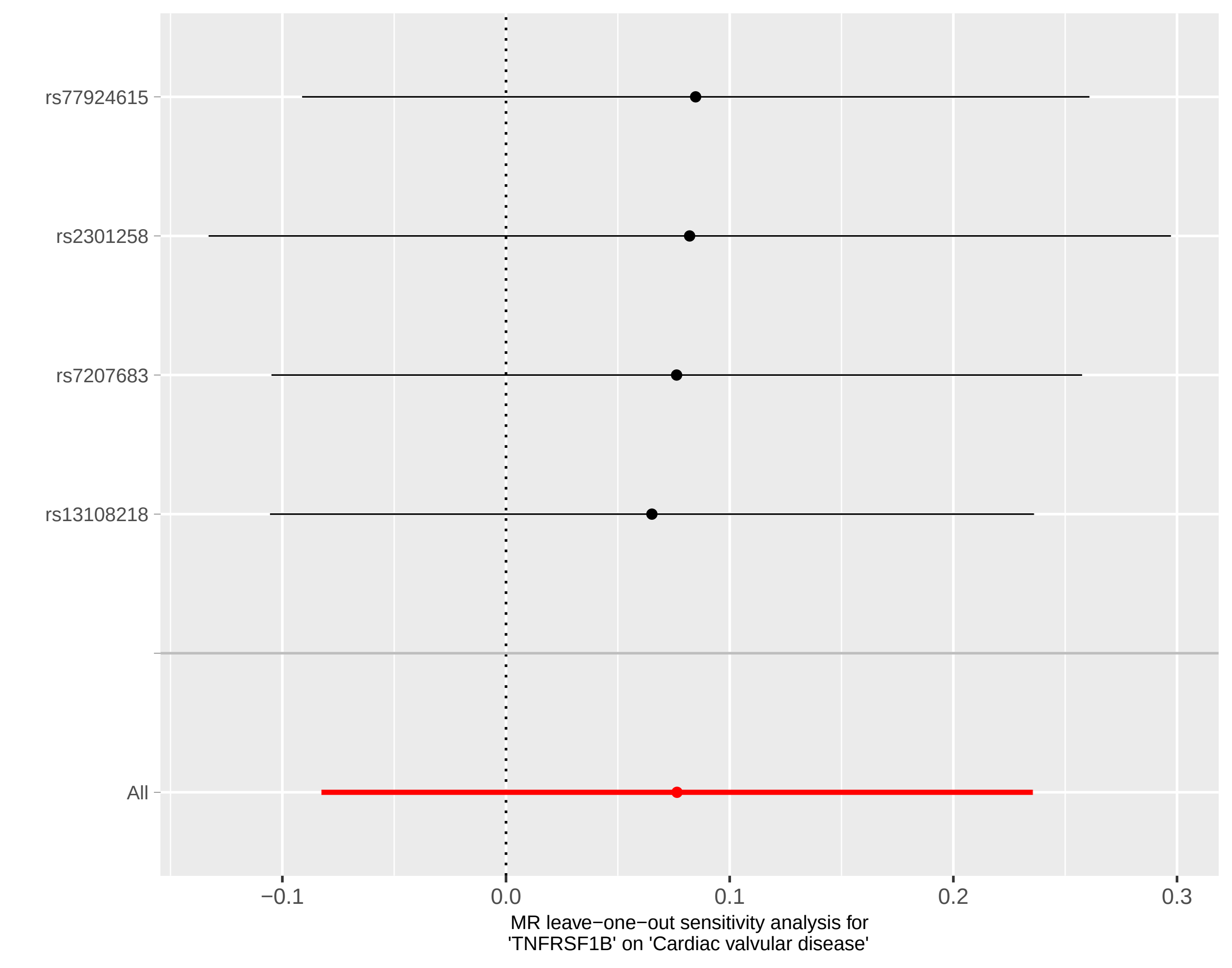
rs77924615
rs2301258
rs7207683
rs13108218
All
−0.1
0.0
0.1
0.2
0.3
MR leave−one−out sensitivity analysis for
'TNFRSF1B' on 'Cardiac valvular disease'

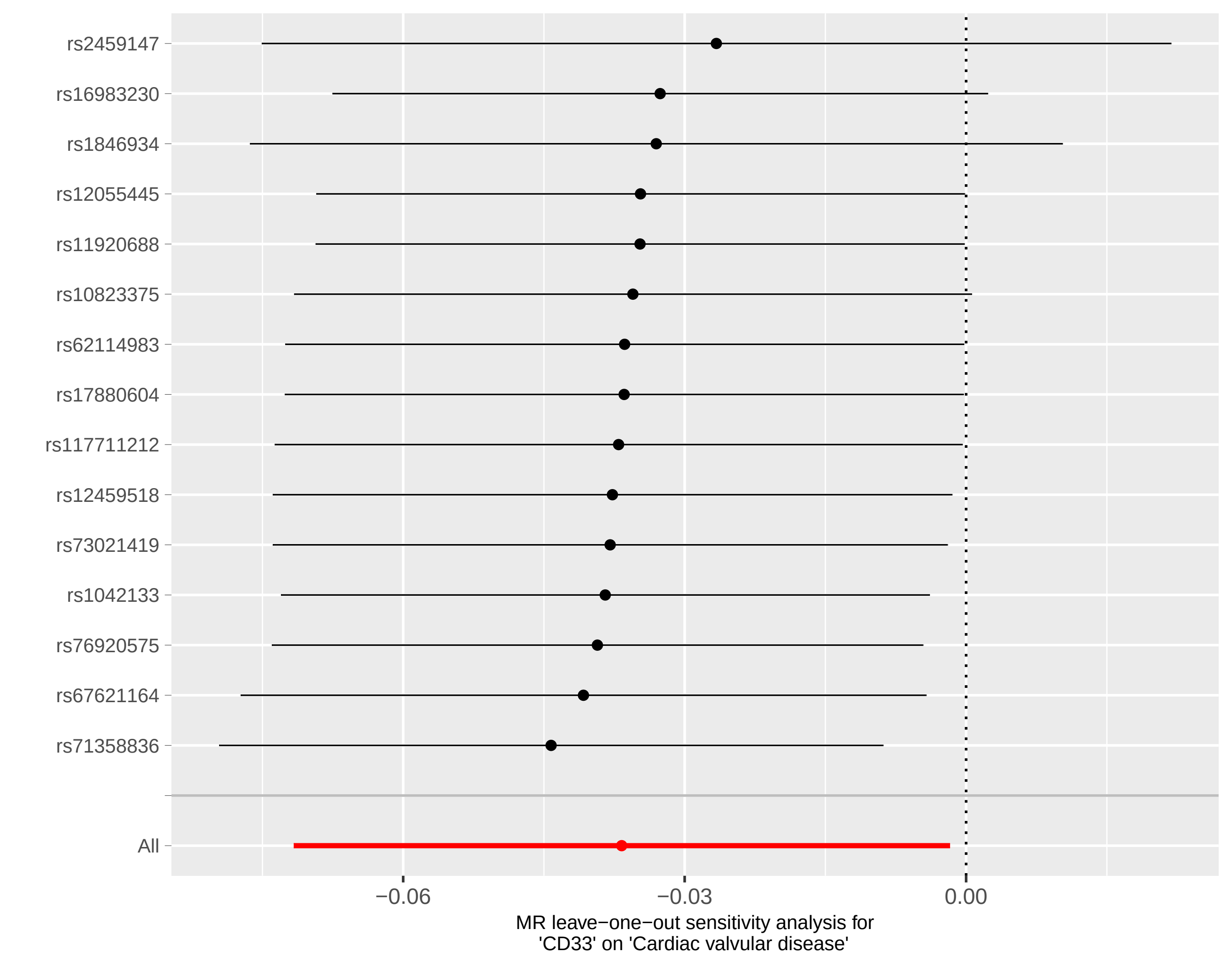

rs2459147
rs16983230
rs1846934
rs12055445
rs11920688
rs10823375
rs62114983
rs17880604
rs117711212
rs12459518
rs73021419
rs1042133
rs76920575
rs67621164
rs71358836
All
−0.06
−0.03
0.00
MR leave−one−out sensitivity analysis for
'CD33' on 'Cardiac valvular disease'

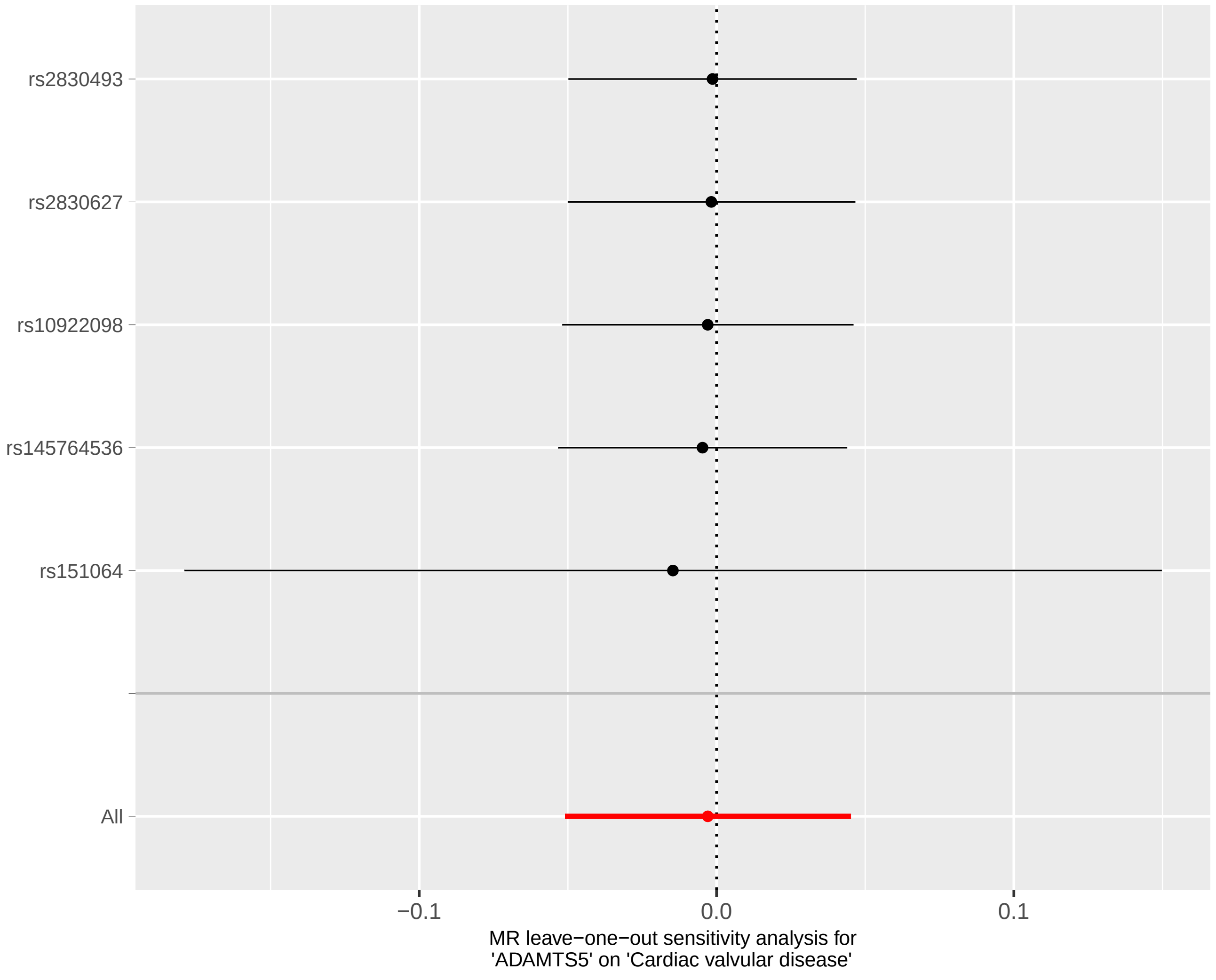
rs2830493
rs2830627
rs10922098
rs145764536
rs151064
All
−0.1
0.0
0.1
MR leave−one−out sensitivity analysis for
'ADAMTS5' on 'Cardiac valvular disease'

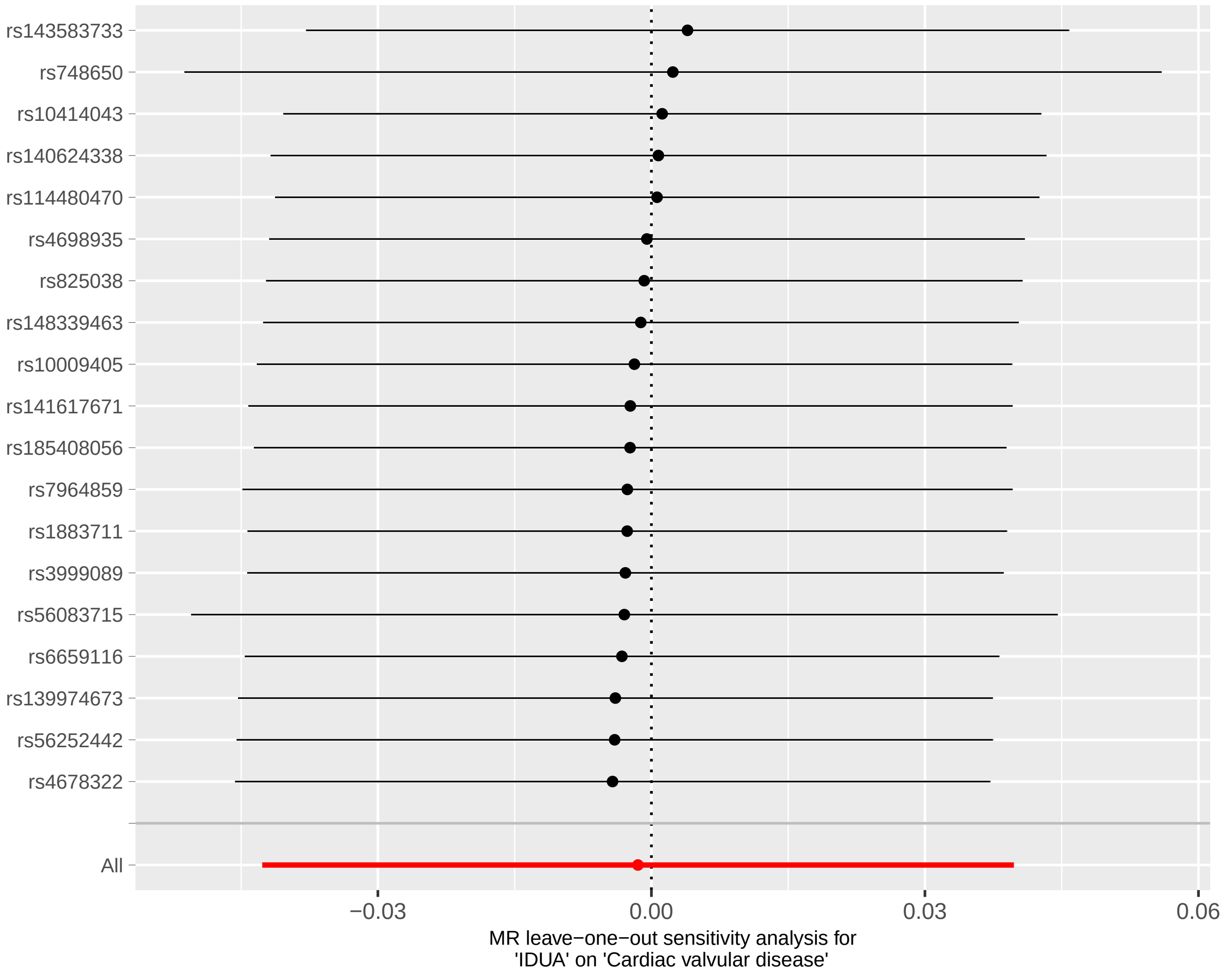
rs143583733
rs748650
rs10414043
rs140624338
rs114480470
rs4698935
rs825038
rs148339463
rs10009405
rs141617671
rs185408056
rs7964859
rs1883711
rs3999089
rs56083715
rs6659116
rs139974673
rs56252442
rs4678322
All
−0.03
0.00
0.03
0.06
MR leave−one−out sensitivity analysis for
'IDUA' on 'Cardiac valvular disease'

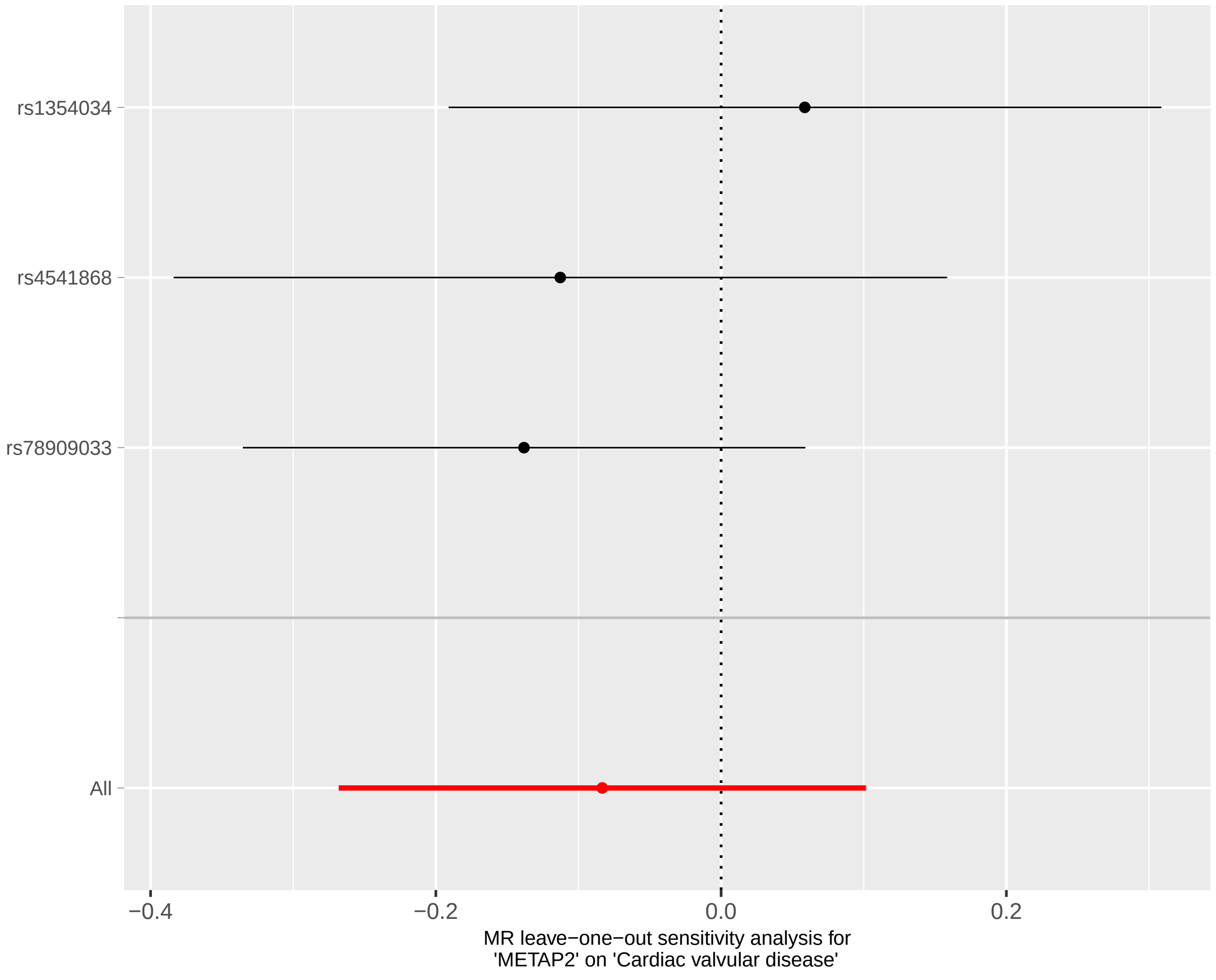
rs1354034
rs4541868
rs78909033
All
−0.4
−0.2
0.0
0.2
MR leave−one−out sensitivity analysis for
'METAP2' on 'Cardiac valvular disease'

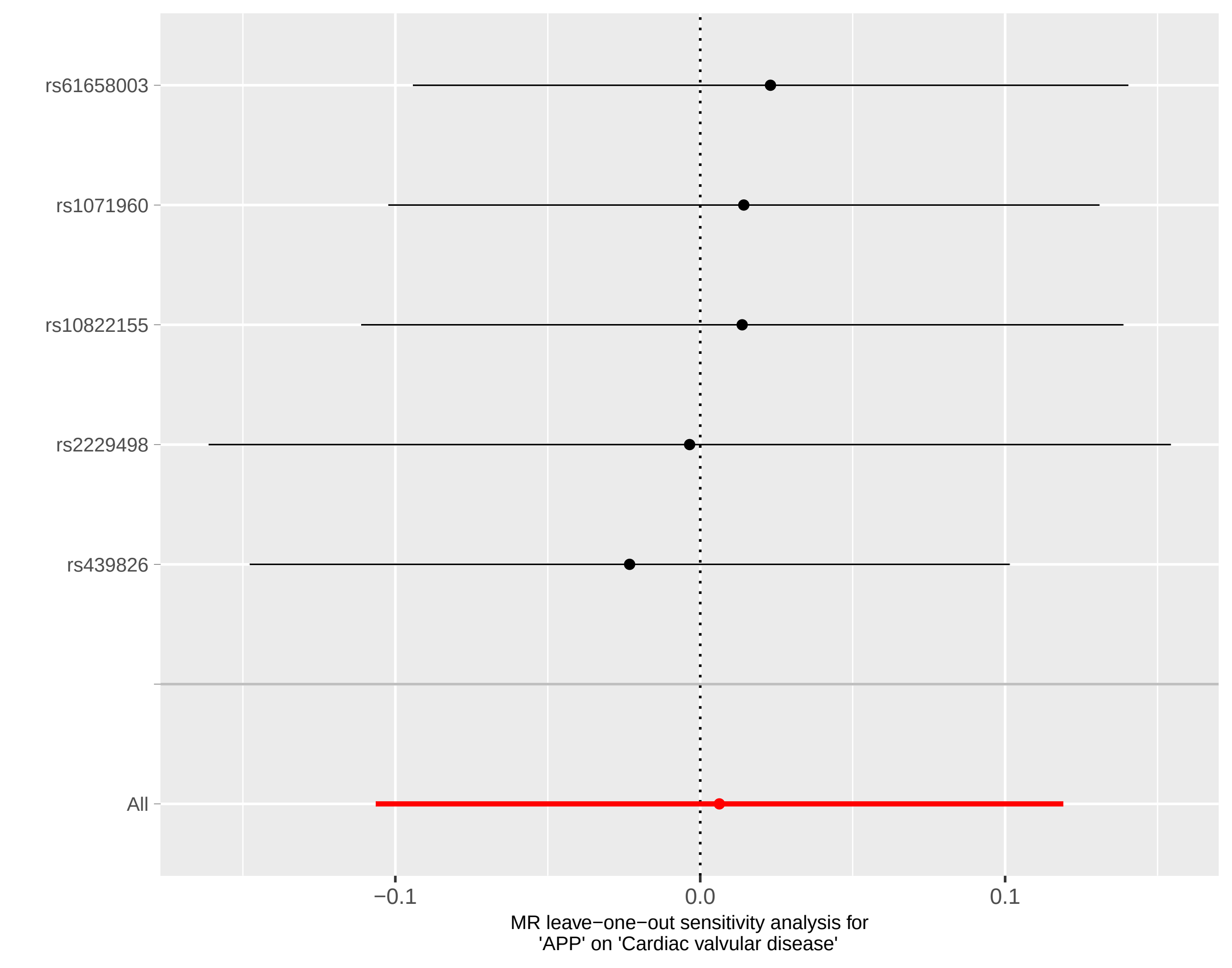

rs61658003
rs1071960
rs10822155
rs2229498
rs439826
All
−0.1
0.0
0.1
MR leave−one−out sensitivity analysis for
'APP' on 'Cardiac valvular disease'

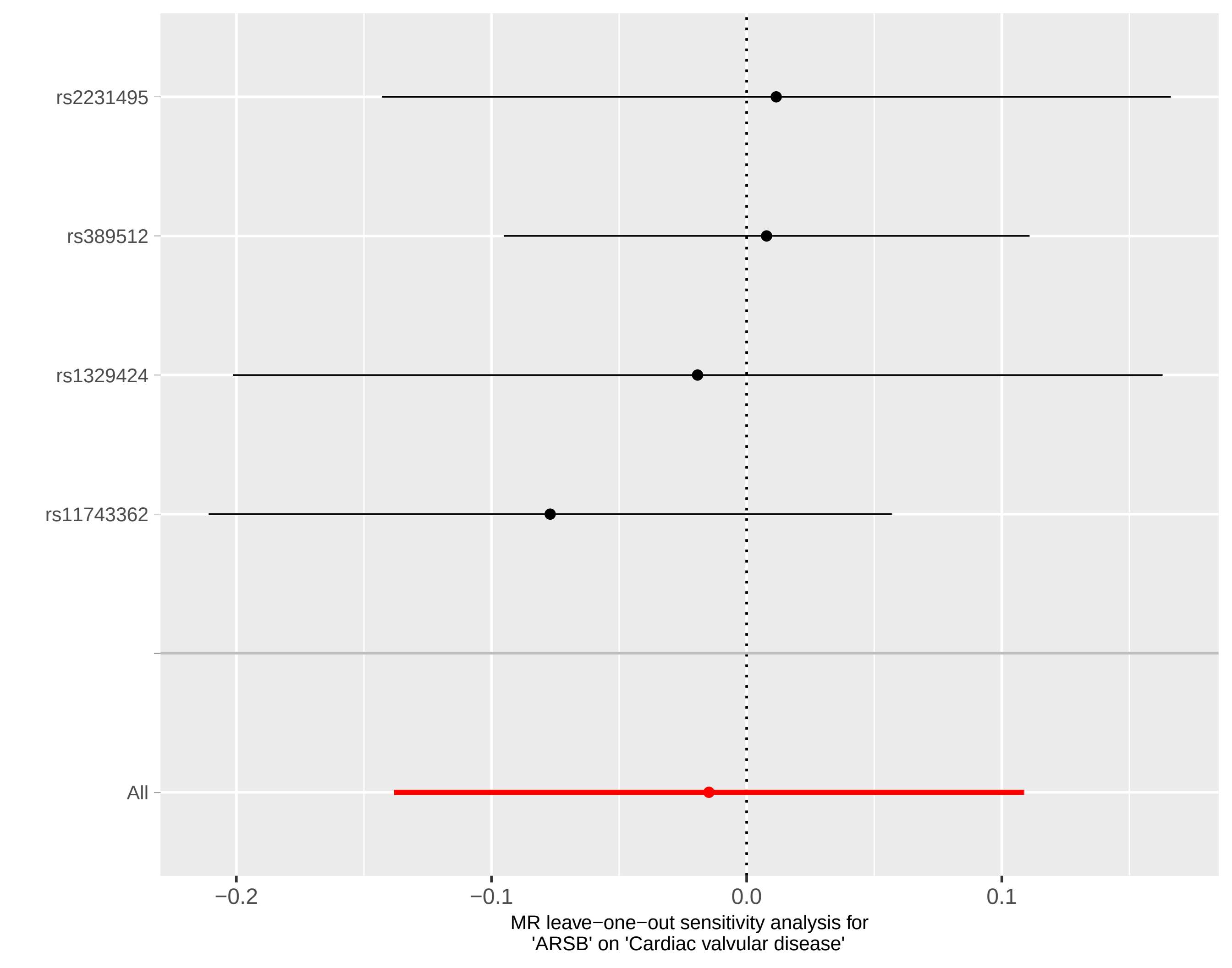
rs2231495
rs389512
rs1329424
rs11743362
All
−0.2
−0.1
0.0
0.1
MR leave−one−out sensitivity analysis for
'ARSB' on 'Cardiac valvular disease'

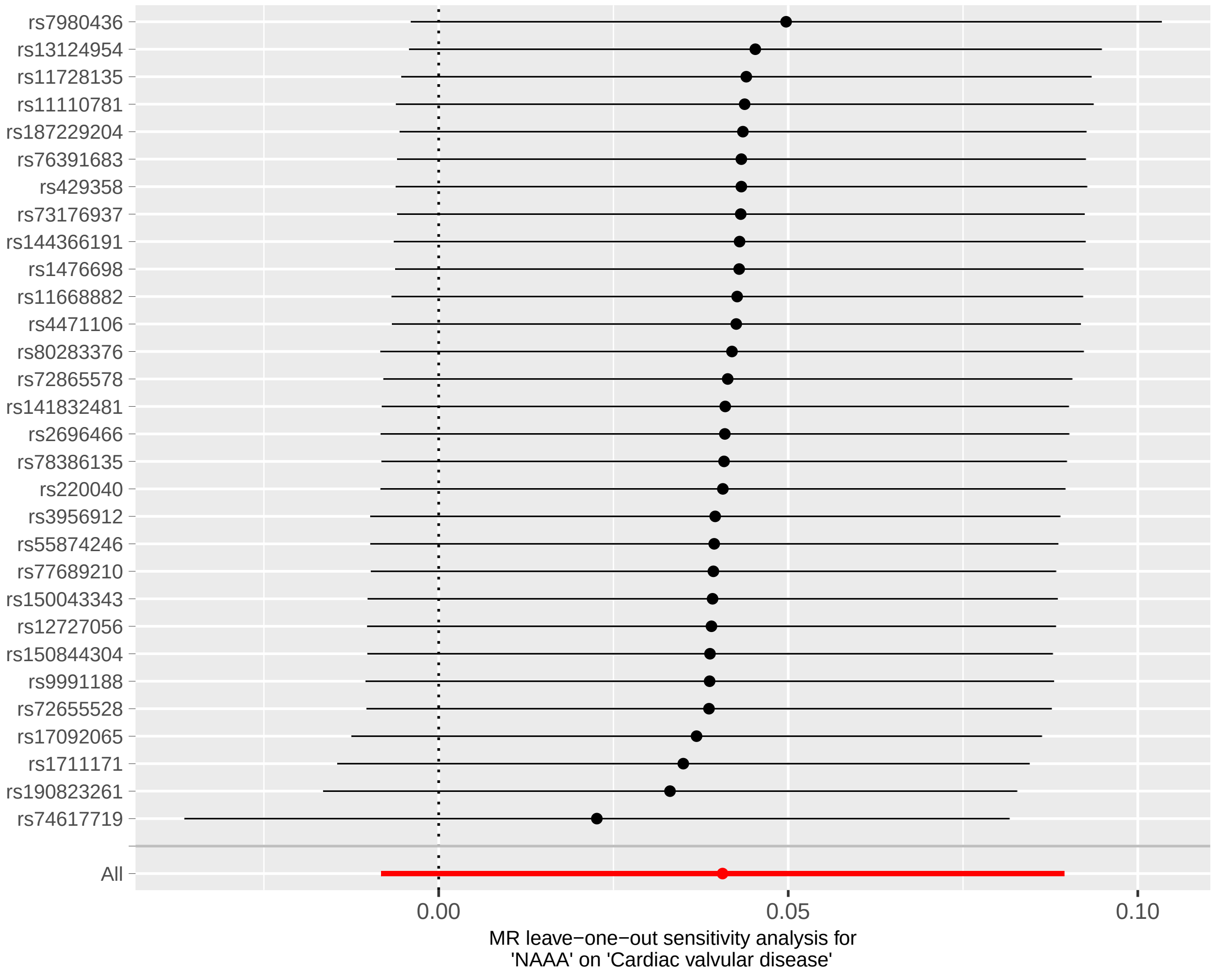
rs7980436
rs13124954
rs11728135
rs11110781
rs187229204
rs76391683
rs429358
rs73176937
rs144366191
rs1476698
rs11668882
rs4471106
rs80283376
rs72865578
rs141832481
rs2696466
rs78386135
rs220040
rs3956912
rs55874246
rs77689210
rs150043343
rs12727056
rs150844304
rs9991188
rs72655528
rs17092065
rs1711171
rs190823261
rs74617719
All
0.00
0.05
0.10
MR leave−one−out sensitivity analysis for
'NAAA' on 'Cardiac valvular disease'

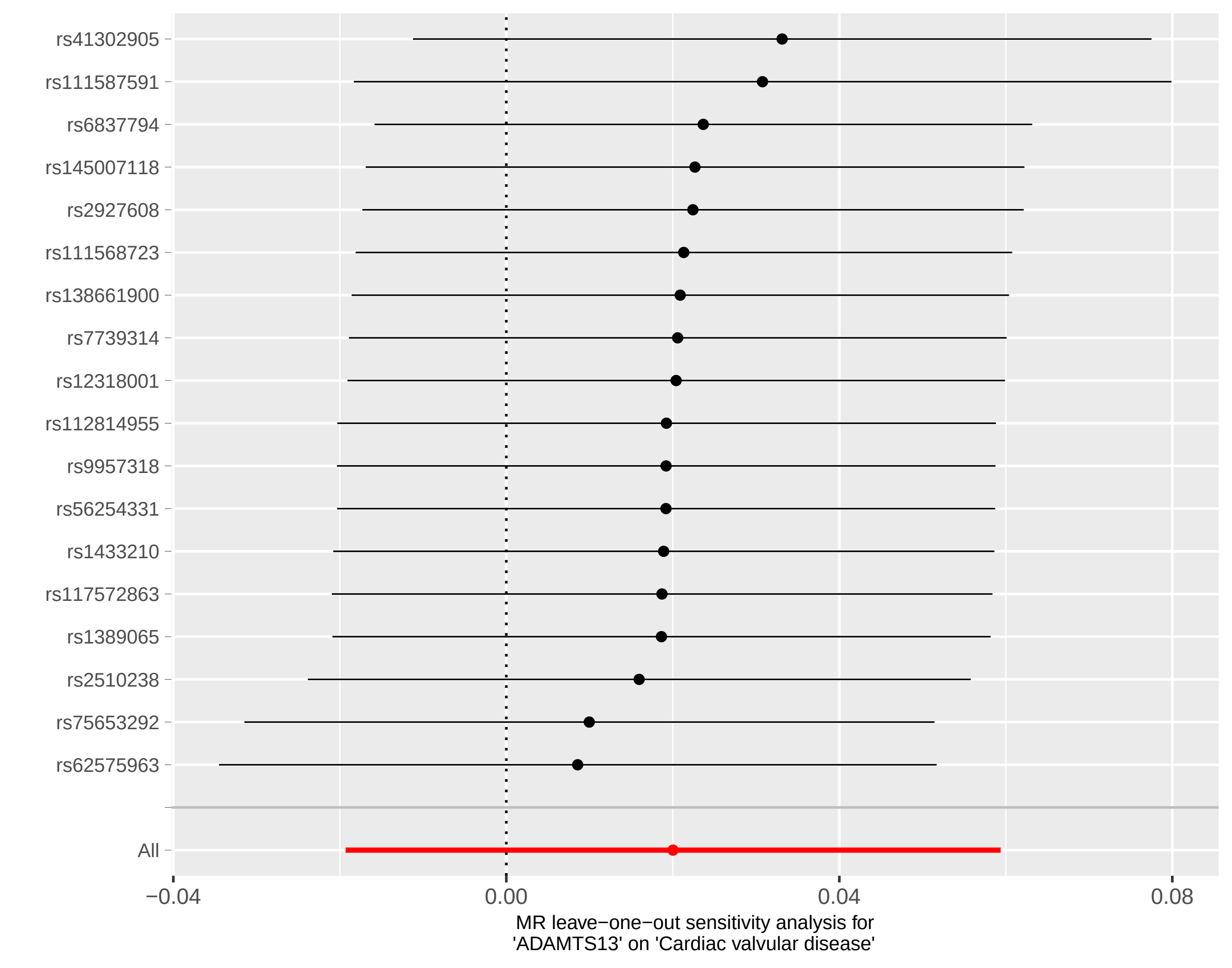

rs41302905
rs111587591
rs6837794
rs145007118
rs2927608
rs111568723
rs138661900
rs7739314
rs12318001
rs112814955
rs9957318
rs56254331
rs1433210
rs117572863
rs1389065
rs2510238
rs75653292
rs62575963
All
−0.04
0.00
0.04
0.08
MR leave−one−out sensitivity analysis for
'ADAMTS13' on 'Cardiac valvular disease'

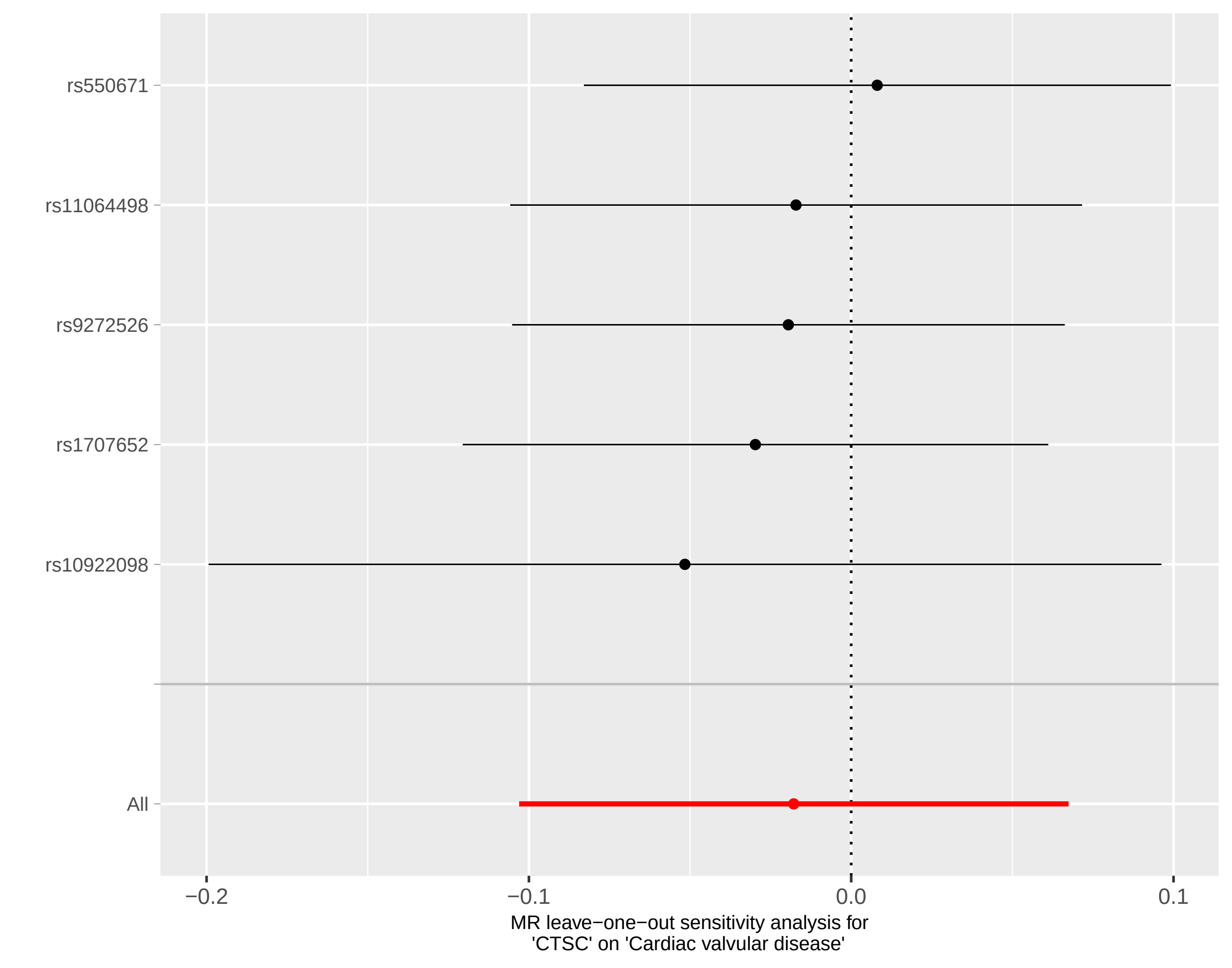
rs550671
rs11064498
rs9272526
rs1707652
rs10922098
All
−0.2
−0.1
0.0
0.1
MR leave−one−out sensitivity analysis for
'CTSC' on 'Cardiac valvular disease'

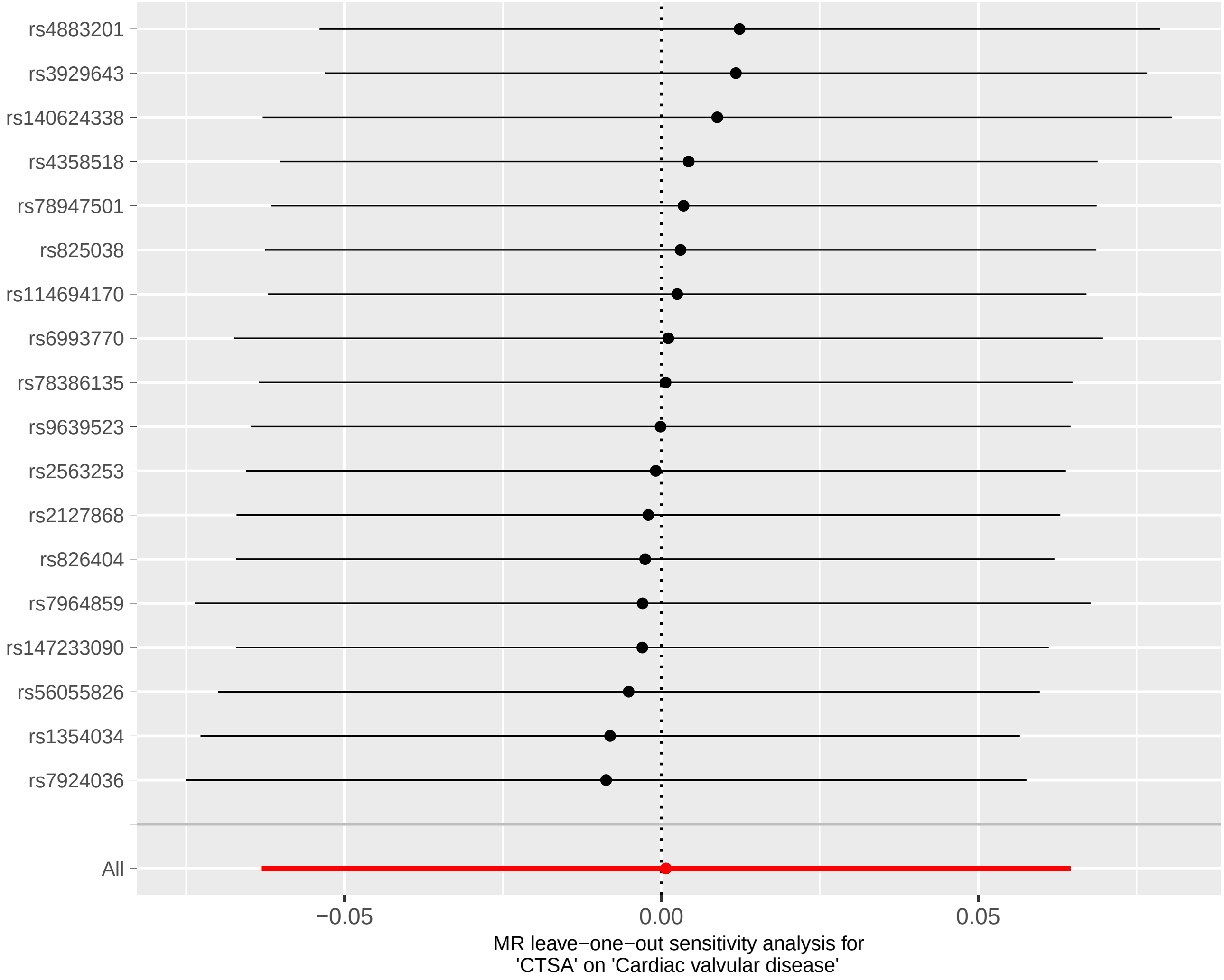
rs4883201
rs3929643
rs140624338
rs4358518
rs78947501
rs825038
rs114694170
rs6993770
rs78386135
rs9639523
rs2563253
rs2127868
rs826404
rs7964859
rs147233090
rs56055826
rs1354034
rs7924036
All
−0.05
0.00
0.05
MR leave−one−out sensitivity analysis for
'CTSA' on 'Cardiac valvular disease'

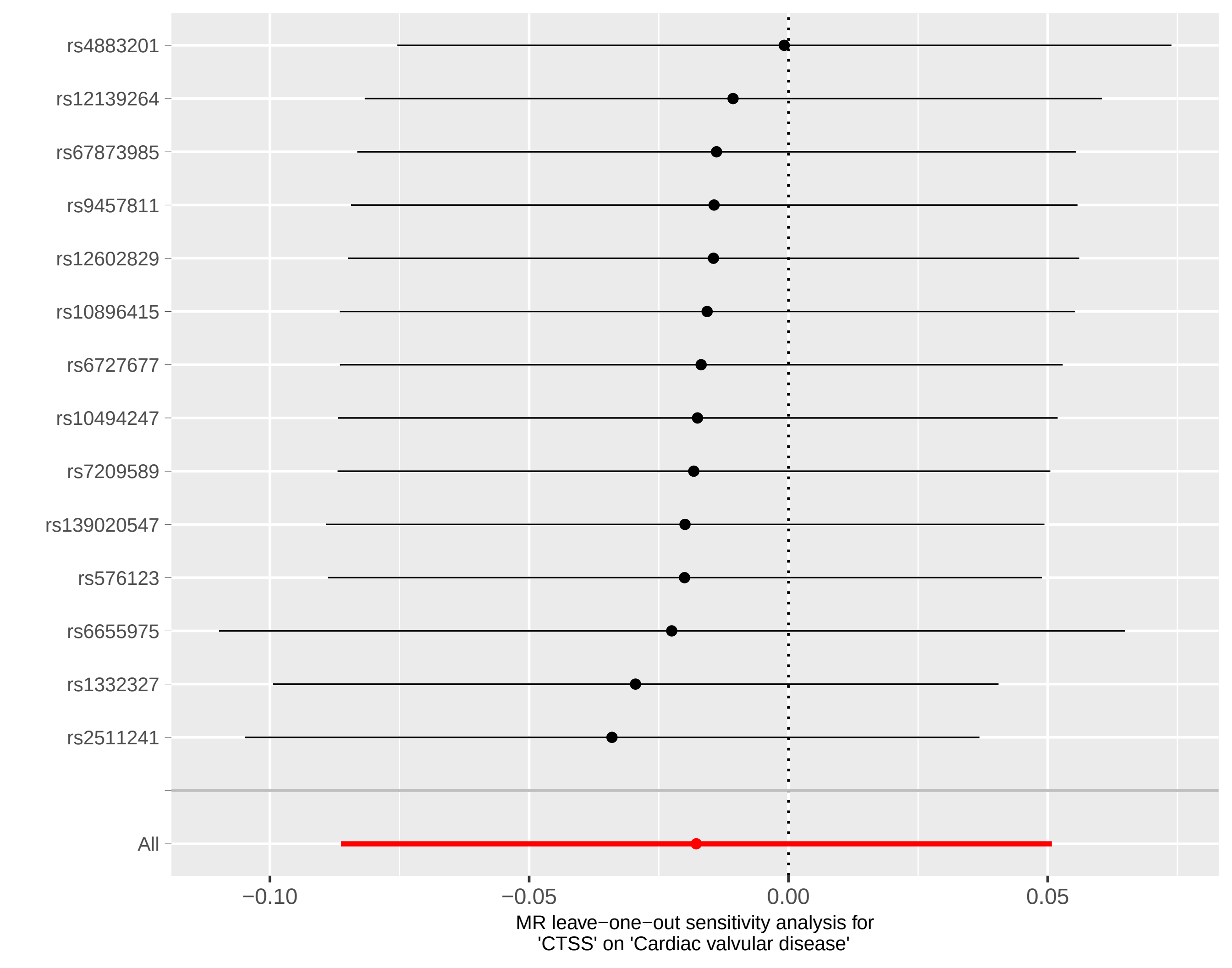

rs4883201
rs12139264
rs67873985
rs9457811
rs12602829
rs10896415
rs6727677
rs10494247
rs7209589
rs139020547
rs576123
rs6655975
rs1332327
rs2511241
All
−0.10
−0.05
0.00
0.05
MR leave−one−out sensitivity analysis for
'CTSS' on 'Cardiac valvular disease'

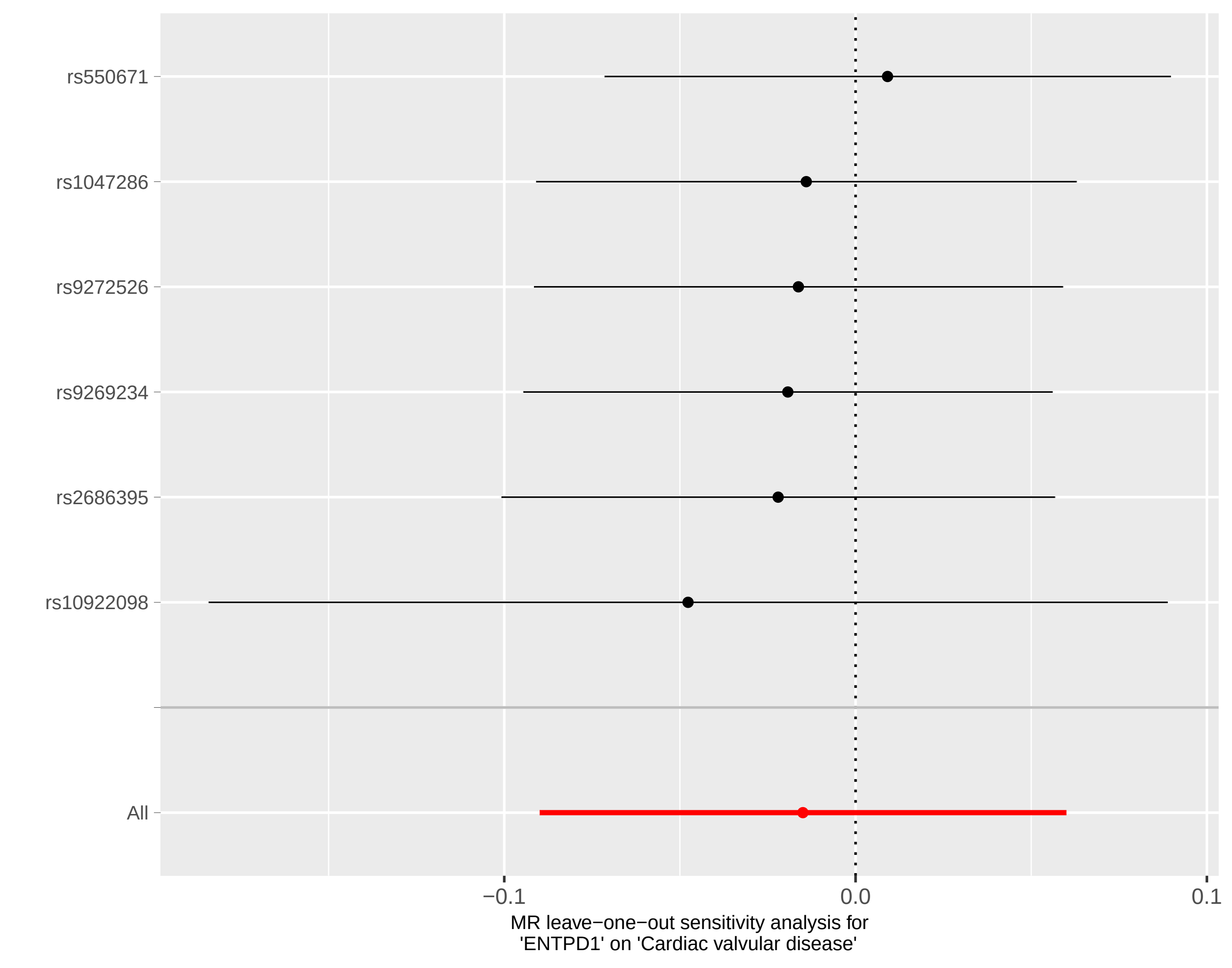
rs550671
rs1047286
rs9272526
rs9269234
rs2686395
rs10922098
All
−0.1
0.0
0.1
MR leave−one−out sensitivity analysis for
'ENTPD1' on 'Cardiac valvular disease'

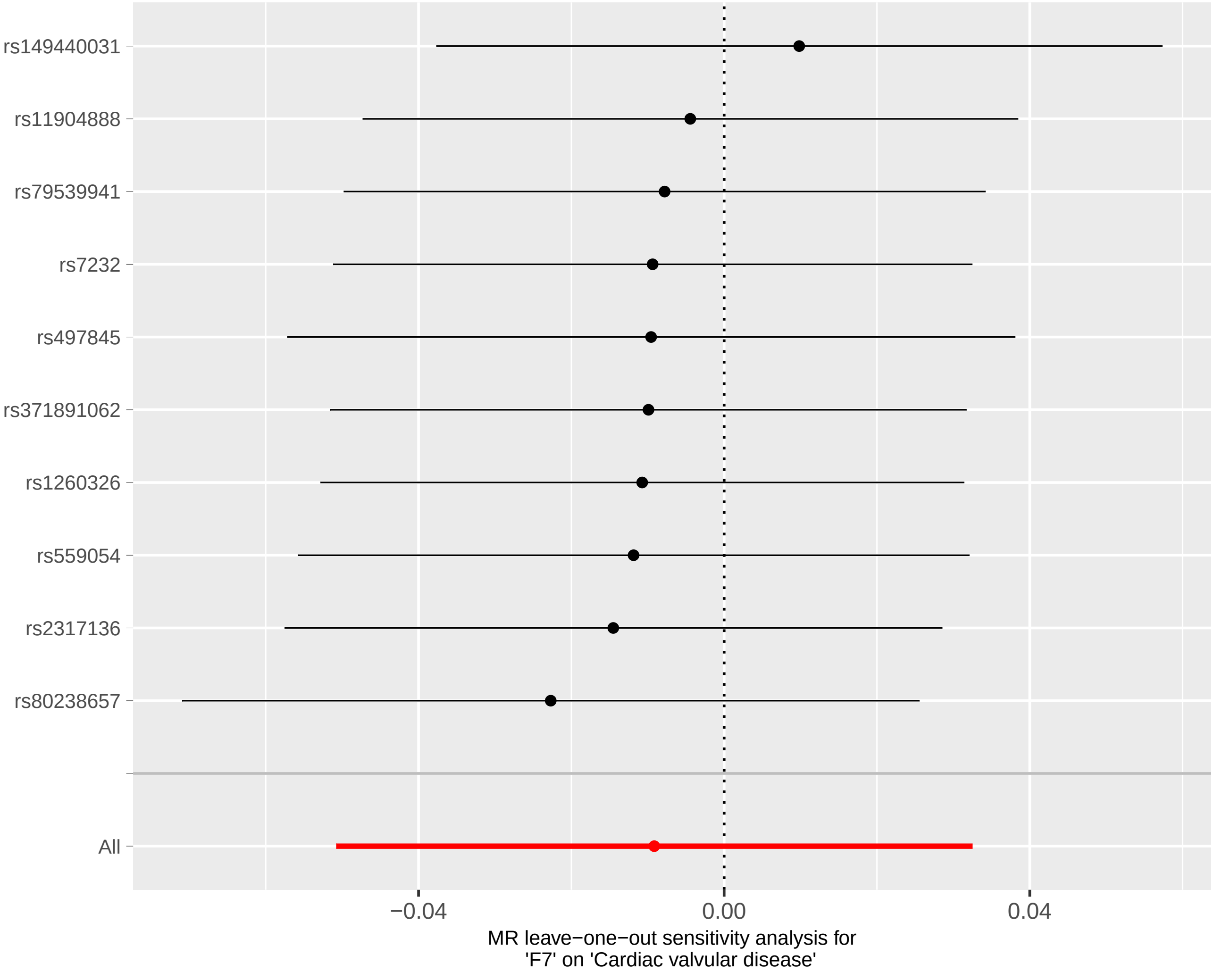

rs149440031
rs11904888
rs79539941
rs7232
rs497845
rs371891062
rs1260326
rs559054
rs2317136
rs80238657
All
−0.04
0.00
0.04
MR leave−one−out sensitivity analysis for
'F7' on 'Cardiac valvular disease'

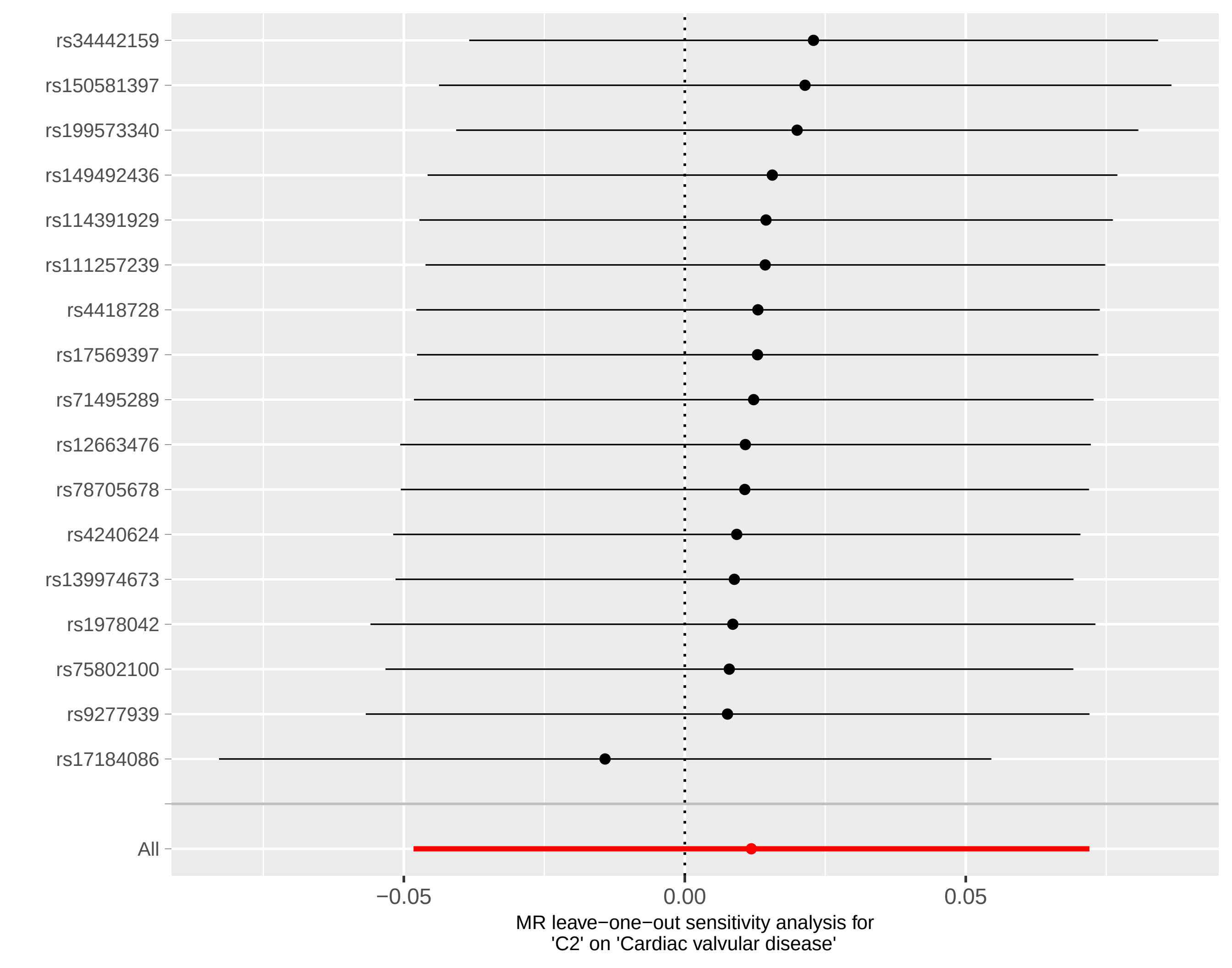

rs34442159
rs150581397
rs199573340
rs149492436
rs114391929
rs111257239
rs4418728
rs17569397
rs71495289
rs12663476
rs78705678
rs4240624
rs139974673
rs1978042
rs75802100
rs9277939
rs17184086
All
−0.05
0.00
0.05
MR leave−one−out sensitivity analysis for
'C2' on 'Cardiac valvular disease'

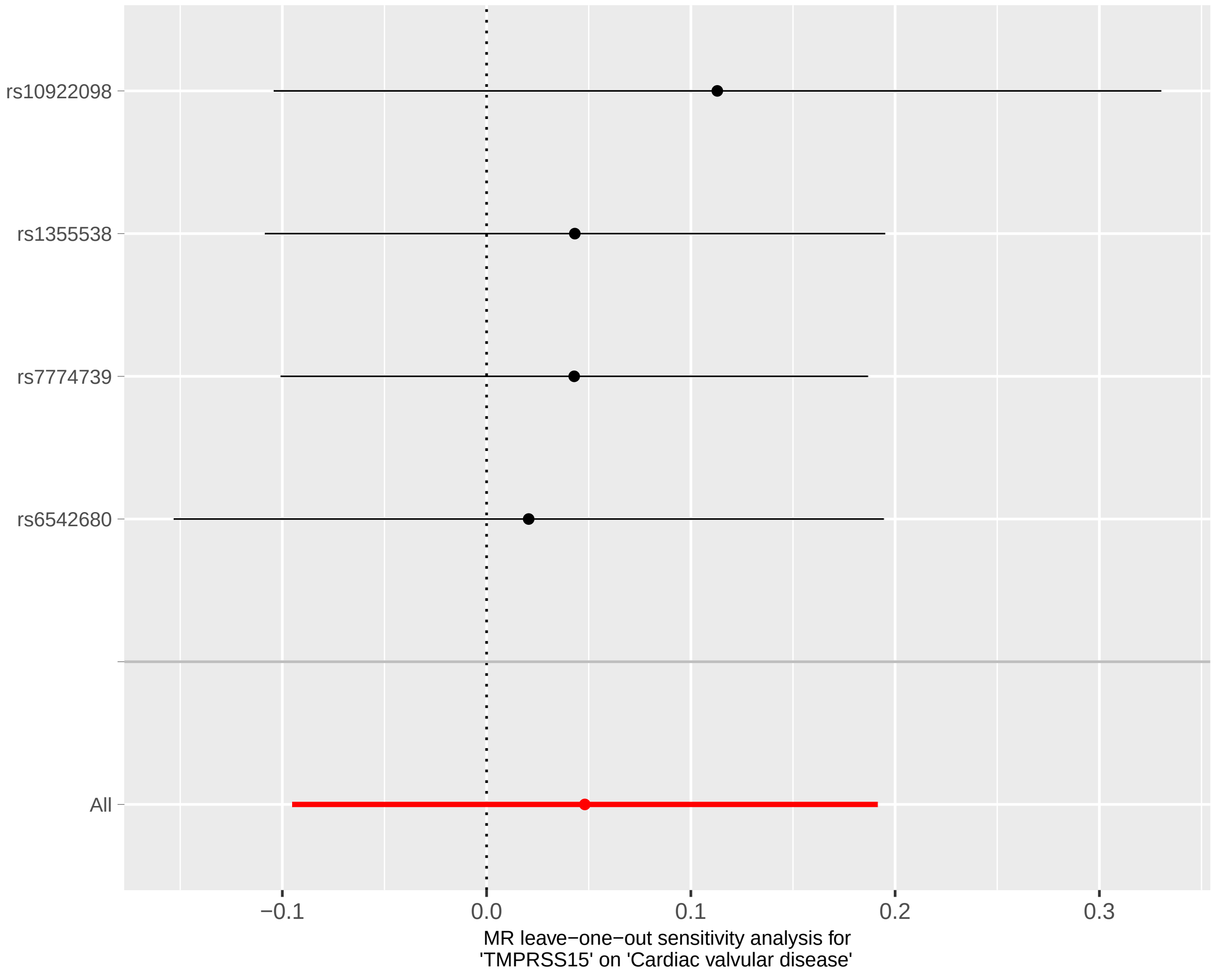
rs10922098
rs1355538
rs7774739
rs6542680
All
−0.1
0.0
0.1
0.2
0.3
MR leave−one−out sensitivity analysis for
'TMPRSS15' on 'Cardiac valvular disease'

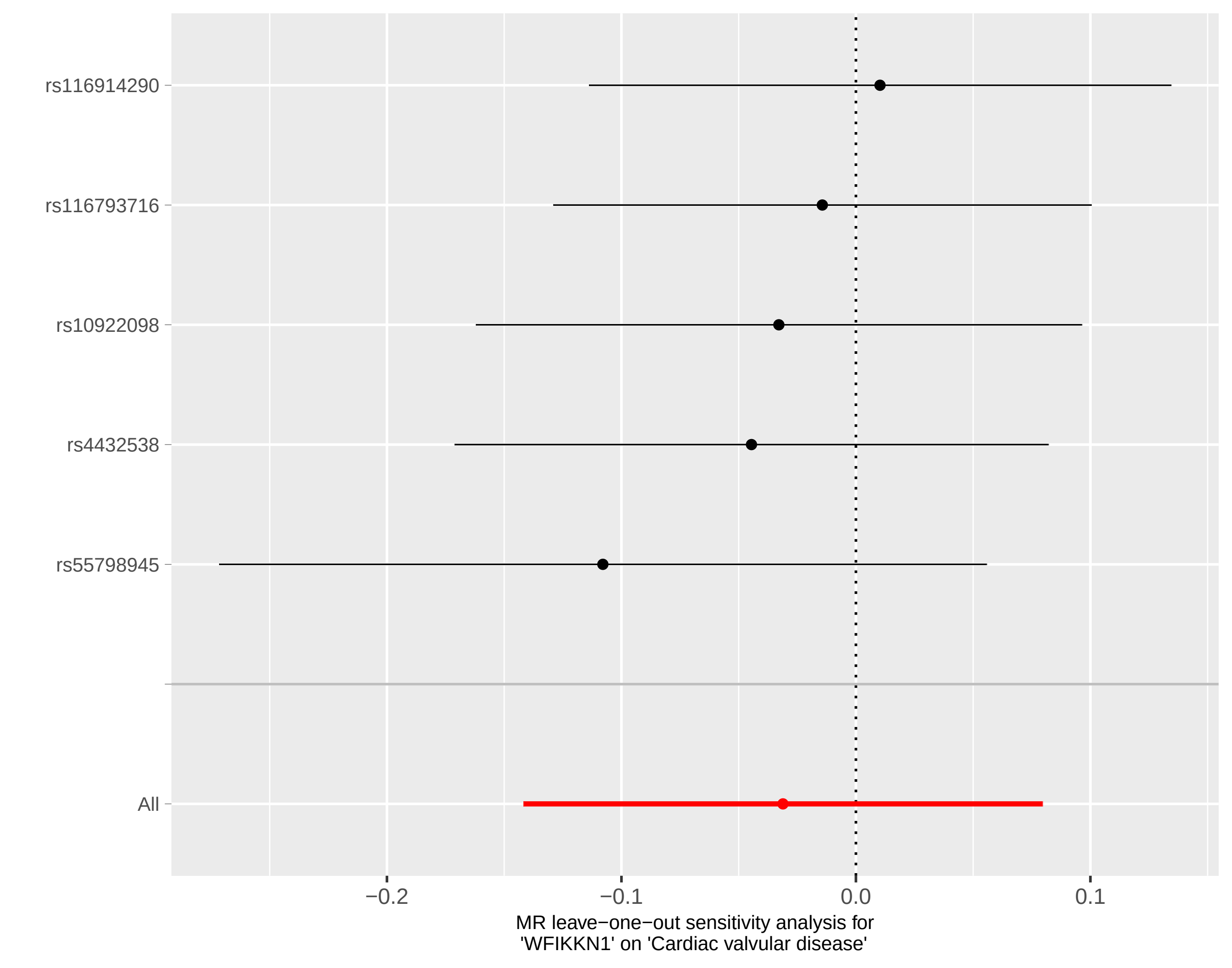
rs116914290
rs116793716
rs10922098
rs4432538
rs55798945
All
−0.2
−0.1
0.0
0.1
MR leave−one−out sensitivity analysis for
'WFIKKN1' on 'Cardiac valvular disease'

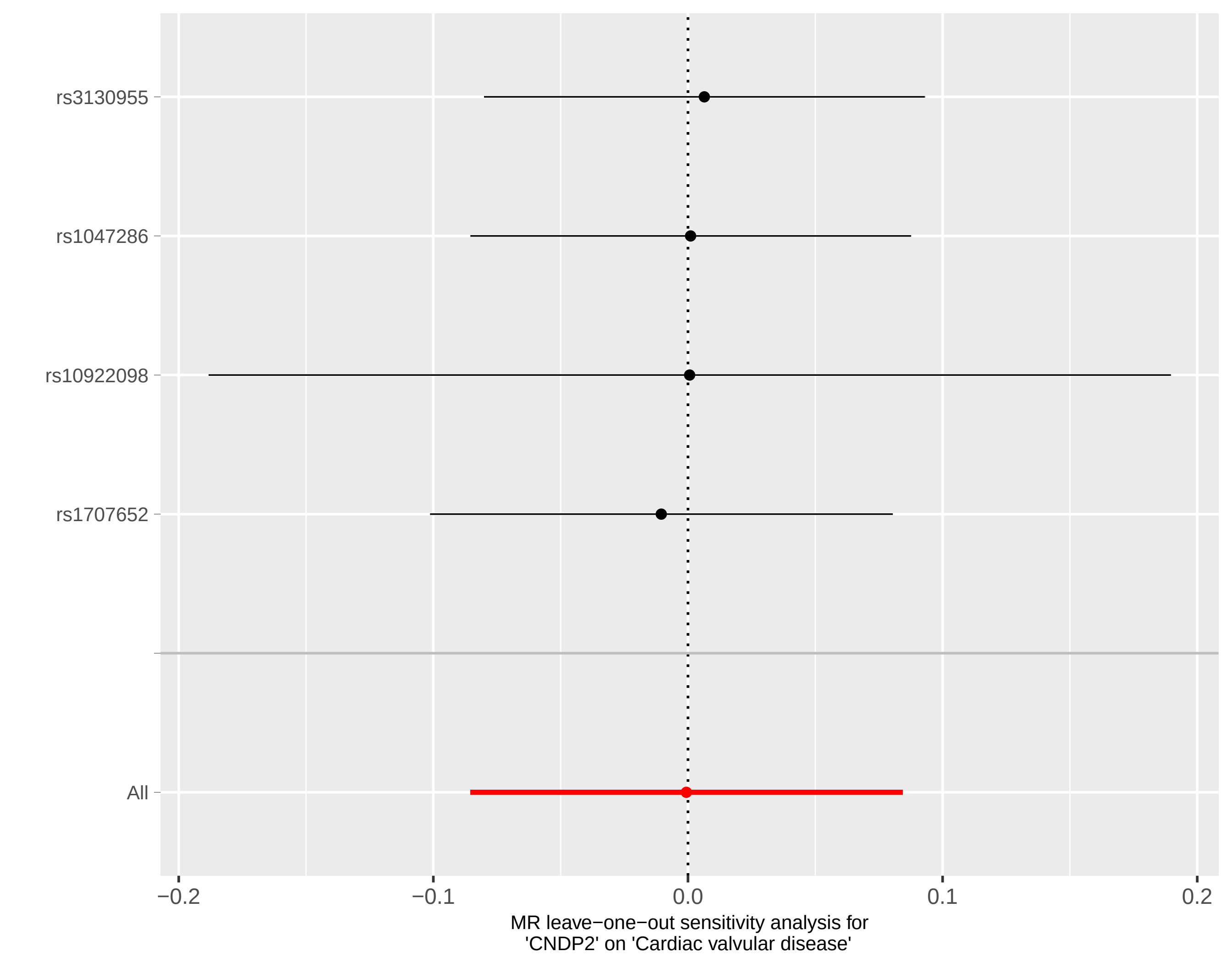
rs3130955
rs1047286
rs10922098
rs1707652
All
−0.2
−0.1
0.0
0.1
0.2
MR leave−one−out sensitivity analysis for
'CNDP2' on 'Cardiac valvular disease'

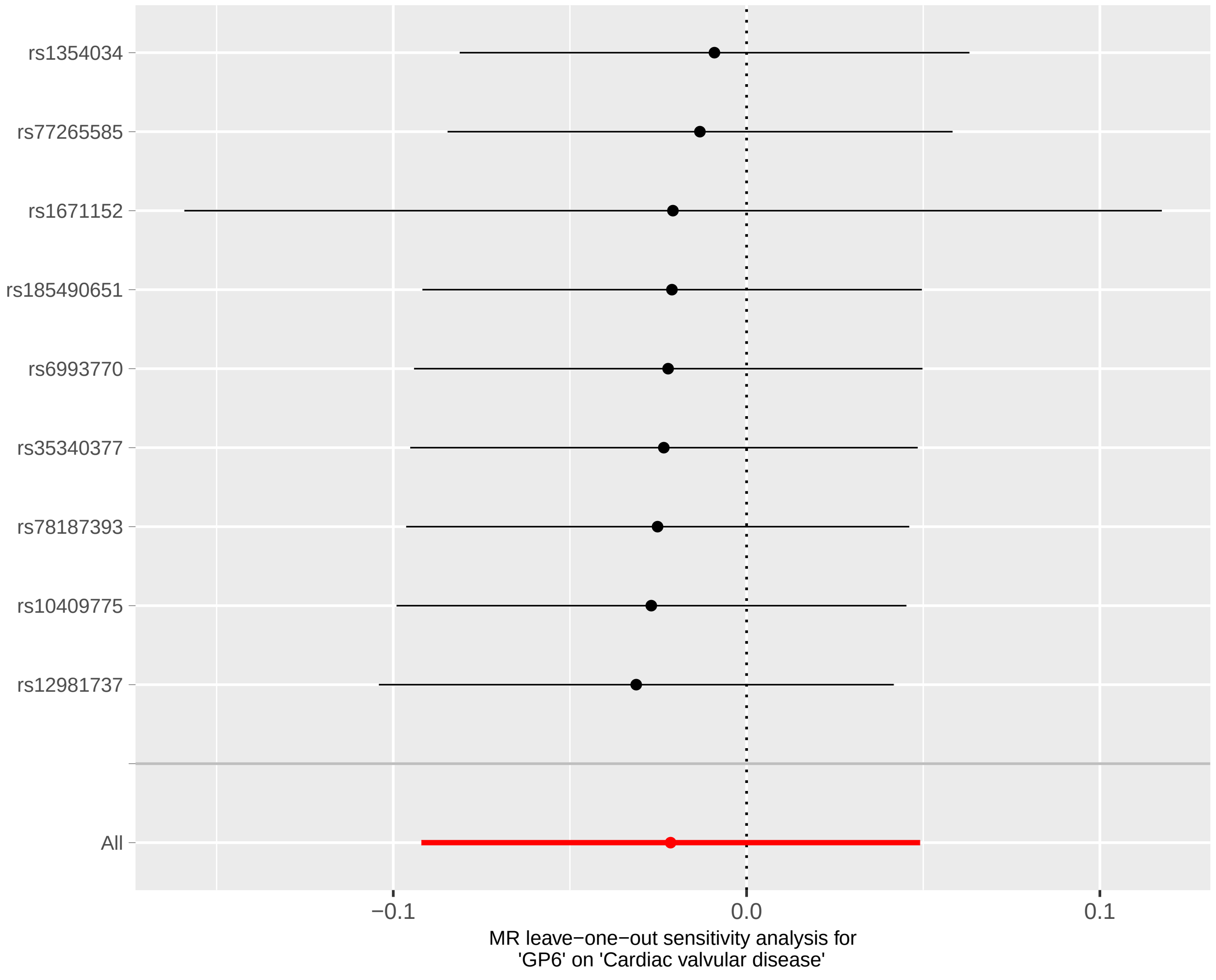
rs1354034
rs77265585
rs1671152
rs185490651
rs6993770
rs35340377
rs78187393
rs10409775
rs12981737
All
−0.1
0.0
0.1
MR leave−one−out sensitivity analysis for
'GP6' on 'Cardiac valvular disease'

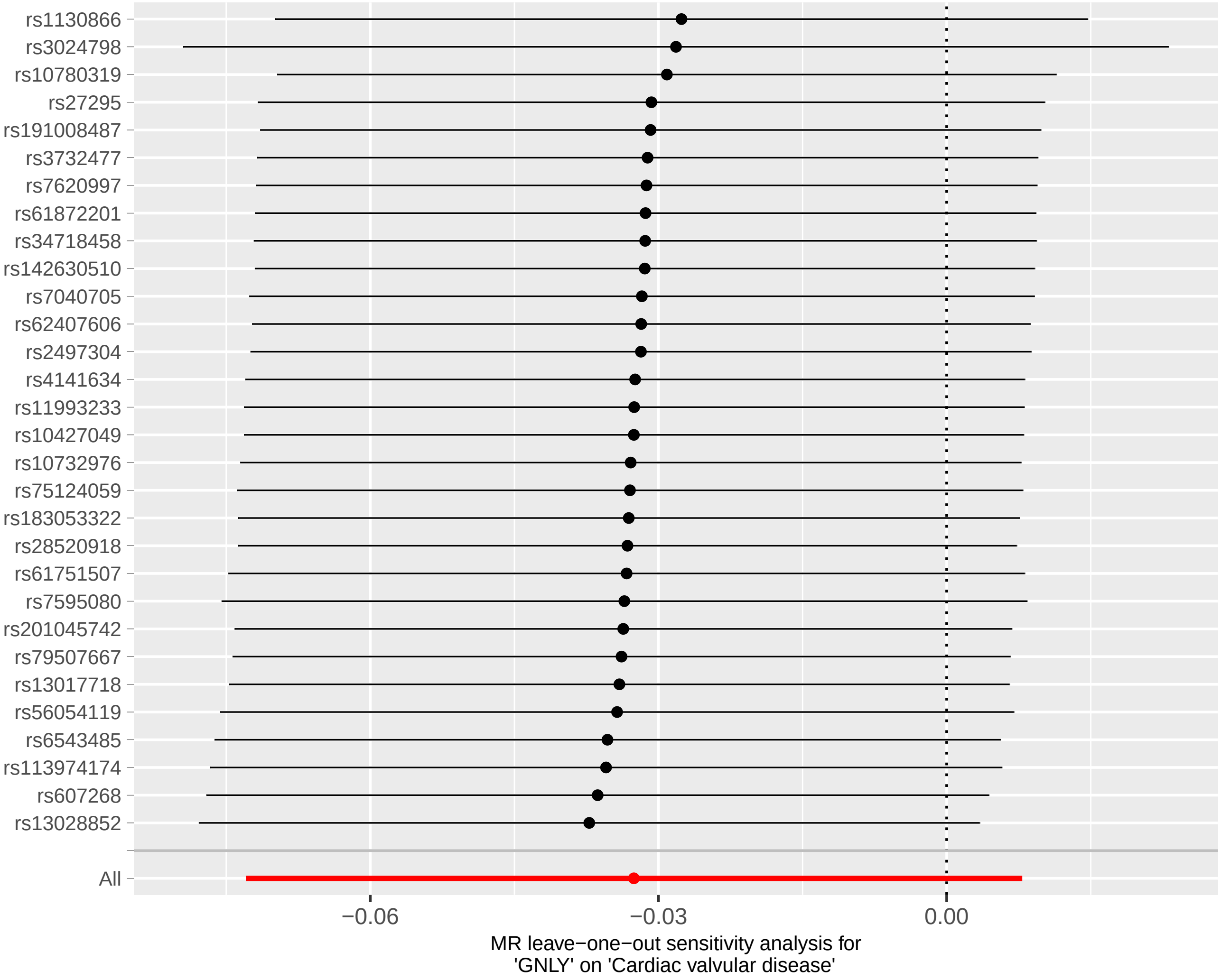
rs1130866
rs3024798
rs10780319
rs27295
rs191008487
rs3732477
rs7620997
rs61872201
rs34718458
rs142630510
rs7040705
rs62407606
rs2497304
rs4141634
rs11993233
rs10427049
rs10732976
rs75124059
rs183053322
rs28520918
rs61751507
rs7595080
rs201045742
rs79507667
rs13017718
rs56054119
rs6543485
rs113974174
rs607268
rs13028852
All
−0.06
−0.03
0.00
MR leave−one−out sensitivity analysis for
'GNLY' on 'Cardiac valvular disease'

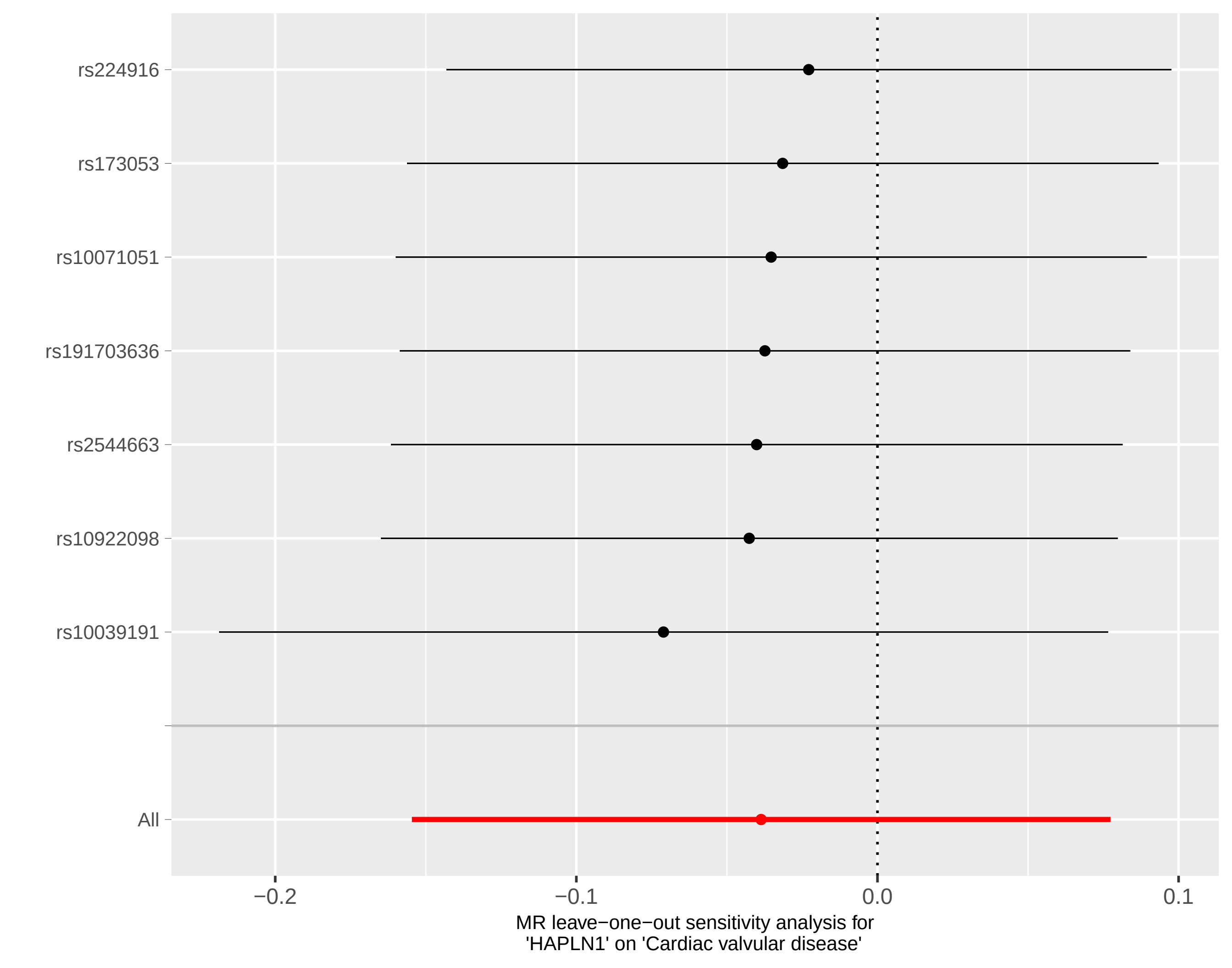

rs224916
rs173053
rs10071051
rs191703636
rs2544663
rs10922098
rs10039191
All
−0.2
−0.1
0.0
0.1
MR leave−one−out sensitivity analysis for
'HAPLN1' on 'Cardiac valvular disease'

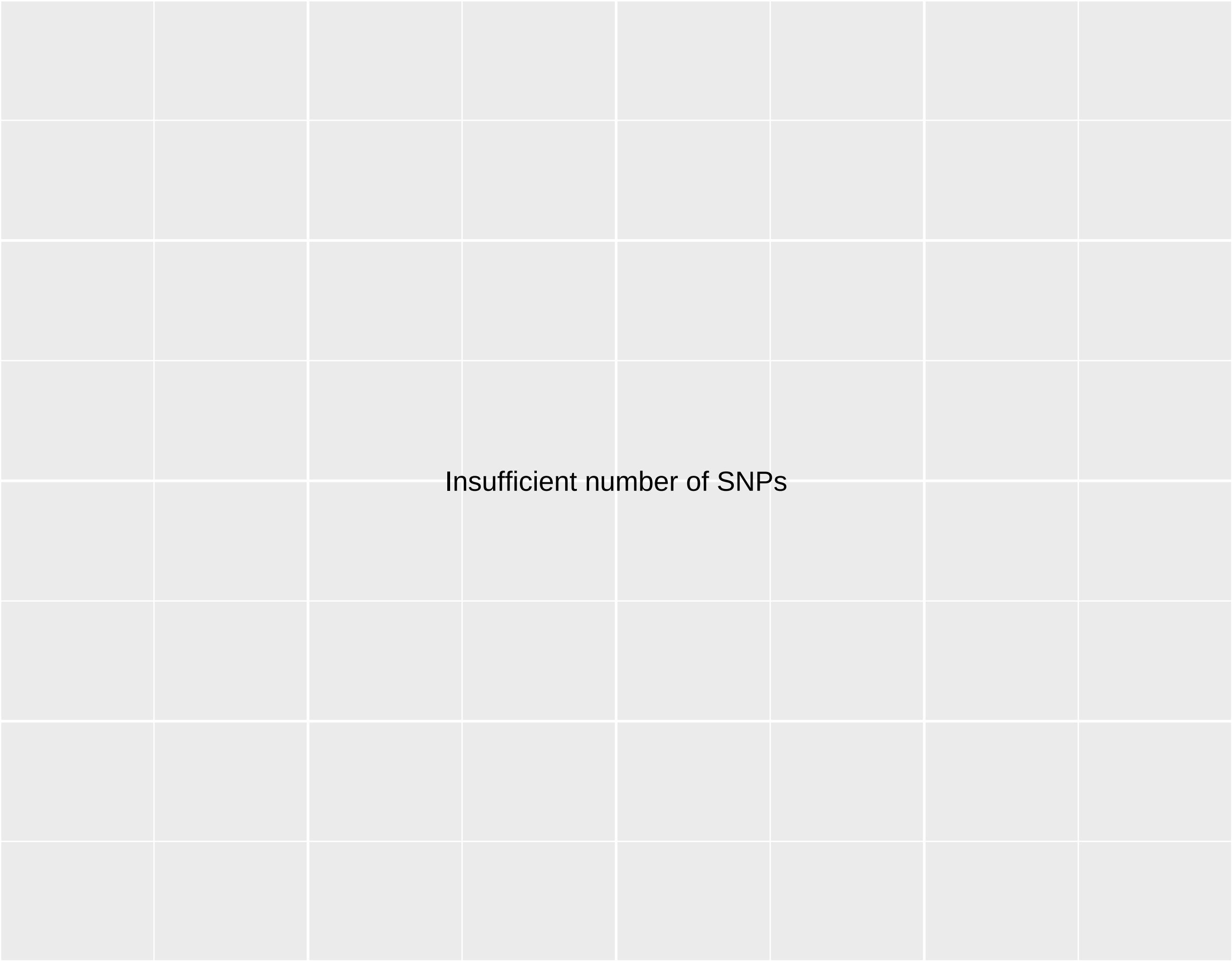
Insufficient number of SNPs

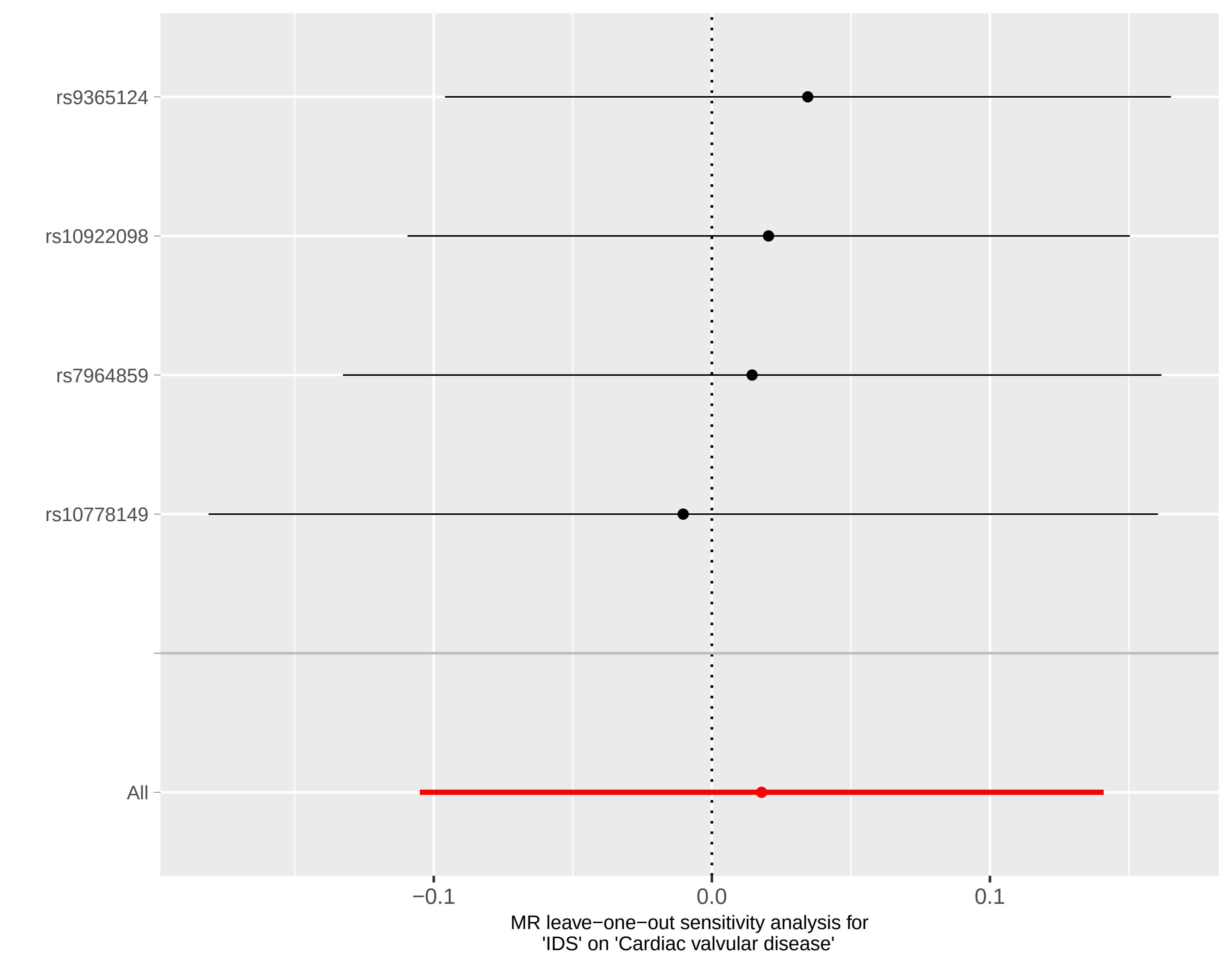
rs9365124
rs10922098
rs7964859
rs10778149
All
−0.1
0.0
0.1
MR leave−one−out sensitivity analysis for
'IDS' on 'Cardiac valvular disease'

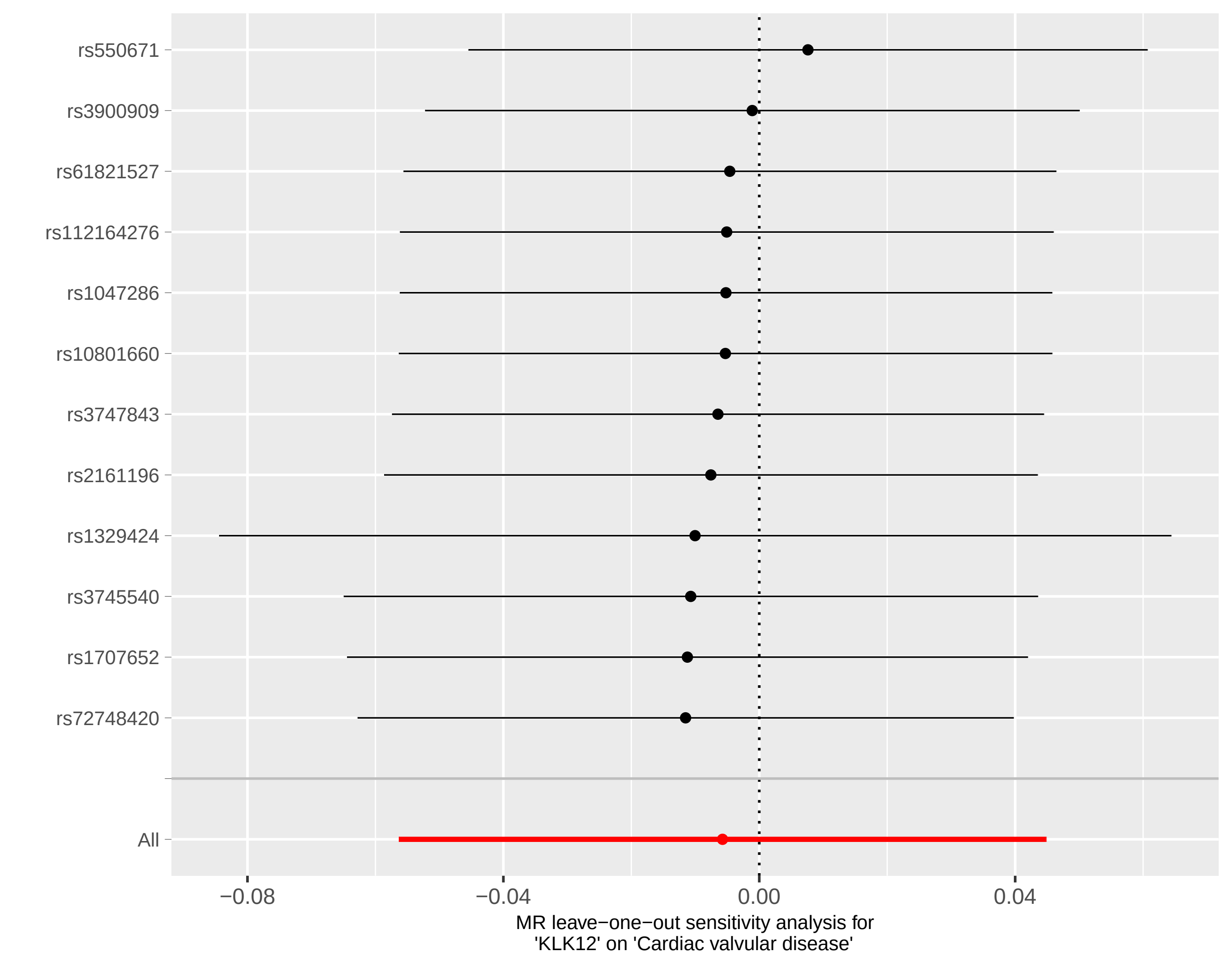
rs550671
rs3900909
rs61821527
rs112164276
rs1047286
rs10801660
rs3747843
rs2161196
rs1329424
rs3745540
rs1707652
rs72748420
All
−0.08
−0.04
0.00
0.04
MR leave−one−out sensitivity analysis for
'KLK12' on 'Cardiac valvular disease'

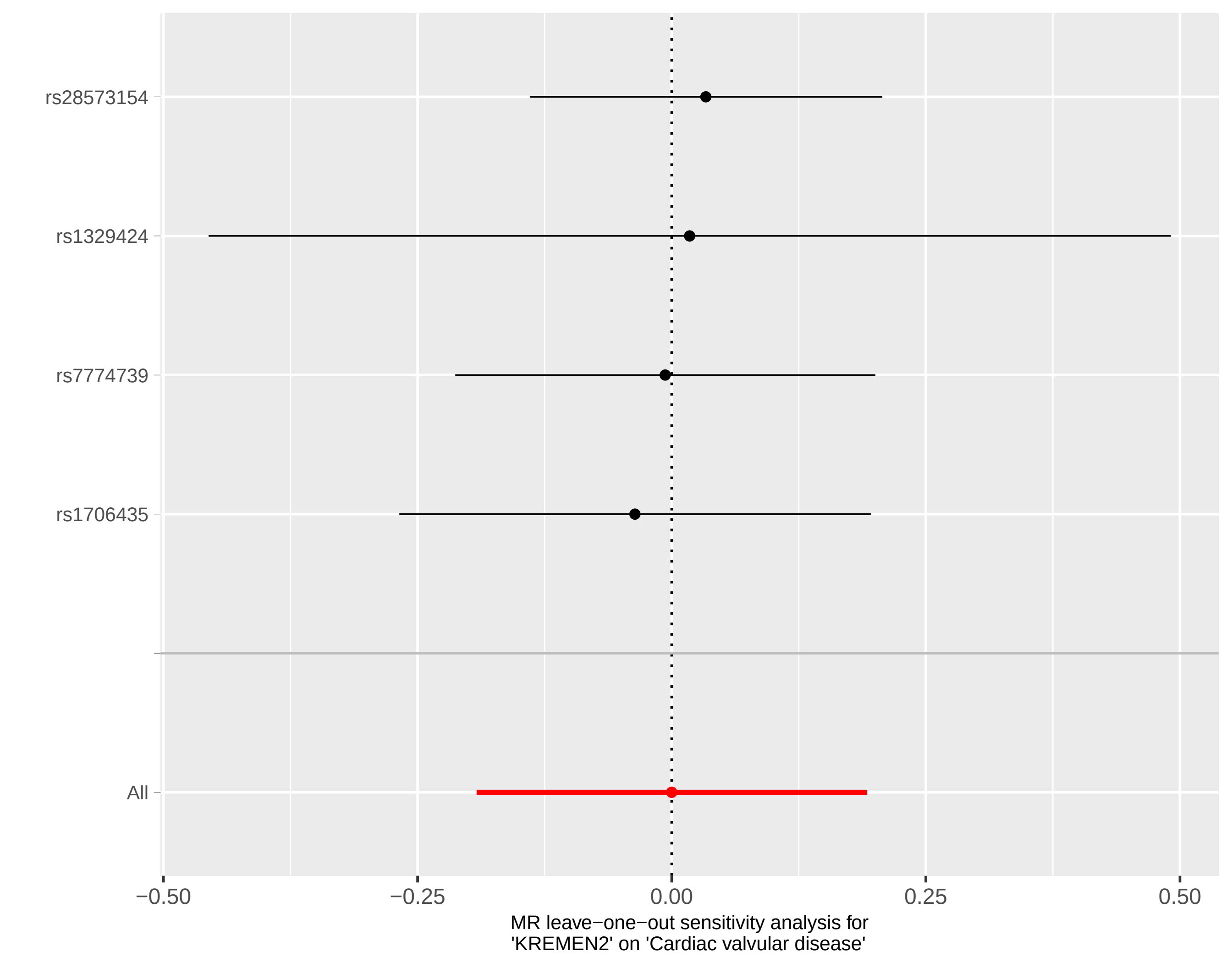

rs28573154
rs1329424
rs7774739
rs1706435
All
−0.50
−0.25
0.00
0.25
0.50
MR leave−one−out sensitivity analysis for
'KREMEN2' on 'Cardiac valvular disease'

Insufficient number of SNPs

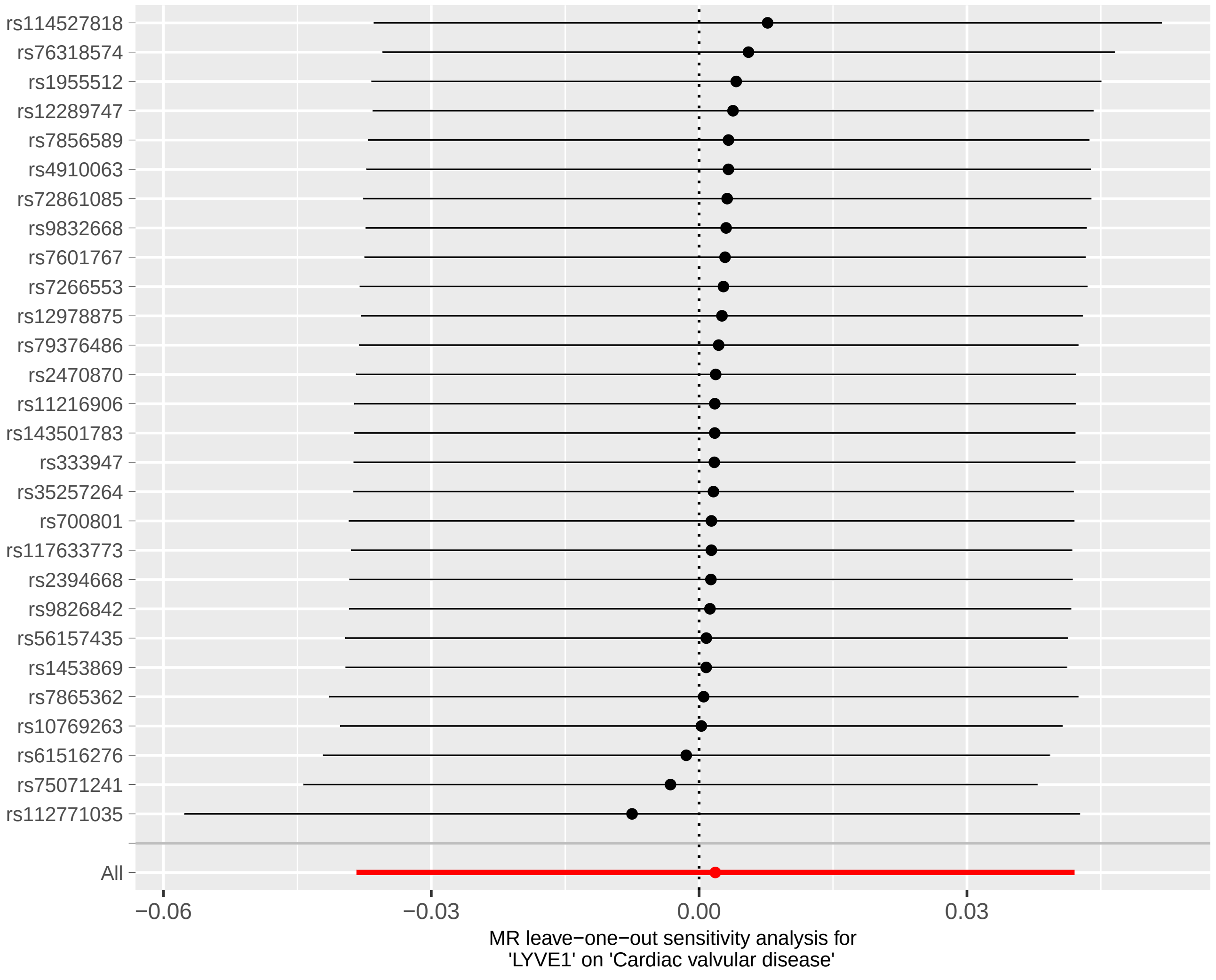
rs114527818
rs76318574
rs1955512
rs12289747
rs7856589
rs4910063
rs72861085
rs9832668
rs7601767
rs7266553
rs12978875
rs79376486
rs2470870
rs11216906
rs143501783
rs333947
rs35257264
rs700801
rs117633773
rs2394668
rs9826842
rs56157435
rs1453869
rs7865362
rs10769263
rs61516276
rs75071241
rs112771035
All
−0.06
−0.03
0.00
0.03
MR leave−one−out sensitivity analysis for
'LYVE1' on 'Cardiac valvular disease'

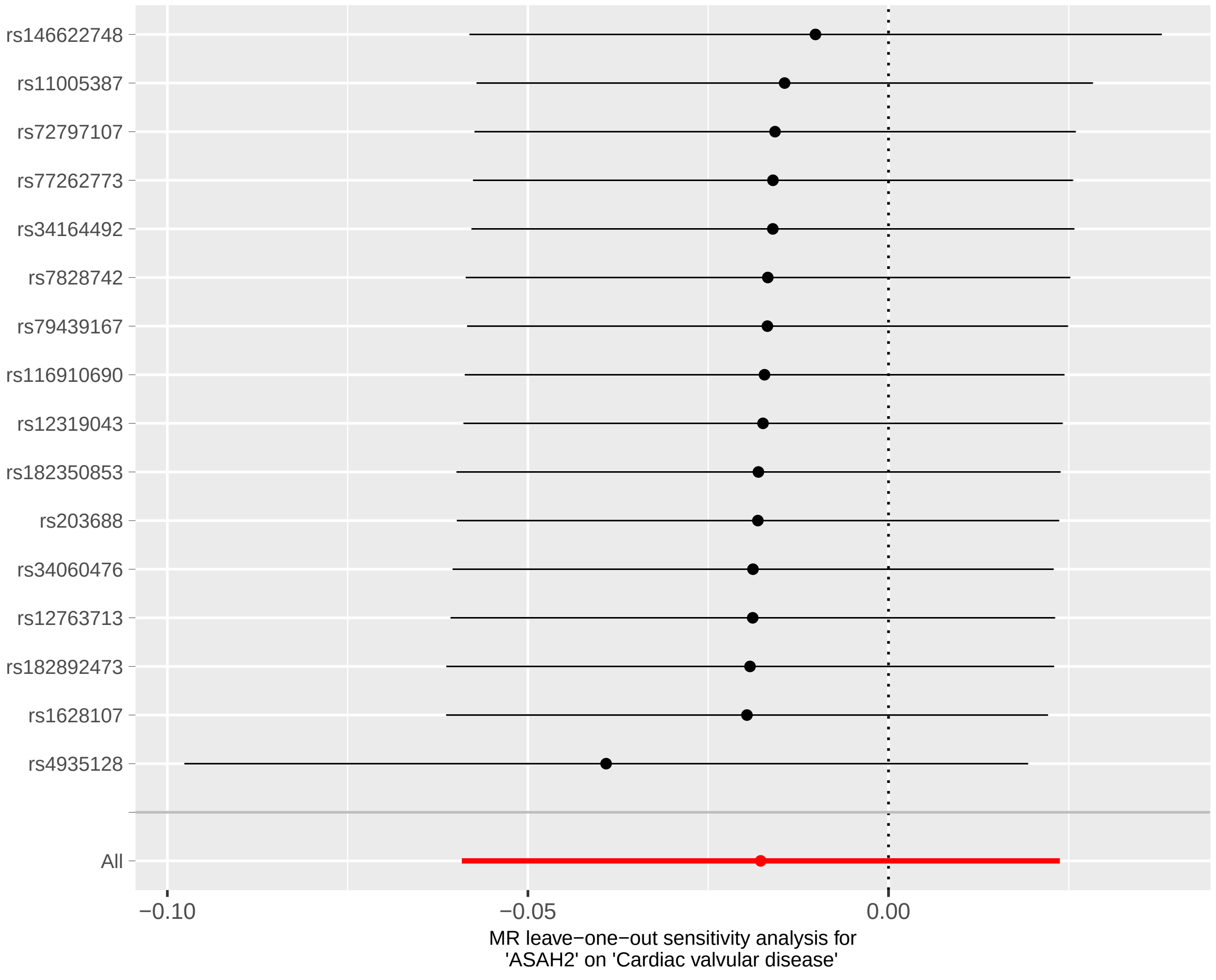
rs146622748
rs11005387
rs72797107
rs77262773
rs34164492
rs7828742
rs79439167
rs116910690
rs12319043
rs182350853
rs203688
rs34060476
rs12763713
rs182892473
rs1628107
rs4935128
All
−0.10
−0.05
0.00
MR leave−one−out sensitivity analysis for
'ASAH2' on 'Cardiac valvular disease'

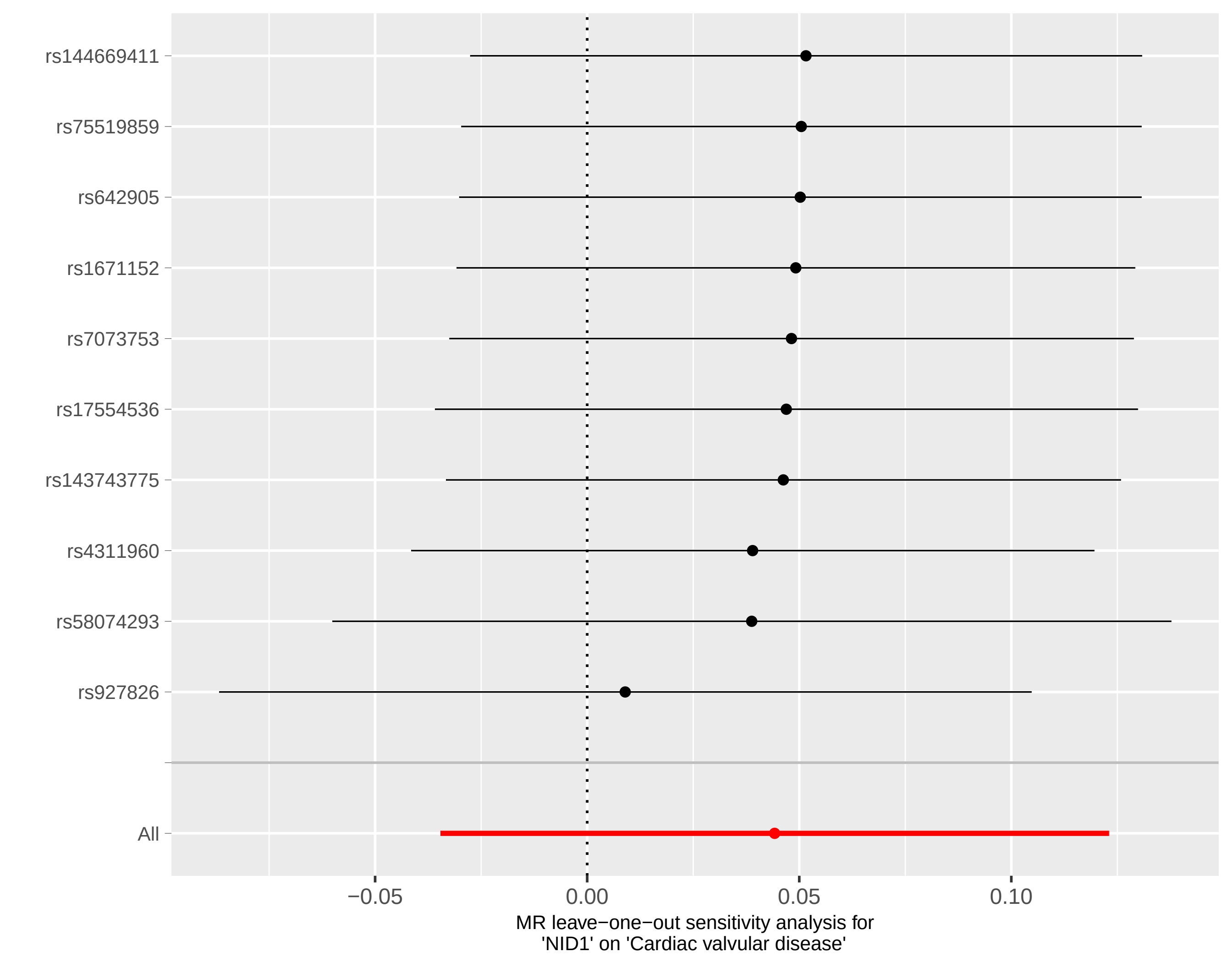
rs144669411
rs75519859
rs642905
rs1671152
rs7073753
rs17554536
rs143743775
rs4311960
rs58074293
rs927826
All
−0.05
0.00
0.05
0.10
MR leave−one−out sensitivity analysis for
'NID1' on 'Cardiac valvular disease'

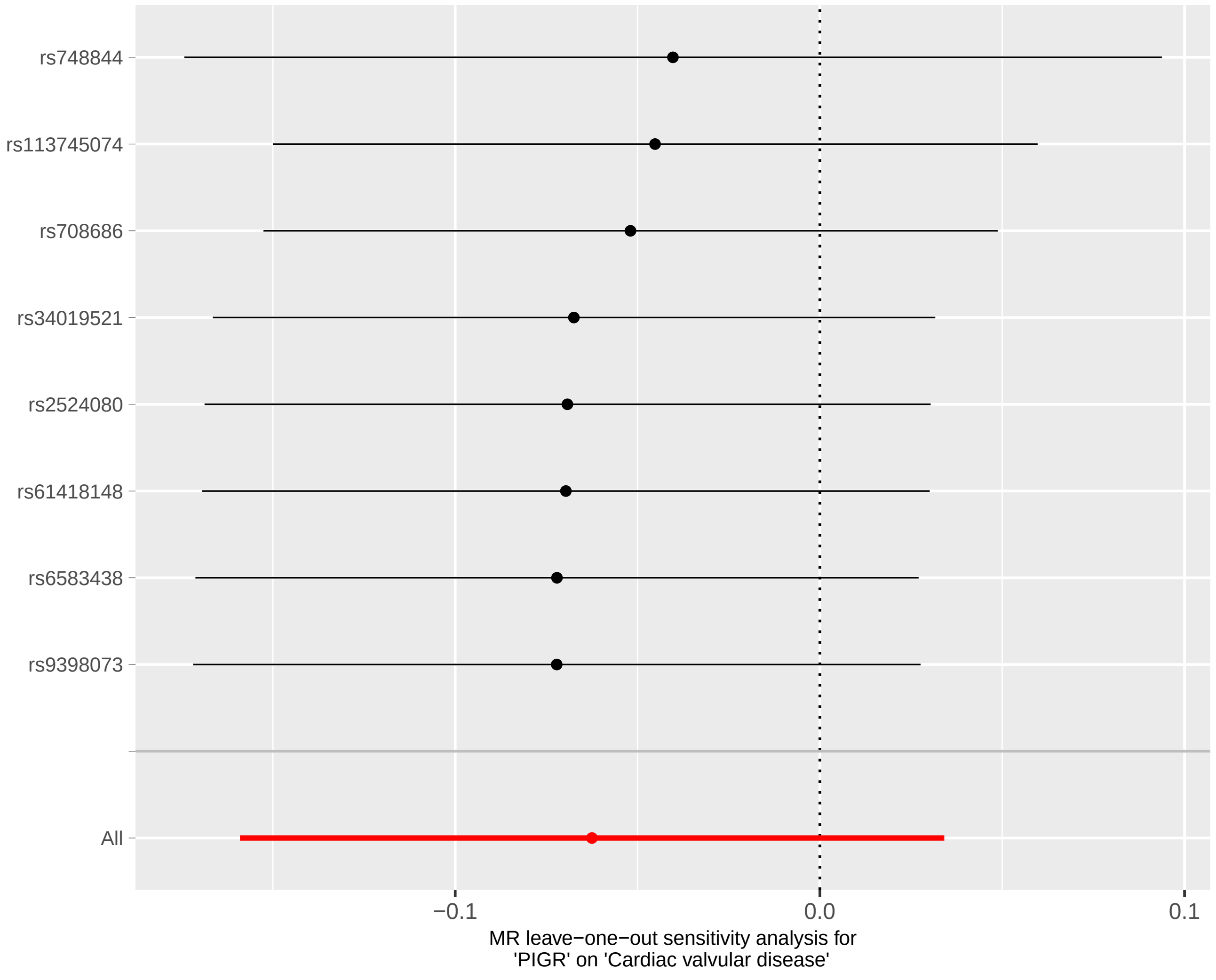
rs748844
rs113745074
rs708686
rs34019521
rs2524080
rs61418148
rs6583438
rs9398073
All
−0.1
0.0
0.1
MR leave−one−out sensitivity analysis for
'PIGR' on 'Cardiac valvular disease'

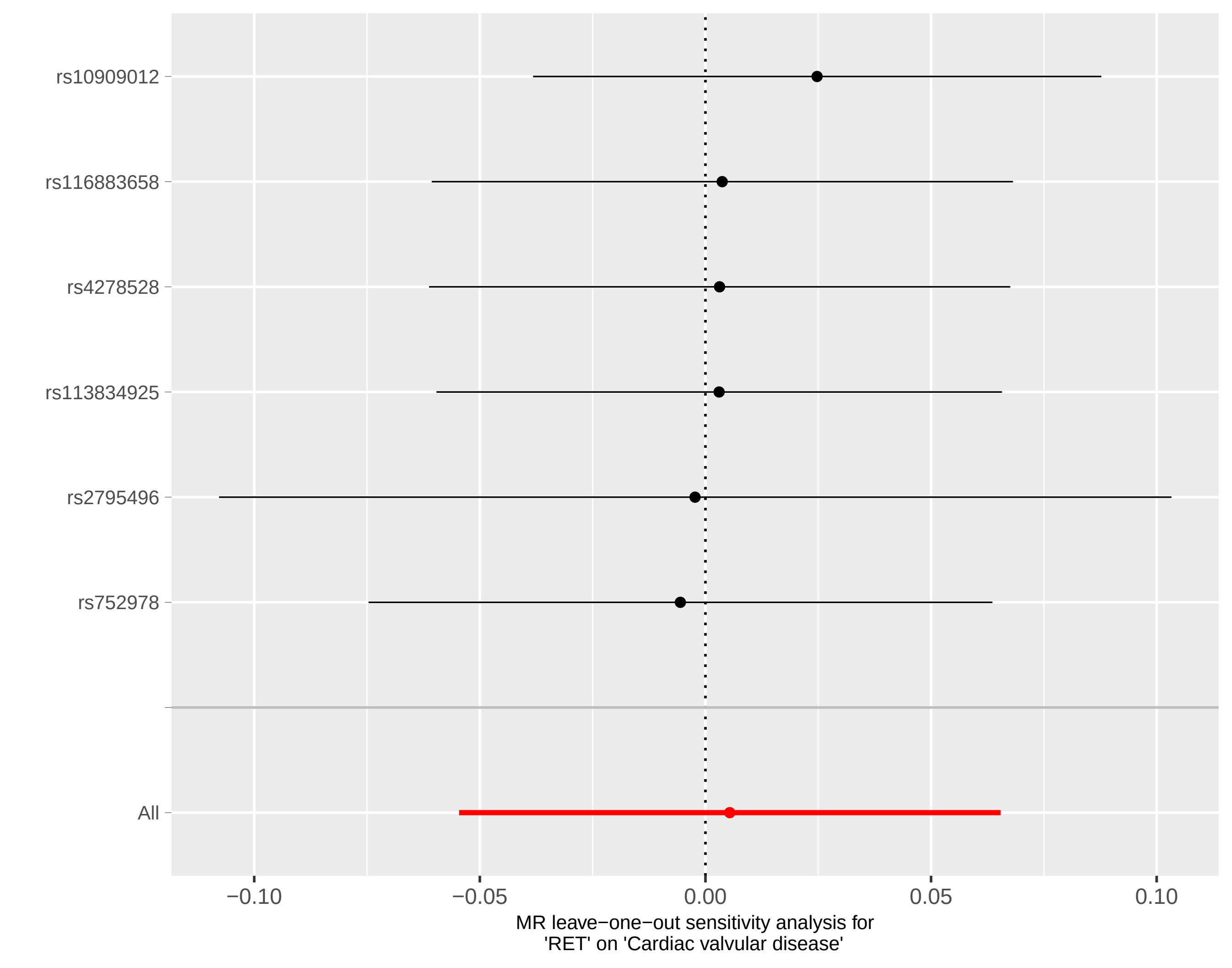
rs10909012
rs116883658
rs4278528
rs113834925
rs2795496
rs752978
All
−0.10
−0.05
0.00
0.05
0.10
MR leave−one−out sensitivity analysis for
'RET' on 'Cardiac valvular disease'

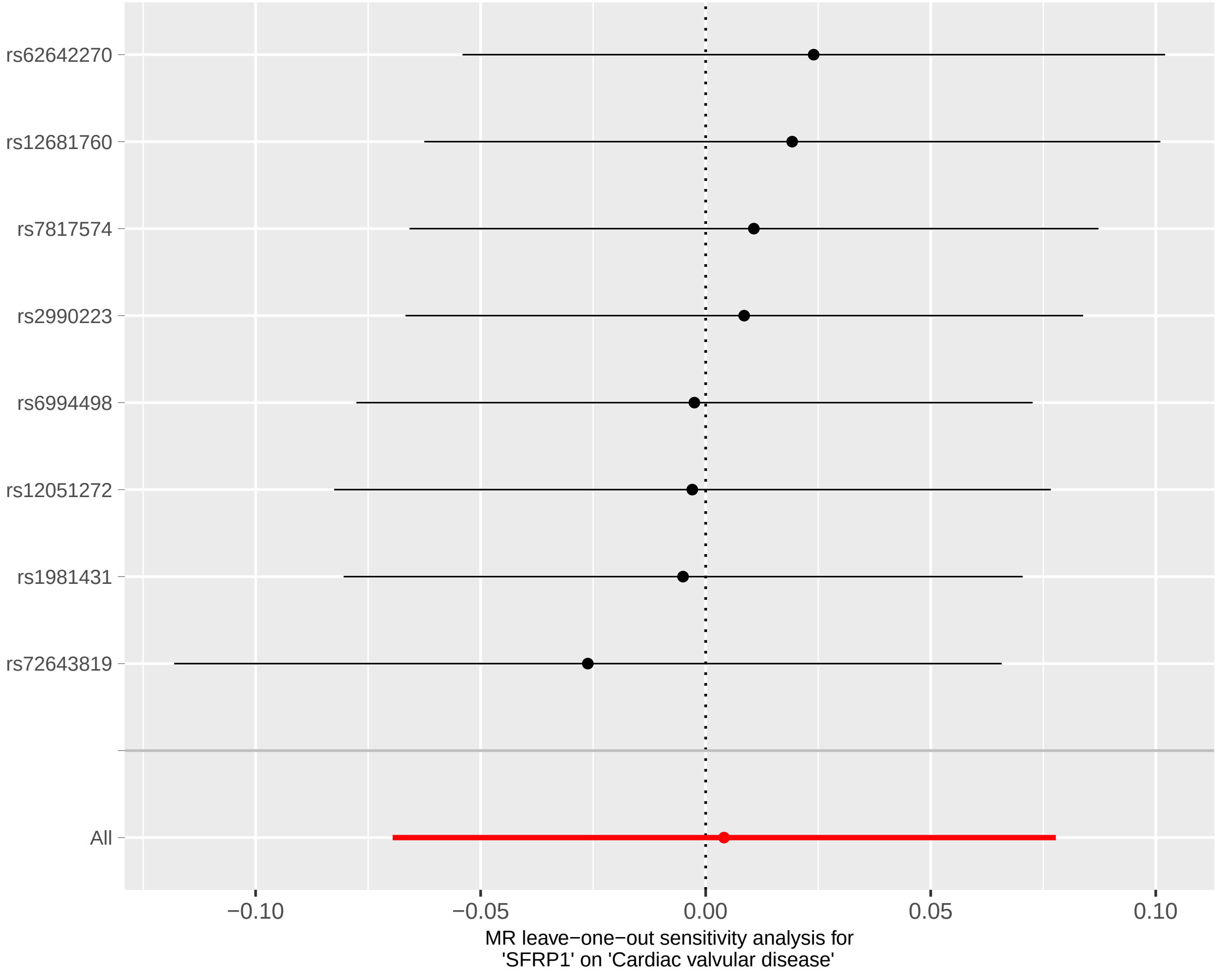

rs62642270
rs12681760
rs7817574
rs2990223
rs6994498
rs12051272
rs1981431
rs72643819
All
−0.10
−0.05
0.00
0.05
0.10
MR leave−one−out sensitivity analysis for
'SFRP1' on 'Cardiac valvular disease'

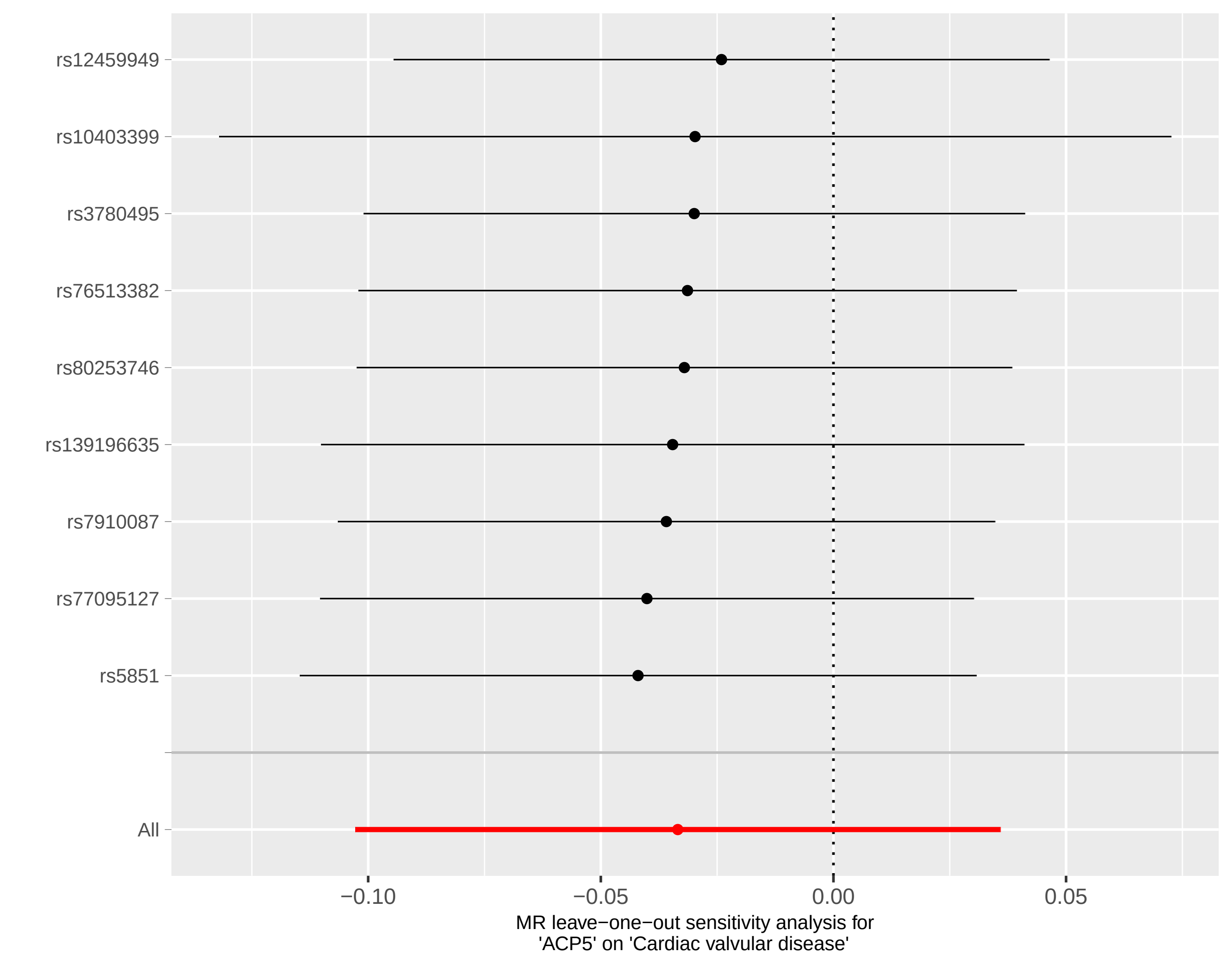
rs12459949
rs10403399
rs3780495
rs76513382
rs80253746
rs139196635
rs7910087
rs77095127
rs5851
All
−0.10
−0.05
0.00
0.05
MR leave−one−out sensitivity analysis for
'ACP5' on 'Cardiac valvular disease'

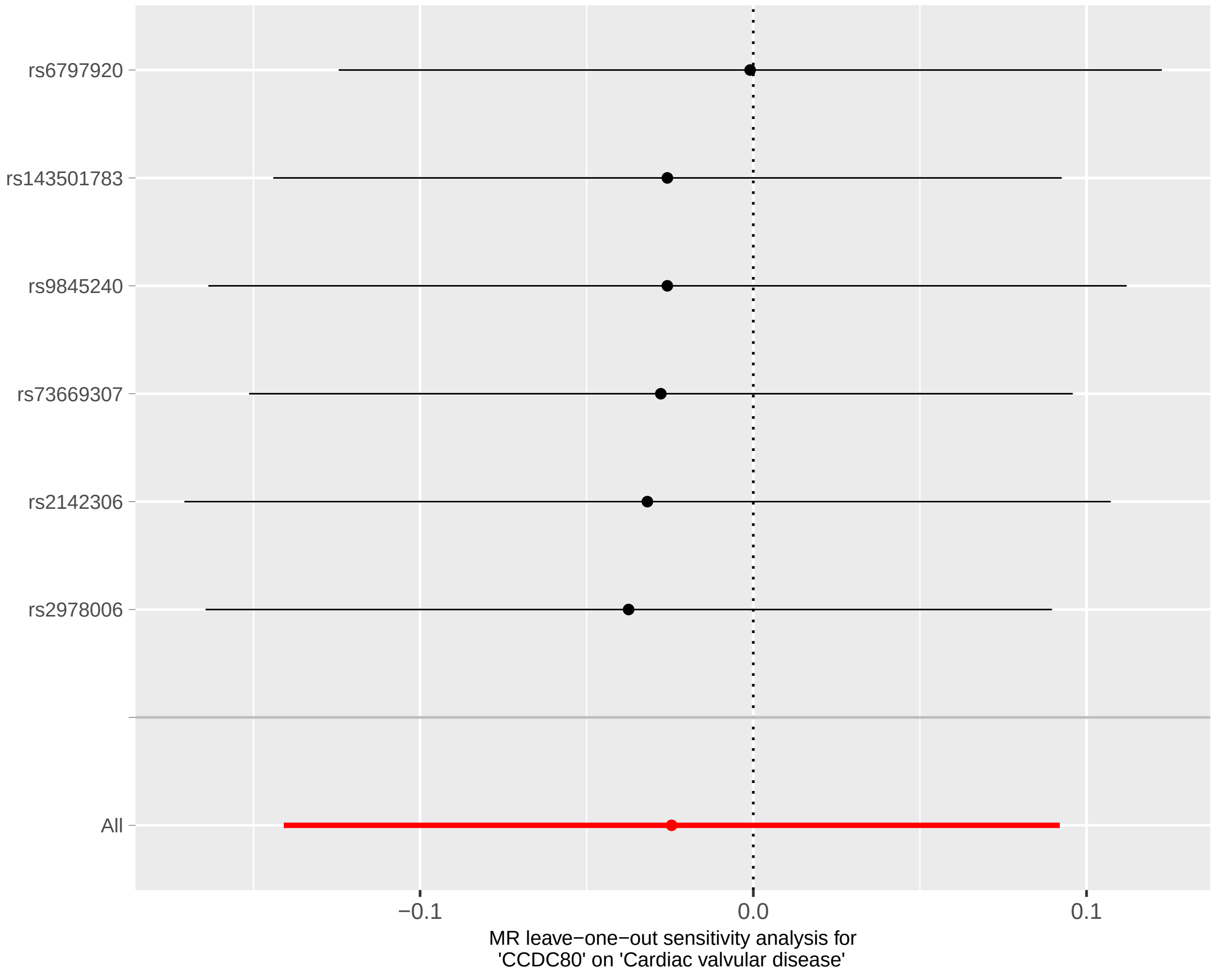
rs6797920
rs143501783
rs9845240
rs73669307
rs2142306
rs2978006
All
−0.1
0.0
0.1
MR leave−one−out sensitivity analysis for
'CCDC80' on 'Cardiac valvular disease'

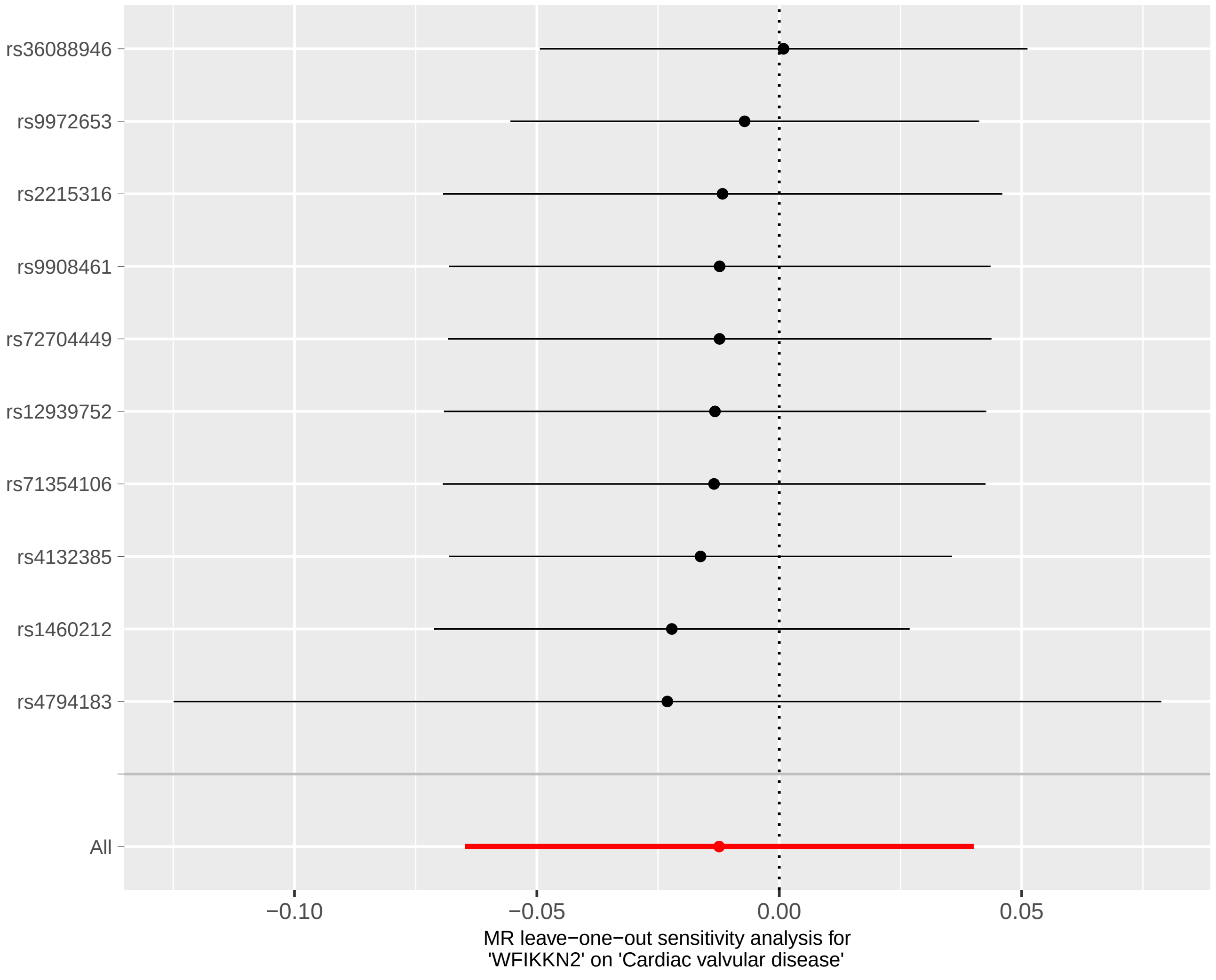
rs36088946
rs9972653
rs2215316
rs9908461
rs72704449
rs12939752
rs71354106
rs4132385
rs1460212
rs4794183
All
−0.10
−0.05
0.00
0.05
MR leave−one−out sensitivity analysis for
'WFIKKN2' on 'Cardiac valvular disease'

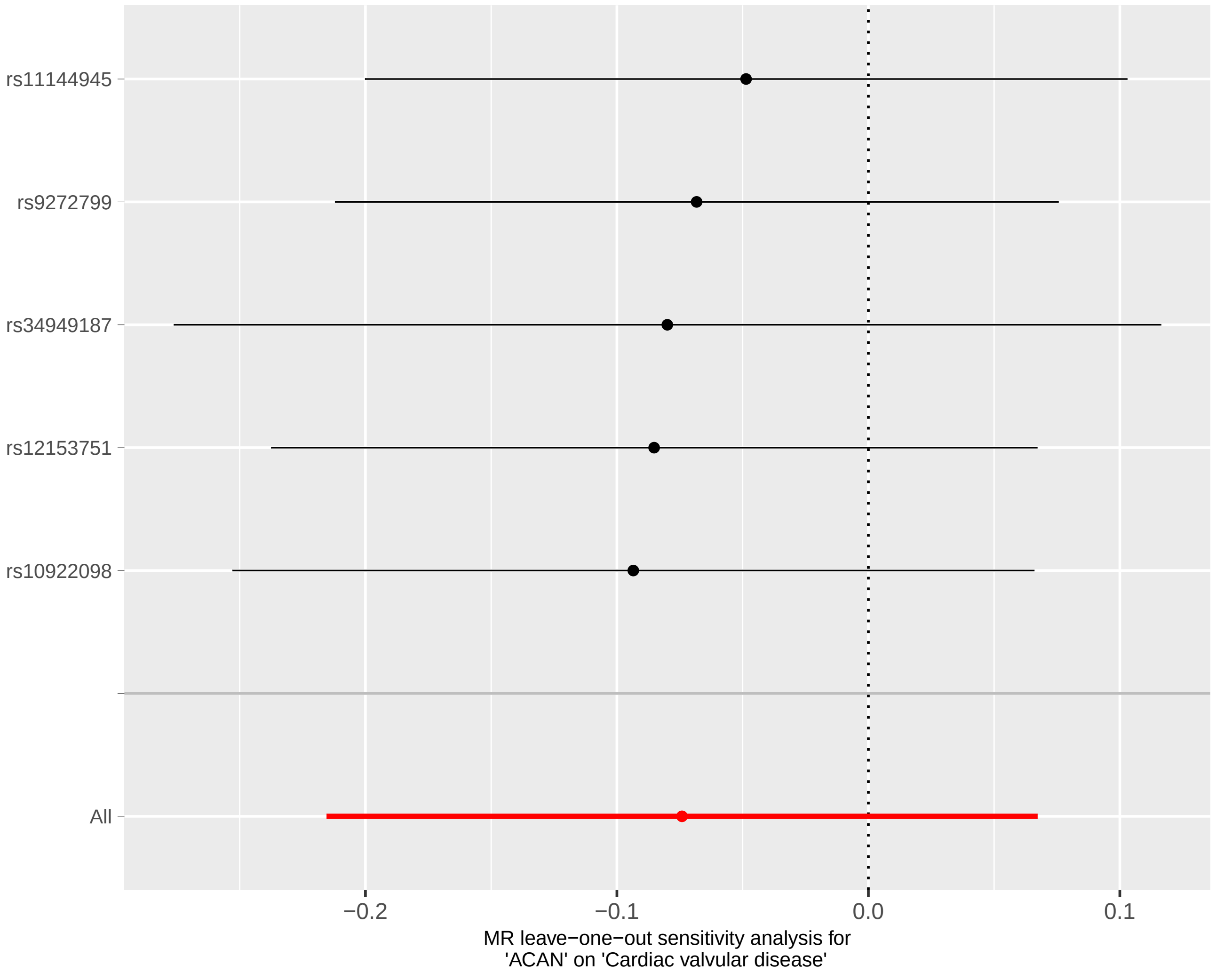
rs11144945
rs9272799
rs34949187
rs12153751
rs10922098
All
−0.2
−0.1
0.0
0.1
MR leave−one−out sensitivity analysis for
'ACAN' on 'Cardiac valvular disease'

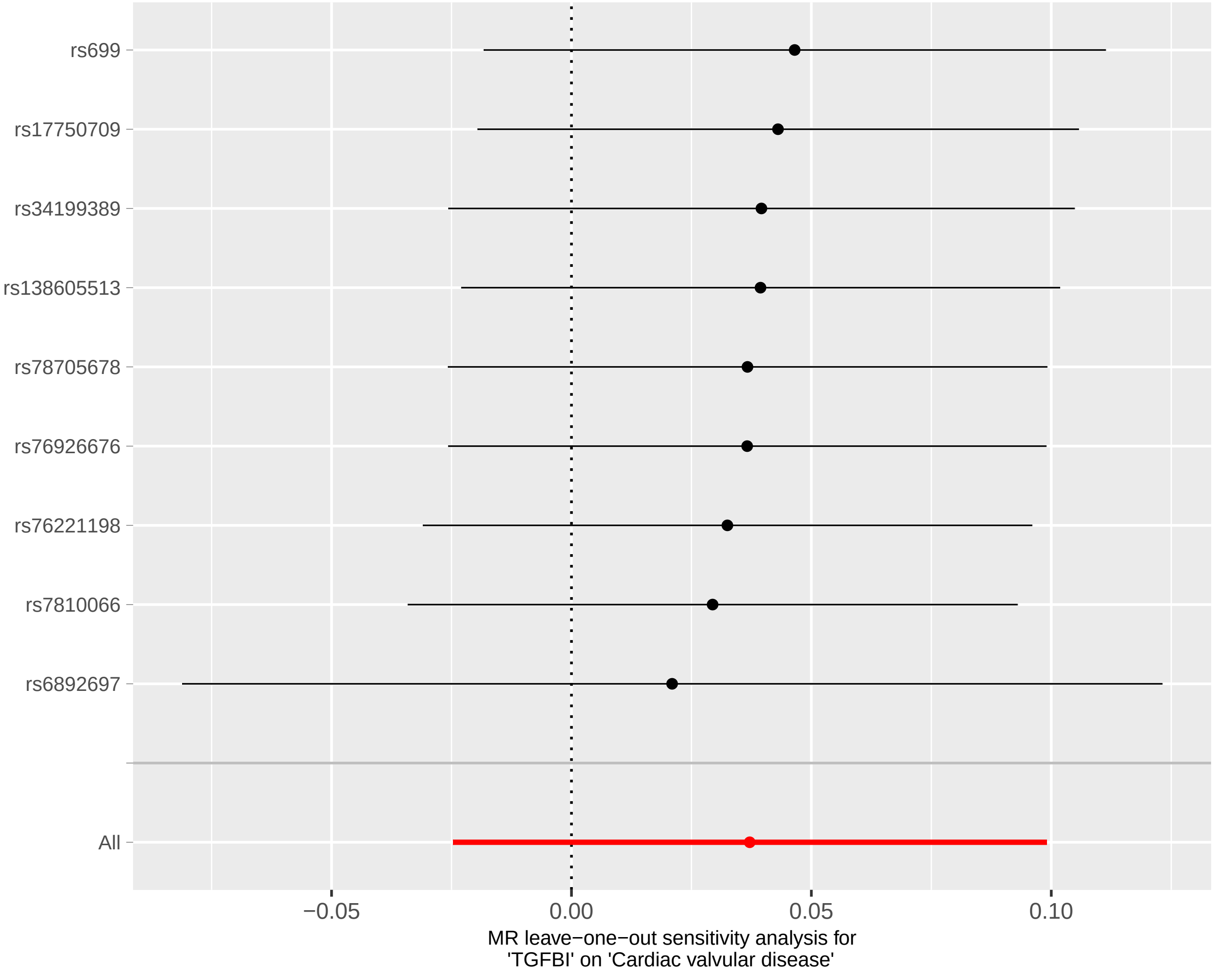

rs699
rs17750709
rs34199389
rs138605513
rs78705678
rs76926676
rs76221198
rs7810066
rs6892697
All
−0.05
0.00
0.05
0.10
MR leave−one−out sensitivity analysis for
'TGFBI' on 'Cardiac valvular disease'

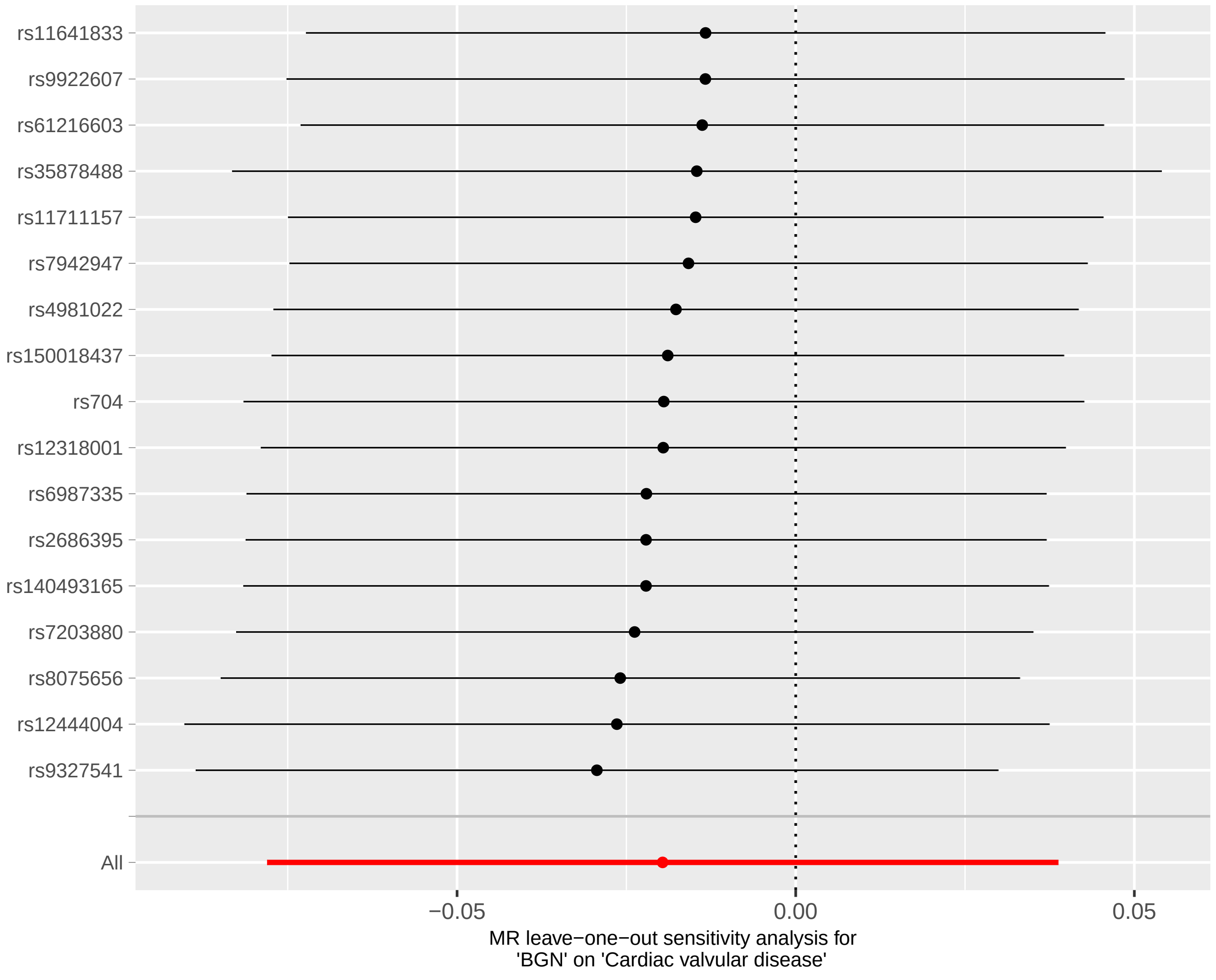
rs11641833
rs9922607
rs61216603
rs35878488
rs11711157
rs7942947
rs4981022
rs150018437
rs704
rs12318001
rs6987335
rs2686395
rs140493165
rs7203880
rs8075656
rs12444004
rs9327541
All
−0.05
0.00
0.05
MR leave−one−out sensitivity analysis for
'BGN' on 'Cardiac valvular disease'

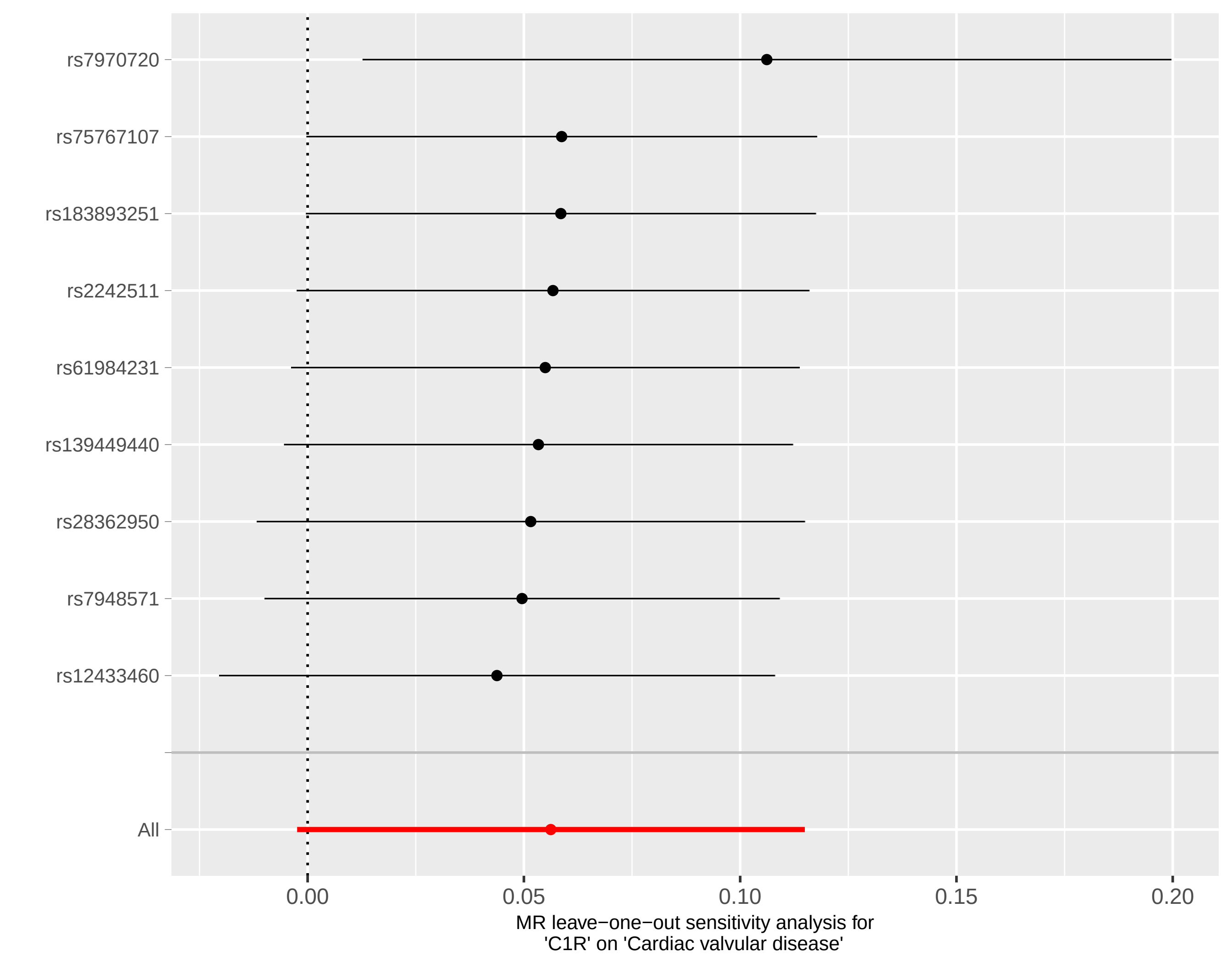
rs7970720
rs75767107
rs183893251
rs2242511
rs61984231
rs139449440
rs28362950
rs7948571
rs12433460
All
0.00
0.05
0.10
0.15
0.20
MR leave−one−out sensitivity analysis for
'C1R' on 'Cardiac valvular disease'

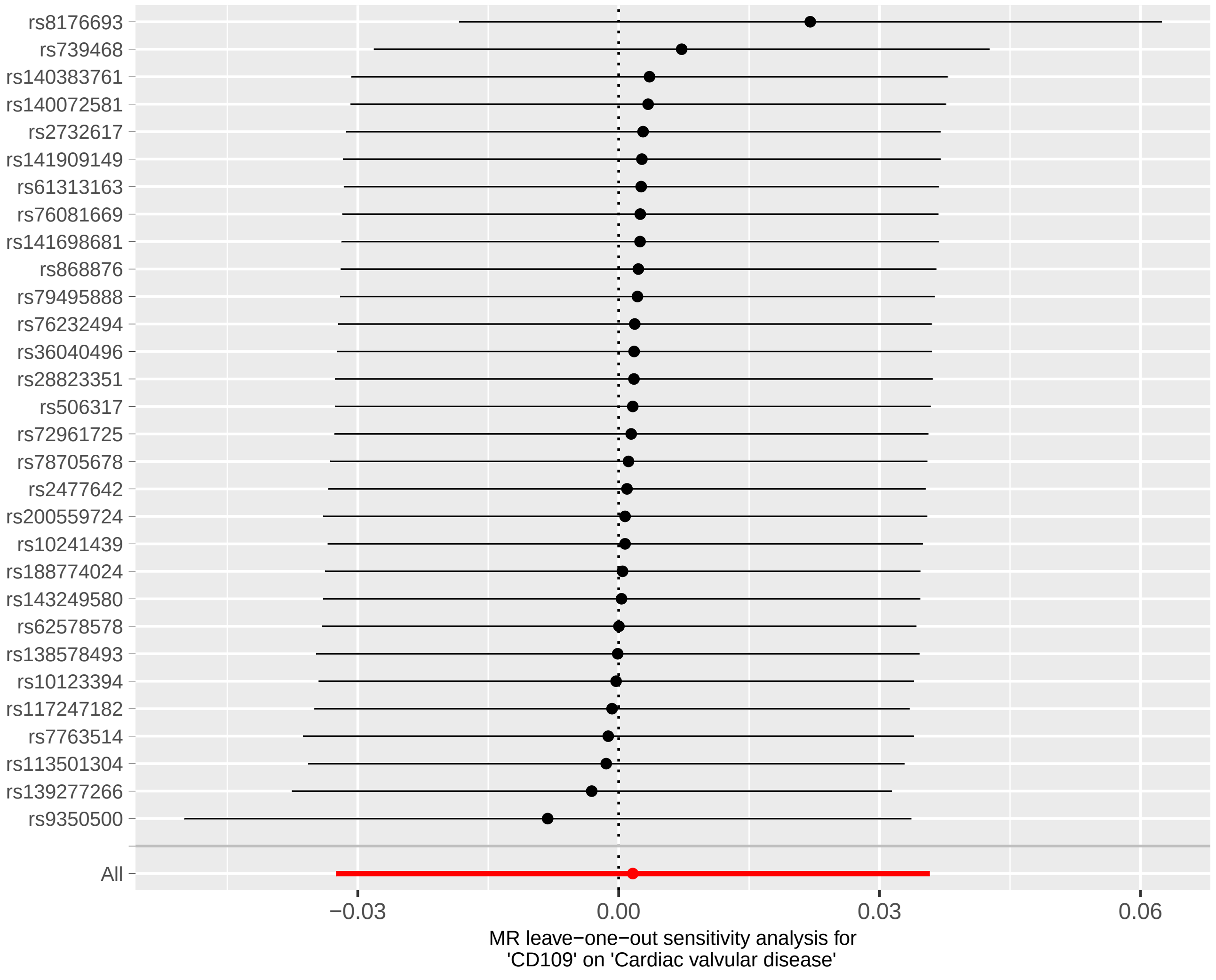
rs8176693
rs739468
rs140383761
rs140072581
rs2732617
rs141909149
rs61313163
rs76081669
rs141698681
rs868876
rs79495888
rs76232494
rs36040496
rs28823351
rs506317
rs72961725
rs78705678
rs2477642
rs200559724
rs10241439
rs188774024
rs143249580
rs62578578
rs138578493
rs10123394
rs117247182
rs7763514
rs113501304
rs139277266
rs9350500
All
−0.03
0.00
0.03
0.06
MR leave−one−out sensitivity analysis for
'CD109' on 'Cardiac valvular disease'

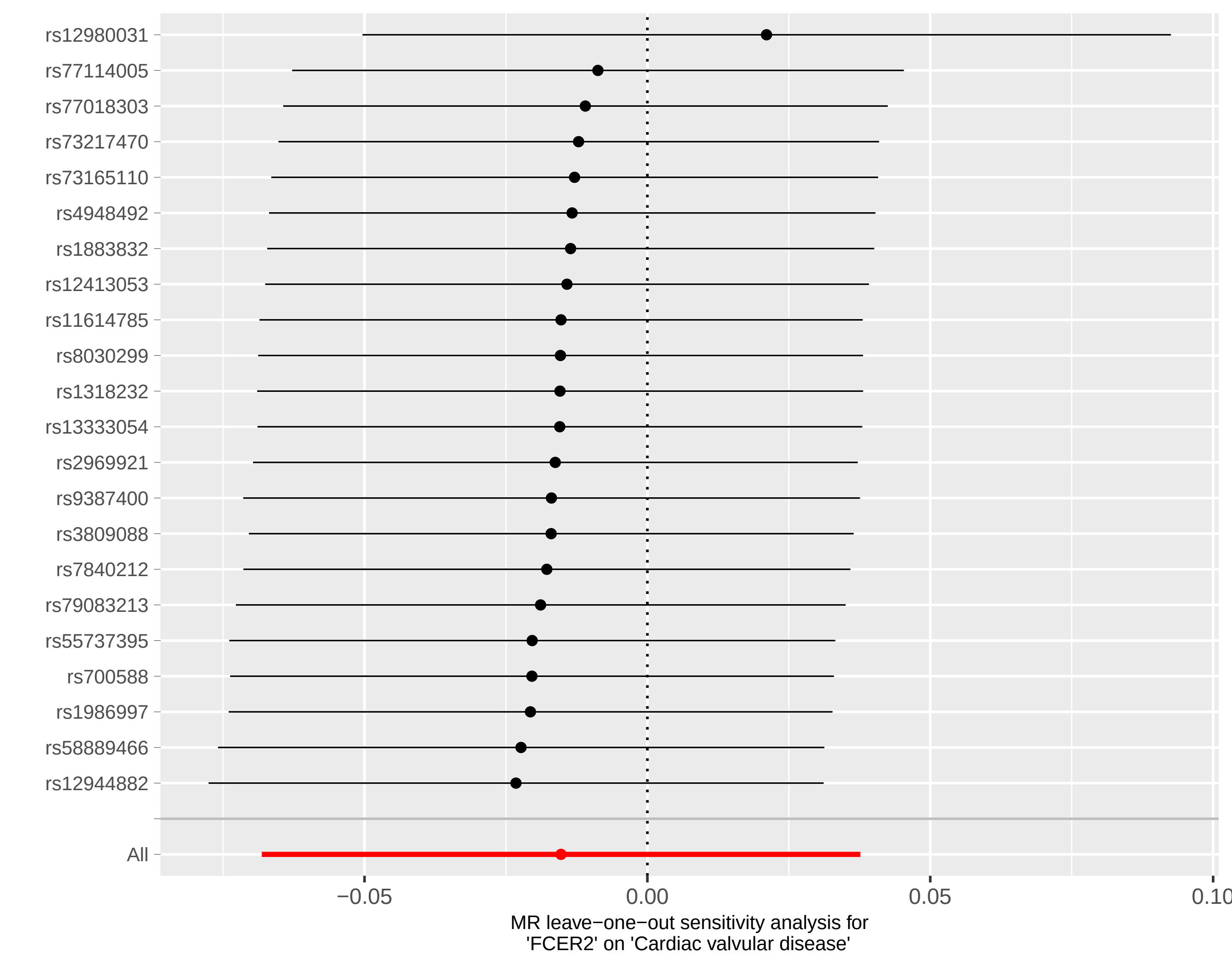
rs12980031
rs77114005
rs77018303
rs73217470
rs73165110
rs4948492
rs1883832
rs12413053
rs11614785
rs8030299
rs1318232
rs13333054
rs2969921
rs9387400
rs3809088
rs7840212
rs79083213
rs55737395
rs700588
rs1986997
rs58889466
rs12944882
All
−0.05
0.00
0.05
0.10
MR leave−one−out sensitivity analysis for
'FCER2' on 'Cardiac valvular disease'

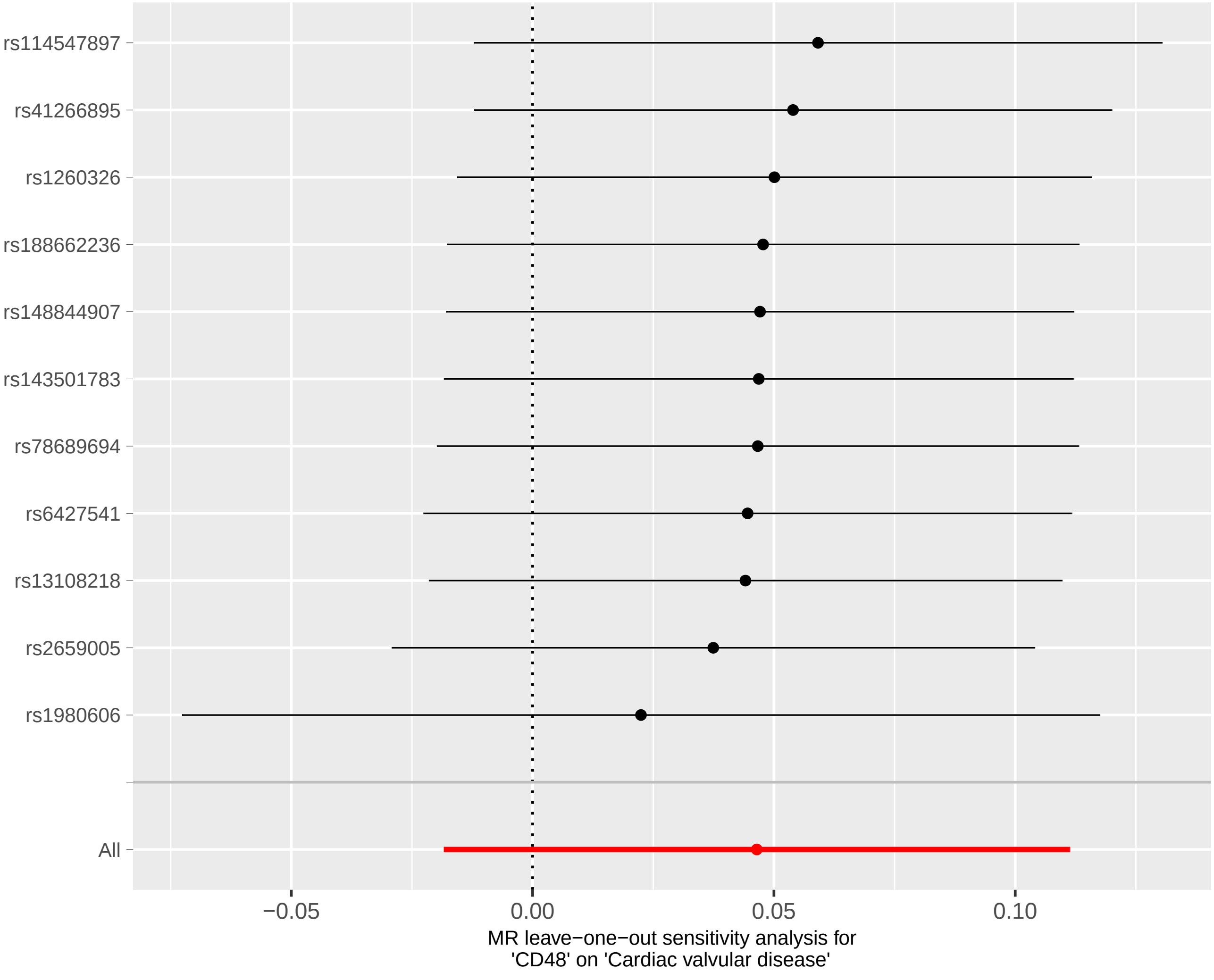
rs114547897
rs41266895
rs1260326
rs188662236
rs148844907
rs143501783
rs78689694
rs6427541
rs13108218
rs2659005
rs1980606
All
−0.05
0.00
0.05
0.10
MR leave−one−out sensitivity analysis for
'CD48' on 'Cardiac valvular disease'

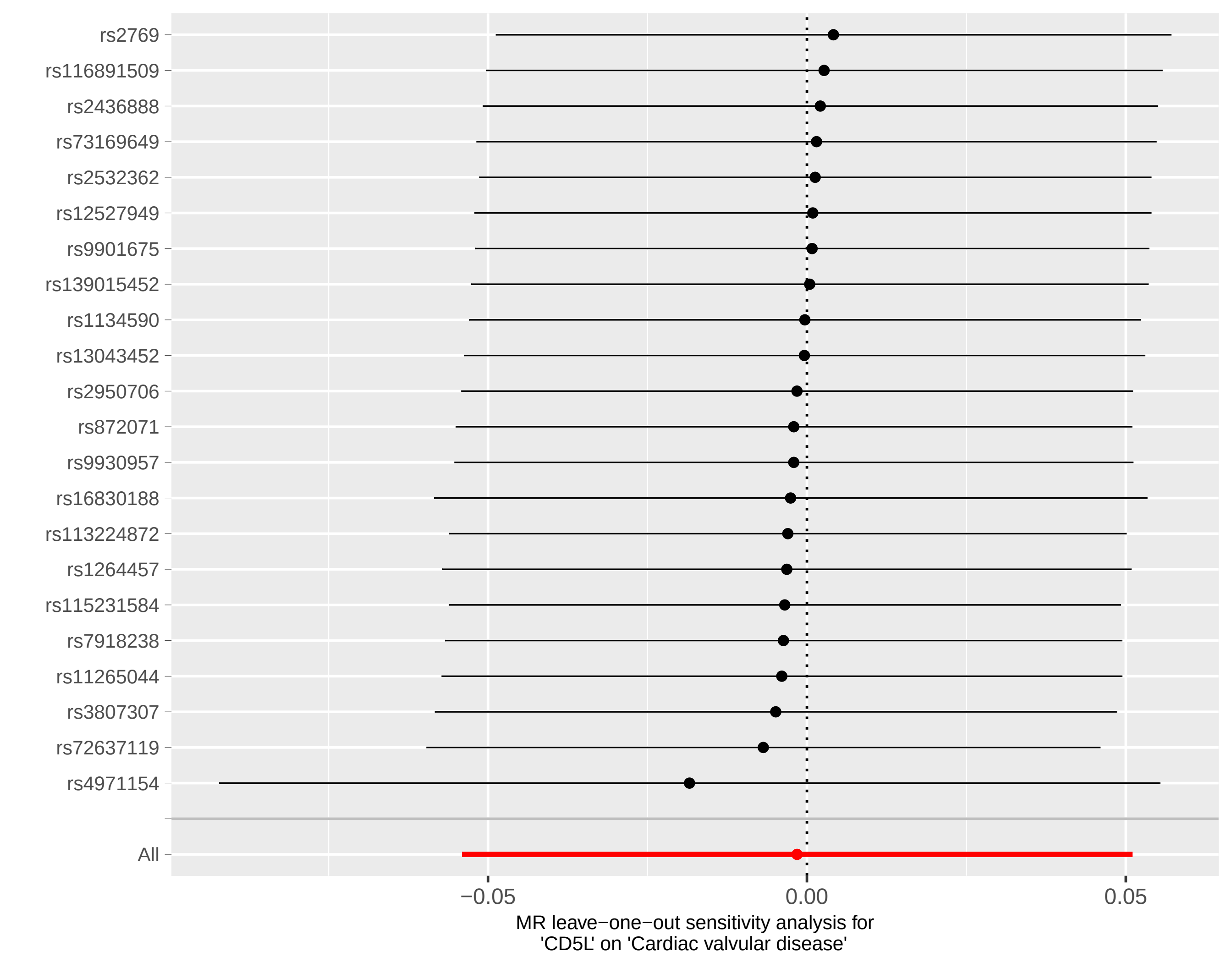
rs2769
rs116891509
rs2436888
rs73169649
rs2532362
rs12527949
rs9901675
rs139015452
rs1134590
rs13043452
rs2950706
rs872071
rs9930957
rs16830188
rs113224872
rs1264457
rs115231584
rs7918238
rs11265044
rs3807307
rs72637119
rs4971154
All
−0.05
0.00
0.05
MR leave−one−out sensitivity analysis for
'CD5L' on 'Cardiac valvular disease'

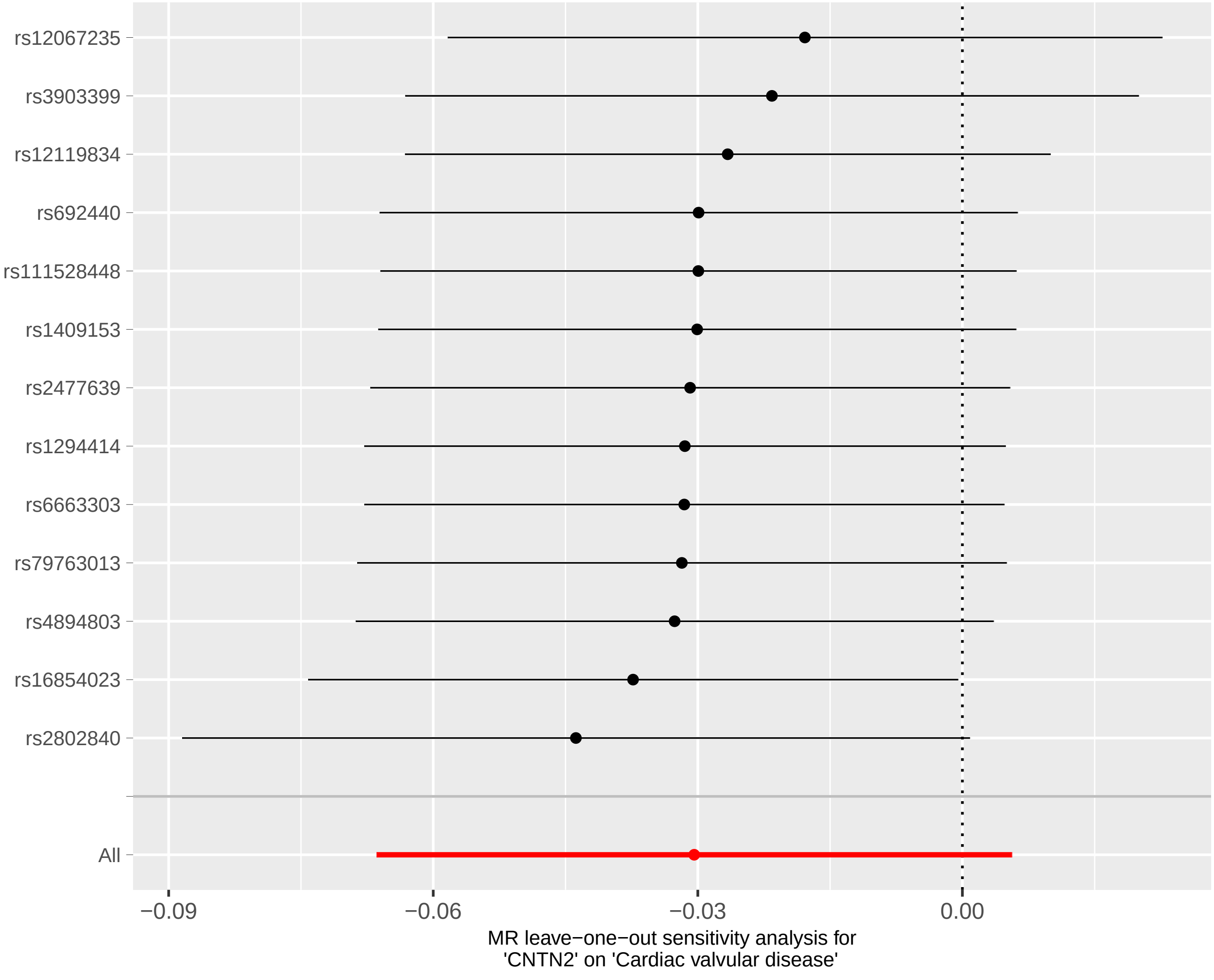
rs12067235
rs3903399
rs12119834
rs692440
rs111528448
rs1409153
rs2477639
rs1294414
rs6663303
rs79763013
rs4894803
rs16854023
rs2802840
All
−0.09
−0.06
−0.03
0.00
MR leave−one−out sensitivity analysis for
'CNTN2' on 'Cardiac valvular disease'

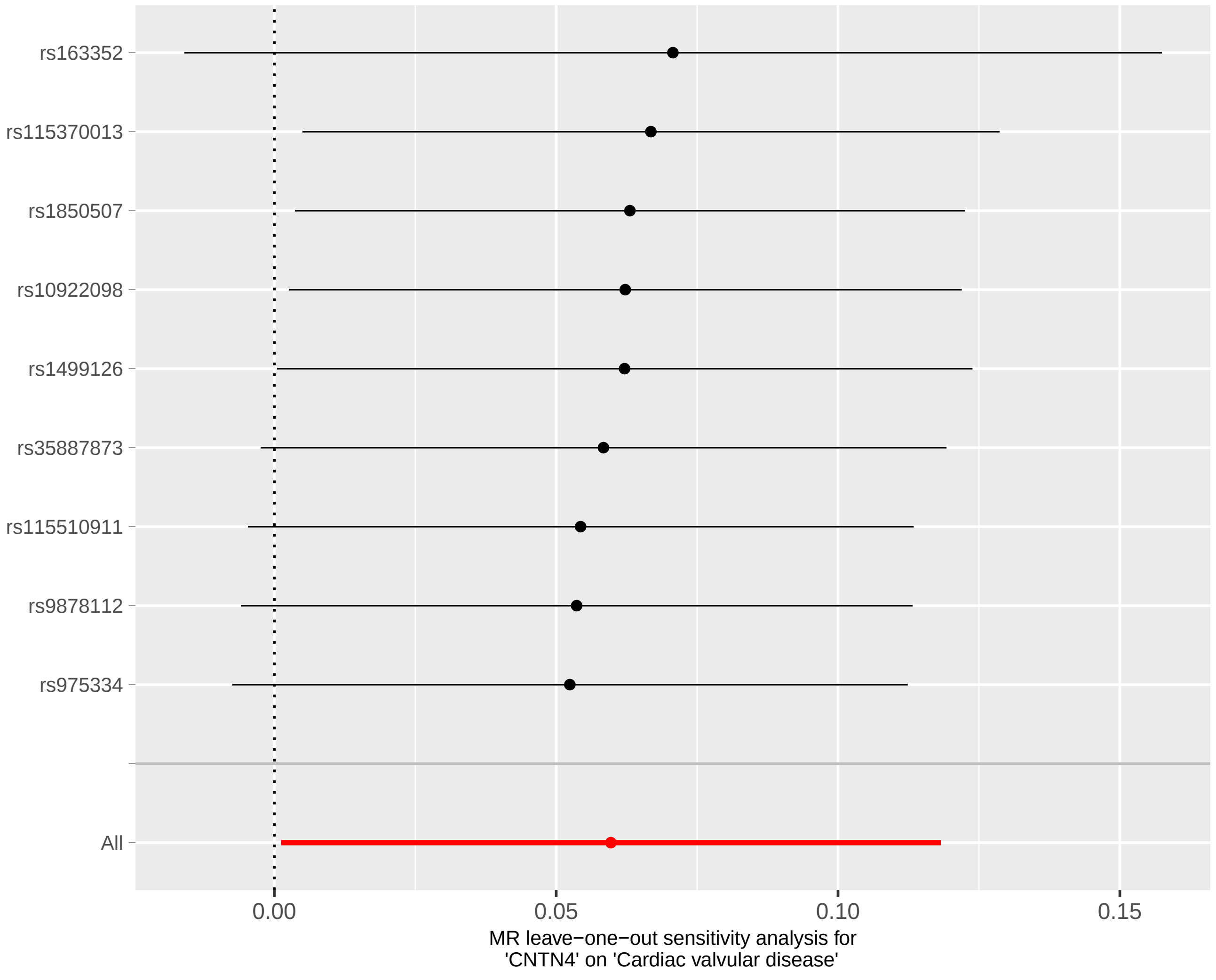
rs163352
rs115370013
rs1850507
rs10922098
rs1499126
rs35887873
rs115510911
rs9878112
rs975334
All
0.00
0.05
0.10
0.15
MR leave−one−out sensitivity analysis for
'CNTN4' on 'Cardiac valvular disease'

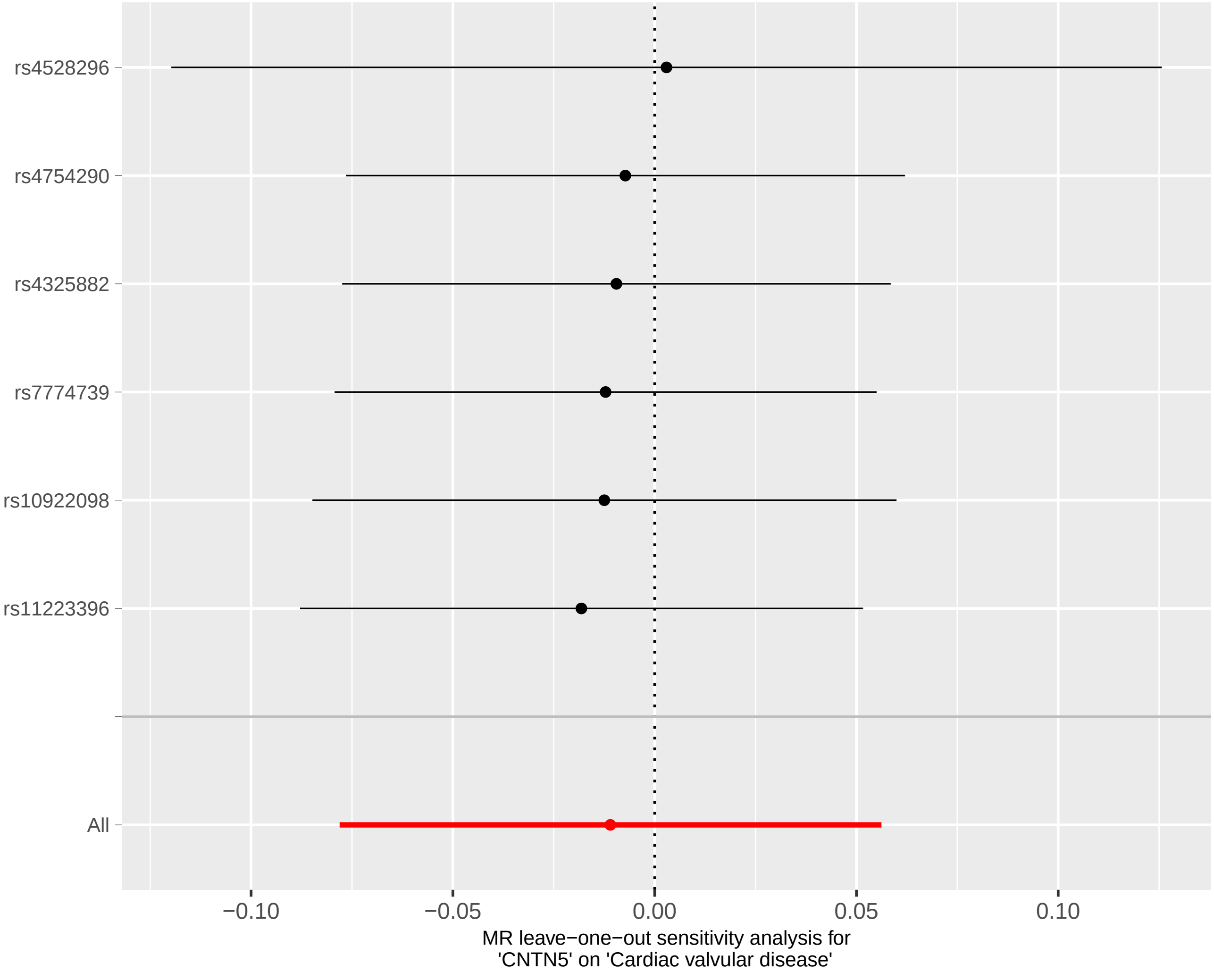
rs4528296
rs4754290
rs4325882
rs7774739
rs10922098
rs11223396
All
−0.10
−0.05
0.00
0.05
0.10
MR leave−one−out sensitivity analysis for
'CNTN5' on 'Cardiac valvular disease'

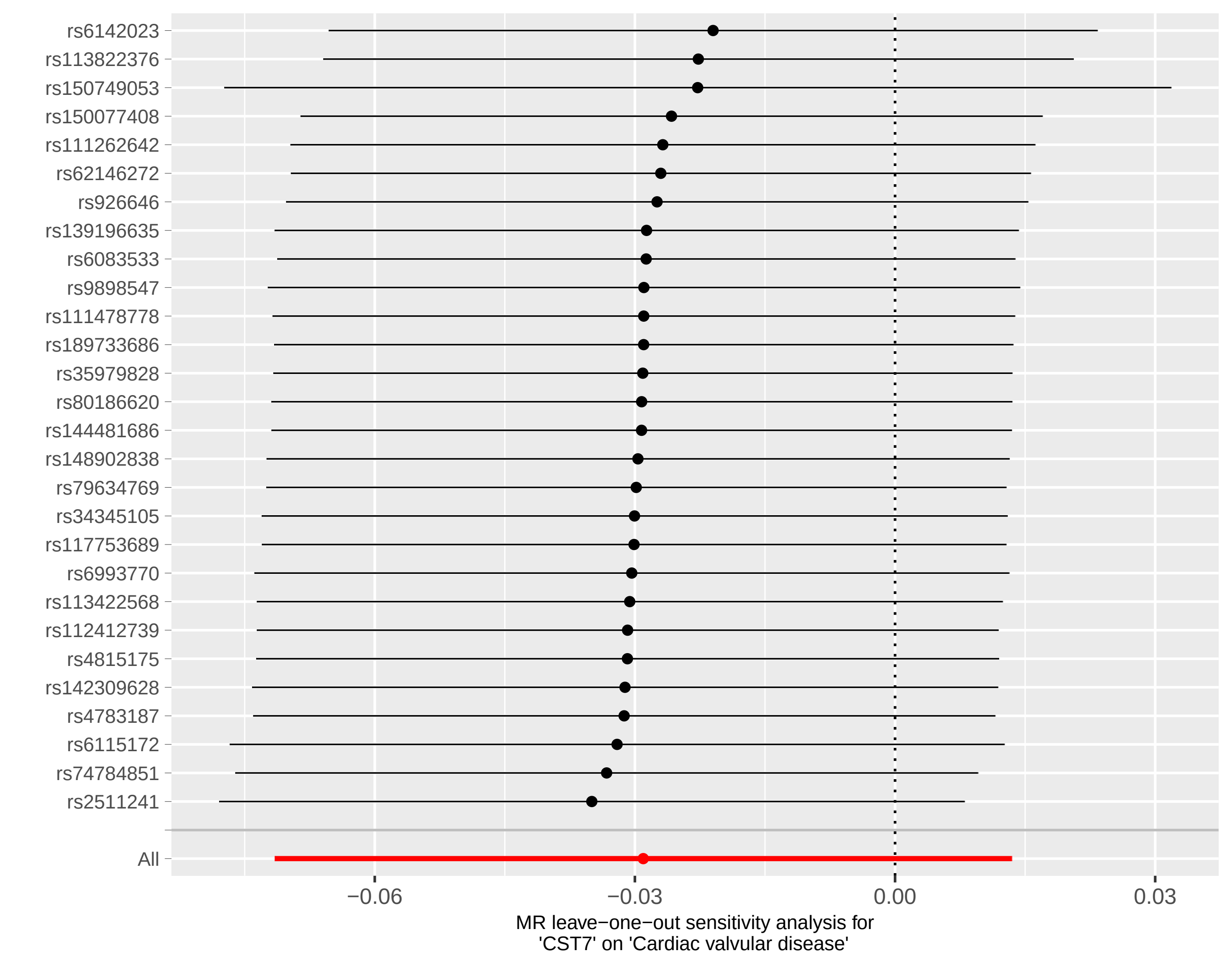
rs6142023
rs113822376
rs150749053
rs150077408
rs111262642
rs62146272
rs926646
rs139196635
rs6083533
rs9898547
rs111478778
rs189733686
rs35979828
rs80186620
rs144481686
rs148902838
rs79634769
rs34345105
rs117753689
rs6993770
rs113422568
rs112412739
rs4815175
rs142309628
rs4783187
rs6115172
rs74784851
rs2511241
All
−0.06
−0.03
0.00
0.03
MR leave−one−out sensitivity analysis for
'CST7' on 'Cardiac valvular disease'

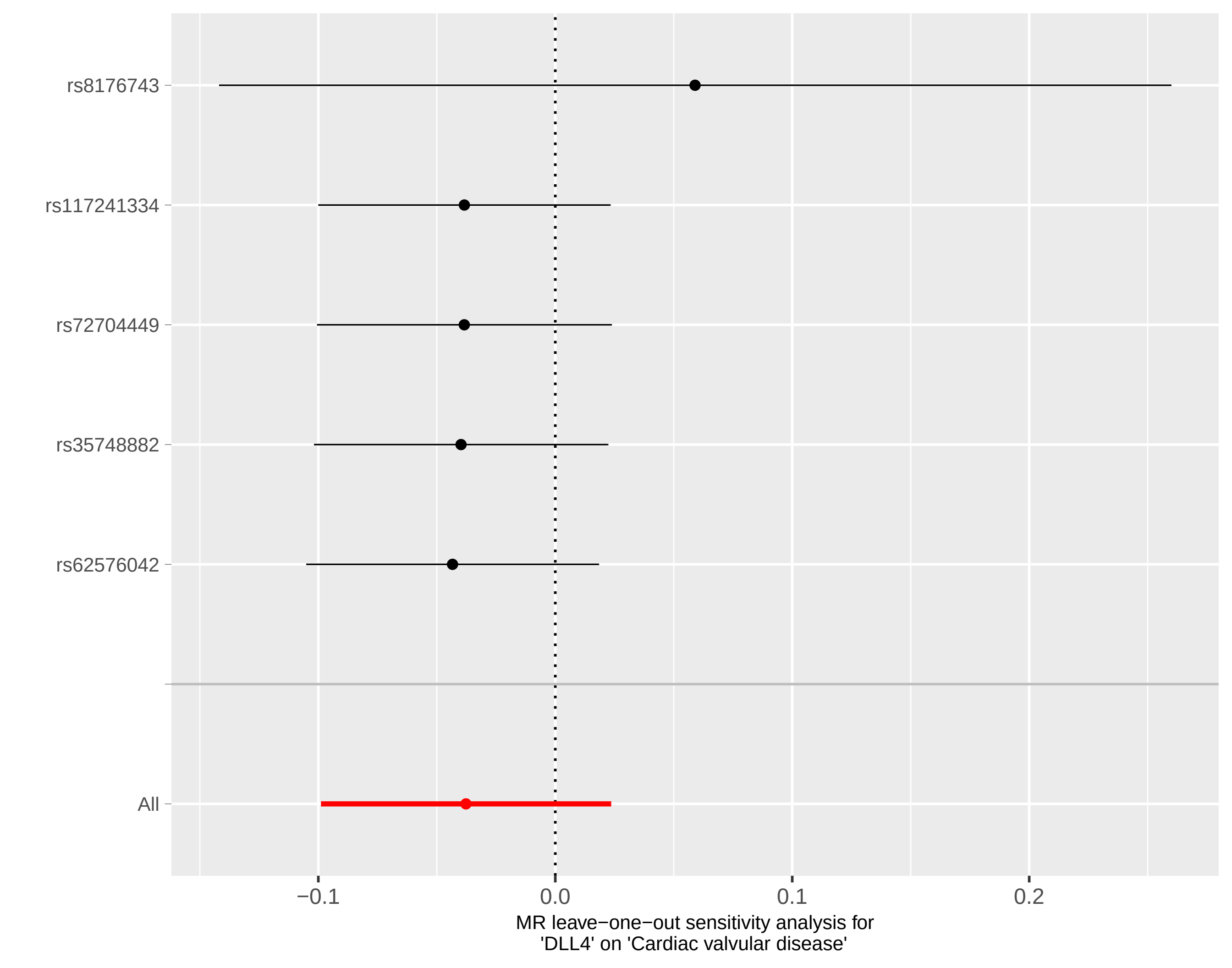
rs8176743
rs117241334
rs72704449
rs35748882
rs62576042
All
−0.1
0.0
0.1
0.2
MR leave−one−out sensitivity analysis for
'DLL4' on 'Cardiac valvular disease'

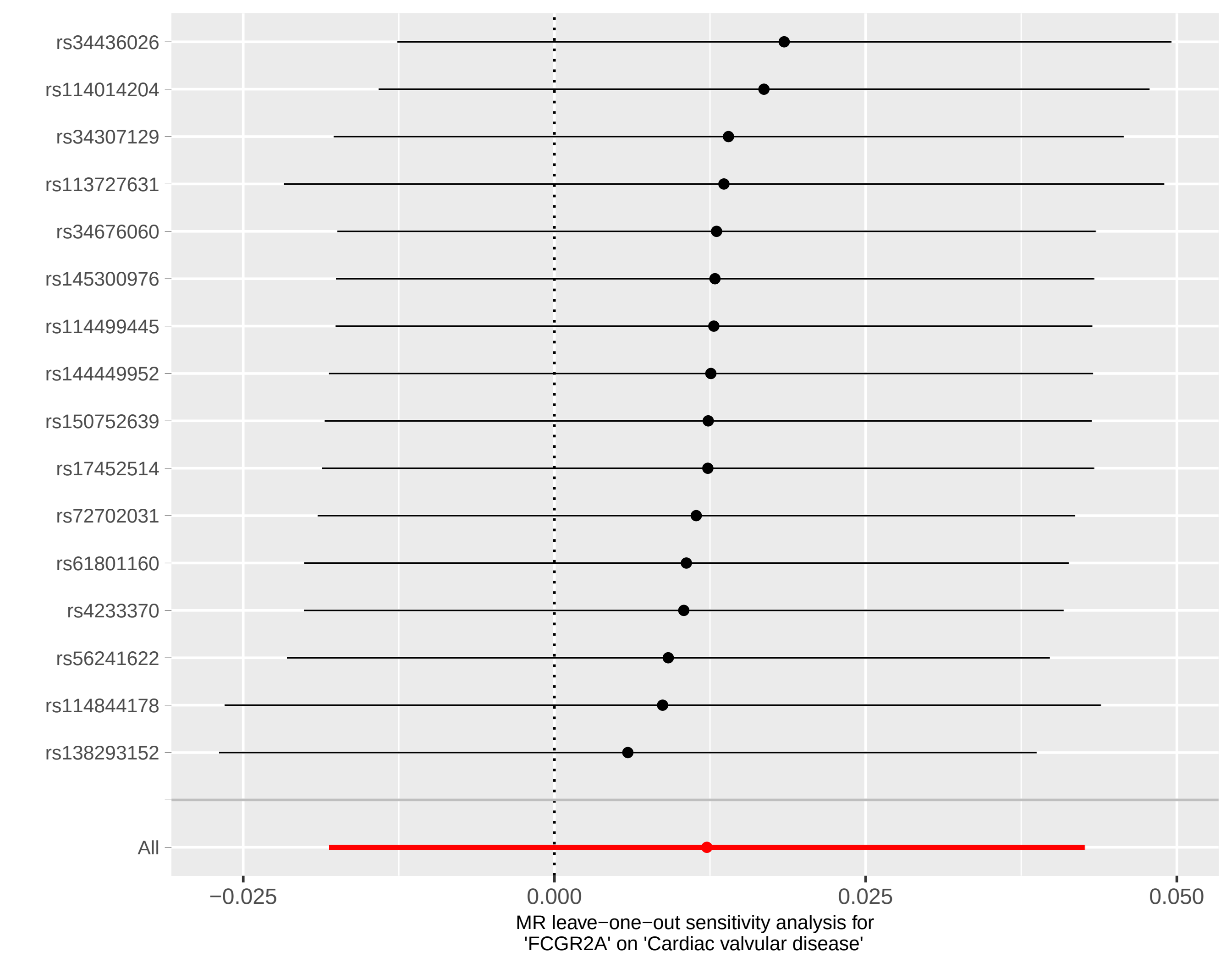
rs34436026
rs114014204
rs34307129
rs113727631
rs34676060
rs145300976
rs114499445
rs144449952
rs150752639
rs17452514
rs72702031
rs61801160
rs4233370
rs56241622
rs114844178
rs138293152
All
−0.025
0.000
0.025
0.050
MR leave−one−out sensitivity analysis for
'FCGR2A' on 'Cardiac valvular disease'

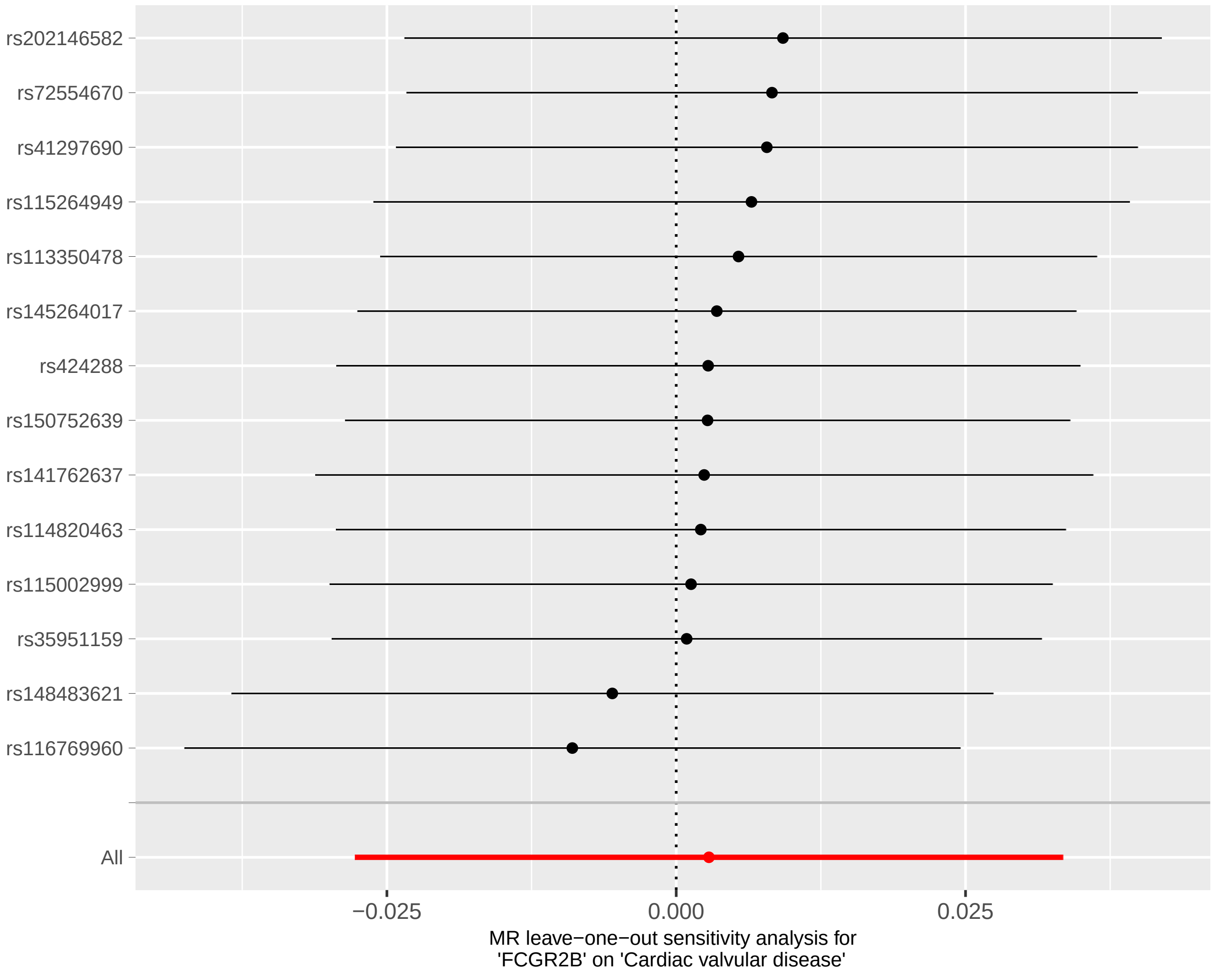
rs202146582
rs72554670
rs41297690
rs115264949
rs113350478
rs145264017
rs424288
rs150752639
rs141762637
rs114820463
rs115002999
rs35951159
rs148483621
rs116769960
All
−0.025
0.000
0.025
MR leave−one−out sensitivity analysis for
'FCGR2B' on 'Cardiac valvular disease'

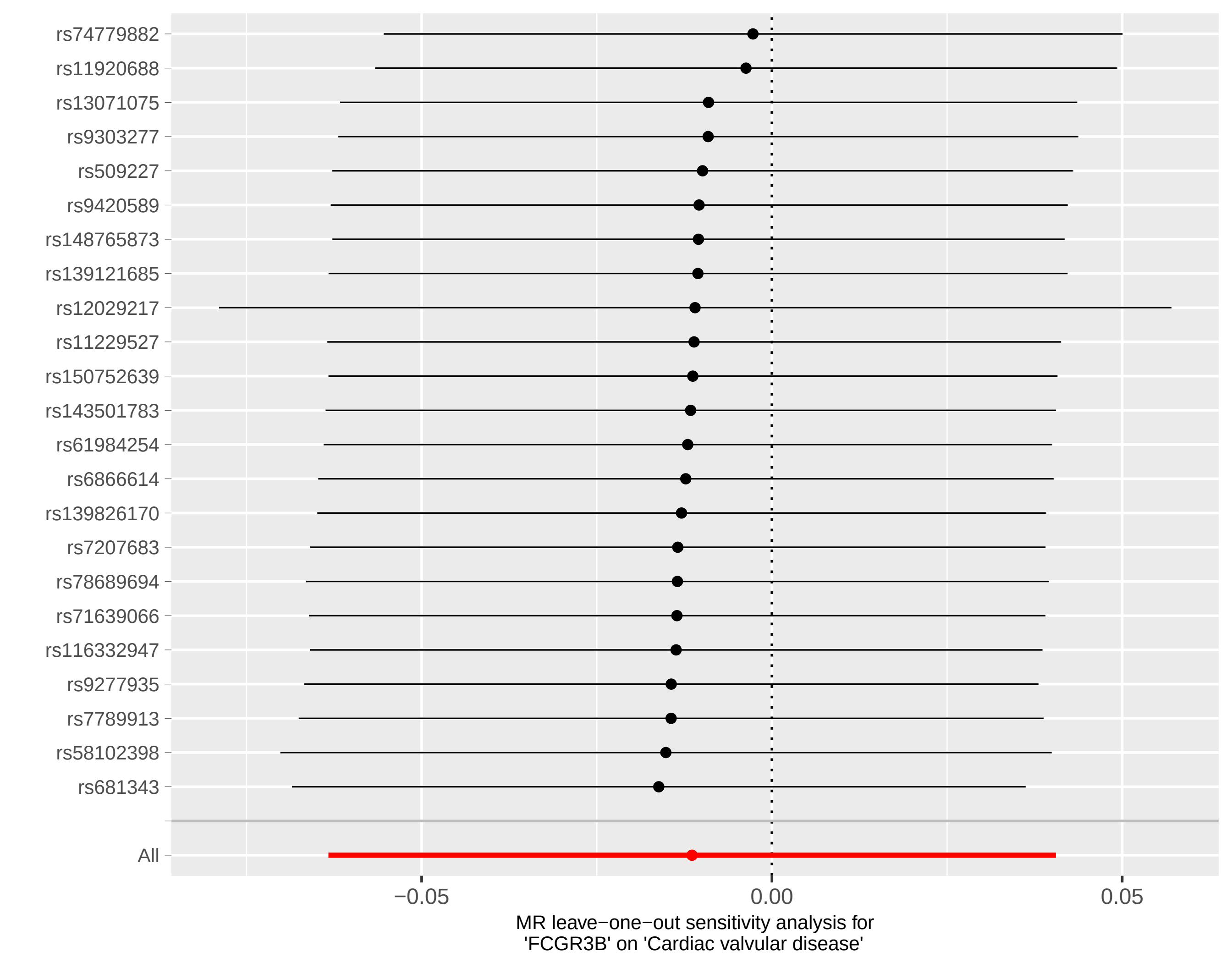
rs74779882
rs11920688
rs13071075
rs9303277
rs509227
rs9420589
rs148765873
rs139121685
rs12029217
rs11229527
rs150752639
rs143501783
rs61984254
rs6866614
rs139826170
rs7207683
rs78689694
rs71639066
rs116332947
rs9277935
rs7789913
rs58102398
rs681343
All
−0.05
0.00
0.05
MR leave−one−out sensitivity analysis for
'FCGR3B' on 'Cardiac valvular disease'

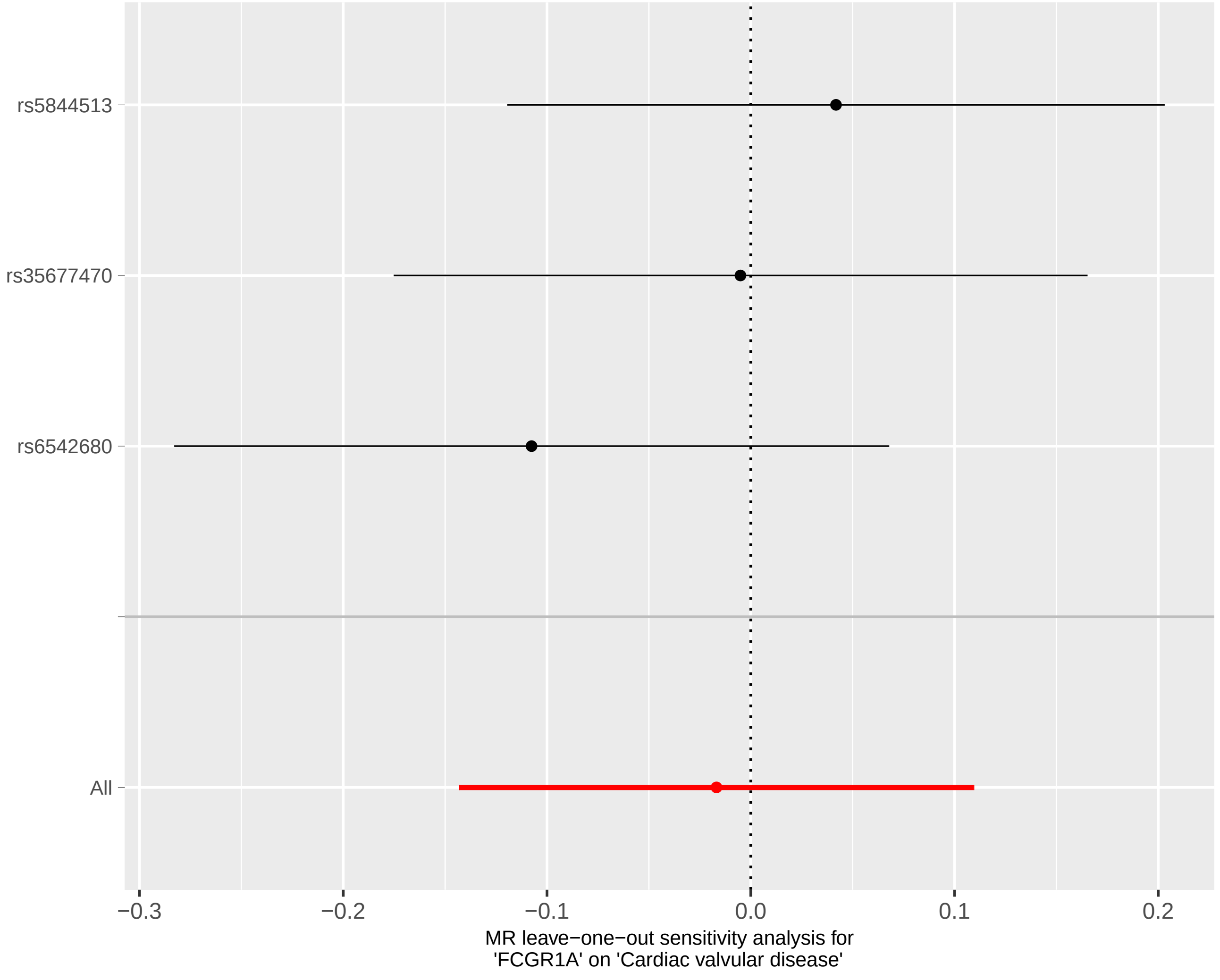
rs5844513
rs35677470
rs6542680
All
−0.3
−0.2
−0.1
0.0
0.1
0.2
MR leave−one−out sensitivity analysis for
'FCGR1A' on 'Cardiac valvular disease'

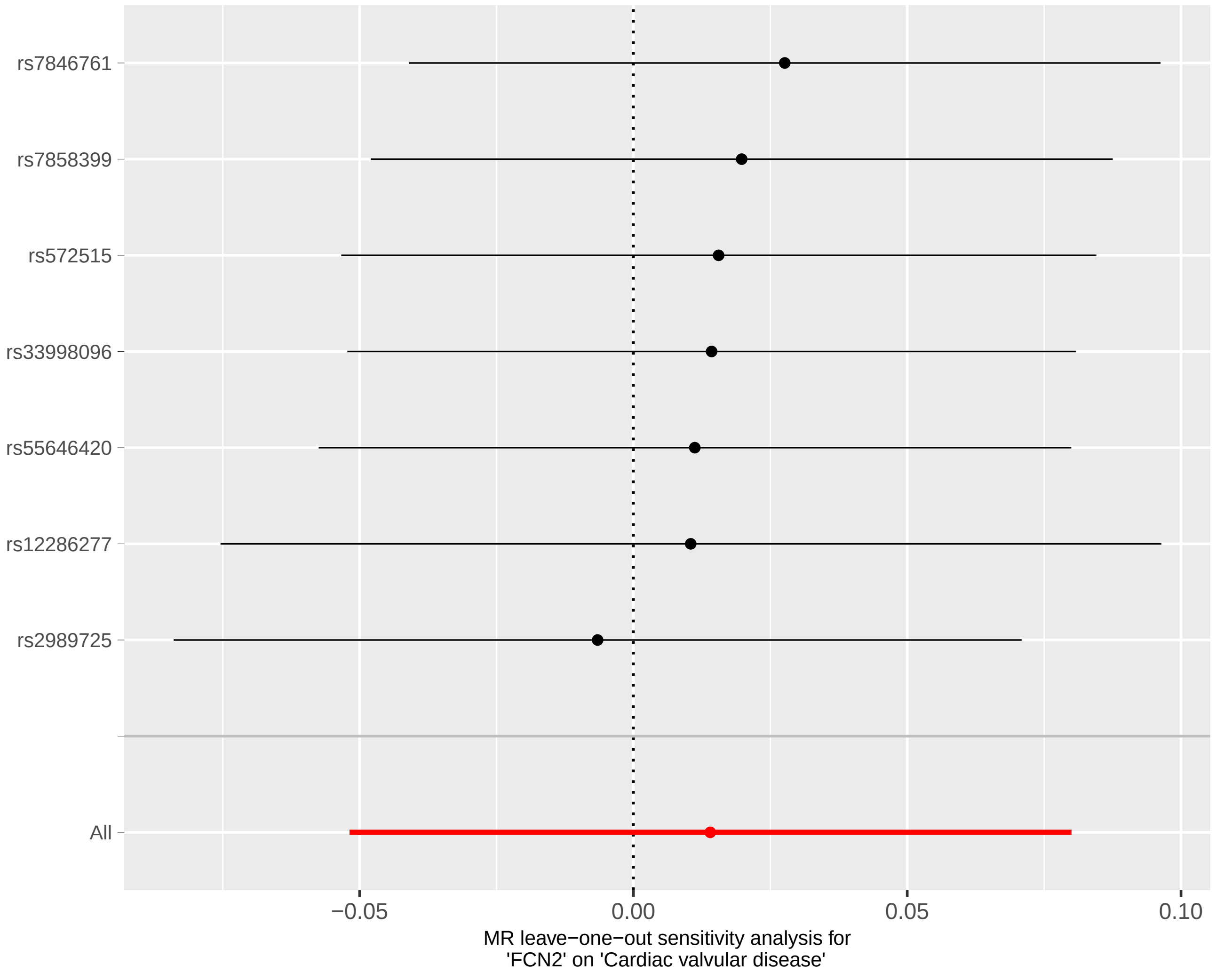

rs7846761
rs7858399
rs572515
rs33998096
rs55646420
rs12286277
rs2989725
All
−0.05
0.00
0.05
0.10
MR leave−one−out sensitivity analysis for
'FCN2' on 'Cardiac valvular disease'

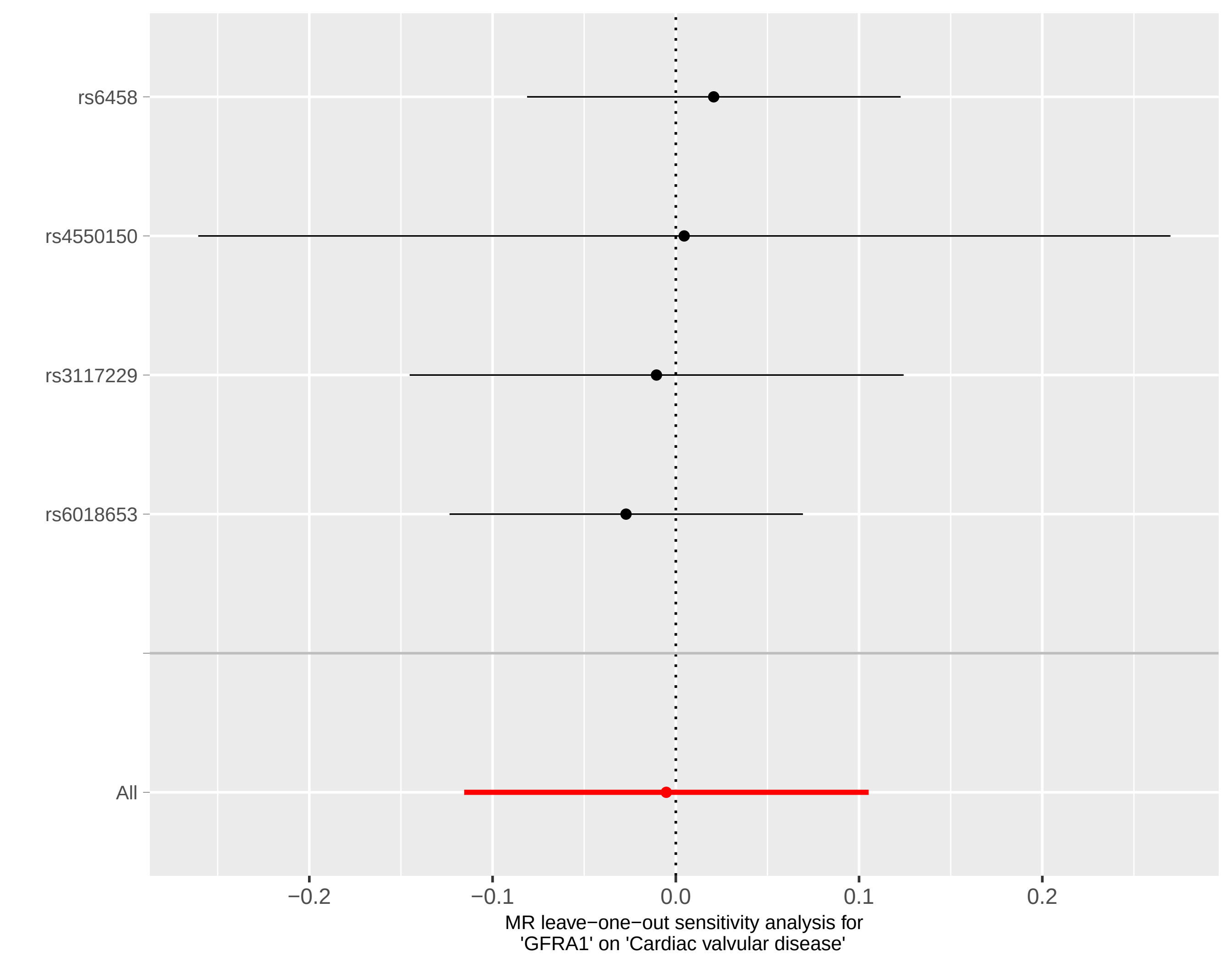
rs6458
rs4550150
rs3117229
rs6018653
All
−0.2
−0.1
0.0
0.1
0.2
MR leave−one−out sensitivity analysis for
'GFRA1' on 'Cardiac valvular disease'

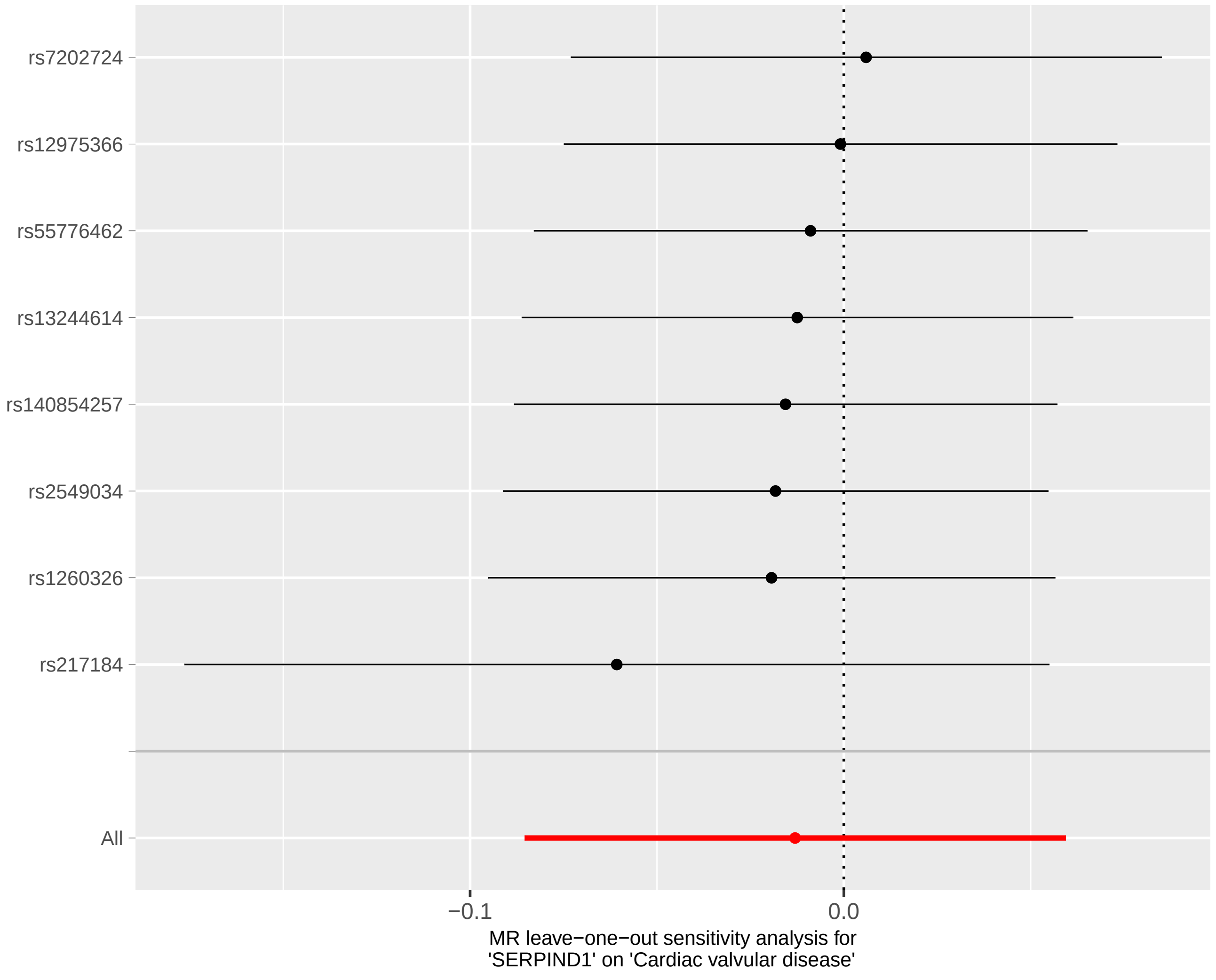
rs7202724
rs12975366
rs55776462
rs13244614
rs140854257
rs2549034
rs1260326
rs217184
All
−0.1
0.0
MR leave−one−out sensitivity analysis for
'SERPIND1' on 'Cardiac valvular disease'

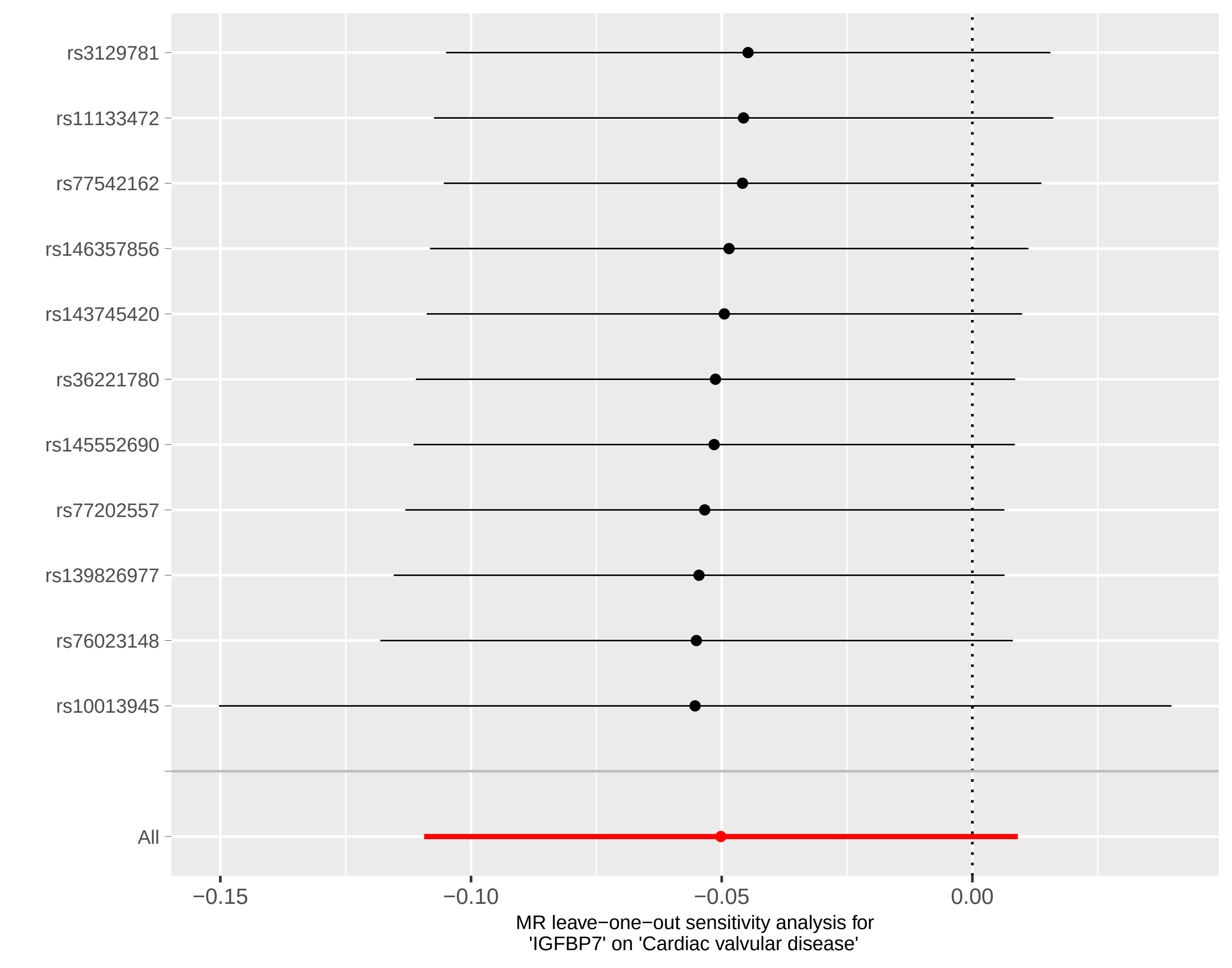

rs3129781
rs11133472
rs77542162
rs146357856
rs143745420
rs36221780
rs145552690
rs77202557
rs139826977
rs76023148
rs10013945
All
−0.15
−0.10
−0.05
0.00
MR leave−one−out sensitivity analysis for
'IGFBP7' on 'Cardiac valvular disease'

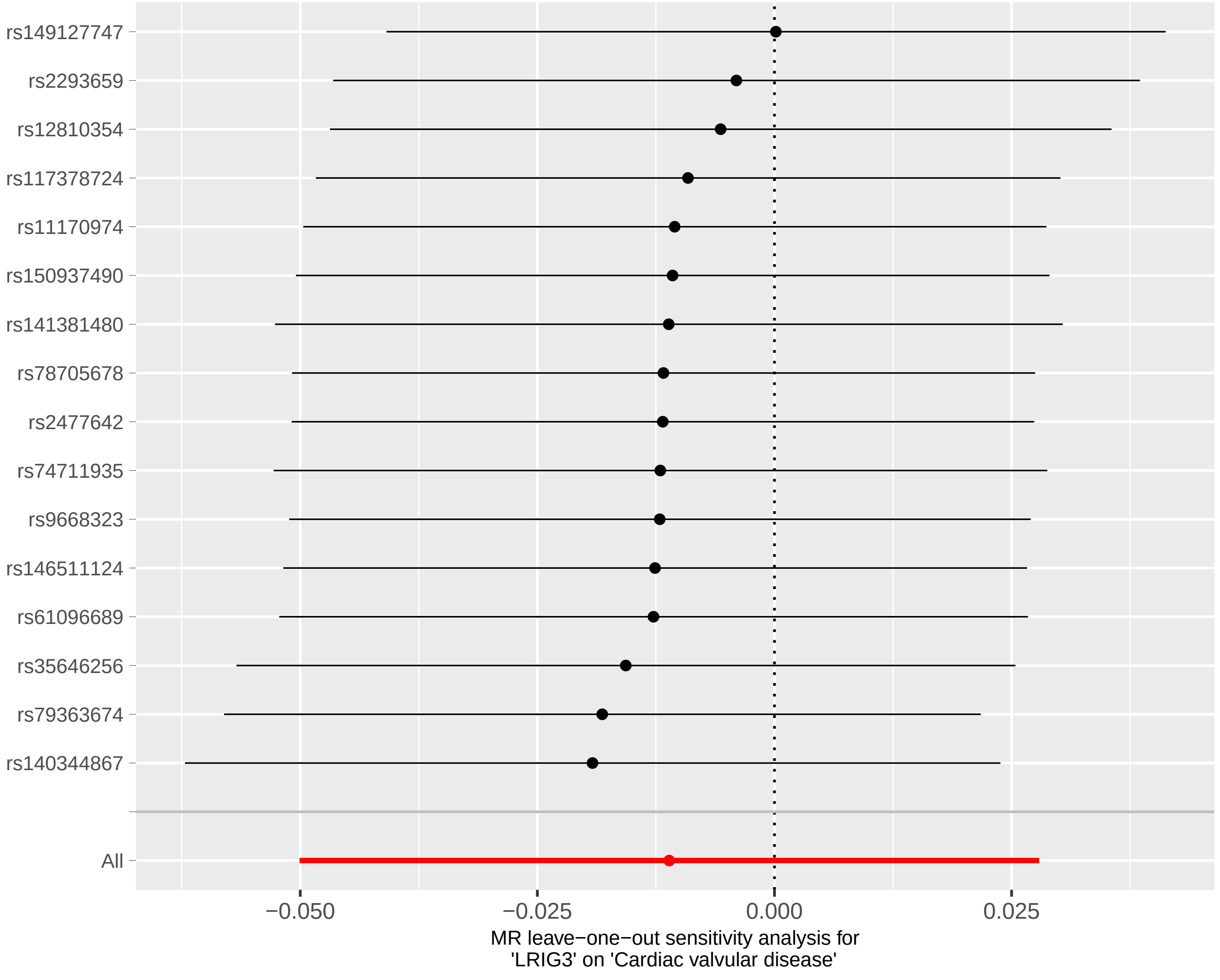
rs149127747
rs2293659
rs12810354
rs117378724
rs11170974
rs150937490
rs141381480
rs78705678
rs2477642
rs74711935
rs9668323
rs146511124
rs61096689
rs35646256
rs79363674
rs140344867
All
−0.050
−0.025
0.000
0.025
MR leave−one−out sensitivity analysis for
'LRIG3' on 'Cardiac valvular disease'

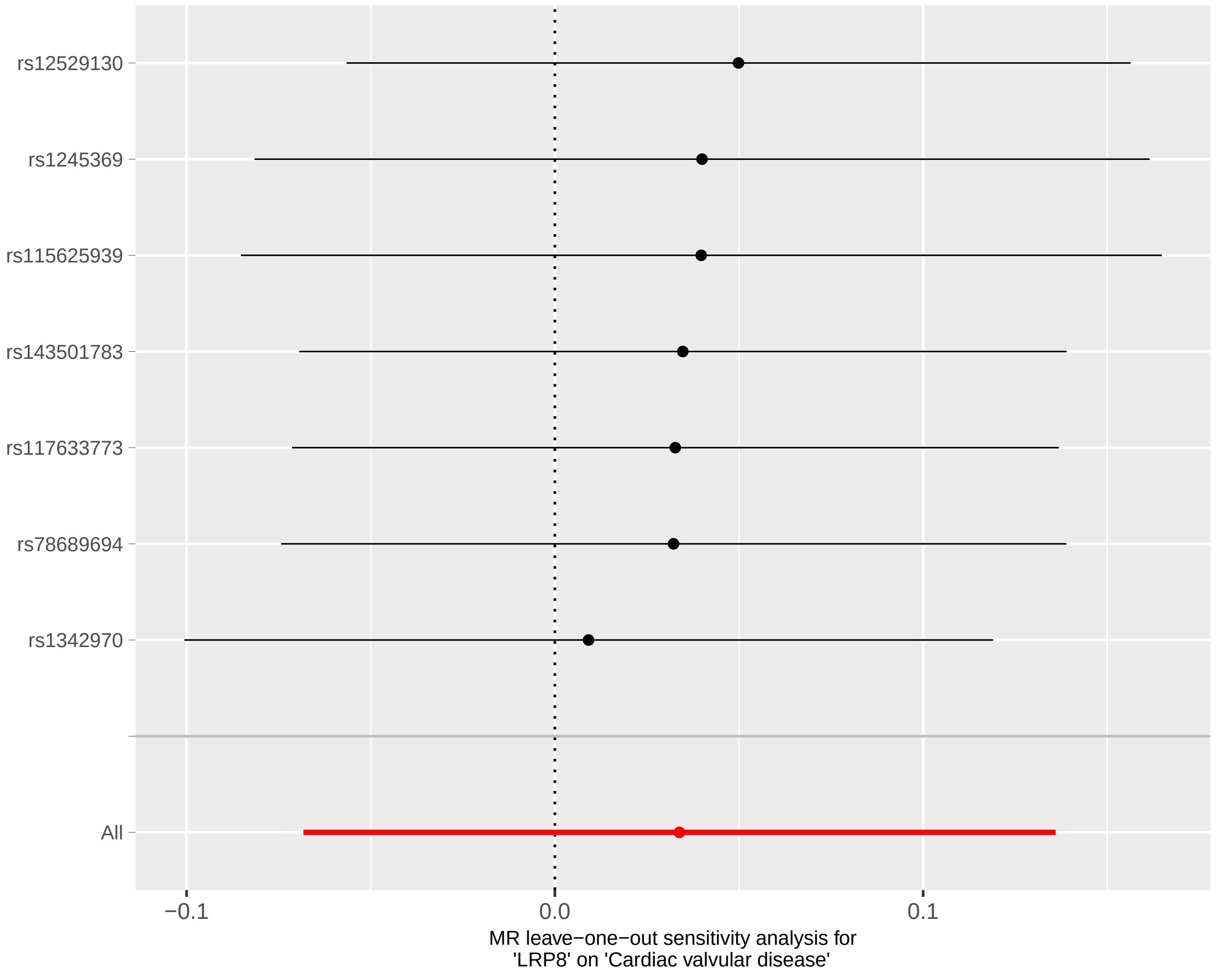
rs12529130
rs1245369
rs115625939
rs143501783
rs117633773
rs78689694
rs1342970
All
−0.1
0.0
0.1
MR leave−one−out sensitivity analysis for
'LRP8' on 'Cardiac valvular disease'

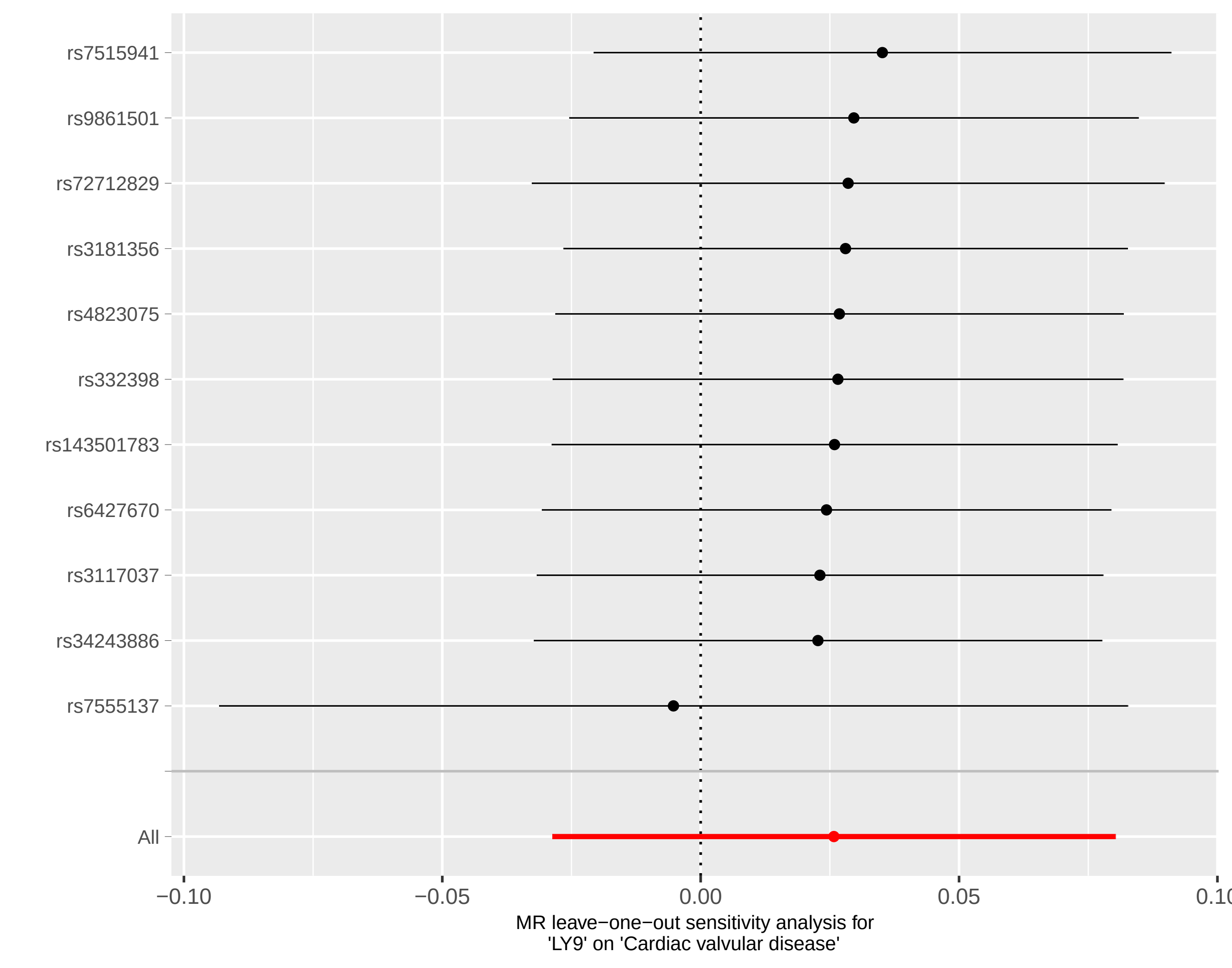

rs7515941
rs9861501
rs72712829
rs3181356
rs4823075
rs332398
rs143501783
rs6427670
rs3117037
rs34243886
rs7555137
All
−0.10
−0.05
0.00
0.05
0.10
MR leave−one−out sensitivity analysis for
'LY9' on 'Cardiac valvular disease'

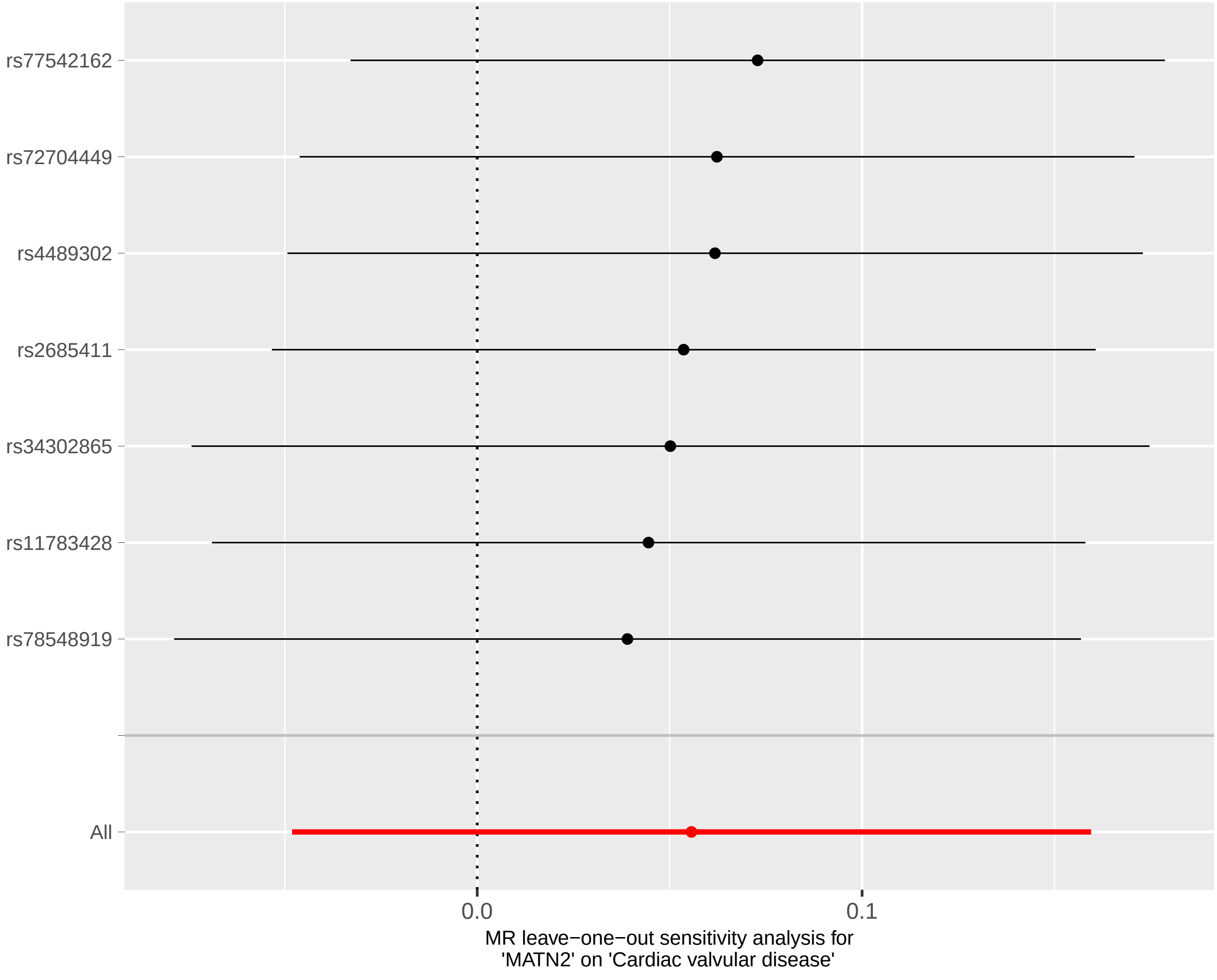
rs77542162
rs72704449
rs4489302
rs2685411
rs34302865
rs11783428
rs78548919
All
0.0
0.1
MR leave−one−out sensitivity analysis for
'MATN2' on 'Cardiac valvular disease'

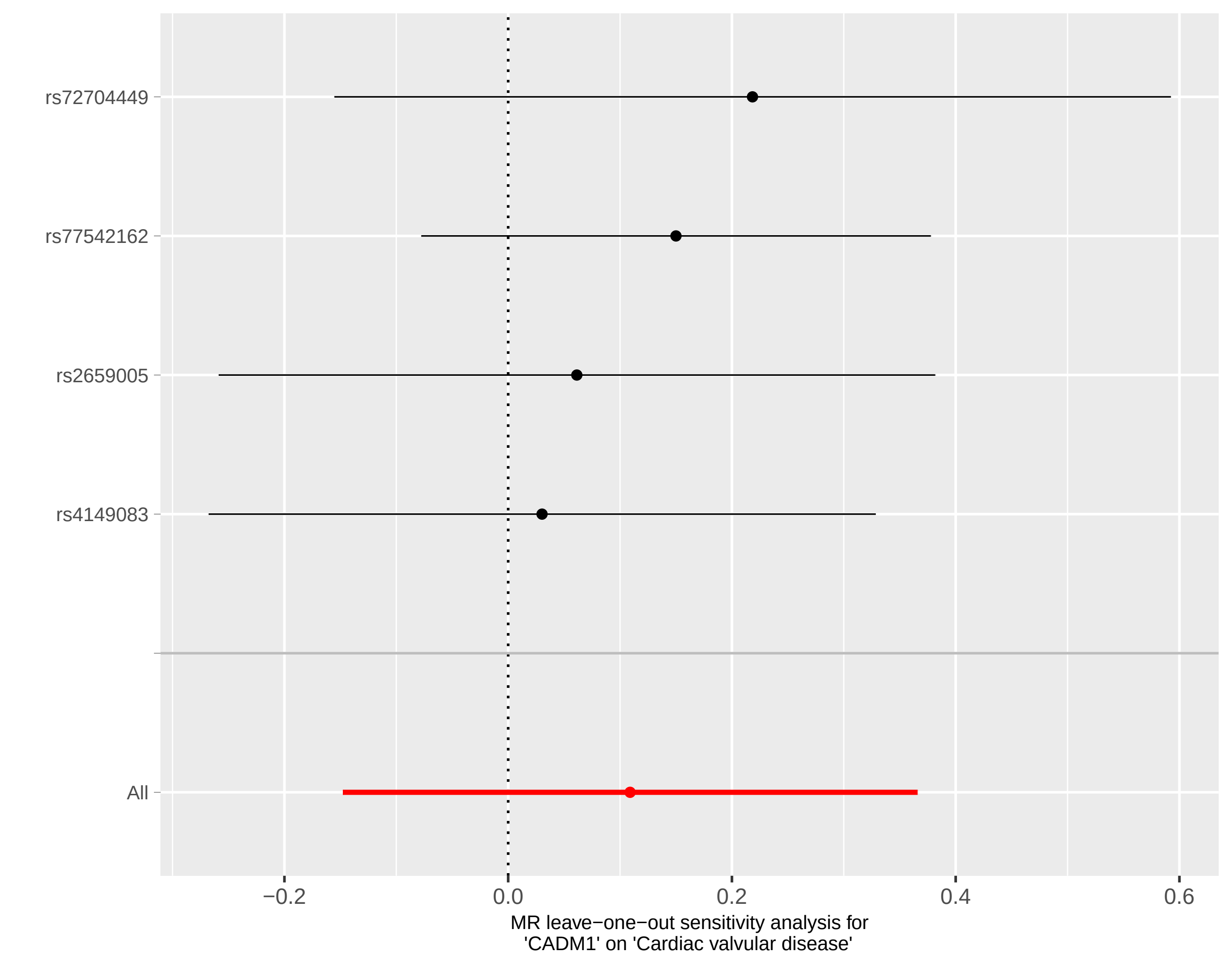
rs72704449
rs77542162
rs2659005
rs4149083
All
−0.2
0.0
0.2
0.4
0.6
MR leave−one−out sensitivity analysis for
'CADM1' on 'Cardiac valvular disease'

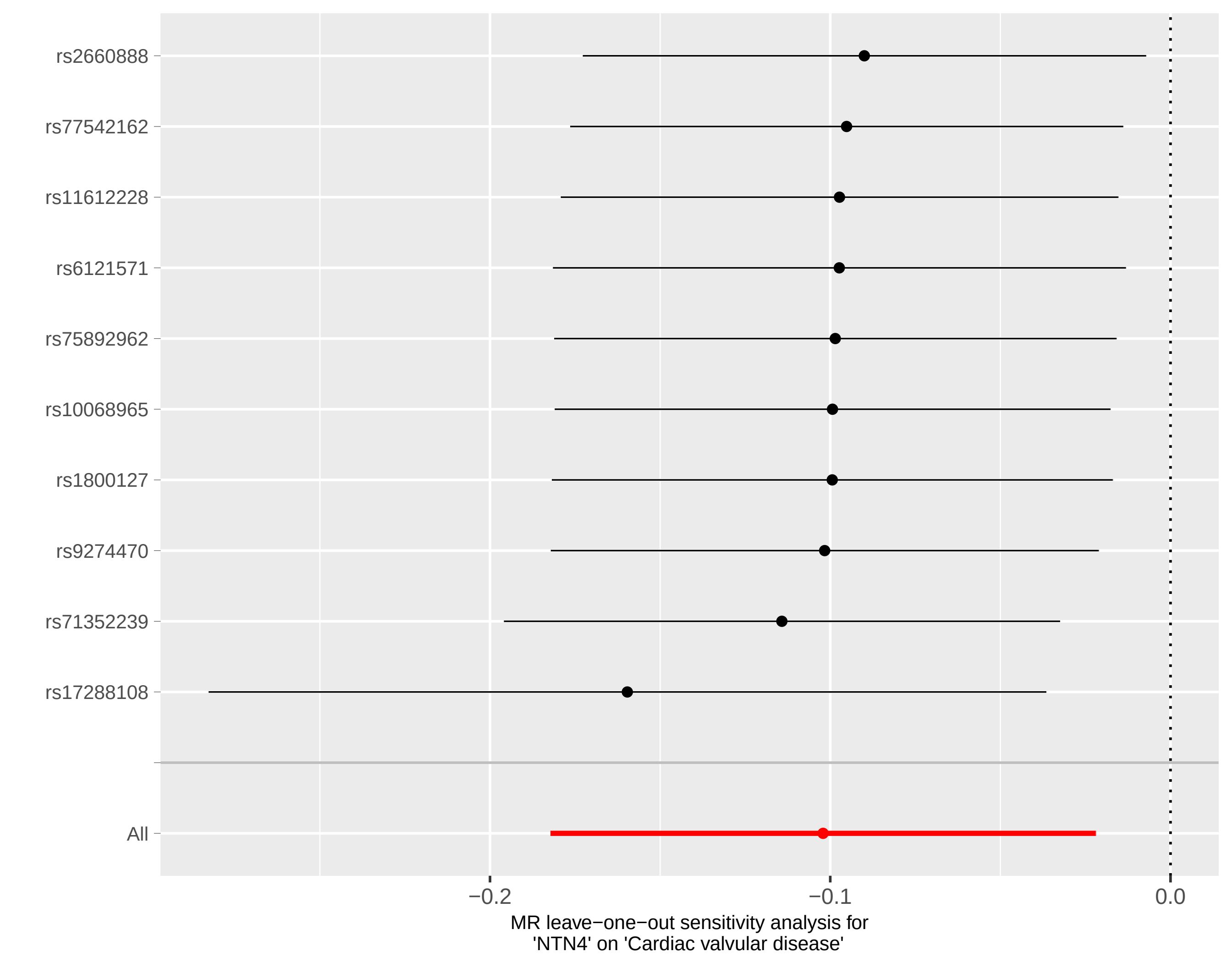
rs2660888
rs77542162
rs11612228
rs6121571
rs75892962
rs10068965
rs1800127
rs9274470
rs71352239
rs17288108
All
−0.2
−0.1
0.0
MR leave−one−out sensitivity analysis for
'NTN4' on 'Cardiac valvular disease'

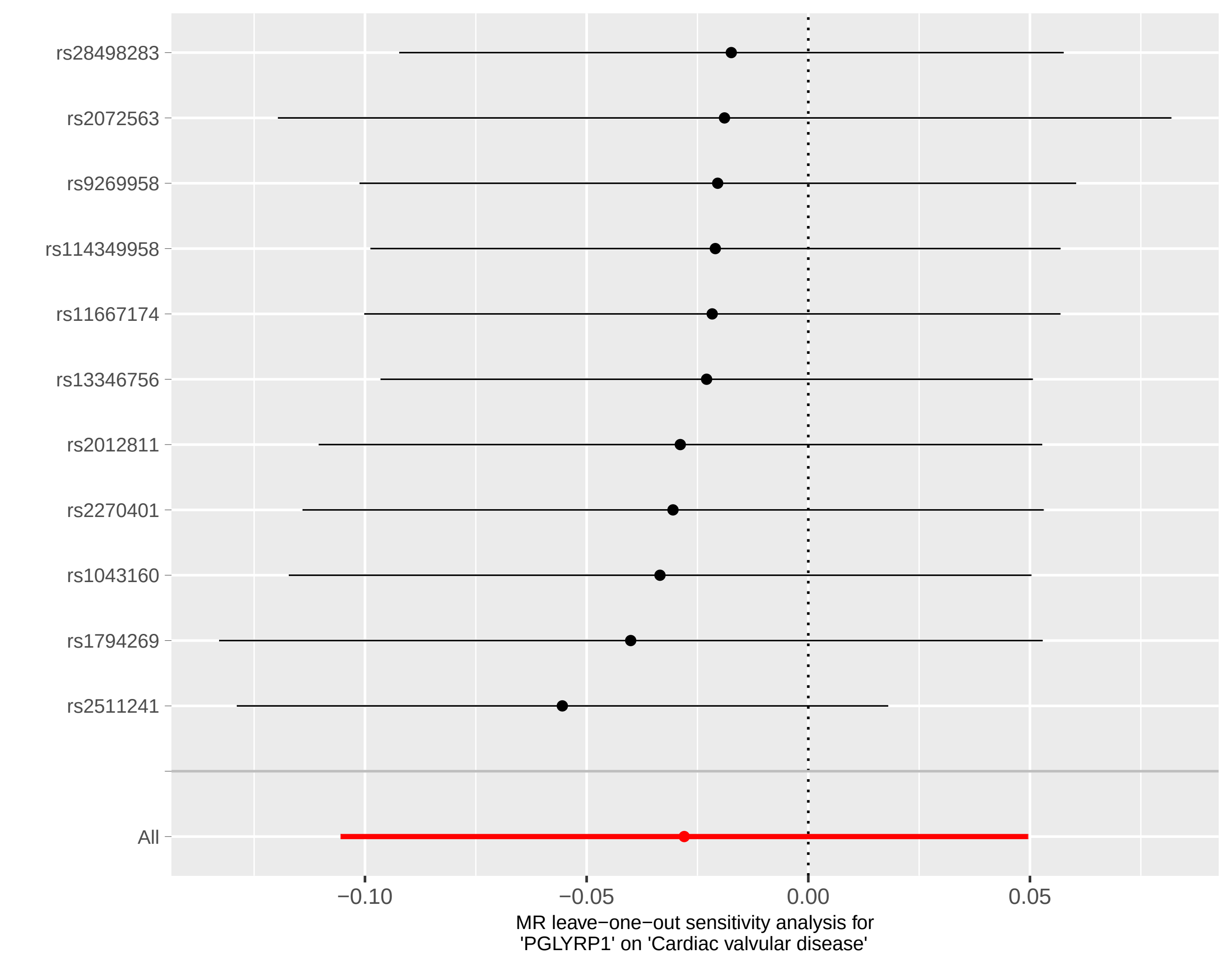

rs28498283
rs2072563
rs9269958
rs114349958
rs11667174
rs13346756
rs2012811
rs2270401
rs1043160
rs1794269
rs2511241
All
−0.10
−0.05
0.00
0.05
MR leave−one−out sensitivity analysis for
'PGLYRP1' on 'Cardiac valvular disease'

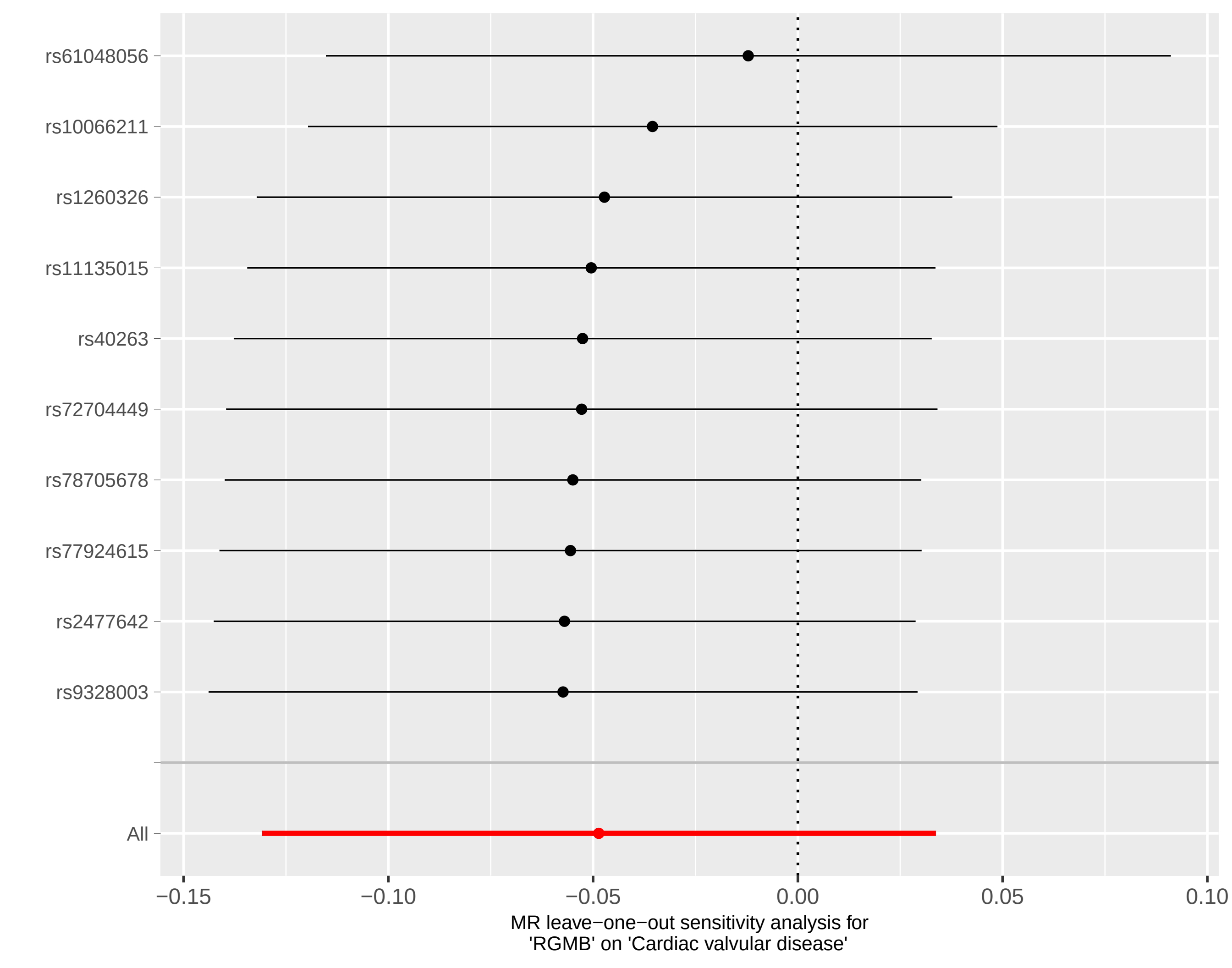
rs61048056
rs10066211
rs1260326
rs11135015
rs40263
rs72704449
rs78705678
rs77924615
rs2477642
rs9328003
All
−0.15
−0.10
−0.05
0.00
0.05
0.10
MR leave−one−out sensitivity analysis for
'RGMB' on 'Cardiac valvular disease'

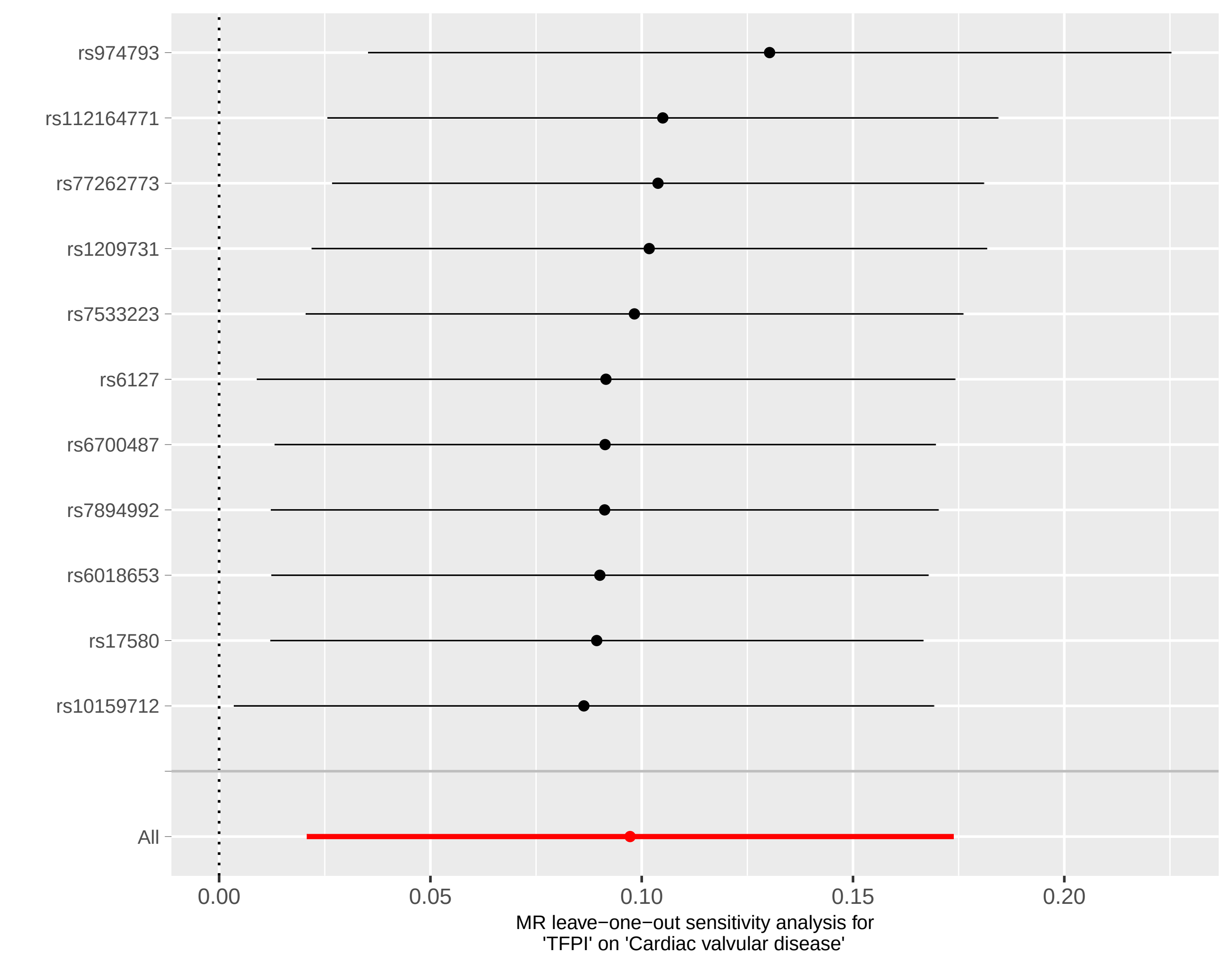
rs974793
rs112164771
rs77262773
rs1209731
rs7533223
rs6127
rs6700487
rs7894992
rs6018653
rs17580
rs10159712
All
0.00
0.05
0.10
0.15
0.20
MR leave−one−out sensitivity analysis for
'TFPI' on 'Cardiac valvular disease'

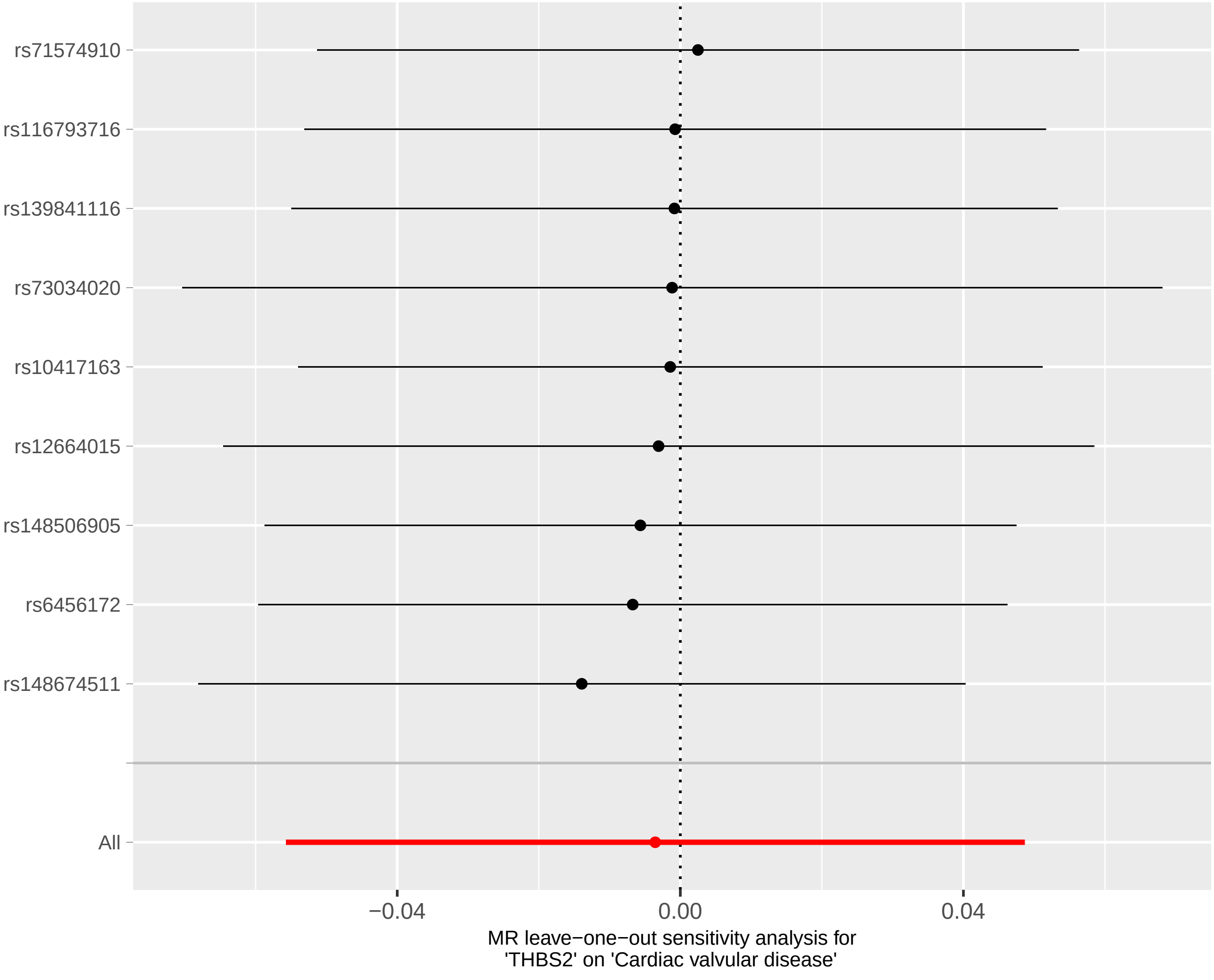
rs71574910
rs116793716
rs139841116
rs73034020
rs10417163
rs12664015
rs148506905
rs6456172
rs148674511
All
−0.04
0.00
0.04
MR leave−one−out sensitivity analysis for
'THBS2' on 'Cardiac valvular disease'

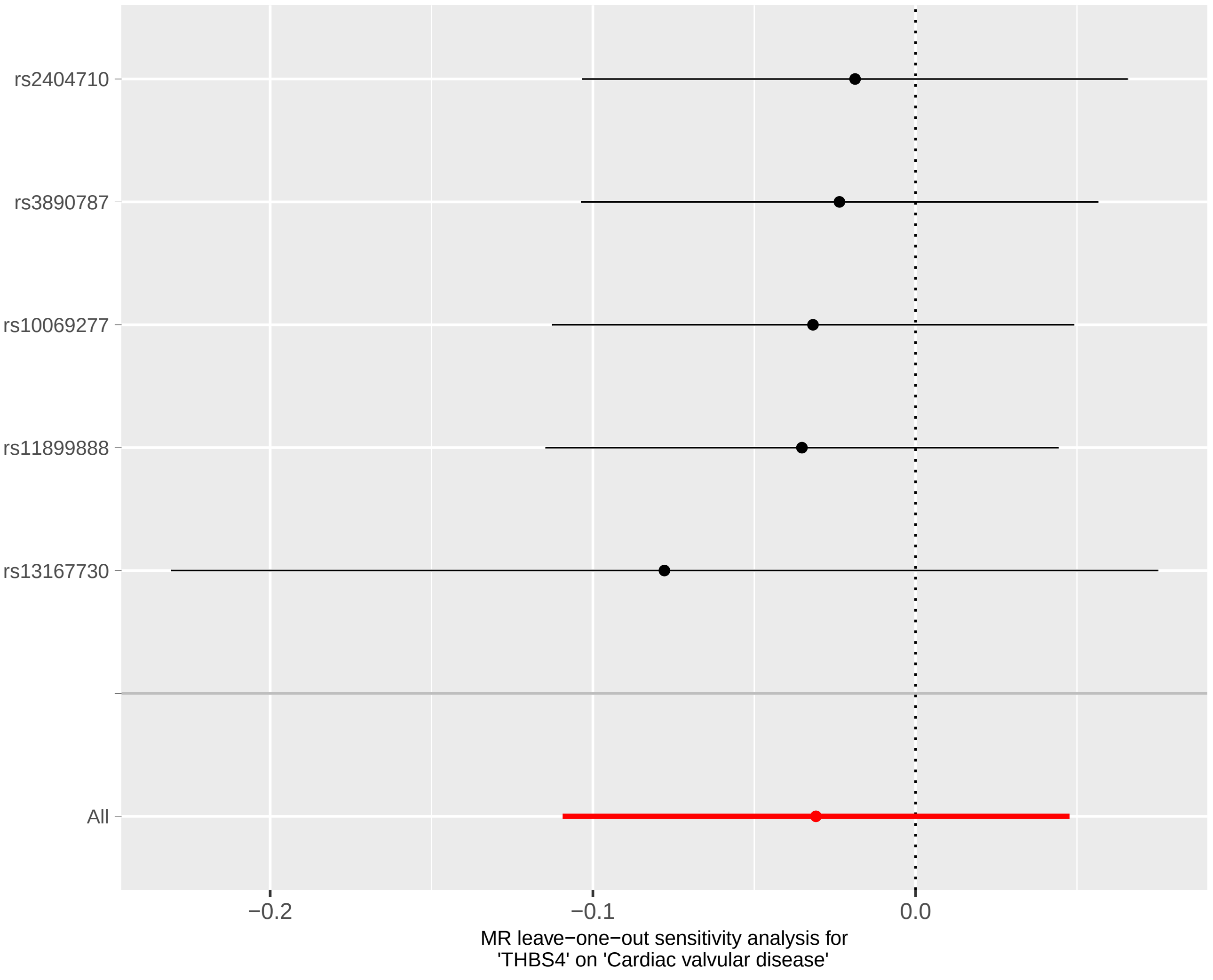

rs2404710
rs3890787
rs10069277
rs11899888
rs13167730
All
−0.2
−0.1
0.0
MR leave−one−out sensitivity analysis for
'THBS4' on 'Cardiac valvular disease'

Insufficient number of SNPs

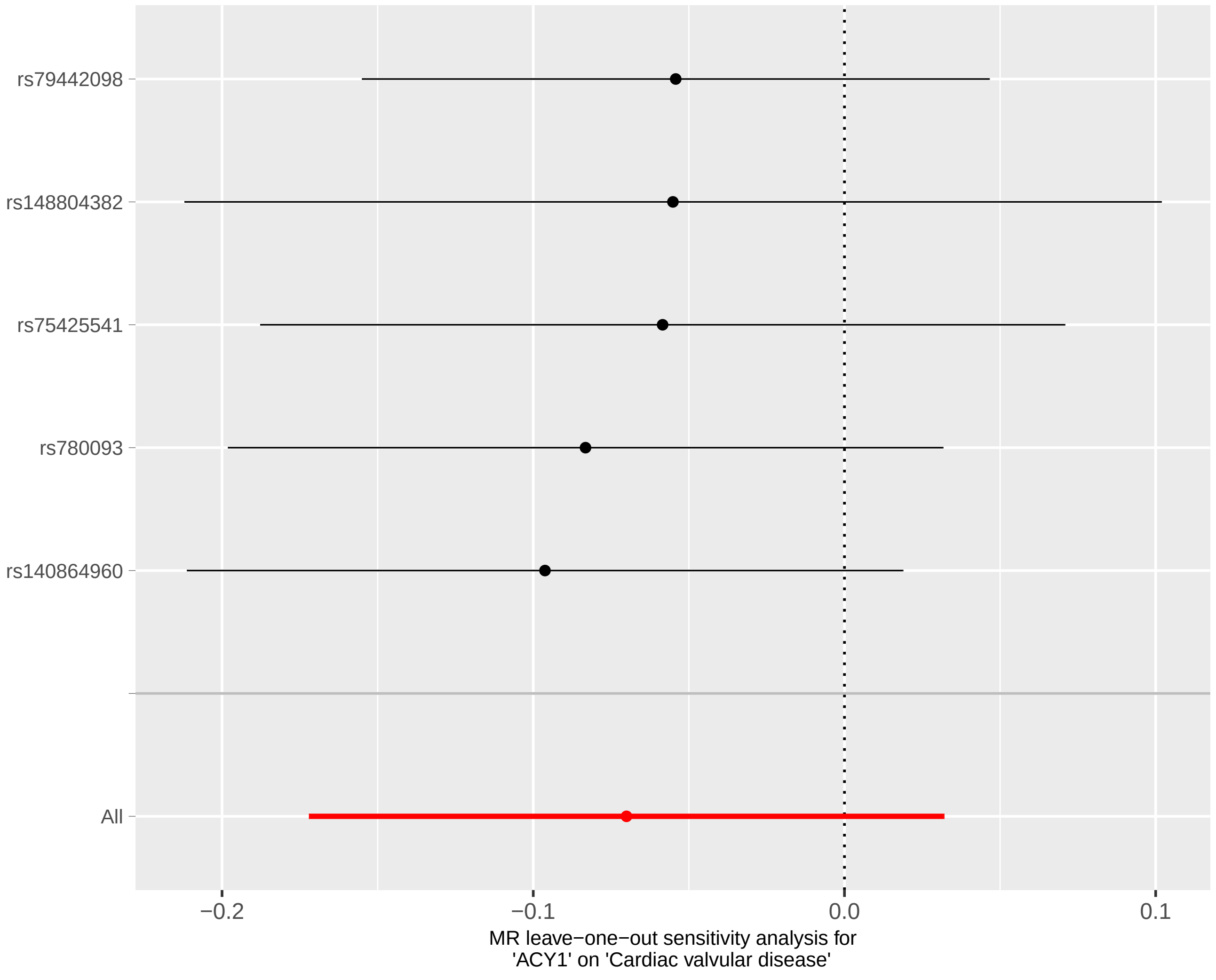
rs79442098
rs148804382
rs75425541
rs780093
rs140864960
All
−0.2
−0.1
0.0
0.1
MR leave−one−out sensitivity analysis for
'ACY1' on 'Cardiac valvular disease'

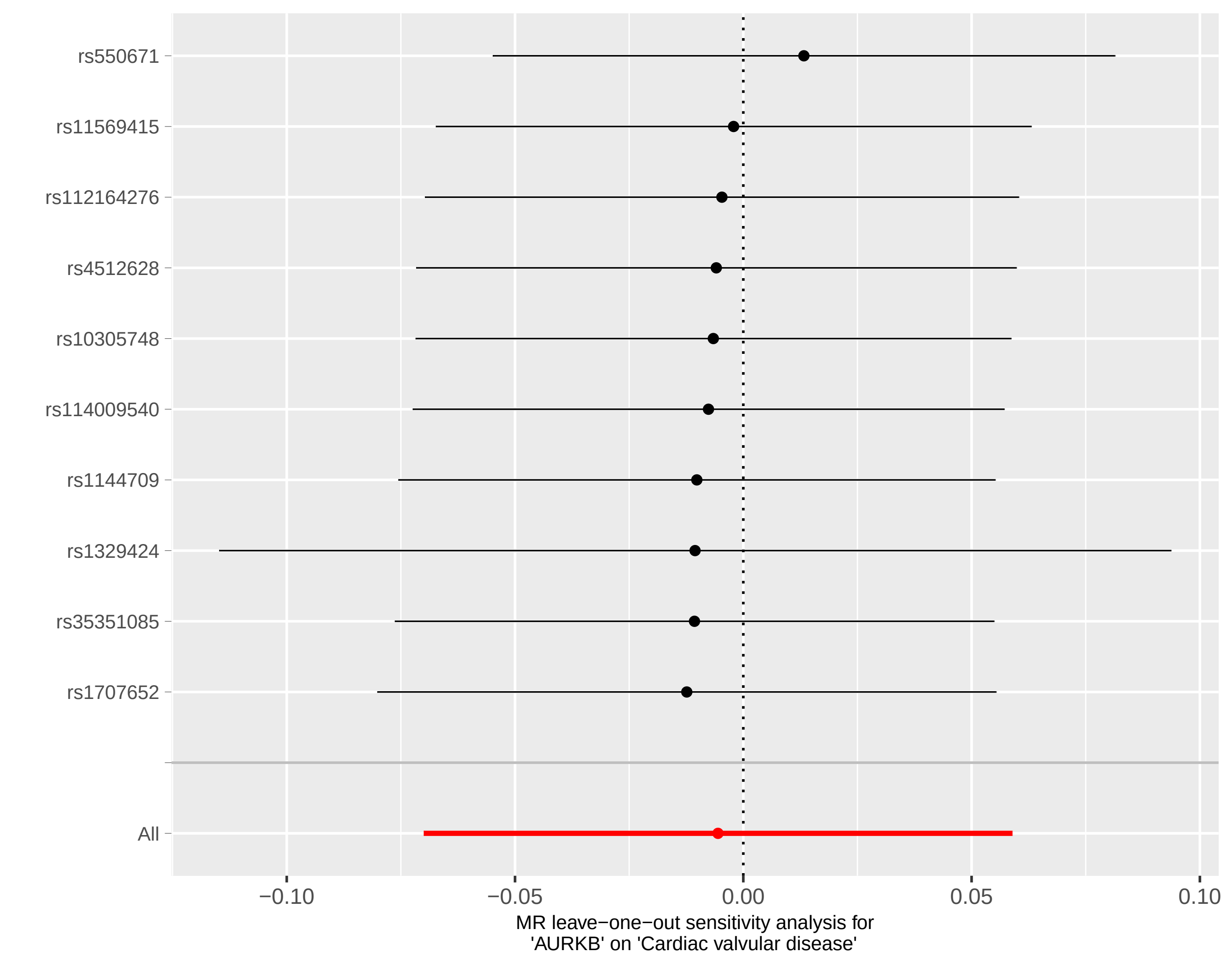
rs550671
rs11569415
rs112164276
rs4512628
rs10305748
rs114009540
rs1144709
rs1329424
rs35351085
rs1707652
All
−0.10
−0.05
0.00
0.05
0.10
MR leave−one−out sensitivity analysis for
'AURKB' on 'Cardiac valvular disease'

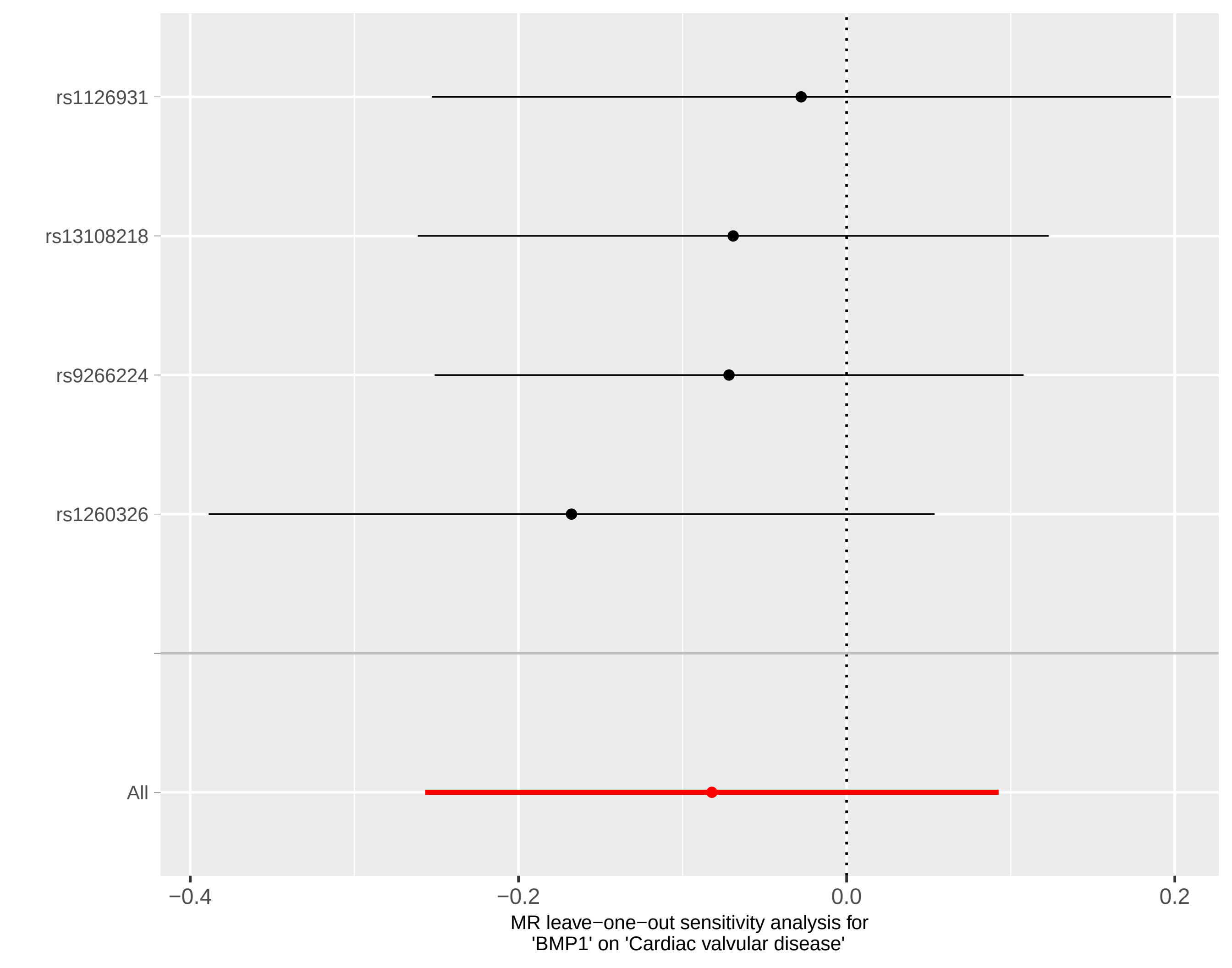
rs1126931
rs13108218
rs9266224
rs1260326
All
−0.4
−0.2
0.0
0.2
MR leave−one−out sensitivity analysis for
'BMP1' on 'Cardiac valvular disease'

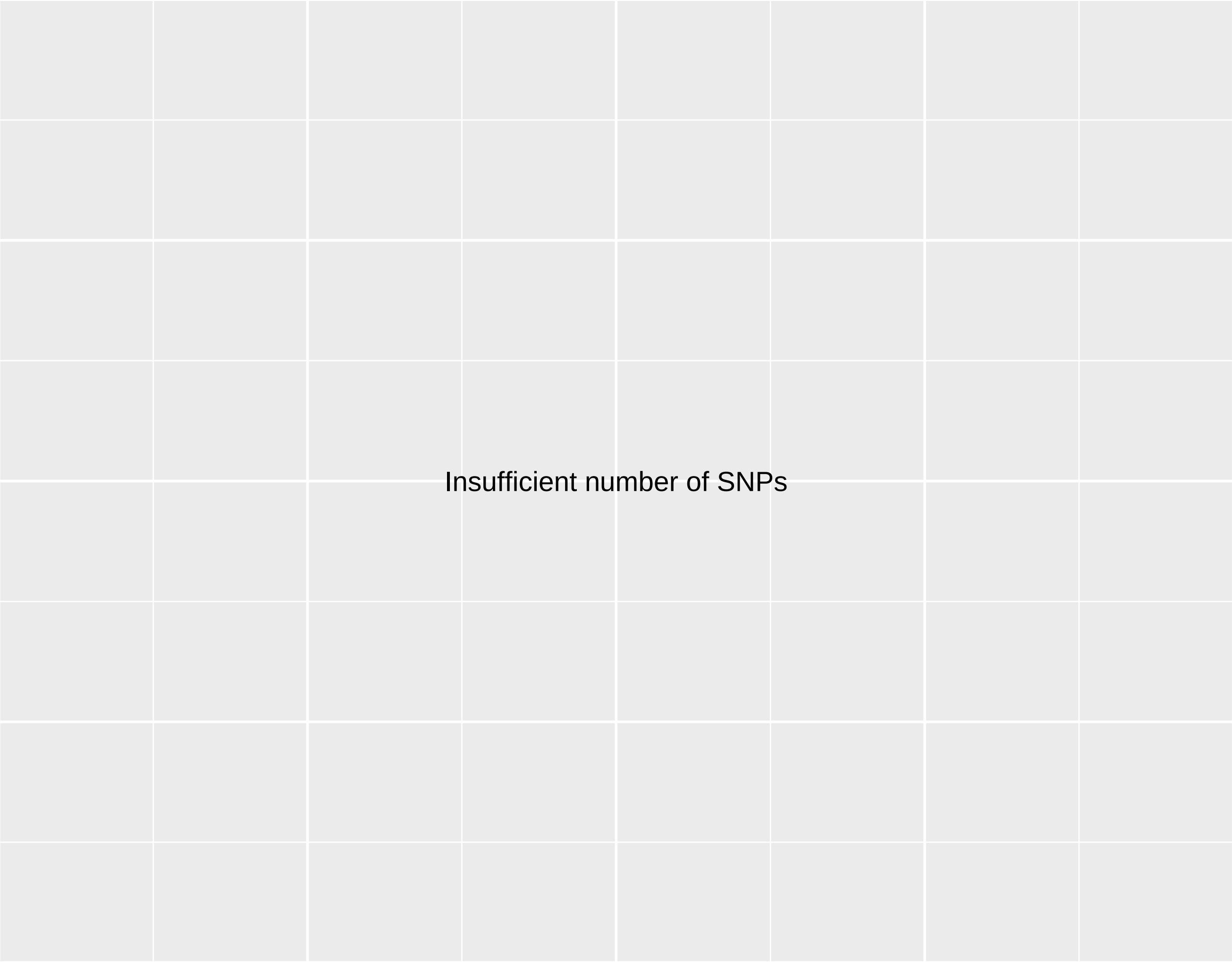
Insufficient number of SNPs

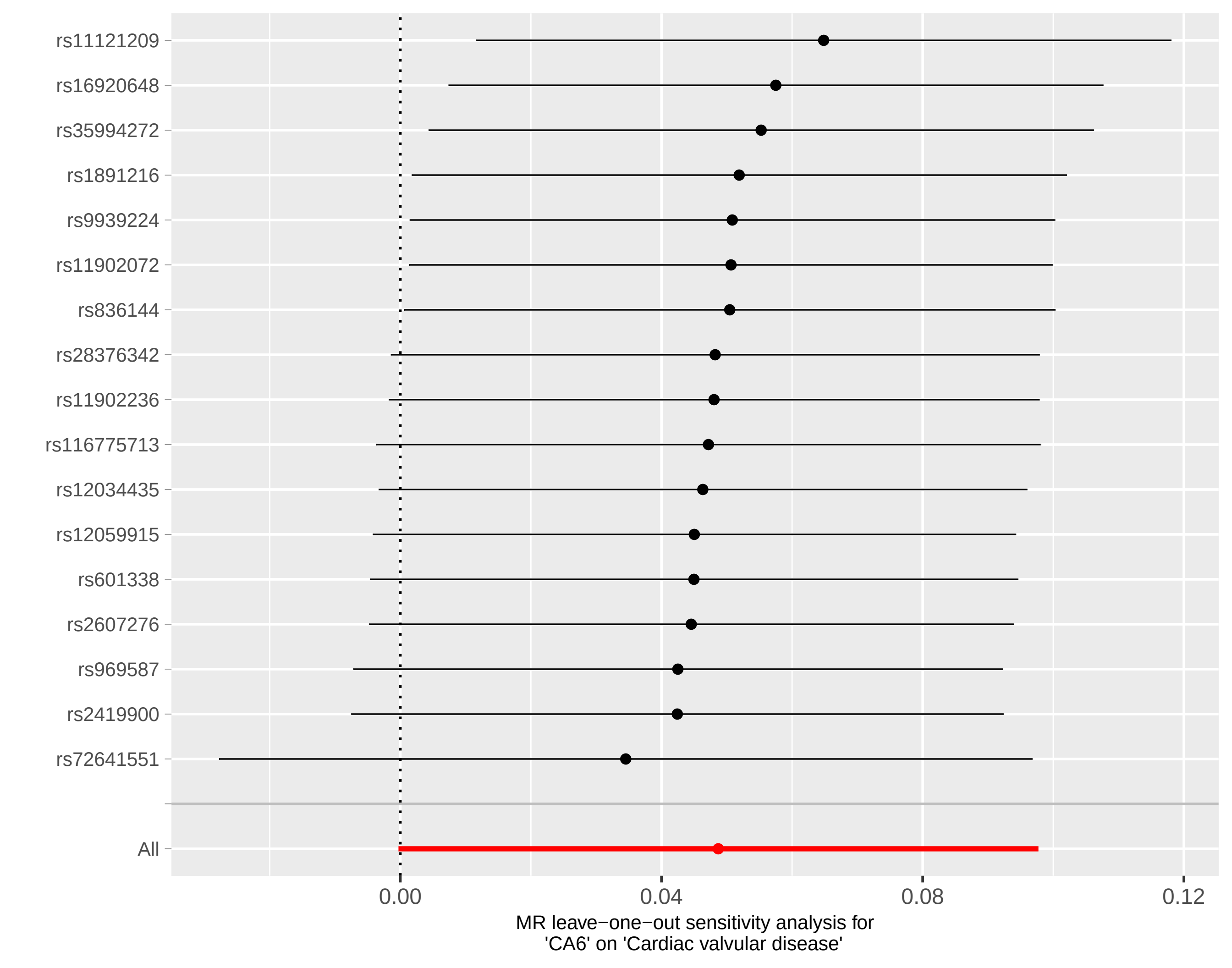
rs11121209
rs16920648
rs35994272
rs1891216
rs9939224
rs11902072
rs836144
rs28376342
rs11902236
rs116775713
rs12034435
rs12059915
rs601338
rs2607276
rs969587
rs2419900
rs72641551
All
0.00
0.04
0.08
0.12
MR leave−one−out sensitivity analysis for
'CA6' on 'Cardiac valvular disease'

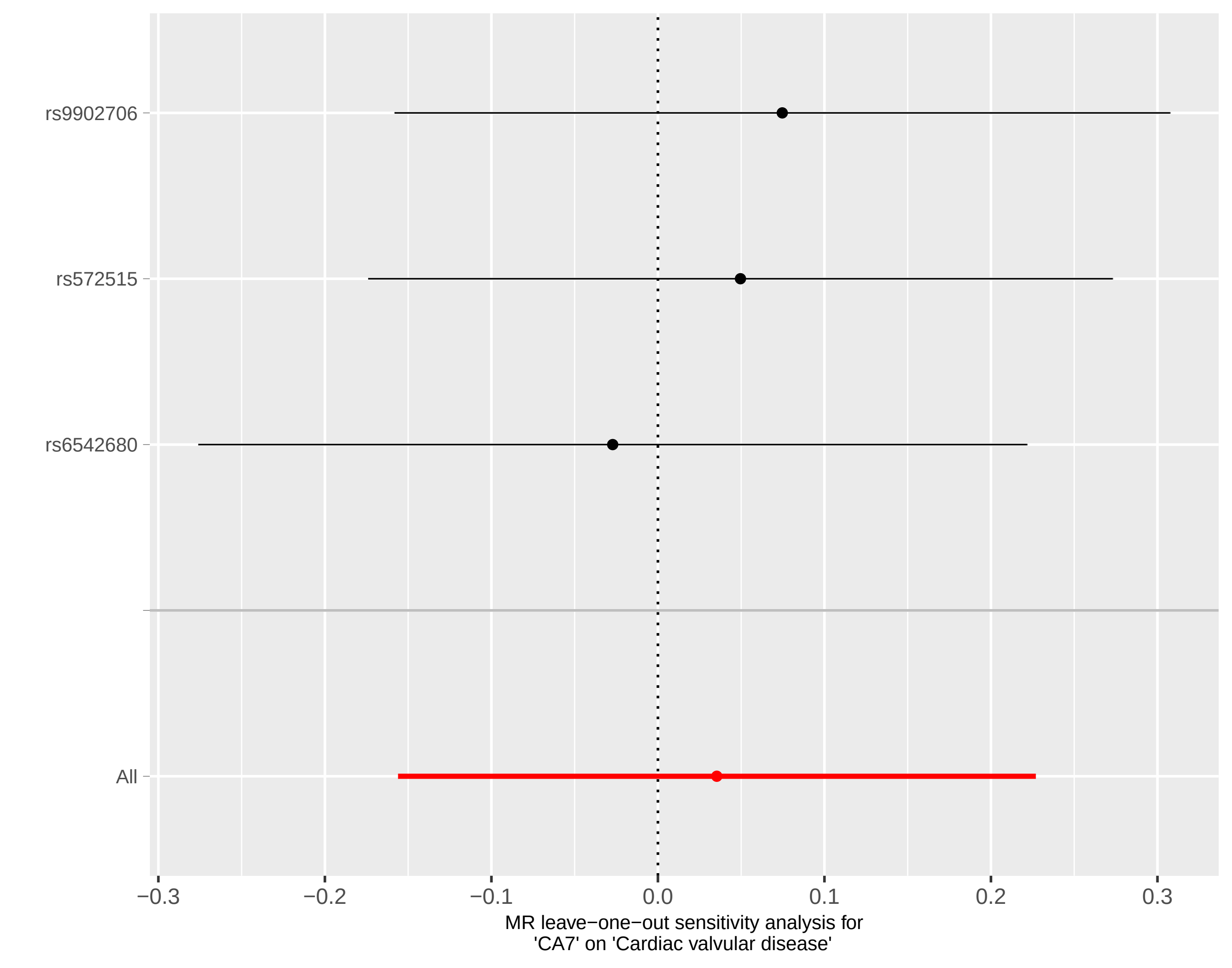

rs9902706
rs572515
rs6542680
All
−0.3
−0.2
−0.1
0.0
0.1
0.2
0.3
MR leave−one−out sensitivity analysis for
'CA7' on 'Cardiac valvular disease'

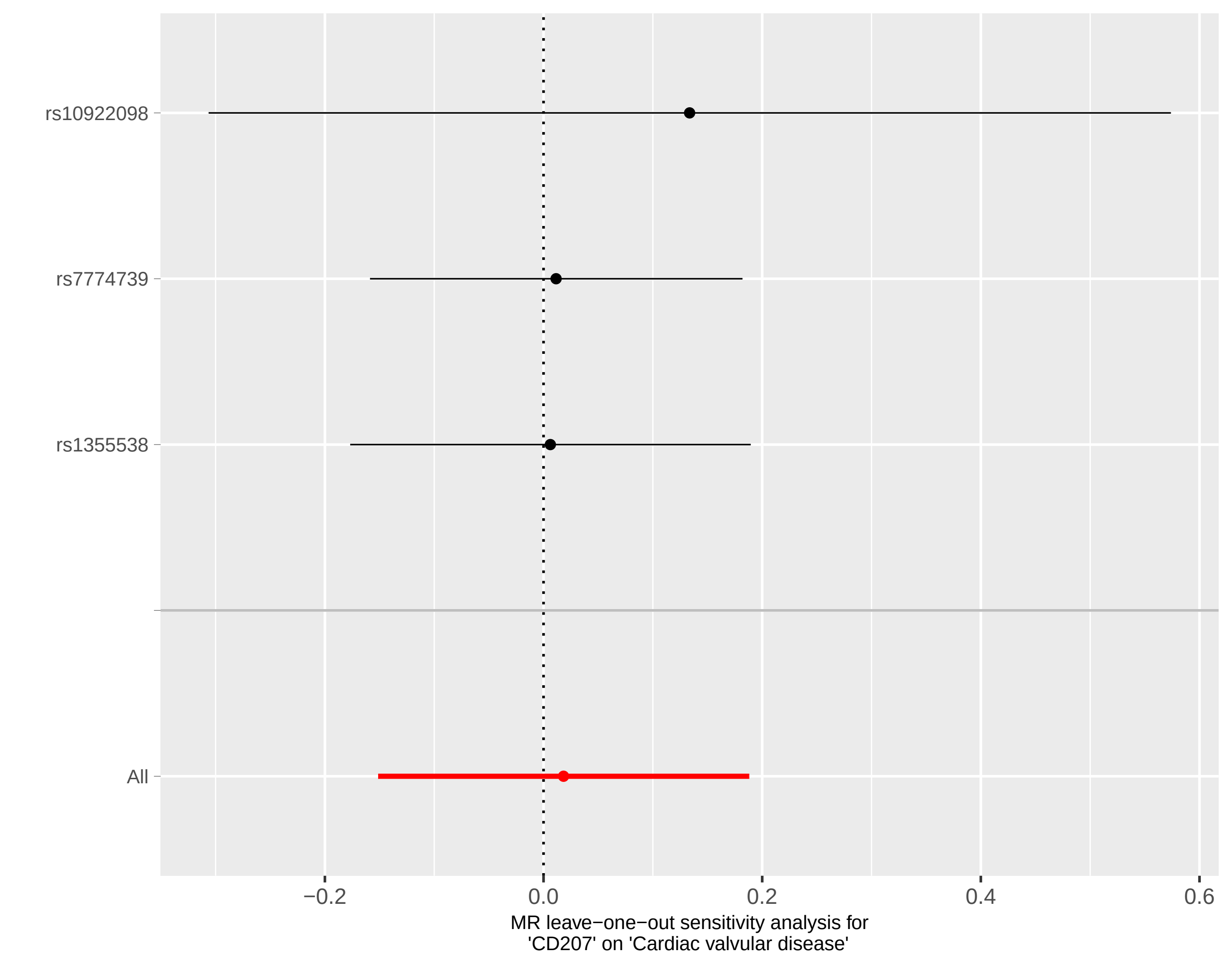
rs10922098
rs7774739
rs1355538
All
−0.2
0.0
0.2
0.4
0.6
MR leave−one−out sensitivity analysis for
'CD207' on 'Cardiac valvular disease'

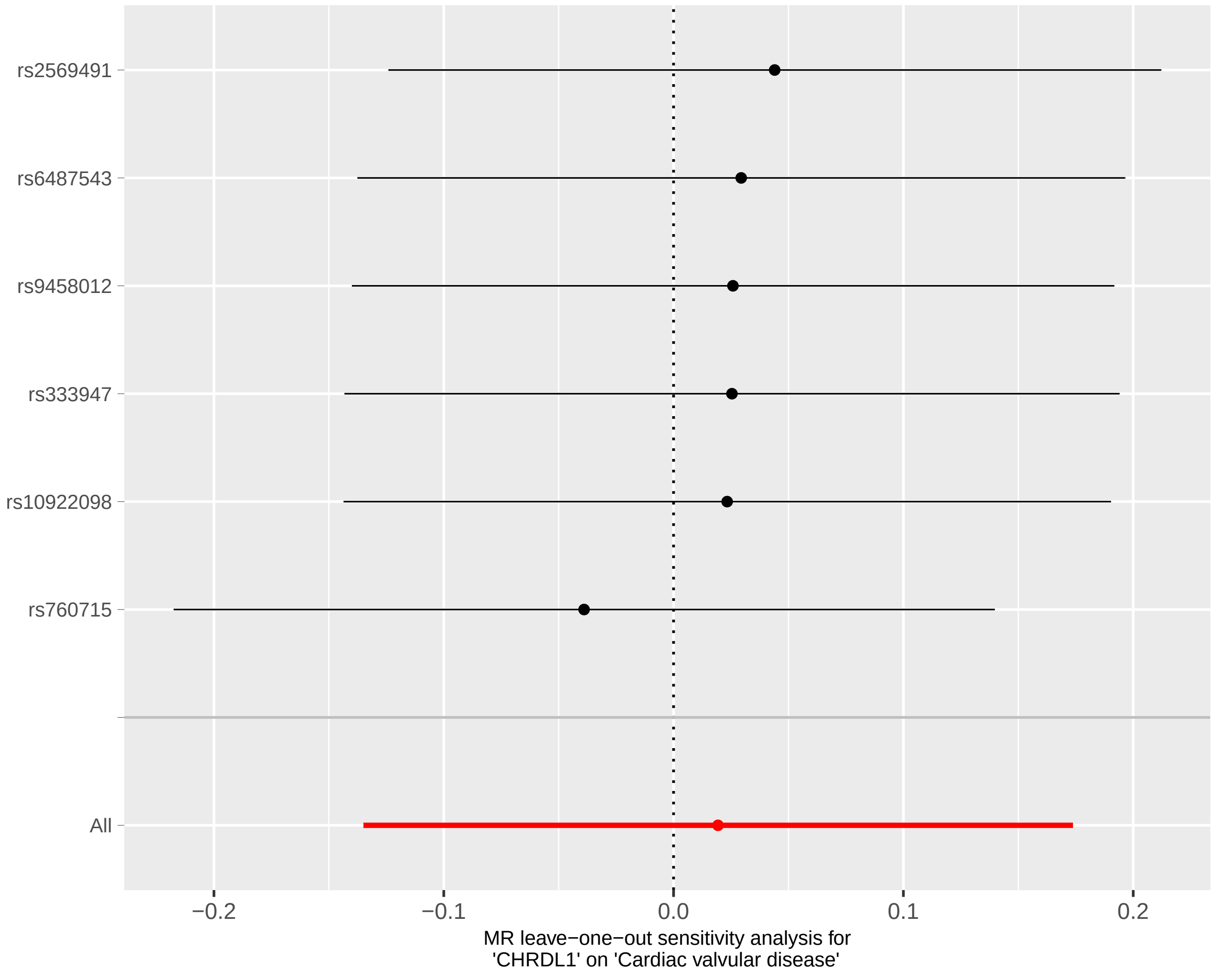
rs2569491
rs6487543
rs9458012
rs333947
rs10922098
rs760715
All
−0.2
−0.1
0.0
0.1
0.2
MR leave−one−out sensitivity analysis for
'CHRDL1' on 'Cardiac valvular disease'

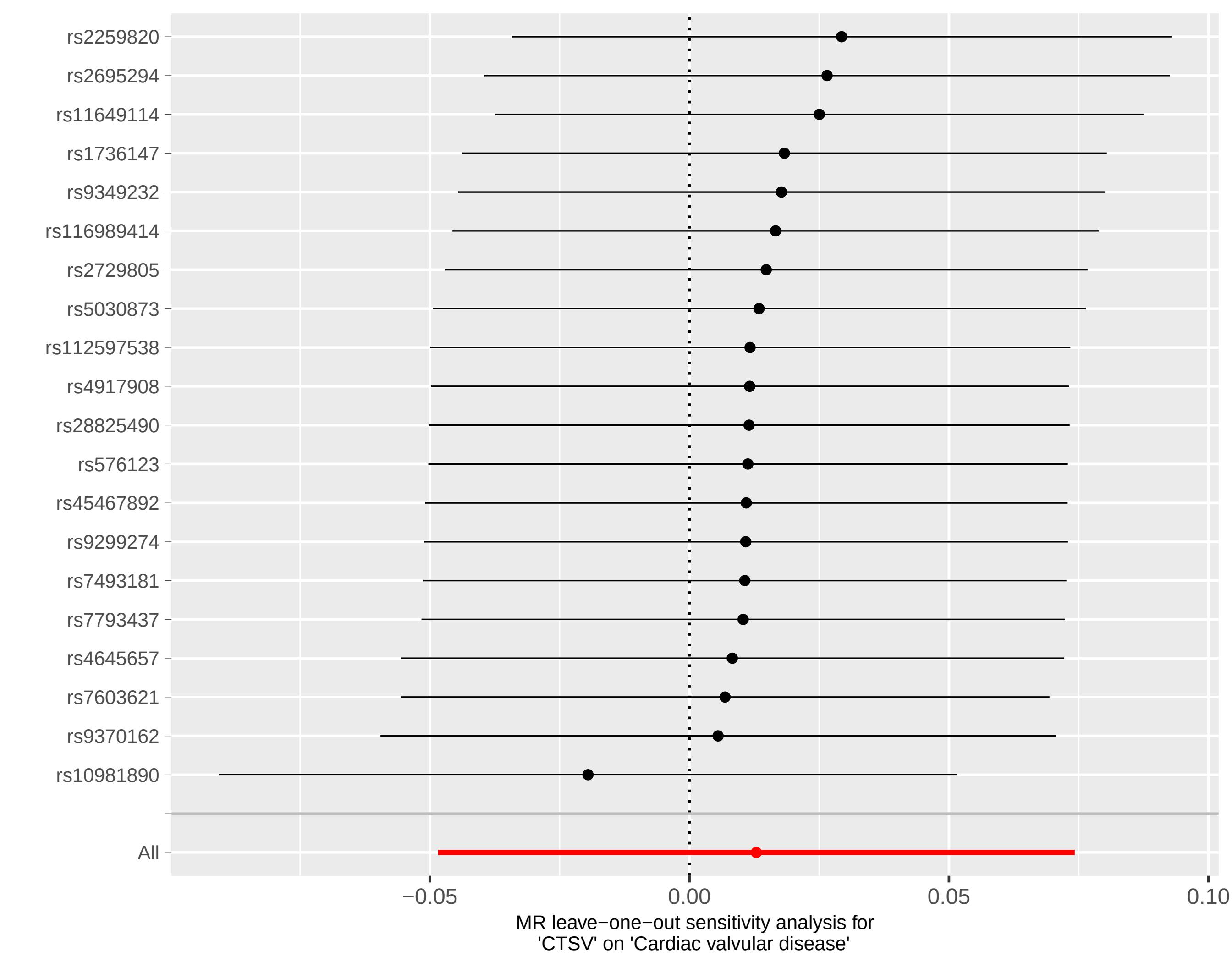
rs2259820
rs2695294
rs11649114
rs1736147
rs9349232
rs116989414
rs2729805
rs5030873
rs112597538
rs4917908
rs28825490
rs576123
rs45467892
rs9299274
rs7493181
rs7793437
rs4645657
rs7603621
rs9370162
rs10981890
All
−0.05
0.00
0.05
0.10
MR leave−one−out sensitivity analysis for
'CTSV' on 'Cardiac valvular disease'

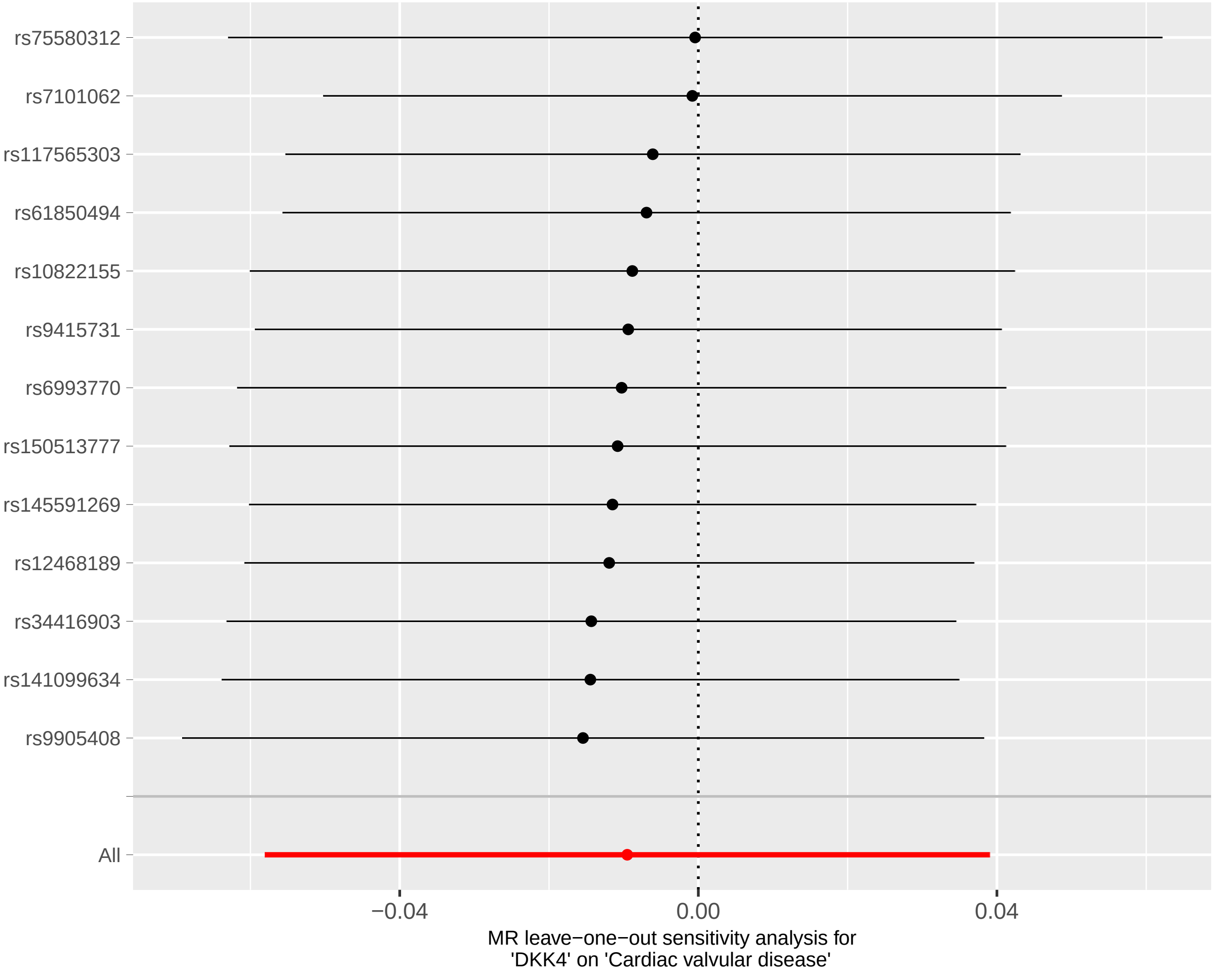
rs75580312
rs7101062
rs117565303
rs61850494
rs10822155
rs9415731
rs6993770
rs150513777
rs145591269
rs12468189
rs34416903
rs141099634
rs9905408
All
−0.04
0.00
0.04
MR leave−one−out sensitivity analysis for
'DKK4' on 'Cardiac valvular disease'

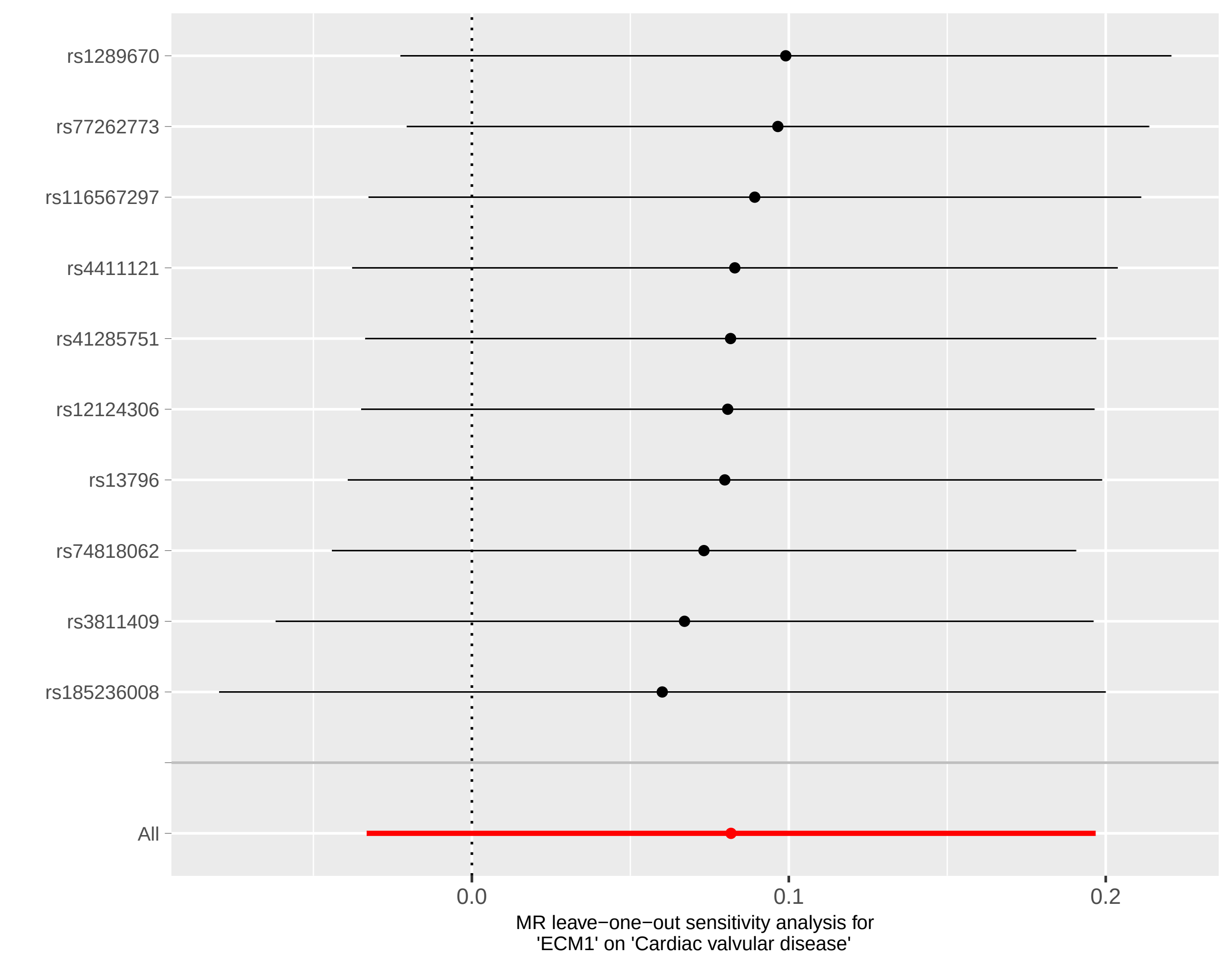
rs1289670
rs77262773
rs116567297
rs4411121
rs41285751
rs12124306
rs13796
rs74818062
rs3811409
rs185236008
All
0.0
0.1
0.2
MR leave−one−out sensitivity analysis for
'ECM1' on 'Cardiac valvular disease'

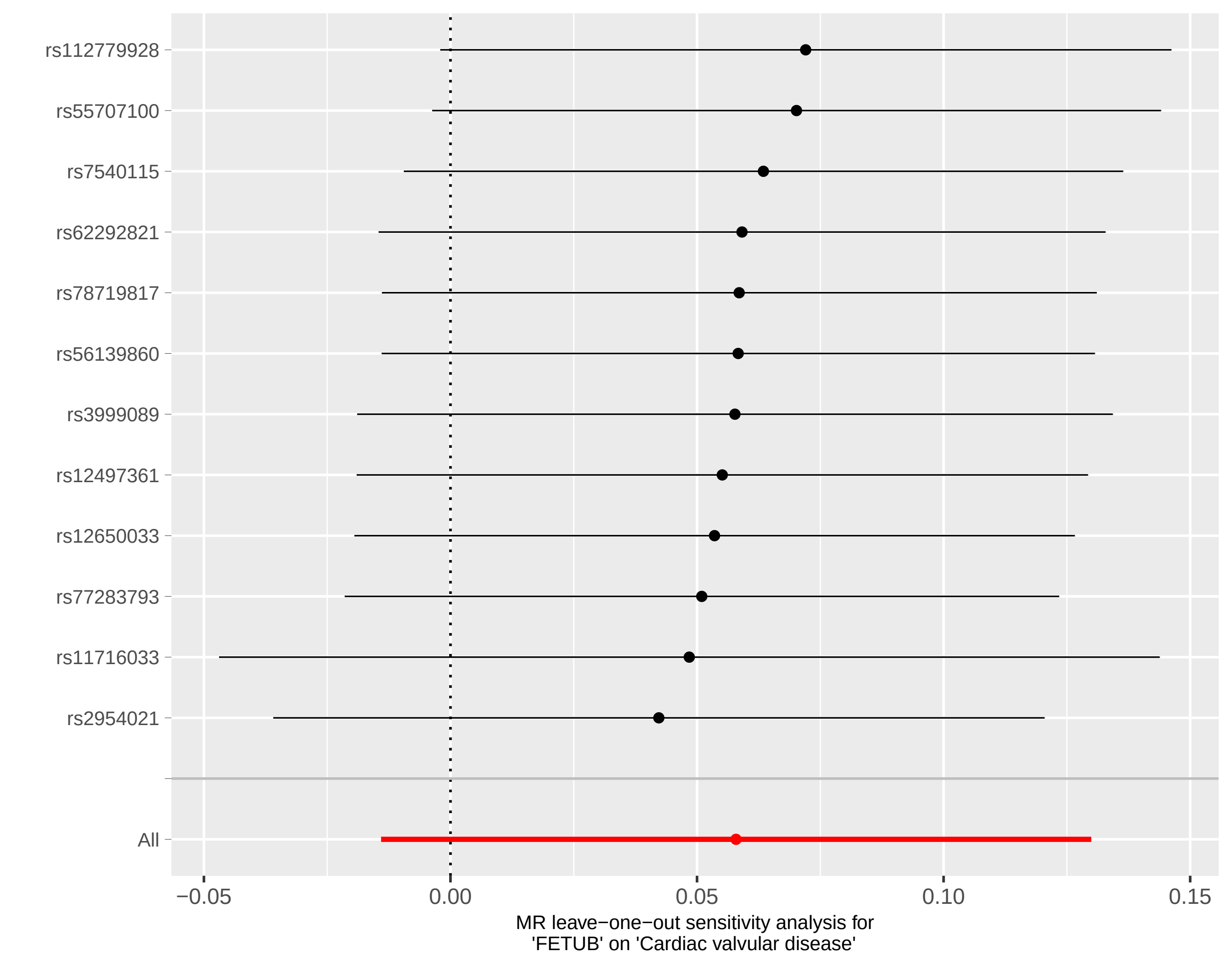

rs112779928
rs55707100
rs7540115
rs62292821
rs78719817
rs56139860
rs3999089
rs12497361
rs12650033
rs77283793
rs11716033
rs2954021
All
−0.05
0.00
0.05
0.10
0.15
MR leave−one−out sensitivity analysis for
'FETUB' on 'Cardiac valvular disease'

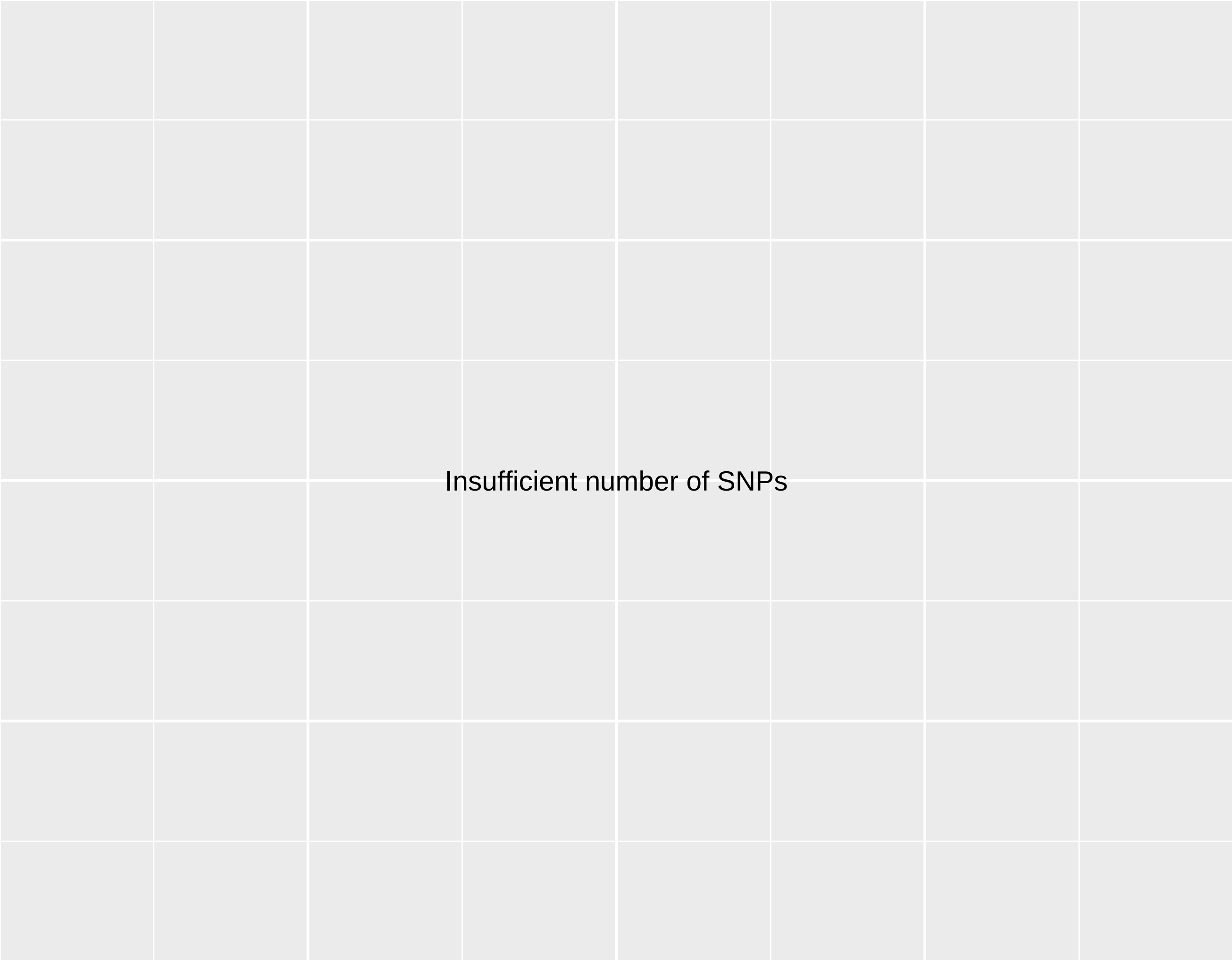
Insufficient number of SNPs

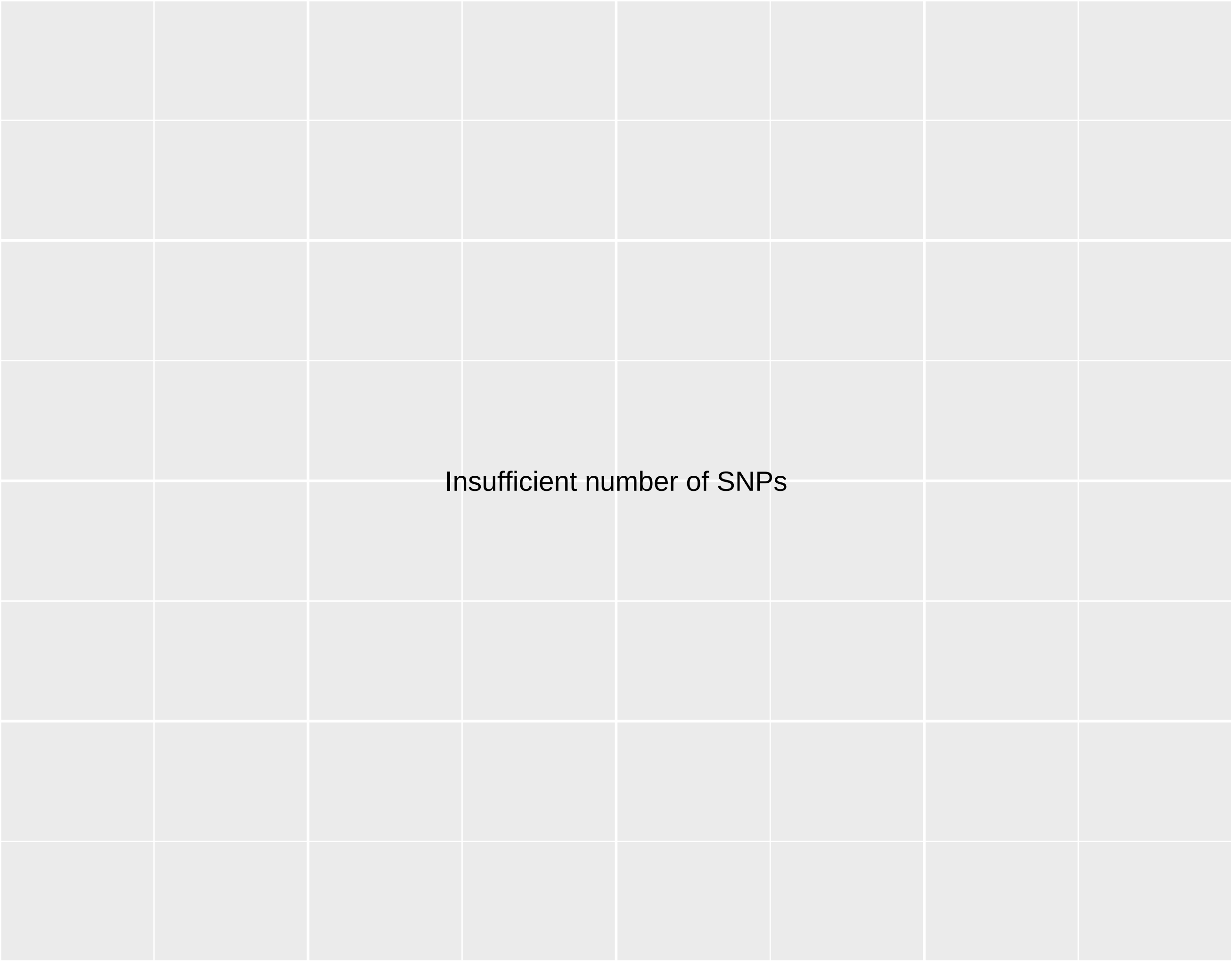
Insufficient number of SNPs

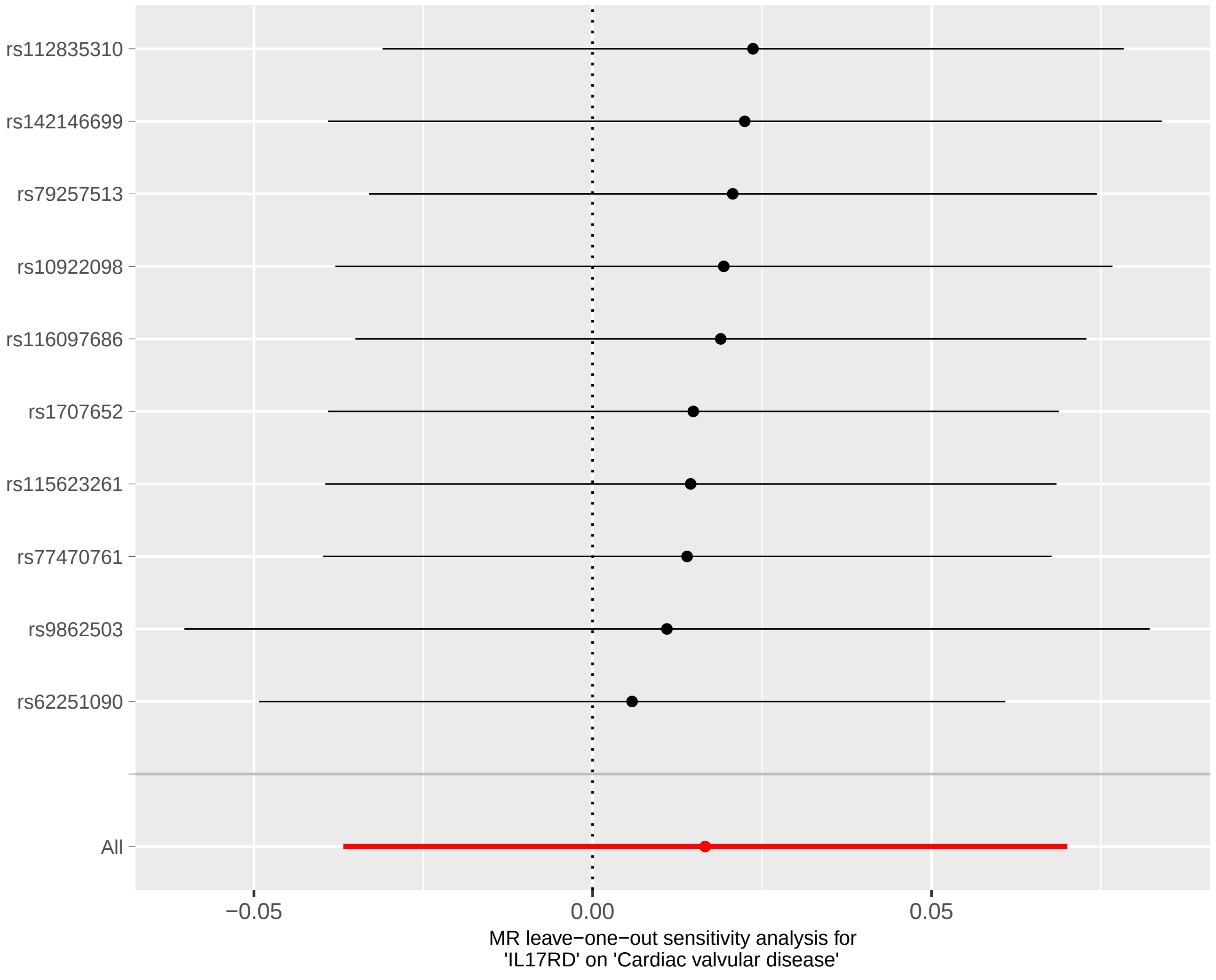

rs112835310
rs142146699
rs79257513
rs10922098
rs116097686
rs1707652
rs115623261
rs77470761
rs9862503
rs62251090
All
−0.05
0.00
0.05
MR leave−one−out sensitivity analysis for
'IL17RD' on 'Cardiac valvular disease'

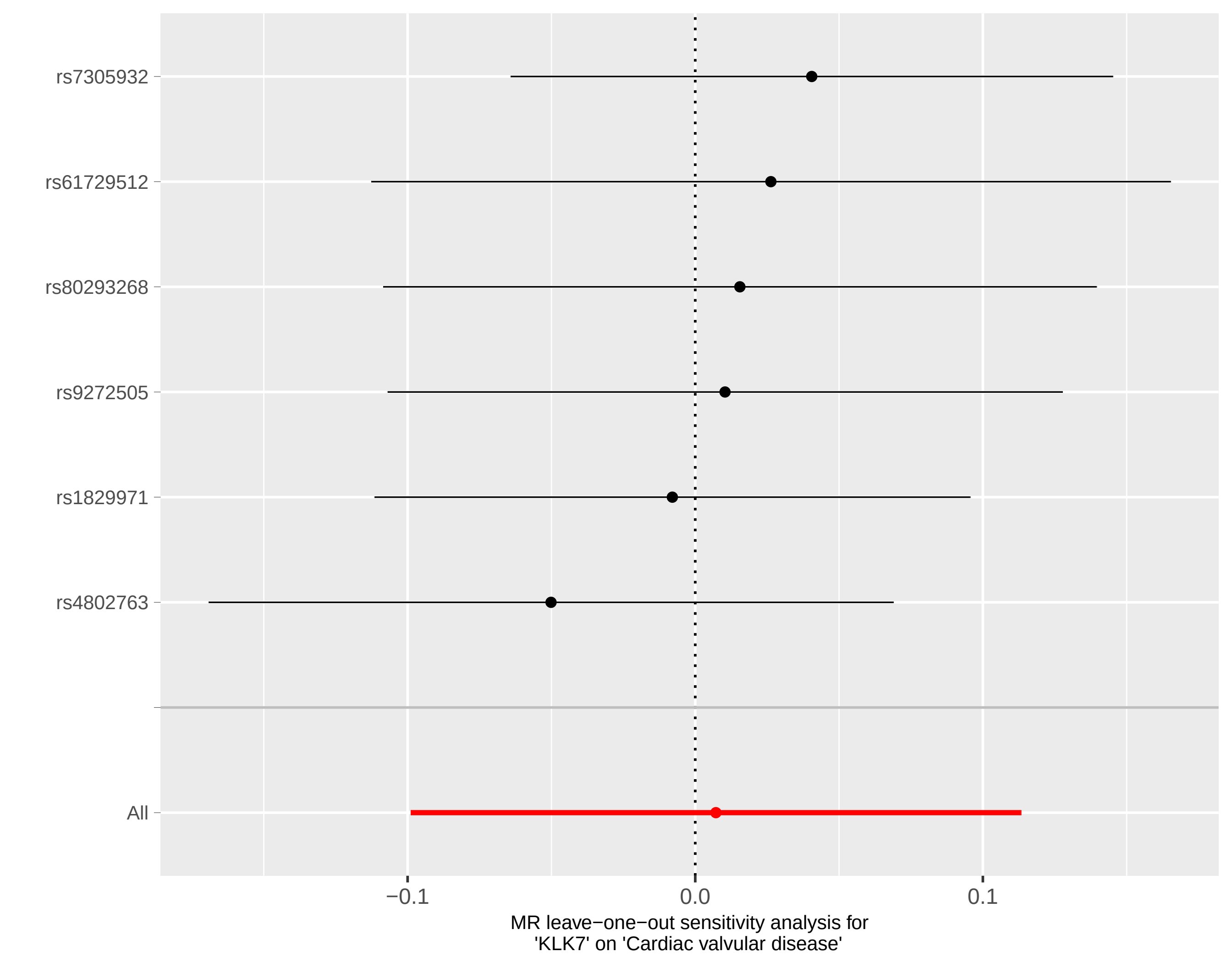

rs7305932
rs61729512
rs80293268
rs9272505
rs1829971
rs4802763
All
−0.1
0.0
0.1
MR leave−one−out sensitivity analysis for
'KLK7' on 'Cardiac valvular disease'

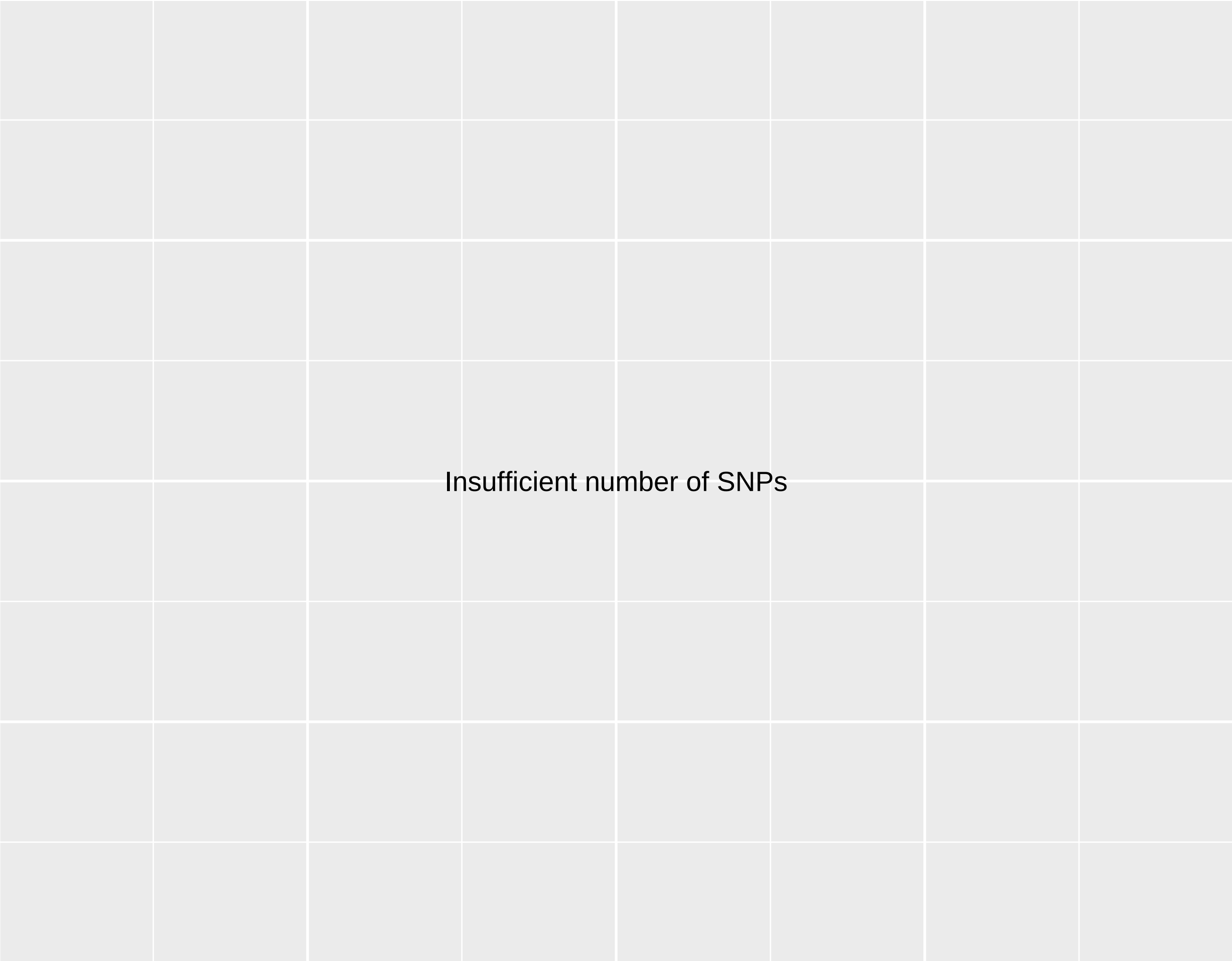
Insufficient number of SNPs

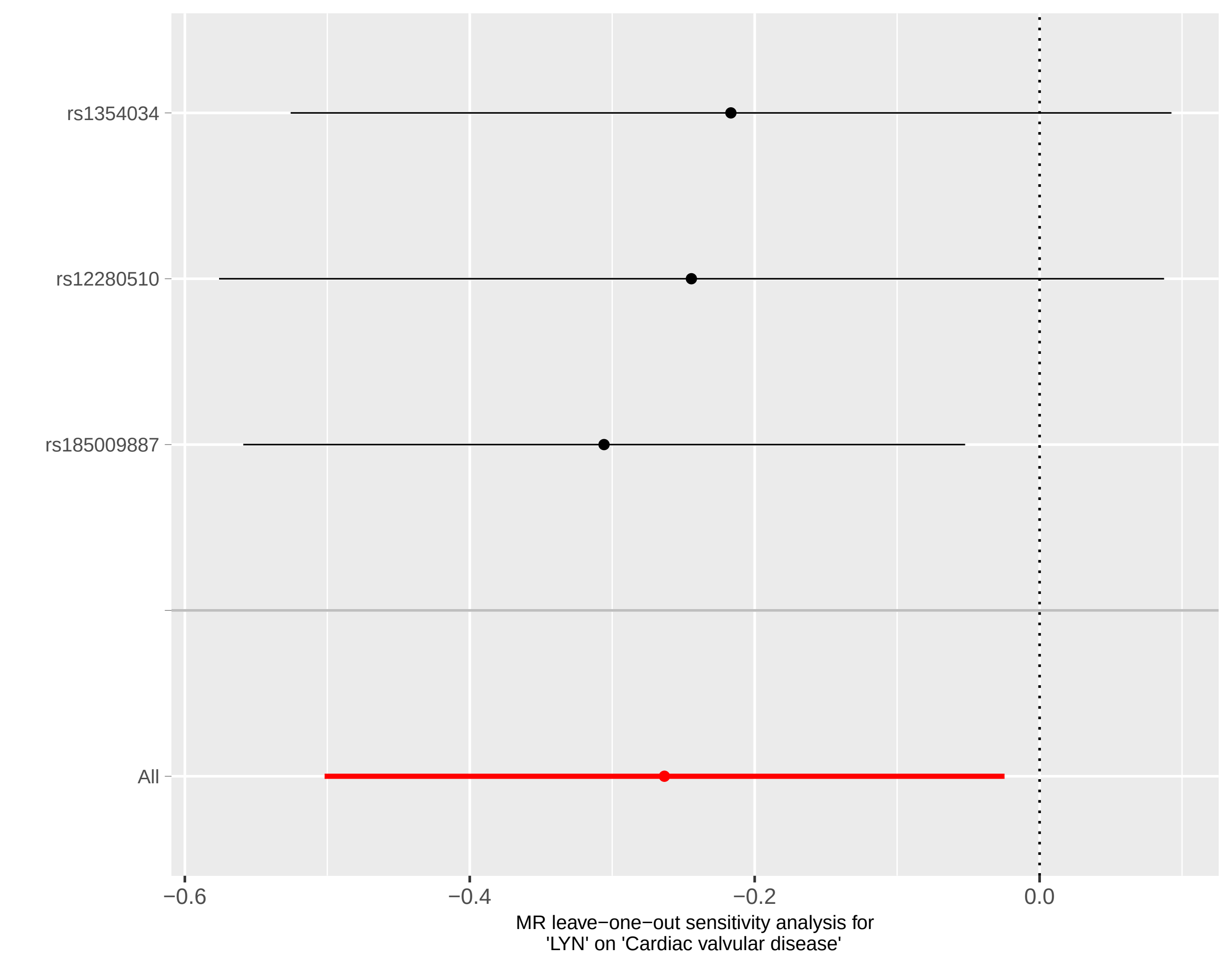

rs1354034
rs12280510
rs185009887
All
−0.6
−0.4
−0.2
0.0
MR leave−one−out sensitivity analysis for
'LYN' on 'Cardiac valvular disease'

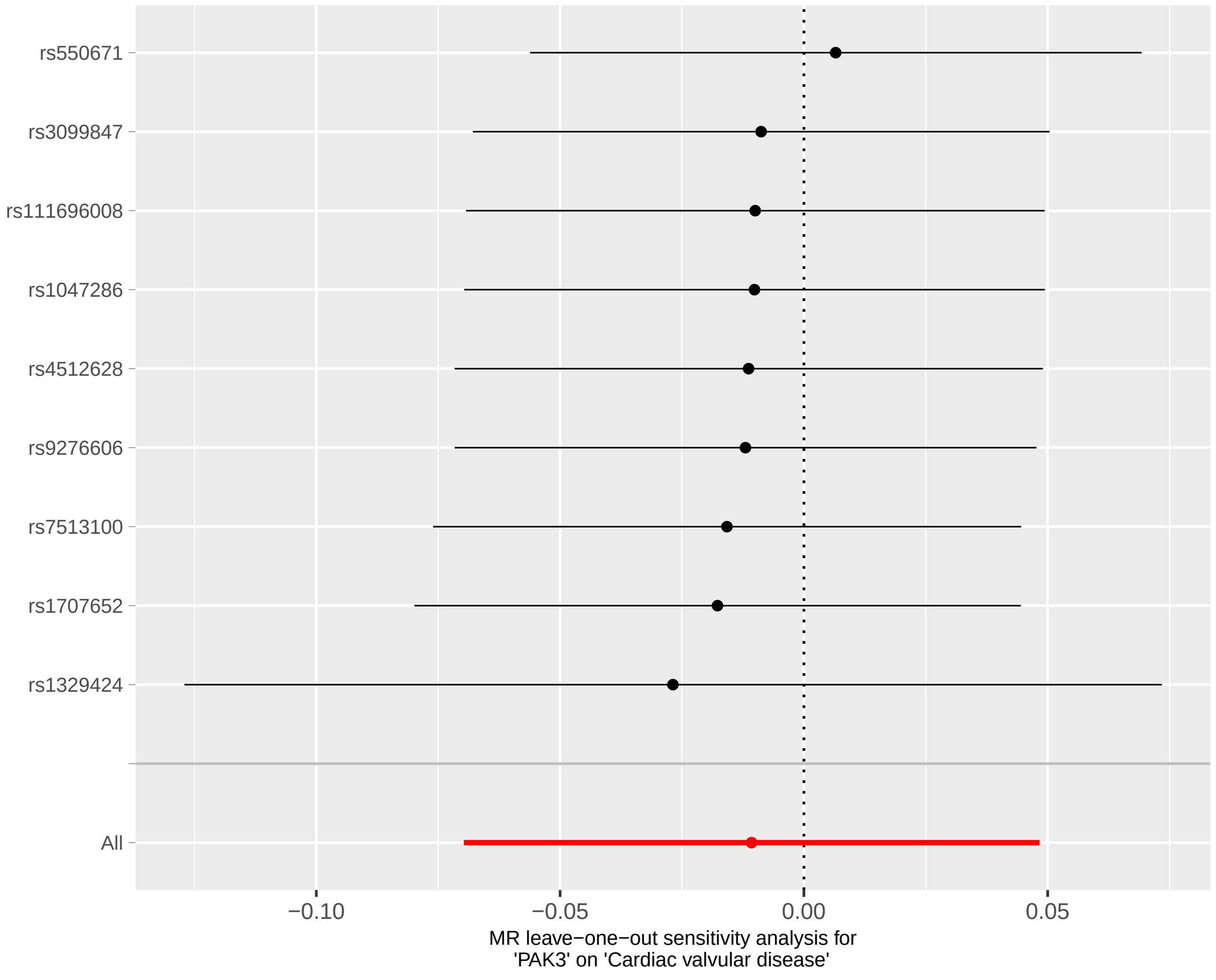
rs550671
rs3099847
rs111696008
rs1047286
rs4512628
rs9276606
rs7513100
rs1707652
rs1329424
All
−0.10
−0.05
0.00
0.05
MR leave−one−out sensitivity analysis for
'PAK3' on 'Cardiac valvular disease'

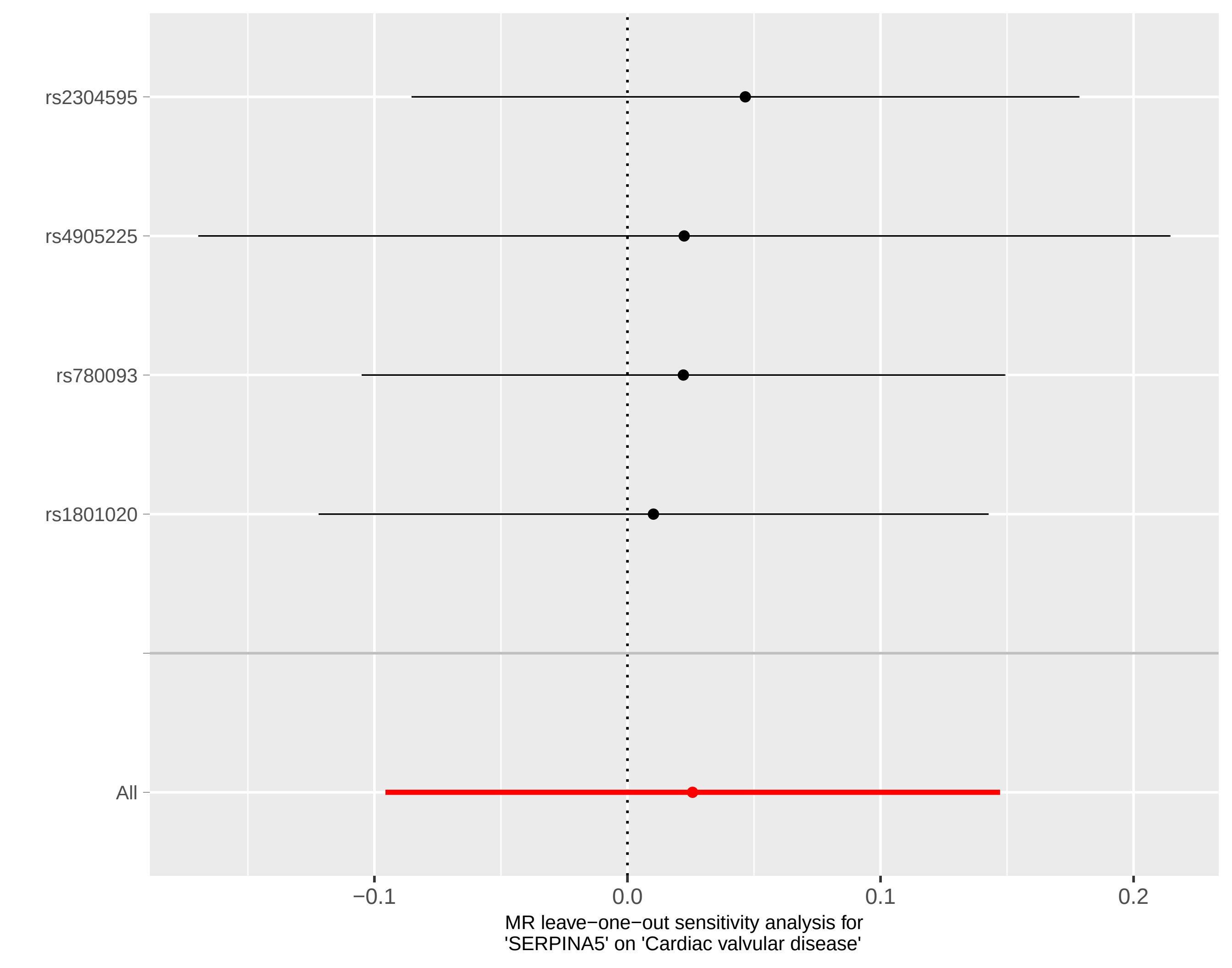
rs2304595
rs4905225
rs780093
rs1801020
All
−0.1
0.0
0.1
0.2
MR leave−one−out sensitivity analysis for
'SERPINA5' on 'Cardiac valvular disease'

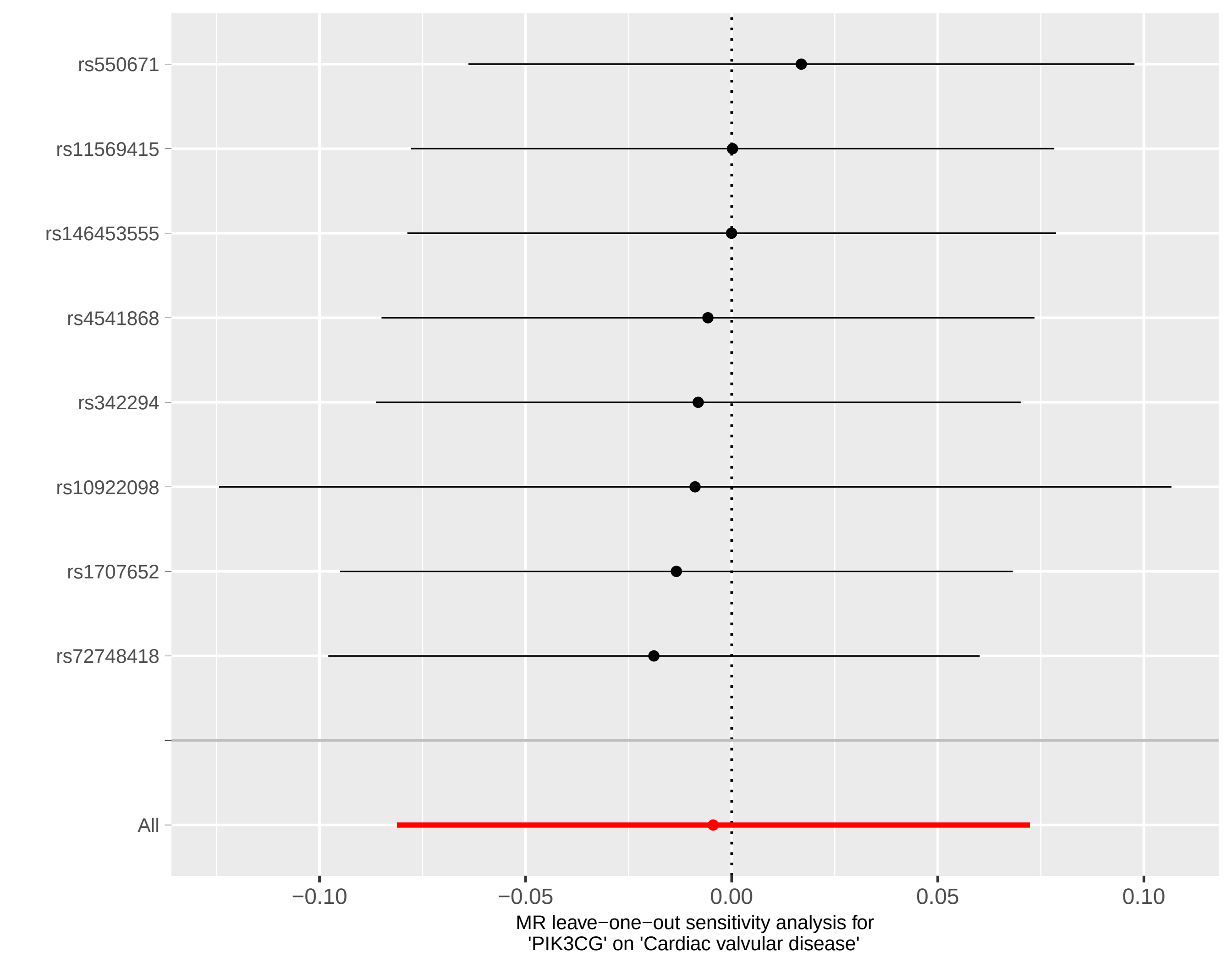
rs550671
rs11569415
rs146453555
rs4541868
rs342294
rs10922098
rs1707652
rs72748418
All
−0.10
−0.05
0.00
0.05
0.10
MR leave−one−out sensitivity analysis for
'PIK3CG' on 'Cardiac valvular disease'

Insufficient number of SNPs

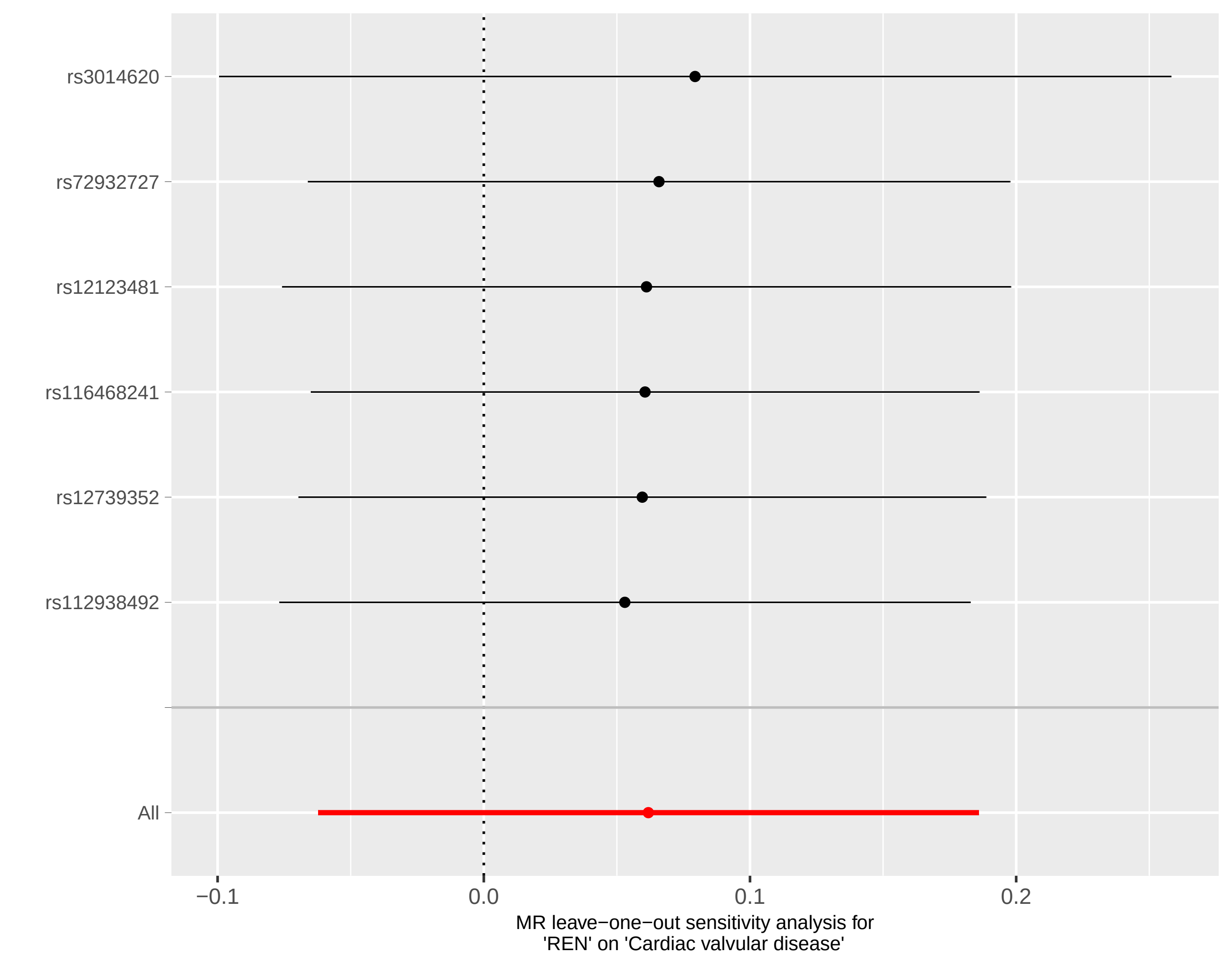
rs3014620
rs72932727
rs12123481
rs116468241
rs12739352
rs112938492
All
−0.1
0.0
0.1
0.2
MR leave−one−out sensitivity analysis for
'REN' on 'Cardiac valvular disease'

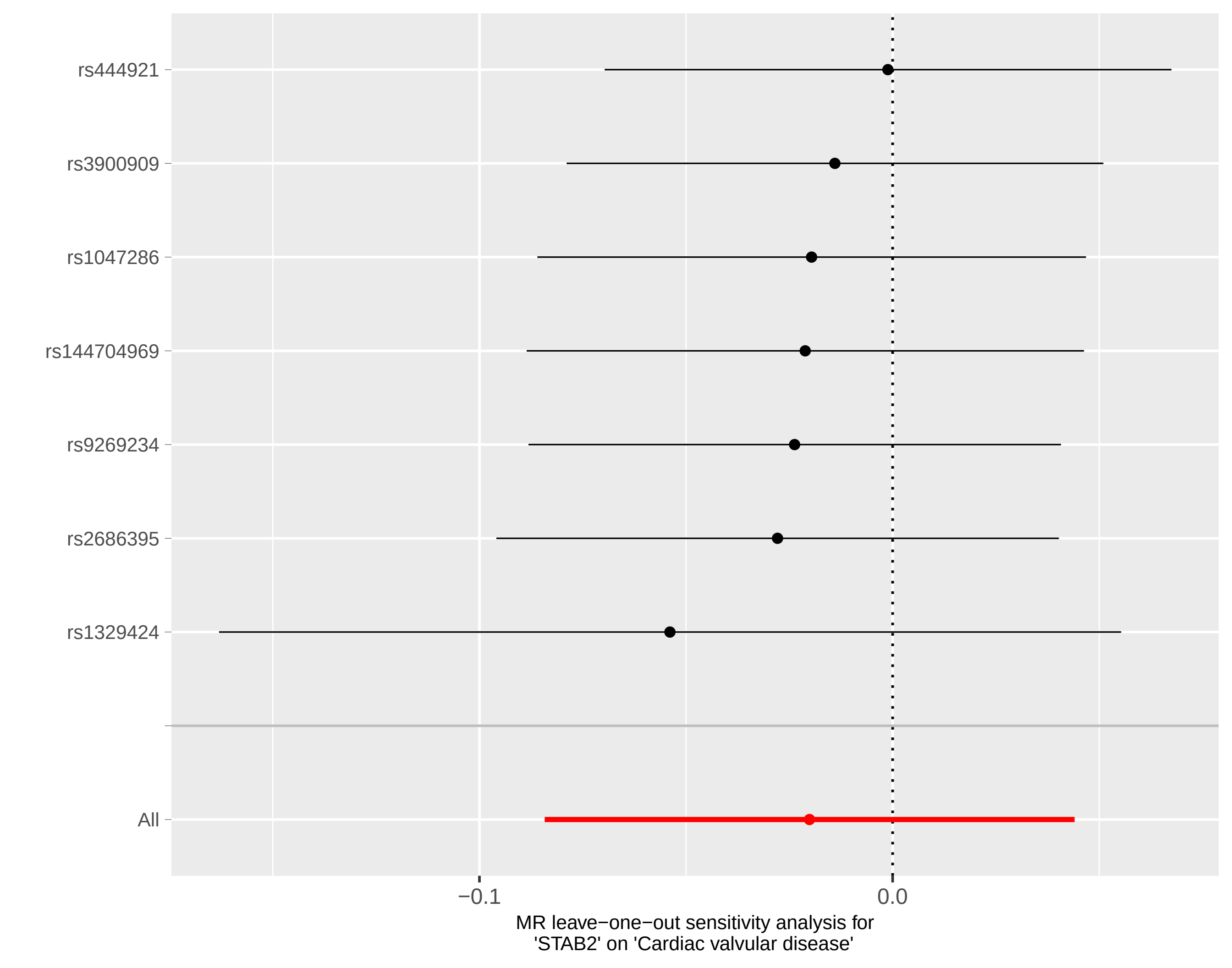
rs444921
rs3900909
rs1047286
rs144704969
rs9269234
rs2686395
rs1329424
All
−0.1
0.0
MR leave−one−out sensitivity analysis for
'STAB2' on 'Cardiac valvular disease'

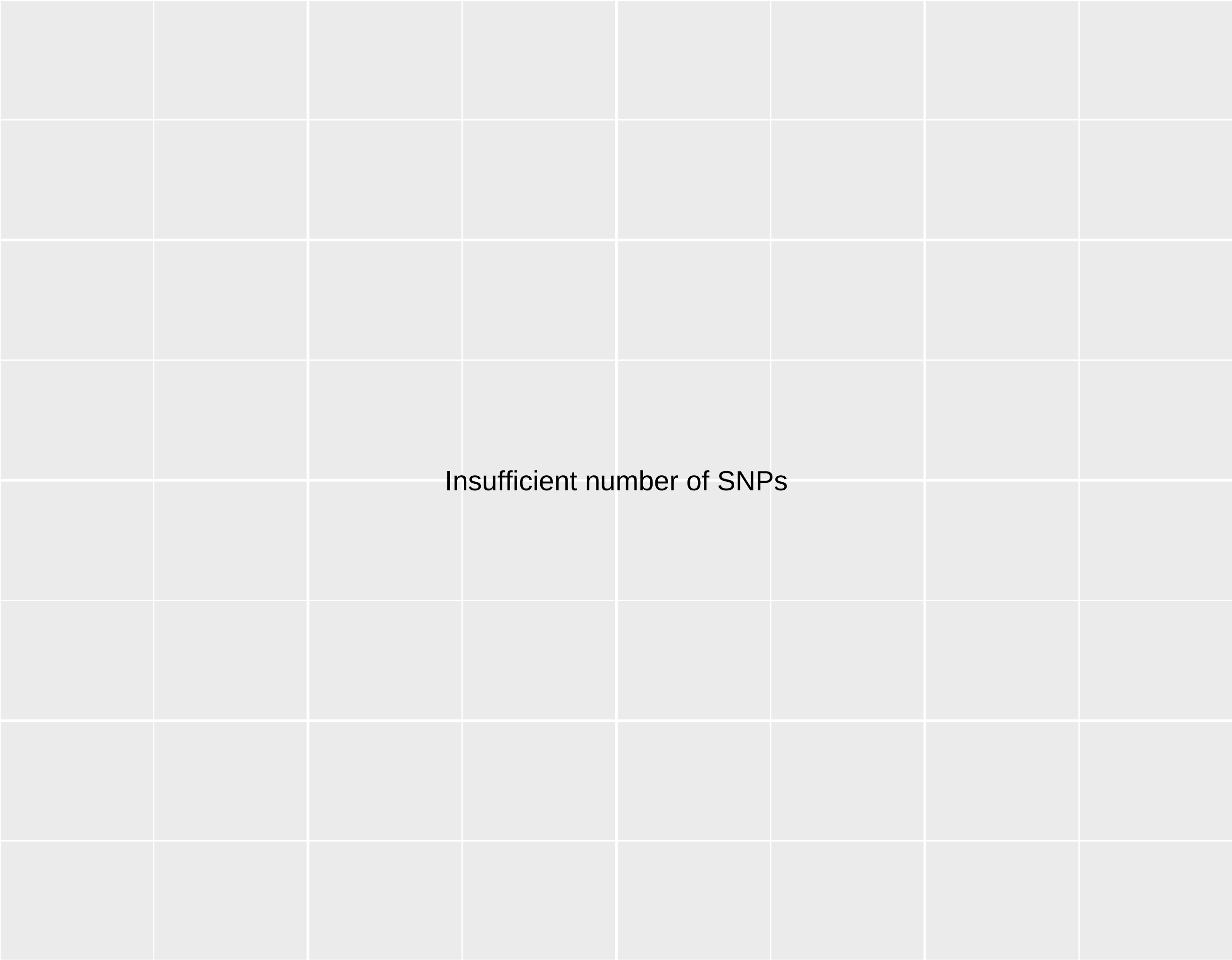
Insufficient number of SNPs

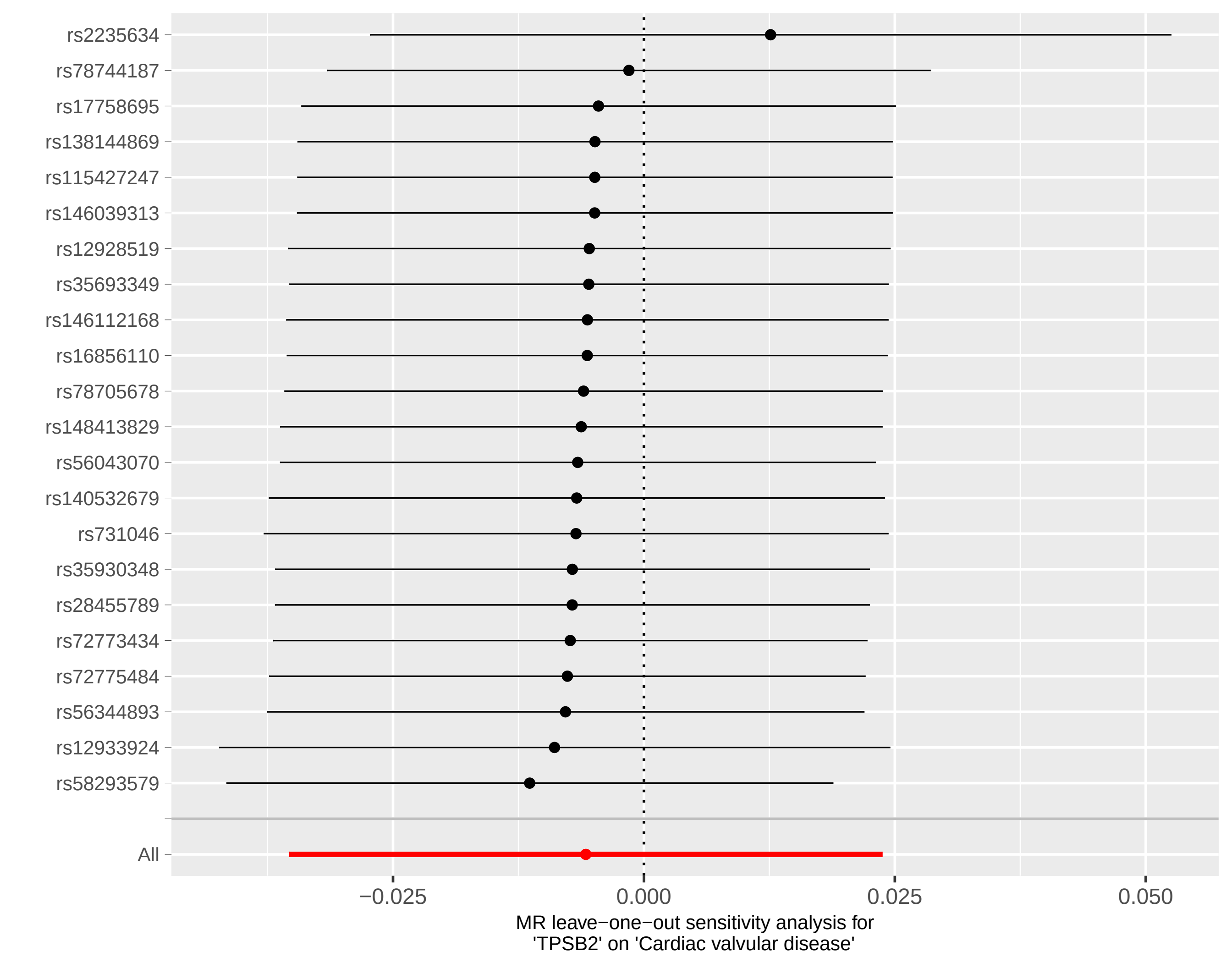
rs2235634
rs78744187
rs17758695
rs138144869
rs115427247
rs146039313
rs12928519
rs35693349
rs146112168
rs16856110
rs78705678
rs148413829
rs56043070
rs140532679
rs731046
rs35930348
rs28455789
rs72773434
rs72775484
rs56344893
rs12933924
rs58293579
All
−0.025
0.000
0.025
0.050
MR leave−one−out sensitivity analysis for
'TPSB2' on 'Cardiac valvular disease'

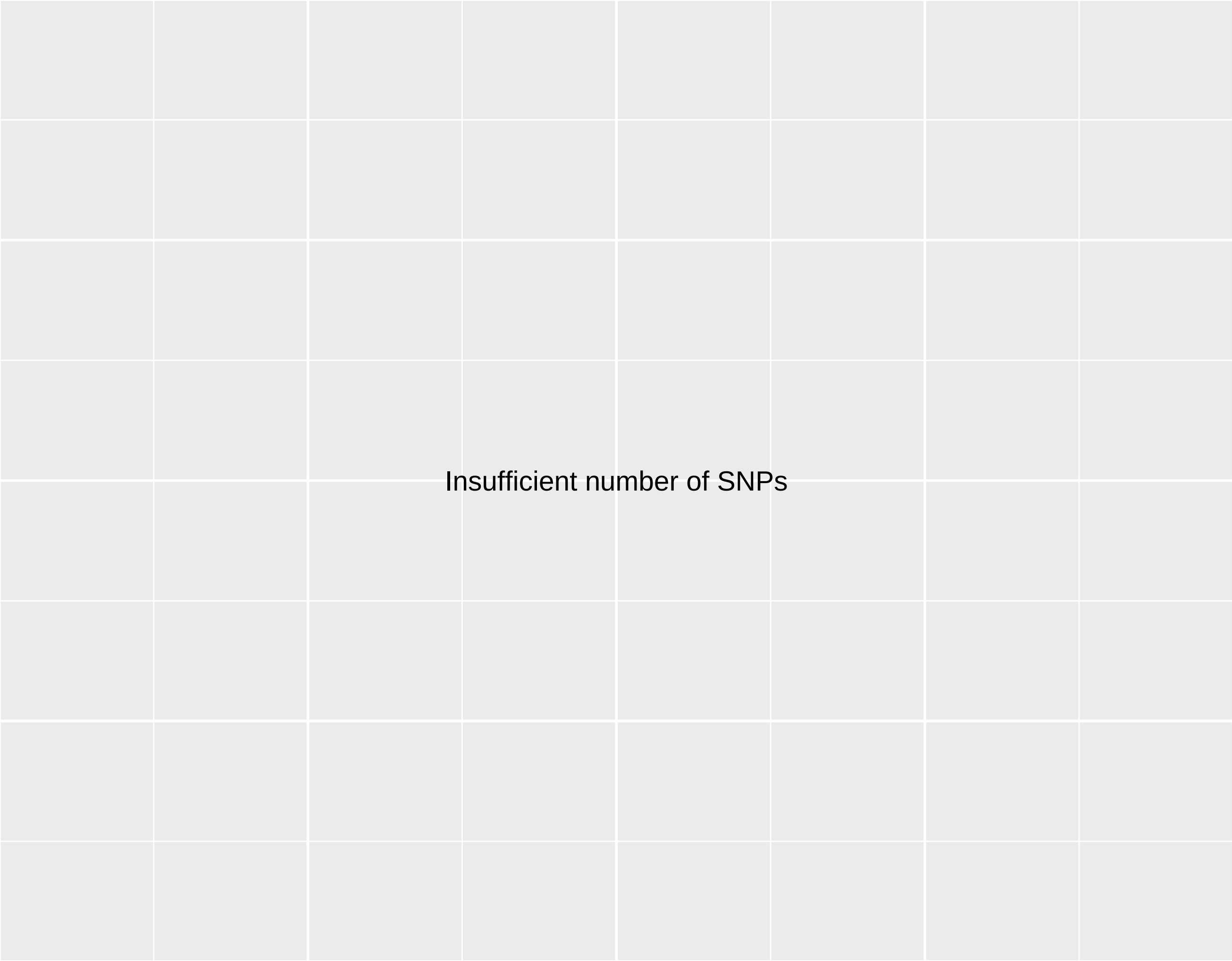
Insufficient number of SNPs

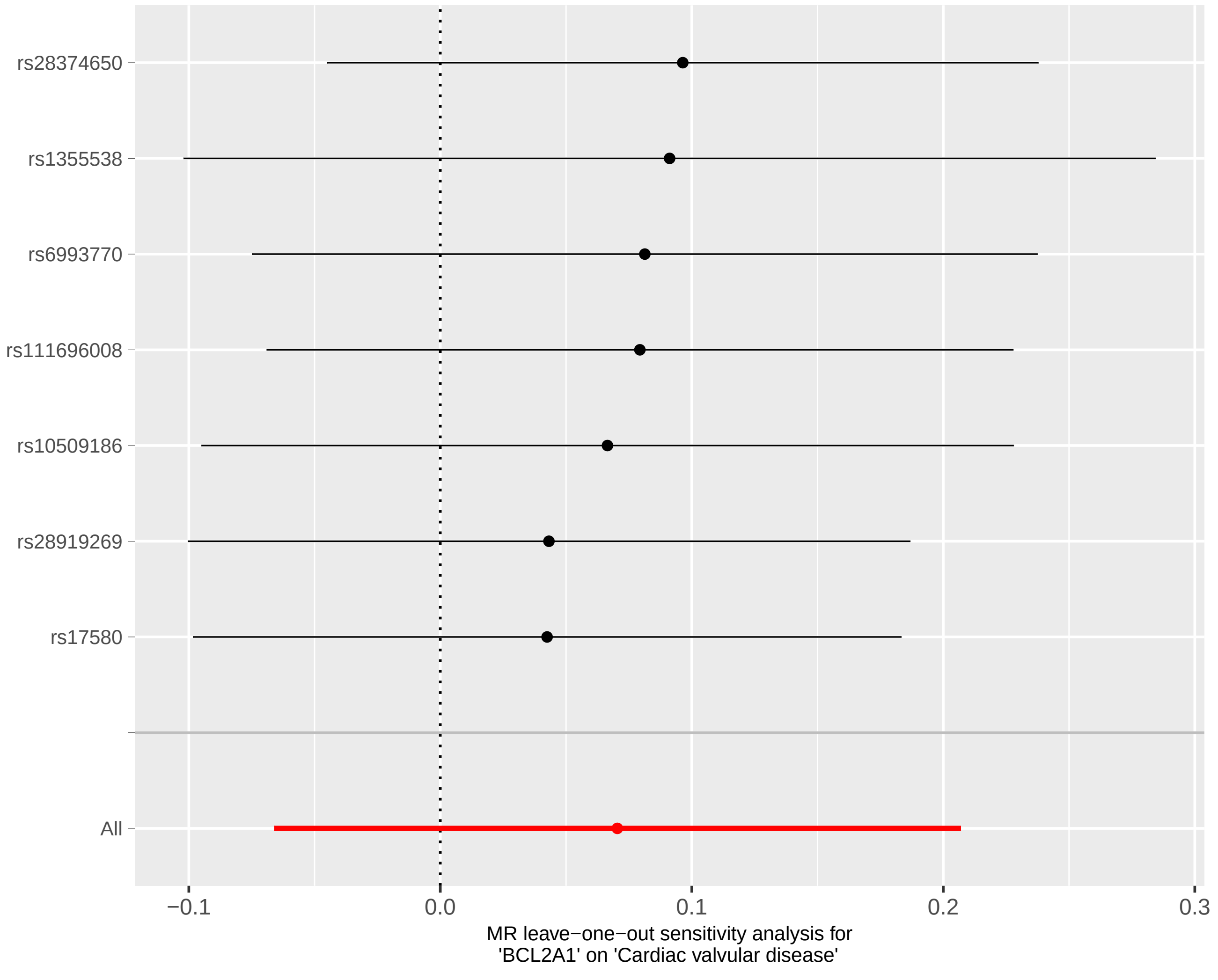

rs28374650
rs1355538
rs6993770
rs111696008
rs10509186
rs28919269
rs17580
All
−0.1
0.0
0.1
0.2
0.3
MR leave−one−out sensitivity analysis for
'BCL2A1' on 'Cardiac valvular disease'

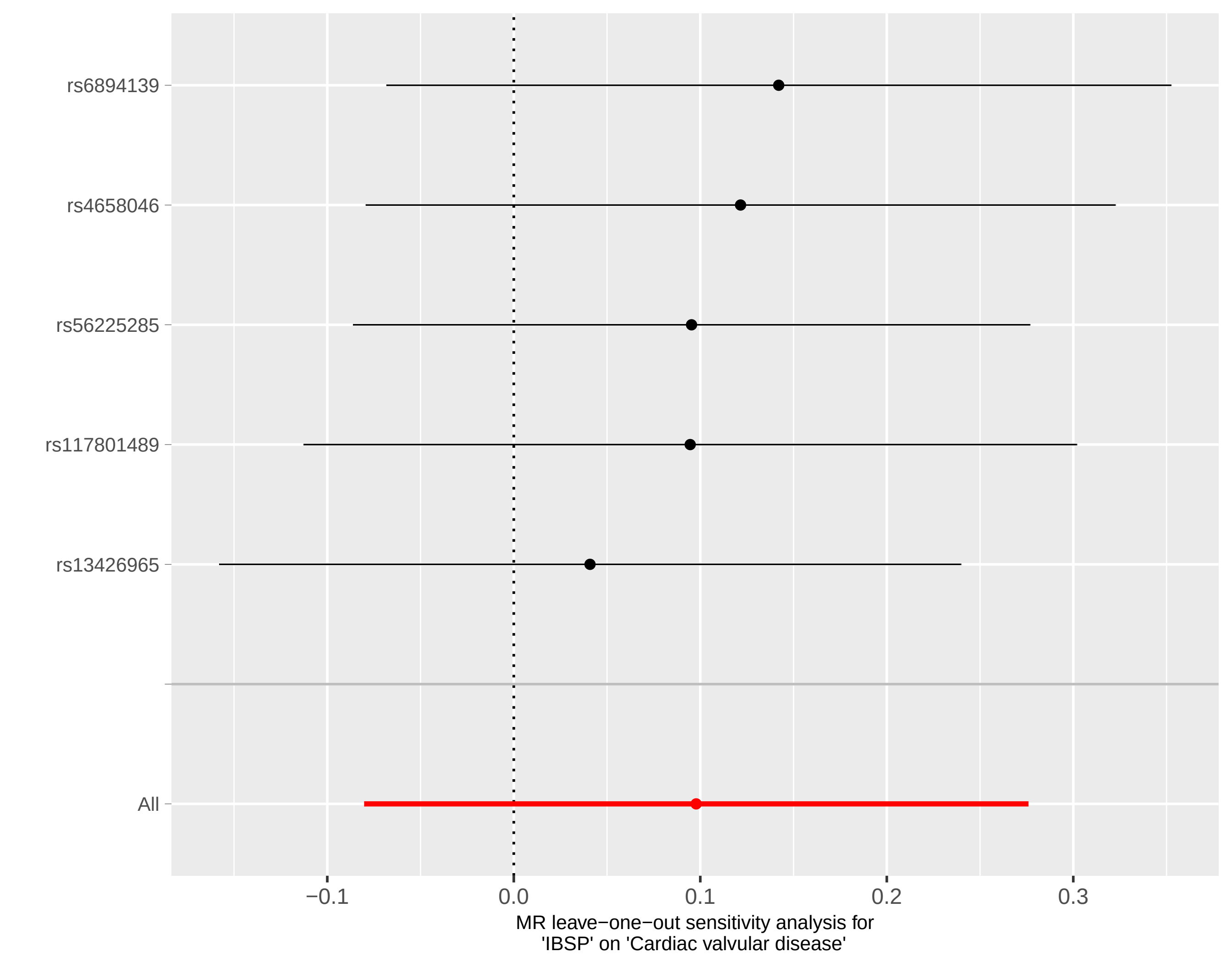

rs6894139
rs4658046
rs56225285
rs117801489
rs13426965
All
−0.1
0.0
0.1
0.2
0.3
MR leave−one−out sensitivity analysis for
'IBSP' on 'Cardiac valvular disease'

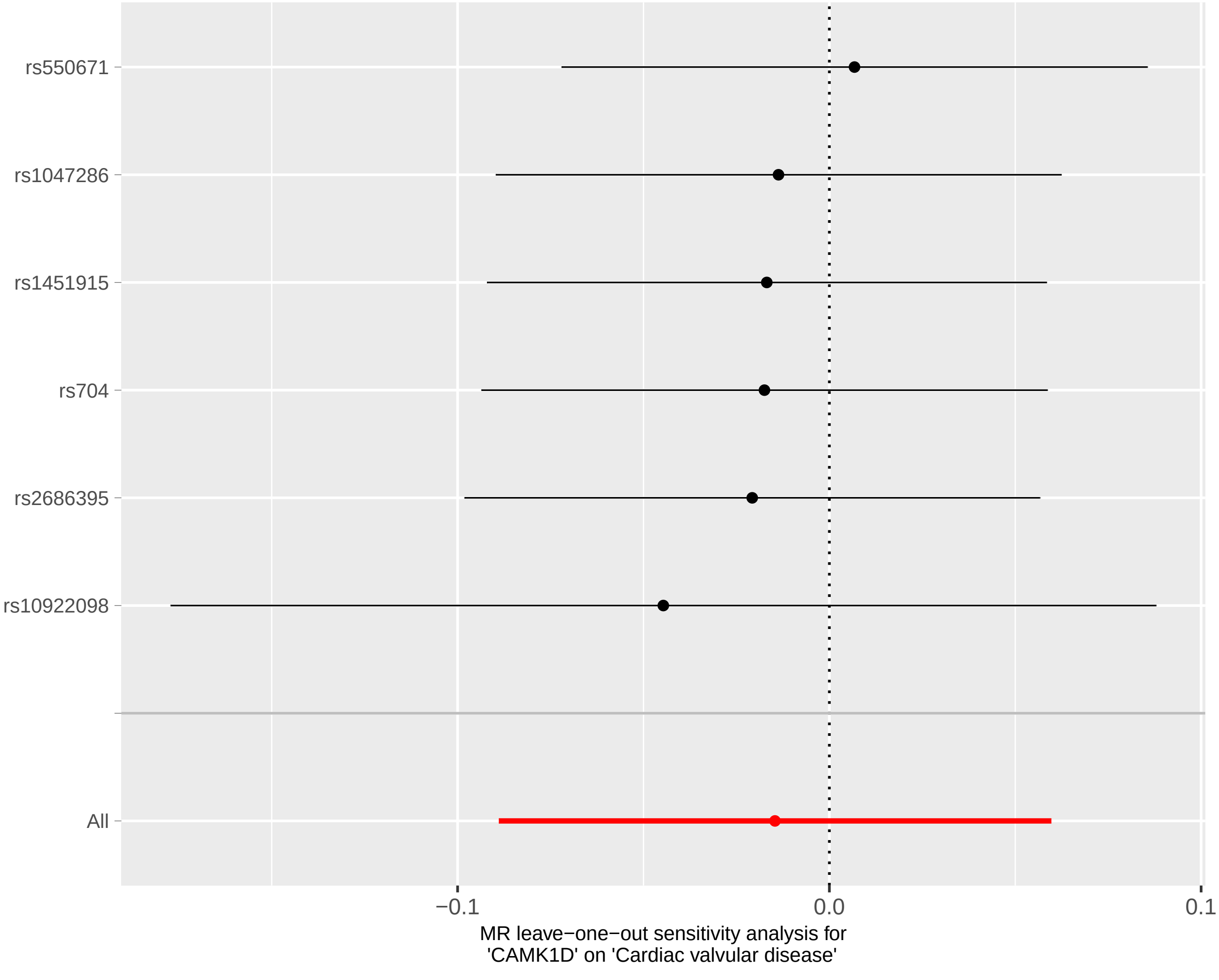
rs550671
rs1047286
rs1451915
rs704
rs2686395
rs10922098
All
−0.1
0.0
0.1
MR leave−one−out sensitivity analysis for
'CAMK1D' on 'Cardiac valvular disease'

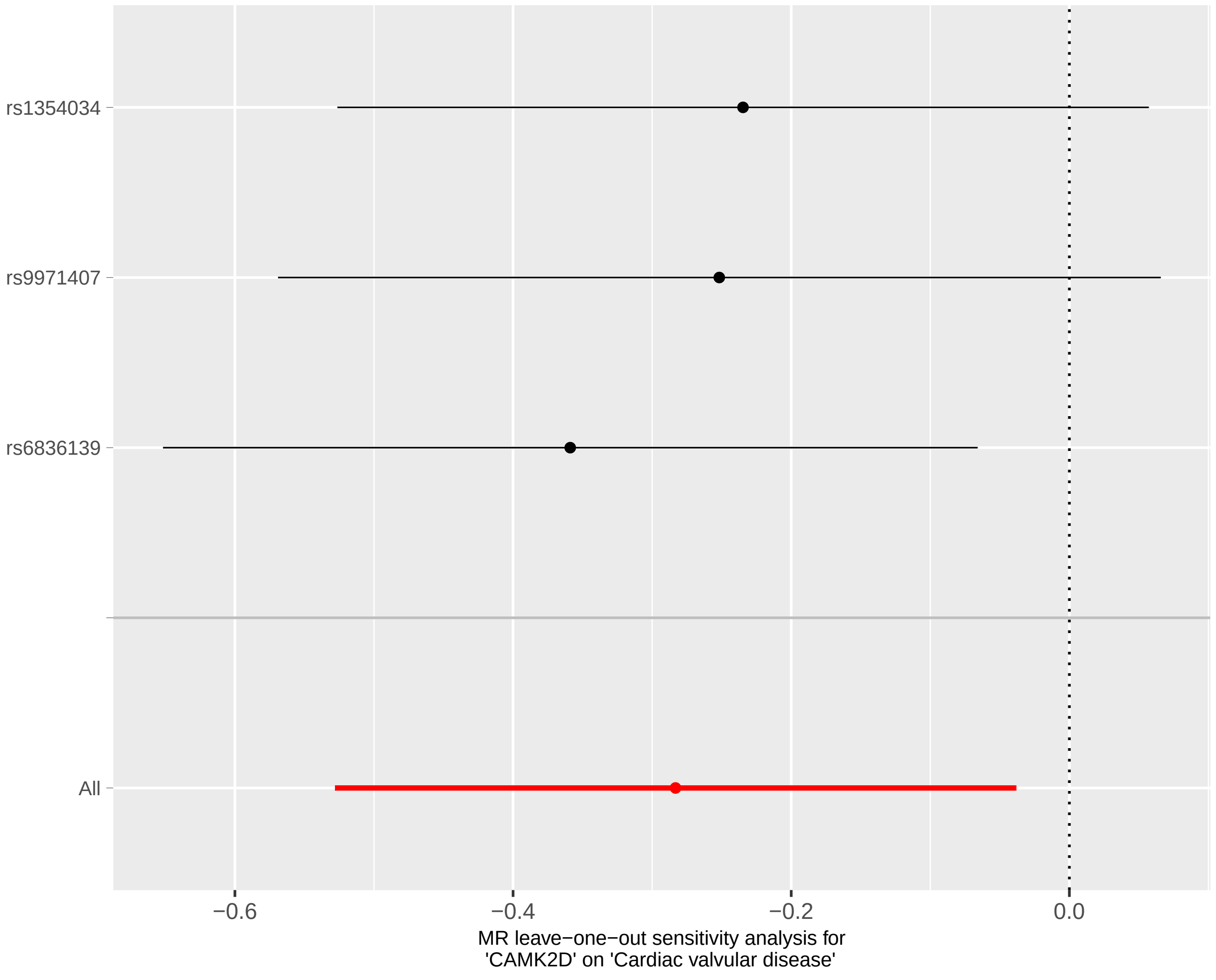
rs1354034
rs9971407
rs6836139
All
−0.6
−0.4
−0.2
0.0
MR leave−one−out sensitivity analysis for
'CAMK2D' on 'Cardiac valvular disease'

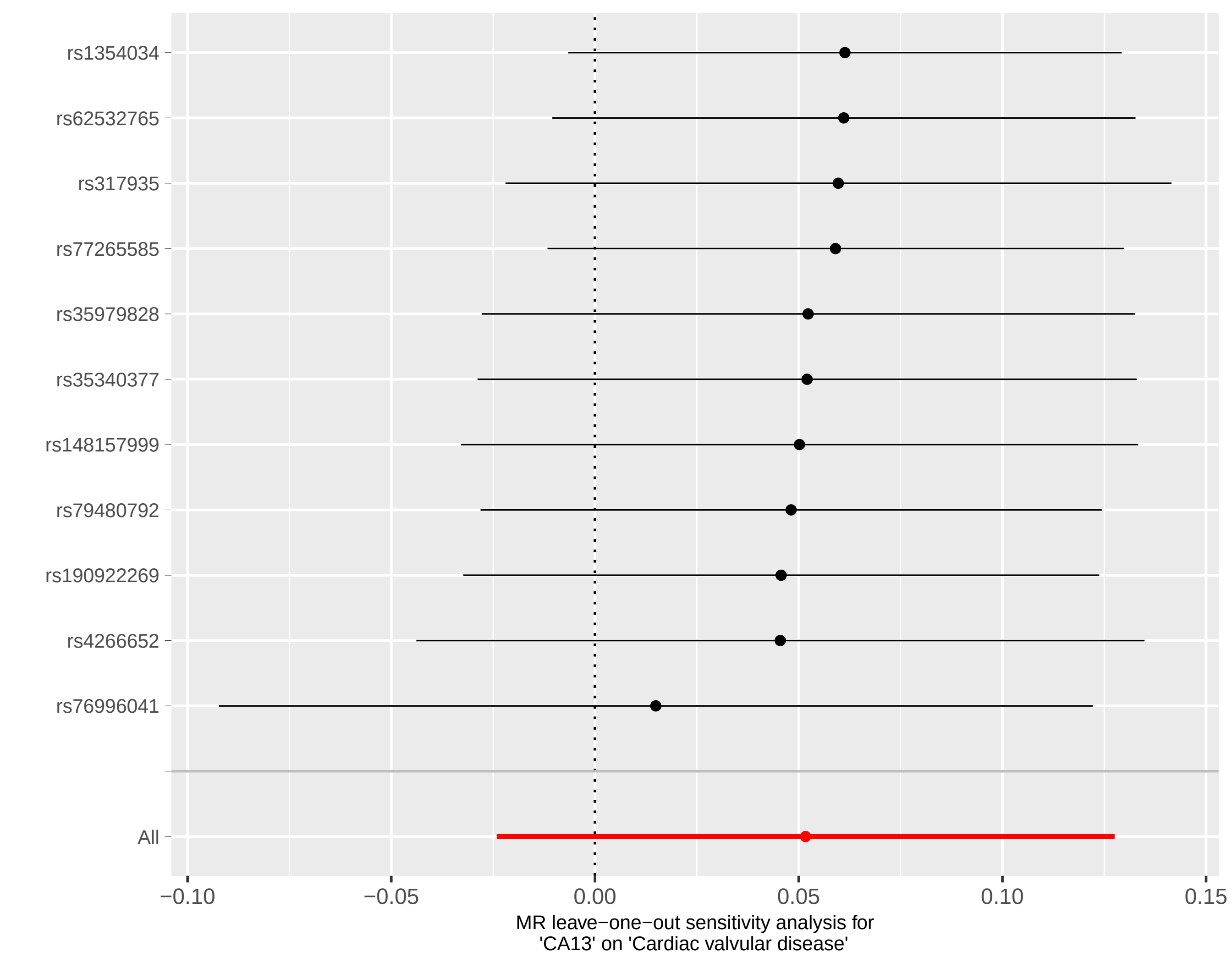

rs1354034
rs62532765
rs317935
rs77265585
rs35979828
rs35340377
rs148157999
rs79480792
rs190922269
rs4266652
rs76996041
All
−0.10
−0.05
0.00
0.05
0.10
0.15
MR leave−one−out sensitivity analysis for
'CA13' on 'Cardiac valvular disease'

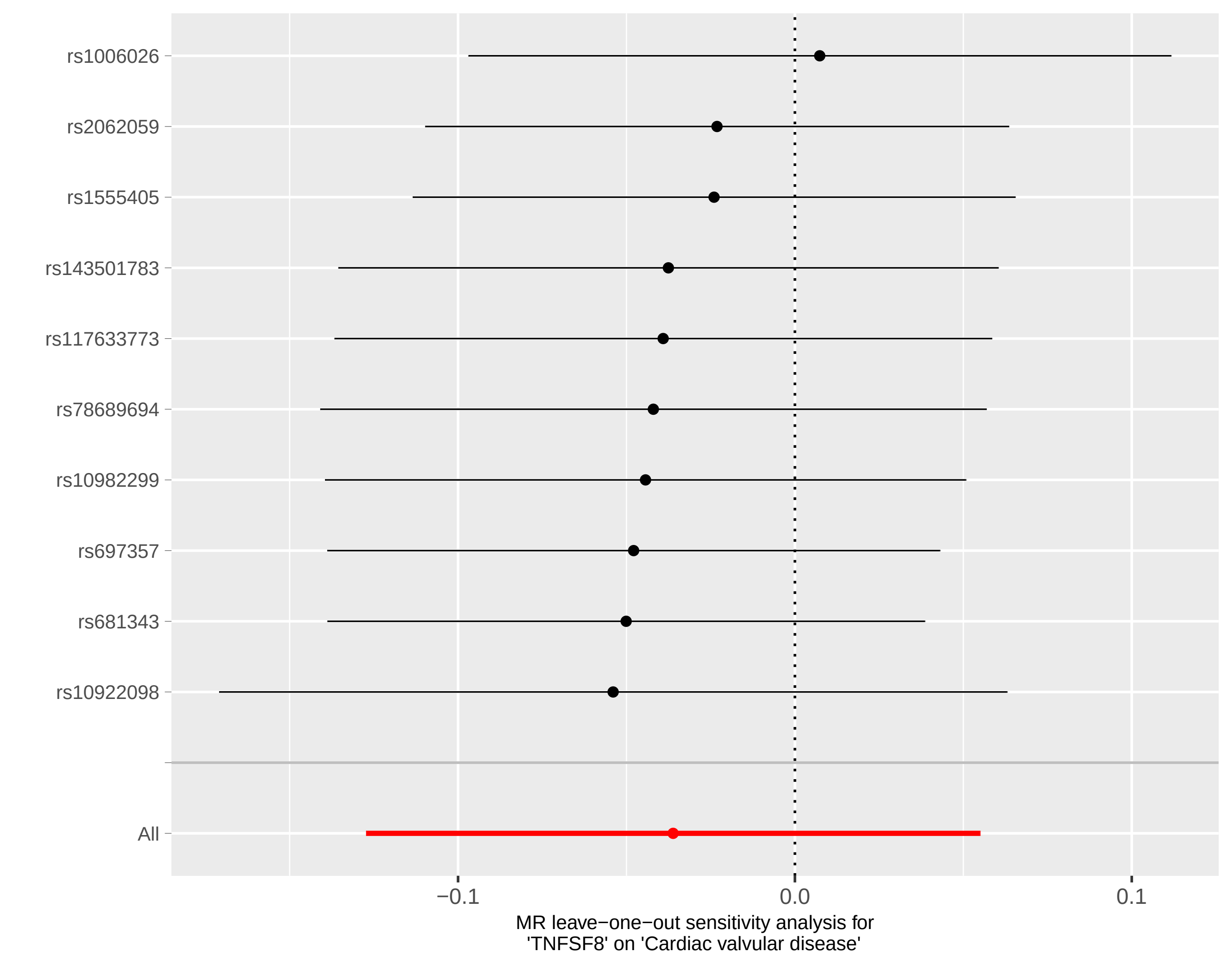
rs1006026
rs2062059
rs1555405
rs143501783
rs117633773
rs78689694
rs10982299
rs697357
rs681343
rs10922098
All
−0.1
0.0
0.1
MR leave−one−out sensitivity analysis for
'TNFSF8' on 'Cardiac valvular disease'

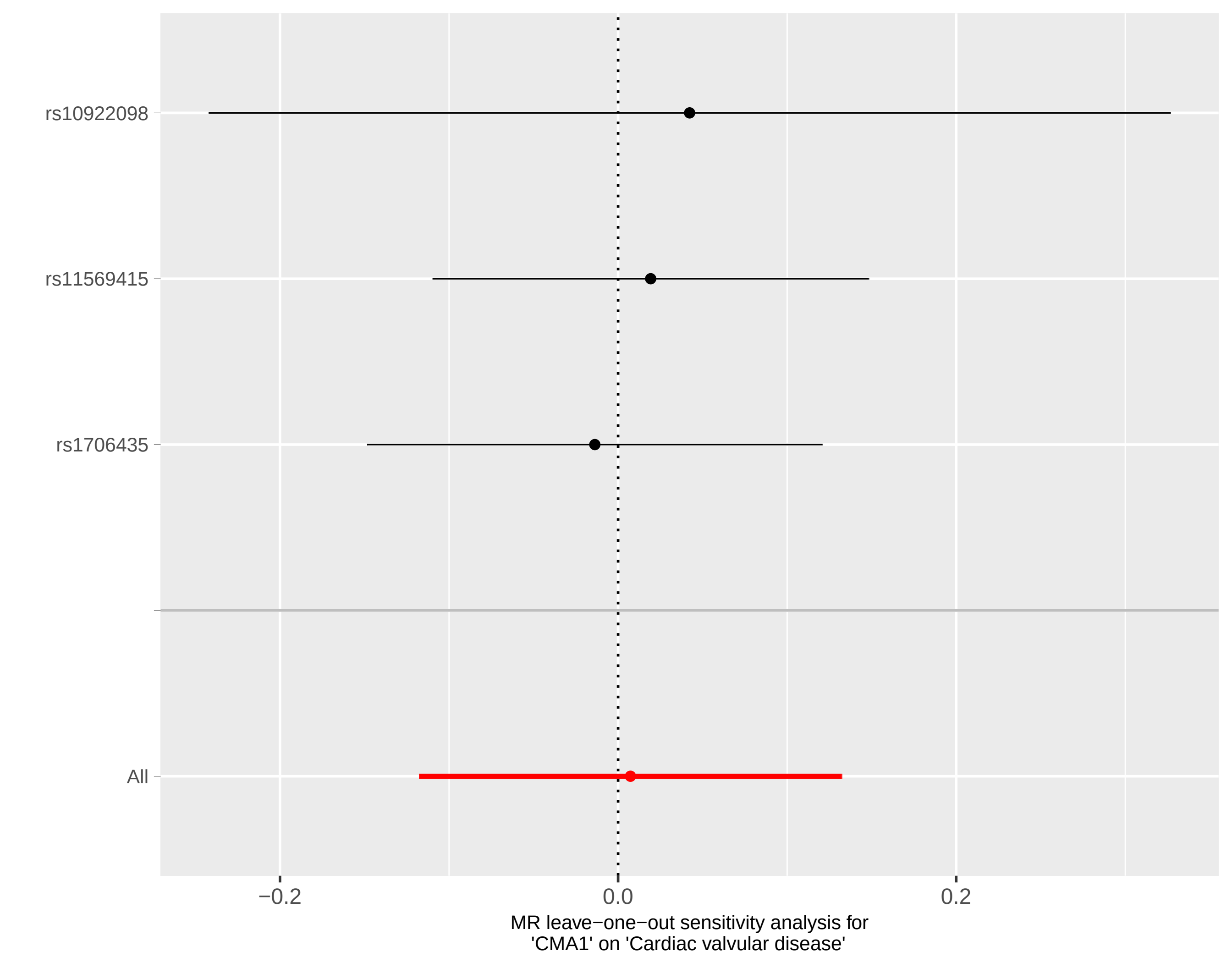

rs10922098
rs11569415
rs1706435
All
−0.2
0.0
0.2
MR leave−one−out sensitivity analysis for
'CMA1' on 'Cardiac valvular disease'

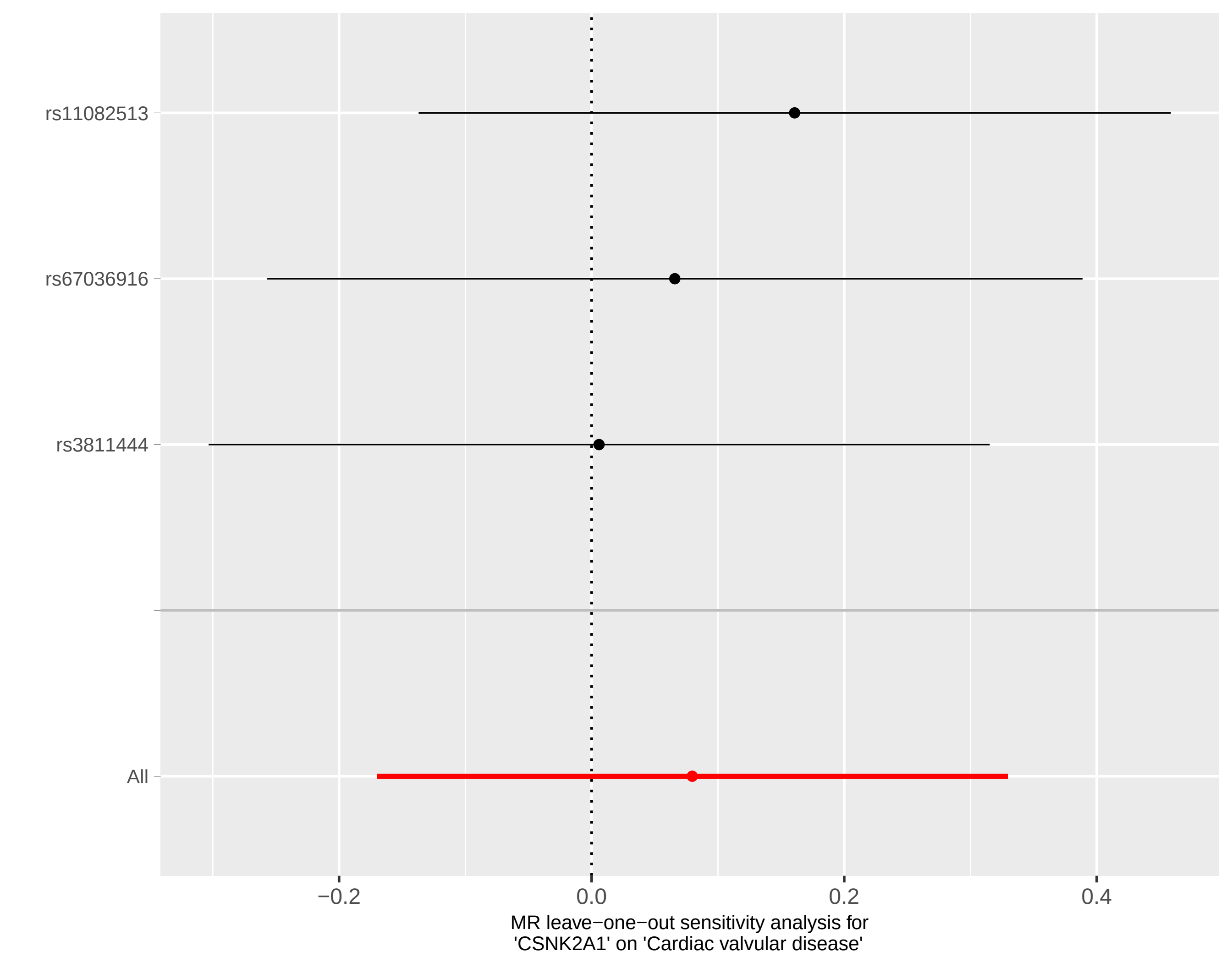
rs11082513
rs67036916
rs3811444
All
−0.2
0.0
0.2
0.4
MR leave−one−out sensitivity analysis for
'CSNK2A1' on 'Cardiac valvular disease'

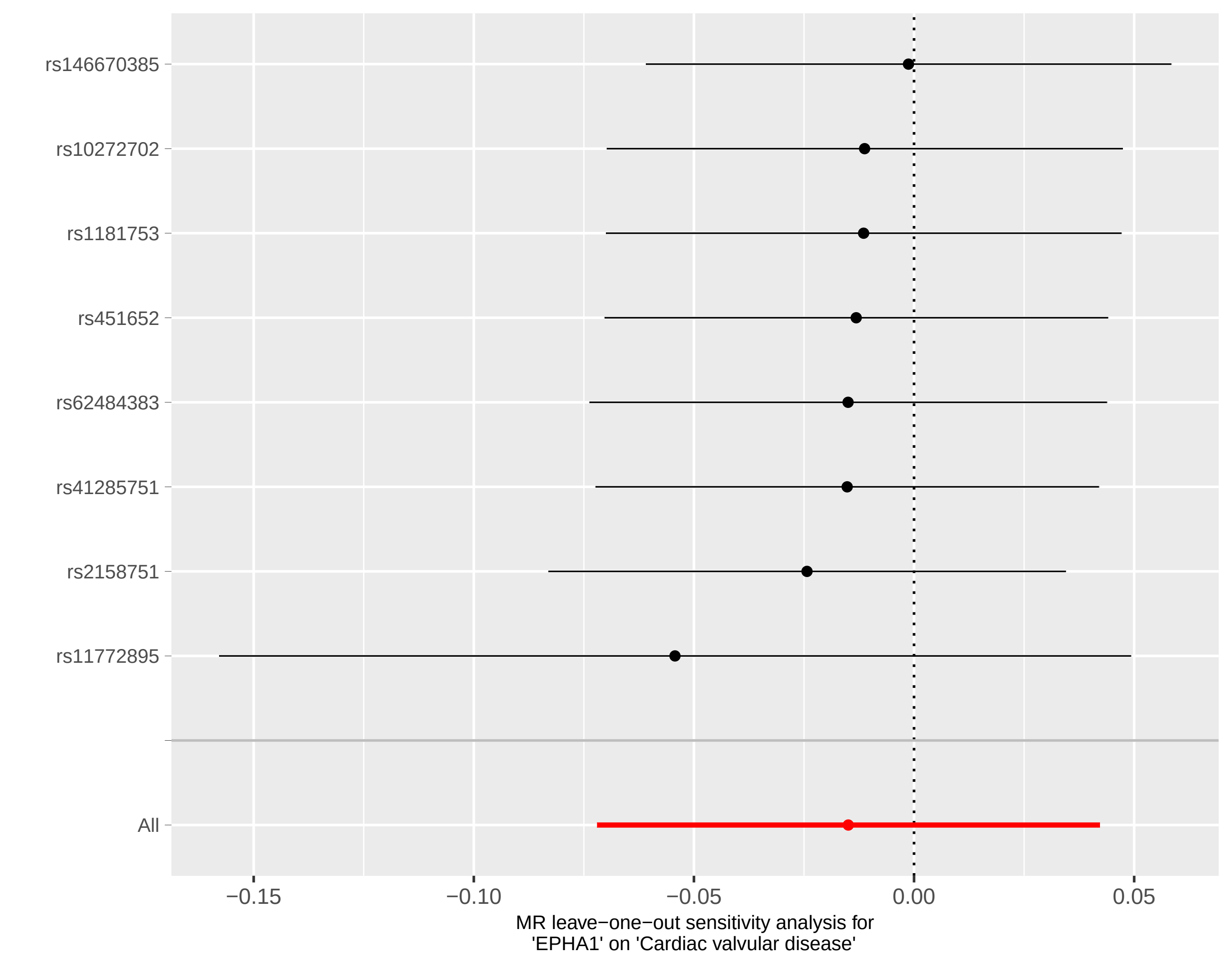

rs146670385
rs10272702
rs1181753
rs451652
rs62484383
rs41285751
rs2158751
rs11772895
All
−0.15
−0.10
−0.05
0.00
0.05
MR leave−one−out sensitivity analysis for
'EPHA1' on 'Cardiac valvular disease'

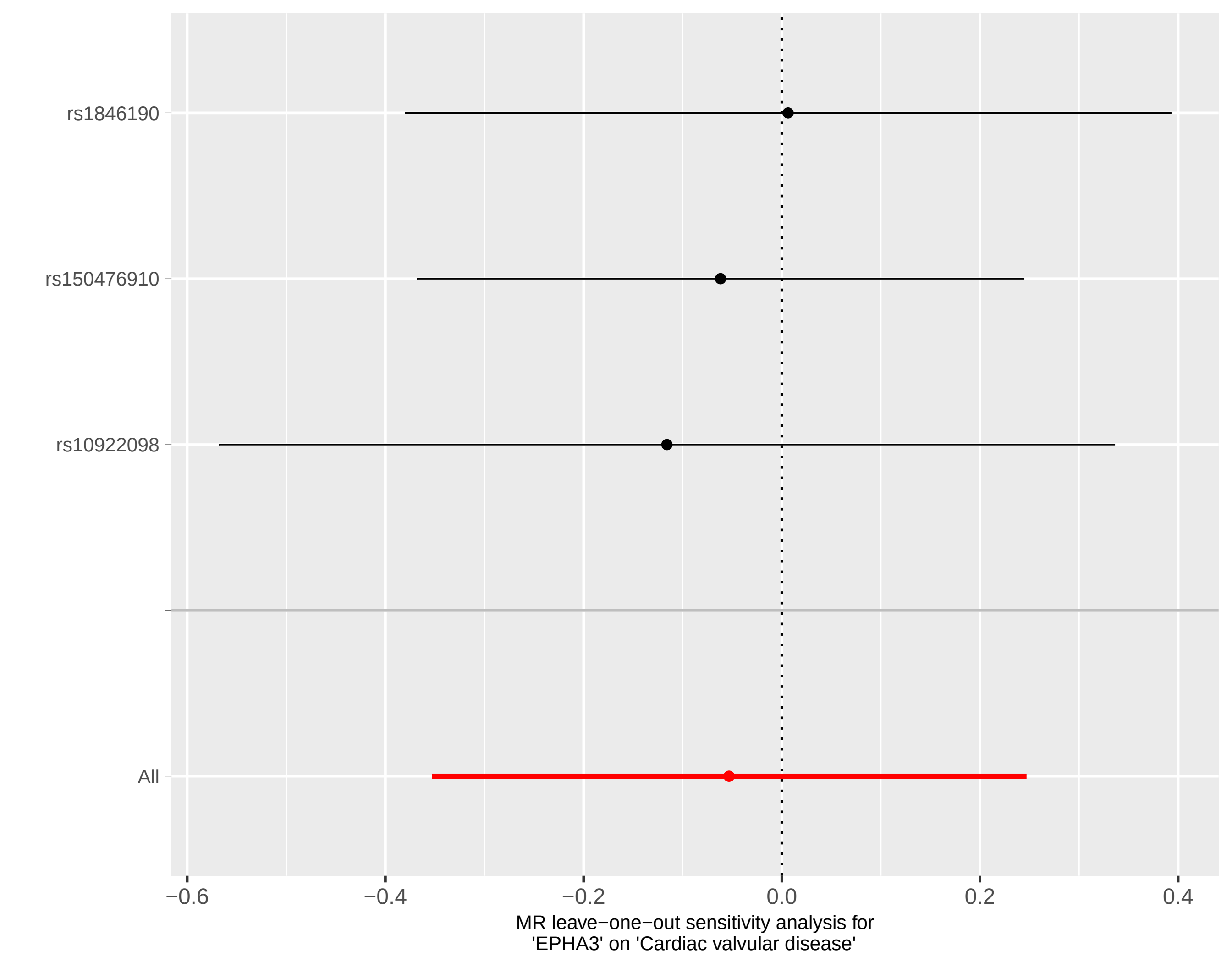
rs1846190
rs150476910
rs10922098
All
−0.6
−0.4
−0.2
0.0
0.2
0.4
MR leave−one−out sensitivity analysis for
'EPHA3' on 'Cardiac valvular disease'

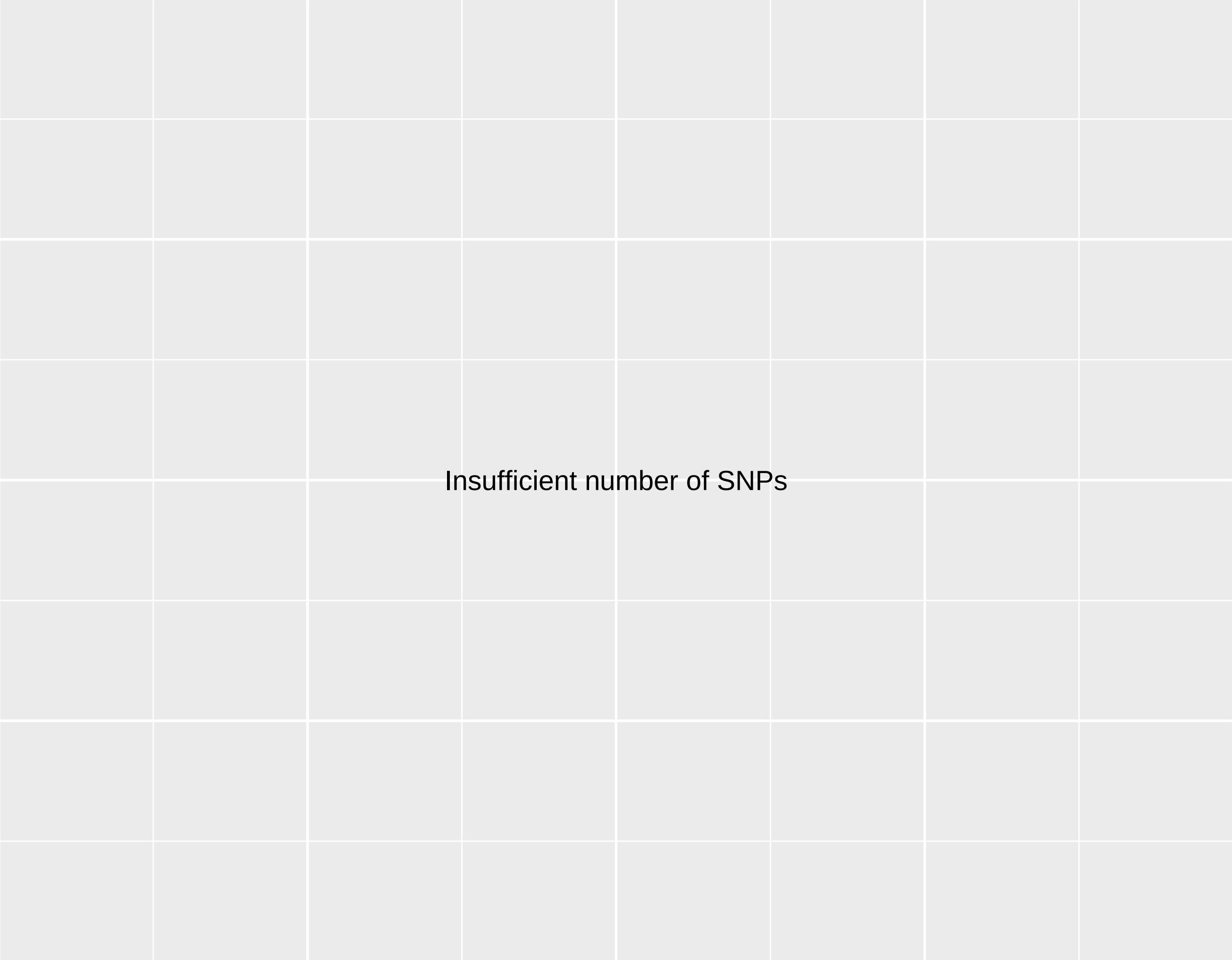
Insufficient number of SNPs

Insufficient number of SNPs

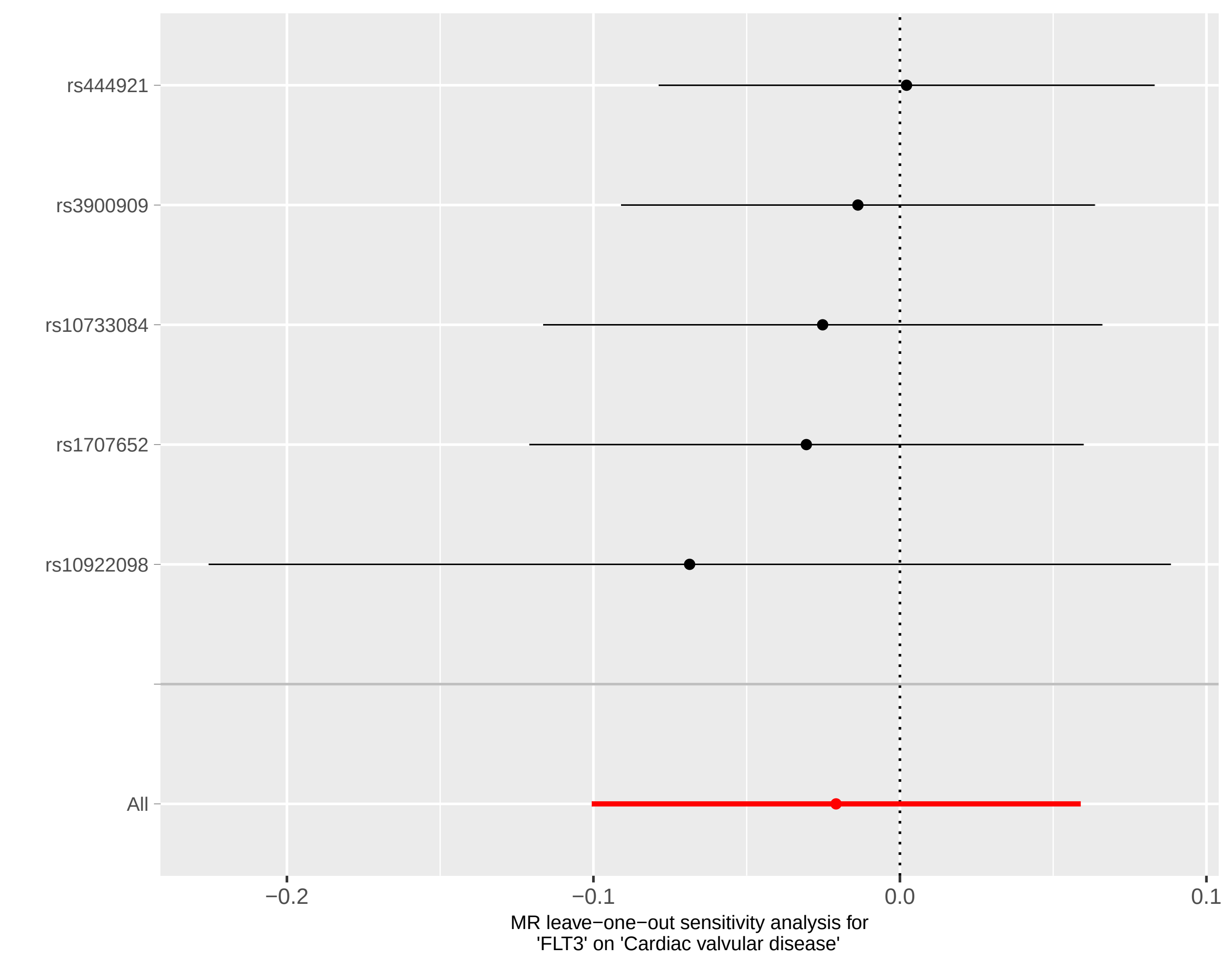
rs444921
rs3900909
rs10733084
rs1707652
rs10922098
All
−0.2
−0.1
0.0
0.1
MR leave−one−out sensitivity analysis for
'FLT3' on 'Cardiac valvular disease'

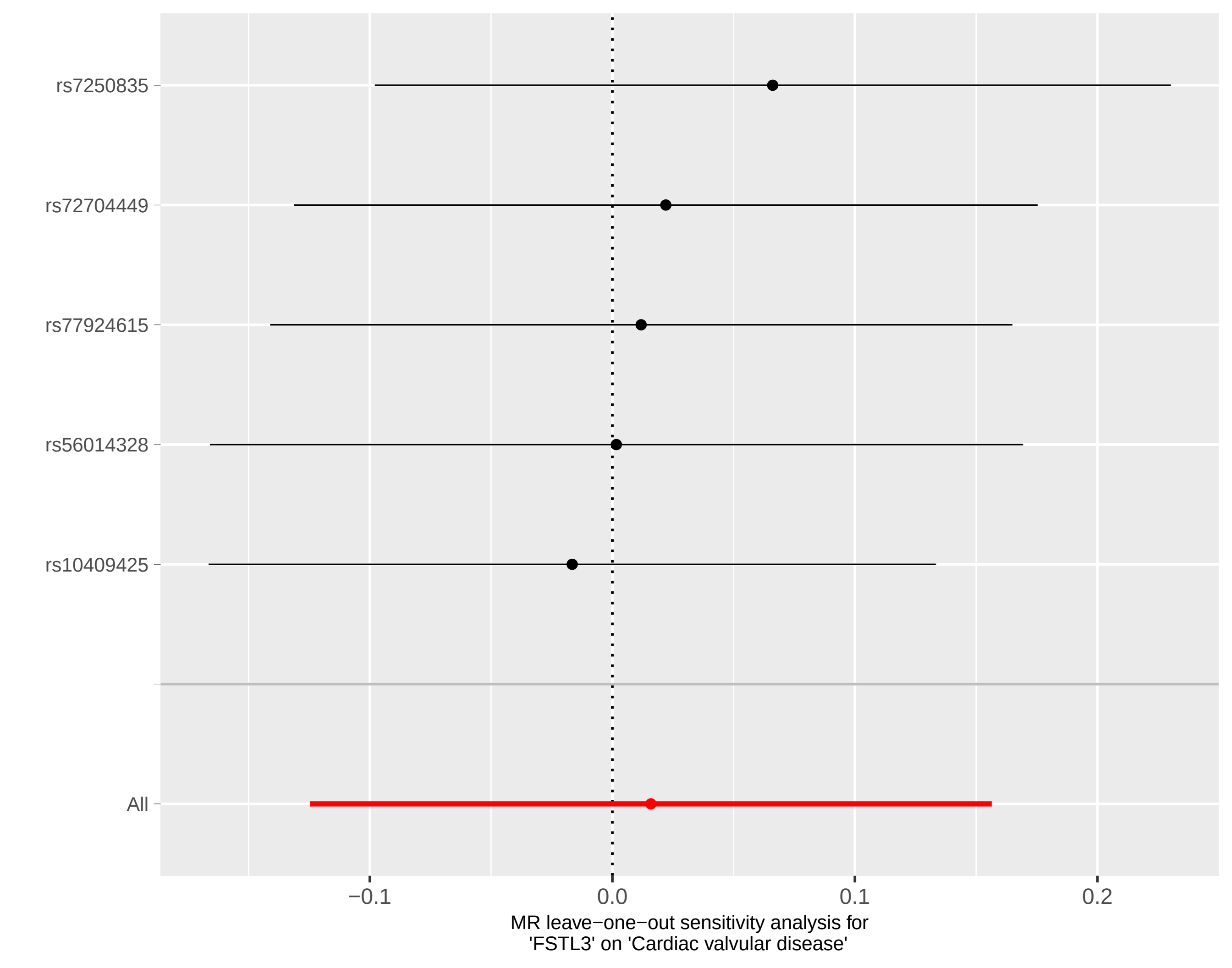

rs7250835
rs72704449
rs77924615
rs56014328
rs10409425
All
−0.1
0.0
0.1
0.2
MR leave−one−out sensitivity analysis for
'FSTL3' on 'Cardiac valvular disease'

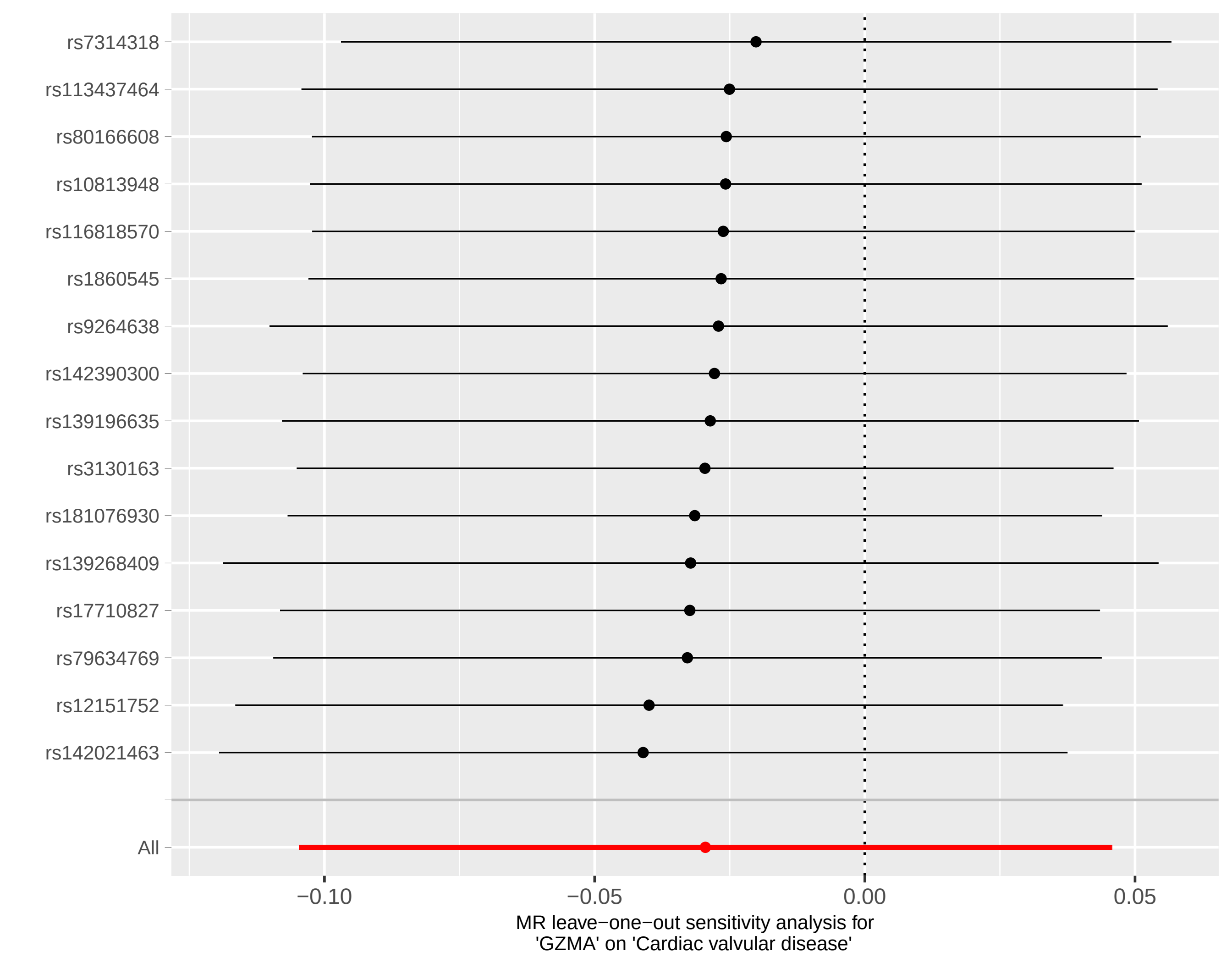
rs7314318
rs113437464
rs80166608
rs10813948
rs116818570
rs1860545
rs9264638
rs142390300
rs139196635
rs3130163
rs181076930
rs139268409
rs17710827
rs79634769
rs12151752
rs142021463
All
−0.10
−0.05
0.00
0.05
MR leave−one−out sensitivity analysis for
'GZMA' on 'Cardiac valvular disease'

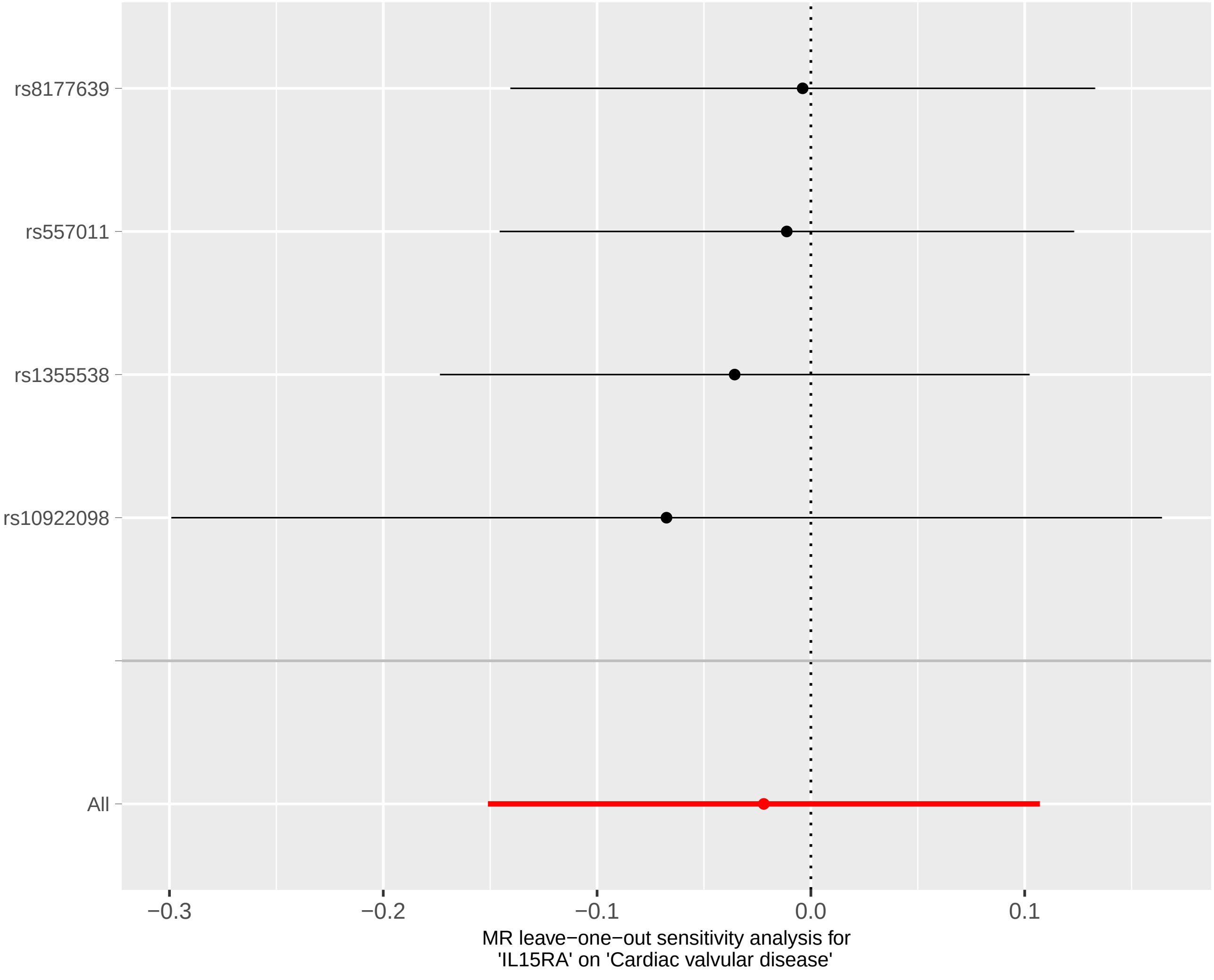
rs8177639
rs557011
rs1355538
rs10922098
All
−0.3
−0.2
−0.1
0.0
0.1
MR leave−one−out sensitivity analysis for
'IL15RA' on 'Cardiac valvular disease'

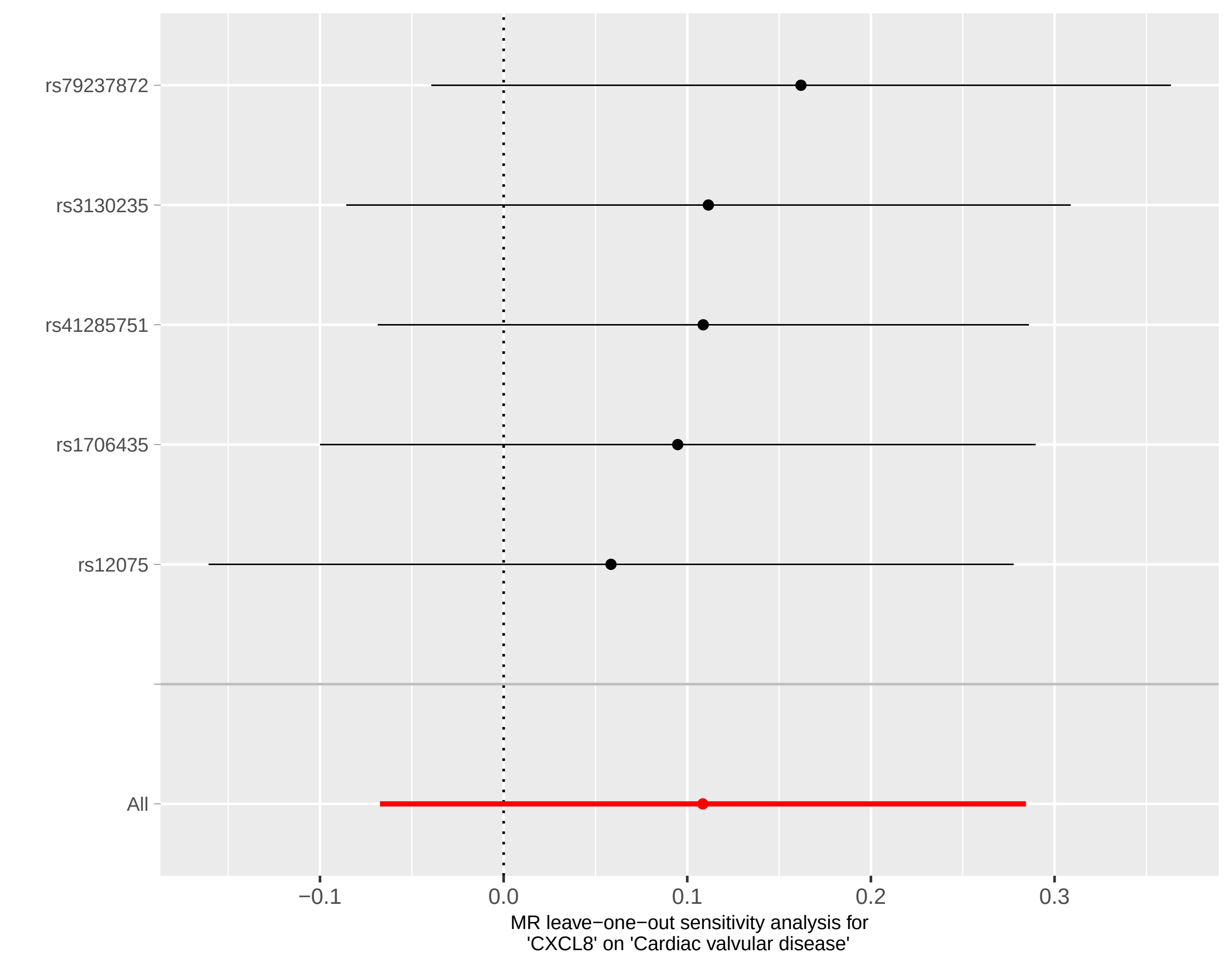
rs79237872
rs3130235
rs41285751
rs1706435
rs12075
All
−0.1
0.0
0.1
0.2
0.3
MR leave−one−out sensitivity analysis for
'CXCL8' on 'Cardiac valvular disease'

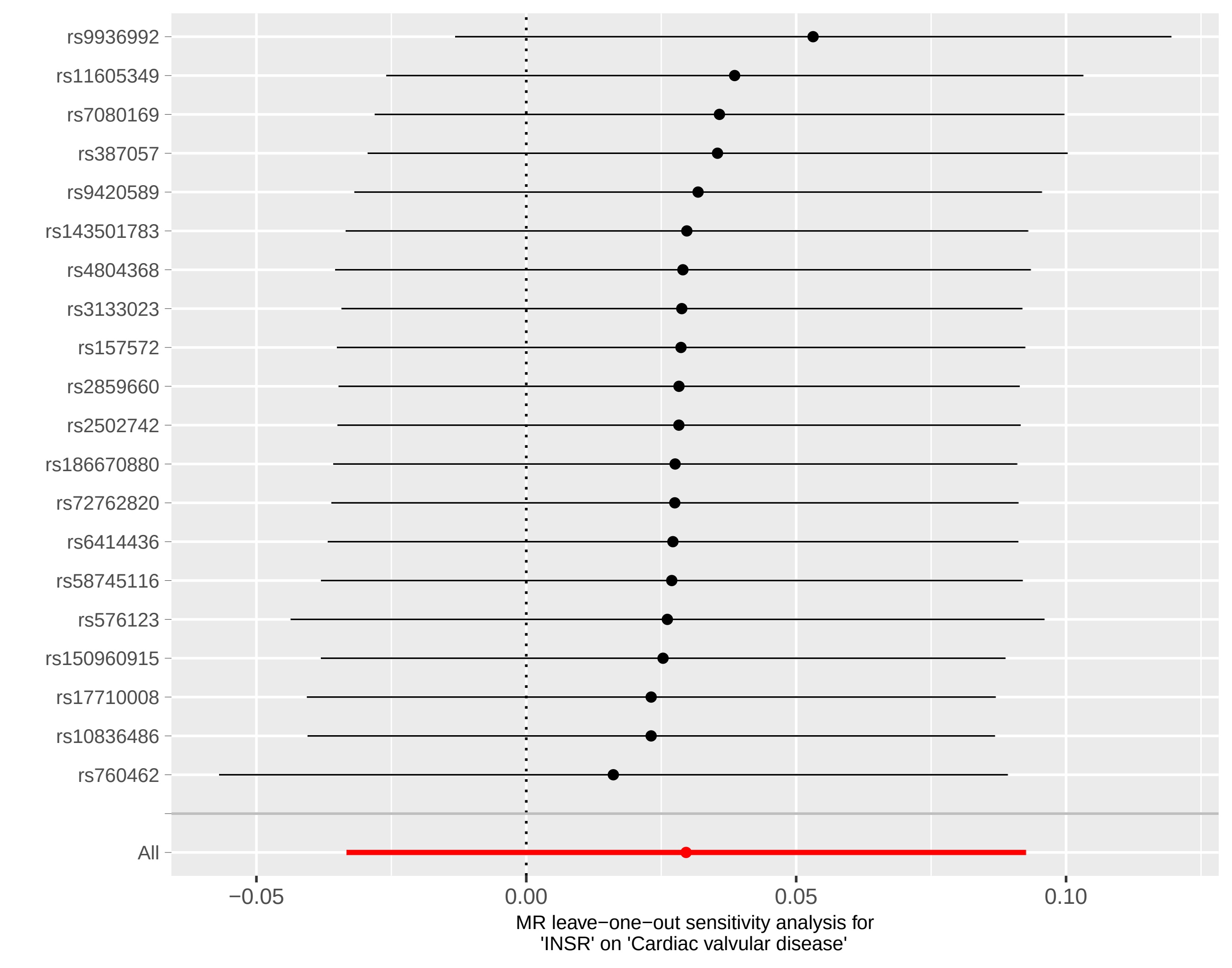
rs9936992
rs11605349
rs7080169
rs387057
rs9420589
rs143501783
rs4804368
rs3133023
rs157572
rs2859660
rs2502742
rs186670880
rs72762820
rs6414436
rs58745116
rs576123
rs150960915
rs17710008
rs10836486
rs760462
All
−0.05
0.00
0.05
0.10
MR leave−one−out sensitivity analysis for
'INSR' on 'Cardiac valvular disease'

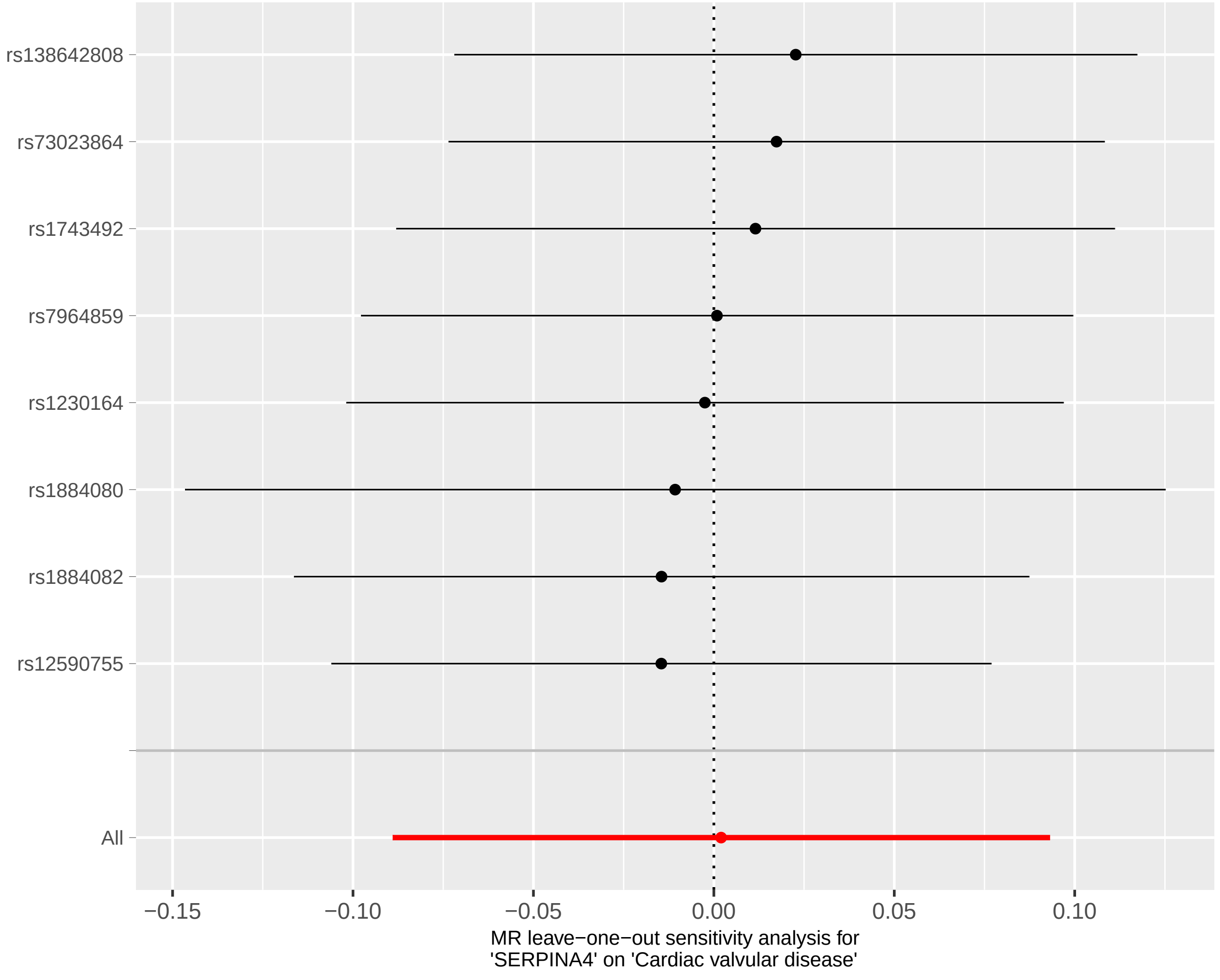

rs138642808
rs73023864
rs1743492
rs7964859
rs1230164
rs1884080
rs1884082
rs12590755
All
−0.15
−0.10
−0.05
0.00
0.05
0.10
MR leave−one−out sensitivity analysis for
'SERPINA4' on 'Cardiac valvular disease'

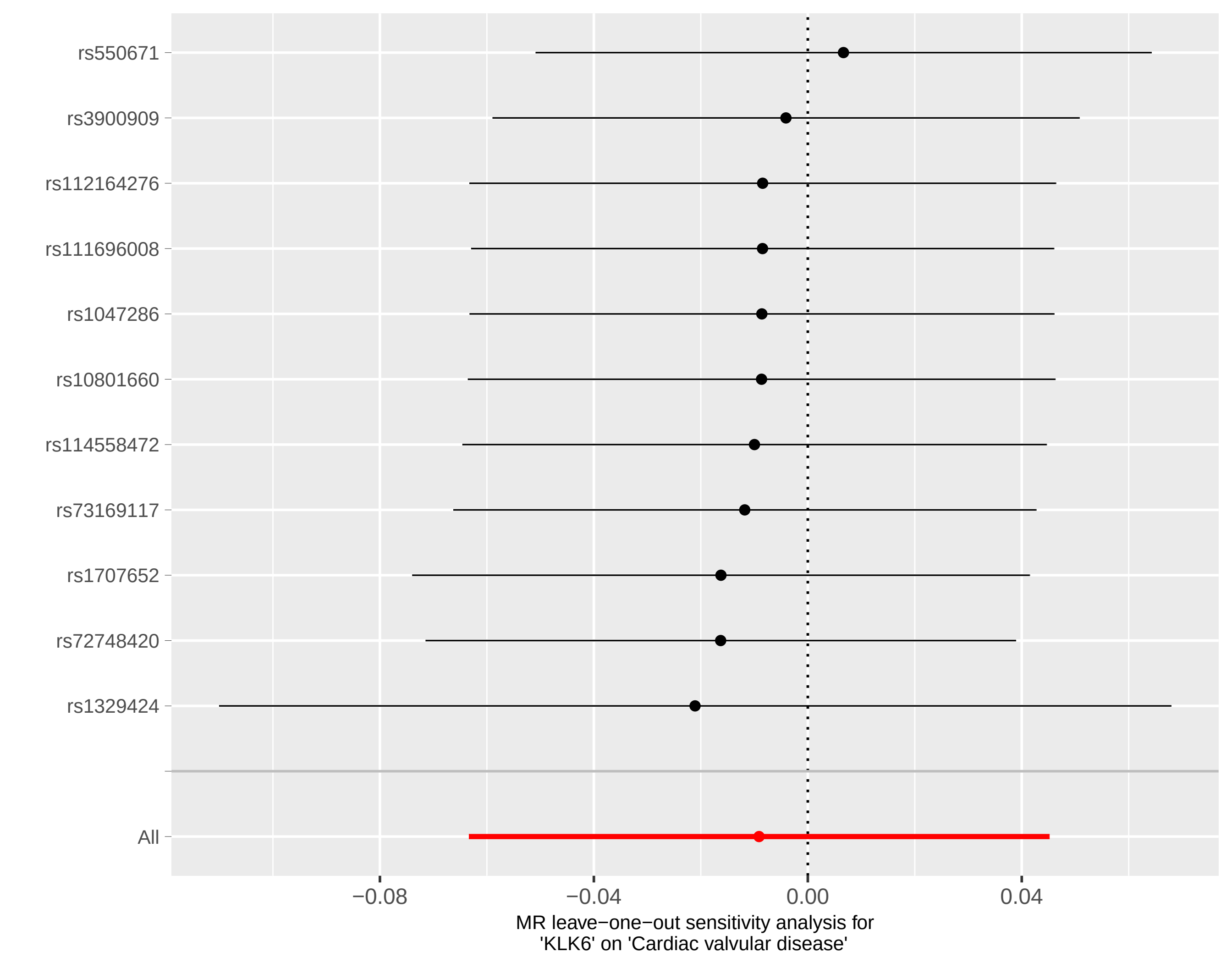
rs550671
rs3900909
rs112164276
rs111696008
rs1047286
rs10801660
rs114558472
rs73169117
rs1707652
rs72748420
rs1329424
All
−0.08
−0.04
0.00
0.04
MR leave−one−out sensitivity analysis for
'KLK6' on 'Cardiac valvular disease'

Insufficient number of SNPs

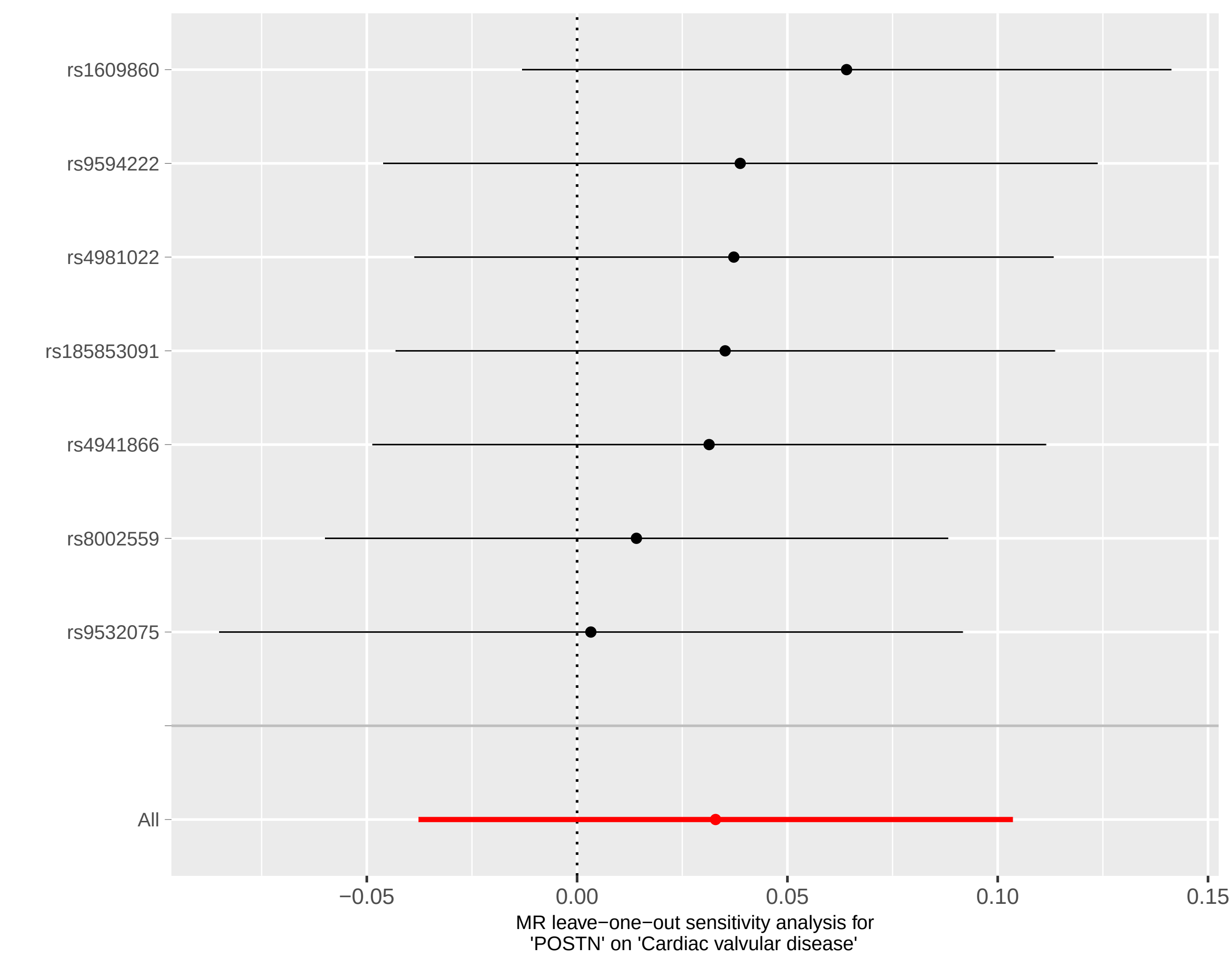
rs1609860
rs9594222
rs4981022
rs185853091
rs4941866
rs8002559
rs9532075
All
−0.05
0.00
0.05
0.10
0.15
MR leave−one−out sensitivity analysis for
'POSTN' on 'Cardiac valvular disease'

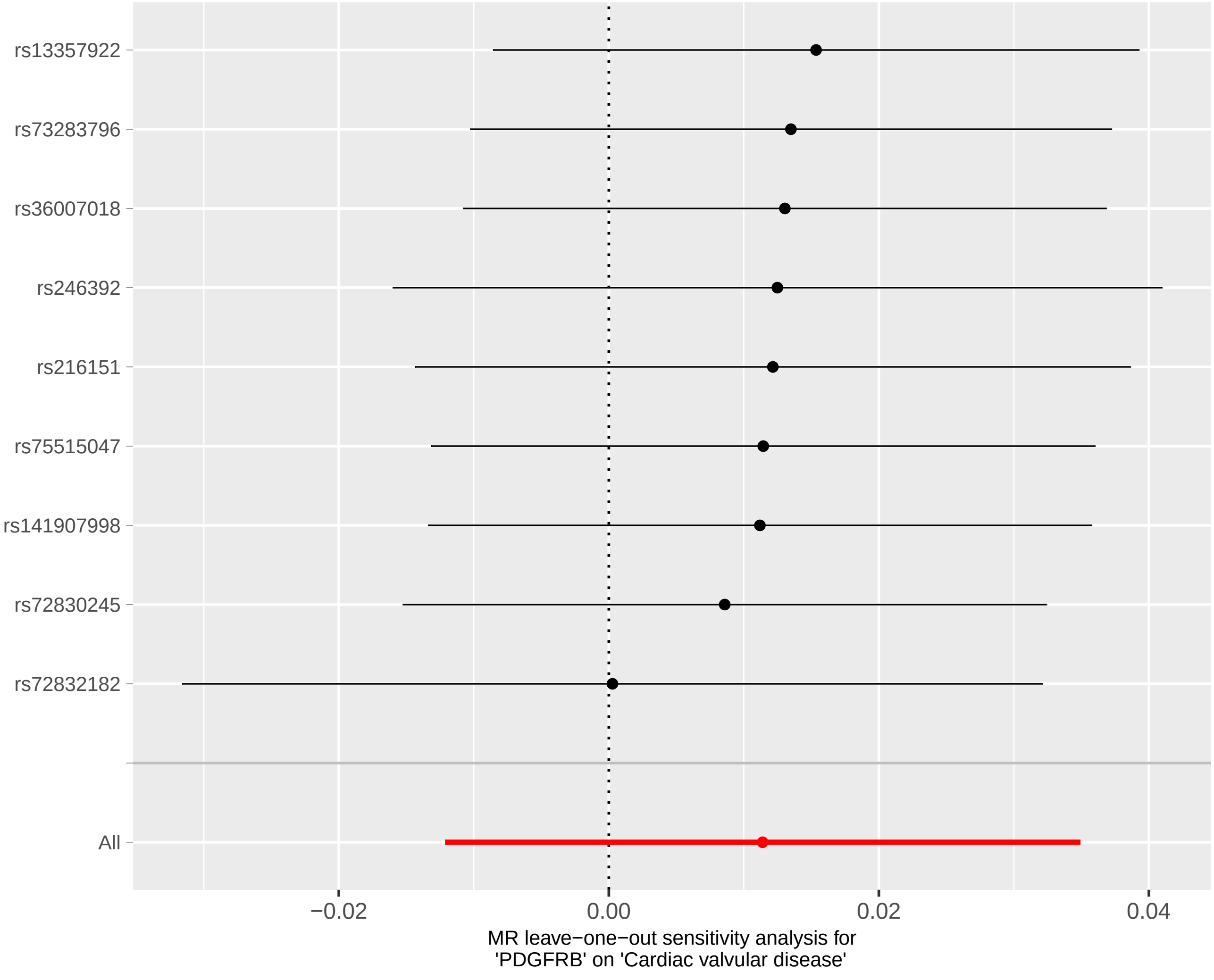
rs13357922
rs73283796
rs36007018
rs246392
rs216151
rs75515047
rs141907998
rs72830245
rs72832182
All
−0.02
0.00
0.02
0.04
MR leave−one−out sensitivity analysis for
'PDGFRB' on 'Cardiac valvular disease'

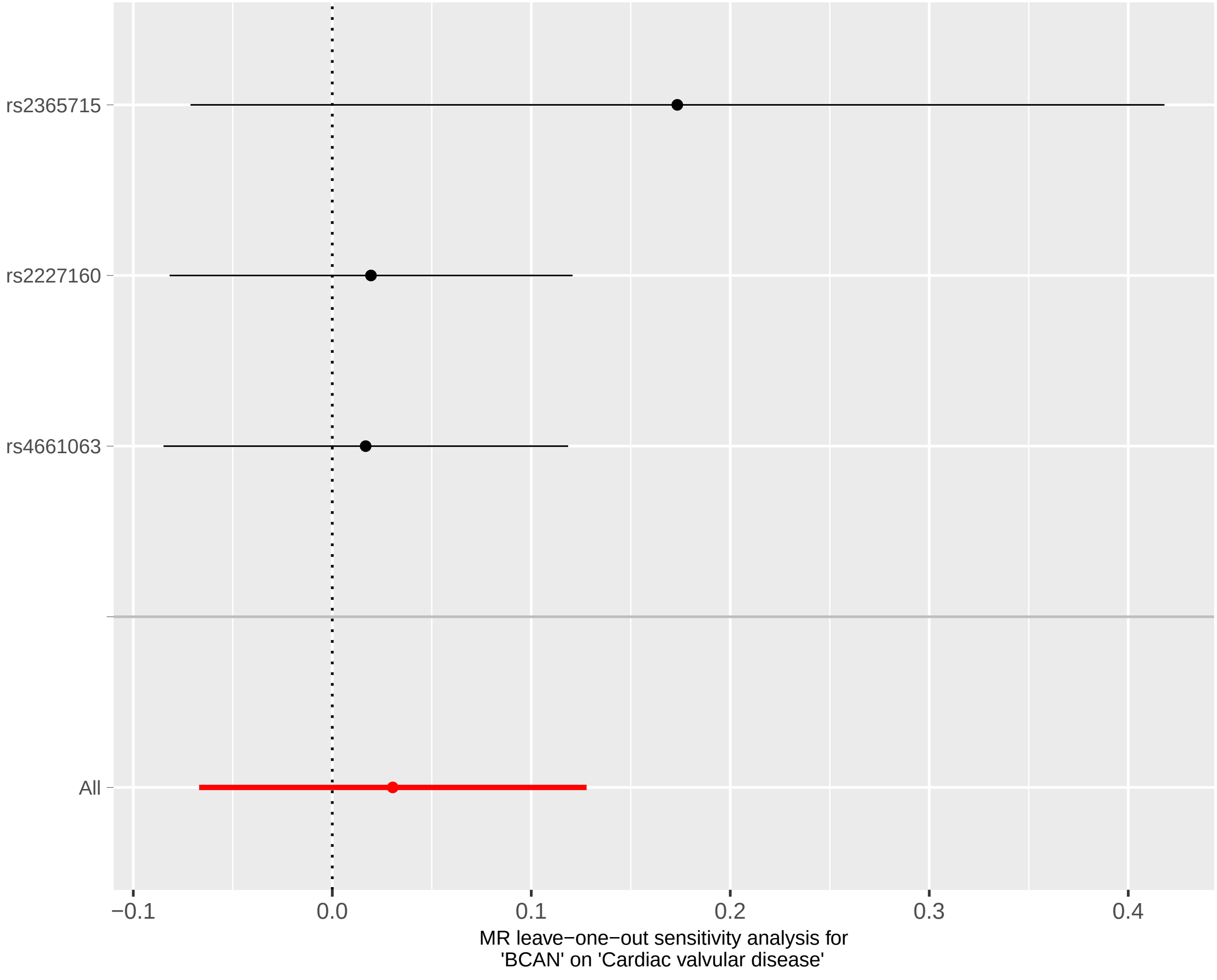

rs2365715
rs2227160
rs4661063
All
−0.1
0.0
0.1
0.2
0.3
0.4
MR leave−one−out sensitivity analysis for
'BCAN' on 'Cardiac valvular disease'

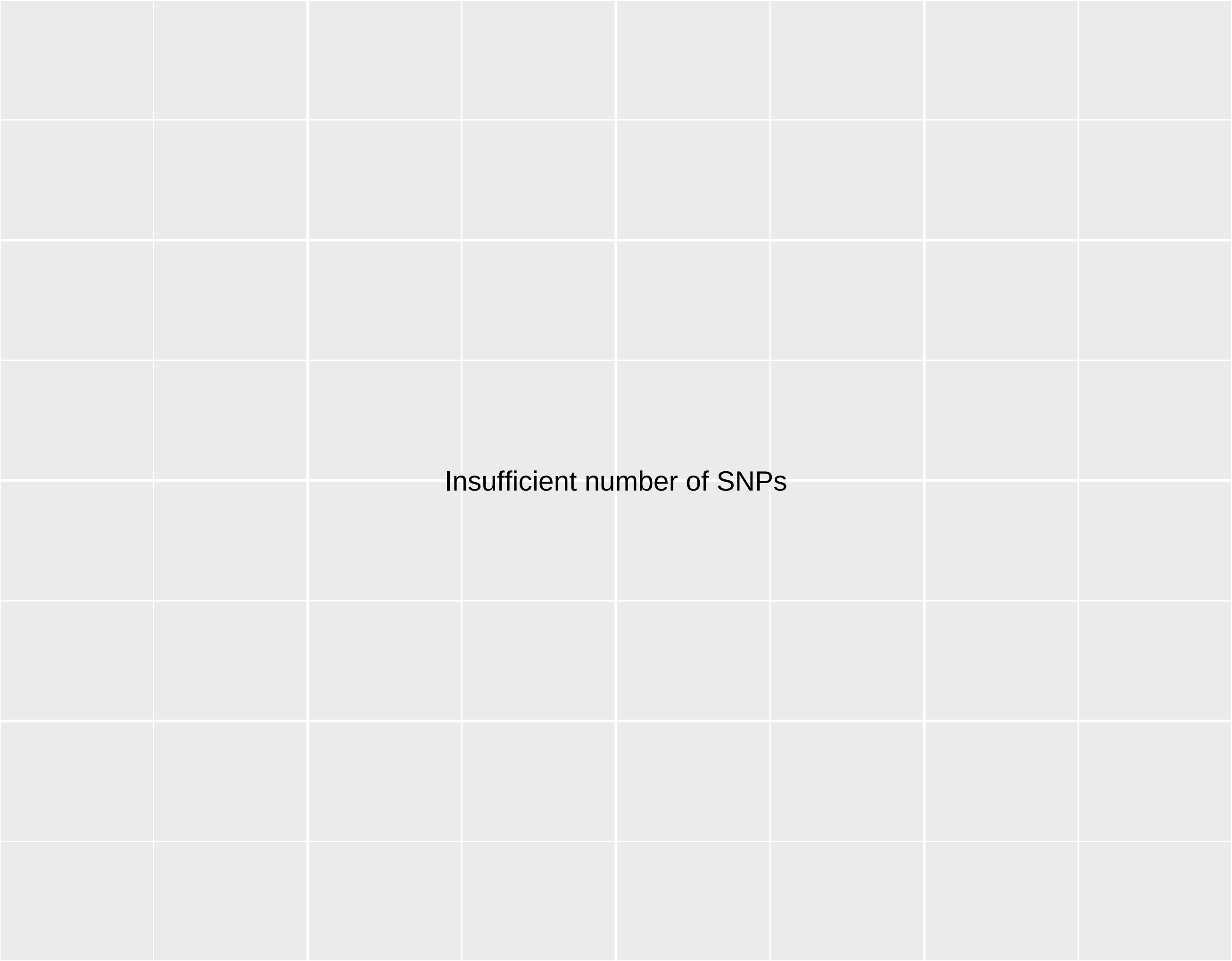
Insufficient number of SNPs

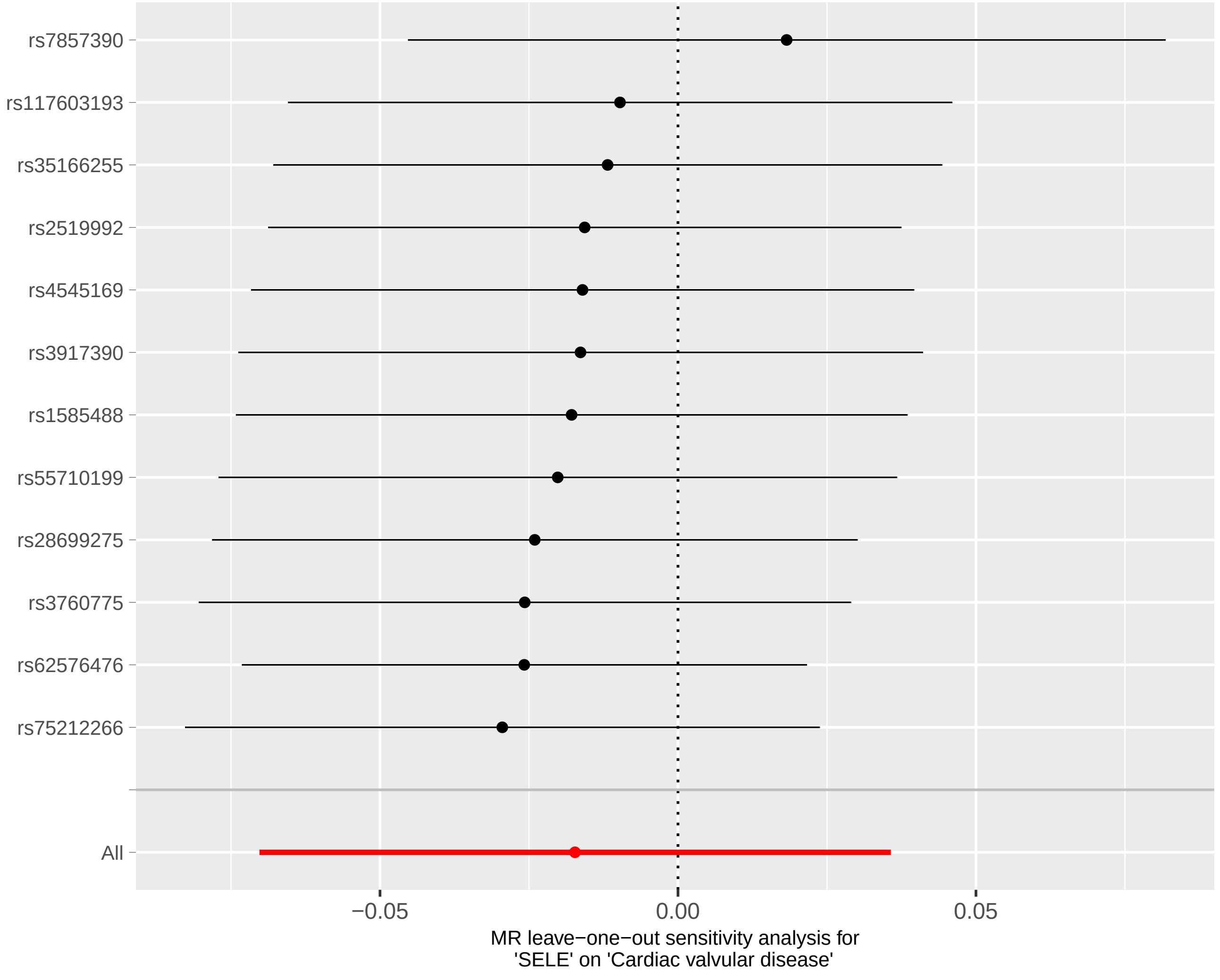
rs7857390
rs117603193
rs35166255
rs2519992
rs4545169
rs3917390
rs1585488
rs55710199
rs28699275
rs3760775
rs62576476
rs75212266
All
−0.05
0.00
0.05
MR leave−one−out sensitivity analysis for
'SELE' on 'Cardiac valvular disease'

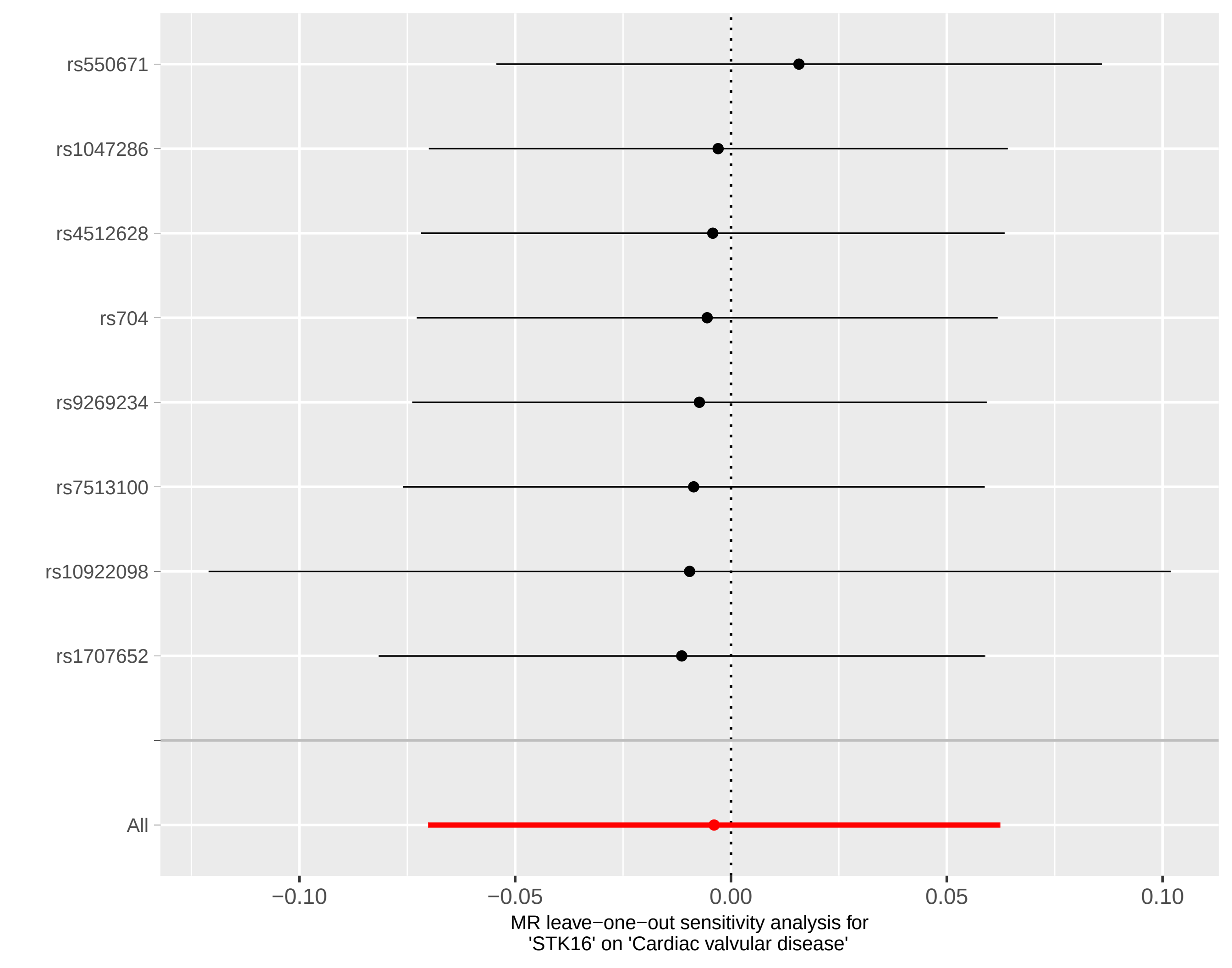
rs550671
rs1047286
rs4512628
rs704
rs9269234
rs7513100
rs10922098
rs1707652
All
−0.10
−0.05
0.00
0.05
0.10
MR leave−one−out sensitivity analysis for
'STK16' on 'Cardiac valvular disease'

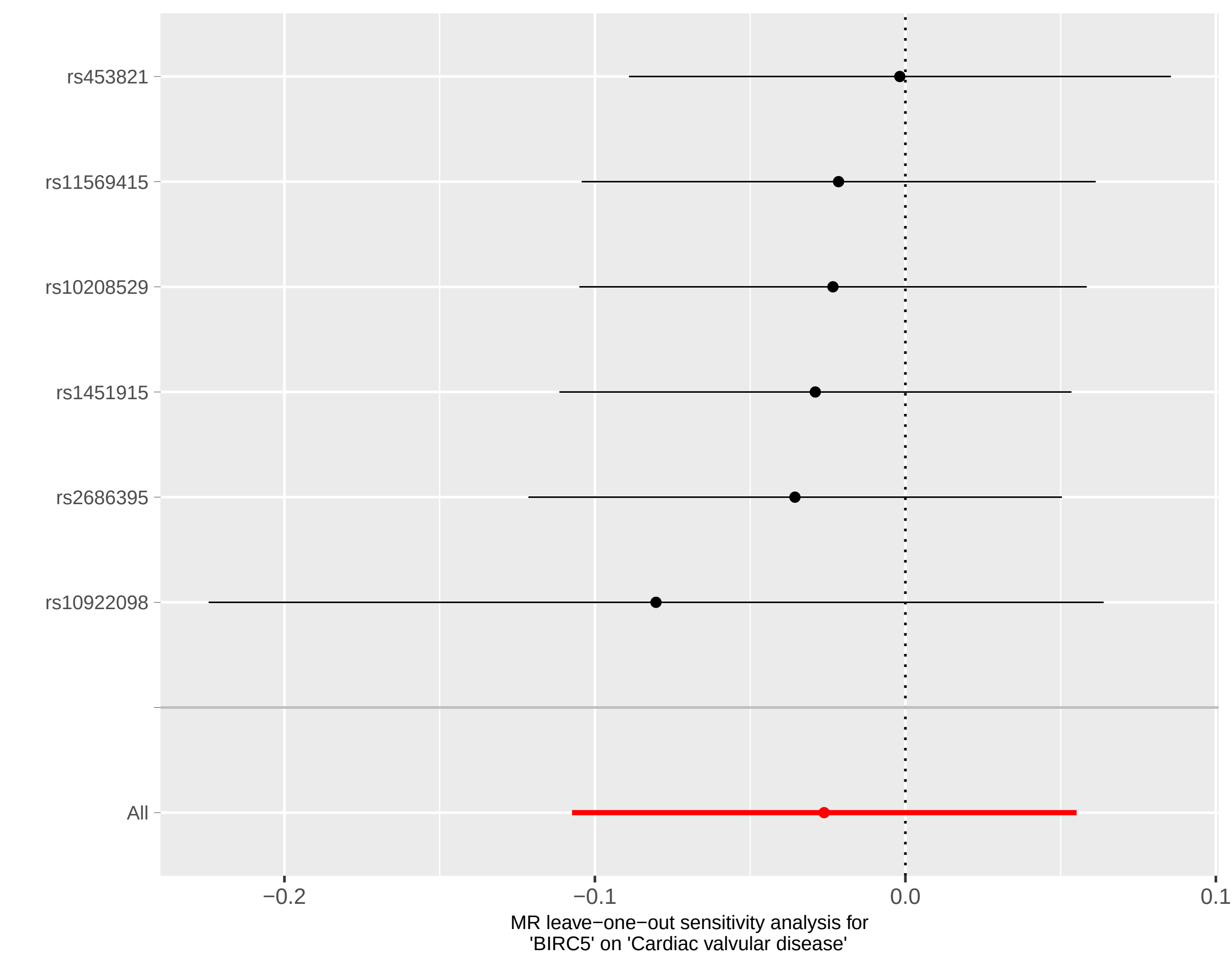

rs453821
rs11569415
rs10208529
rs1451915
rs2686395
rs10922098
All
−0.2
−0.1
0.0
0.1
MR leave−one−out sensitivity analysis for
'BIRC5' on 'Cardiac valvular disease'

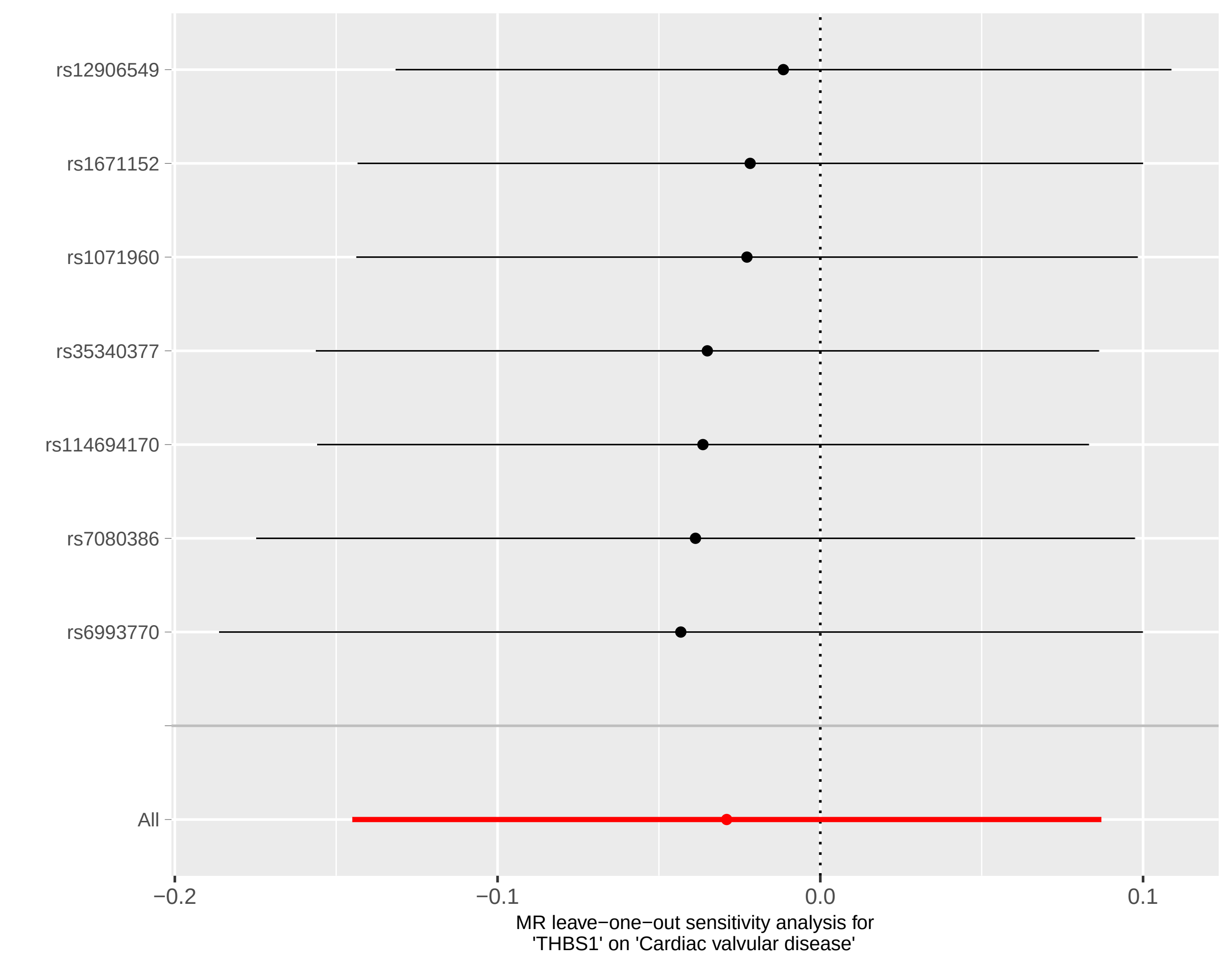
rs12906549
rs1671152
rs1071960
rs35340377
rs114694170
rs7080386
rs6993770
All
−0.2
−0.1
0.0
0.1
MR leave−one−out sensitivity analysis for
'THBS1' on 'Cardiac valvular disease'

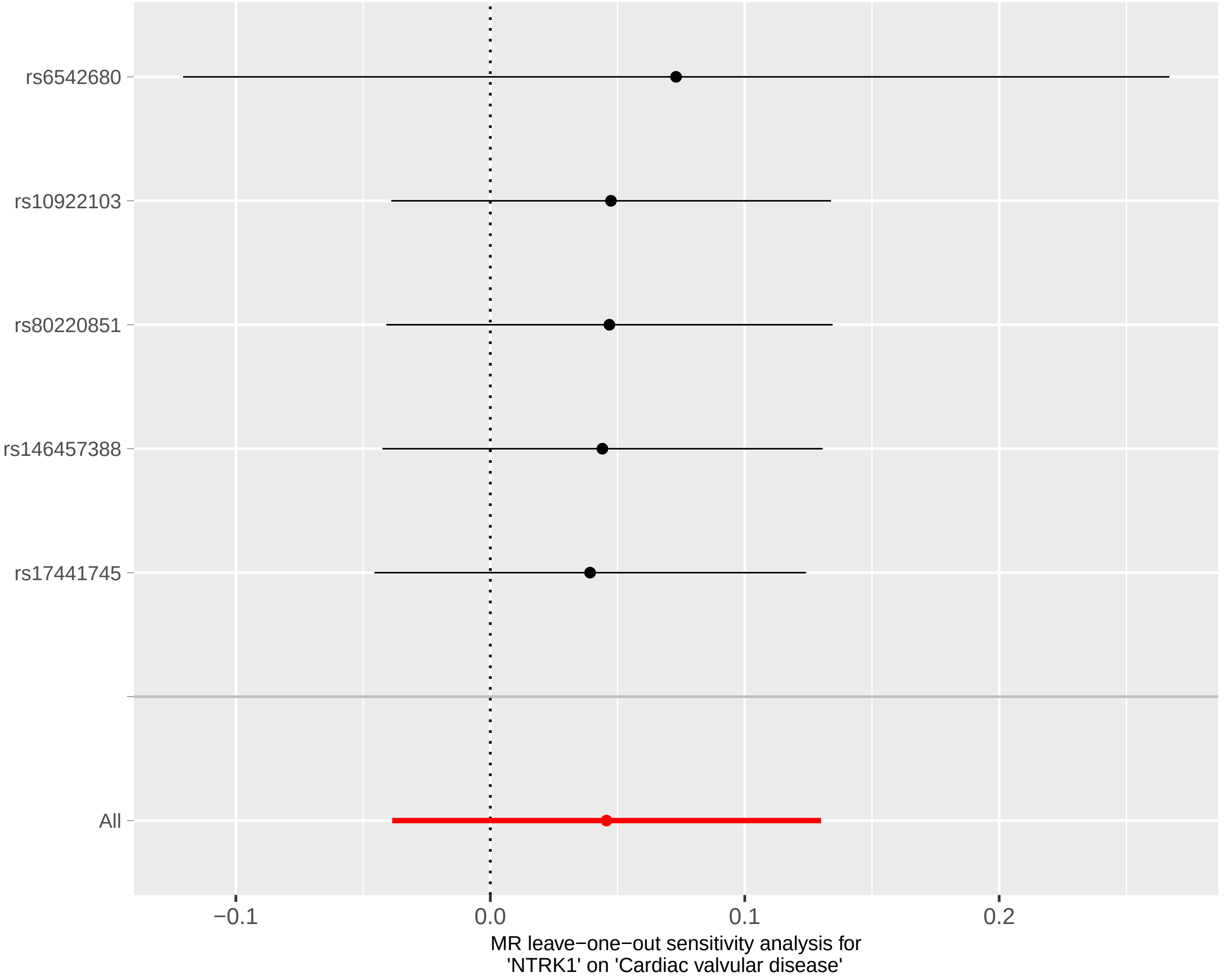
rs6542680
rs10922103
rs80220851
rs146457388
rs17441745
All
−0.1
0.0
0.1
0.2
MR leave−one−out sensitivity analysis for
'NTRK1' on 'Cardiac valvular disease'

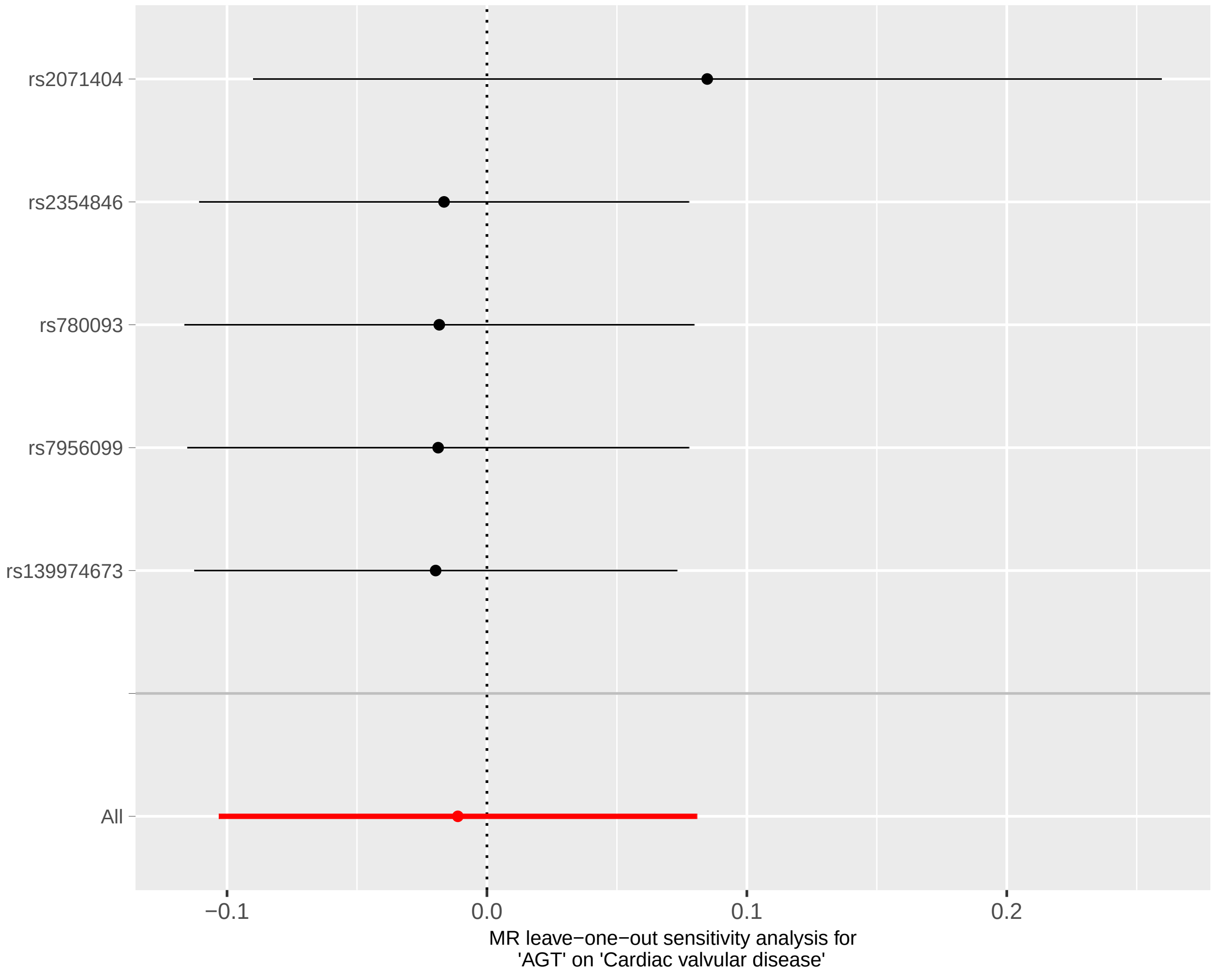
rs2071404
rs2354846
rs780093
rs7956099
rs139974673
All
−0.1
0.0
0.1
0.2
MR leave−one−out sensitivity analysis for
'AGT' on 'Cardiac valvular disease'

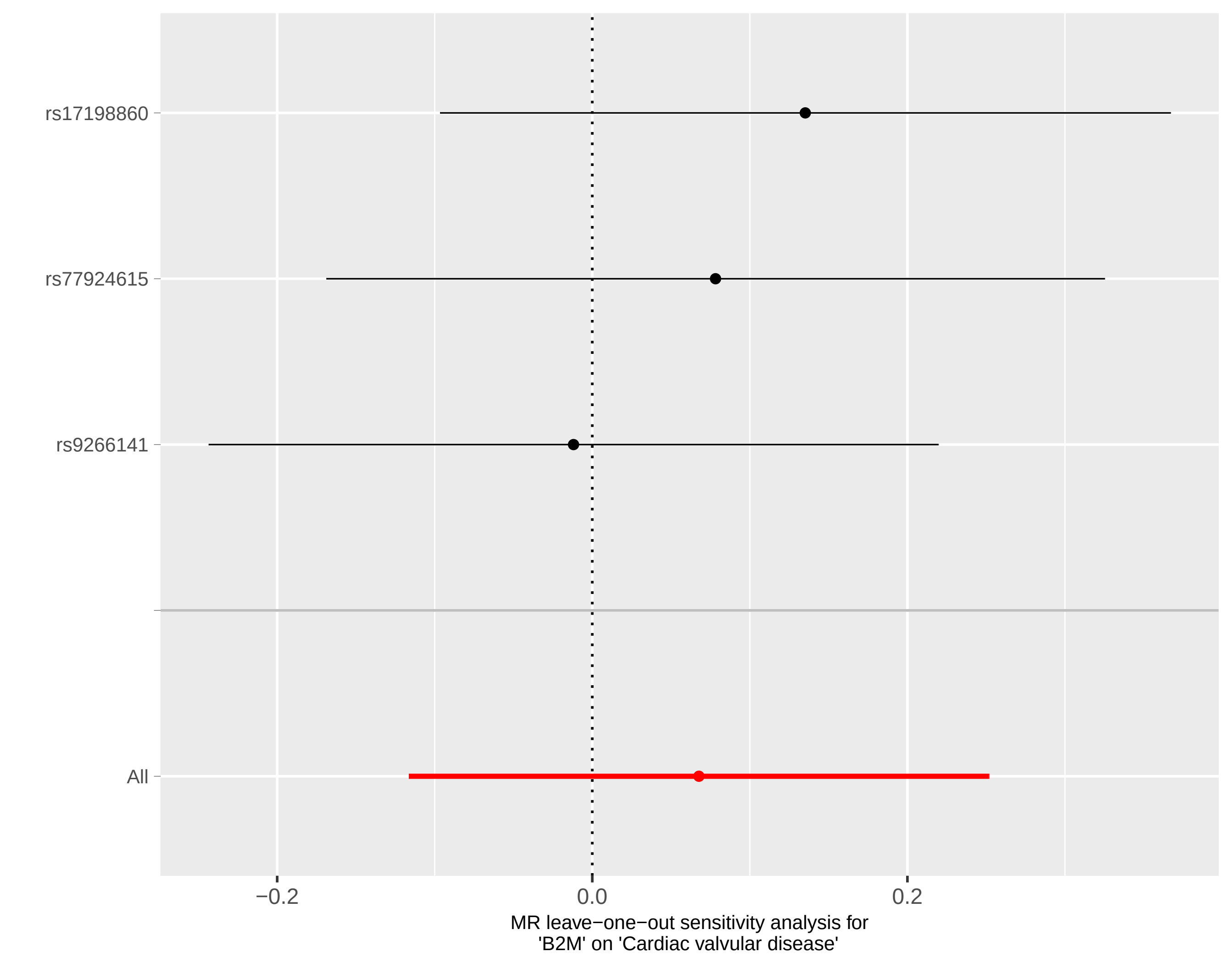
rs17198860
rs77924615
rs9266141
All
−0.2
0.0
0.2
MR leave−one−out sensitivity analysis for
'B2M' on 'Cardiac valvular disease'

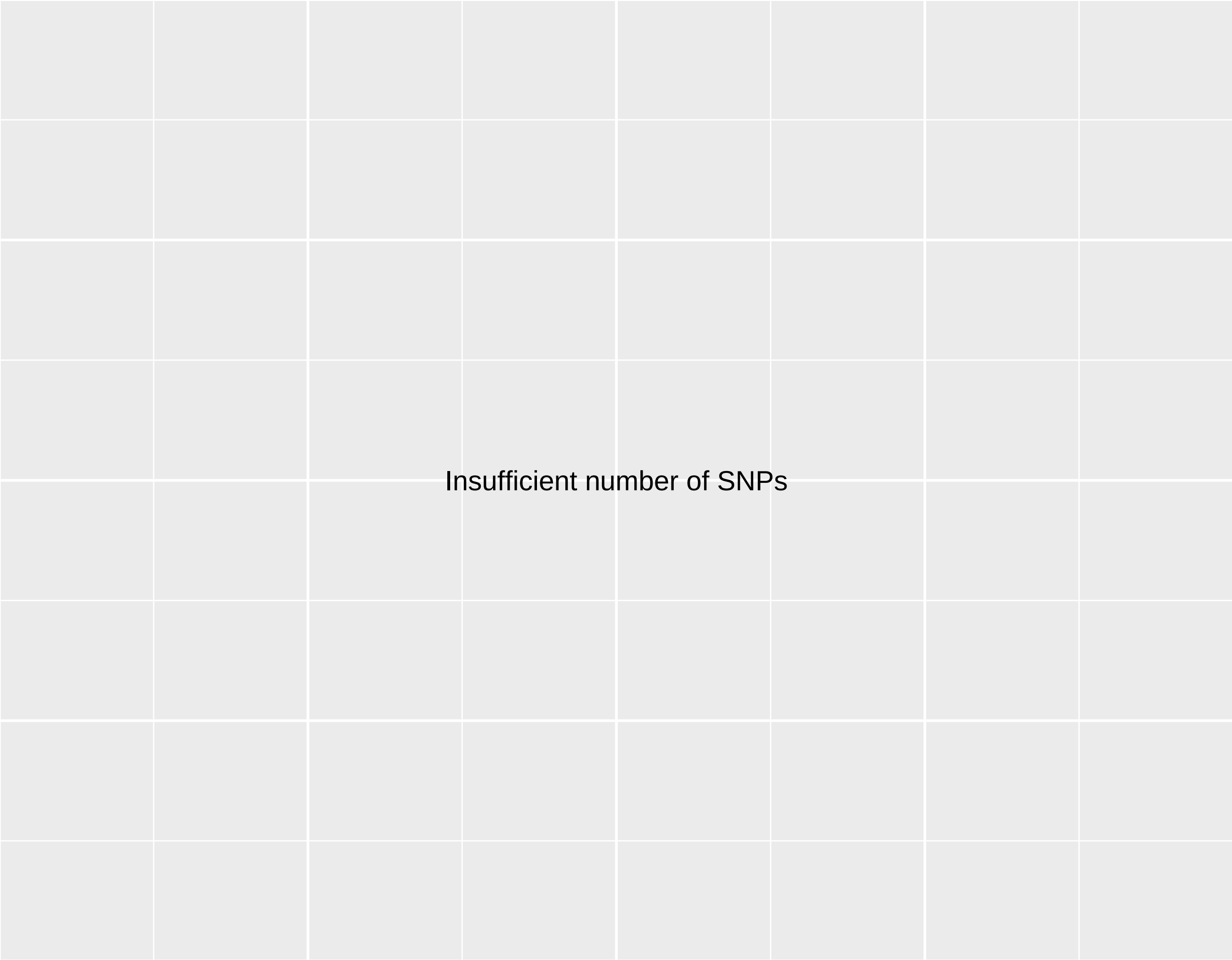
Insufficient number of SNPs

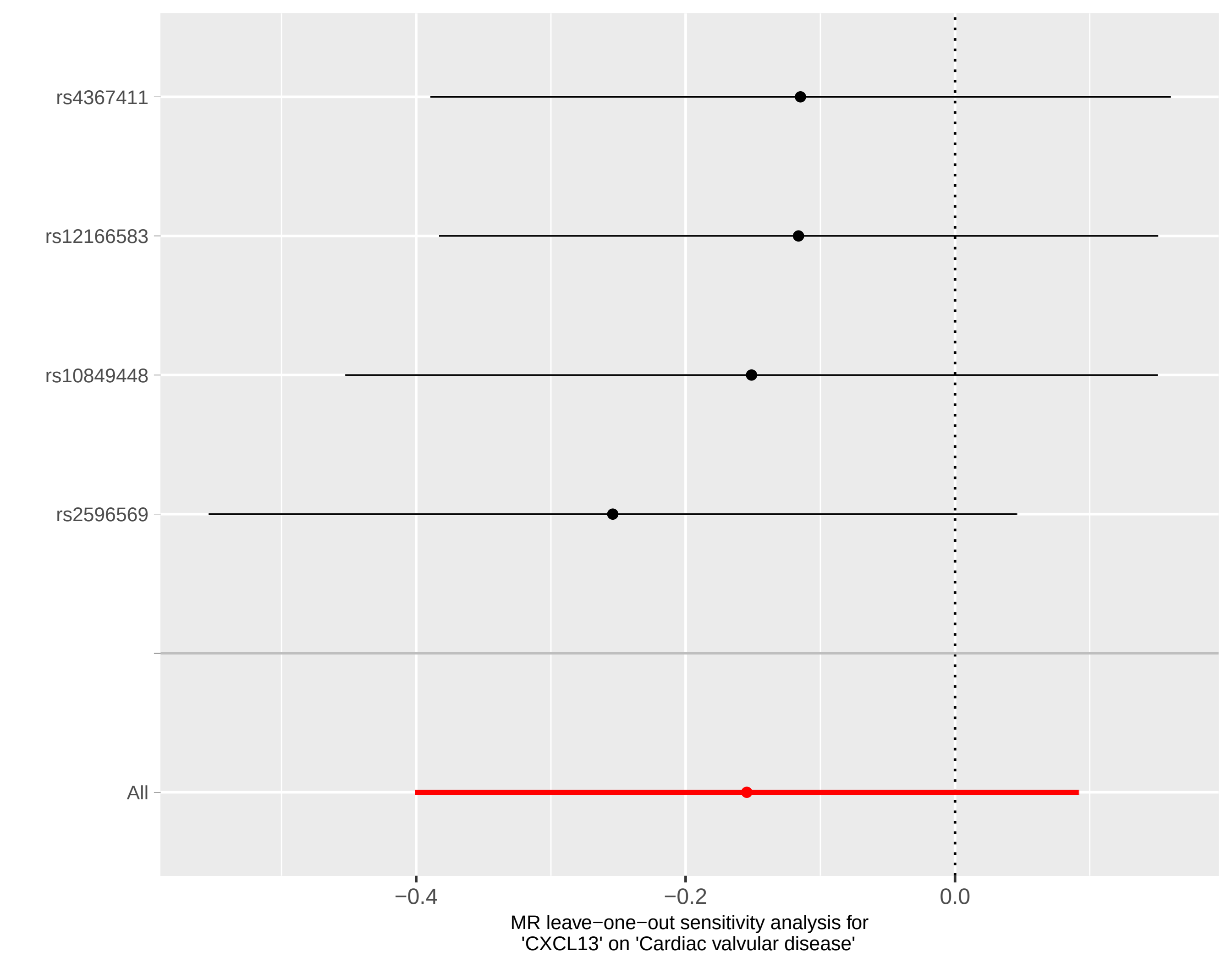

rs4367411
rs12166583
rs10849448
rs2596569
All
−0.4
−0.2
0.0
MR leave−one−out sensitivity analysis for
'CXCL13' on 'Cardiac valvular disease'

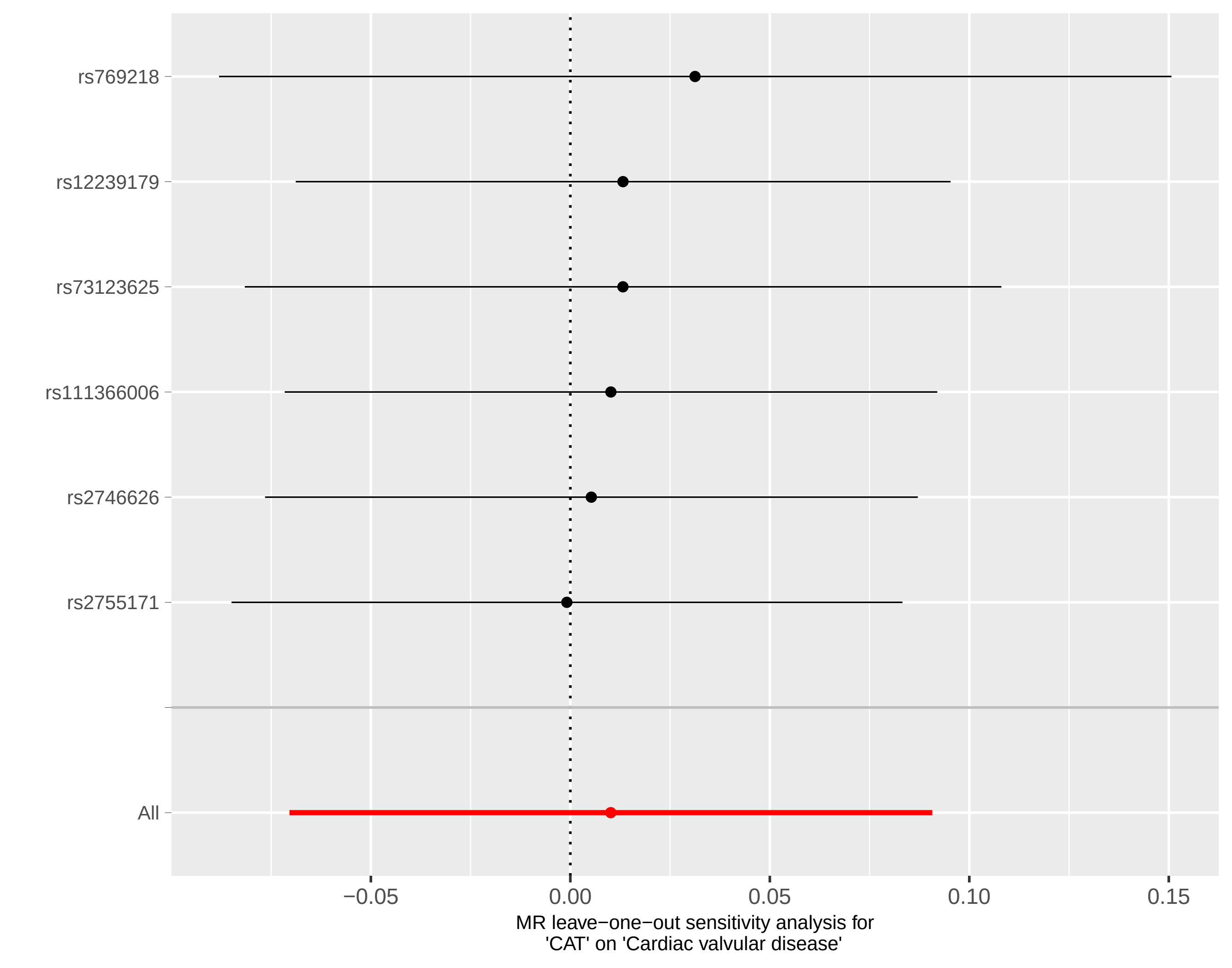
rs769218
rs12239179
rs73123625
rs111366006
rs2746626
rs2755171
All
−0.05
0.00
0.05
0.10
0.15
MR leave−one−out sensitivity analysis for
'CAT' on 'Cardiac valvular disease'

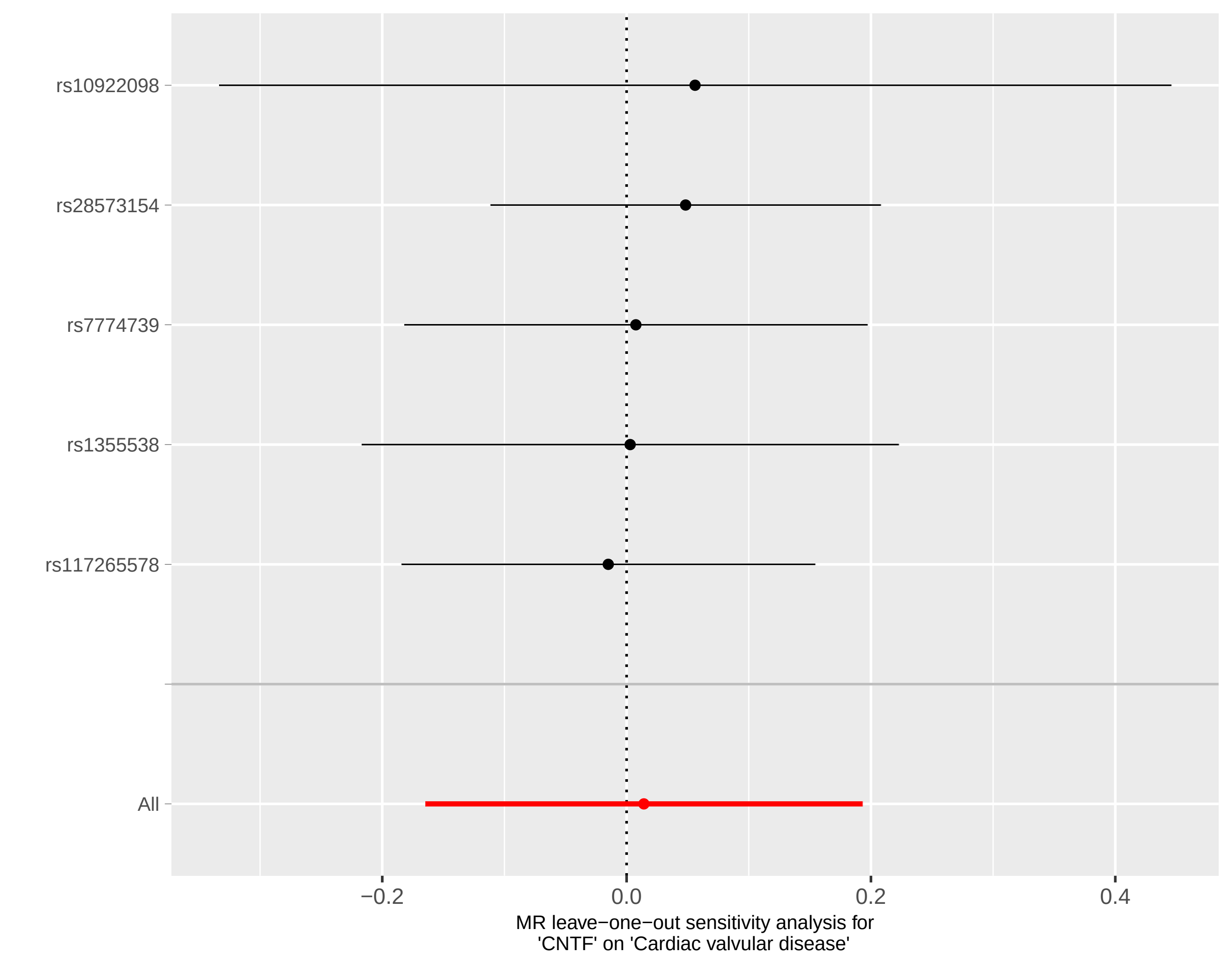
rs10922098
rs28573154
rs7774739
rs1355538
rs117265578
All
−0.2
0.0
0.2
0.4
MR leave−one−out sensitivity analysis for
'CNTF' on 'Cardiac valvular disease'

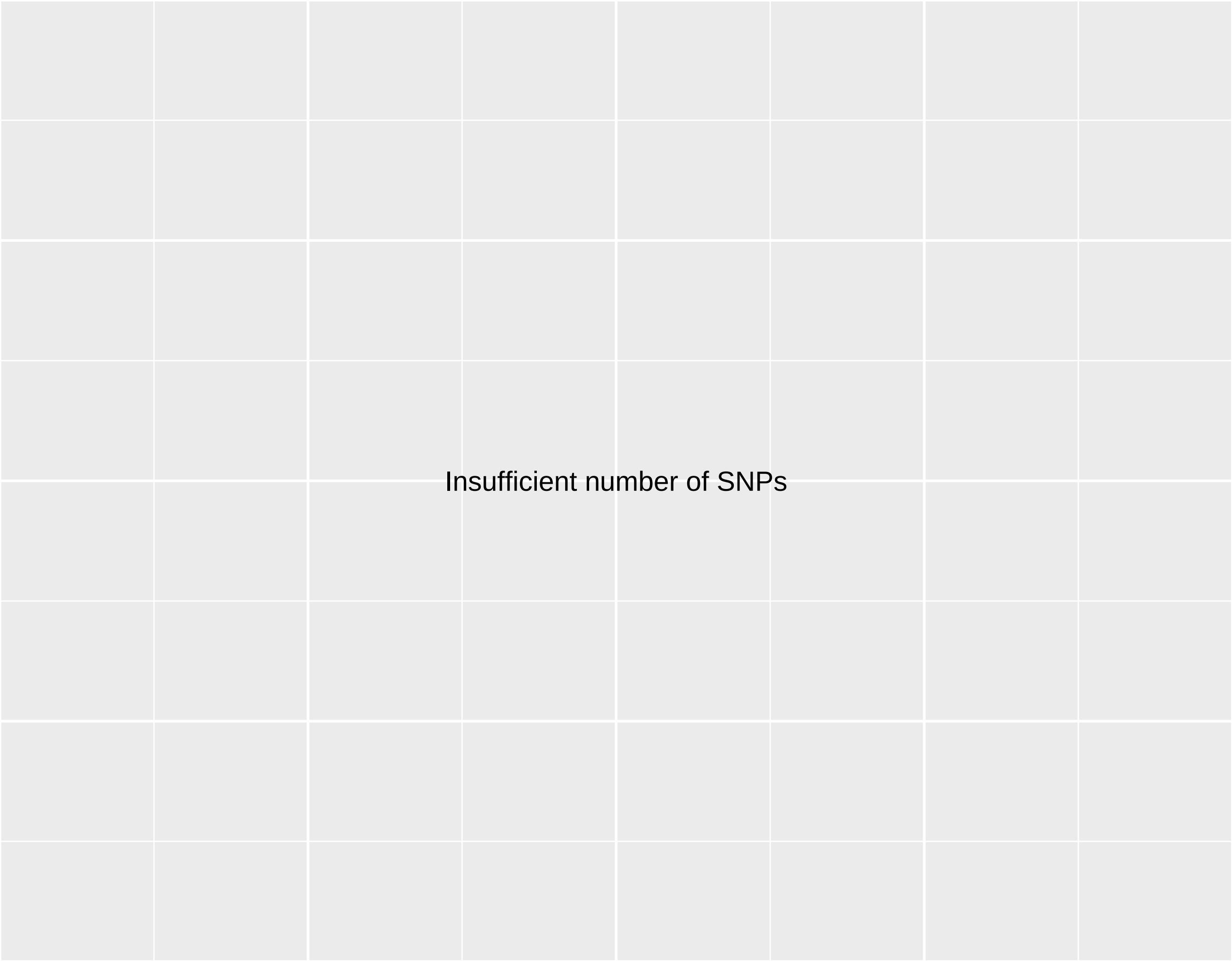
Insufficient number of SNPs

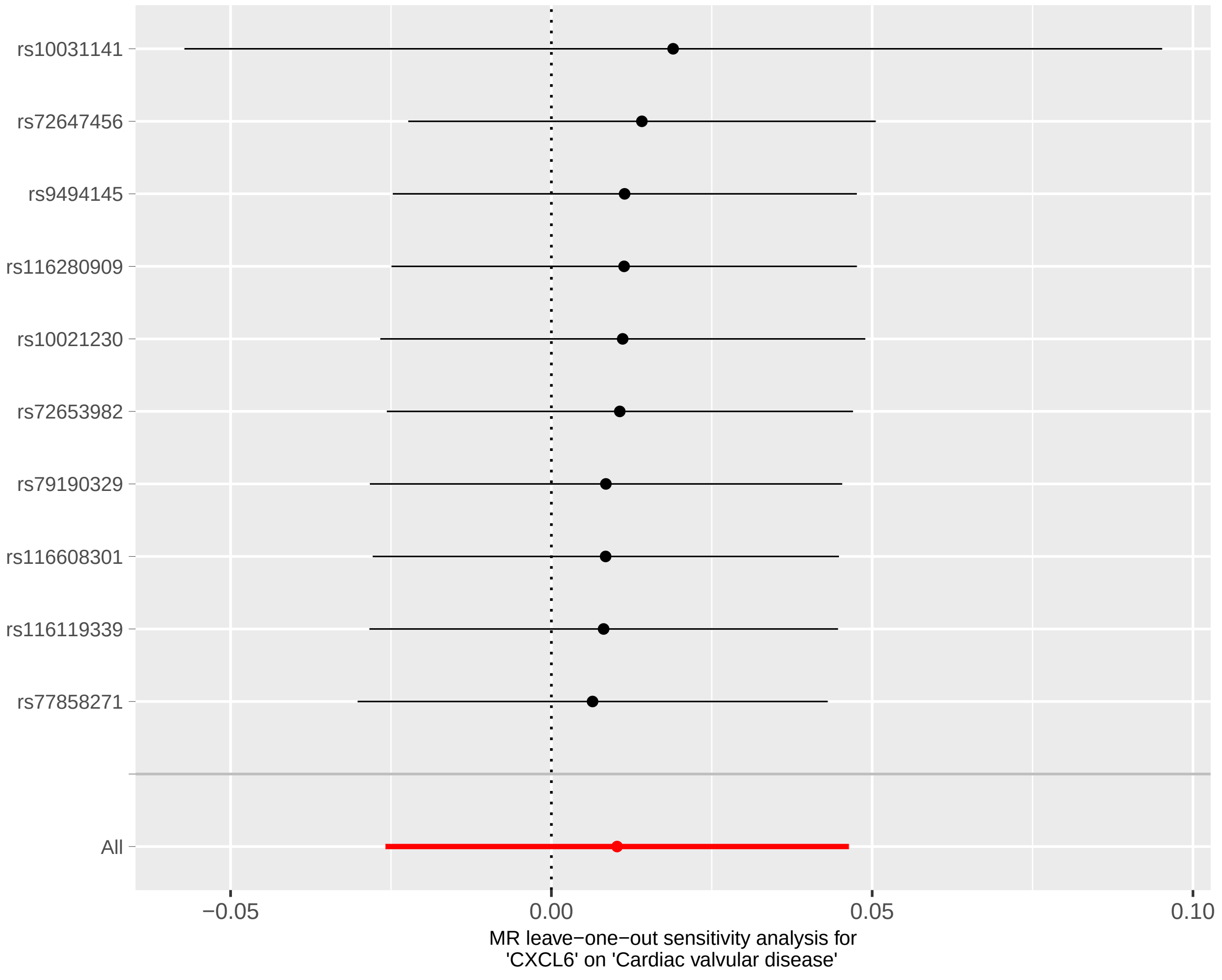
rs10031141
rs72647456
rs9494145
rs116280909
rs10021230
rs72653982
rs79190329
rs116608301
rs116119339
rs77858271
All
−0.05
0.00
0.05
0.10
MR leave−one−out sensitivity analysis for
'CXCL6' on 'Cardiac valvular disease'

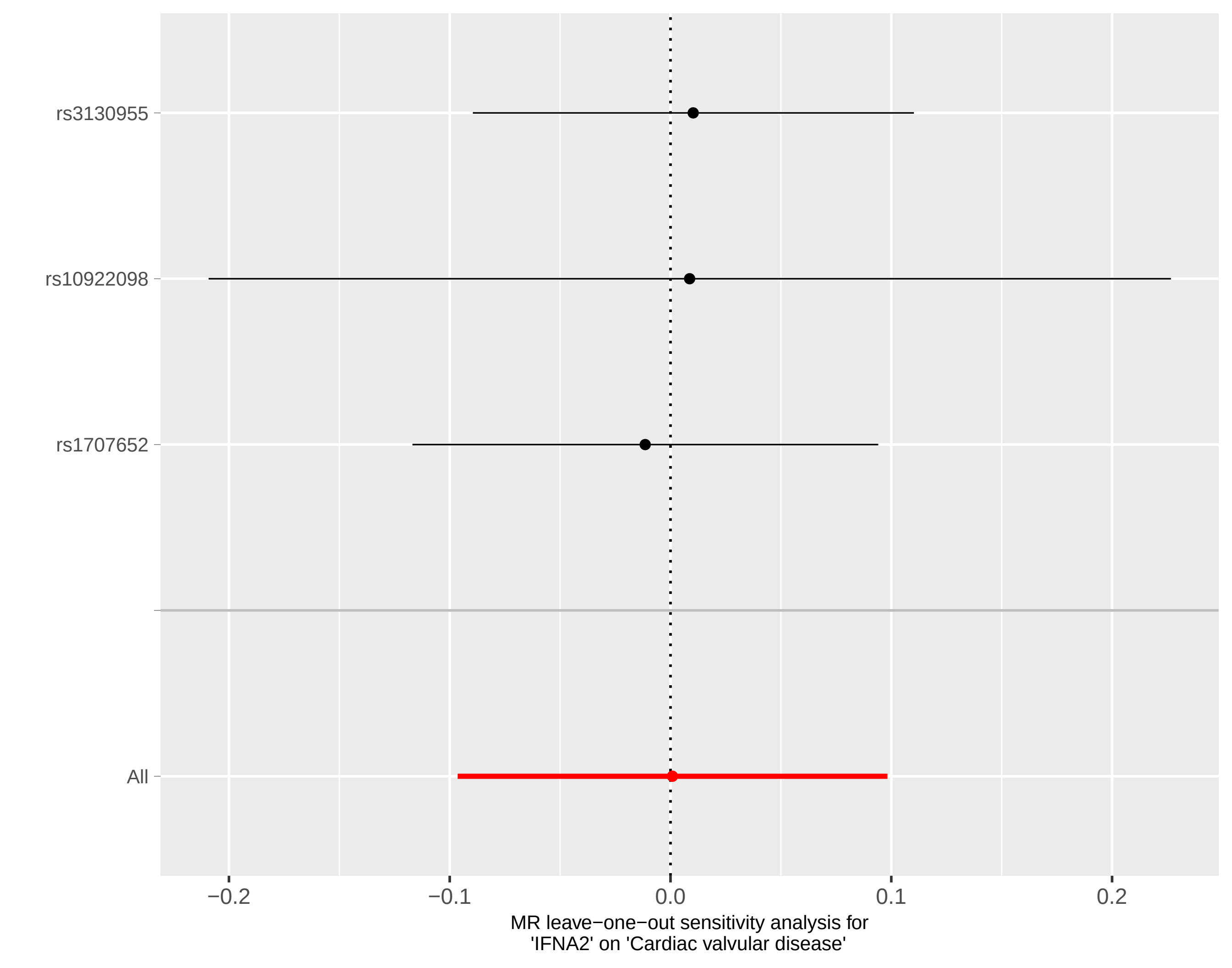
rs3130955
rs10922098
rs1707652
All
−0.2
−0.1
0.0
0.1
0.2
MR leave−one−out sensitivity analysis for
'IFNA2' on 'Cardiac valvular disease'

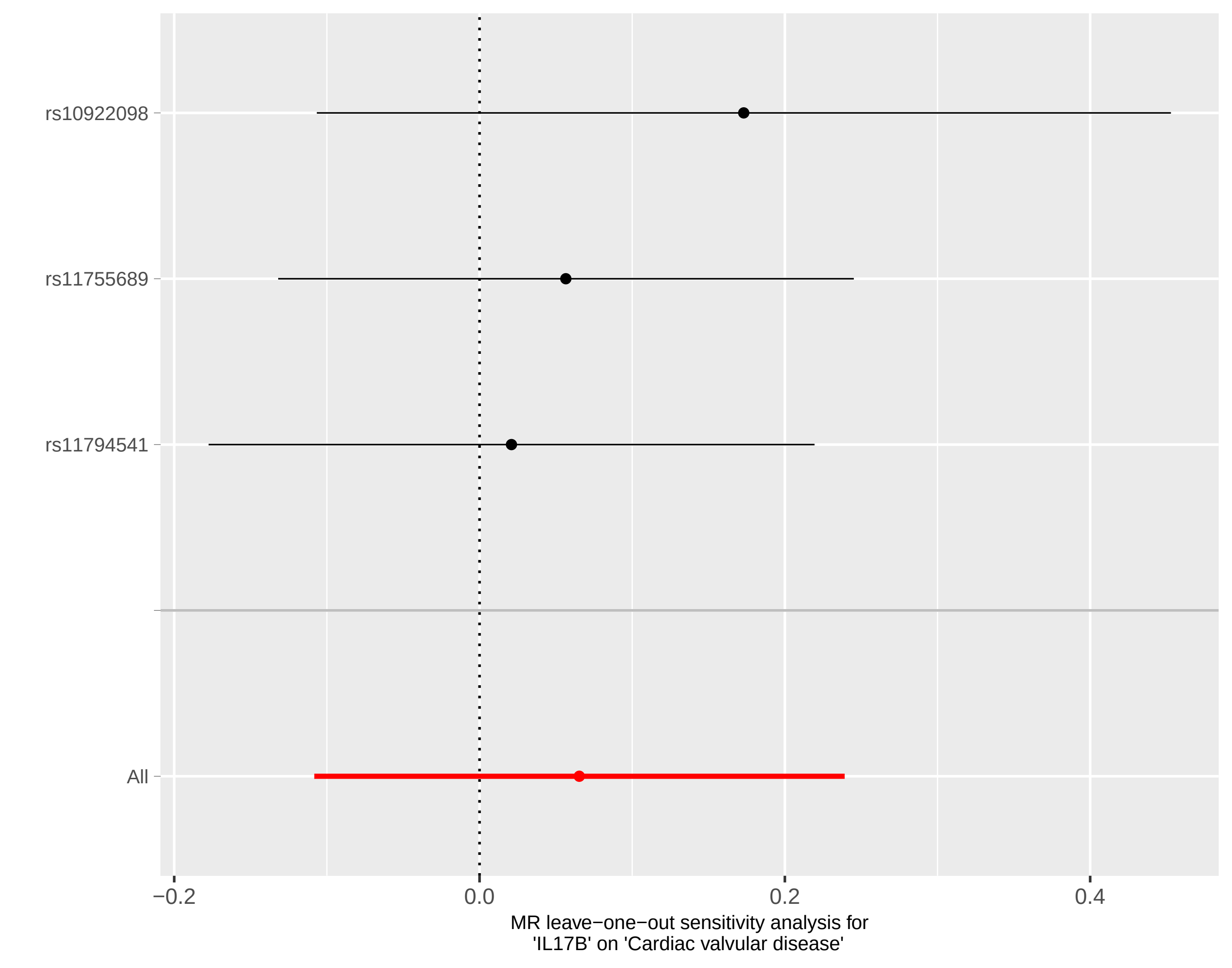
rs10922098
rs11755689
rs11794541
All
−0.2
0.0
0.2
0.4
MR leave−one−out sensitivity analysis for
'IL17B' on 'Cardiac valvular disease'

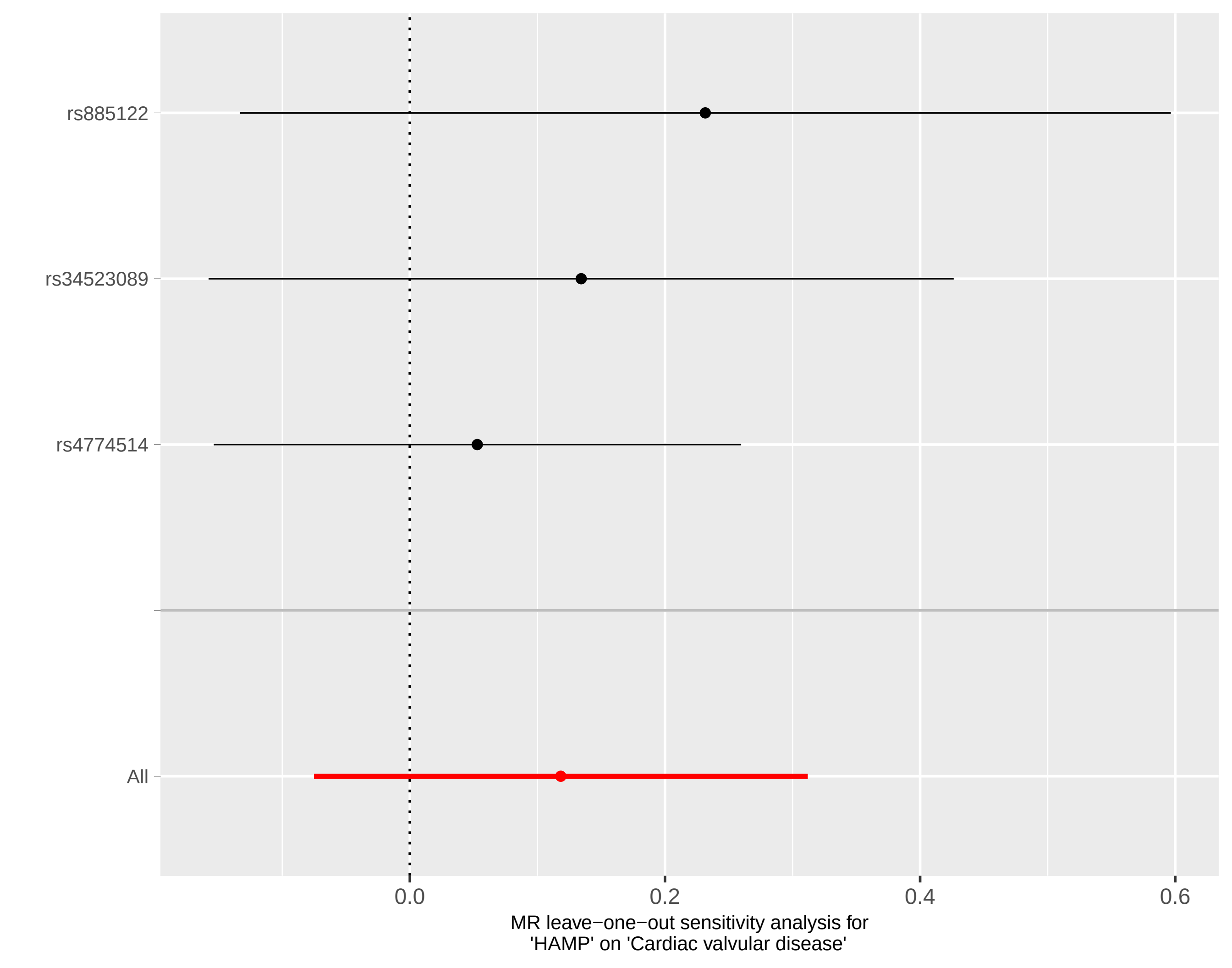

rs885122
rs34523089
rs4774514
All
0.0
0.2
0.4
0.6
MR leave−one−out sensitivity analysis for
'HAMP' on 'Cardiac valvular disease'

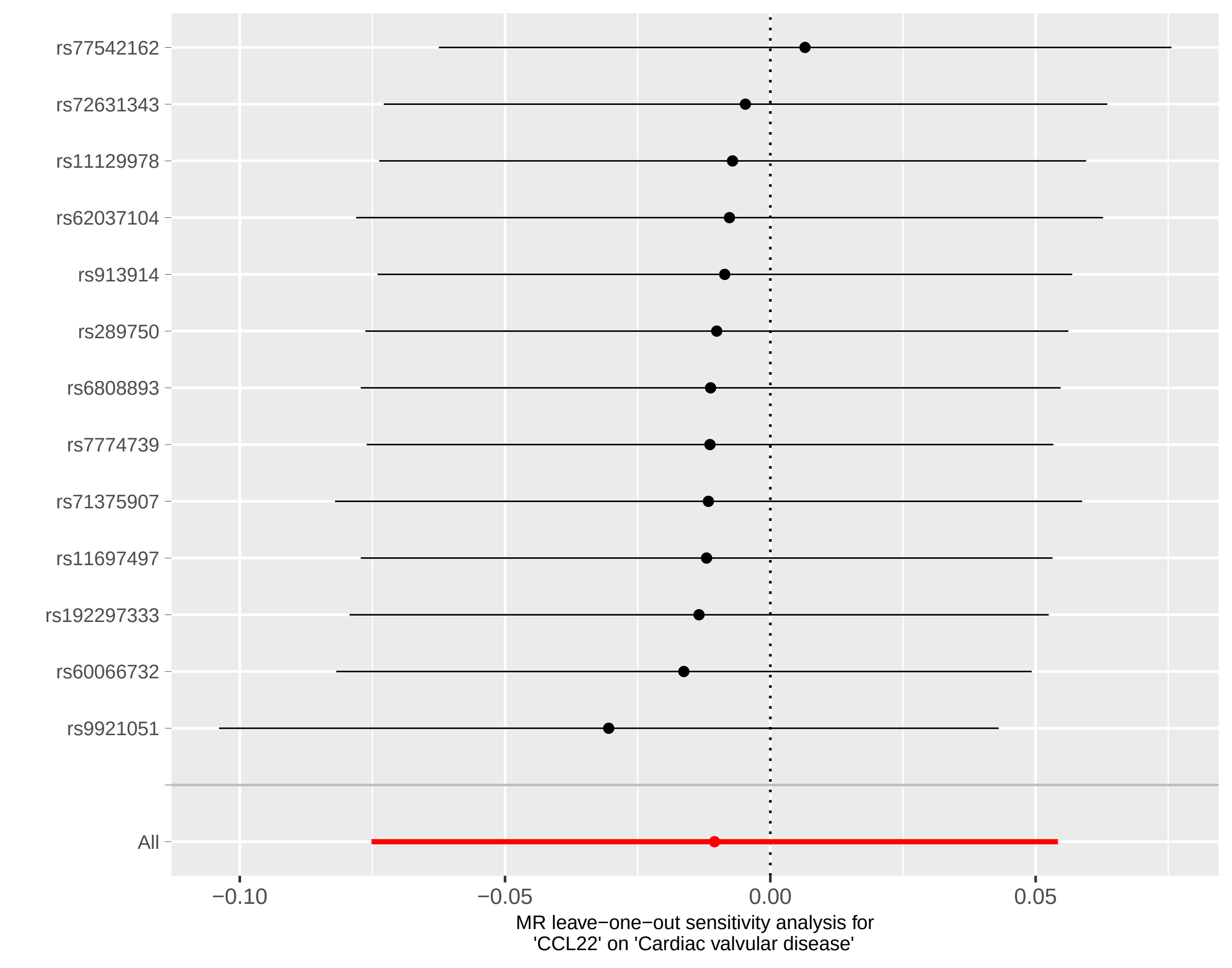
rs77542162
rs72631343
rs11129978
rs62037104
rs913914
rs289750
rs6808893
rs7774739
rs71375907
rs11697497
rs192297333
rs60066732
rs9921051
All
−0.10
−0.05
0.00
0.05
MR leave−one−out sensitivity analysis for
'CCL22' on 'Cardiac valvular disease'

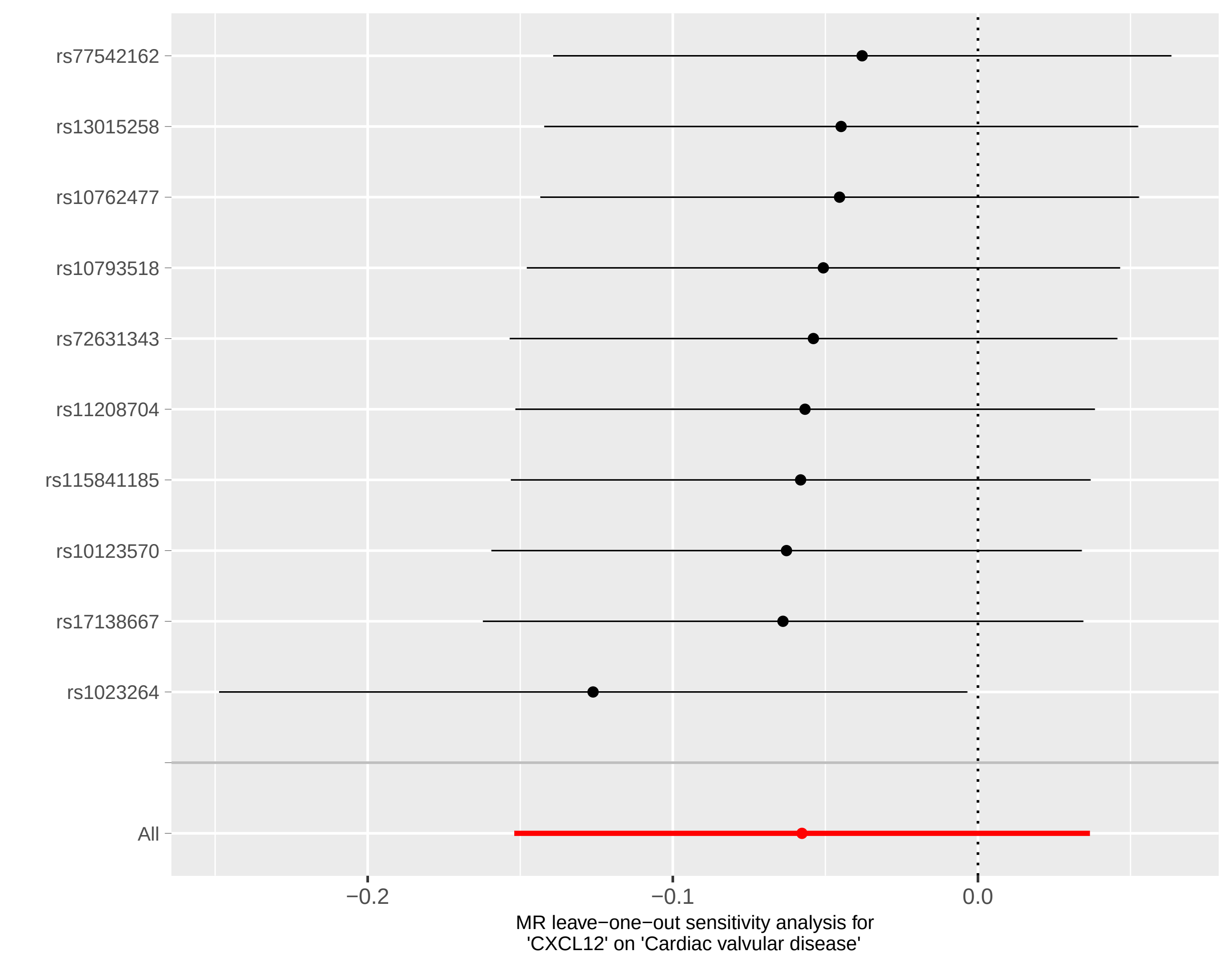
rs77542162
rs13015258
rs10762477
rs10793518
rs72631343
rs11208704
rs115841185
rs10123570
rs17138667
rs1023264
All
−0.2
−0.1
0.0
MR leave−one−out sensitivity analysis for
'CXCL12' on 'Cardiac valvular disease'

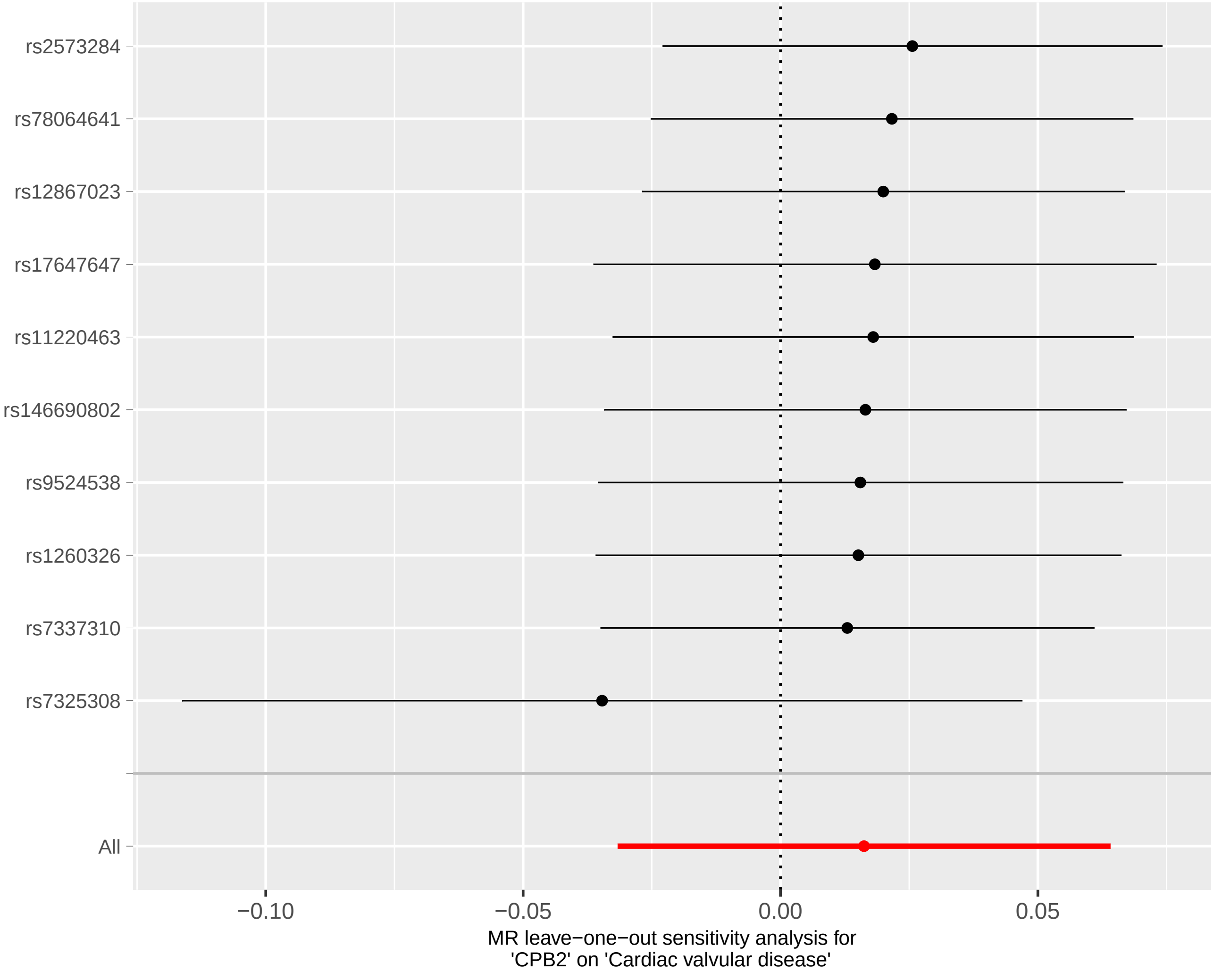
rs2573284
rs78064641
rs12867023
rs17647647
rs11220463
rs146690802
rs9524538
rs1260326
rs7337310
rs7325308
All
−0.10
−0.05
0.00
0.05
MR leave−one−out sensitivity analysis for
'CPB2' on 'Cardiac valvular disease'

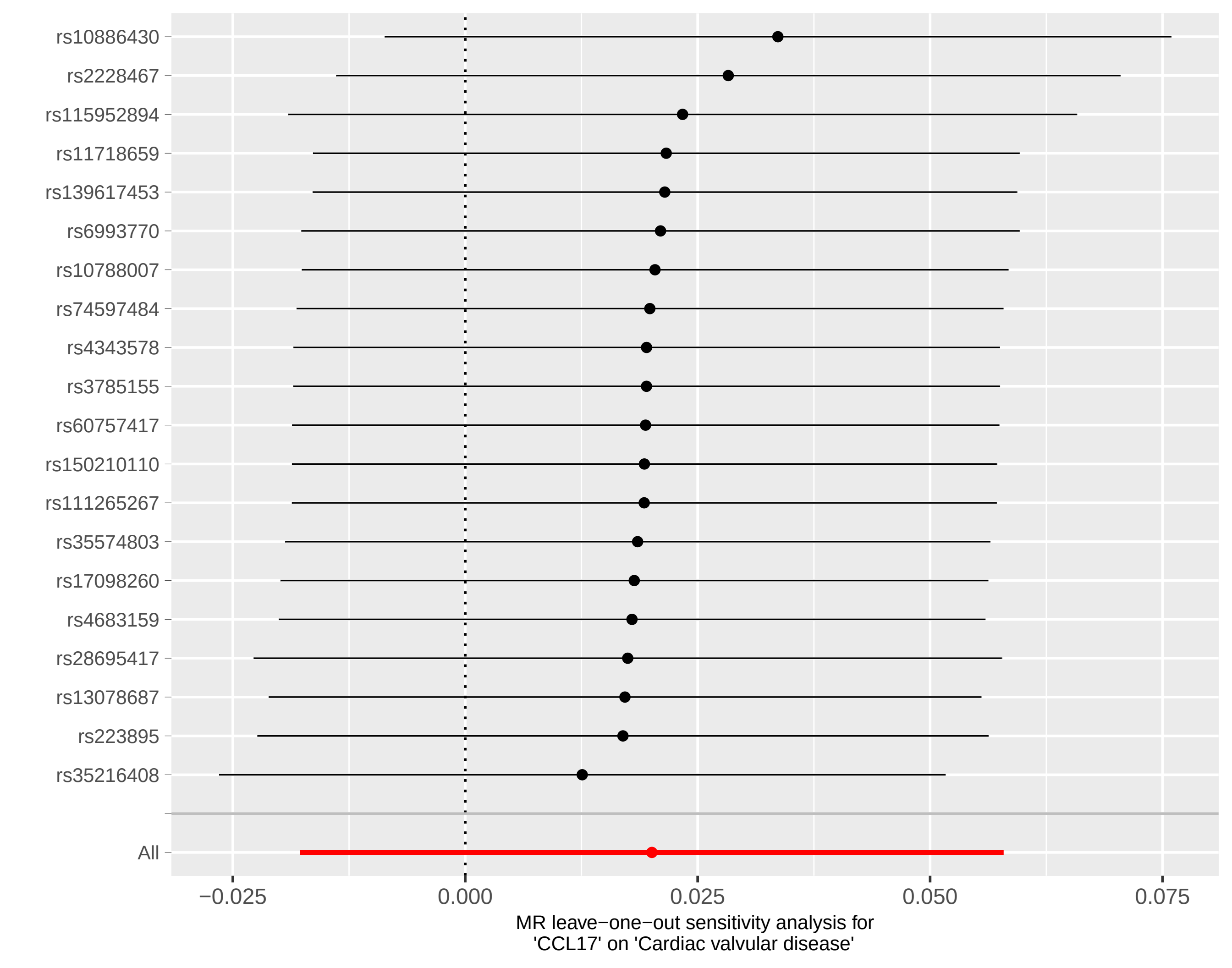
rs10886430
rs2228467
rs115952894
rs11718659
rs139617453
rs6993770
rs10788007
rs74597484
rs4343578
rs3785155
rs60757417
rs150210110
rs111265267
rs35574803
rs17098260
rs4683159
rs28695417
rs13078687
rs223895
rs35216408
All
−0.025
0.000
0.025
0.050
0.075
MR leave−one−out sensitivity analysis for
'CCL17' on 'Cardiac valvular disease'

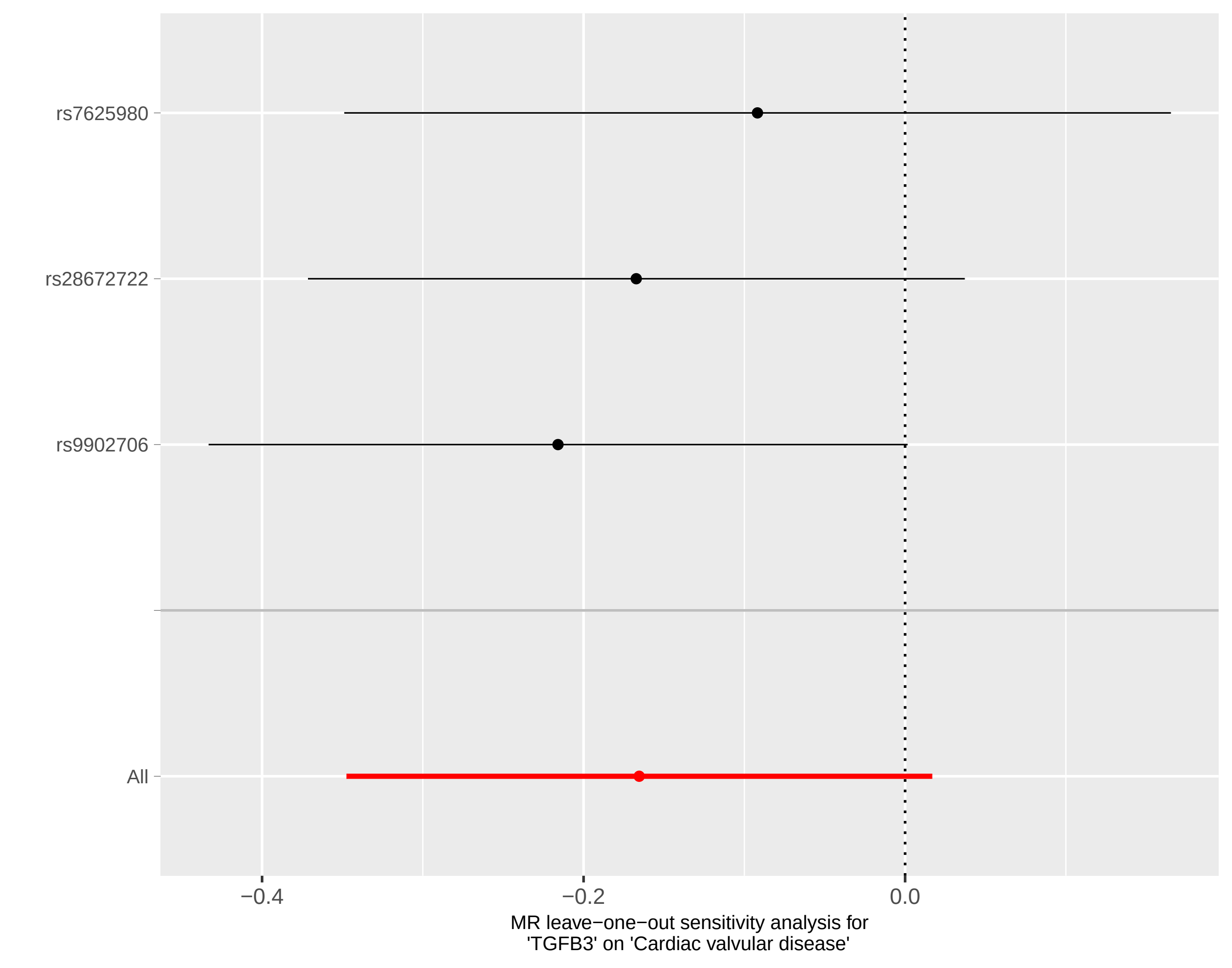

rs7625980
rs28672722
rs9902706
All
−0.4
−0.2
0.0
MR leave−one−out sensitivity analysis for
'TGFB3' on 'Cardiac valvular disease'

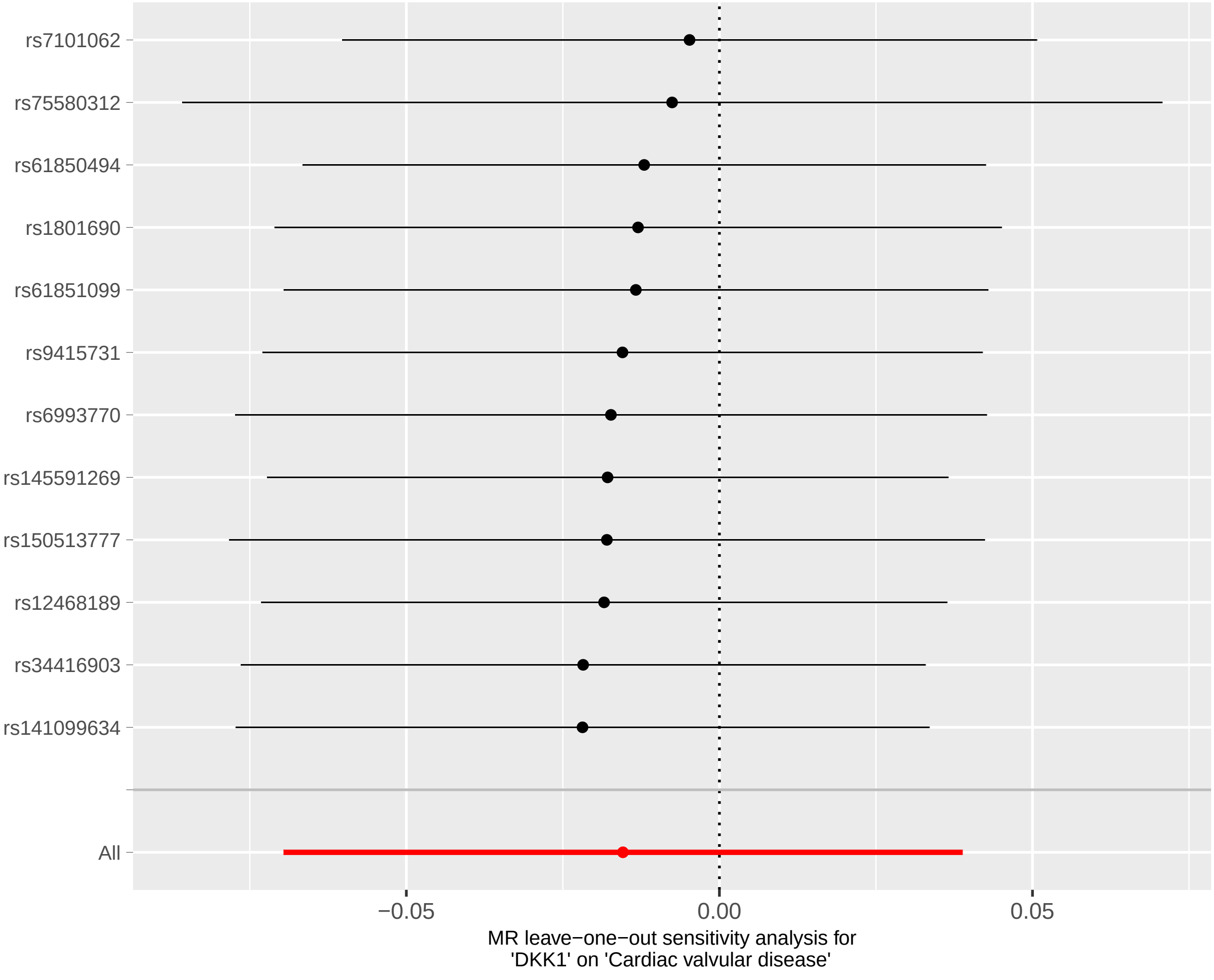

rs7101062
rs75580312
rs61850494
rs1801690
rs61851099
rs9415731
rs6993770
rs145591269
rs150513777
rs12468189
rs34416903
rs141099634
All
−0.05
0.00
0.05
MR leave−one−out sensitivity analysis for
'DKK1' on 'Cardiac valvular disease'

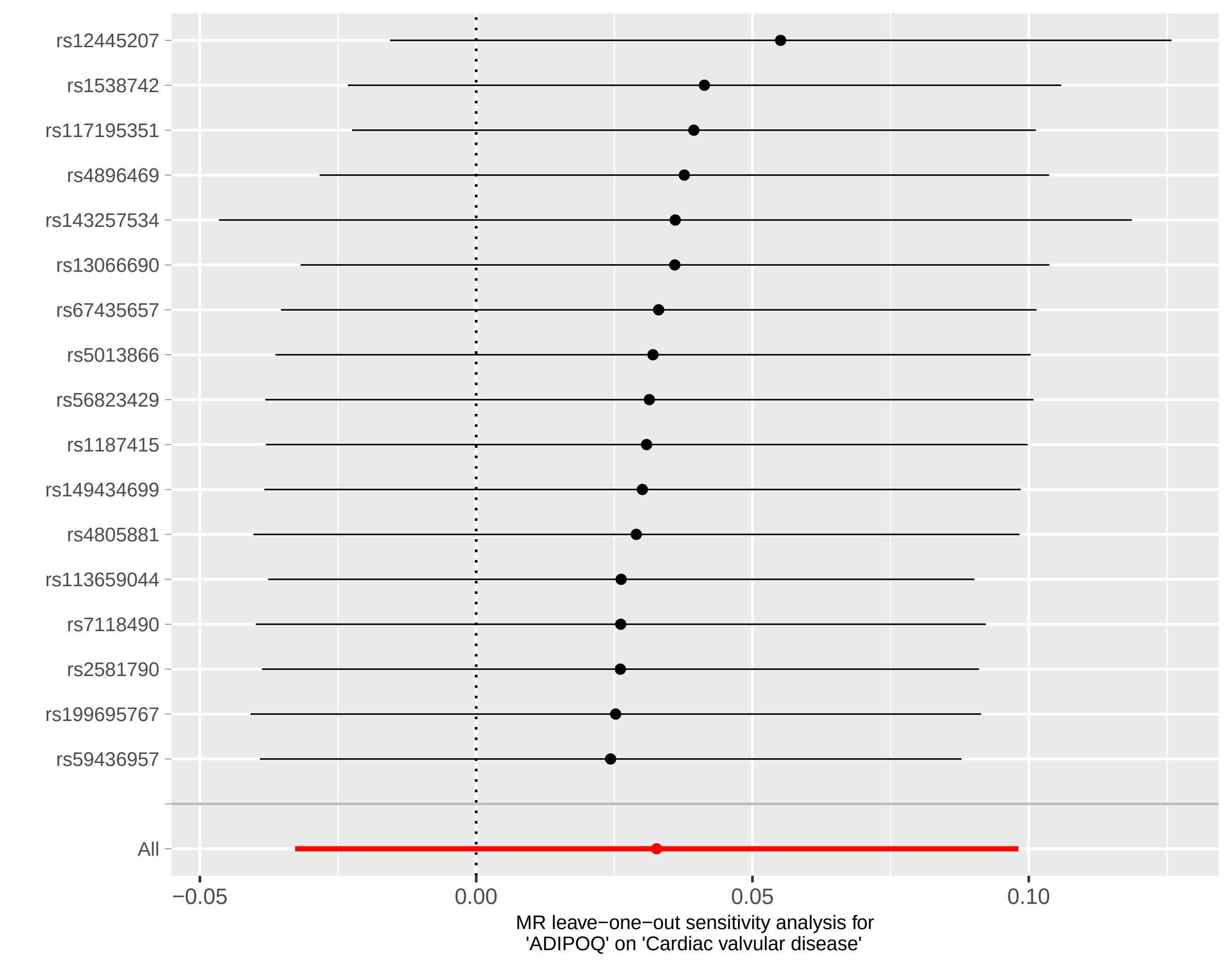

rs12445207
rs1538742
rs117195351
rs4896469
rs143257534
rs13066690
rs67435657
rs5013866
rs56823429
rs1187415
rs149434699
rs4805881
rs113659044
rs7118490
rs2581790
rs199695767
rs59436957
All
−0.05
0.00
0.05
0.10
MR leave−one−out sensitivity analysis for
'ADIPOQ' on 'Cardiac valvular disease'

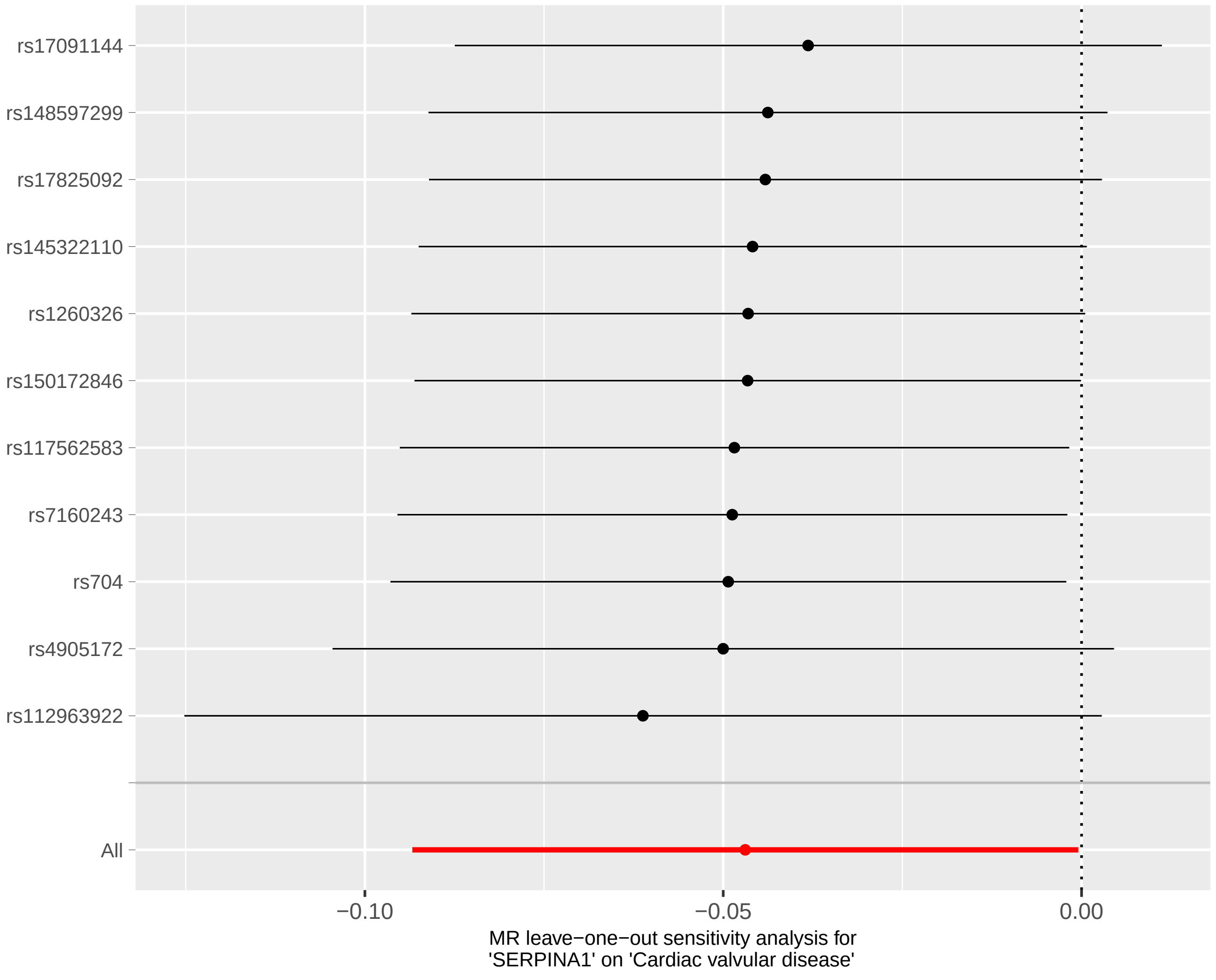
rs17091144
rs148597299
rs17825092
rs145322110
rs1260326
rs150172846
rs117562583
rs7160243
rs704
rs4905172
rs112963922
All
−0.10
−0.05
0.00
MR leave−one−out sensitivity analysis for
'SERPINA1' on 'Cardiac valvular disease'

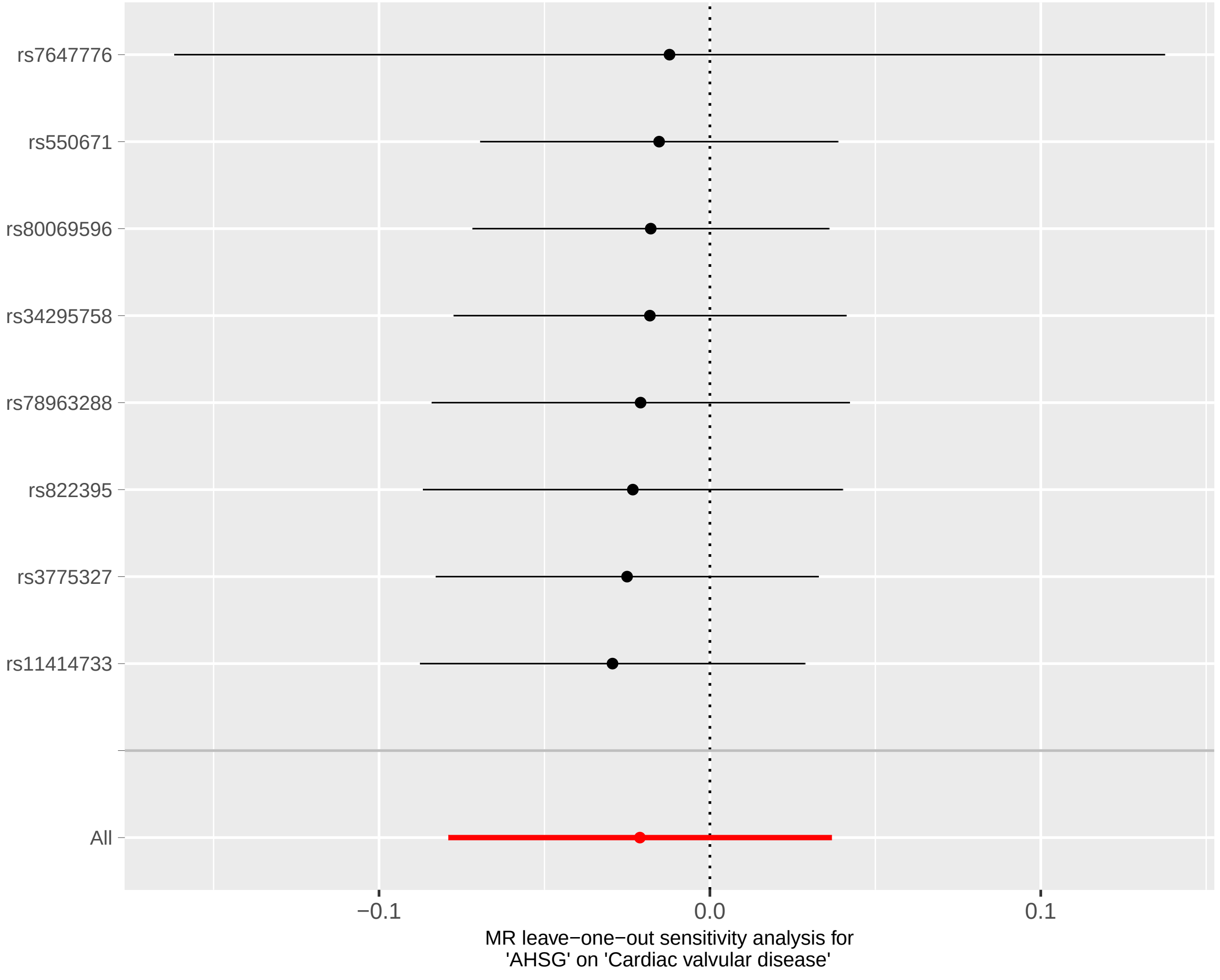
rs7647776
rs550671
rs80069596
rs34295758
rs78963288
rs822395
rs3775327
rs11414733
All
−0.1
0.0
0.1
MR leave−one−out sensitivity analysis for
'AHSG' on 'Cardiac valvular disease'

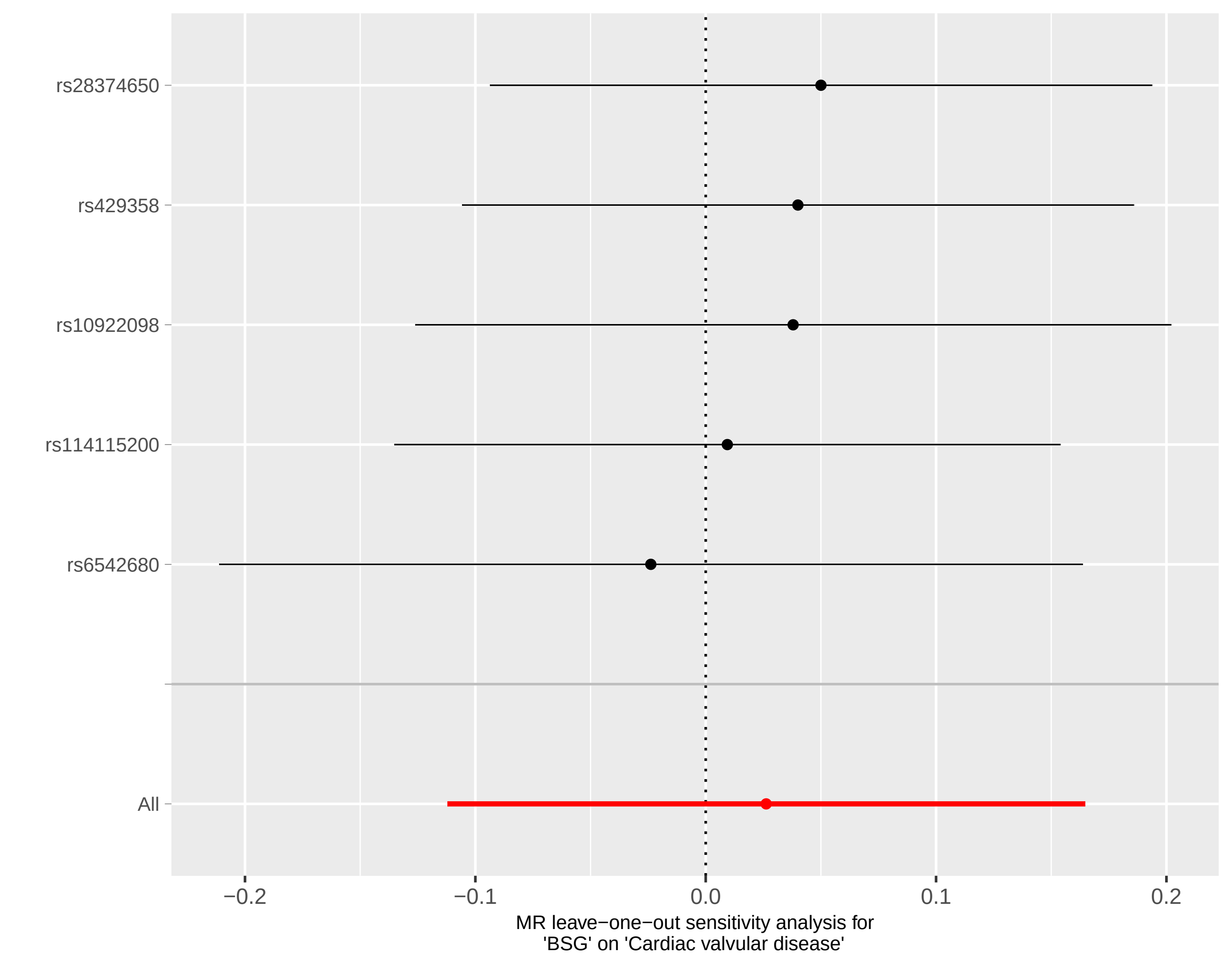
rs28374650
rs429358
rs10922098
rs114115200
rs6542680
All
−0.2
−0.1
0.0
0.1
0.2
MR leave−one−out sensitivity analysis for
'BSG' on 'Cardiac valvular disease'

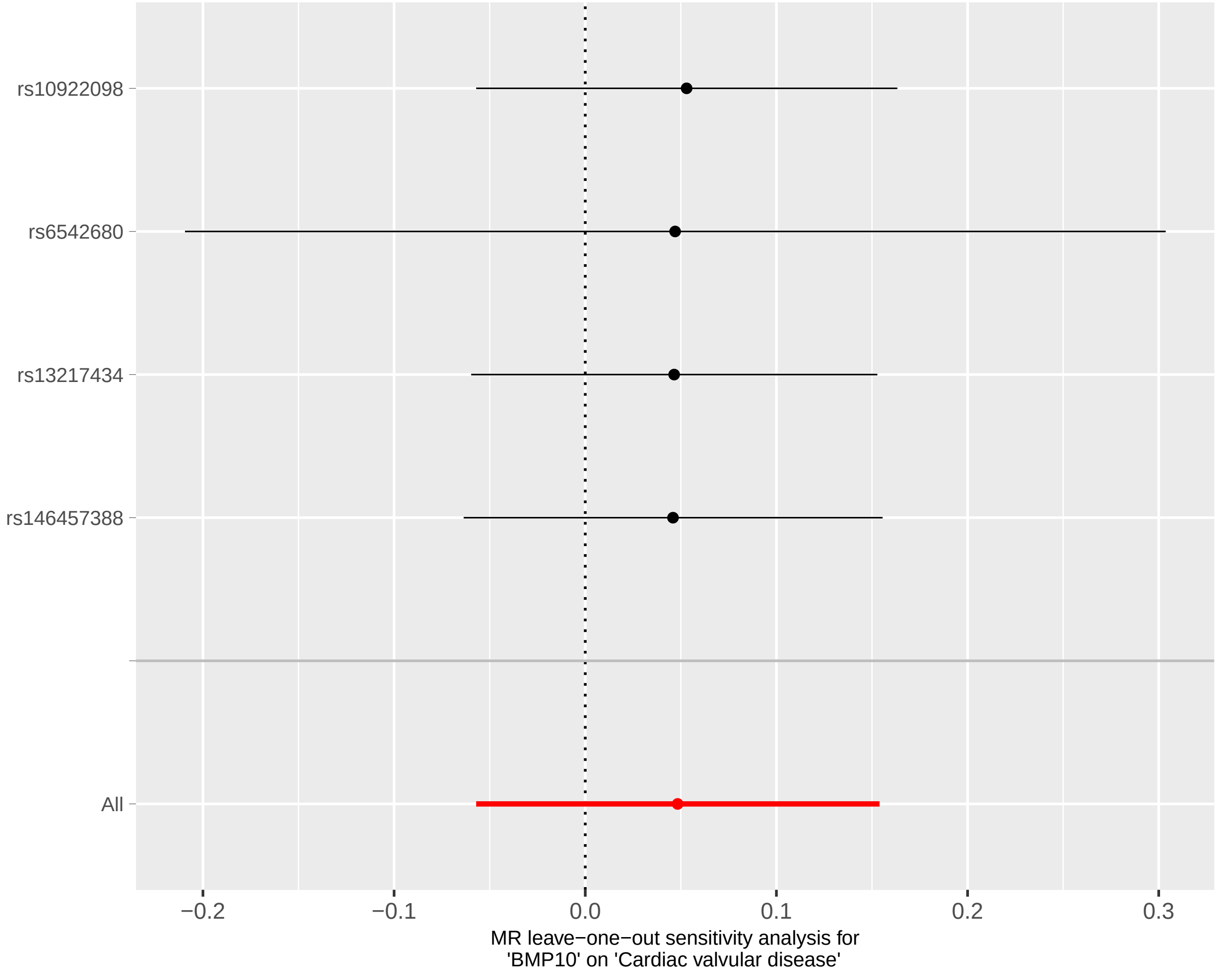
rs10922098
rs6542680
rs13217434
rs146457388
All
−0.2
−0.1
0.0
0.1
0.2
0.3
MR leave−one−out sensitivity analysis for
'BMP10' on 'Cardiac valvular disease'

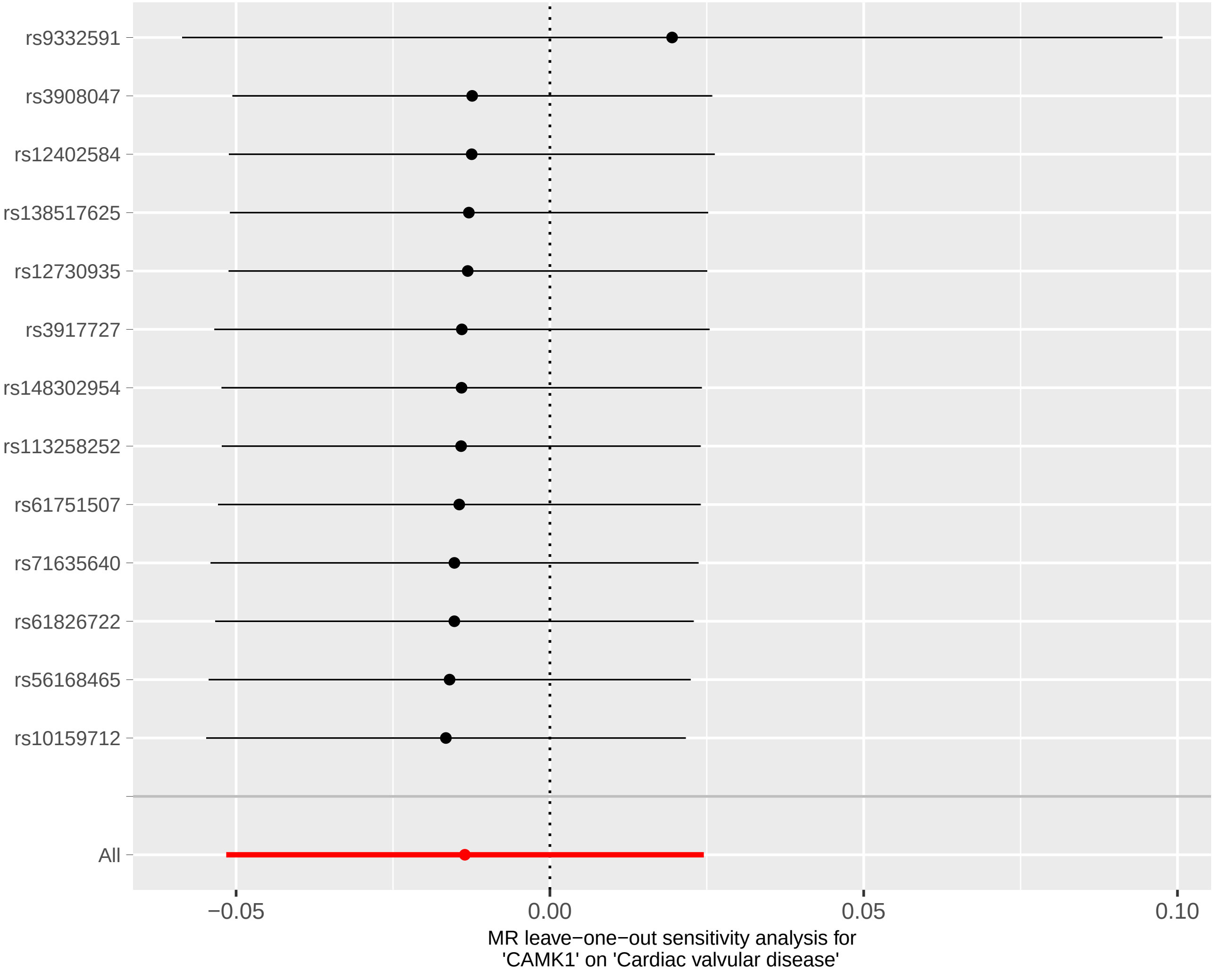

rs9332591
rs3908047
rs12402584
rs138517625
rs12730935
rs3917727
rs148302954
rs113258252
rs61751507
rs71635640
rs61826722
rs56168465
rs10159712
All
−0.05
0.00
0.05
0.10
MR leave−one−out sensitivity analysis for
'CAMK1' on 'Cardiac valvular disease'

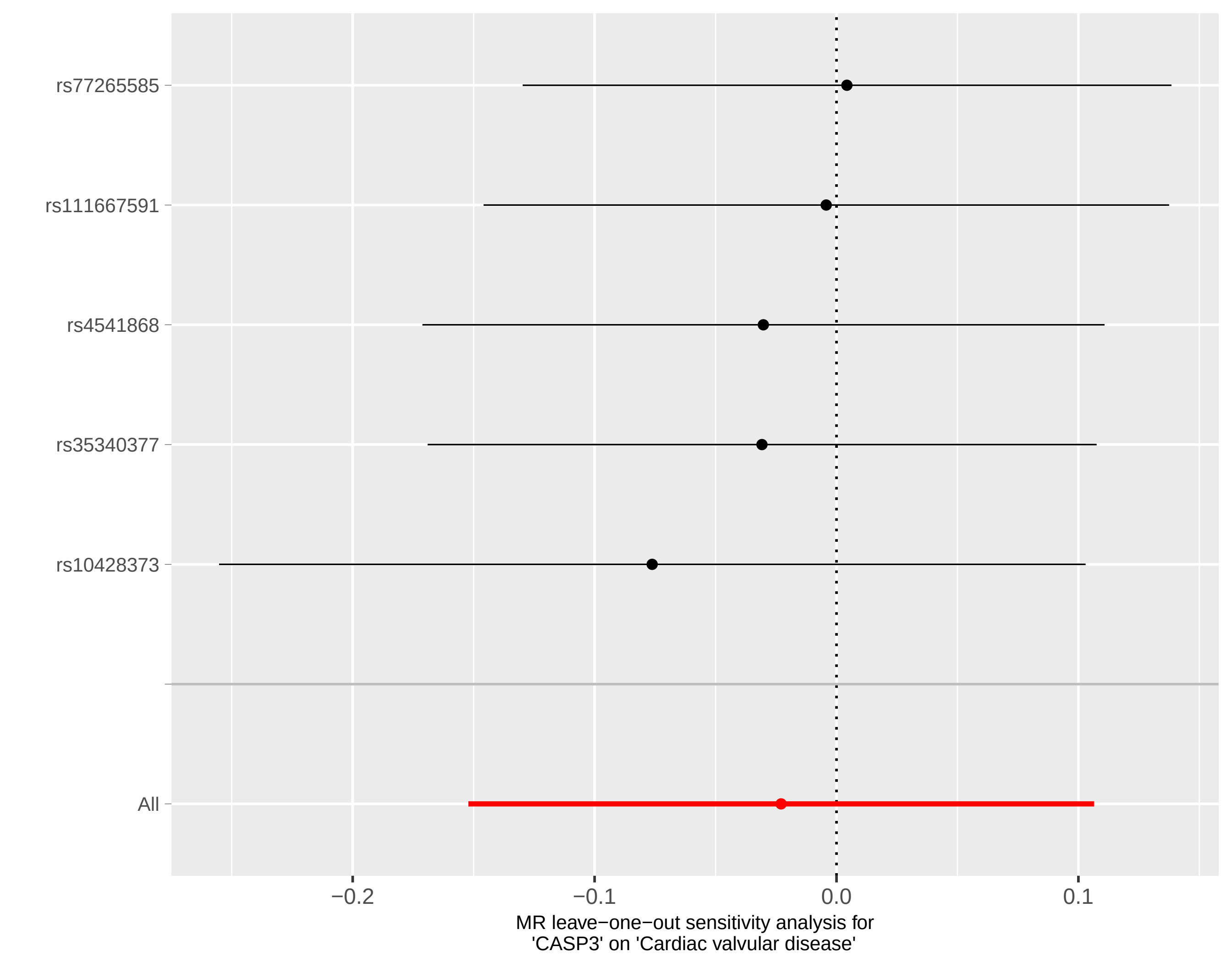
rs77265585
rs111667591
rs4541868
rs35340377
rs10428373
All
−0.2
−0.1
0.0
0.1
MR leave−one−out sensitivity analysis for
'CASP3' on 'Cardiac valvular disease'

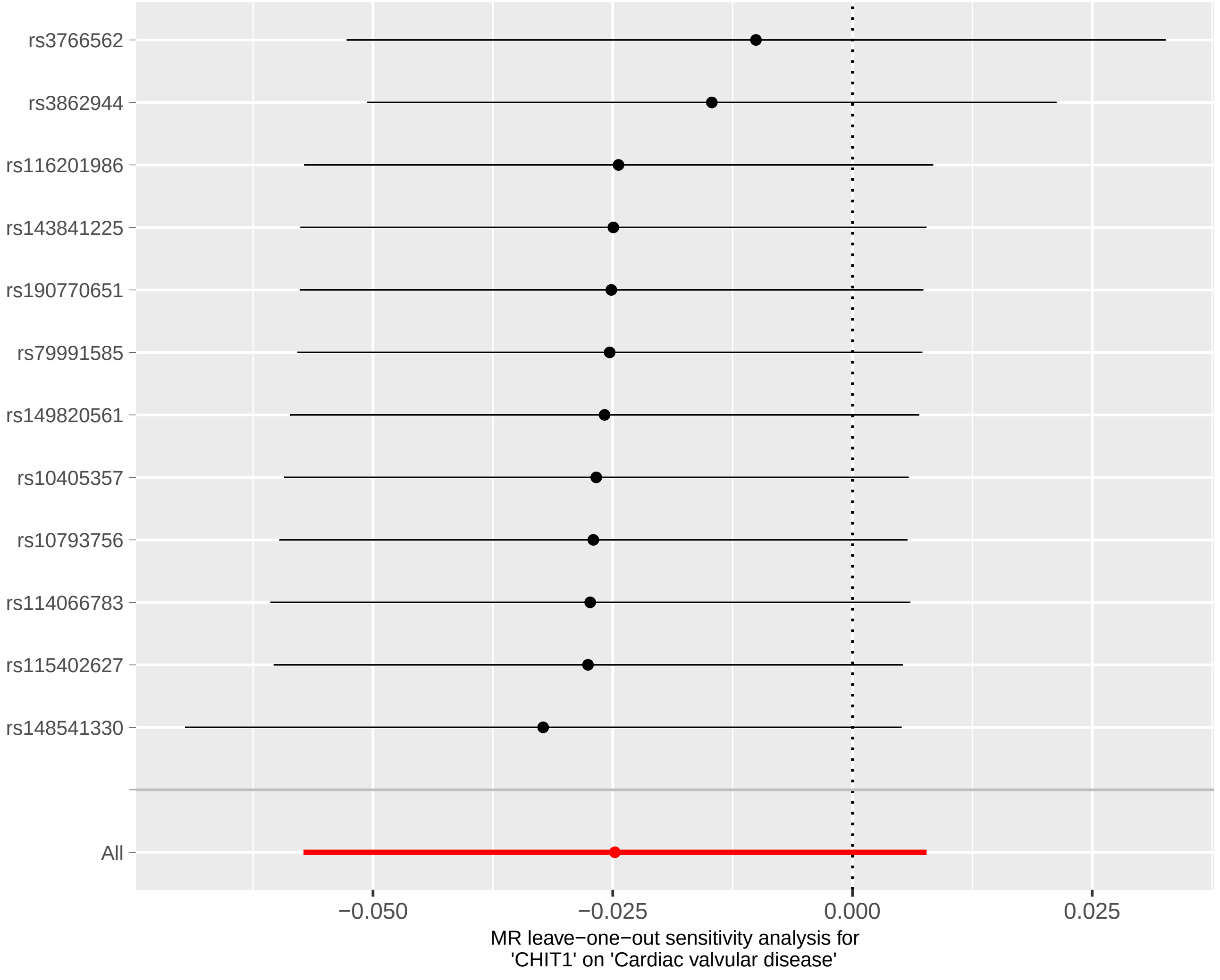
rs3766562
rs3862944
rs116201986
rs143841225
rs190770651
rs79991585
rs149820561
rs10405357
rs10793756
rs114066783
rs115402627
rs148541330
All
−0.050
−0.025
0.000
0.025
MR leave−one−out sensitivity analysis for
'CHIT1' on 'Cardiac valvular disease'

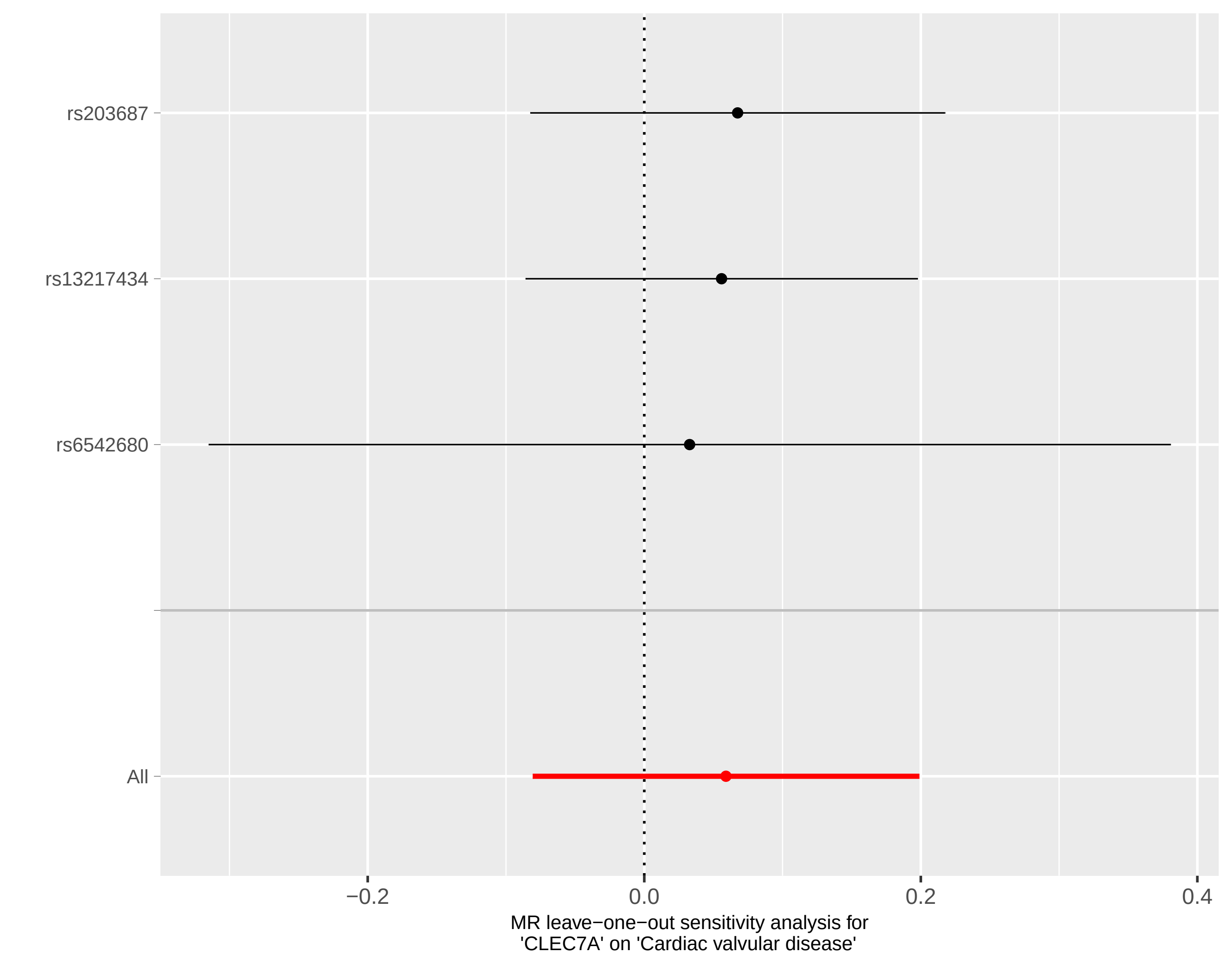
rs203687
rs13217434
rs6542680
All
−0.2
0.0
0.2
0.4
MR leave−one−out sensitivity analysis for
'CLEC7A' on 'Cardiac valvular disease'

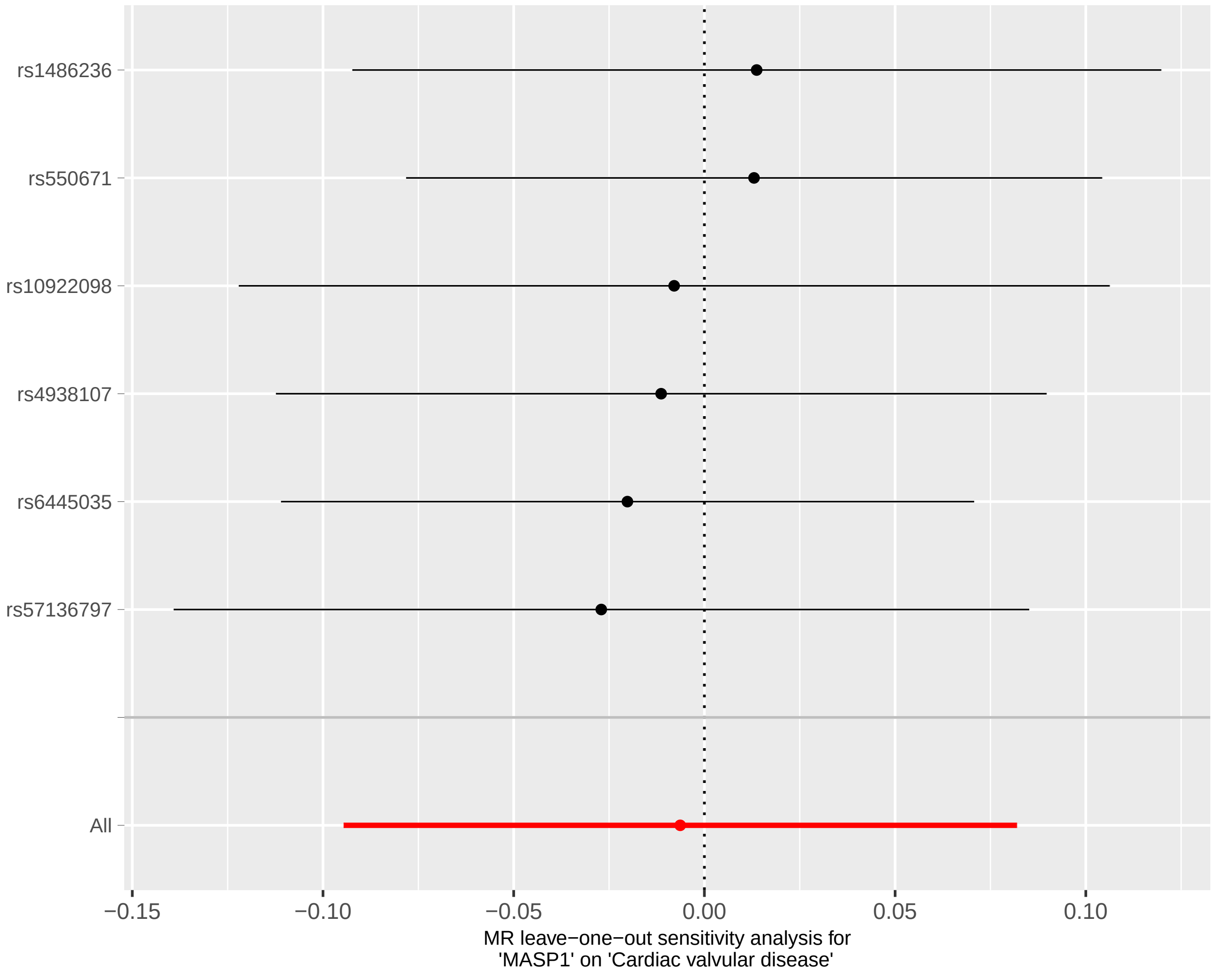
rs1486236
rs550671
rs10922098
rs4938107
rs6445035
rs57136797
All
−0.15
−0.10
−0.05
0.00
0.05
0.10
MR leave−one−out sensitivity analysis for
'MASP1' on 'Cardiac valvular disease'

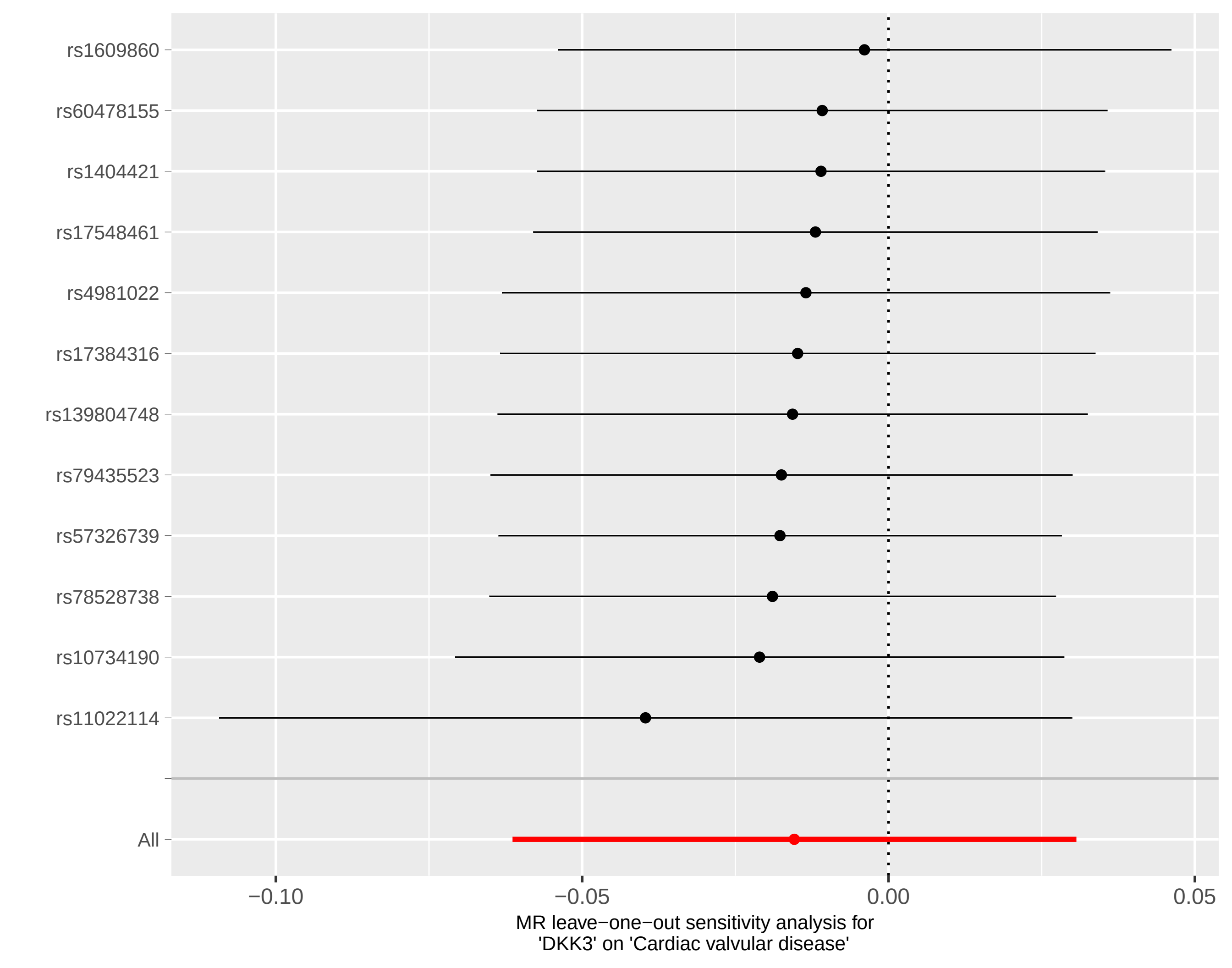
rs1609860
rs60478155
rs1404421
rs17548461
rs4981022
rs17384316
rs139804748
rs79435523
rs57326739
rs78528738
rs10734190
rs11022114
All
−0.10
−0.05
0.00
0.05
MR leave−one−out sensitivity analysis for
'DKK3' on 'Cardiac valvular disease'

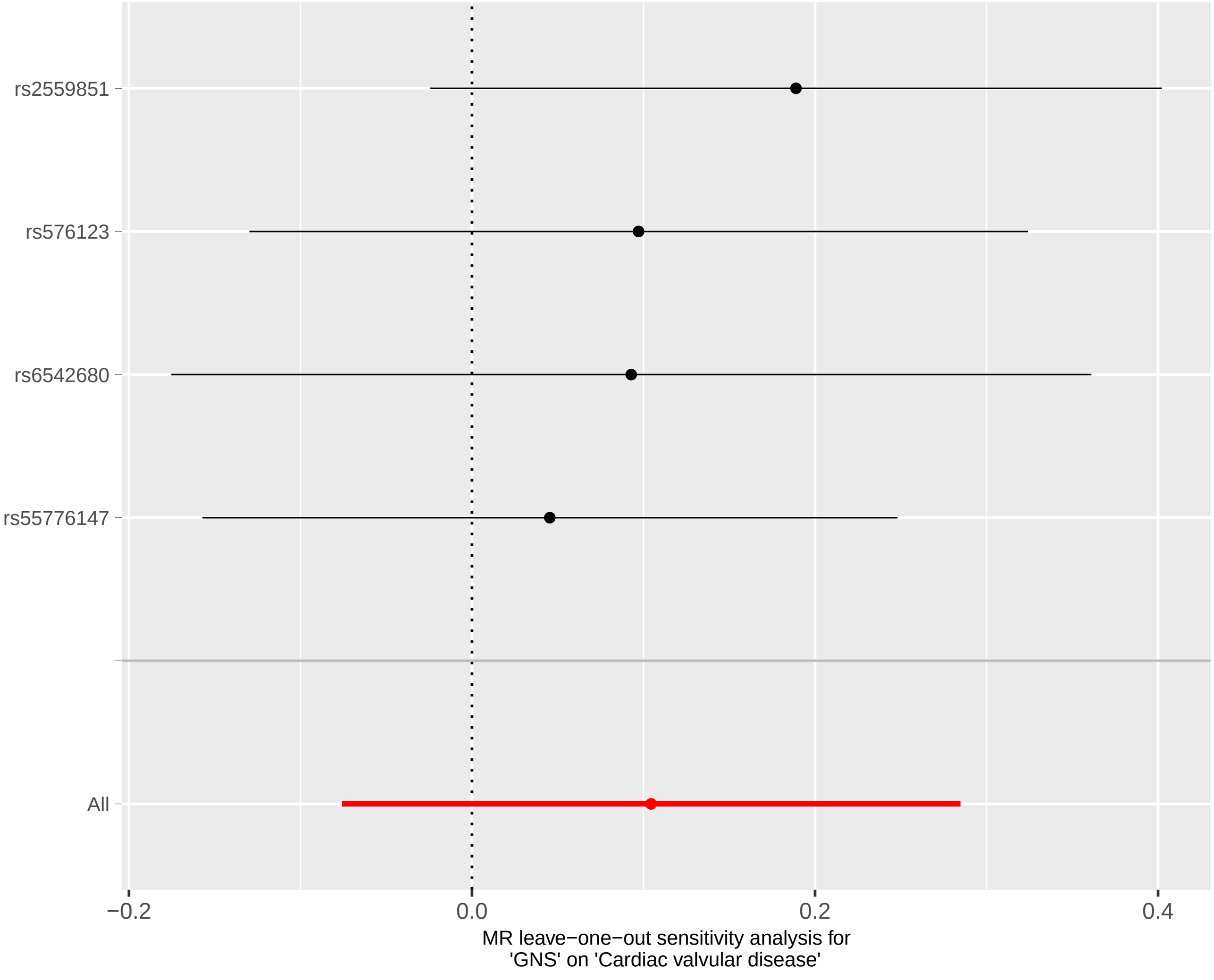
rs2559851
rs576123
rs6542680
rs55776147
All
−0.2
0.0
0.2
0.4
MR leave−one−out sensitivity analysis for
'GNS' on 'Cardiac valvular disease'

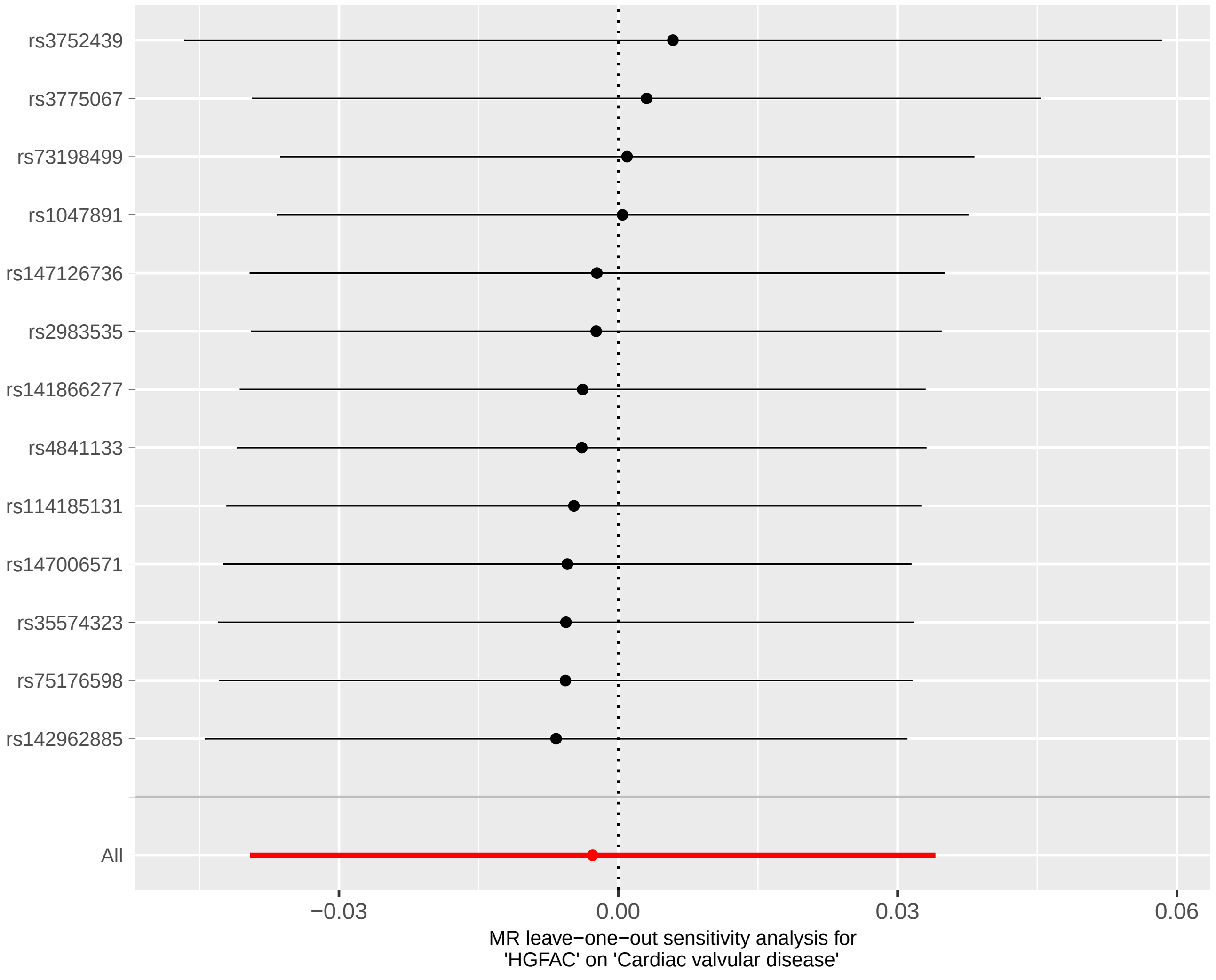
rs3752439
rs3775067
rs73198499
rs1047891
rs147126736
rs2983535
rs141866277
rs4841133
rs114185131
rs147006571
rs35574323
rs75176598
rs142962885
All
−0.03
0.00
0.03
0.06
MR leave−one−out sensitivity analysis for
'HGFAC' on 'Cardiac valvular disease'

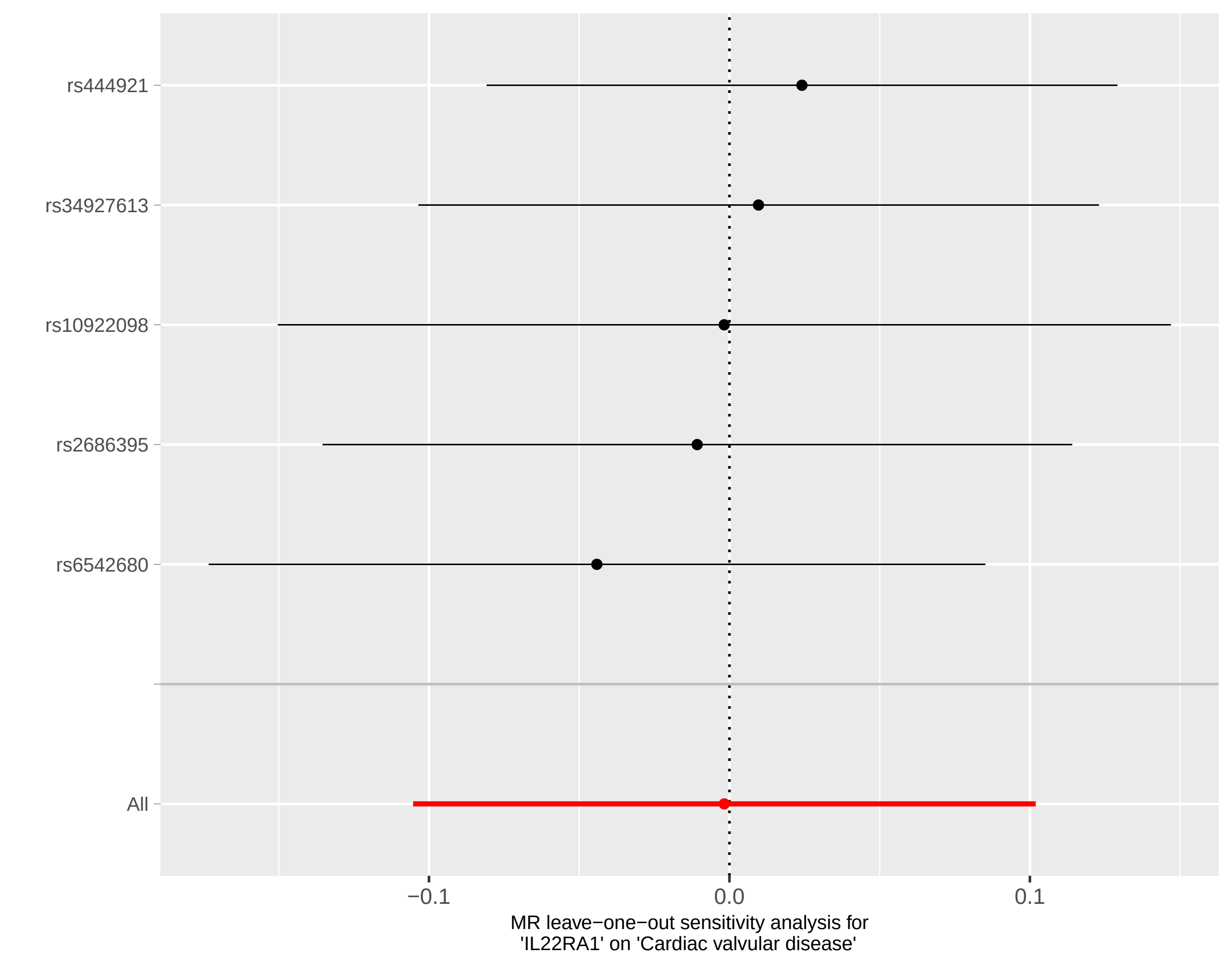
rs444921
rs34927613
rs10922098
rs2686395
rs6542680
All
−0.1
0.0
0.1
MR leave−one−out sensitivity analysis for
'IL22RA1' on 'Cardiac valvular disease'

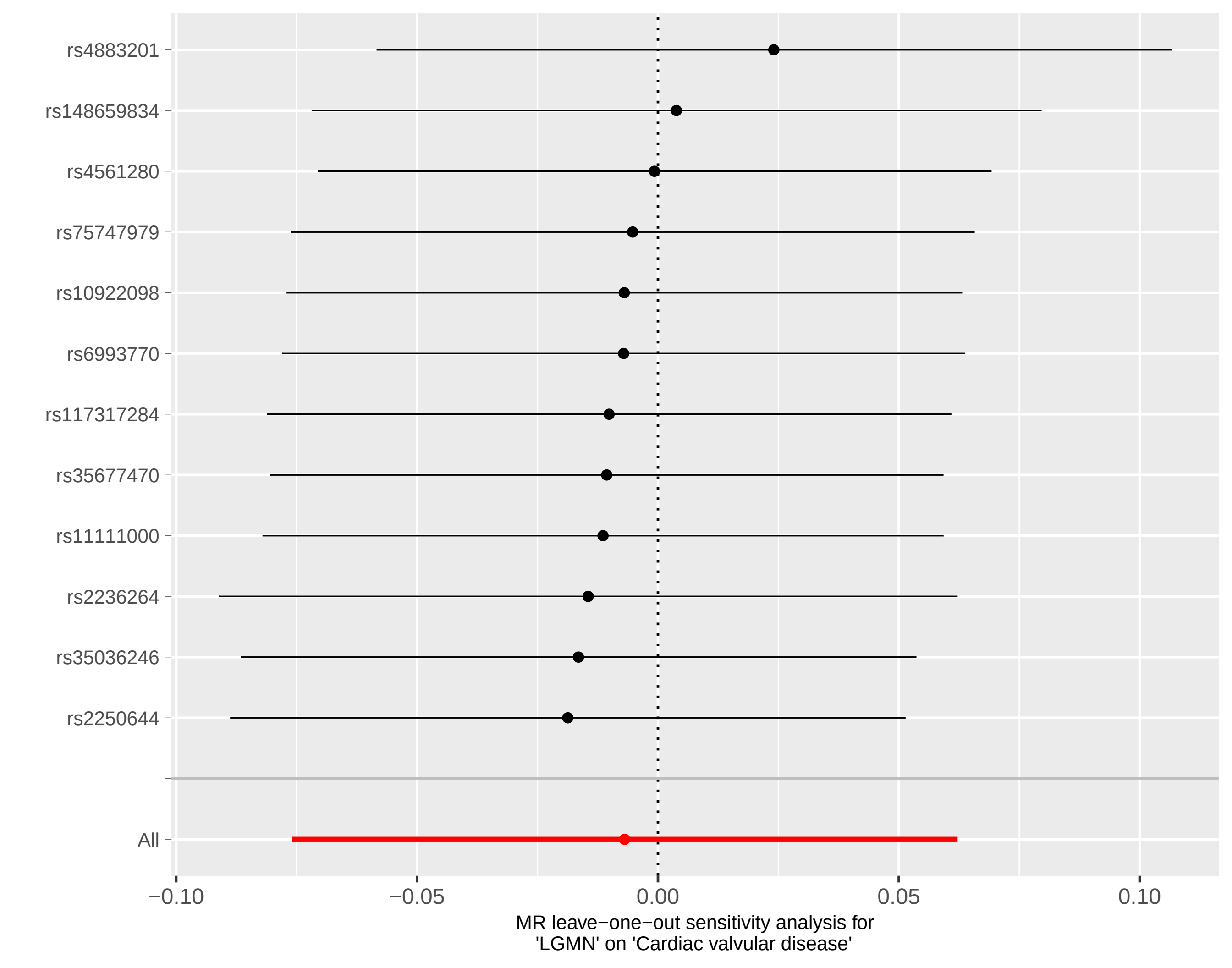
rs4883201
rs148659834
rs4561280
rs75747979
rs10922098
rs6993770
rs117317284
rs35677470
rs11111000
rs2236264
rs35036246
rs2250644
All
−0.10
−0.05
0.00
0.05
0.10
MR leave−one−out sensitivity analysis for
'LGMN' on 'Cardiac valvular disease'

Insufficient number of SNPs

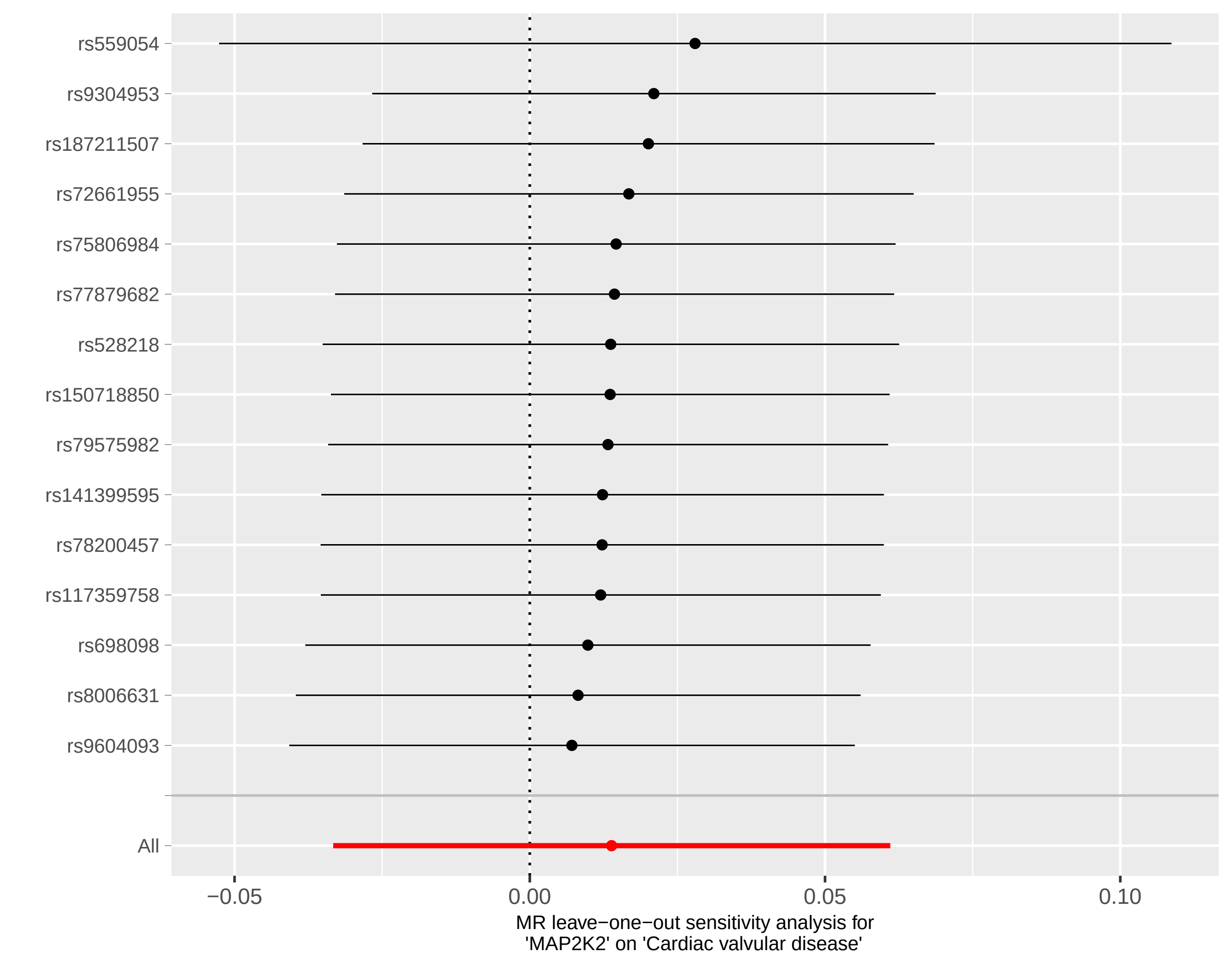

rs559054
rs9304953
rs187211507
rs72661955
rs75806984
rs77879682
rs528218
rs150718850
rs79575982
rs141399595
rs78200457
rs117359758
rs698098
rs8006631
rs9604093
All
−0.05
0.00
0.05
0.10
MR leave−one−out sensitivity analysis for
'MAP2K2' on 'Cardiac valvular disease'

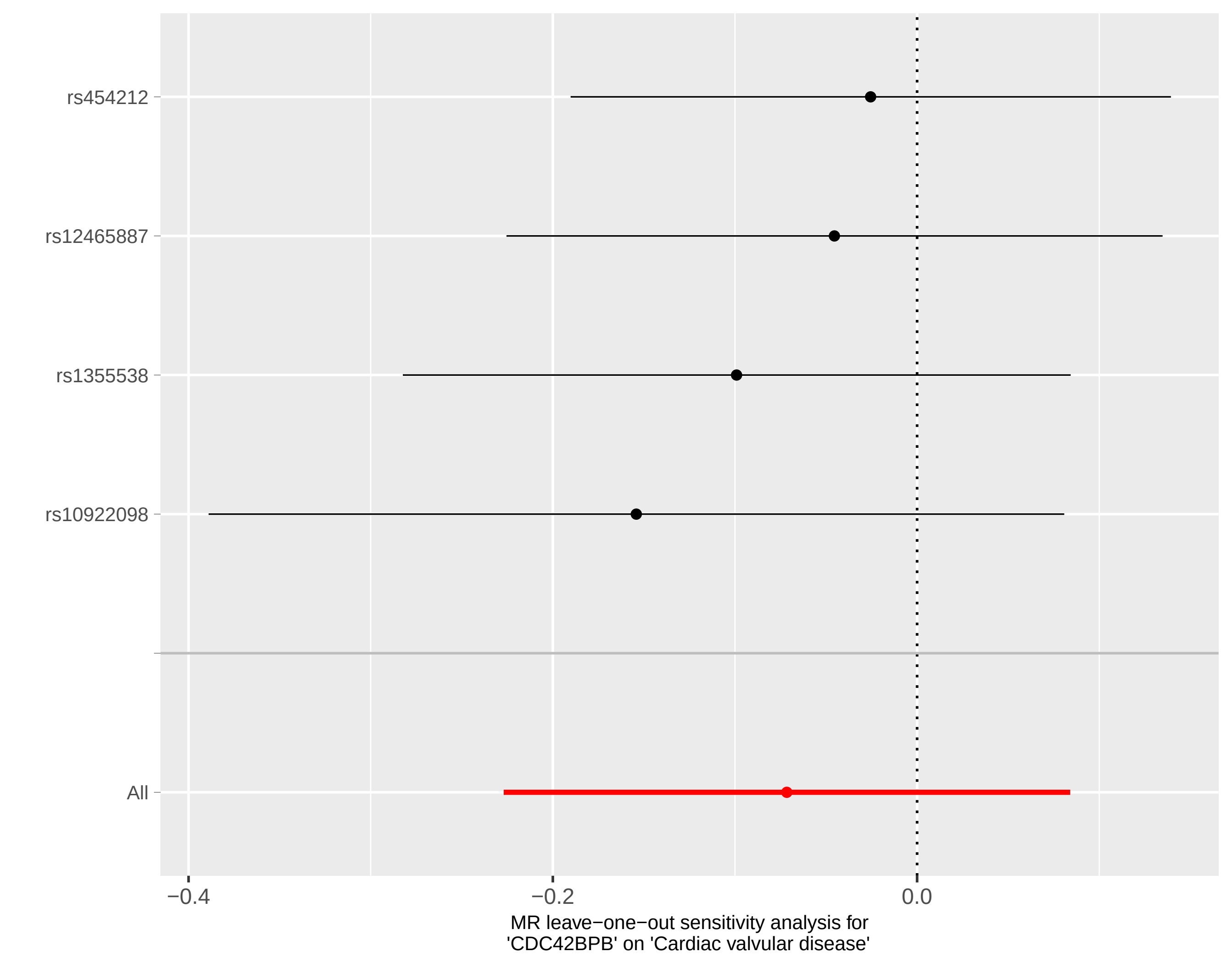

rs454212
rs12465887
rs1355538
rs10922098
All
−0.4
−0.2
0.0
MR leave−one−out sensitivity analysis for
'CDC42BPB' on 'Cardiac valvular disease'

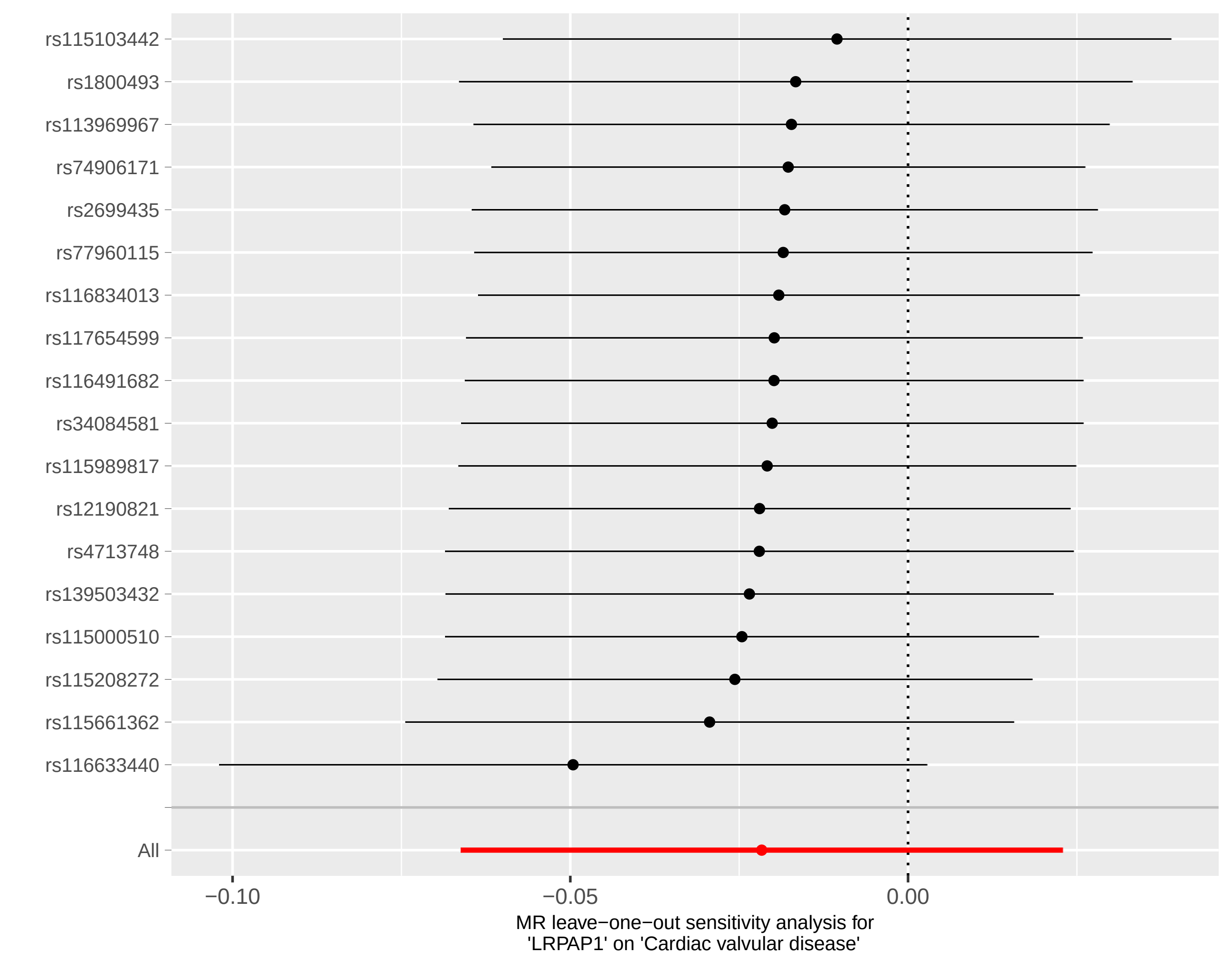
rs115103442
rs1800493
rs113969967
rs74906171
rs2699435
rs77960115
rs116834013
rs117654599
rs116491682
rs34084581
rs115989817
rs12190821
rs4713748
rs139503432
rs115000510
rs115208272
rs115661362
rs116633440
All
−0.10
−0.05
0.00
MR leave−one−out sensitivity analysis for
'LRPAP1' on 'Cardiac valvular disease'

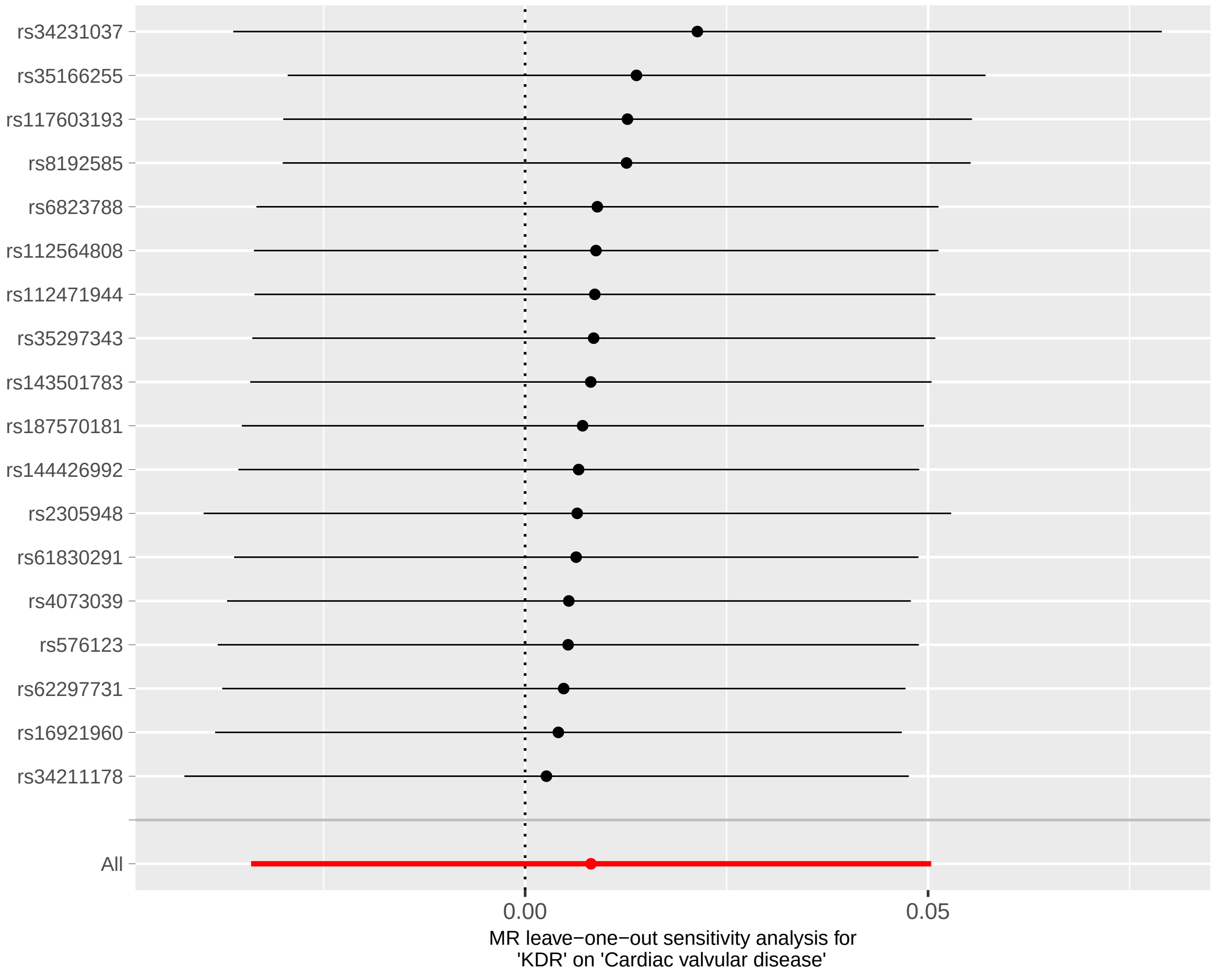

rs34231037
rs35166255
rs117603193
rs8192585
rs6823788
rs112564808
rs112471944
rs35297343
rs143501783
rs187570181
rs144426992
rs2305948
rs61830291
rs4073039
rs576123
rs62297731
rs16921960
rs34211178
All
0.00
0.05
MR leave−one−out sensitivity analysis for
'KDR' on 'Cardiac valvular disease'

Insufficient number of SNPs

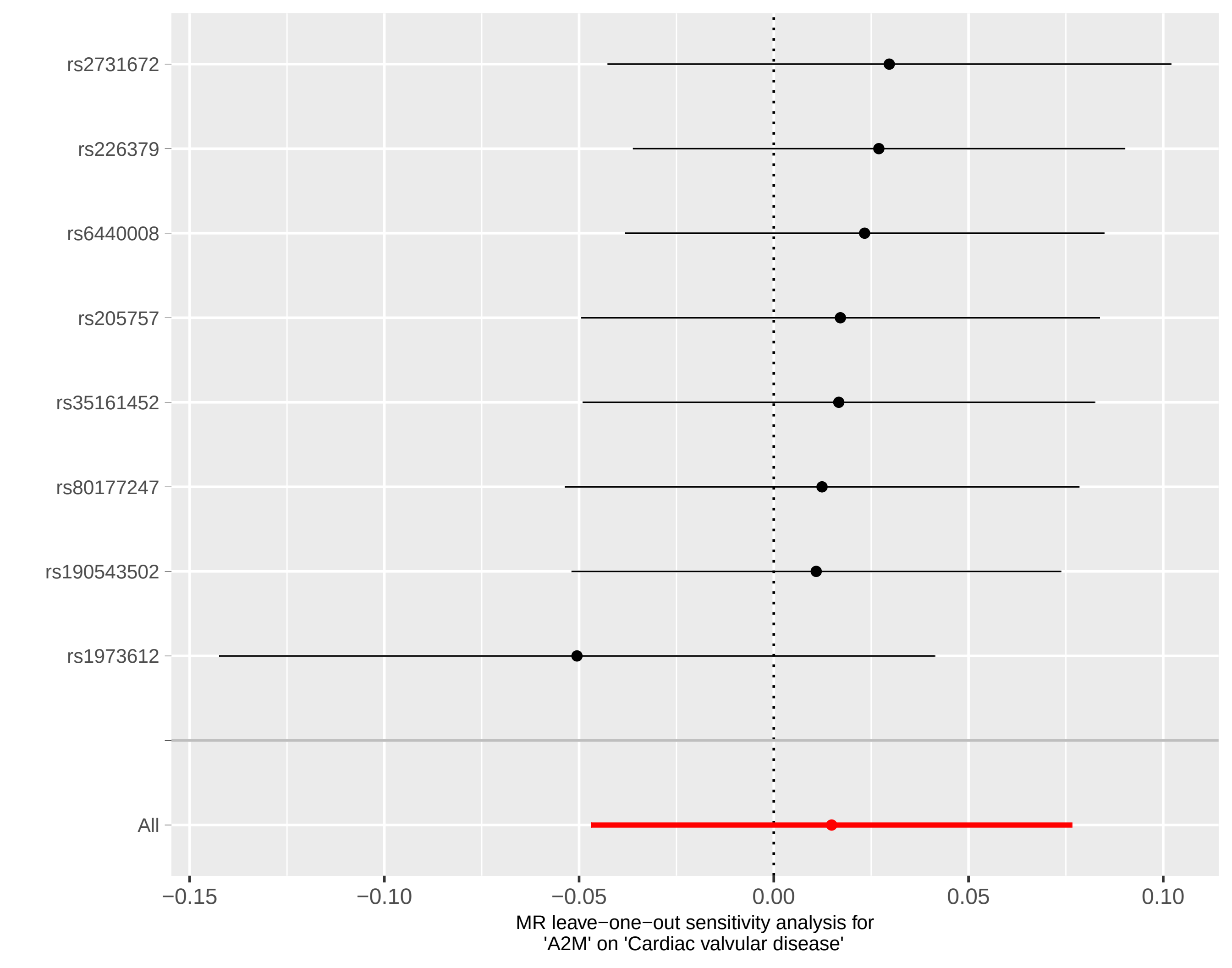
rs2731672
rs226379
rs6440008
rs205757
rs35161452
rs80177247
rs190543502
rs1973612
All
−0.15
−0.10
−0.05
0.00
0.05
0.10
MR leave−one−out sensitivity analysis for
'A2M' on 'Cardiac valvular disease'

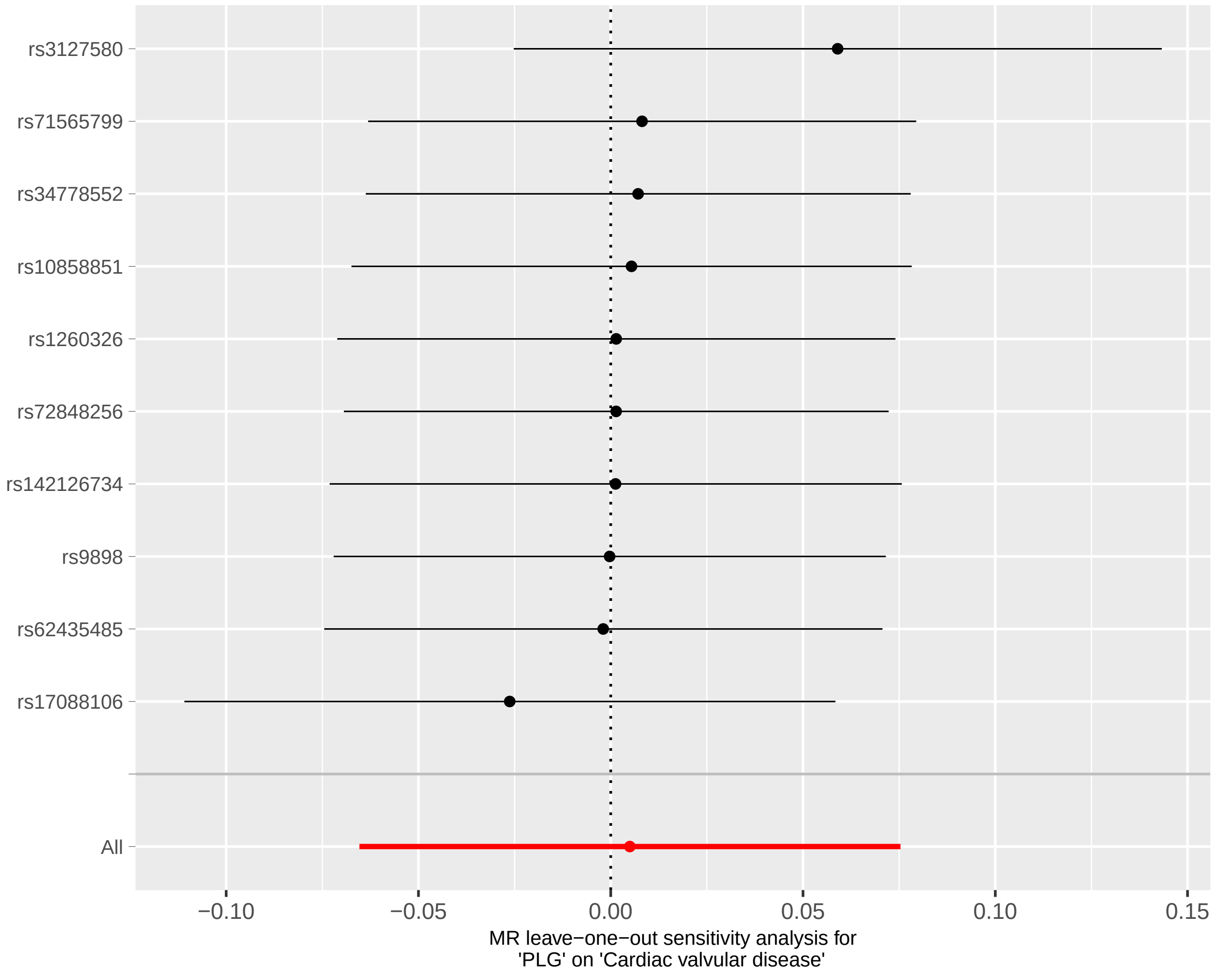

rs3127580
rs71565799
rs34778552
rs10858851
rs1260326
rs72848256
rs142126734
rs9898
rs62435485
rs17088106
All
−0.10
−0.05
0.00
0.05
0.10
0.15
MR leave−one−out sensitivity analysis for
'PLG' on 'Cardiac valvular disease'

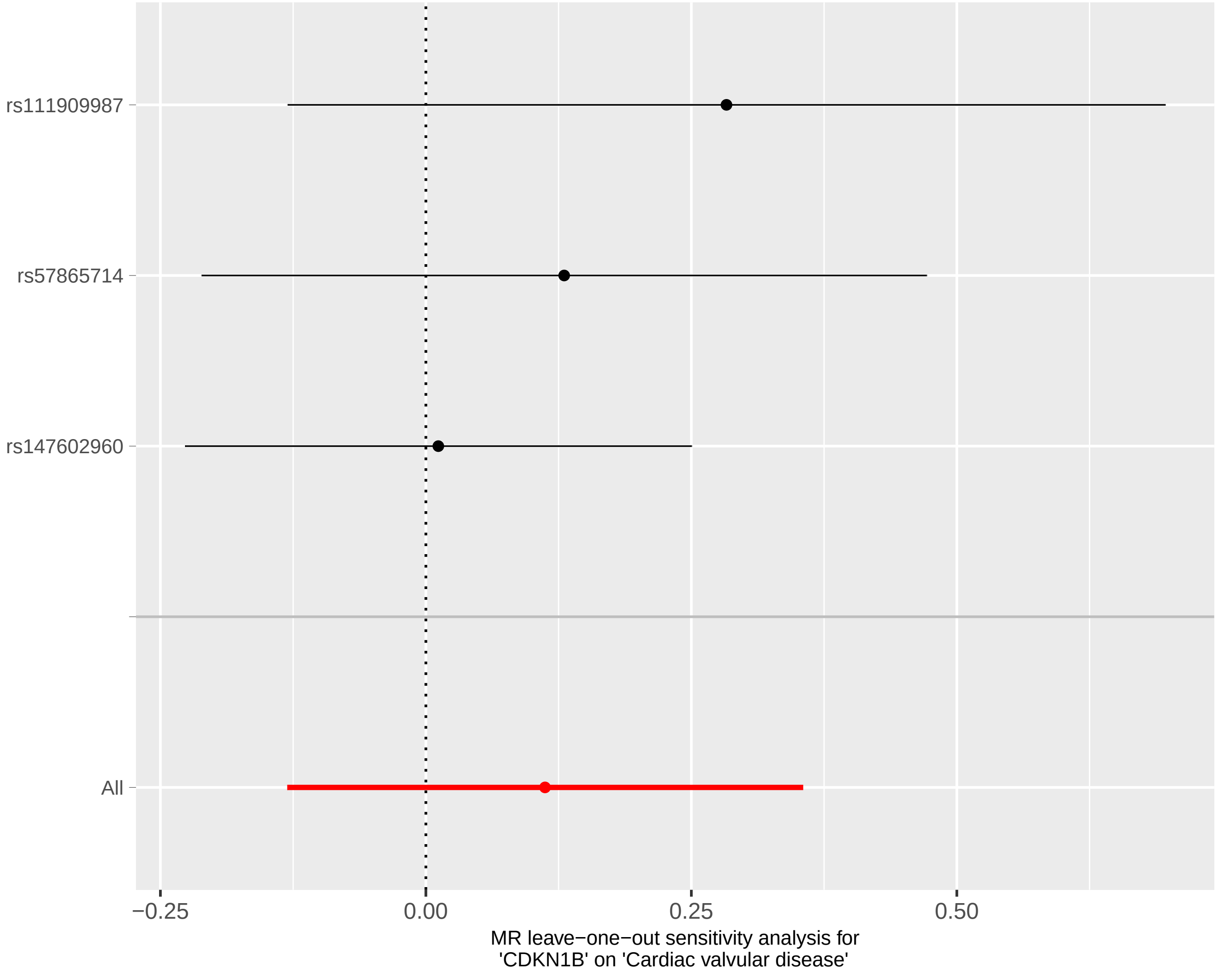
rs111909987
rs57865714
rs147602960
All
−0.25
0.00
0.25
0.50
MR leave−one−out sensitivity analysis for
'CDKN1B' on 'Cardiac valvular disease'

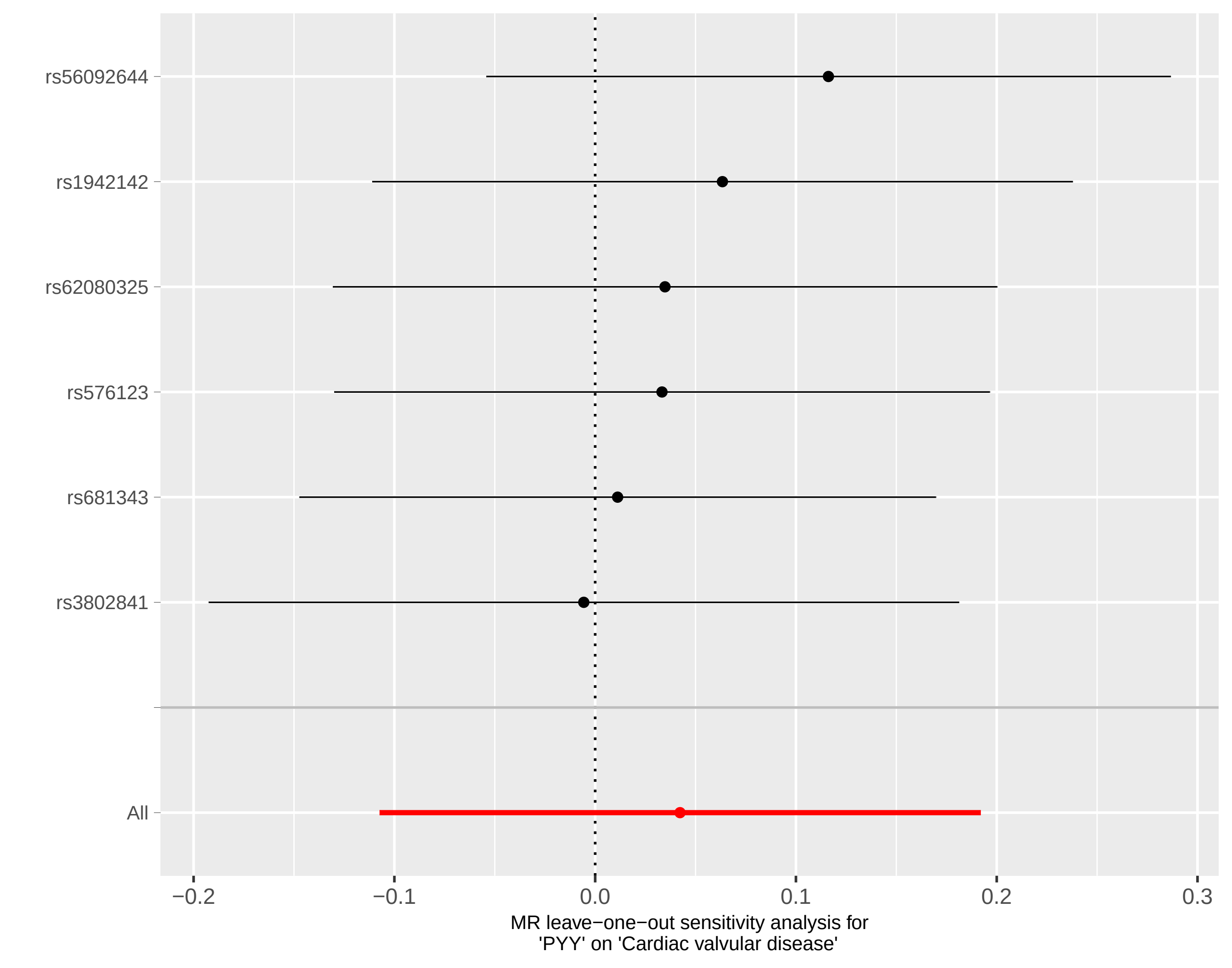

rs56092644
rs1942142
rs62080325
rs576123
rs681343
rs3802841
All
−0.2
−0.1
0.0
0.1
0.2
0.3
MR leave−one−out sensitivity analysis for
'PYY' on 'Cardiac valvular disease'

Insufficient number of SNPs

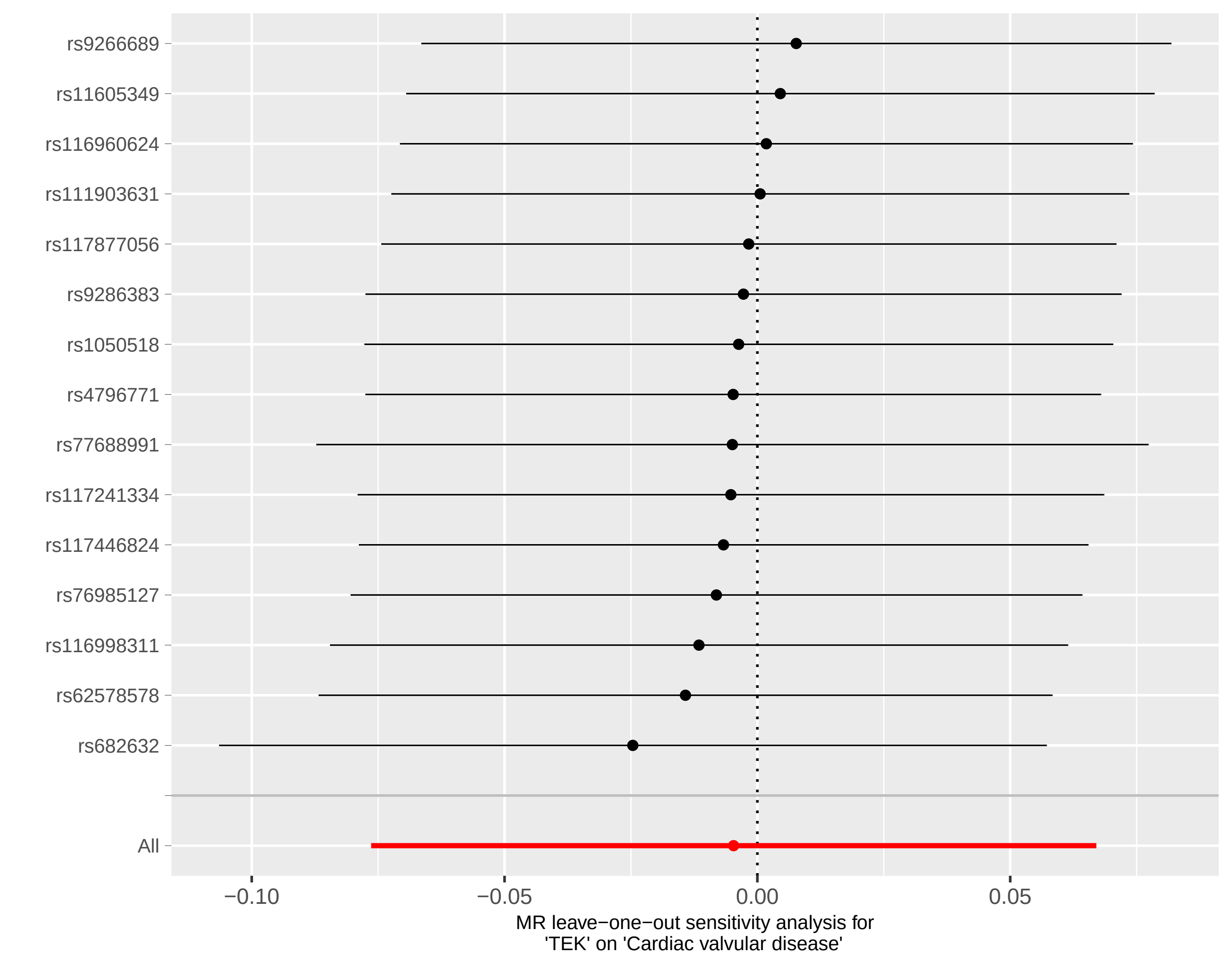
rs9266689
rs11605349
rs116960624
rs111903631
rs117877056
rs9286383
rs1050518
rs4796771
rs77688991
rs117241334
rs117446824
rs76985127
rs116998311
rs62578578
rs682632
All
−0.10
−0.05
0.00
0.05
MR leave−one−out sensitivity analysis for
'TEK' on 'Cardiac valvular disease'

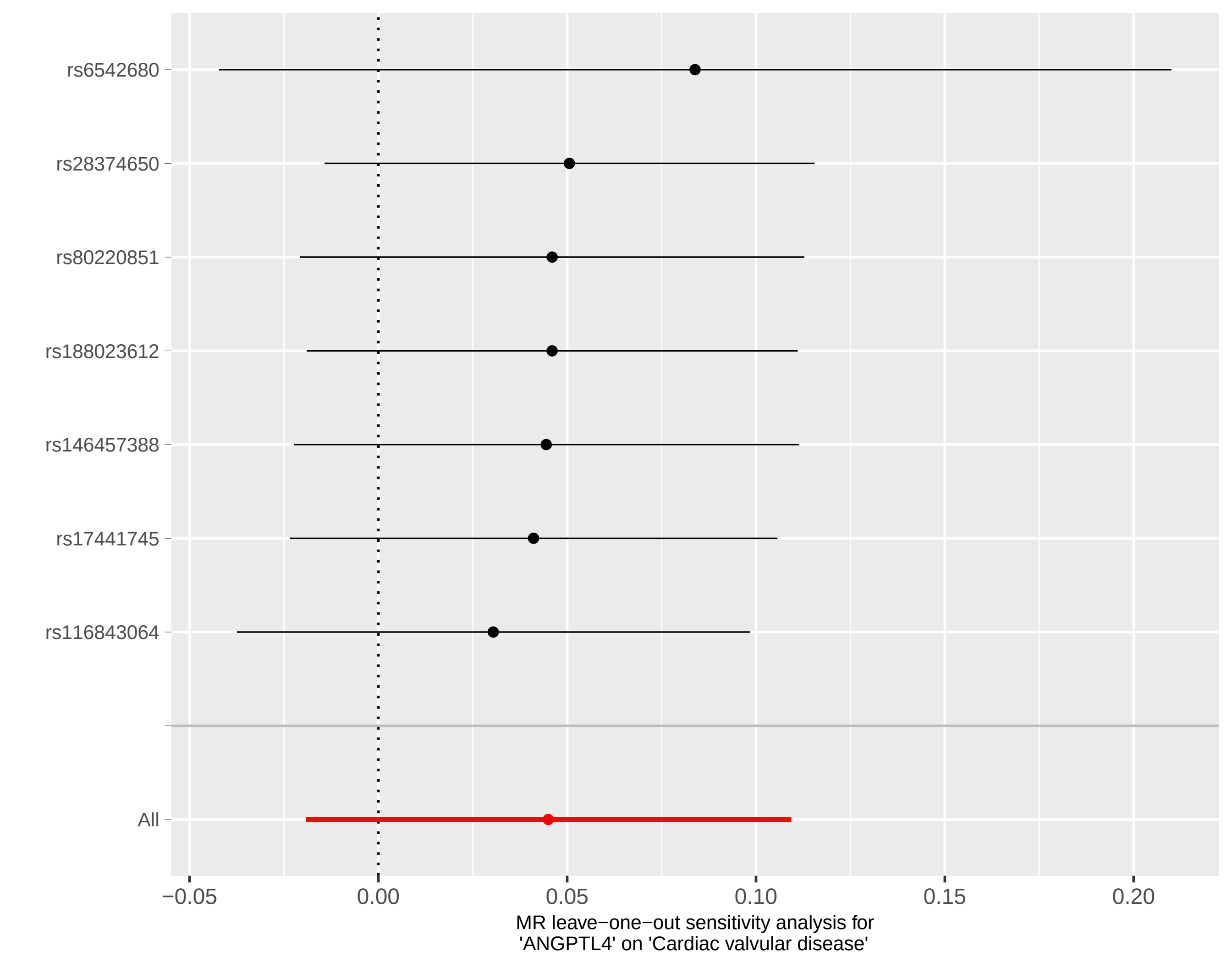

rs6542680
rs28374650
rs80220851
rs188023612
rs146457388
rs17441745
rs116843064
All
−0.05
0.00
0.05
0.10
0.15
0.20
MR leave−one−out sensitivity analysis for
'ANGPTL4' on 'Cardiac valvular disease'

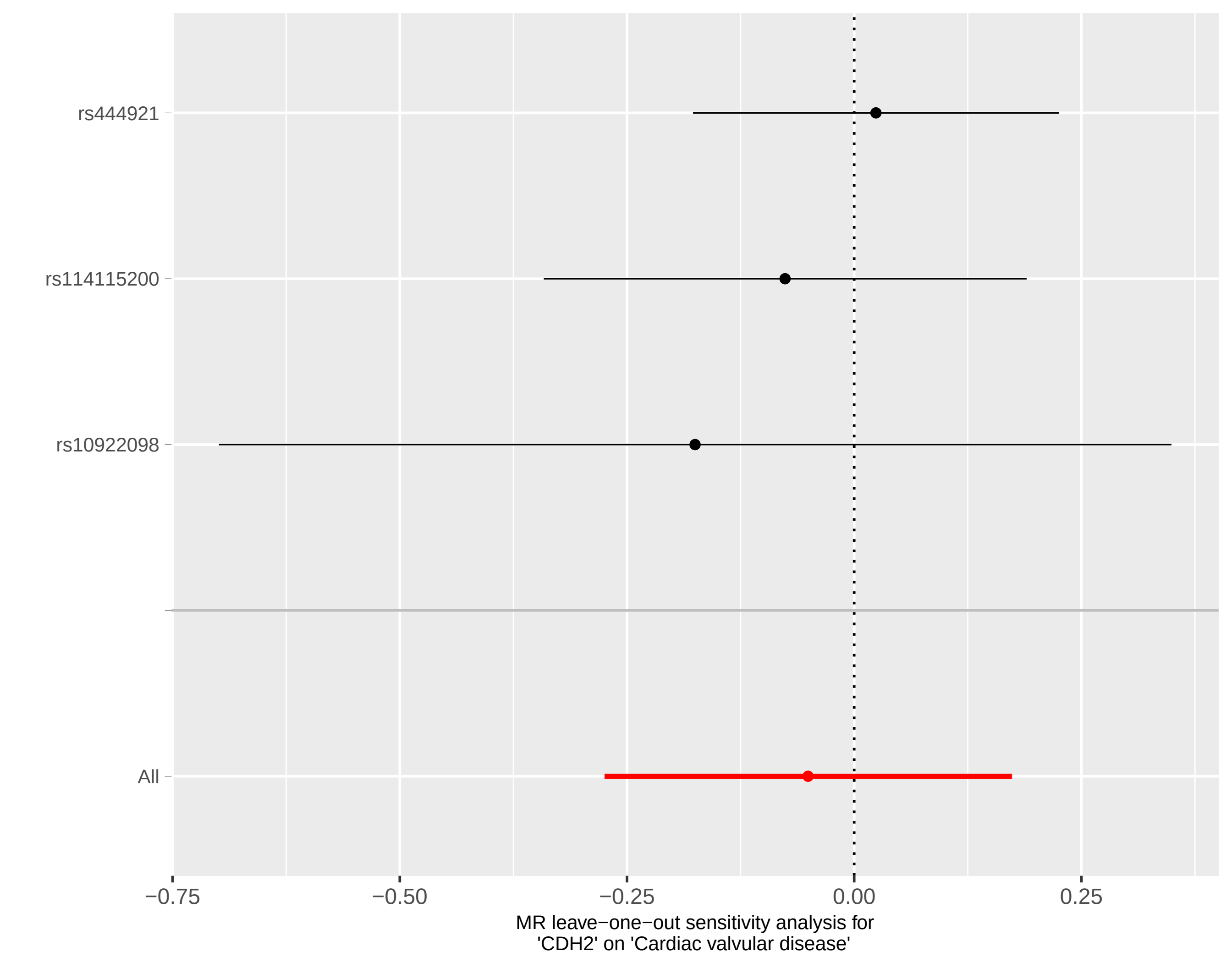
rs444921
rs114115200
rs10922098
All
−0.75
−0.50
−0.25
0.00
0.25
MR leave−one−out sensitivity analysis for
'CDH2' on 'Cardiac valvular disease'

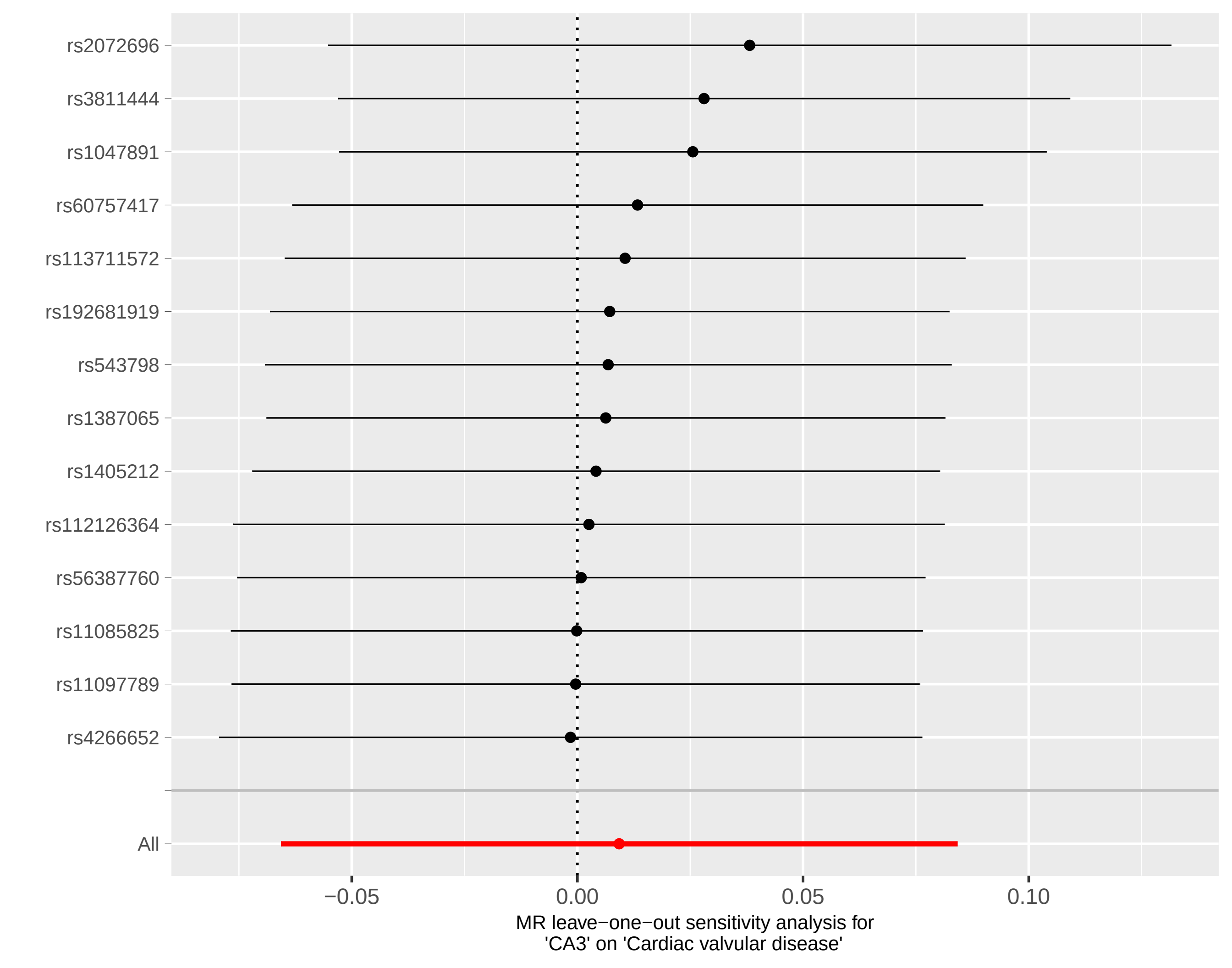

rs2072696
rs3811444
rs1047891
rs60757417
rs113711572
rs192681919
rs543798
rs1387065
rs1405212
rs112126364
rs56387760
rs11085825
rs11097789
rs4266652
All
−0.05
0.00
0.05
0.10
MR leave−one−out sensitivity analysis for
'CA3' on 'Cardiac valvular disease'

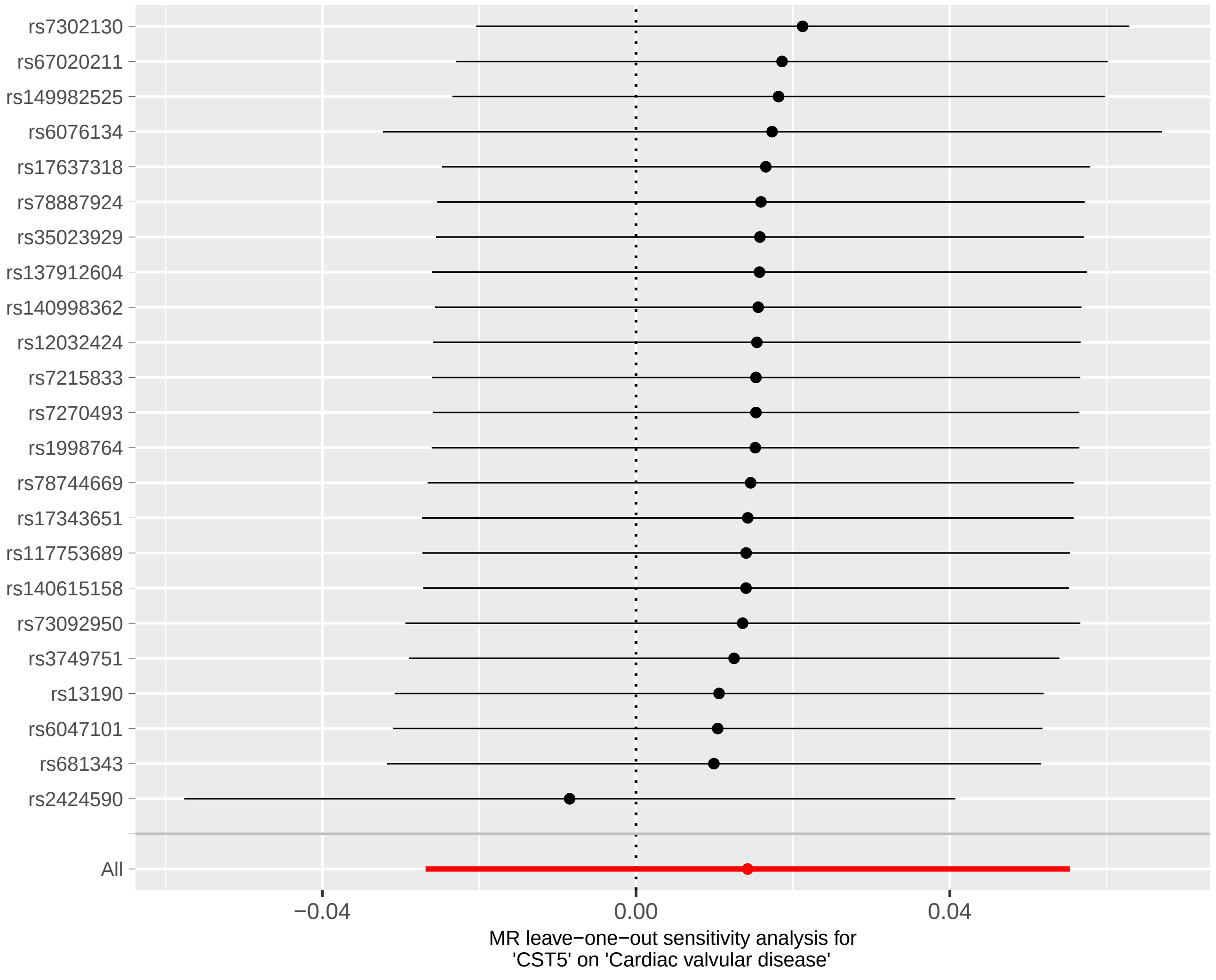

rs7302130
rs67020211
rs149982525
rs6076134
rs17637318
rs78887924
rs35023929
rs137912604
rs140998362
rs12032424
rs7215833
rs7270493
rs1998764
rs78744669
rs17343651
rs117753689
rs140615158
rs73092950
rs3749751
rs13190
rs6047101
rs681343
rs2424590
All
−0.04
0.00
0.04
MR leave−one−out sensitivity analysis for
'CST5' on 'Cardiac valvular disease'

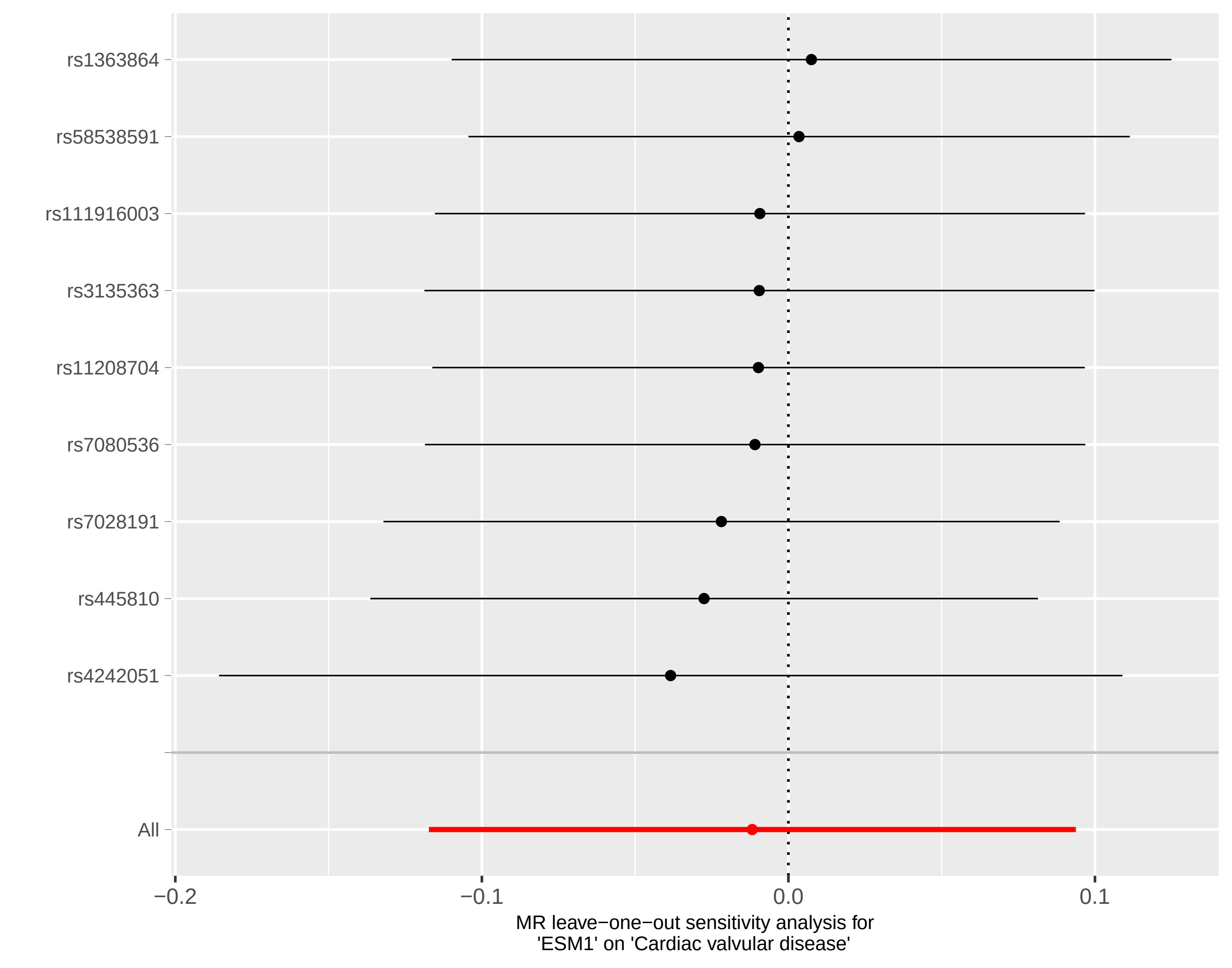
rs1363864
rs58538591
rs111916003
rs3135363
rs11208704
rs7080536
rs7028191
rs445810
rs4242051
All
−0.2
−0.1
0.0
0.1
MR leave−one−out sensitivity analysis for
'ESM1' on 'Cardiac valvular disease'

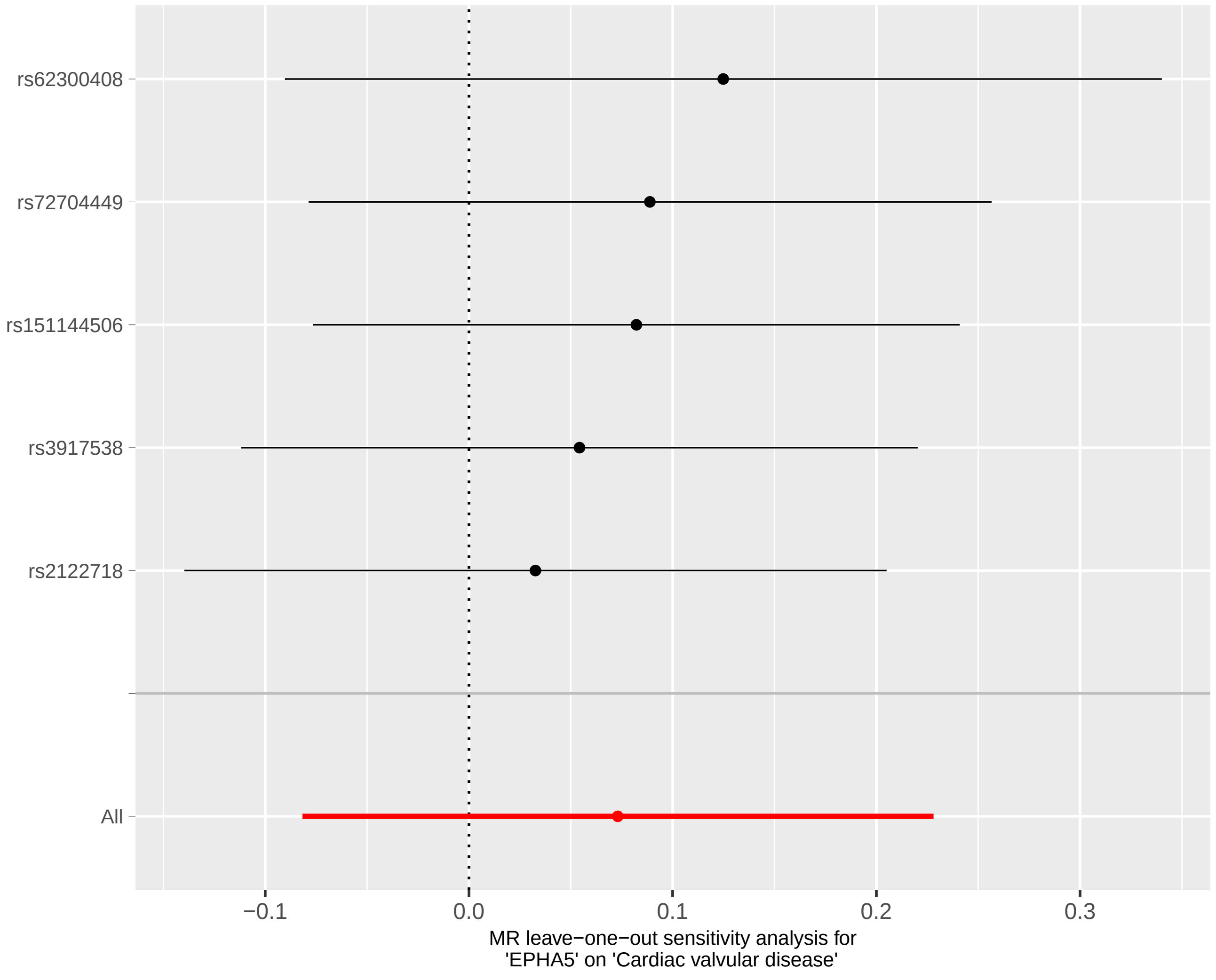
rs62300408
rs72704449
rs151144506
rs3917538
rs2122718
All
−0.1
0.0
0.1
0.2
0.3
MR leave−one−out sensitivity analysis for
'EPHA5' on 'Cardiac valvular disease'

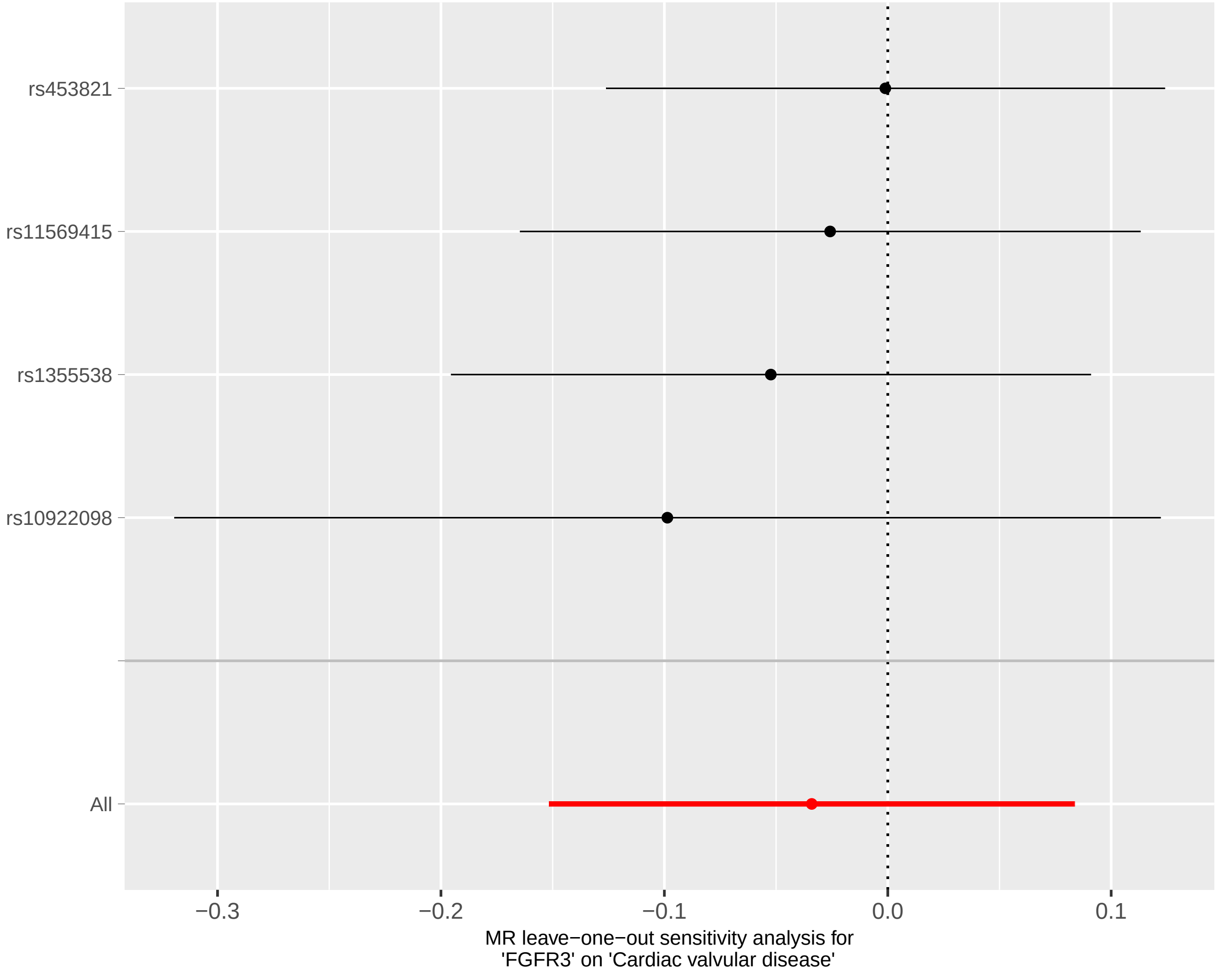
rs453821
rs11569415
rs1355538
rs10922098
All
−0.3
−0.2
−0.1
0.0
0.1
MR leave−one−out sensitivity analysis for
'FGFR3' on 'Cardiac valvular disease'

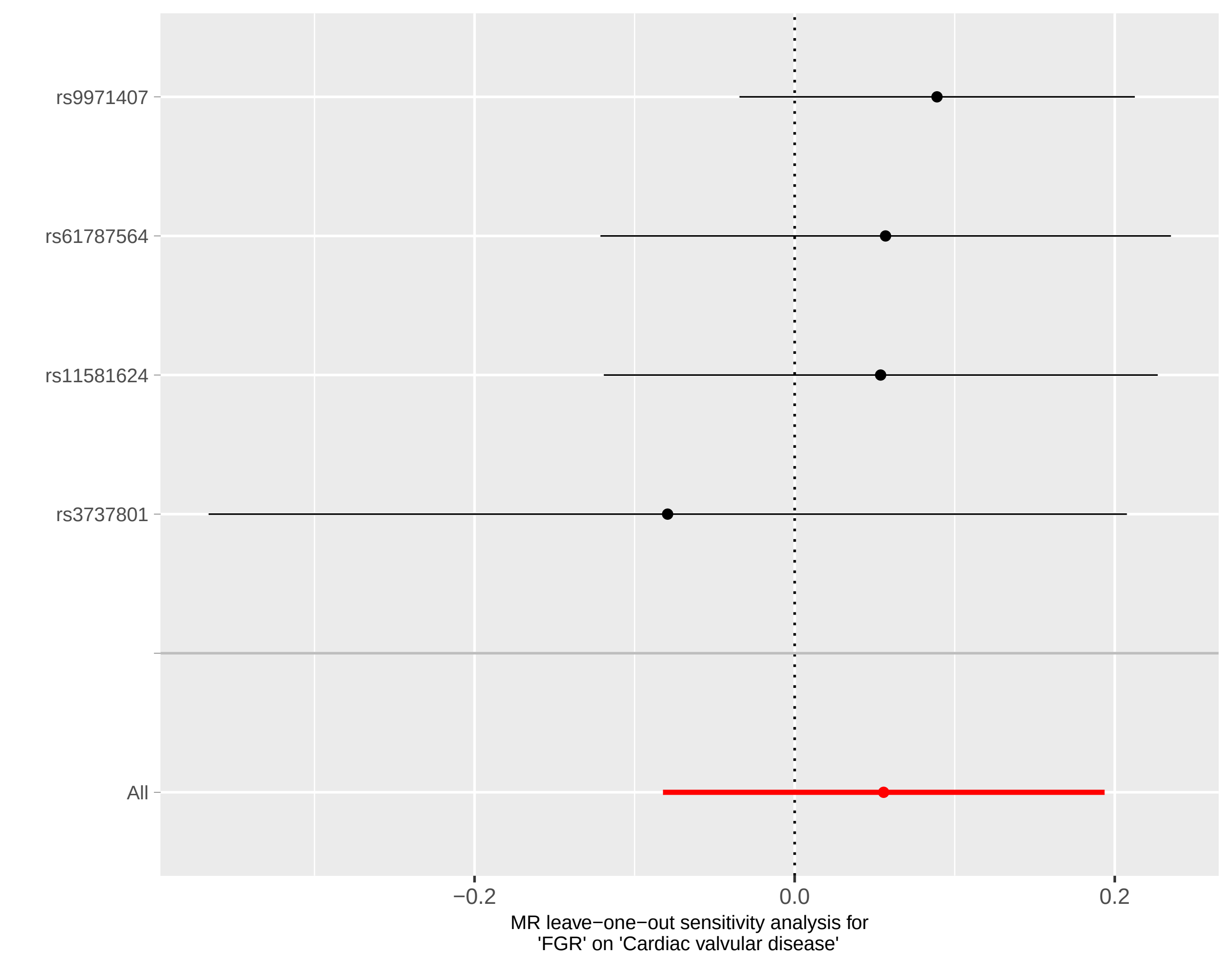
rs9971407
rs61787564
rs11581624
rs3737801
All
−0.2
0.0
0.2
MR leave−one−out sensitivity analysis for
'FGR' on 'Cardiac valvular disease'

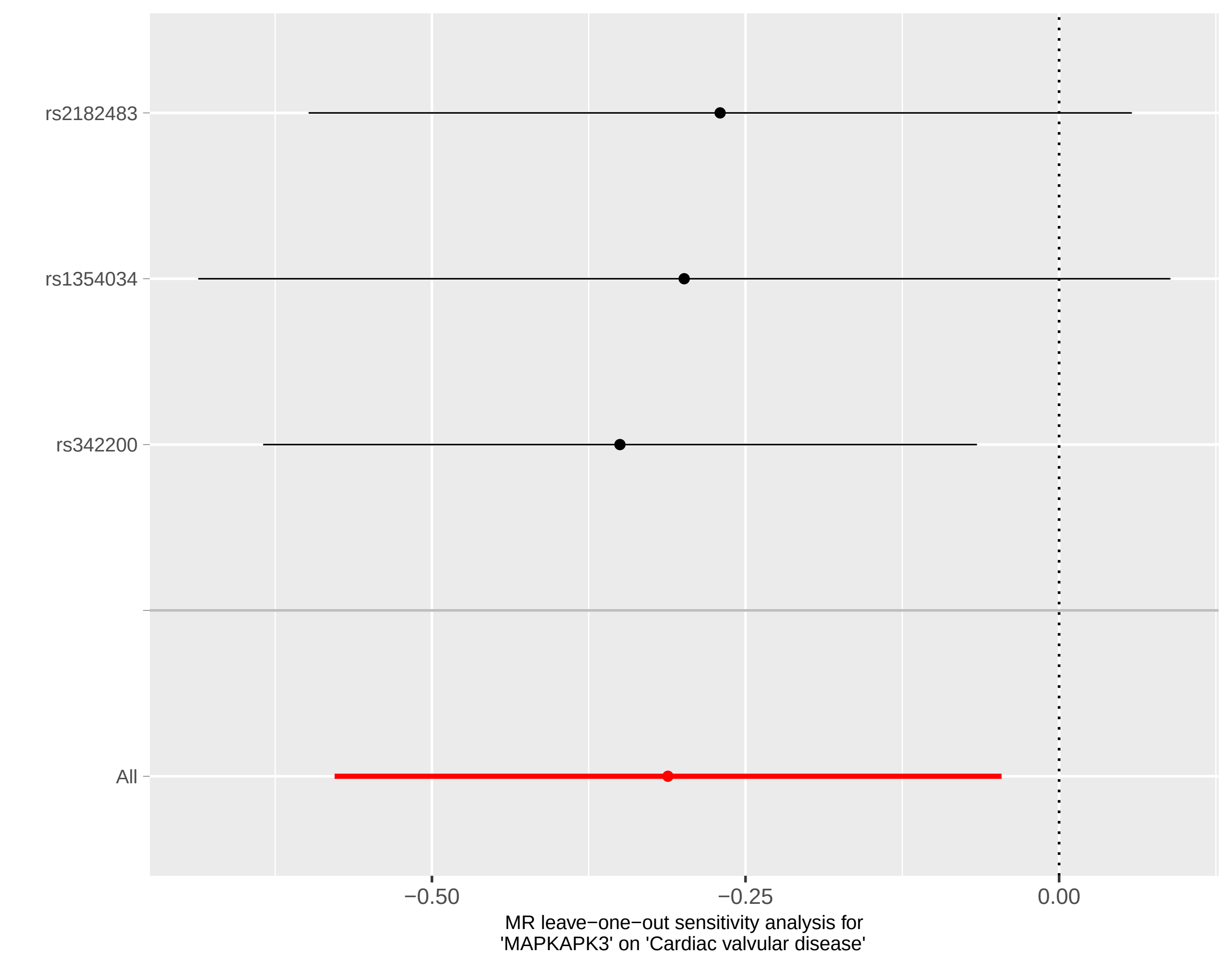
rs2182483
rs1354034
rs342200
All
−0.50
−0.25
0.00
MR leave−one−out sensitivity analysis for
'MAPKAPK3' on 'Cardiac valvular disease'

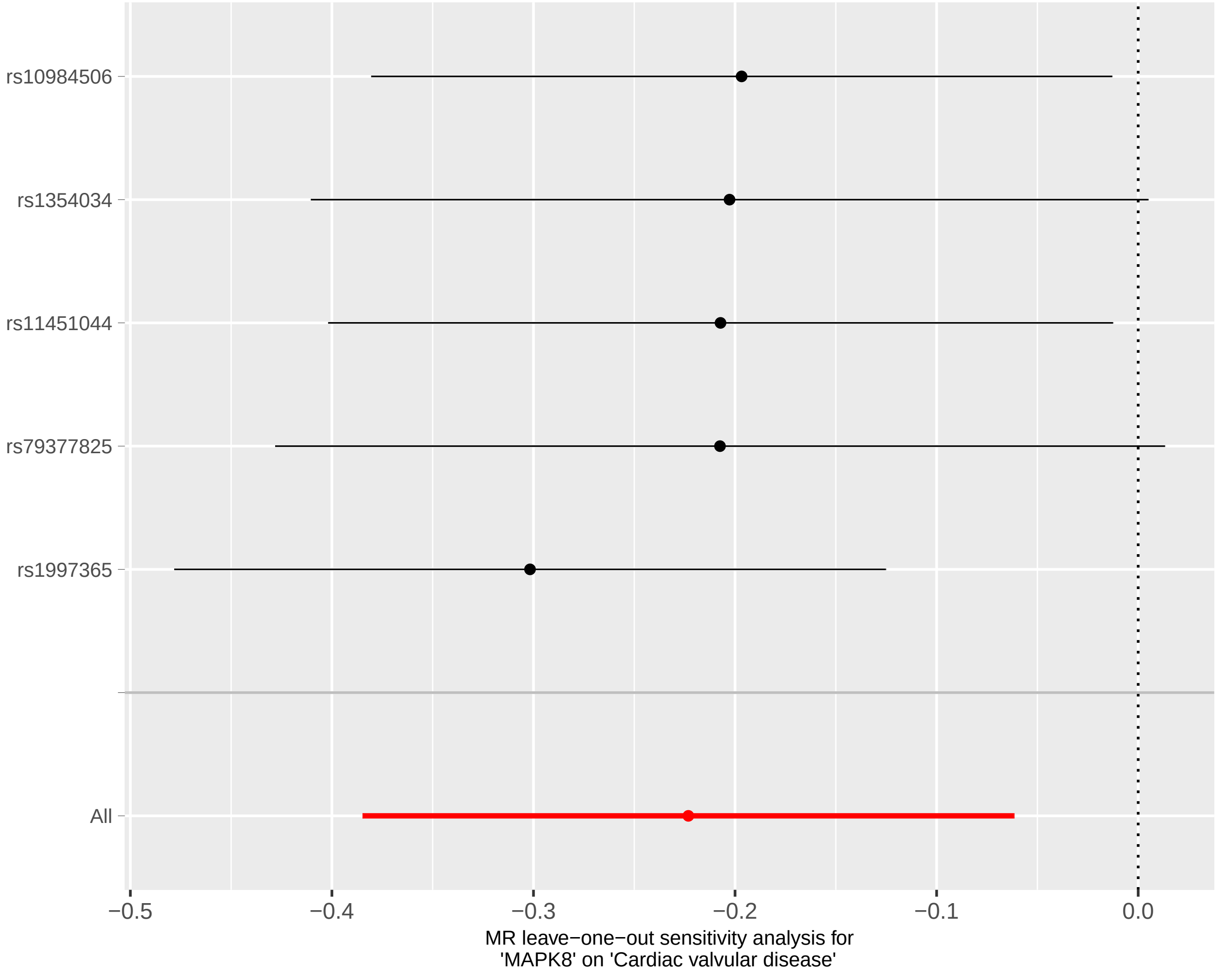

rs10984506
rs1354034
rs11451044
rs79377825
rs1997365
All
−0.5
−0.4
−0.3
−0.2
−0.1
0.0
MR leave−one−out sensitivity analysis for
'MAPK8' on 'Cardiac valvular disease'

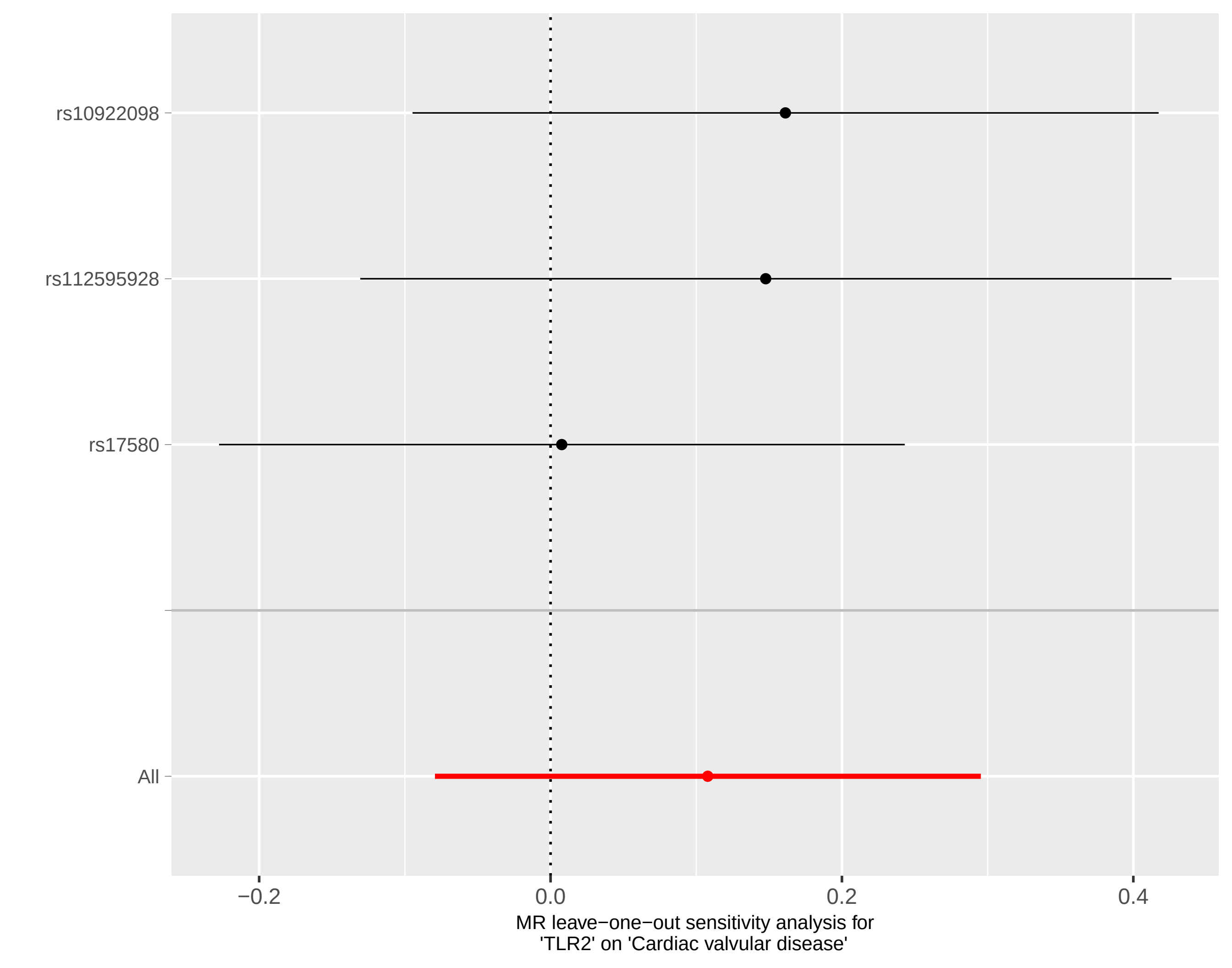
rs10922098
rs112595928
rs17580
All
−0.2
0.0
0.2
0.4
MR leave−one−out sensitivity analysis for
'TLR2' on 'Cardiac valvular disease'

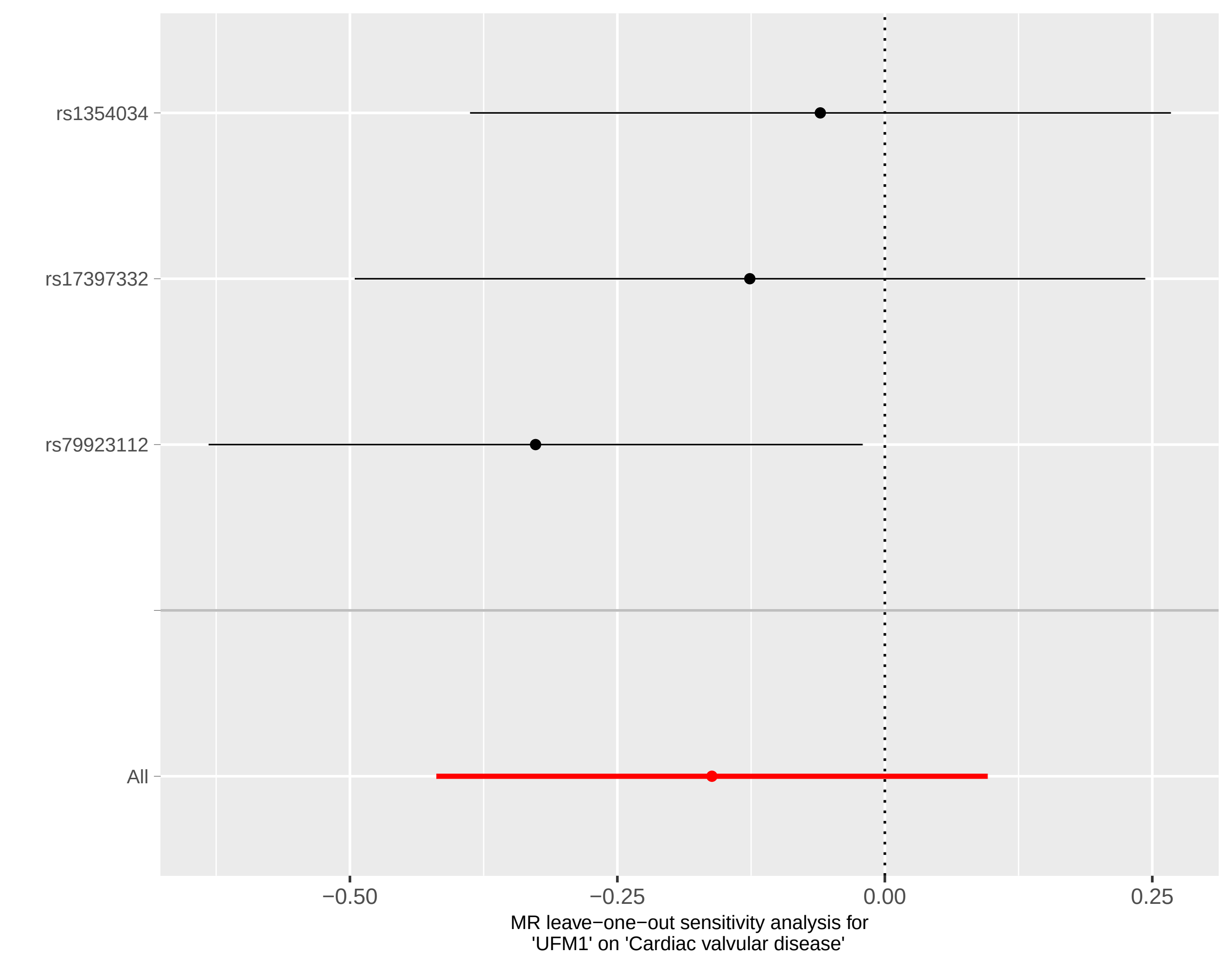

rs1354034
rs17397332
rs79923112
All
−0.50
−0.25
0.00
0.25
MR leave−one−out sensitivity analysis for
'UFM1' on 'Cardiac valvular disease'

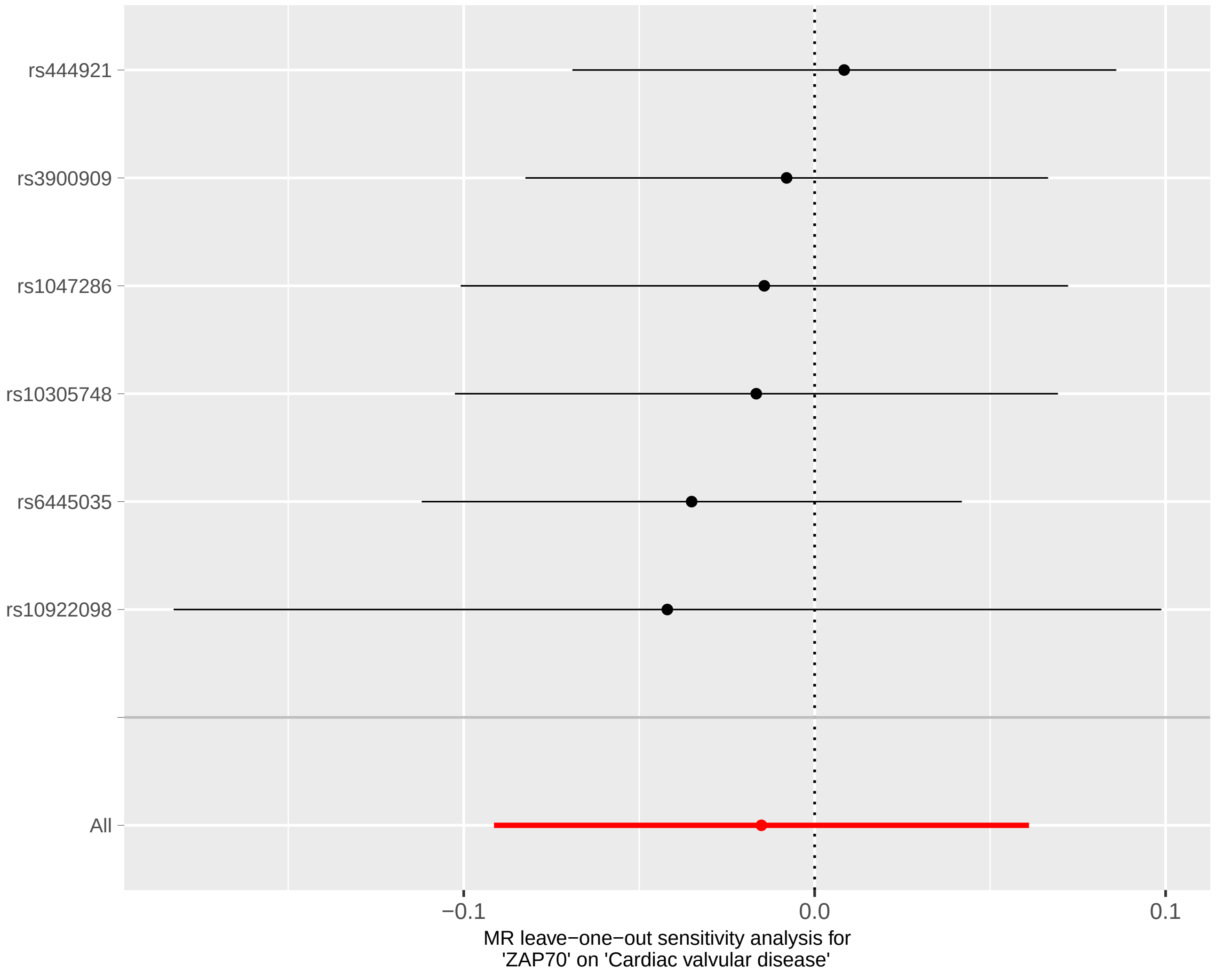

rs444921
rs3900909
rs1047286
rs10305748
rs6445035
rs10922098
All
−0.1
0.0
0.1
MR leave−one−out sensitivity analysis for
'ZAP70' on 'Cardiac valvular disease'

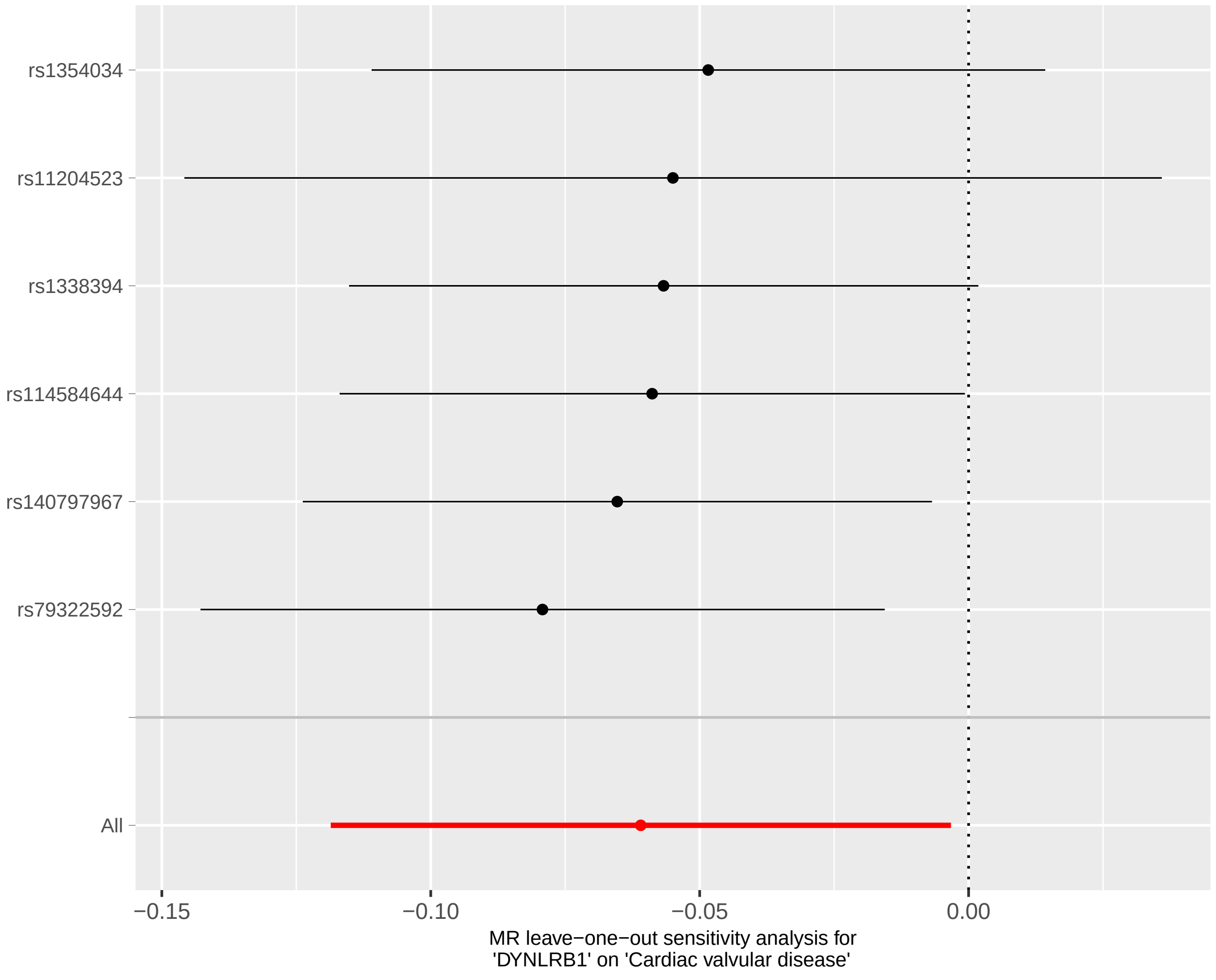

rs1354034
rs11204523
rs1338394
rs114584644
rs140797967
rs79322592
All
−0.15
−0.10
−0.05
0.00
MR leave−one−out sensitivity analysis for
'DYNLRB1' on 'Cardiac valvular disease'

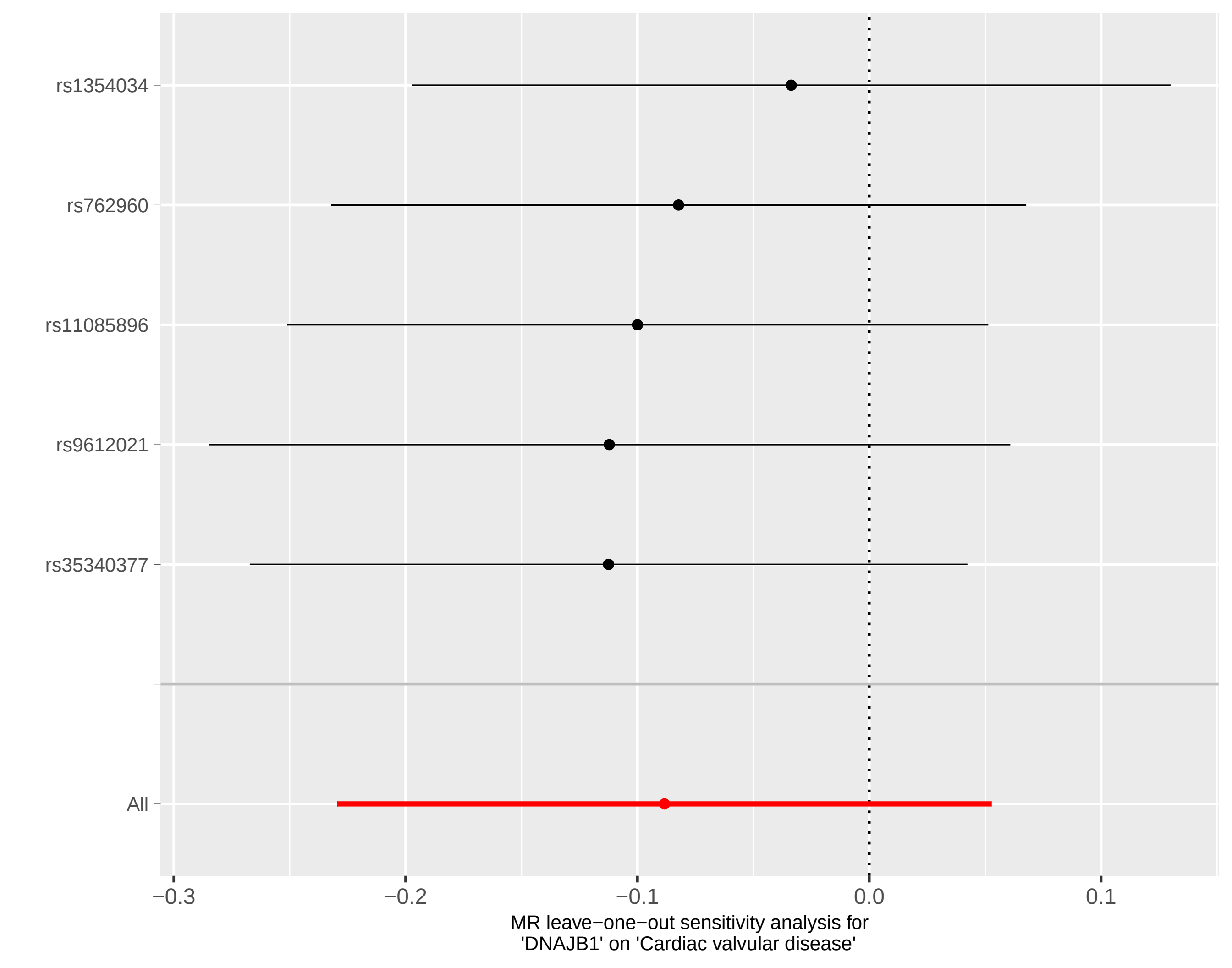
rs1354034
rs762960
rs11085896
rs9612021
rs35340377
All
−0.3
−0.2
−0.1
0.0
0.1
MR leave−one−out sensitivity analysis for
'DNAJB1' on 'Cardiac valvular disease'

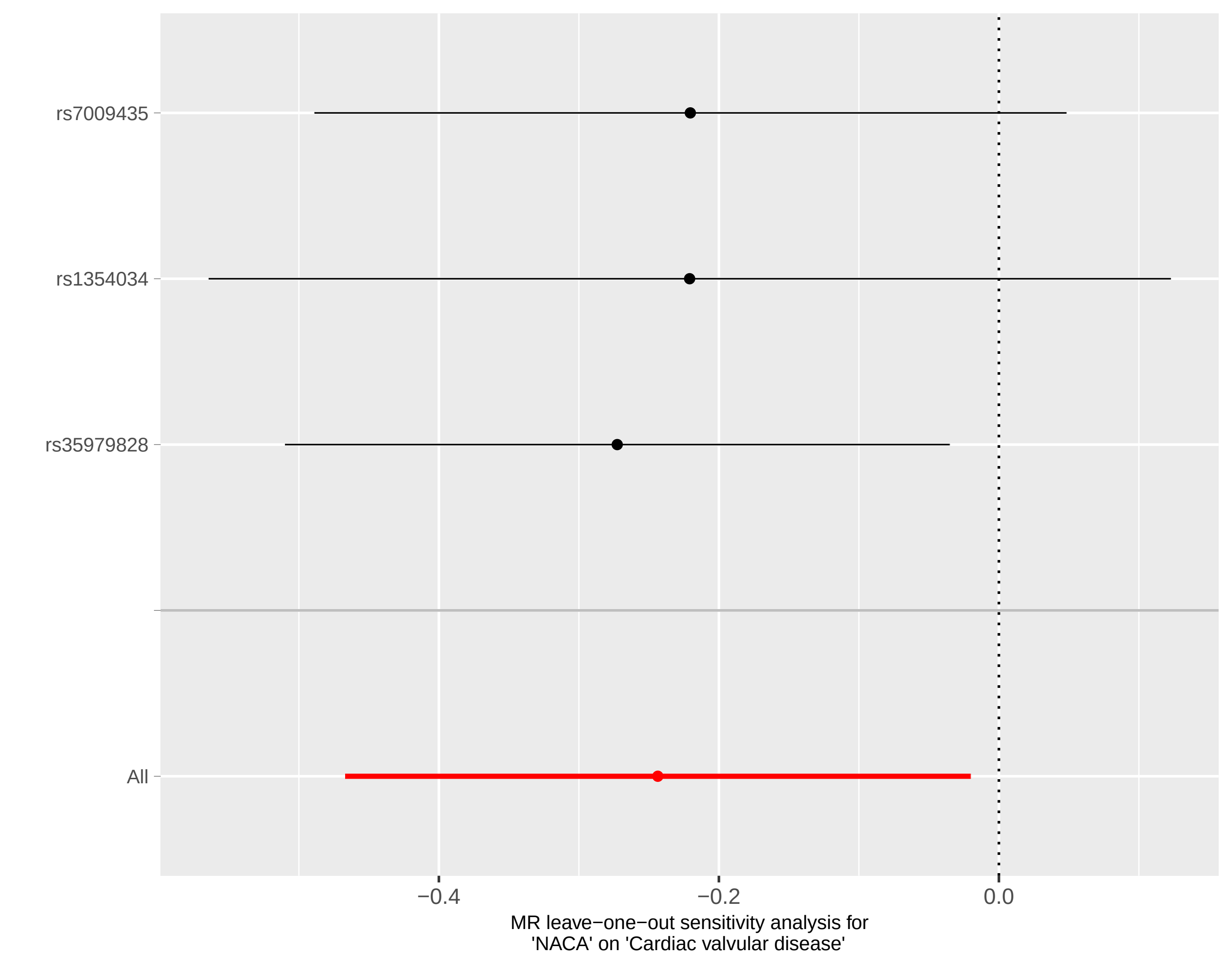
rs7009435
rs1354034
rs35979828
All
−0.4
−0.2
0.0
MR leave−one−out sensitivity analysis for
'NACA' on 'Cardiac valvular disease'

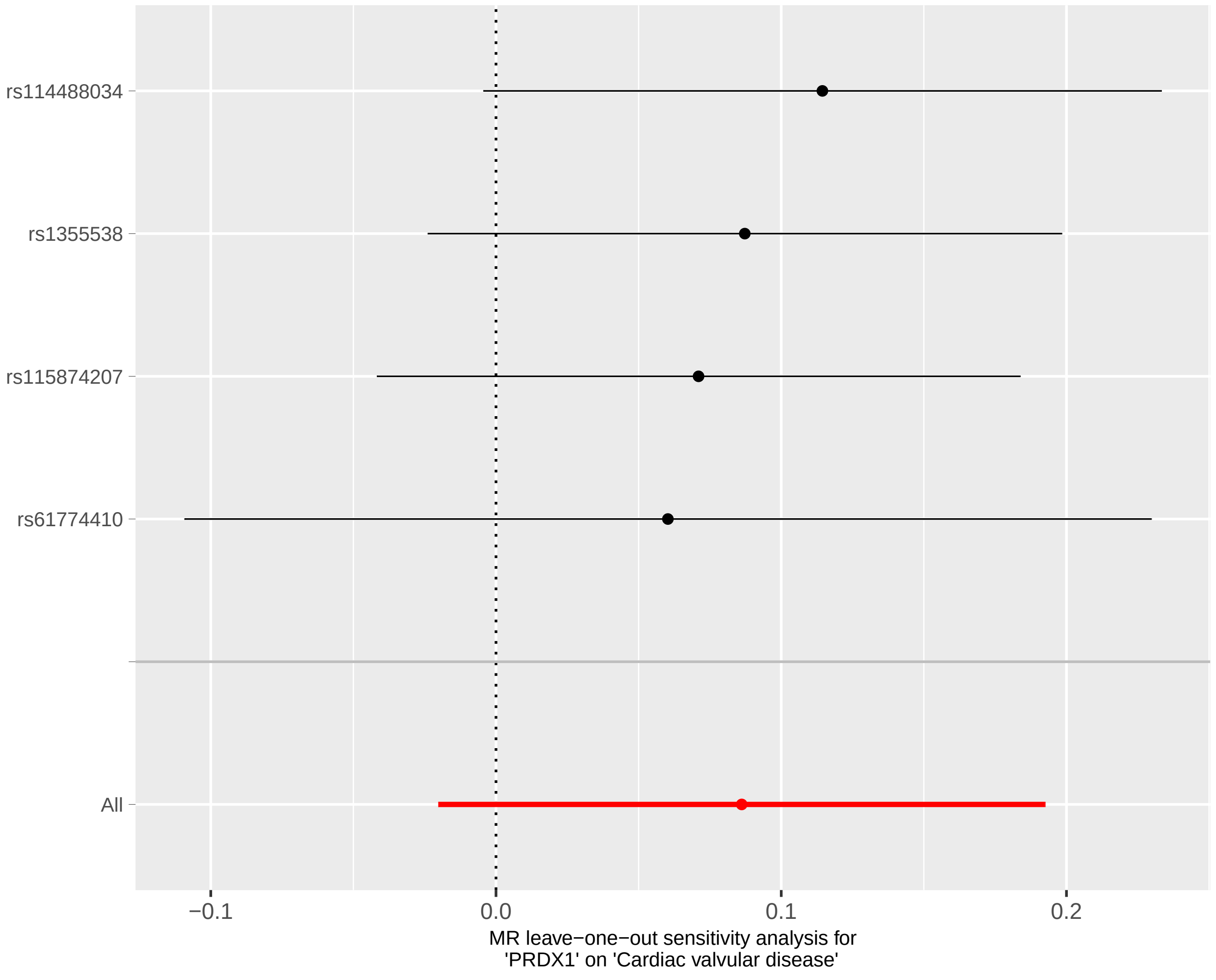
rs114488034
rs1355538
rs115874207
rs61774410
All
−0.1
0.0
0.1
0.2
MR leave−one−out sensitivity analysis for
'PRDX1' on 'Cardiac valvular disease'

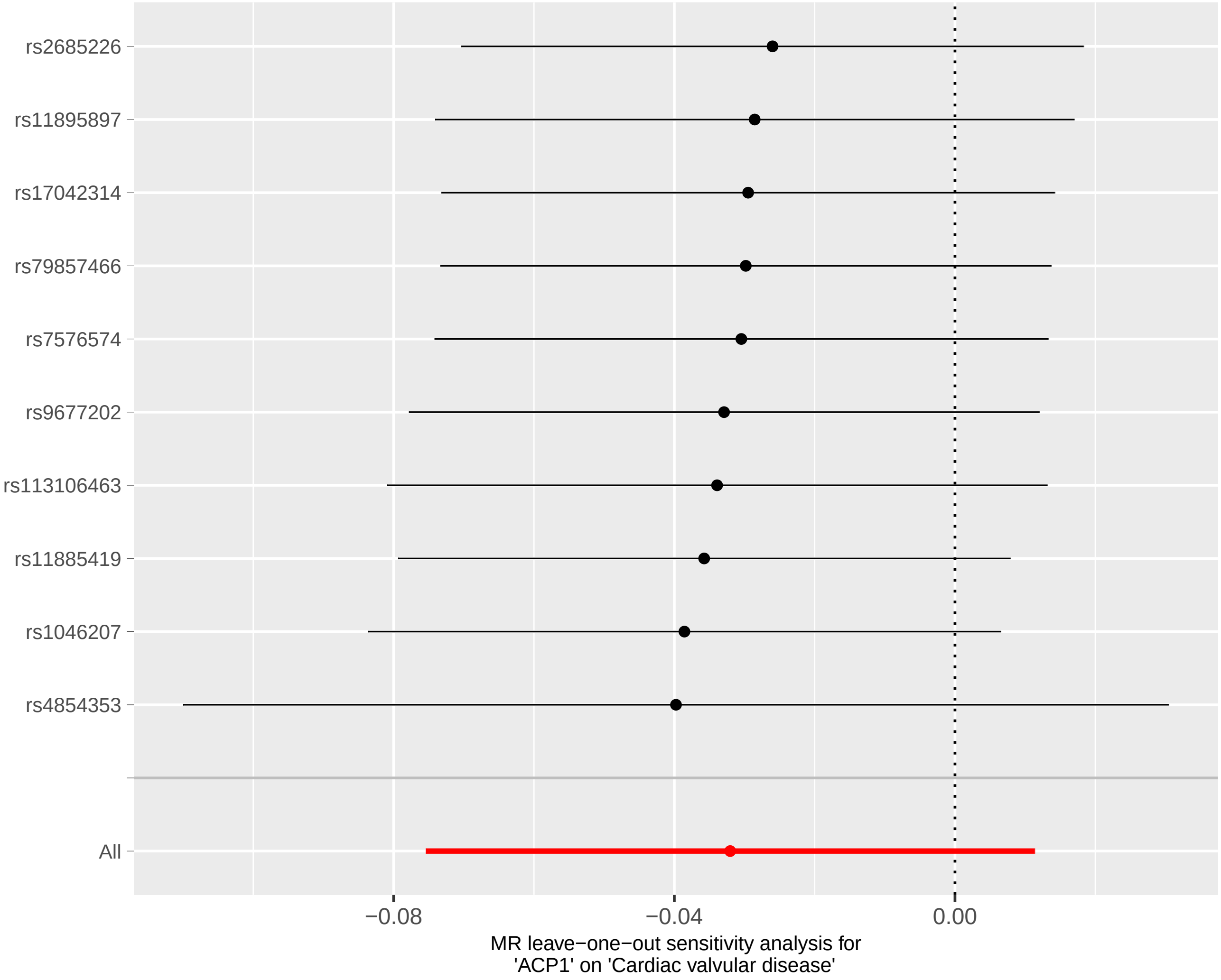
rs2685226
rs11895897
rs17042314
rs79857466
rs7576574
rs9677202
rs113106463
rs11885419
rs1046207
rs4854353
All
−0.08
−0.04
0.00
MR leave−one−out sensitivity analysis for
'ACP1' on 'Cardiac valvular disease'

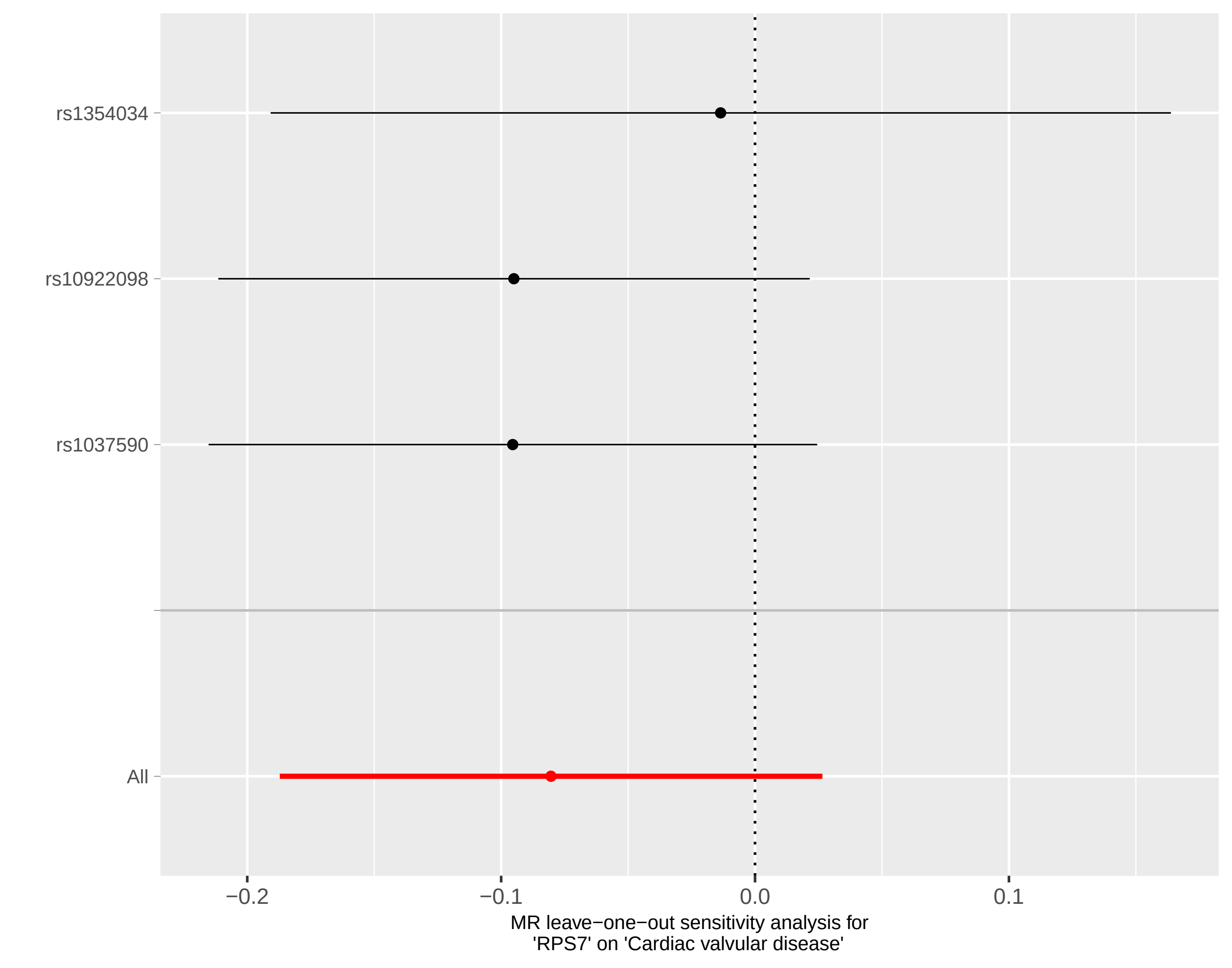
rs1354034
rs10922098
rs1037590
All
−0.2
−0.1
0.0
0.1
MR leave−one−out sensitivity analysis for
'RPS7' on 'Cardiac valvular disease'

Insufficient number of SNPs

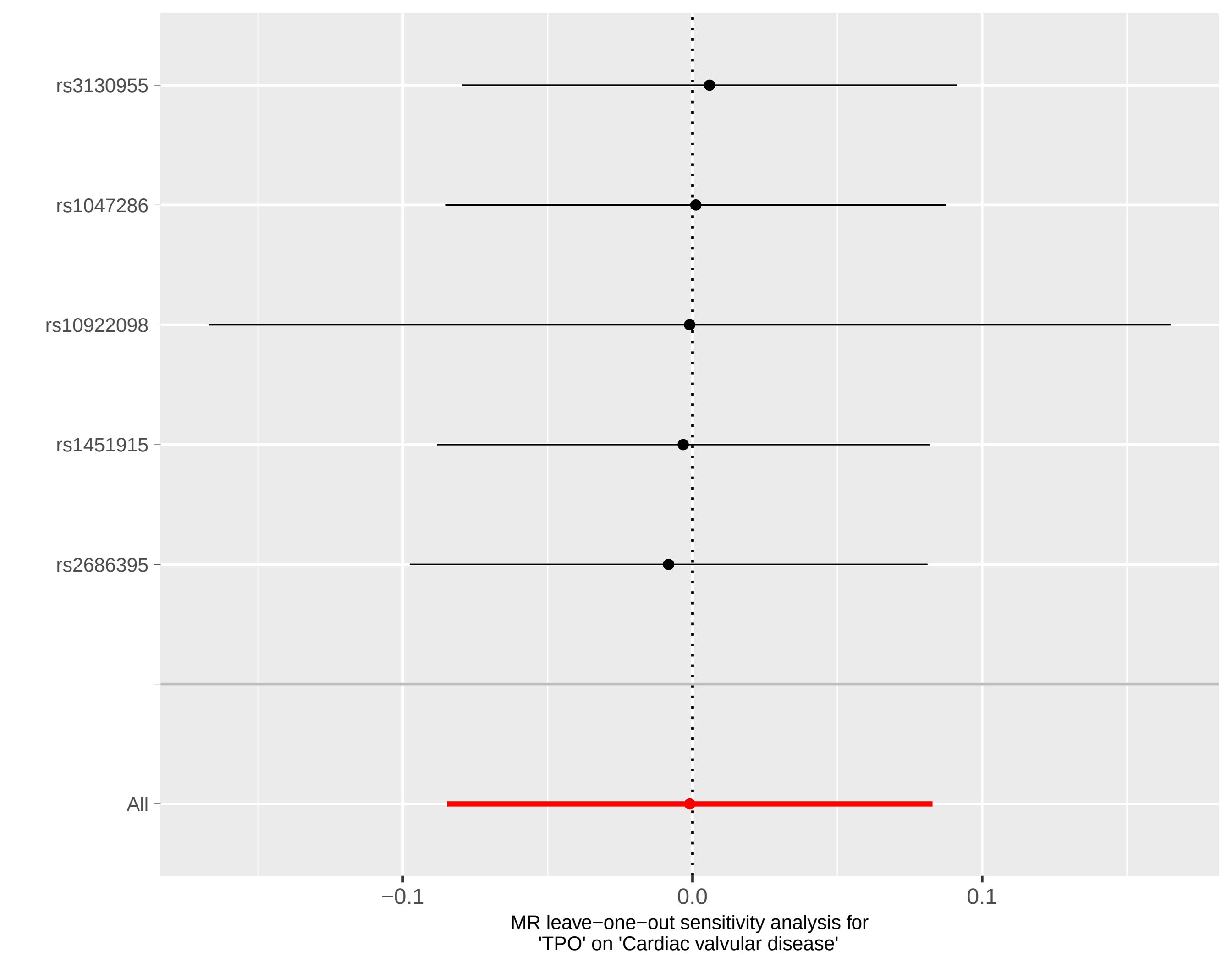
rs3130955
rs1047286
rs10922098
rs1451915
rs2686395
All
−0.1
0.0
0.1
MR leave−one−out sensitivity analysis for
'TPO' on 'Cardiac valvular disease'

Insufficient number of SNPs

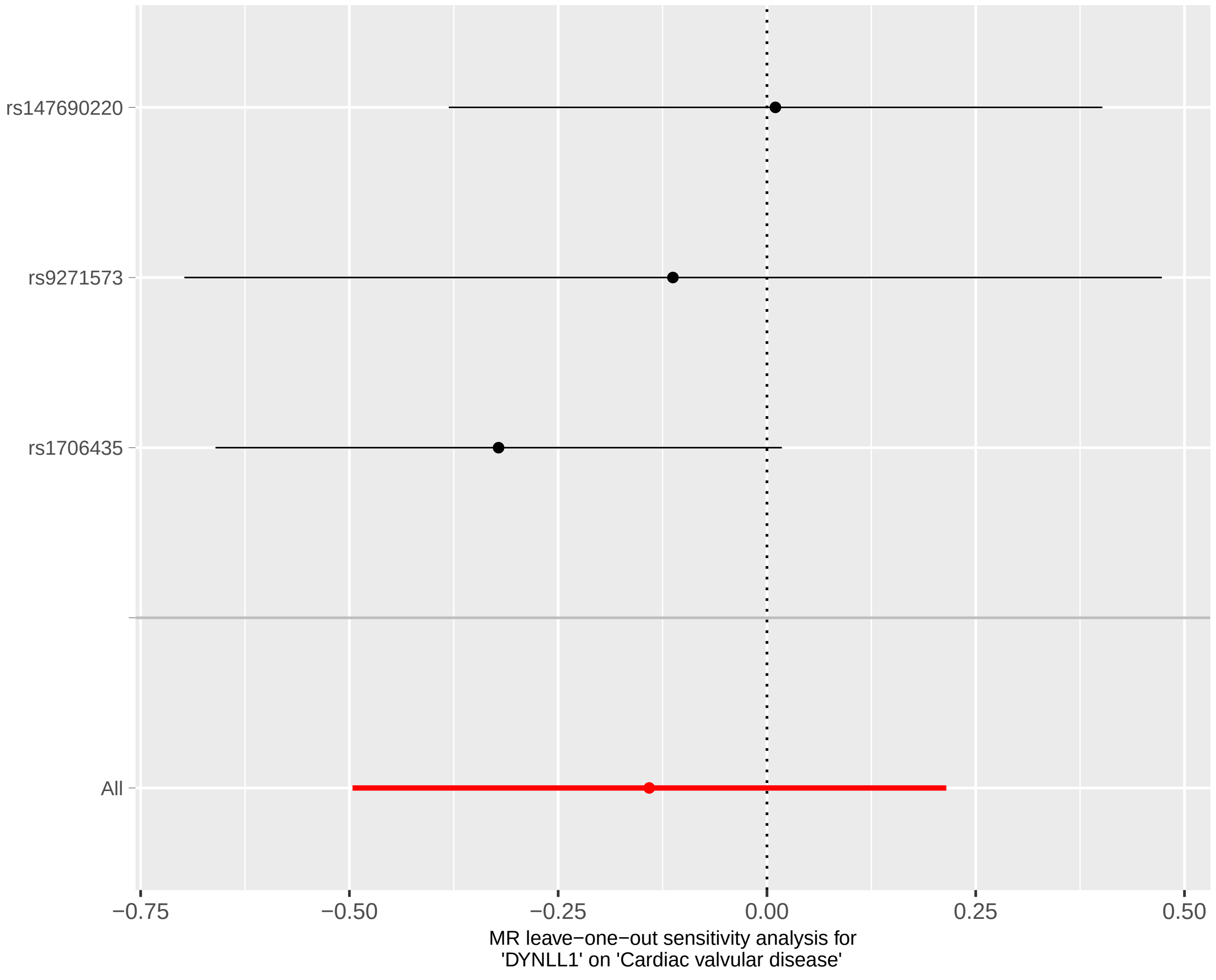
rs147690220
rs9271573
rs1706435
All
−0.75
−0.50
−0.25
0.00
0.25
0.50
MR leave−one−out sensitivity analysis for
'DYNLL1' on 'Cardiac valvular disease'

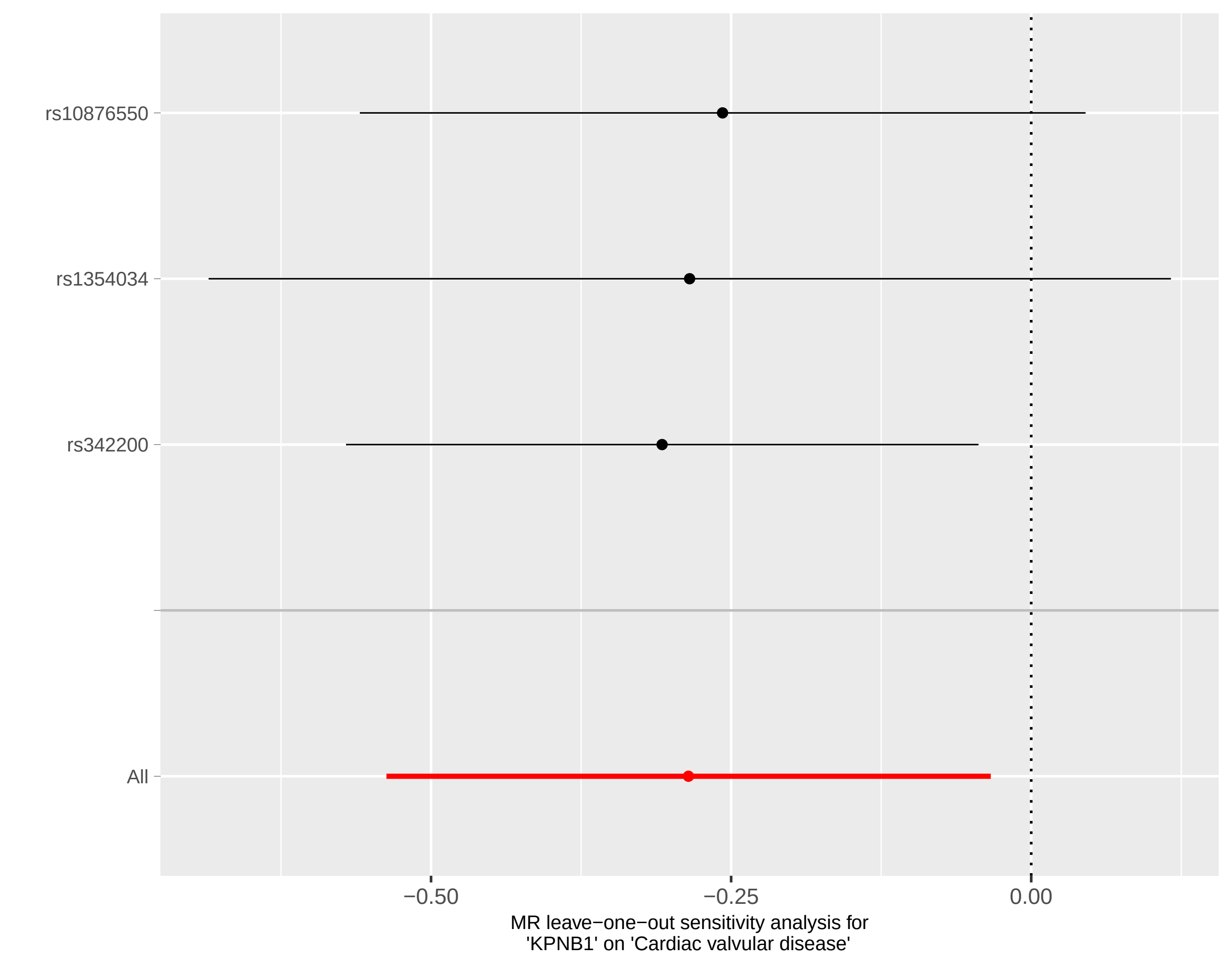
rs10876550
rs1354034
rs342200
All
−0.50
−0.25
0.00
MR leave−one−out sensitivity analysis for
'KPNB1' on 'Cardiac valvular disease'

Insufficient number of SNPs

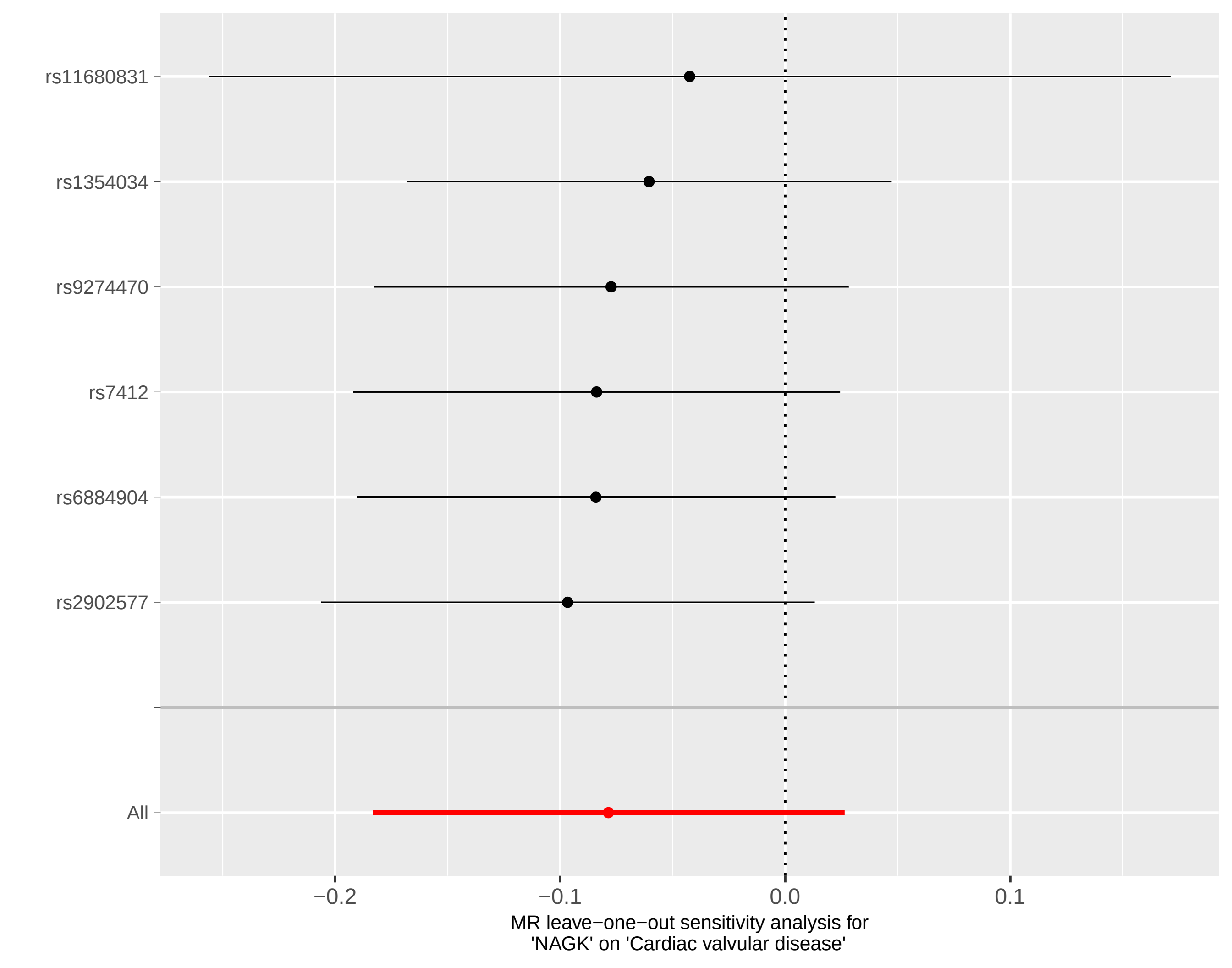

rs11680831
rs1354034
rs9274470
rs7412
rs6884904
rs2902577
All
−0.2
−0.1
0.0
0.1
MR leave−one−out sensitivity analysis for
'NAGK' on 'Cardiac valvular disease'

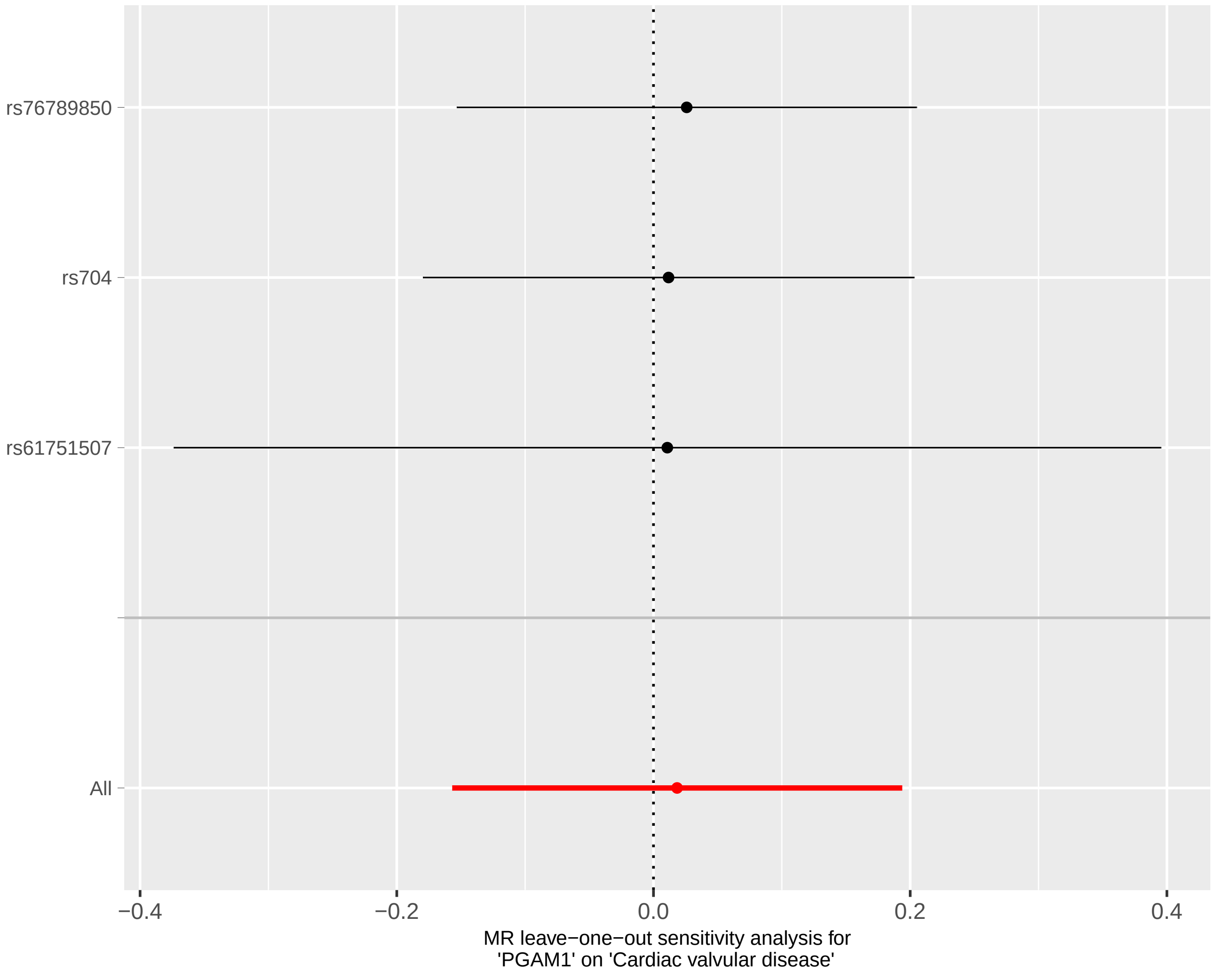
rs76789850
rs704
rs61751507
All
−0.4
−0.2
0.0
0.2
0.4
MR leave−one−out sensitivity analysis for
'PGAM1' on 'Cardiac valvular disease'

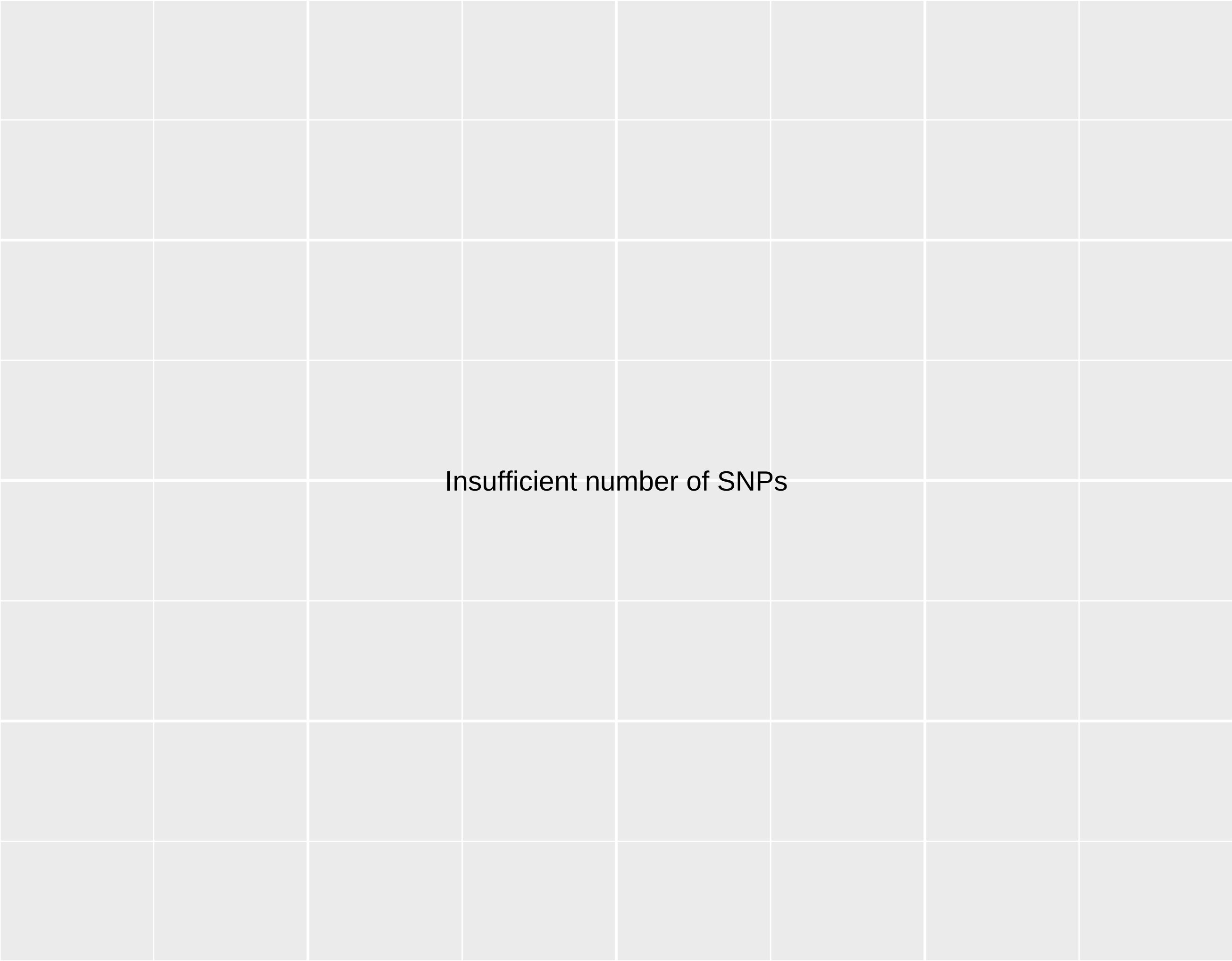
Insufficient number of SNPs

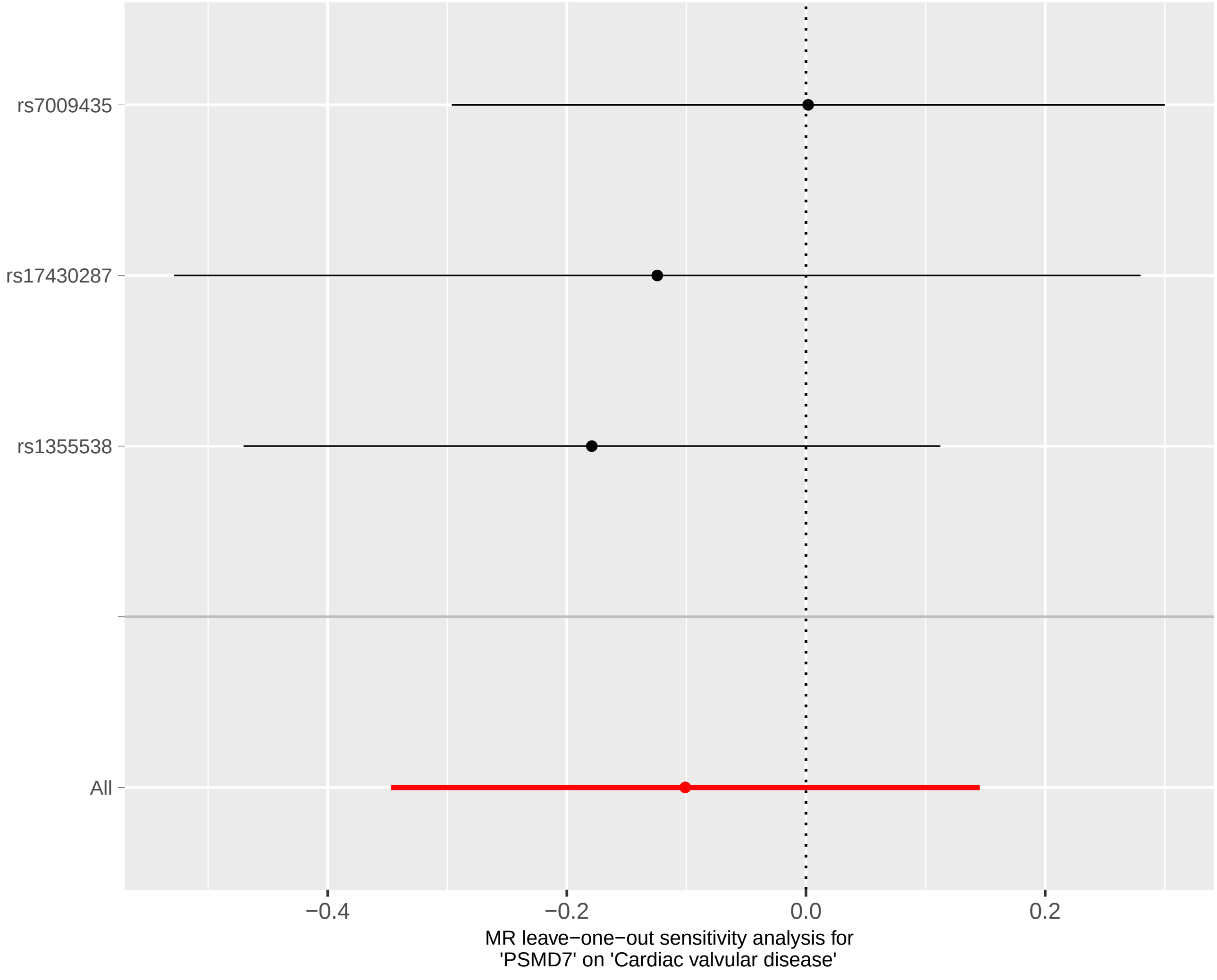

rs7009435
rs17430287
rs1355538
All
−0.4
−0.2
0.0
0.2
MR leave−one−out sensitivity analysis for
'PSMD7' on 'Cardiac valvular disease'

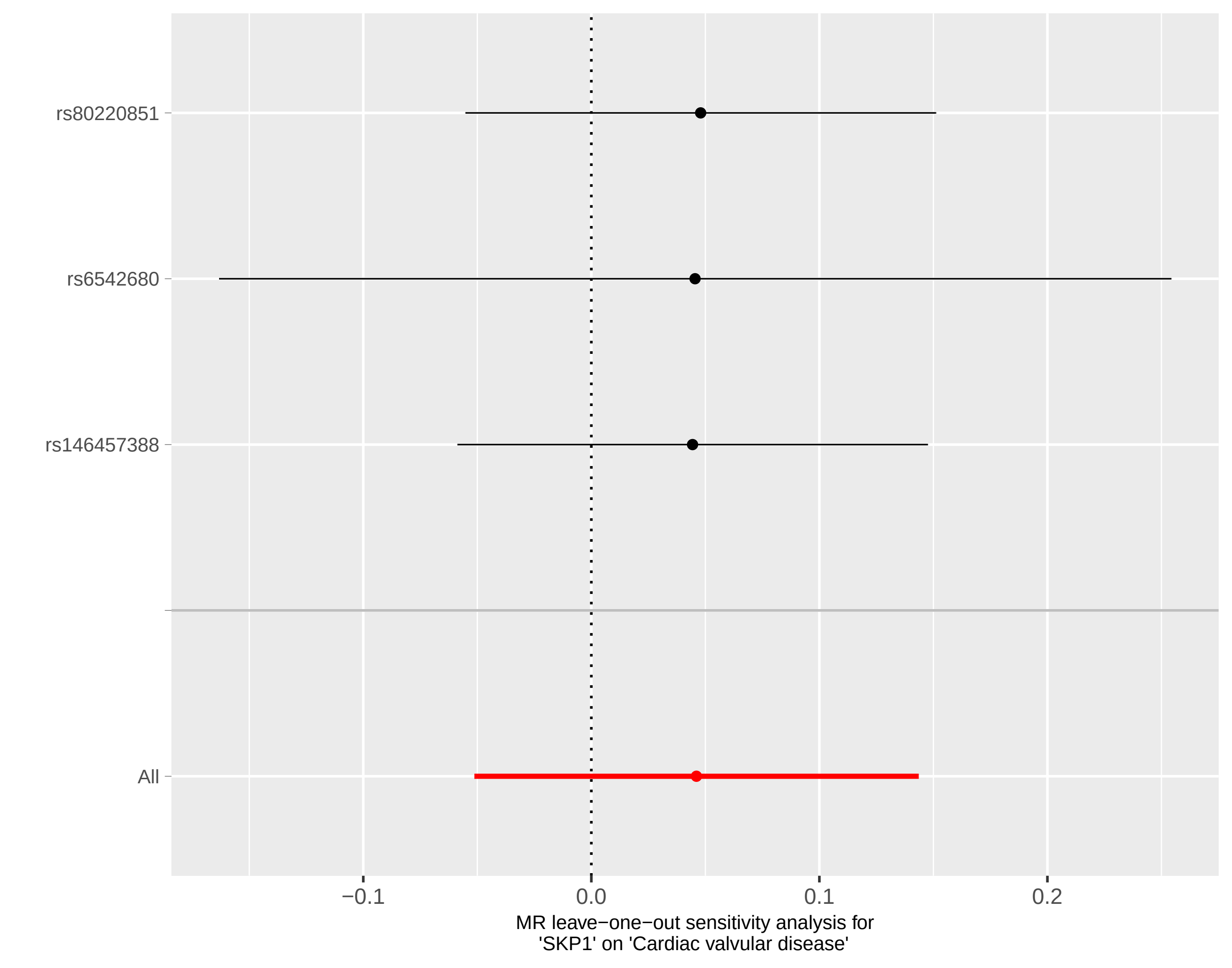
rs80220851
rs6542680
rs146457388
All
−0.1
0.0
0.1
0.2
MR leave−one−out sensitivity analysis for
'SKP1' on 'Cardiac valvular disease'

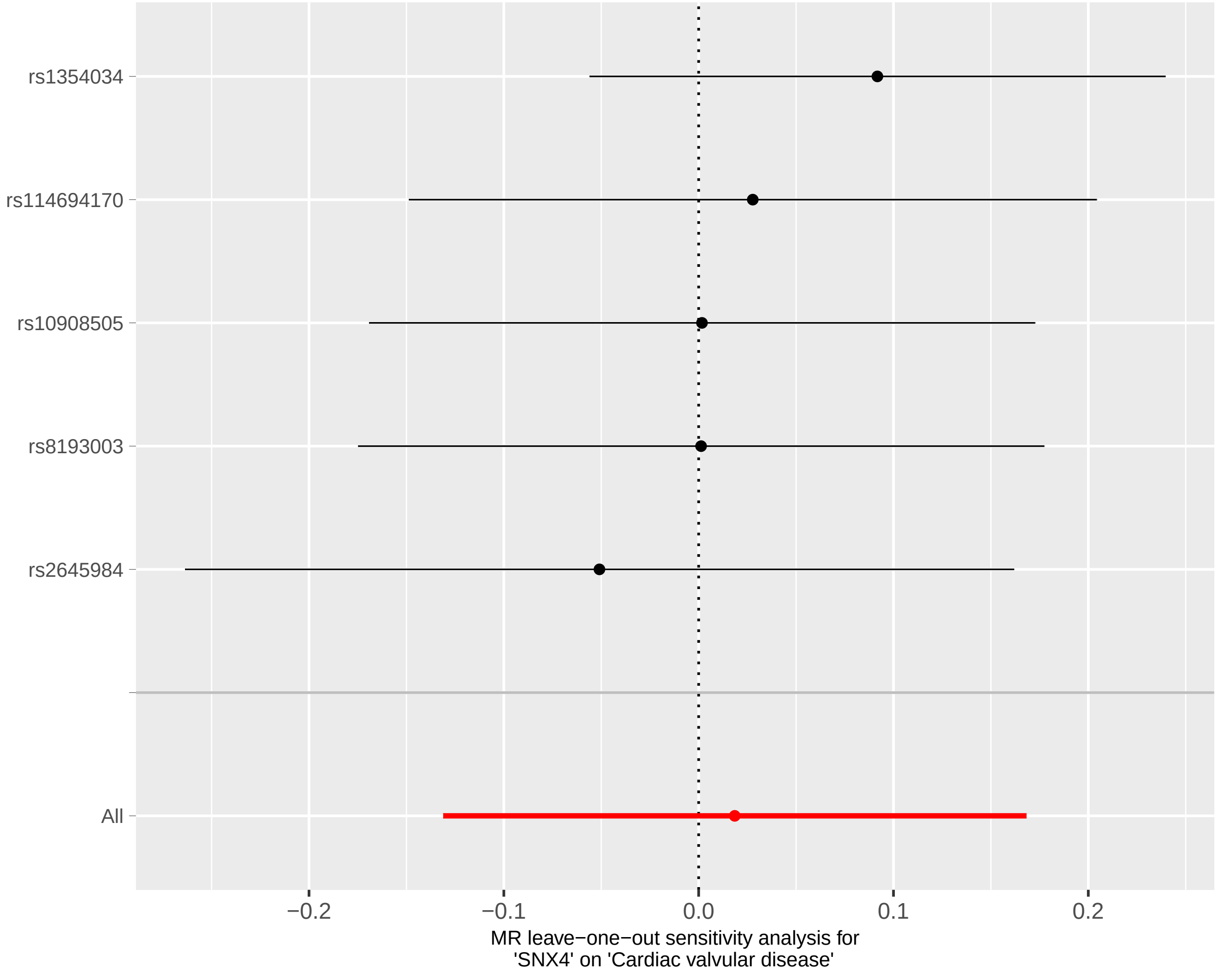
rs1354034
rs114694170
rs10908505
rs8193003
rs2645984
All
−0.2
−0.1
0.0
0.1
0.2
MR leave−one−out sensitivity analysis for
'SNX4' on 'Cardiac valvular disease'

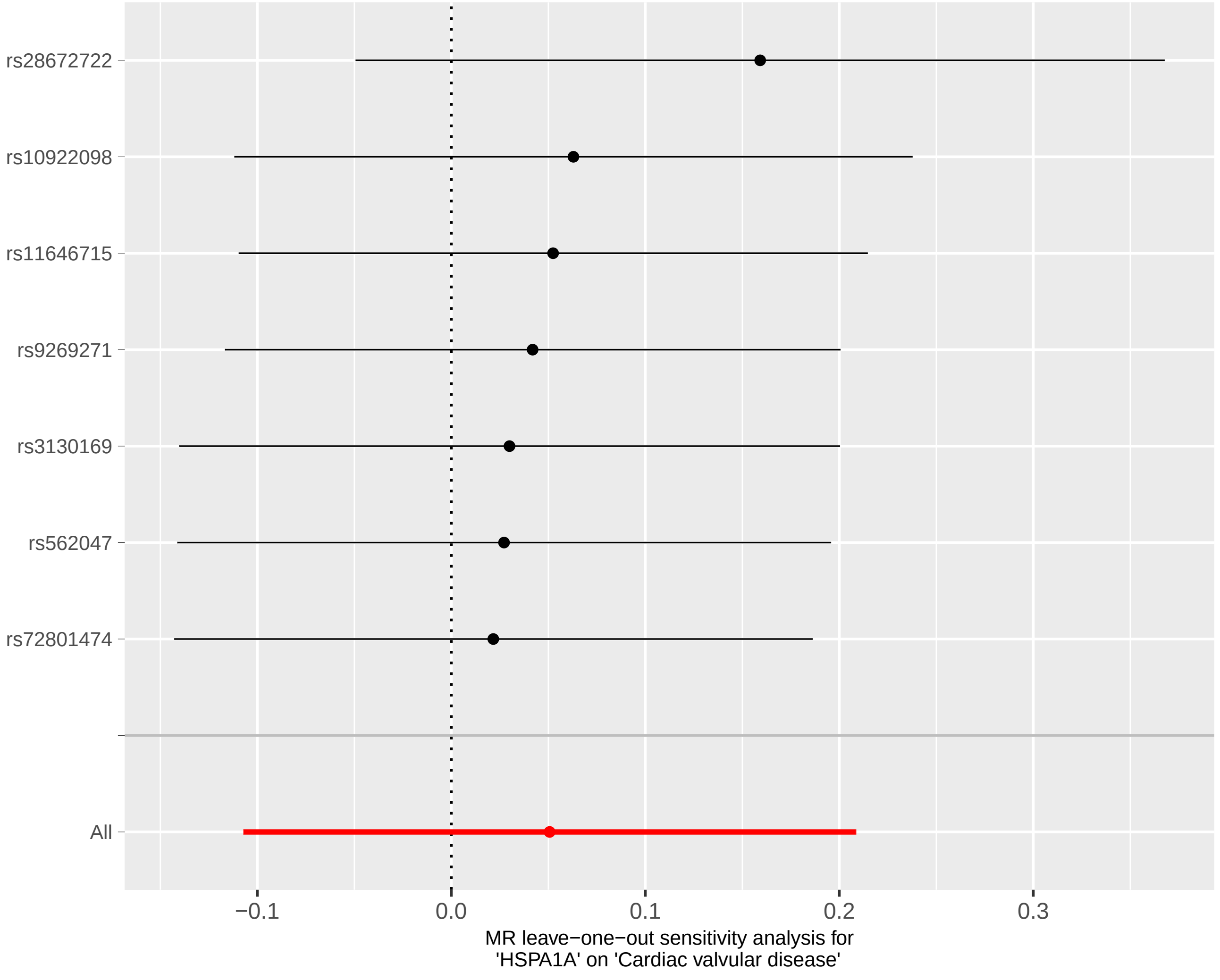
rs28672722
rs10922098
rs11646715
rs9269271
rs3130169
rs562047
rs72801474
All
−0.1
0.0
0.1
0.2
0.3
MR leave−one−out sensitivity analysis for
'HSPA1A' on 'Cardiac valvular disease'

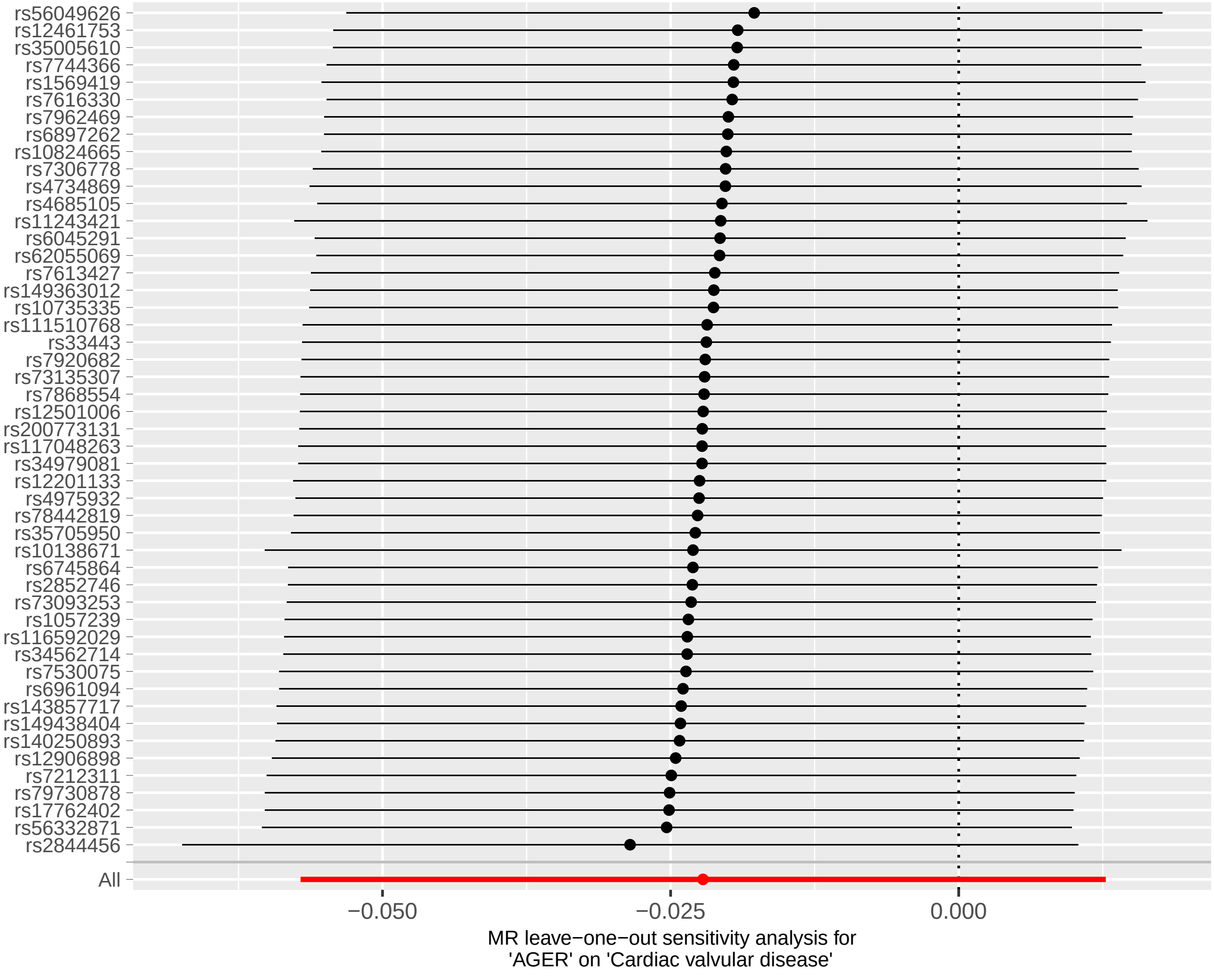
rs56049626
rs12461753
rs35005610
rs7744366
rs1569419
rs7616330
rs7962469
rs6897262
rs10824665
rs7306778
rs4734869
rs4685105
rs11243421
rs6045291
rs62055069
rs7613427
rs149363012
rs10735335
rs111510768
rs33443
rs7920682
rs73135307
rs7868554
rs12501006
rs200773131
rs117048263
rs34979081
rs12201133
rs4975932
rs78442819
rs35705950
rs10138671
rs6745864
rs2852746
rs73093253
rs1057239
rs116592029
rs34562714
rs7530075
rs6961094
rs143857717
rs149438404
rs140250893
rs12906898
rs7212311
rs79730878
rs17762402
rs56332871
rs2844456
All
−0.050
−0.025
0.000
MR leave−one−out sensitivity analysis for
'AGER' on 'Cardiac valvular disease'

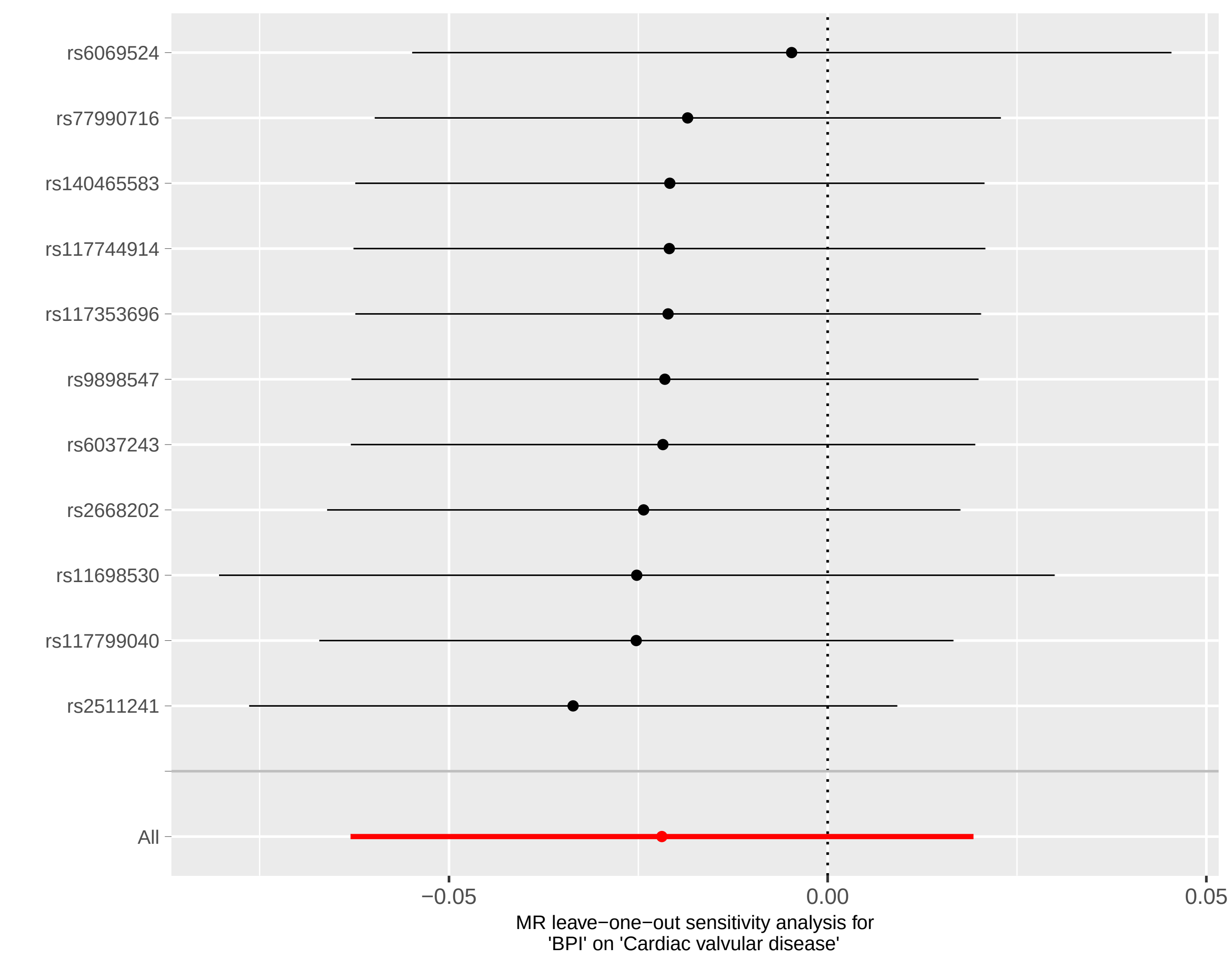
rs6069524
rs77990716
rs140465583
rs117744914
rs117353696
rs9898547
rs6037243
rs2668202
rs11698530
rs117799040
rs2511241
All
−0.05
0.00
0.05
MR leave−one−out sensitivity analysis for
'BPI' on 'Cardiac valvular disease'

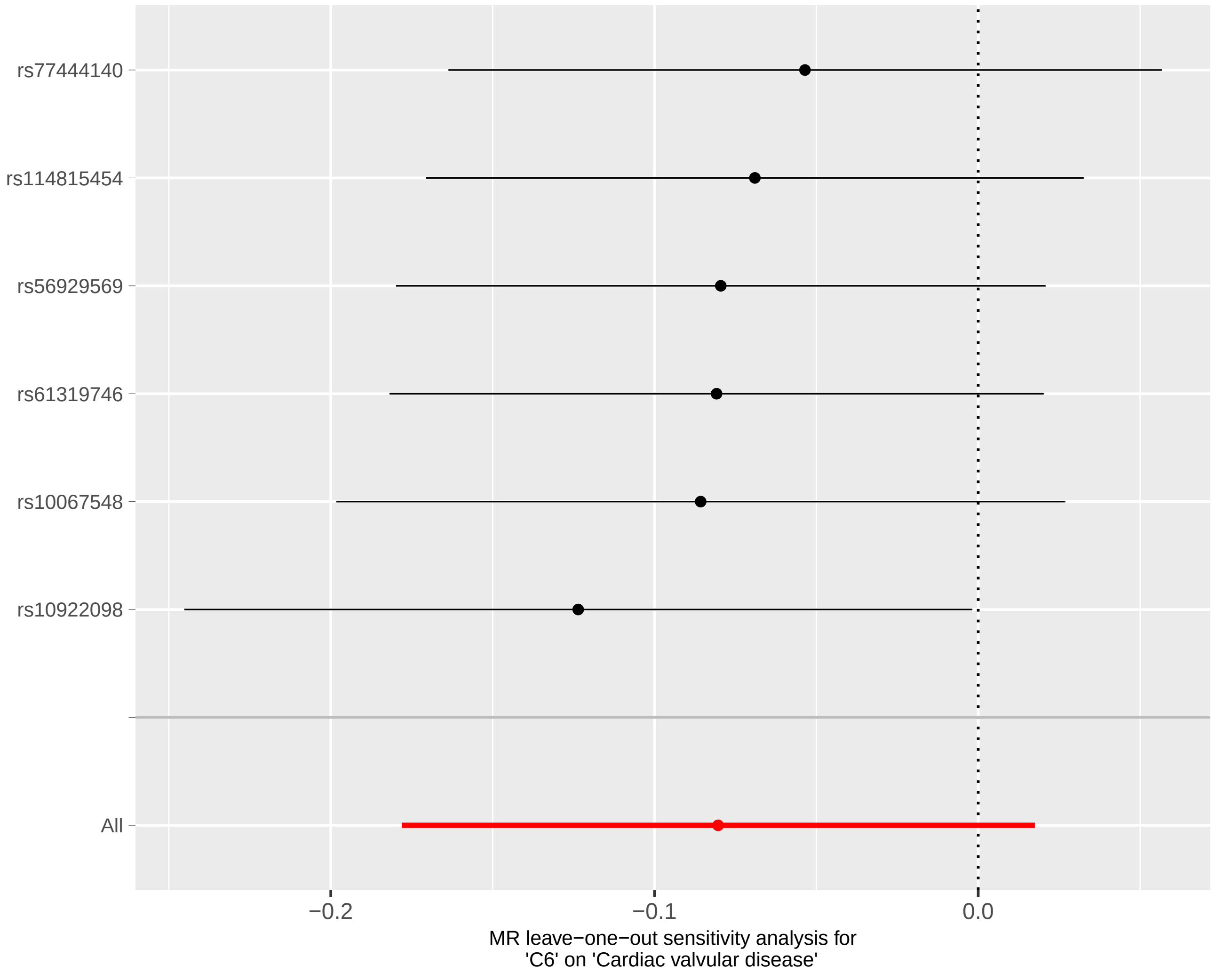
rs77444140
rs114815454
rs56929569
rs61319746
rs10067548
rs10922098
All
−0.2
−0.1
0.0
MR leave−one−out sensitivity analysis for
'C6' on 'Cardiac valvular disease'

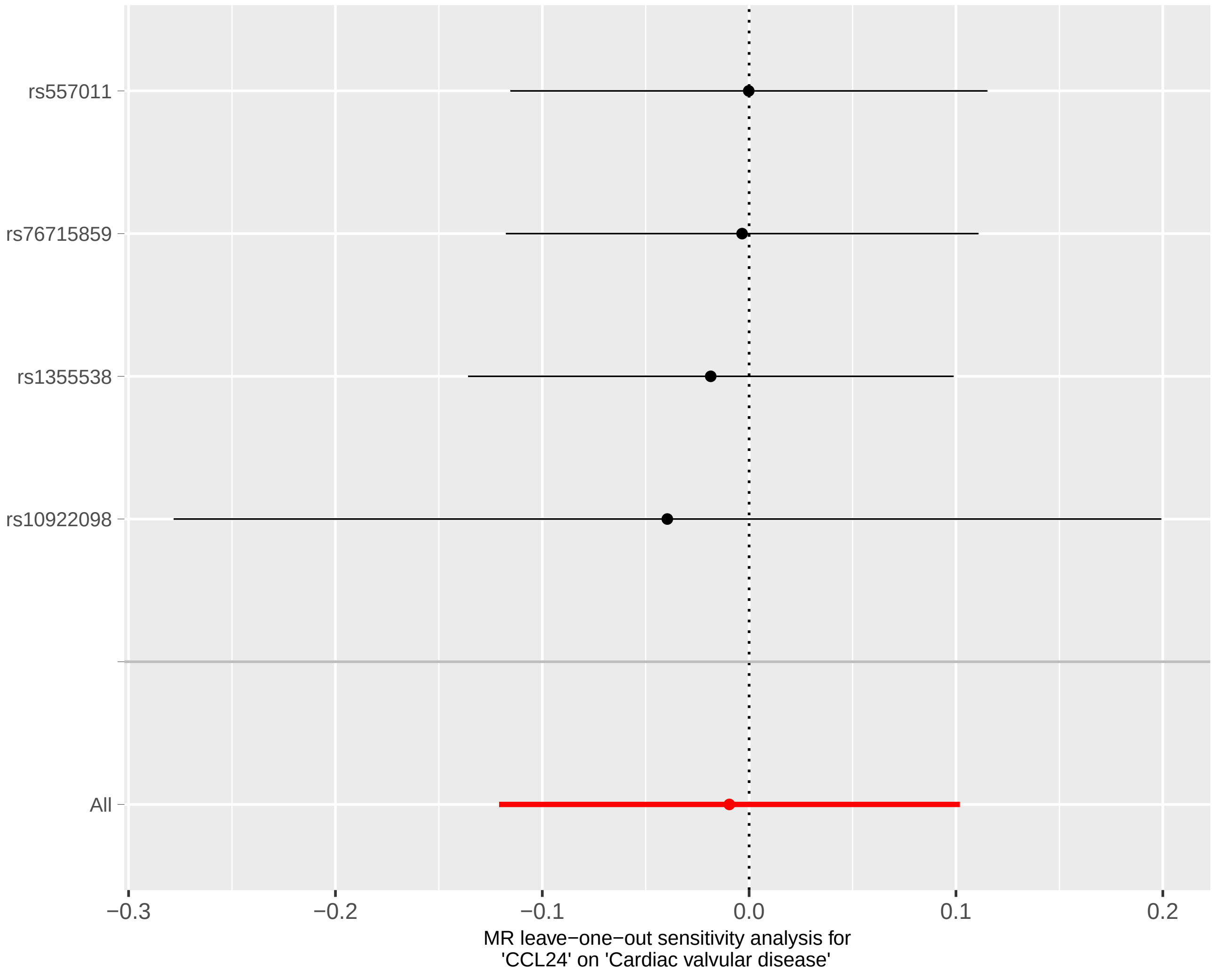
rs557011
rs76715859
rs1355538
rs10922098
All
−0.3
−0.2
−0.1
0.0
0.1
0.2
MR leave−one−out sensitivity analysis for
'CCL24' on 'Cardiac valvular disease'

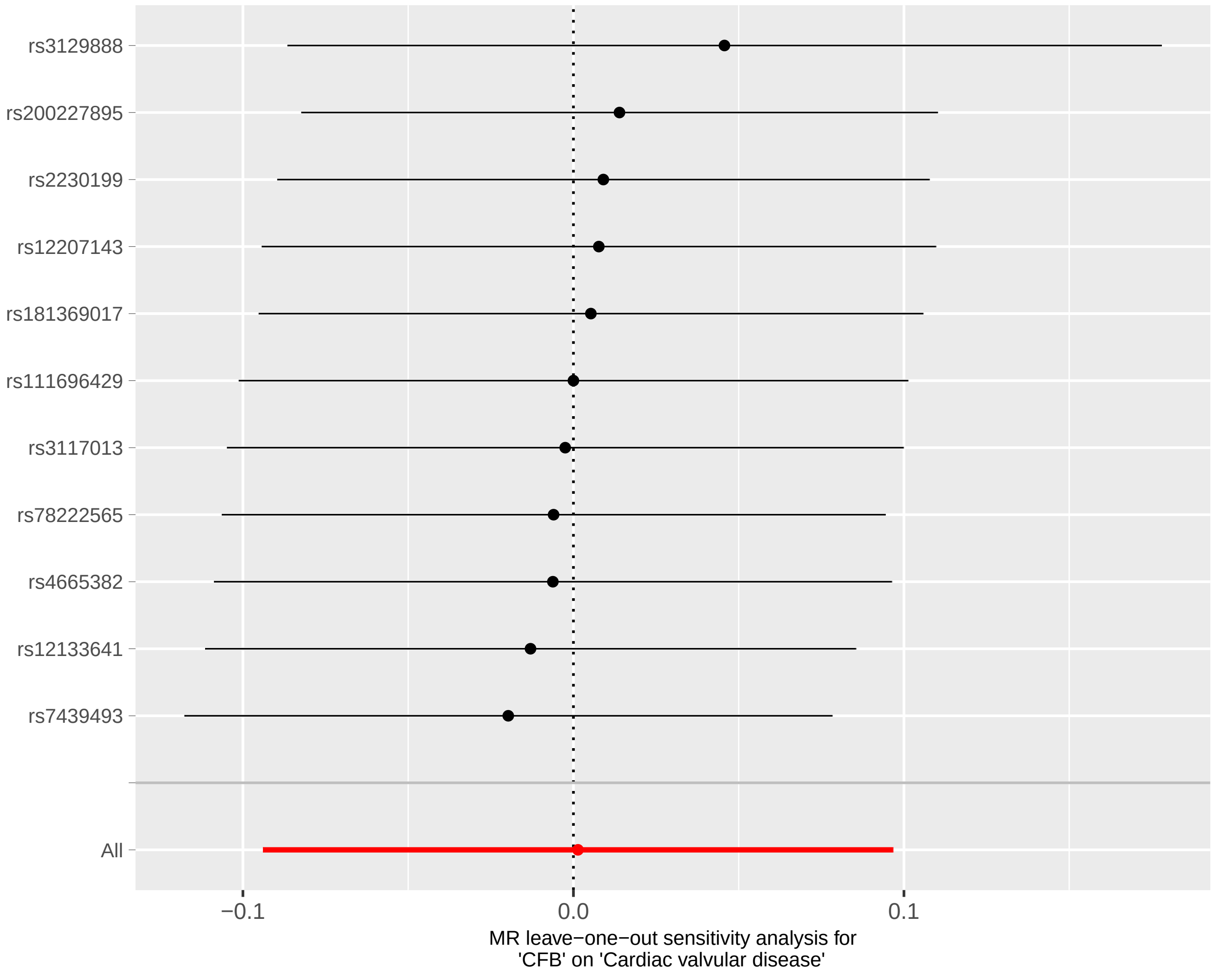
rs3129888
rs200227895
rs2230199
rs12207143
rs181369017
rs111696429
rs3117013
rs78222565
rs4665382
rs12133641
rs7439493
All
−0.1
0.0
0.1
MR leave−one−out sensitivity analysis for
'CFB' on 'Cardiac valvular disease'

Insufficient number of SNPs

Insufficient number of SNPs

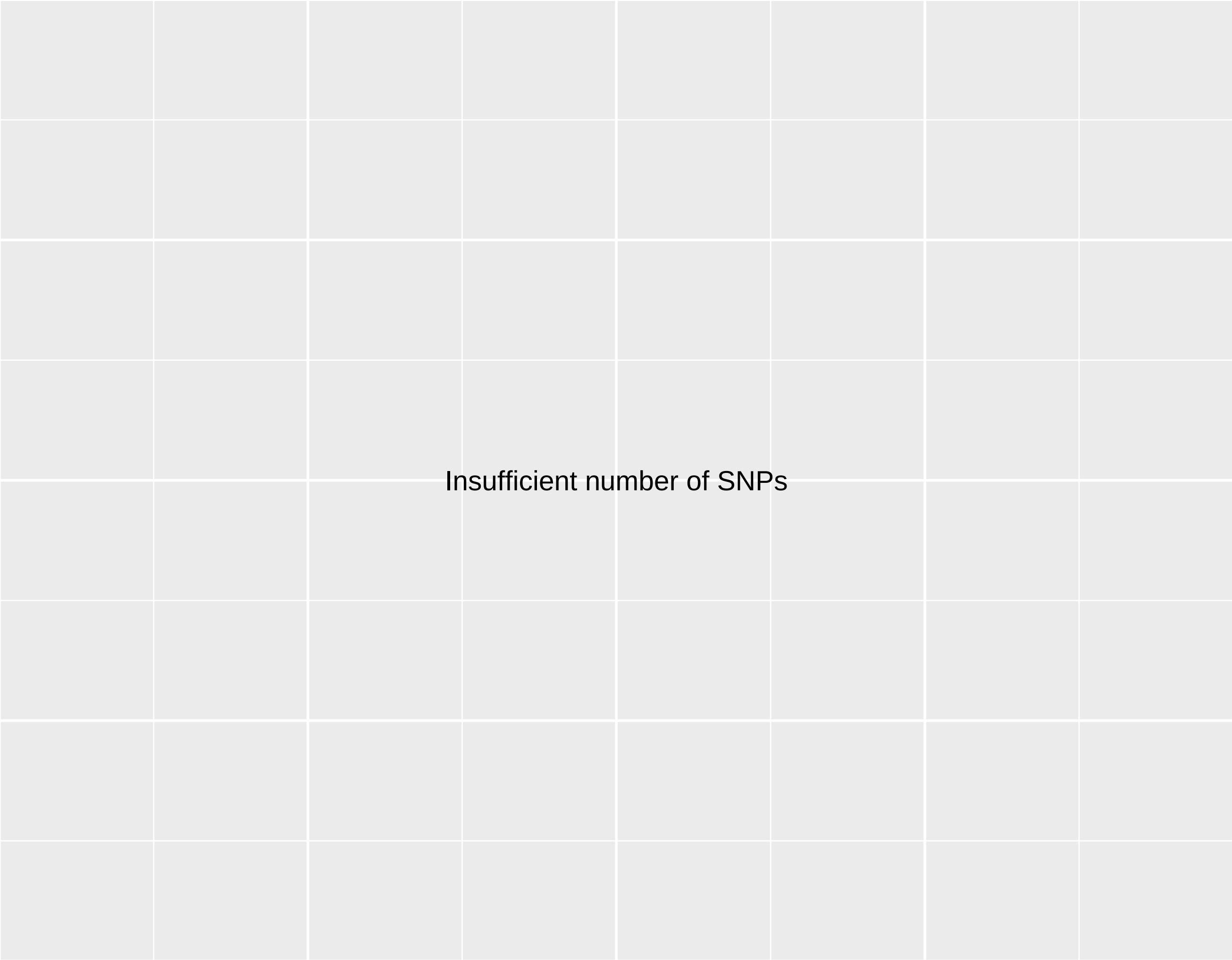
Insufficient number of SNPs

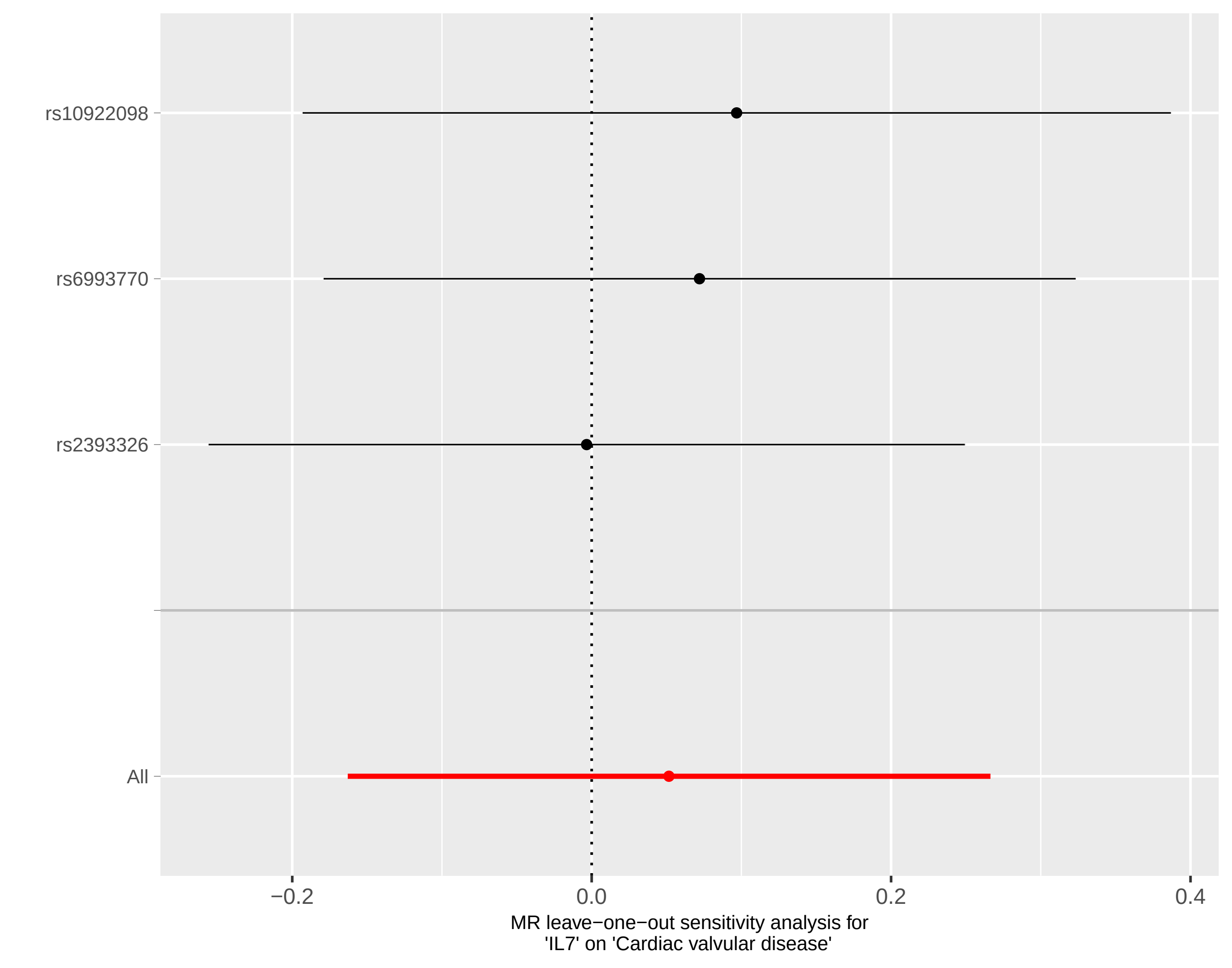
rs10922098
rs6993770
rs2393326
All
−0.2
0.0
0.2
0.4
MR leave−one−out sensitivity analysis for
'IL7' on 'Cardiac valvular disease'

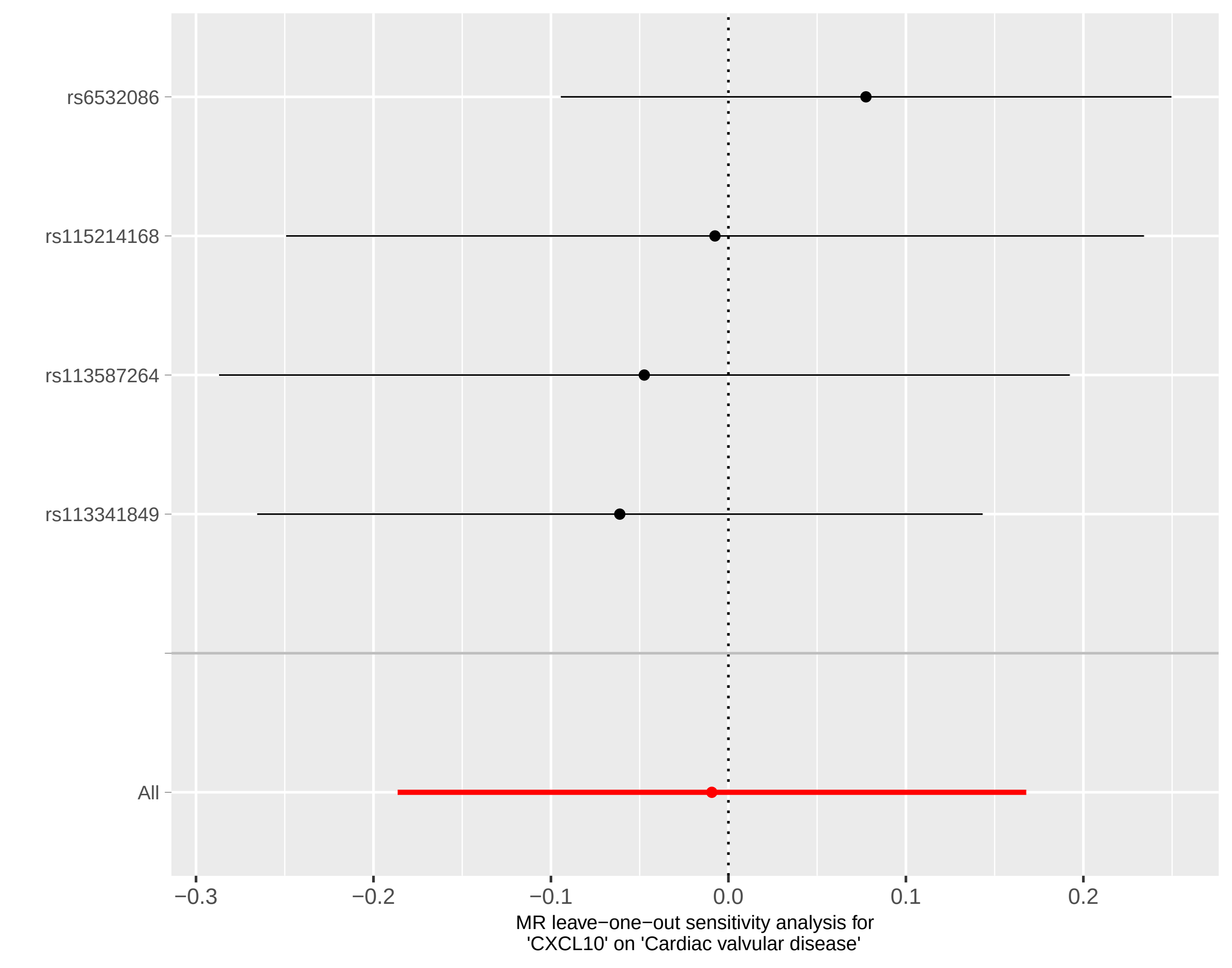
rs6532086
rs115214168
rs113587264
rs113341849
All
−0.3
−0.2
−0.1
0.0
0.1
0.2
MR leave−one−out sensitivity analysis for
'CXCL10' on 'Cardiac valvular disease'

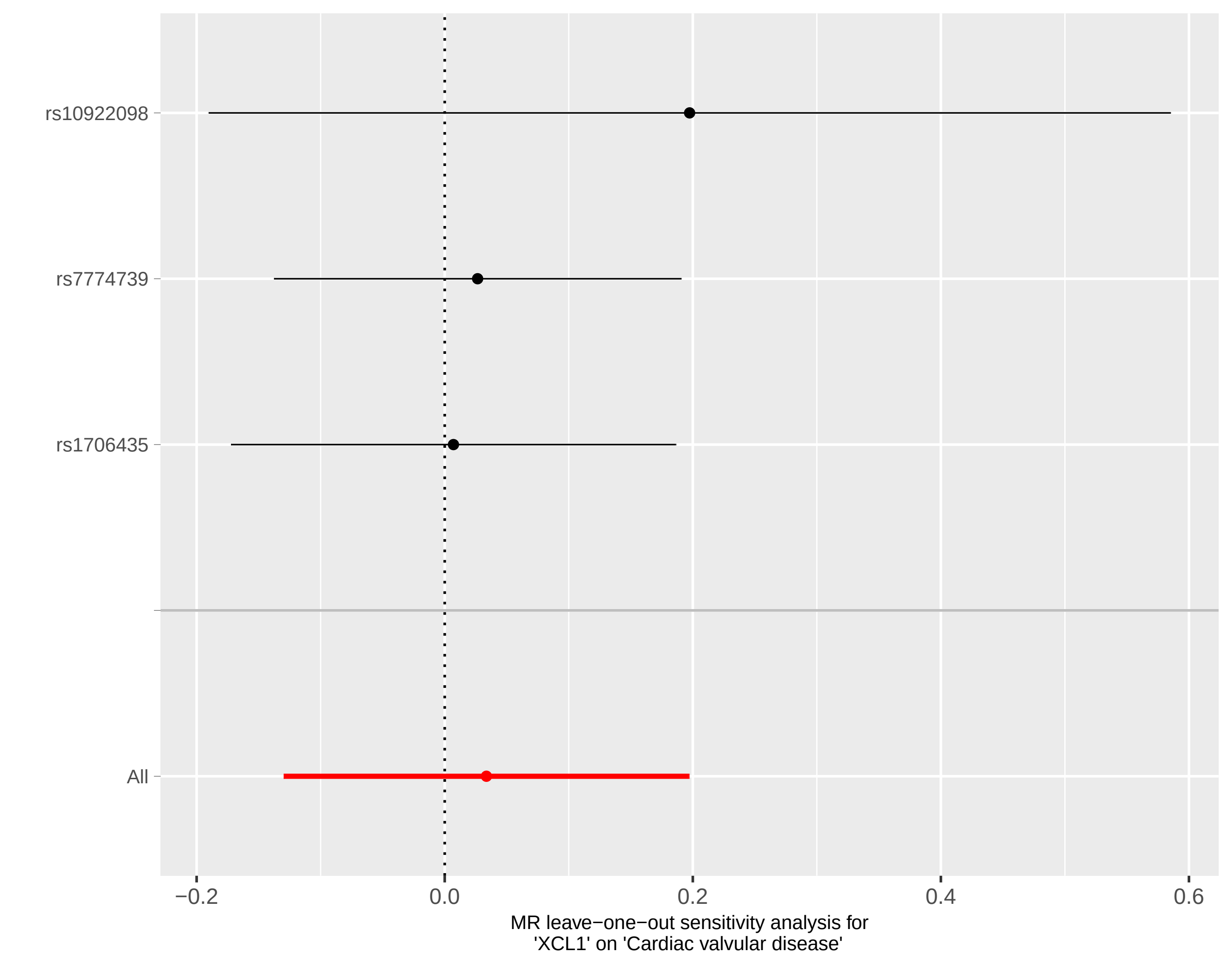

rs10922098
rs7774739
rs1706435
All
−0.2
0.0
0.2
0.4
0.6
MR leave−one−out sensitivity analysis for
'XCL1' on 'Cardiac valvular disease'

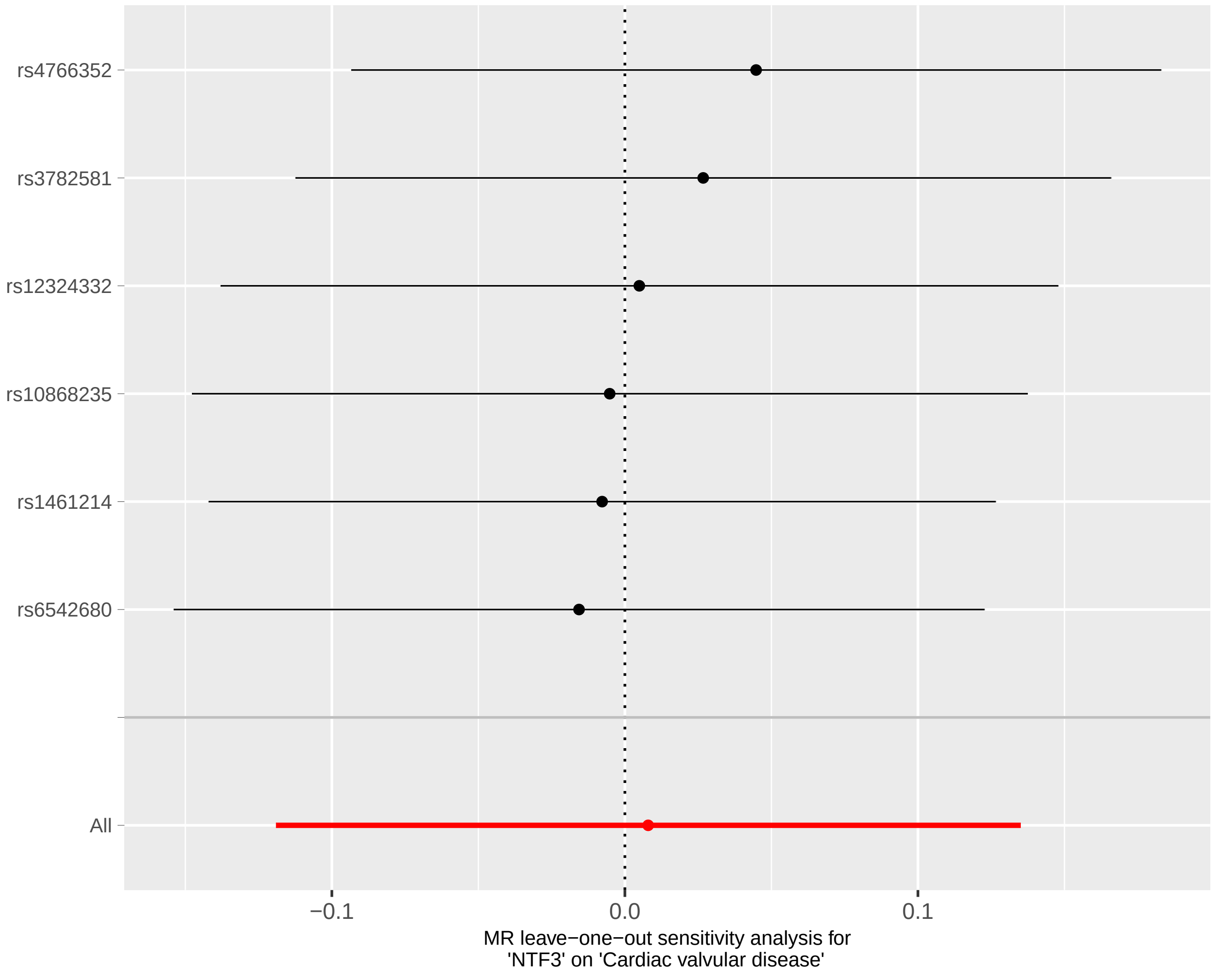

rs4766352
rs3782581
rs12324332
rs10868235
rs1461214
rs6542680
All
−0.1
0.0
0.1
MR leave−one−out sensitivity analysis for
'NTF3' on 'Cardiac valvular disease'

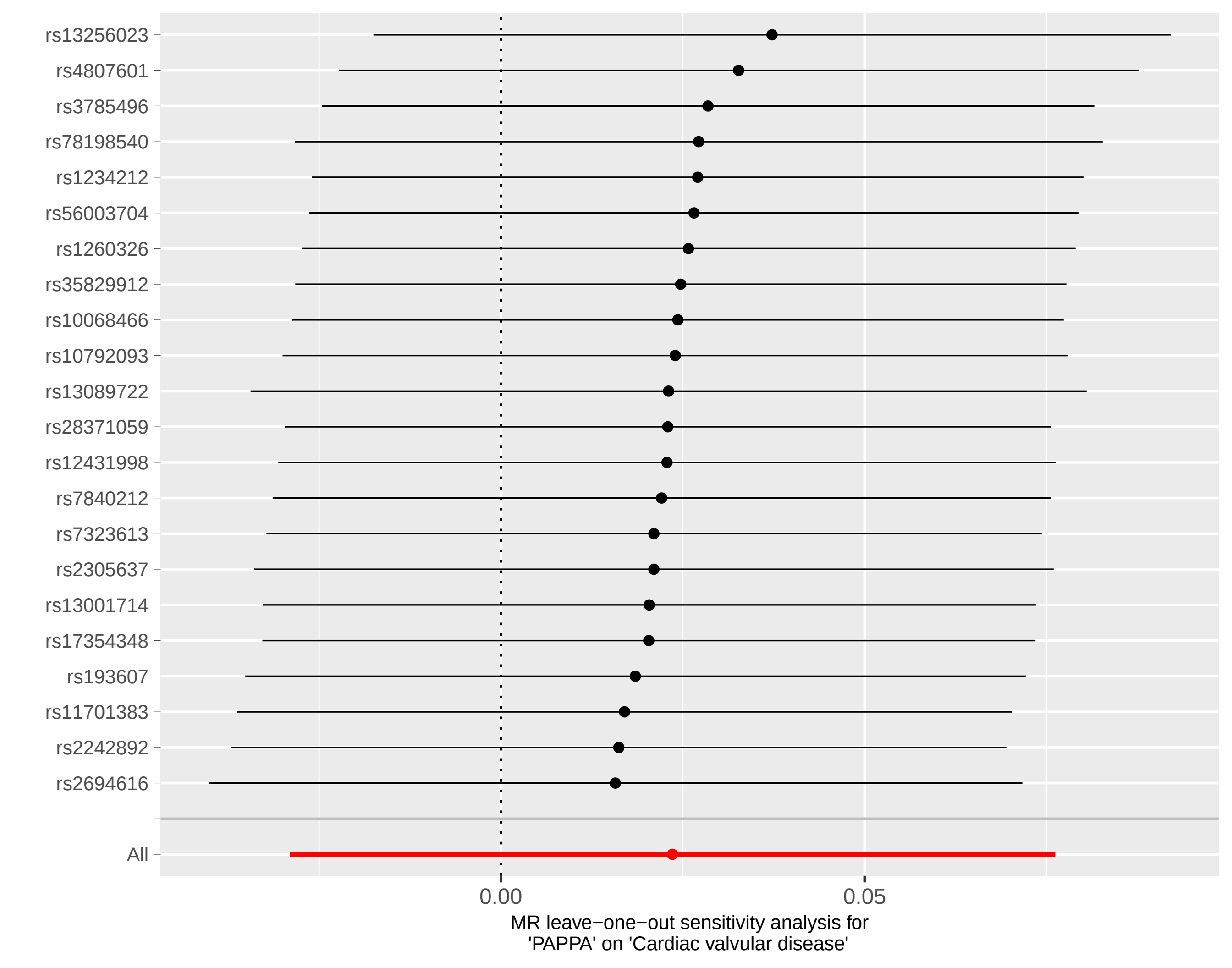
rs13256023
rs4807601
rs3785496
rs78198540
rs1234212
rs56003704
rs1260326
rs35829912
rs10068466
rs10792093
rs13089722
rs28371059
rs12431998
rs7840212
rs7323613
rs2305637
rs13001714
rs17354348
rs193607
rs11701383
rs2242892
rs2694616
All
0.00
0.05
MR leave−one−out sensitivity analysis for
'PAPPA' on 'Cardiac valvular disease'

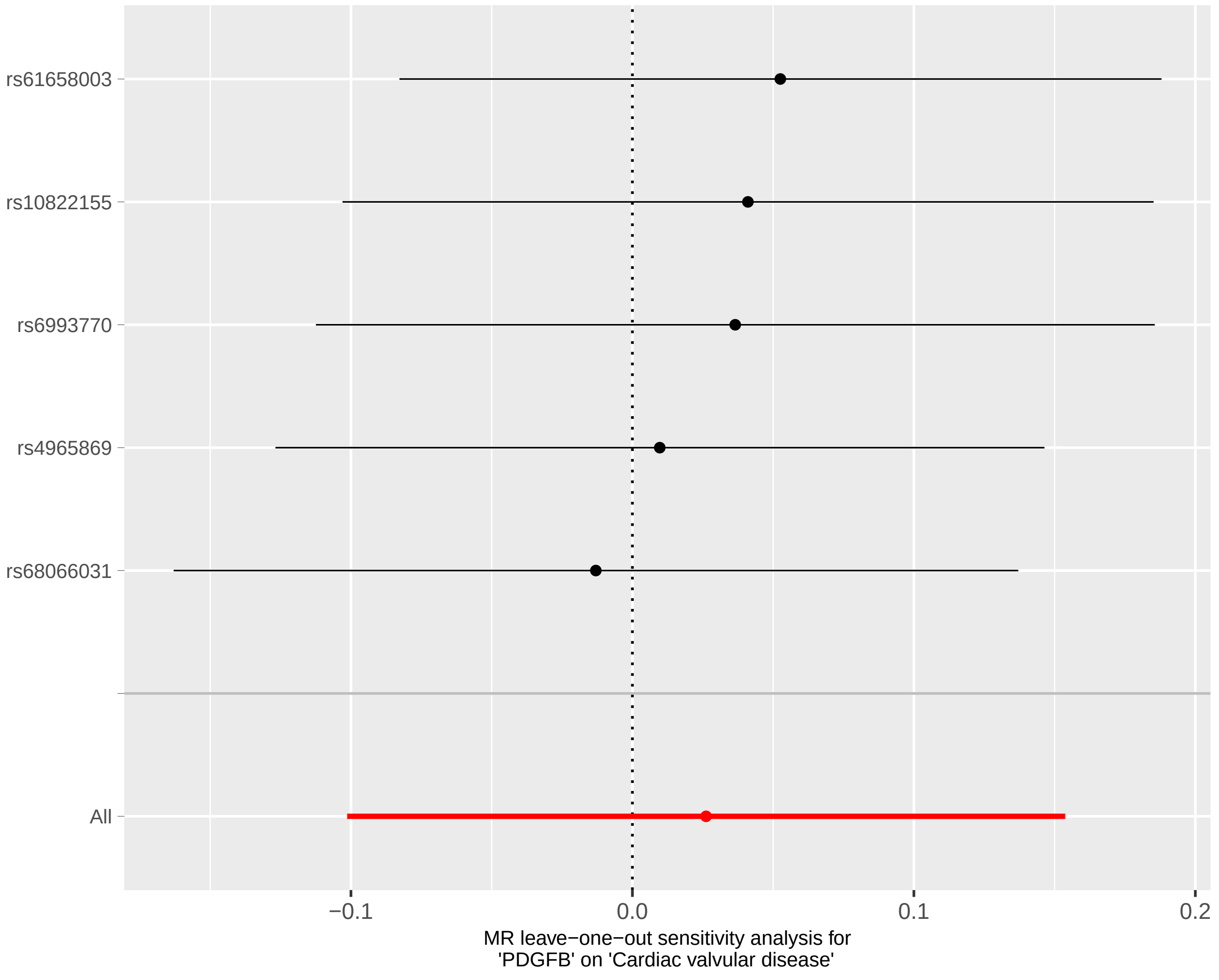
rs61658003
rs10822155
rs6993770
rs4965869
rs68066031
All
−0.1
0.0
0.1
0.2
MR leave−one−out sensitivity analysis for
'PDGFB' on 'Cardiac valvular disease'

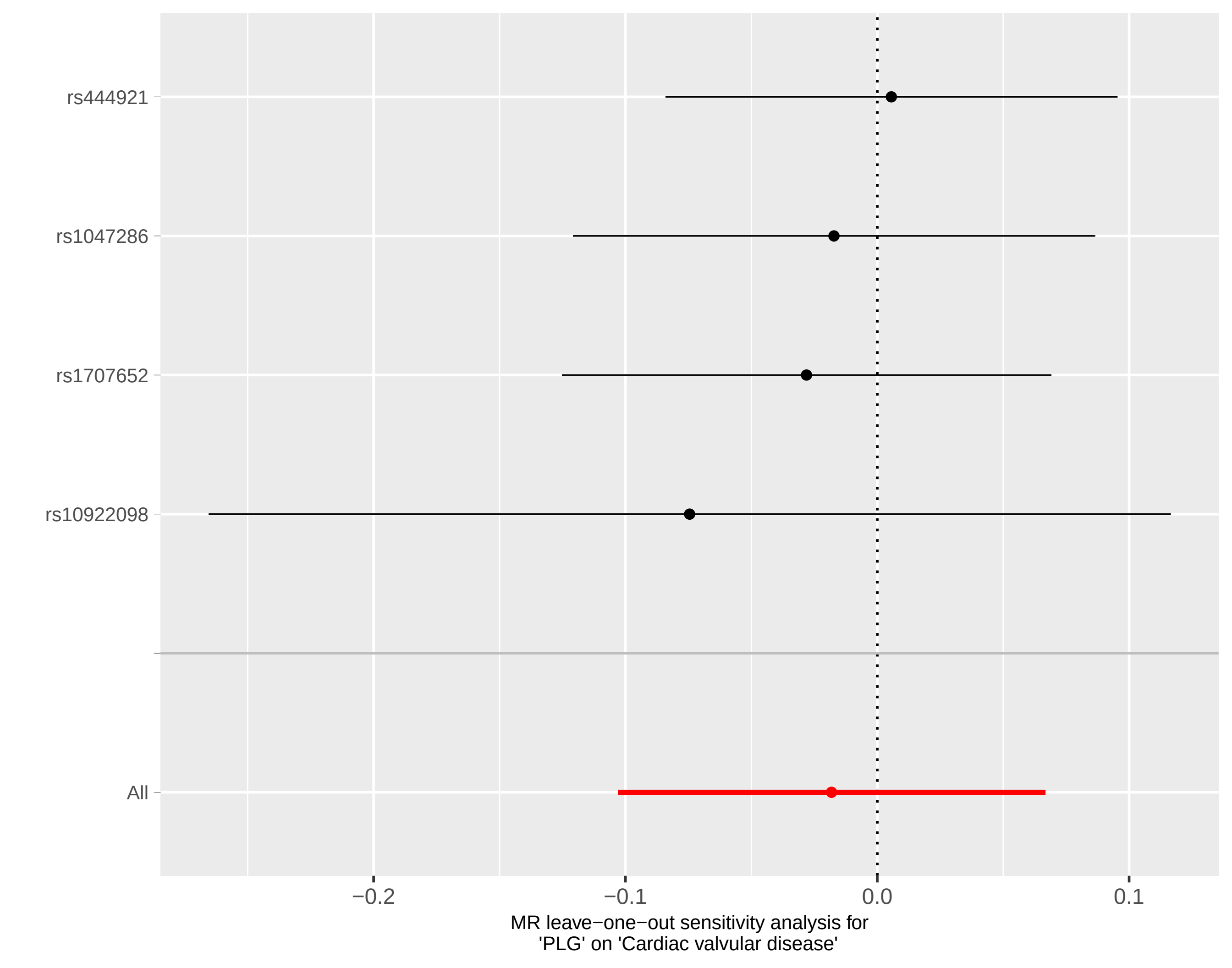
rs444921
rs1047286
rs1707652
rs10922098
All
−0.2
−0.1
0.0
0.1
MR leave−one−out sensitivity analysis for
'PLG' on 'Cardiac valvular disease'

Insufficient number of SNPs

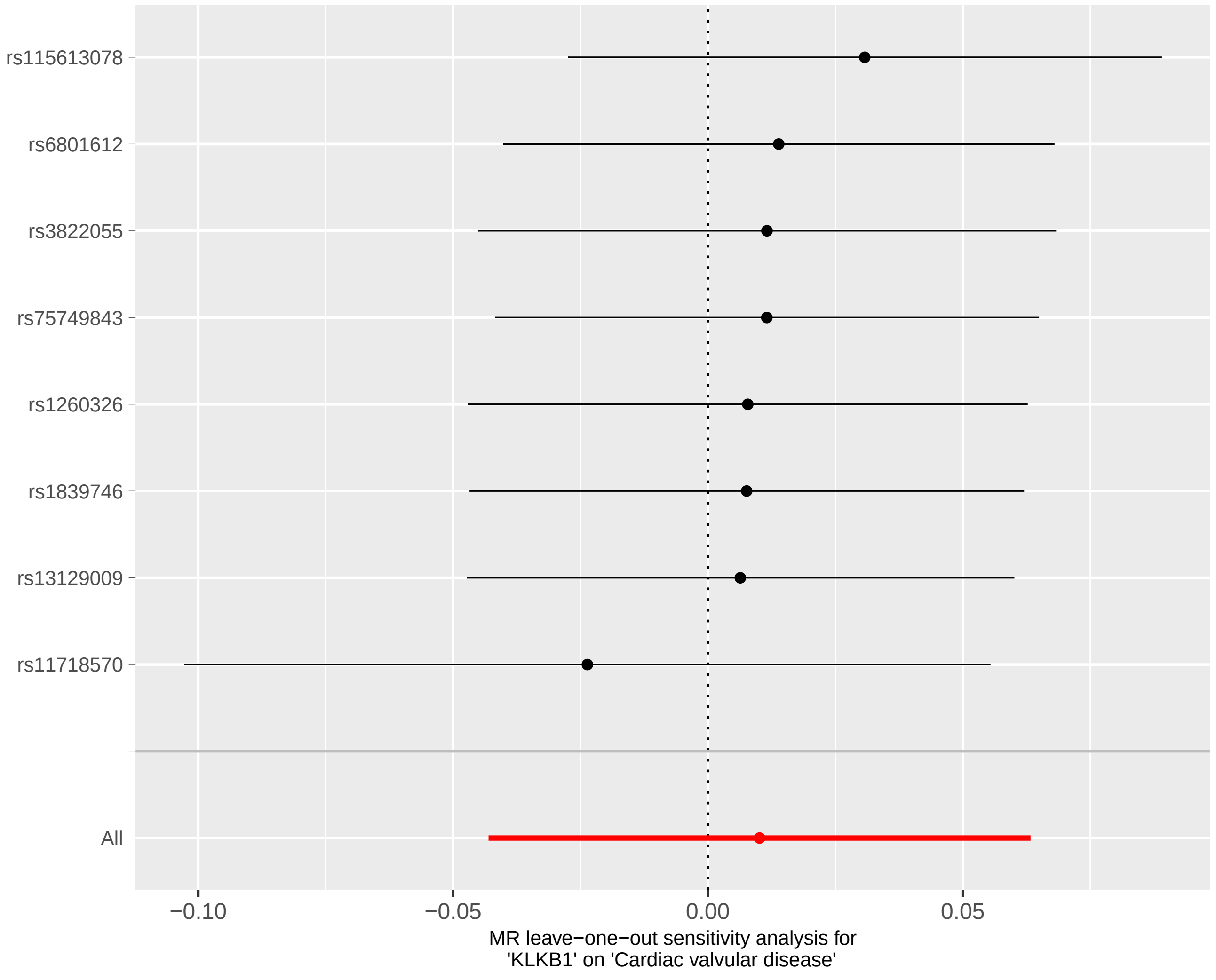

rs115613078
rs6801612
rs3822055
rs75749843
rs1260326
rs1839746
rs13129009
rs11718570
All
−0.10
−0.05
0.00
0.05
MR leave−one−out sensitivity analysis for
'KLKB1' on 'Cardiac valvular disease'

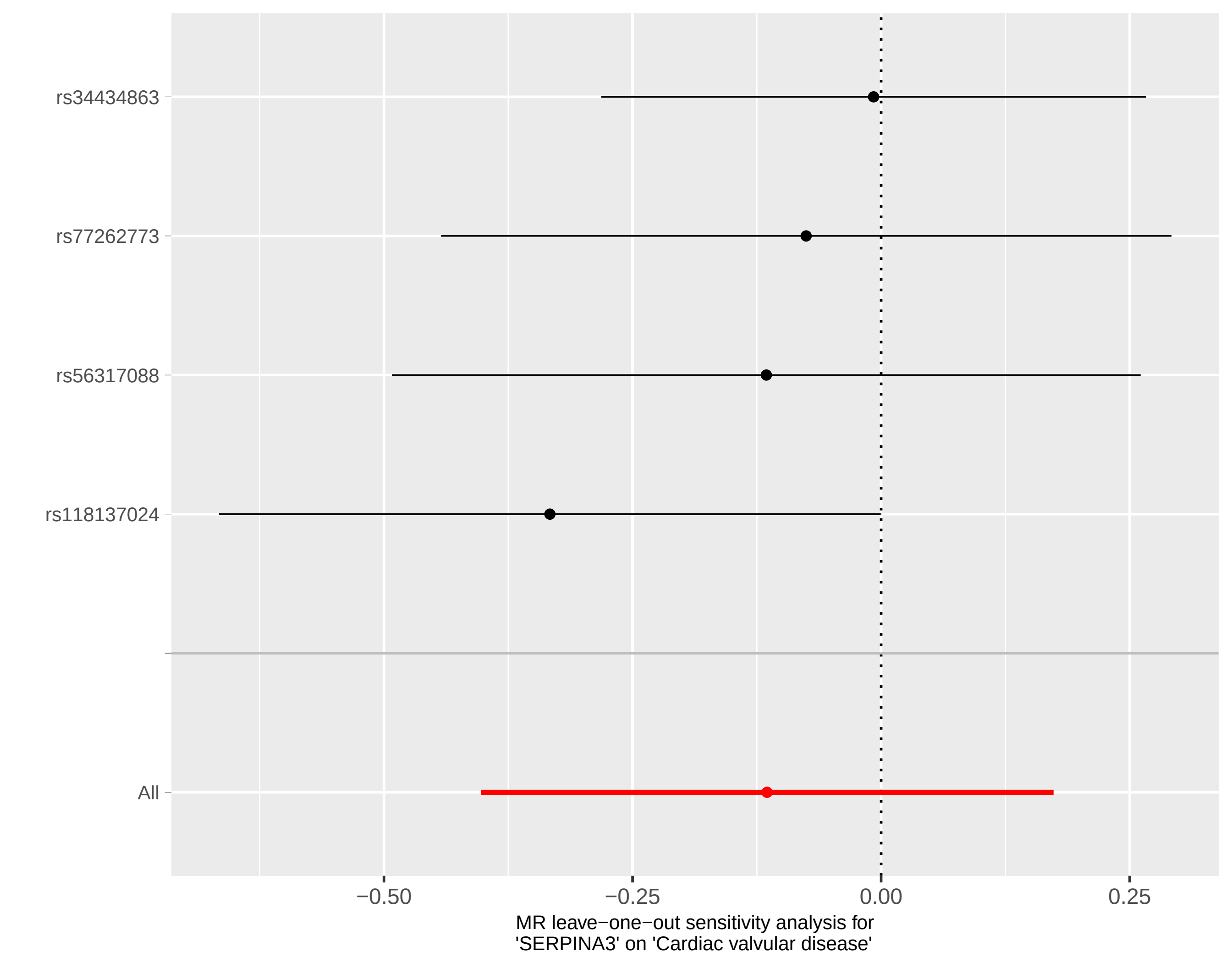
rs34434863
rs77262773
rs56317088
rs118137024
All
−0.50
−0.25
0.00
0.25
MR leave−one−out sensitivity analysis for
'SERPINA3' on 'Cardiac valvular disease'

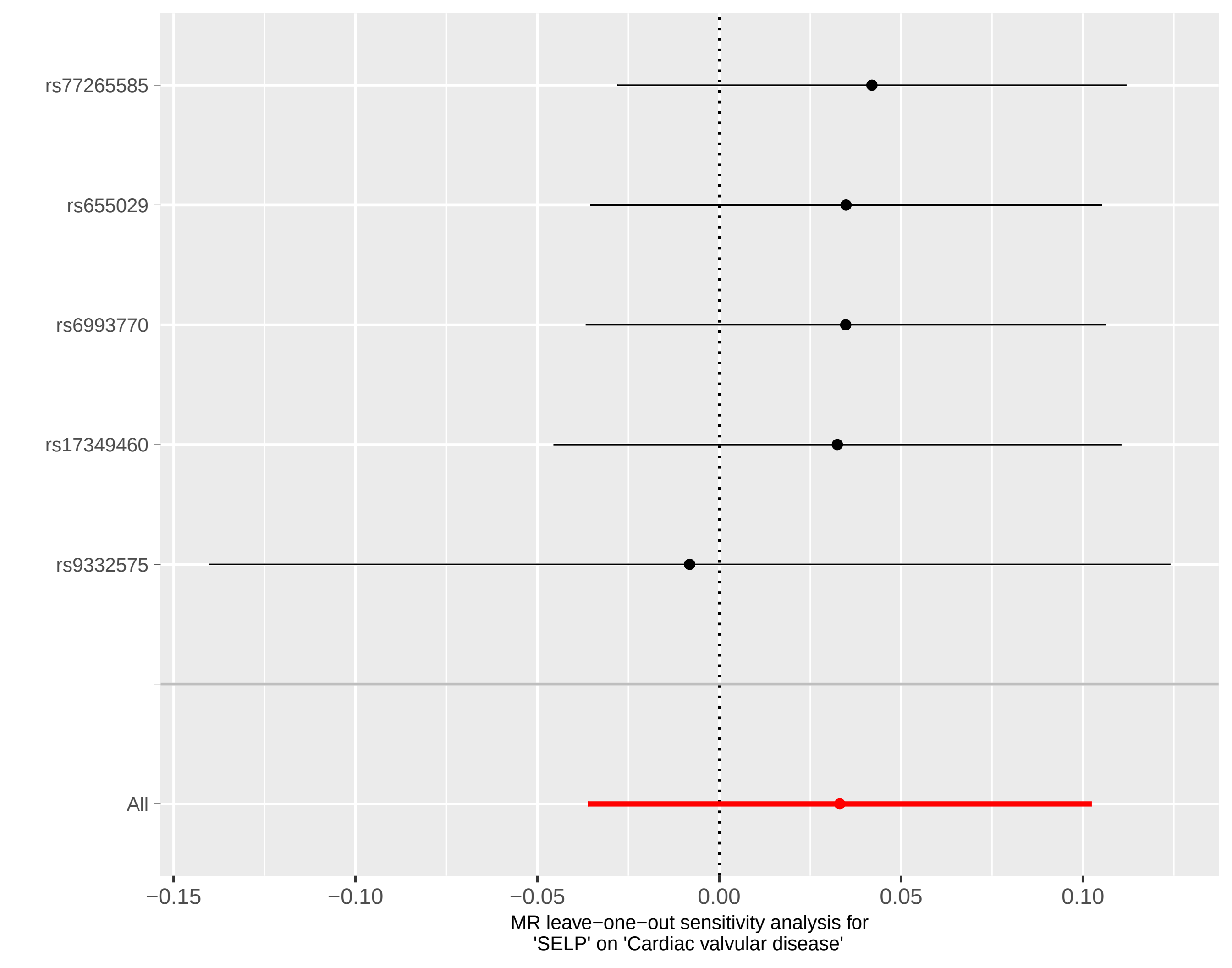

rs77265585
rs655029
rs6993770
rs17349460
rs9332575
All
−0.15
−0.10
−0.05
0.00
0.05
0.10
MR leave−one−out sensitivity analysis for
'SELP' on 'Cardiac valvular disease'

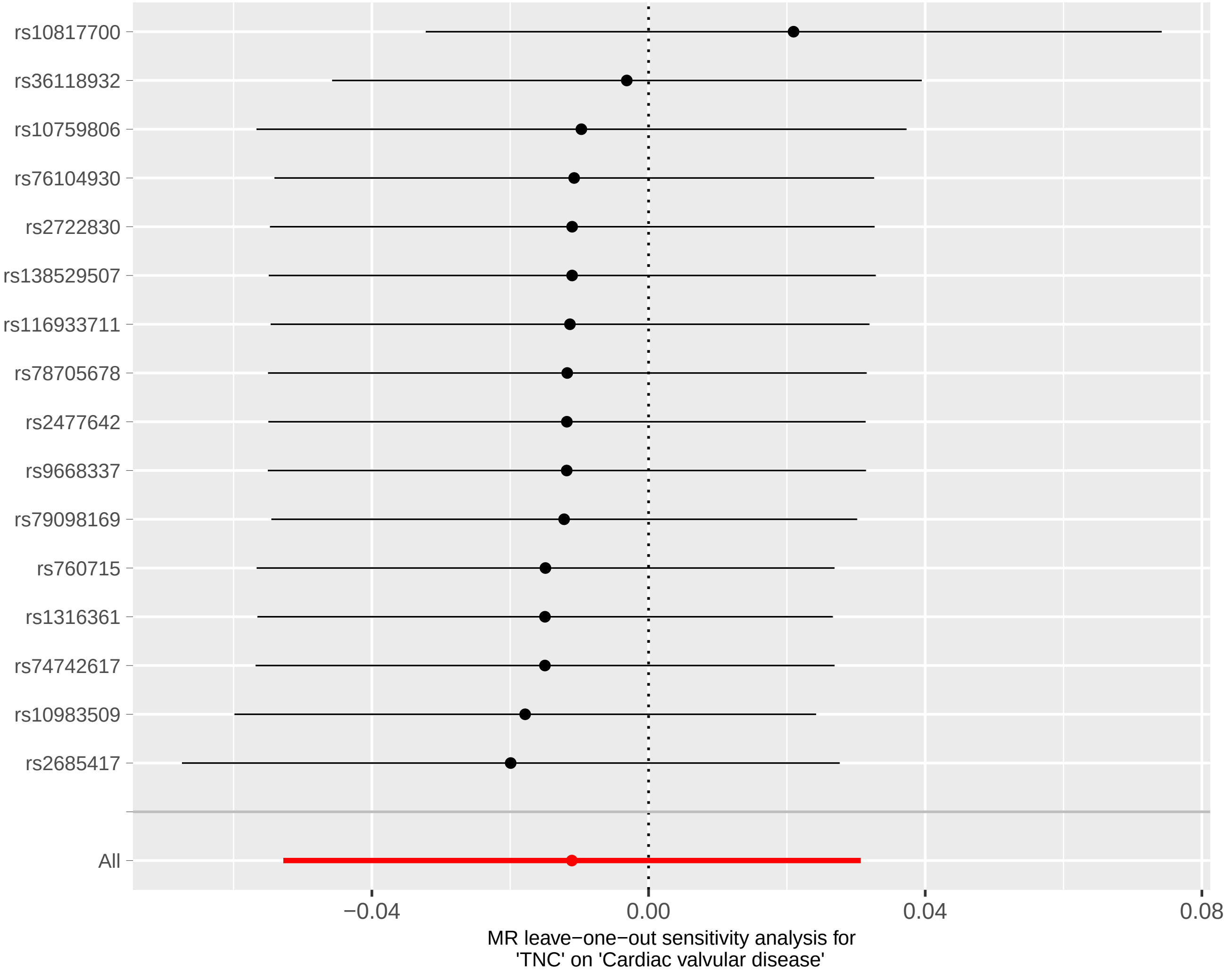

rs10817700
rs36118932
rs10759806
rs76104930
rs2722830
rs138529507
rs116933711
rs78705678
rs2477642
rs9668337
rs79098169
rs760715
rs1316361
rs74742617
rs10983509
rs2685417
All
−0.04
0.00
0.04
0.08
MR leave−one−out sensitivity analysis for
'TNC' on 'Cardiac valvular disease'

Insufficient number of SNPs

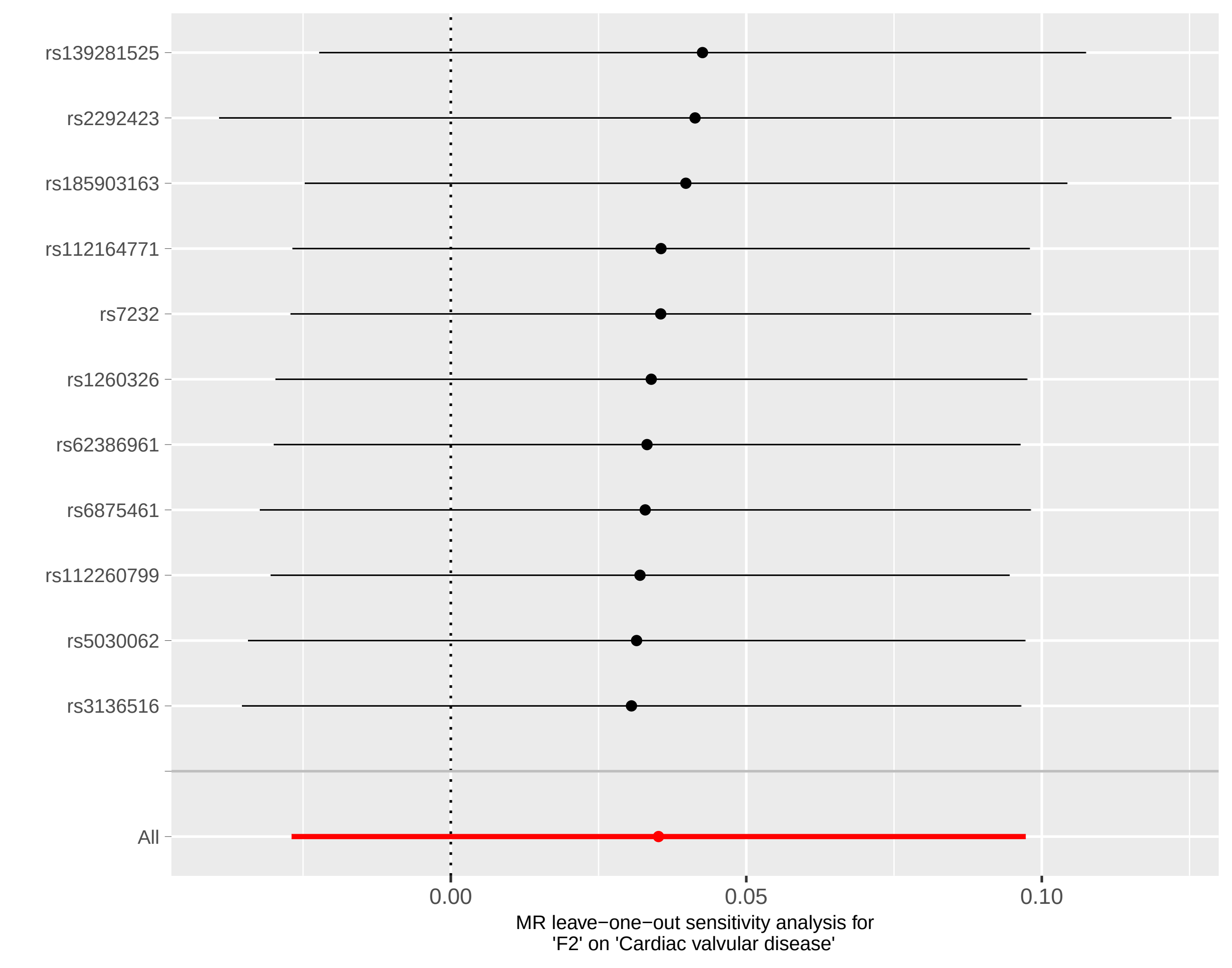
rs139281525
rs2292423
rs185903163
rs112164771
rs7232
rs1260326
rs62386961
rs6875461
rs112260799
rs5030062
rs3136516
All
0.00
0.05
0.10
MR leave−one−out sensitivity analysis for
'F2' on 'Cardiac valvular disease'

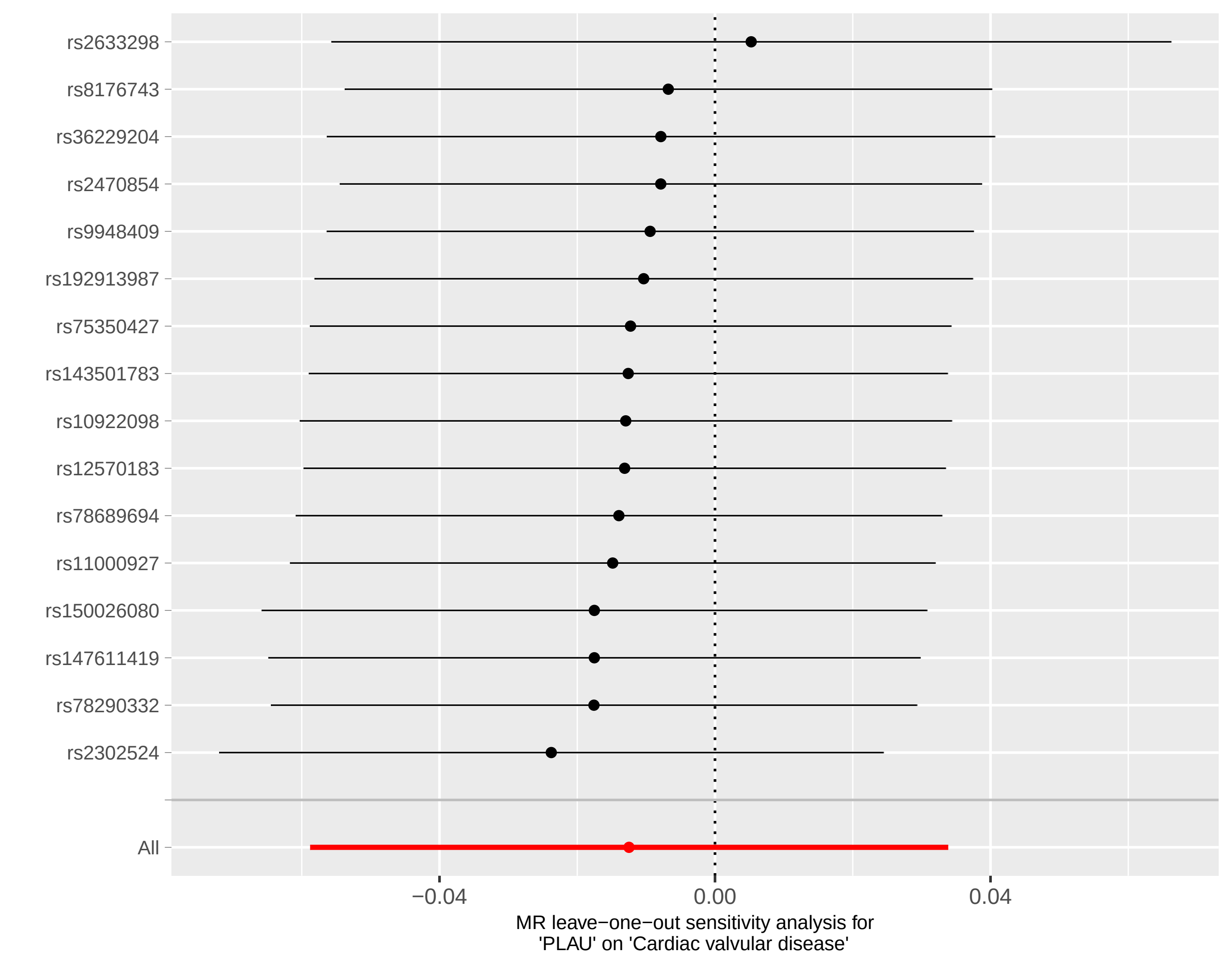

rs2633298
rs8176743
rs36229204
rs2470854
rs9948409
rs192913987
rs75350427
rs143501783
rs10922098
rs12570183
rs78689694
rs11000927
rs150026080
rs147611419
rs78290332
rs2302524
All
−0.04
0.00
0.04
MR leave−one−out sensitivity analysis for
'PLAU' on 'Cardiac valvular disease'

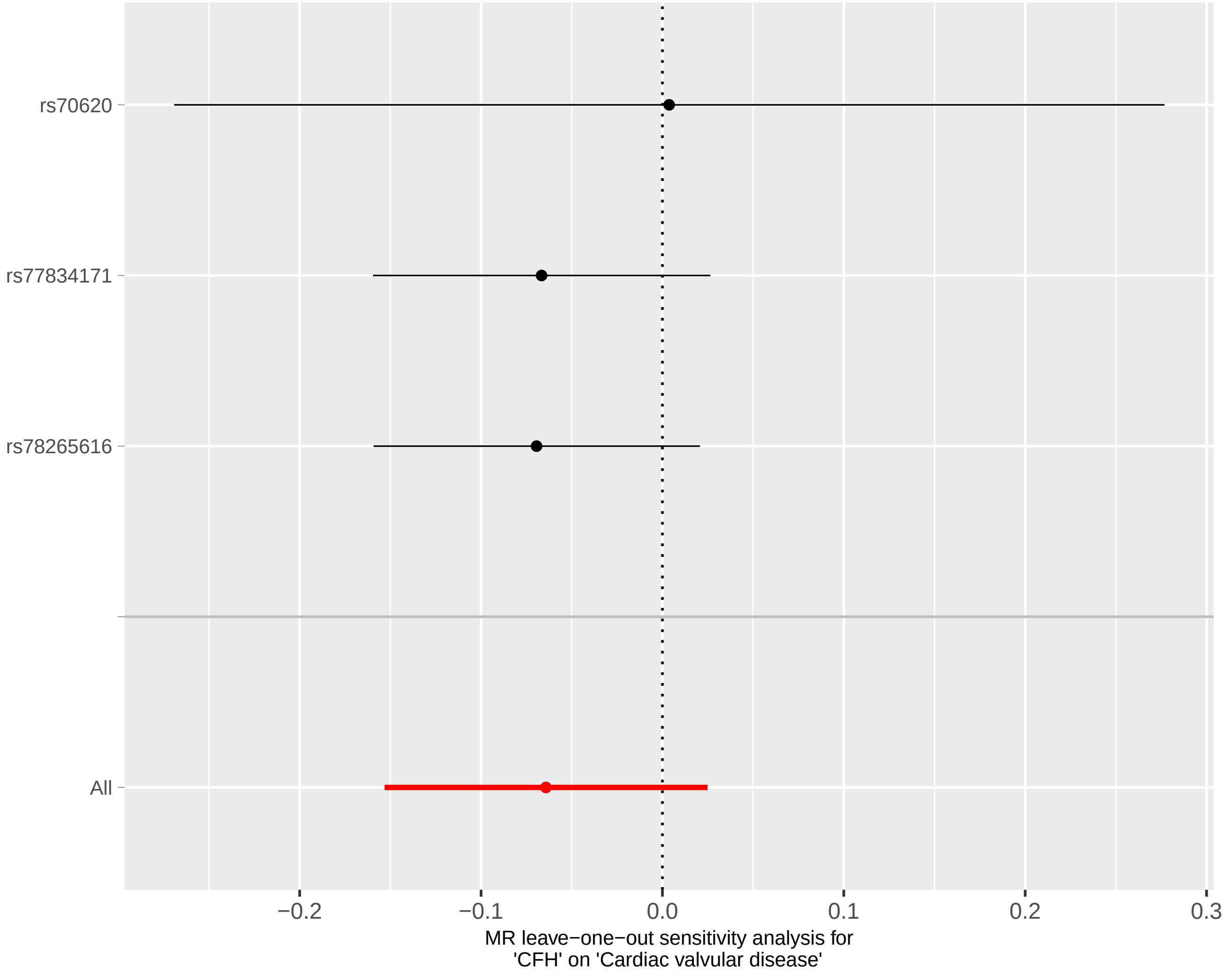
rs70620
rs77834171
rs78265616
All
−0.2
−0.1
0.0
0.1
0.2
0.3
MR leave−one−out sensitivity analysis for
'CFH' on 'Cardiac valvular disease'

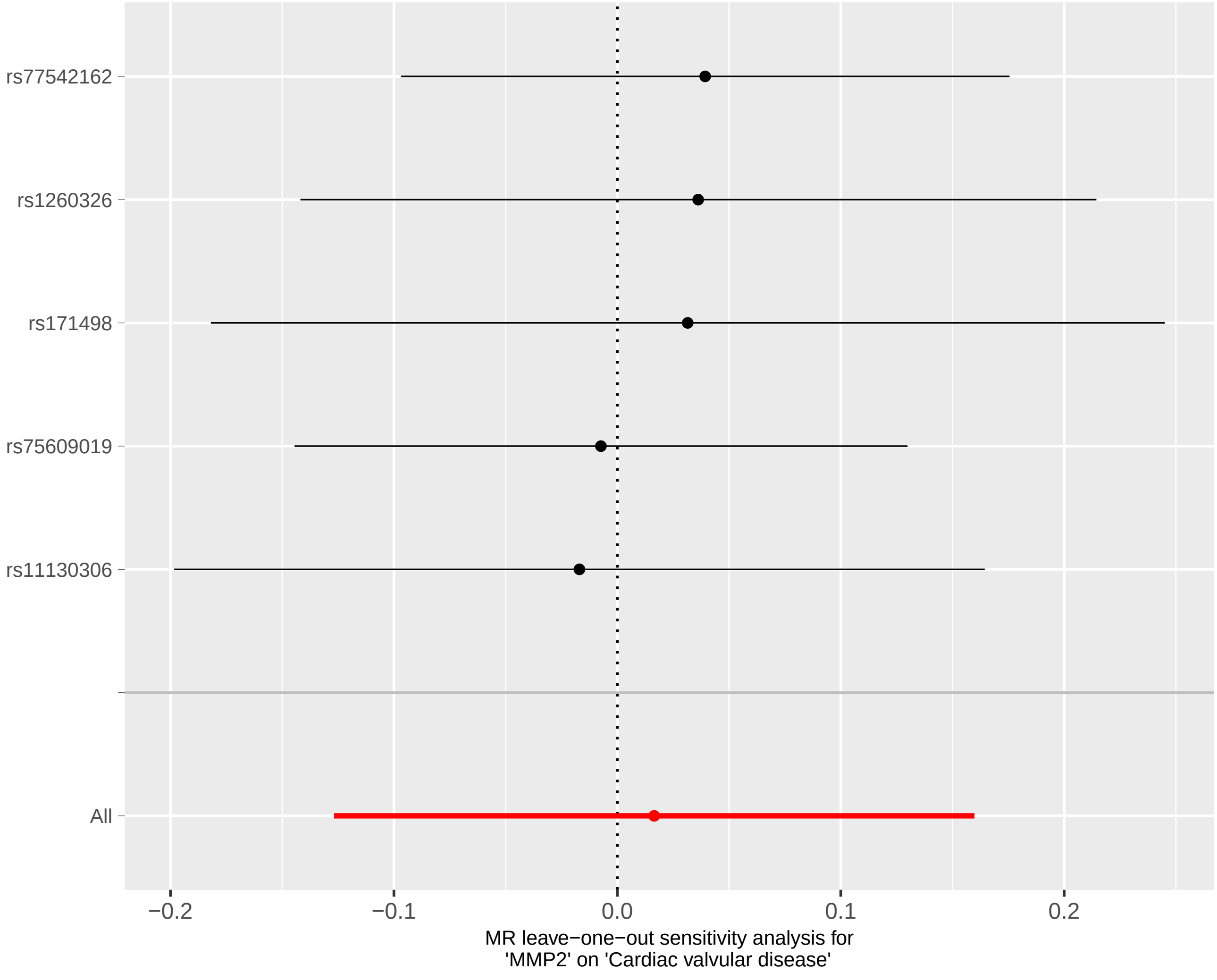

rs77542162
rs1260326
rs171498
rs75609019
rs11130306
All
−0.2
−0.1
0.0
0.1
0.2
MR leave−one−out sensitivity analysis for
'MMP2' on 'Cardiac valvular disease'

Insufficient number of SNPs

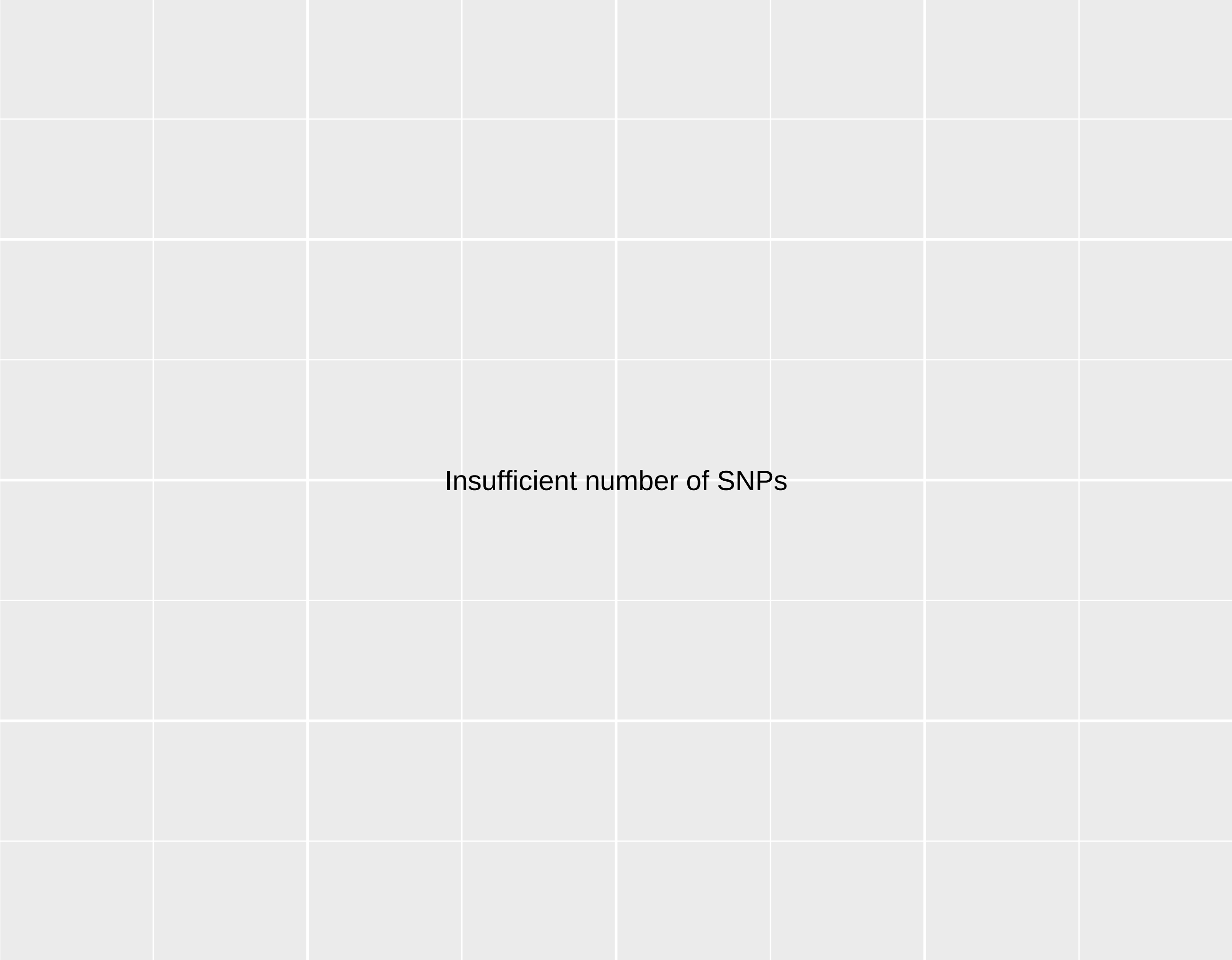
Insufficient number of SNPs

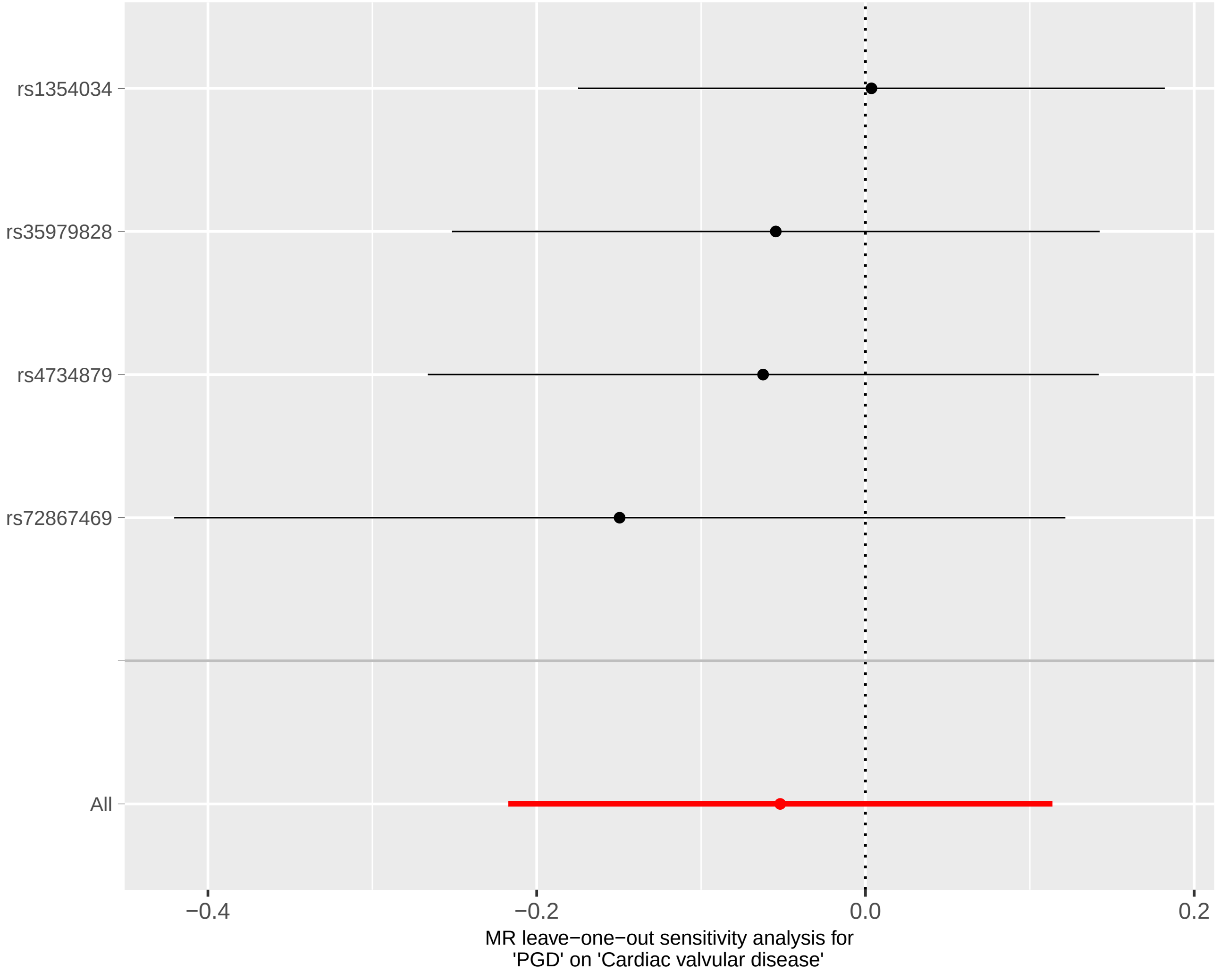

rs1354034
rs35979828
rs4734879
rs72867469
All
−0.4
−0.2
0.0
0.2
MR leave−one−out sensitivity analysis for
'PGD' on 'Cardiac valvular disease'

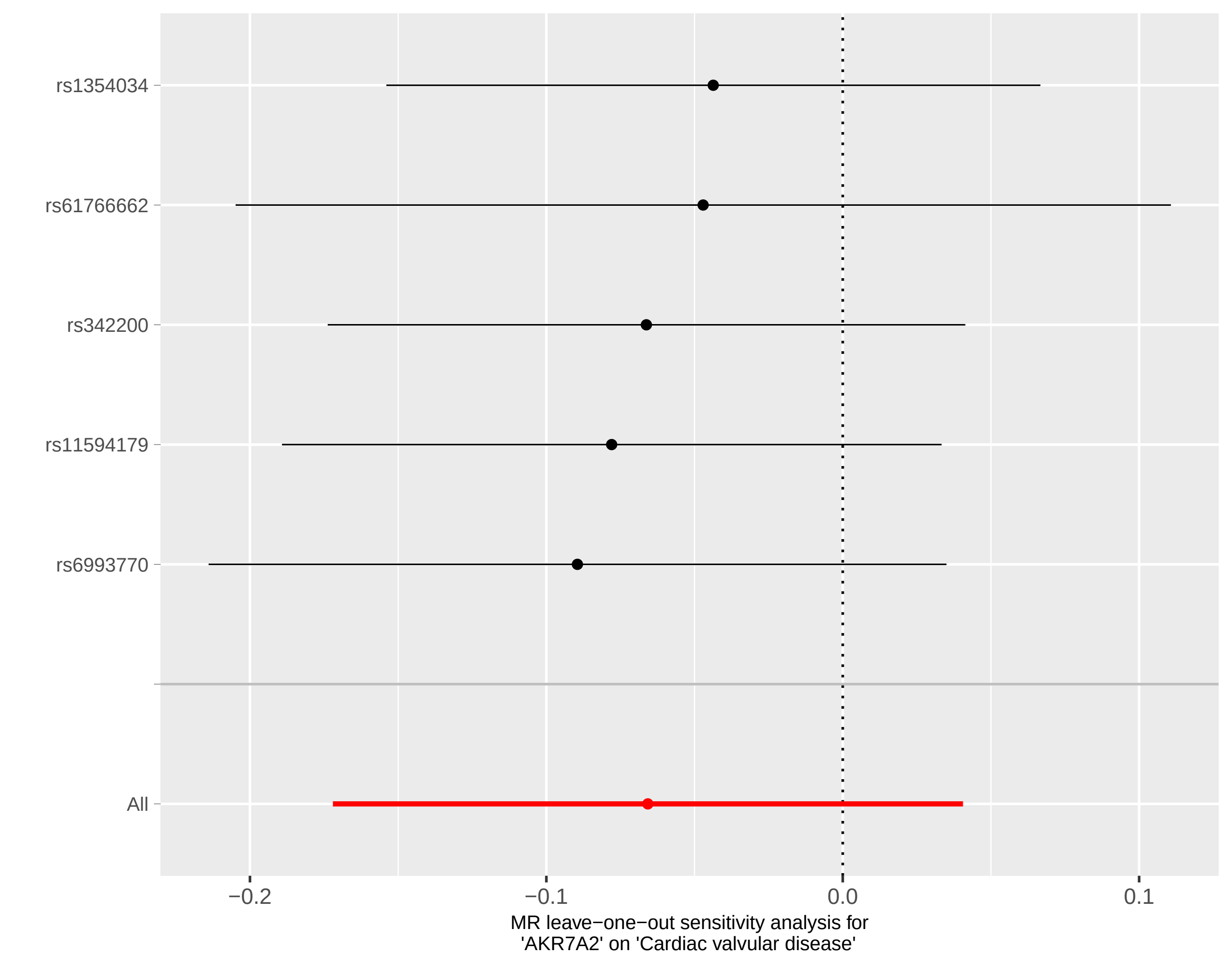
rs1354034
rs61766662
rs342200
rs11594179
rs6993770
All
−0.2
−0.1
0.0
0.1
MR leave−one−out sensitivity analysis for
'AKR7A2' on 'Cardiac valvular disease'

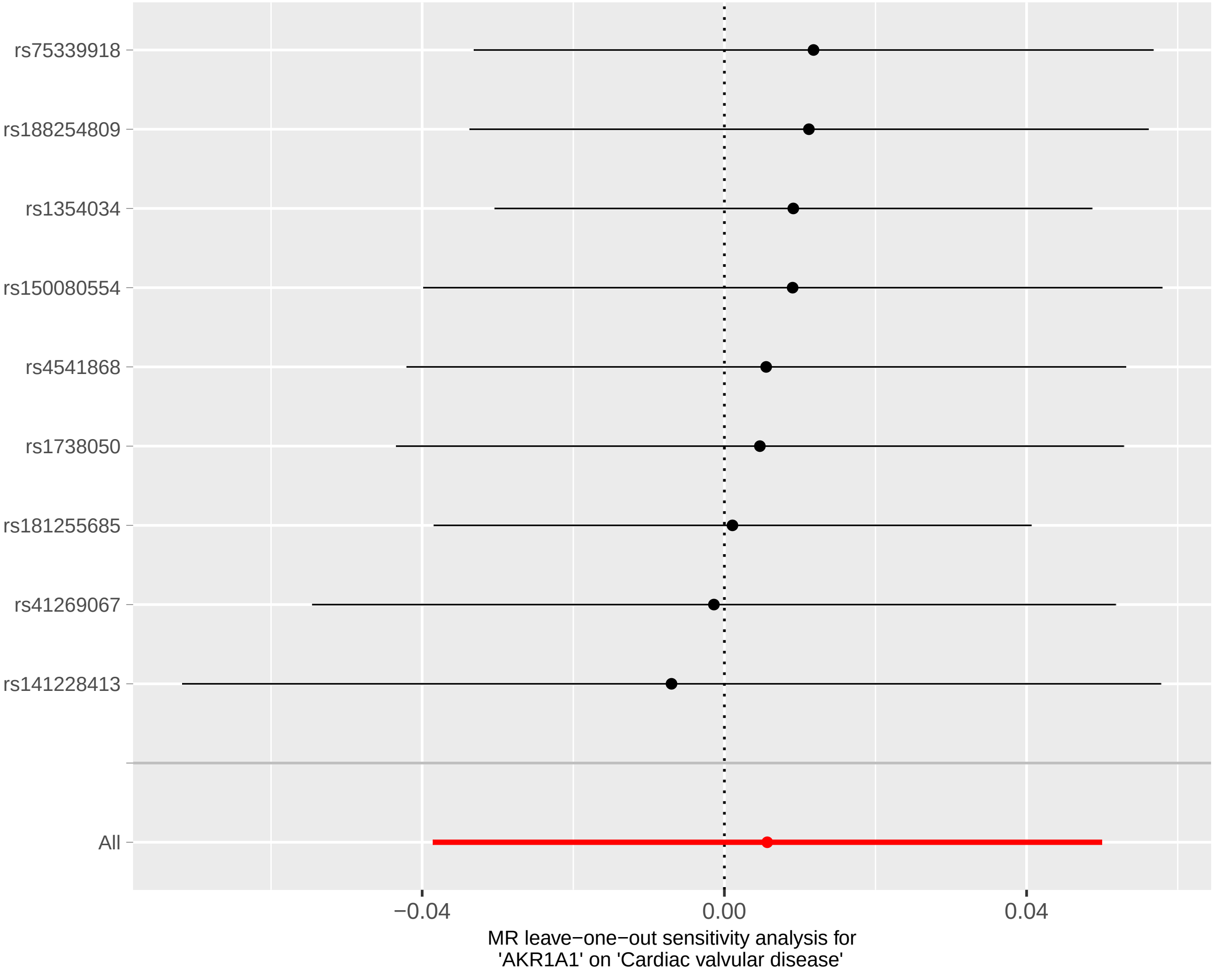

rs75339918
rs188254809
rs1354034
rs150080554
rs4541868
rs1738050
rs181255685
rs41269067
rs141228413
All
−0.04
0.00
0.04
MR leave−one−out sensitivity analysis for
'AKR1A1' on 'Cardiac valvular disease'

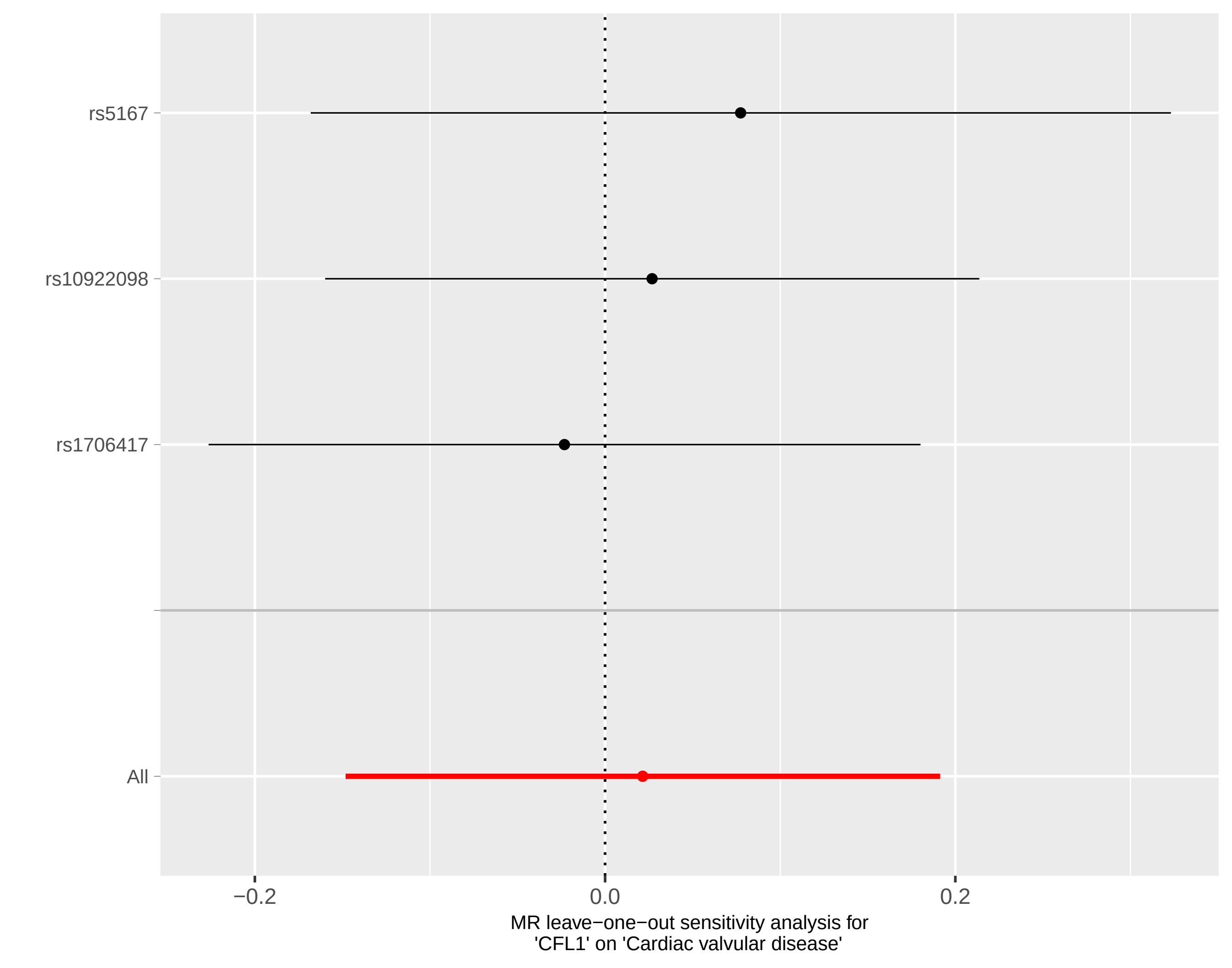
rs5167
rs10922098
rs1706417
All
−0.2
0.0
0.2
MR leave−one−out sensitivity analysis for
'CFL1' on 'Cardiac valvular disease'

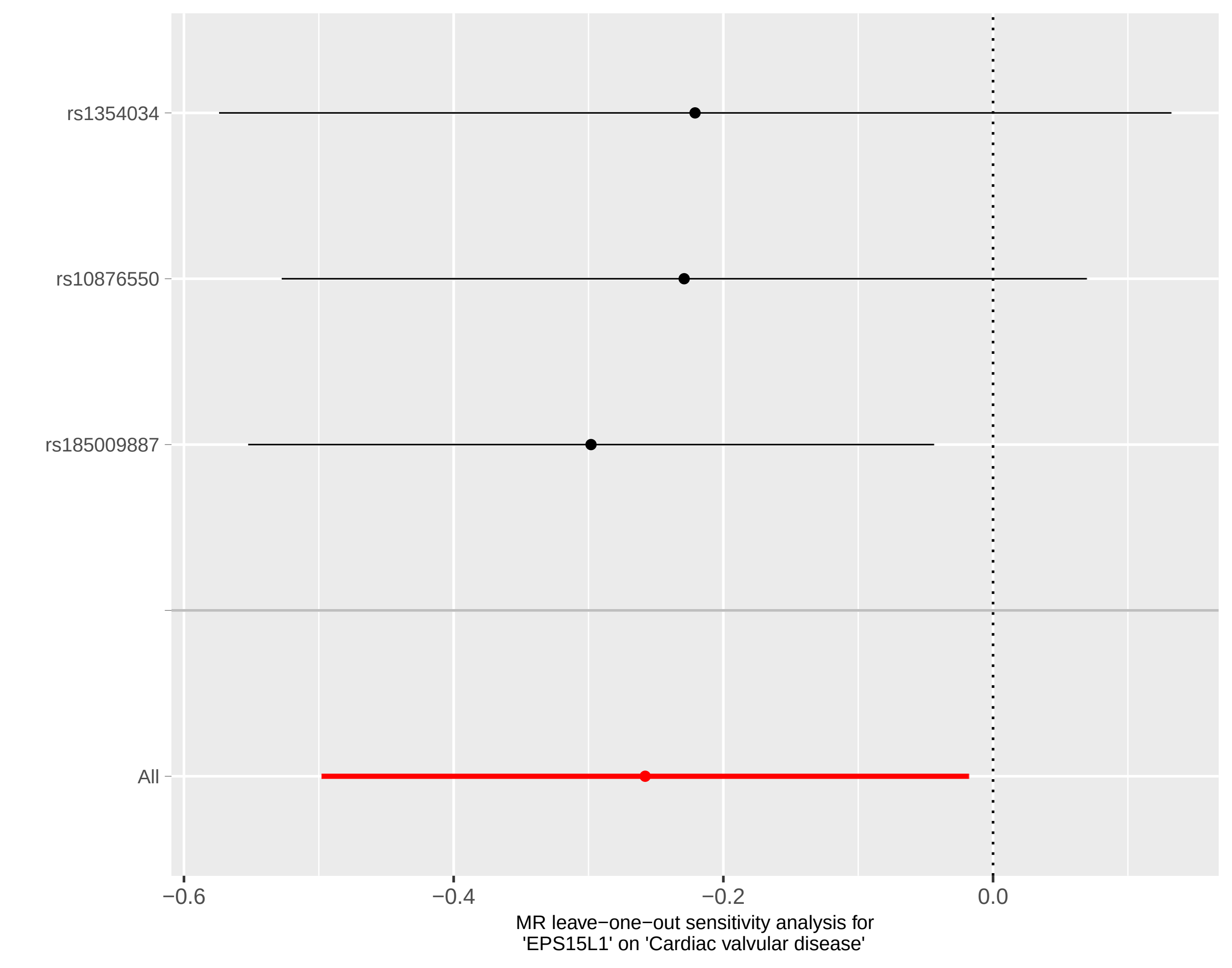
rs1354034
rs10876550
rs185009887
All
−0.6
−0.4
−0.2
0.0
MR leave−one−out sensitivity analysis for
'EPS15L1' on 'Cardiac valvular disease'

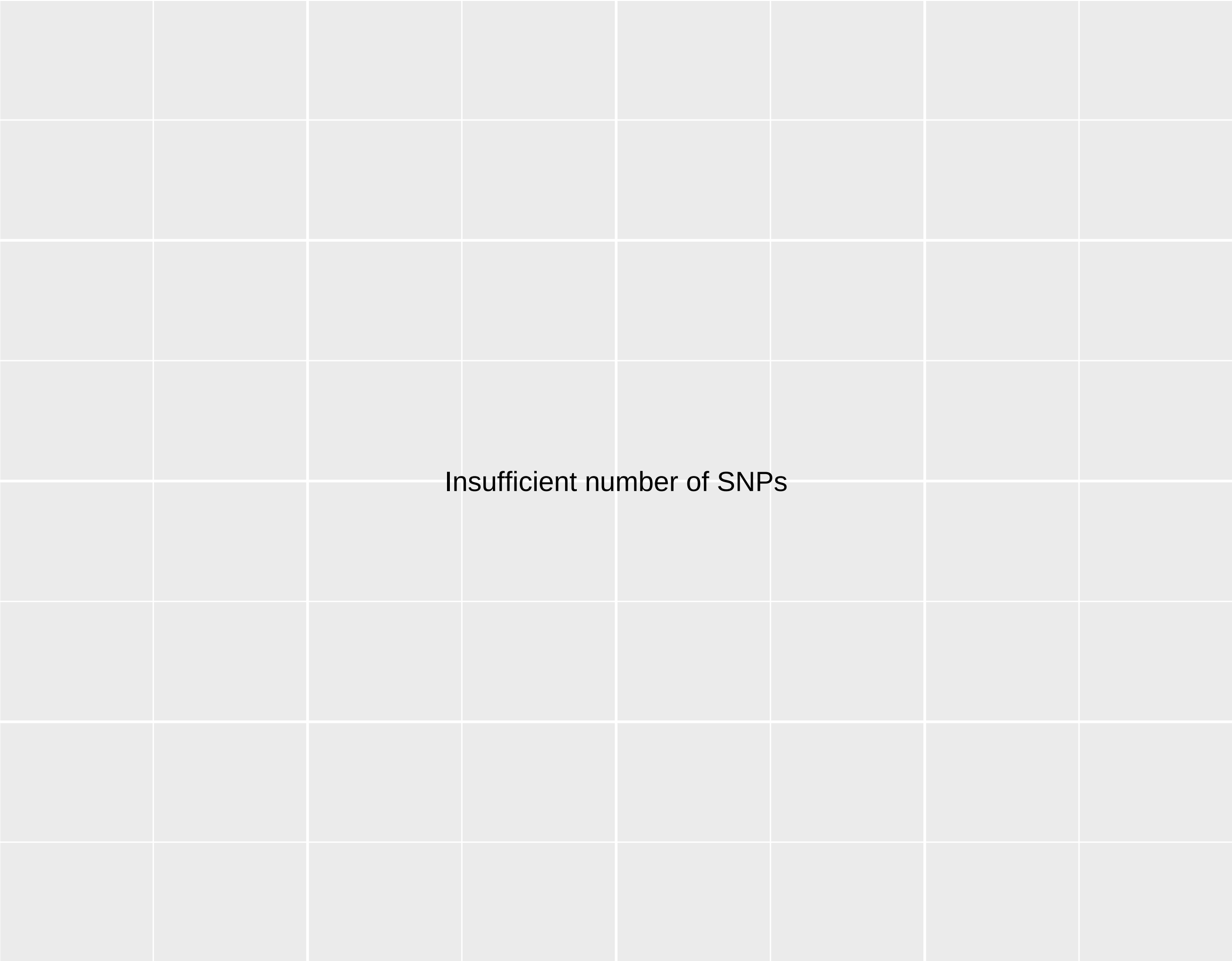
Insufficient number of SNPs

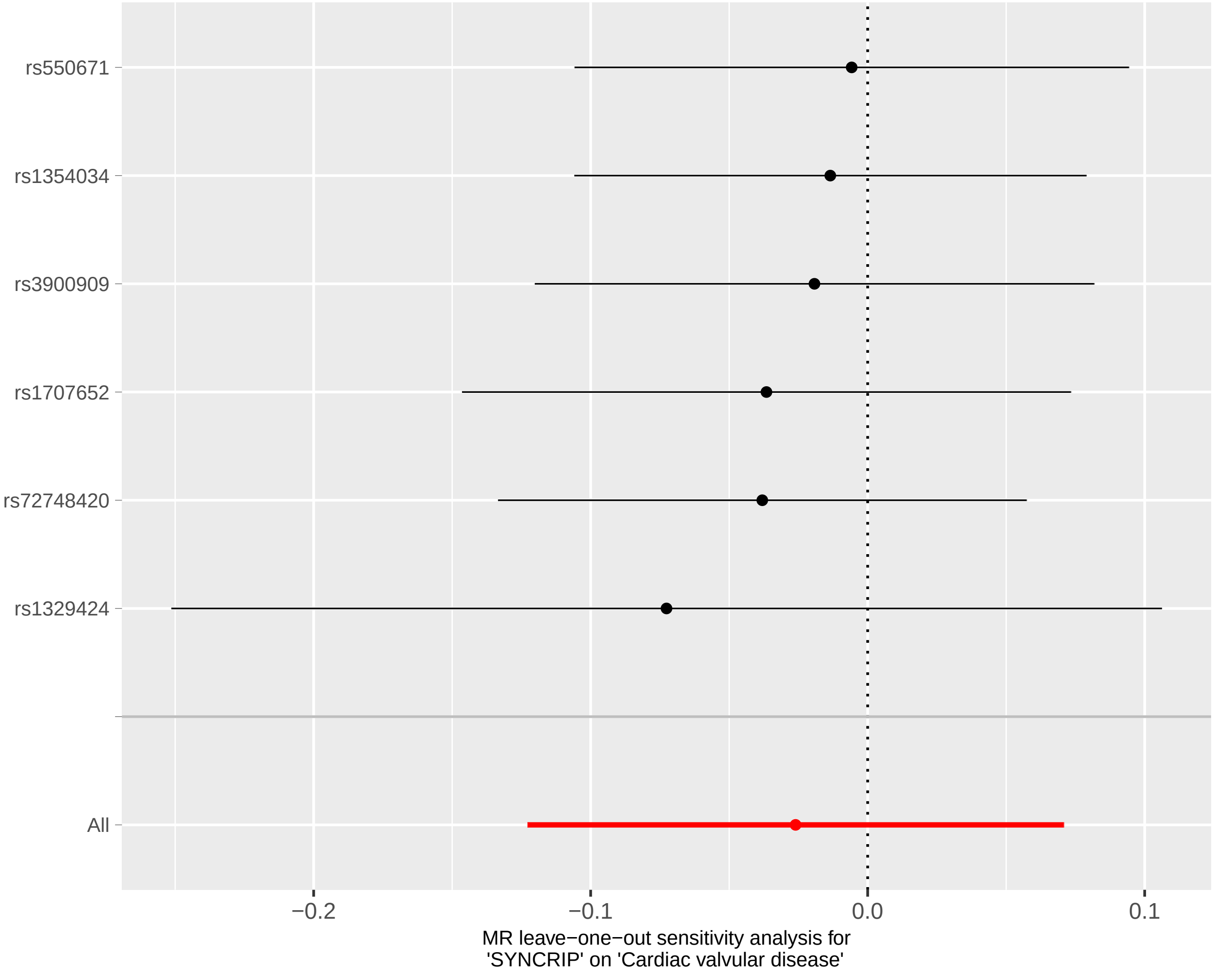

rs550671
rs1354034
rs3900909
rs1707652
rs72748420
rs1329424
All
−0.2
−0.1
0.0
0.1
MR leave−one−out sensitivity analysis for
'SYNCRIP' on 'Cardiac valvular disease'

Insufficient number of SNPs

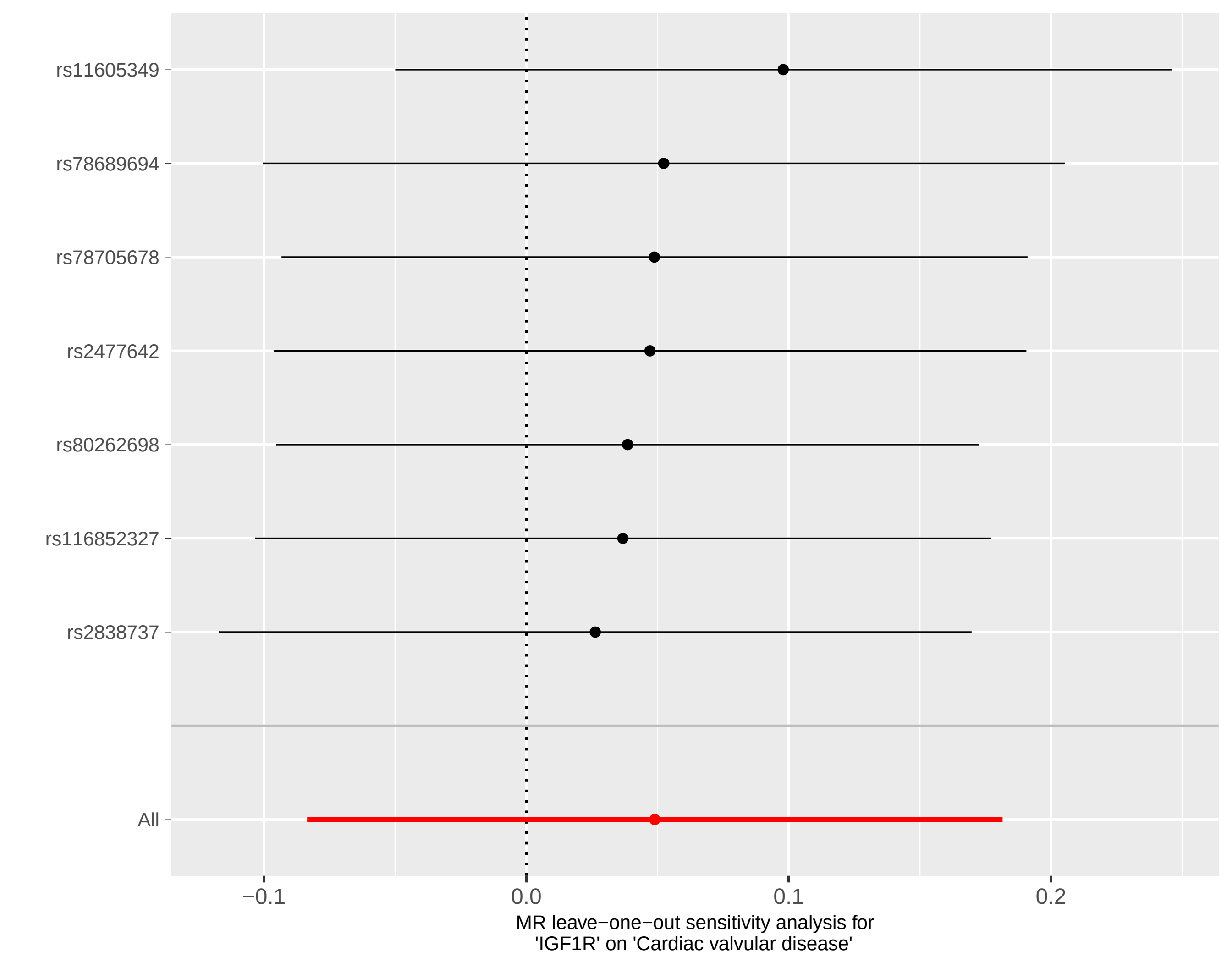

rs11605349
rs78689694
rs78705678
rs2477642
rs80262698
rs116852327
rs2838737
All
−0.1
0.0
0.1
0.2
MR leave−one−out sensitivity analysis for
'IGF1R' on 'Cardiac valvular disease'

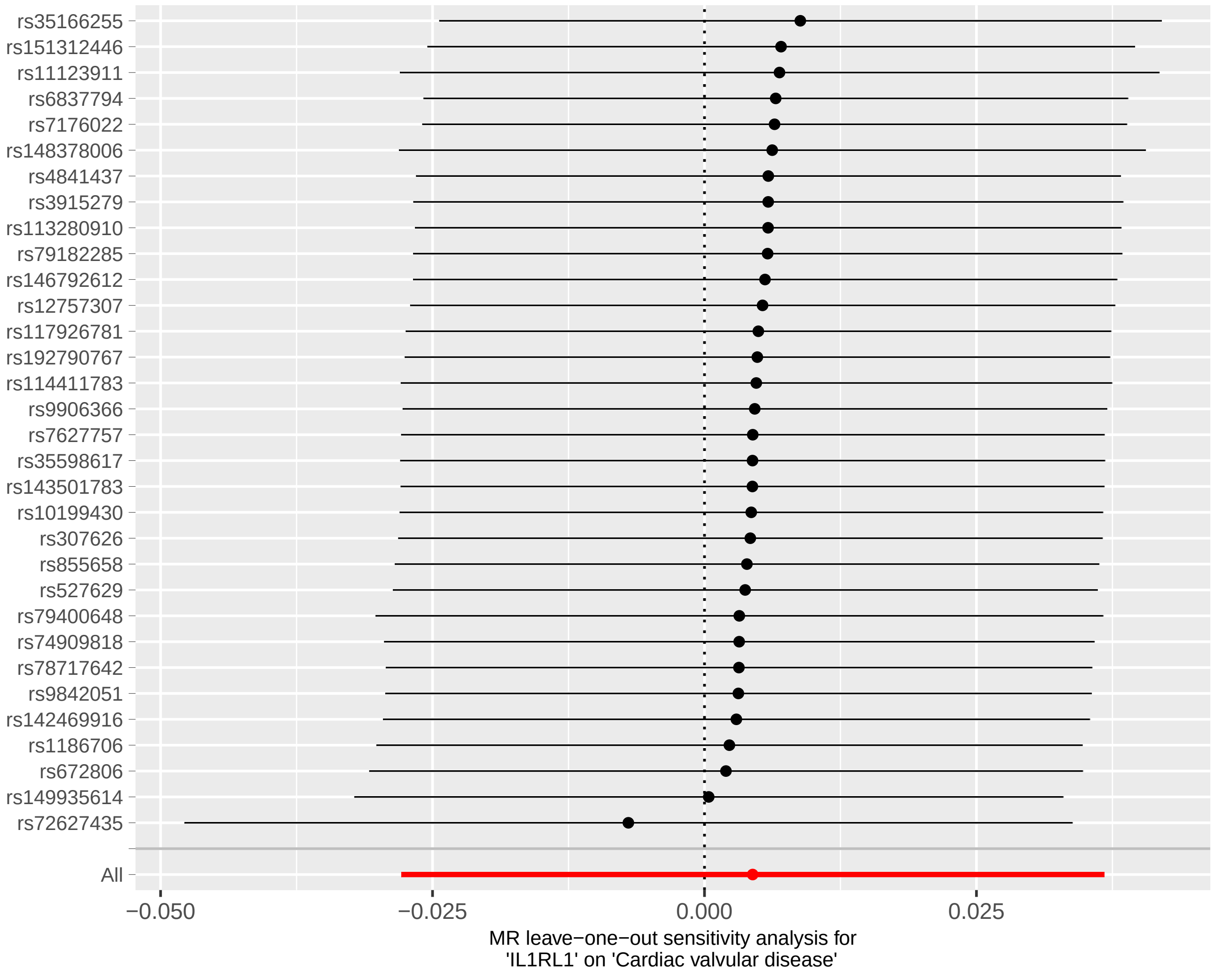
rs35166255
rs151312446
rs11123911
rs6837794
rs7176022
rs148378006
rs4841437
rs3915279
rs113280910
rs79182285
rs146792612
rs12757307
rs117926781
rs192790767
rs114411783
rs9906366
rs7627757
rs35598617
rs143501783
rs10199430
rs307626
rs855658
rs527629
rs79400648
rs74909818
rs78717642
rs9842051
rs142469916
rs1186706
rs672806
rs149935614
rs72627435
All
−0.050
−0.025
0.000
0.025
MR leave−one−out sensitivity analysis for
'IL1RL1' on 'Cardiac valvular disease'

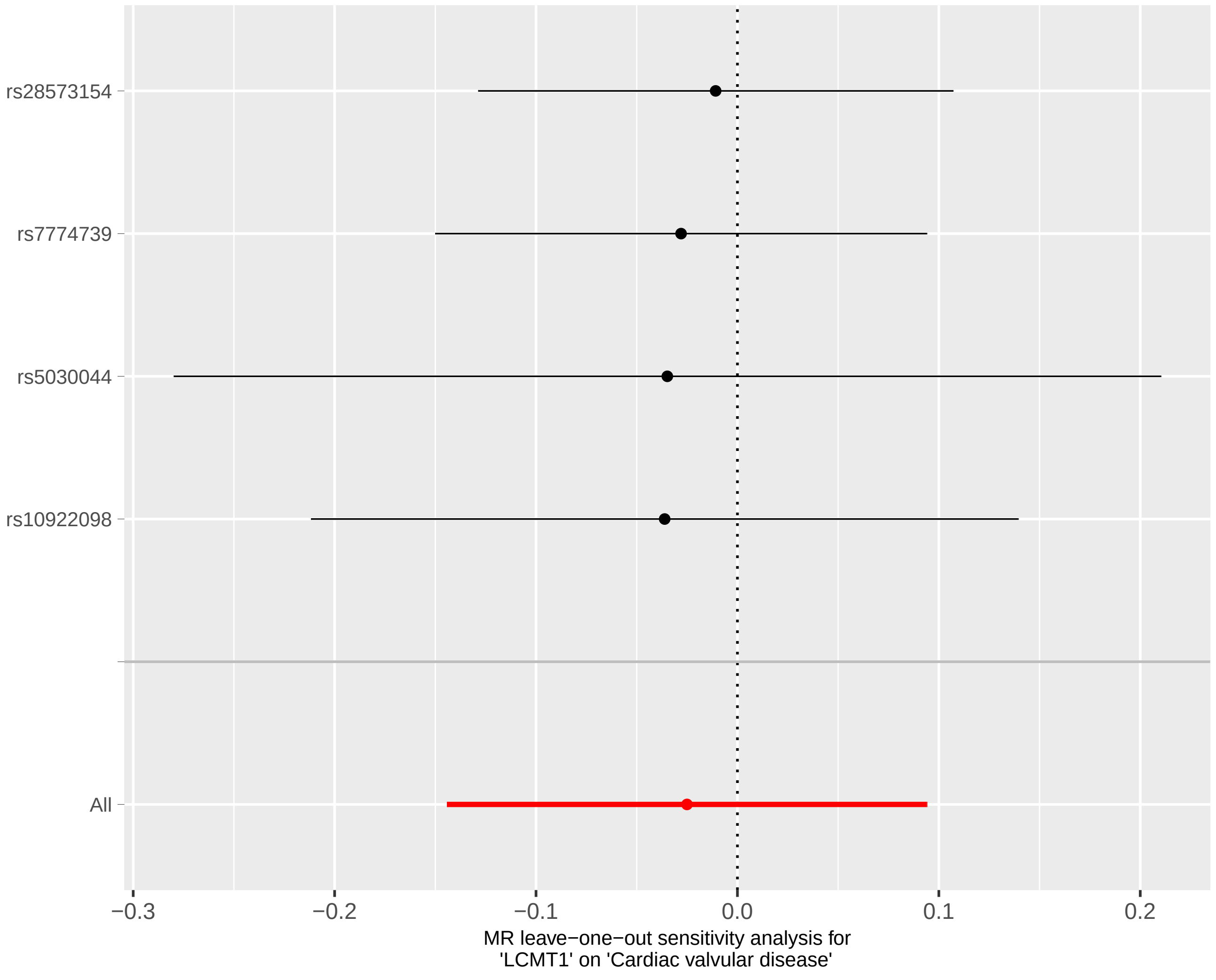
rs28573154
rs7774739
rs5030044
rs10922098
All
−0.3
−0.2
−0.1
0.0
0.1
0.2
MR leave−one−out sensitivity analysis for
'LCMT1' on 'Cardiac valvular disease'

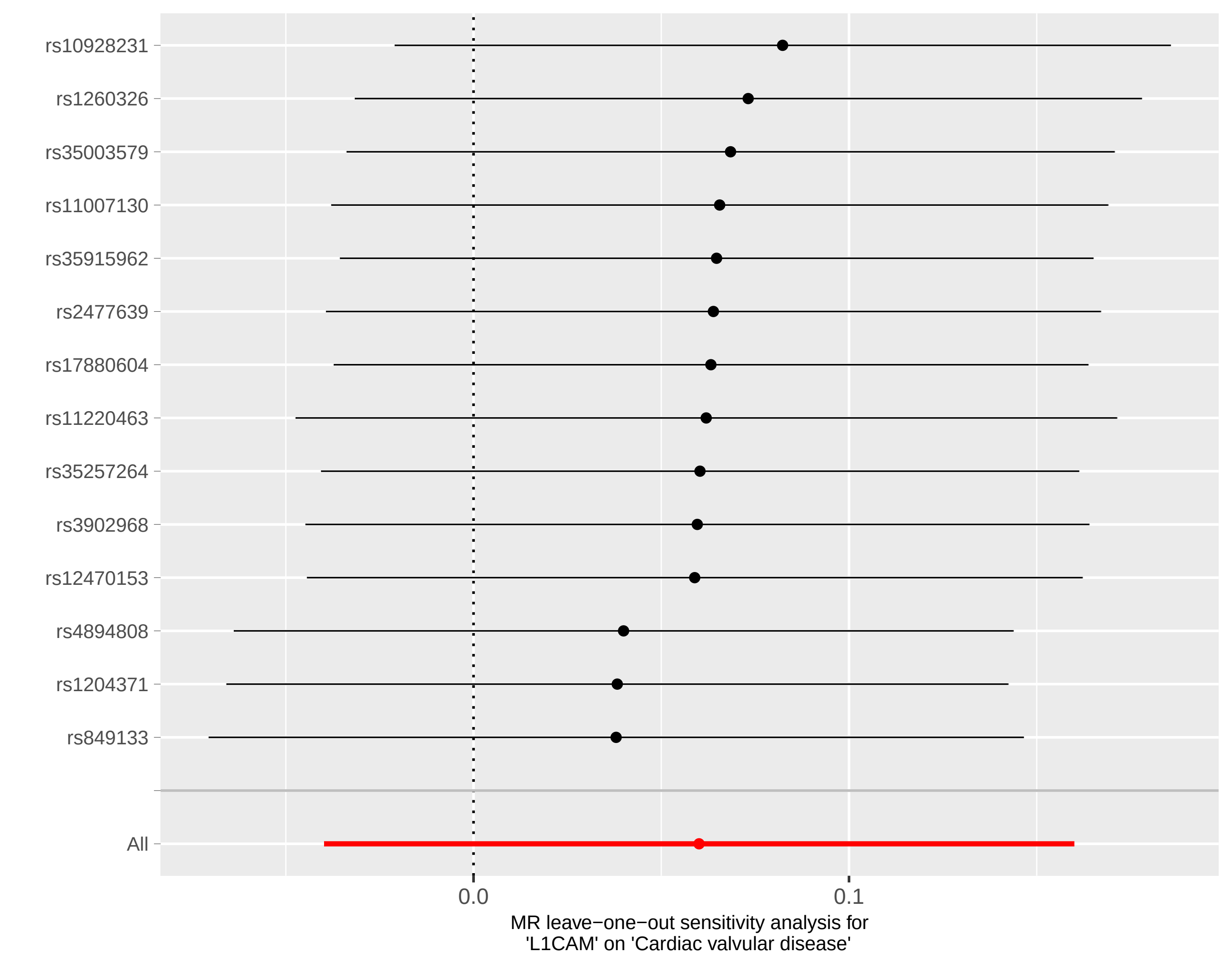
rs10928231
rs1260326
rs35003579
rs11007130
rs35915962
rs2477639
rs17880604
rs11220463
rs35257264
rs3902968
rs12470153
rs4894808
rs1204371
rs849133
All
0.0
0.1
MR leave−one−out sensitivity analysis for
'L1CAM' on 'Cardiac valvular disease'

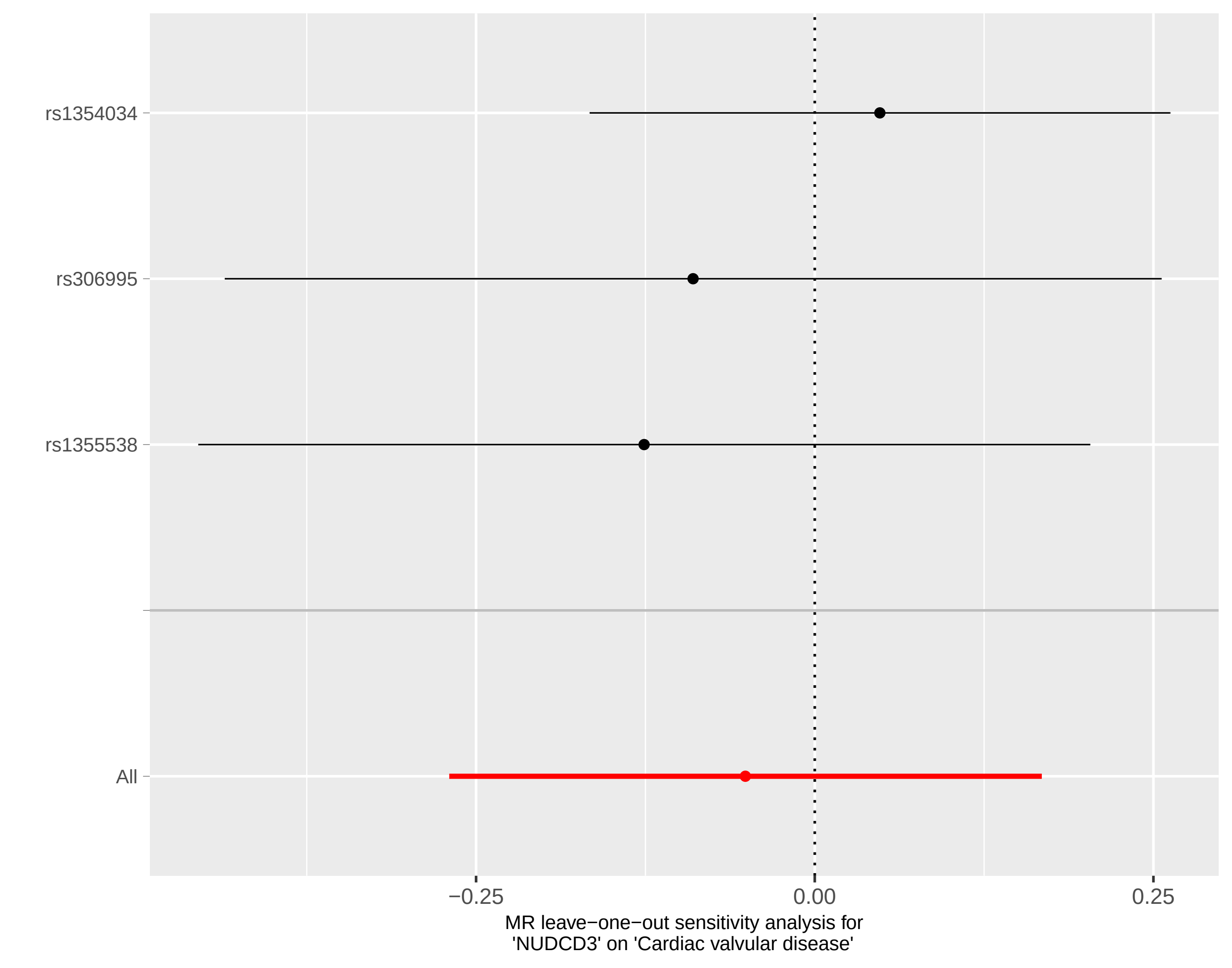
rs1354034
rs306995
rs1355538
All
−0.25
0.00
0.25
MR leave−one−out sensitivity analysis for
'NUDCD3' on 'Cardiac valvular disease'

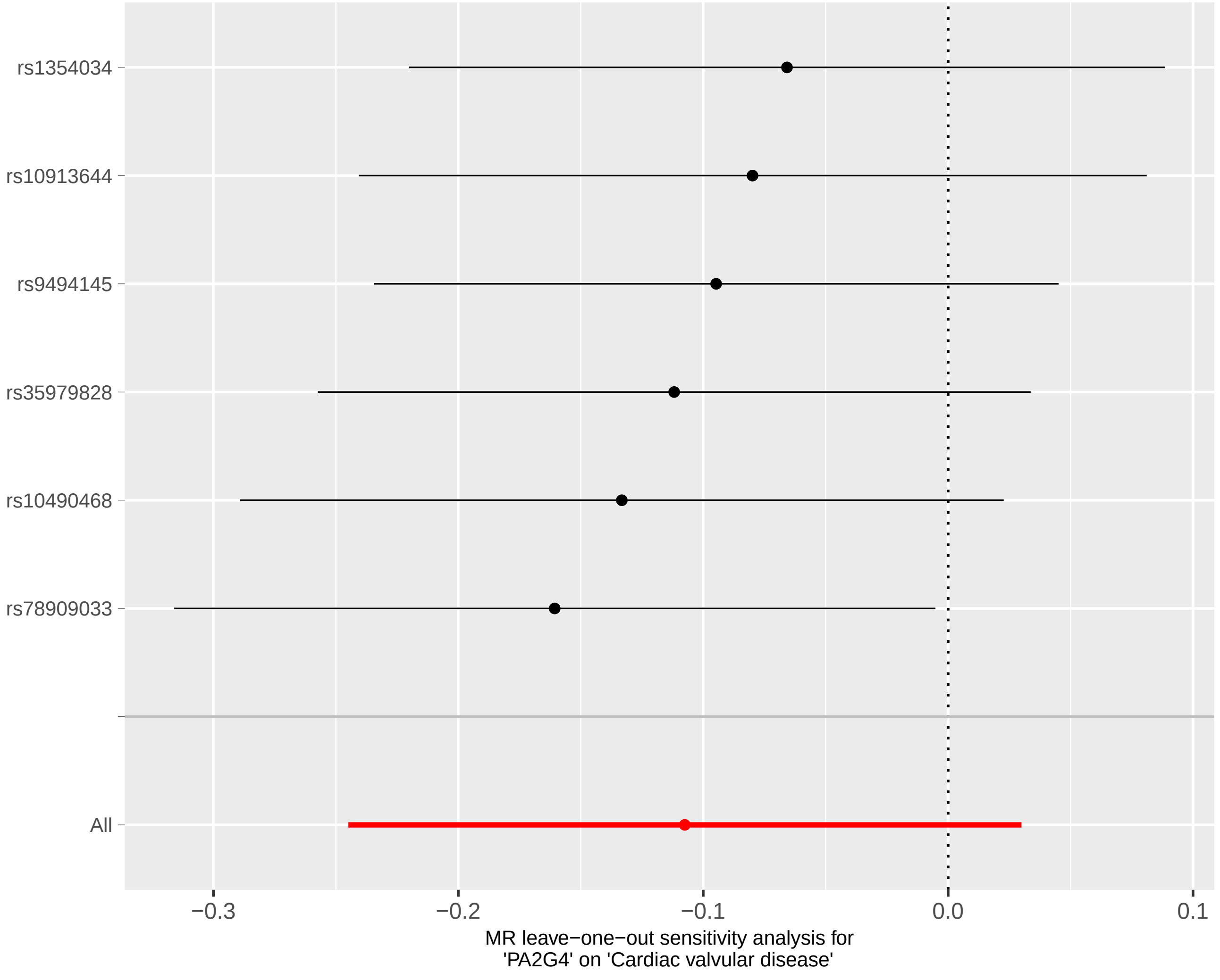
rs1354034
rs10913644
rs9494145
rs35979828
rs10490468
rs78909033
All
−0.3
−0.2
−0.1
0.0
0.1
MR leave−one−out sensitivity analysis for
'PA2G4' on 'Cardiac valvular disease'

Insufficient number of SNPs

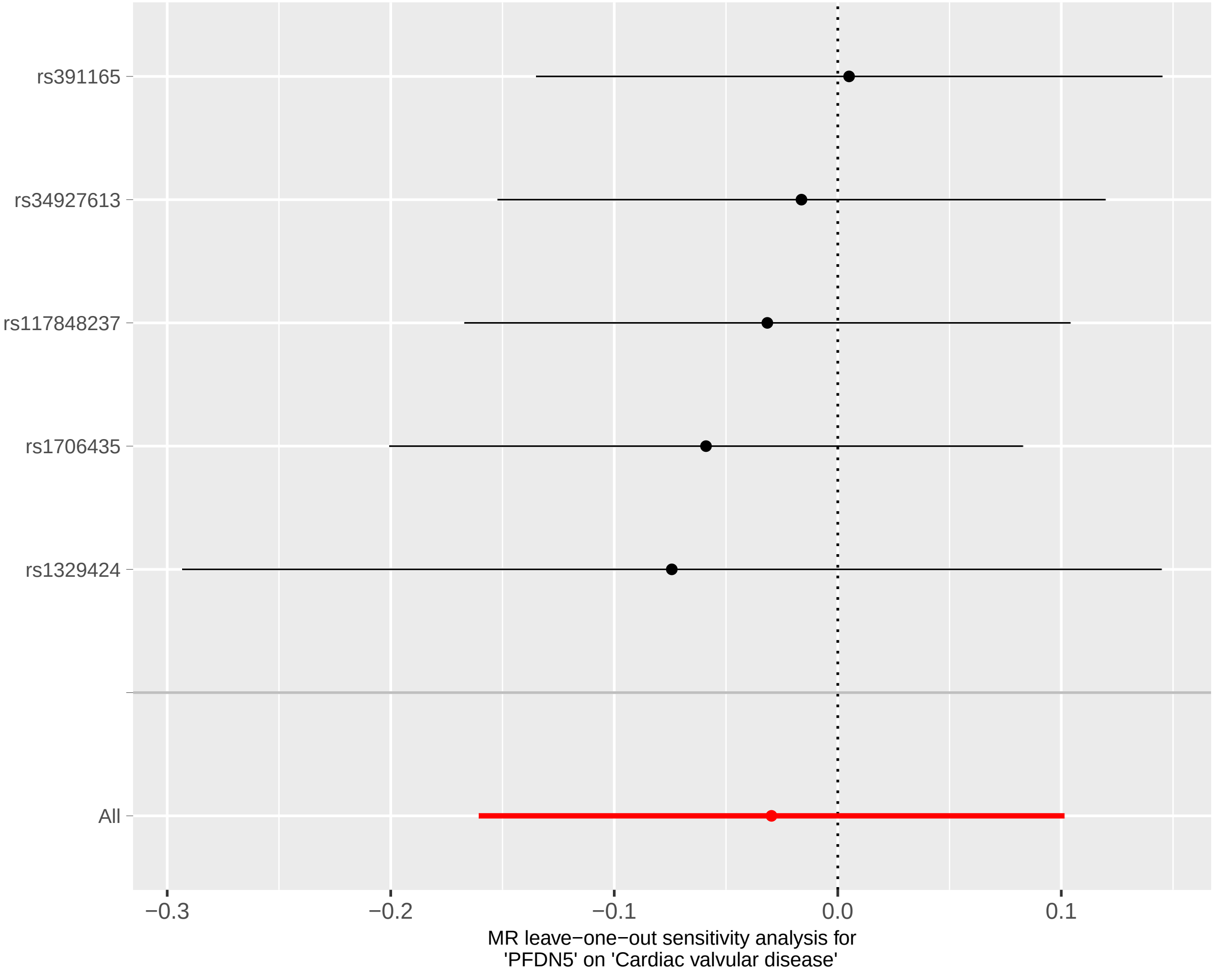

rs391165
rs34927613
rs117848237
rs1706435
rs1329424
All
−0.3
−0.2
−0.1
0.0
0.1
MR leave−one−out sensitivity analysis for
'PFDN5' on 'Cardiac valvular disease'

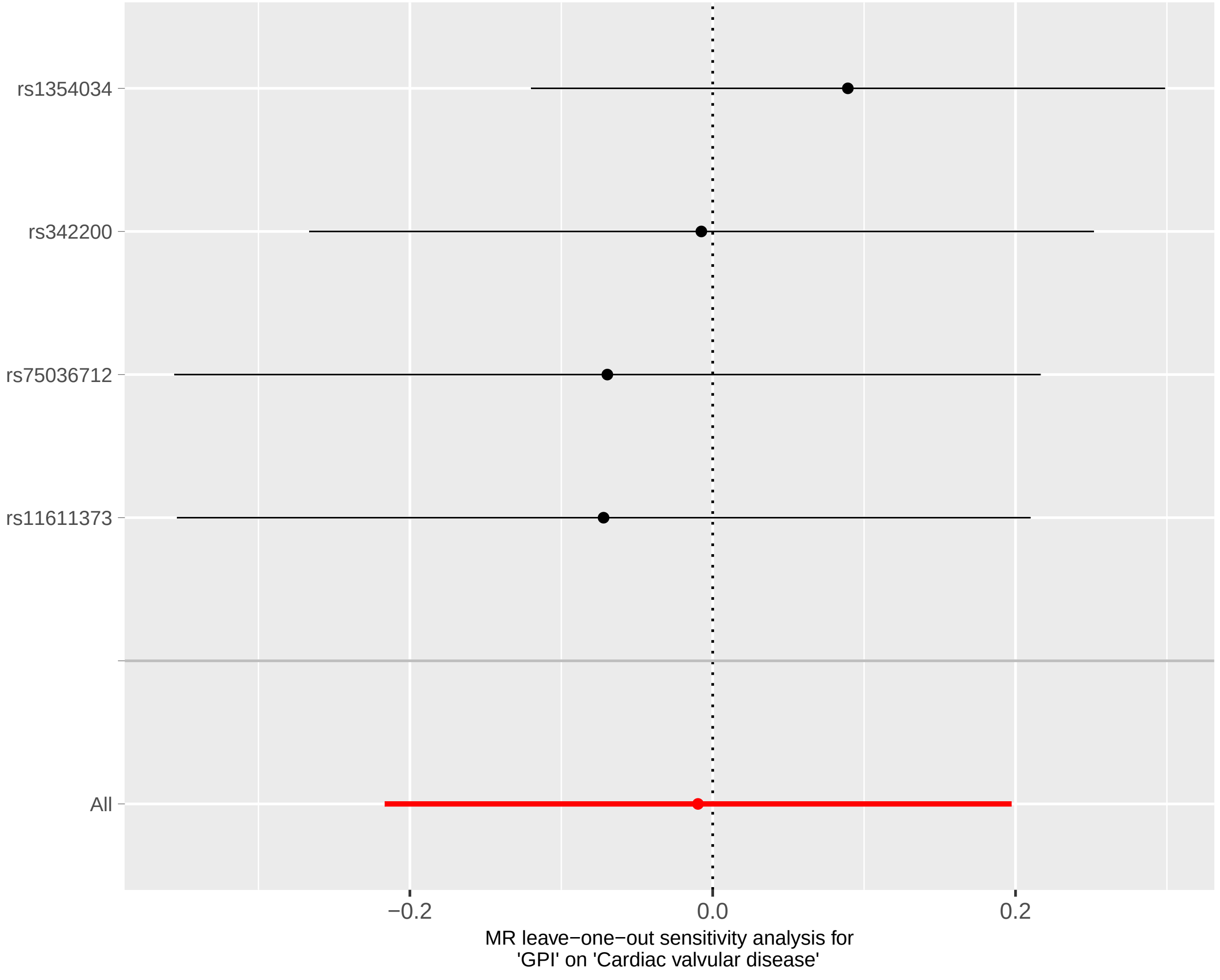
rs1354034
rs342200
rs75036712
rs11611373
All
−0.2
0.0
0.2
MR leave−one−out sensitivity analysis for
'GPI' on 'Cardiac valvular disease'

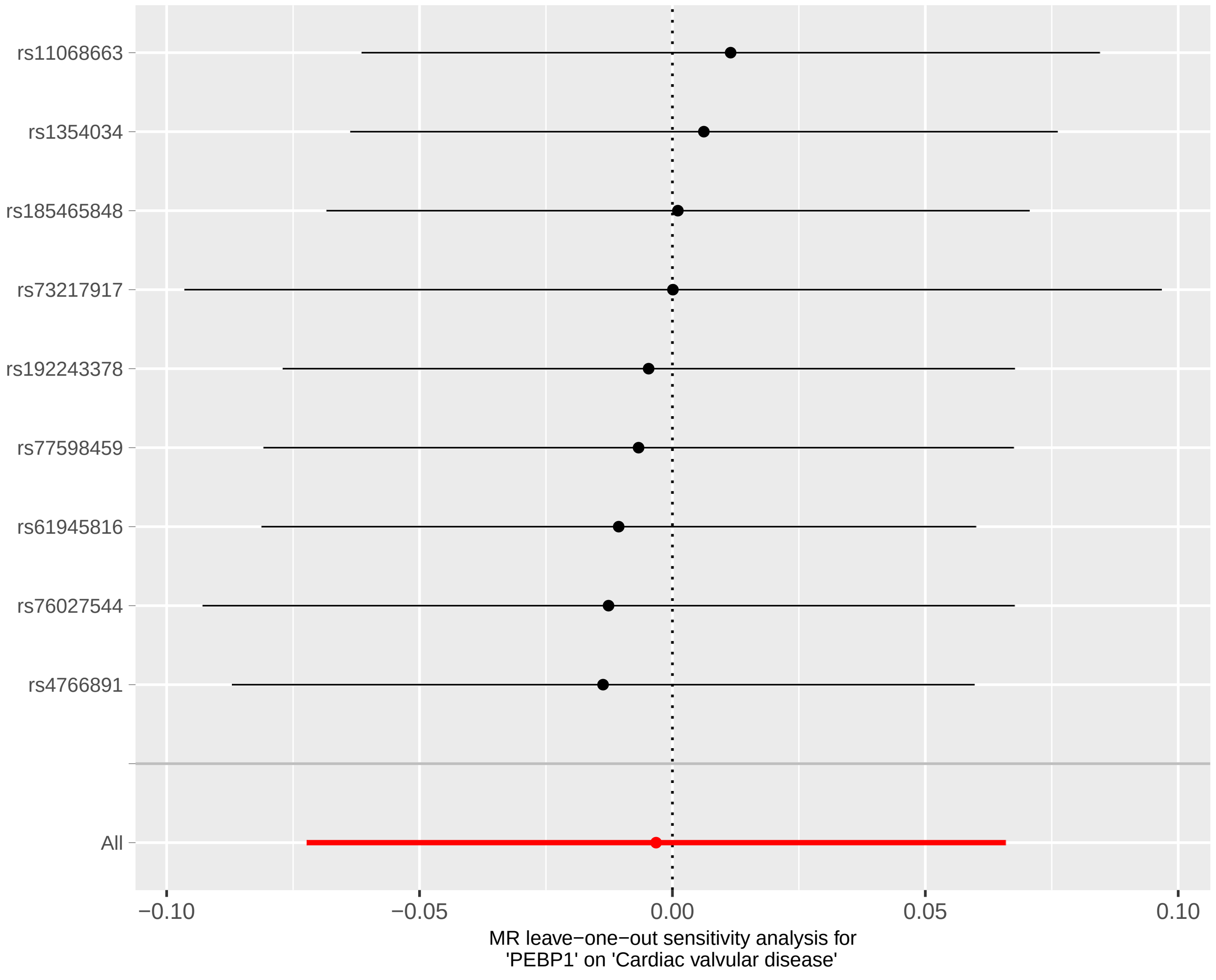
rs11068663
rs1354034
rs185465848
rs73217917
rs192243378
rs77598459
rs61945816
rs76027544
rs4766891
All
−0.10
−0.05
0.00
0.05
0.10
MR leave−one−out sensitivity analysis for
'PEBP1' on 'Cardiac valvular disease'

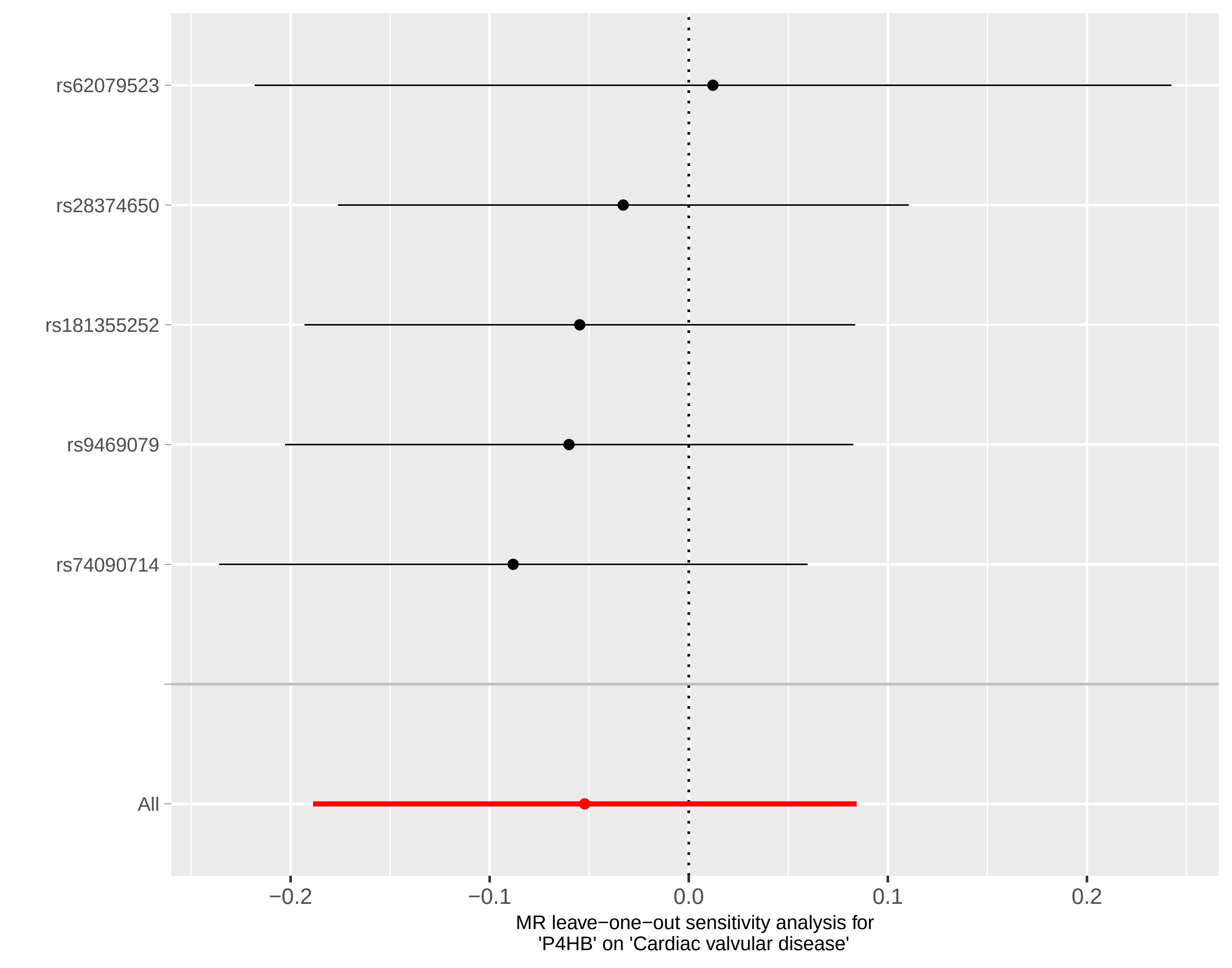
rs62079523
rs28374650
rs181355252
rs9469079
rs74090714
All
−0.2
−0.1
0.0
0.1
0.2
MR leave−one−out sensitivity analysis for
'P4HB' on 'Cardiac valvular disease'

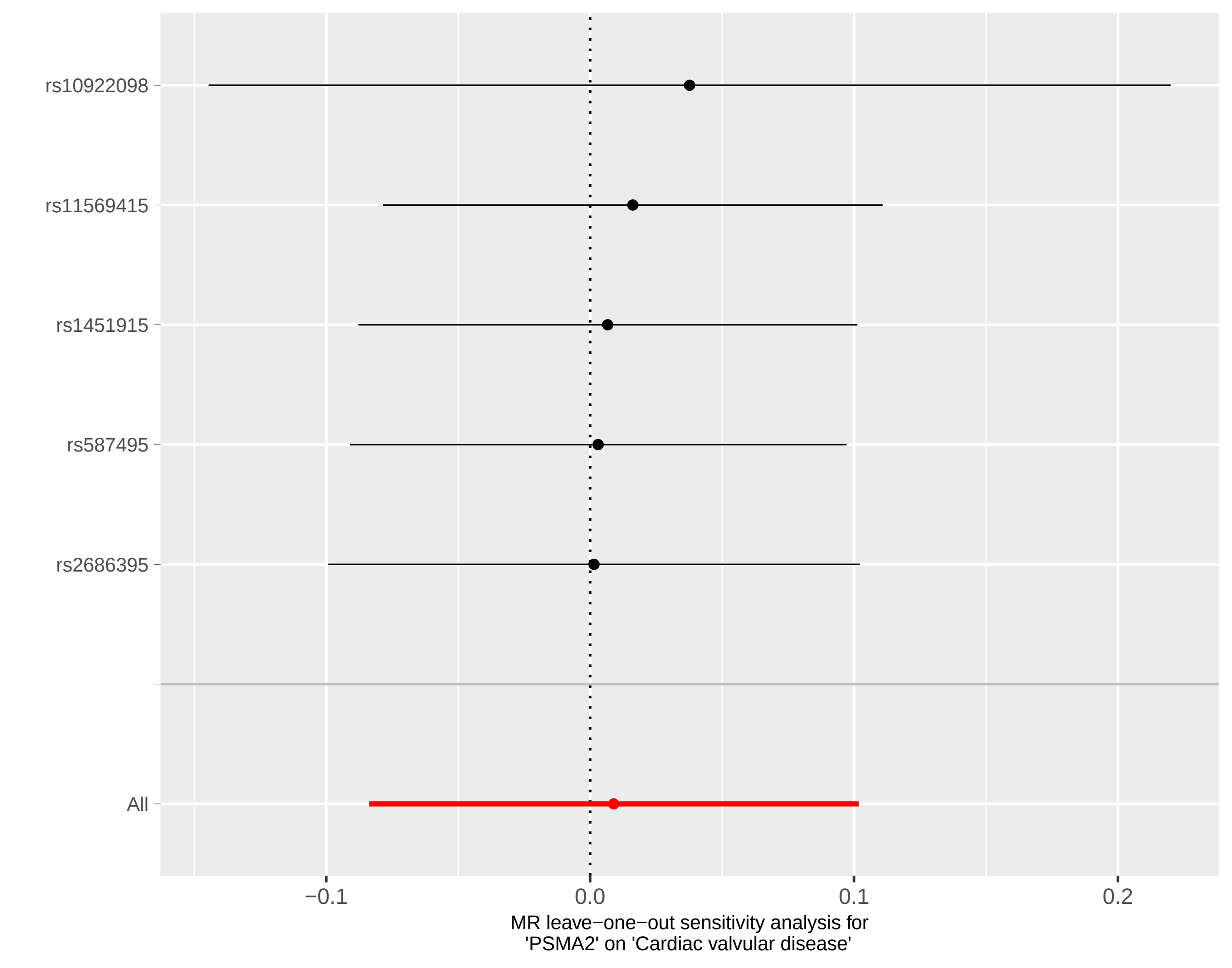

rs10922098
rs11569415
rs1451915
rs587495
rs2686395
All
−0.1
0.0
0.1
0.2
MR leave−one−out sensitivity analysis for
'PSMA2' on 'Cardiac valvular disease'

Insufficient number of SNPs

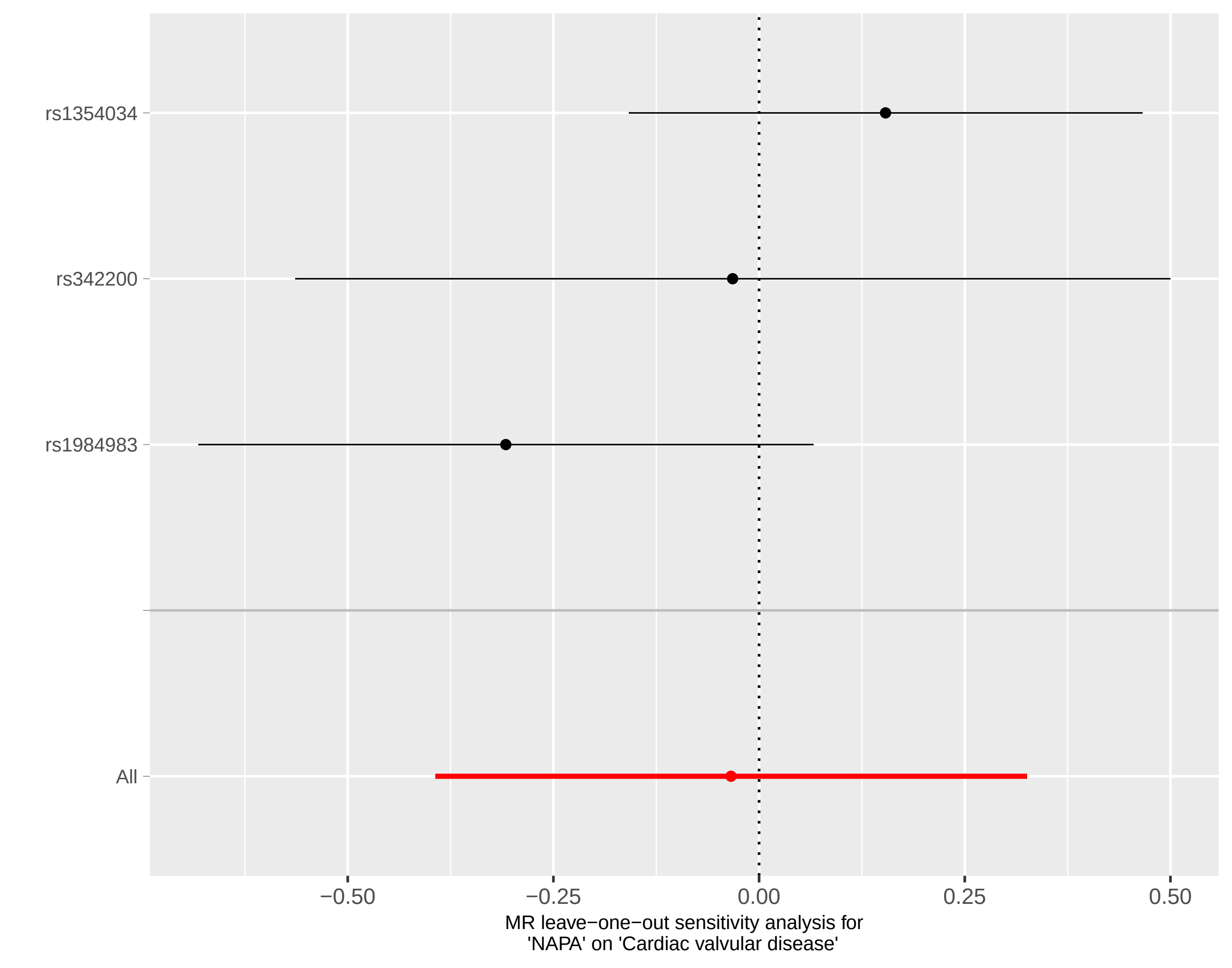
rs1354034
rs342200
rs1984983
All
−0.50
−0.25
0.00
0.25
0.50
MR leave−one−out sensitivity analysis for
'NAPA' on 'Cardiac valvular disease'

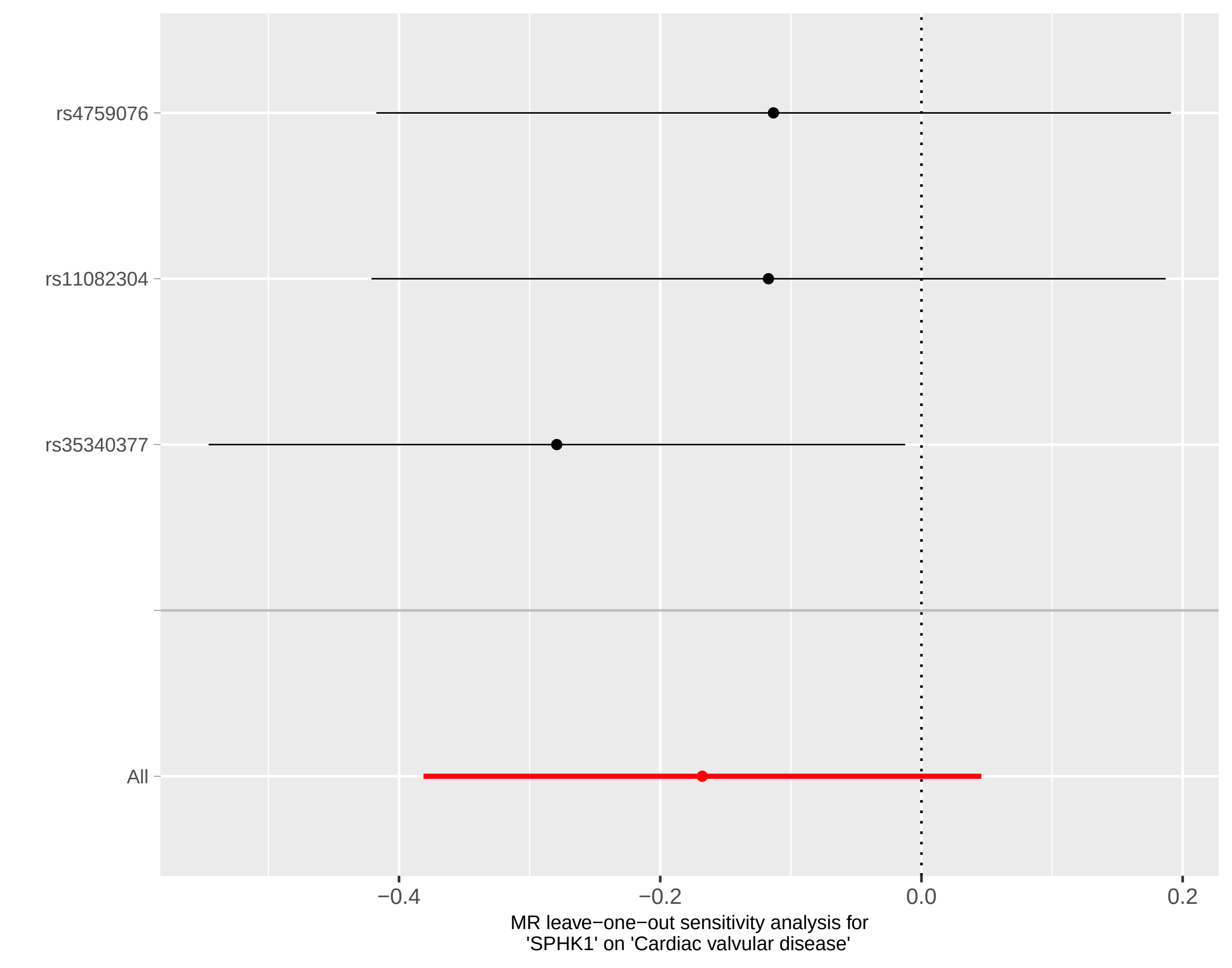
rs4759076
rs11082304
rs35340377
All
−0.4
−0.2
0.0
0.2
MR leave−one−out sensitivity analysis for
'SPHK1' on 'Cardiac valvular disease'

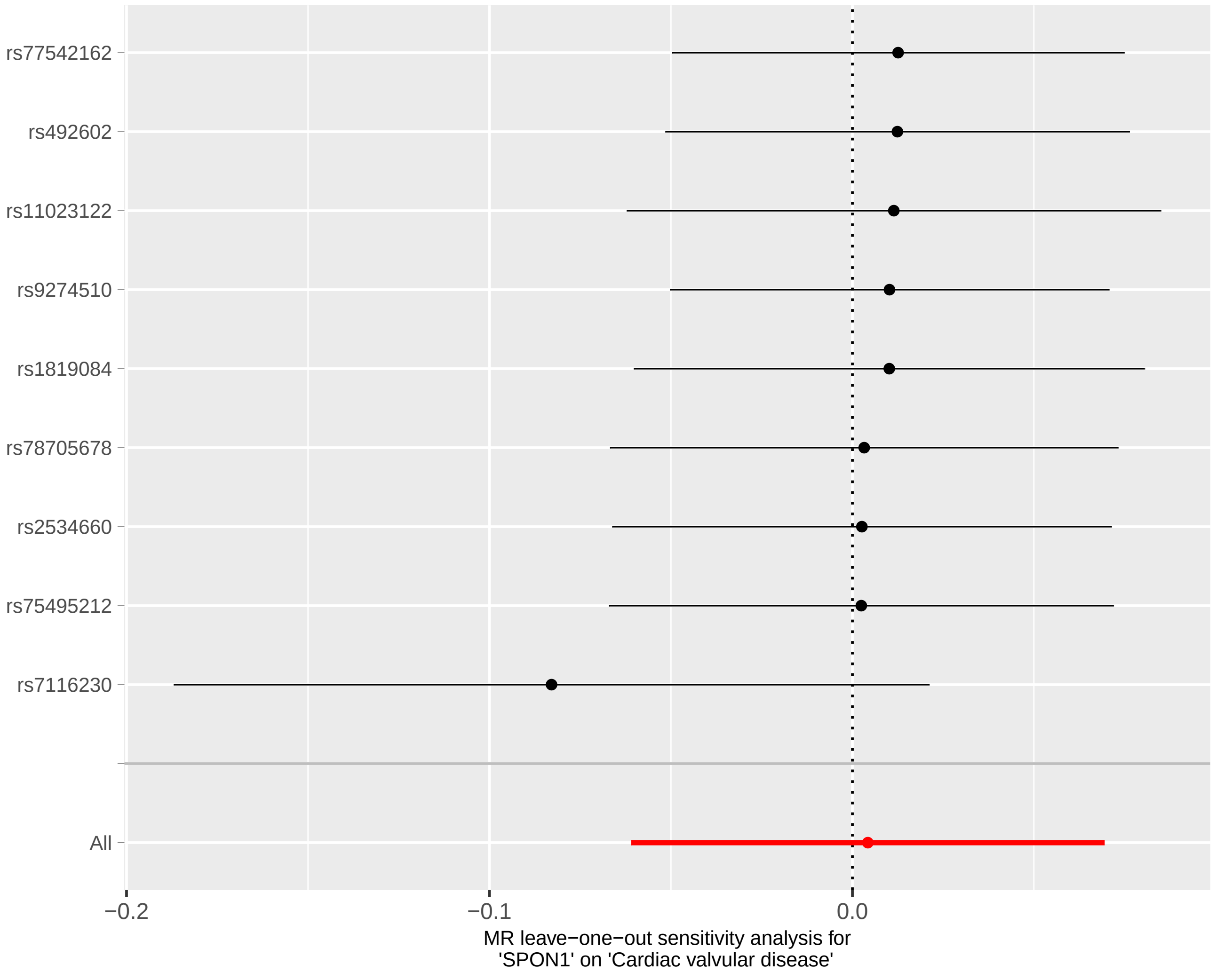

rs77542162
rs492602
rs11023122
rs9274510
rs1819084
rs78705678
rs2534660
rs75495212
rs7116230
All
−0.2
−0.1
0.0
MR leave−one−out sensitivity analysis for
'SPON1' on 'Cardiac valvular disease'

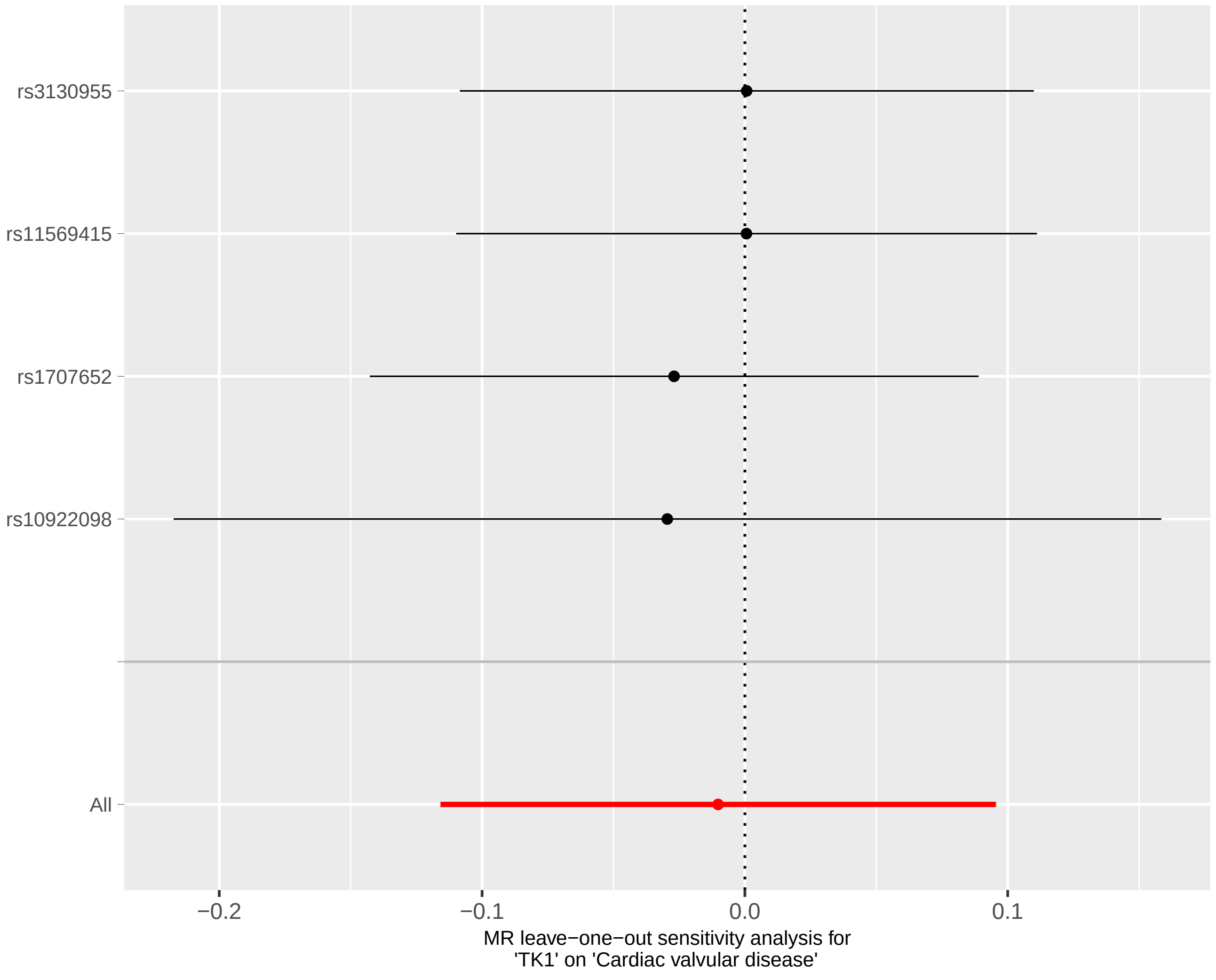
rs3130955
rs11569415
rs1707652
rs10922098
All
−0.2
−0.1
0.0
0.1
MR leave−one−out sensitivity analysis for
'TK1' on 'Cardiac valvular disease'

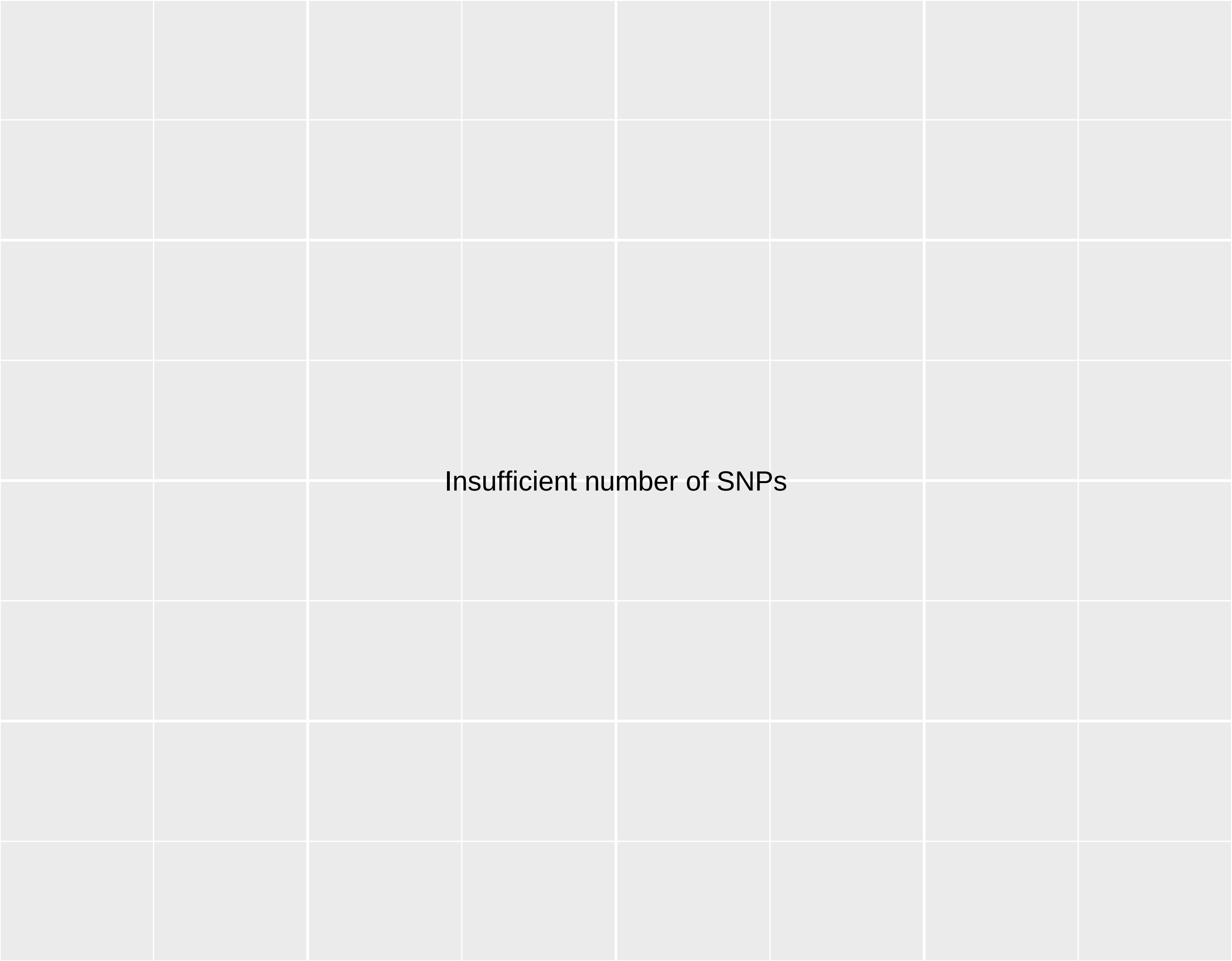
Insufficient number of SNPs

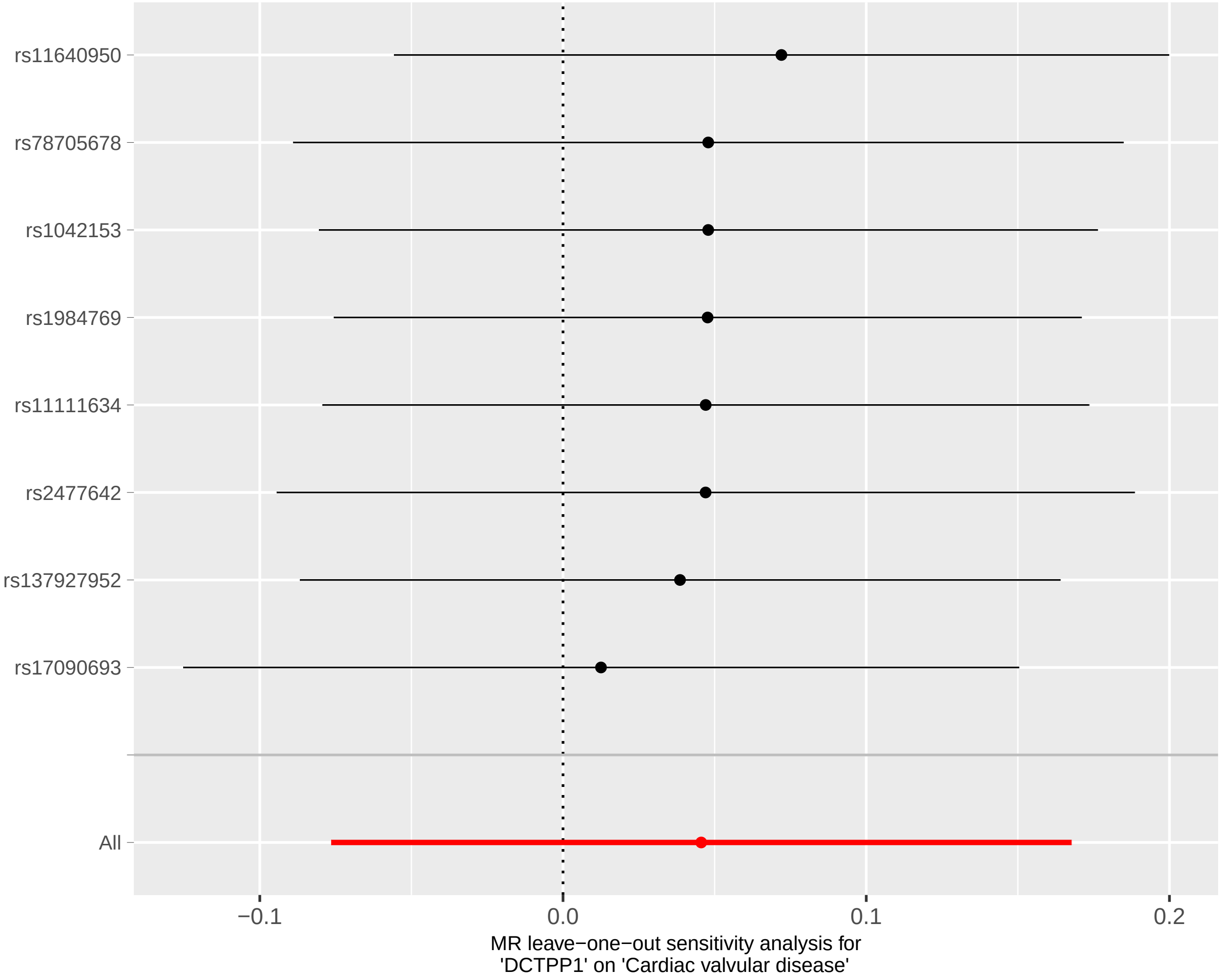
rs11640950
rs78705678
rs1042153
rs1984769
rs11111634
rs2477642
rs137927952
rs17090693
All
−0.1
0.0
0.1
0.2
MR leave−one−out sensitivity analysis for
'DCTPP1' on 'Cardiac valvular disease'

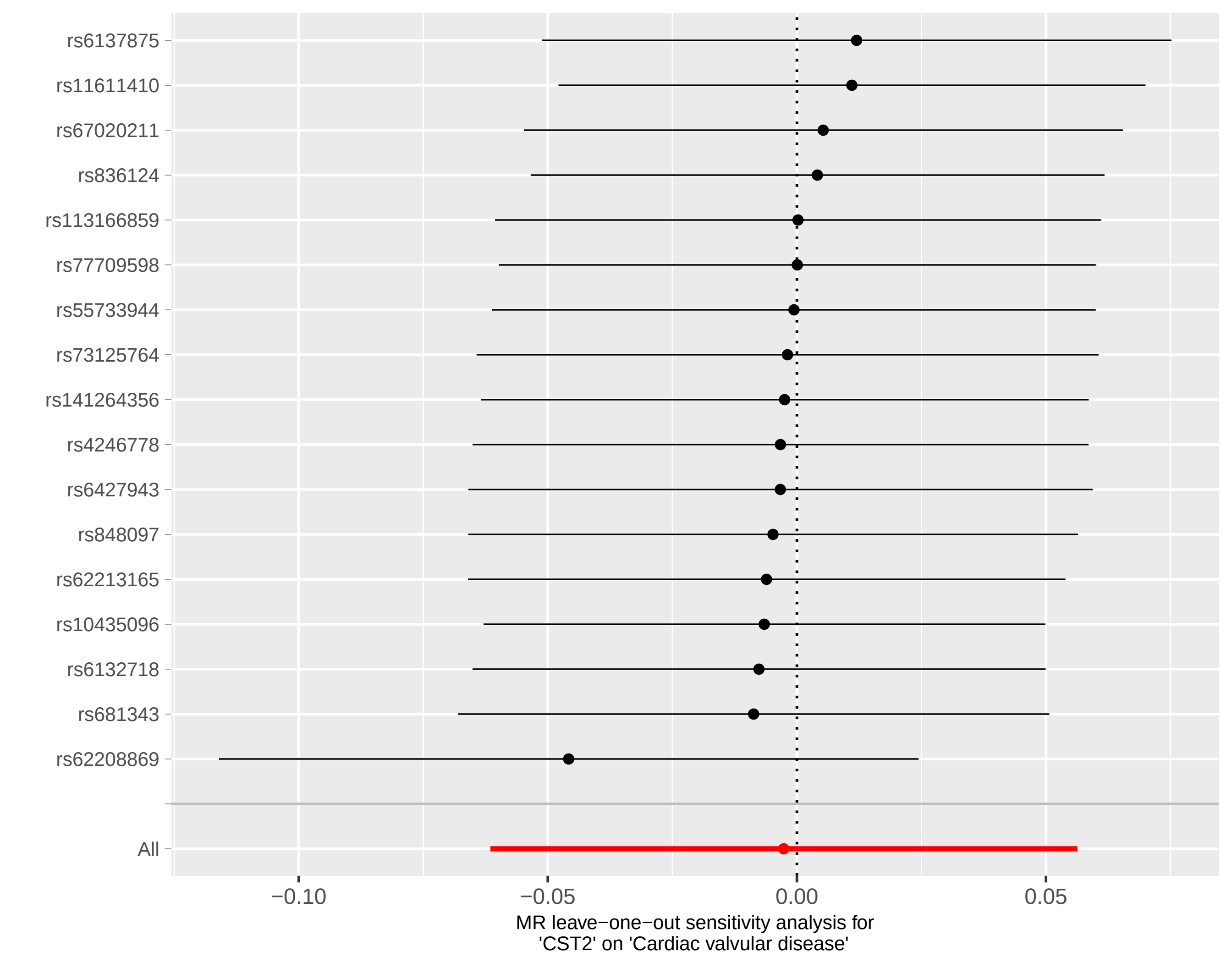

rs6137875
rs11611410
rs67020211
rs836124
rs113166859
rs77709598
rs55733944
rs73125764
rs141264356
rs4246778
rs6427943
rs848097
rs62213165
rs10435096
rs6132718
rs681343
rs62208869
All
−0.10
−0.05
0.00
0.05
MR leave−one−out sensitivity analysis for
'CST2' on 'Cardiac valvular disease'

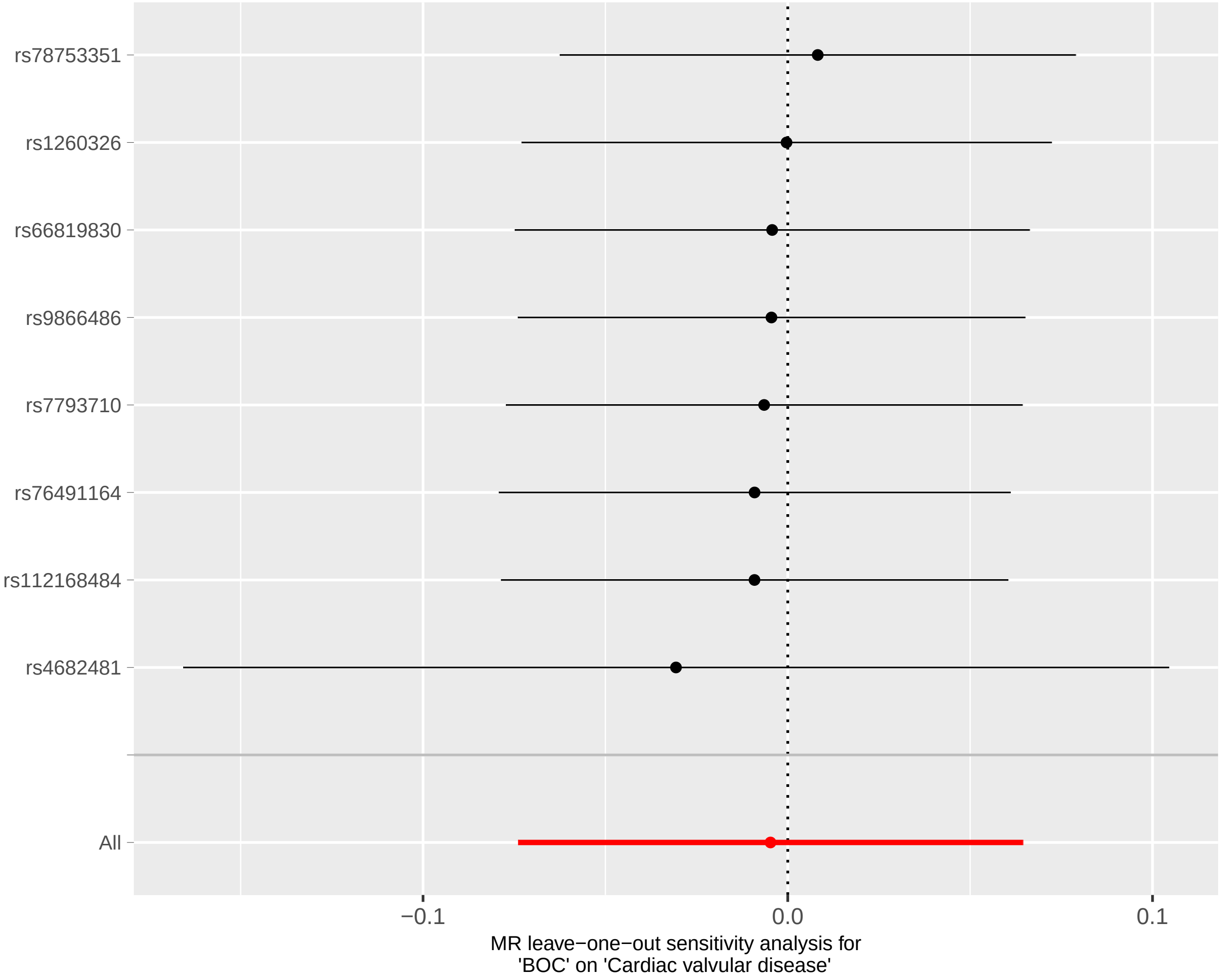

rs78753351
rs1260326
rs66819830
rs9866486
rs7793710
rs76491164
rs112168484
rs4682481
All
−0.1
0.0
0.1
MR leave−one−out sensitivity analysis for
'BOC' on 'Cardiac valvular disease'

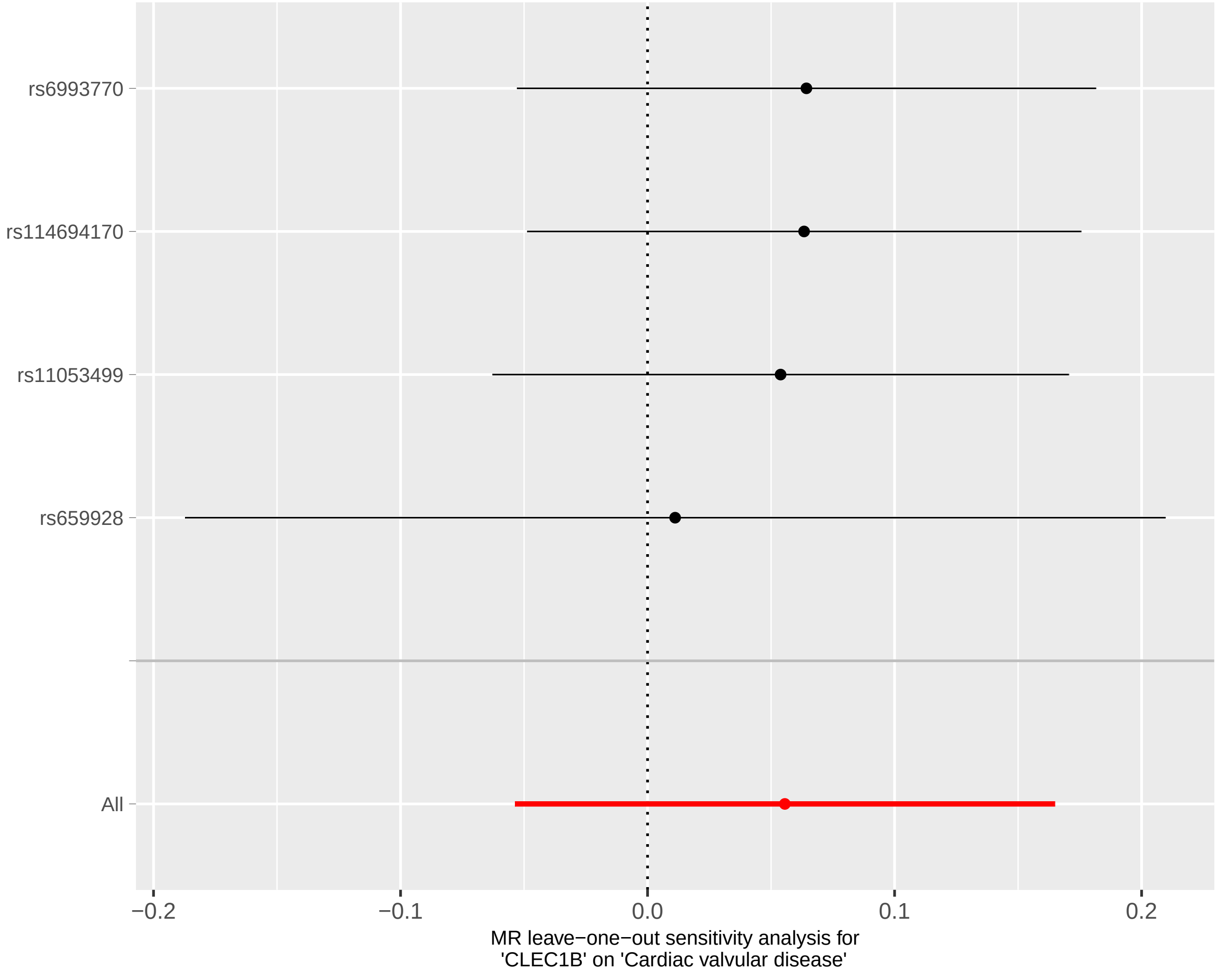

rs6993770
rs114694170
rs11053499
rs659928
All
−0.2
−0.1
0.0
0.1
0.2
MR leave−one−out sensitivity analysis for
'CLEC1B' on 'Cardiac valvular disease'

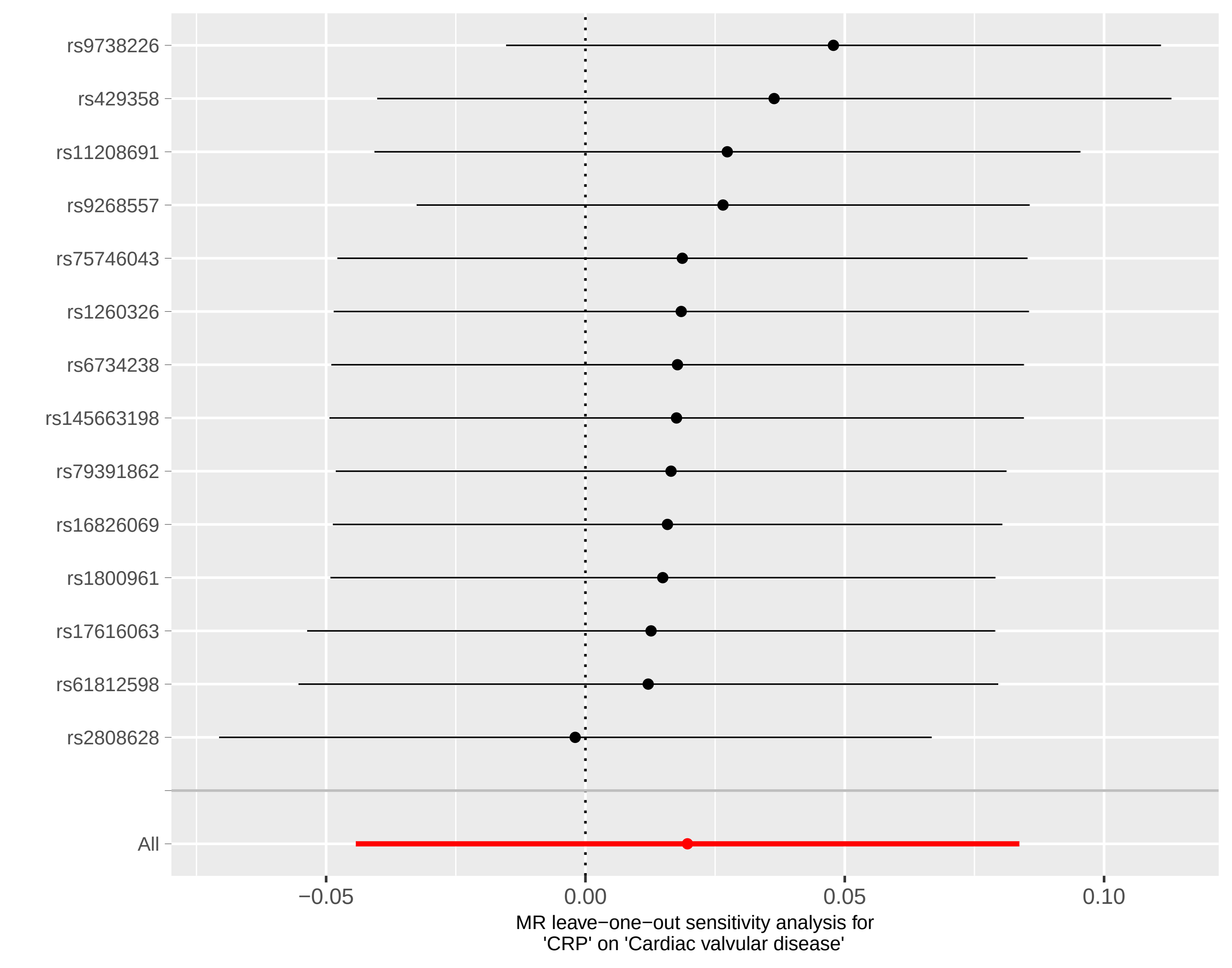
rs9738226
rs429358
rs11208691
rs9268557
rs75746043
rs1260326
rs6734238
rs145663198
rs79391862
rs16826069
rs1800961
rs17616063
rs61812598
rs2808628
All
−0.05
0.00
0.05
0.10
MR leave−one−out sensitivity analysis for
'CRP' on 'Cardiac valvular disease'

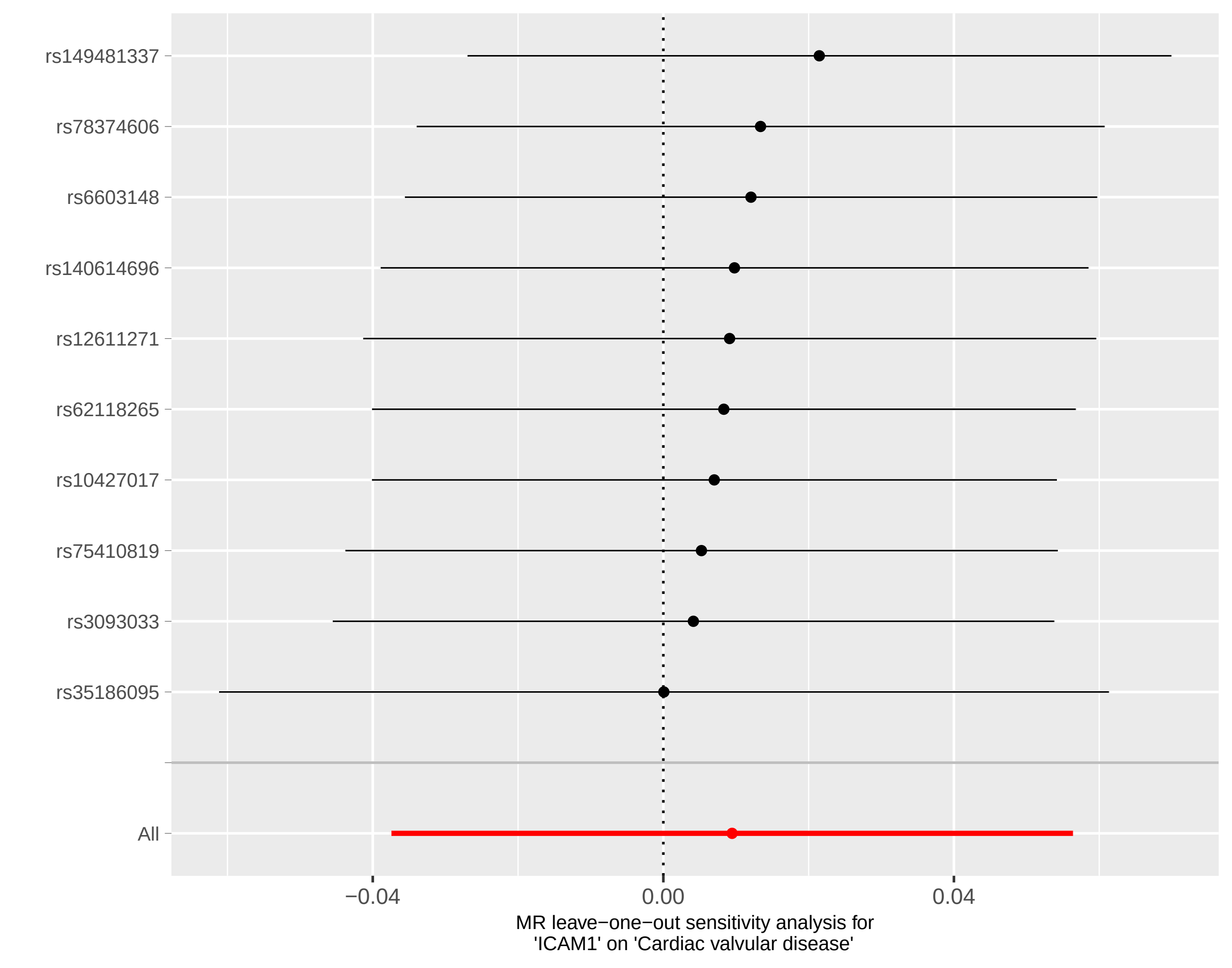
rs149481337
rs78374606
rs6603148
rs140614696
rs12611271
rs62118265
rs10427017
rs75410819
rs3093033
rs35186095
All
−0.04
0.00
0.04
MR leave−one−out sensitivity analysis for
'ICAM1' on 'Cardiac valvular disease'

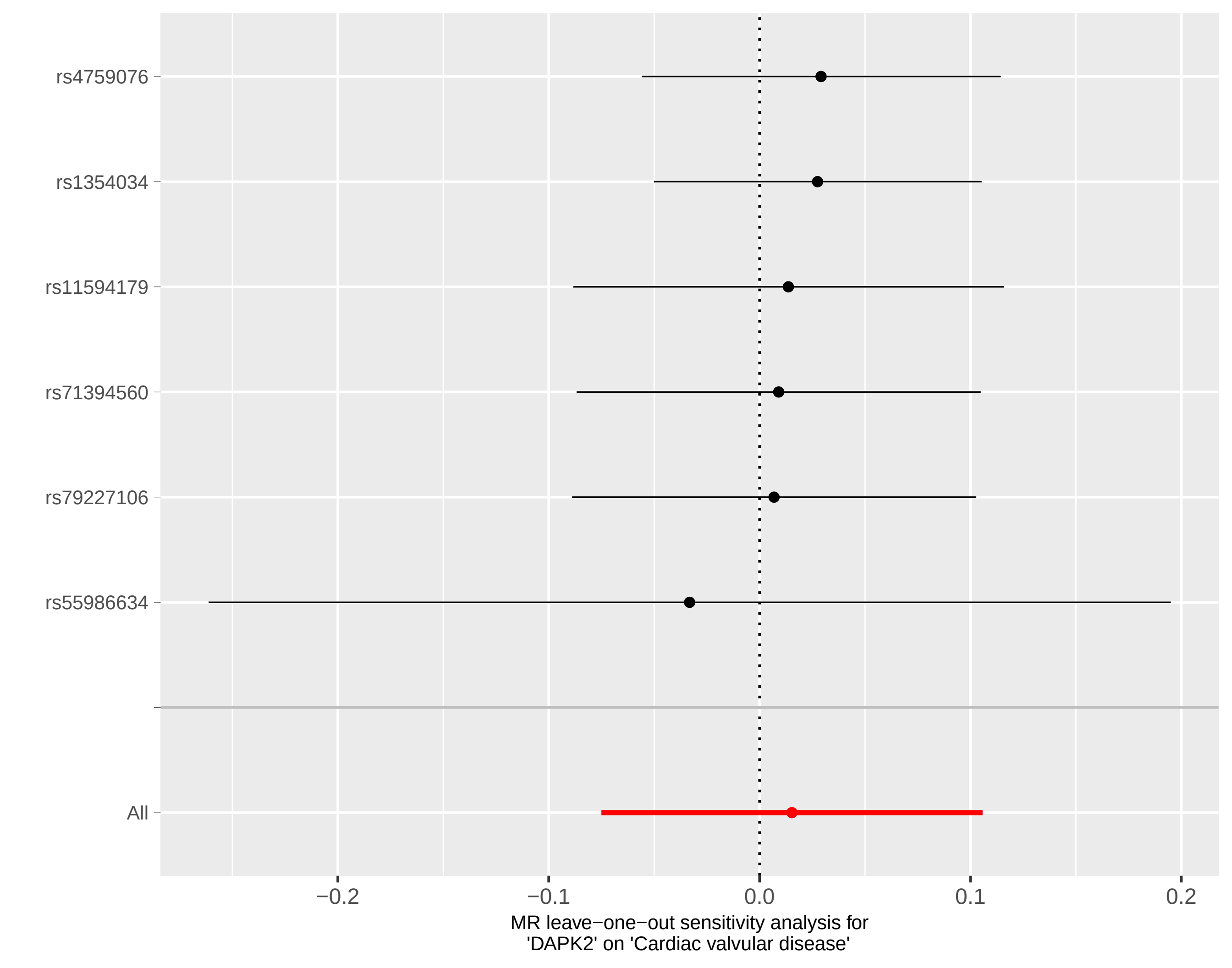

rs4759076
rs1354034
rs11594179
rs71394560
rs79227106
rs55986634
All
−0.2
−0.1
0.0
0.1
0.2
MR leave−one−out sensitivity analysis for
'DAPK2' on 'Cardiac valvular disease'

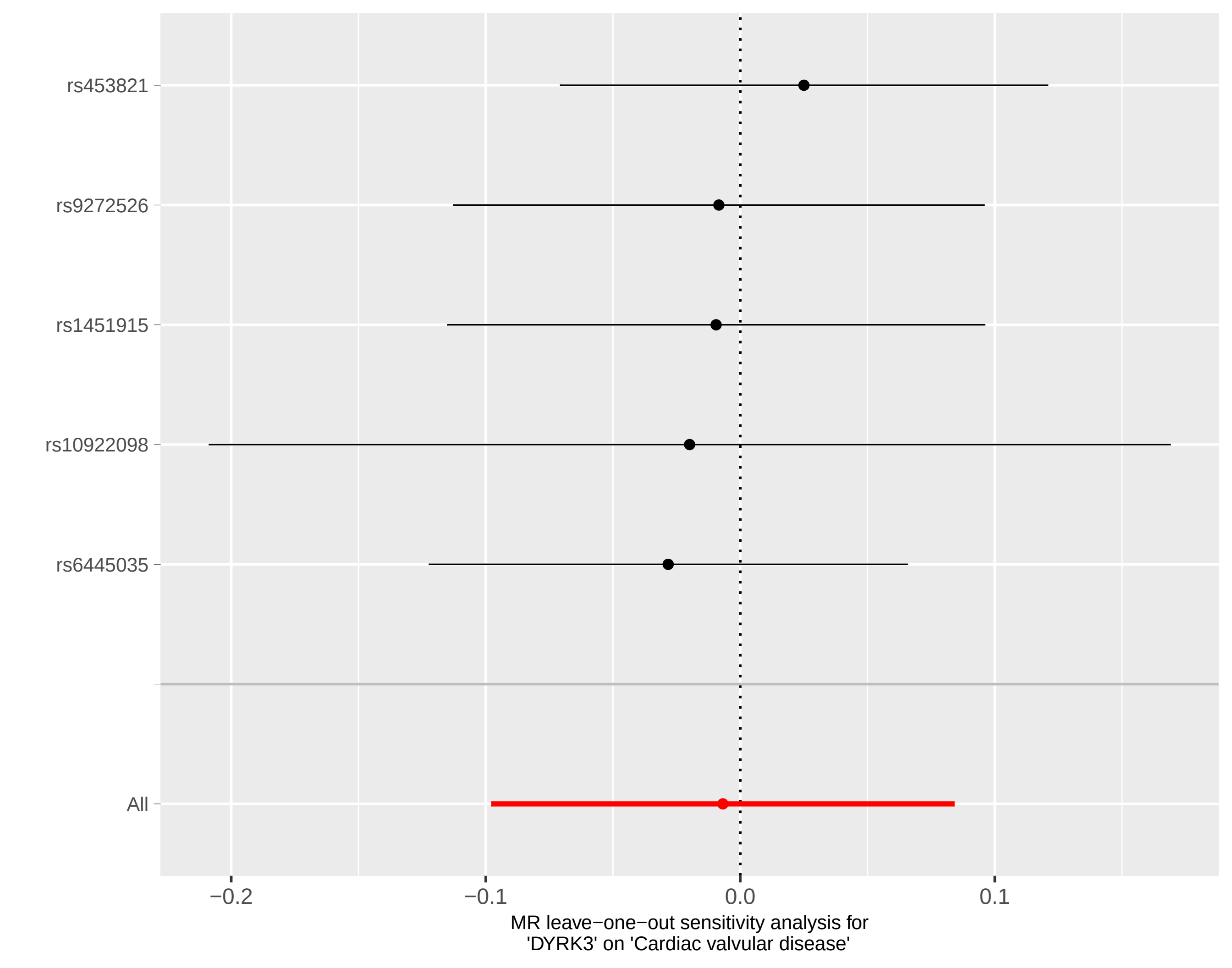
rs453821
rs9272526
rs1451915
rs10922098
rs6445035
All
−0.2
−0.1
0.0
0.1
MR leave−one−out sensitivity analysis for
'DYRK3' on 'Cardiac valvular disease'

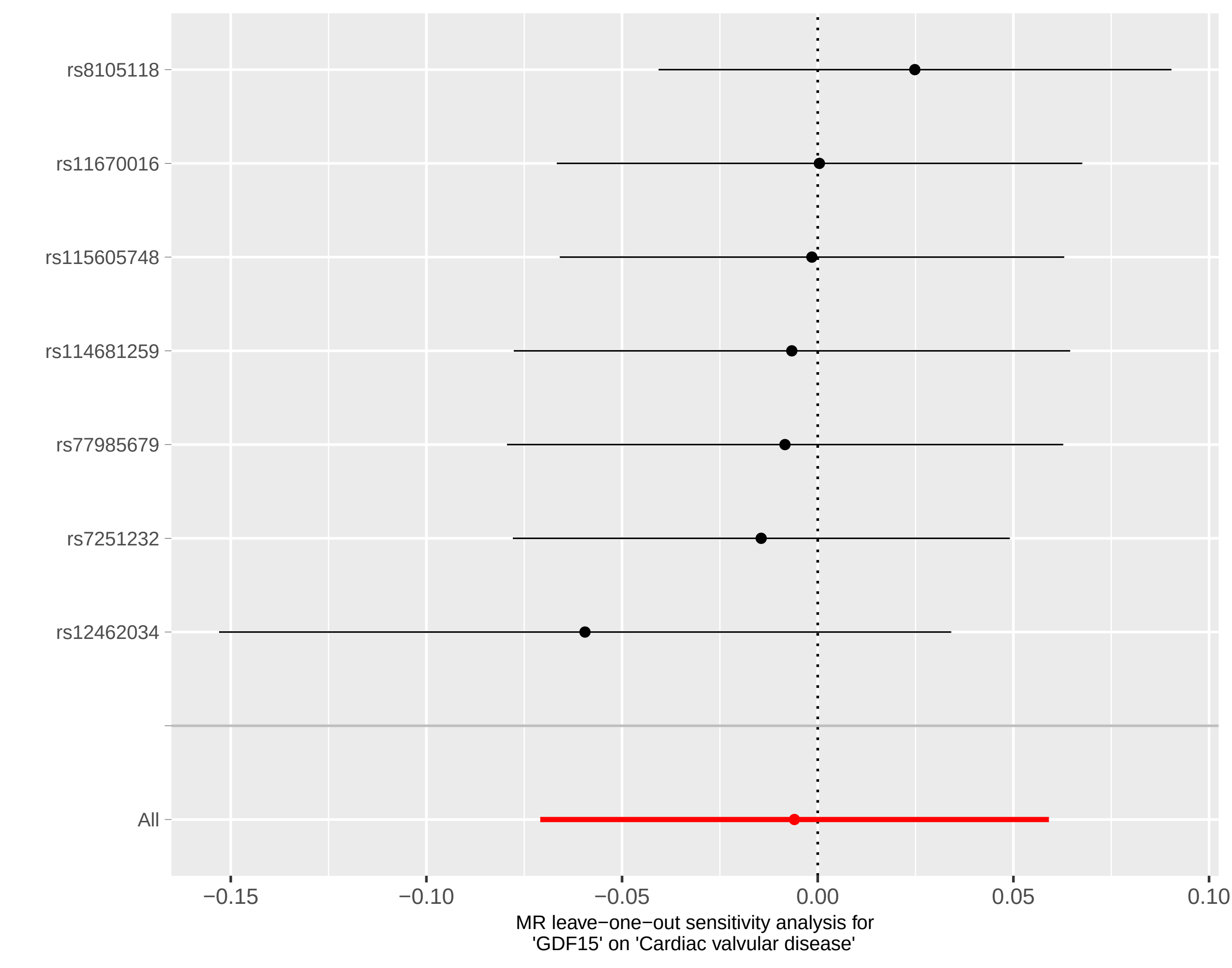

rs8105118
rs11670016
rs115605748
rs114681259
rs77985679
rs7251232
rs12462034
All
−0.15
−0.10
−0.05
0.00
0.05
0.10
MR leave−one−out sensitivity analysis for
'GDF15' on 'Cardiac valvular disease'

Insufficient number of SNPs

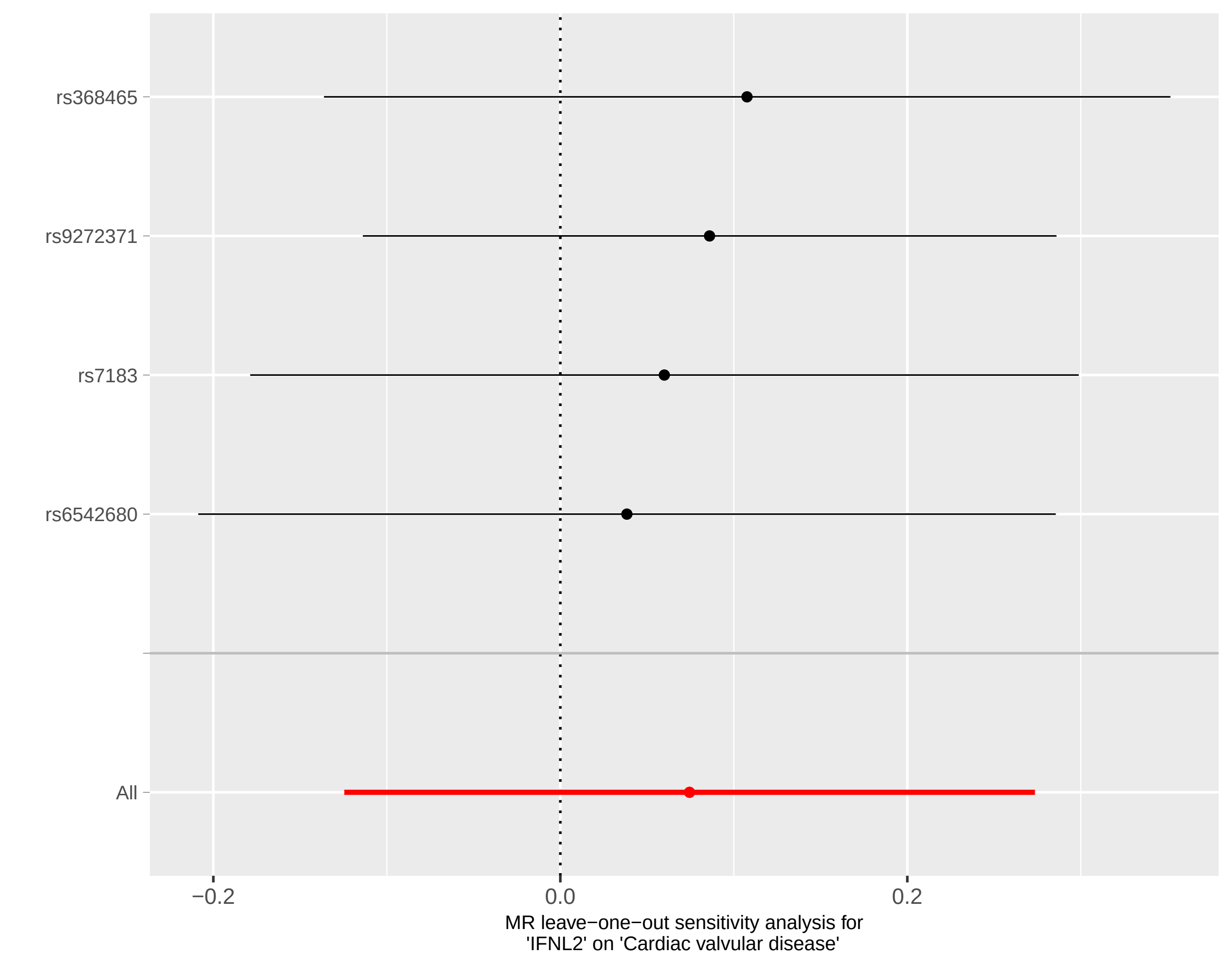

rs368465
rs9272371
rs7183
rs6542680
All
−0.2
0.0
0.2
MR leave−one−out sensitivity analysis for
'IFNL2' on 'Cardiac valvular disease'

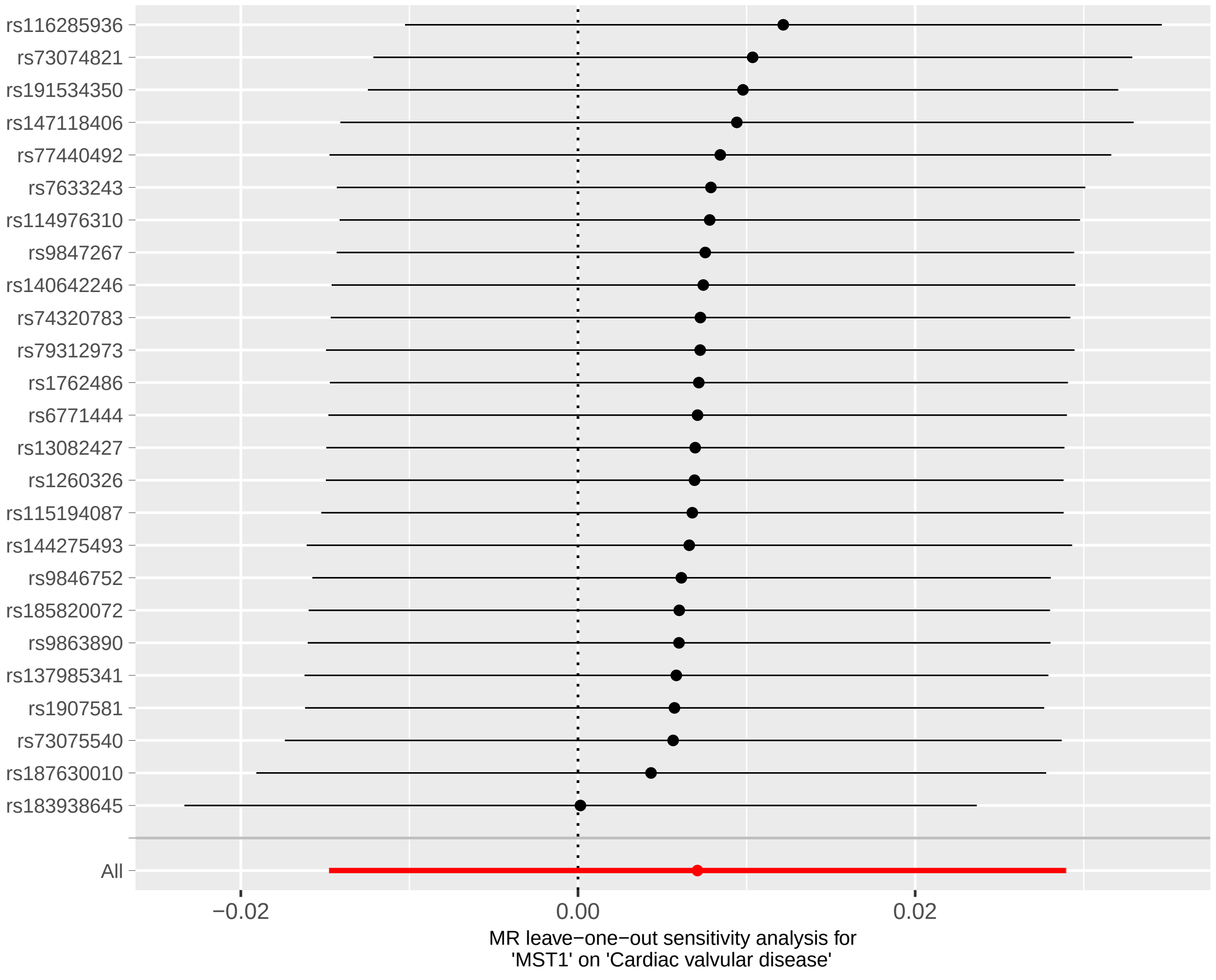
rs116285936
rs73074821
rs191534350
rs147118406
rs77440492
rs7633243
rs114976310
rs9847267
rs140642246
rs74320783
rs79312973
rs1762486
rs6771444
rs13082427
rs1260326
rs115194087
rs144275493
rs9846752
rs185820072
rs9863890
rs137985341
rs1907581
rs73075540
rs187630010
rs183938645
All
−0.02
0.00
0.02
MR leave−one−out sensitivity analysis for
'MST1' on 'Cardiac valvular disease'

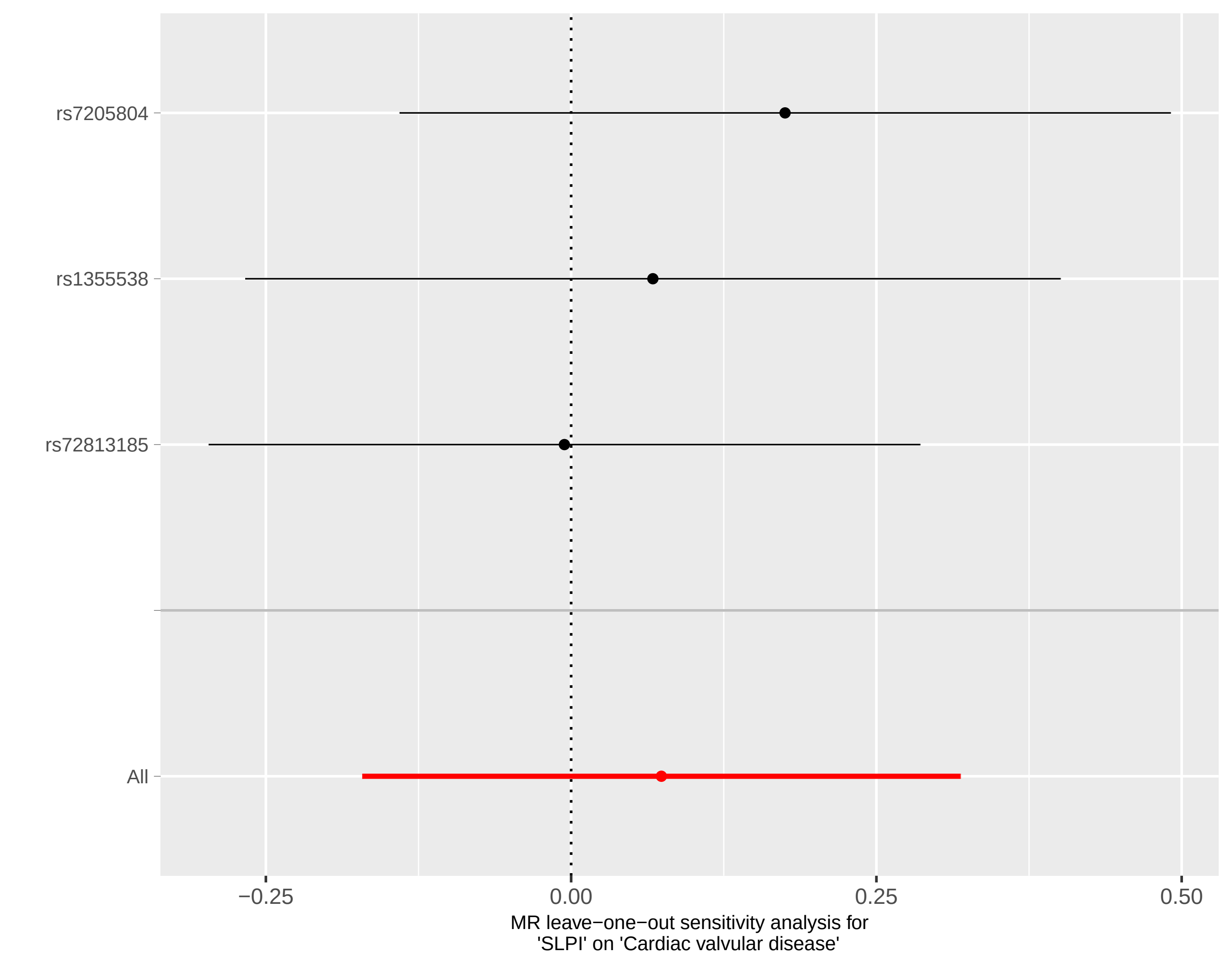
rs7205804
rs1355538
rs72813185
All
−0.25
0.00
0.25
0.50
MR leave−one−out sensitivity analysis for
'SLPI' on 'Cardiac valvular disease'

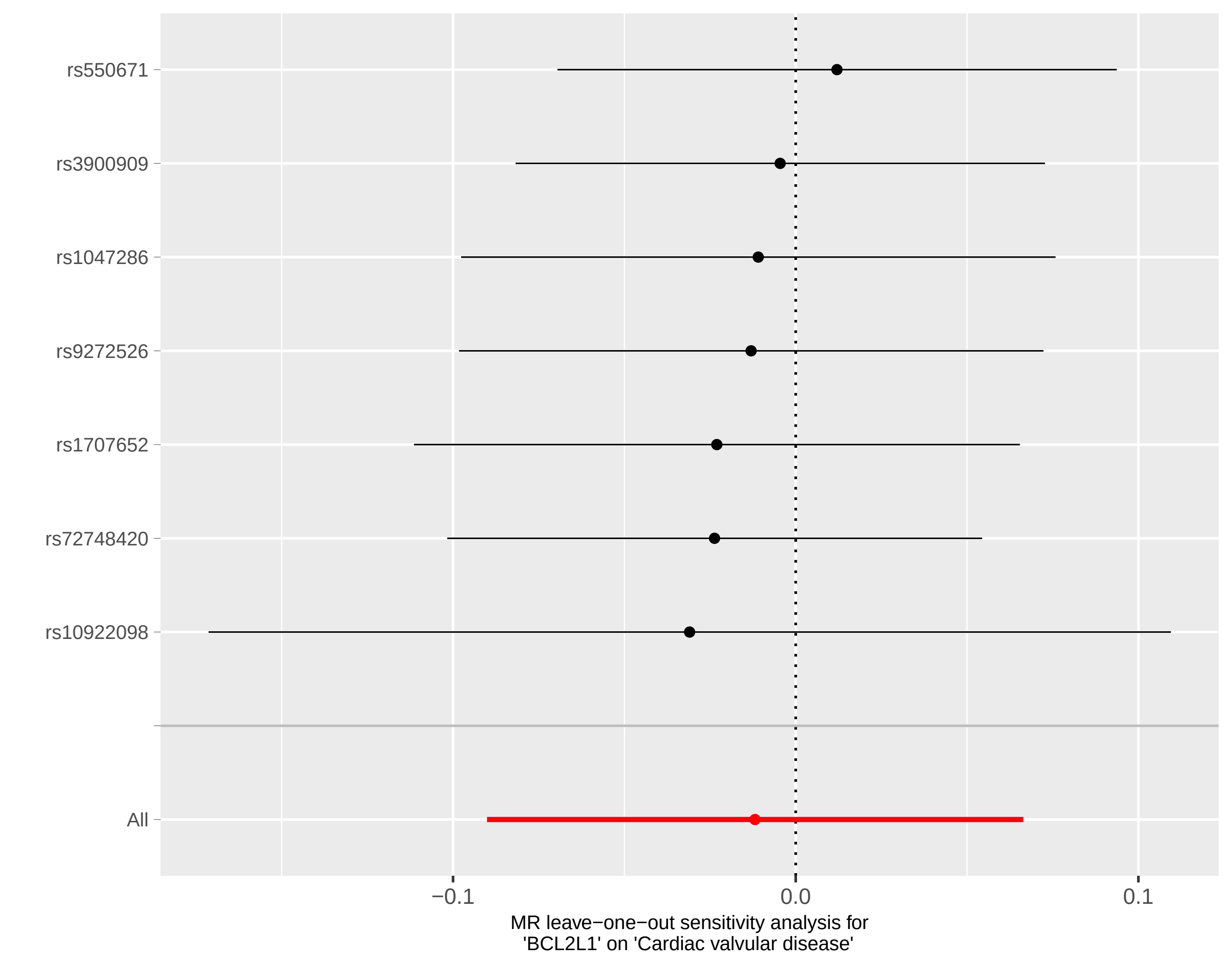

rs550671
rs3900909
rs1047286
rs9272526
rs1707652
rs72748420
rs10922098
All
−0.1
0.0
0.1
MR leave−one−out sensitivity analysis for
'BCL2L1' on 'Cardiac valvular disease'

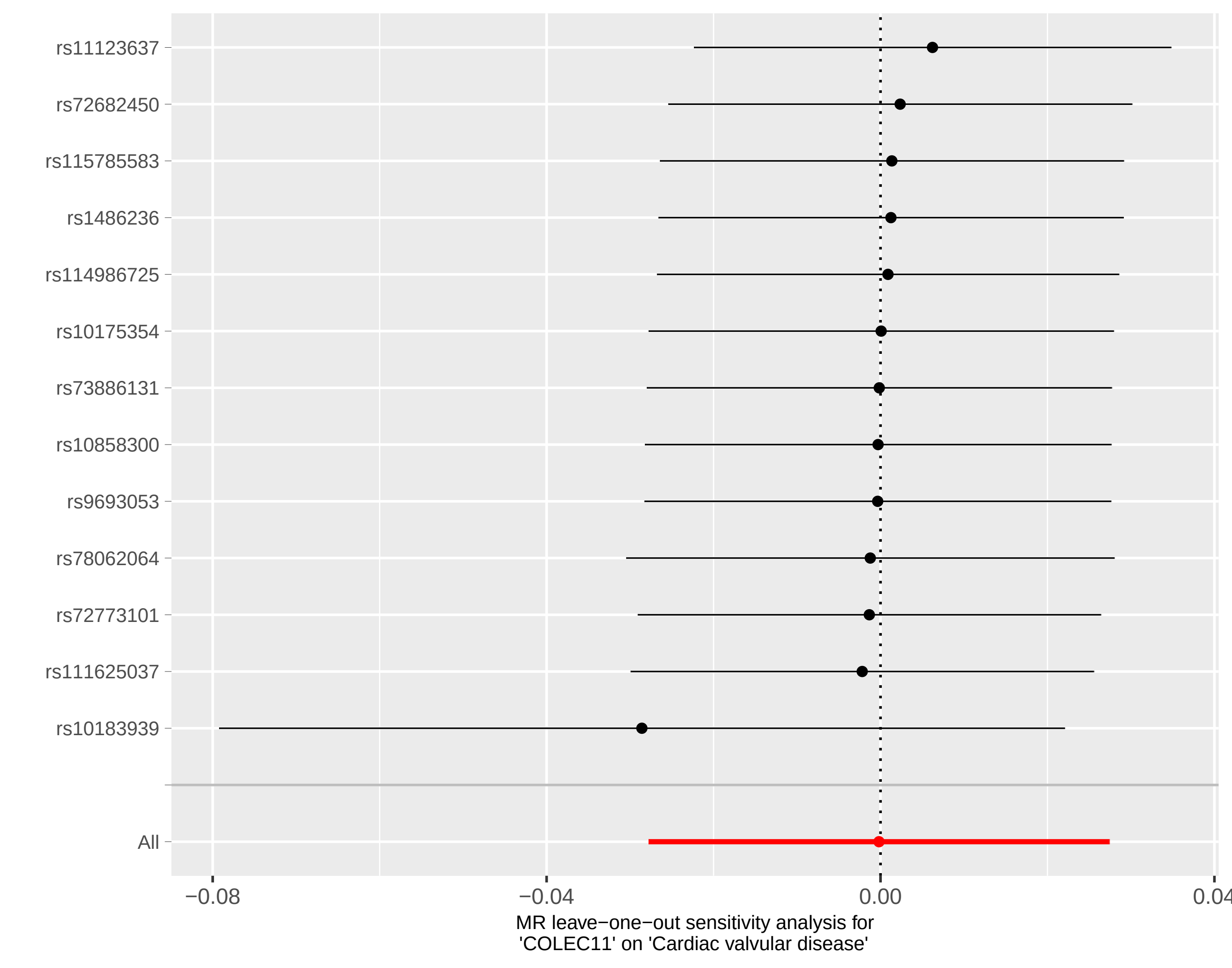

rs11123637
rs72682450
rs115785583
rs1486236
rs114986725
rs10175354
rs73886131
rs10858300
rs9693053
rs78062064
rs72773101
rs111625037
rs10183939
All
−0.08
−0.04
0.00
0.04
MR leave−one−out sensitivity analysis for
'COLEC11' on 'Cardiac valvular disease'

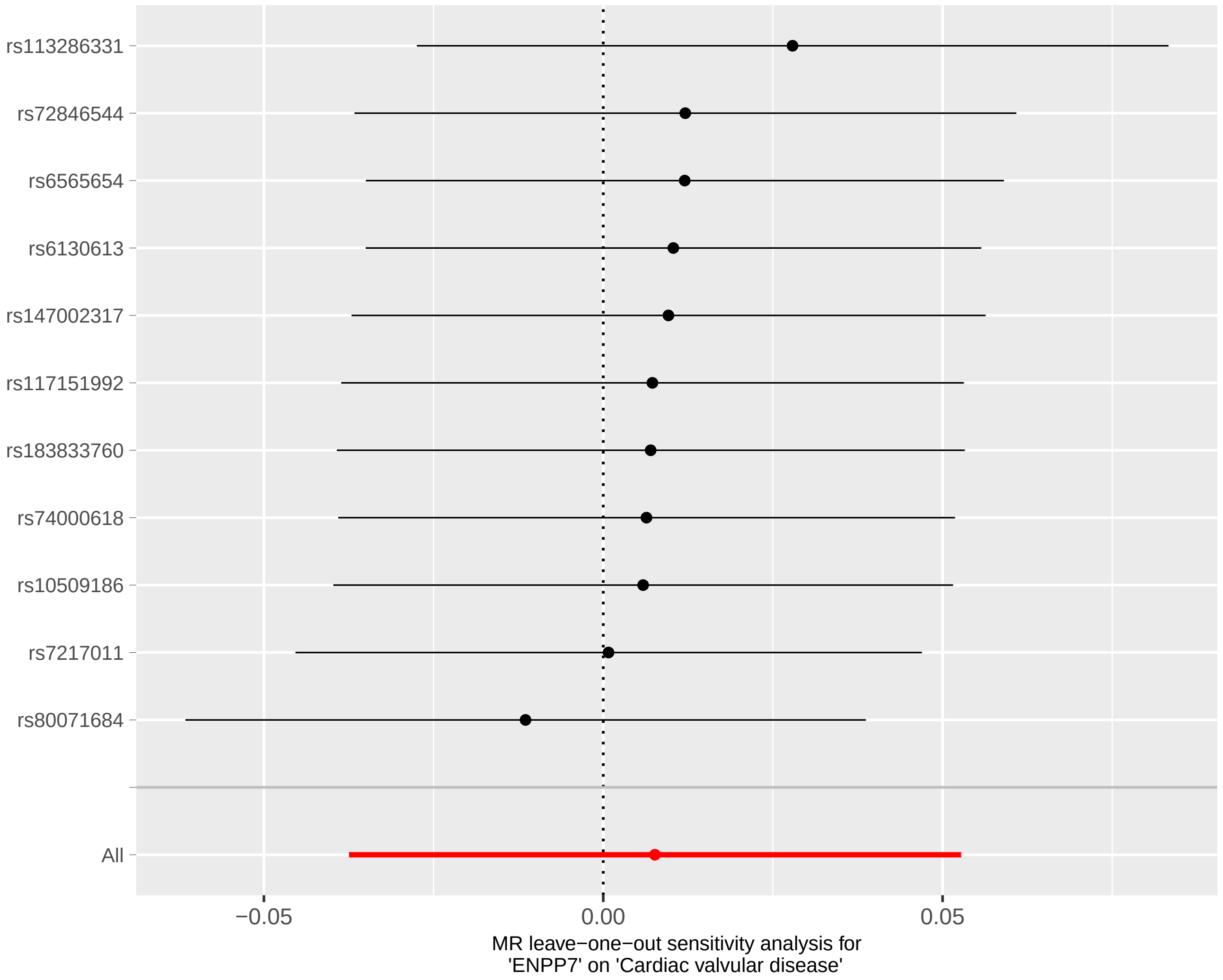

rs113286331
rs72846544
rs6565654
rs6130613
rs147002317
rs117151992
rs183833760
rs74000618
rs10509186
rs7217011
rs80071684
All
−0.05
0.00
0.05
MR leave−one−out sensitivity analysis for
'ENPP7' on 'Cardiac valvular disease'

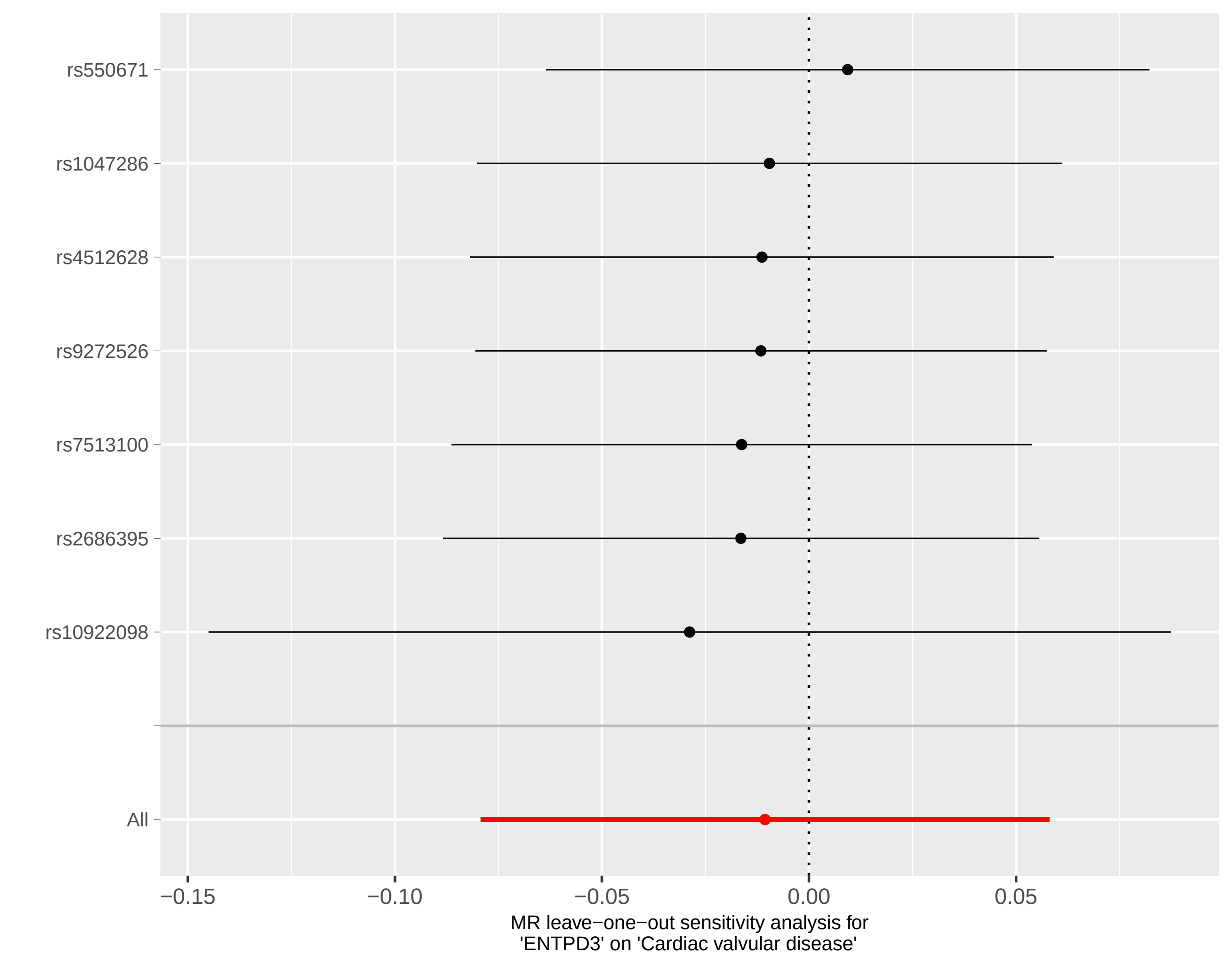
rs550671
rs1047286
rs4512628
rs9272526
rs7513100
rs2686395
rs10922098
All
−0.15
−0.10
−0.05
0.00
0.05
MR leave−one−out sensitivity analysis for
'ENTPD3' on 'Cardiac valvular disease'

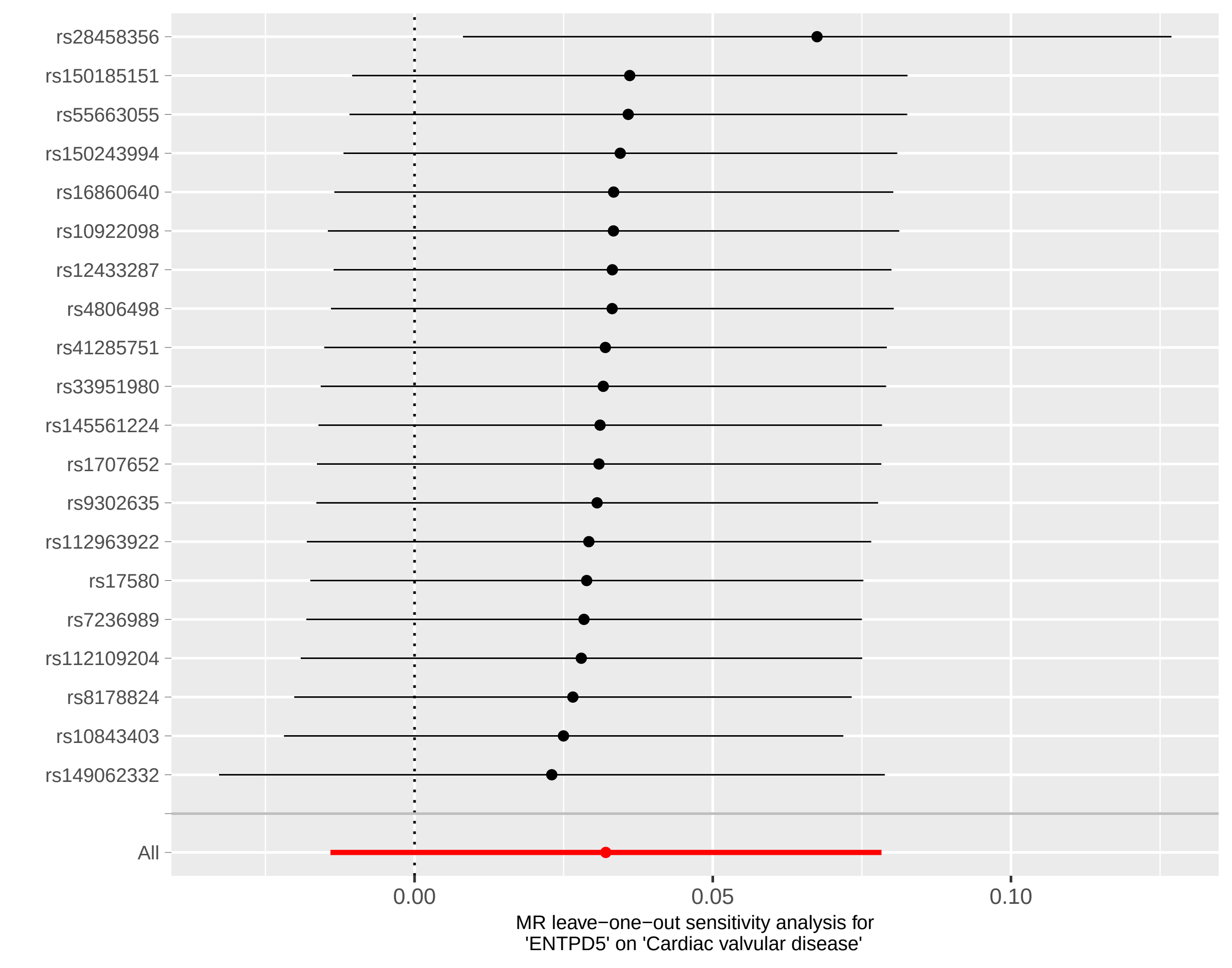
rs28458356
rs150185151
rs55663055
rs150243994
rs16860640
rs10922098
rs12433287
rs4806498
rs41285751
rs33951980
rs145561224
rs1707652
rs9302635
rs112963922
rs17580
rs7236989
rs112109204
rs8178824
rs10843403
rs149062332
All
0.00
0.05
0.10
MR leave−one−out sensitivity analysis for
'ENTPD5' on 'Cardiac valvular disease'

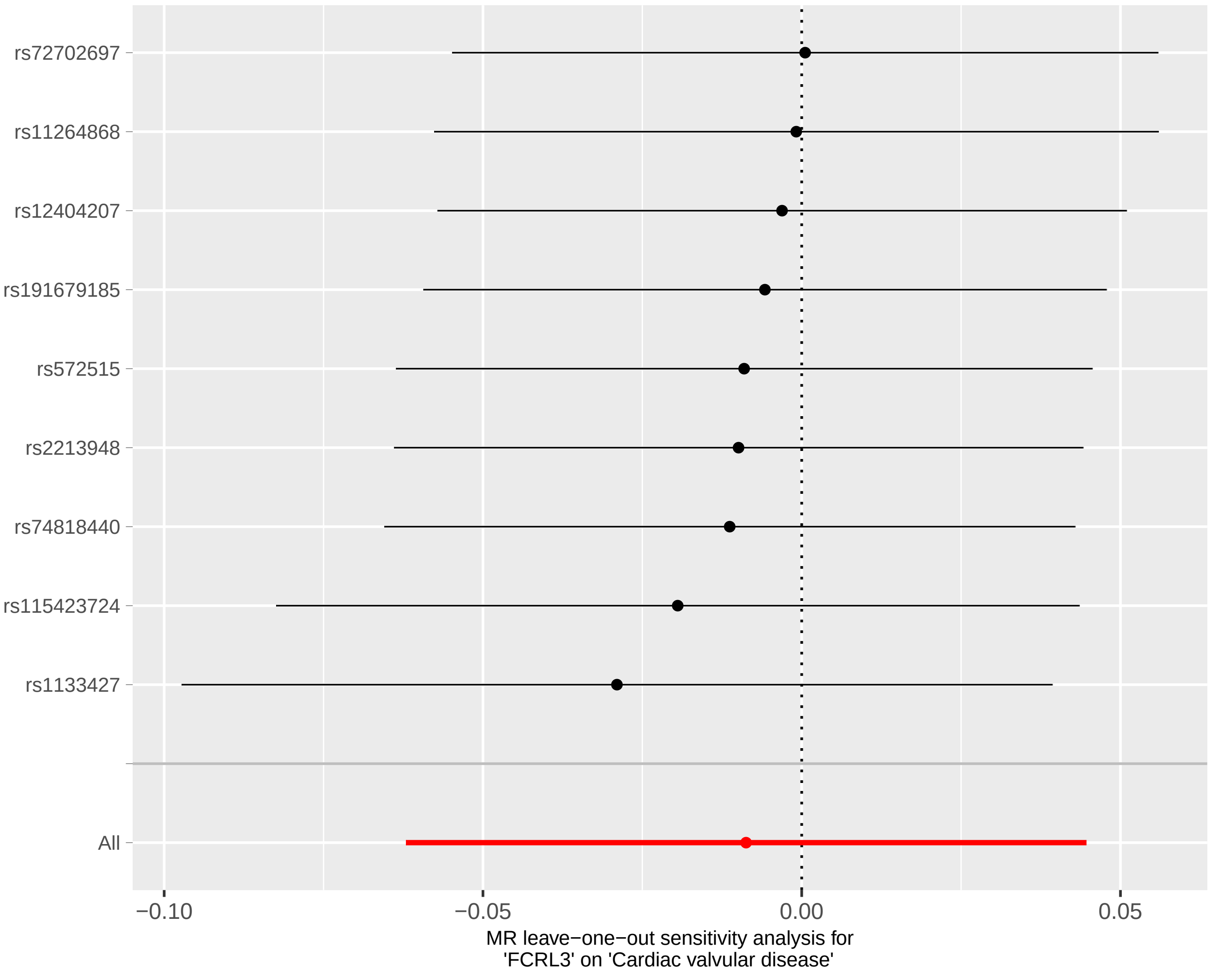

rs72702697
rs11264868
rs12404207
rs191679185
rs572515
rs2213948
rs74818440
rs115423724
rs1133427
All
−0.10
−0.05
0.00
0.05
MR leave−one−out sensitivity analysis for
'FCRL3' on 'Cardiac valvular disease'

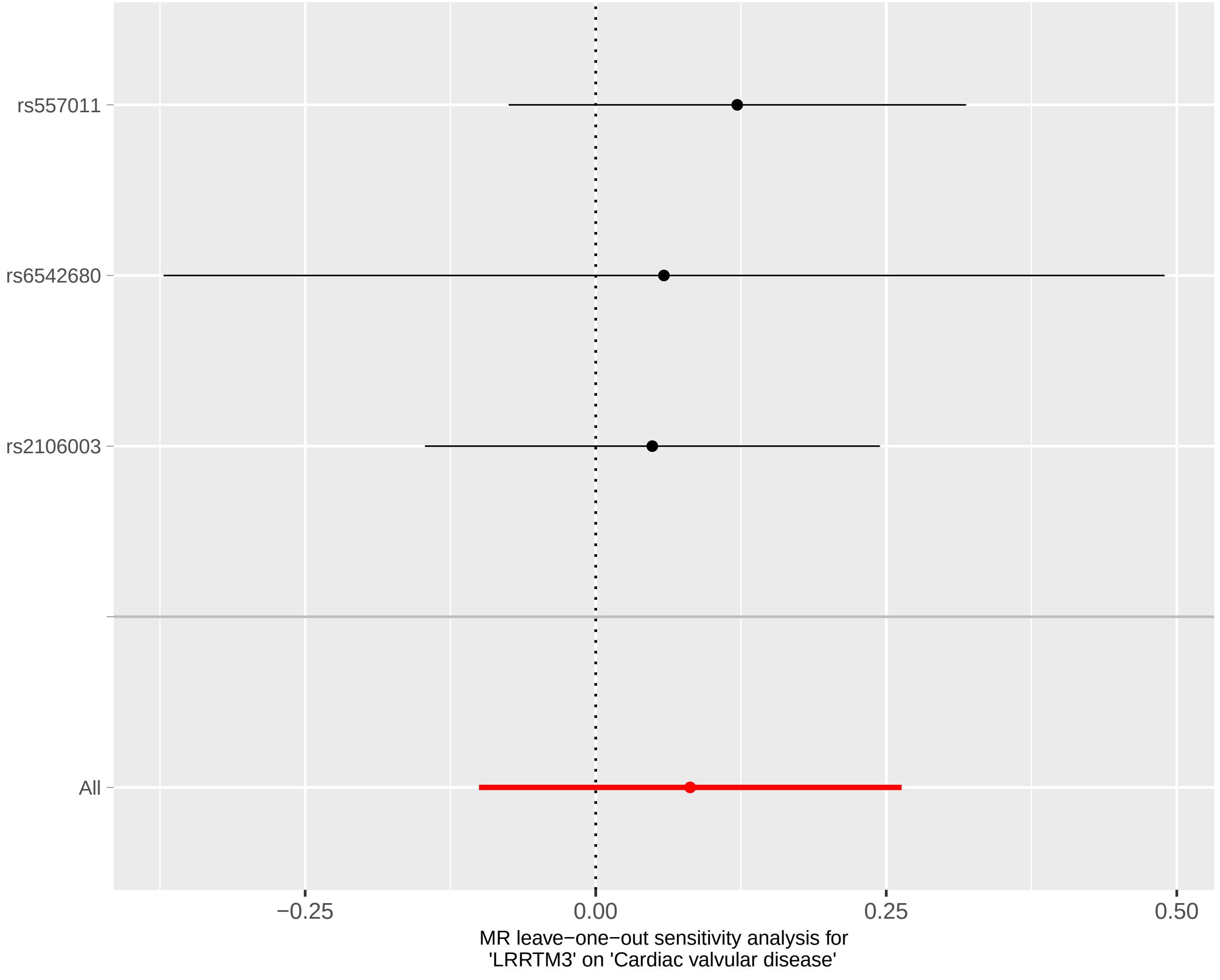
rs557011
rs6542680
rs2106003
All
−0.25
0.00
0.25
0.50
MR leave−one−out sensitivity analysis for
'LRRTM3' on 'Cardiac valvular disease'

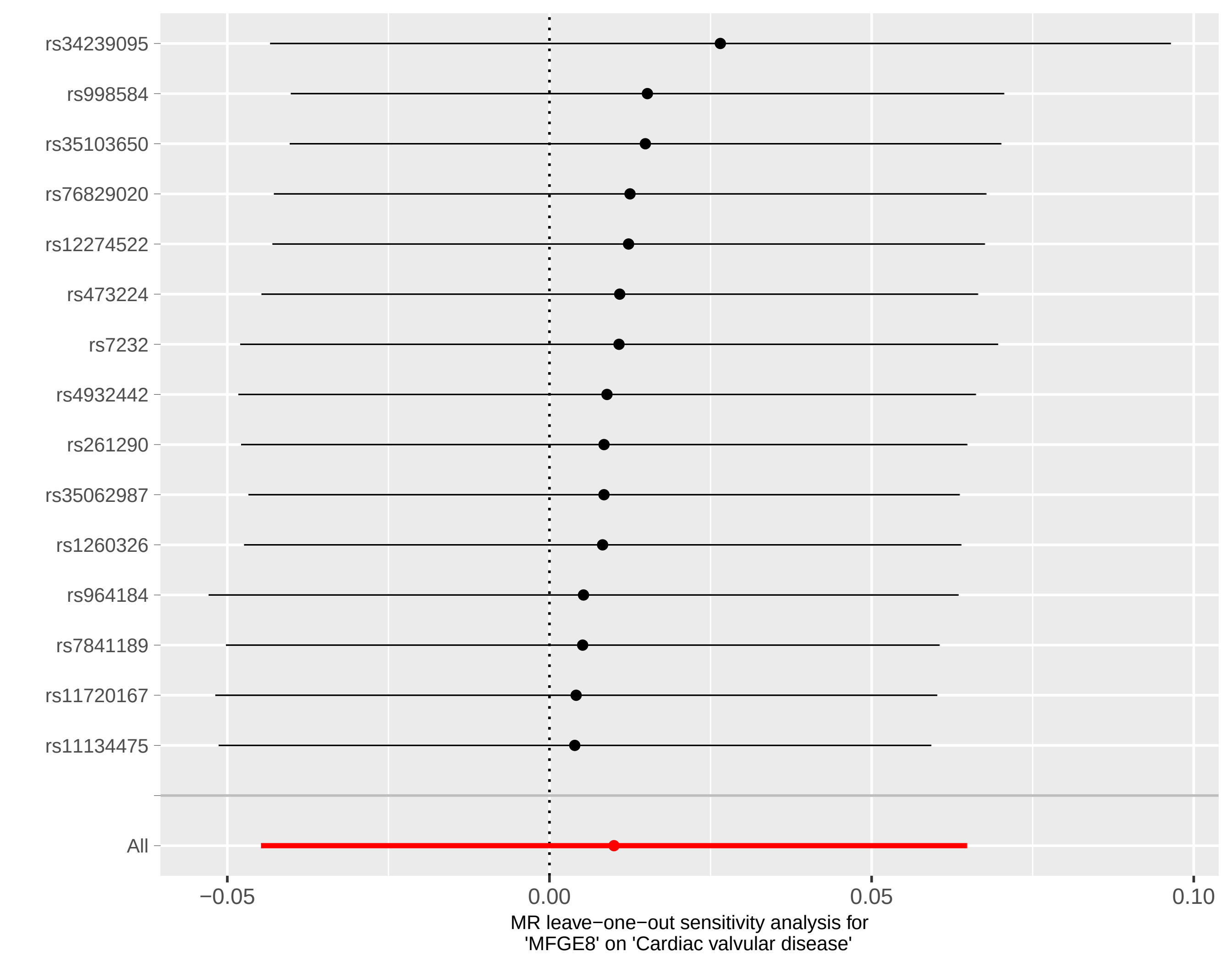

rs34239095
rs998584
rs35103650
rs76829020
rs12274522
rs473224
rs7232
rs4932442
rs261290
rs35062987
rs1260326
rs964184
rs7841189
rs11720167
rs11134475
All
−0.05
0.00
0.05
0.10
MR leave−one−out sensitivity analysis for
'MFGE8' on 'Cardiac valvular disease'

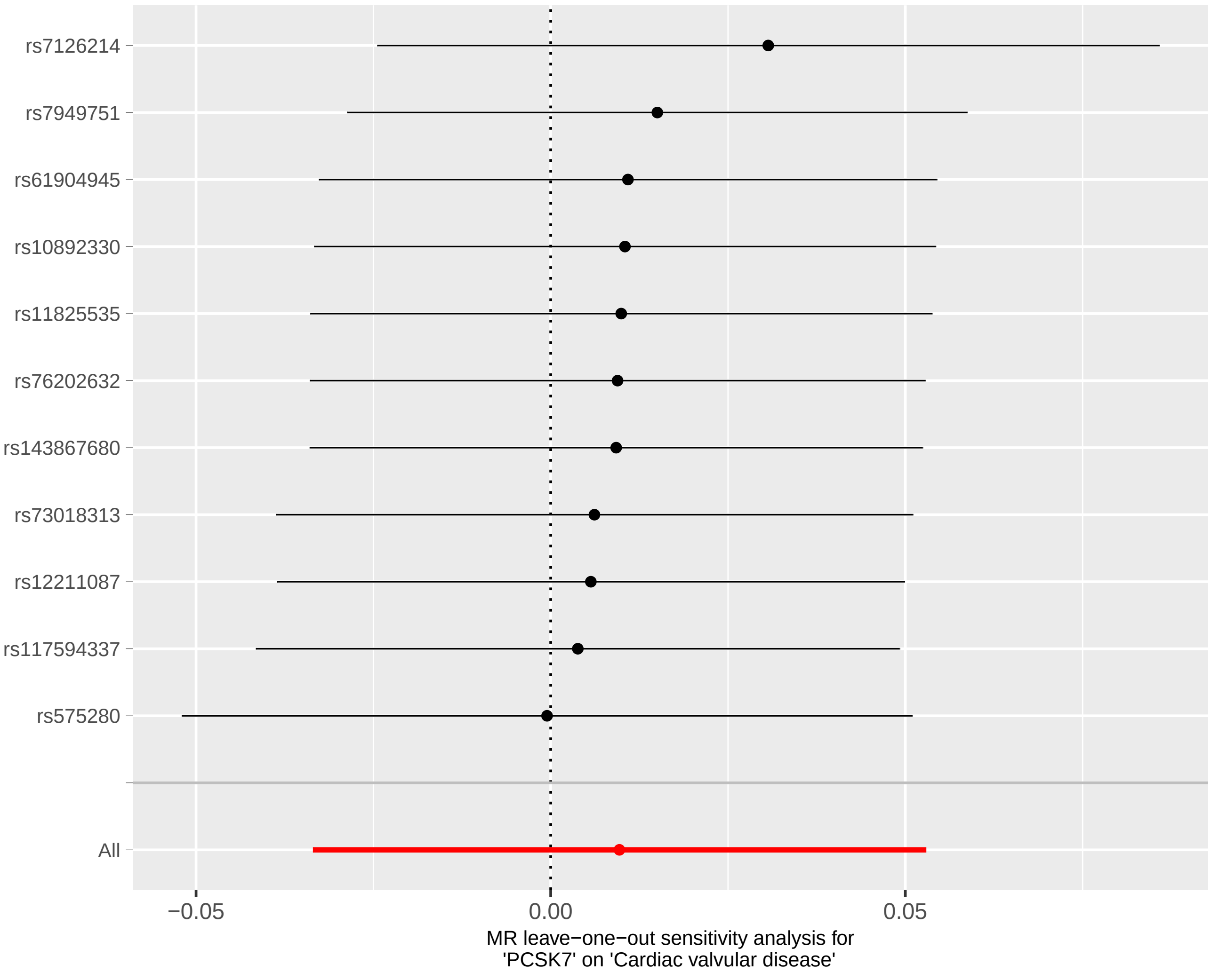
rs7126214
rs7949751
rs61904945
rs10892330
rs11825535
rs76202632
rs143867680
rs73018313
rs12211087
rs117594337
rs575280
All
−0.05
0.00
0.05
MR leave−one−out sensitivity analysis for
'PCSK7' on 'Cardiac valvular disease'

Insufficient number of SNPs

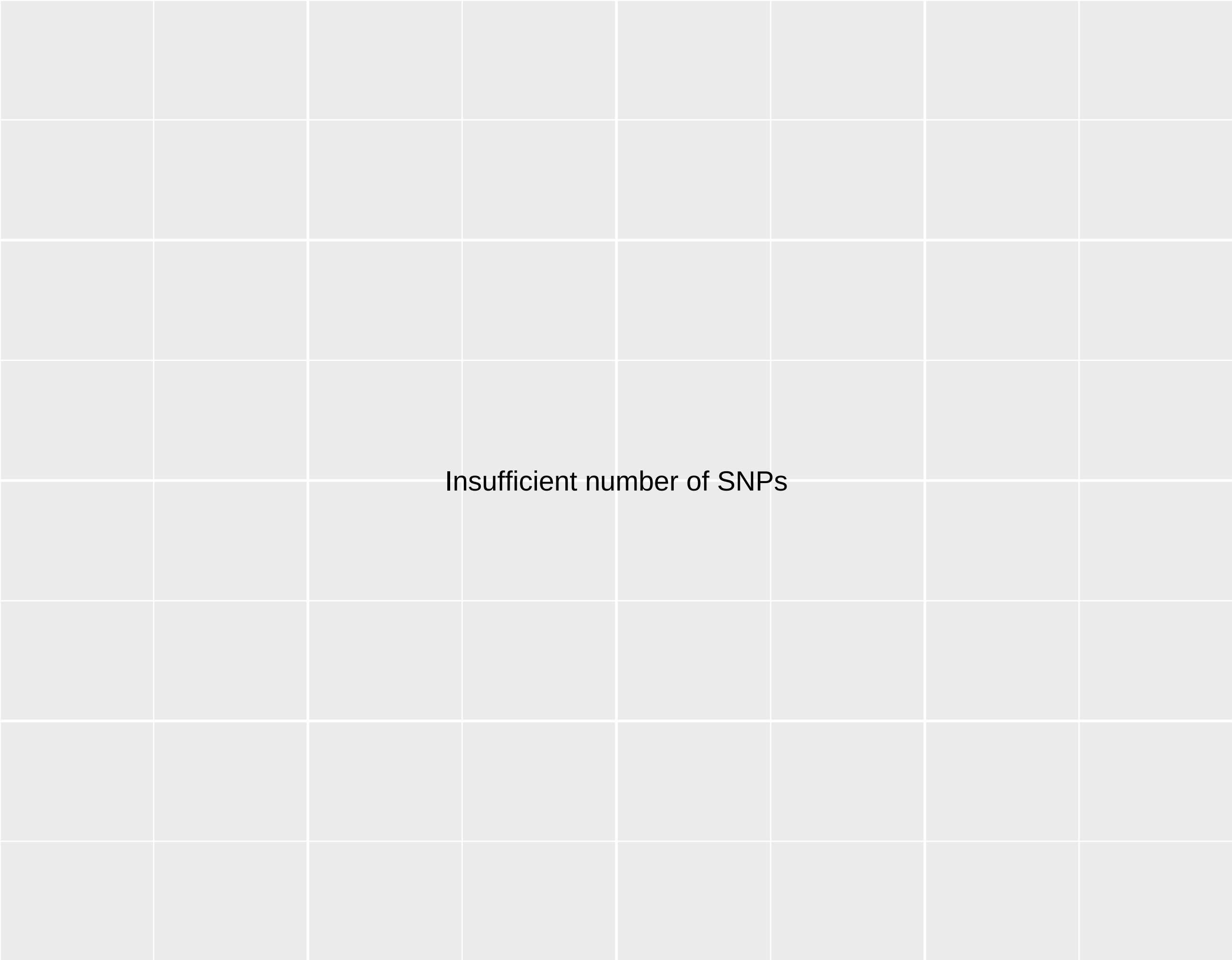
Insufficient number of SNPs

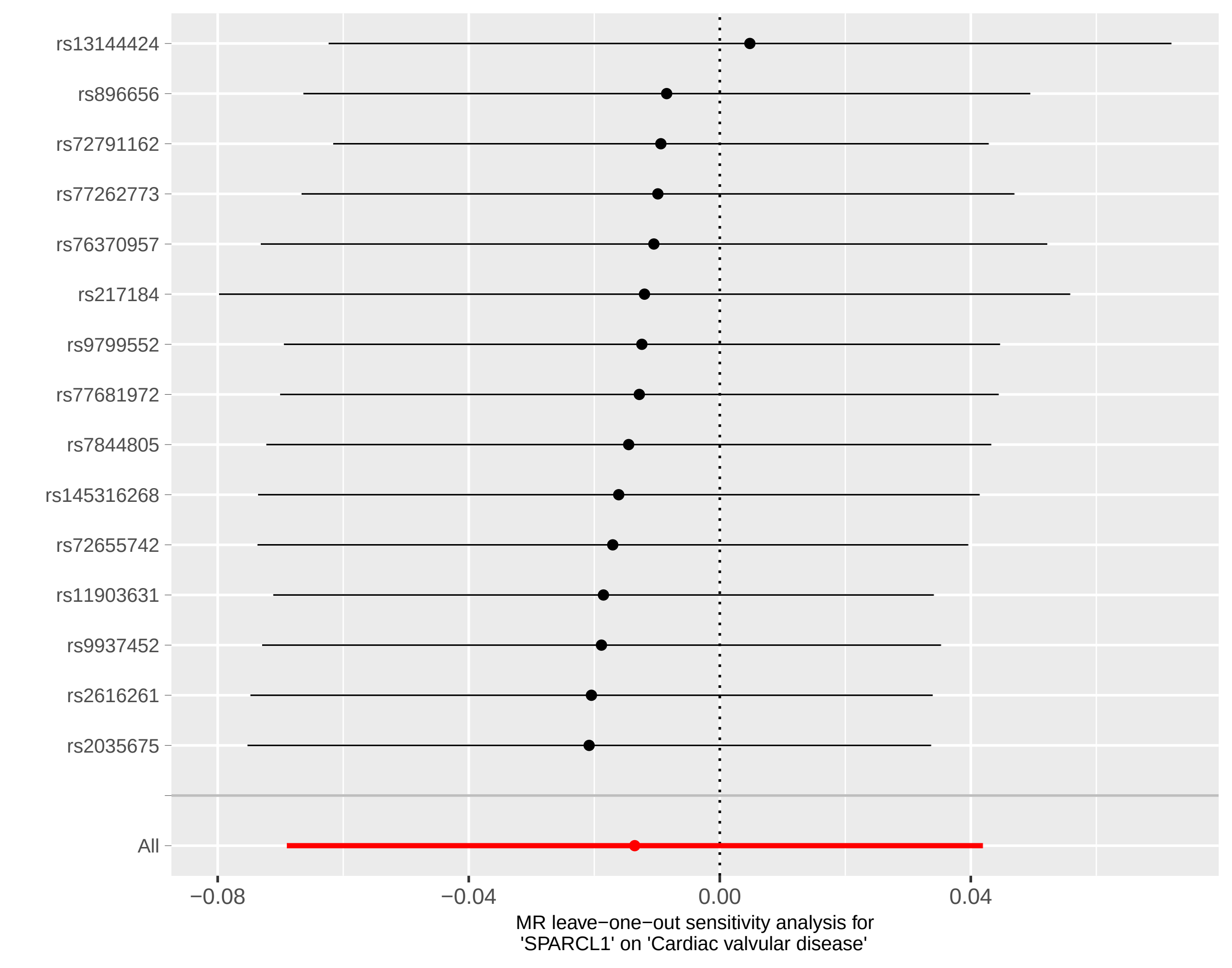
rs13144424
rs896656
rs72791162
rs77262773
rs76370957
rs217184
rs9799552
rs77681972
rs7844805
rs145316268
rs72655742
rs11903631
rs9937452
rs2616261
rs2035675
All
−0.08
−0.04
0.00
0.04
MR leave−one−out sensitivity analysis for
'SPARCL1' on 'Cardiac valvular disease'

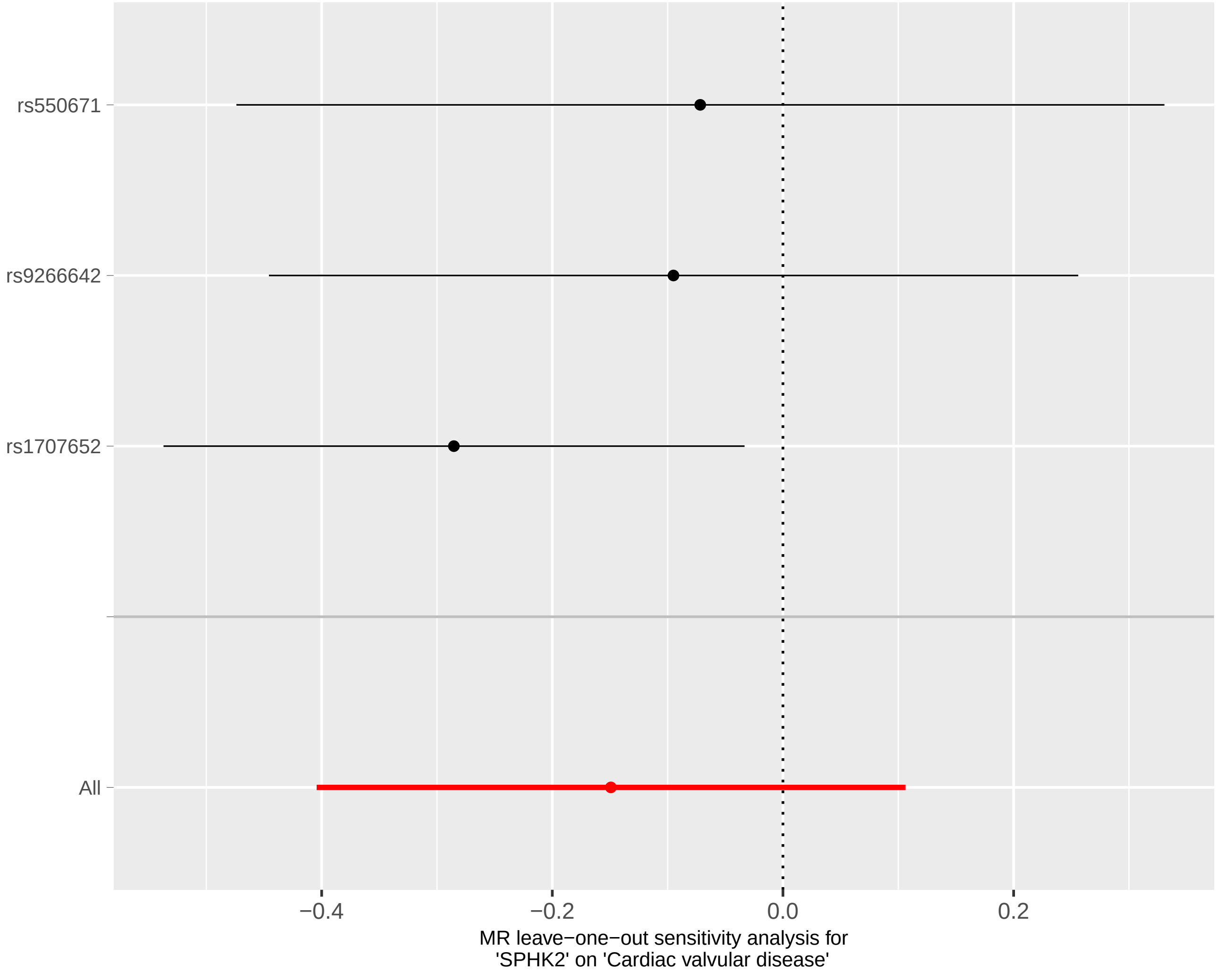
rs550671
rs9266642
rs1707652
All
−0.4
−0.2
0.0
0.2
MR leave−one−out sensitivity analysis for
'SPHK2' on 'Cardiac valvular disease'

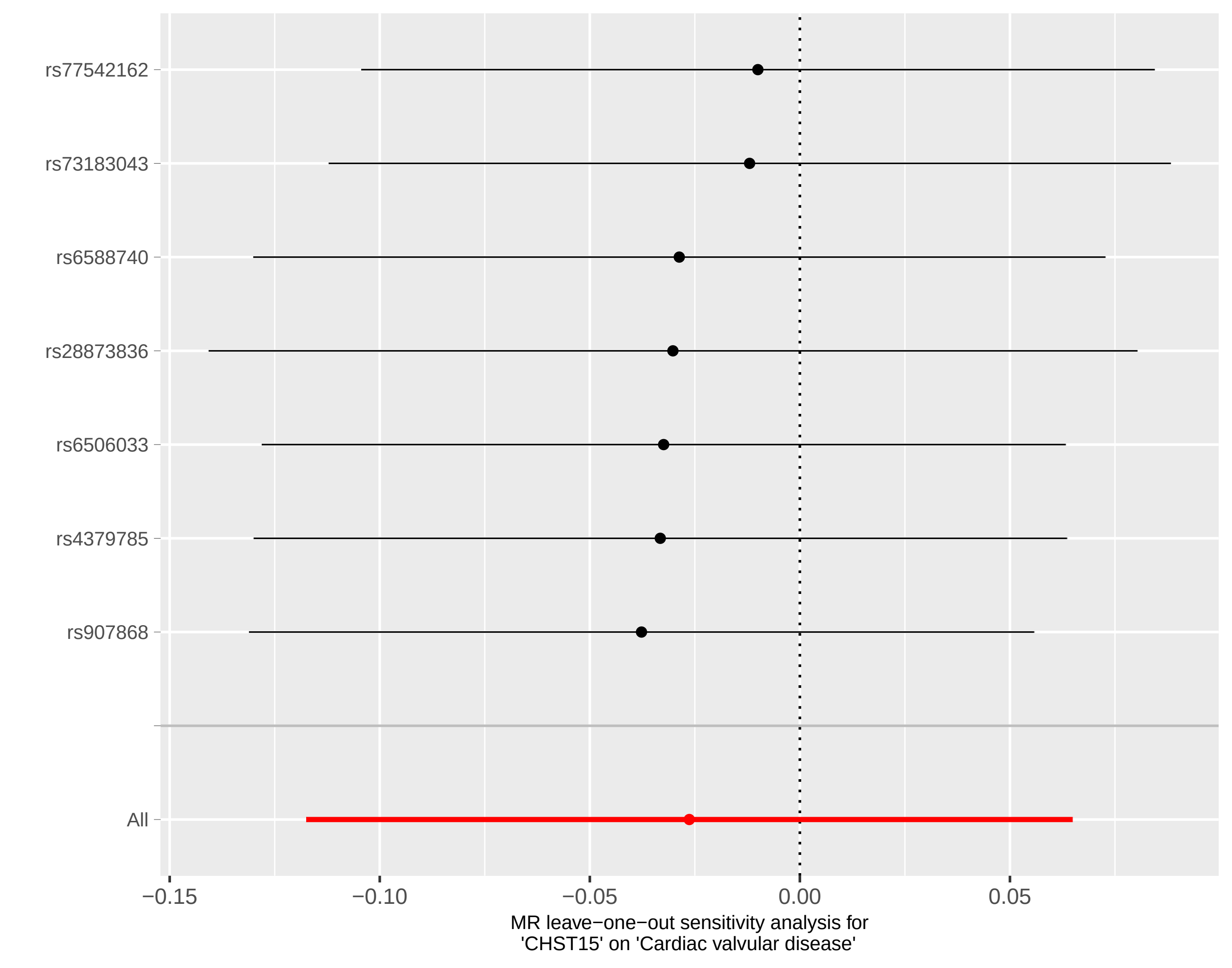
rs77542162
rs73183043
rs6588740
rs28873836
rs6506033
rs4379785
rs907868
All
−0.15
−0.10
−0.05
0.00
0.05
MR leave−one−out sensitivity analysis for
'CHST15' on 'Cardiac valvular disease'

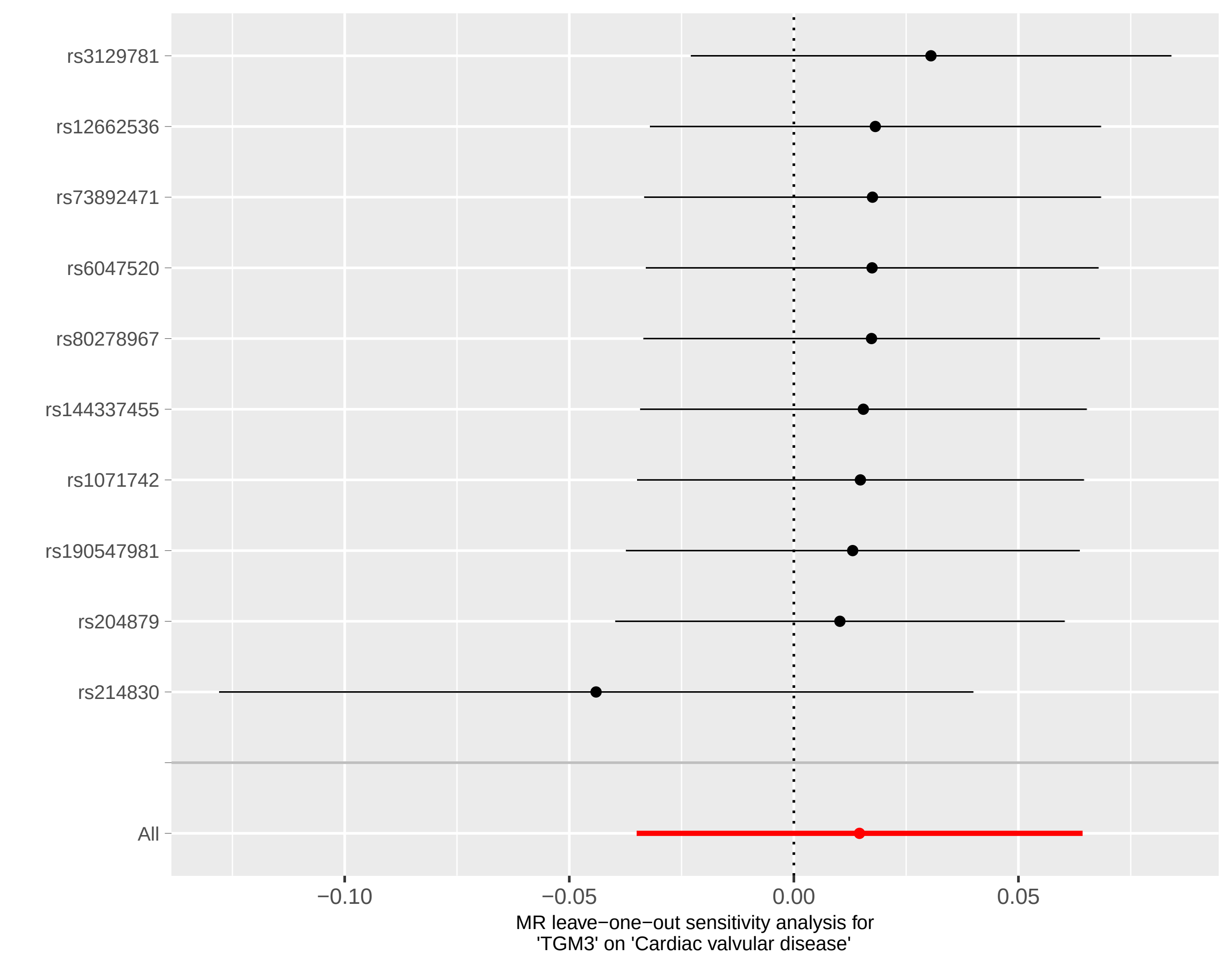
rs3129781
rs12662536
rs73892471
rs6047520
rs80278967
rs144337455
rs1071742
rs190547981
rs204879
rs214830
All
−0.10
−0.05
0.00
0.05
MR leave−one−out sensitivity analysis for
'TGM3' on 'Cardiac valvular disease'

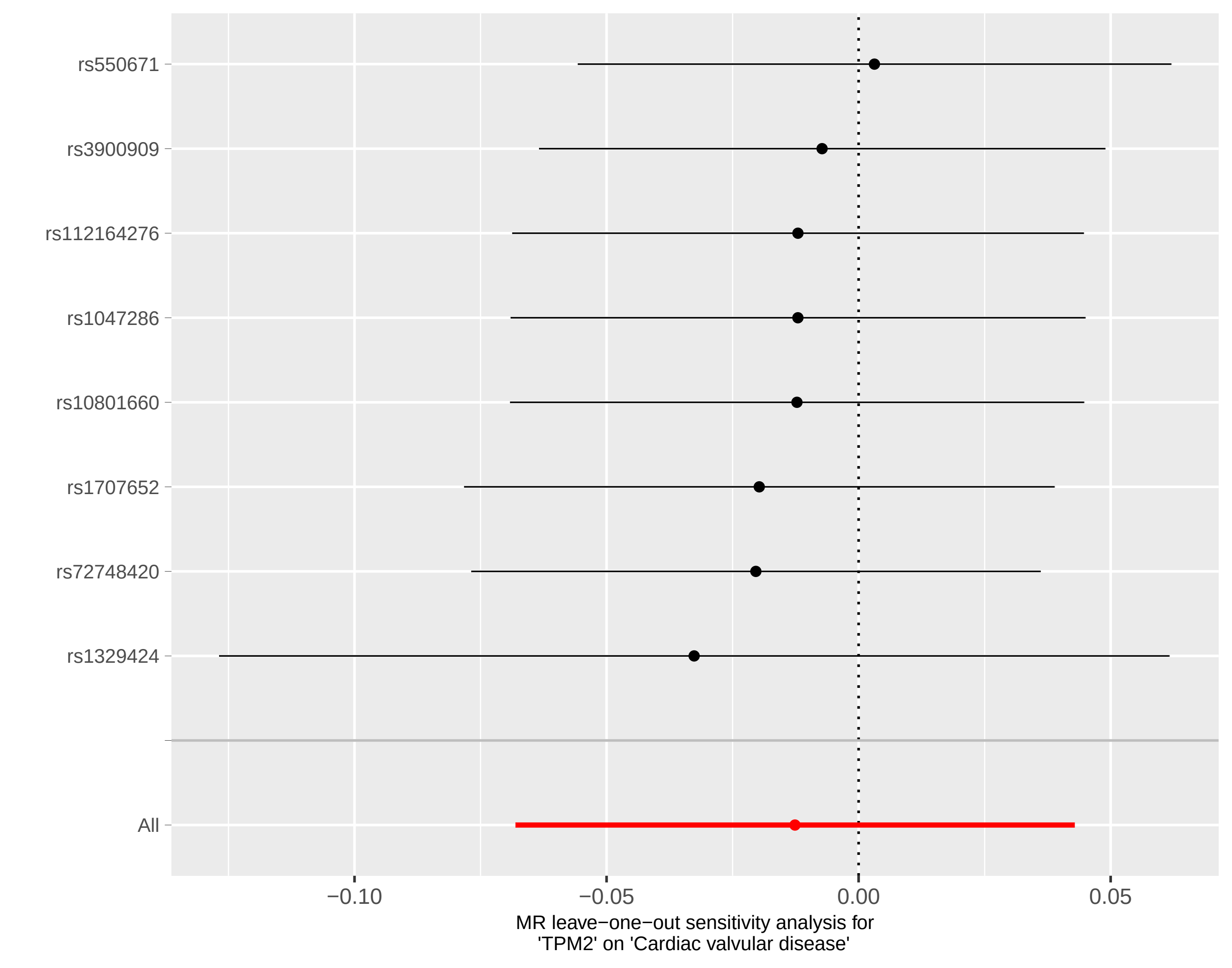
rs550671
rs3900909
rs112164276
rs1047286
rs10801660
rs1707652
rs72748420
rs1329424
All
−0.10
−0.05
0.00
0.05
MR leave−one−out sensitivity analysis for
'TPM2' on 'Cardiac valvular disease'

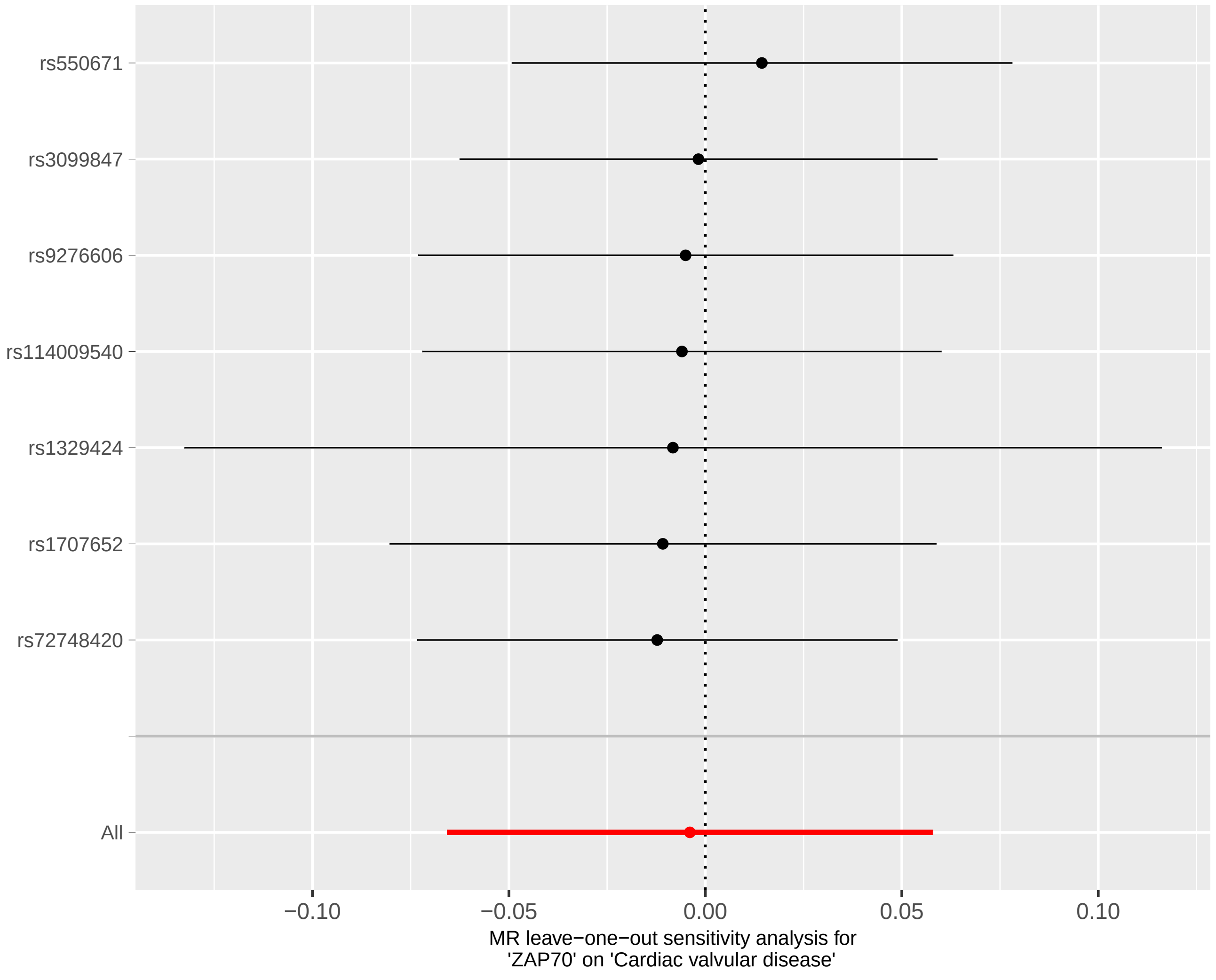
rs550671
rs3099847
rs9276606
rs114009540
rs1329424
rs1707652
rs72748420
All
−0.10
−0.05
0.00
0.05
0.10
MR leave−one−out sensitivity analysis for
'ZAP70' on 'Cardiac valvular disease'

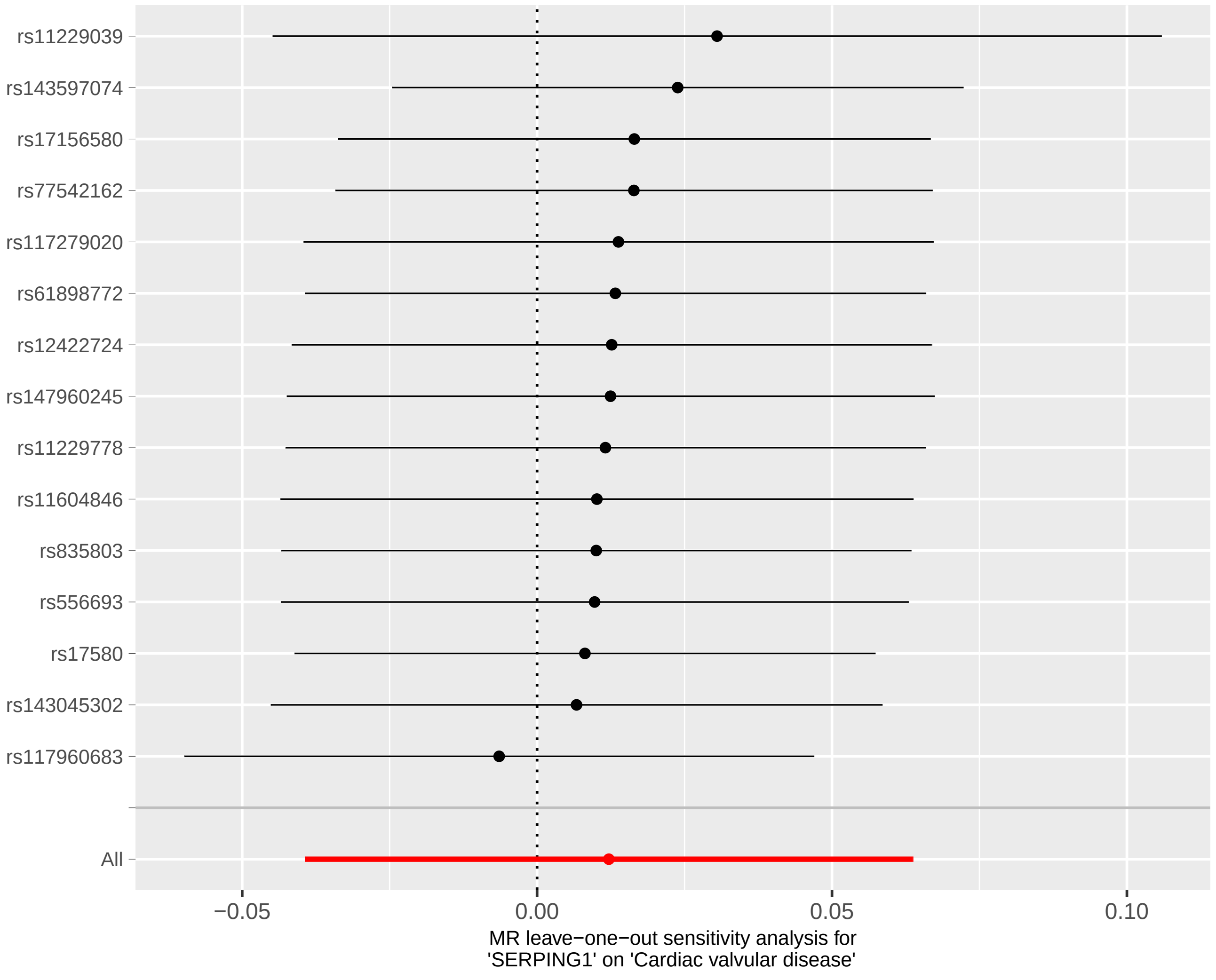
rs11229039
rs143597074
rs17156580
rs77542162
rs117279020
rs61898772
rs12422724
rs147960245
rs11229778
rs11604846
rs835803
rs556693
rs17580
rs143045302
rs117960683
All
−0.05
0.00
0.05
0.10
MR leave−one−out sensitivity analysis for
'SERPING1' on 'Cardiac valvular disease'

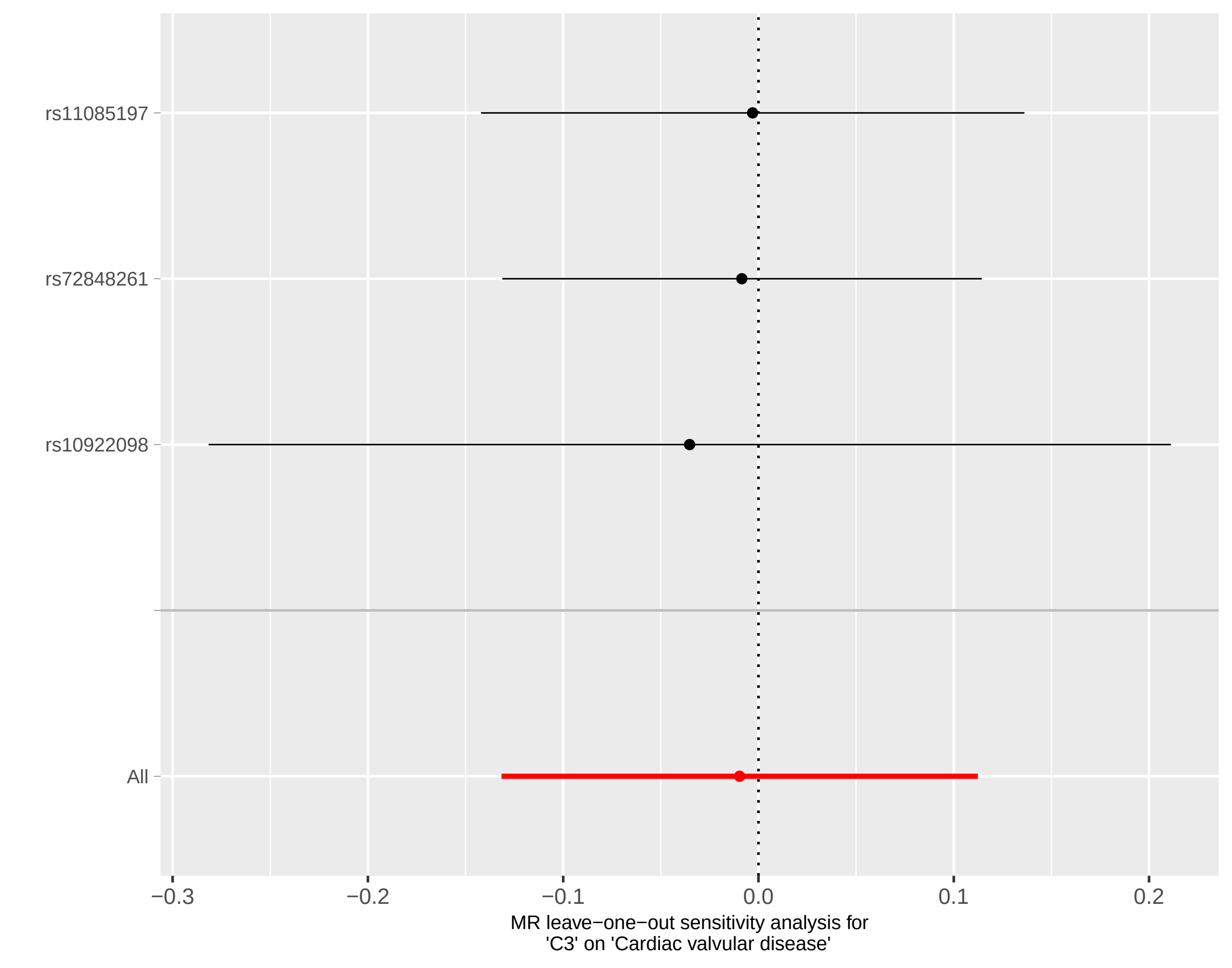
rs11085197
rs72848261
rs10922098
All
−0.3
−0.2
−0.1
0.0
0.1
0.2
MR leave−one−out sensitivity analysis for
'C3' on 'Cardiac valvular disease'

Insufficient number of SNPs

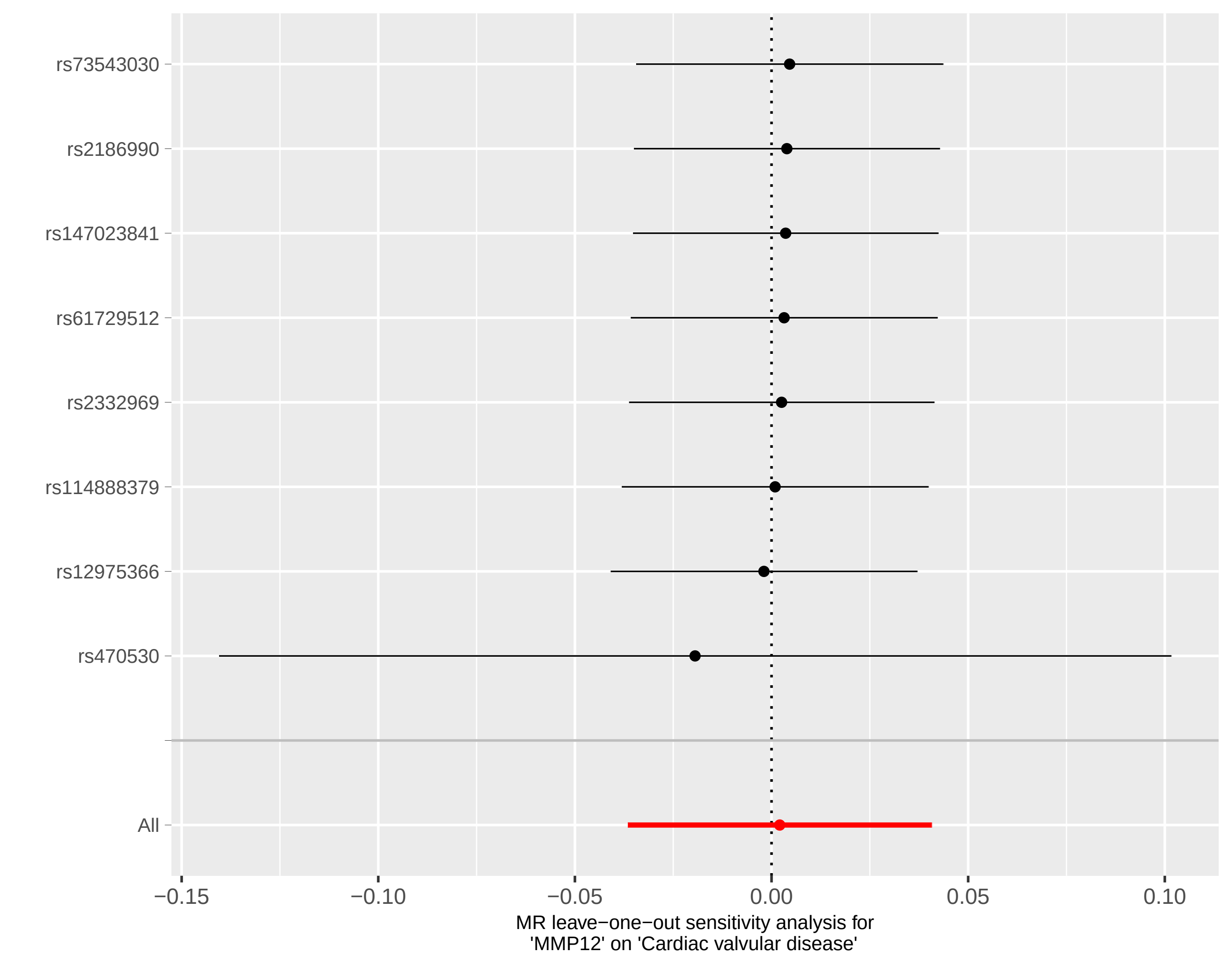
rs73543030
rs2186990
rs147023841
rs61729512
rs2332969
rs114888379
rs12975366
rs470530
All
−0.15
−0.10
−0.05
0.00
0.05
0.10
MR leave−one−out sensitivity analysis for
'MMP12' on 'Cardiac valvular disease'

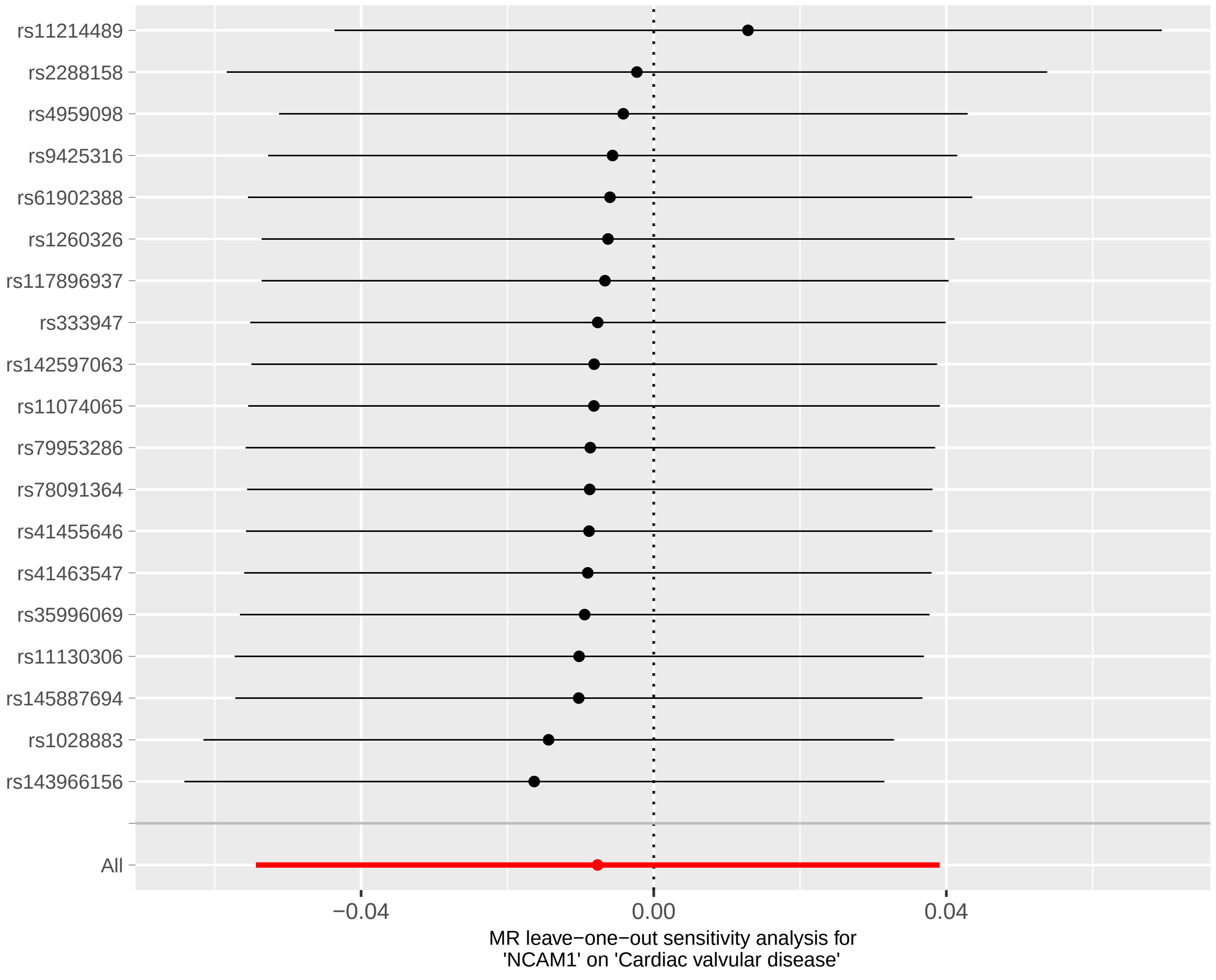
rs11214489
rs2288158
rs4959098
rs9425316
rs61902388
rs1260326
rs117896937
rs333947
rs142597063
rs11074065
rs79953286
rs78091364
rs41455646
rs41463547
rs35996069
rs11130306
rs145887694
rs1028883
rs143966156
All
−0.04
0.00
0.04
MR leave−one−out sensitivity analysis for
'NCAM1' on 'Cardiac valvular disease'

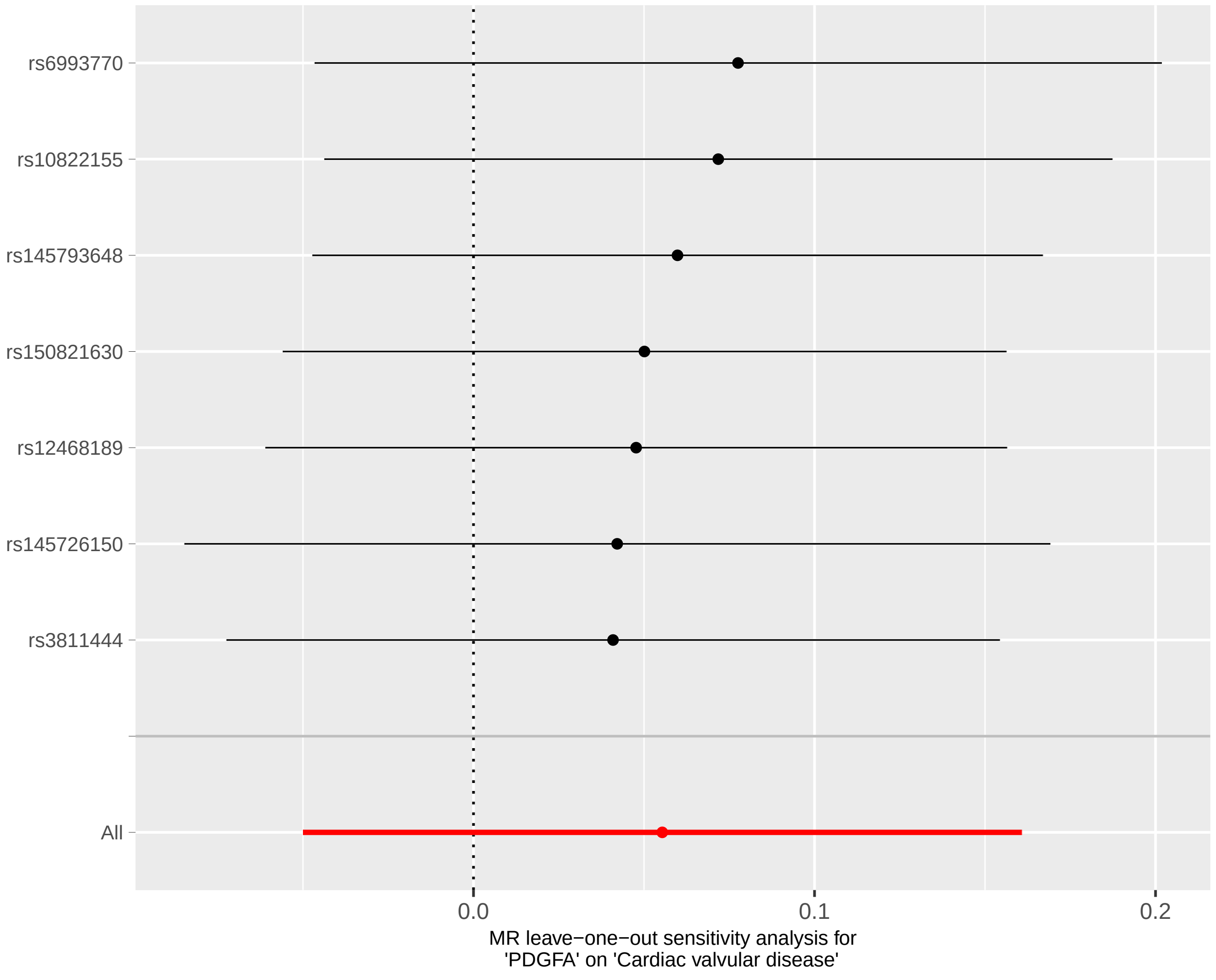
rs6993770
rs10822155
rs145793648
rs150821630
rs12468189
rs145726150
rs3811444
All
0.0
0.1
0.2
MR leave−one−out sensitivity analysis for
'PDGFA' on 'Cardiac valvular disease'

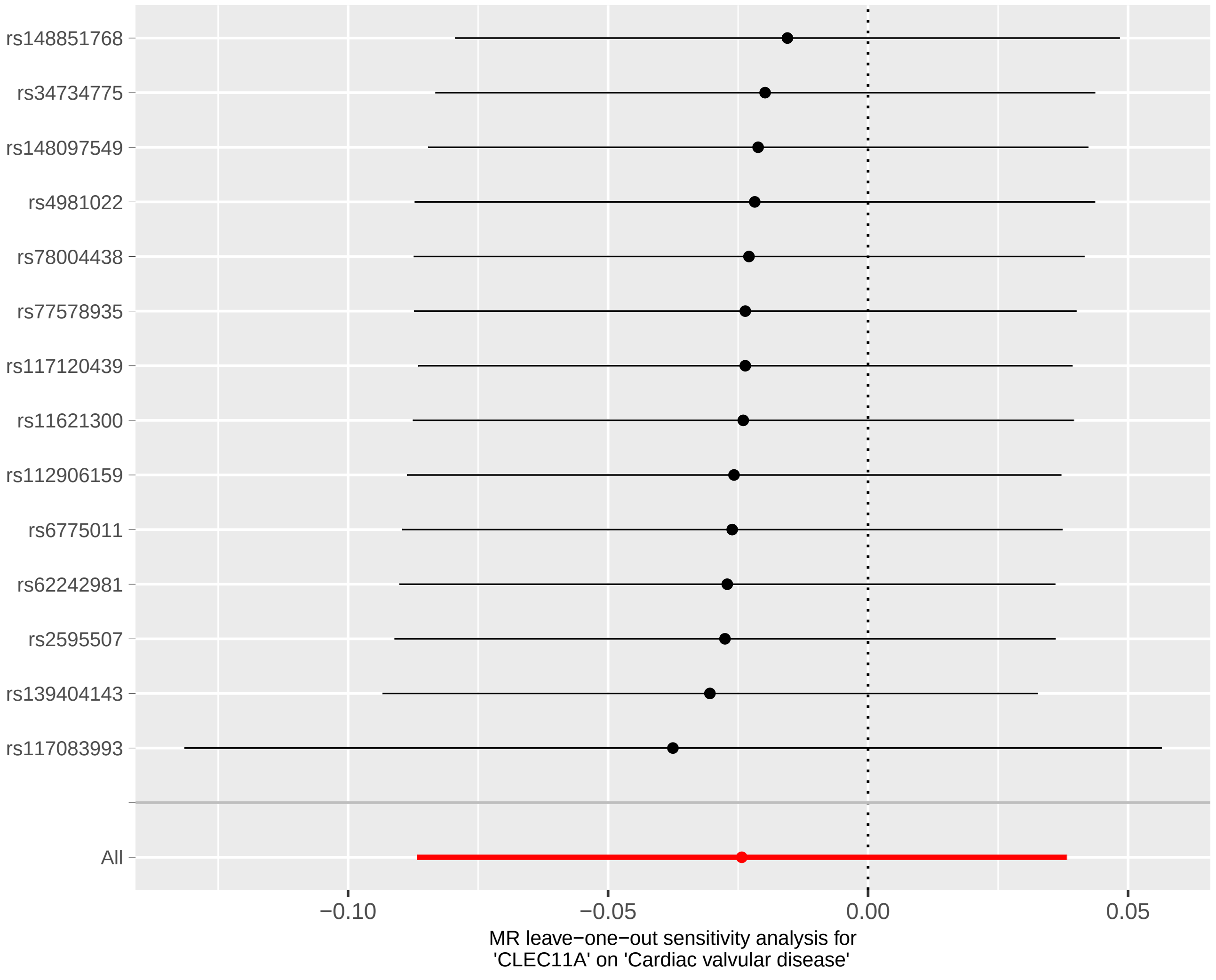

rs148851768
rs34734775
rs148097549
rs4981022
rs78004438
rs77578935
rs117120439
rs11621300
rs112906159
rs6775011
rs62242981
rs2595507
rs139404143
rs117083993
All
−0.10
−0.05
0.00
0.05
MR leave−one−out sensitivity analysis for
'CLEC11A' on 'Cardiac valvular disease'

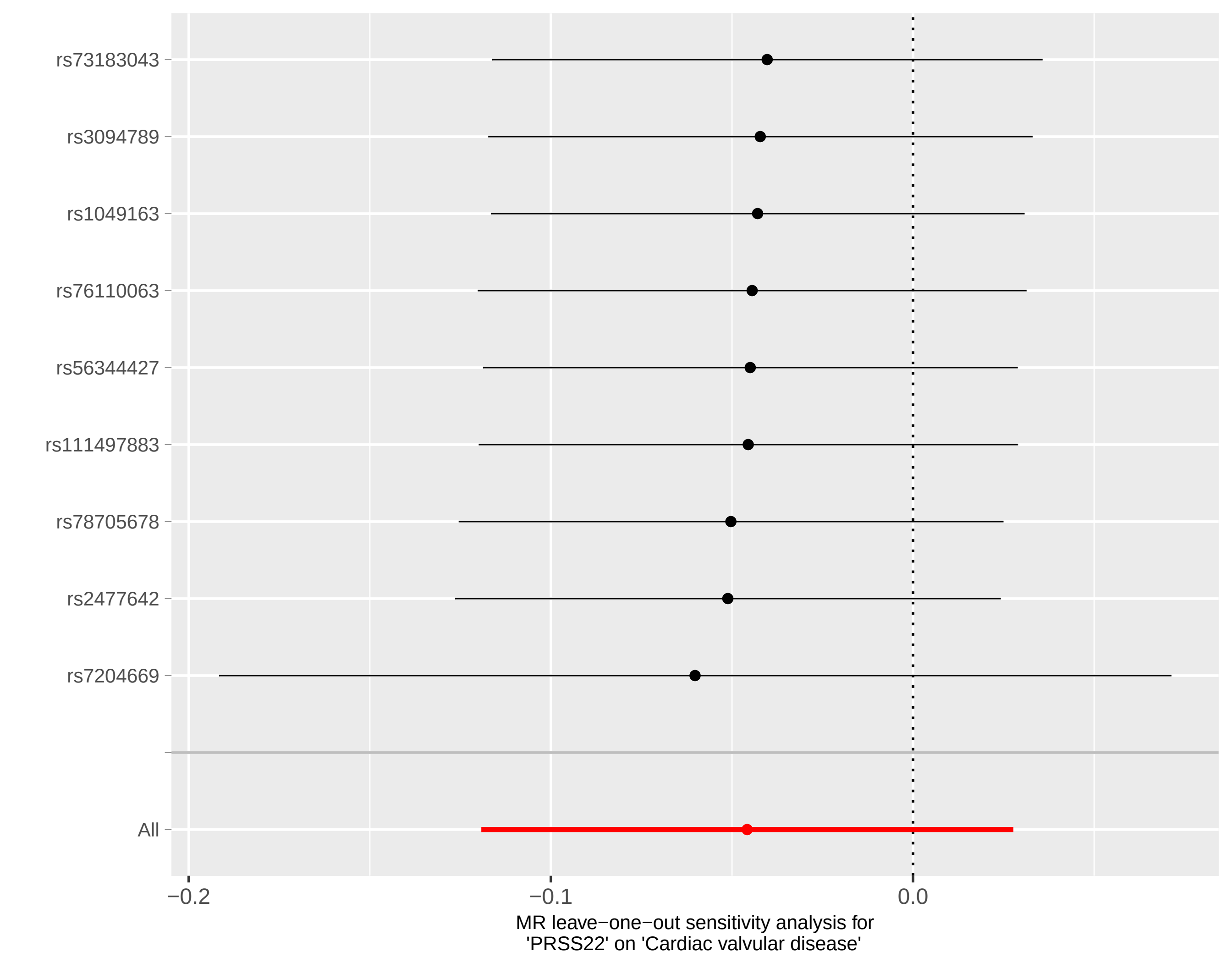
rs73183043
rs3094789
rs1049163
rs76110063
rs56344427
rs111497883
rs78705678
rs2477642
rs7204669
All
−0.2
−0.1
0.0
MR leave−one−out sensitivity analysis for
'PRSS22' on 'Cardiac valvular disease'

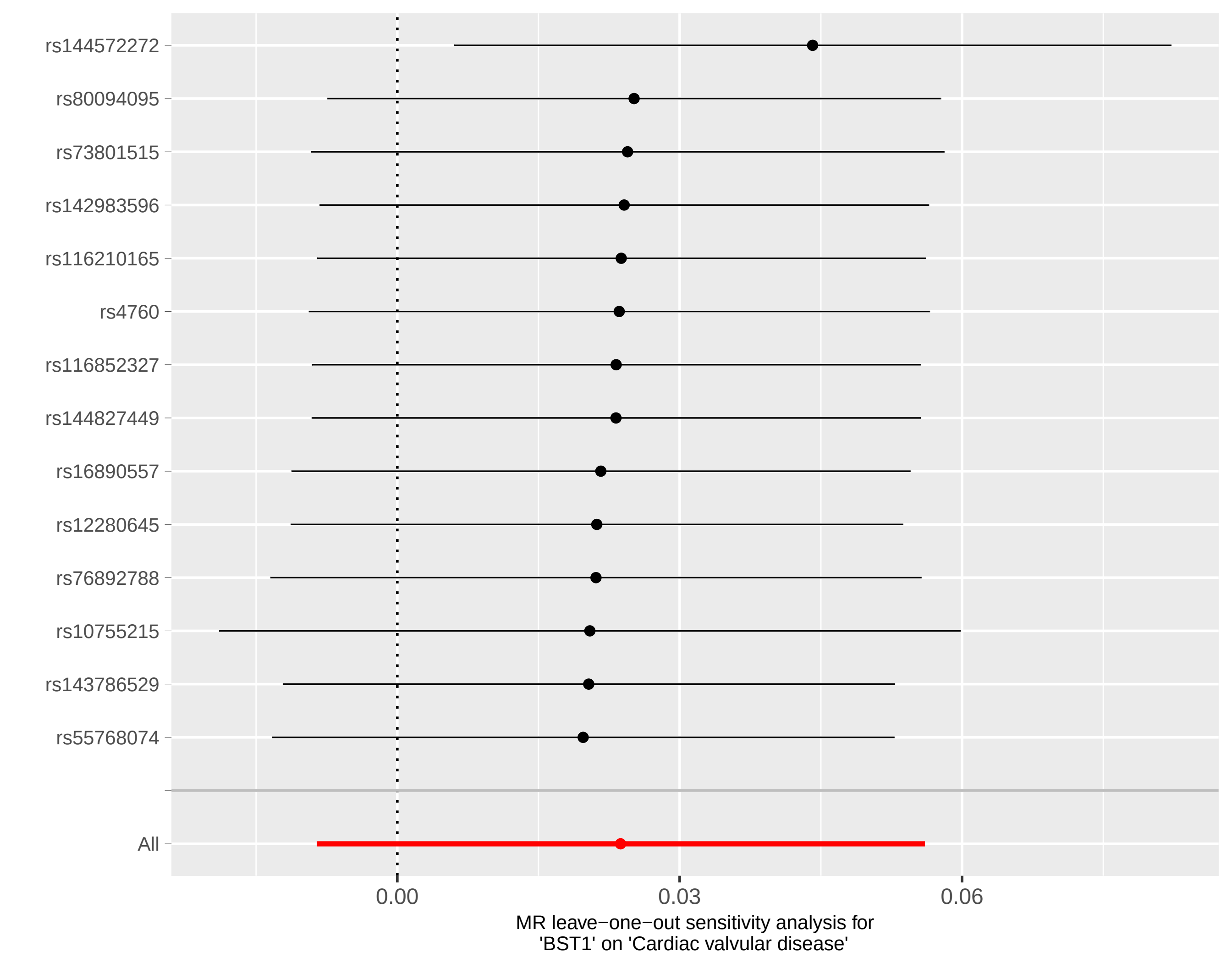
rs144572272
rs80094095
rs73801515
rs142983596
rs116210165
rs4760
rs116852327
rs144827449
rs16890557
rs12280645
rs76892788
rs10755215
rs143786529
rs55768074
All
0.00
0.03
0.06
MR leave−one−out sensitivity analysis for
'BST1' on 'Cardiac valvular disease'

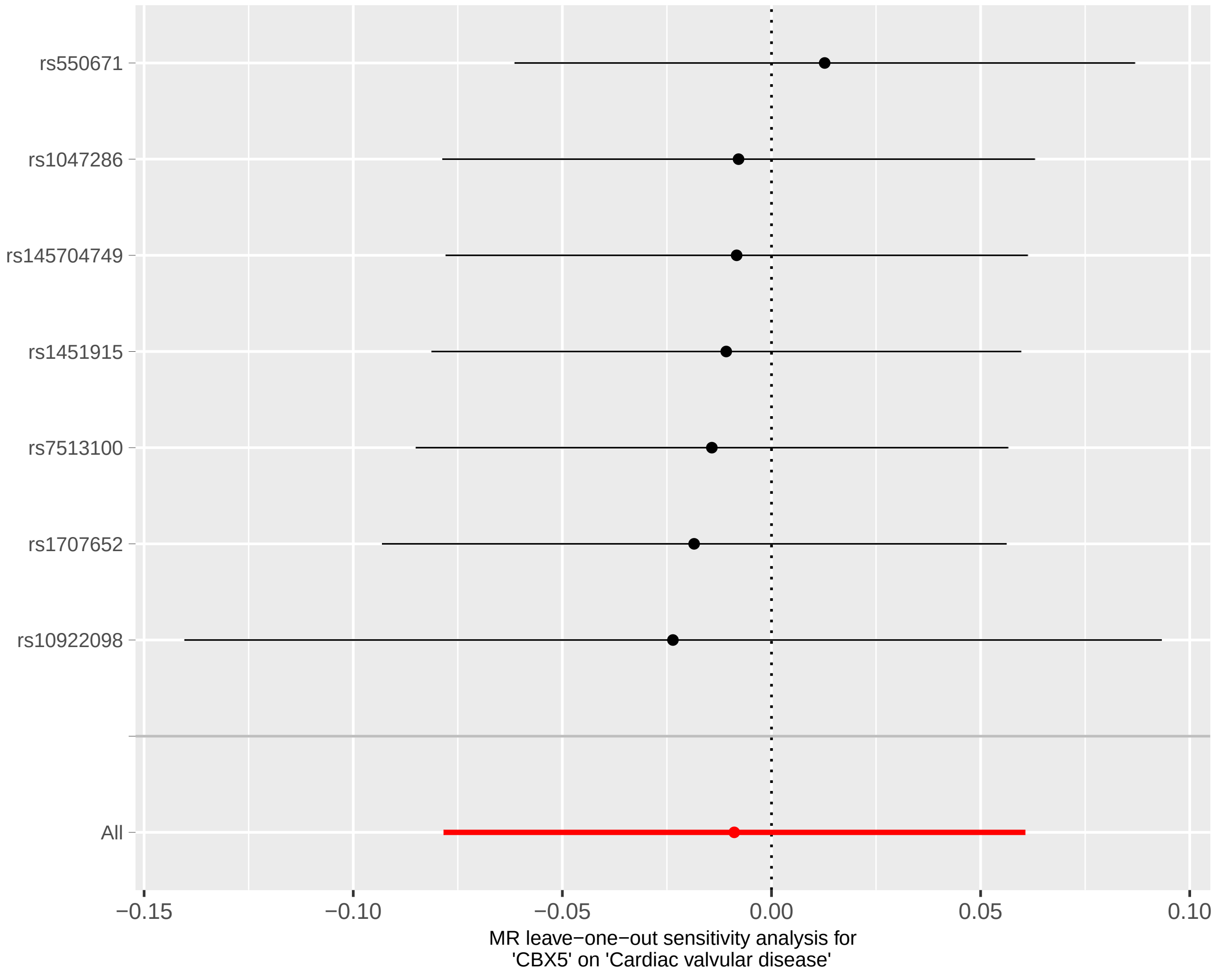
rs550671
rs1047286
rs145704749
rs1451915
rs7513100
rs1707652
rs10922098
All
−0.15
−0.10
−0.05
0.00
0.05
0.10
MR leave−one−out sensitivity analysis for
'CBX5' on 'Cardiac valvular disease'

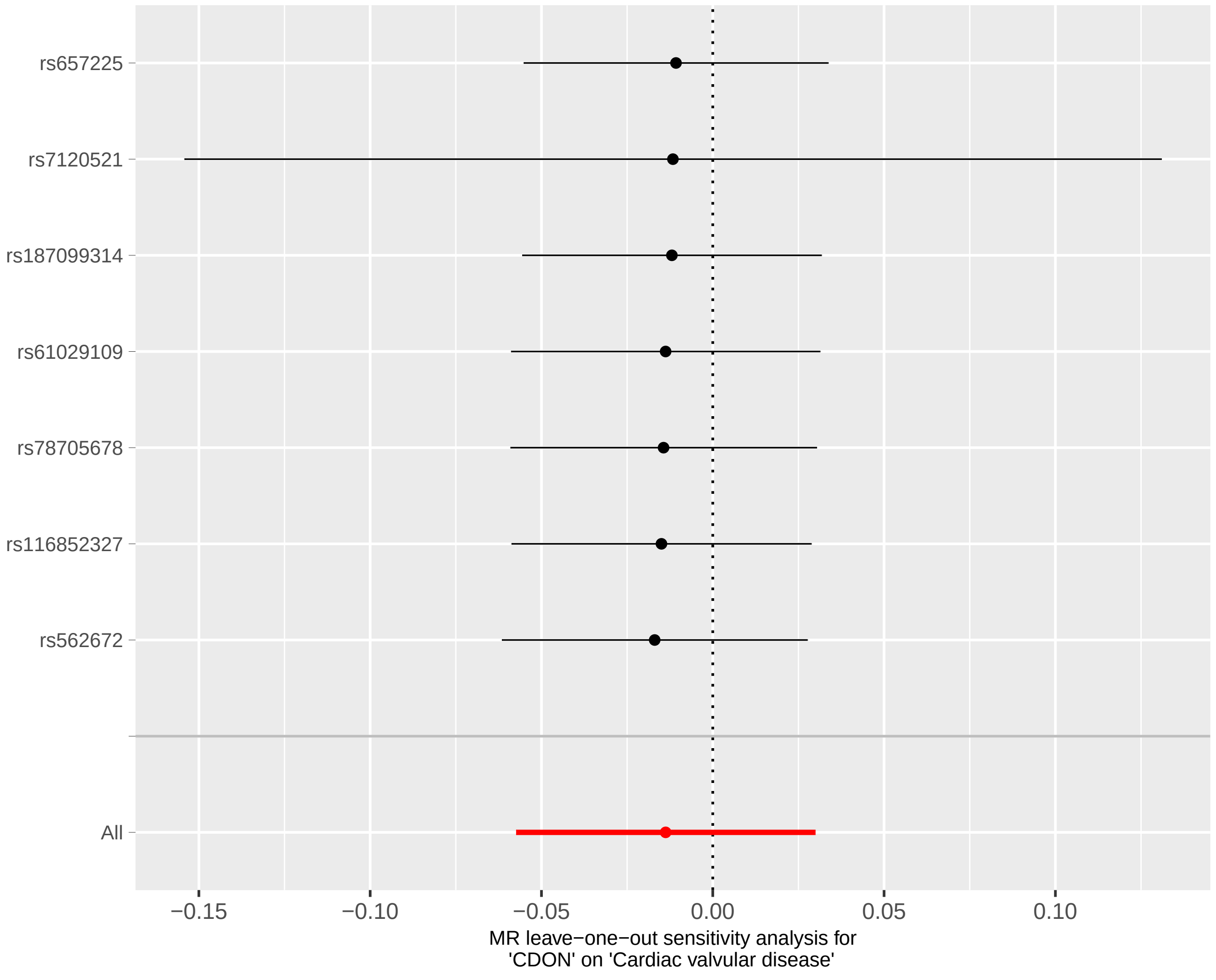
rs657225
rs7120521
rs187099314
rs61029109
rs78705678
rs116852327
rs562672
All
−0.15
−0.10
−0.05
0.00
0.05
0.10
MR leave−one−out sensitivity analysis for
'CDON' on 'Cardiac valvular disease'

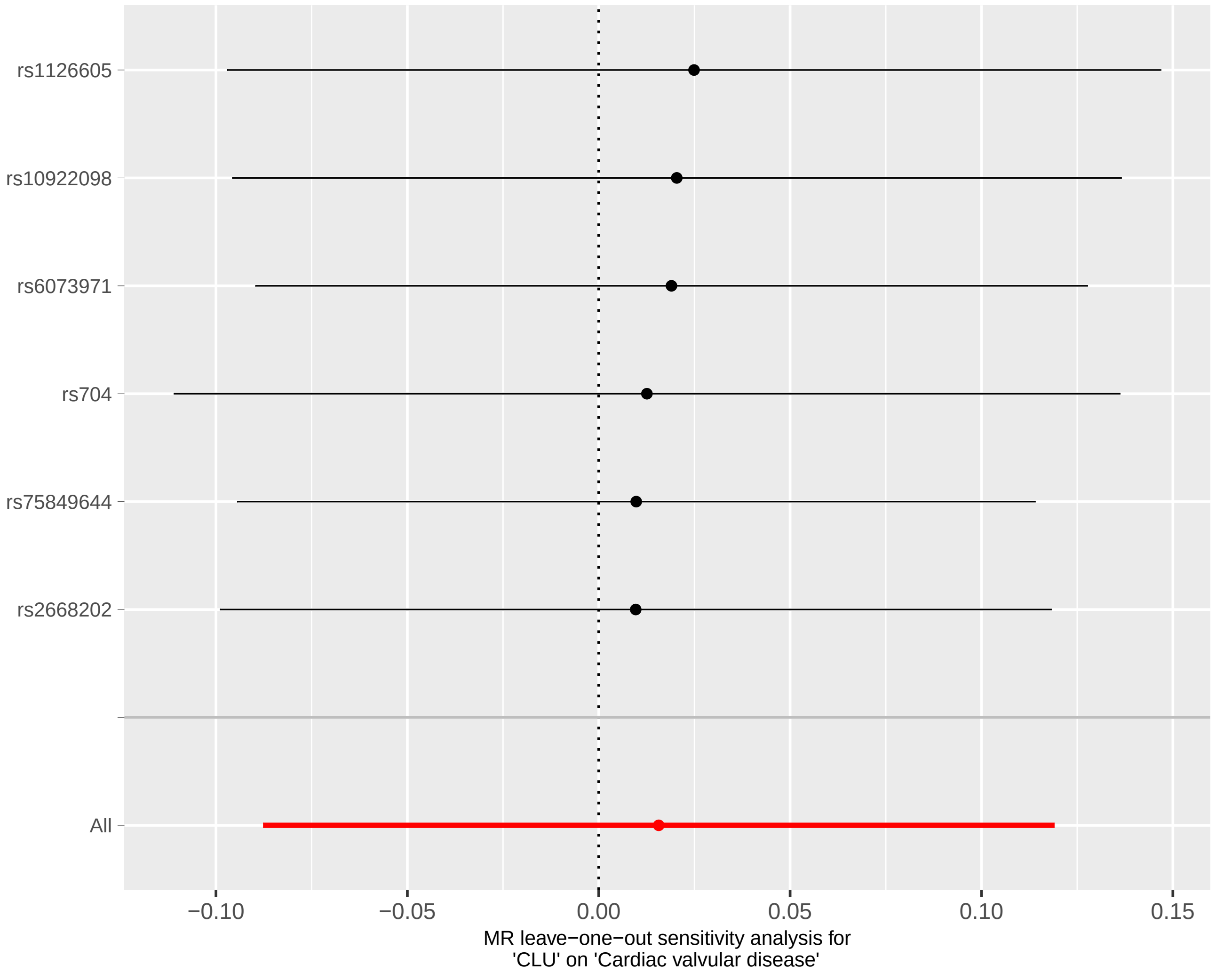

rs1126605
rs10922098
rs6073971
rs704
rs75849644
rs2668202
All
−0.10
−0.05
0.00
0.05
0.10
0.15
MR leave−one−out sensitivity analysis for
'CLU' on 'Cardiac valvular disease'

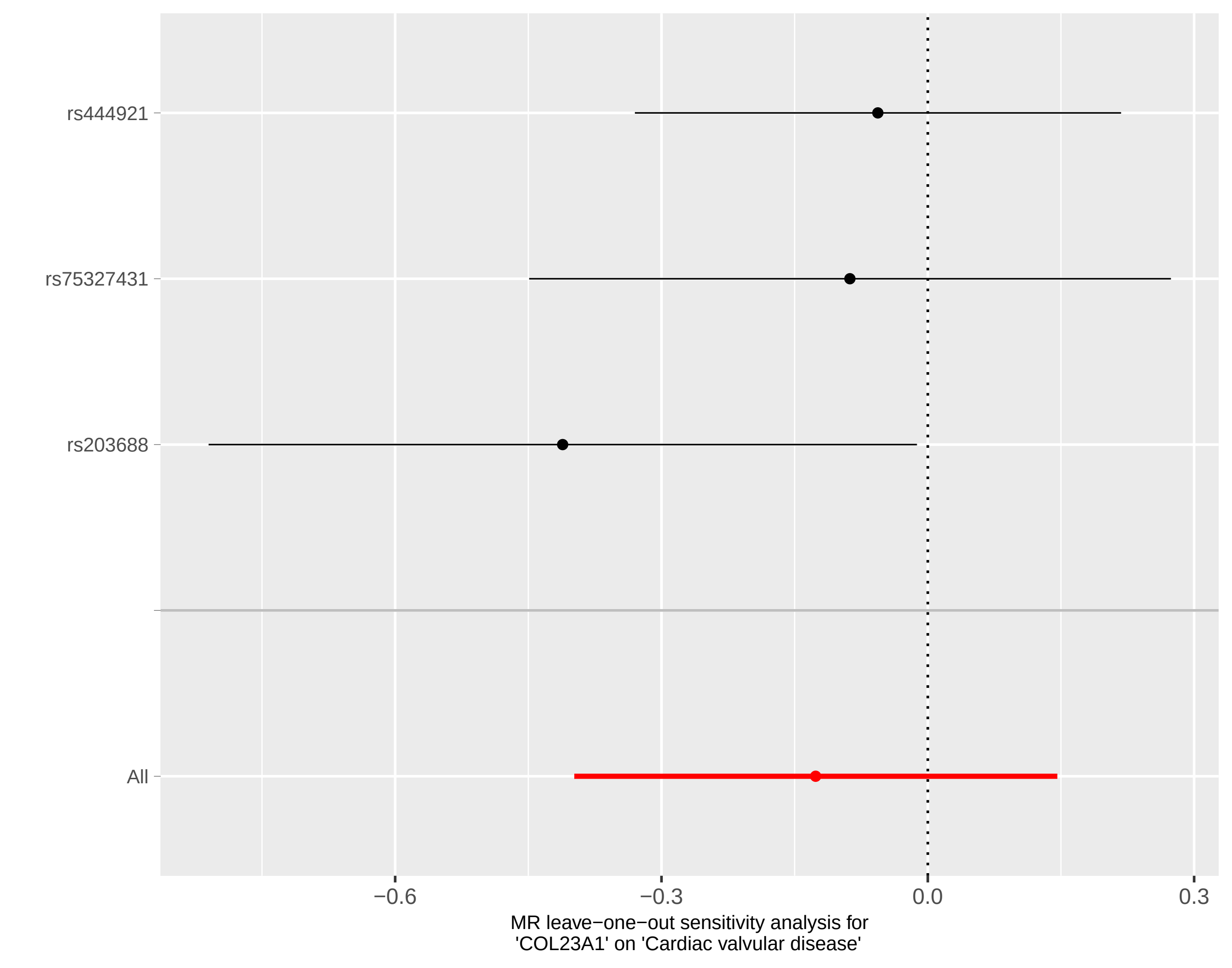
rs444921
rs75327431
rs203688
All
−0.6
−0.3
0.0
0.3
MR leave−one−out sensitivity analysis for
'COL23A1' on 'Cardiac valvular disease'

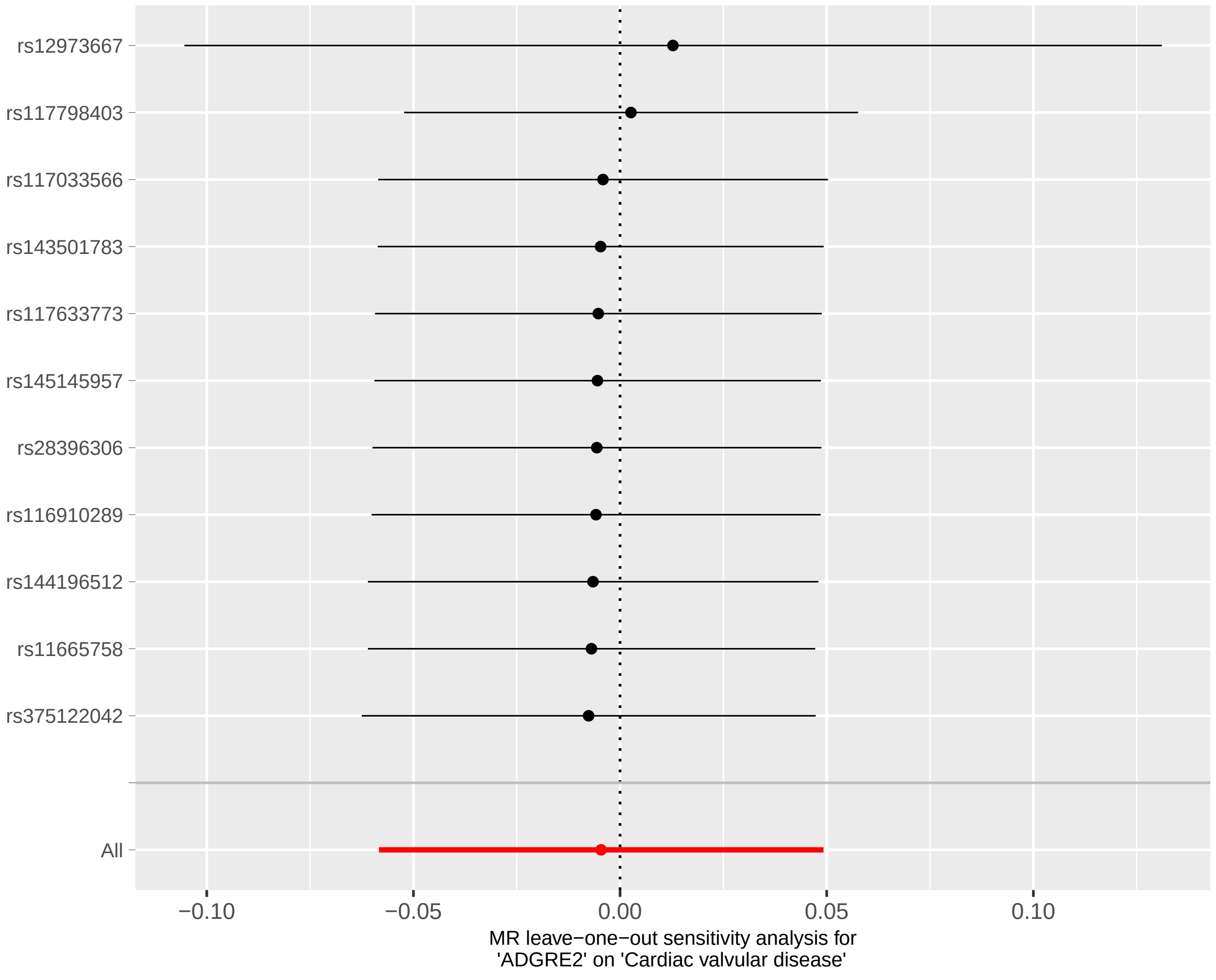

rs12973667
rs117798403
rs117033566
rs143501783
rs117633773
rs145145957
rs28396306
rs116910289
rs144196512
rs11665758
rs375122042
All
−0.10
−0.05
0.00
0.05
0.10
MR leave−one−out sensitivity analysis for
'ADGRE2' on 'Cardiac valvular disease'

Insufficient number of SNPs

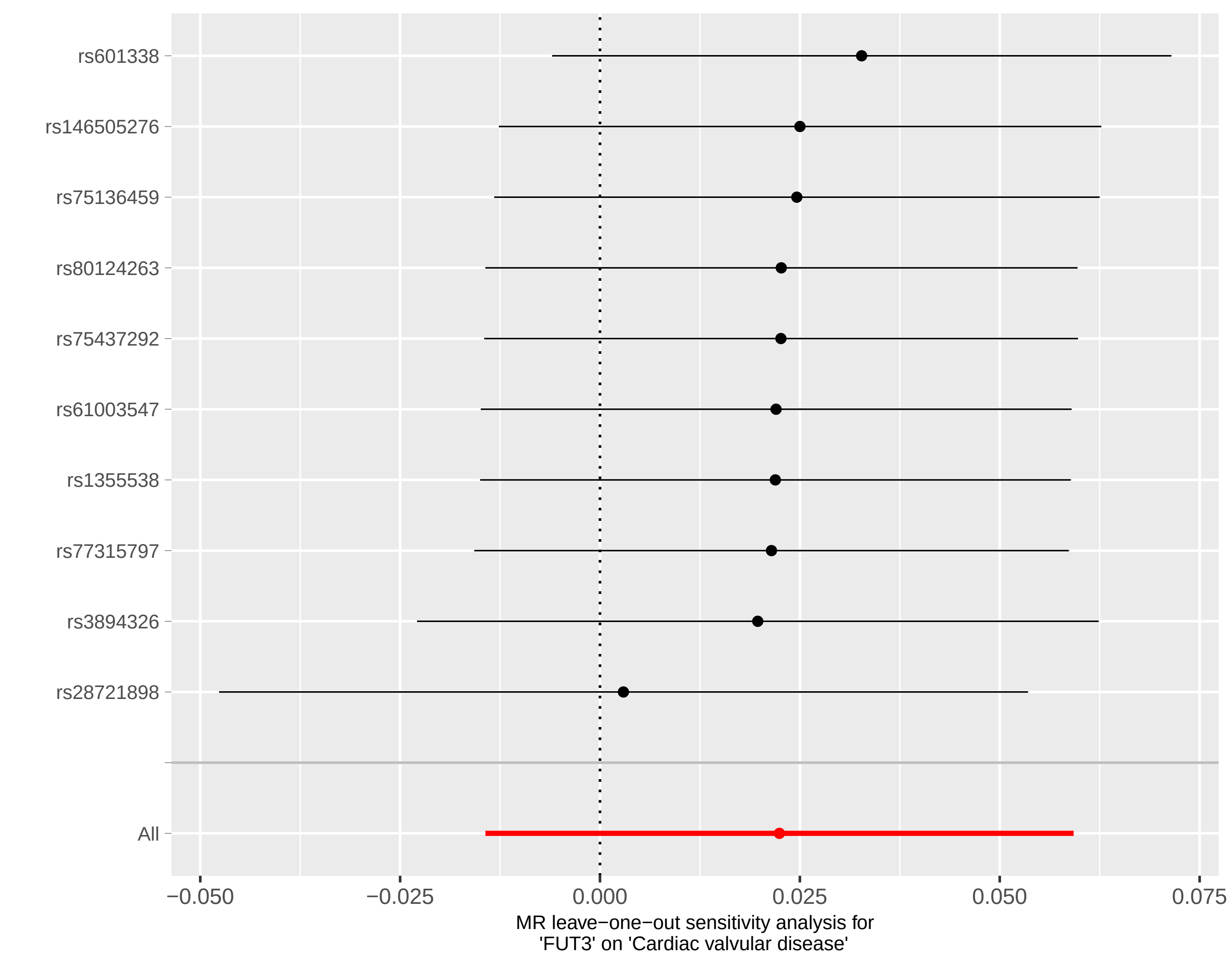

rs601338
rs146505276
rs75136459
rs80124263
rs75437292
rs61003547
rs1355538
rs77315797
rs3894326
rs28721898
All
−0.050
−0.025
0.000
0.025
0.050
0.075
MR leave−one−out sensitivity analysis for
'FUT3' on 'Cardiac valvular disease'

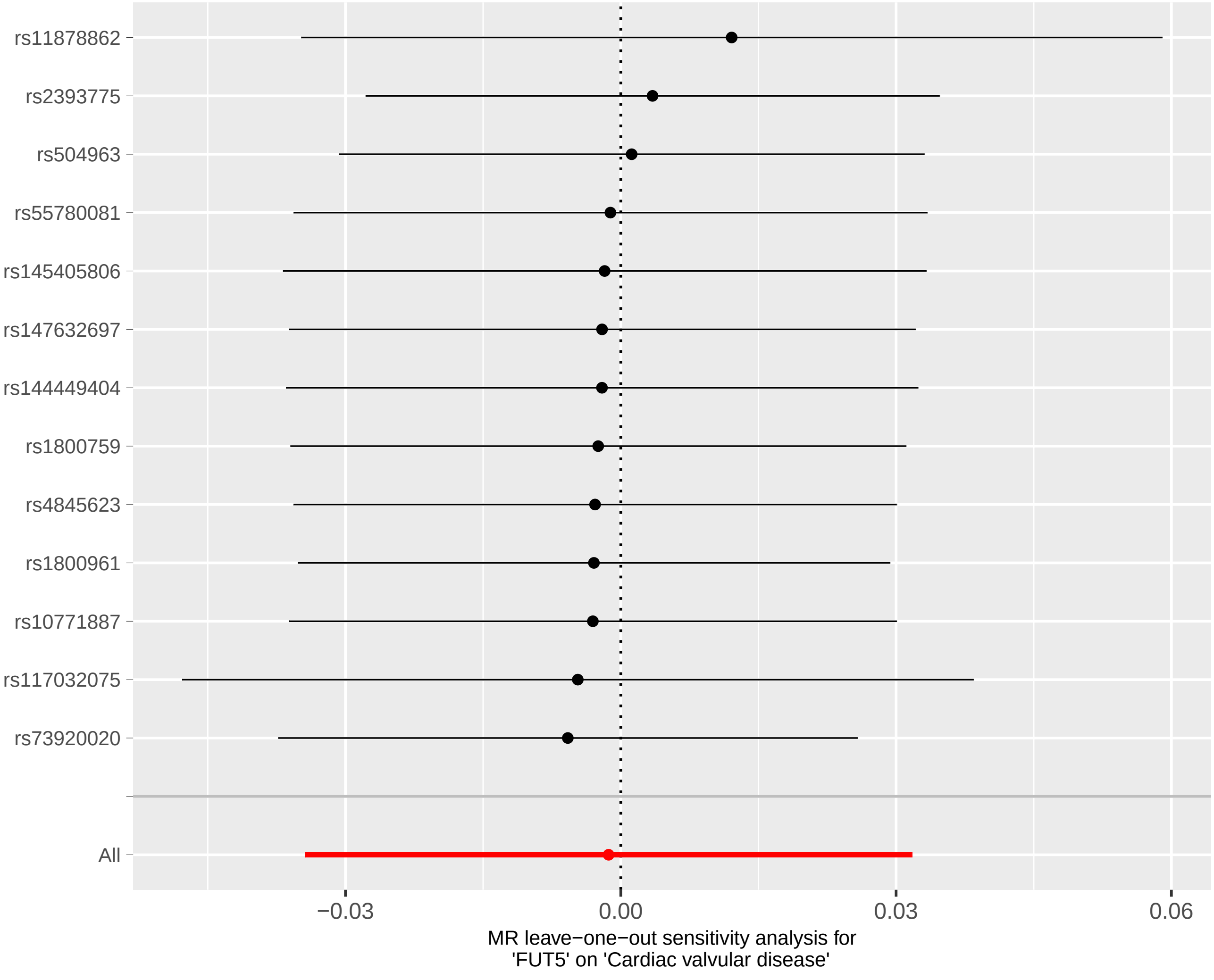
rs11878862
rs2393775
rs504963
rs55780081
rs145405806
rs147632697
rs144449404
rs1800759
rs4845623
rs1800961
rs10771887
rs117032075
rs73920020
All
−0.03
0.00
0.03
0.06
MR leave−one−out sensitivity analysis for
'FUT5' on 'Cardiac valvular disease'

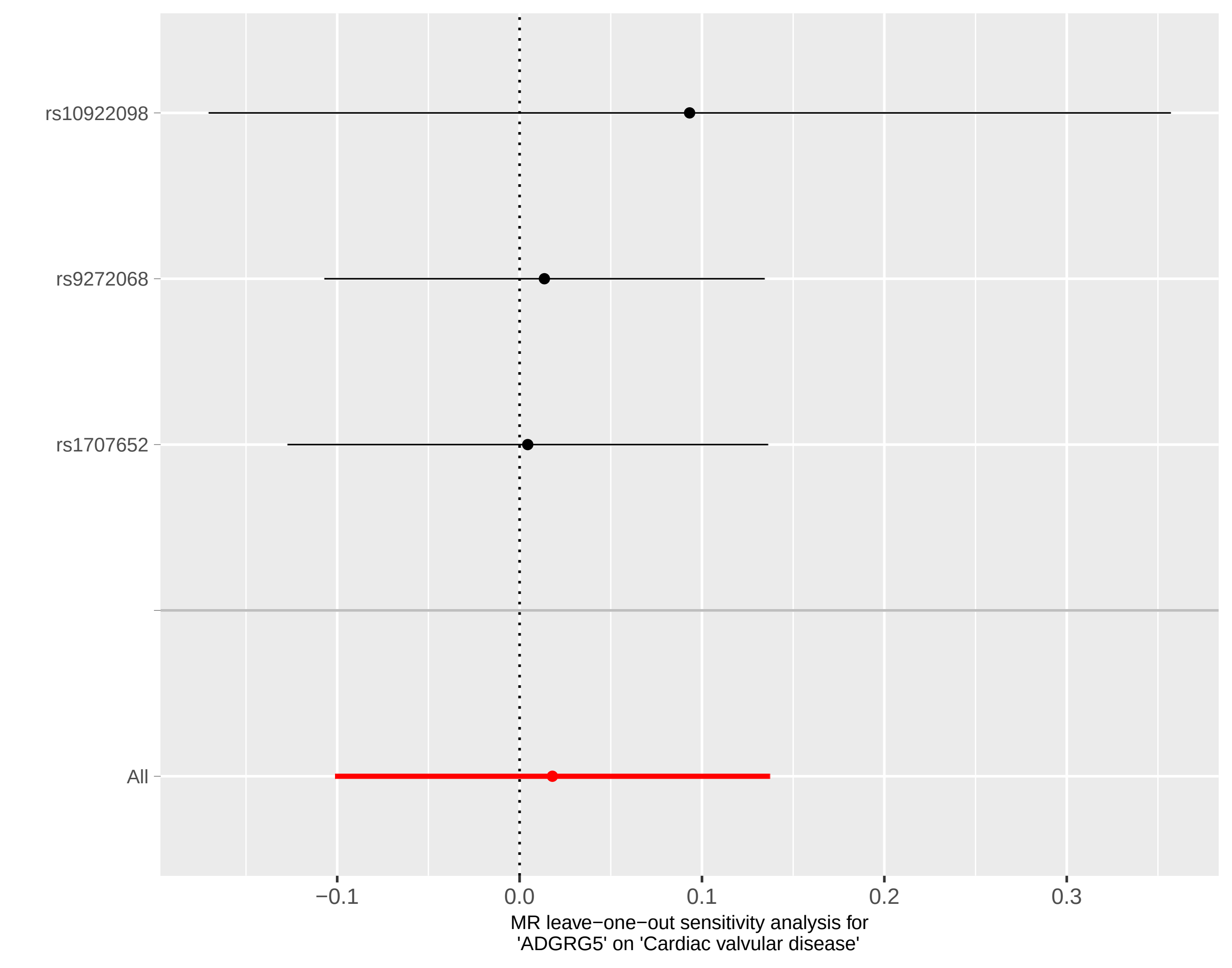
rs10922098
rs9272068
rs1707652
All
−0.1
0.0
0.1
0.2
0.3
MR leave−one−out sensitivity analysis for
'ADGRG5' on 'Cardiac valvular disease'

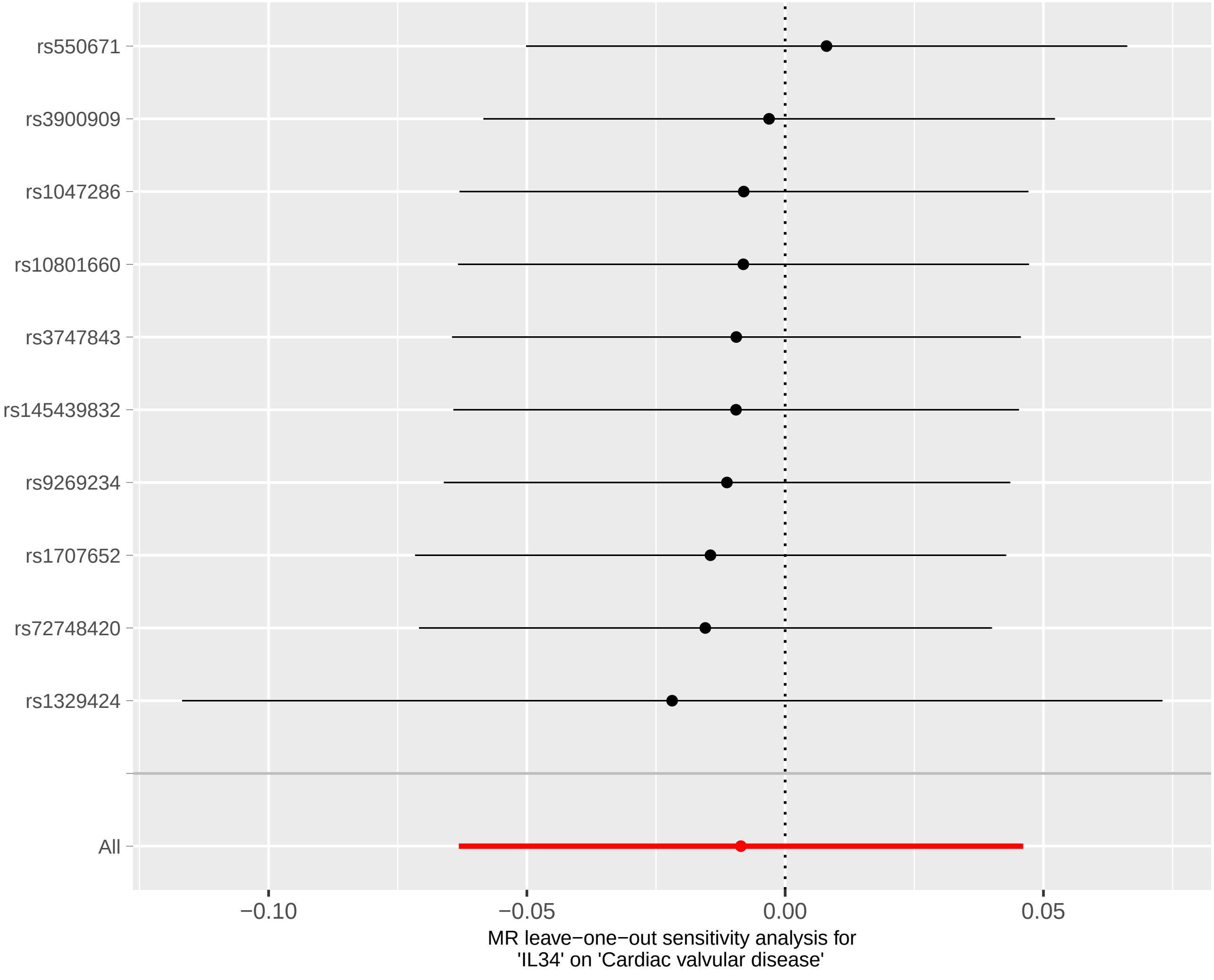
rs550671
rs3900909
rs1047286
rs10801660
rs3747843
rs145439832
rs9269234
rs1707652
rs72748420
rs1329424
All
−0.10
−0.05
0.00
0.05
MR leave−one−out sensitivity analysis for
'IL34' on 'Cardiac valvular disease'

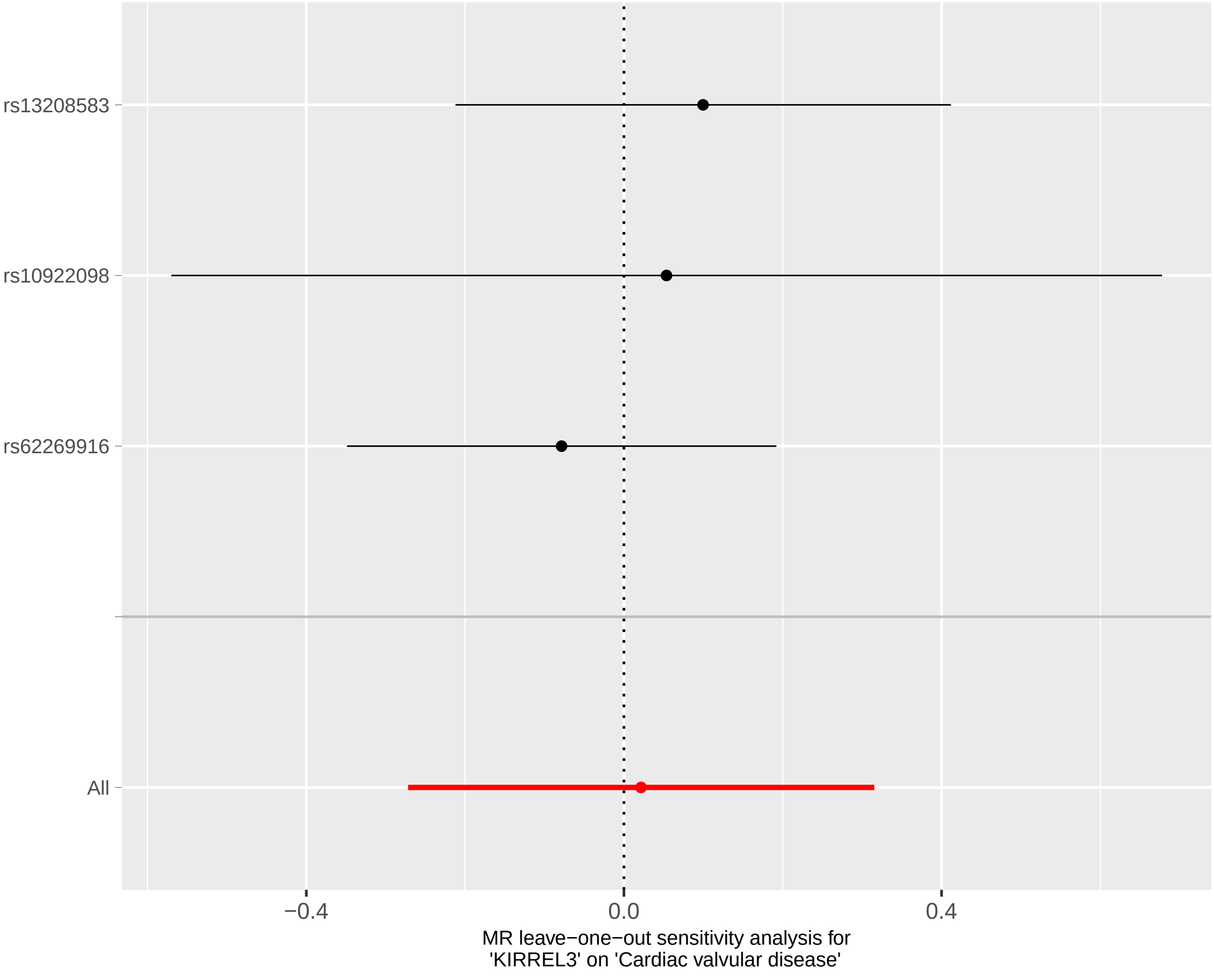
rs13208583
rs10922098
rs62269916
All
−0.4
0.0
0.4
MR leave−one−out sensitivity analysis for
'KIRREL3' on 'Cardiac valvular disease'

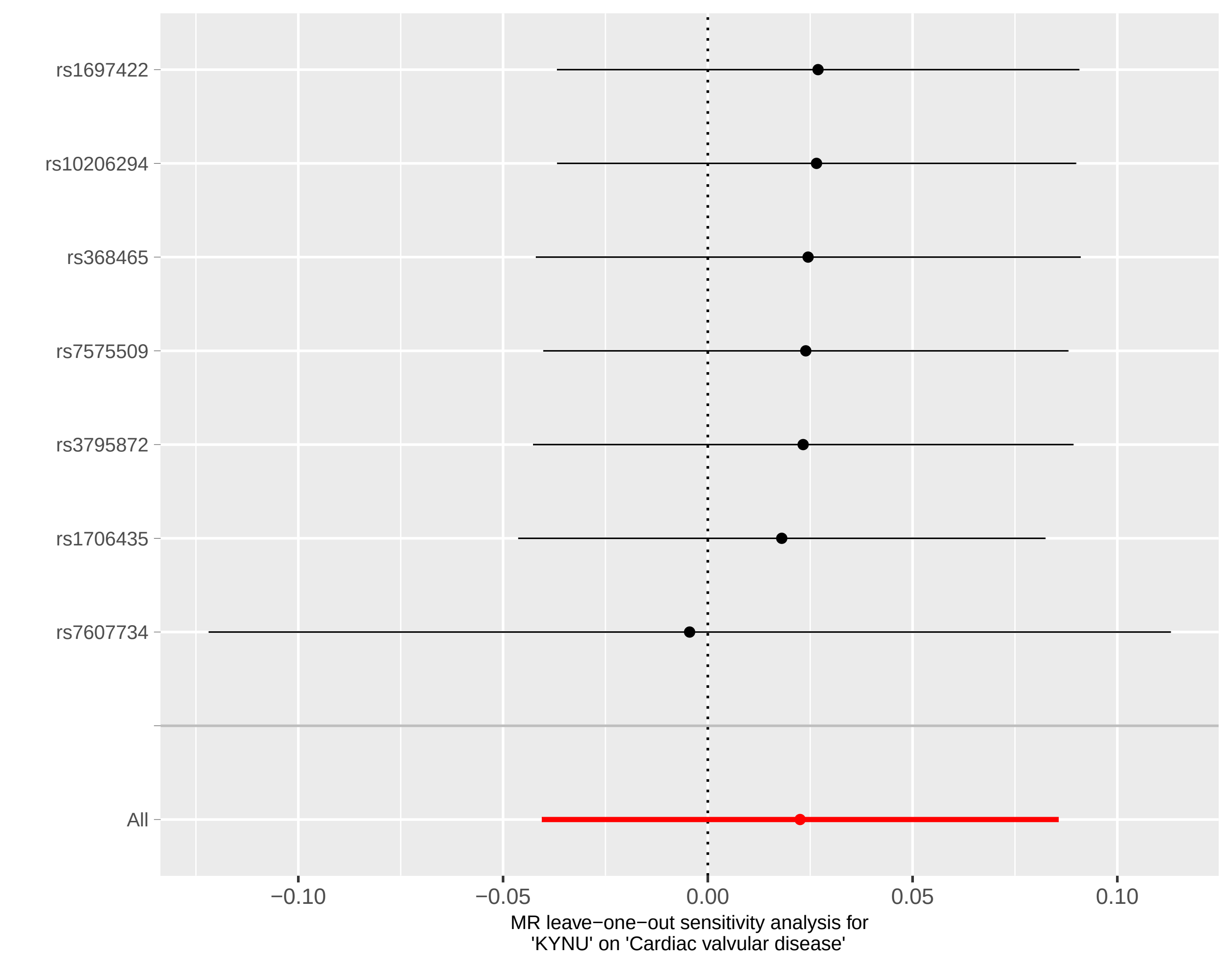
rs1697422
rs10206294
rs368465
rs7575509
rs3795872
rs1706435
rs7607734
All
−0.10
−0.05
0.00
0.05
0.10
MR leave−one−out sensitivity analysis for
'KYNU' on 'Cardiac valvular disease'

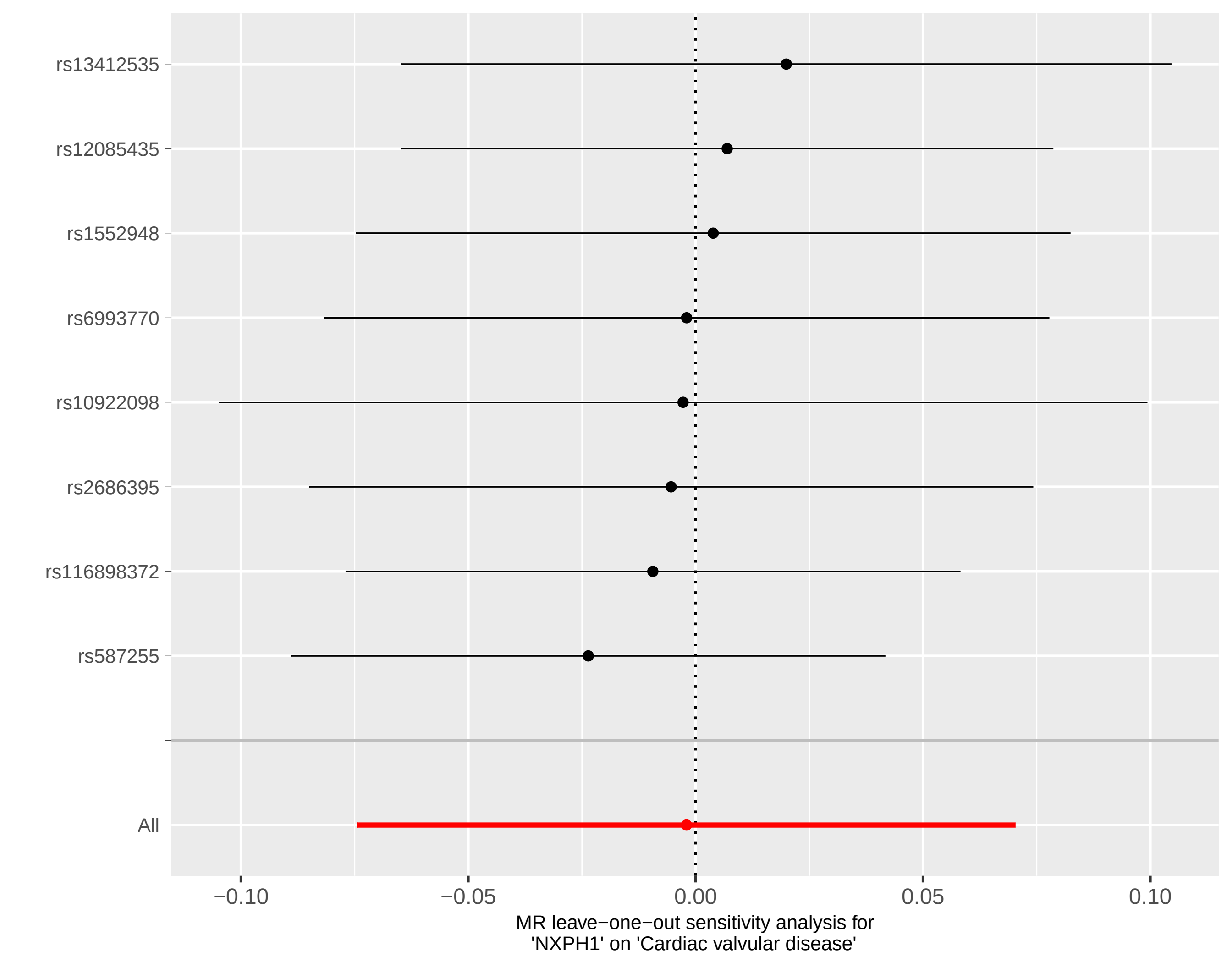
rs13412535
rs12085435
rs1552948
rs6993770
rs10922098
rs2686395
rs116898372
rs587255
All
−0.10
−0.05
0.00
0.05
0.10
MR leave−one−out sensitivity analysis for
'NXPH1' on 'Cardiac valvular disease'

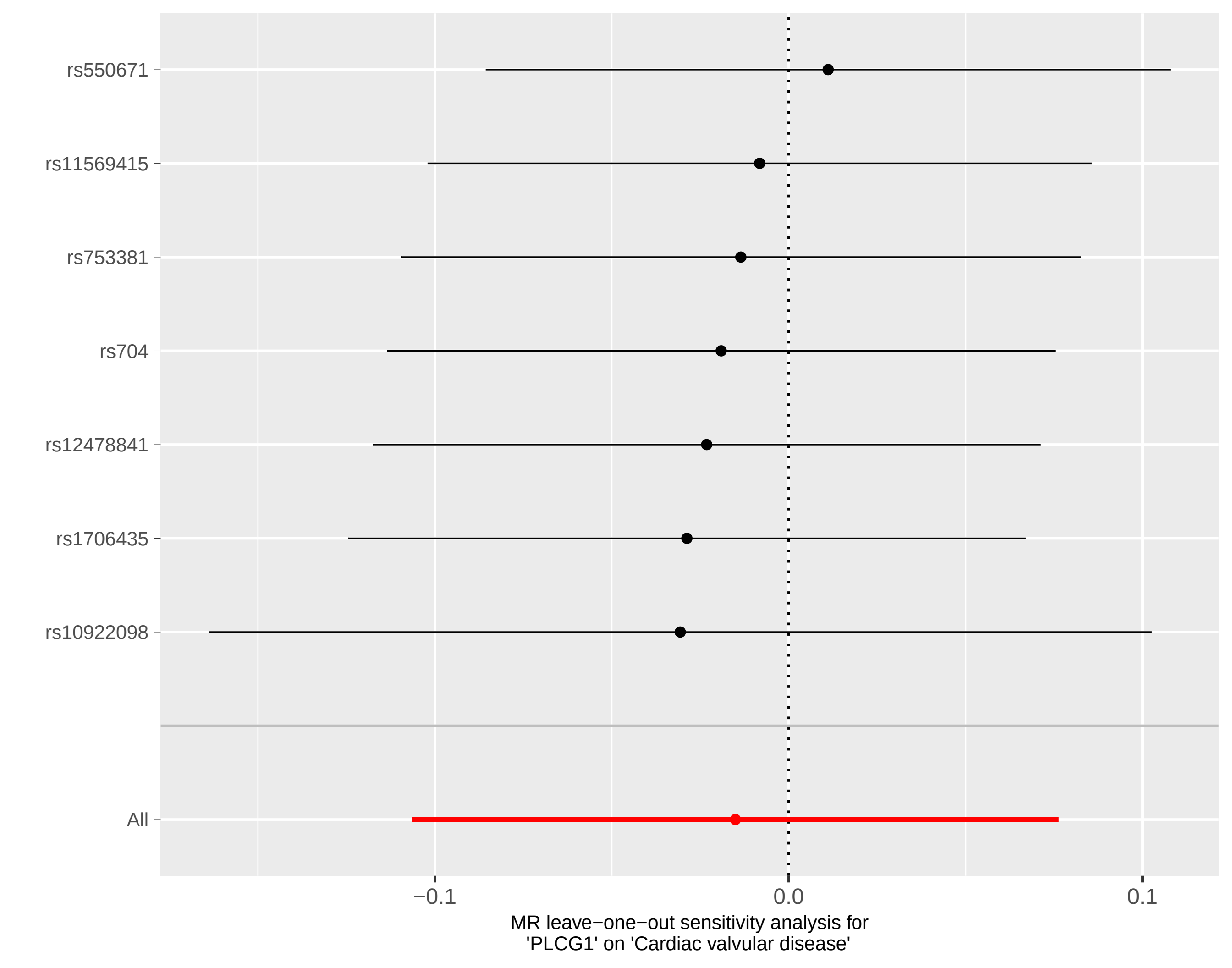

rs550671
rs11569415
rs753381
rs704
rs12478841
rs1706435
rs10922098
All
−0.1
0.0
0.1
MR leave−one−out sensitivity analysis for
'PLCG1' on 'Cardiac valvular disease'

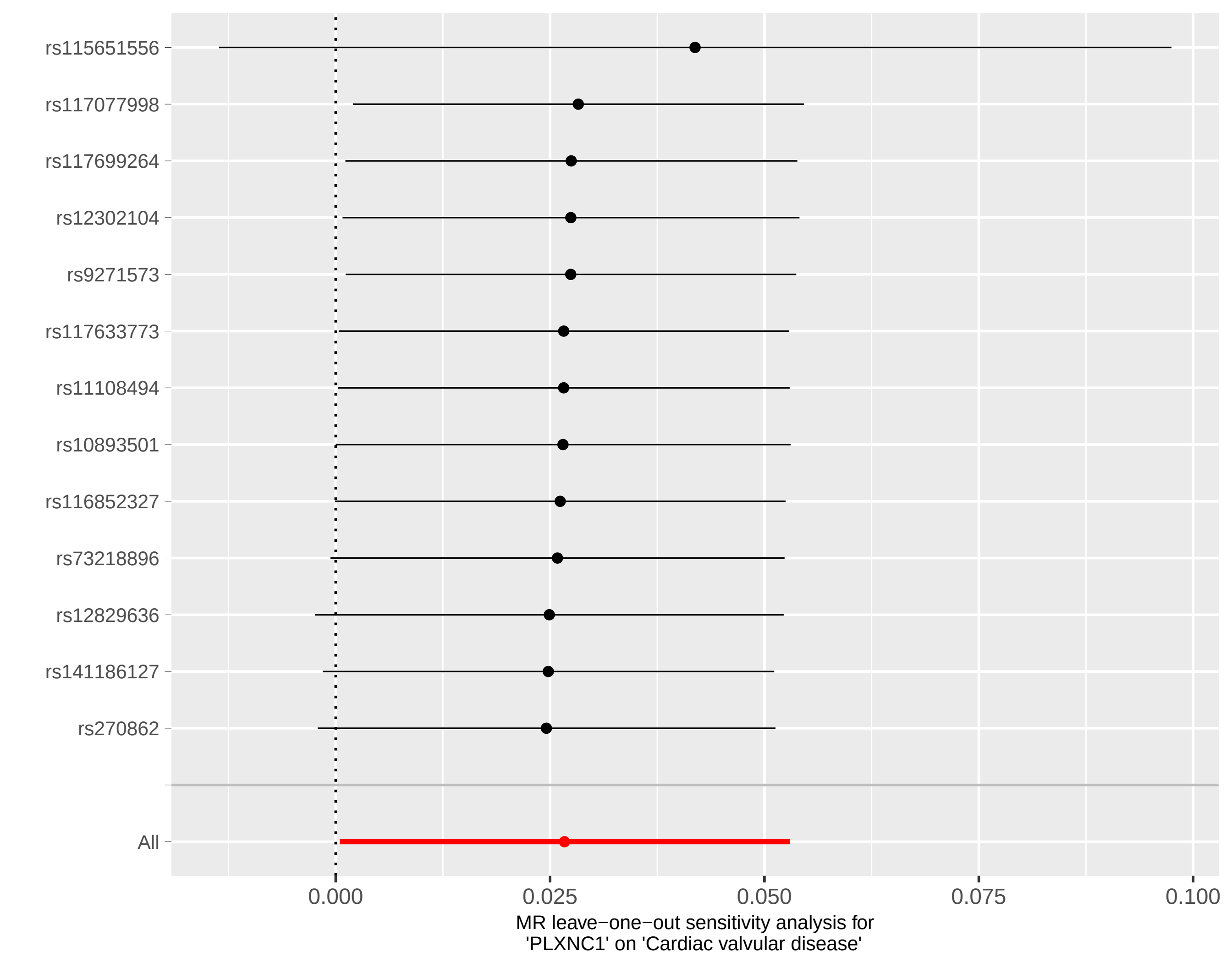

rs115651556
rs117077998
rs117699264
rs12302104
rs9271573
rs117633773
rs11108494
rs10893501
rs116852327
rs73218896
rs12829636
rs141186127
rs270862
All
0.000
0.025
0.050
0.075
0.100
MR leave−one−out sensitivity analysis for
'PLXNC1' on 'Cardiac valvular disease'

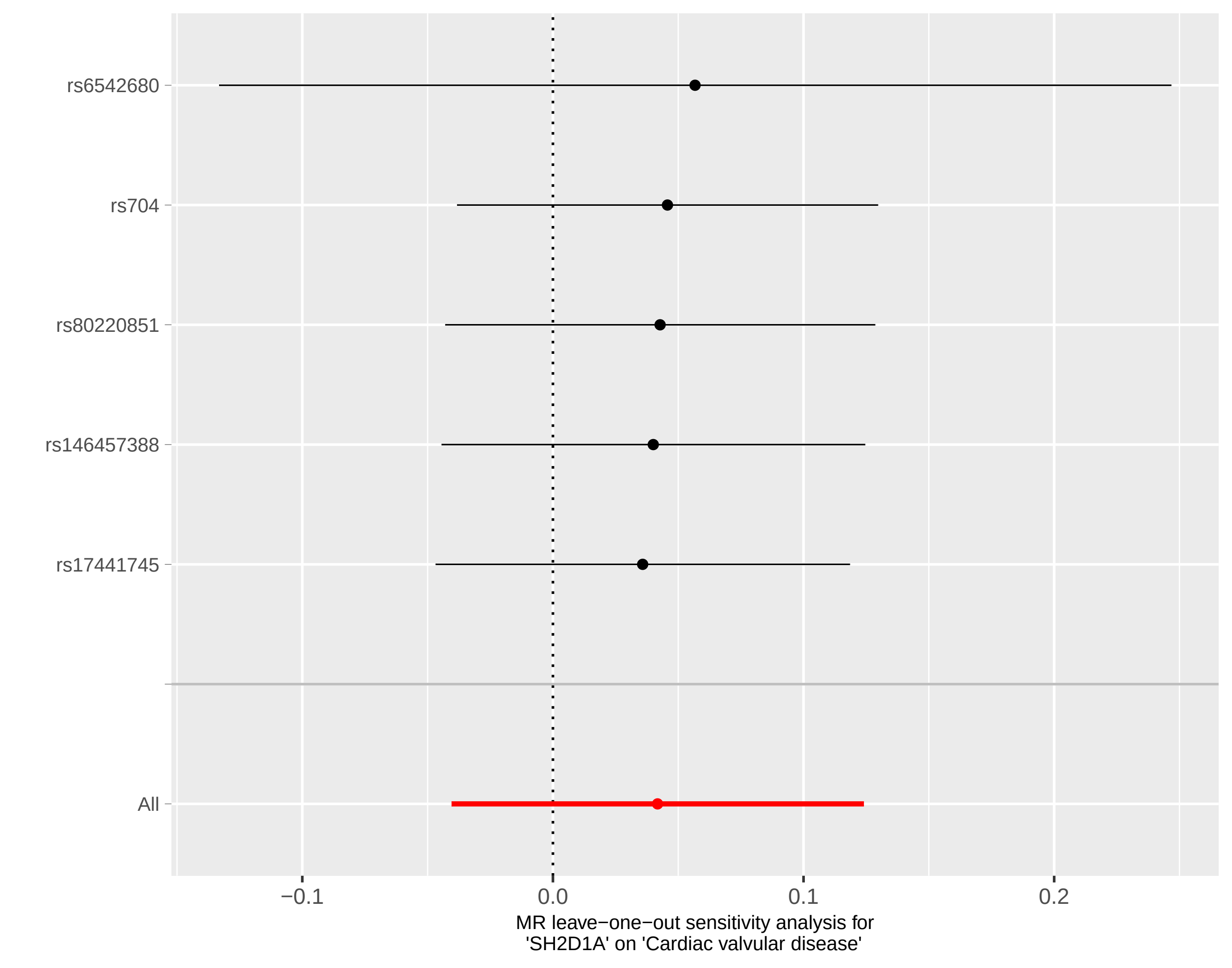
rs6542680
rs704
rs80220851
rs146457388
rs17441745
All
−0.1
0.0
0.1
0.2
MR leave−one−out sensitivity analysis for
'SH2D1A' on 'Cardiac valvular disease'

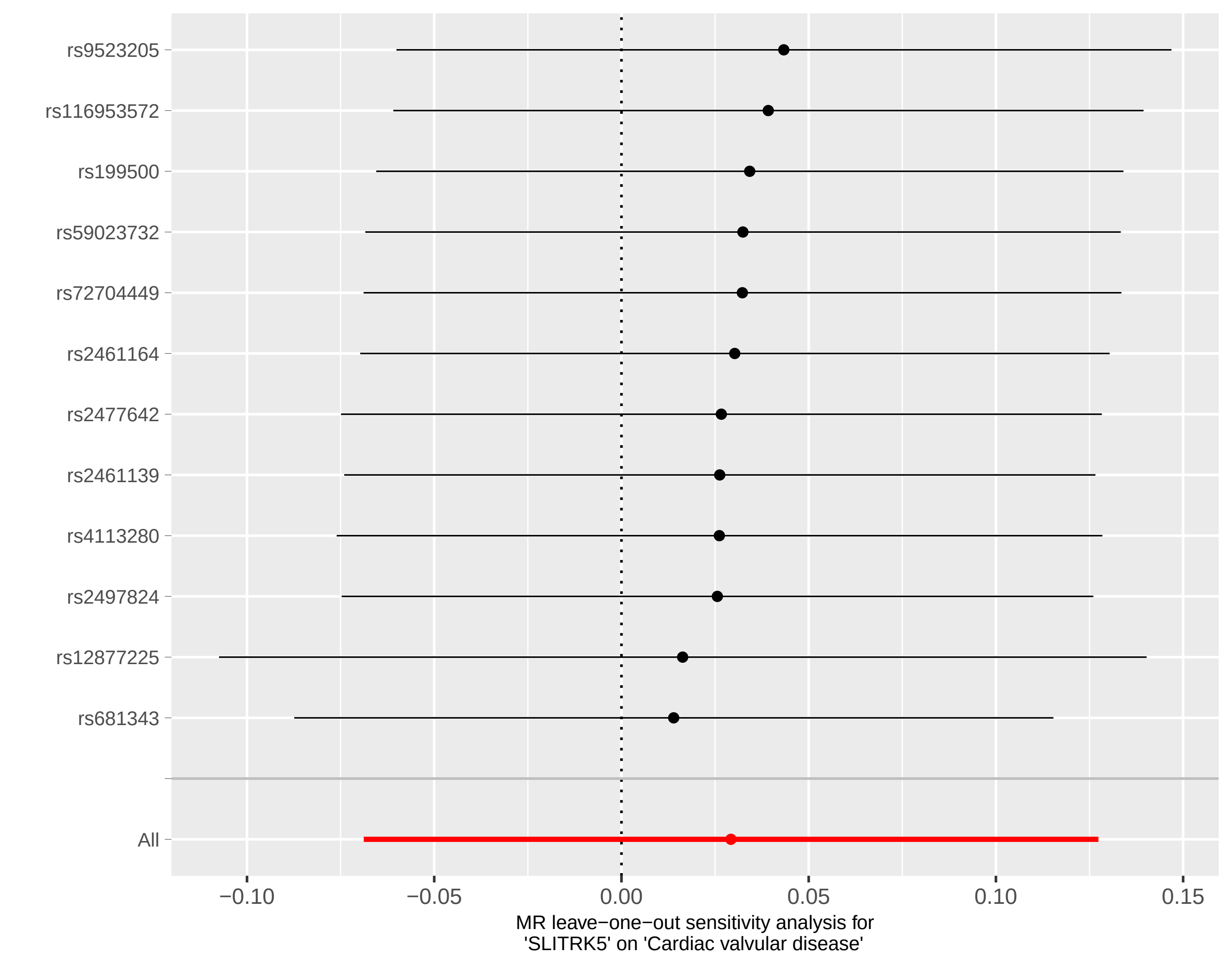

rs9523205
rs116953572
rs199500
rs59023732
rs72704449
rs2461164
rs2477642
rs2461139
rs4113280
rs2497824
rs12877225
rs681343
All
−0.10
−0.05
0.00
0.05
0.10
0.15
MR leave−one−out sensitivity analysis for
'SLITRK5' on 'Cardiac valvular disease'

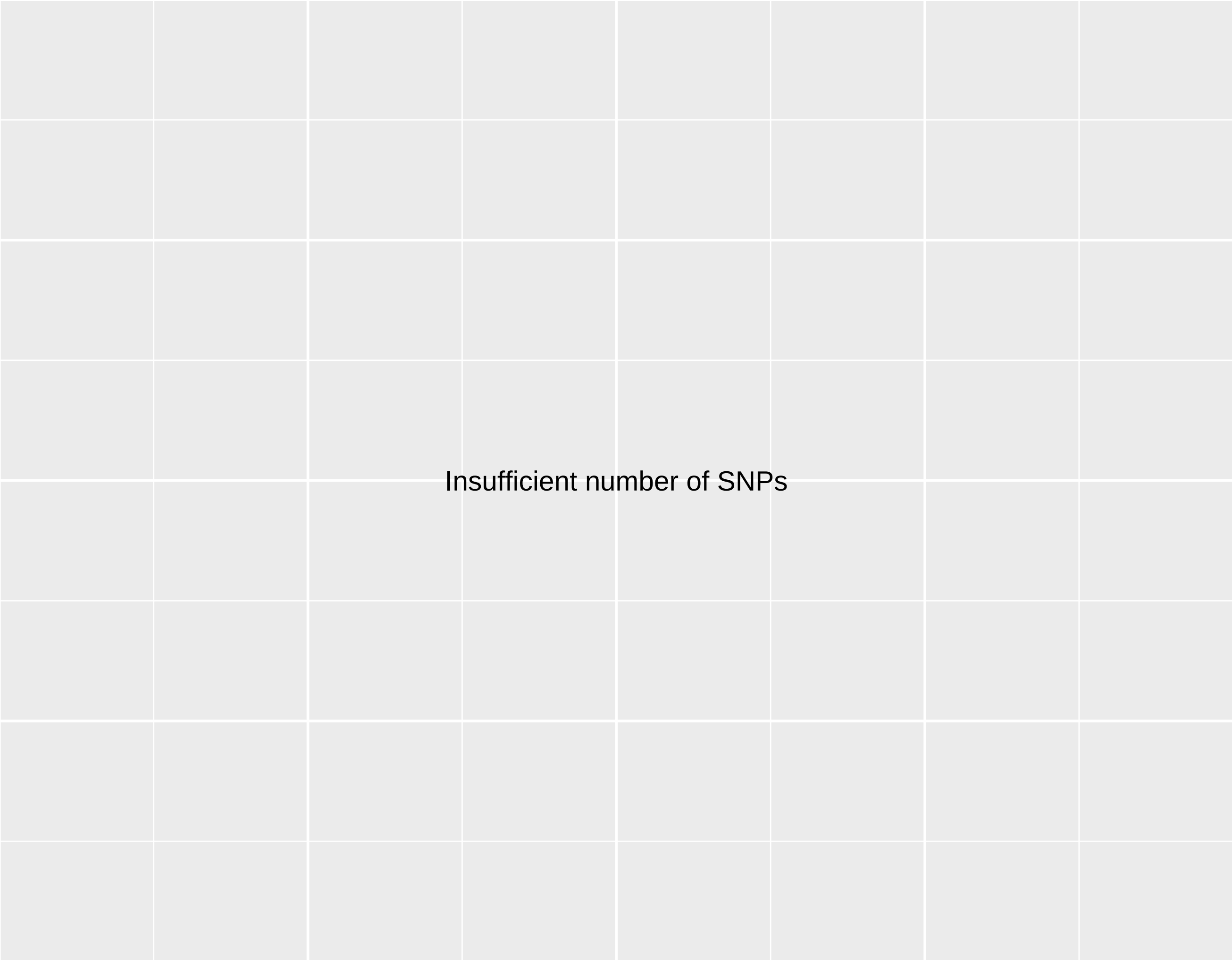
Insufficient number of SNPs

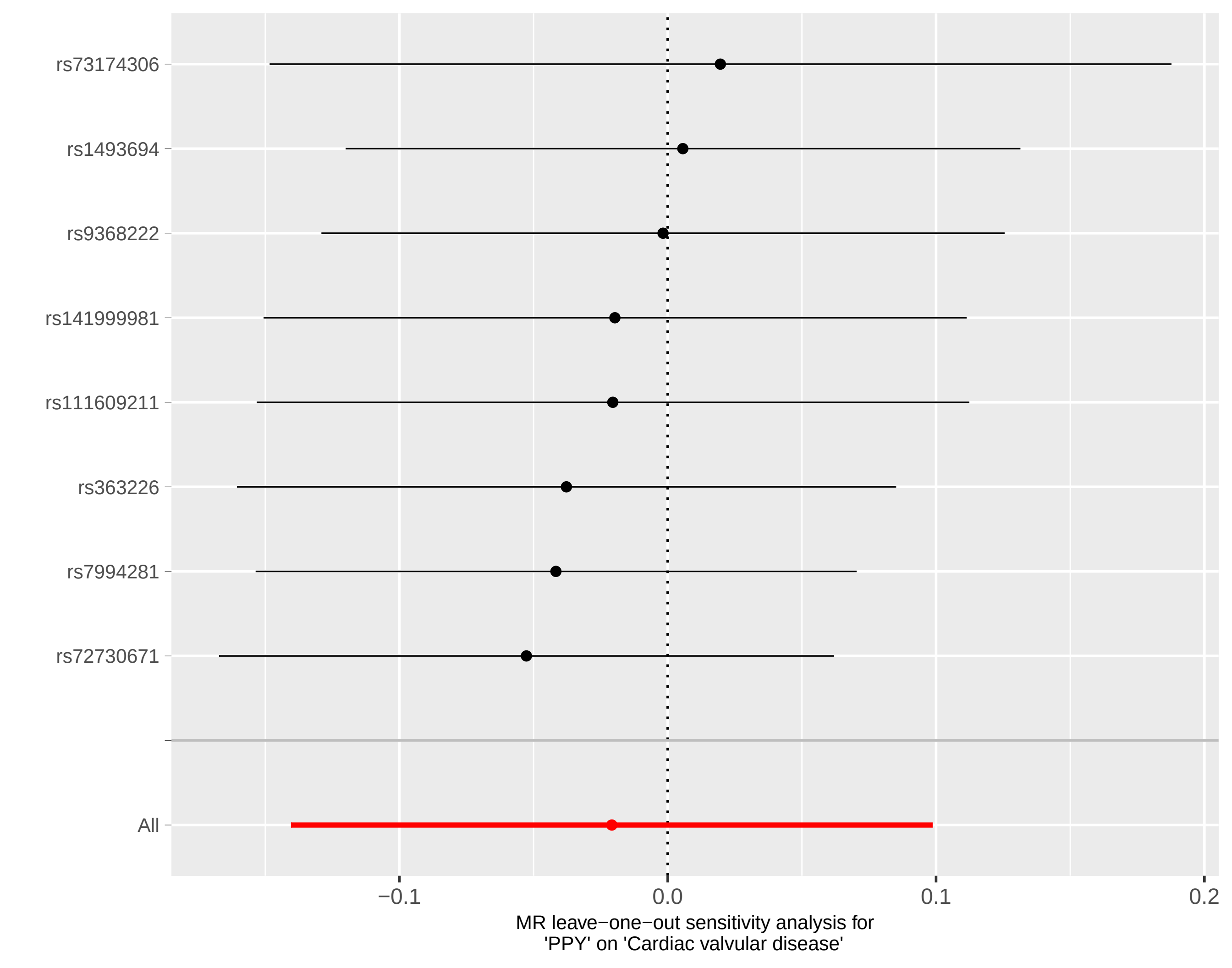

rs73174306
rs1493694
rs9368222
rs141999981
rs111609211
rs363226
rs7994281
rs72730671
All
−0.1
0.0
0.1
0.2
MR leave−one−out sensitivity analysis for
'PPY' on 'Cardiac valvular disease'

Insufficient number of SNPs

Insufficient number of SNPs

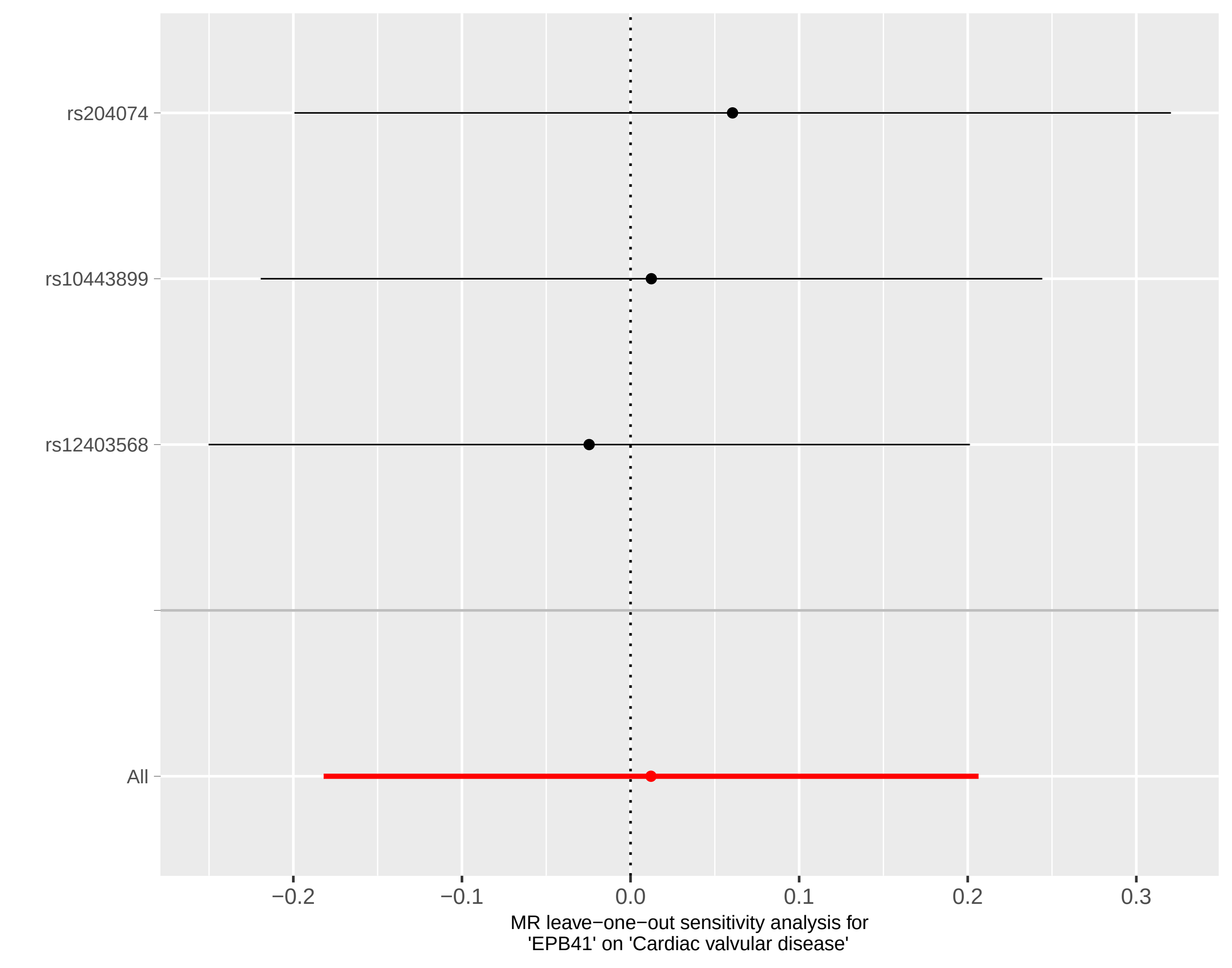
rs204074
rs10443899
rs12403568
All
−0.2
−0.1
0.0
0.1
0.2
0.3
MR leave−one−out sensitivity analysis for
'EPB41' on 'Cardiac valvular disease'

Insufficient number of SNPs

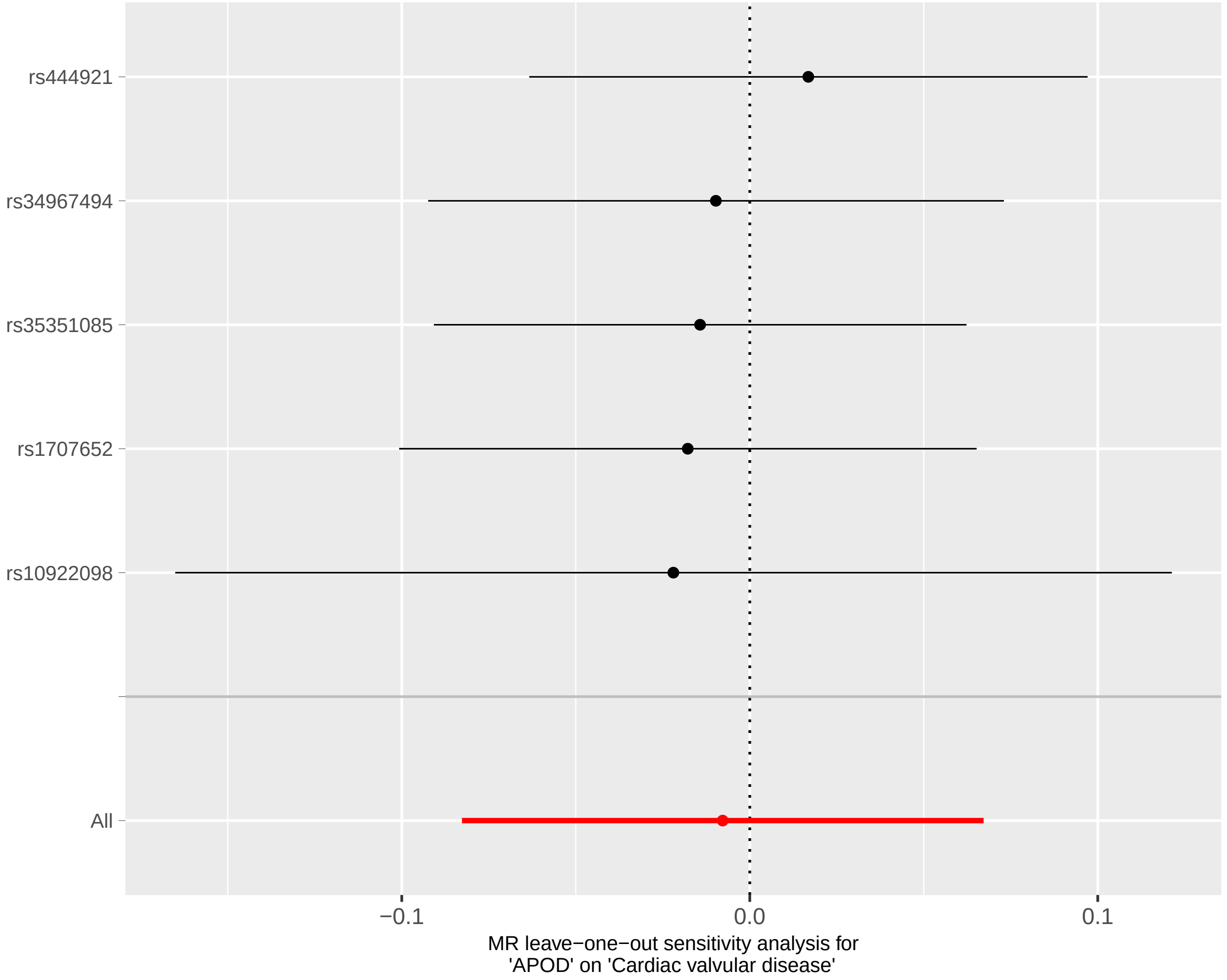
rs444921
rs34967494
rs35351085
rs1707652
rs10922098
All
−0.1
0.0
0.1
MR leave−one−out sensitivity analysis for
'APOD' on 'Cardiac valvular disease'

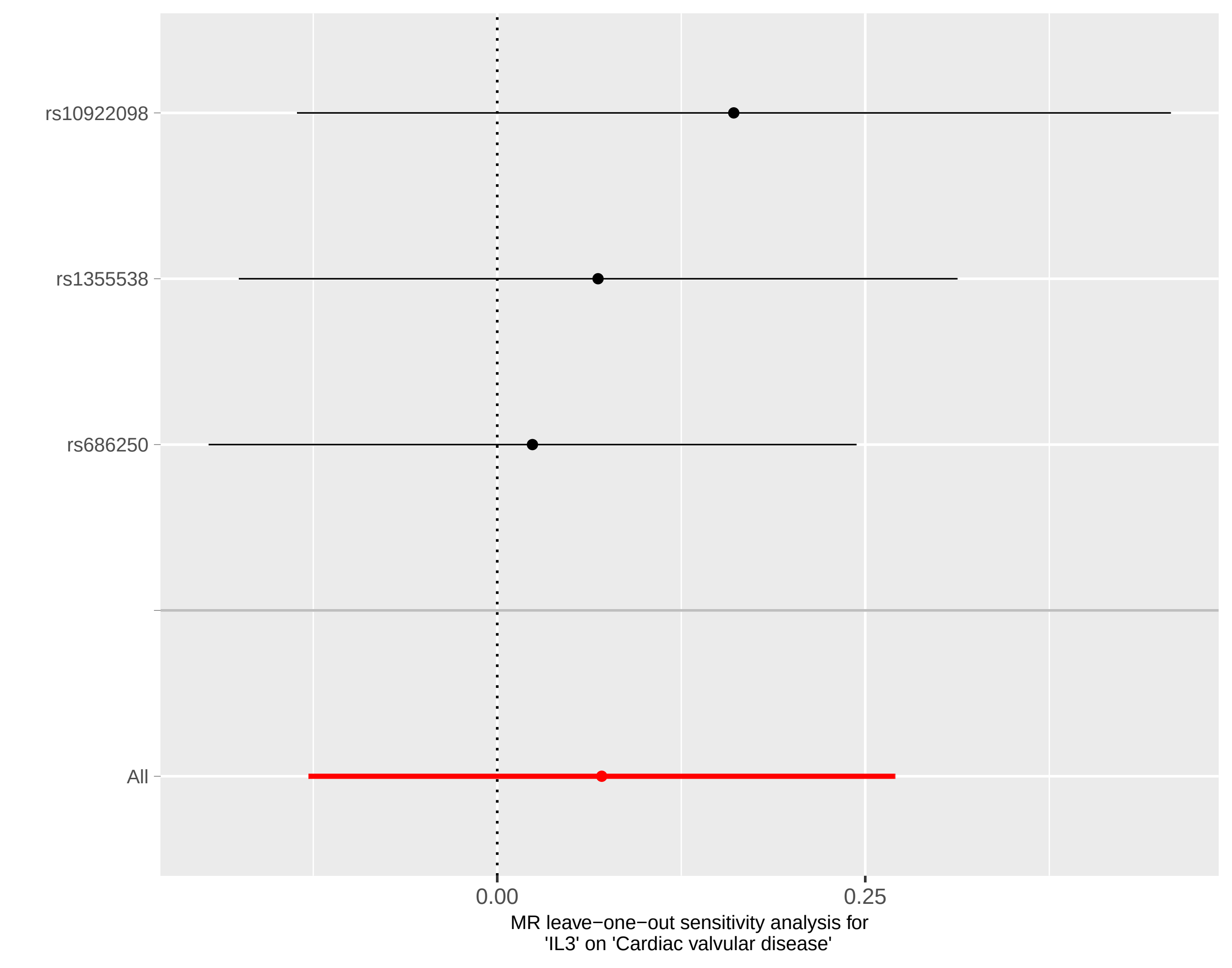
rs10922098
rs1355538
rs686250
All
0.00
0.25
MR leave−one−out sensitivity analysis for
'IL3' on 'Cardiac valvular disease'

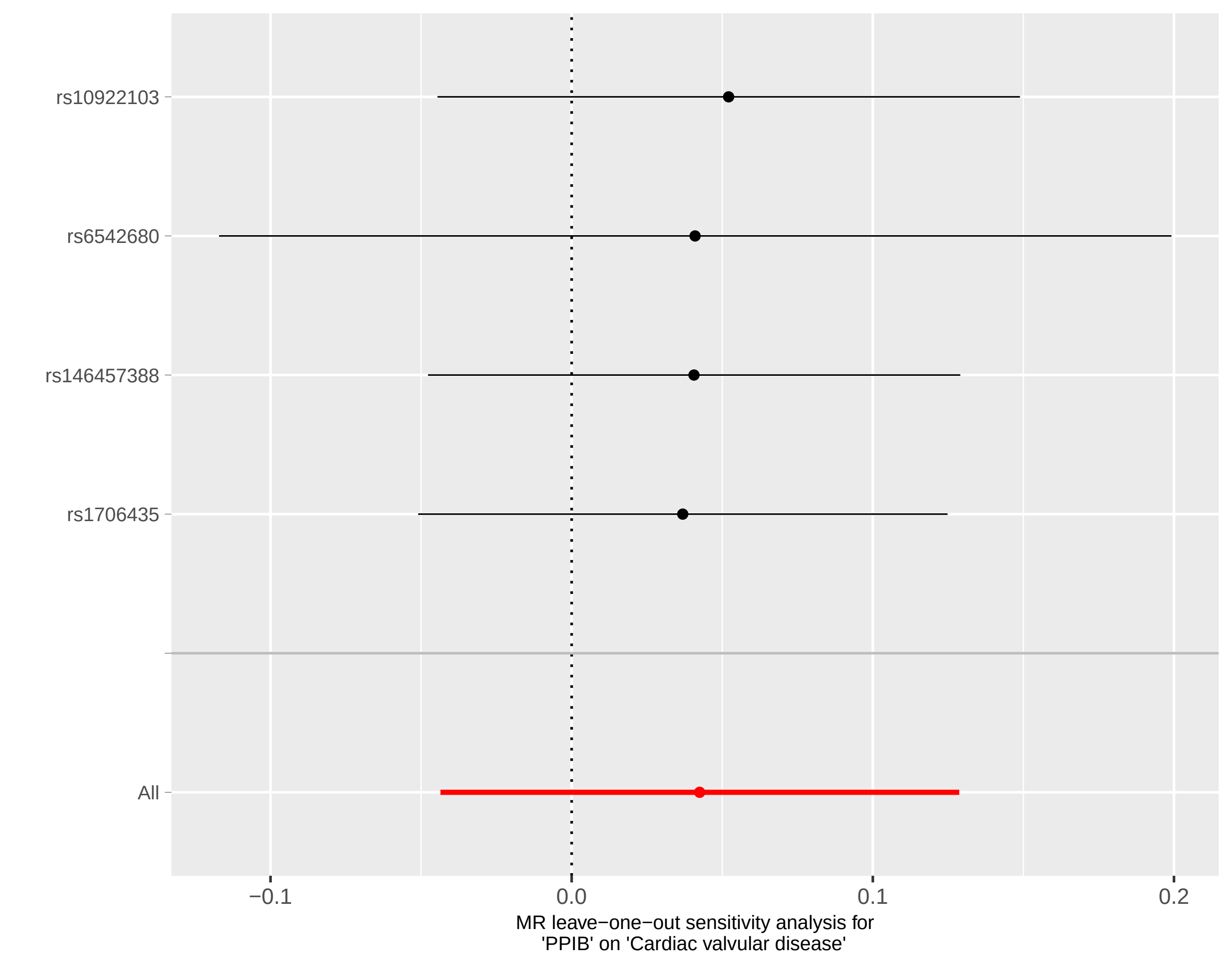
rs10922103
rs6542680
rs146457388
rs1706435
All
−0.1
0.0
0.1
0.2
MR leave−one−out sensitivity analysis for
'PPIB' on 'Cardiac valvular disease'

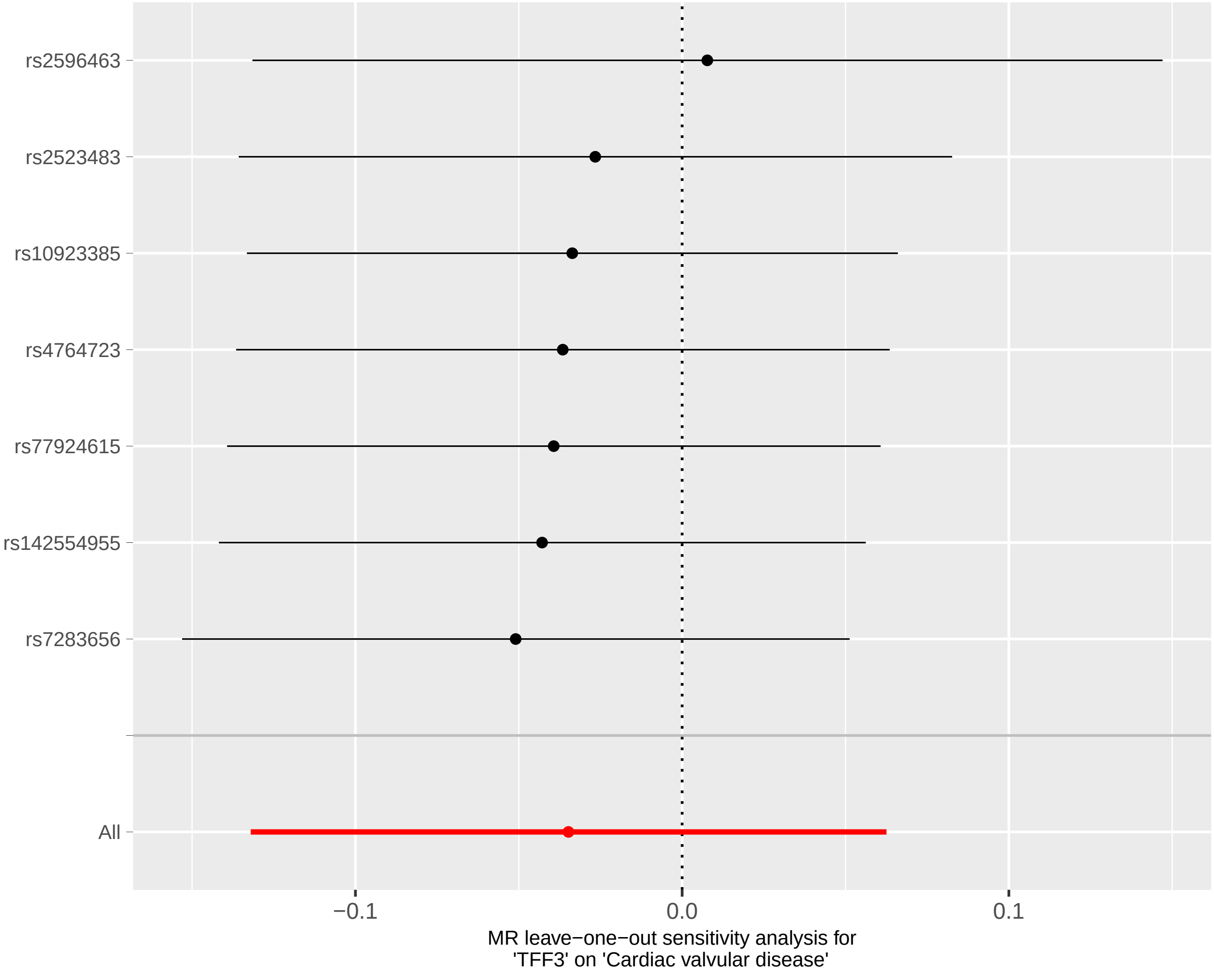
rs2596463
rs2523483
rs10923385
rs4764723
rs77924615
rs142554955
rs7283656
All
−0.1
0.0
0.1
MR leave−one−out sensitivity analysis for
'TFF3' on 'Cardiac valvular disease'

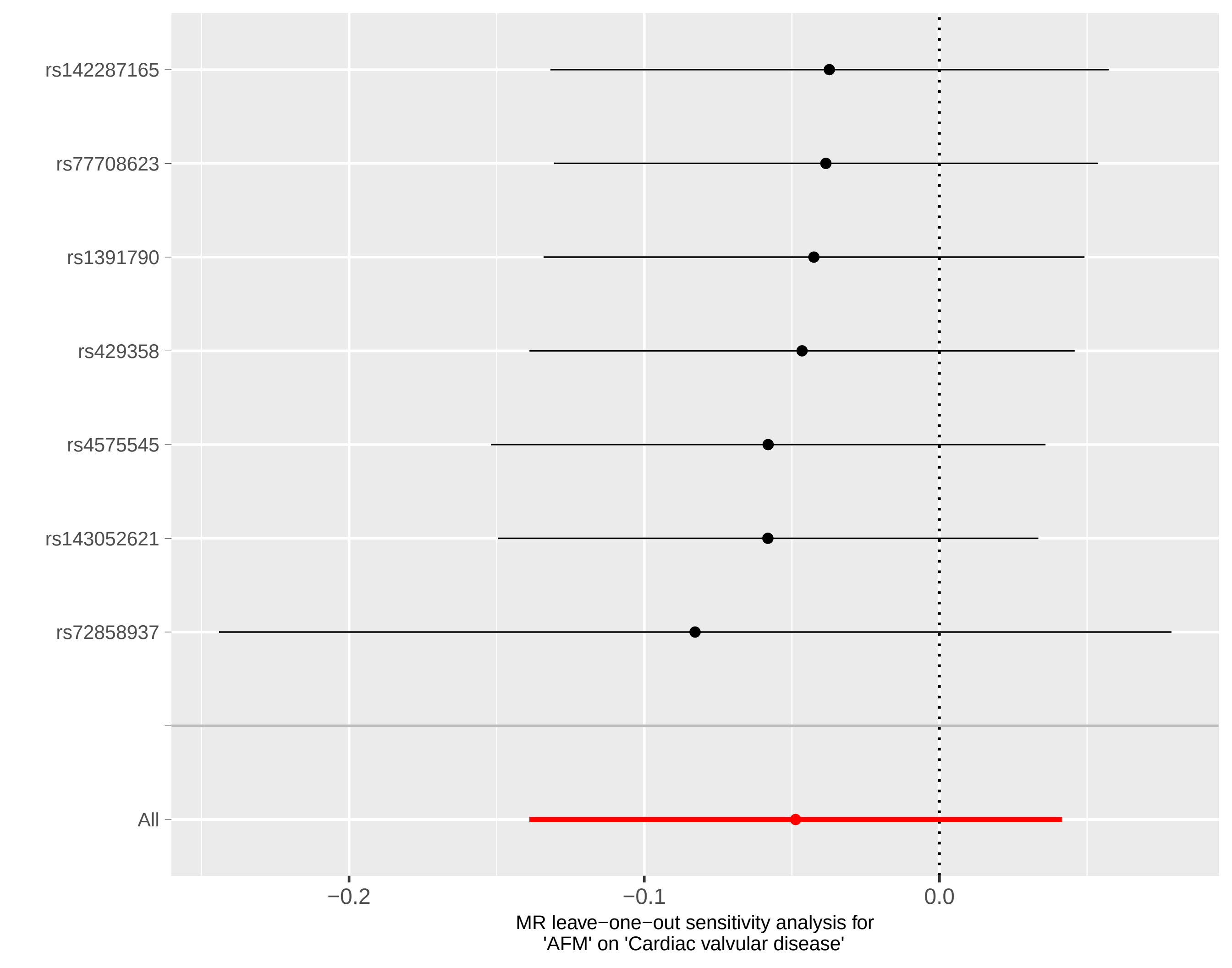
rs142287165
rs77708623
rs1391790
rs429358
rs4575545
rs143052621
rs72858937
All
−0.2
−0.1
0.0
MR leave−one−out sensitivity analysis for
'AFM' on 'Cardiac valvular disease'

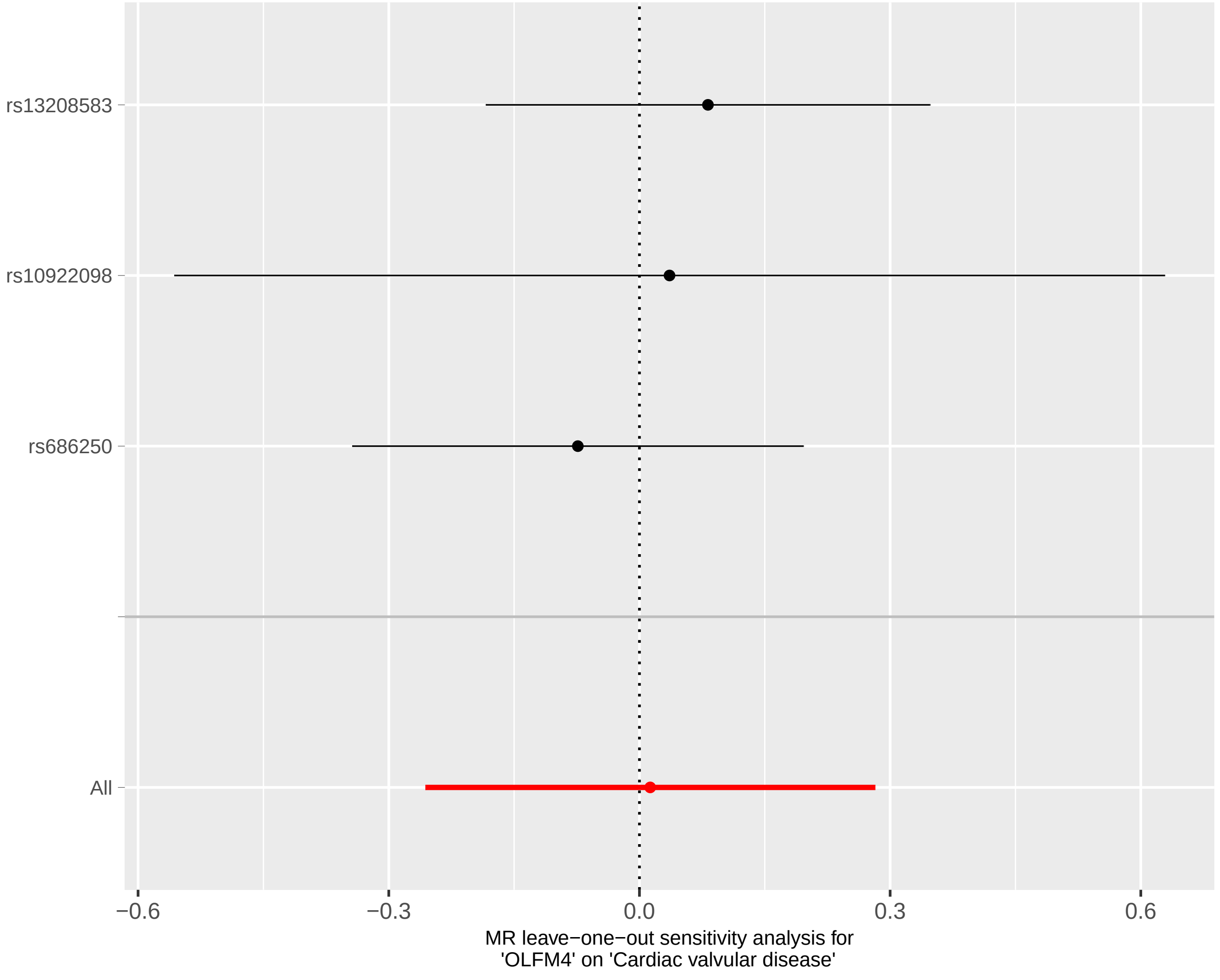
rs13208583
rs10922098
rs686250
All
−0.6
−0.3
0.0
0.3
0.6
MR leave−one−out sensitivity analysis for
'OLFM4' on 'Cardiac valvular disease'

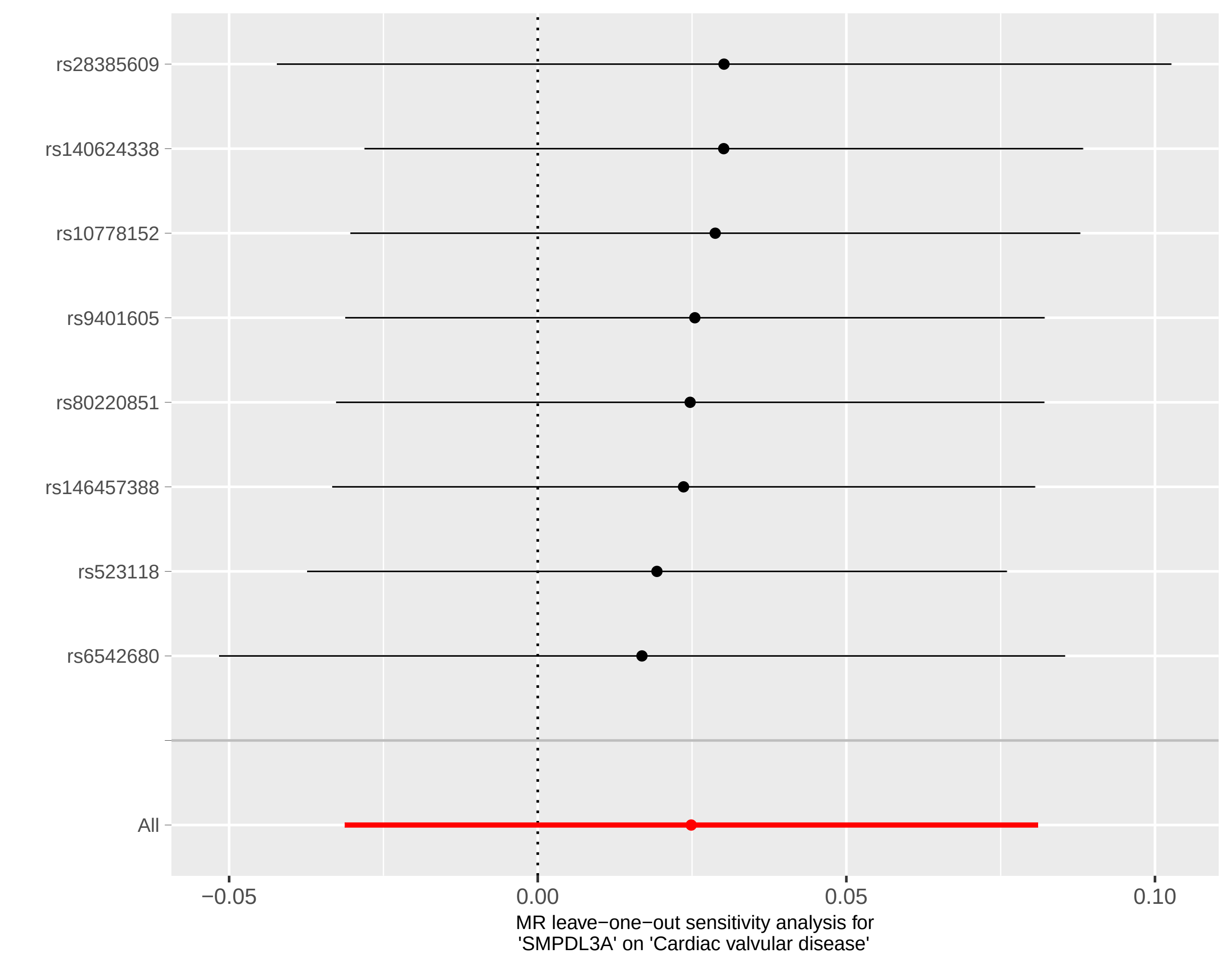

rs28385609
rs140624338
rs10778152
rs9401605
rs80220851
rs146457388
rs523118
rs6542680
All
−0.05
0.00
0.05
0.10
MR leave−one−out sensitivity analysis for
'SMPDL3A' on 'Cardiac valvular disease'

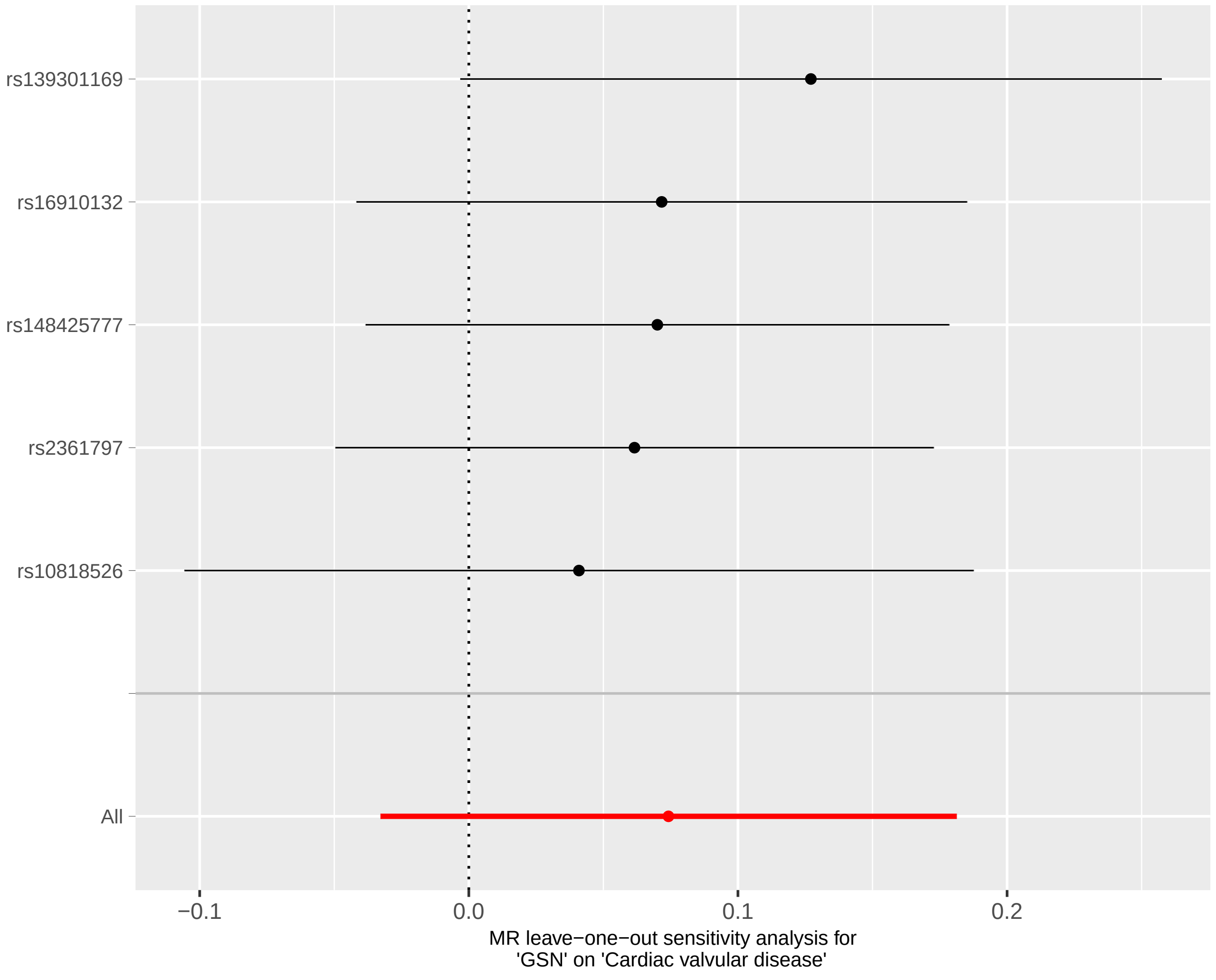
rs139301169
rs16910132
rs148425777
rs2361797
rs10818526
All
−0.1
0.0
0.1
0.2
MR leave−one−out sensitivity analysis for
'GSN' on 'Cardiac valvular disease'

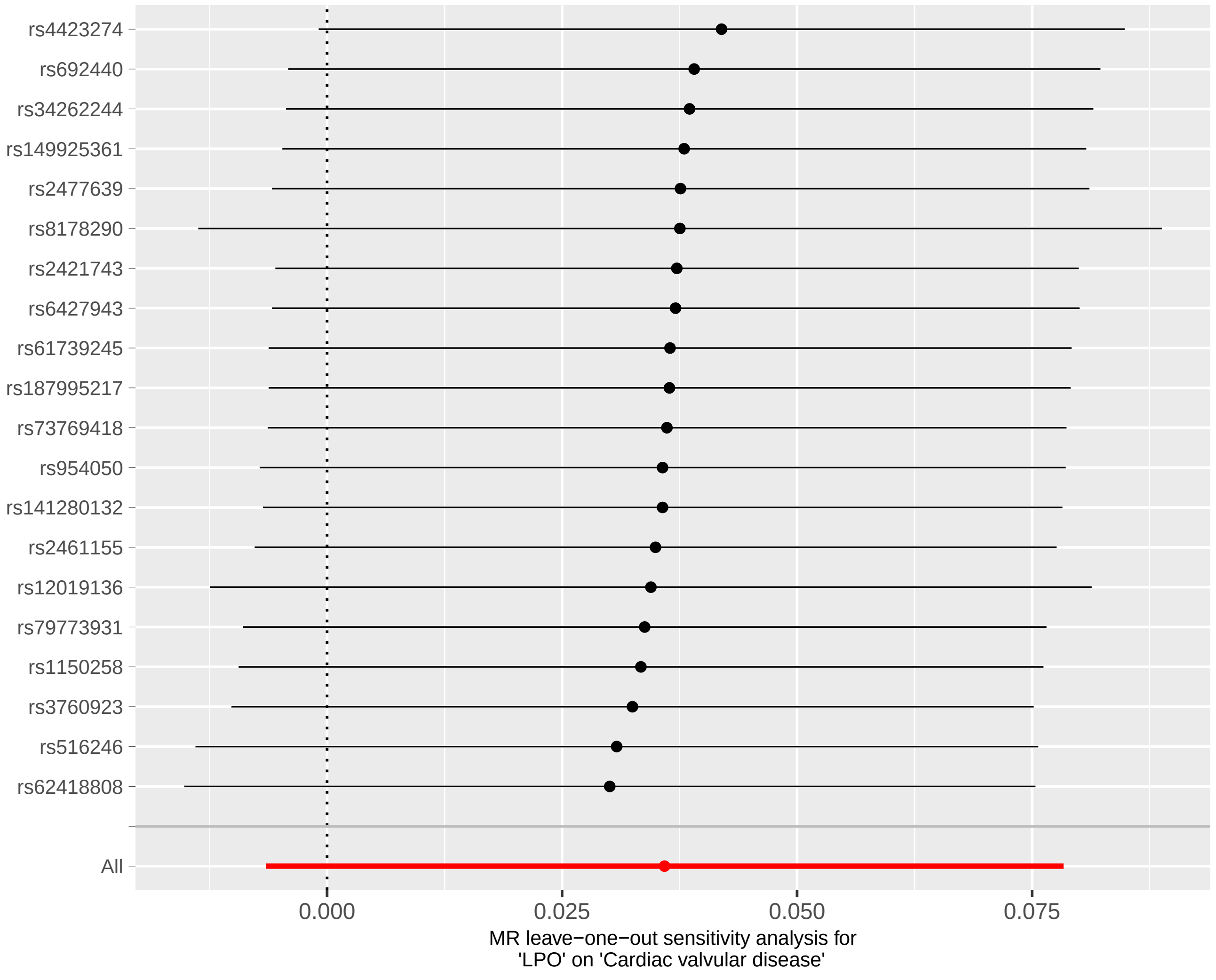

rs4423274
rs692440
rs34262244
rs149925361
rs2477639
rs8178290
rs2421743
rs6427943
rs61739245
rs187995217
rs73769418
rs954050
rs141280132
rs2461155
rs12019136
rs79773931
rs1150258
rs3760923
rs516246
rs62418808
All
0.000
0.025
0.050
0.075
MR leave−one−out sensitivity analysis for
'LPO' on 'Cardiac valvular disease'

Insufficient number of SNPs

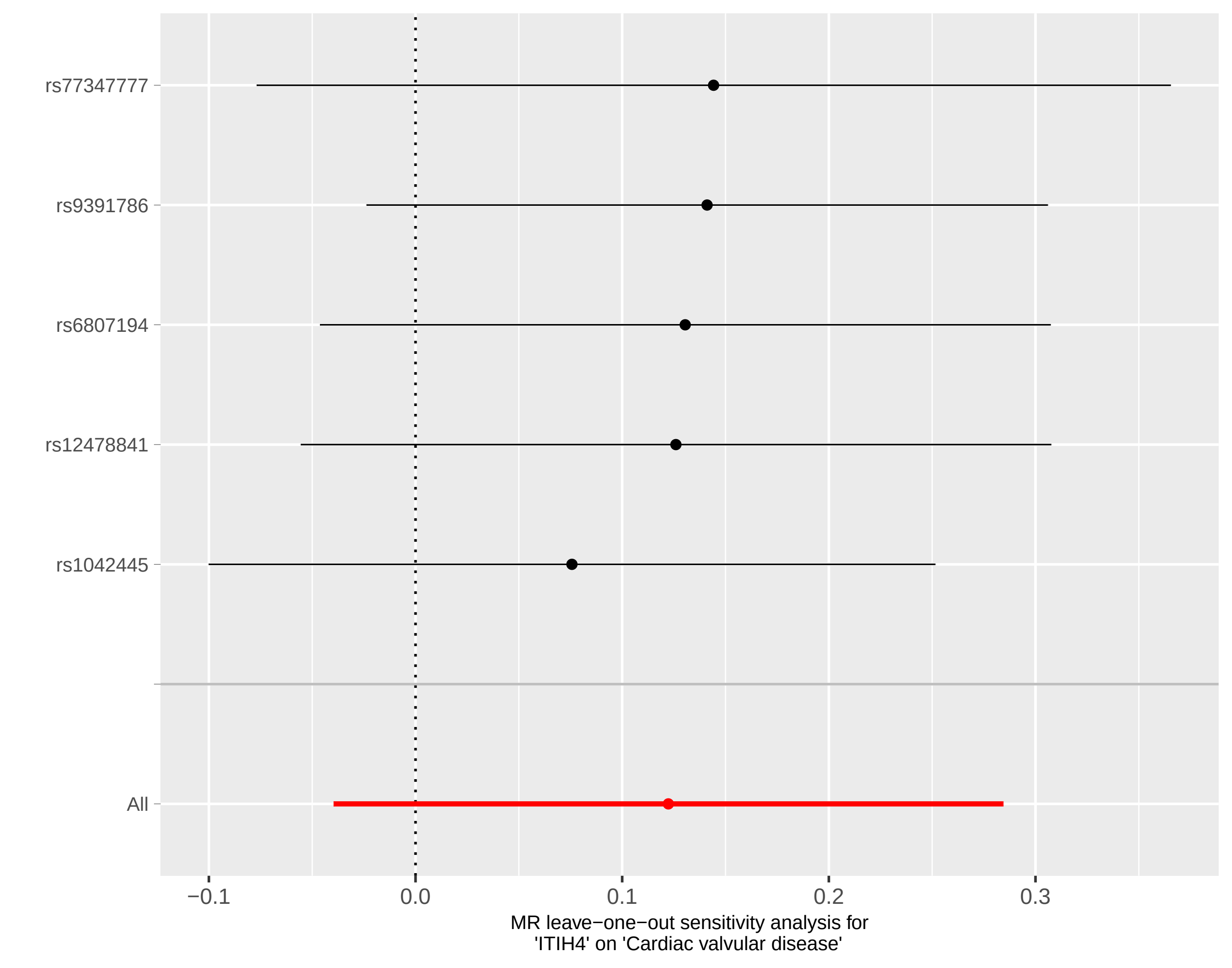
rs77347777
rs9391786
rs6807194
rs12478841
rs1042445
All
−0.1
0.0
0.1
0.2
0.3
MR leave−one−out sensitivity analysis for
'ITIH4' on 'Cardiac valvular disease'

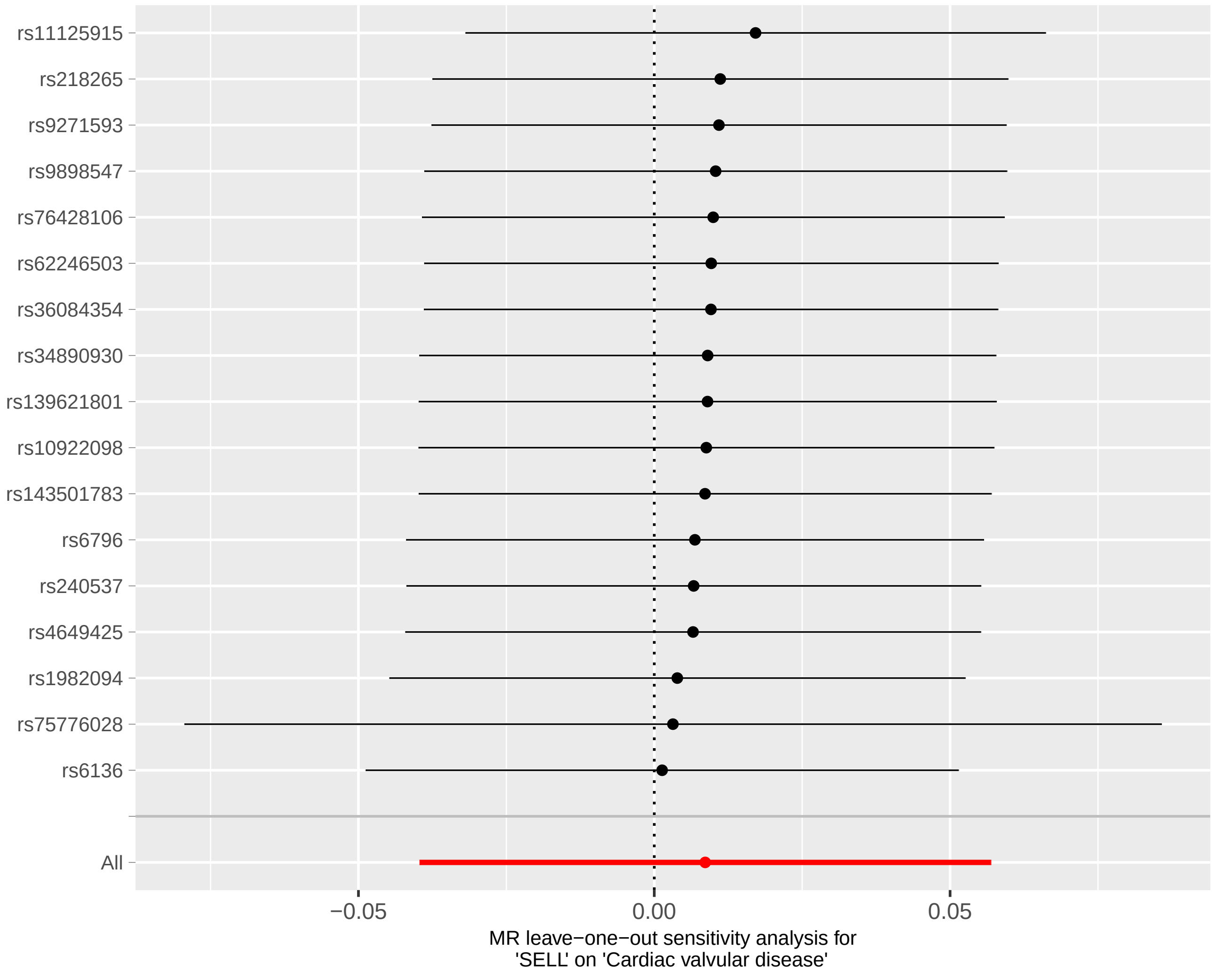
rs11125915
rs218265
rs9271593
rs9898547
rs76428106
rs62246503
rs36084354
rs34890930
rs139621801
rs10922098
rs143501783
rs6796
rs240537
rs4649425
rs1982094
rs75776028
rs6136
All
−0.05
0.00
0.05
MR leave−one−out sensitivity analysis for
'SELL' on 'Cardiac valvular disease'

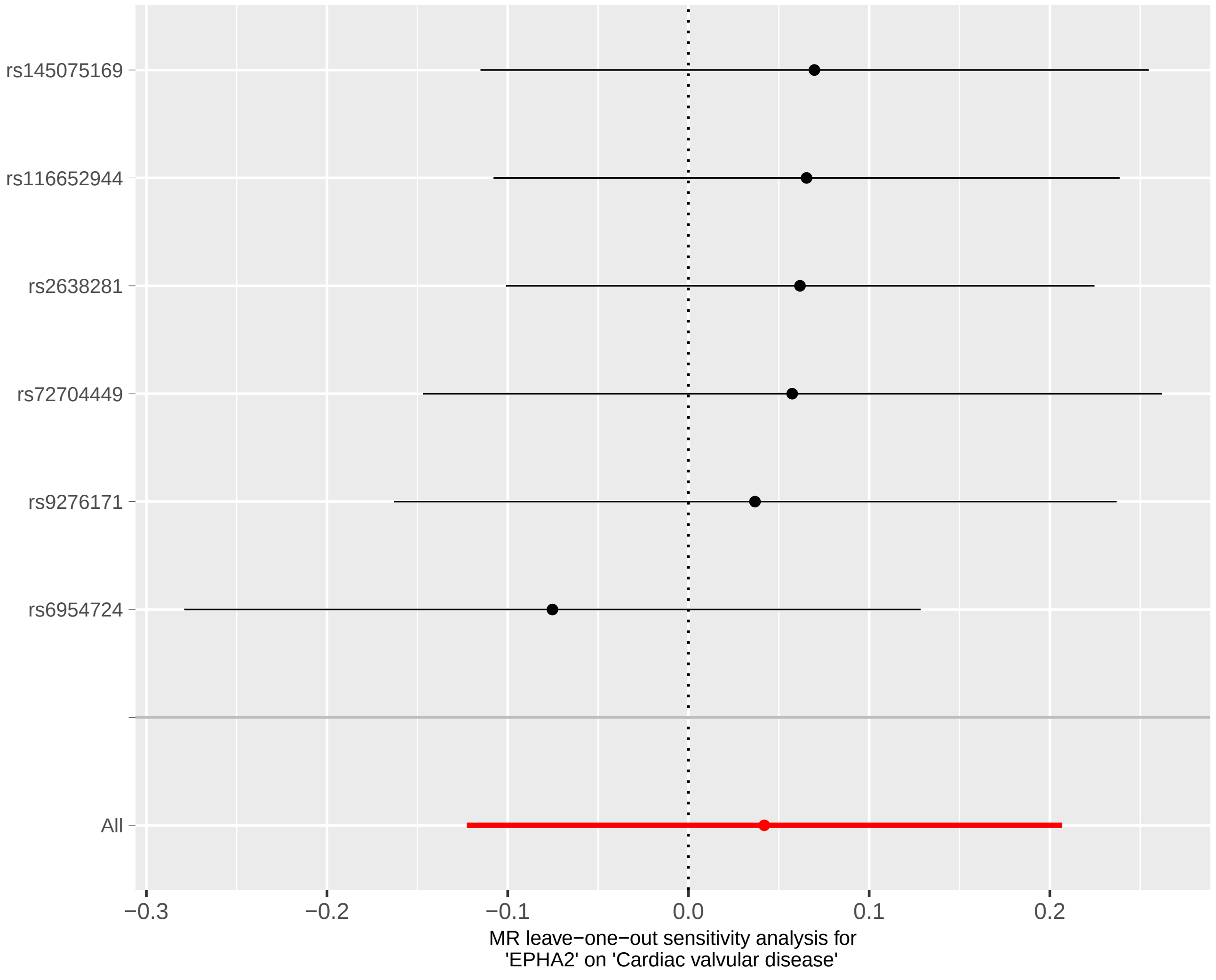

rs145075169
rs116652944
rs2638281
rs72704449
rs9276171
rs6954724
All
−0.3
−0.2
−0.1
0.0
0.1
0.2
MR leave−one−out sensitivity analysis for
'EPHA2' on 'Cardiac valvular disease'

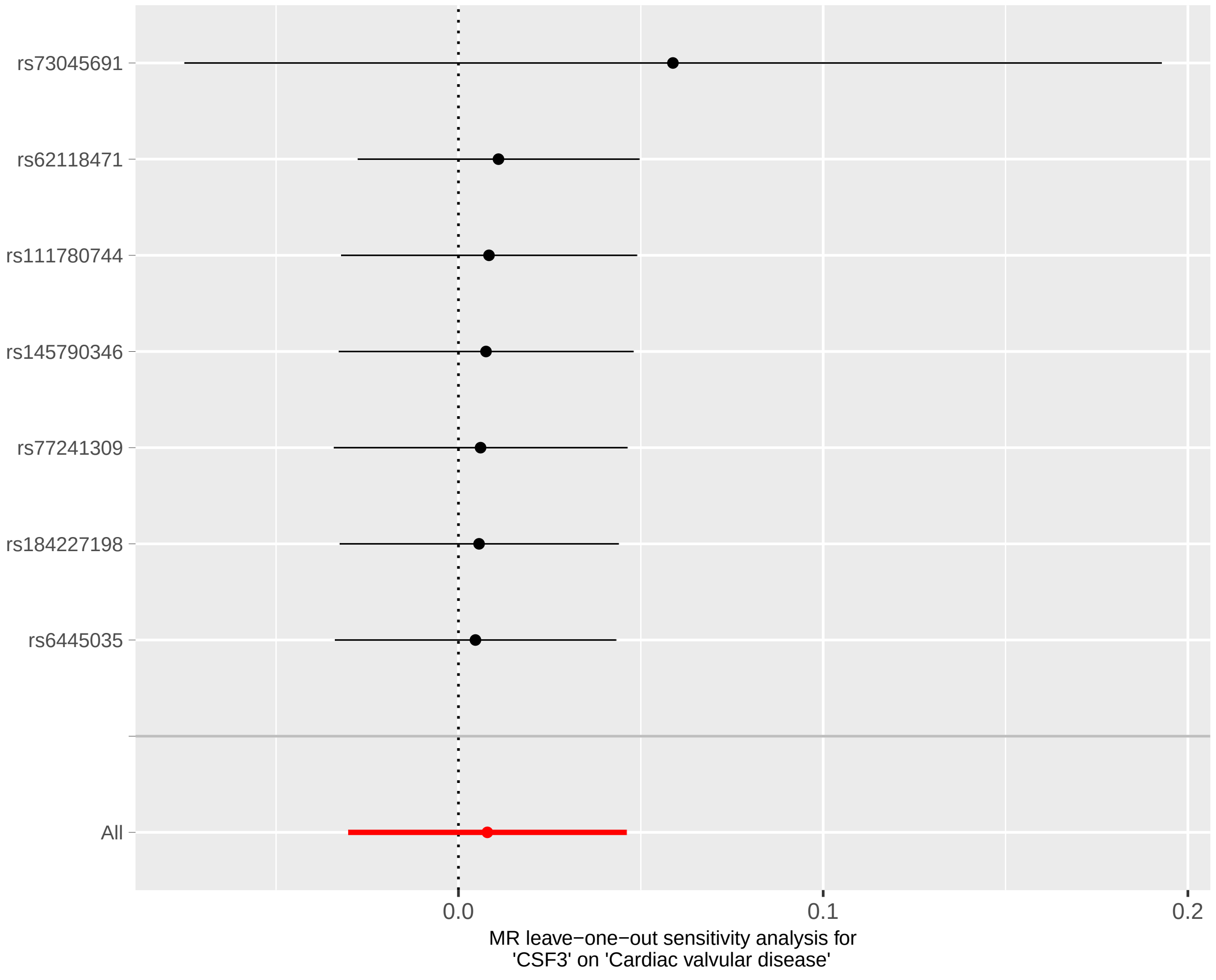
rs73045691
rs62118471
rs111780744
rs145790346
rs77241309
rs184227198
rs6445035
All
0.0
0.1
0.2
MR leave−one−out sensitivity analysis for
'CSF3' on 'Cardiac valvular disease'

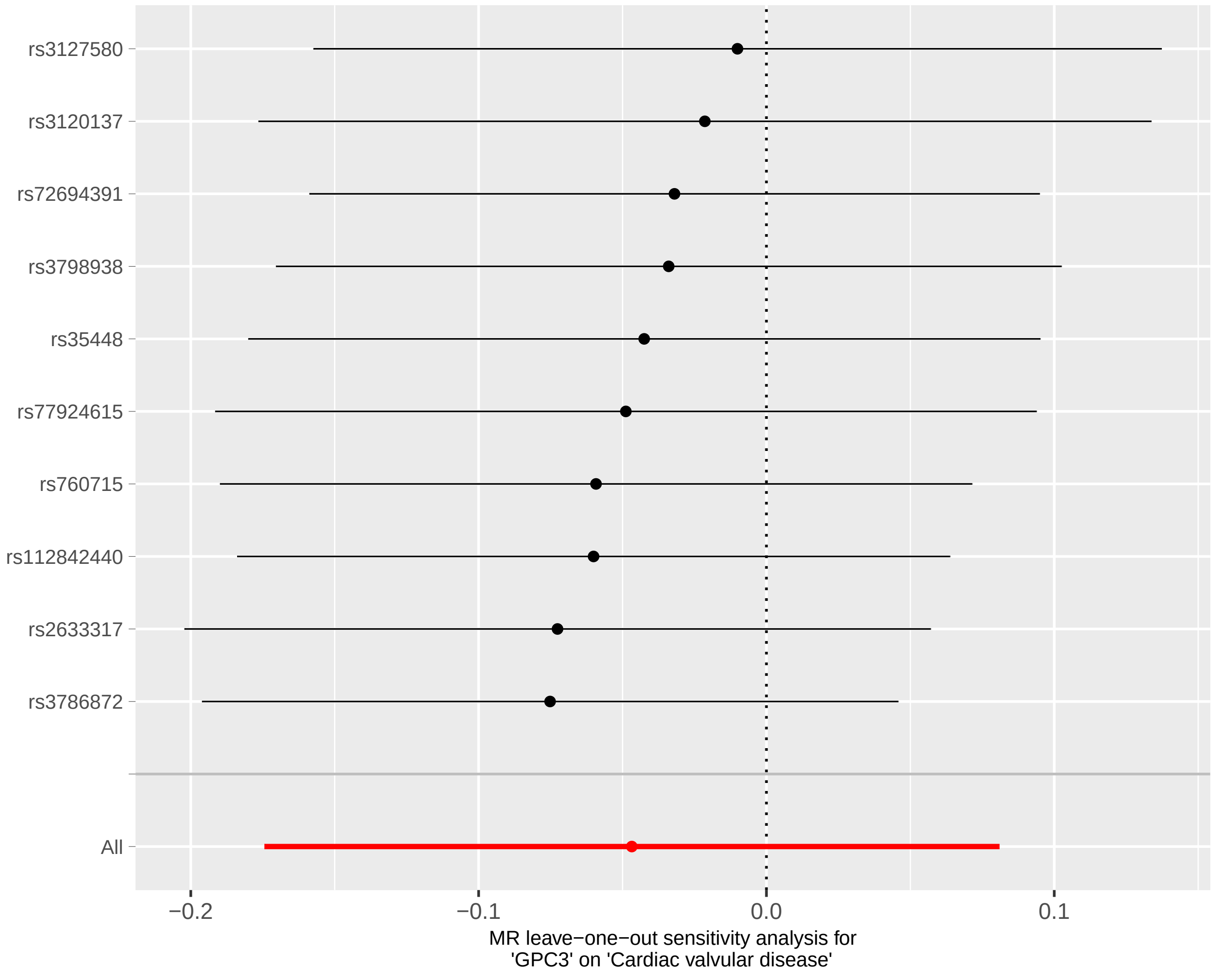
rs3127580
rs3120137
rs72694391
rs3798938
rs35448
rs77924615
rs760715
rs112842440
rs2633317
rs3786872
All
−0.2
−0.1
0.0
0.1
MR leave−one−out sensitivity analysis for
'GPC3' on 'Cardiac valvular disease'

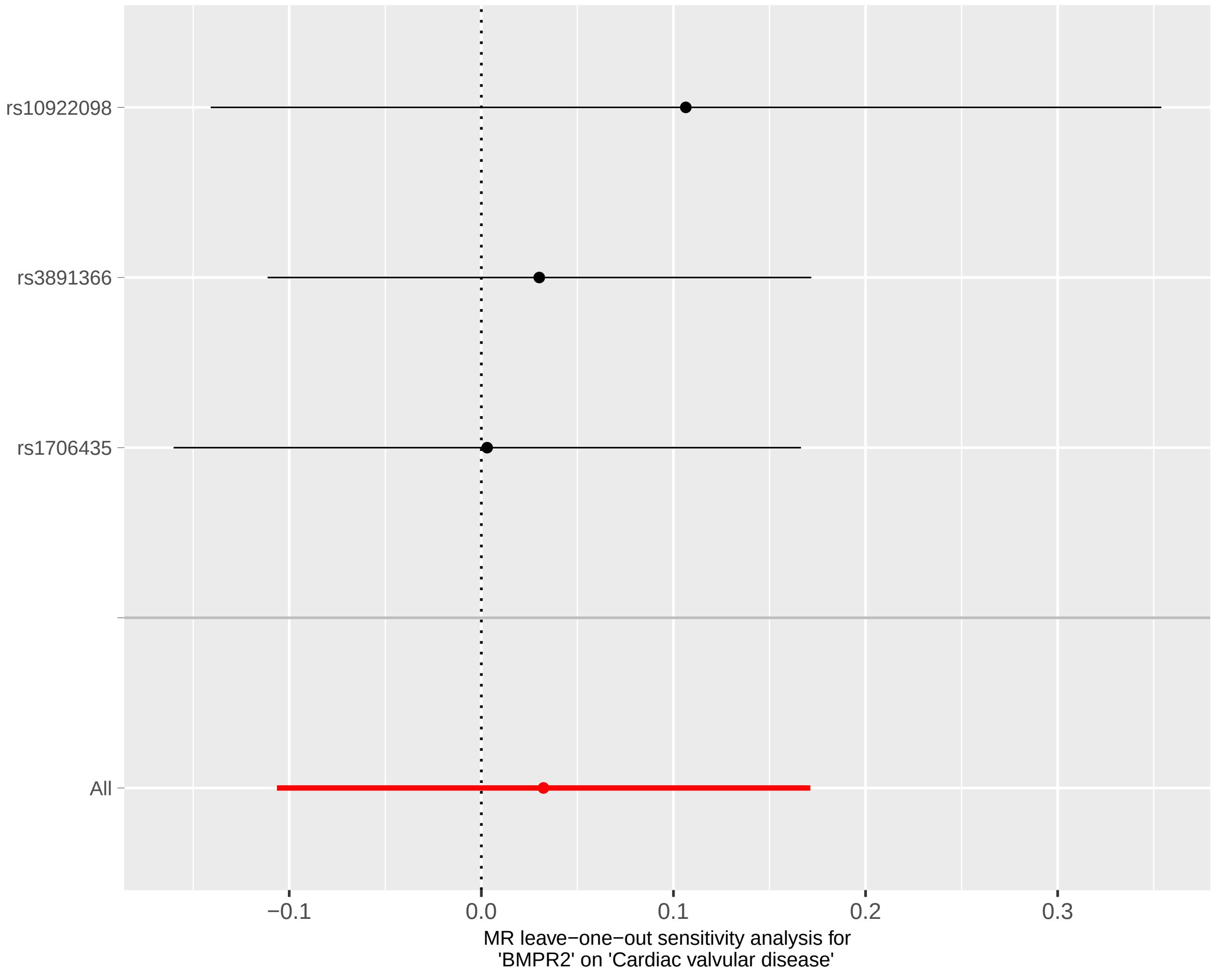
rs10922098
rs3891366
rs1706435
All
−0.1
0.0
0.1
0.2
0.3
MR leave−one−out sensitivity analysis for
'BMPR2' on 'Cardiac valvular disease'

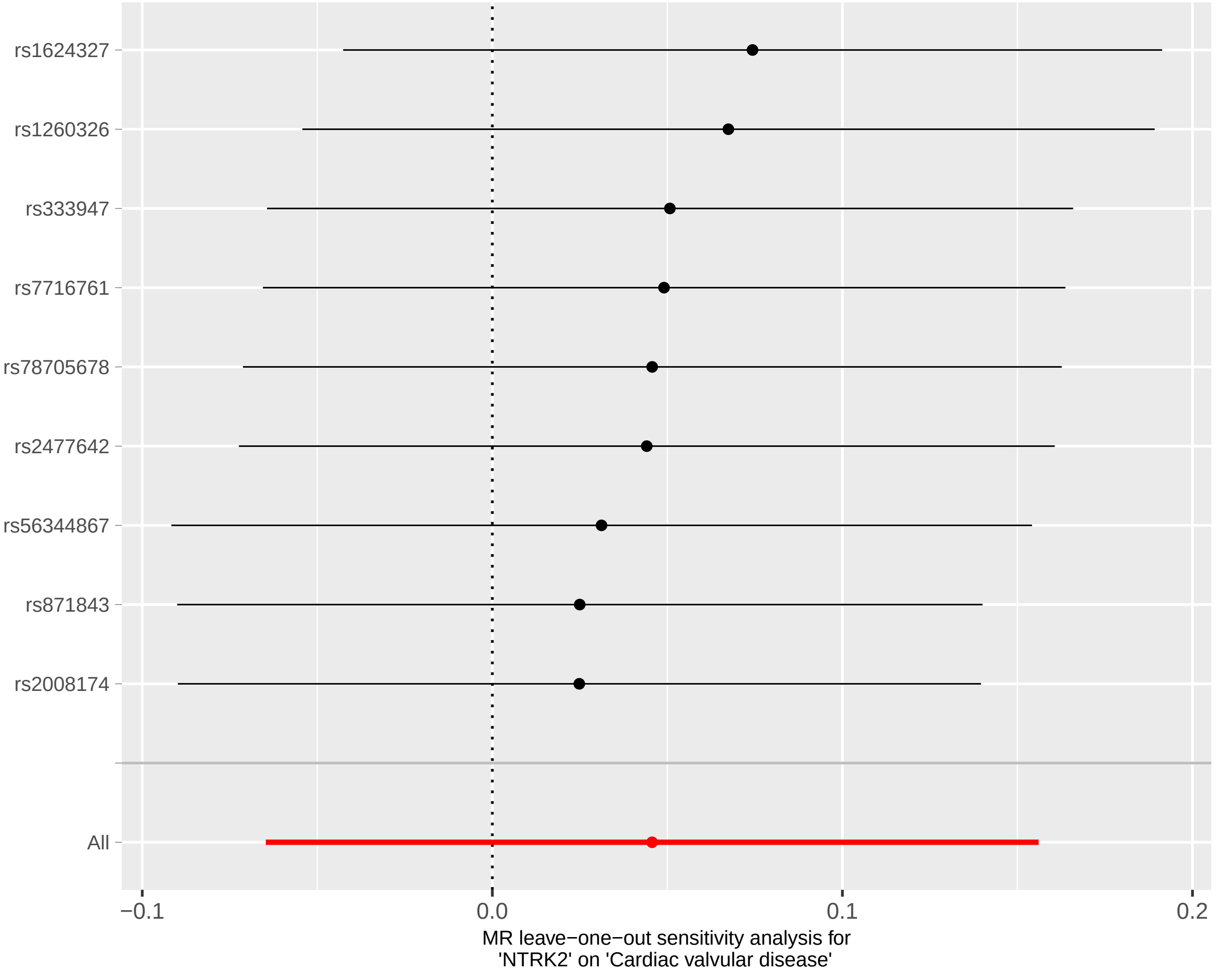
rs1624327
rs1260326
rs333947
rs7716761
rs78705678
rs2477642
rs56344867
rs871843
rs2008174
All
−0.1
0.0
0.1
0.2
MR leave−one−out sensitivity analysis for
'NTRK2' on 'Cardiac valvular disease'

Insufficient number of SNPs

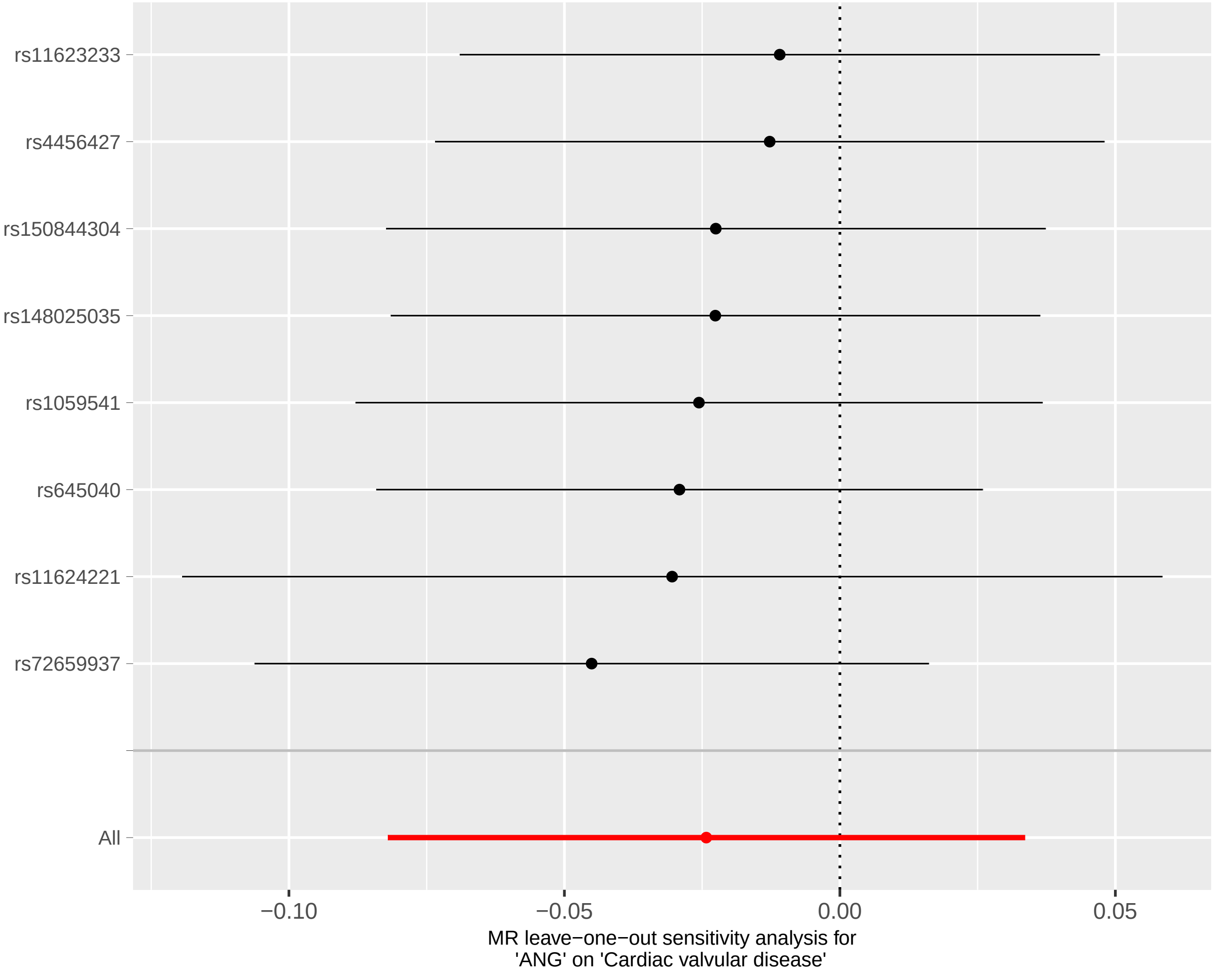
rs11623233
rs4456427
rs150844304
rs148025035
rs1059541
rs645040
rs11624221
rs72659937
All
−0.10
−0.05
0.00
0.05
MR leave−one−out sensitivity analysis for
'ANG' on 'Cardiac valvular disease'

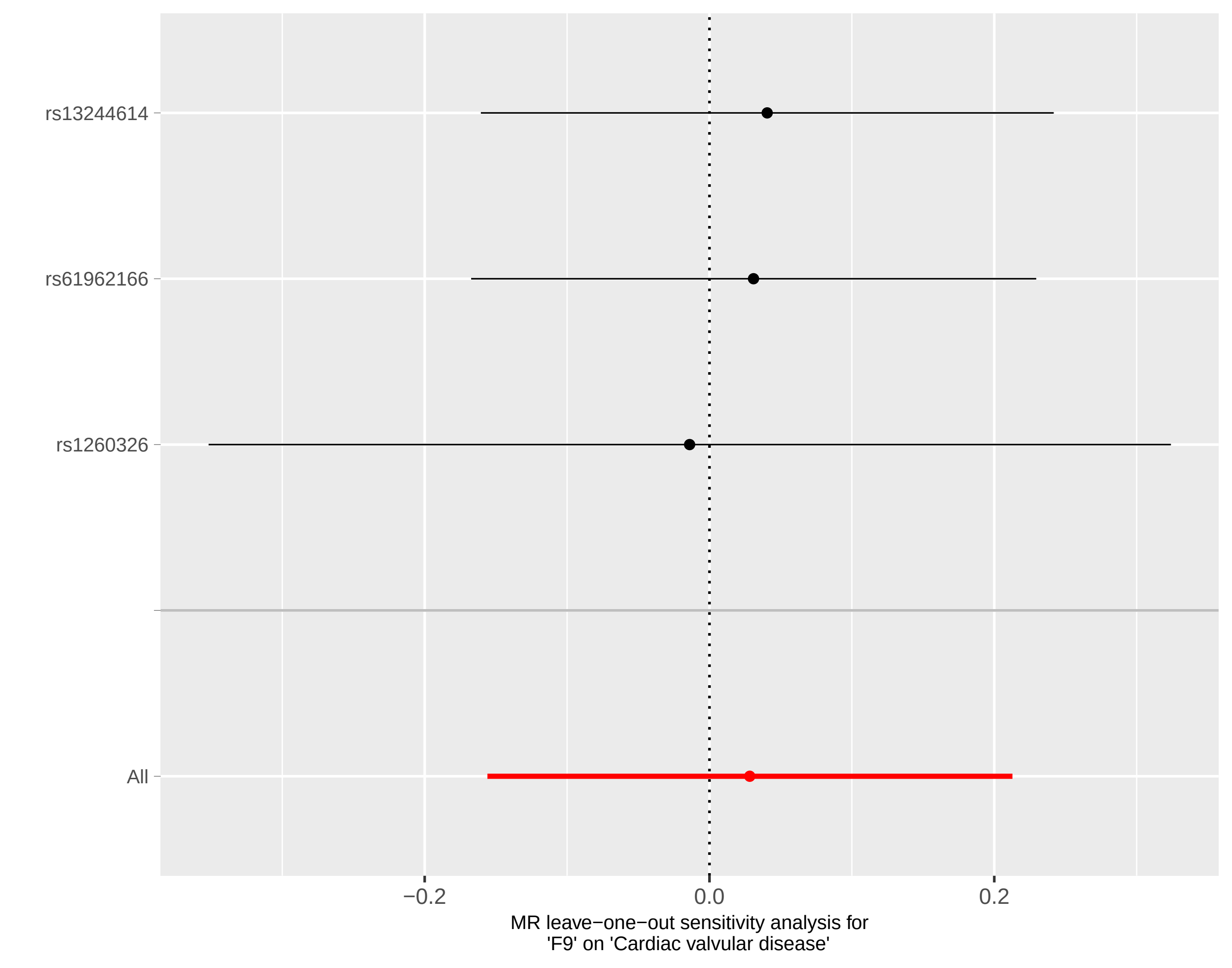

rs13244614
rs61962166
rs1260326
All
−0.2
0.0
0.2
MR leave−one−out sensitivity analysis for
'F9' on 'Cardiac valvular disease'

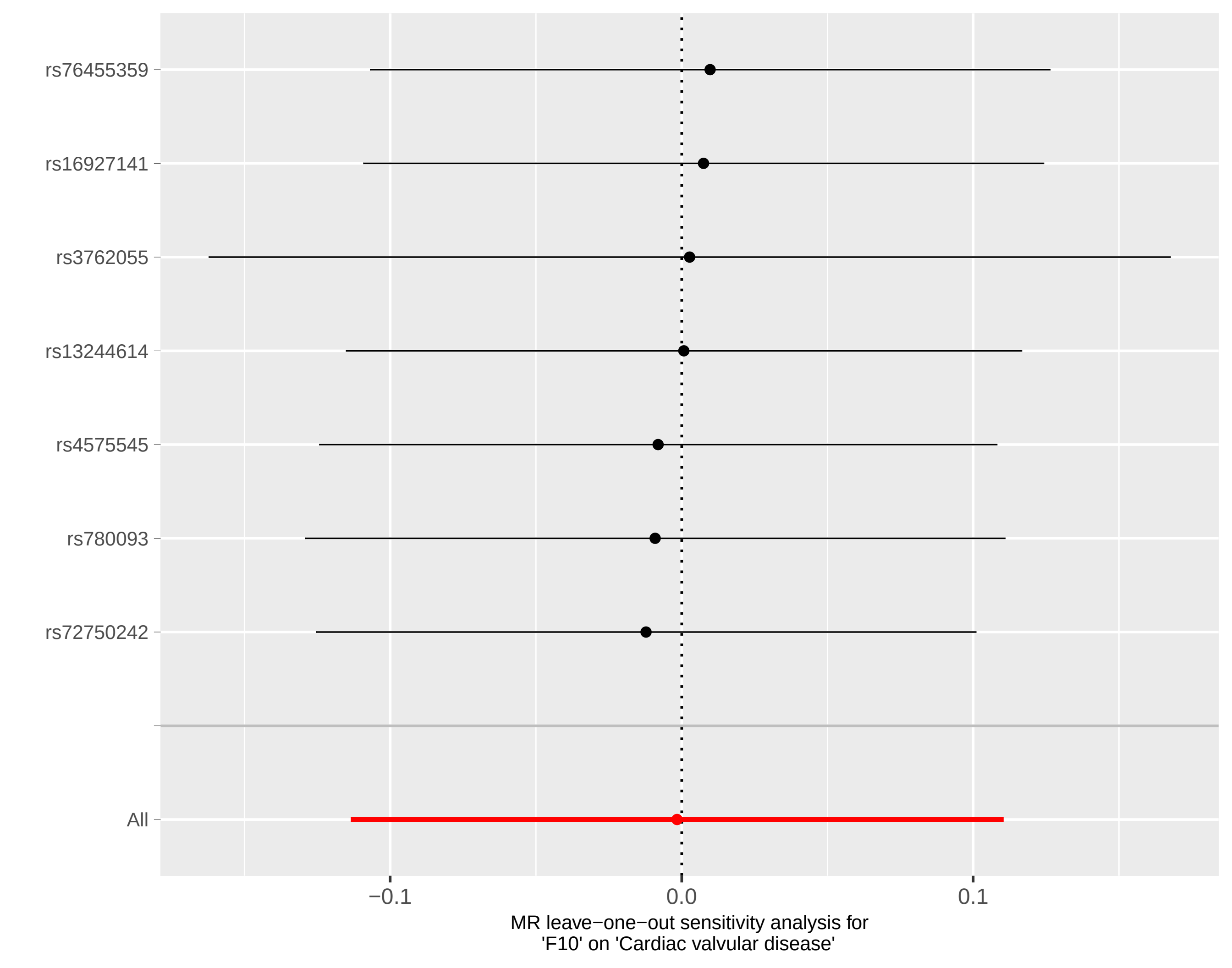
rs76455359
rs16927141
rs3762055
rs13244614
rs4575545
rs780093
rs72750242
All
−0.1
0.0
0.1
MR leave−one−out sensitivity analysis for
'F10' on 'Cardiac valvular disease'

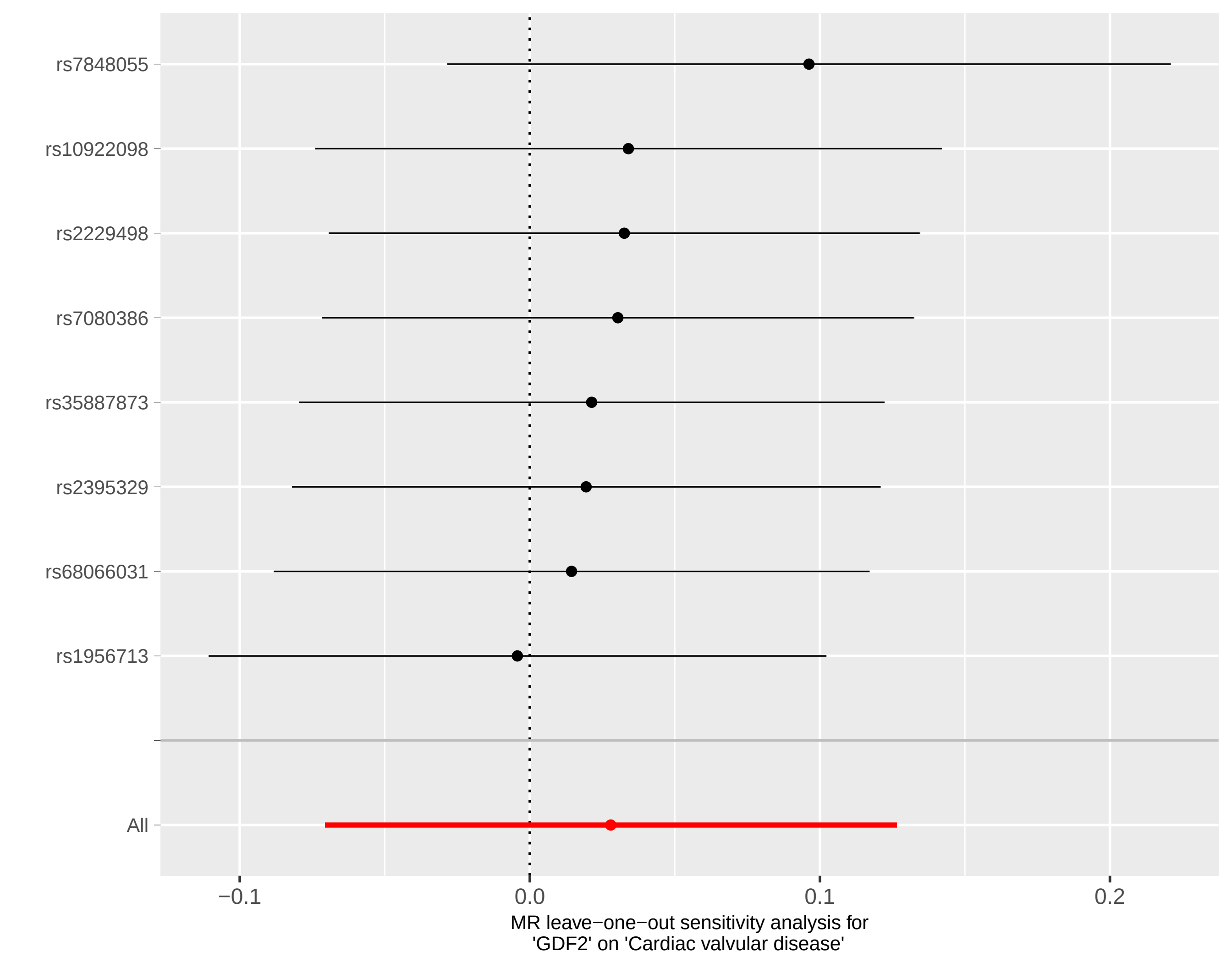

rs7848055
rs10922098
rs2229498
rs7080386
rs35887873
rs2395329
rs68066031
rs1956713
All
−0.1
0.0
0.1
0.2
MR leave−one−out sensitivity analysis for
'GDF2' on 'Cardiac valvular disease'

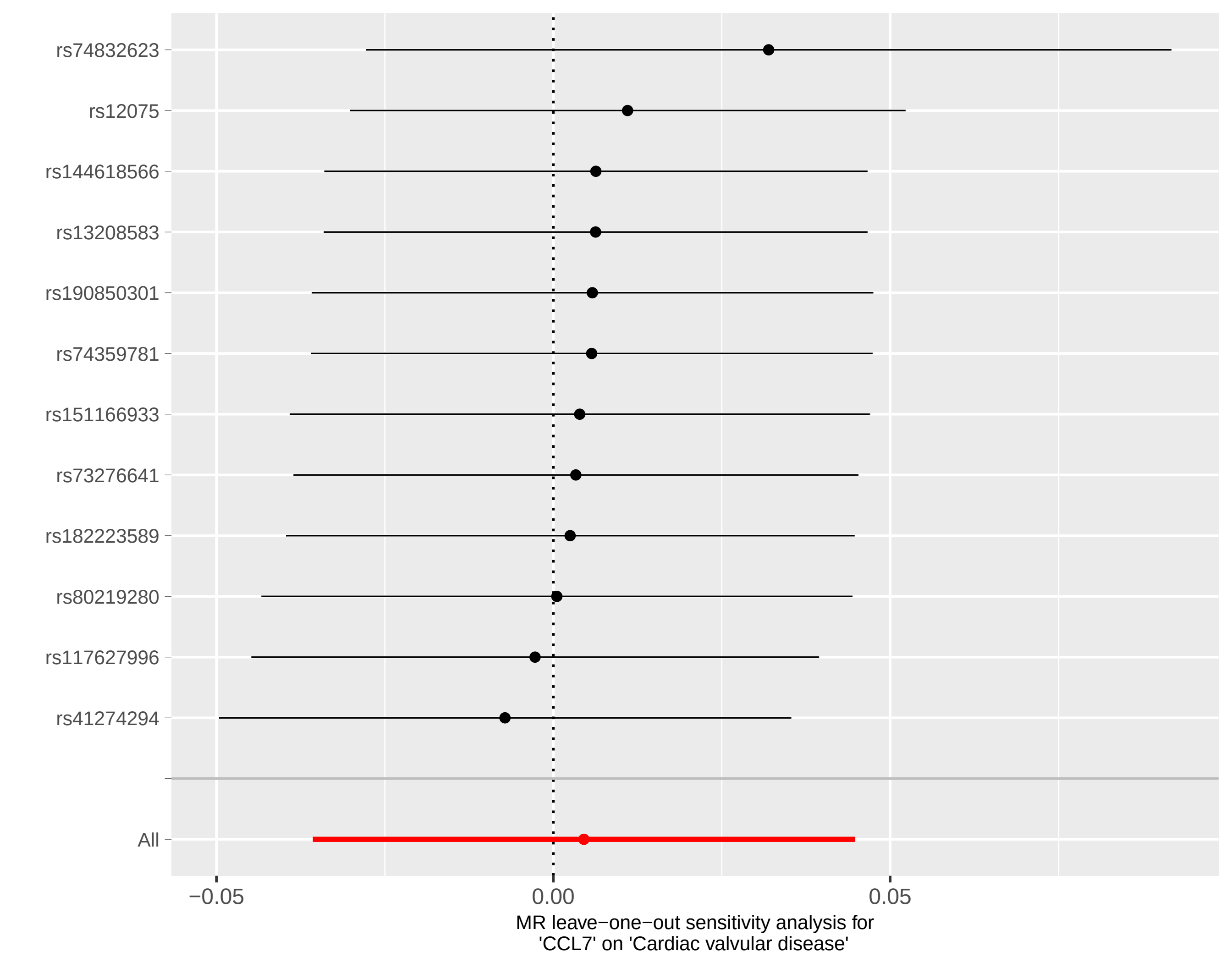

rs74832623
rs12075
rs144618566
rs13208583
rs190850301
rs74359781
rs151166933
rs73276641
rs182223589
rs80219280
rs117627996
rs41274294
All
−0.05
0.00
0.05
MR leave−one−out sensitivity analysis for
'CCL7' on 'Cardiac valvular disease'

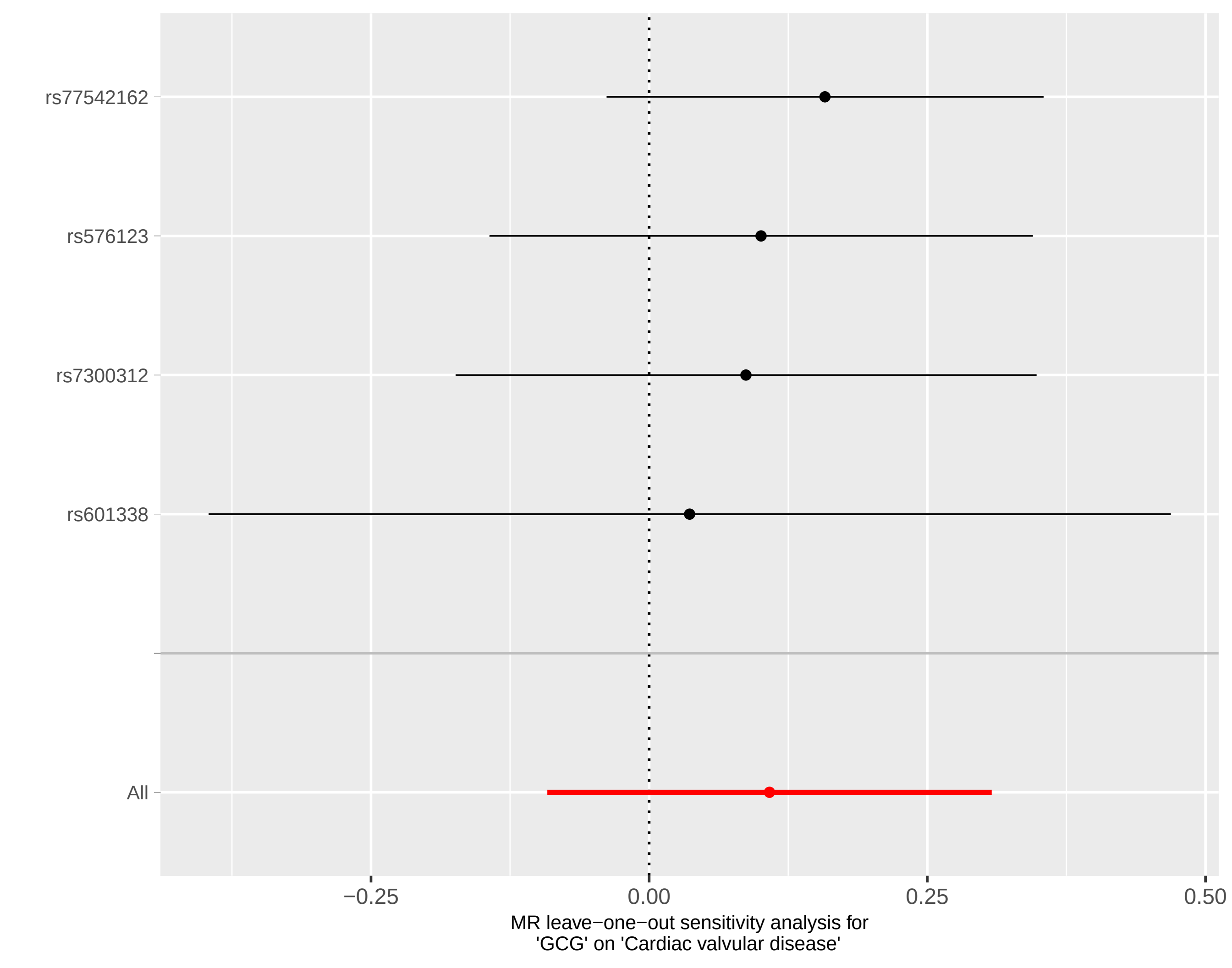

rs77542162
rs576123
rs7300312
rs601338
All
−0.25
0.00
0.25
0.50
MR leave−one−out sensitivity analysis for
'GCG' on 'Cardiac valvular disease'

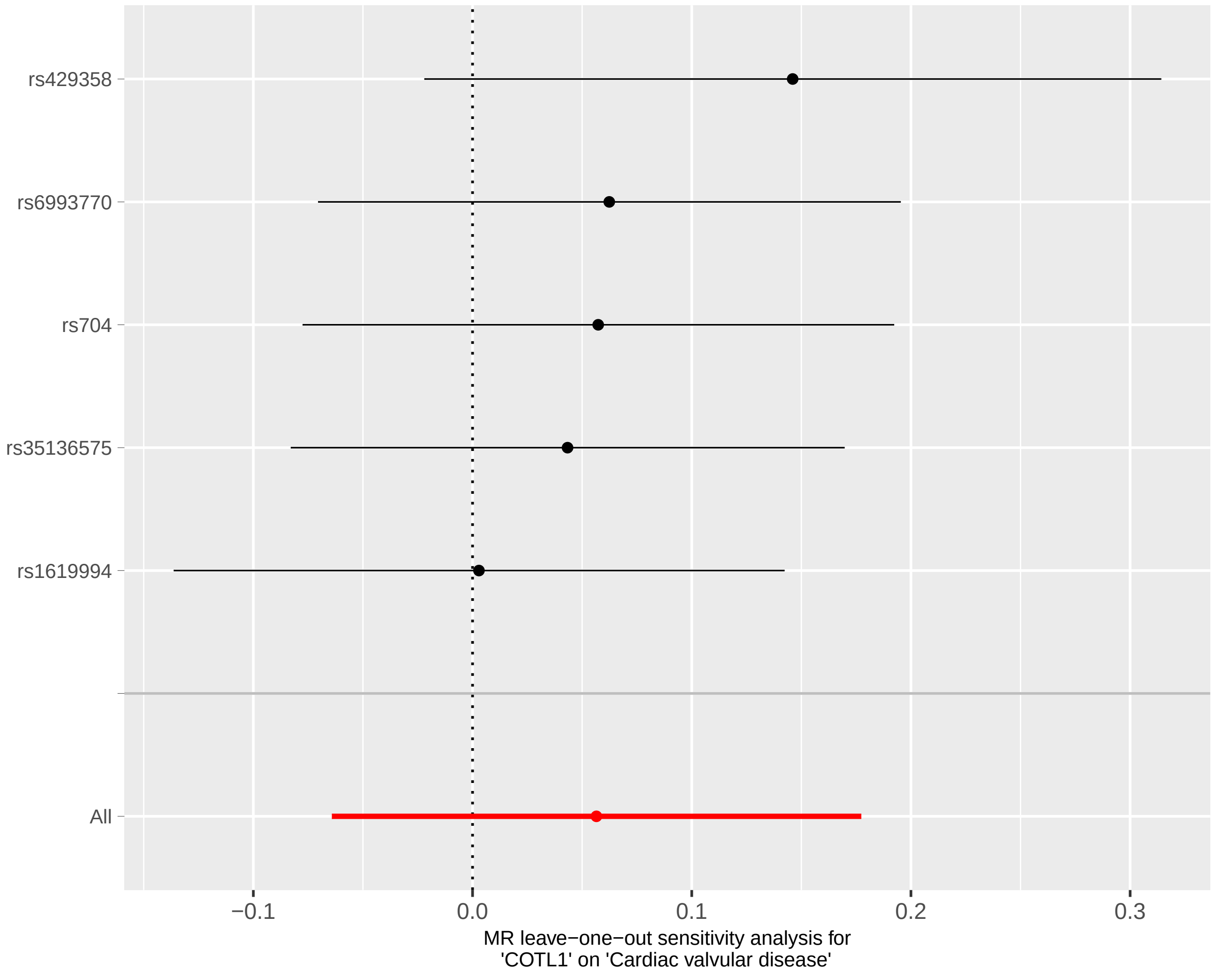
rs429358
rs6993770
rs704
rs35136575
rs1619994
All
−0.1
0.0
0.1
0.2
0.3
MR leave−one−out sensitivity analysis for
'COTL1' on 'Cardiac valvular disease'

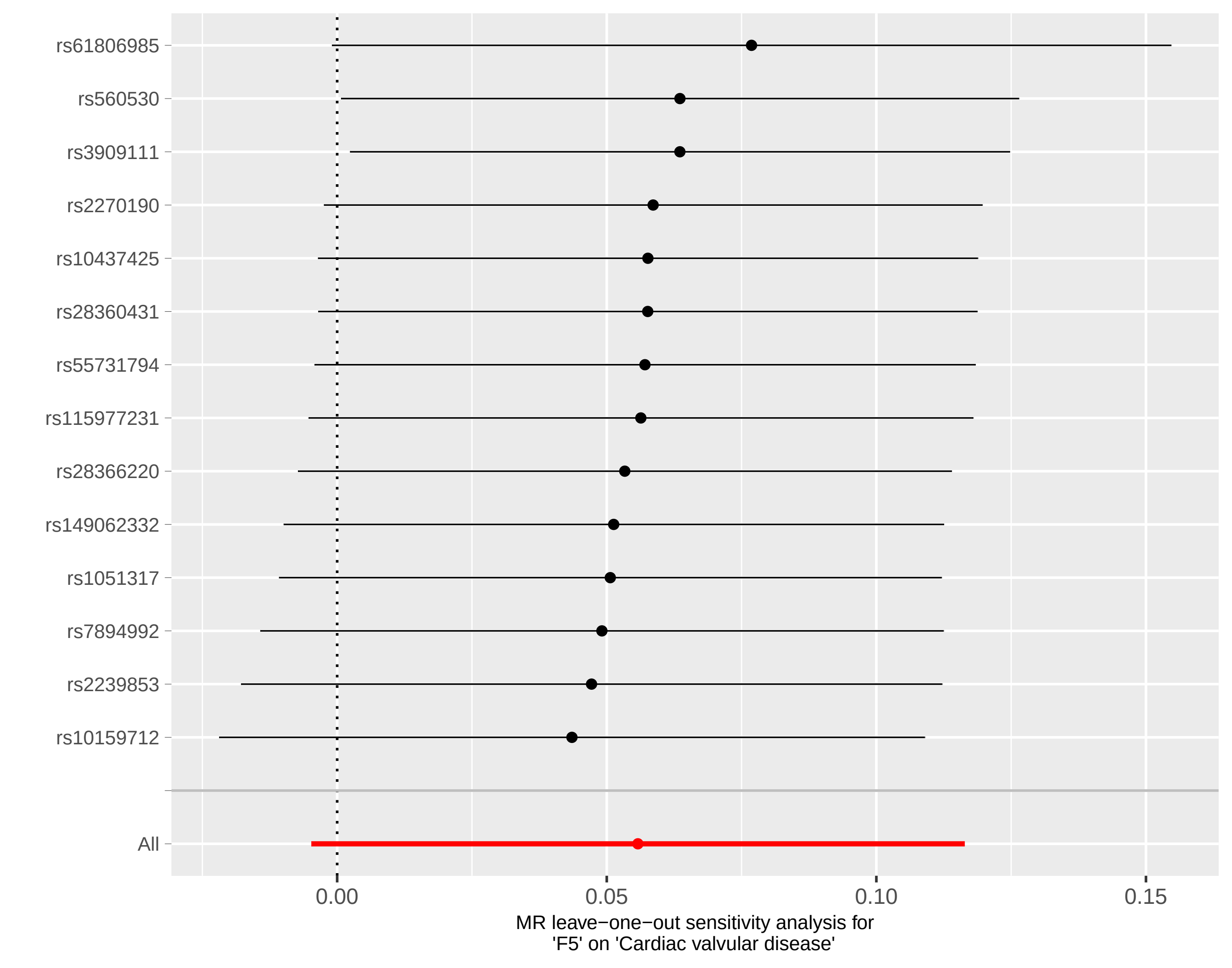
rs61806985
rs560530
rs3909111
rs2270190
rs10437425
rs28360431
rs55731794
rs115977231
rs28366220
rs149062332
rs1051317
rs7894992
rs2239853
rs10159712
All
0.00
0.05
0.10
0.15
MR leave−one−out sensitivity analysis for
'F5' on 'Cardiac valvular disease'

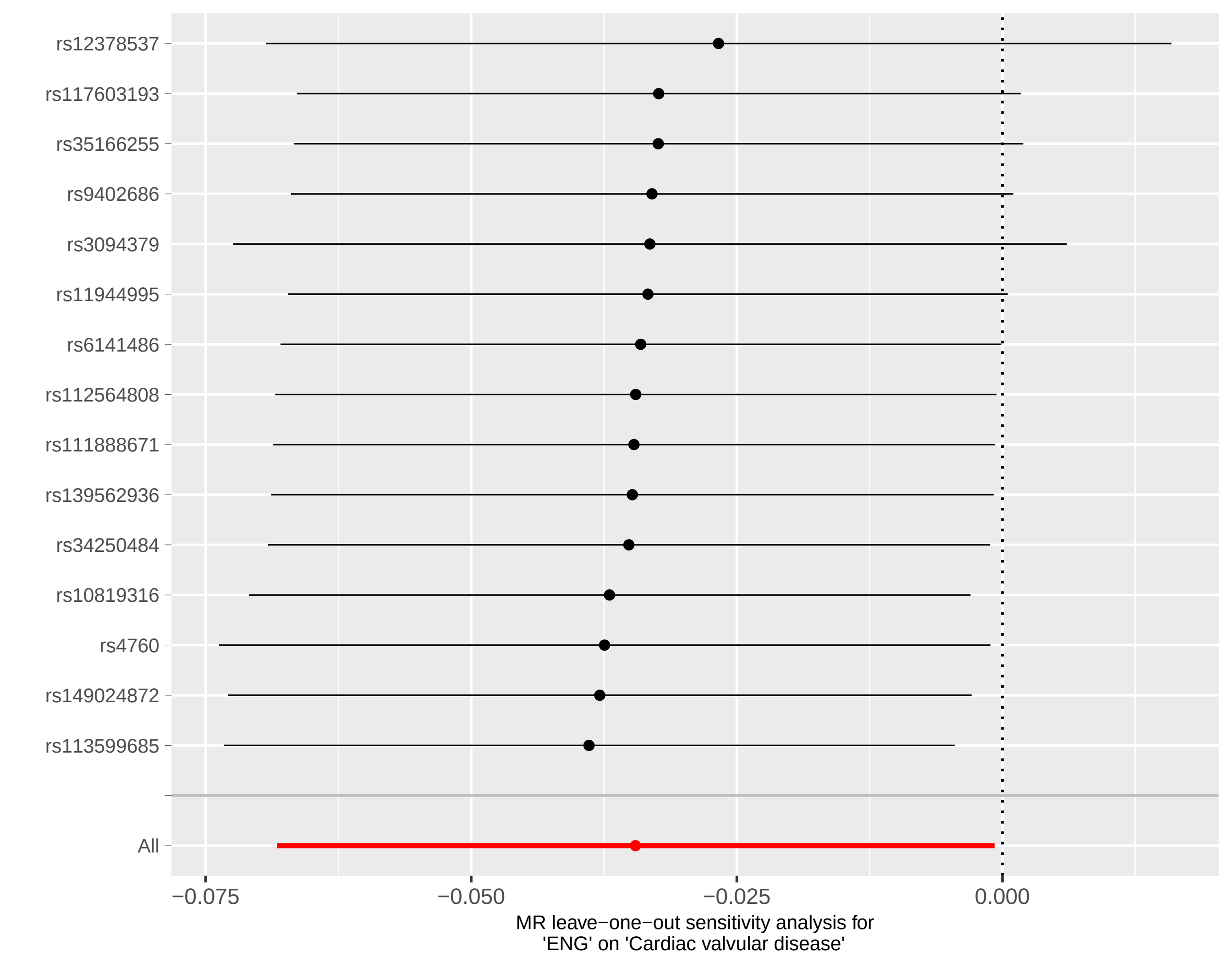
rs12378537
rs117603193
rs35166255
rs9402686
rs3094379
rs11944995
rs6141486
rs112564808
rs111888671
rs139562936
rs34250484
rs10819316
rs4760
rs149024872
rs113599685
All
−0.075
−0.050
−0.025
0.000
MR leave−one−out sensitivity analysis for
'ENG' on 'Cardiac valvular disease'

Insufficient number of SNPs

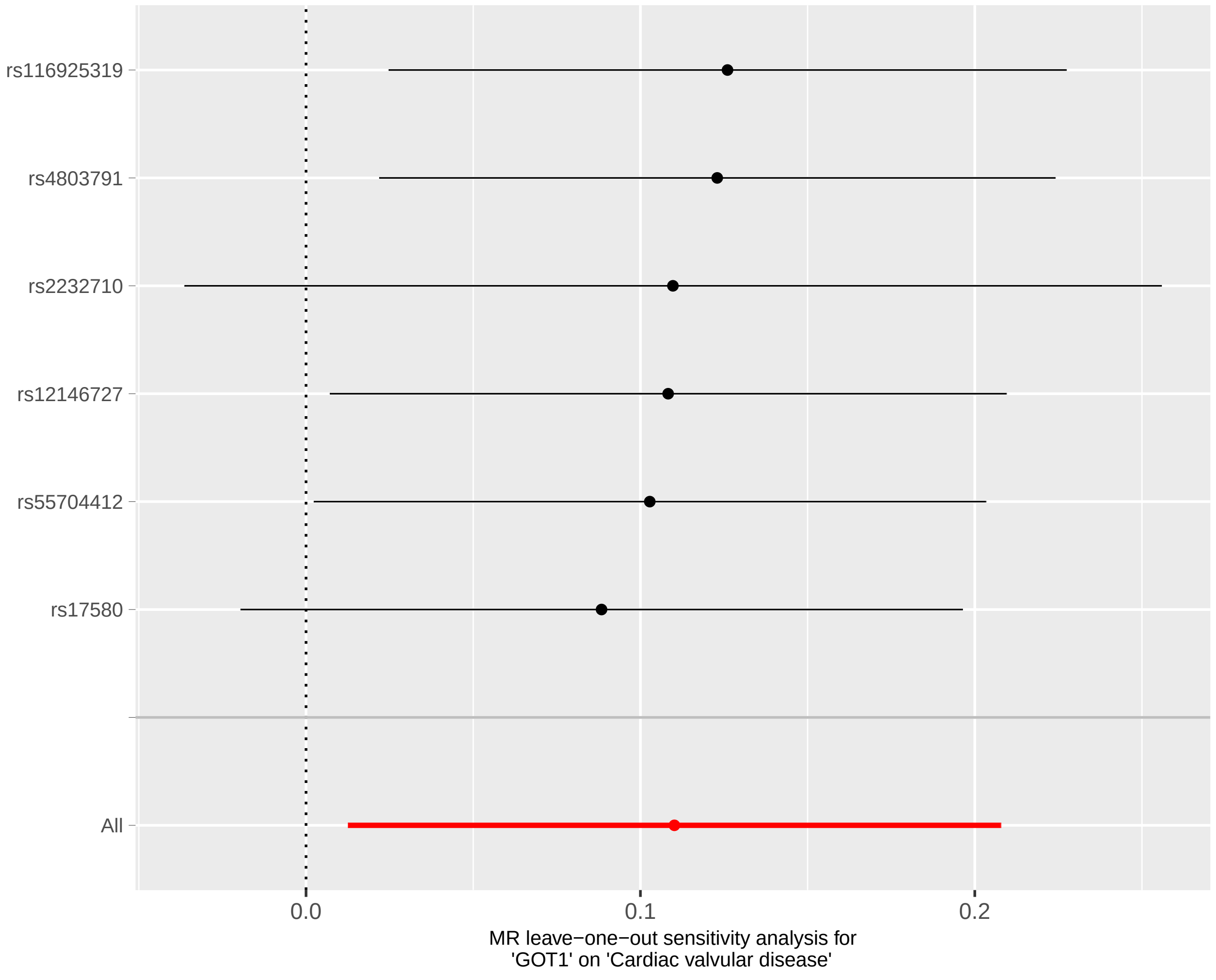

rs116925319
rs4803791
rs2232710
rs12146727
rs55704412
rs17580
All
0.0
0.1
0.2
MR leave−one−out sensitivity analysis for
'GOT1' on 'Cardiac valvular disease'

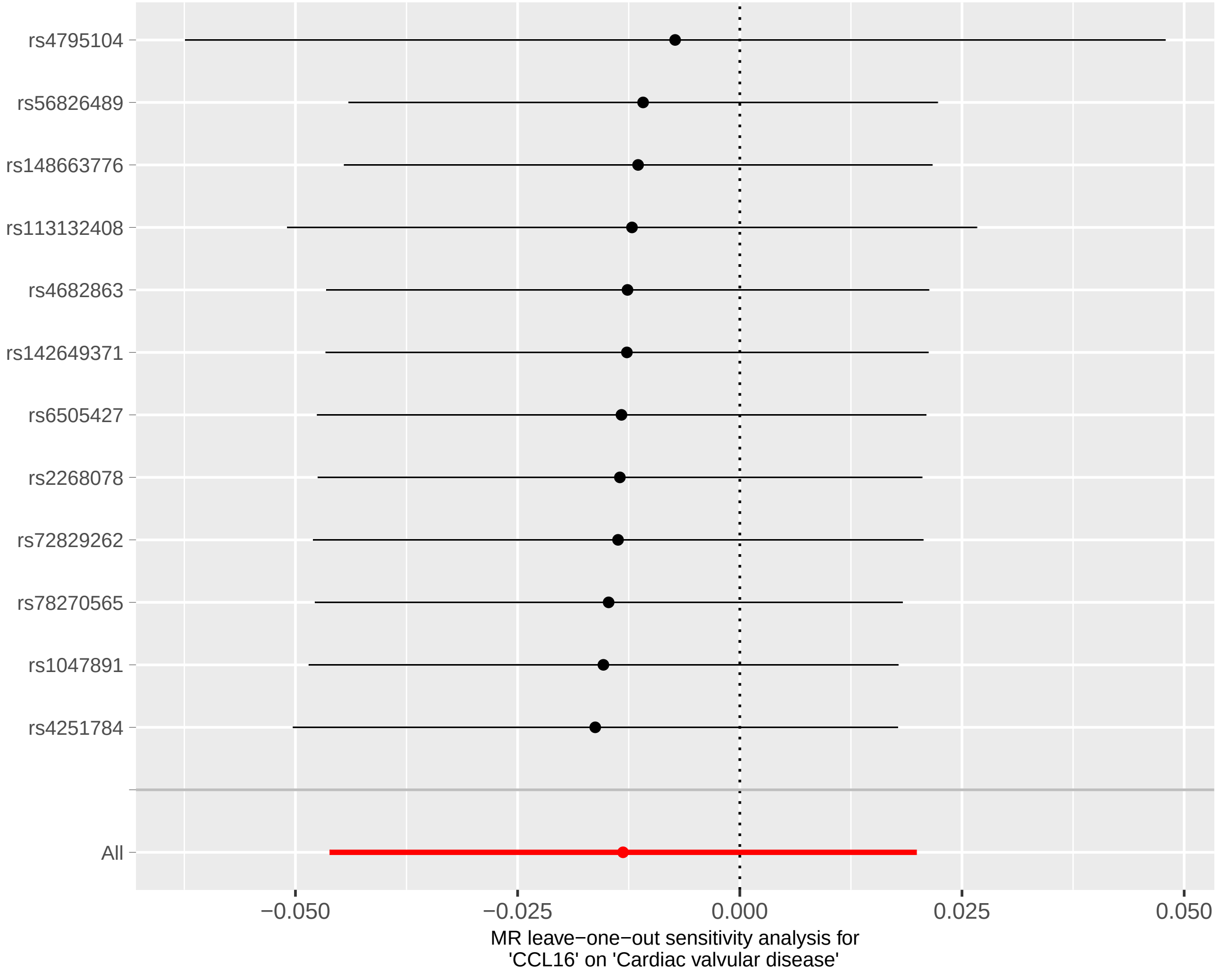
rs4795104
rs56826489
rs148663776
rs113132408
rs4682863
rs142649371
rs6505427
rs2268078
rs72829262
rs78270565
rs1047891
rs4251784
All
−0.050
−0.025
0.000
0.025
0.050
MR leave−one−out sensitivity analysis for
'CCL16' on 'Cardiac valvular disease'

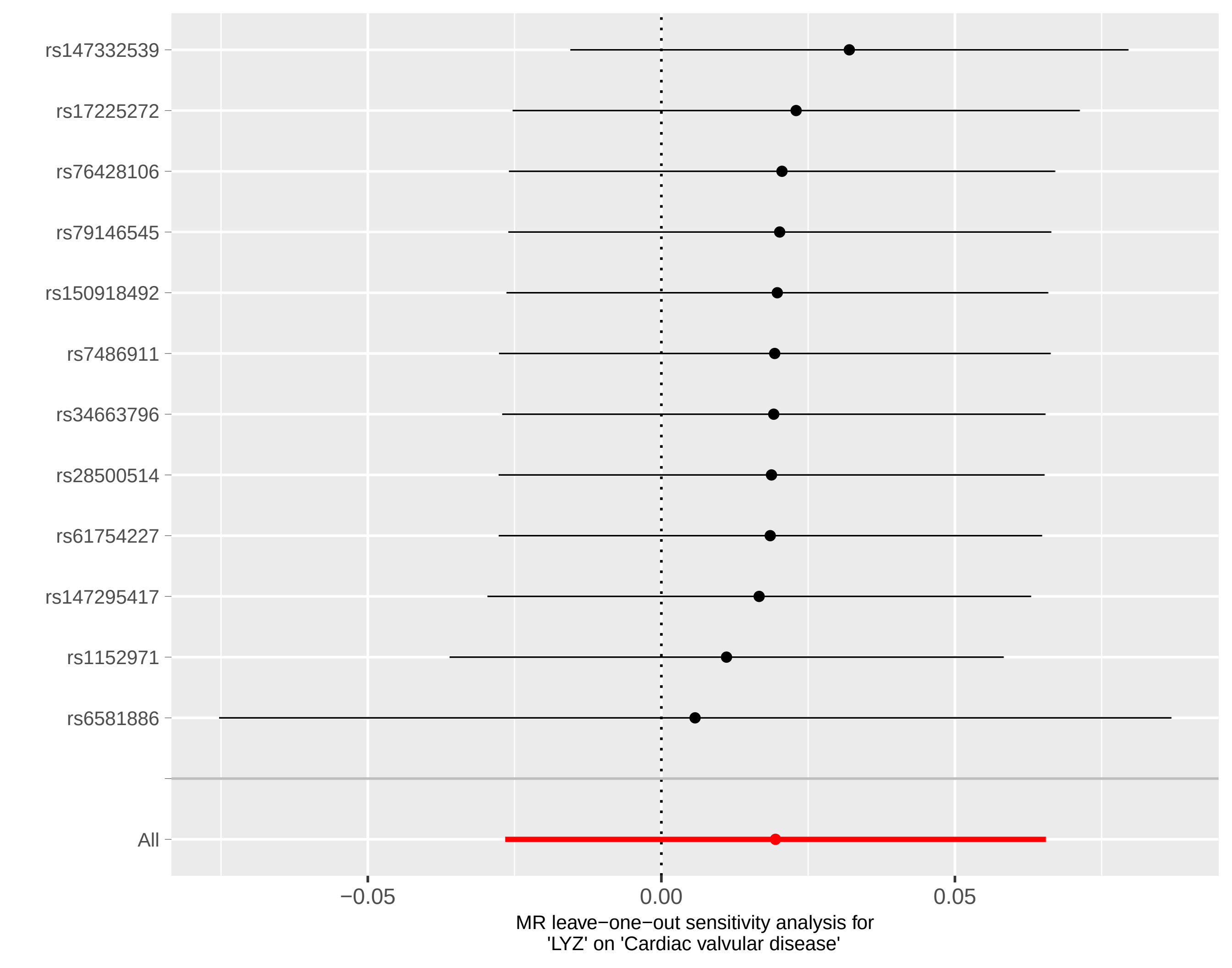
rs147332539
rs17225272
rs76428106
rs79146545
rs150918492
rs7486911
rs34663796
rs28500514
rs61754227
rs147295417
rs1152971
rs6581886
All
−0.05
0.00
0.05
MR leave−one−out sensitivity analysis for
'LYZ' on 'Cardiac valvular disease'

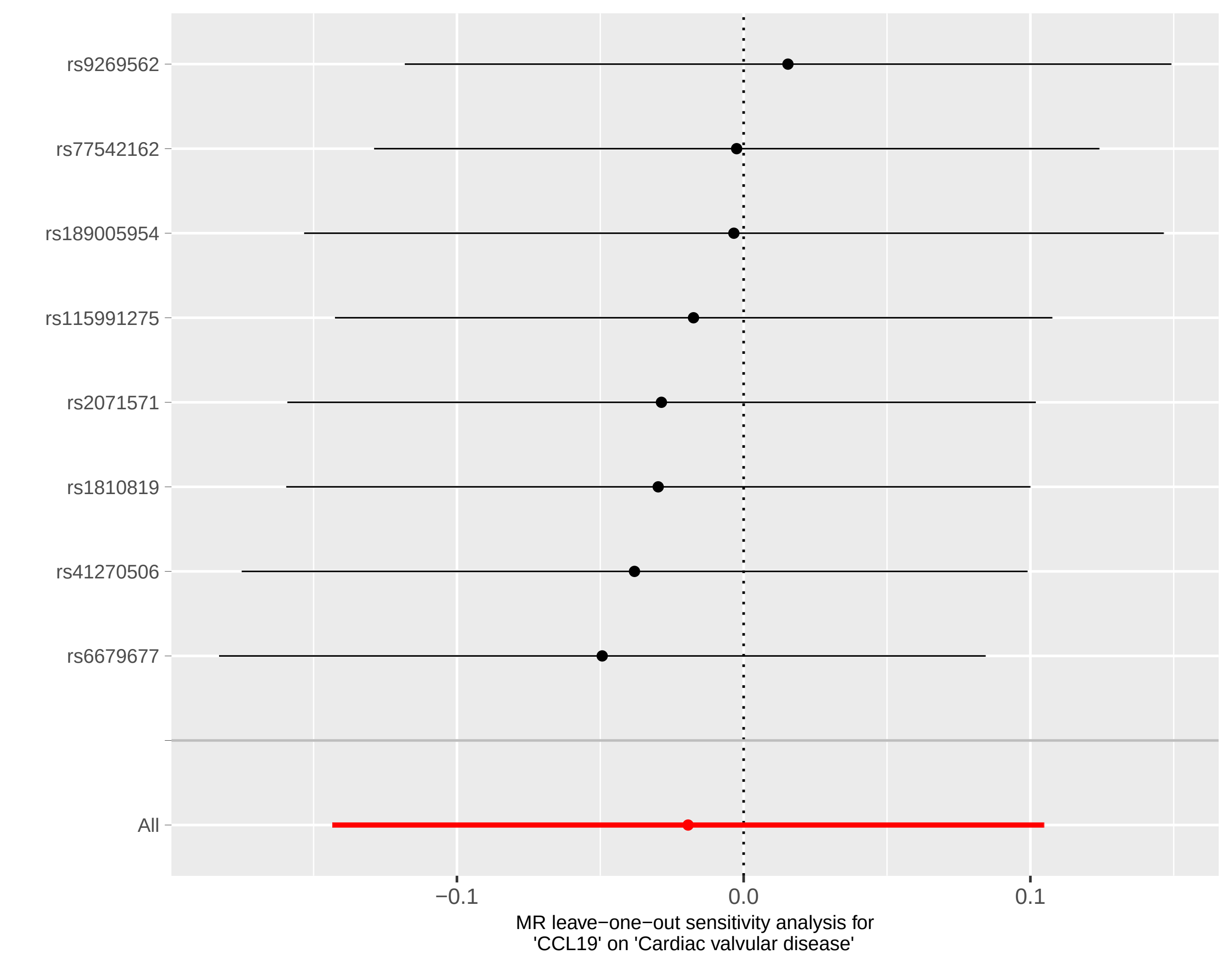
rs9269562
rs77542162
rs189005954
rs115991275
rs2071571
rs1810819
rs41270506
rs6679677
All
−0.1
0.0
0.1
MR leave−one−out sensitivity analysis for
'CCL19' on 'Cardiac valvular disease'

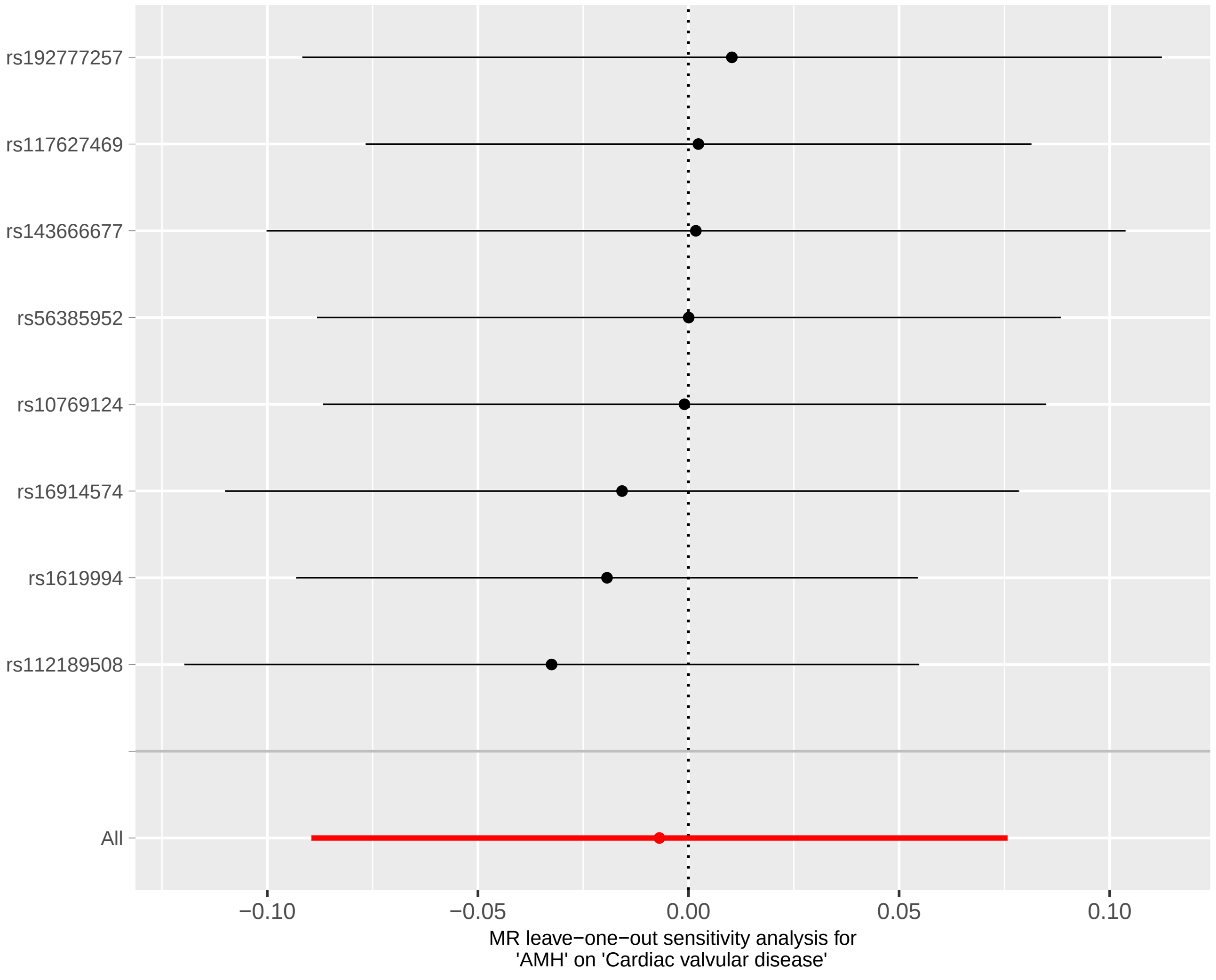
rs192777257
rs117627469
rs143666677
rs56385952
rs10769124
rs16914574
rs1619994
rs112189508
All
−0.10
−0.05
0.00
0.05
0.10
MR leave−one−out sensitivity analysis for
'AMH' on 'Cardiac valvular disease'

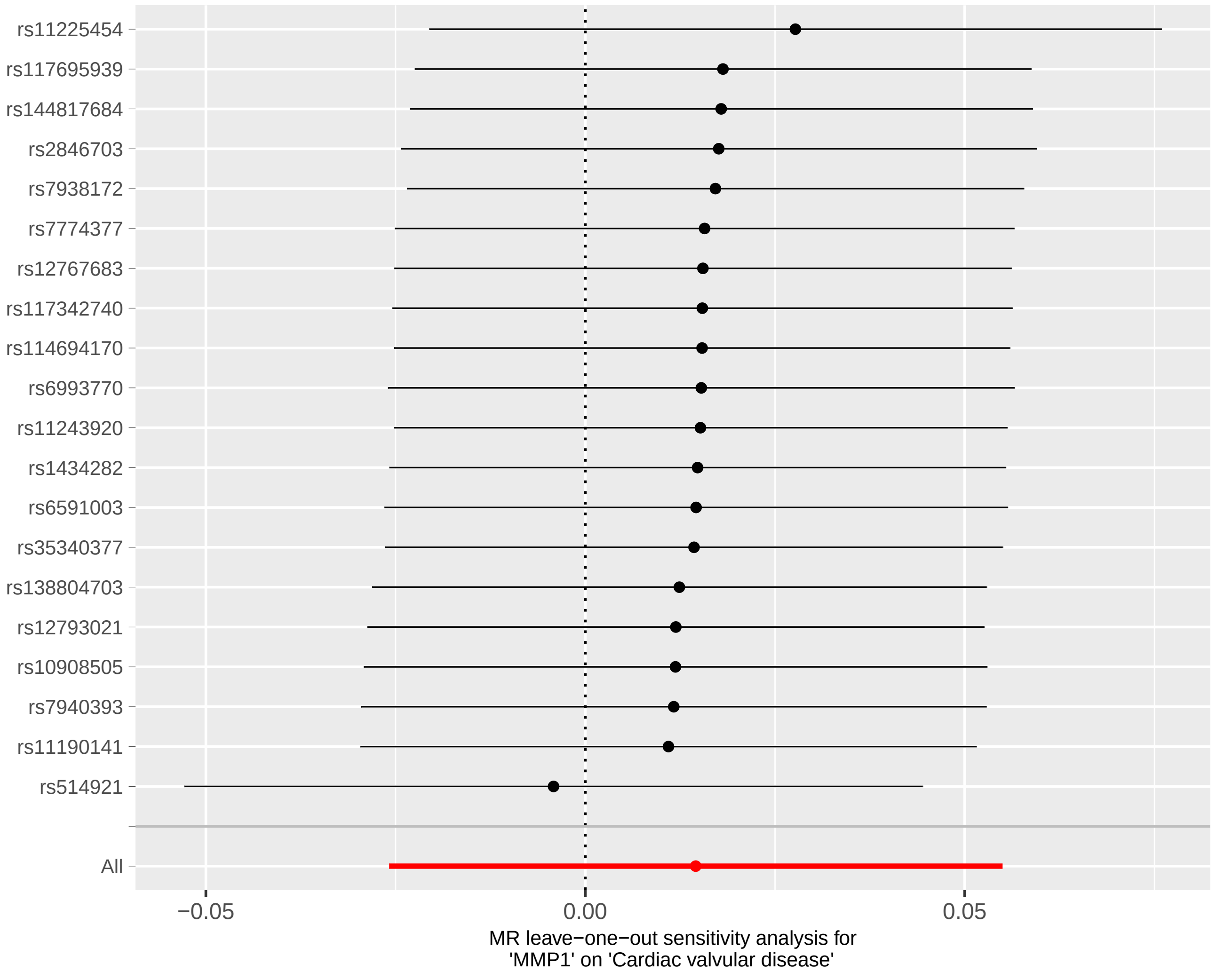
rs11225454
rs117695939
rs144817684
rs2846703
rs7938172
rs7774377
rs12767683
rs117342740
rs114694170
rs6993770
rs11243920
rs1434282
rs6591003
rs35340377
rs138804703
rs12793021
rs10908505
rs7940393
rs11190141
rs514921
All
−0.05
0.00
0.05
MR leave−one−out sensitivity analysis for
'MMP1' on 'Cardiac valvular disease'

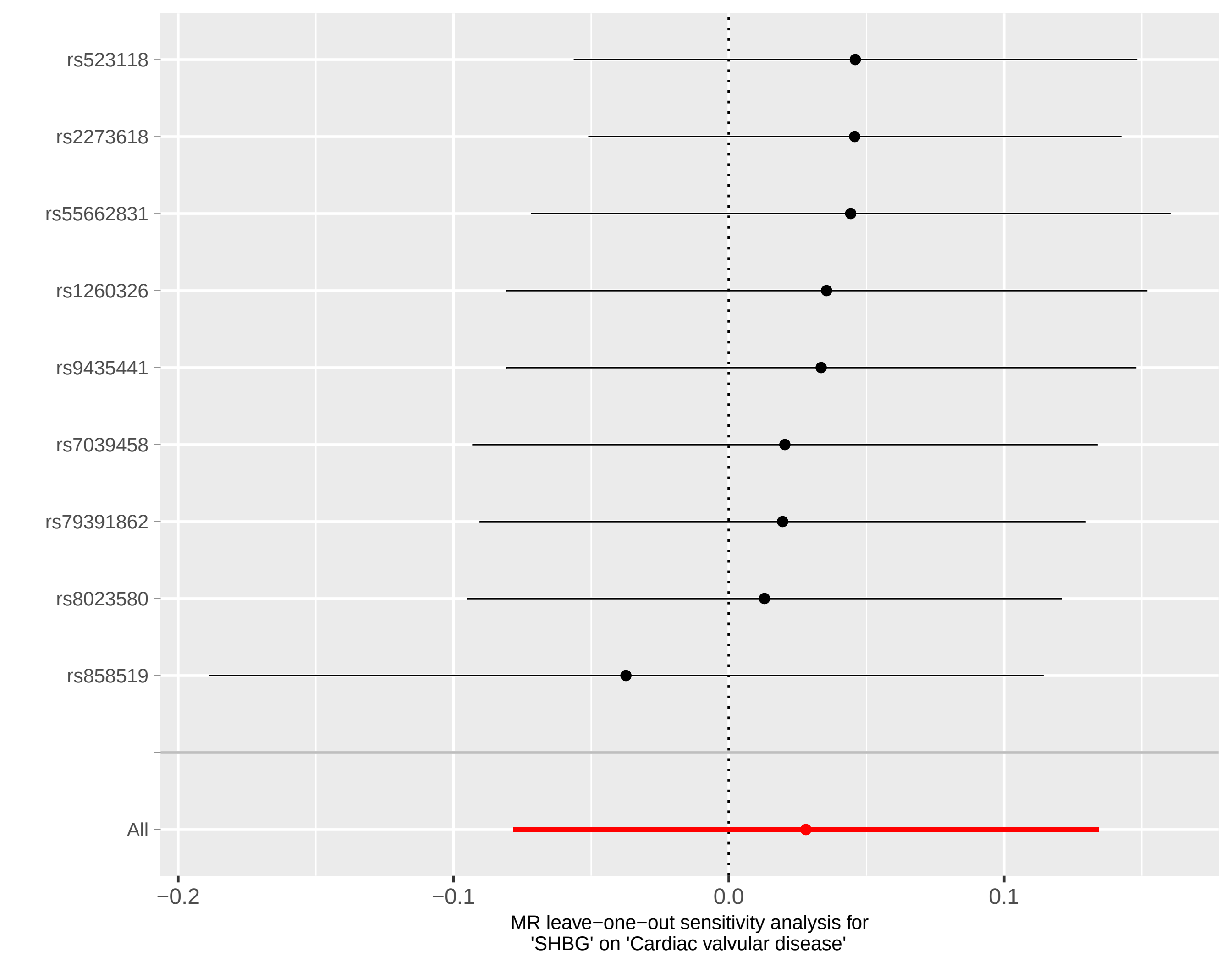

rs523118
rs2273618
rs55662831
rs1260326
rs9435441
rs7039458
rs79391862
rs8023580
rs858519
All
−0.2
−0.1
0.0
0.1
MR leave−one−out sensitivity analysis for
'SHBG' on 'Cardiac valvular disease'

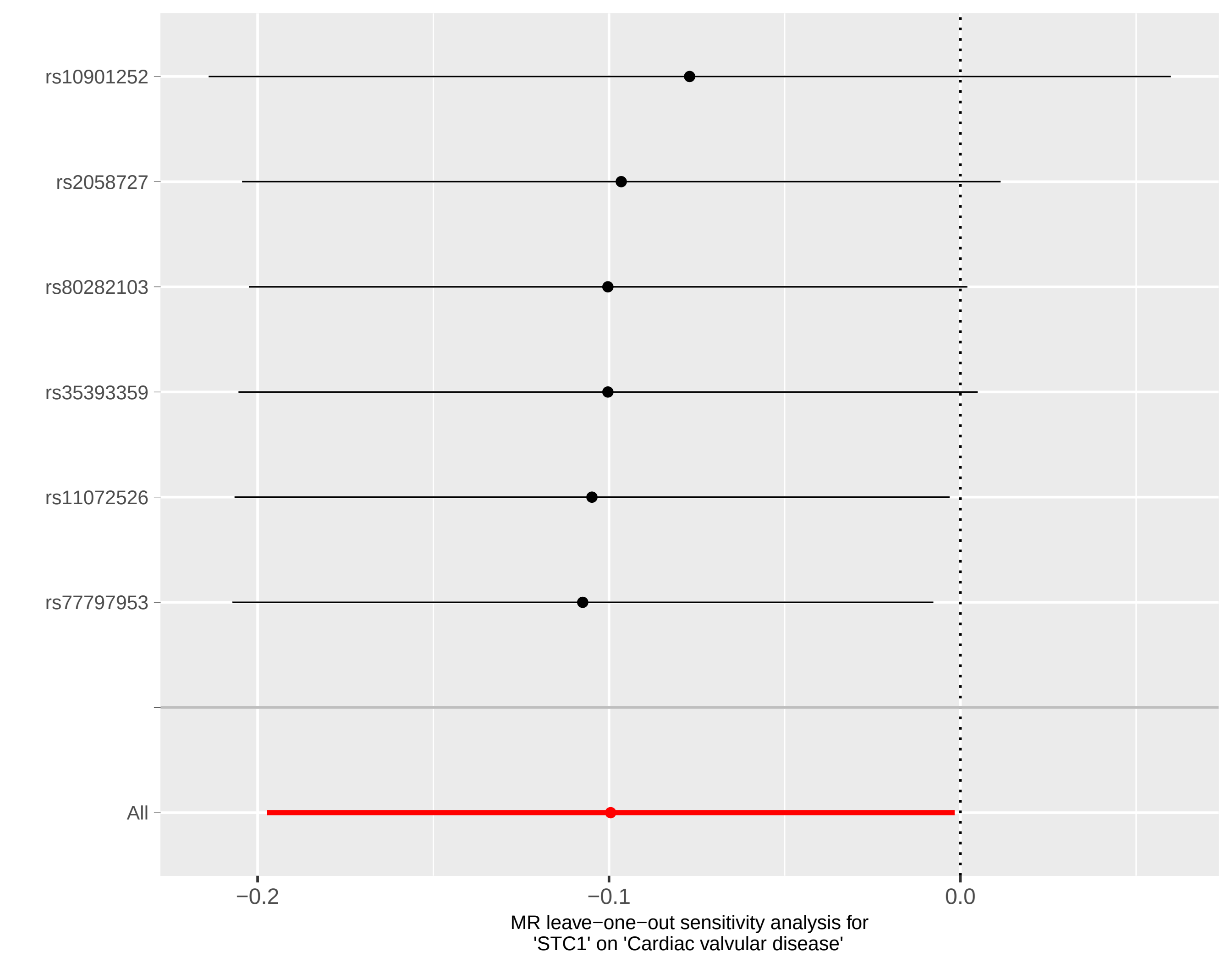

rs10901252
rs2058727
rs80282103
rs35393359
rs11072526
rs77797953
All
−0.2
−0.1
0.0
MR leave−one−out sensitivity analysis for
'STC1' on 'Cardiac valvular disease'

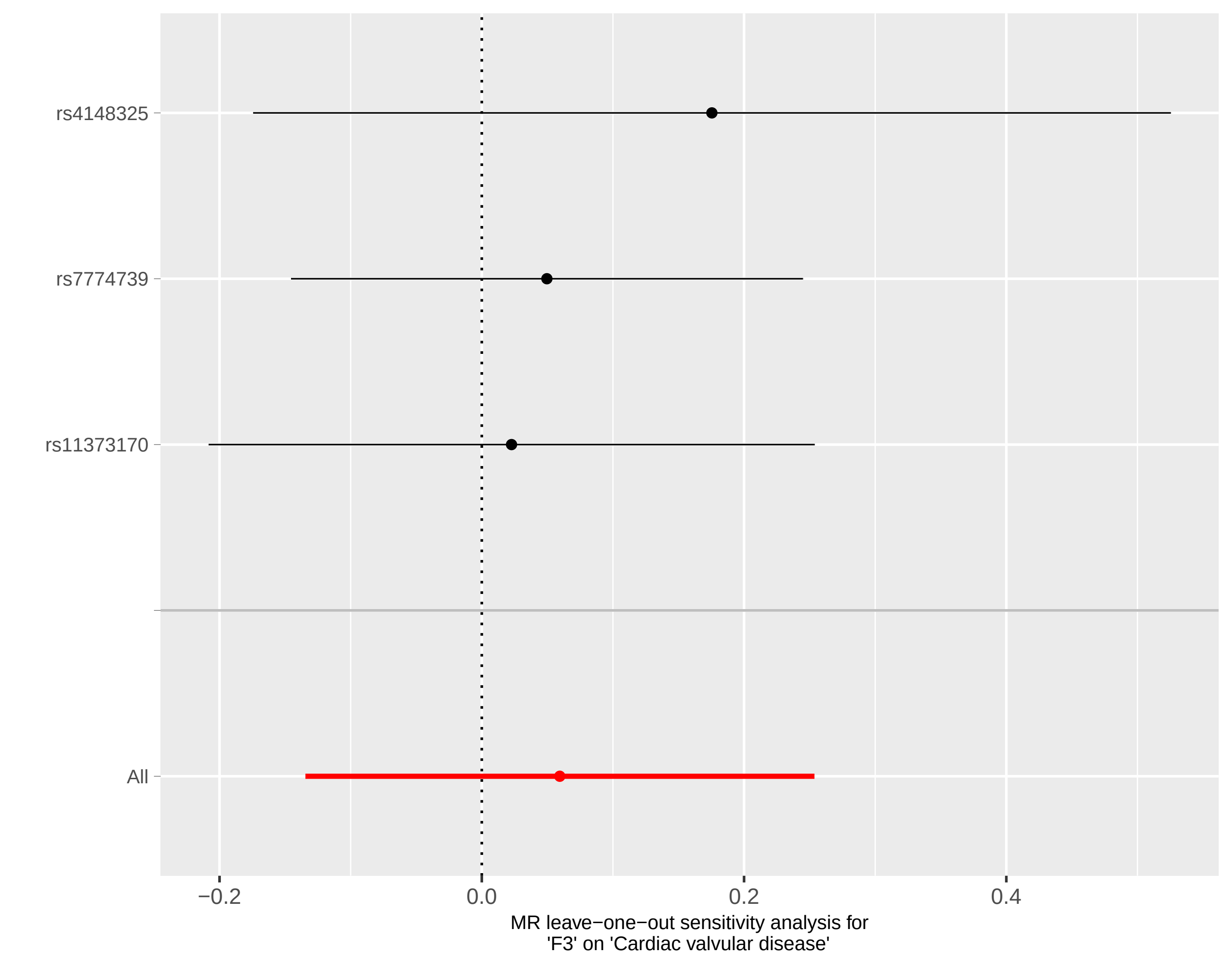
rs4148325
rs7774739
rs11373170
All
−0.2
0.0
0.2
0.4
MR leave−one−out sensitivity analysis for
'F3' on 'Cardiac valvular disease'

Insufficient number of SNPs

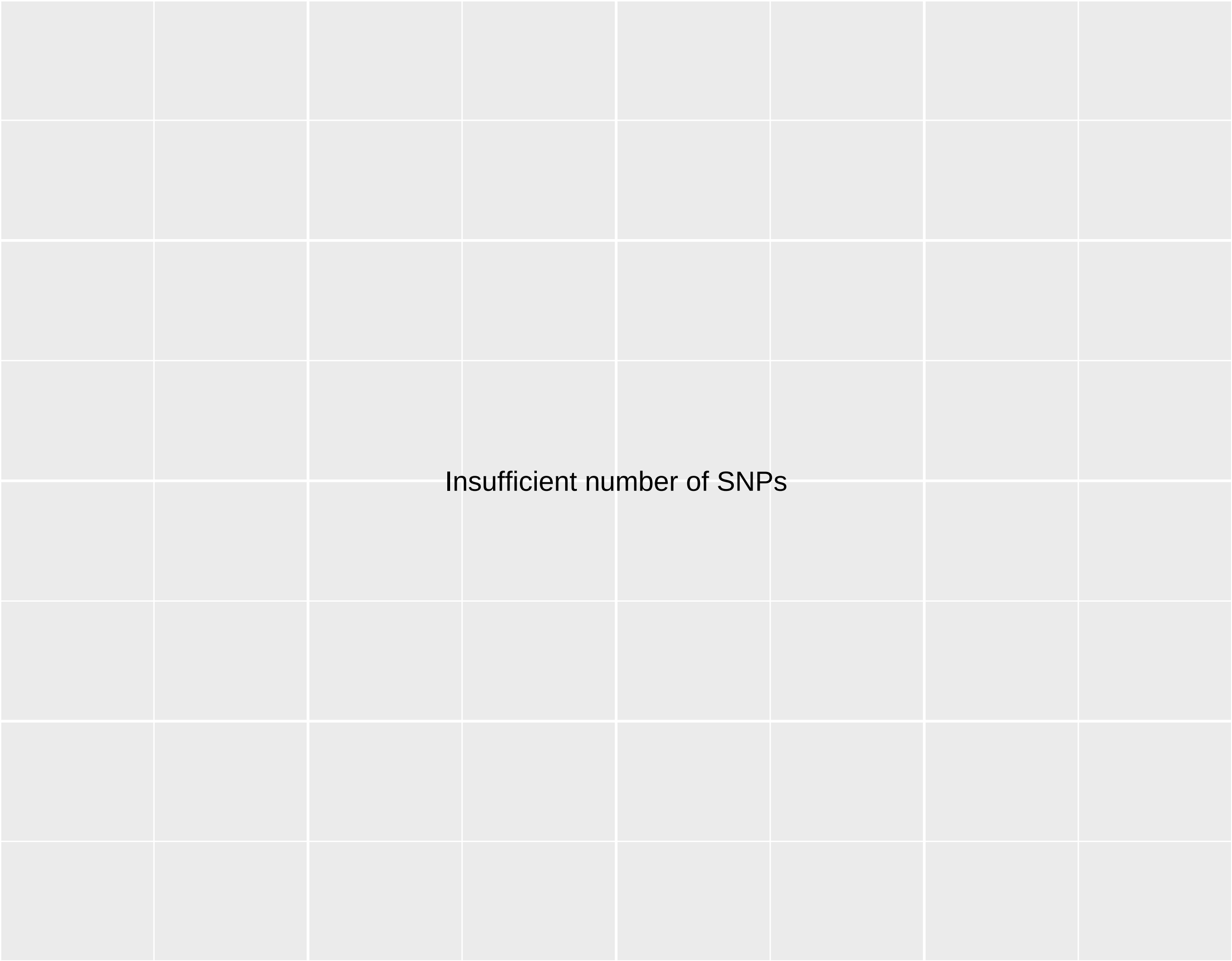
Insufficient number of SNPs

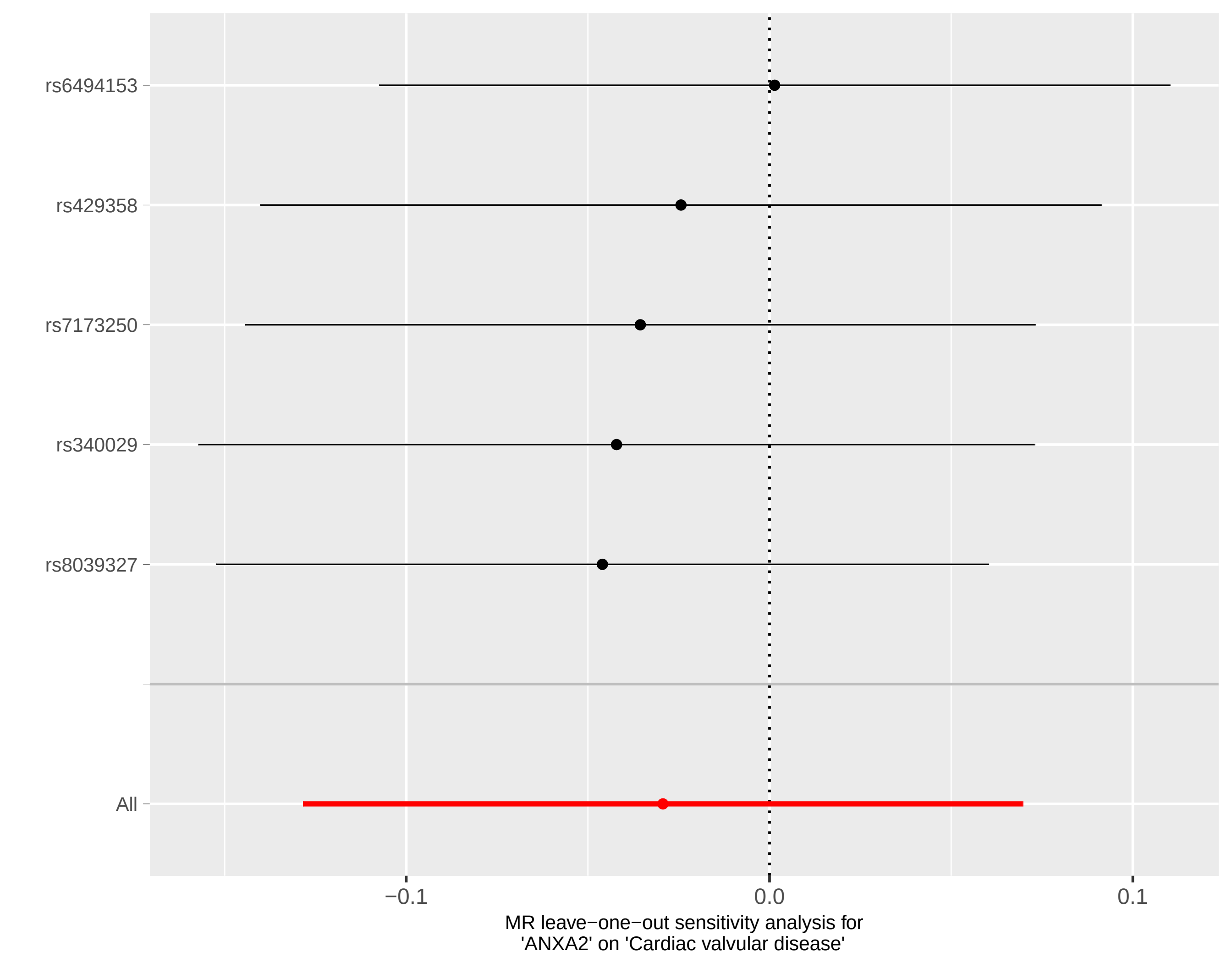
rs6494153
rs429358
rs7173250
rs340029
rs8039327
All
−0.1
0.0
0.1
MR leave−one−out sensitivity analysis for
'ANXA2' on 'Cardiac valvular disease'

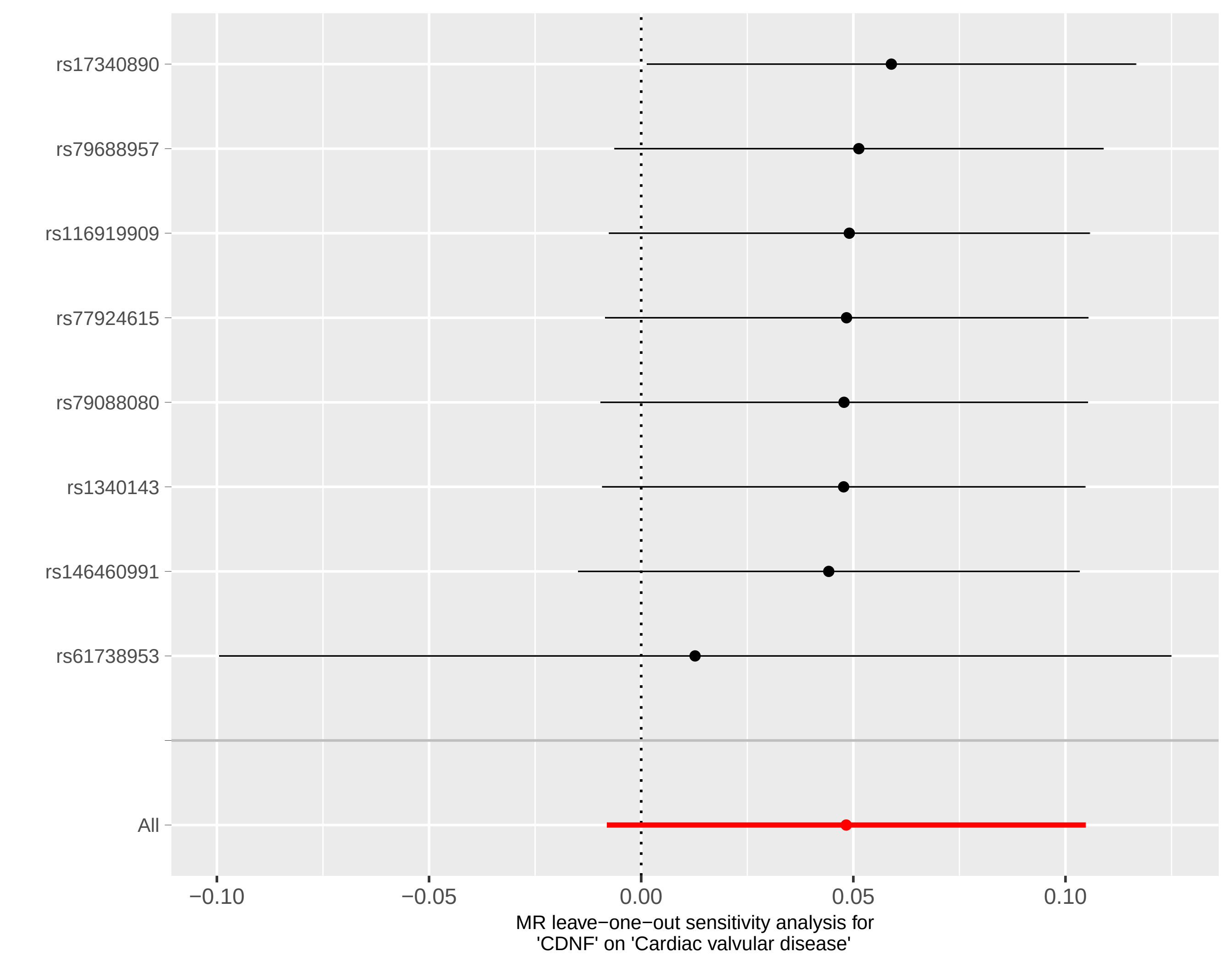
rs17340890
rs79688957
rs116919909
rs77924615
rs79088080
rs1340143
rs146460991
rs61738953
All
−0.10
−0.05
0.00
0.05
0.10
MR leave−one−out sensitivity analysis for
'CDNF' on 'Cardiac valvular disease'

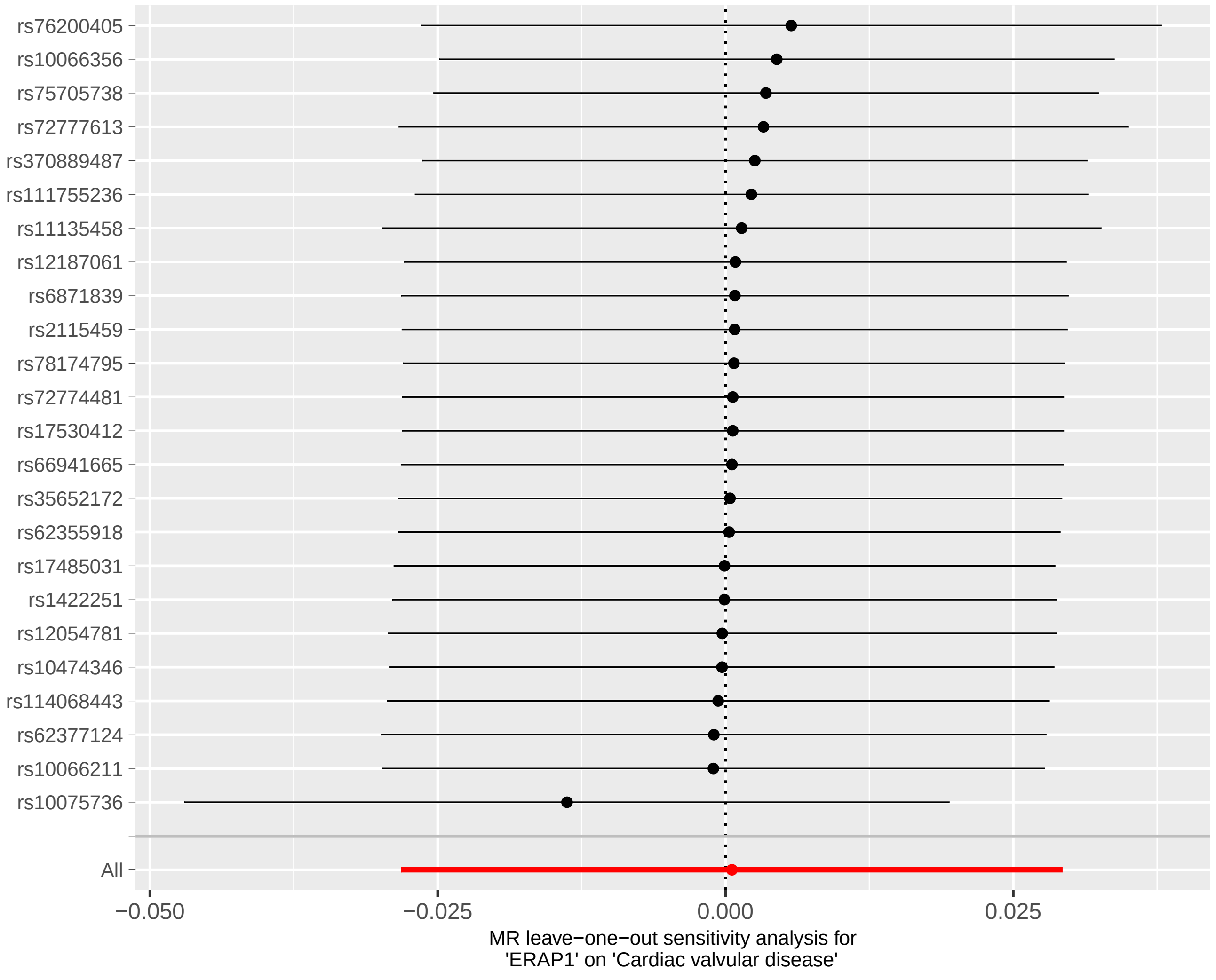
rs76200405
rs10066356
rs75705738
rs72777613
rs370889487
rs111755236
rs11135458
rs12187061
rs6871839
rs2115459
rs78174795
rs72774481
rs17530412
rs66941665
rs35652172
rs62355918
rs17485031
rs1422251
rs12054781
rs10474346
rs114068443
rs62377124
rs10066211
rs10075736
All
−0.050
−0.025
0.000
0.025
MR leave−one−out sensitivity analysis for
'ERAP1' on 'Cardiac valvular disease'

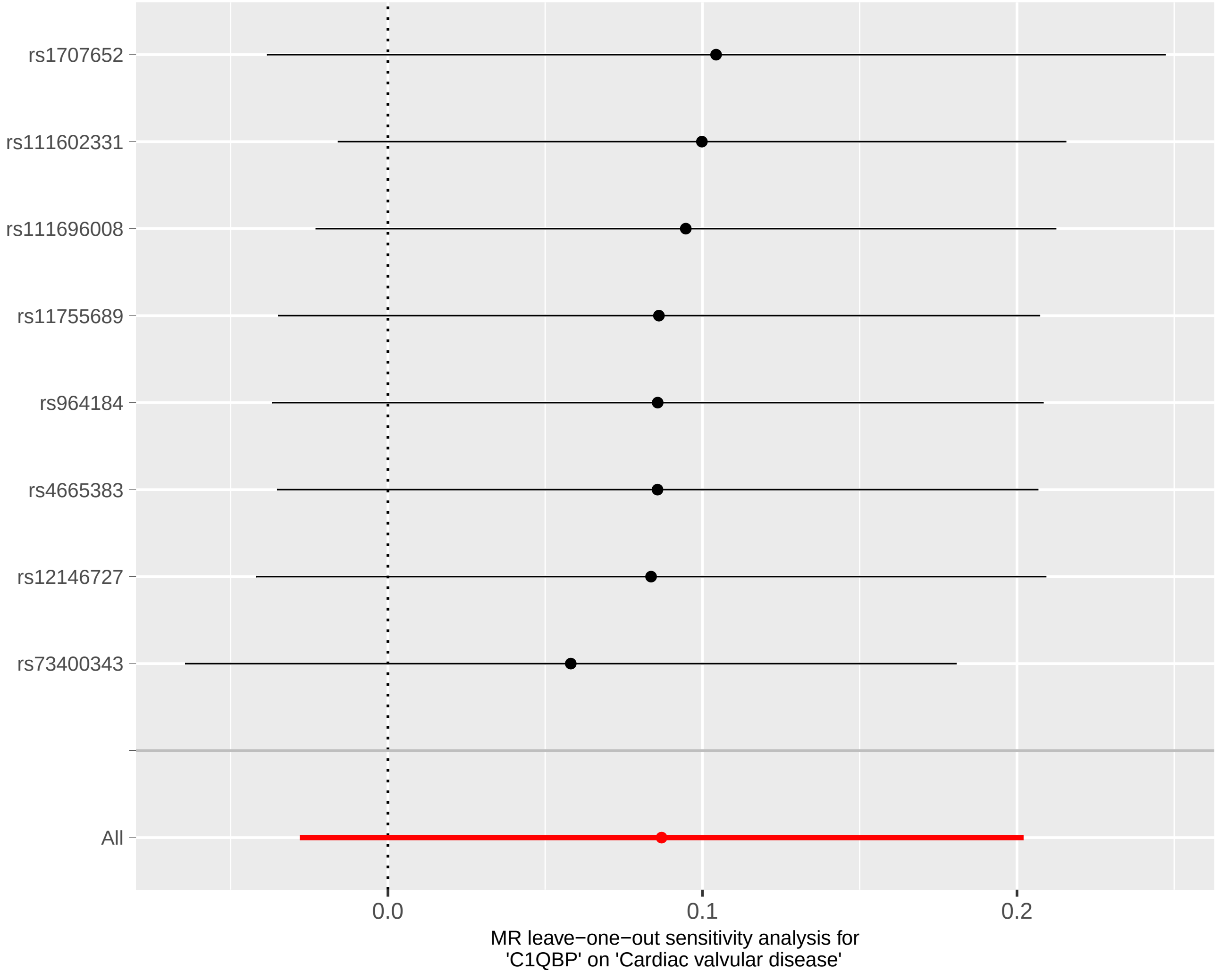
rs1707652
rs111602331
rs111696008
rs11755689
rs964184
rs4665383
rs12146727
rs73400343
All
0.0
0.1
0.2
MR leave−one−out sensitivity analysis for
'C1QBP' on 'Cardiac valvular disease'

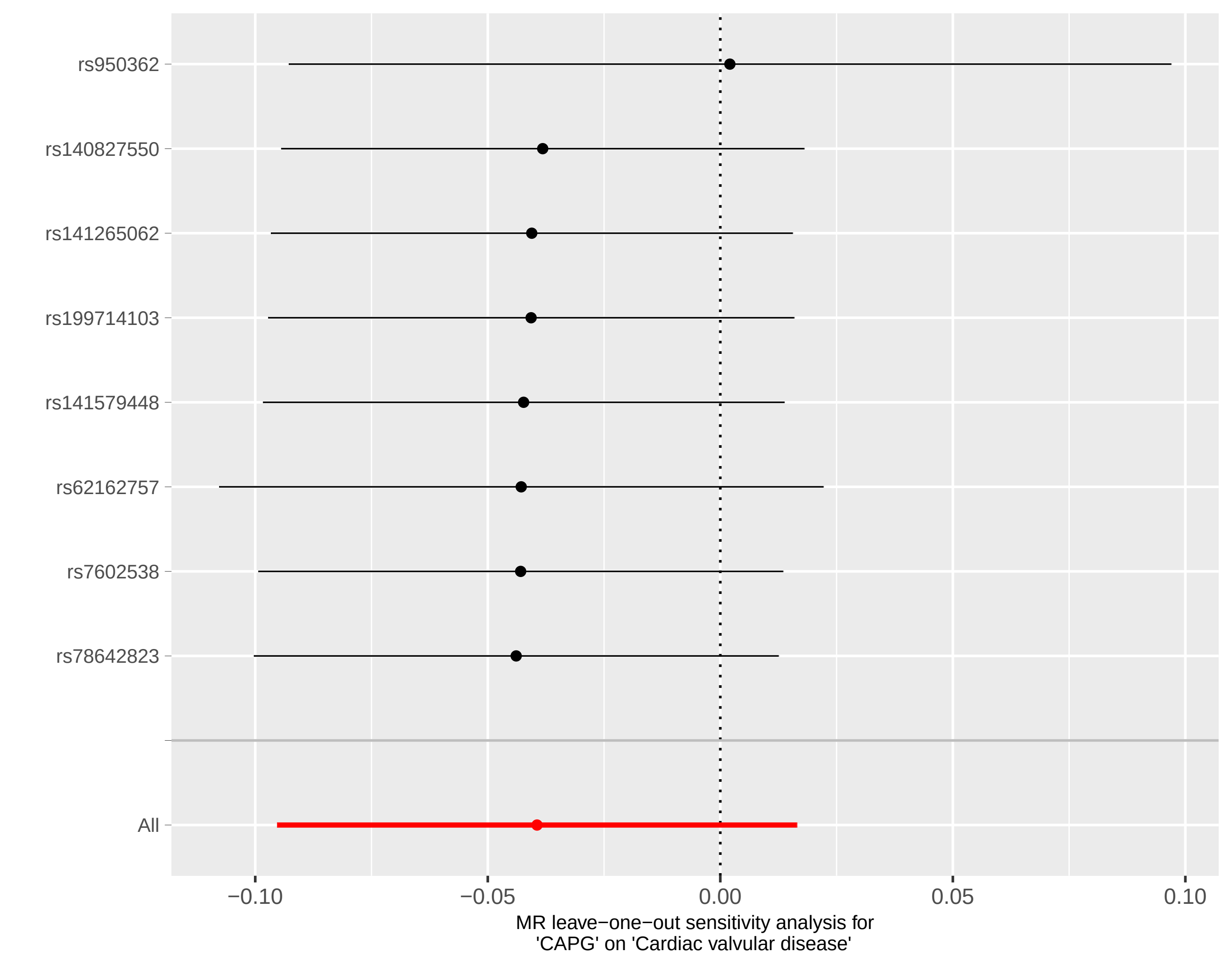
rs950362
rs140827550
rs141265062
rs199714103
rs141579448
rs62162757
rs7602538
rs78642823
All
−0.10
−0.05
0.00
0.05
0.10
MR leave−one−out sensitivity analysis for
'CAPG' on 'Cardiac valvular disease'

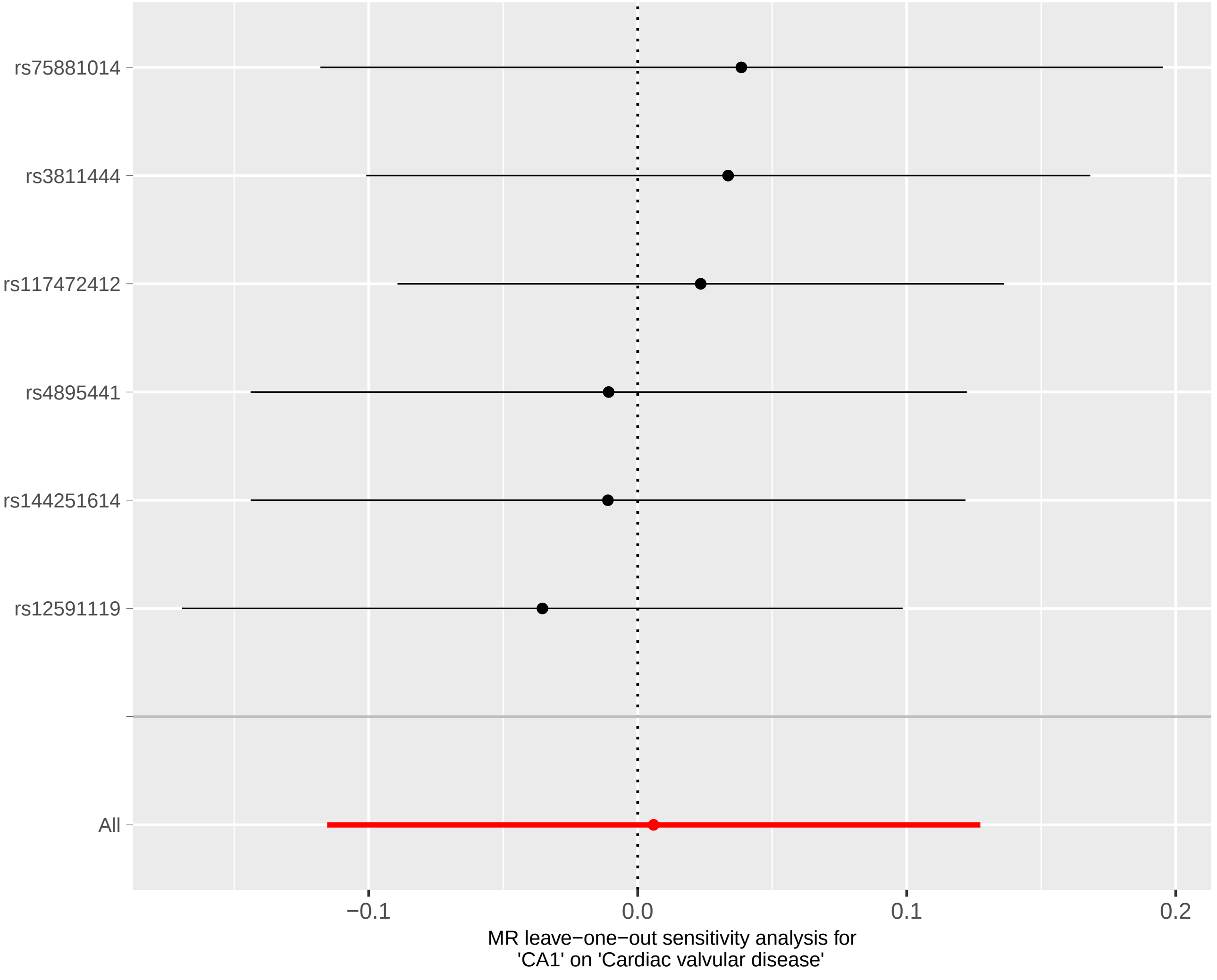

rs75881014
rs3811444
rs117472412
rs4895441
rs144251614
rs12591119
All
−0.1
0.0
0.1
0.2
MR leave−one−out sensitivity analysis for
'CA1' on 'Cardiac valvular disease'

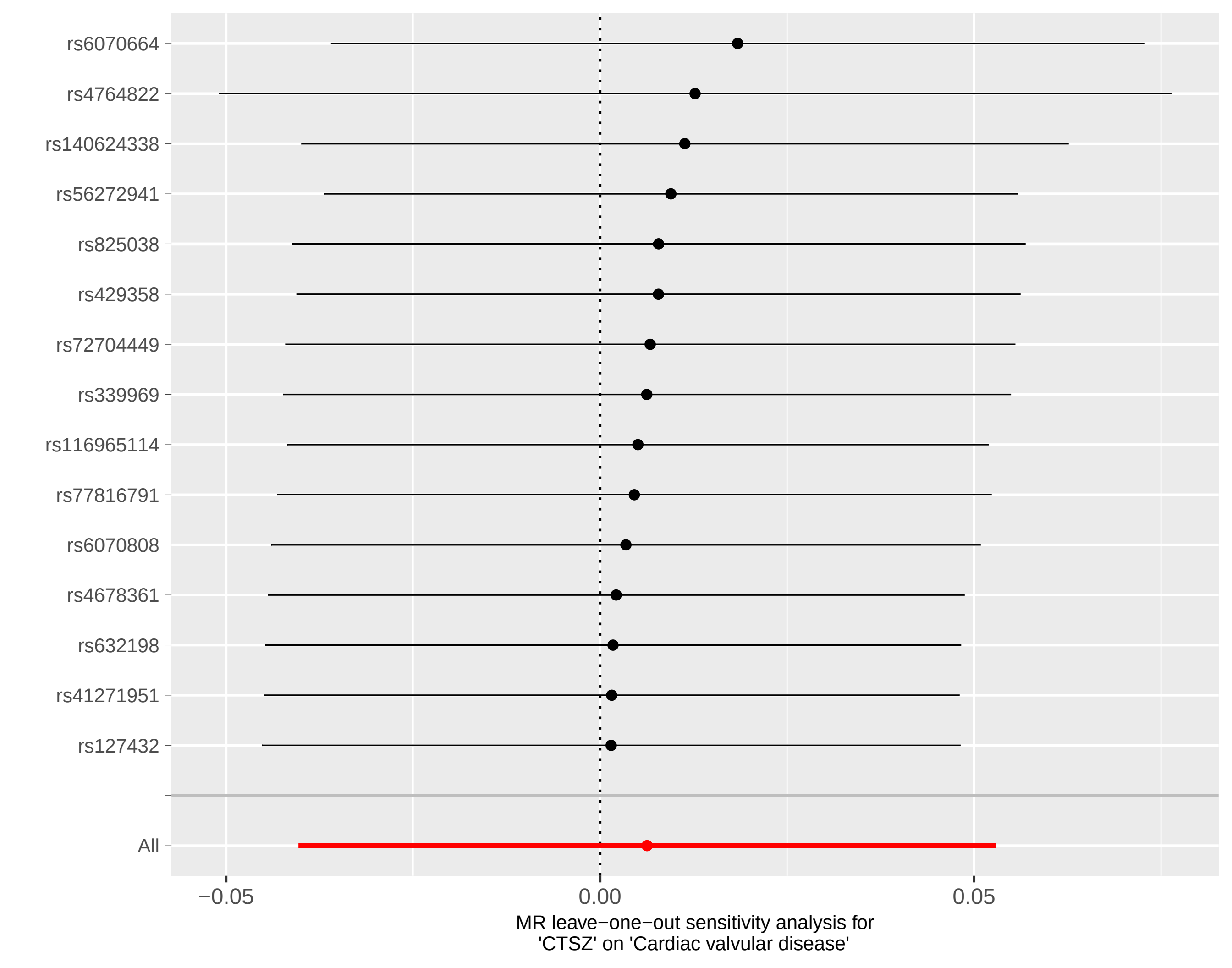

rs6070664
rs4764822
rs140624338
rs56272941
rs825038
rs429358
rs72704449
rs339969
rs116965114
rs77816791
rs6070808
rs4678361
rs632198
rs41271951
rs127432
All
−0.05
0.00
0.05
MR leave−one−out sensitivity analysis for
'CTSZ' on 'Cardiac valvular disease'

Insufficient number of SNPs

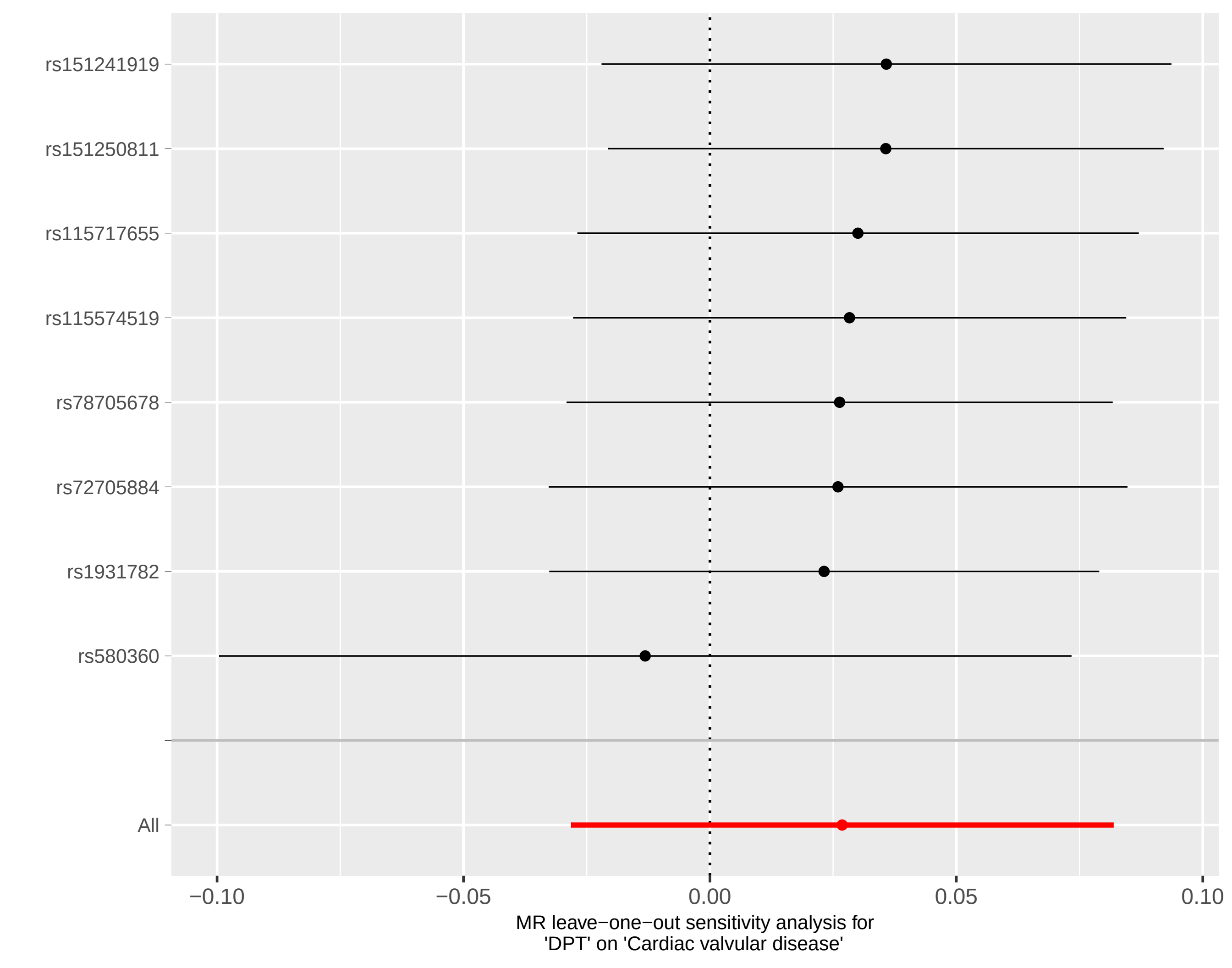
rs151241919
rs151250811
rs115717655
rs115574519
rs78705678
rs72705884
rs1931782
rs580360
All
−0.10
−0.05
0.00
0.05
0.10
MR leave−one−out sensitivity analysis for
'DPT' on 'Cardiac valvular disease'

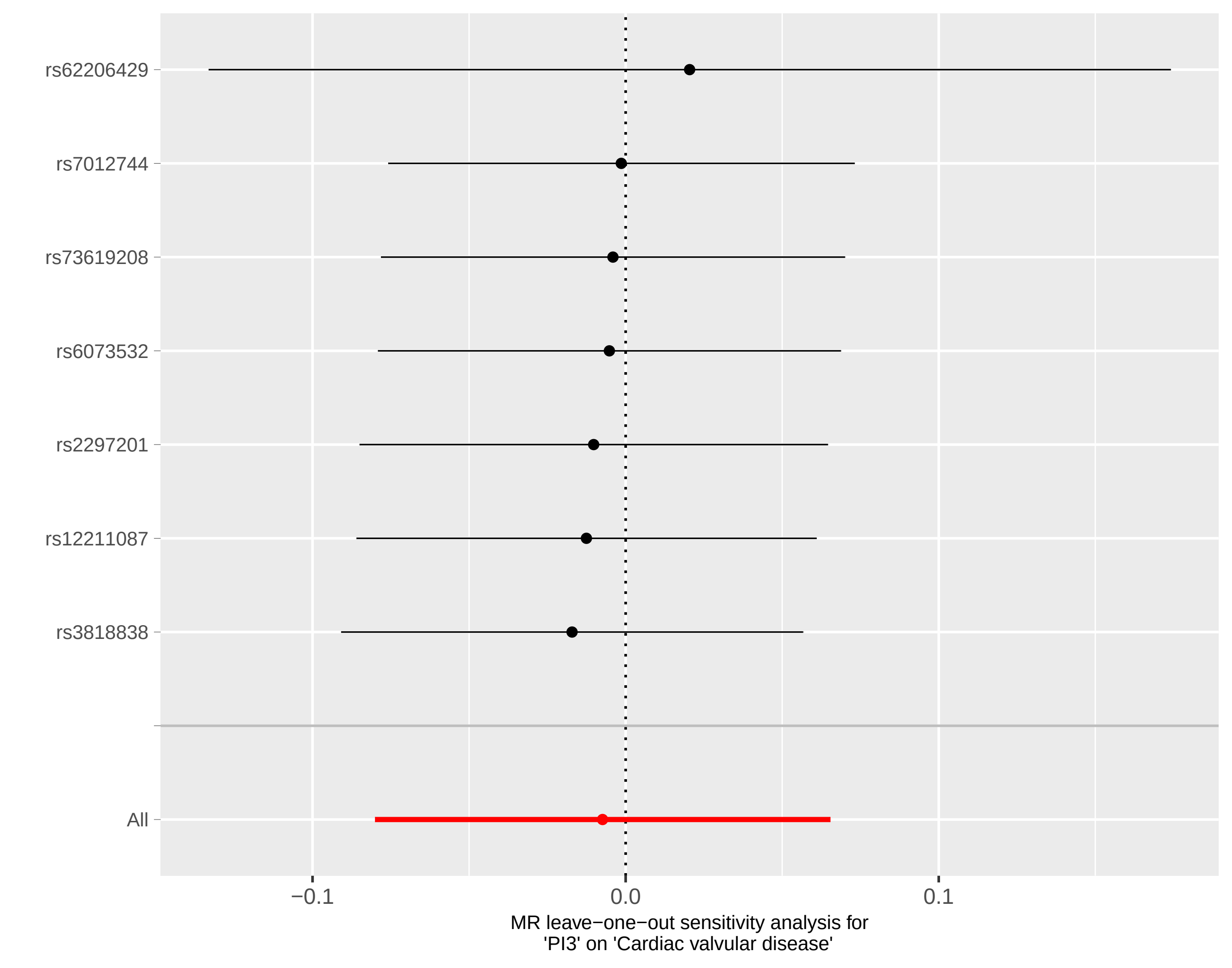
rs62206429
rs7012744
rs73619208
rs6073532
rs2297201
rs12211087
rs3818838
All
−0.1
0.0
0.1
MR leave−one−out sensitivity analysis for
'PI3' on 'Cardiac valvular disease'

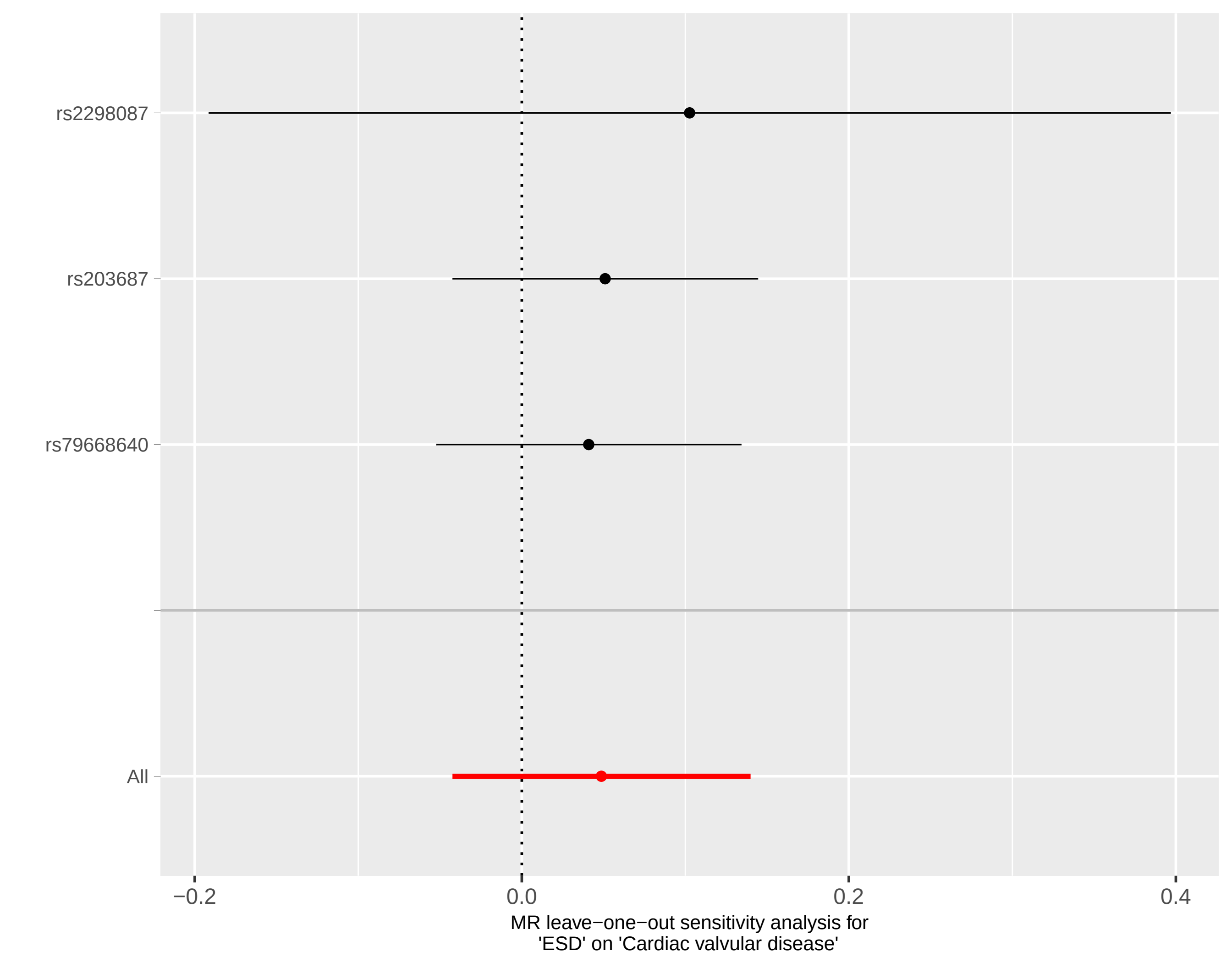
rs2298087
rs203687
rs79668640
All
−0.2
0.0
0.2
0.4
MR leave−one−out sensitivity analysis for
'ESD' on 'Cardiac valvular disease'

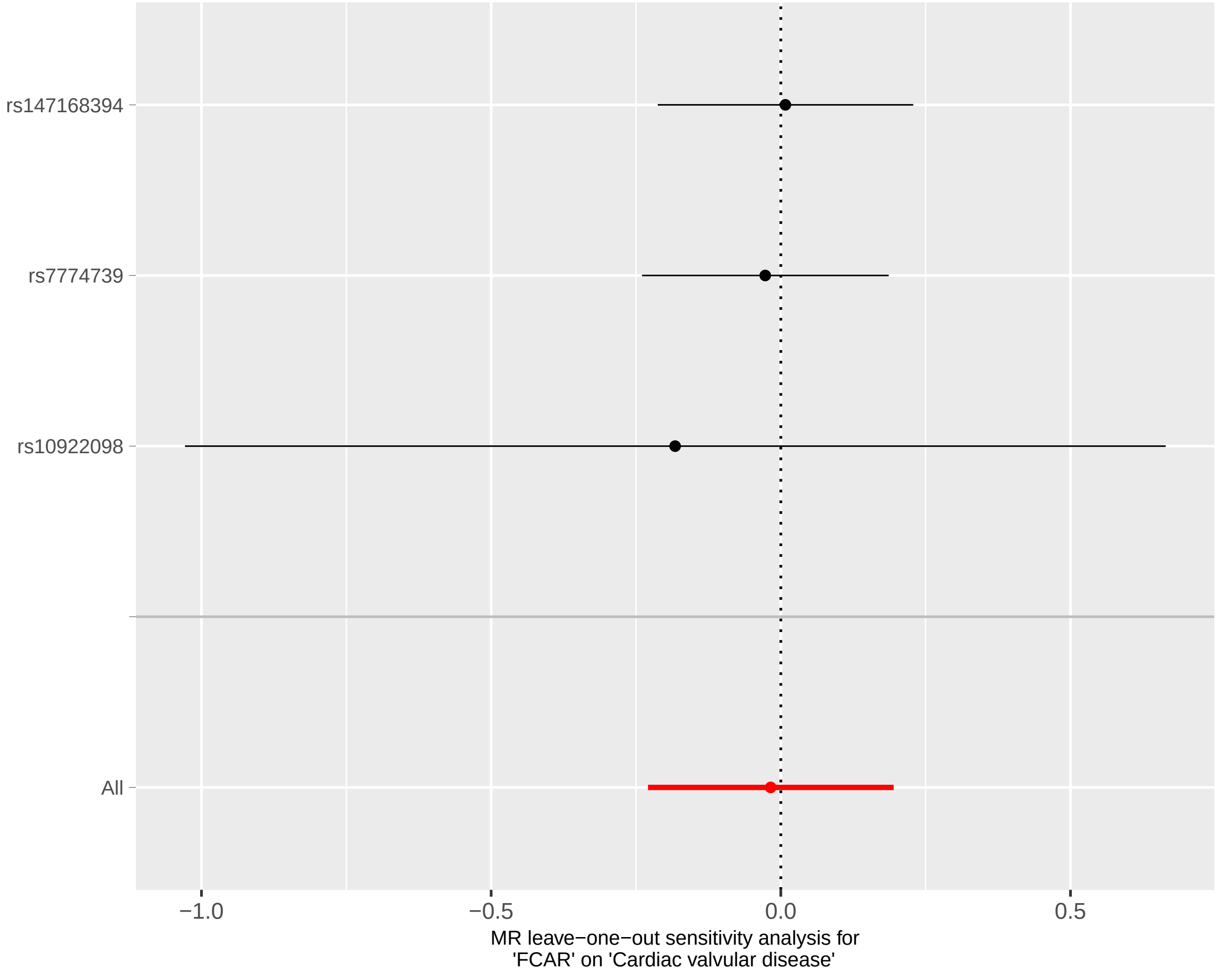
rs147168394
rs7774739
rs10922098
All
−1.0
−0.5
0.0
0.5
MR leave−one−out sensitivity analysis for
'FCAR' on 'Cardiac valvular disease'

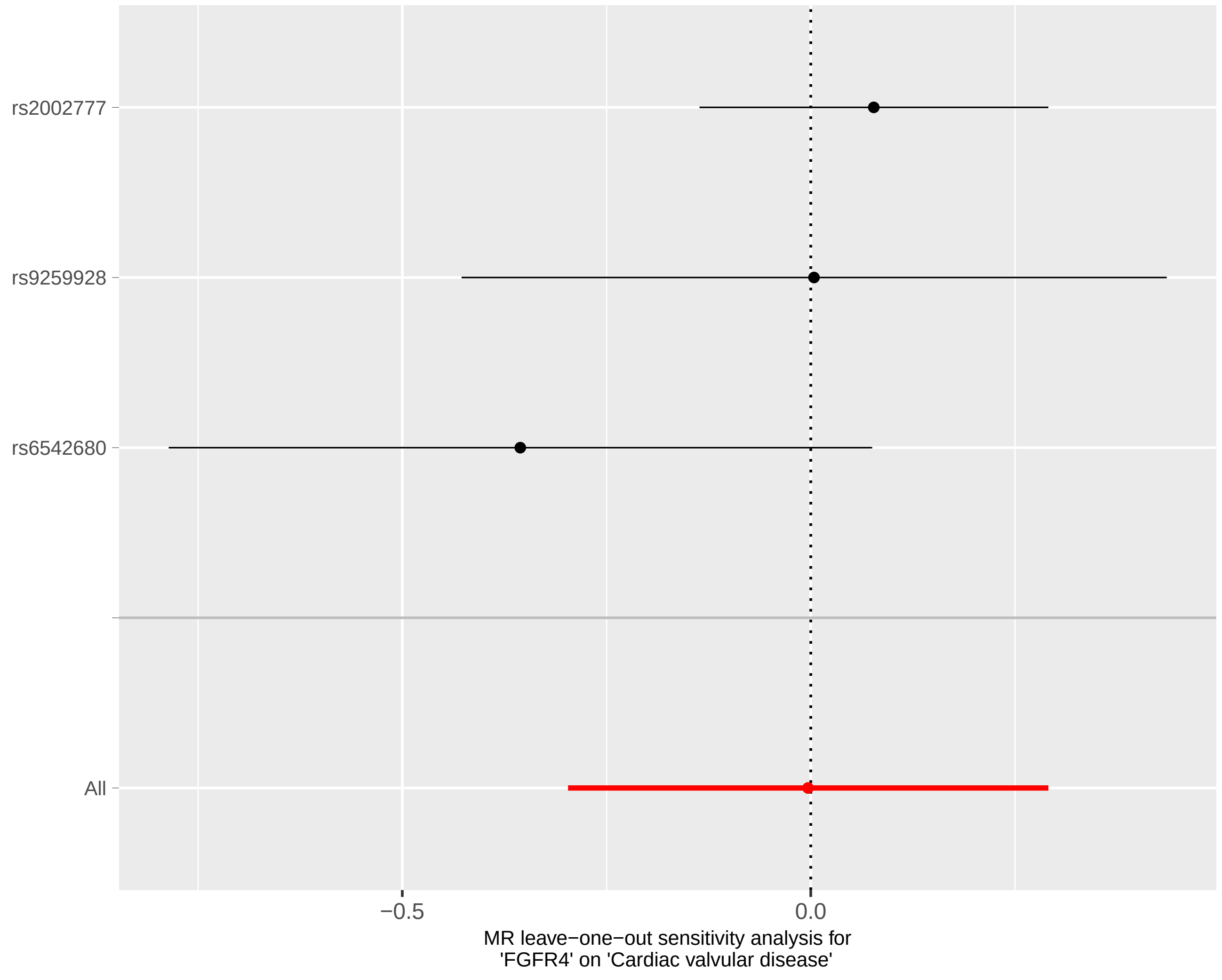

rs2002777
rs9259928
rs6542680
All
−0.5
0.0
MR leave−one−out sensitivity analysis for
'FGFR4' on 'Cardiac valvular disease'

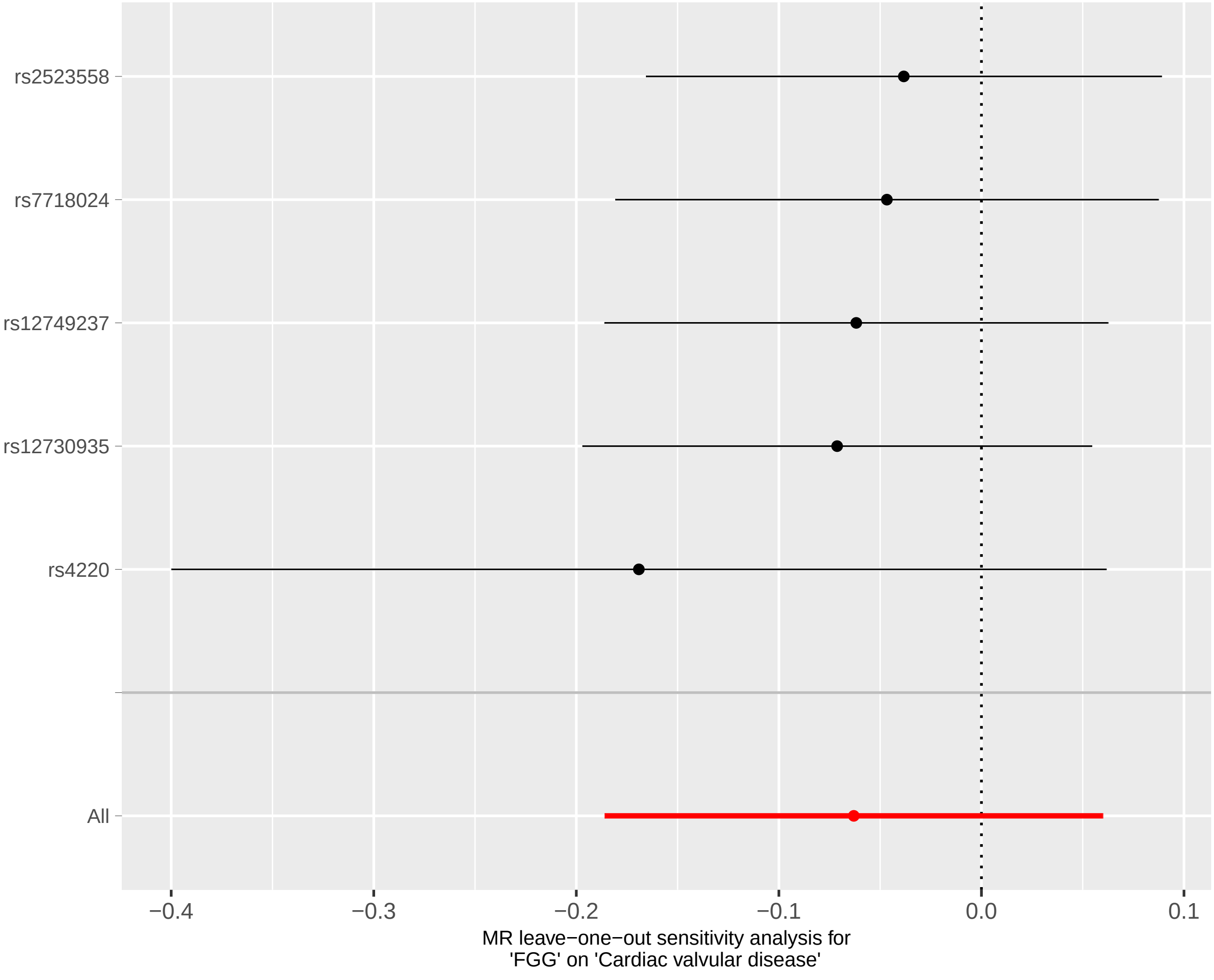
rs2523558
rs7718024
rs12749237
rs12730935
rs4220
All
−0.4
−0.3
−0.2
−0.1
0.0
0.1
MR leave−one−out sensitivity analysis for
'FGG' on 'Cardiac valvular disease'

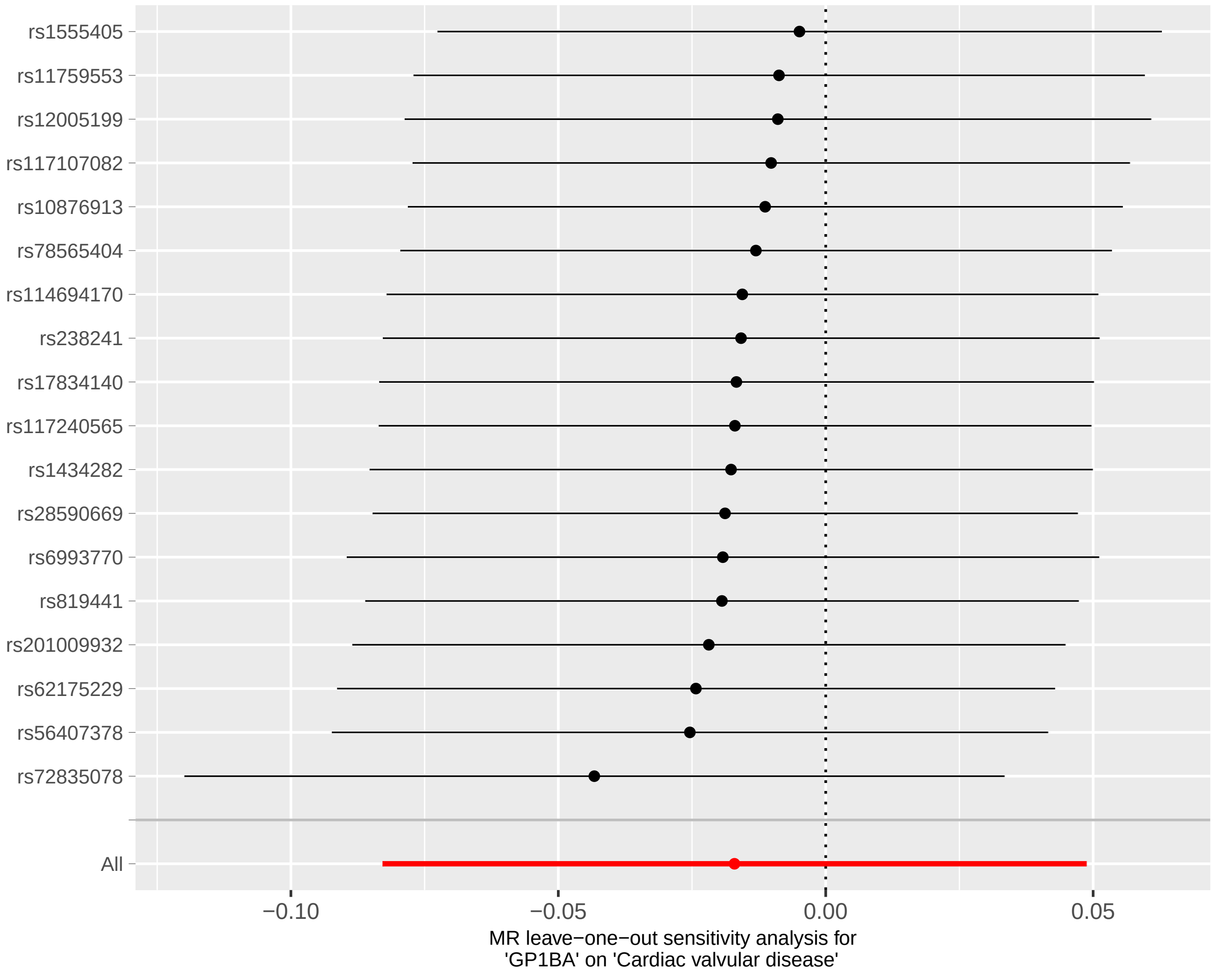
rs1555405
rs11759553
rs12005199
rs117107082
rs10876913
rs78565404
rs114694170
rs238241
rs17834140
rs117240565
rs1434282
rs28590669
rs6993770
rs819441
rs201009932
rs62175229
rs56407378
rs72835078
All
−0.10
−0.05
0.00
0.05
MR leave−one−out sensitivity analysis for
'GP1BA' on 'Cardiac valvular disease'

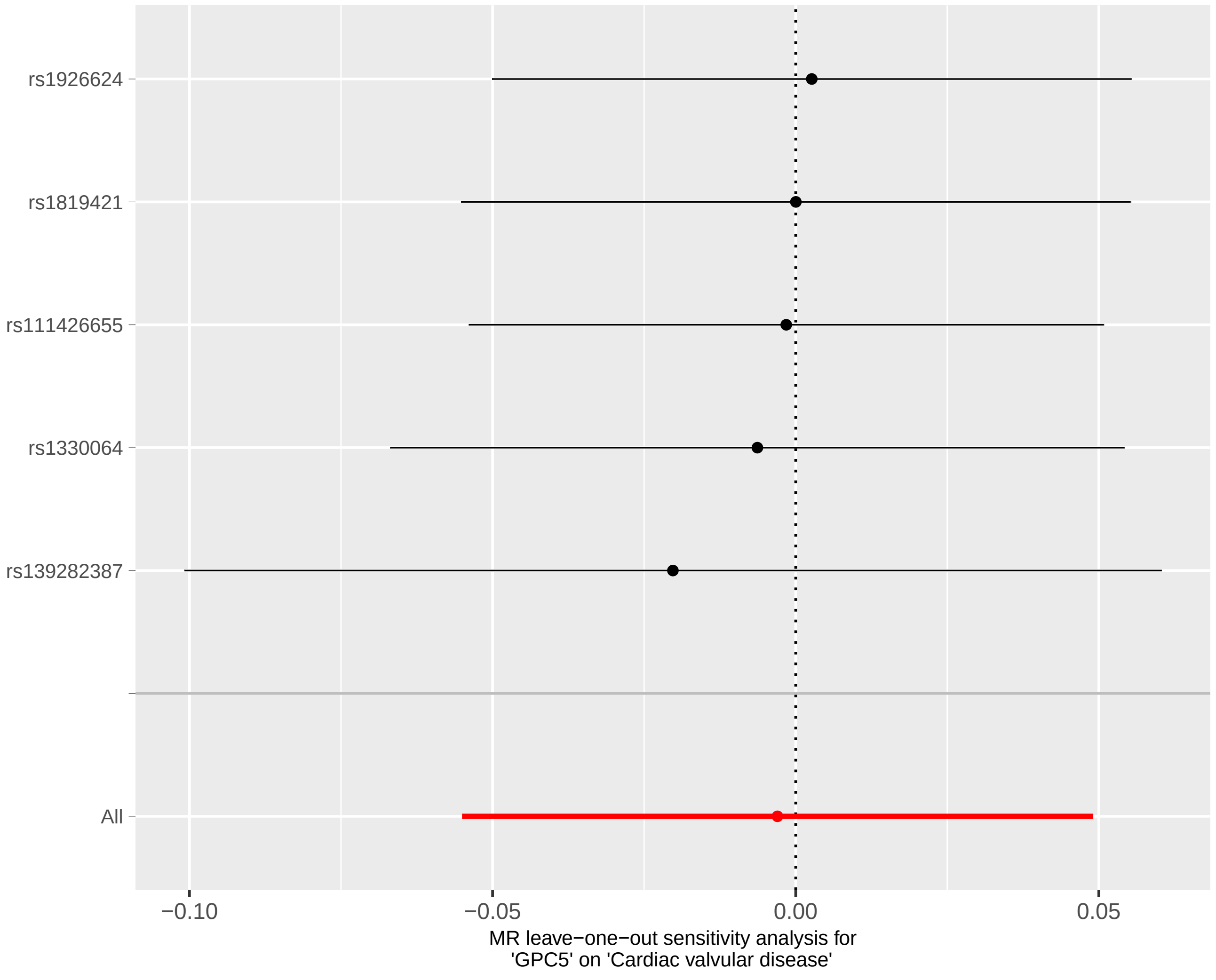
rs1926624
rs1819421
rs111426655
rs1330064
rs139282387
All
−0.10
−0.05
0.00
0.05
MR leave−one−out sensitivity analysis for
'GPC5' on 'Cardiac valvular disease'

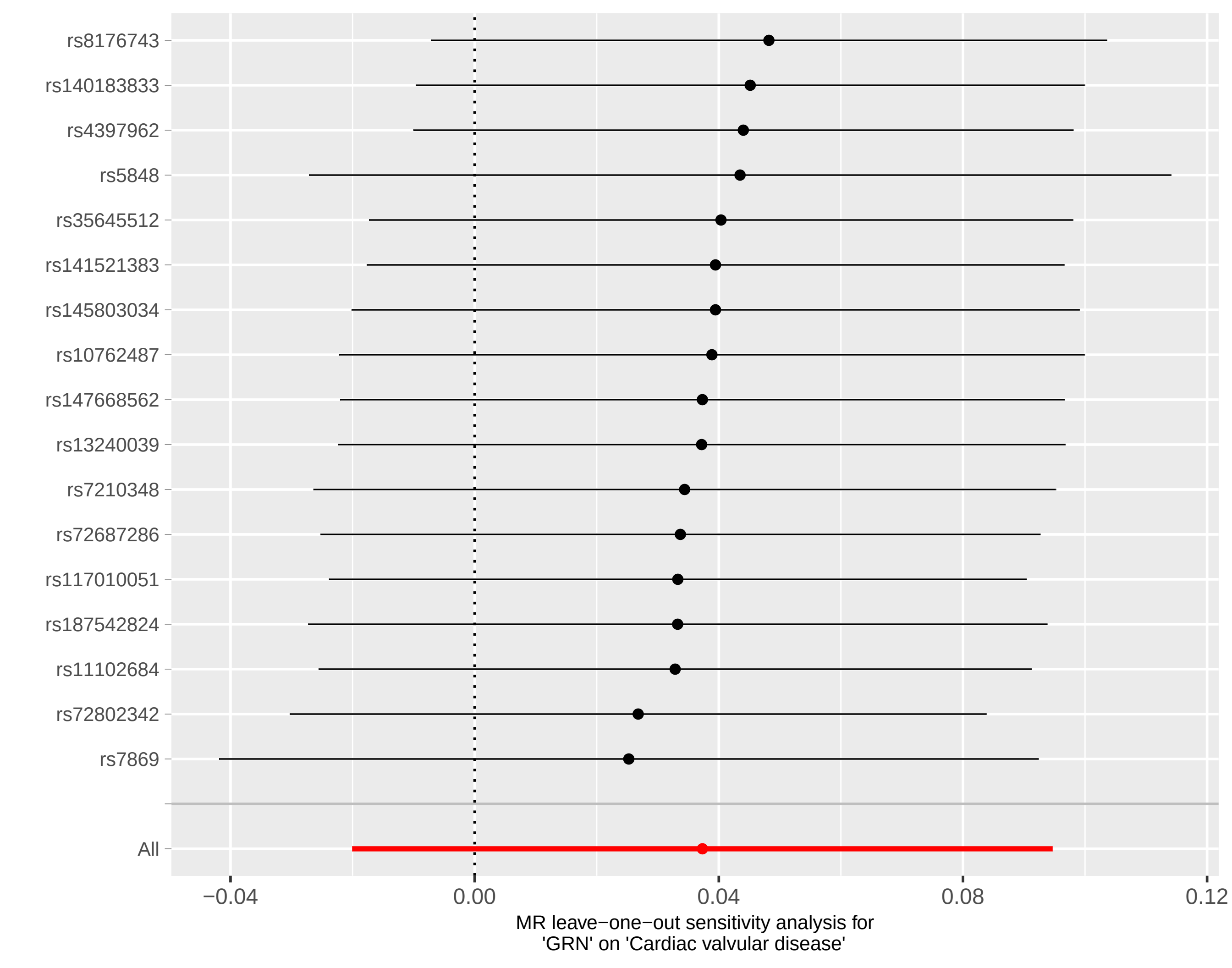

rs8176743
rs140183833
rs4397962
rs5848
rs35645512
rs141521383
rs145803034
rs10762487
rs147668562
rs13240039
rs7210348
rs72687286
rs117010051
rs187542824
rs11102684
rs72802342
rs7869
All
−0.04
0.00
0.04
0.08
0.12
MR leave−one−out sensitivity analysis for
'GRN' on 'Cardiac valvular disease'

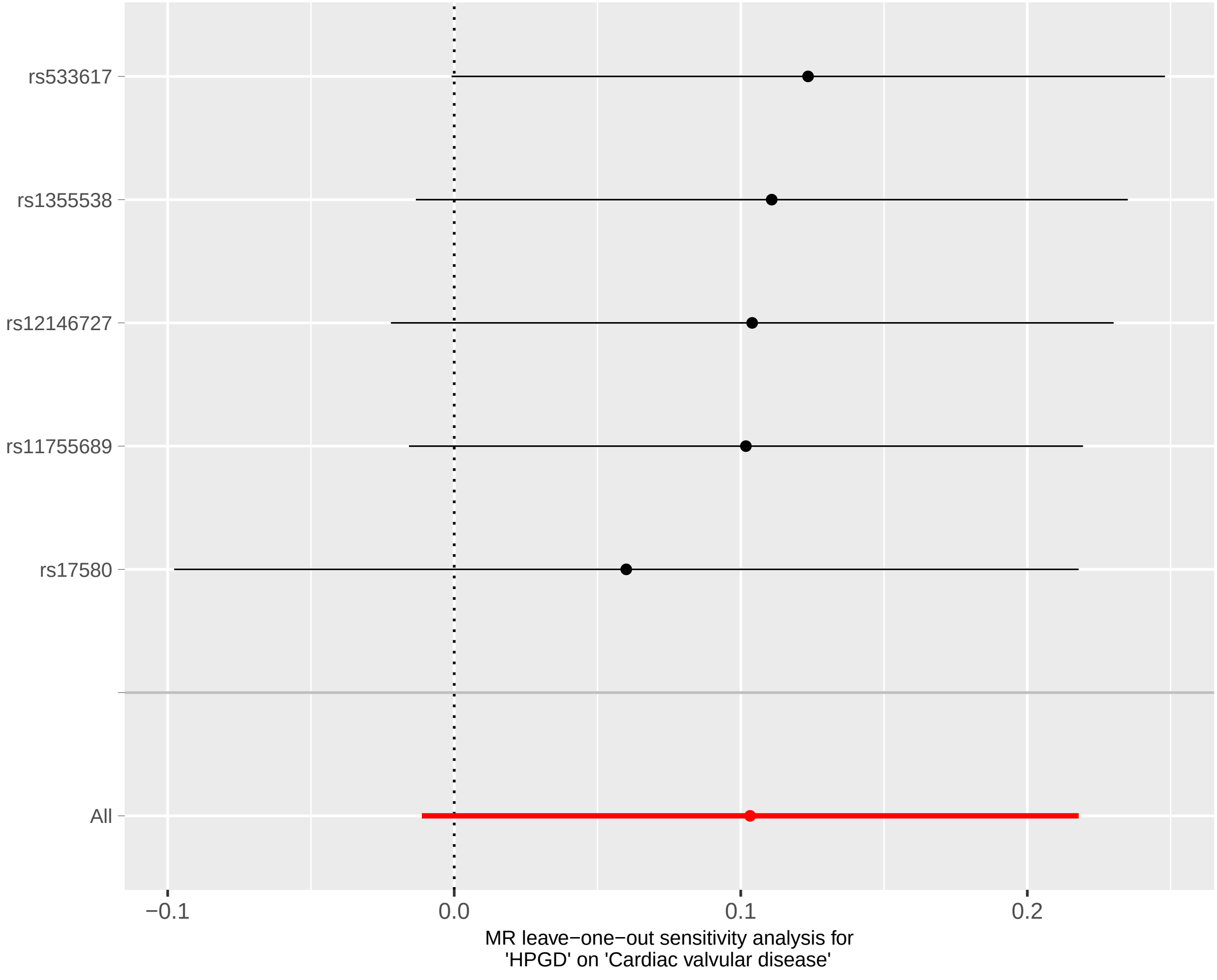

rs533617
rs1355538
rs12146727
rs11755689
rs17580
All
−0.1
0.0
0.1
0.2
MR leave−one−out sensitivity analysis for
'HPGD' on 'Cardiac valvular disease'

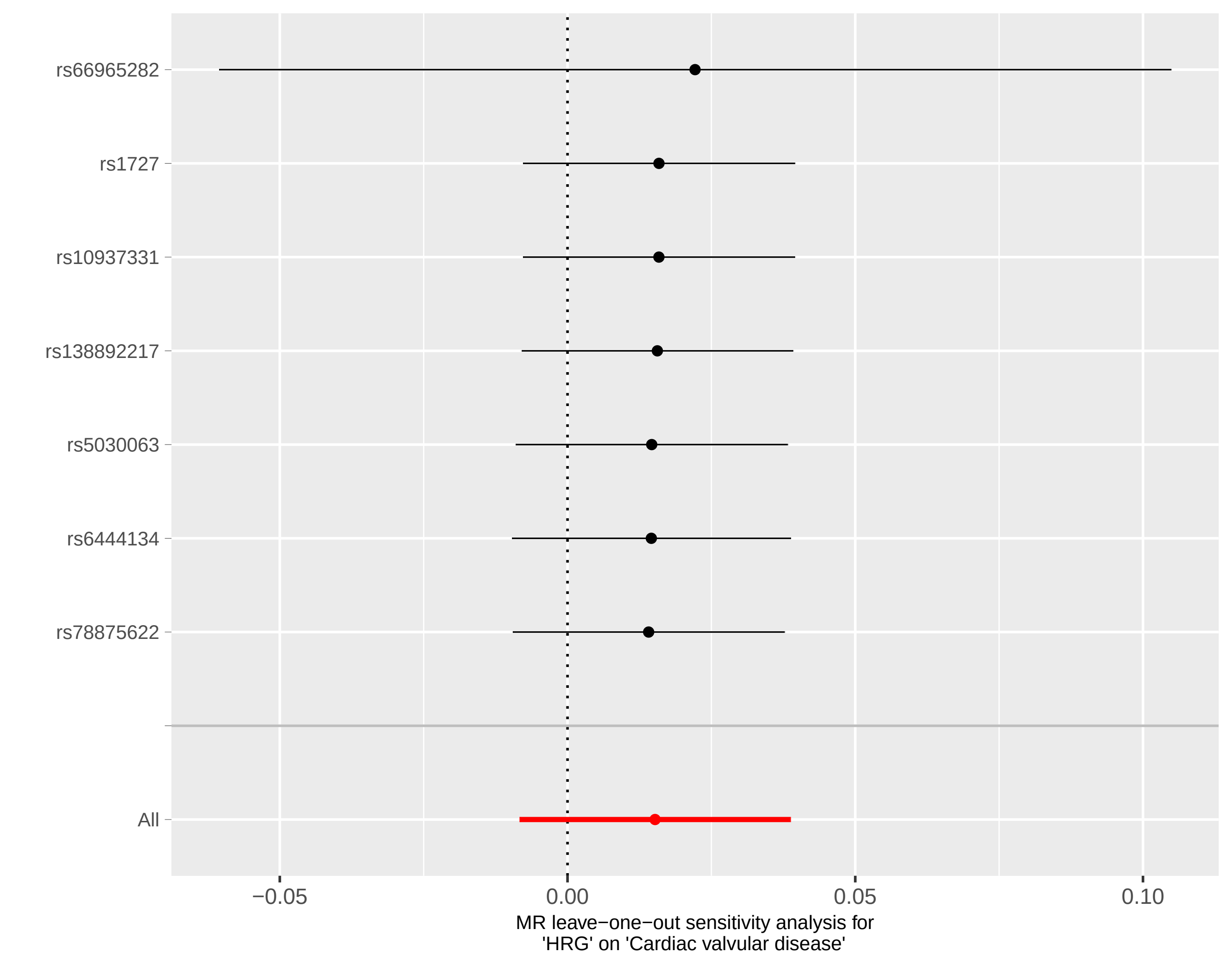
rs66965282
rs1727
rs10937331
rs138892217
rs5030063
rs6444134
rs78875622
All
−0.05
0.00
0.05
0.10
MR leave−one−out sensitivity analysis for
'HRG' on 'Cardiac valvular disease'

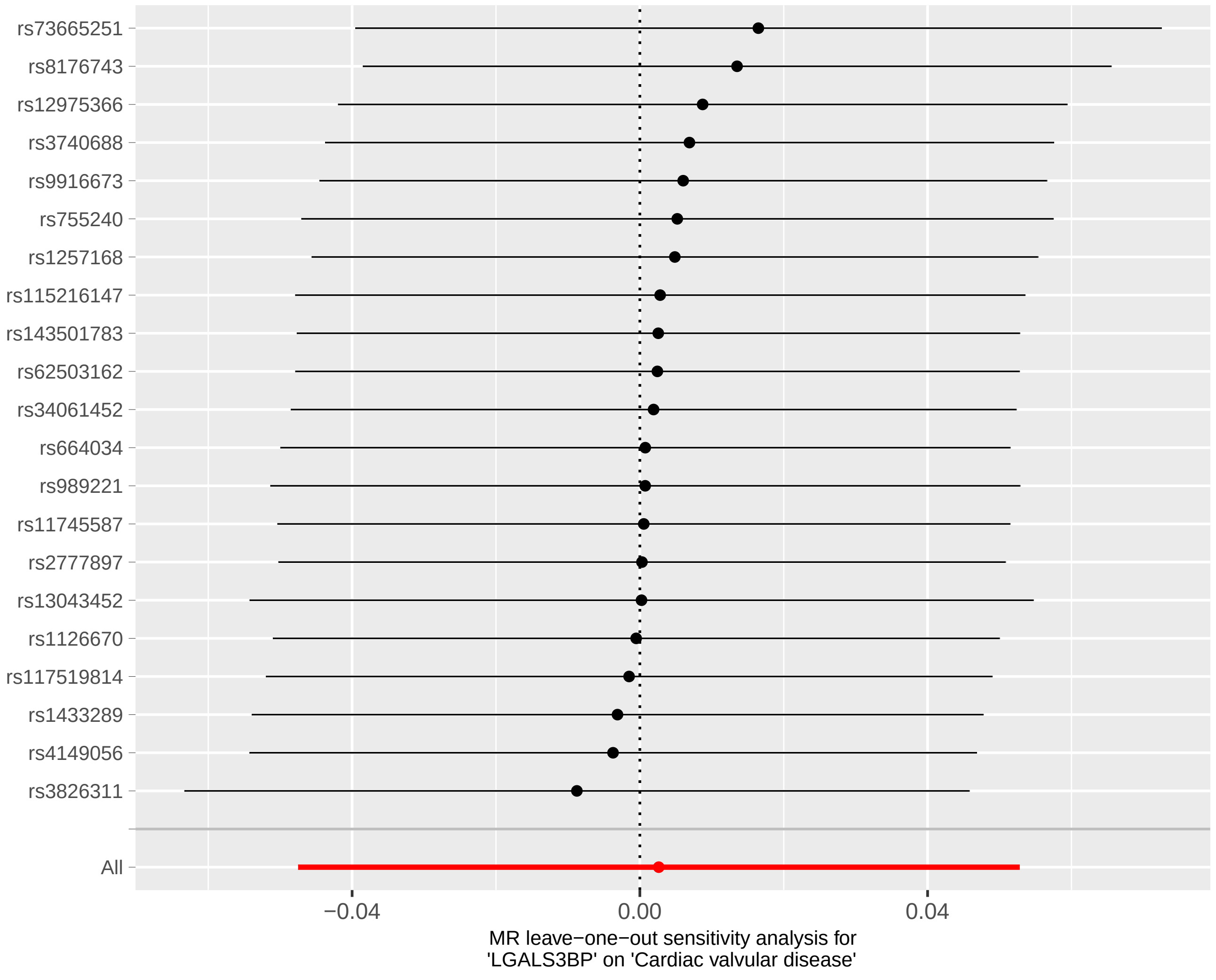
rs73665251
rs8176743
rs12975366
rs3740688
rs9916673
rs755240
rs1257168
rs115216147
rs143501783
rs62503162
rs34061452
rs664034
rs989221
rs11745587
rs2777897
rs13043452
rs1126670
rs117519814
rs1433289
rs4149056
rs3826311
All
−0.04
0.00
0.04
MR leave−one−out sensitivity analysis for
'LGALS3BP' on 'Cardiac valvular disease'

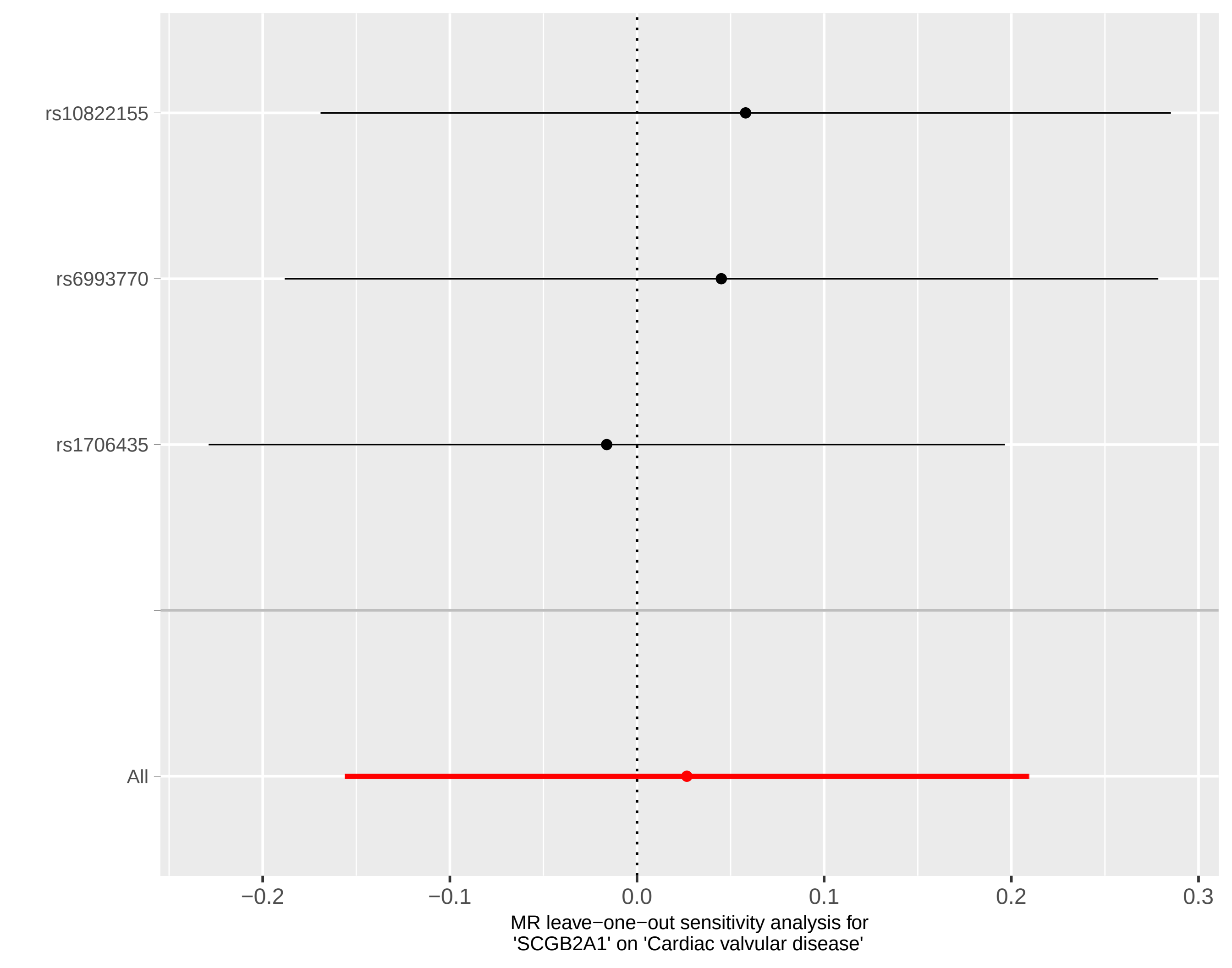

rs10822155
rs6993770
rs1706435
All
−0.2
−0.1
0.0
0.1
0.2
0.3
MR leave−one−out sensitivity analysis for
'SCGB2A1' on 'Cardiac valvular disease'

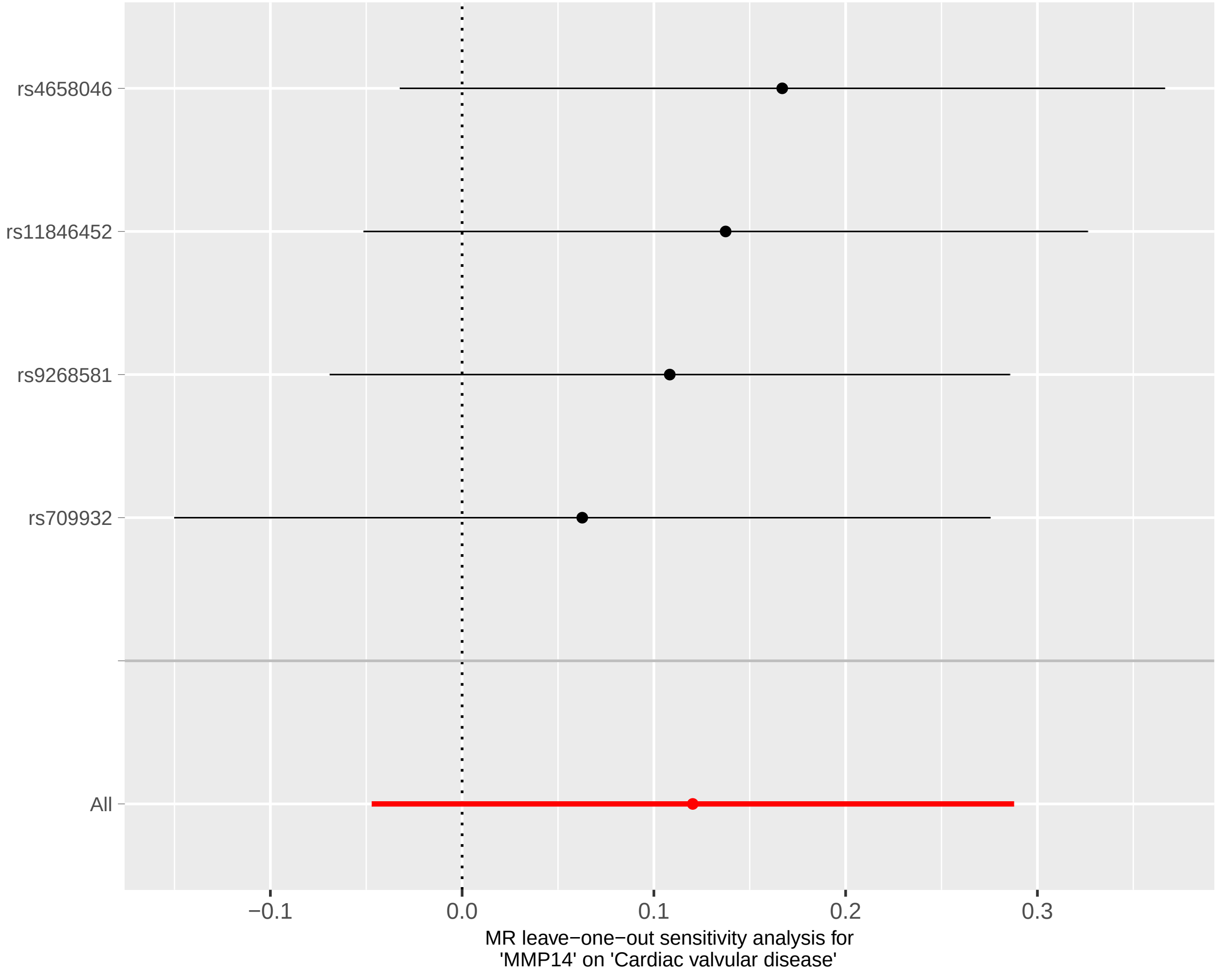
rs4658046
rs11846452
rs9268581
rs709932
All
−0.1
0.0
0.1
0.2
0.3
MR leave−one−out sensitivity analysis for
'MMP14' on 'Cardiac valvular disease'

Insufficient number of SNPs

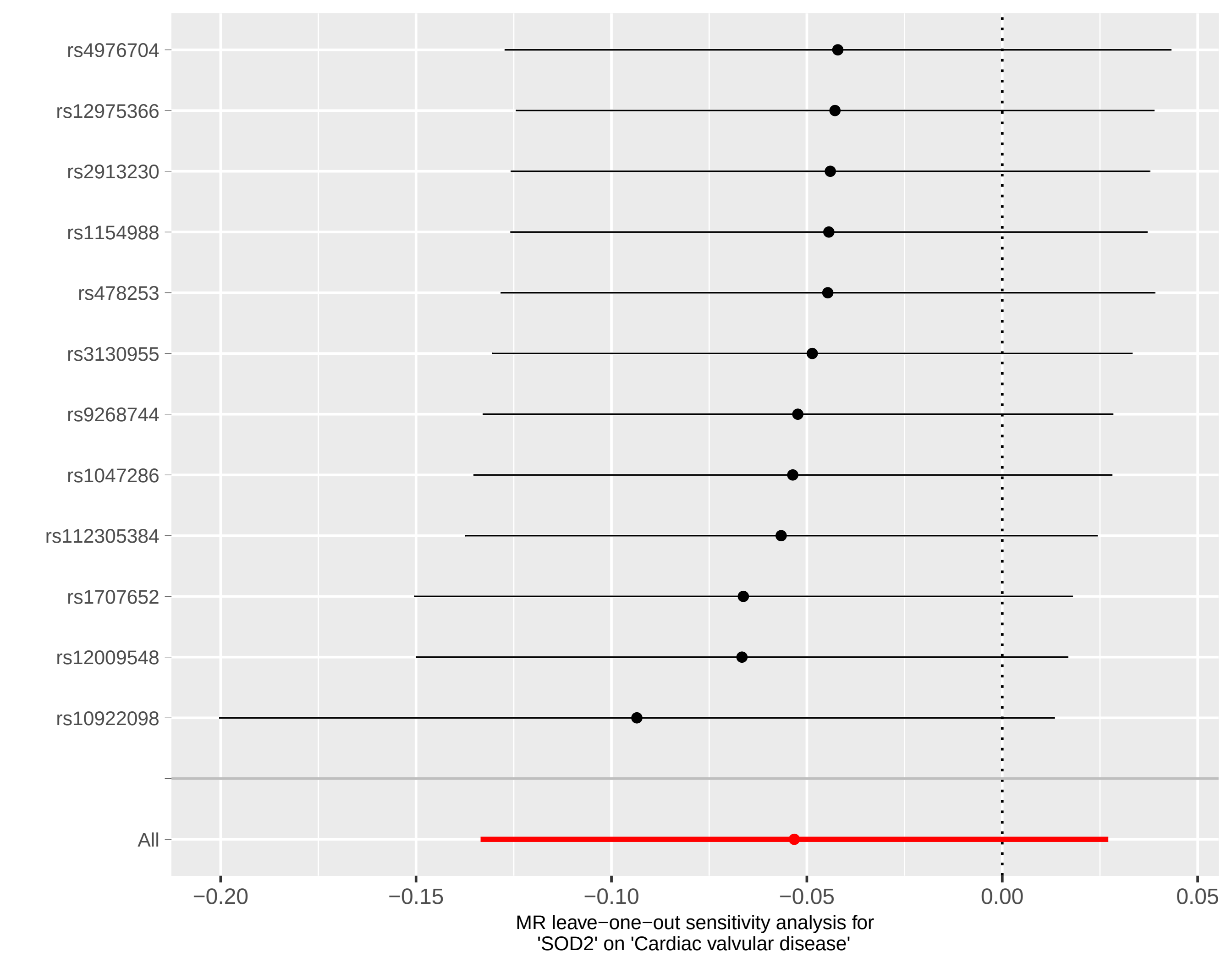
rs4976704
rs12975366
rs2913230
rs1154988
rs478253
rs3130955
rs9268744
rs1047286
rs112305384
rs1707652
rs12009548
rs10922098
All
−0.20
−0.15
−0.10
−0.05
0.00
0.05
MR leave−one−out sensitivity analysis for
'SOD2' on 'Cardiac valvular disease'

Insufficient number of SNPs

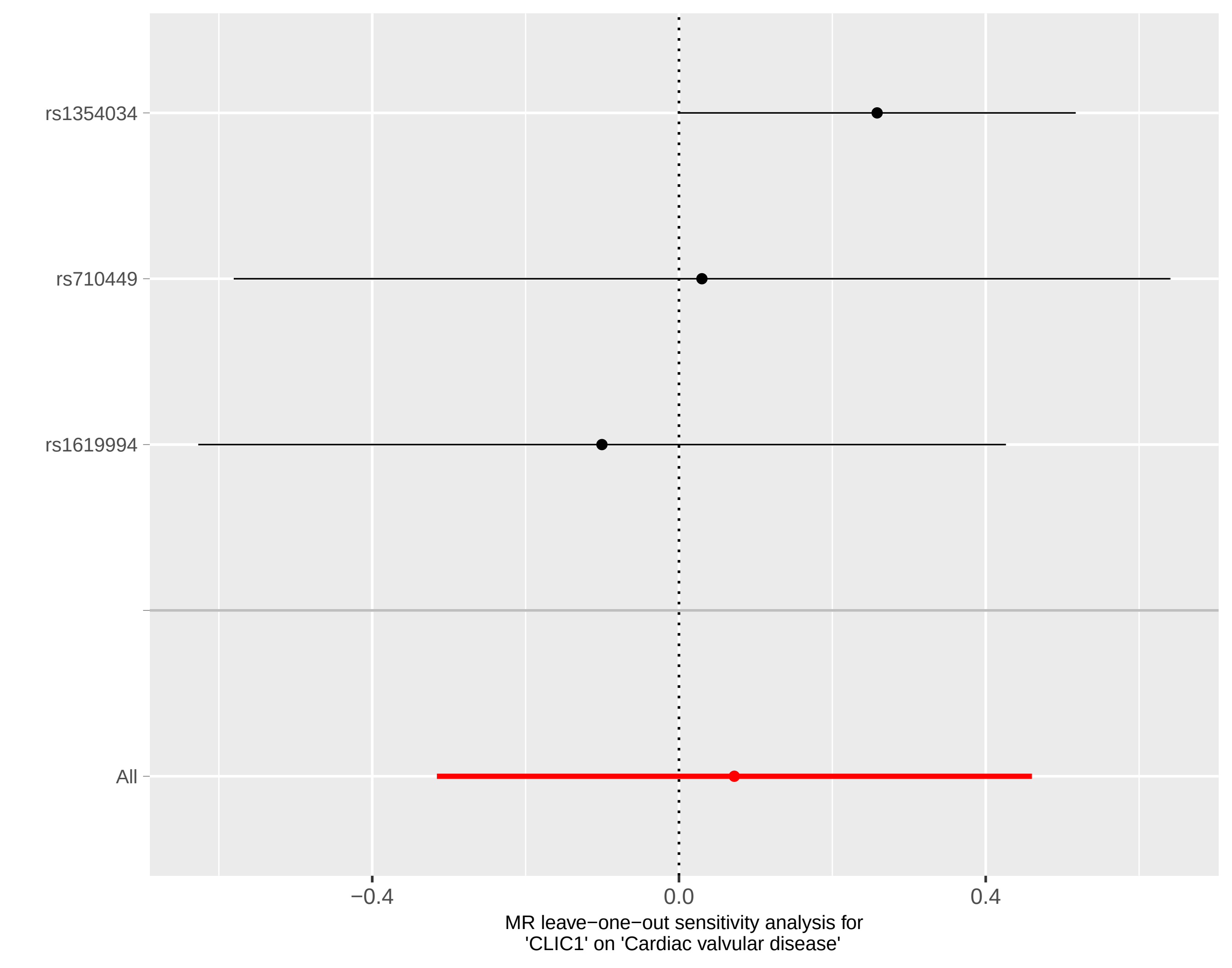

rs1354034
rs710449
rs1619994
All
−0.4
0.0
0.4
MR leave−one−out sensitivity analysis for
'CLIC1' on 'Cardiac valvular disease'

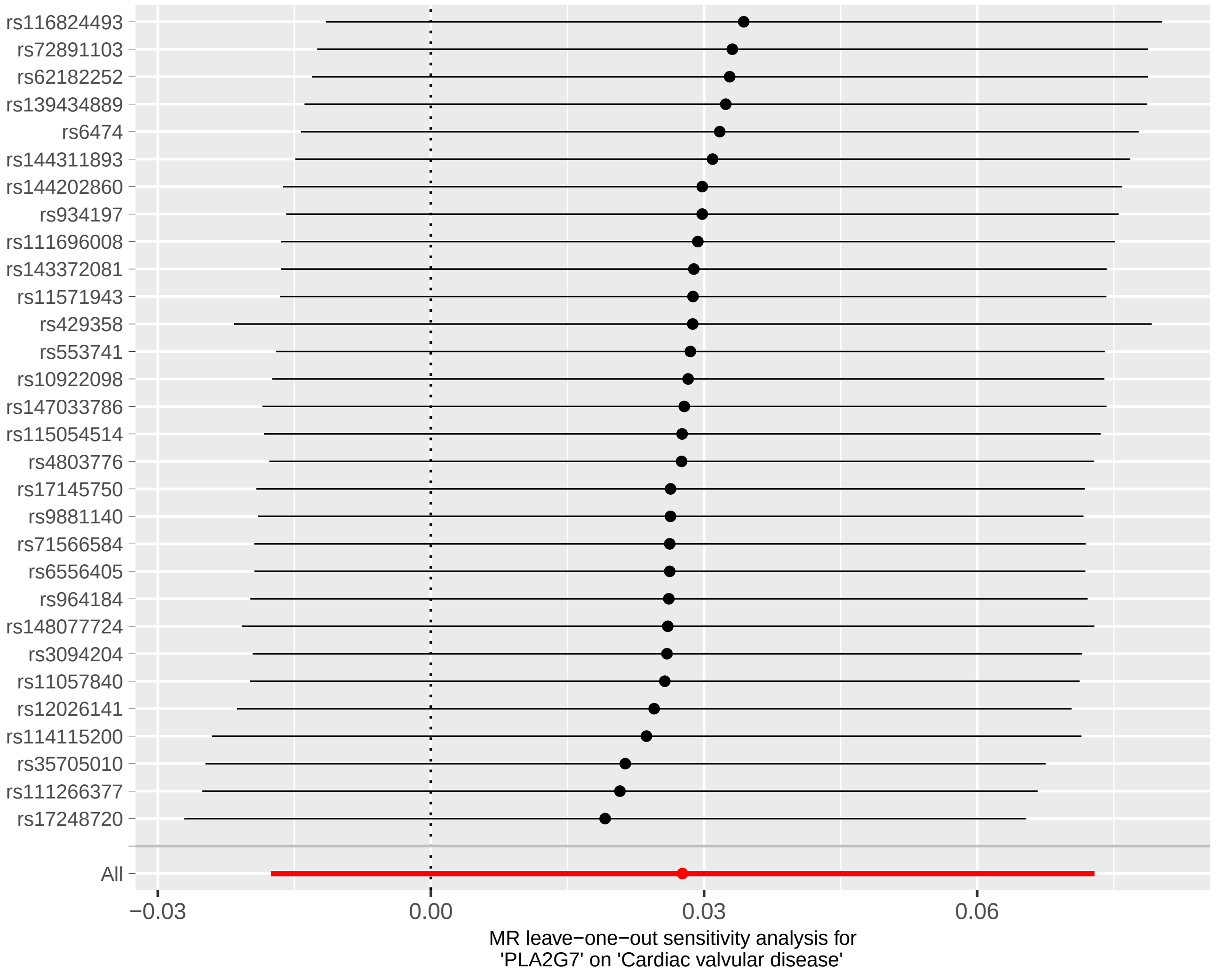
rs116824493
rs72891103
rs62182252
rs139434889
rs6474
rs144311893
rs144202860
rs934197
rs111696008
rs143372081
rs11571943
rs429358
rs553741
rs10922098
rs147033786
rs115054514
rs4803776
rs17145750
rs9881140
rs71566584
rs6556405
rs964184
rs148077724
rs3094204
rs11057840
rs12026141
rs114115200
rs35705010
rs111266377
rs17248720
All
−0.03
0.00
0.03
0.06
MR leave−one−out sensitivity analysis for
'PLA2G7' on 'Cardiac valvular disease'

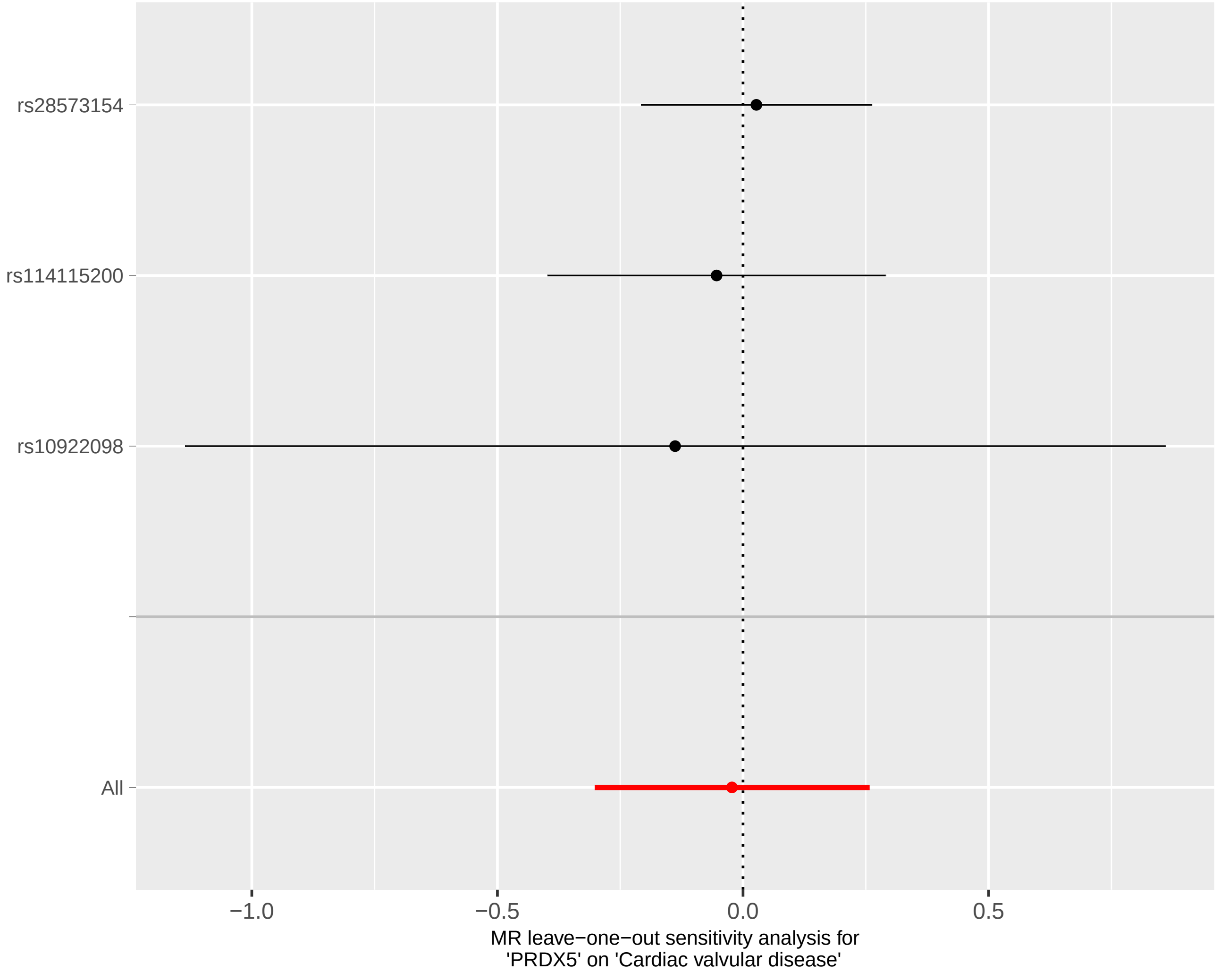

rs28573154
rs114115200
rs10922098
All
−1.0
−0.5
0.0
0.5
MR leave−one−out sensitivity analysis for
'PRDX5' on 'Cardiac valvular disease'

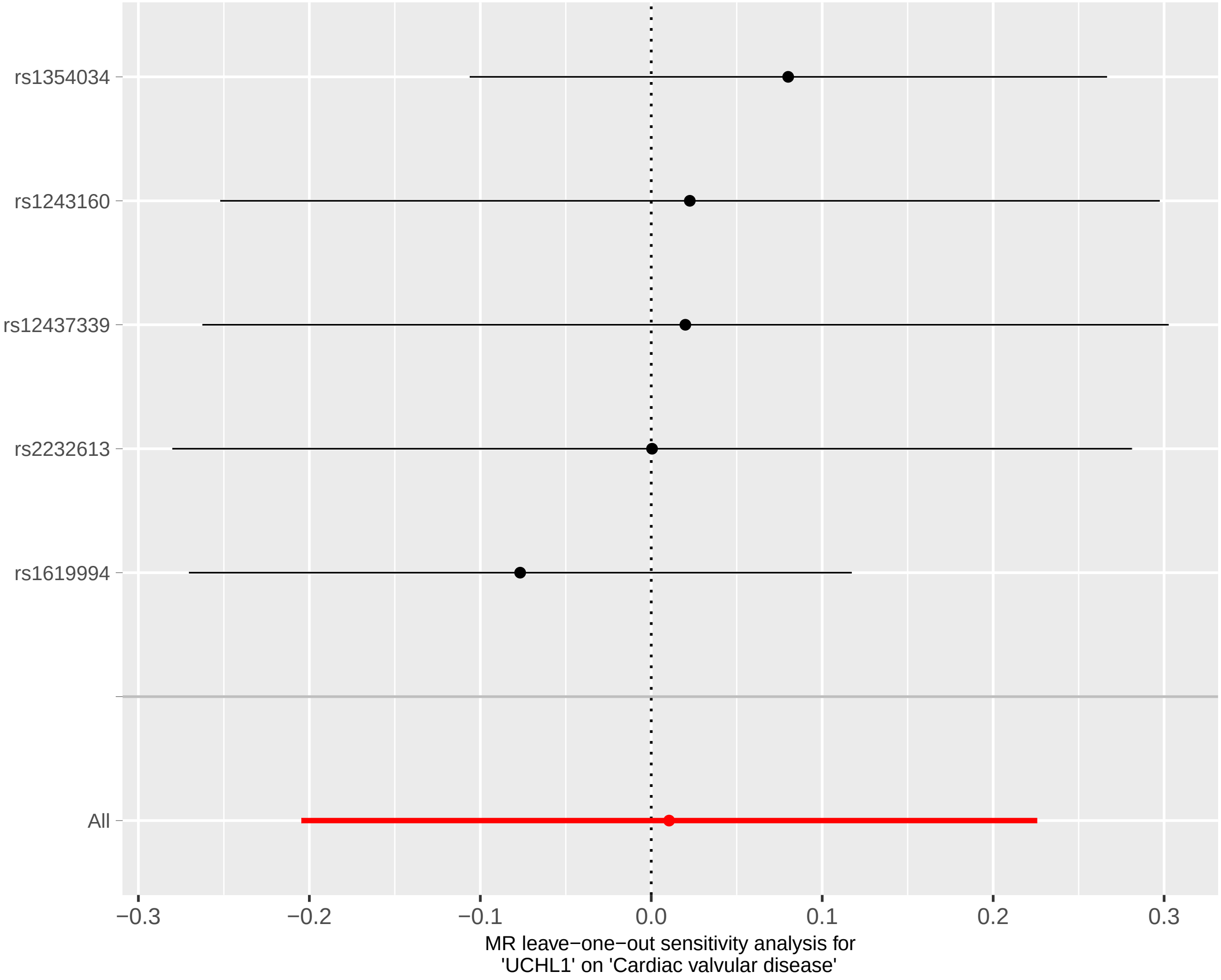
rs1354034
rs1243160
rs12437339
rs2232613
rs1619994
All
−0.3
−0.2
−0.1
0.0
0.1
0.2
0.3
MR leave−one−out sensitivity analysis for
'UCHL1' on 'Cardiac valvular disease'

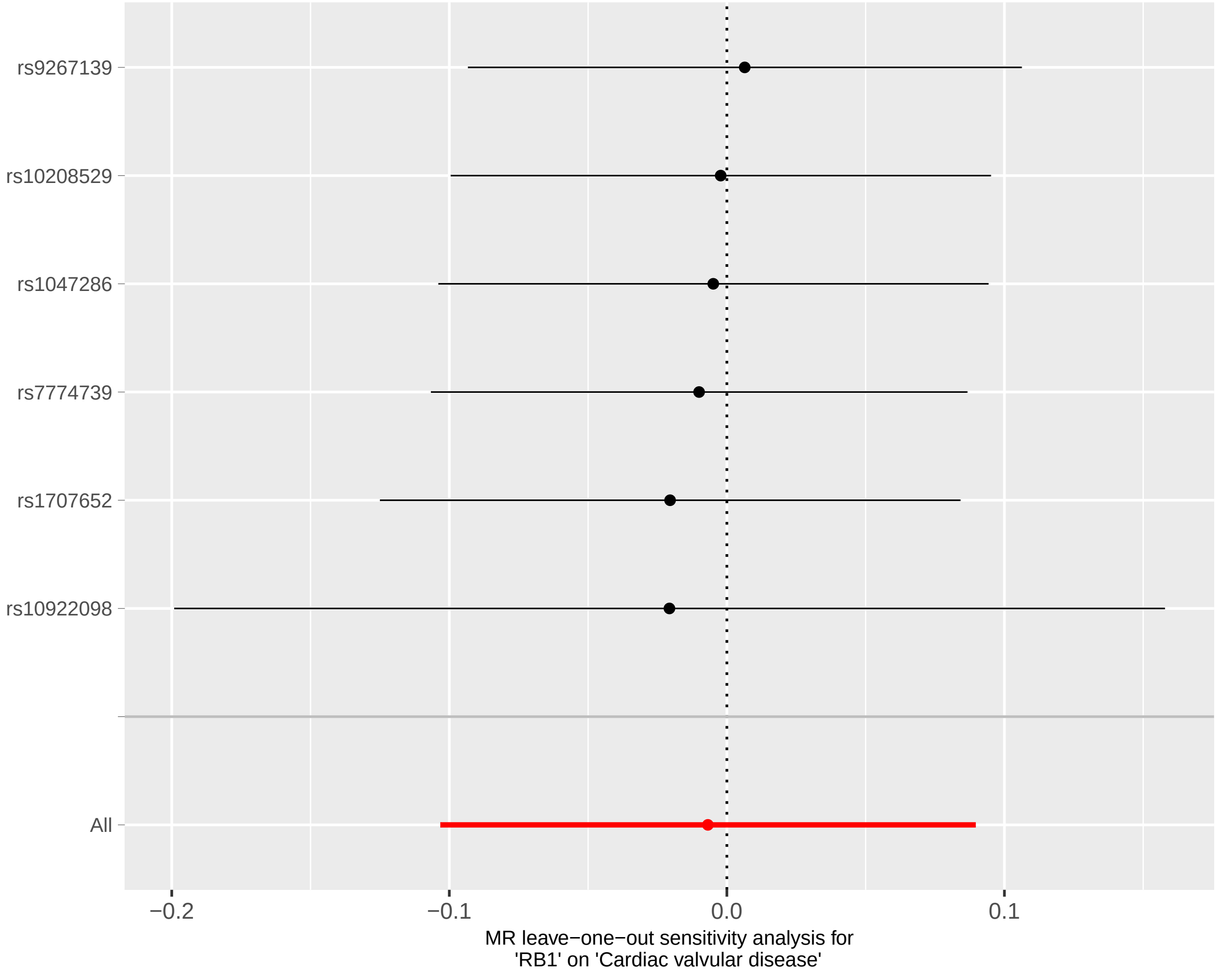

rs9267139
rs10208529
rs1047286
rs7774739
rs1707652
rs10922098
All
−0.2
−0.1
0.0
0.1
MR leave−one−out sensitivity analysis for
'RB1' on 'Cardiac valvular disease'

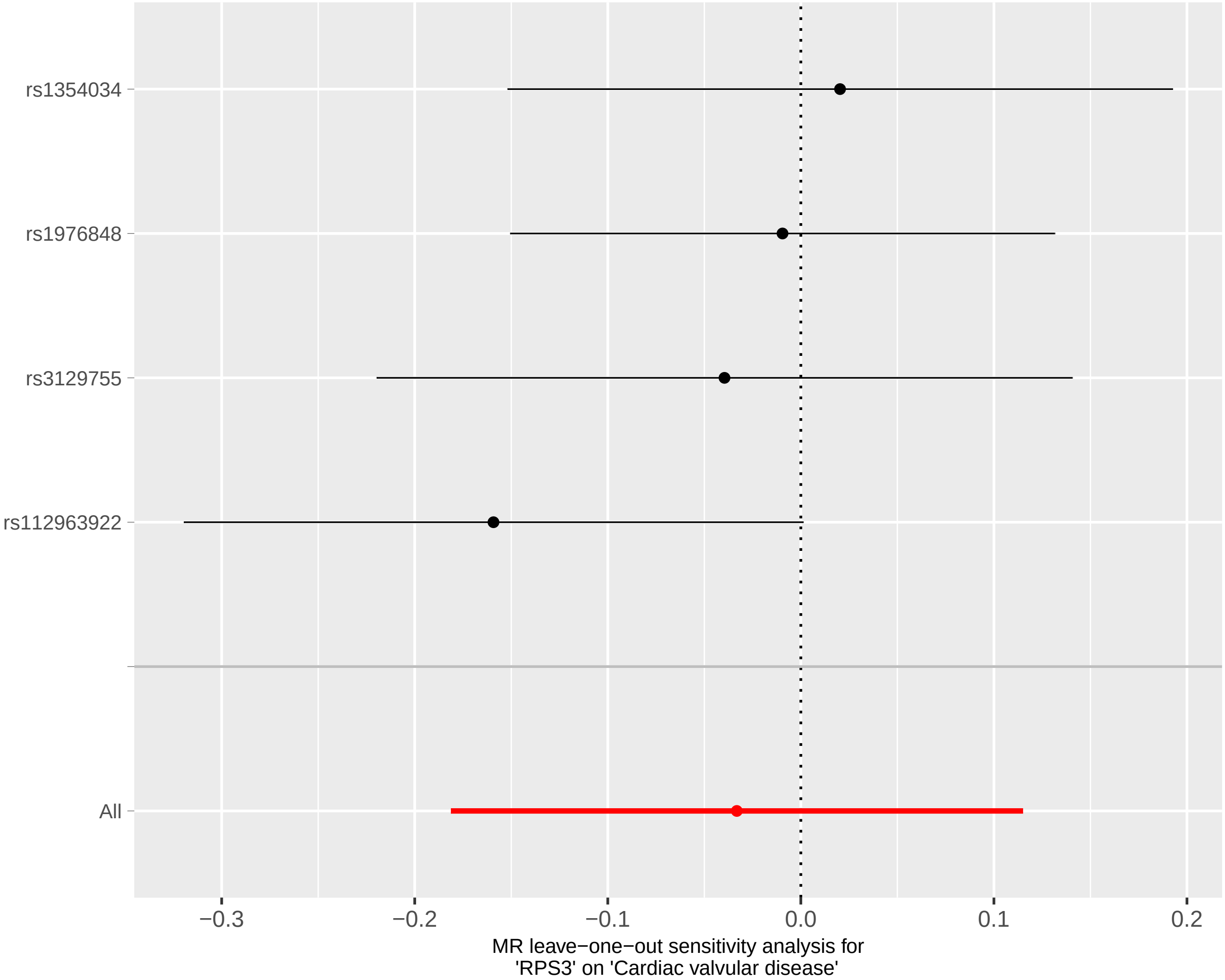

rs1354034
rs1976848
rs3129755
rs112963922
All
−0.3
−0.2
−0.1
0.0
0.1
0.2
MR leave−one−out sensitivity analysis for
'RPS3' on 'Cardiac valvular disease'

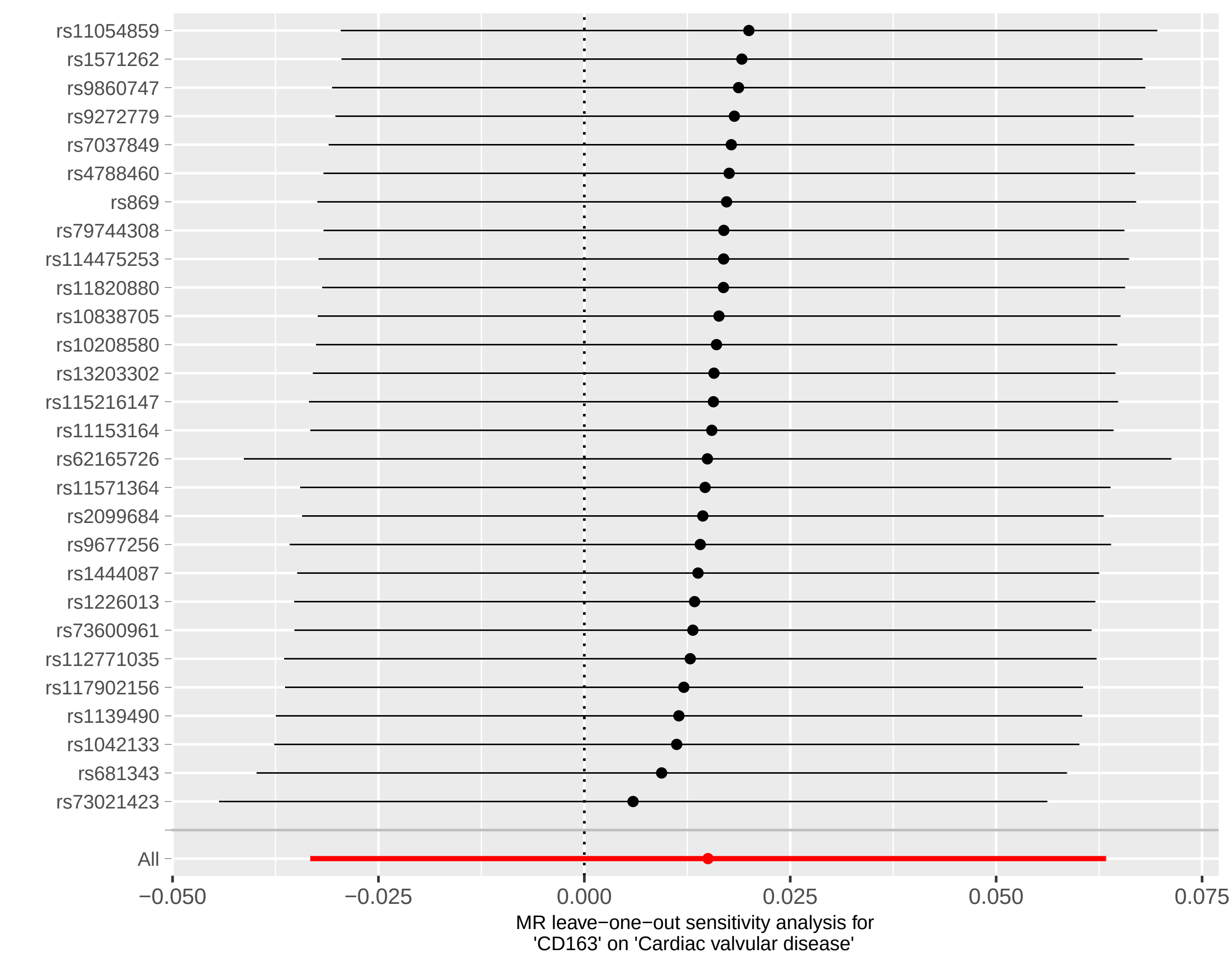

rs11054859
rs1571262
rs9860747
rs9272779
rs7037849
rs4788460
rs869
rs79744308
rs114475253
rs11820880
rs10838705
rs10208580
rs13203302
rs115216147
rs11153164
rs62165726
rs11571364
rs2099684
rs9677256
rs1444087
rs1226013
rs73600961
rs112771035
rs117902156
rs1139490
rs1042133
rs681343
rs73021423
All
−0.050
−0.025
0.000
0.025
0.050
0.075
MR leave−one−out sensitivity analysis for
'CD163' on 'Cardiac valvular disease'

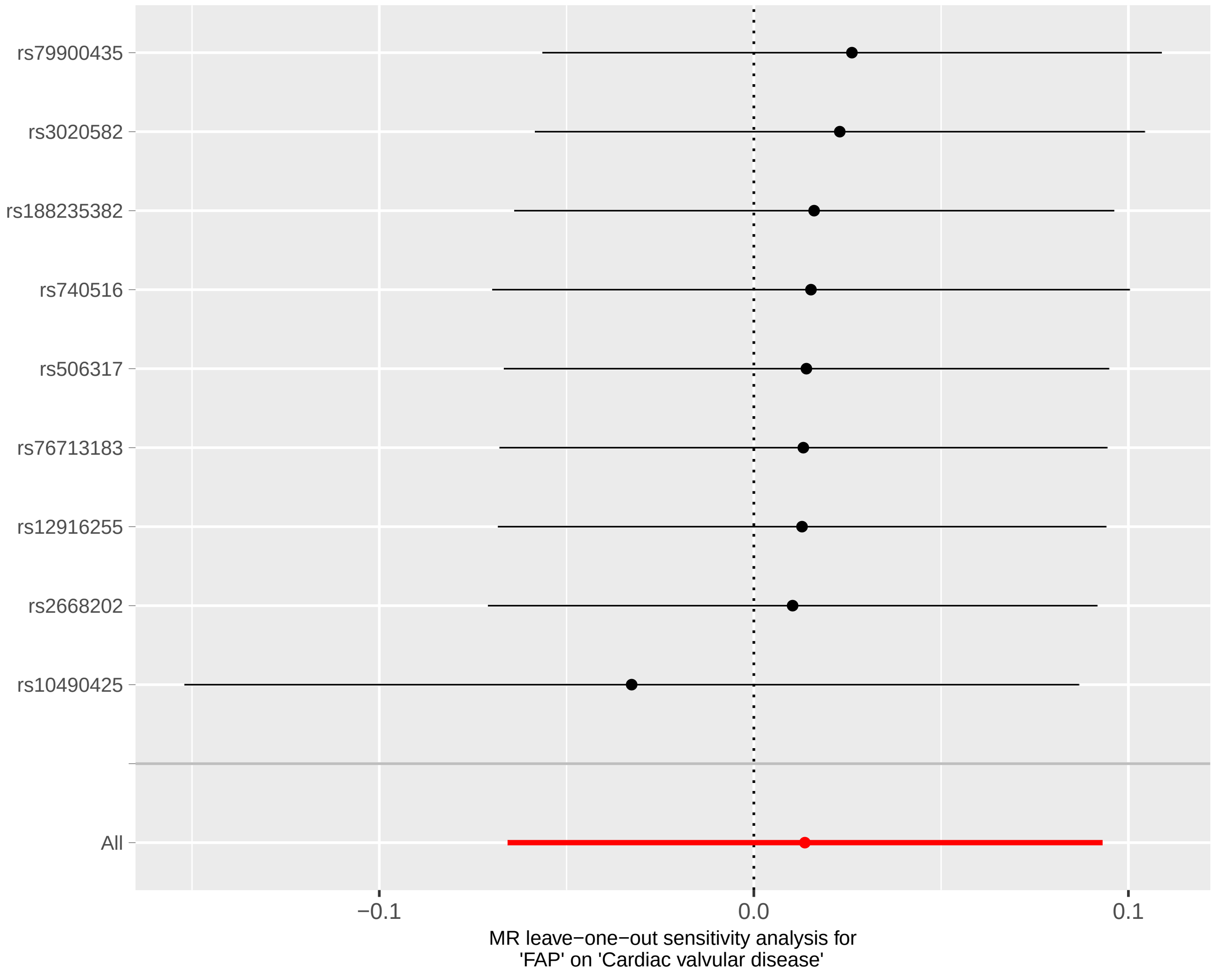

rs79900435
rs3020582
rs188235382
rs740516
rs506317
rs76713183
rs12916255
rs2668202
rs10490425
All
−0.1
0.0
0.1
MR leave−one−out sensitivity analysis for
'FAP' on 'Cardiac valvular disease'

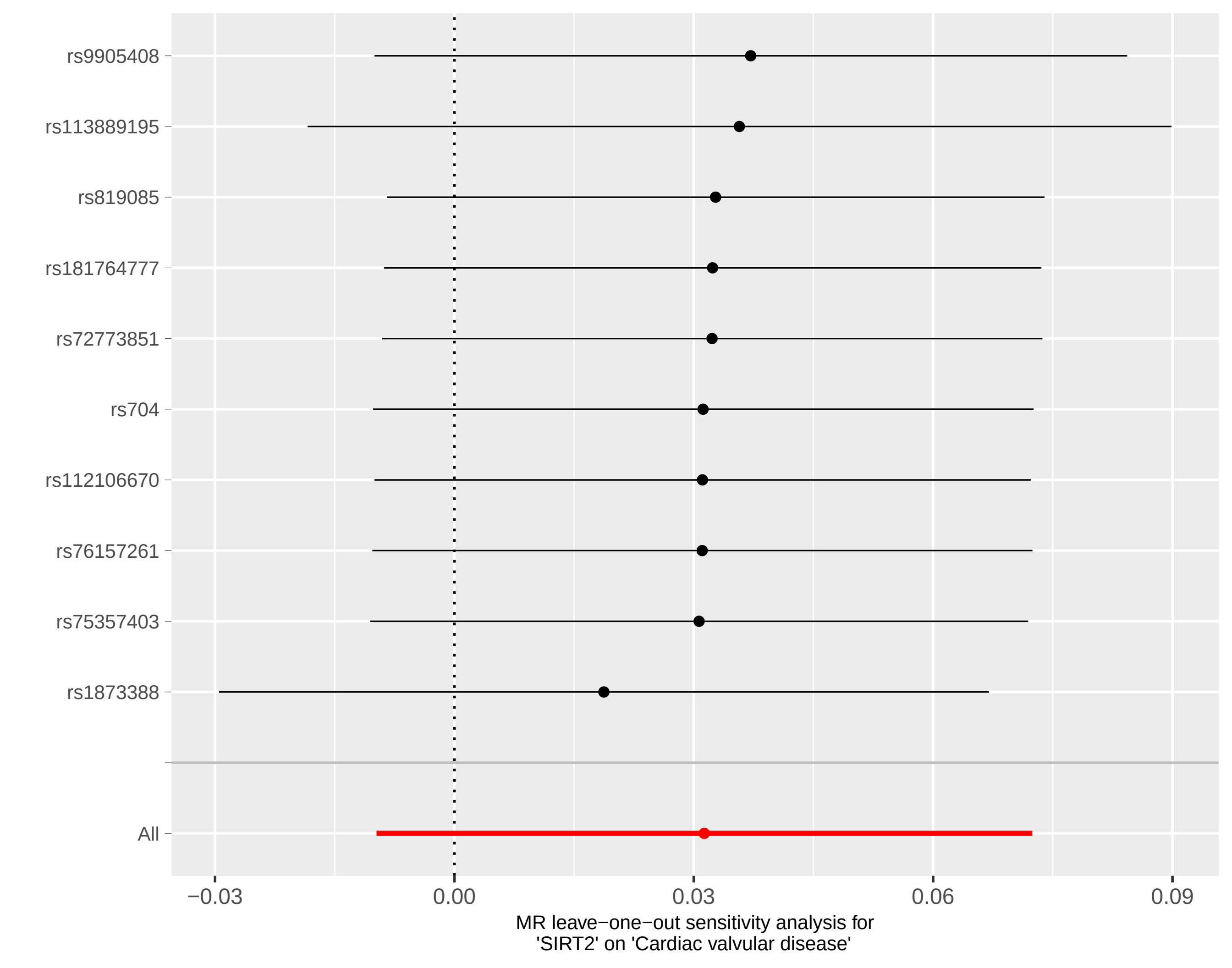
rs9905408
rs113889195
rs819085
rs181764777
rs72773851
rs704
rs112106670
rs76157261
rs75357403
rs1873388
All
−0.03
0.00
0.03
0.06
0.09
MR leave−one−out sensitivity analysis for
'SIRT2' on 'Cardiac valvular disease'

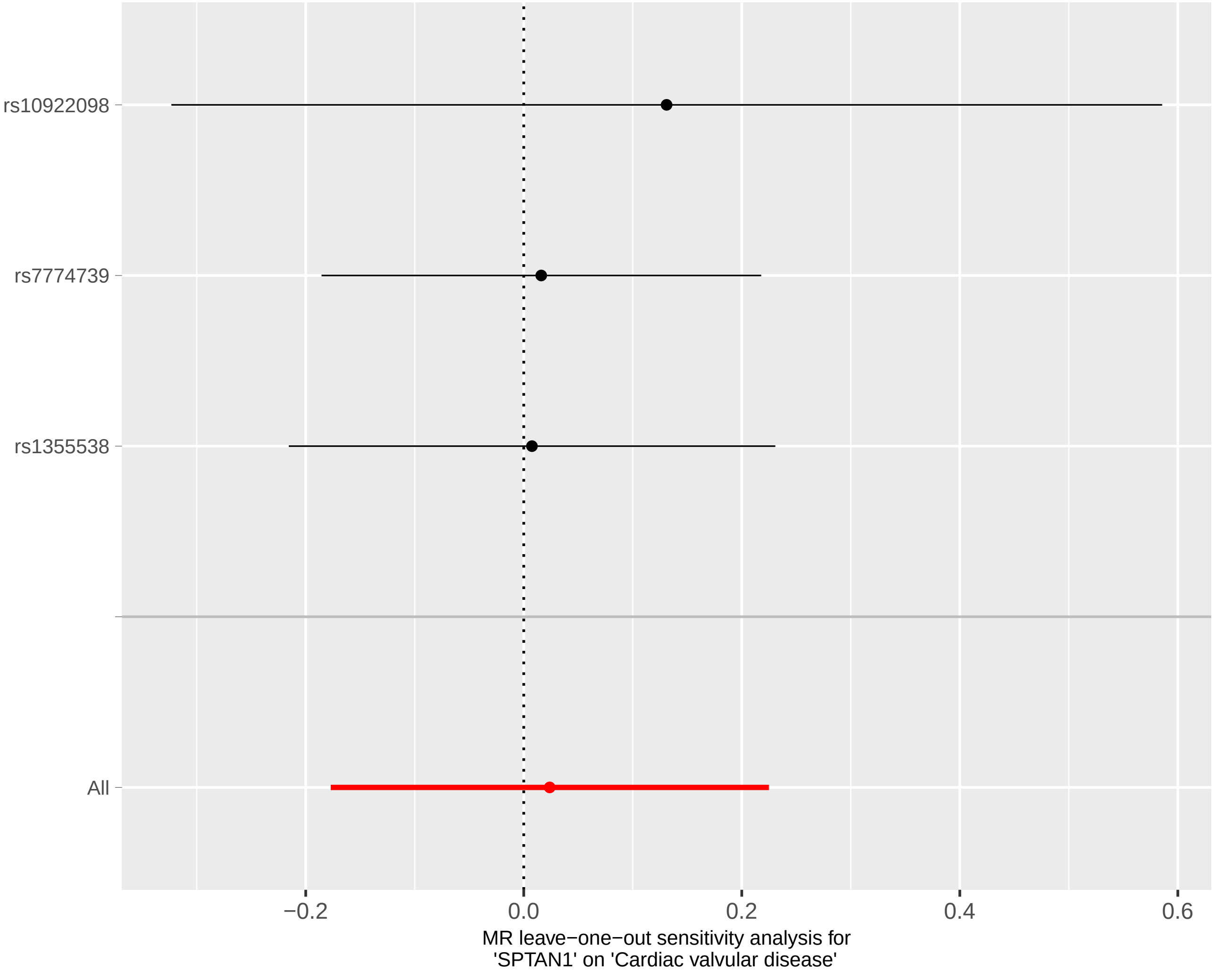
rs10922098
rs7774739
rs1355538
All
−0.2
0.0
0.2
0.4
0.6
MR leave−one−out sensitivity analysis for
'SPTAN1' on 'Cardiac valvular disease'

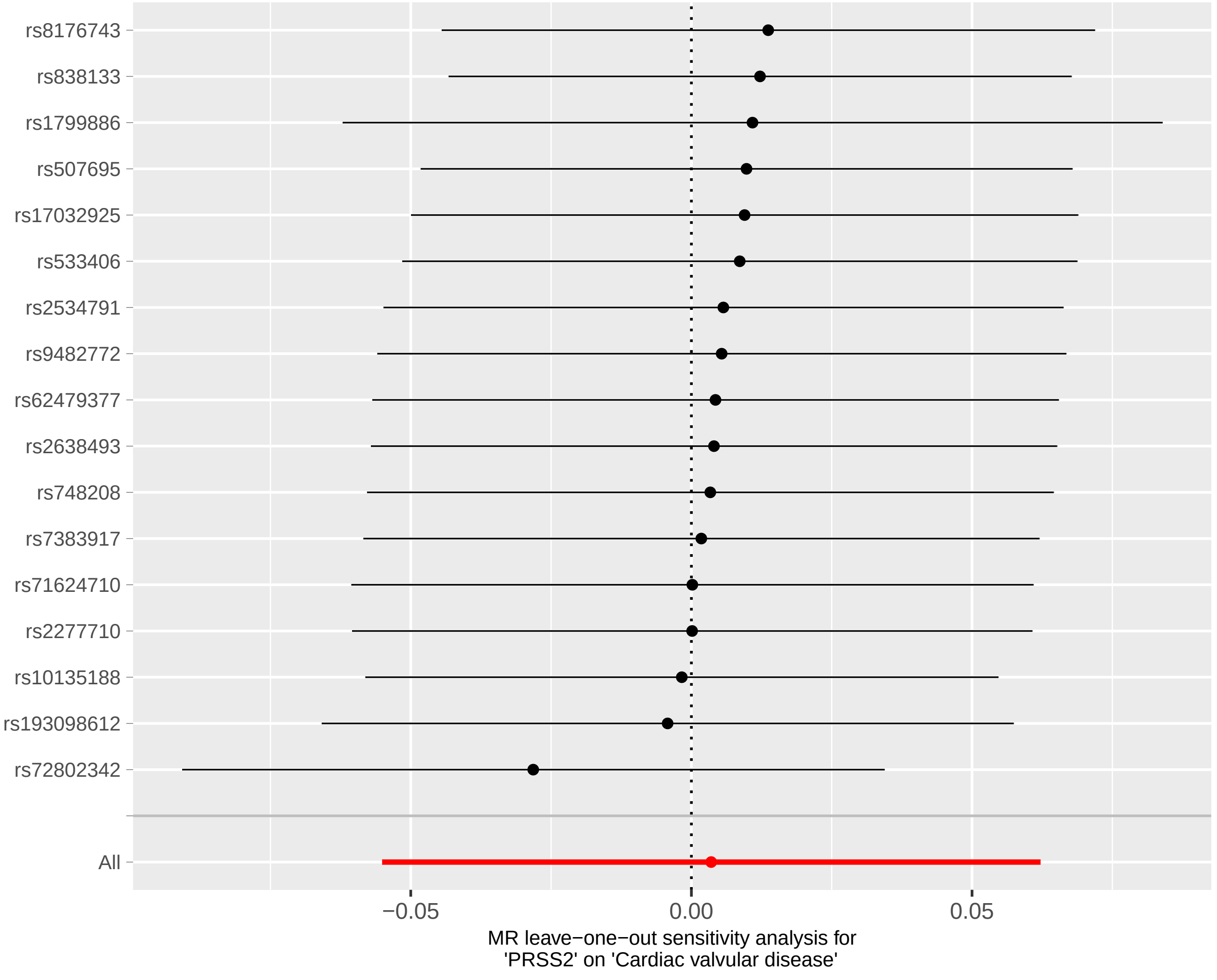

rs8176743
rs838133
rs1799886
rs507695
rs17032925
rs533406
rs2534791
rs9482772
rs62479377
rs2638493
rs748208
rs7383917
rs71624710
rs2277710
rs10135188
rs193098612
rs72802342
All
−0.05
0.00
0.05
MR leave−one−out sensitivity analysis for
'PRSS2' on 'Cardiac valvular disease'

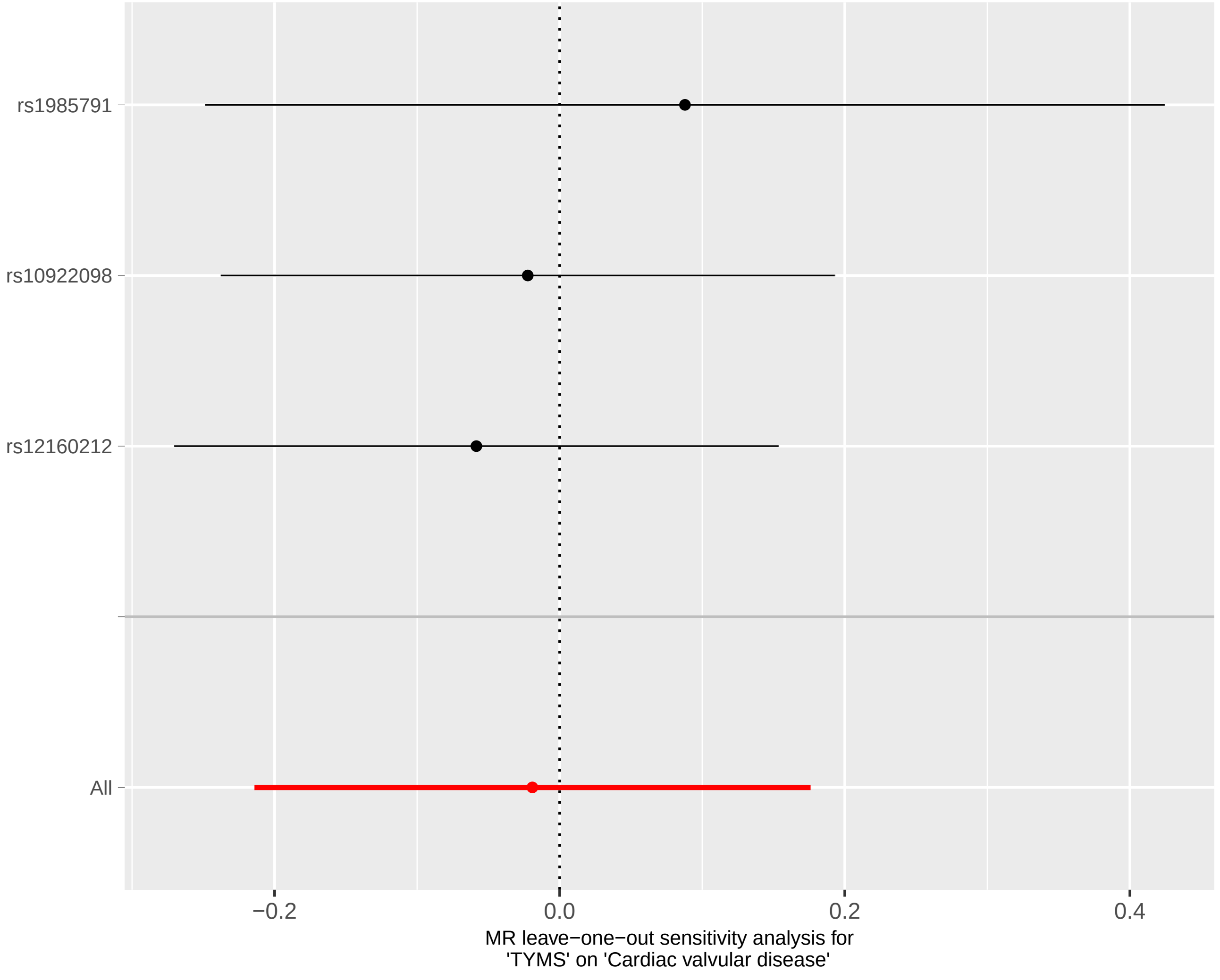
rs1985791
rs10922098
rs12160212
All
−0.2
0.0
0.2
0.4
MR leave−one−out sensitivity analysis for
'TYMS' on 'Cardiac valvular disease'

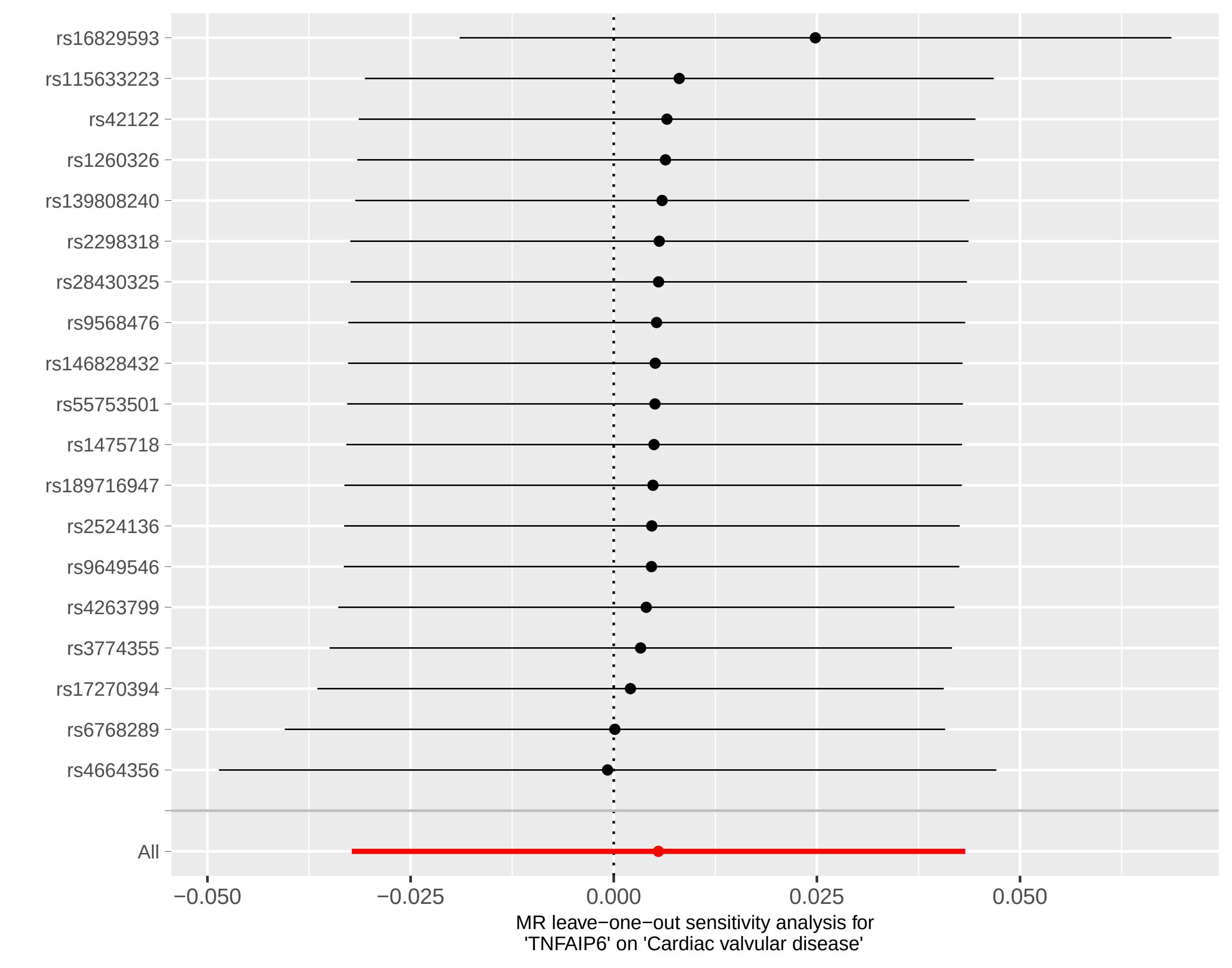
rs16829593
rs115633223
rs42122
rs1260326
rs139808240
rs2298318
rs28430325
rs9568476
rs146828432
rs55753501
rs1475718
rs189716947
rs2524136
rs9649546
rs4263799
rs3774355
rs17270394
rs6768289
rs4664356
All
−0.050
−0.025
0.000
0.025
0.050
MR leave−one−out sensitivity analysis for
'TNFAIP6' on 'Cardiac valvular disease'

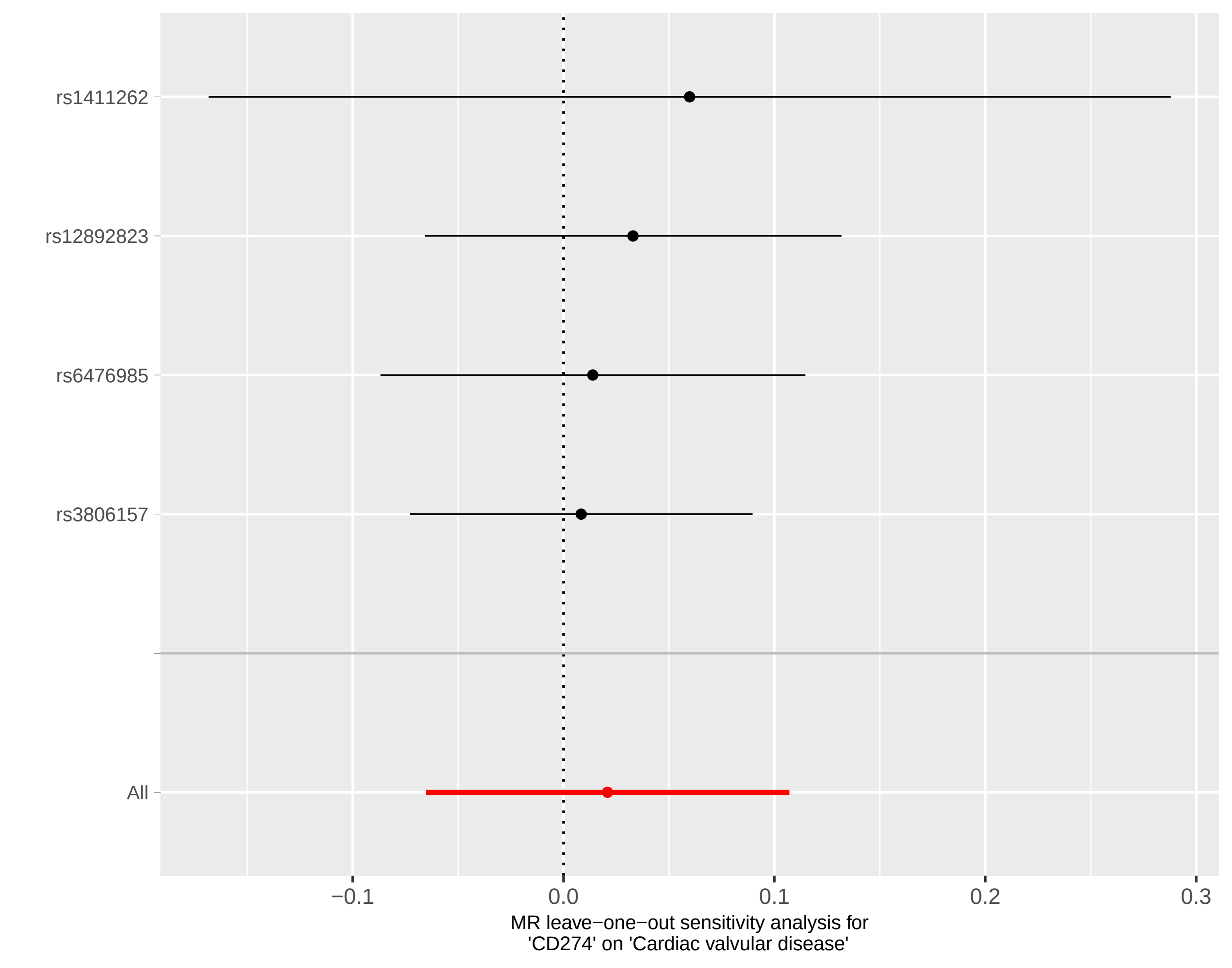
rs1411262
rs12892823
rs6476985
rs3806157
All
−0.1
0.0
0.1
0.2
0.3
MR leave−one−out sensitivity analysis for
'CD274' on 'Cardiac valvular disease'

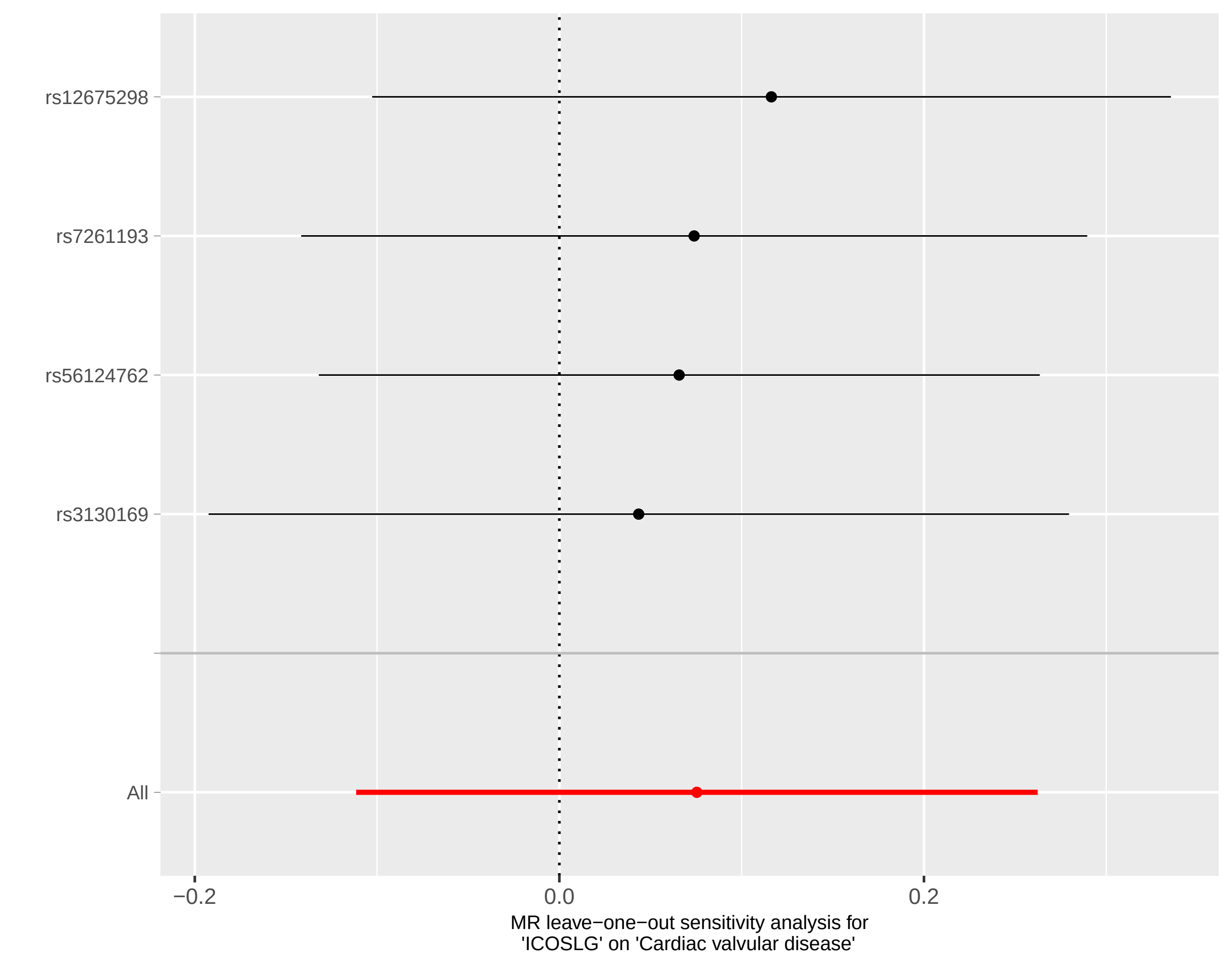
rs12675298
rs7261193
rs56124762
rs3130169
All
−0.2
0.0
0.2
MR leave−one−out sensitivity analysis for
'ICOSLG' on 'Cardiac valvular disease'

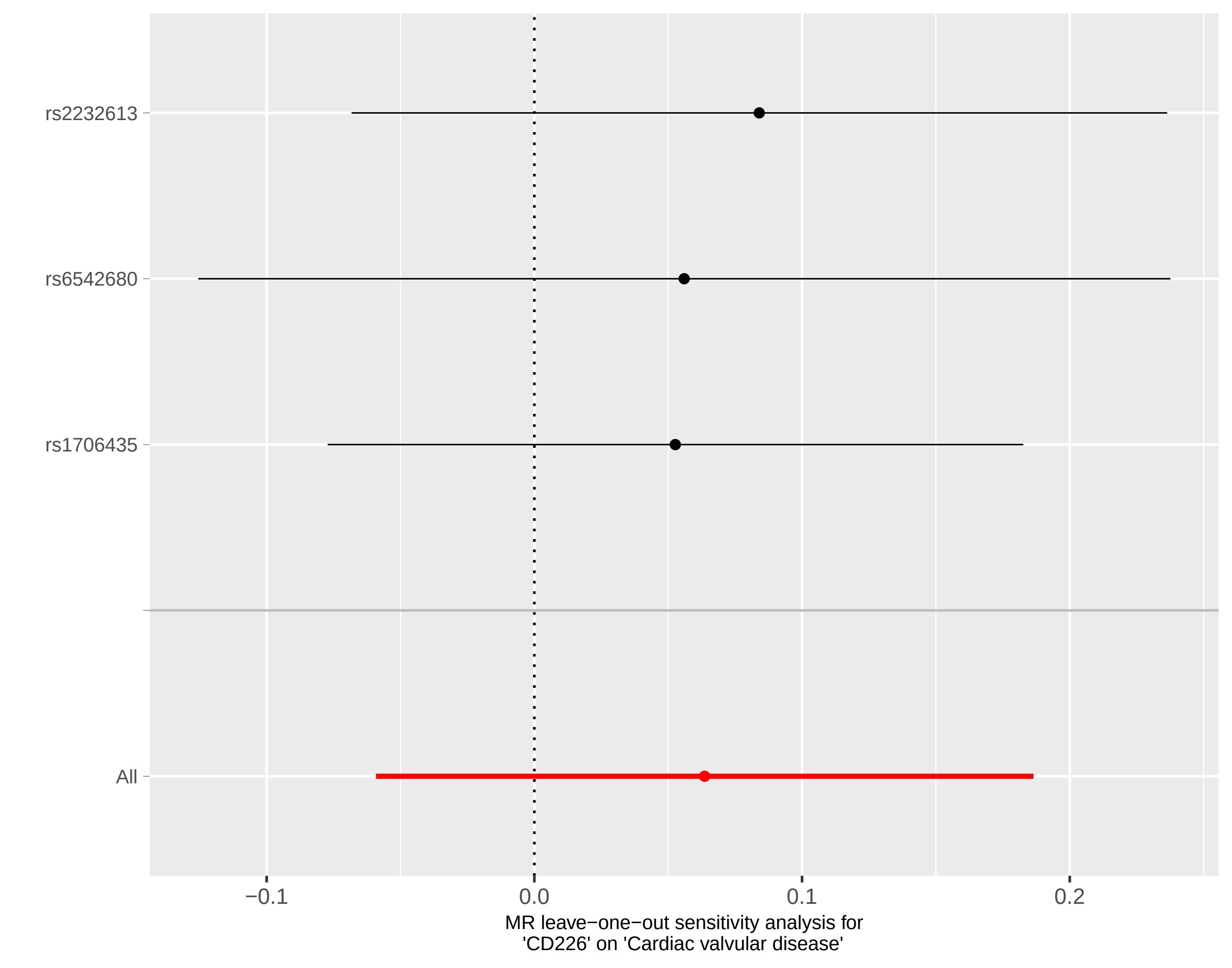
rs2232613
rs6542680
rs1706435
All
−0.1
0.0
0.1
0.2
MR leave−one−out sensitivity analysis for
'CD226' on 'Cardiac valvular disease'

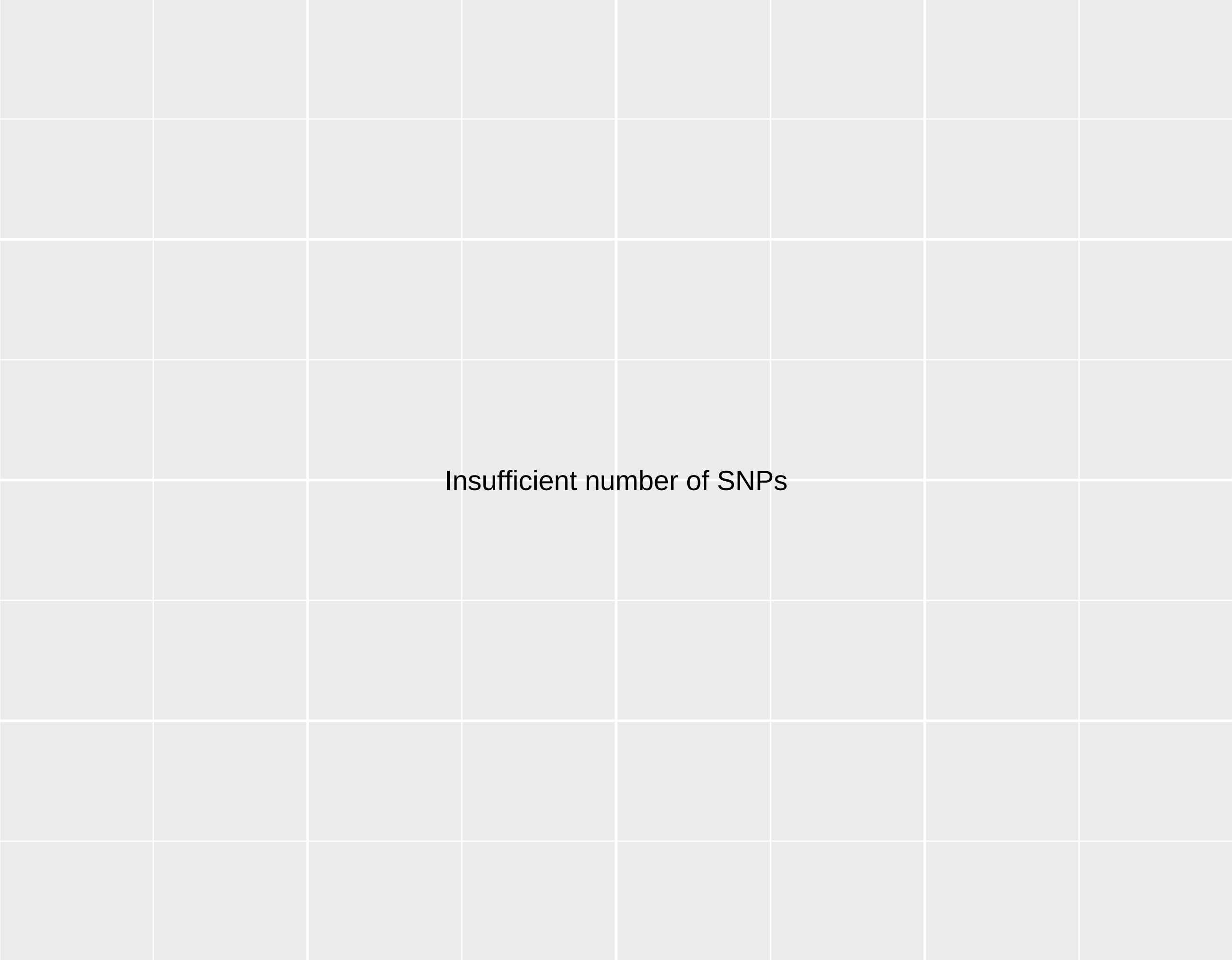
Insufficient number of SNPs

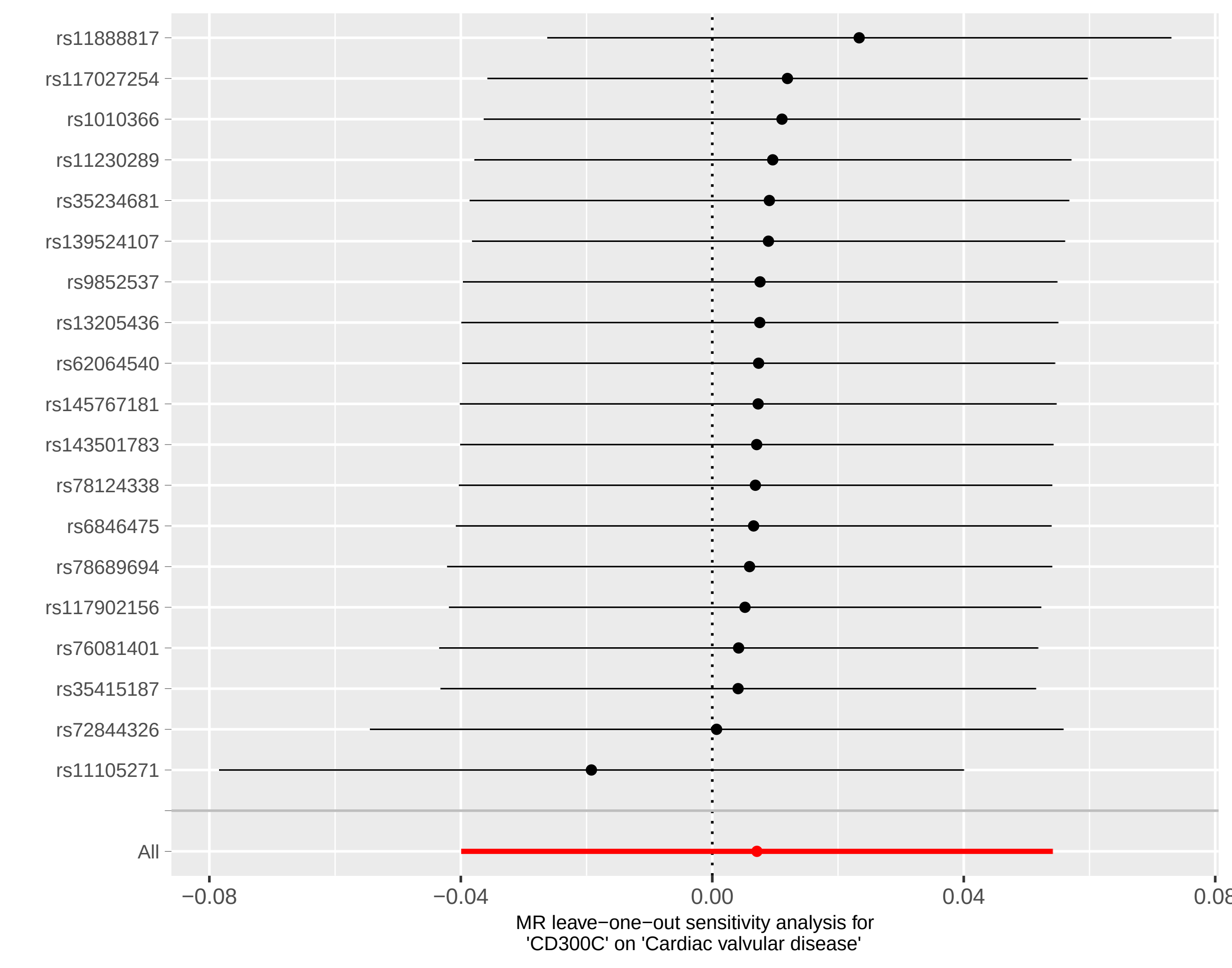

rs11888817
rs117027254
rs1010366
rs11230289
rs35234681
rs139524107
rs9852537
rs13205436
rs62064540
rs145767181
rs143501783
rs78124338
rs6846475
rs78689694
rs117902156
rs76081401
rs35415187
rs72844326
rs11105271
All
−0.08
−0.04
0.00
0.04
0.08
MR leave−one−out sensitivity analysis for
'CD300C' on 'Cardiac valvular disease'

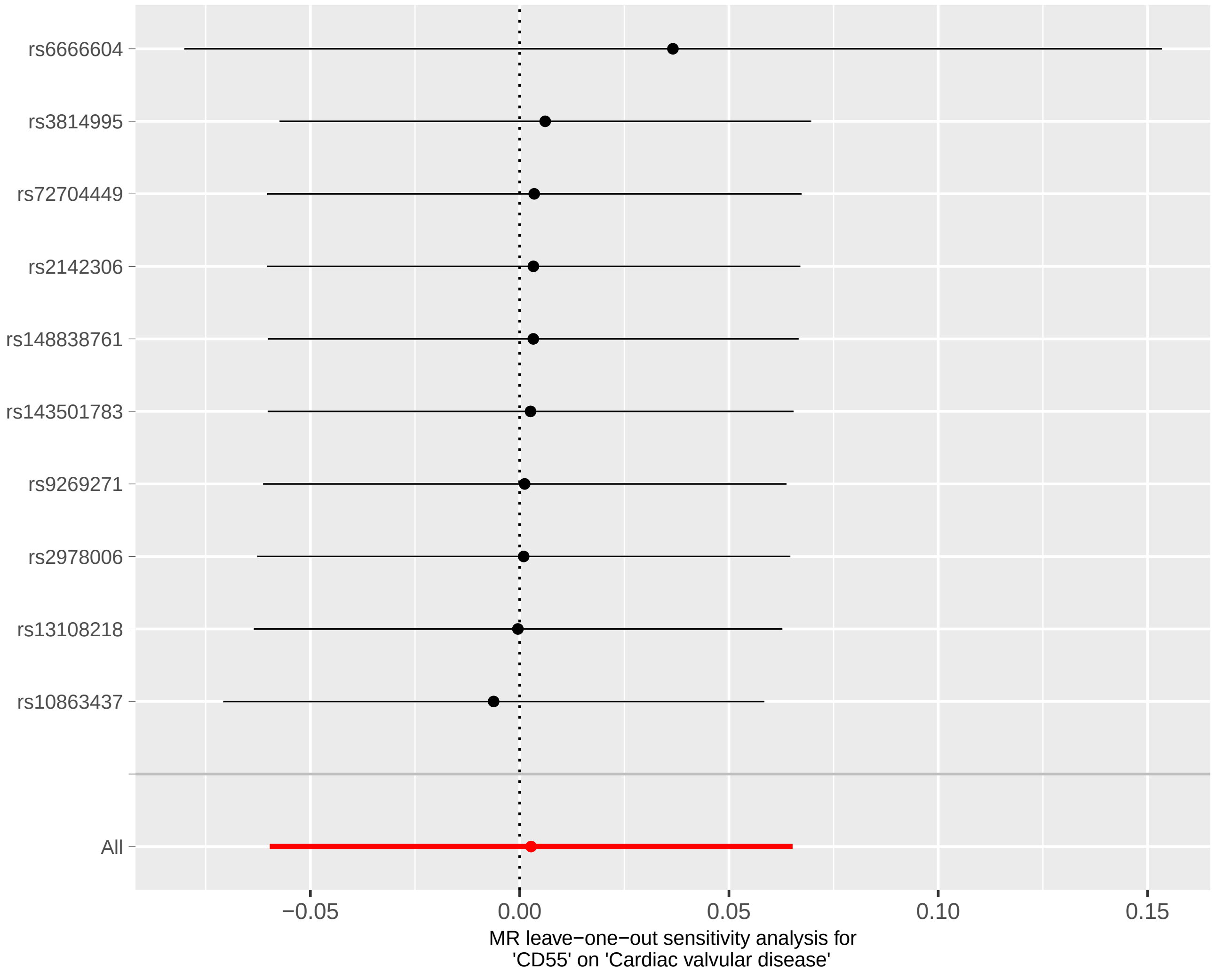
rs6666604
rs3814995
rs72704449
rs2142306
rs148838761
rs143501783
rs9269271
rs2978006
rs13108218
rs10863437
All
−0.05
0.00
0.05
0.10
0.15
MR leave−one−out sensitivity analysis for
'CD55' on 'Cardiac valvular disease'

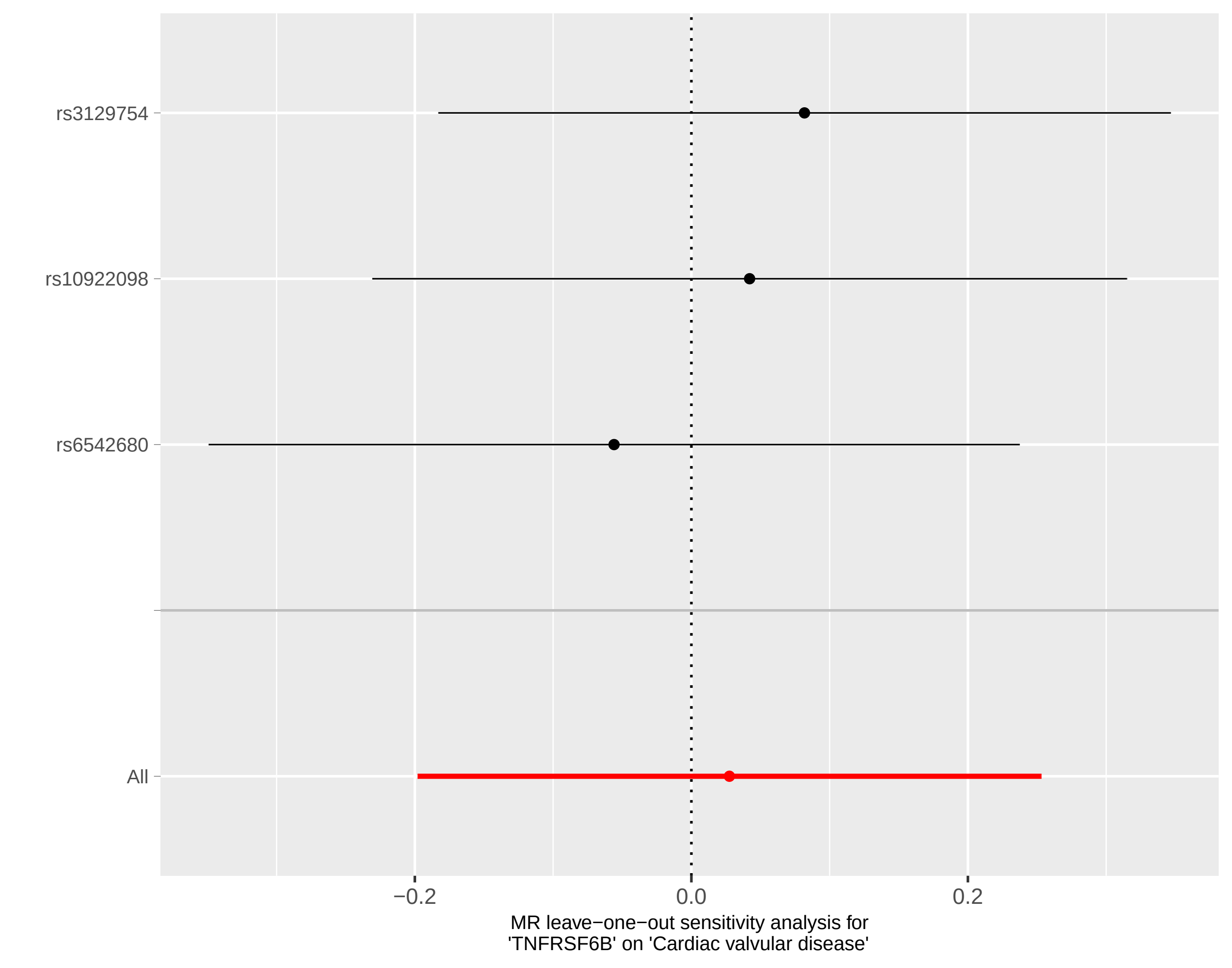
rs3129754
rs10922098
rs6542680
All
−0.2
0.0
0.2
MR leave−one−out sensitivity analysis for
'TNFRSF6B' on 'Cardiac valvular disease'

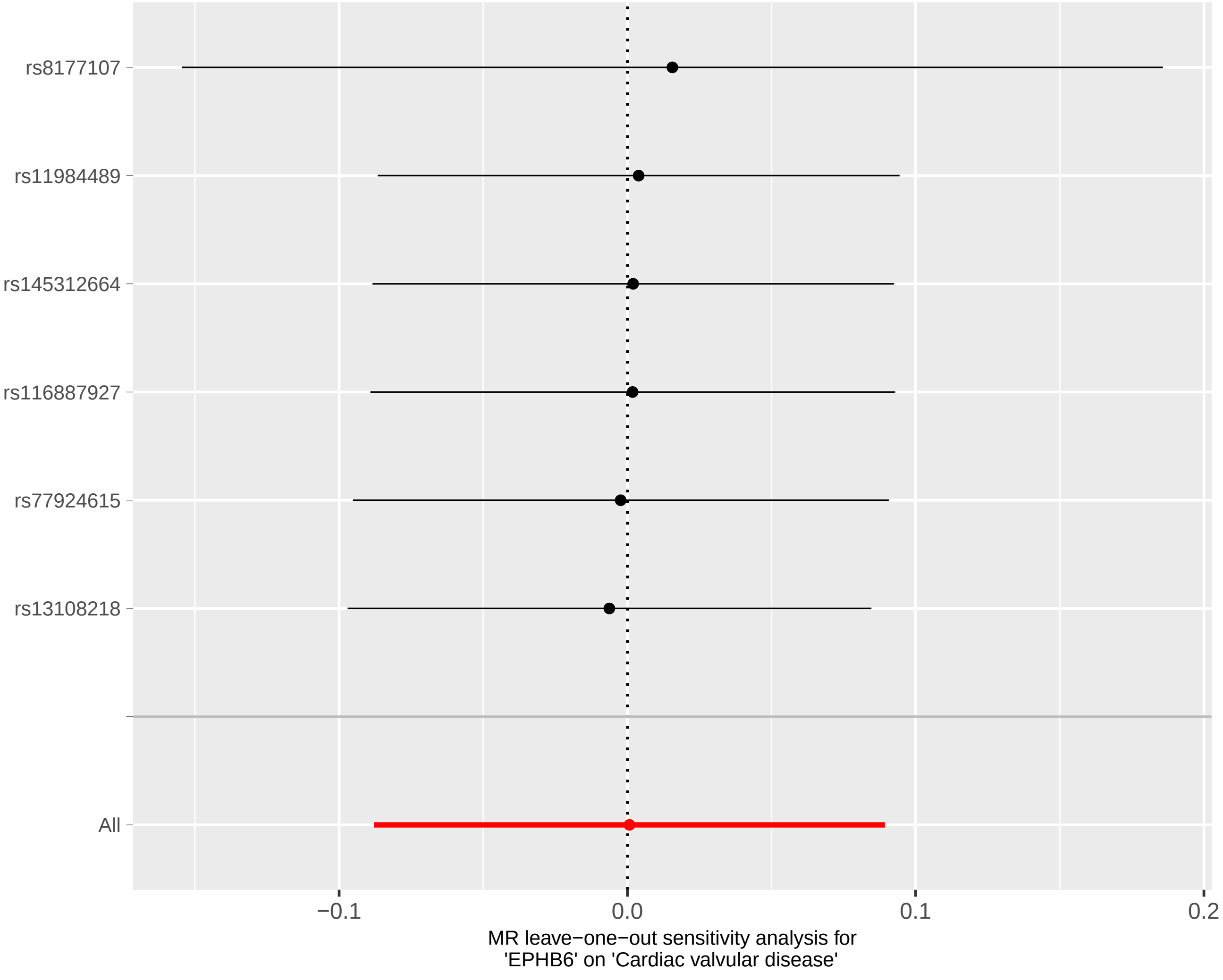
rs8177107
rs11984489
rs145312664
rs116887927
rs77924615
rs13108218
All
−0.1
0.0
0.1
0.2
MR leave−one−out sensitivity analysis for
'EPHB6' on 'Cardiac valvular disease'

Insufficient number of SNPs

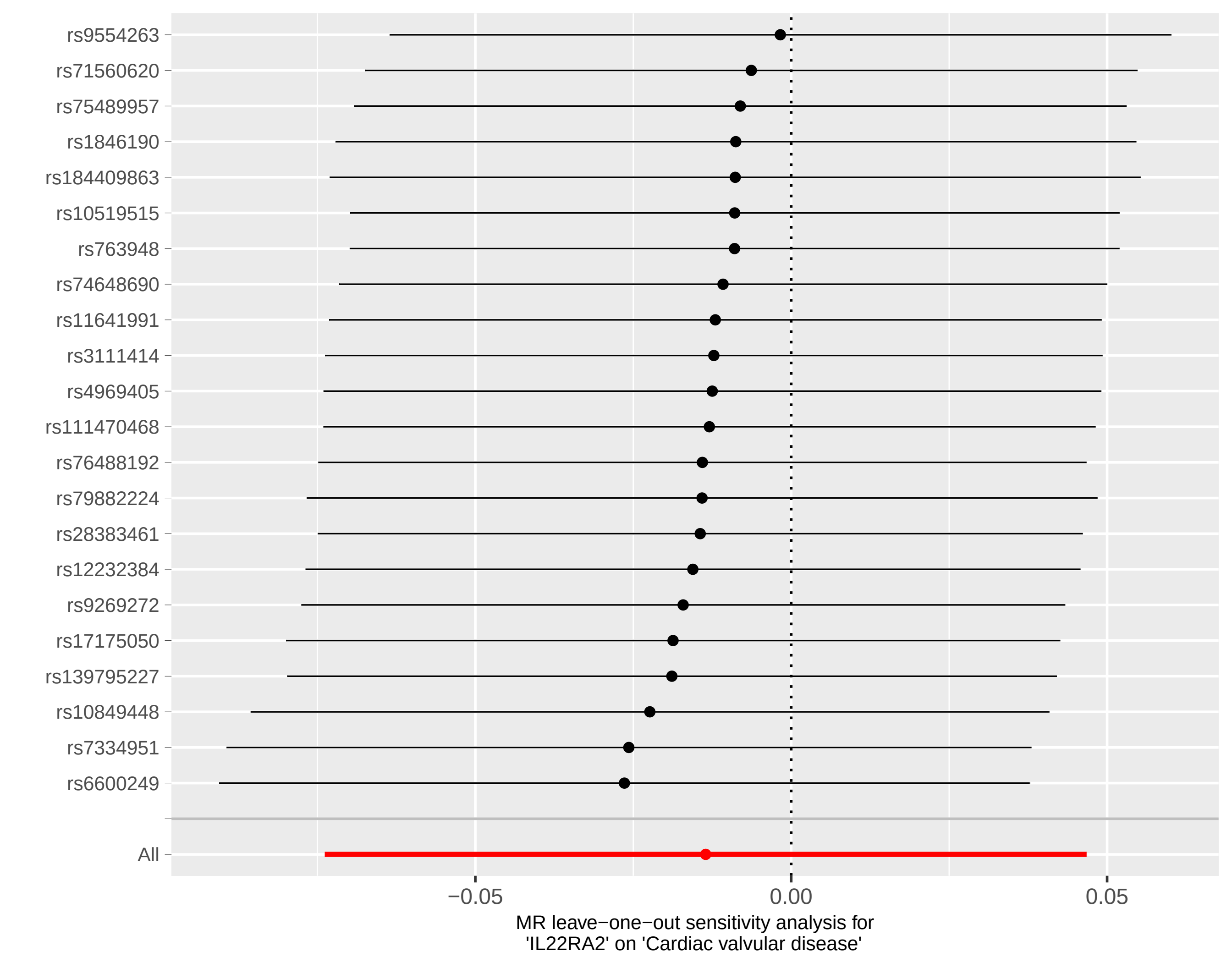
rs9554263
rs71560620
rs75489957
rs1846190
rs184409863
rs10519515
rs763948
rs74648690
rs11641991
rs3111414
rs4969405
rs111470468
rs76488192
rs79882224
rs28383461
rs12232384
rs9269272
rs17175050
rs139795227
rs10849448
rs7334951
rs6600249
All
−0.05
0.00
0.05
MR leave−one−out sensitivity analysis for
'IL22RA2' on 'Cardiac valvular disease'

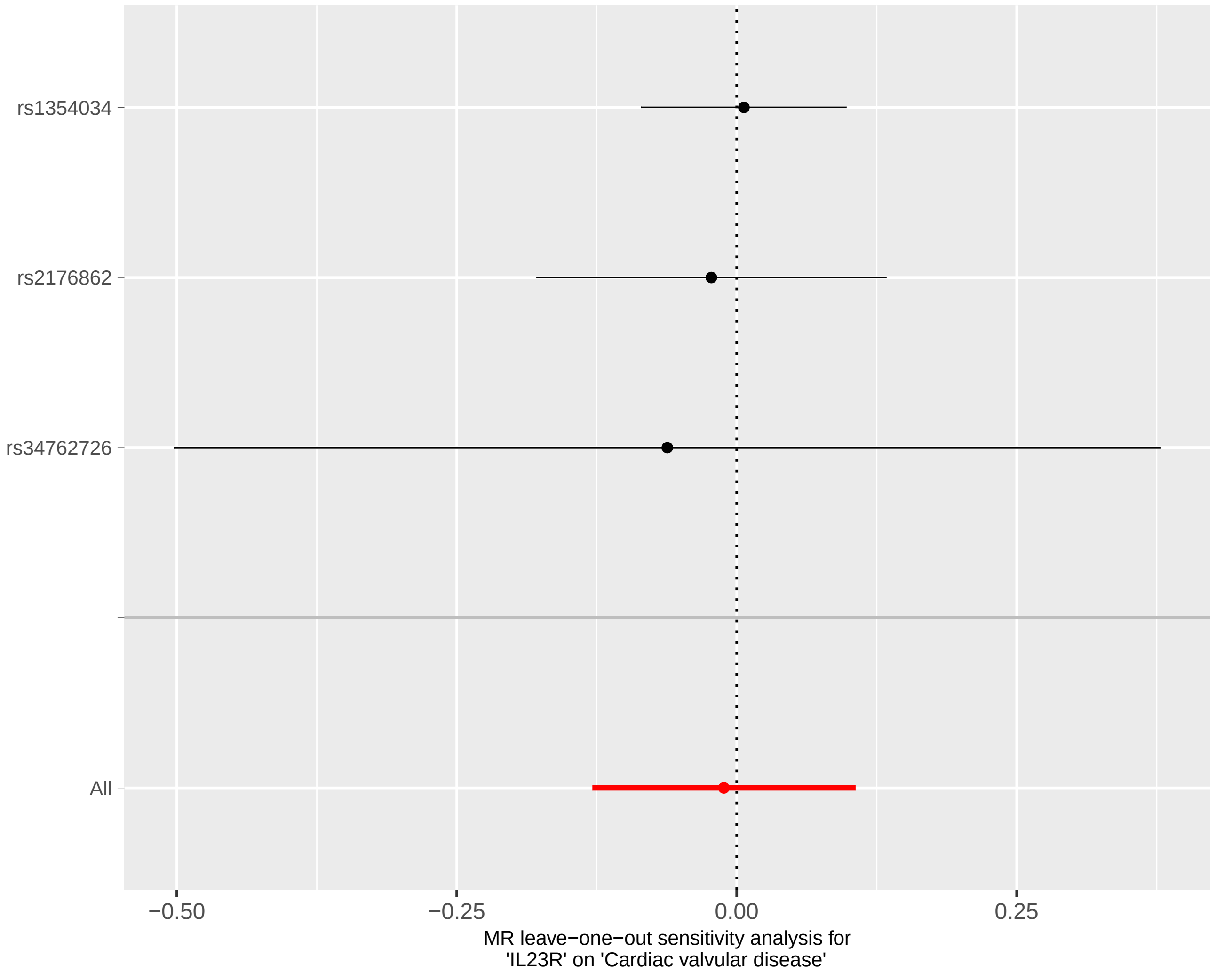
rs1354034
rs2176862
rs34762726
All
−0.50
−0.25
0.00
0.25
MR leave−one−out sensitivity analysis for
'IL23R' on 'Cardiac valvular disease'

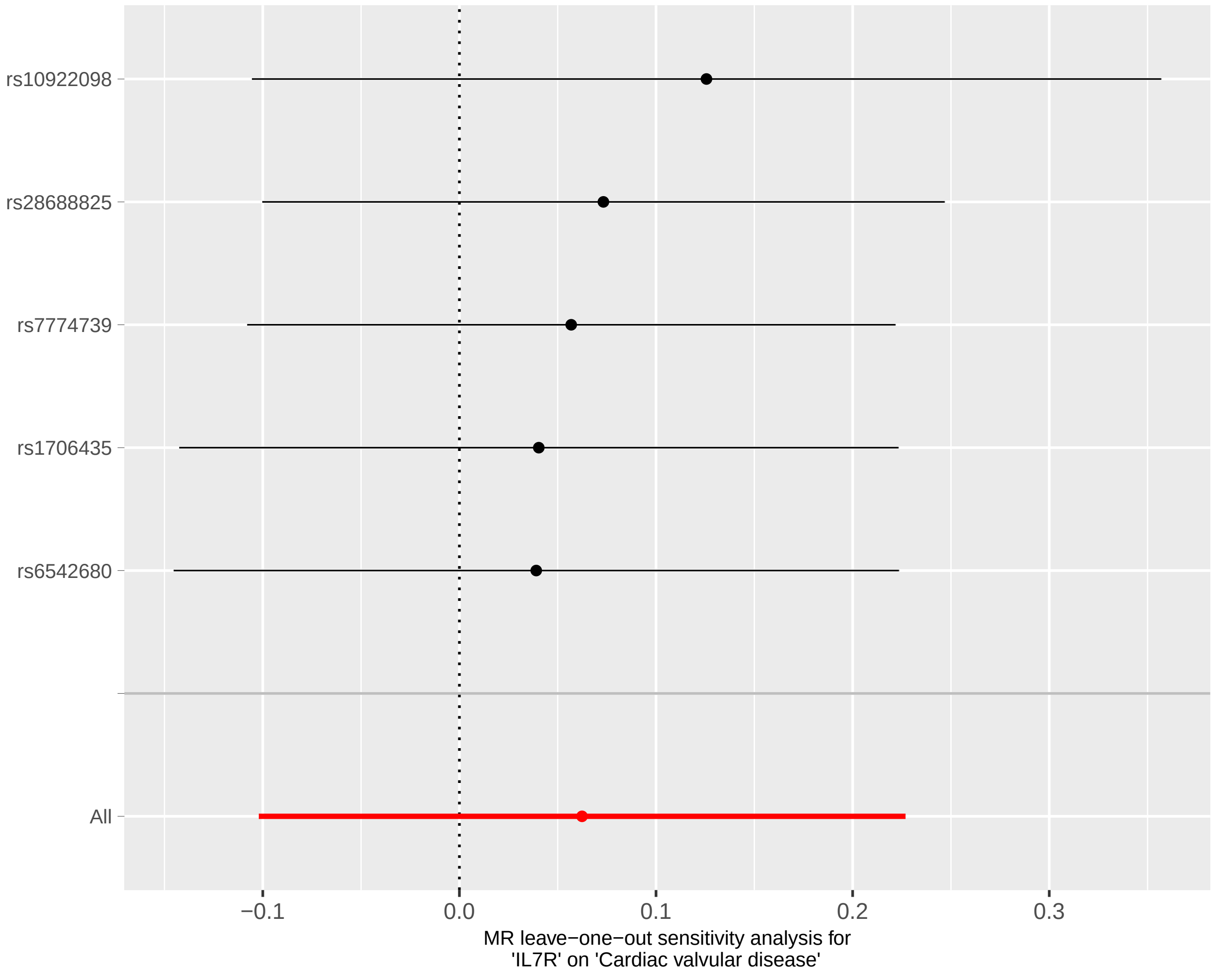

rs10922098
rs28688825
rs7774739
rs1706435
rs6542680
All
−0.1
0.0
0.1
0.2
0.3
MR leave−one−out sensitivity analysis for
'IL7R' on 'Cardiac valvular disease'

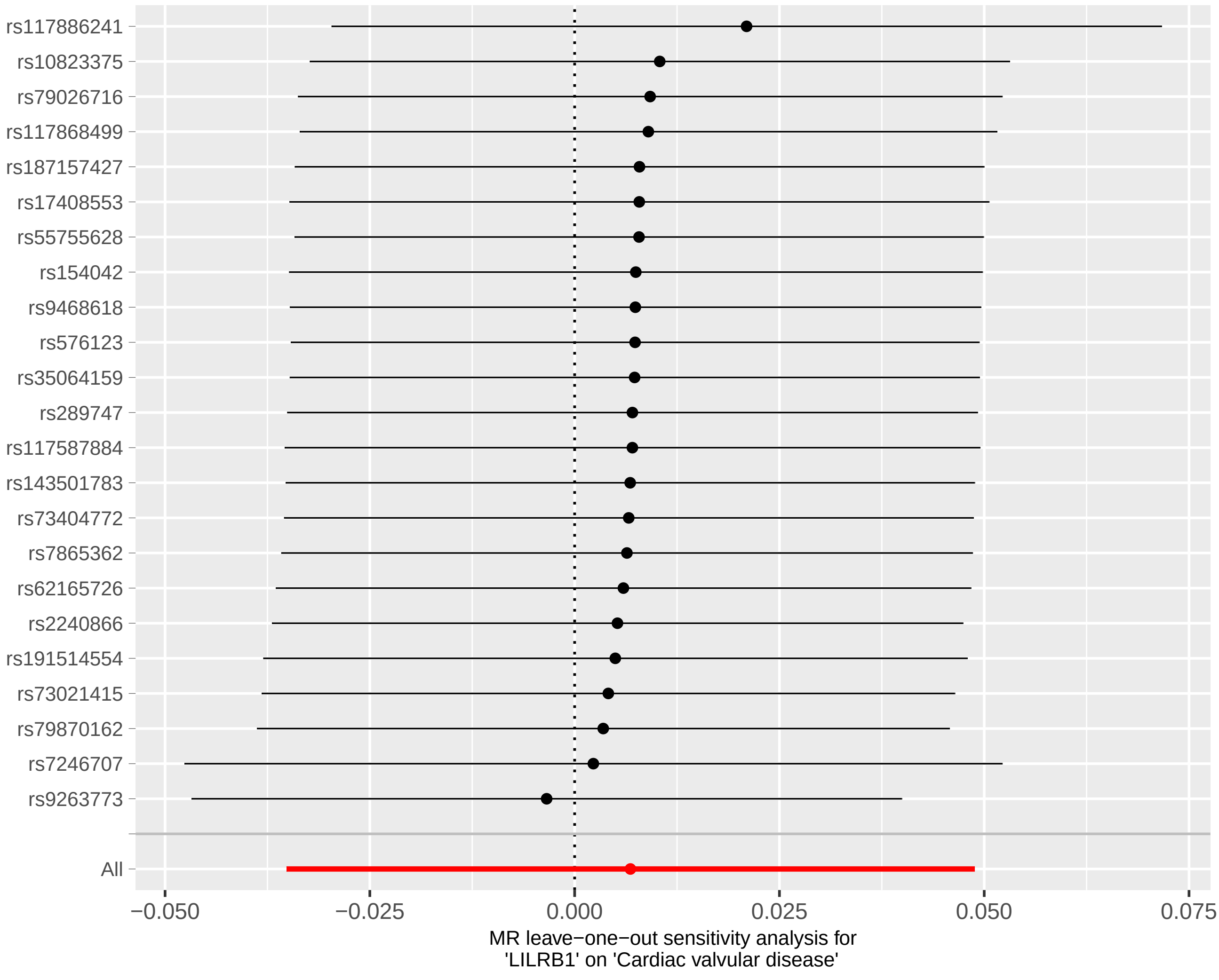
rs117886241
rs10823375
rs79026716
rs117868499
rs187157427
rs17408553
rs55755628
rs154042
rs9468618
rs576123
rs35064159
rs289747
rs117587884
rs143501783
rs73404772
rs7865362
rs62165726
rs2240866
rs191514554
rs73021415
rs79870162
rs7246707
rs9263773
All
−0.050
−0.025
0.000
0.025
0.050
0.075
MR leave−one−out sensitivity analysis for
'LILRB1' on 'Cardiac valvular disease'

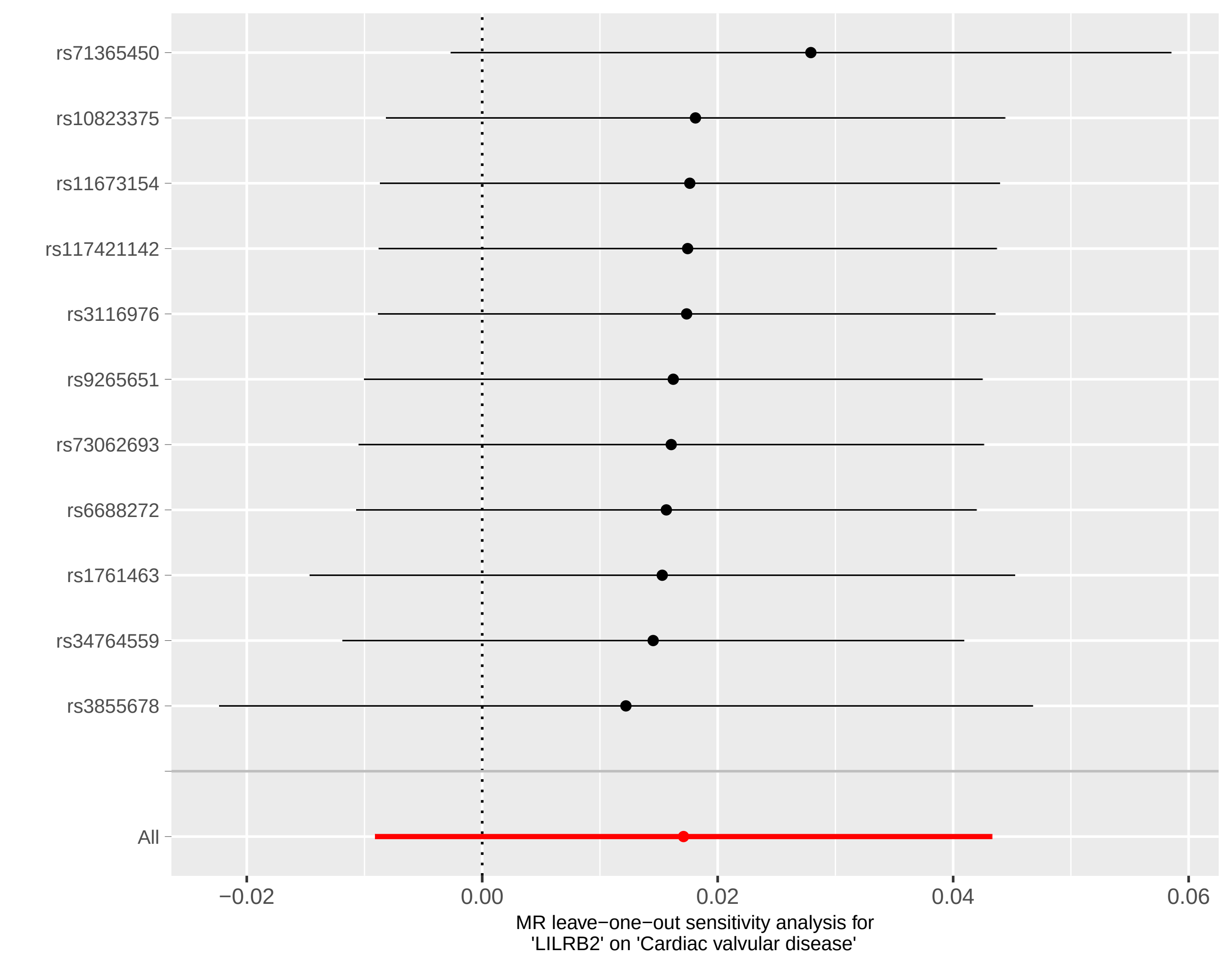
rs71365450
rs10823375
rs11673154
rs117421142
rs3116976
rs9265651
rs73062693
rs6688272
rs1761463
rs34764559
rs3855678
All
−0.02
0.00
0.02
0.04
0.06
MR leave−one−out sensitivity analysis for
'LILRB2' on 'Cardiac valvular disease'

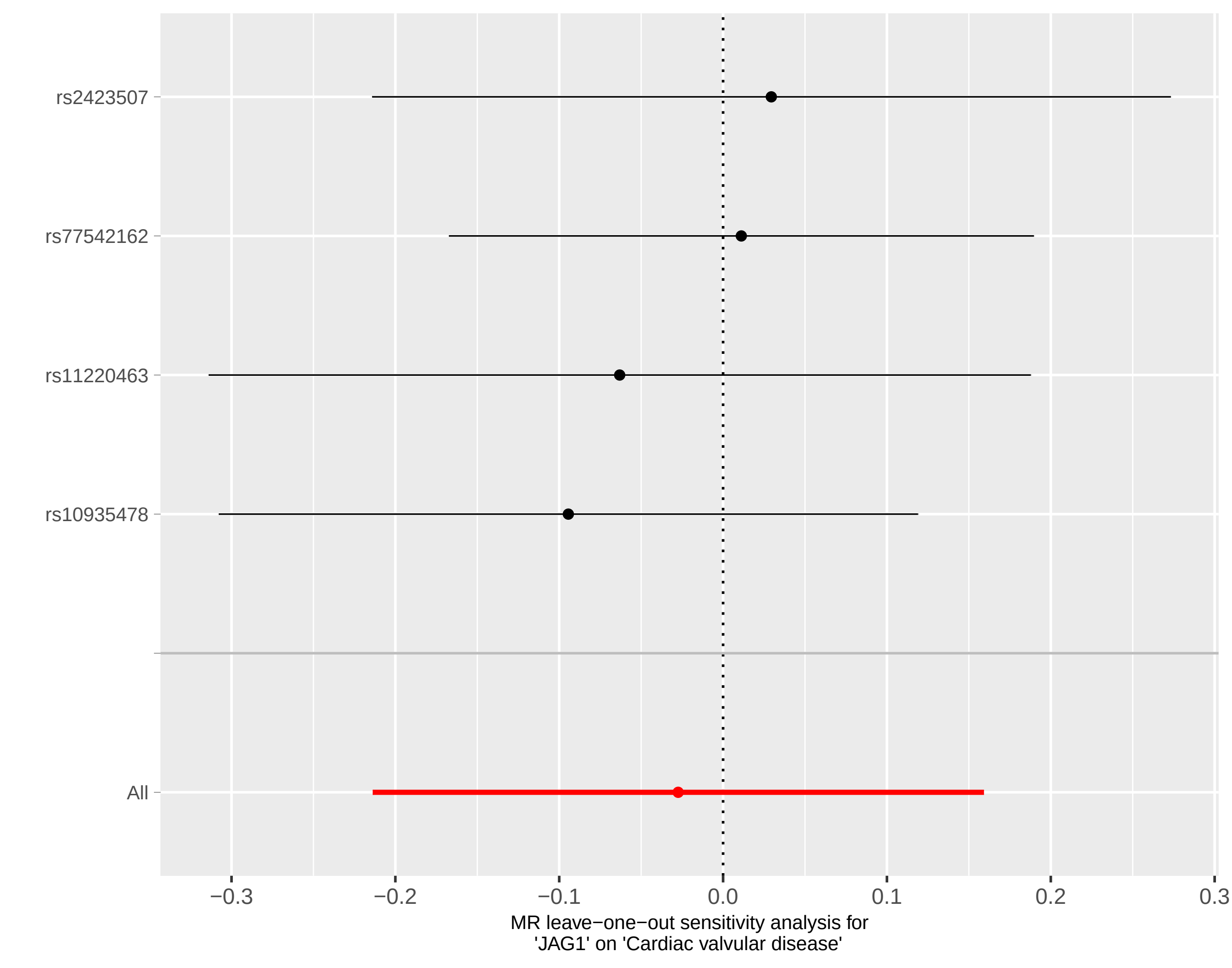

rs2423507
rs77542162
rs11220463
rs10935478
All
−0.3
−0.2
−0.1
0.0
0.1
0.2
0.3
MR leave−one−out sensitivity analysis for
'JAG1' on 'Cardiac valvular disease'

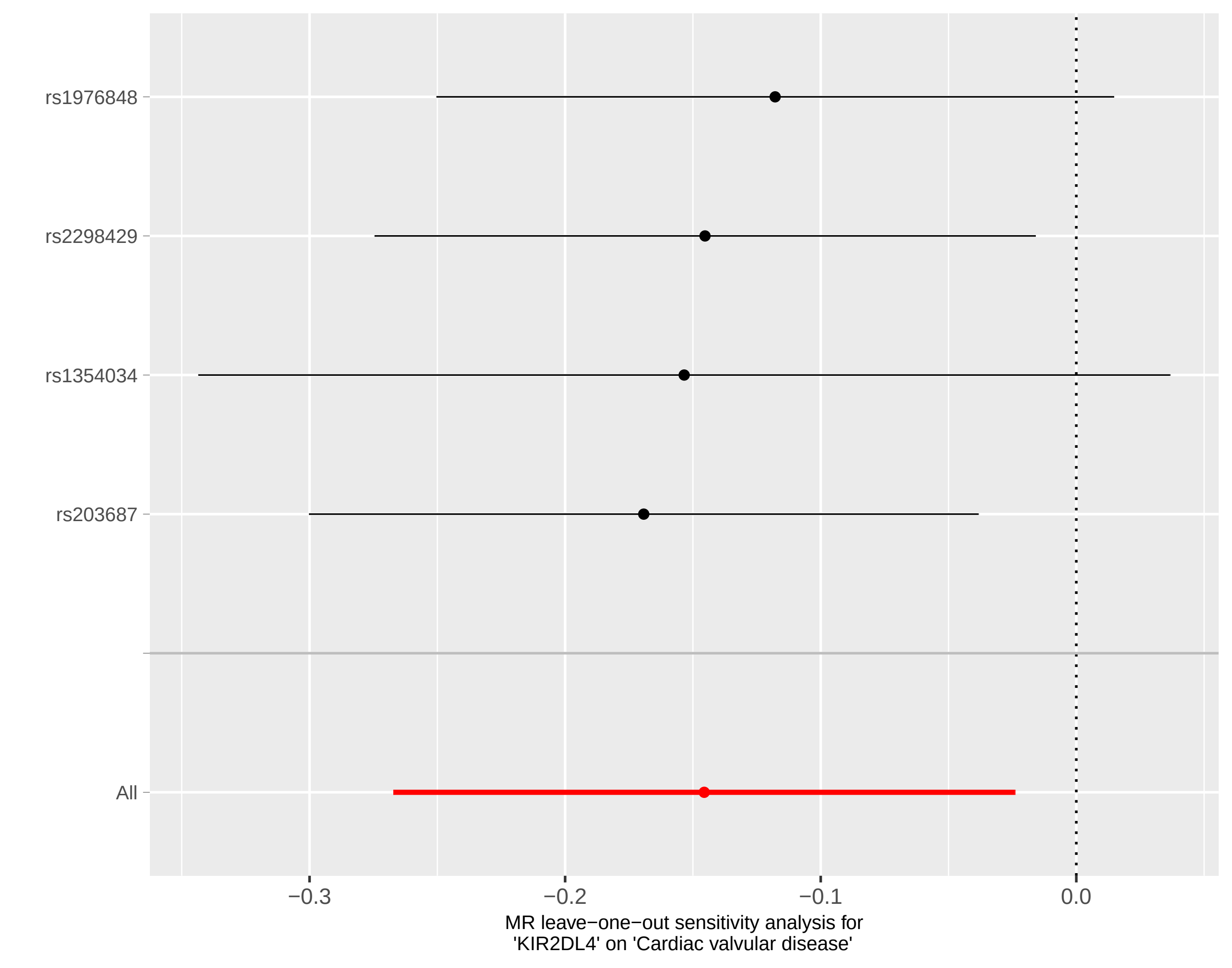
rs1976848
rs2298429
rs1354034
rs203687
All
−0.3
−0.2
−0.1
0.0
MR leave−one−out sensitivity analysis for
'KIR2DL4' on 'Cardiac valvular disease'

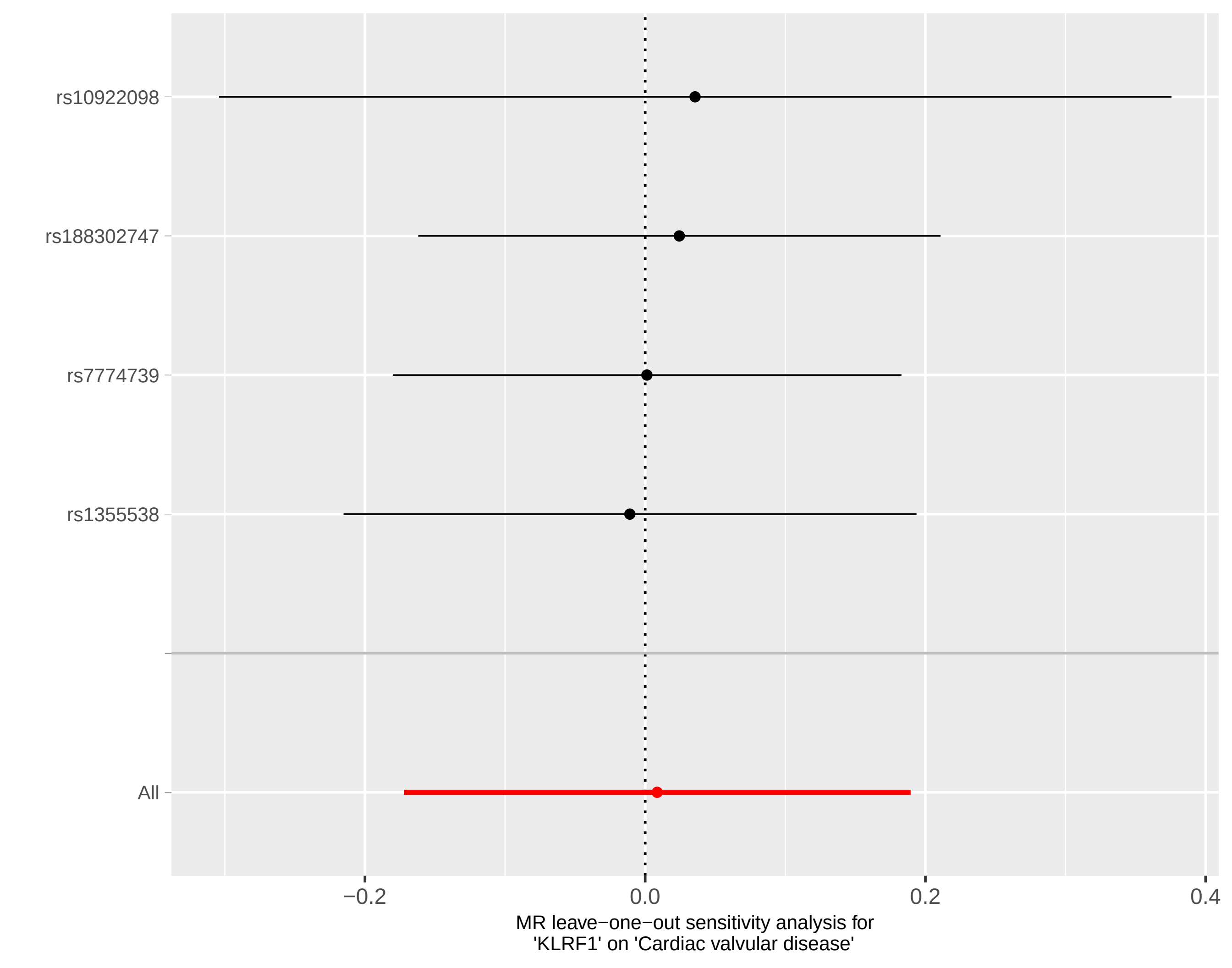
rs10922098
rs188302747
rs7774739
rs1355538
All
−0.2
0.0
0.2
0.4
MR leave−one−out sensitivity analysis for
'KLRF1' on 'Cardiac valvular disease'

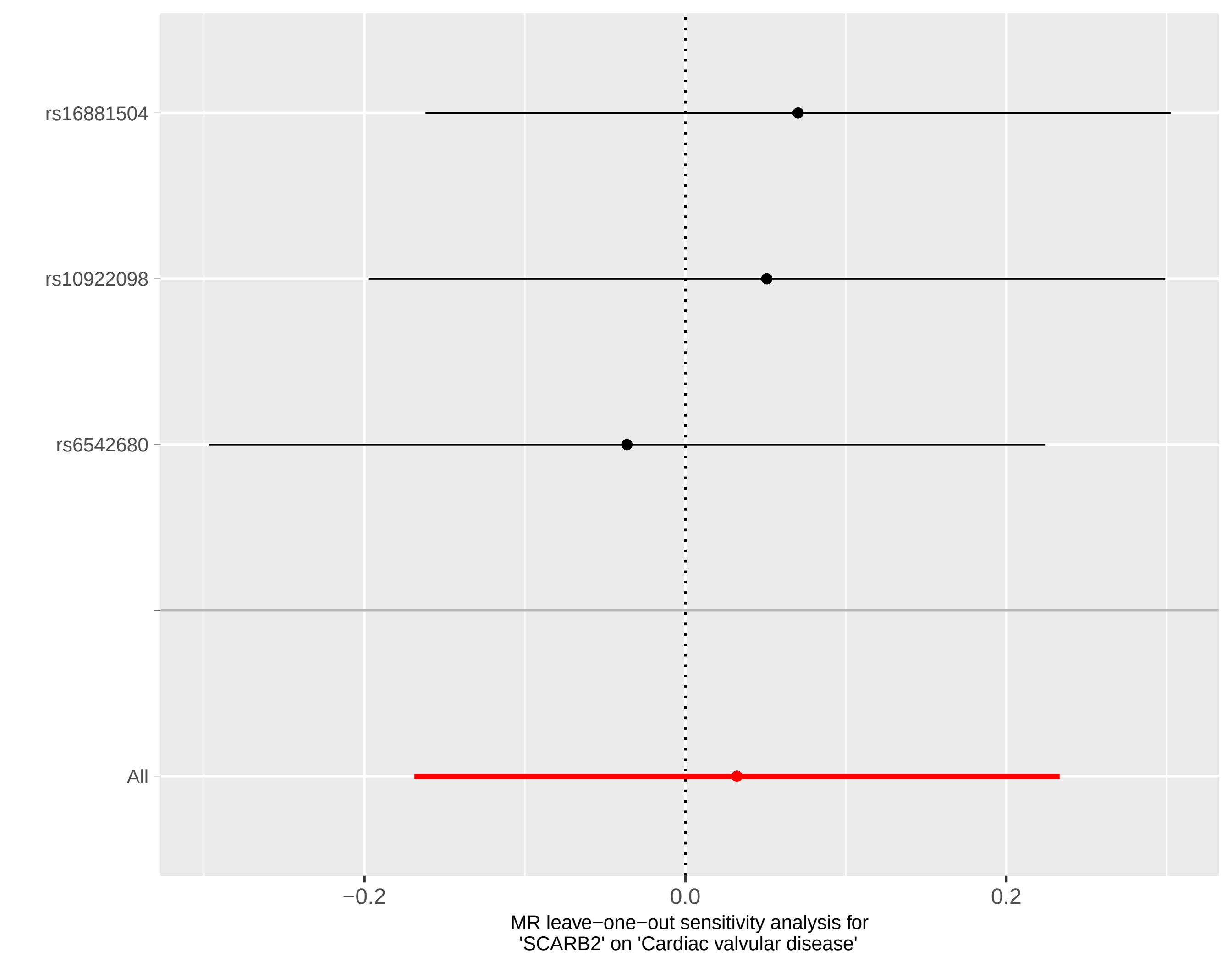

rs16881504
rs10922098
rs6542680
All
−0.2
0.0
0.2
MR leave−one−out sensitivity analysis for
'SCARB2' on 'Cardiac valvular disease'

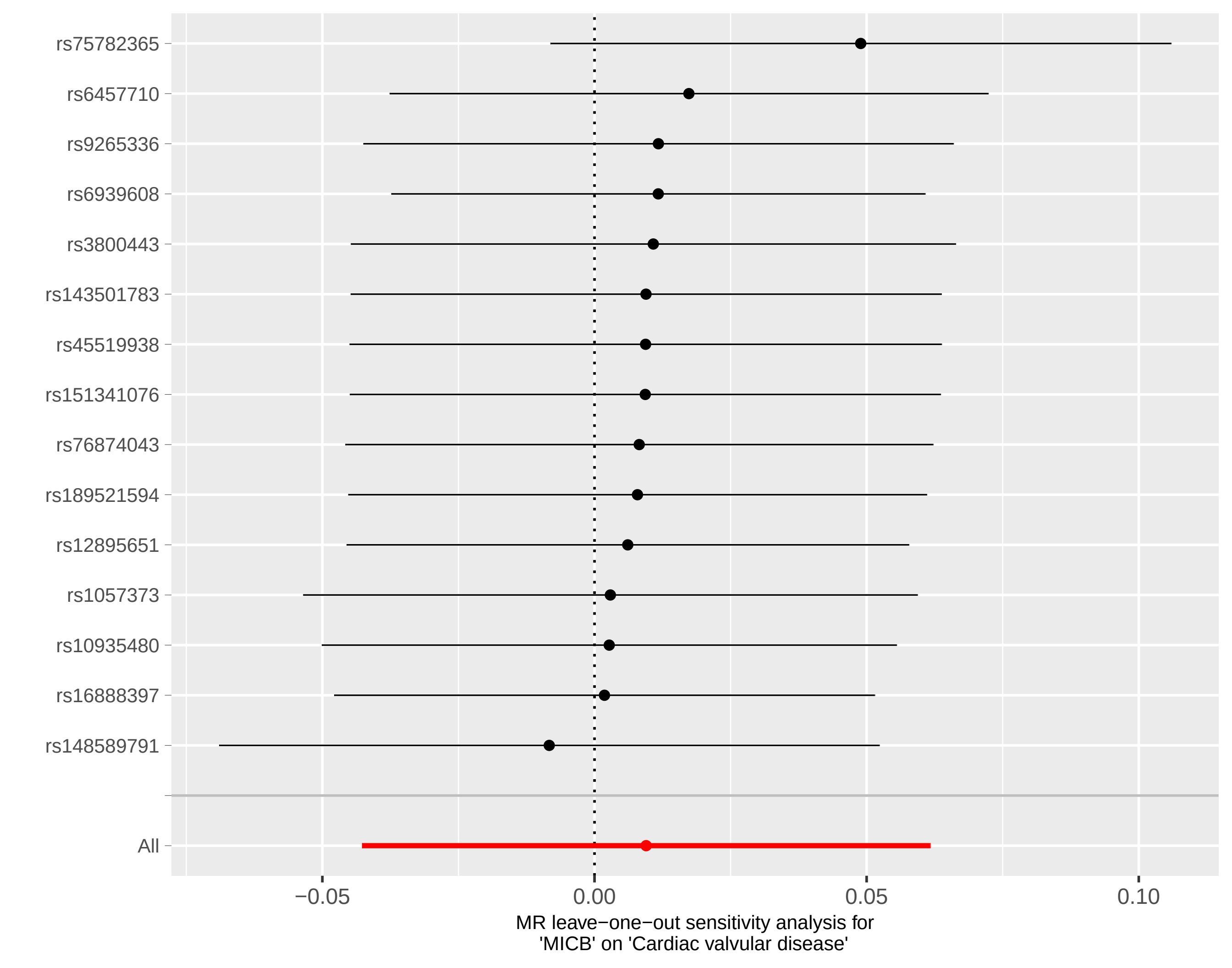
rs75782365
rs6457710
rs9265336
rs6939608
rs3800443
rs143501783
rs45519938
rs151341076
rs76874043
rs189521594
rs12895651
rs1057373
rs10935480
rs16888397
rs148589791
All
−0.05
0.00
0.05
0.10
MR leave−one−out sensitivity analysis for
'MICB' on 'Cardiac valvular disease'

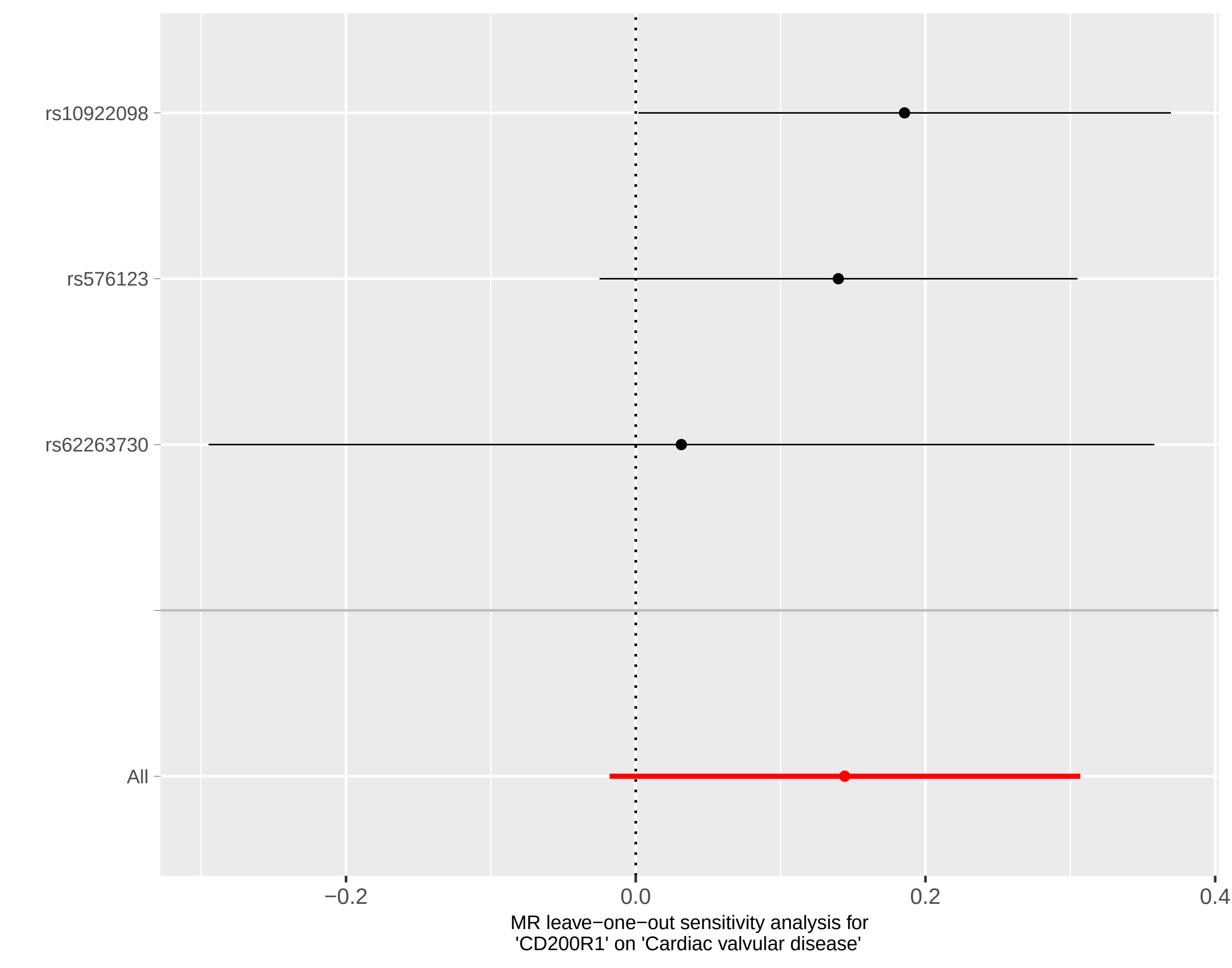

rs10922098
rs576123
rs62263730
All
−0.2
0.0
0.2
0.4
MR leave−one−out sensitivity analysis for
'CD200R1' on 'Cardiac valvular disease'

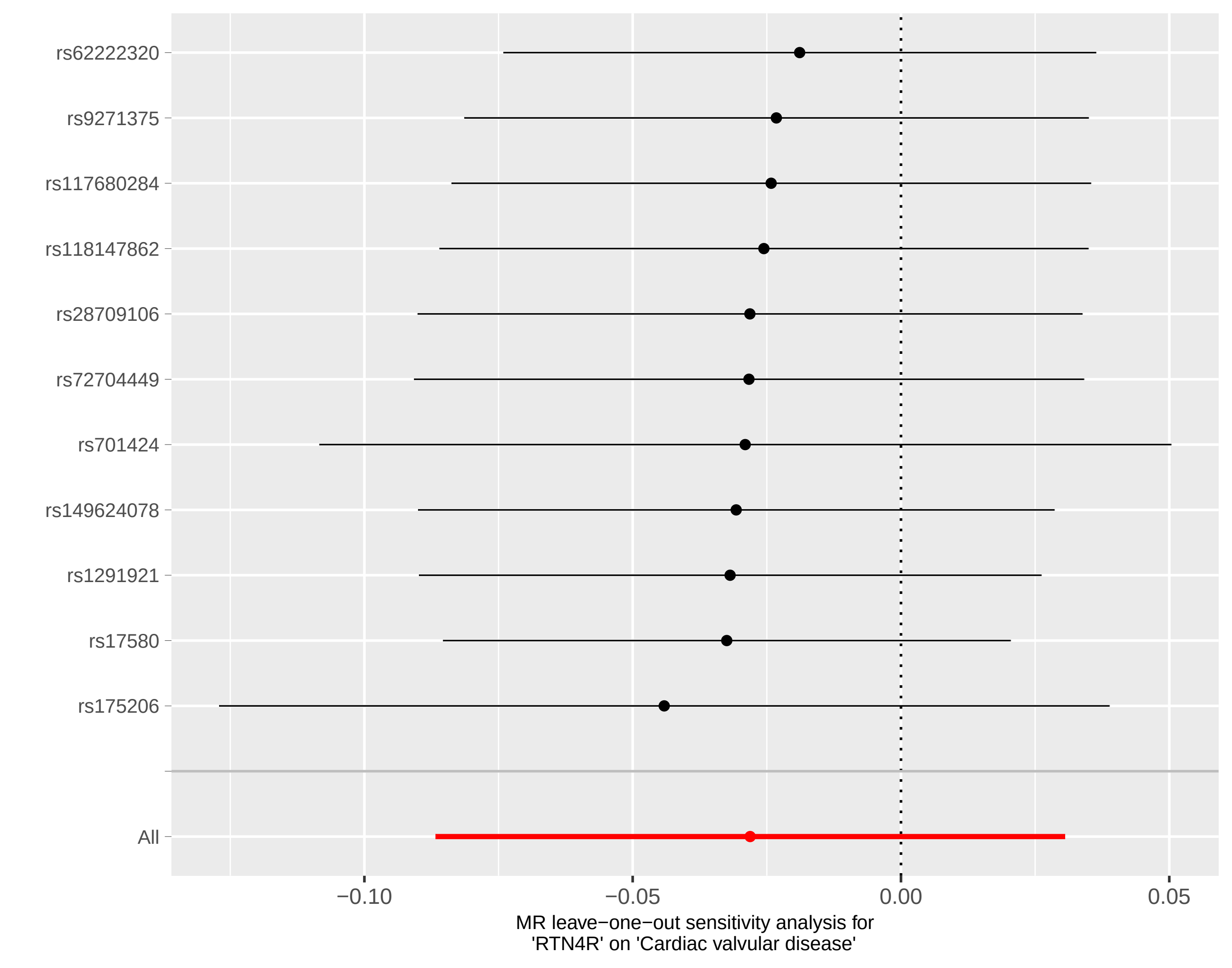
rs62222320
rs9271375
rs117680284
rs118147862
rs28709106
rs72704449
rs701424
rs149624078
rs1291921
rs17580
rs175206
All
−0.10
−0.05
0.00
0.05
MR leave−one−out sensitivity analysis for
'RTN4R' on 'Cardiac valvular disease'

Insufficient number of SNPs

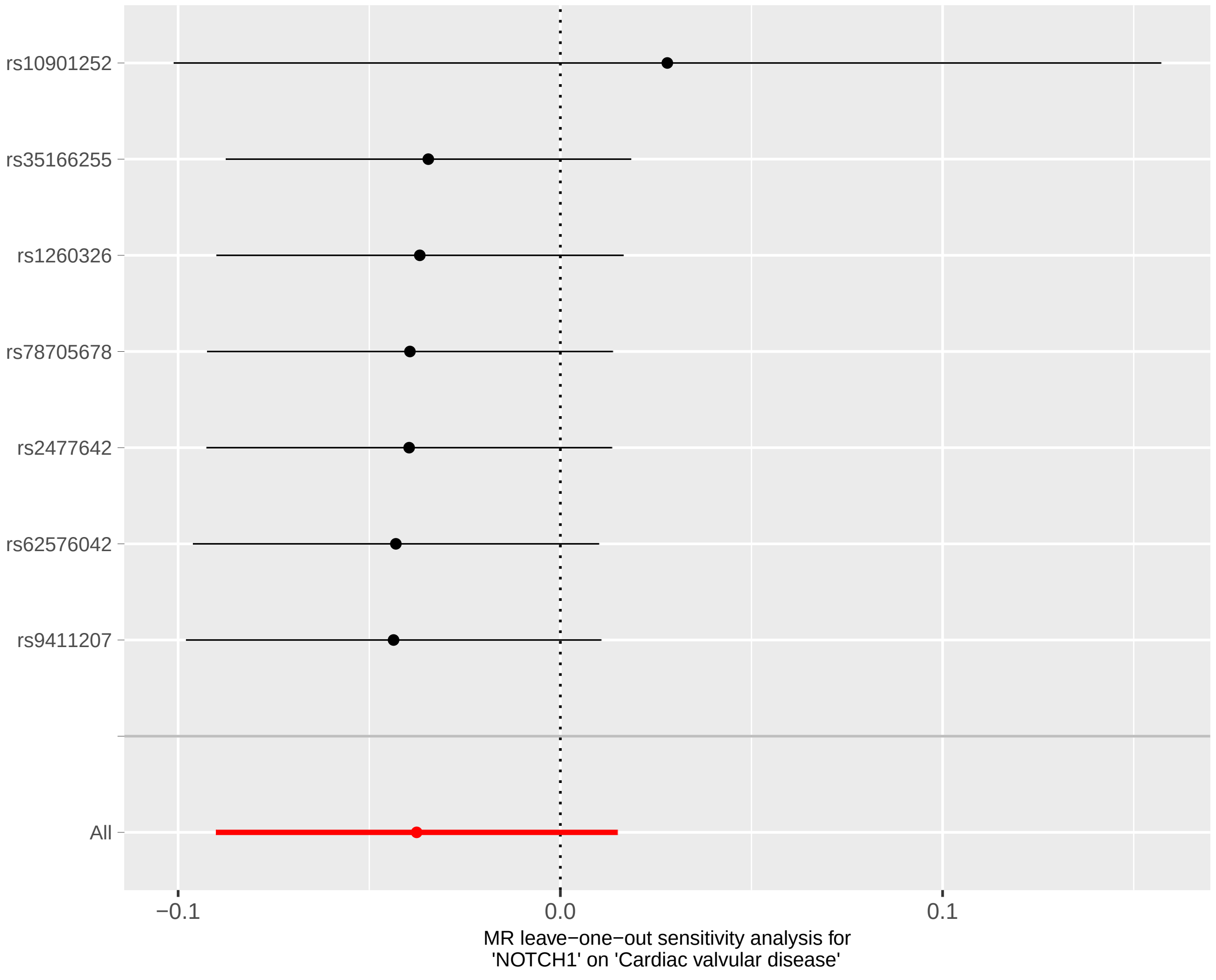
rs10901252
rs35166255
rs1260326
rs78705678
rs2477642
rs62576042
rs9411207
All
−0.1
0.0
0.1
MR leave−one−out sensitivity analysis for
'NOTCH1' on 'Cardiac valvular disease'

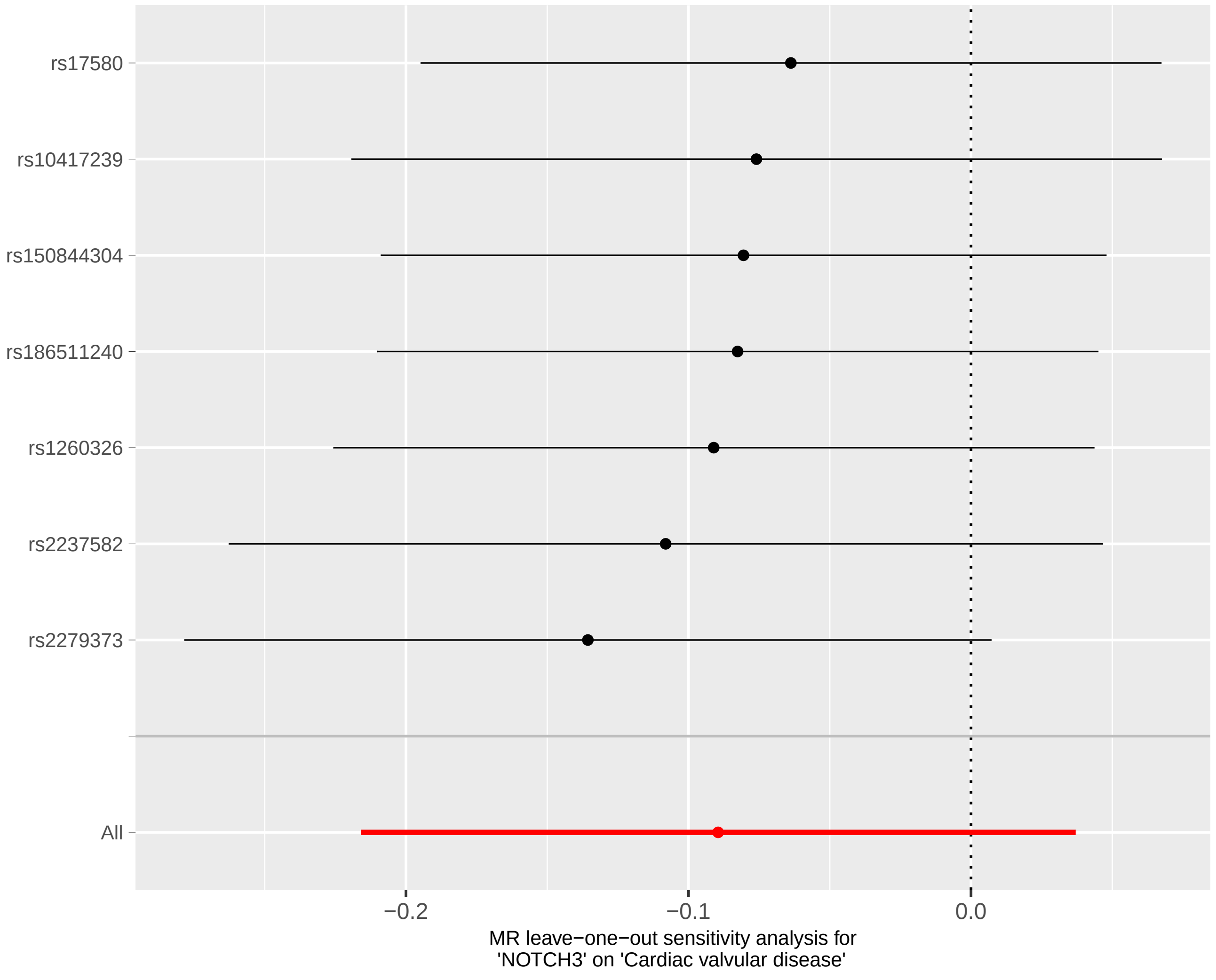

rs17580
rs10417239
rs150844304
rs186511240
rs1260326
rs2237582
rs2279373
All
−0.2
−0.1
0.0
MR leave−one−out sensitivity analysis for
'NOTCH3' on 'Cardiac valvular disease'

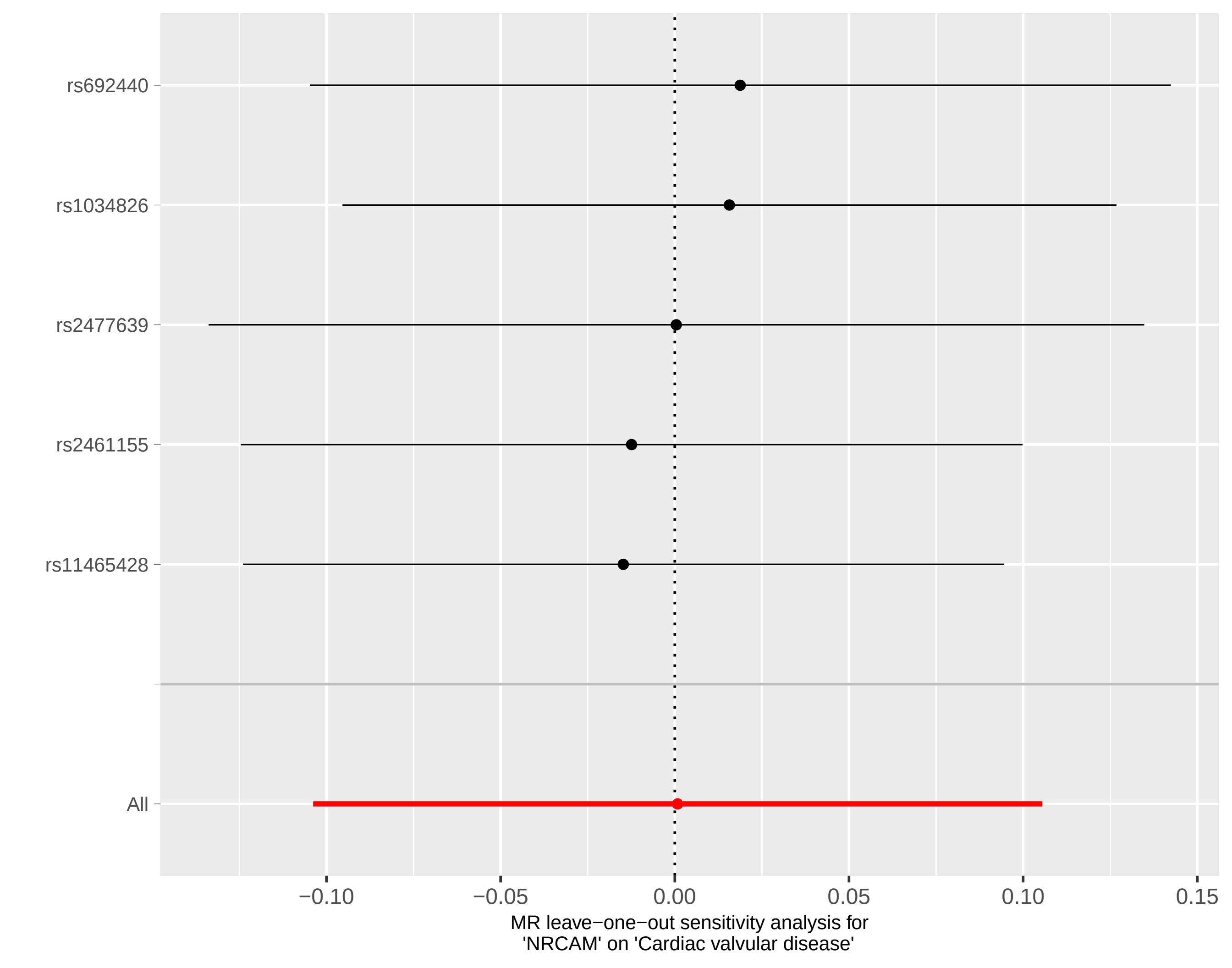

rs692440
rs1034826
rs2477639
rs2461155
rs11465428
All
−0.10
−0.05
0.00
0.05
0.10
0.15
MR leave−one−out sensitivity analysis for
'NRCAM' on 'Cardiac valvular disease'

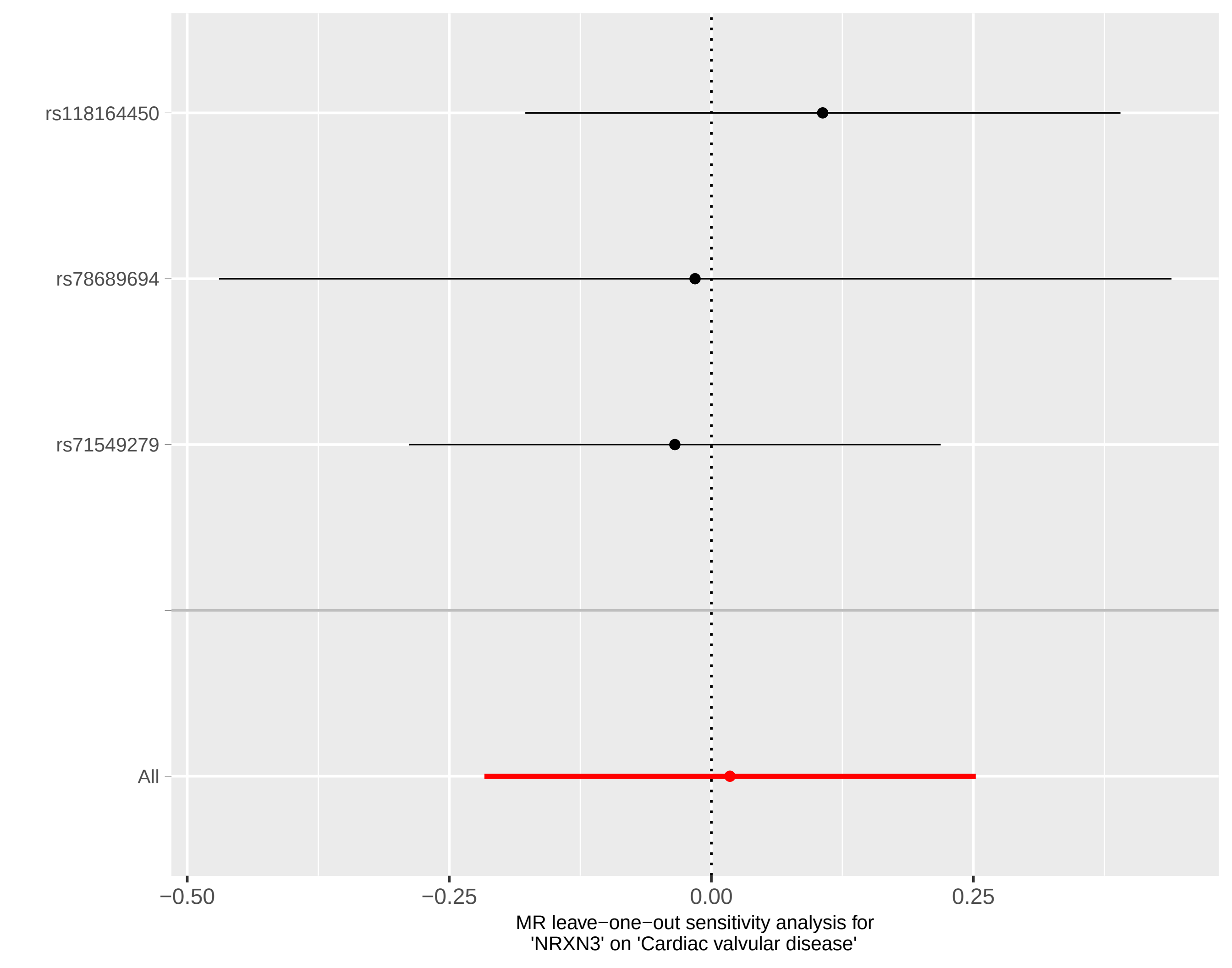

rs118164450
rs78689694
rs71549279
All
−0.50
−0.25
0.00
0.25
MR leave−one−out sensitivity analysis for
'NRXN3' on 'Cardiac valvular disease'

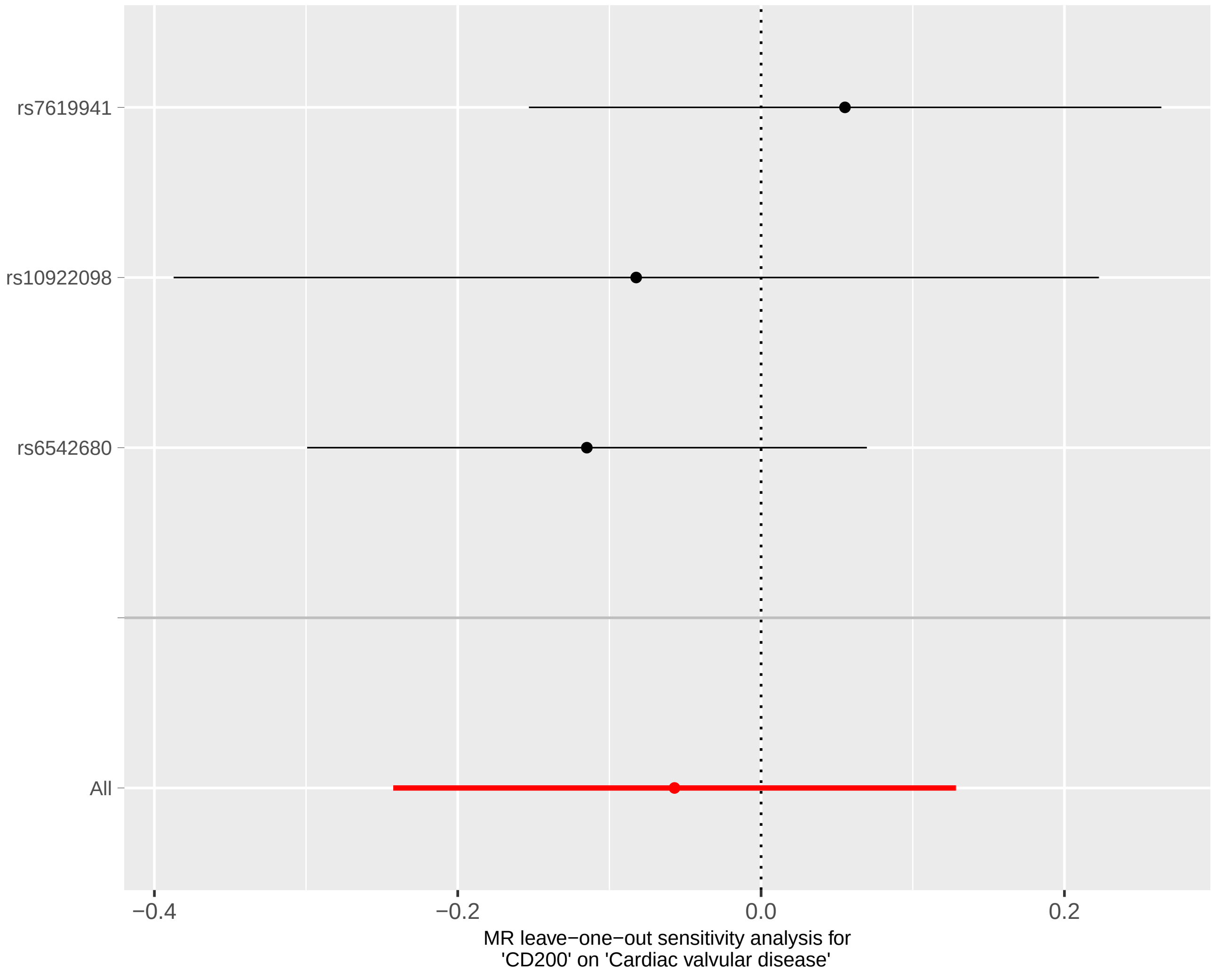
rs7619941
rs10922098
rs6542680
All
−0.4
−0.2
0.0
0.2
MR leave−one−out sensitivity analysis for
'CD200' on 'Cardiac valvular disease'

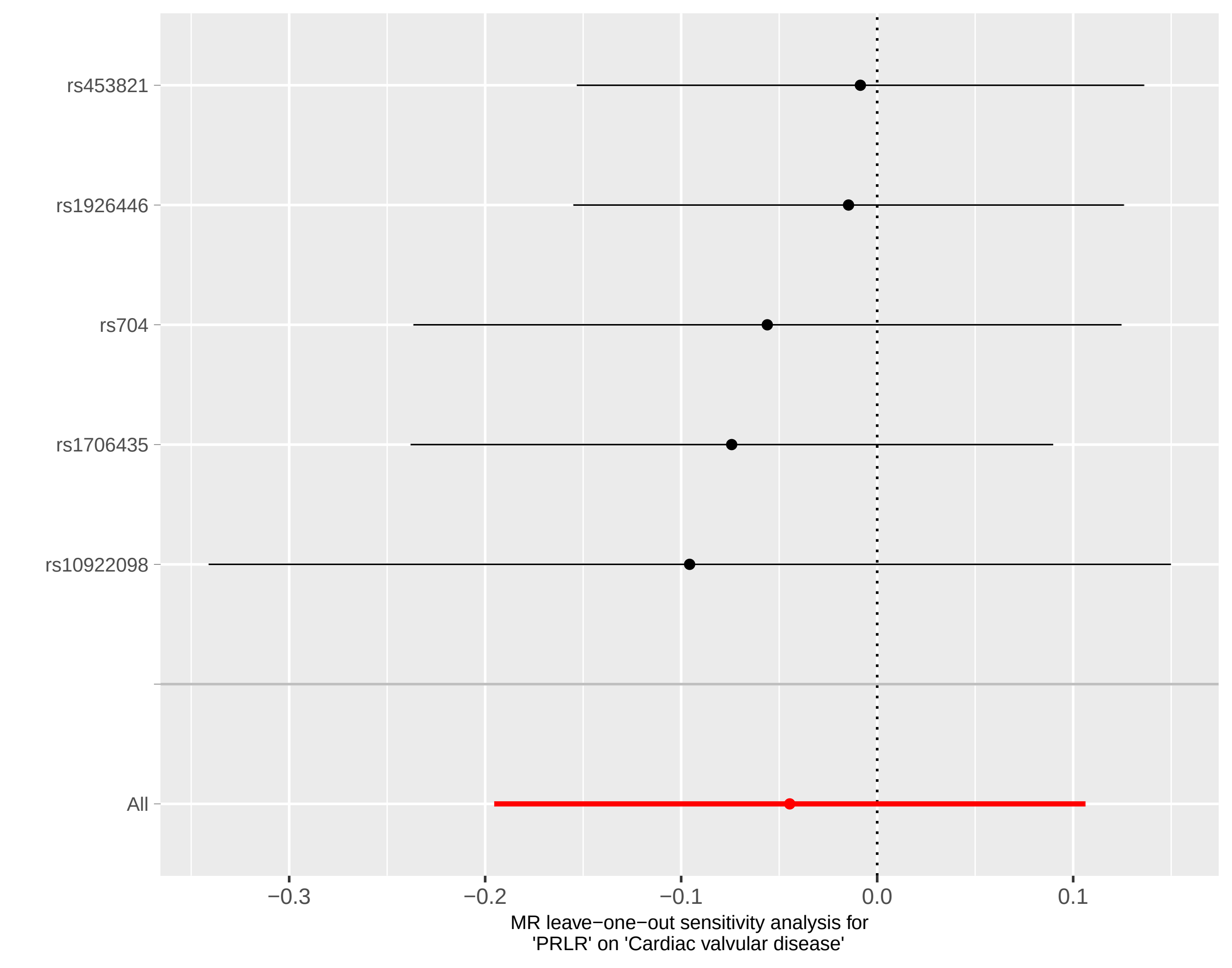
rs453821
rs1926446
rs704
rs1706435
rs10922098
All
−0.3
−0.2
−0.1
0.0
0.1
MR leave−one−out sensitivity analysis for
'PRLR' on 'Cardiac valvular disease'

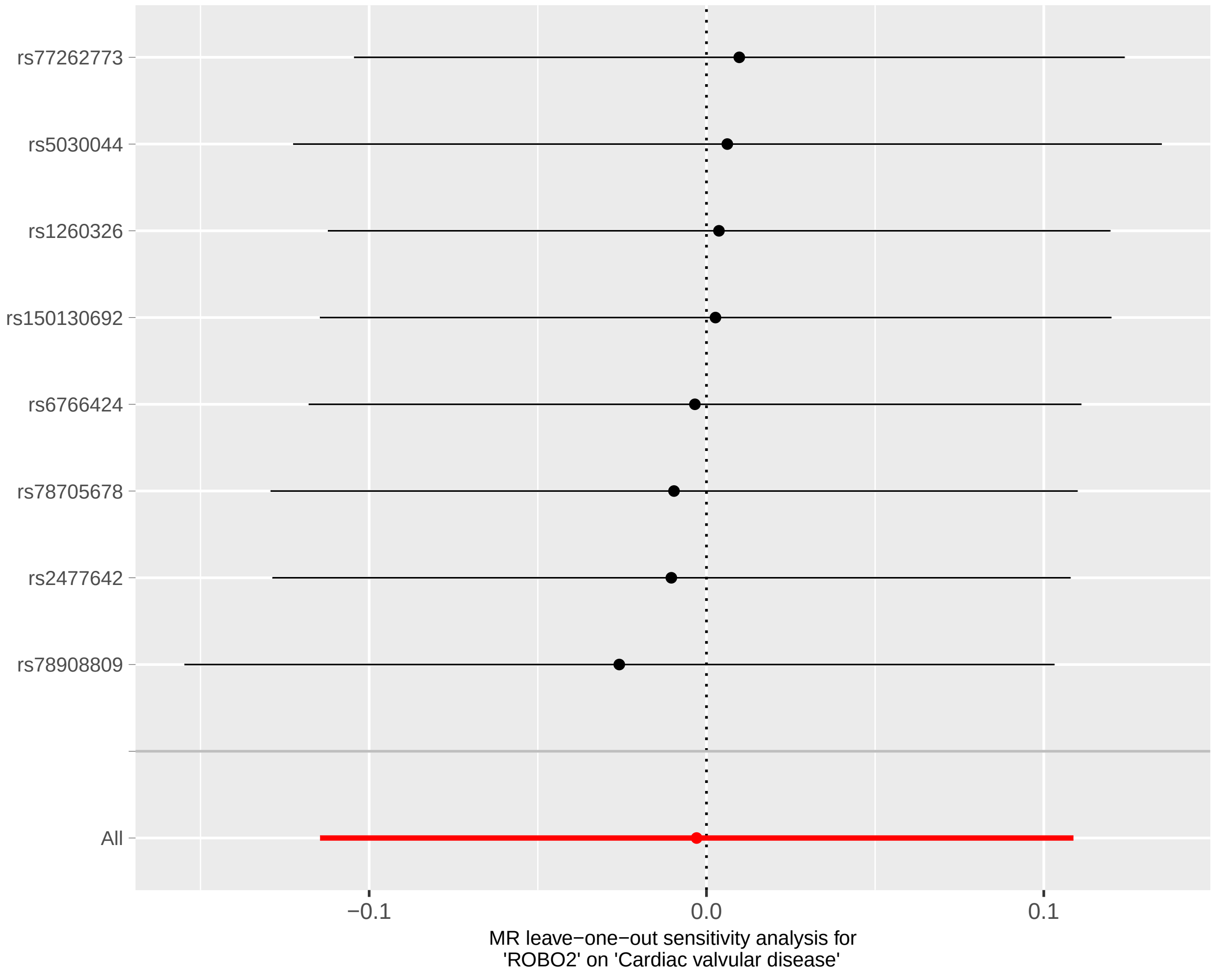

rs77262773
rs5030044
rs1260326
rs150130692
rs6766424
rs78705678
rs2477642
rs78908809
All
−0.1
0.0
0.1
MR leave−one−out sensitivity analysis for
'ROBO2' on 'Cardiac valvular disease'

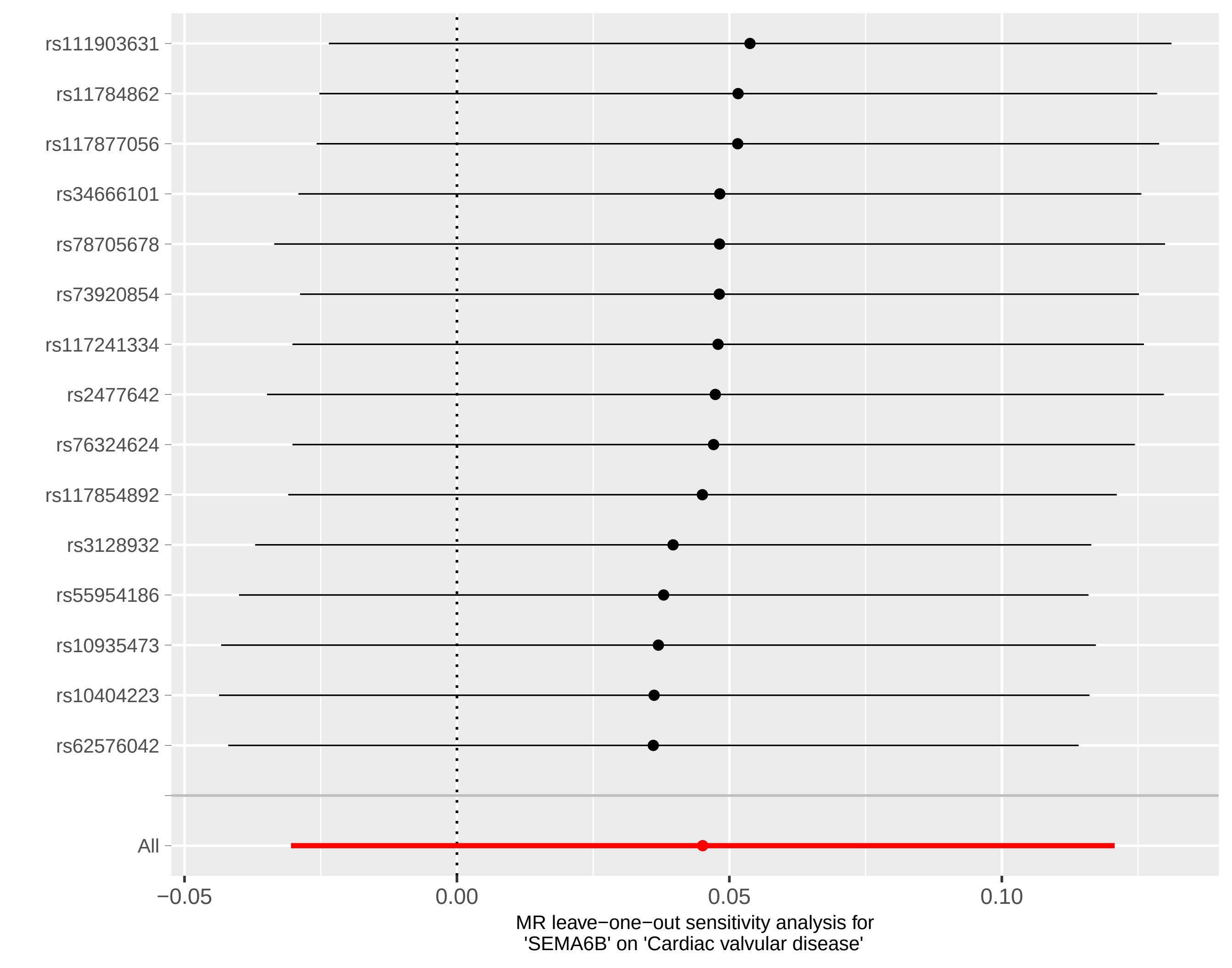
rs111903631
rs11784862
rs117877056
rs34666101
rs78705678
rs73920854
rs117241334
rs2477642
rs76324624
rs117854892
rs3128932
rs55954186
rs10935473
rs10404223
rs62576042
All
−0.05
0.00
0.05
0.10
MR leave−one−out sensitivity analysis for
'SEMA6B' on 'Cardiac valvular disease'

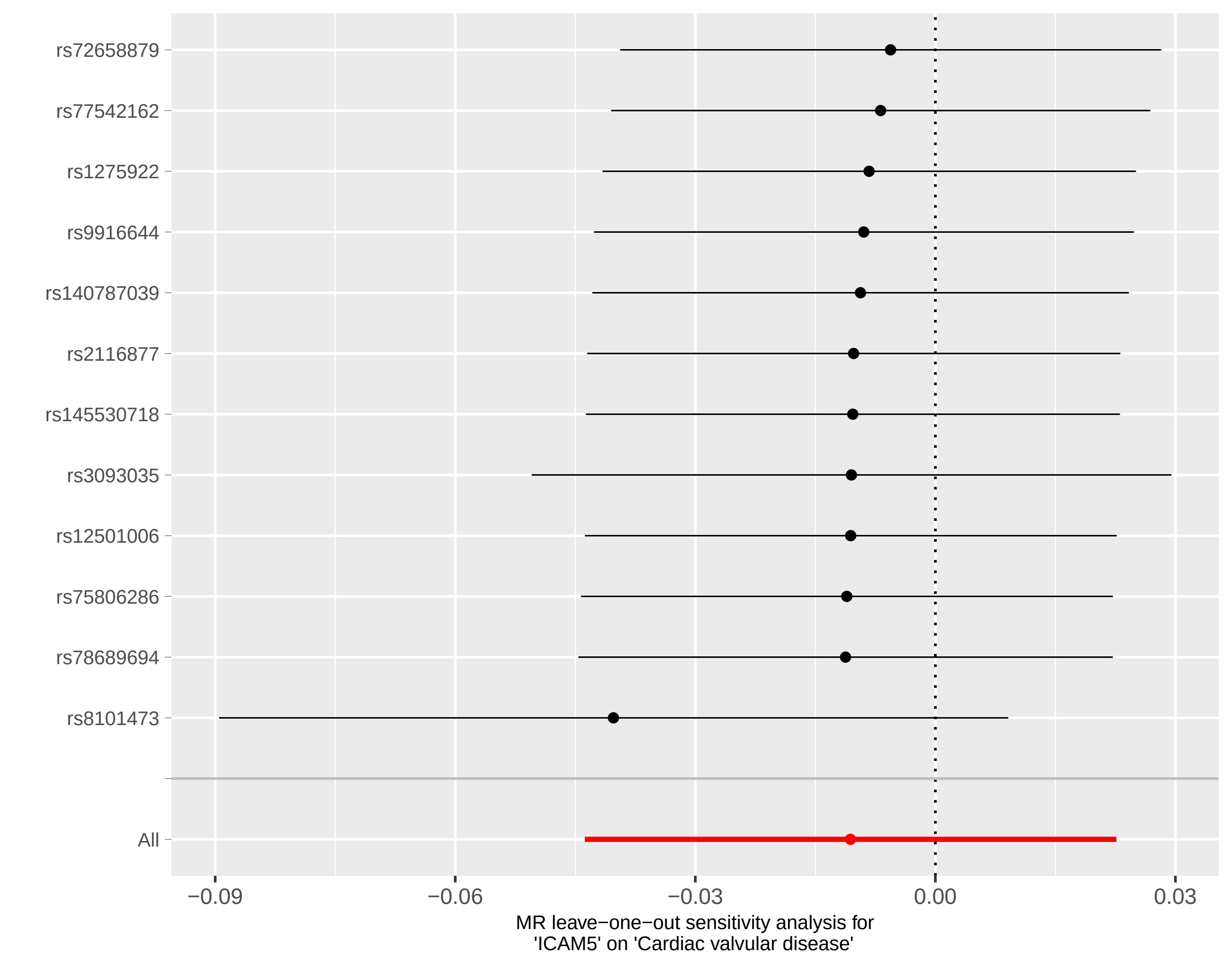
rs72658879
rs77542162
rs1275922
rs9916644
rs140787039
rs2116877
rs145530718
rs3093035
rs12501006
rs75806286
rs78689694
rs8101473
All
−0.09
−0.06
−0.03
0.00
0.03
MR leave−one−out sensitivity analysis for
'ICAM5' on 'Cardiac valvular disease'

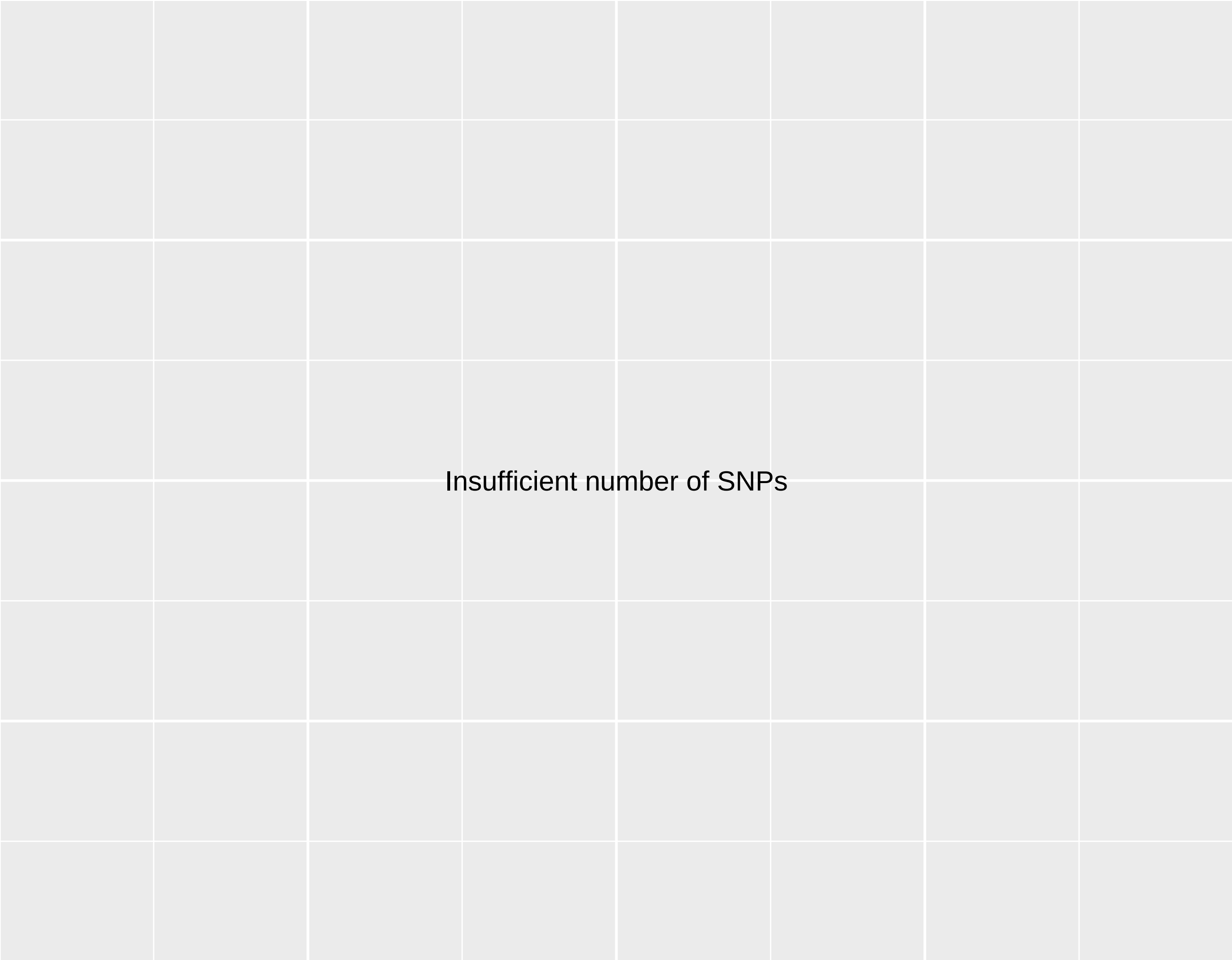
Insufficient number of SNPs

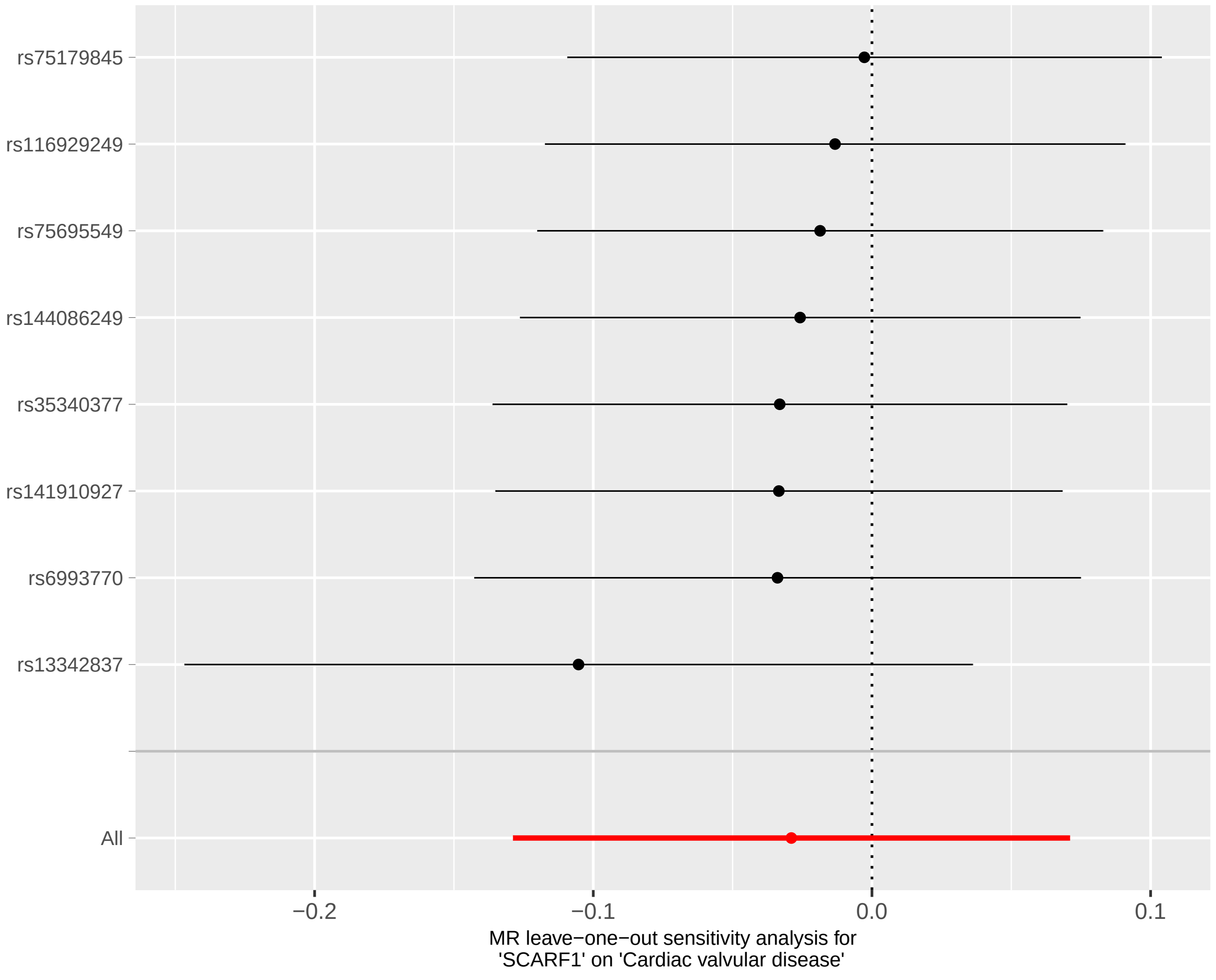
rs75179845
rs116929249
rs75695549
rs144086249
rs35340377
rs141910927
rs6993770
rs13342837
All
−0.2
−0.1
0.0
0.1
MR leave−one−out sensitivity analysis for
'SCARF1' on 'Cardiac valvular disease'

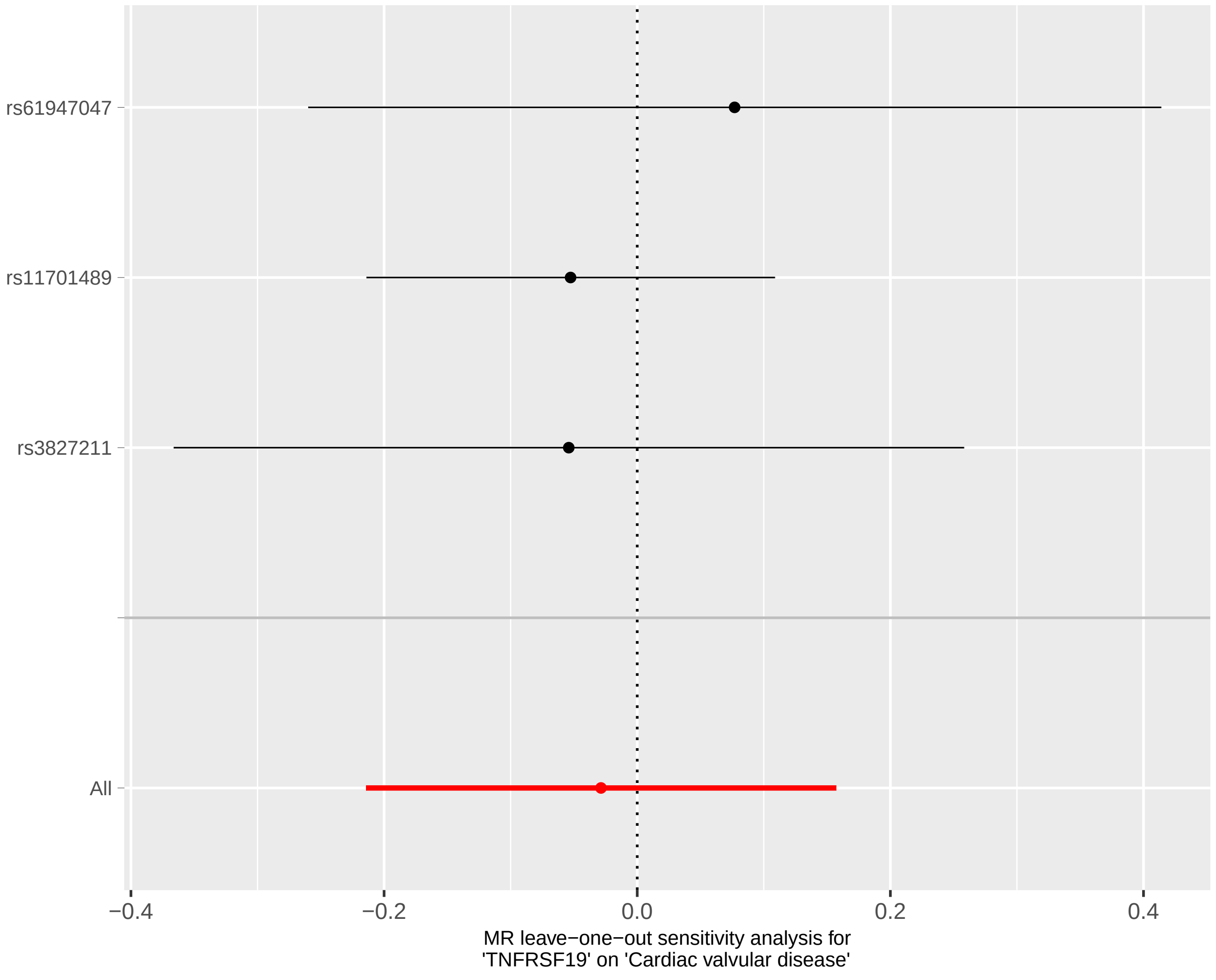
rs61947047
rs11701489
rs3827211
All
−0.4
−0.2
0.0
0.2
0.4
MR leave−one−out sensitivity analysis for
'TNFRSF19' on 'Cardiac valvular disease'

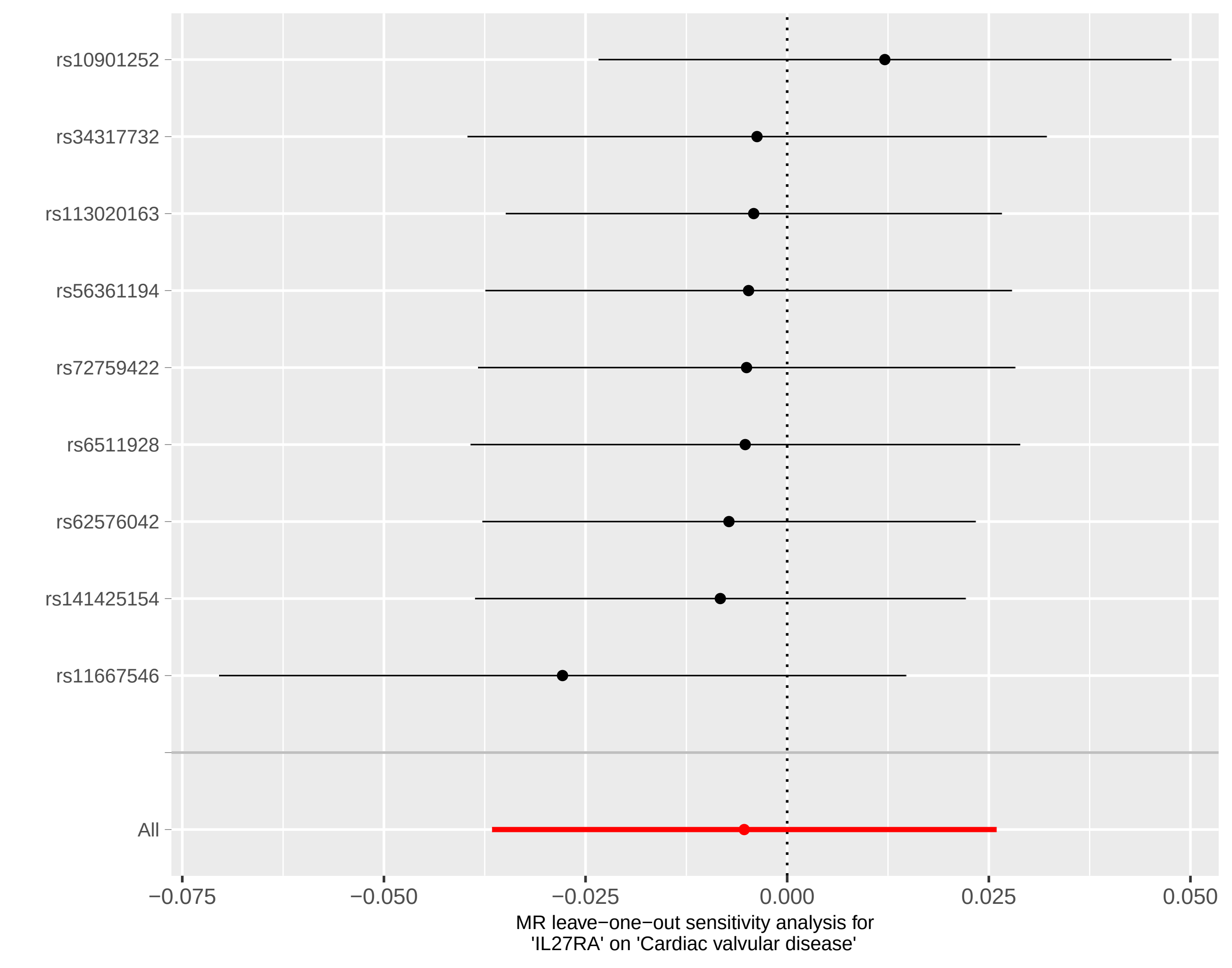
rs10901252
rs34317732
rs113020163
rs56361194
rs72759422
rs6511928
rs62576042
rs141425154
rs11667546
All
−0.075
−0.050
−0.025
0.000
0.025
0.050
MR leave−one−out sensitivity analysis for
'IL27RA' on 'Cardiac valvular disease'

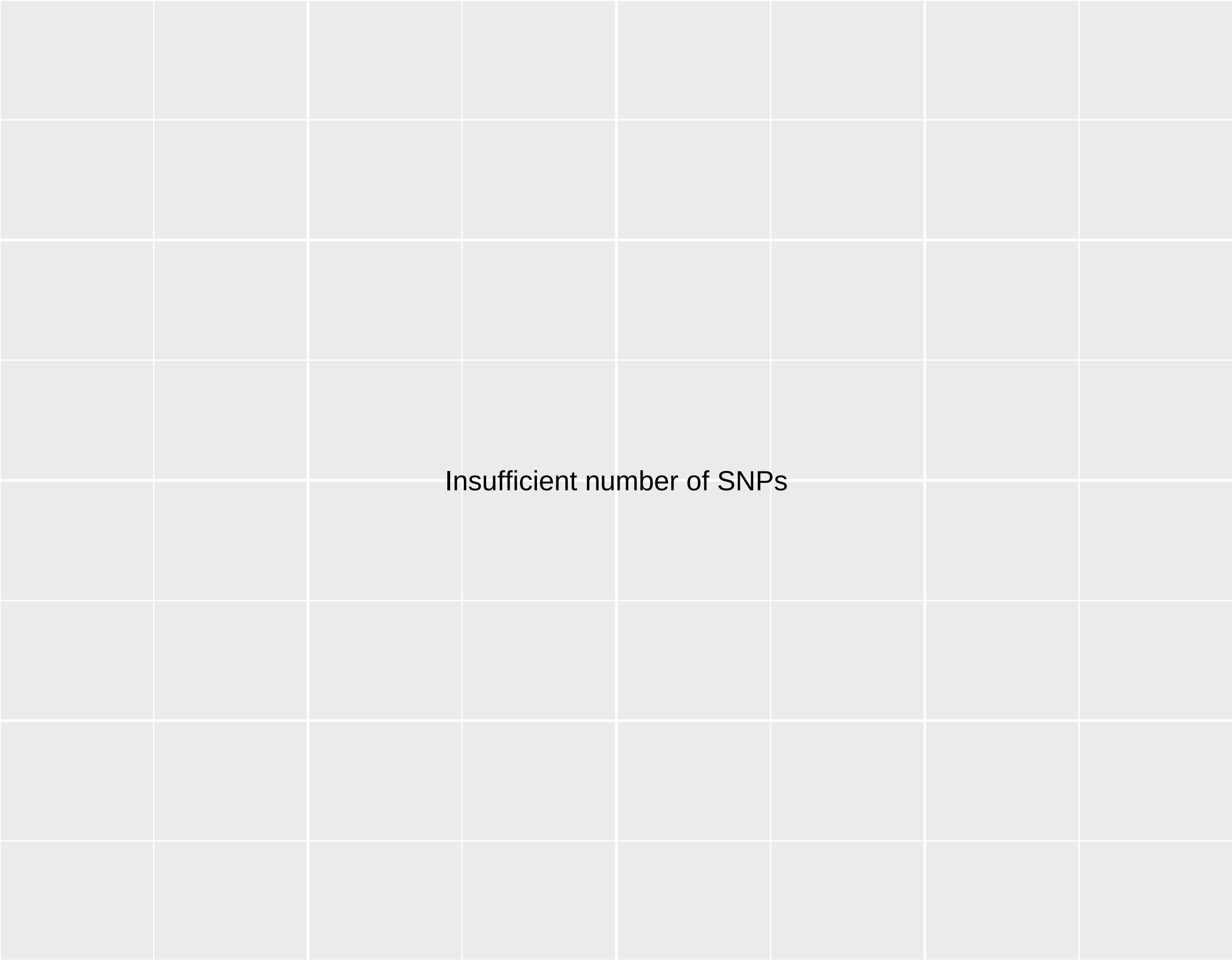
Insufficient number of SNPs

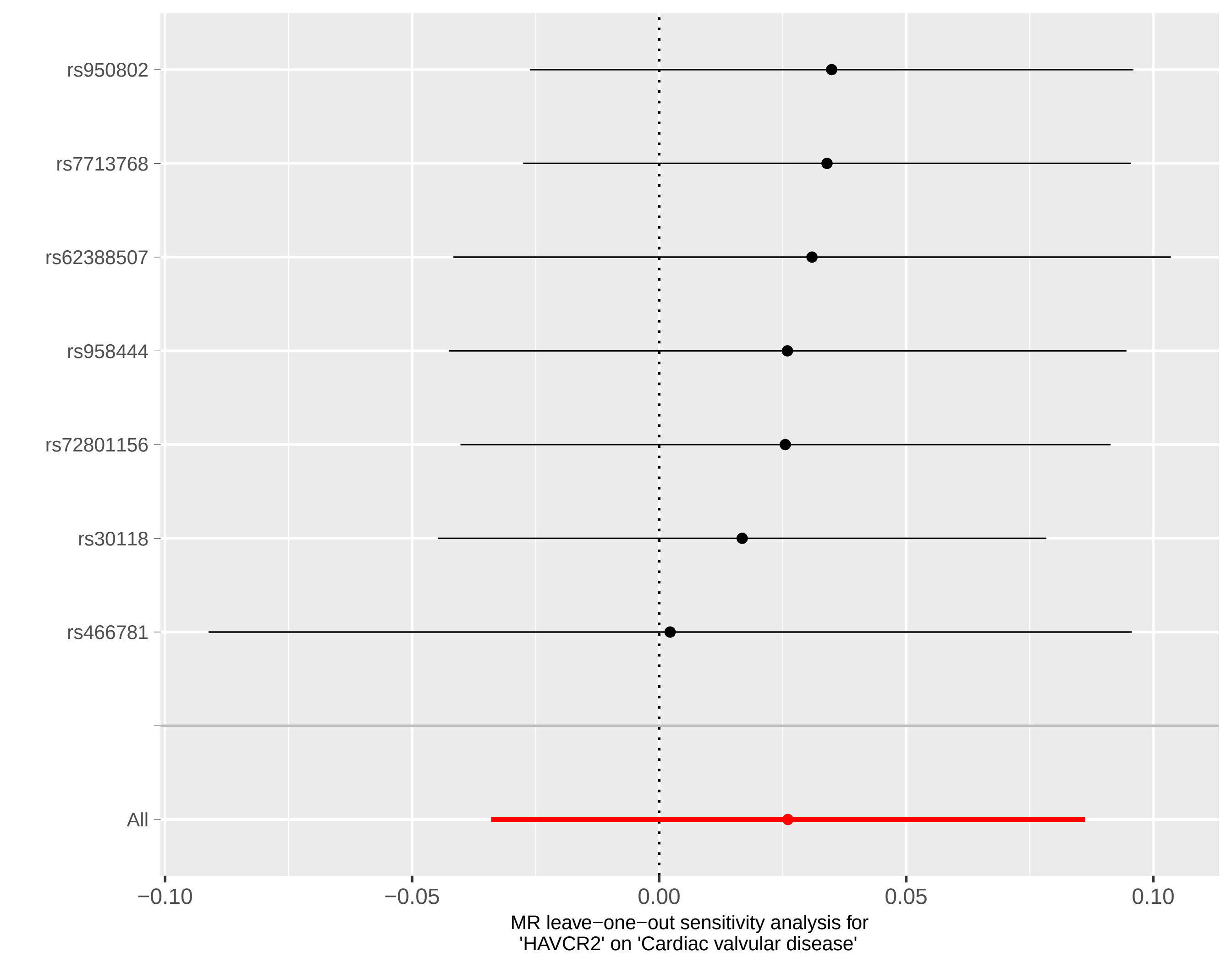
rs950802
rs7713768
rs62388507
rs958444
rs72801156
rs30118
rs466781
All
−0.10
−0.05
0.00
0.05
0.10
MR leave−one−out sensitivity analysis for
'HAVCR2' on 'Cardiac valvular disease'

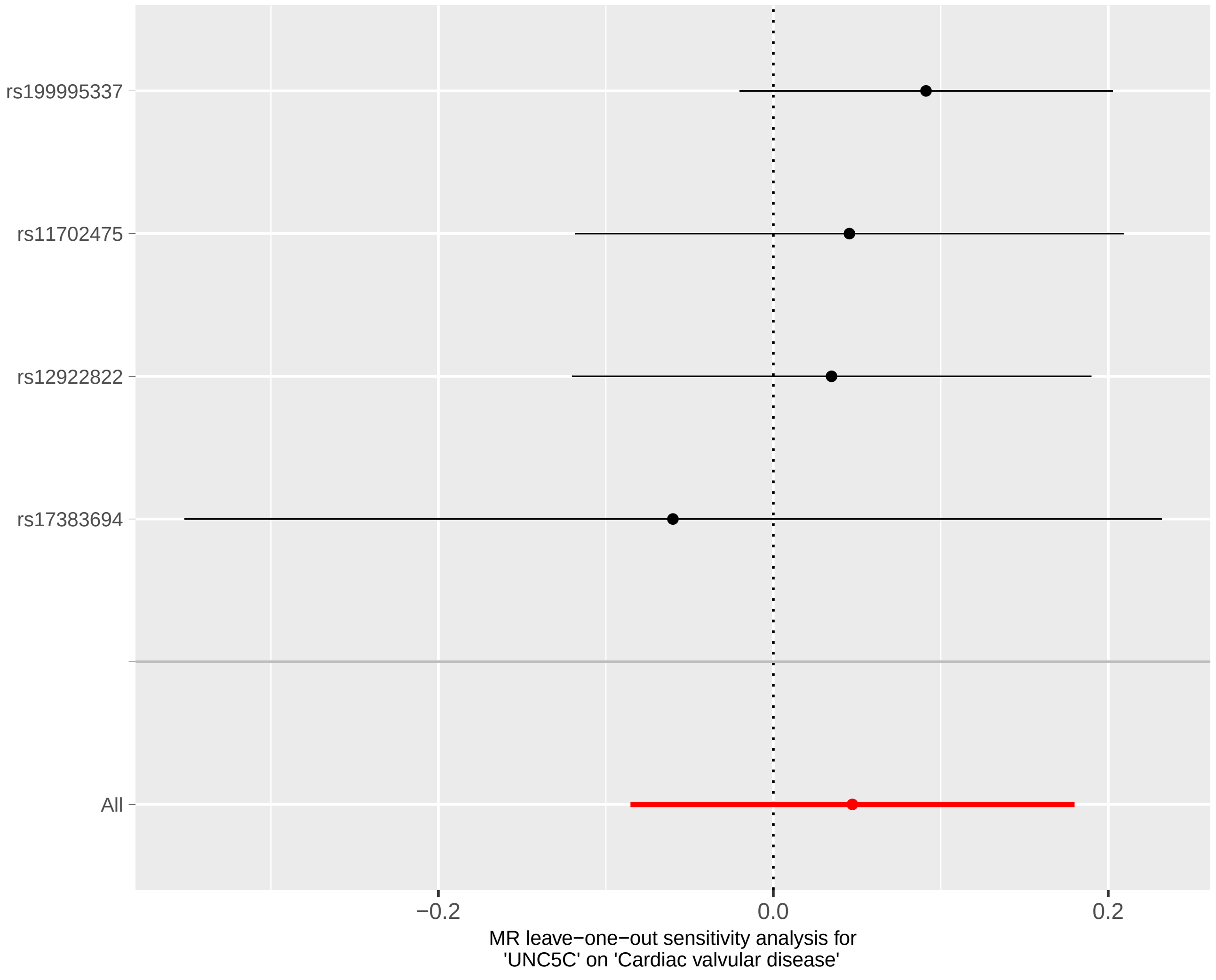

rs199995337
rs11702475
rs12922822
rs17383694
All
−0.2
0.0
0.2
MR leave−one−out sensitivity analysis for
'UNC5C' on 'Cardiac valvular disease'

Insufficient number of SNPs

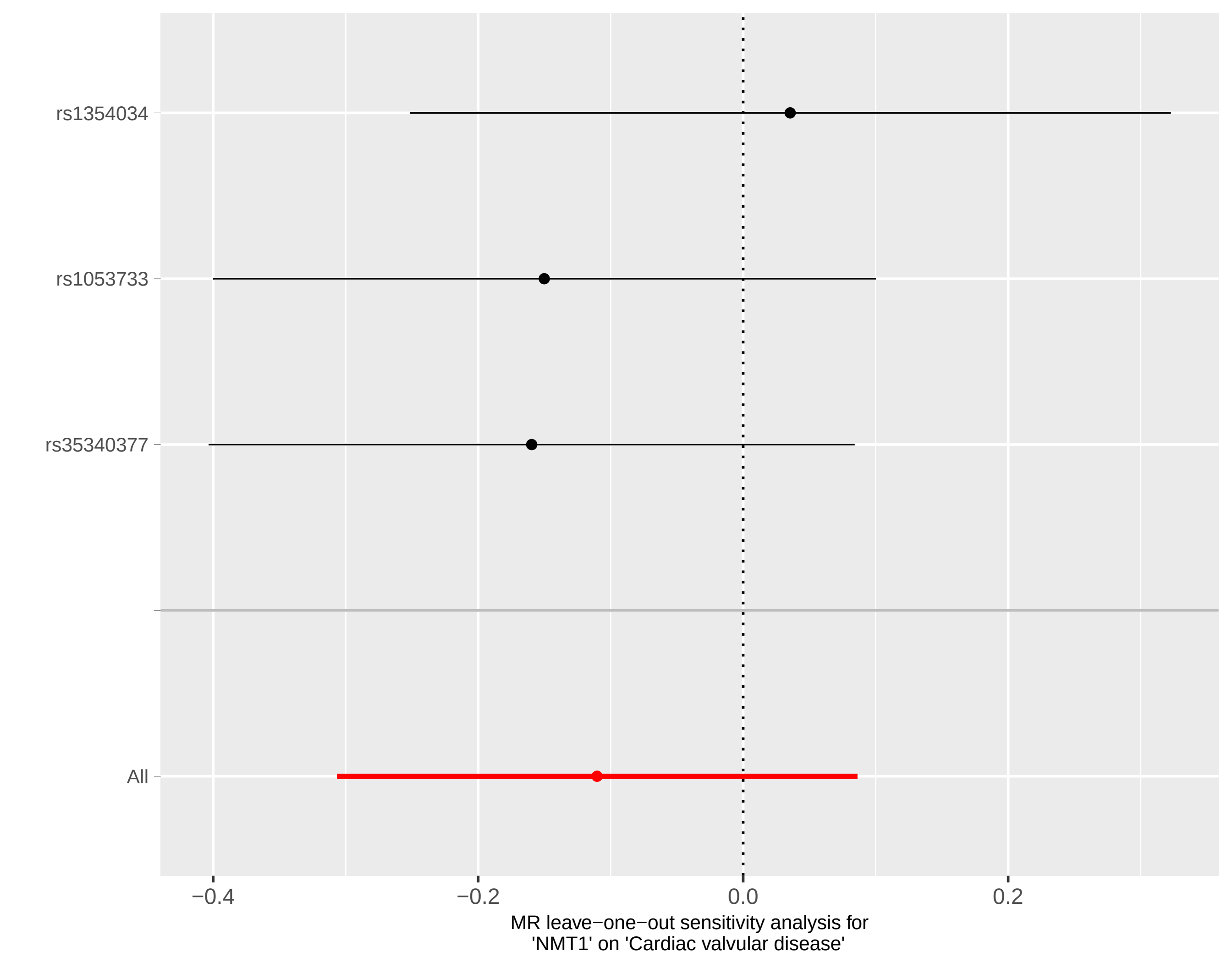
rs1354034
rs1053733
rs35340377
All
−0.4
−0.2
0.0
0.2
MR leave−one−out sensitivity analysis for
'NMT1' on 'Cardiac valvular disease'

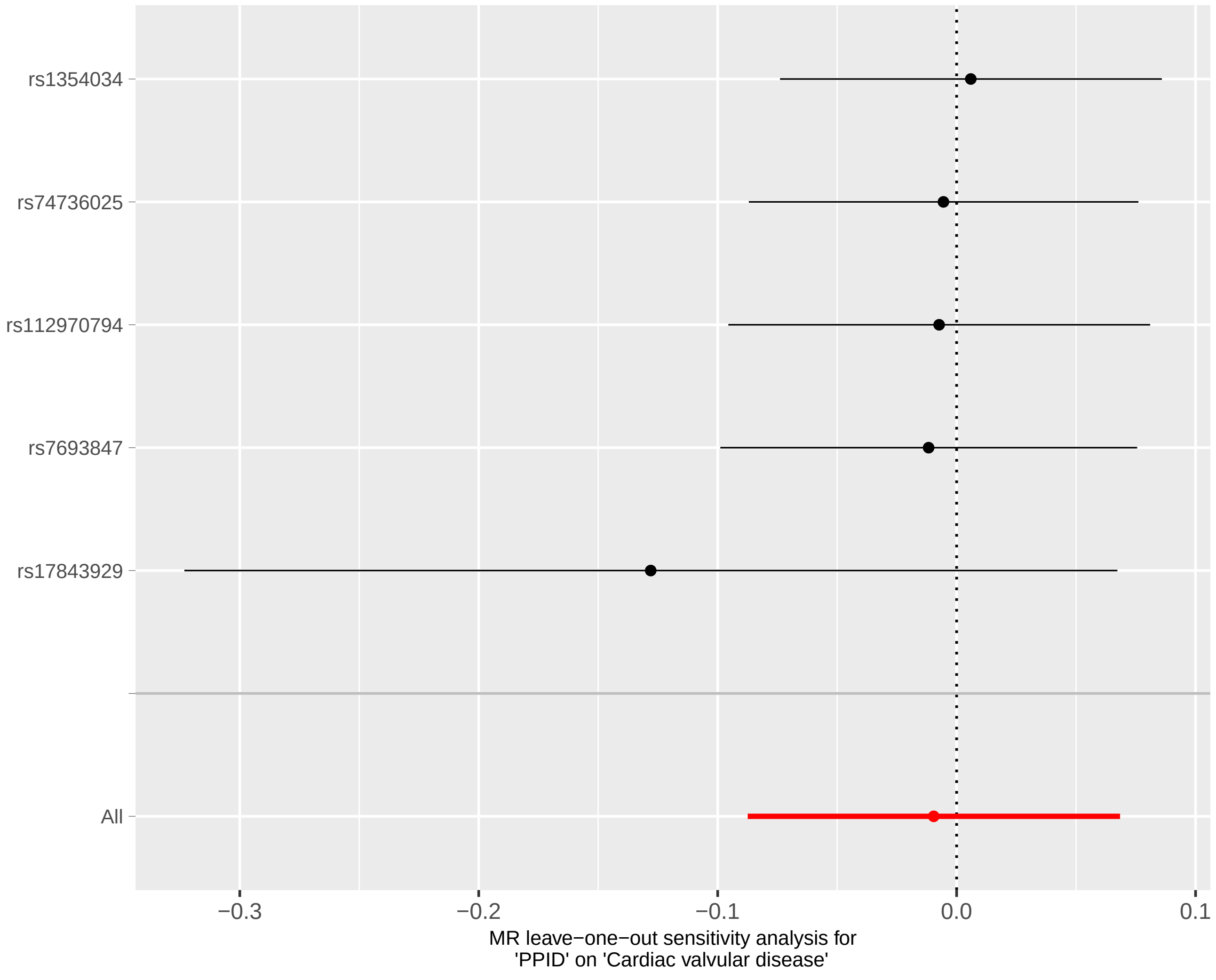
rs1354034
rs74736025
rs112970794
rs7693847
rs17843929
All
−0.3
−0.2
−0.1
0.0
0.1
MR leave−one−out sensitivity analysis for
'PPID' on 'Cardiac valvular disease'

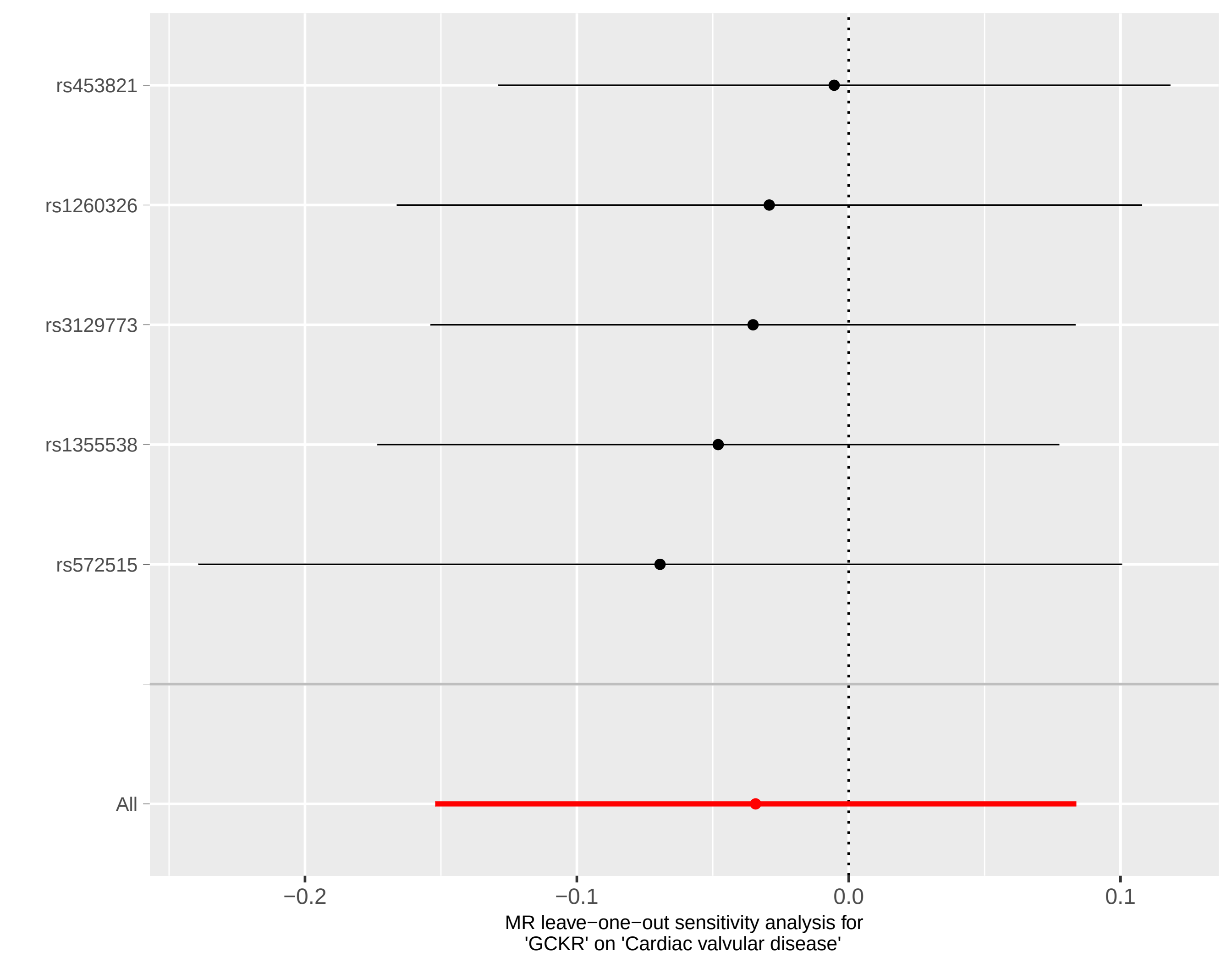
rs453821
rs1260326
rs3129773
rs1355538
rs572515
All
−0.2
−0.1
0.0
0.1
MR leave−one−out sensitivity analysis for
'GCKR' on 'Cardiac valvular disease'

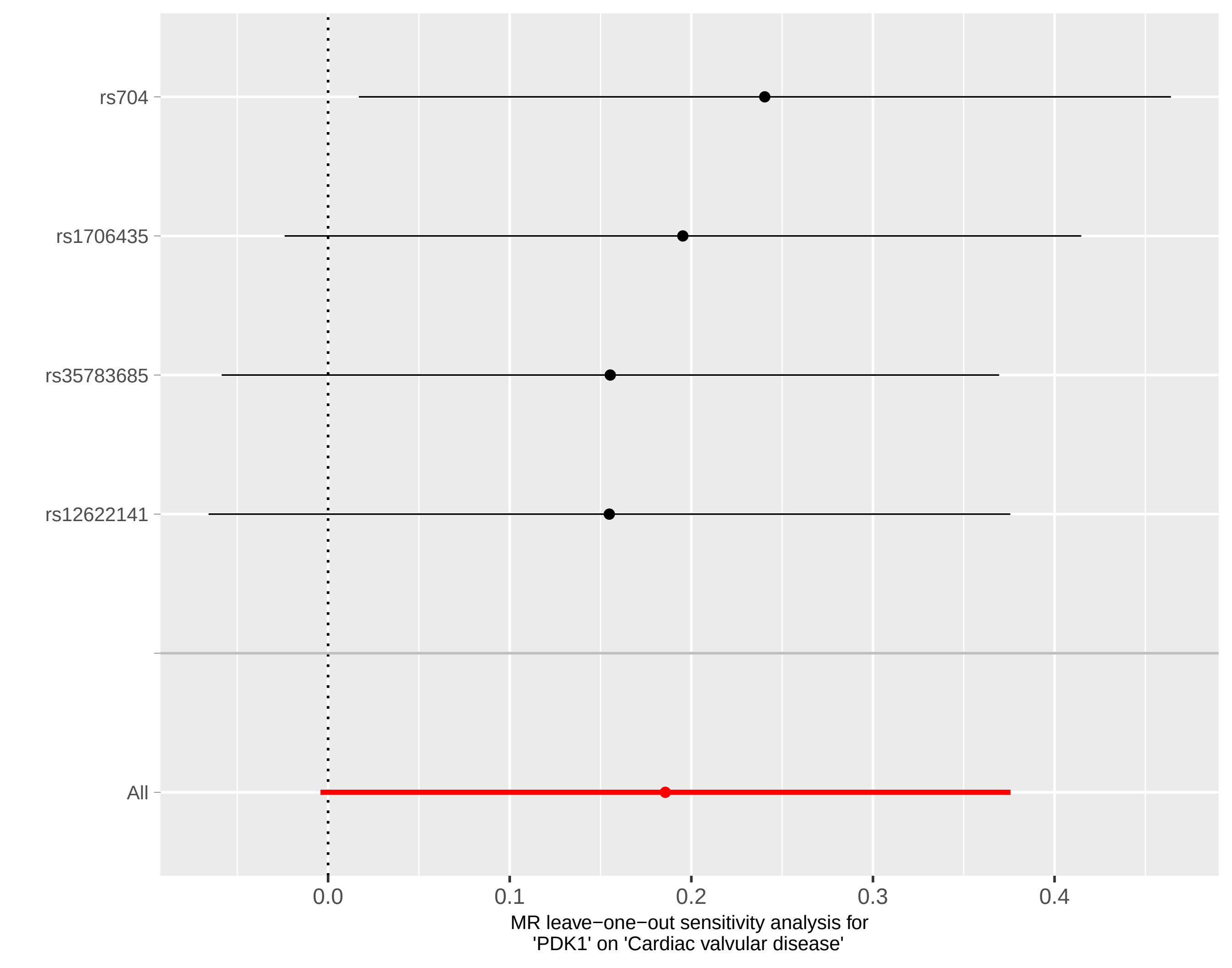

rs704
rs1706435
rs35783685
rs12622141
All
0.0
0.1
0.2
0.3
0.4
MR leave−one−out sensitivity analysis for
'PDK1' on 'Cardiac valvular disease'

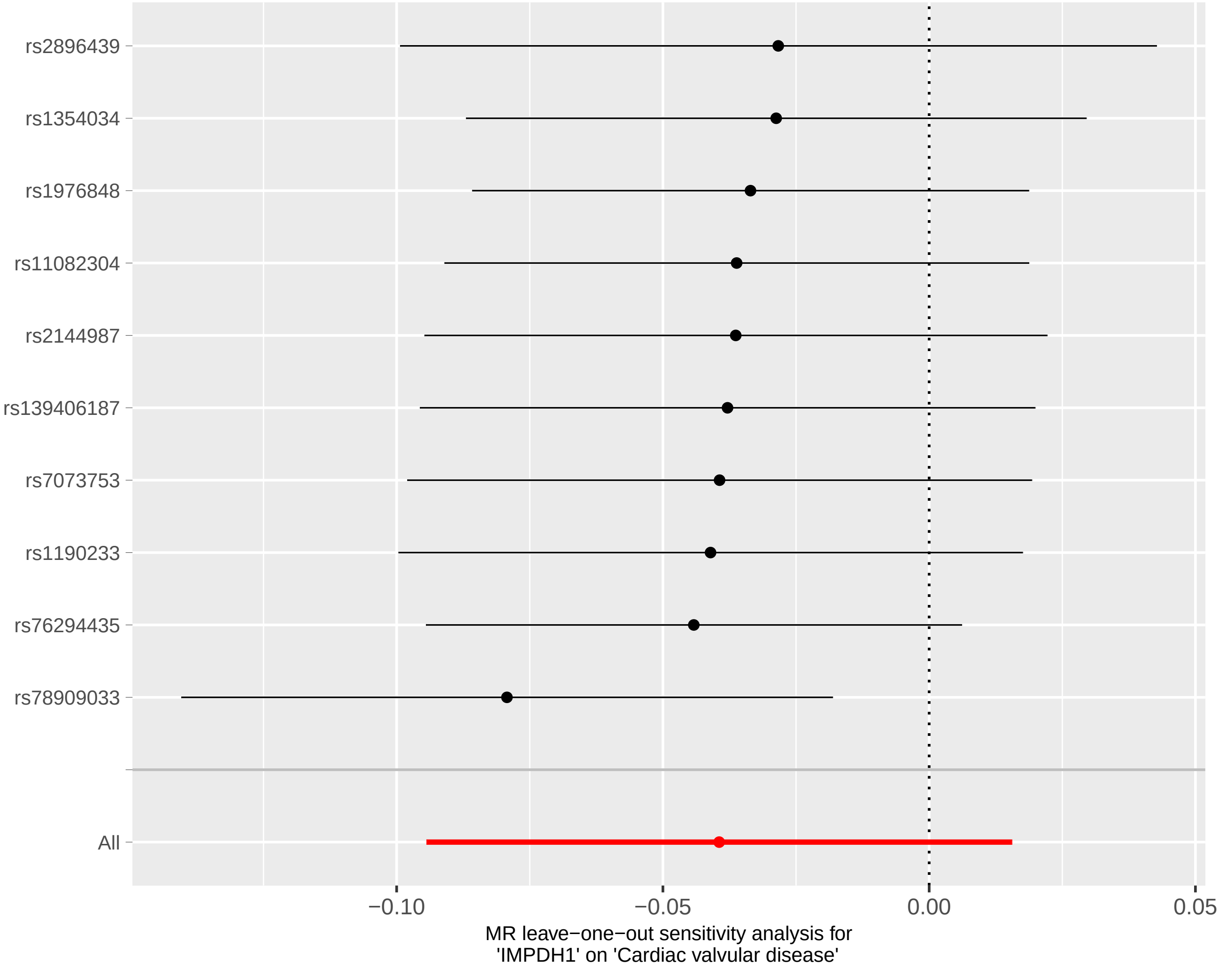
rs2896439
rs1354034
rs1976848
rs11082304
rs2144987
rs139406187
rs7073753
rs1190233
rs76294435
rs78909033
All
−0.10
−0.05
0.00
0.05
MR leave−one−out sensitivity analysis for
'IMPDH1' on 'Cardiac valvular disease'

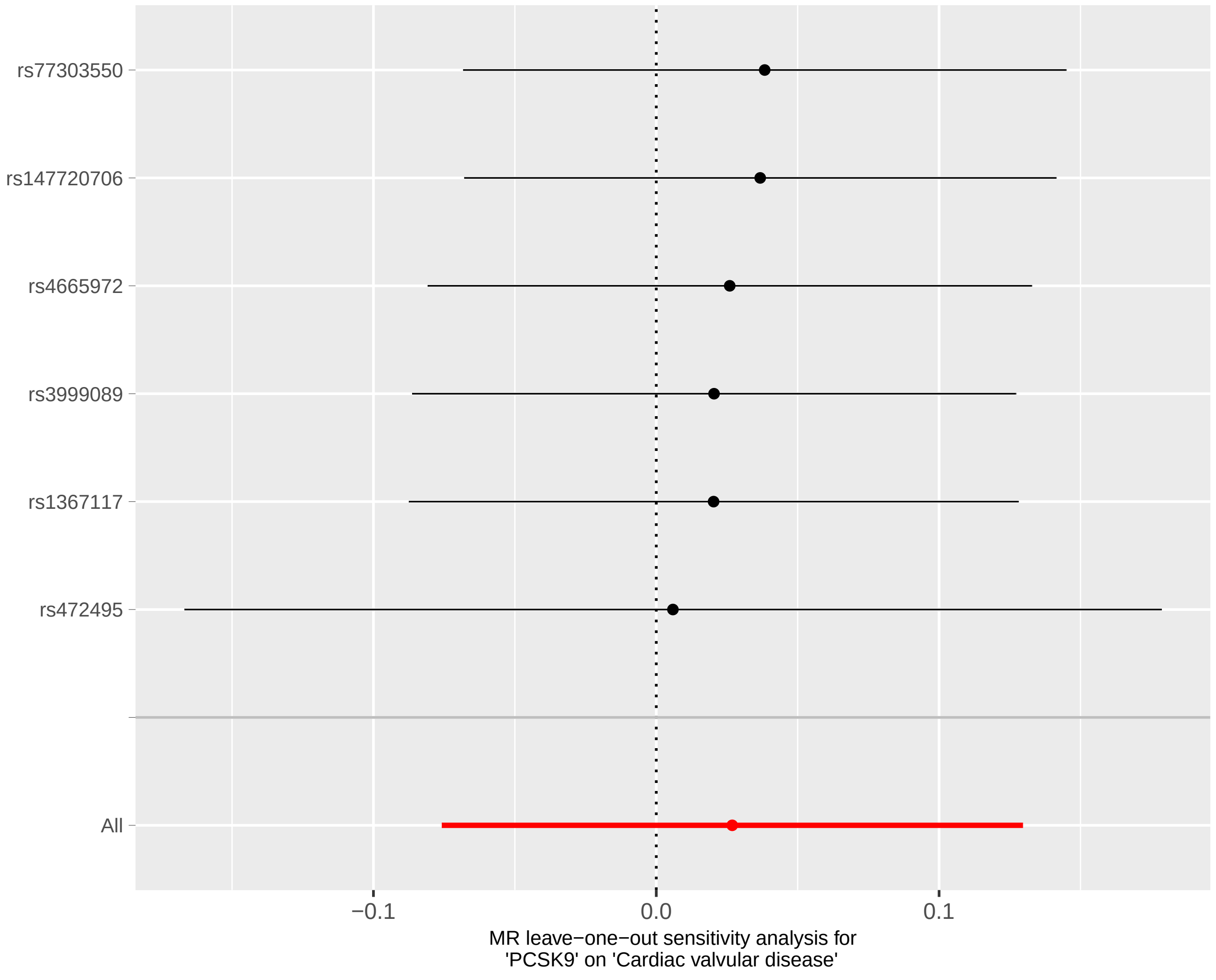
rs77303550
rs147720706
rs4665972
rs3999089
rs1367117
rs472495
All
−0.1
0.0
0.1
MR leave−one−out sensitivity analysis for
'PCSK9' on 'Cardiac valvular disease'

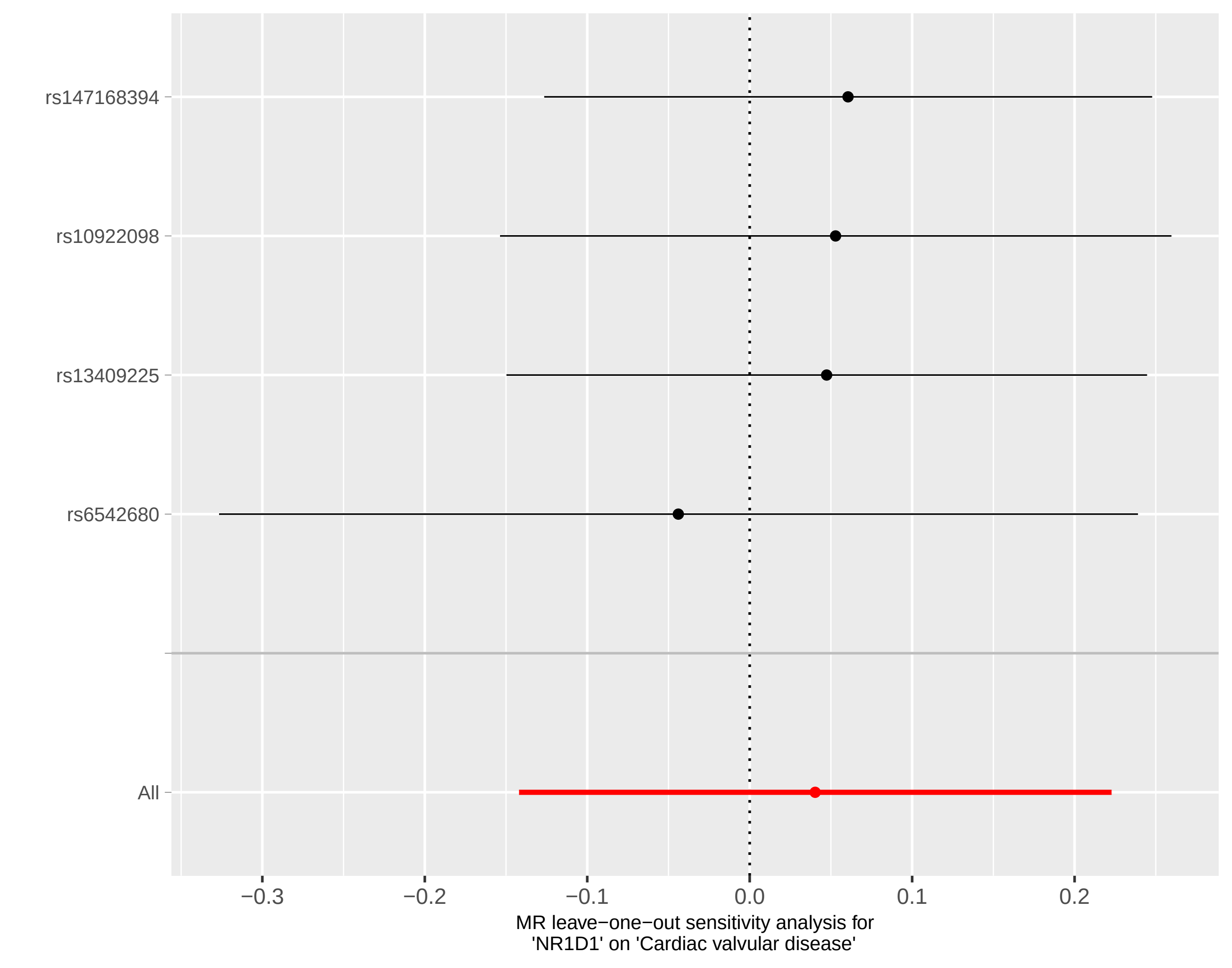

rs147168394
rs10922098
rs13409225
rs6542680
All
−0.3
−0.2
−0.1
0.0
0.1
0.2
MR leave−one−out sensitivity analysis for
'NR1D1' on 'Cardiac valvular disease'

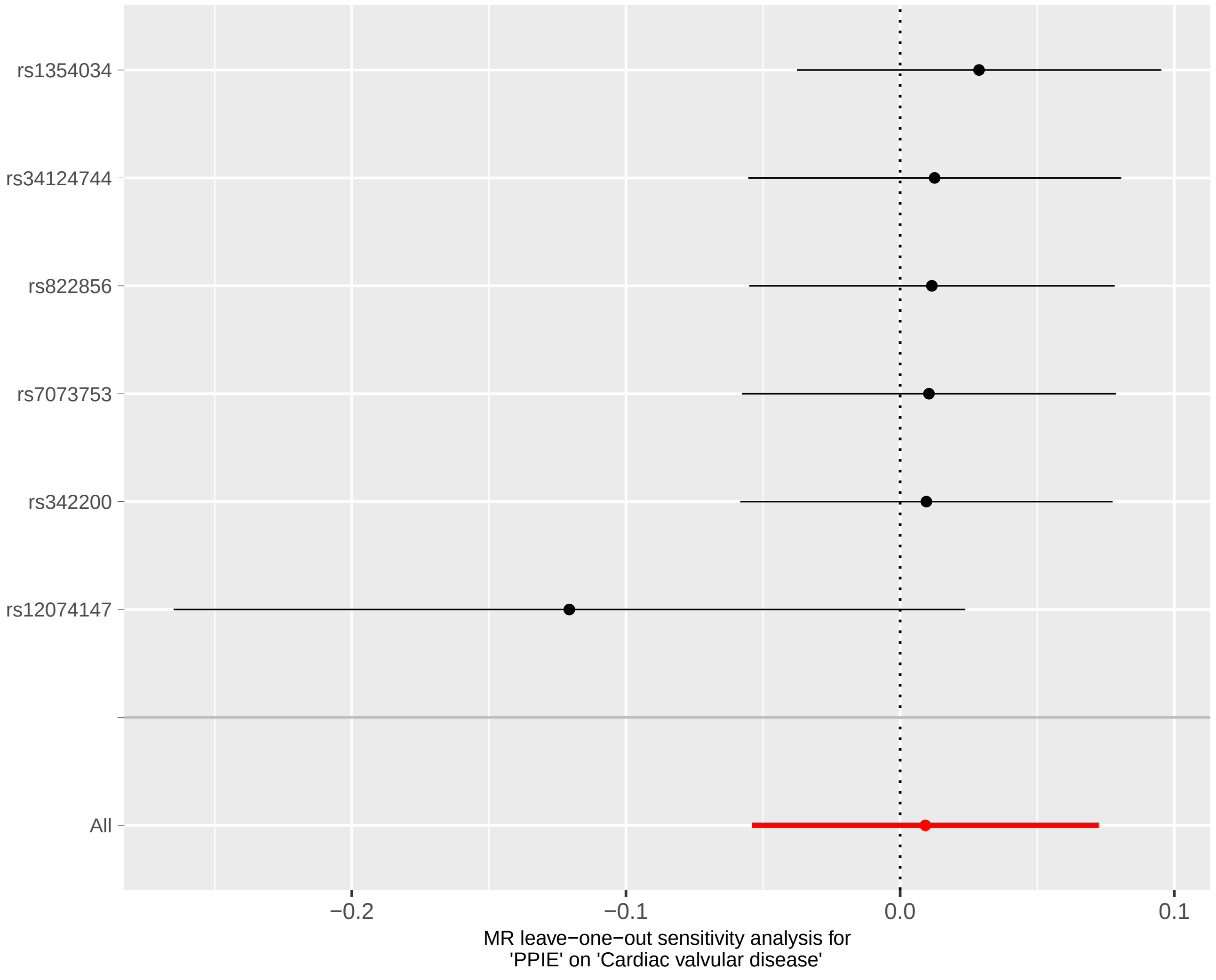

rs1354034
rs34124744
rs822856
rs7073753
rs342200
rs12074147
All
−0.2
−0.1
0.0
0.1
MR leave−one−out sensitivity analysis for
'PPIE' on 'Cardiac valvular disease'

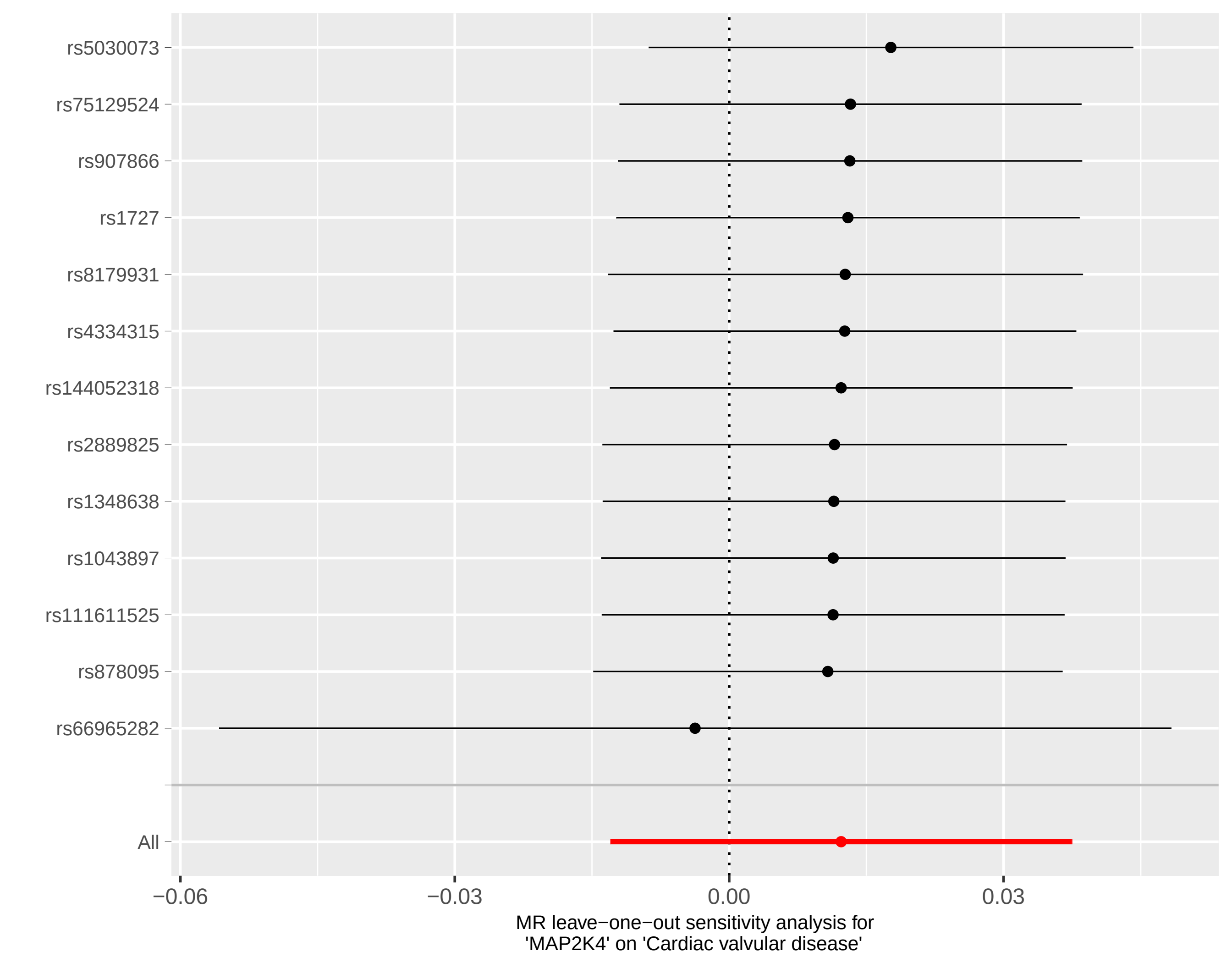
rs5030073
rs75129524
rs907866
rs1727
rs8179931
rs4334315
rs144052318
rs2889825
rs1348638
rs1043897
rs111611525
rs878095
rs66965282
All
−0.06
−0.03
0.00
0.03
MR leave−one−out sensitivity analysis for
'MAP2K4' on 'Cardiac valvular disease'

Insufficient number of SNPs

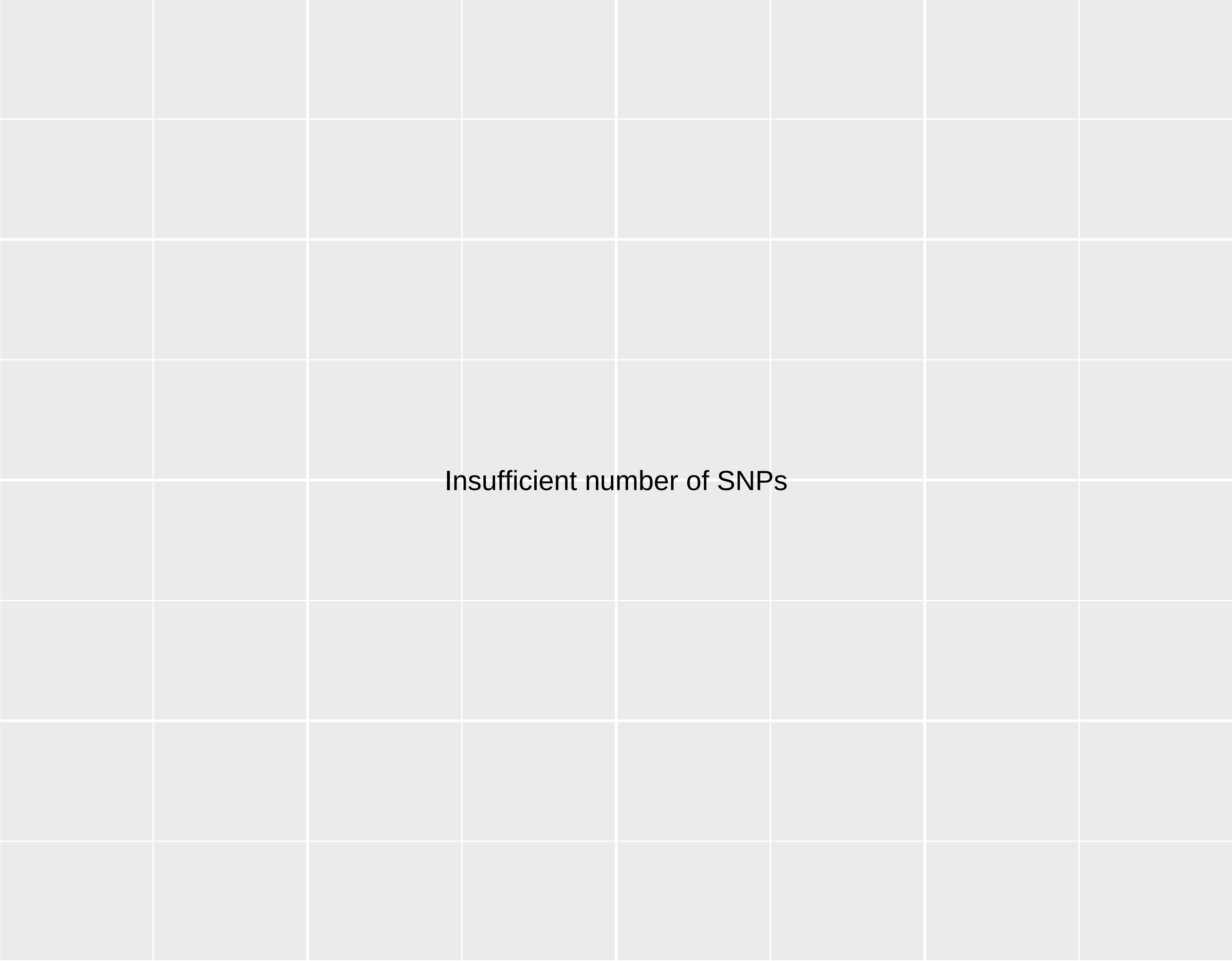
Insufficient number of SNPs

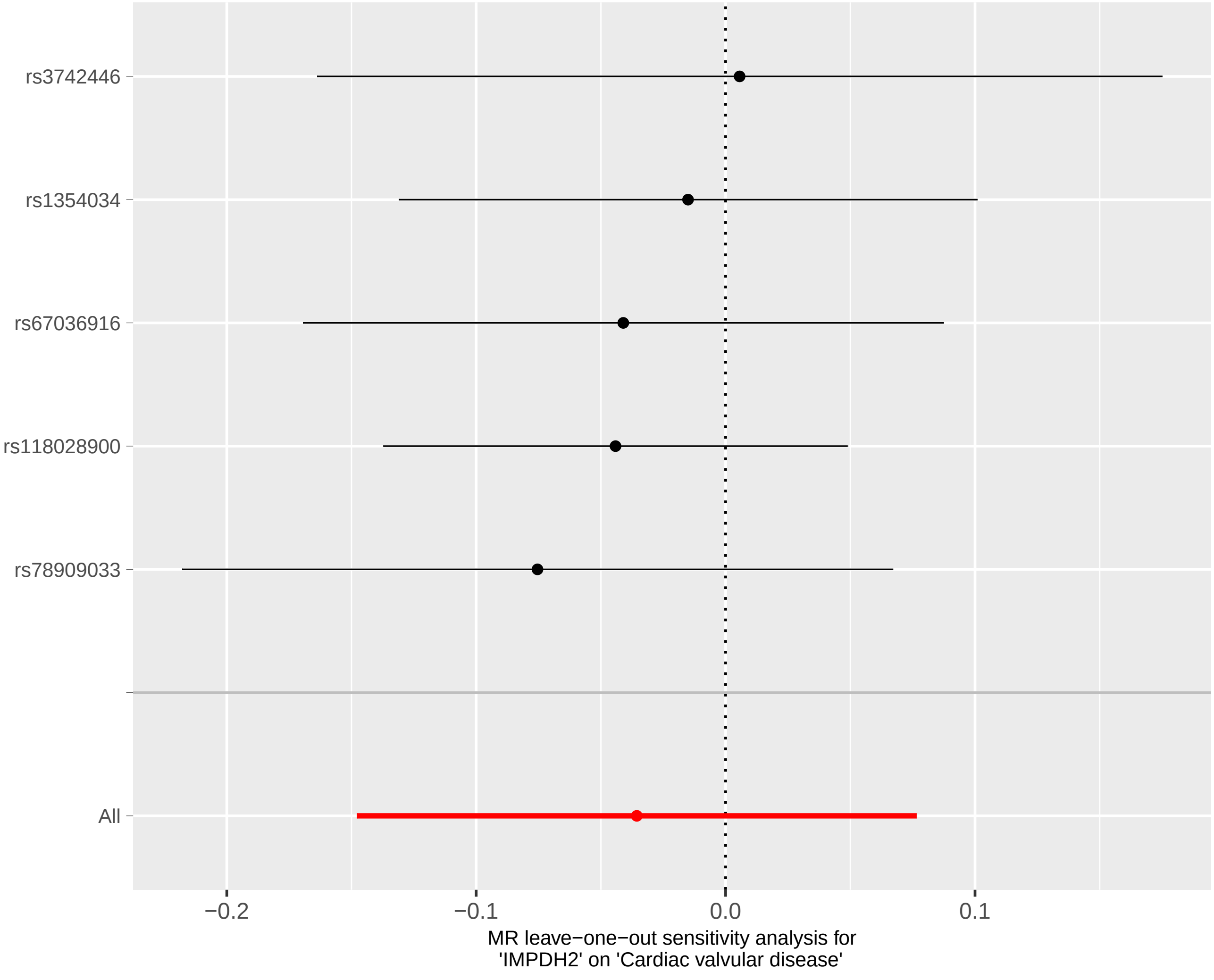

rs3742446
rs1354034
rs67036916
rs118028900
rs78909033
All
−0.2
−0.1
0.0
0.1
MR leave−one−out sensitivity analysis for
'IMPDH2' on 'Cardiac valvular disease'

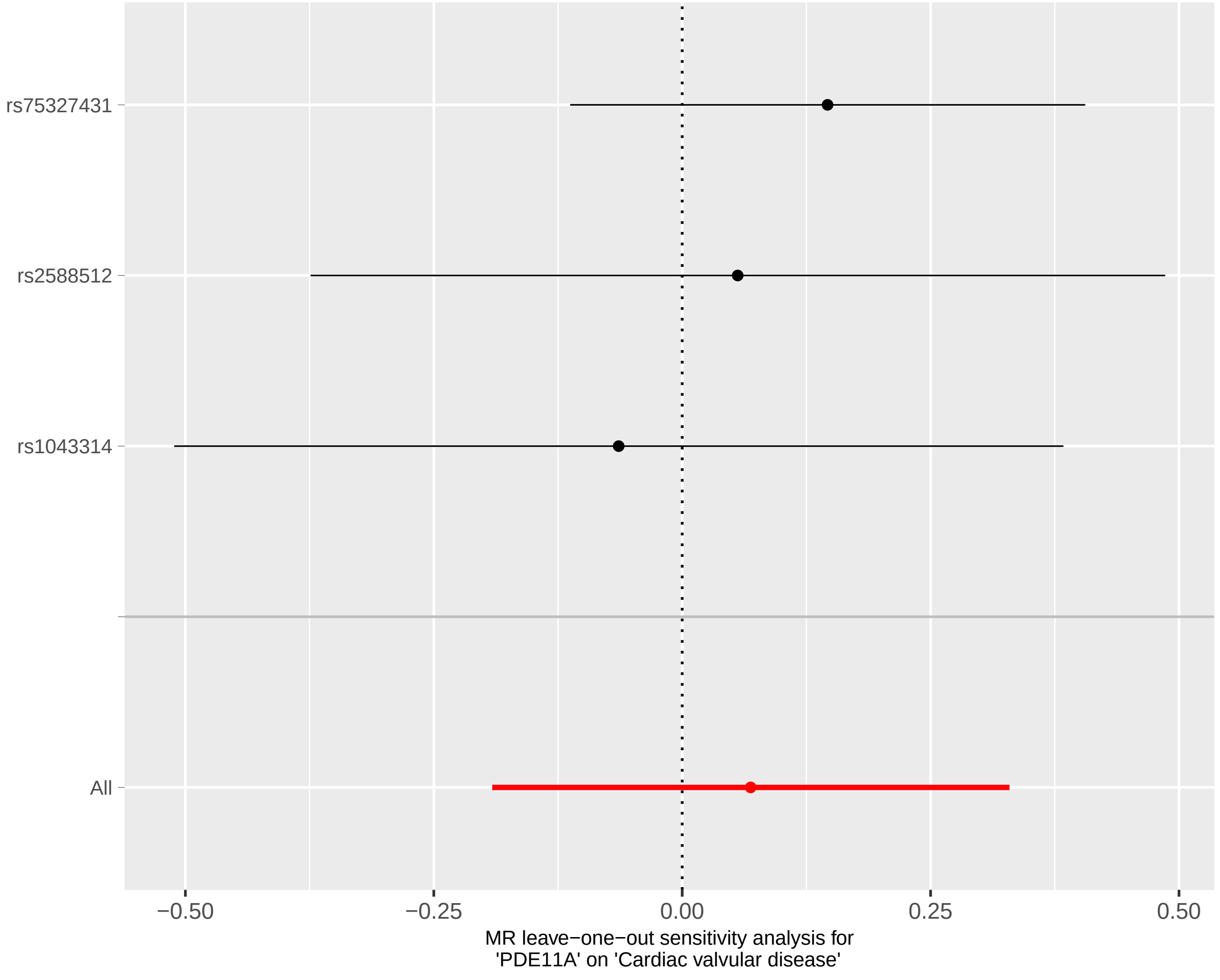
rs75327431
rs2588512
rs1043314
All
−0.50
−0.25
0.00
0.25
0.50
MR leave−one−out sensitivity analysis for
'PDE11A' on 'Cardiac valvular disease'

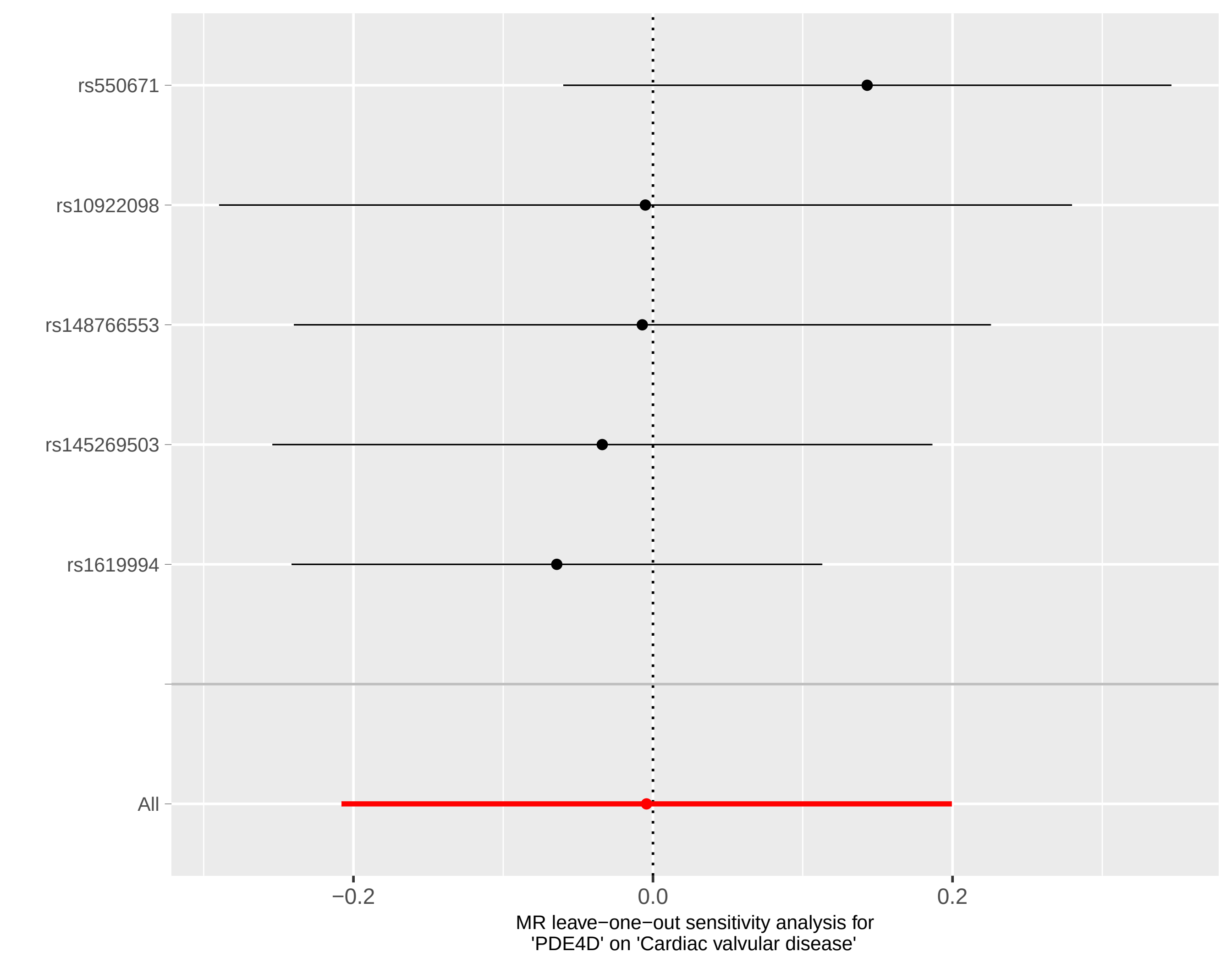
rs550671
rs10922098
rs148766553
rs145269503
rs1619994
All
−0.2
0.0
0.2
MR leave−one−out sensitivity analysis for
'PDE4D' on 'Cardiac valvular disease'

Insufficient number of SNPs

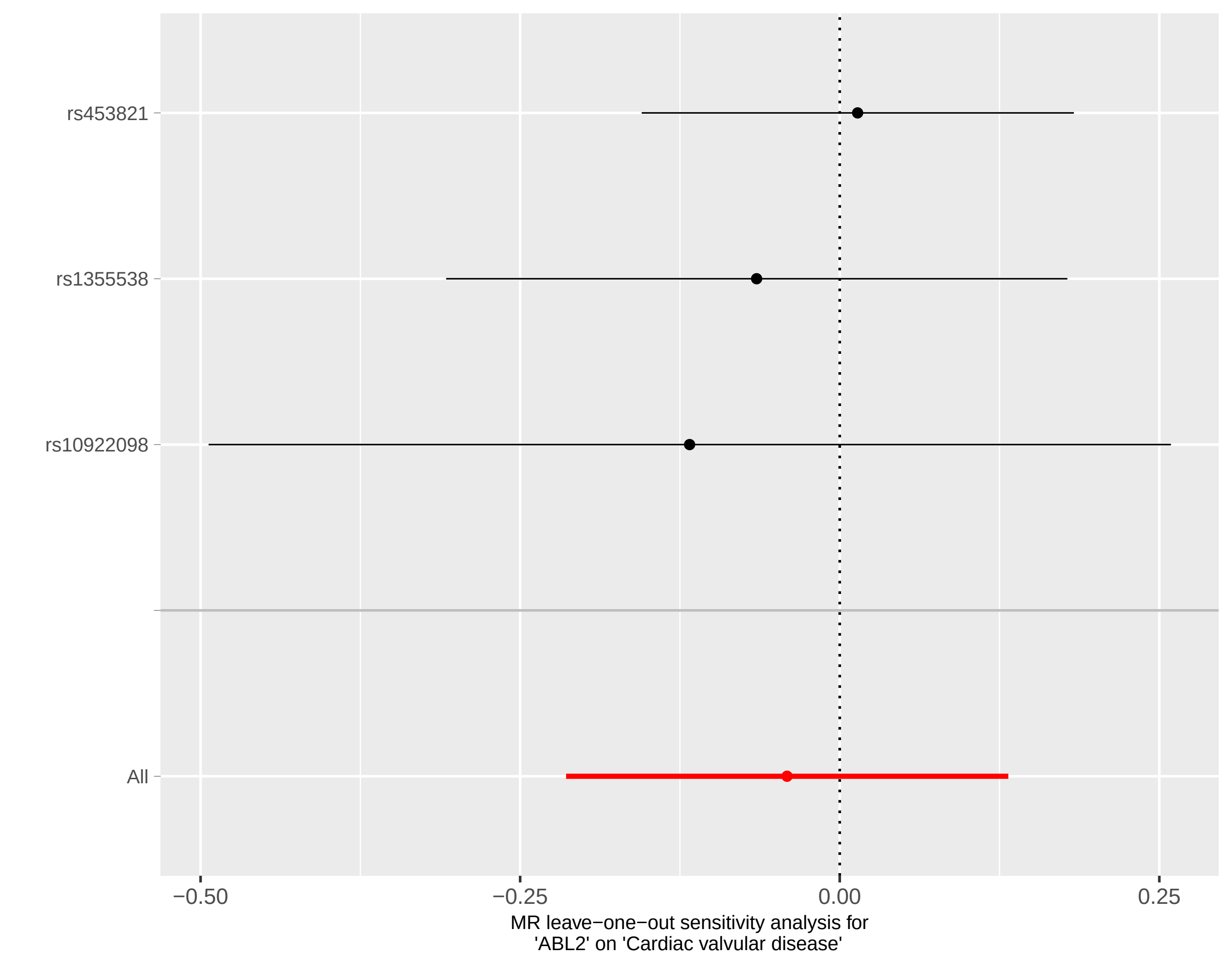
rs453821
rs1355538
rs10922098
All
−0.50
−0.25
0.00
0.25
MR leave−one−out sensitivity analysis for
'ABL2' on 'Cardiac valvular disease'

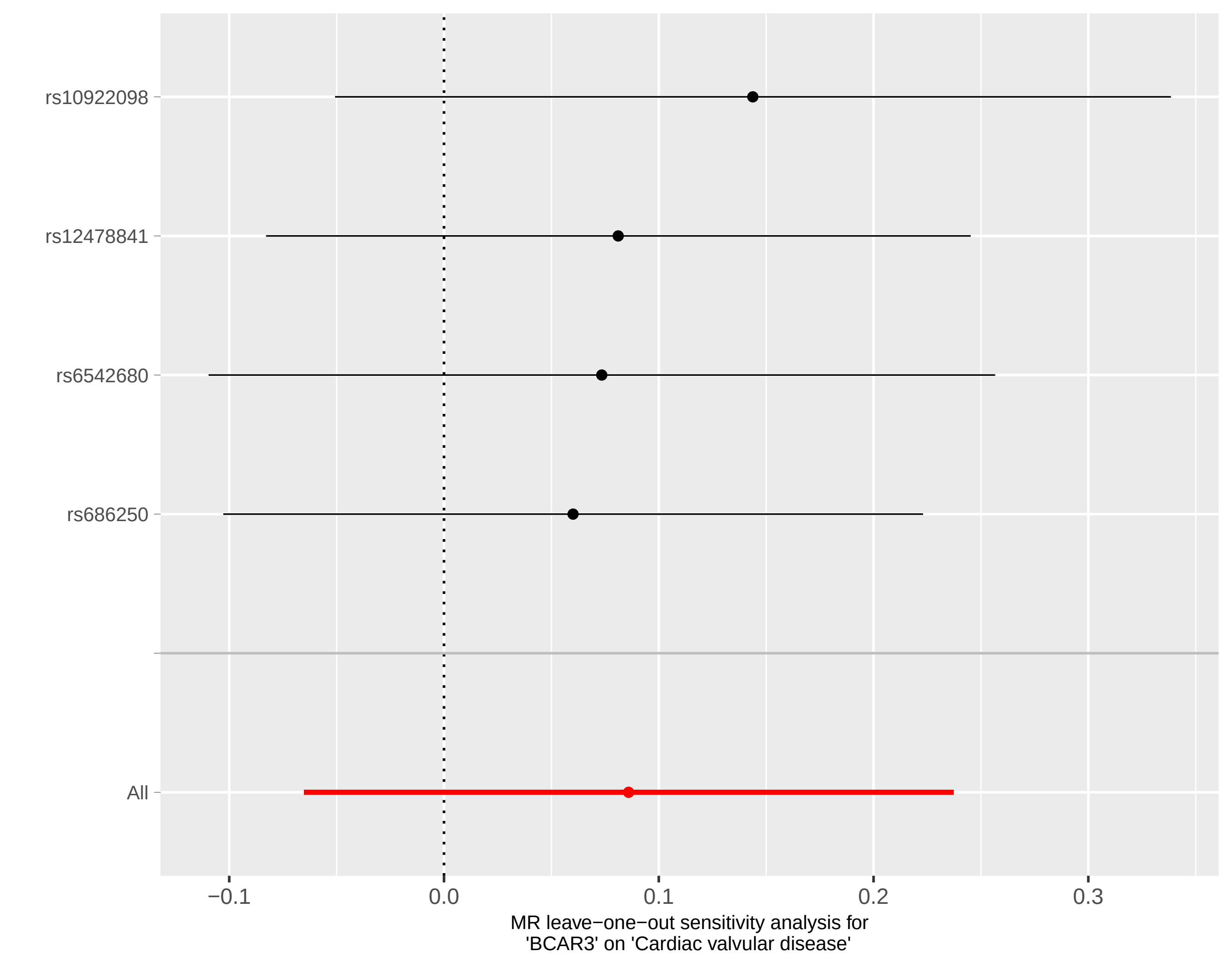
rs10922098
rs12478841
rs6542680
rs686250
All
−0.1
0.0
0.1
0.2
0.3
MR leave−one−out sensitivity analysis for
'BCAR3' on 'Cardiac valvular disease'

Insufficient number of SNPs

Insufficient number of SNPs

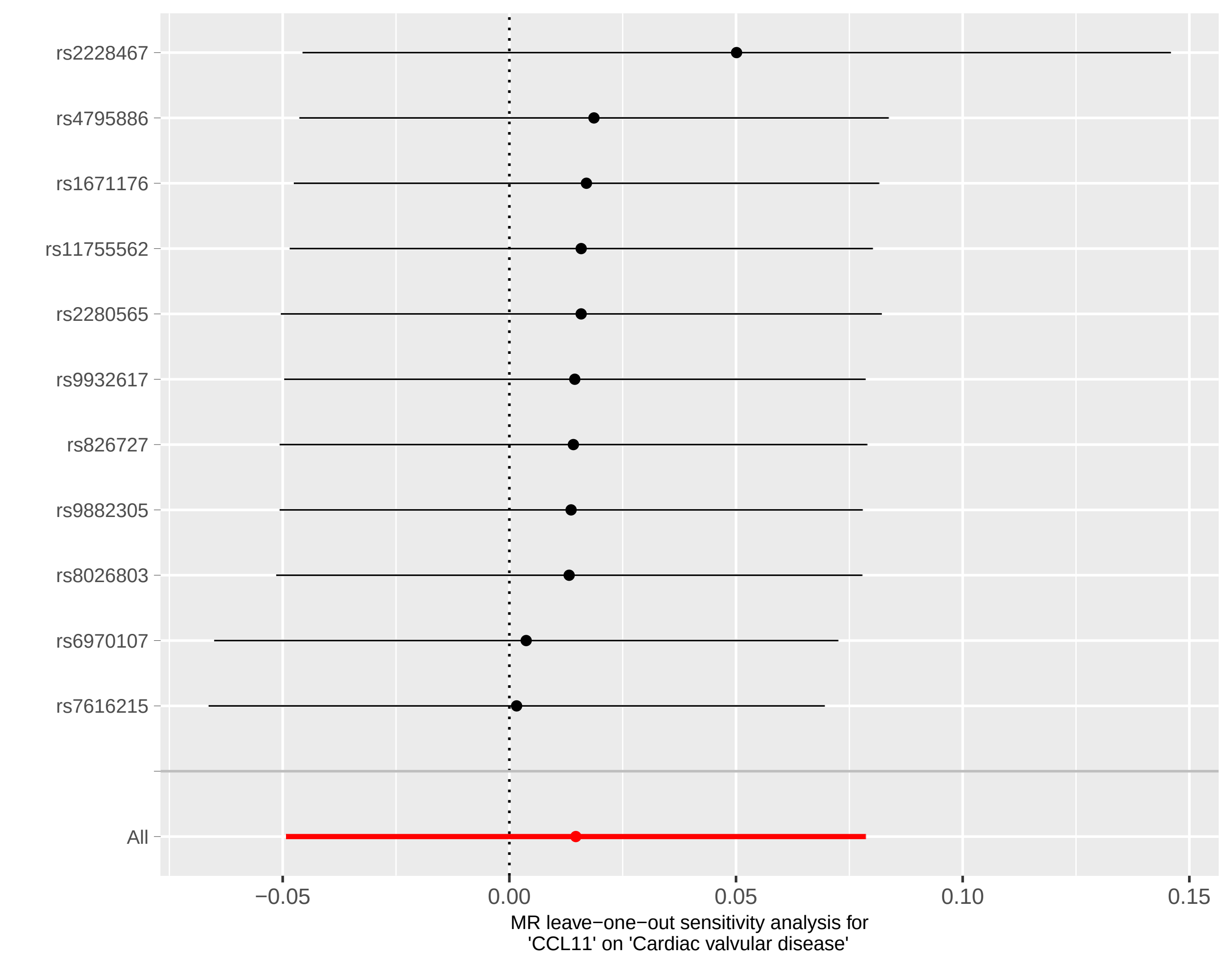
rs2228467
rs4795886
rs1671176
rs11755562
rs2280565
rs9932617
rs826727
rs9882305
rs8026803
rs6970107
rs7616215
All
−0.05
0.00
0.05
0.10
0.15
MR leave−one−out sensitivity analysis for
'CCL11' on 'Cardiac valvular disease'

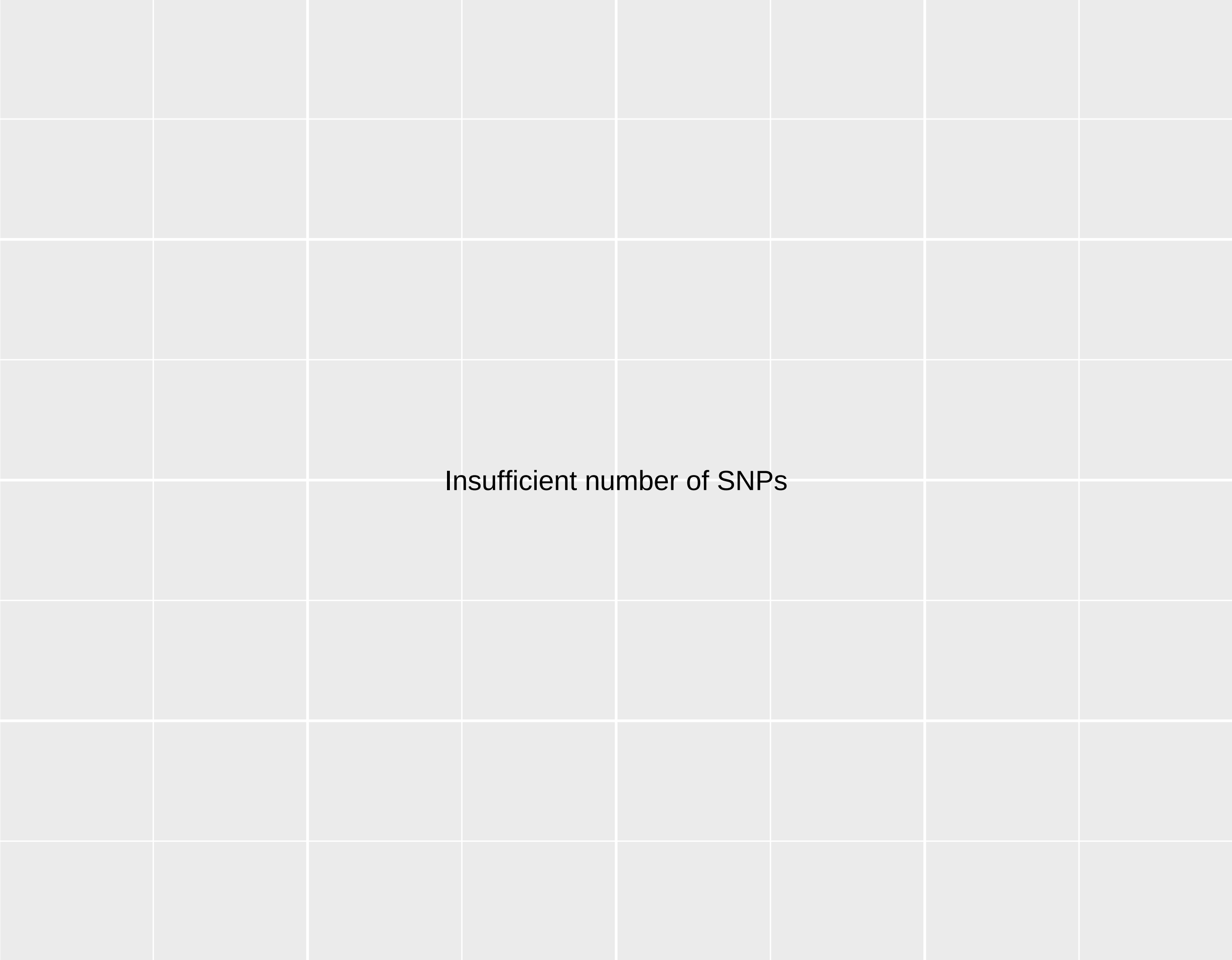
Insufficient number of SNPs

Insufficient number of SNPs

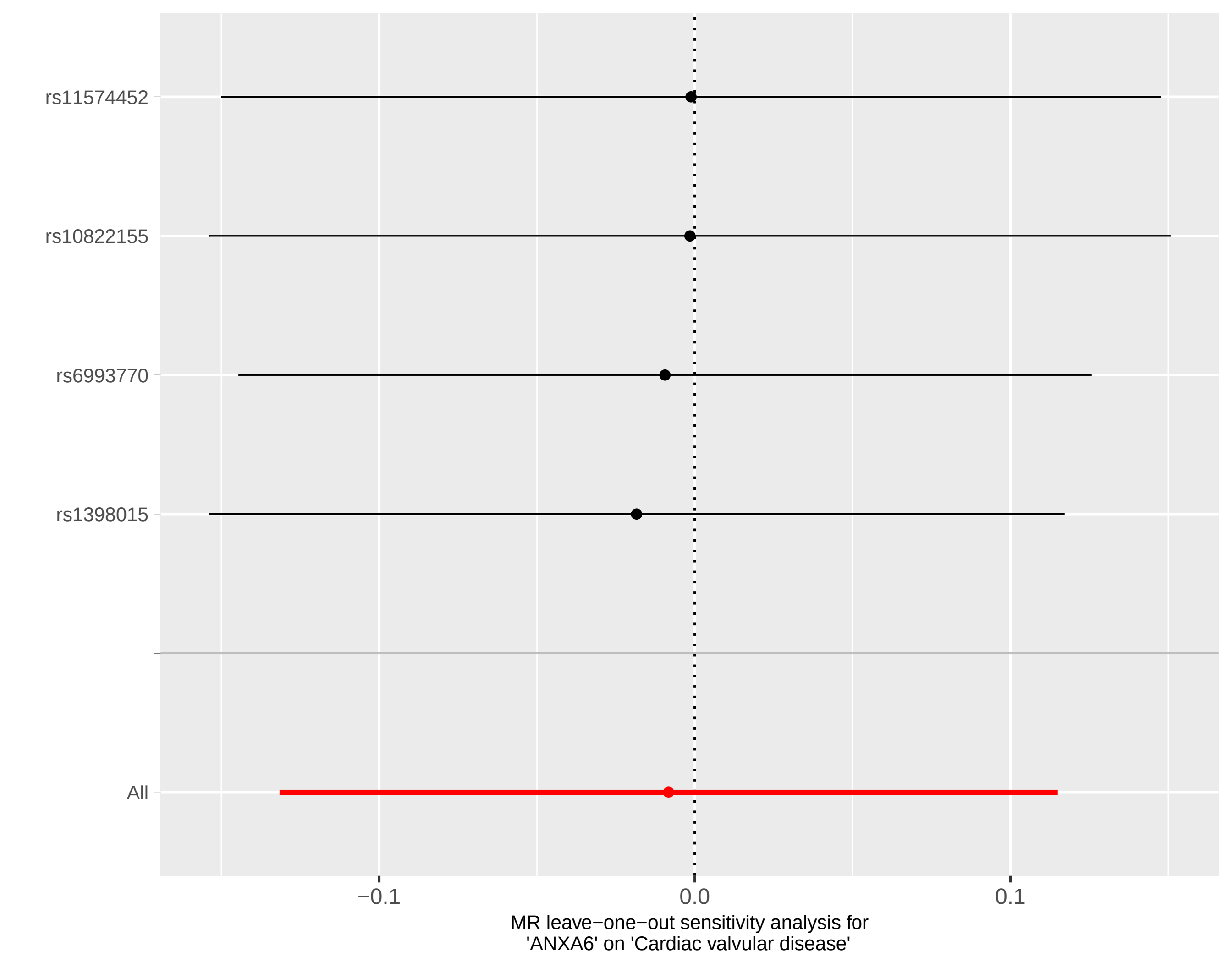
rs11574452
rs10822155
rs6993770
rs1398015
All
−0.1
0.0
0.1
MR leave−one−out sensitivity analysis for
'ANXA6' on 'Cardiac valvular disease'

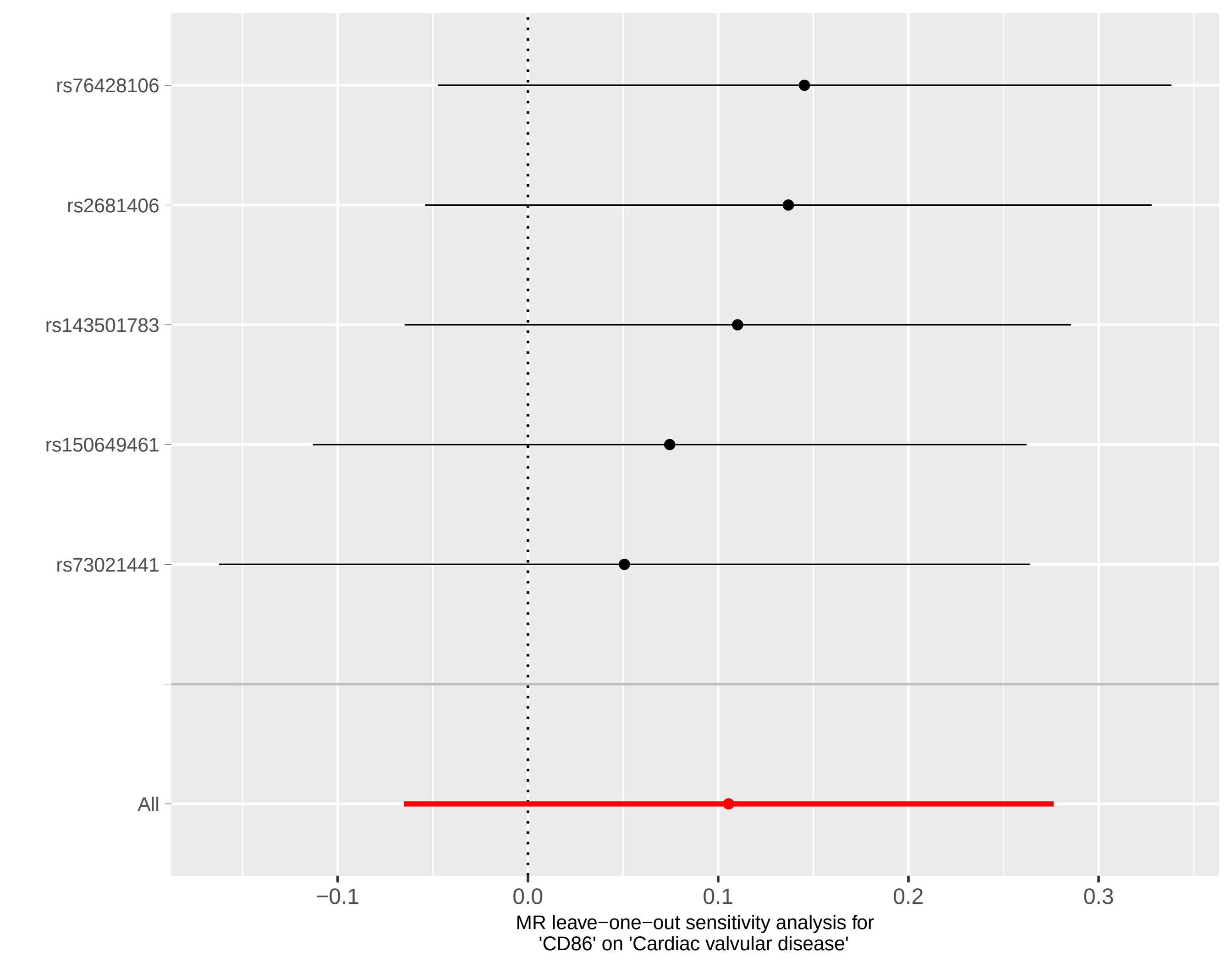
rs76428106
rs2681406
rs143501783
rs150649461
rs73021441
All
−0.1
0.0
0.1
0.2
0.3
MR leave−one−out sensitivity analysis for
'CD86' on 'Cardiac valvular disease'

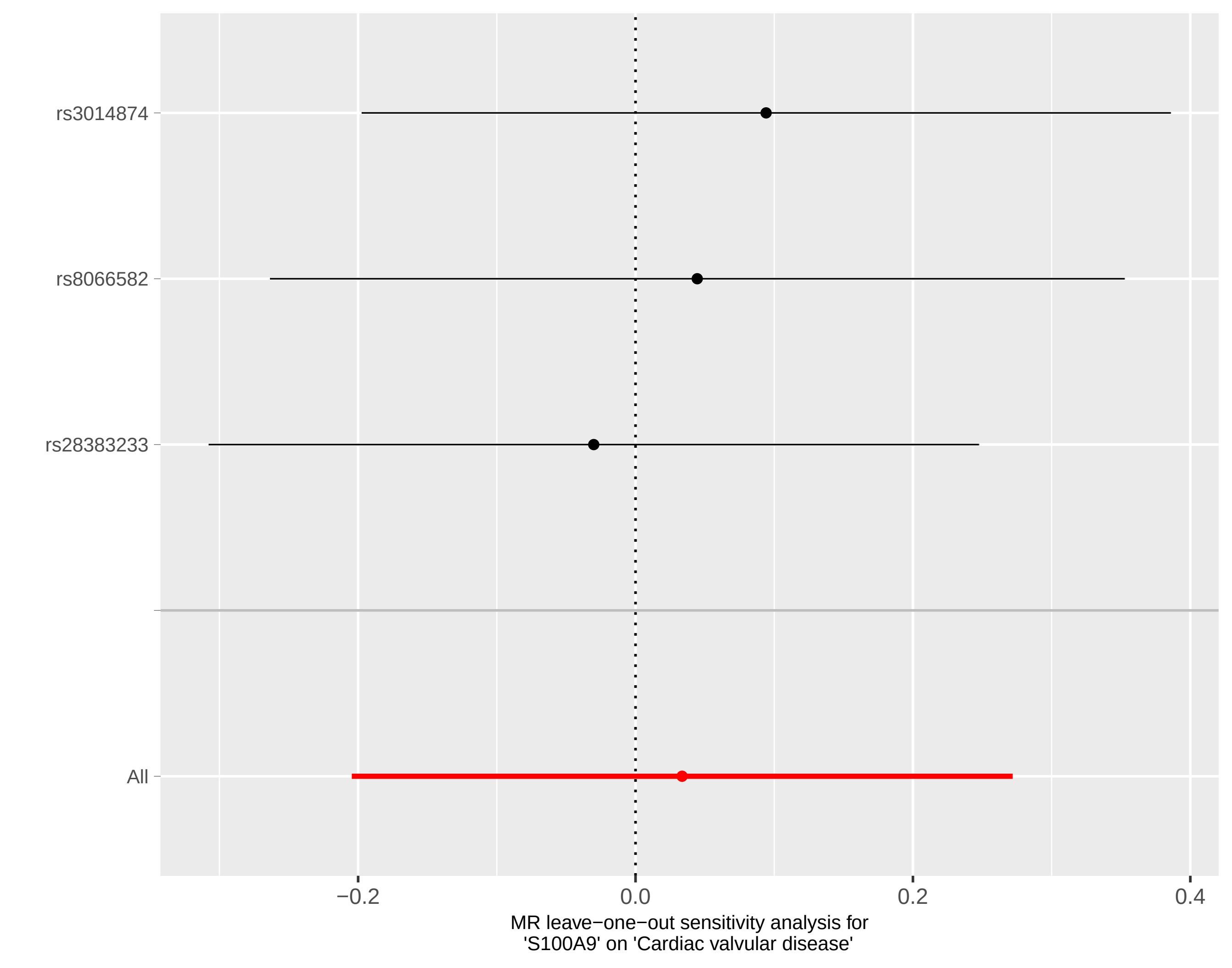
rs3014874
rs8066582
rs28383233
All
−0.2
0.0
0.2
0.4
MR leave−one−out sensitivity analysis for
'S100A9' on 'Cardiac valvular disease'

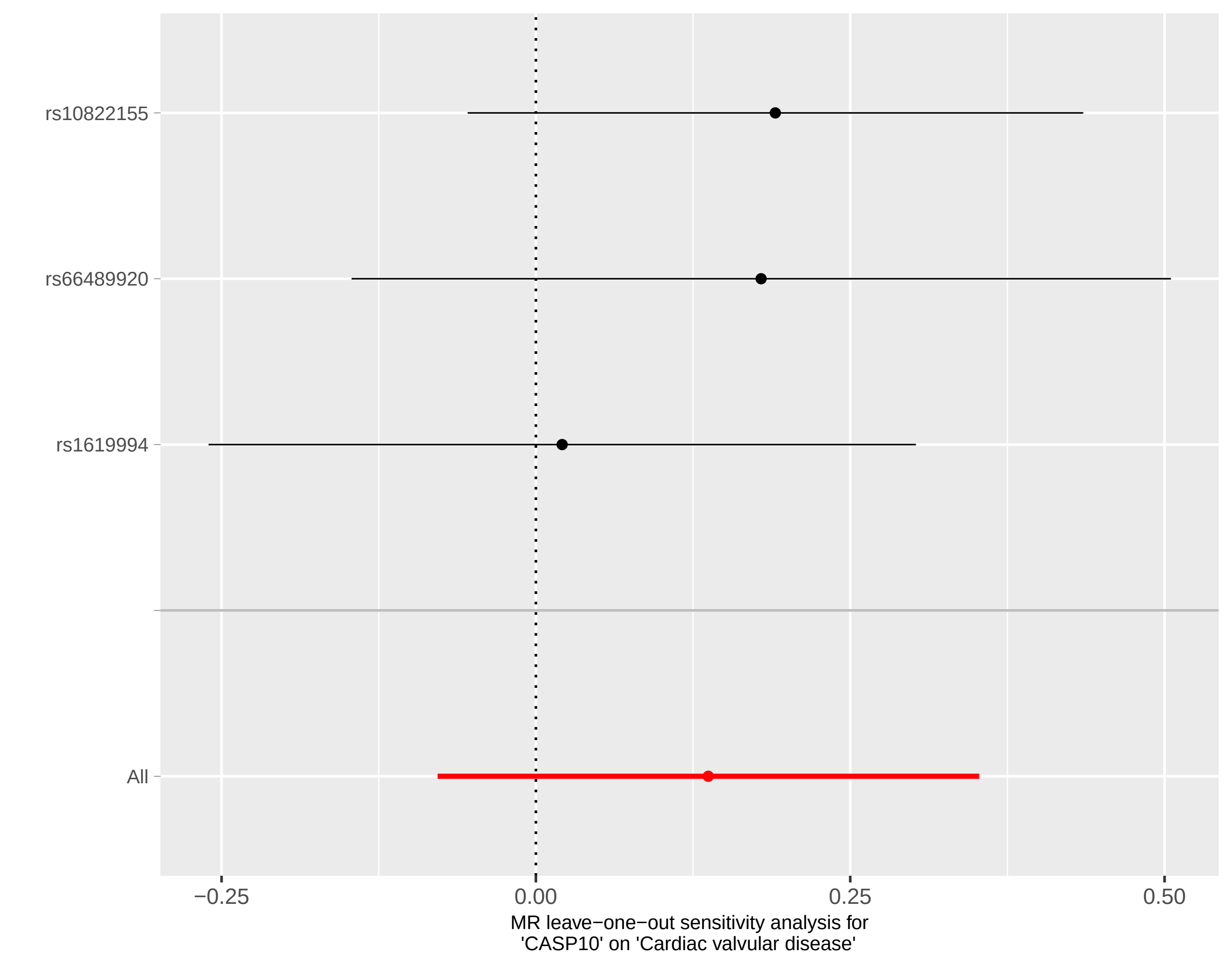
rs10822155
rs66489920
rs1619994
All
−0.25
0.00
0.25
0.50
MR leave−one−out sensitivity analysis for
'CASP10' on 'Cardiac valvular disease'

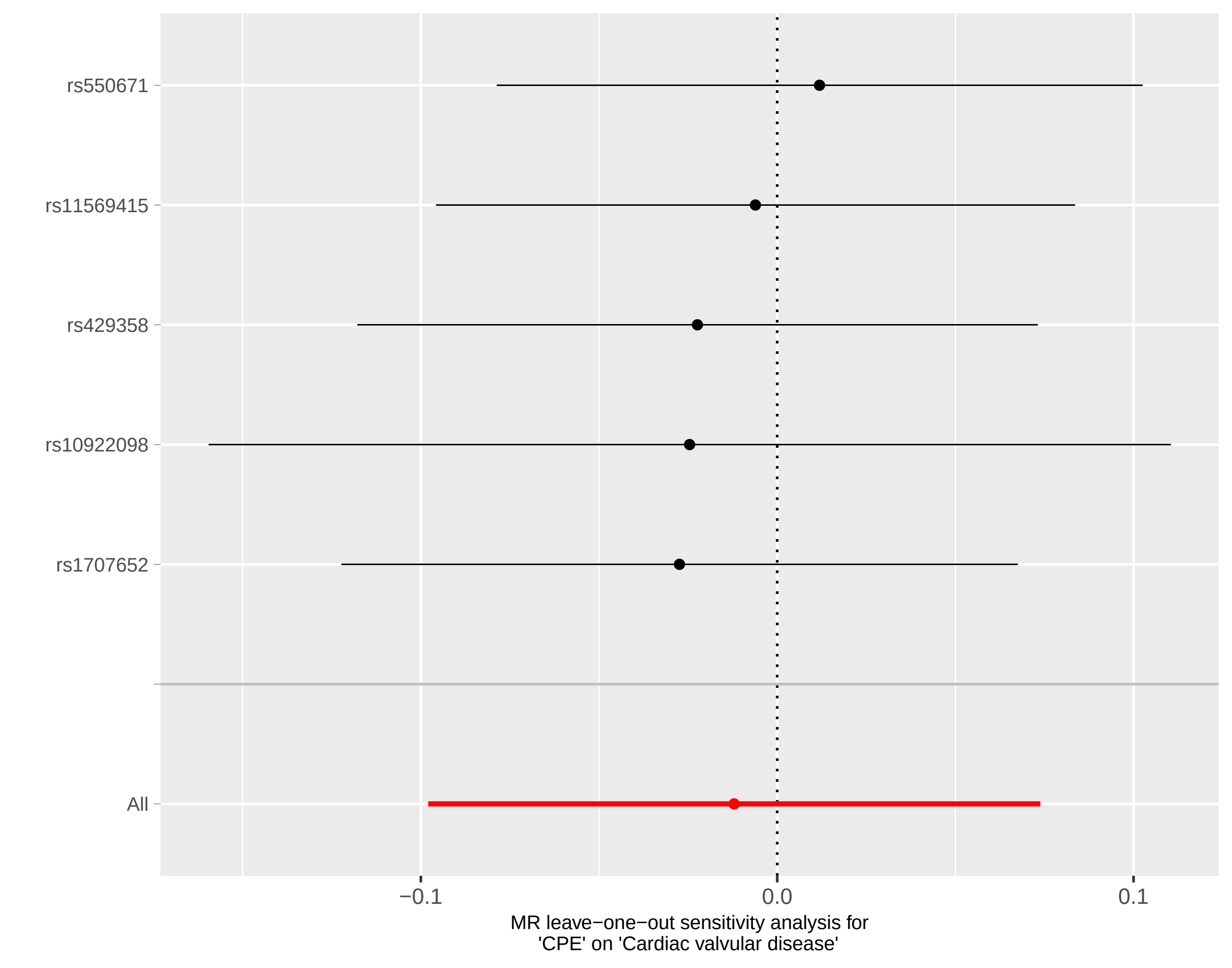
rs550671
rs11569415
rs429358
rs10922098
rs1707652
All
−0.1
0.0
0.1
MR leave−one−out sensitivity analysis for
'CPE' on 'Cardiac valvular disease'

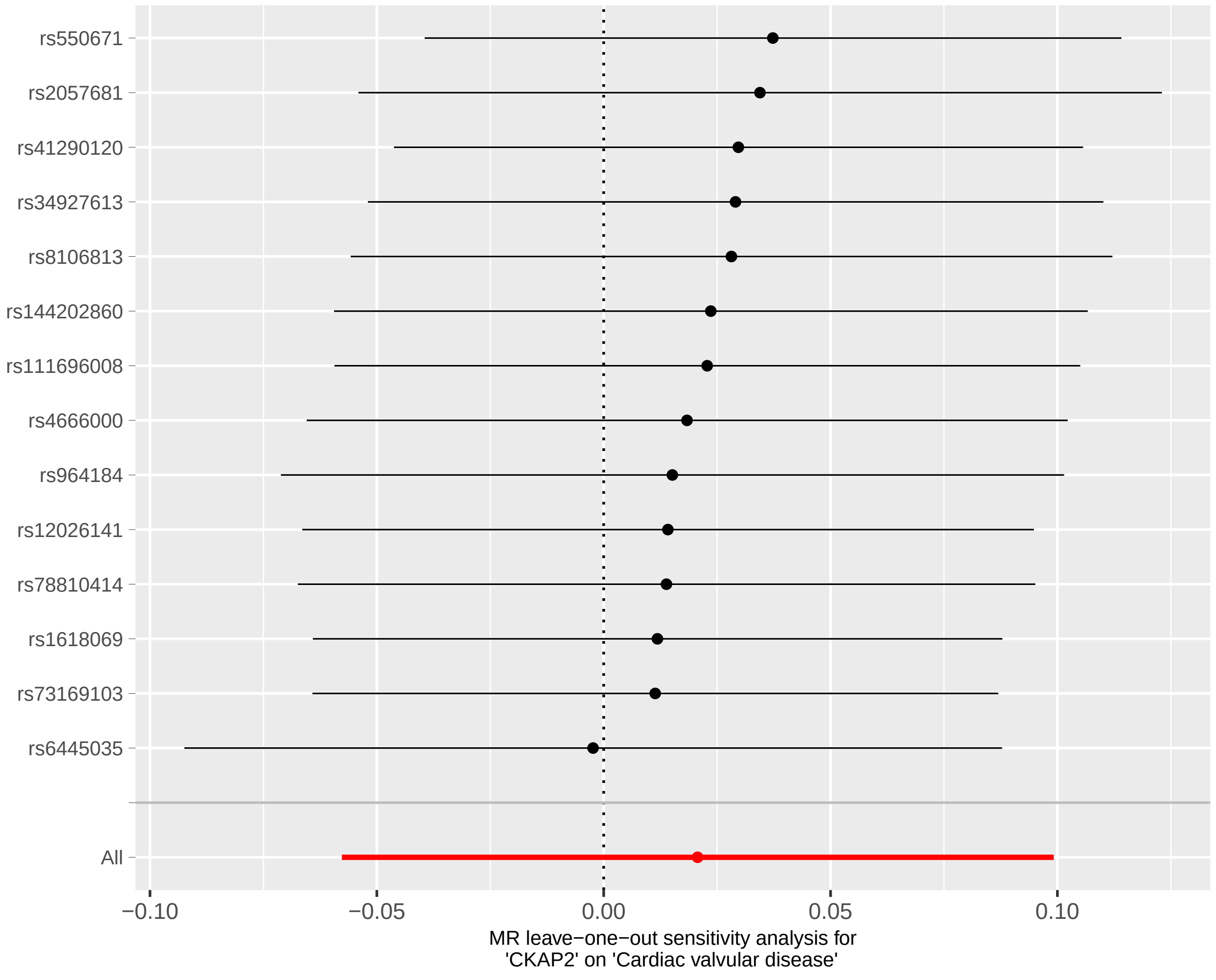

rs550671
rs2057681
rs41290120
rs34927613
rs8106813
rs144202860
rs111696008
rs4666000
rs964184
rs12026141
rs78810414
rs1618069
rs73169103
rs6445035
All
−0.10
−0.05
0.00
0.05
0.10
MR leave−one−out sensitivity analysis for
'CKAP2' on 'Cardiac valvular disease'

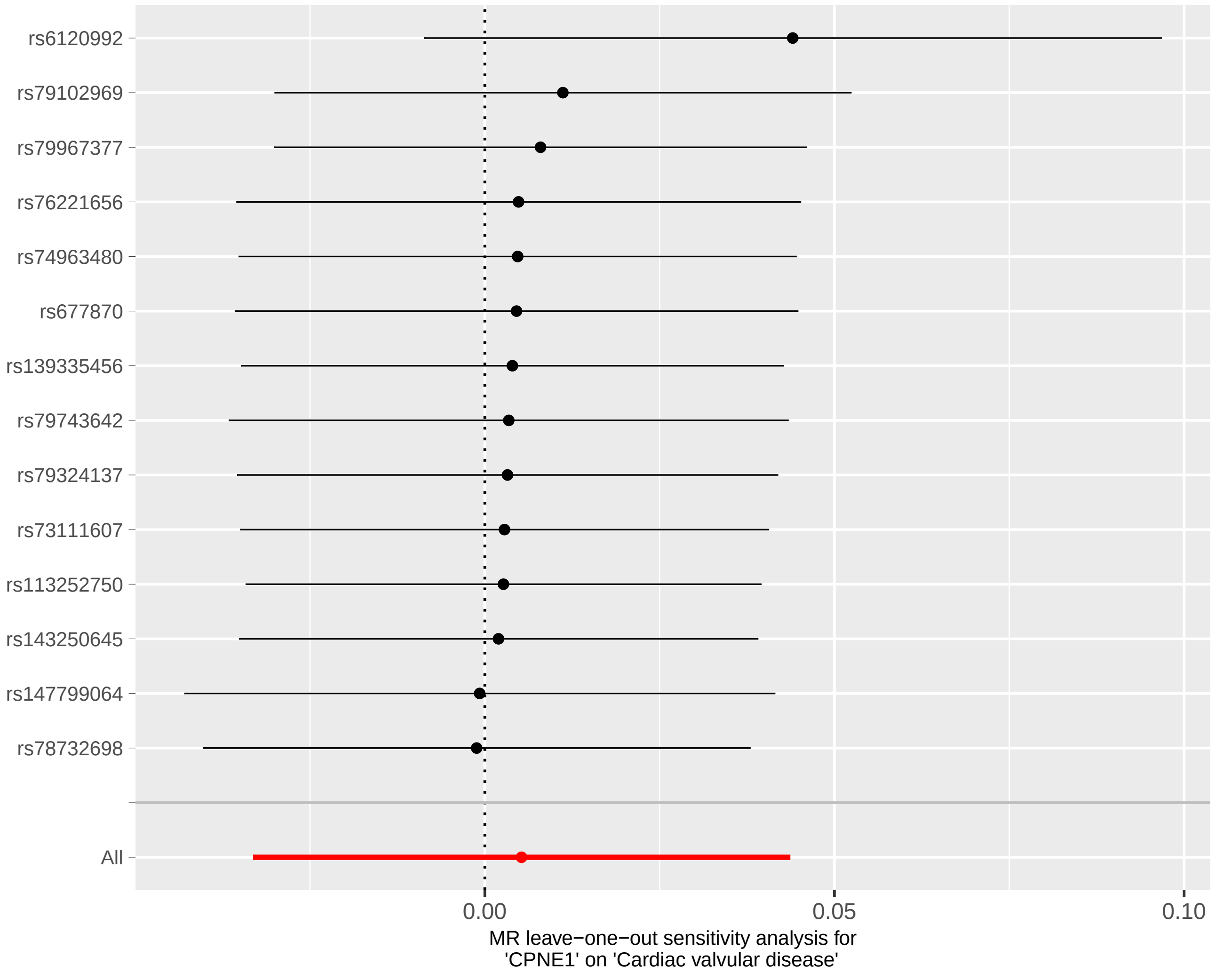
rs6120992
rs79102969
rs79967377
rs76221656
rs74963480
rs677870
rs139335456
rs79743642
rs79324137
rs73111607
rs113252750
rs143250645
rs147799064
rs78732698
All
0.00
0.05
0.10
MR leave−one−out sensitivity analysis for
'CPNE1' on 'Cardiac valvular disease'

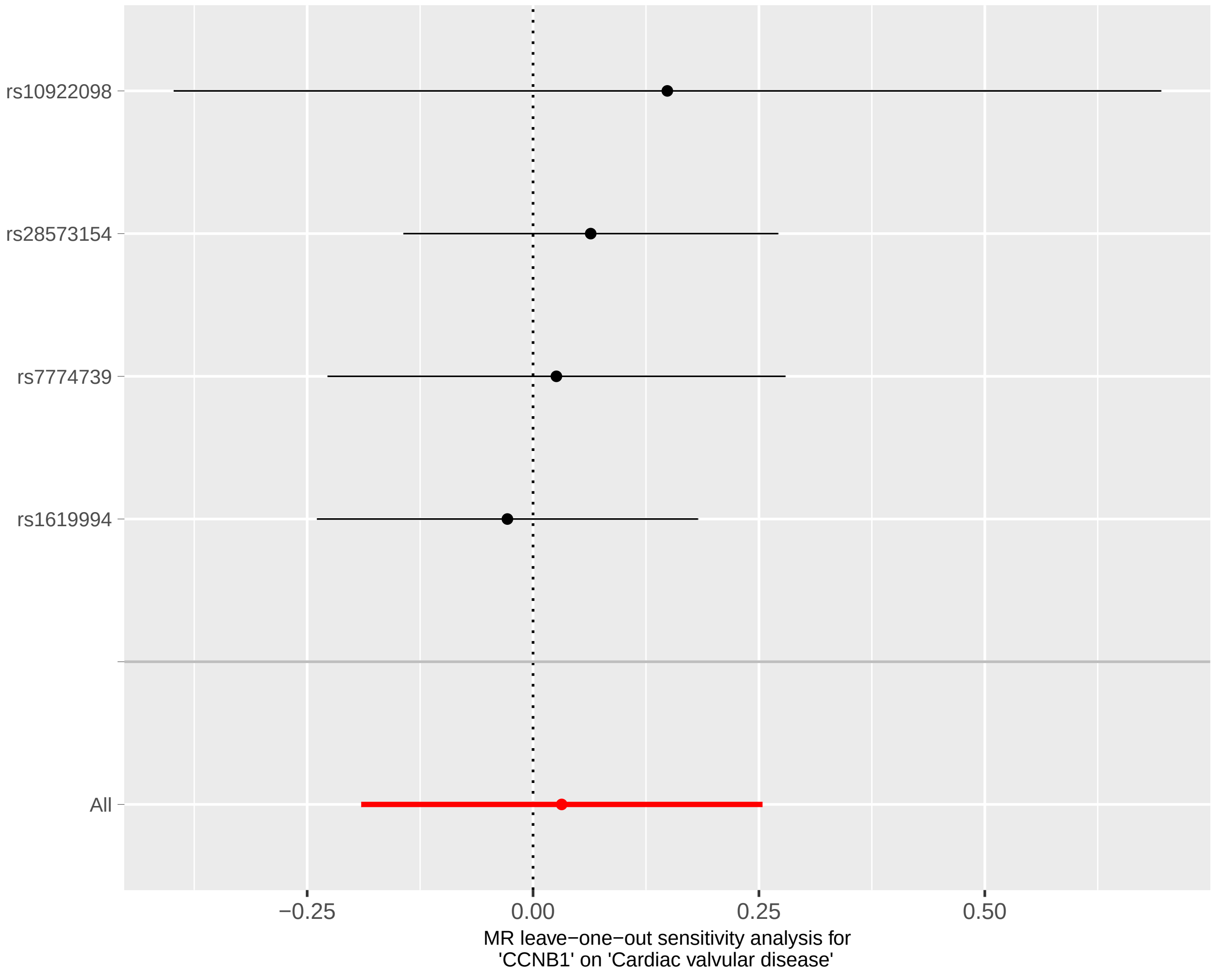
rs10922098
rs28573154
rs7774739
rs1619994
All
−0.25
0.00
0.25
0.50
MR leave−one−out sensitivity analysis for
'CCNB1' on 'Cardiac valvular disease'

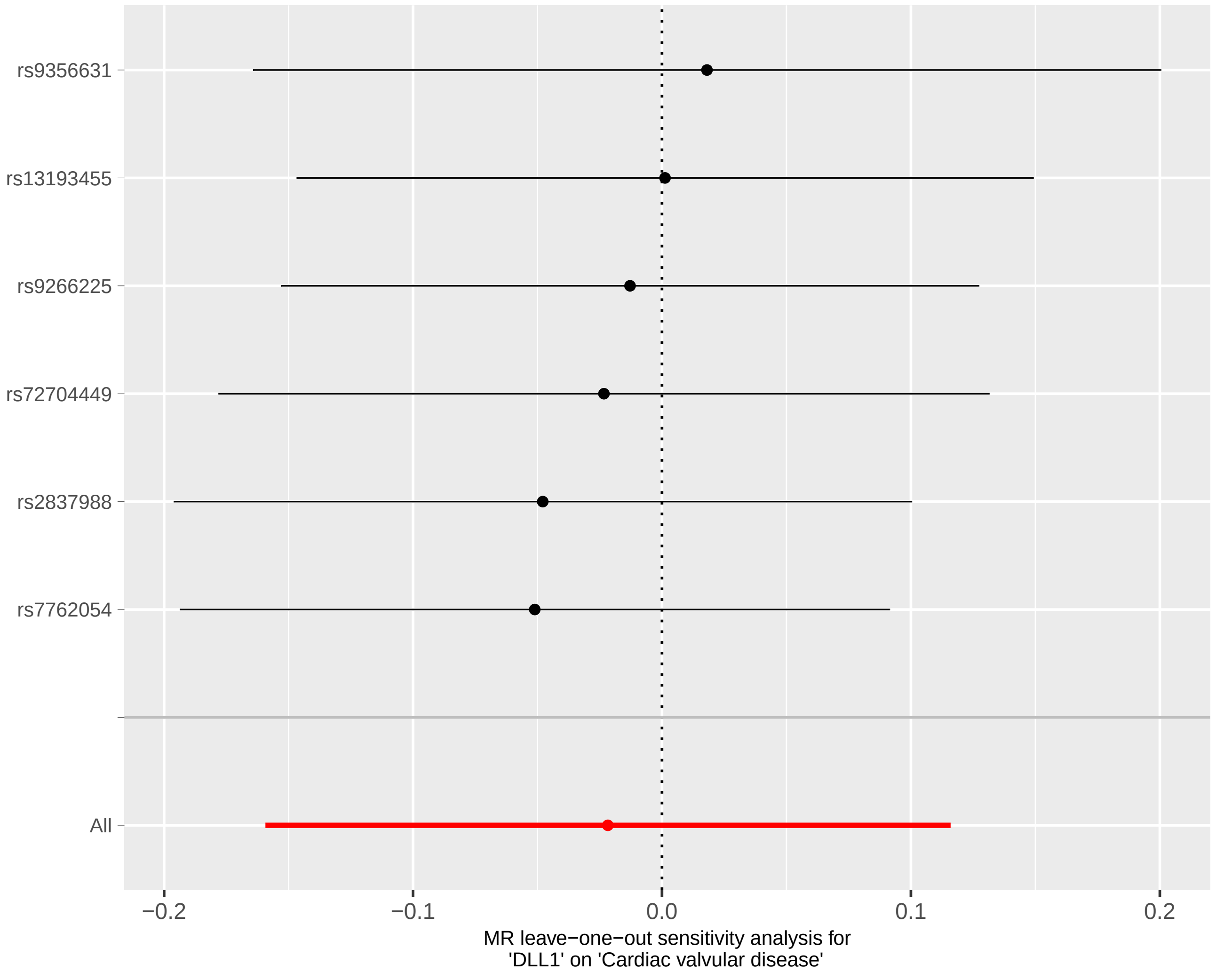
rs9356631
rs13193455
rs9266225
rs72704449
rs2837988
rs7762054
All
−0.2
−0.1
0.0
0.1
0.2
MR leave−one−out sensitivity analysis for
'DLL1' on 'Cardiac valvular disease'

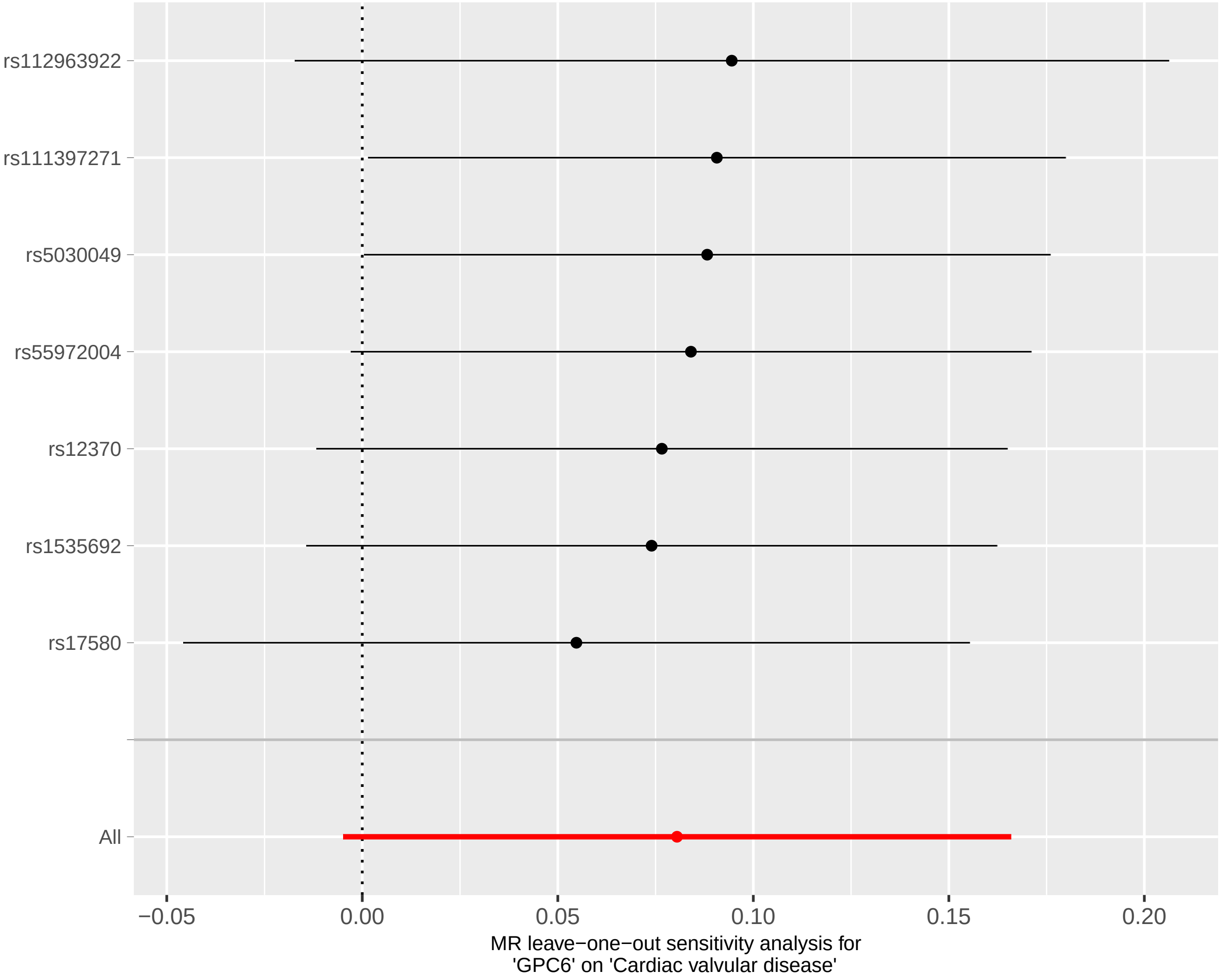
rs112963922
rs111397271
rs5030049
rs55972004
rs12370
rs1535692
rs17580
All
−0.05
0.00
0.05
0.10
0.15
0.20
MR leave−one−out sensitivity analysis for
'GPC6' on 'Cardiac valvular disease'

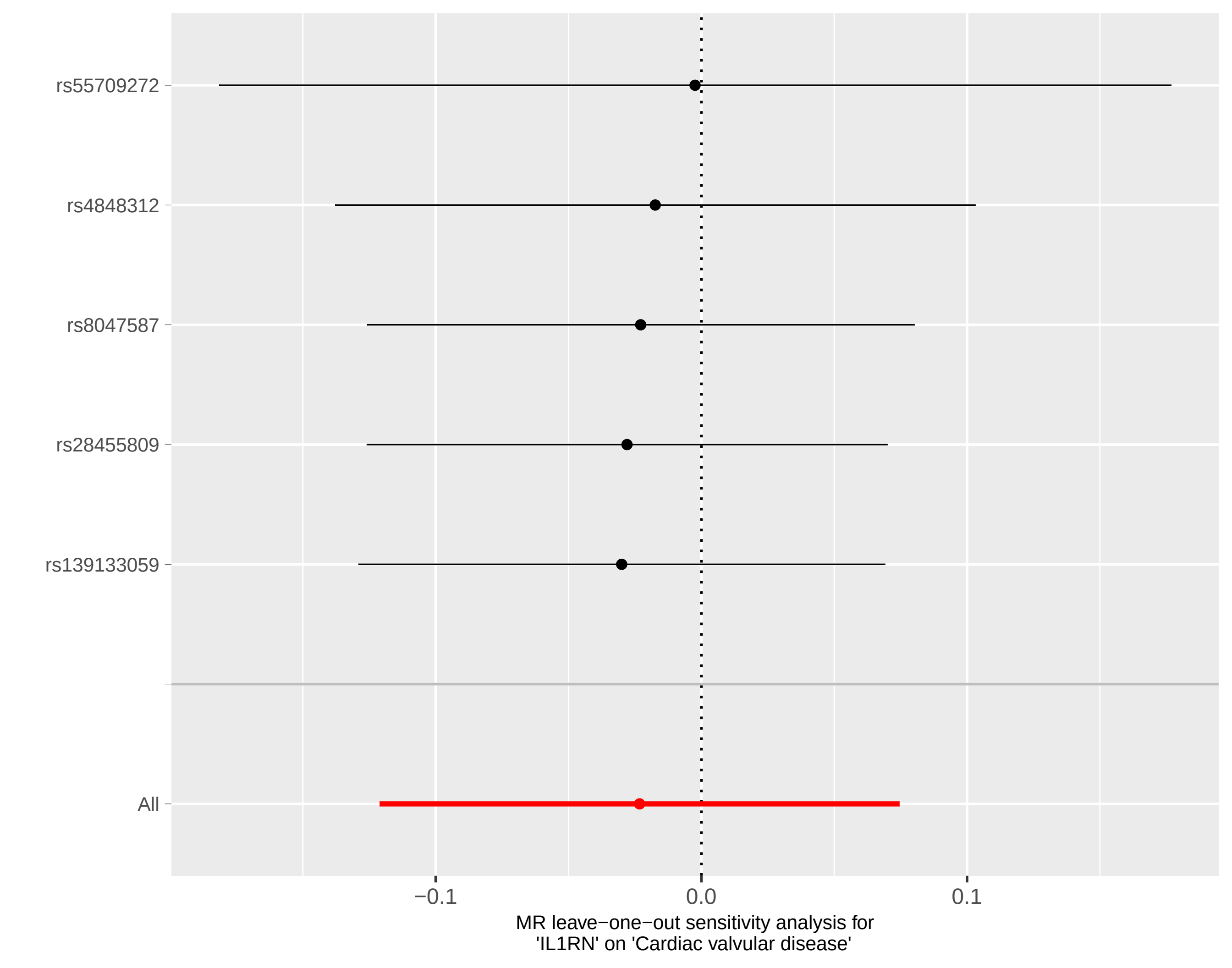

rs55709272
rs4848312
rs8047587
rs28455809
rs139133059
All
−0.1
0.0
0.1
MR leave−one−out sensitivity analysis for
'IL1RN' on 'Cardiac valvular disease'

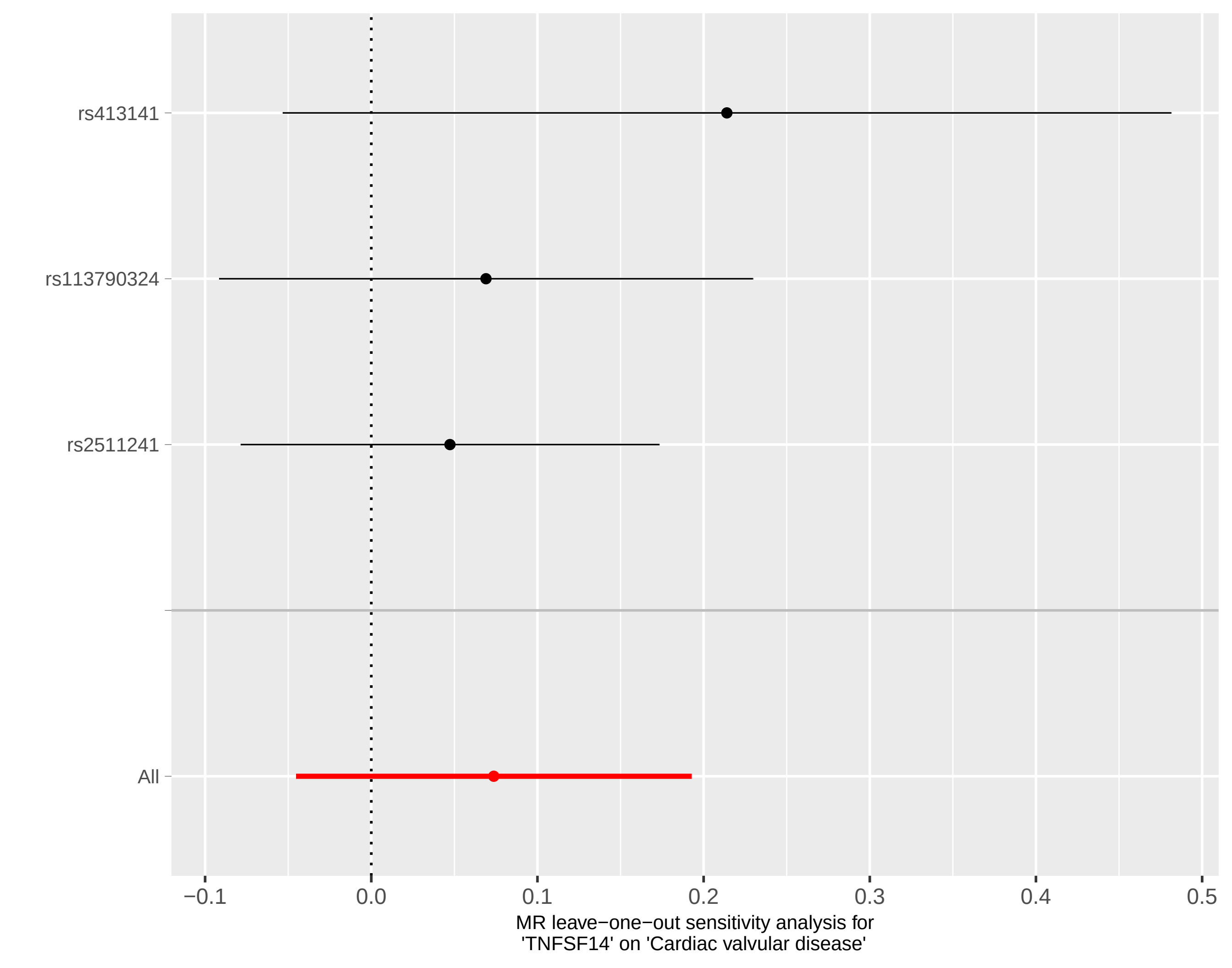

rs413141
rs113790324
rs2511241
All
−0.1
0.0
0.1
0.2
0.3
0.4
0.5
MR leave−one−out sensitivity analysis for
'TNFSF14' on 'Cardiac valvular disease'

Insufficient number of SNPs

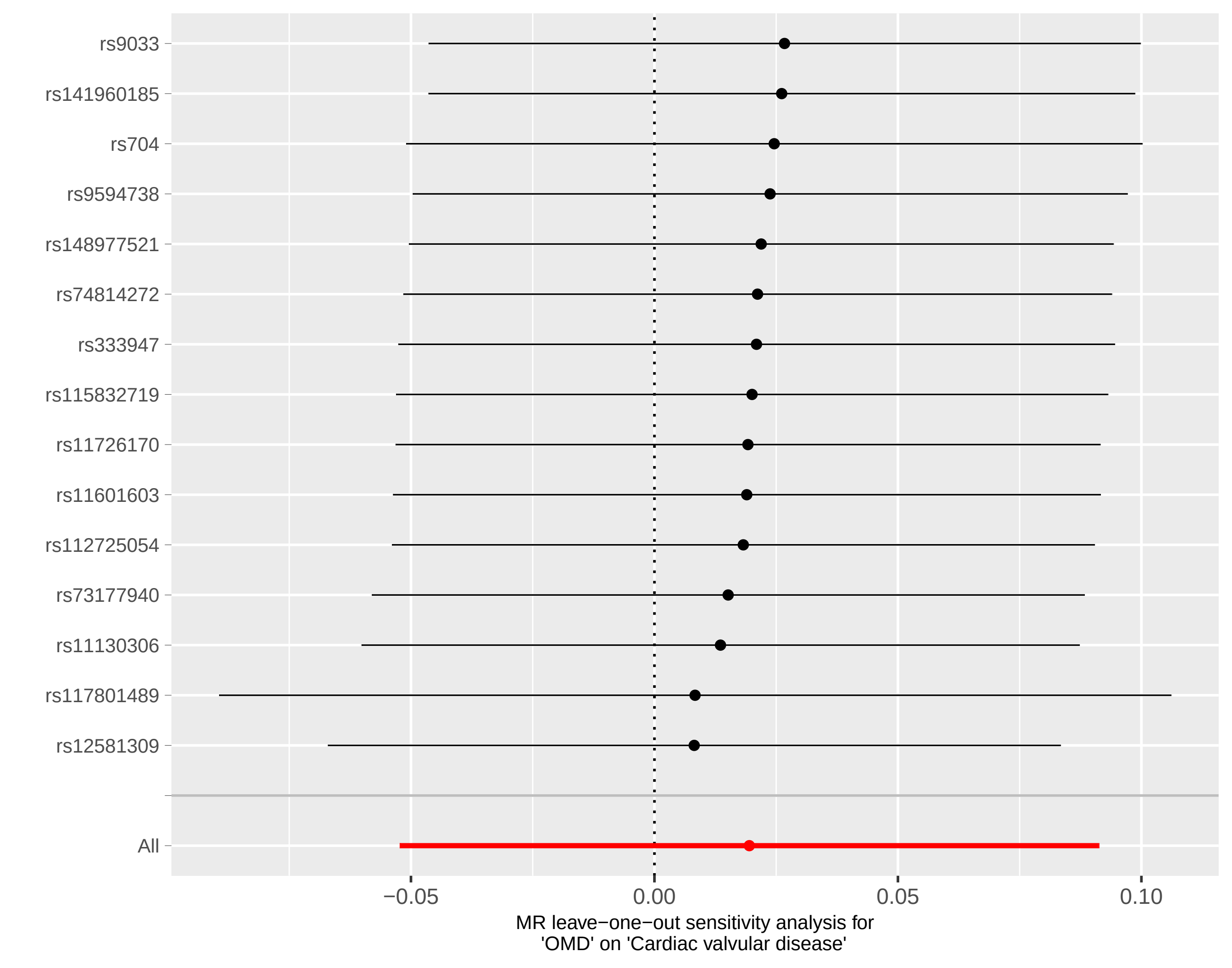
rs9033
rs141960185
rs704
rs9594738
rs148977521
rs74814272
rs333947
rs115832719
rs11726170
rs11601603
rs112725054
rs73177940
rs11130306
rs117801489
rs12581309
All
−0.05
0.00
0.05
0.10
MR leave−one−out sensitivity analysis for
'OMD' on 'Cardiac valvular disease'

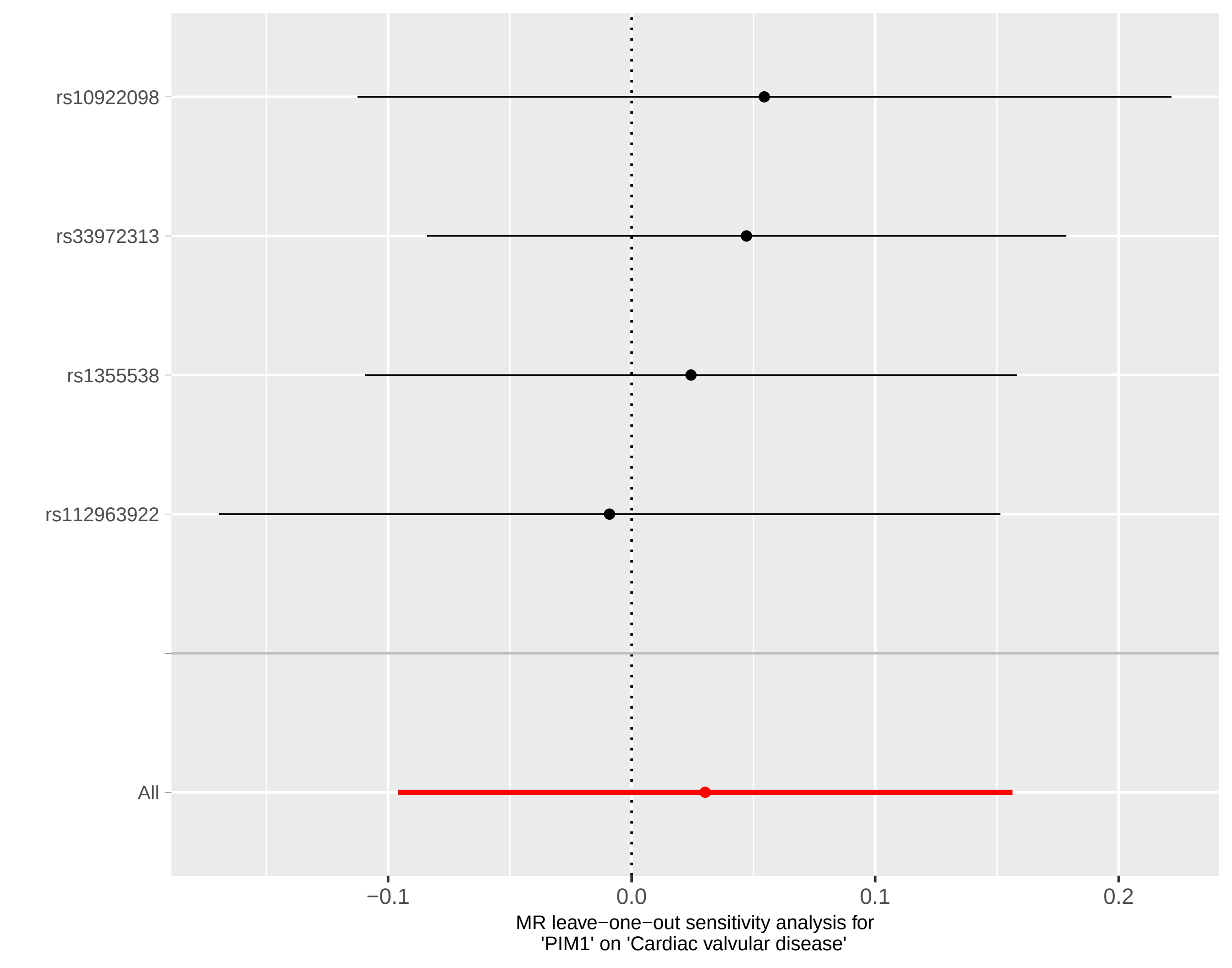
rs10922098
rs33972313
rs1355538
rs112963922
All
−0.1
0.0
0.1
0.2
MR leave−one−out sensitivity analysis for
'PIM1' on 'Cardiac valvular disease'

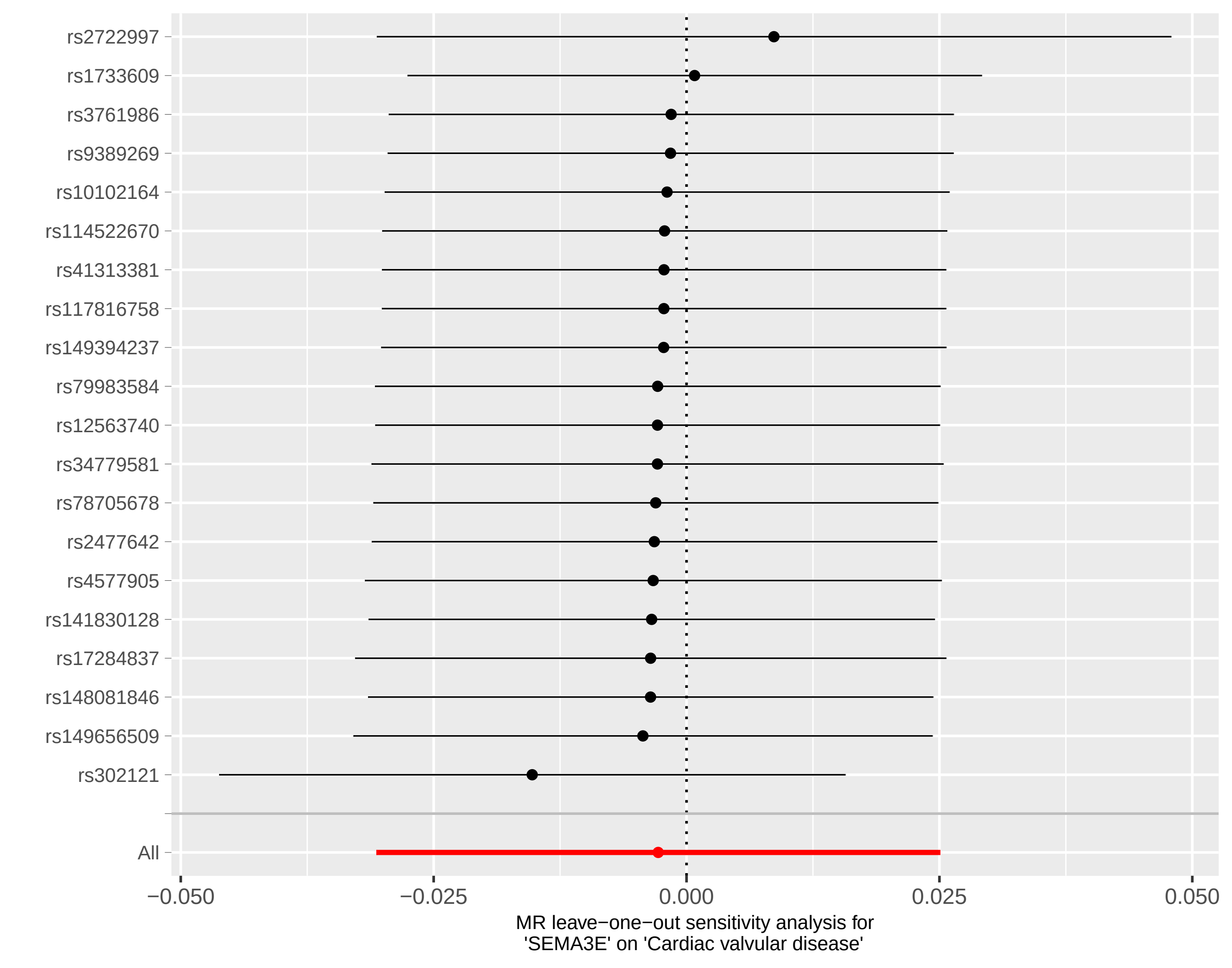

rs2722997
rs1733609
rs3761986
rs9389269
rs10102164
rs114522670
rs41313381
rs117816758
rs149394237
rs79983584
rs12563740
rs34779581
rs78705678
rs2477642
rs4577905
rs141830128
rs17284837
rs148081846
rs149656509
rs302121
All
−0.050
−0.025
0.000
0.025
0.050
MR leave−one−out sensitivity analysis for
'SEMA3E' on 'Cardiac valvular disease'

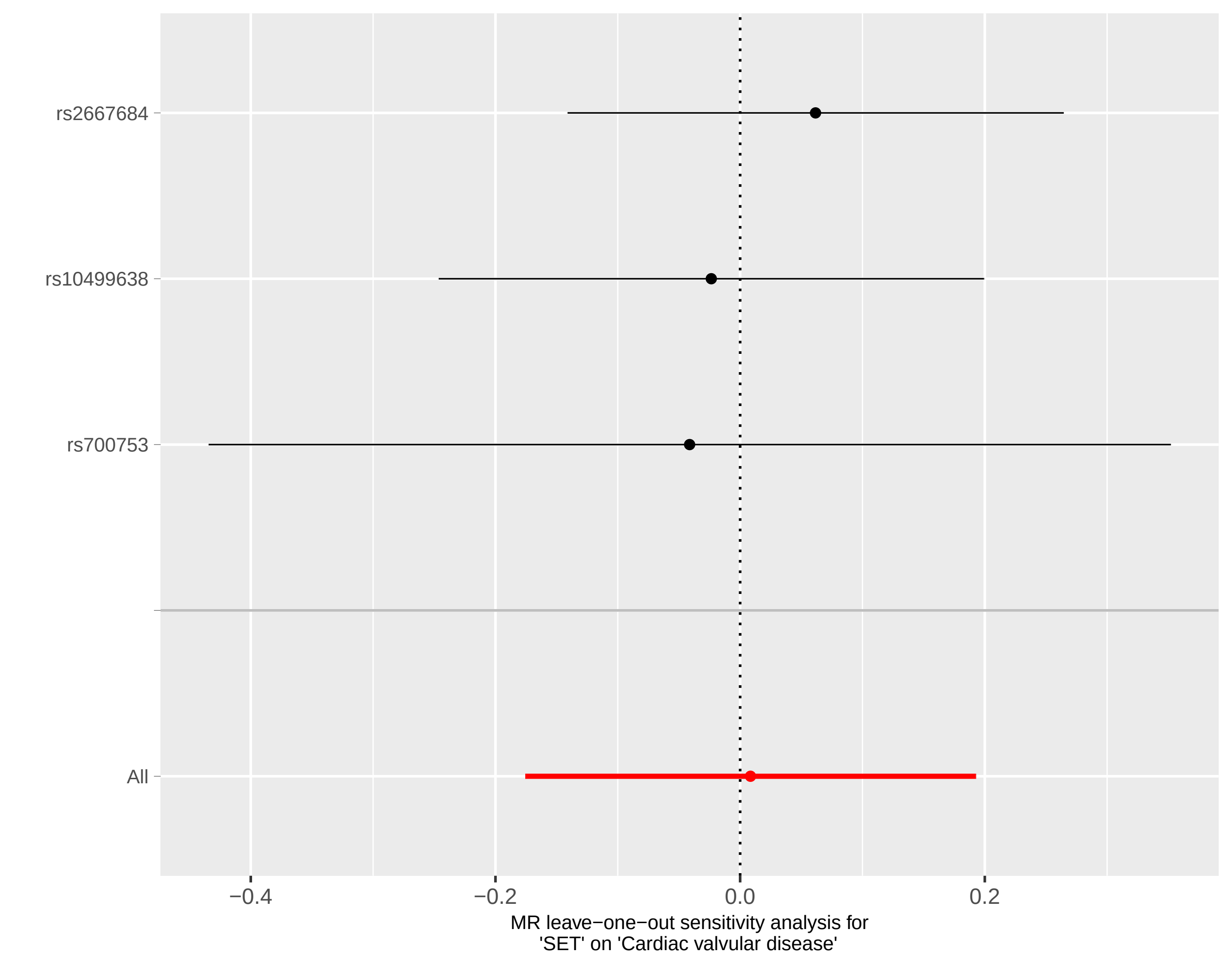
rs2667684
rs10499638
rs700753
All
−0.4
−0.2
0.0
0.2
MR leave−one−out sensitivity analysis for
'SET' on 'Cardiac valvular disease'

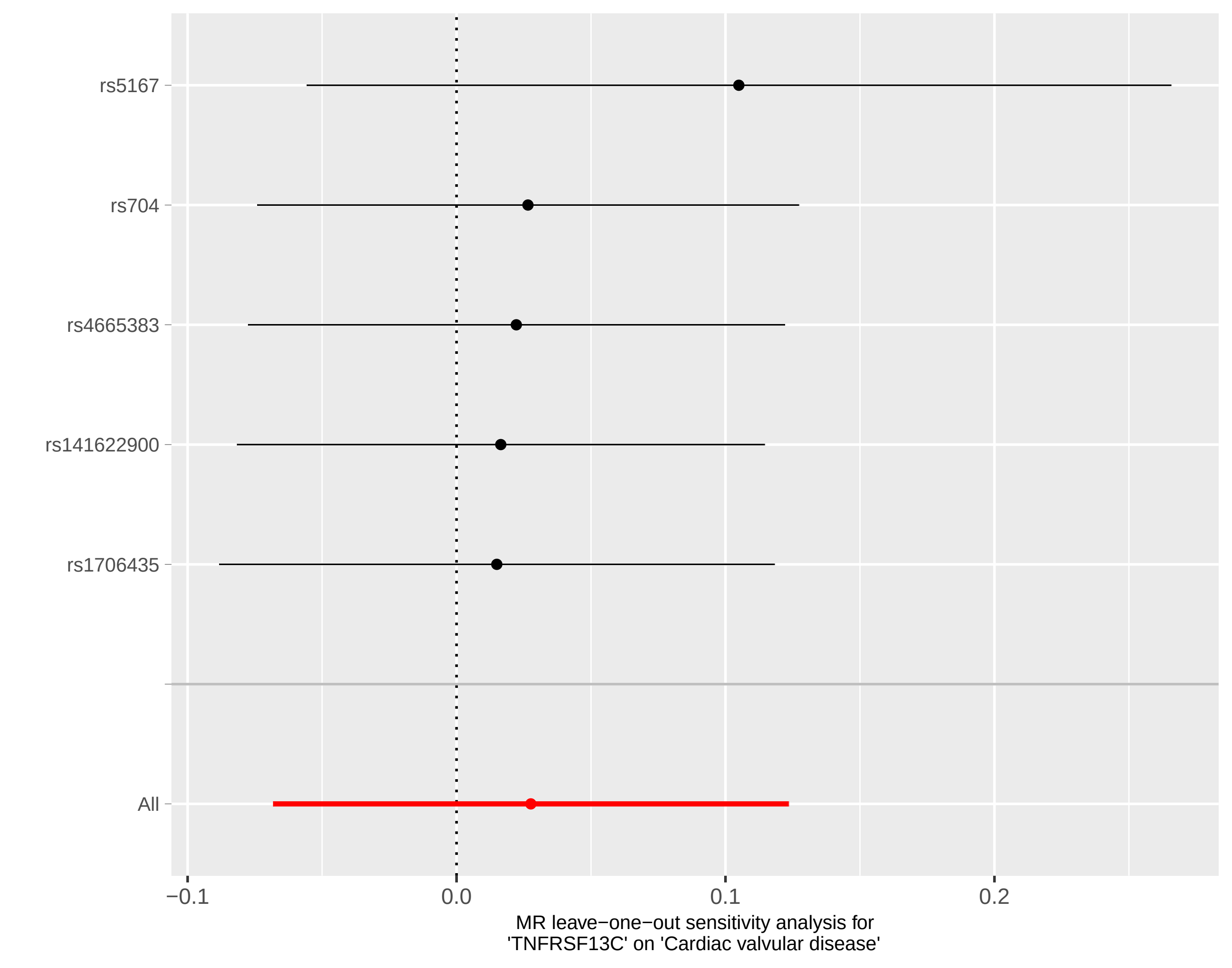
rs5167
rs704
rs4665383
rs141622900
rs1706435
All
−0.1
0.0
0.1
0.2
MR leave−one−out sensitivity analysis for
'TNFRSF13C' on 'Cardiac valvular disease'

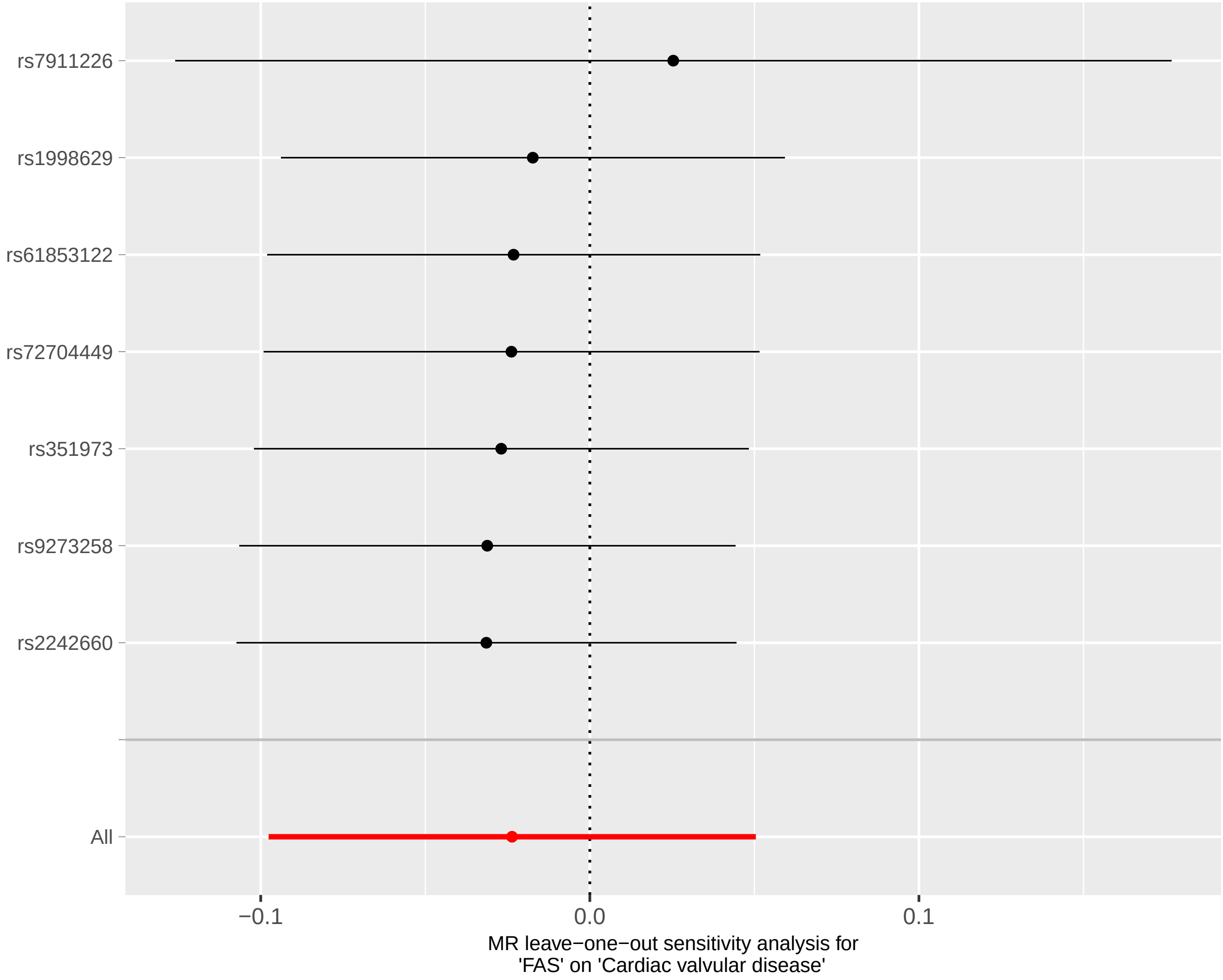
rs7911226
rs1998629
rs61853122
rs72704449
rs351973
rs9273258
rs2242660
All
−0.1
0.0
0.1
MR leave−one−out sensitivity analysis for
'FAS' on 'Cardiac valvular disease'

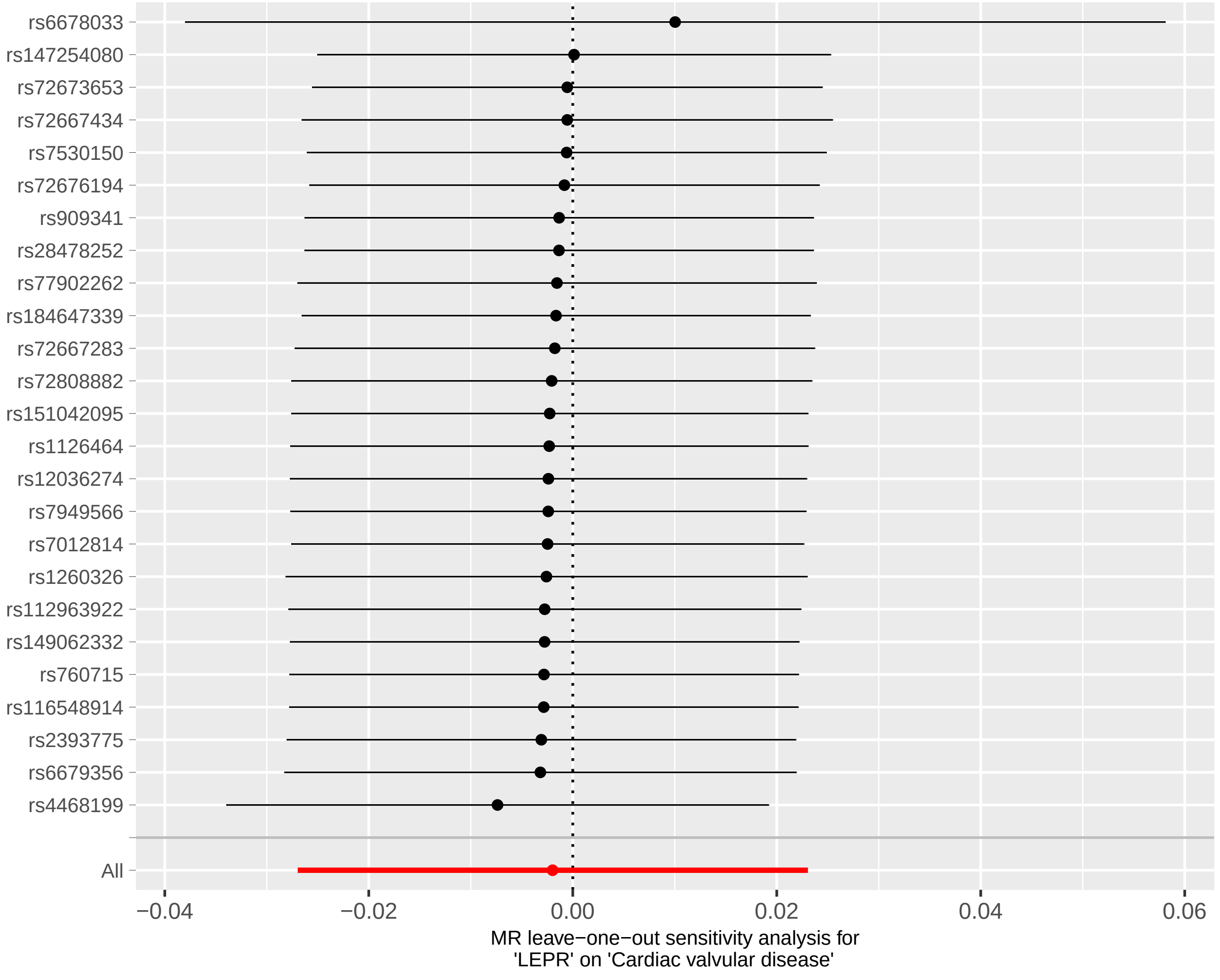

rs6678033
rs147254080
rs72673653
rs72667434
rs7530150
rs72676194
rs909341
rs28478252
rs77902262
rs184647339
rs72667283
rs72808882
rs151042095
rs1126464
rs12036274
rs7949566
rs7012814
rs1260326
rs112963922
rs149062332
rs760715
rs116548914
rs2393775
rs6679356
rs4468199
All
−0.04
−0.02
0.00
0.02
0.04
0.06
MR leave−one−out sensitivity analysis for
'LEPR' on 'Cardiac valvular disease'

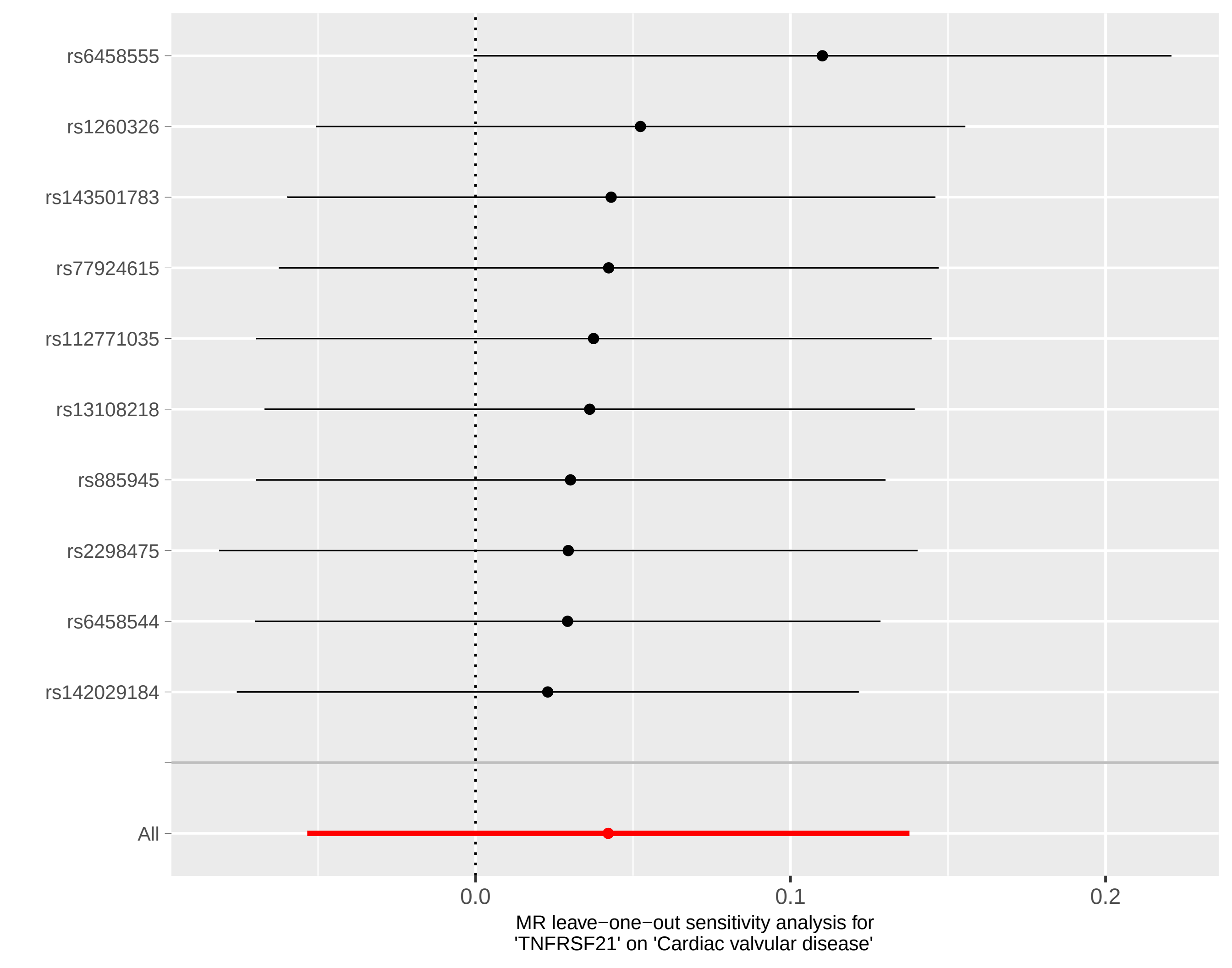

rs6458555
rs1260326
rs143501783
rs77924615
rs112771035
rs13108218
rs885945
rs2298475
rs6458544
rs142029184
All
0.0
0.1
0.2
MR leave−one−out sensitivity analysis for
'TNFRSF21' on 'Cardiac valvular disease'

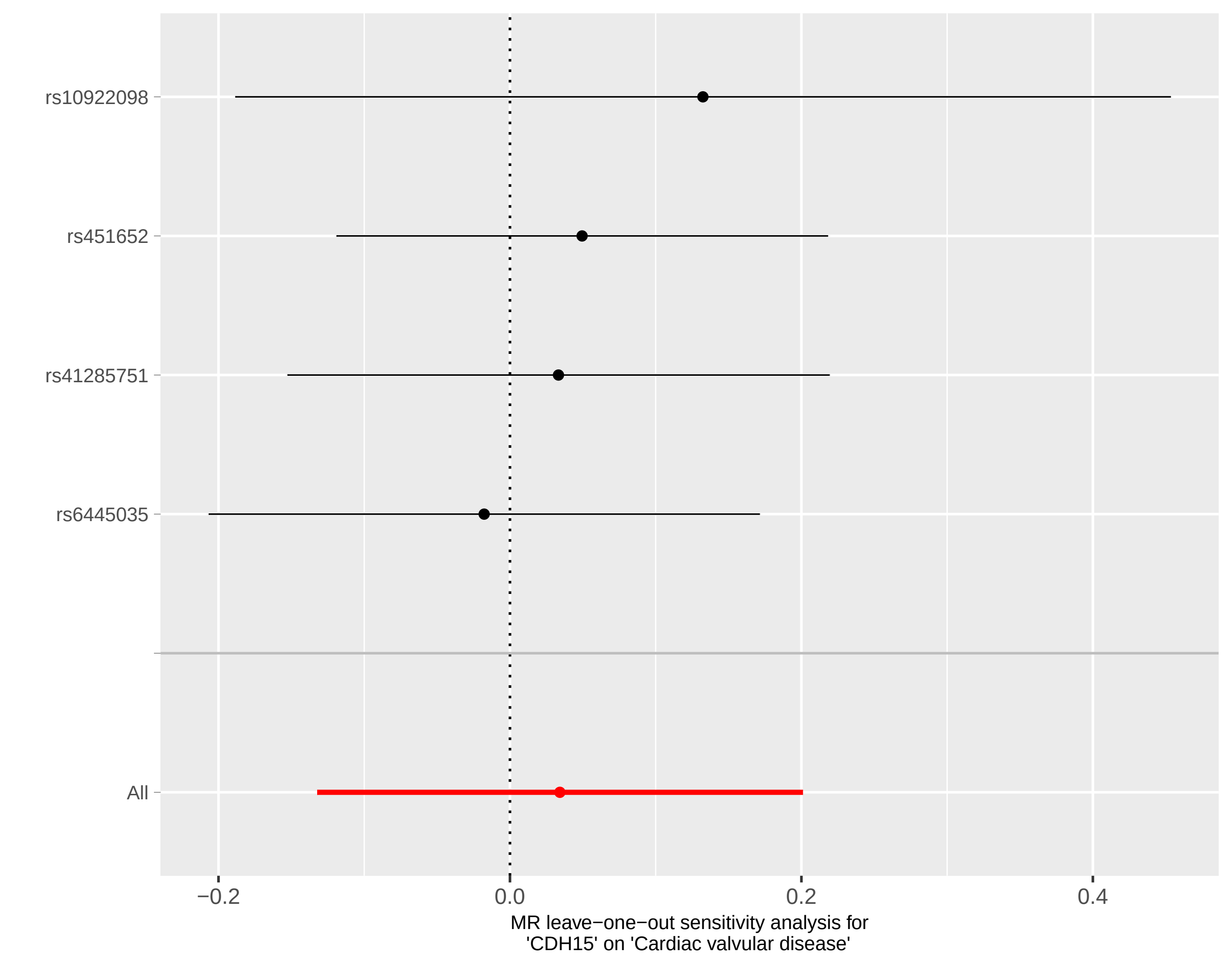

rs10922098
rs451652
rs41285751
rs6445035
All
−0.2
0.0
0.2
0.4
MR leave−one−out sensitivity analysis for
'CDH15' on 'Cardiac valvular disease'

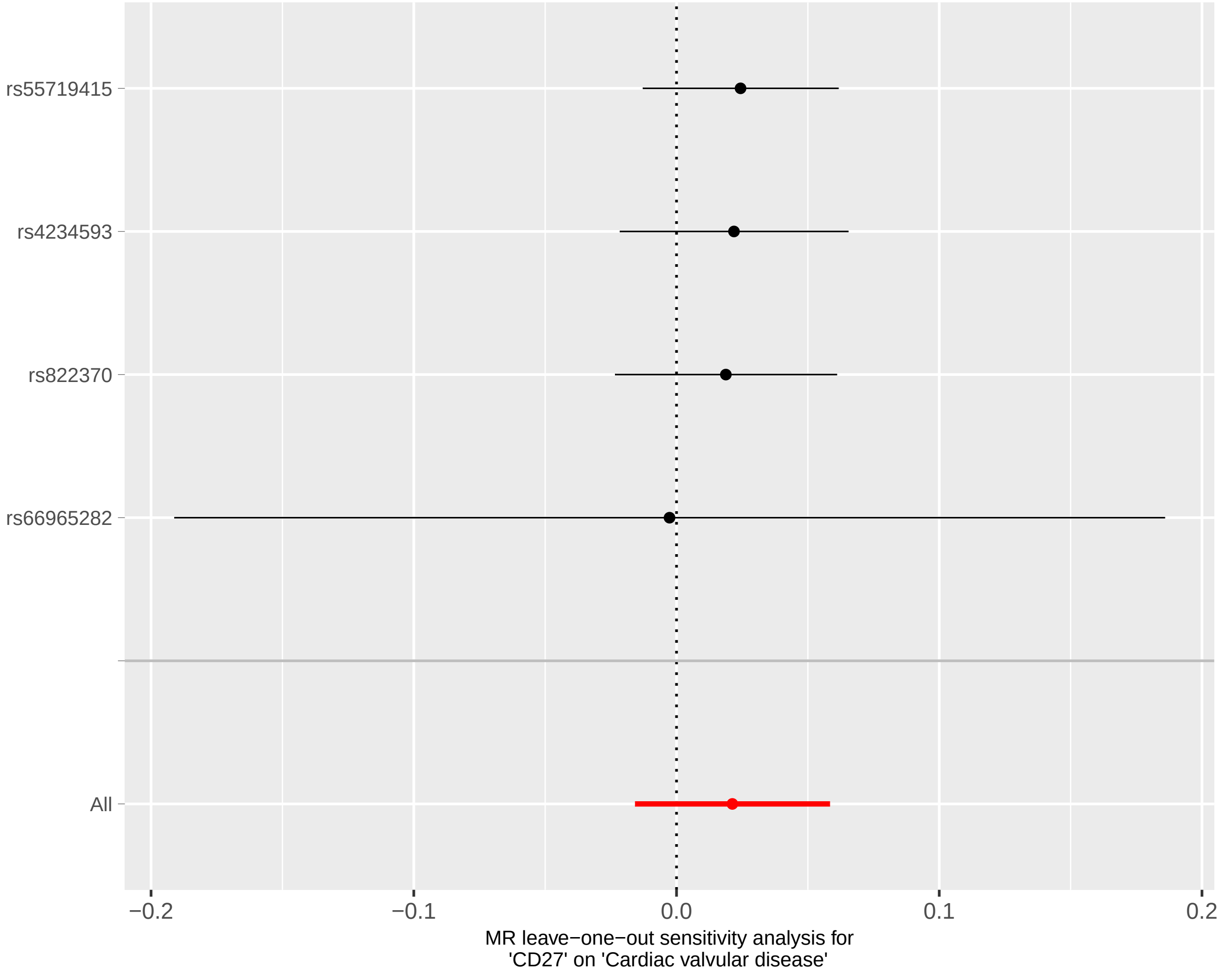
rs55719415
rs4234593
rs822370
rs66965282
All
−0.2
−0.1
0.0
0.1
0.2
MR leave−one−out sensitivity analysis for
'CD27' on 'Cardiac valvular disease'

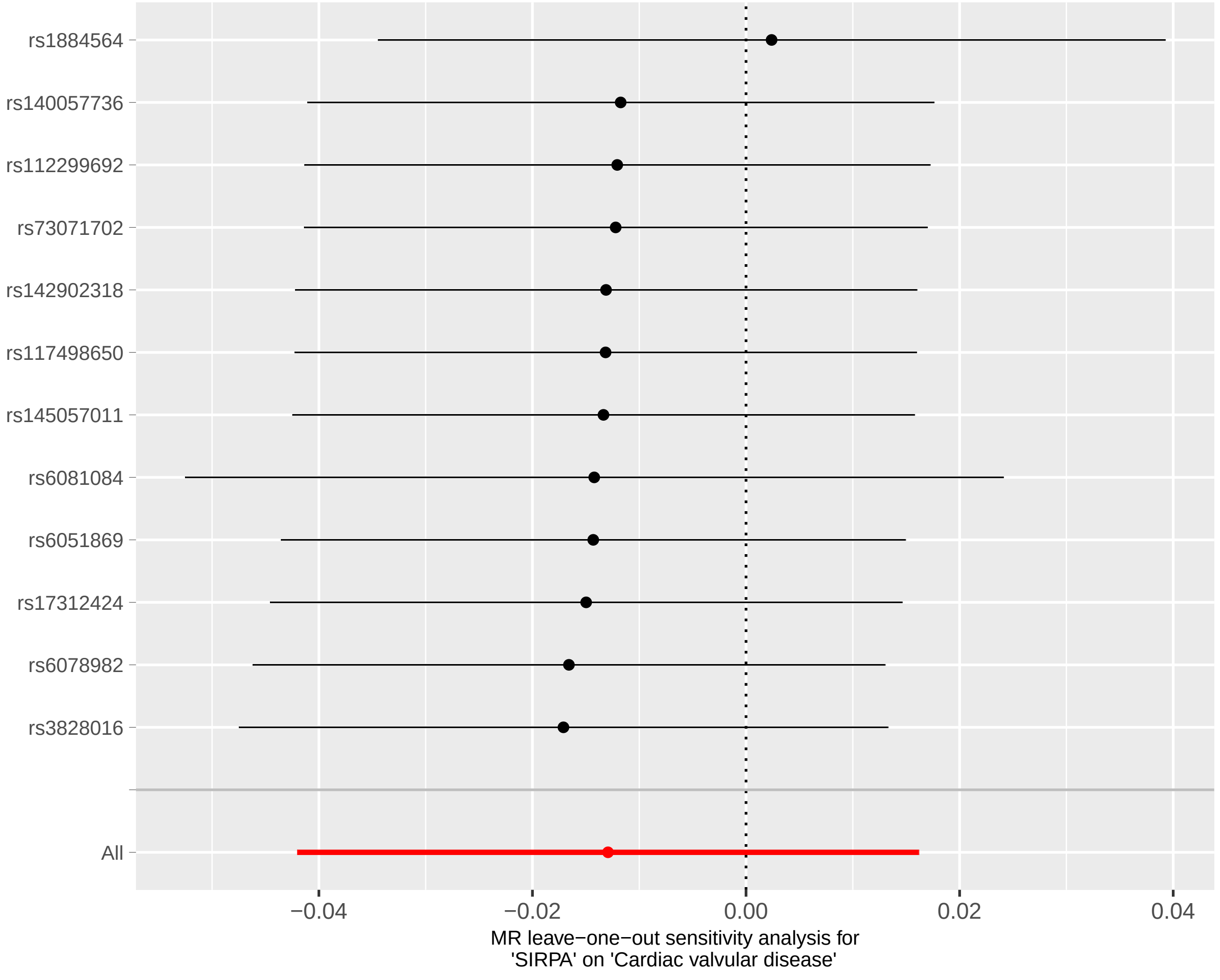
rs1884564
rs140057736
rs112299692
rs73071702
rs142902318
rs117498650
rs145057011
rs6081084
rs6051869
rs17312424
rs6078982
rs3828016
All
−0.04
−0.02
0.00
0.02
0.04
MR leave−one−out sensitivity analysis for
'SIRPA' on 'Cardiac valvular disease'

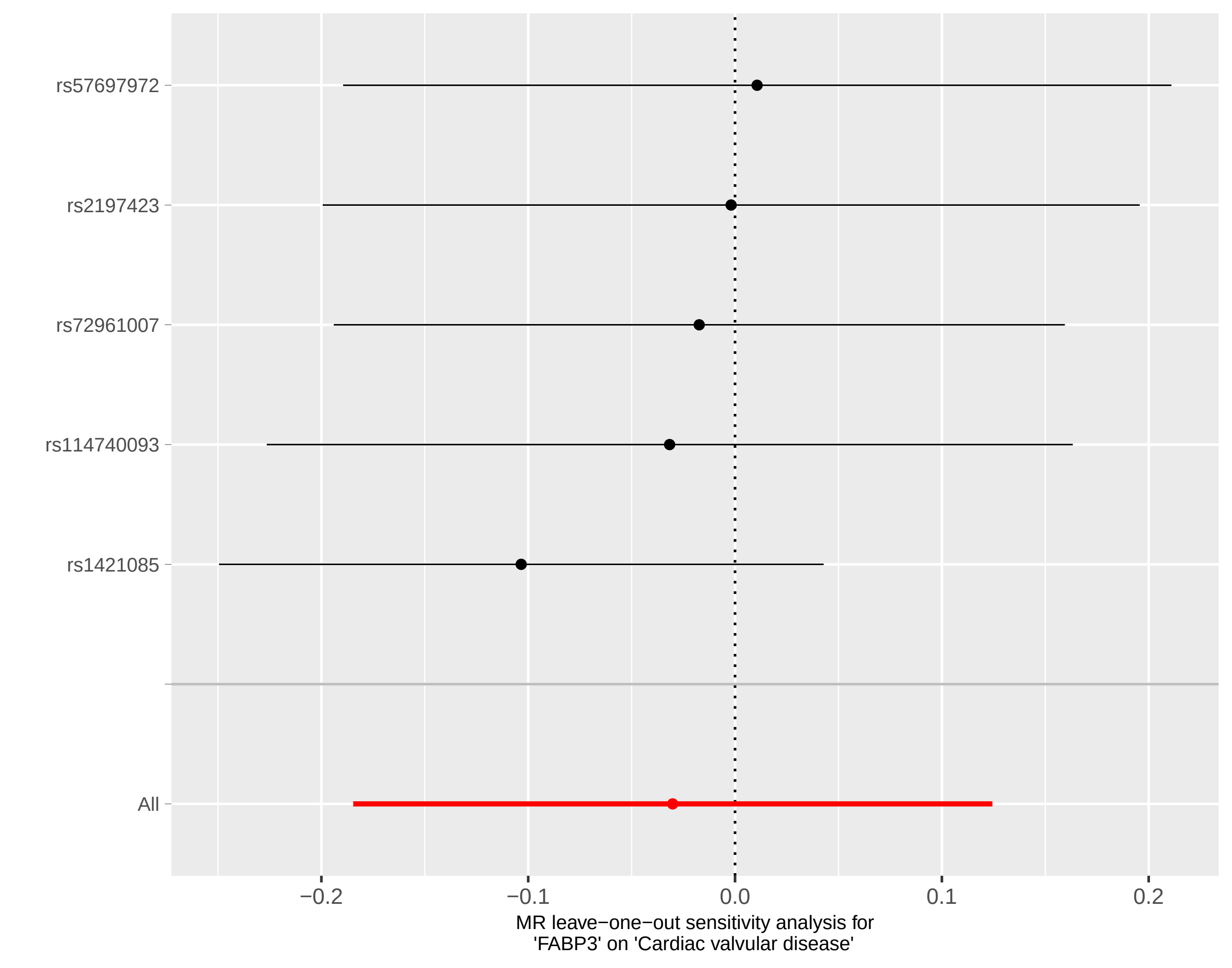

rs57697972
rs2197423
rs72961007
rs114740093
rs1421085
All
−0.2
−0.1
0.0
0.1
0.2
MR leave−one−out sensitivity analysis for
'FABP3' on 'Cardiac valvular disease'

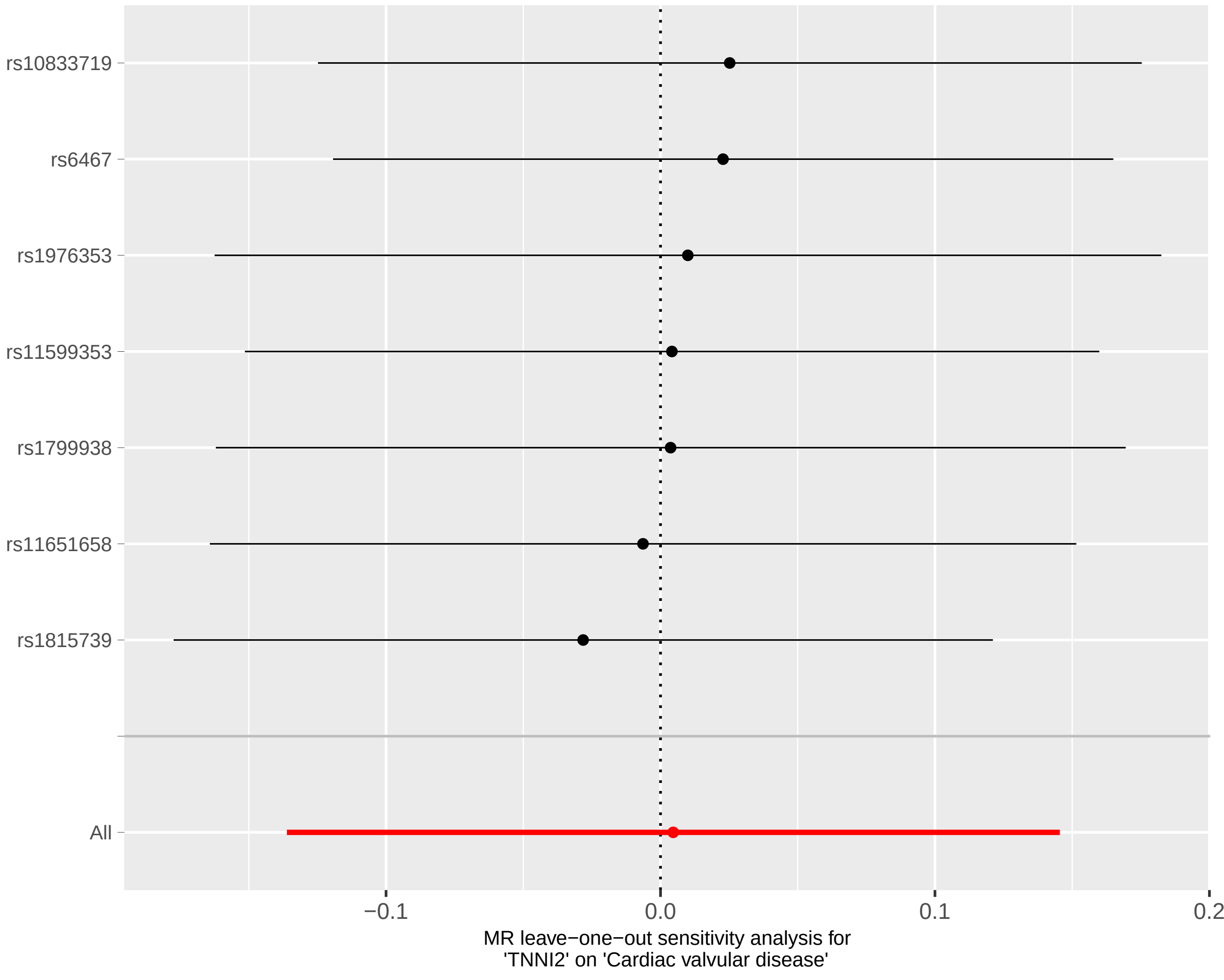

rs10833719
rs6467
rs1976353
rs11599353
rs1799938
rs11651658
rs1815739
All
−0.1
0.0
0.1
0.2
MR leave−one−out sensitivity analysis for
'TNNI2' on 'Cardiac valvular disease'

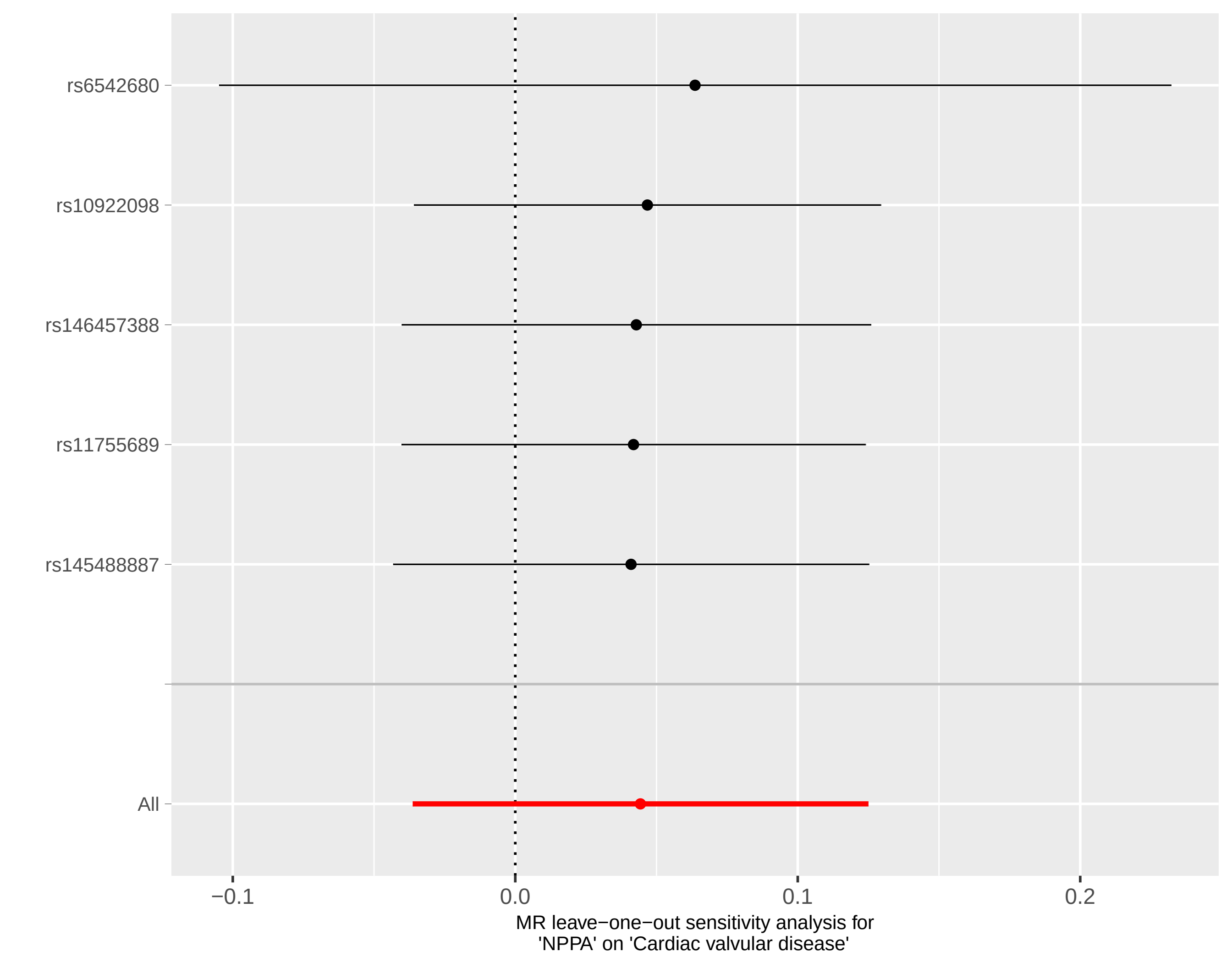

rs6542680
rs10922098
rs146457388
rs11755689
rs145488887
All
−0.1
0.0
0.1
0.2
MR leave−one−out sensitivity analysis for
'NPPA' on 'Cardiac valvular disease'

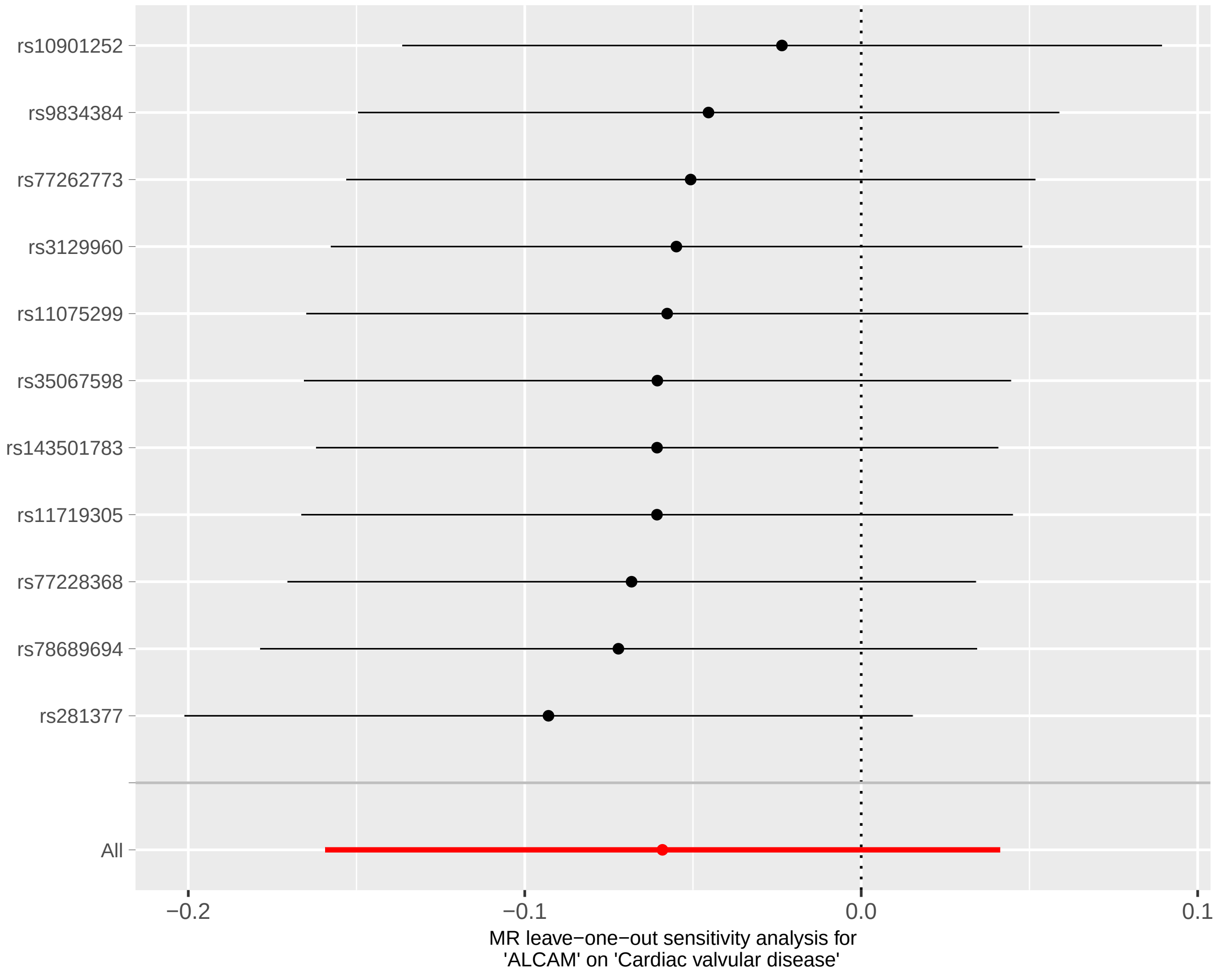

rs10901252
rs9834384
rs77262773
rs3129960
rs11075299
rs35067598
rs143501783
rs11719305
rs77228368
rs78689694
rs281377
All
−0.2
−0.1
0.0
0.1
MR leave−one−out sensitivity analysis for
'ALCAM' on 'Cardiac valvular disease'

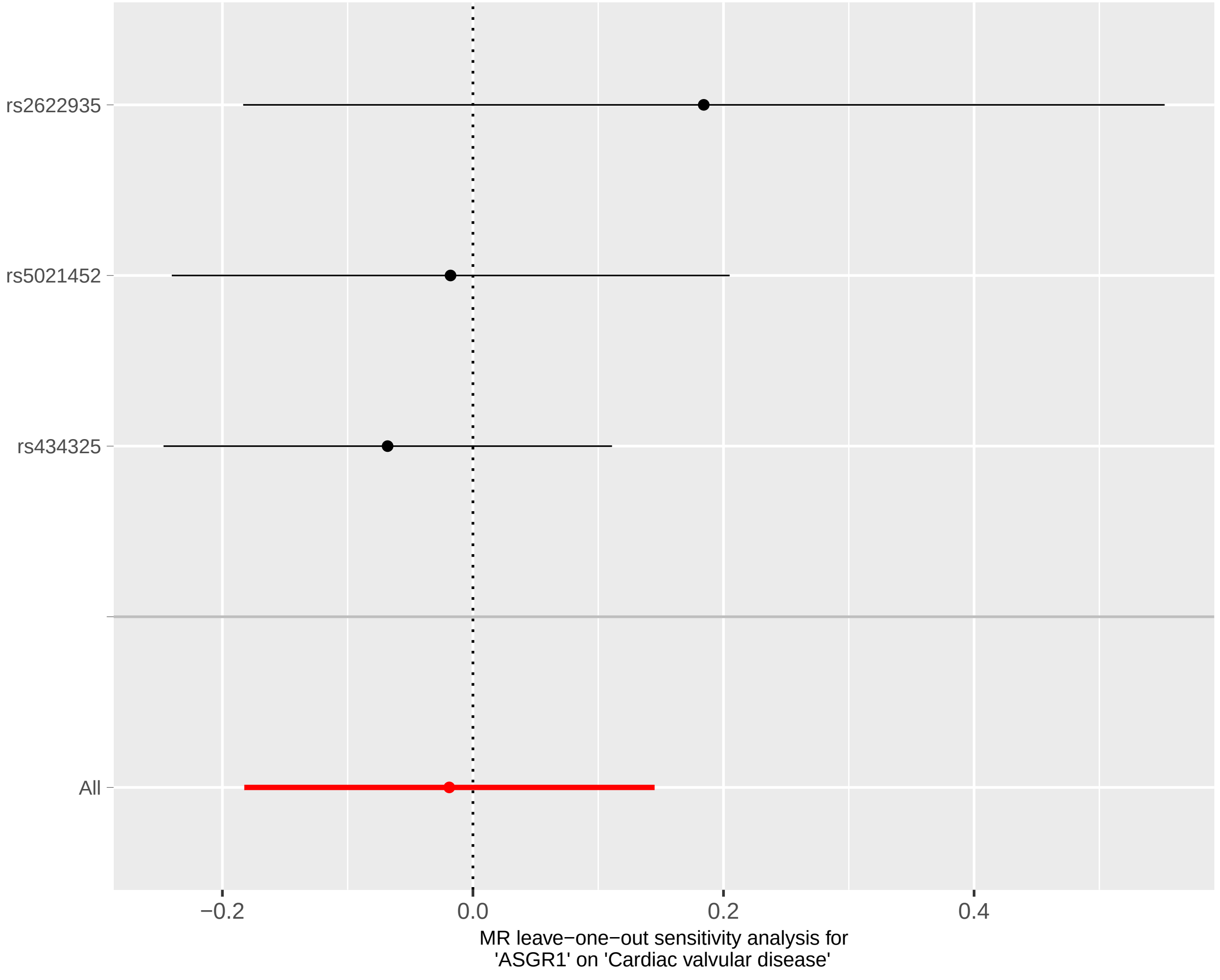
rs2622935
rs5021452
rs434325
All
−0.2
0.0
0.2
0.4
MR leave−one−out sensitivity analysis for
'ASGR1' on 'Cardiac valvular disease'

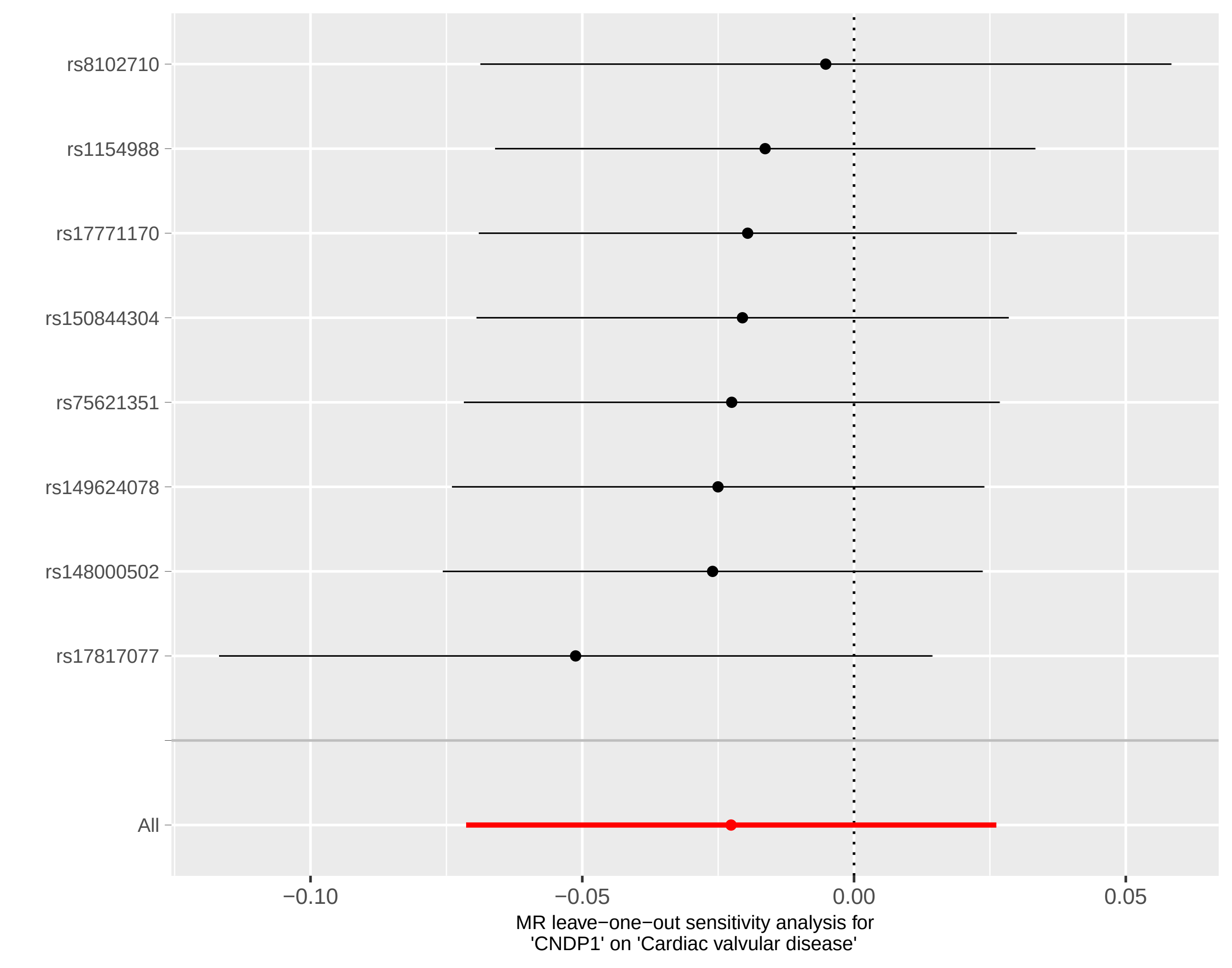
rs8102710
rs1154988
rs17771170
rs150844304
rs75621351
rs149624078
rs148000502
rs17817077
All
−0.10
−0.05
0.00
0.05
MR leave−one−out sensitivity analysis for
'CNDP1' on 'Cardiac valvular disease'

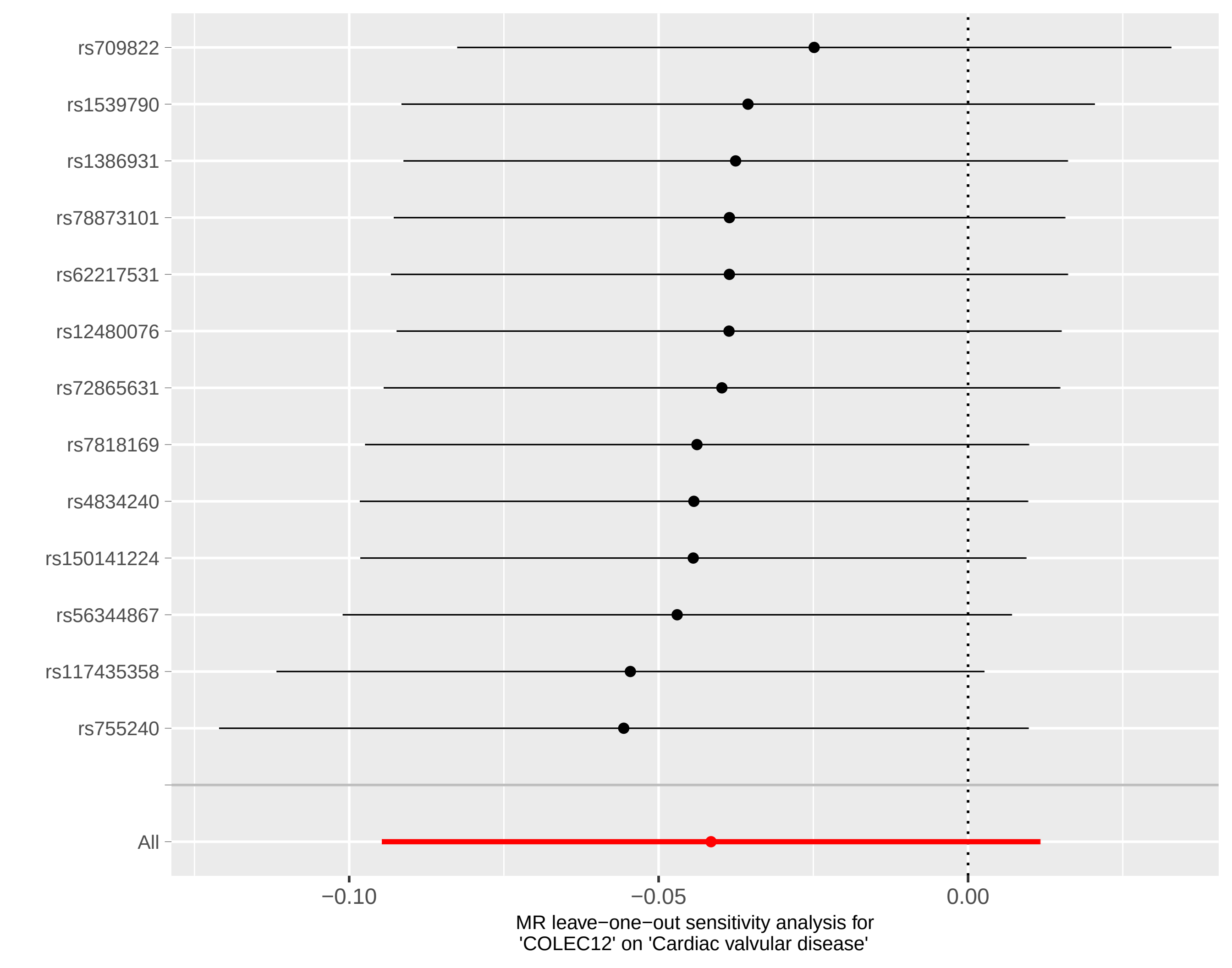

rs709822
rs1539790
rs1386931
rs78873101
rs62217531
rs12480076
rs72865631
rs7818169
rs4834240
rs150141224
rs56344867
rs117435358
rs755240
All
−0.10
−0.05
0.00
MR leave−one−out sensitivity analysis for
'COLEC12' on 'Cardiac valvular disease'

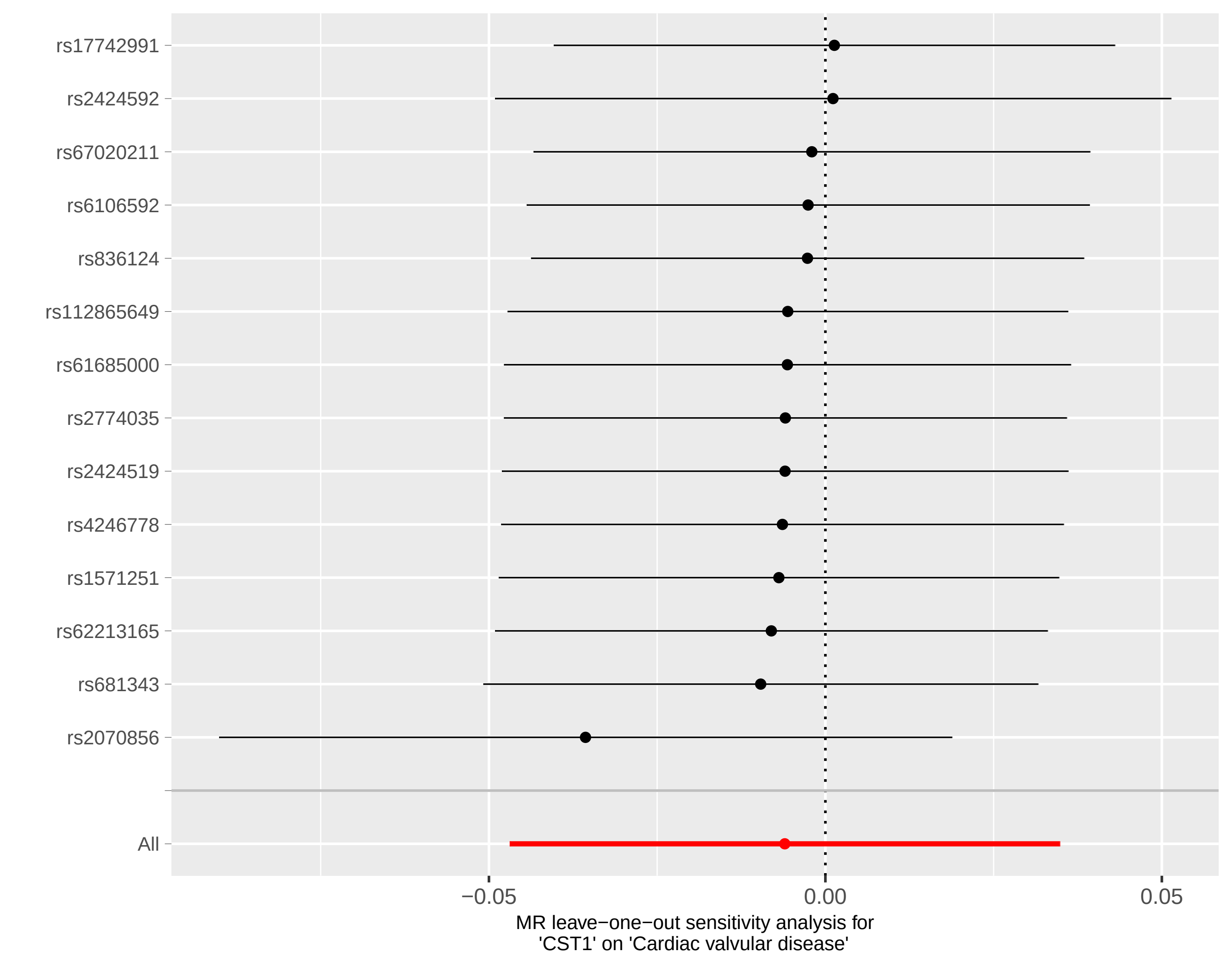
rs17742991
rs2424592
rs67020211
rs6106592
rs836124
rs112865649
rs61685000
rs2774035
rs2424519
rs4246778
rs1571251
rs62213165
rs681343
rs2070856
All
−0.05
0.00
0.05
MR leave−one−out sensitivity analysis for
'CST1' on 'Cardiac valvular disease'

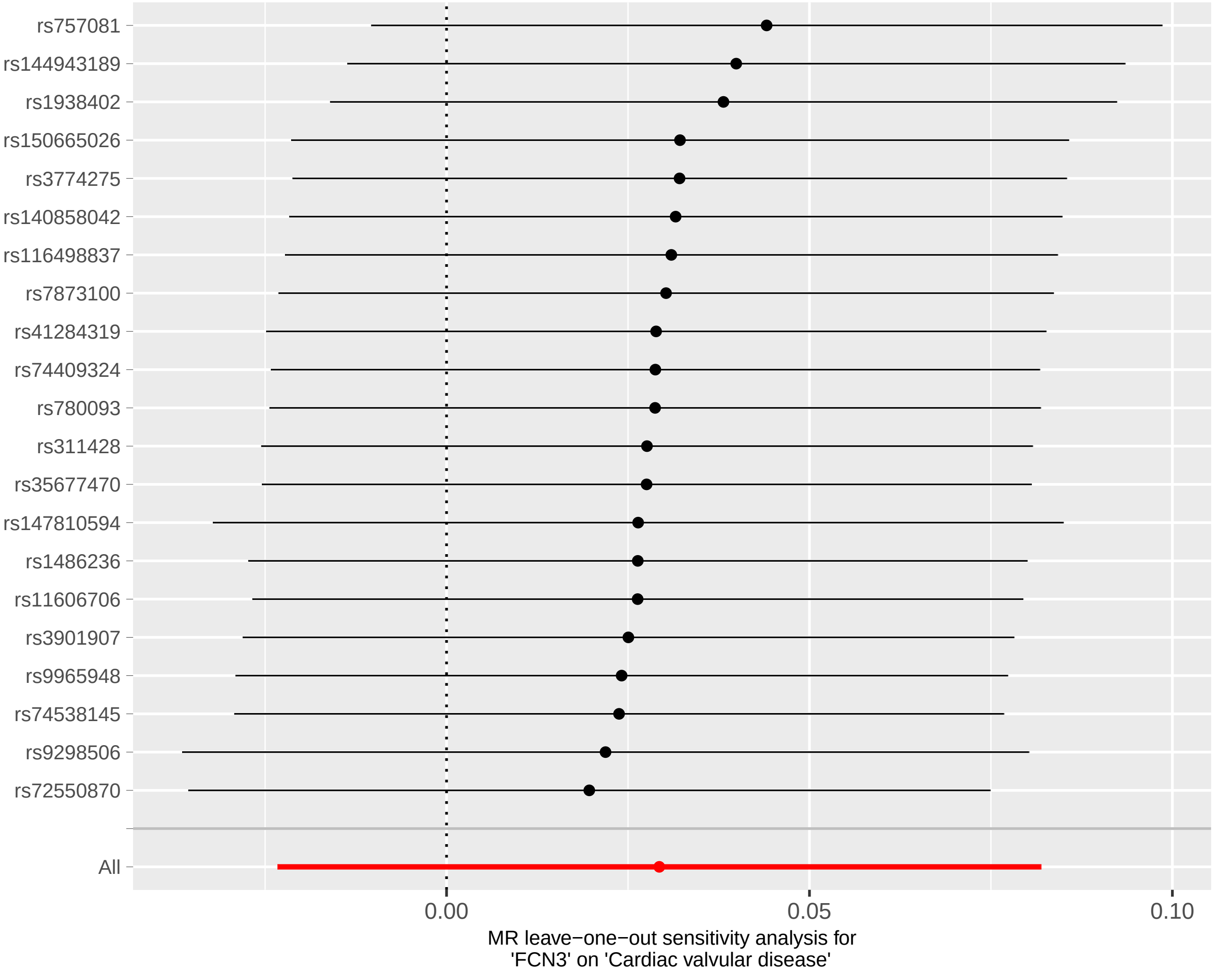
rs757081
rs144943189
rs1938402
rs150665026
rs3774275
rs140858042
rs116498837
rs7873100
rs41284319
rs74409324
rs780093
rs311428
rs35677470
rs147810594
rs1486236
rs11606706
rs3901907
rs9965948
rs74538145
rs9298506
rs72550870
All
0.00
0.05
0.10
MR leave−one−out sensitivity analysis for
'FCN3' on 'Cardiac valvular disease'

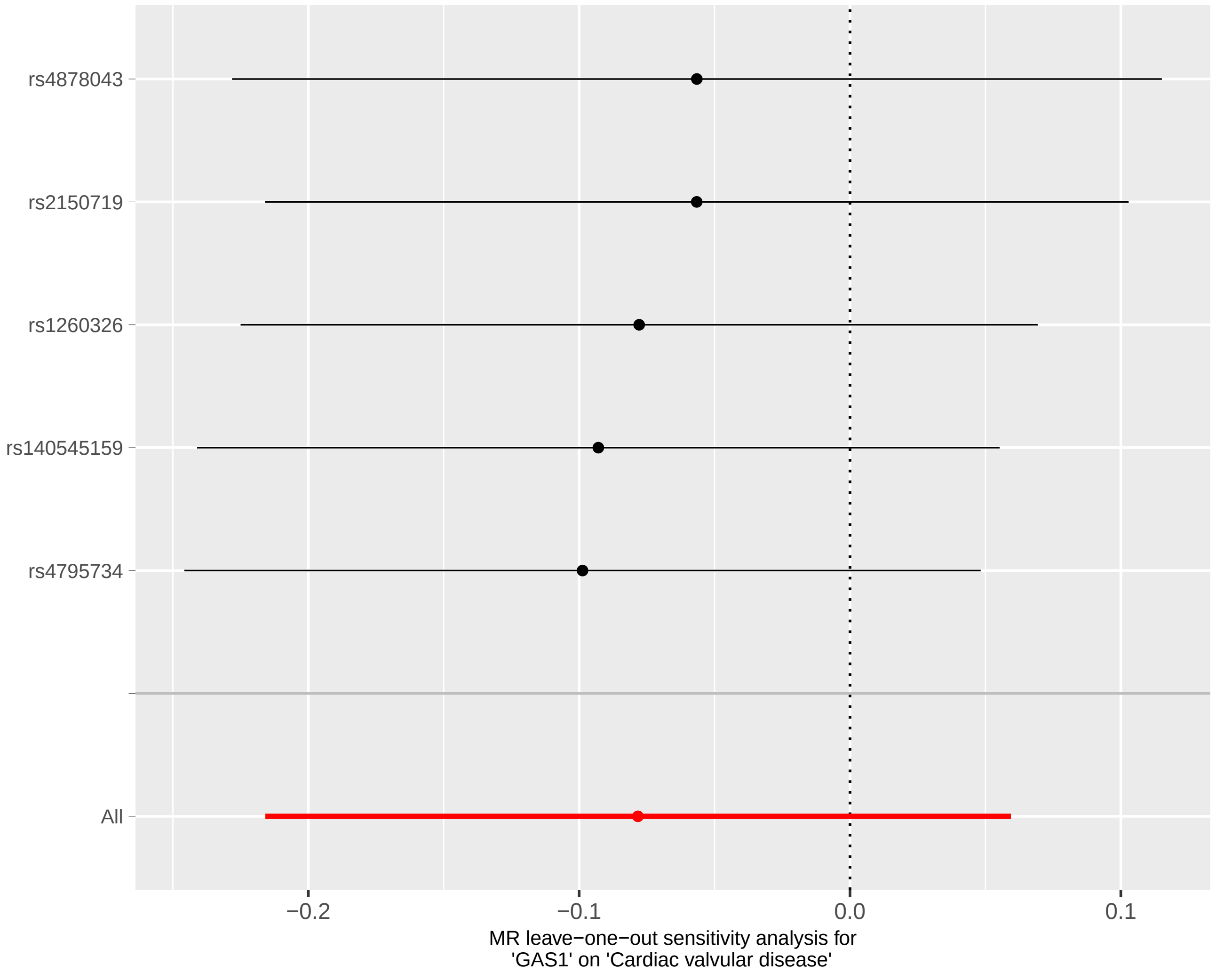

rs4878043
rs2150719
rs1260326
rs140545159
rs4795734
All
−0.2
−0.1
0.0
0.1
MR leave−one−out sensitivity analysis for
'GAS1' on 'Cardiac valvular disease'

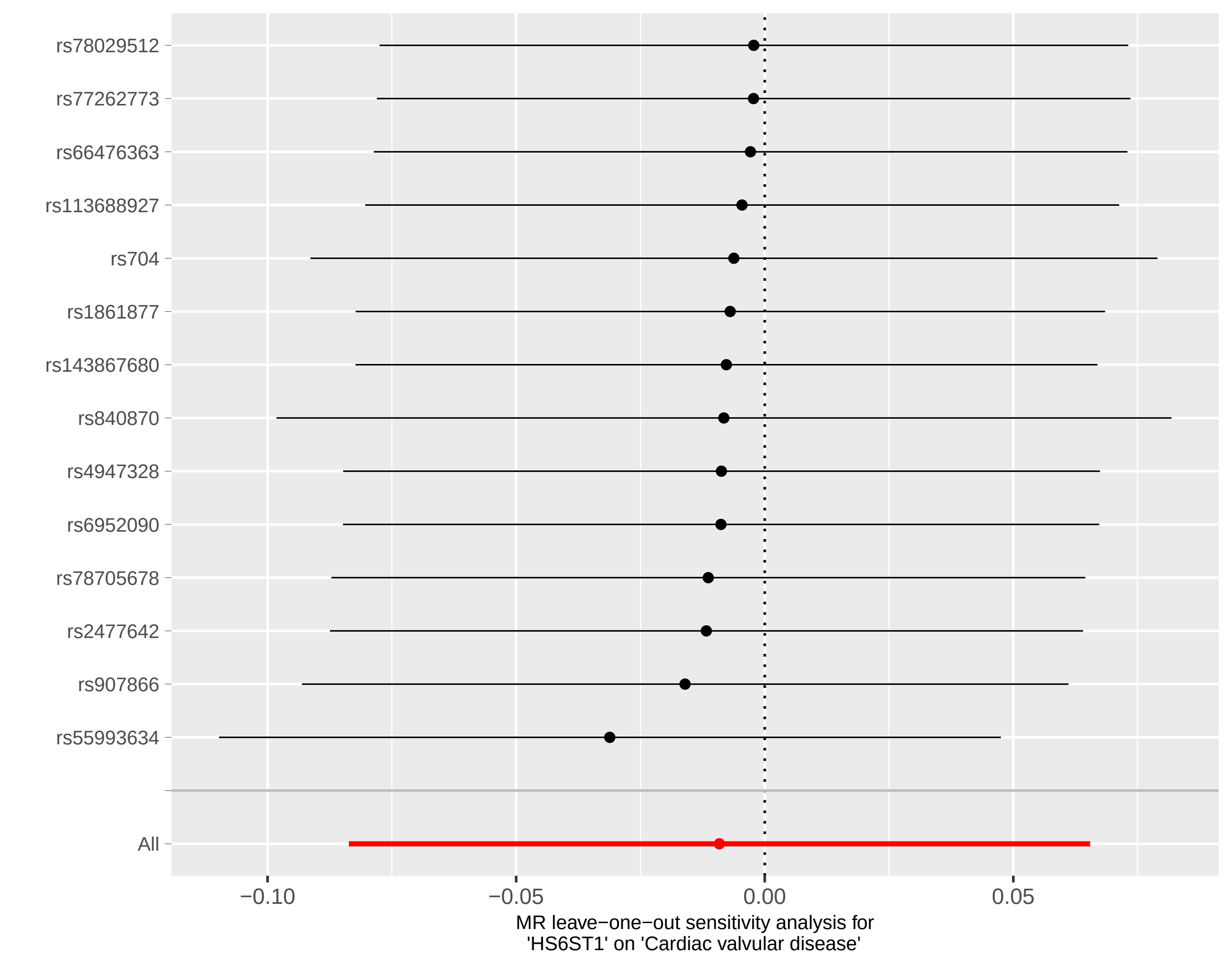
rs78029512
rs77262773
rs66476363
rs113688927
rs704
rs1861877
rs143867680
rs840870
rs4947328
rs6952090
rs78705678
rs2477642
rs907866
rs55993634
All
−0.10
−0.05
0.00
0.05
MR leave−one−out sensitivity analysis for
'HS6ST1' on 'Cardiac valvular disease'

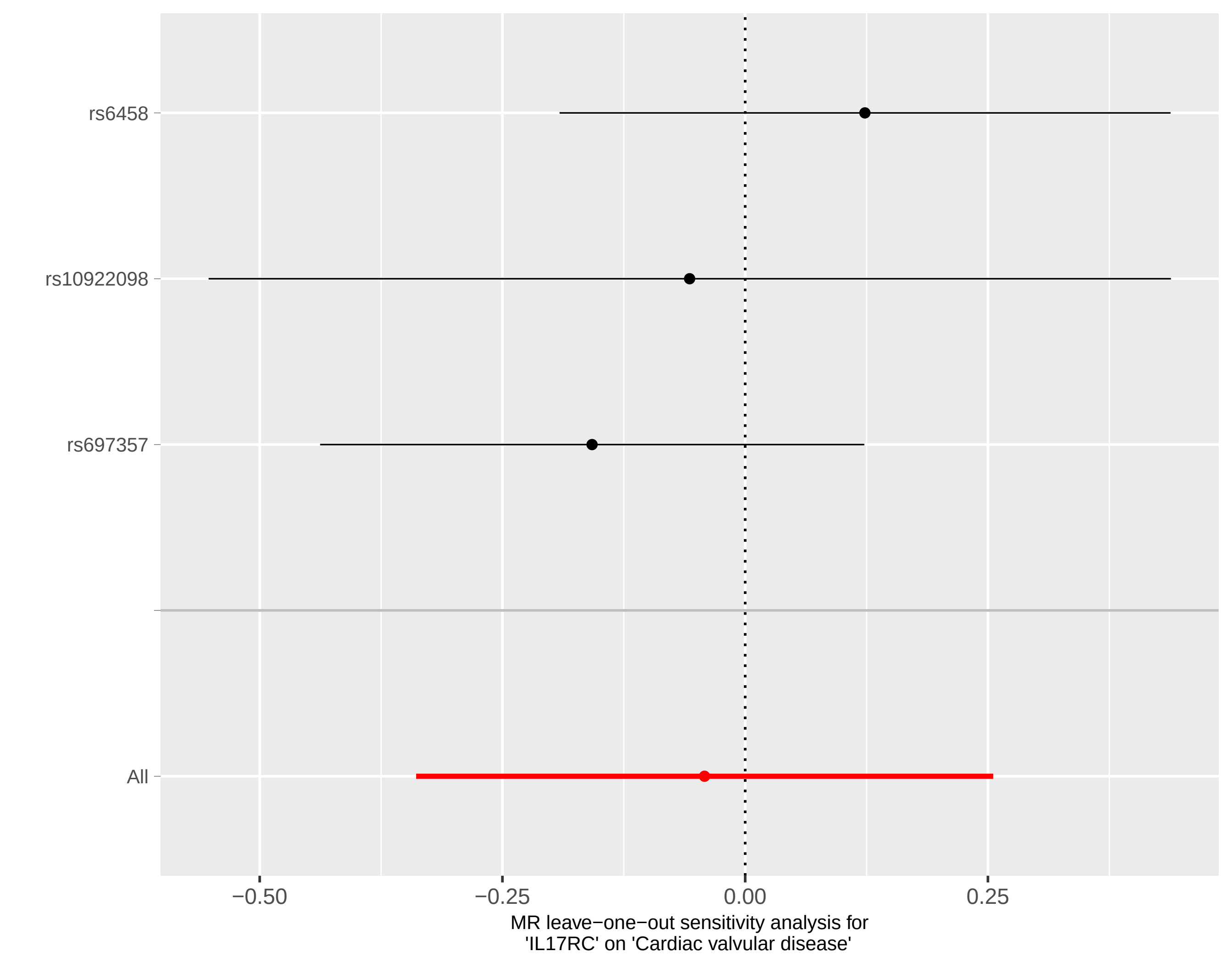
rs6458
rs10922098
rs697357
All
−0.50
−0.25
0.00
0.25
MR leave−one−out sensitivity analysis for
'IL17RC' on 'Cardiac valvular disease'

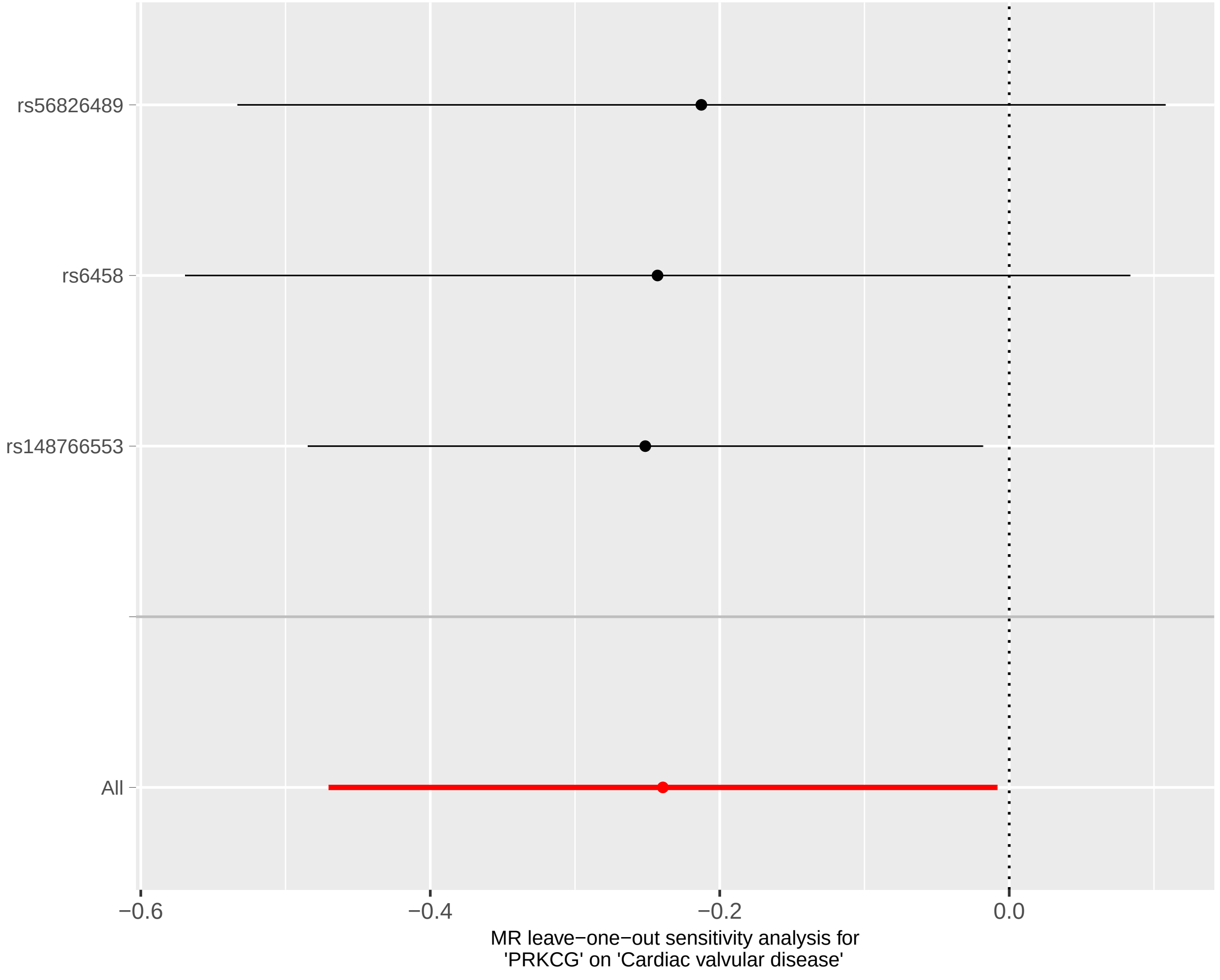

rs56826489
rs6458
rs148766553
All
−0.6
−0.4
−0.2
0.0
MR leave−one−out sensitivity analysis for
'PRKCG' on 'Cardiac valvular disease'

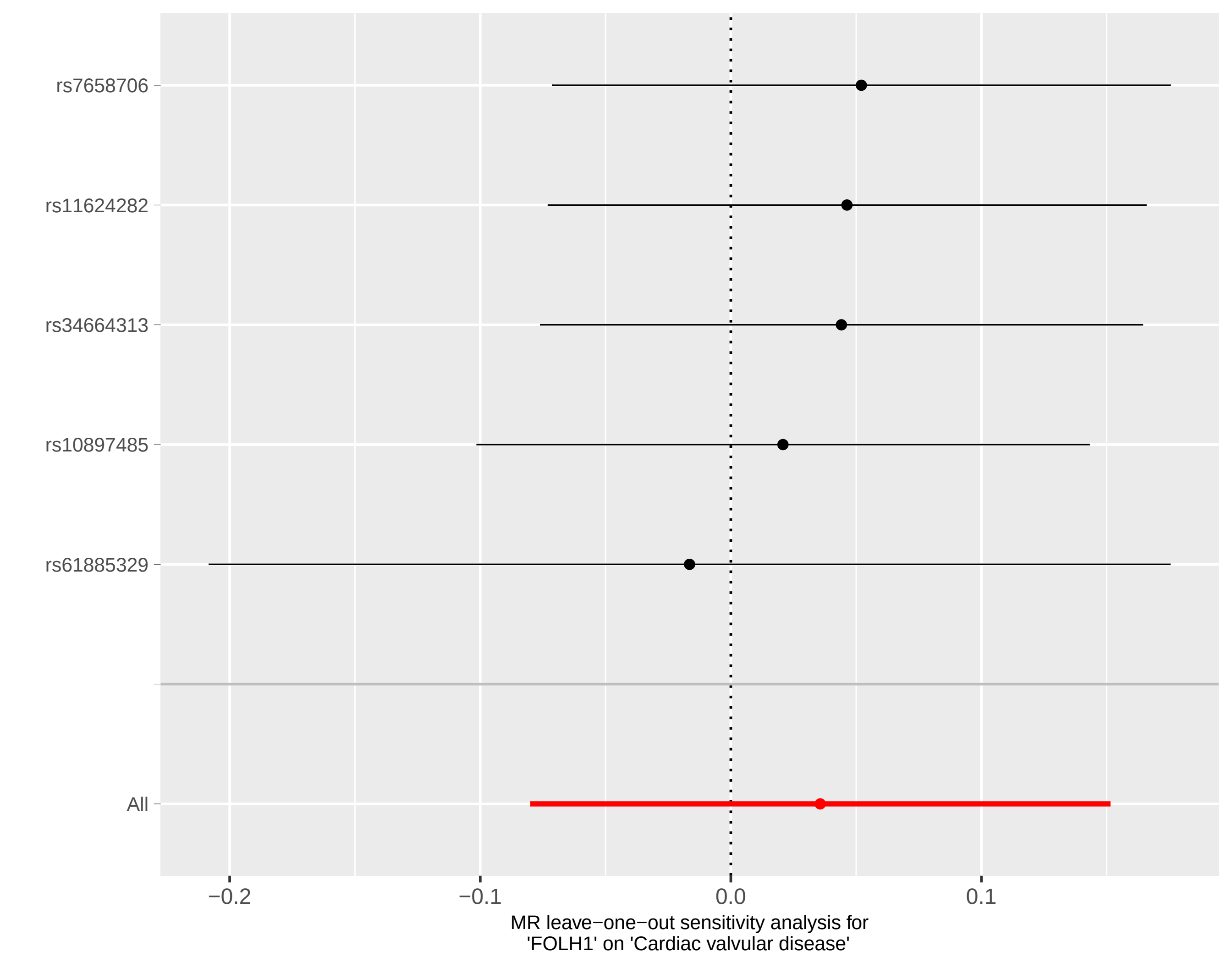
rs7658706
rs11624282
rs34664313
rs10897485
rs61885329
All
−0.2
−0.1
0.0
0.1
MR leave−one−out sensitivity analysis for
'FOLH1' on 'Cardiac valvular disease'

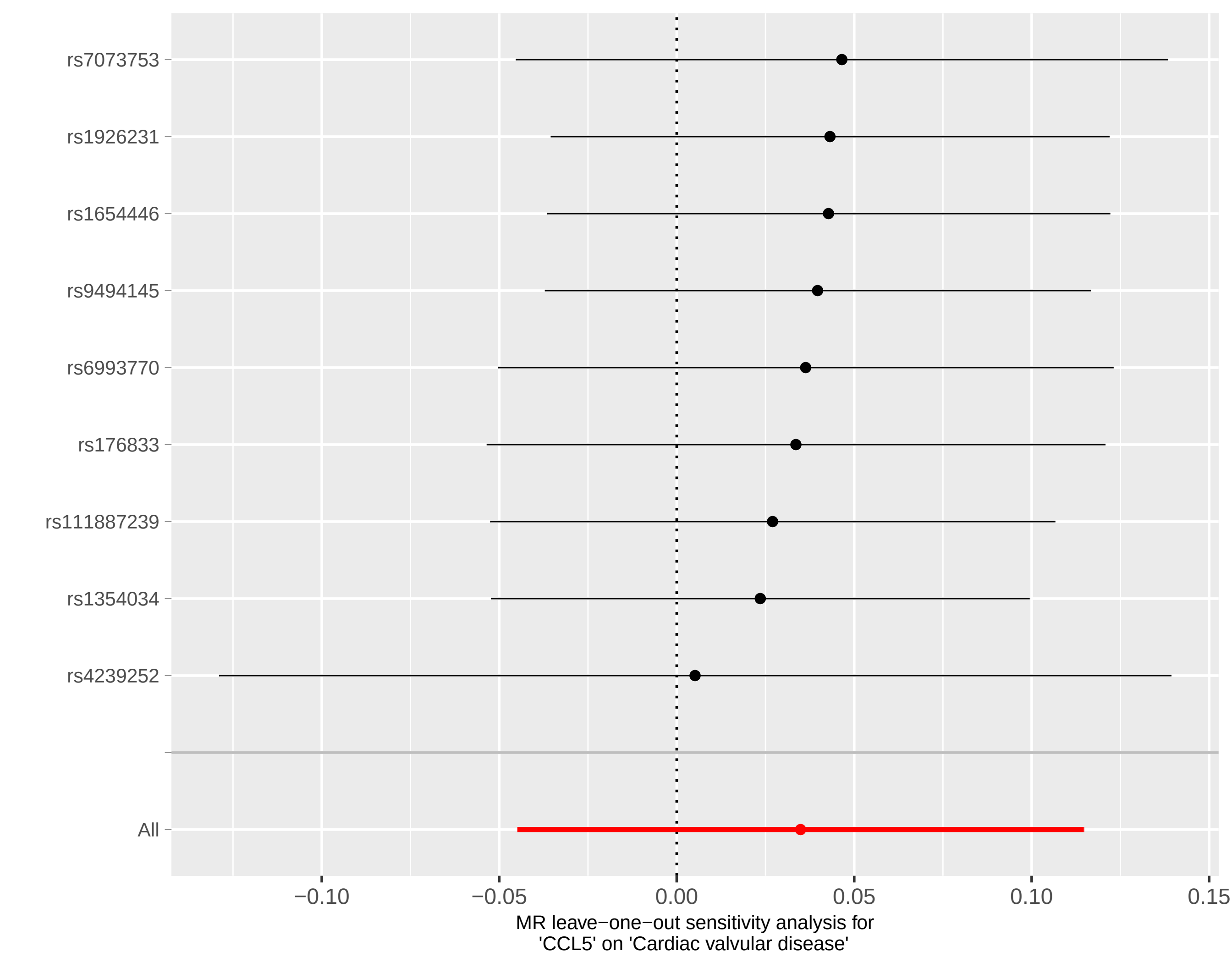
rs7073753
rs1926231
rs1654446
rs9494145
rs6993770
rs176833
rs111887239
rs1354034
rs4239252
All
−0.10
−0.05
0.00
0.05
0.10
0.15
MR leave−one−out sensitivity analysis for
'CCL5' on 'Cardiac valvular disease'

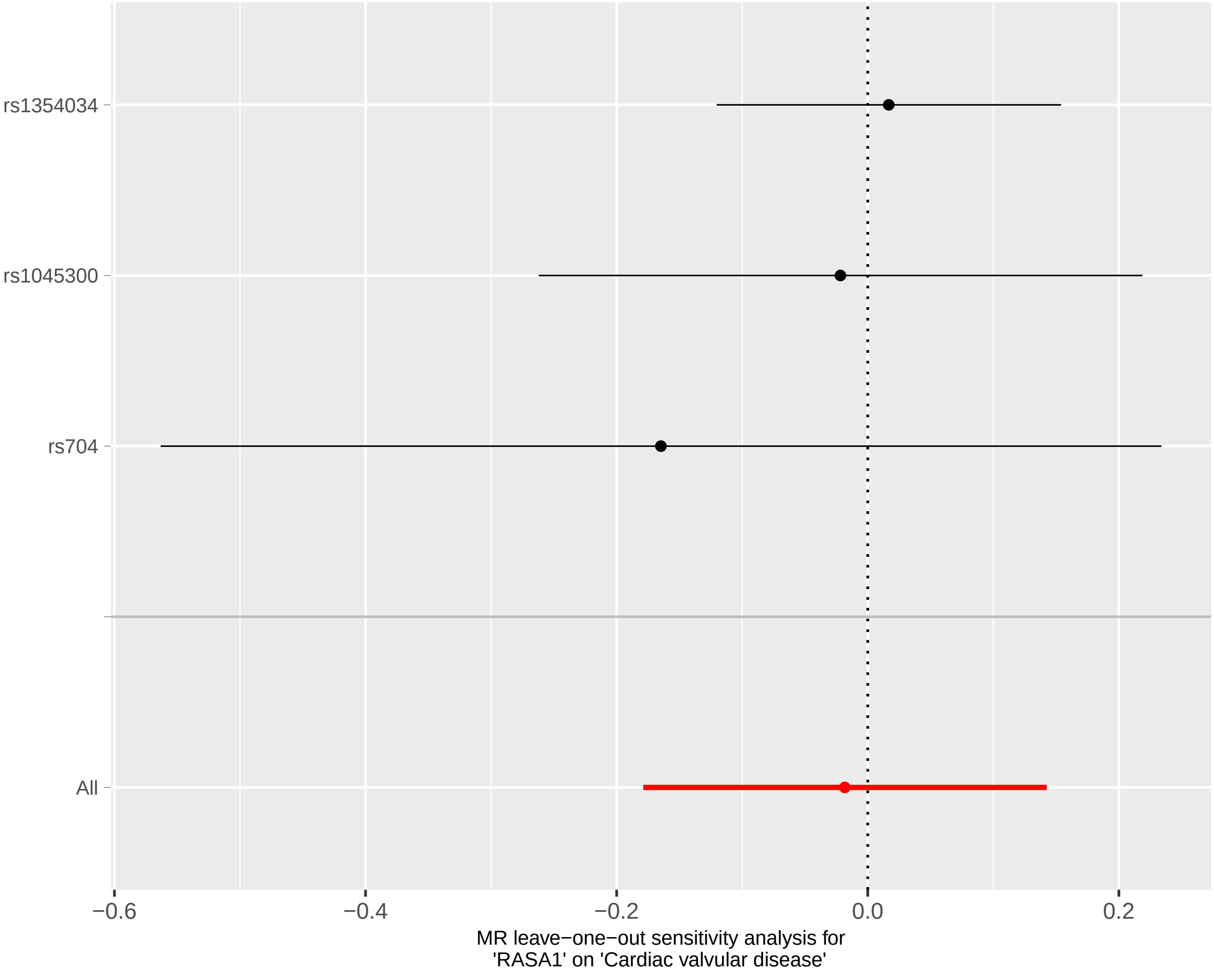

rs1354034
rs1045300
rs704
All
−0.6
−0.4
−0.2
0.0
0.2
MR leave−one−out sensitivity analysis for
'RASA1' on 'Cardiac valvular disease'

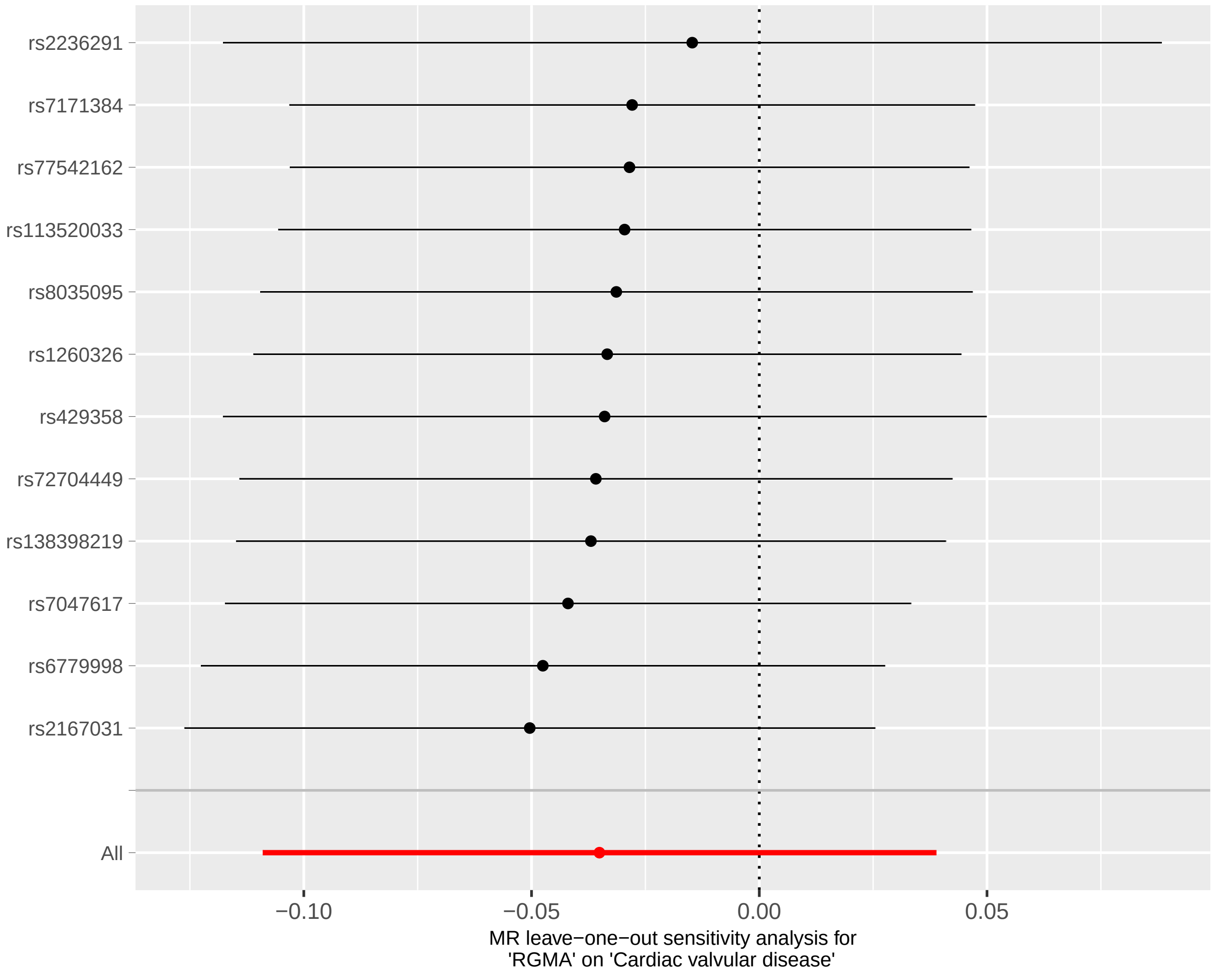
rs2236291
rs7171384
rs77542162
rs113520033
rs8035095
rs1260326
rs429358
rs72704449
rs138398219
rs7047617
rs6779998
rs2167031
All
−0.10
−0.05
0.00
0.05
MR leave−one−out sensitivity analysis for
'RGMA' on 'Cardiac valvular disease'

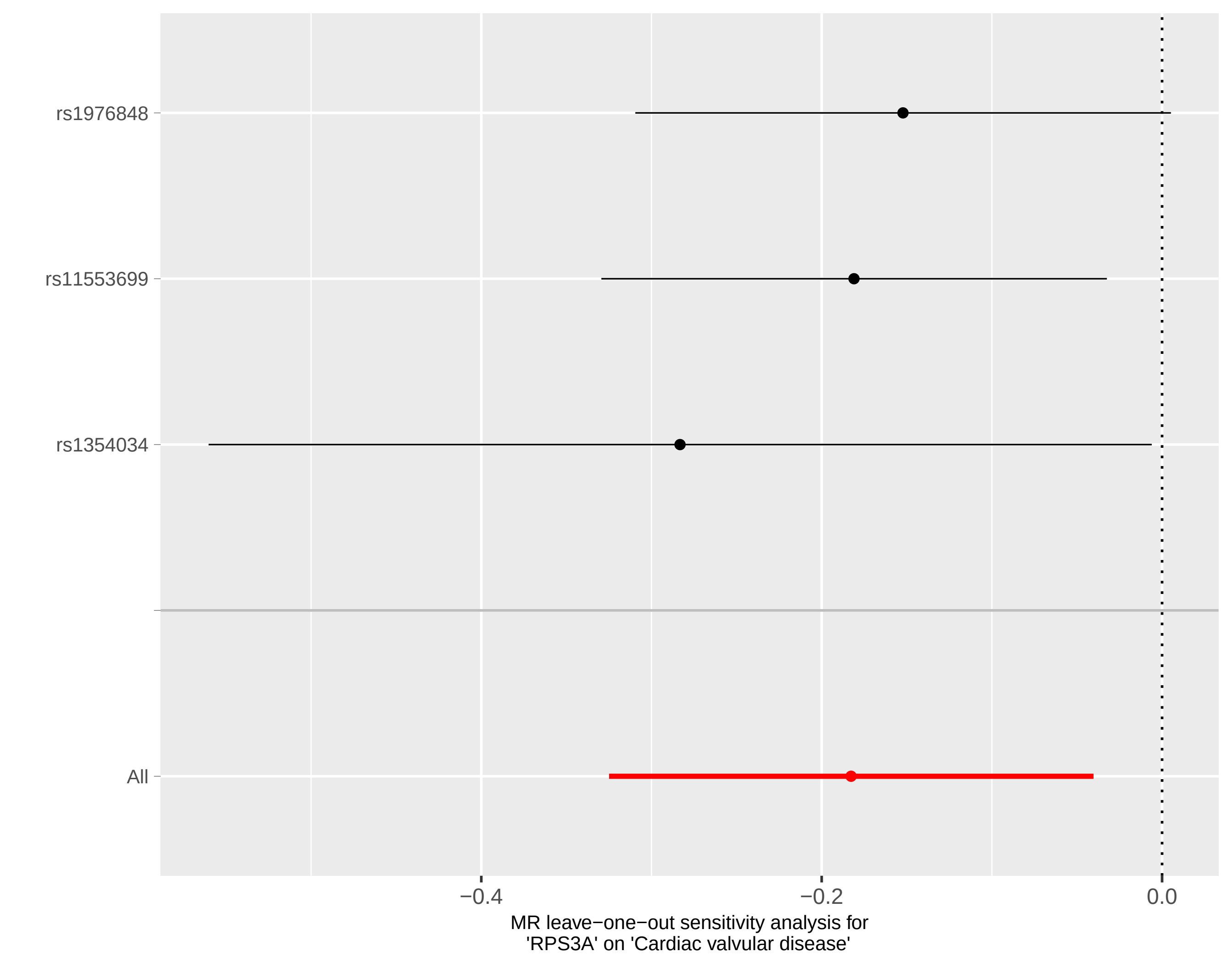
rs1976848
rs11553699
rs1354034
All
−0.4
−0.2
0.0
MR leave−one−out sensitivity analysis for
'RPS3A' on 'Cardiac valvular disease'

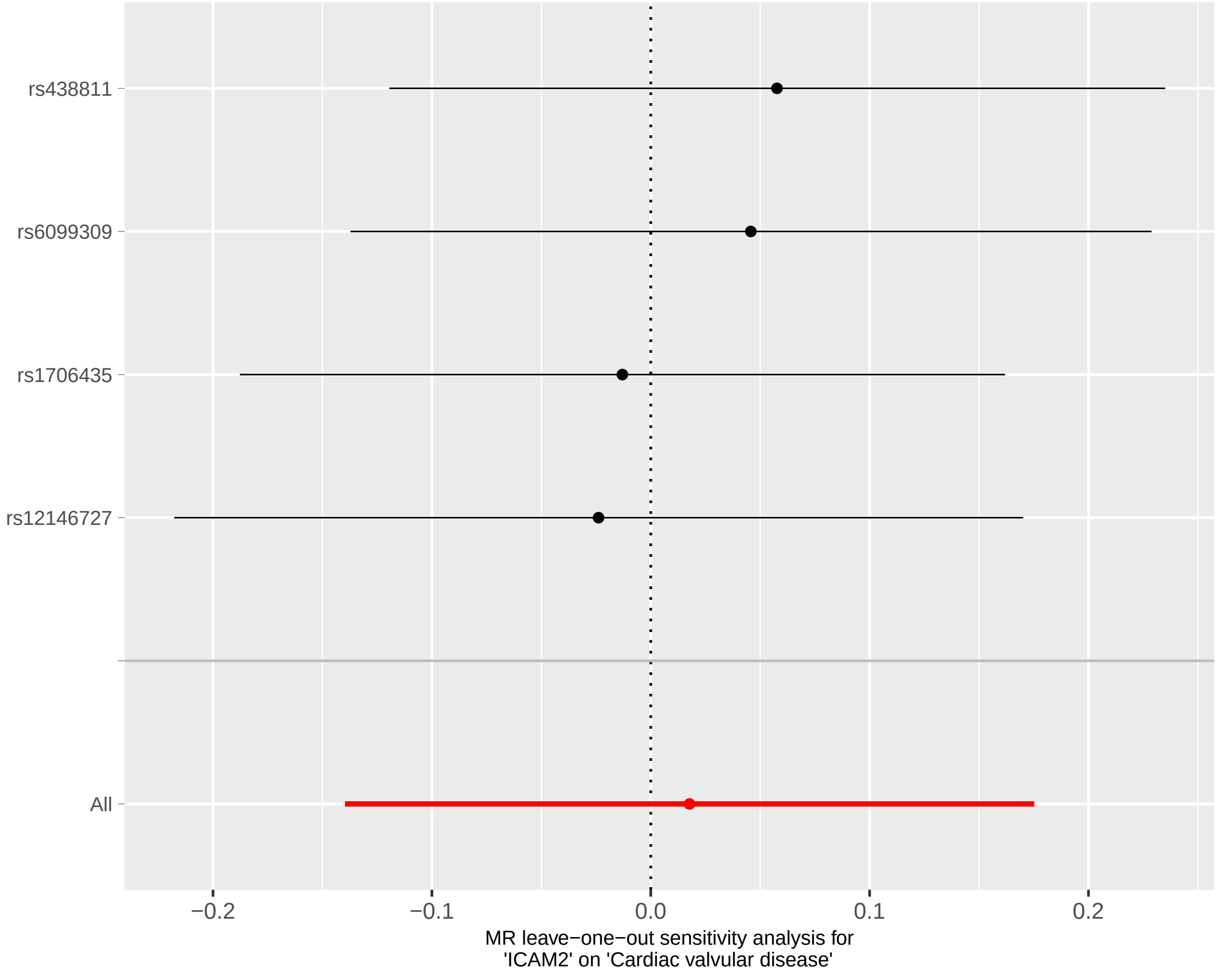
rs438811
rs6099309
rs1706435
rs12146727
All
−0.2
−0.1
0.0
0.1
0.2
MR leave−one−out sensitivity analysis for
'ICAM2' on 'Cardiac valvular disease'

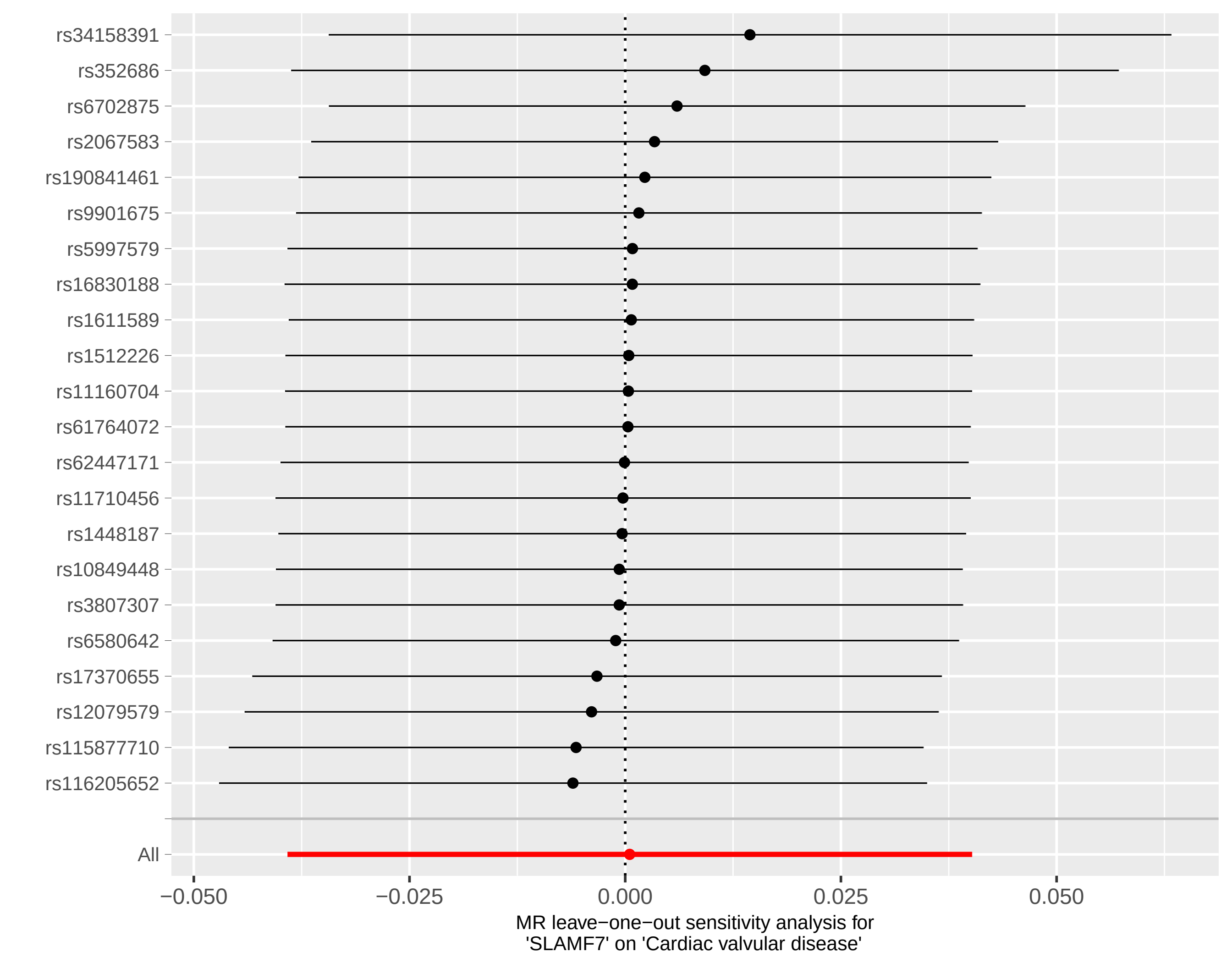
rs34158391
rs352686
rs6702875
rs2067583
rs190841461
rs9901675
rs5997579
rs16830188
rs1611589
rs1512226
rs11160704
rs61764072
rs62447171
rs11710456
rs1448187
rs10849448
rs3807307
rs6580642
rs17370655
rs12079579
rs115877710
rs116205652
All
−0.050
−0.025
0.000
0.025
0.050
MR leave−one−out sensitivity analysis for
'SLAMF7' on 'Cardiac valvular disease'

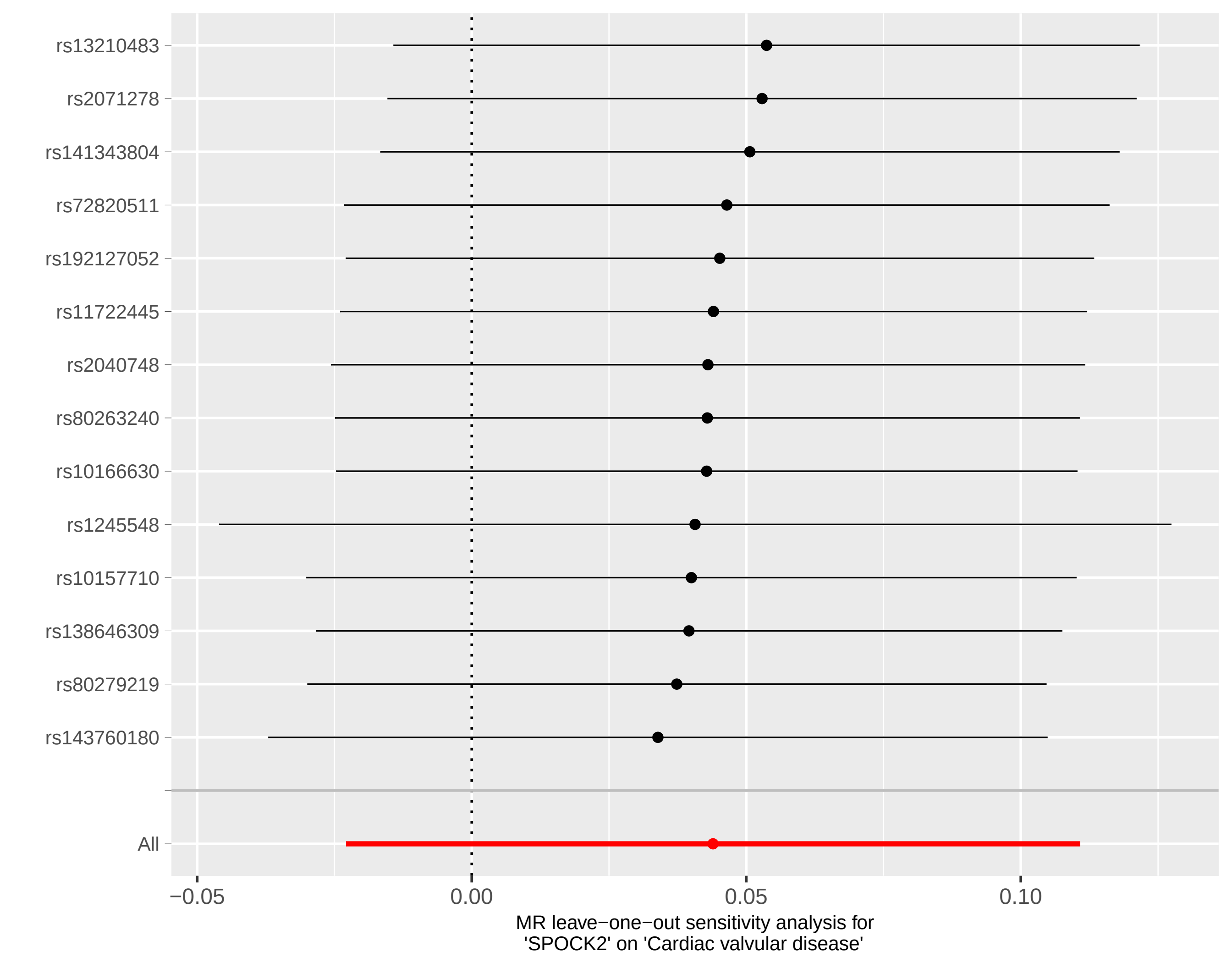

rs13210483
rs2071278
rs141343804
rs72820511
rs192127052
rs11722445
rs2040748
rs80263240
rs10166630
rs1245548
rs10157710
rs138646309
rs80279219
rs143760180
All
−0.05
0.00
0.05
0.10
MR leave−one−out sensitivity analysis for
'SPOCK2' on 'Cardiac valvular disease'

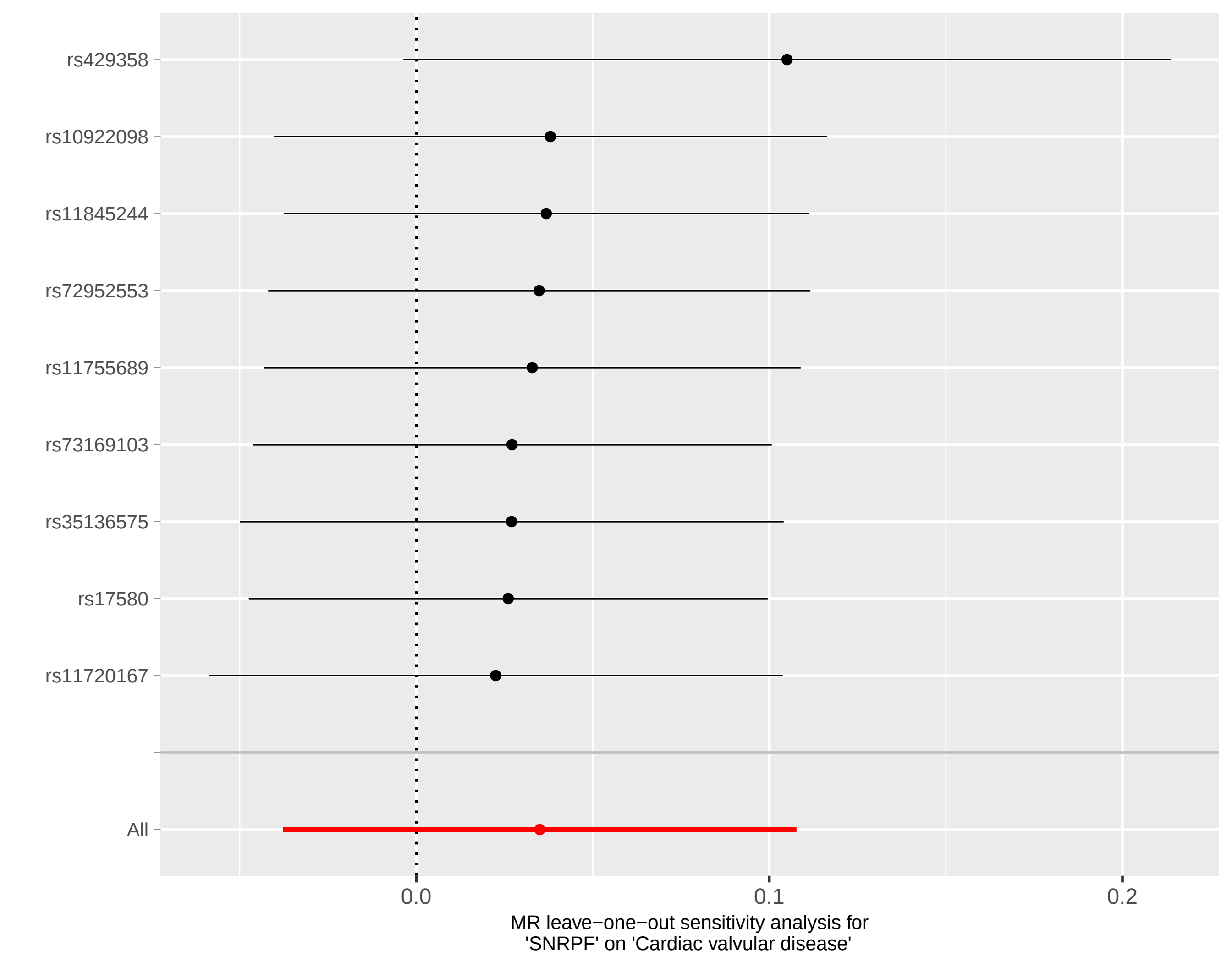
rs429358
rs10922098
rs11845244
rs72952553
rs11755689
rs73169103
rs35136575
rs17580
rs11720167
All
0.0
0.1
0.2
MR leave−one−out sensitivity analysis for
'SNRPF' on 'Cardiac valvular disease'

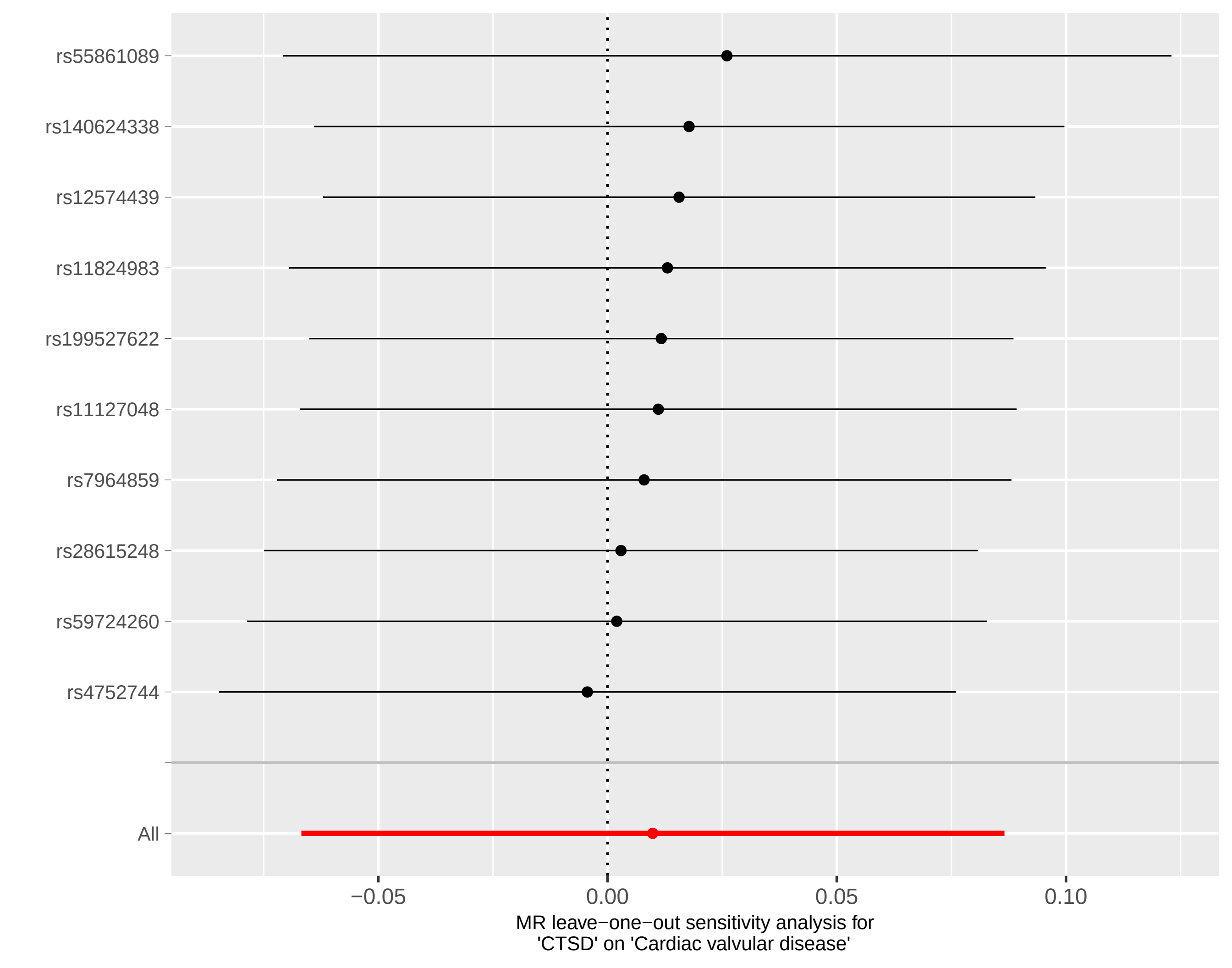
rs55861089
rs140624338
rs12574439
rs11824983
rs199527622
rs11127048
rs7964859
rs28615248
rs59724260
rs4752744
All
−0.05
0.00
0.05
0.10
MR leave−one−out sensitivity analysis for
'CTSD' on 'Cardiac valvular disease'

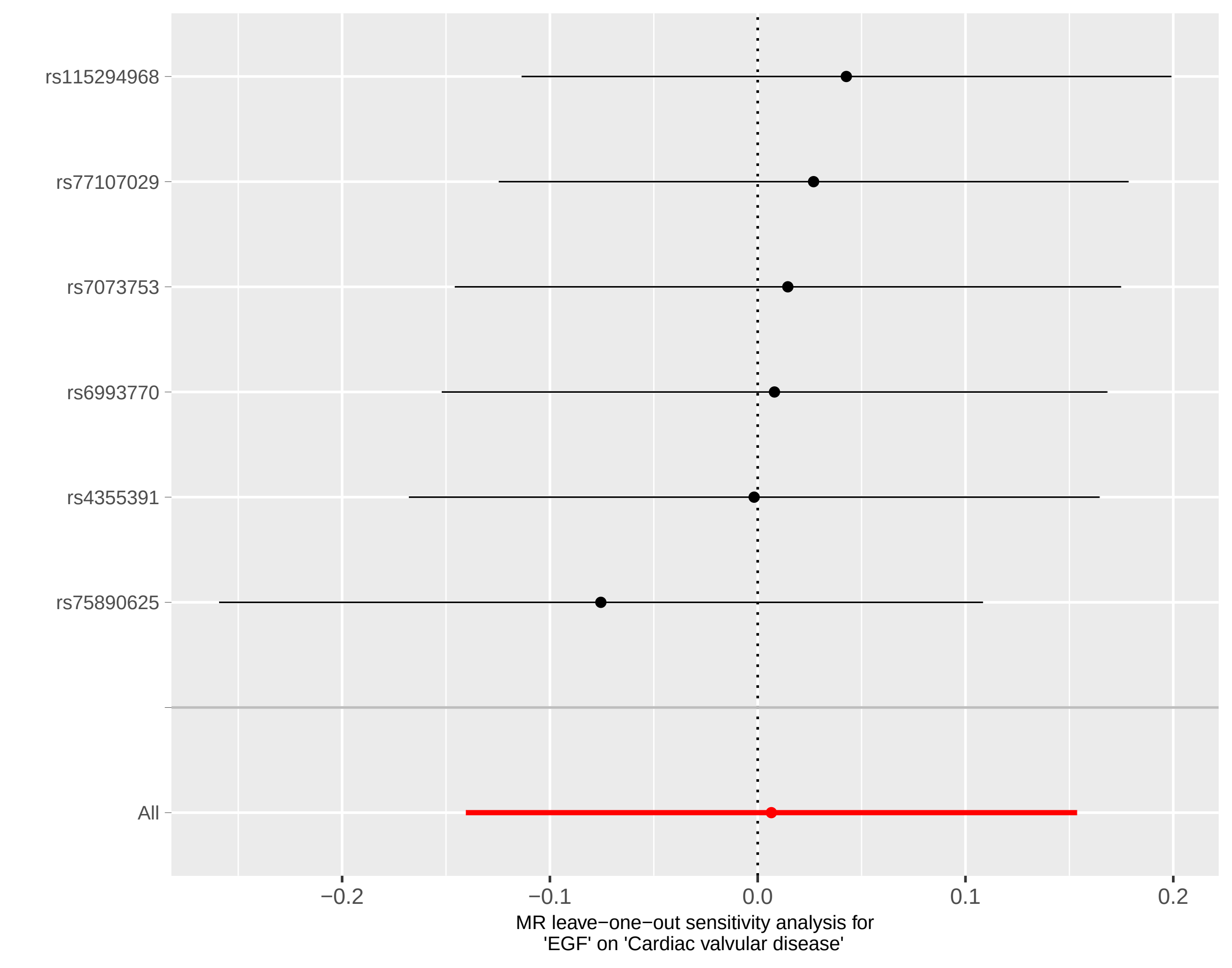
rs115294968
rs77107029
rs7073753
rs6993770
rs4355391
rs75890625
All
−0.2
−0.1
0.0
0.1
0.2
MR leave−one−out sensitivity analysis for
'EGF' on 'Cardiac valvular disease'

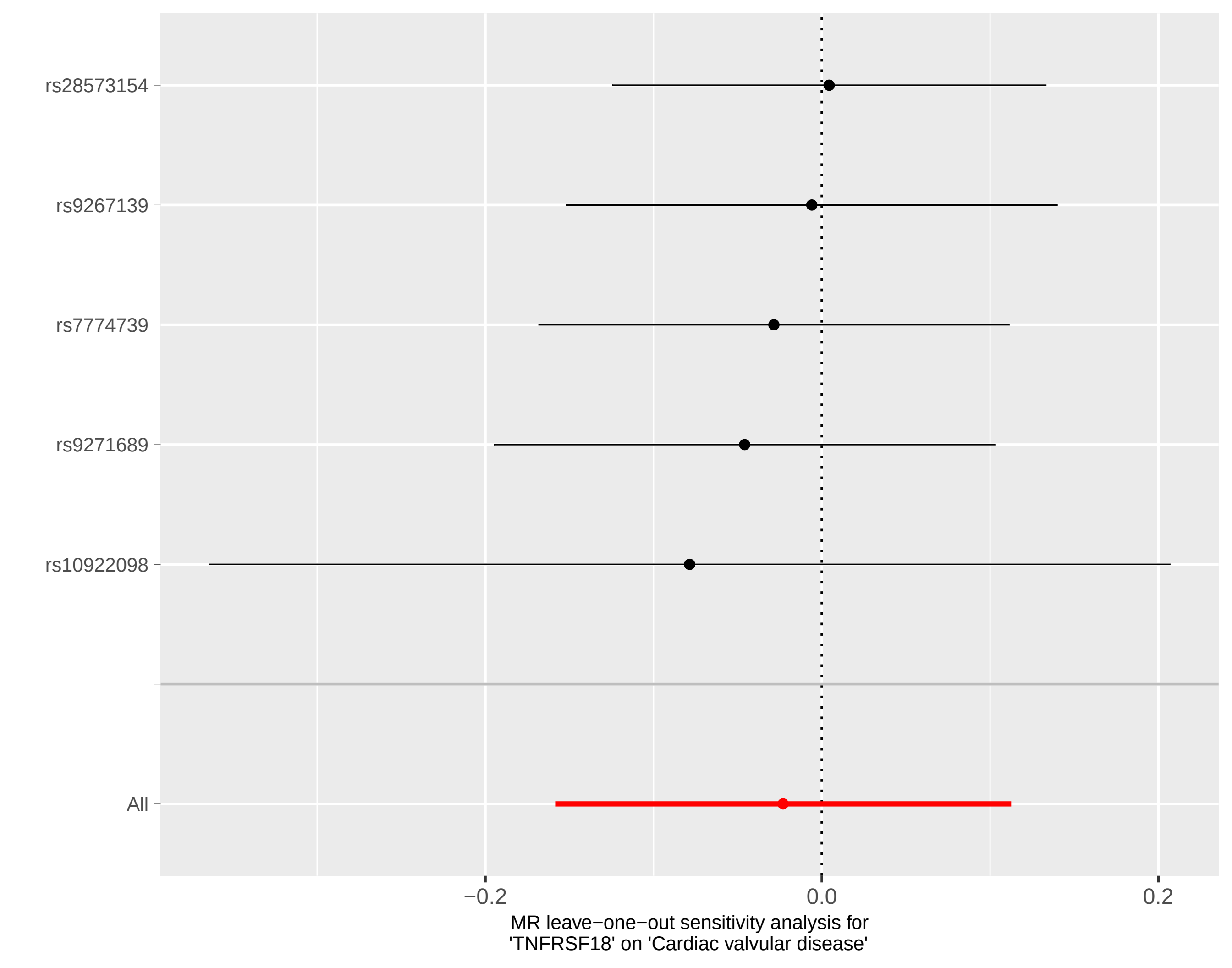
rs28573154
rs9267139
rs7774739
rs9271689
rs10922098
All
−0.2
0.0
0.2
MR leave−one−out sensitivity analysis for
'TNFRSF18' on 'Cardiac valvular disease'

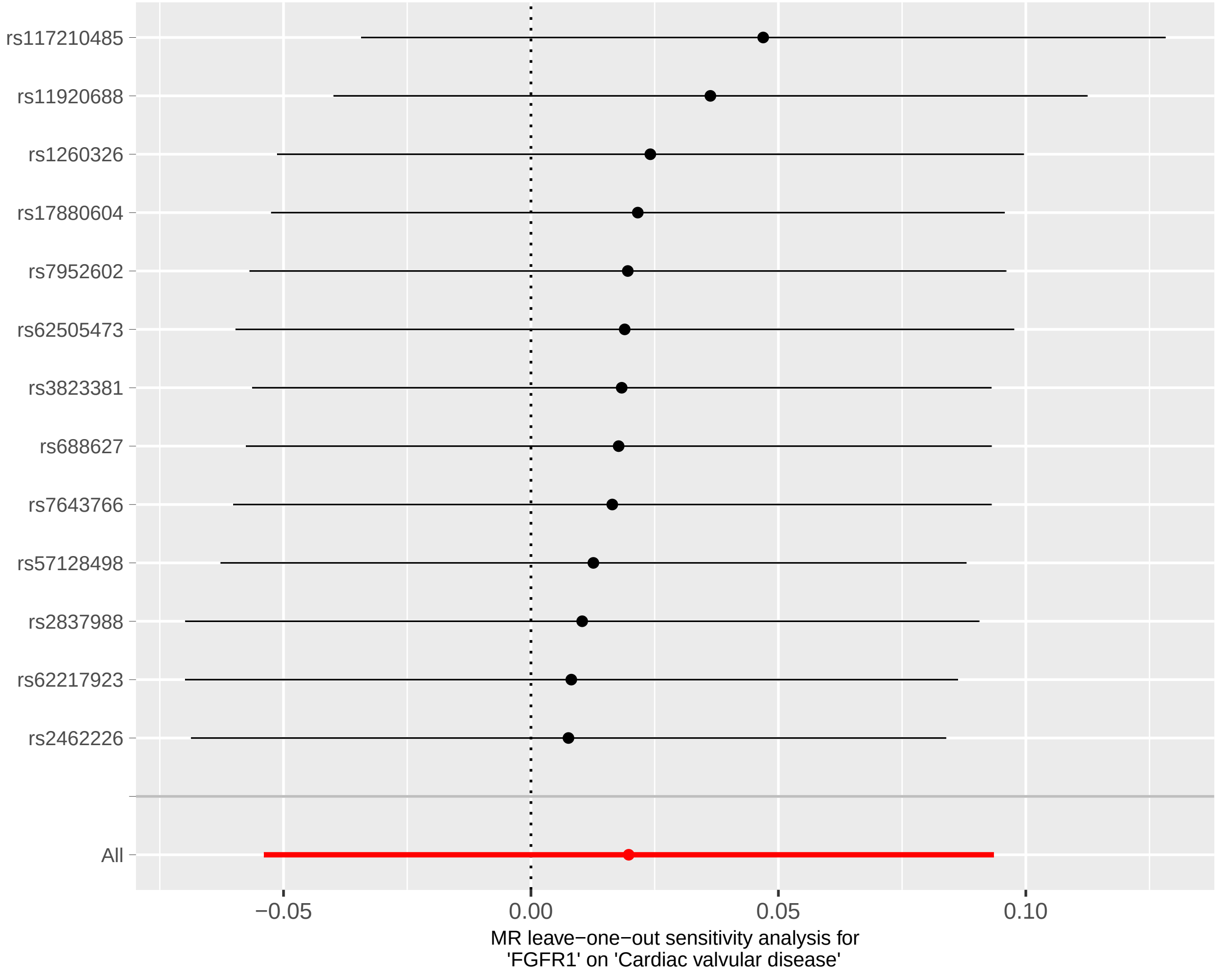
rs117210485
rs11920688
rs1260326
rs17880604
rs7952602
rs62505473
rs3823381
rs688627
rs7643766
rs57128498
rs2837988
rs62217923
rs2462226
All
−0.05
0.00
0.05
0.10
MR leave−one−out sensitivity analysis for
'FGFR1' on 'Cardiac valvular disease'

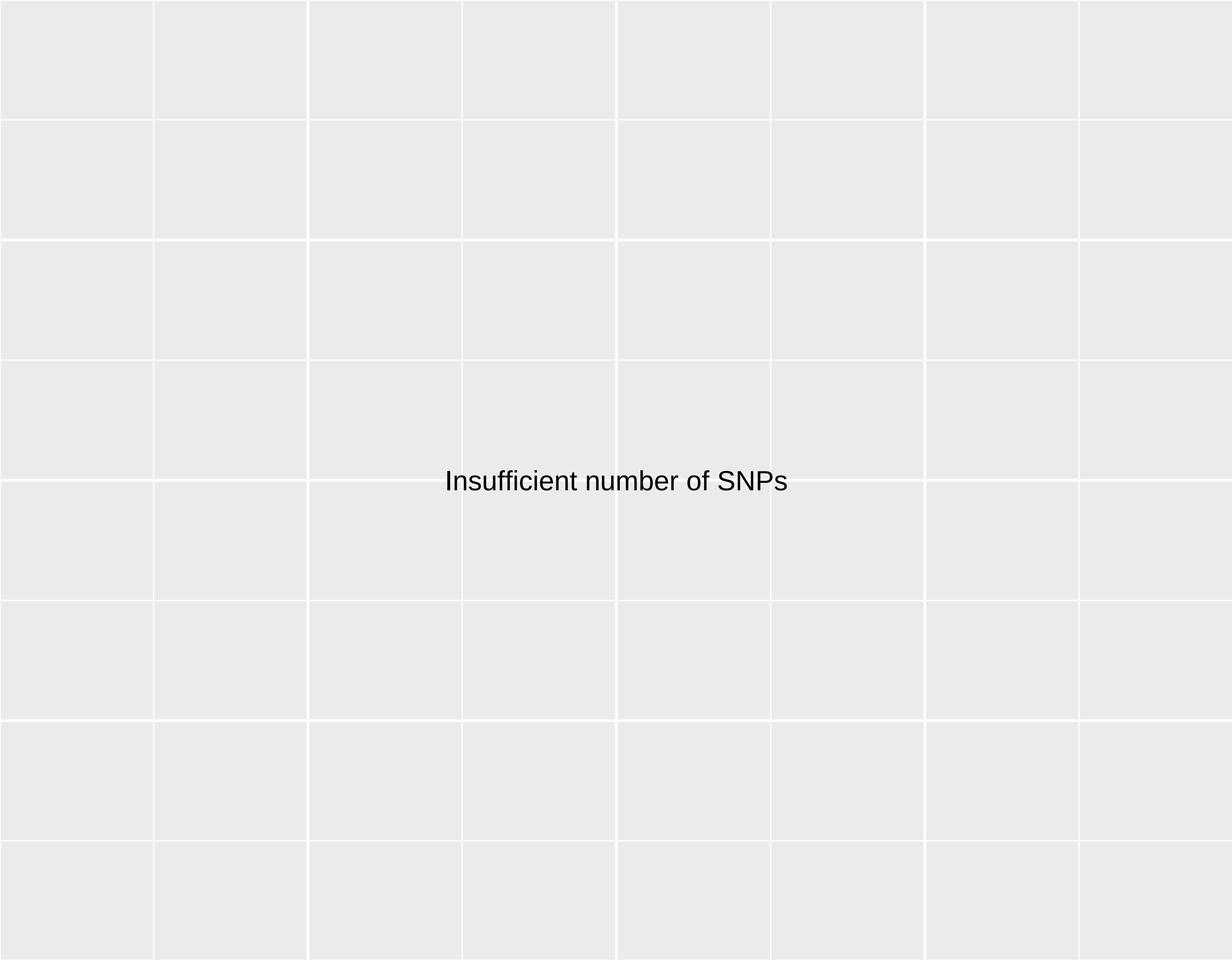
Insufficient number of SNPs

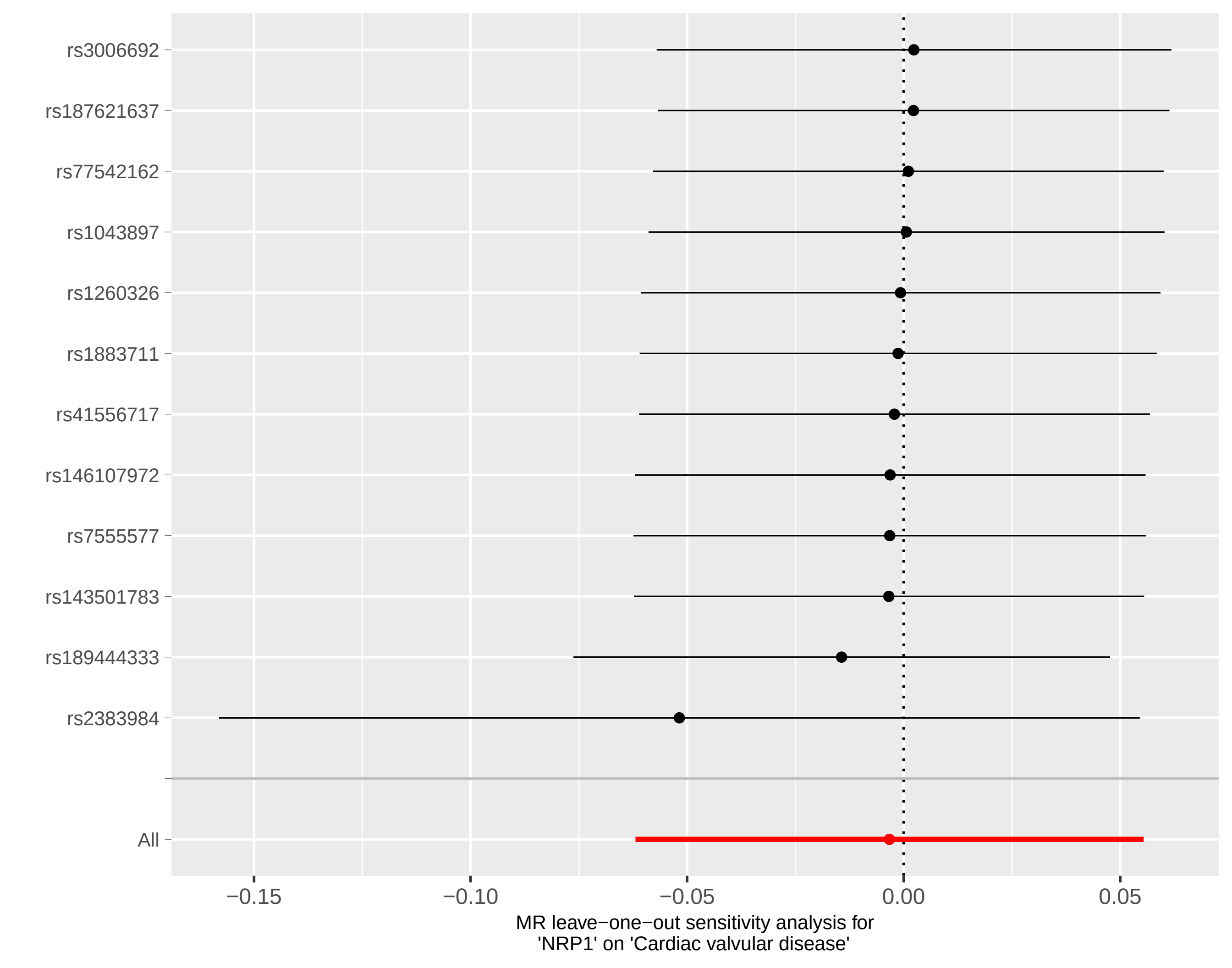

rs3006692
rs187621637
rs77542162
rs1043897
rs1260326
rs1883711
rs41556717
rs146107972
rs7555577
rs143501783
rs189444333
rs2383984
All
−0.15
−0.10
−0.05
0.00
0.05
MR leave−one−out sensitivity analysis for
'NRP1' on 'Cardiac valvular disease'

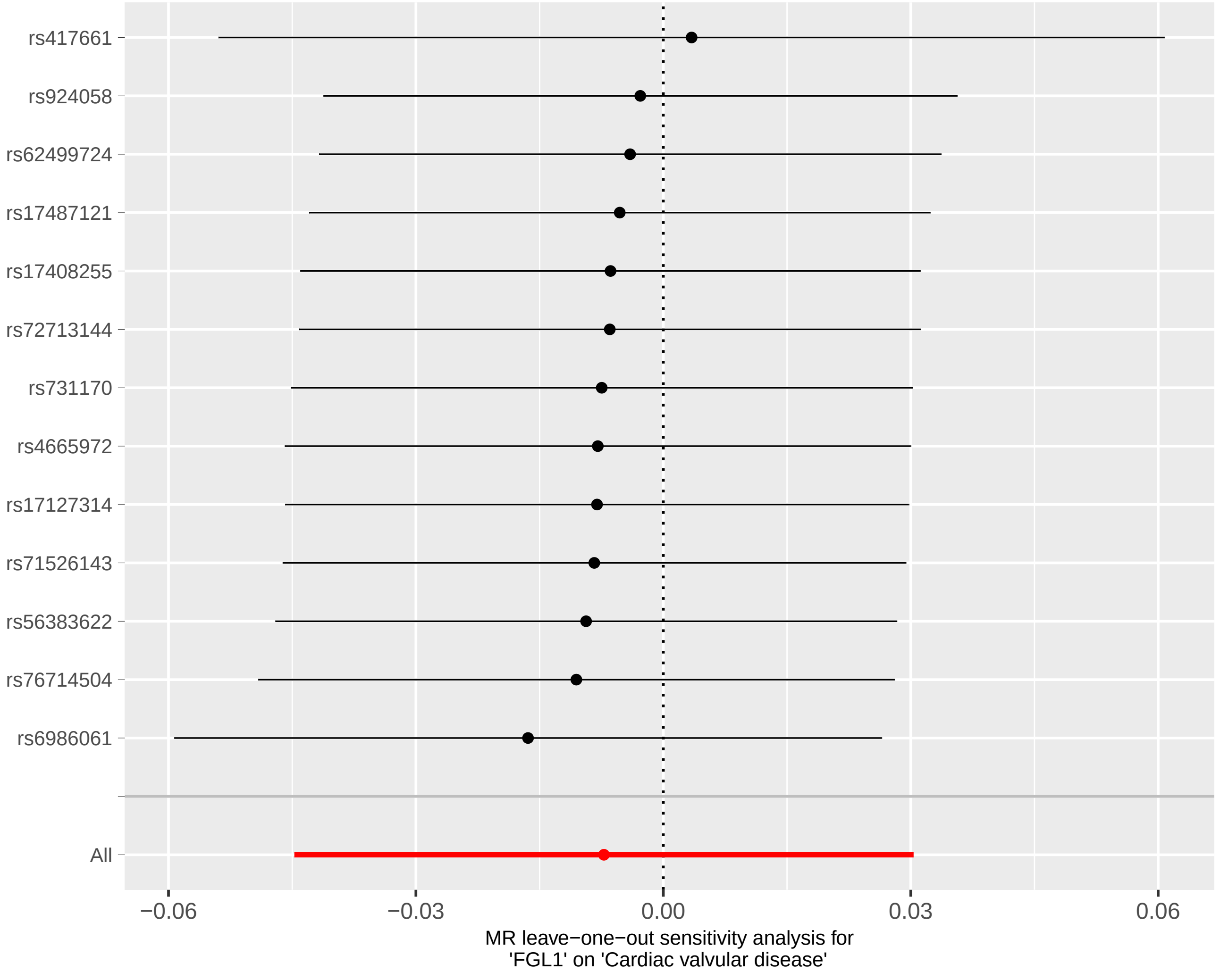
rs417661
rs924058
rs62499724
rs17487121
rs17408255
rs72713144
rs731170
rs4665972
rs17127314
rs71526143
rs56383622
rs76714504
rs6986061
All
−0.06
−0.03
0.00
0.03
0.06
MR leave−one−out sensitivity analysis for
'FGL1' on 'Cardiac valvular disease'

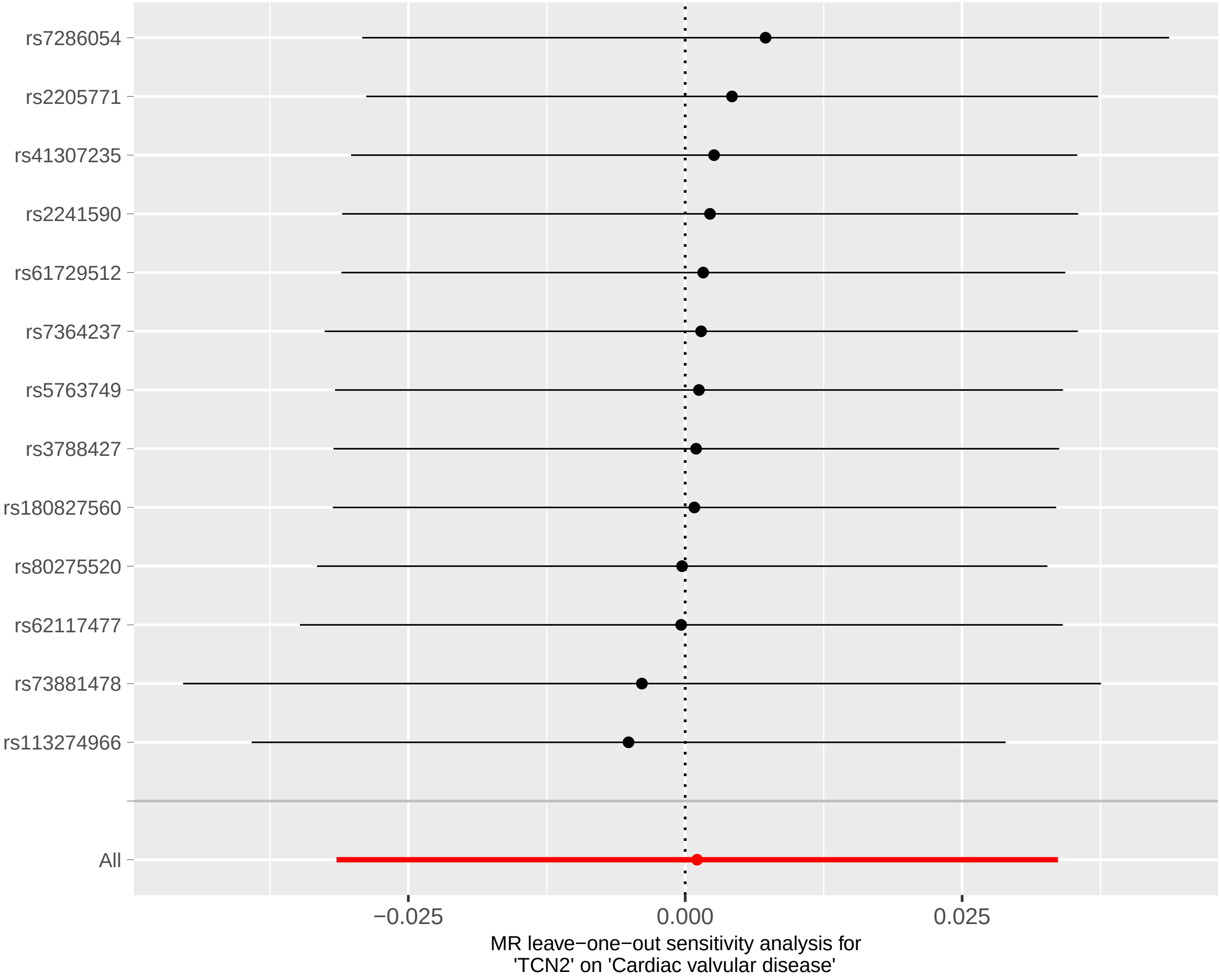

rs7286054
rs2205771
rs41307235
rs2241590
rs61729512
rs7364237
rs5763749
rs3788427
rs180827560
rs80275520
rs62117477
rs73881478
rs113274966
All
−0.025
0.000
0.025
MR leave−one−out sensitivity analysis for
'TCN2' on 'Cardiac valvular disease'

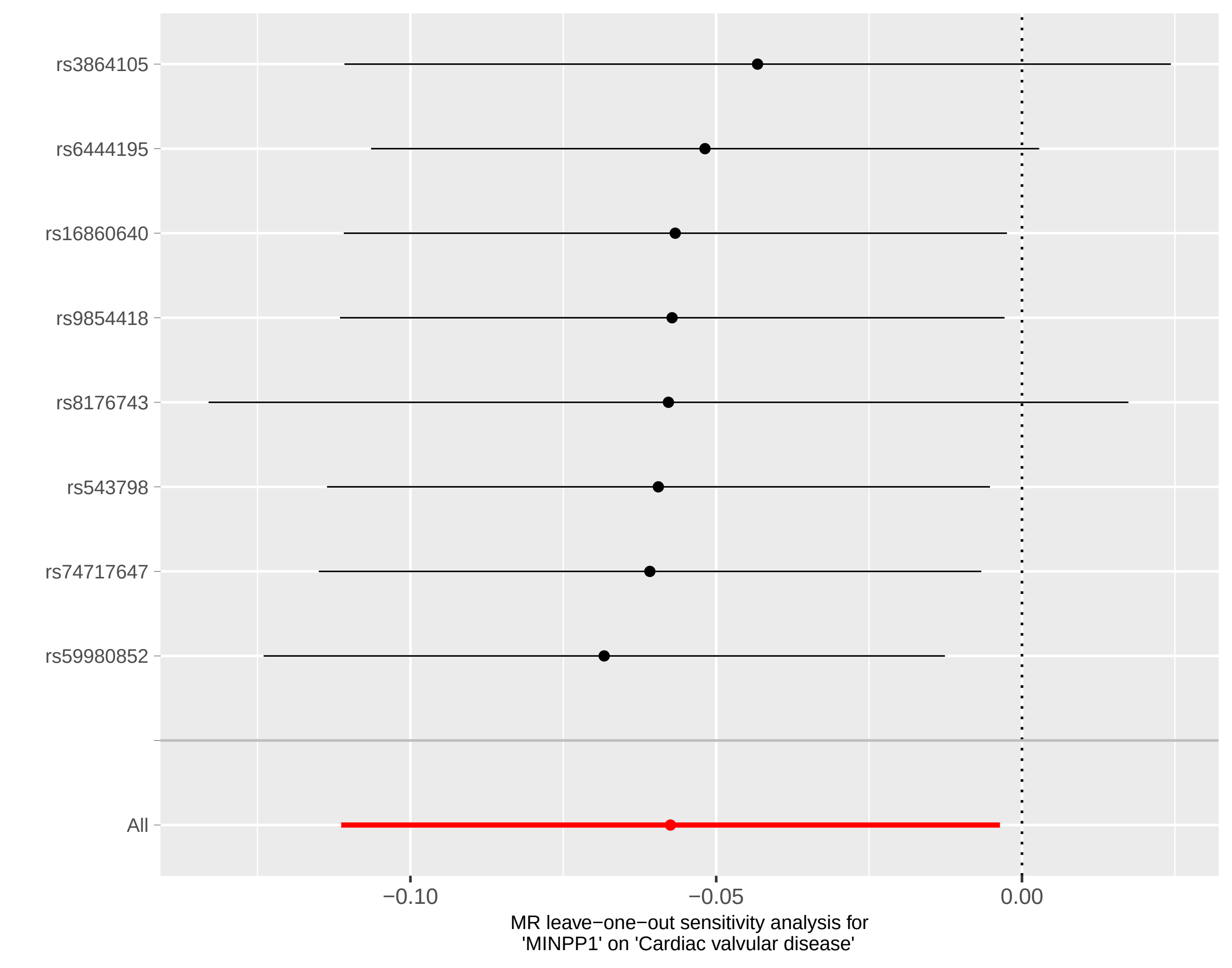
rs3864105
rs6444195
rs16860640
rs9854418
rs8176743
rs543798
rs74717647
rs59980852
All
−0.10
−0.05
0.00
MR leave−one−out sensitivity analysis for
'MINPP1' on 'Cardiac valvular disease'

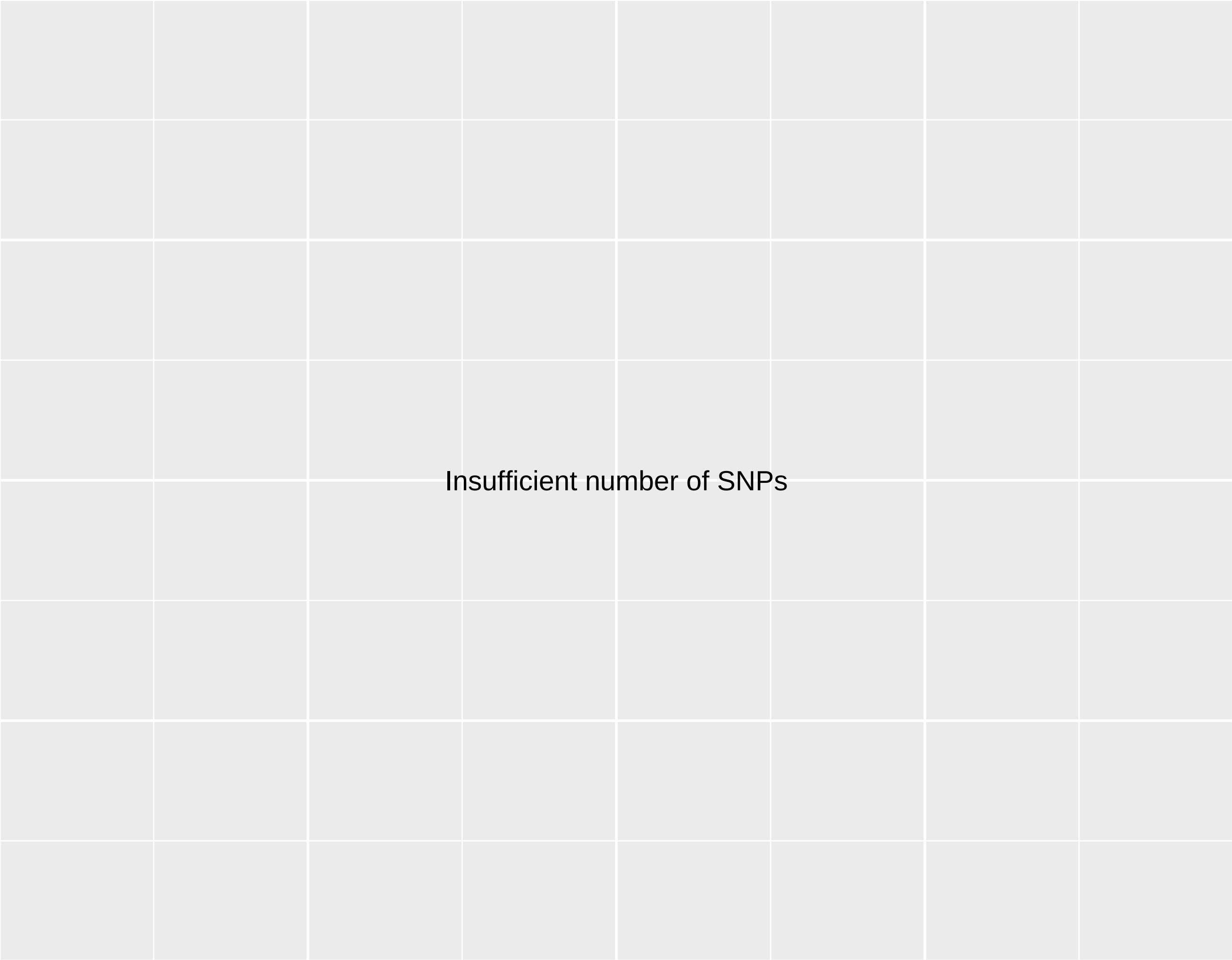
Insufficient number of SNPs

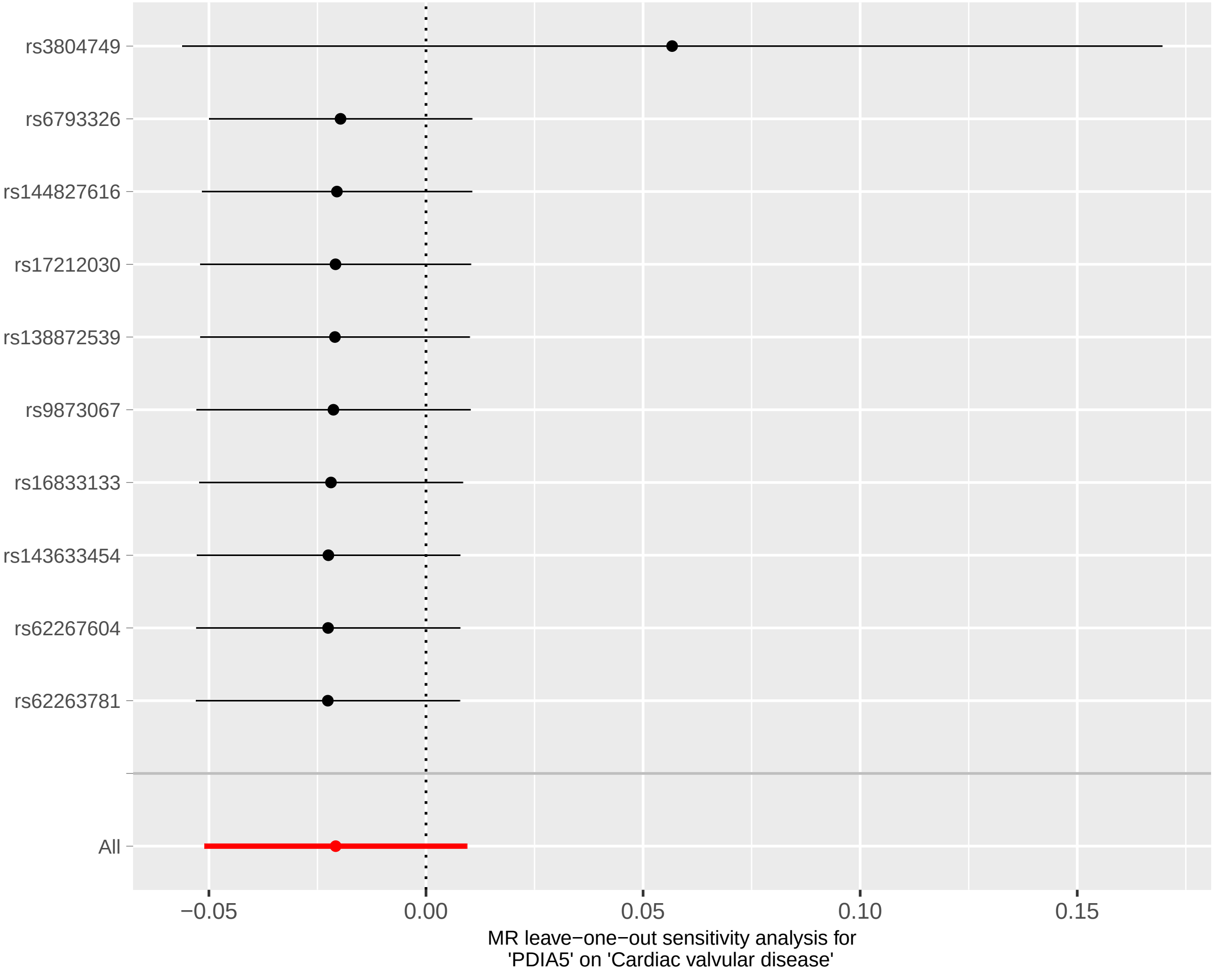
rs3804749
rs6793326
rs144827616
rs17212030
rs138872539
rs9873067
rs16833133
rs143633454
rs62267604
rs62263781
All
−0.05
0.00
0.05
0.10
0.15
MR leave−one−out sensitivity analysis for
'PDIA5' on 'Cardiac valvular disease'

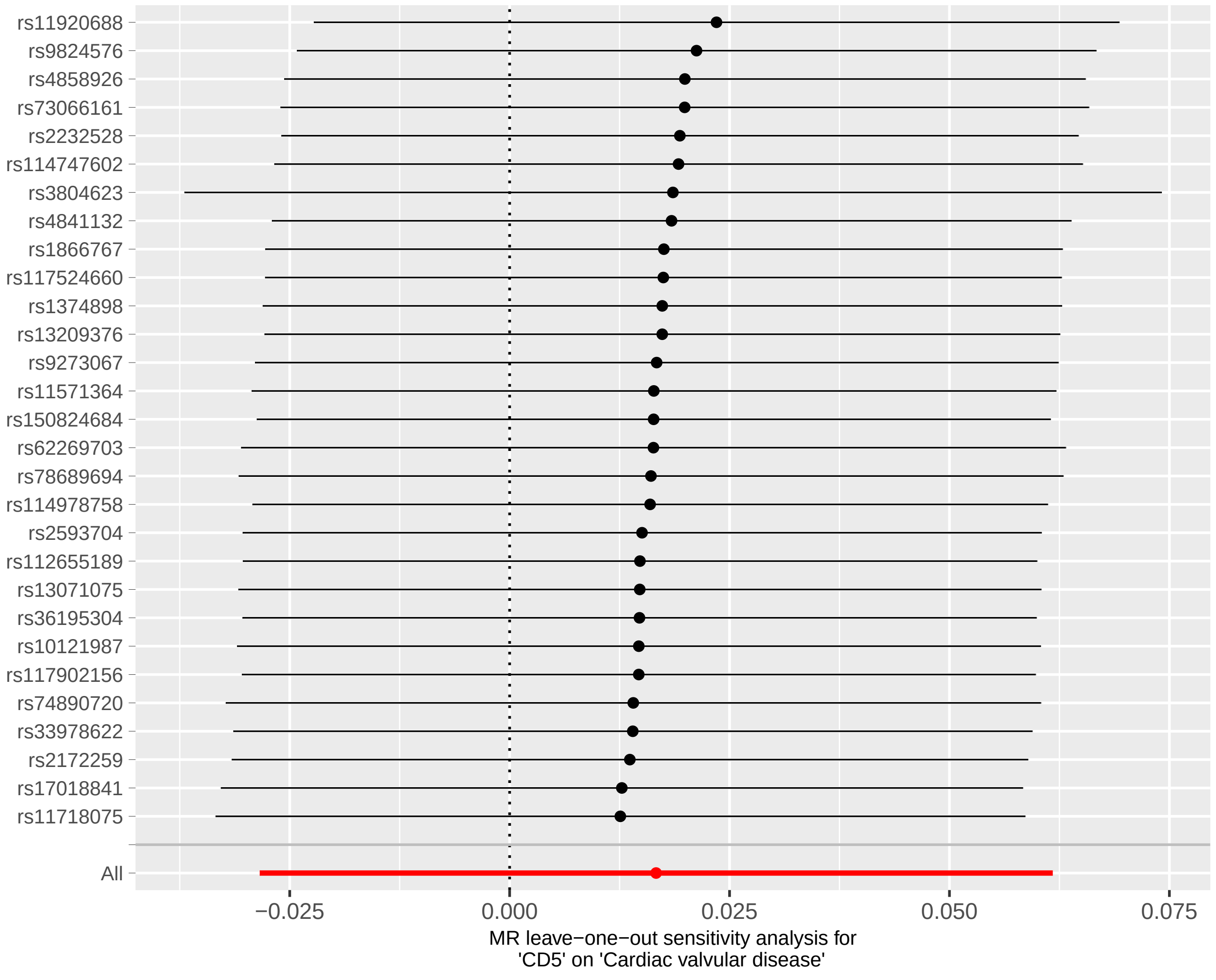
rs11920688
rs9824576
rs4858926
rs73066161
rs2232528
rs114747602
rs3804623
rs4841132
rs1866767
rs117524660
rs1374898
rs13209376
rs9273067
rs11571364
rs150824684
rs62269703
rs78689694
rs114978758
rs2593704
rs112655189
rs13071075
rs36195304
rs10121987
rs117902156
rs74890720
rs33978622
rs2172259
rs17018841
rs11718075
All
−0.025
0.000
0.025
0.050
0.075
MR leave−one−out sensitivity analysis for
'CD5' on 'Cardiac valvular disease'

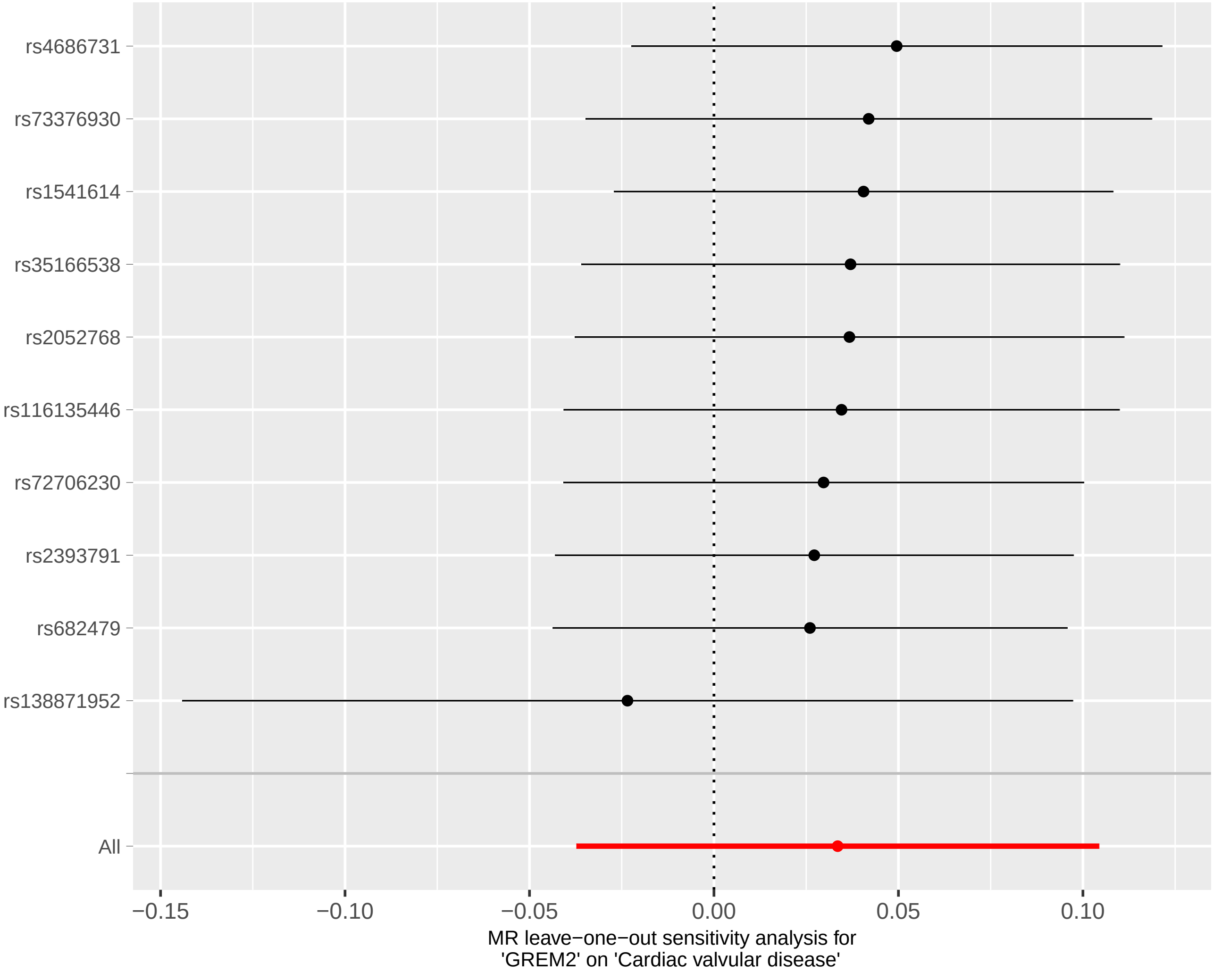

rs4686731
rs73376930
rs1541614
rs35166538
rs2052768
rs116135446
rs72706230
rs2393791
rs682479
rs138871952
All
−0.15
−0.10
−0.05
0.00
0.05
0.10
MR leave−one−out sensitivity analysis for
'GREM2' on 'Cardiac valvular disease'

Insufficient number of SNPs

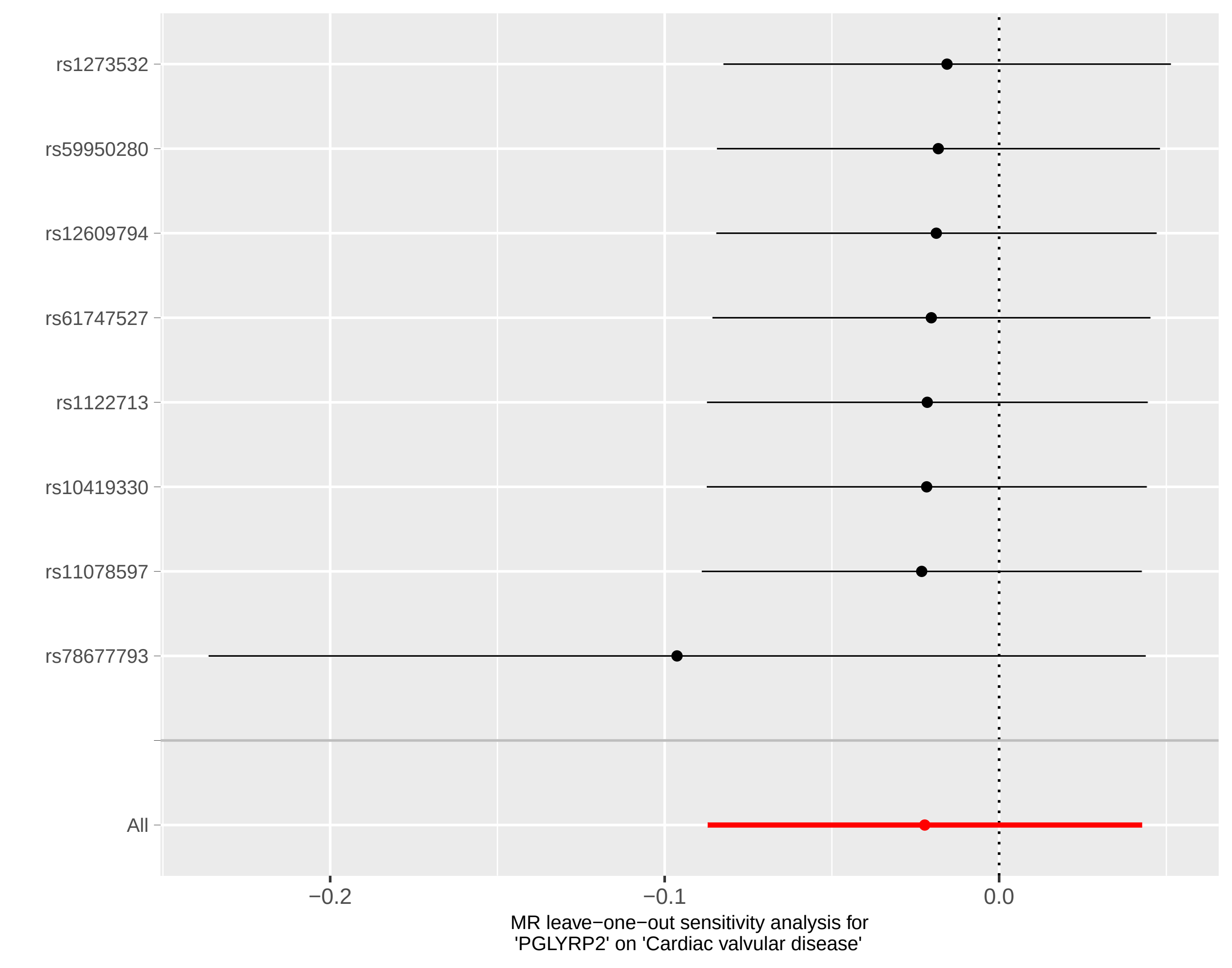

rs1273532
rs59950280
rs12609794
rs61747527
rs1122713
rs10419330
rs11078597
rs78677793
All
−0.2
−0.1
0.0
MR leave−one−out sensitivity analysis for
'PGLYRP2' on 'Cardiac valvular disease'

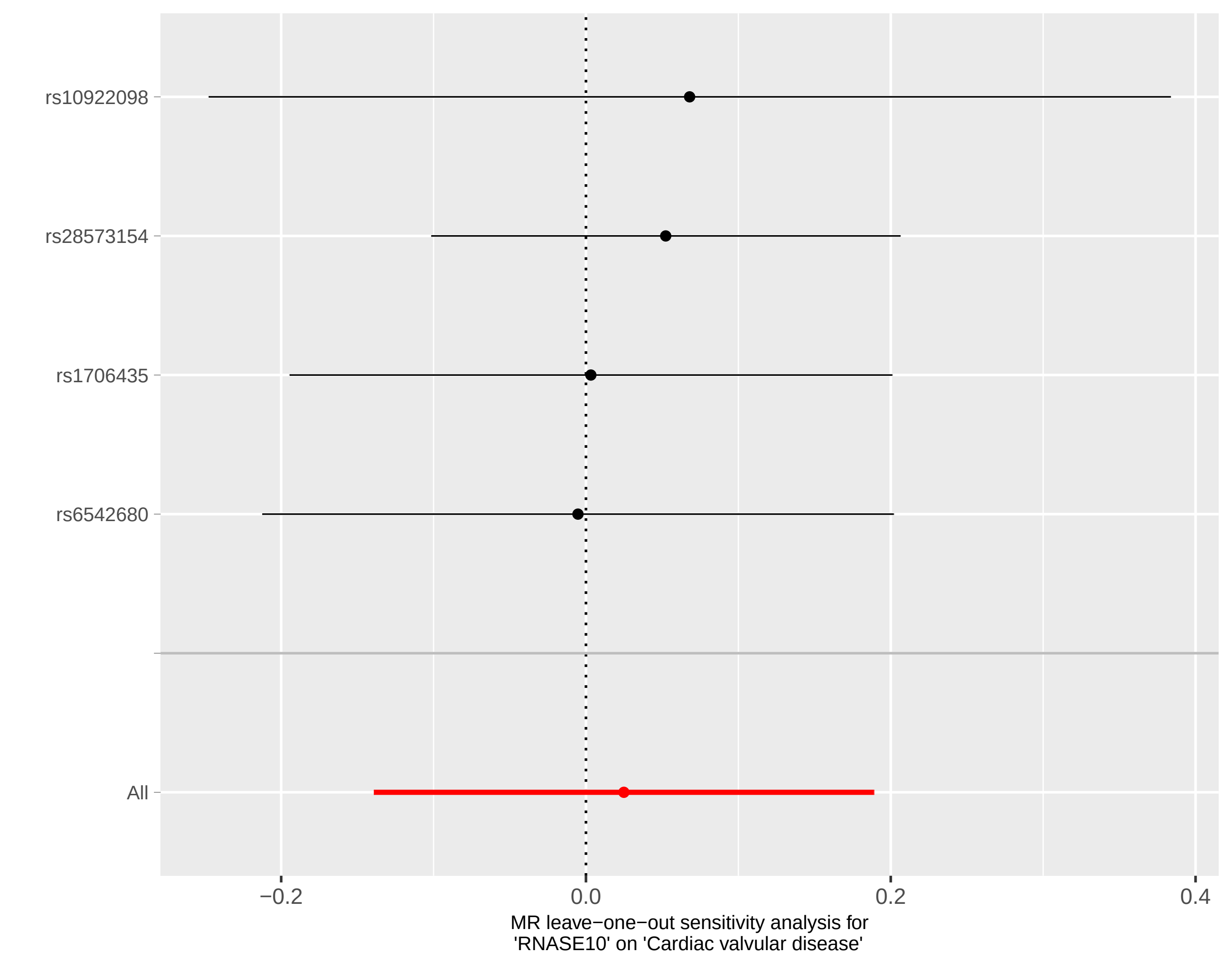
rs10922098
rs28573154
rs1706435
rs6542680
All
−0.2
0.0
0.2
0.4
MR leave−one−out sensitivity analysis for
'RNASE10' on 'Cardiac valvular disease'

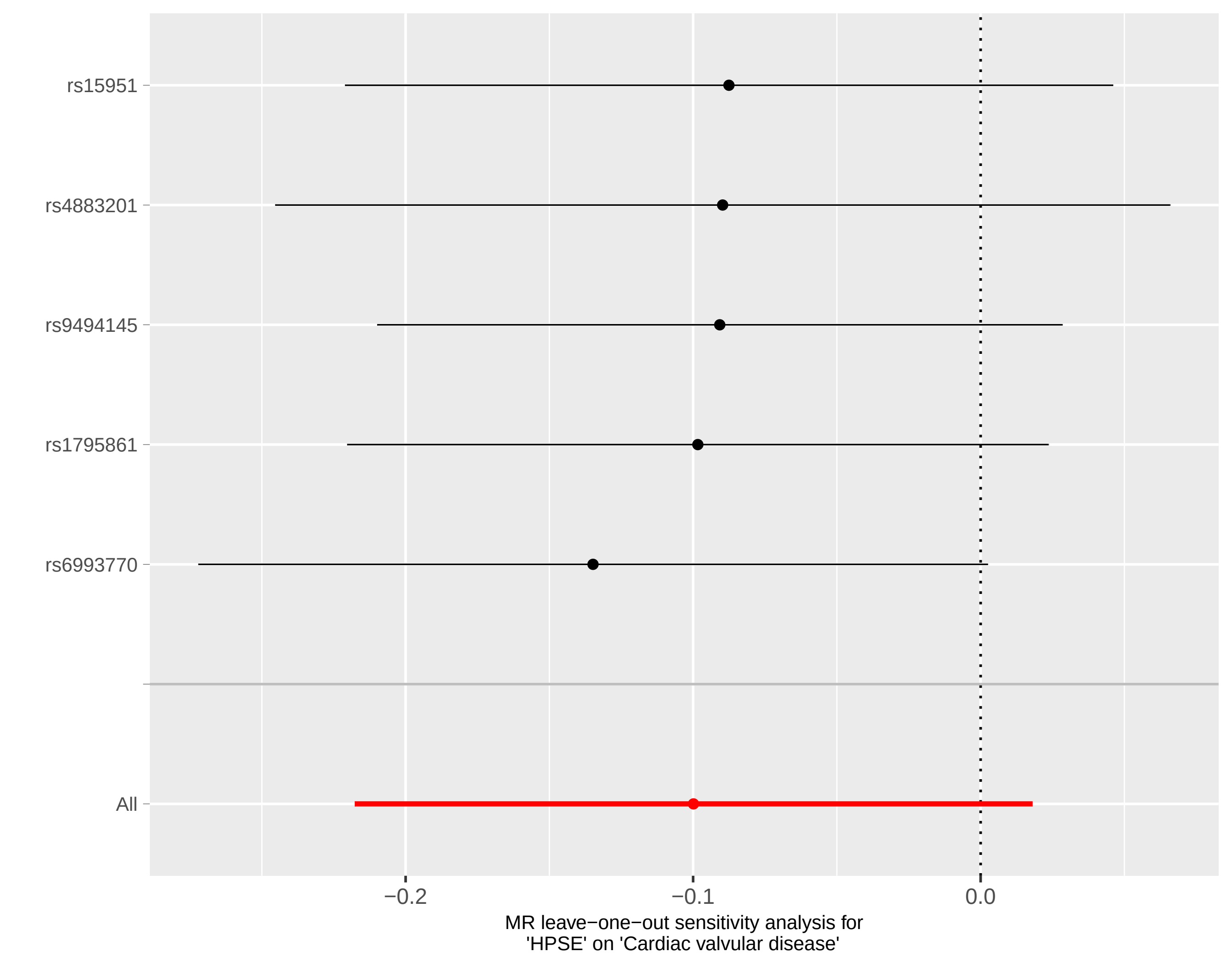
rs15951
rs4883201
rs9494145
rs1795861
rs6993770
All
−0.2
−0.1
0.0
MR leave−one−out sensitivity analysis for
'HPSE' on 'Cardiac valvular disease'

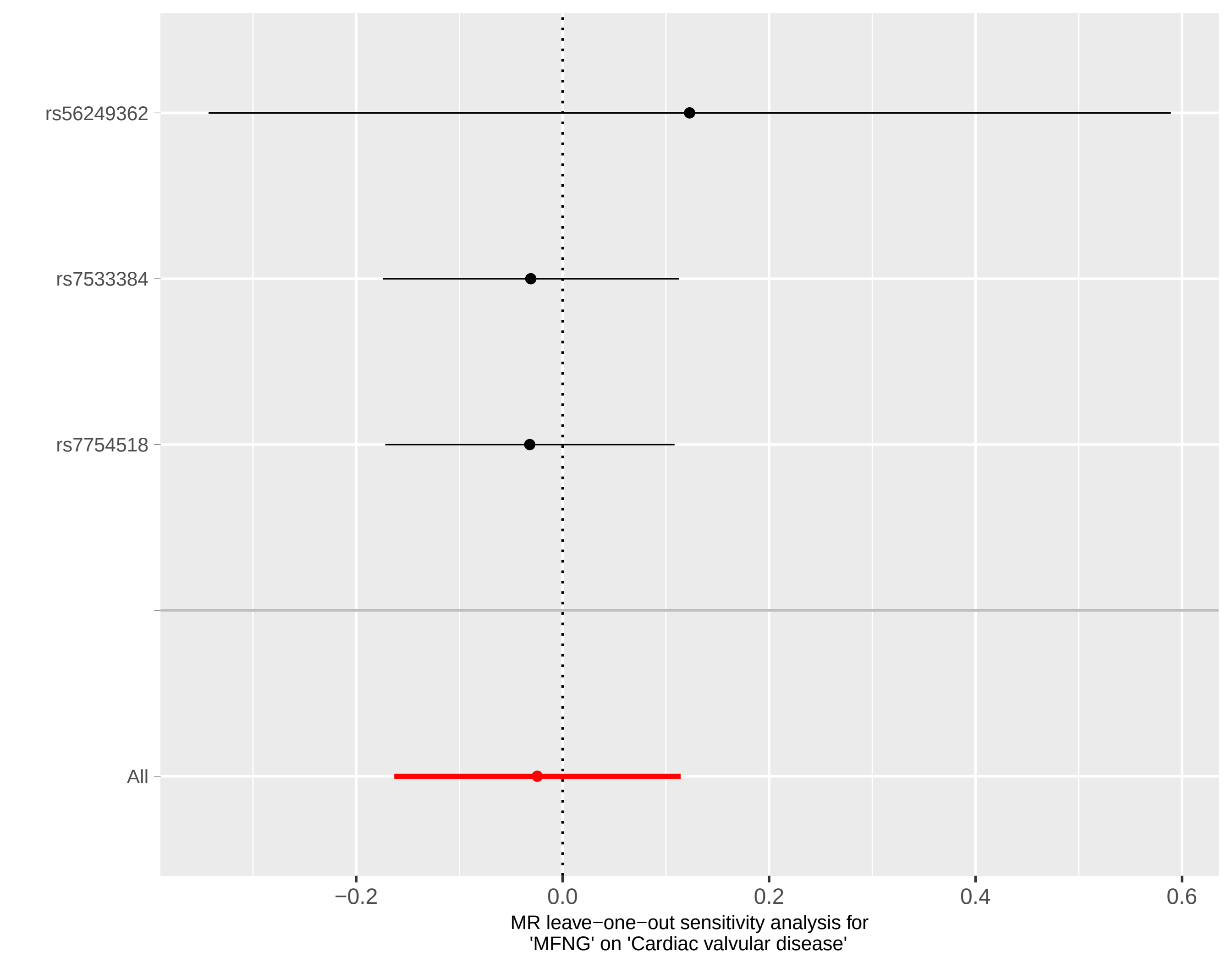
rs56249362
rs7533384
rs7754518
All
−0.2
0.0
0.2
0.4
0.6
MR leave−one−out sensitivity analysis for
'MFNG' on 'Cardiac valvular disease'

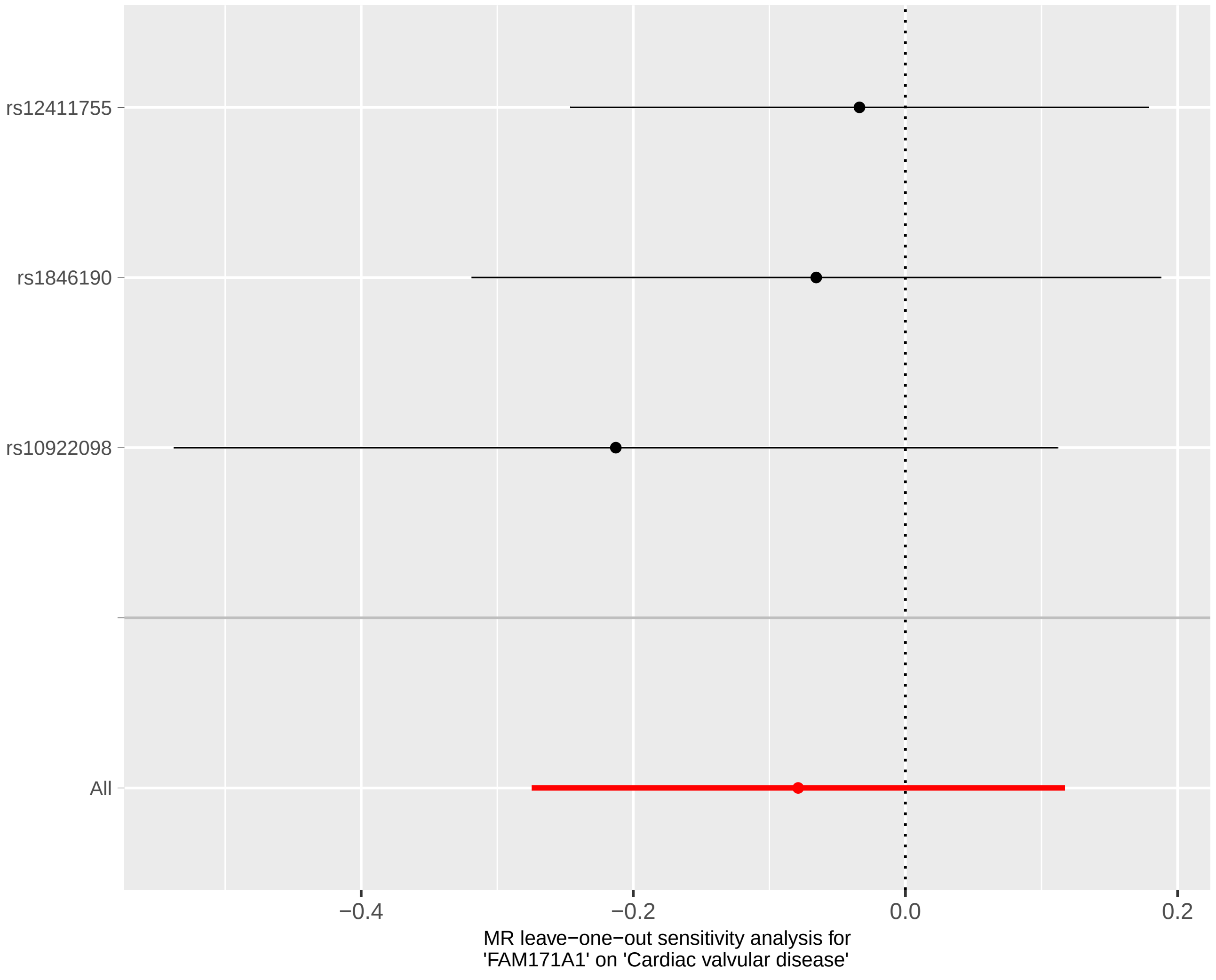
rs12411755
rs1846190
rs10922098
All
−0.4
−0.2
0.0
0.2
MR leave−one−out sensitivity analysis for
'FAM171A1' on 'Cardiac valvular disease'

Insufficient number of SNPs

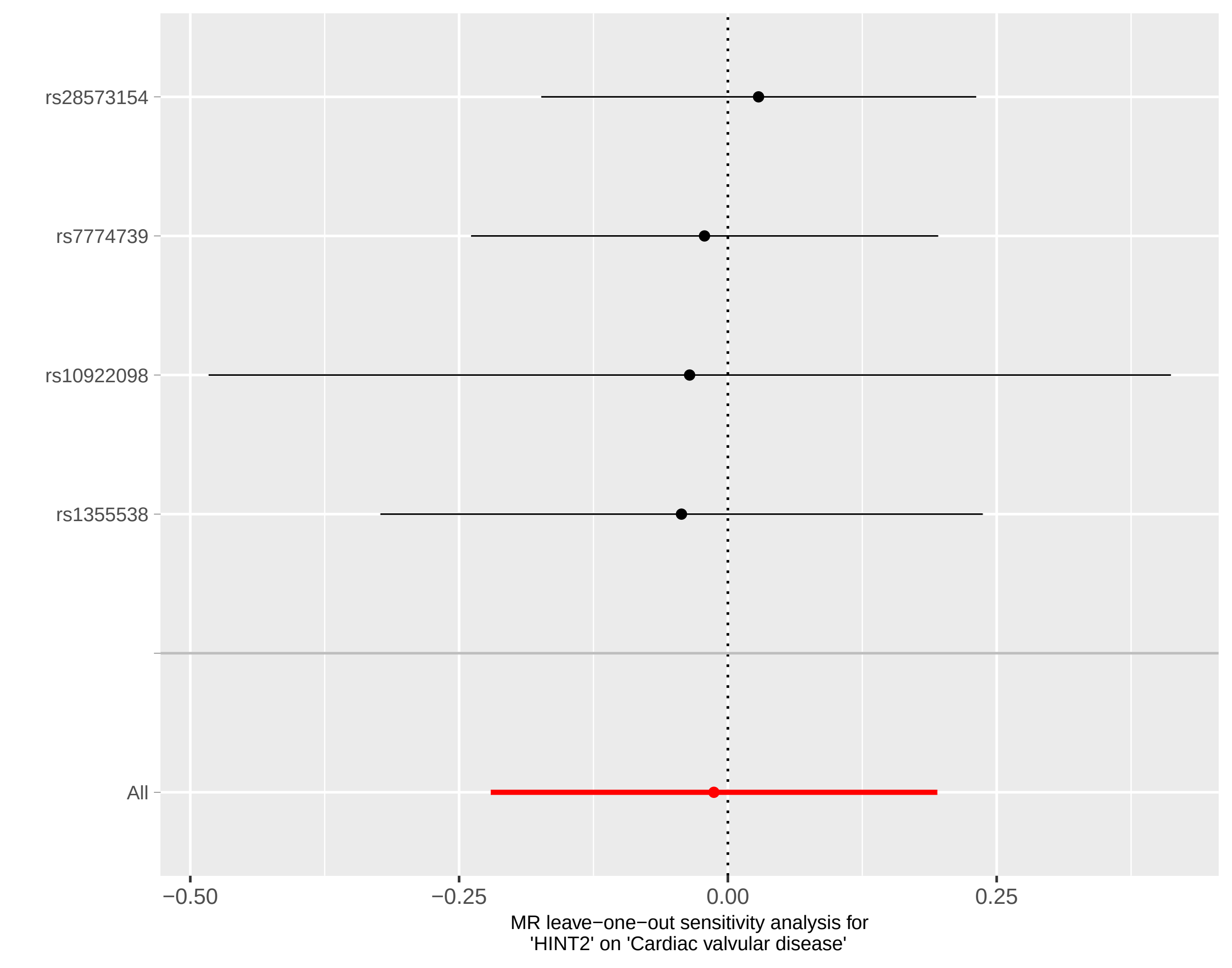
rs28573154
rs7774739
rs10922098
rs1355538
All
−0.50
−0.25
0.00
0.25
MR leave−one−out sensitivity analysis for
'HINT2' on 'Cardiac valvular disease'

Insufficient number of SNPs

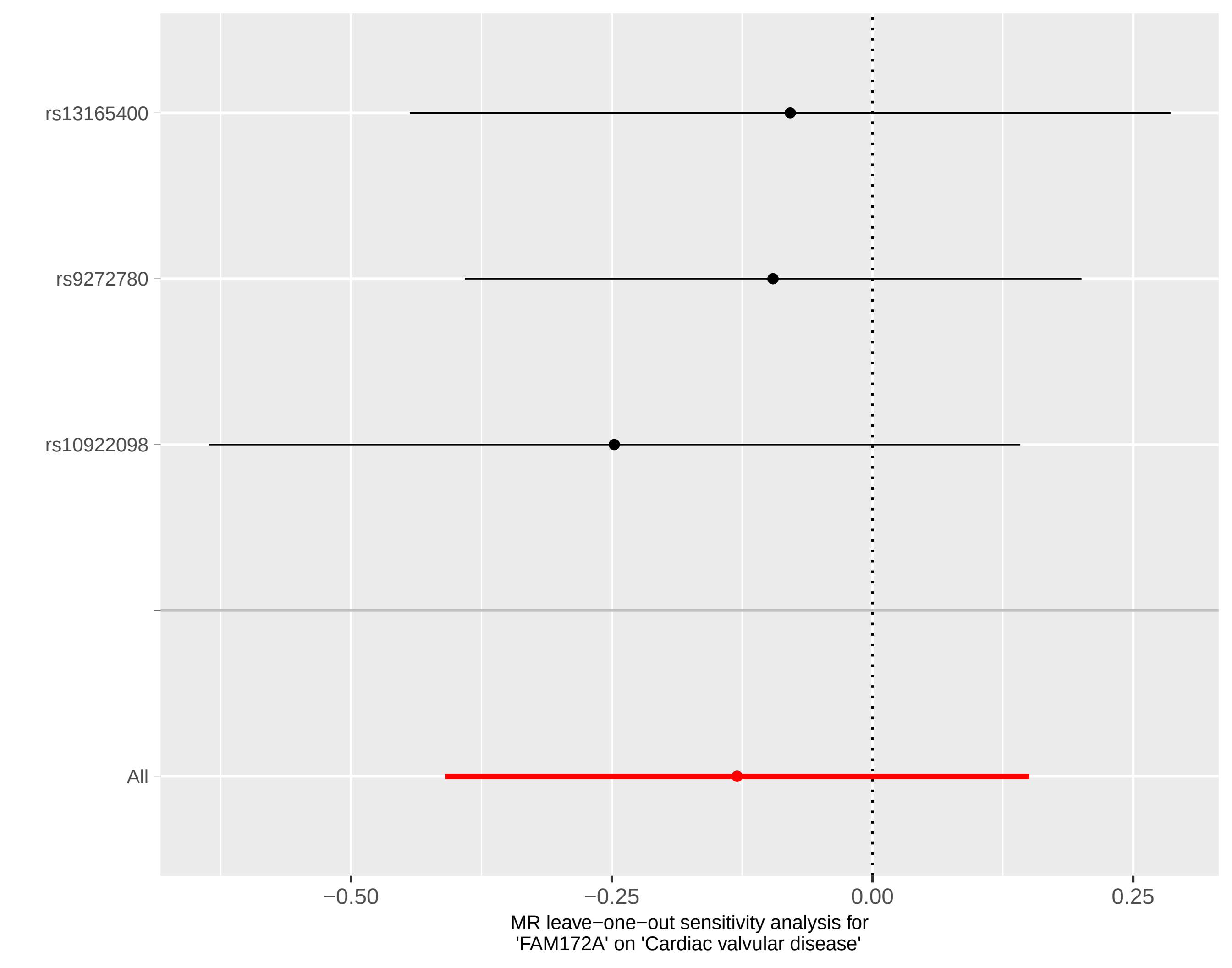
rs13165400
rs9272780
rs10922098
All
−0.50
−0.25
0.00
0.25
MR leave−one−out sensitivity analysis for
'FAM172A' on 'Cardiac valvular disease'

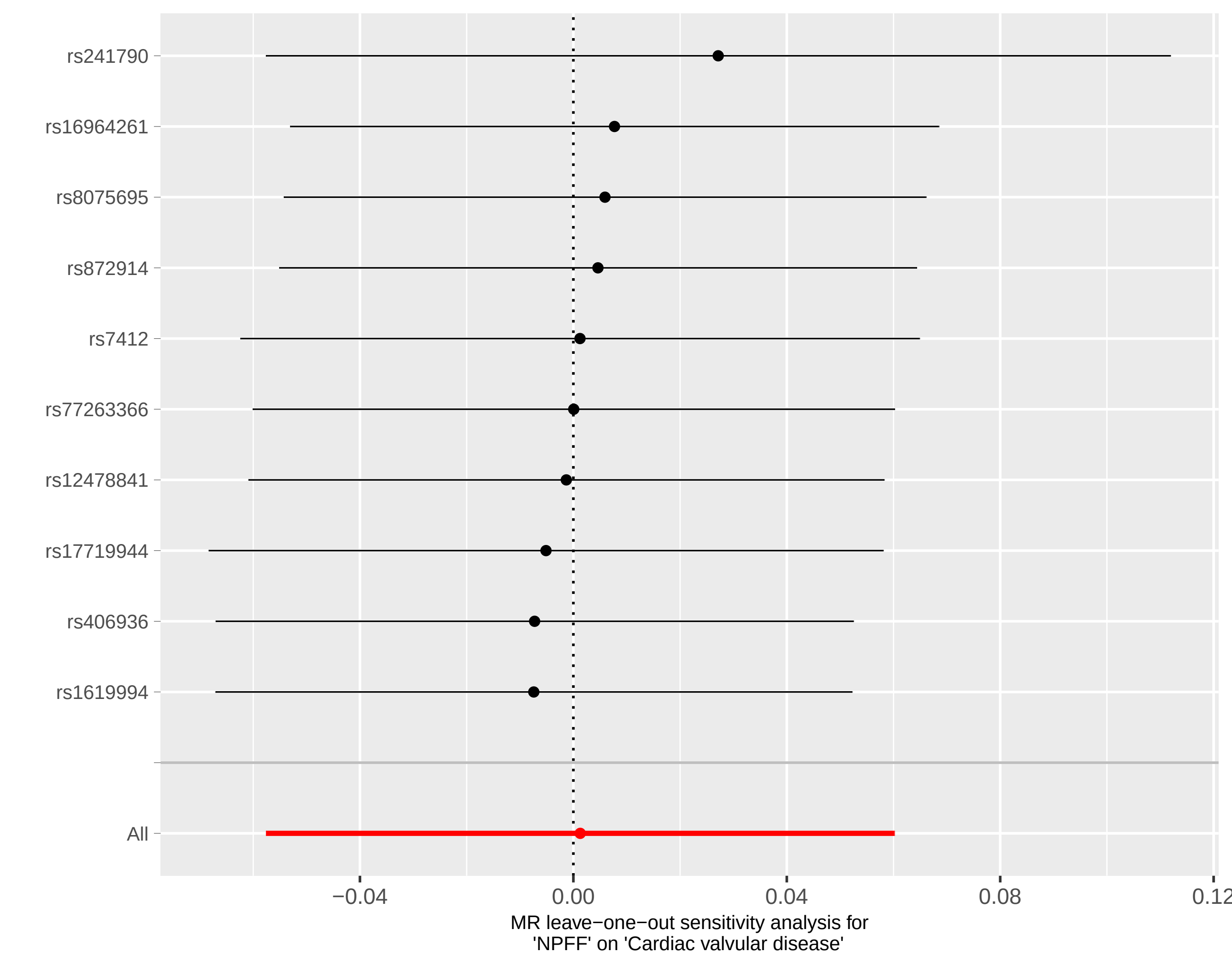

rs241790
rs16964261
rs8075695
rs872914
rs7412
rs77263366
rs12478841
rs17719944
rs406936
rs1619994
All
−0.04
0.00
0.04
0.08
0.12
MR leave−one−out sensitivity analysis for
'NPFF' on 'Cardiac valvular disease'

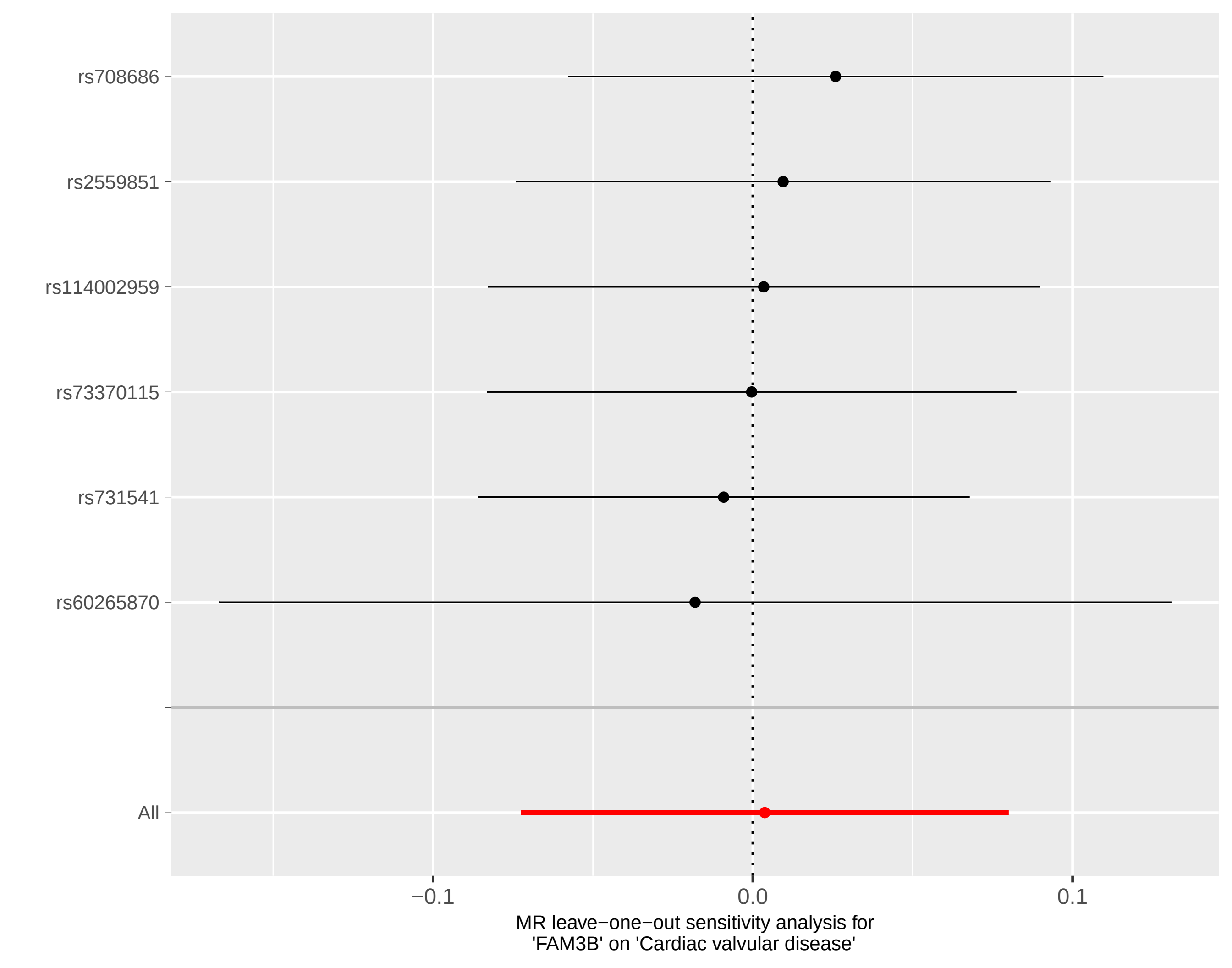
rs708686
rs2559851
rs114002959
rs73370115
rs731541
rs60265870
All
−0.1
0.0
0.1
MR leave−one−out sensitivity analysis for
'FAM3B' on 'Cardiac valvular disease'

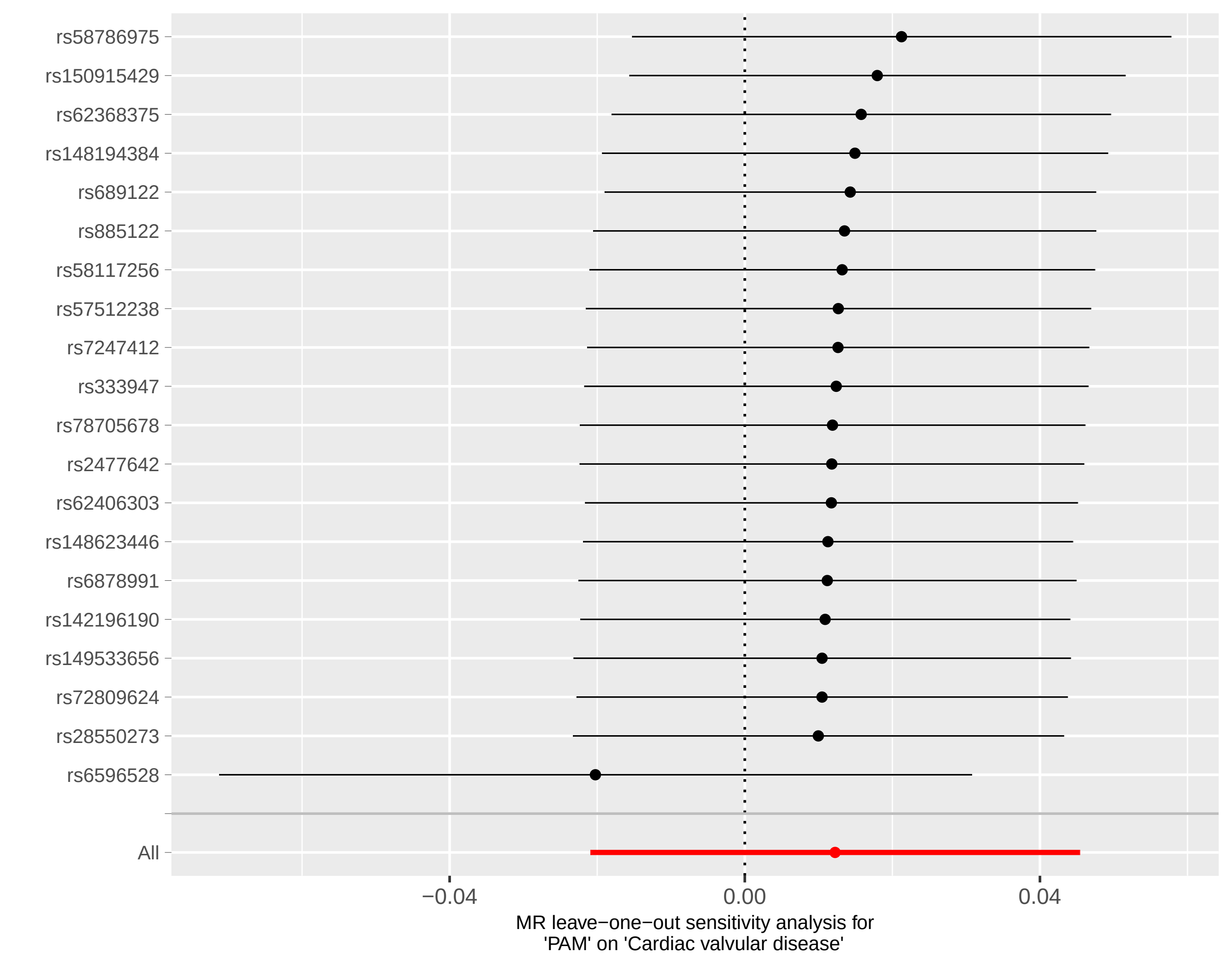
rs58786975
rs150915429
rs62368375
rs148194384
rs689122
rs885122
rs58117256
rs57512238
rs7247412
rs333947
rs78705678
rs2477642
rs62406303
rs148623446
rs6878991
rs142196190
rs149533656
rs72809624
rs28550273
rs6596528
All
−0.04
0.00
0.04
MR leave−one−out sensitivity analysis for
'PAM' on 'Cardiac valvular disease'

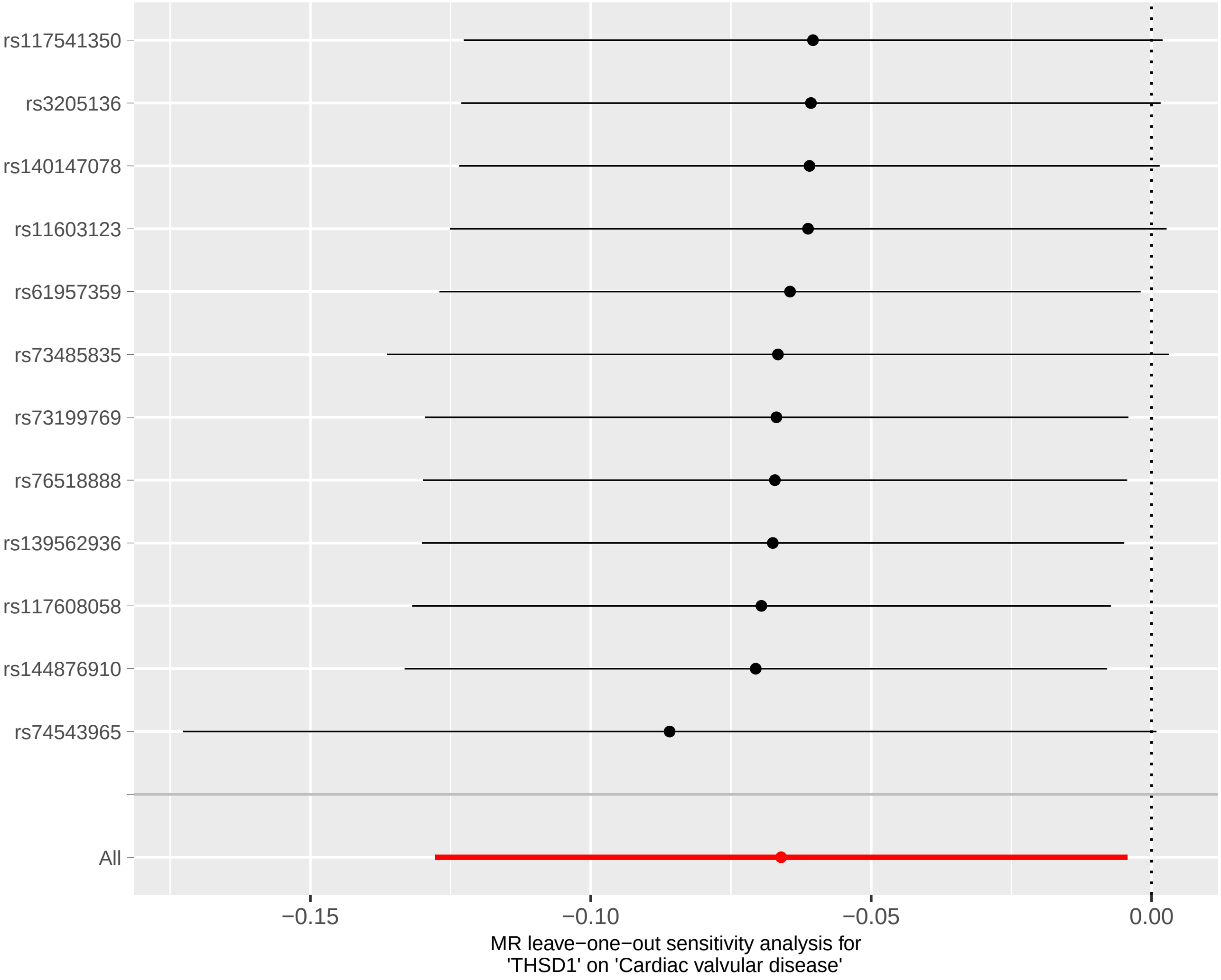

rs117541350
rs3205136
rs140147078
rs11603123
rs61957359
rs73485835
rs73199769
rs76518888
rs139562936
rs117608058
rs144876910
rs74543965
All
−0.15
−0.10
−0.05
0.00
MR leave−one−out sensitivity analysis for
'THSD1' on 'Cardiac valvular disease'

Insufficient number of SNPs

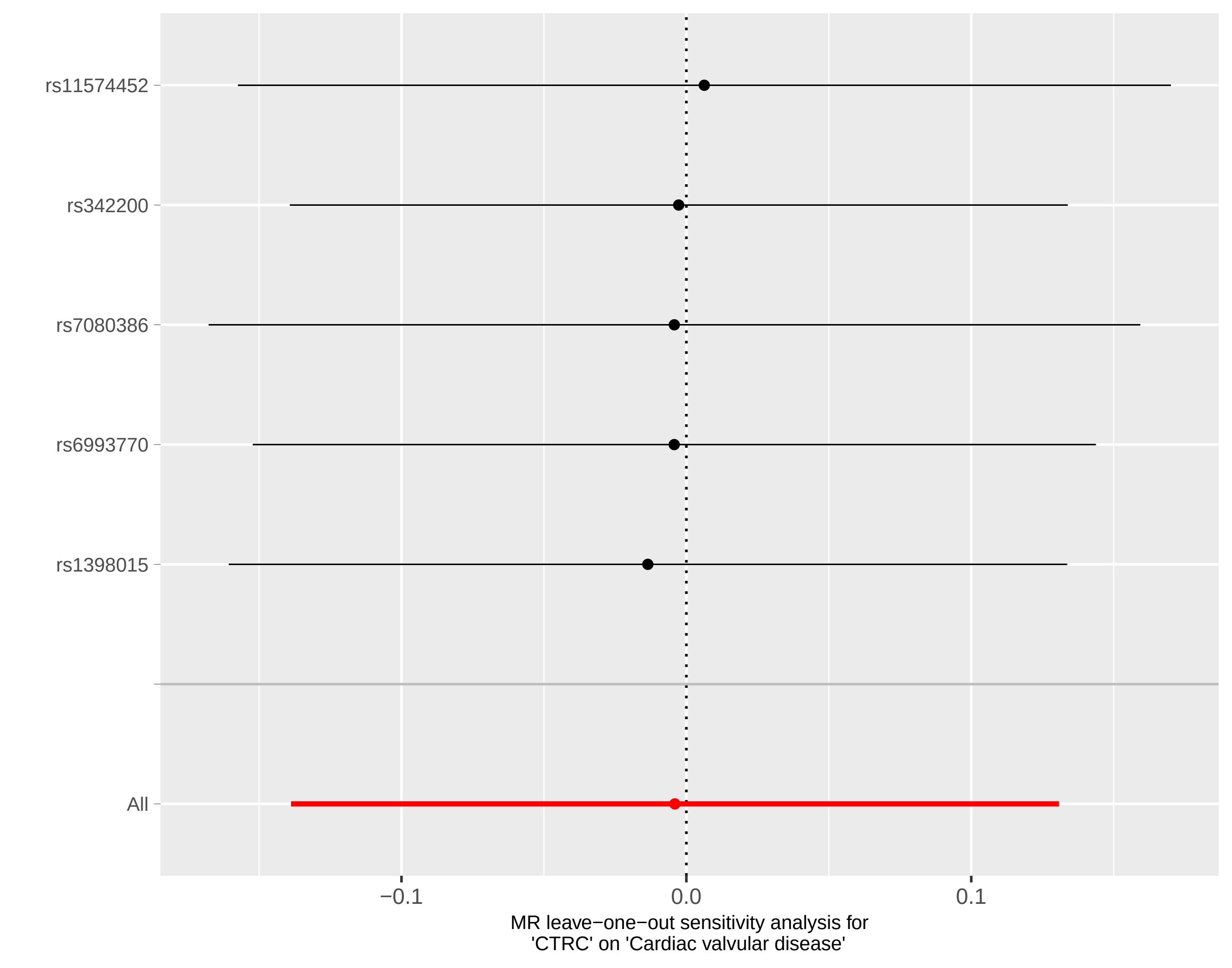
rs11574452
rs342200
rs7080386
rs6993770
rs1398015
All
−0.1
0.0
0.1
MR leave−one−out sensitivity analysis for
'CTRC' on 'Cardiac valvular disease'

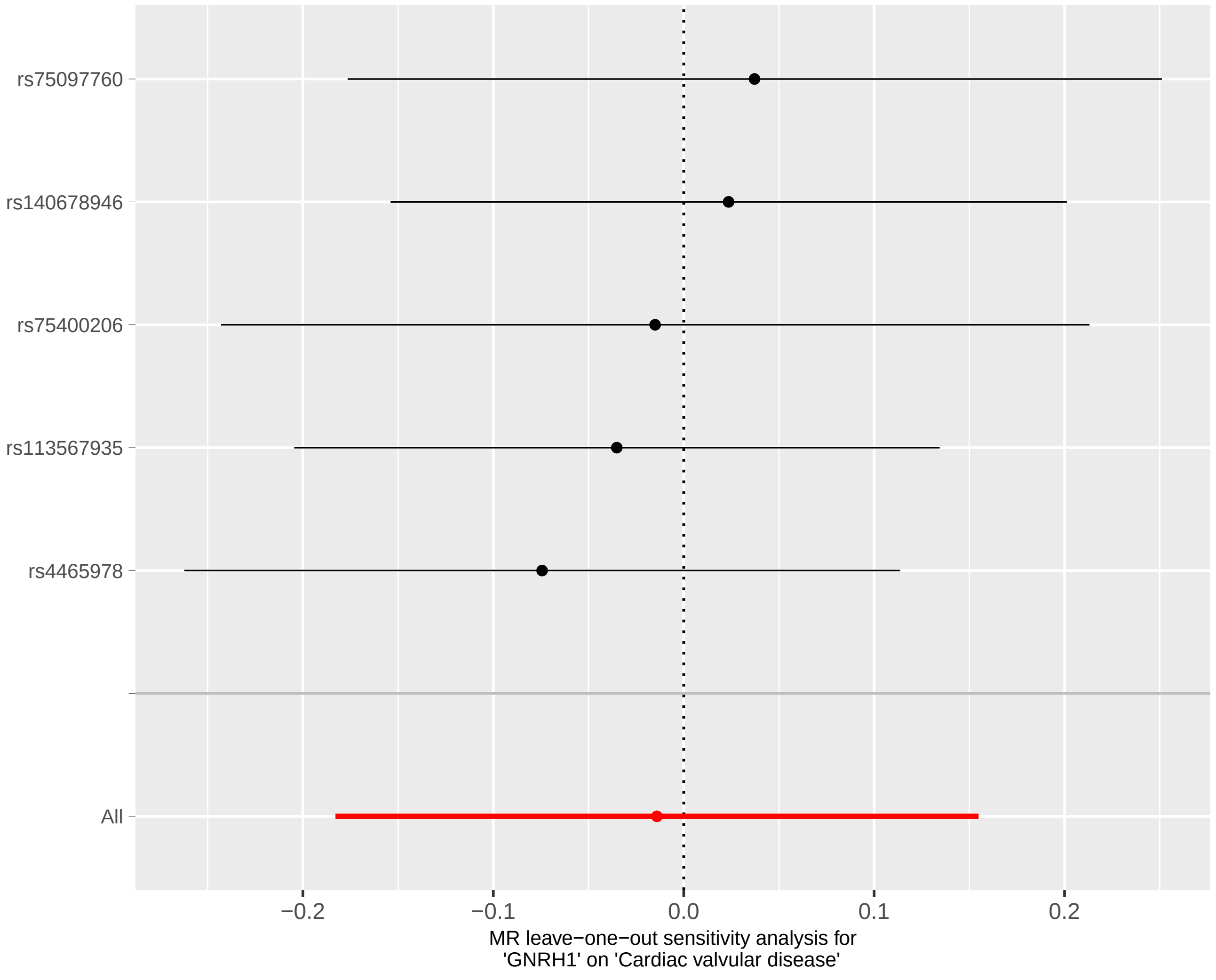

rs75097760
rs140678946
rs75400206
rs113567935
rs4465978
All
−0.2
−0.1
0.0
0.1
0.2
MR leave−one−out sensitivity analysis for
'GNRH1' on 'Cardiac valvular disease'

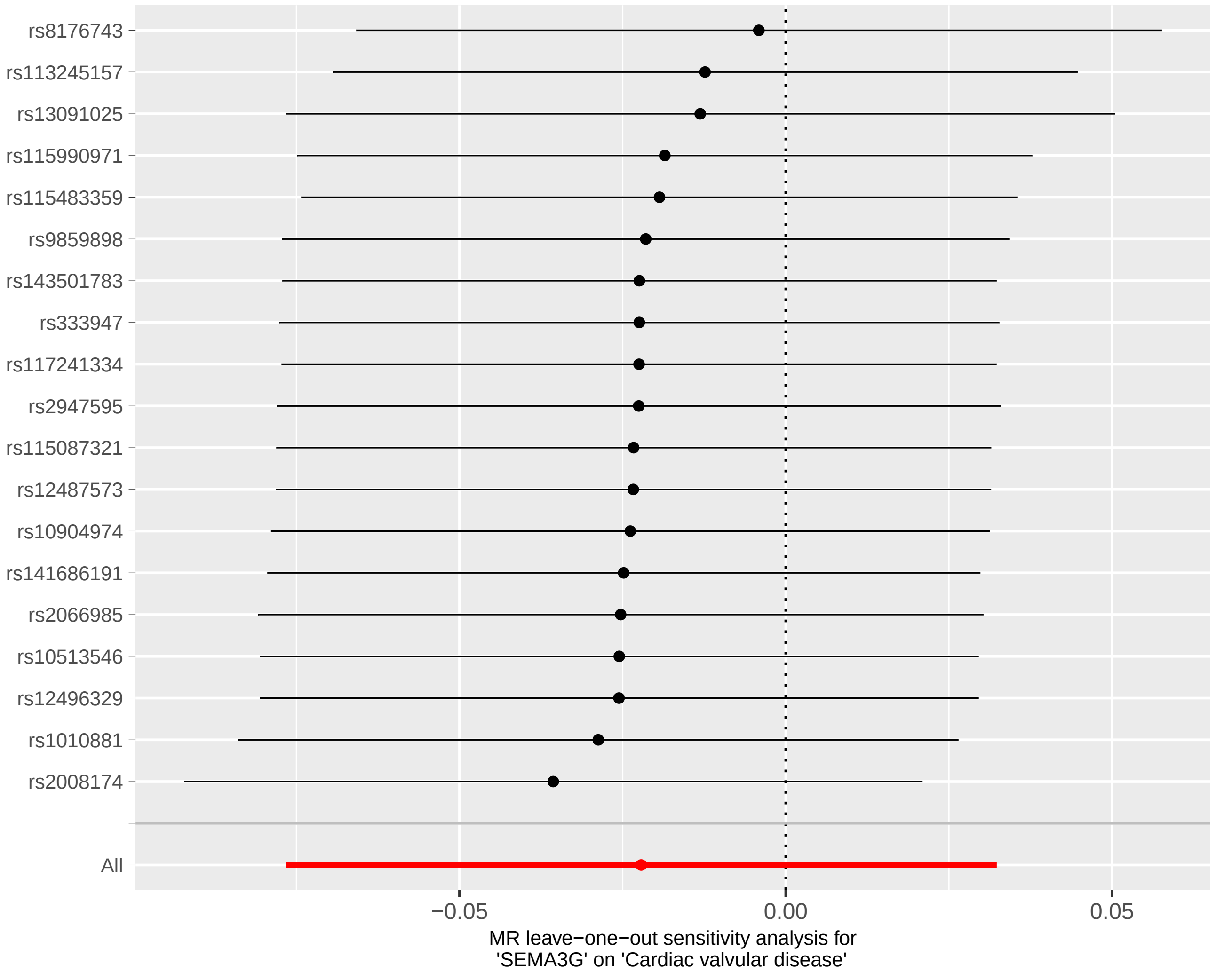
rs8176743
rs113245157
rs13091025
rs115990971
rs115483359
rs9859898
rs143501783
rs333947
rs117241334
rs2947595
rs115087321
rs12487573
rs10904974
rs141686191
rs2066985
rs10513546
rs12496329
rs1010881
rs2008174
All
−0.05
0.00
0.05
MR leave−one−out sensitivity analysis for
'SEMA3G' on 'Cardiac valvular disease'

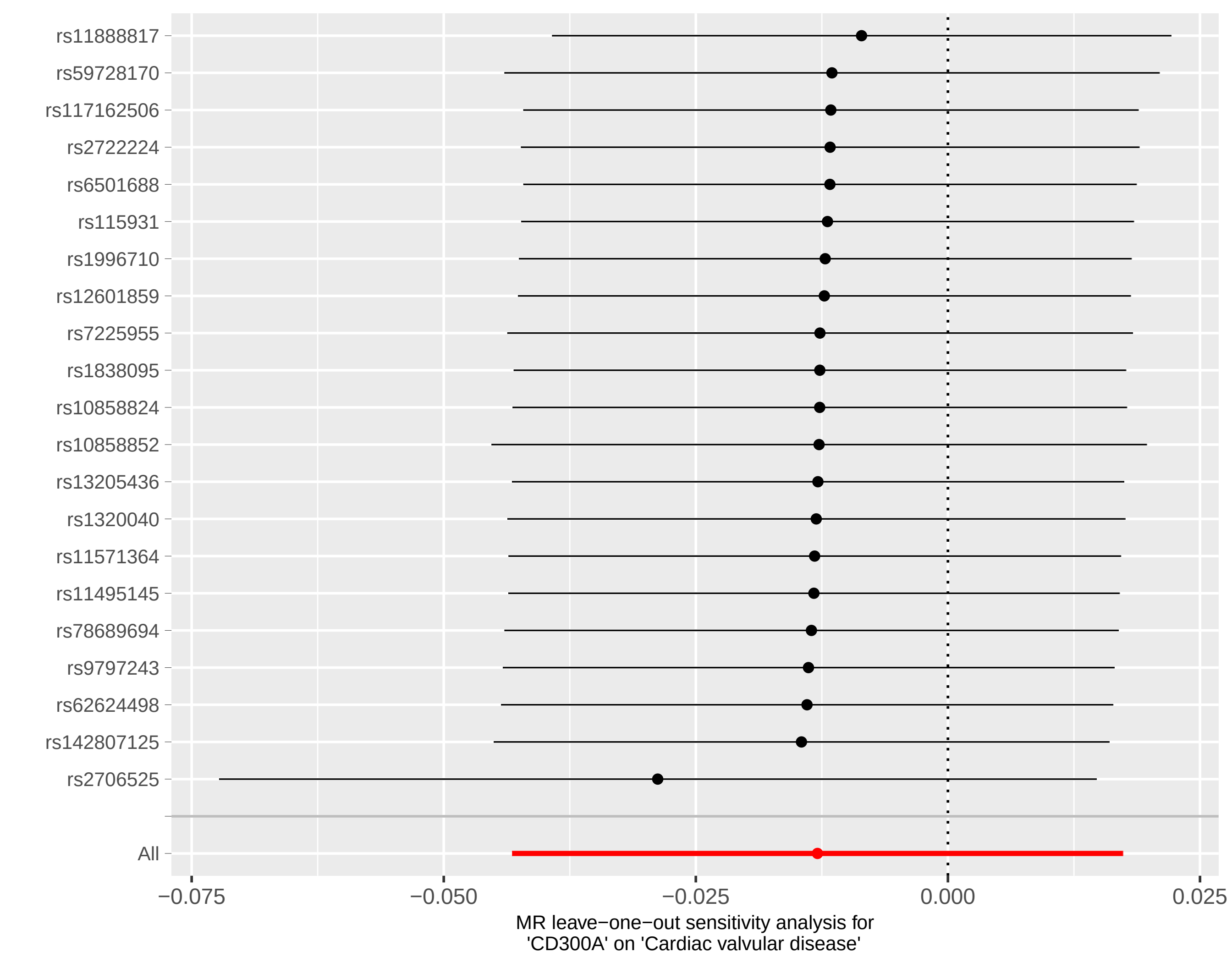
rs11888817
rs59728170
rs117162506
rs2722224
rs6501688
rs115931
rs1996710
rs12601859
rs7225955
rs1838095
rs10858824
rs10858852
rs13205436
rs1320040
rs11571364
rs11495145
rs78689694
rs9797243
rs62624498
rs142807125
rs2706525
All
−0.075
−0.050
−0.025
0.000
0.025
MR leave−one−out sensitivity analysis for
'CD300A' on 'Cardiac valvular disease'

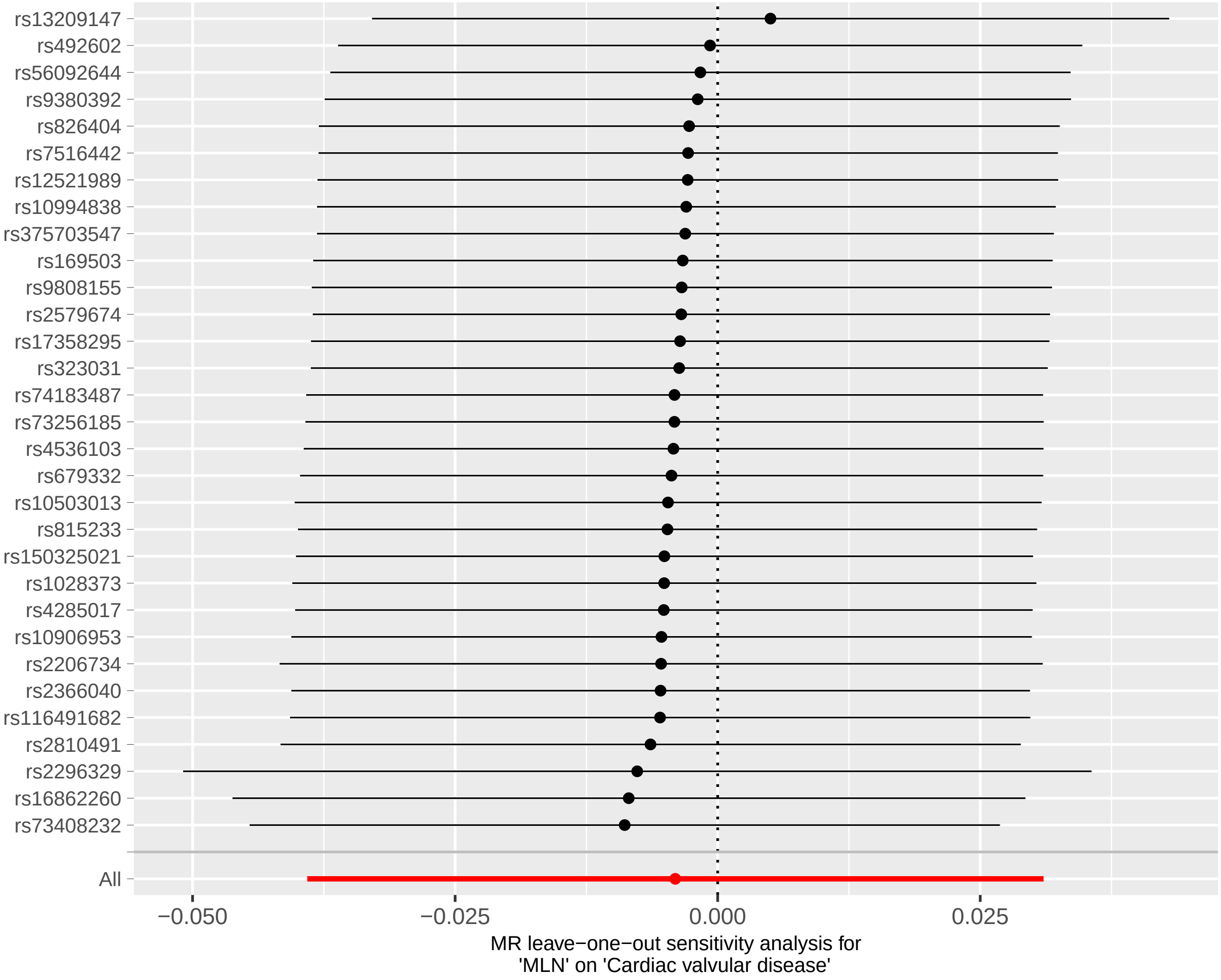

rs13209147
rs492602
rs56092644
rs9380392
rs826404
rs7516442
rs12521989
rs10994838
rs375703547
rs169503
rs9808155
rs2579674
rs17358295
rs323031
rs74183487
rs73256185
rs4536103
rs679332
rs10503013
rs815233
rs150325021
rs1028373
rs4285017
rs10906953
rs2206734
rs2366040
rs116491682
rs2810491
rs2296329
rs16862260
rs73408232
All
−0.050
−0.025
0.000
0.025
MR leave−one−out sensitivity analysis for
'MLN' on 'Cardiac valvular disease'

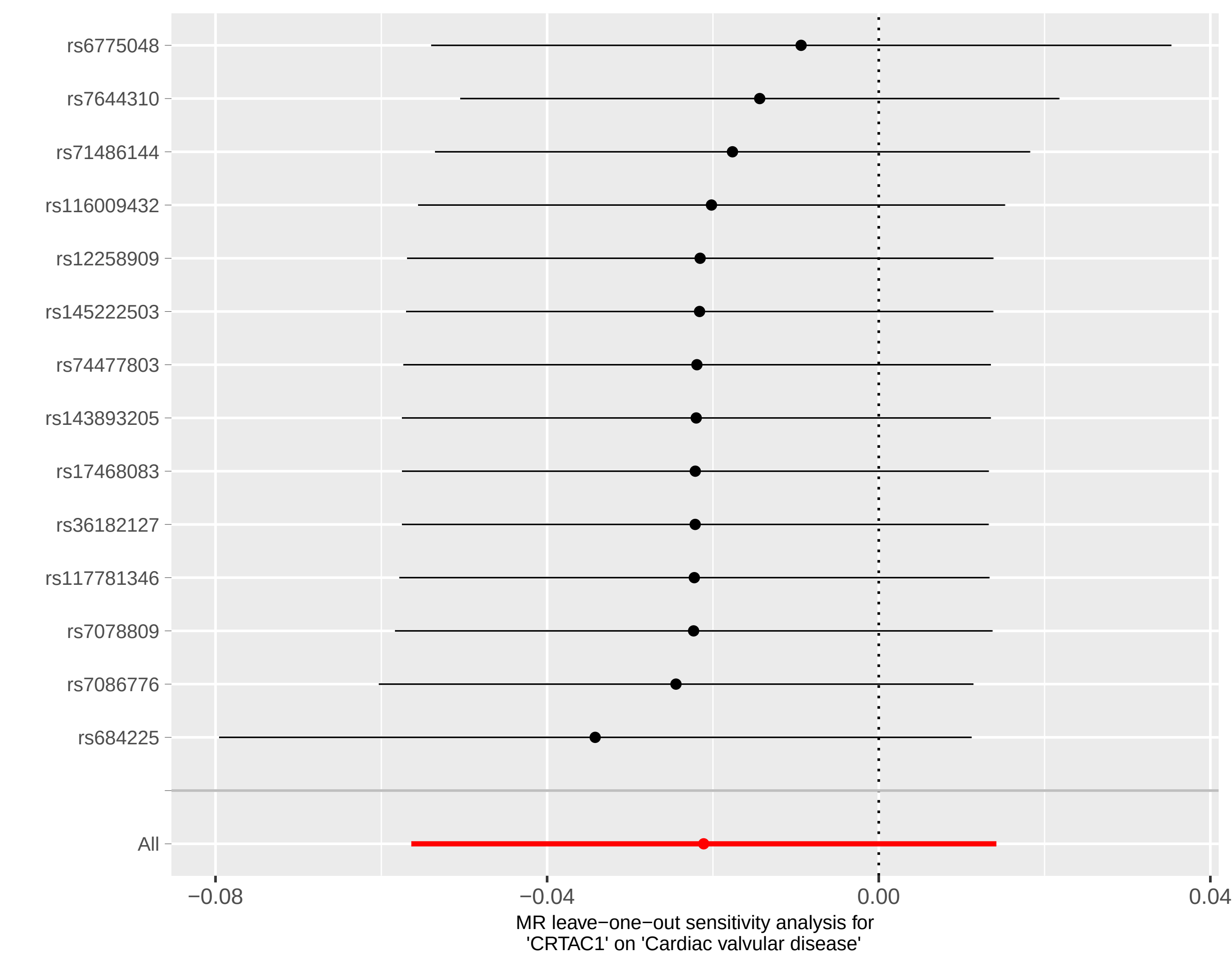
rs6775048
rs7644310
rs71486144
rs116009432
rs12258909
rs145222503
rs74477803
rs143893205
rs17468083
rs36182127
rs117781346
rs7078809
rs7086776
rs684225
All
−0.08
−0.04
0.00
0.04
MR leave−one−out sensitivity analysis for
'CRTAC1' on 'Cardiac valvular disease'

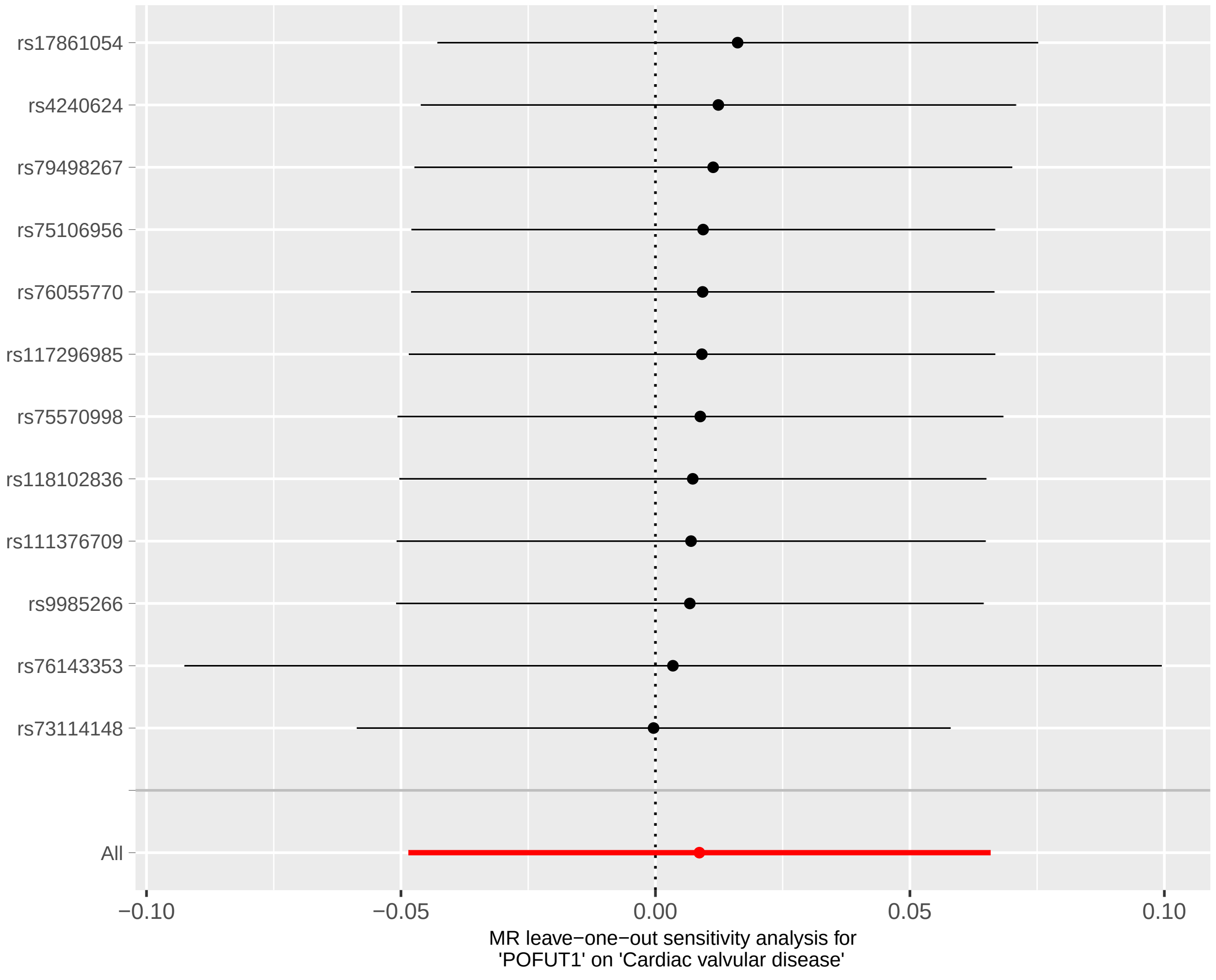

rs17861054
rs4240624
rs79498267
rs75106956
rs76055770
rs117296985
rs75570998
rs118102836
rs111376709
rs9985266
rs76143353
rs73114148
All
−0.10
−0.05
0.00
0.05
0.10
MR leave−one−out sensitivity analysis for
'POFUT1' on 'Cardiac valvular disease'

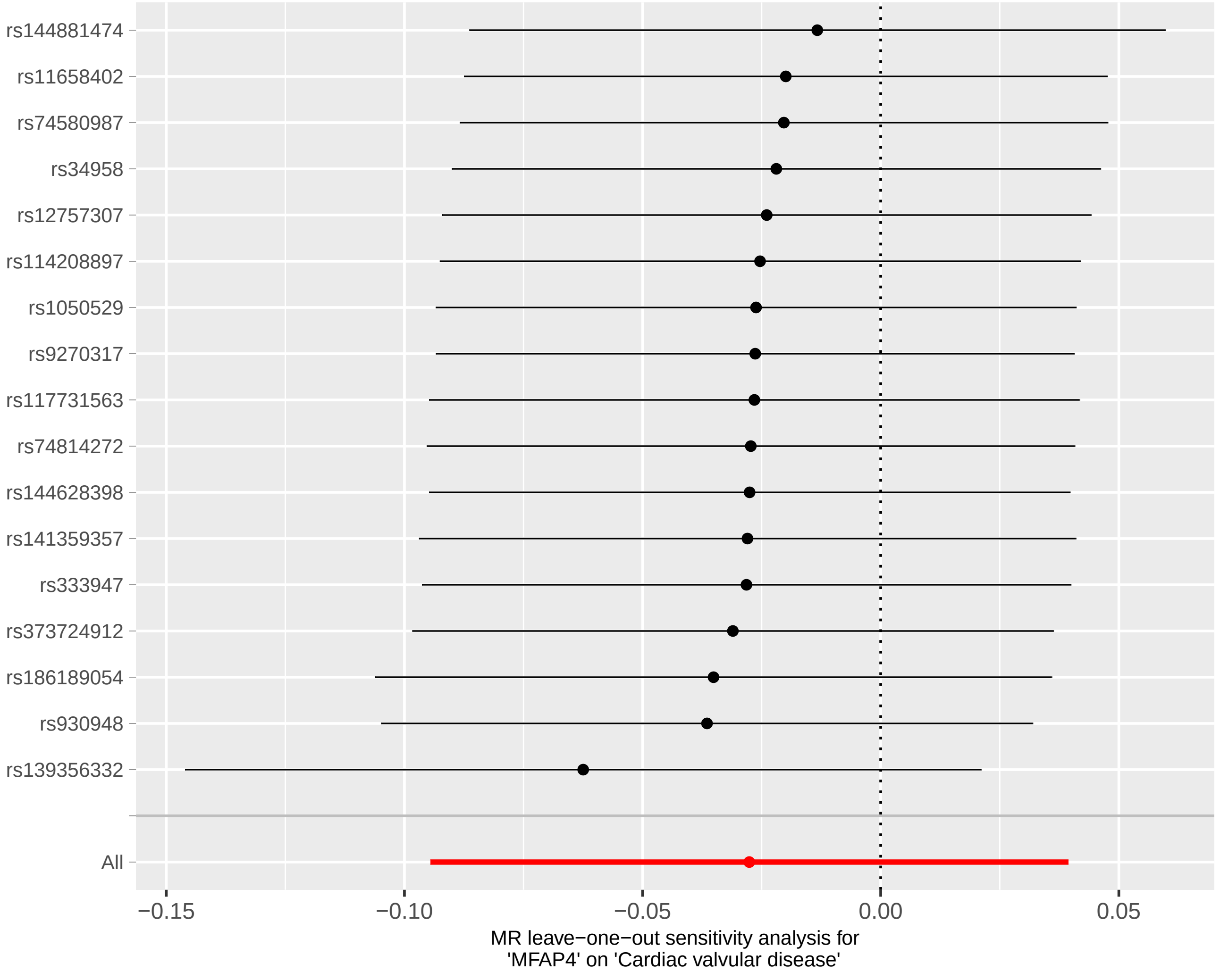
rs144881474
rs11658402
rs74580987
rs34958
rs12757307
rs114208897
rs1050529
rs9270317
rs117731563
rs74814272
rs144628398
rs141359357
rs333947
rs373724912
rs186189054
rs930948
rs139356332
All
−0.15
−0.10
−0.05
0.00
0.05
MR leave−one−out sensitivity analysis for
'MFAP4' on 'Cardiac valvular disease'

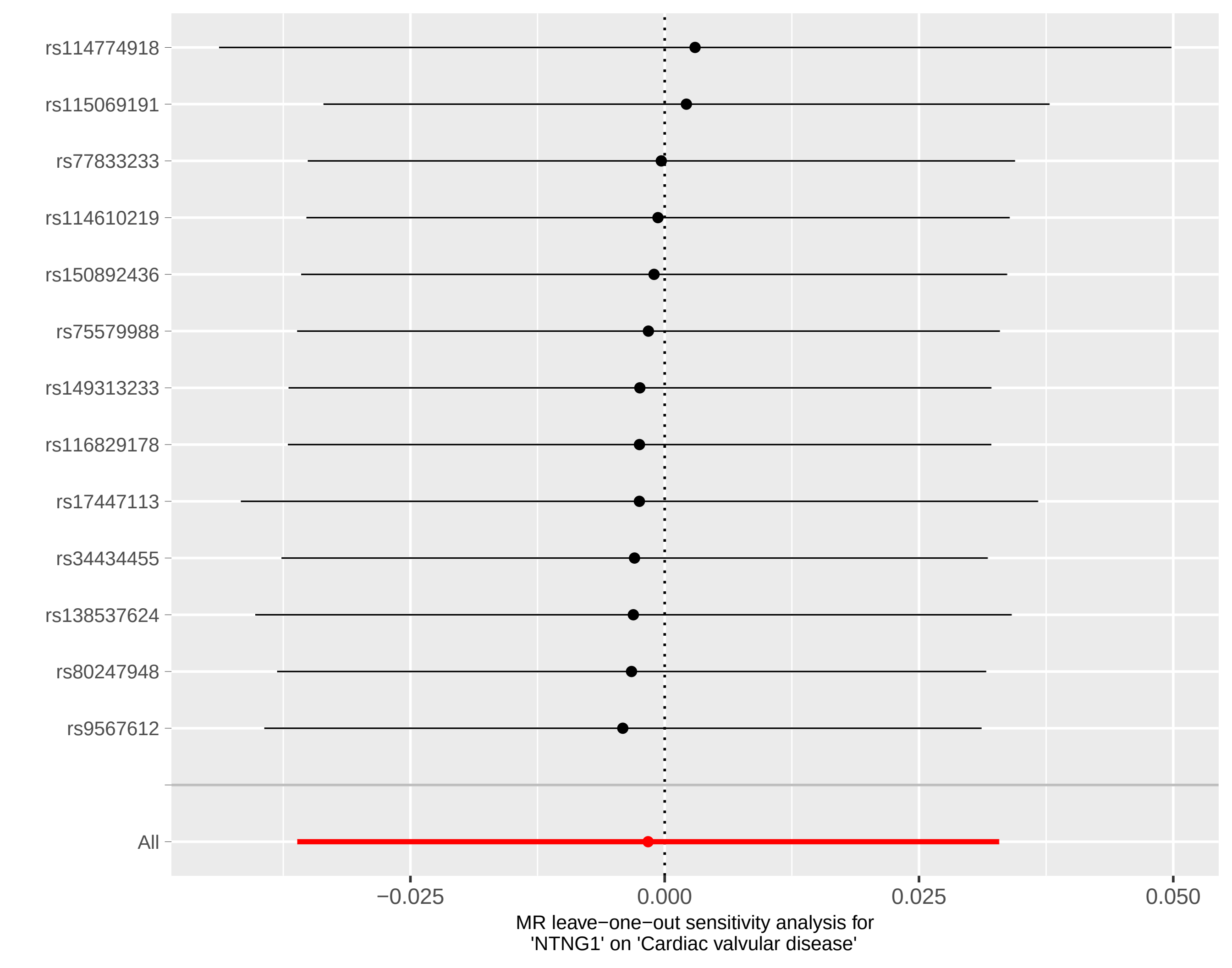
rs114774918
rs115069191
rs77833233
rs114610219
rs150892436
rs75579988
rs149313233
rs116829178
rs17447113
rs34434455
rs138537624
rs80247948
rs9567612
All
−0.025
0.000
0.025
0.050
MR leave−one−out sensitivity analysis for
'NTNG1' on 'Cardiac valvular disease'

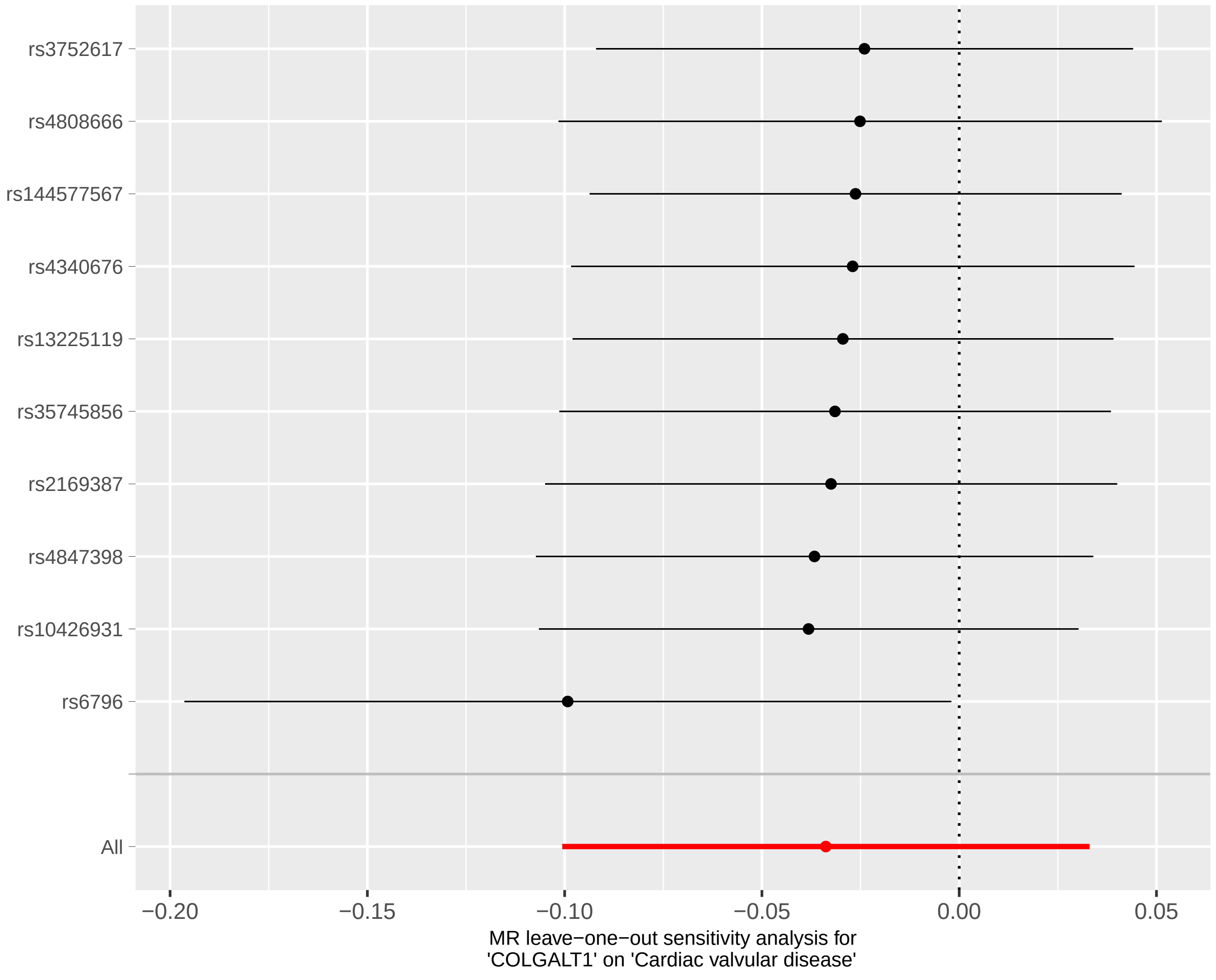

rs3752617
rs4808666
rs144577567
rs4340676
rs13225119
rs35745856
rs2169387
rs4847398
rs10426931
rs6796
All
−0.20
−0.15
−0.10
−0.05
0.00
0.05
MR leave−one−out sensitivity analysis for
'COLGALT1' on 'Cardiac valvular disease'

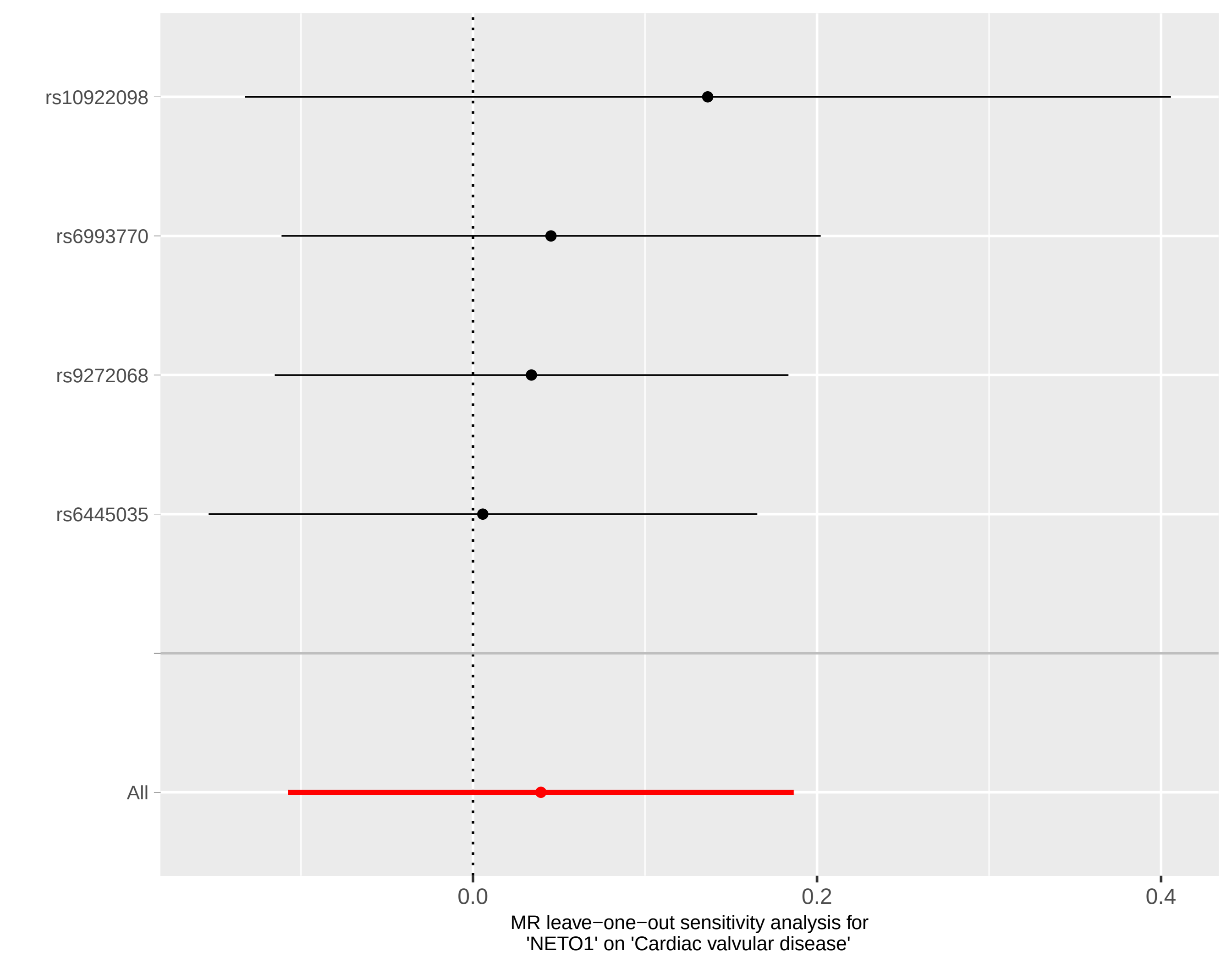

rs10922098
rs6993770
rs9272068
rs6445035
All
0.0
0.2
0.4
MR leave−one−out sensitivity analysis for
'NETO1' on 'Cardiac valvular disease'

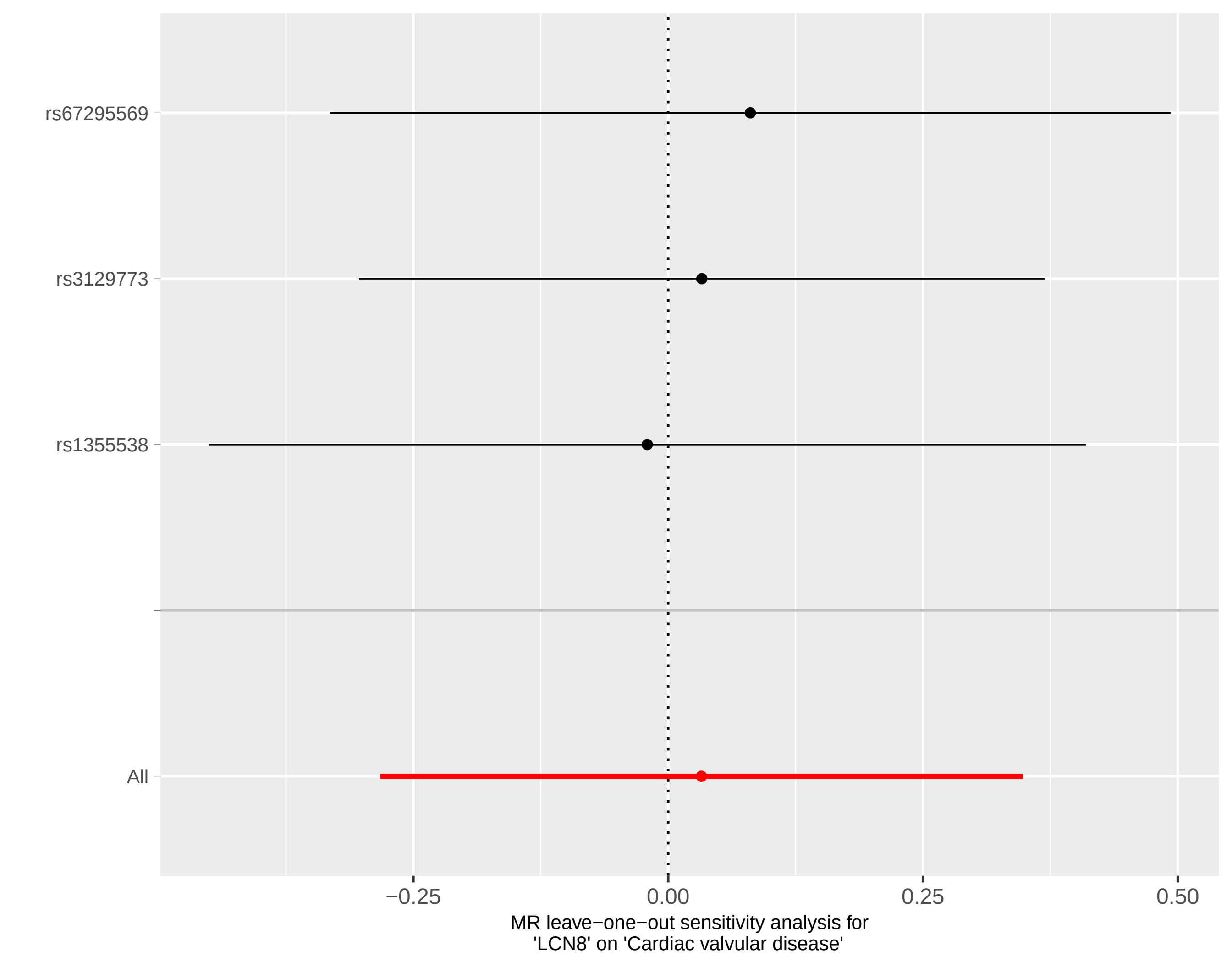
rs67295569
rs3129773
rs1355538
All
−0.25
0.00
0.25
0.50
MR leave−one−out sensitivity analysis for
'LCN8' on 'Cardiac valvular disease'

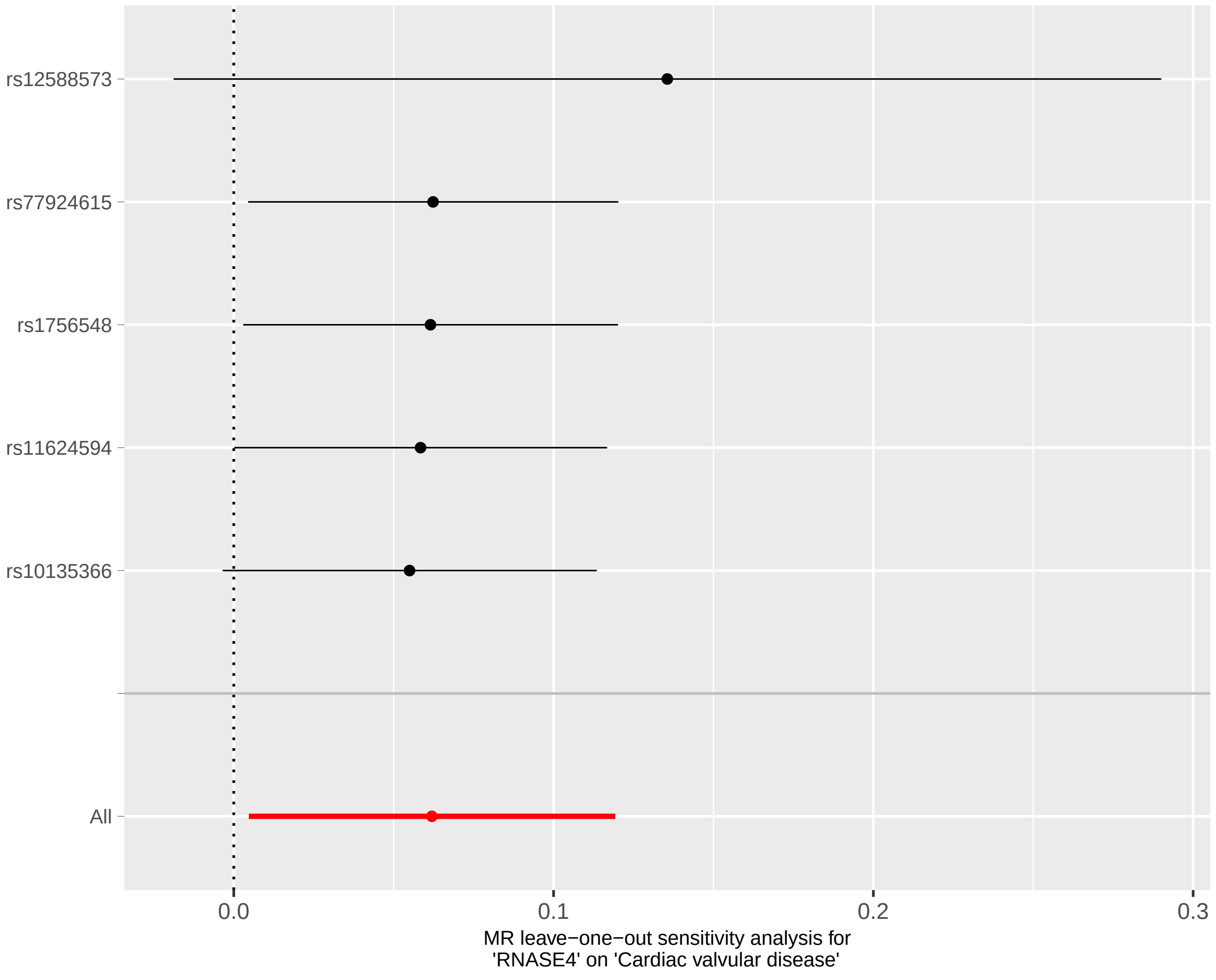
rs12588573
rs77924615
rs1756548
rs11624594
rs10135366
All
0.0
0.1
0.2
0.3
MR leave−one−out sensitivity analysis for
'RNASE4' on 'Cardiac valvular disease'

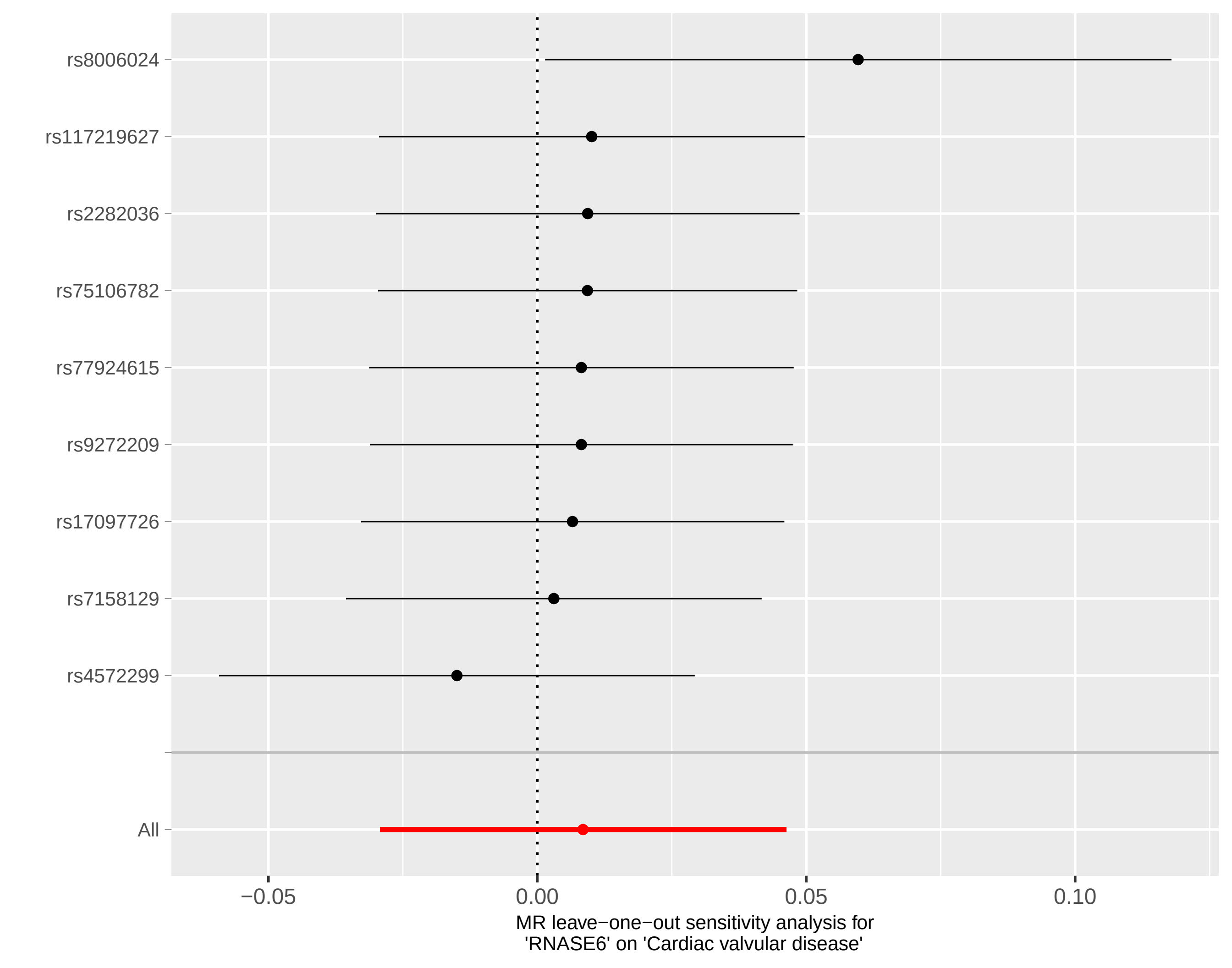

rs8006024
rs117219627
rs2282036
rs75106782
rs77924615
rs9272209
rs17097726
rs7158129
rs4572299
All
−0.05
0.00
0.05
0.10
MR leave−one−out sensitivity analysis for
'RNASE6' on 'Cardiac valvular disease'

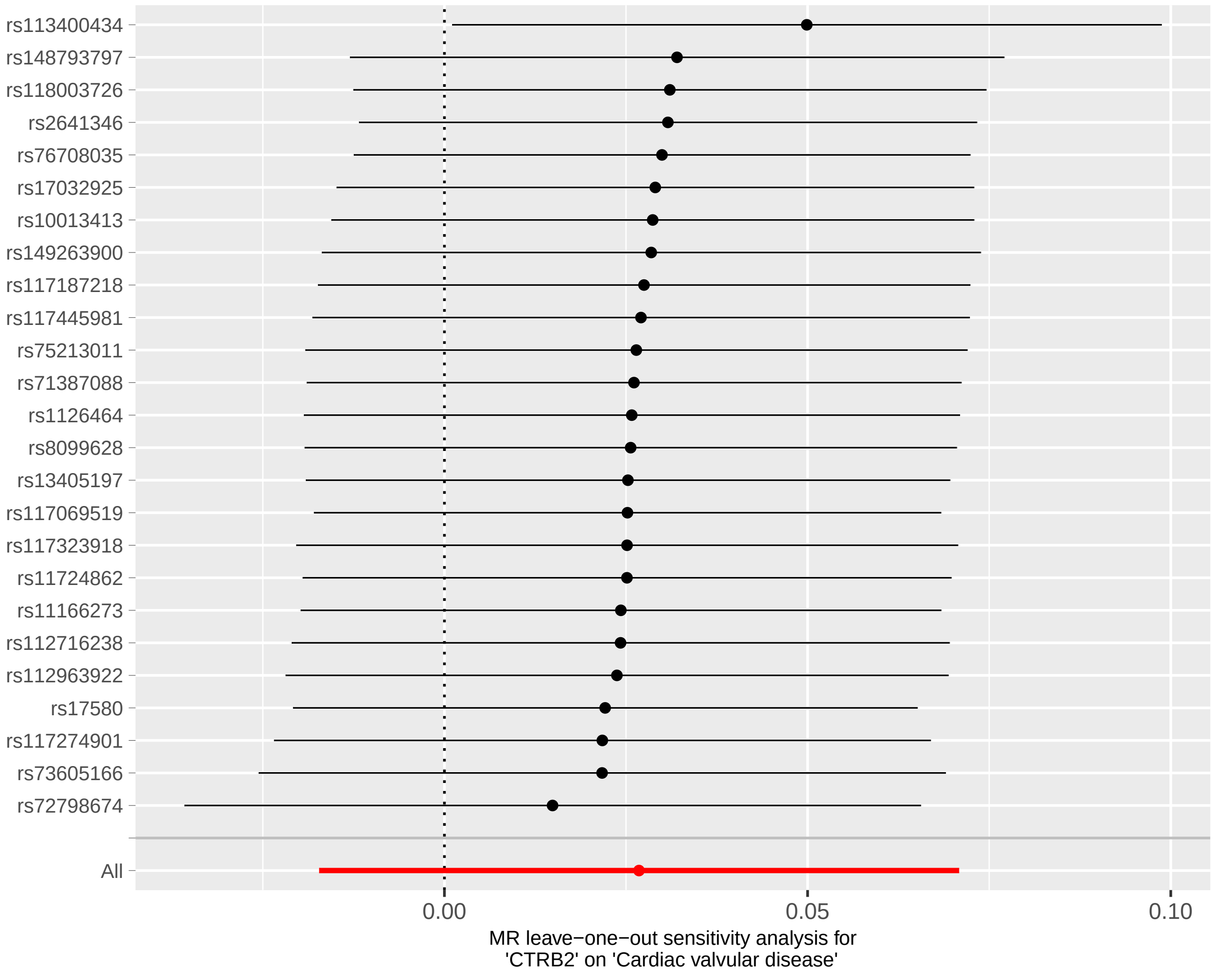
rs113400434
rs148793797
rs118003726
rs2641346
rs76708035
rs17032925
rs10013413
rs149263900
rs117187218
rs117445981
rs75213011
rs71387088
rs1126464
rs8099628
rs13405197
rs117069519
rs117323918
rs11724862
rs11166273
rs112716238
rs112963922
rs17580
rs117274901
rs73605166
rs72798674
All
0.00
0.05
0.10
MR leave−one−out sensitivity analysis for
'CTRB2' on 'Cardiac valvular disease'

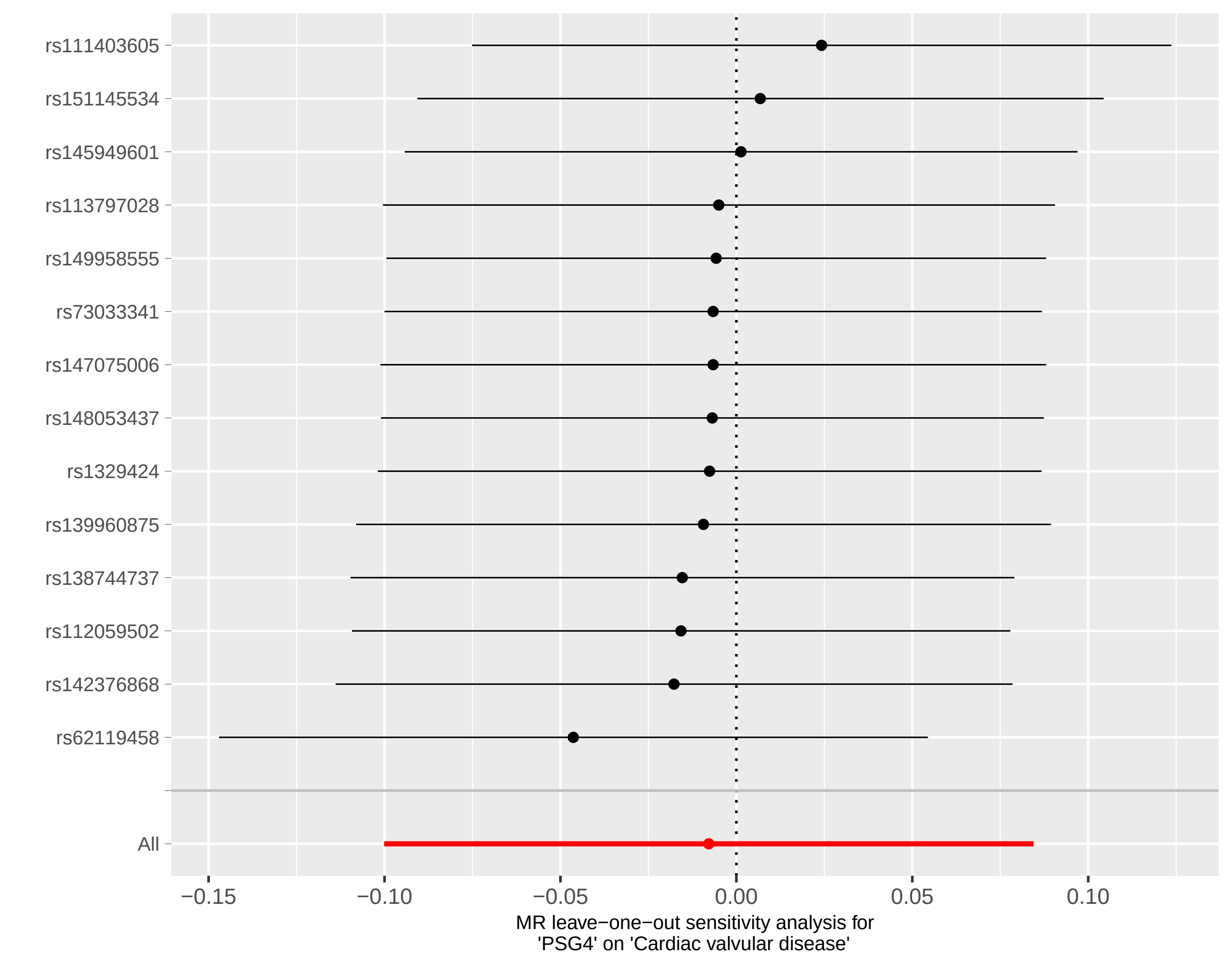
rs111403605
rs151145534
rs145949601
rs113797028
rs149958555
rs73033341
rs147075006
rs148053437
rs1329424
rs139960875
rs138744737
rs112059502
rs142376868
rs62119458
All
−0.15
−0.10
−0.05
0.00
0.05
0.10
MR leave−one−out sensitivity analysis for
'PSG4' on 'Cardiac valvular disease'

Insufficient number of SNPs

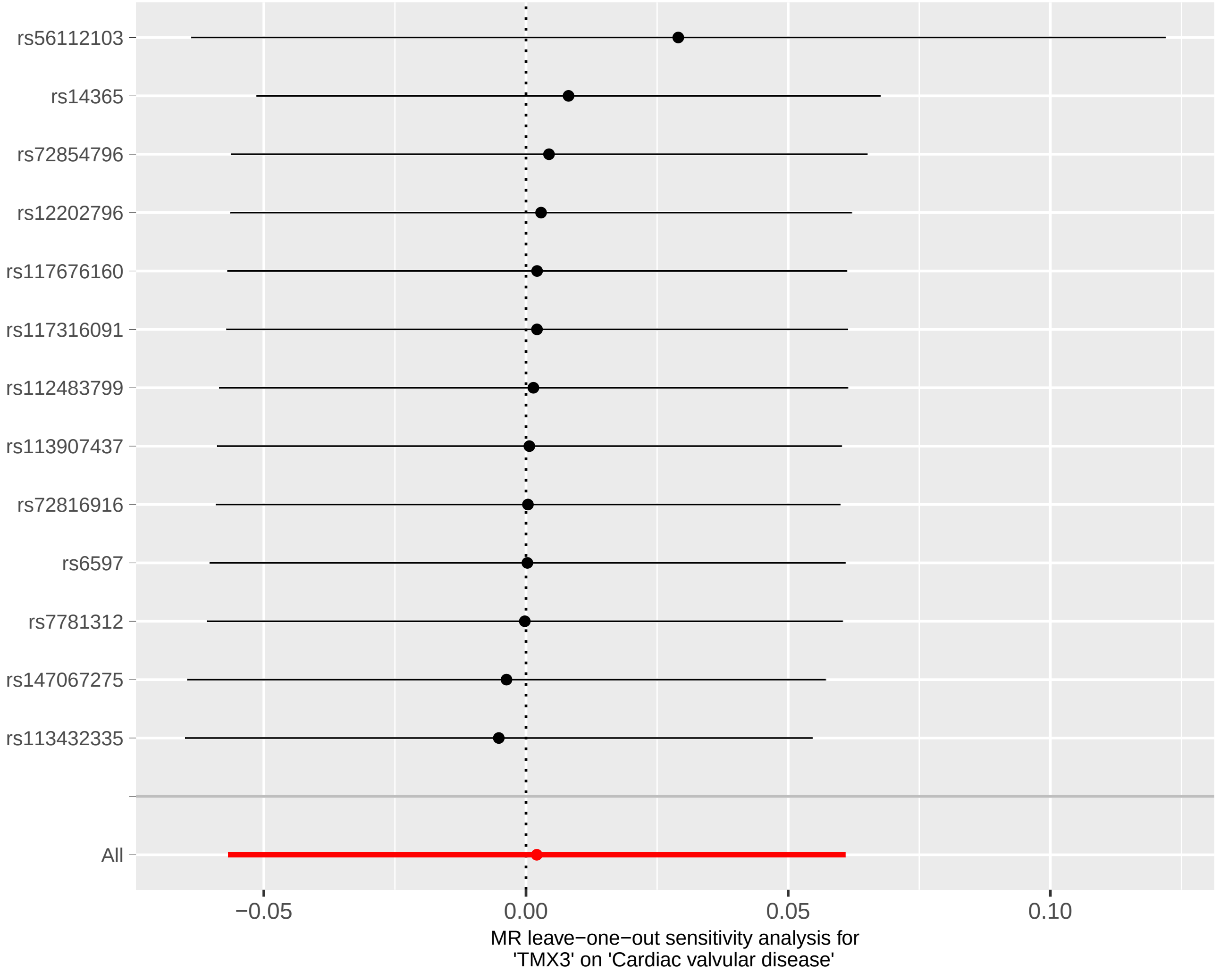

rs56112103
rs14365
rs72854796
rs12202796
rs117676160
rs117316091
rs112483799
rs113907437
rs72816916
rs6597
rs7781312
rs147067275
rs113432335
All
−0.05
0.00
0.05
0.10
MR leave−one−out sensitivity analysis for
'TMX3' on 'Cardiac valvular disease'

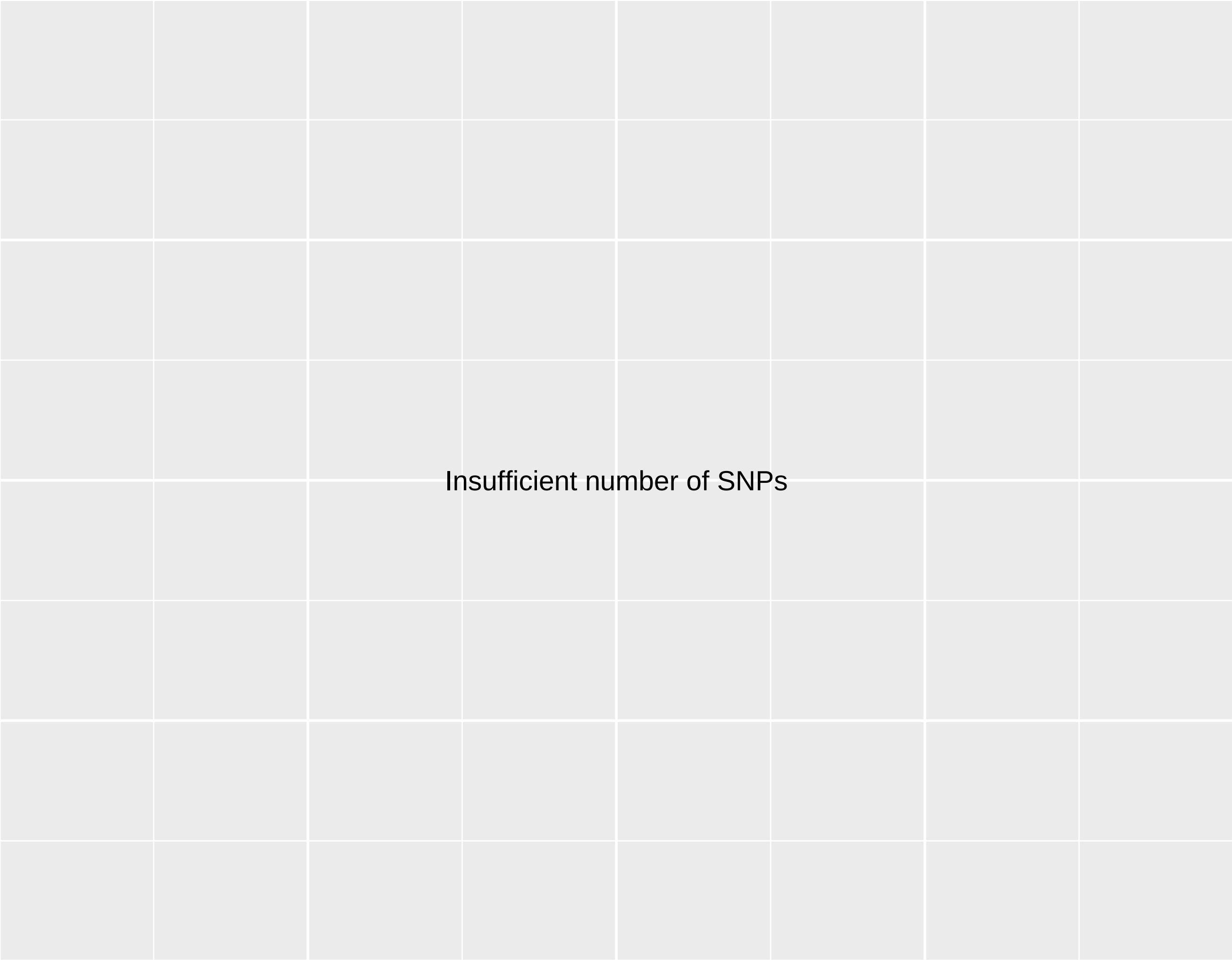
Insufficient number of SNPs

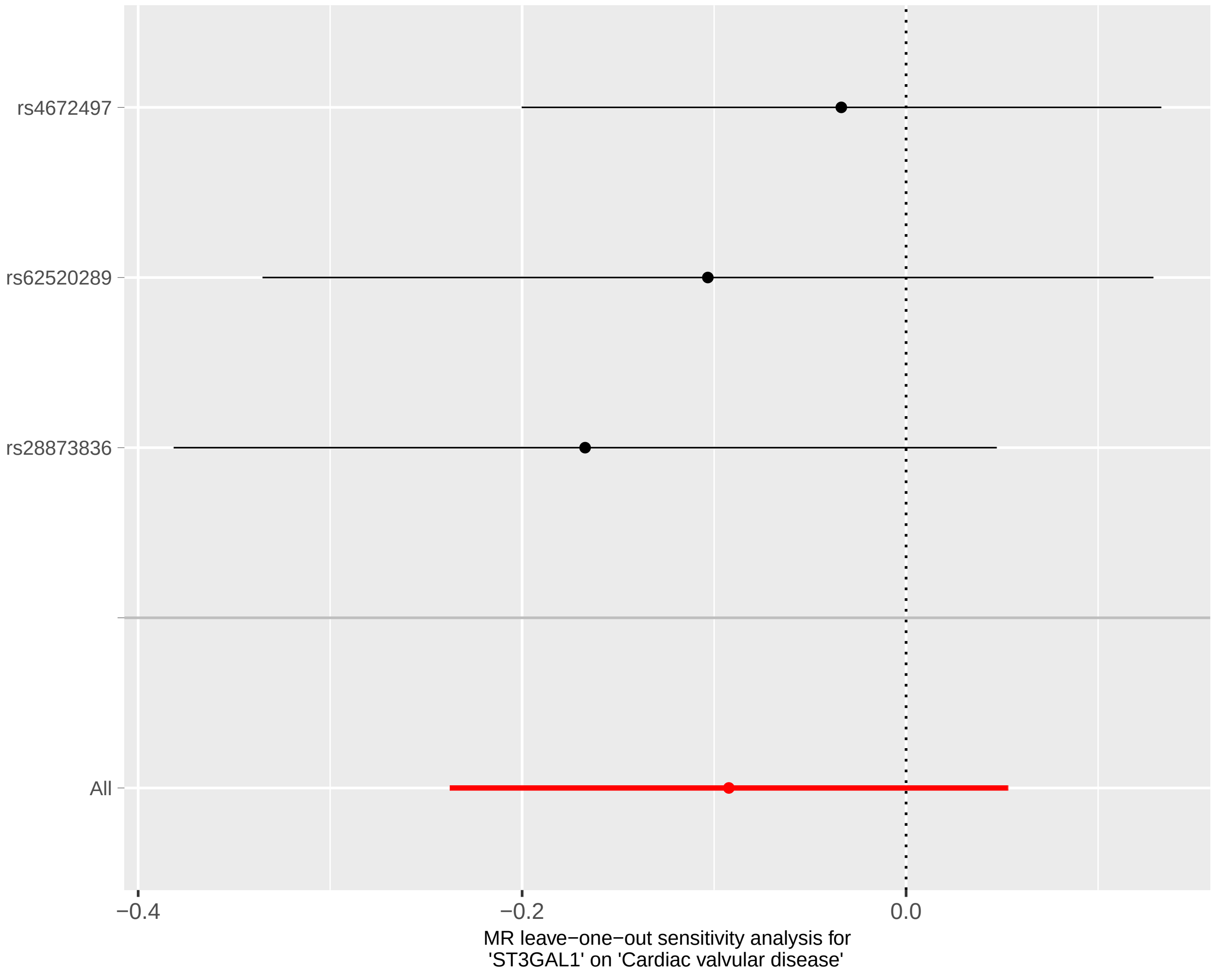
rs4672497
rs62520289
rs28873836
All
−0.4
−0.2
0.0
MR leave−one−out sensitivity analysis for
'ST3GAL1' on 'Cardiac valvular disease'

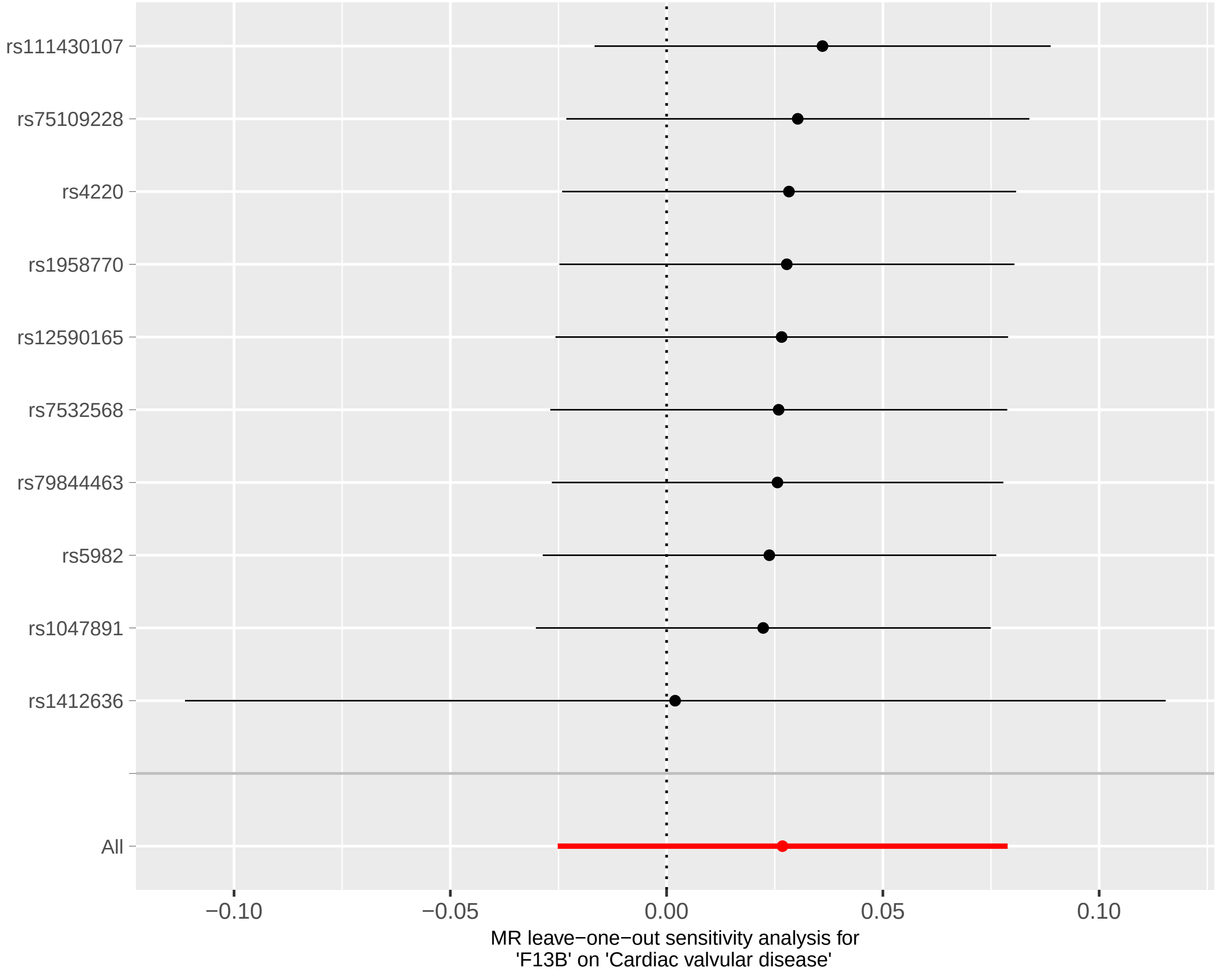

rs111430107
rs75109228
rs4220
rs1958770
rs12590165
rs7532568
rs79844463
rs5982
rs1047891
rs1412636
All
−0.10
−0.05
0.00
0.05
0.10
MR leave−one−out sensitivity analysis for
'F13B' on 'Cardiac valvular disease'

Insufficient number of SNPs

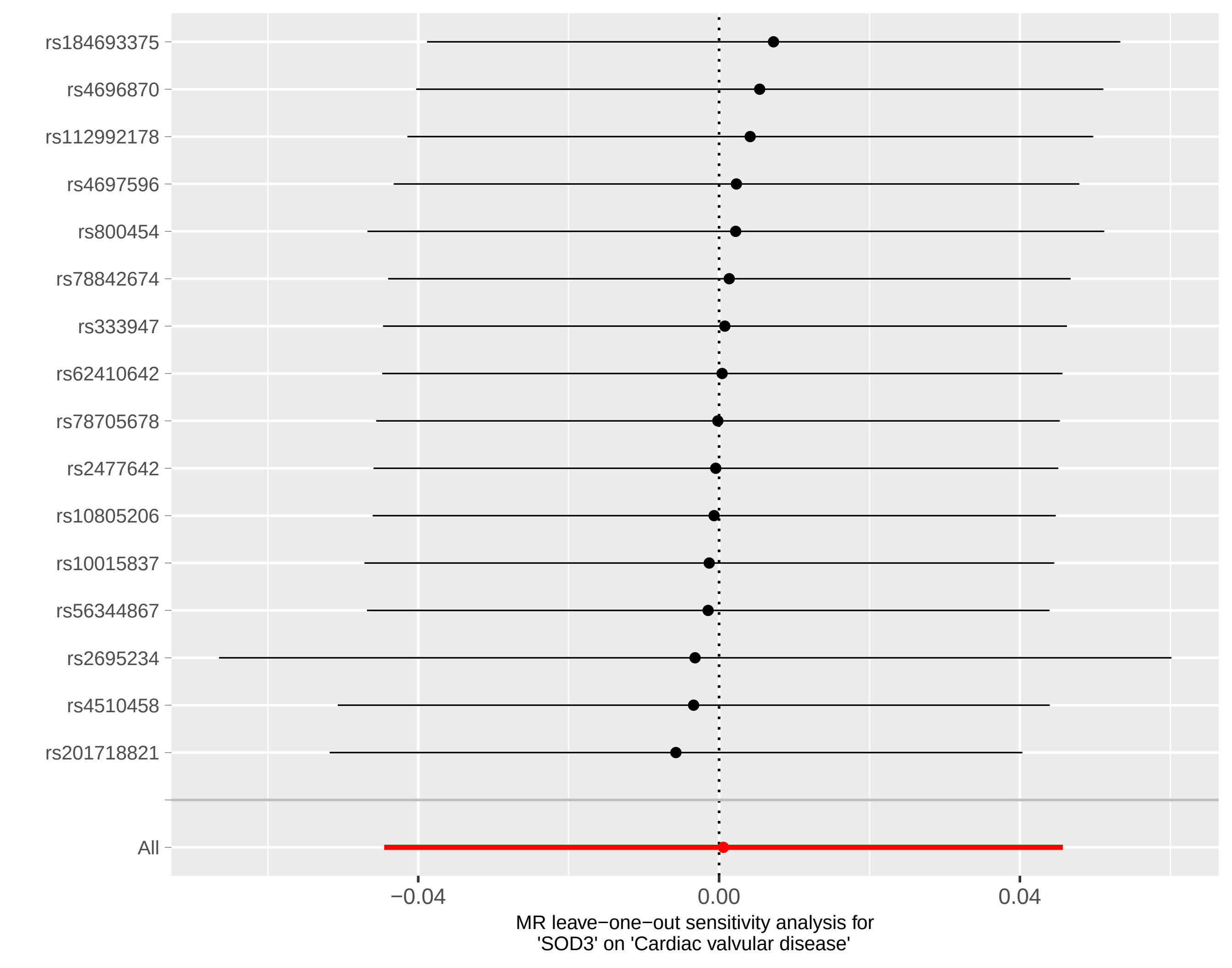
rs184693375
rs4696870
rs112992178
rs4697596
rs800454
rs78842674
rs333947
rs62410642
rs78705678
rs2477642
rs10805206
rs10015837
rs56344867
rs2695234
rs4510458
rs201718821
All
−0.04
0.00
0.04
MR leave−one−out sensitivity analysis for
'SOD3' on 'Cardiac valvular disease'

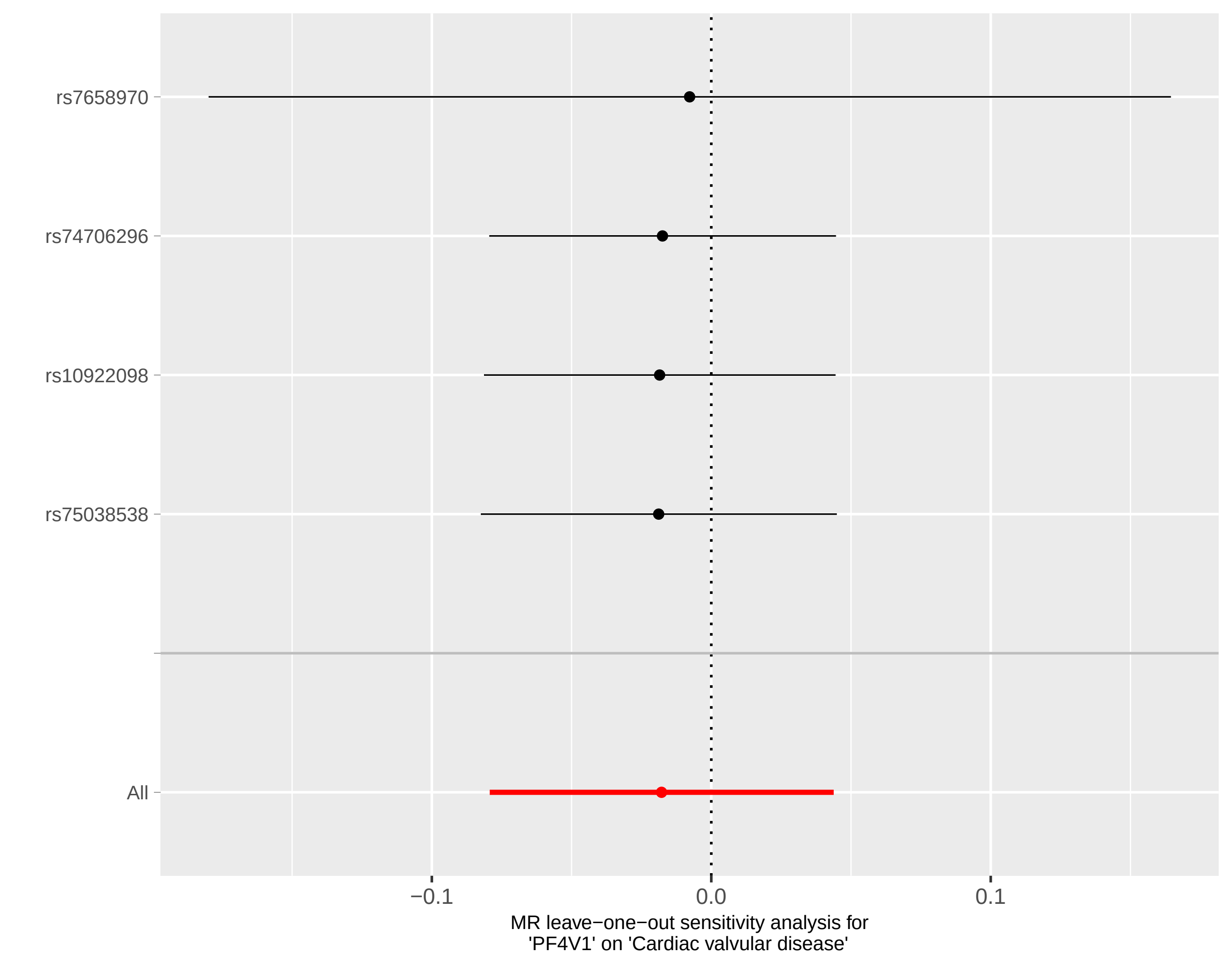
rs7658970
rs74706296
rs10922098
rs75038538
All
−0.1
0.0
0.1
MR leave−one−out sensitivity analysis for
'PF4V1' on 'Cardiac valvular disease'

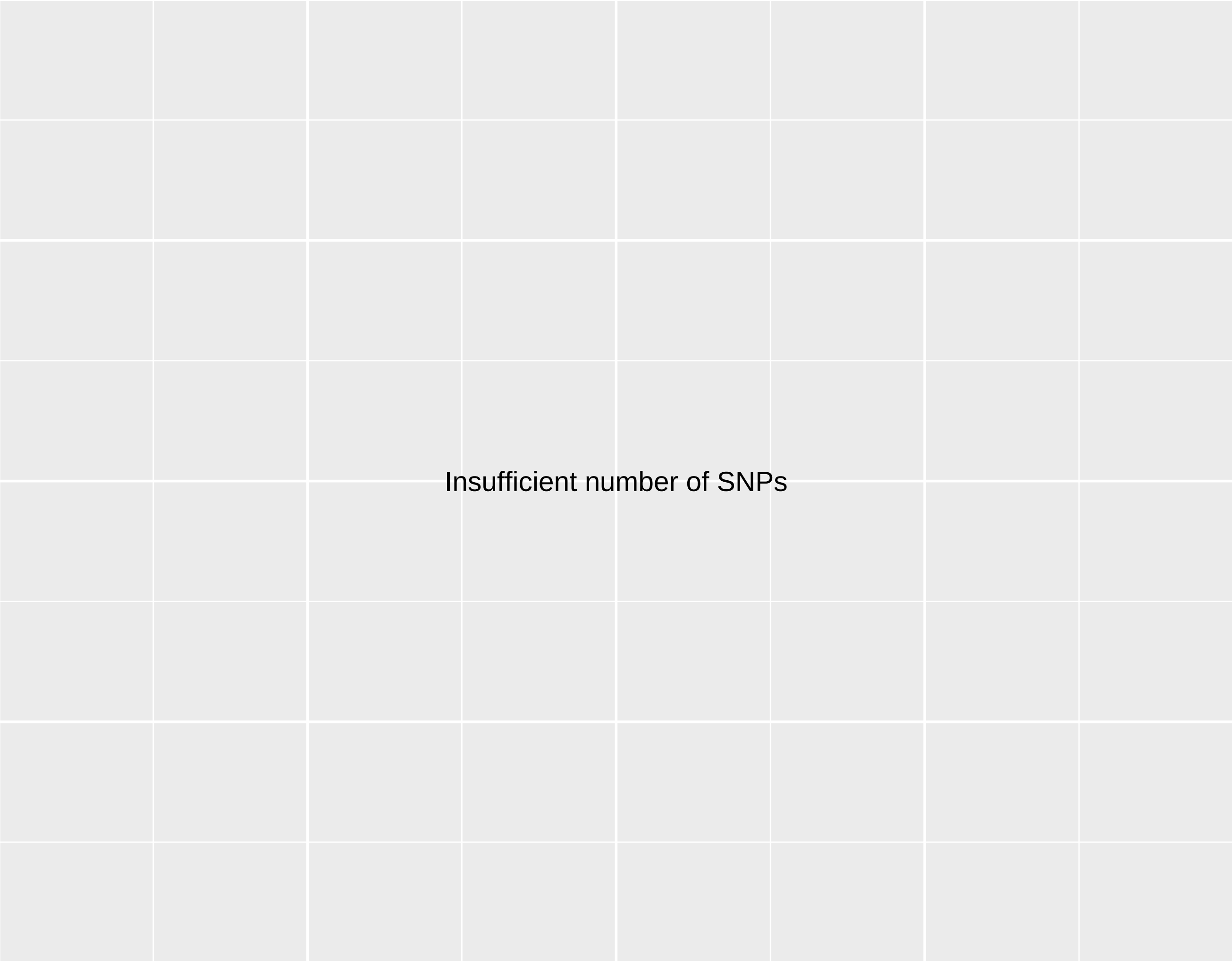
Insufficient number of SNPs

Insufficient number of SNPs

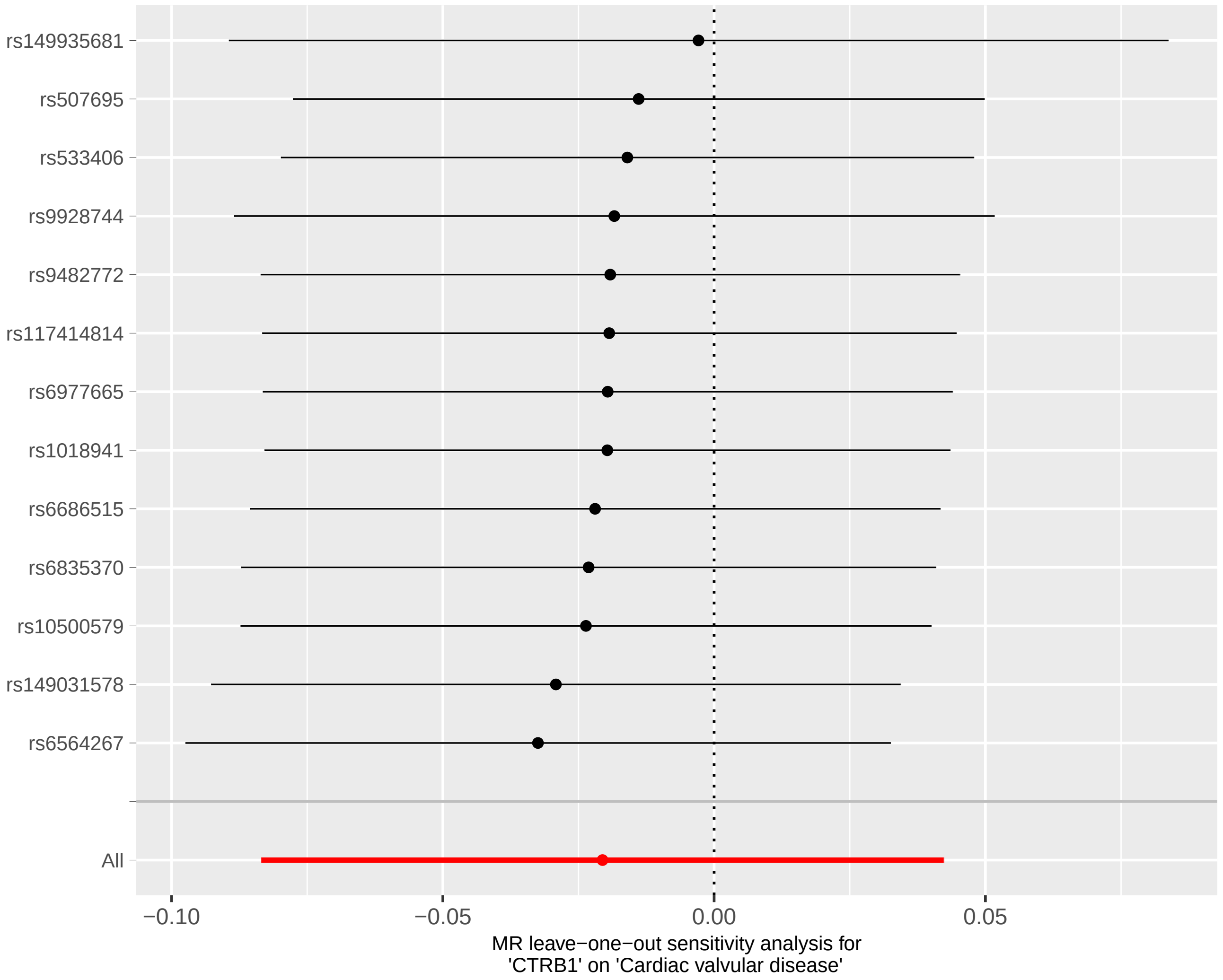
rs149935681
rs507695
rs533406
rs9928744
rs9482772
rs117414814
rs6977665
rs1018941
rs6686515
rs6835370
rs10500579
rs149031578
rs6564267
All
−0.10
−0.05
0.00
0.05
MR leave−one−out sensitivity analysis for
'CTRB1' on 'Cardiac valvular disease'

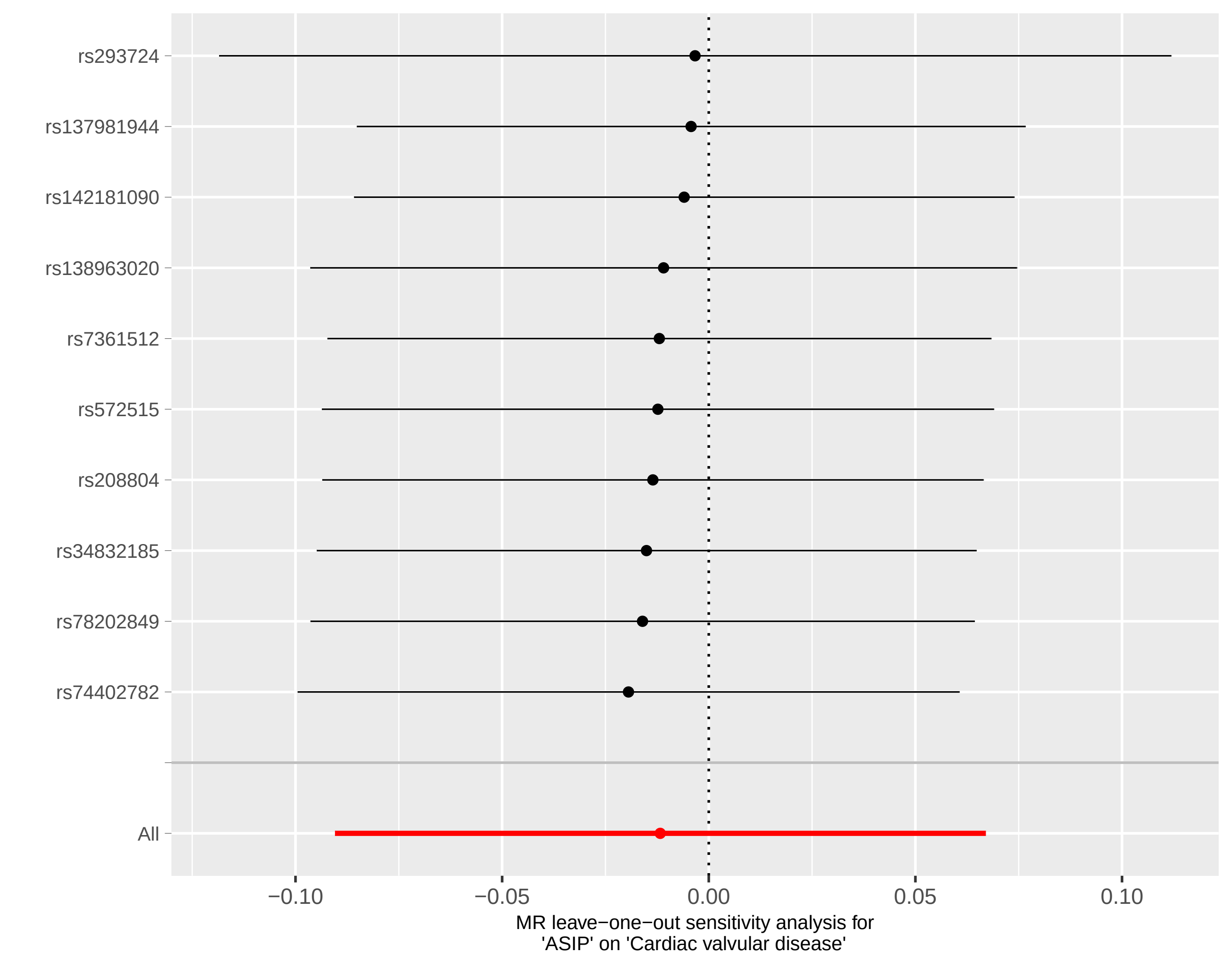

rs293724
rs137981944
rs142181090
rs138963020
rs7361512
rs572515
rs208804
rs34832185
rs78202849
rs74402782
All
−0.10
−0.05
0.00
0.05
0.10
MR leave−one−out sensitivity analysis for
'ASIP' on 'Cardiac valvular disease'

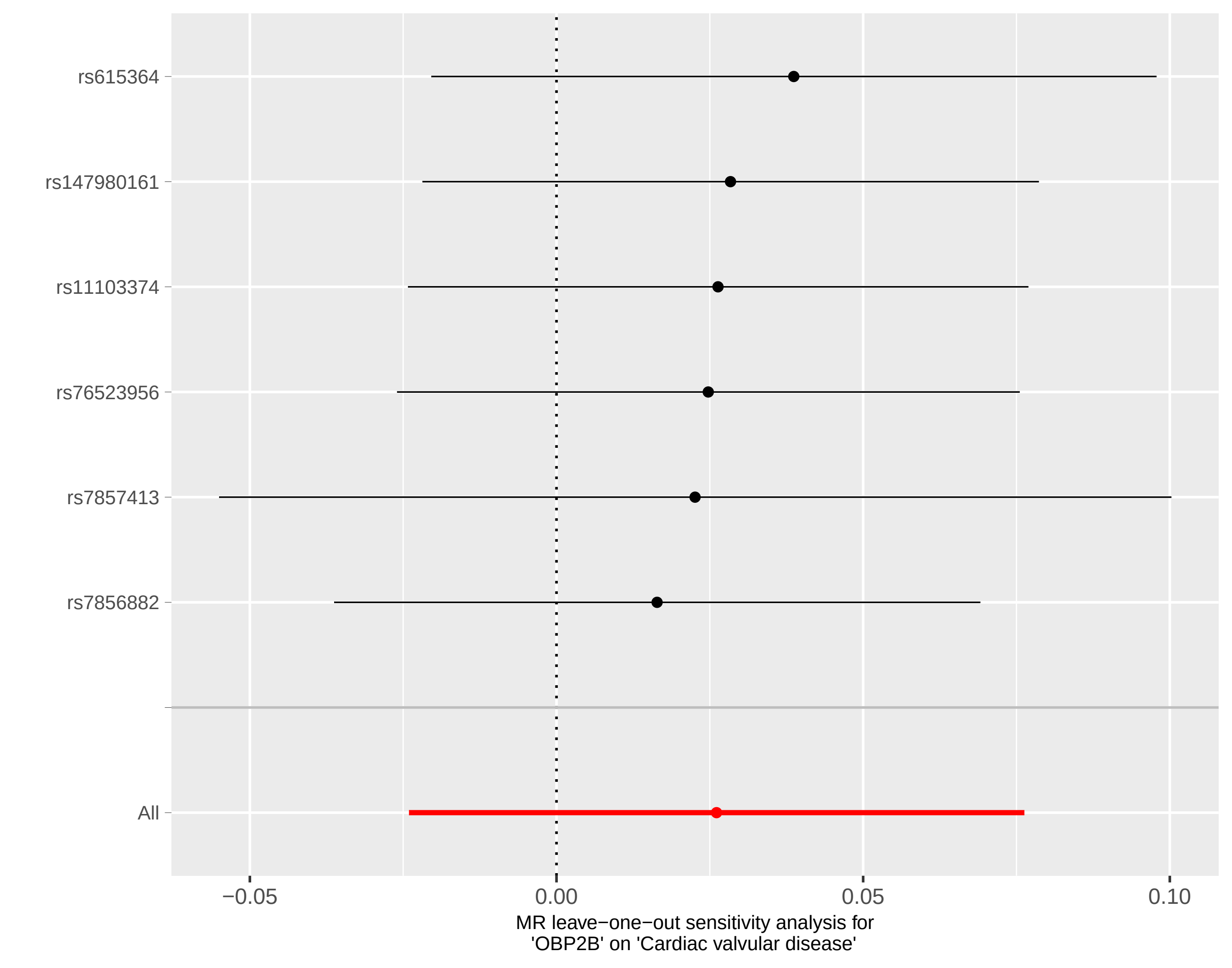
rs615364
rs147980161
rs11103374
rs76523956
rs7857413
rs7856882
All
−0.05
0.00
0.05
0.10
MR leave−one−out sensitivity analysis for
'OBP2B' on 'Cardiac valvular disease'

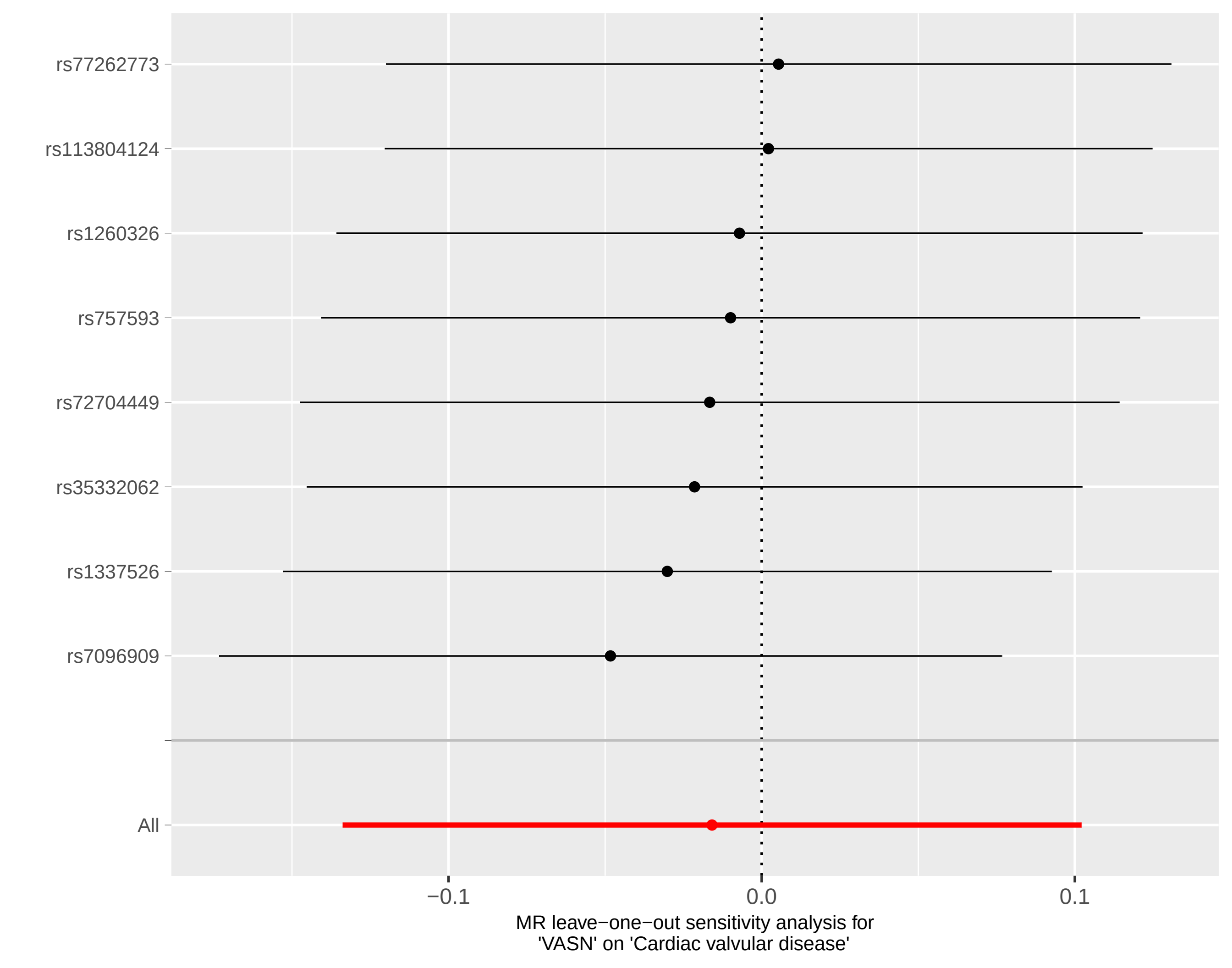
rs77262773
rs113804124
rs1260326
rs757593
rs72704449
rs35332062
rs1337526
rs7096909
All
−0.1
0.0
0.1
MR leave−one−out sensitivity analysis for
'VASN' on 'Cardiac valvular disease'

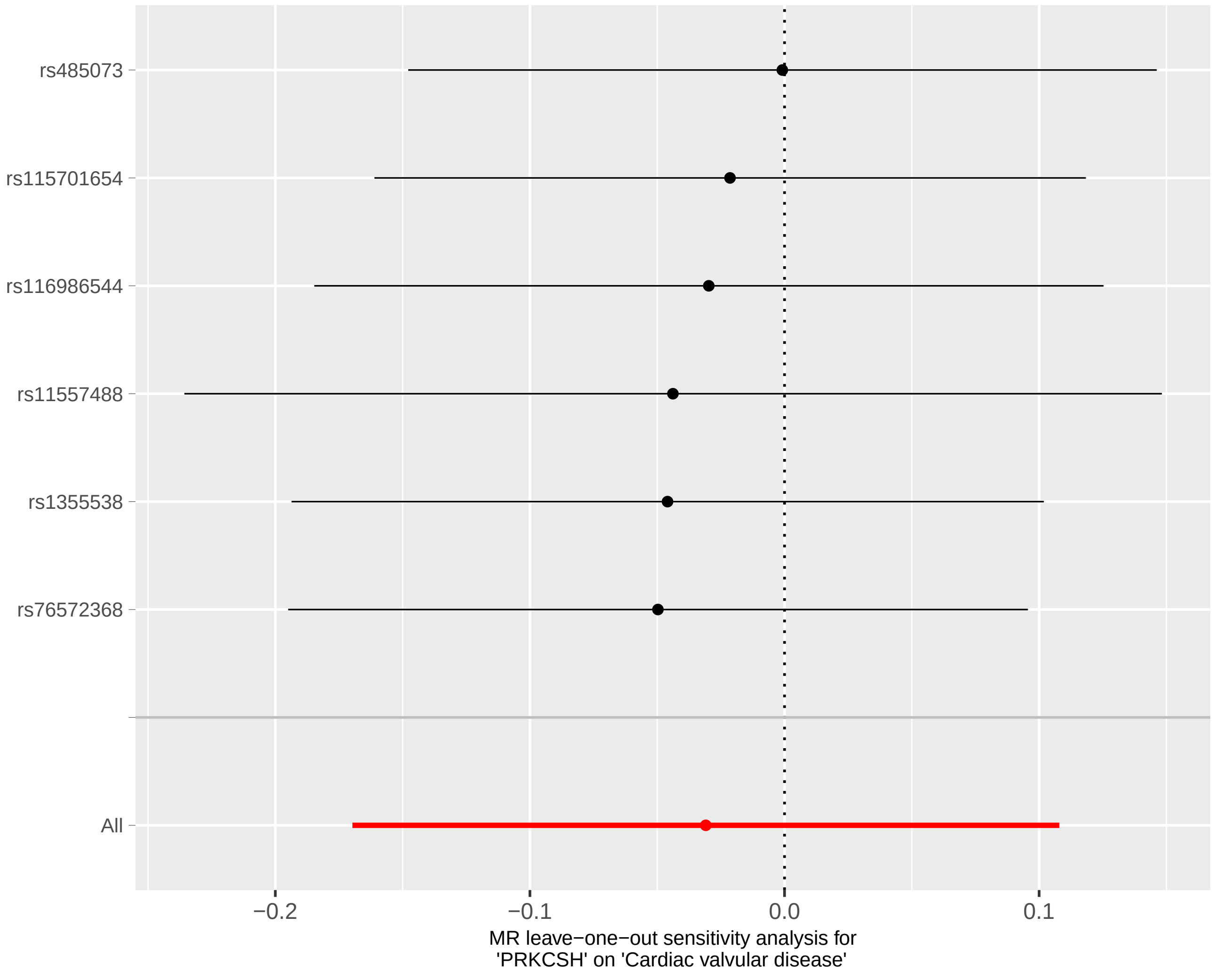

rs485073
rs115701654
rs116986544
rs11557488
rs1355538
rs76572368
All
−0.2
−0.1
0.0
0.1
MR leave−one−out sensitivity analysis for
'PRKCSH' on 'Cardiac valvular disease'

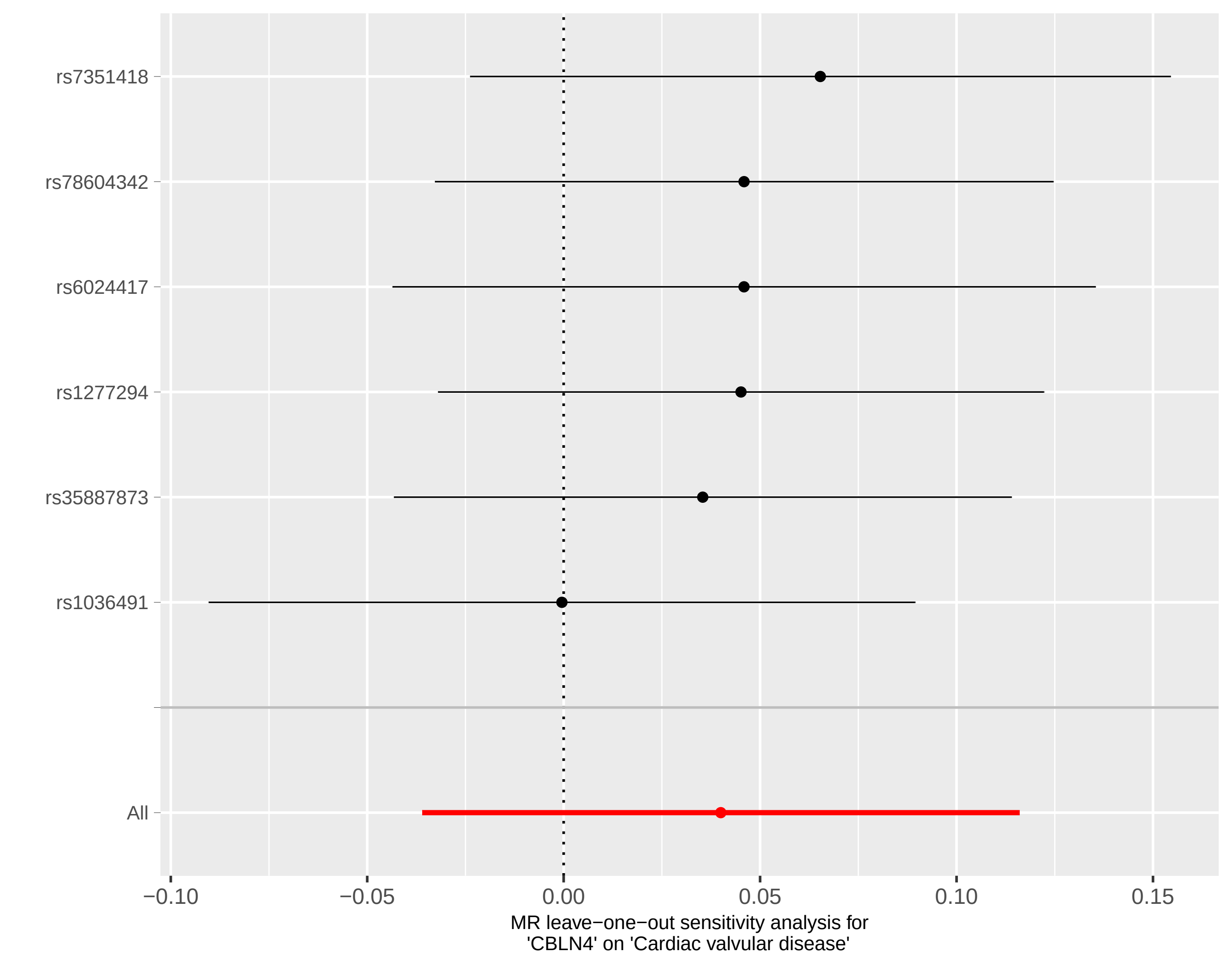

rs7351418
rs78604342
rs6024417
rs1277294
rs35887873
rs1036491
All
−0.10
−0.05
0.00
0.05
0.10
0.15
MR leave−one−out sensitivity analysis for
'CBLN4' on 'Cardiac valvular disease'

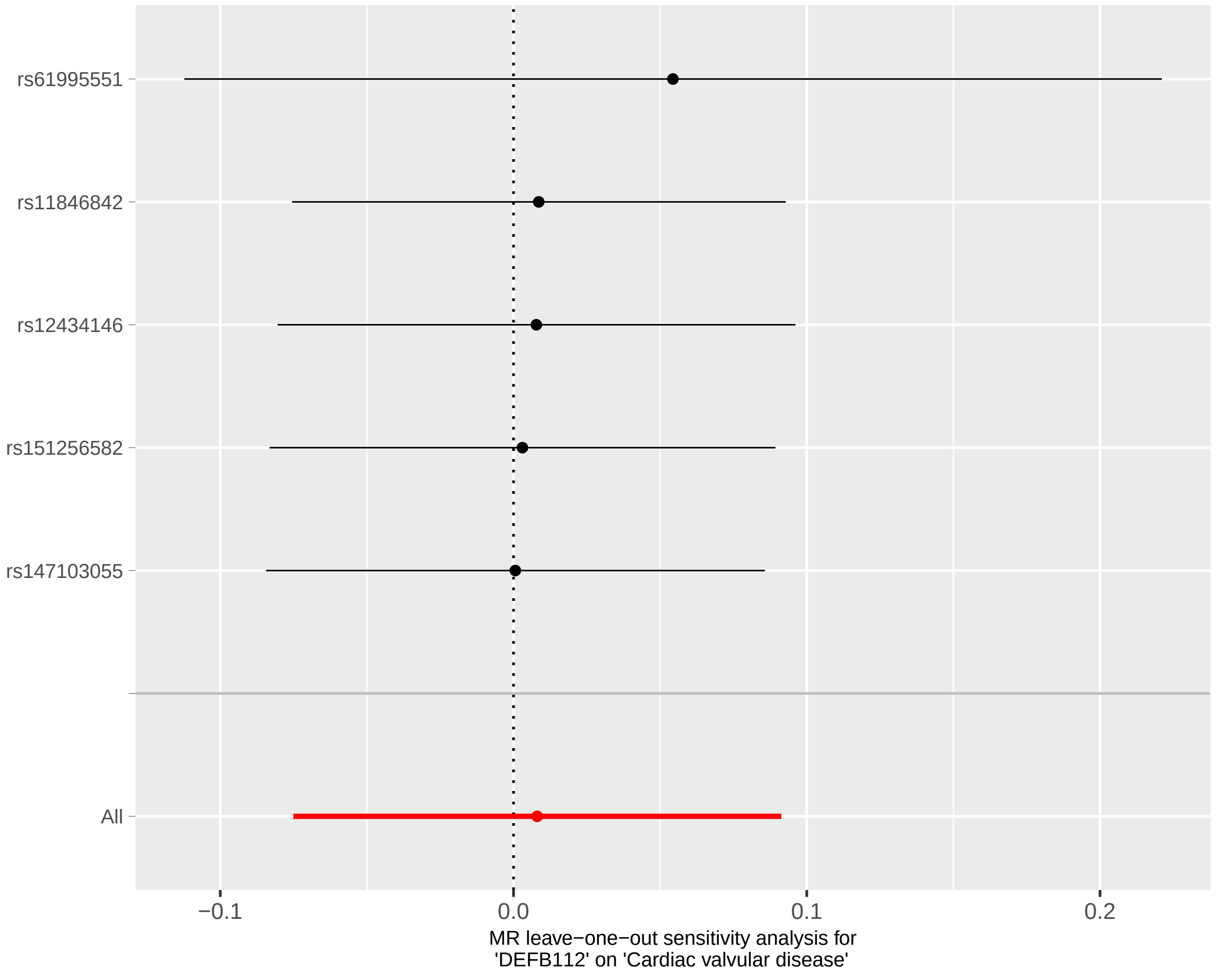

rs61995551
rs11846842
rs12434146
rs151256582
rs147103055
All
−0.1
0.0
0.1
0.2
MR leave−one−out sensitivity analysis for
'DEFB112' on 'Cardiac valvular disease'

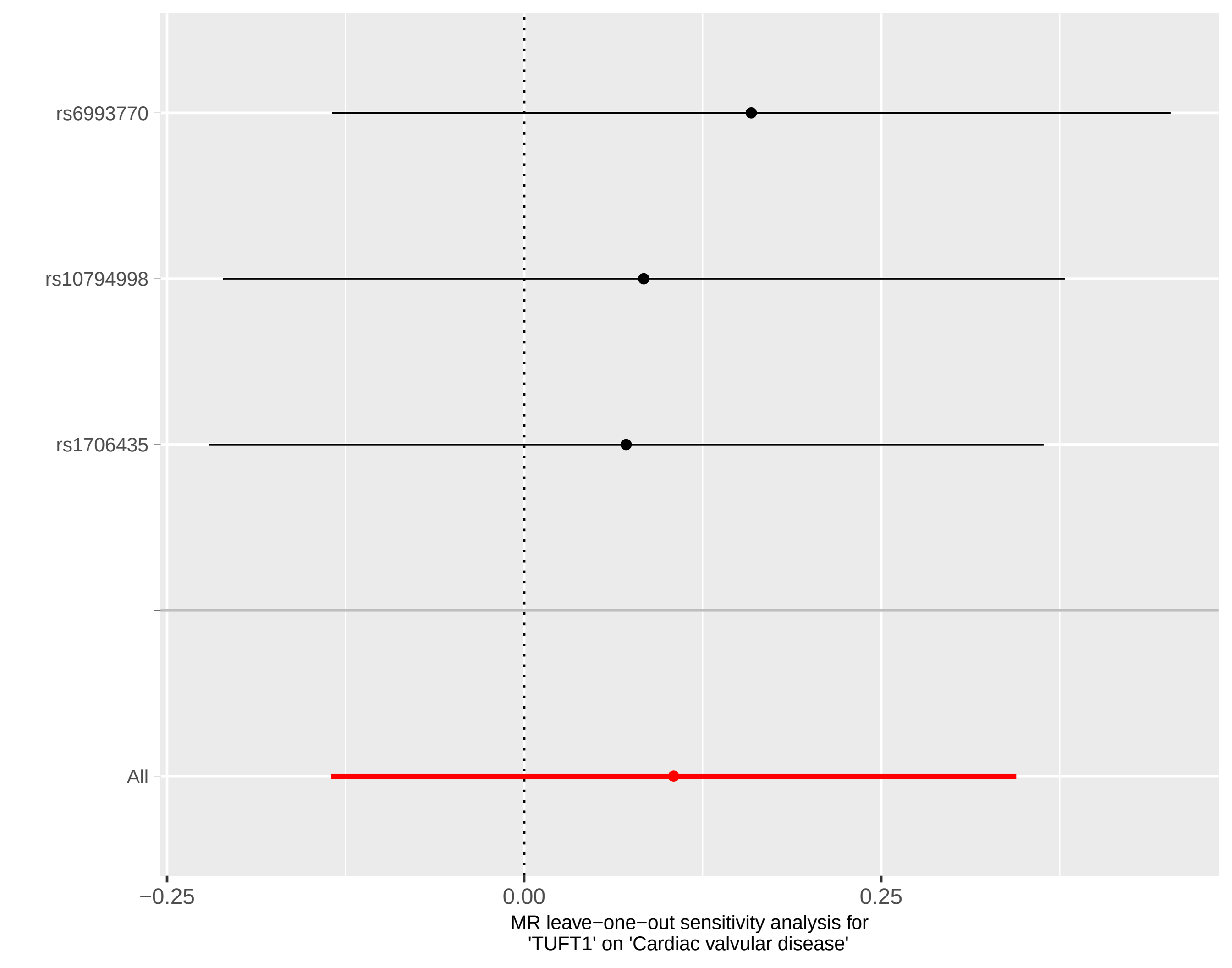
rs6993770
rs10794998
rs1706435
All
−0.25
0.00
0.25
MR leave−one−out sensitivity analysis for
'TUFT1' on 'Cardiac valvular disease'

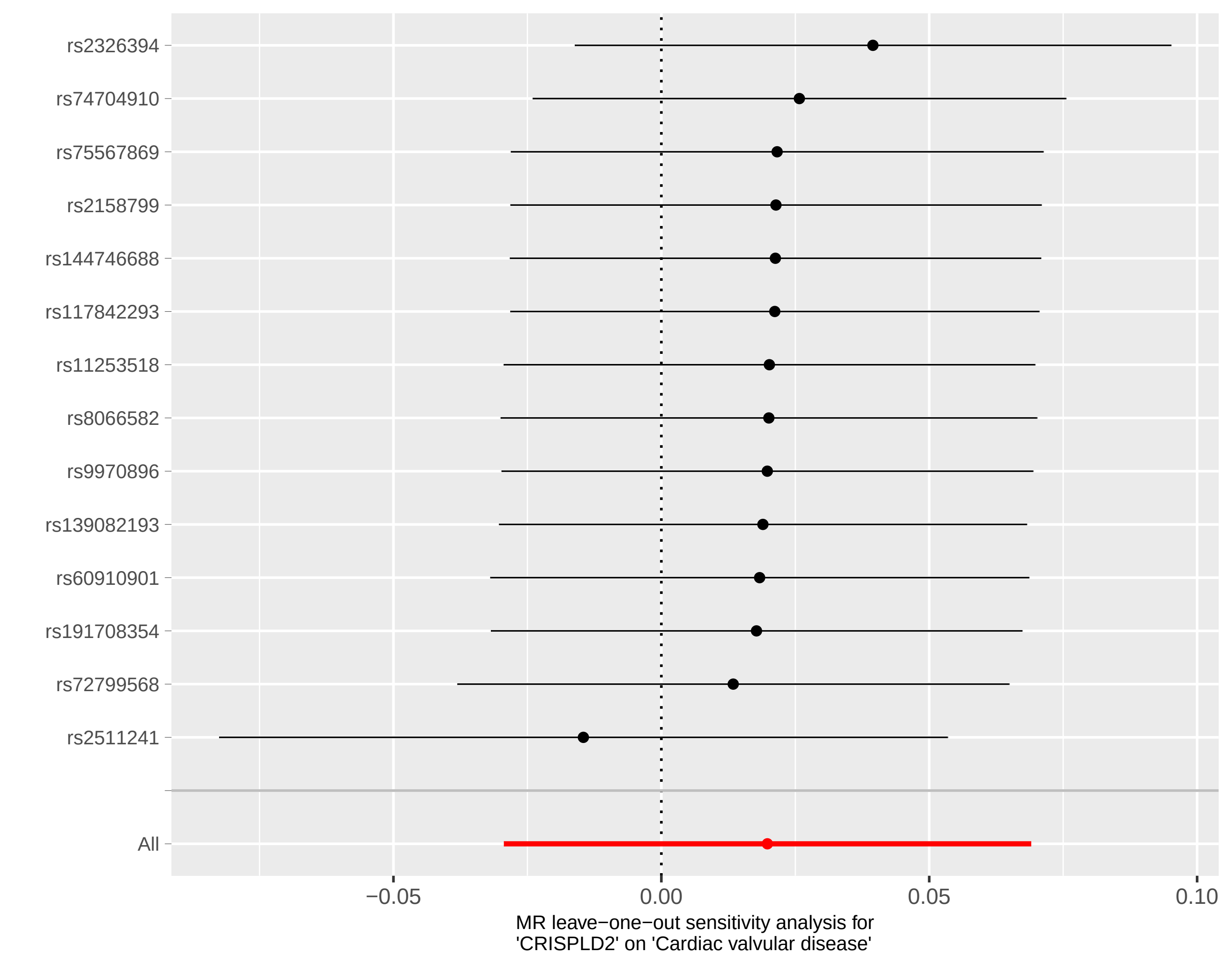

rs2326394
rs74704910
rs75567869
rs2158799
rs144746688
rs117842293
rs11253518
rs8066582
rs9970896
rs139082193
rs60910901
rs191708354
rs72799568
rs2511241
All
−0.05
0.00
0.05
0.10
MR leave−one−out sensitivity analysis for
'CRISPLD2' on 'Cardiac valvular disease'

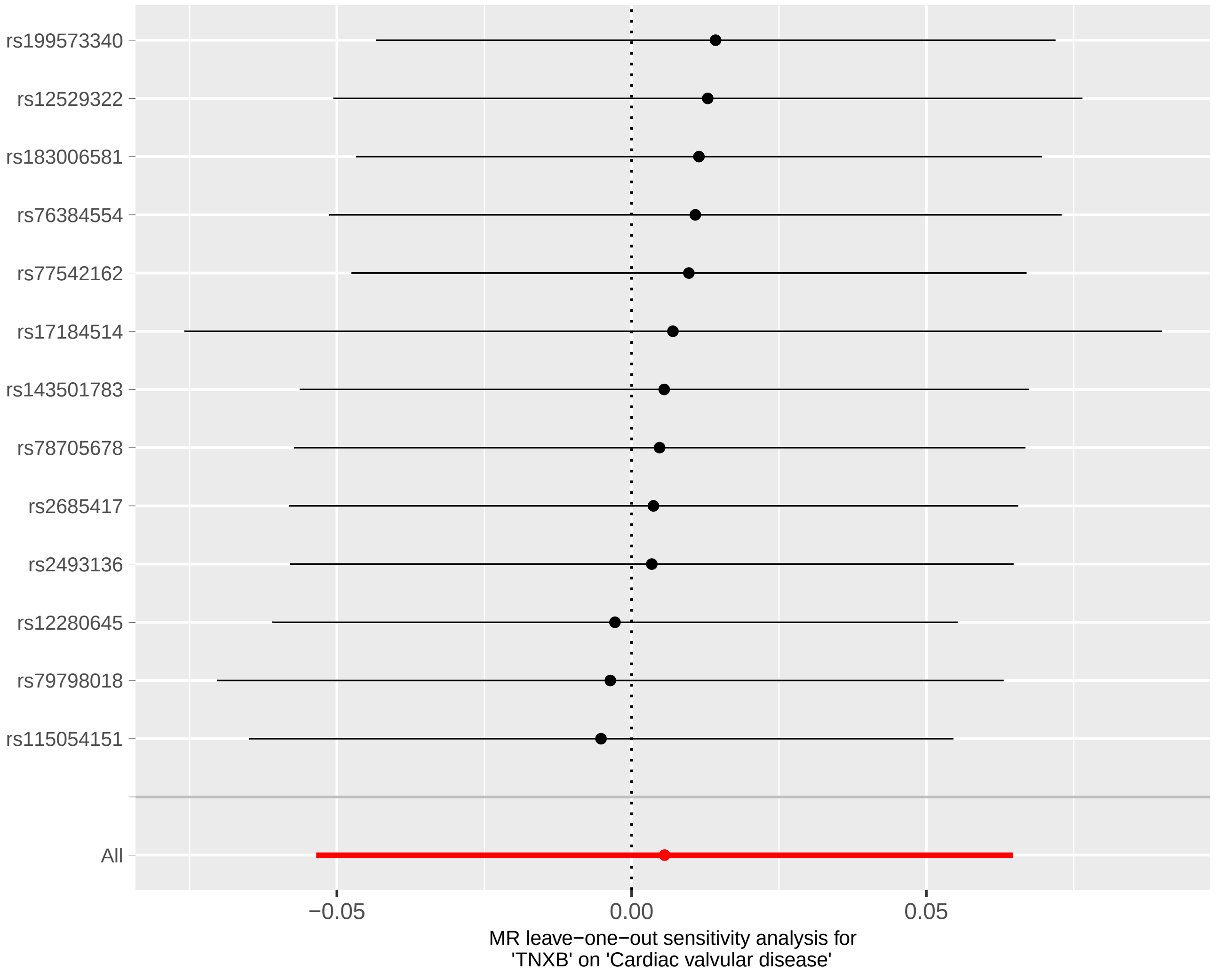

rs199573340
rs12529322
rs183006581
rs76384554
rs77542162
rs17184514
rs143501783
rs78705678
rs2685417
rs2493136
rs12280645
rs79798018
rs115054151
All
−0.05
0.00
0.05
MR leave−one−out sensitivity analysis for
'TNXB' on 'Cardiac valvular disease'

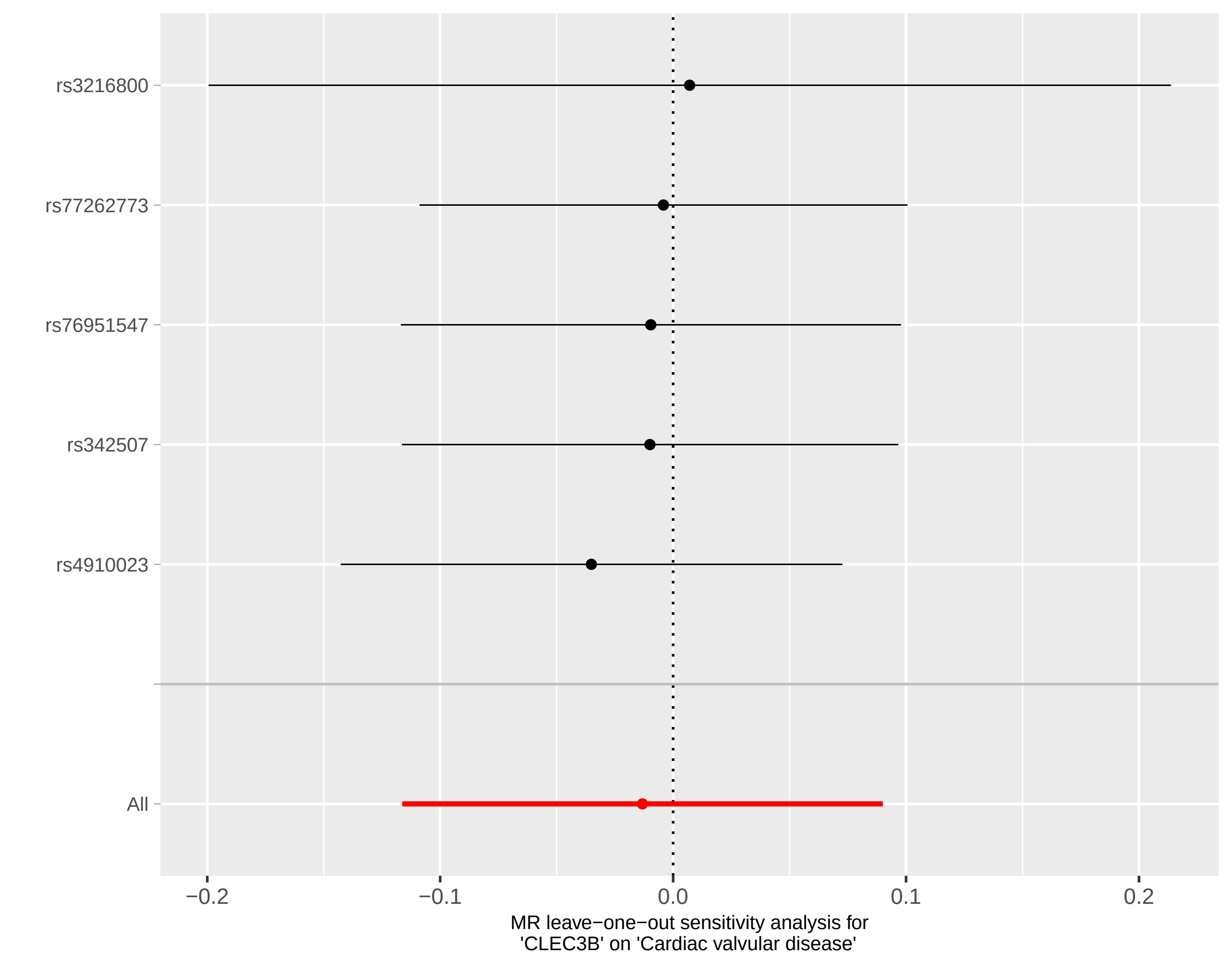

rs3216800
rs77262773
rs76951547
rs342507
rs4910023
All
−0.2
−0.1
0.0
0.1
0.2
MR leave−one−out sensitivity analysis for
'CLEC3B' on 'Cardiac valvular disease'

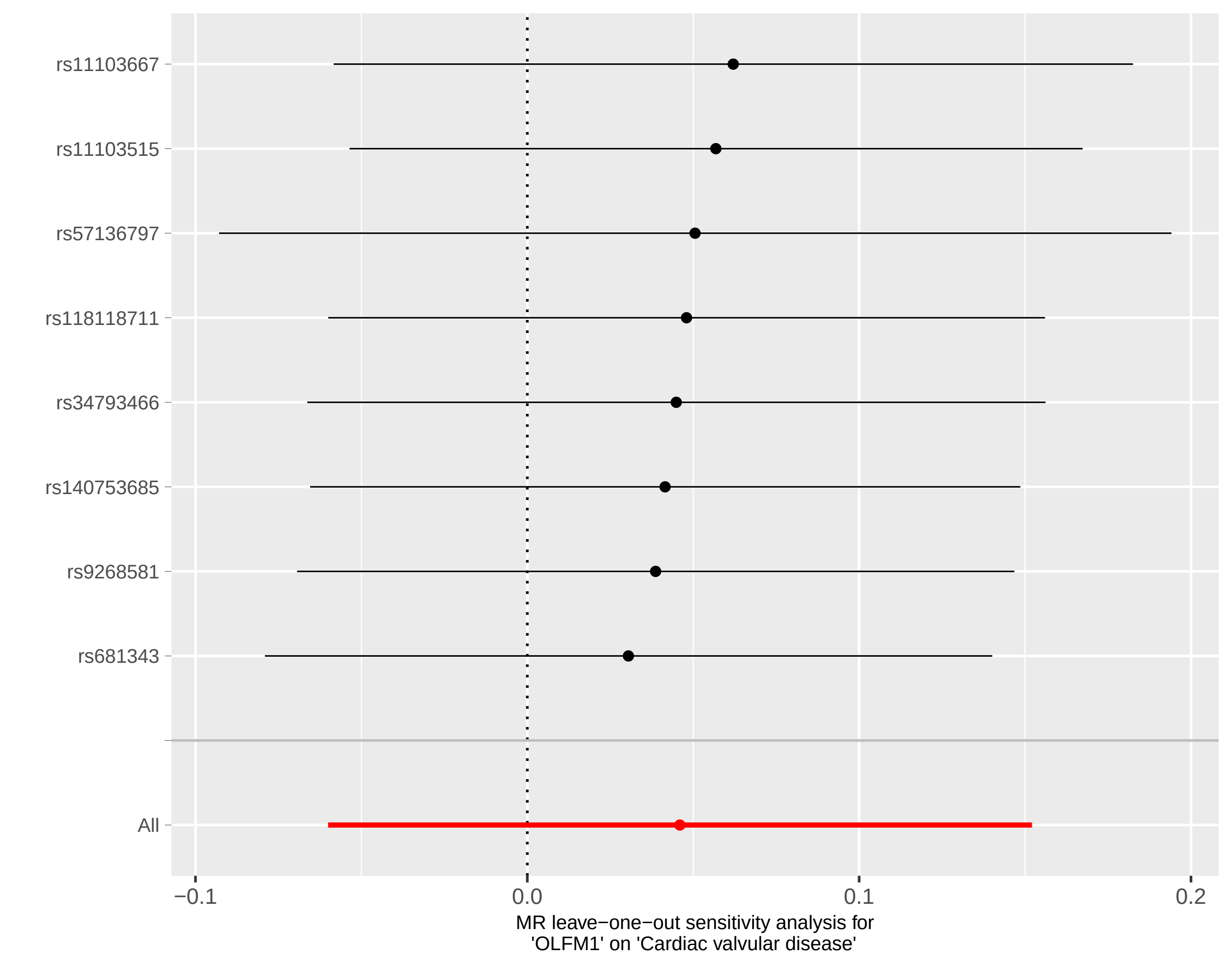

rs11103667
rs11103515
rs57136797
rs118118711
rs34793466
rs140753685
rs9268581
rs681343
All
−0.1
0.0
0.1
0.2
MR leave−one−out sensitivity analysis for
'OLFM1' on 'Cardiac valvular disease'

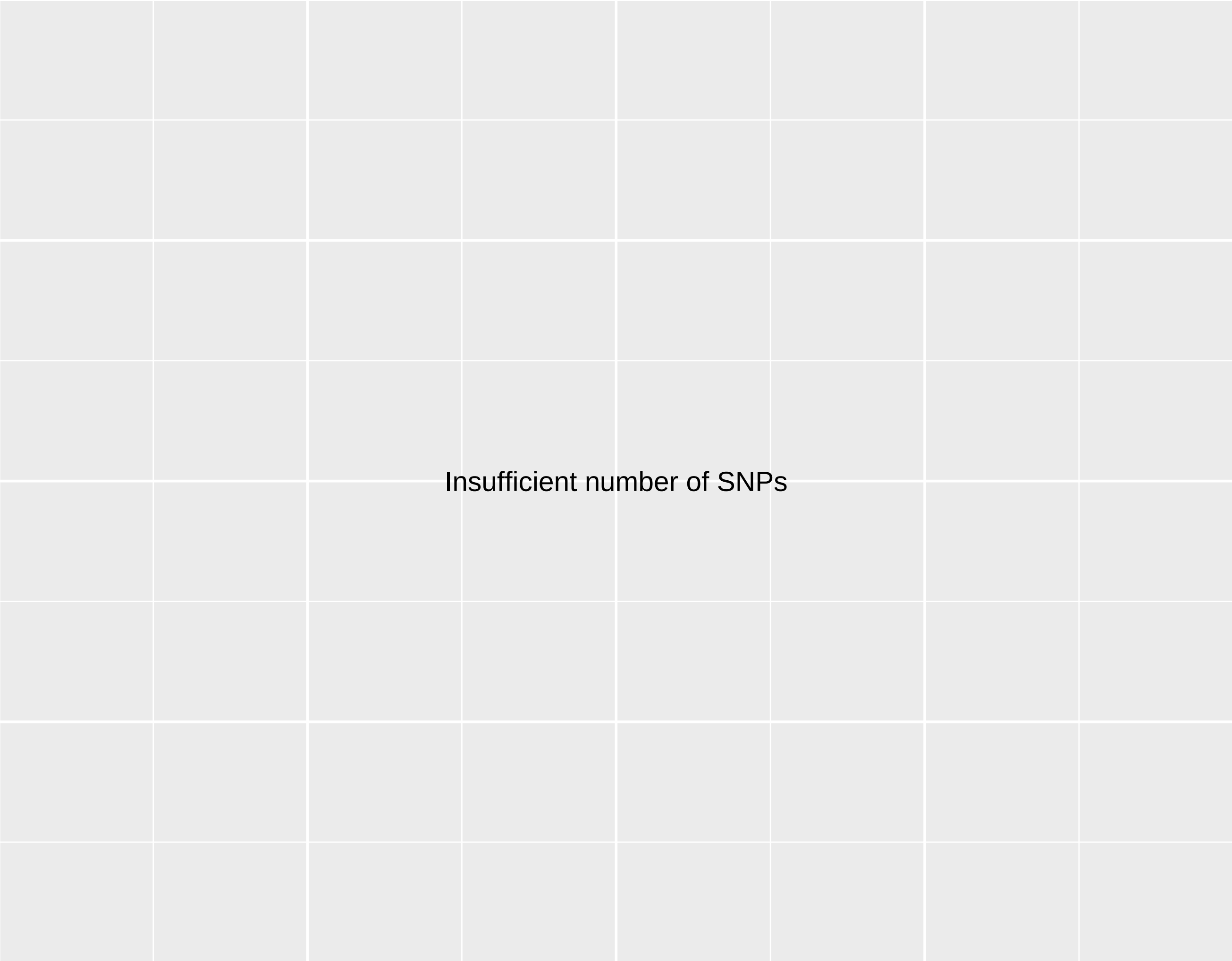
Insufficient number of SNPs

Insufficient number of SNPs

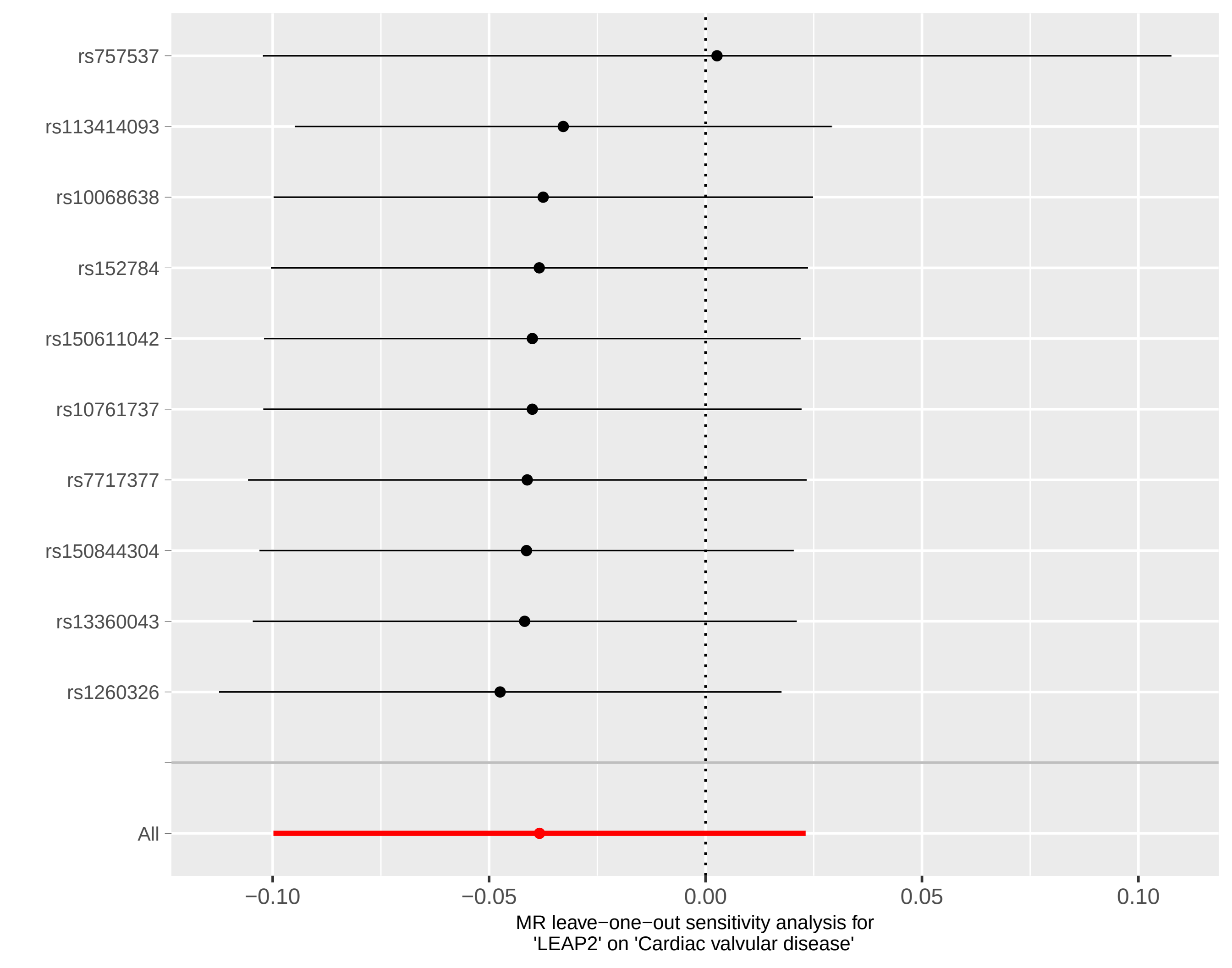
rs757537
rs113414093
rs10068638
rs152784
rs150611042
rs10761737
rs7717377
rs150844304
rs13360043
rs1260326
All
−0.10
−0.05
0.00
0.05
0.10
MR leave−one−out sensitivity analysis for
'LEAP2' on 'Cardiac valvular disease'

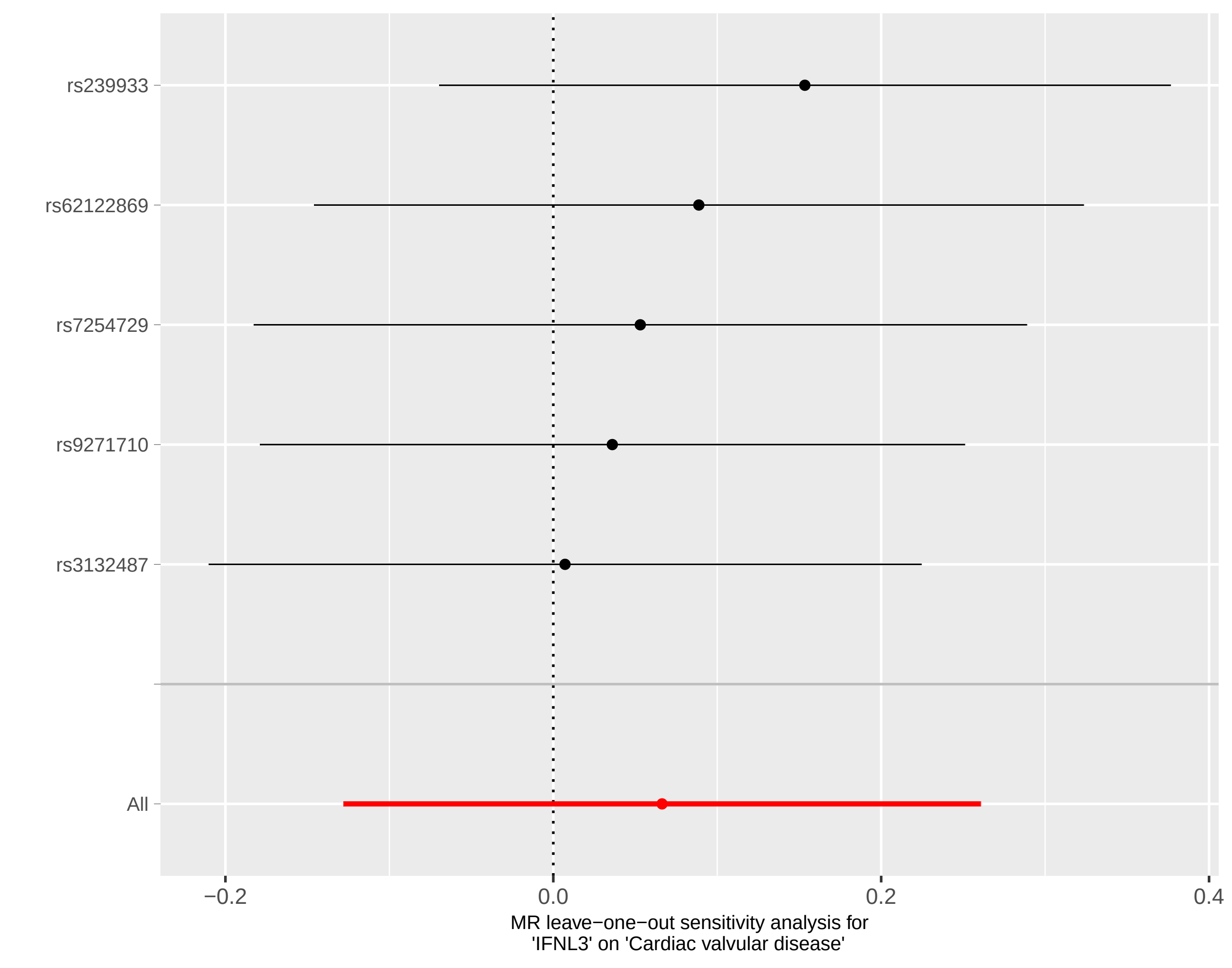

rs239933
rs62122869
rs7254729
rs9271710
rs3132487
All
−0.2
0.0
0.2
0.4
MR leave−one−out sensitivity analysis for
'IFNL3' on 'Cardiac valvular disease'

Insufficient number of SNPs

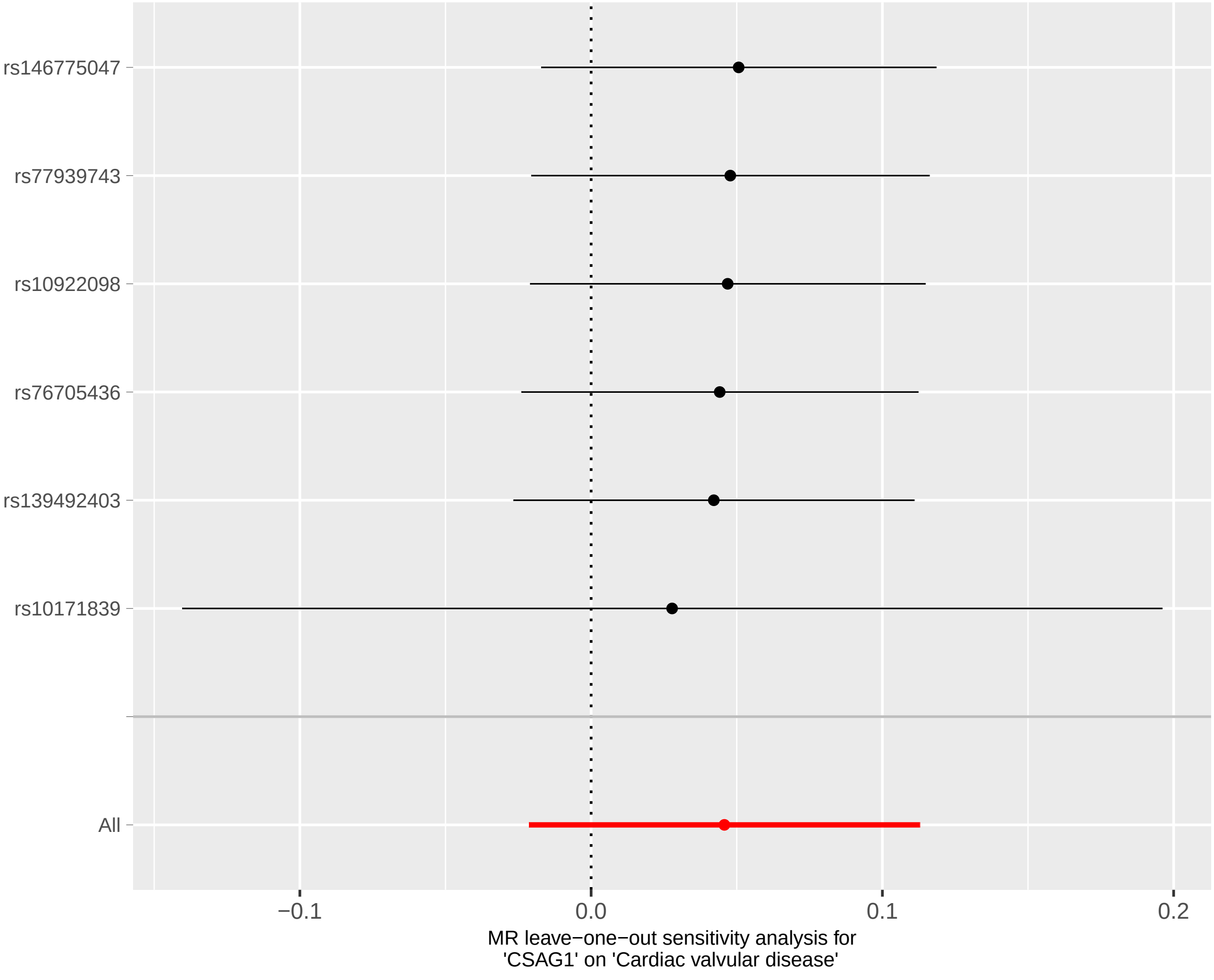
rs146775047
rs77939743
rs10922098
rs76705436
rs139492403
rs10171839
All
−0.1
0.0
0.1
0.2
MR leave−one−out sensitivity analysis for
'CSAG1' on 'Cardiac valvular disease'

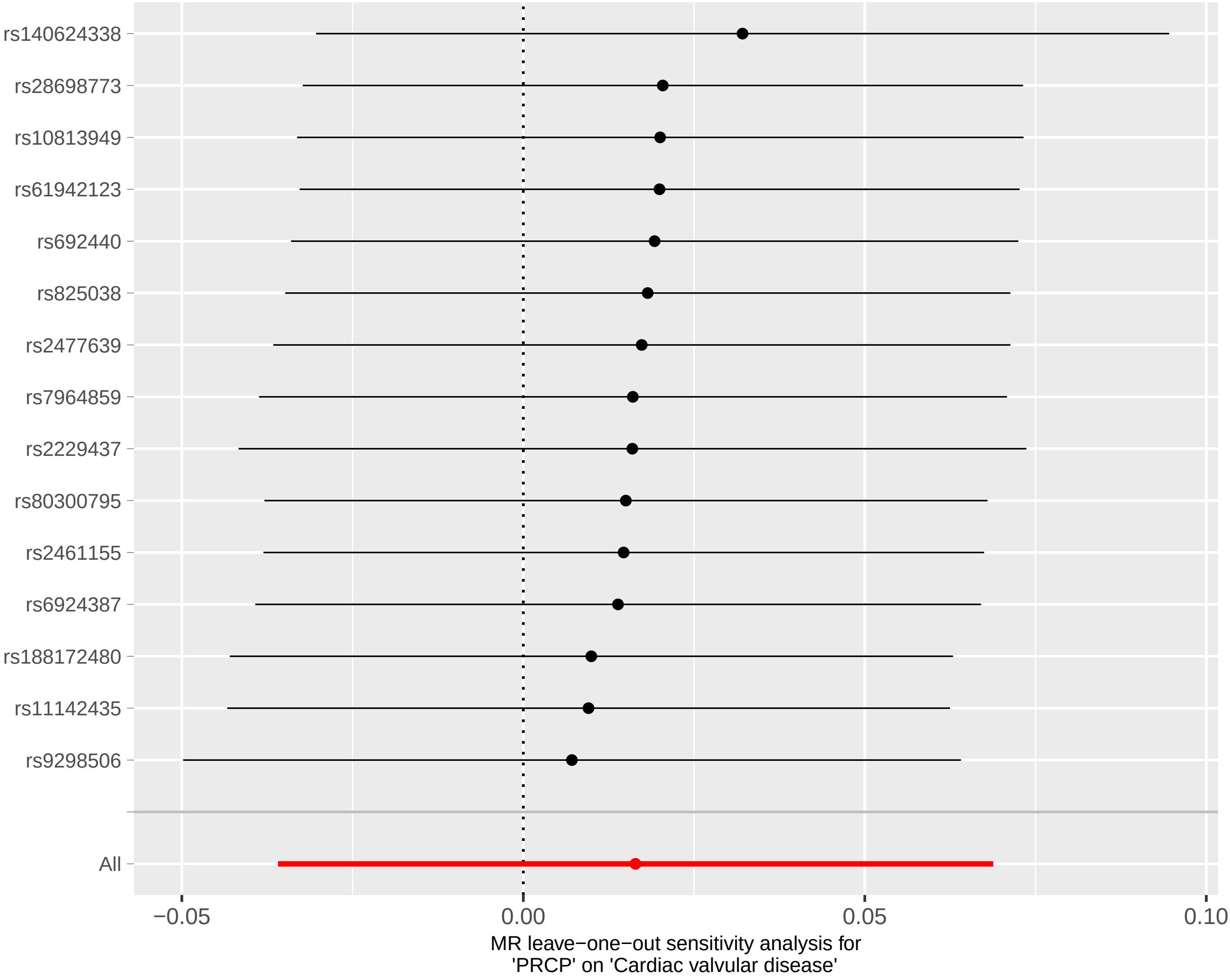
rs140624338
rs28698773
rs10813949
rs61942123
rs692440
rs825038
rs2477639
rs7964859
rs2229437
rs80300795
rs2461155
rs6924387
rs188172480
rs11142435
rs9298506
All
−0.05
0.00
0.05
0.10
MR leave−one−out sensitivity analysis for
'PRCP' on 'Cardiac valvular disease'

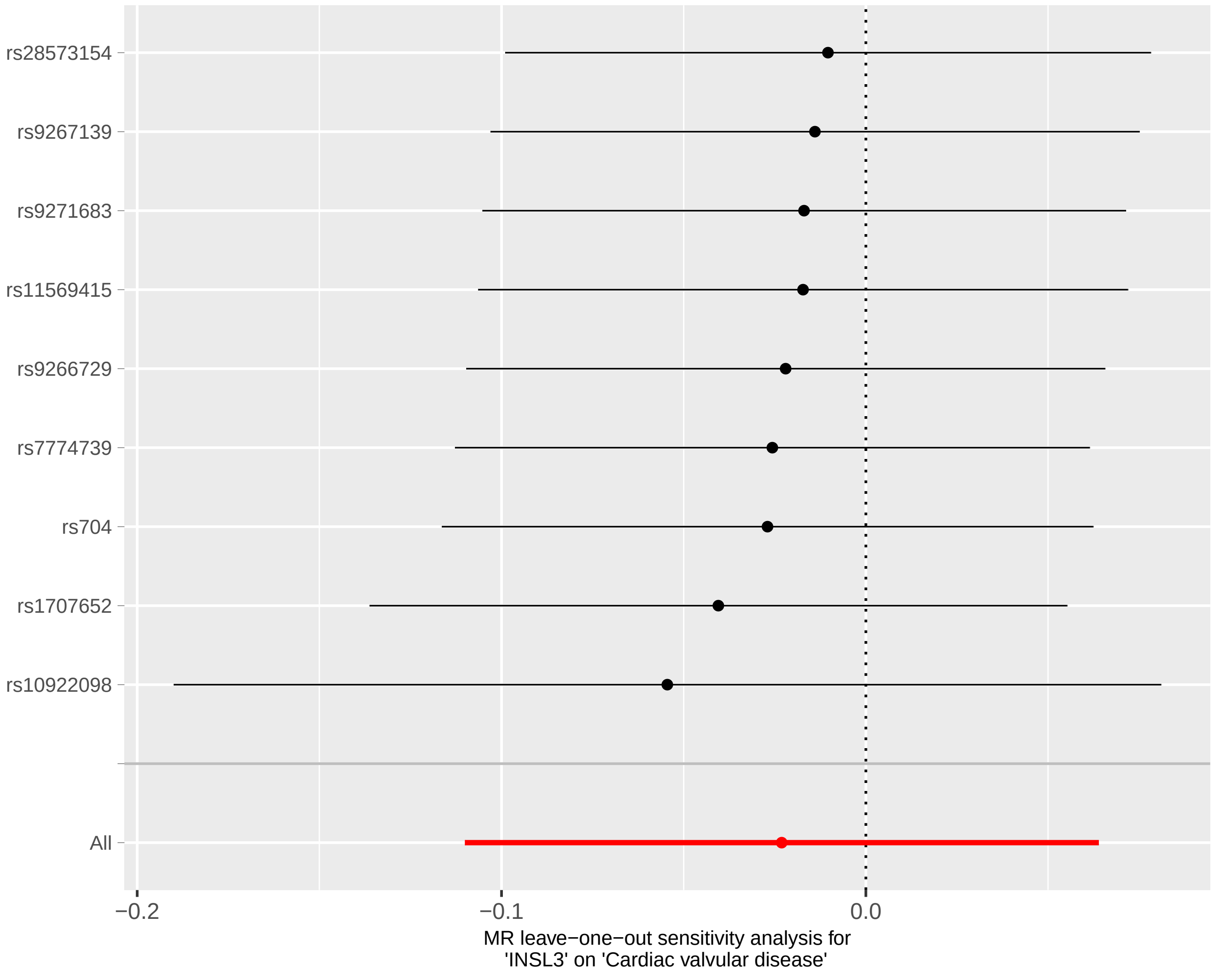

rs28573154
rs9267139
rs9271683
rs11569415
rs9266729
rs7774739
rs704
rs1707652
rs10922098
All
−0.2
−0.1
0.0
MR leave−one−out sensitivity analysis for
'INSL3' on 'Cardiac valvular disease'

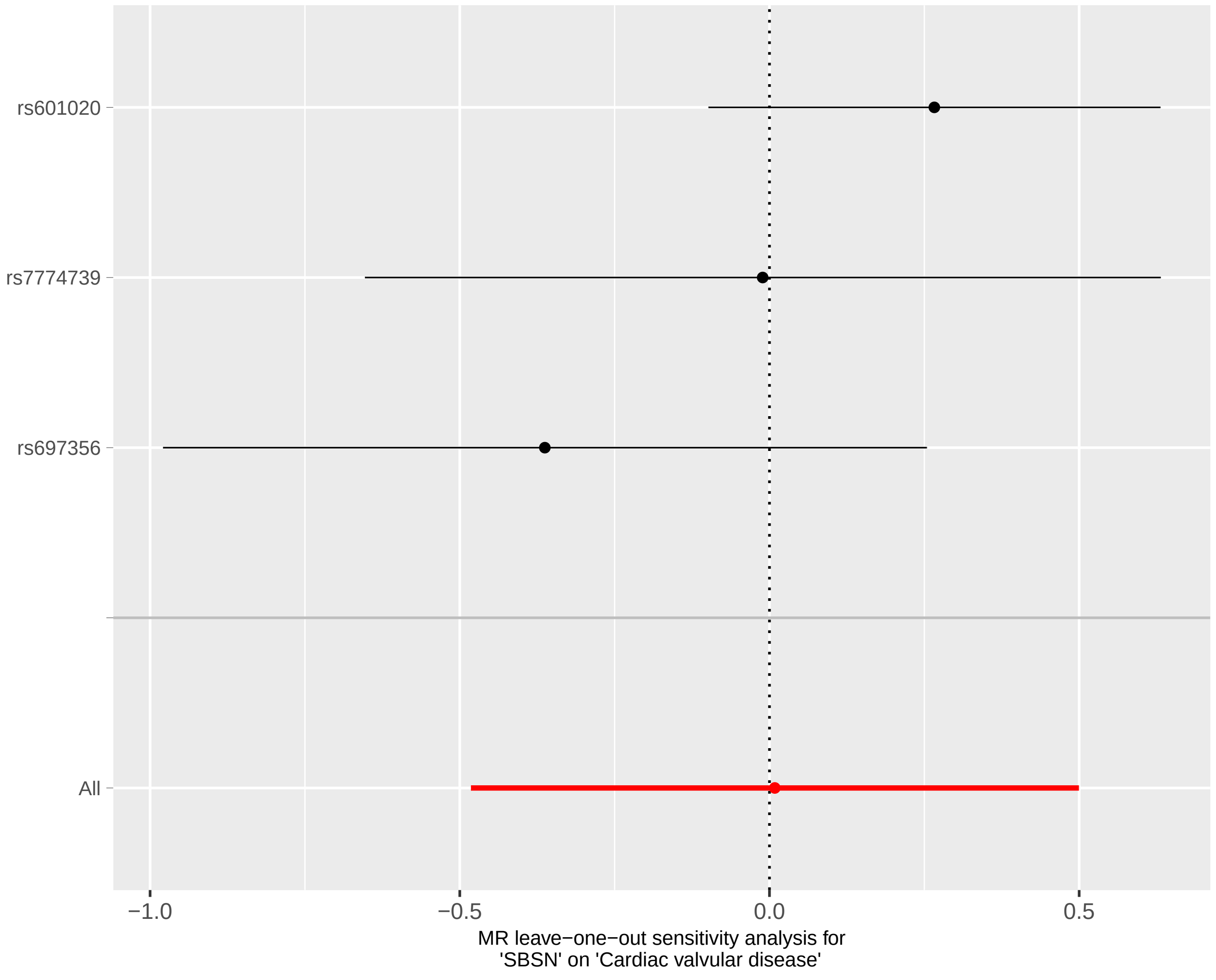
rs601020
rs7774739
rs697356
All
−1.0
−0.5
0.0
0.5
MR leave−one−out sensitivity analysis for
'SBSN' on 'Cardiac valvular disease'

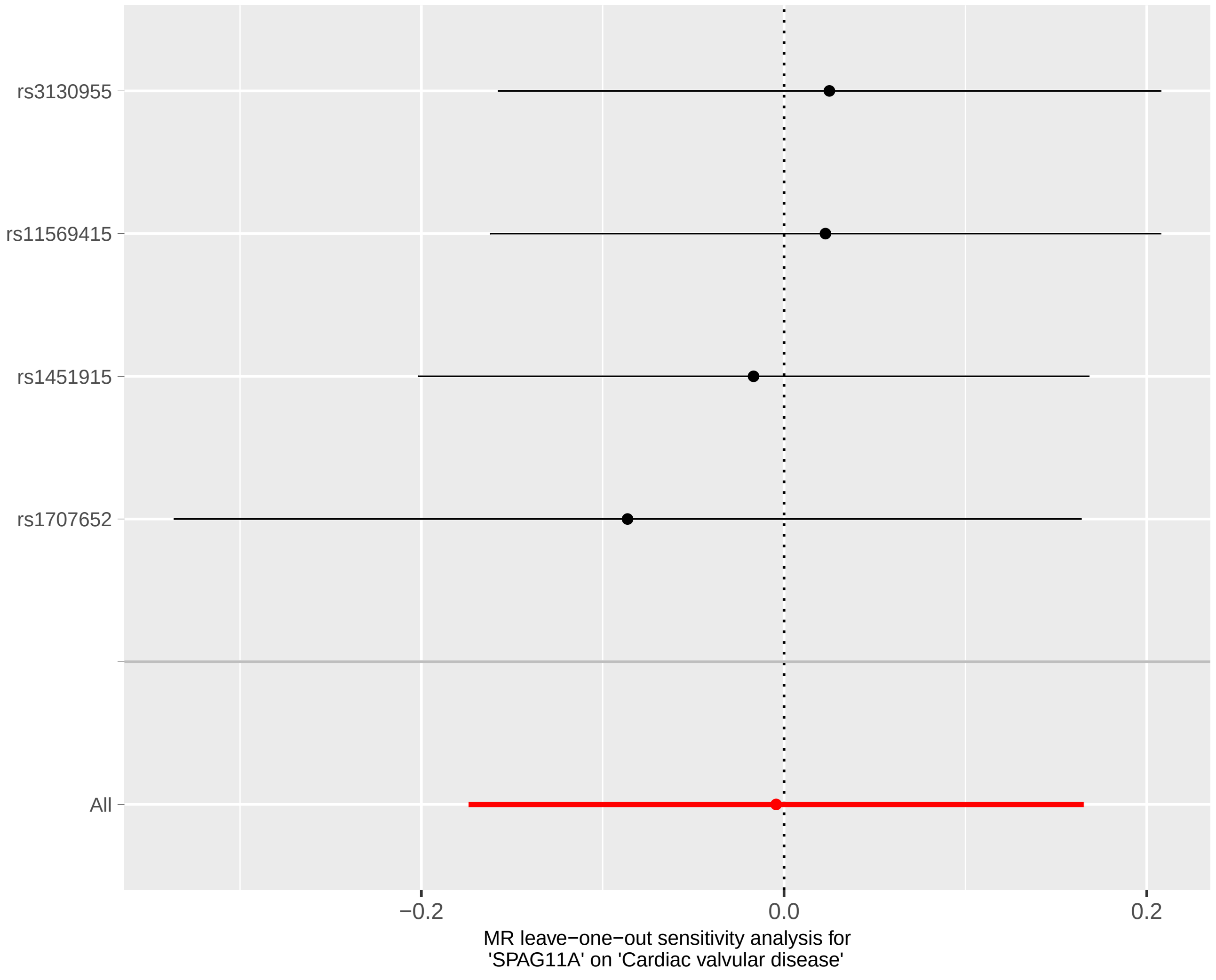
rs3130955
rs11569415
rs1451915
rs1707652
All
−0.2
0.0
0.2
MR leave−one−out sensitivity analysis for
'SPAG11A' on 'Cardiac valvular disease'

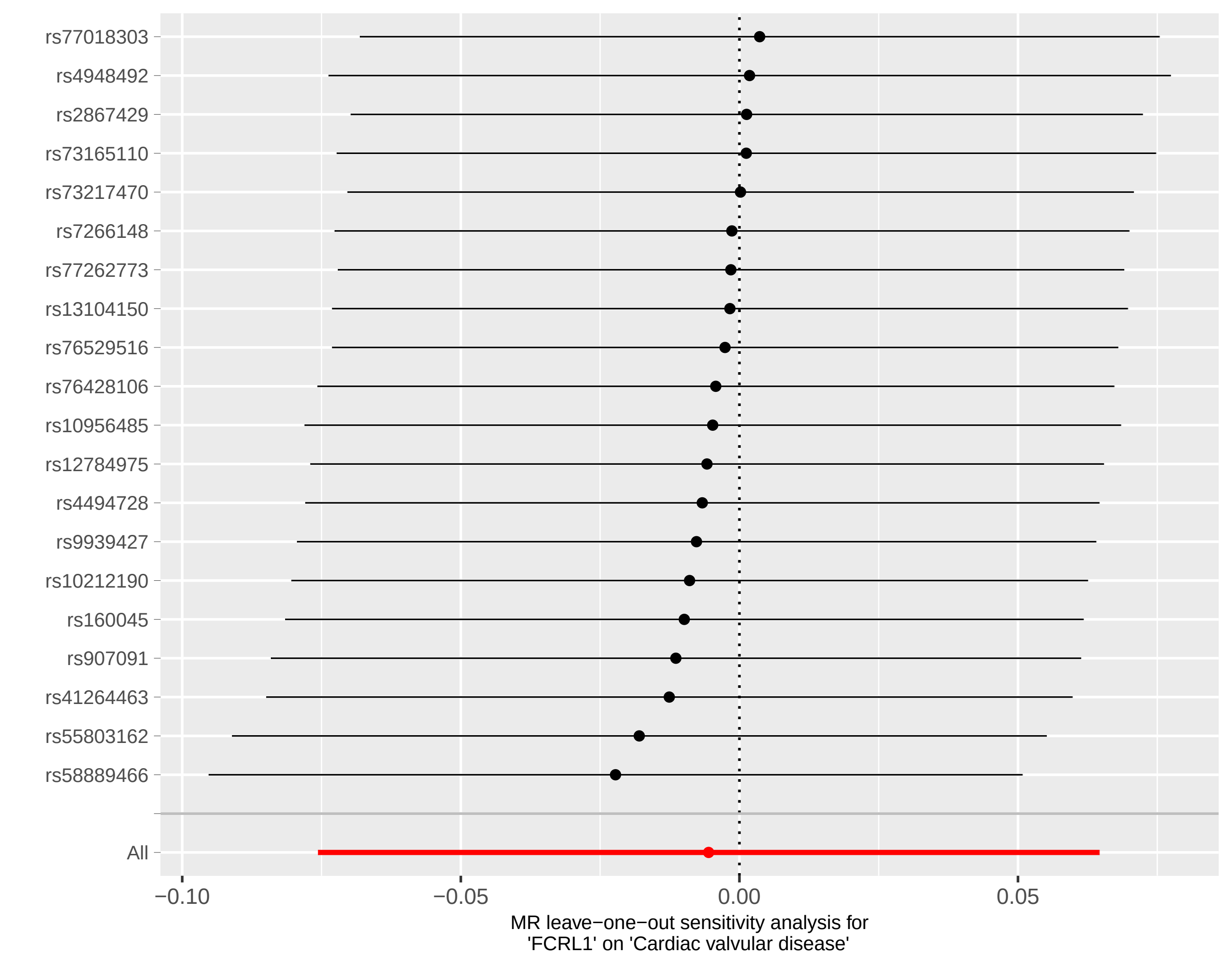
rs77018303
rs4948492
rs2867429
rs73165110
rs73217470
rs7266148
rs77262773
rs13104150
rs76529516
rs76428106
rs10956485
rs12784975
rs4494728
rs9939427
rs10212190
rs160045
rs907091
rs41264463
rs55803162
rs58889466
All
−0.10
−0.05
0.00
0.05
MR leave−one−out sensitivity analysis for
'FCRL1' on 'Cardiac valvular disease'

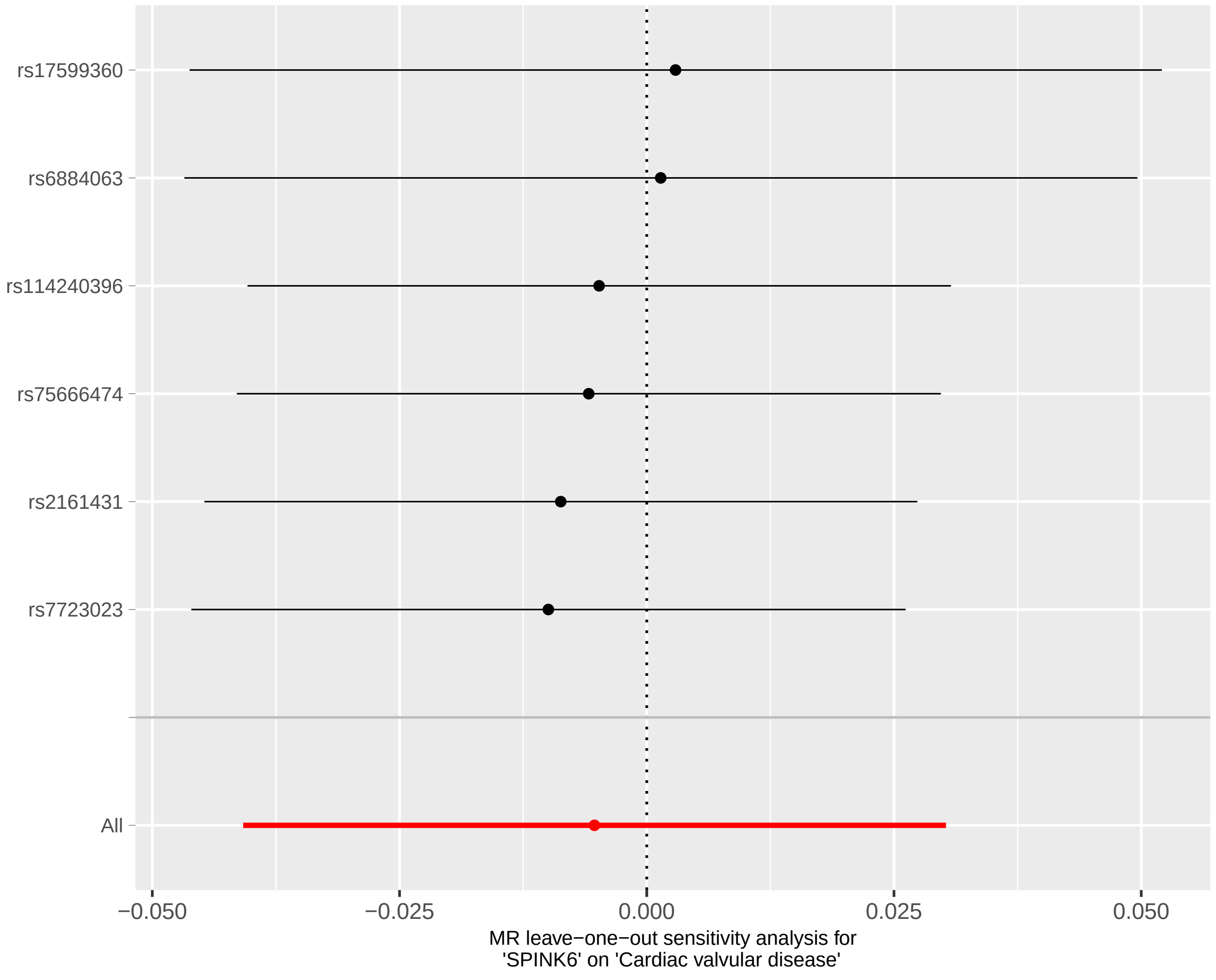
rs17599360
rs6884063
rs114240396
rs75666474
rs2161431
rs7723023
All
−0.050
−0.025
0.000
0.025
0.050
MR leave−one−out sensitivity analysis for
'SPINK6' on 'Cardiac valvular disease'

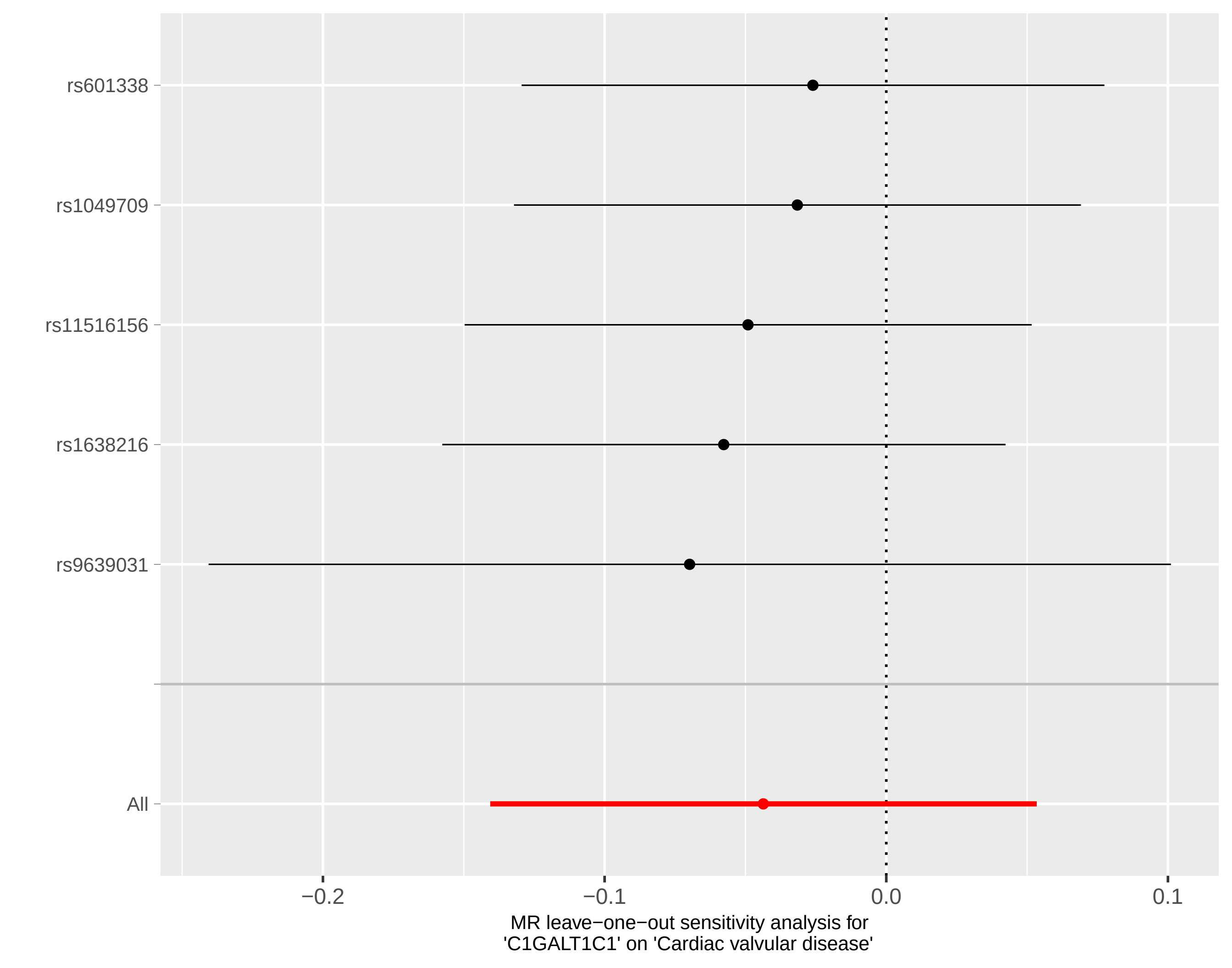
rs601338
rs1049709
rs11516156
rs1638216
rs9639031
All
−0.2
−0.1
0.0
0.1
MR leave−one−out sensitivity analysis for
'C1GALT1C1' on 'Cardiac valvular disease'

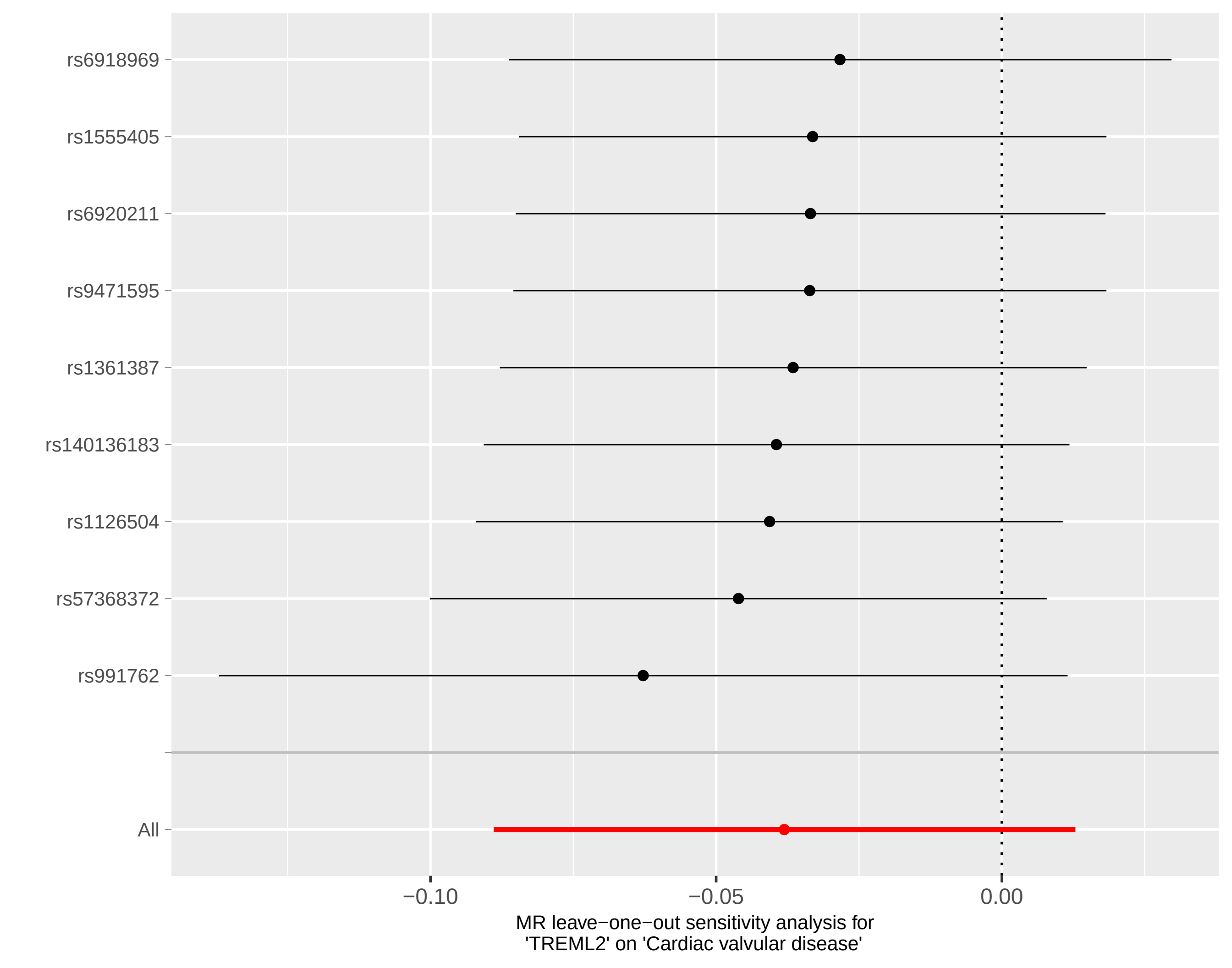
rs6918969
rs1555405
rs6920211
rs9471595
rs1361387
rs140136183
rs1126504
rs57368372
rs991762
All
−0.10
−0.05
0.00
MR leave−one−out sensitivity analysis for
'TREML2' on 'Cardiac valvular disease'

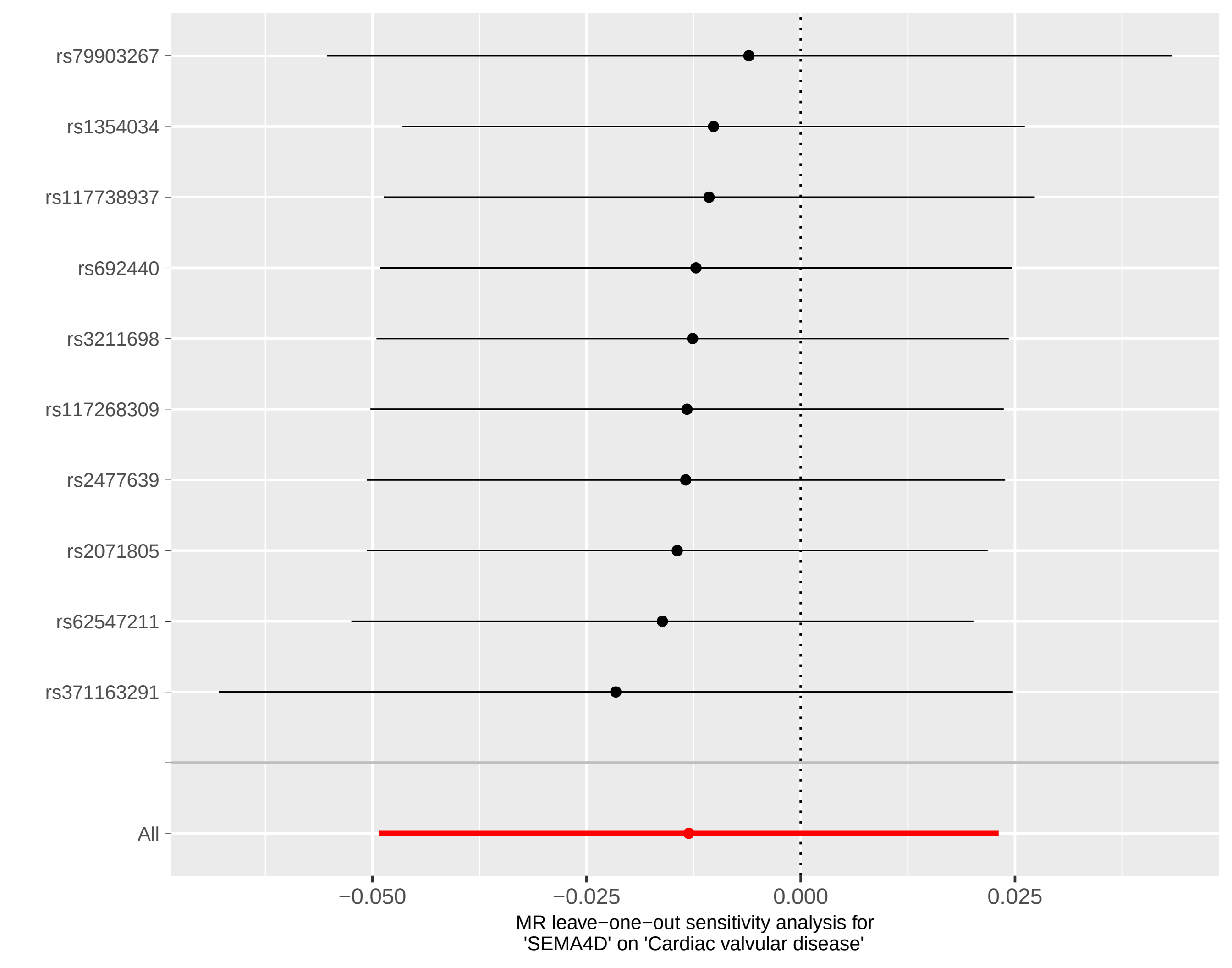
rs79903267
rs1354034
rs117738937
rs692440
rs3211698
rs117268309
rs2477639
rs2071805
rs62547211
rs371163291
All
−0.050
−0.025
0.000
0.025
MR leave−one−out sensitivity analysis for
'SEMA4D' on 'Cardiac valvular disease'

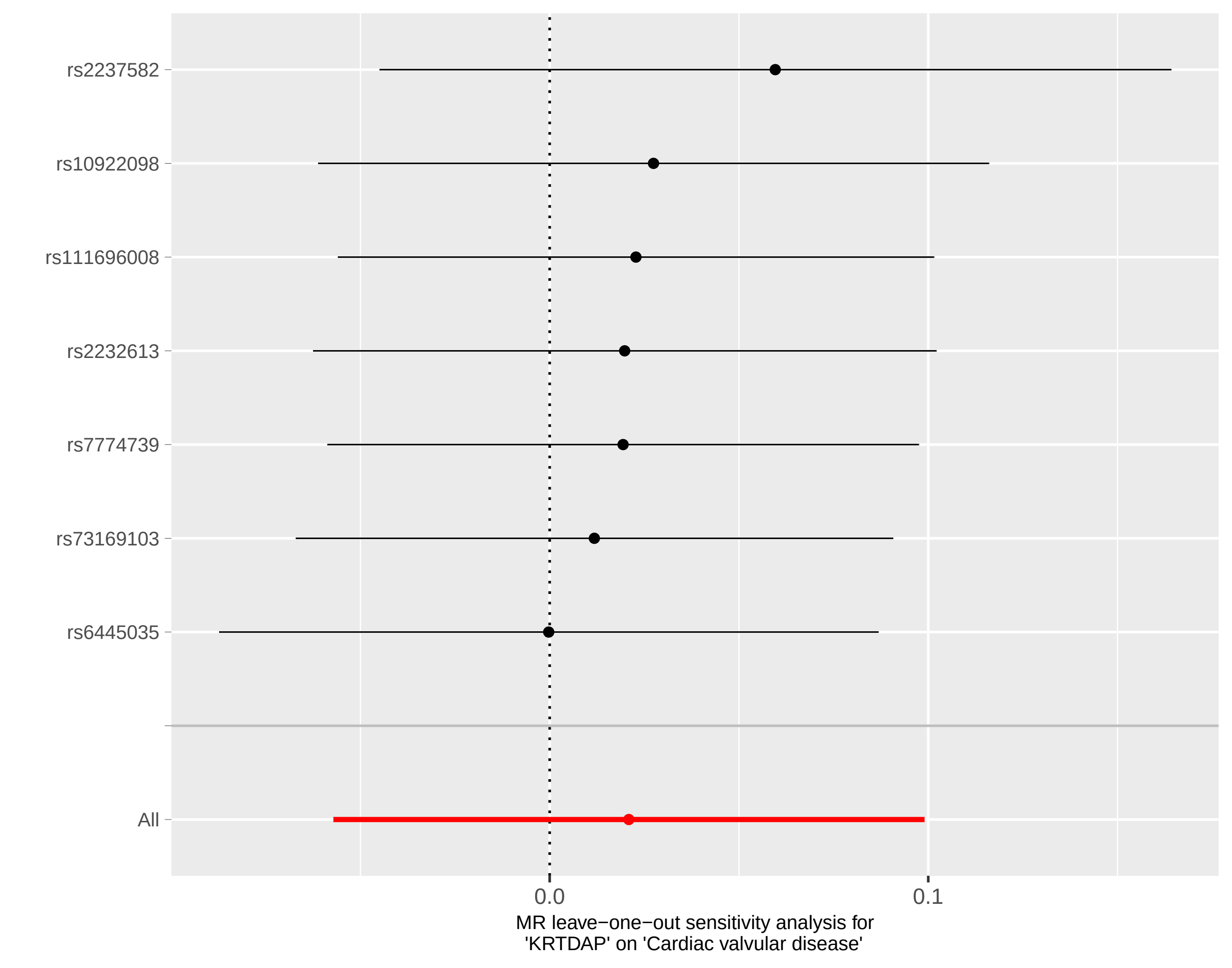
rs2237582
rs10922098
rs111696008
rs2232613
rs7774739
rs73169103
rs6445035
All
0.0
0.1
MR leave−one−out sensitivity analysis for
'KRTDAP' on 'Cardiac valvular disease'

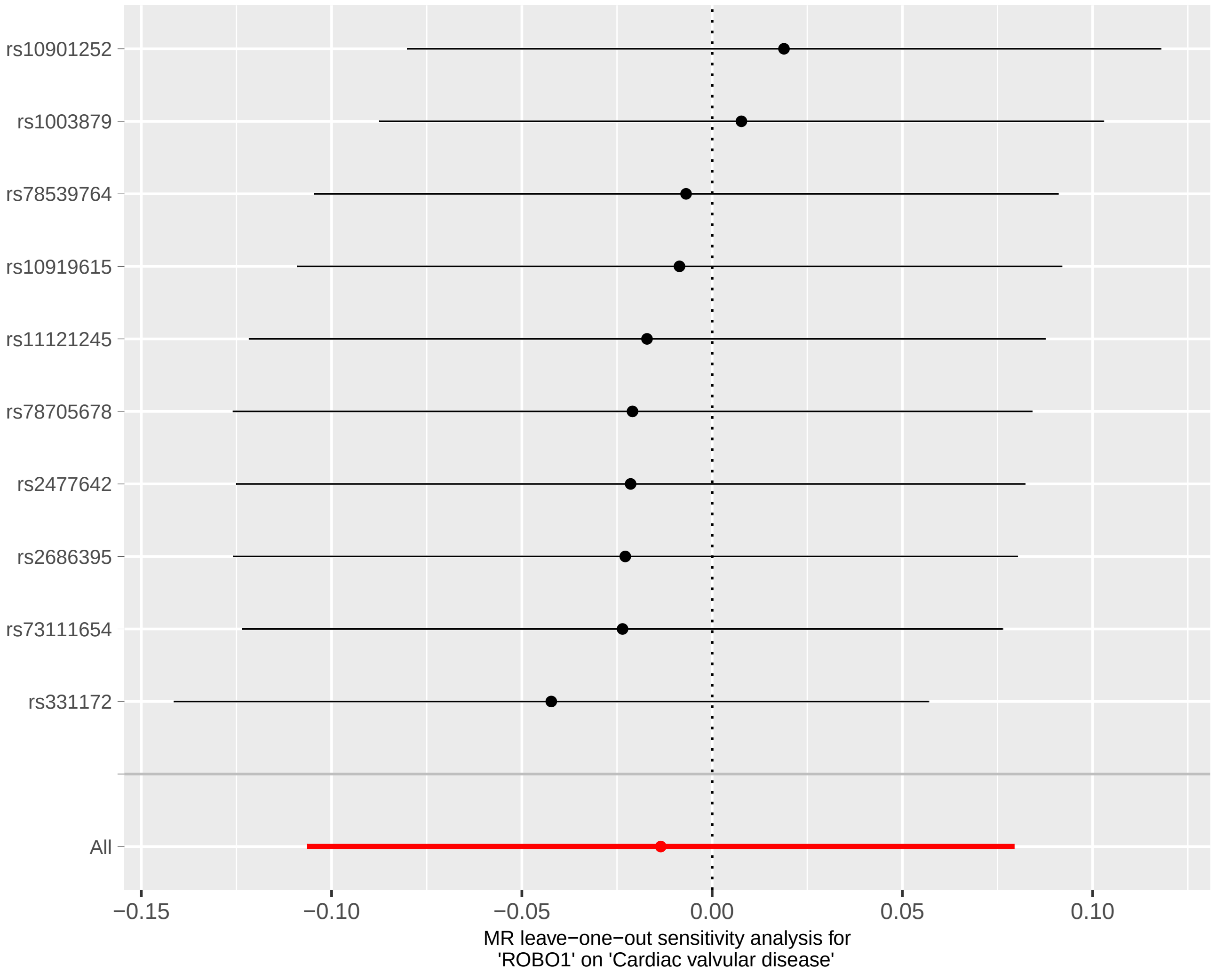
rs10901252
rs1003879
rs78539764
rs10919615
rs11121245
rs78705678
rs2477642
rs2686395
rs73111654
rs331172
All
−0.15
−0.10
−0.05
0.00
0.05
0.10
MR leave−one−out sensitivity analysis for
'ROBO1' on 'Cardiac valvular disease'

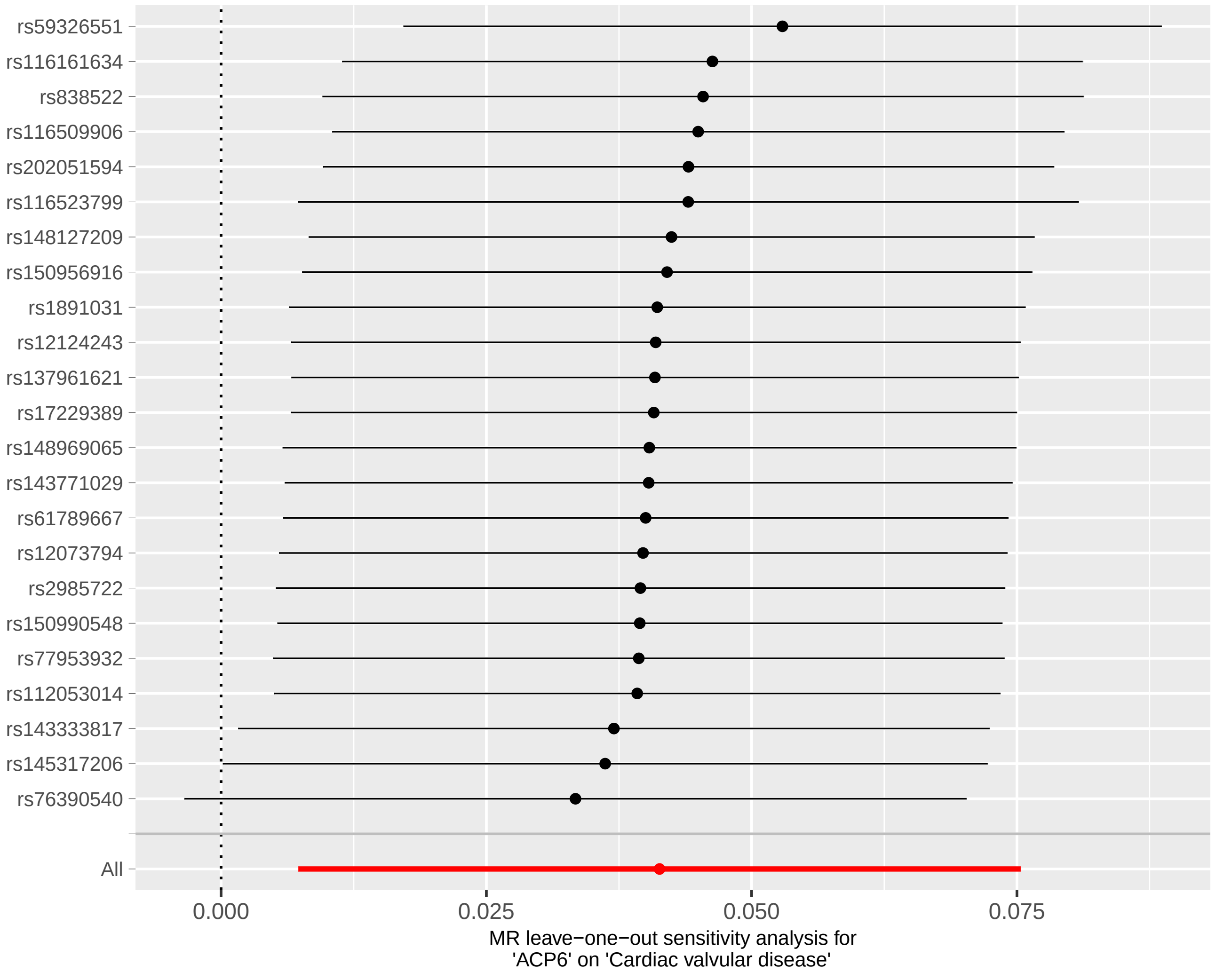
rs59326551
rs116161634
rs838522
rs116509906
rs202051594
rs116523799
rs148127209
rs150956916
rs1891031
rs12124243
rs137961621
rs17229389
rs148969065
rs143771029
rs61789667
rs12073794
rs2985722
rs150990548
rs77953932
rs112053014
rs143333817
rs145317206
rs76390540
All
0.000
0.025
0.050
0.075
MR leave−one−out sensitivity analysis for
'ACP6' on 'Cardiac valvular disease'

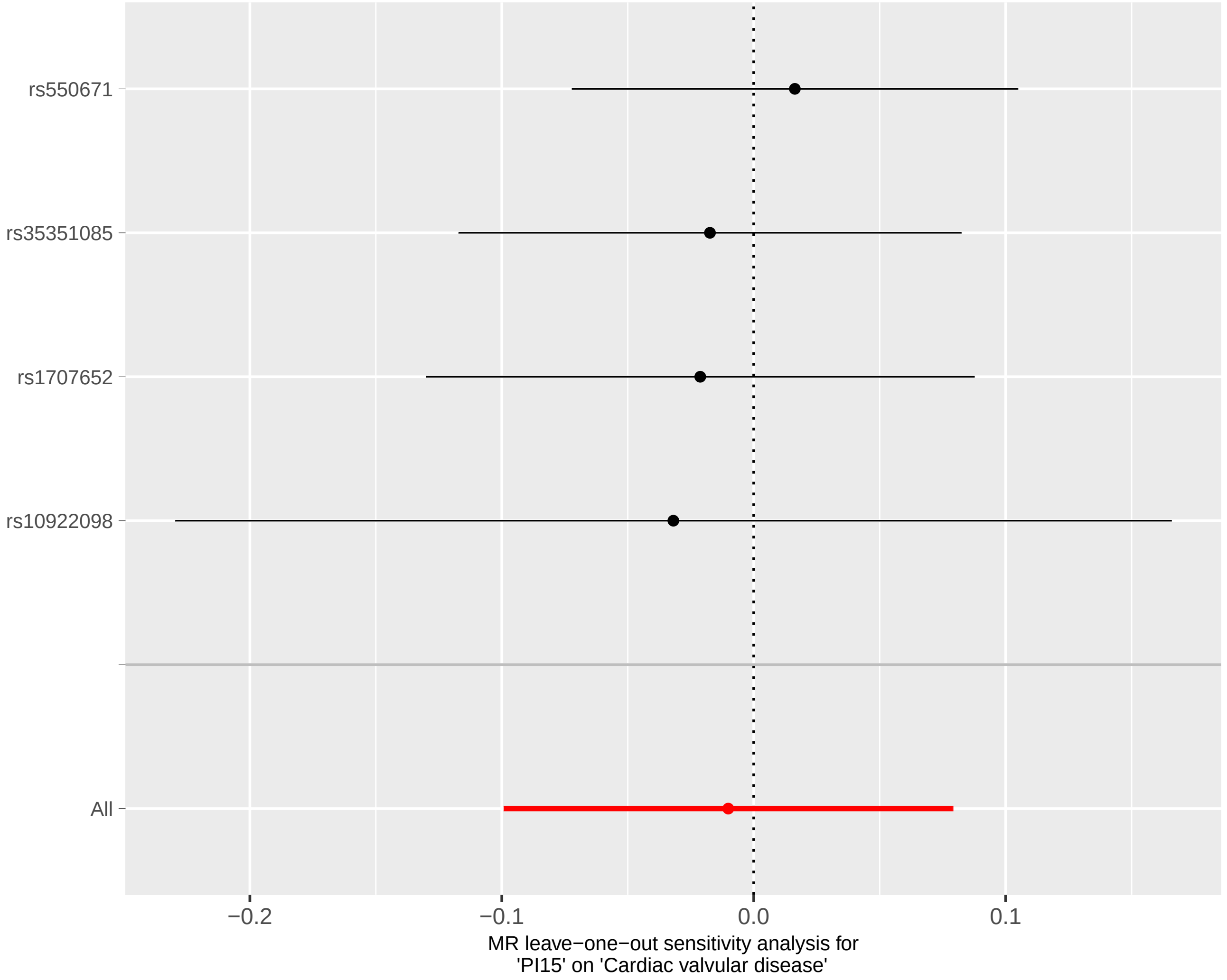
rs550671
rs35351085
rs1707652
rs10922098
All
−0.2
−0.1
0.0
0.1
MR leave−one−out sensitivity analysis for
'PI15' on 'Cardiac valvular disease'

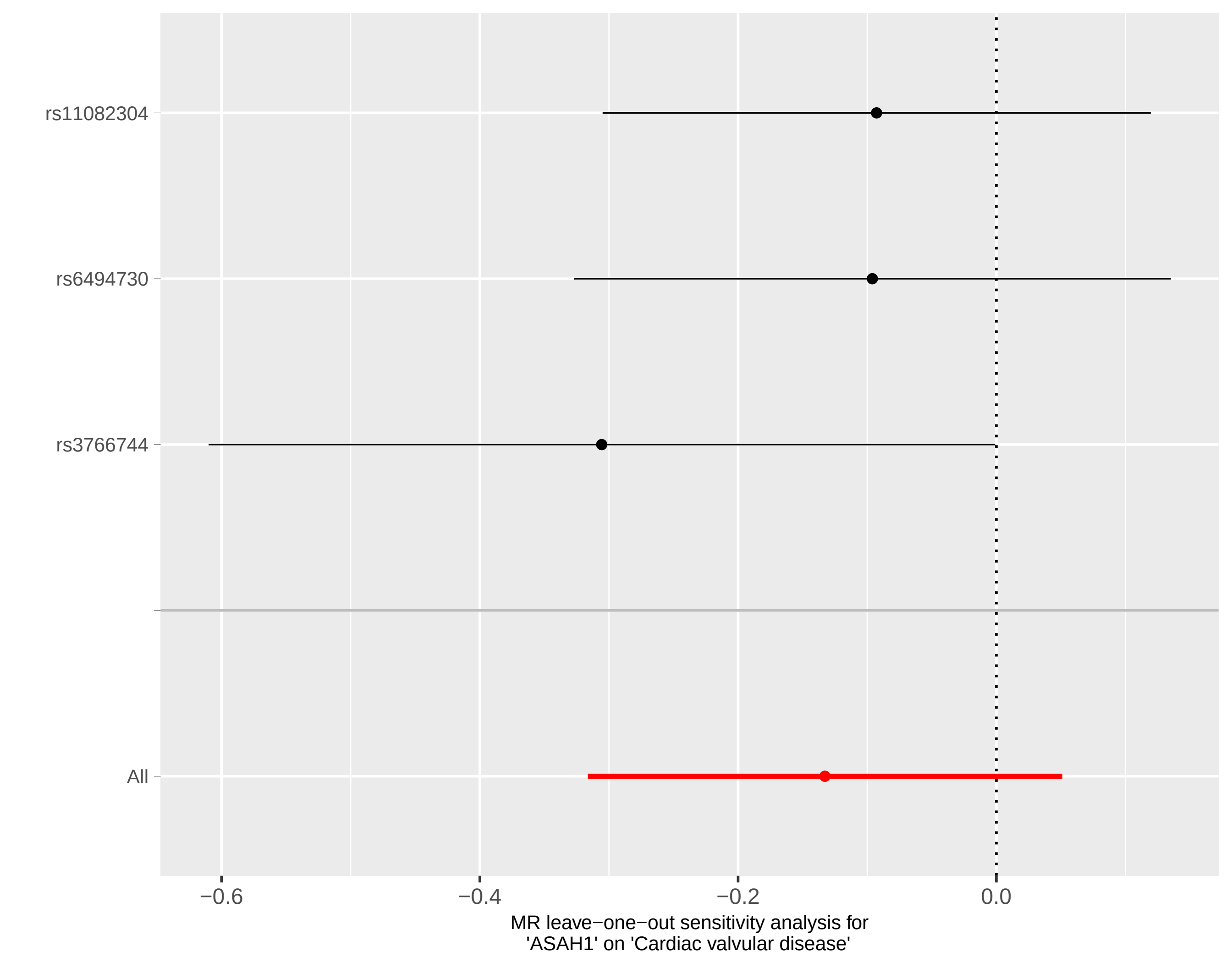
rs11082304
rs6494730
rs3766744
All
−0.6
−0.4
−0.2
0.0
MR leave−one−out sensitivity analysis for
'ASAH1' on 'Cardiac valvular disease'

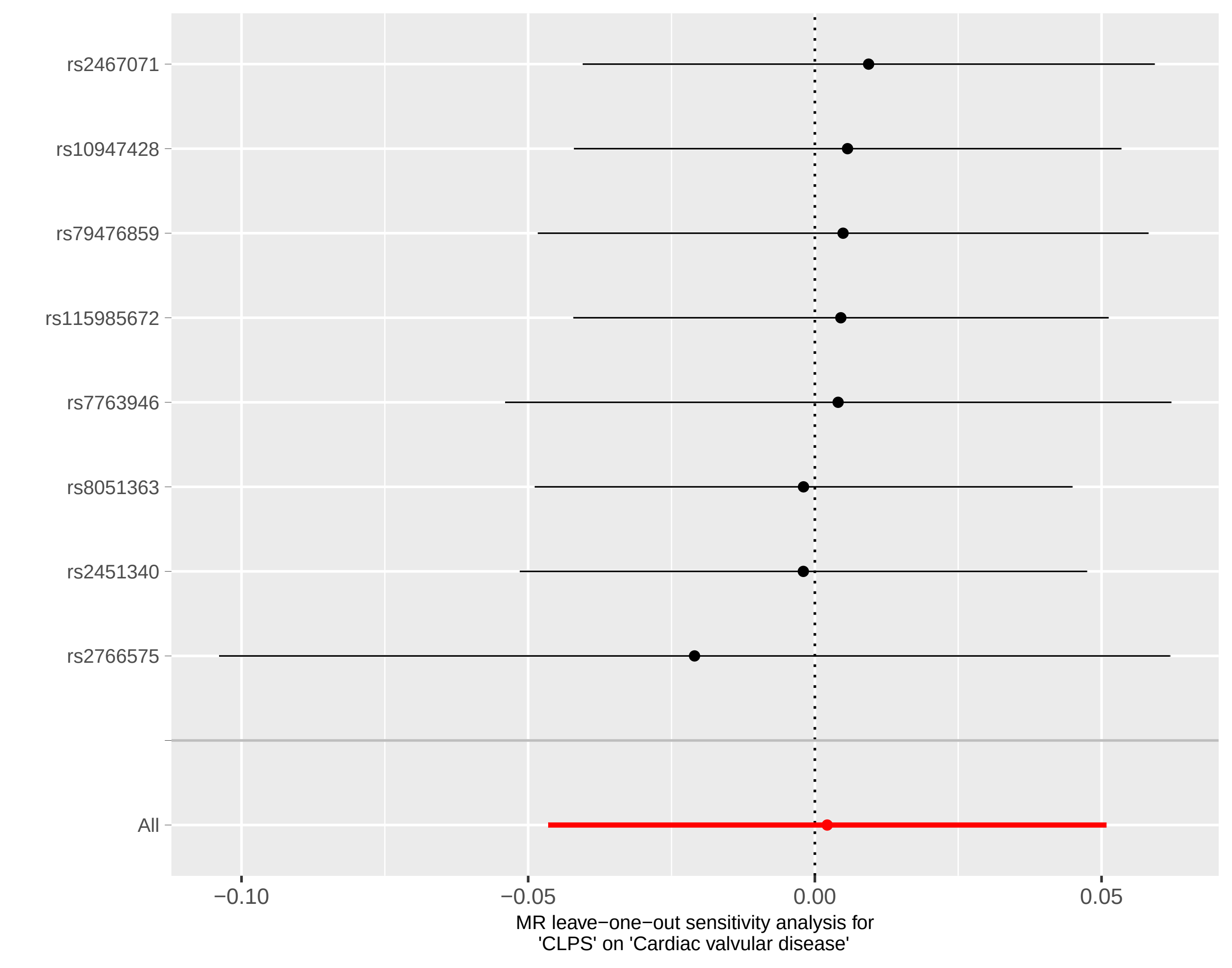

rs2467071
rs10947428
rs79476859
rs115985672
rs7763946
rs8051363
rs2451340
rs2766575
All
−0.10
−0.05
0.00
0.05
MR leave−one−out sensitivity analysis for
'CLPS' on 'Cardiac valvular disease'

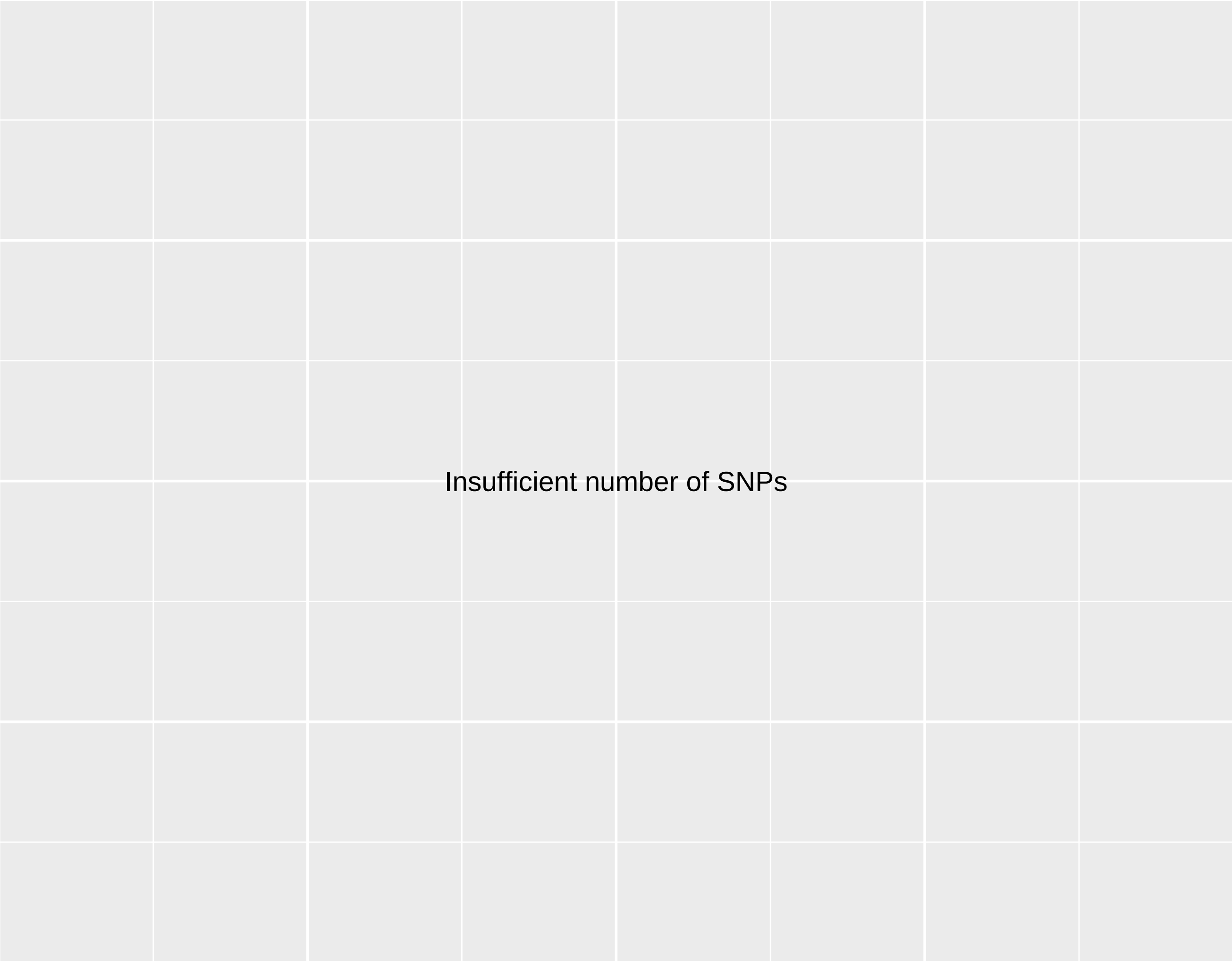
Insufficient number of SNPs

Insufficient number of SNPs

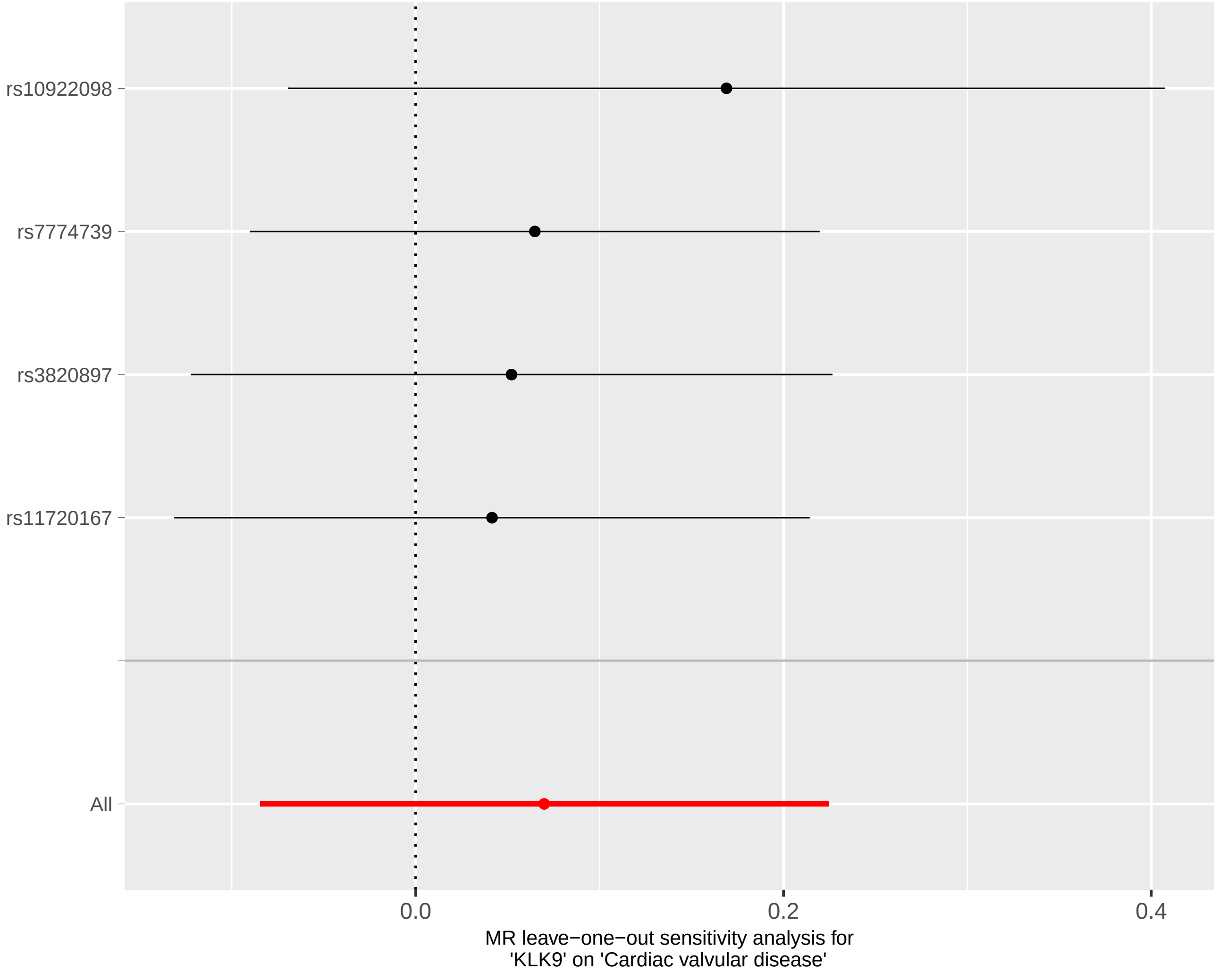
rs10922098
rs7774739
rs3820897
rs11720167
All
0.0
0.2
0.4
MR leave−one−out sensitivity analysis for
'KLK9' on 'Cardiac valvular disease'

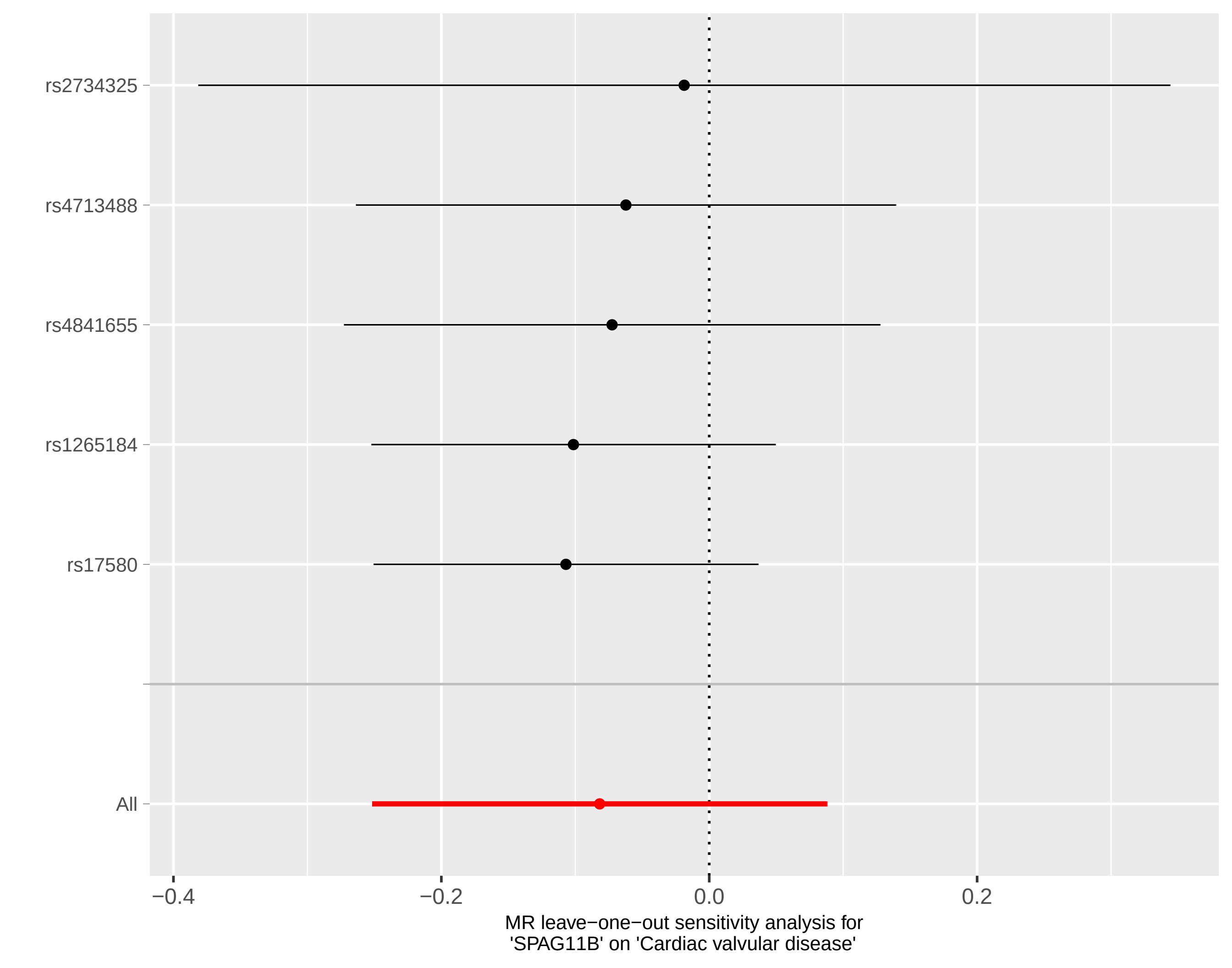

rs2734325
rs4713488
rs4841655
rs1265184
rs17580
All
−0.4
−0.2
0.0
0.2
MR leave−one−out sensitivity analysis for
'SPAG11B' on 'Cardiac valvular disease'

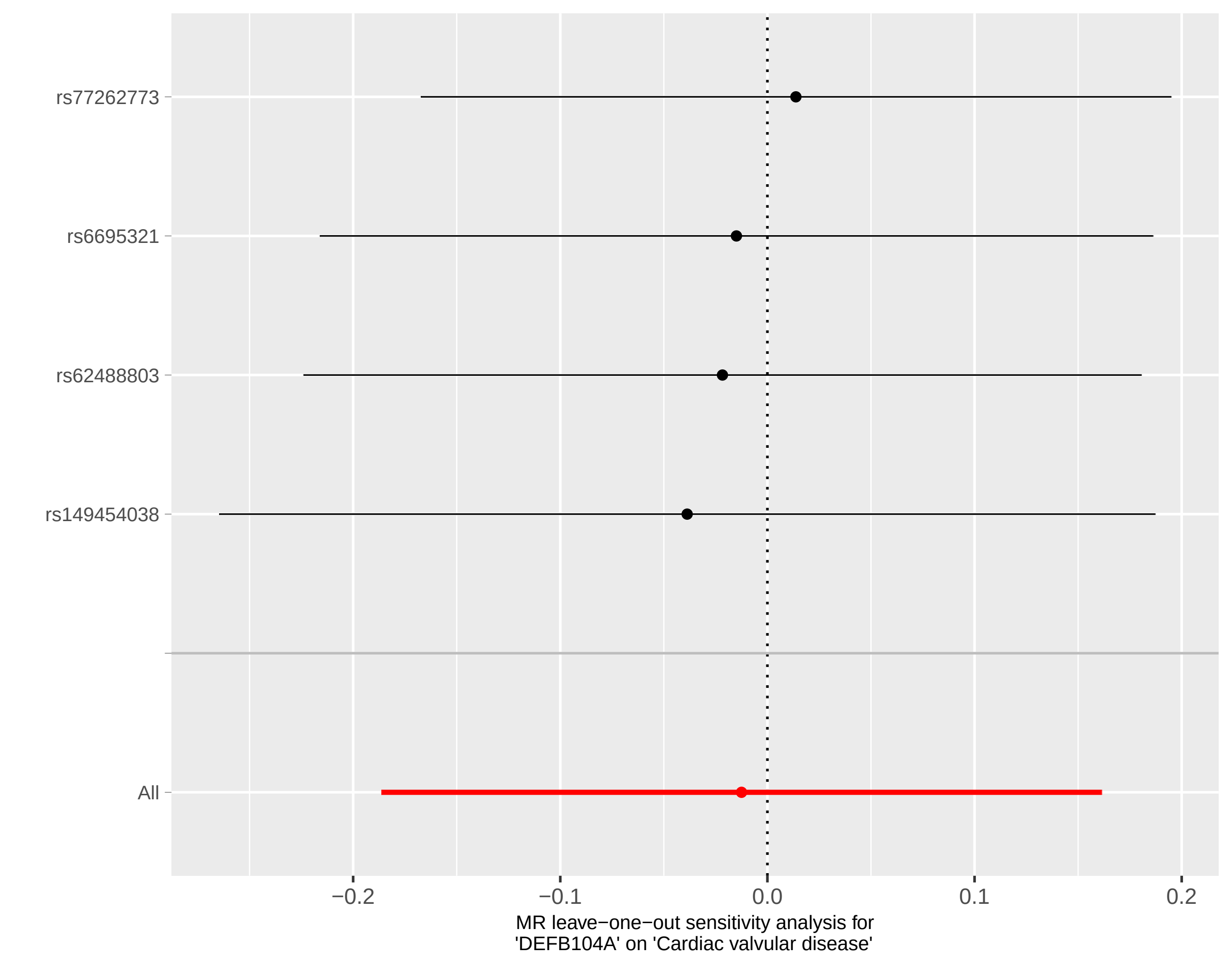
rs77262773
rs6695321
rs62488803
rs149454038
All
−0.2
−0.1
0.0
0.1
0.2
MR leave−one−out sensitivity analysis for
'DEFB104A' on 'Cardiac valvular disease'

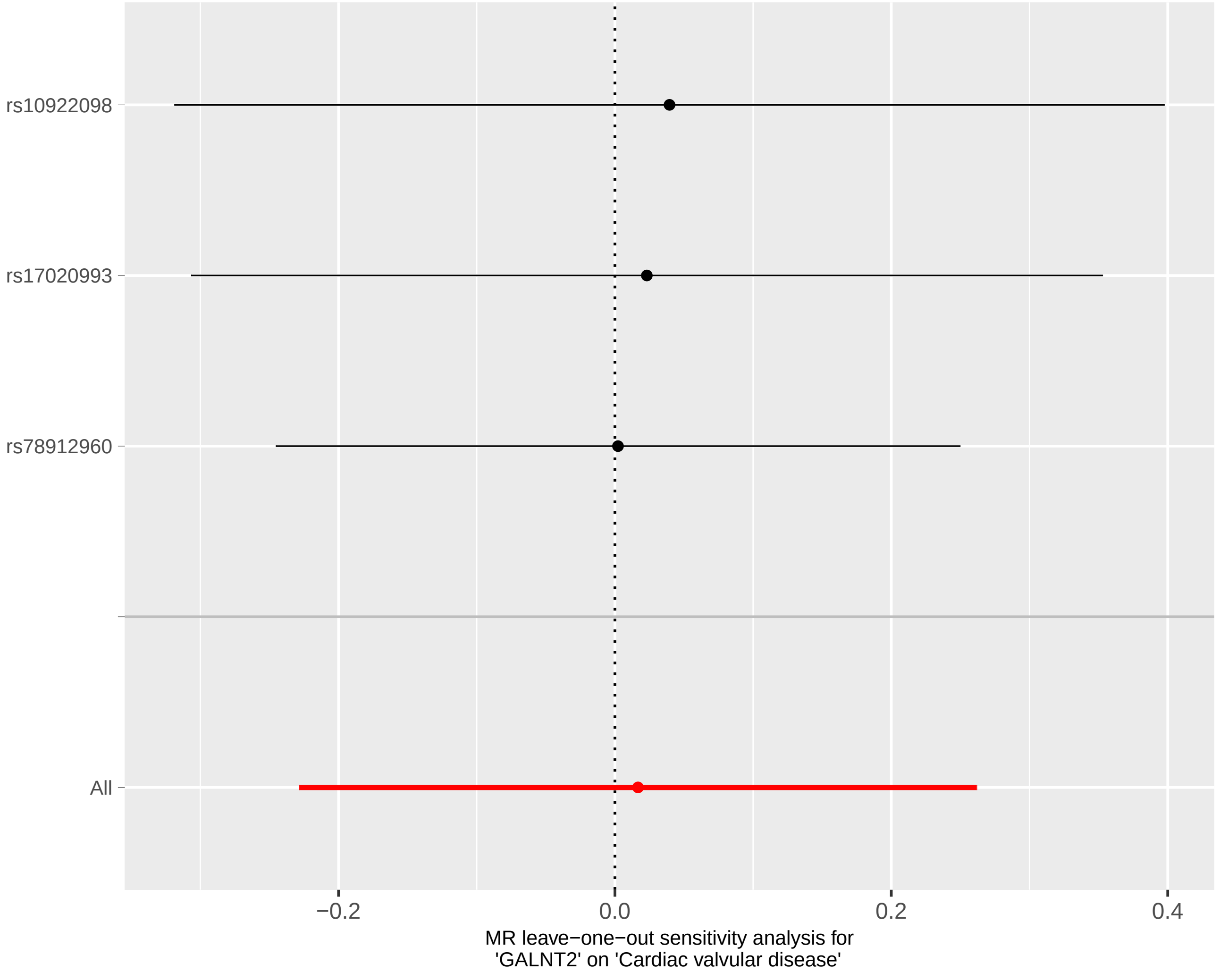
rs10922098
rs17020993
rs78912960
All
−0.2
0.0
0.2
0.4
MR leave−one−out sensitivity analysis for
'GALNT2' on 'Cardiac valvular disease'

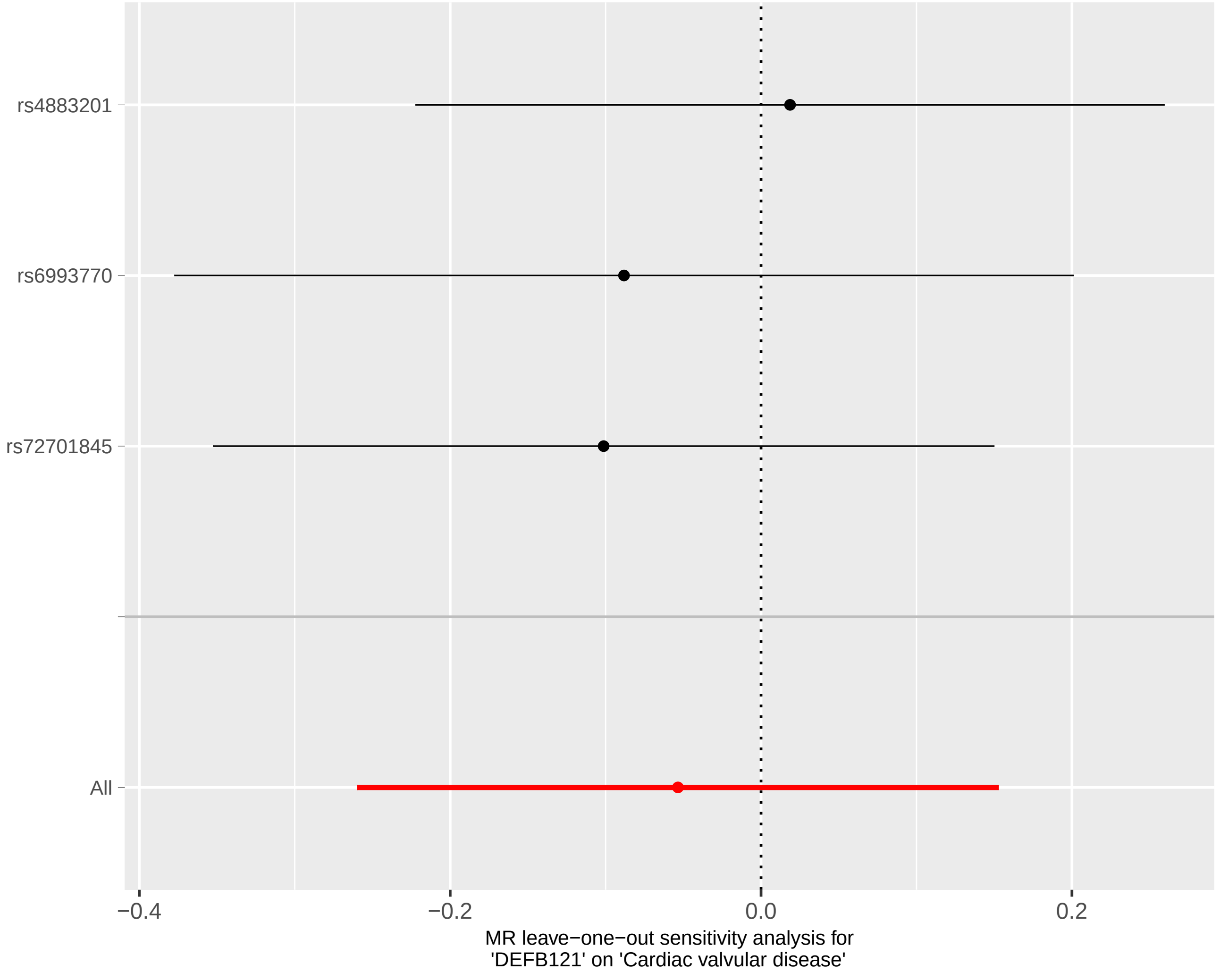

rs4883201
rs6993770
rs72701845
All
−0.4
−0.2
0.0
0.2
MR leave−one−out sensitivity analysis for
'DEFB121' on 'Cardiac valvular disease'

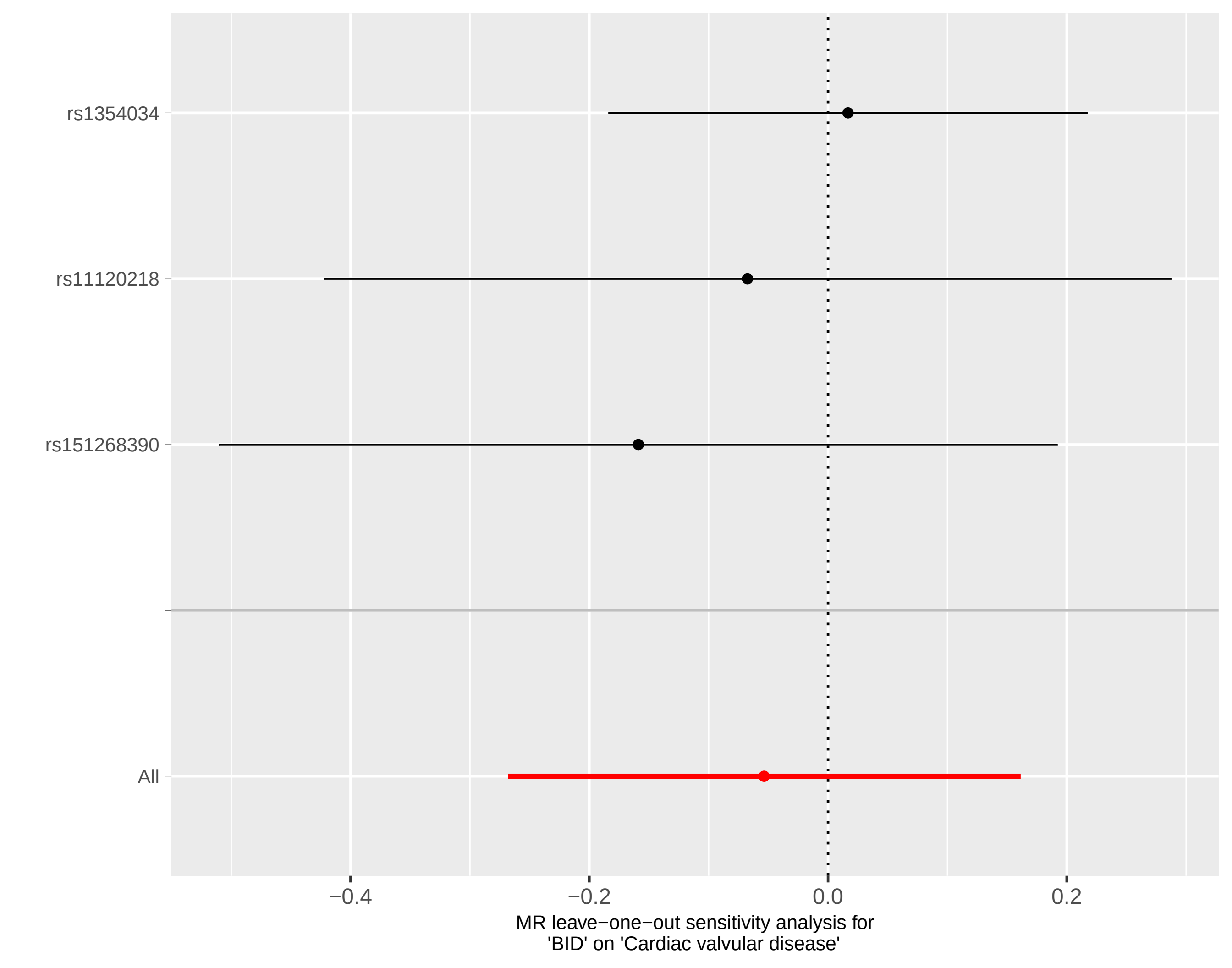

rs1354034
rs11120218
rs151268390
All
−0.4
−0.2
0.0
0.2
MR leave−one−out sensitivity analysis for
'BID' on 'Cardiac valvular disease'

Insufficient number of SNPs

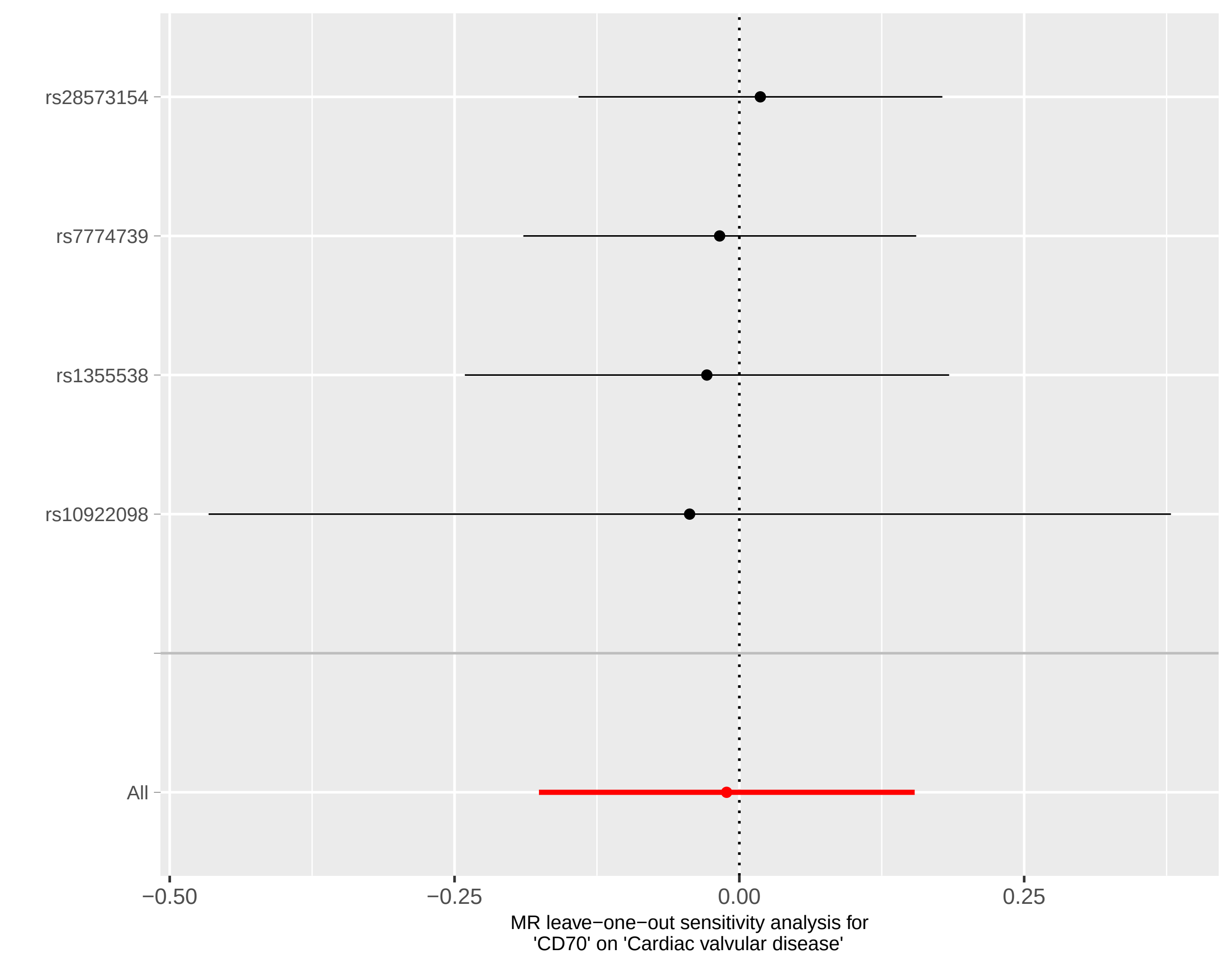
rs28573154
rs7774739
rs1355538
rs10922098
All
−0.50
−0.25
0.00
0.25
MR leave−one−out sensitivity analysis for
'CD70' on 'Cardiac valvular disease'

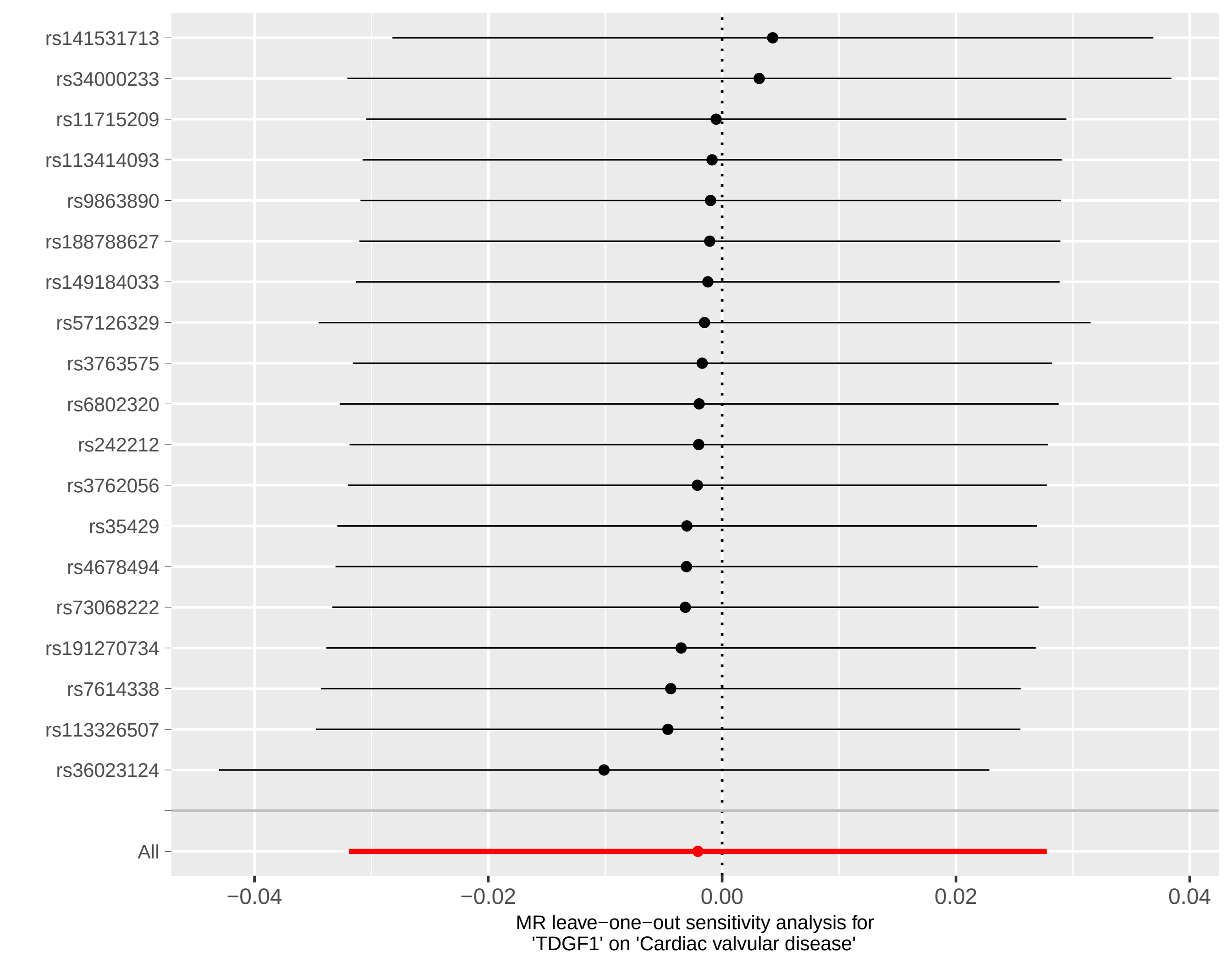
rs141531713
rs34000233
rs11715209
rs113414093
rs9863890
rs188788627
rs149184033
rs57126329
rs3763575
rs6802320
rs242212
rs3762056
rs35429
rs4678494
rs73068222
rs191270734
rs7614338
rs113326507
rs36023124
All
−0.04
−0.02
0.00
0.02
0.04
MR leave−one−out sensitivity analysis for
'TDGF1' on 'Cardiac valvular disease'

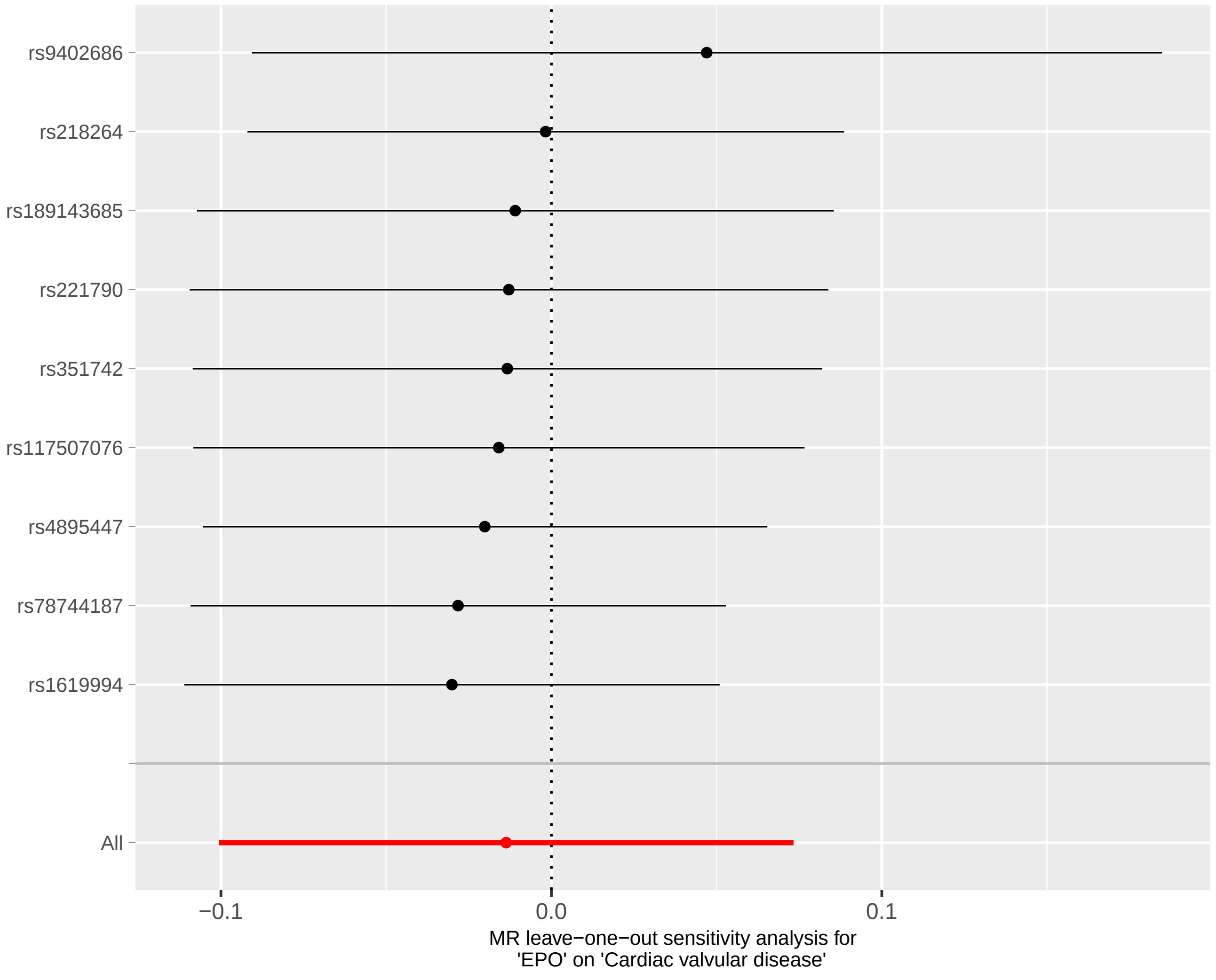
rs9402686
rs218264
rs189143685
rs221790
rs351742
rs117507076
rs4895447
rs78744187
rs1619994
All
−0.1
0.0
0.1
MR leave−one−out sensitivity analysis for
'EPO' on 'Cardiac valvular disease'

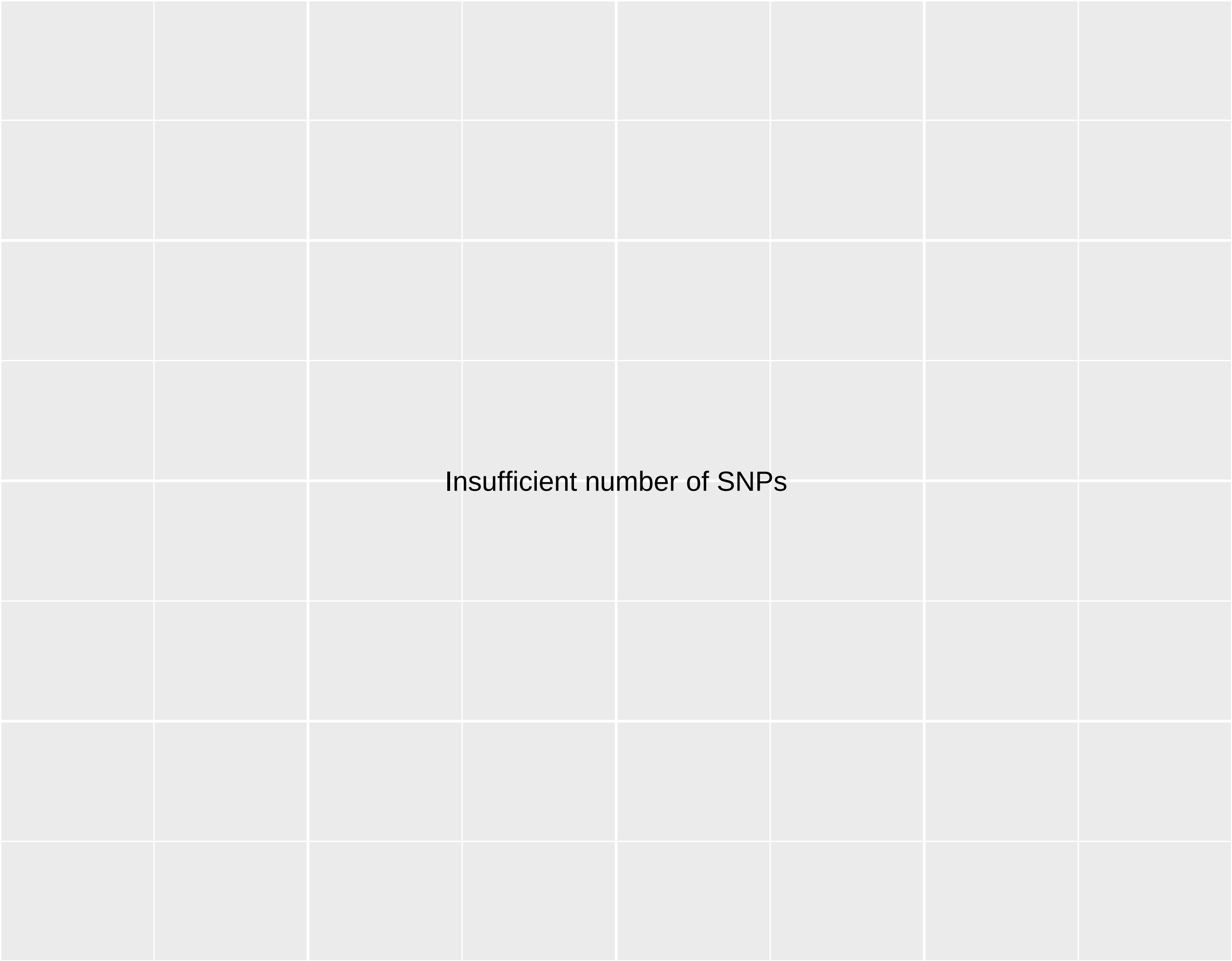
Insufficient number of SNPs

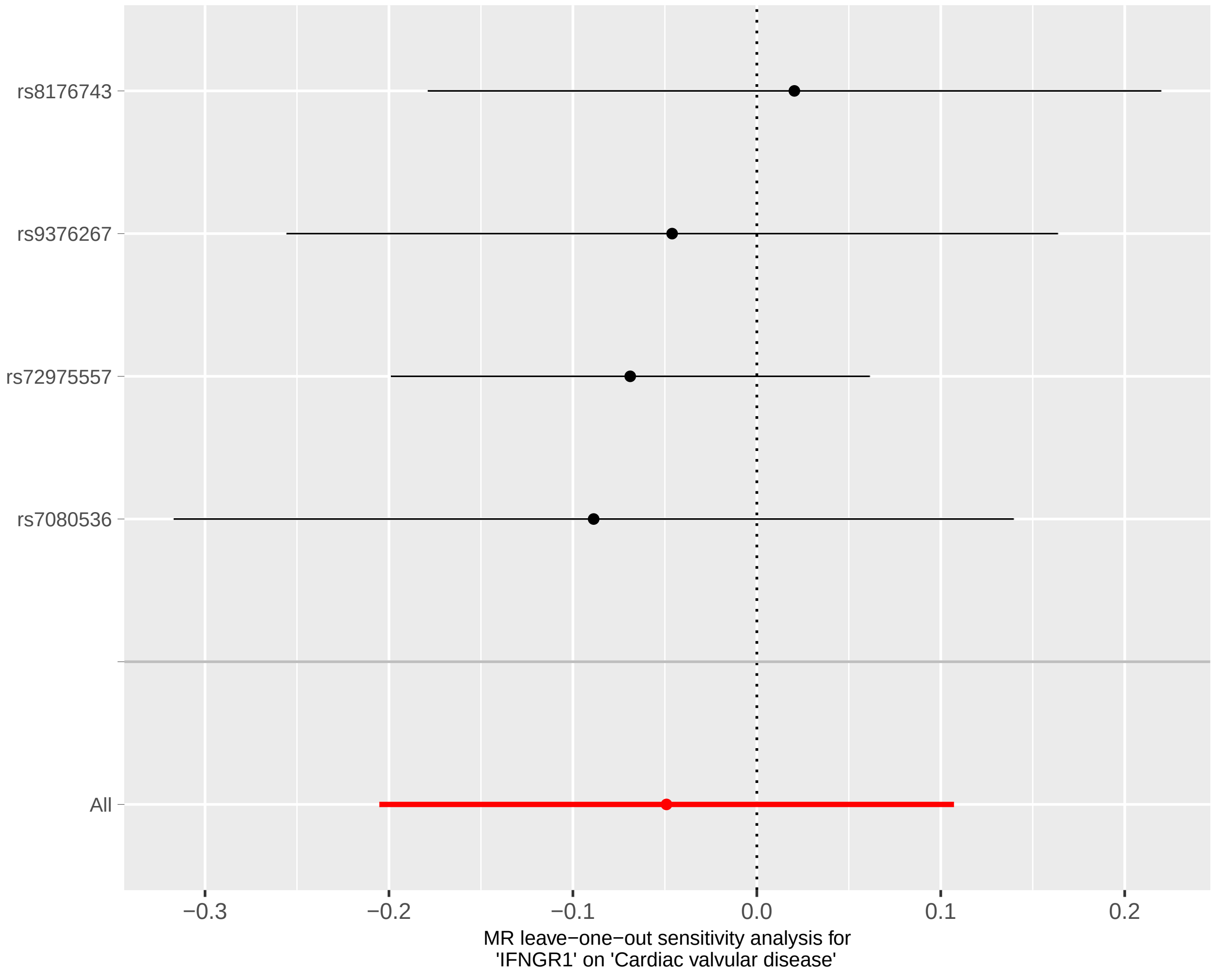

rs8176743
rs9376267
rs72975557
rs7080536
All
−0.3
−0.2
−0.1
0.0
0.1
0.2
MR leave−one−out sensitivity analysis for
'IFNGR1' on 'Cardiac valvular disease'

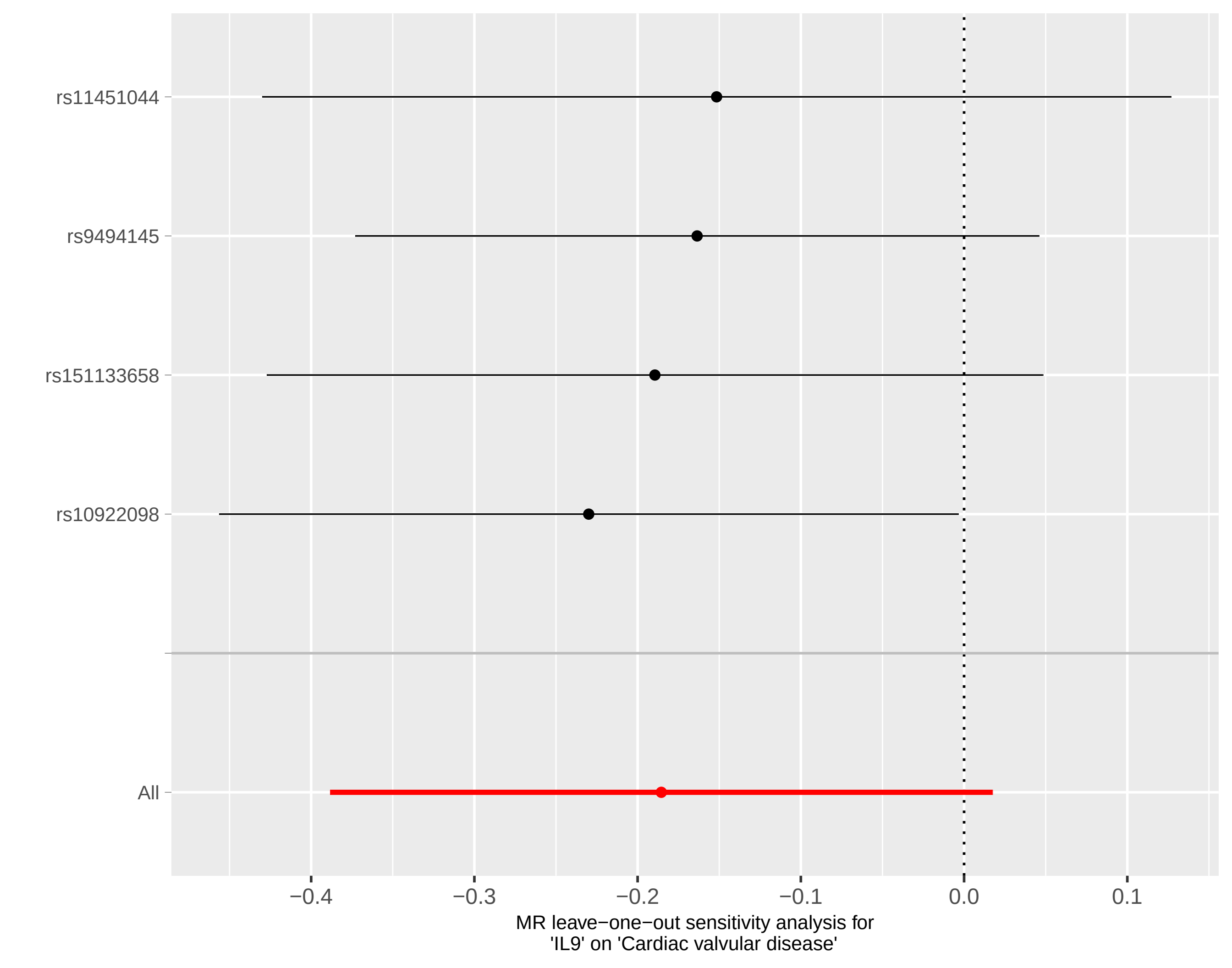

rs11451044
rs9494145
rs151133658
rs10922098
All
−0.4
−0.3
−0.2
−0.1
0.0
0.1
MR leave−one−out sensitivity analysis for
'IL9' on 'Cardiac valvular disease'

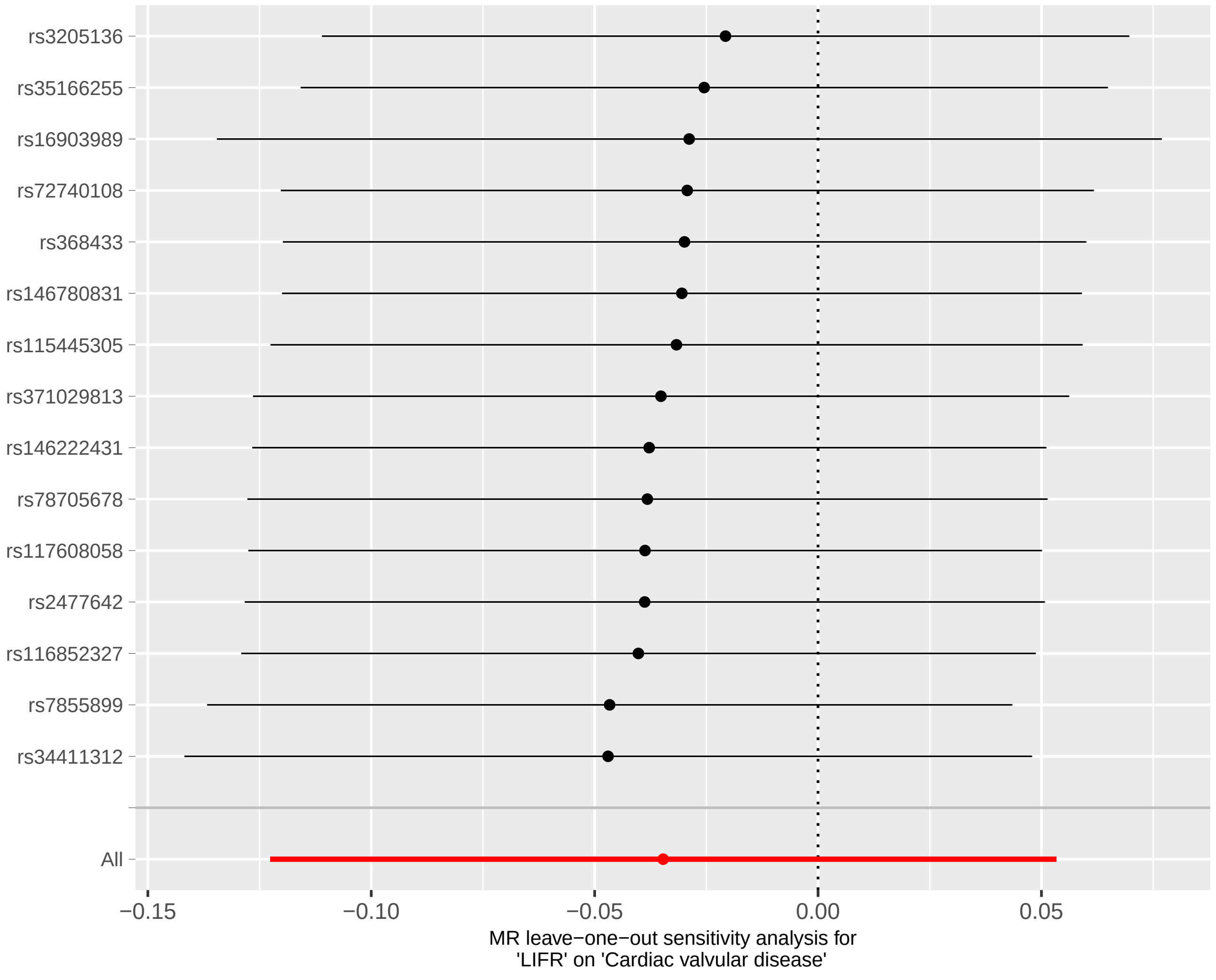
rs3205136
rs35166255
rs16903989
rs72740108
rs368433
rs146780831
rs115445305
rs371029813
rs146222431
rs78705678
rs117608058
rs2477642
rs116852327
rs7855899
rs34411312
All
−0.15
−0.10
−0.05
0.00
0.05
MR leave−one−out sensitivity analysis for
'LIFR' on 'Cardiac valvular disease'

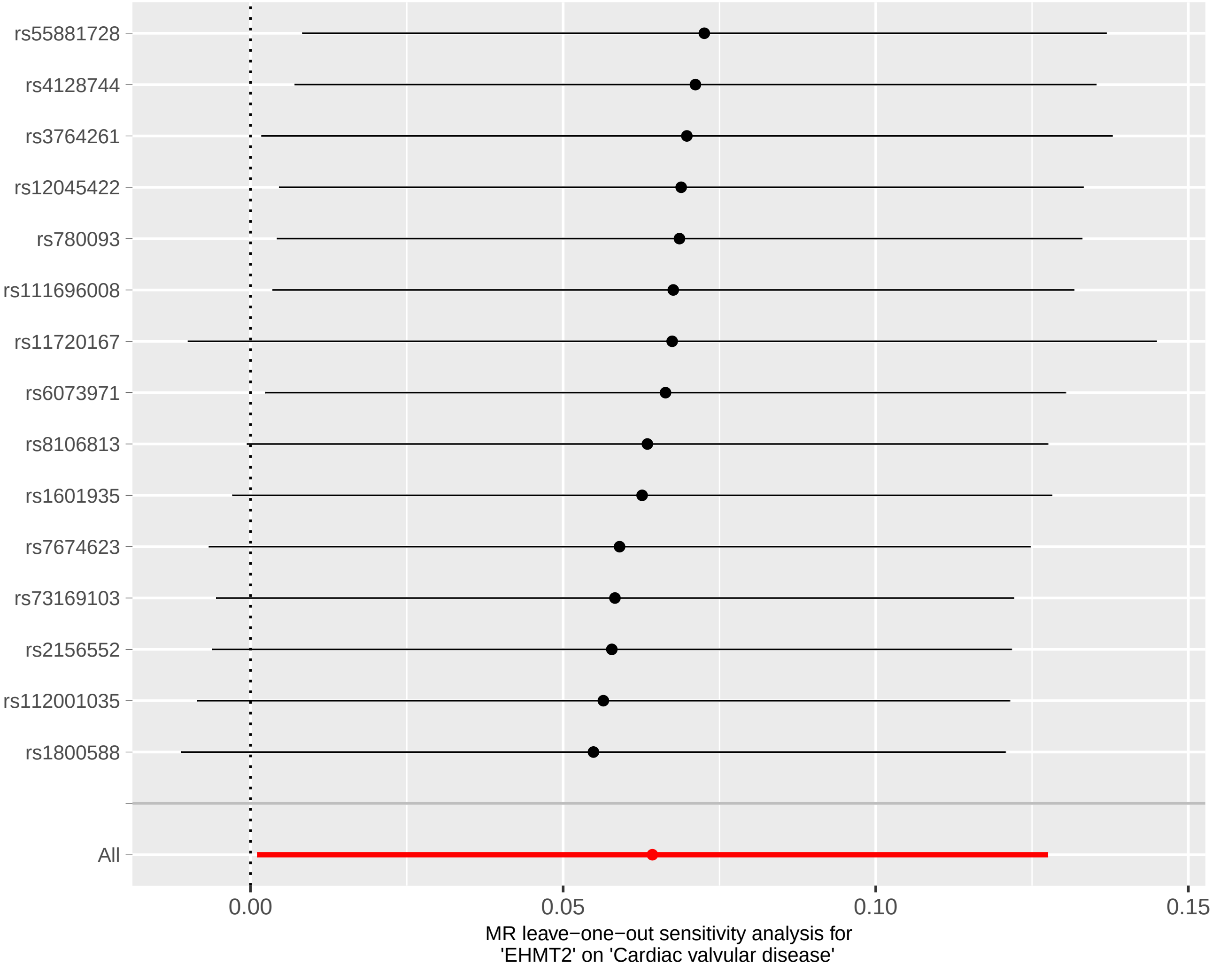
rs55881728
rs4128744
rs3764261
rs12045422
rs780093
rs111696008
rs11720167
rs6073971
rs8106813
rs1601935
rs7674623
rs73169103
rs2156552
rs112001035
rs1800588
All
0.00
0.05
0.10
0.15
MR leave−one−out sensitivity analysis for
'EHMT2' on 'Cardiac valvular disease'

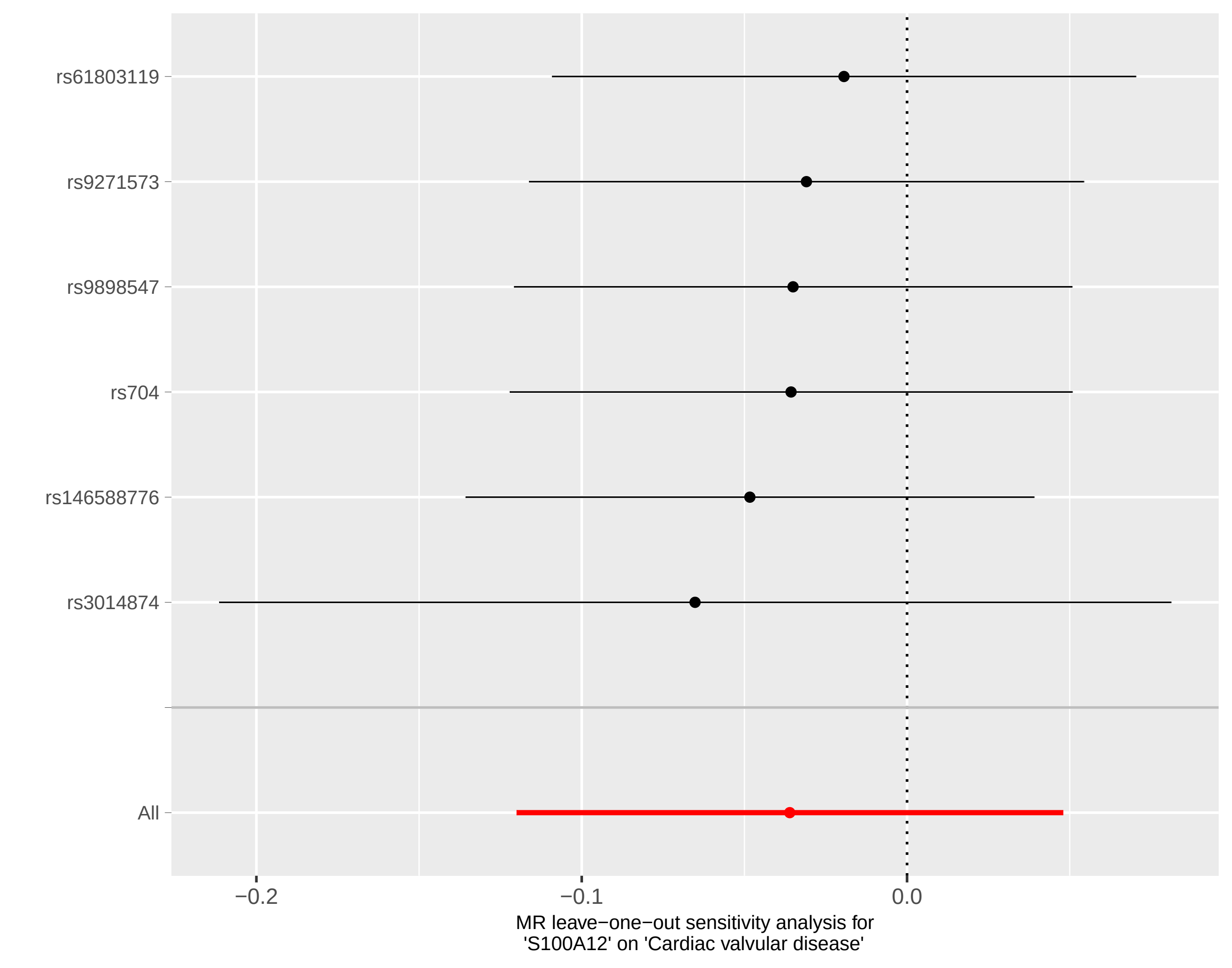

rs61803119
rs9271573
rs9898547
rs704
rs146588776
rs3014874
All
−0.2
−0.1
0.0
MR leave−one−out sensitivity analysis for
'S100A12' on 'Cardiac valvular disease'

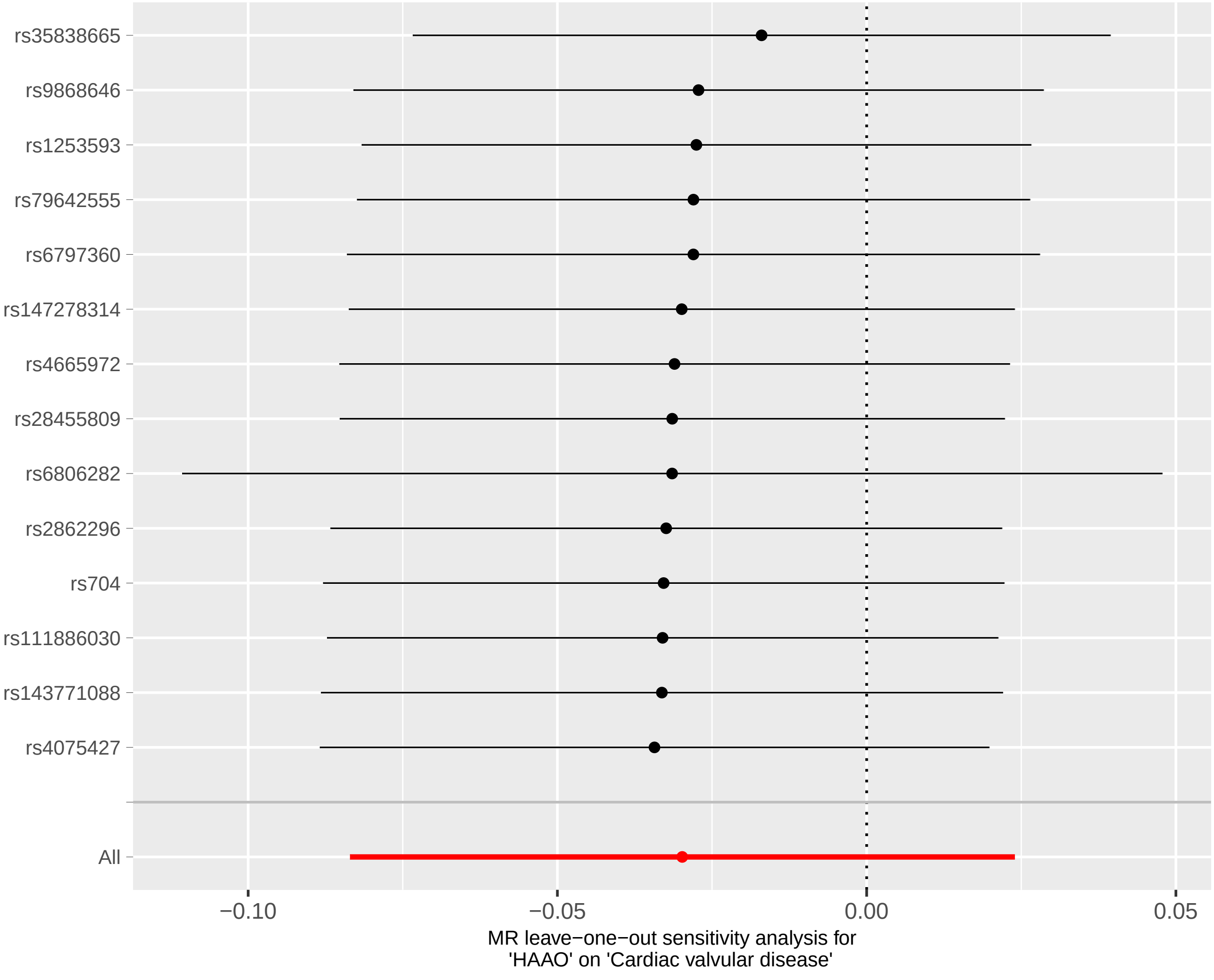
rs35838665
rs9868646
rs1253593
rs79642555
rs6797360
rs147278314
rs4665972
rs28455809
rs6806282
rs2862296
rs704
rs111886030
rs143771088
rs4075427
All
−0.10
−0.05
0.00
0.05
MR leave−one−out sensitivity analysis for
'HAAO' on 'Cardiac valvular disease'

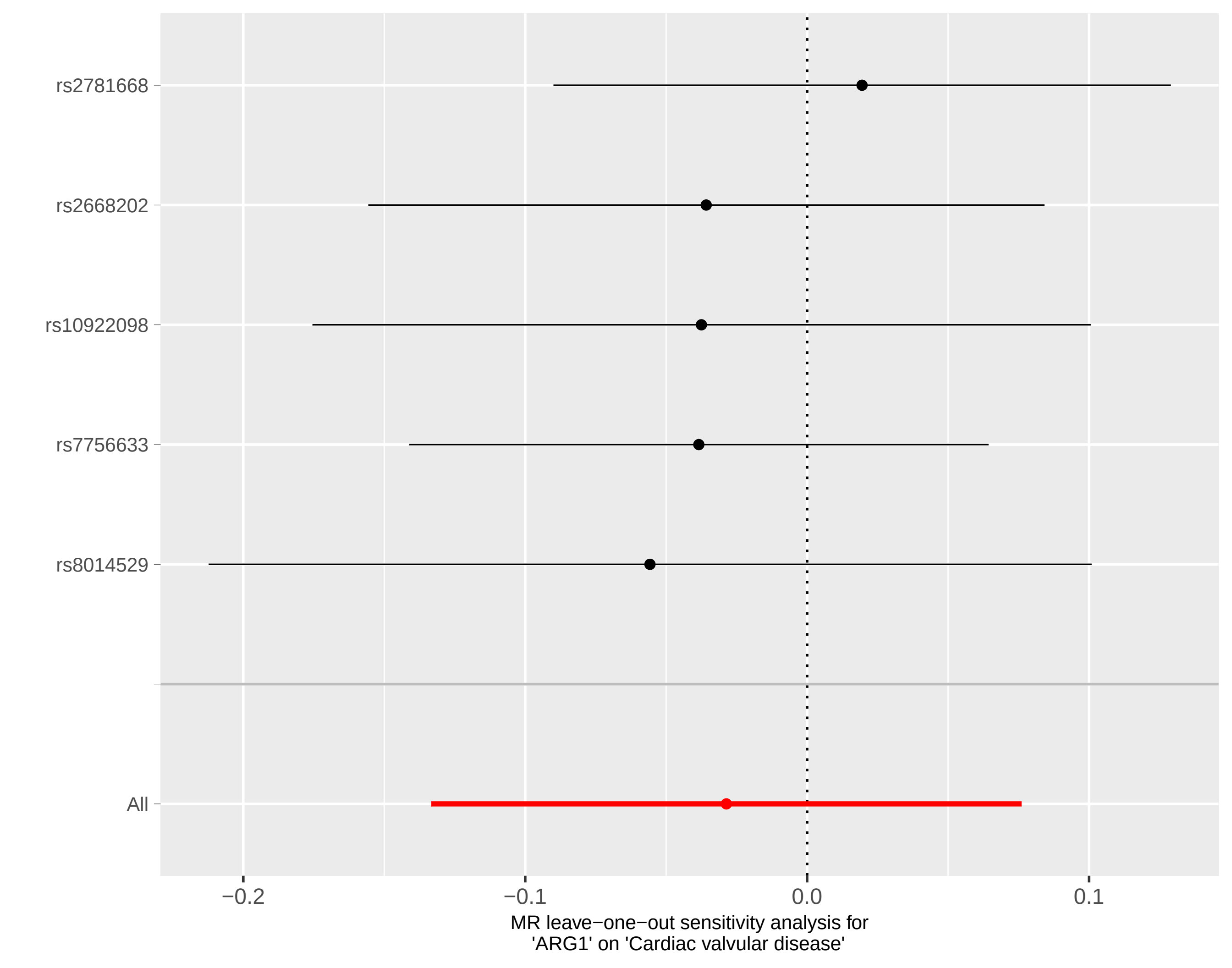
rs2781668
rs2668202
rs10922098
rs7756633
rs8014529
All
−0.2
−0.1
0.0
0.1
MR leave−one−out sensitivity analysis for
'ARG1' on 'Cardiac valvular disease'

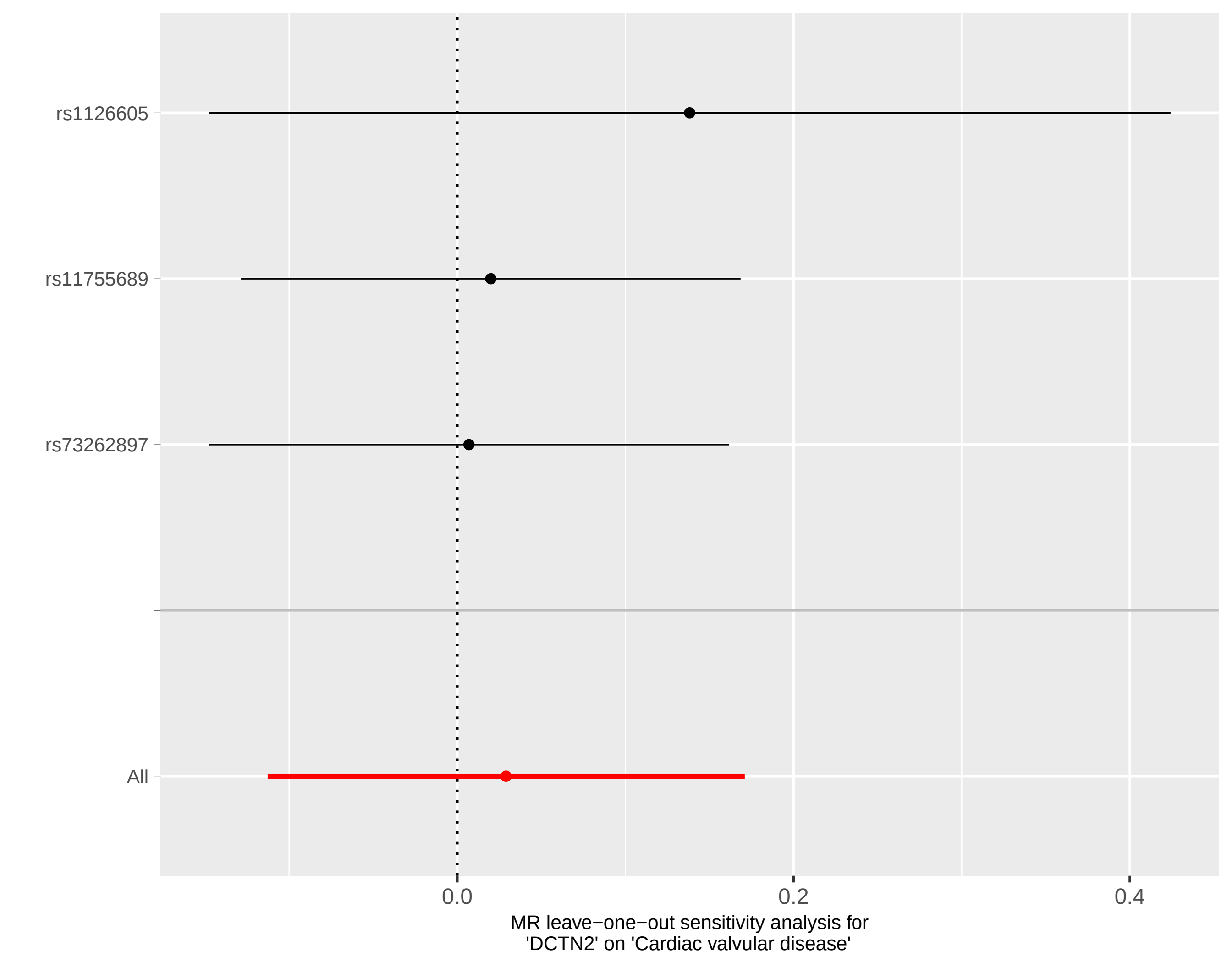

rs1126605
rs11755689
rs73262897
All
0.0
0.2
0.4
MR leave−one−out sensitivity analysis for
'DCTN2' on 'Cardiac valvular disease'

Insufficient number of SNPs

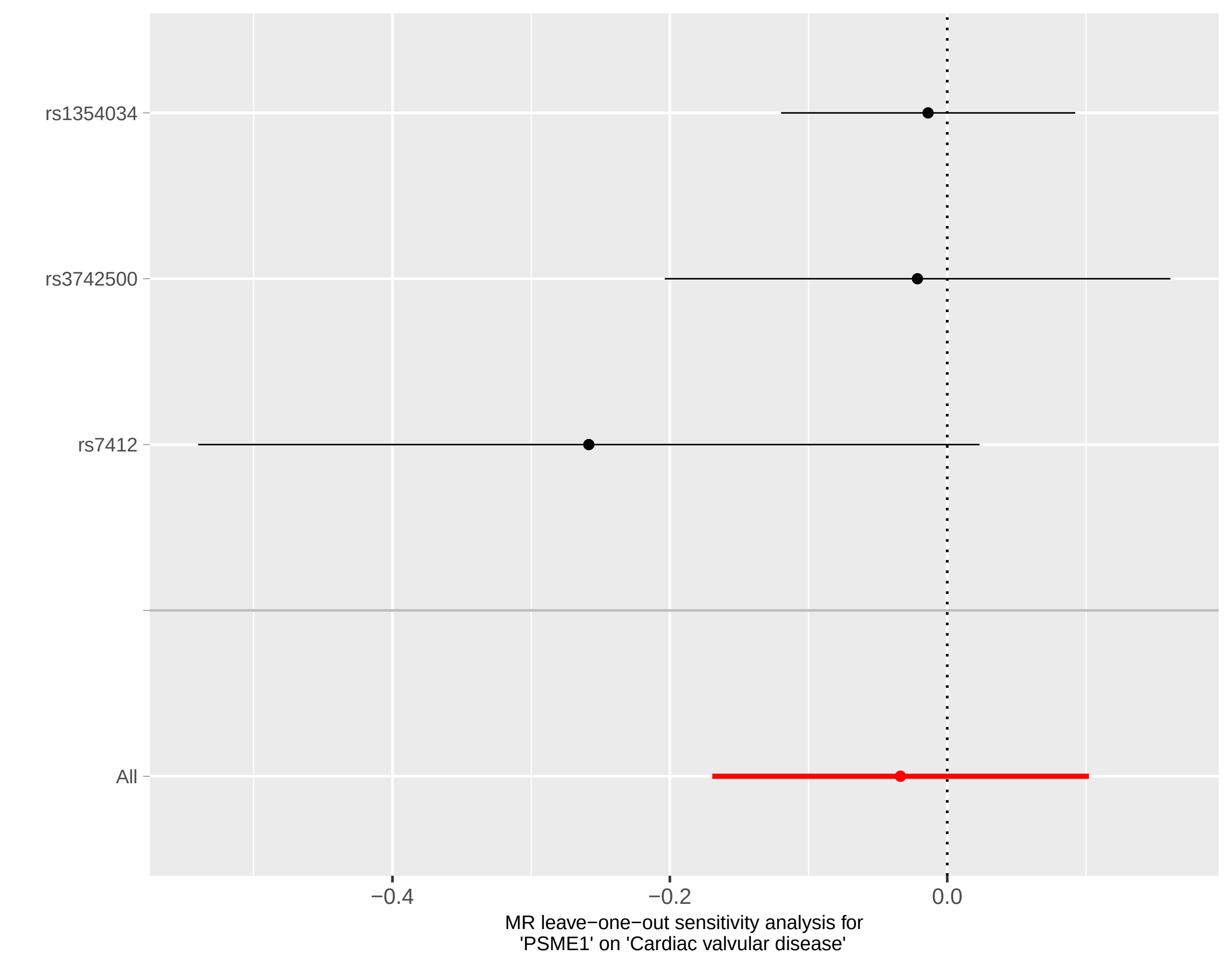

rs1354034
rs3742500
rs7412
All
−0.4
−0.2
0.0
MR leave−one−out sensitivity analysis for
'PSME1' on 'Cardiac valvular disease'

Insufficient number of SNPs

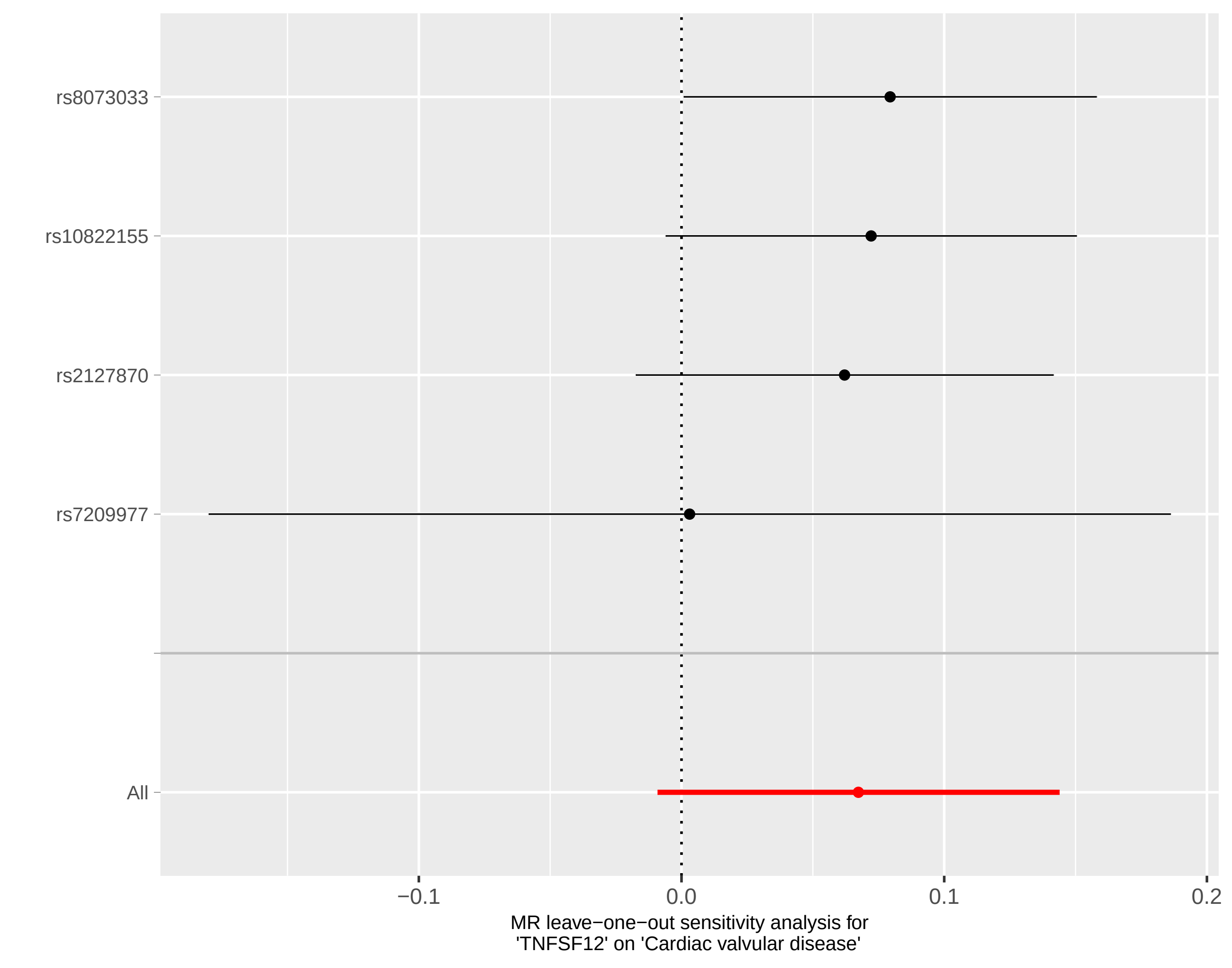
rs8073033
rs10822155
rs2127870
rs7209977
All
−0.1
0.0
0.1
0.2
MR leave−one−out sensitivity analysis for
'TNFSF12' on 'Cardiac valvular disease'

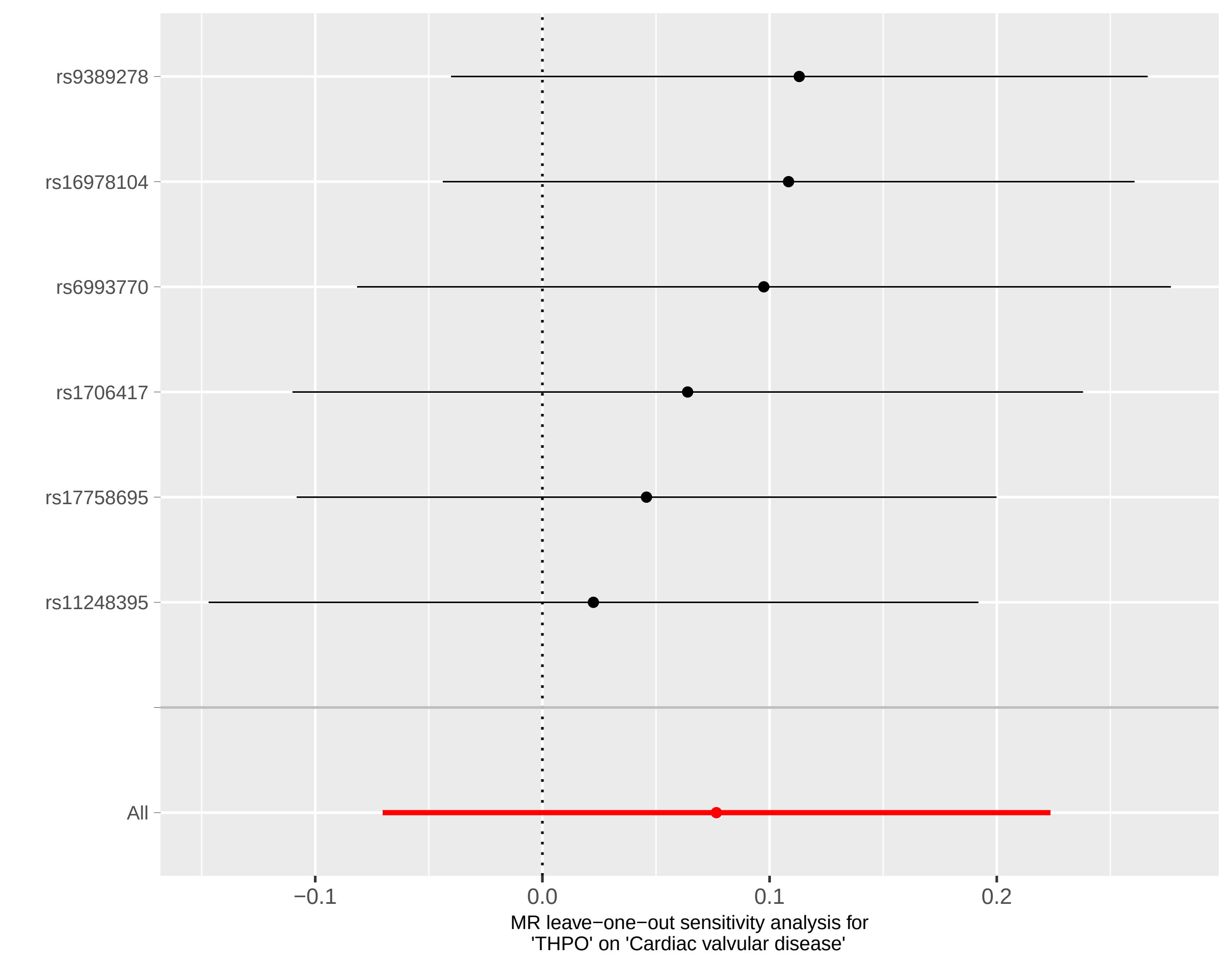
rs9389278
rs16978104
rs6993770
rs1706417
rs17758695
rs11248395
All
−0.1
0.0
0.1
0.2
MR leave−one−out sensitivity analysis for
'THPO' on 'Cardiac valvular disease'

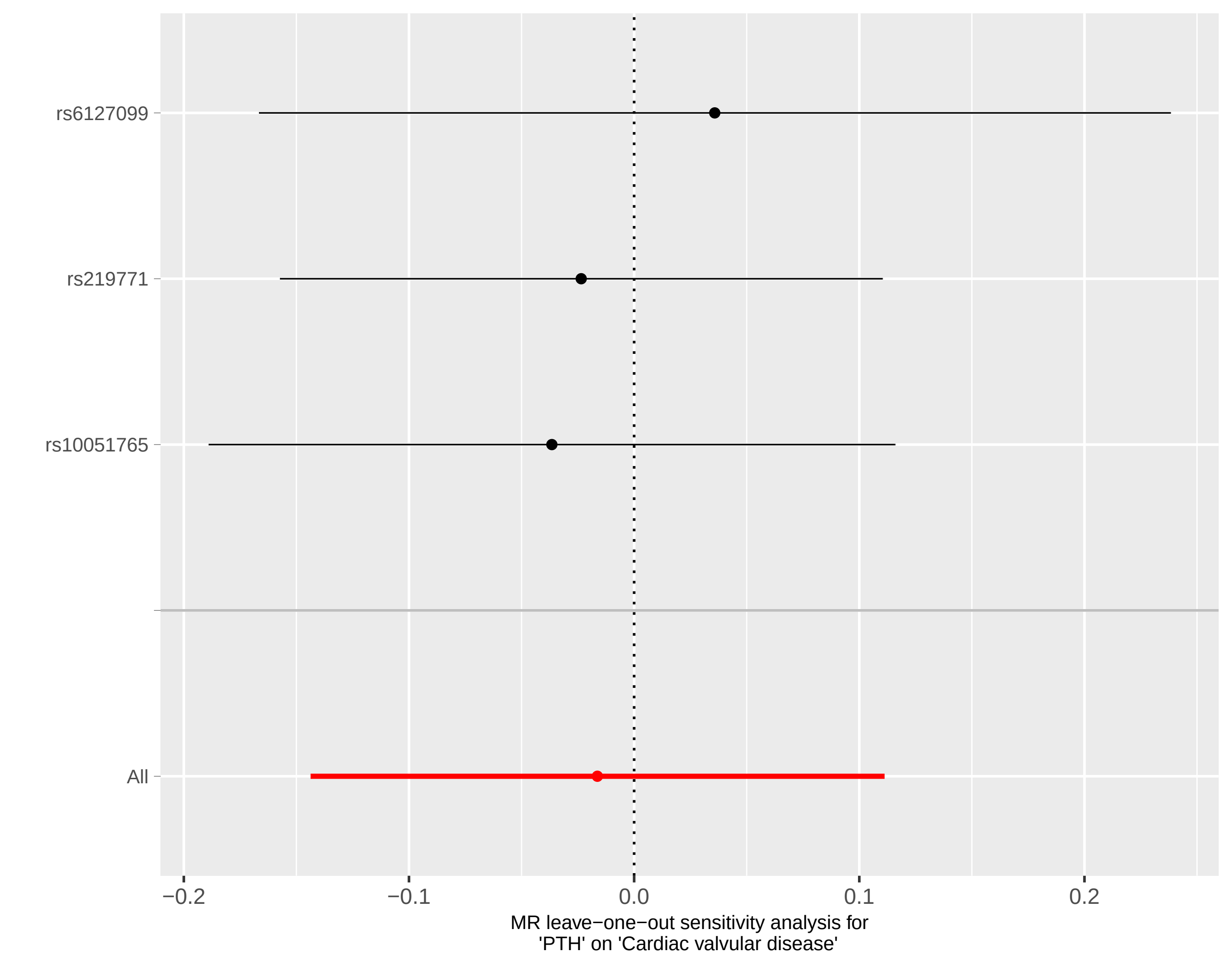

rs6127099
rs219771
rs10051765
All
−0.2
−0.1
0.0
0.1
0.2
MR leave−one−out sensitivity analysis for
'PTH' on 'Cardiac valvular disease'

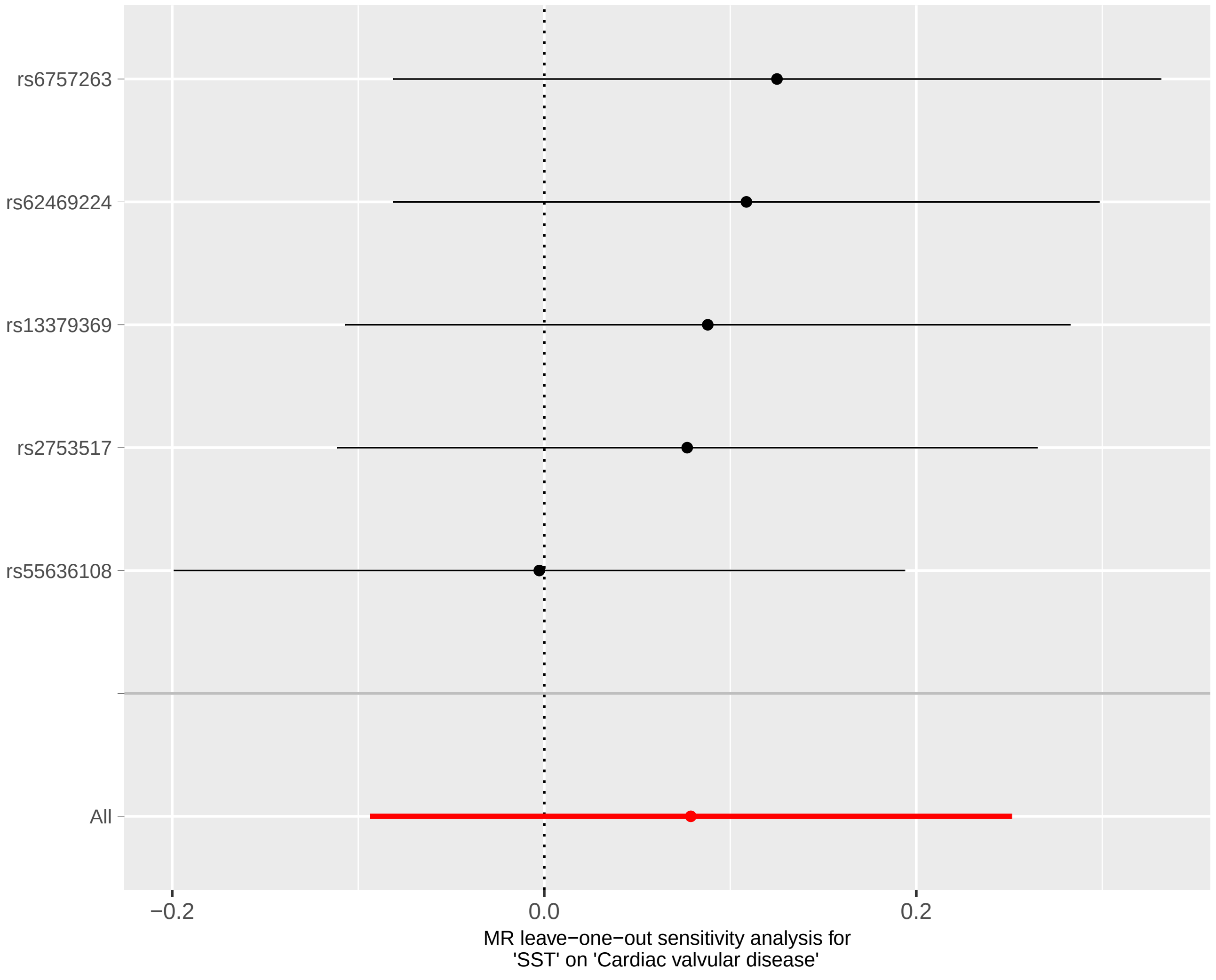
rs6757263
rs62469224
rs13379369
rs2753517
rs55636108
All
−0.2
0.0
0.2
MR leave−one−out sensitivity analysis for
'SST' on 'Cardiac valvular disease'

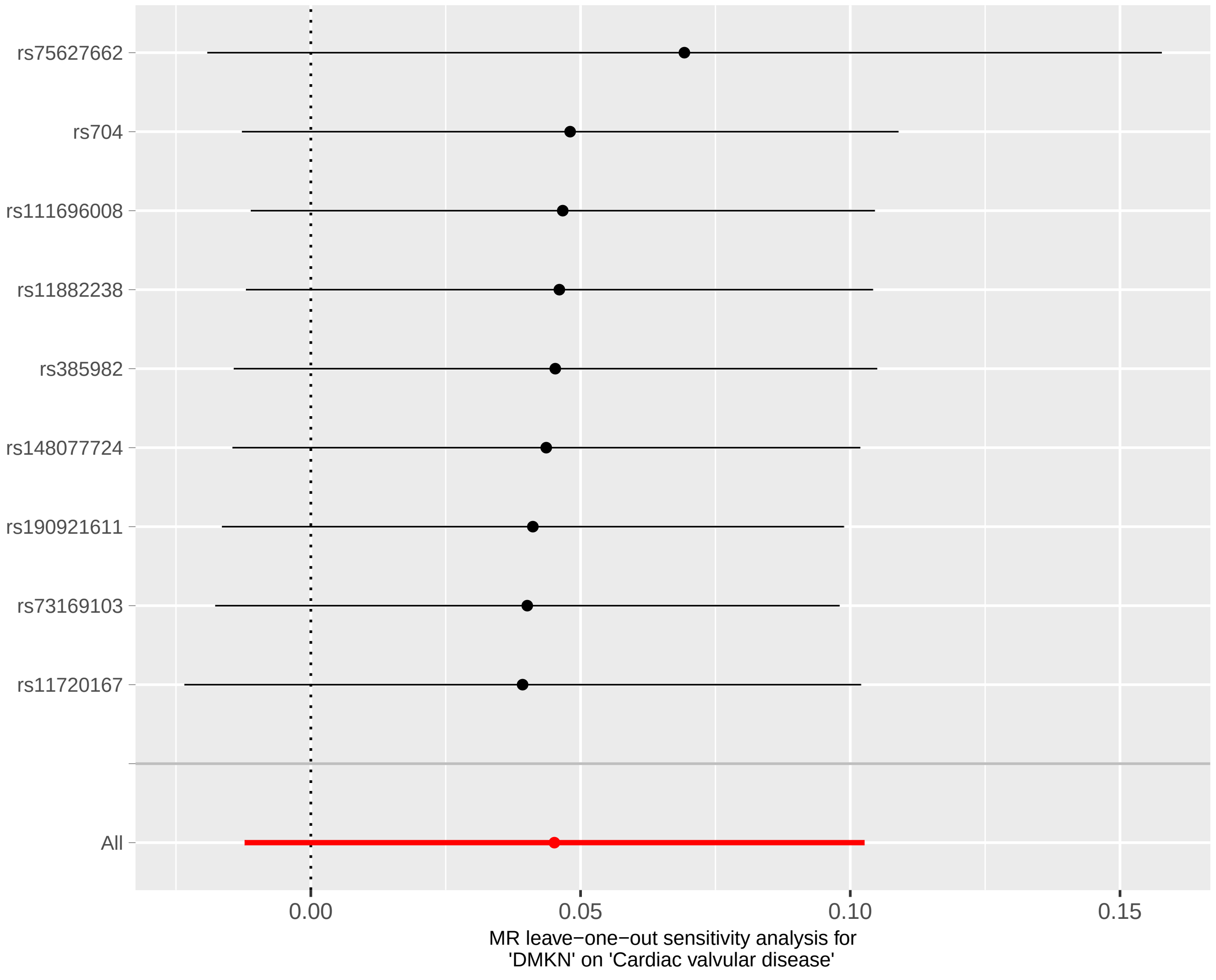

rs75627662
rs704
rs111696008
rs11882238
rs385982
rs148077724
rs190921611
rs73169103
rs11720167
All
0.00
0.05
0.10
0.15
MR leave−one−out sensitivity analysis for
'DMKN' on 'Cardiac valvular disease'

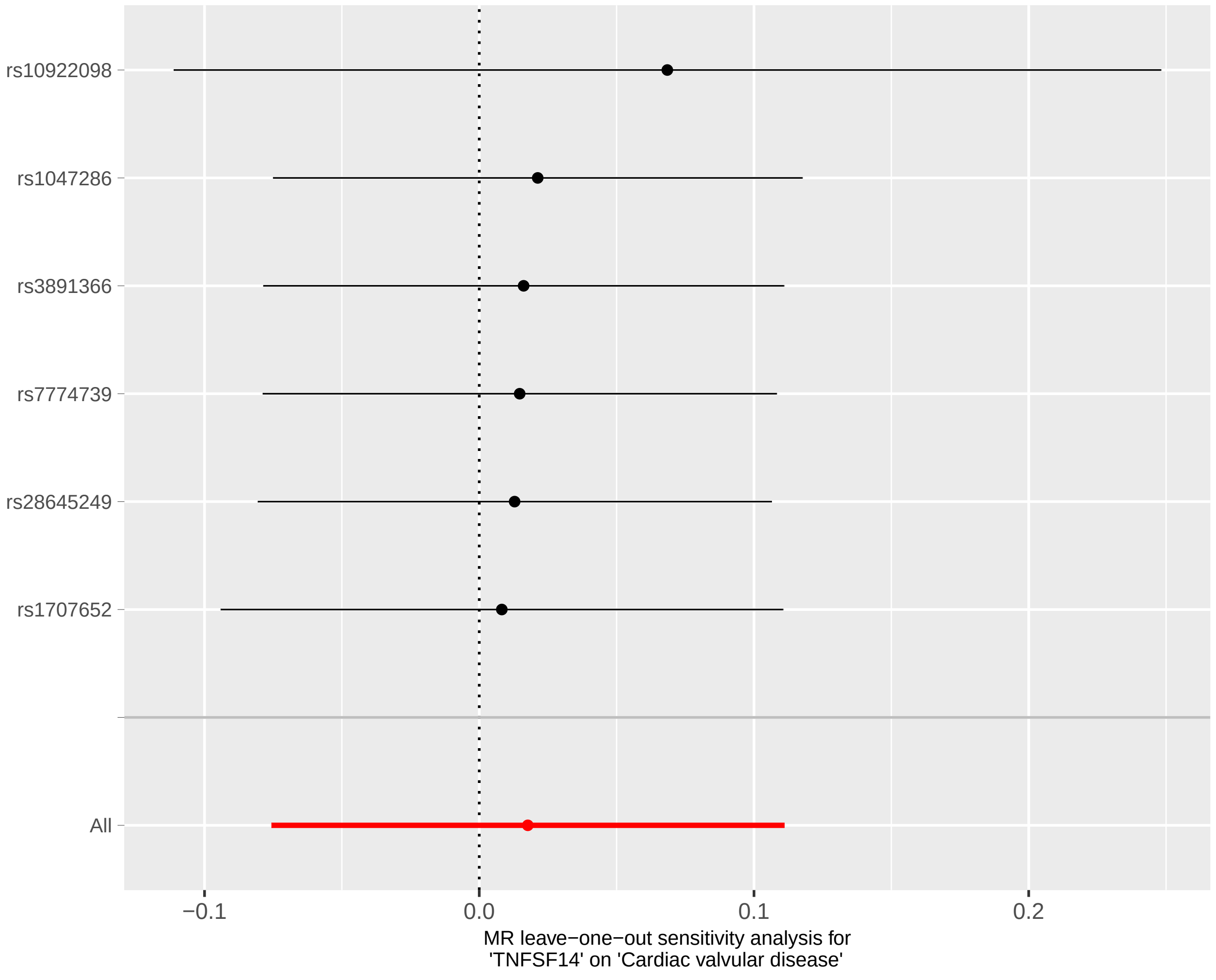
rs10922098
rs1047286
rs3891366
rs7774739
rs28645249
rs1707652
All
−0.1
0.0
0.1
0.2
MR leave−one−out sensitivity analysis for
'TNFSF14' on 'Cardiac valvular disease'

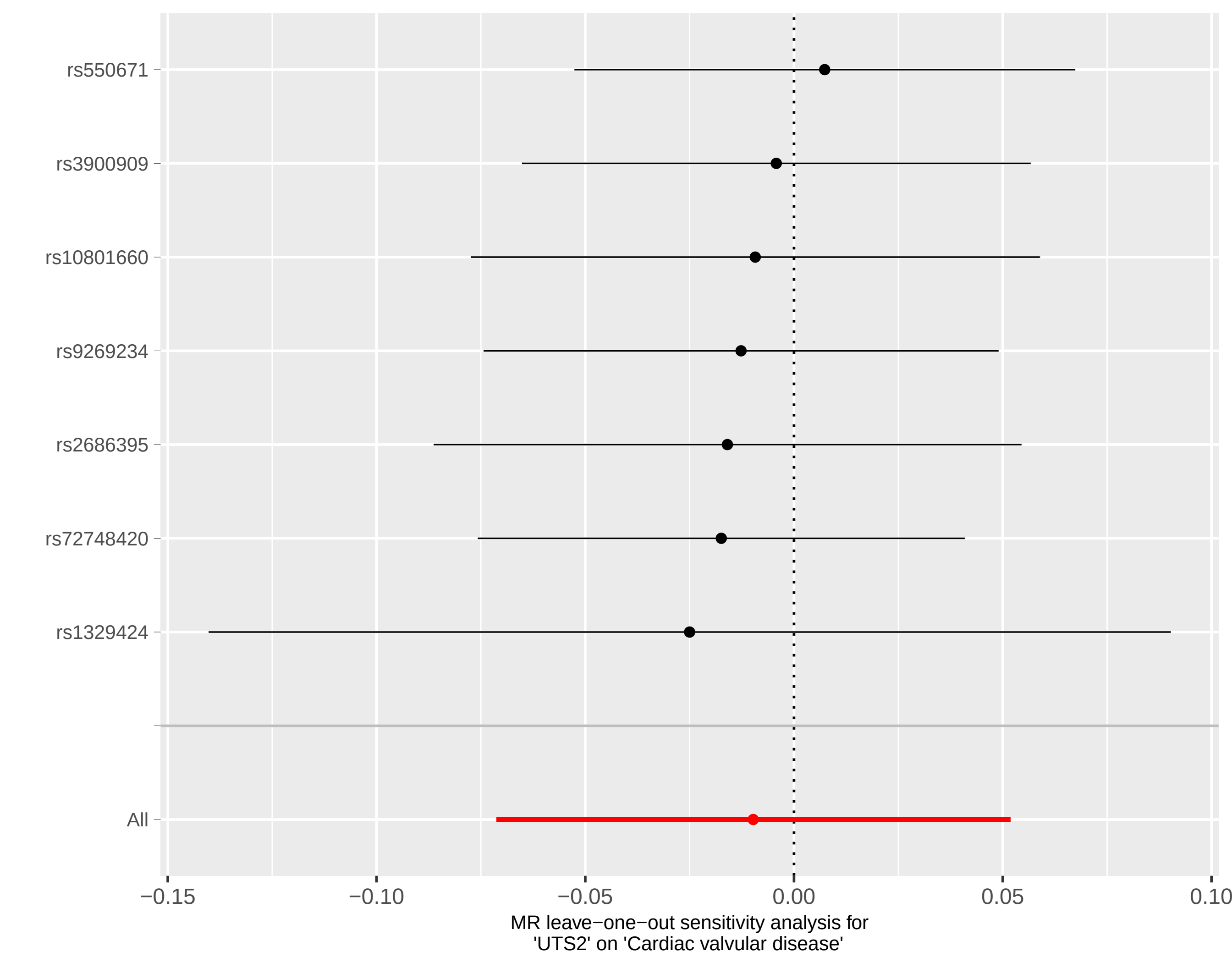
rs550671
rs3900909
rs10801660
rs9269234
rs2686395
rs72748420
rs1329424
All
−0.15
−0.10
−0.05
0.00
0.05
0.10
MR leave−one−out sensitivity analysis for
'UTS2' on 'Cardiac valvular disease'

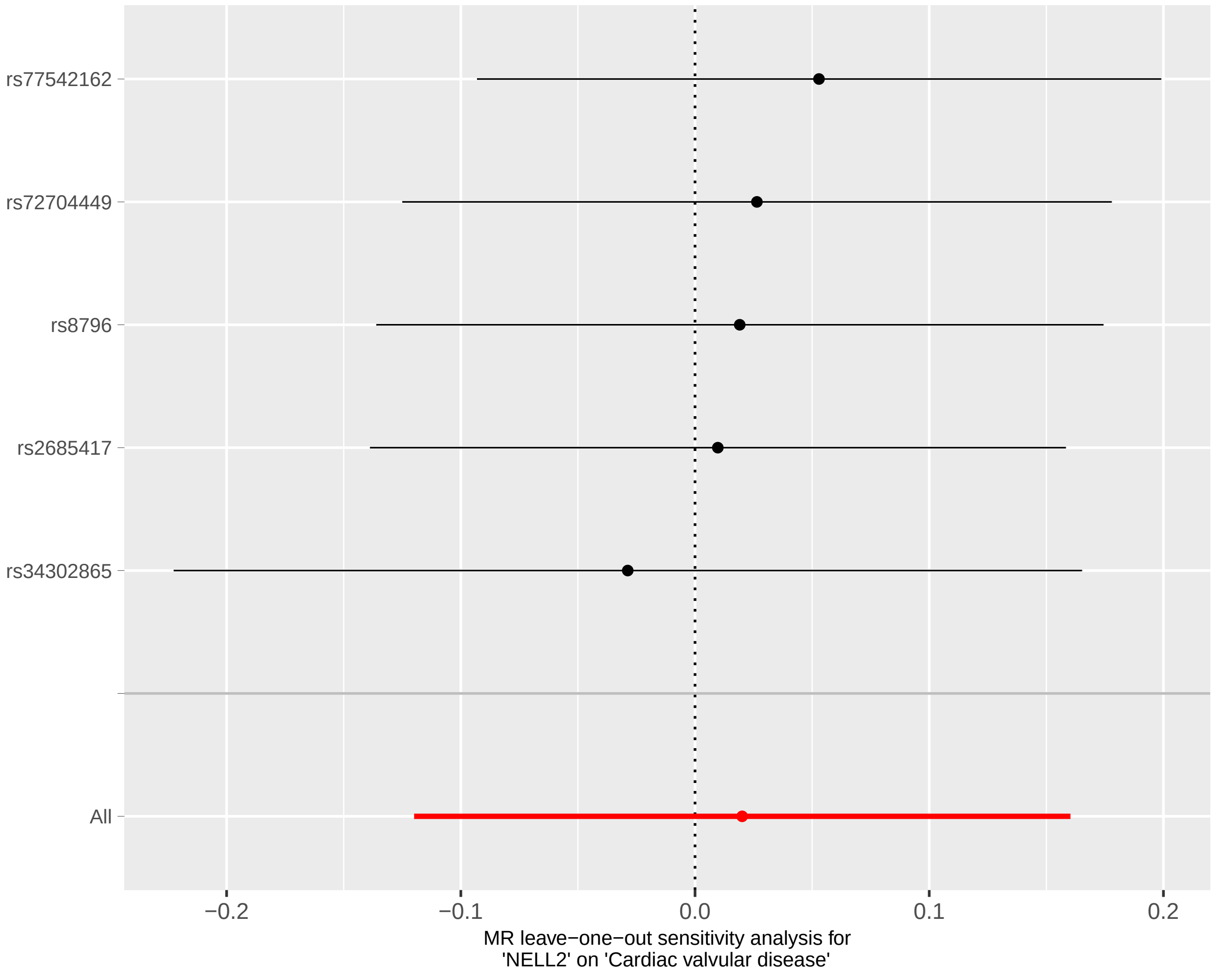

rs77542162
rs72704449
rs8796
rs2685417
rs34302865
All
−0.2
−0.1
0.0
0.1
0.2
MR leave−one−out sensitivity analysis for
'NELL2' on 'Cardiac valvular disease'

Insufficient number of SNPs

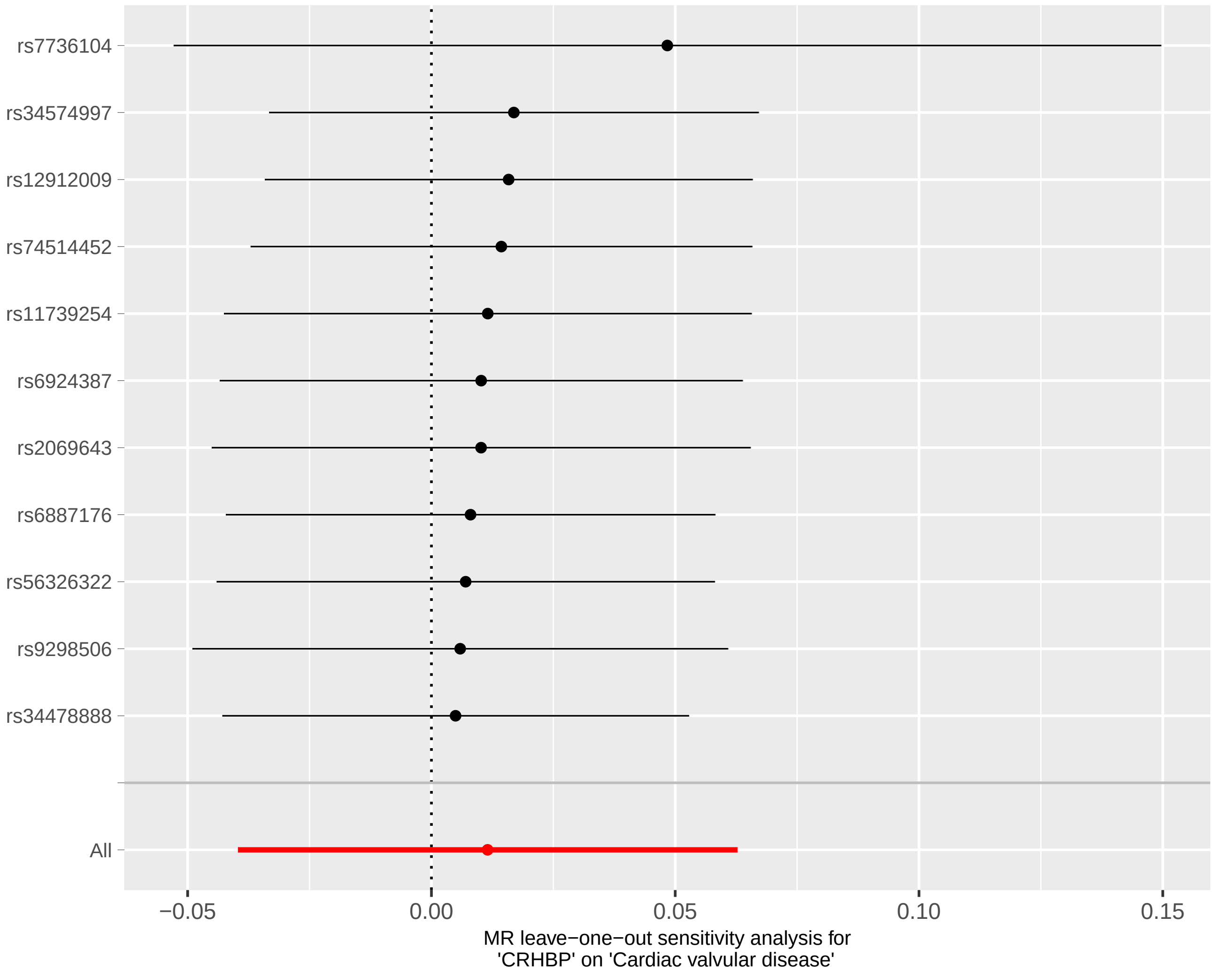
rs7736104
rs34574997
rs12912009
rs74514452
rs11739254
rs6924387
rs2069643
rs6887176
rs56326322
rs9298506
rs34478888
All
−0.05
0.00
0.05
0.10
0.15
MR leave−one−out sensitivity analysis for
'CRHBP' on 'Cardiac valvular disease'

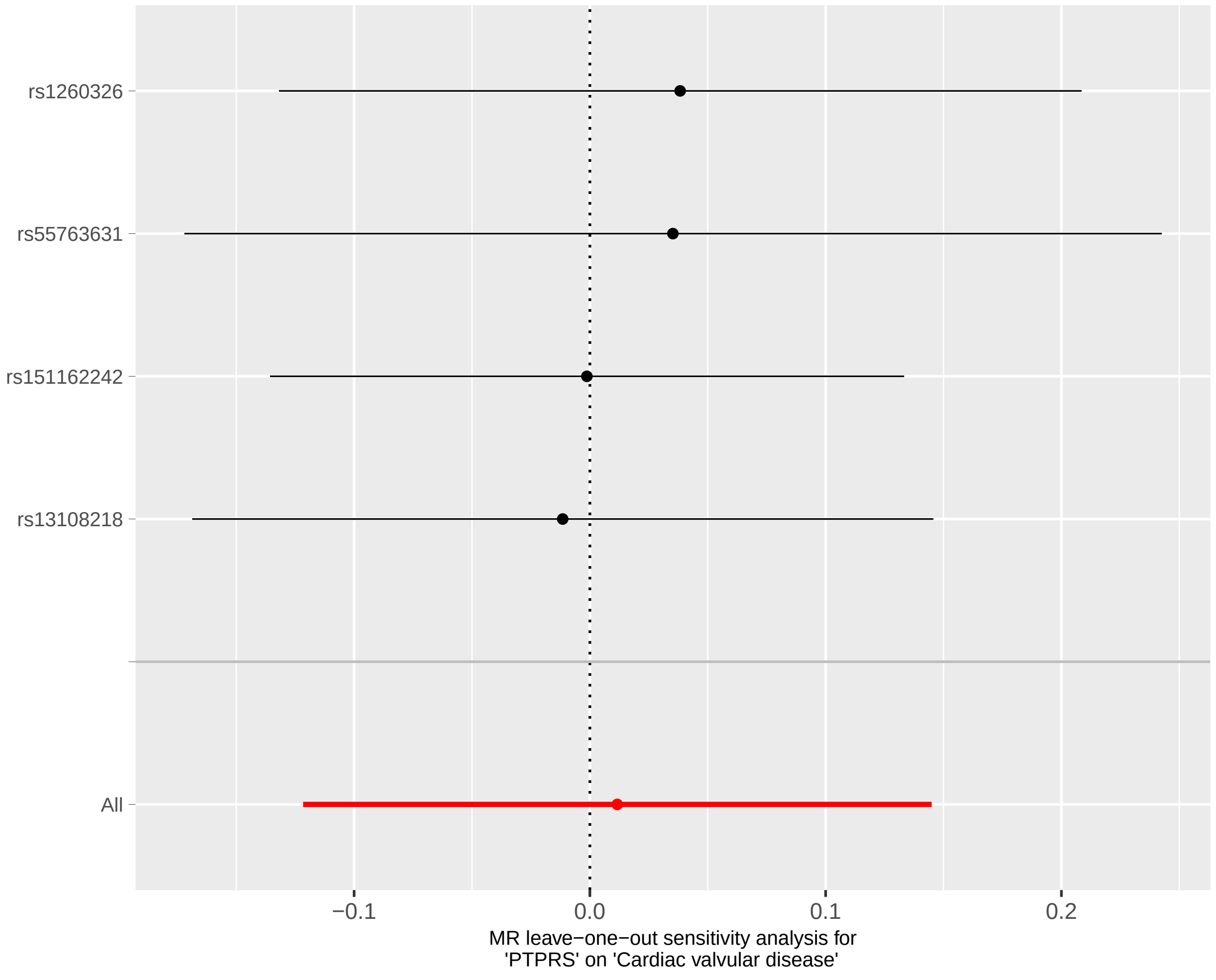
rs1260326
rs55763631
rs151162242
rs13108218
All
−0.1
0.0
0.1
0.2
MR leave−one−out sensitivity analysis for
'PTPRS' on 'Cardiac valvular disease'

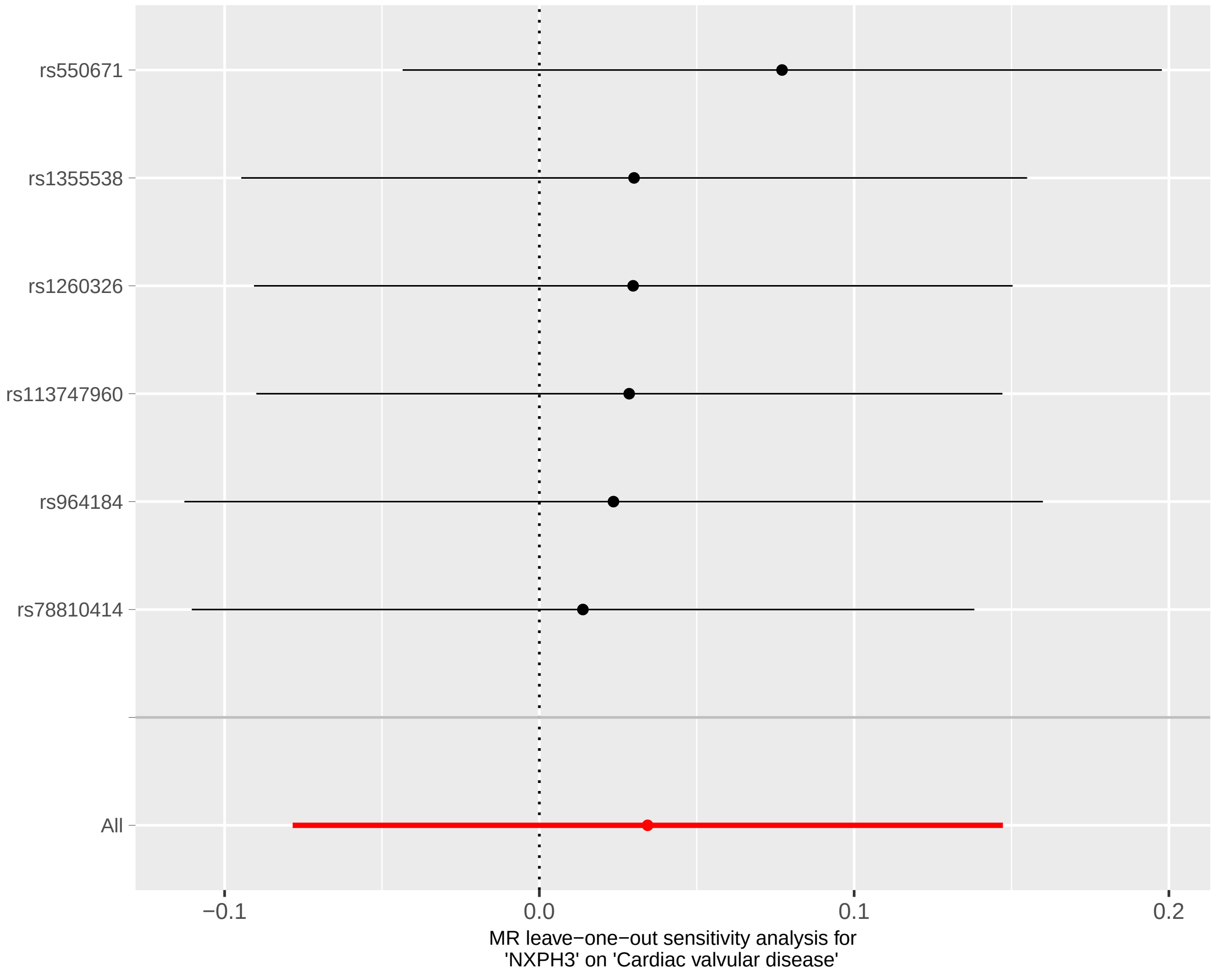
rs550671
rs1355538
rs1260326
rs113747960
rs964184
rs78810414
All
−0.1
0.0
0.1
0.2
MR leave−one−out sensitivity analysis for
'NXPH3' on 'Cardiac valvular disease'

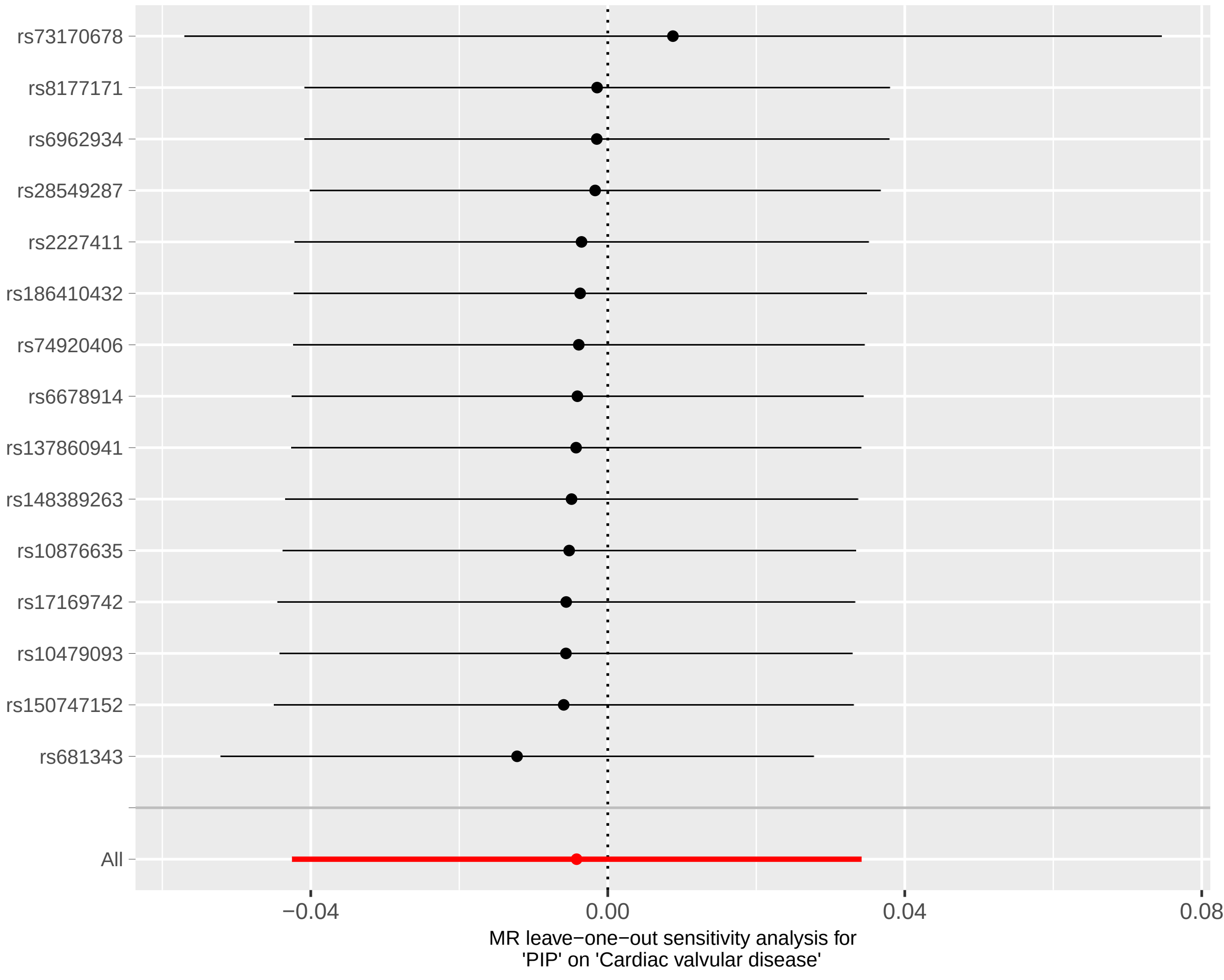
rs73170678
rs8177171
rs6962934
rs28549287
rs2227411
rs186410432
rs74920406
rs6678914
rs137860941
rs148389263
rs10876635
rs17169742
rs10479093
rs150747152
rs681343
All
−0.04
0.00
0.04
0.08
MR leave−one−out sensitivity analysis for
'PIP' on 'Cardiac valvular disease'

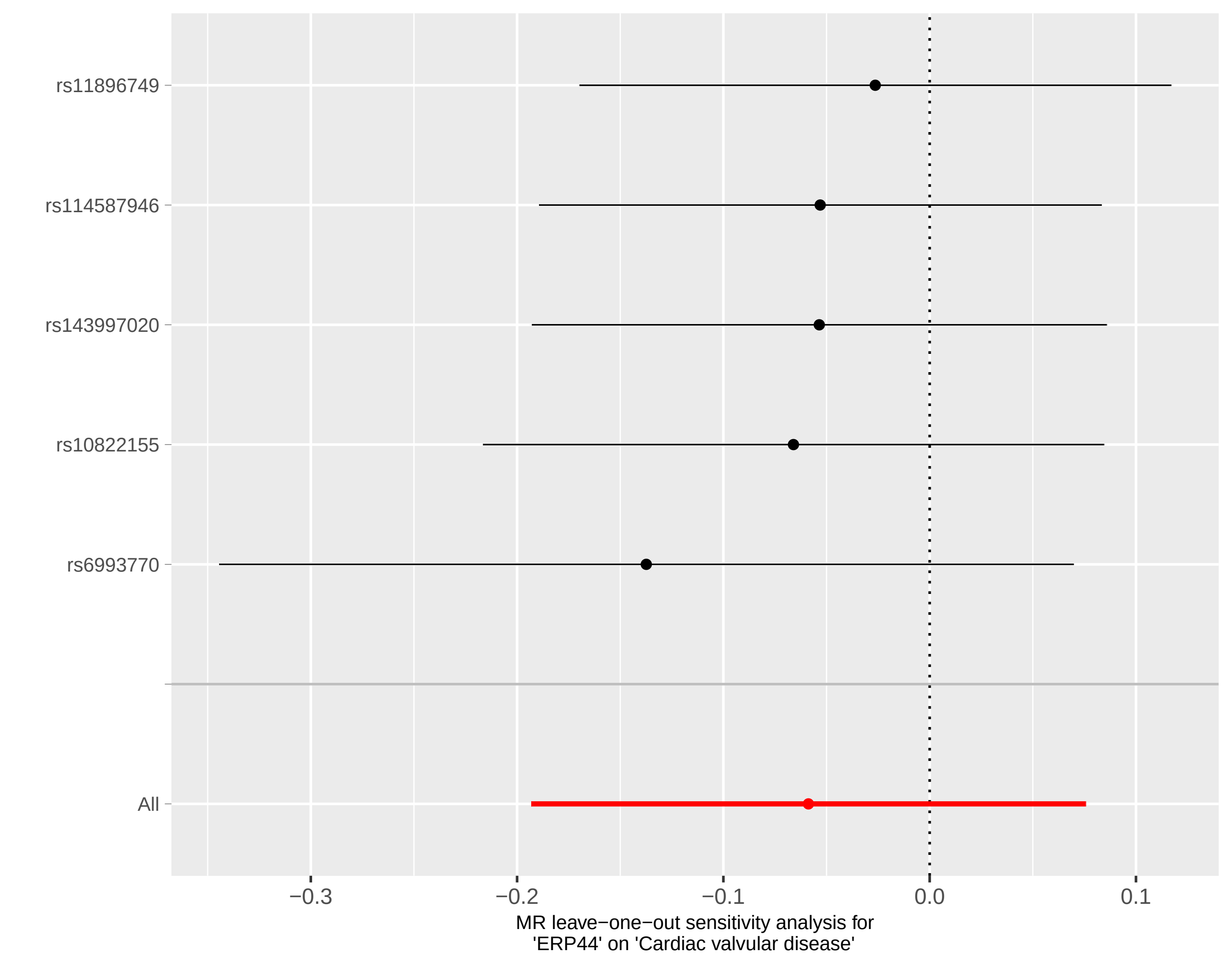
rs11896749
rs114587946
rs143997020
rs10822155
rs6993770
All
−0.3
−0.2
−0.1
0.0
0.1
MR leave−one−out sensitivity analysis for
'ERP44' on 'Cardiac valvular disease'

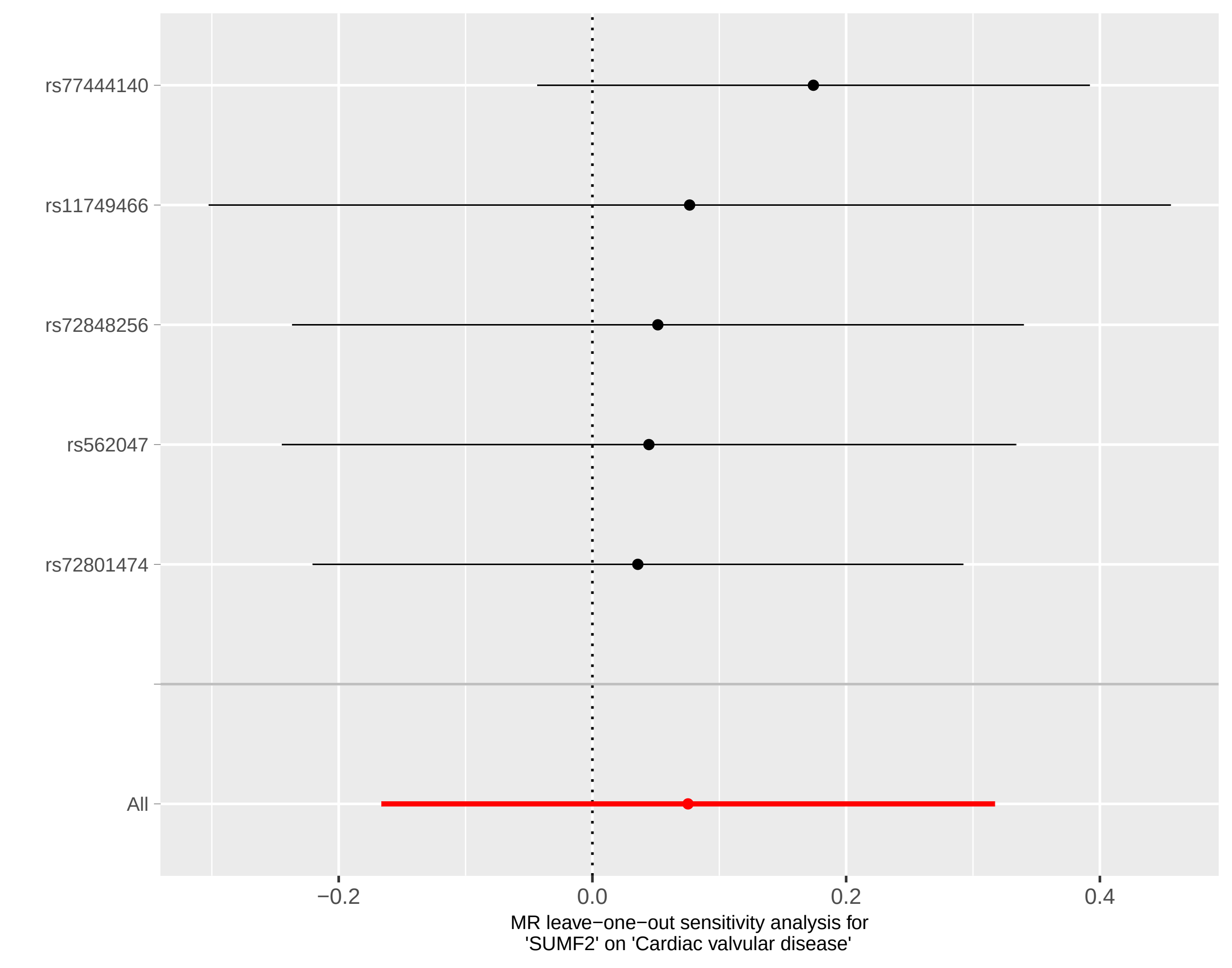
rs77444140
rs11749466
rs72848256
rs562047
rs72801474
All
−0.2
0.0
0.2
0.4
MR leave−one−out sensitivity analysis for
'SUMF2' on 'Cardiac valvular disease'

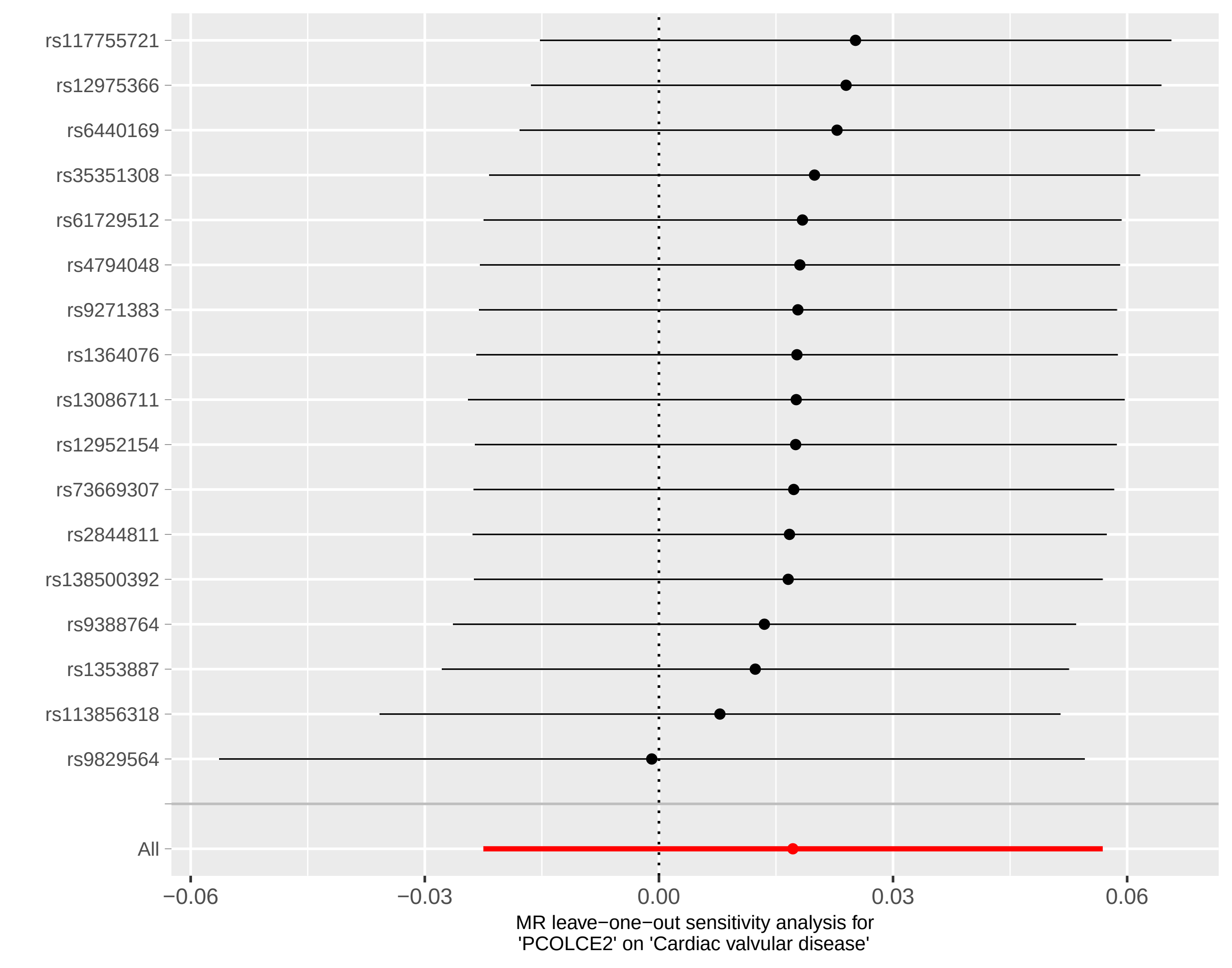
rs117755721
rs12975366
rs6440169
rs35351308
rs61729512
rs4794048
rs9271383
rs1364076
rs13086711
rs12952154
rs73669307
rs2844811
rs138500392
rs9388764
rs1353887
rs113856318
rs9829564
All
−0.06
−0.03
0.00
0.03
0.06
MR leave−one−out sensitivity analysis for
'PCOLCE2' on 'Cardiac valvular disease'

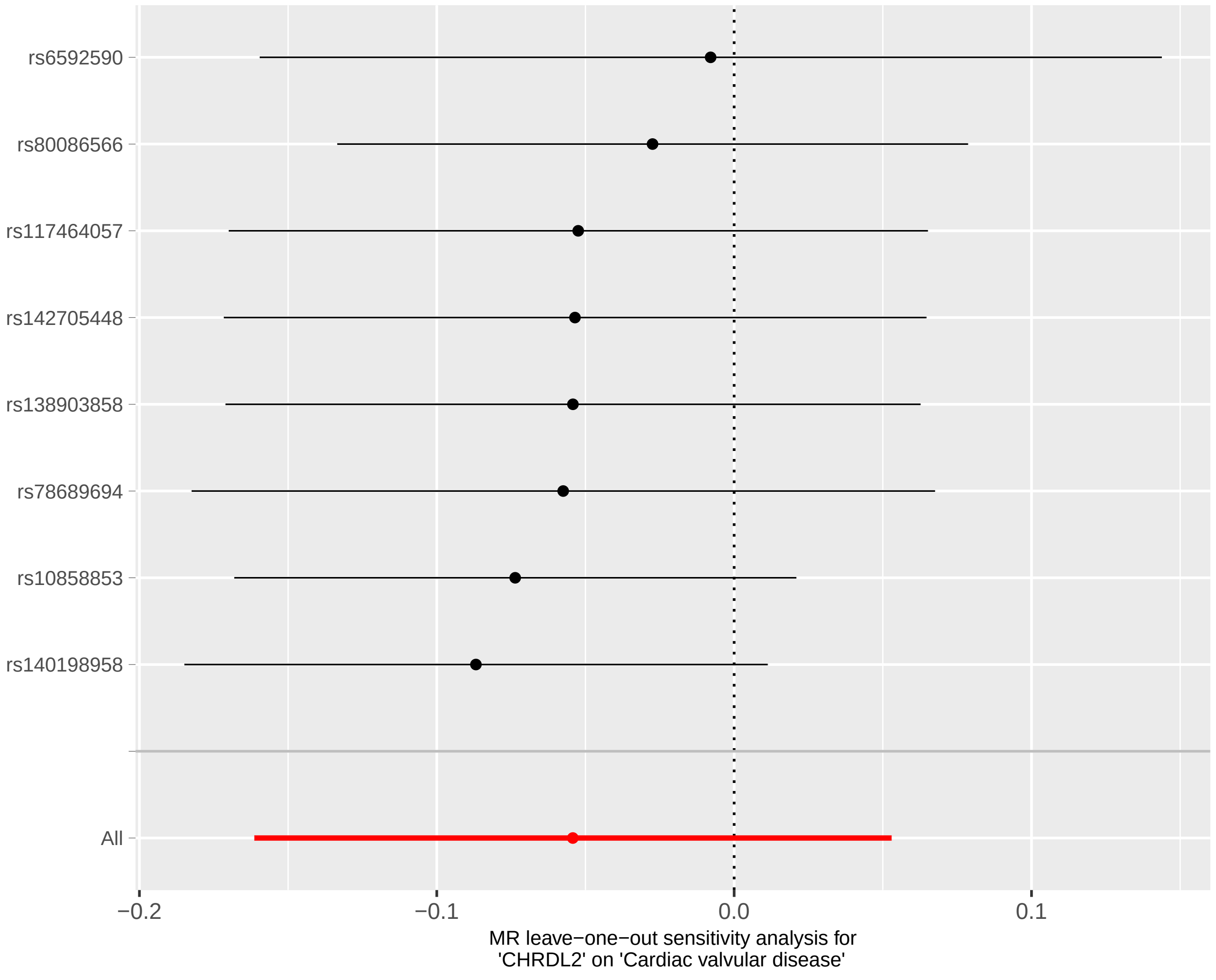
rs6592590
rs80086566
rs117464057
rs142705448
rs138903858
rs78689694
rs10858853
rs140198958
All
−0.2
−0.1
0.0
0.1
MR leave−one−out sensitivity analysis for
'CHRDL2' on 'Cardiac valvular disease'

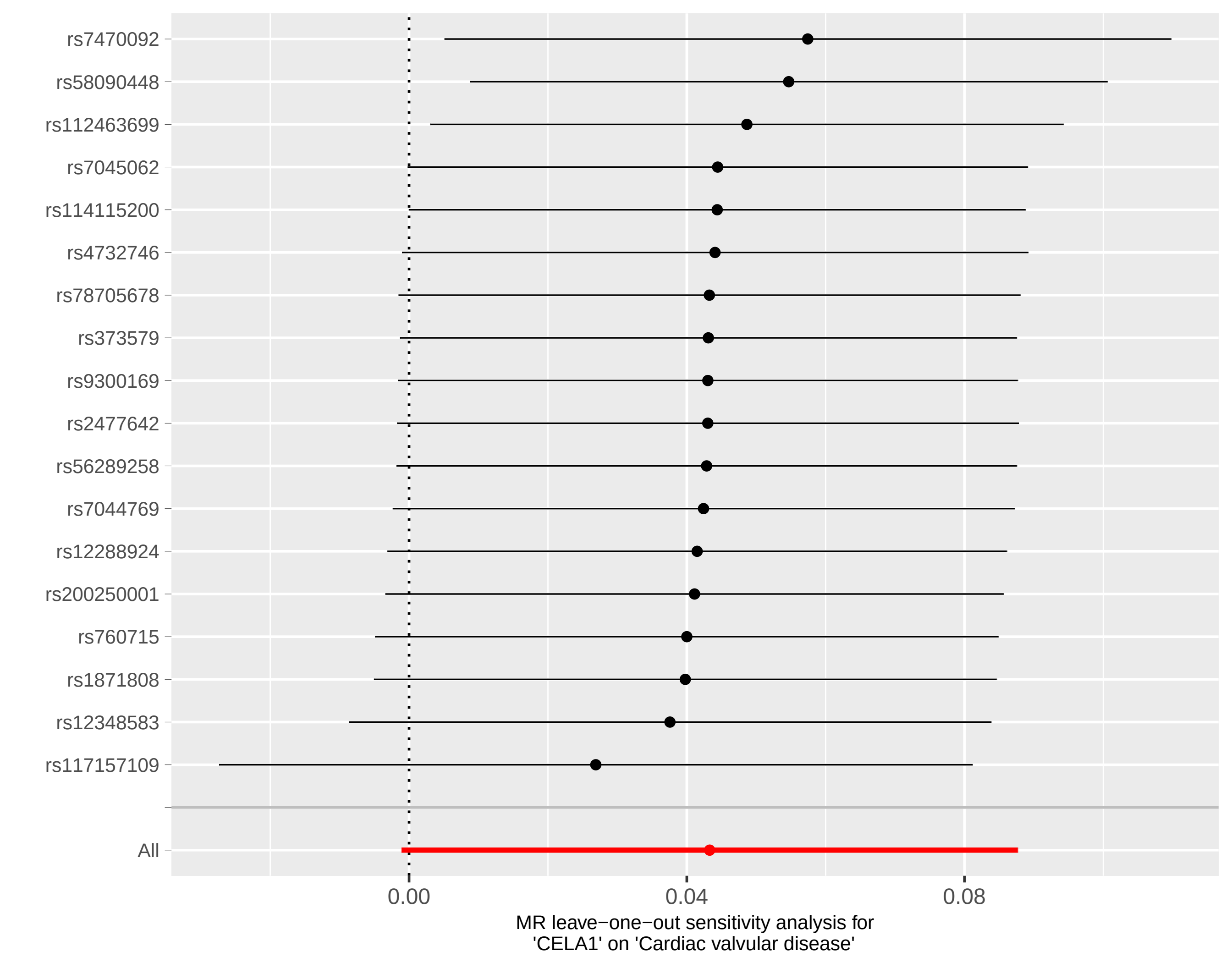
rs7470092
rs58090448
rs112463699
rs7045062
rs114115200
rs4732746
rs78705678
rs373579
rs9300169
rs2477642
rs56289258
rs7044769
rs12288924
rs200250001
rs760715
rs1871808
rs12348583
rs117157109
All
0.00
0.04
0.08
MR leave−one−out sensitivity analysis for
'CELA1' on 'Cardiac valvular disease'

Insufficient number of SNPs

Insufficient number of SNPs

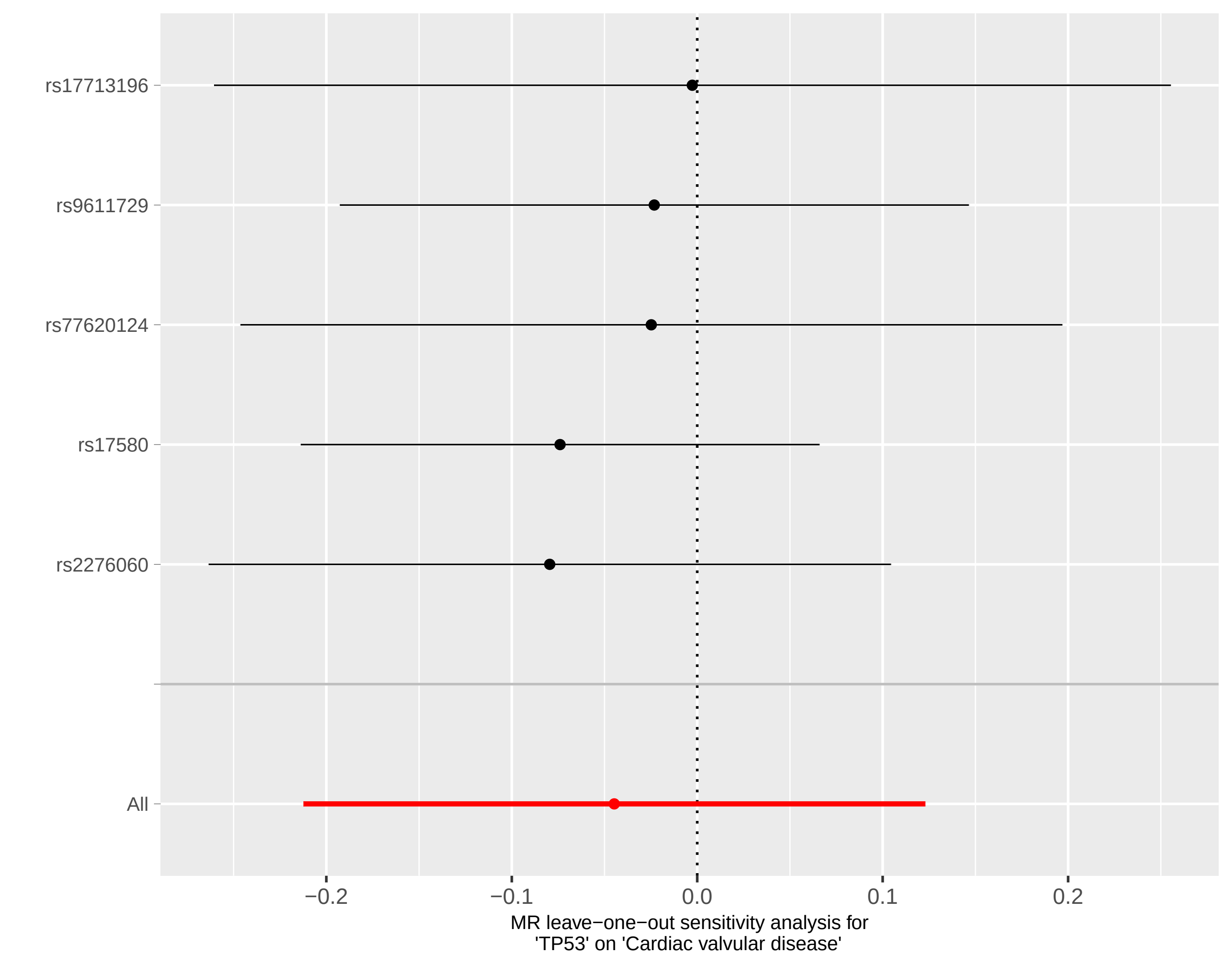
rs17713196
rs9611729
rs77620124
rs17580
rs2276060
All
−0.2
−0.1
0.0
0.1
0.2
MR leave−one−out sensitivity analysis for
'TP53' on 'Cardiac valvular disease'

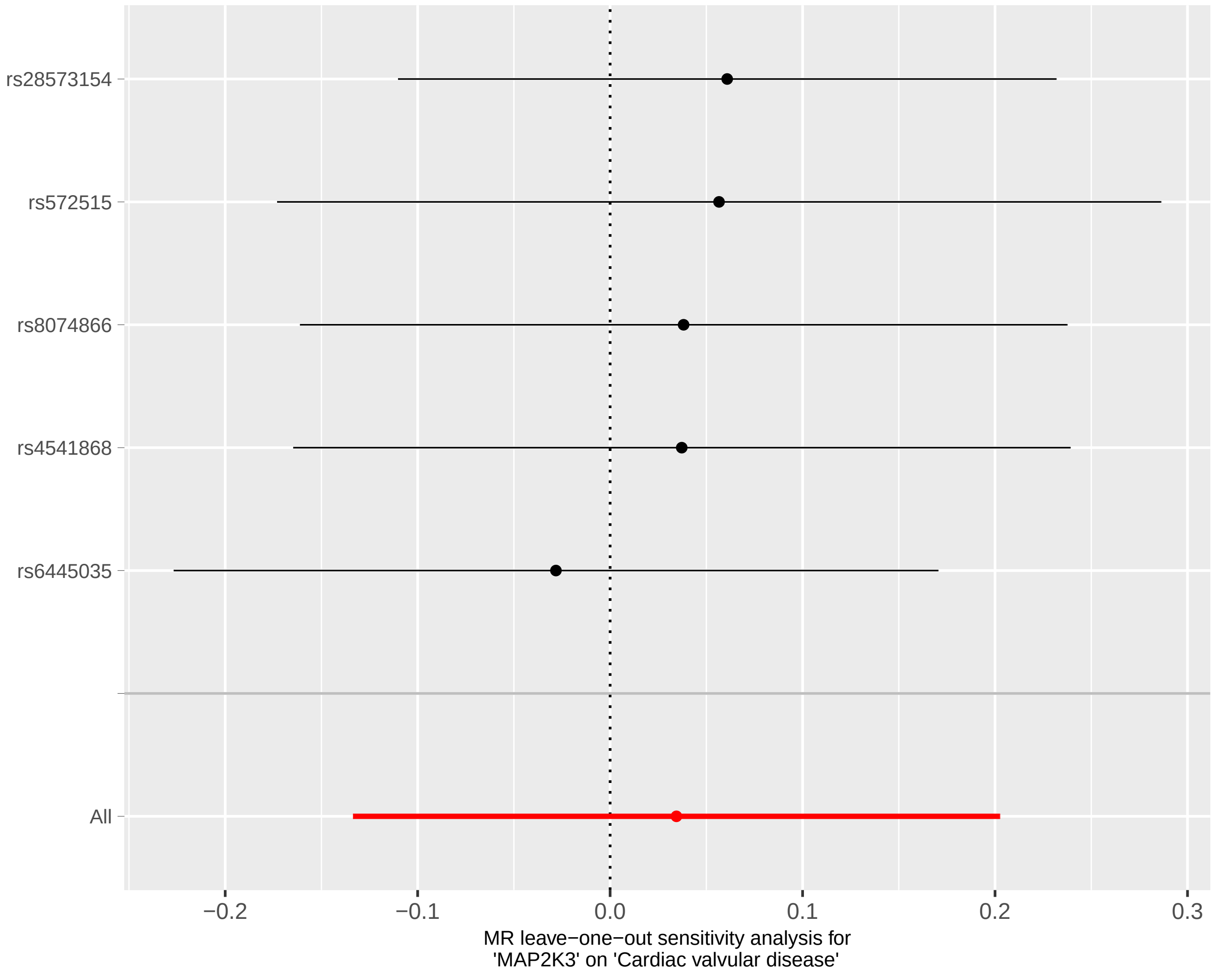
rs28573154
rs572515
rs8074866
rs4541868
rs6445035
All
−0.2
−0.1
0.0
0.1
0.2
0.3
MR leave−one−out sensitivity analysis for
'MAP2K3' on 'Cardiac valvular disease'

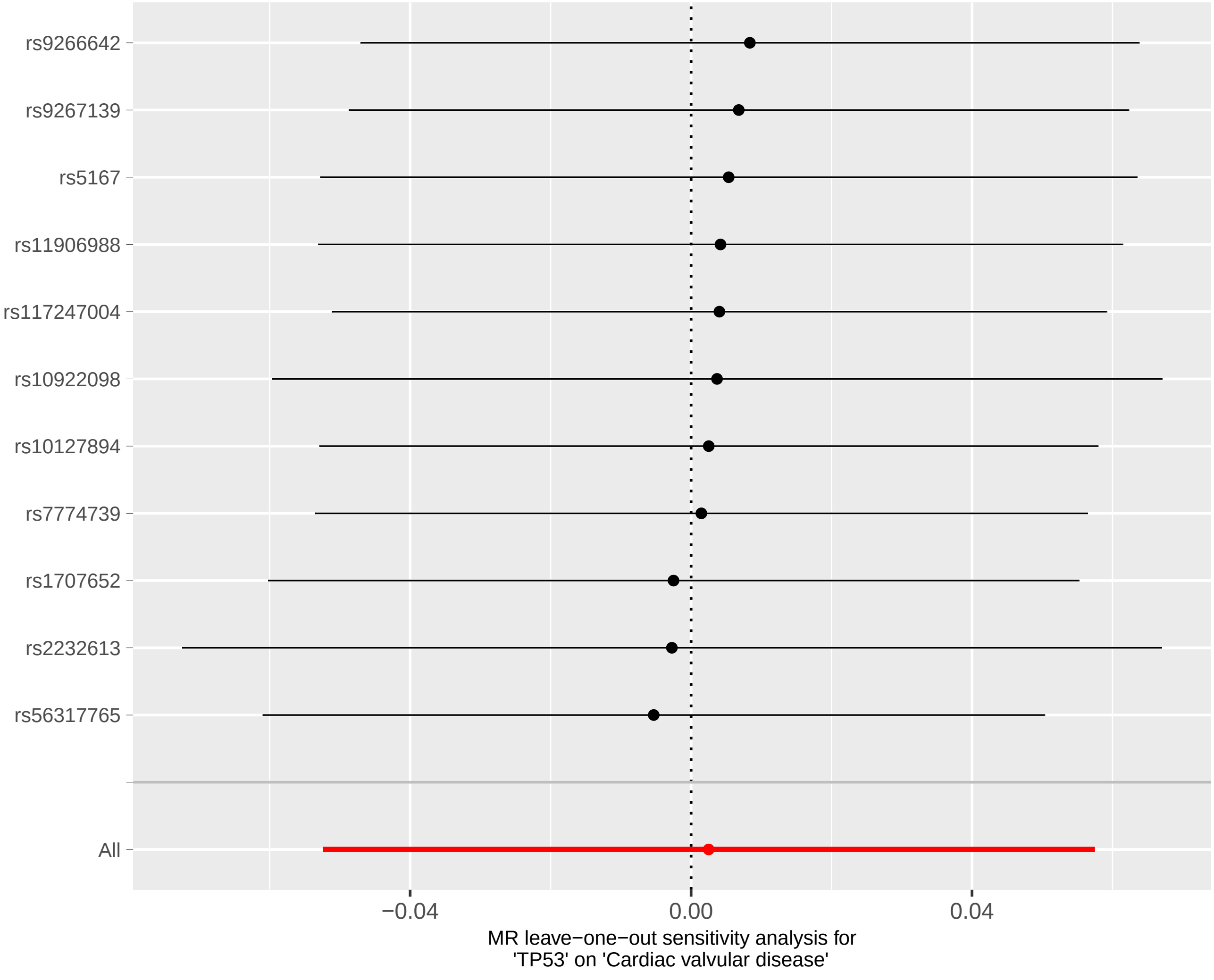
rs9266642
rs9267139
rs5167
rs11906988
rs117247004
rs10922098
rs10127894
rs7774739
rs1707652
rs2232613
rs56317765
All
−0.04
0.00
0.04
MR leave−one−out sensitivity analysis for
'TP53' on 'Cardiac valvular disease'

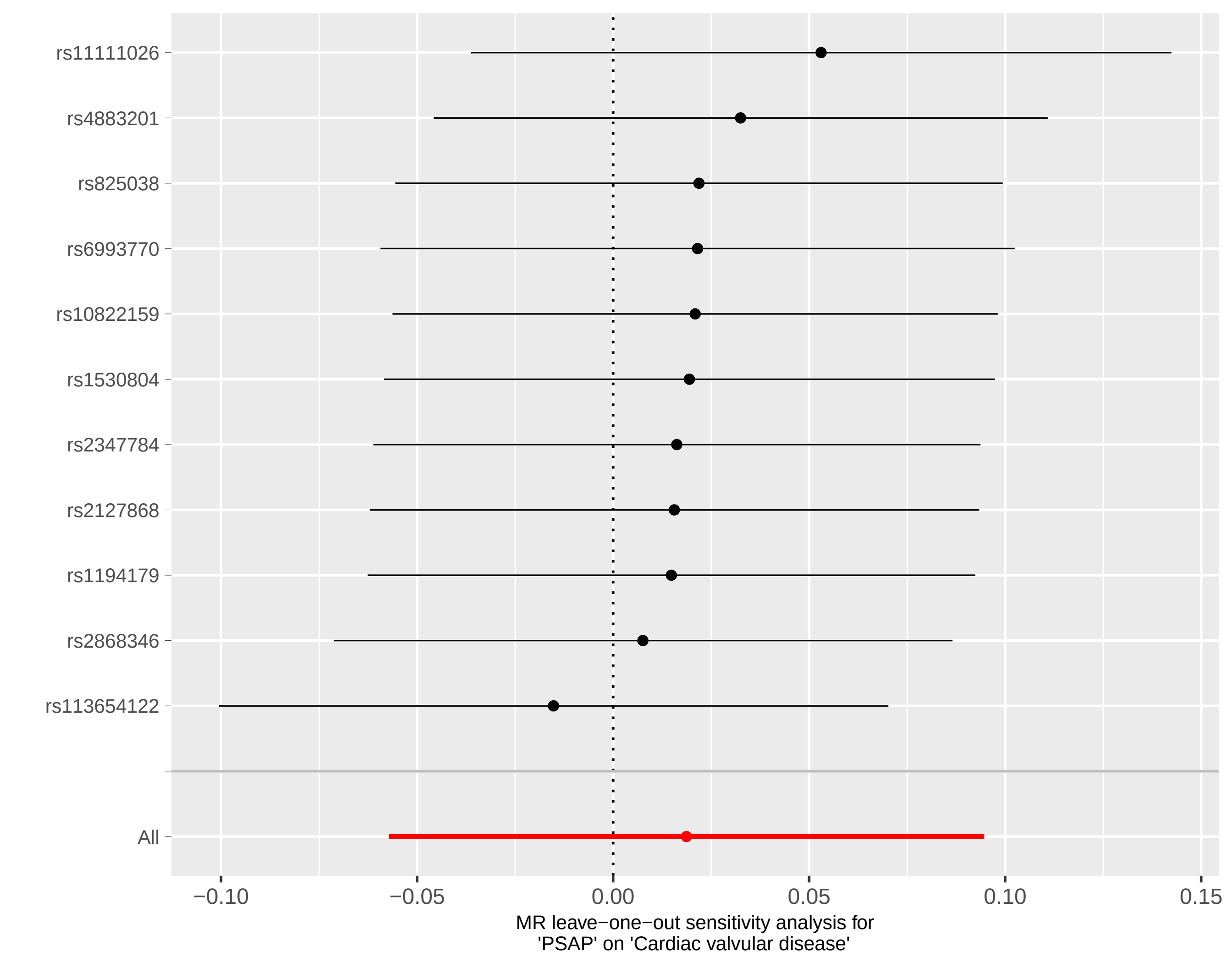
rs11111026
rs4883201
rs825038
rs6993770
rs10822159
rs1530804
rs2347784
rs2127868
rs1194179
rs2868346
rs113654122
All
−0.10
−0.05
0.00
0.05
0.10
0.15
MR leave−one−out sensitivity analysis for
'PSAP' on 'Cardiac valvular disease'

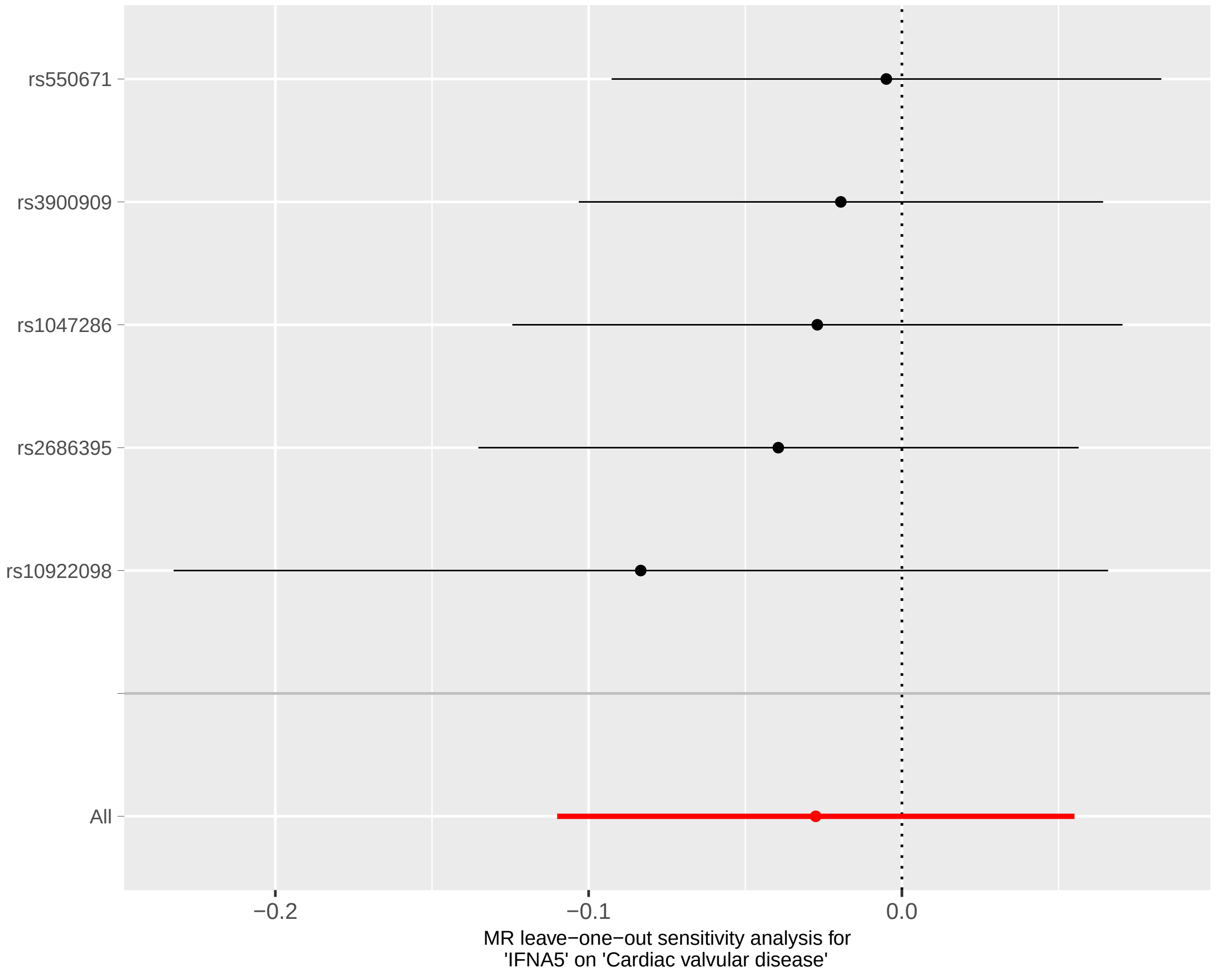
rs550671
rs3900909
rs1047286
rs2686395
rs10922098
All
−0.2
−0.1
0.0
MR leave−one−out sensitivity analysis for
'IFNA5' on 'Cardiac valvular disease'

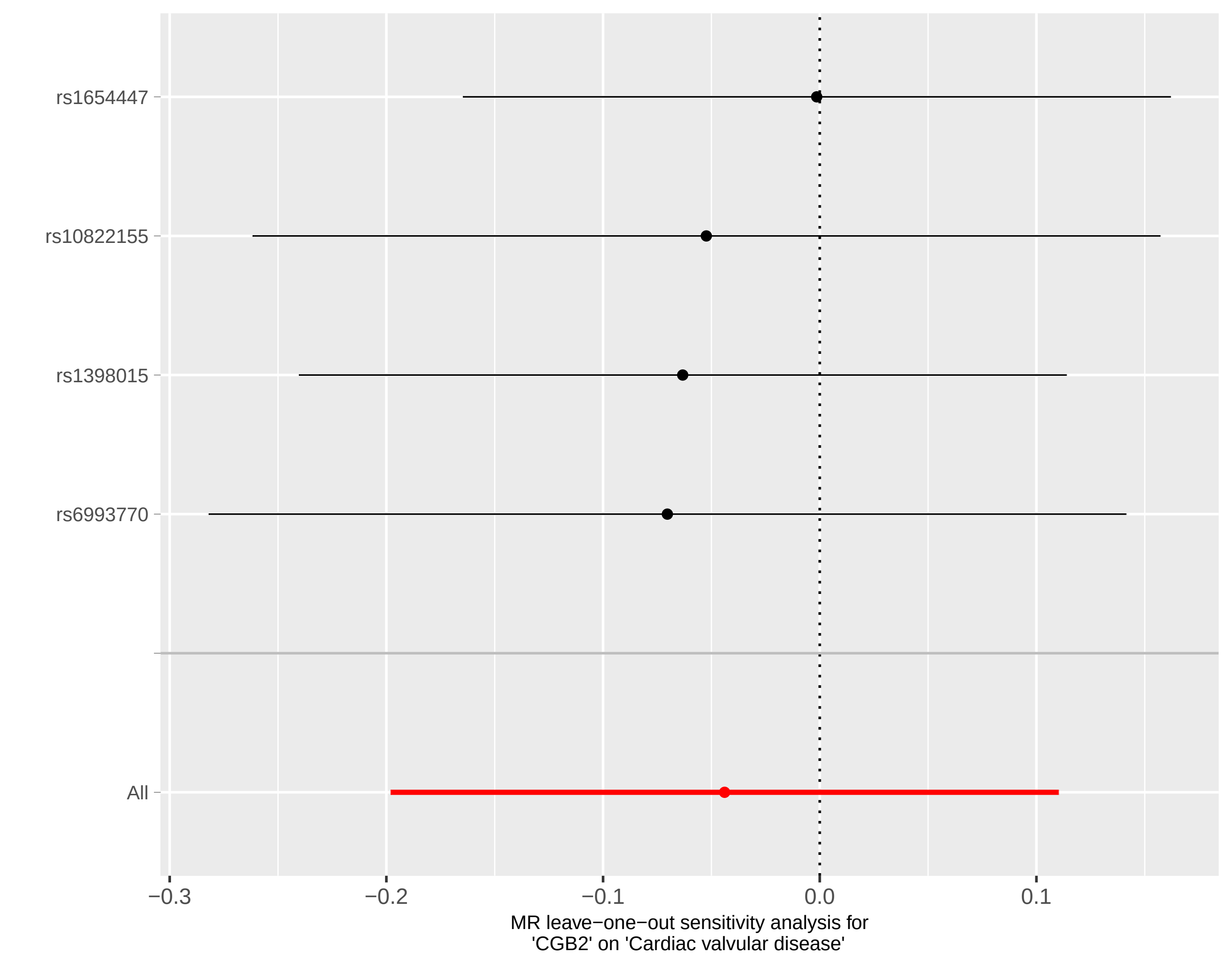
rs1654447
rs10822155
rs1398015
rs6993770
All
−0.3
−0.2
−0.1
0.0
0.1
MR leave−one−out sensitivity analysis for
'CGB2' on 'Cardiac valvular disease'

Insufficient number of SNPs

Insufficient number of SNPs

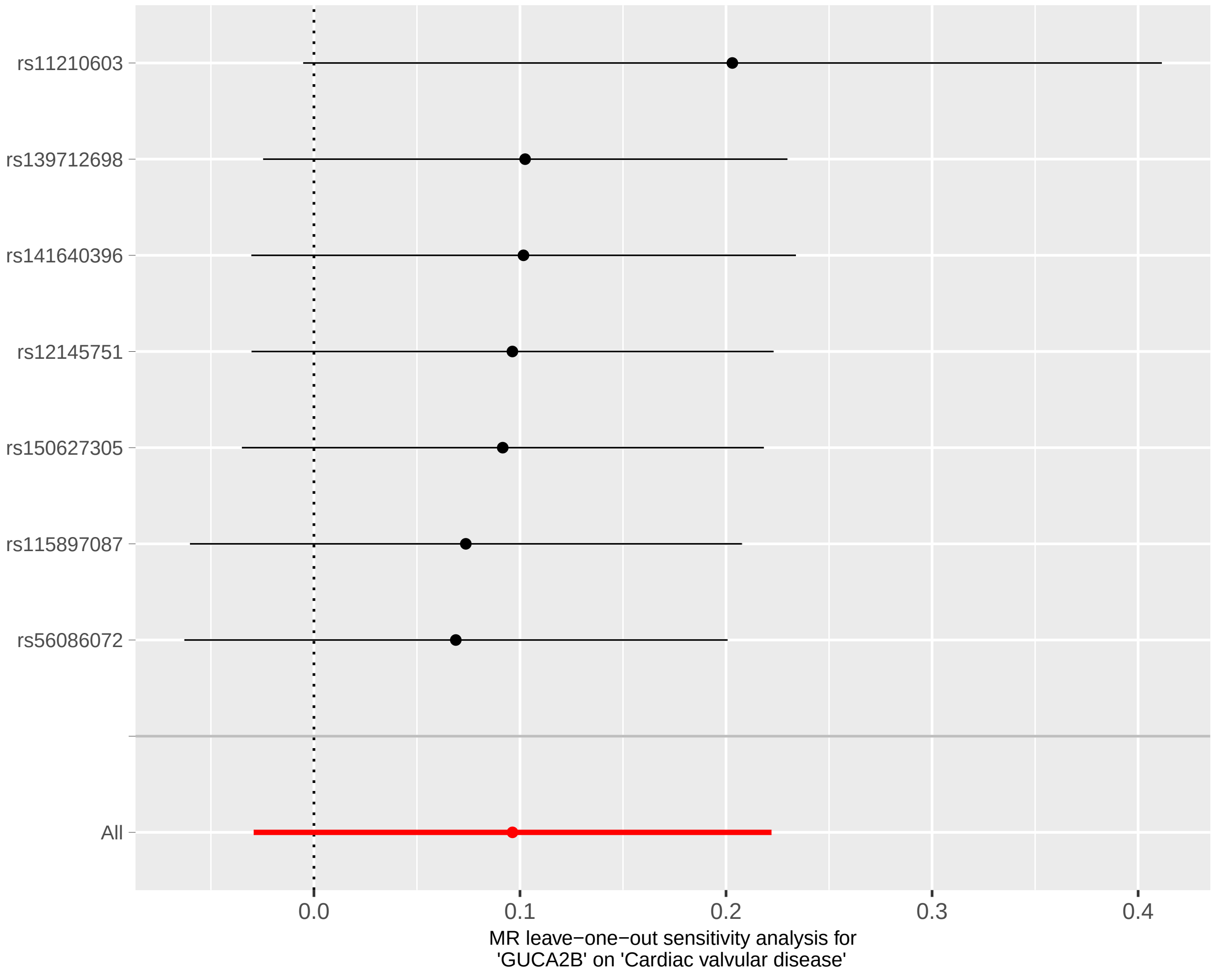

rs11210603
rs139712698
rs141640396
rs12145751
rs150627305
rs115897087
rs56086072
All
0.0
0.1
0.2
0.3
0.4
MR leave−one−out sensitivity analysis for
'GUCA2B' on 'Cardiac valvular disease'

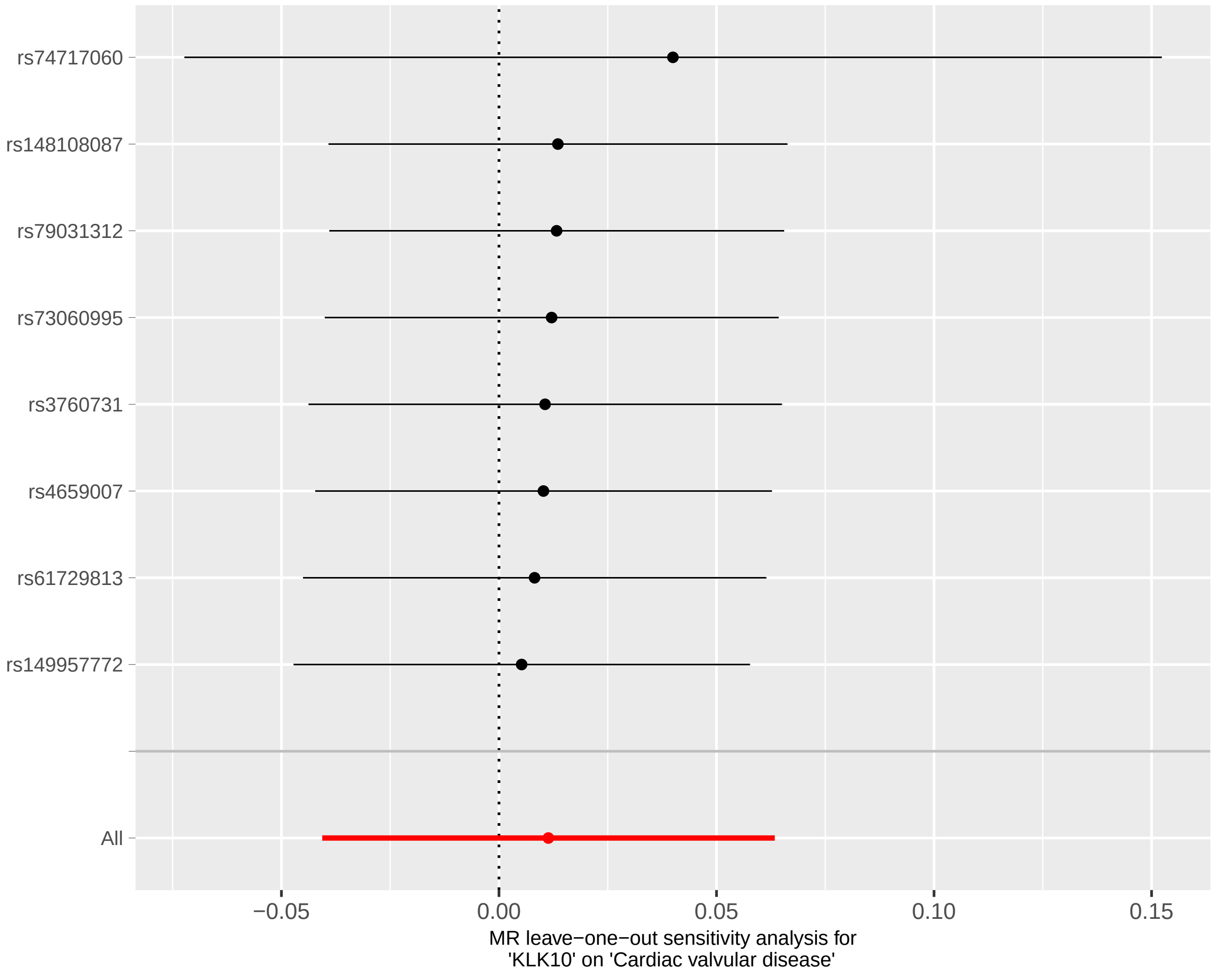
rs74717060
rs148108087
rs79031312
rs73060995
rs3760731
rs4659007
rs61729813
rs149957772
All
−0.05
0.00
0.05
0.10
0.15
MR leave−one−out sensitivity analysis for
'KLK10' on 'Cardiac valvular disease'

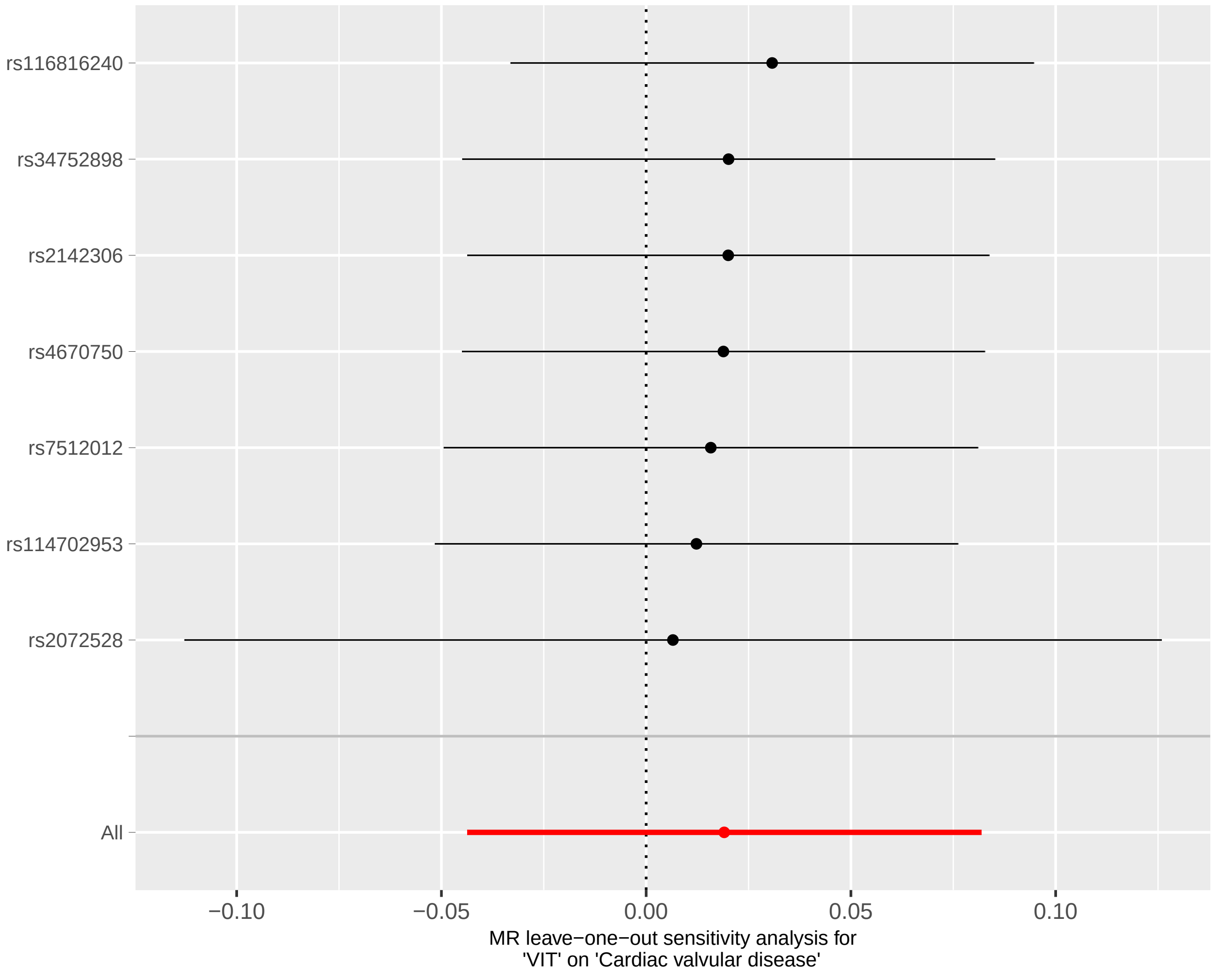
rs116816240
rs34752898
rs2142306
rs4670750
rs7512012
rs114702953
rs2072528
All
−0.10
−0.05
0.00
0.05
0.10
MR leave−one−out sensitivity analysis for
'VIT' on 'Cardiac valvular disease'

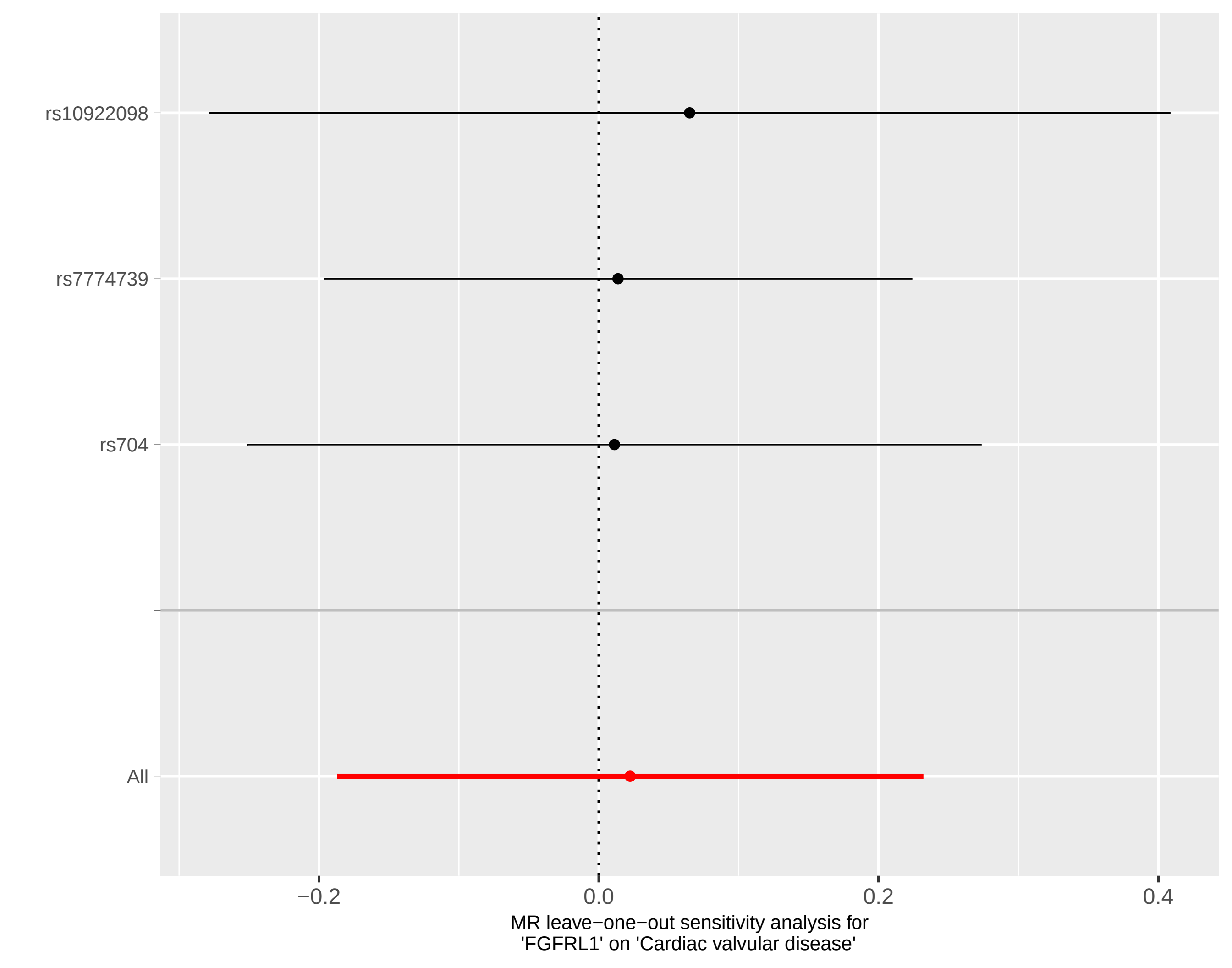
rs10922098
rs7774739
rs704
All
−0.2
0.0
0.2
0.4
MR leave−one−out sensitivity analysis for
'FGFRL1' on 'Cardiac valvular disease'

Insufficient number of SNPs

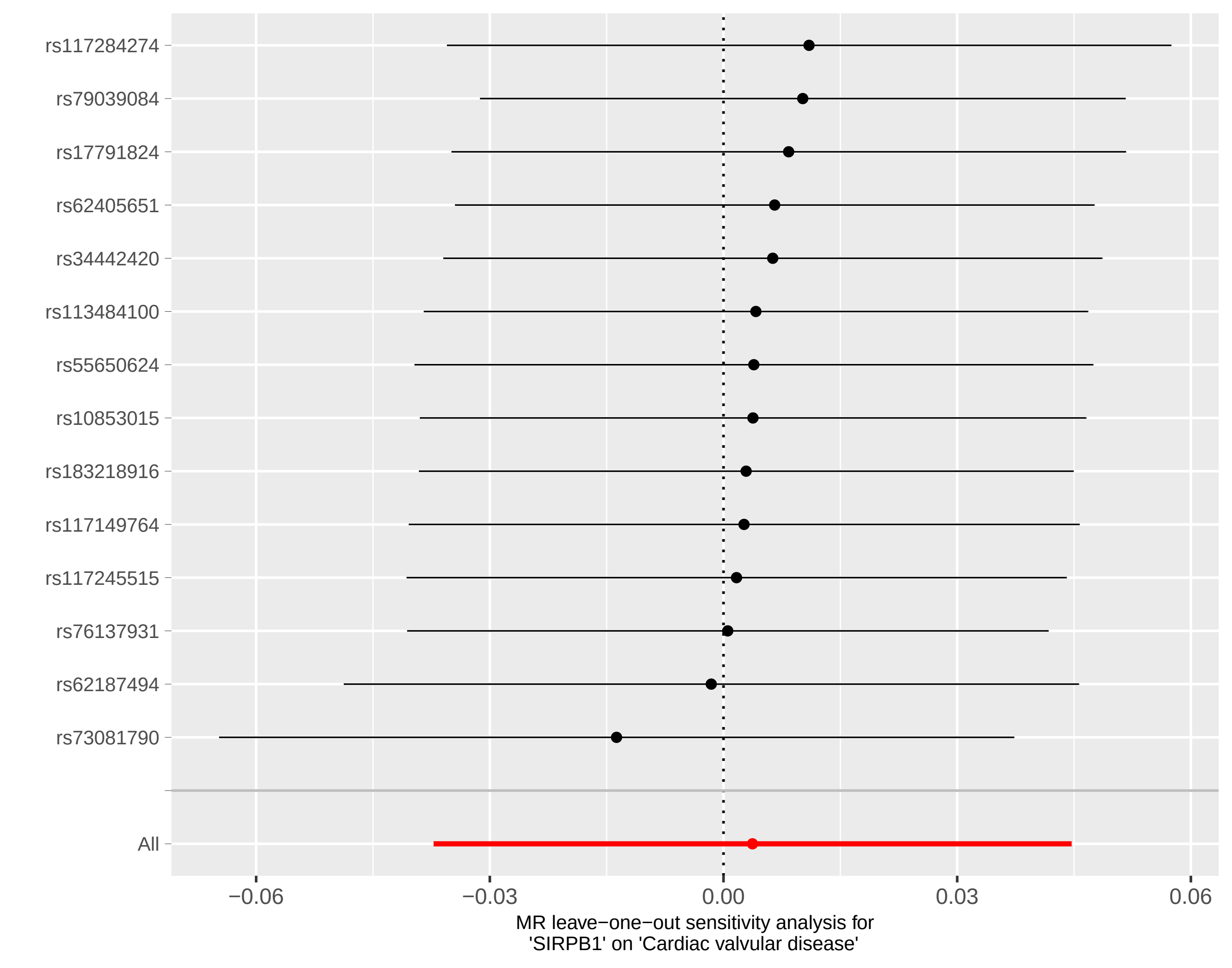
rs117284274
rs79039084
rs17791824
rs62405651
rs34442420
rs113484100
rs55650624
rs10853015
rs183218916
rs117149764
rs117245515
rs76137931
rs62187494
rs73081790
All
−0.06
−0.03
0.00
0.03
0.06
MR leave−one−out sensitivity analysis for
'SIRPB1' on 'Cardiac valvular disease'

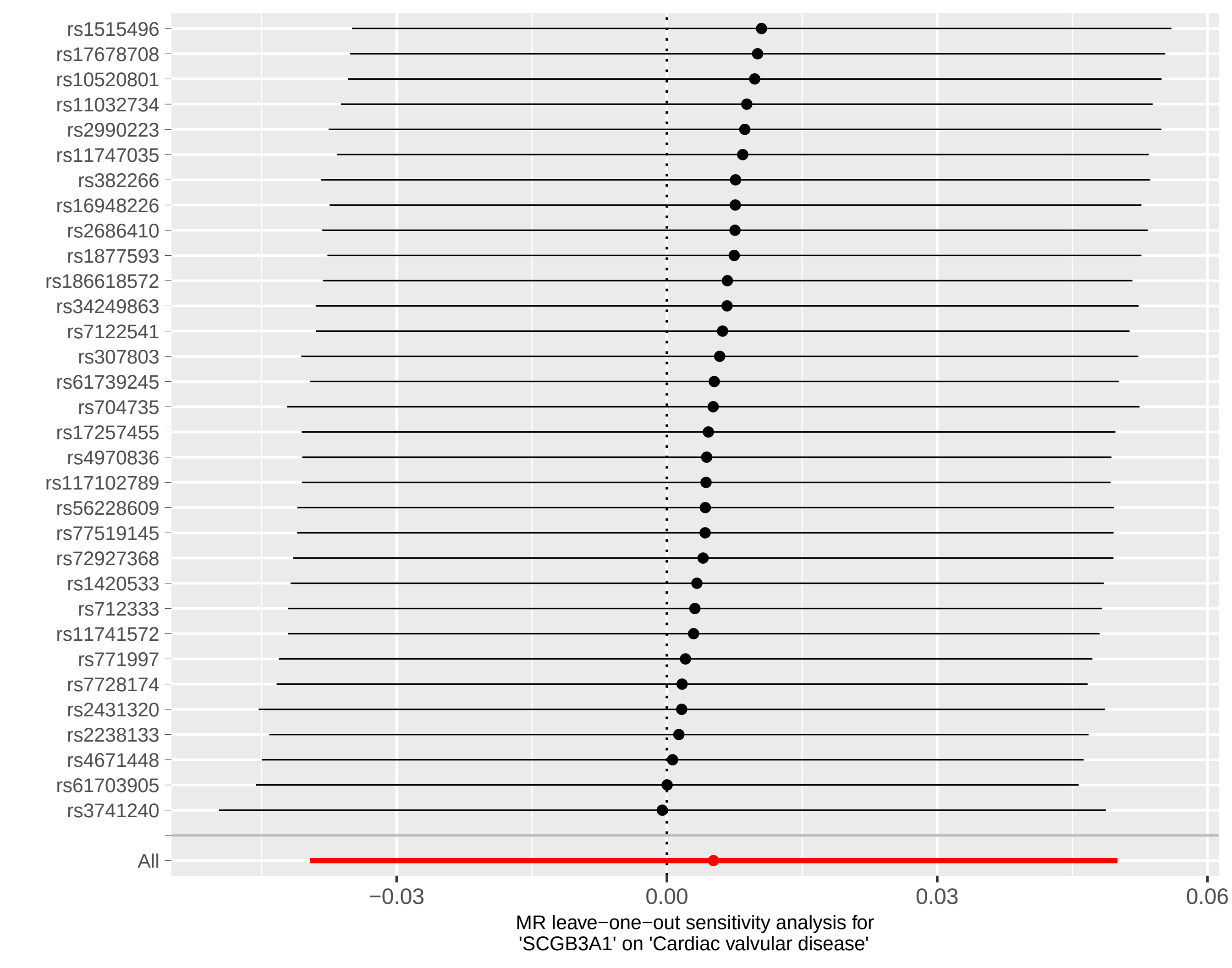
rs1515496
rs17678708
rs10520801
rs11032734
rs2990223
rs11747035
rs382266
rs16948226
rs2686410
rs1877593
rs186618572
rs34249863
rs7122541
rs307803
rs61739245
rs704735
rs17257455
rs4970836
rs117102789
rs56228609
rs77519145
rs72927368
rs1420533
rs712333
rs11741572
rs771997
rs7728174
rs2431320
rs2238133
rs4671448
rs61703905
rs3741240
All
−0.03
0.00
0.03
0.06
MR leave−one−out sensitivity analysis for
'SCGB3A1' on 'Cardiac valvular disease'

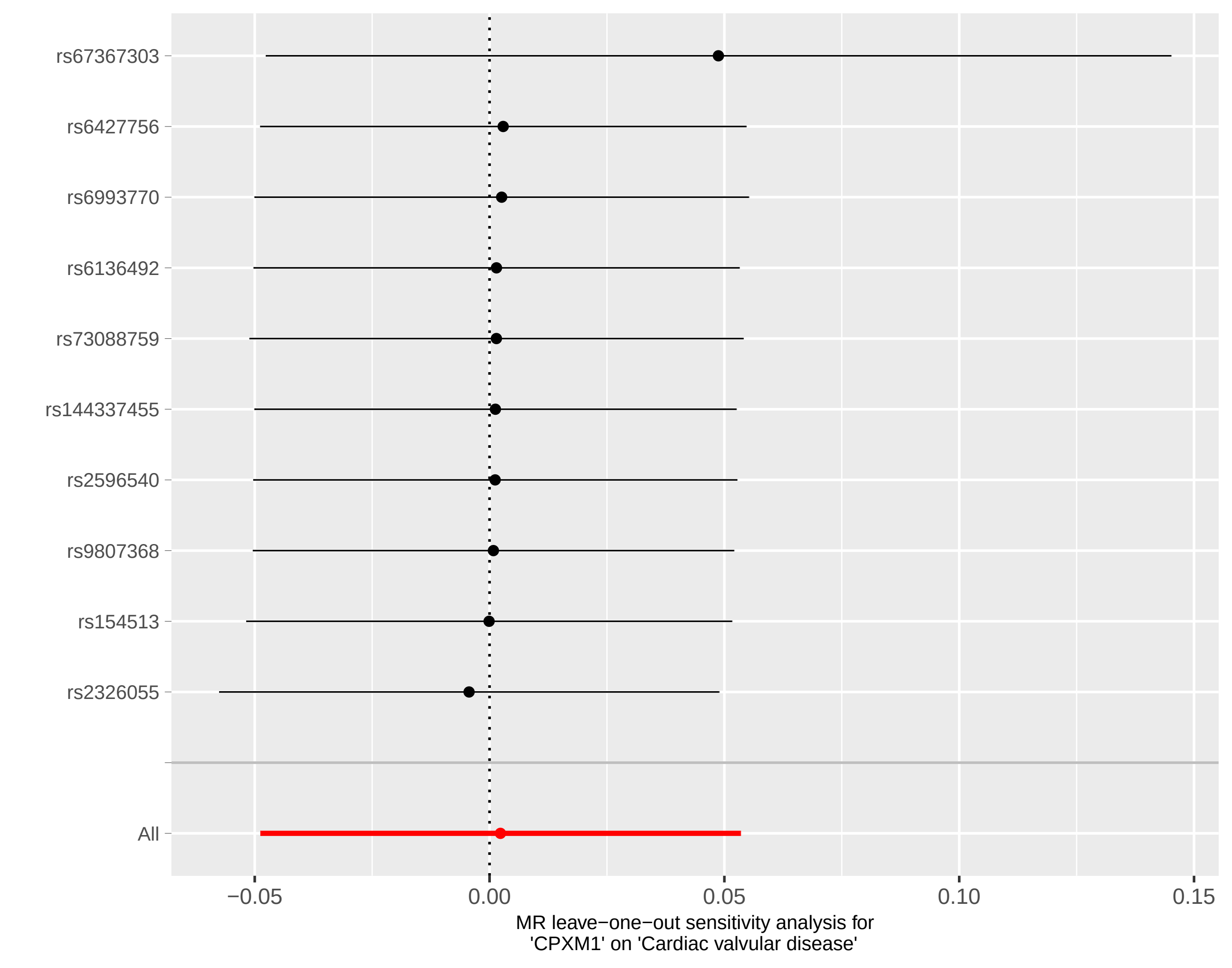
rs67367303
rs6427756
rs6993770
rs6136492
rs73088759
rs144337455
rs2596540
rs9807368
rs154513
rs2326055
All
−0.05
0.00
0.05
0.10
0.15
MR leave−one−out sensitivity analysis for
'CPXM1' on 'Cardiac valvular disease'

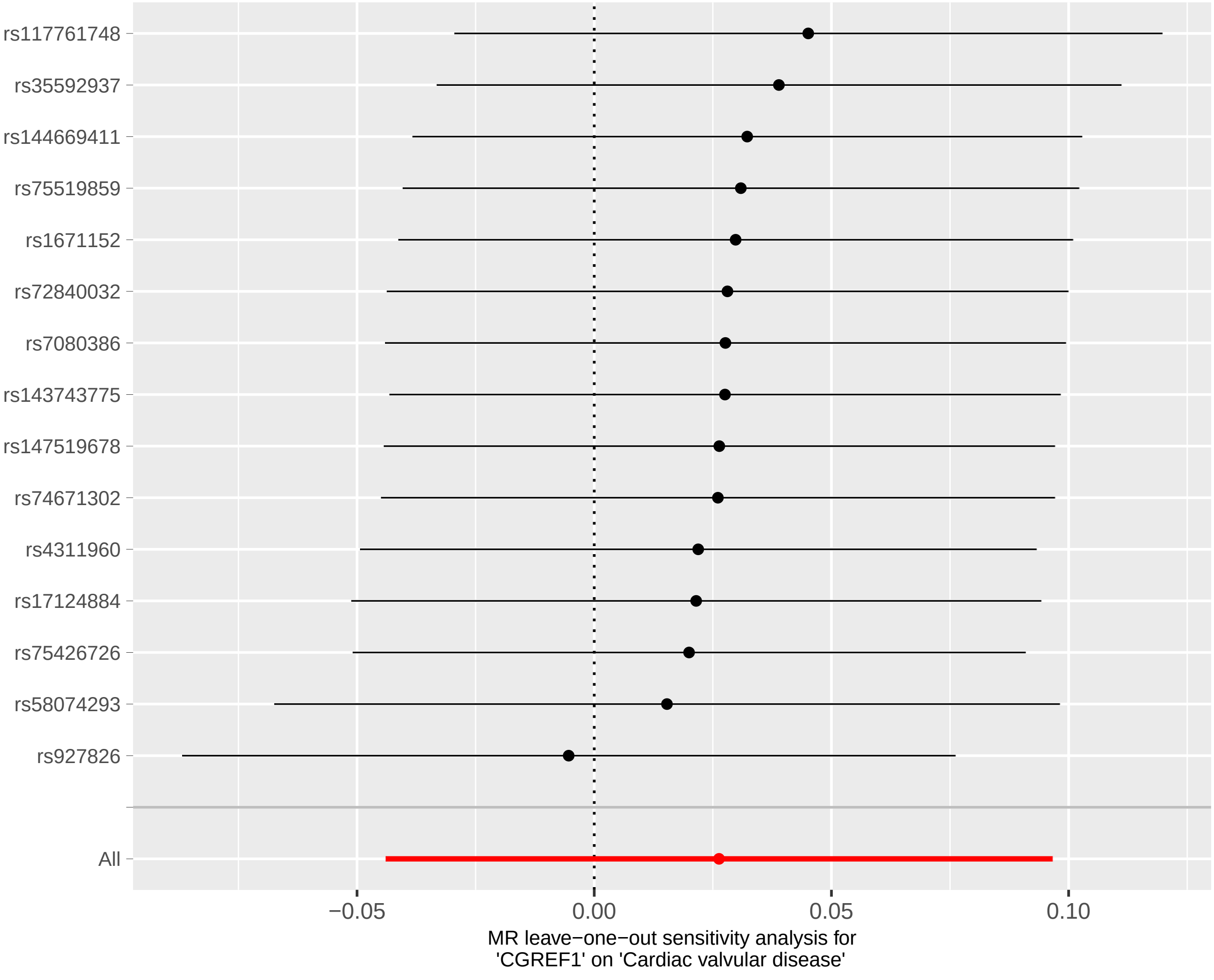
rs117761748
rs35592937
rs144669411
rs75519859
rs1671152
rs72840032
rs7080386
rs143743775
rs147519678
rs74671302
rs4311960
rs17124884
rs75426726
rs58074293
rs927826
All
−0.05
0.00
0.05
0.10
MR leave−one−out sensitivity analysis for
'CGREF1' on 'Cardiac valvular disease'

Insufficient number of SNPs

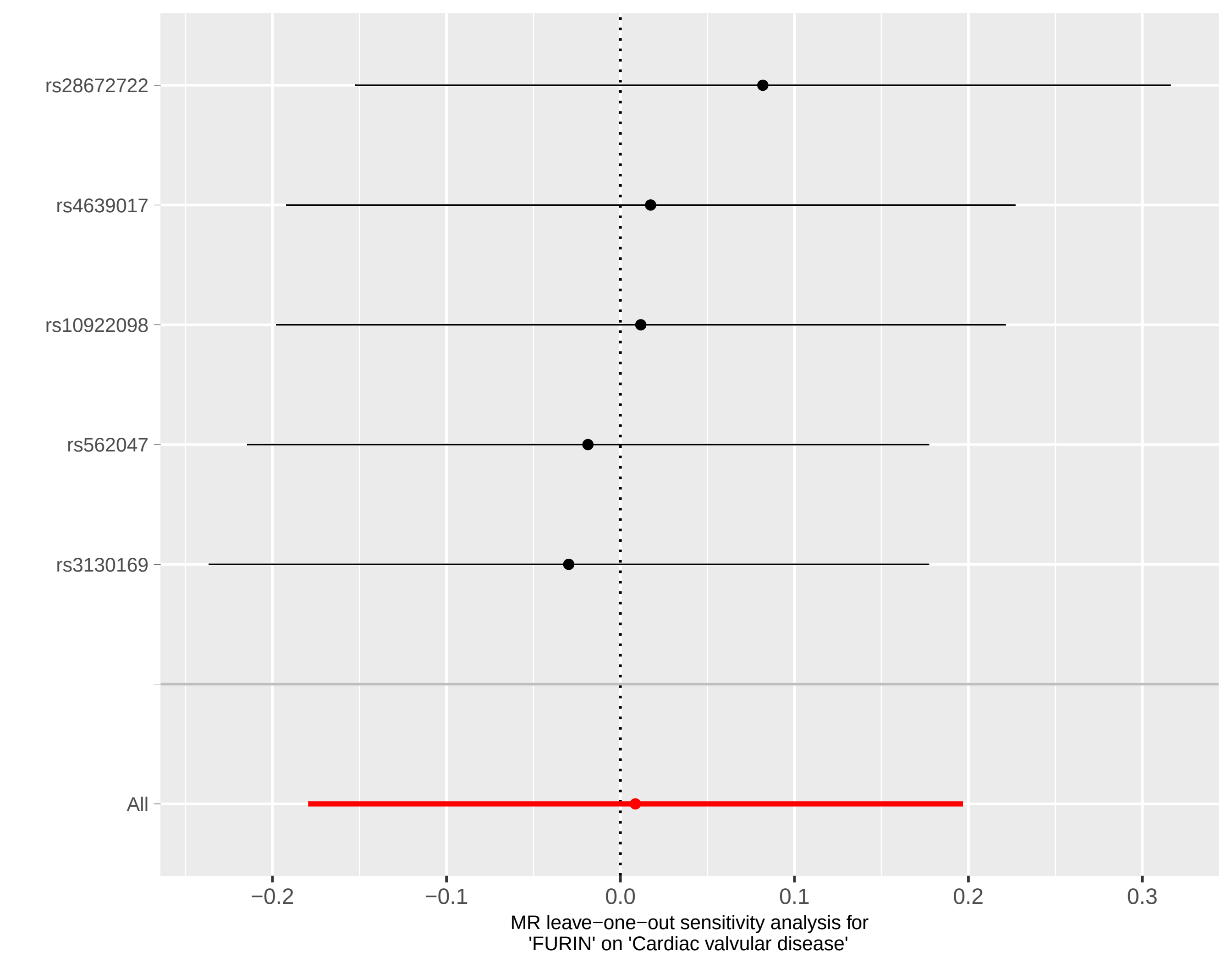
rs28672722
rs4639017
rs10922098
rs562047
rs3130169
All
−0.2
−0.1
0.0
0.1
0.2
0.3
MR leave−one−out sensitivity analysis for
'FURIN' on 'Cardiac valvular disease'

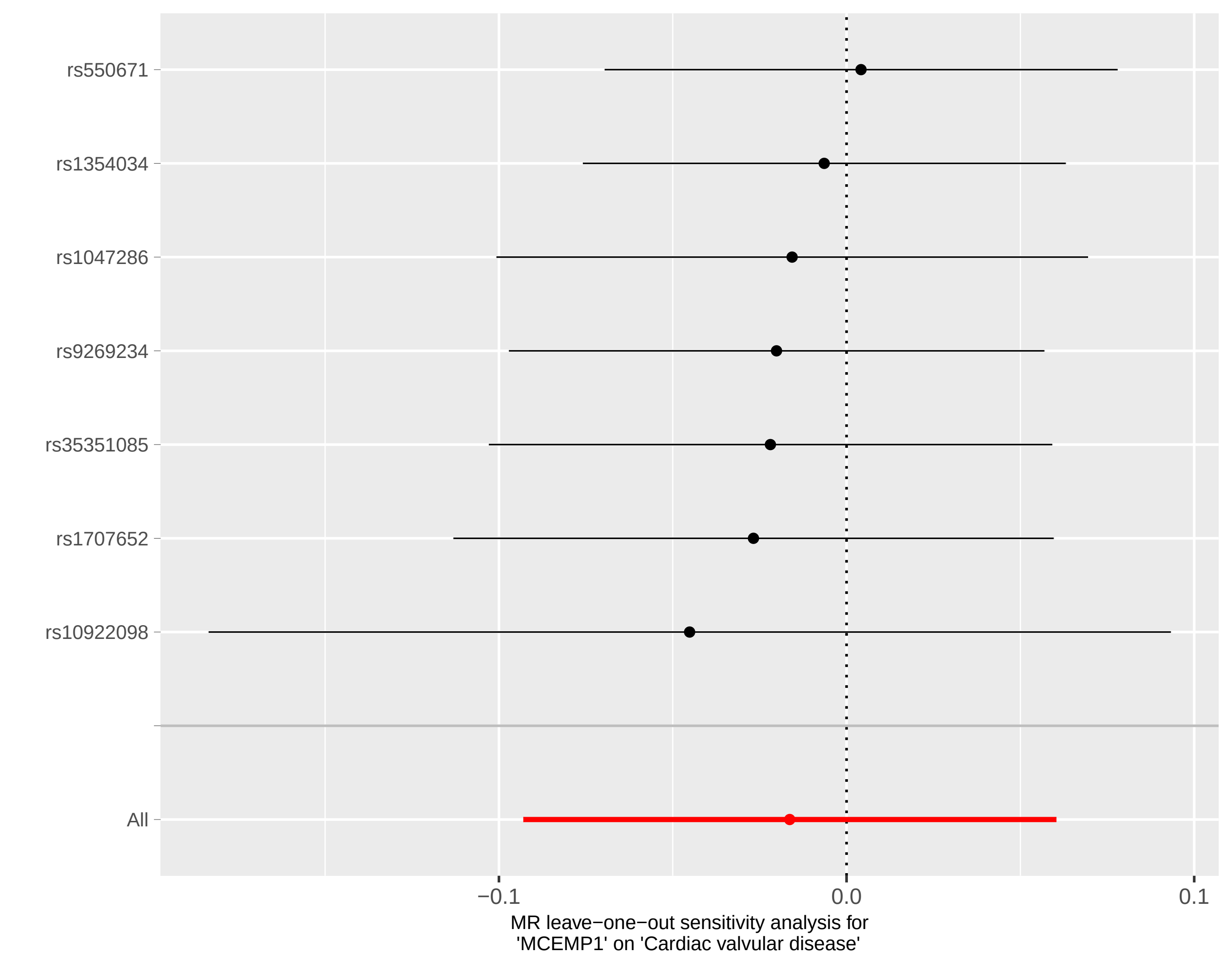
rs550671
rs1354034
rs1047286
rs9269234
rs35351085
rs1707652
rs10922098
All
−0.1
0.0
0.1
MR leave−one−out sensitivity analysis for
'MCEMP1' on 'Cardiac valvular disease'

Insufficient number of SNPs

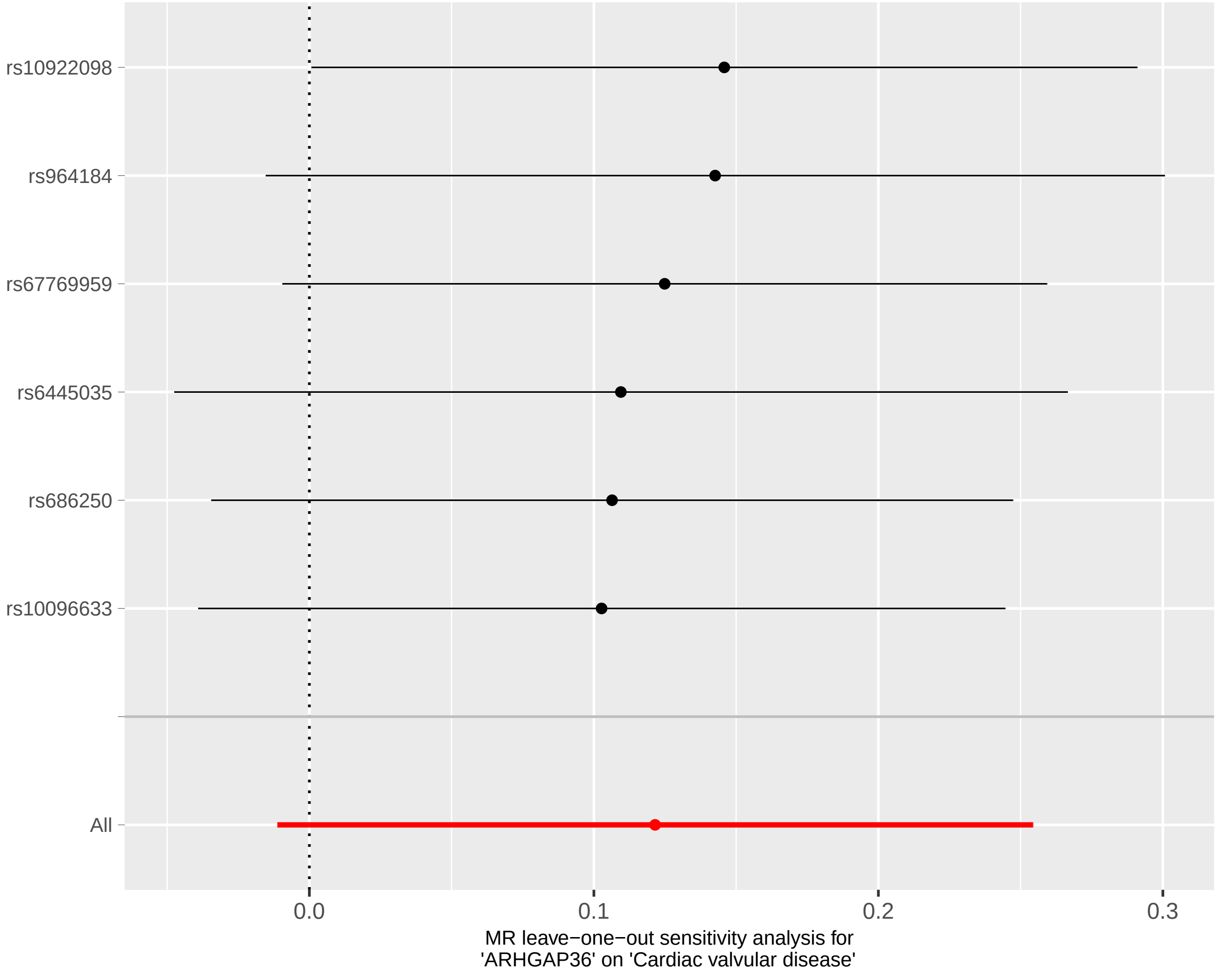

rs10922098
rs964184
rs67769959
rs6445035
rs686250
rs10096633
All
0.0
0.1
0.2
0.3
MR leave−one−out sensitivity analysis for
'ARHGAP36' on 'Cardiac valvular disease'

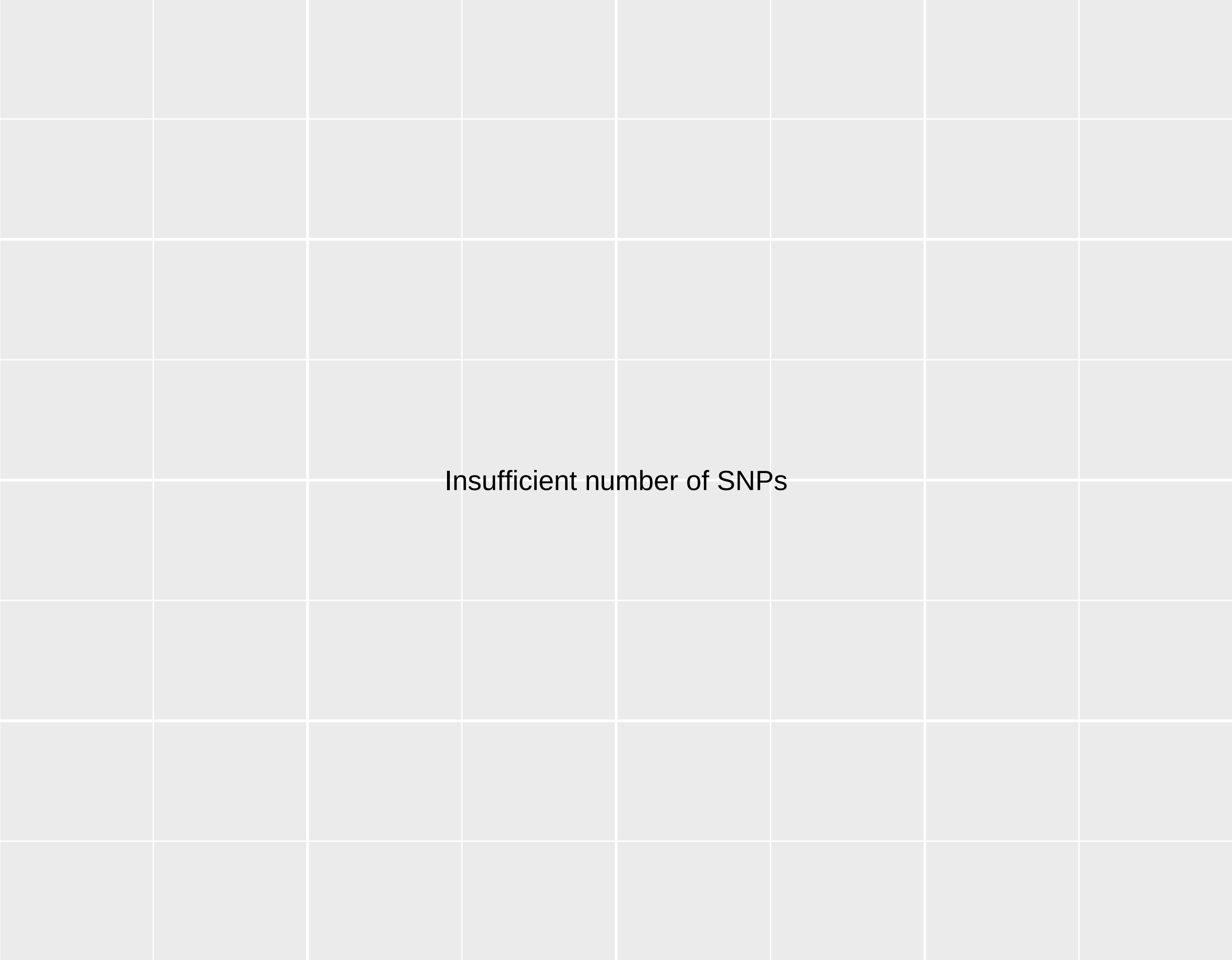
Insufficient number of SNPs

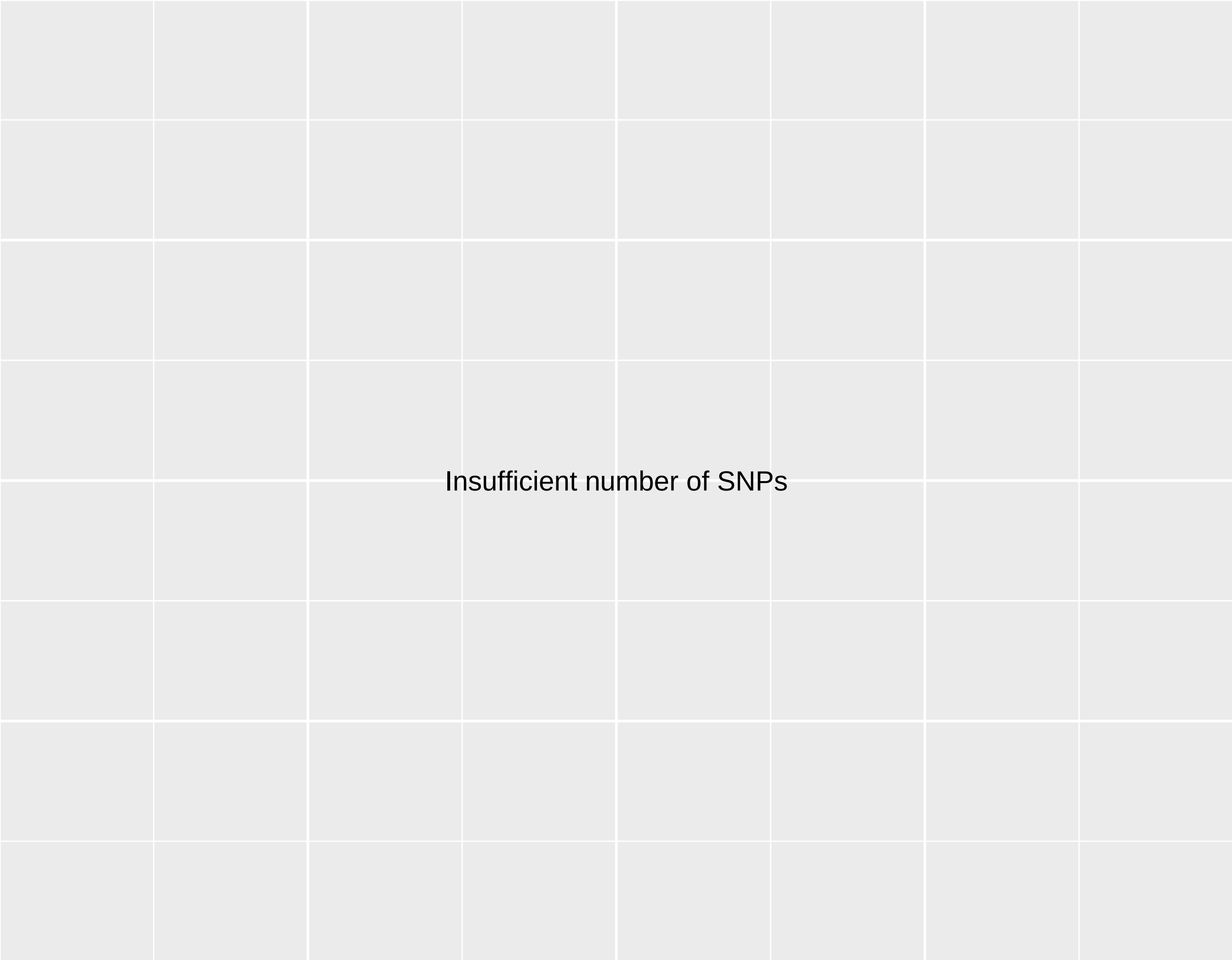
Insufficient number of SNPs

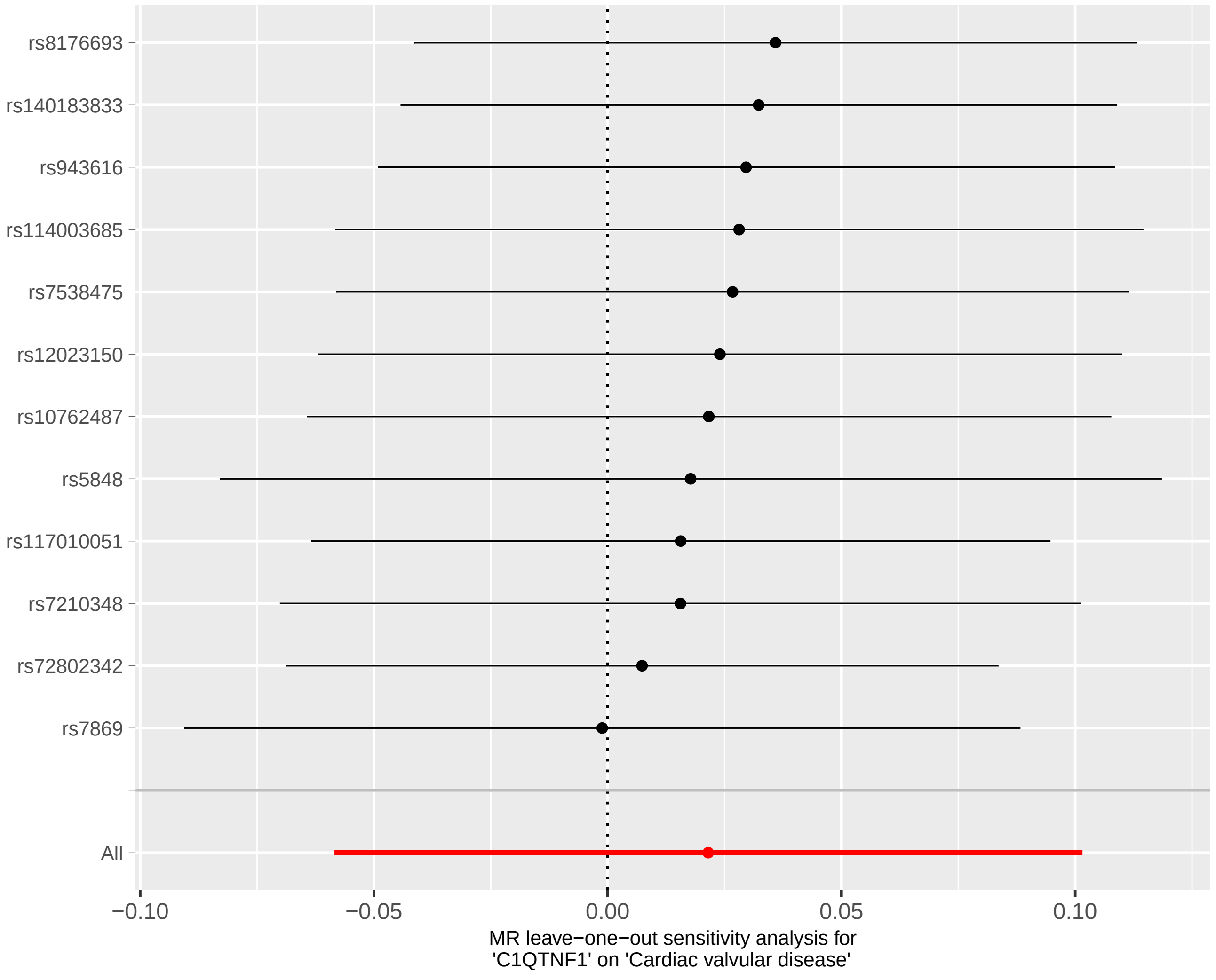
rs8176693
rs140183833
rs943616
rs114003685
rs7538475
rs12023150
rs10762487
rs5848
rs117010051
rs7210348
rs72802342
rs7869
All
−0.10
−0.05
0.00
0.05
0.10
MR leave−one−out sensitivity analysis for
'C1QTNF1' on 'Cardiac valvular disease'

Insufficient number of SNPs

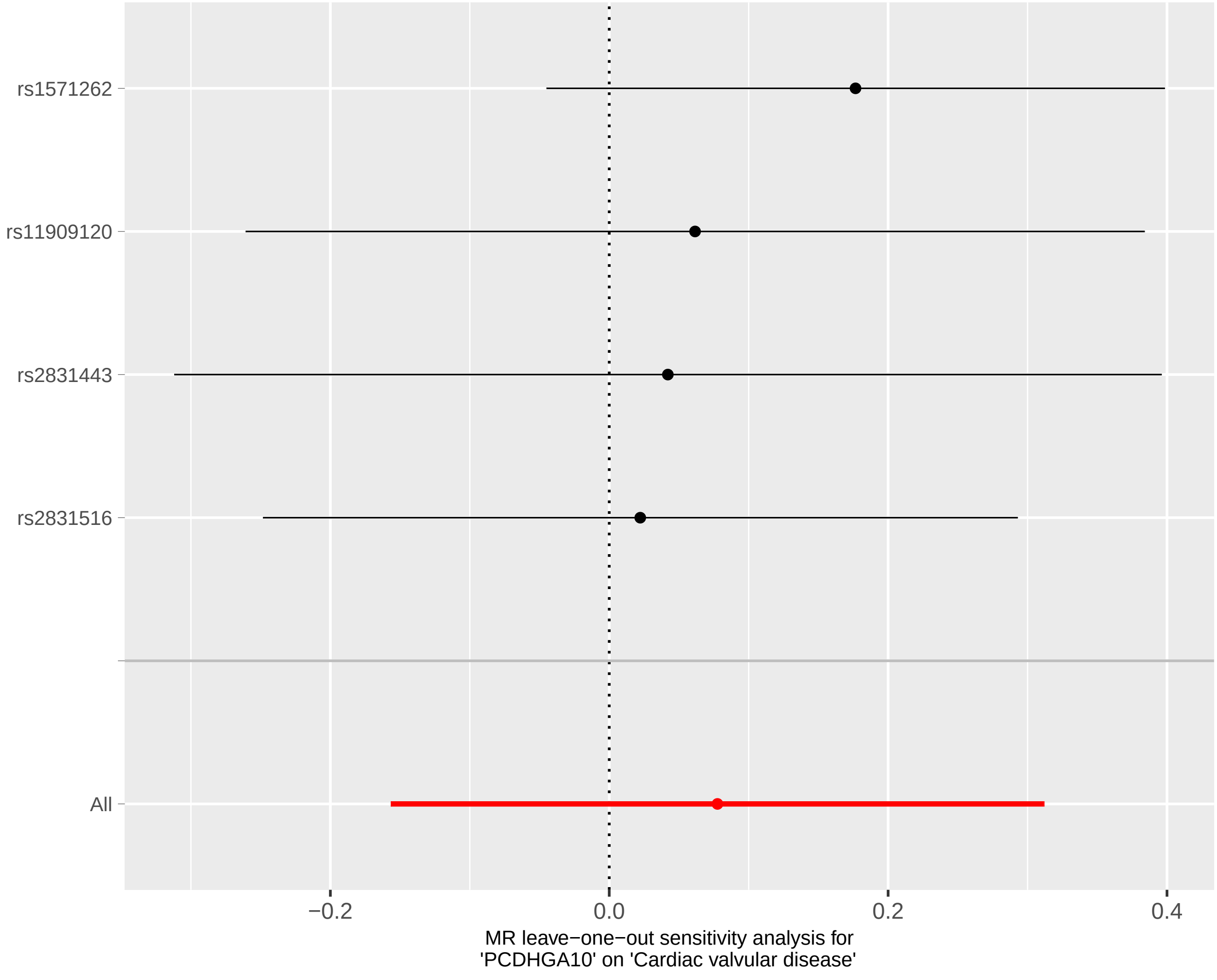
rs1571262
rs11909120
rs2831443
rs2831516
All
−0.2
0.0
0.2
0.4
MR leave−one−out sensitivity analysis for
'PCDHGA10' on 'Cardiac valvular disease'

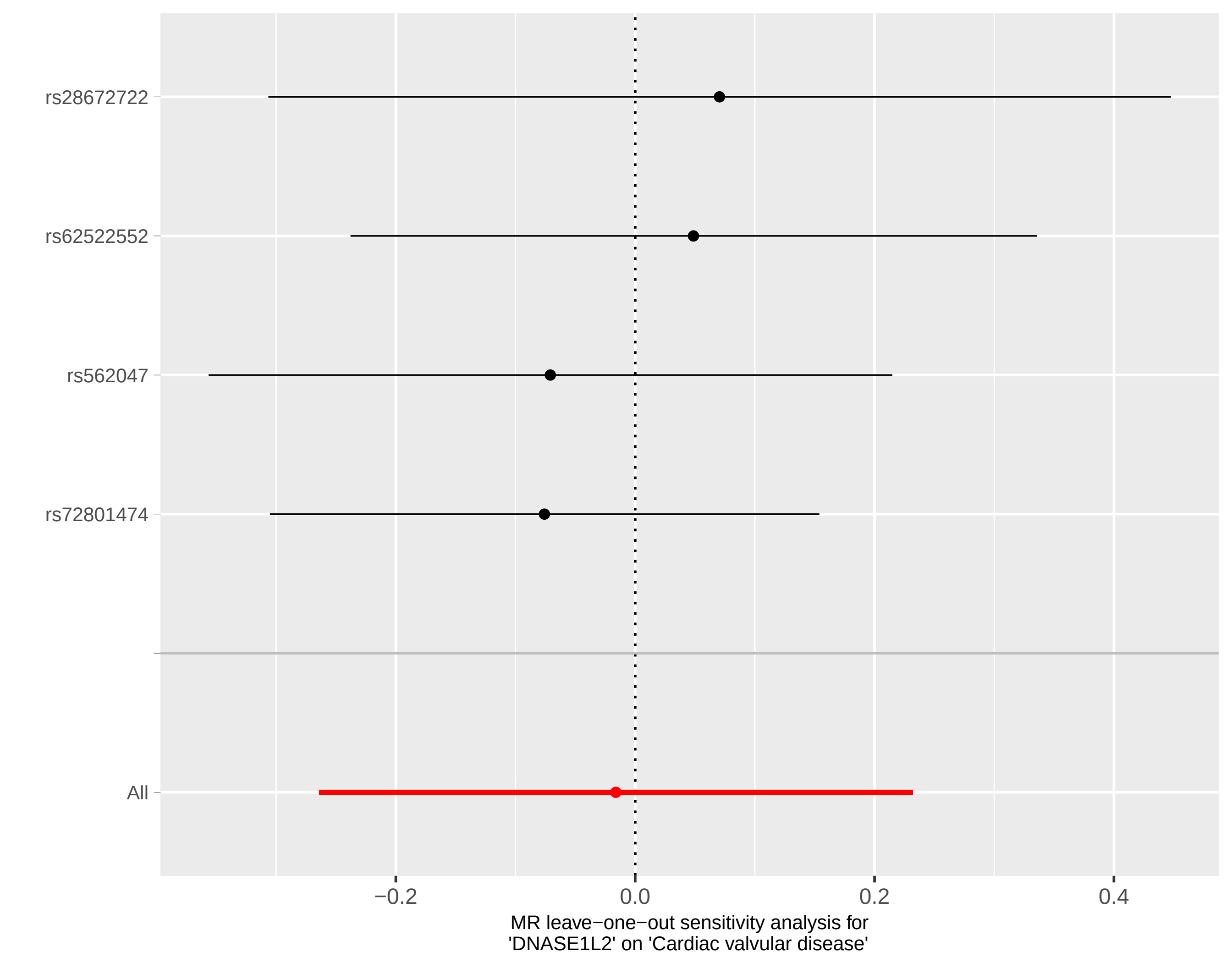

rs28672722
rs62522552
rs562047
rs72801474
All
−0.2
0.0
0.2
0.4
MR leave−one−out sensitivity analysis for
'DNASE1L2' on 'Cardiac valvular disease'

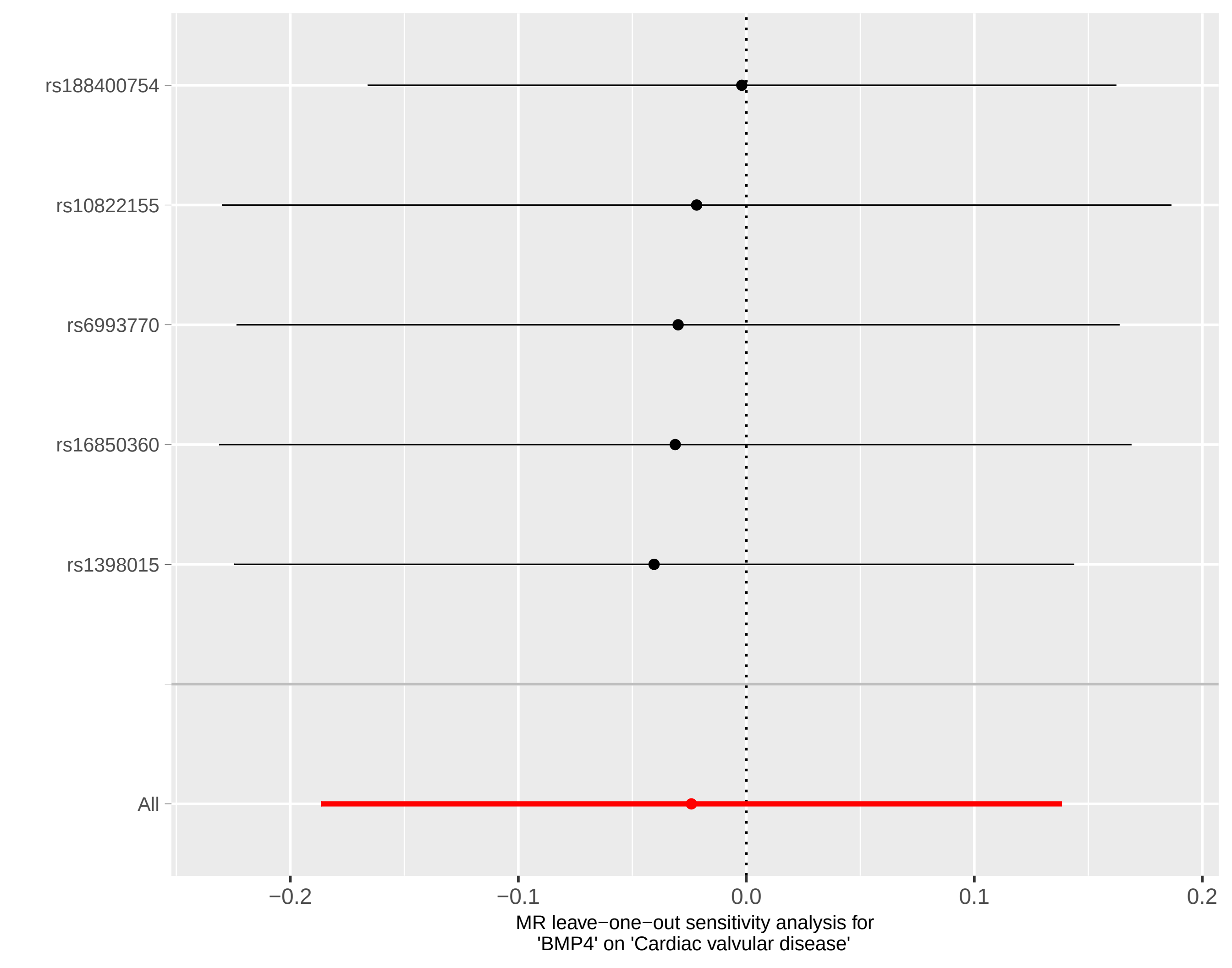
rs188400754
rs10822155
rs6993770
rs16850360
rs1398015
All
−0.2
−0.1
0.0
0.1
0.2
MR leave−one−out sensitivity analysis for
'BMP4' on 'Cardiac valvular disease'

Insufficient number of SNPs

Insufficient number of SNPs

Insufficient number of SNPs

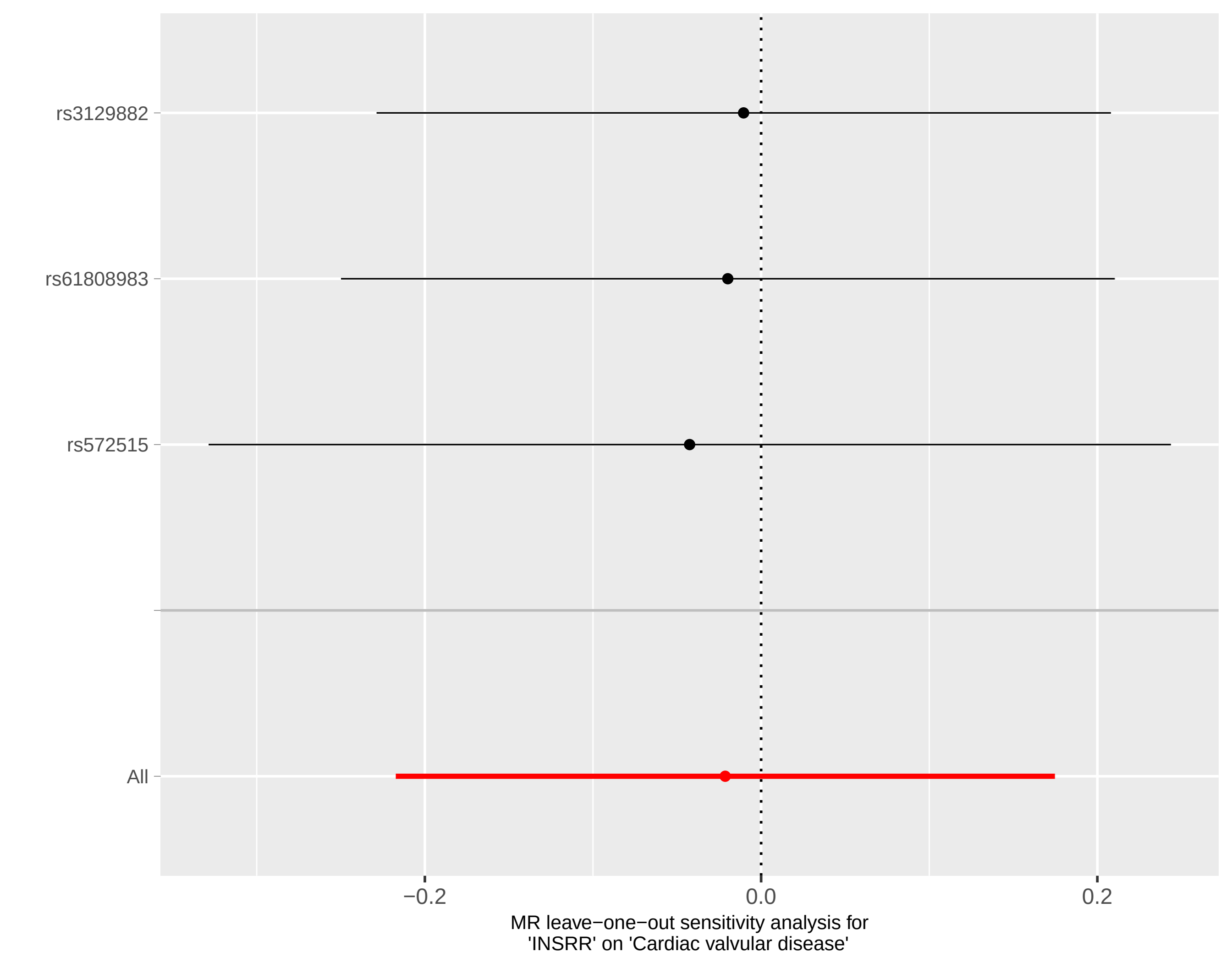

rs3129882
rs61808983
rs572515
All
−0.2
0.0
0.2
MR leave−one−out sensitivity analysis for
'INSRR' on 'Cardiac valvular disease'

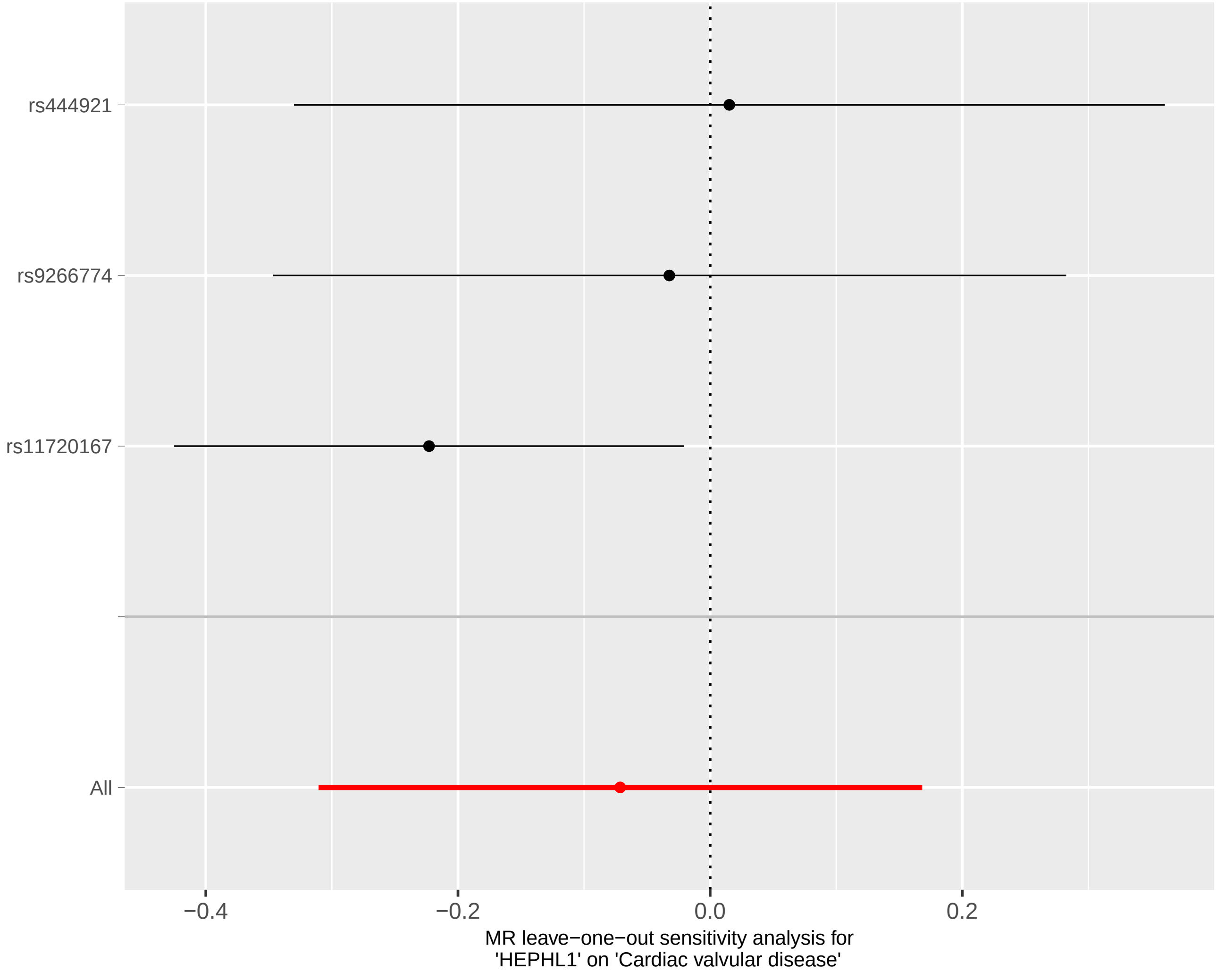
rs444921
rs9266774
rs11720167
All
−0.4
−0.2
0.0
0.2
MR leave−one−out sensitivity analysis for
'HEPHL1' on 'Cardiac valvular disease'

Insufficient number of SNPs

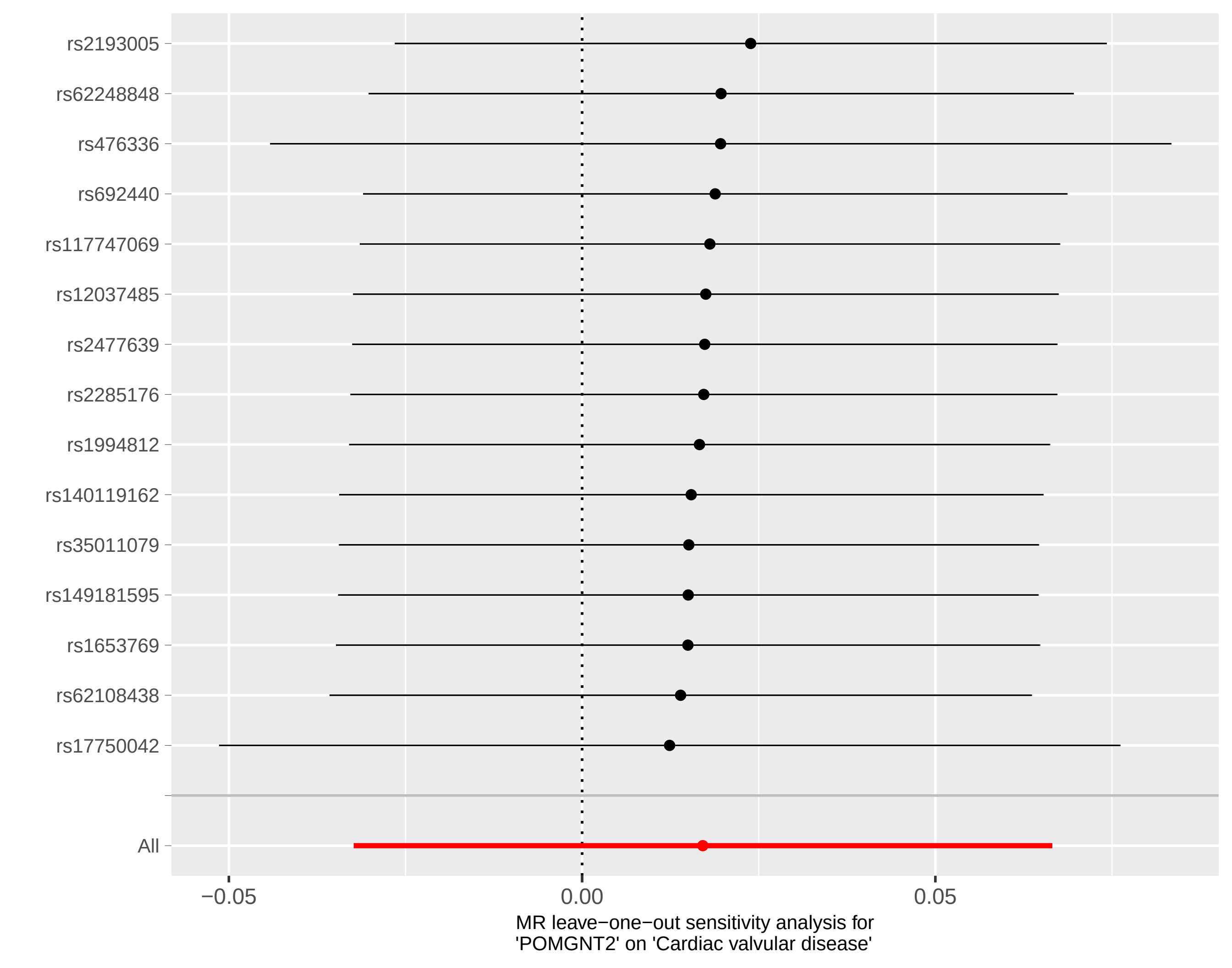
rs2193005
rs62248848
rs476336
rs692440
rs117747069
rs12037485
rs2477639
rs2285176
rs1994812
rs140119162
rs35011079
rs149181595
rs1653769
rs62108438
rs17750042
All
−0.05
0.00
0.05
MR leave−one−out sensitivity analysis for
'POMGNT2' on 'Cardiac valvular disease'

Insufficient number of SNPs

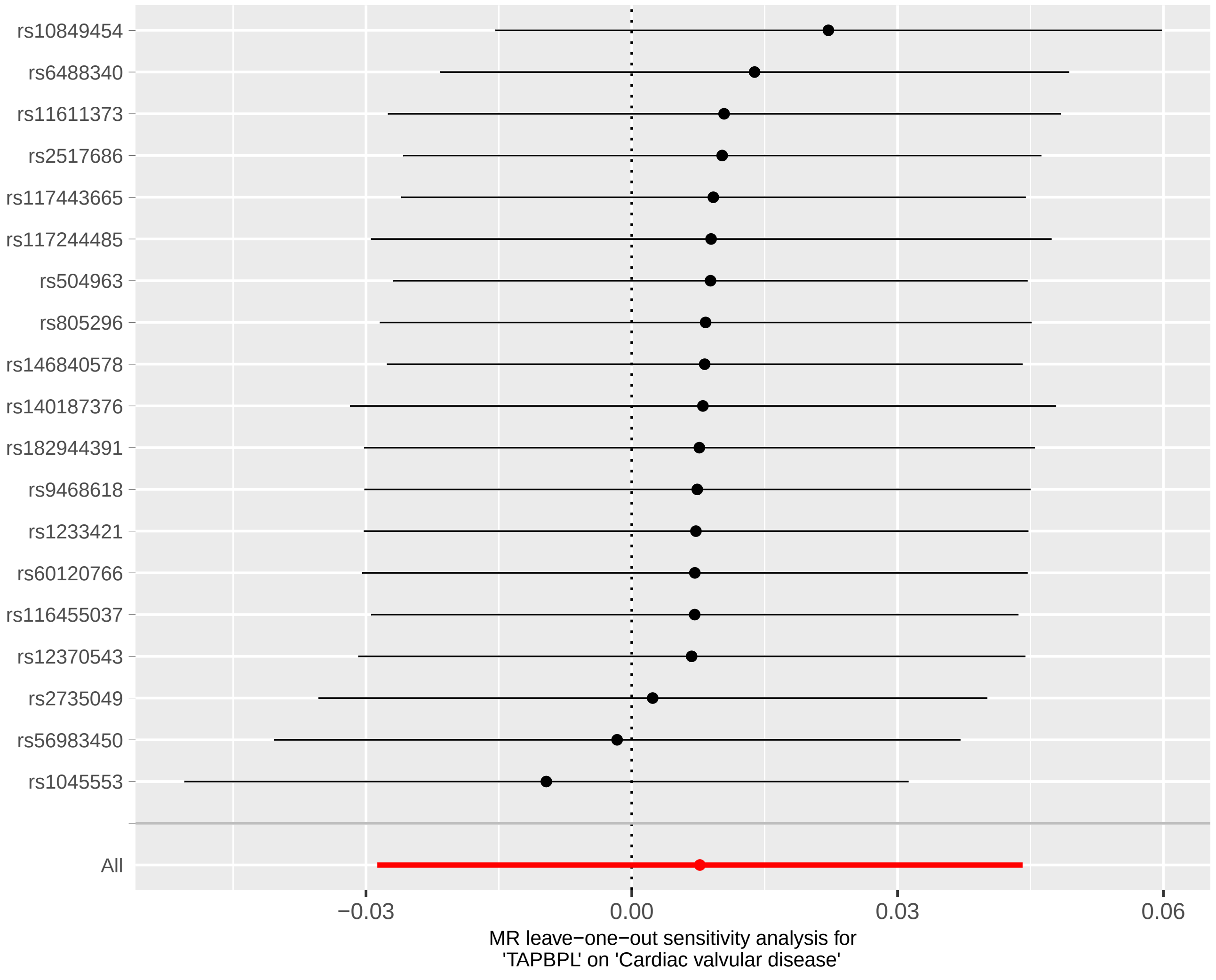
rs10849454
rs6488340
rs11611373
rs2517686
rs117443665
rs117244485
rs504963
rs805296
rs146840578
rs140187376
rs182944391
rs9468618
rs1233421
rs60120766
rs116455037
rs12370543
rs2735049
rs56983450
rs1045553
All
−0.03
0.00
0.03
0.06
MR leave−one−out sensitivity analysis for
'TAPBPL' on 'Cardiac valvular disease'

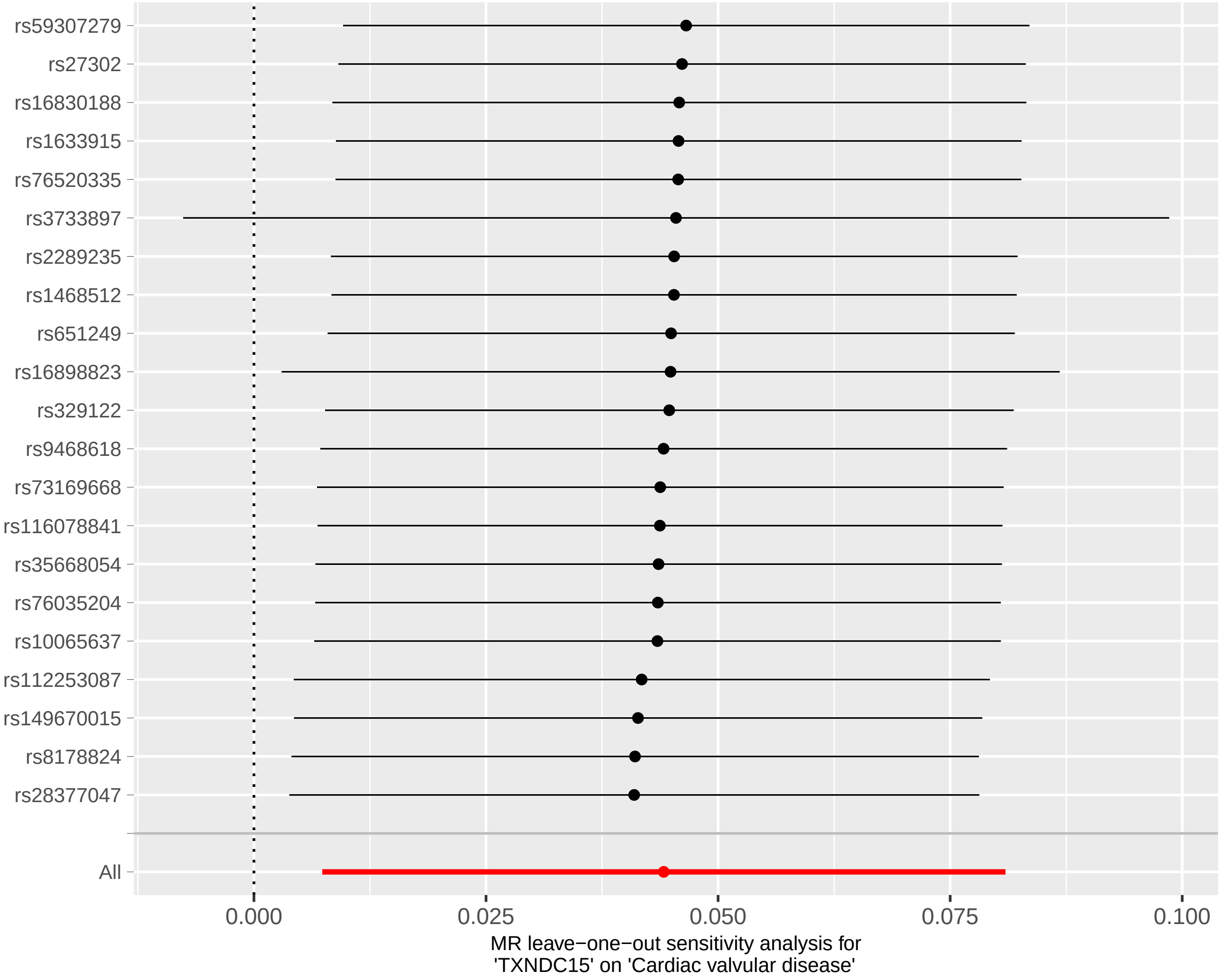
rs59307279
rs27302
rs16830188
rs1633915
rs76520335
rs3733897
rs2289235
rs1468512
rs651249
rs16898823
rs329122
rs9468618
rs73169668
rs116078841
rs35668054
rs76035204
rs10065637
rs112253087
rs149670015
rs8178824
rs28377047
All
0.000
0.025
0.050
0.075
0.100
MR leave−one−out sensitivity analysis for
'TXNDC15' on 'Cardiac valvular disease'

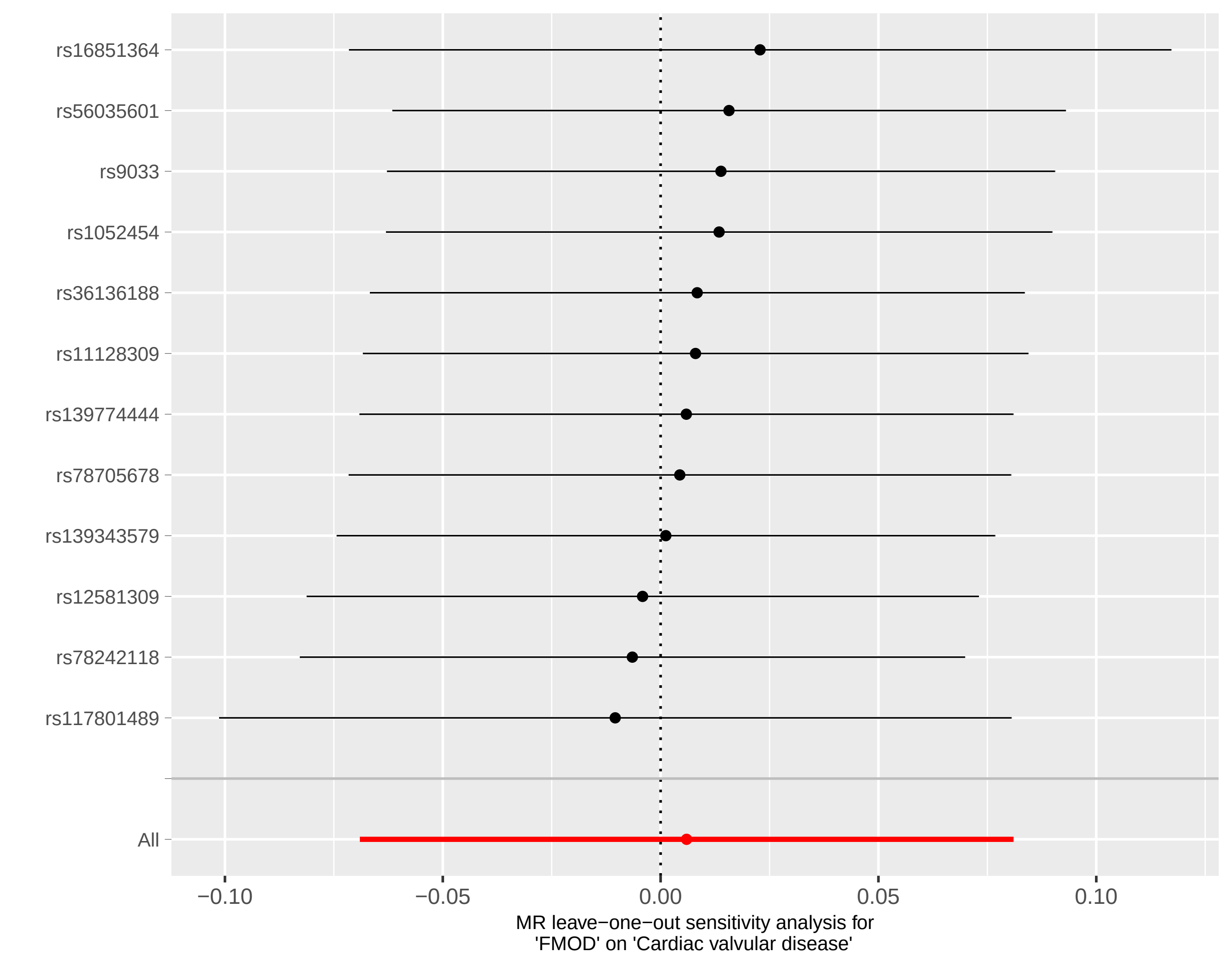
rs16851364
rs56035601
rs9033
rs1052454
rs36136188
rs11128309
rs139774444
rs78705678
rs139343579
rs12581309
rs78242118
rs117801489
All
−0.10
−0.05
0.00
0.05
0.10
MR leave−one−out sensitivity analysis for
'FMOD' on 'Cardiac valvular disease'

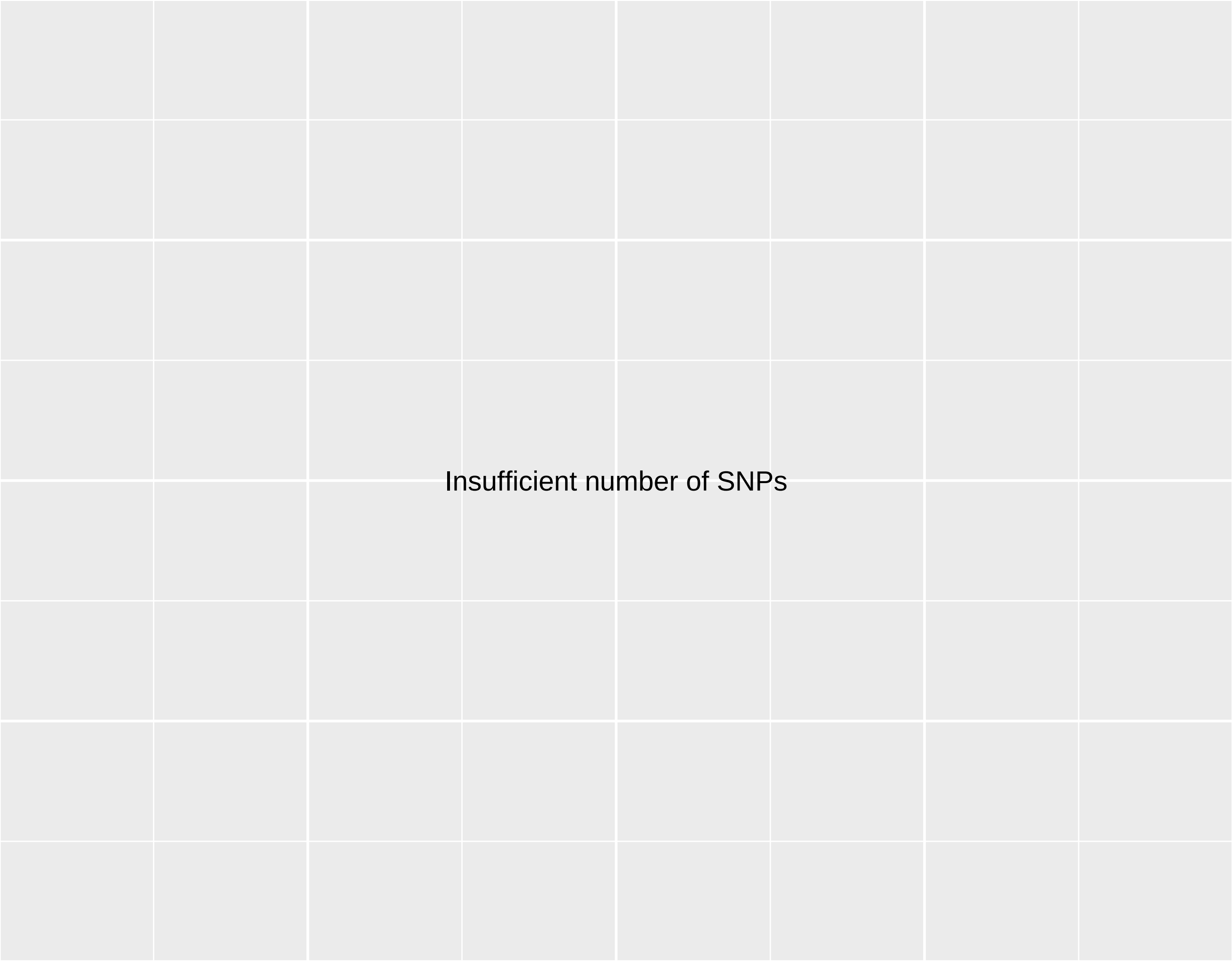
Insufficient number of SNPs

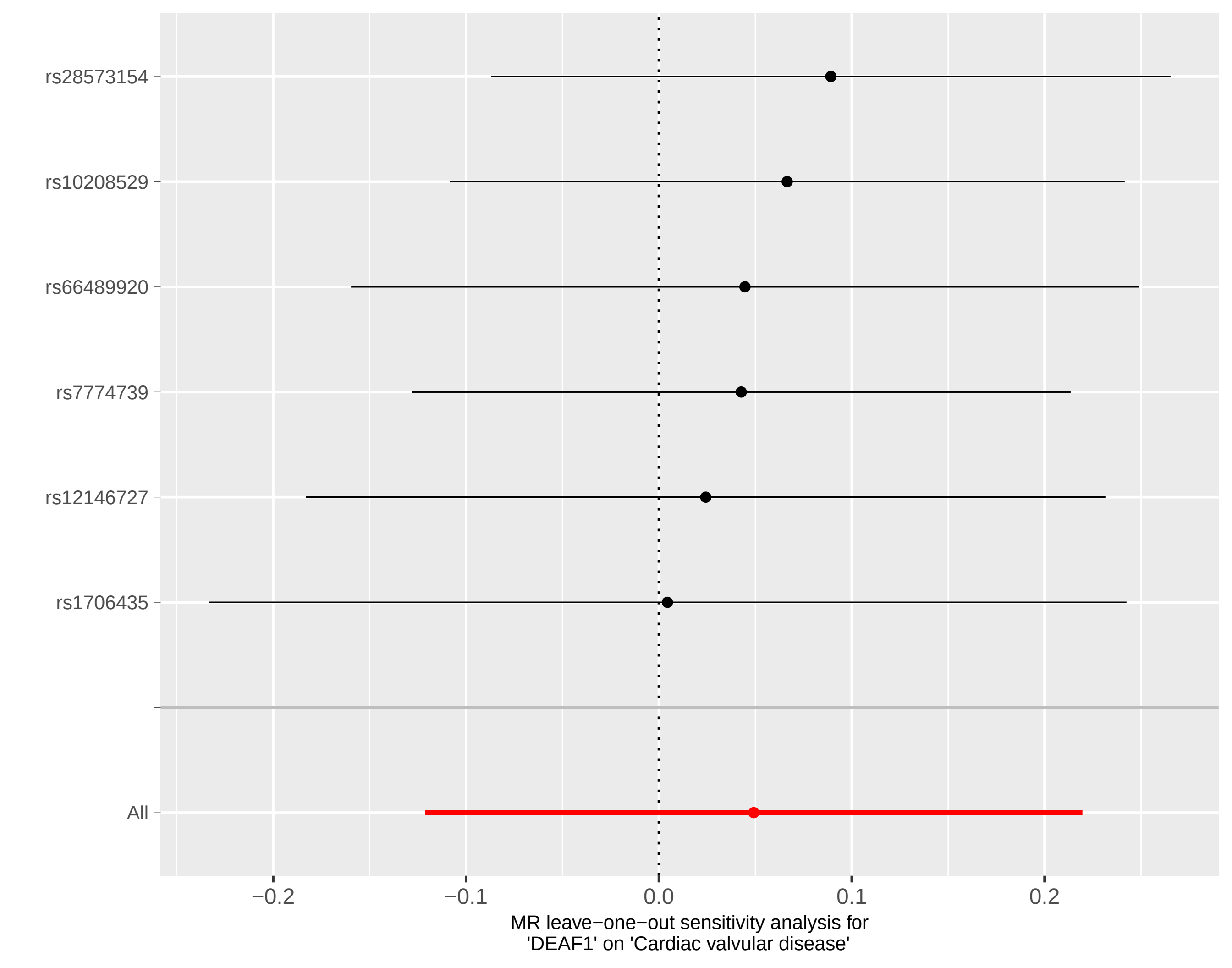

rs28573154
rs10208529
rs66489920
rs7774739
rs12146727
rs1706435
All
−0.2
−0.1
0.0
0.1
0.2
MR leave−one−out sensitivity analysis for
'DEAF1' on 'Cardiac valvular disease'

Insufficient number of SNPs

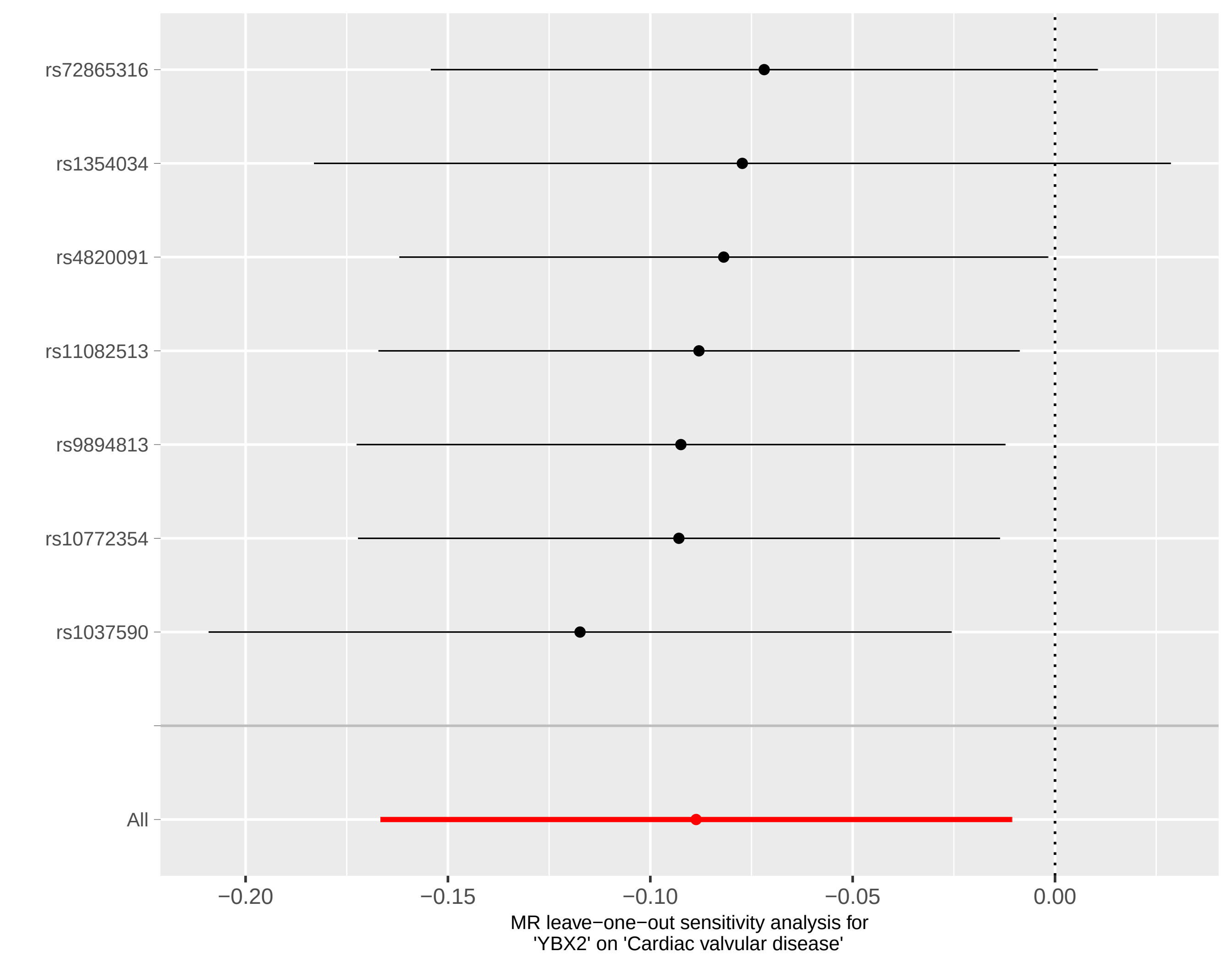
rs72865316
rs1354034
rs4820091
rs11082513
rs9894813
rs10772354
rs1037590
All
−0.20
−0.15
−0.10
−0.05
0.00
MR leave−one−out sensitivity analysis for
'YBX2' on 'Cardiac valvular disease'

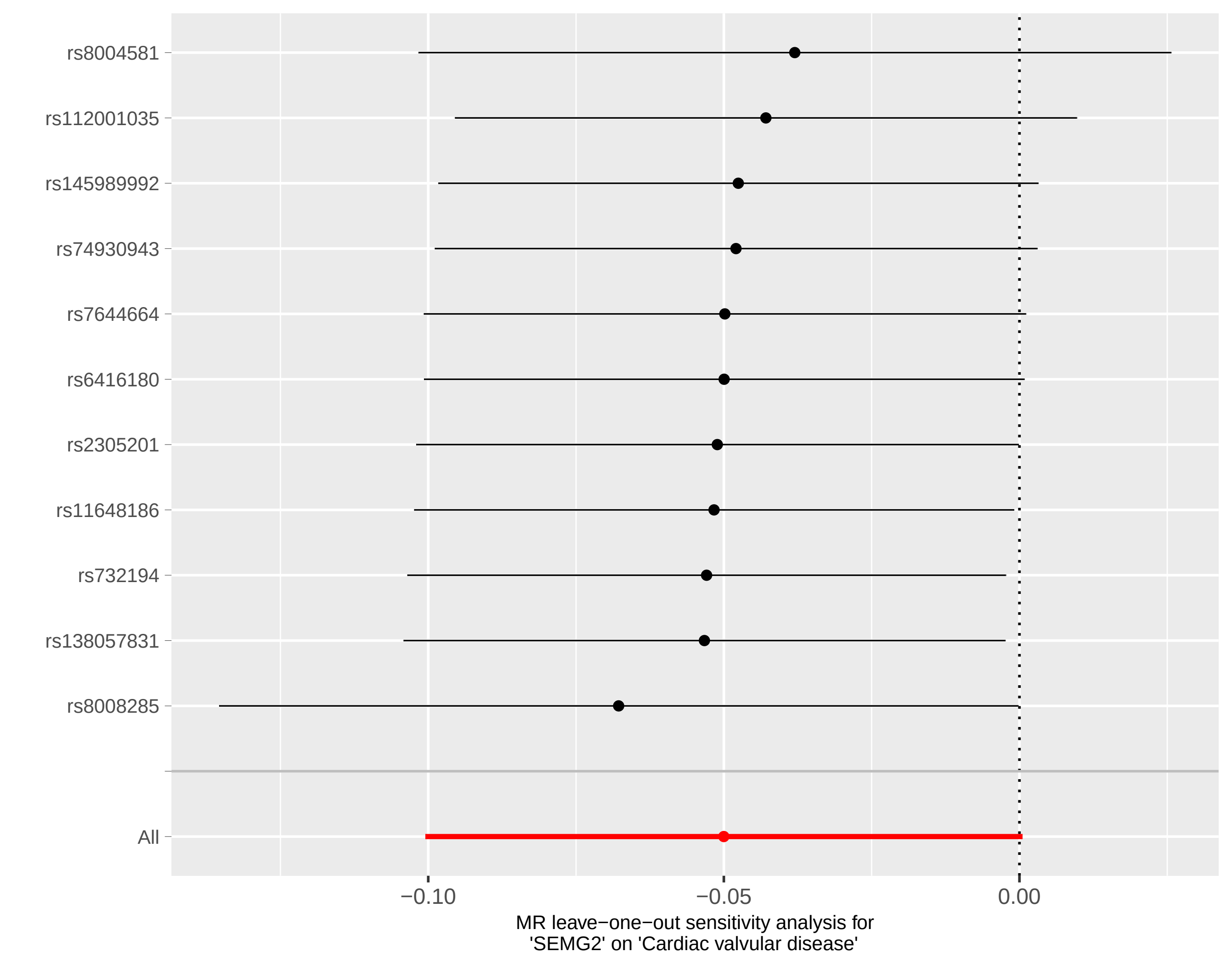
rs8004581
rs112001035
rs145989992
rs74930943
rs7644664
rs6416180
rs2305201
rs11648186
rs732194
rs138057831
rs8008285
All
−0.10
−0.05
0.00
MR leave−one−out sensitivity analysis for
'SEMG2' on 'Cardiac valvular disease'

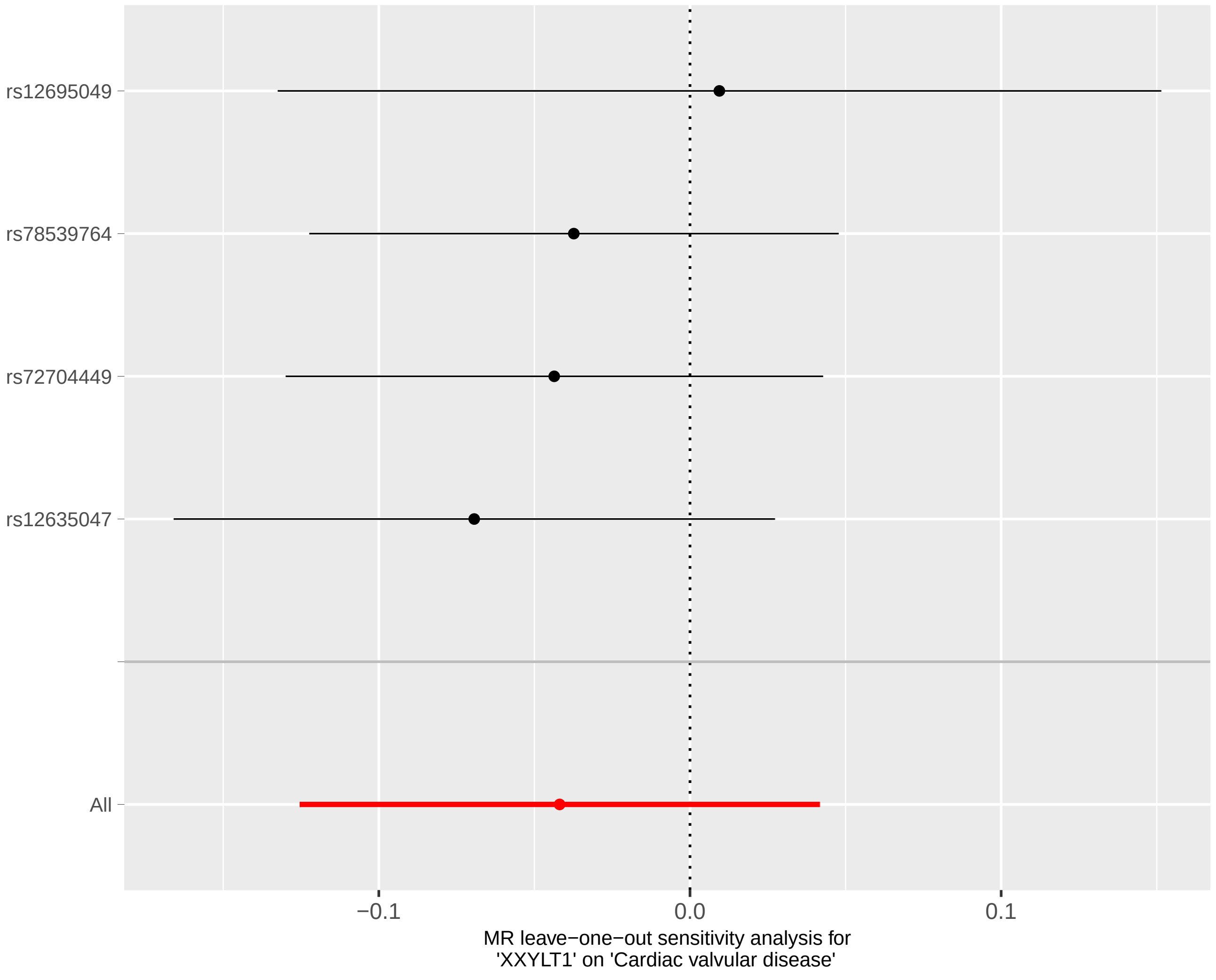
rs12695049
rs78539764
rs72704449
rs12635047
All
−0.1
0.0
0.1
MR leave−one−out sensitivity analysis for
'XXYLT1' on 'Cardiac valvular disease'

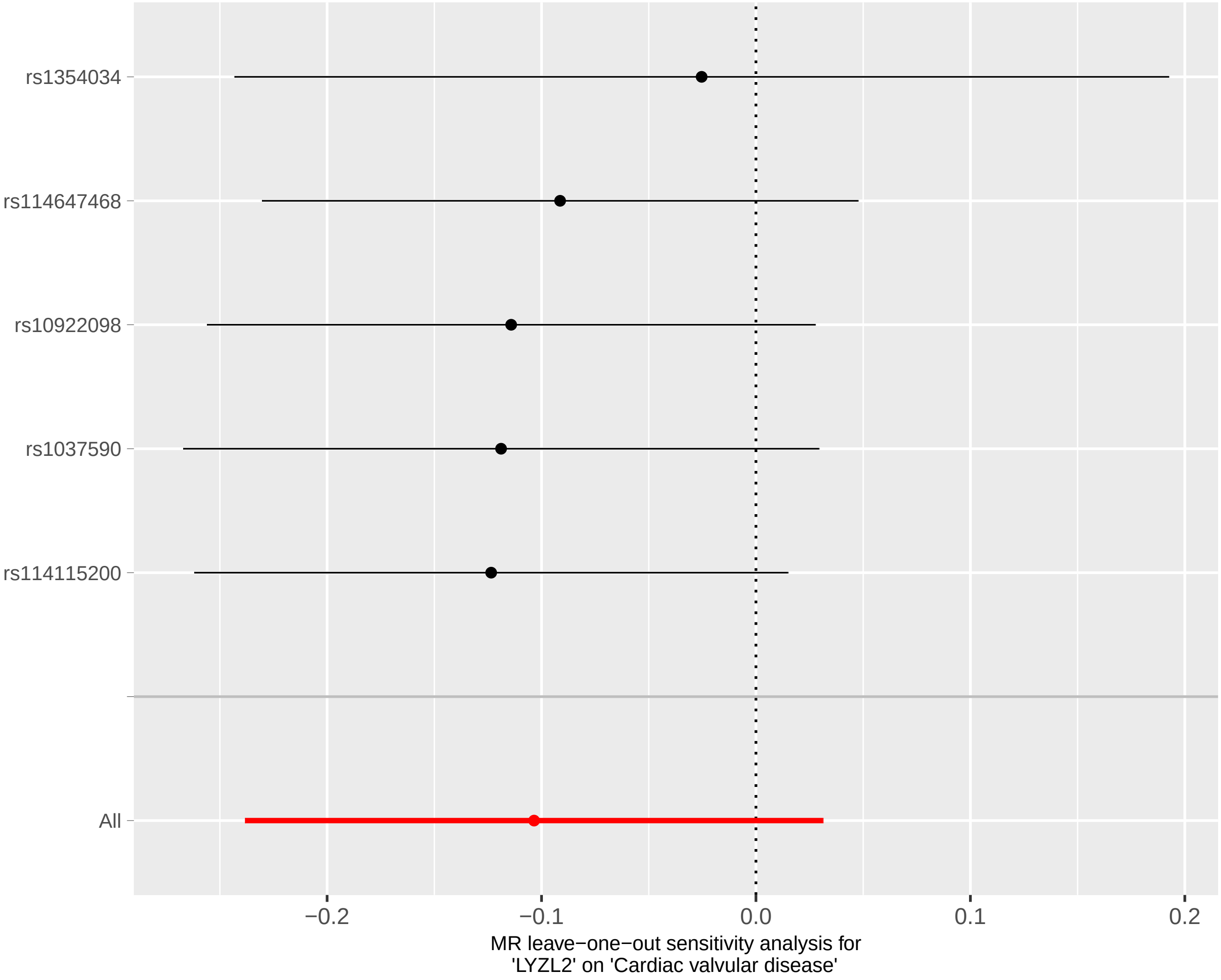

rs1354034
rs114647468
rs10922098
rs1037590
rs114115200
All
−0.2
−0.1
0.0
0.1
0.2
MR leave−one−out sensitivity analysis for
'LYZL2' on 'Cardiac valvular disease'

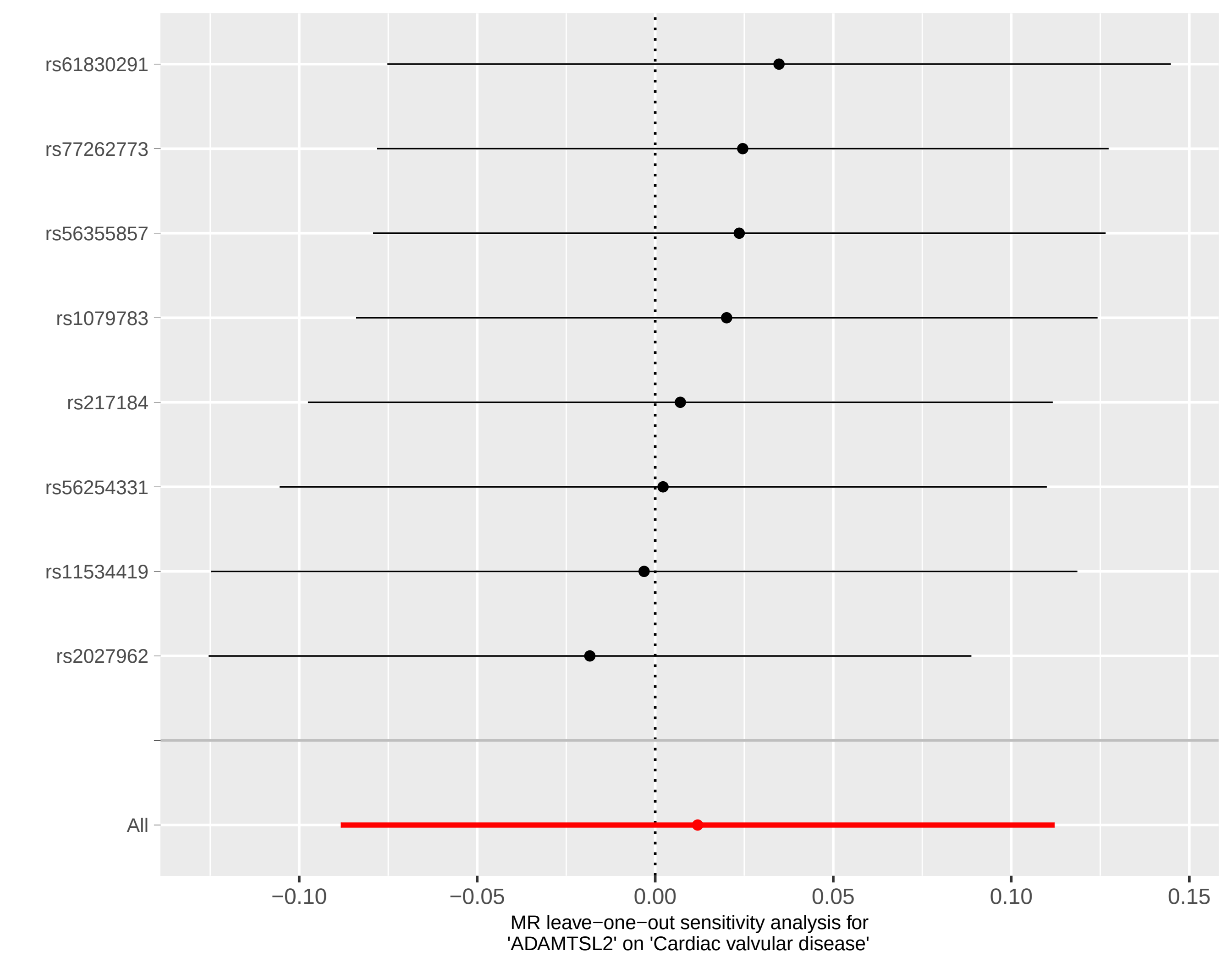

rs61830291
rs77262773
rs56355857
rs1079783
rs217184
rs56254331
rs11534419
rs2027962
All
−0.10
−0.05
0.00
0.05
0.10
0.15
MR leave−one−out sensitivity analysis for
'ADAMTSL2' on 'Cardiac valvular disease'

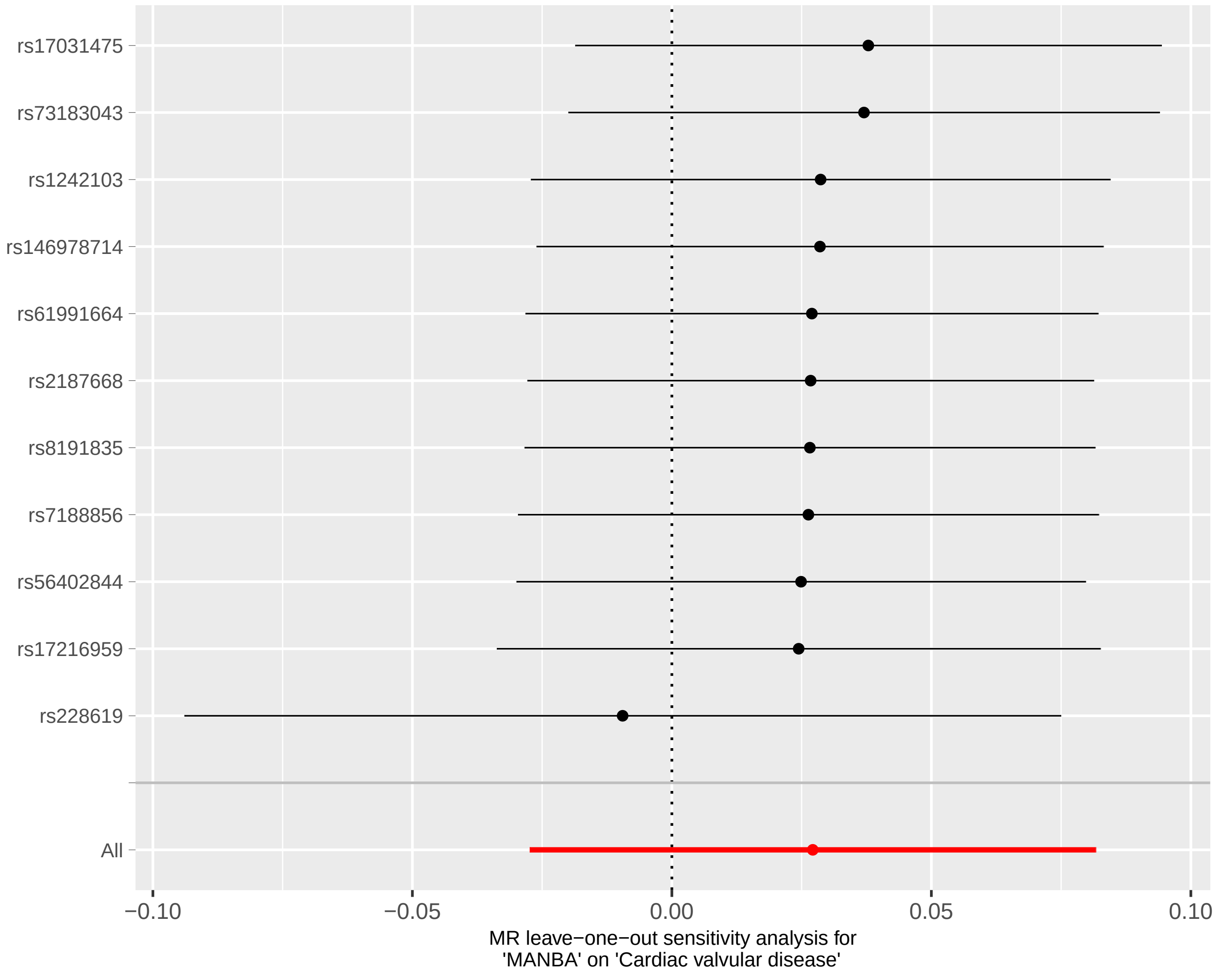

rs17031475
rs73183043
rs1242103
rs146978714
rs61991664
rs2187668
rs8191835
rs7188856
rs56402844
rs17216959
rs228619
All
−0.10
−0.05
0.00
0.05
0.10
MR leave−one−out sensitivity analysis for
'MANBA' on 'Cardiac valvular disease'

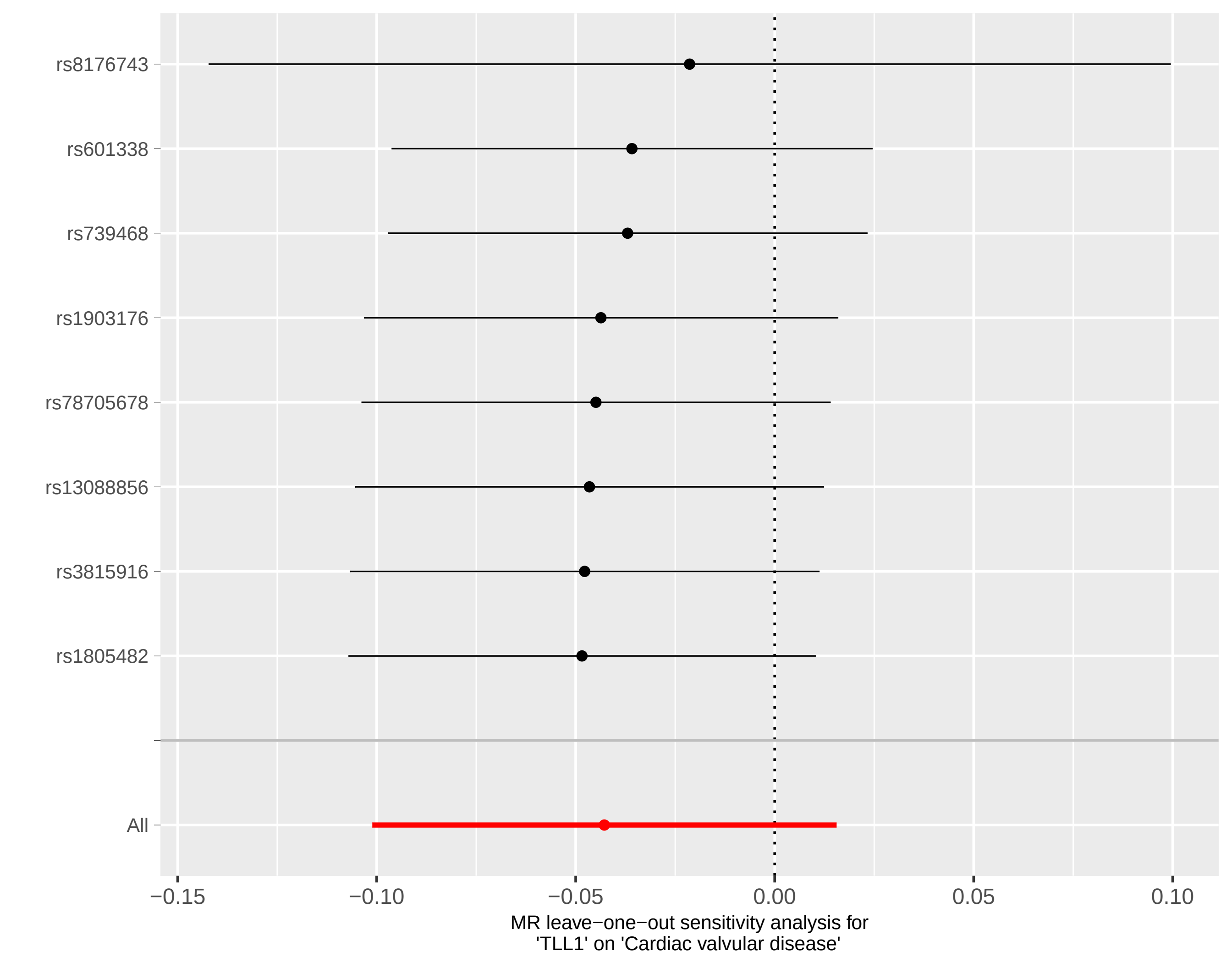
rs8176743
rs601338
rs739468
rs1903176
rs78705678
rs13088856
rs3815916
rs1805482
All
−0.15
−0.10
−0.05
0.00
0.05
0.10
MR leave−one−out sensitivity analysis for
'TLL1' on 'Cardiac valvular disease'

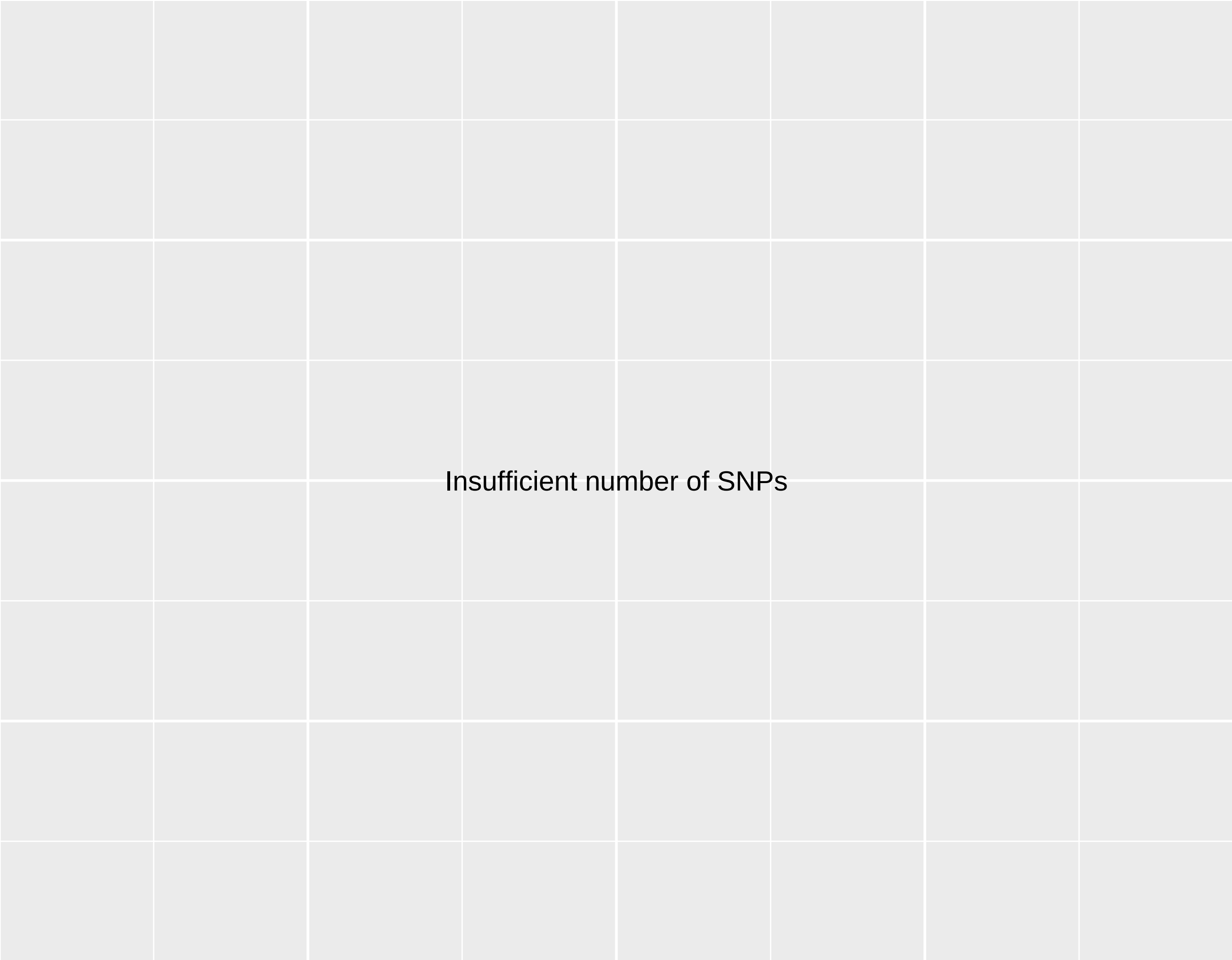
Insufficient number of SNPs

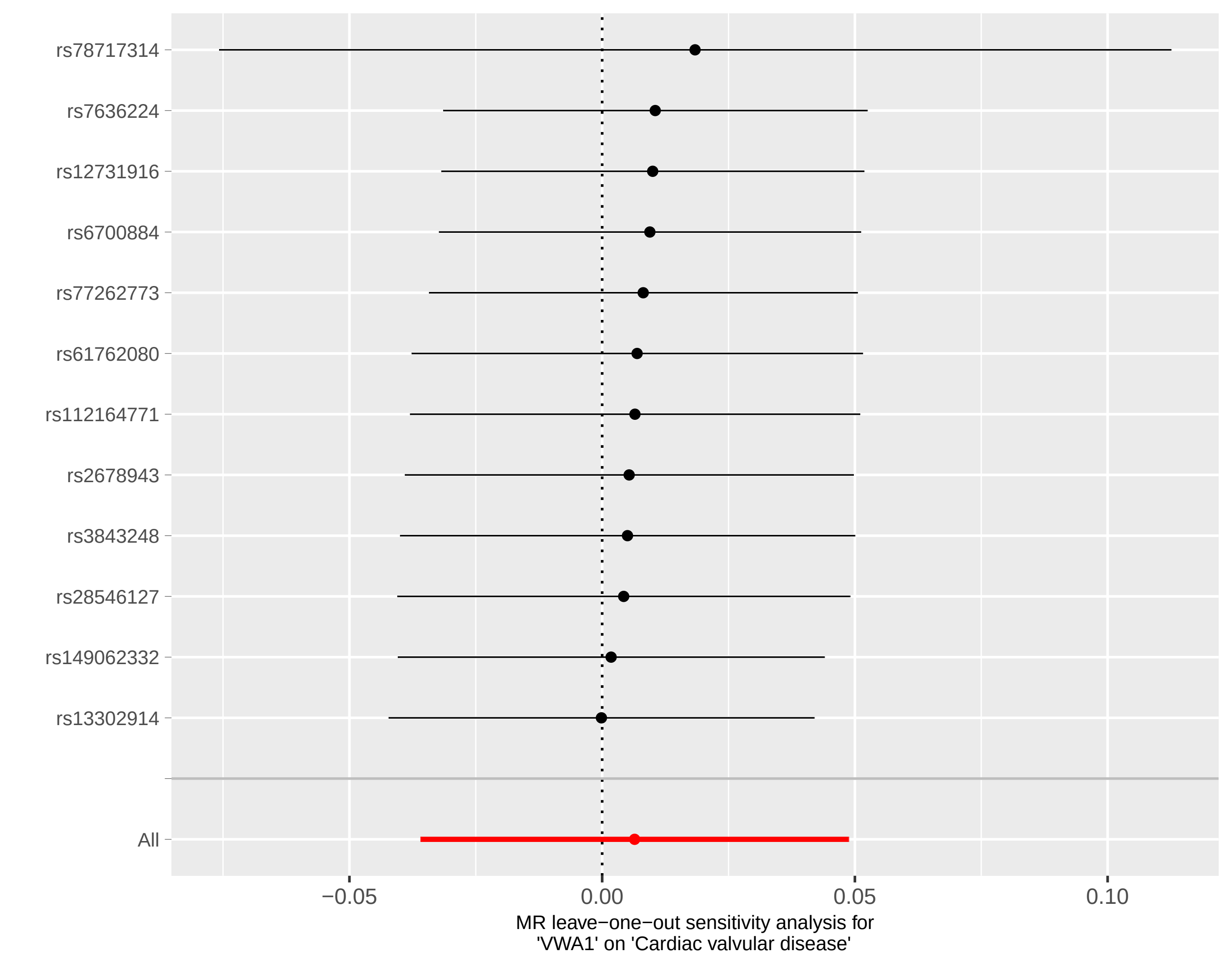
rs78717314
rs7636224
rs12731916
rs6700884
rs77262773
rs61762080
rs112164771
rs2678943
rs3843248
rs28546127
rs149062332
rs13302914
All
−0.05
0.00
0.05
0.10
MR leave−one−out sensitivity analysis for
'VWA1' on 'Cardiac valvular disease'

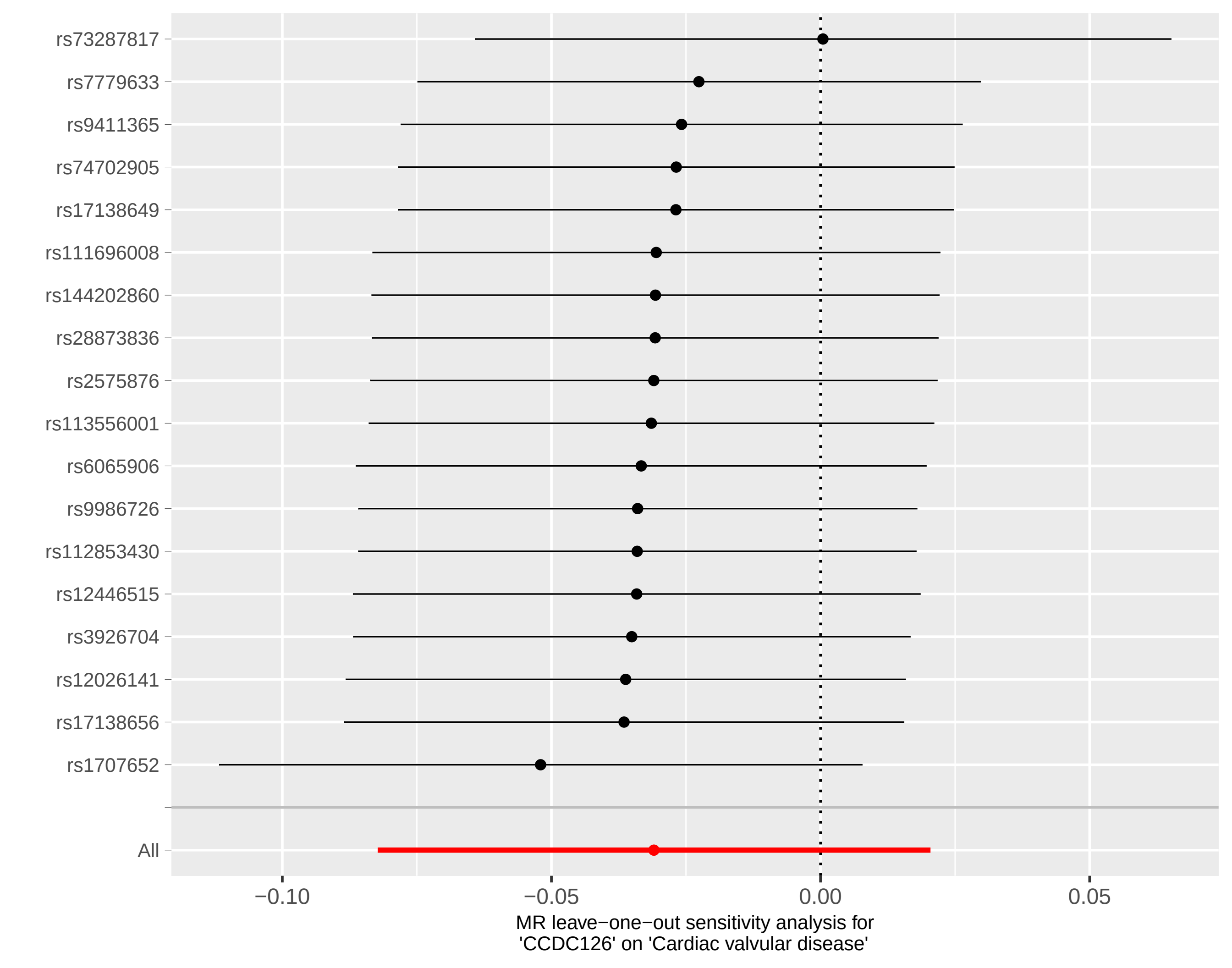
rs73287817
rs7779633
rs9411365
rs74702905
rs17138649
rs111696008
rs144202860
rs28873836
rs2575876
rs113556001
rs6065906
rs9986726
rs112853430
rs12446515
rs3926704
rs12026141
rs17138656
rs1707652
All
−0.10
−0.05
0.00
0.05
MR leave−one−out sensitivity analysis for
'CCDC126' on 'Cardiac valvular disease'

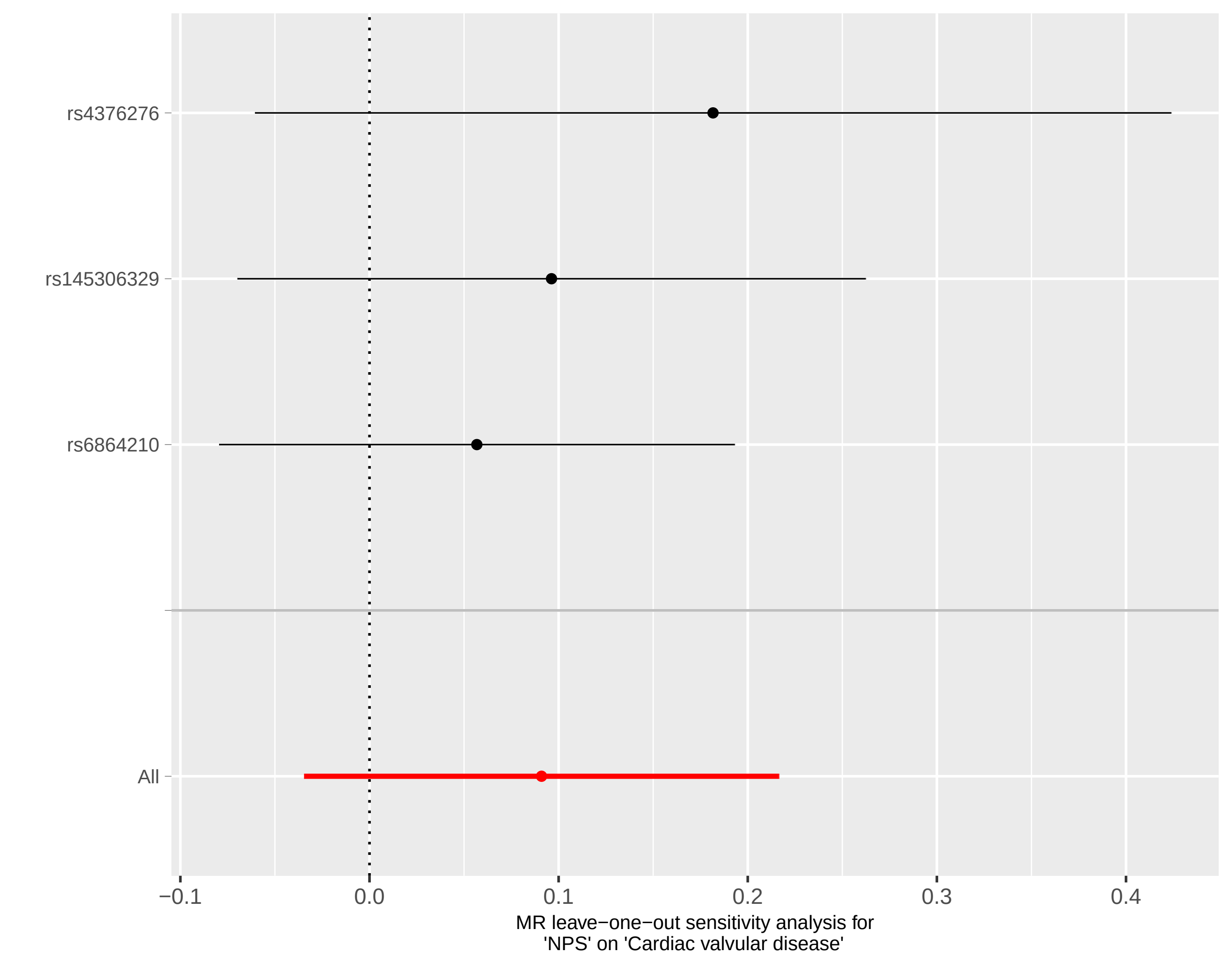
rs4376276
rs145306329
rs6864210
All
−0.1
0.0
0.1
0.2
0.3
0.4
MR leave−one−out sensitivity analysis for
'NPS' on 'Cardiac valvular disease'

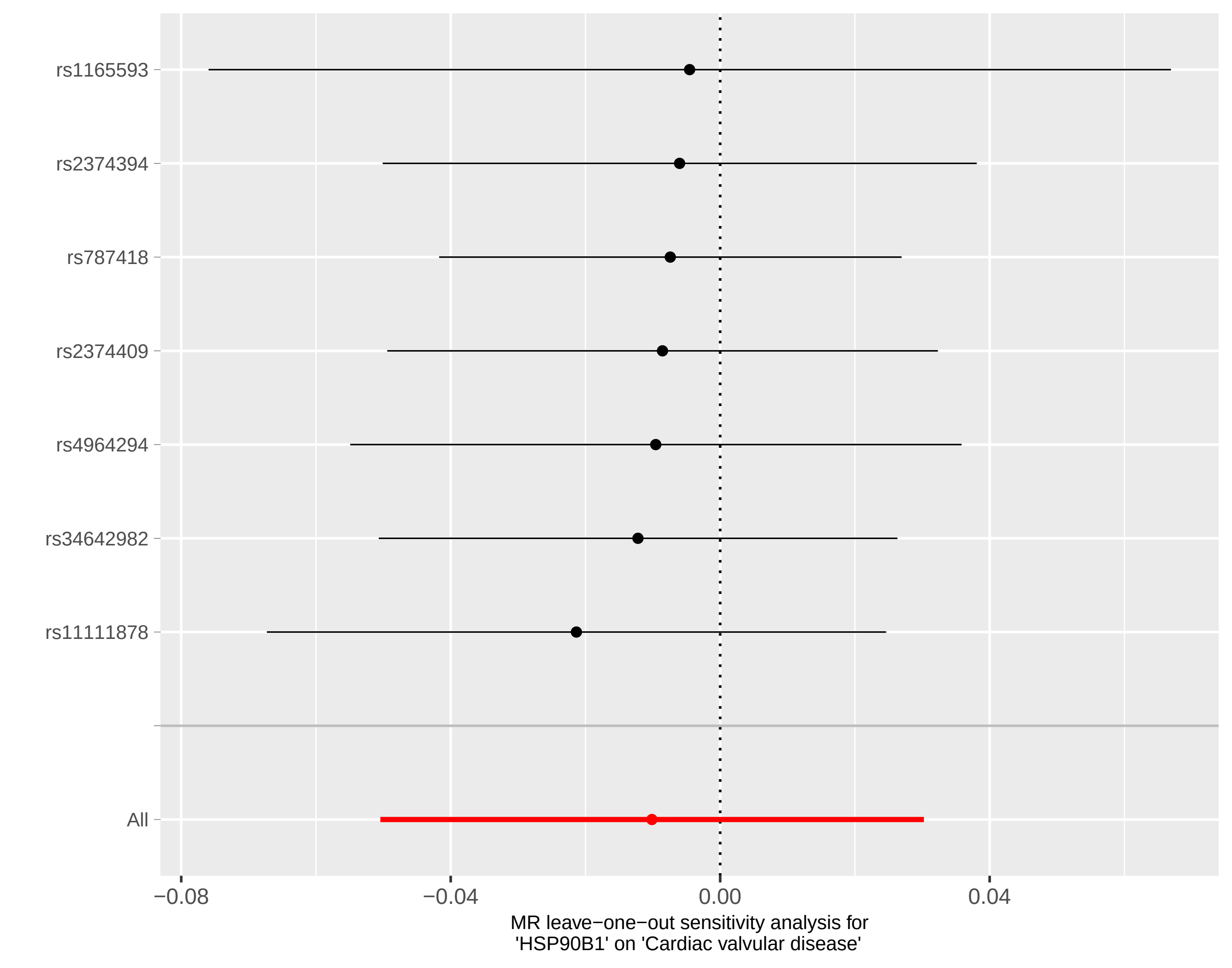
rs1165593
rs2374394
rs787418
rs2374409
rs4964294
rs34642982
rs11111878
All
−0.08
−0.04
0.00
0.04
MR leave−one−out sensitivity analysis for
'HSP90B1' on 'Cardiac valvular disease'

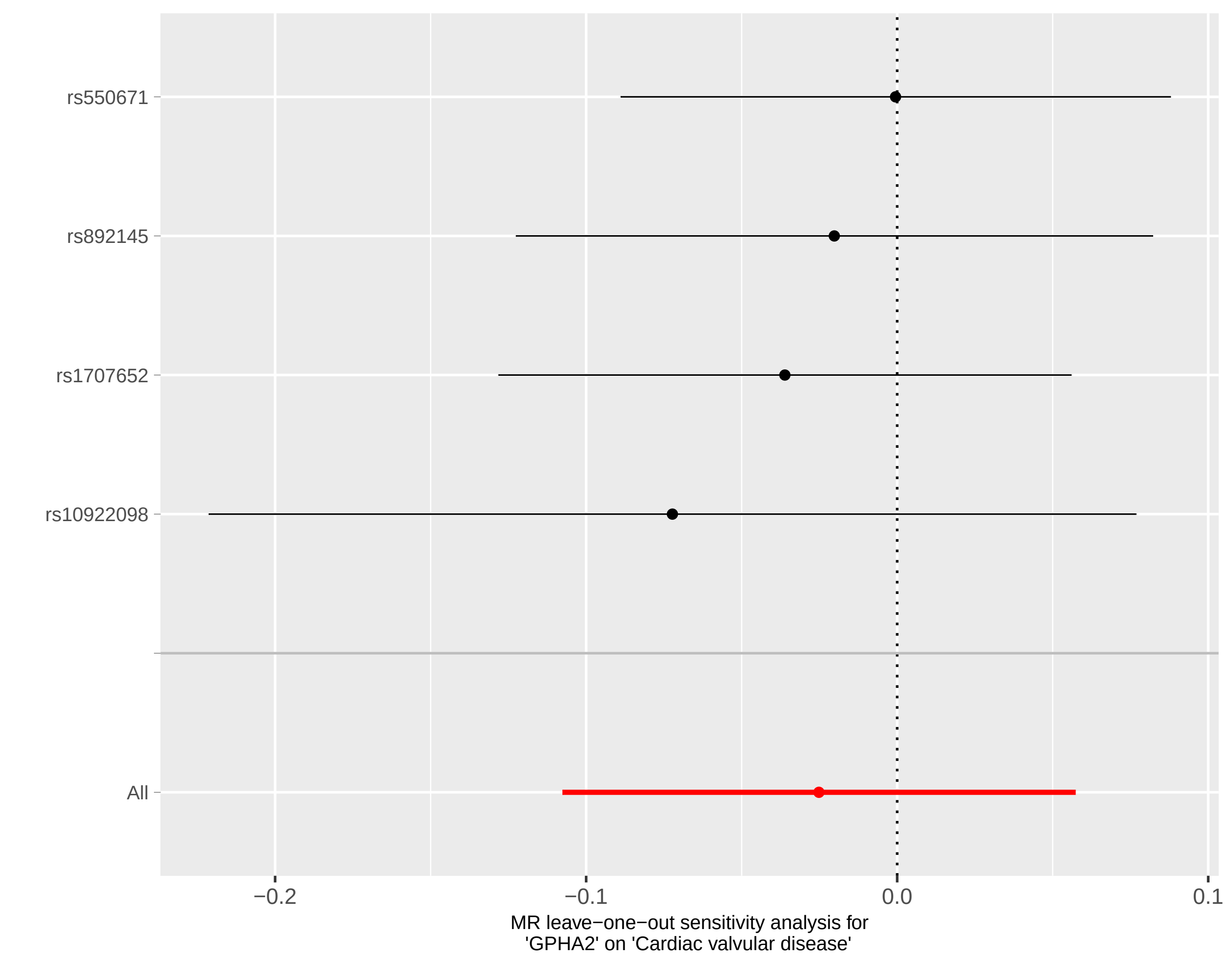
rs550671
rs892145
rs1707652
rs10922098
All
−0.2
−0.1
0.0
0.1
MR leave−one−out sensitivity analysis for
'GPHA2' on 'Cardiac valvular disease'

Insufficient number of SNPs

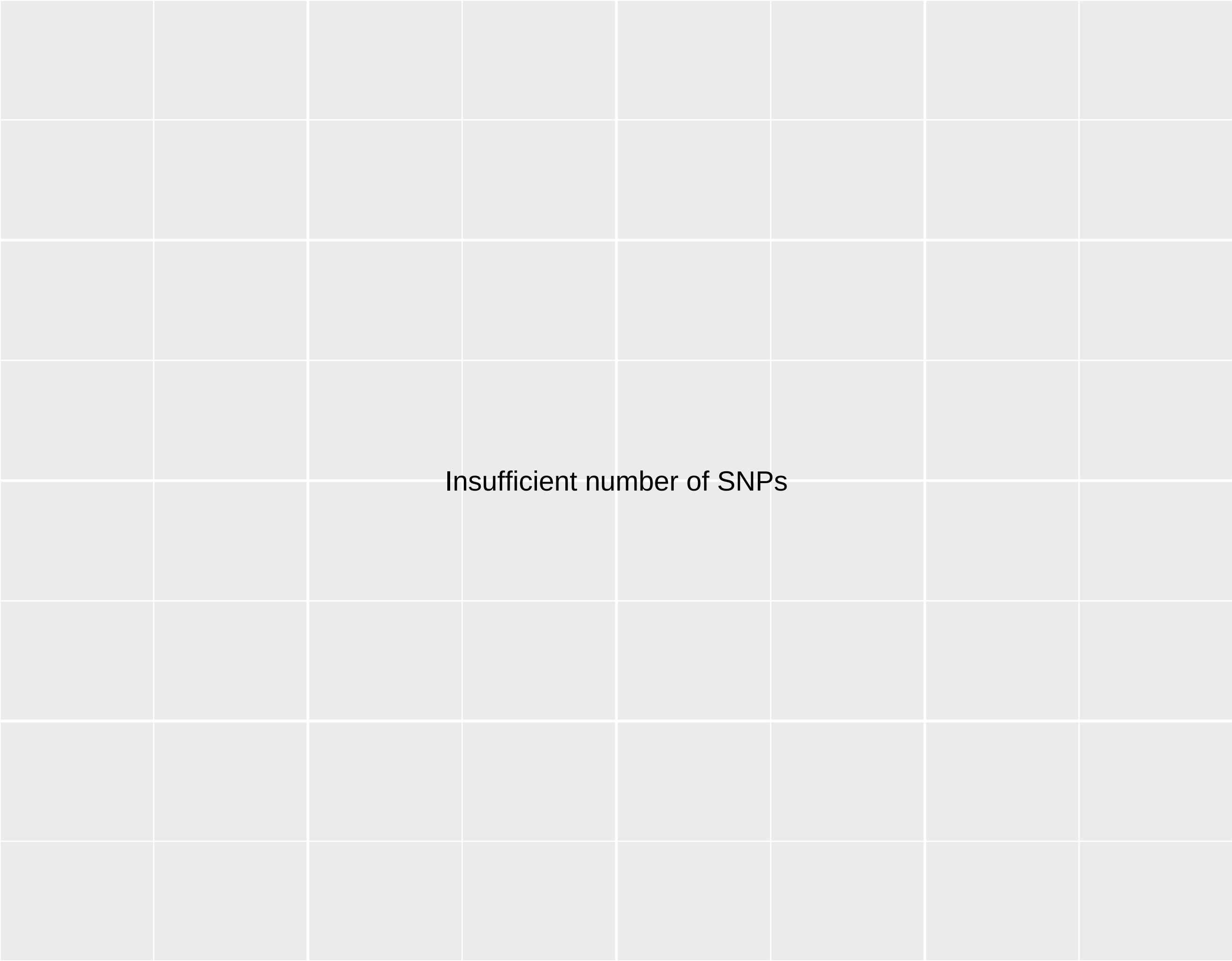
Insufficient number of SNPs

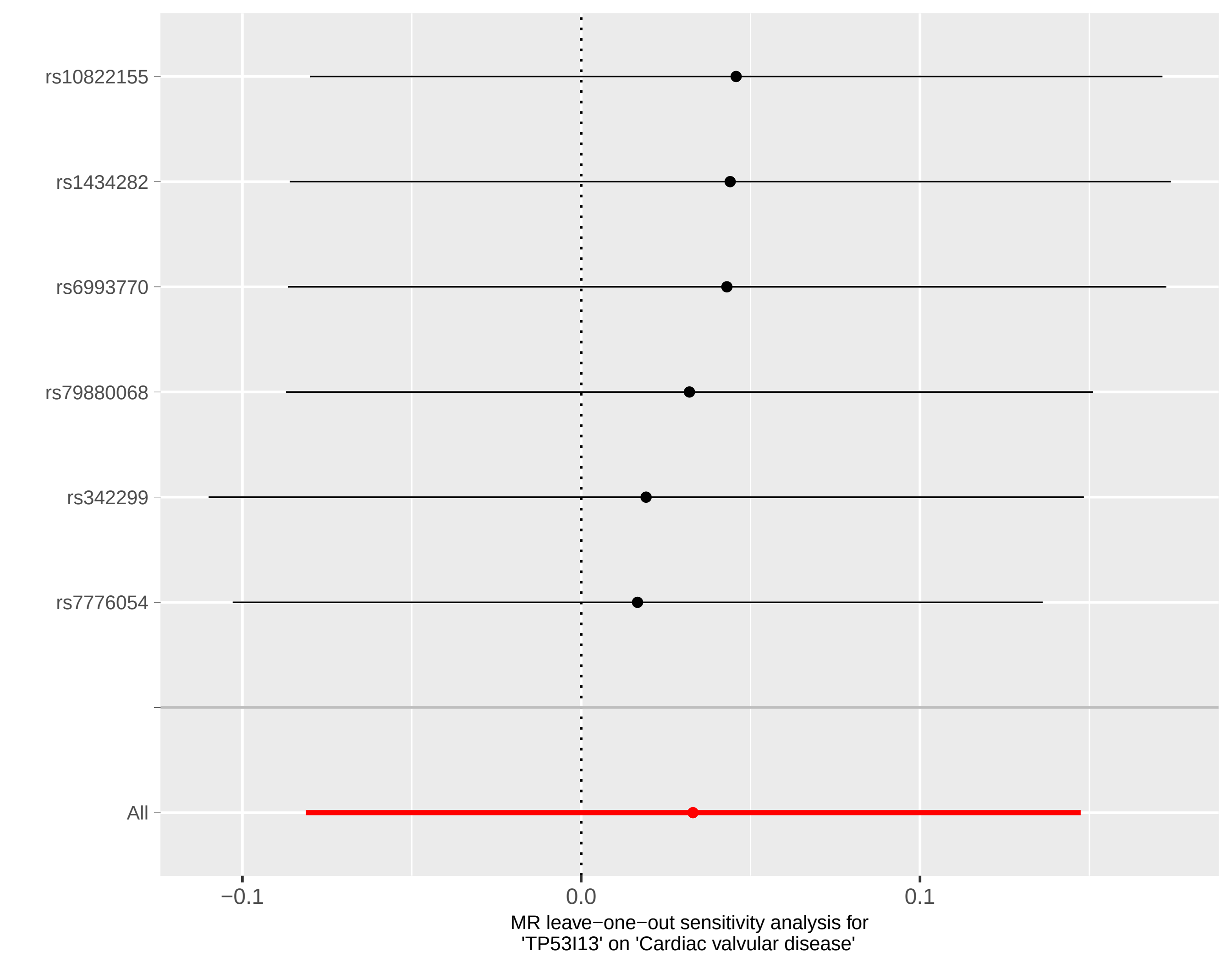
rs10822155
rs1434282
rs6993770
rs79880068
rs342299
rs7776054
All
−0.1
0.0
0.1
MR leave−one−out sensitivity analysis for
'TP53I13' on 'Cardiac valvular disease'

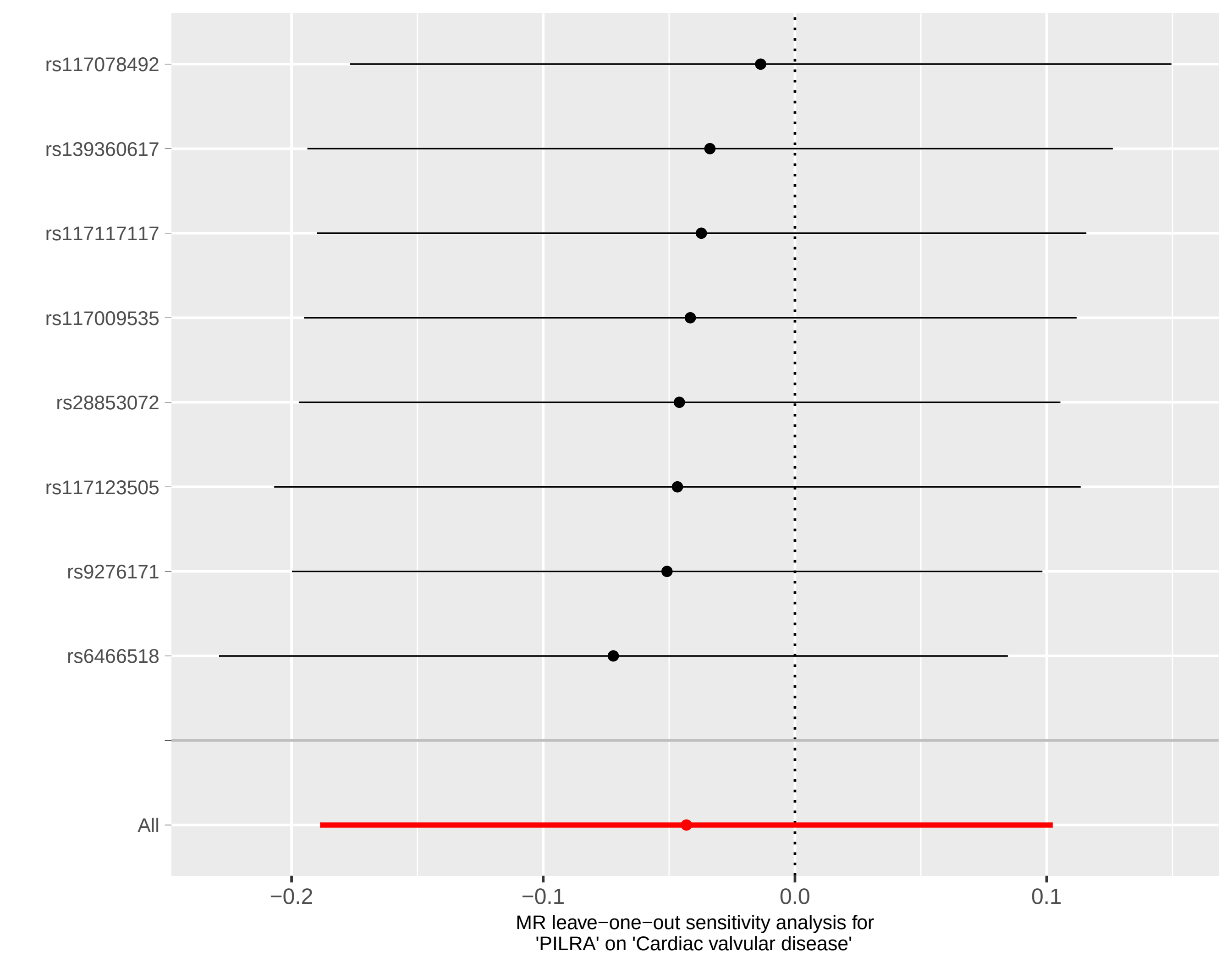

rs117078492
rs139360617
rs117117117
rs117009535
rs28853072
rs117123505
rs9276171
rs6466518
All
−0.2
−0.1
0.0
0.1
MR leave−one−out sensitivity analysis for
'PILRA' on 'Cardiac valvular disease'

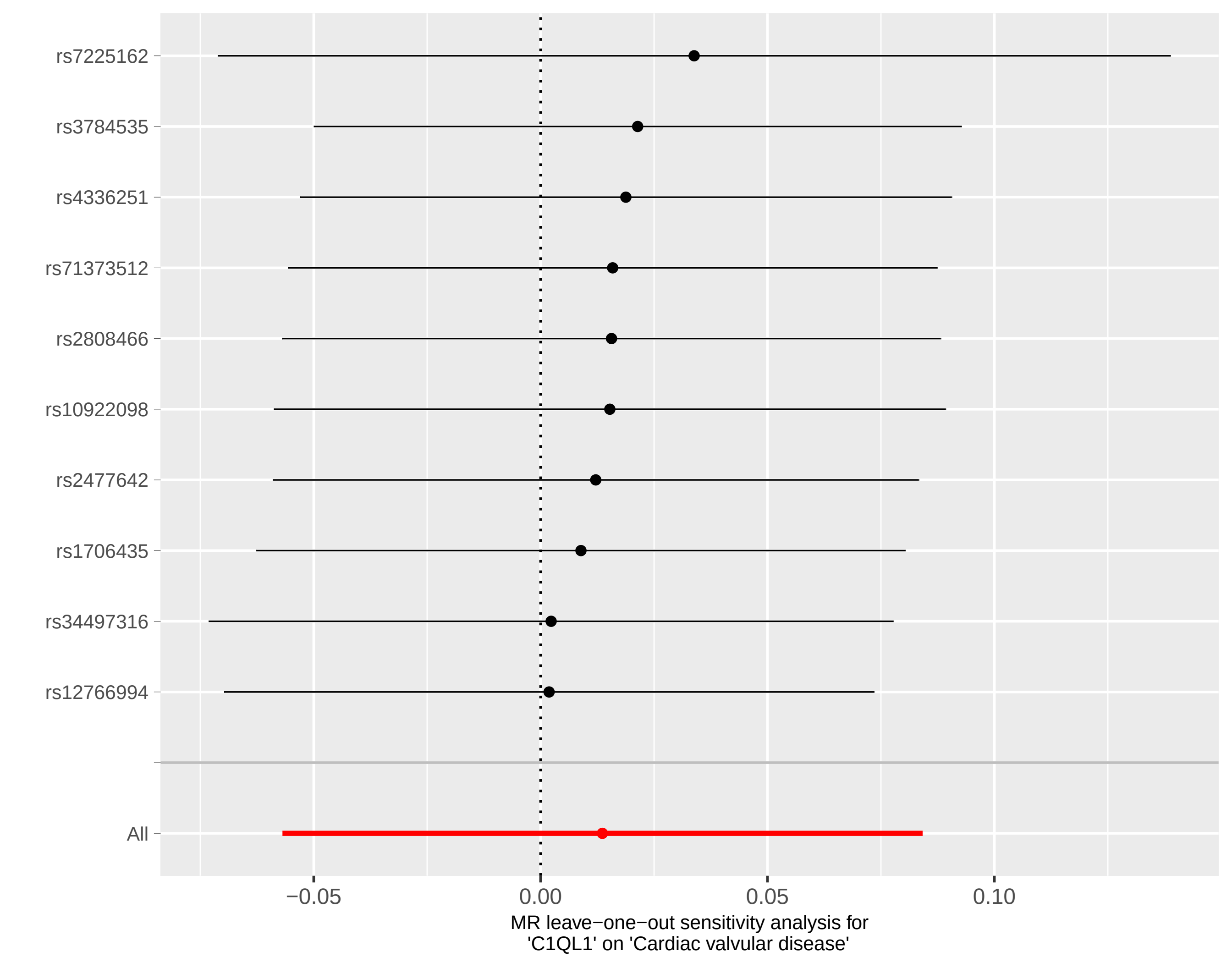
rs7225162
rs3784535
rs4336251
rs71373512
rs2808466
rs10922098
rs2477642
rs1706435
rs34497316
rs12766994
All
−0.05
0.00
0.05
0.10
MR leave−one−out sensitivity analysis for
'C1QL1' on 'Cardiac valvular disease'

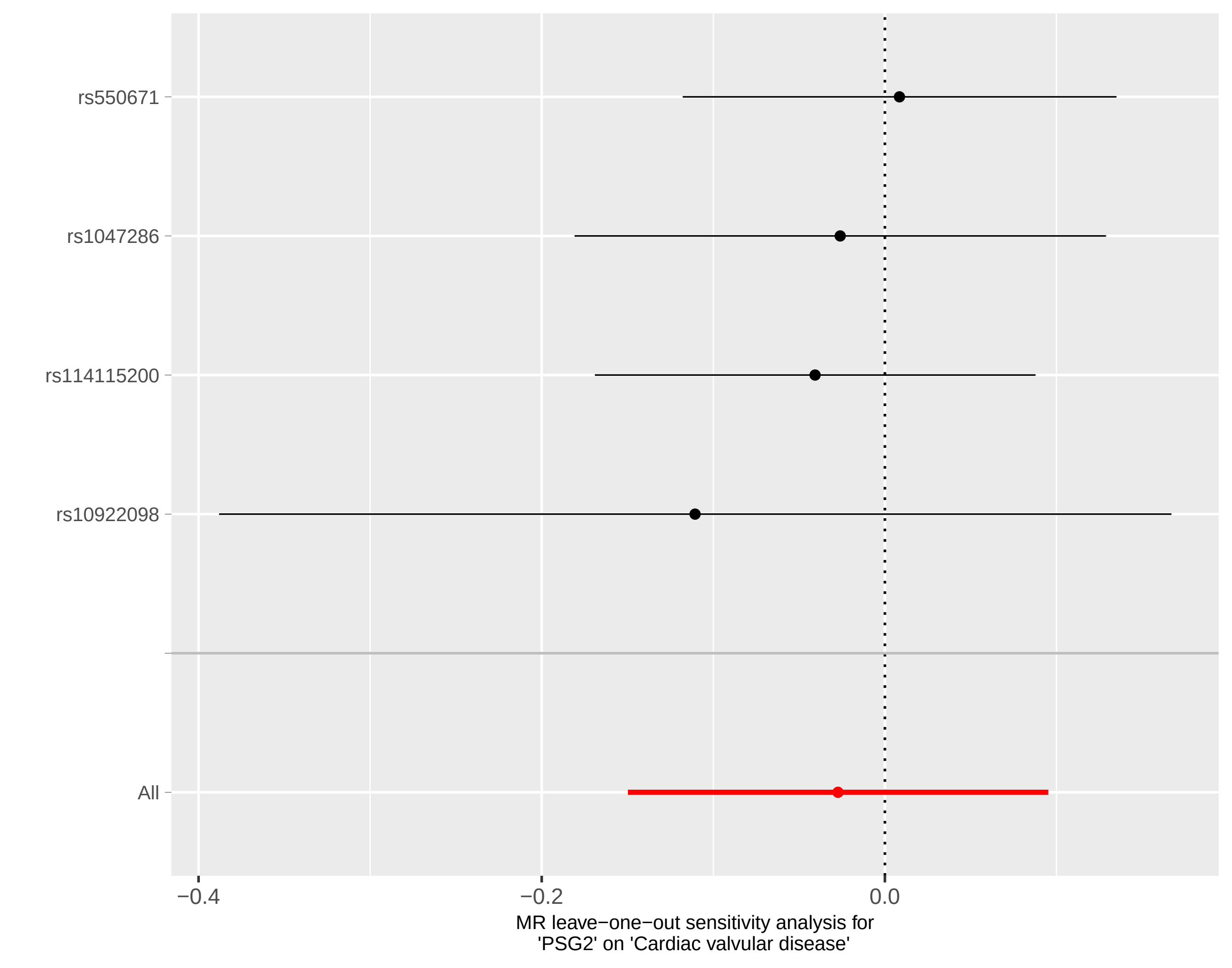
rs550671
rs1047286
rs114115200
rs10922098
All
−0.4
−0.2
0.0
MR leave−one−out sensitivity analysis for
'PSG2' on 'Cardiac valvular disease'

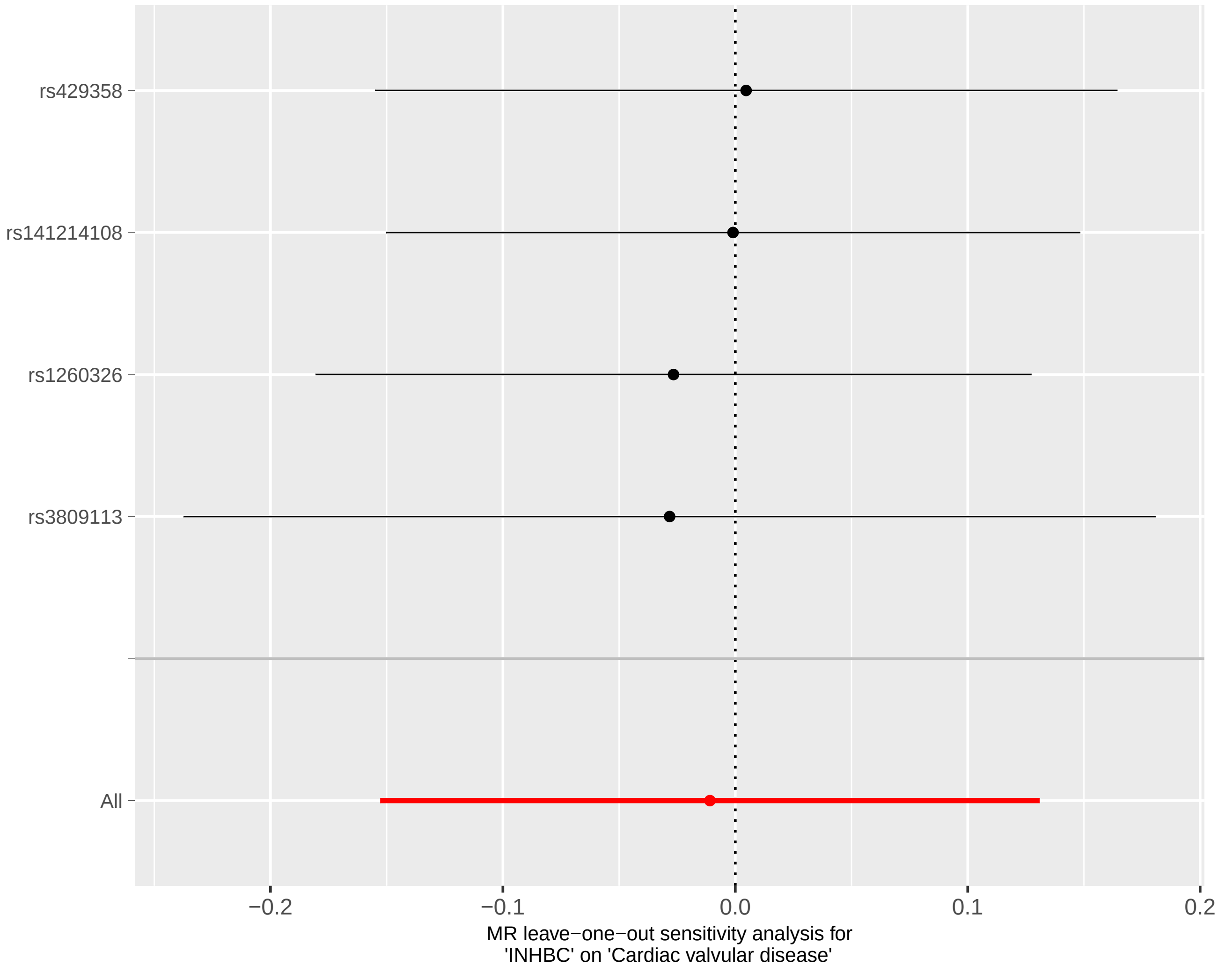
rs429358
rs141214108
rs1260326
rs3809113
All
−0.2
−0.1
0.0
0.1
0.2
MR leave−one−out sensitivity analysis for
'INHBC' on 'Cardiac valvular disease'

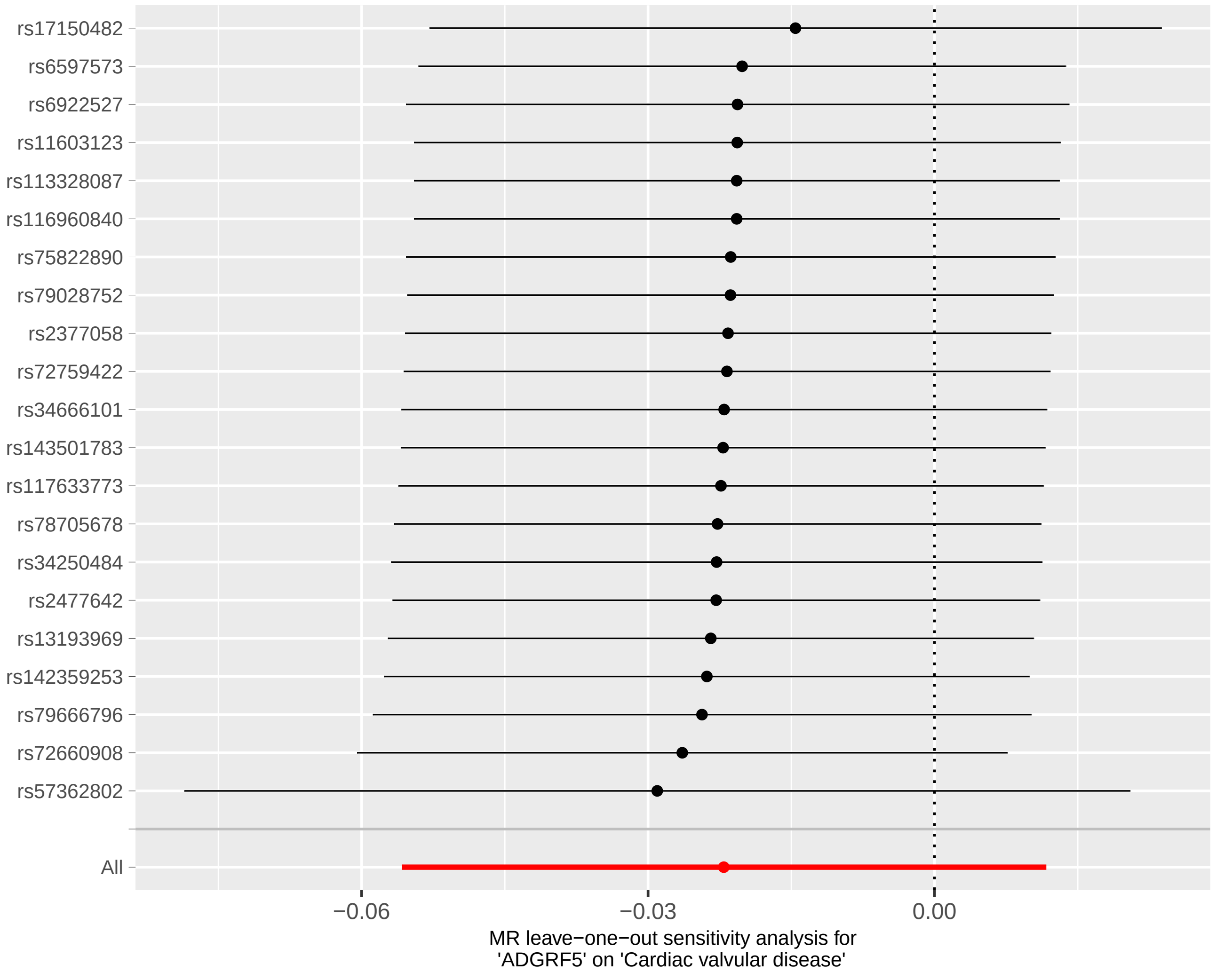
rs17150482
rs6597573
rs6922527
rs11603123
rs113328087
rs116960840
rs75822890
rs79028752
rs2377058
rs72759422
rs34666101
rs143501783
rs117633773
rs78705678
rs34250484
rs2477642
rs13193969
rs142359253
rs79666796
rs72660908
rs57362802
All
−0.06
−0.03
0.00
MR leave−one−out sensitivity analysis for
'ADGRF5' on 'Cardiac valvular disease'

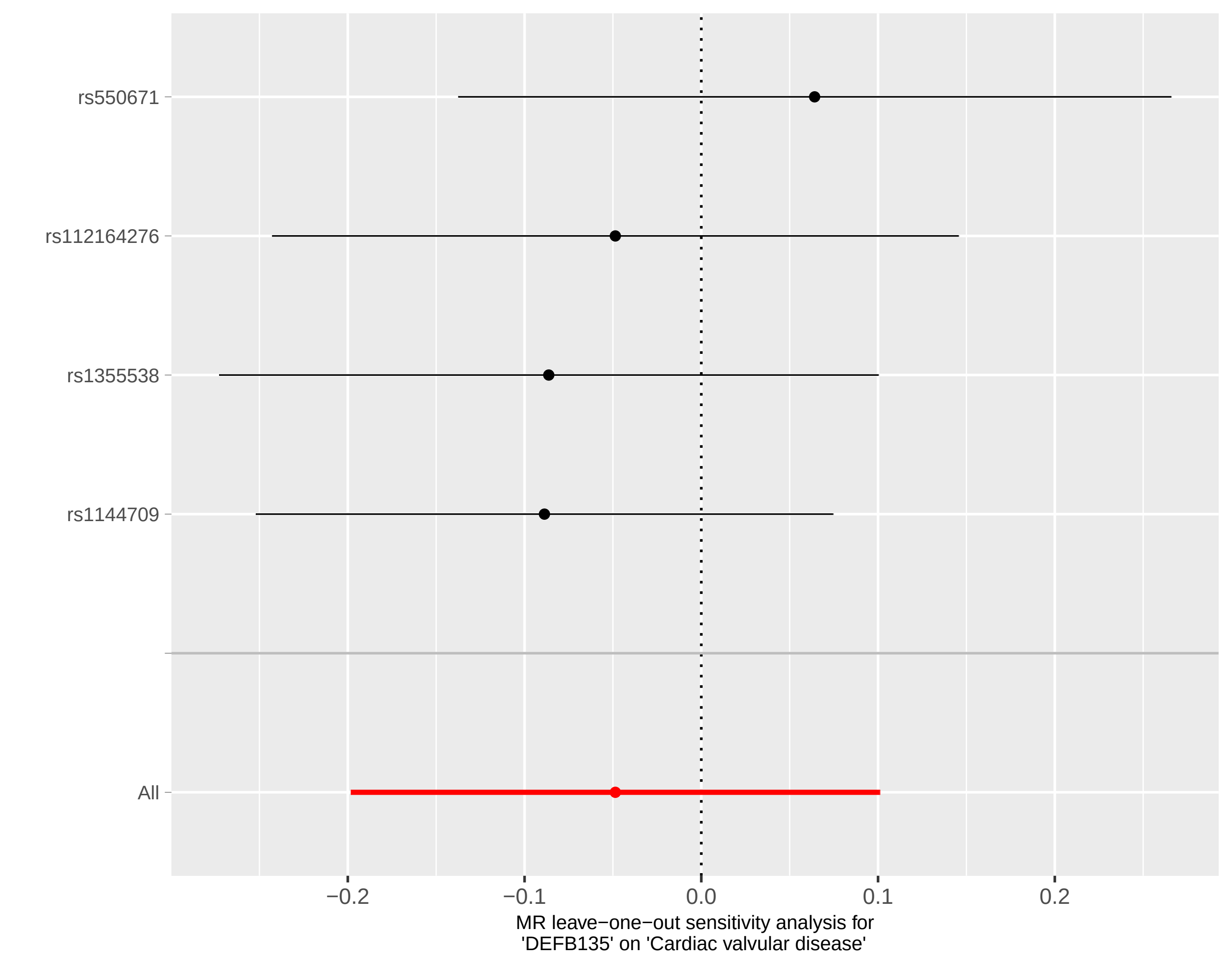
rs550671
rs112164276
rs1355538
rs1144709
All
−0.2
−0.1
0.0
0.1
0.2
MR leave−one−out sensitivity analysis for
'DEFB135' on 'Cardiac valvular disease'

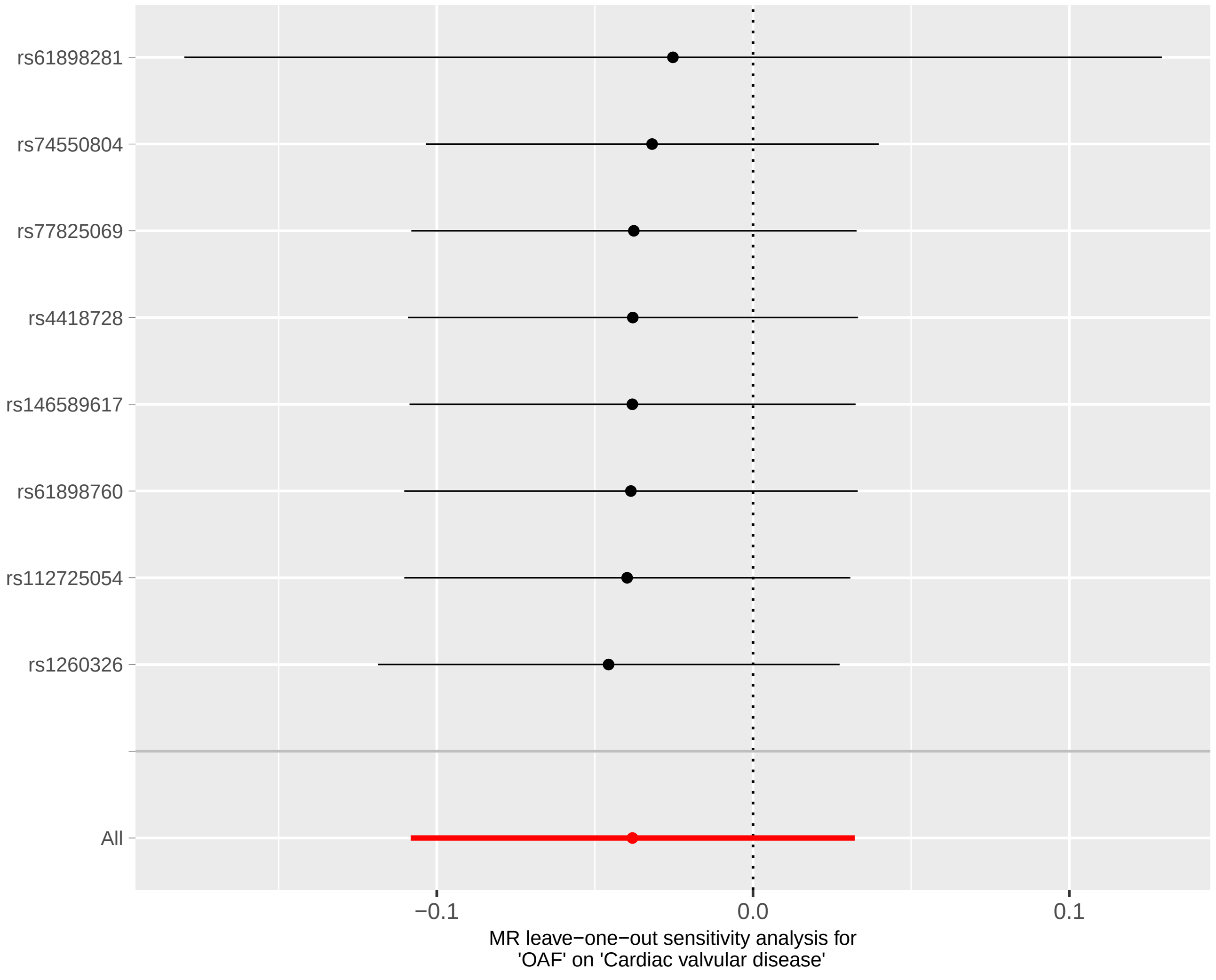

rs61898281
rs74550804
rs77825069
rs4418728
rs146589617
rs61898760
rs112725054
rs1260326
All
−0.1
0.0
0.1
MR leave−one−out sensitivity analysis for
'OAF' on 'Cardiac valvular disease'

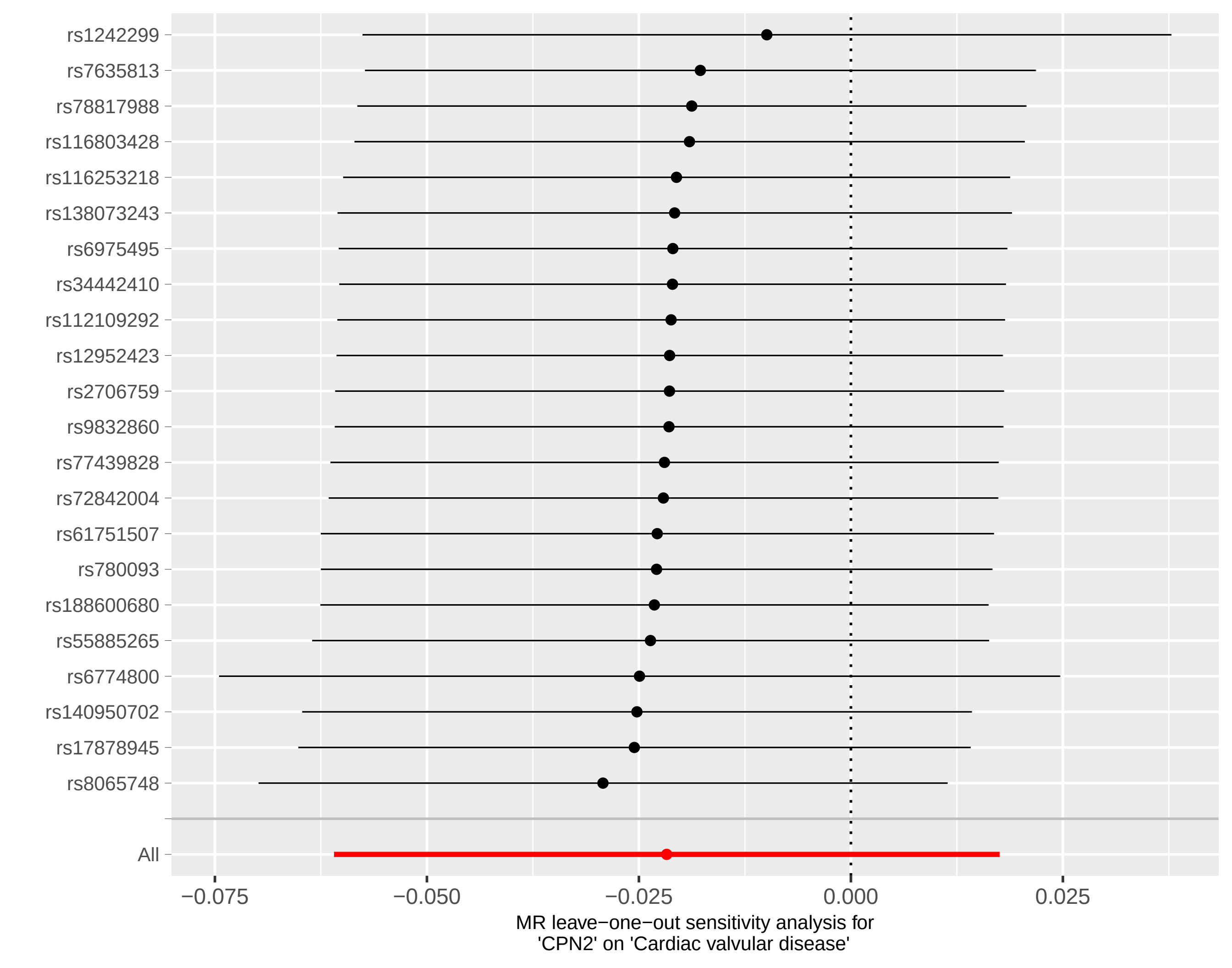

rs1242299
rs7635813
rs78817988
rs116803428
rs116253218
rs138073243
rs6975495
rs34442410
rs112109292
rs12952423
rs2706759
rs9832860
rs77439828
rs72842004
rs61751507
rs780093
rs188600680
rs55885265
rs6774800
rs140950702
rs17878945
rs8065748
All
−0.075
−0.050
−0.025
0.000
0.025
MR leave−one−out sensitivity analysis for
'CPN2' on 'Cardiac valvular disease'

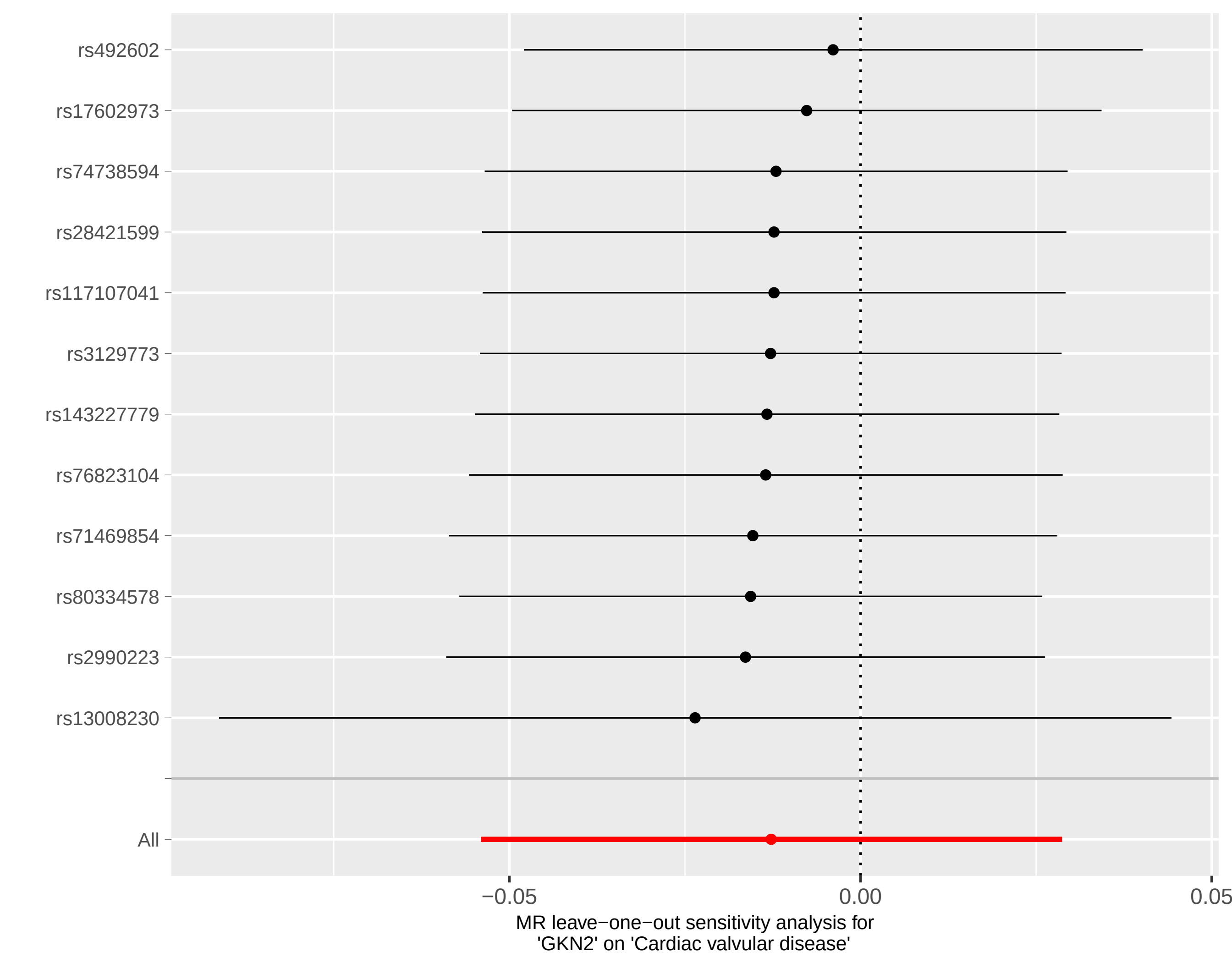
rs492602
rs17602973
rs74738594
rs28421599
rs117107041
rs3129773
rs143227779
rs76823104
rs71469854
rs80334578
rs2990223
rs13008230
All
−0.05
0.00
0.05
MR leave−one−out sensitivity analysis for
'GKN2' on 'Cardiac valvular disease'

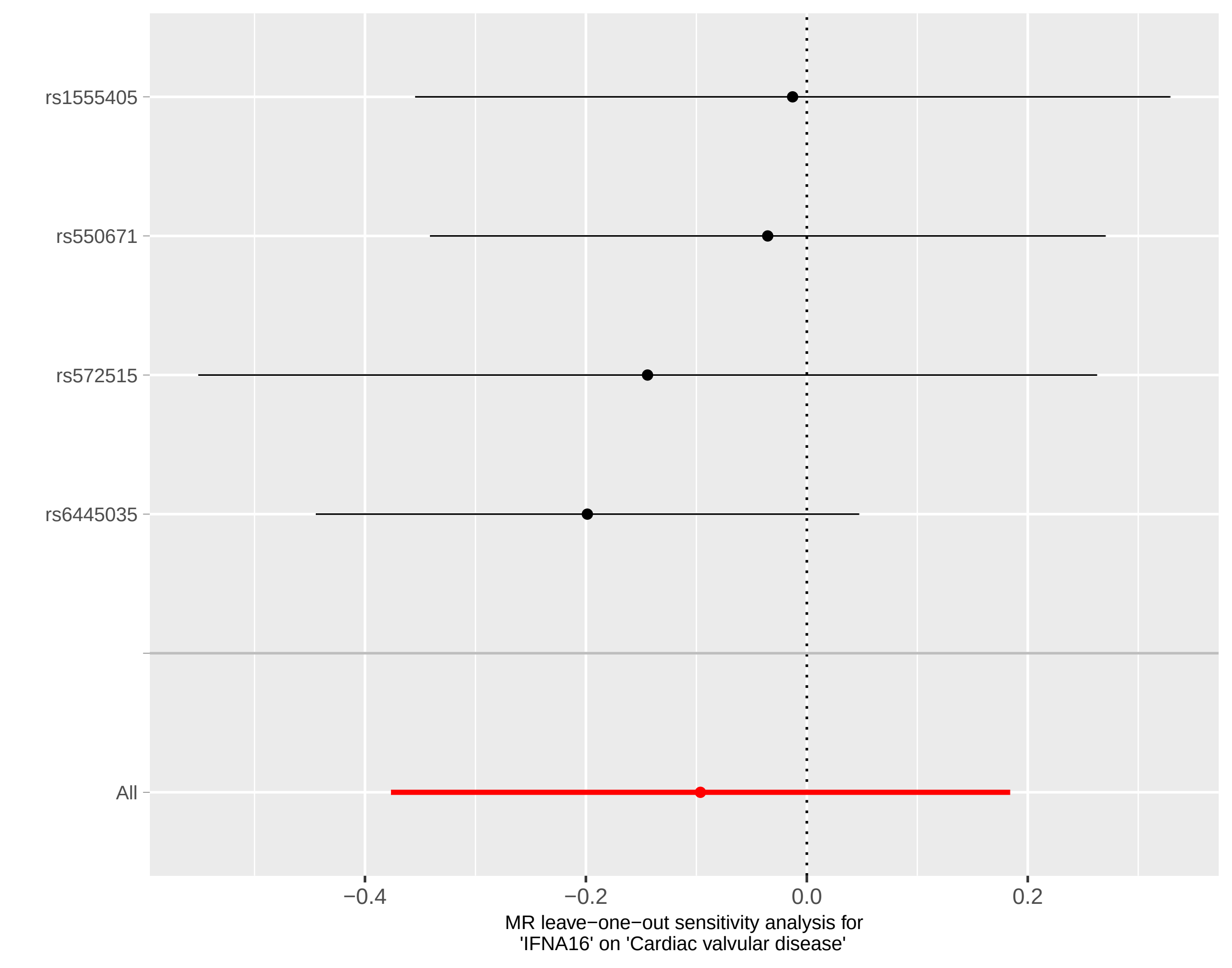
rs1555405
rs550671
rs572515
rs6445035
All
−0.4
−0.2
0.0
0.2
MR leave−one−out sensitivity analysis for
'IFNA16' on 'Cardiac valvular disease'

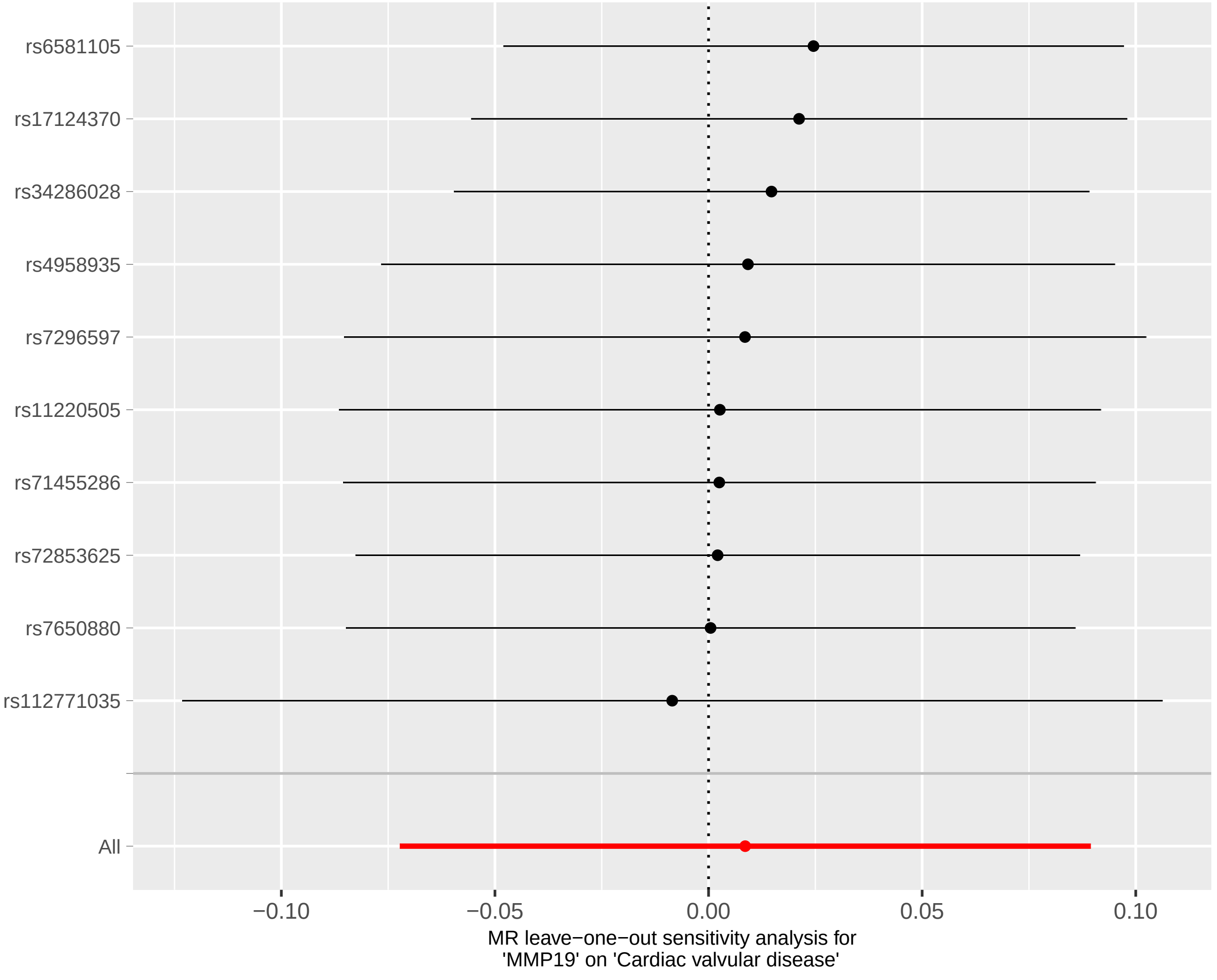
rs6581105
rs17124370
rs34286028
rs4958935
rs7296597
rs11220505
rs71455286
rs72853625
rs7650880
rs112771035
All
−0.10
−0.05
0.00
0.05
0.10
MR leave−one−out sensitivity analysis for
'MMP19' on 'Cardiac valvular disease'

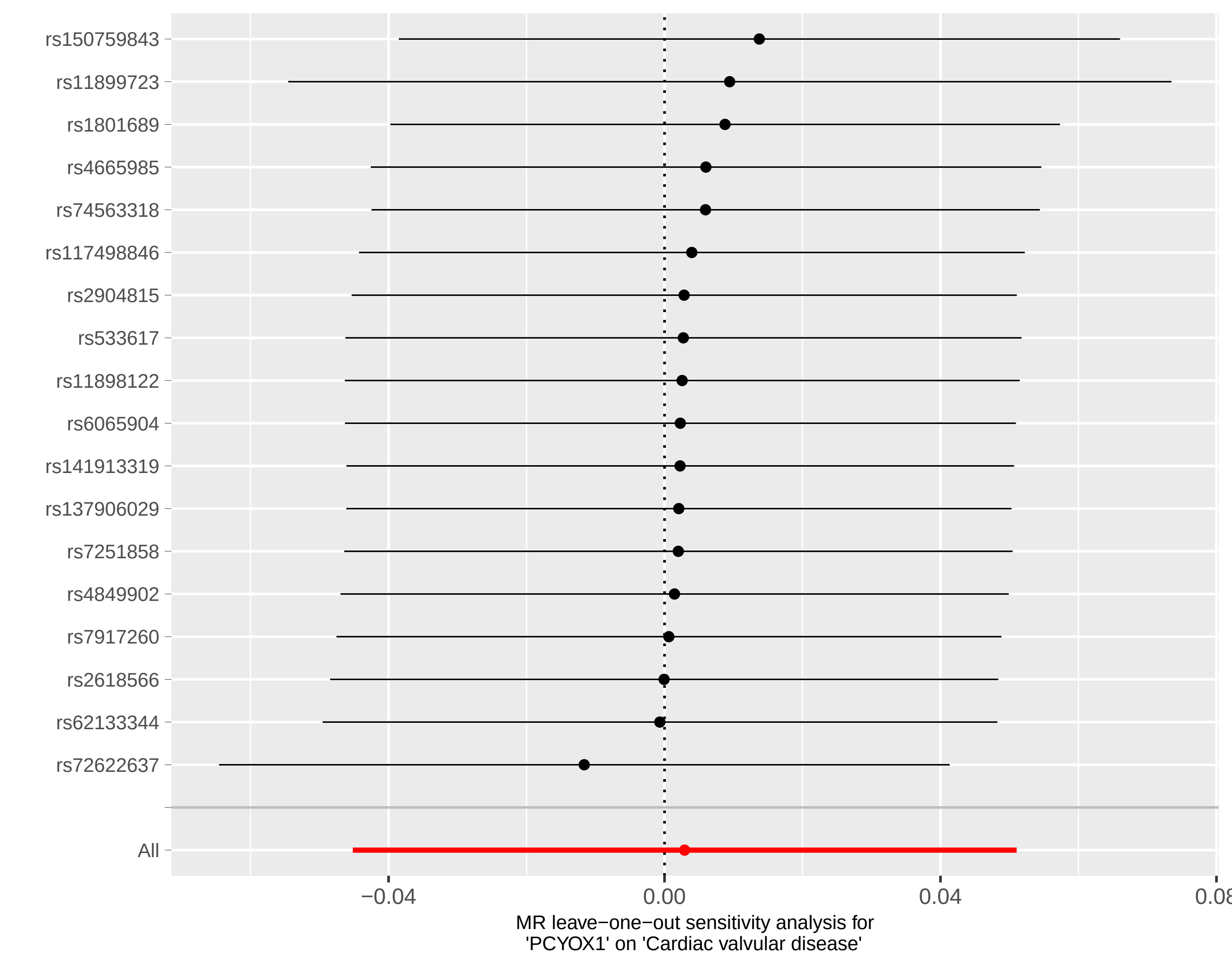

rs150759843
rs11899723
rs1801689
rs4665985
rs74563318
rs117498846
rs2904815
rs533617
rs11898122
rs6065904
rs141913319
rs137906029
rs7251858
rs4849902
rs7917260
rs2618566
rs62133344
rs72622637
All
−0.04
0.00
0.04
0.08
MR leave−one−out sensitivity analysis for
'PCYOX1' on 'Cardiac valvular disease'

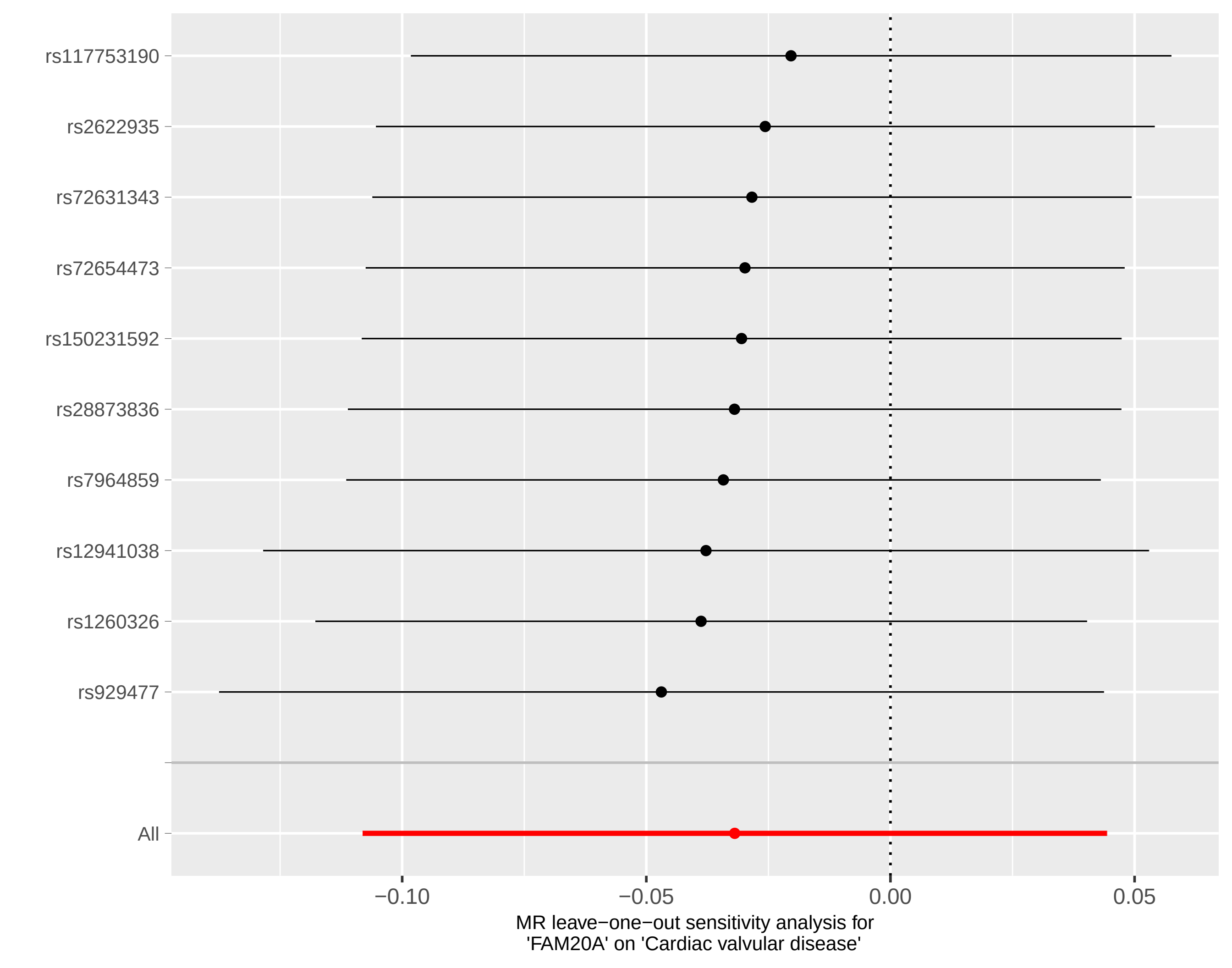

rs117753190
rs2622935
rs72631343
rs72654473
rs150231592
rs28873836
rs7964859
rs12941038
rs1260326
rs929477
All
−0.10
−0.05
0.00
0.05
MR leave−one−out sensitivity analysis for
'FAM20A' on 'Cardiac valvular disease'

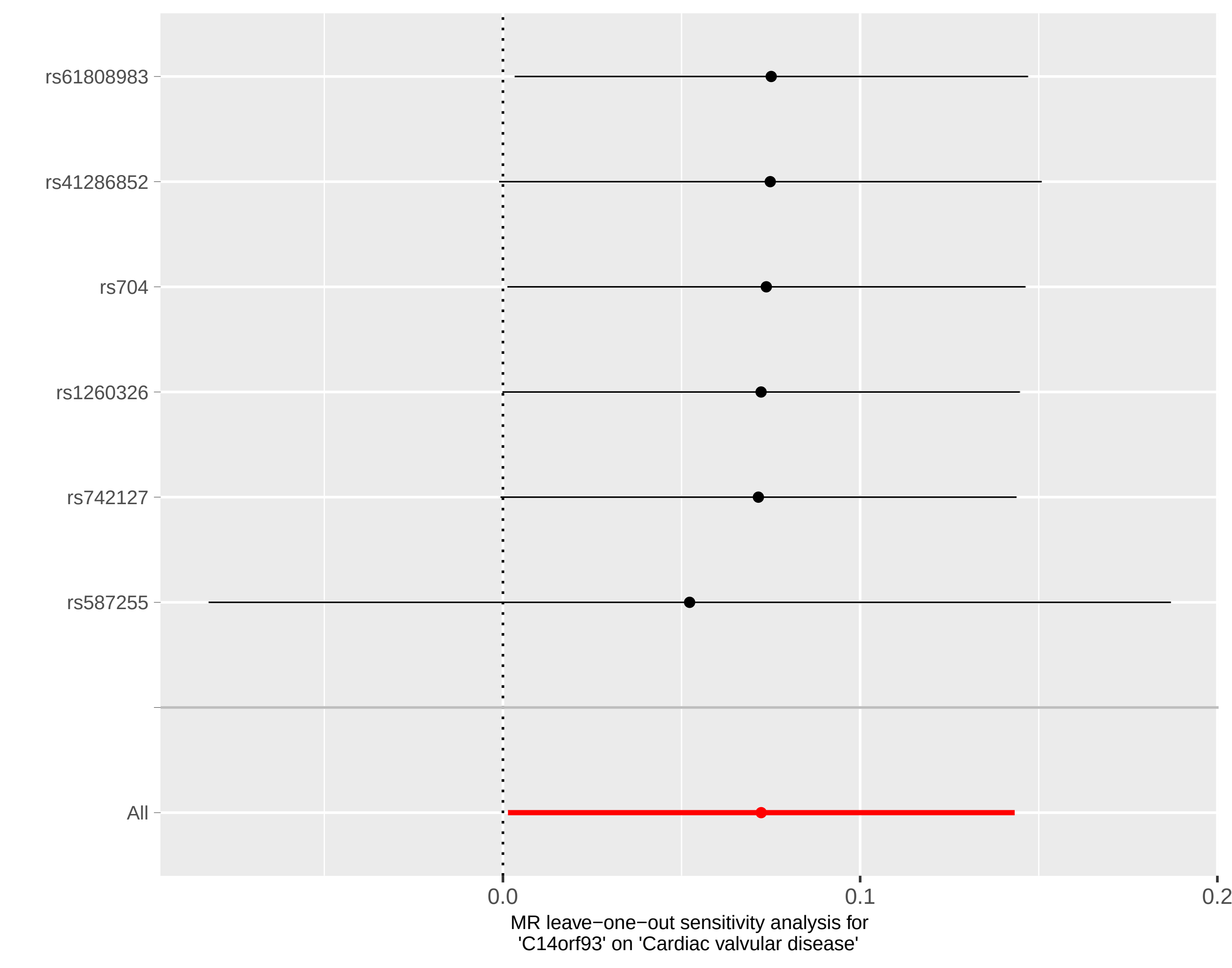
rs61808983
rs41286852
rs704
rs1260326
rs742127
rs587255
All
0.0
0.1
0.2
MR leave−one−out sensitivity analysis for
'C14orf93' on 'Cardiac valvular disease'

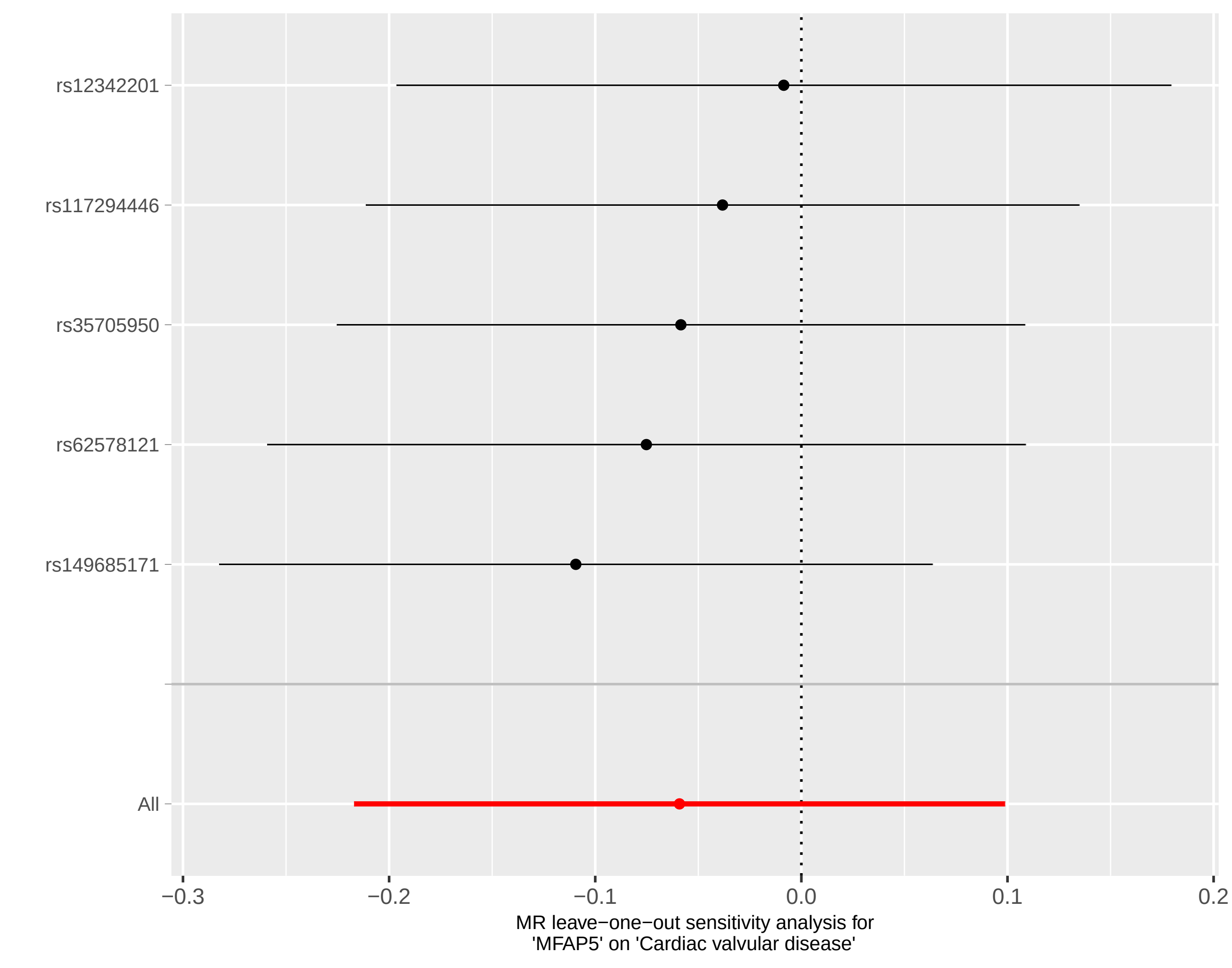

rs12342201
rs117294446
rs35705950
rs62578121
rs149685171
All
−0.3
−0.2
−0.1
0.0
0.1
0.2
MR leave−one−out sensitivity analysis for
'MFAP5' on 'Cardiac valvular disease'

Insufficient number of SNPs

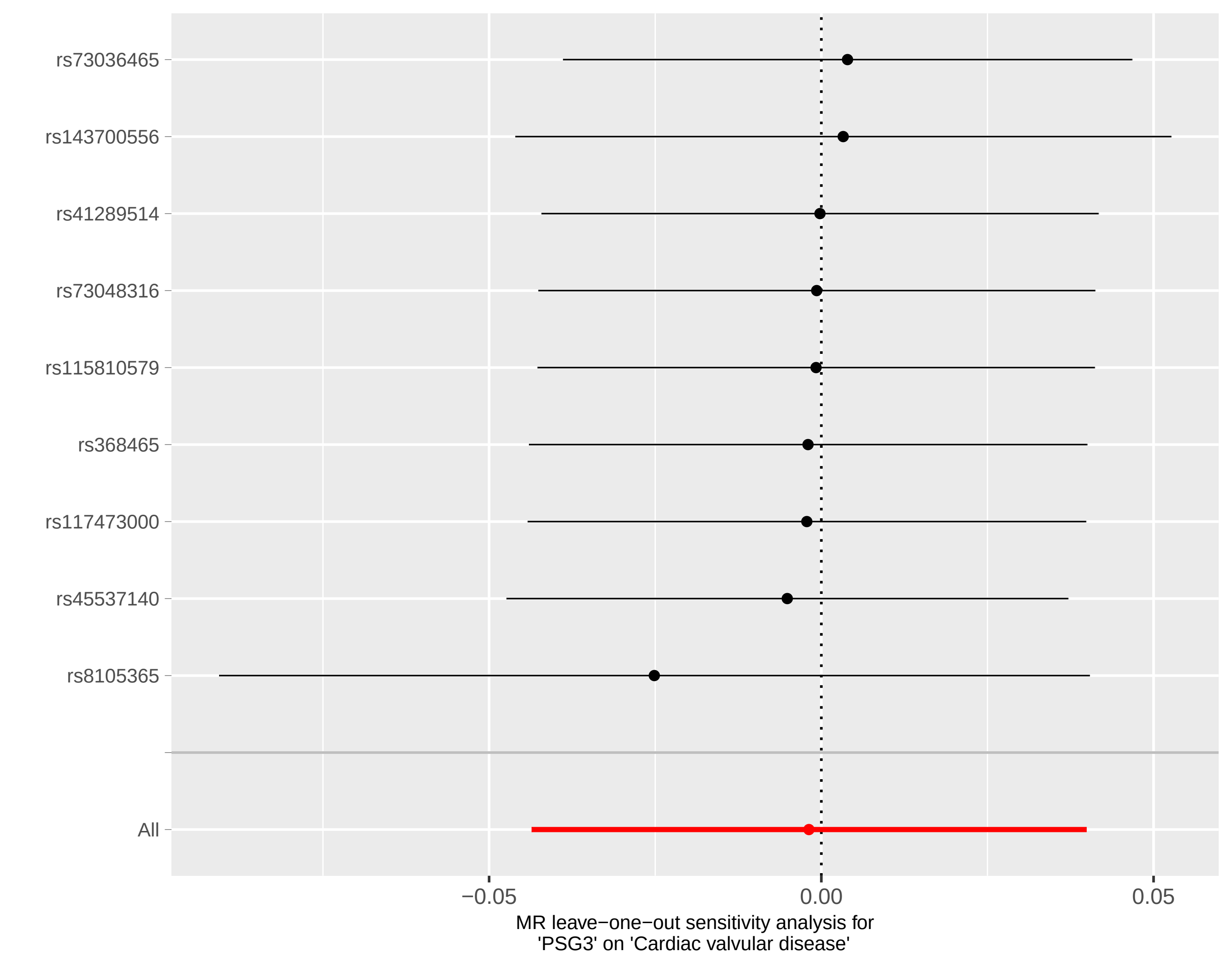
rs73036465
rs143700556
rs41289514
rs73048316
rs115810579
rs368465
rs117473000
rs45537140
rs8105365
All
−0.05
0.00
0.05
MR leave−one−out sensitivity analysis for
'PSG3' on 'Cardiac valvular disease'

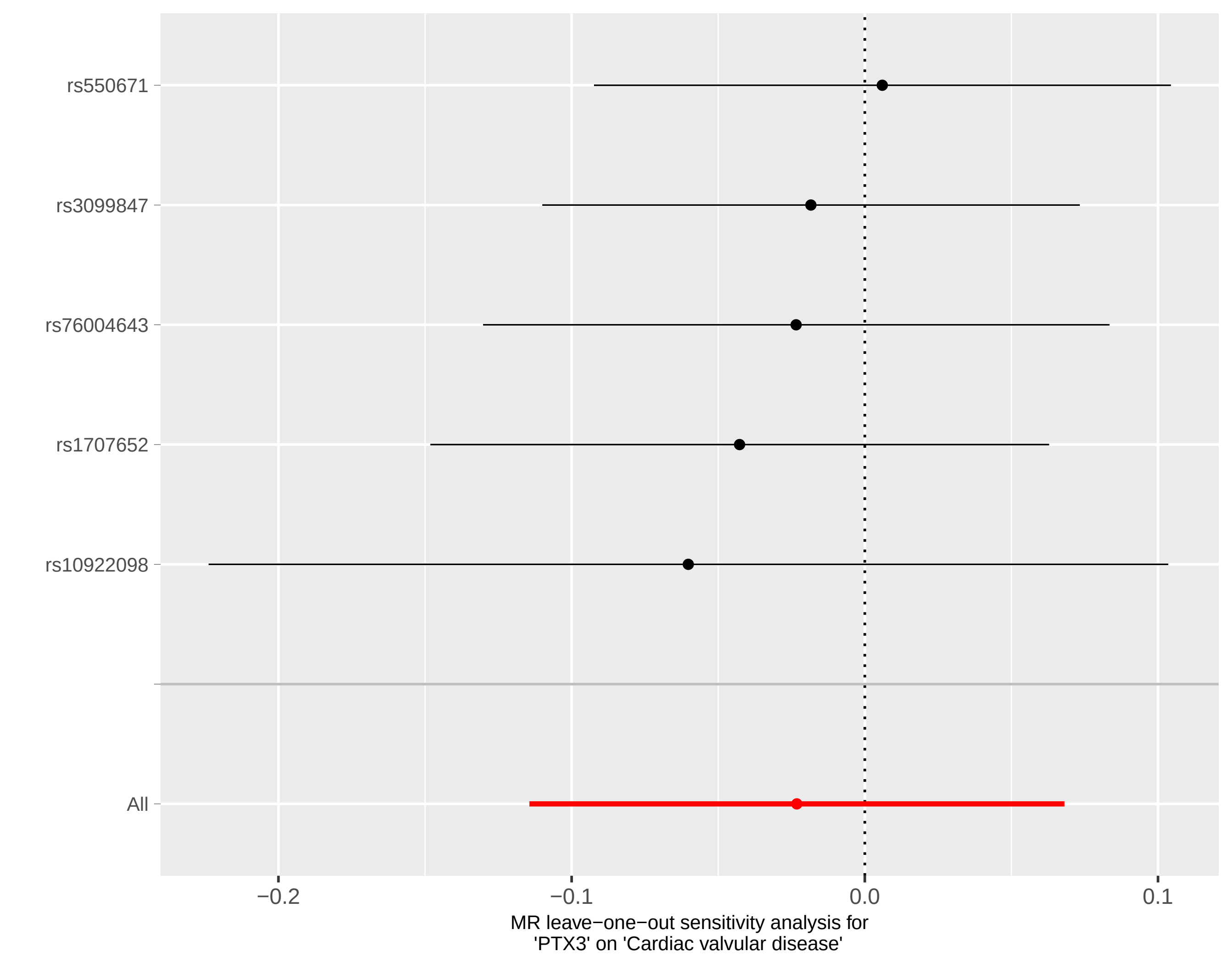
rs550671
rs3099847
rs76004643
rs1707652
rs10922098
All
−0.2
−0.1
0.0
0.1
MR leave−one−out sensitivity analysis for
'PTX3' on 'Cardiac valvular disease'

Insufficient number of SNPs

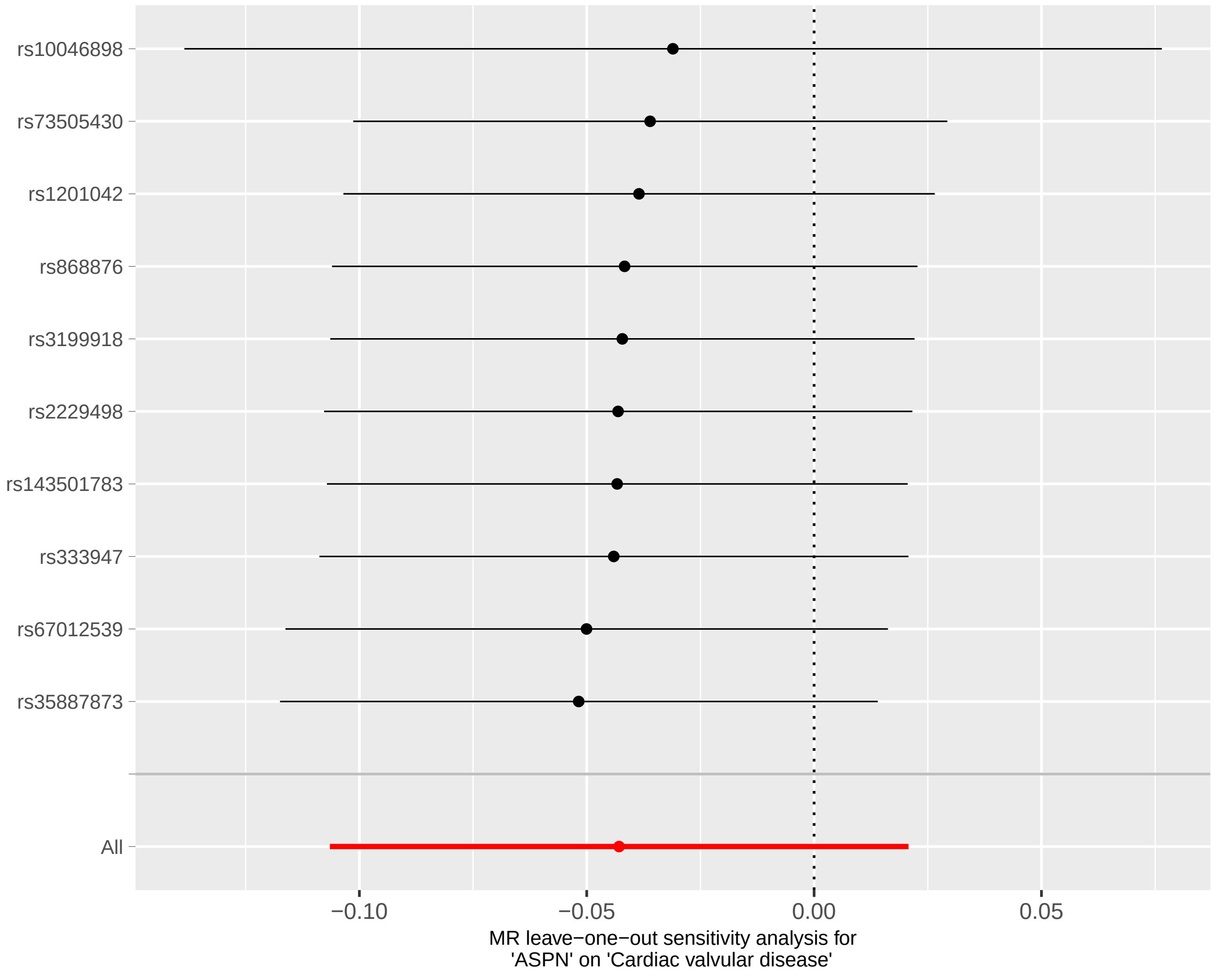
rs10046898
rs73505430
rs1201042
rs868876
rs3199918
rs2229498
rs143501783
rs333947
rs67012539
rs35887873
All
−0.10
−0.05
0.00
0.05
MR leave−one−out sensitivity analysis for
'ASPN' on 'Cardiac valvular disease'

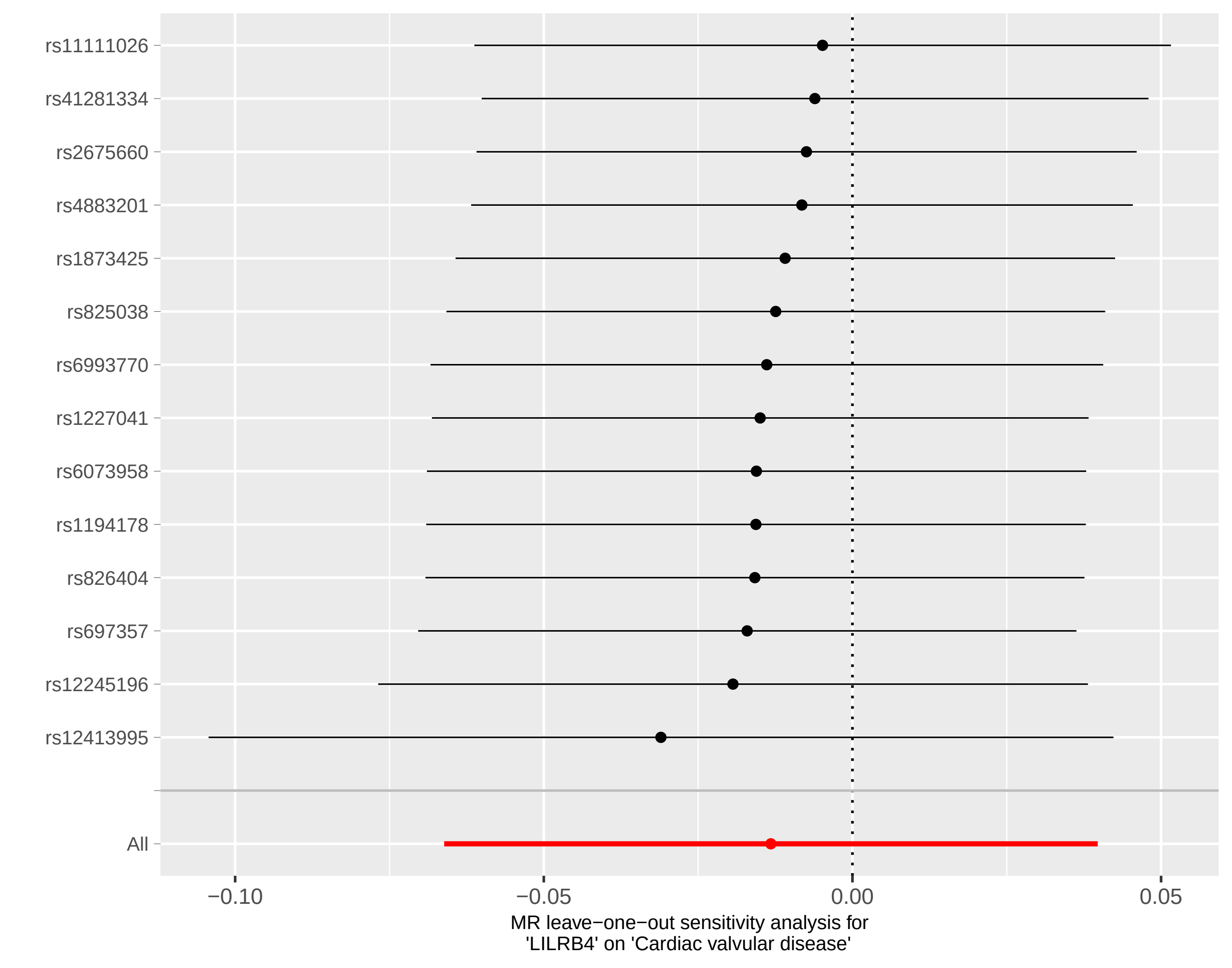

rs11111026
rs41281334
rs2675660
rs4883201
rs1873425
rs825038
rs6993770
rs1227041
rs6073958
rs1194178
rs826404
rs697357
rs12245196
rs12413995
All
−0.10
−0.05
0.00
0.05
MR leave−one−out sensitivity analysis for
'LILRB4' on 'Cardiac valvular disease'

Insufficient number of SNPs

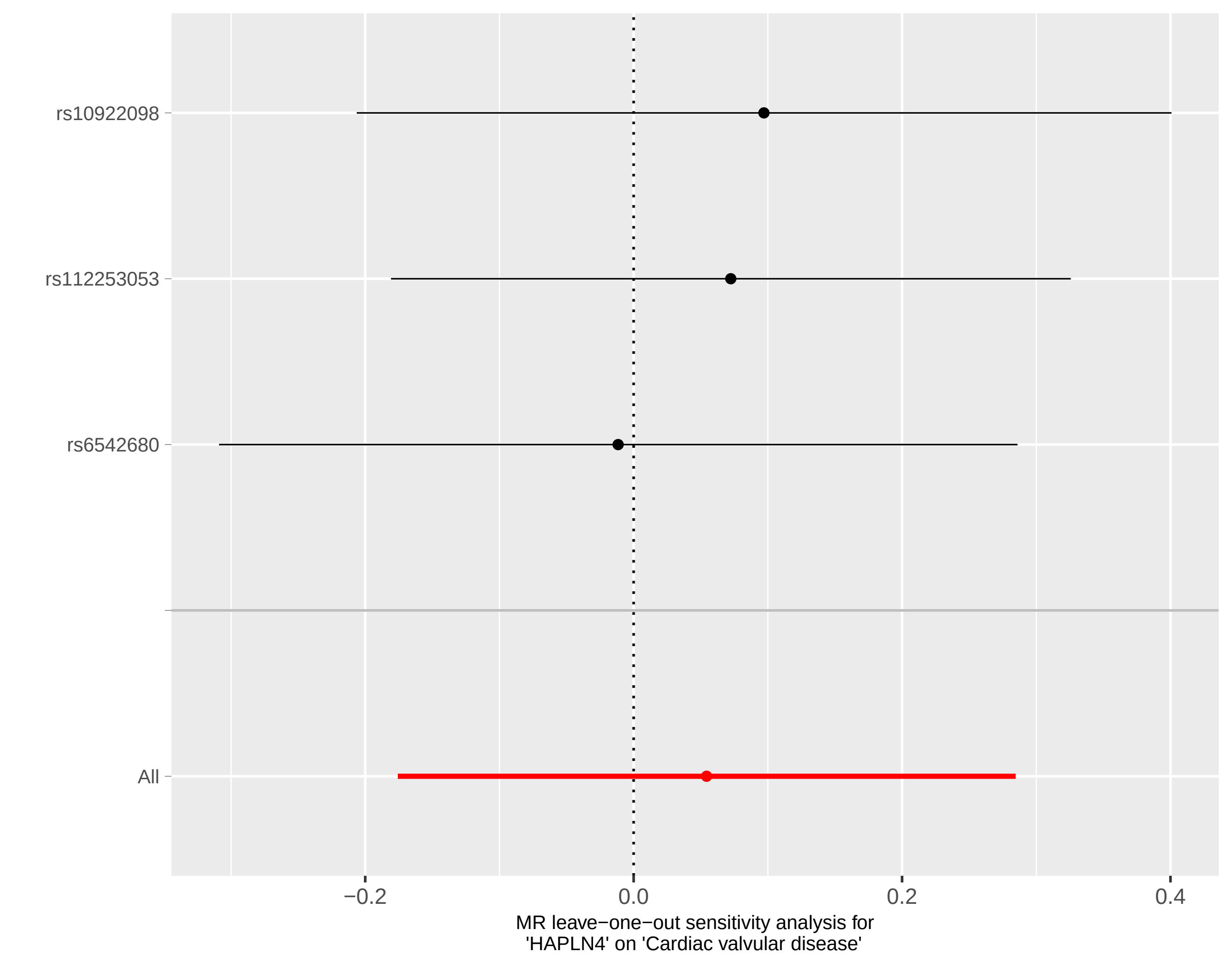

rs10922098
rs112253053
rs6542680
All
−0.2
0.0
0.2
0.4
MR leave−one−out sensitivity analysis for
'HAPLN4' on 'Cardiac valvular disease'

Insufficient number of SNPs

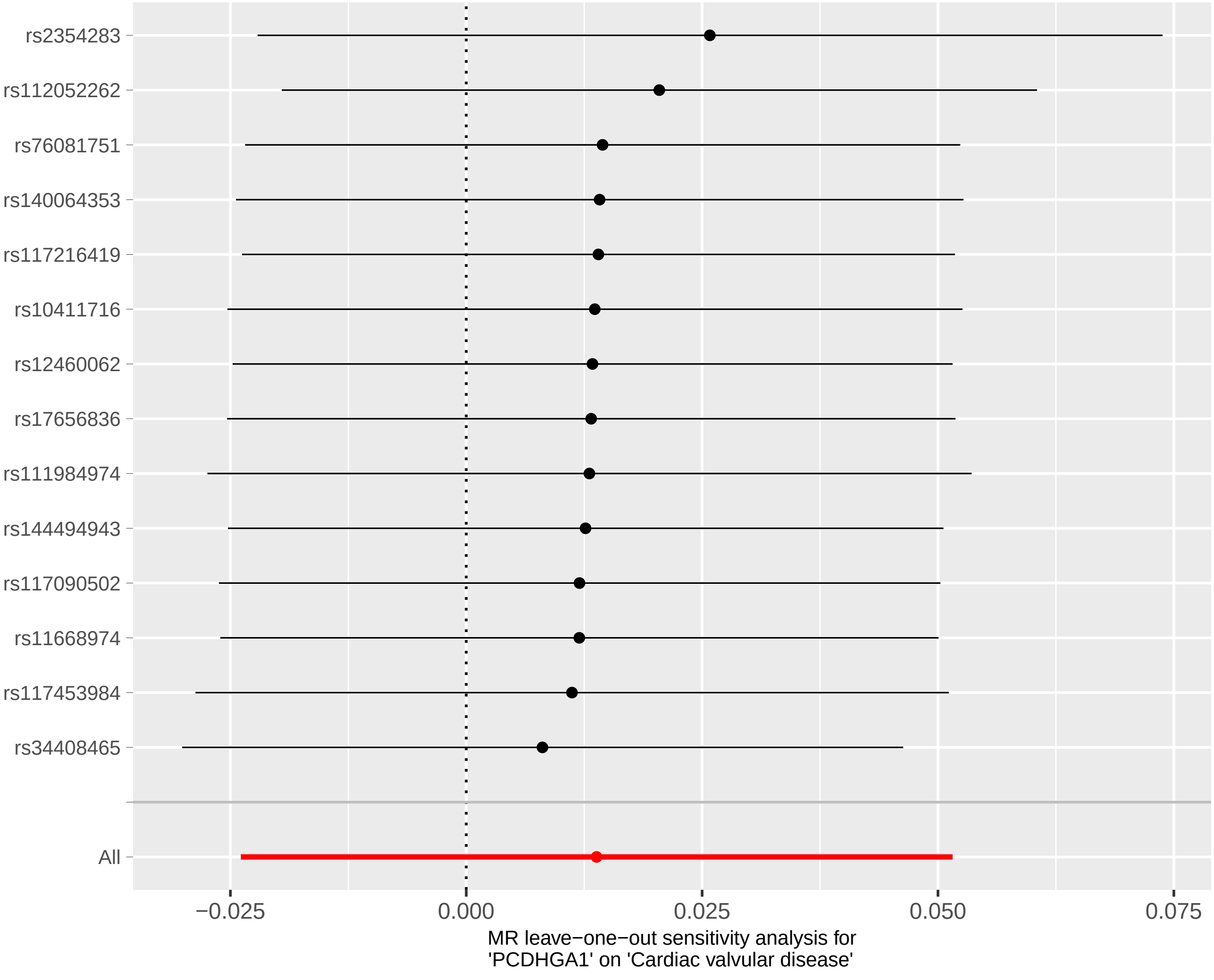

rs2354283
rs112052262
rs76081751
rs140064353
rs117216419
rs10411716
rs12460062
rs17656836
rs111984974
rs144494943
rs117090502
rs11668974
rs117453984
rs34408465
All
−0.025
0.000
0.025
0.050
0.075
MR leave−one−out sensitivity analysis for
'PCDHGA1' on 'Cardiac valvular disease'

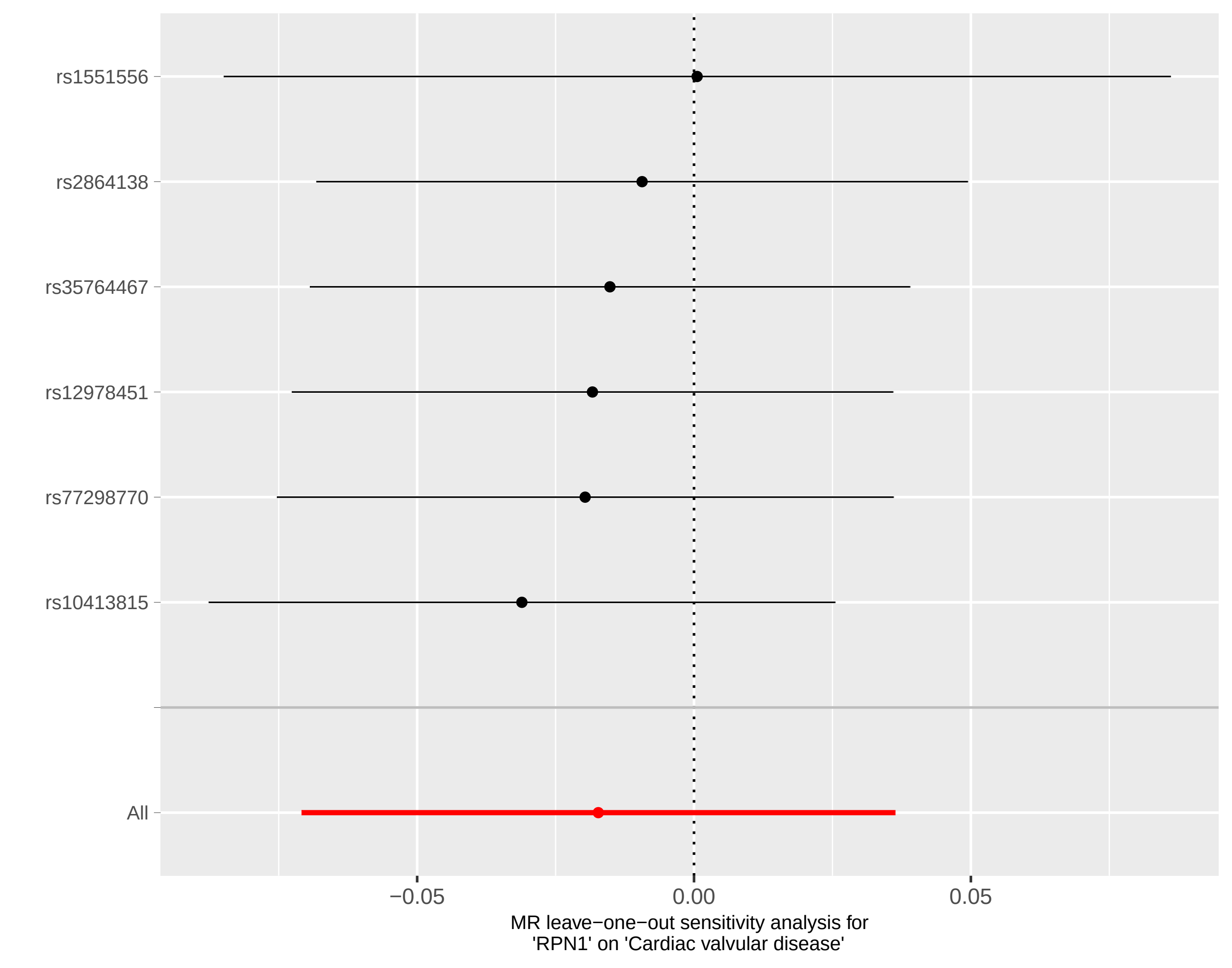

rs1551556
rs2864138
rs35764467
rs12978451
rs77298770
rs10413815
All
−0.05
0.00
0.05
MR leave−one−out sensitivity analysis for
'RPN1' on 'Cardiac valvular disease'

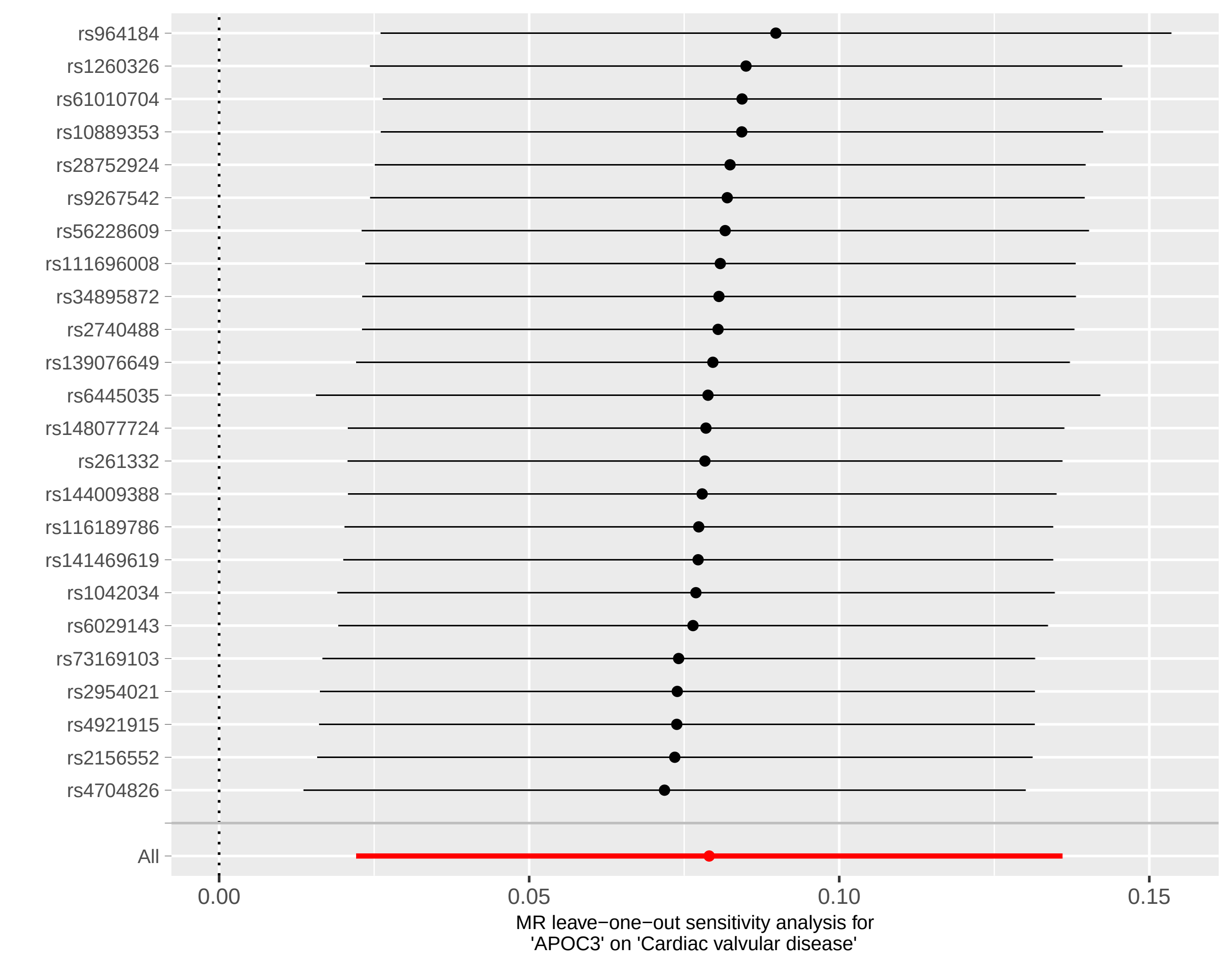
rs964184
rs1260326
rs61010704
rs10889353
rs28752924
rs9267542
rs56228609
rs111696008
rs34895872
rs2740488
rs139076649
rs6445035
rs148077724
rs261332
rs144009388
rs116189786
rs141469619
rs1042034
rs6029143
rs73169103
rs2954021
rs4921915
rs2156552
rs4704826
All
0.00
0.05
0.10
0.15
MR leave−one−out sensitivity analysis for
'APOC3' on 'Cardiac valvular disease'

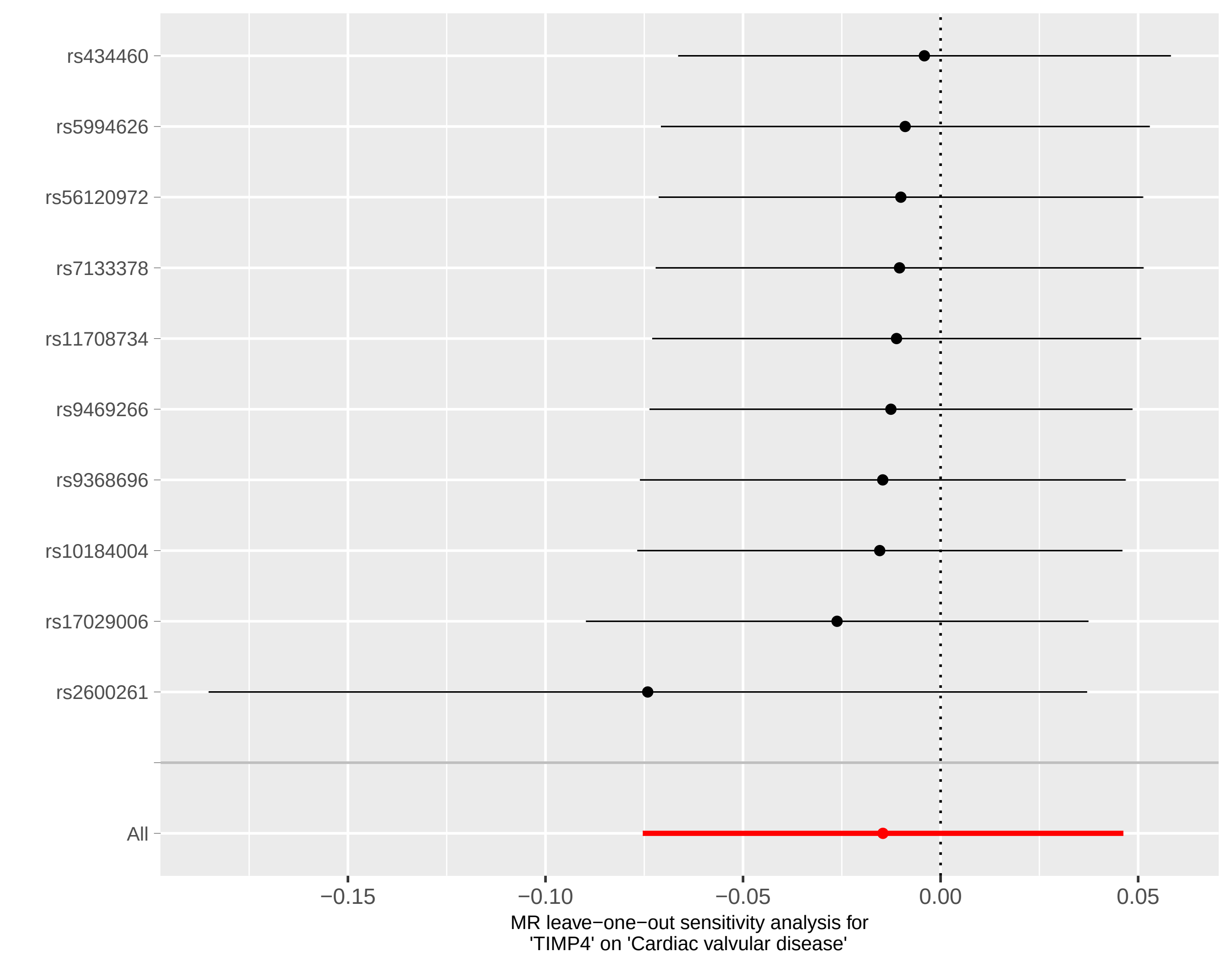
rs434460
rs5994626
rs56120972
rs7133378
rs11708734
rs9469266
rs9368696
rs10184004
rs17029006
rs2600261
All
−0.15
−0.10
−0.05
0.00
0.05
MR leave−one−out sensitivity analysis for
'TIMP4' on 'Cardiac valvular disease'

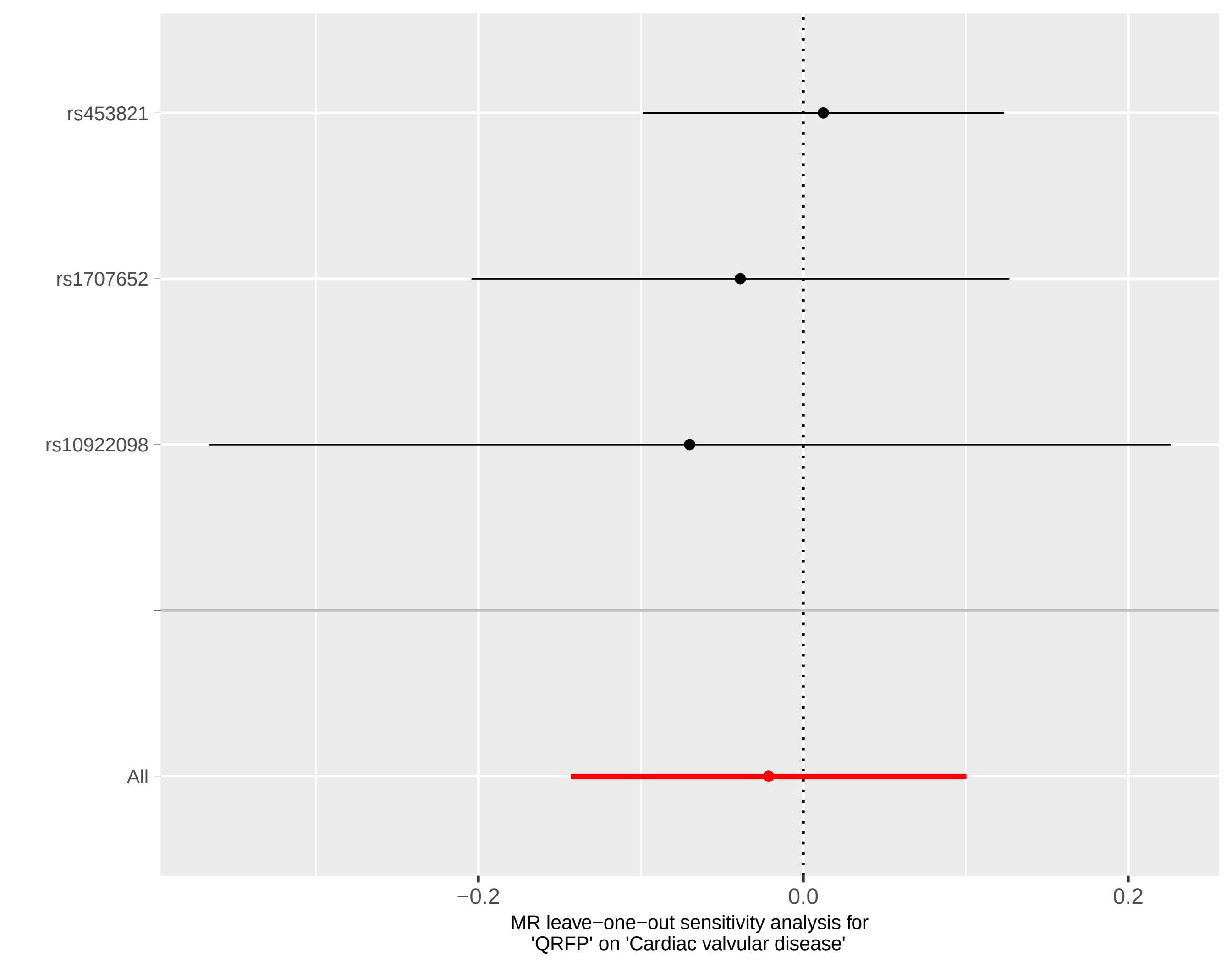

rs453821
rs1707652
rs10922098
All
−0.2
0.0
0.2
MR leave−one−out sensitivity analysis for
'QRFP' on 'Cardiac valvular disease'

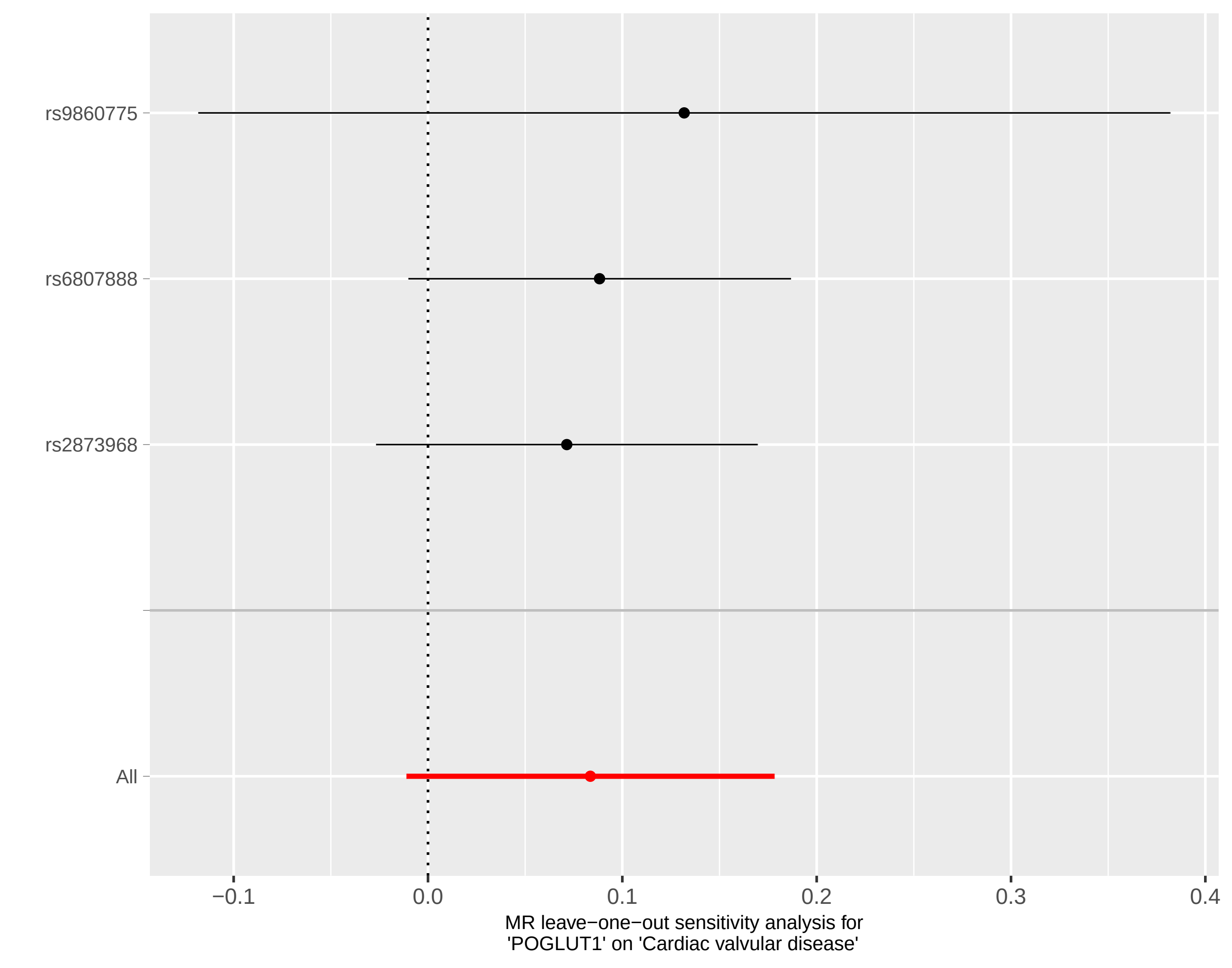
rs9860775
rs6807888
rs2873968
All
−0.1
0.0
0.1
0.2
0.3
0.4
MR leave−one−out sensitivity analysis for
'POGLUT1' on 'Cardiac valvular disease'

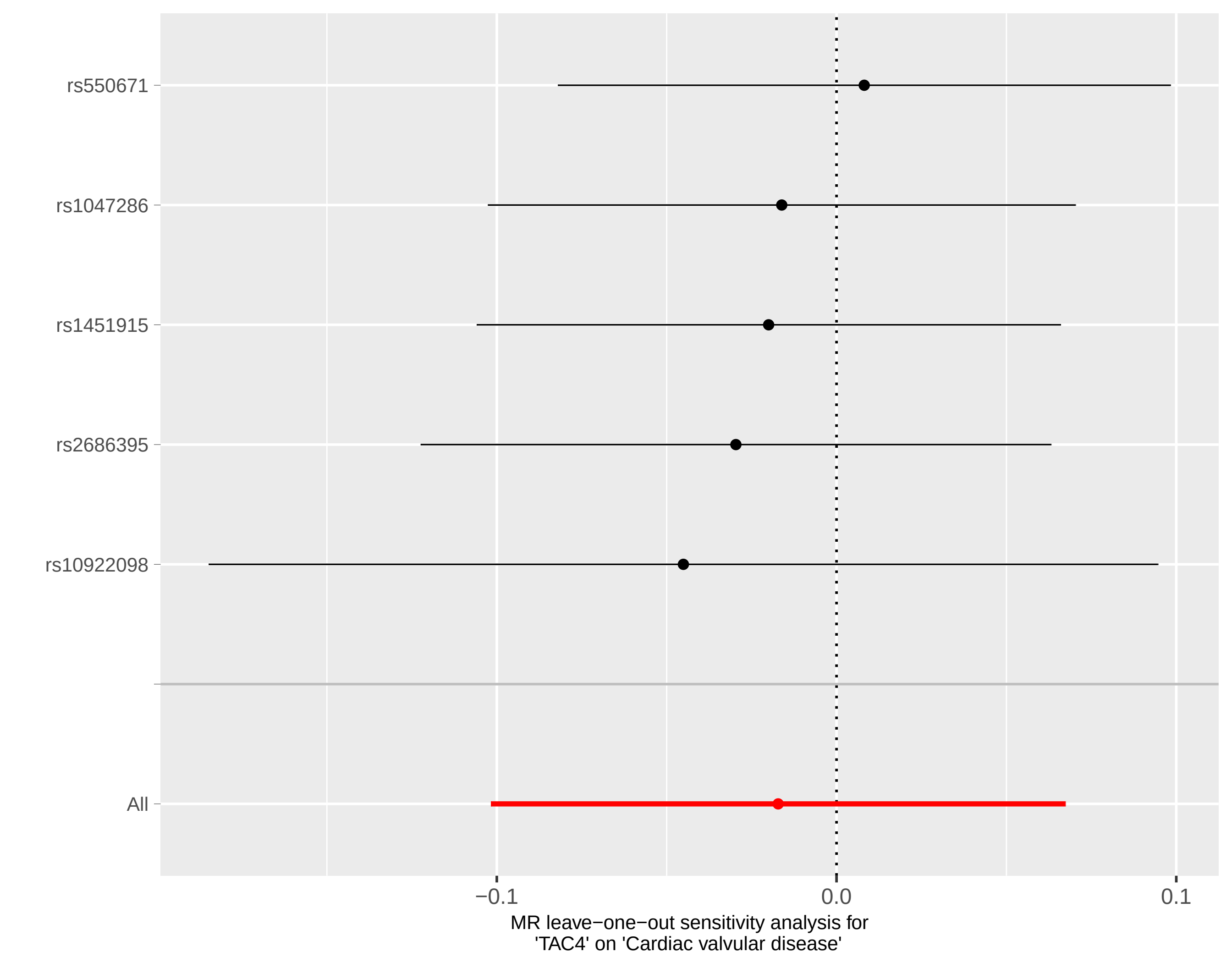
rs550671
rs1047286
rs1451915
rs2686395
rs10922098
All
−0.1
0.0
0.1
MR leave−one−out sensitivity analysis for
'TAC4' on 'Cardiac valvular disease'

Insufficient number of SNPs

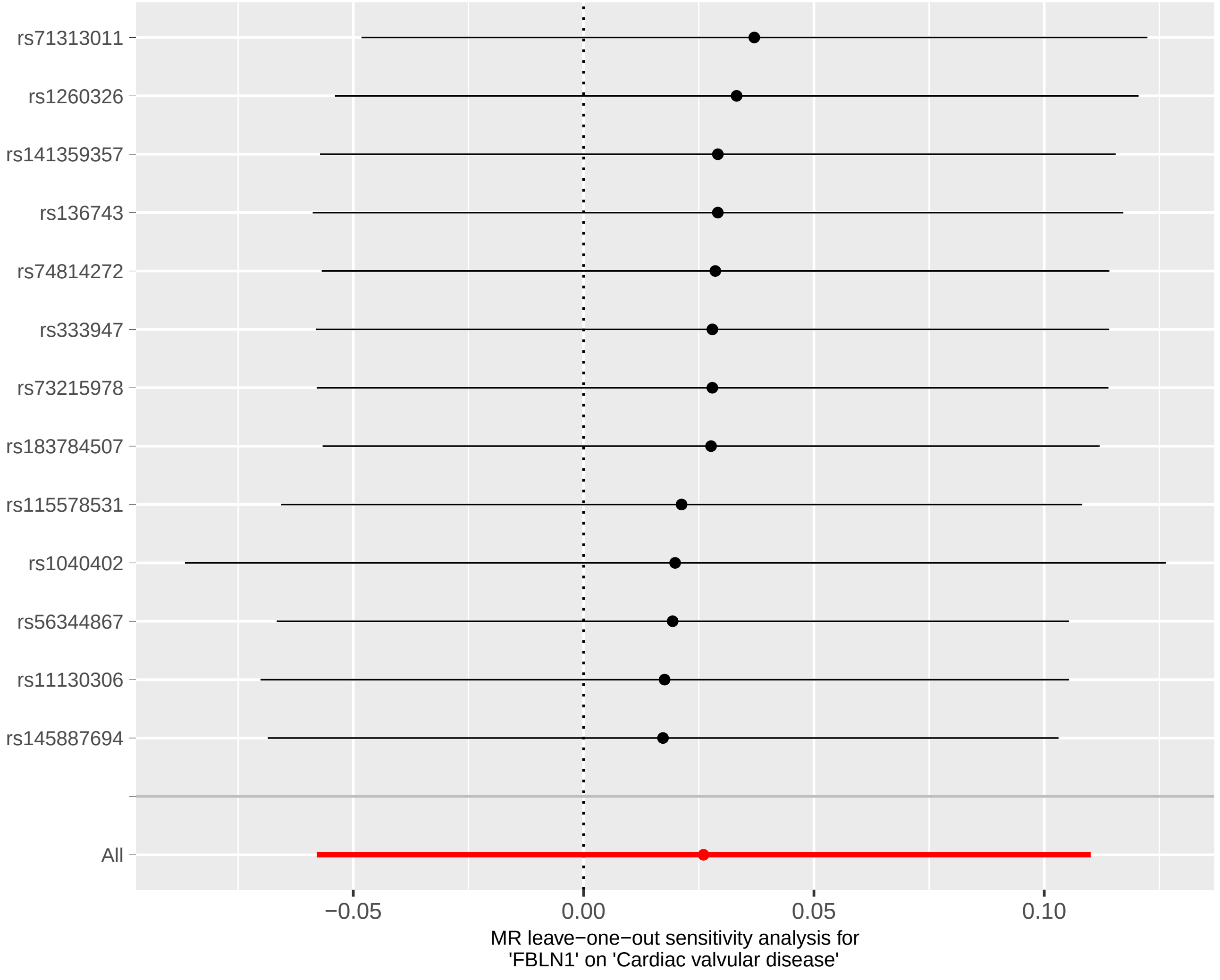

rs71313011
rs1260326
rs141359357
rs136743
rs74814272
rs333947
rs73215978
rs183784507
rs115578531
rs1040402
rs56344867
rs11130306
rs145887694
All
−0.05
0.00
0.05
0.10
MR leave−one−out sensitivity analysis for
'FBLN1' on 'Cardiac valvular disease'

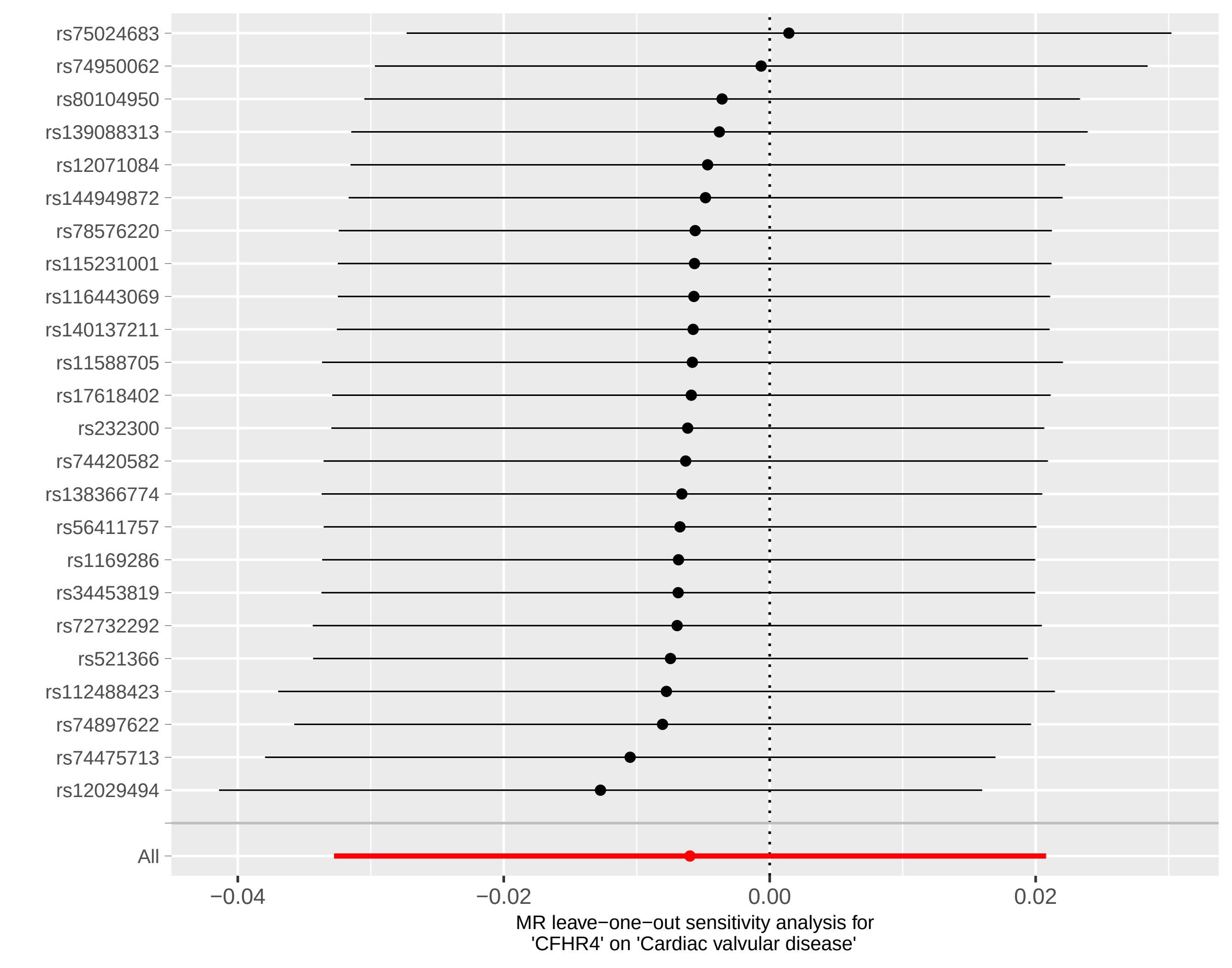
rs75024683
rs74950062
rs80104950
rs139088313
rs12071084
rs144949872
rs78576220
rs115231001
rs116443069
rs140137211
rs11588705
rs17618402
rs232300
rs74420582
rs138366774
rs56411757
rs1169286
rs34453819
rs72732292
rs521366
rs112488423
rs74897622
rs74475713
rs12029494
All
−0.04
−0.02
0.00
0.02
MR leave−one−out sensitivity analysis for
'CFHR4' on 'Cardiac valvular disease'

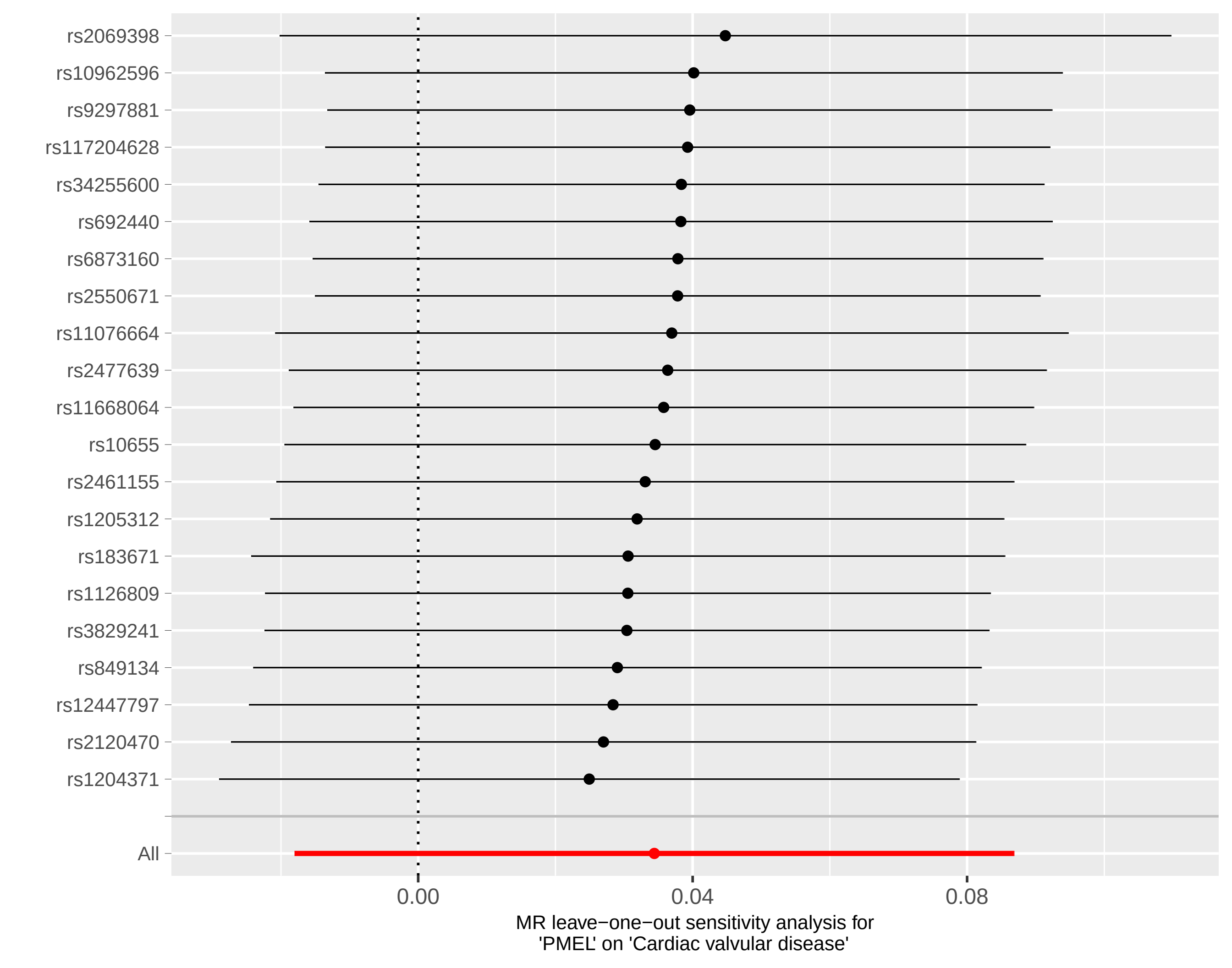
rs2069398
rs10962596
rs9297881
rs117204628
rs34255600
rs692440
rs6873160
rs2550671
rs11076664
rs2477639
rs11668064
rs10655
rs2461155
rs1205312
rs183671
rs1126809
rs3829241
rs849134
rs12447797
rs2120470
rs1204371
All
0.00
0.04
0.08
MR leave−one−out sensitivity analysis for
'PMEL' on 'Cardiac valvular disease'

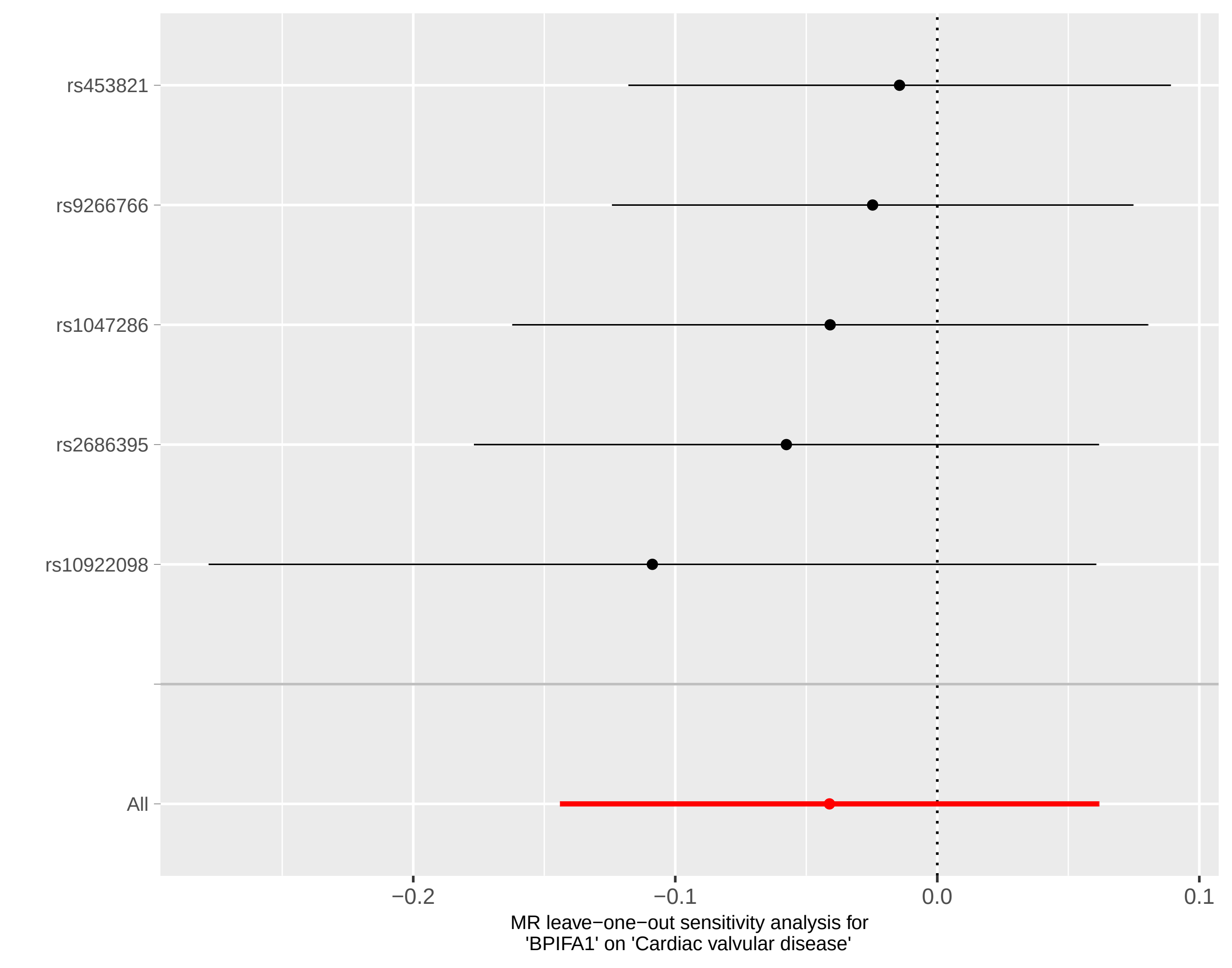
rs453821
rs9266766
rs1047286
rs2686395
rs10922098
All
−0.2
−0.1
0.0
0.1
MR leave−one−out sensitivity analysis for
'BPIFA1' on 'Cardiac valvular disease'

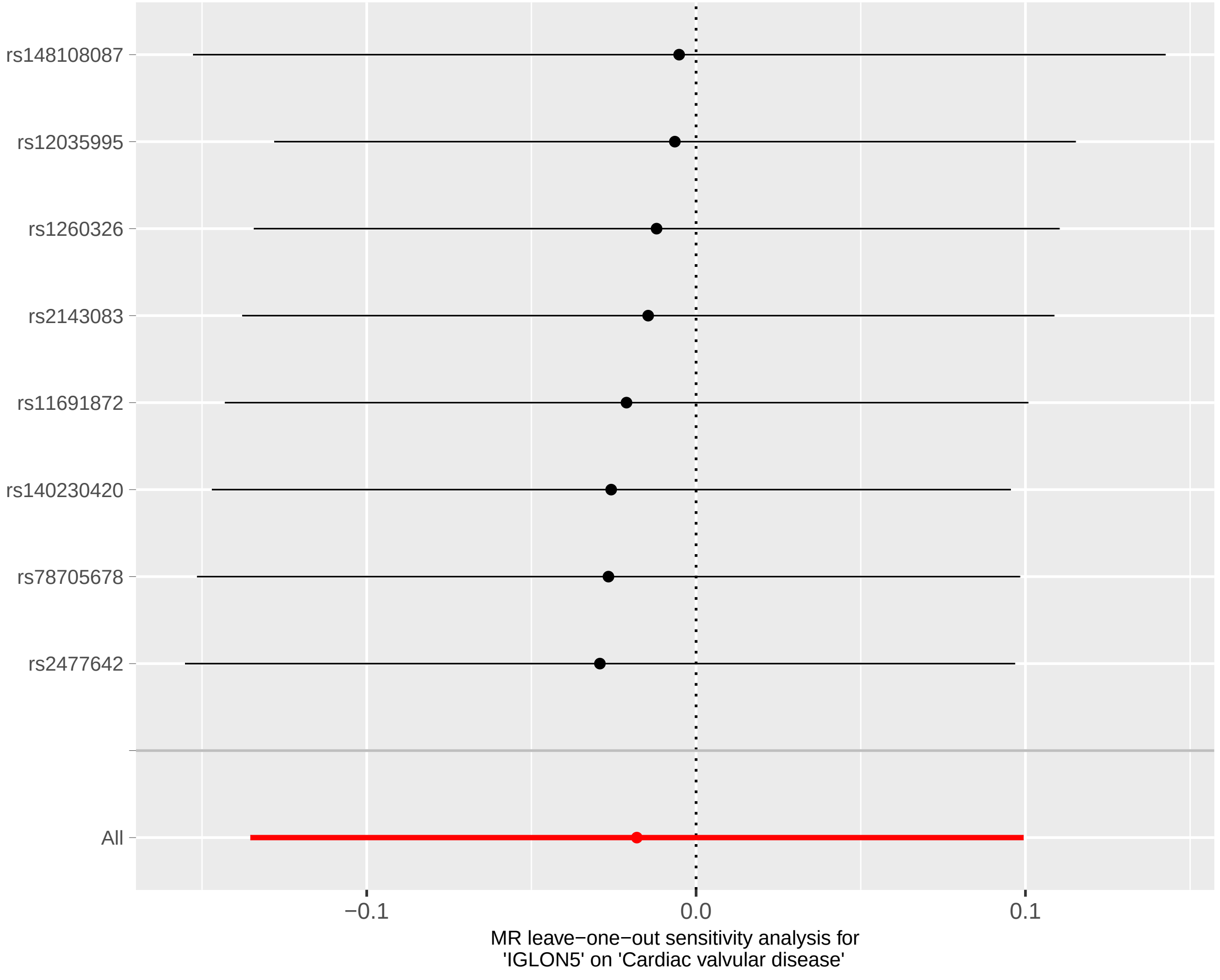

rs148108087
rs12035995
rs1260326
rs2143083
rs11691872
rs140230420
rs78705678
rs2477642
All
−0.1
0.0
0.1
MR leave−one−out sensitivity analysis for
'IGLON5' on 'Cardiac valvular disease'

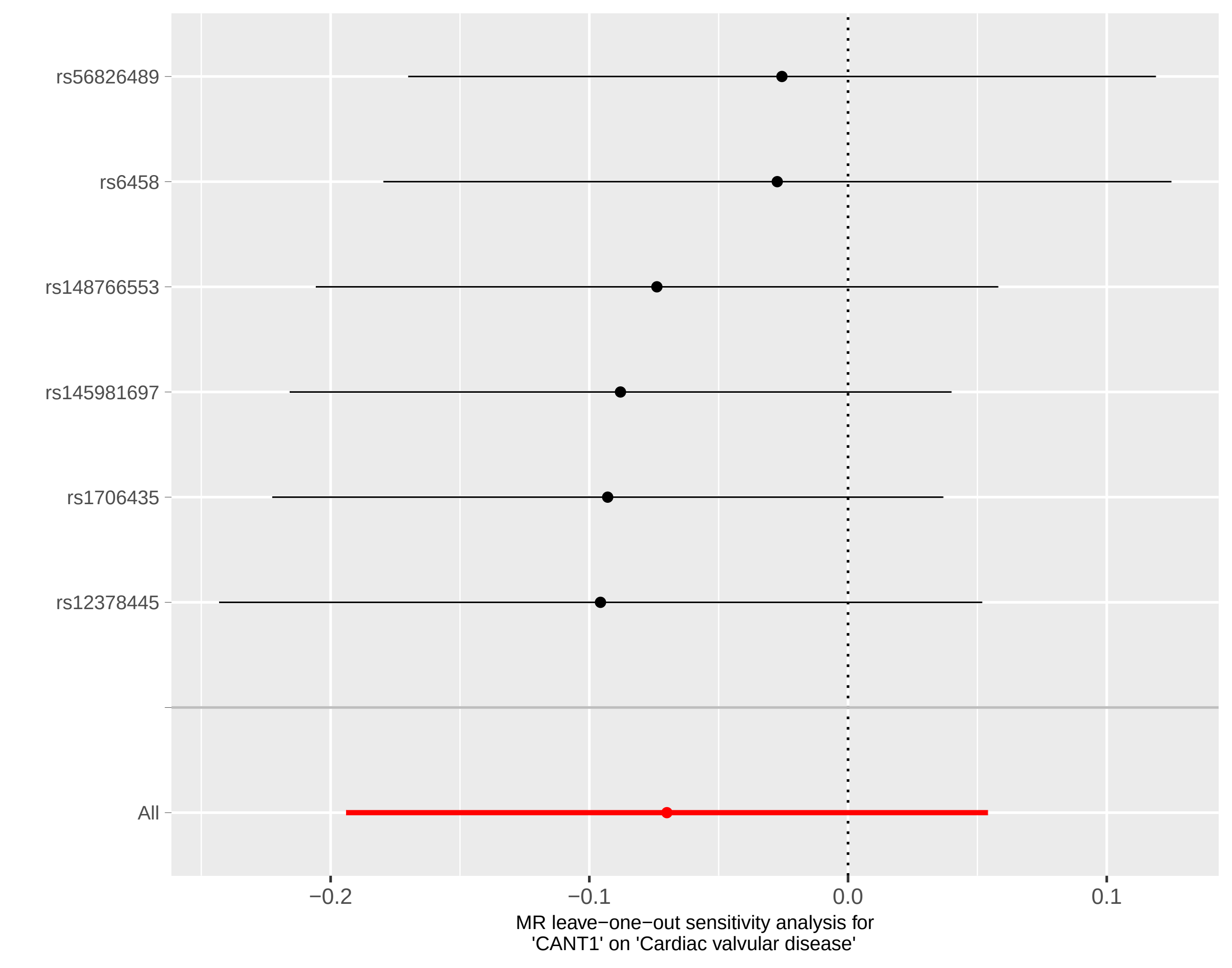

rs56826489
rs6458
rs148766553
rs145981697
rs1706435
rs12378445
All
−0.2
−0.1
0.0
0.1
MR leave−one−out sensitivity analysis for
'CANT1' on 'Cardiac valvular disease'

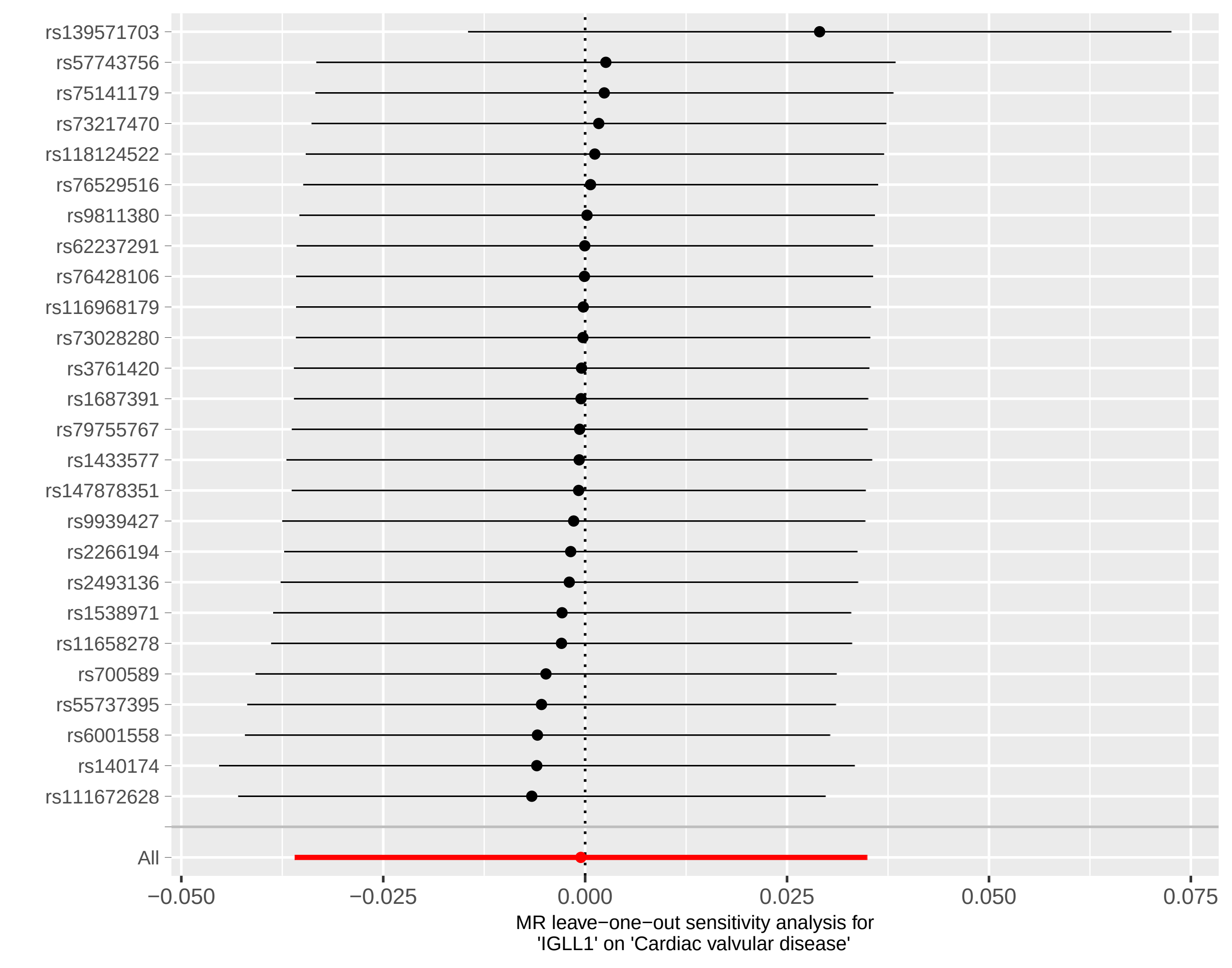
rs139571703
rs57743756
rs75141179
rs73217470
rs118124522
rs76529516
rs9811380
rs62237291
rs76428106
rs116968179
rs73028280
rs3761420
rs1687391
rs79755767
rs1433577
rs147878351
rs9939427
rs2266194
rs2493136
rs1538971
rs11658278
rs700589
rs55737395
rs6001558
rs140174
rs111672628
All
−0.050
−0.025
0.000
0.025
0.050
0.075
MR leave−one−out sensitivity analysis for
'IGLL1' on 'Cardiac valvular disease'

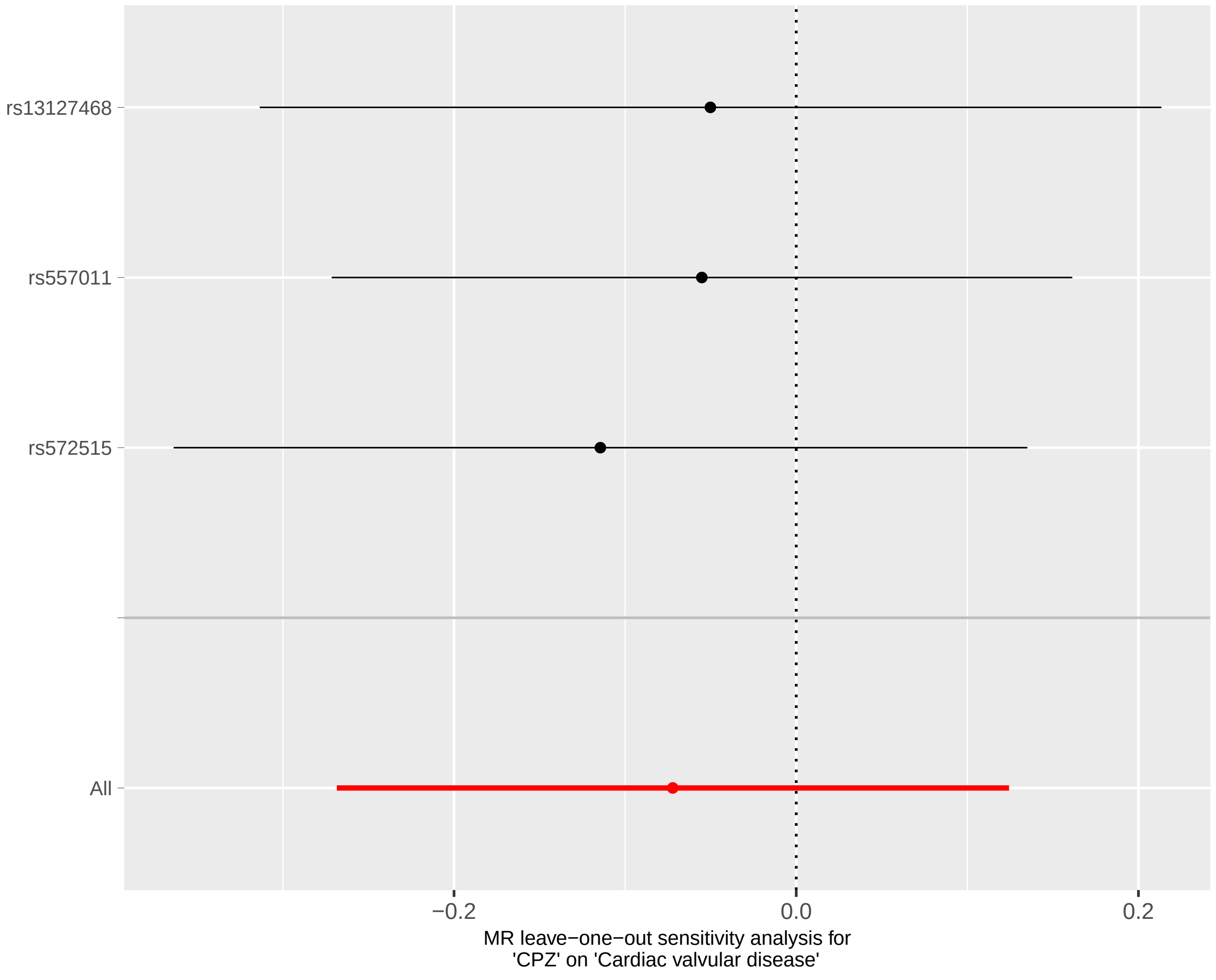
rs13127468
rs557011
rs572515
All
−0.2
0.0
0.2
MR leave−one−out sensitivity analysis for
'CPZ' on 'Cardiac valvular disease'

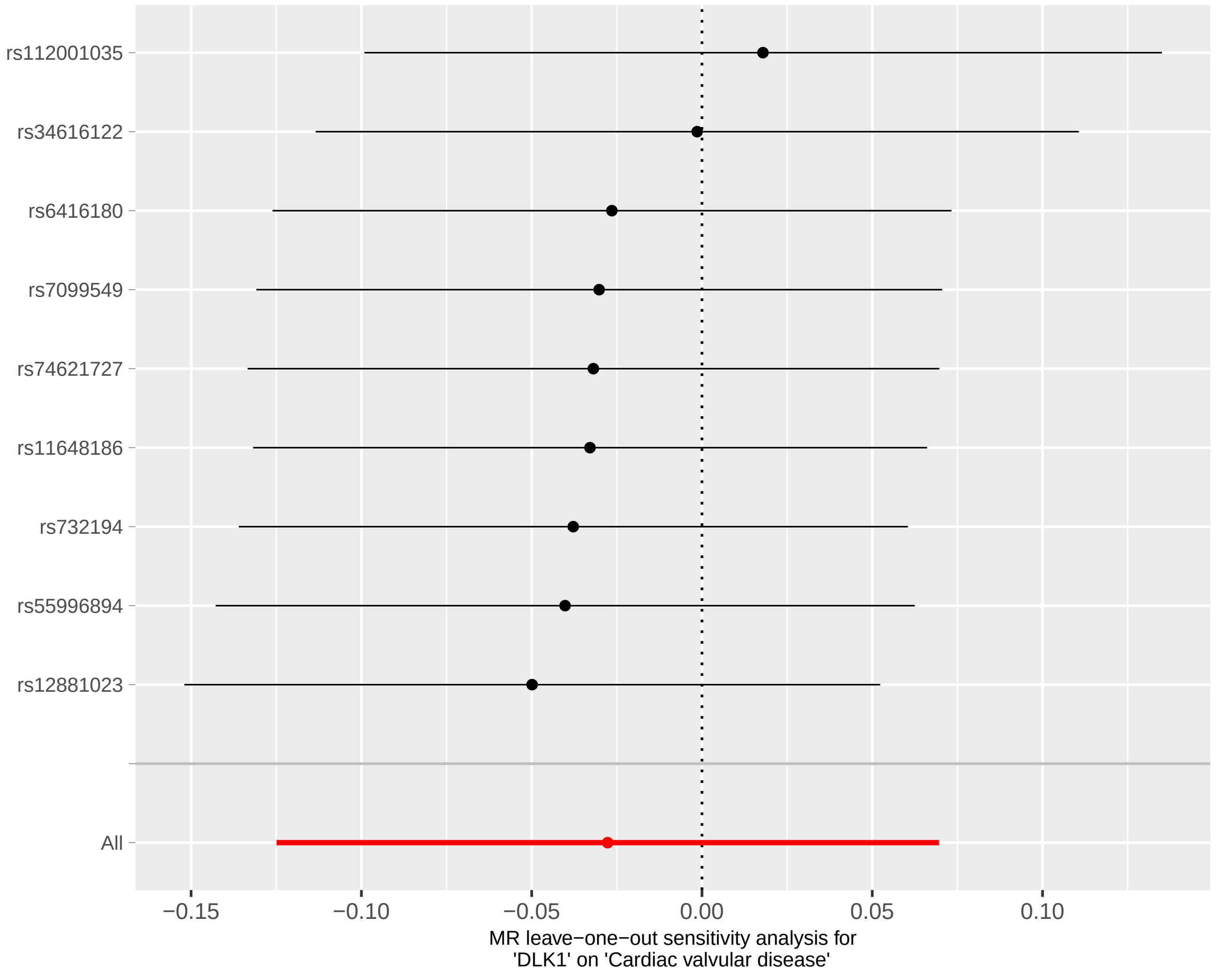

rs112001035
rs34616122
rs6416180
rs7099549
rs74621727
rs11648186
rs732194
rs55996894
rs12881023
All
−0.15
−0.10
−0.05
0.00
0.05
0.10
MR leave−one−out sensitivity analysis for
'DLK1' on 'Cardiac valvular disease'

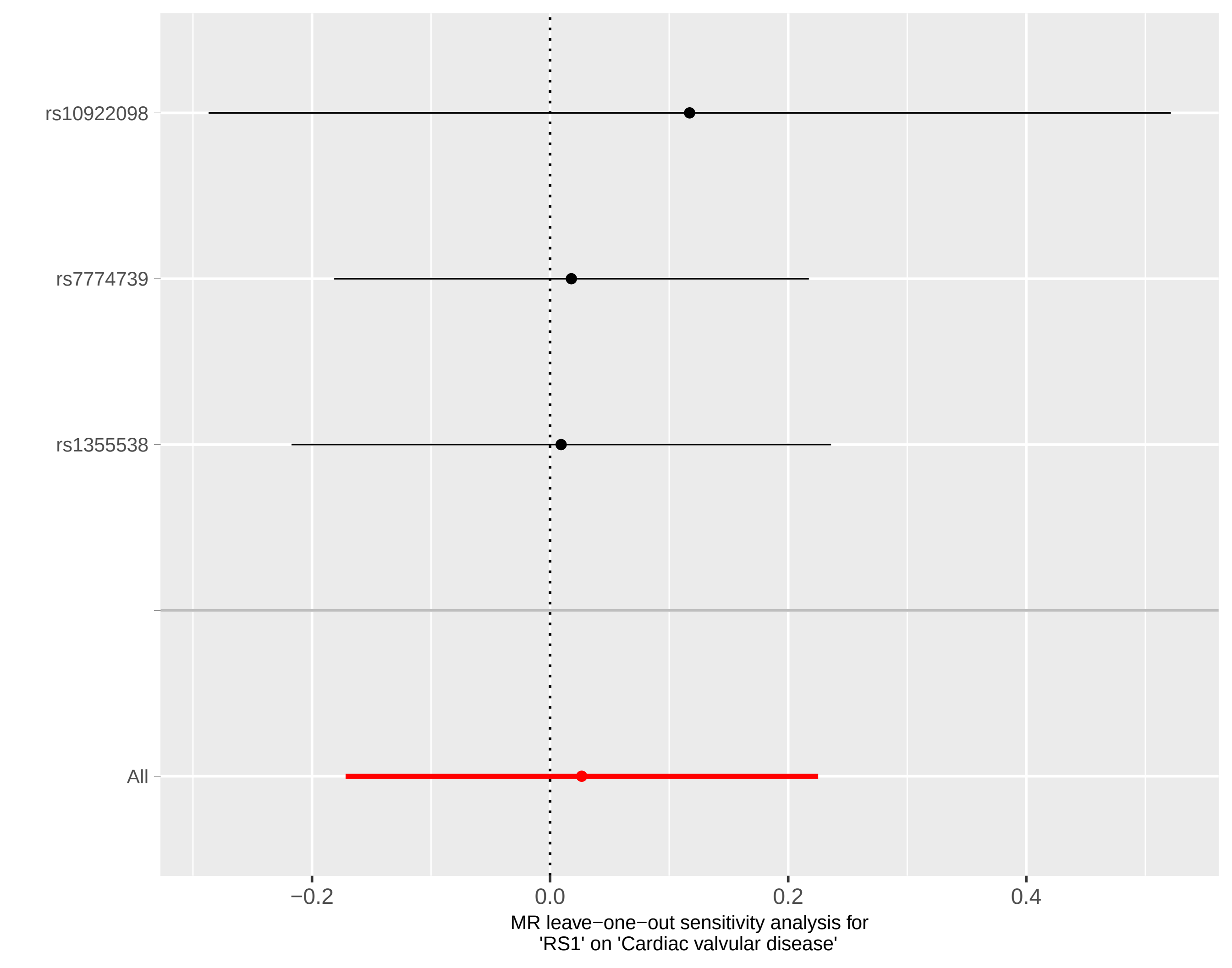
rs10922098
rs7774739
rs1355538
All
−0.2
0.0
0.2
0.4
MR leave−one−out sensitivity analysis for
'RS1' on 'Cardiac valvular disease'

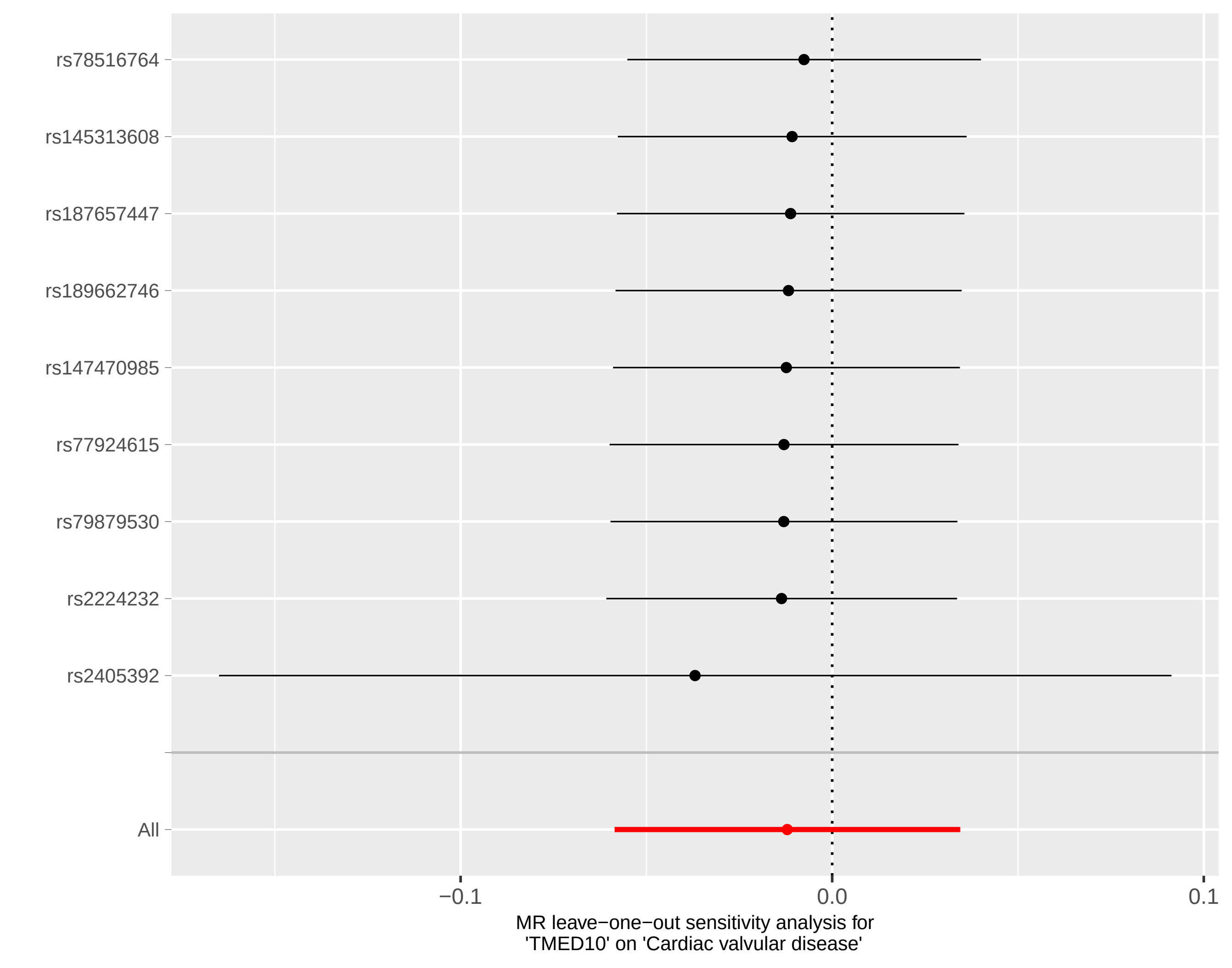
rs78516764
rs145313608
rs187657447
rs189662746
rs147470985
rs77924615
rs79879530
rs2224232
rs2405392
All
−0.1
0.0
0.1
MR leave−one−out sensitivity analysis for
'TMED10' on 'Cardiac valvular disease'

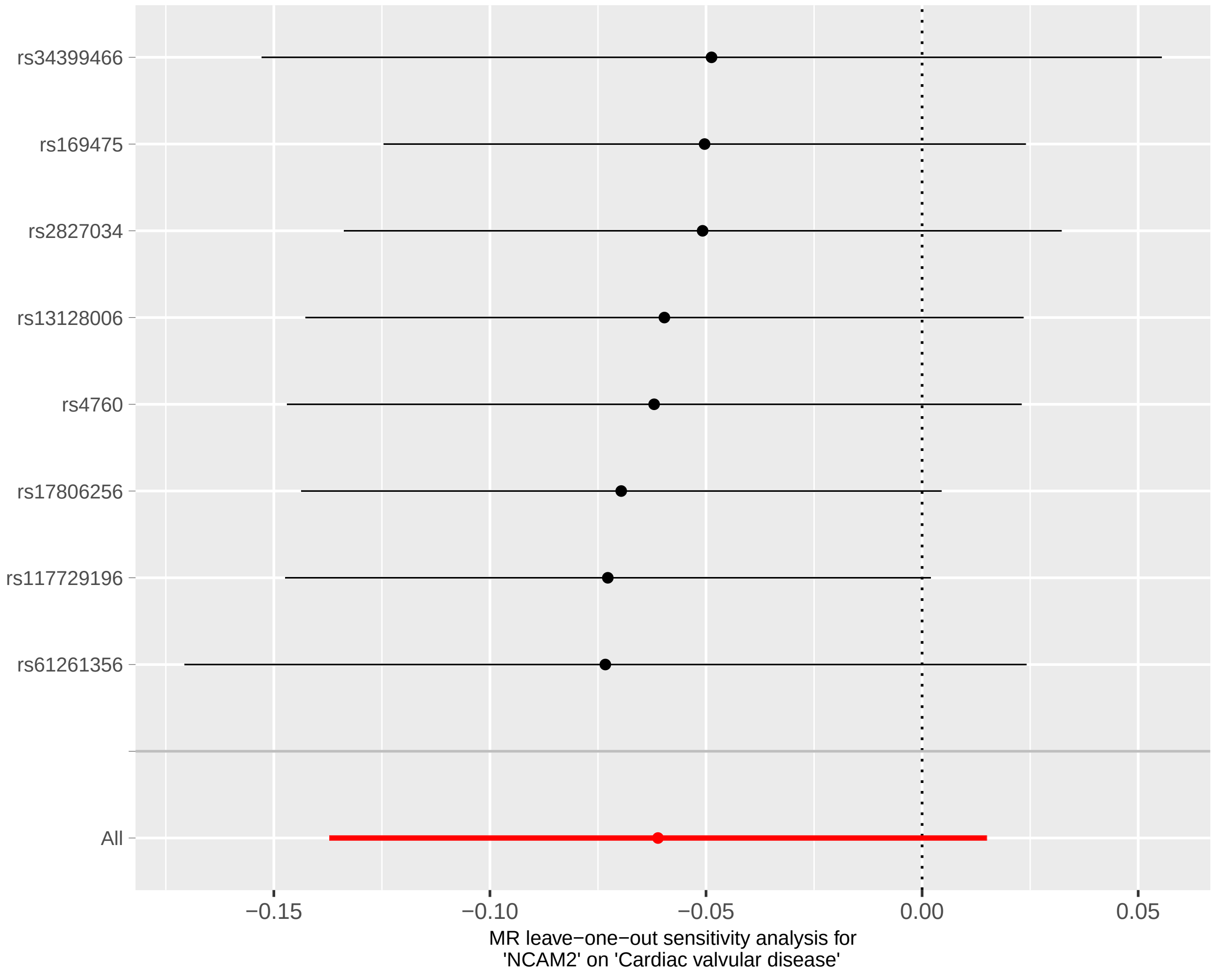
rs34399466
rs169475
rs2827034
rs13128006
rs4760
rs17806256
rs117729196
rs61261356
All
−0.15
−0.10
−0.05
0.00
0.05
MR leave−one−out sensitivity analysis for
'NCAM2' on 'Cardiac valvular disease'

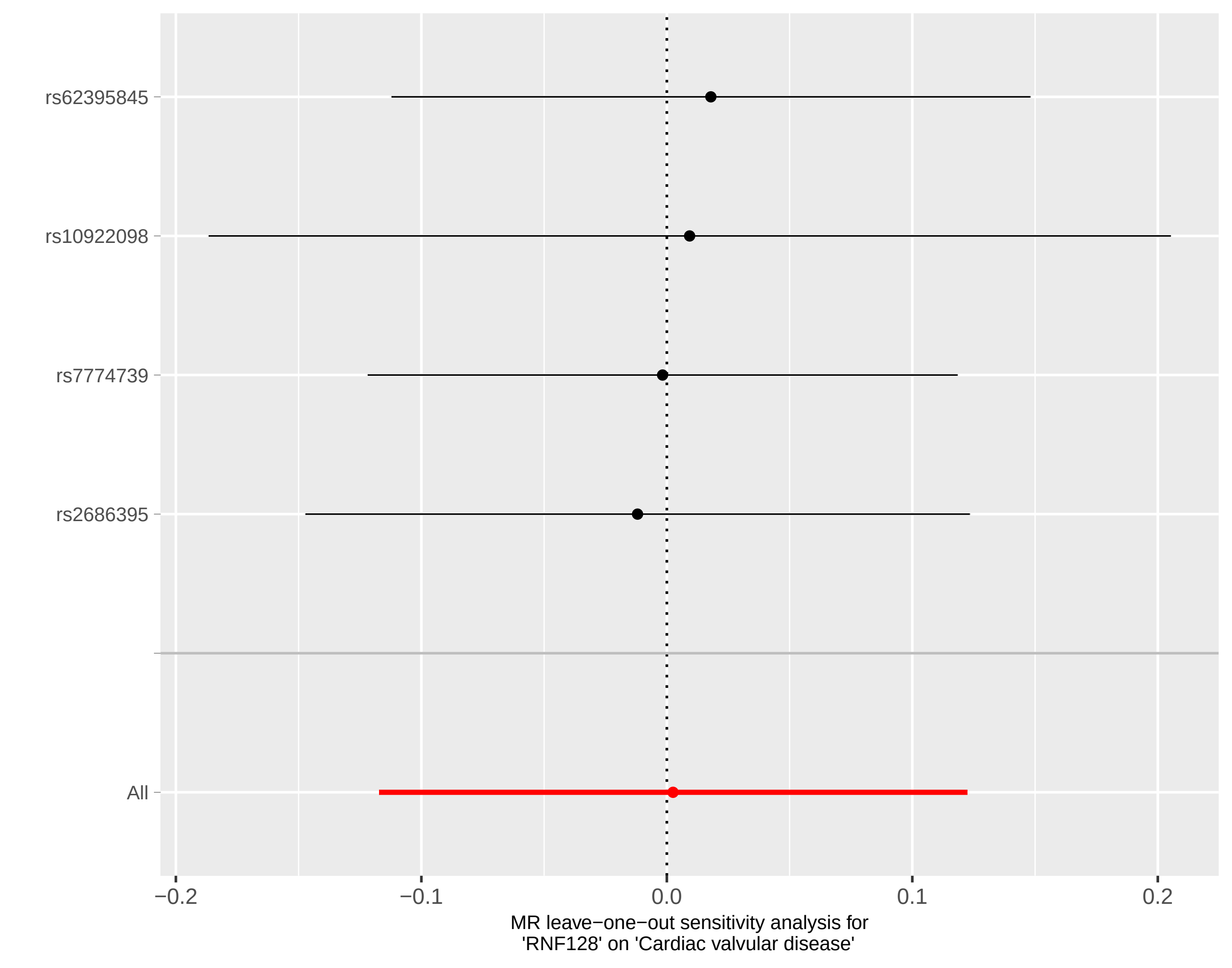

rs62395845
rs10922098
rs7774739
rs2686395
All
−0.2
−0.1
0.0
0.1
0.2
MR leave−one−out sensitivity analysis for
'RNF128' on 'Cardiac valvular disease'

Insufficient number of SNPs

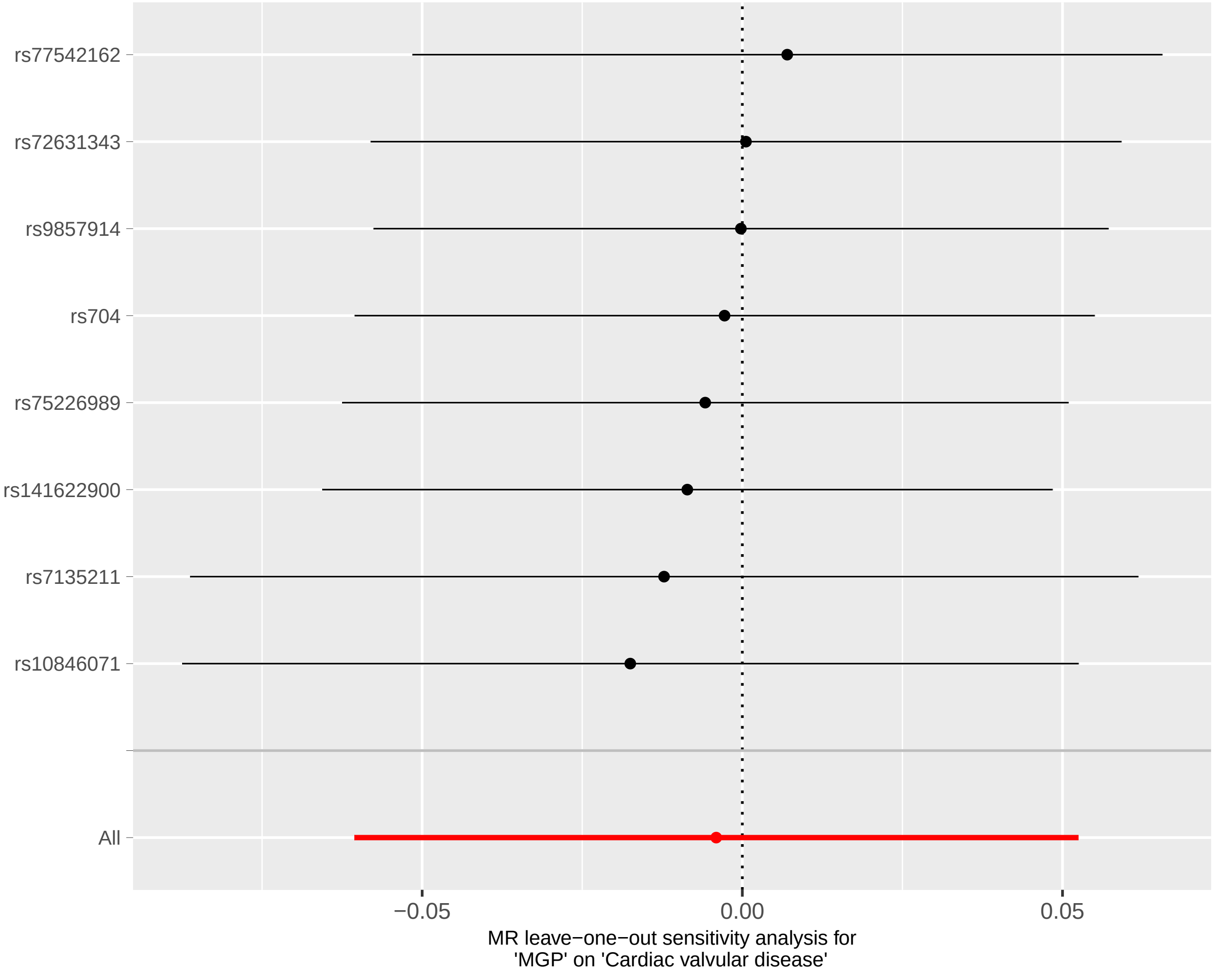
rs77542162
rs72631343
rs9857914
rs704
rs75226989
rs141622900
rs7135211
rs10846071
All
−0.05
0.00
0.05
MR leave−one−out sensitivity analysis for
'MGP' on 'Cardiac valvular disease'

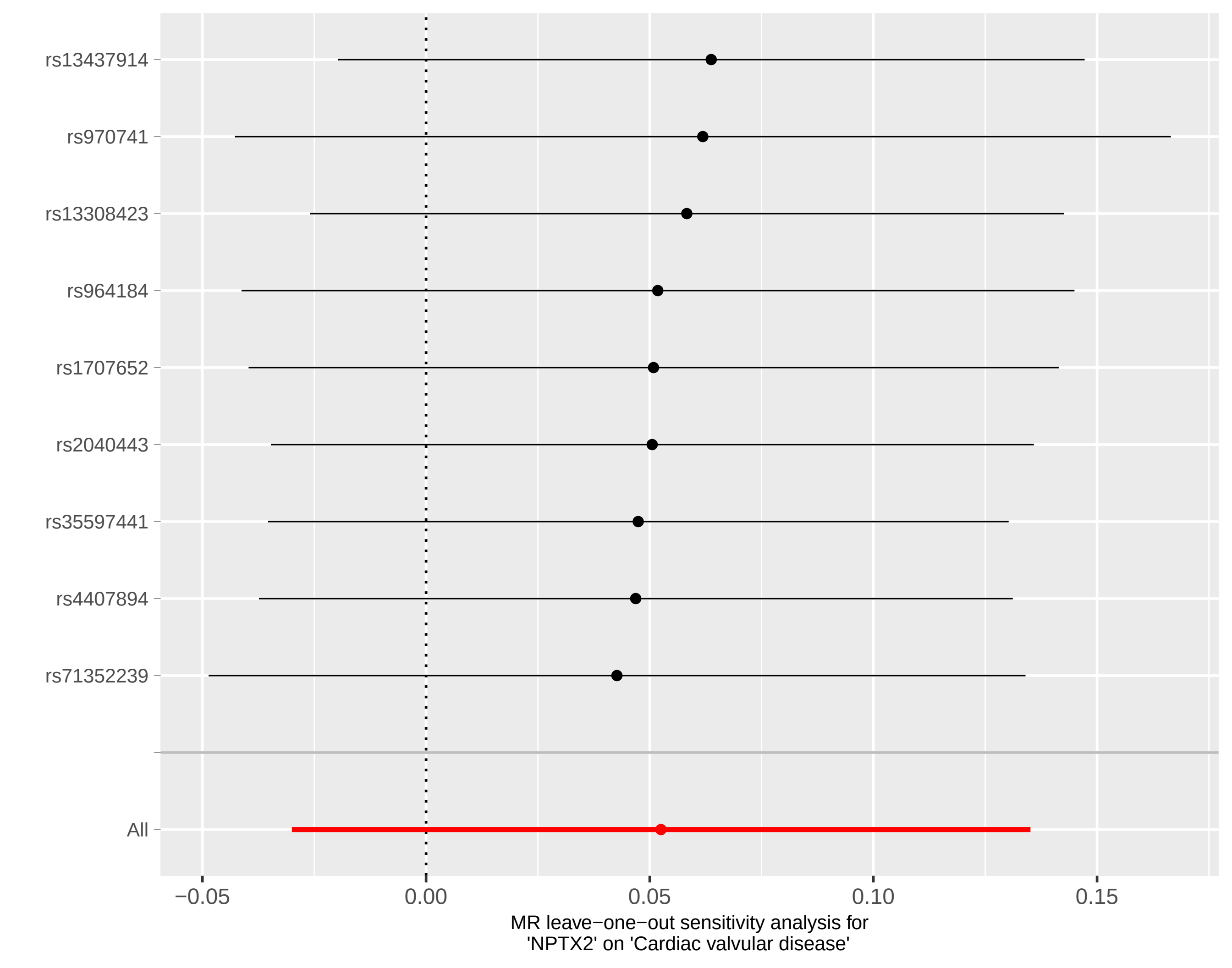

rs13437914
rs970741
rs13308423
rs964184
rs1707652
rs2040443
rs35597441
rs4407894
rs71352239
All
−0.05
0.00
0.05
0.10
0.15
MR leave−one−out sensitivity analysis for
'NPTX2' on 'Cardiac valvular disease'

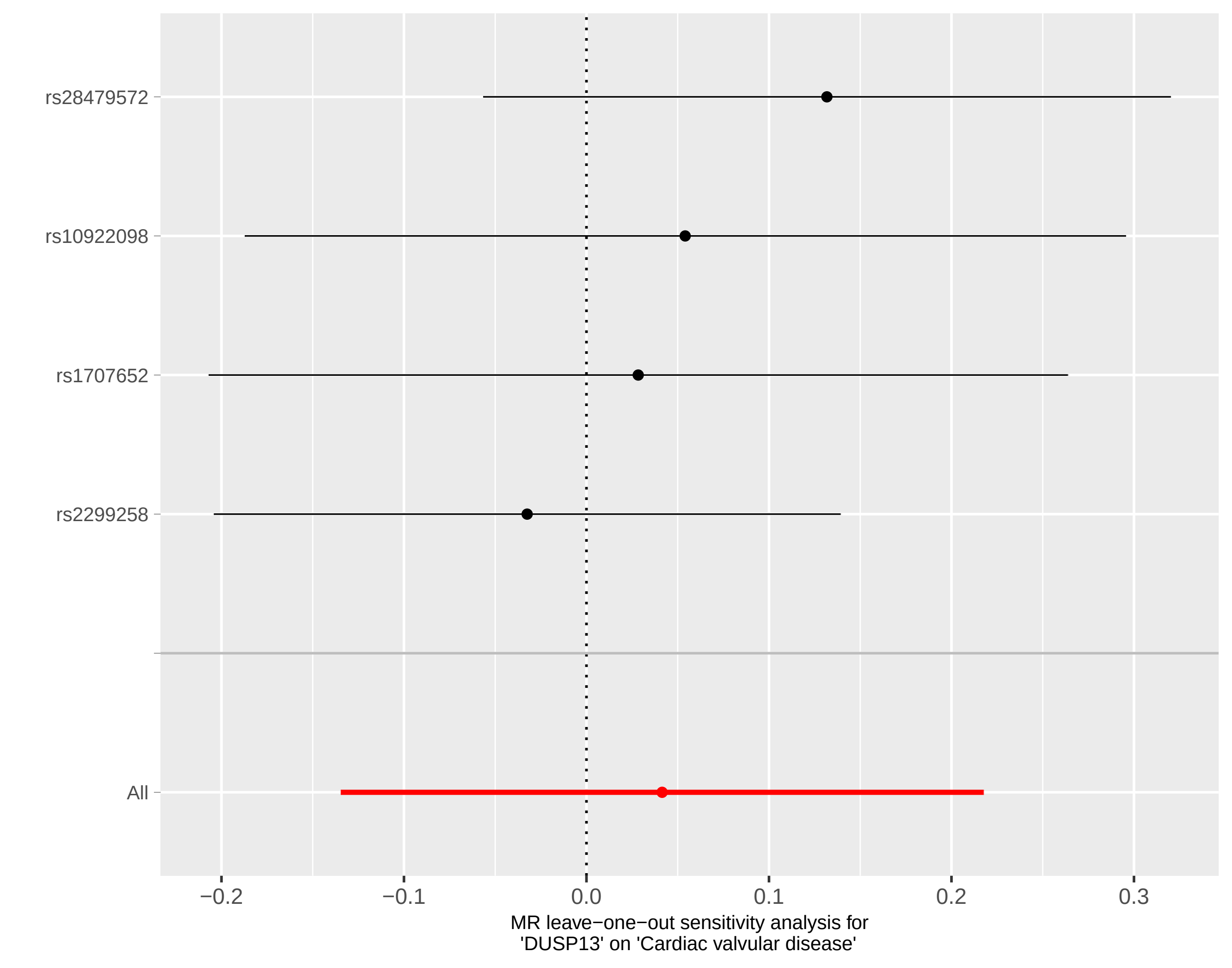
rs28479572
rs10922098
rs1707652
rs2299258
All
−0.2
−0.1
0.0
0.1
0.2
0.3
MR leave−one−out sensitivity analysis for
'DUSP13' on 'Cardiac valvular disease'

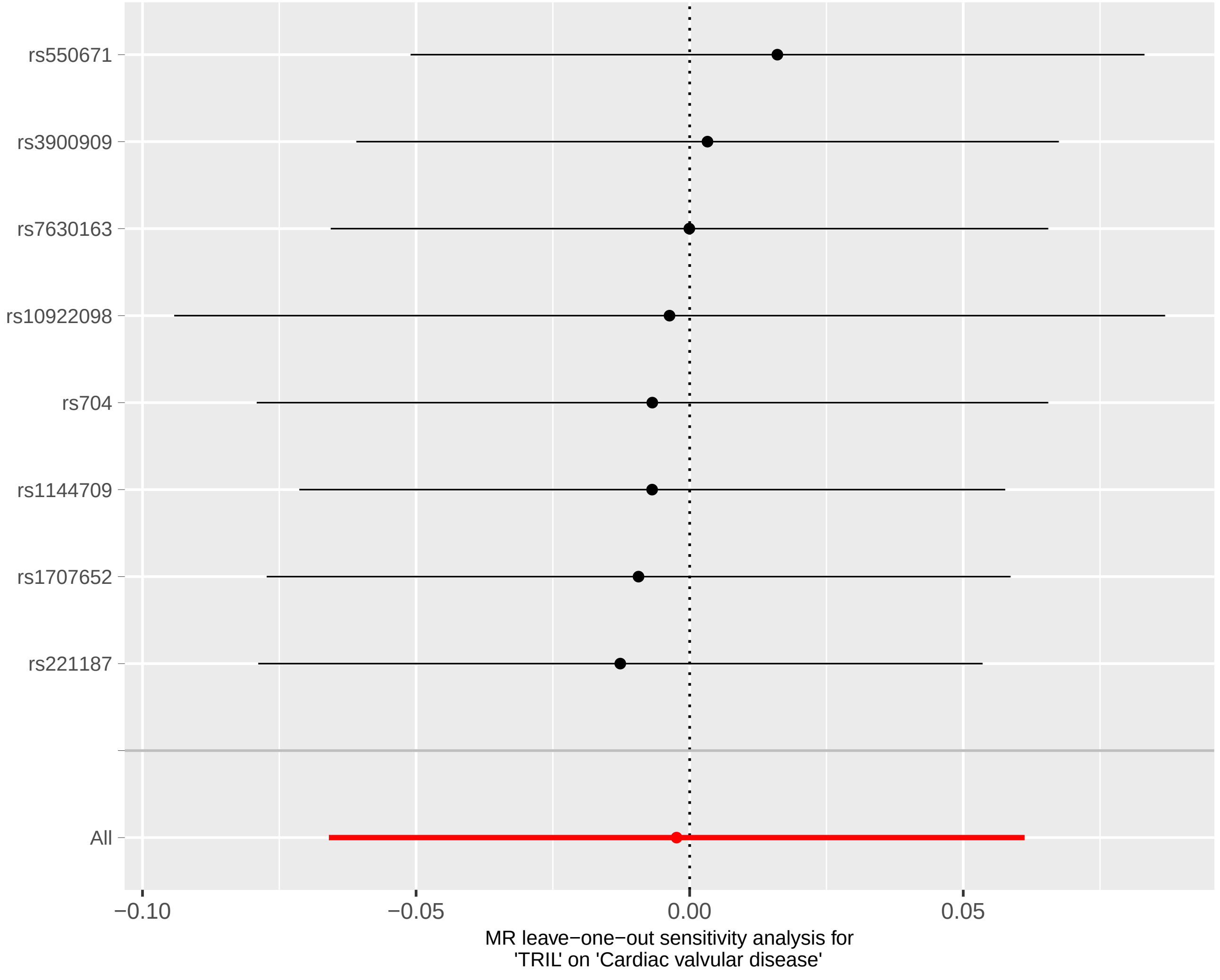
rs550671
rs3900909
rs7630163
rs10922098
rs704
rs1144709
rs1707652
rs221187
All
−0.10
−0.05
0.00
0.05
MR leave−one−out sensitivity analysis for
'TRIL' on 'Cardiac valvular disease'

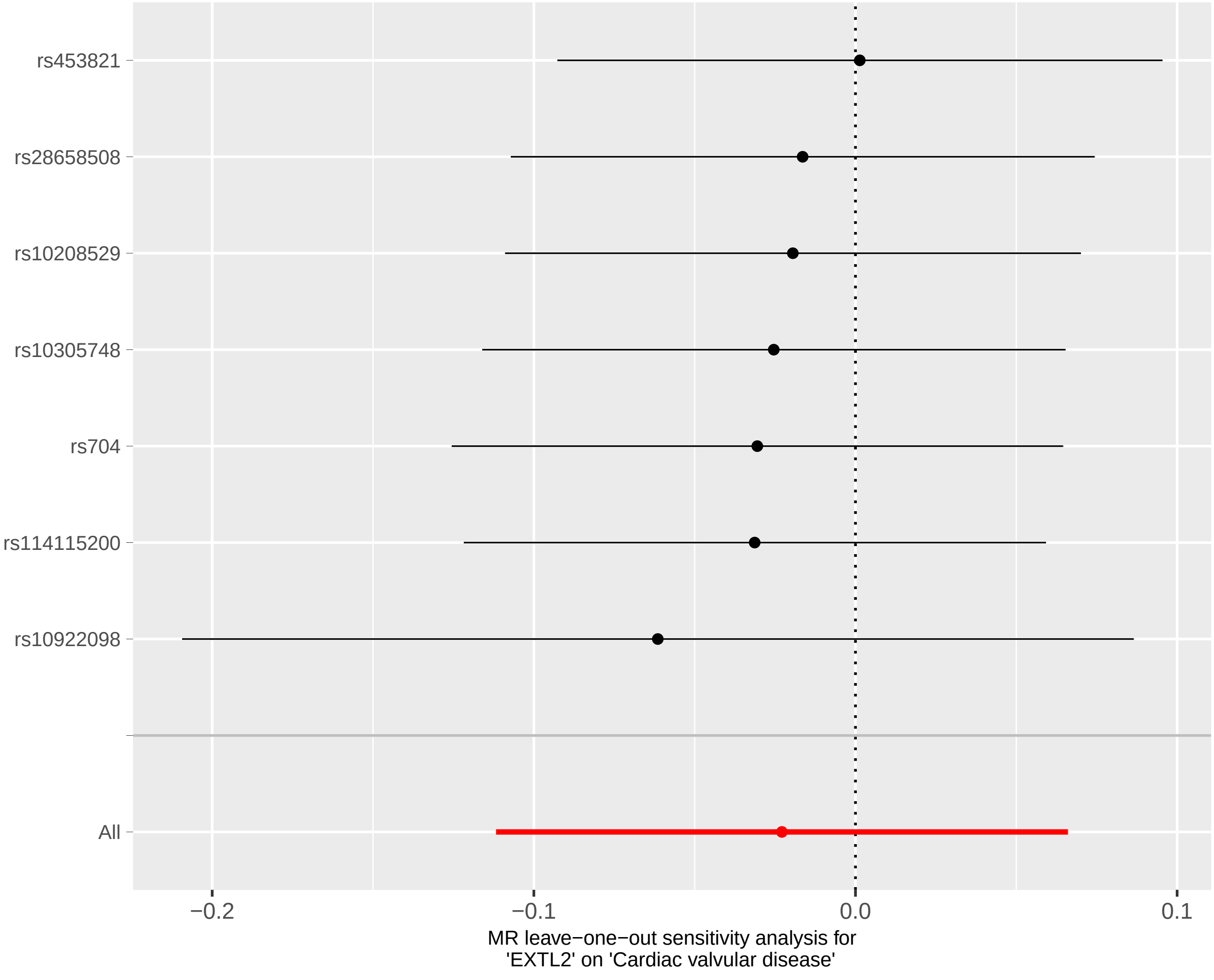

rs453821
rs28658508
rs10208529
rs10305748
rs704
rs114115200
rs10922098
All
−0.2
−0.1
0.0
0.1
MR leave−one−out sensitivity analysis for
'EXTL2' on 'Cardiac valvular disease'

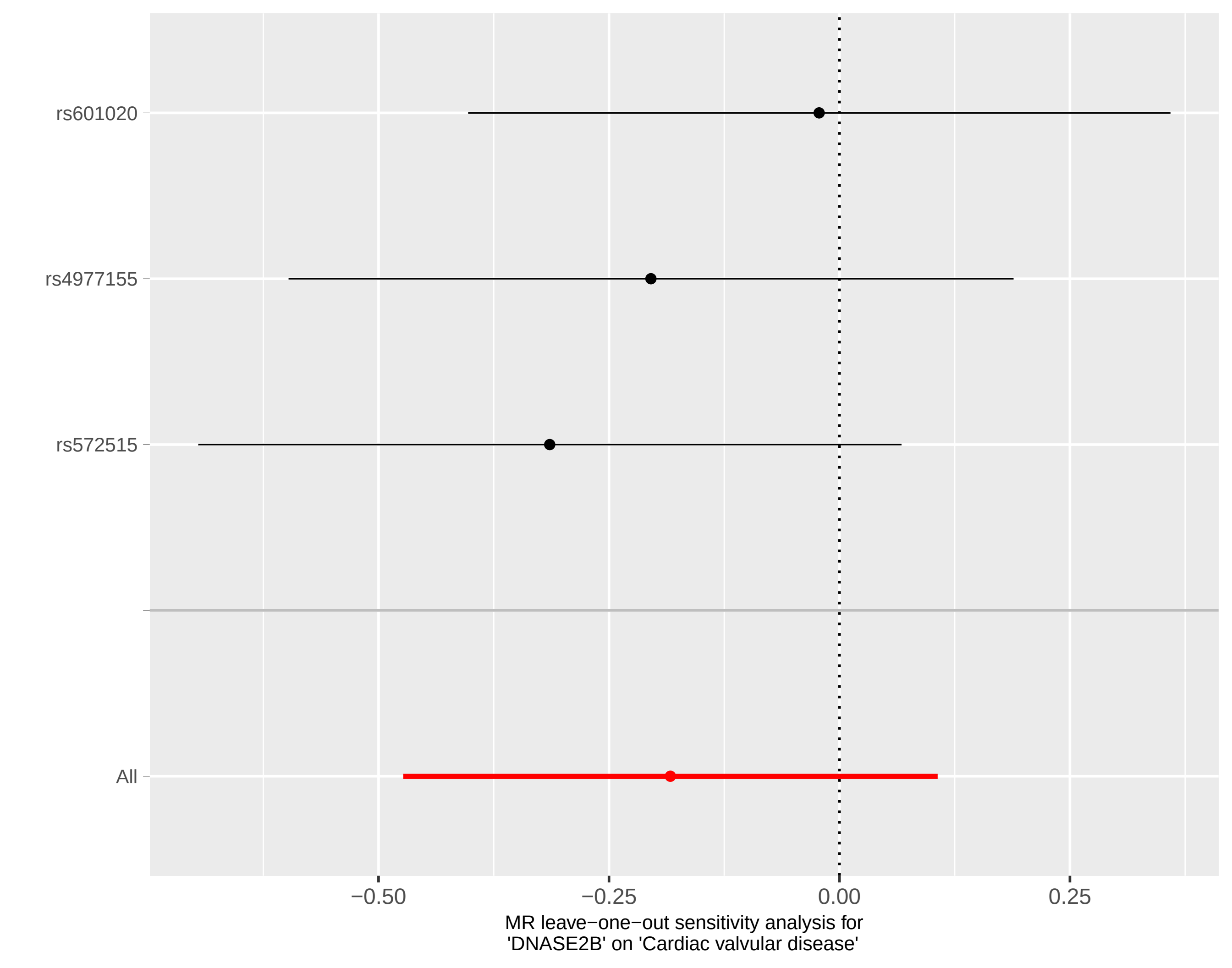
rs601020
rs4977155
rs572515
All
−0.50
−0.25
0.00
0.25
MR leave−one−out sensitivity analysis for
'DNASE2B' on 'Cardiac valvular disease'

Insufficient number of SNPs

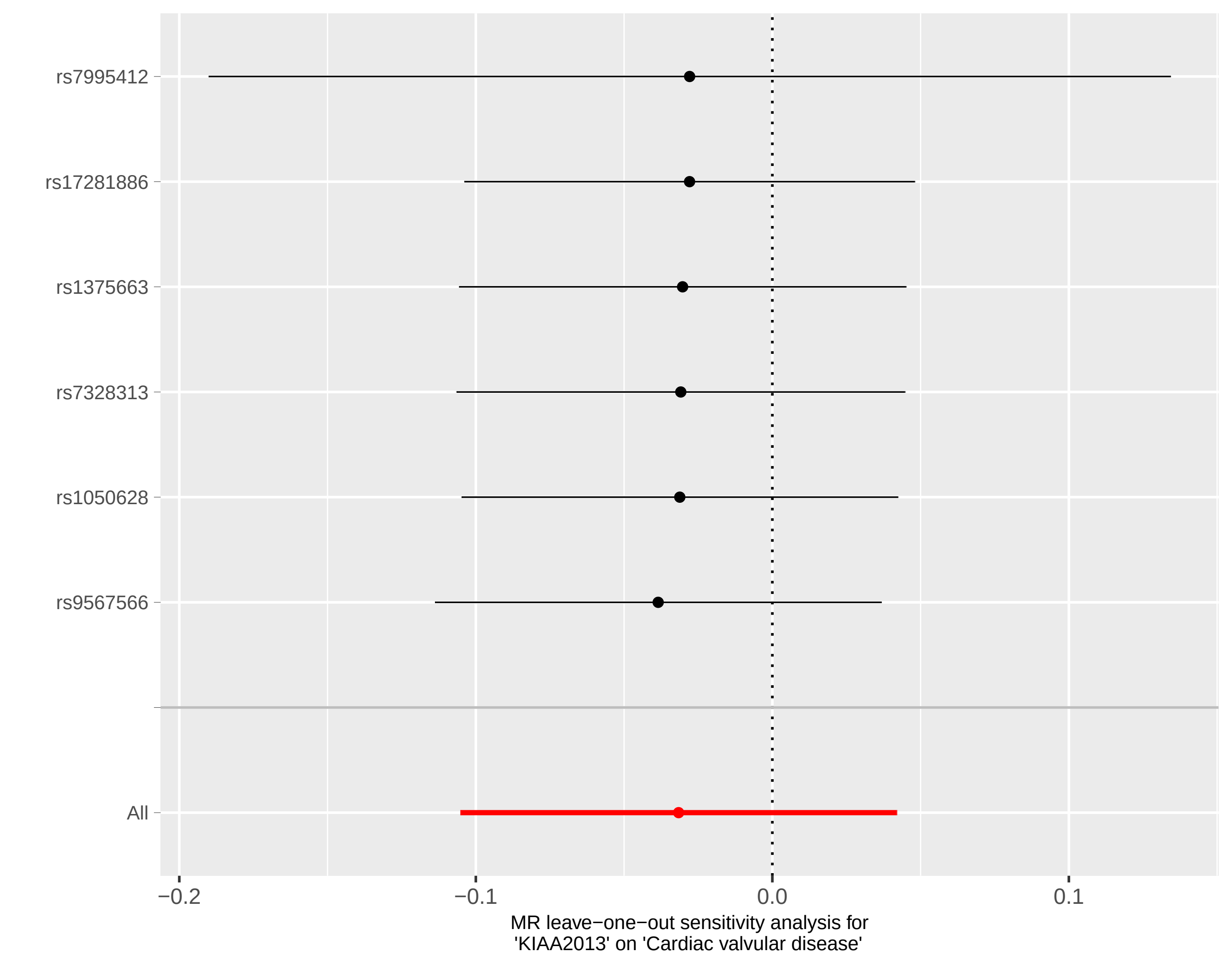
rs7995412
rs17281886
rs1375663
rs7328313
rs1050628
rs9567566
All
−0.2
−0.1
0.0
0.1
MR leave−one−out sensitivity analysis for
'KIAA2013' on 'Cardiac valvular disease'

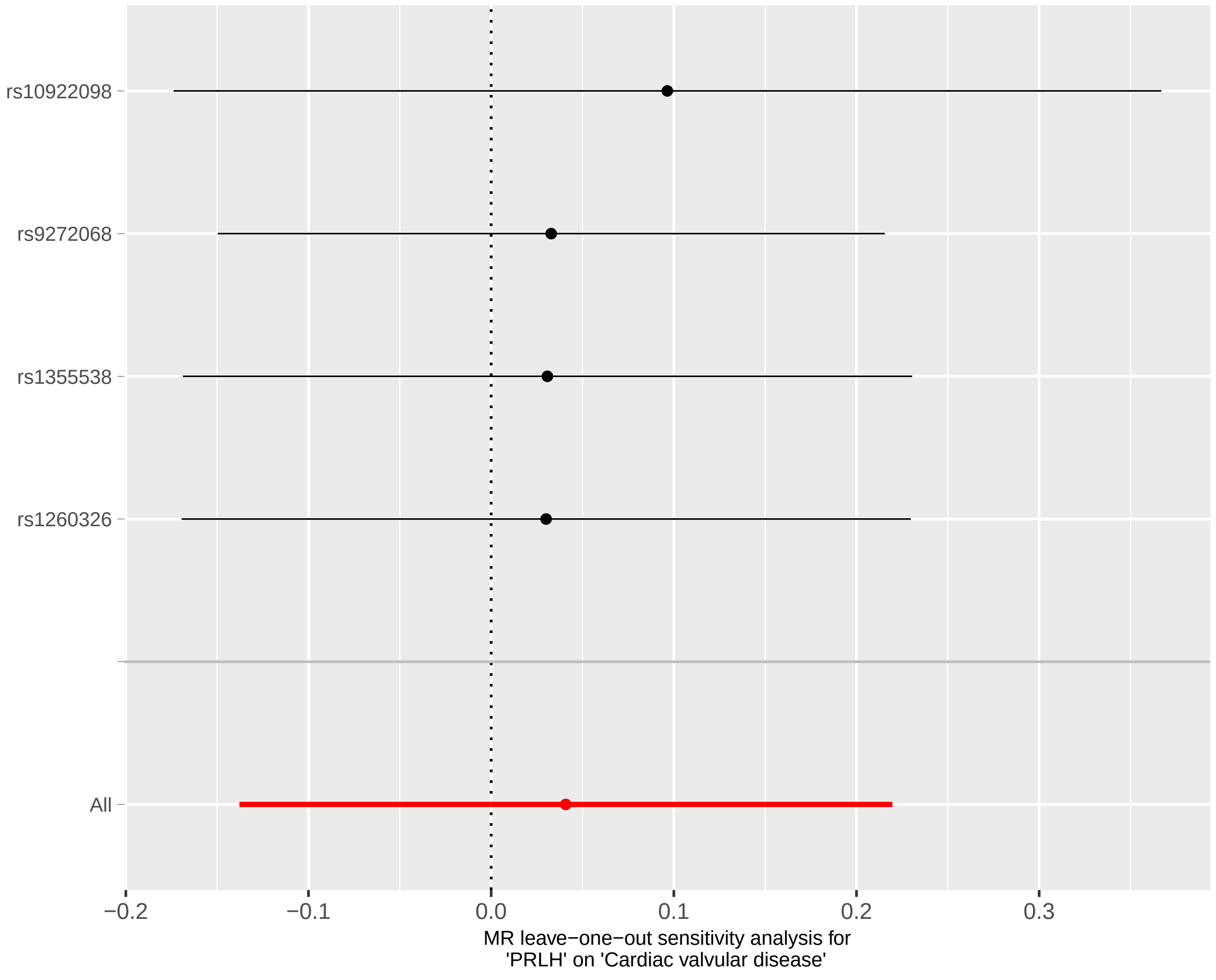

rs10922098
rs9272068
rs1355538
rs1260326
All
−0.2
−0.1
0.0
0.1
0.2
0.3
MR leave−one−out sensitivity analysis for
'PRLH' on 'Cardiac valvular disease'

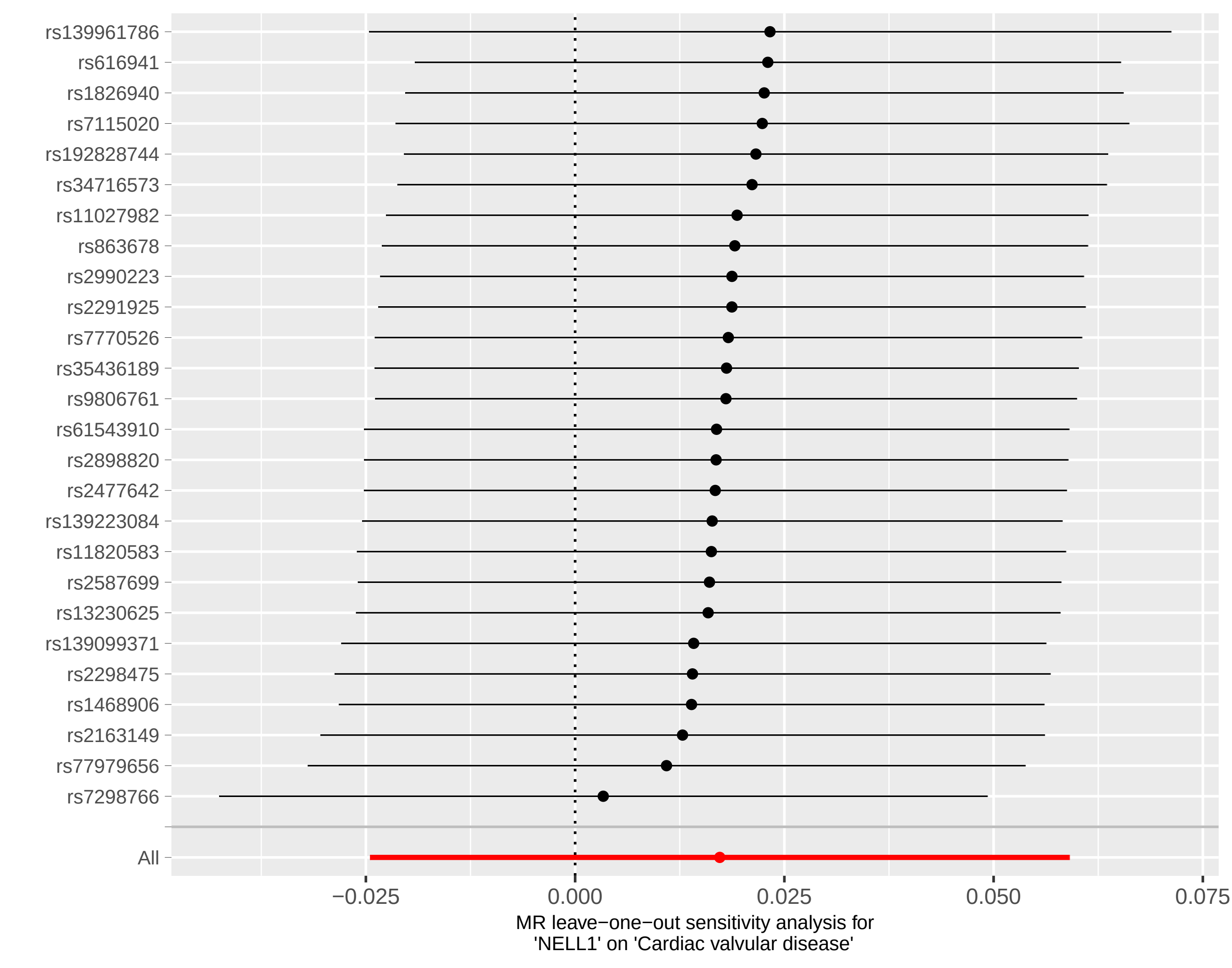

rs139961786
rs616941
rs1826940
rs7115020
rs192828744
rs34716573
rs11027982
rs863678
rs2990223
rs2291925
rs7770526
rs35436189
rs9806761
rs61543910
rs2898820
rs2477642
rs139223084
rs11820583
rs2587699
rs13230625
rs139099371
rs2298475
rs1468906
rs2163149
rs77979656
rs7298766
All
−0.025
0.000
0.025
0.050
0.075
MR leave−one−out sensitivity analysis for
'NELL1' on 'Cardiac valvular disease'

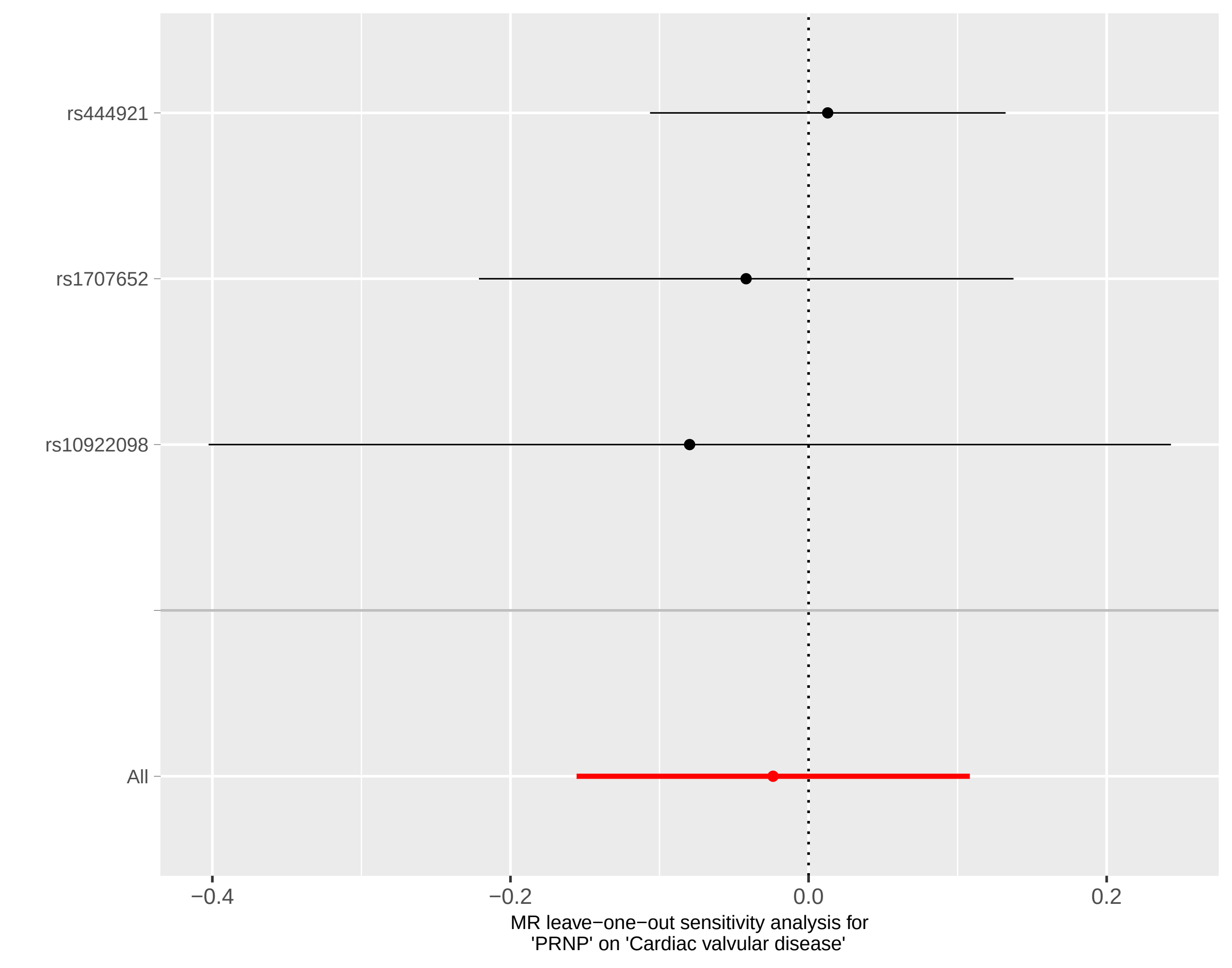
rs444921
rs1707652
rs10922098
All
−0.4
−0.2
0.0
0.2
MR leave−one−out sensitivity analysis for
'PRNP' on 'Cardiac valvular disease'

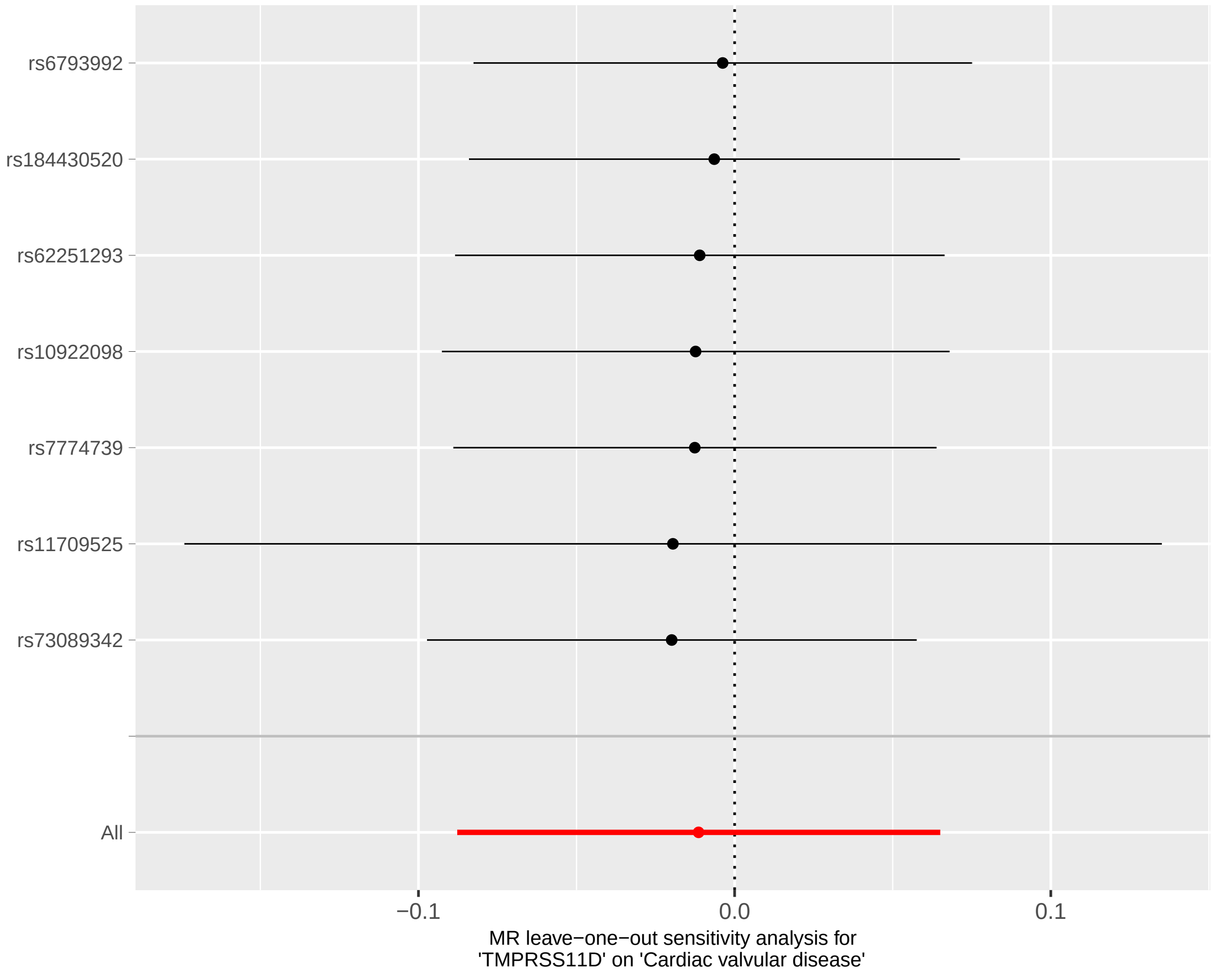

rs6793992
rs184430520
rs62251293
rs10922098
rs7774739
rs11709525
rs73089342
All
−0.1
0.0
0.1
MR leave−one−out sensitivity analysis for
'TMPRSS11D' on 'Cardiac valvular disease'

Insufficient number of SNPs

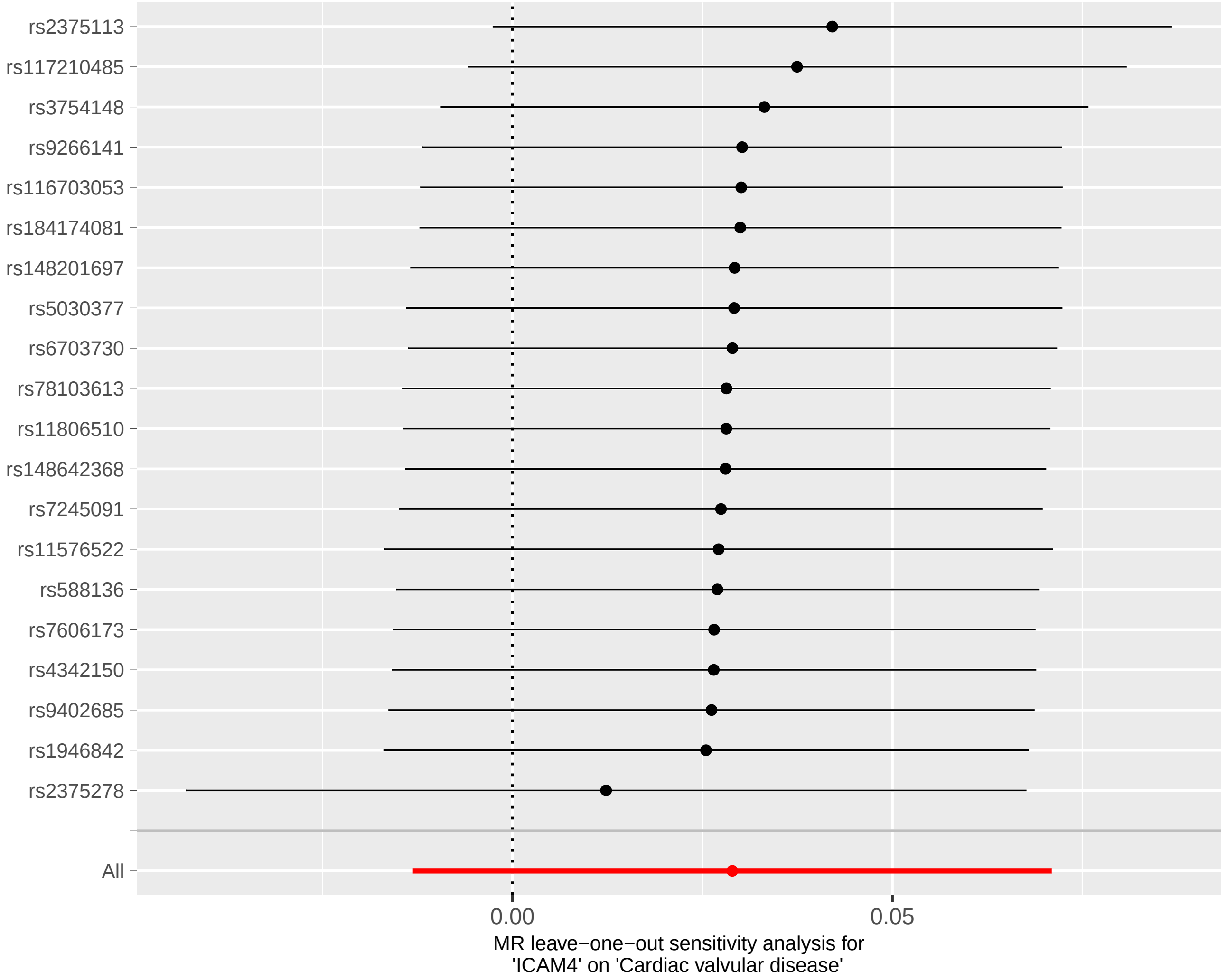
rs2375113
rs117210485
rs3754148
rs9266141
rs116703053
rs184174081
rs148201697
rs5030377
rs6703730
rs78103613
rs11806510
rs148642368
rs7245091
rs11576522
rs588136
rs7606173
rs4342150
rs9402685
rs1946842
rs2375278
All
0.00
0.05
MR leave−one−out sensitivity analysis for
'ICAM4' on 'Cardiac valvular disease'

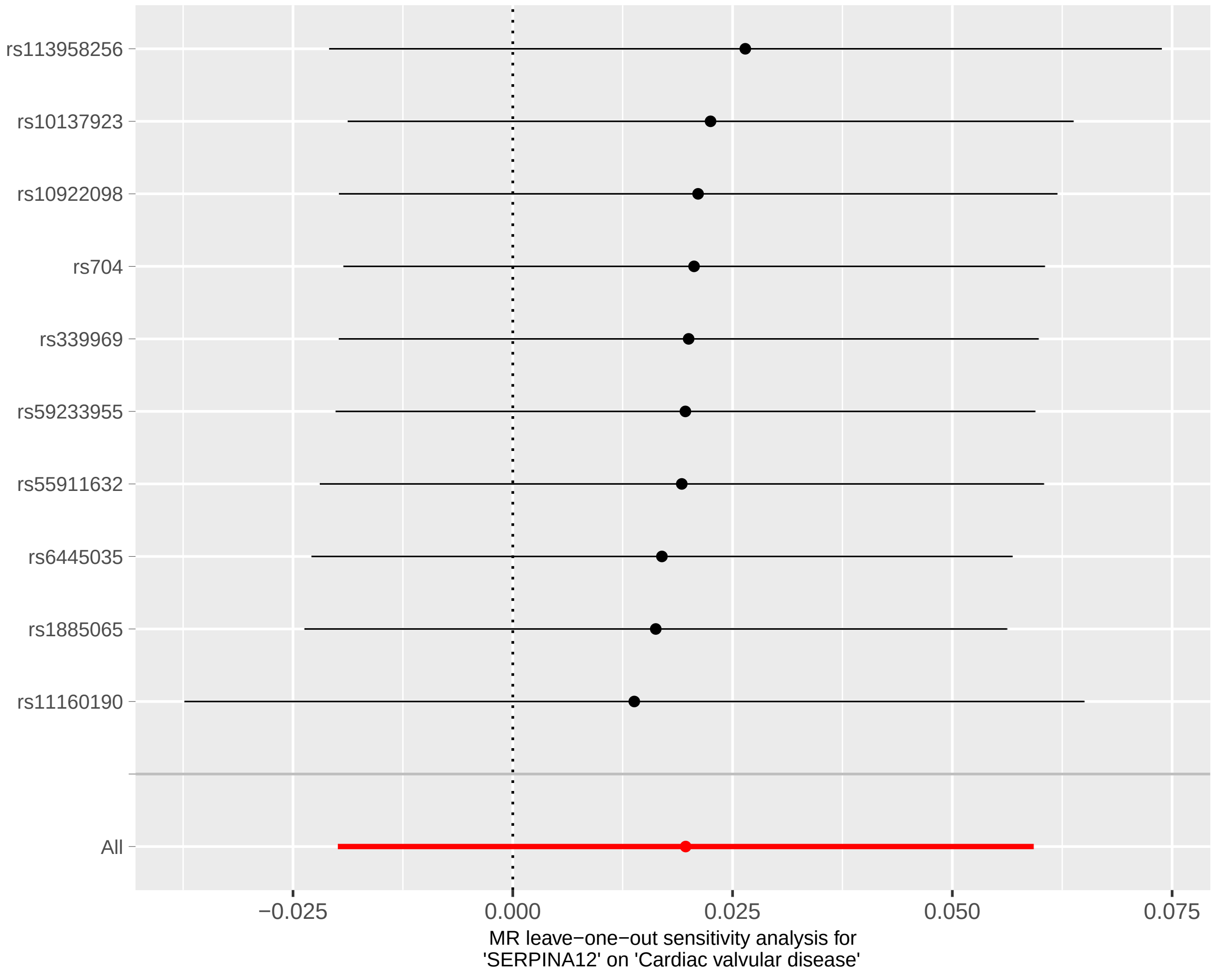
rs113958256
rs10137923
rs10922098
rs704
rs339969
rs59233955
rs55911632
rs6445035
rs1885065
rs11160190
All
−0.025
0.000
0.025
0.050
0.075
MR leave−one−out sensitivity analysis for
'SERPINA12' on 'Cardiac valvular disease'

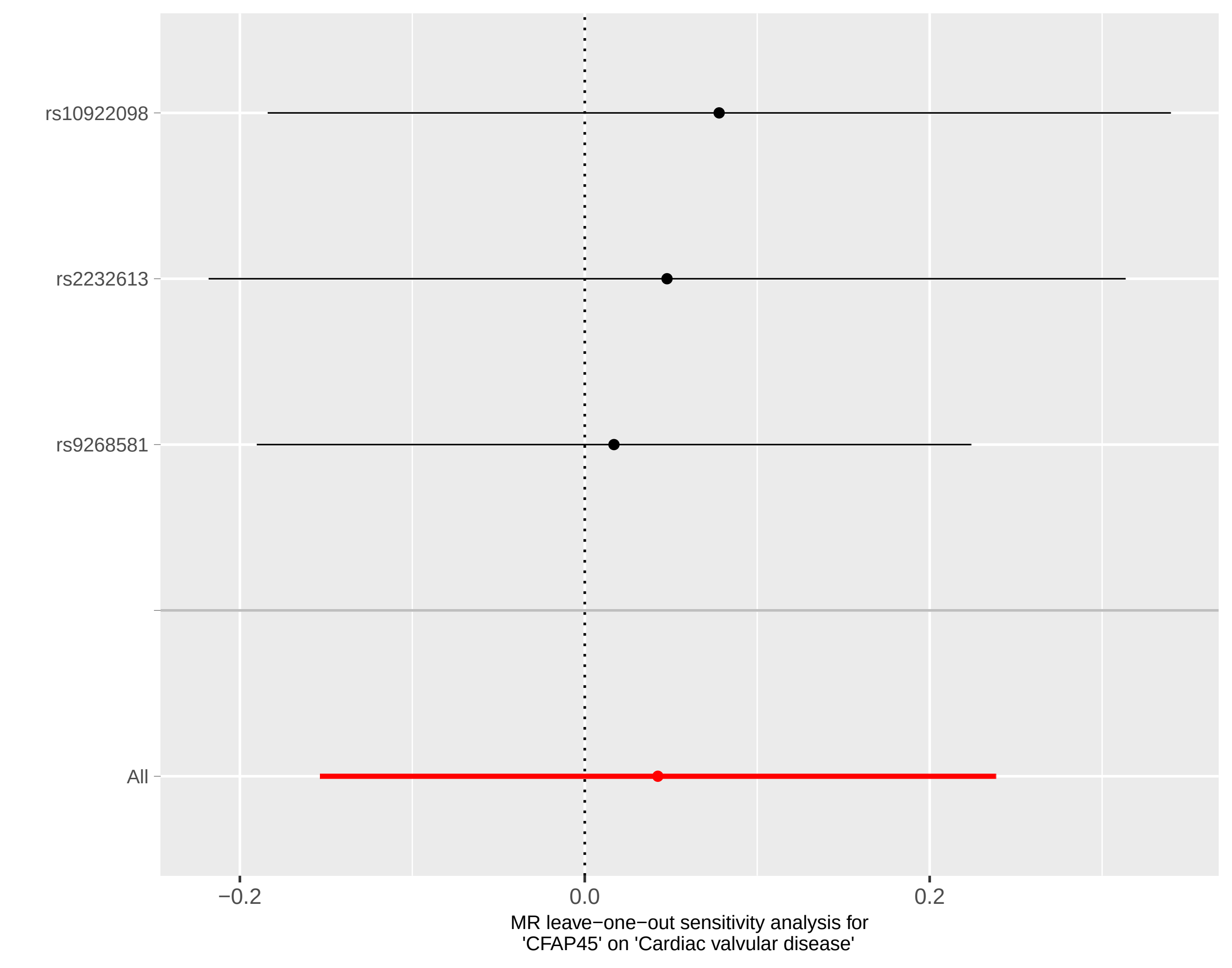
rs10922098
rs2232613
rs9268581
All
−0.2
0.0
0.2
MR leave−one−out sensitivity analysis for
'CFAP45' on 'Cardiac valvular disease'

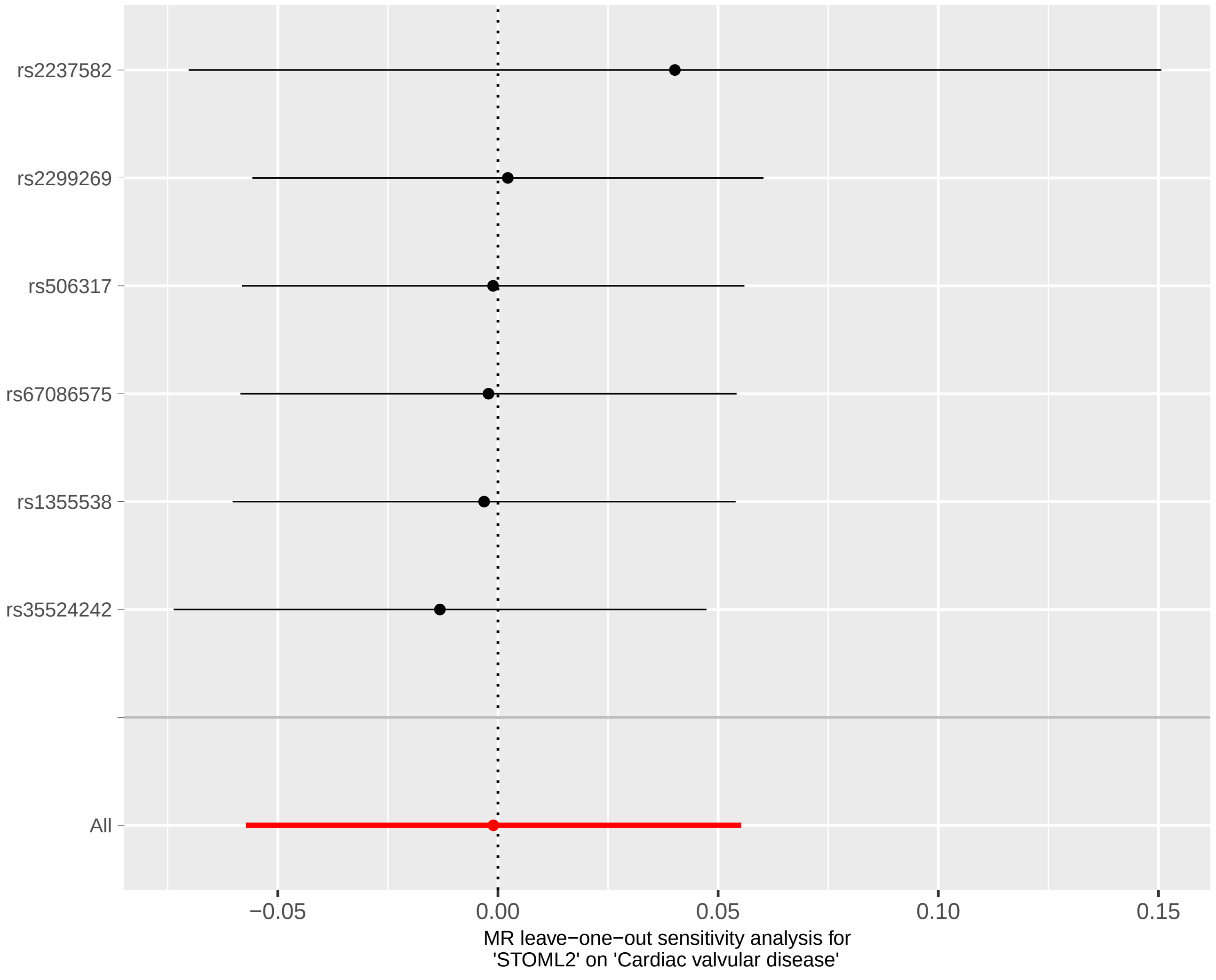
rs2237582
rs2299269
rs506317
rs67086575
rs1355538
rs35524242
All
−0.05
0.00
0.05
0.10
0.15
MR leave−one−out sensitivity analysis for
'STOML2' on 'Cardiac valvular disease'

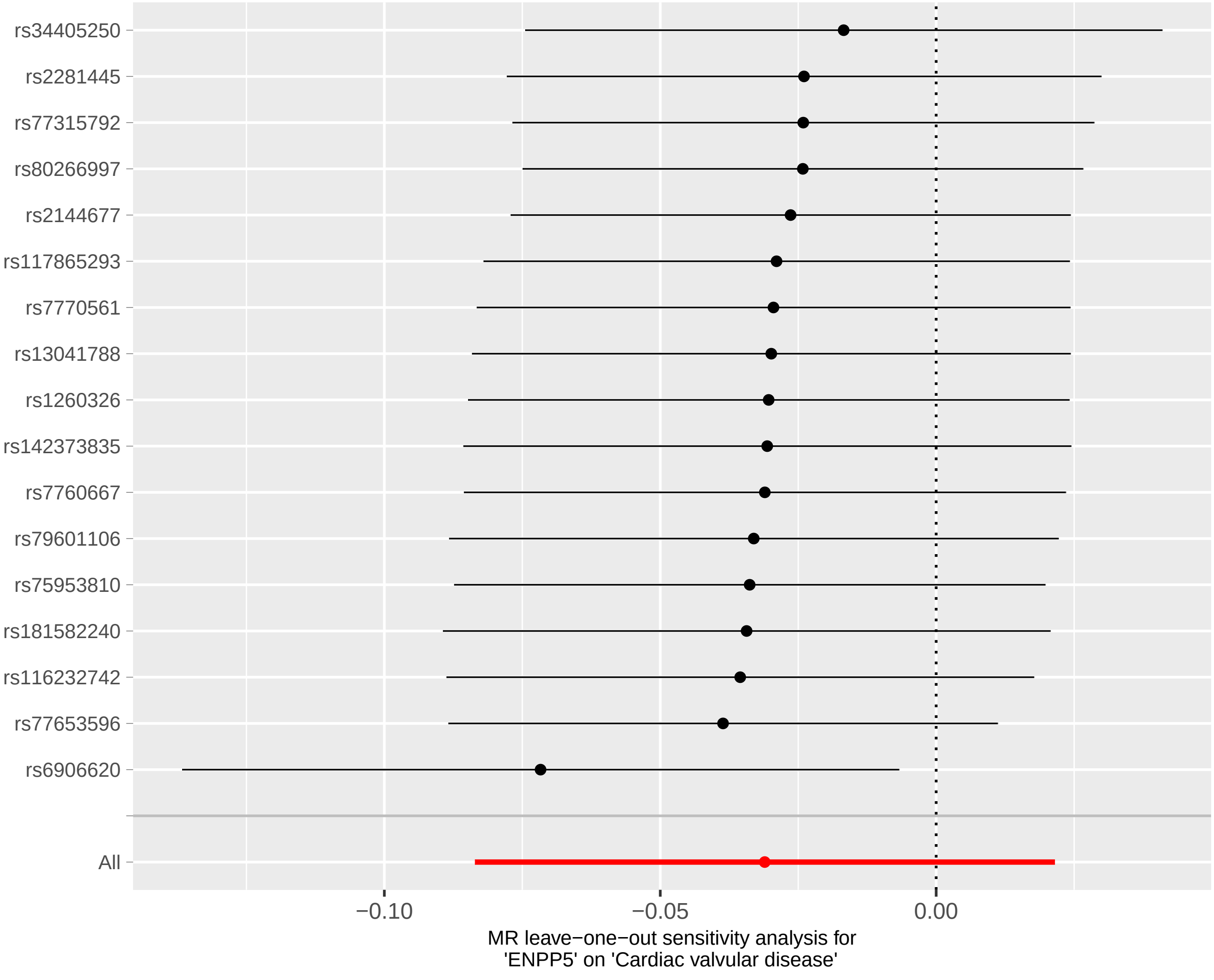
rs34405250
rs2281445
rs77315792
rs80266997
rs2144677
rs117865293
rs7770561
rs13041788
rs1260326
rs142373835
rs7760667
rs79601106
rs75953810
rs181582240
rs116232742
rs77653596
rs6906620
All
−0.10
−0.05
0.00
MR leave−one−out sensitivity analysis for
'ENPP5' on 'Cardiac valvular disease'

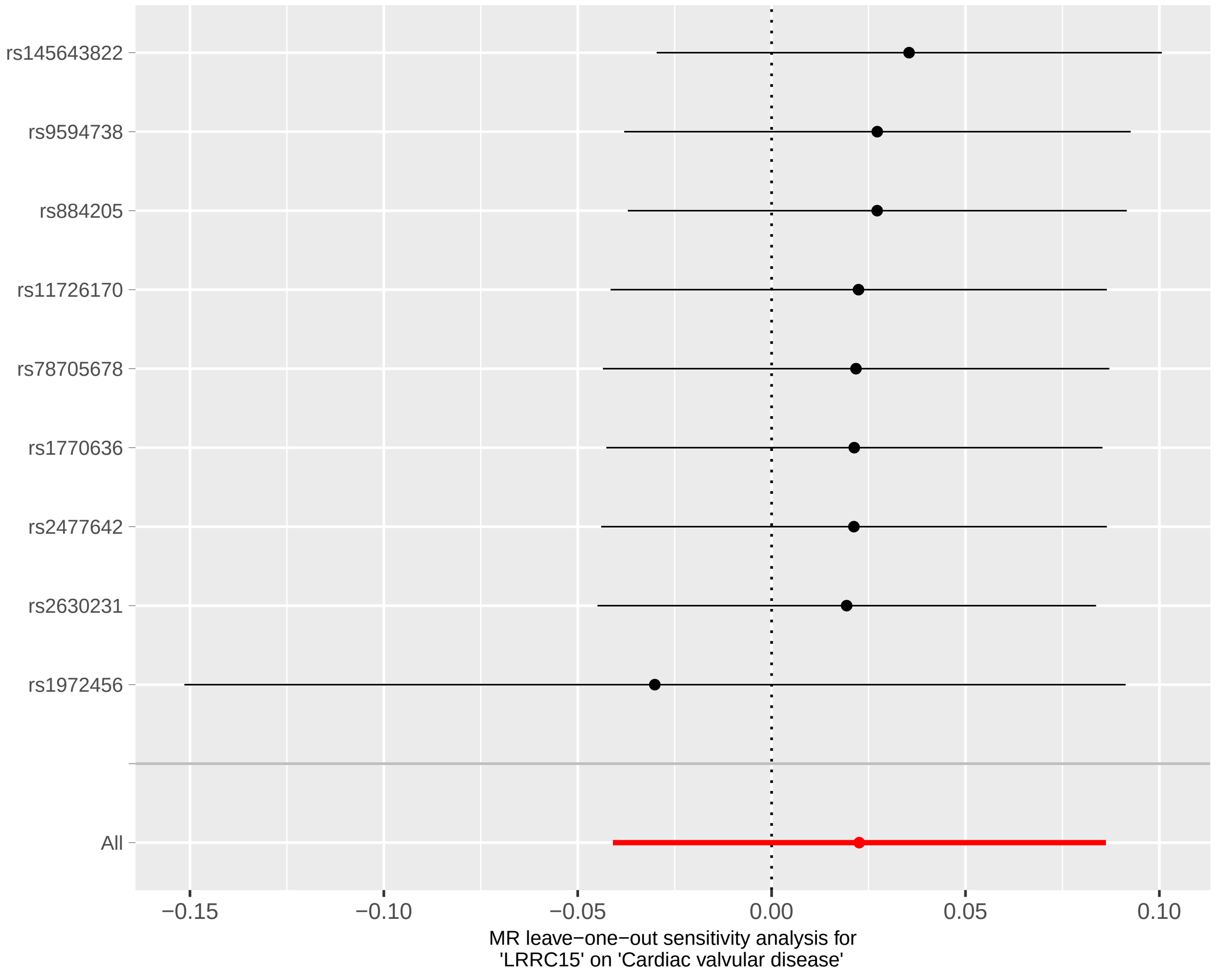
rs145643822
rs9594738
rs884205
rs11726170
rs78705678
rs1770636
rs2477642
rs2630231
rs1972456
All
−0.15
−0.10
−0.05
0.00
0.05
0.10
MR leave−one−out sensitivity analysis for
'LRRC15' on 'Cardiac valvular disease'

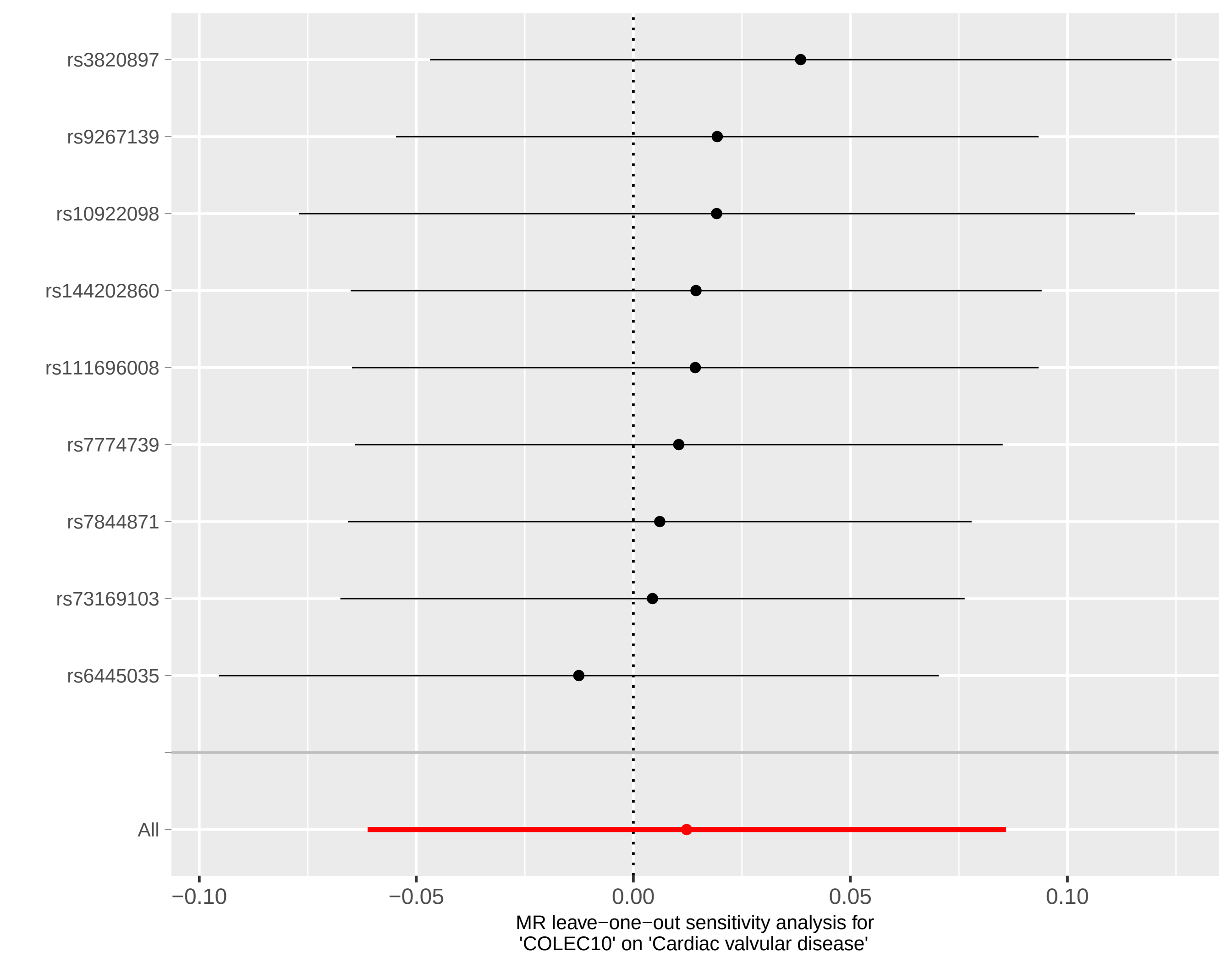
rs3820897
rs9267139
rs10922098
rs144202860
rs111696008
rs7774739
rs7844871
rs73169103
rs6445035
All
−0.10
−0.05
0.00
0.05
0.10
MR leave−one−out sensitivity analysis for
'COLEC10' on 'Cardiac valvular disease'

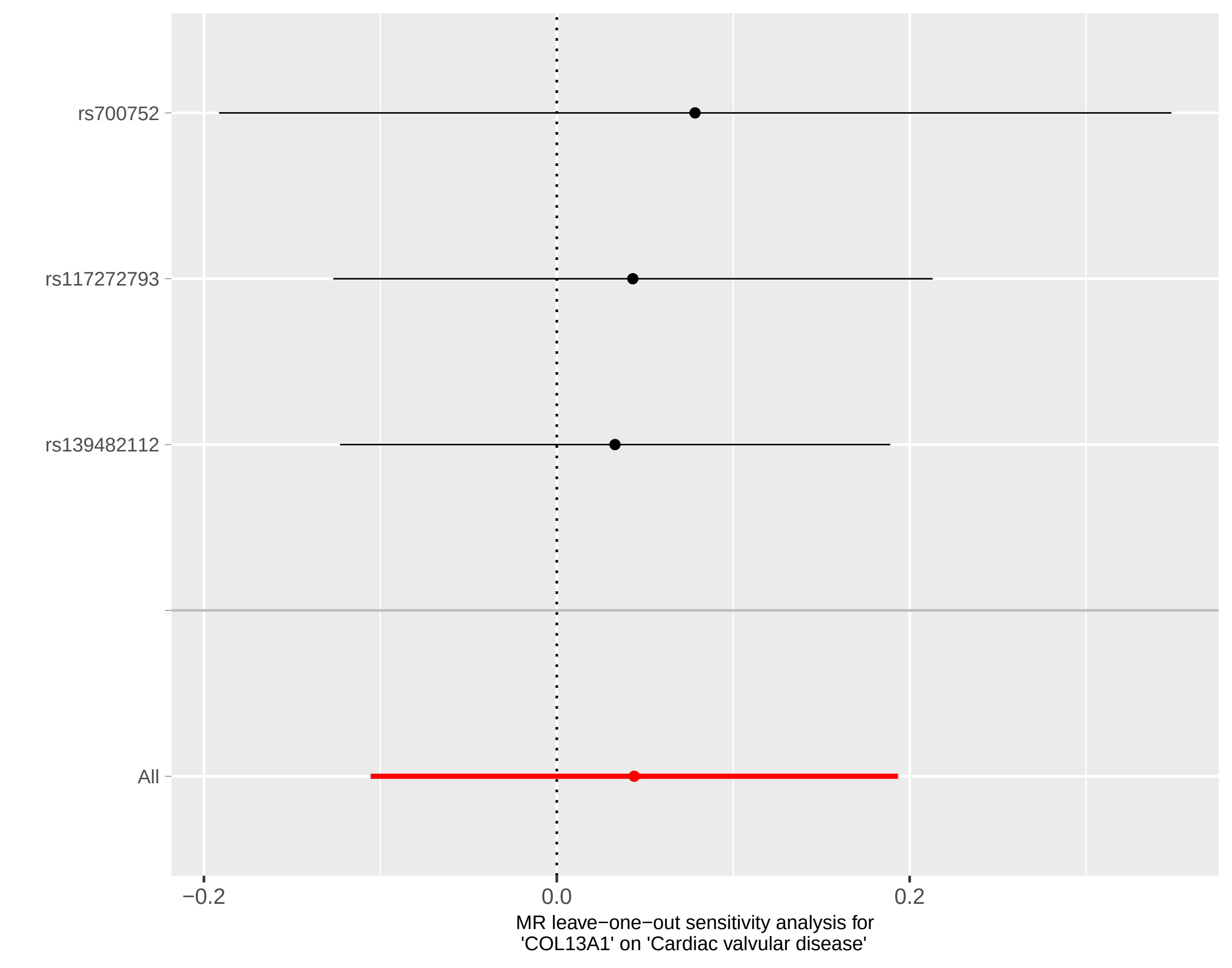

rs700752
rs117272793
rs139482112
All
−0.2
0.0
0.2
MR leave−one−out sensitivity analysis for
'COL13A1' on 'Cardiac valvular disease'

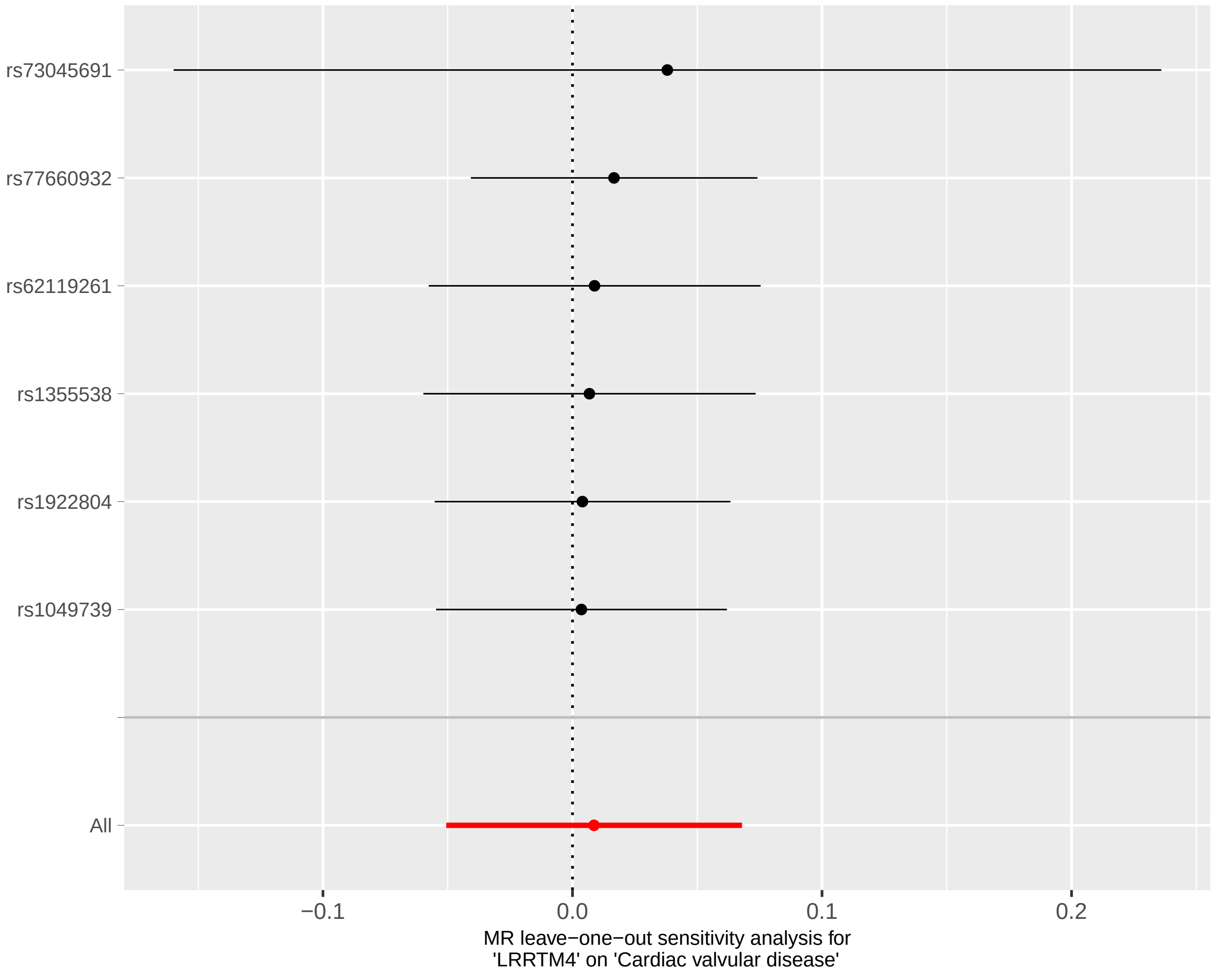
rs73045691
rs77660932
rs62119261
rs1355538
rs1922804
rs1049739
All
−0.1
0.0
0.1
0.2
MR leave−one−out sensitivity analysis for
'LRRTM4' on 'Cardiac valvular disease'

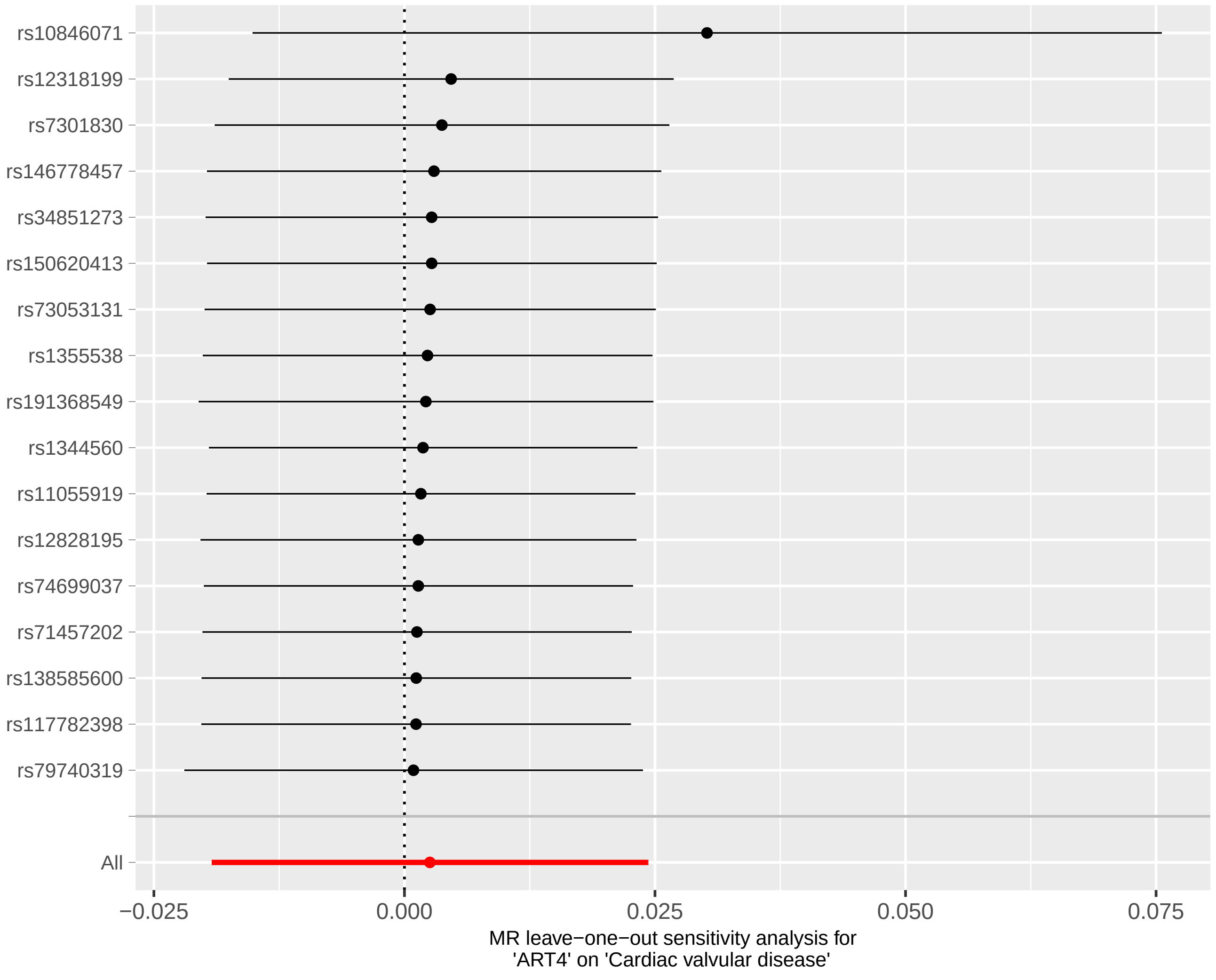
rs10846071
rs12318199
rs7301830
rs146778457
rs34851273
rs150620413
rs73053131
rs1355538
rs191368549
rs1344560
rs11055919
rs12828195
rs74699037
rs71457202
rs138585600
rs117782398
rs79740319
All
−0.025
0.000
0.025
0.050
0.075
MR leave−one−out sensitivity analysis for
'ART4' on 'Cardiac valvular disease'

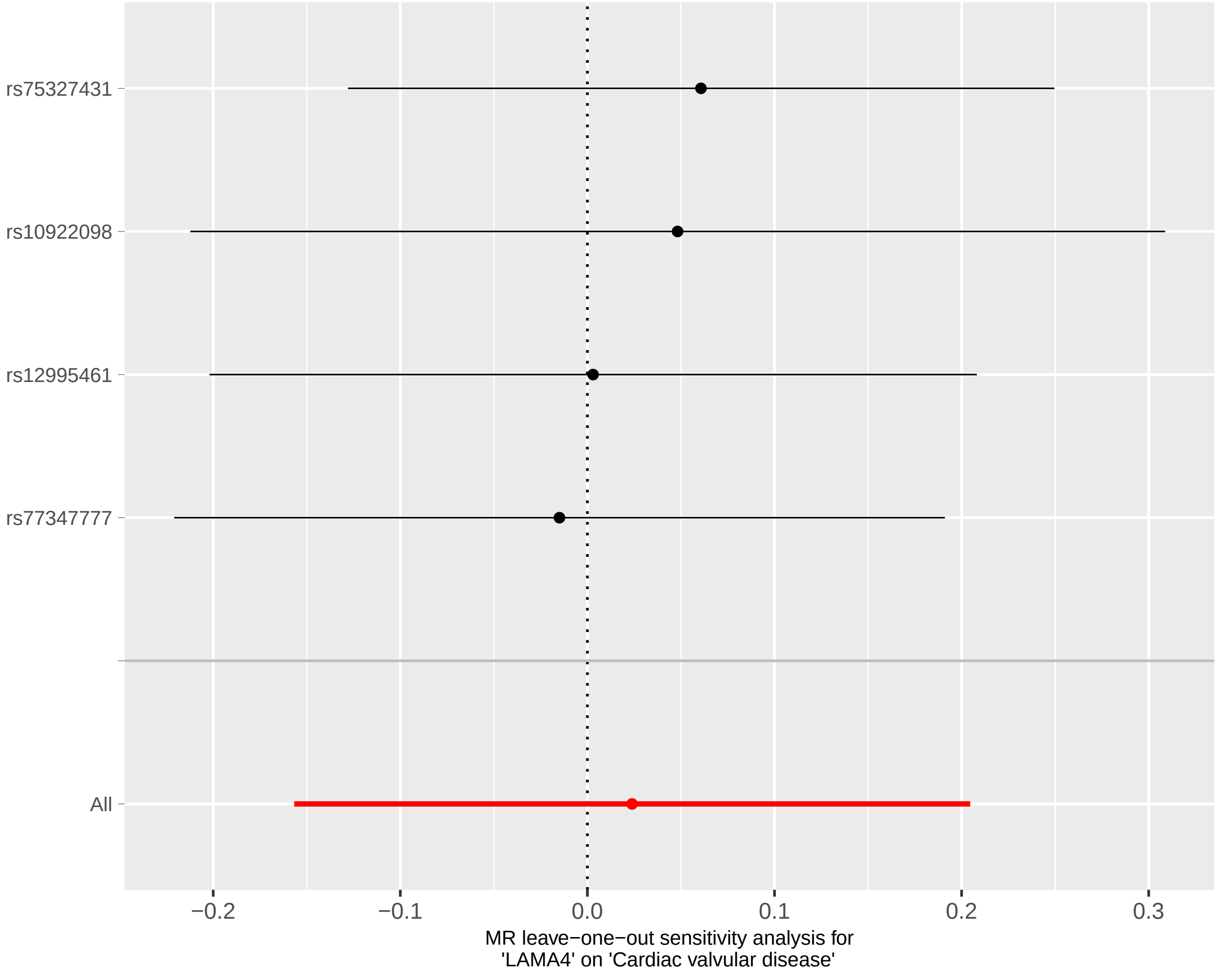

rs75327431
rs10922098
rs12995461
rs77347777
All
−0.2
−0.1
0.0
0.1
0.2
0.3
MR leave−one−out sensitivity analysis for
'LAMA4' on 'Cardiac valvular disease'

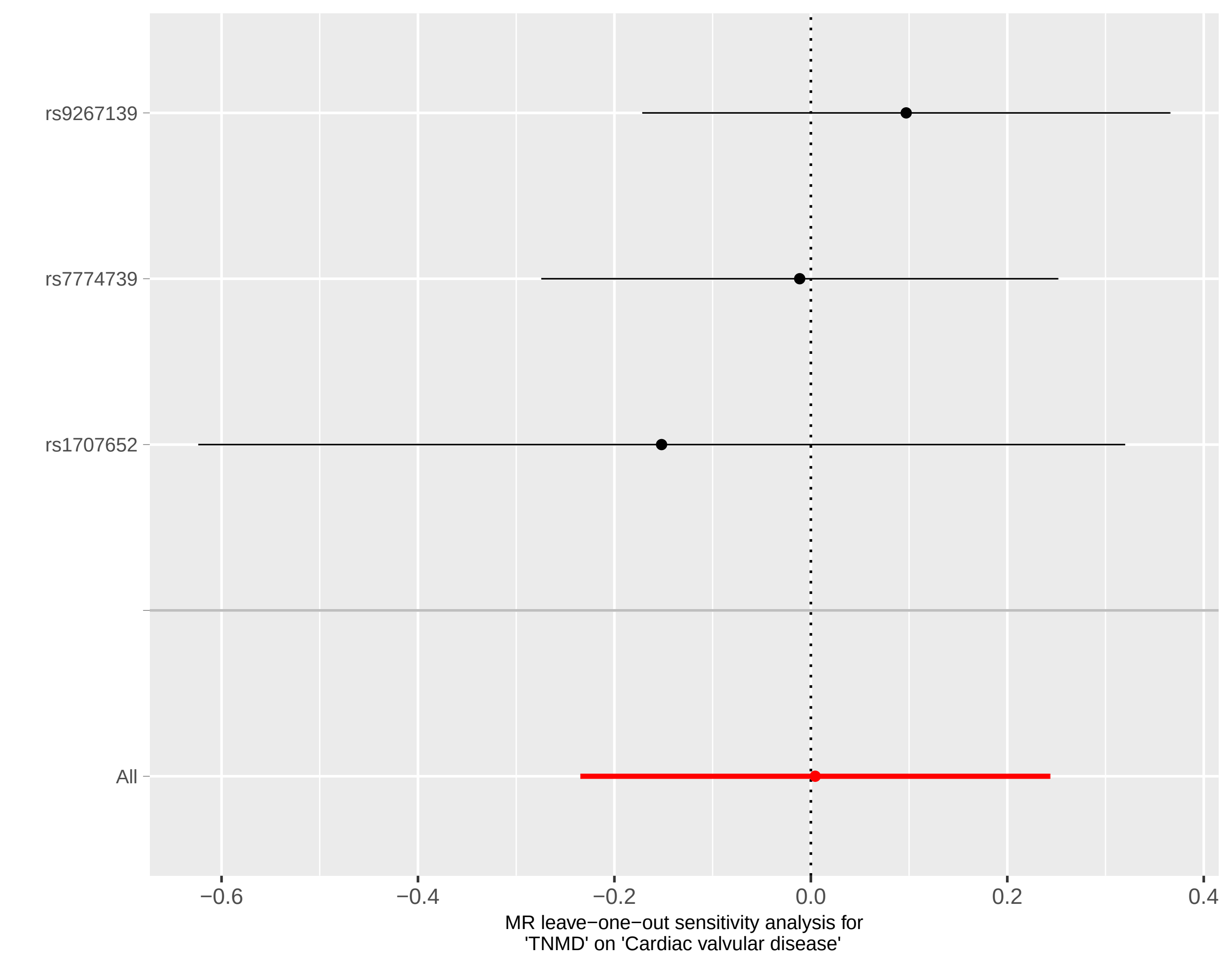
rs9267139
rs7774739
rs1707652
All
−0.6
−0.4
−0.2
0.0
0.2
0.4
MR leave−one−out sensitivity analysis for
'TNMD' on 'Cardiac valvular disease'

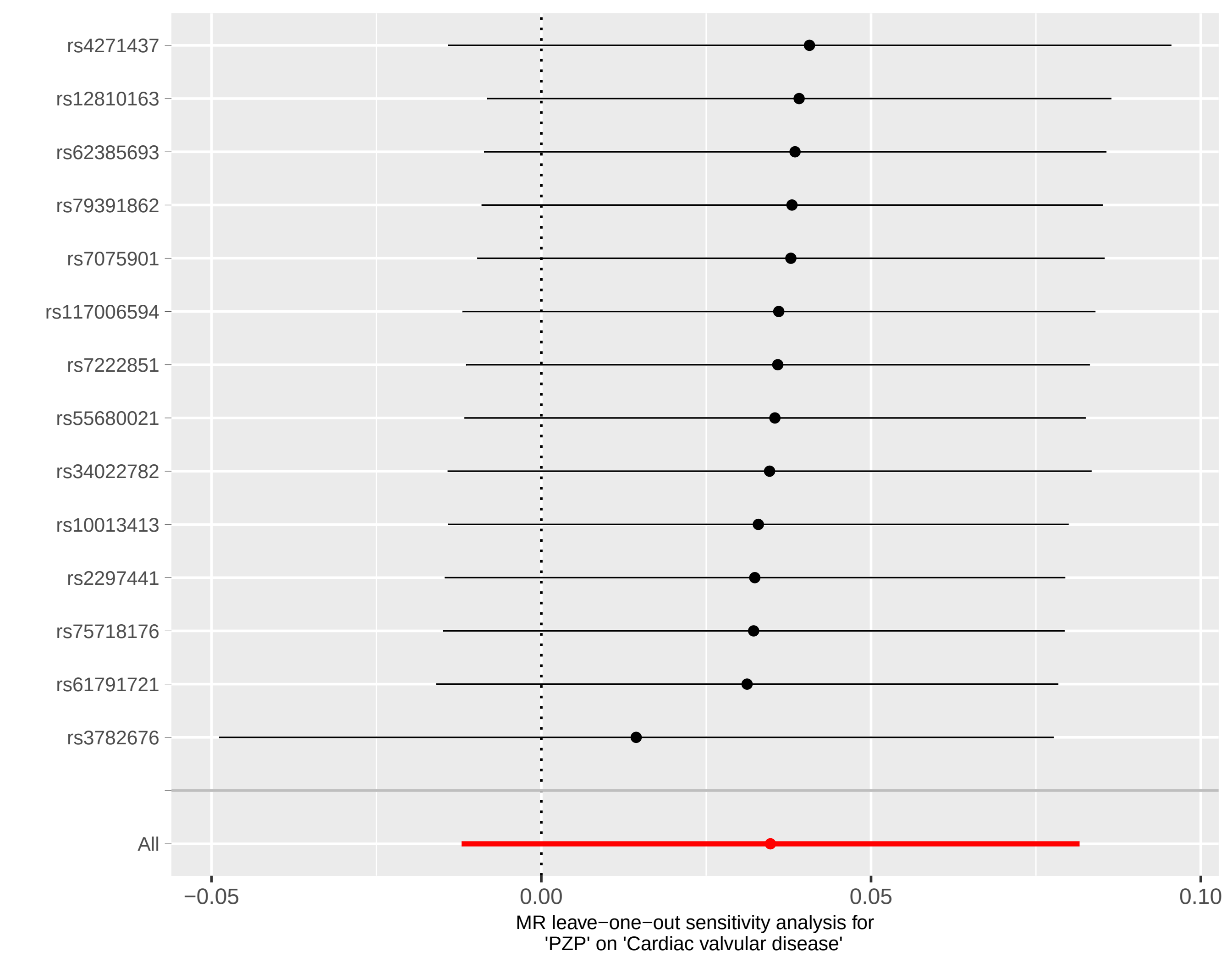

rs4271437
rs12810163
rs62385693
rs79391862
rs7075901
rs117006594
rs7222851
rs55680021
rs34022782
rs10013413
rs2297441
rs75718176
rs61791721
rs3782676
All
−0.05
0.00
0.05
0.10
MR leave−one−out sensitivity analysis for
'PZP' on 'Cardiac valvular disease'

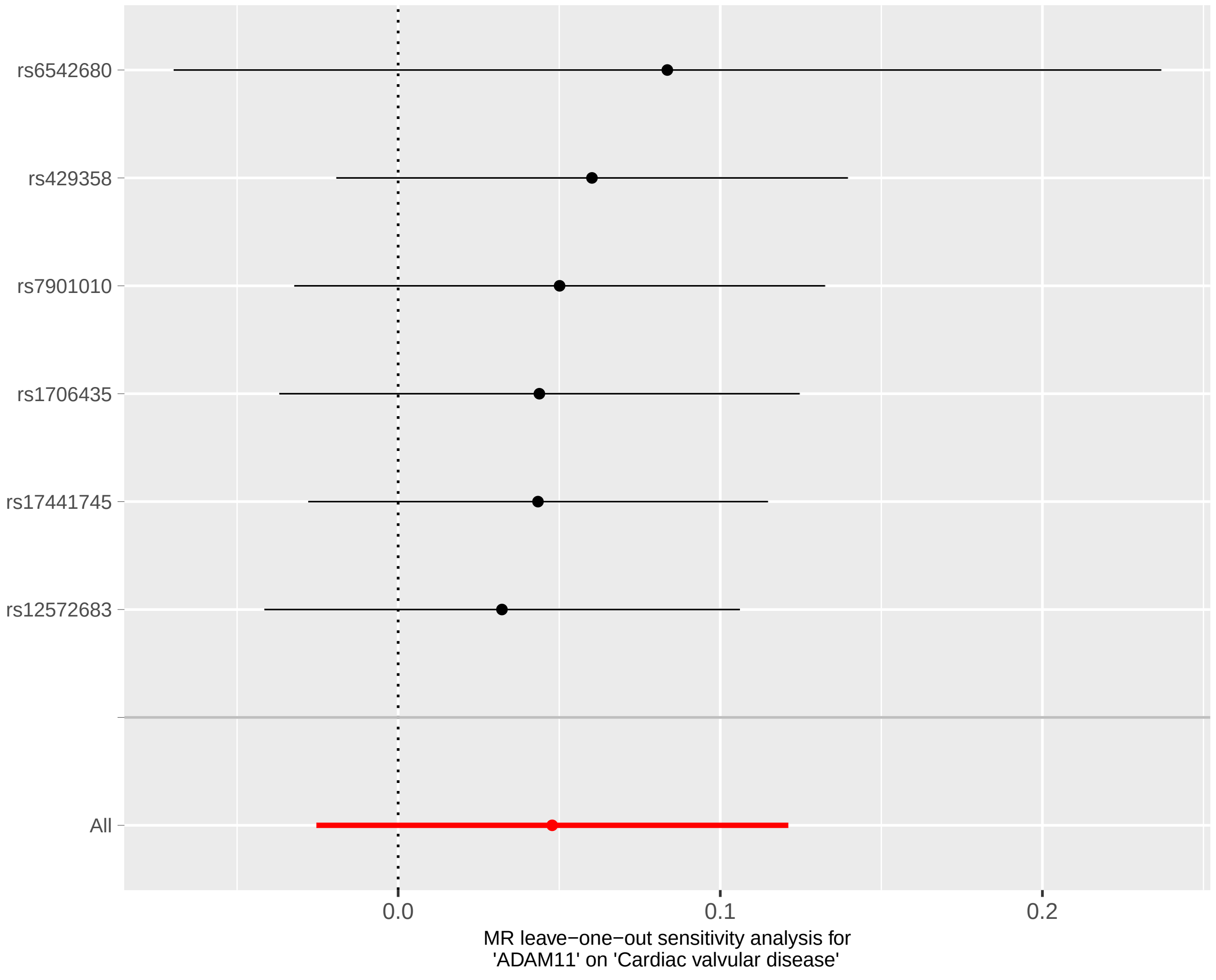

rs6542680
rs429358
rs7901010
rs1706435
rs17441745
rs12572683
All
0.0
0.1
0.2
MR leave−one−out sensitivity analysis for
'ADAM11' on 'Cardiac valvular disease'

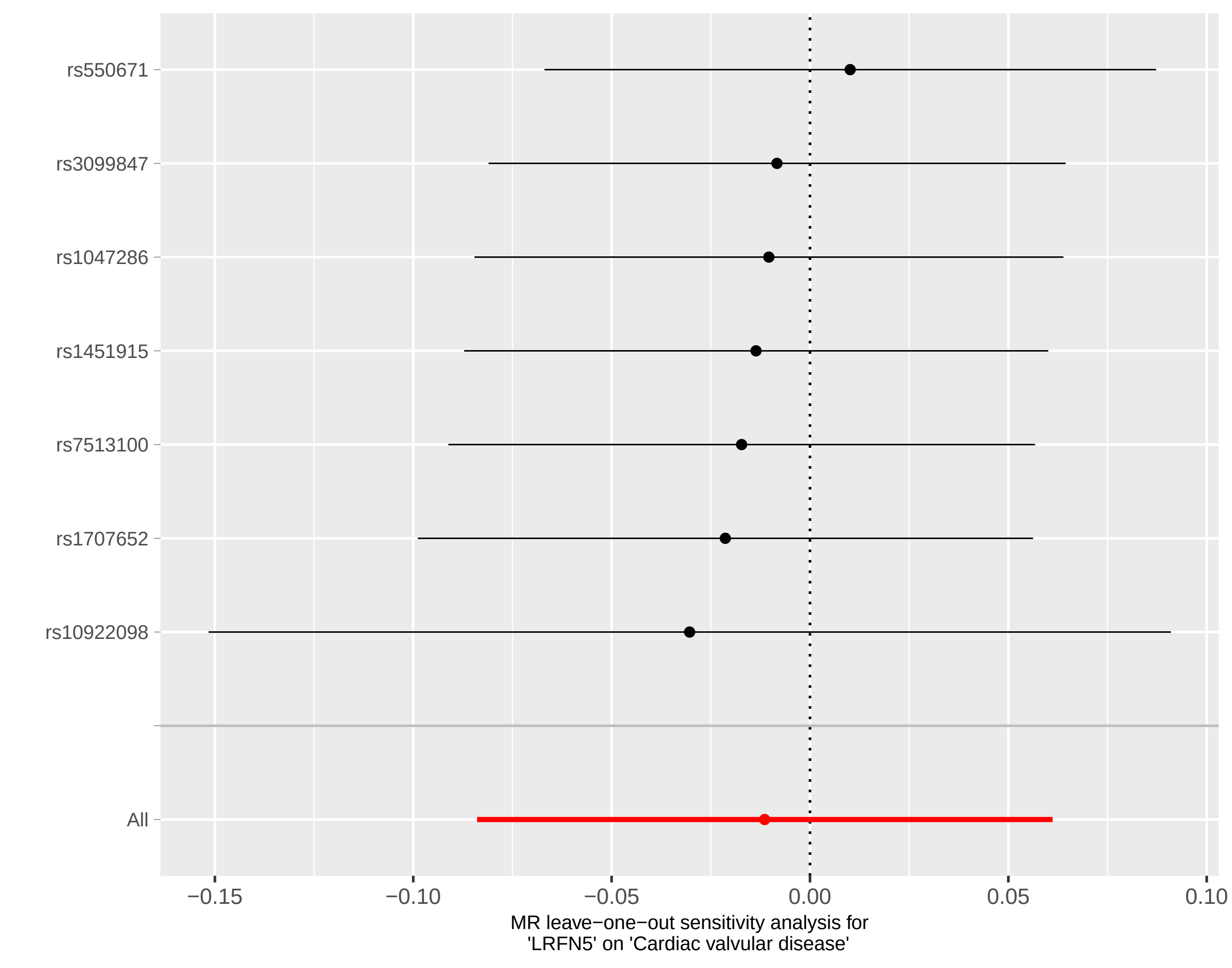
rs550671
rs3099847
rs1047286
rs1451915
rs7513100
rs1707652
rs10922098
All
−0.15
−0.10
−0.05
0.00
0.05
0.10
MR leave−one−out sensitivity analysis for
'LRFN5' on 'Cardiac valvular disease'

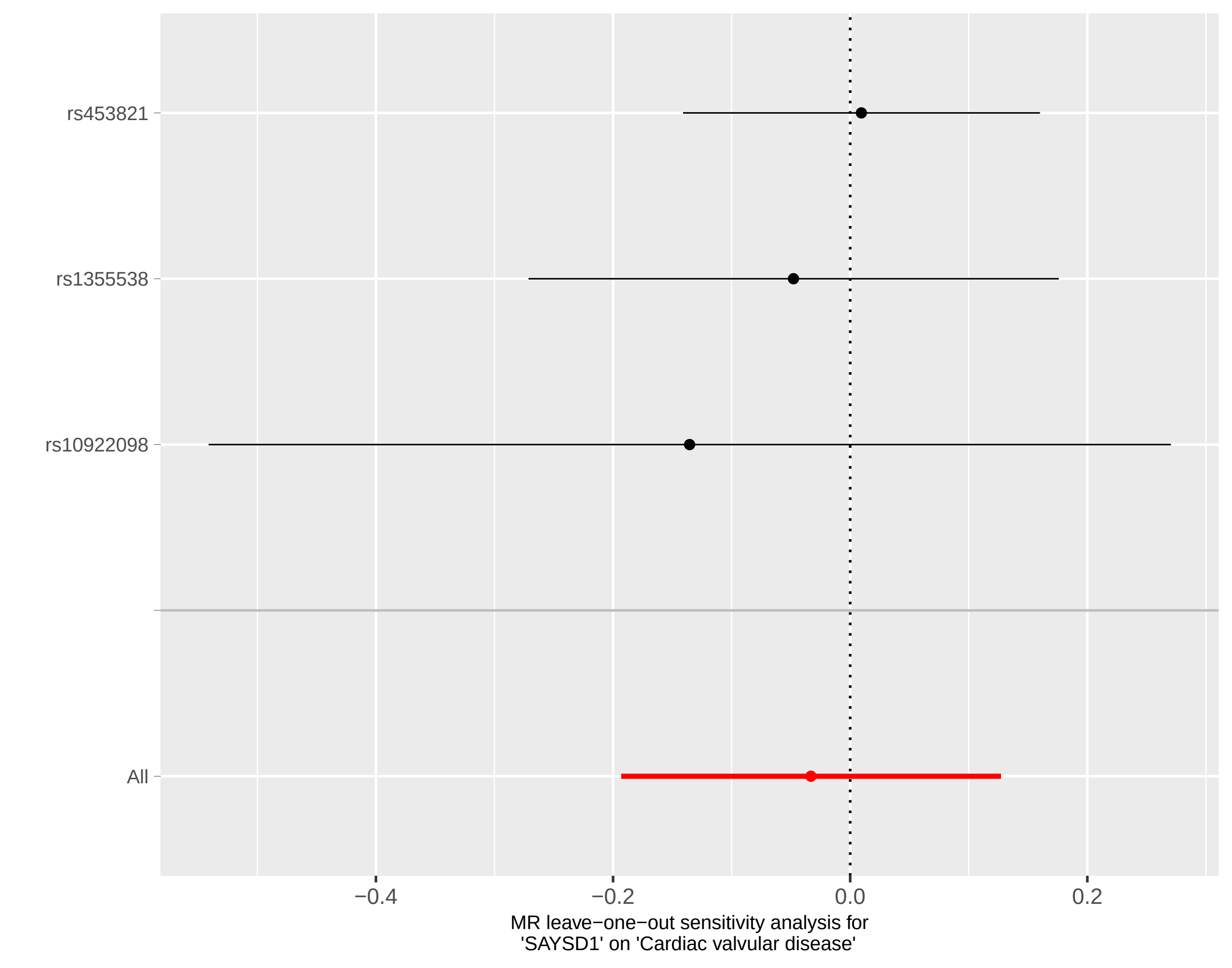
rs453821
rs1355538
rs10922098
All
−0.4
−0.2
0.0
0.2
MR leave−one−out sensitivity analysis for
'SAYSD1' on 'Cardiac valvular disease'

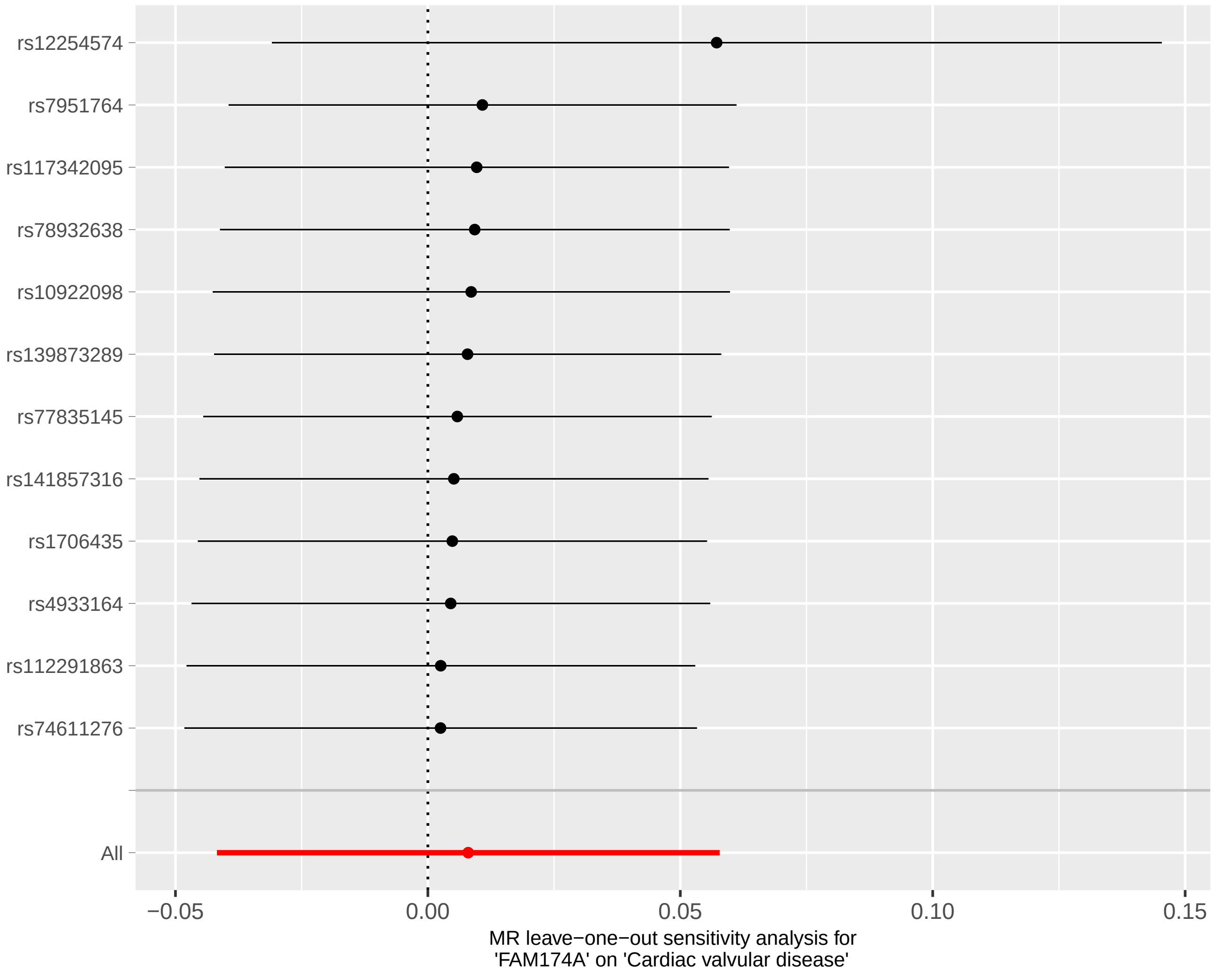

rs12254574
rs7951764
rs117342095
rs78932638
rs10922098
rs139873289
rs77835145
rs141857316
rs1706435
rs4933164
rs112291863
rs74611276
All
−0.05
0.00
0.05
0.10
0.15
MR leave−one−out sensitivity analysis for
'FAM174A' on 'Cardiac valvular disease'

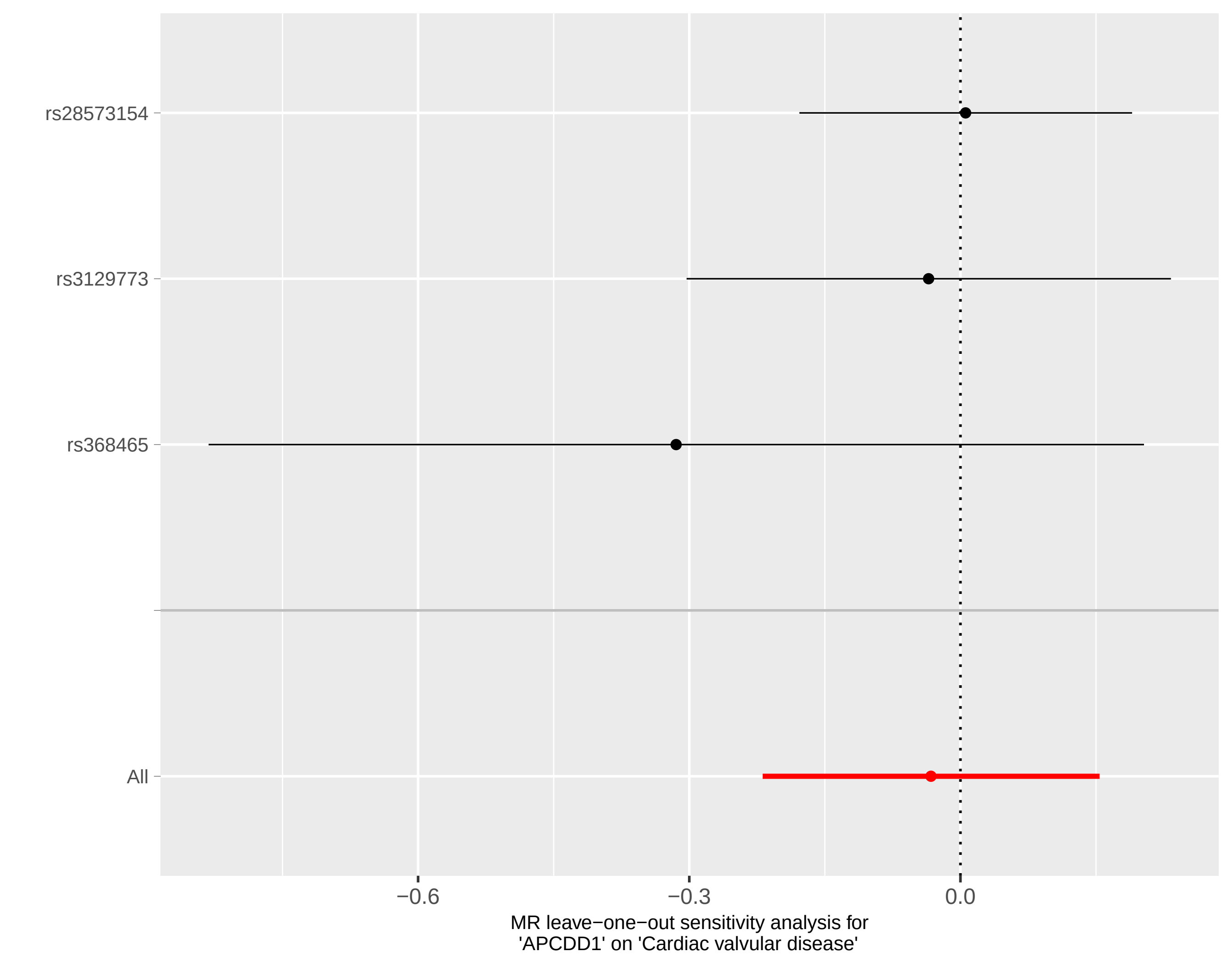
rs28573154
rs3129773
rs368465
All
−0.6
−0.3
0.0
MR leave−one−out sensitivity analysis for
'APCDD1' on 'Cardiac valvular disease'

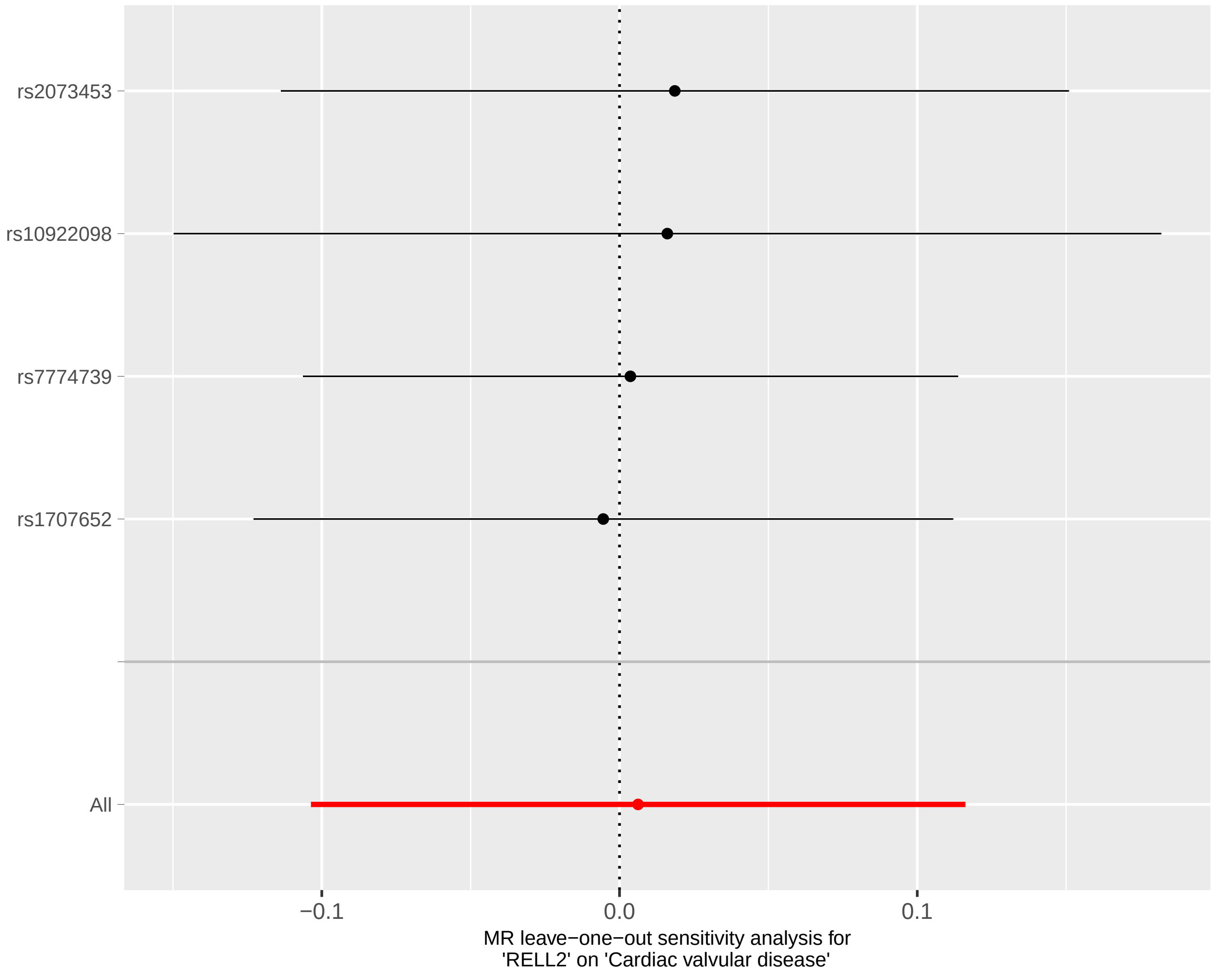
rs2073453
rs10922098
rs7774739
rs1707652
All
−0.1
0.0
0.1
MR leave−one−out sensitivity analysis for
'RELL2' on 'Cardiac valvular disease'

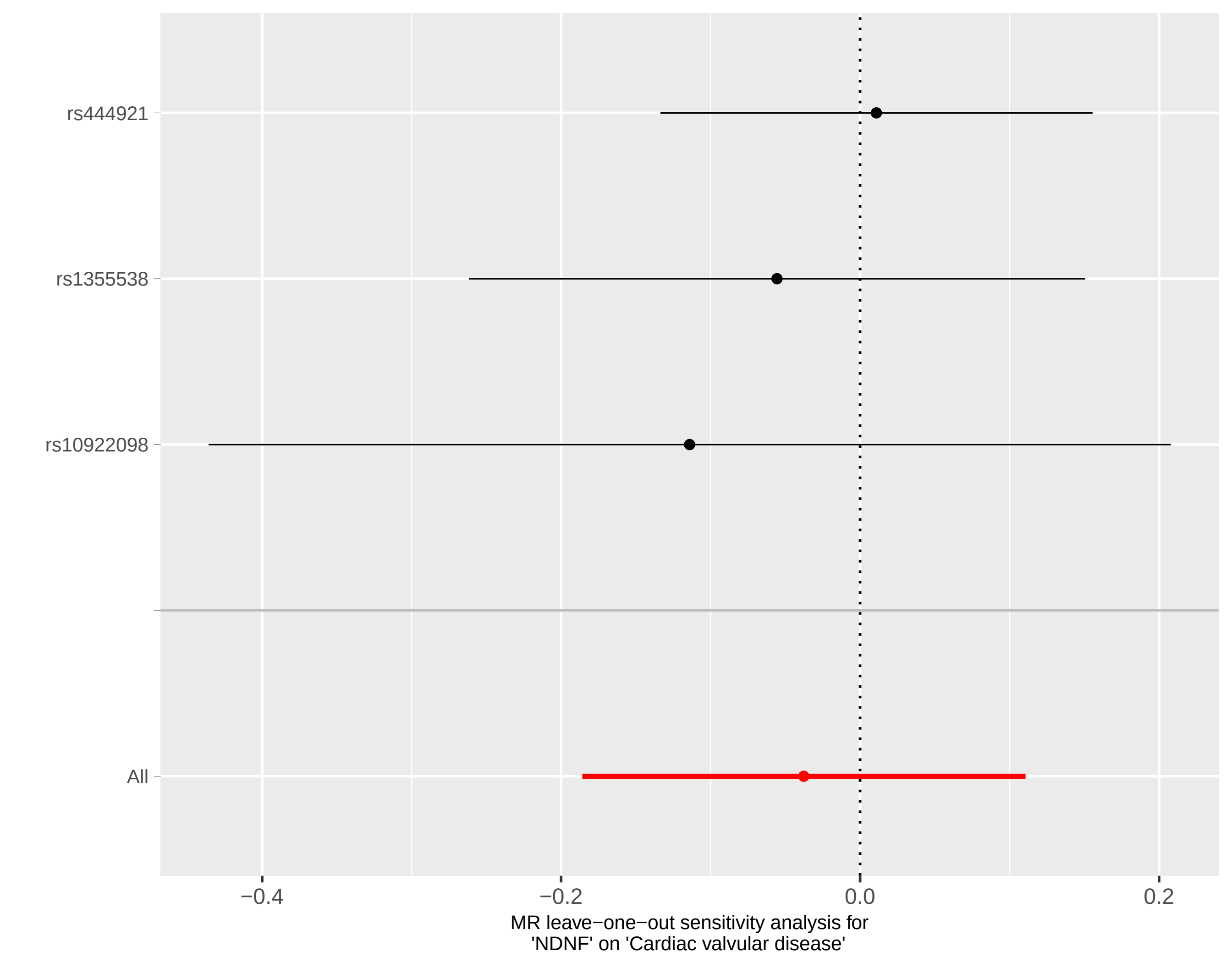
rs444921
rs1355538
rs10922098
All
−0.4
−0.2
0.0
0.2
MR leave−one−out sensitivity analysis for
'NDNF' on 'Cardiac valvular disease'

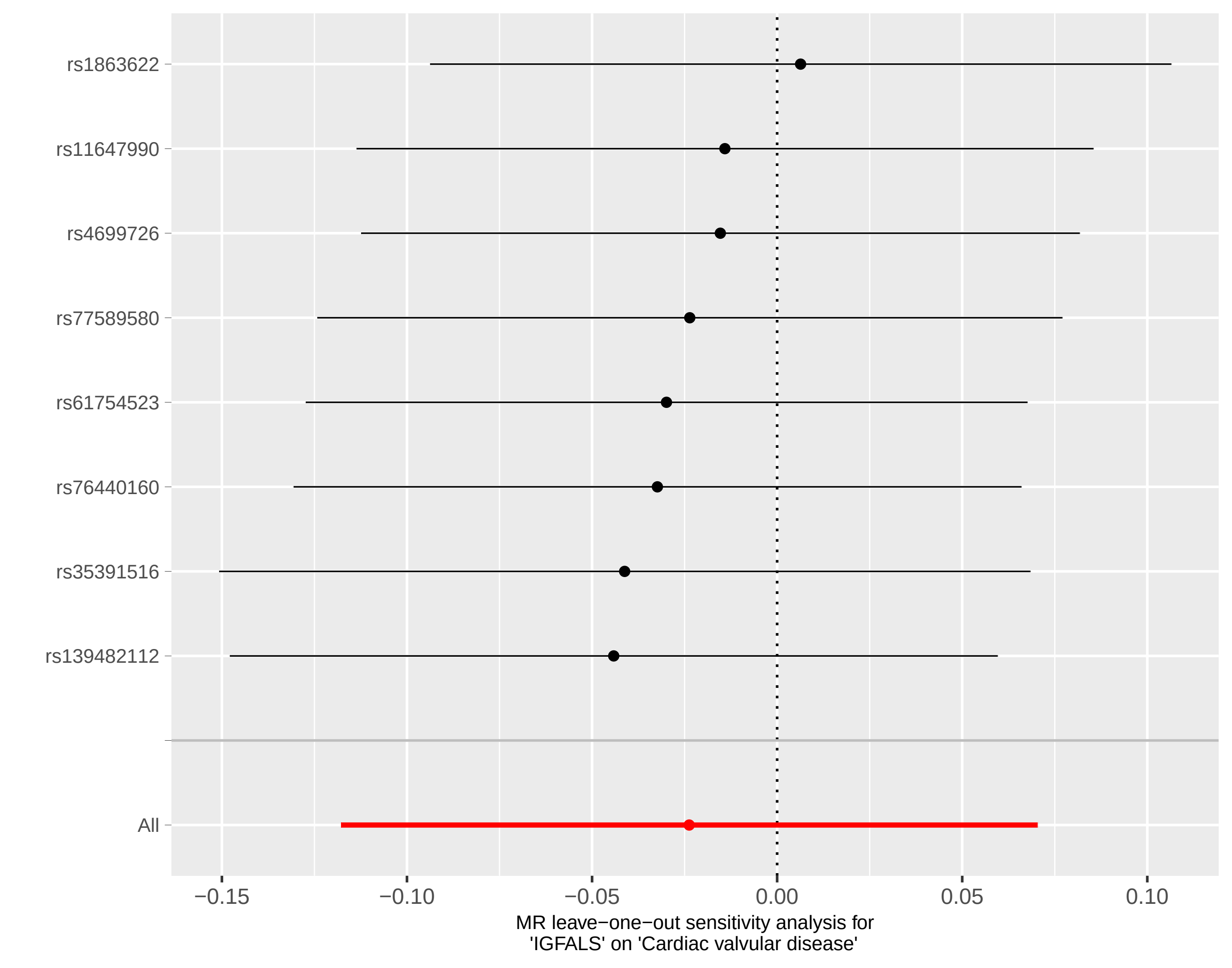
rs1863622
rs11647990
rs4699726
rs77589580
rs61754523
rs76440160
rs35391516
rs139482112
All
−0.15
−0.10
−0.05
0.00
0.05
0.10
MR leave−one−out sensitivity analysis for
'IGFALS' on 'Cardiac valvular disease'

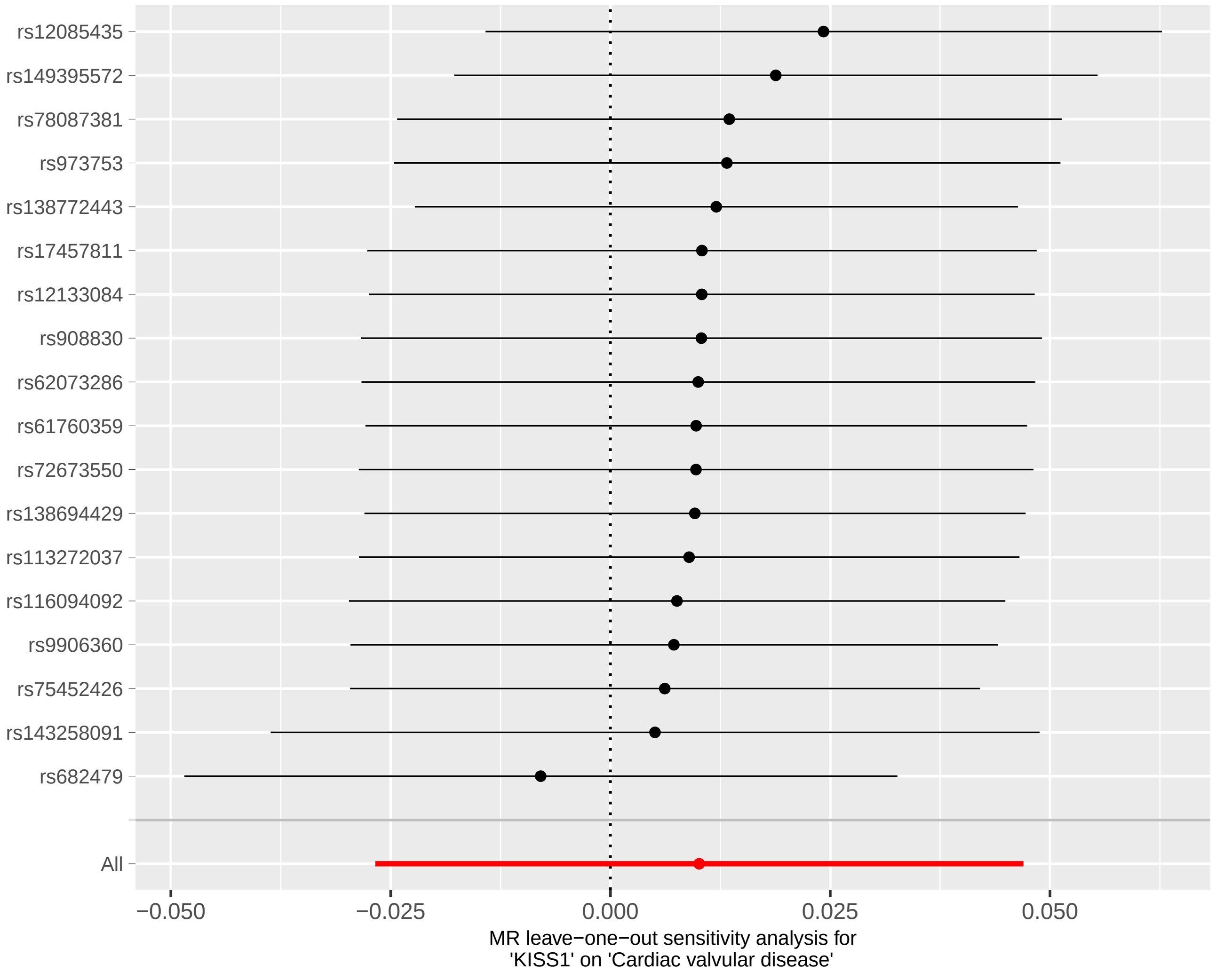
rs12085435
rs149395572
rs78087381
rs973753
rs138772443
rs17457811
rs12133084
rs908830
rs62073286
rs61760359
rs72673550
rs138694429
rs113272037
rs116094092
rs9906360
rs75452426
rs143258091
rs682479
All
−0.050
−0.025
0.000
0.025
0.050
MR leave−one−out sensitivity analysis for
'KISS1' on 'Cardiac valvular disease'

Insufficient number of SNPs

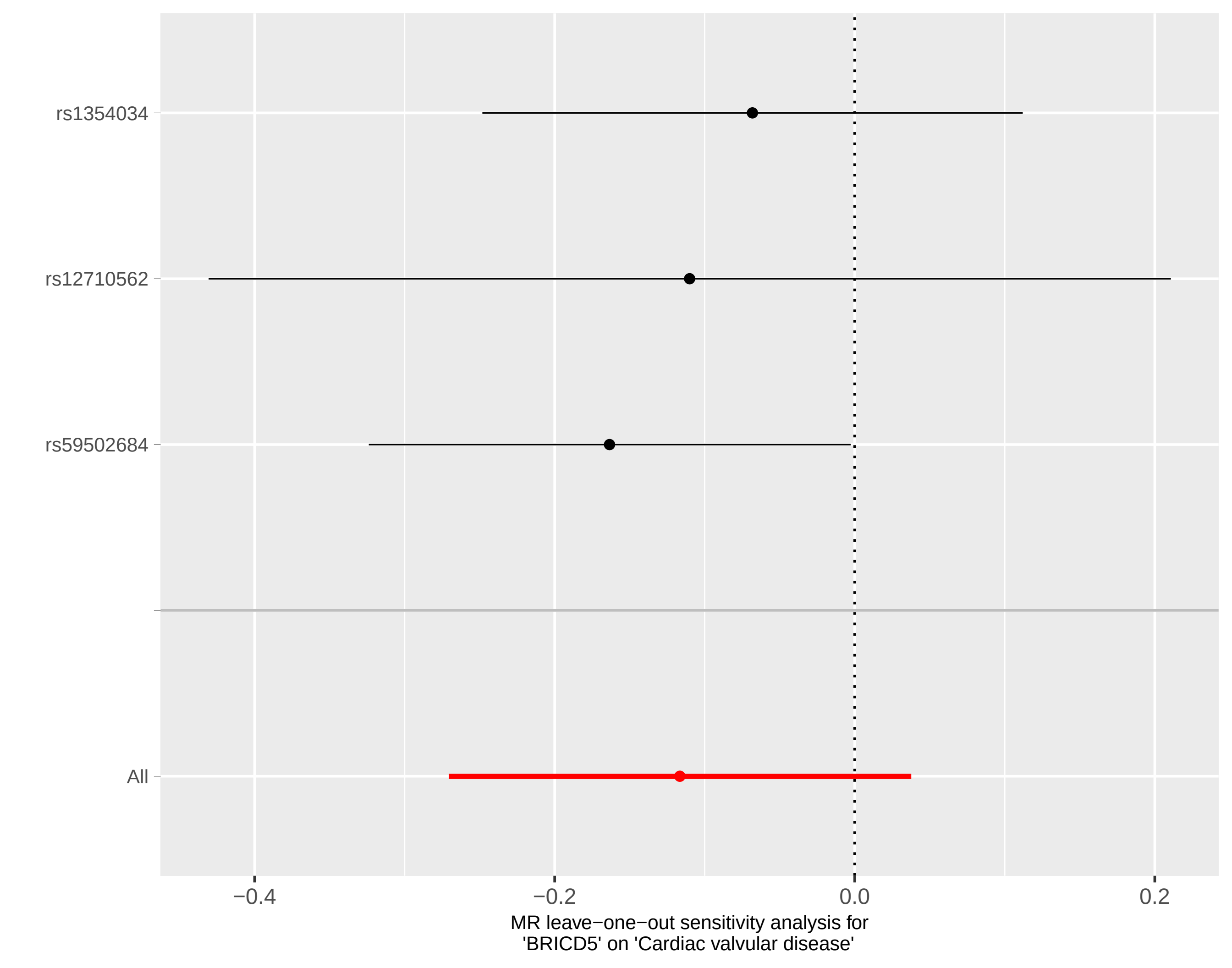

rs1354034
rs12710562
rs59502684
All
−0.4
−0.2
0.0
0.2
MR leave−one−out sensitivity analysis for
'BRICD5' on 'Cardiac valvular disease'

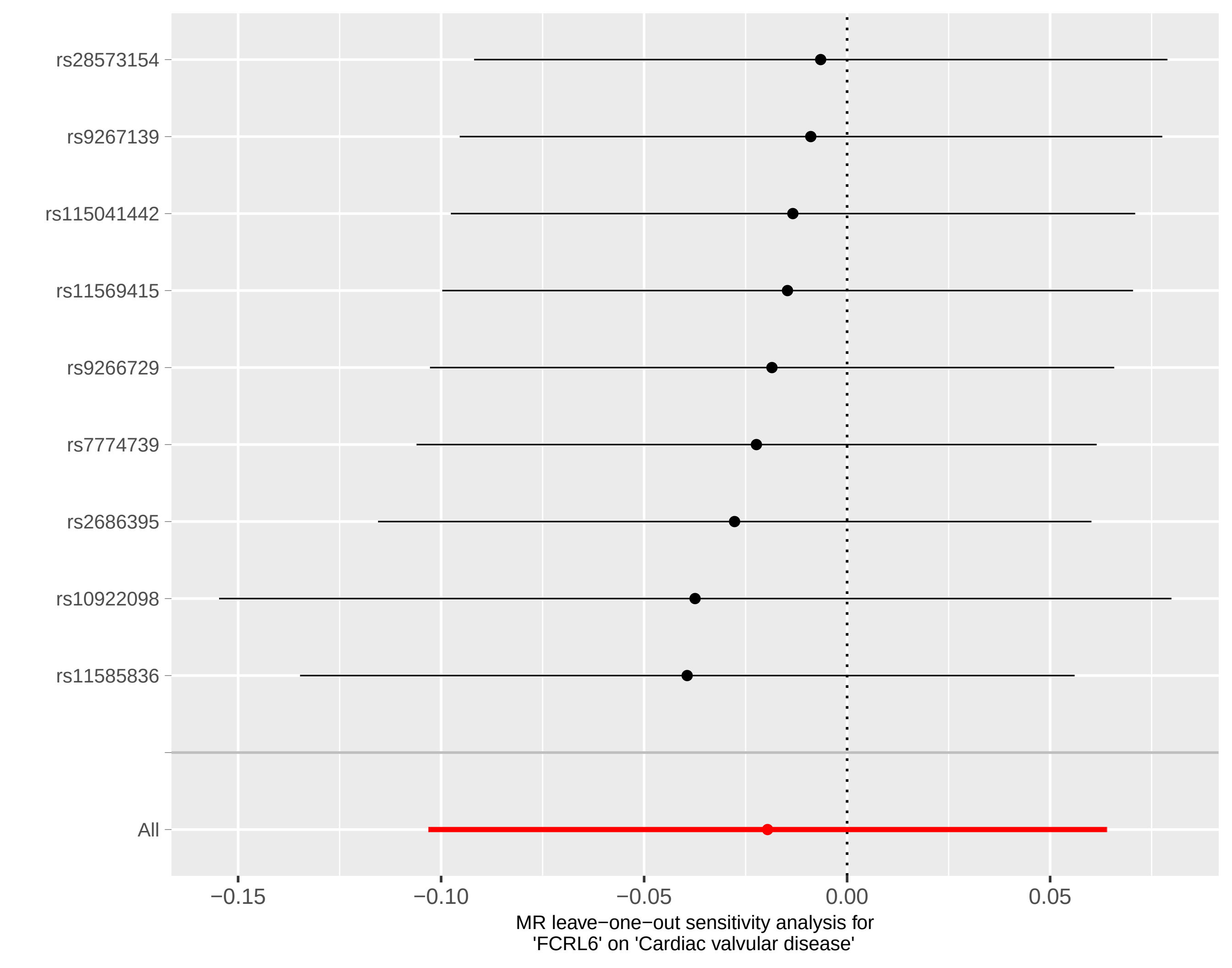

rs28573154
rs9267139
rs115041442
rs11569415
rs9266729
rs7774739
rs2686395
rs10922098
rs11585836
All
−0.15
−0.10
−0.05
0.00
0.05
MR leave−one−out sensitivity analysis for
'FCRL6' on 'Cardiac valvular disease'

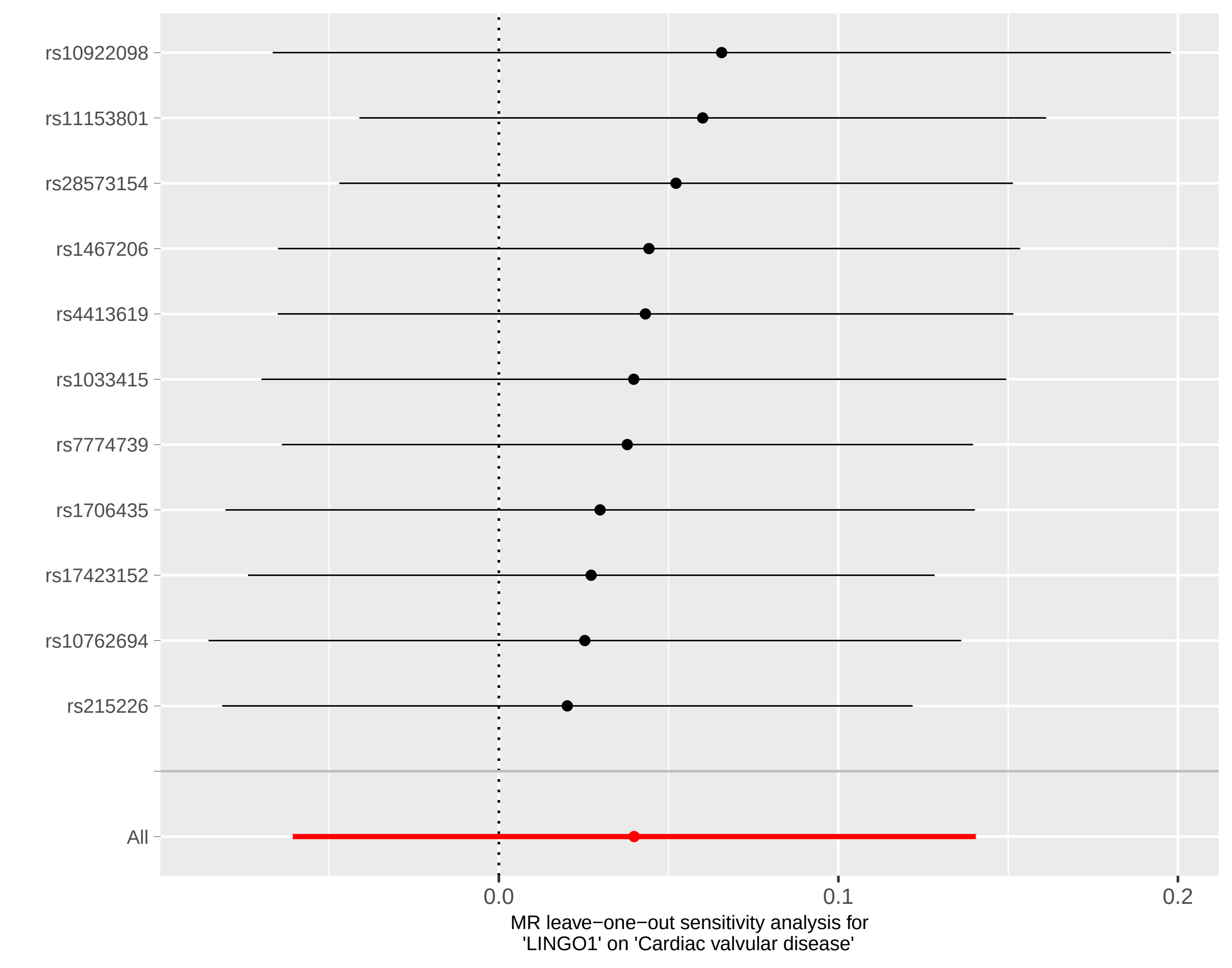

rs10922098
rs11153801
rs28573154
rs1467206
rs4413619
rs1033415
rs7774739
rs1706435
rs17423152
rs10762694
rs215226
All
0.0
0.1
0.2
MR leave−one−out sensitivity analysis for
'LINGO1' on 'Cardiac valvular disease'

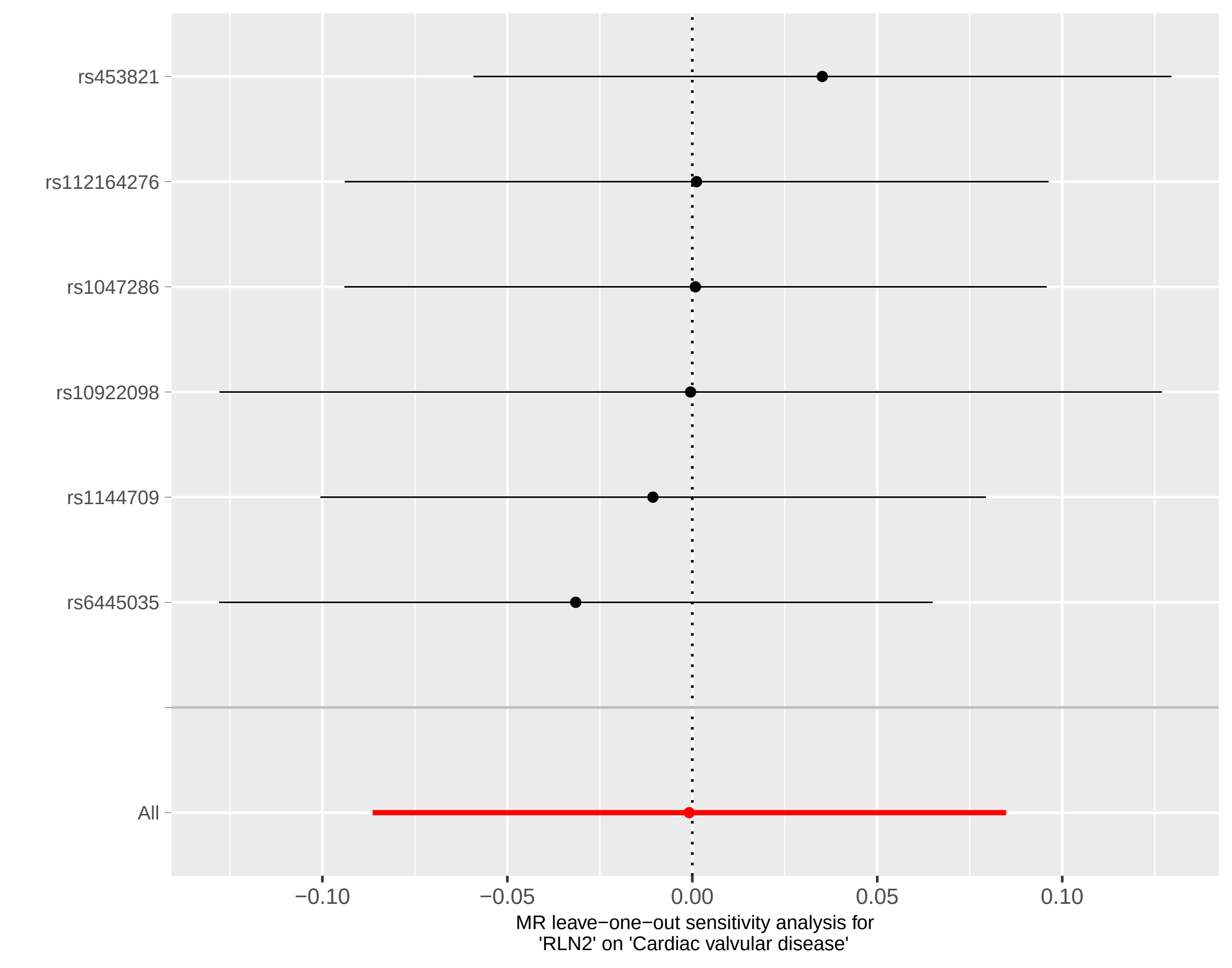

rs453821
rs112164276
rs1047286
rs10922098
rs1144709
rs6445035
All
−0.10
−0.05
0.00
0.05
0.10
MR leave−one−out sensitivity analysis for
'RLN2' on 'Cardiac valvular disease'

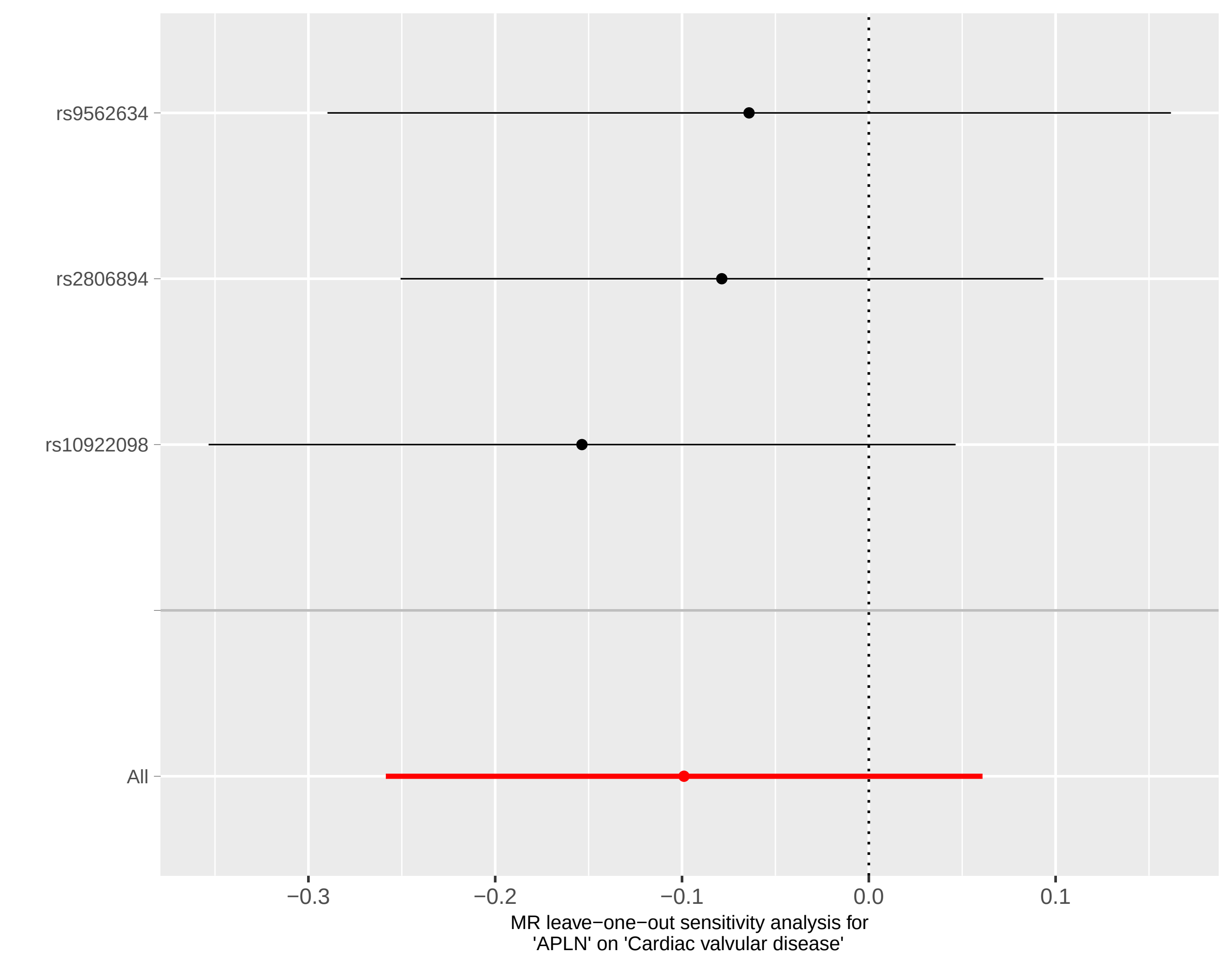
rs9562634
rs2806894
rs10922098
All
−0.3
−0.2
−0.1
0.0
0.1
MR leave−one−out sensitivity analysis for
'APLN' on 'Cardiac valvular disease'

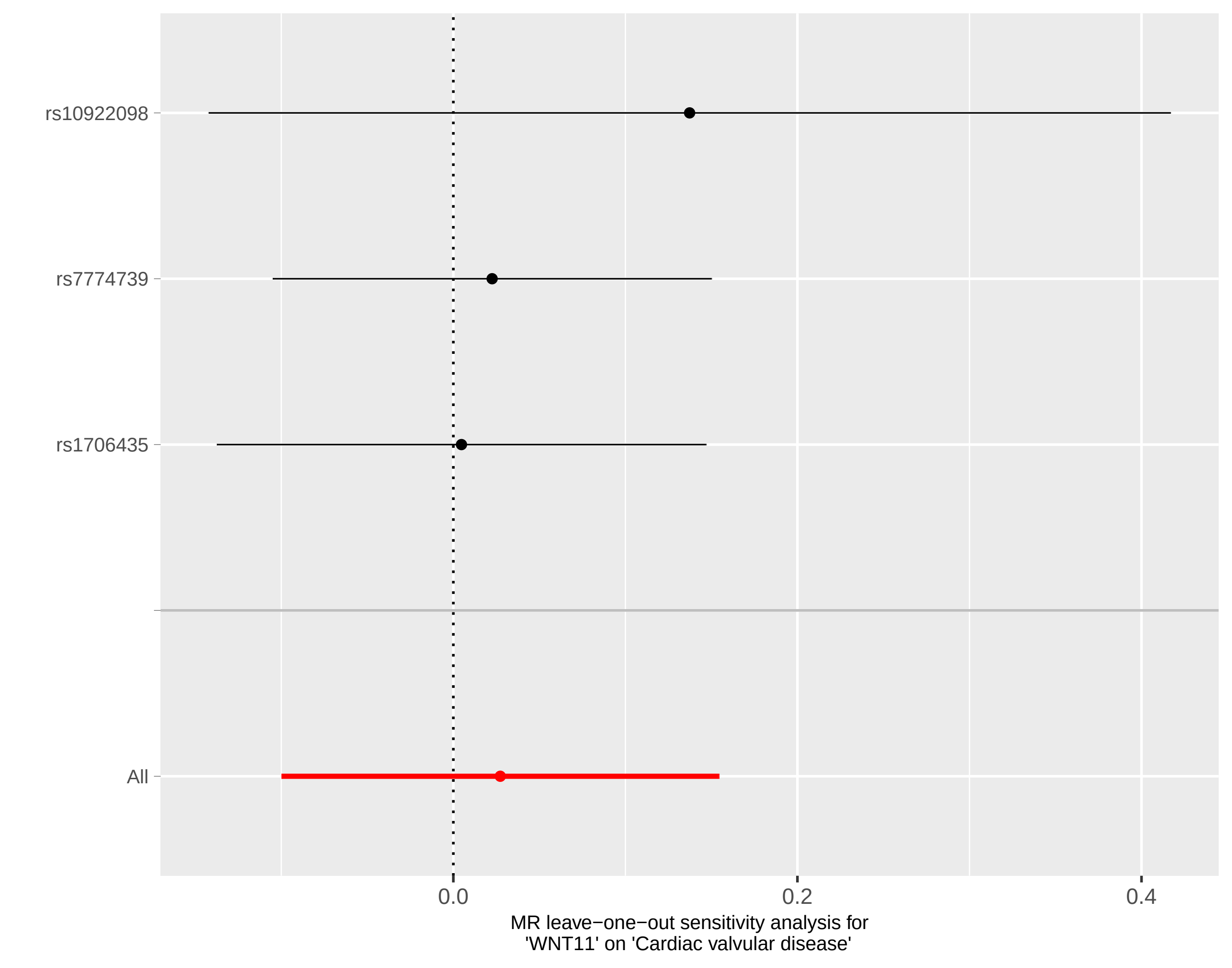
rs10922098
rs7774739
rs1706435
All
0.0
0.2
0.4
MR leave−one−out sensitivity analysis for
'WNT11' on 'Cardiac valvular disease'

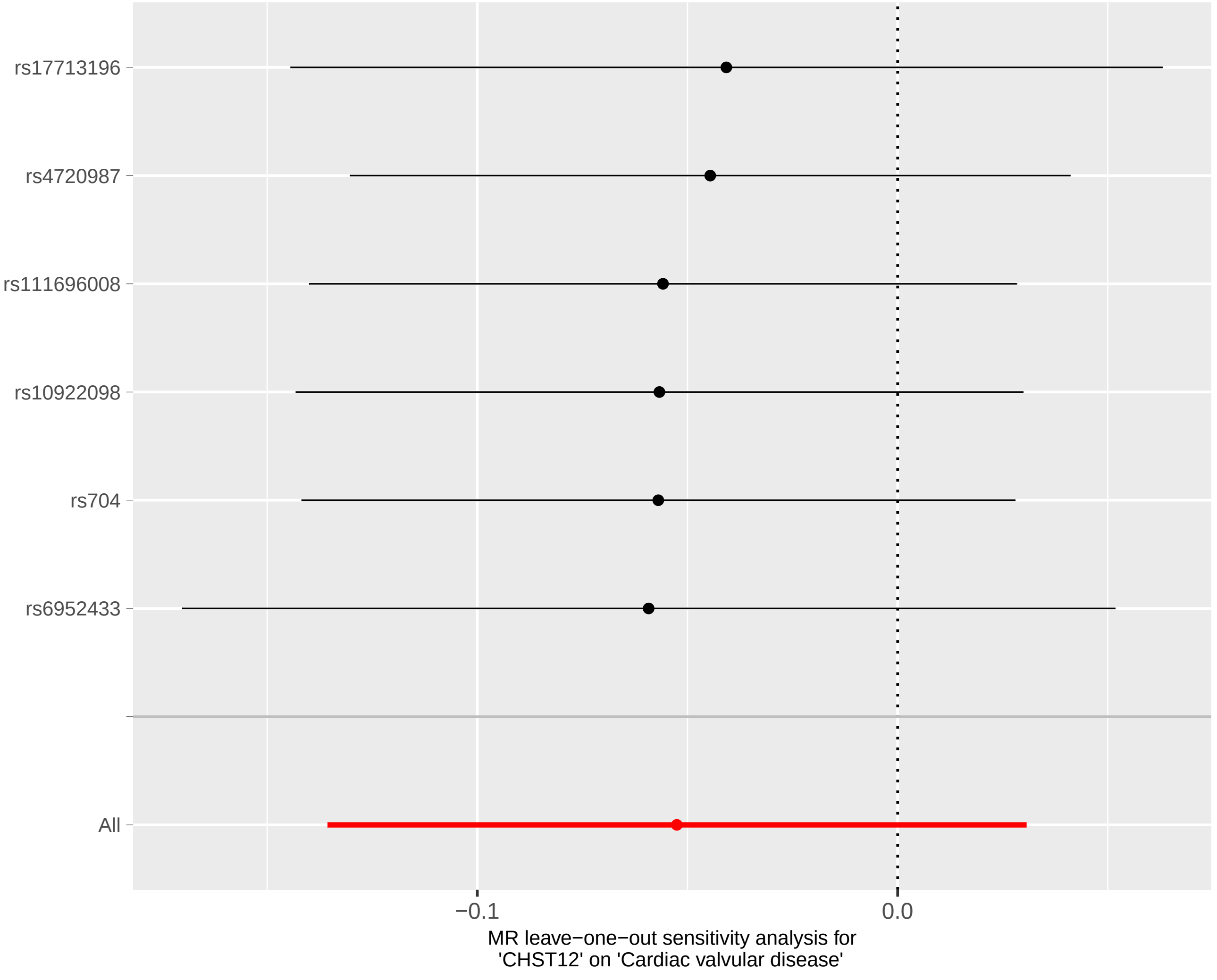
rs17713196
rs4720987
rs111696008
rs10922098
rs704
rs6952433
All
−0.1
0.0
MR leave−one−out sensitivity analysis for
'CHST12' on 'Cardiac valvular disease'

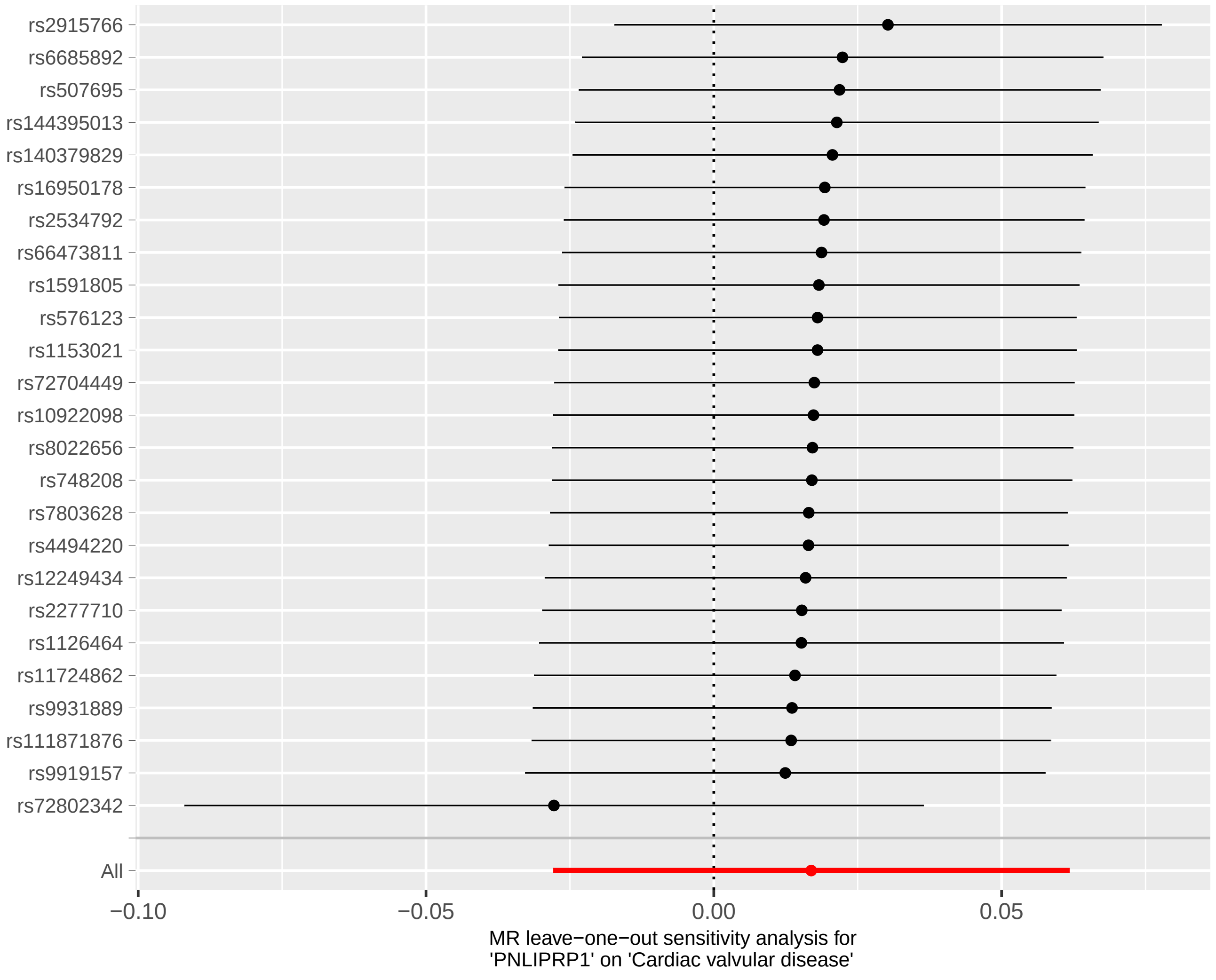
rs2915766
rs6685892
rs507695
rs144395013
rs140379829
rs16950178
rs2534792
rs66473811
rs1591805
rs576123
rs1153021
rs72704449
rs10922098
rs8022656
rs748208
rs7803628
rs4494220
rs12249434
rs2277710
rs1126464
rs11724862
rs9931889
rs111871876
rs9919157
rs72802342
All
−0.10
−0.05
0.00
0.05
MR leave−one−out sensitivity analysis for
'PNLIPRP1' on 'Cardiac valvular disease'

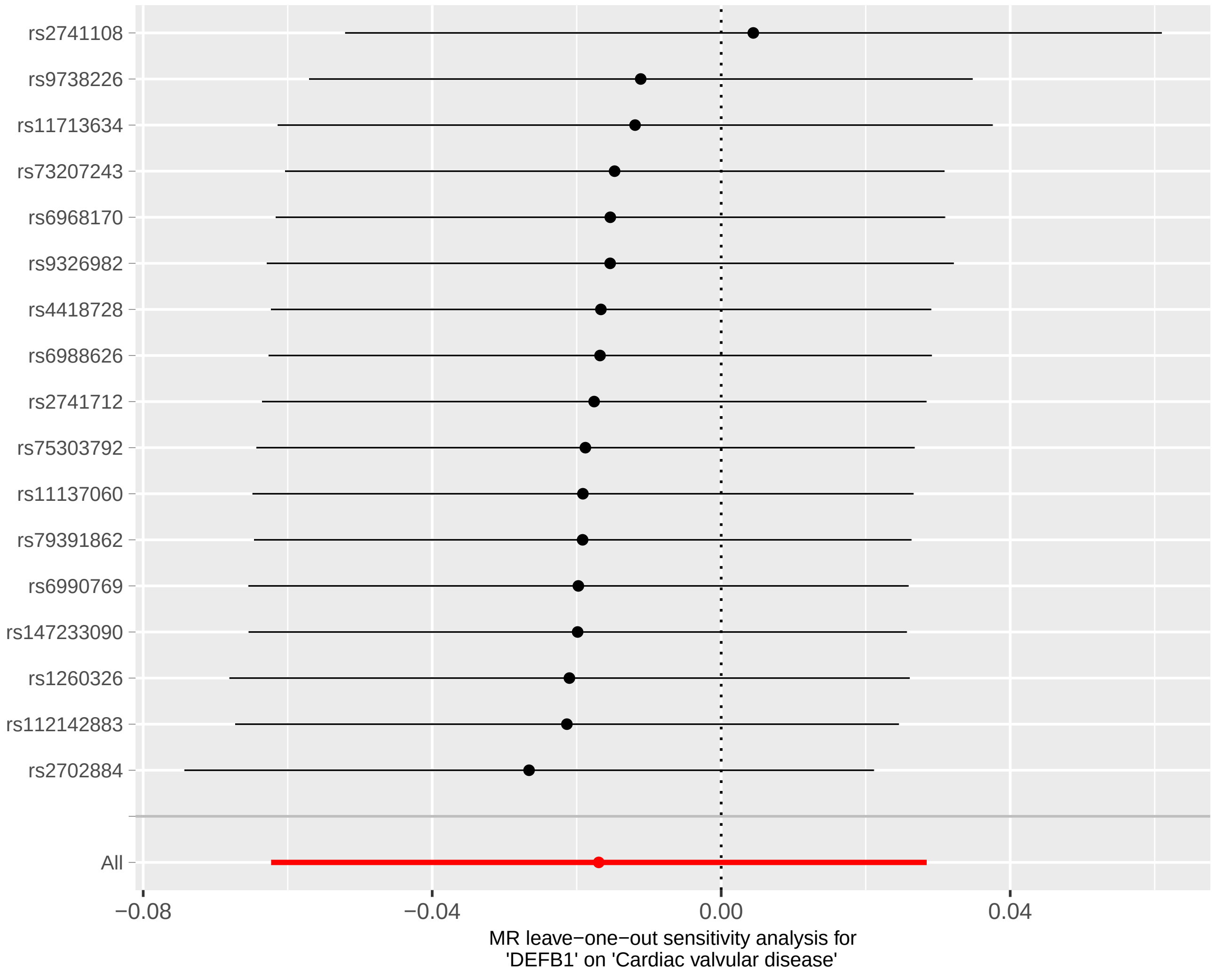
rs2741108
rs9738226
rs11713634
rs73207243
rs6968170
rs9326982
rs4418728
rs6988626
rs2741712
rs75303792
rs11137060
rs79391862
rs6990769
rs147233090
rs1260326
rs112142883
rs2702884
All
−0.08
−0.04
0.00
0.04
MR leave−one−out sensitivity analysis for
'DEFB1' on 'Cardiac valvular disease'

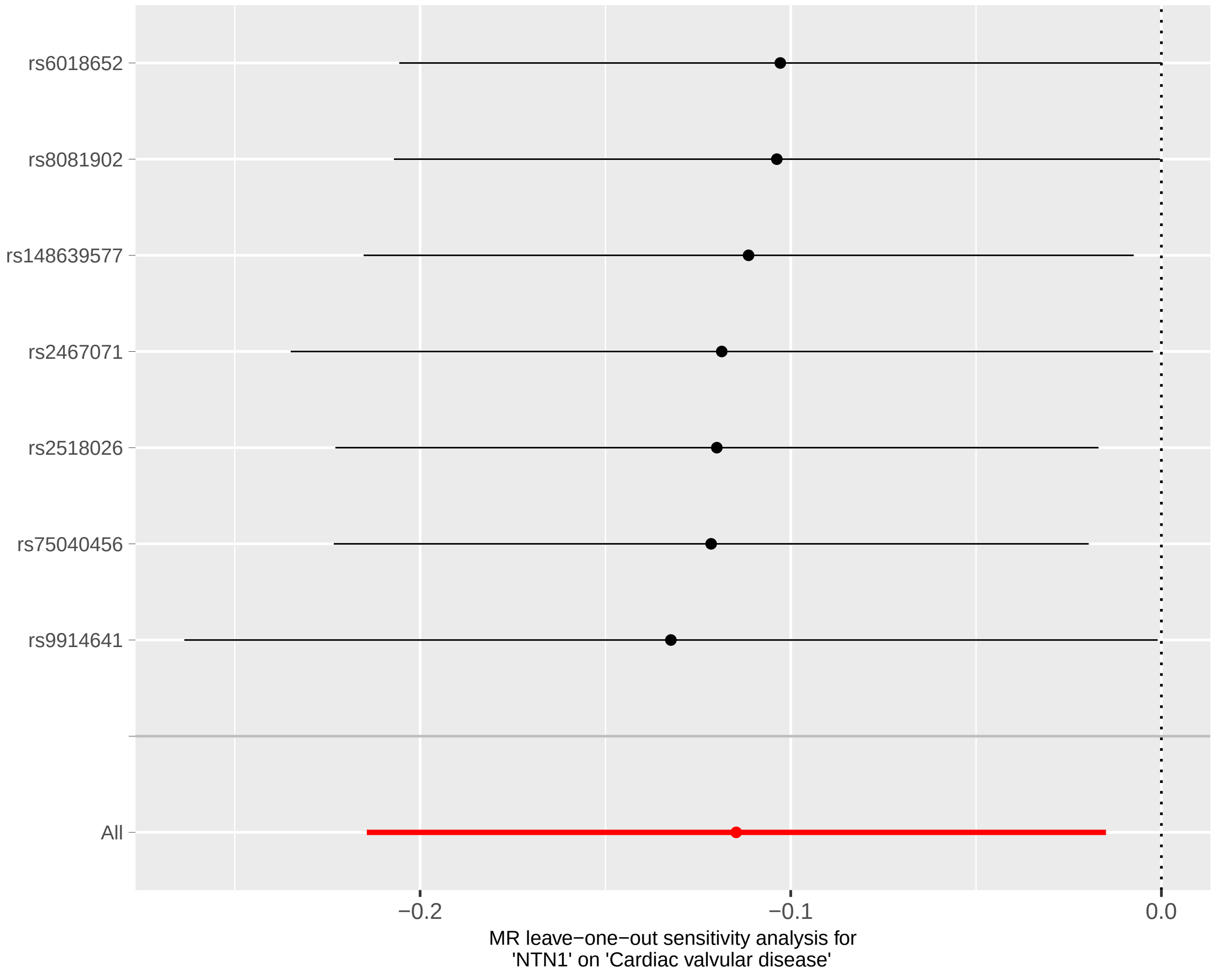
rs6018652
rs8081902
rs148639577
rs2467071
rs2518026
rs75040456
rs9914641
All
−0.2
−0.1
0.0
MR leave−one−out sensitivity analysis for
'NTN1' on 'Cardiac valvular disease'

Insufficient number of SNPs

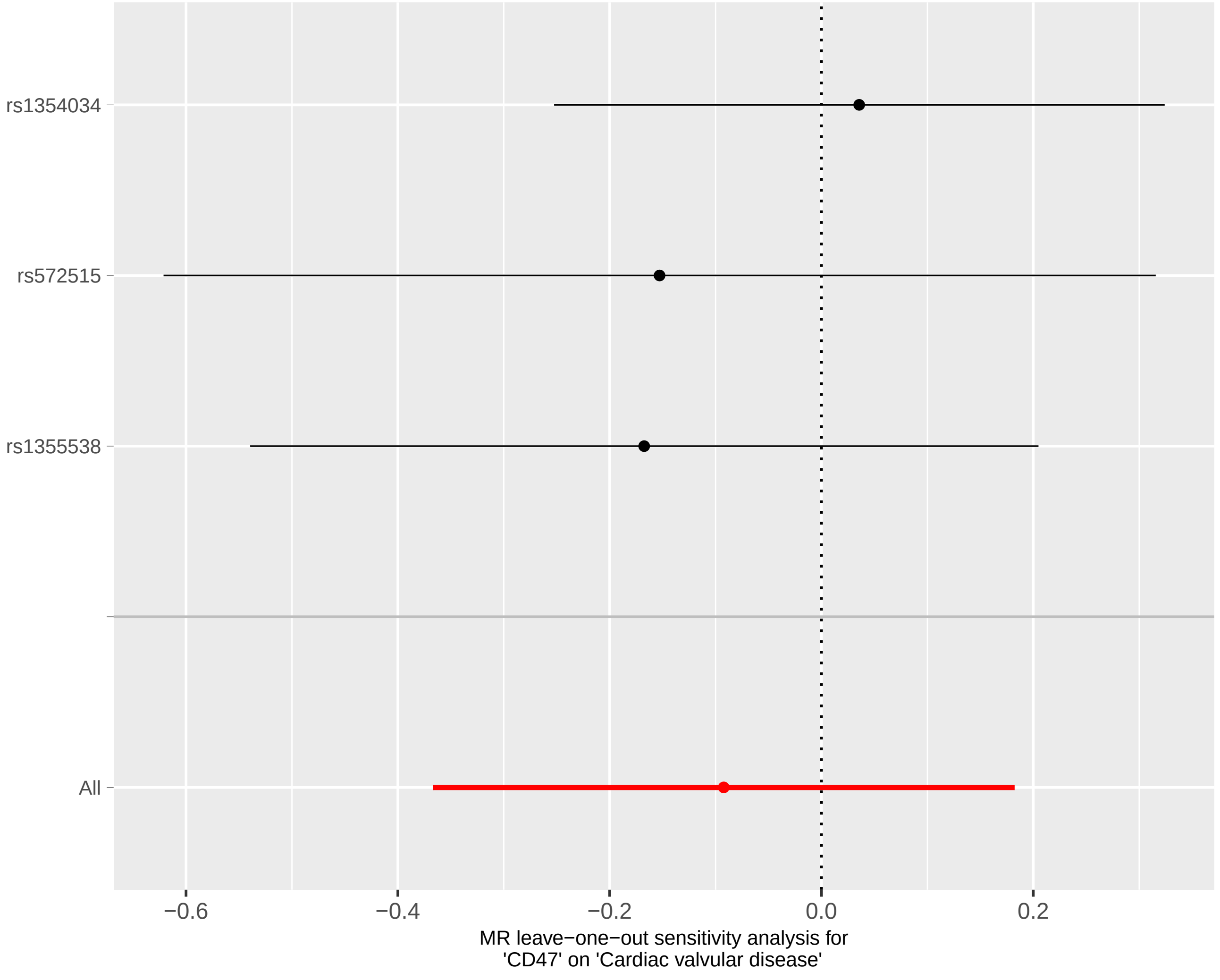

rs1354034
rs572515
rs1355538
All
−0.6
−0.4
−0.2
0.0
0.2
MR leave−one−out sensitivity analysis for
'CD47' on 'Cardiac valvular disease'

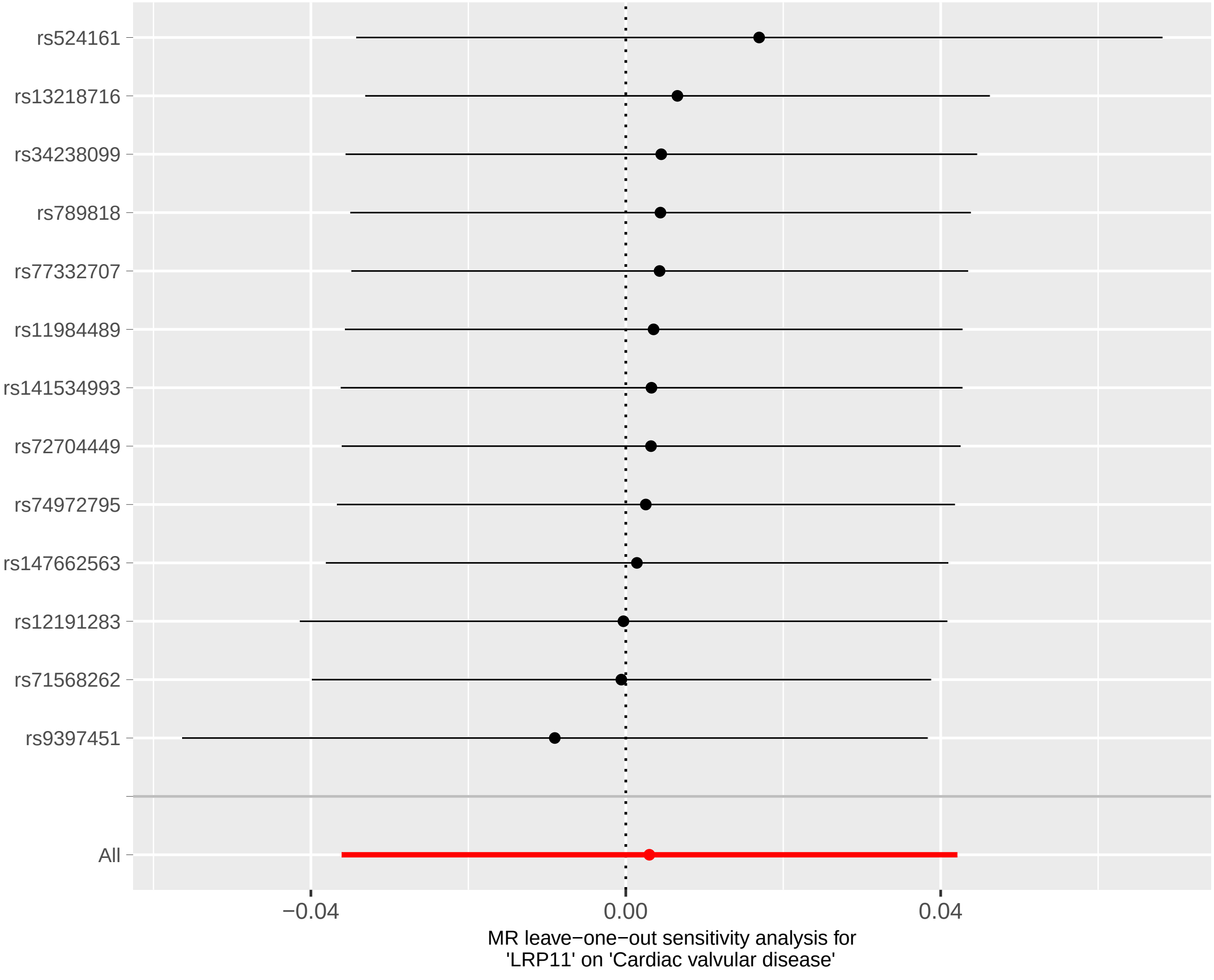
rs524161
rs13218716
rs34238099
rs789818
rs77332707
rs11984489
rs141534993
rs72704449
rs74972795
rs147662563
rs12191283
rs71568262
rs9397451
All
−0.04
0.00
0.04
MR leave−one−out sensitivity analysis for
'LRP11' on 'Cardiac valvular disease'

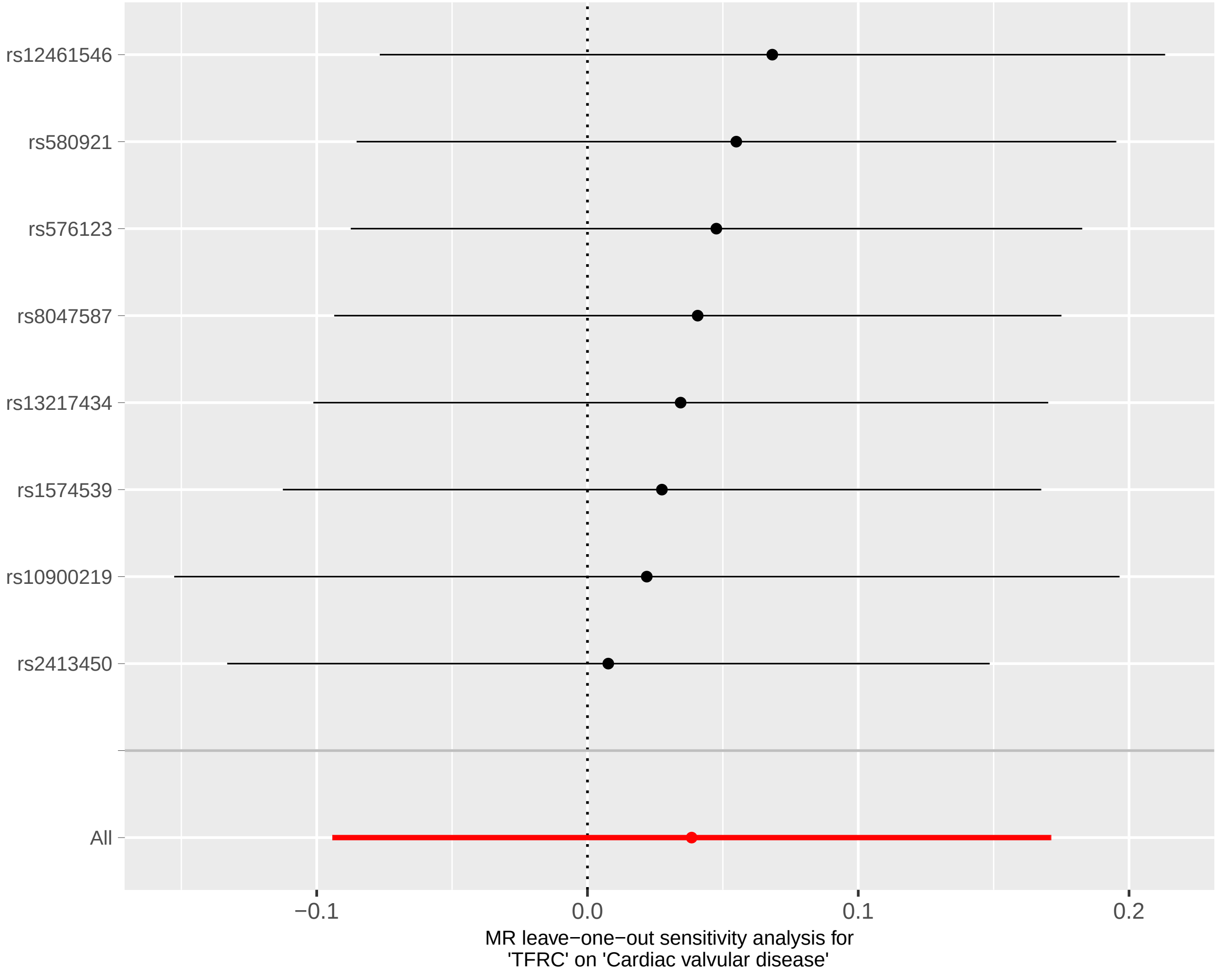
rs12461546
rs580921
rs576123
rs8047587
rs13217434
rs1574539
rs10900219
rs2413450
All
−0.1
0.0
0.1
0.2
MR leave−one−out sensitivity analysis for
'TFRC' on 'Cardiac valvular disease'

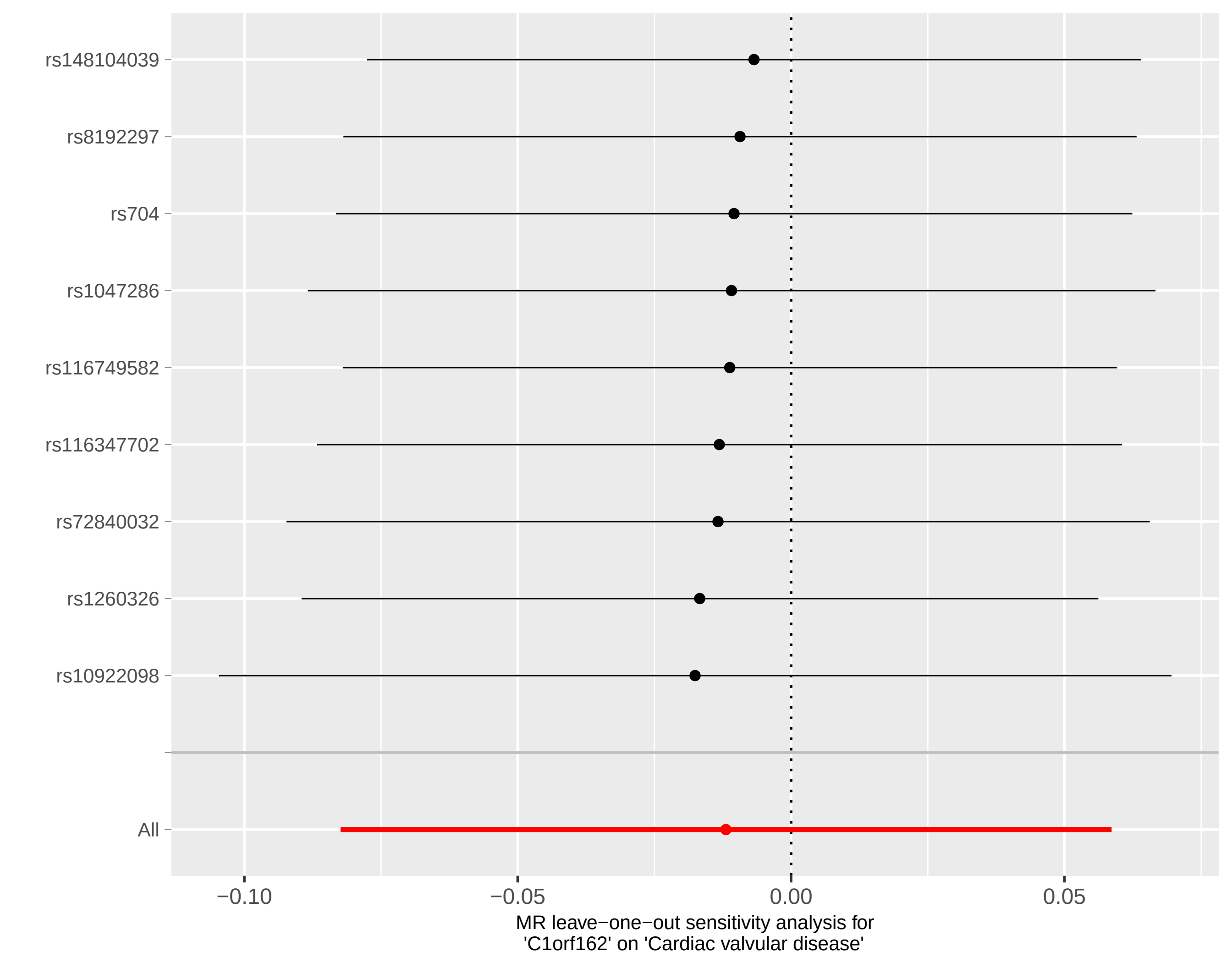

rs148104039
rs8192297
rs704
rs1047286
rs116749582
rs116347702
rs72840032
rs1260326
rs10922098
All
−0.10
−0.05
0.00
0.05
MR leave−one−out sensitivity analysis for
'C1orf162' on 'Cardiac valvular disease'

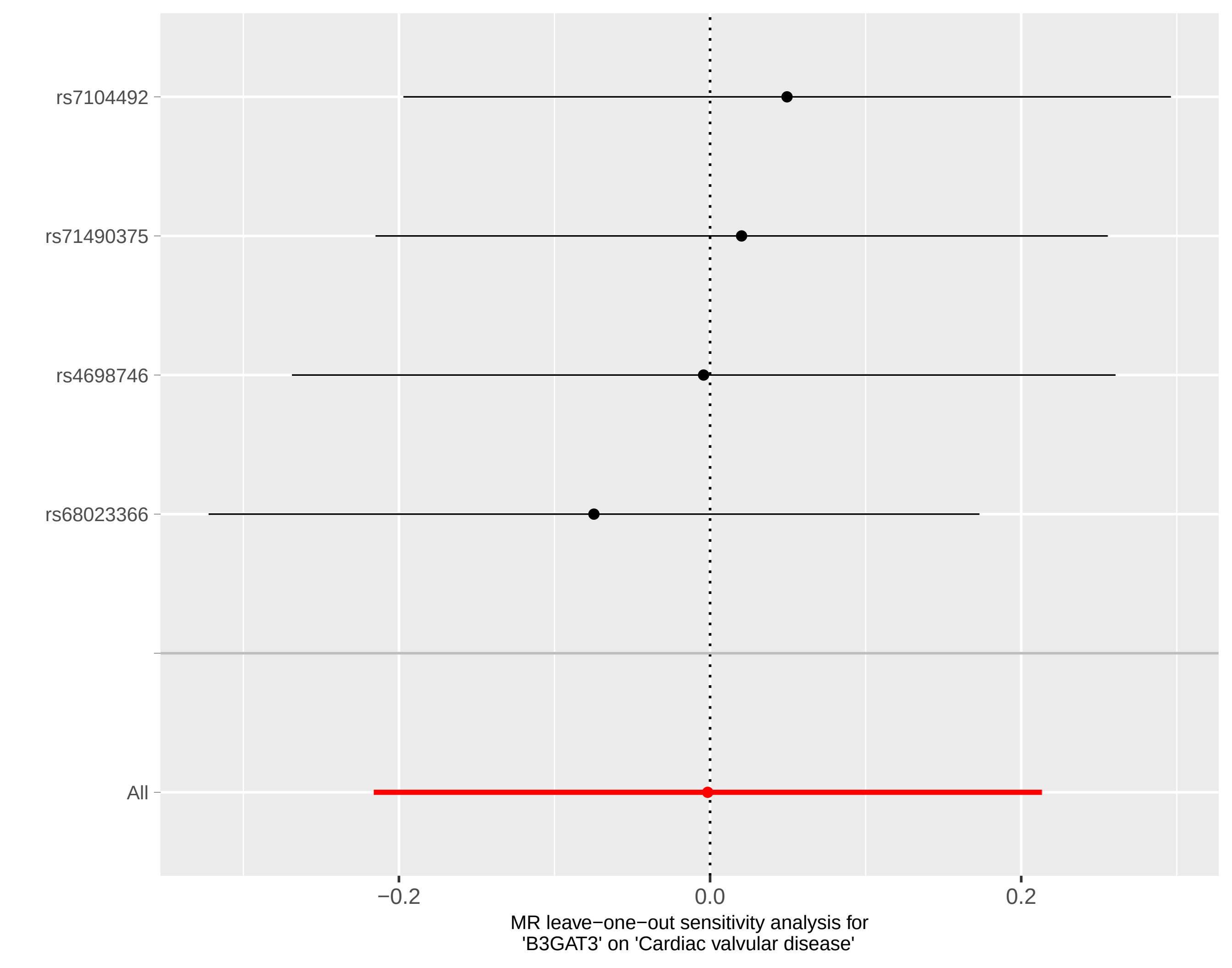
rs7104492
rs71490375
rs4698746
rs68023366
All
−0.2
0.0
0.2
MR leave−one−out sensitivity analysis for
'B3GAT3' on 'Cardiac valvular disease'

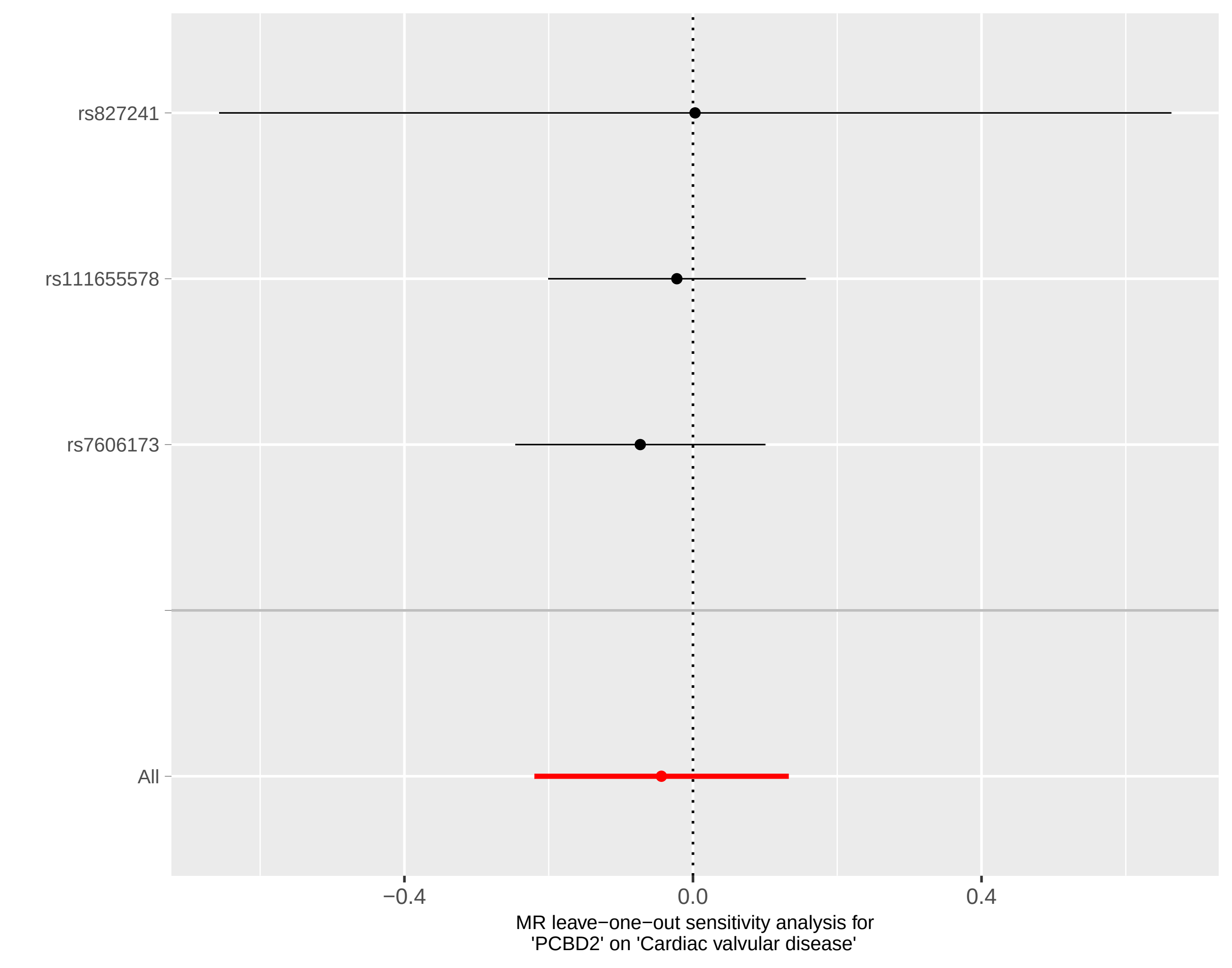

rs827241
rs111655578
rs7606173
All
−0.4
0.0
0.4
MR leave−one−out sensitivity analysis for
'PCBD2' on 'Cardiac valvular disease'

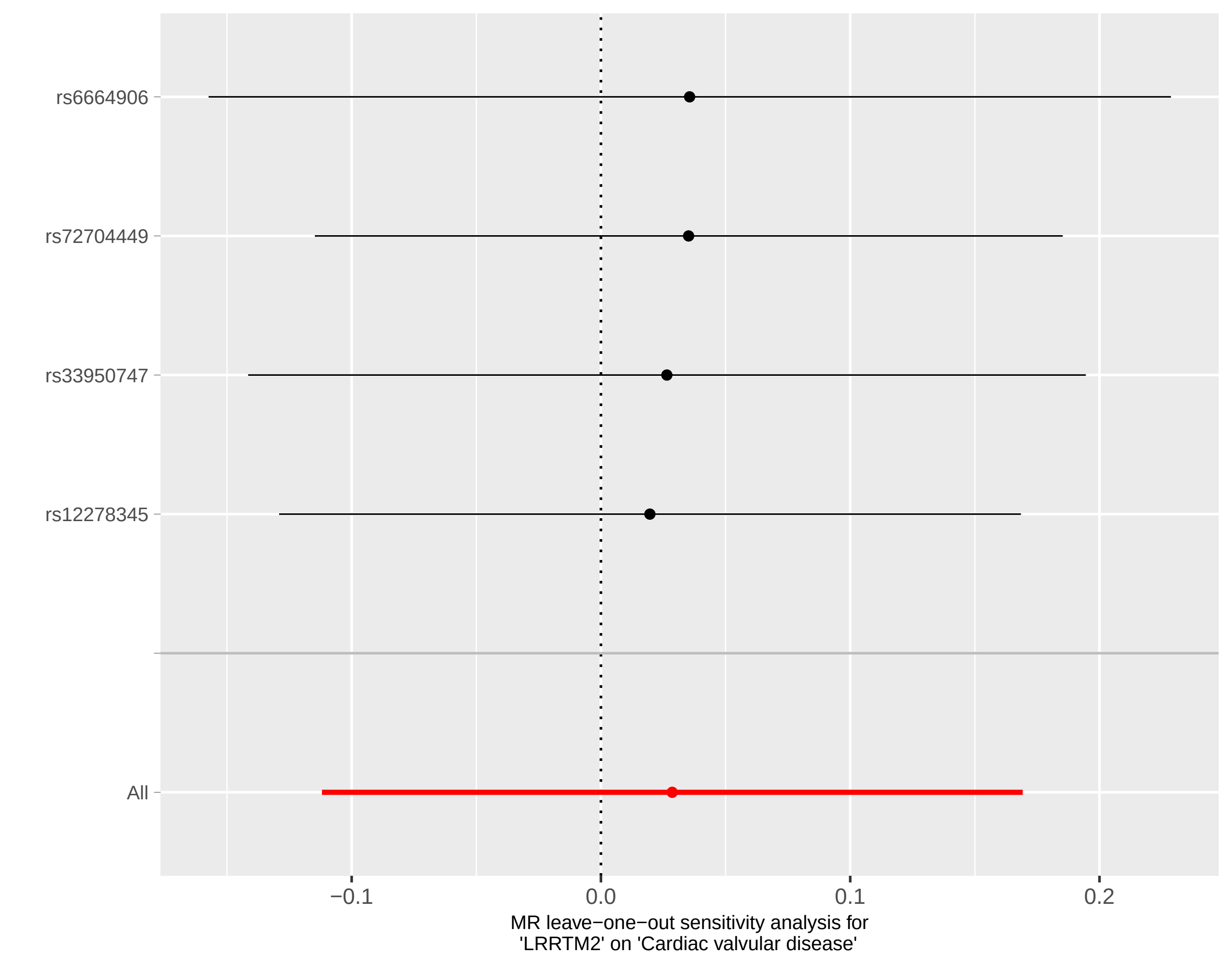
rs6664906
rs72704449
rs33950747
rs12278345
All
−0.1
0.0
0.1
0.2
MR leave−one−out sensitivity analysis for
'LRRTM2' on 'Cardiac valvular disease'

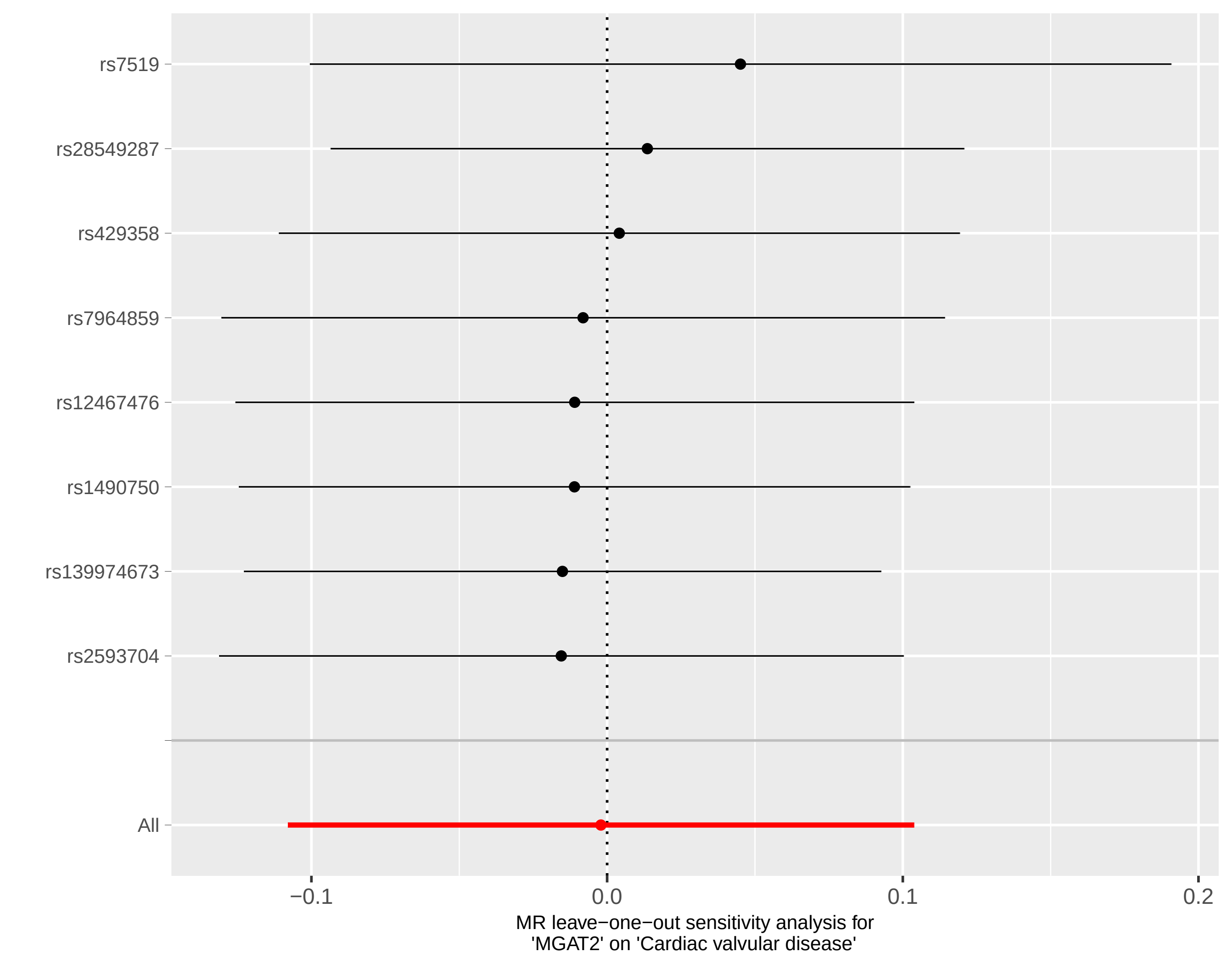
rs7519
rs28549287
rs429358
rs7964859
rs12467476
rs1490750
rs139974673
rs2593704
All
−0.1
0.0
0.1
0.2
MR leave−one−out sensitivity analysis for
'MGAT2' on 'Cardiac valvular disease'

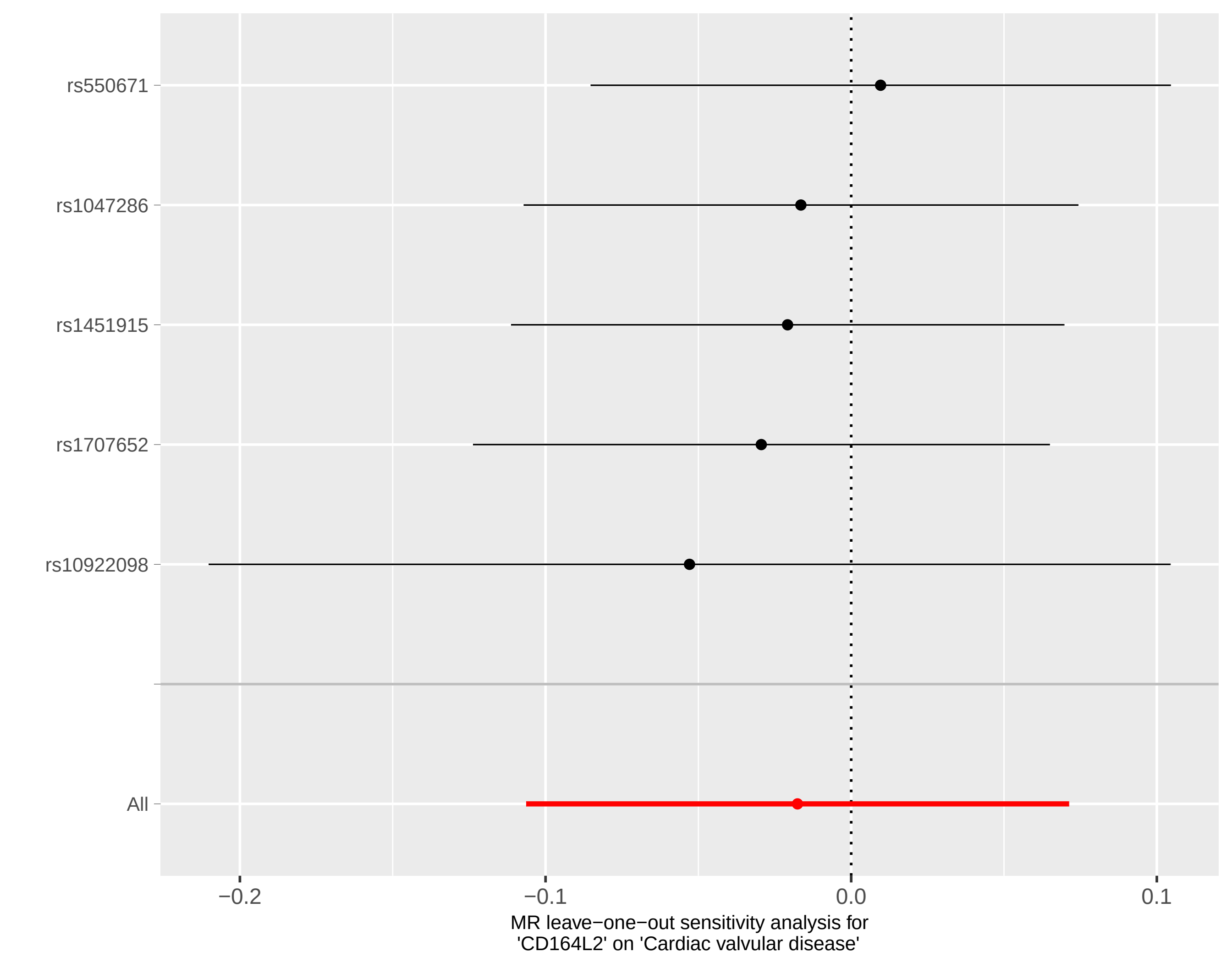
rs550671
rs1047286
rs1451915
rs1707652
rs10922098
All
−0.2
−0.1
0.0
0.1
MR leave−one−out sensitivity analysis for
'CD164L2' on 'Cardiac valvular disease'

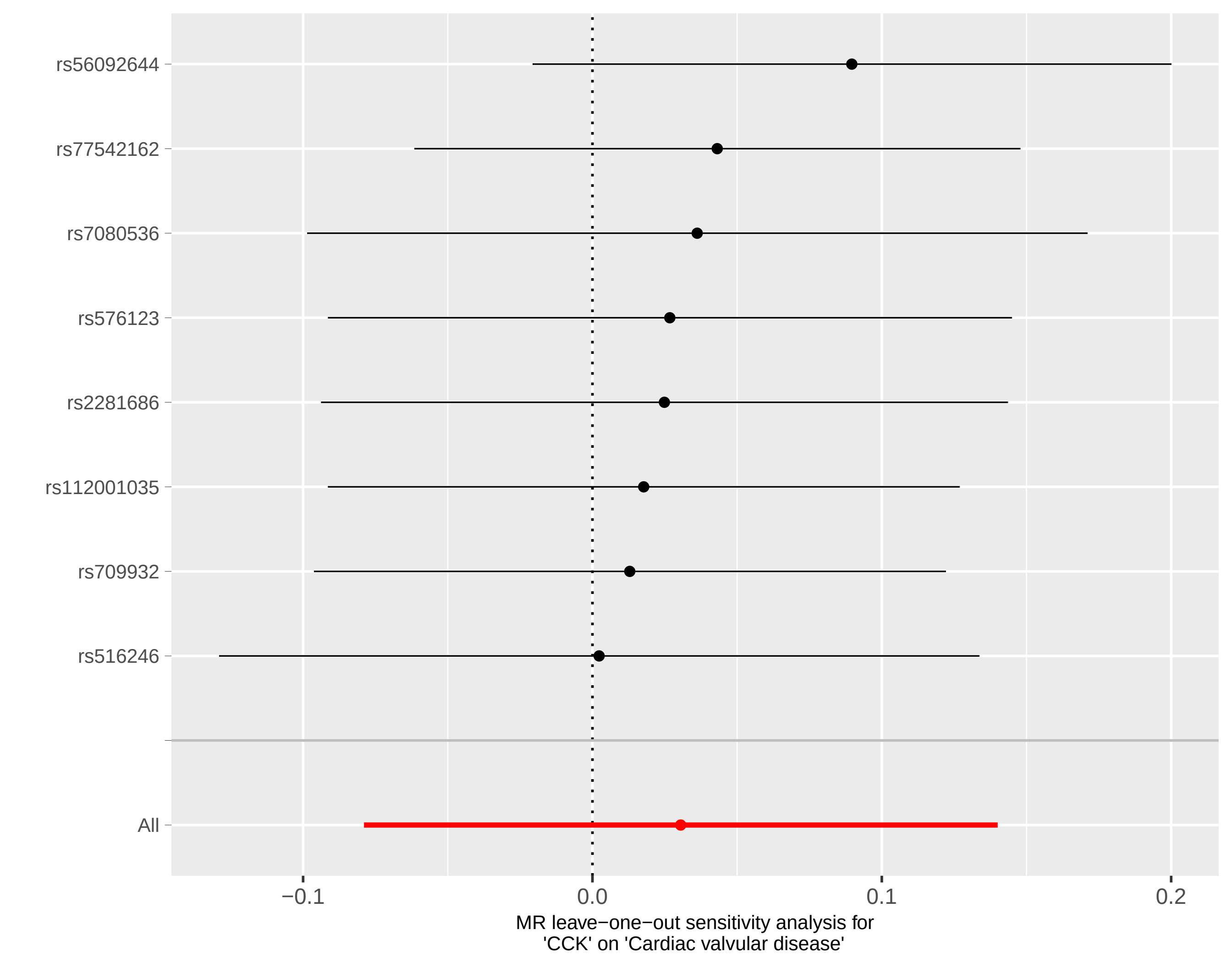
rs56092644
rs77542162
rs7080536
rs576123
rs2281686
rs112001035
rs709932
rs516246
All
−0.1
0.0
0.1
0.2
MR leave−one−out sensitivity analysis for
'CCK' on 'Cardiac valvular disease'

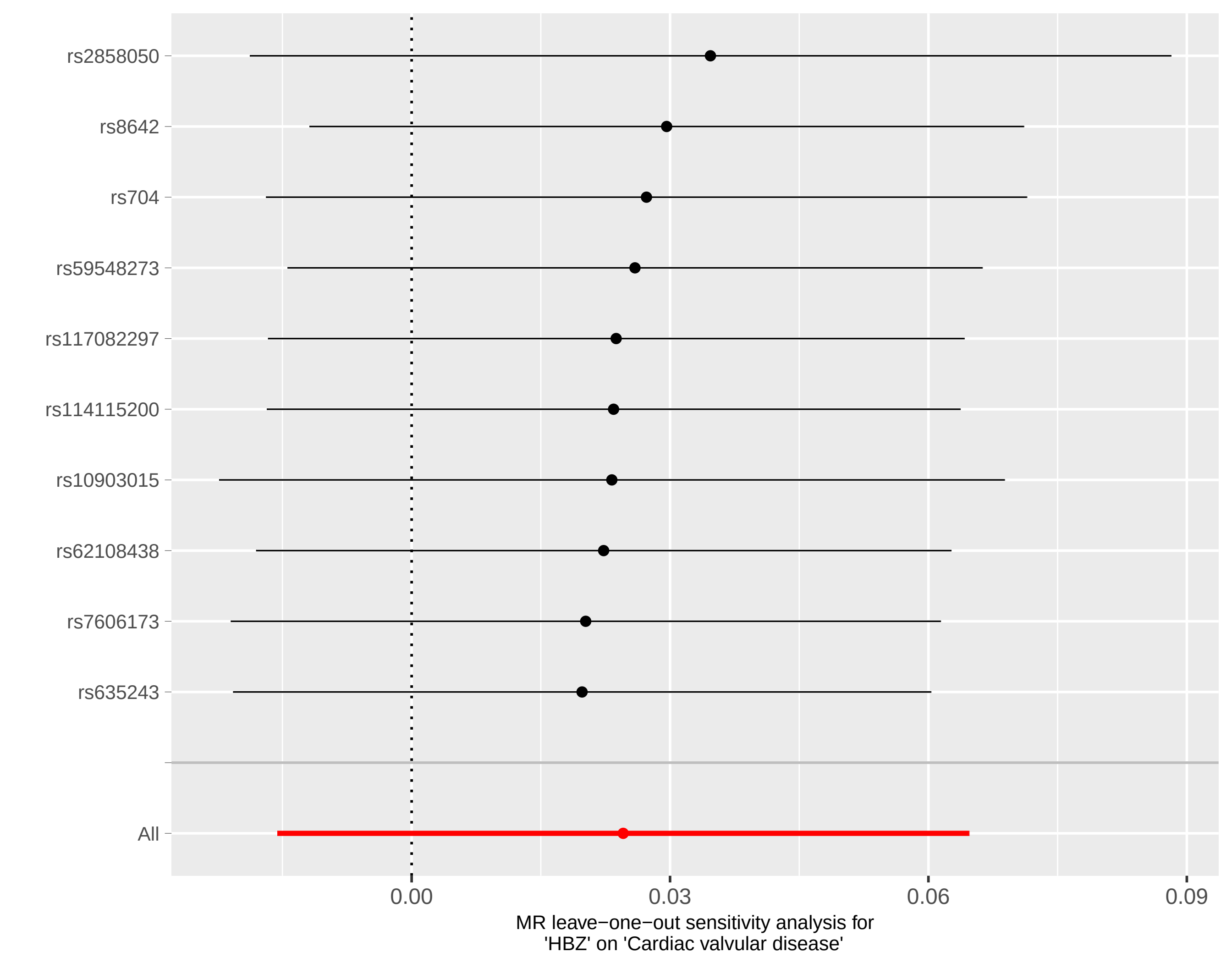
rs2858050
rs8642
rs704
rs59548273
rs117082297
rs114115200
rs10903015
rs62108438
rs7606173
rs635243
All
0.00
0.03
0.06
0.09
MR leave−one−out sensitivity analysis for
'HBZ' on 'Cardiac valvular disease'

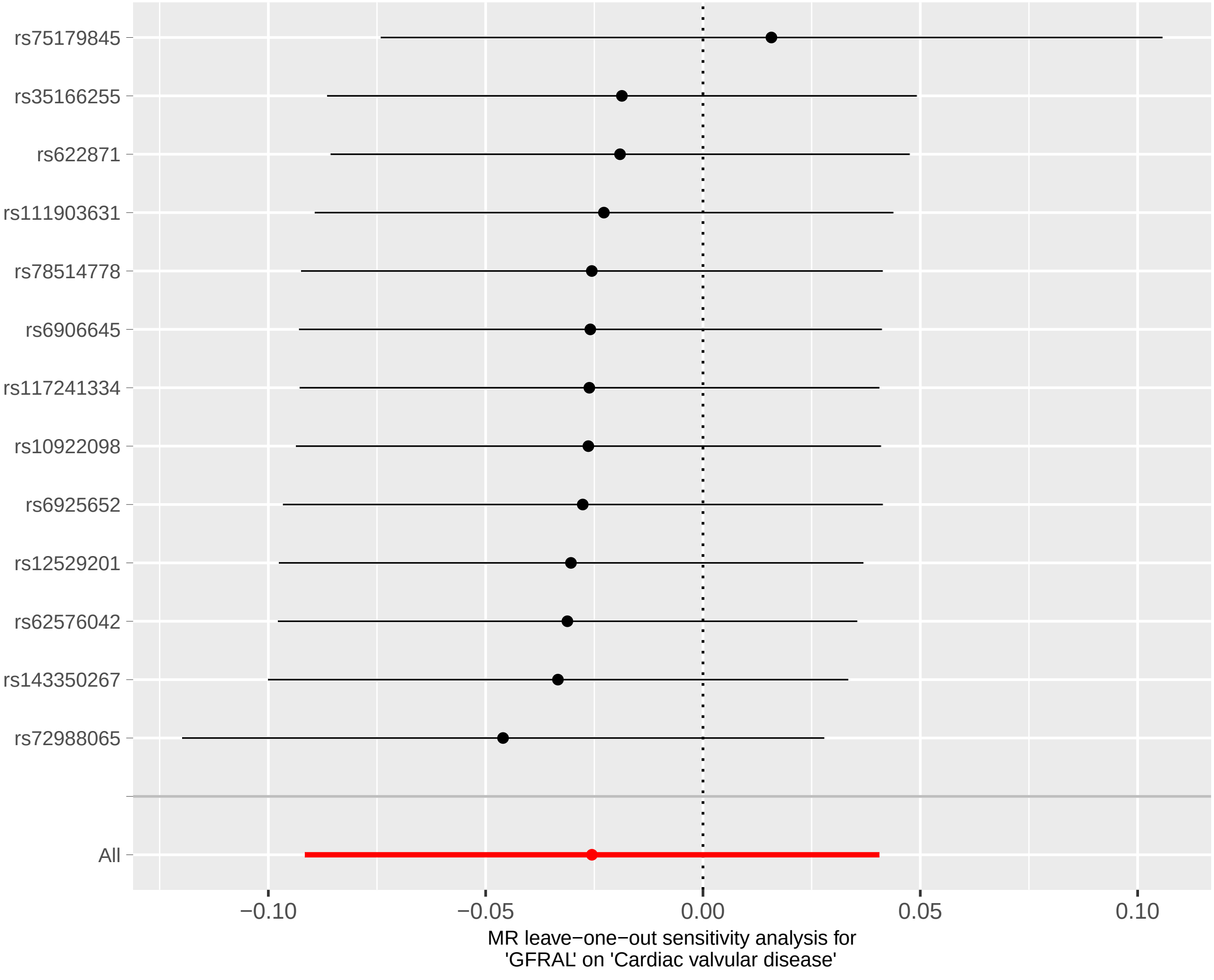

rs75179845
rs35166255
rs622871
rs111903631
rs78514778
rs6906645
rs117241334
rs10922098
rs6925652
rs12529201
rs62576042
rs143350267
rs72988065
All
−0.10
−0.05
0.00
0.05
0.10
MR leave−one−out sensitivity analysis for
'GFRAL' on 'Cardiac valvular disease'

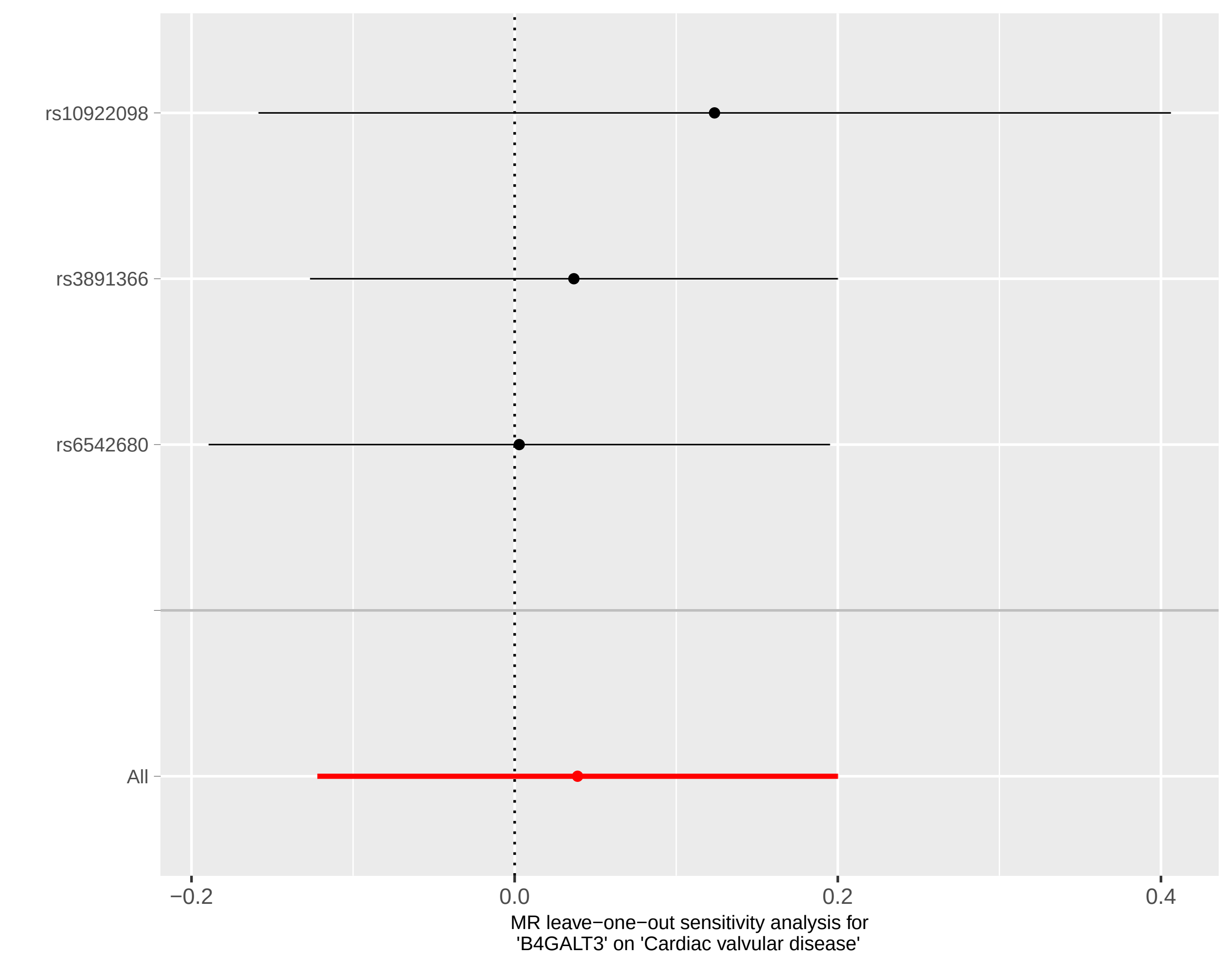
rs10922098
rs3891366
rs6542680
All
−0.2
0.0
0.2
0.4
MR leave−one−out sensitivity analysis for
'B4GALT3' on 'Cardiac valvular disease'

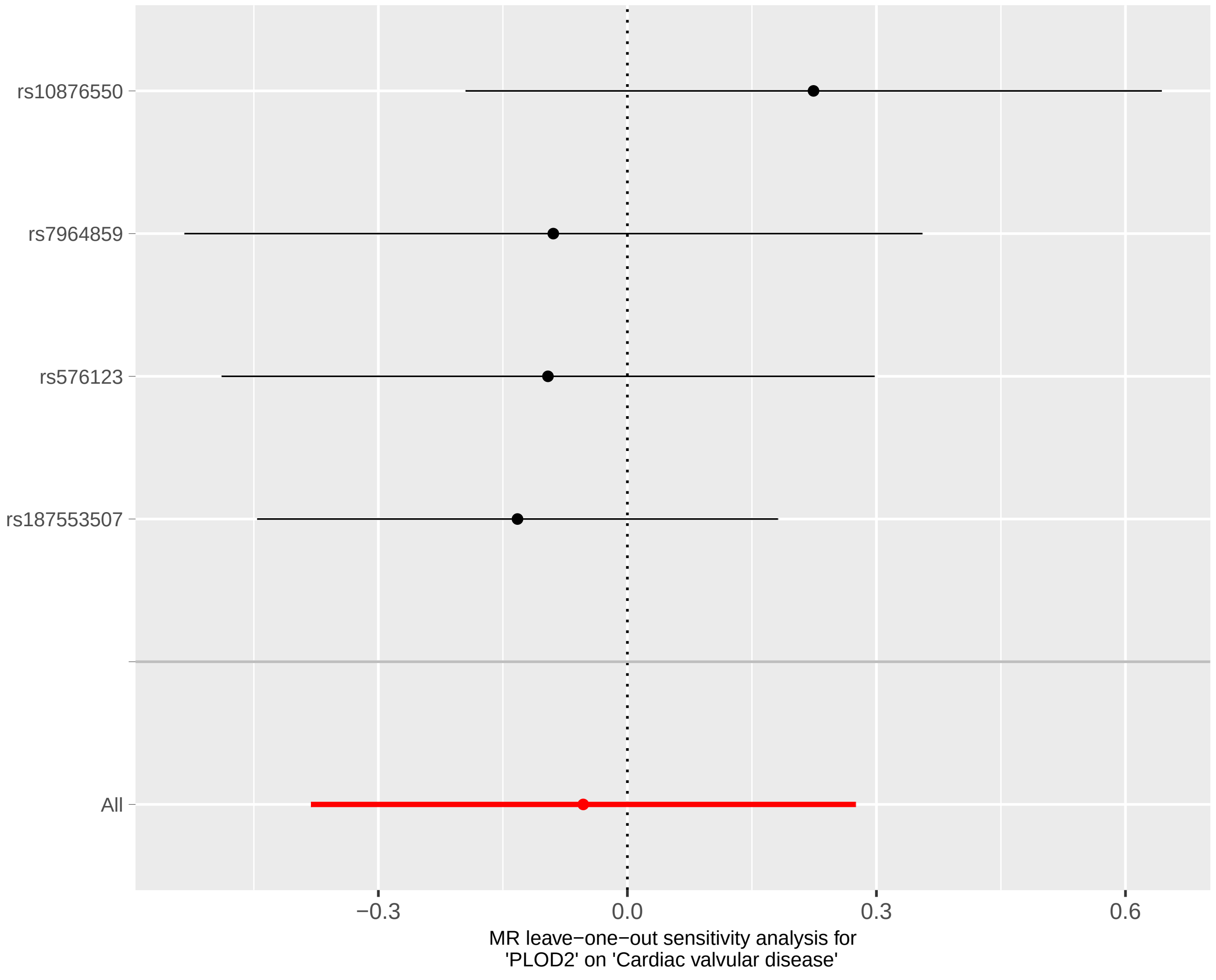
rs10876550
rs7964859
rs576123
rs187553507
All
−0.3
0.0
0.3
0.6
MR leave−one−out sensitivity analysis for
'PLOD2' on 'Cardiac valvular disease'

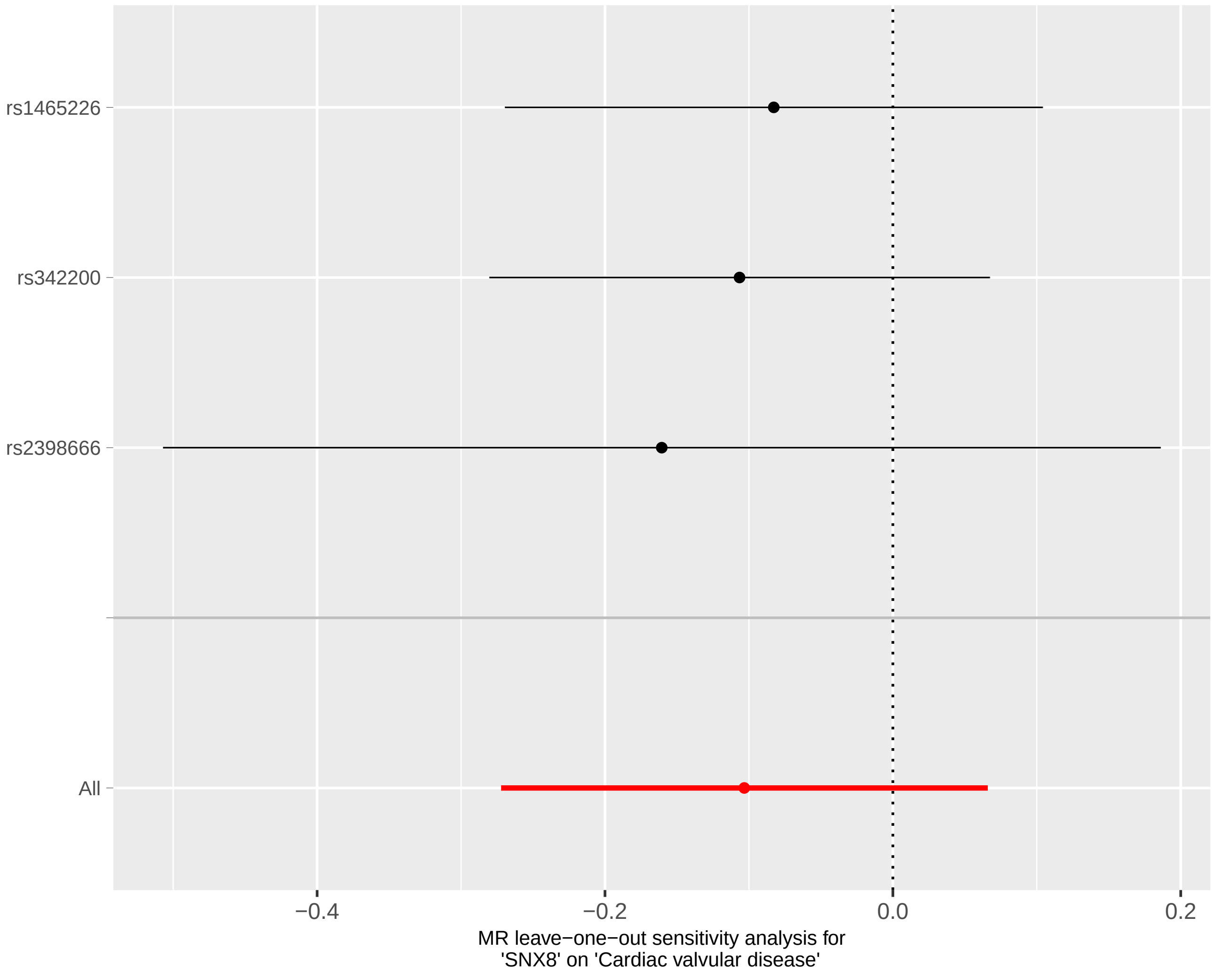
rs1465226
rs342200
rs2398666
All
−0.4
−0.2
0.0
0.2
MR leave−one−out sensitivity analysis for
'SNX8' on 'Cardiac valvular disease'

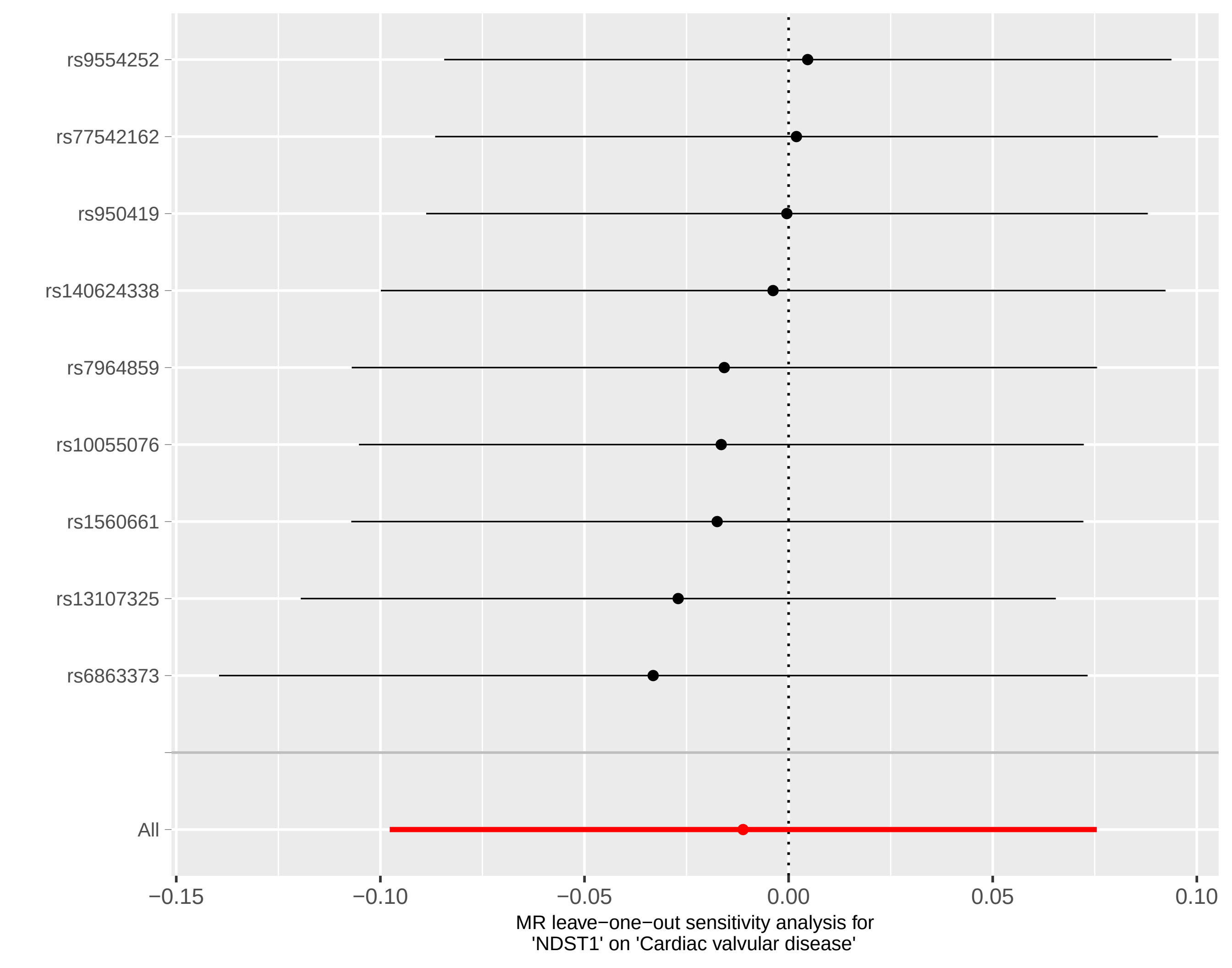
rs9554252
rs77542162
rs950419
rs140624338
rs7964859
rs10055076
rs1560661
rs13107325
rs6863373
All
−0.15
−0.10
−0.05
0.00
0.05
0.10
MR leave−one−out sensitivity analysis for
'NDST1' on 'Cardiac valvular disease'

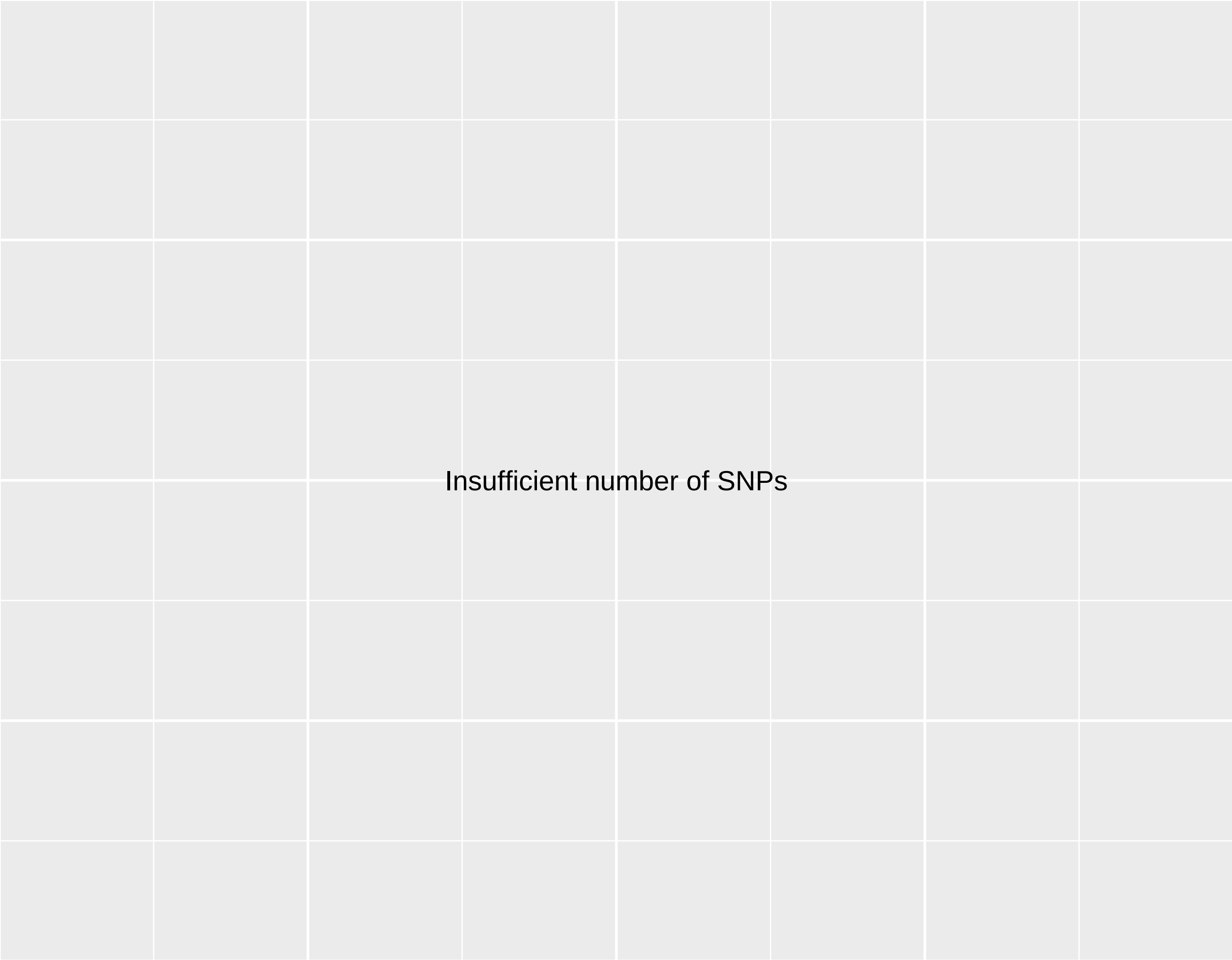
Insufficient number of SNPs

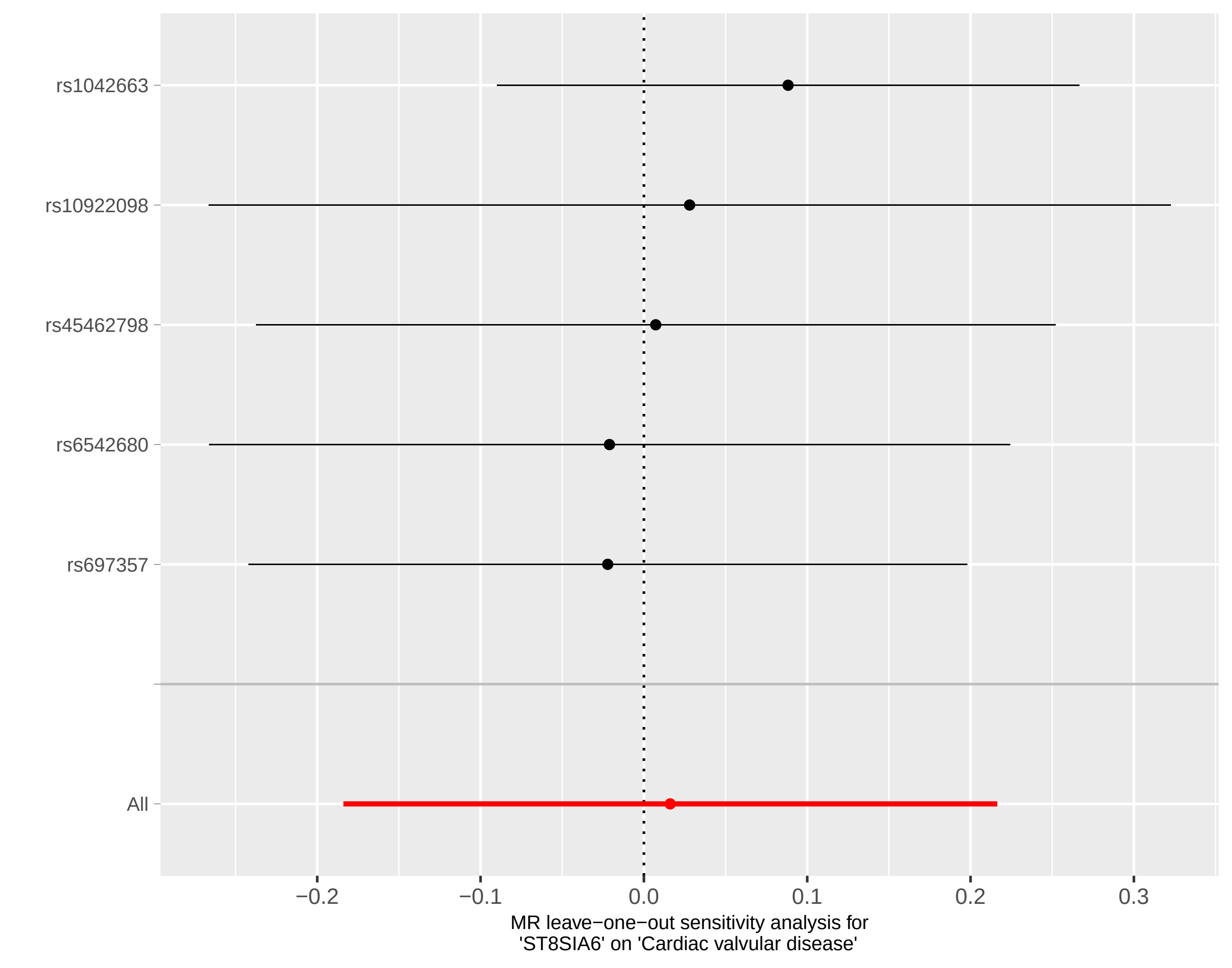
rs1042663
rs10922098
rs45462798
rs6542680
rs697357
All
−0.2
−0.1
0.0
0.1
0.2
0.3
MR leave−one−out sensitivity analysis for
'ST8SIA6' on 'Cardiac valvular disease'

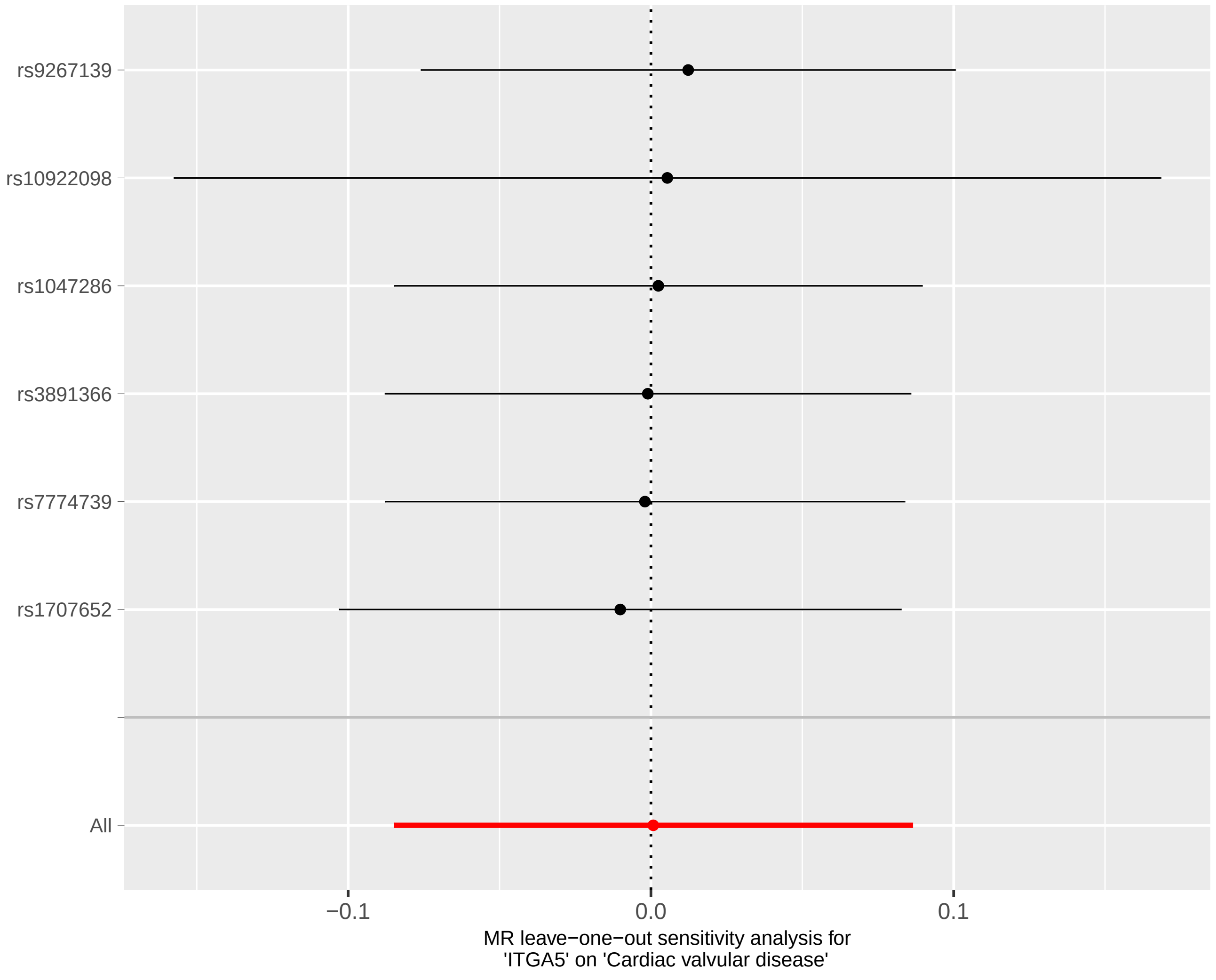

rs9267139
rs10922098
rs1047286
rs3891366
rs7774739
rs1707652
All
−0.1
0.0
0.1
MR leave−one−out sensitivity analysis for
'ITGA5' on 'Cardiac valvular disease'

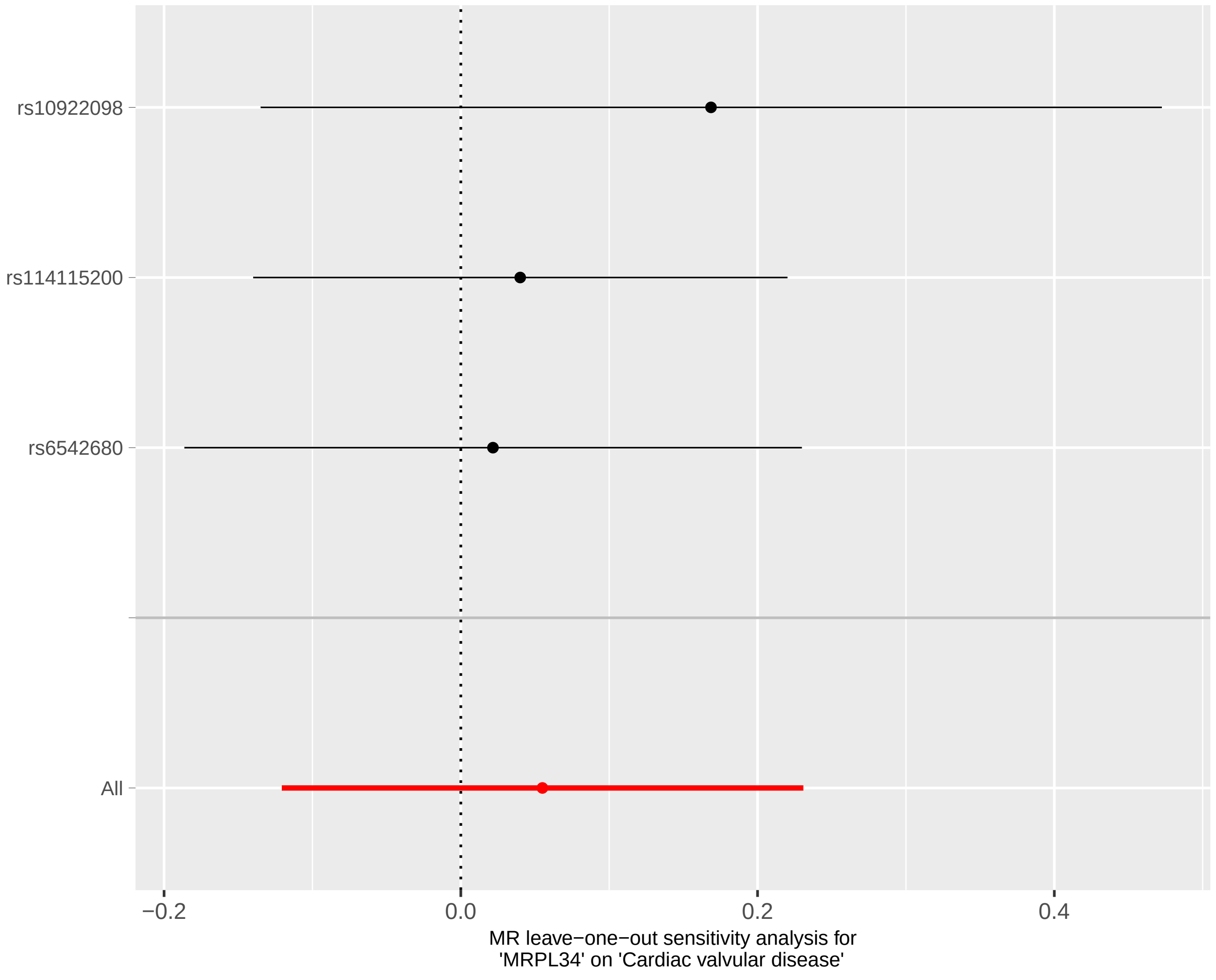

rs10922098
rs114115200
rs6542680
All
−0.2
0.0
0.2
0.4
MR leave−one−out sensitivity analysis for
'MRPL34' on 'Cardiac valvular disease'

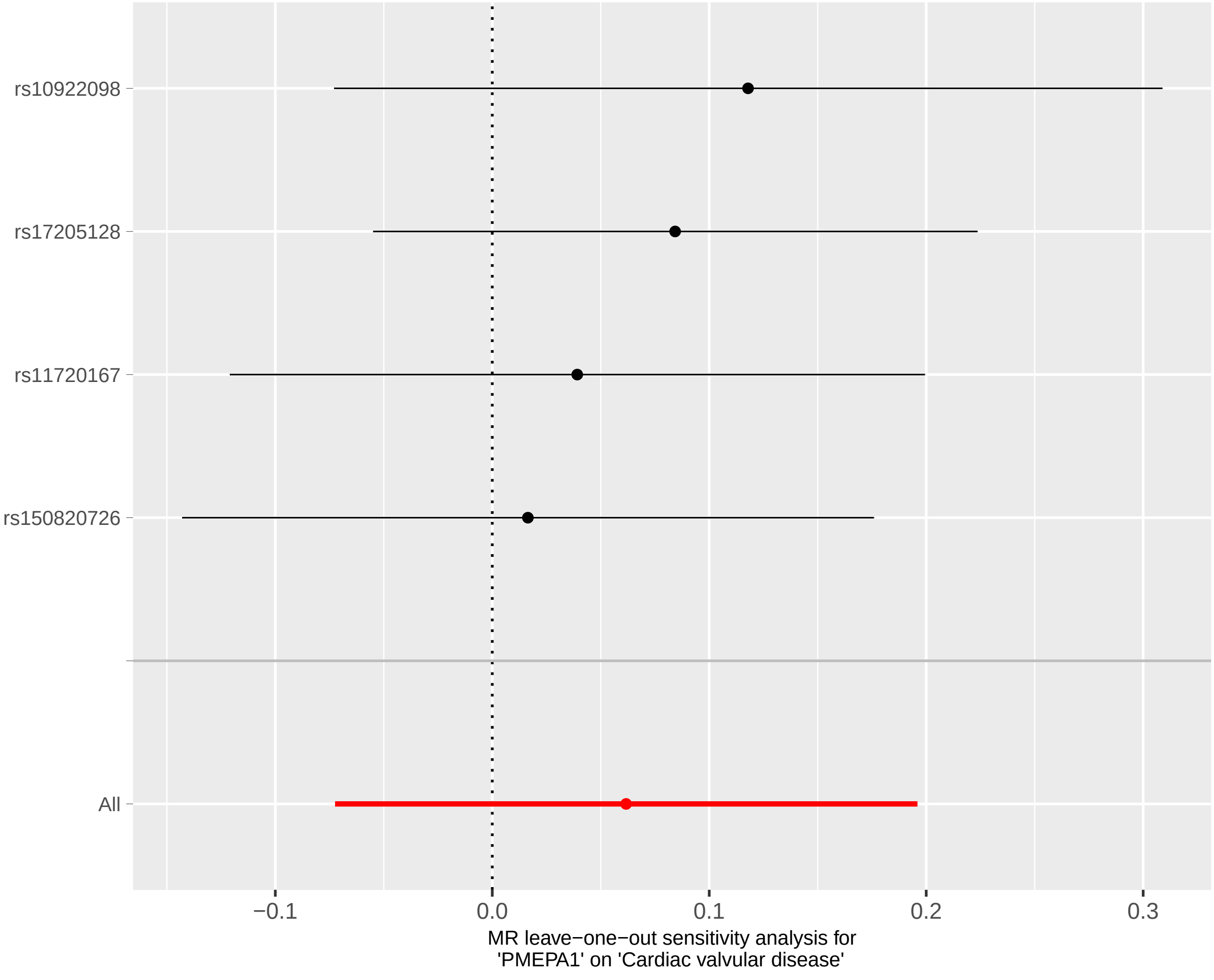
rs10922098
rs17205128
rs11720167
rs150820726
All
−0.1
0.0
0.1
0.2
0.3
MR leave−one−out sensitivity analysis for
'PMEPA1' on 'Cardiac valvular disease'

Insufficient number of SNPs

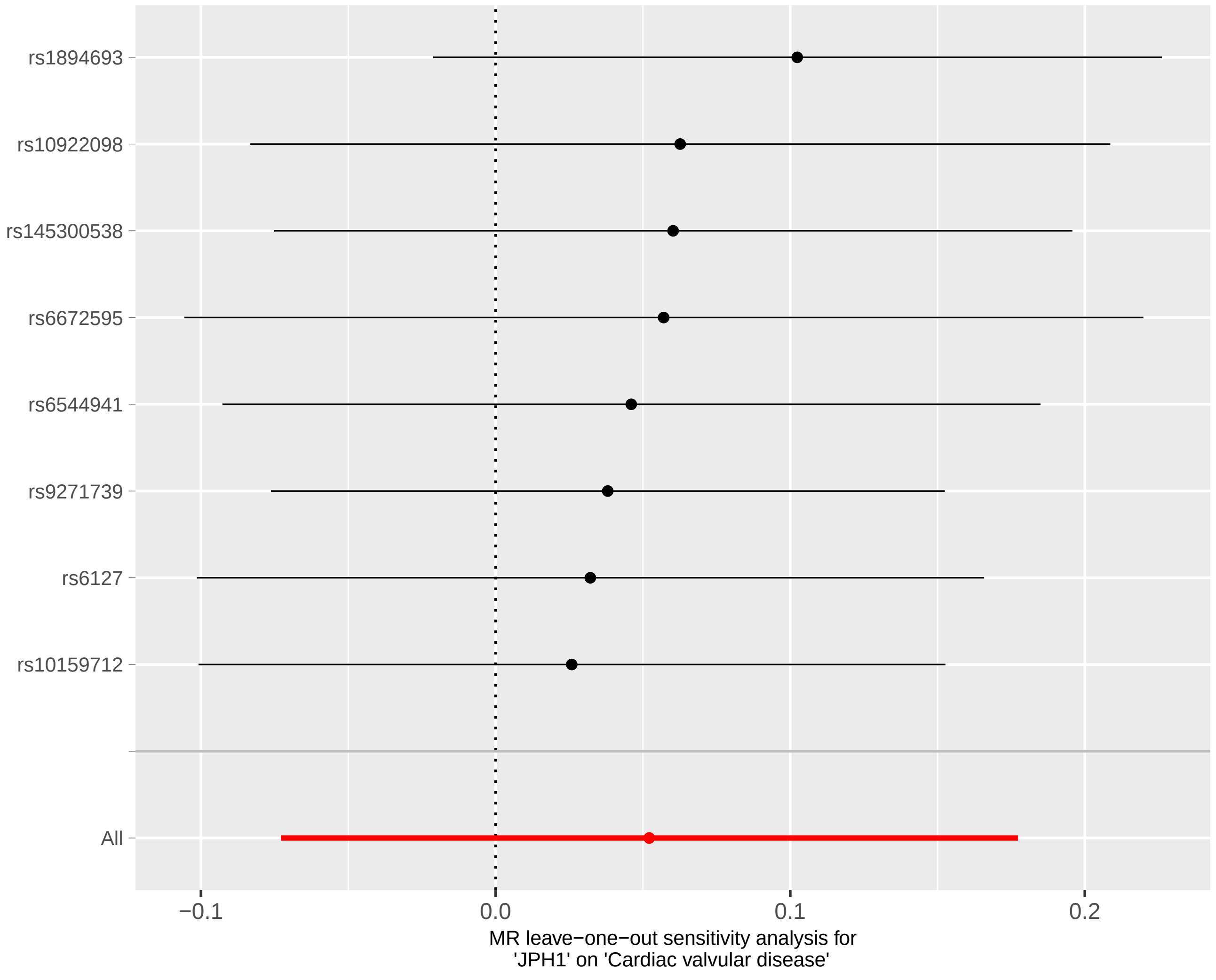
rs1894693
rs10922098
rs145300538
rs6672595
rs6544941
rs9271739
rs6127
rs10159712
All
−0.1
0.0
0.1
0.2
MR leave−one−out sensitivity analysis for
'JPH1' on 'Cardiac valvular disease'

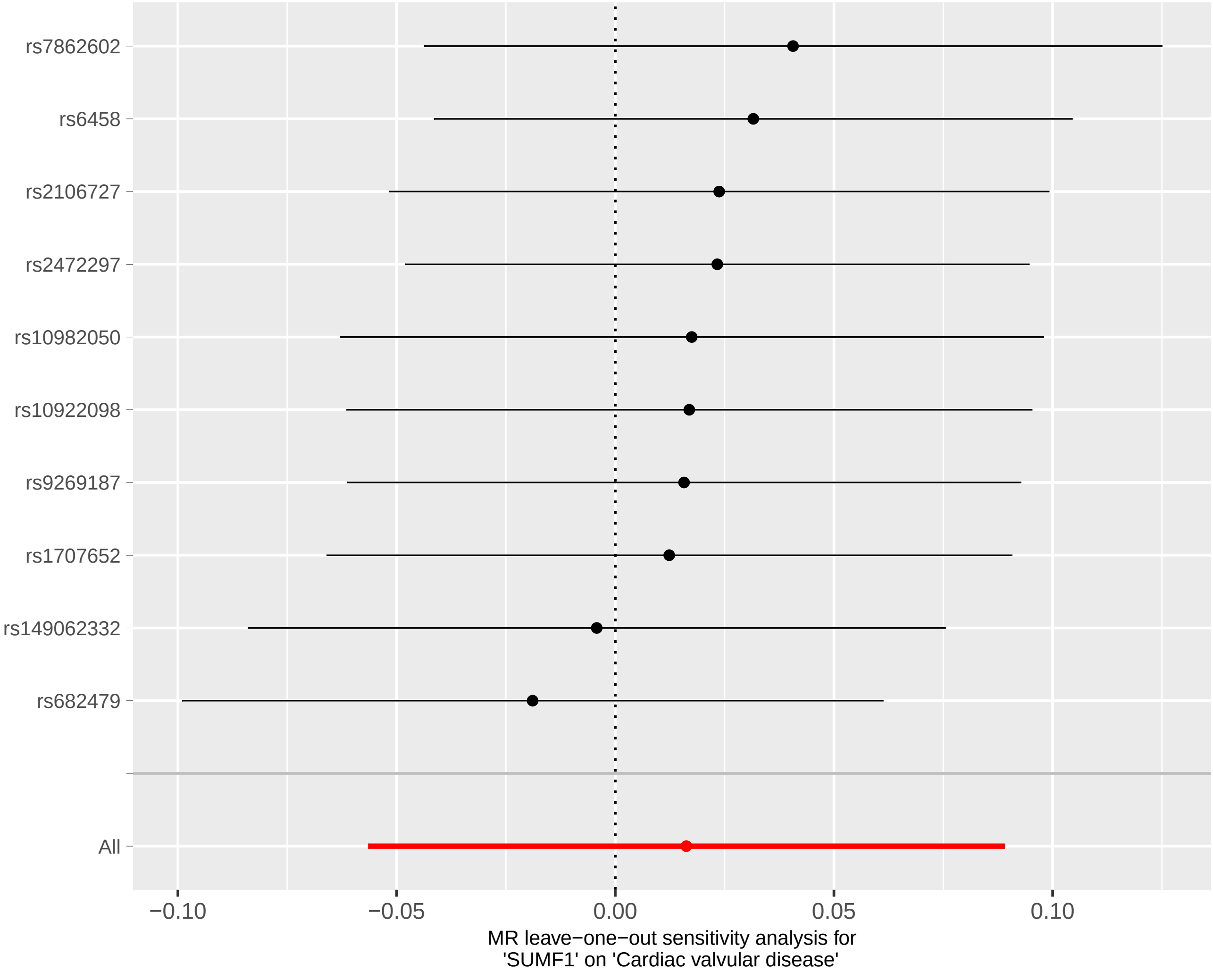
rs7862602
rs6458
rs2106727
rs2472297
rs10982050
rs10922098
rs9269187
rs1707652
rs149062332
rs682479
All
−0.10
−0.05
0.00
0.05
0.10
MR leave−one−out sensitivity analysis for
'SUMF1' on 'Cardiac valvular disease'

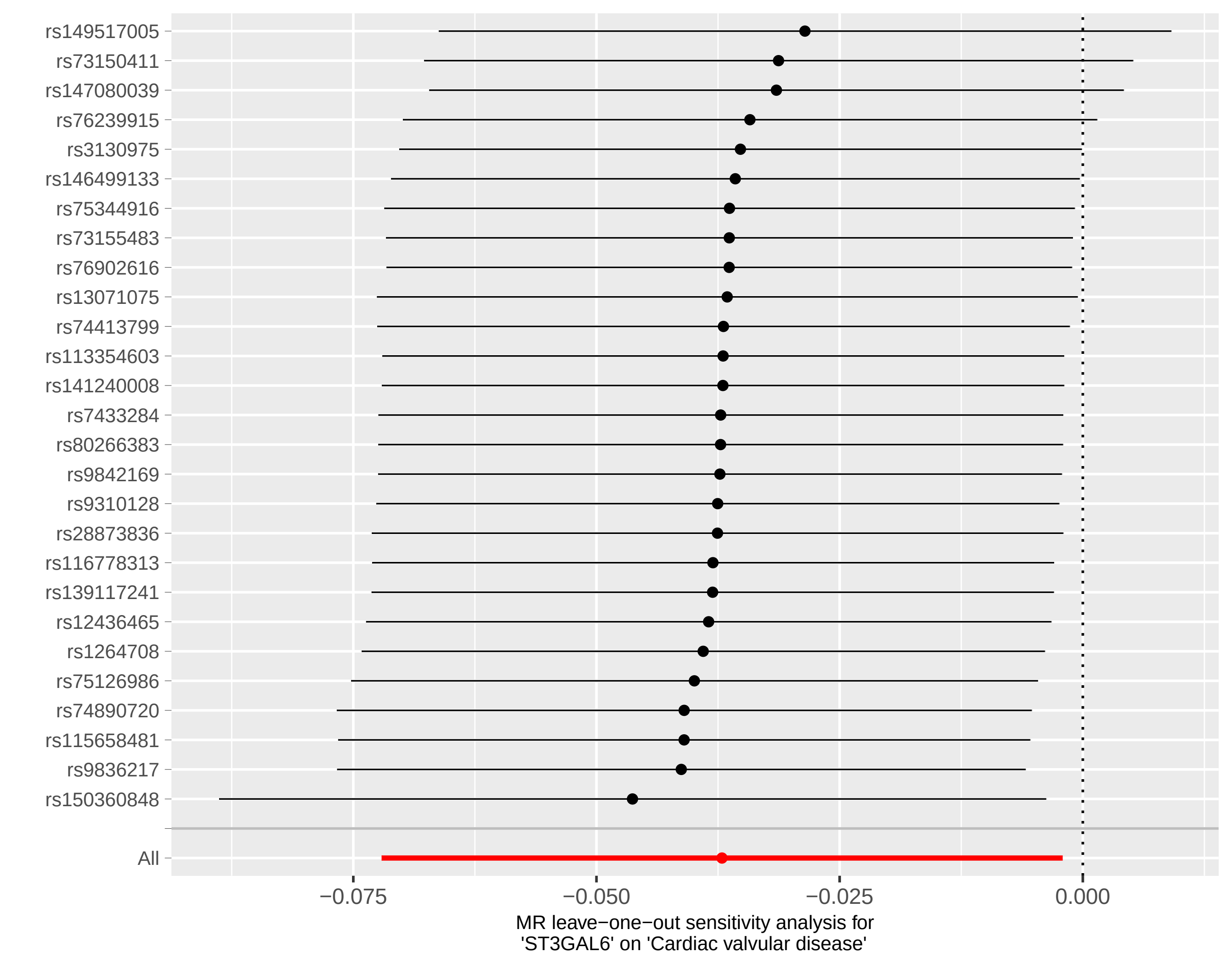

rs149517005
rs73150411
rs147080039
rs76239915
rs3130975
rs146499133
rs75344916
rs73155483
rs76902616
rs13071075
rs74413799
rs113354603
rs141240008
rs7433284
rs80266383
rs9842169
rs9310128
rs28873836
rs116778313
rs139117241
rs12436465
rs1264708
rs75126986
rs74890720
rs115658481
rs9836217
rs150360848
All
−0.075
−0.050
−0.025
0.000
MR leave−one−out sensitivity analysis for
'ST3GAL6' on 'Cardiac valvular disease'

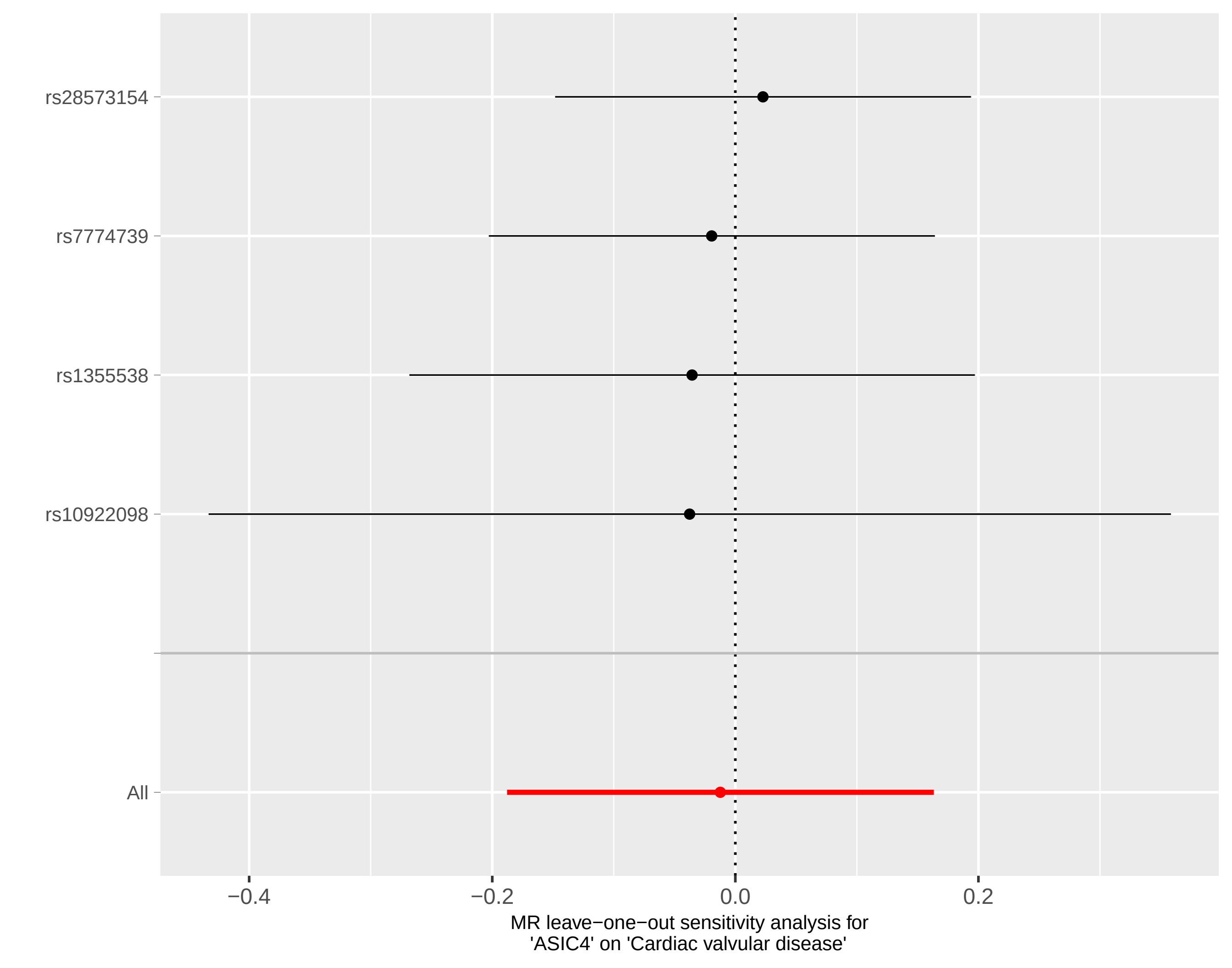

rs28573154
rs7774739
rs1355538
rs10922098
All
−0.4
−0.2
0.0
0.2
MR leave−one−out sensitivity analysis for
'ASIC4' on 'Cardiac valvular disease'

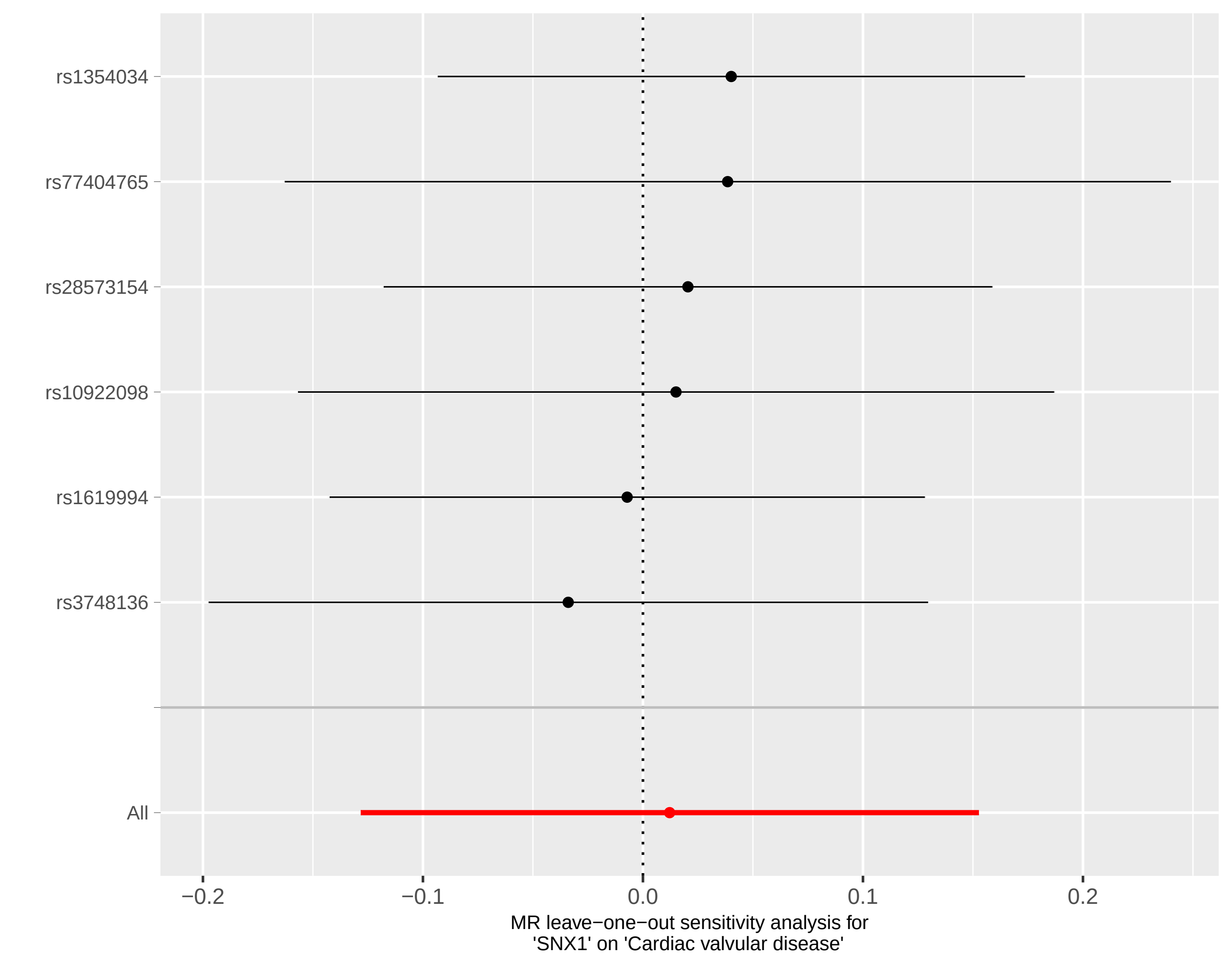

rs1354034
rs77404765
rs28573154
rs10922098
rs1619994
rs3748136
All
−0.2
−0.1
0.0
0.1
0.2
MR leave−one−out sensitivity analysis for
'SNX1' on 'Cardiac valvular disease'

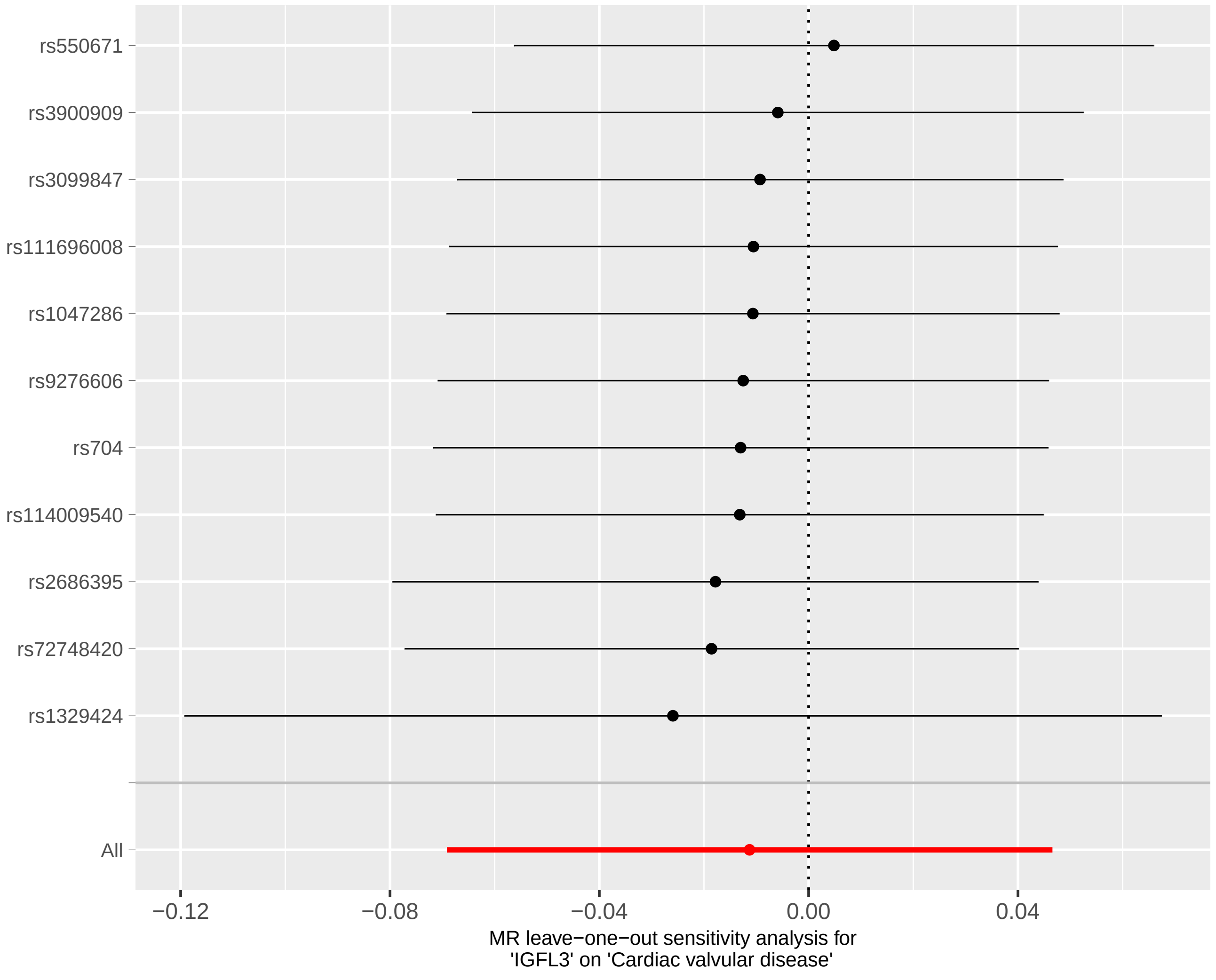

rs550671
rs3900909
rs3099847
rs111696008
rs1047286
rs9276606
rs704
rs114009540
rs2686395
rs72748420
rs1329424
All
−0.12
−0.08
−0.04
0.00
0.04
MR leave−one−out sensitivity analysis for
'IGFL3' on 'Cardiac valvular disease'

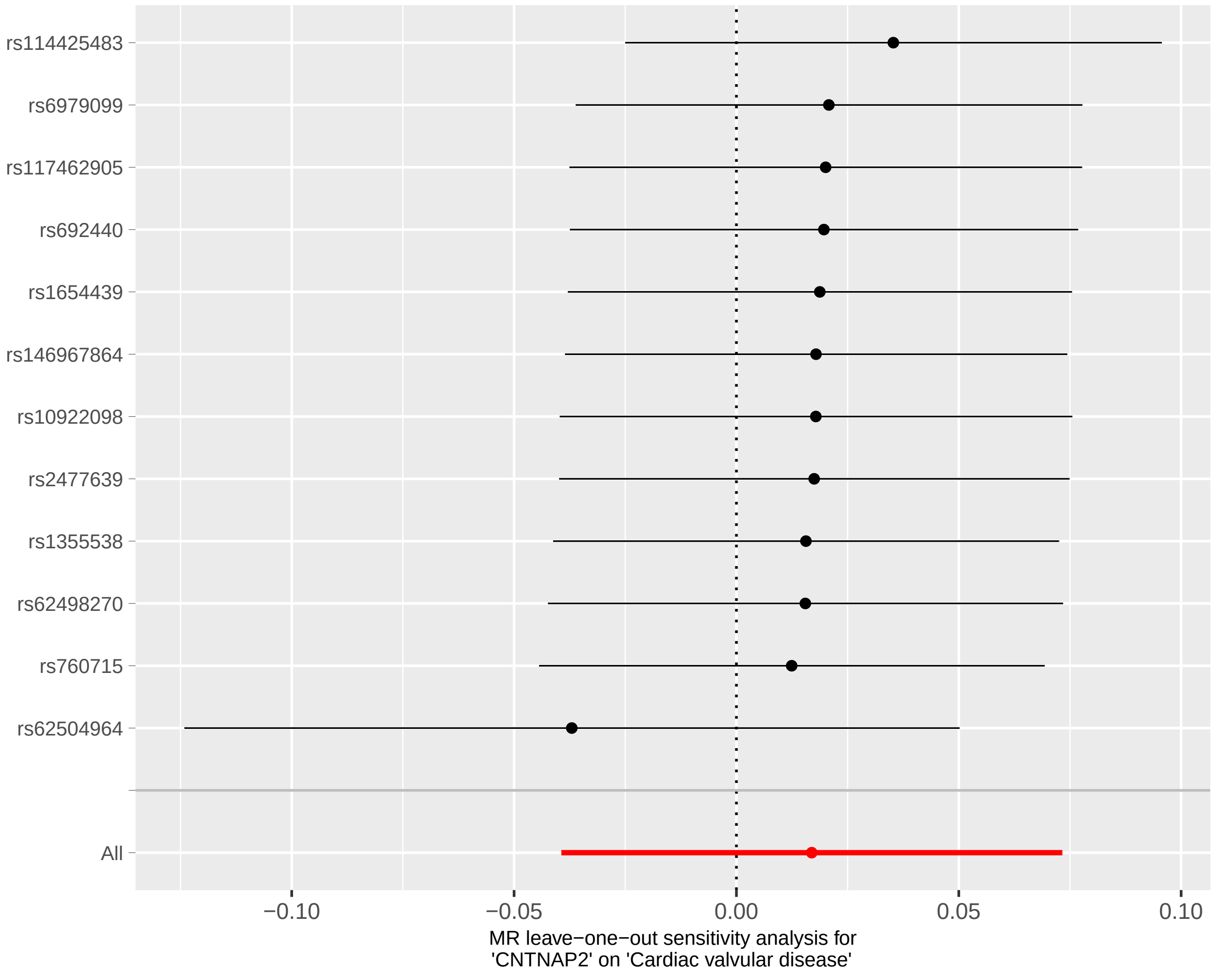

rs114425483
rs6979099
rs117462905
rs692440
rs1654439
rs146967864
rs10922098
rs2477639
rs1355538
rs62498270
rs760715
rs62504964
All
−0.10
−0.05
0.00
0.05
0.10
MR leave−one−out sensitivity analysis for
'CNTNAP2' on 'Cardiac valvular disease'

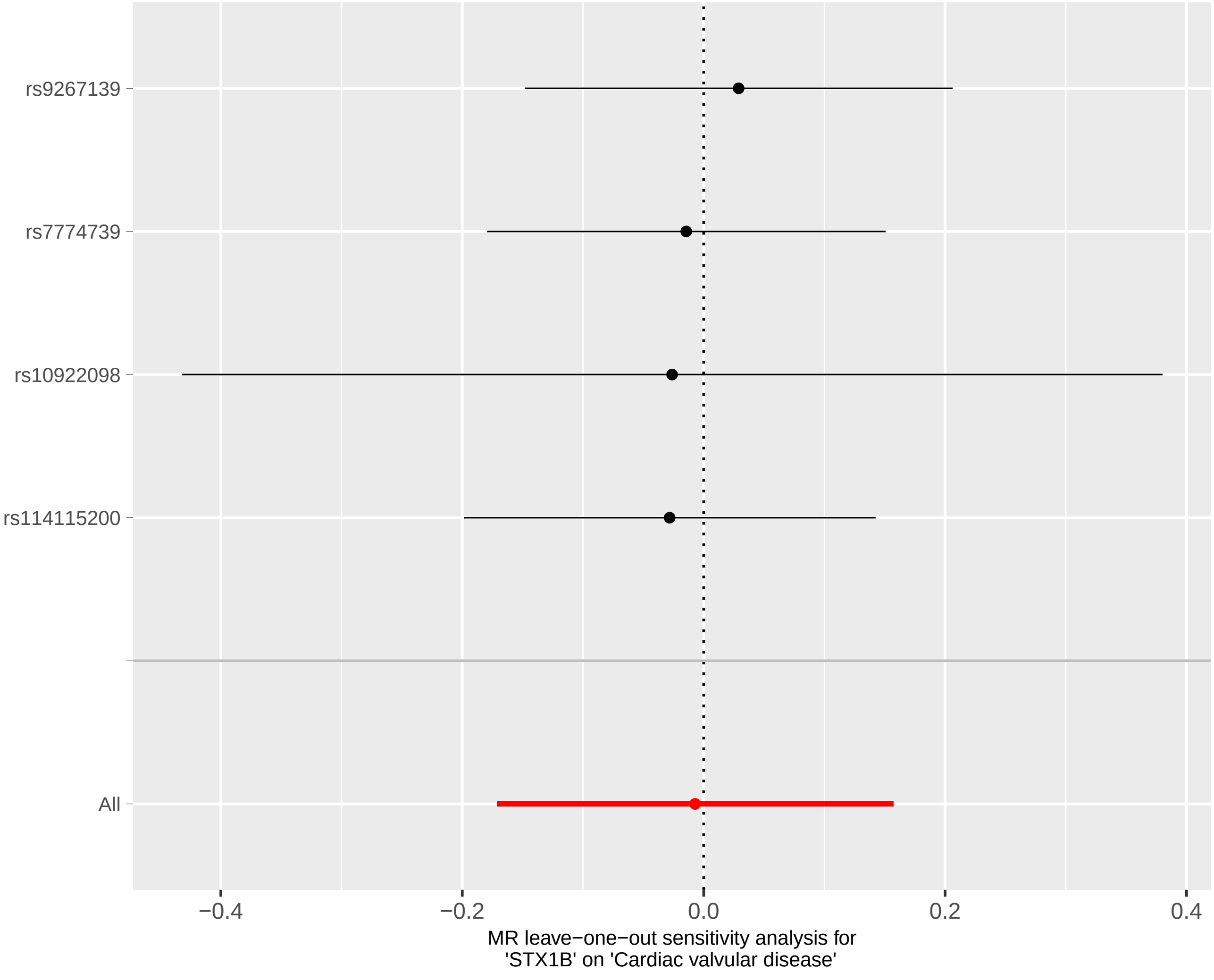

rs9267139
rs7774739
rs10922098
rs114115200
All
−0.4
−0.2
0.0
0.2
0.4
MR leave−one−out sensitivity analysis for
'STX1B' on 'Cardiac valvular disease'

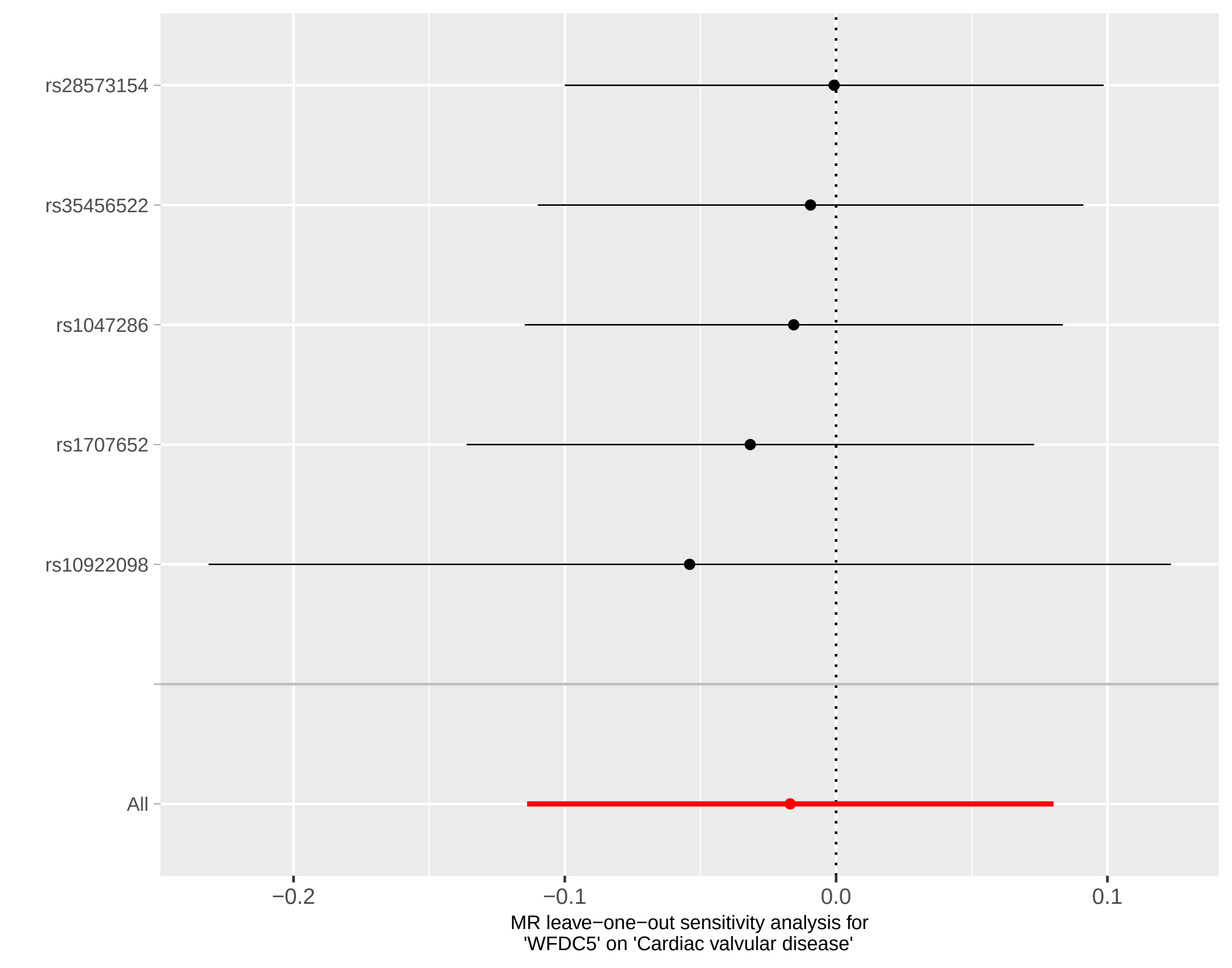

rs28573154
rs35456522
rs1047286
rs1707652
rs10922098
All
−0.2
−0.1
0.0
0.1
MR leave−one−out sensitivity analysis for
'WFDC5' on 'Cardiac valvular disease'

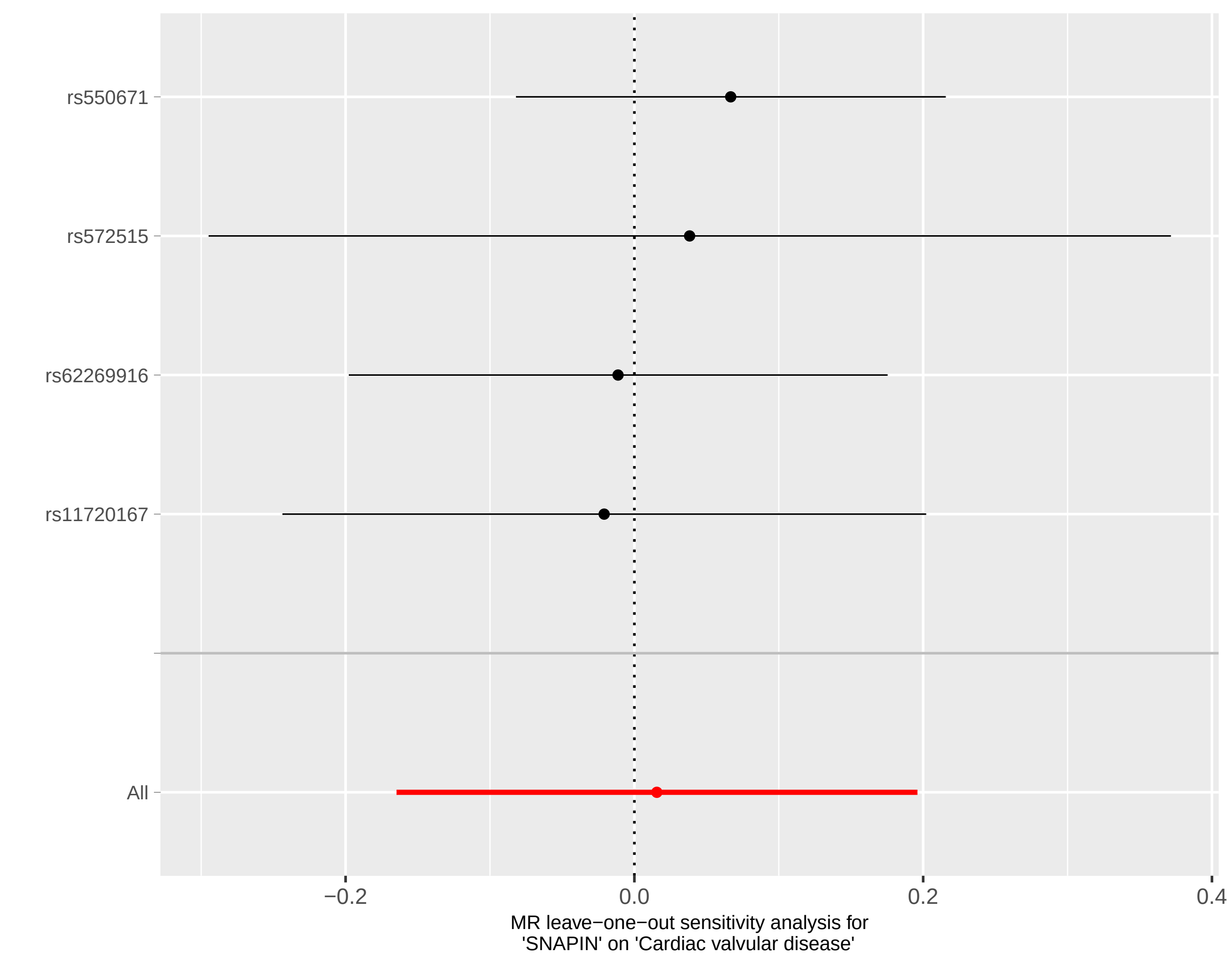

rs550671
rs572515
rs62269916
rs11720167
All
−0.2
0.0
0.2
0.4
MR leave−one−out sensitivity analysis for
'SNAPIN' on 'Cardiac valvular disease'

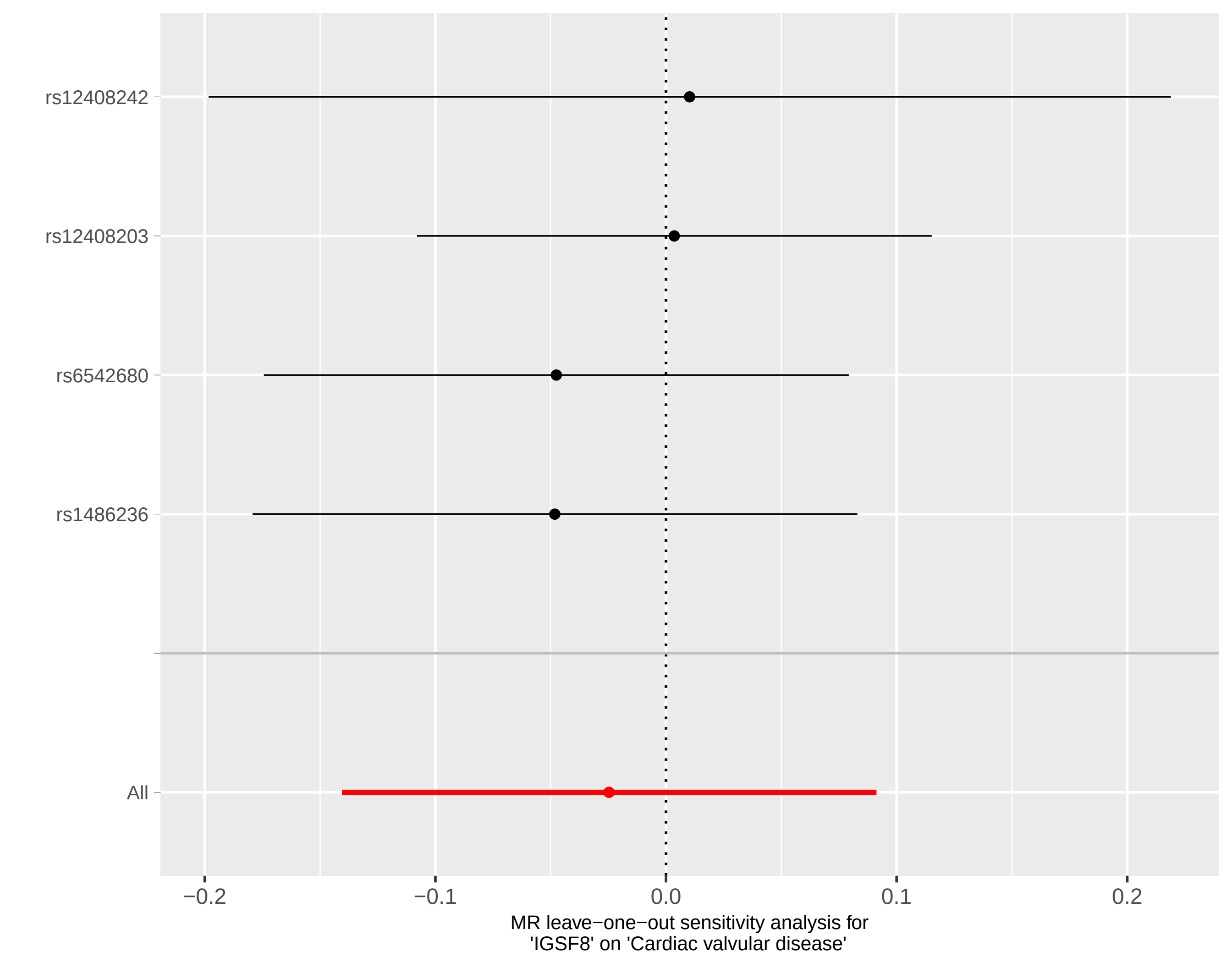

rs12408242
rs12408203
rs6542680
rs1486236
All
−0.2
−0.1
0.0
0.1
0.2
MR leave−one−out sensitivity analysis for
'IGSF8' on 'Cardiac valvular disease'

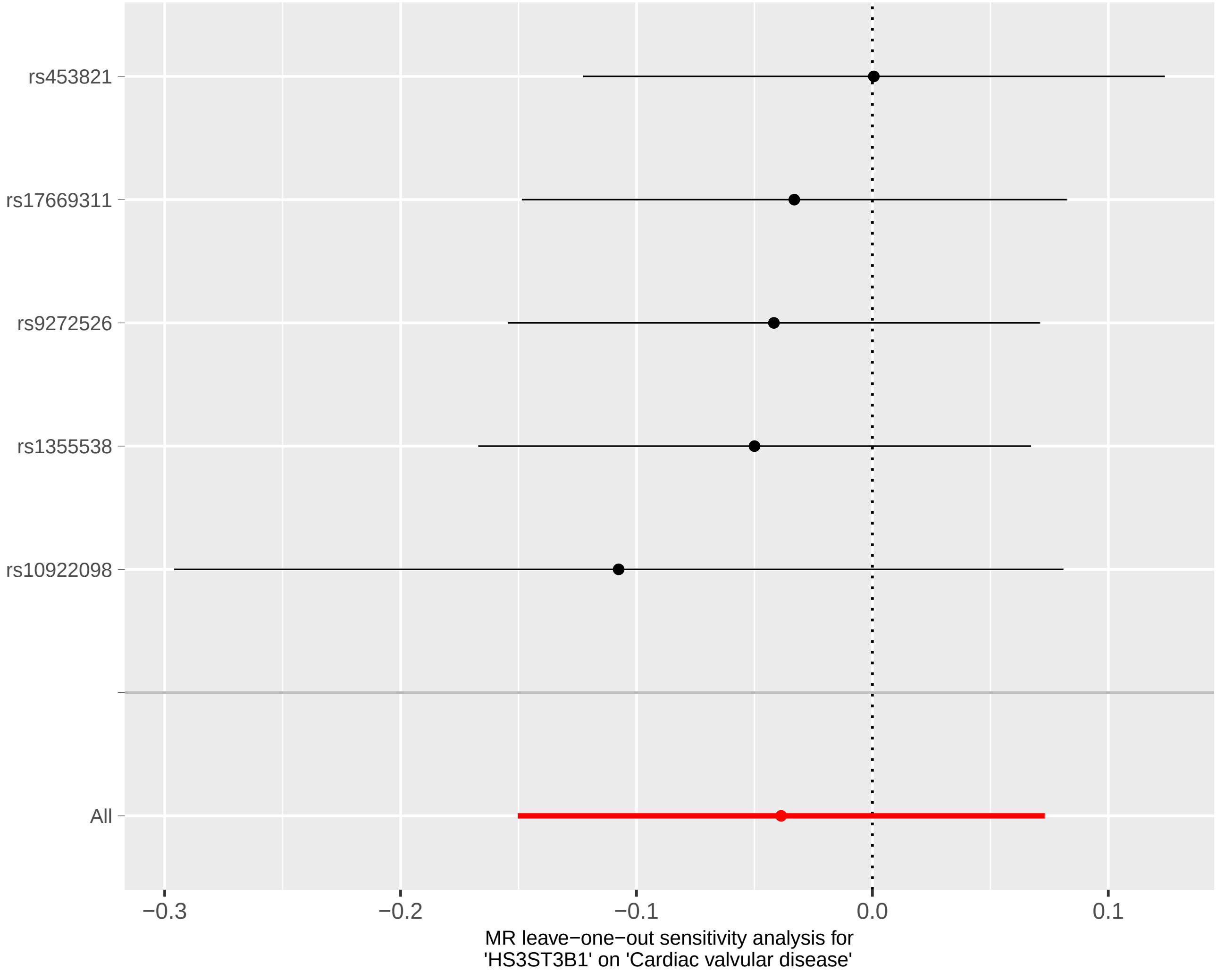
rs453821
rs17669311
rs9272526
rs1355538
rs10922098
All
−0.3
−0.2
−0.1
0.0
0.1
MR leave−one−out sensitivity analysis for
'HS3ST3B1' on 'Cardiac valvular disease'

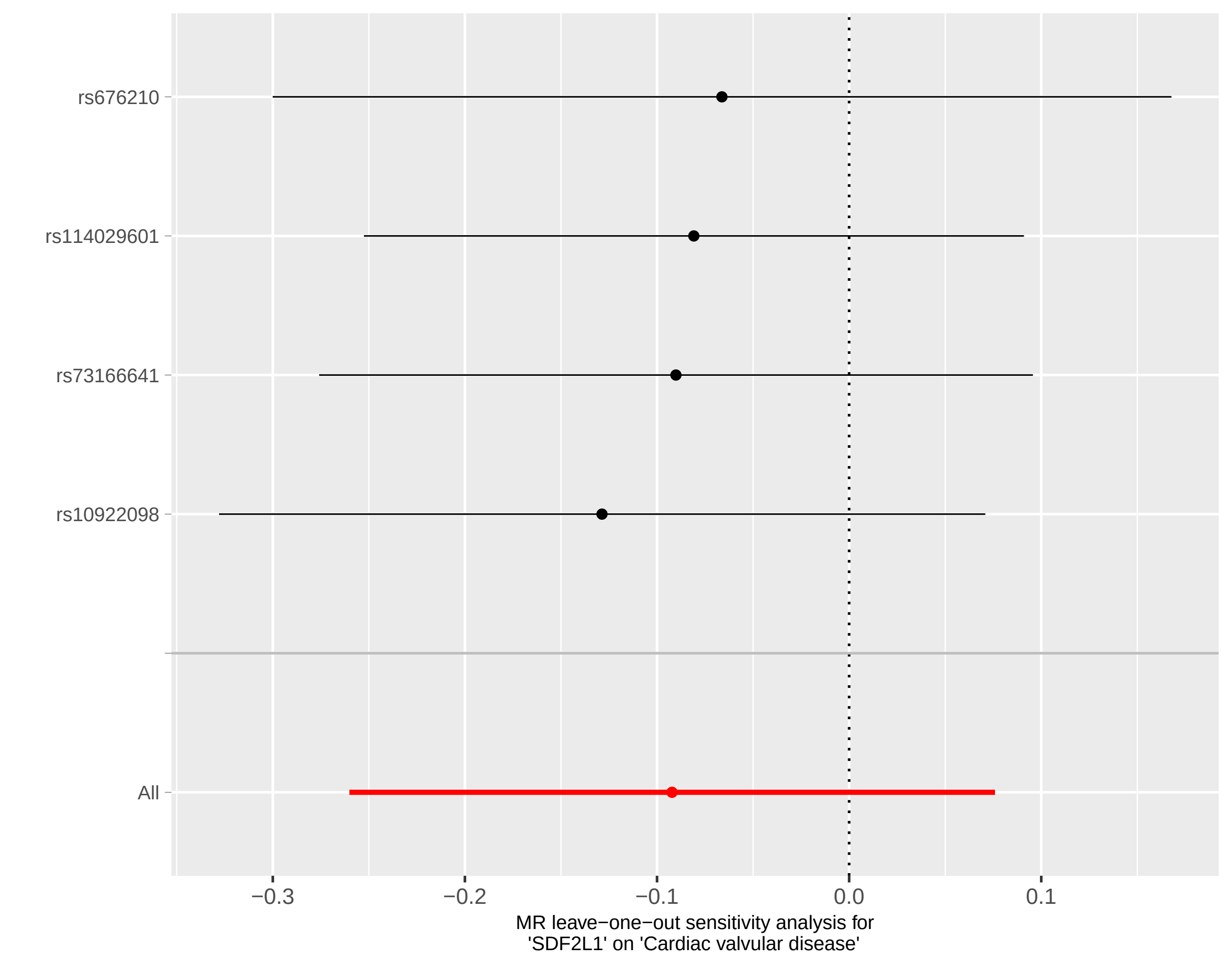
rs676210
rs114029601
rs73166641
rs10922098
All
−0.3
−0.2
−0.1
0.0
0.1
MR leave−one−out sensitivity analysis for
'SDF2L1' on 'Cardiac valvular disease'

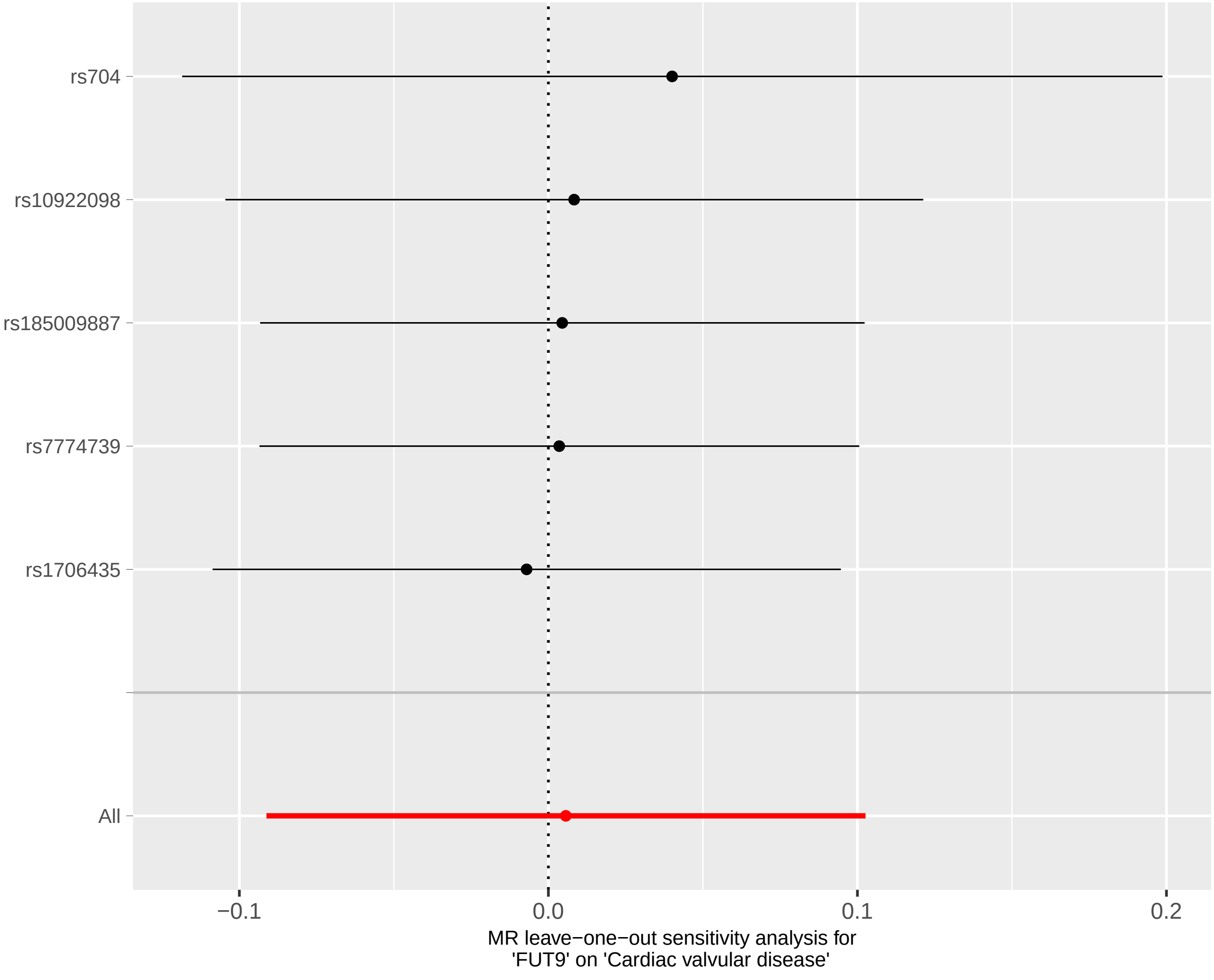
rs704
rs10922098
rs185009887
rs7774739
rs1706435
All
−0.1
0.0
0.1
0.2
MR leave−one−out sensitivity analysis for
'FUT9' on 'Cardiac valvular disease'

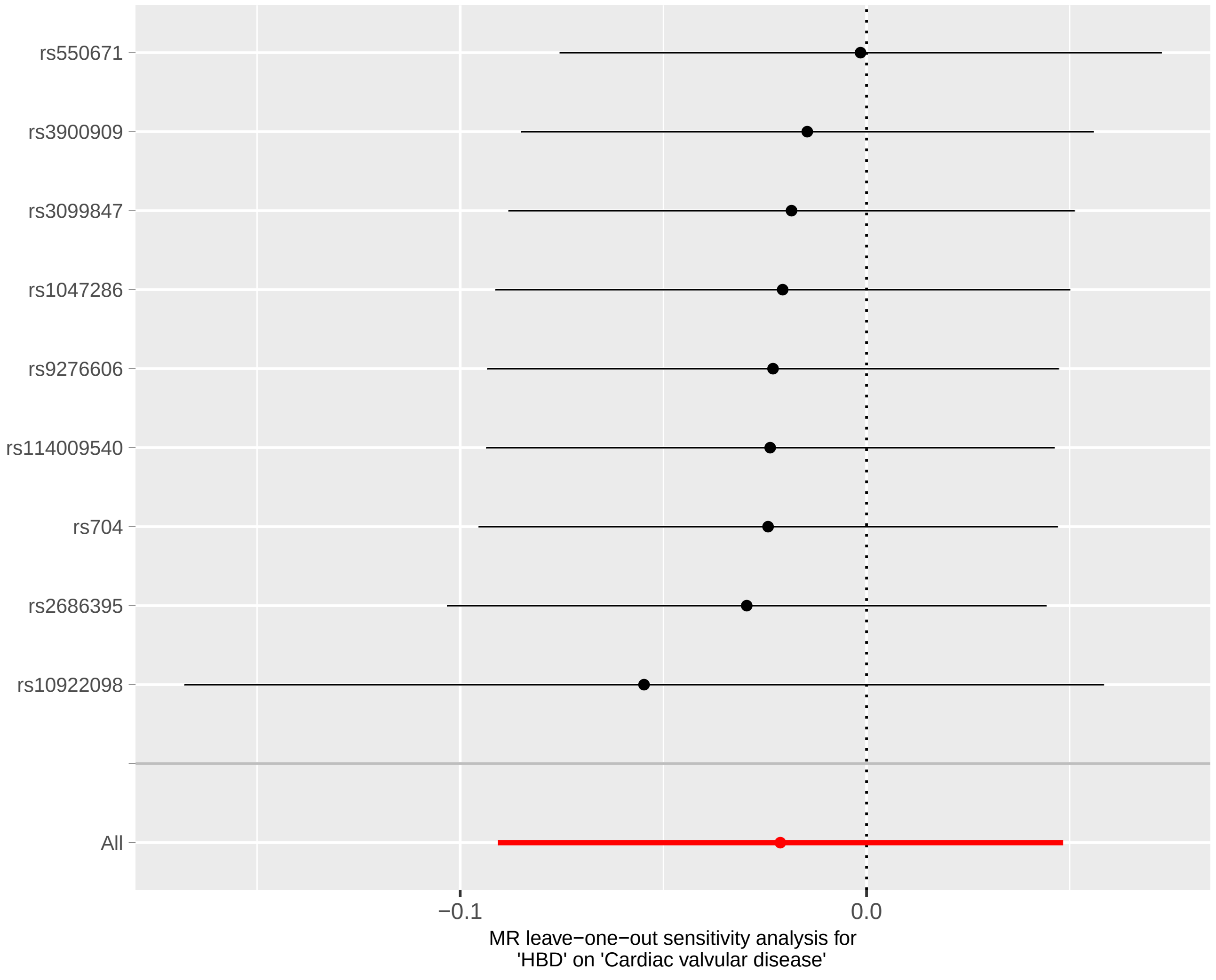
rs550671
rs3900909
rs3099847
rs1047286
rs9276606
rs114009540
rs704
rs2686395
rs10922098
All
−0.1
0.0
MR leave−one−out sensitivity analysis for
'HBD' on 'Cardiac valvular disease'

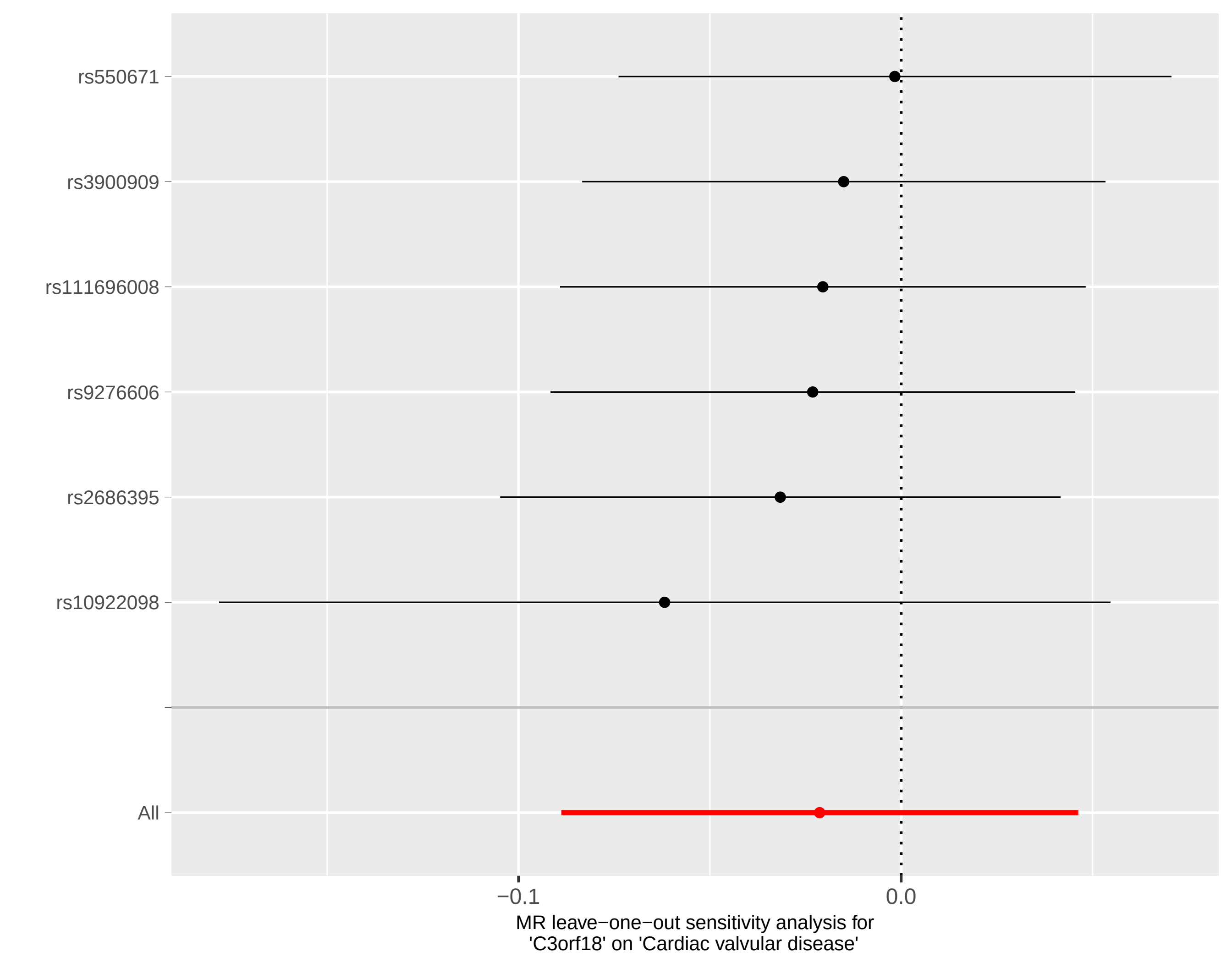
rs550671
rs3900909
rs111696008
rs9276606
rs2686395
rs10922098
All
−0.1
0.0
MR leave−one−out sensitivity analysis for
'C3orf18' on 'Cardiac valvular disease'

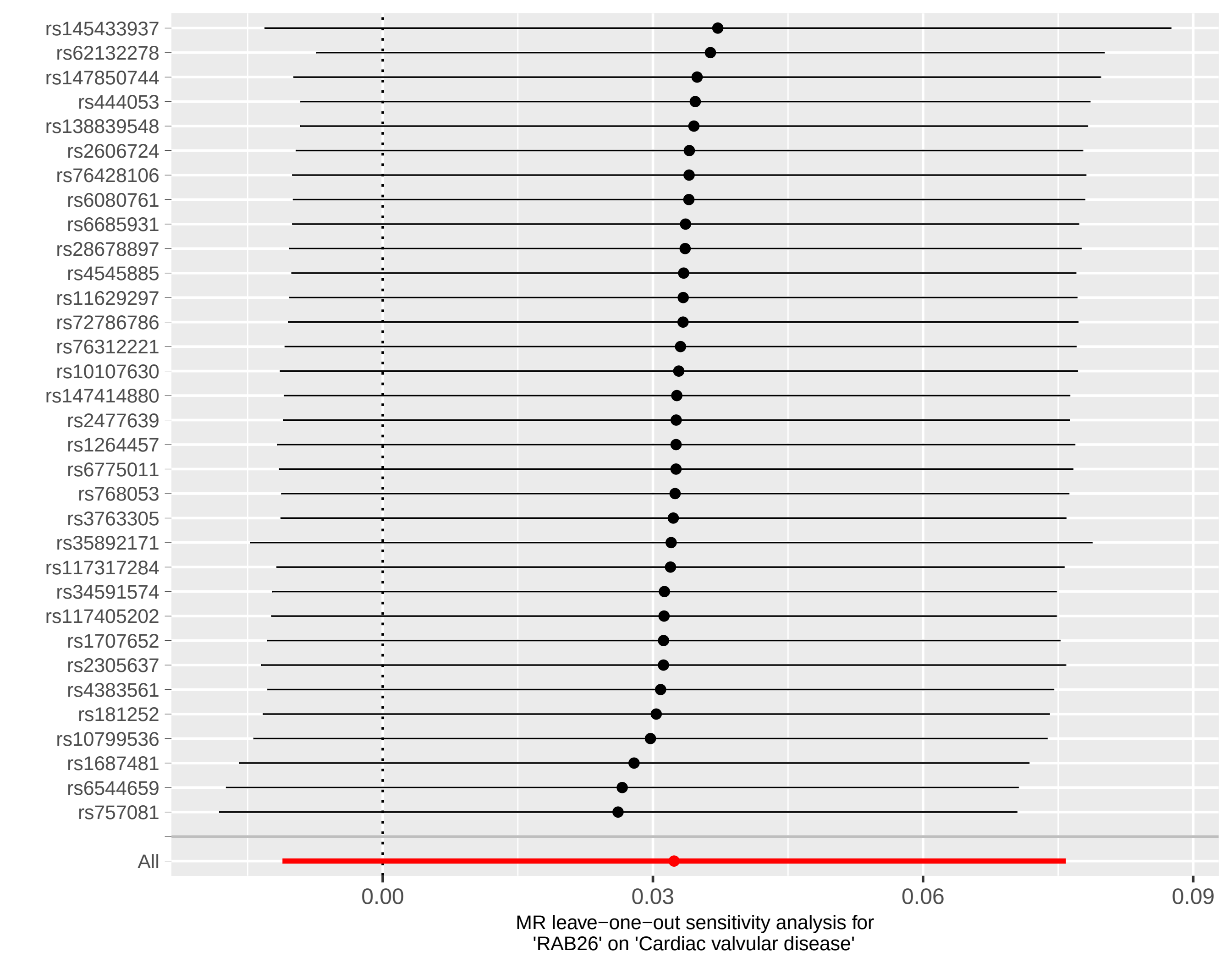

rs145433937
rs62132278
rs147850744
rs444053
rs138839548
rs2606724
rs76428106
rs6080761
rs6685931
rs28678897
rs4545885
rs11629297
rs72786786
rs76312221
rs10107630
rs147414880
rs2477639
rs1264457
rs6775011
rs768053
rs3763305
rs35892171
rs117317284
rs34591574
rs117405202
rs1707652
rs2305637
rs4383561
rs181252
rs10799536
rs1687481
rs6544659
rs757081
All
0.00
0.03
0.06
0.09
MR leave−one−out sensitivity analysis for
'RAB26' on 'Cardiac valvular disease'

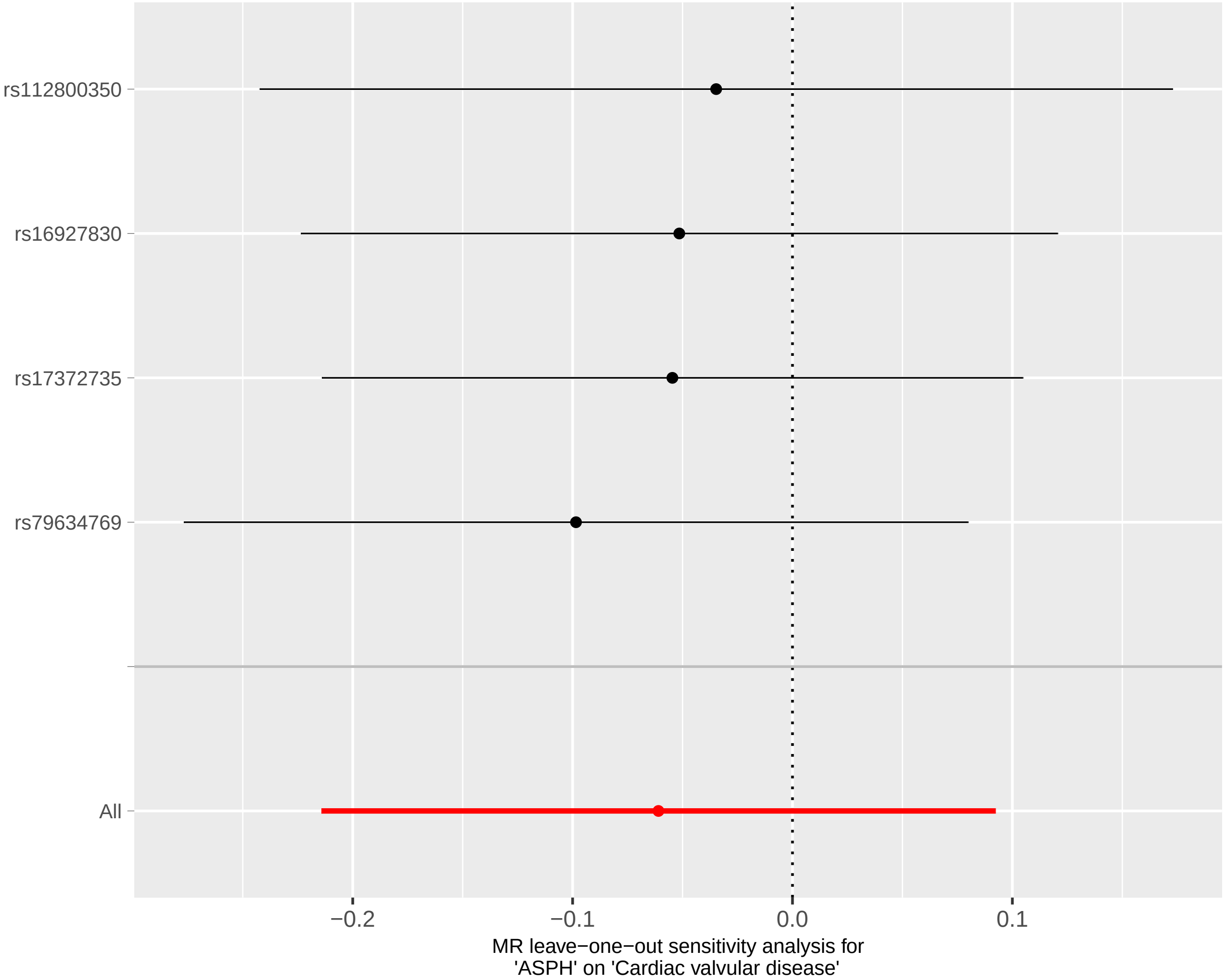

rs112800350
rs16927830
rs17372735
rs79634769
All
−0.2
−0.1
0.0
0.1
MR leave−one−out sensitivity analysis for
'ASPH' on 'Cardiac valvular disease'

Insufficient number of SNPs

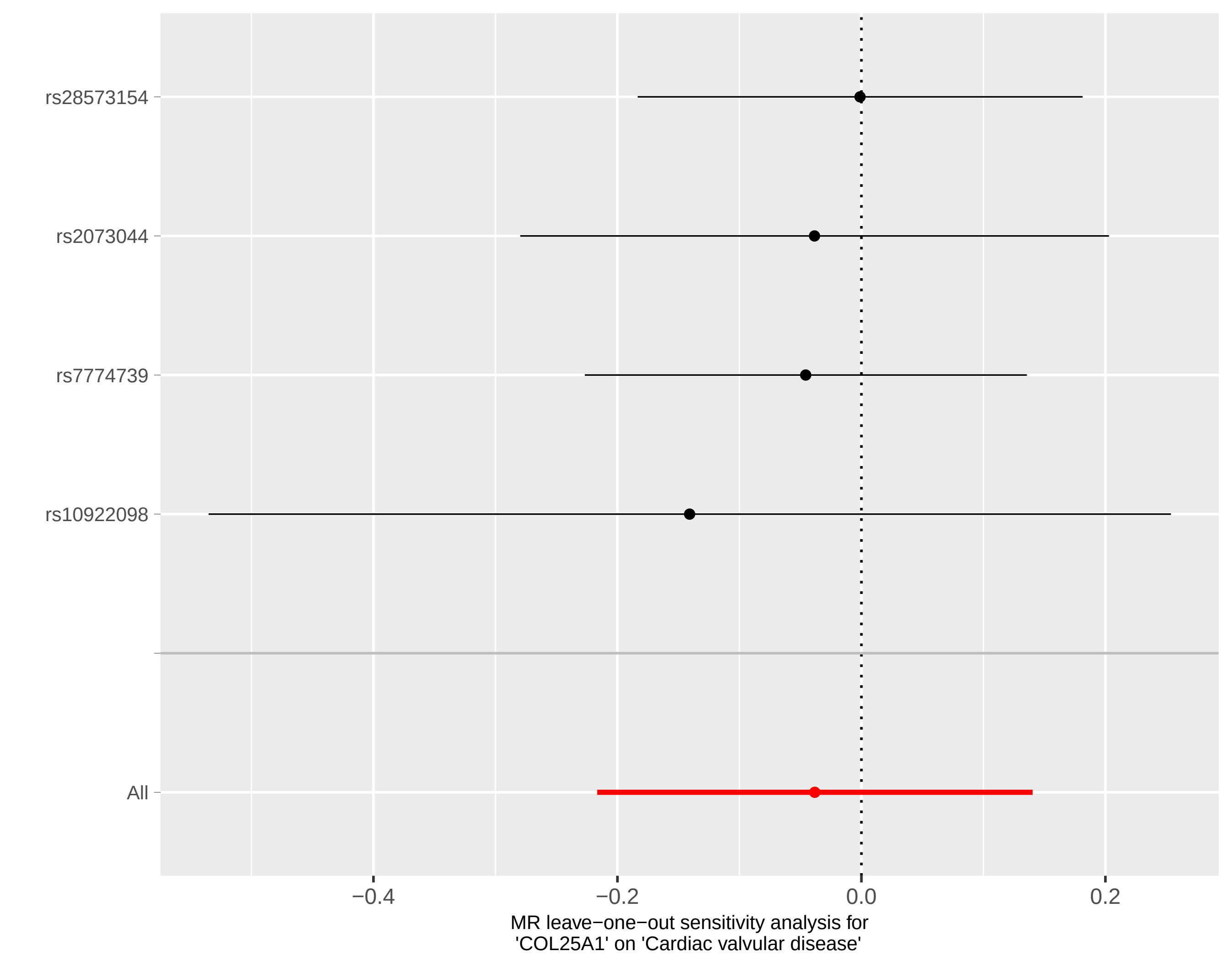

rs28573154
rs2073044
rs7774739
rs10922098
All
−0.4
−0.2
0.0
0.2
MR leave−one−out sensitivity analysis for
'COL25A1' on 'Cardiac valvular disease'

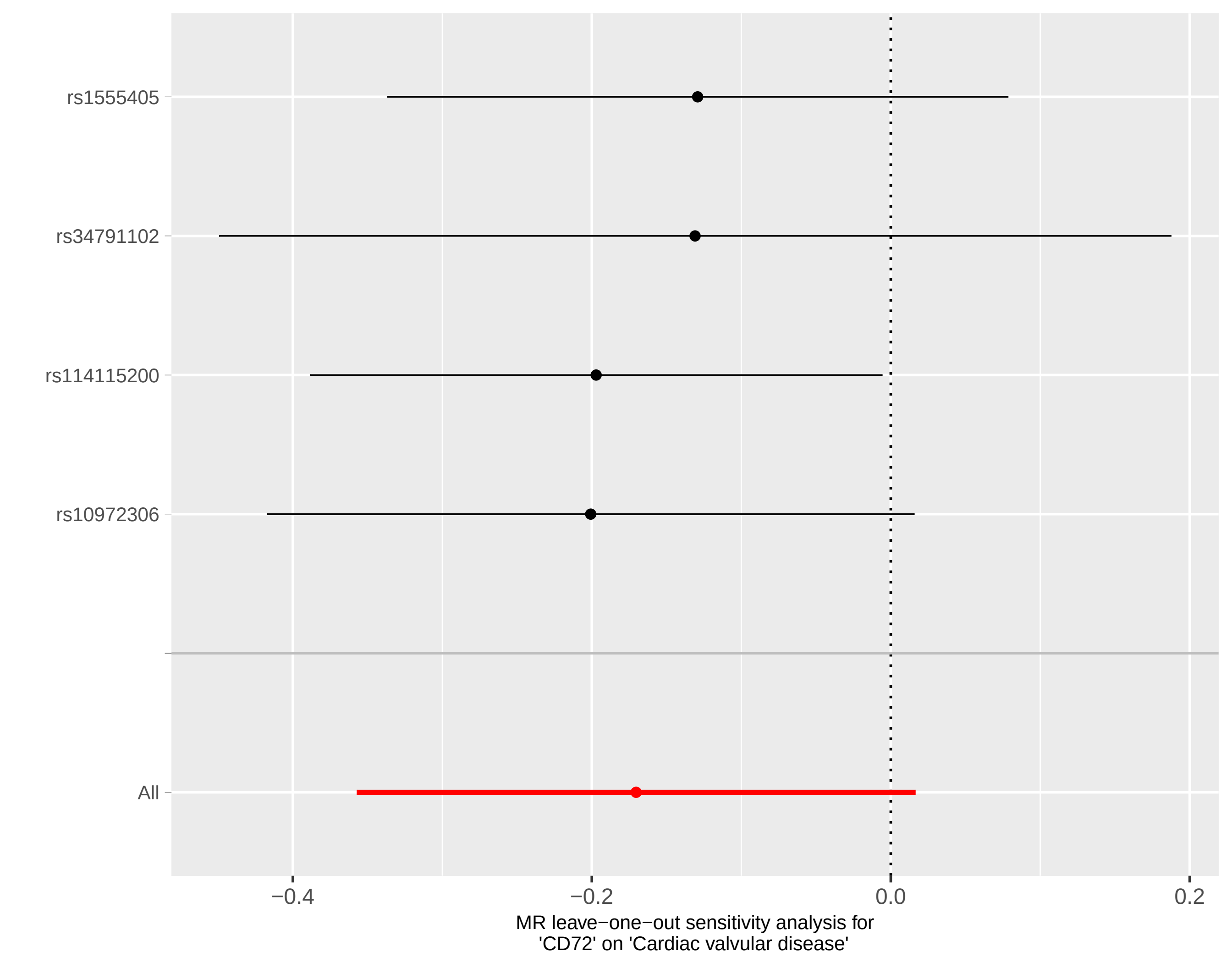

rs1555405
rs34791102
rs114115200
rs10972306
All
−0.4
−0.2
0.0
0.2
MR leave−one−out sensitivity analysis for
'CD72' on 'Cardiac valvular disease'

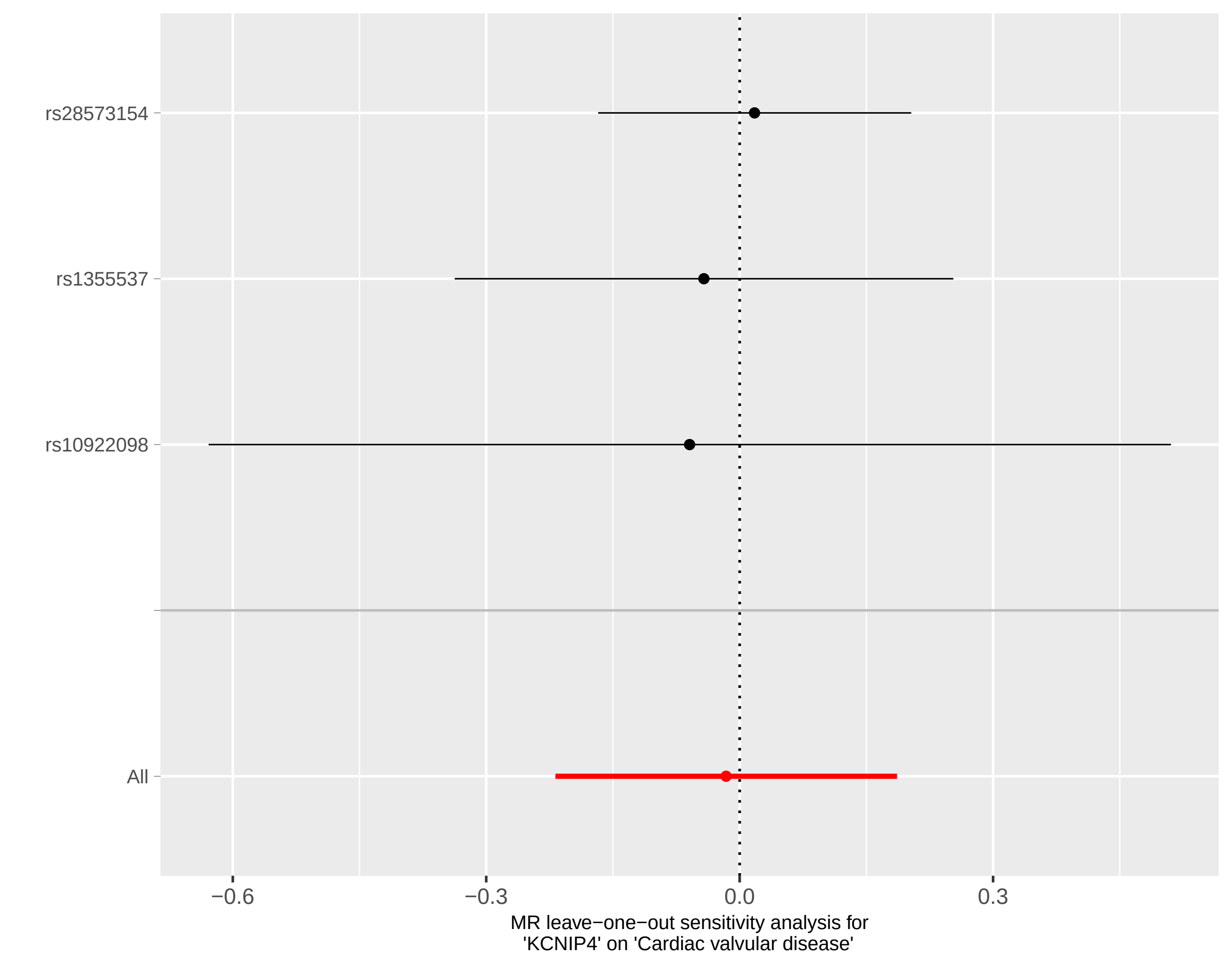

rs28573154
rs1355537
rs10922098
All
−0.6
−0.3
0.0
0.3
MR leave−one−out sensitivity analysis for
'KCNIP4' on 'Cardiac valvular disease'

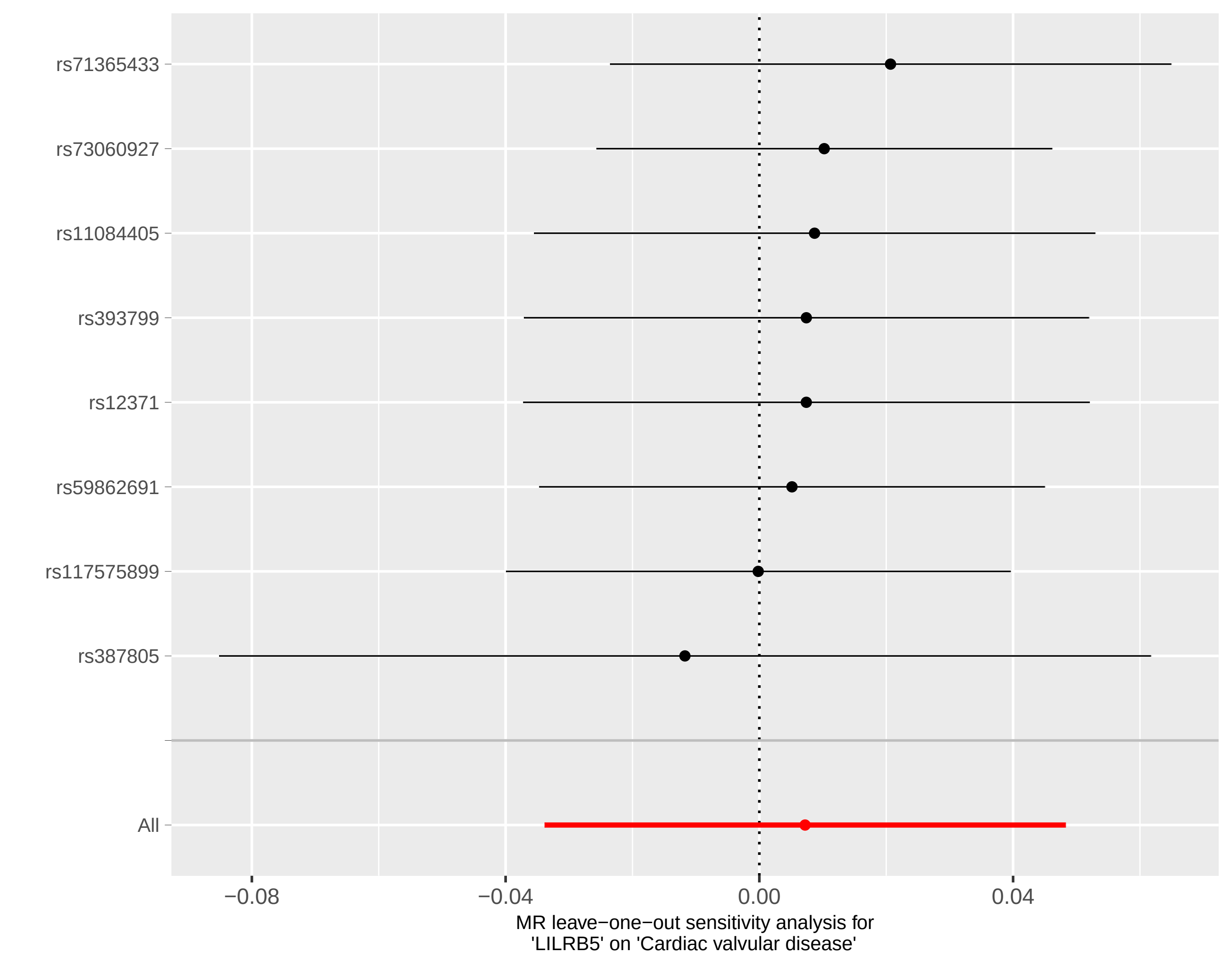

rs71365433
rs73060927
rs11084405
rs393799
rs12371
rs59862691
rs117575899
rs387805
All
−0.08
−0.04
0.00
0.04
MR leave−one−out sensitivity analysis for
'LILRB5' on 'Cardiac valvular disease'

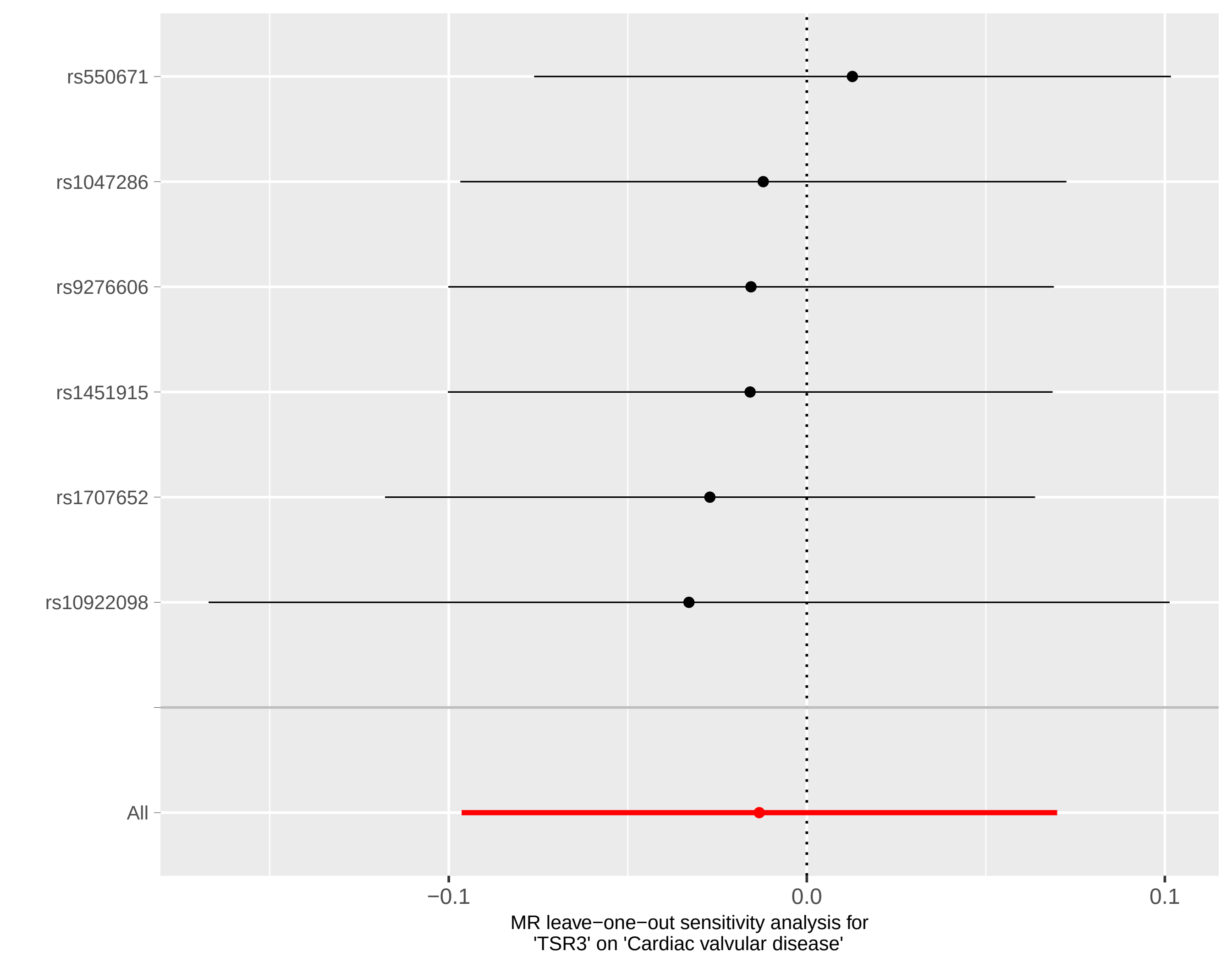

rs550671
rs1047286
rs9276606
rs1451915
rs1707652
rs10922098
All
−0.1
0.0
0.1
MR leave−one−out sensitivity analysis for
'TSR3' on 'Cardiac valvular disease'

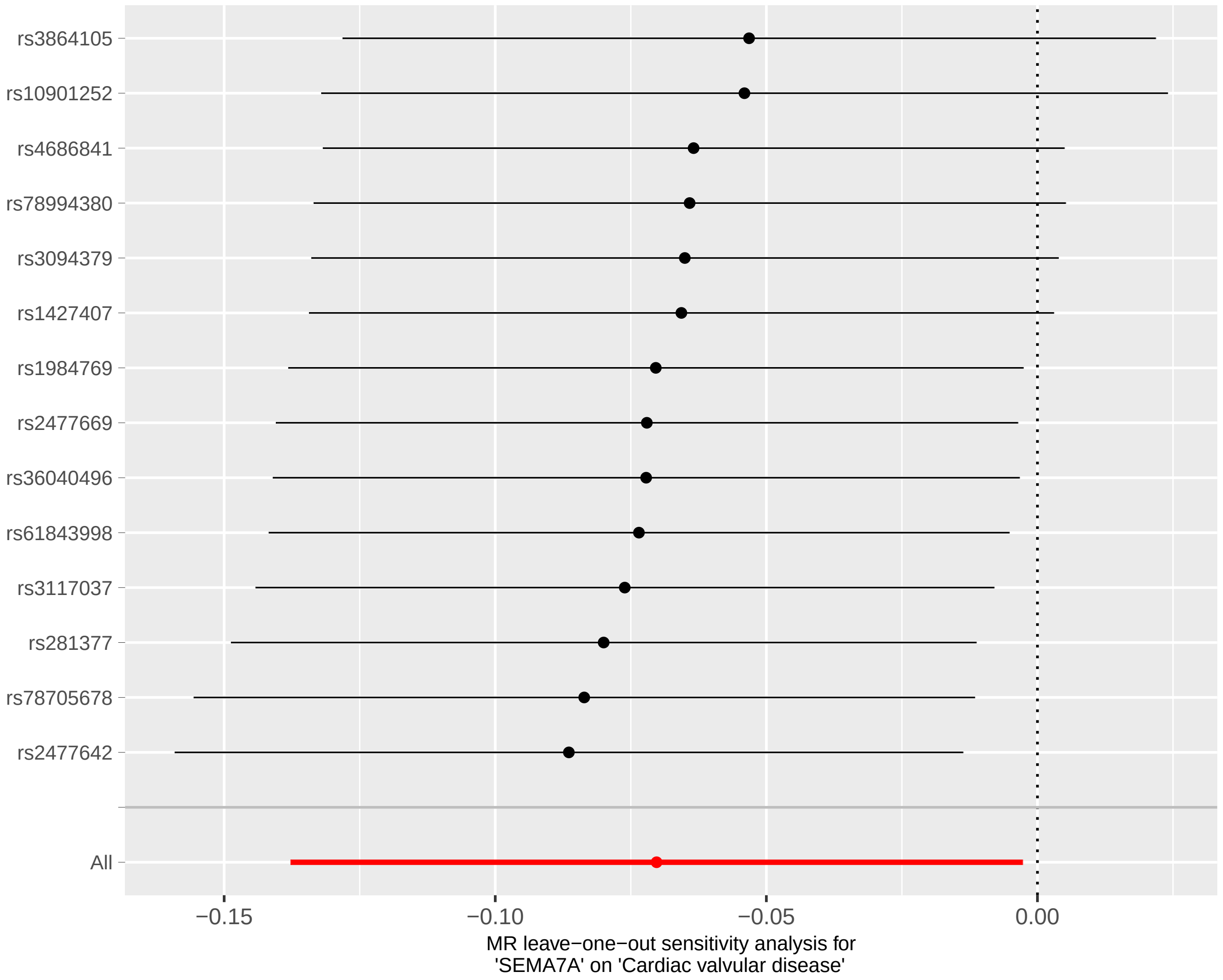

rs3864105
rs10901252
rs4686841
rs78994380
rs3094379
rs1427407
rs1984769
rs2477669
rs36040496
rs61843998
rs3117037
rs281377
rs78705678
rs2477642
All
−0.15
−0.10
−0.05
0.00
MR leave−one−out sensitivity analysis for
'SEMA7A' on 'Cardiac valvular disease'

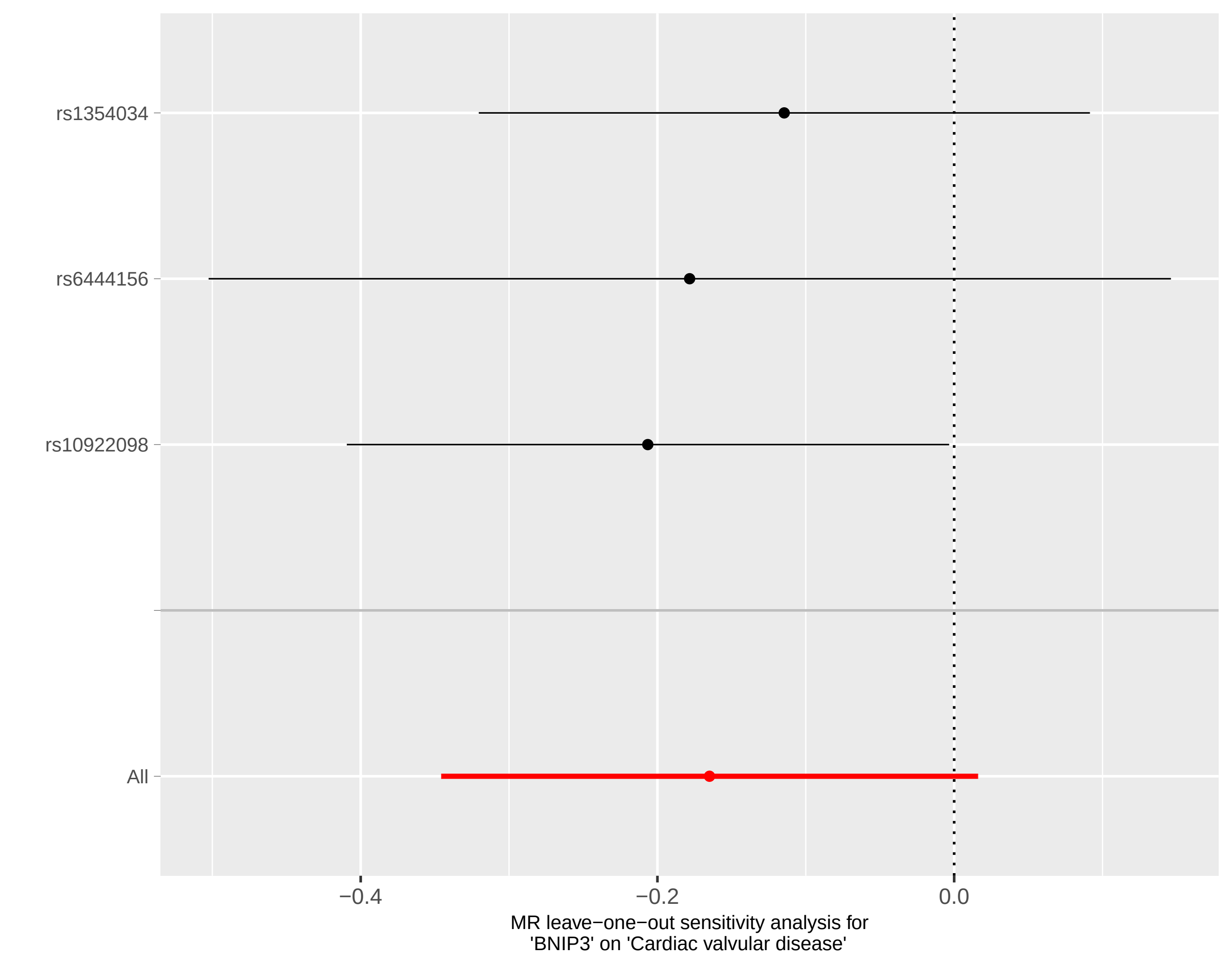
rs1354034
rs6444156
rs10922098
All
−0.4
−0.2
0.0
MR leave−one−out sensitivity analysis for
'BNIP3' on 'Cardiac valvular disease'

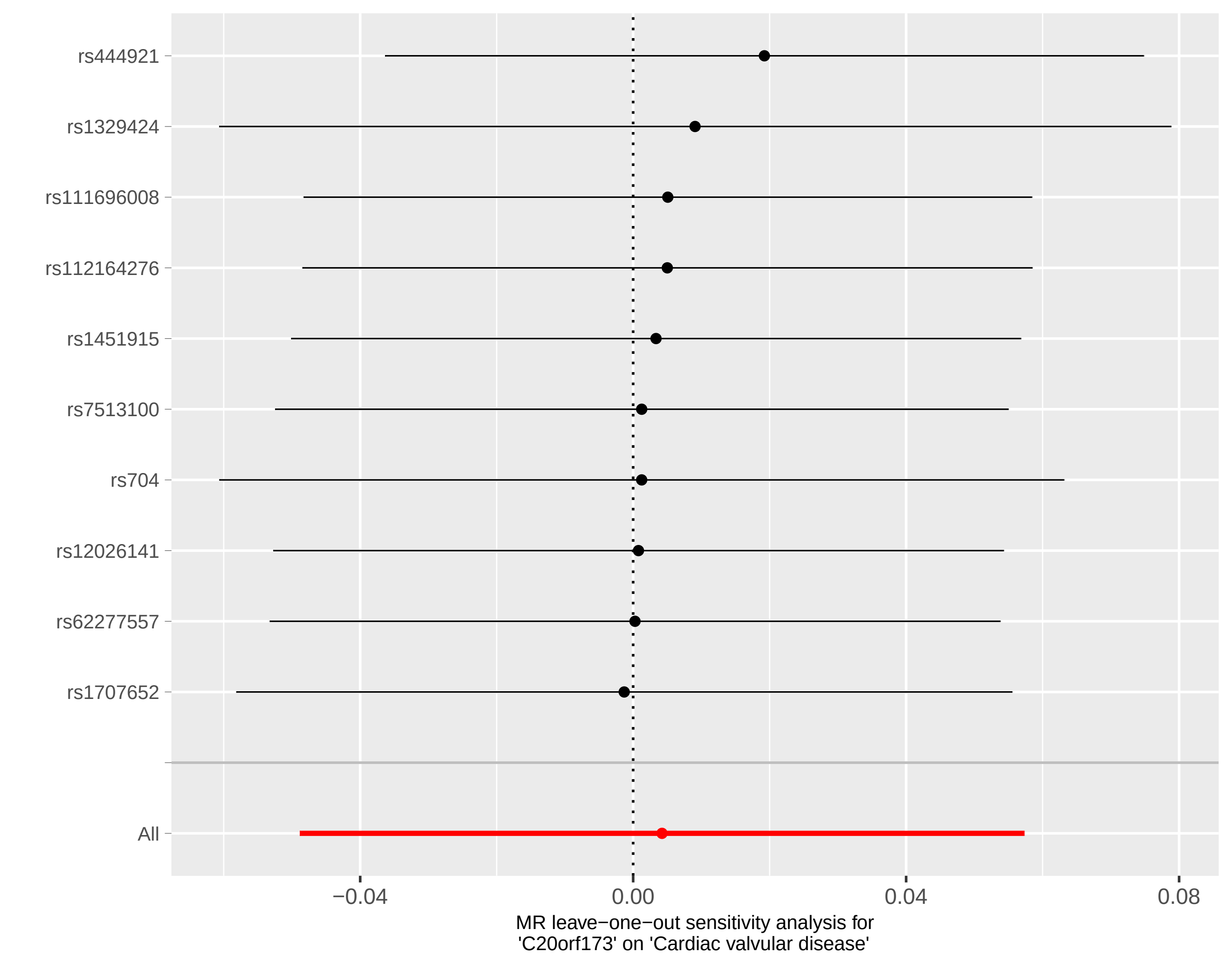

rs444921
rs1329424
rs111696008
rs112164276
rs1451915
rs7513100
rs704
rs12026141
rs62277557
rs1707652
All
−0.04
0.00
0.04
0.08
MR leave−one−out sensitivity analysis for
'C20orf173' on 'Cardiac valvular disease'

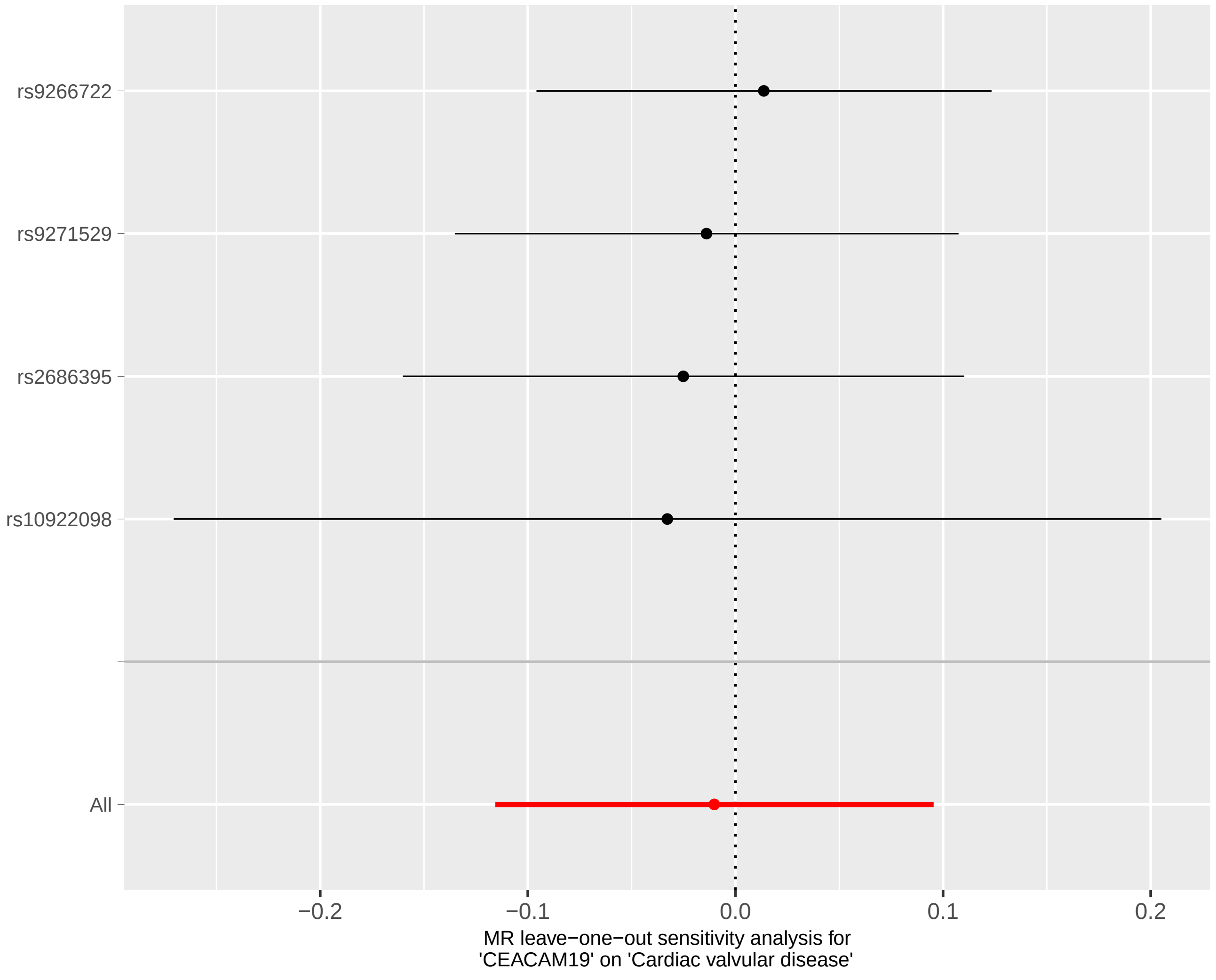
rs9266722
rs9271529
rs2686395
rs10922098
All
−0.2
−0.1
0.0
0.1
0.2
MR leave−one−out sensitivity analysis for
'CEACAM19' on 'Cardiac valvular disease'

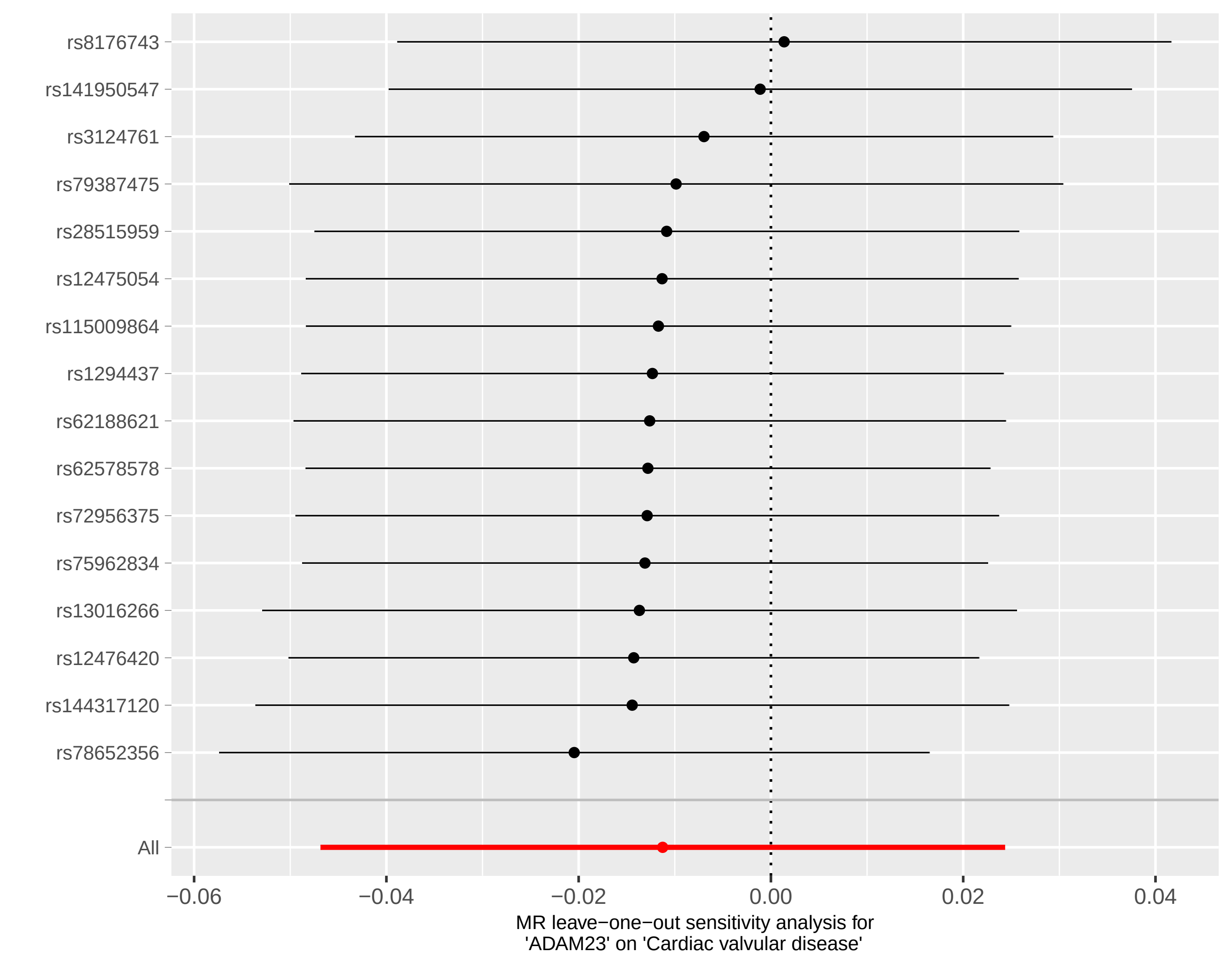
rs8176743
rs141950547
rs3124761
rs79387475
rs28515959
rs12475054
rs115009864
rs1294437
rs62188621
rs62578578
rs72956375
rs75962834
rs13016266
rs12476420
rs144317120
rs78652356
All
−0.06
−0.04
−0.02
0.00
0.02
0.04
MR leave−one−out sensitivity analysis for
'ADAM23' on 'Cardiac valvular disease'

Insufficient number of SNPs

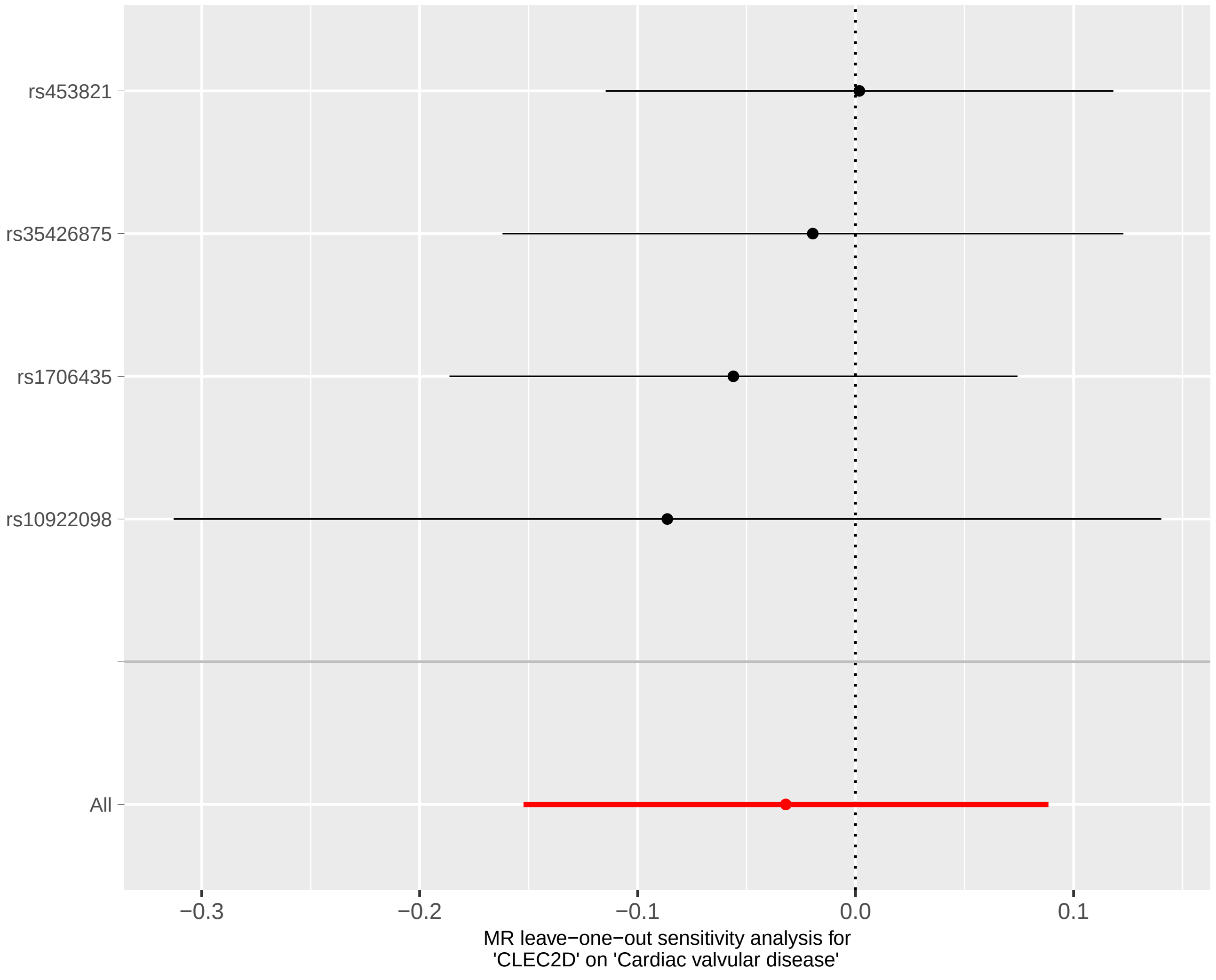
rs453821
rs35426875
rs1706435
rs10922098
All
−0.3
−0.2
−0.1
0.0
0.1
MR leave−one−out sensitivity analysis for
'CLEC2D' on 'Cardiac valvular disease'

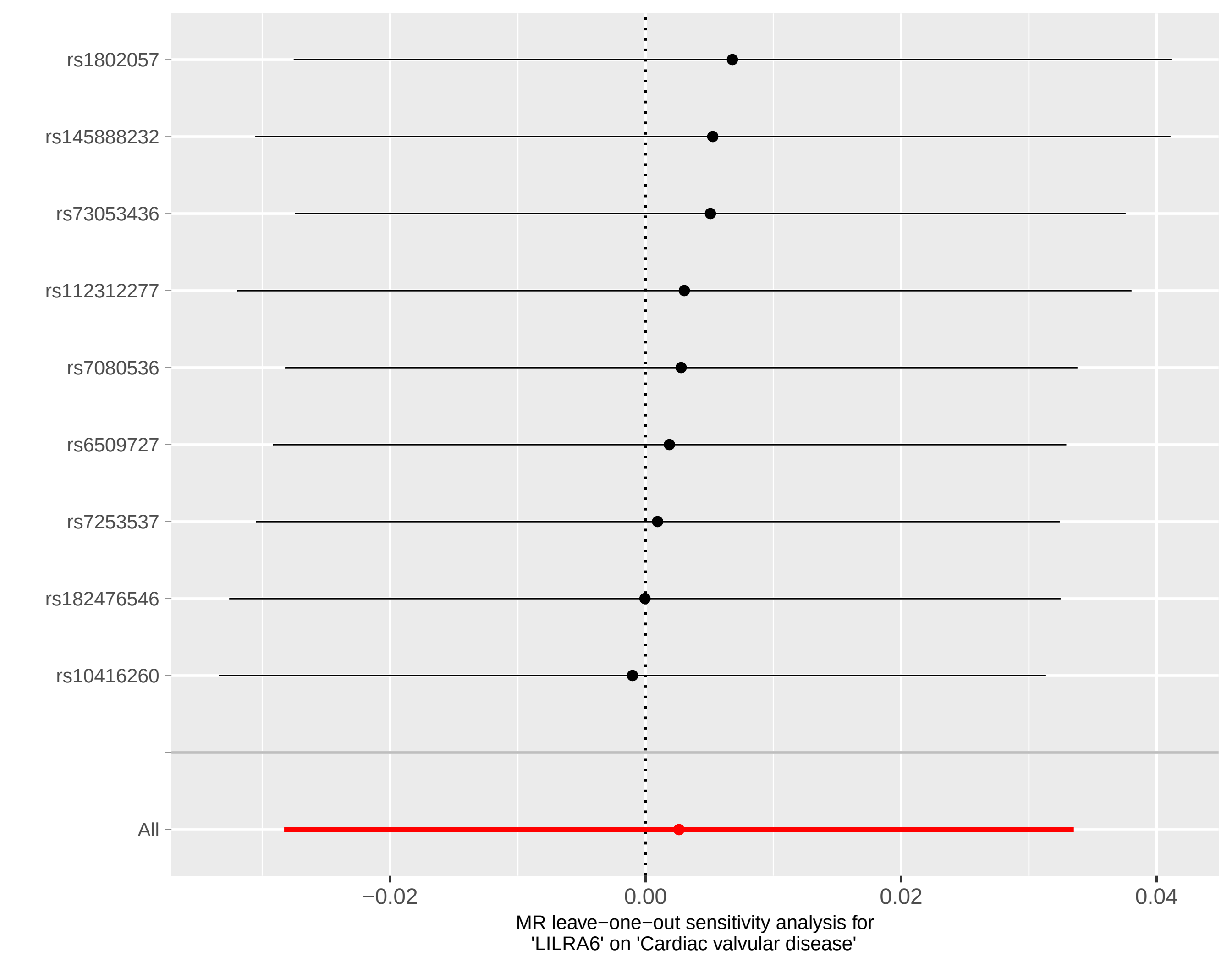

rs1802057
rs145888232
rs73053436
rs112312277
rs7080536
rs6509727
rs7253537
rs182476546
rs10416260
All
−0.02
0.00
0.02
0.04
MR leave−one−out sensitivity analysis for
'LILRA6' on 'Cardiac valvular disease'

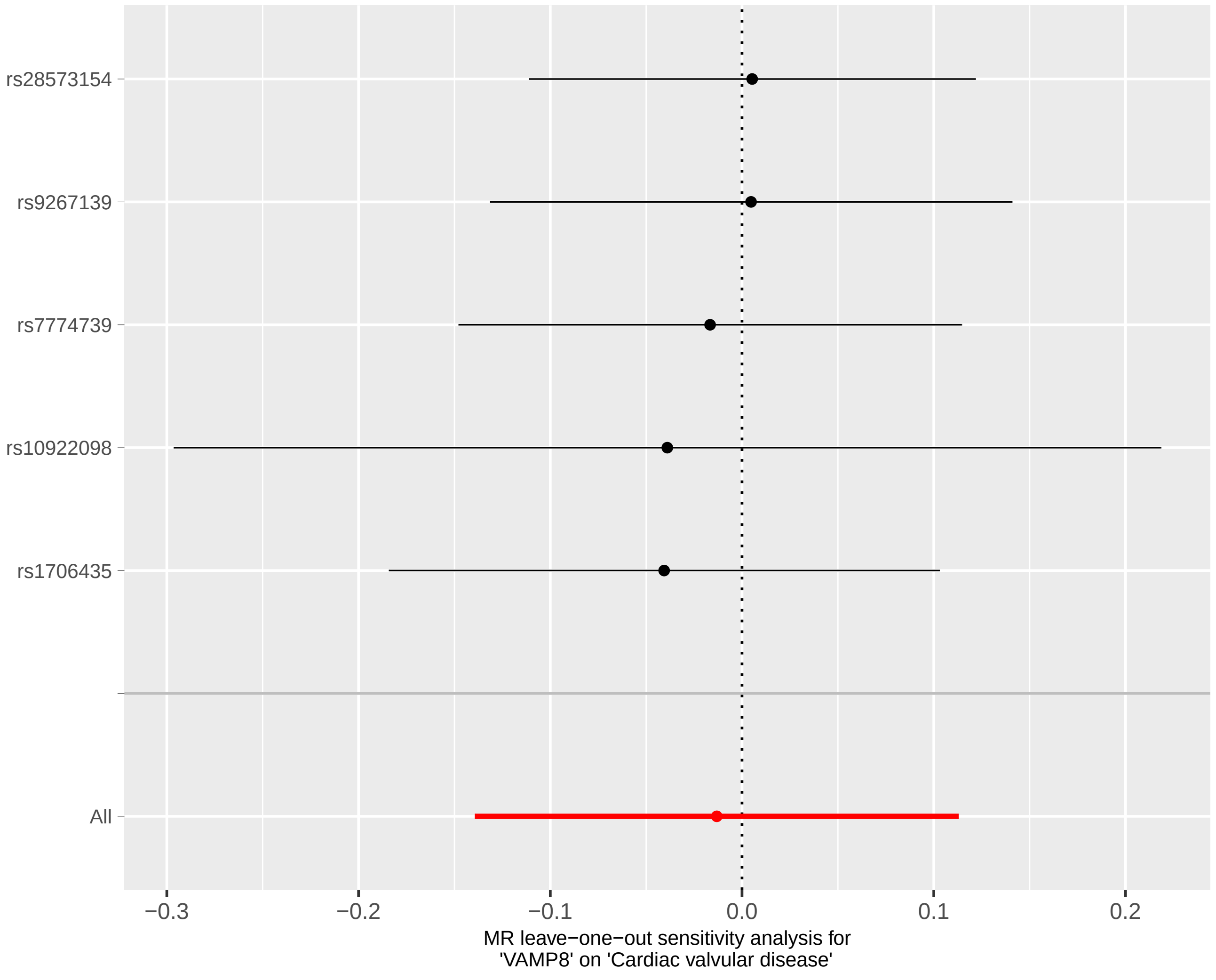

rs28573154
rs9267139
rs7774739
rs10922098
rs1706435
All
−0.3
−0.2
−0.1
0.0
0.1
0.2
MR leave−one−out sensitivity analysis for
'VAMP8' on 'Cardiac valvular disease'

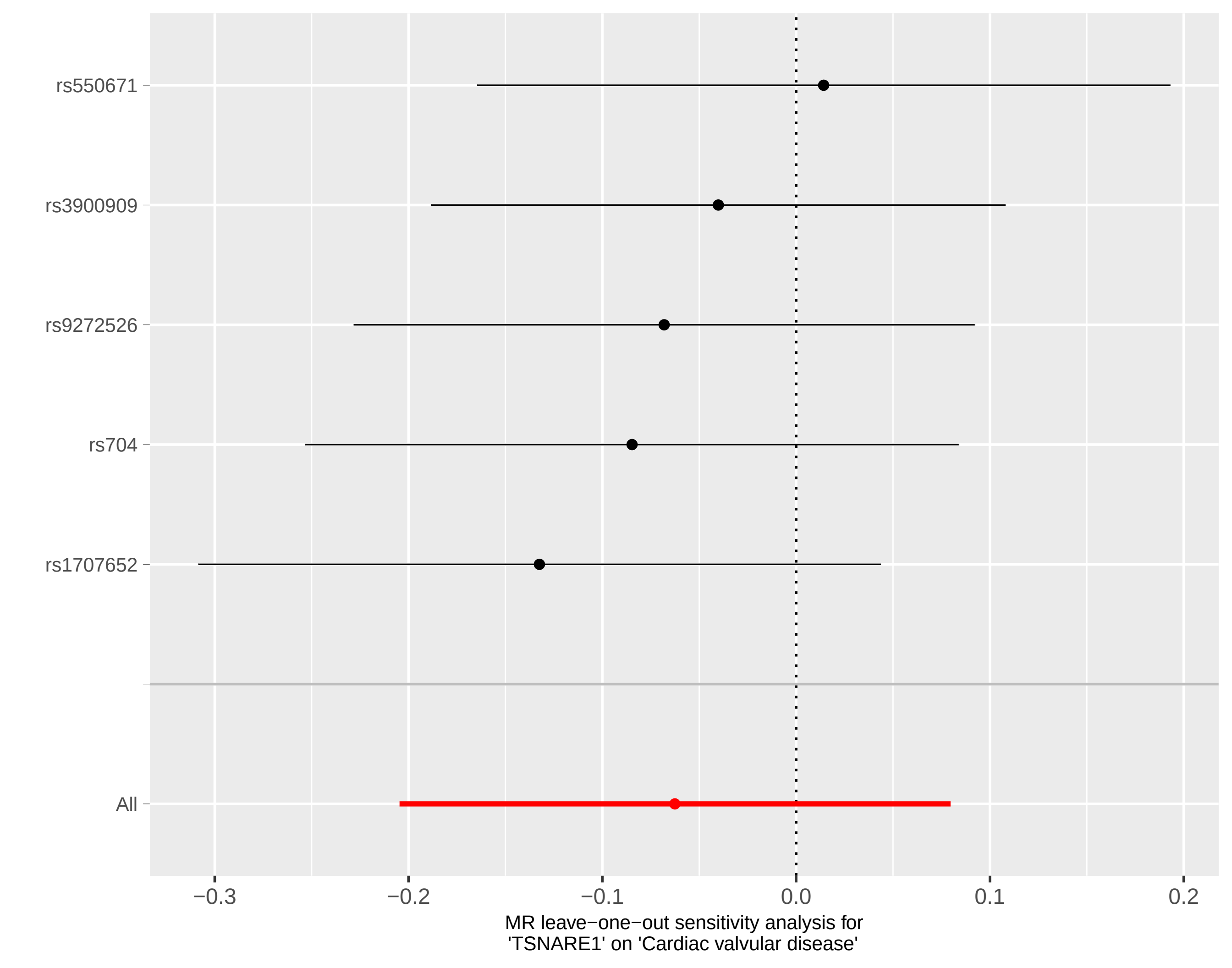

rs550671
rs3900909
rs9272526
rs704
rs1707652
All
−0.3
−0.2
−0.1
0.0
0.1
0.2
MR leave−one−out sensitivity analysis for
'TSNARE1' on 'Cardiac valvular disease'

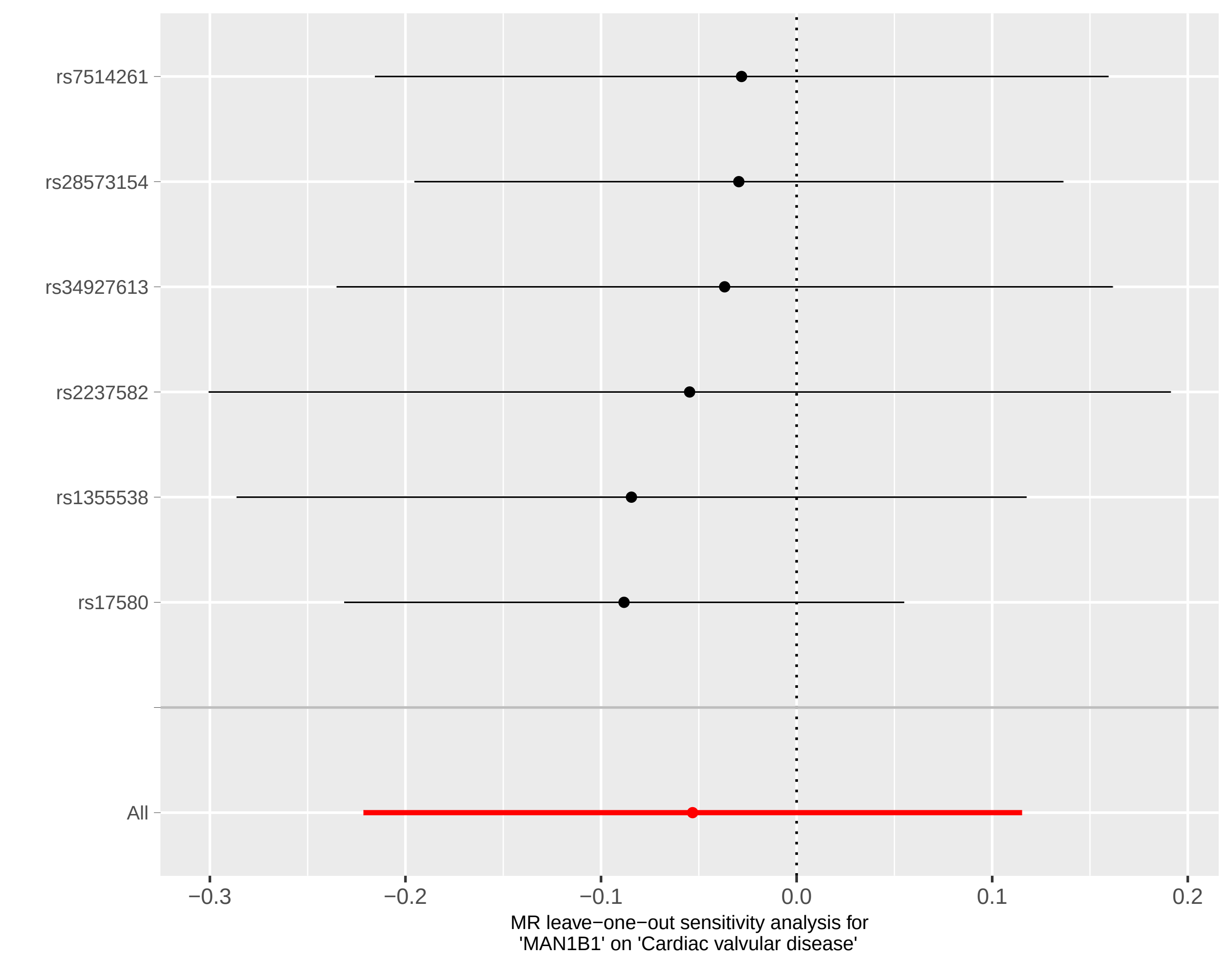

rs7514261
rs28573154
rs34927613
rs2237582
rs1355538
rs17580
All
−0.3
−0.2
−0.1
0.0
0.1
0.2
MR leave−one−out sensitivity analysis for
'MAN1B1' on 'Cardiac valvular disease'

Insufficient number of SNPs

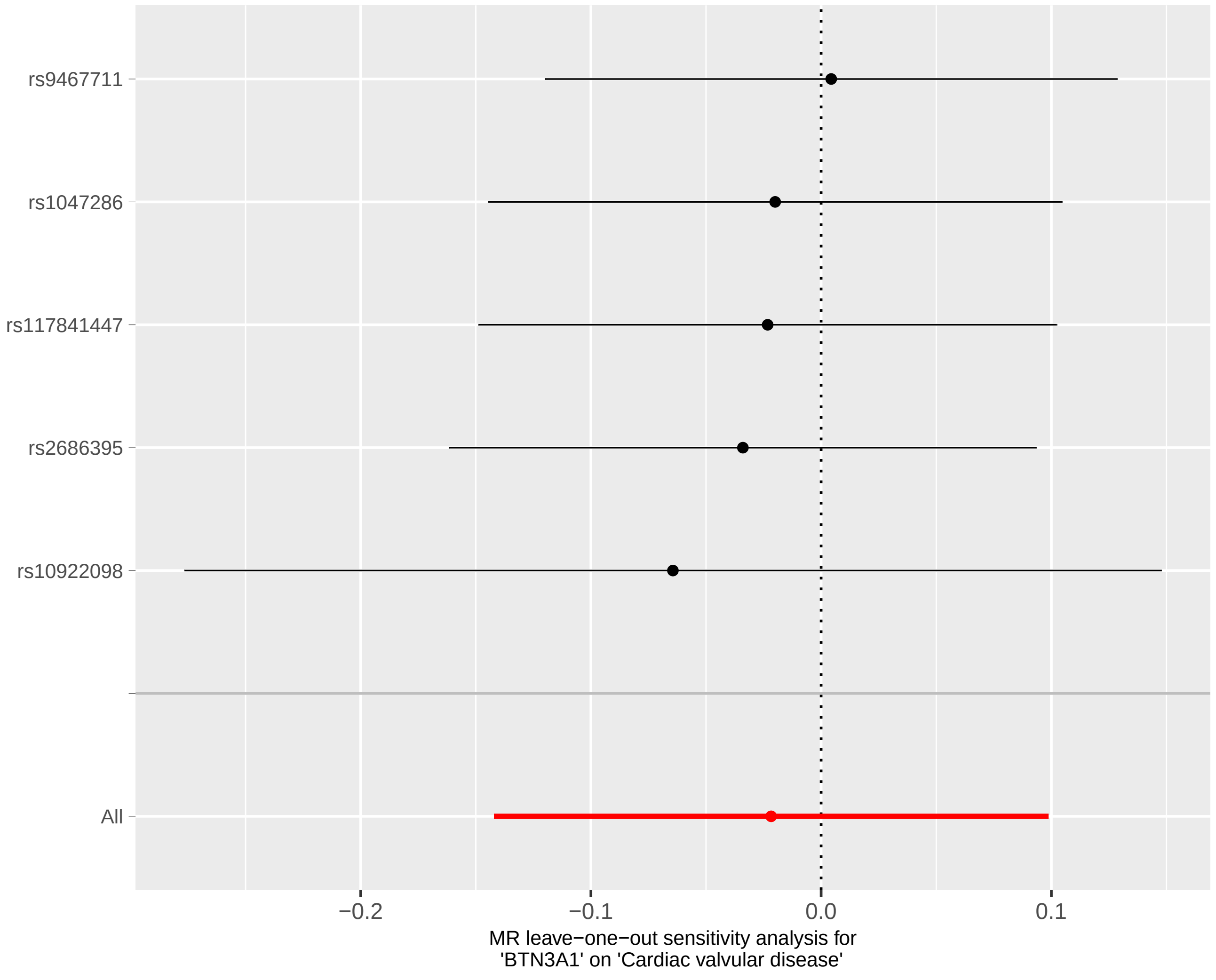

rs9467711
rs1047286
rs117841447
rs2686395
rs10922098
All
−0.2
−0.1
0.0
0.1
MR leave−one−out sensitivity analysis for
'BTN3A1' on 'Cardiac valvular disease'

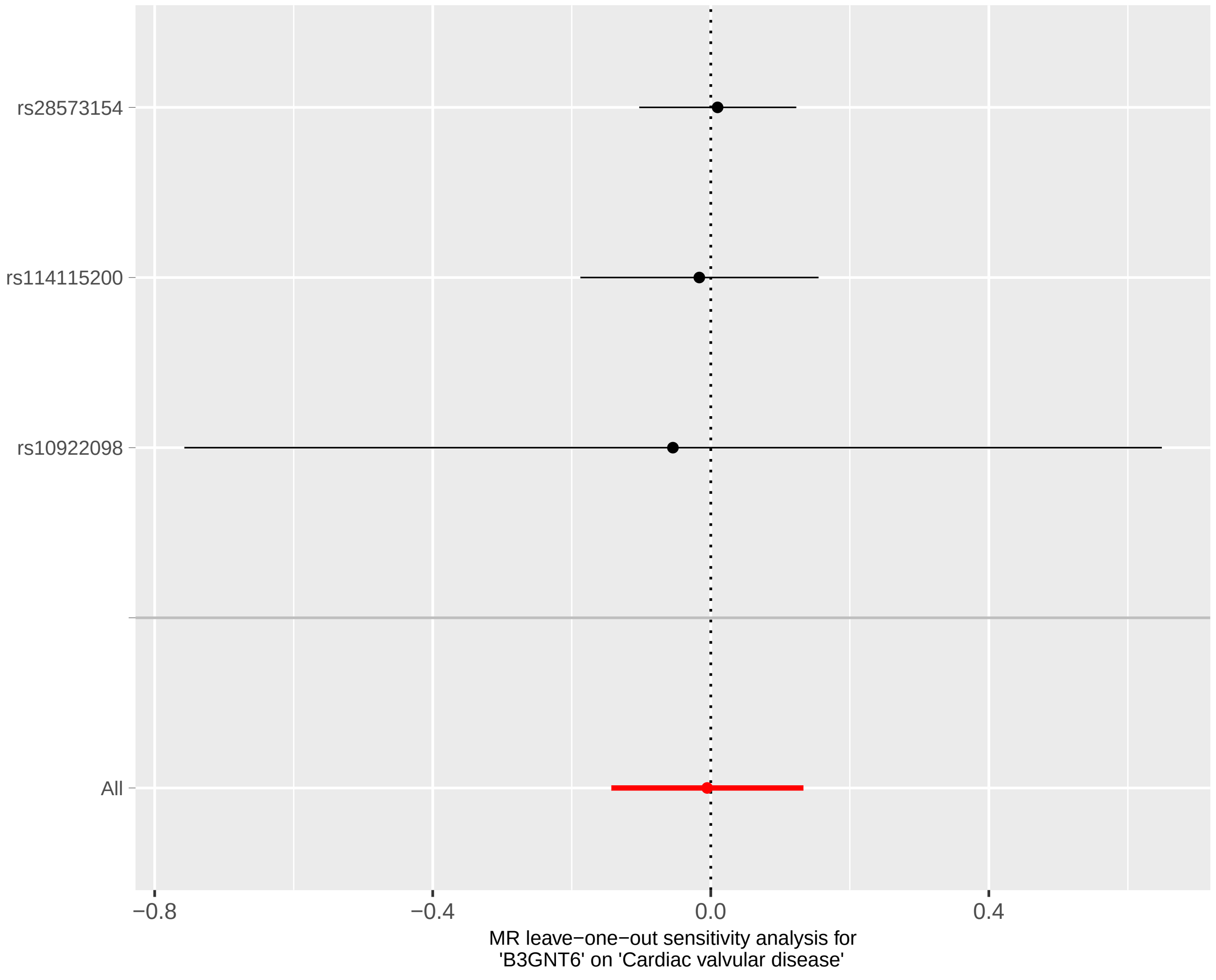
rs28573154
rs114115200
rs10922098
All
−0.8
−0.4
0.0
0.4
MR leave−one−out sensitivity analysis for
'B3GNT6' on 'Cardiac valvular disease'

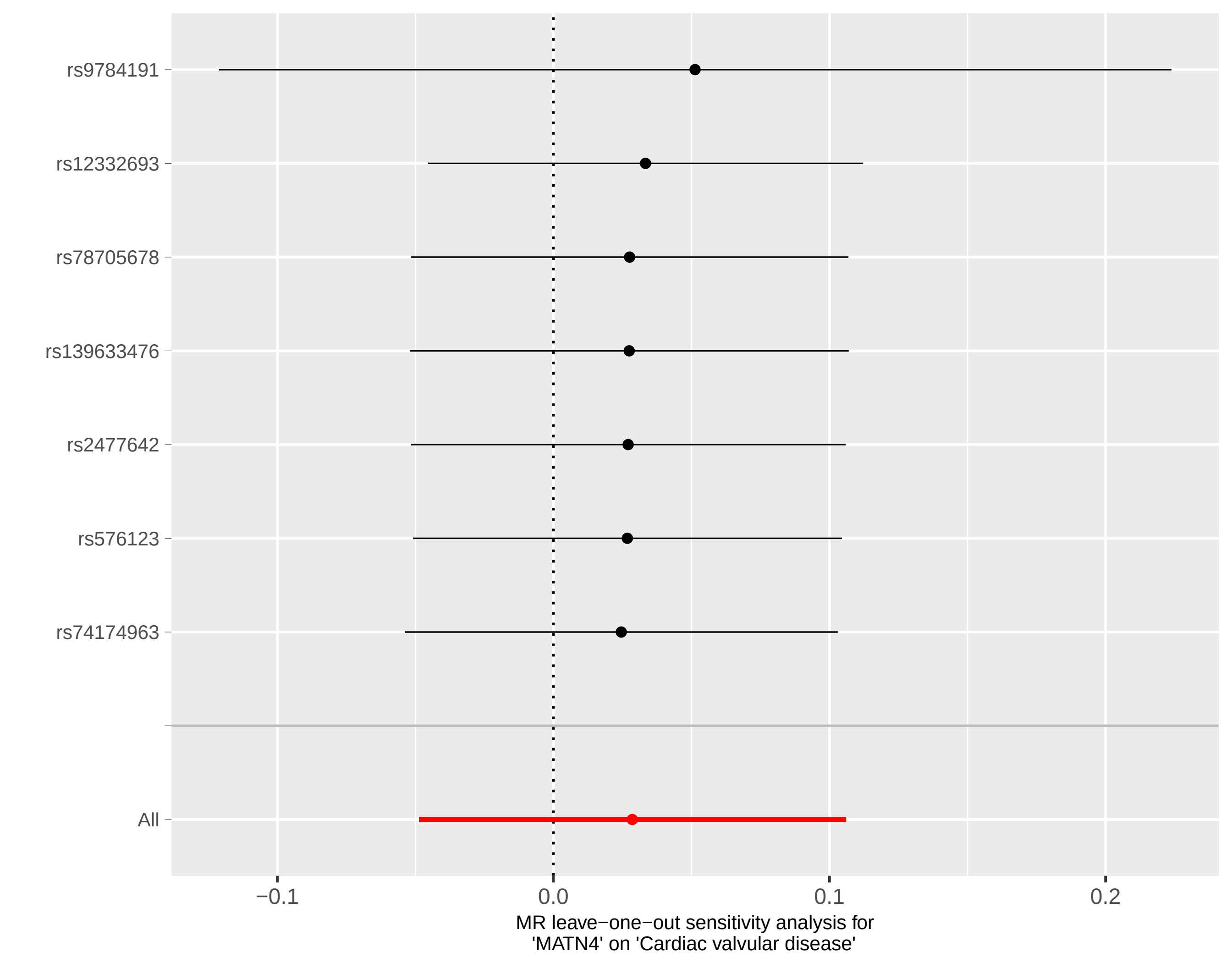

rs9784191
rs12332693
rs78705678
rs139633476
rs2477642
rs576123
rs74174963
All
−0.1
0.0
0.1
0.2
MR leave−one−out sensitivity analysis for
'MATN4' on 'Cardiac valvular disease'

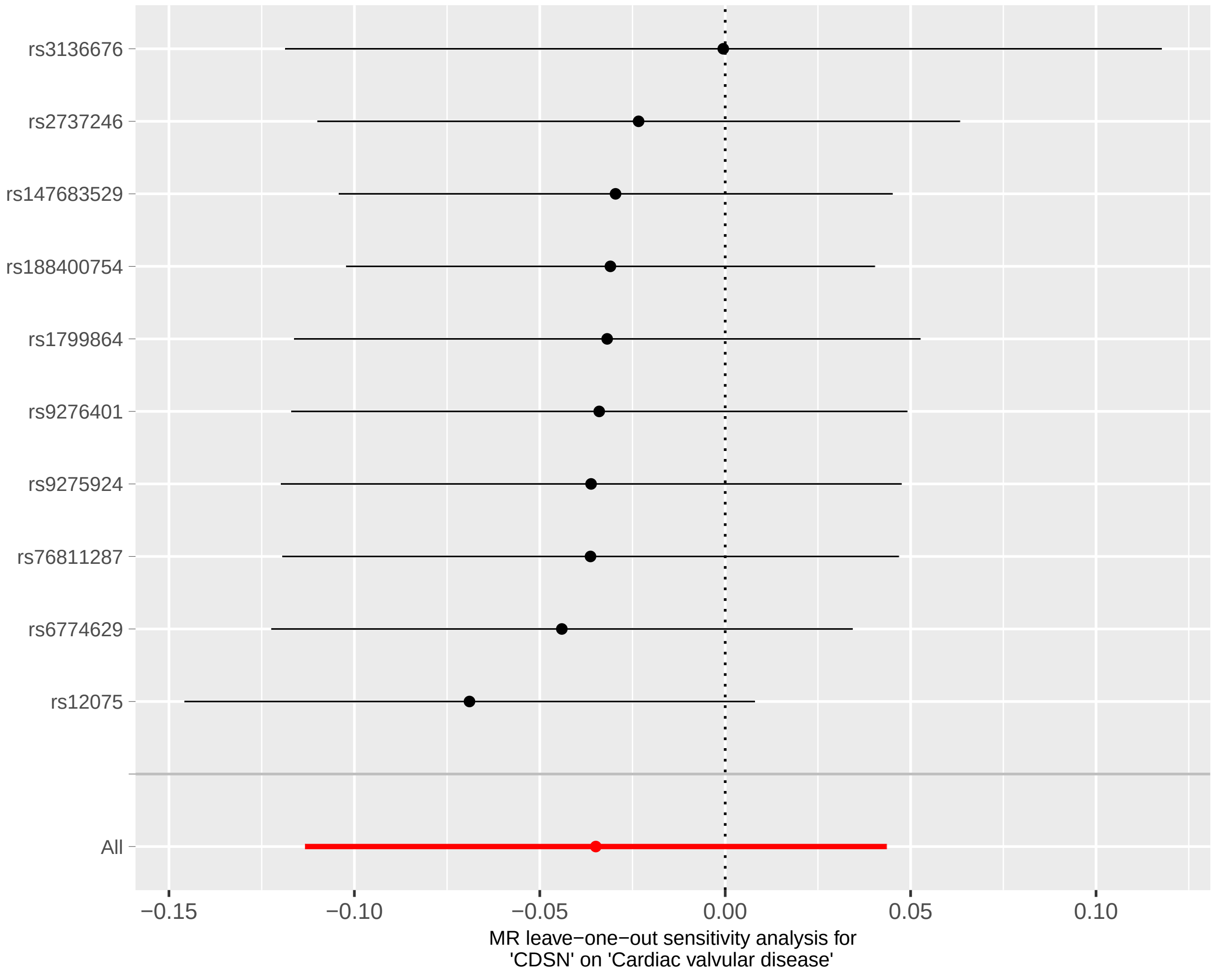
rs3136676
rs2737246
rs147683529
rs188400754
rs1799864
rs9276401
rs9275924
rs76811287
rs6774629
rs12075
All
−0.15
−0.10
−0.05
0.00
0.05
0.10
MR leave−one−out sensitivity analysis for
'CDSN' on 'Cardiac valvular disease'

Insufficient number of SNPs

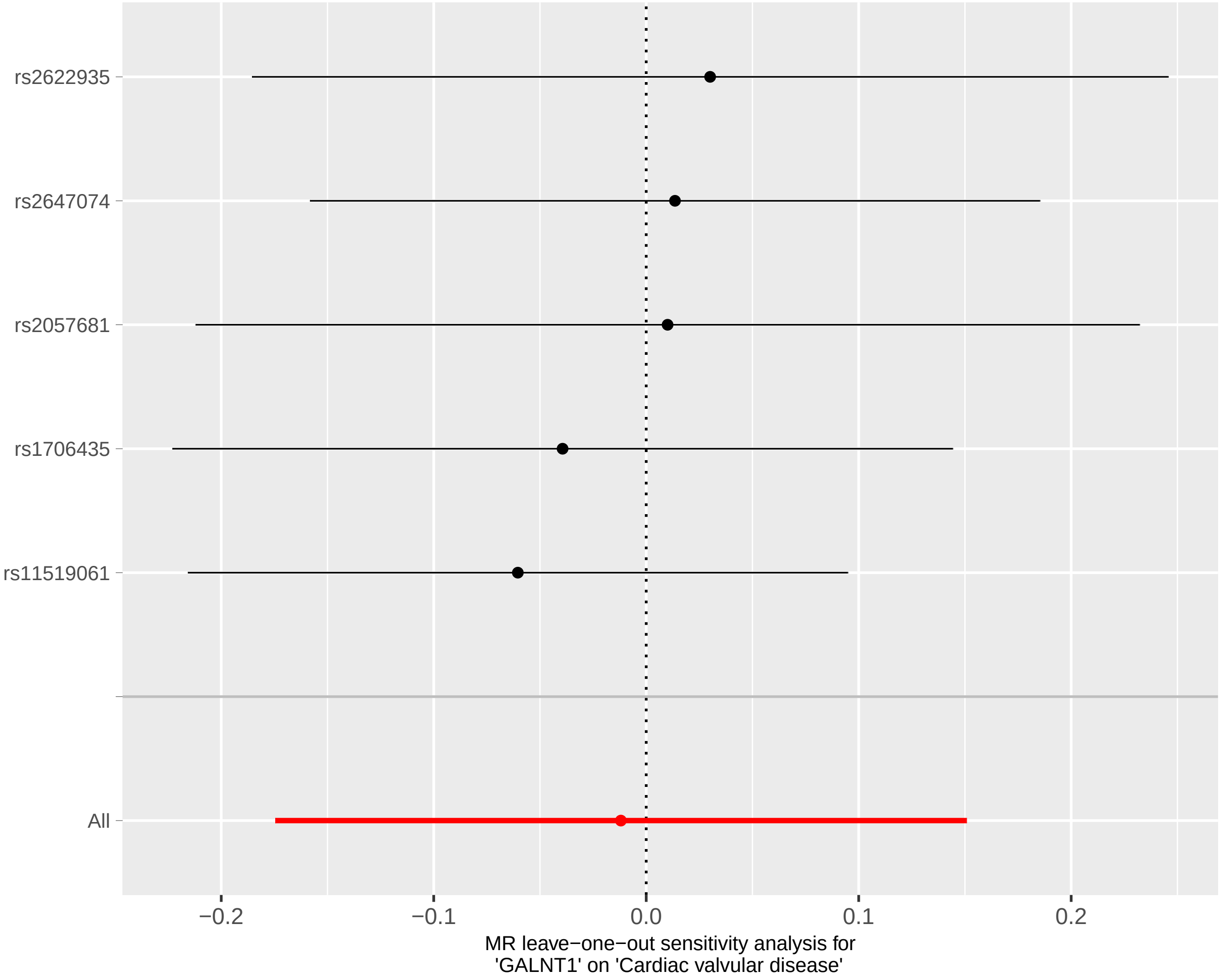
rs2622935
rs2647074
rs2057681
rs1706435
rs11519061
All
−0.2
−0.1
0.0
0.1
0.2
MR leave−one−out sensitivity analysis for
'GALNT1' on 'Cardiac valvular disease'

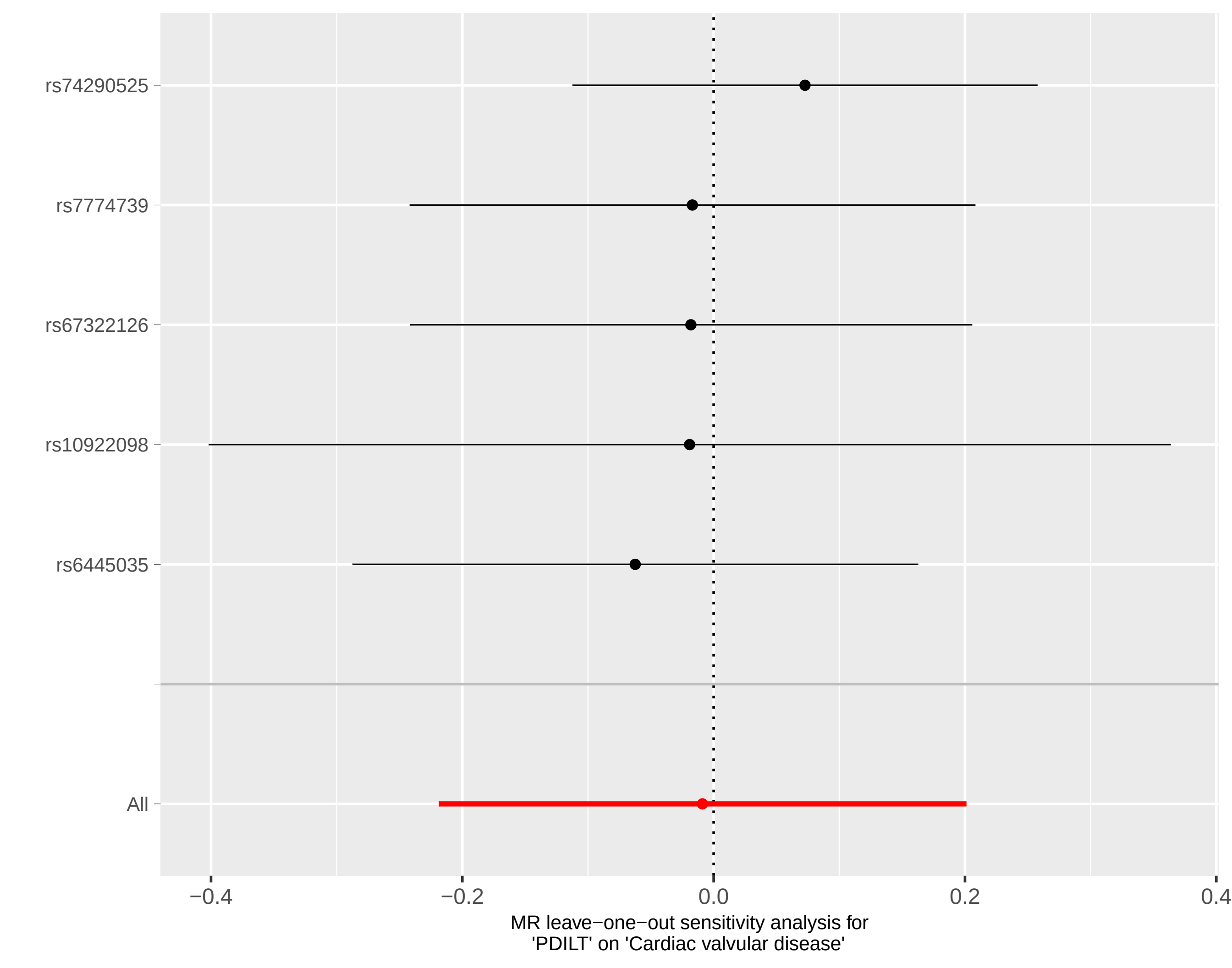
rs74290525
rs7774739
rs67322126
rs10922098
rs6445035
All
−0.4
−0.2
0.0
0.2
0.4
MR leave−one−out sensitivity analysis for
'PDILT' on 'Cardiac valvular disease'

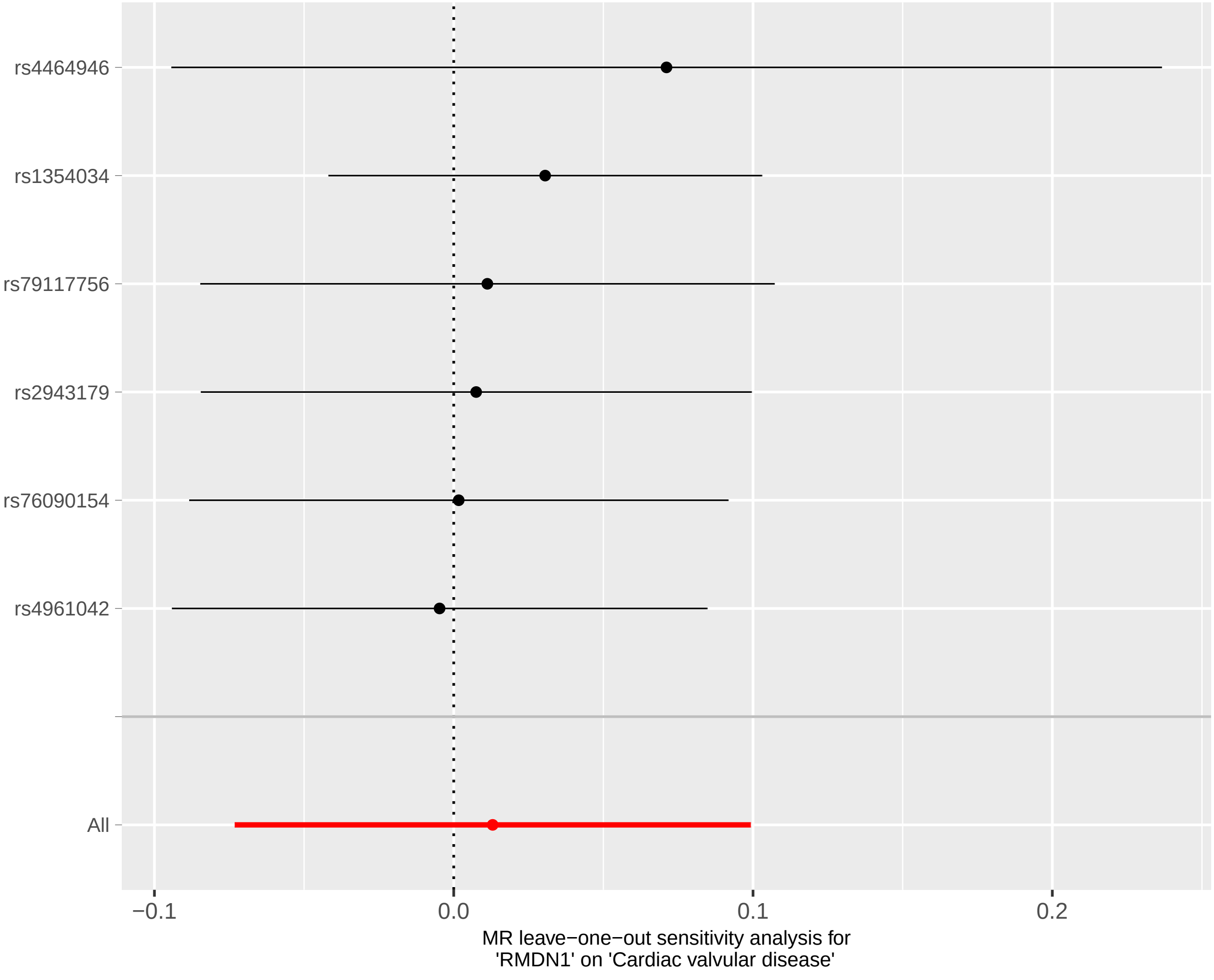

rs4464946
rs1354034
rs79117756
rs2943179
rs76090154
rs4961042
All
−0.1
0.0
0.1
0.2
MR leave−one−out sensitivity analysis for
'RMDN1' on 'Cardiac valvular disease'

Insufficient number of SNPs

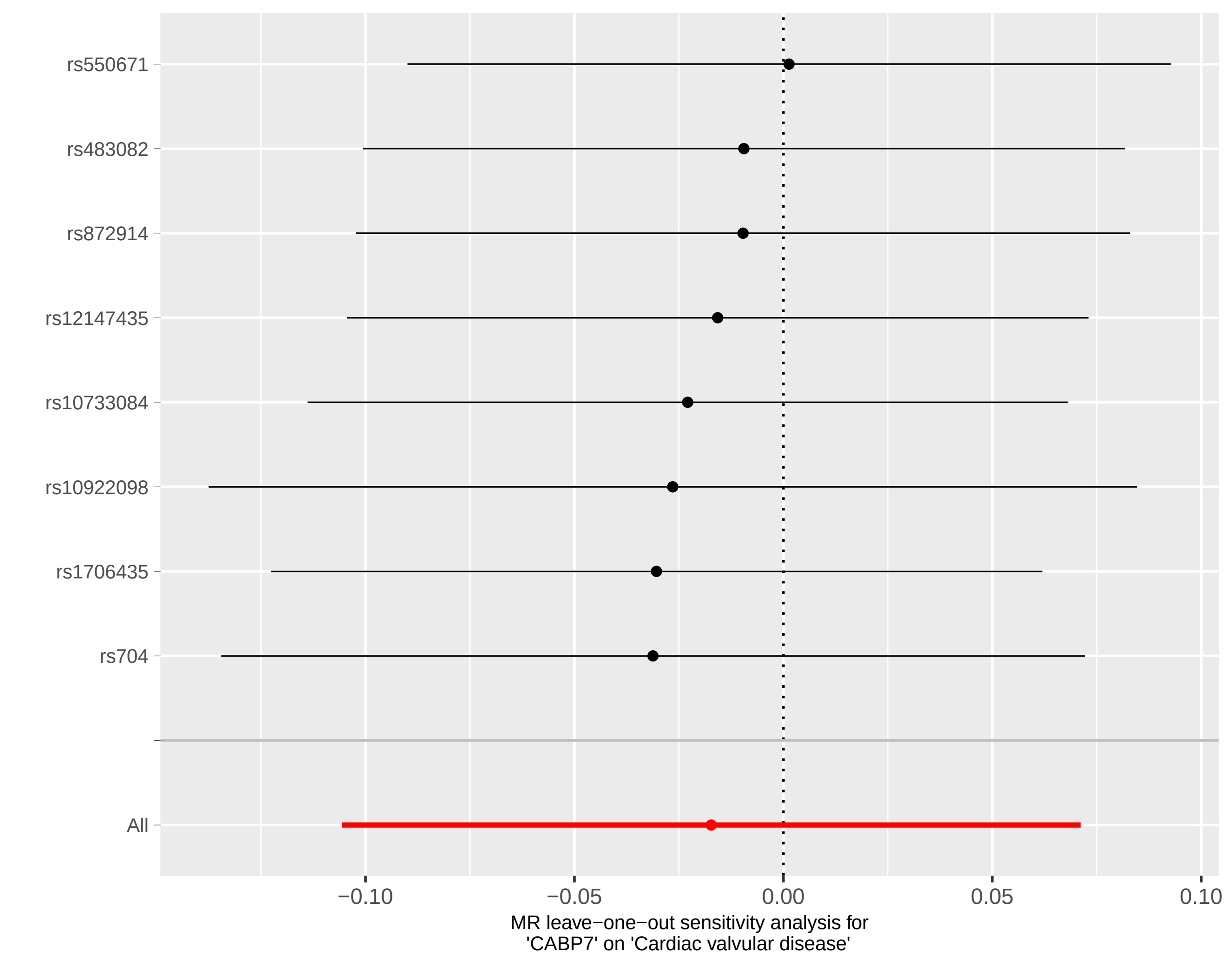
rs550671
rs483082
rs872914
rs12147435
rs10733084
rs10922098
rs1706435
rs704
All
−0.10
−0.05
0.00
0.05
0.10
MR leave−one−out sensitivity analysis for
'CABP7' on 'Cardiac valvular disease'

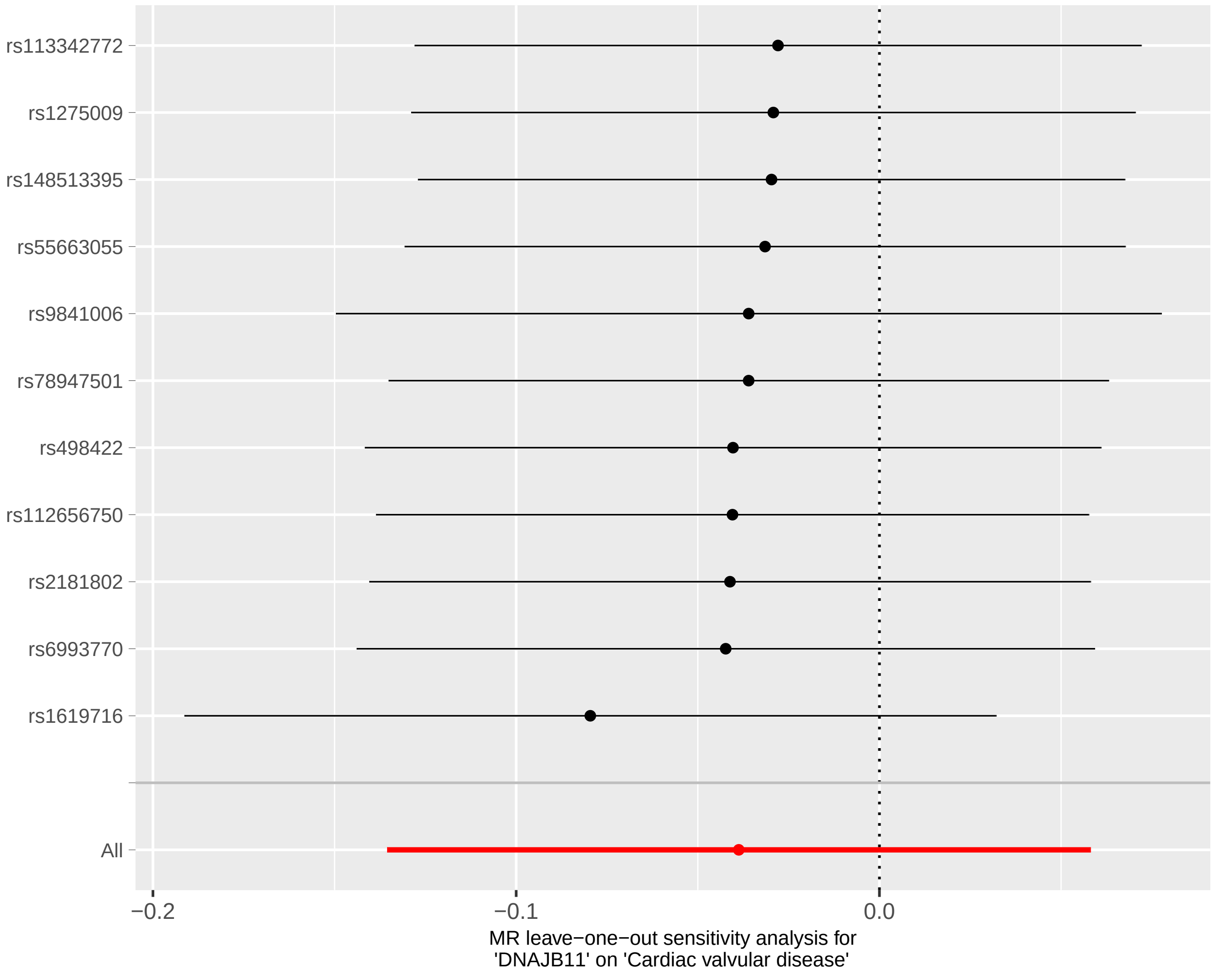
rs113342772
rs1275009
rs148513395
rs55663055
rs9841006
rs78947501
rs498422
rs112656750
rs2181802
rs6993770
rs1619716
All
−0.2
−0.1
0.0
MR leave−one−out sensitivity analysis for
'DNAJB11' on 'Cardiac valvular disease'

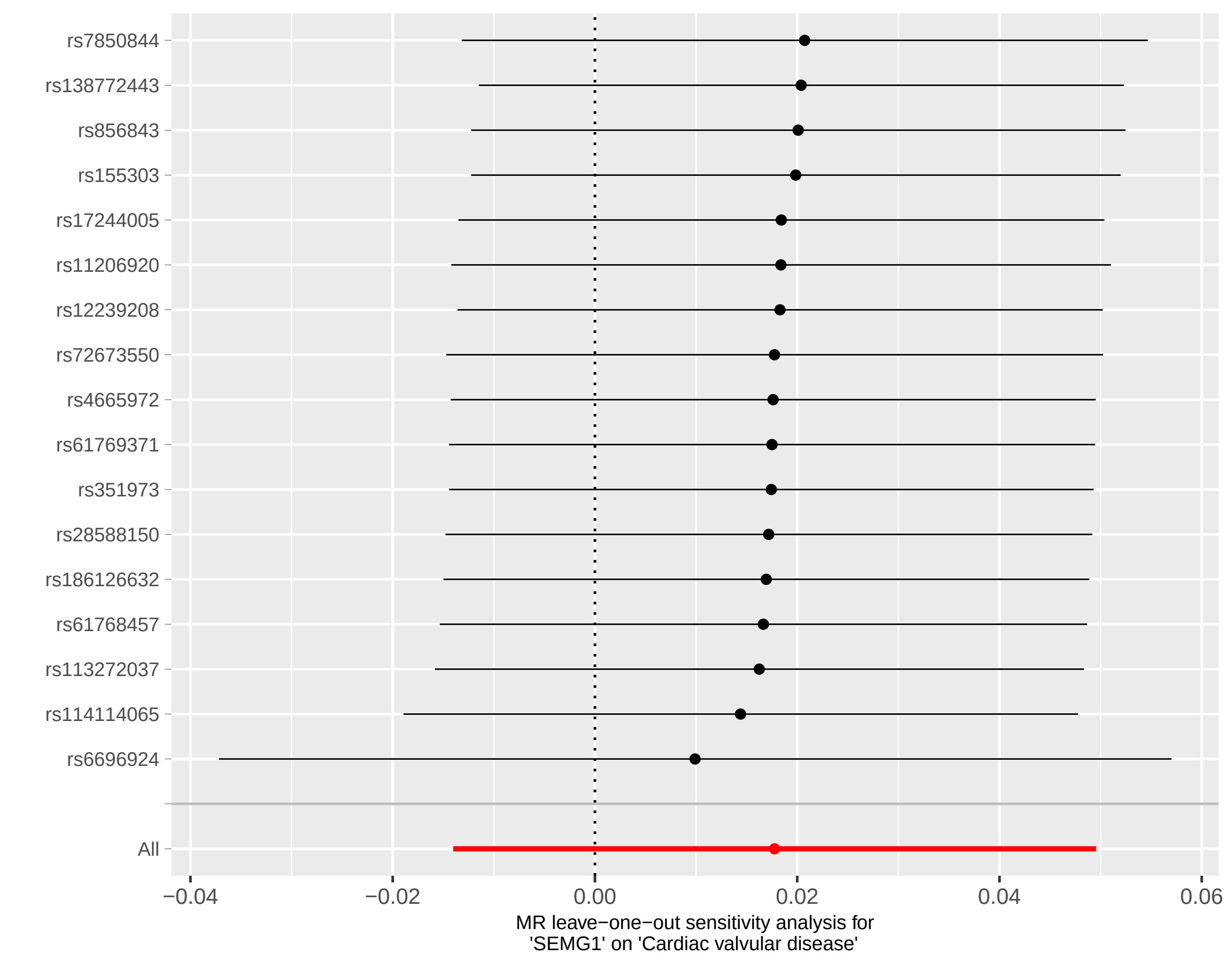
rs7850844
rs138772443
rs856843
rs155303
rs17244005
rs11206920
rs12239208
rs72673550
rs4665972
rs61769371
rs351973
rs28588150
rs186126632
rs61768457
rs113272037
rs114114065
rs6696924
All
−0.04
−0.02
0.00
0.02
0.04
0.06
MR leave−one−out sensitivity analysis for
'SEMG1' on 'Cardiac valvular disease'

Insufficient number of SNPs

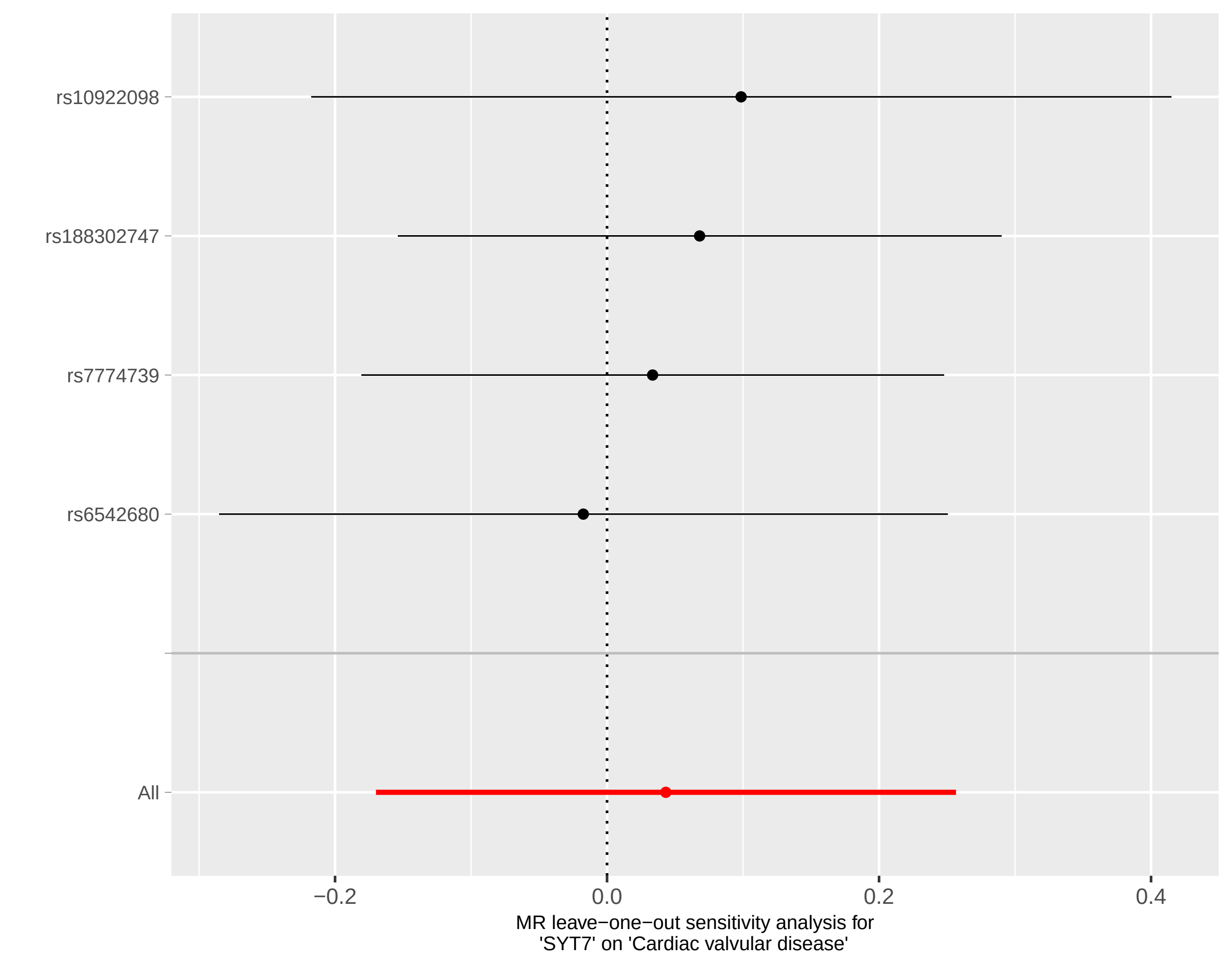

rs10922098
rs188302747
rs7774739
rs6542680
All
−0.2
0.0
0.2
0.4
MR leave−one−out sensitivity analysis for
'SYT7' on 'Cardiac valvular disease'

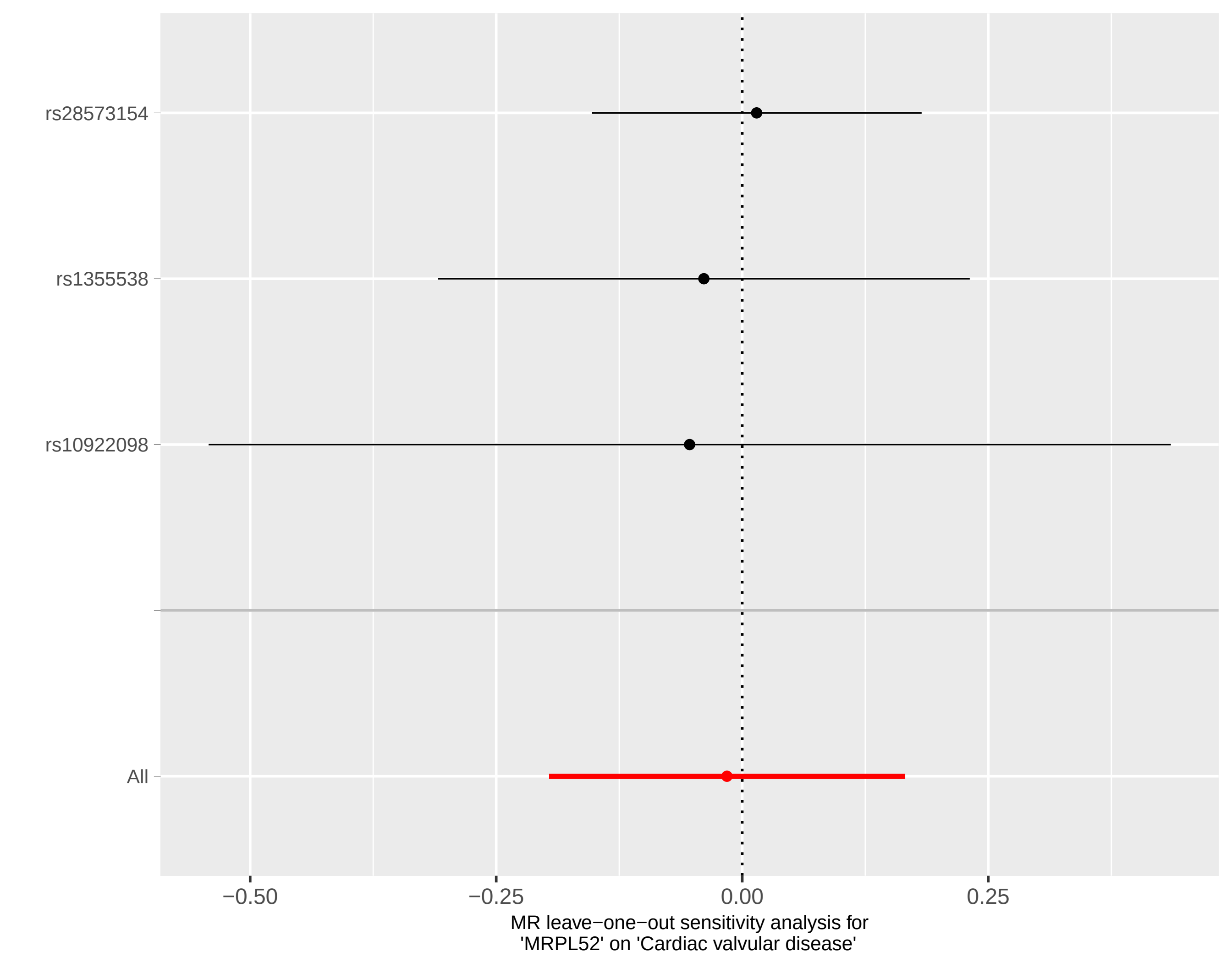
rs28573154
rs1355538
rs10922098
All
−0.50
−0.25
0.00
0.25
MR leave−one−out sensitivity analysis for
'MRPL52' on 'Cardiac valvular disease'

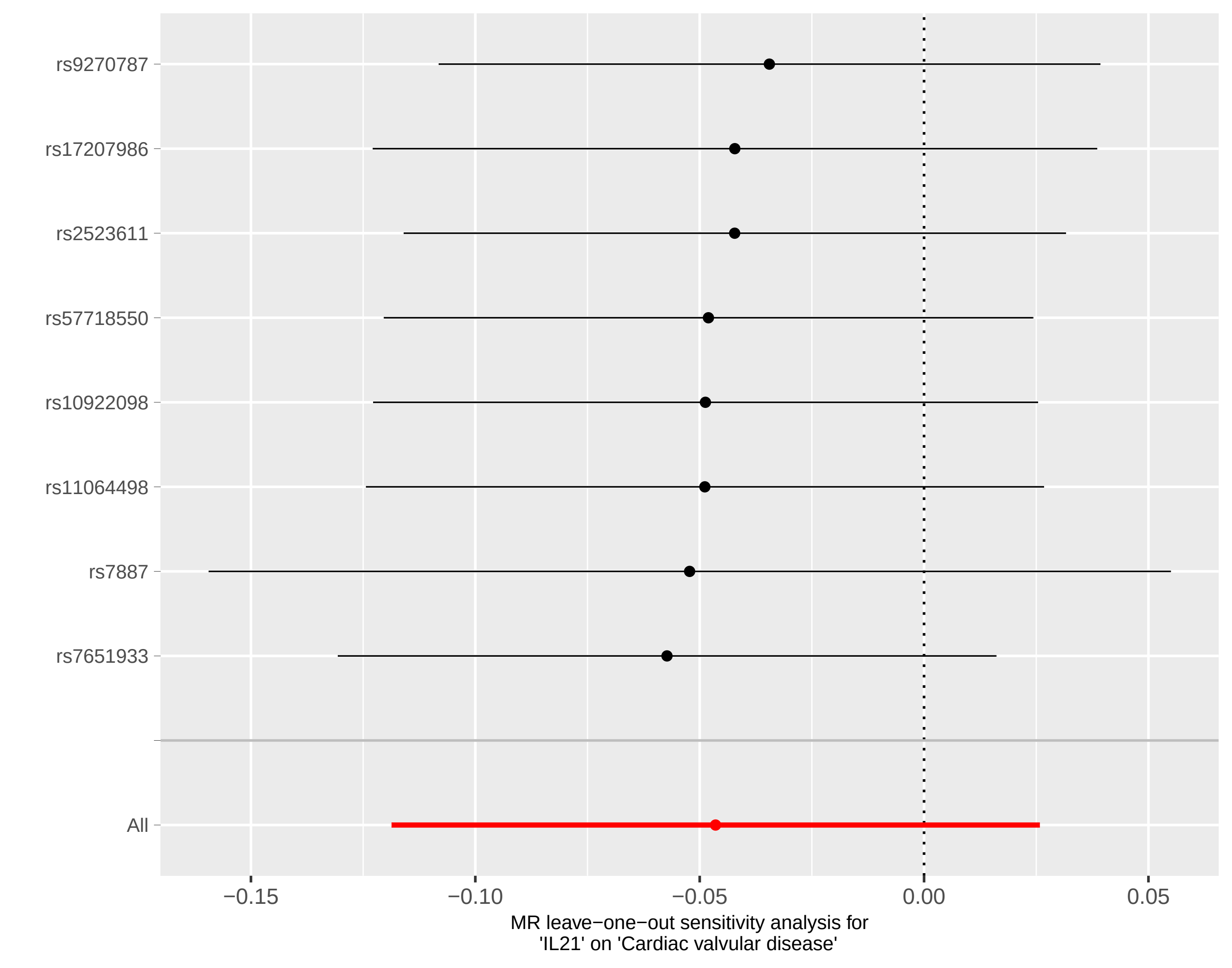
rs9270787
rs17207986
rs2523611
rs57718550
rs10922098
rs11064498
rs7887
rs7651933
All
−0.15
−0.10
−0.05
0.00
0.05
MR leave−one−out sensitivity analysis for
'IL21' on 'Cardiac valvular disease'

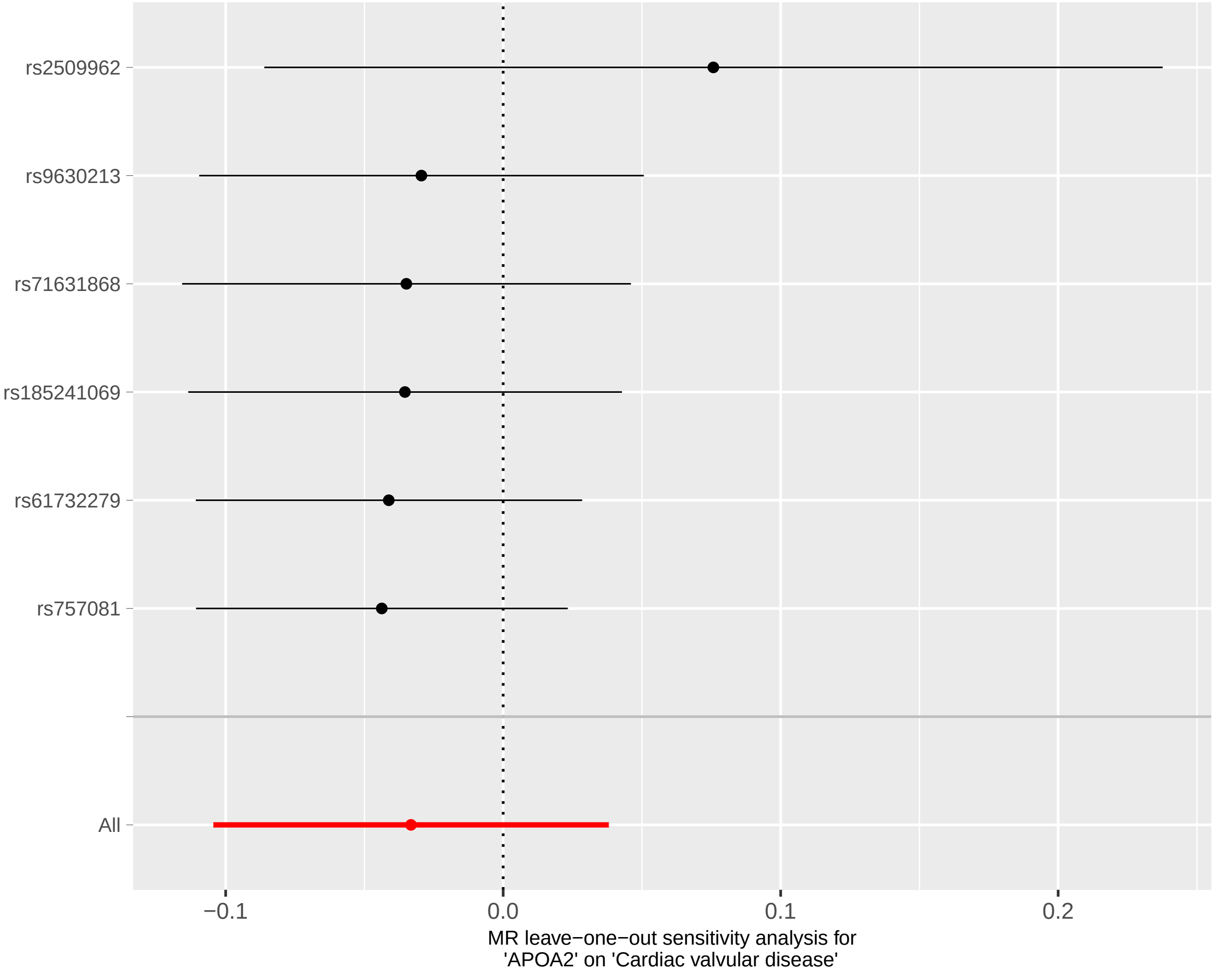
rs2509962
rs9630213
rs71631868
rs185241069
rs61732279
rs757081
All
−0.1
0.0
0.1
0.2
MR leave−one−out sensitivity analysis for
'APOA2' on 'Cardiac valvular disease'

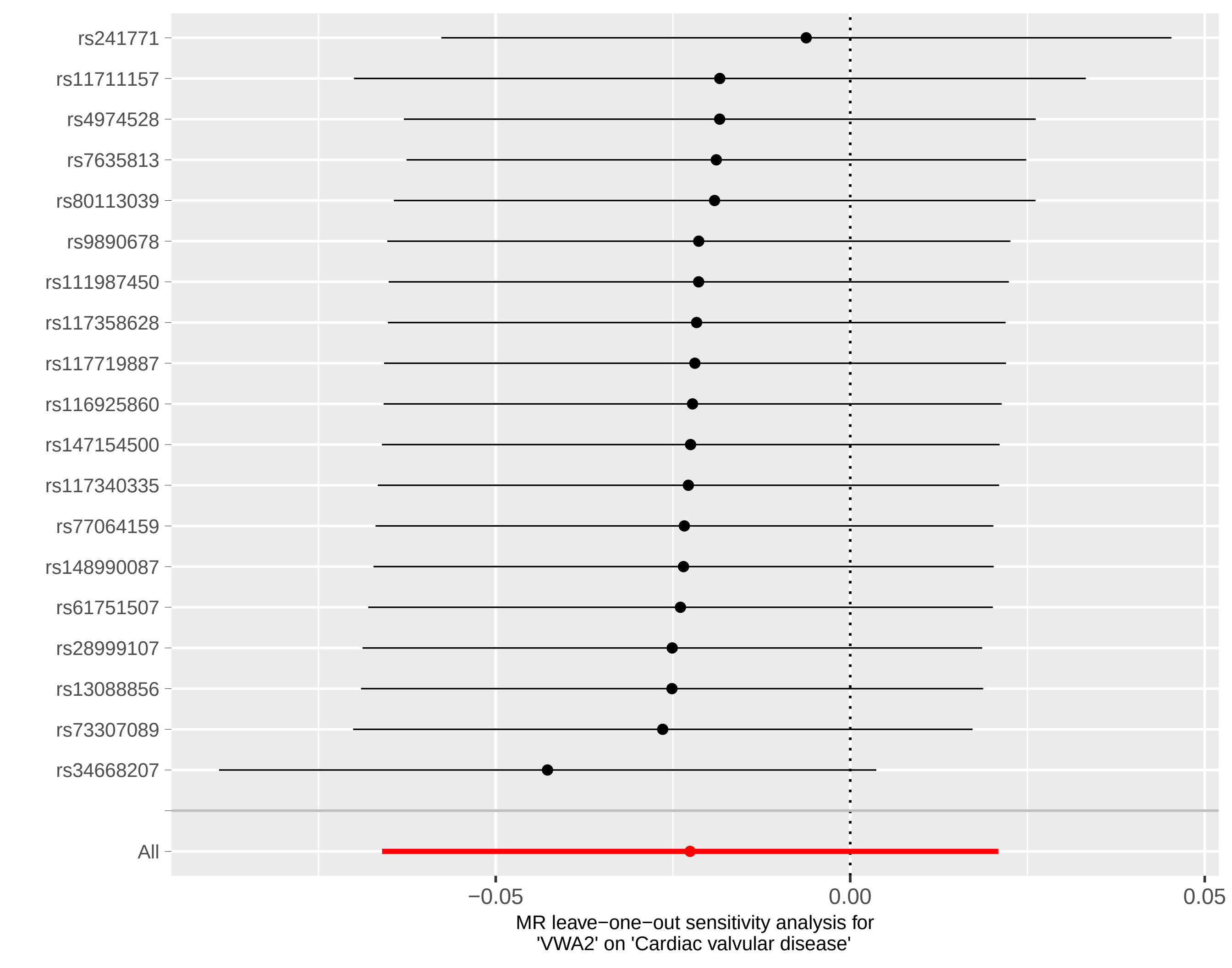

rs241771
rs11711157
rs4974528
rs7635813
rs80113039
rs9890678
rs111987450
rs117358628
rs117719887
rs116925860
rs147154500
rs117340335
rs77064159
rs148990087
rs61751507
rs28999107
rs13088856
rs73307089
rs34668207
All
−0.05
0.00
0.05
MR leave−one−out sensitivity analysis for
'VWA2' on 'Cardiac valvular disease'

Insufficient number of SNPs

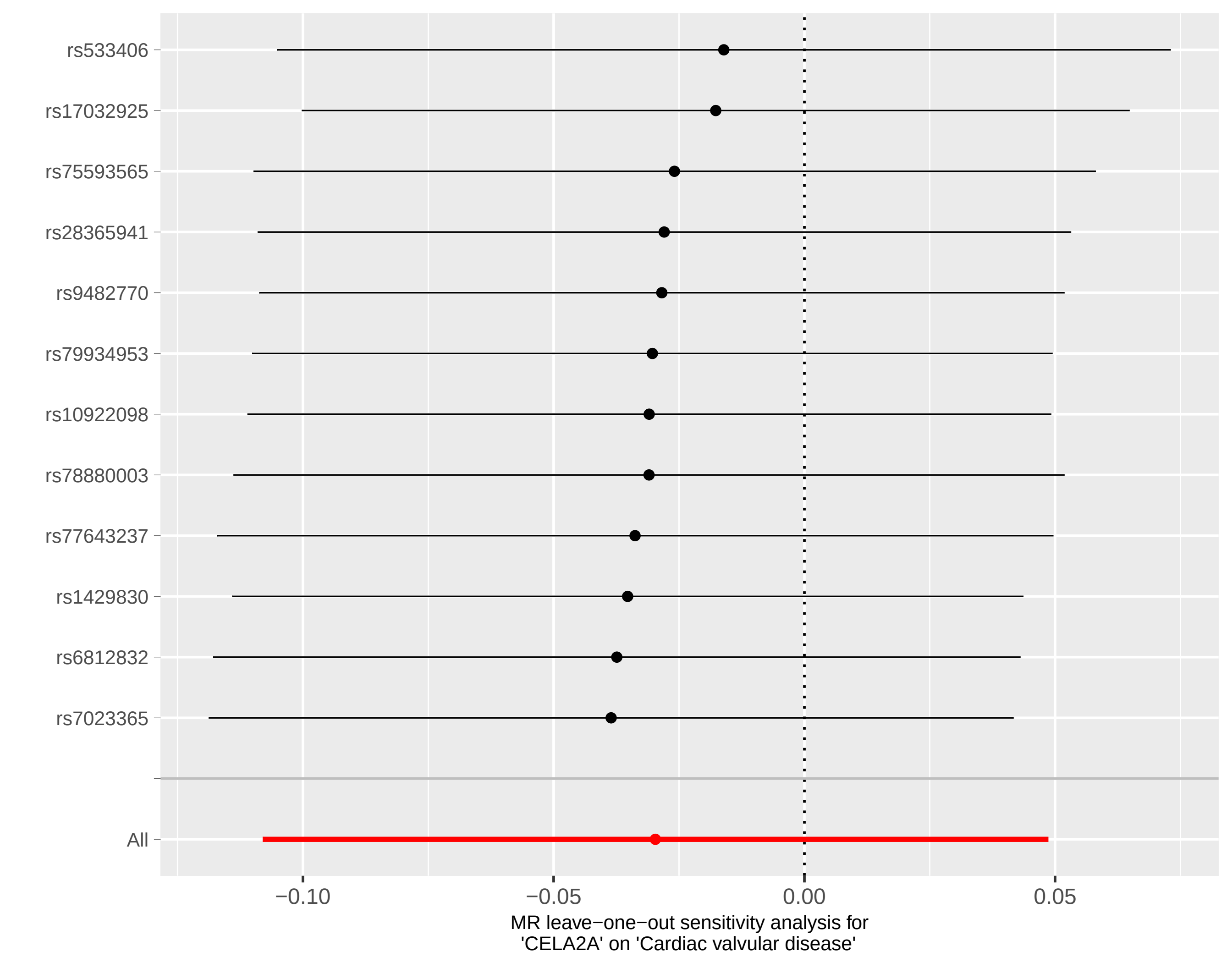
rs533406
rs17032925
rs75593565
rs28365941
rs9482770
rs79934953
rs10922098
rs78880003
rs77643237
rs1429830
rs6812832
rs7023365
All
−0.10
−0.05
0.00
0.05
MR leave−one−out sensitivity analysis for
'CELA2A' on 'Cardiac valvular disease'

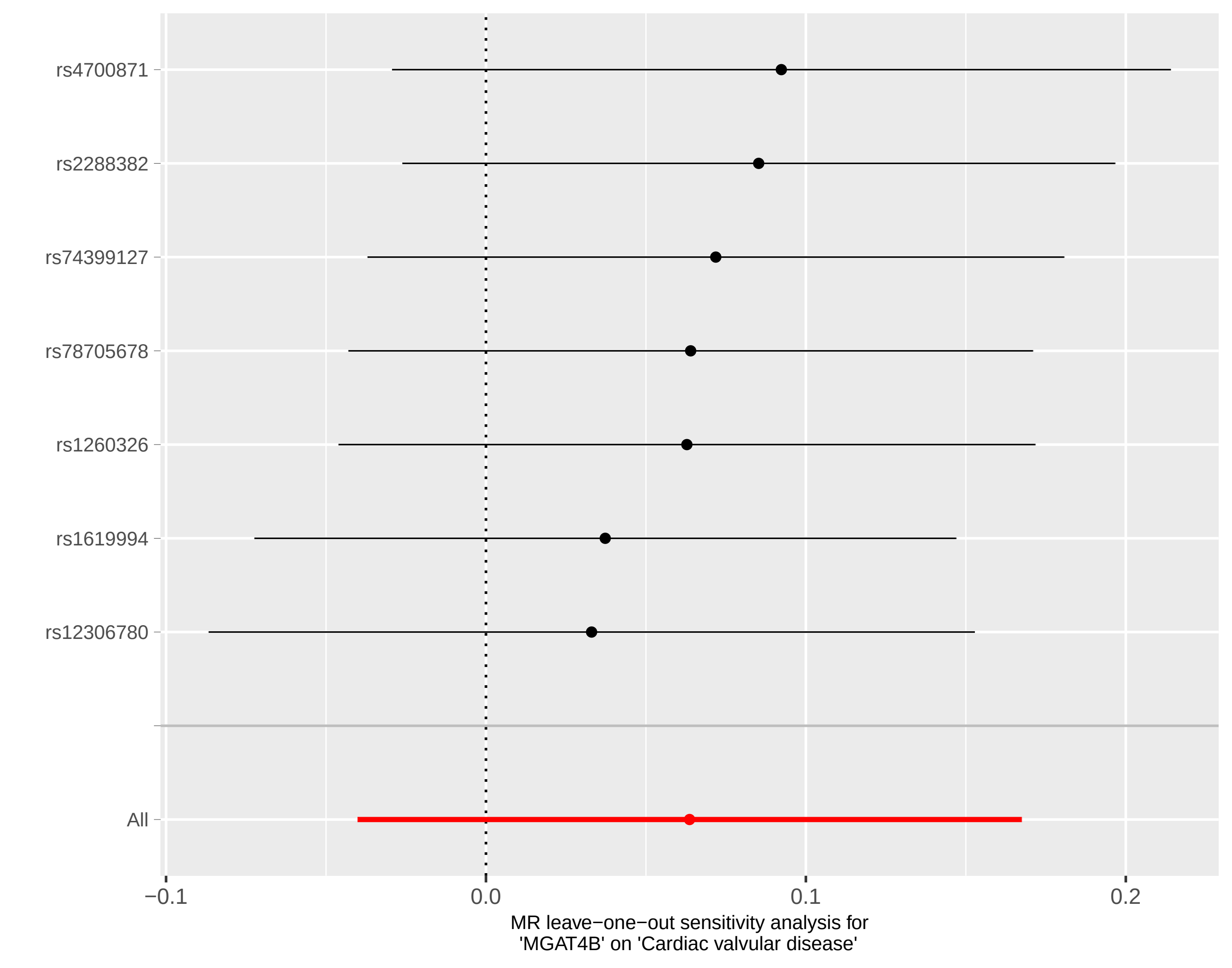

rs4700871
rs2288382
rs74399127
rs78705678
rs1260326
rs1619994
rs12306780
All
−0.1
0.0
0.1
0.2
MR leave−one−out sensitivity analysis for
'MGAT4B' on 'Cardiac valvular disease'

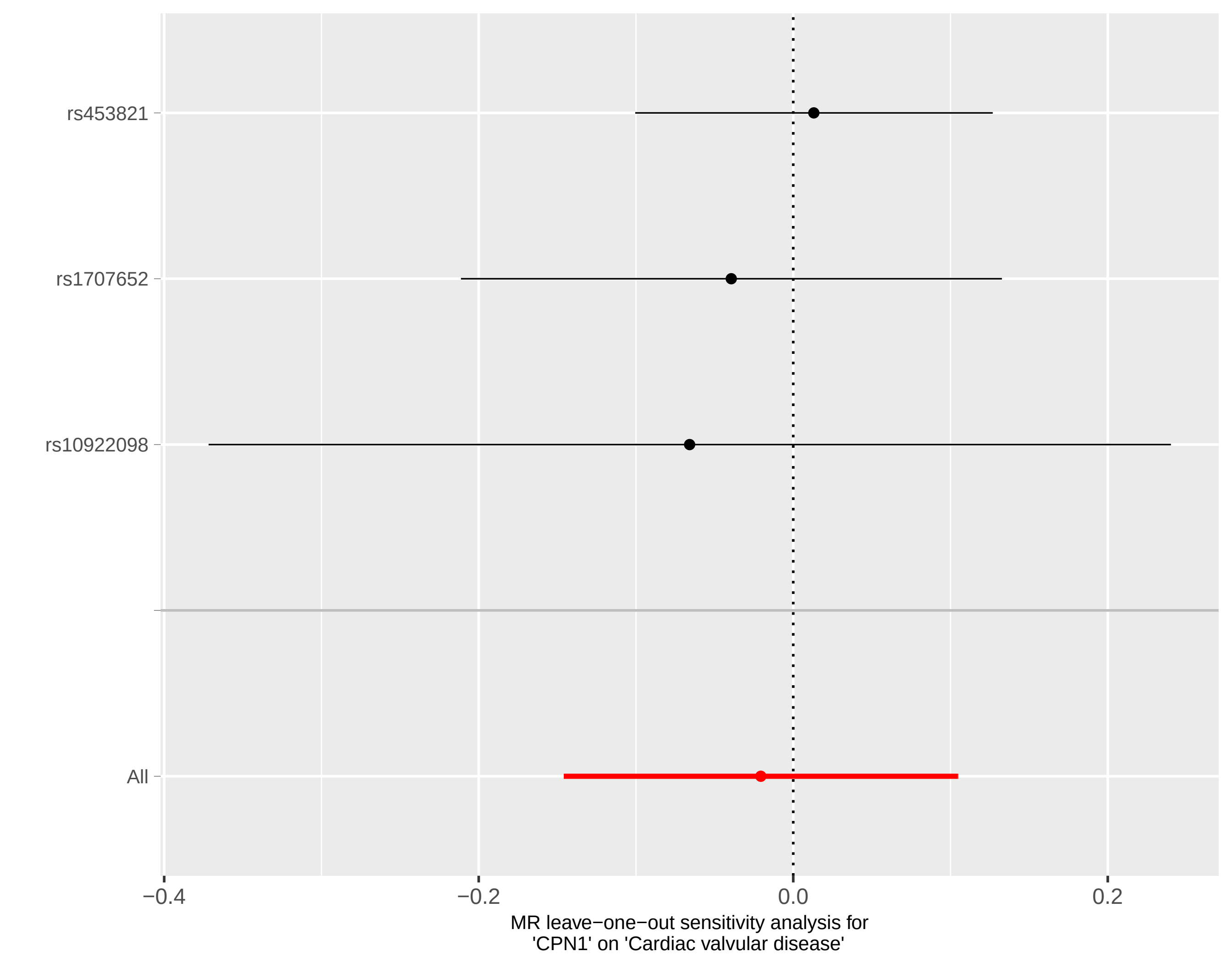
rs453821
rs1707652
rs10922098
All
−0.4
−0.2
0.0
0.2
MR leave−one−out sensitivity analysis for
'CPN1' on 'Cardiac valvular disease'

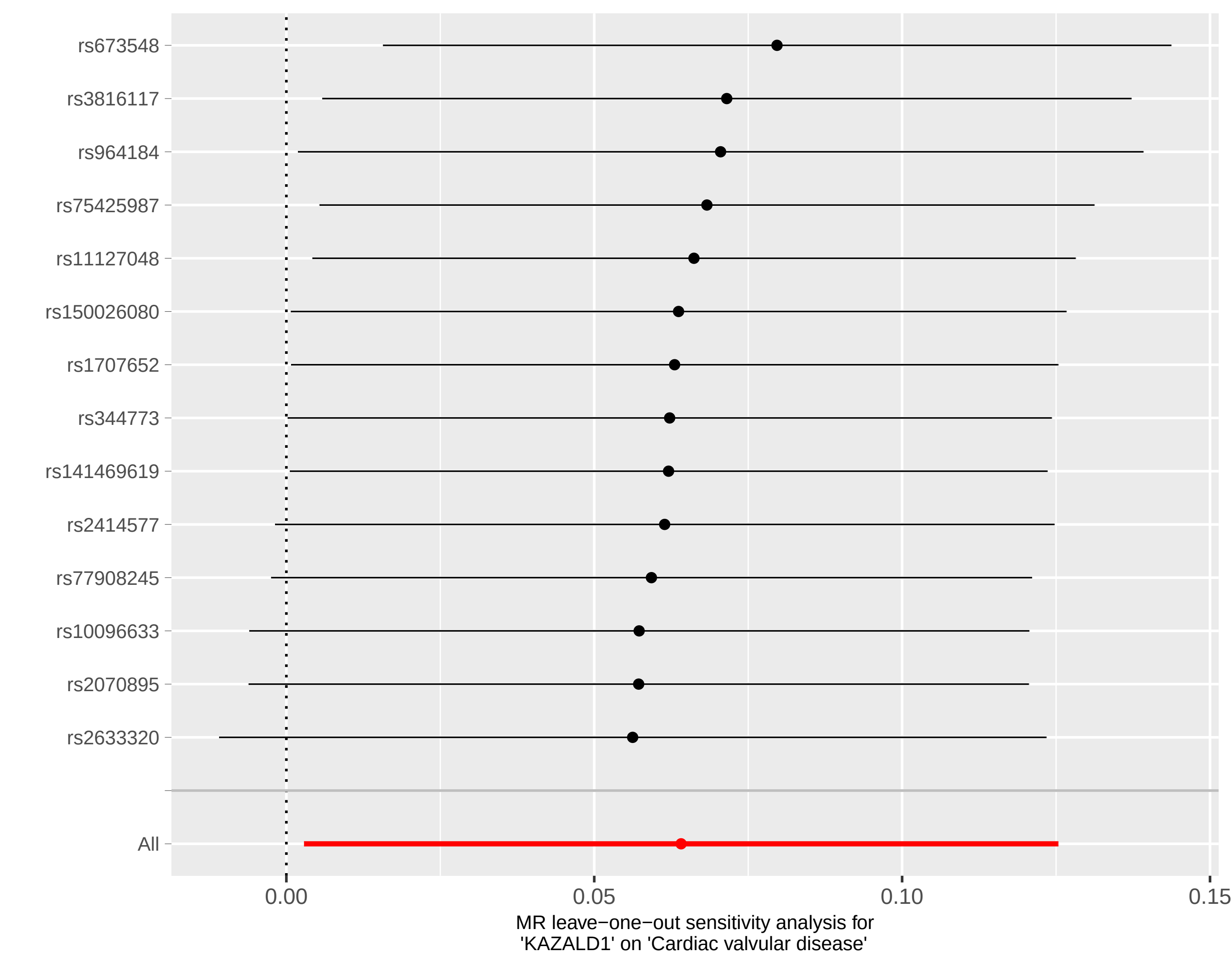
rs673548
rs3816117
rs964184
rs75425987
rs11127048
rs150026080
rs1707652
rs344773
rs141469619
rs2414577
rs77908245
rs10096633
rs2070895
rs2633320
All
0.00
0.05
0.10
0.15
MR leave−one−out sensitivity analysis for
'KAZALD1' on 'Cardiac valvular disease'

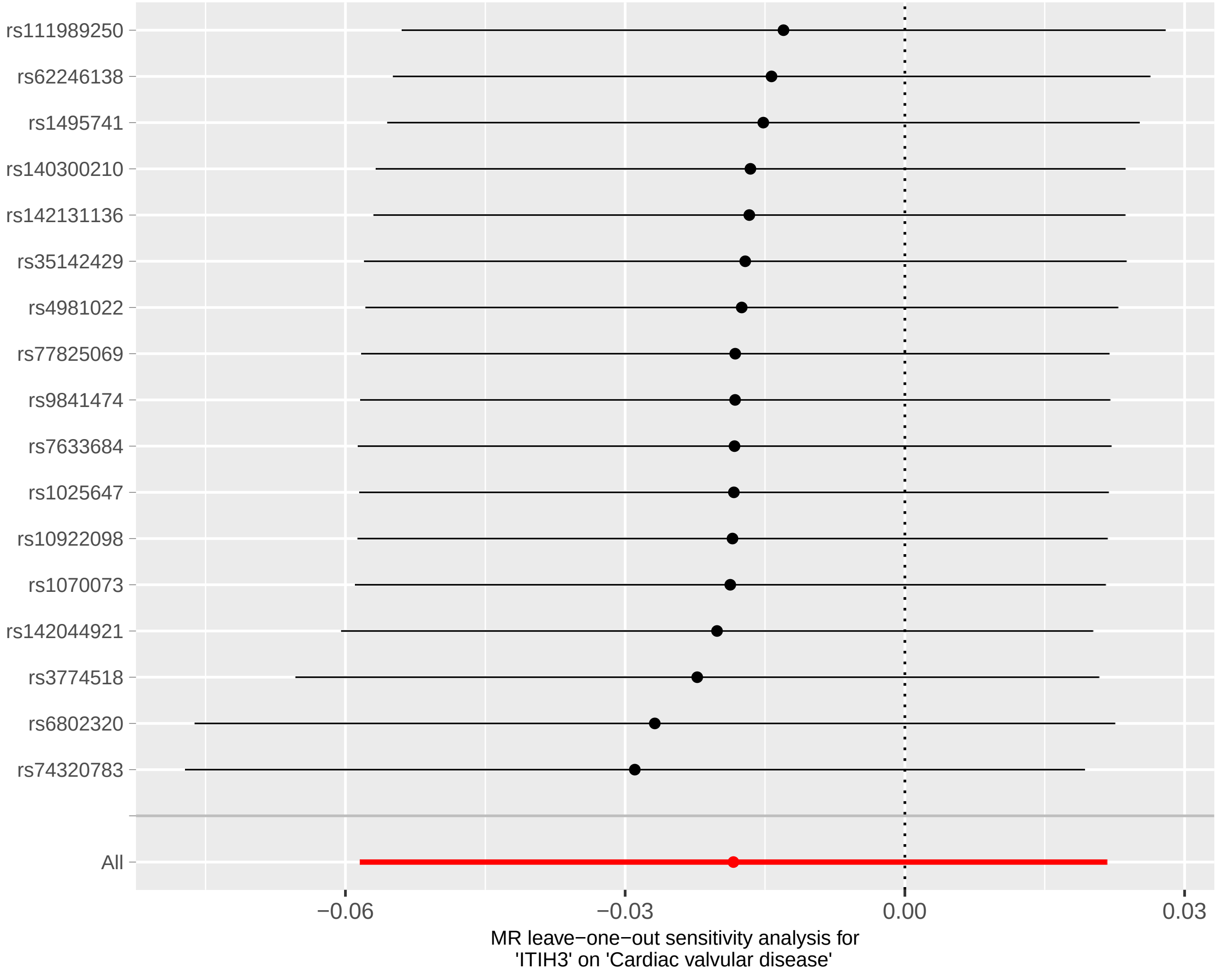
rs111989250
rs62246138
rs1495741
rs140300210
rs142131136
rs35142429
rs4981022
rs77825069
rs9841474
rs7633684
rs1025647
rs10922098
rs1070073
rs142044921
rs3774518
rs6802320
rs74320783
All
−0.06
−0.03
0.00
0.03
MR leave−one−out sensitivity analysis for
'ITIH3' on 'Cardiac valvular disease'

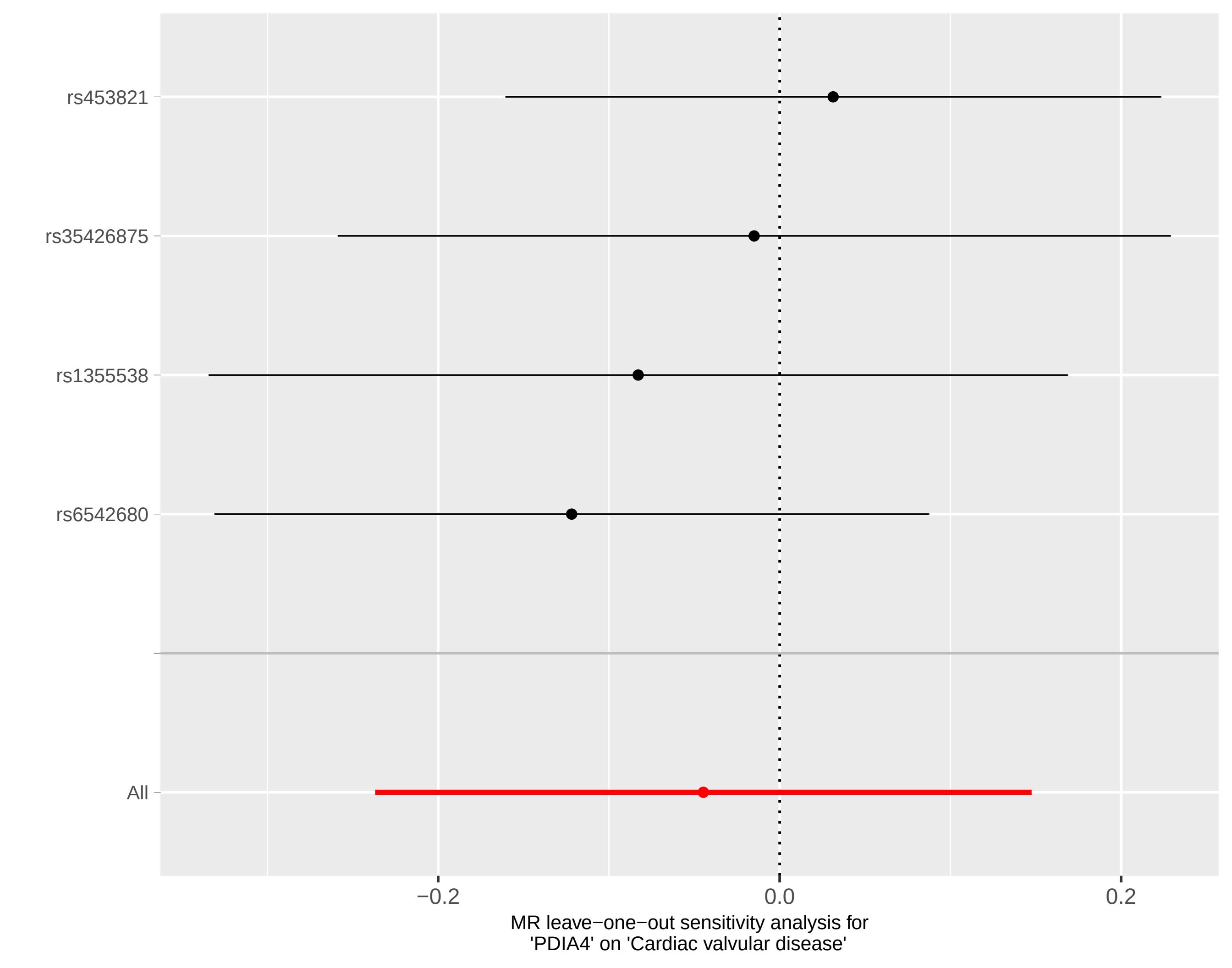

rs453821
rs35426875
rs1355538
rs6542680
All
−0.2
0.0
0.2
MR leave−one−out sensitivity analysis for
'PDIA4' on 'Cardiac valvular disease'

Insufficient number of SNPs

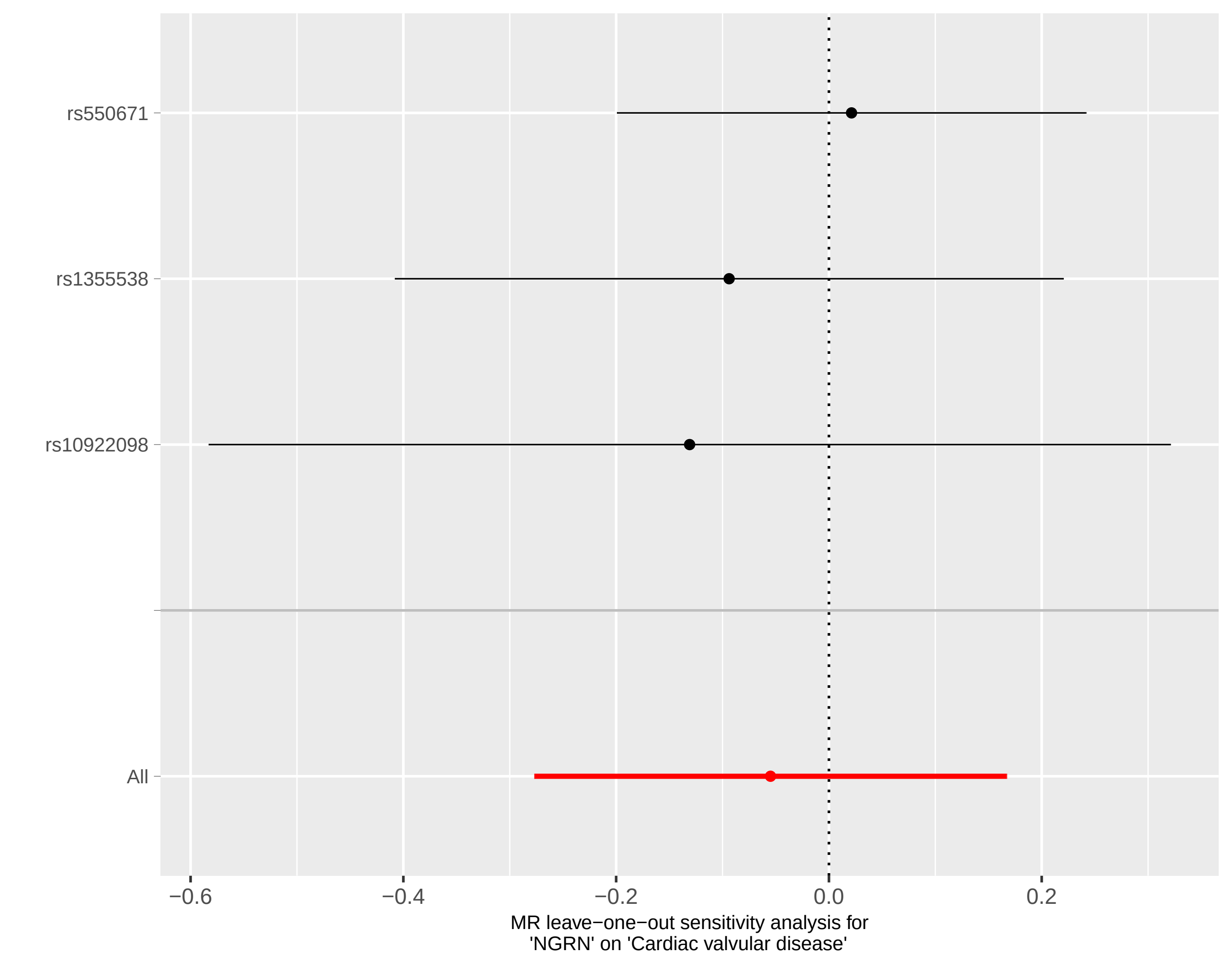

rs550671
rs1355538
rs10922098
All
−0.6
−0.4
−0.2
0.0
0.2
MR leave−one−out sensitivity analysis for
'NGRN' on 'Cardiac valvular disease'

Insufficient number of SNPs

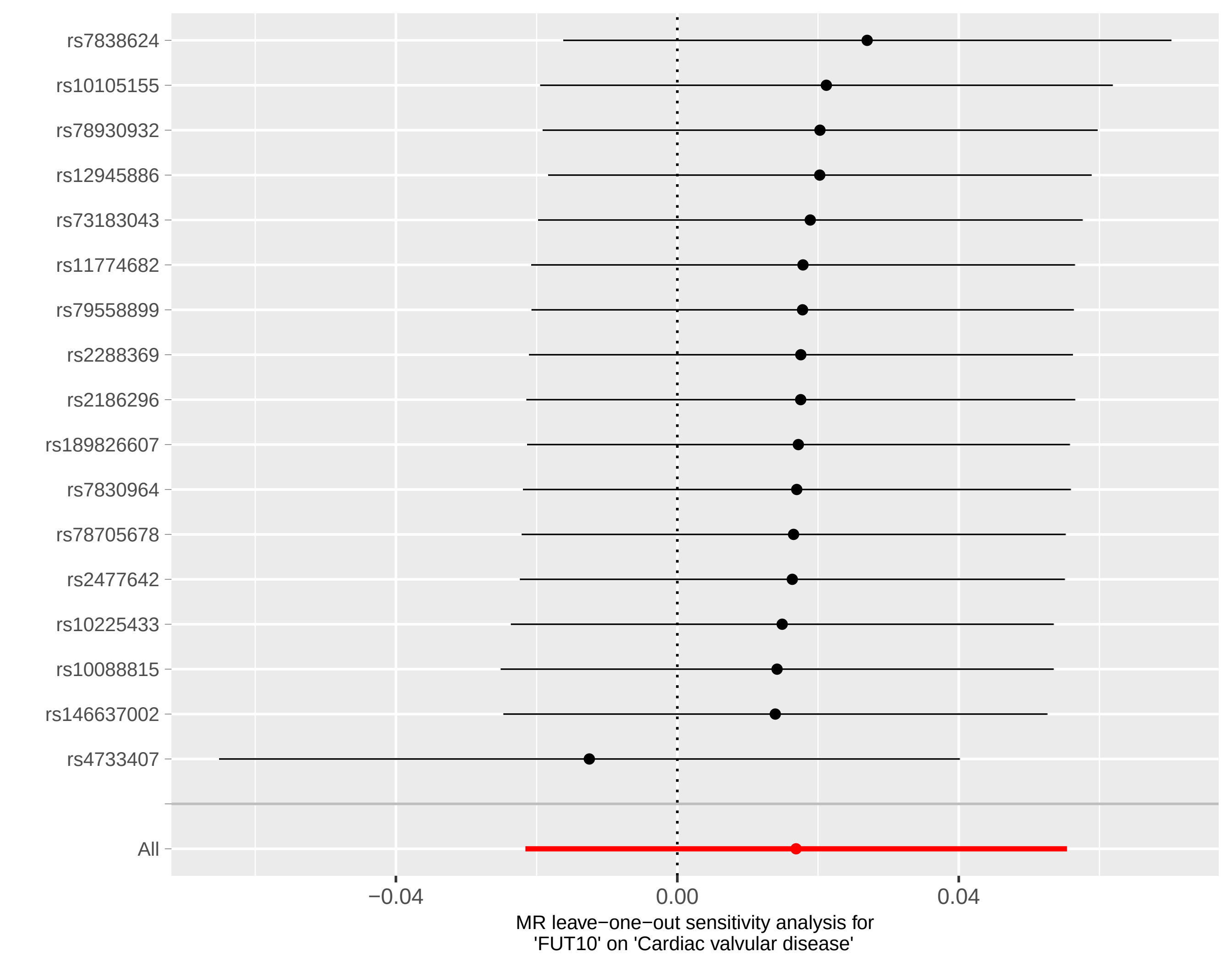
rs7838624
rs10105155
rs78930932
rs12945886
rs73183043
rs11774682
rs79558899
rs2288369
rs2186296
rs189826607
rs7830964
rs78705678
rs2477642
rs10225433
rs10088815
rs146637002
rs4733407
All
−0.04
0.00
0.04
MR leave−one−out sensitivity analysis for
'FUT10' on 'Cardiac valvular disease'

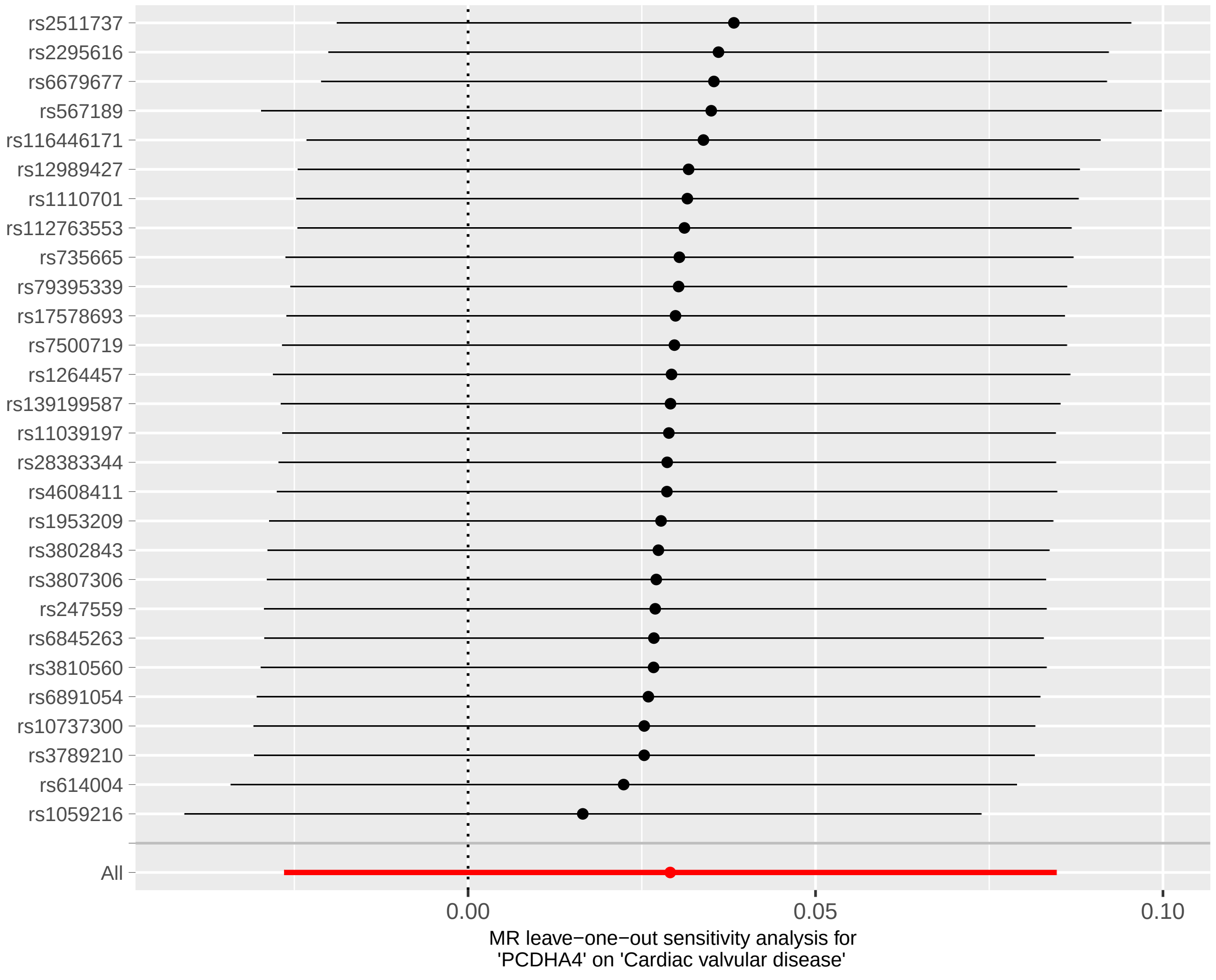
rs2511737
rs2295616
rs6679677
rs567189
rs116446171
rs12989427
rs1110701
rs112763553
rs735665
rs79395339
rs17578693
rs7500719
rs1264457
rs139199587
rs11039197
rs28383344
rs4608411
rs1953209
rs3802843
rs3807306
rs247559
rs6845263
rs3810560
rs6891054
rs10737300
rs3789210
rs614004
rs1059216
All
0.00
0.05
0.10
MR leave−one−out sensitivity analysis for
'PCDHA4' on 'Cardiac valvular disease'

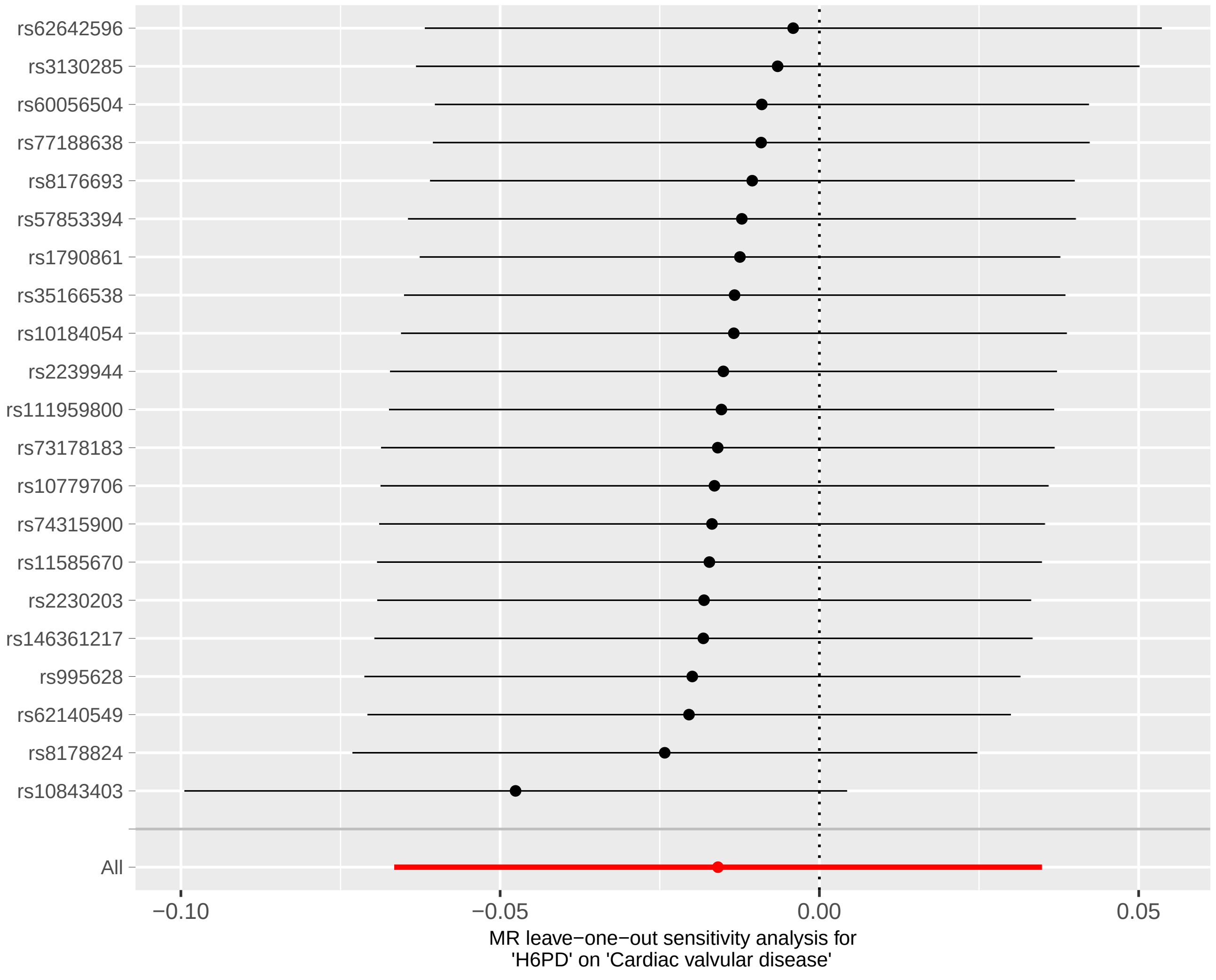

rs62642596
rs3130285
rs60056504
rs77188638
rs8176693
rs57853394
rs1790861
rs35166538
rs10184054
rs2239944
rs111959800
rs73178183
rs10779706
rs74315900
rs11585670
rs2230203
rs146361217
rs995628
rs62140549
rs8178824
rs10843403
All
−0.10
−0.05
0.00
0.05
MR leave−one−out sensitivity analysis for
'H6PD' on 'Cardiac valvular disease'

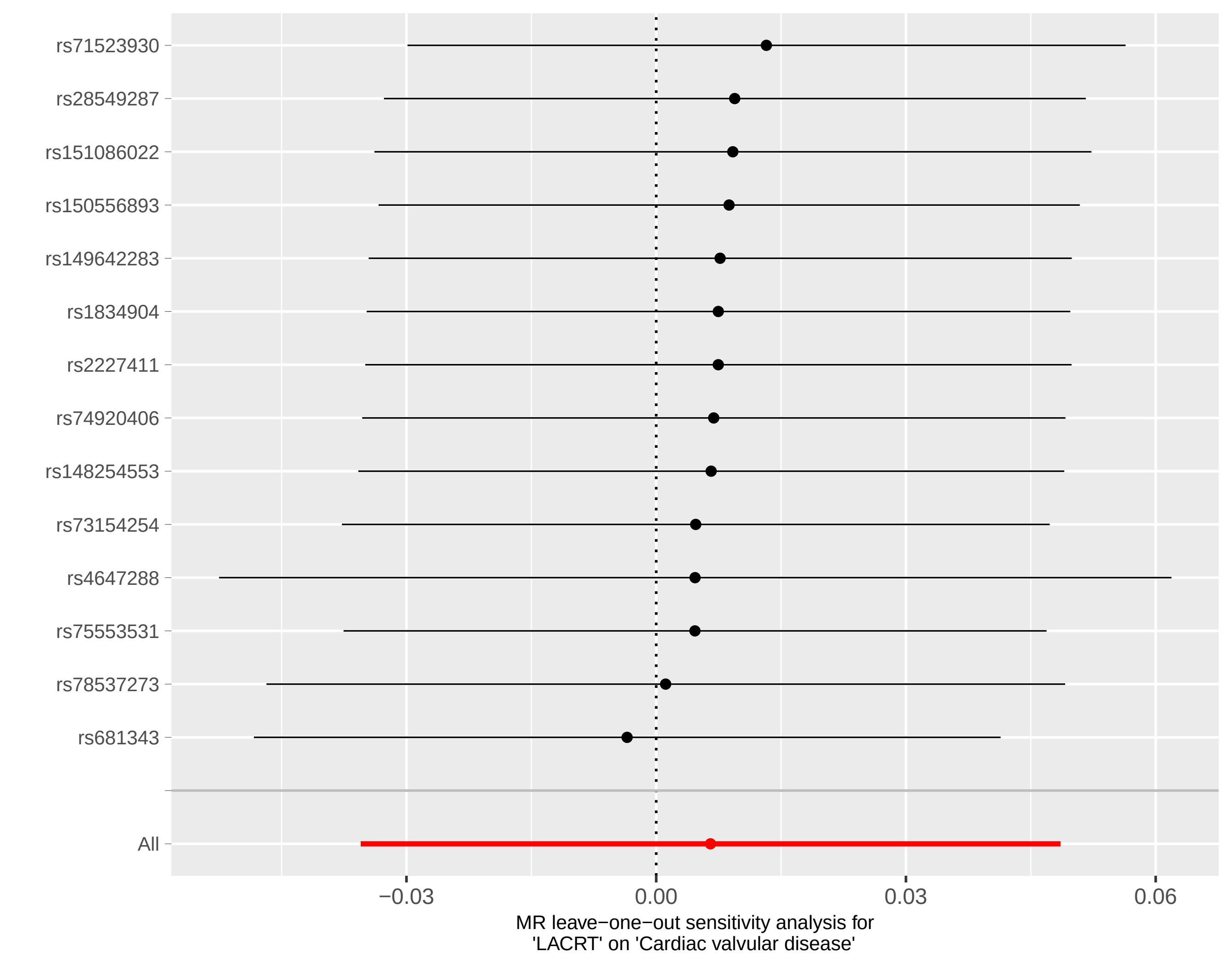
rs71523930
rs28549287
rs151086022
rs150556893
rs149642283
rs1834904
rs2227411
rs74920406
rs148254553
rs73154254
rs4647288
rs75553531
rs78537273
rs681343
All
−0.03
0.00
0.03
0.06
MR leave−one−out sensitivity analysis for
'LACRT' on 'Cardiac valvular disease'

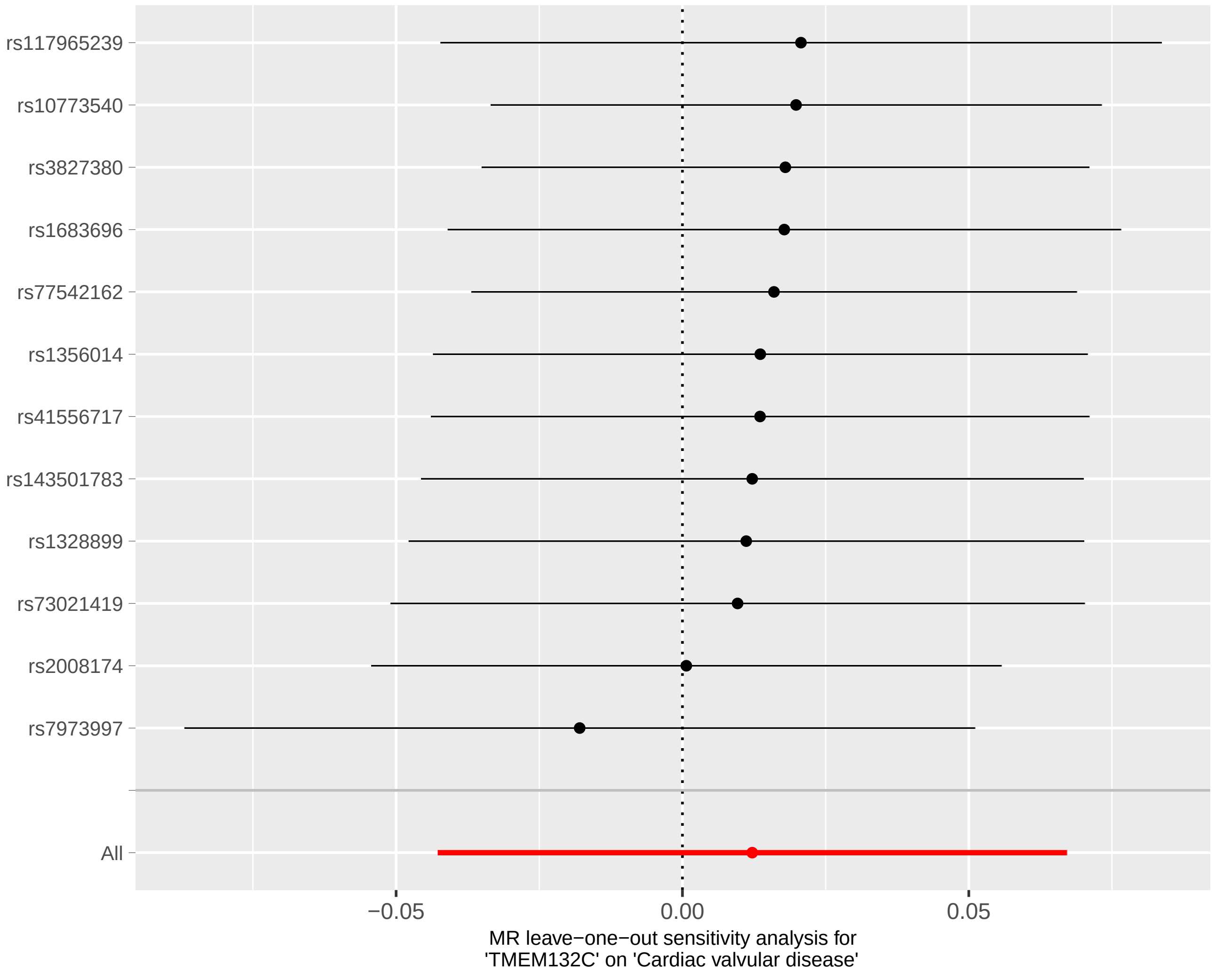
rs117965239
rs10773540
rs3827380
rs1683696
rs77542162
rs1356014
rs41556717
rs143501783
rs1328899
rs73021419
rs2008174
rs7973997
All
−0.05
0.00
0.05
MR leave−one−out sensitivity analysis for
'TMEM132C' on 'Cardiac valvular disease'

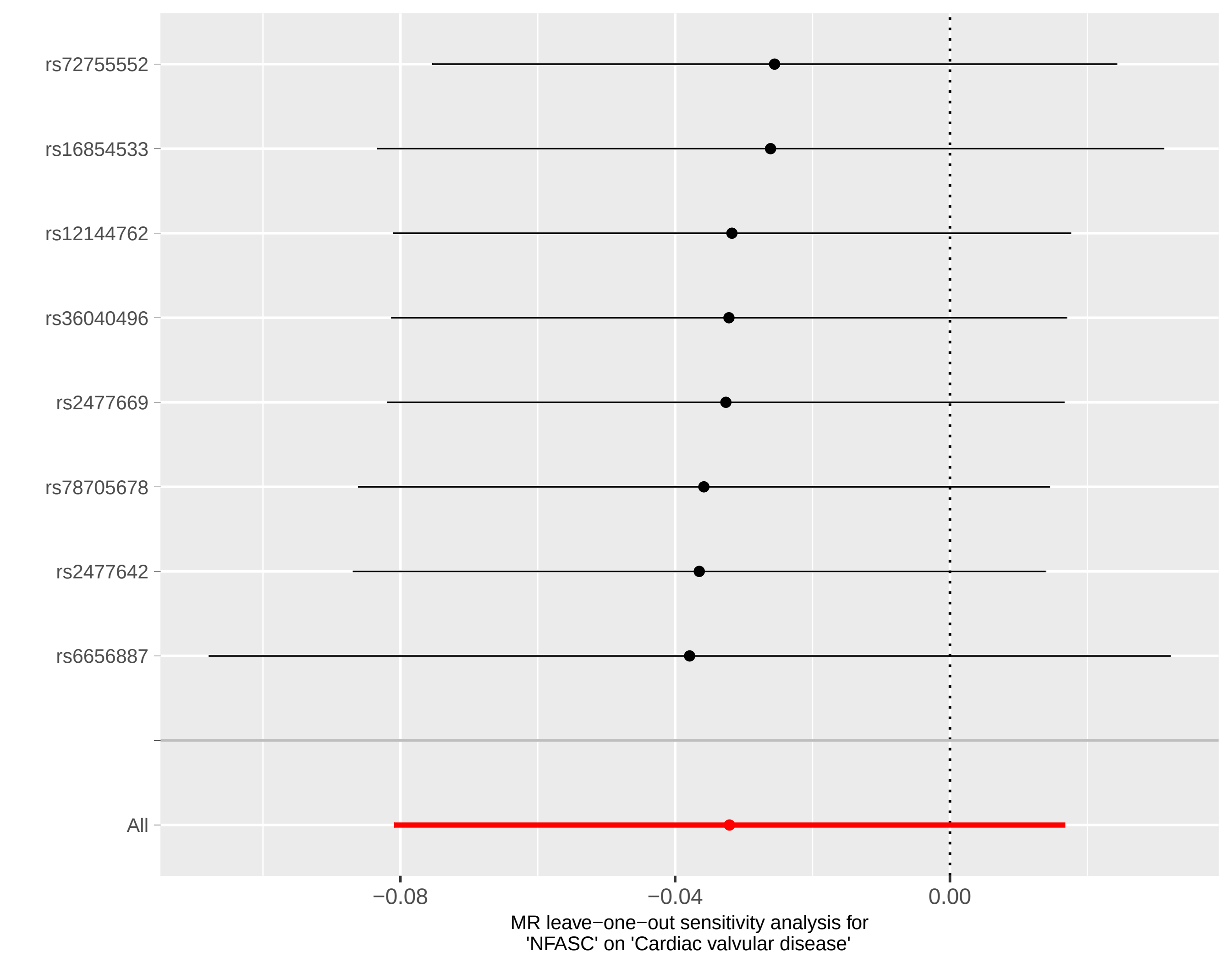
rs72755552
rs16854533
rs12144762
rs36040496
rs2477669
rs78705678
rs2477642
rs6656887
All
−0.08
−0.04
0.00
MR leave−one−out sensitivity analysis for
'NFASC' on 'Cardiac valvular disease'

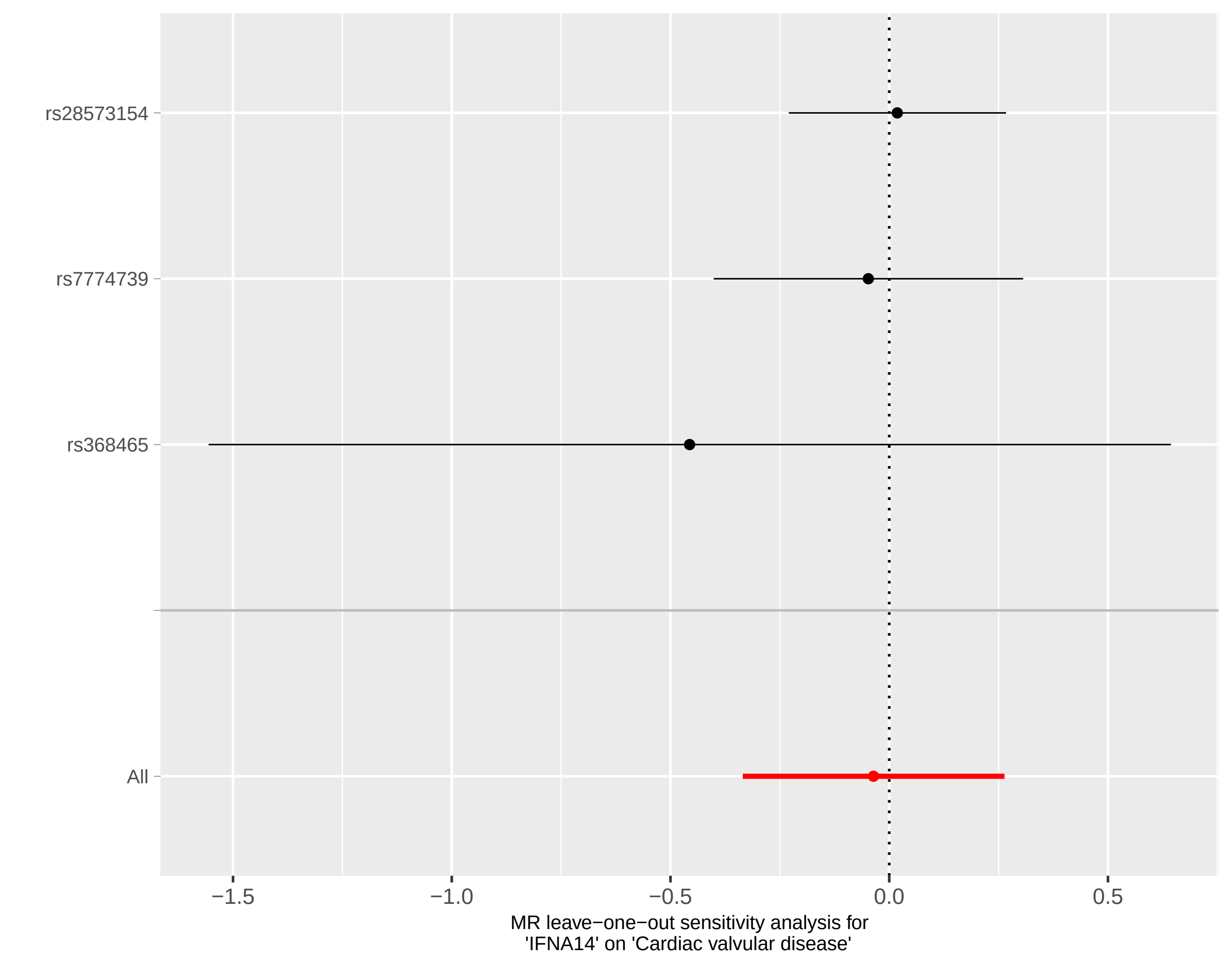
rs28573154
rs7774739
rs368465
All
−1.5
−1.0
−0.5
0.0
0.5
MR leave−one−out sensitivity analysis for
'IFNA14' on 'Cardiac valvular disease'

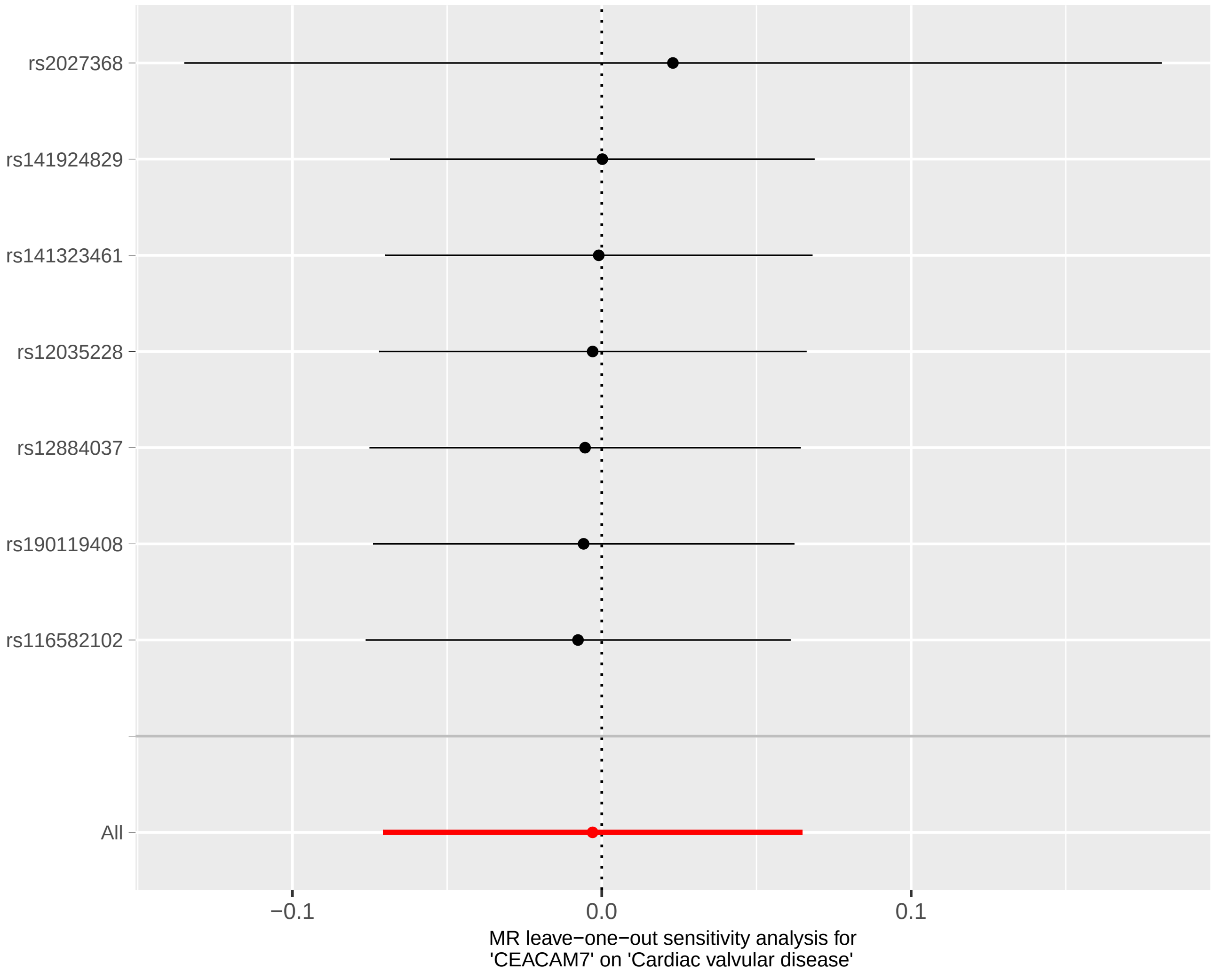
rs2027368
rs141924829
rs141323461
rs12035228
rs12884037
rs190119408
rs116582102
All
−0.1
0.0
0.1
MR leave−one−out sensitivity analysis for
'CEACAM7' on 'Cardiac valvular disease'

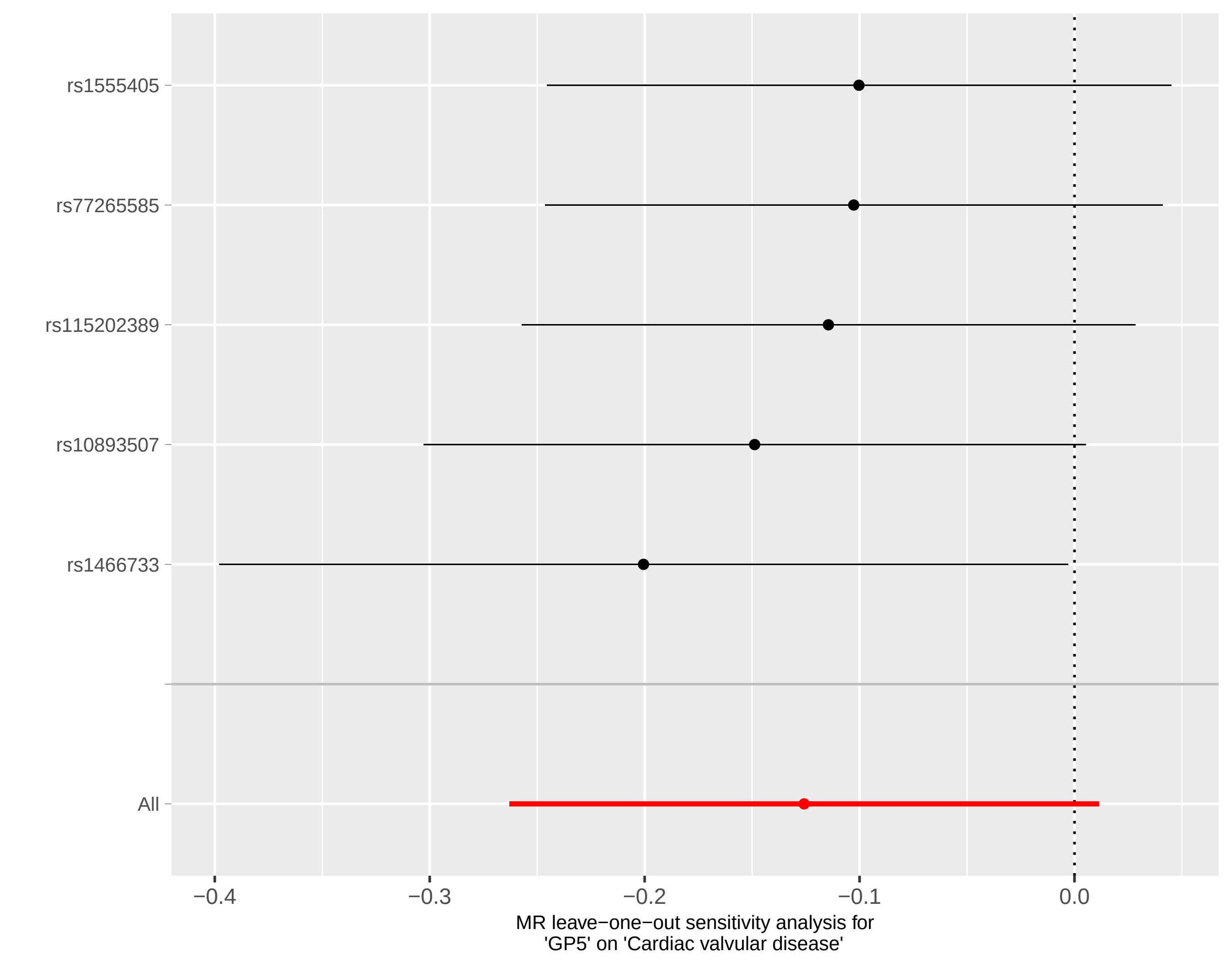
rs1555405
rs77265585
rs115202389
rs10893507
rs1466733
All
−0.4
−0.3
−0.2
−0.1
0.0
MR leave−one−out sensitivity analysis for
'GP5' on 'Cardiac valvular disease'

Insufficient number of SNPs

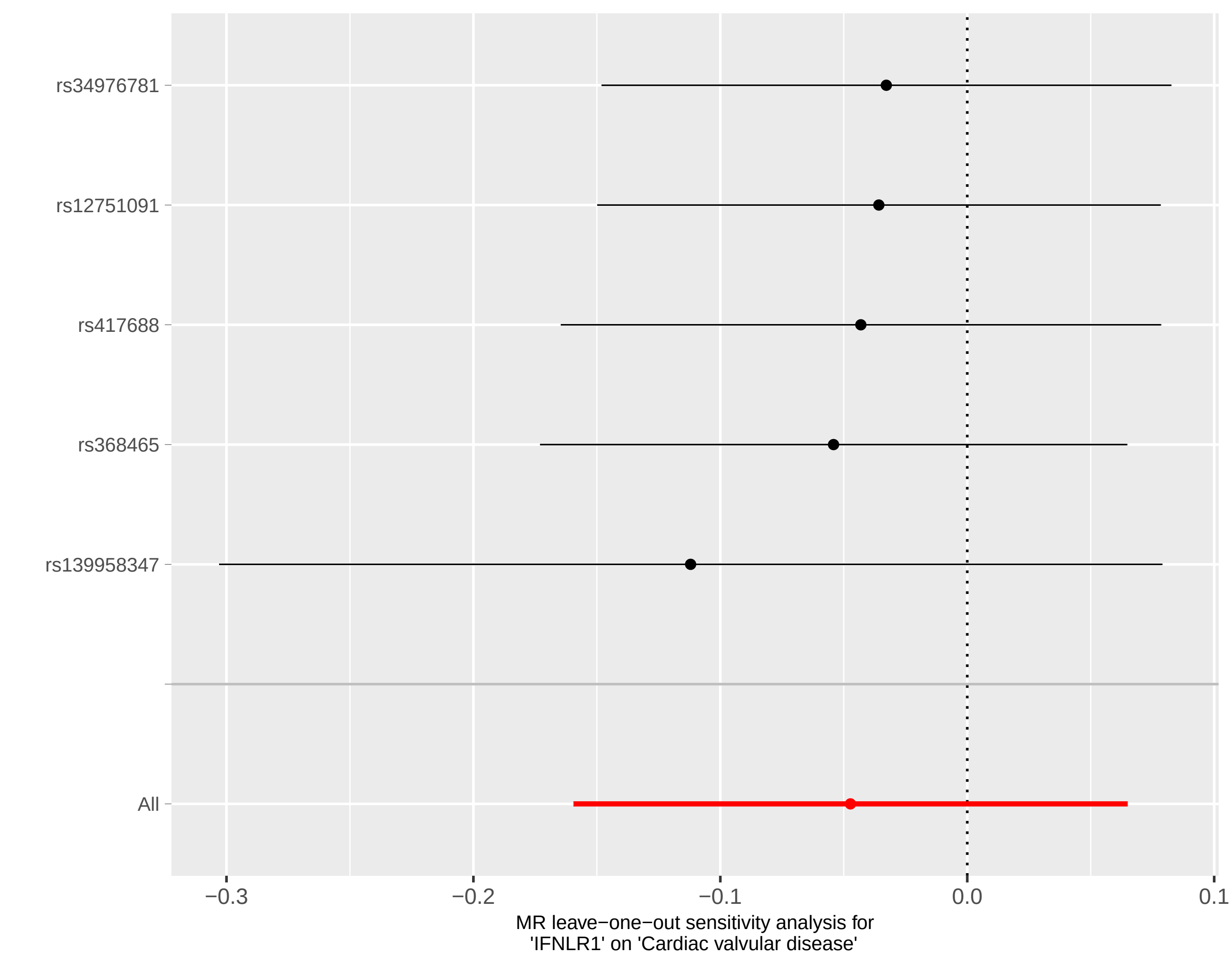

rs34976781
rs12751091
rs417688
rs368465
rs139958347
All
−0.3
−0.2
−0.1
0.0
0.1
MR leave−one−out sensitivity analysis for
'IFNLR1' on 'Cardiac valvular disease'

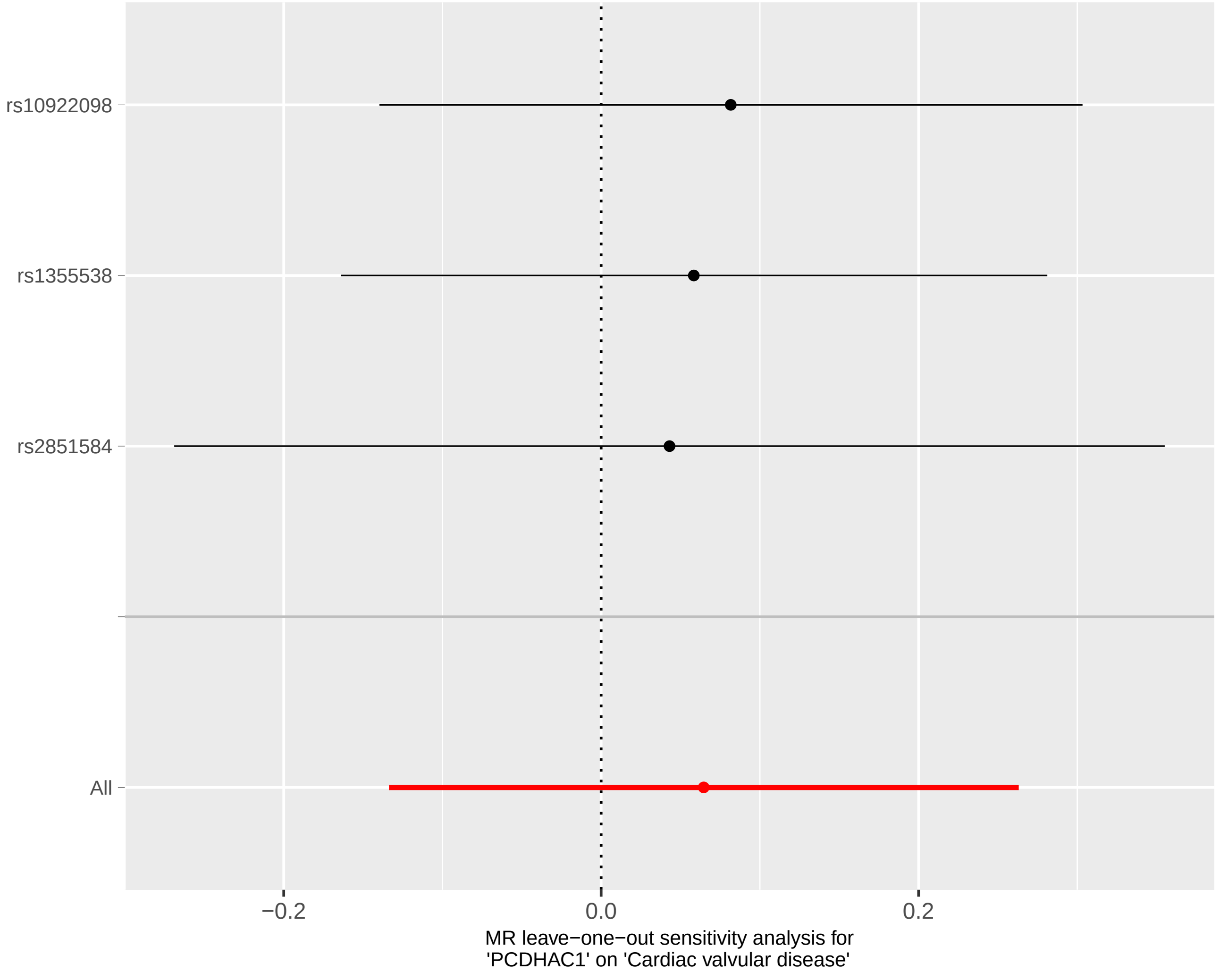

rs10922098
rs1355538
rs2851584
All
−0.2
0.0
0.2
MR leave−one−out sensitivity analysis for
'PCDHAC1' on 'Cardiac valvular disease'

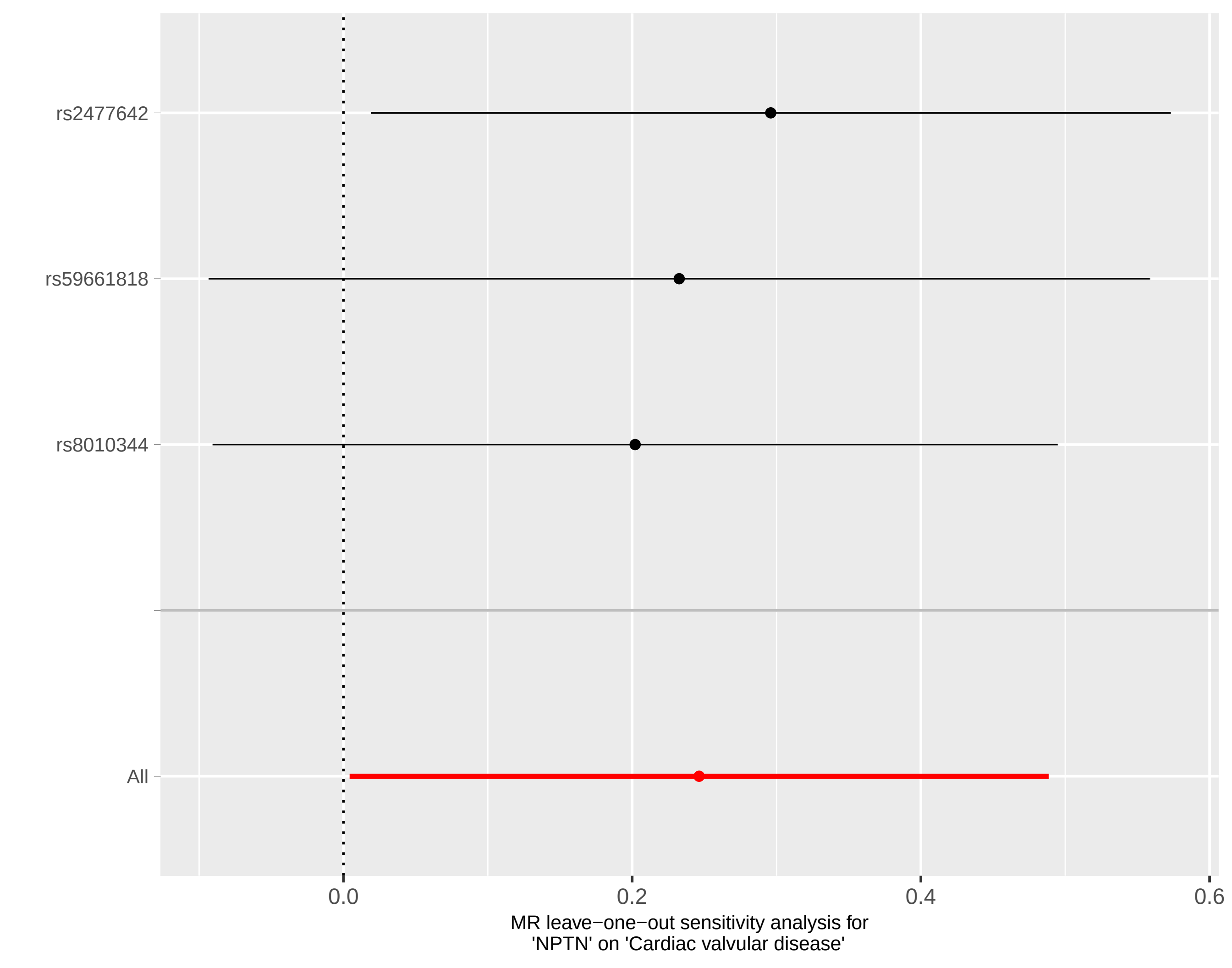
rs2477642
rs59661818
rs8010344
All
0.0
0.2
0.4
0.6
MR leave−one−out sensitivity analysis for
'NPTN' on 'Cardiac valvular disease'

Insufficient number of SNPs

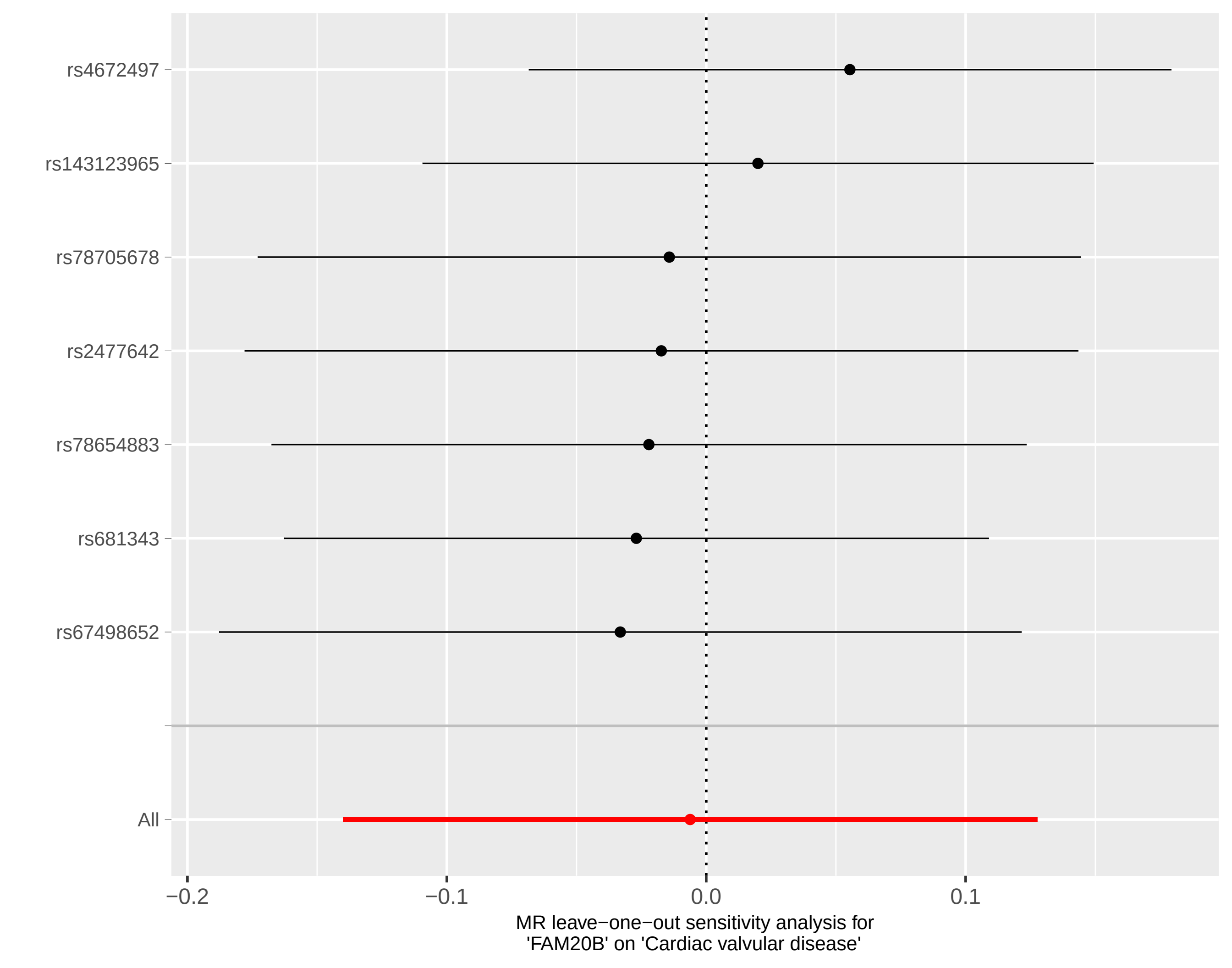
rs4672497
rs143123965
rs78705678
rs2477642
rs78654883
rs681343
rs67498652
All
−0.2
−0.1
0.0
0.1
MR leave−one−out sensitivity analysis for
'FAM20B' on 'Cardiac valvular disease'

Insufficient number of SNPs

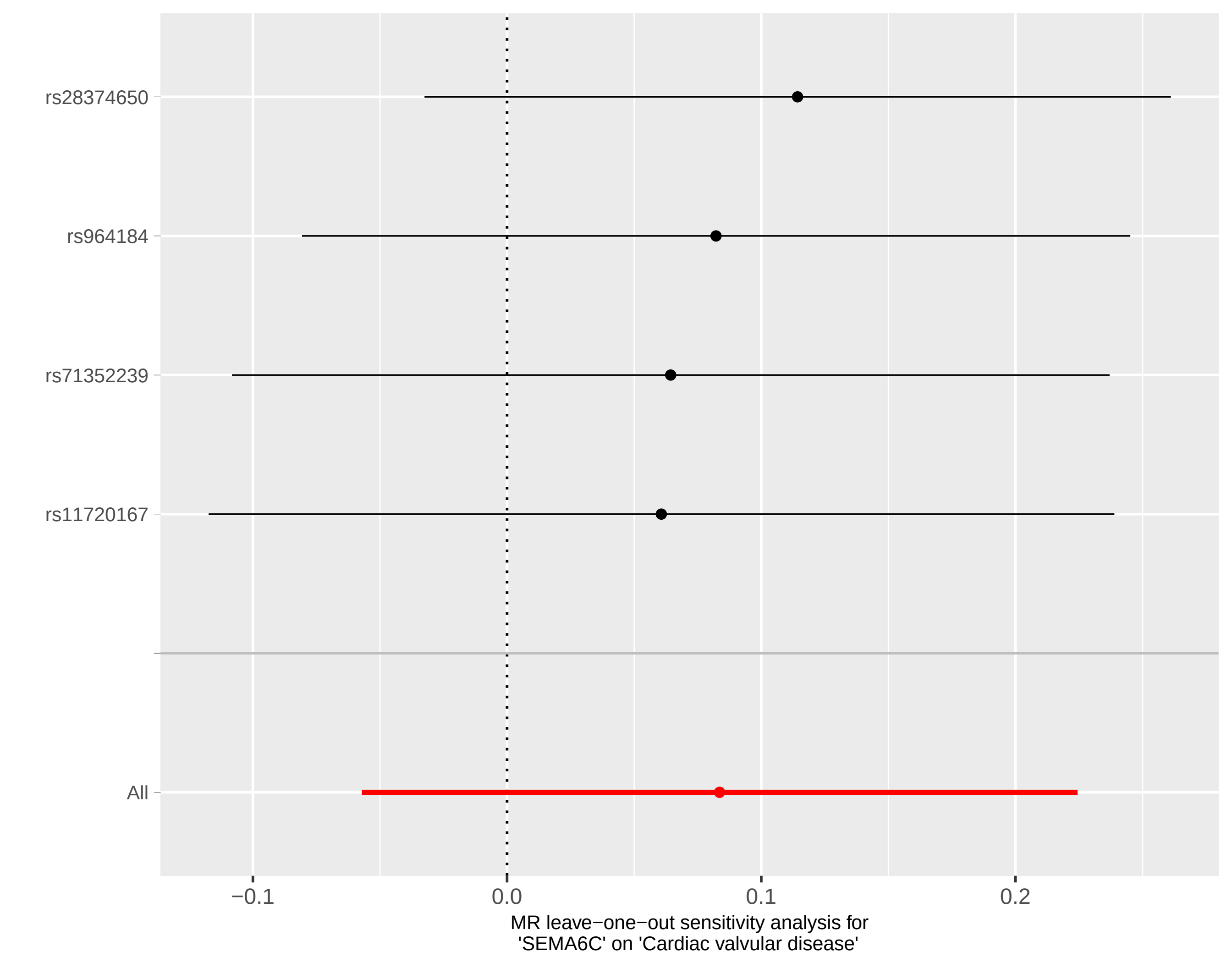
rs28374650
rs964184
rs71352239
rs11720167
All
−0.1
0.0
0.1
0.2
MR leave−one−out sensitivity analysis for
'SEMA6C' on 'Cardiac valvular disease'

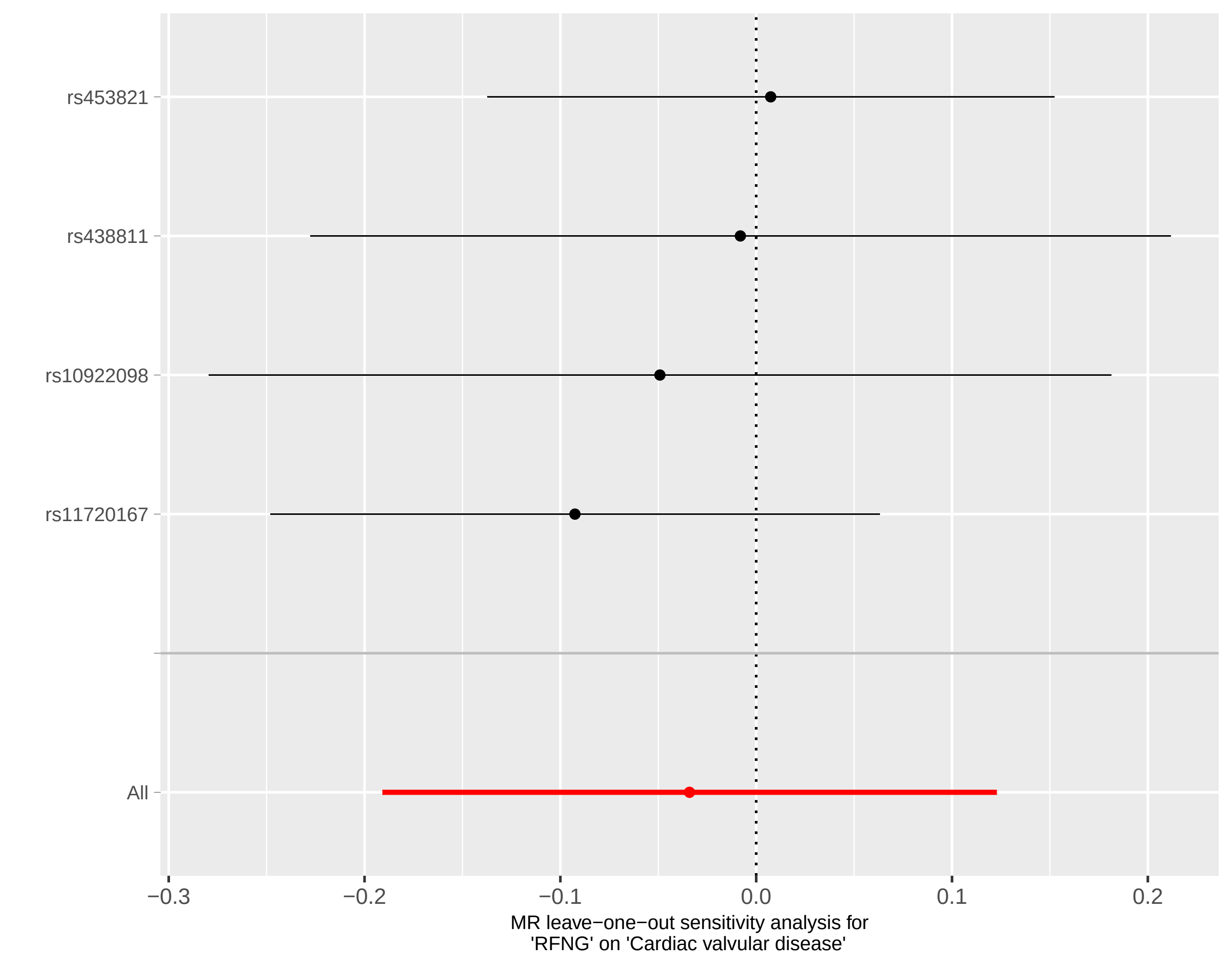
rs453821
rs438811
rs10922098
rs11720167
All
−0.3
−0.2
−0.1
0.0
0.1
0.2
MR leave−one−out sensitivity analysis for
'RFNG' on 'Cardiac valvular disease'

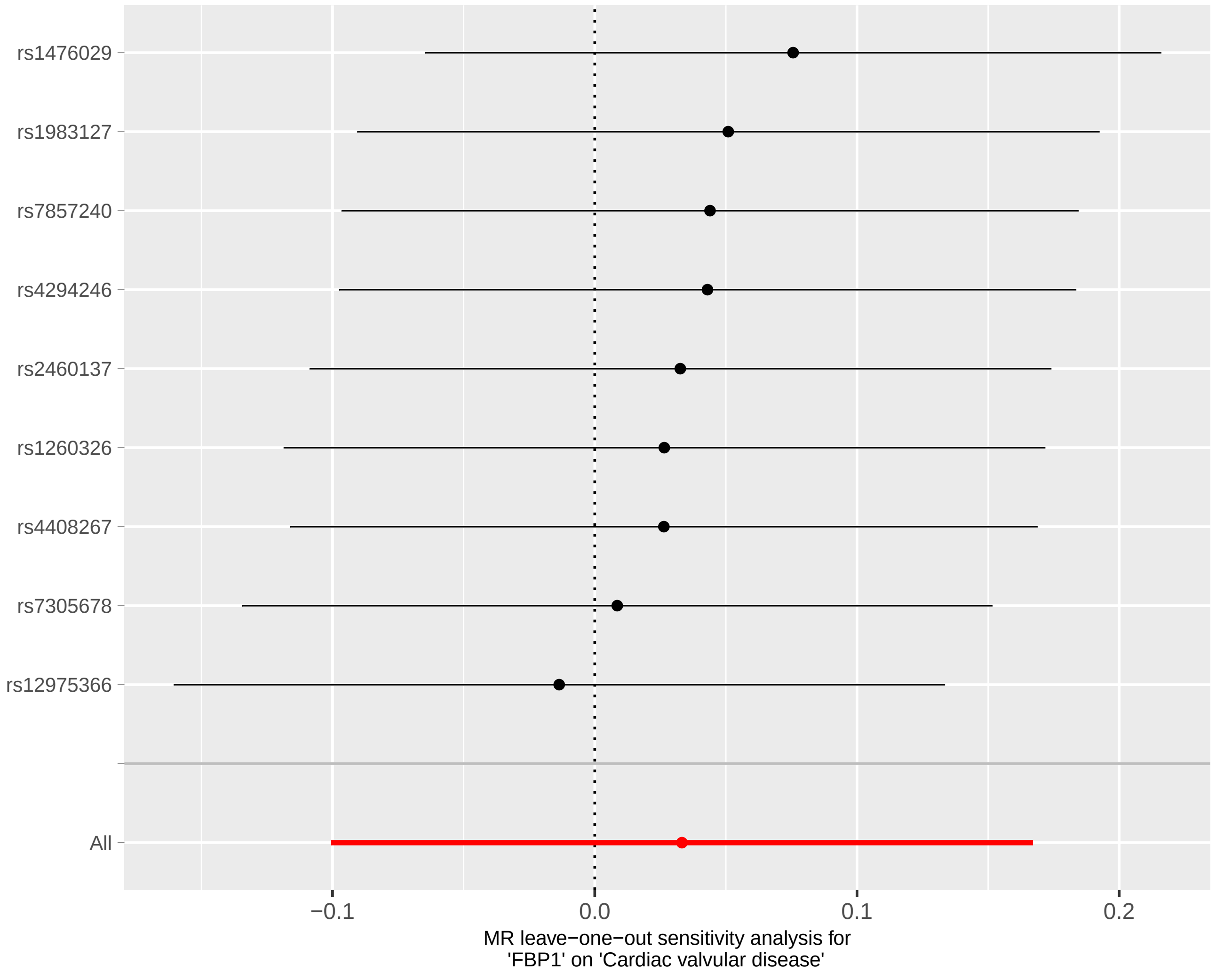
rs1476029
rs1983127
rs7857240
rs4294246
rs2460137
rs1260326
rs4408267
rs7305678
rs12975366
All
−0.1
0.0
0.1
0.2
MR leave−one−out sensitivity analysis for
'FBP1' on 'Cardiac valvular disease'

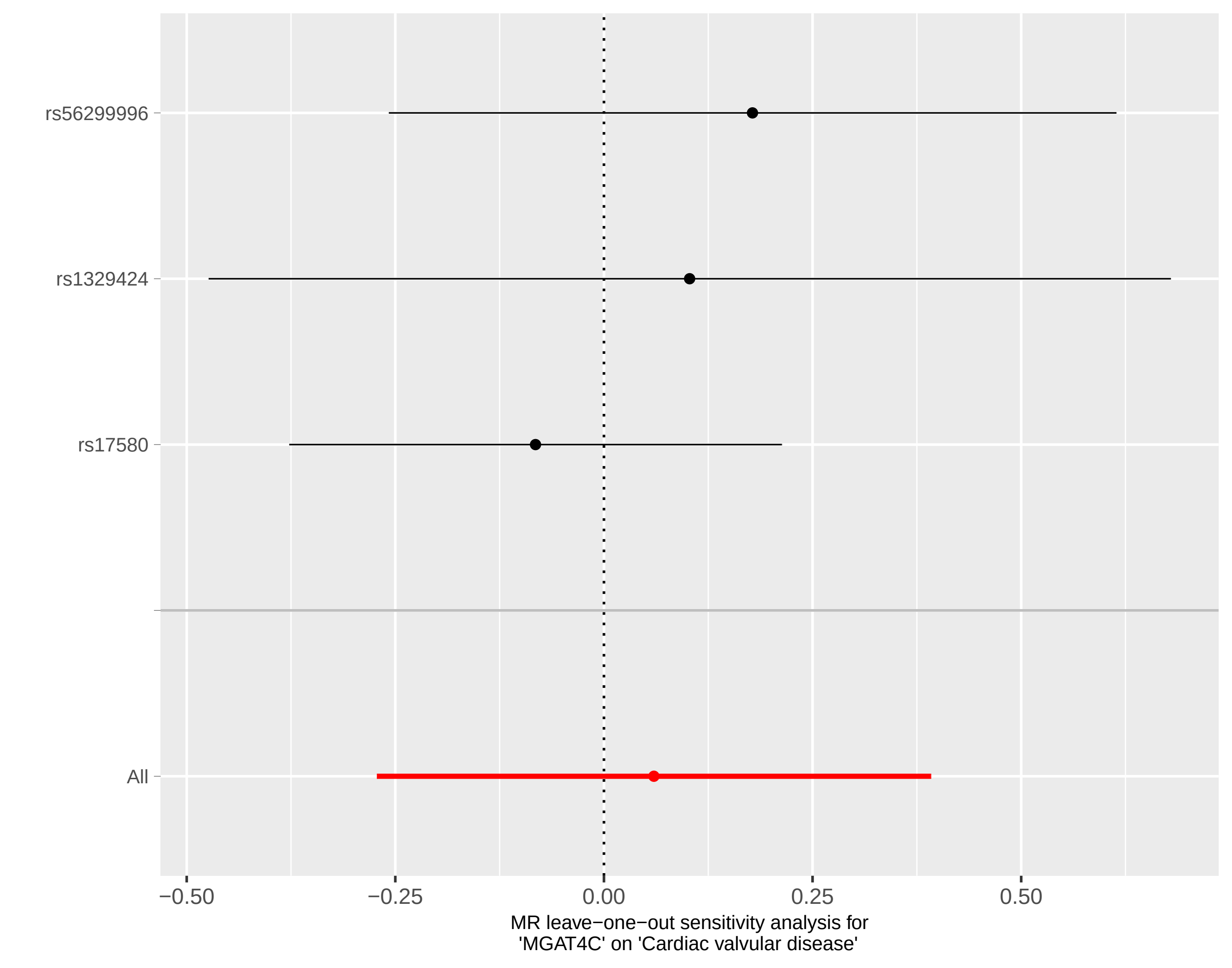

rs56299996
rs1329424
rs17580
All
−0.50
−0.25
0.00
0.25
0.50
MR leave−one−out sensitivity analysis for
'MGAT4C' on 'Cardiac valvular disease'

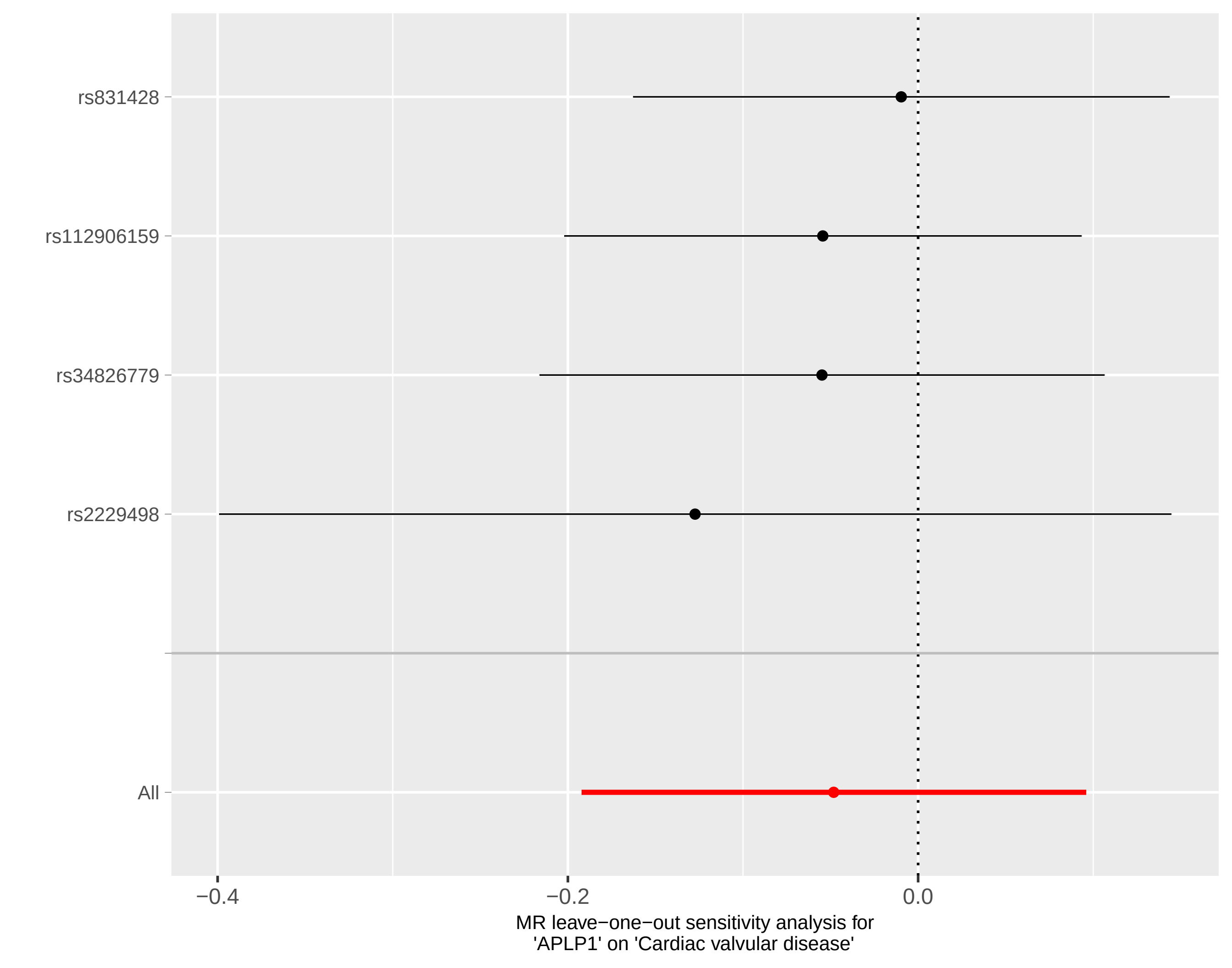
rs831428
rs112906159
rs34826779
rs2229498
All
−0.4
−0.2
0.0
MR leave−one−out sensitivity analysis for
'APLP1' on 'Cardiac valvular disease'

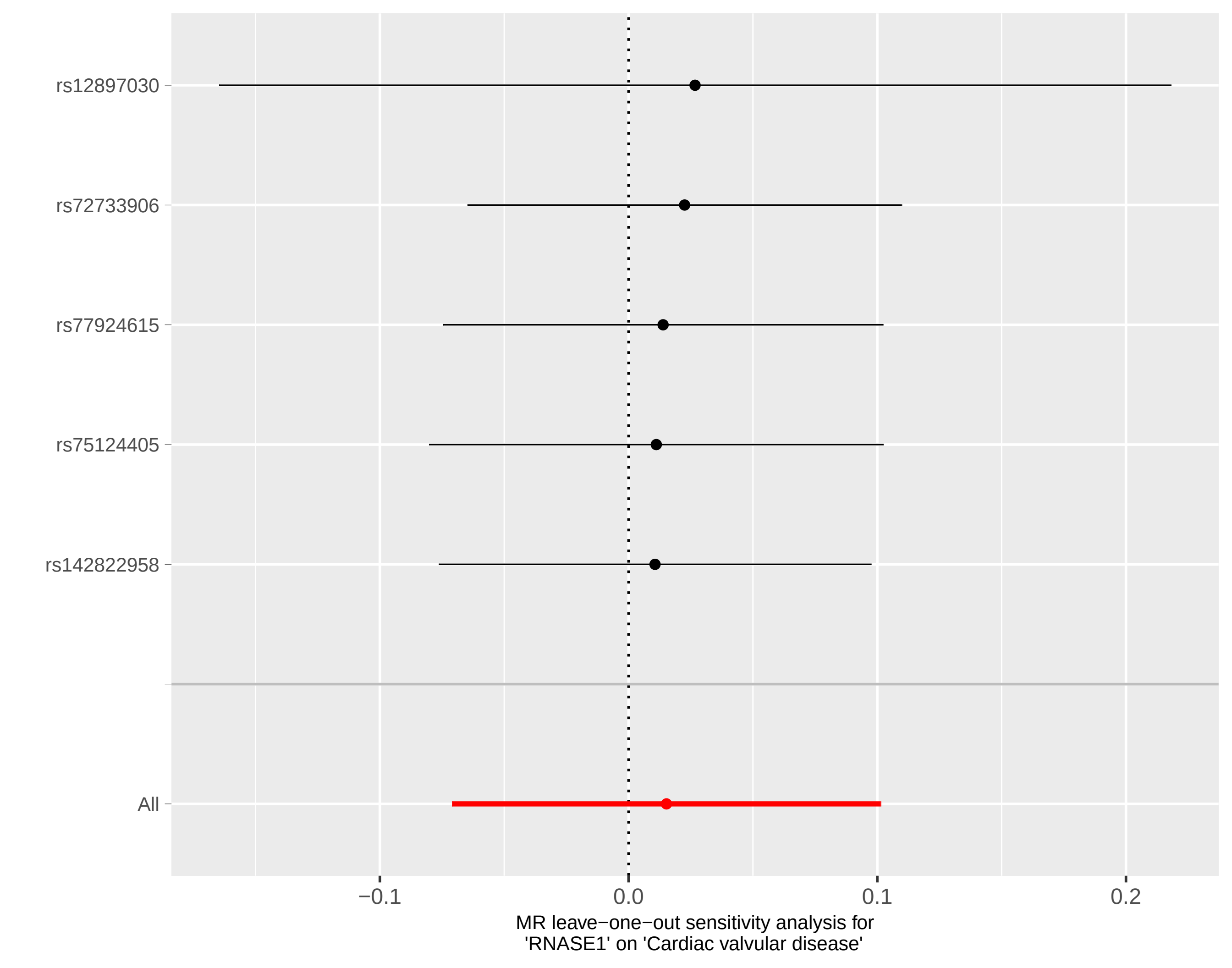
rs12897030
rs72733906
rs77924615
rs75124405
rs142822958
All
−0.1
0.0
0.1
0.2
MR leave−one−out sensitivity analysis for
'RNASE1' on 'Cardiac valvular disease'

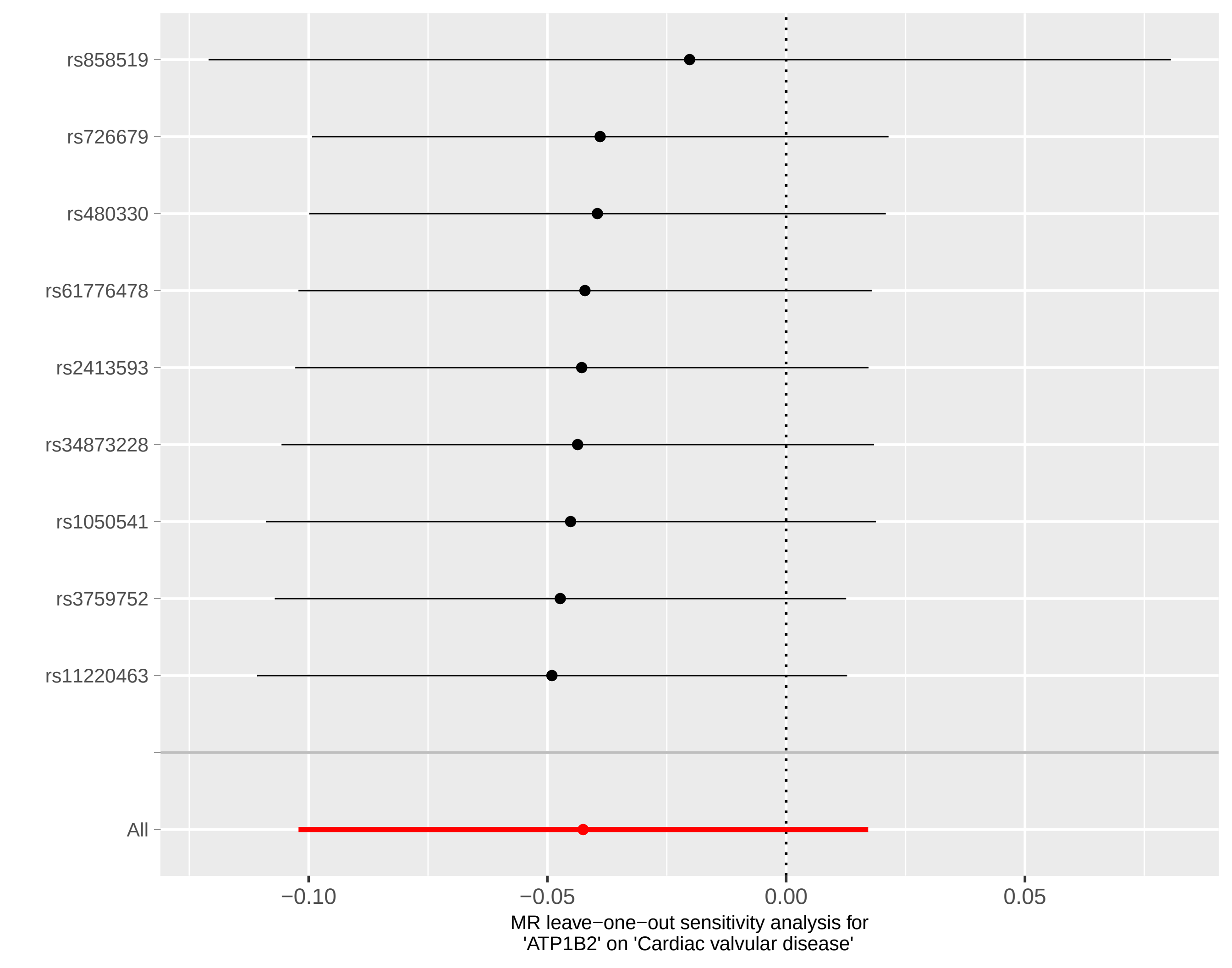
rs858519
rs726679
rs480330
rs61776478
rs2413593
rs34873228
rs1050541
rs3759752
rs11220463
All
−0.10
−0.05
0.00
0.05
MR leave−one−out sensitivity analysis for
'ATP1B2' on 'Cardiac valvular disease'

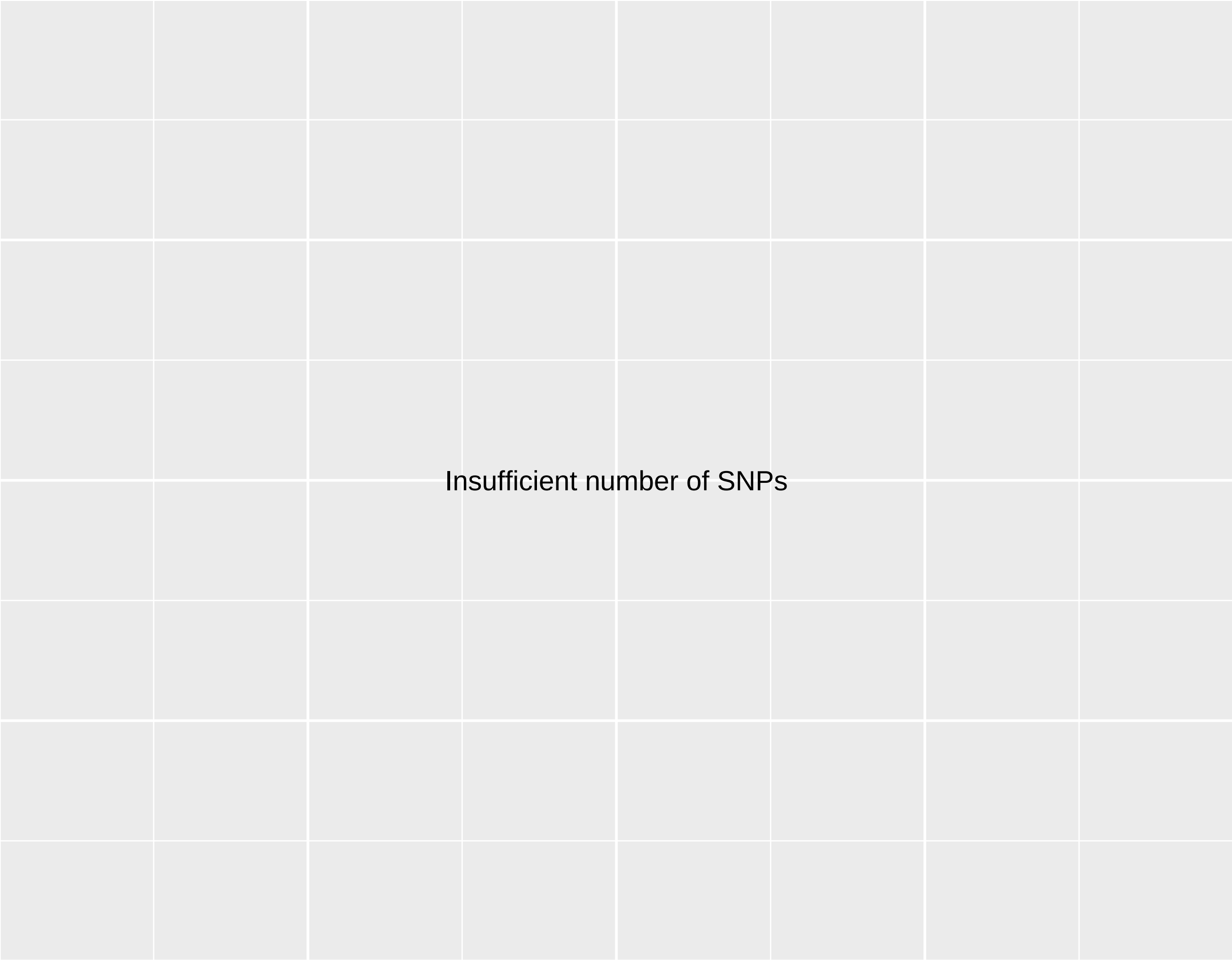
Insufficient number of SNPs

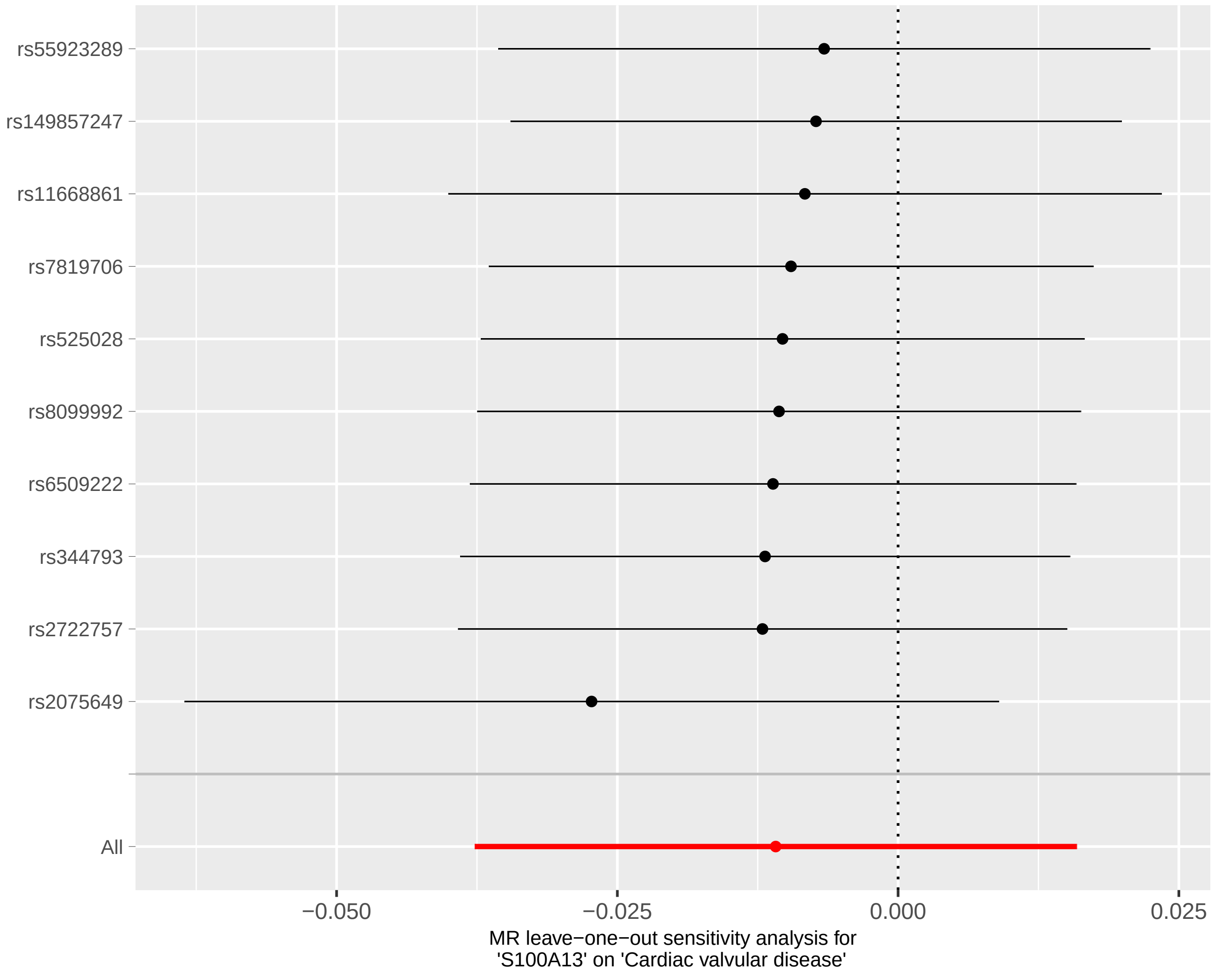
rs55923289
rs149857247
rs11668861
rs7819706
rs525028
rs8099992
rs6509222
rs344793
rs2722757
rs2075649
All
−0.050
−0.025
0.000
0.025
MR leave−one−out sensitivity analysis for
'S100A13' on 'Cardiac valvular disease'

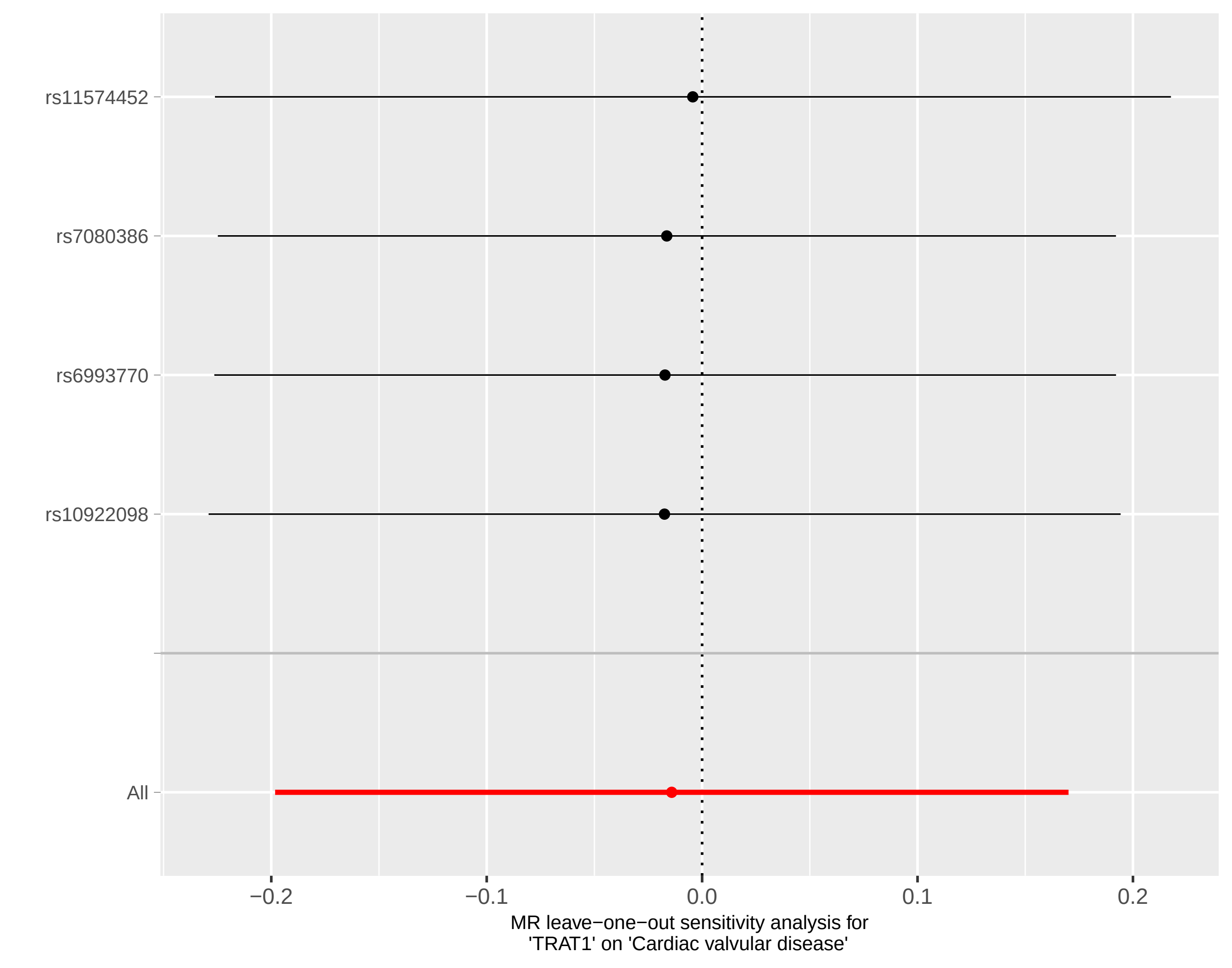
rs11574452
rs7080386
rs6993770
rs10922098
All
−0.2
−0.1
0.0
0.1
0.2
MR leave−one−out sensitivity analysis for
'TRAT1' on 'Cardiac valvular disease'

Insufficient number of SNPs

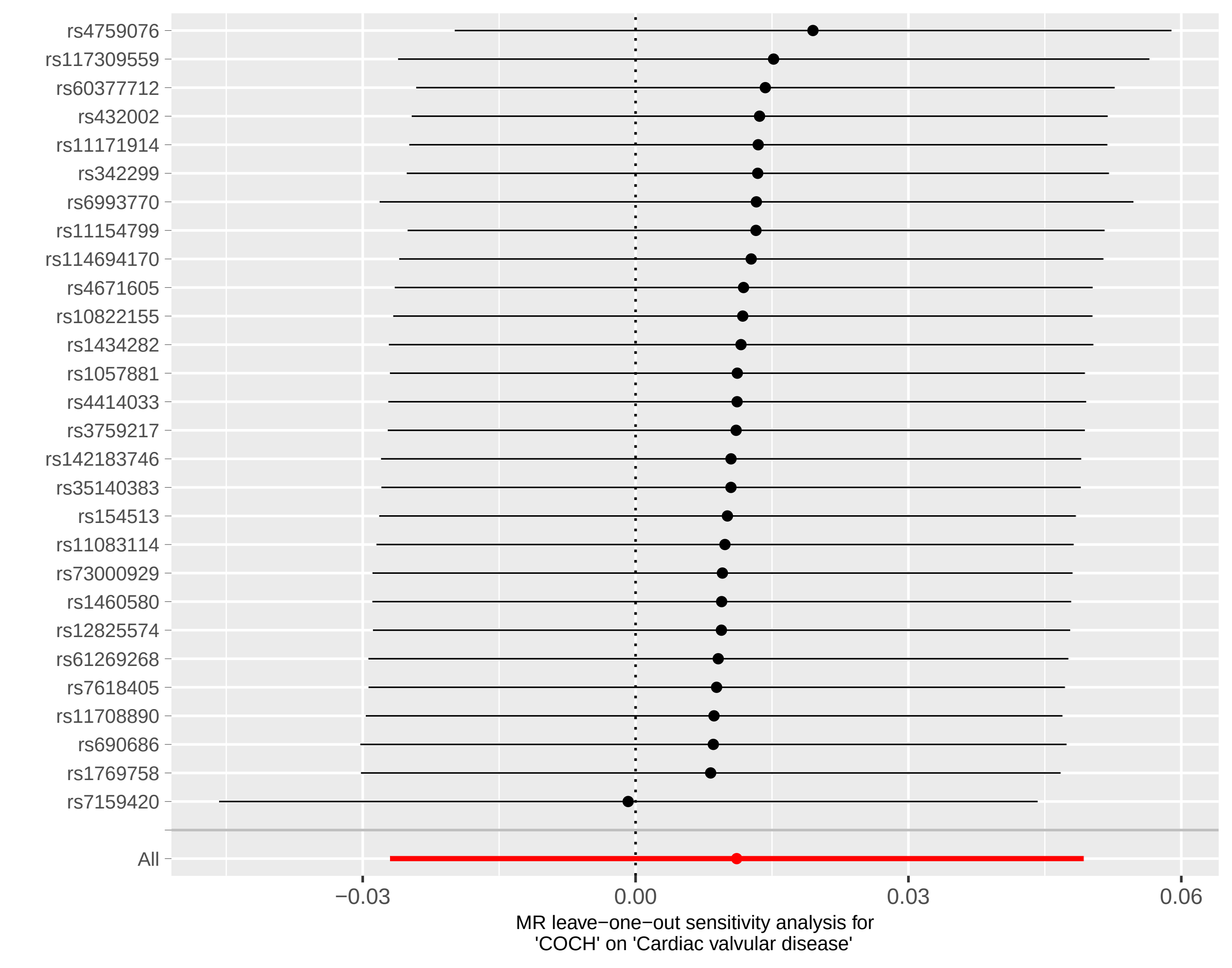
rs4759076
rs117309559
rs60377712
rs432002
rs11171914
rs342299
rs6993770
rs11154799
rs114694170
rs4671605
rs10822155
rs1434282
rs1057881
rs4414033
rs3759217
rs142183746
rs35140383
rs154513
rs11083114
rs73000929
rs1460580
rs12825574
rs61269268
rs7618405
rs11708890
rs690686
rs1769758
rs7159420
All
−0.03
0.00
0.03
0.06
MR leave−one−out sensitivity analysis for
'COCH' on 'Cardiac valvular disease'

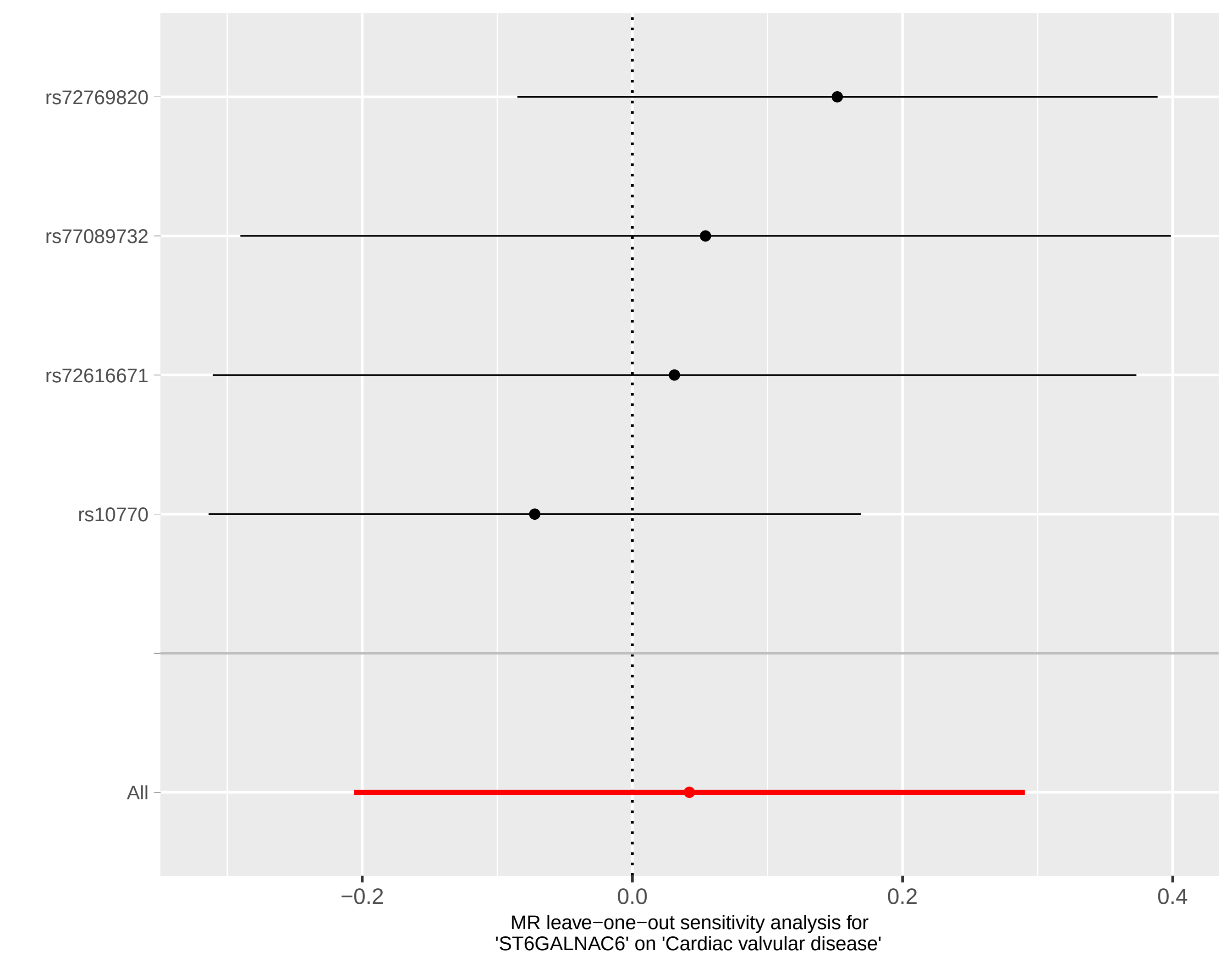
rs72769820
rs77089732
rs72616671
rs10770
All
−0.2
0.0
0.2
0.4
MR leave−one−out sensitivity analysis for
'ST6GALNAC6' on 'Cardiac valvular disease'

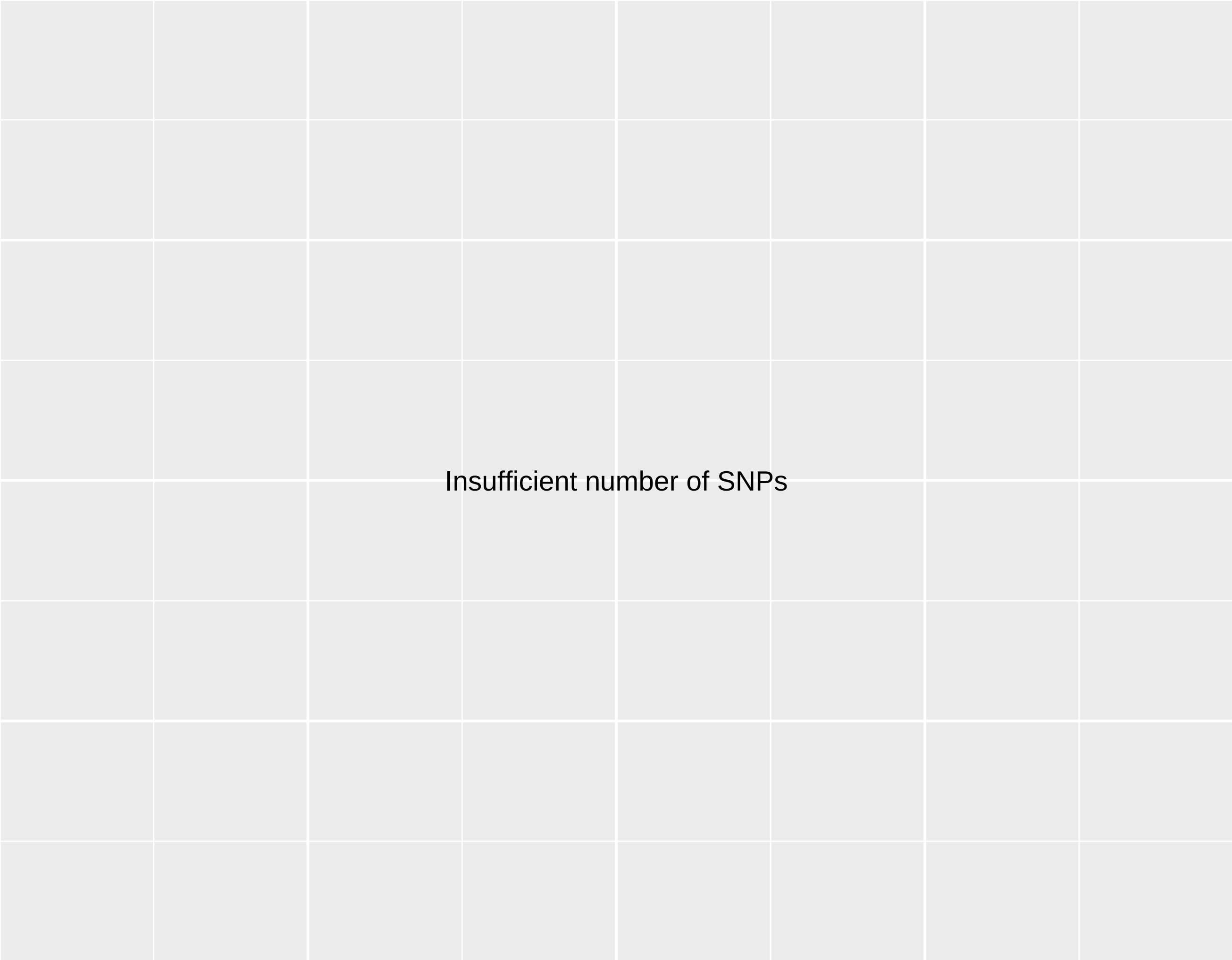
Insufficient number of SNPs

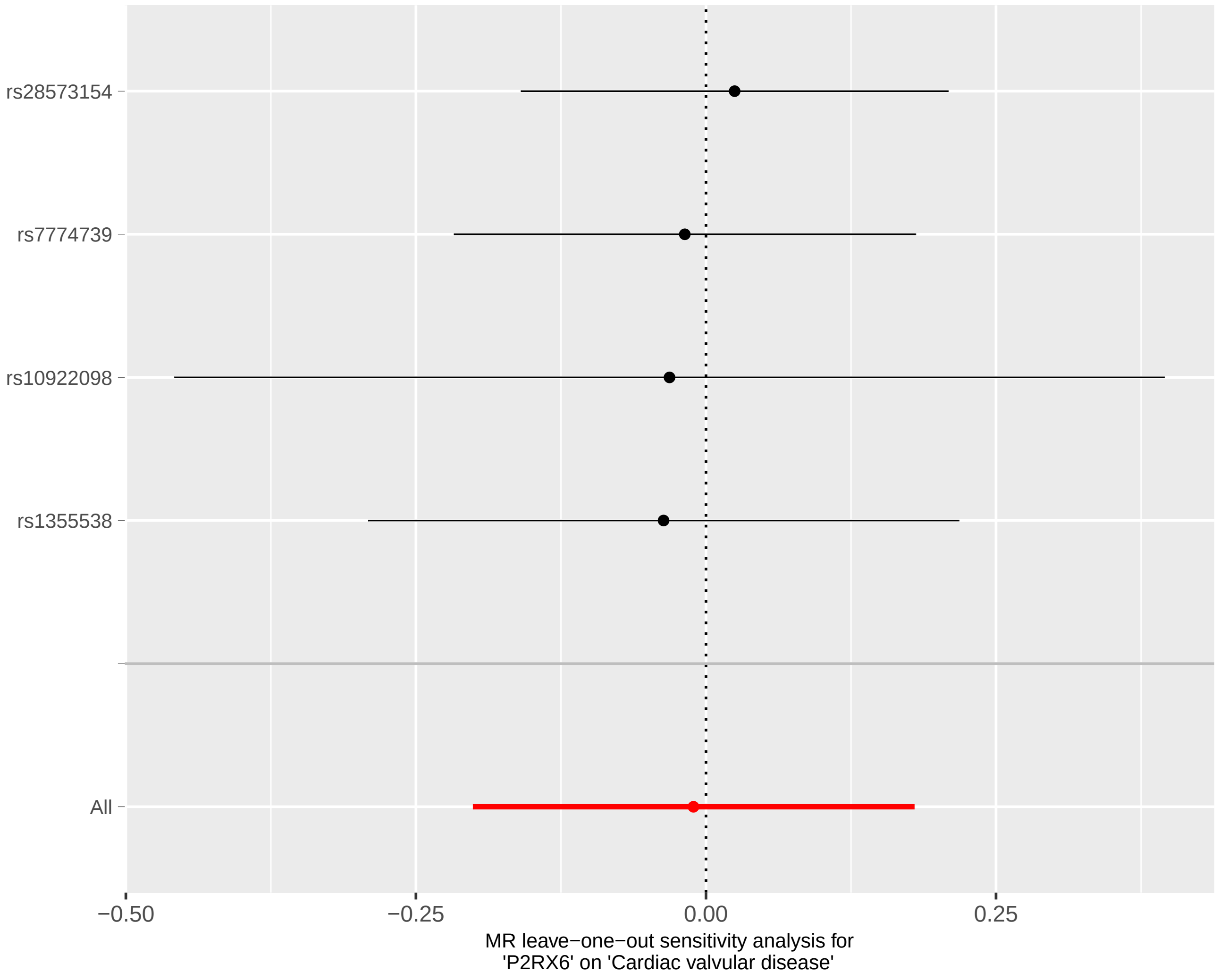
rs28573154
rs7774739
rs10922098
rs1355538
All
−0.50
−0.25
0.00
0.25
MR leave−one−out sensitivity analysis for
'P2RX6' on 'Cardiac valvular disease'

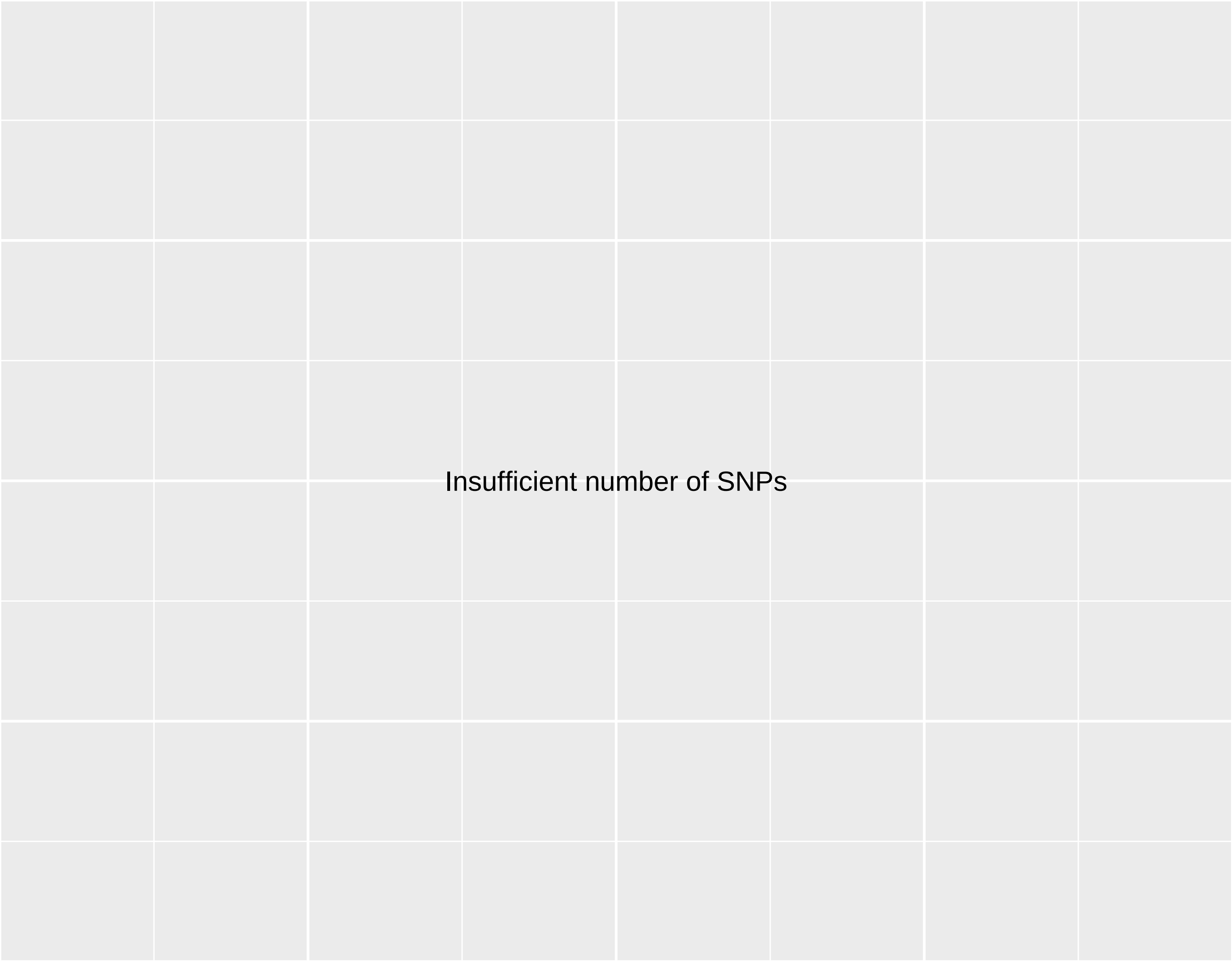
Insufficient number of SNPs

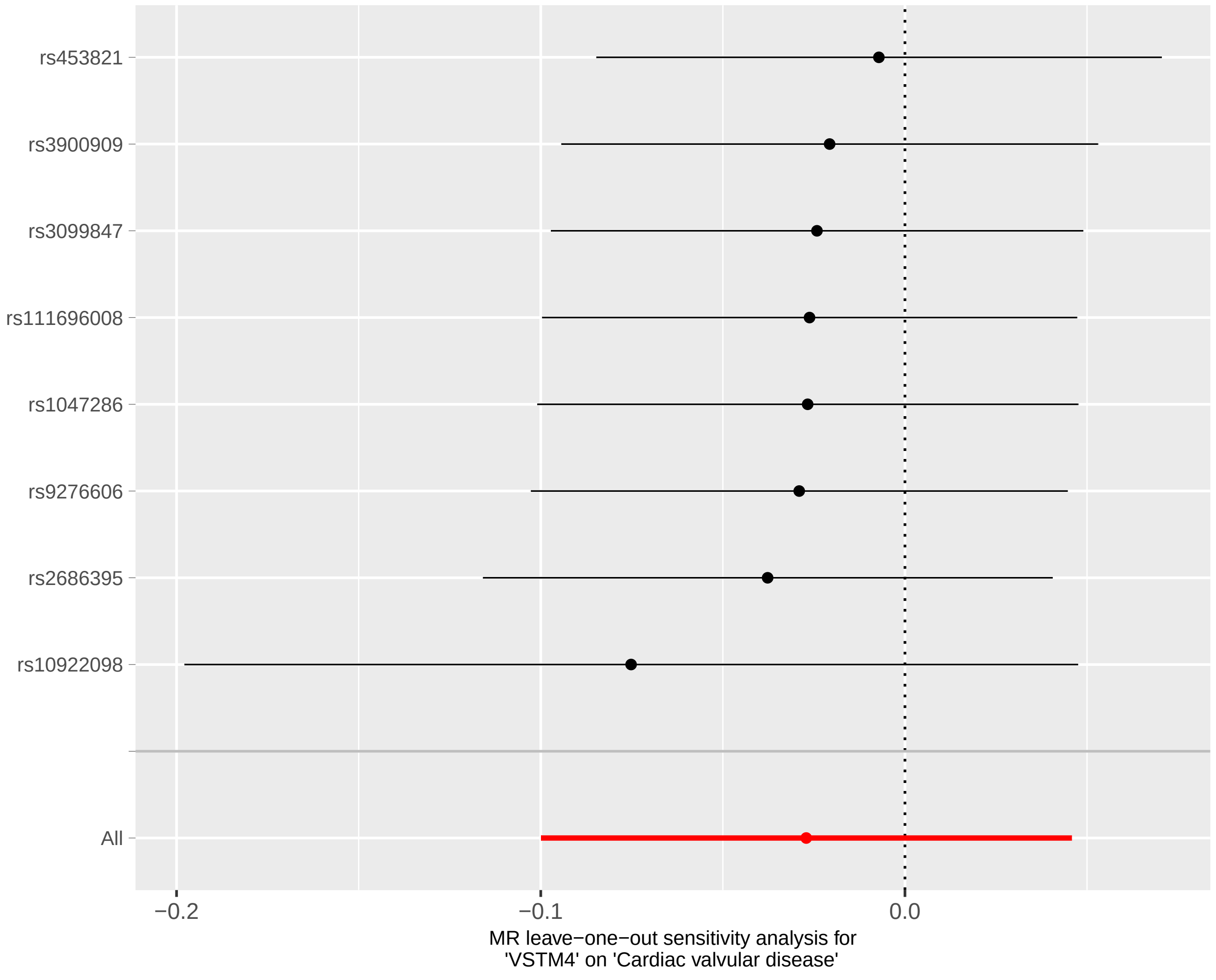

rs453821
rs3900909
rs3099847
rs111696008
rs1047286
rs9276606
rs2686395
rs10922098
All
−0.2
−0.1
0.0
MR leave−one−out sensitivity analysis for
'VSTM4' on 'Cardiac valvular disease'

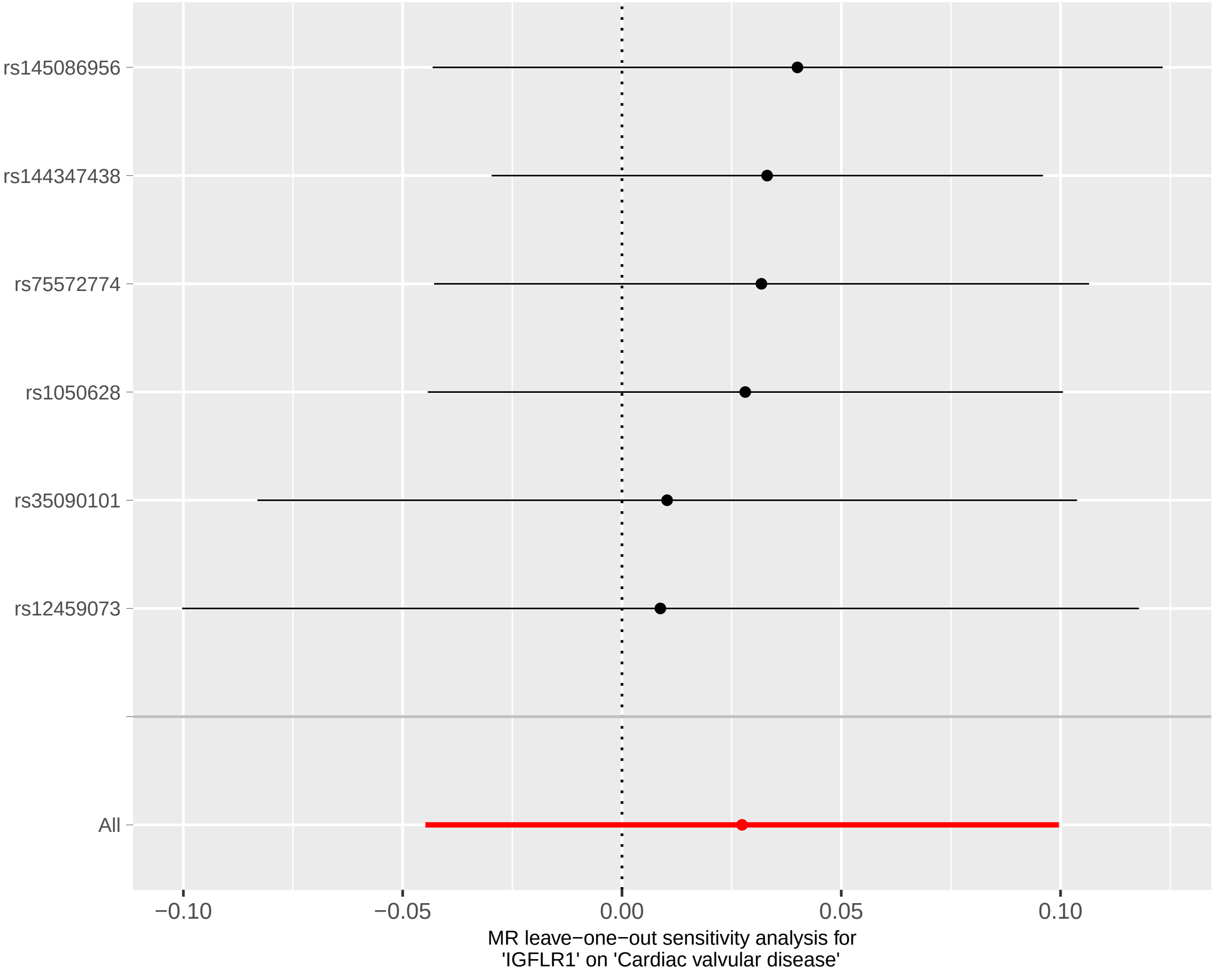

rs145086956
rs144347438
rs75572774
rs1050628
rs35090101
rs12459073
All
−0.10
−0.05
0.00
0.05
0.10
MR leave−one−out sensitivity analysis for
'IGFLR1' on 'Cardiac valvular disease'

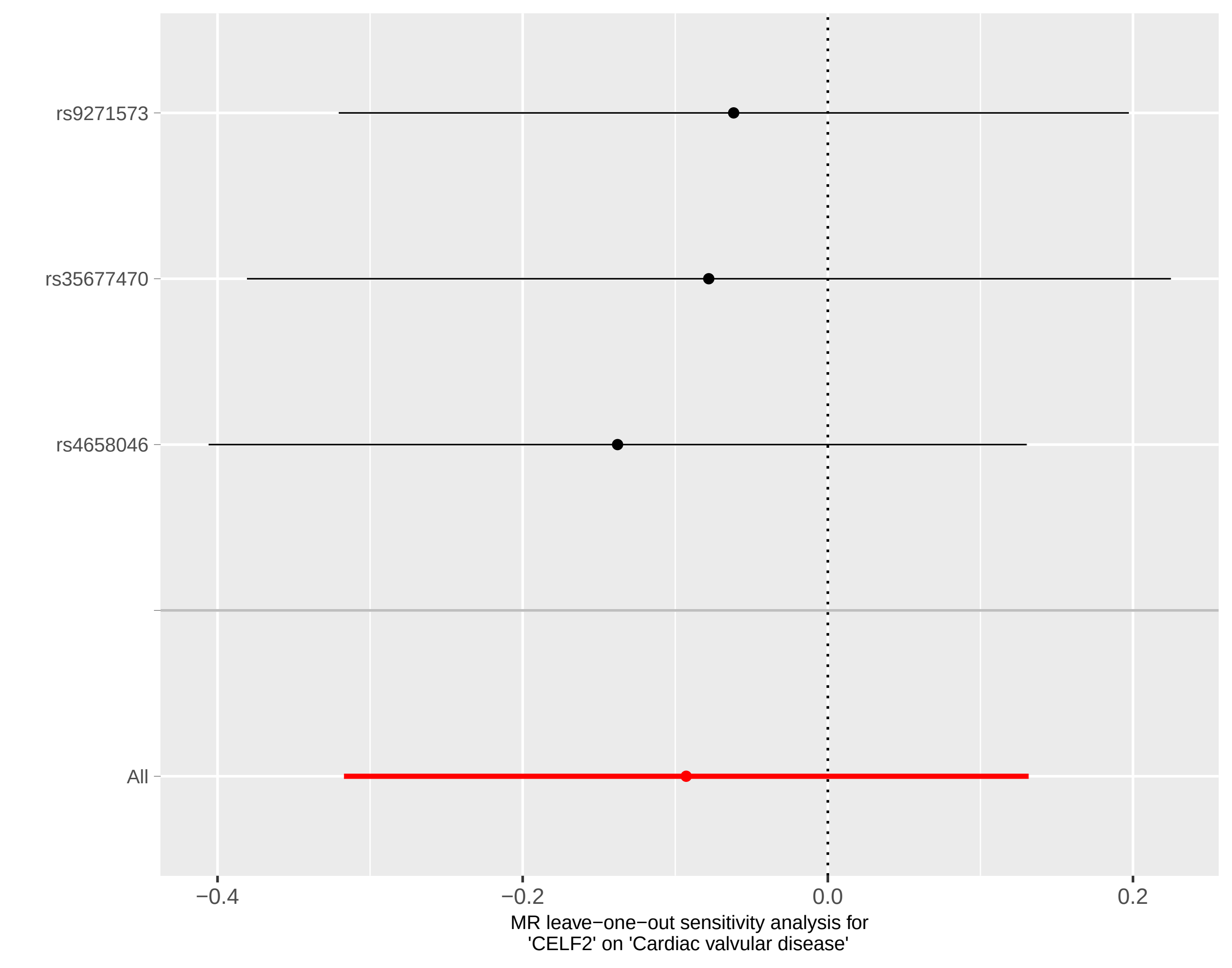
rs9271573
rs35677470
rs4658046
All
−0.4
−0.2
0.0
0.2
MR leave−one−out sensitivity analysis for
'CELF2' on 'Cardiac valvular disease'

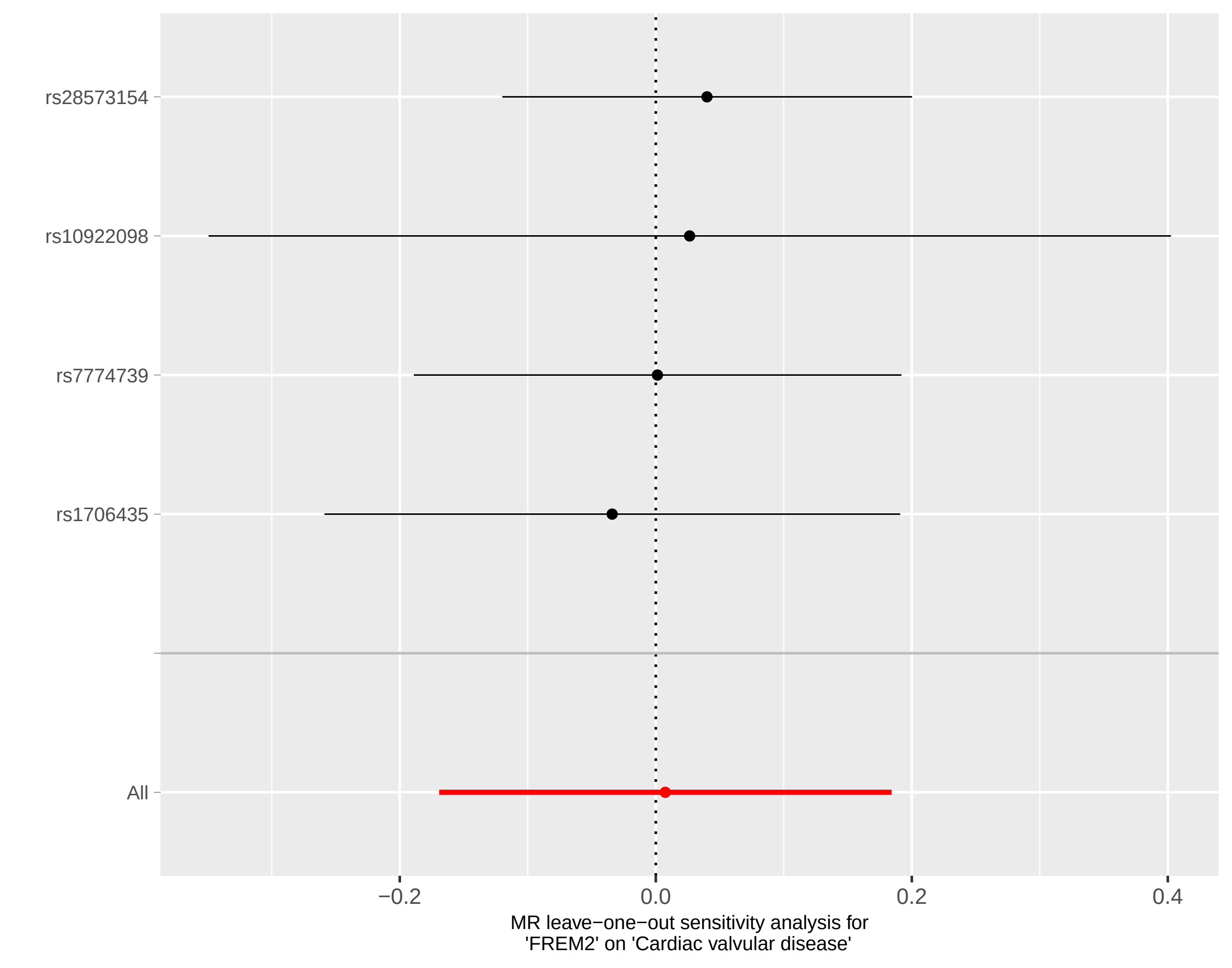
rs28573154
rs10922098
rs7774739
rs1706435
All
−0.2
0.0
0.2
0.4
MR leave−one−out sensitivity analysis for
'FREM2' on 'Cardiac valvular disease'

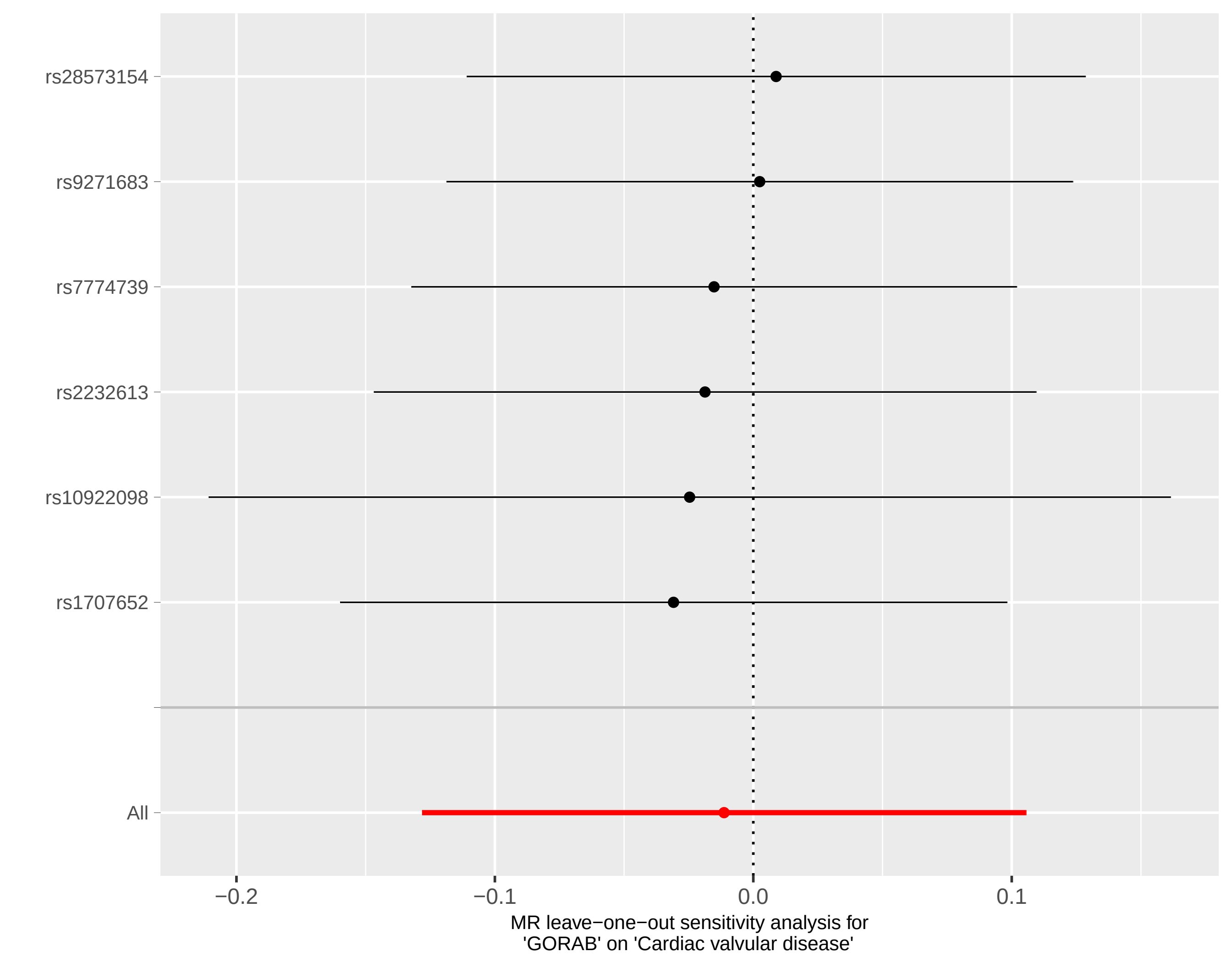

rs28573154
rs9271683
rs7774739
rs2232613
rs10922098
rs1707652
All
−0.2
−0.1
0.0
0.1
MR leave−one−out sensitivity analysis for
'GORAB' on 'Cardiac valvular disease'

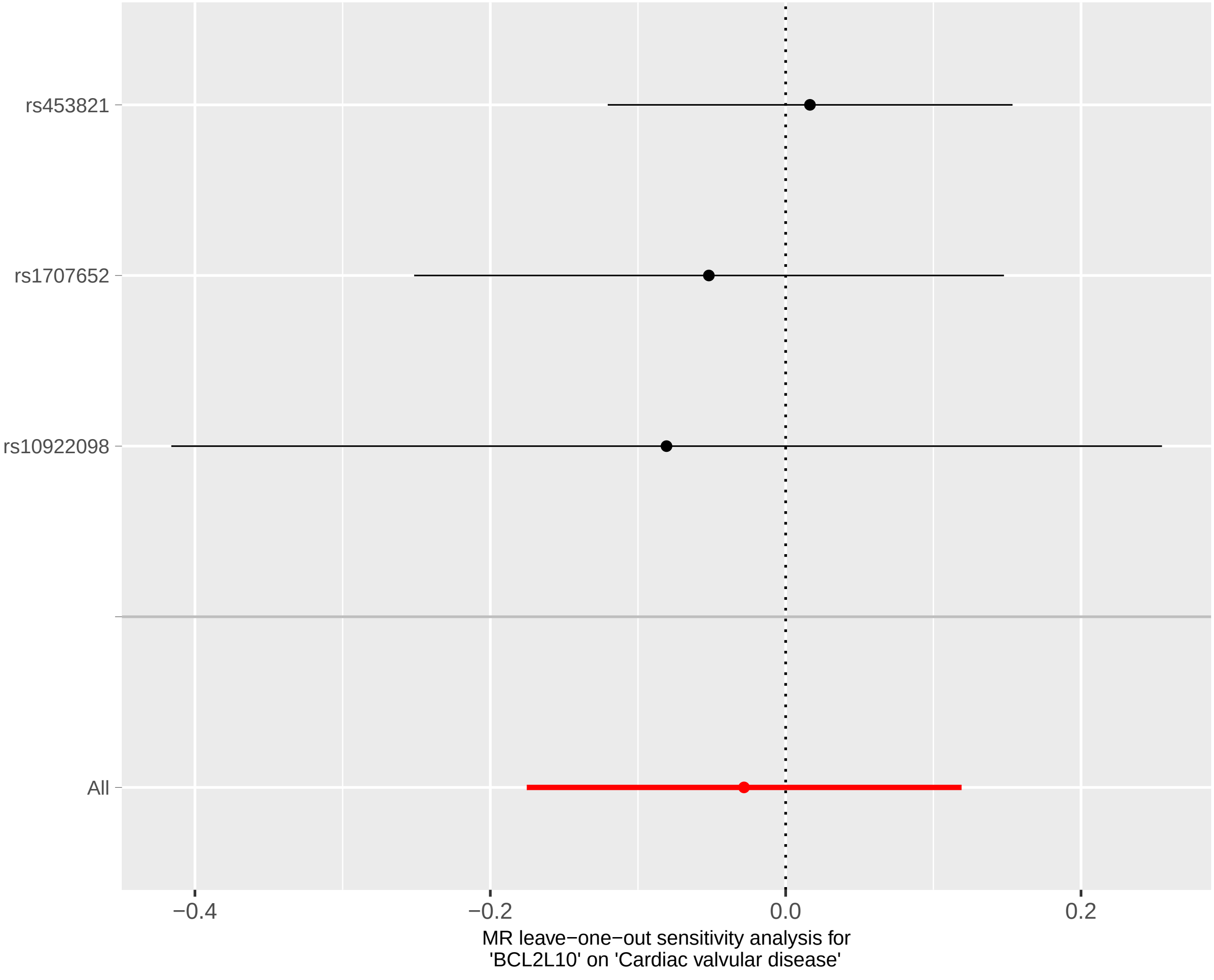
rs453821
rs1707652
rs10922098
All
−0.4
−0.2
0.0
0.2
MR leave−one−out sensitivity analysis for
'BCL2L10' on 'Cardiac valvular disease'

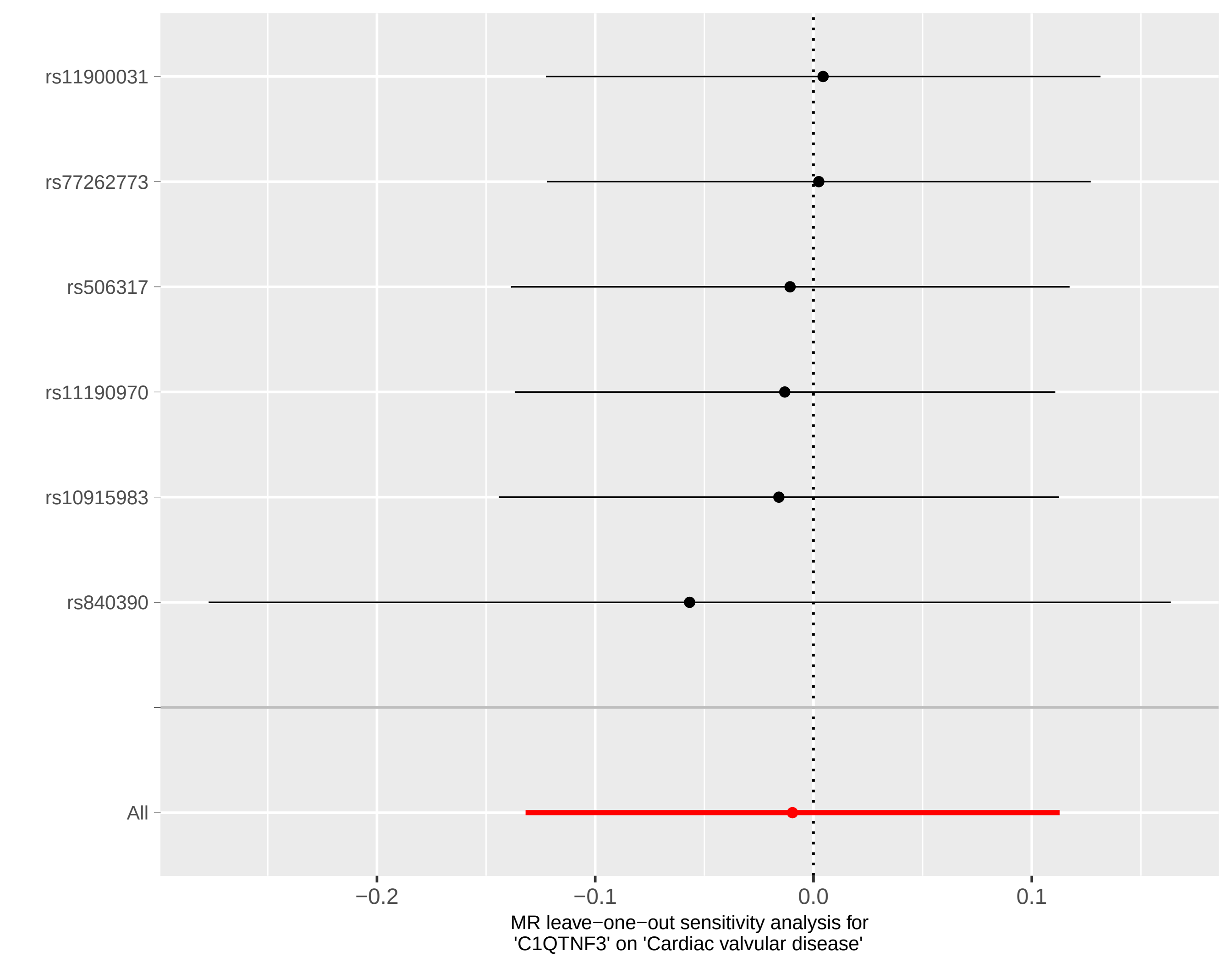
rs11900031
rs77262773
rs506317
rs11190970
rs10915983
rs840390
All
−0.2
−0.1
0.0
0.1
MR leave−one−out sensitivity analysis for
'C1QTNF3' on 'Cardiac valvular disease'

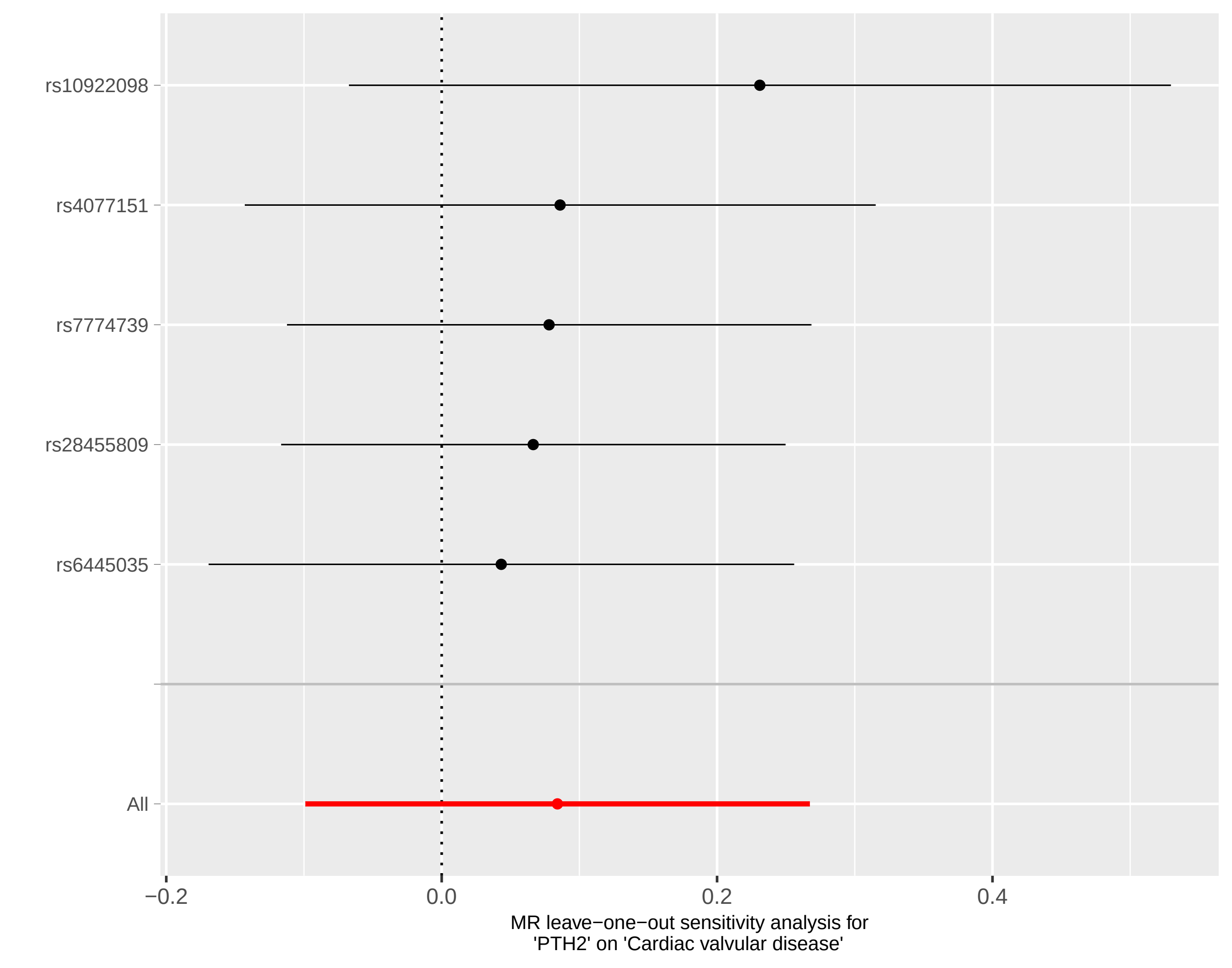
rs10922098
rs4077151
rs7774739
rs28455809
rs6445035
All
−0.2
0.0
0.2
0.4
MR leave−one−out sensitivity analysis for
'PTH2' on 'Cardiac valvular disease'

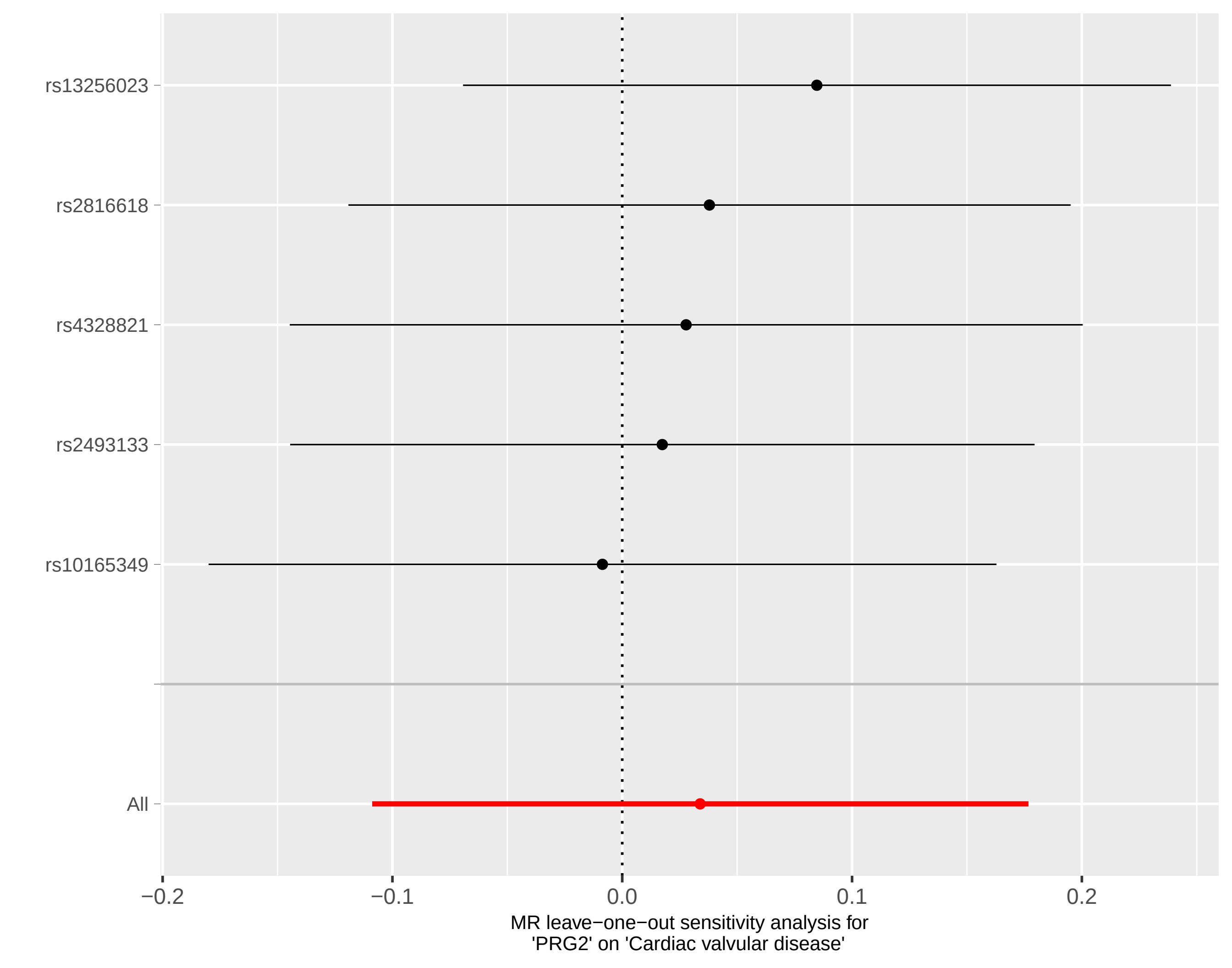
rs13256023
rs2816618
rs4328821
rs2493133
rs10165349
All
−0.2
−0.1
0.0
0.1
0.2
MR leave−one−out sensitivity analysis for
'PRG2' on 'Cardiac valvular disease'

Insufficient number of SNPs

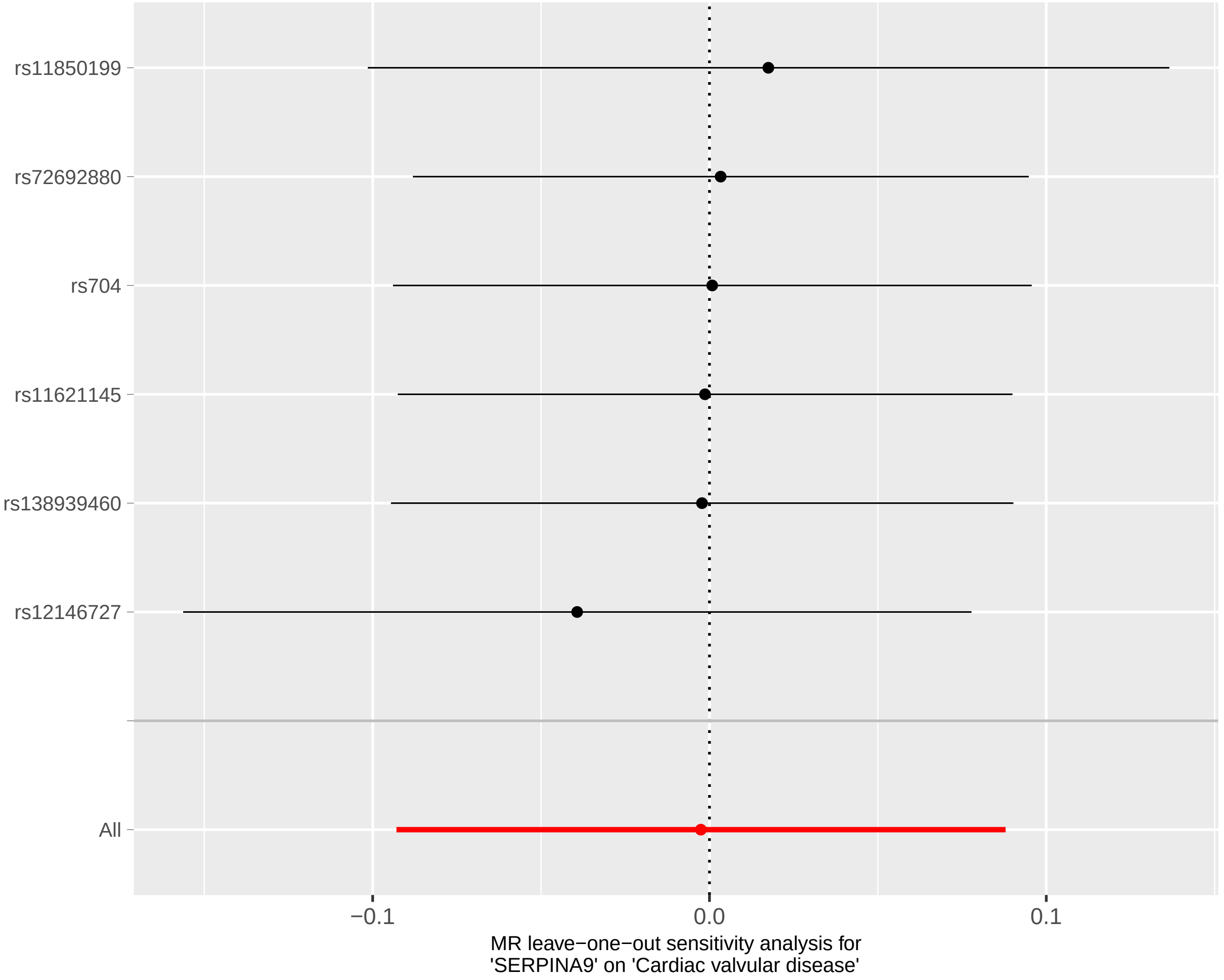
rs11850199
rs72692880
rs704
rs11621145
rs138939460
rs12146727
All
−0.1
0.0
0.1
MR leave−one−out sensitivity analysis for
'SERPINA9' on 'Cardiac valvular disease'

Insufficient number of SNPs

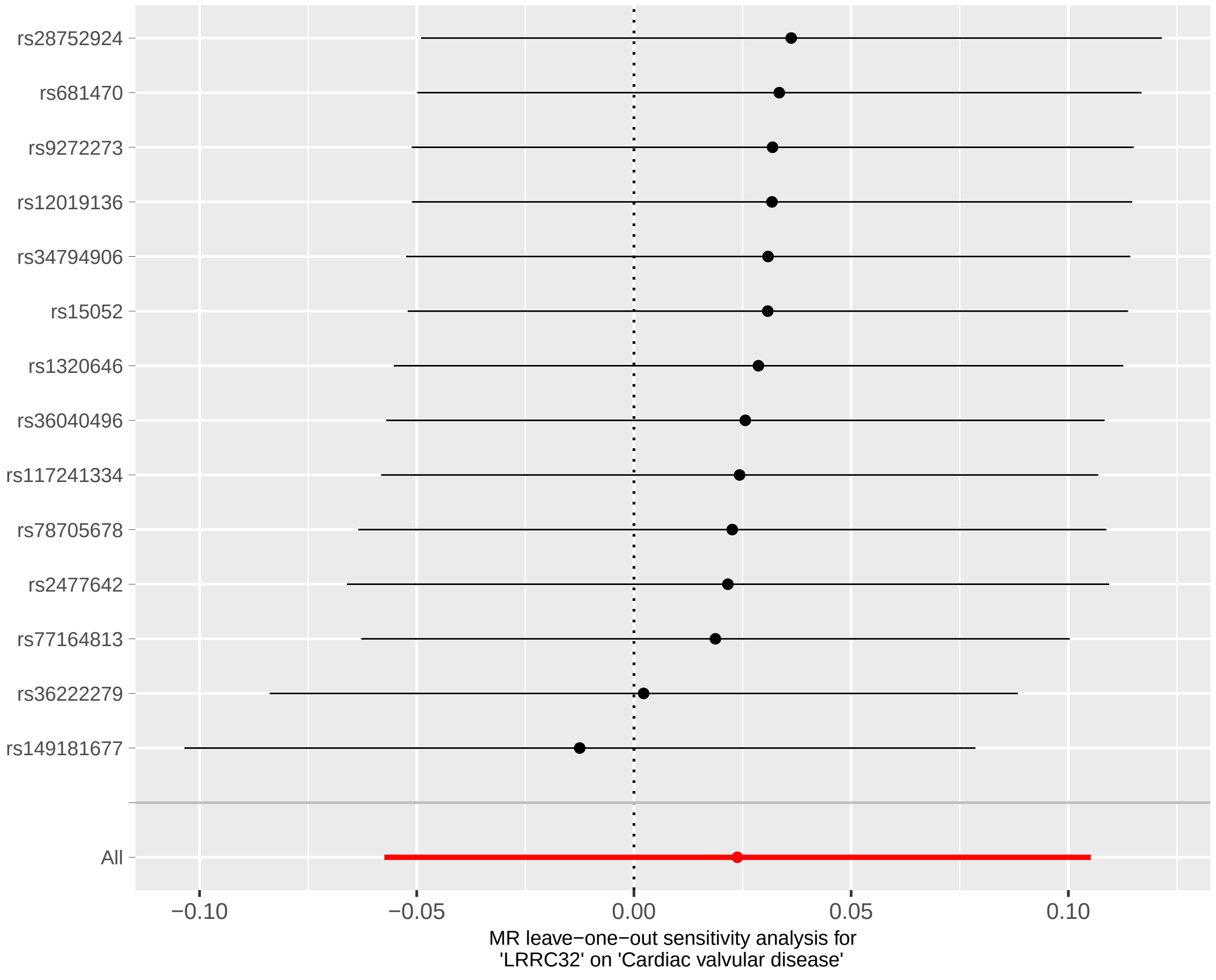
rs28752924
rs681470
rs9272273
rs12019136
rs34794906
rs15052
rs1320646
rs36040496
rs117241334
rs78705678
rs2477642
rs77164813
rs36222279
rs149181677
All
−0.10
−0.05
0.00
0.05
0.10
MR leave−one−out sensitivity analysis for
'LRRC32' on 'Cardiac valvular disease'

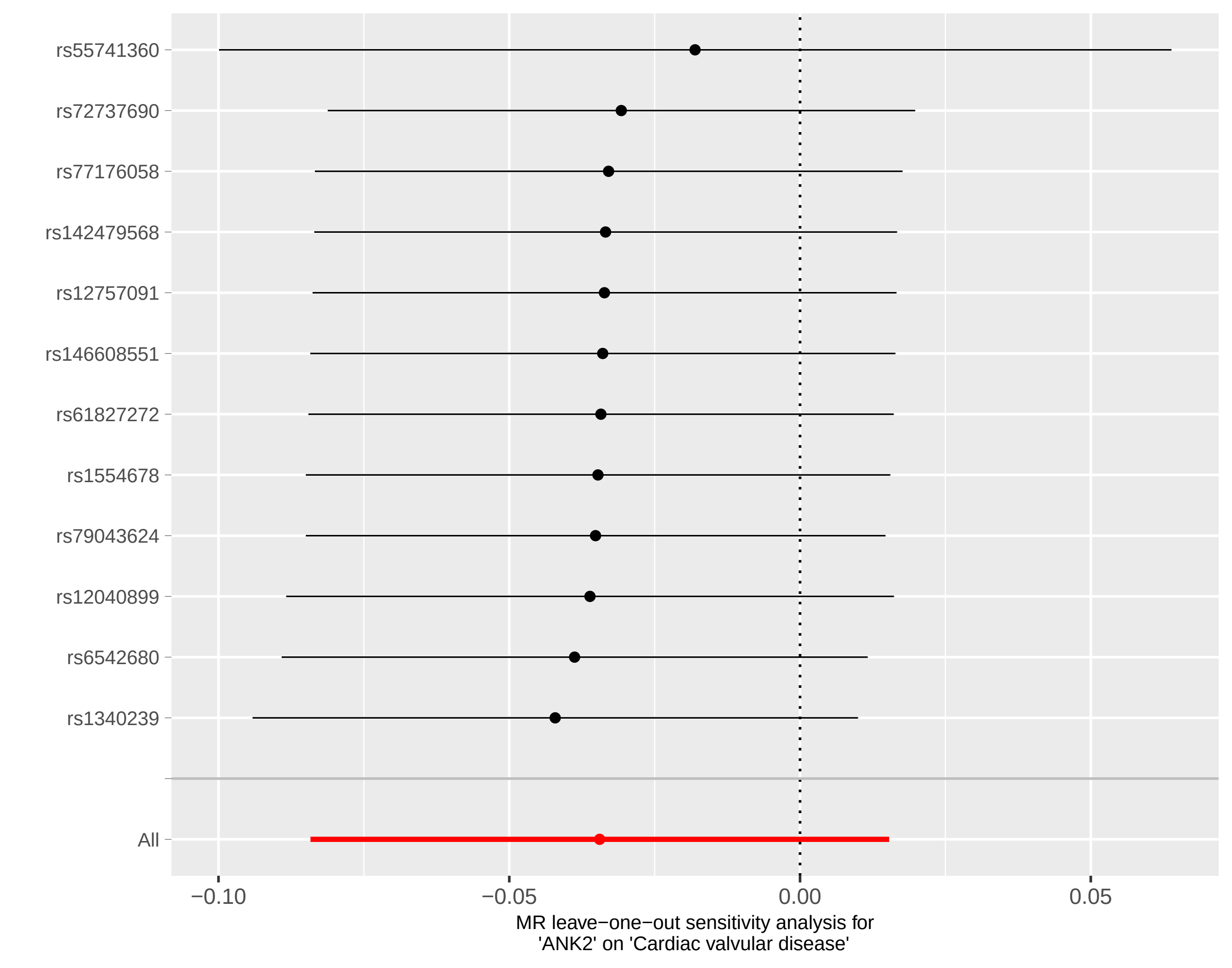

rs55741360
rs72737690
rs77176058
rs142479568
rs12757091
rs146608551
rs61827272
rs1554678
rs79043624
rs12040899
rs6542680
rs1340239
All
−0.10
−0.05
0.00
0.05
MR leave−one−out sensitivity analysis for
'ANK2' on 'Cardiac valvular disease'

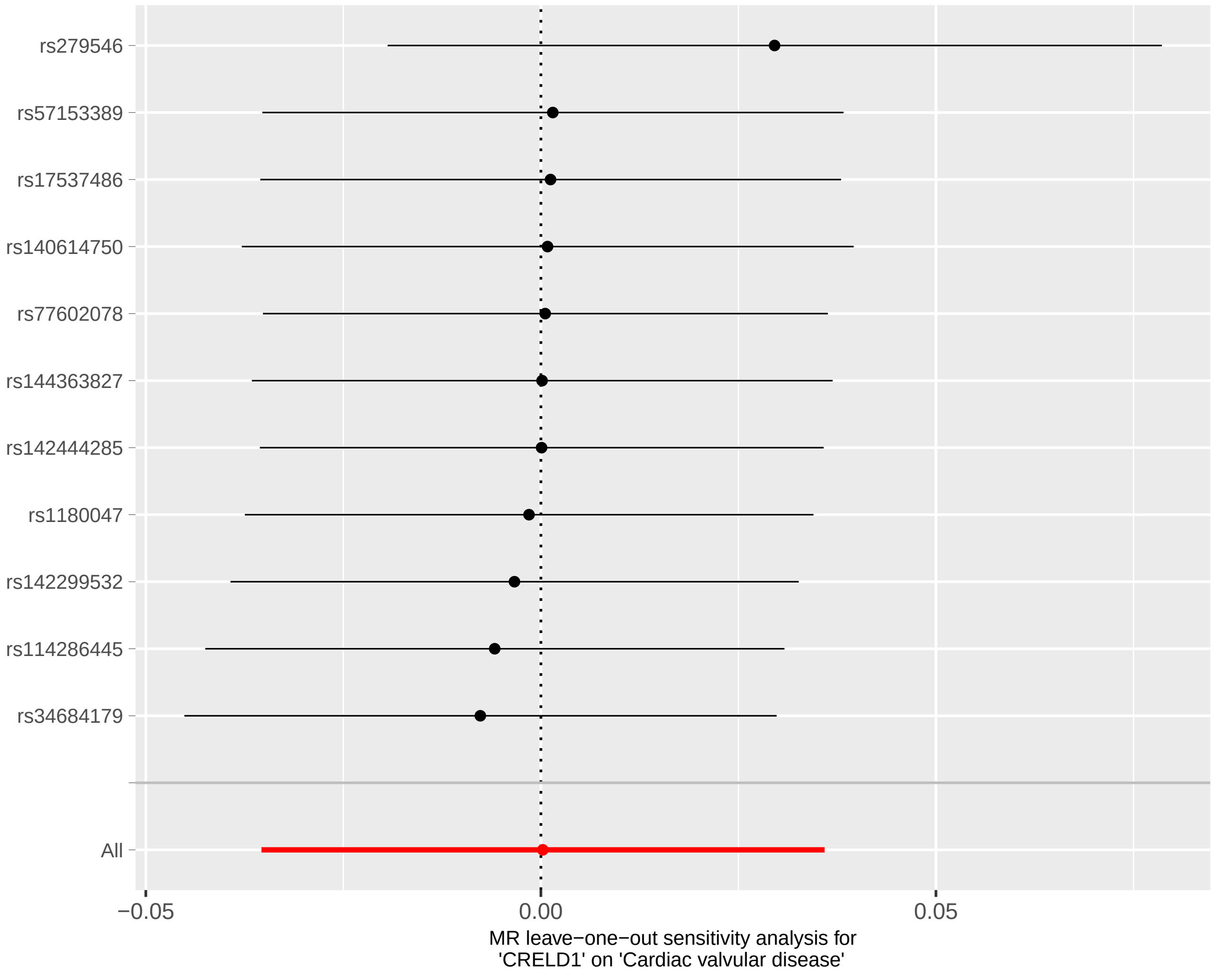
rs279546
rs57153389
rs17537486
rs140614750
rs77602078
rs144363827
rs142444285
rs1180047
rs142299532
rs114286445
rs34684179
All
−0.05
0.00
0.05
MR leave−one−out sensitivity analysis for
'CRELD1' on 'Cardiac valvular disease'

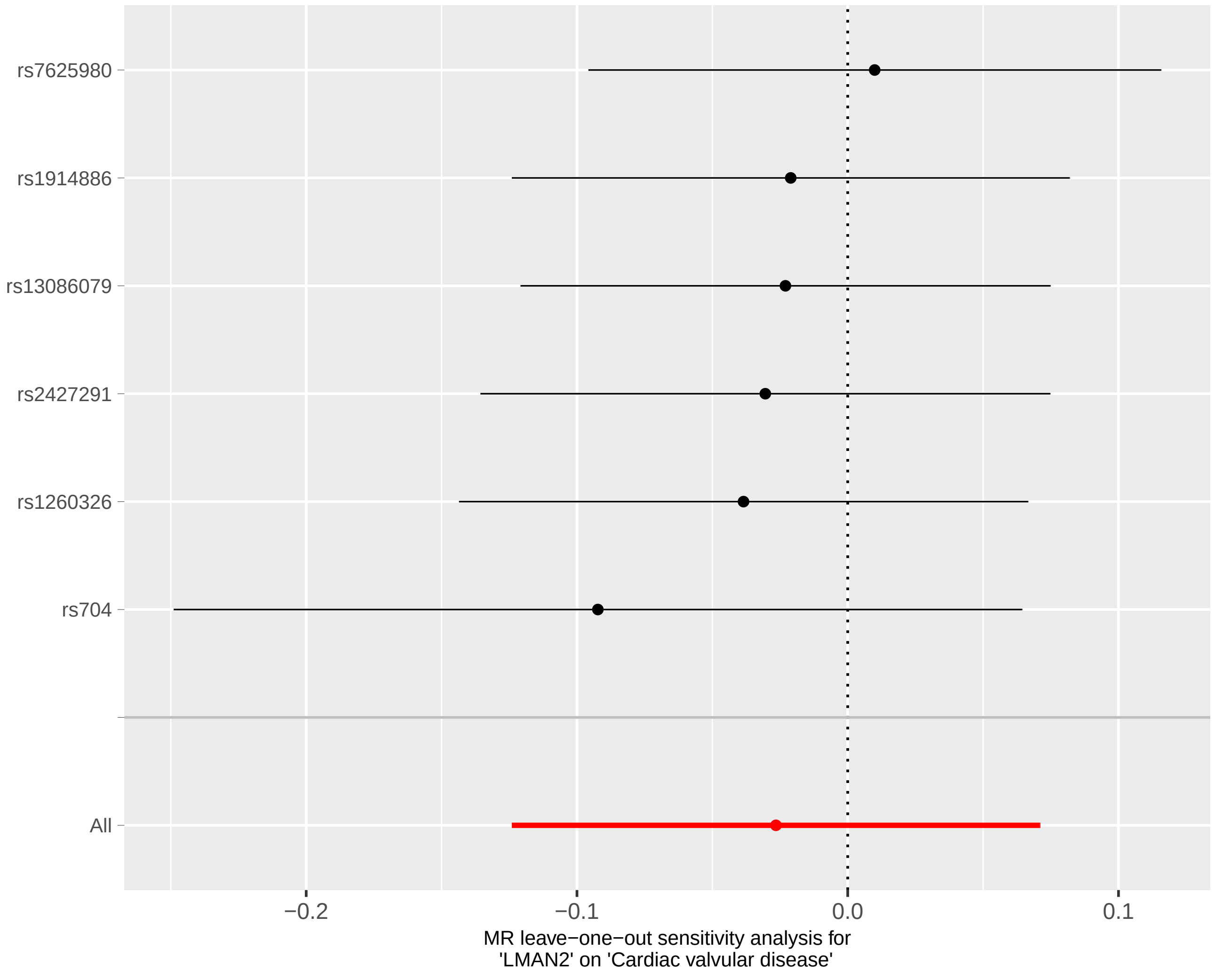
rs7625980
rs1914886
rs13086079
rs2427291
rs1260326
rs704
All
−0.2
−0.1
0.0
0.1
MR leave−one−out sensitivity analysis for
'LMAN2' on 'Cardiac valvular disease'

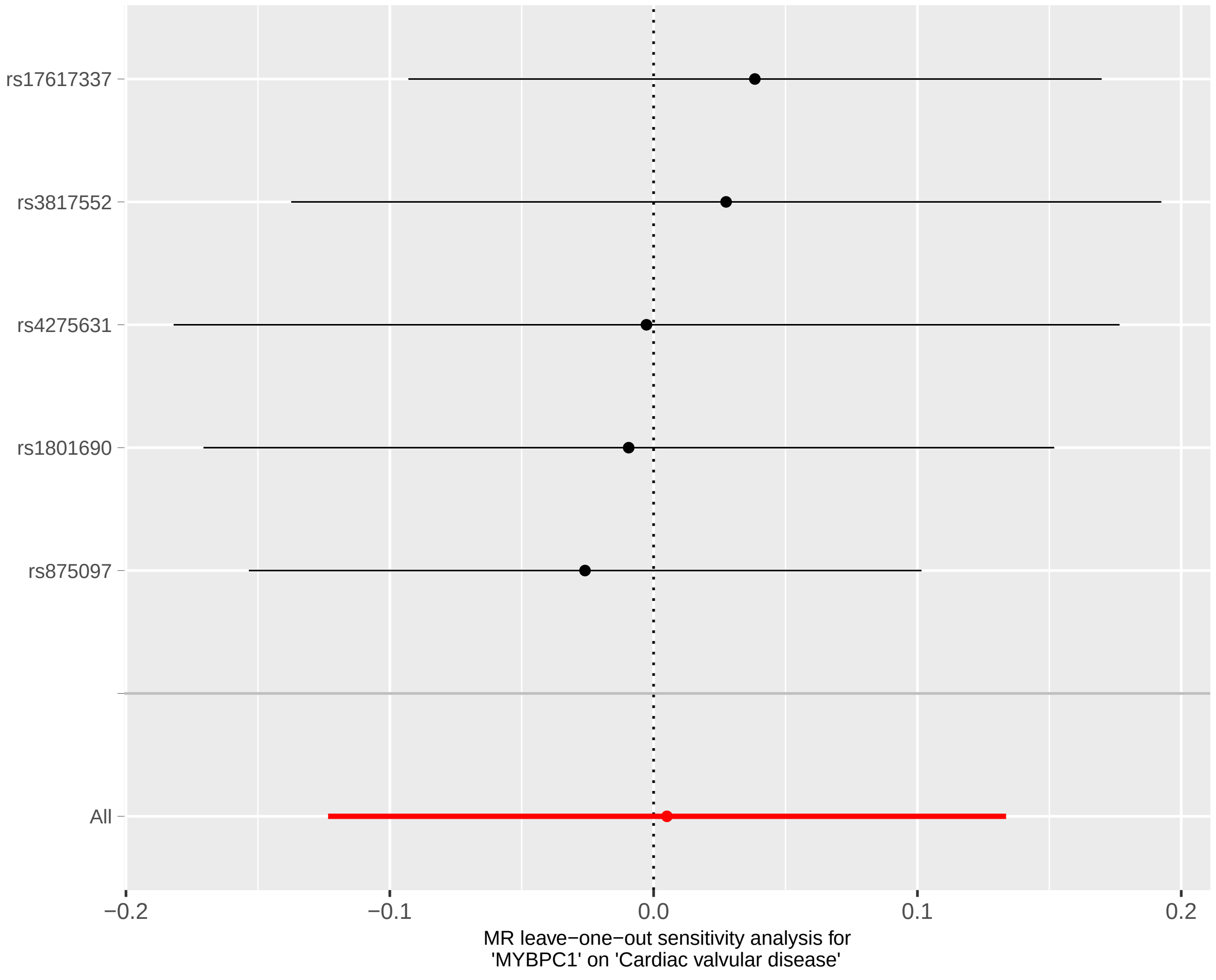

rs17617337
rs3817552
rs4275631
rs1801690
rs875097
All
−0.2
−0.1
0.0
0.1
0.2
MR leave−one−out sensitivity analysis for
'MYBPC1' on 'Cardiac valvular disease'

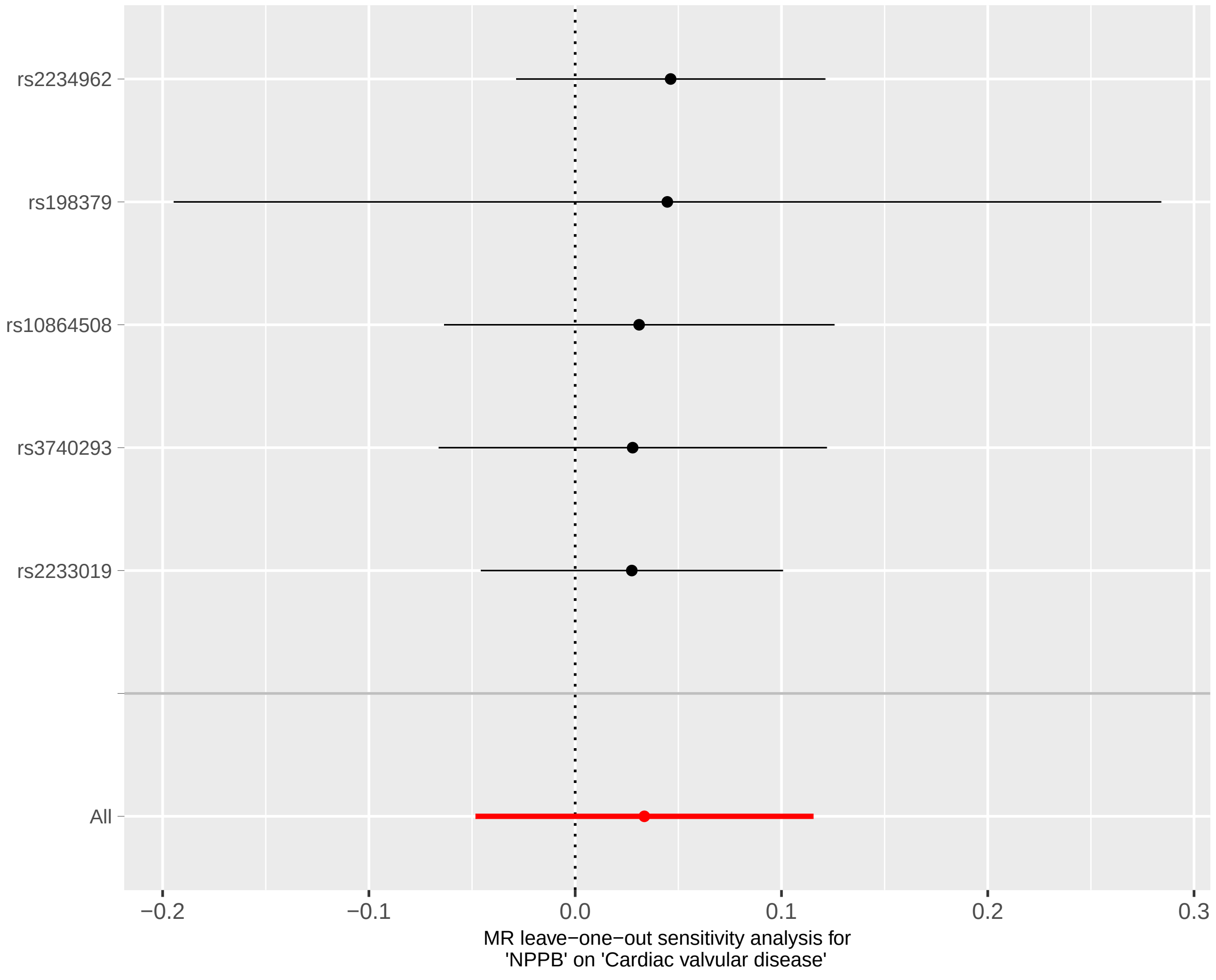

rs2234962
rs198379
rs10864508
rs3740293
rs2233019
All
−0.2
−0.1
0.0
0.1
0.2
0.3
MR leave−one−out sensitivity analysis for
'NPPB' on 'Cardiac valvular disease'

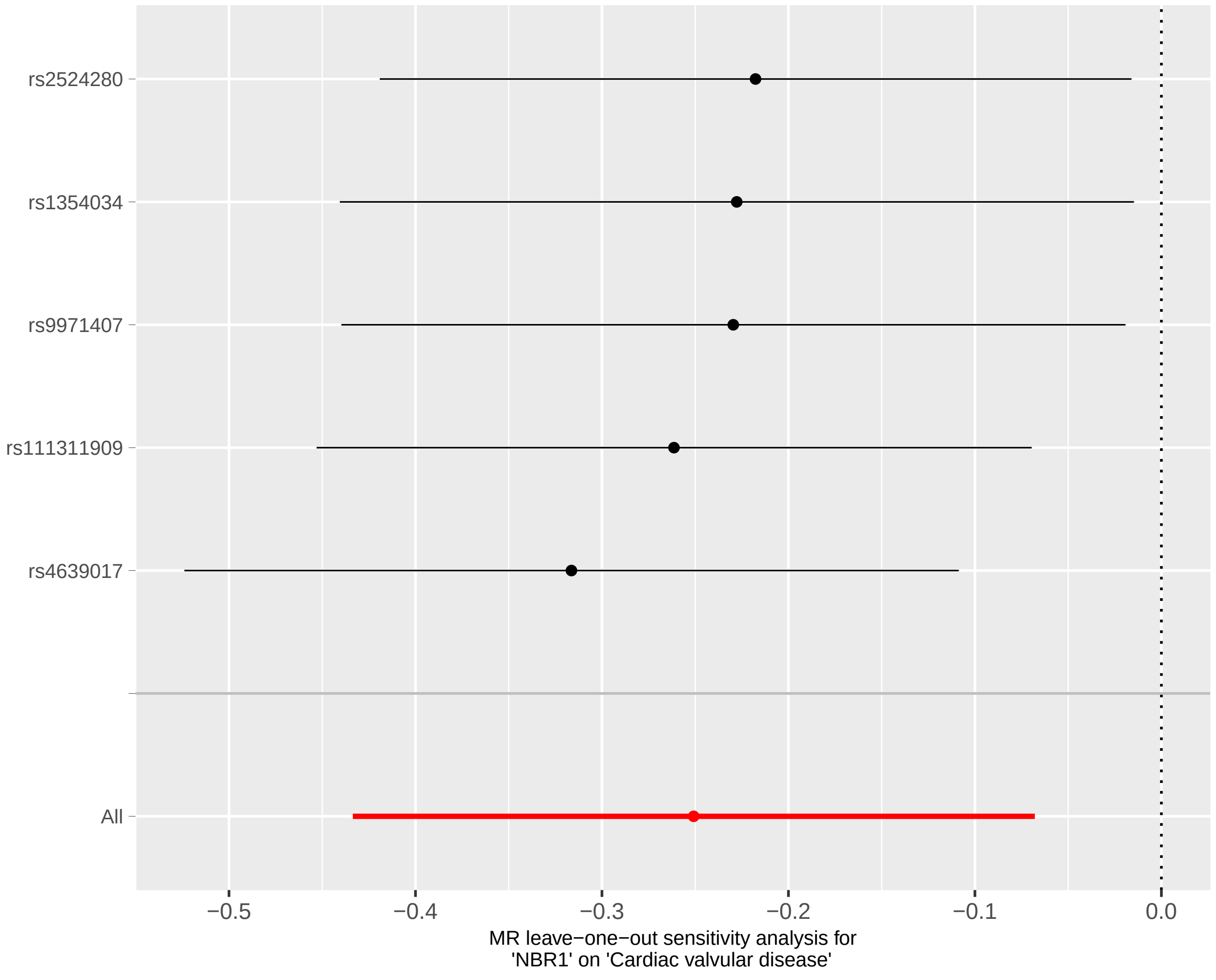
rs2524280
rs1354034
rs9971407
rs111311909
rs4639017
All
−0.5
−0.4
−0.3
−0.2
−0.1
0.0
MR leave−one−out sensitivity analysis for
'NBR1' on 'Cardiac valvular disease'

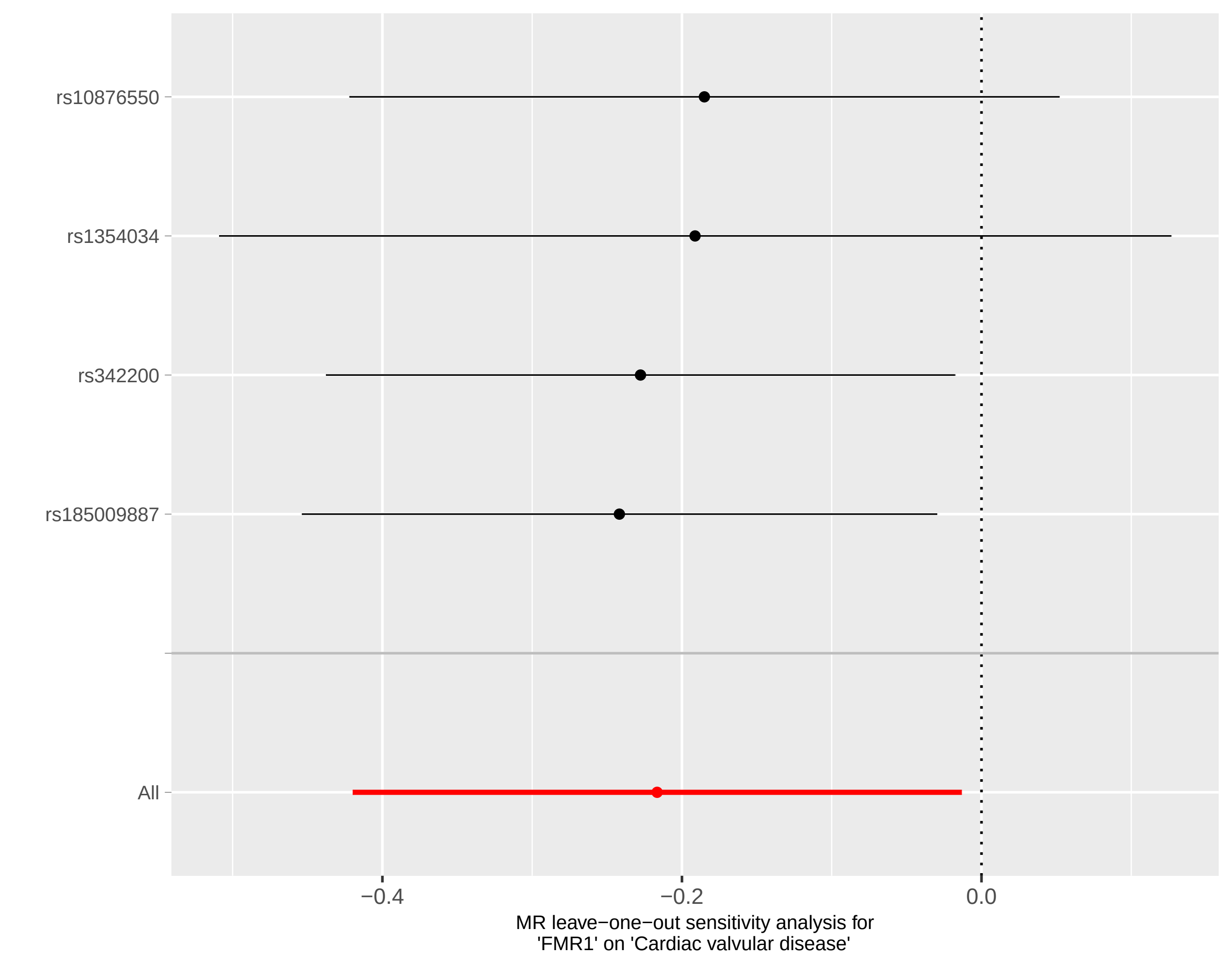

rs10876550
rs1354034
rs342200
rs185009887
All
−0.4
−0.2
0.0
MR leave−one−out sensitivity analysis for
'FMR1' on 'Cardiac valvular disease'

Insufficient number of SNPs

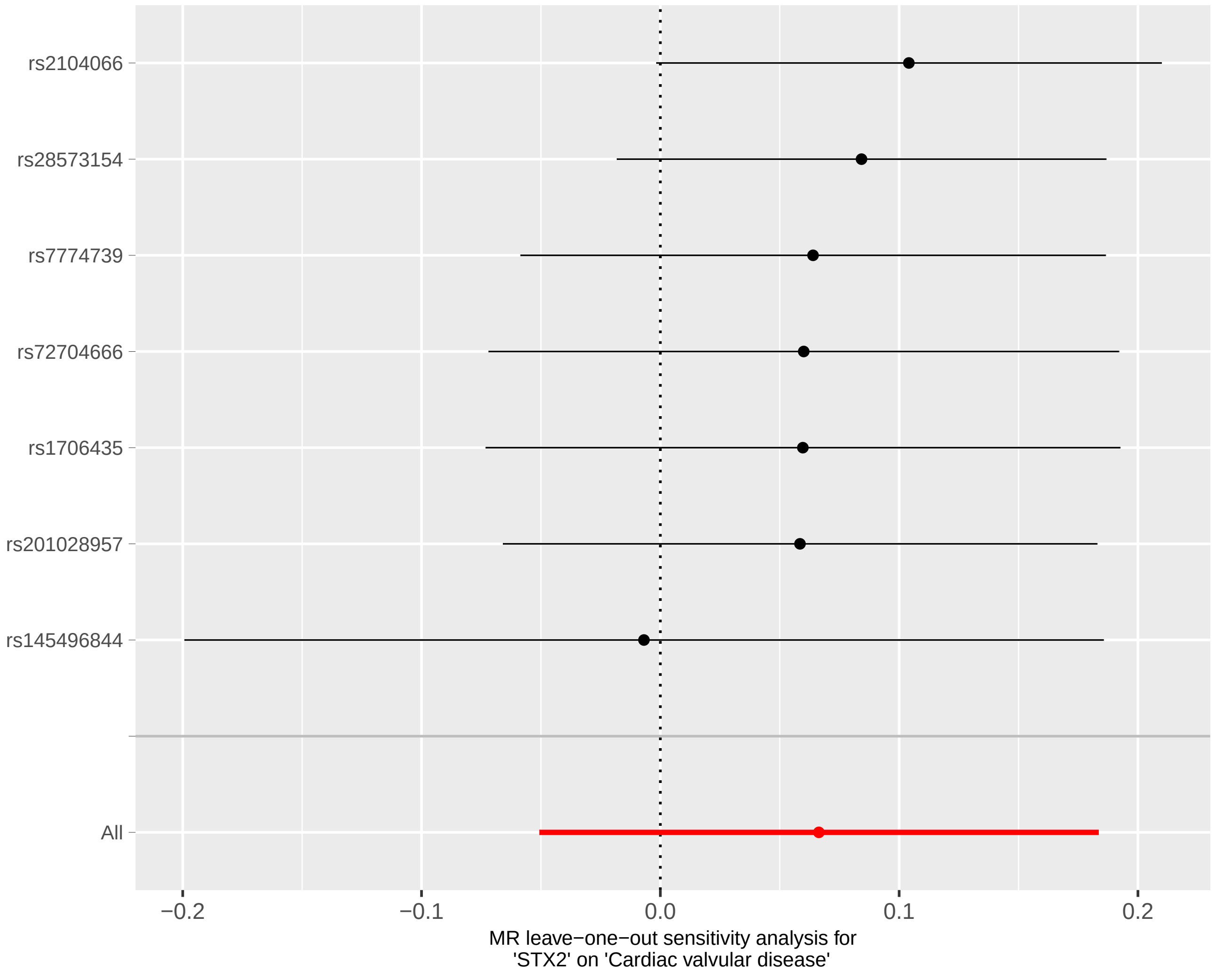
rs2104066
rs28573154
rs7774739
rs72704666
rs1706435
rs201028957
rs145496844
All
−0.2
−0.1
0.0
0.1
0.2
MR leave−one−out sensitivity analysis for
'STX2' on 'Cardiac valvular disease'

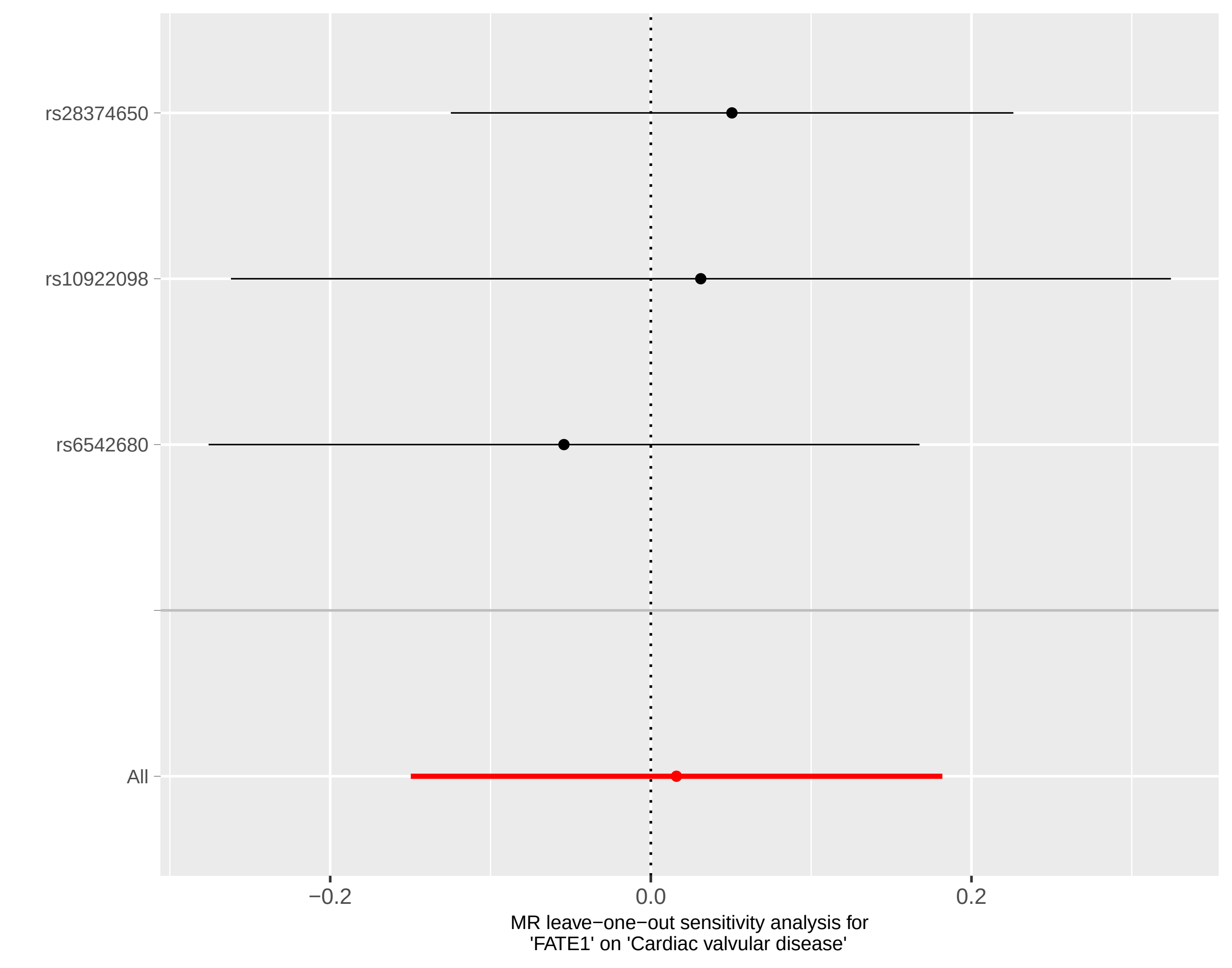

rs28374650
rs10922098
rs6542680
All
−0.2
0.0
0.2
MR leave−one−out sensitivity analysis for
'FATE1' on 'Cardiac valvular disease'

Insufficient number of SNPs

Insufficient number of SNPs

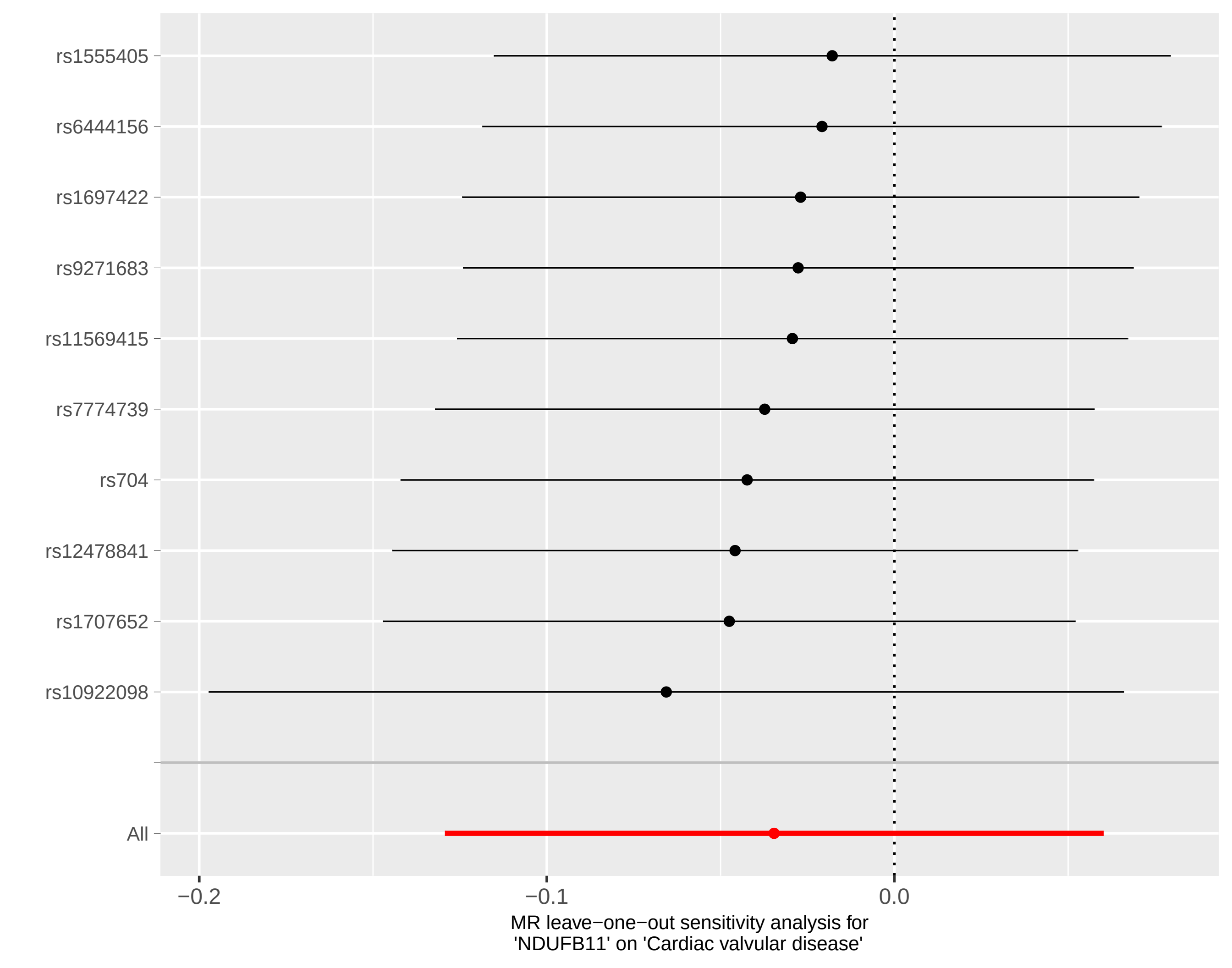

rs1555405
rs6444156
rs1697422
rs9271683
rs11569415
rs7774739
rs704
rs12478841
rs1707652
rs10922098
All
−0.2
−0.1
0.0
MR leave−one−out sensitivity analysis for
'NDUFB11' on 'Cardiac valvular disease'

Insufficient number of SNPs

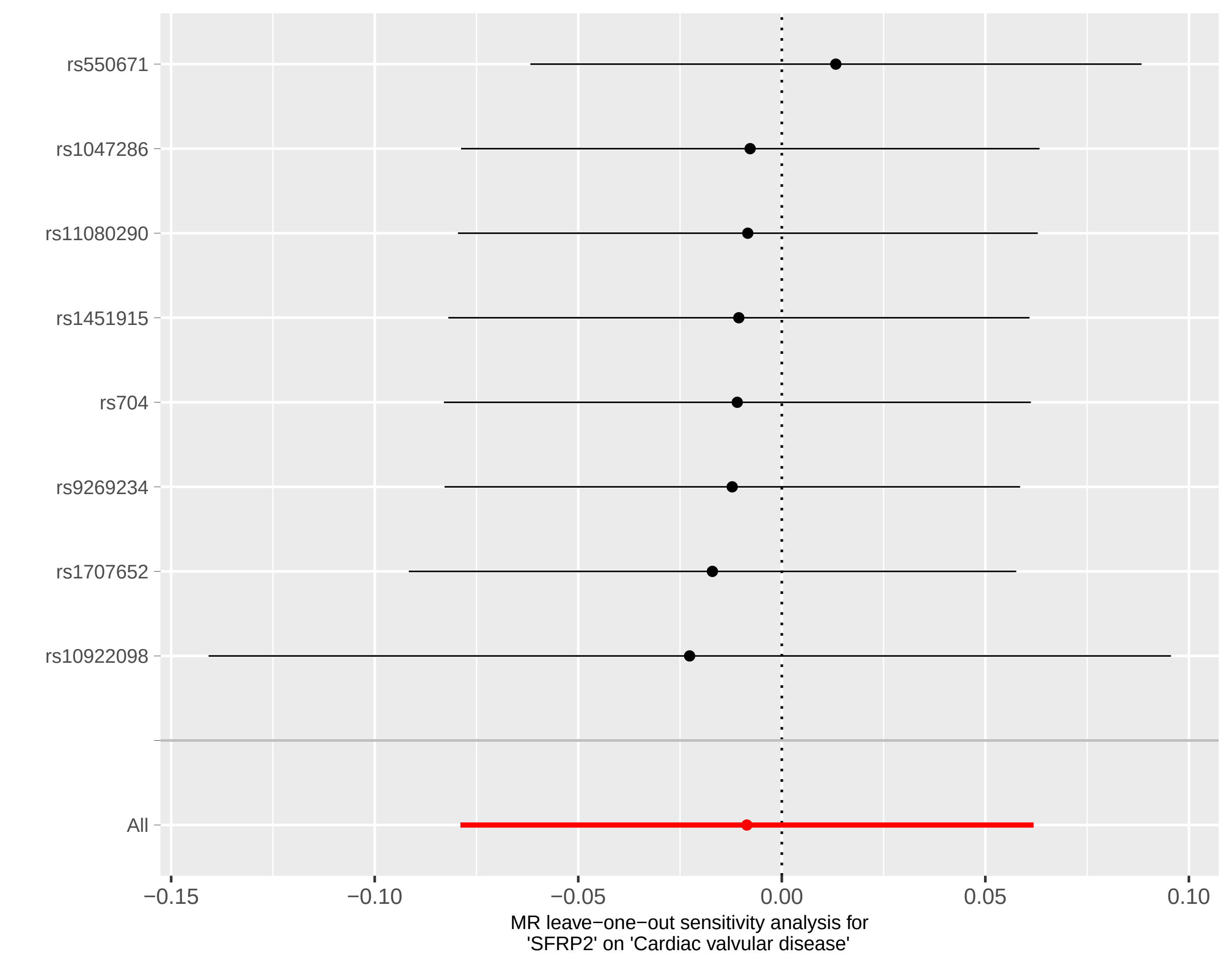

rs550671
rs1047286
rs11080290
rs1451915
rs704
rs9269234
rs1707652
rs10922098
All
−0.15
−0.10
−0.05
0.00
0.05
0.10
MR leave−one−out sensitivity analysis for
'SFRP2' on 'Cardiac valvular disease'

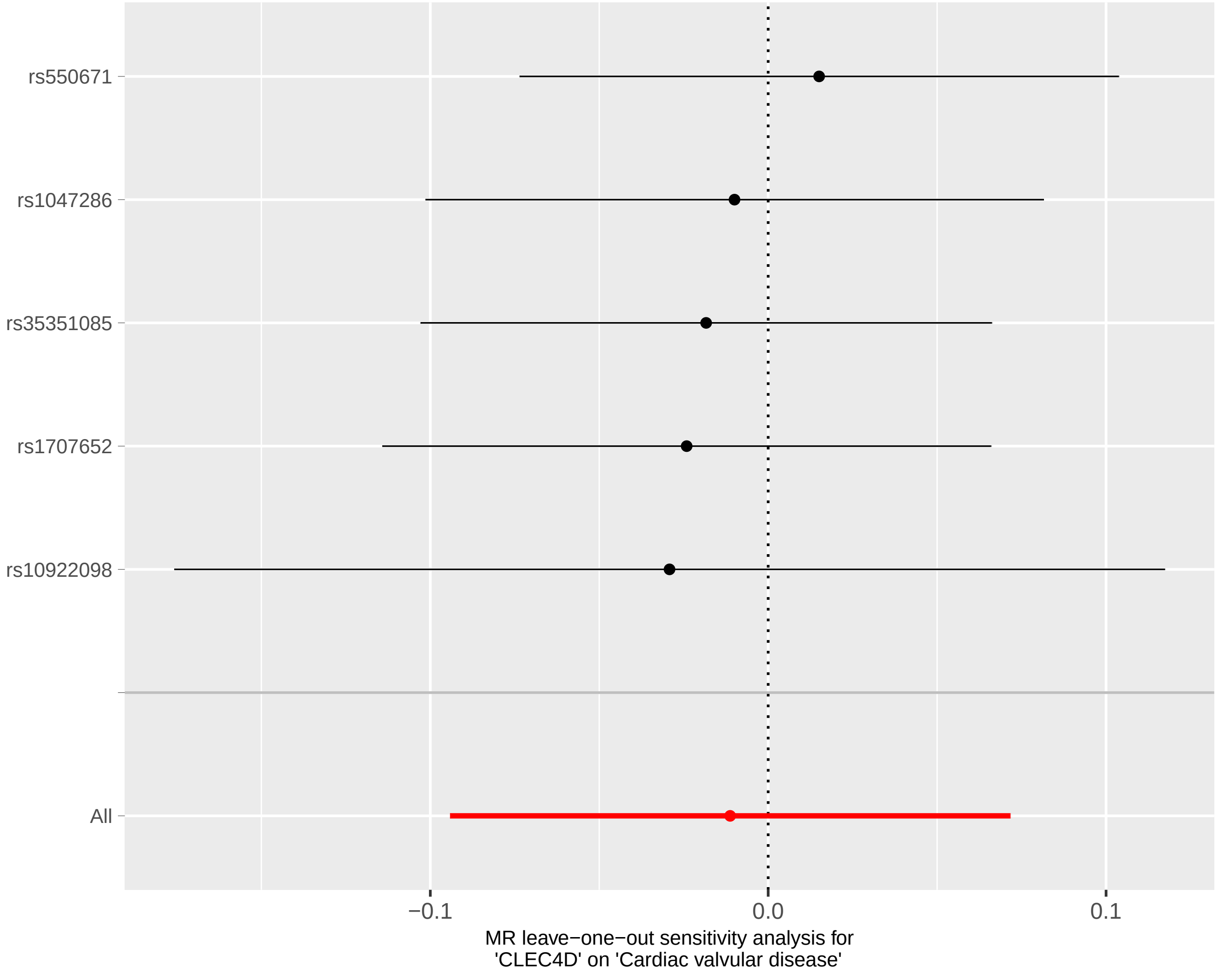
rs550671
rs1047286
rs35351085
rs1707652
rs10922098
All
−0.1
0.0
0.1
MR leave−one−out sensitivity analysis for
'CLEC4D' on 'Cardiac valvular disease'

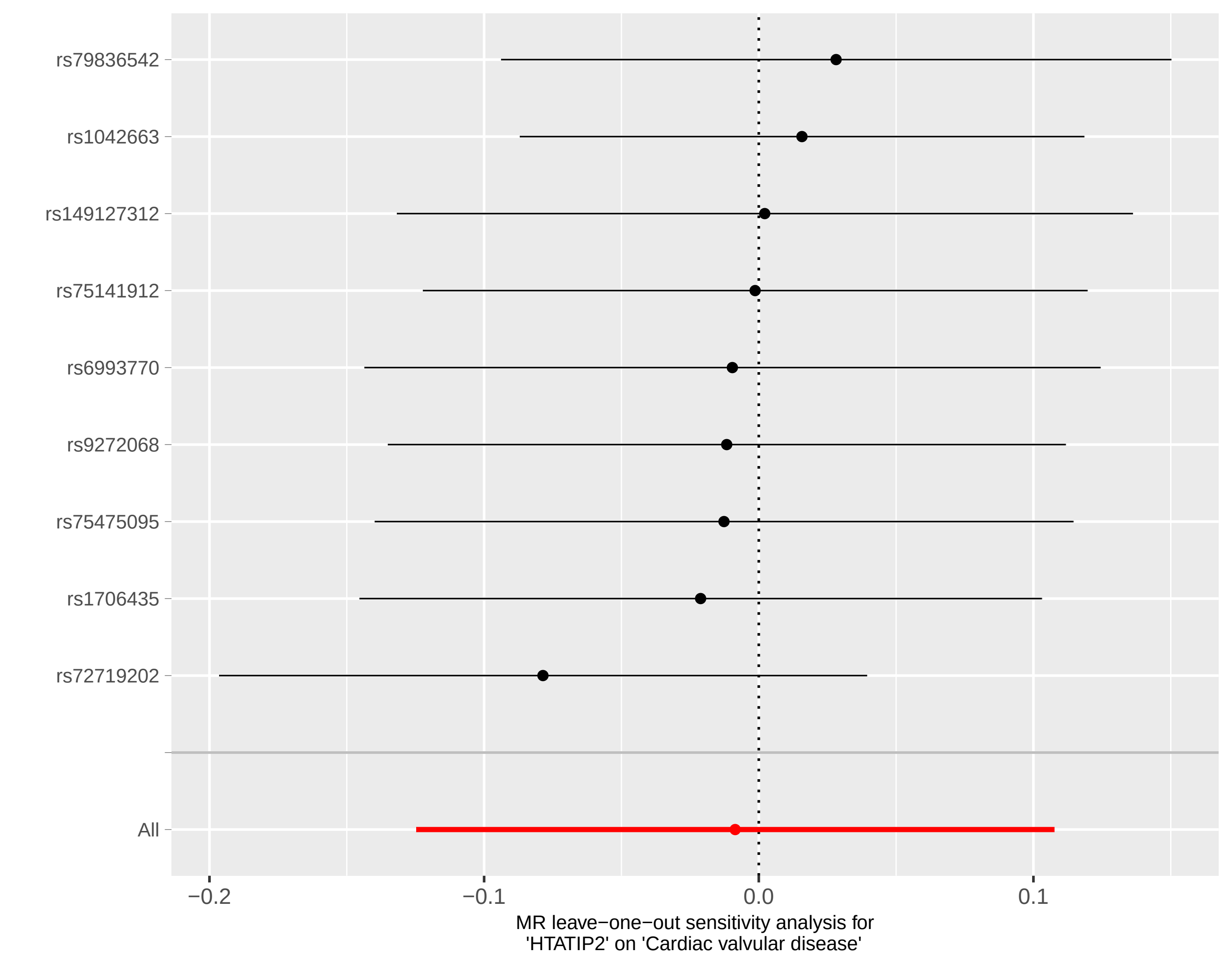
rs79836542
rs1042663
rs149127312
rs75141912
rs6993770
rs9272068
rs75475095
rs1706435
rs72719202
All
−0.2
−0.1
0.0
0.1
MR leave−one−out sensitivity analysis for
'HTATIP2' on 'Cardiac valvular disease'

Insufficient number of SNPs

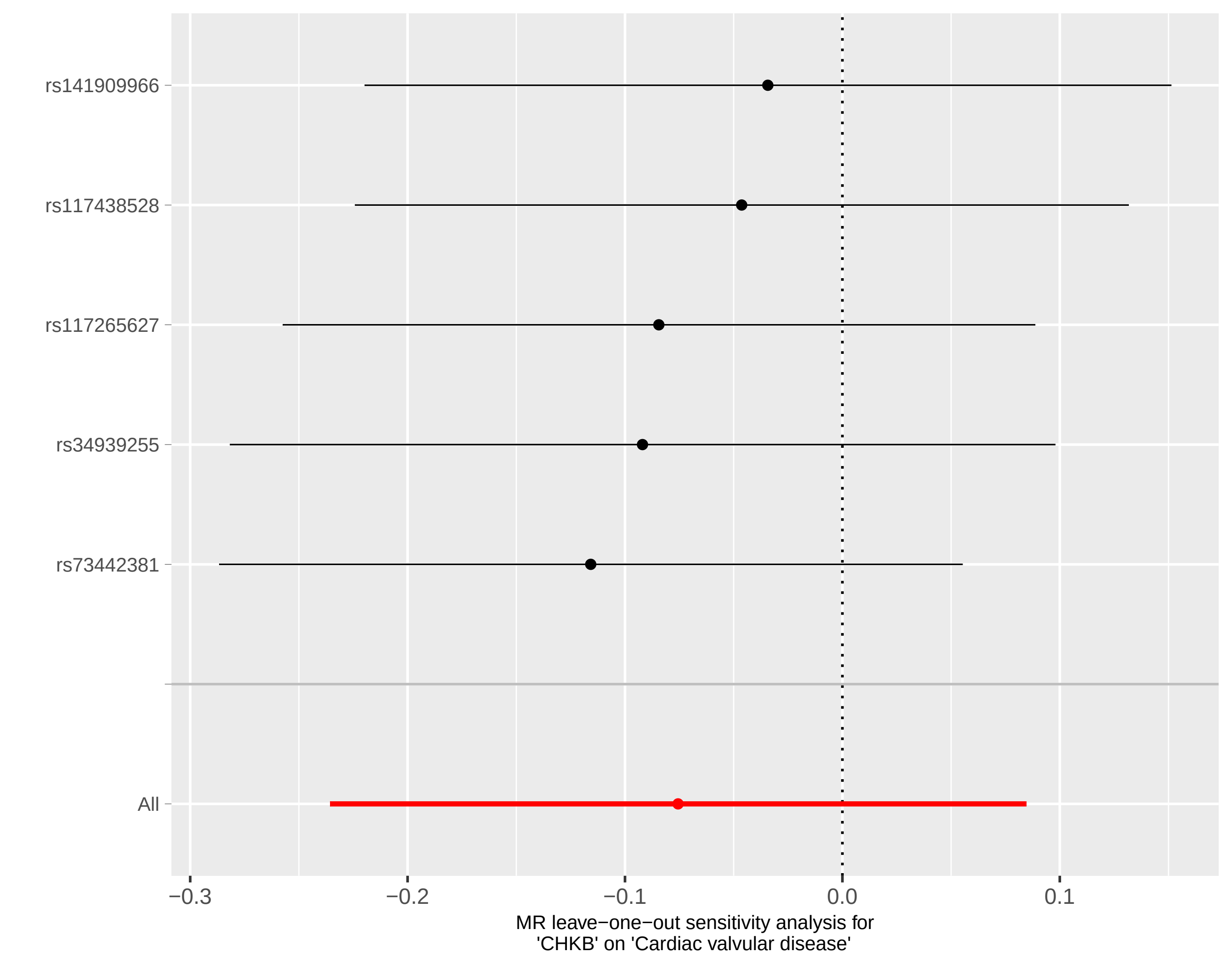
rs141909966
rs117438528
rs117265627
rs34939255
rs73442381
All
−0.3
−0.2
−0.1
0.0
0.1
MR leave−one−out sensitivity analysis for
'CHKB' on 'Cardiac valvular disease'

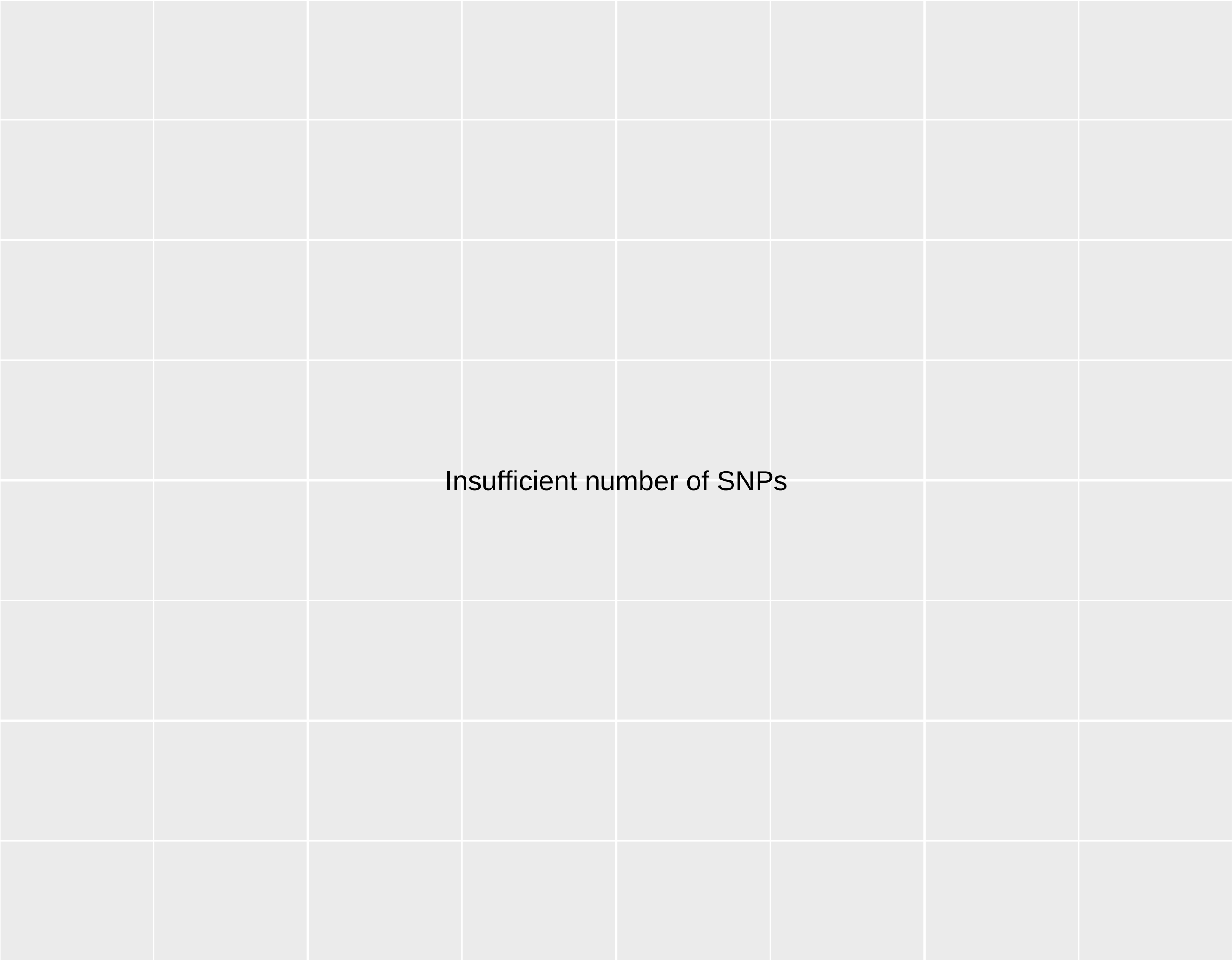
Insufficient number of SNPs

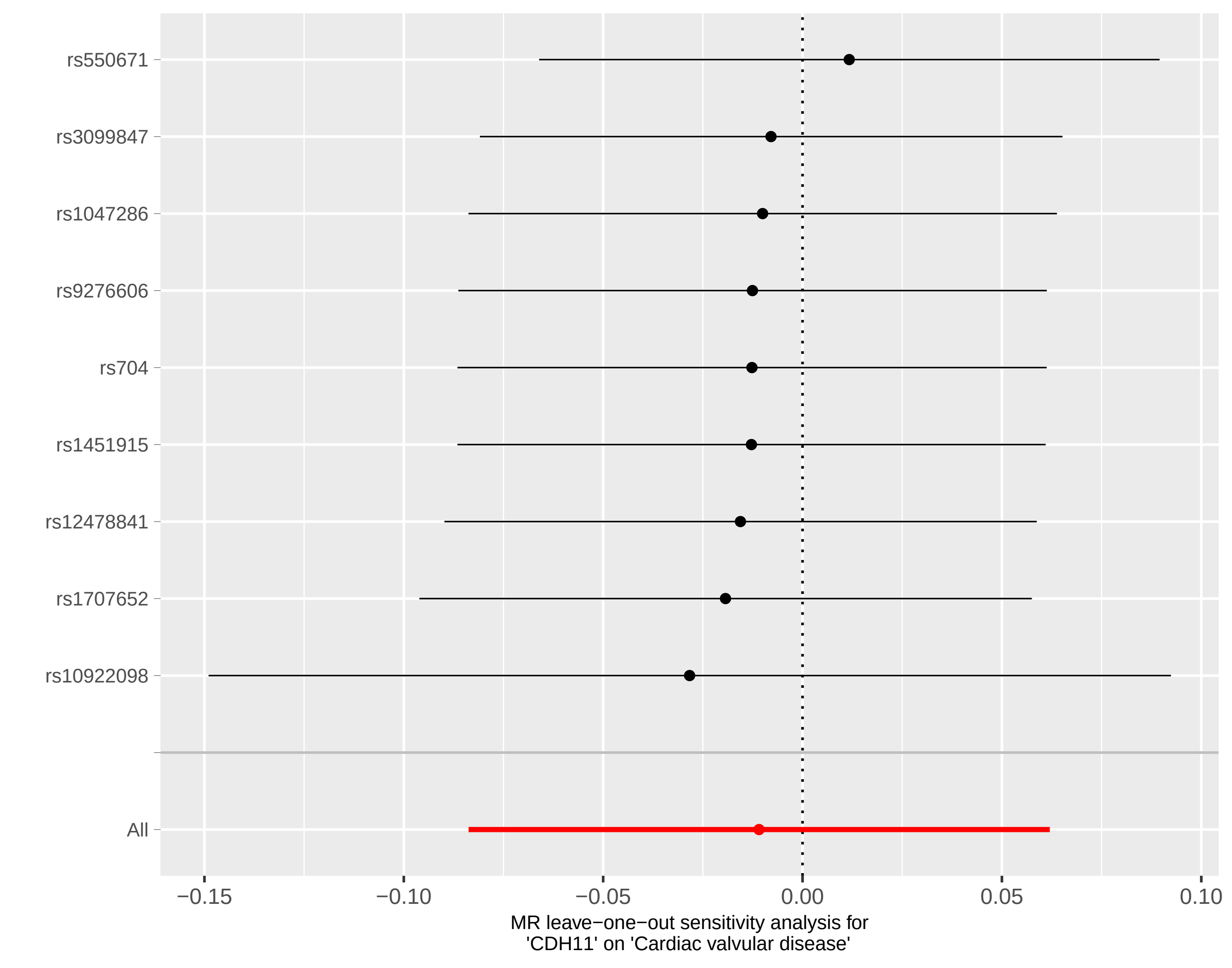

rs550671
rs3099847
rs1047286
rs9276606
rs704
rs1451915
rs12478841
rs1707652
rs10922098
All
−0.15
−0.10
−0.05
0.00
0.05
0.10
MR leave−one−out sensitivity analysis for
'CDH11' on 'Cardiac valvular disease'

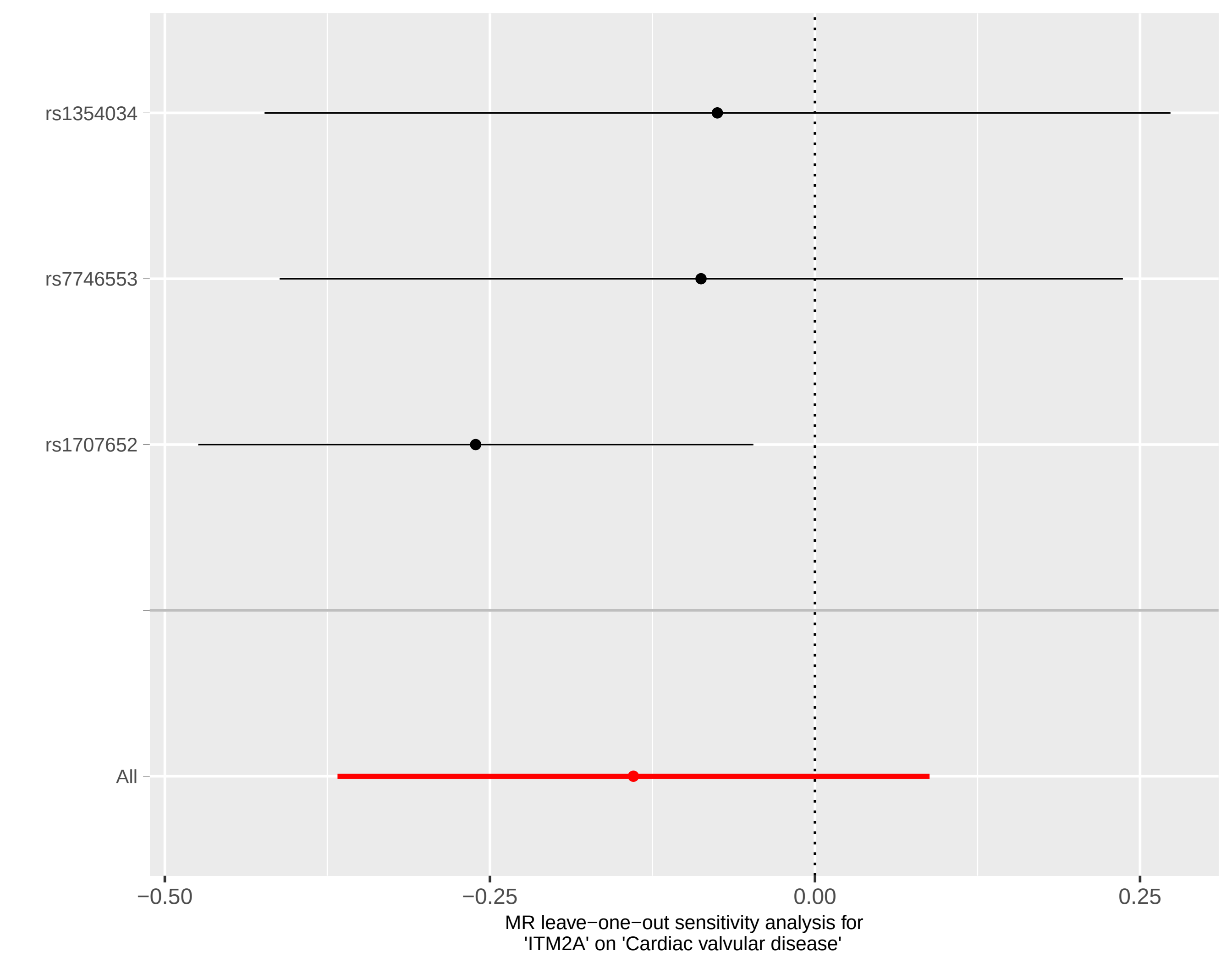
rs1354034
rs7746553
rs1707652
All
−0.50
−0.25
0.00
0.25
MR leave−one−out sensitivity analysis for
'ITM2A' on 'Cardiac valvular disease'

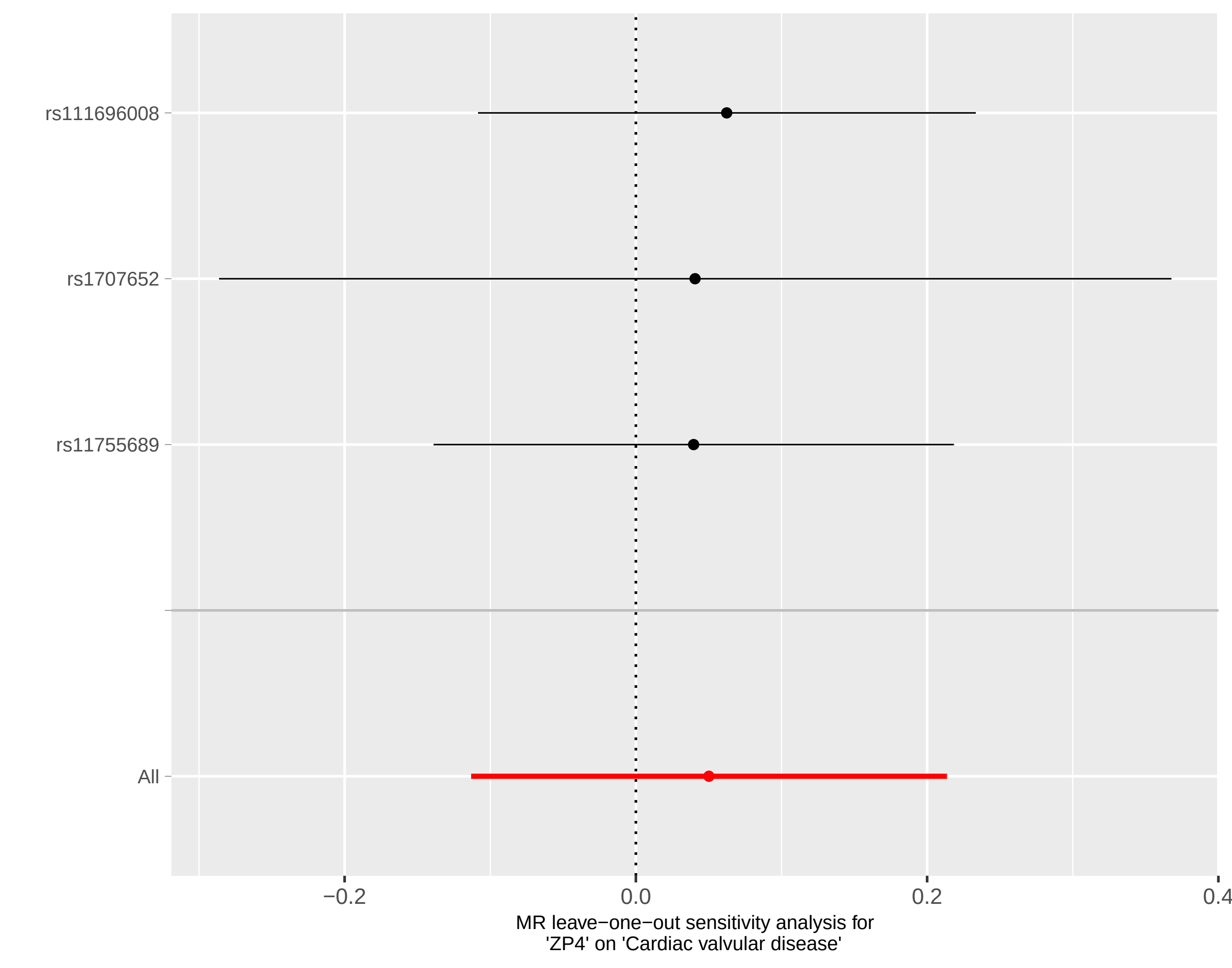
rs111696008
rs1707652
rs11755689
All
−0.2
0.0
0.2
0.4
MR leave−one−out sensitivity analysis for
'ZP4' on 'Cardiac valvular disease'

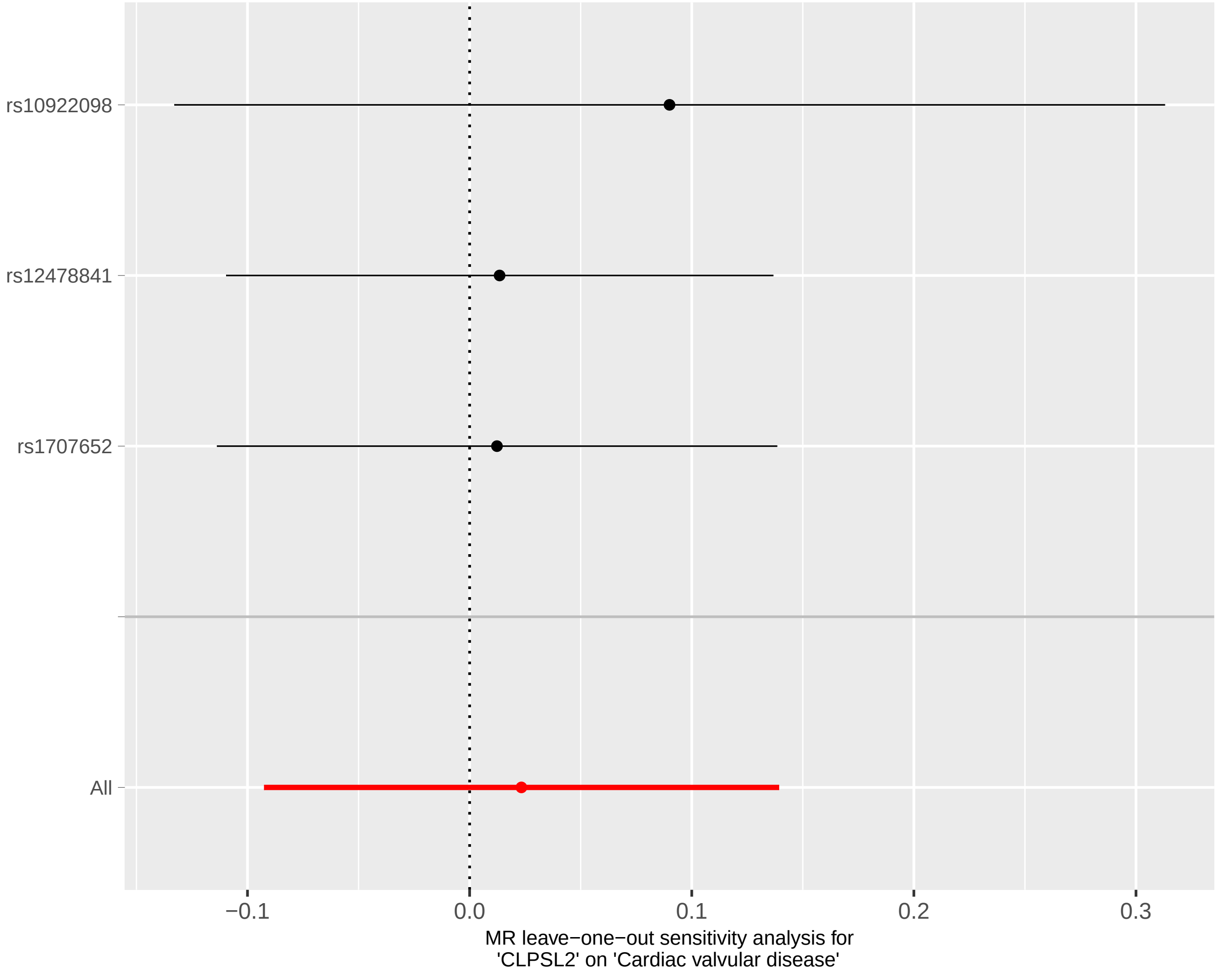

rs10922098
rs12478841
rs1707652
All
−0.1
0.0
0.1
0.2
0.3
MR leave−one−out sensitivity analysis for
'CLPSL2' on 'Cardiac valvular disease'

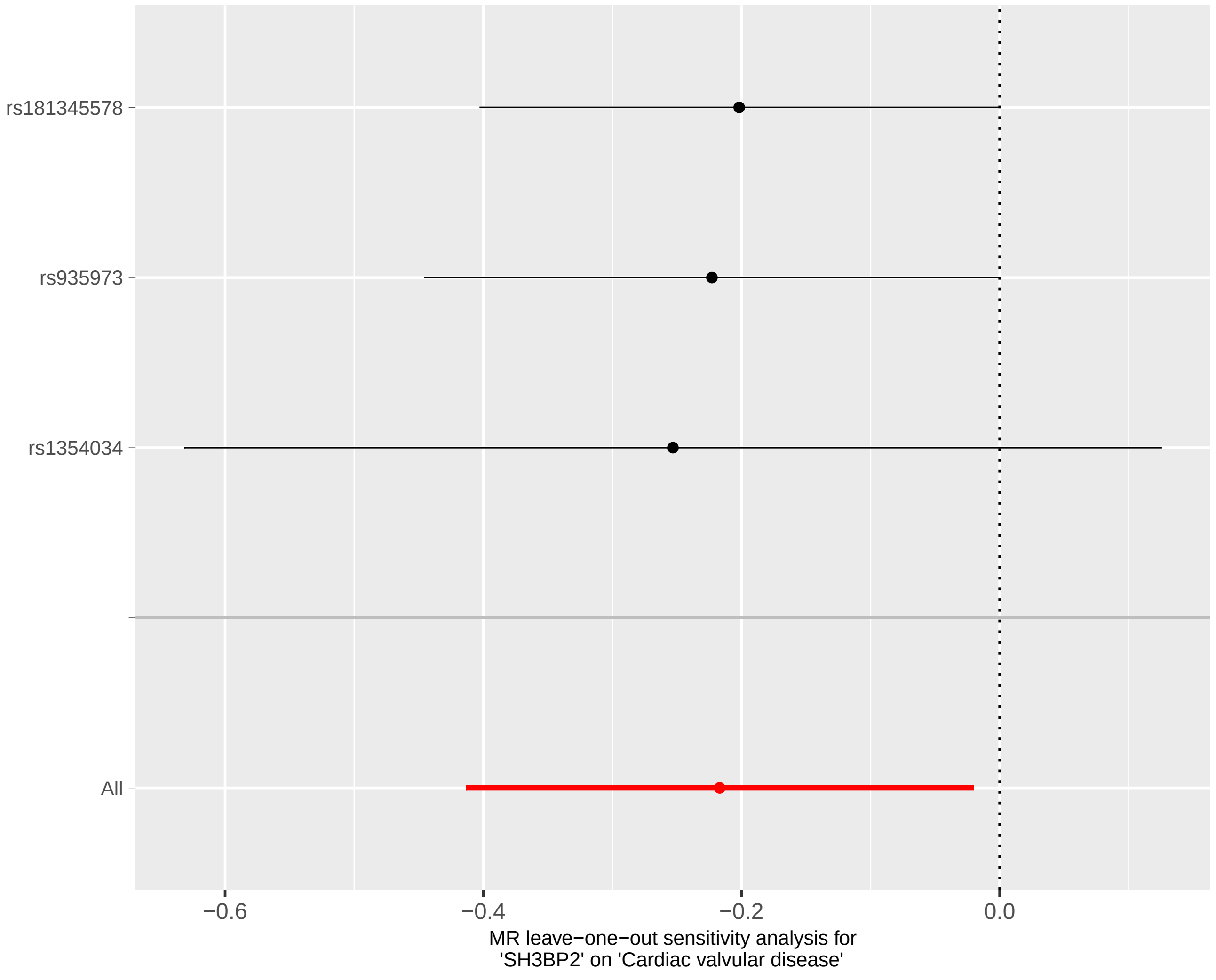

rs181345578
rs935973
rs1354034
All
−0.6
−0.4
−0.2
0.0
MR leave−one−out sensitivity analysis for
'SH3BP2' on 'Cardiac valvular disease'

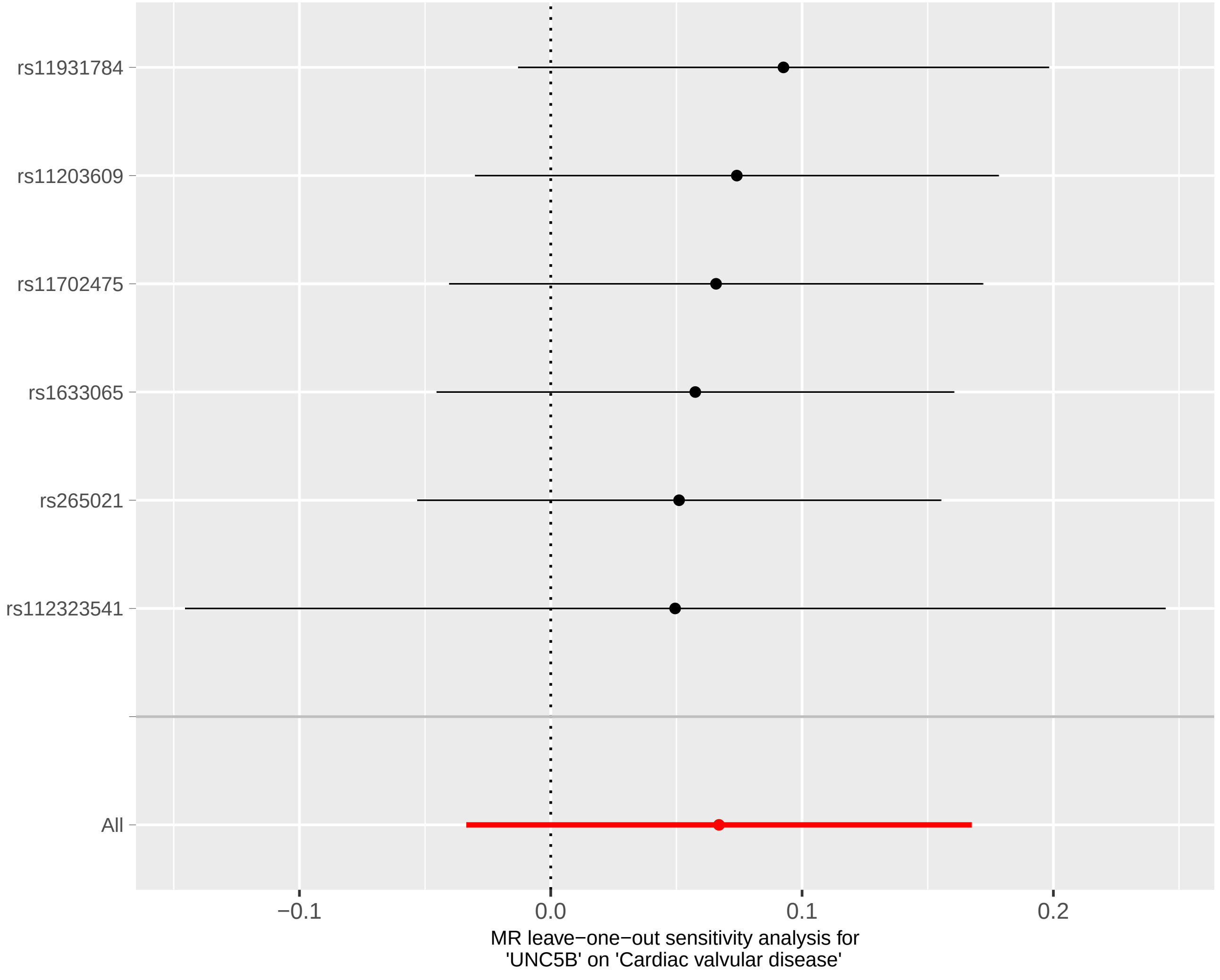
rs11931784
rs11203609
rs11702475
rs1633065
rs265021
rs112323541
All
−0.1
0.0
0.1
0.2
MR leave−one−out sensitivity analysis for
'UNC5B' on 'Cardiac valvular disease'

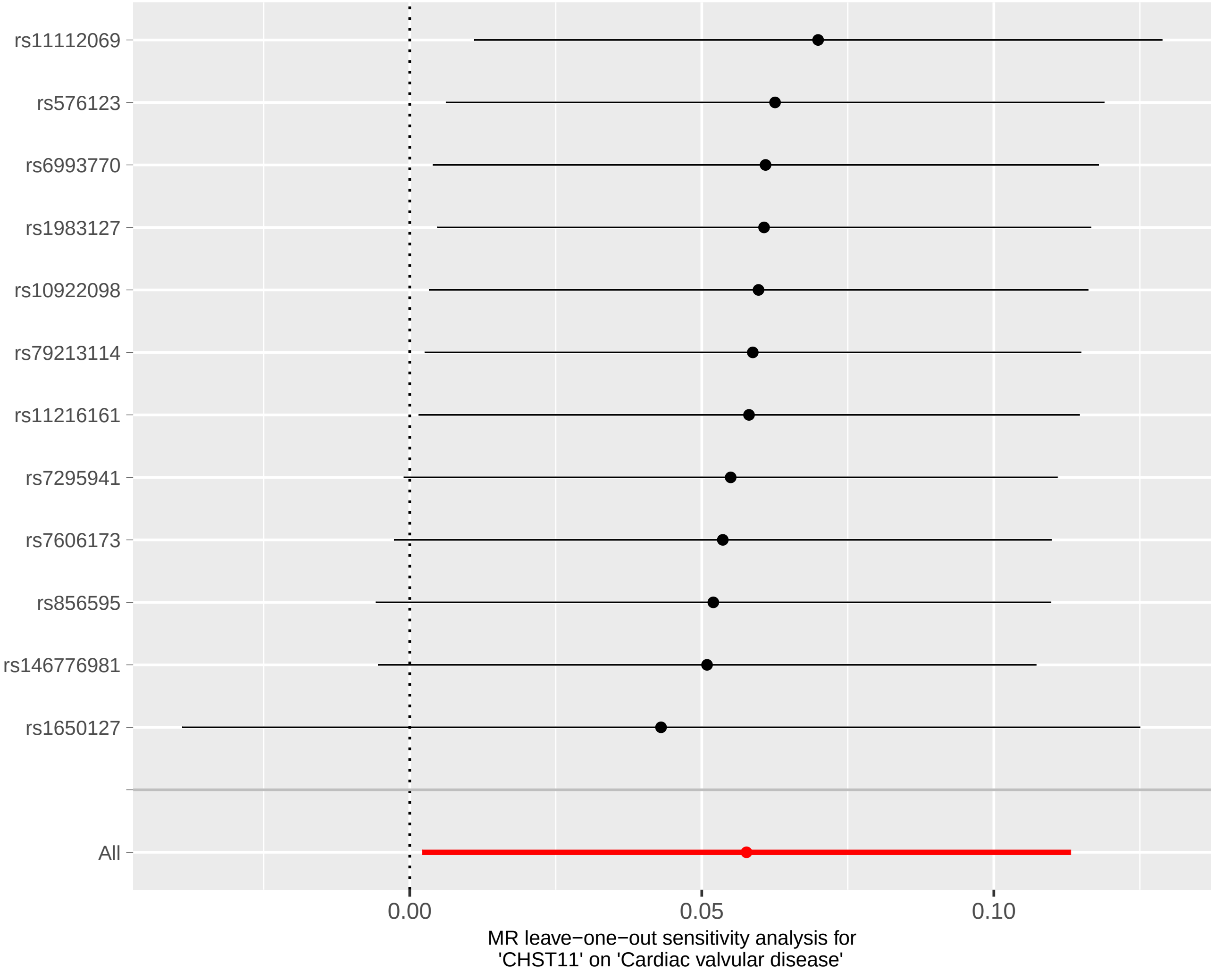
rs11112069
rs576123
rs6993770
rs1983127
rs10922098
rs79213114
rs11216161
rs7295941
rs7606173
rs856595
rs146776981
rs1650127
All
0.00
0.05
0.10
MR leave−one−out sensitivity analysis for
'CHST11' on 'Cardiac valvular disease'

Insufficient number of SNPs

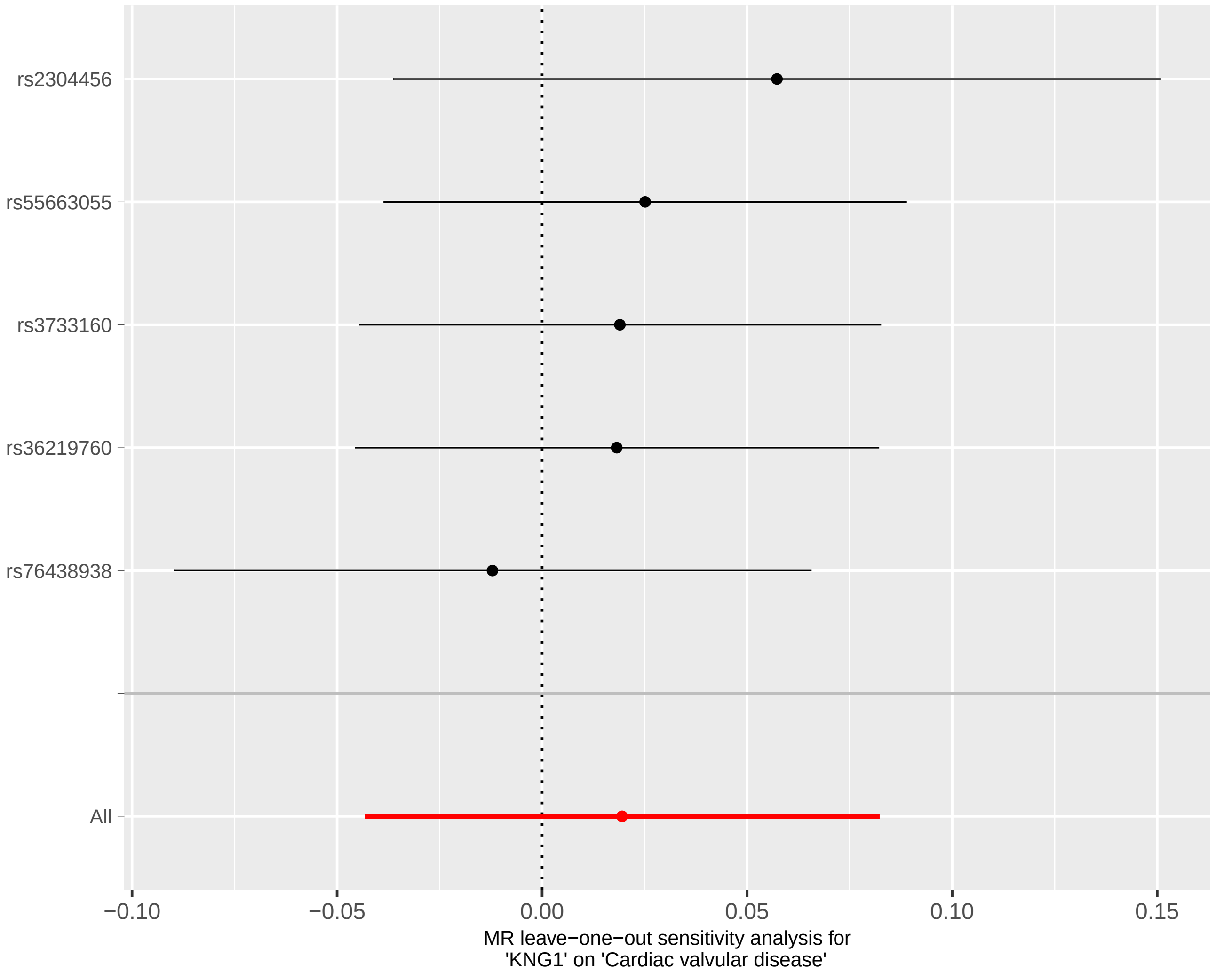

rs2304456
rs55663055
rs3733160
rs36219760
rs76438938
All
−0.10
−0.05
0.00
0.05
0.10
0.15
MR leave−one−out sensitivity analysis for
'KNG1' on 'Cardiac valvular disease'

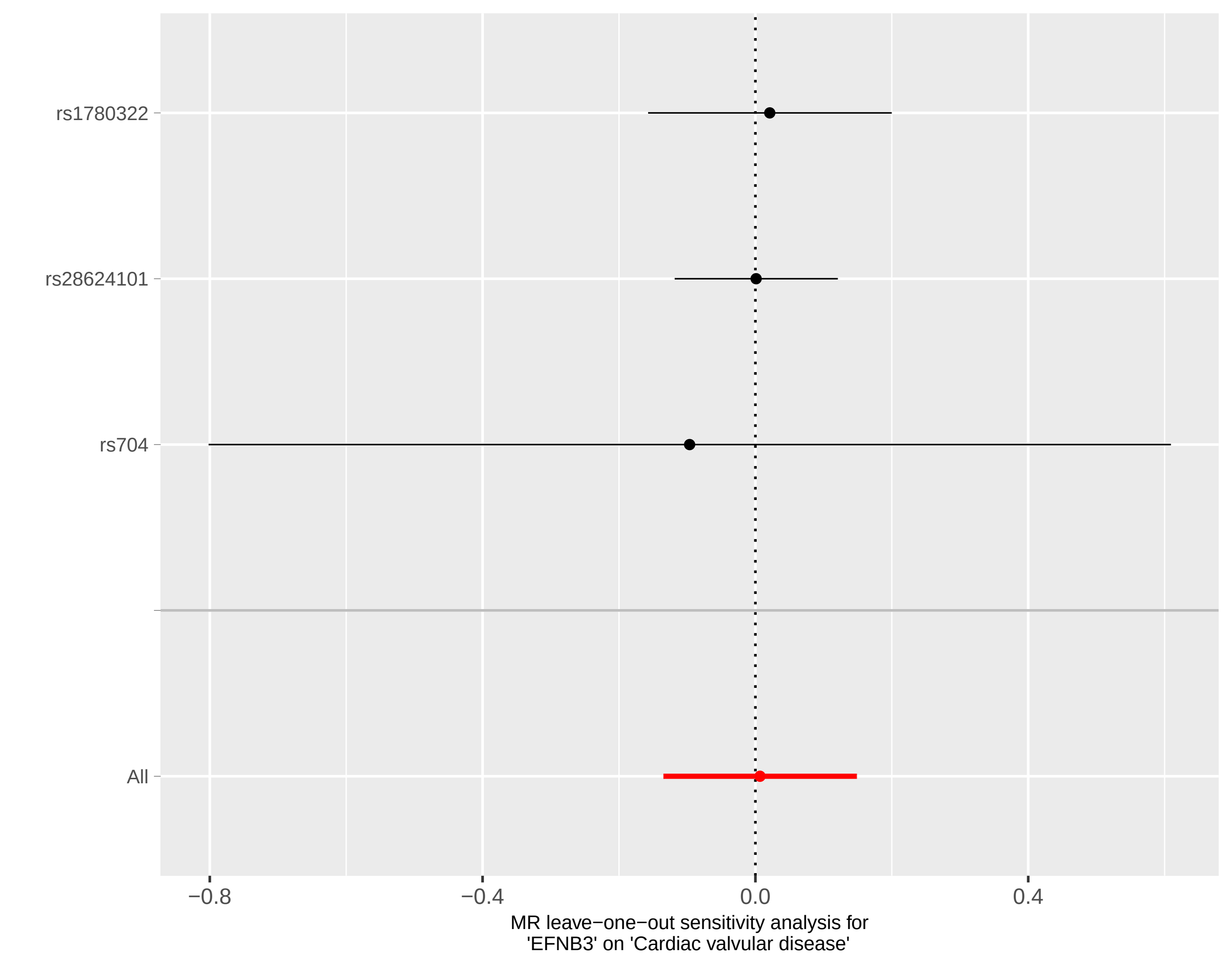

rs1780322
rs28624101
rs704
All
−0.8
−0.4
0.0
0.4
MR leave−one−out sensitivity analysis for
'EFNB3' on 'Cardiac valvular disease'

Insufficient number of SNPs

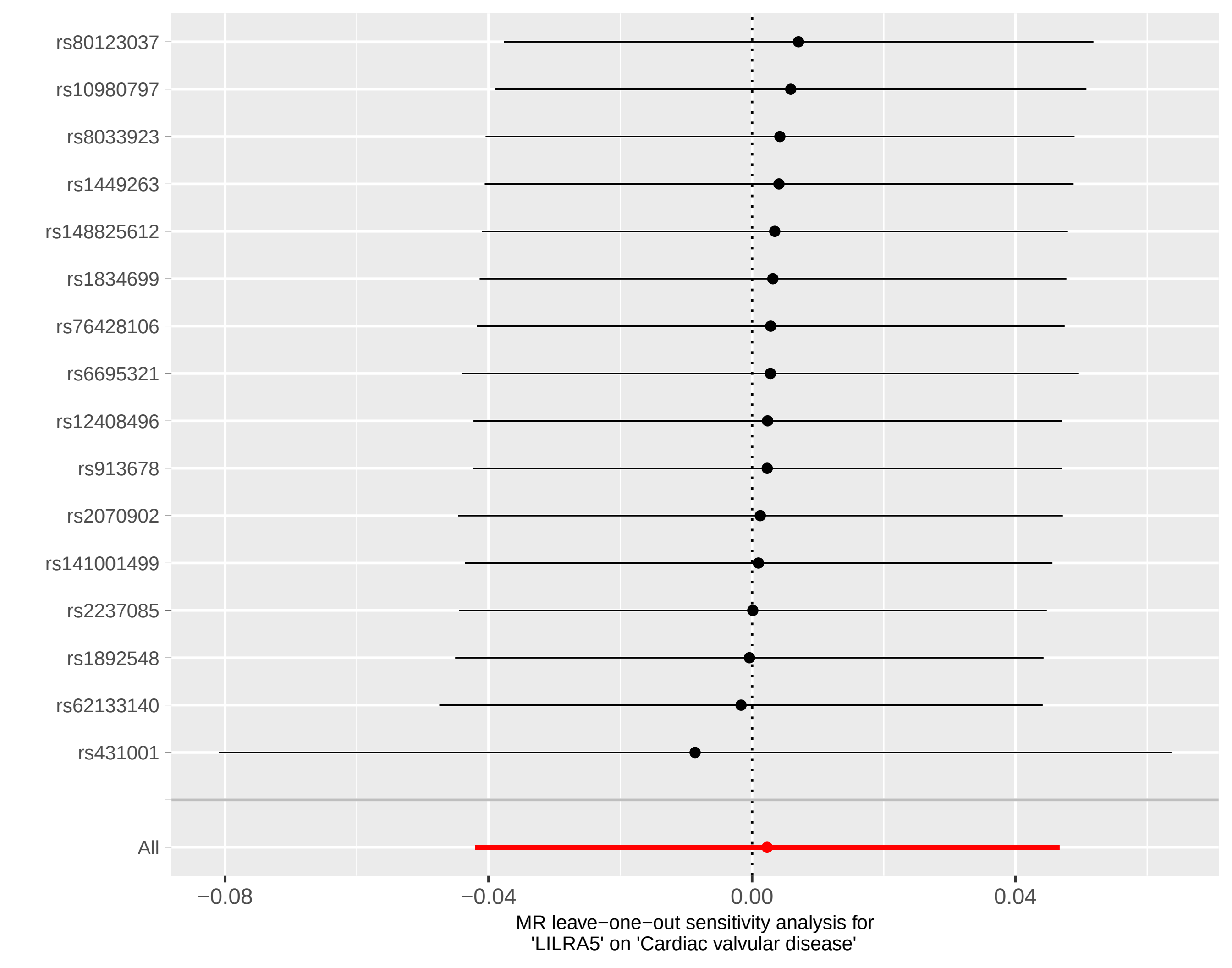
rs80123037
rs10980797
rs8033923
rs1449263
rs148825612
rs1834699
rs76428106
rs6695321
rs12408496
rs913678
rs2070902
rs141001499
rs2237085
rs1892548
rs62133140
rs431001
All
−0.08
−0.04
0.00
0.04
MR leave−one−out sensitivity analysis for
'LILRA5' on 'Cardiac valvular disease'

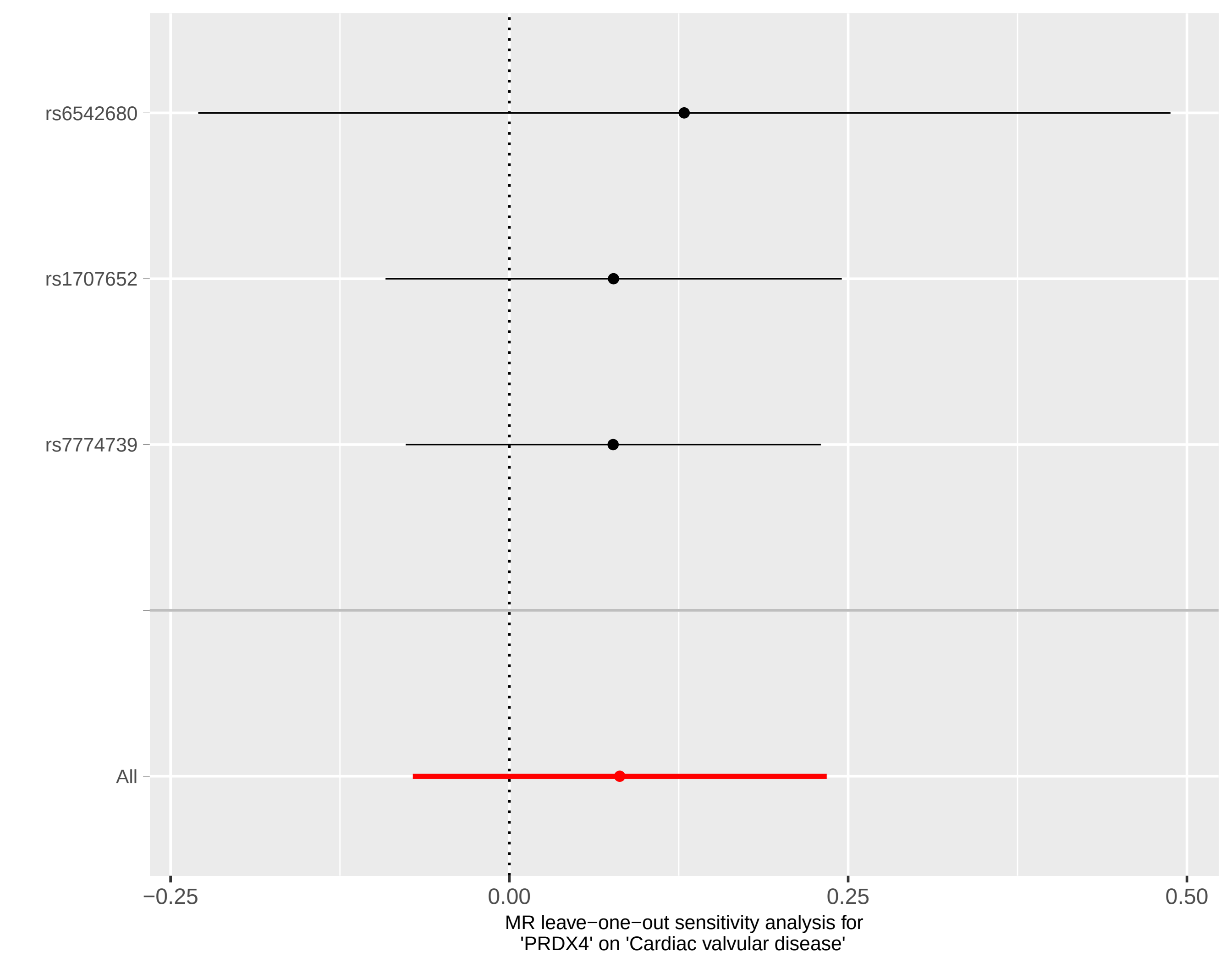
rs6542680
rs1707652
rs7774739
All
−0.25
0.00
0.25
0.50
MR leave−one−out sensitivity analysis for
'PRDX4' on 'Cardiac valvular disease'

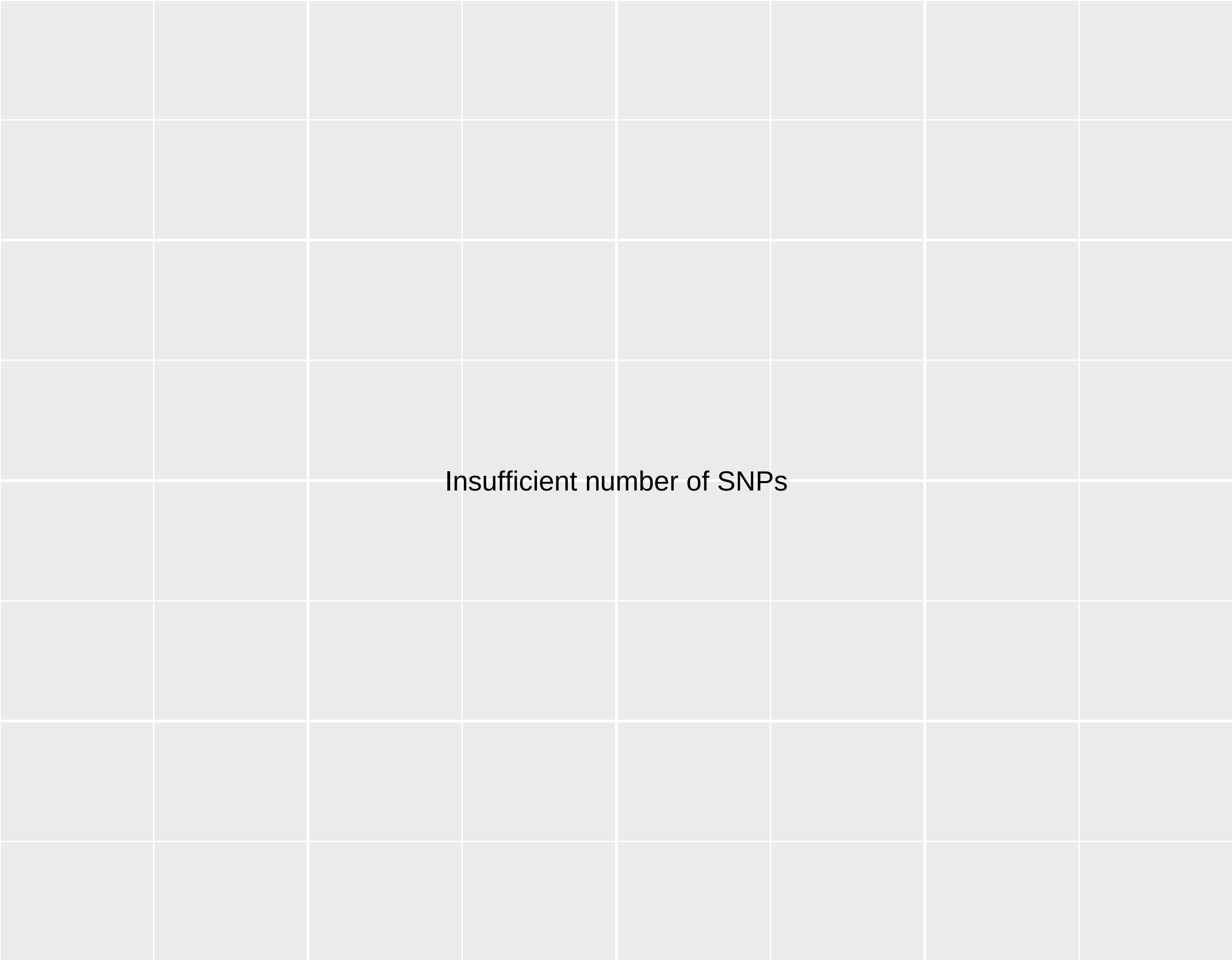
Insufficient number of SNPs

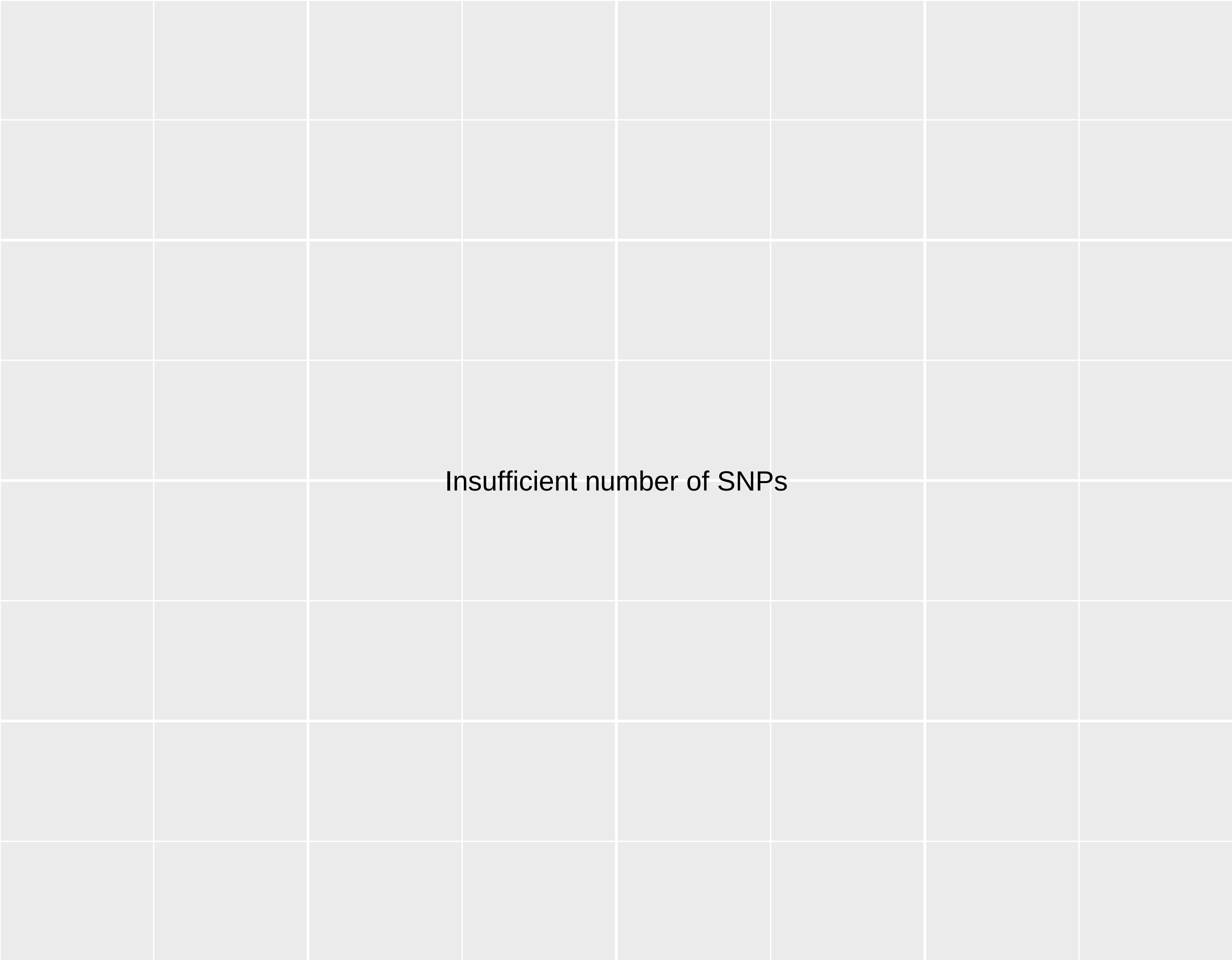
Insufficient number of SNPs

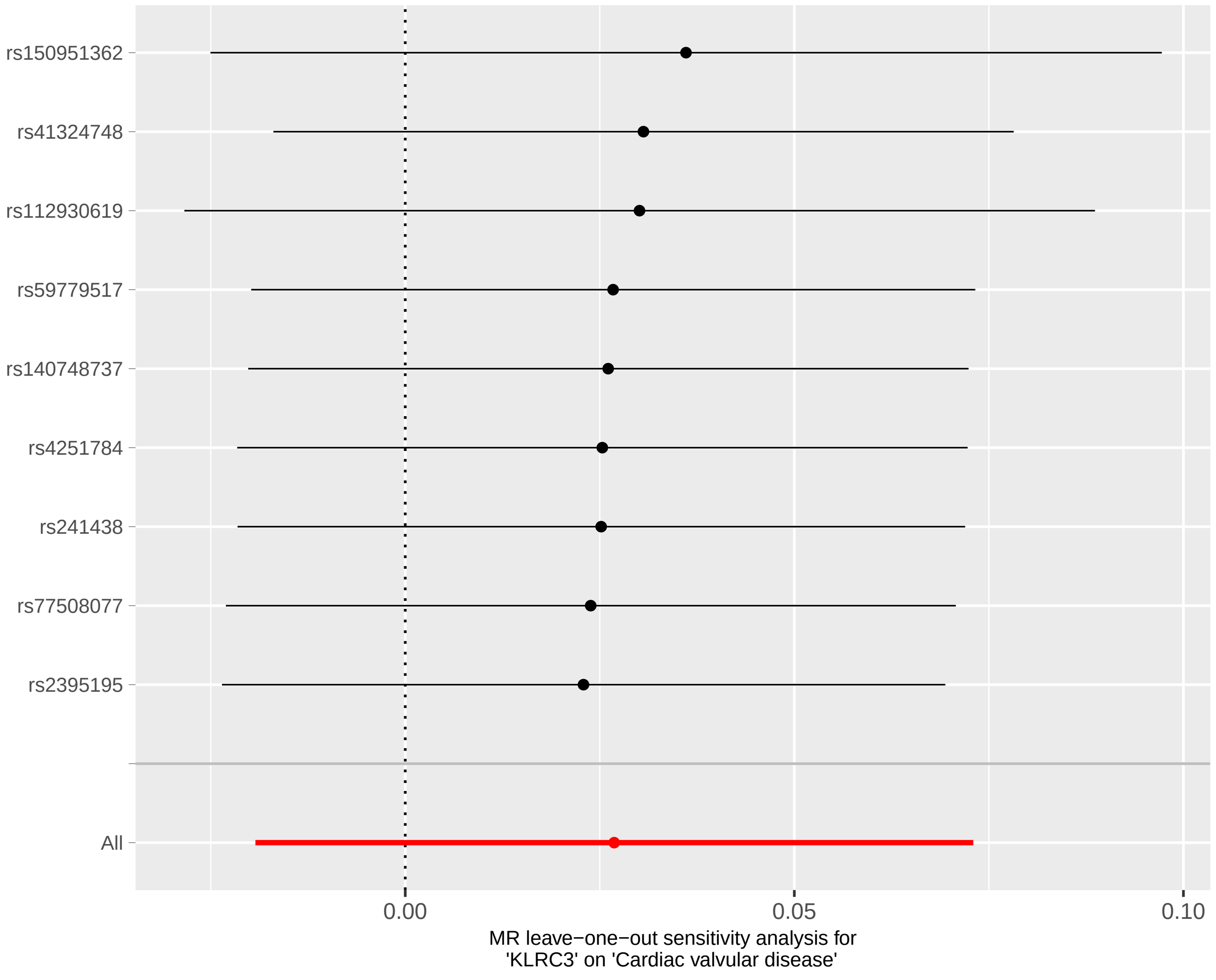
rs150951362
rs41324748
rs112930619
rs59779517
rs140748737
rs4251784
rs241438
rs77508077
rs2395195
All
0.00
0.05
0.10
MR leave−one−out sensitivity analysis for
'KLRC3' on 'Cardiac valvular disease'

Insufficient number of SNPs

Insufficient number of SNPs

Insufficient number of SNPs

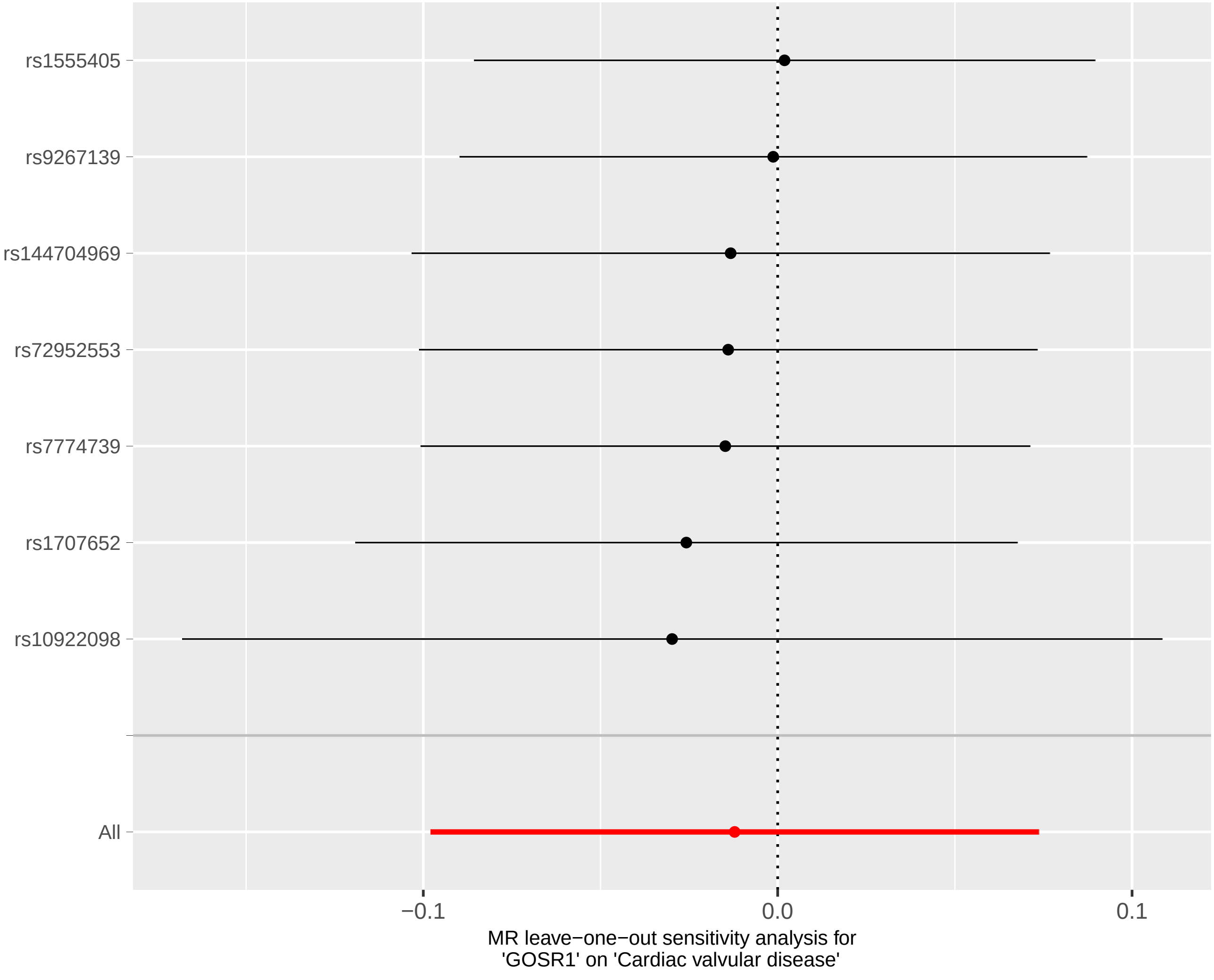
rs1555405
rs9267139
rs144704969
rs72952553
rs7774739
rs1707652
rs10922098
All
−0.1
0.0
0.1
MR leave−one−out sensitivity analysis for
'GOSR1' on 'Cardiac valvular disease'

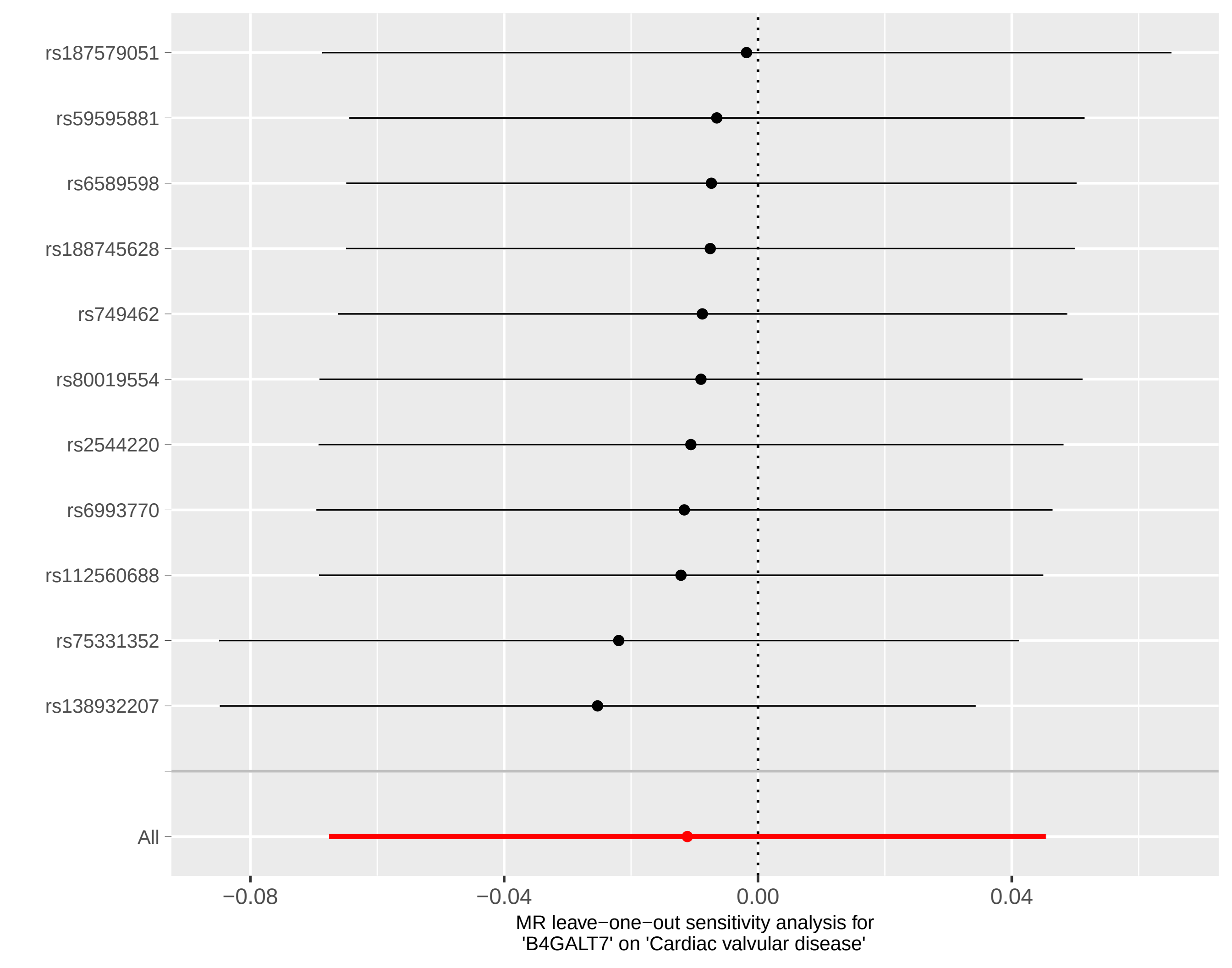
rs187579051
rs59595881
rs6589598
rs188745628
rs749462
rs80019554
rs2544220
rs6993770
rs112560688
rs75331352
rs138932207
All
−0.08
−0.04
0.00
0.04
MR leave−one−out sensitivity analysis for
'B4GALT7' on 'Cardiac valvular disease'

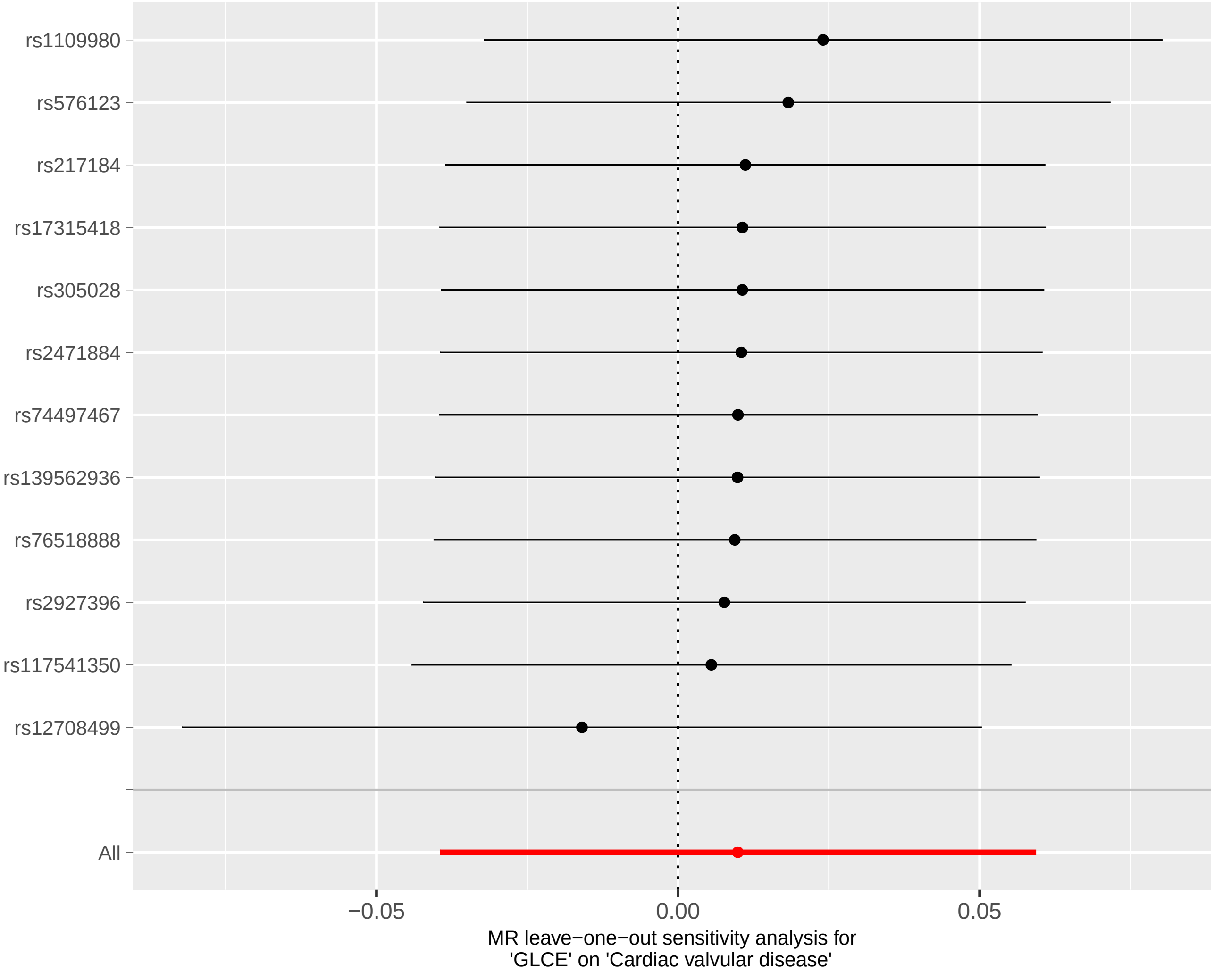
rs1109980
rs576123
rs217184
rs17315418
rs305028
rs2471884
rs74497467
rs139562936
rs76518888
rs2927396
rs117541350
rs12708499
All
−0.05
0.00
0.05
MR leave−one−out sensitivity analysis for
'GLCE' on 'Cardiac valvular disease'

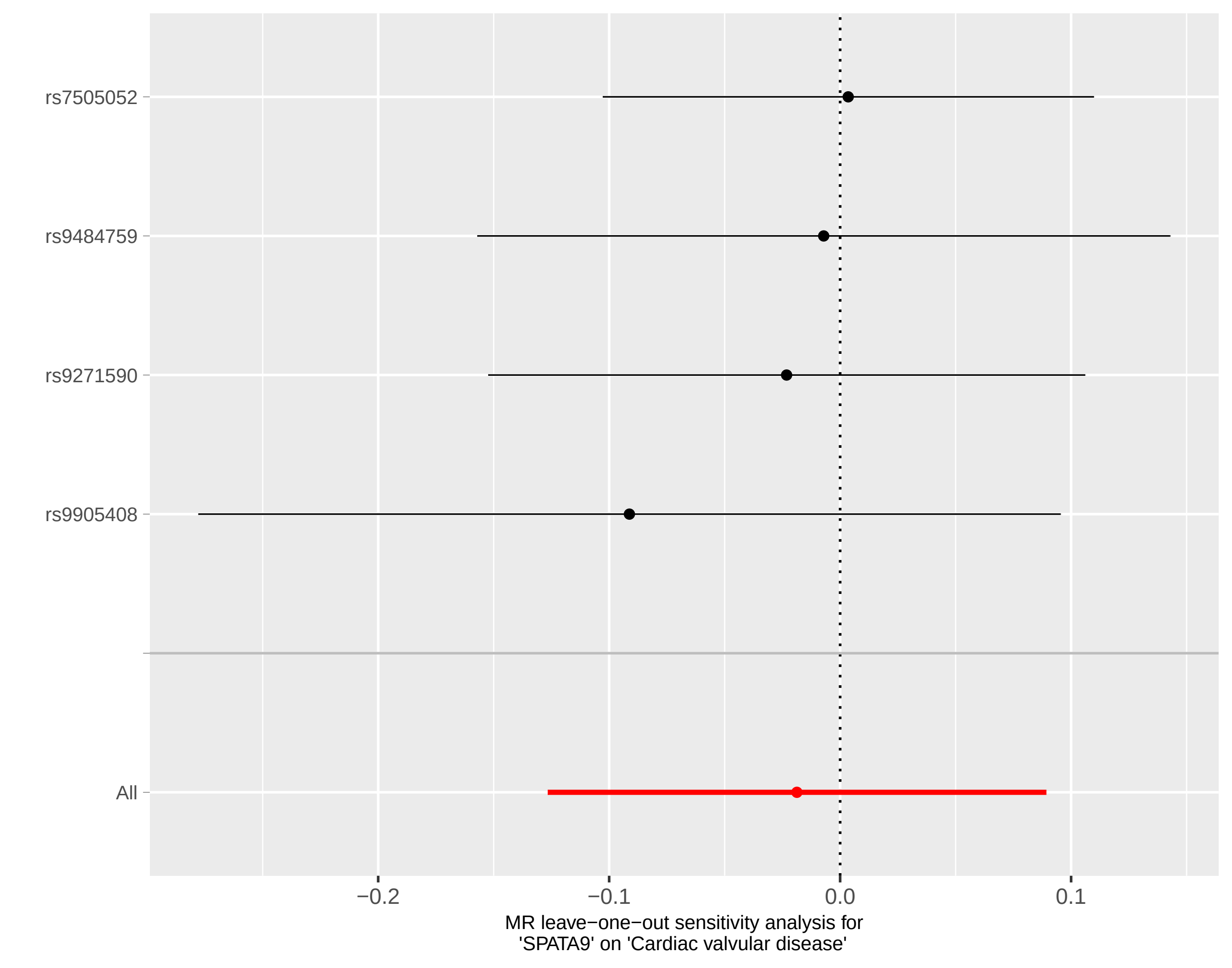
rs7505052
rs9484759
rs9271590
rs9905408
All
−0.2
−0.1
0.0
0.1
MR leave−one−out sensitivity analysis for
'SPATA9' on 'Cardiac valvular disease'

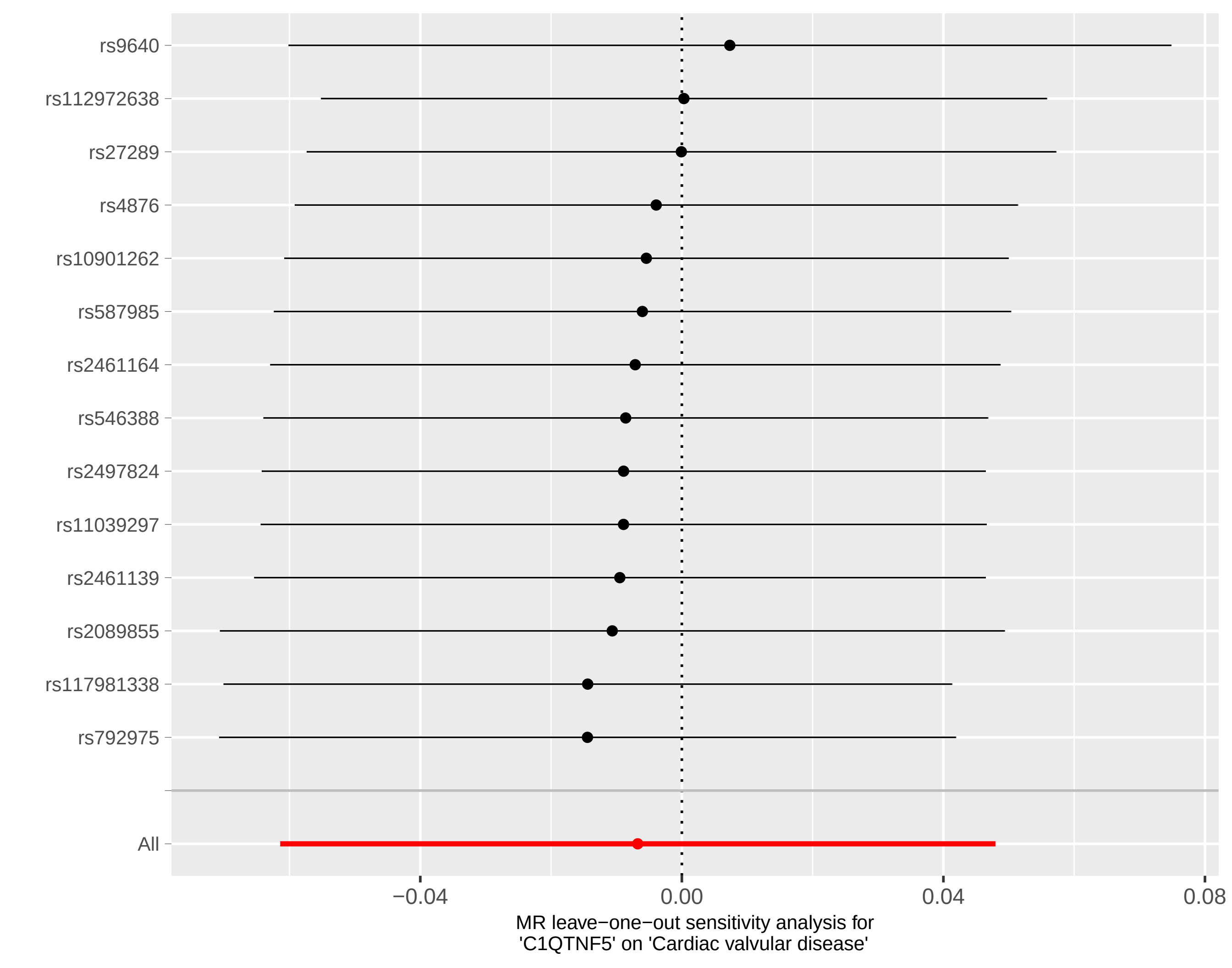
rs9640
rs112972638
rs27289
rs4876
rs10901262
rs587985
rs2461164
rs546388
rs2497824
rs11039297
rs2461139
rs2089855
rs117981338
rs792975
All
−0.04
0.00
0.04
0.08
MR leave−one−out sensitivity analysis for
'C1QTNF5' on 'Cardiac valvular disease'

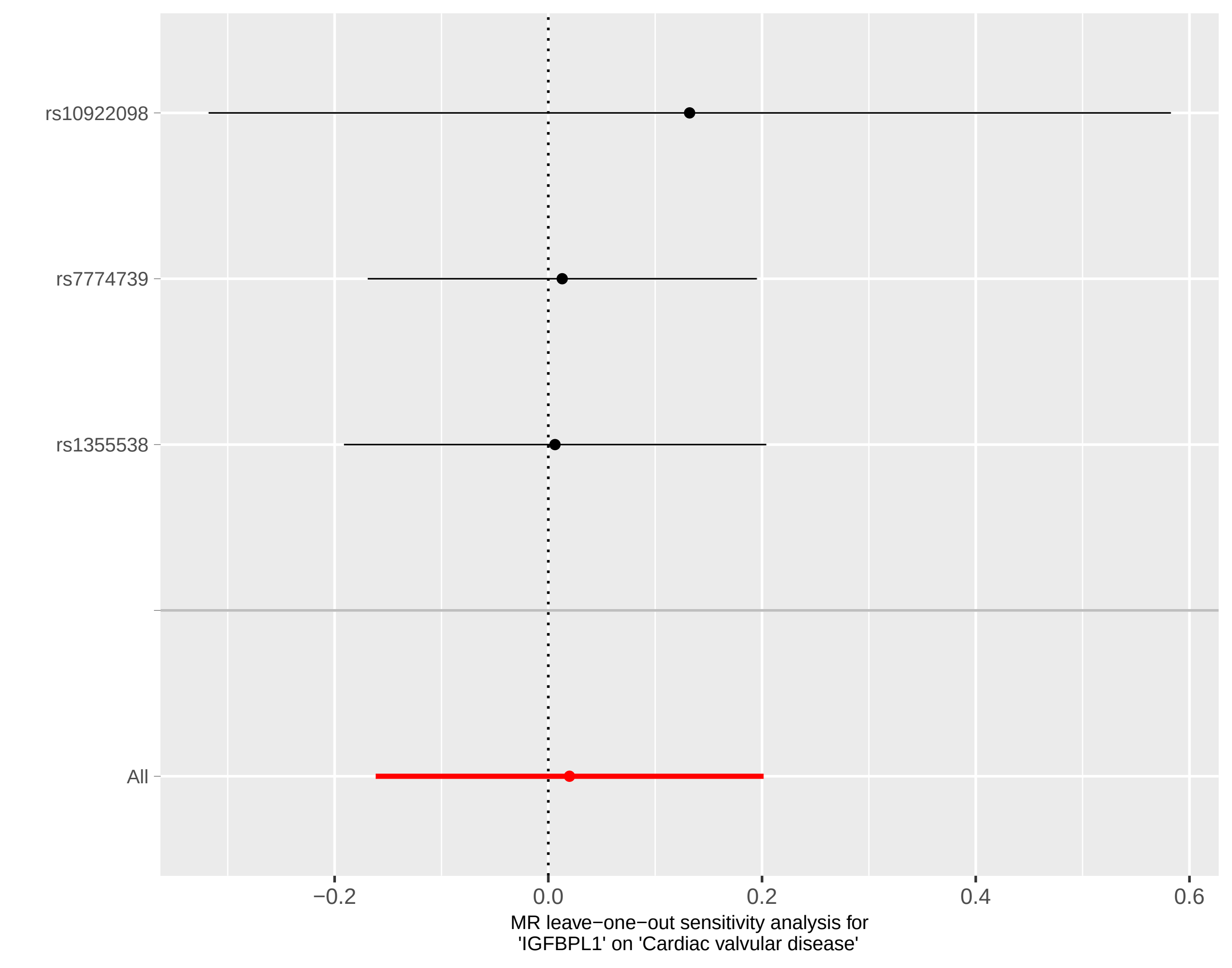

rs10922098
rs7774739
rs1355538
All
−0.2
0.0
0.2
0.4
0.6
MR leave−one−out sensitivity analysis for
'IGFBPL1' on 'Cardiac valvular disease'

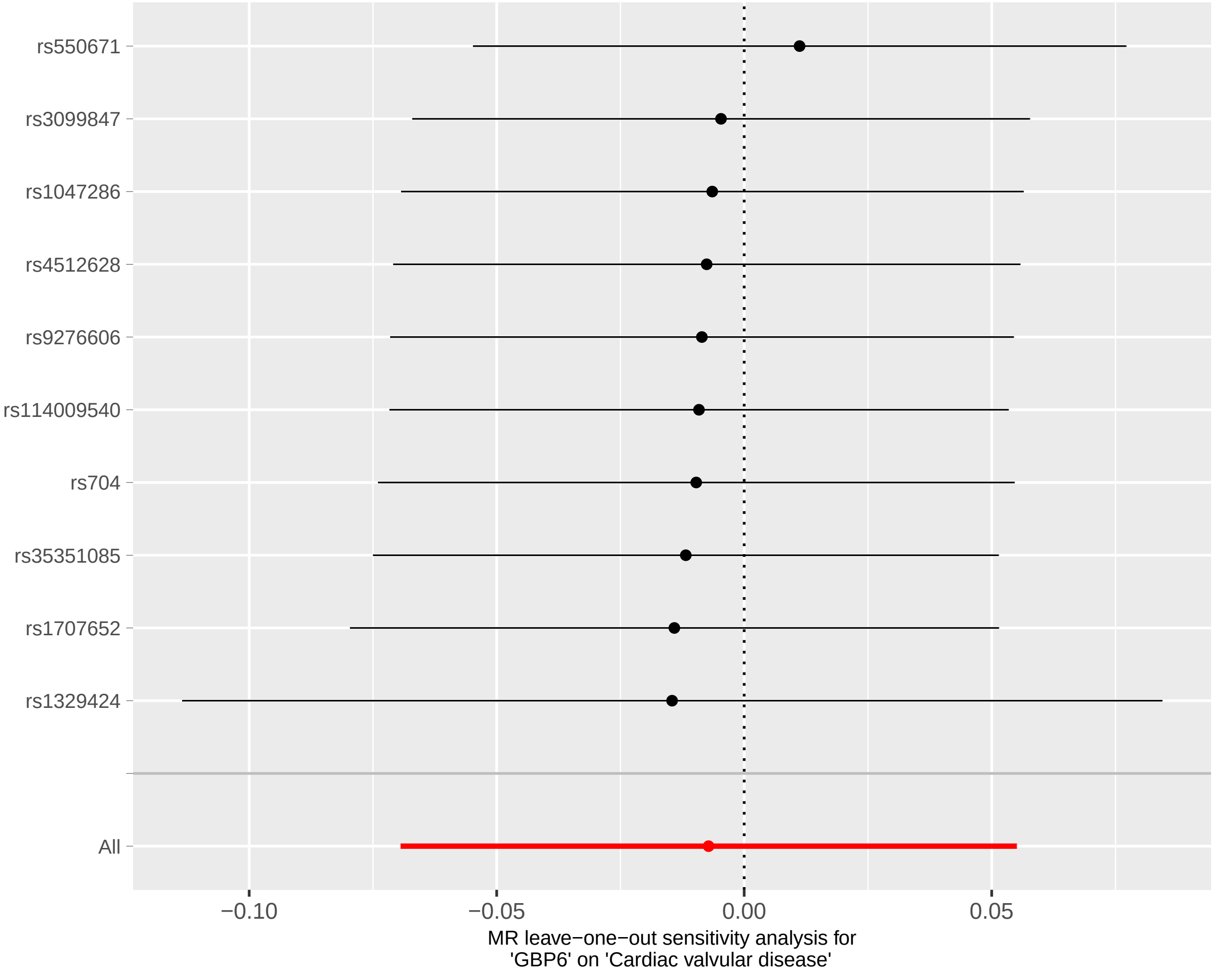

rs550671
rs3099847
rs1047286
rs4512628
rs9276606
rs114009540
rs704
rs35351085
rs1707652
rs1329424
All
−0.10
−0.05
0.00
0.05
MR leave−one−out sensitivity analysis for
'GBP6' on 'Cardiac valvular disease'

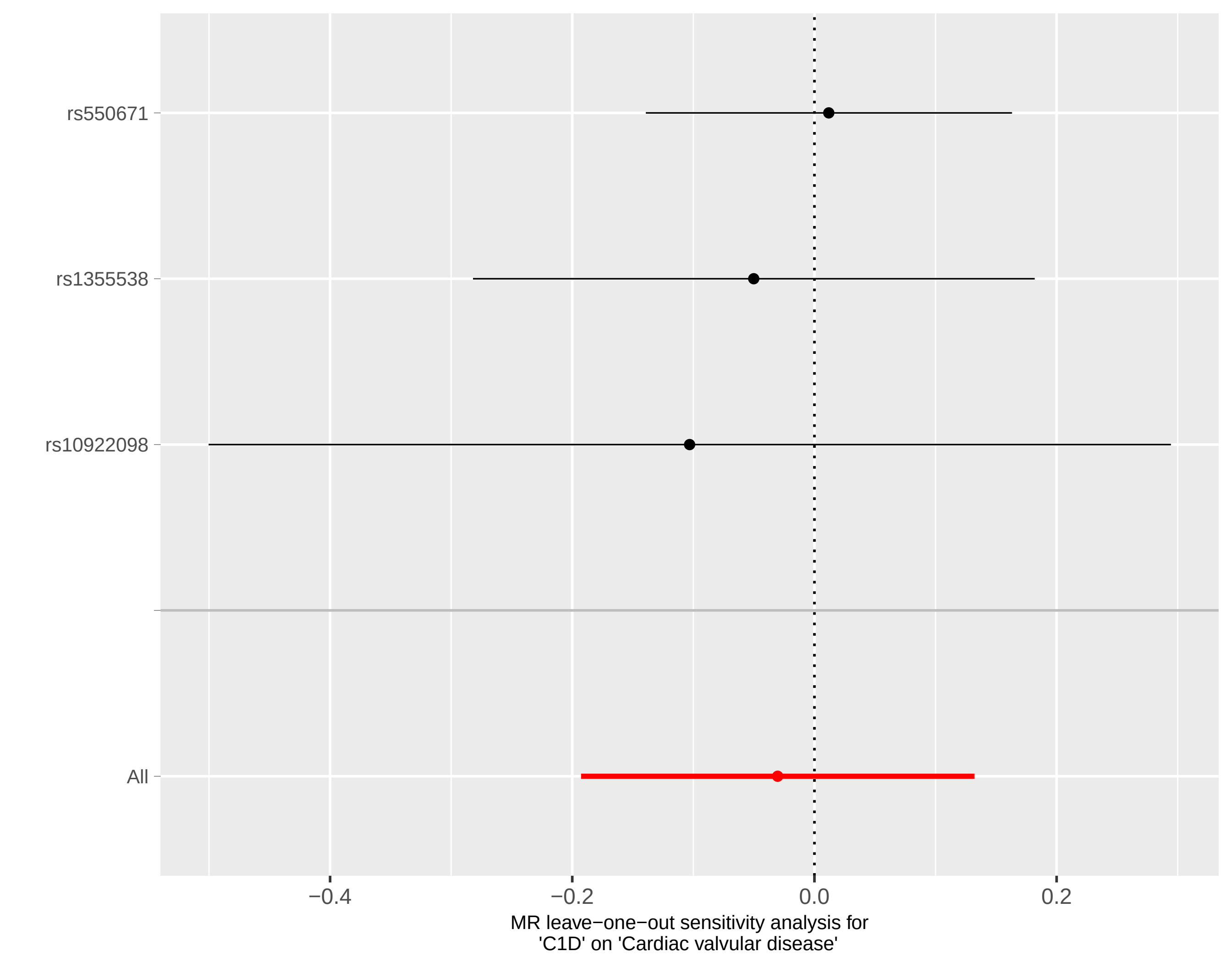

rs550671
rs1355538
rs10922098
All
−0.4
−0.2
0.0
0.2
MR leave−one−out sensitivity analysis for
'C1D' on 'Cardiac valvular disease'

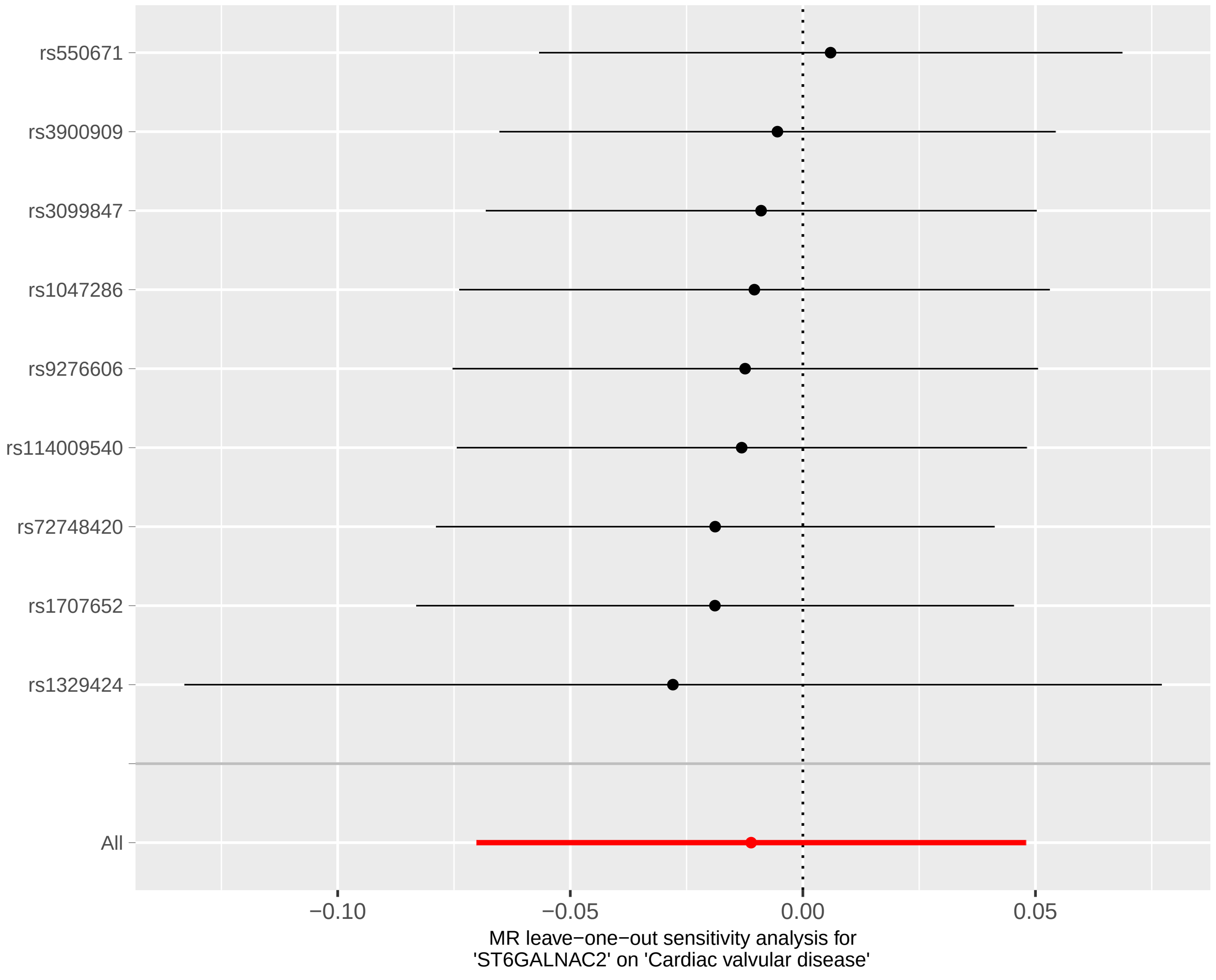
rs550671
rs3900909
rs3099847
rs1047286
rs9276606
rs114009540
rs72748420
rs1707652
rs1329424
All
−0.10
−0.05
0.00
0.05
MR leave−one−out sensitivity analysis for
'ST6GALNAC2' on 'Cardiac valvular disease'

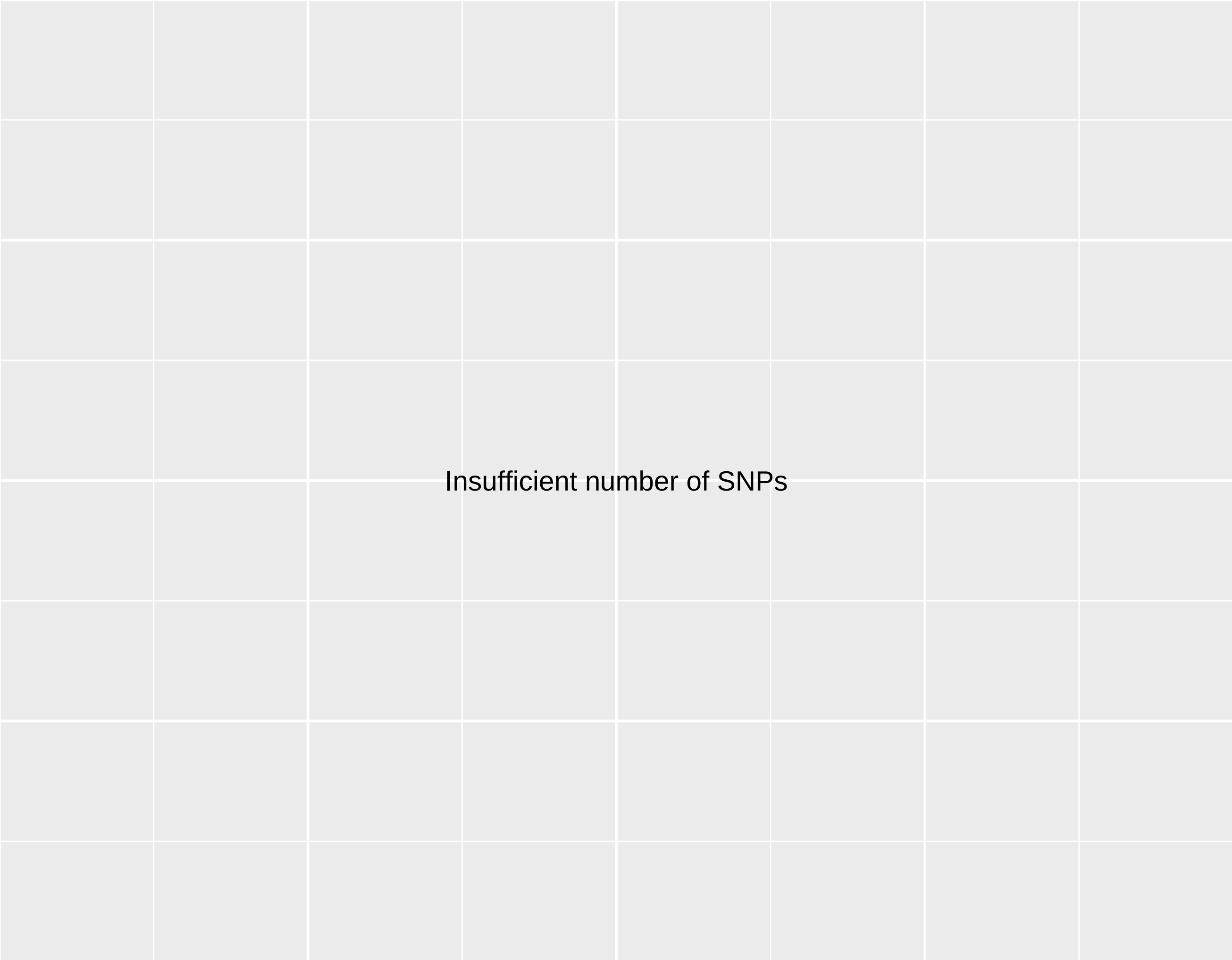
Insufficient number of SNPs

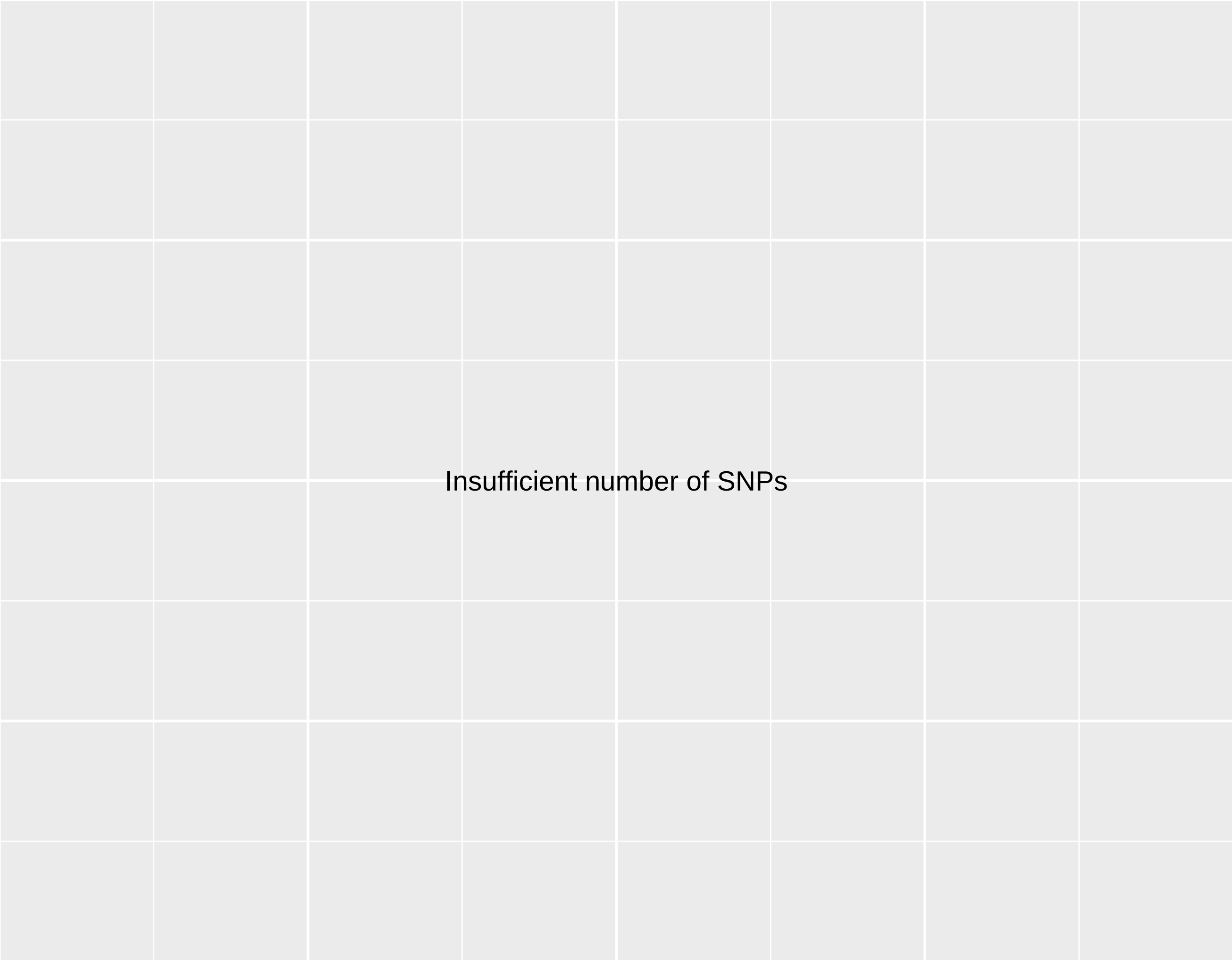
Insufficient number of SNPs

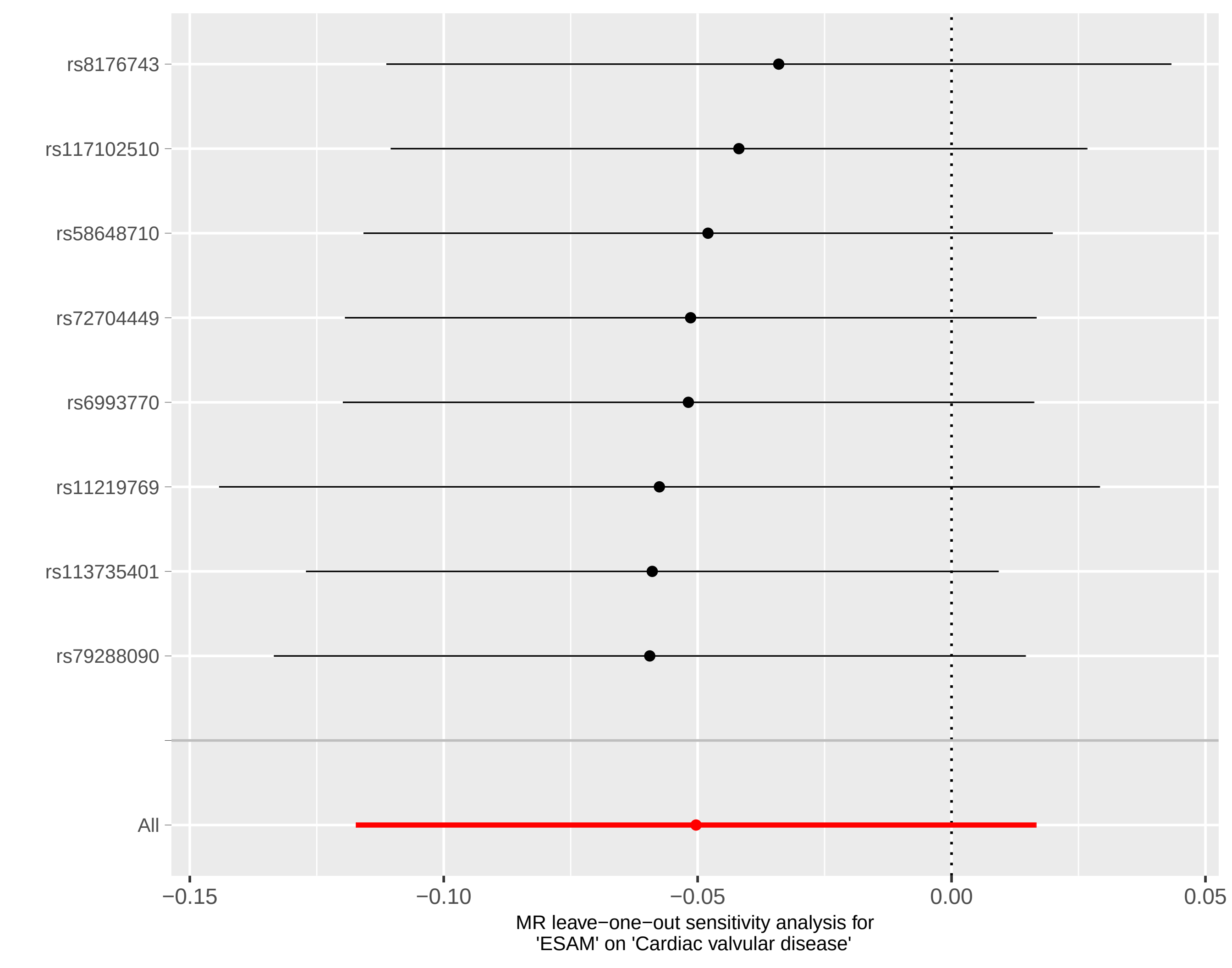
rs8176743
rs117102510
rs58648710
rs72704449
rs6993770
rs11219769
rs113735401
rs79288090
All
−0.15
−0.10
−0.05
0.00
0.05
MR leave−one−out sensitivity analysis for
'ESAM' on 'Cardiac valvular disease'

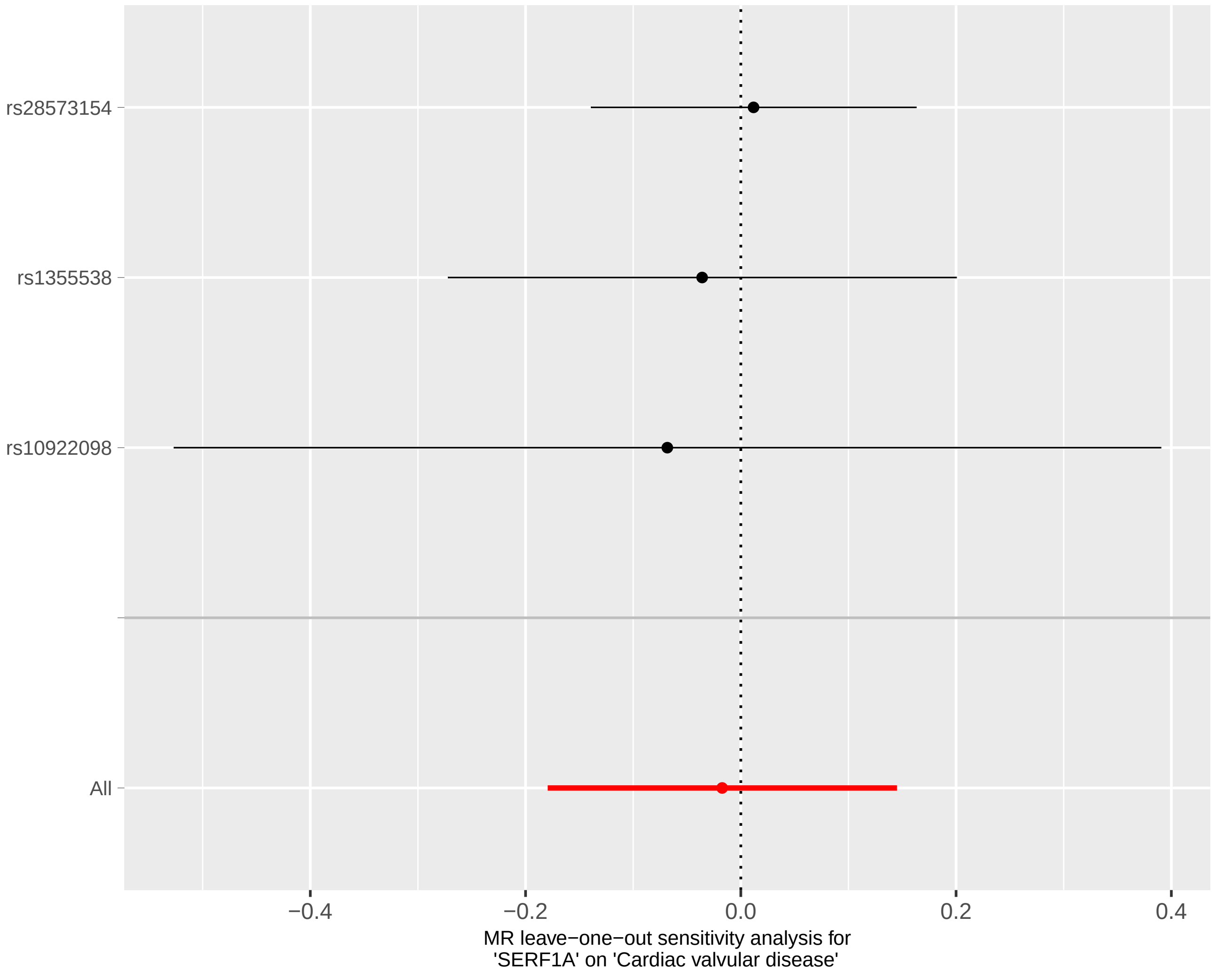
rs28573154
rs1355538
rs10922098
All
−0.4
−0.2
0.0
0.2
0.4
MR leave−one−out sensitivity analysis for
'SERF1A' on 'Cardiac valvular disease'

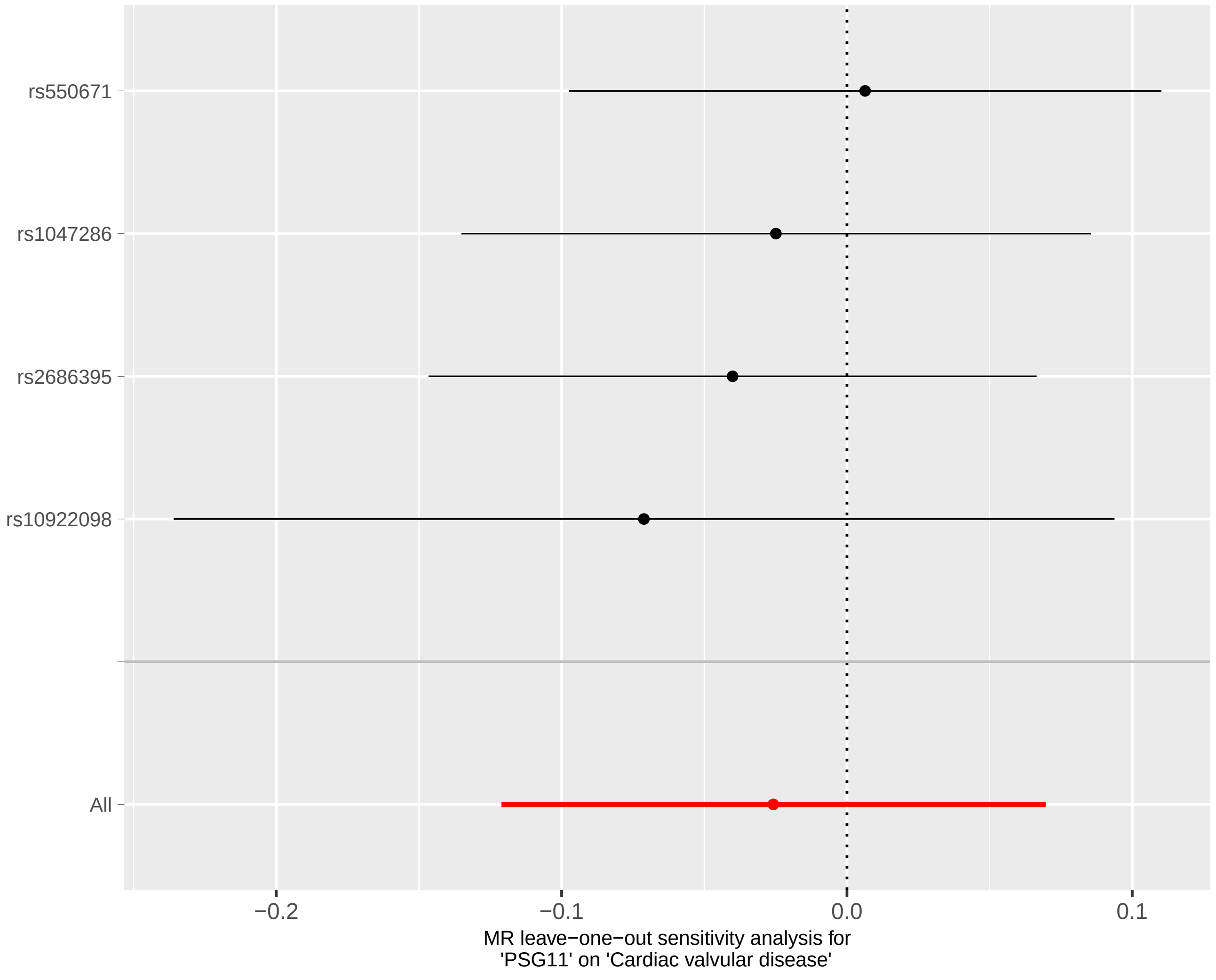
rs550671
rs1047286
rs2686395
rs10922098
All
−0.2
−0.1
0.0
0.1
MR leave−one−out sensitivity analysis for
'PSG11' on 'Cardiac valvular disease'

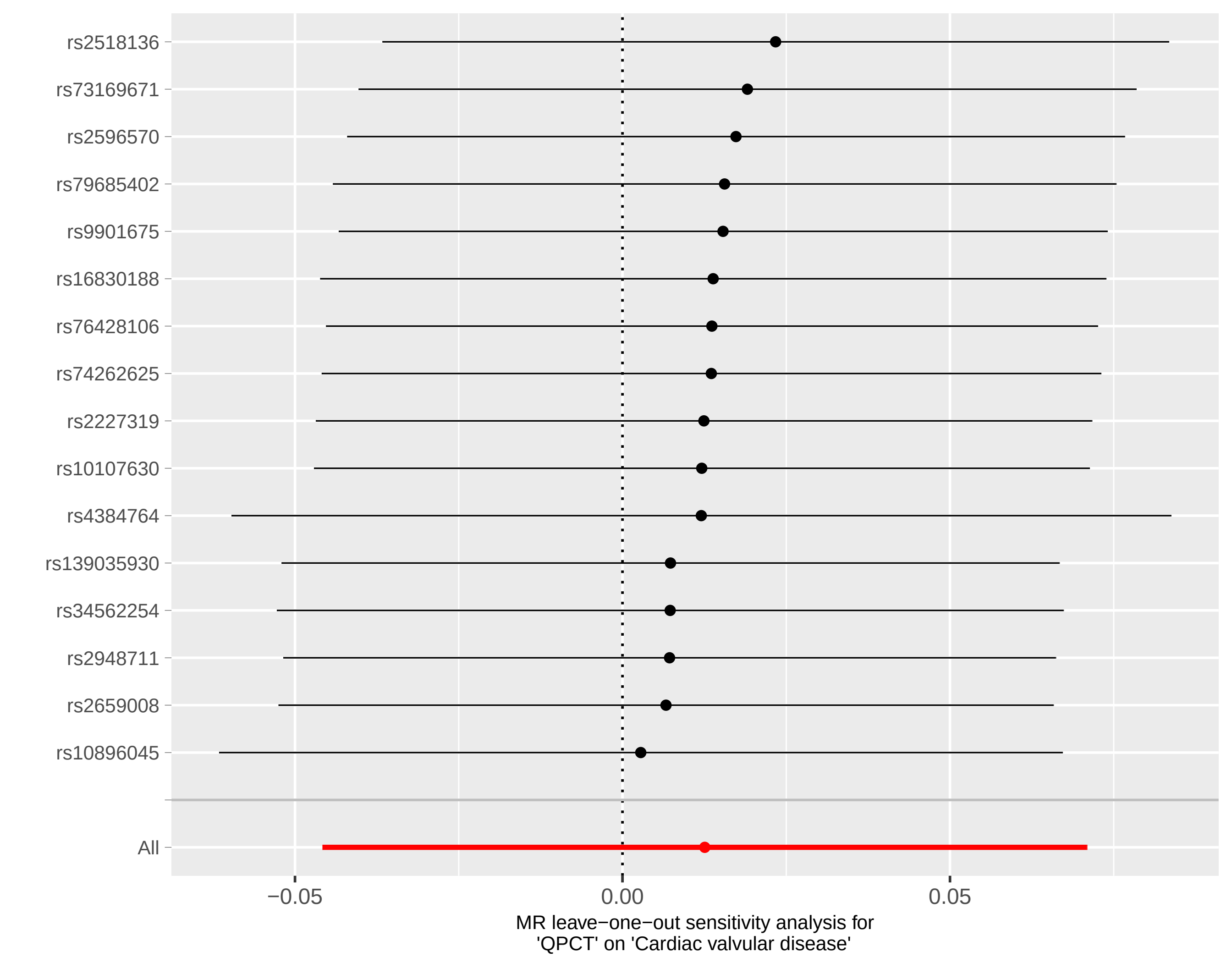
rs2518136
rs73169671
rs2596570
rs79685402
rs9901675
rs16830188
rs76428106
rs74262625
rs2227319
rs10107630
rs4384764
rs139035930
rs34562254
rs2948711
rs2659008
rs10896045
All
−0.05
0.00
0.05
MR leave−one−out sensitivity analysis for
'QPCT' on 'Cardiac valvular disease'

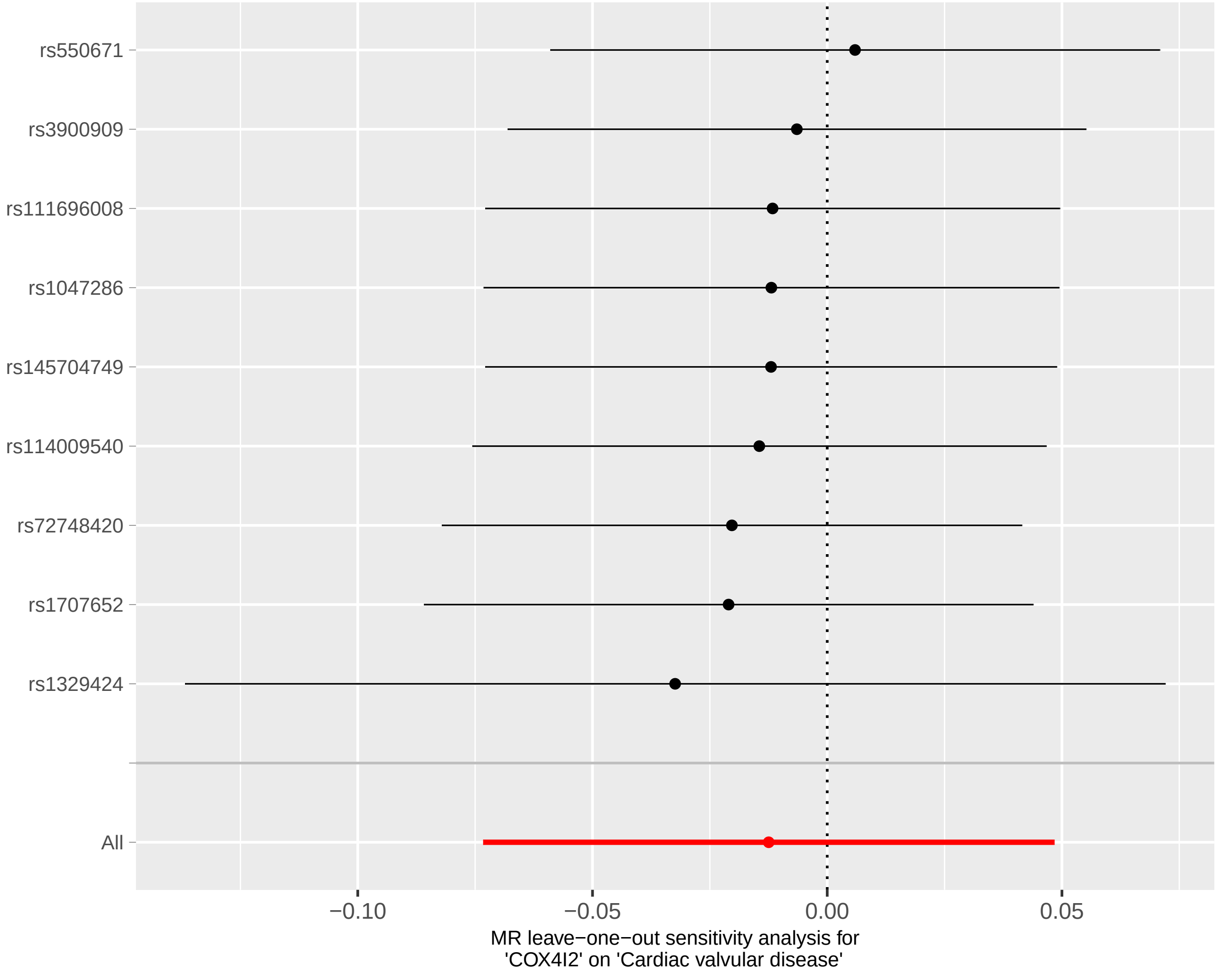
rs550671
rs3900909
rs111696008
rs1047286
rs145704749
rs114009540
rs72748420
rs1707652
rs1329424
All
−0.10
−0.05
0.00
0.05
MR leave−one−out sensitivity analysis for
'COX4I2' on 'Cardiac valvular disease'

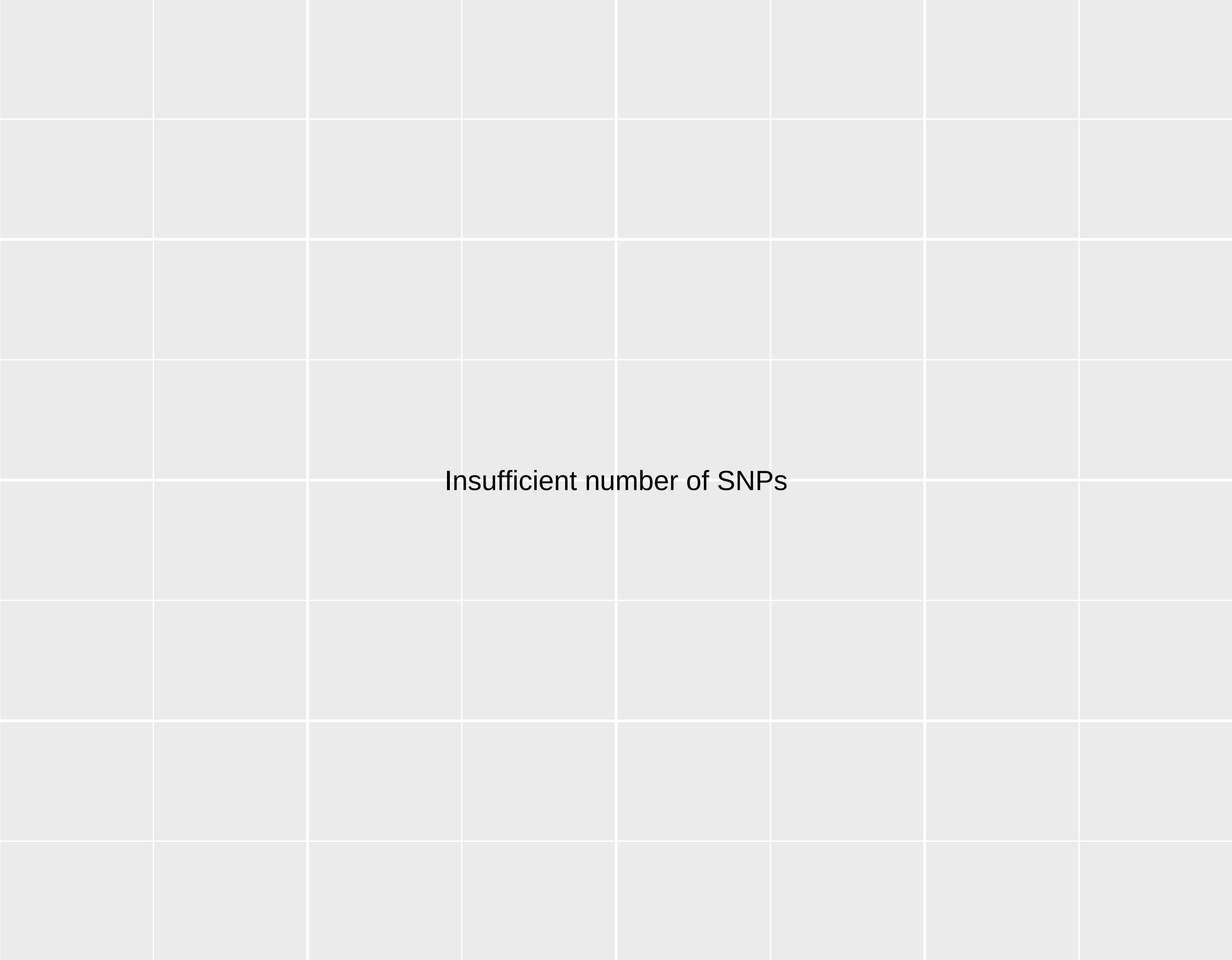
Insufficient number of SNPs

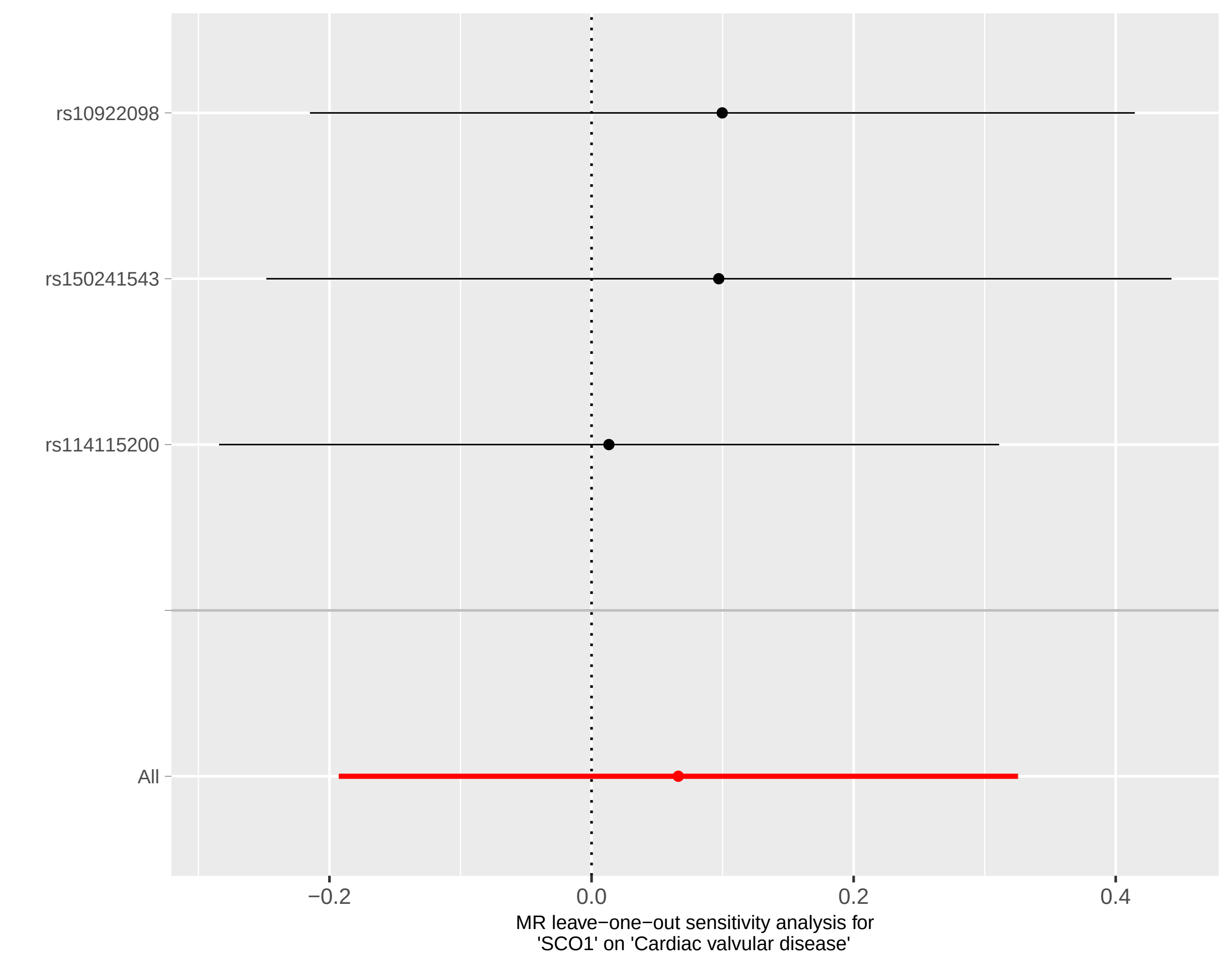

rs10922098
rs150241543
rs114115200
All
−0.2
0.0
0.2
0.4
MR leave−one−out sensitivity analysis for
'SCO1' on 'Cardiac valvular disease'

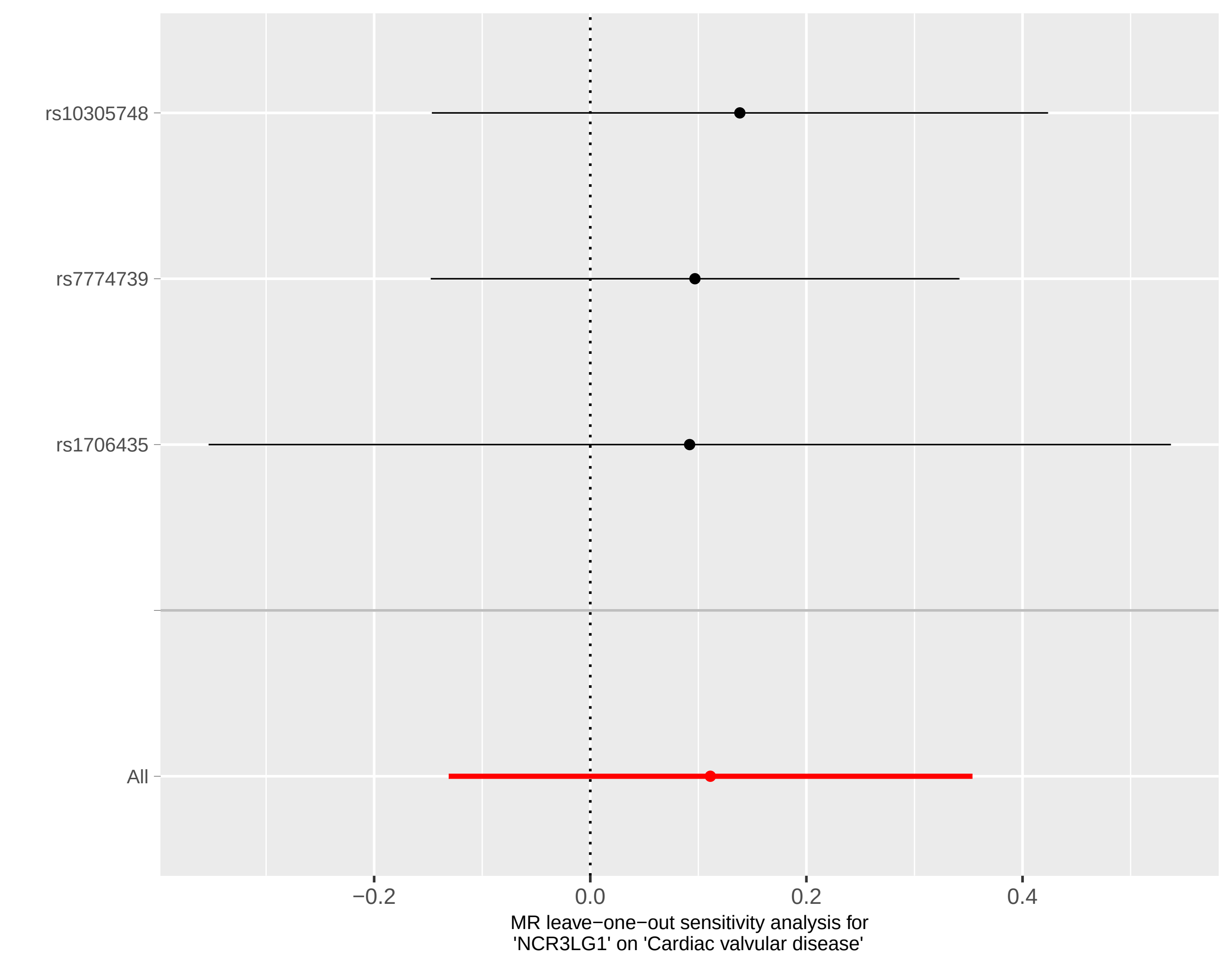
rs10305748
rs7774739
rs1706435
All
−0.2
0.0
0.2
0.4
MR leave−one−out sensitivity analysis for
'NCR3LG1' on 'Cardiac valvular disease'

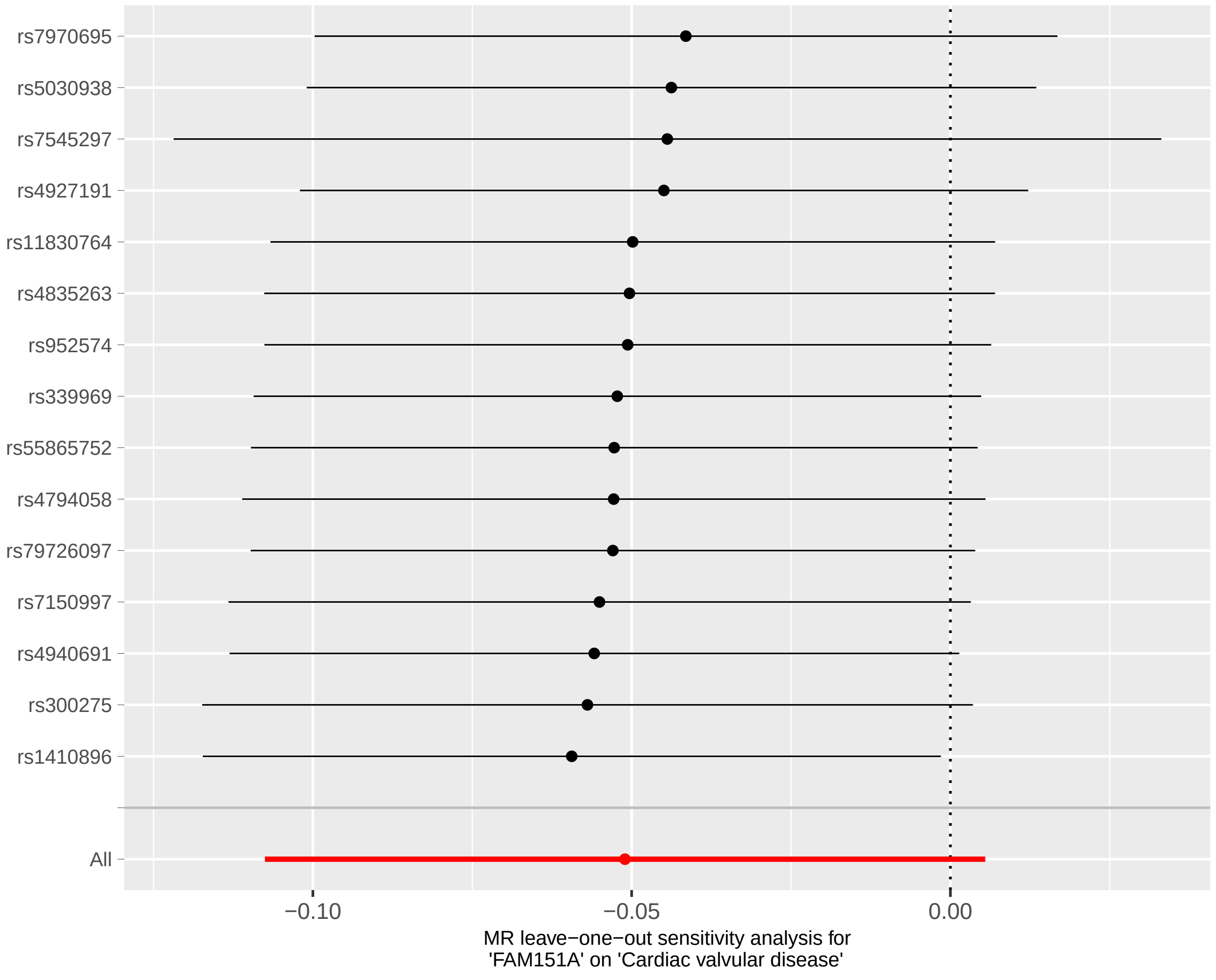

rs7970695
rs5030938
rs7545297
rs4927191
rs11830764
rs4835263
rs952574
rs339969
rs55865752
rs4794058
rs79726097
rs7150997
rs4940691
rs300275
rs1410896
All
−0.10
−0.05
0.00
MR leave−one−out sensitivity analysis for
'FAM151A' on 'Cardiac valvular disease'

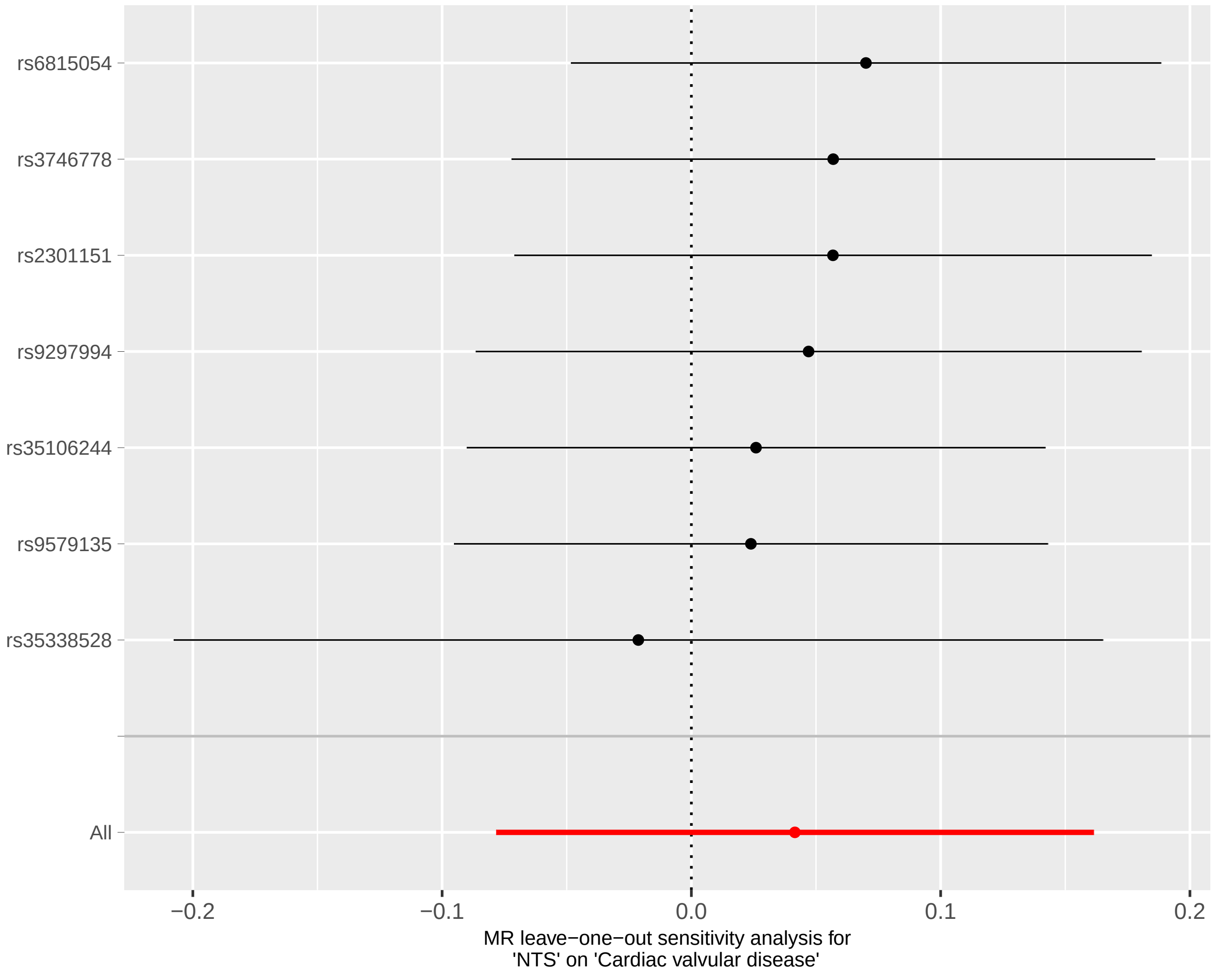
rs6815054
rs3746778
rs2301151
rs9297994
rs35106244
rs9579135
rs35338528
All
−0.2
−0.1
0.0
0.1
0.2
MR leave−one−out sensitivity analysis for
'NTS' on 'Cardiac valvular disease'

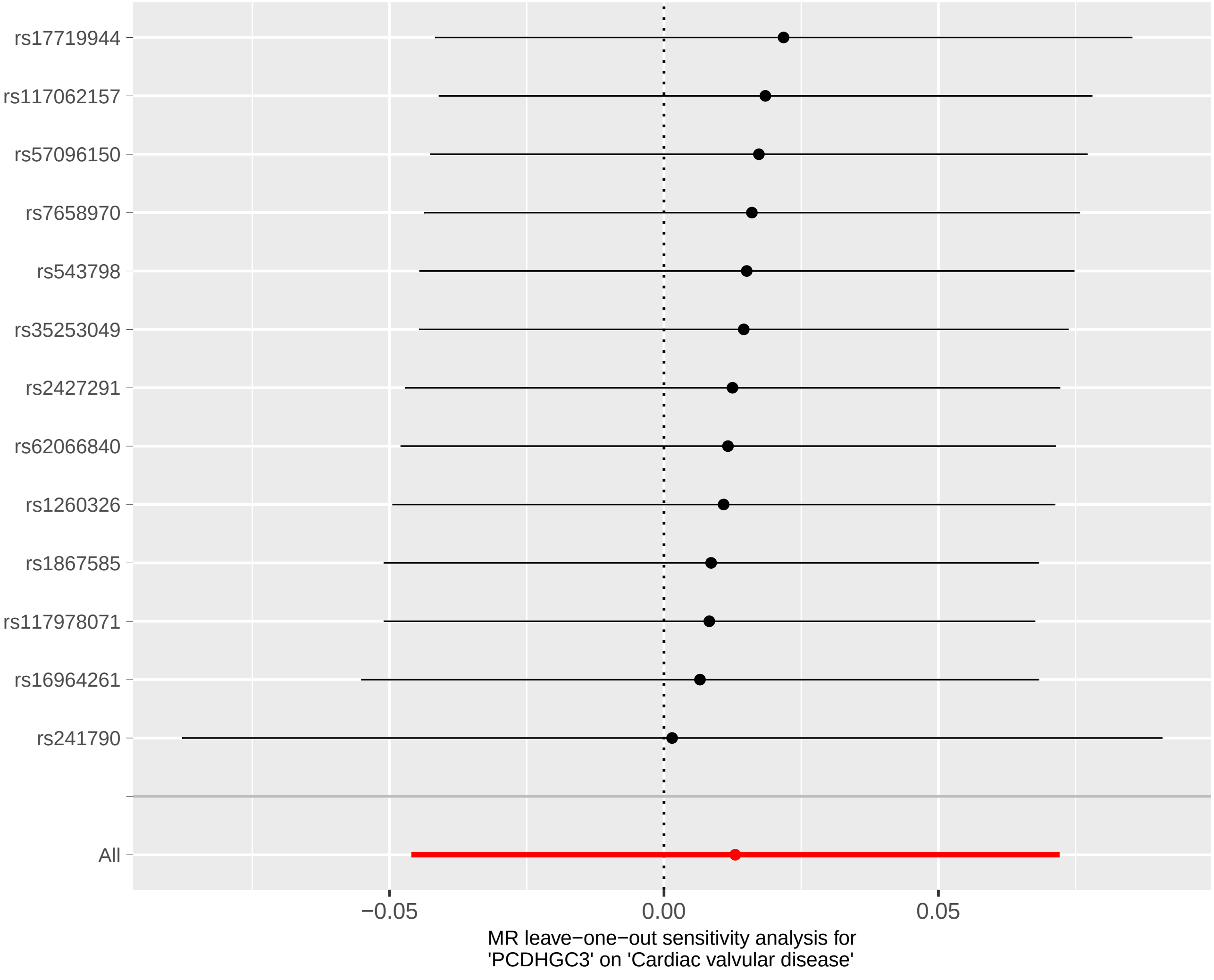

rs17719944
rs117062157
rs57096150
rs7658970
rs543798
rs35253049
rs2427291
rs62066840
rs1260326
rs1867585
rs117978071
rs16964261
rs241790
All
−0.05
0.00
0.05
MR leave−one−out sensitivity analysis for
'PCDHGC3' on 'Cardiac valvular disease'

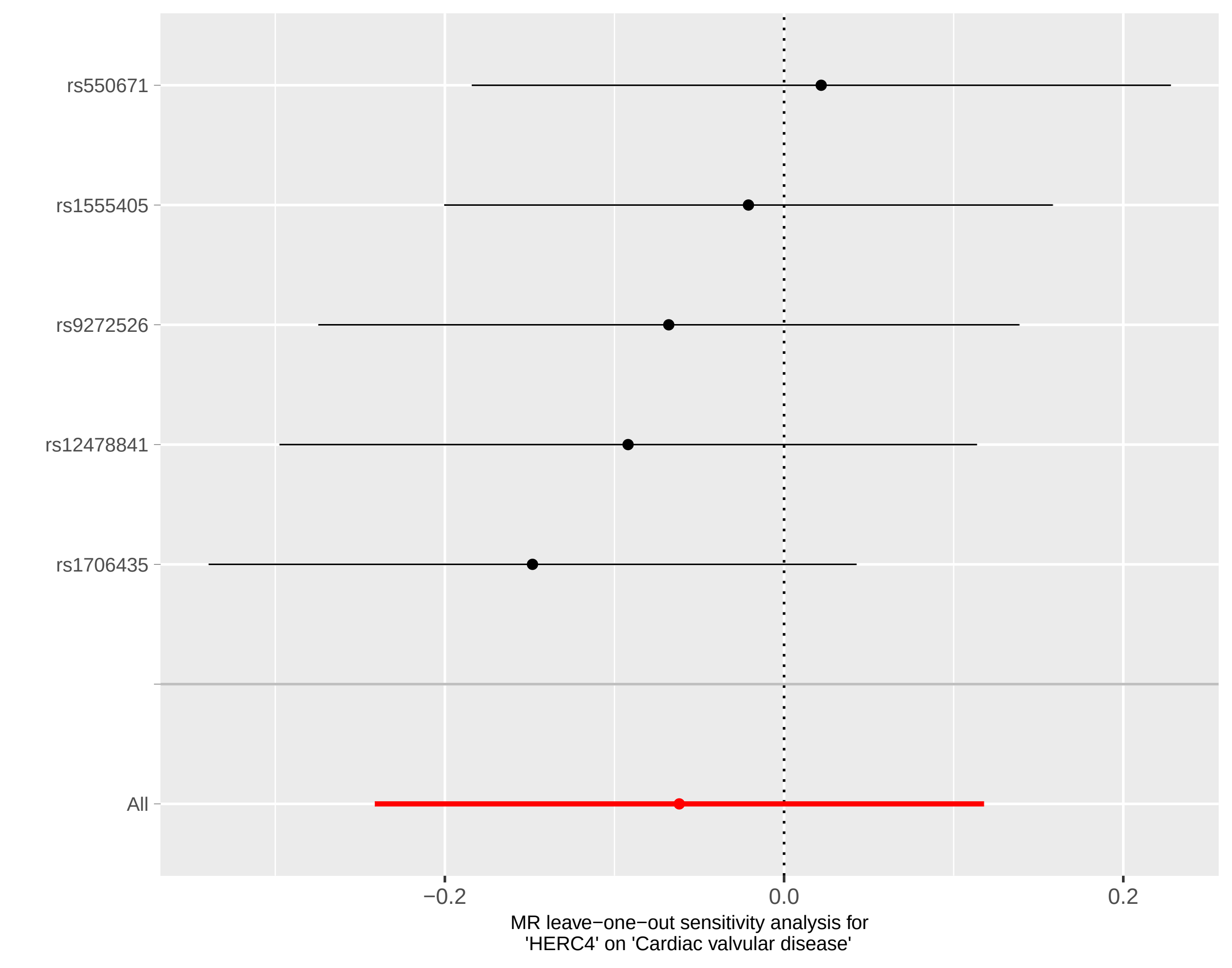
rs550671
rs1555405
rs9272526
rs12478841
rs1706435
All
−0.2
0.0
0.2
MR leave−one−out sensitivity analysis for
'HERC4' on 'Cardiac valvular disease'

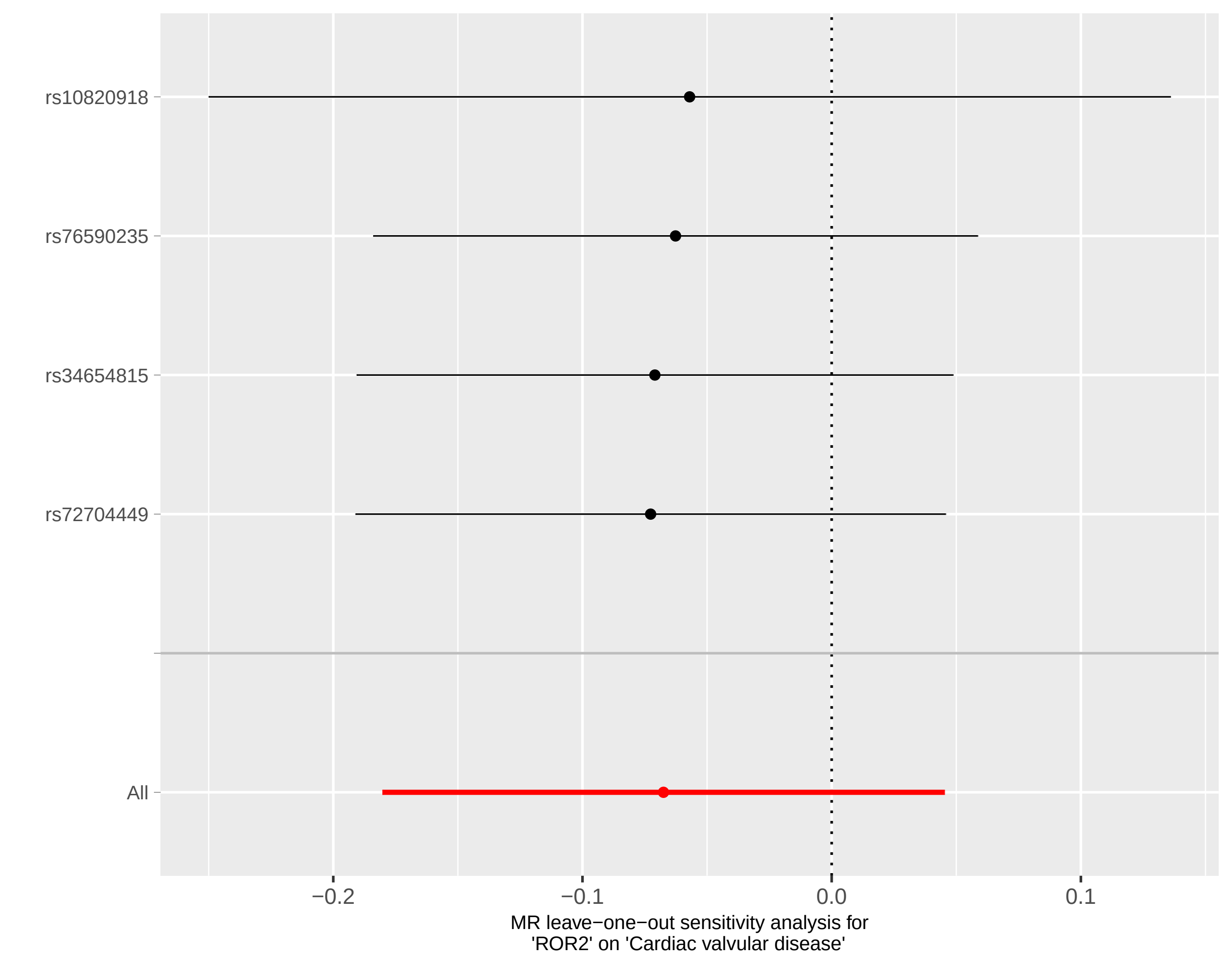
rs10820918
rs76590235
rs34654815
rs72704449
All
−0.2
−0.1
0.0
0.1
MR leave−one−out sensitivity analysis for
'ROR2' on 'Cardiac valvular disease'

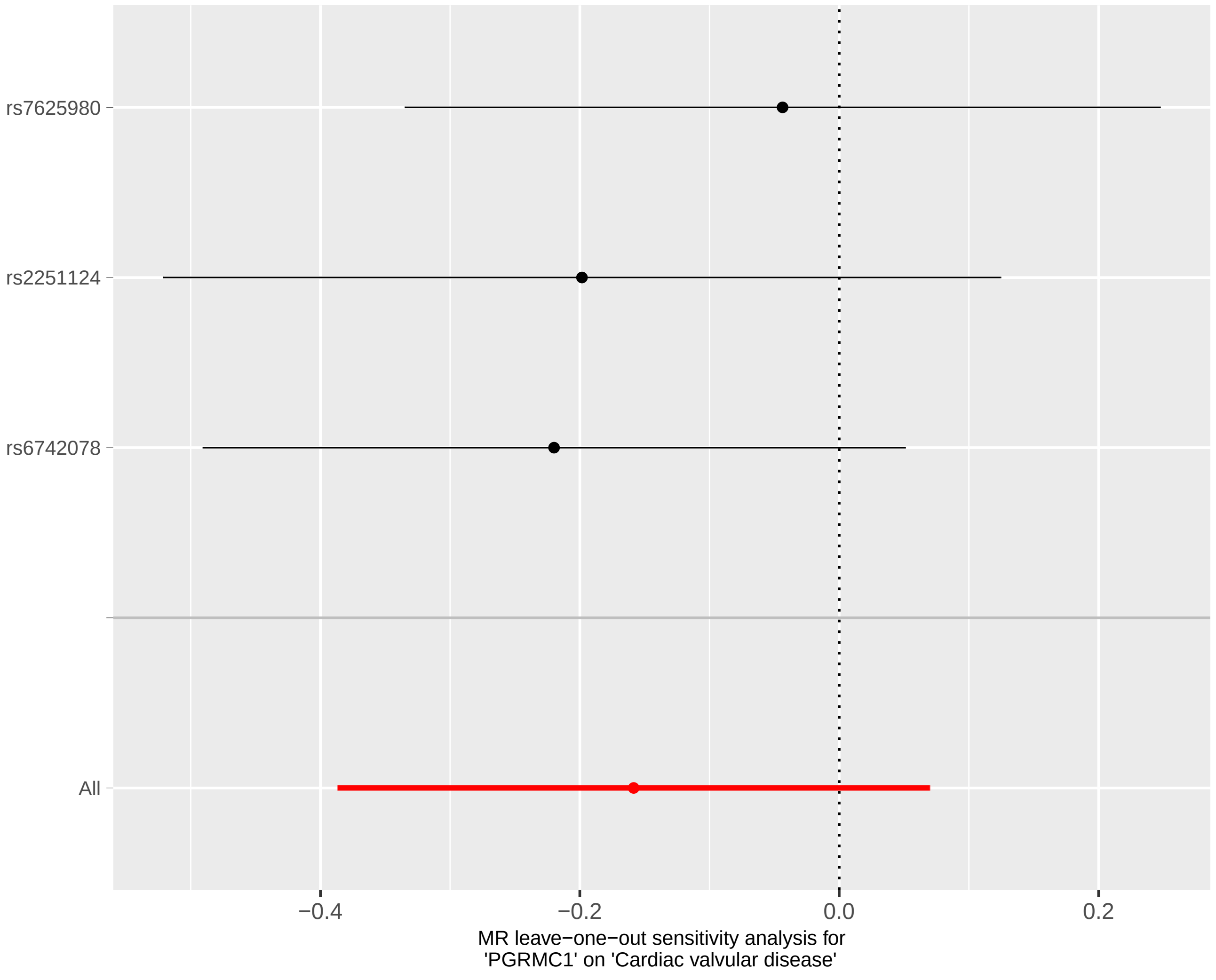
rs7625980
rs2251124
rs6742078
All
−0.4
−0.2
0.0
0.2
MR leave−one−out sensitivity analysis for
'PGRMC1' on 'Cardiac valvular disease'

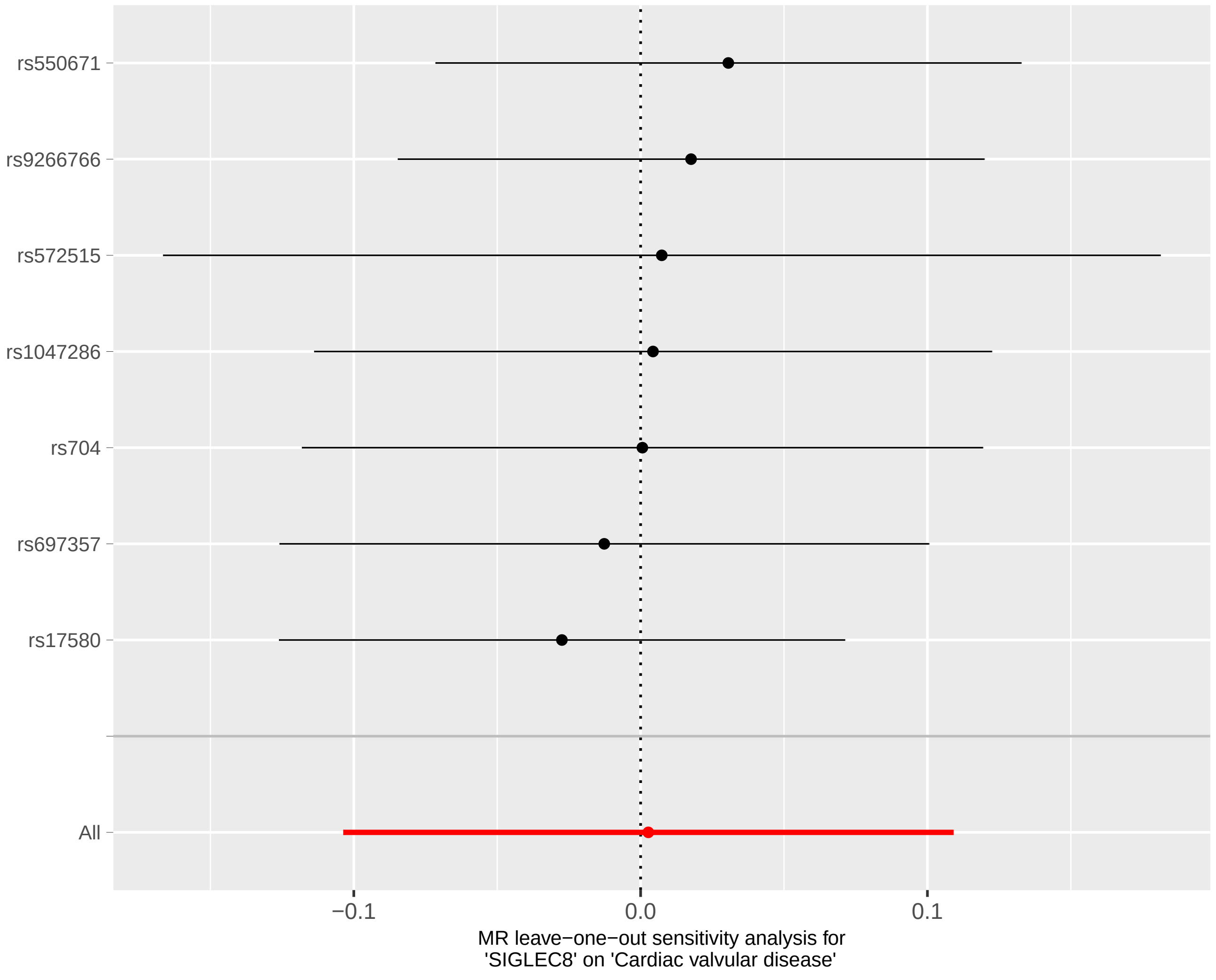

rs550671
rs9266766
rs572515
rs1047286
rs704
rs697357
rs17580
All
−0.1
0.0
0.1
MR leave−one−out sensitivity analysis for
'SIGLEC8' on 'Cardiac valvular disease'

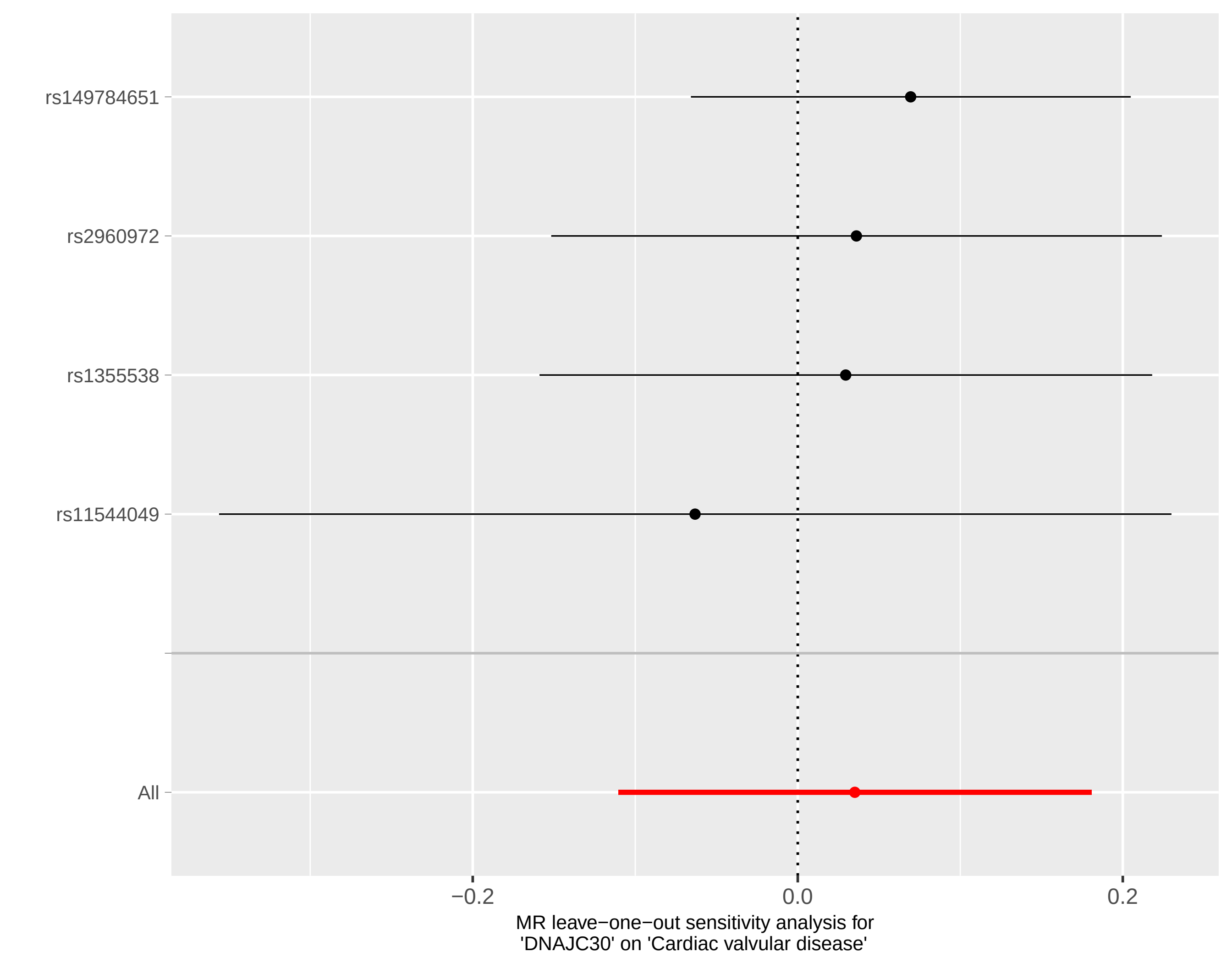
rs149784651
rs2960972
rs1355538
rs11544049
All
−0.2
0.0
0.2
MR leave−one−out sensitivity analysis for
'DNAJC30' on 'Cardiac valvular disease'

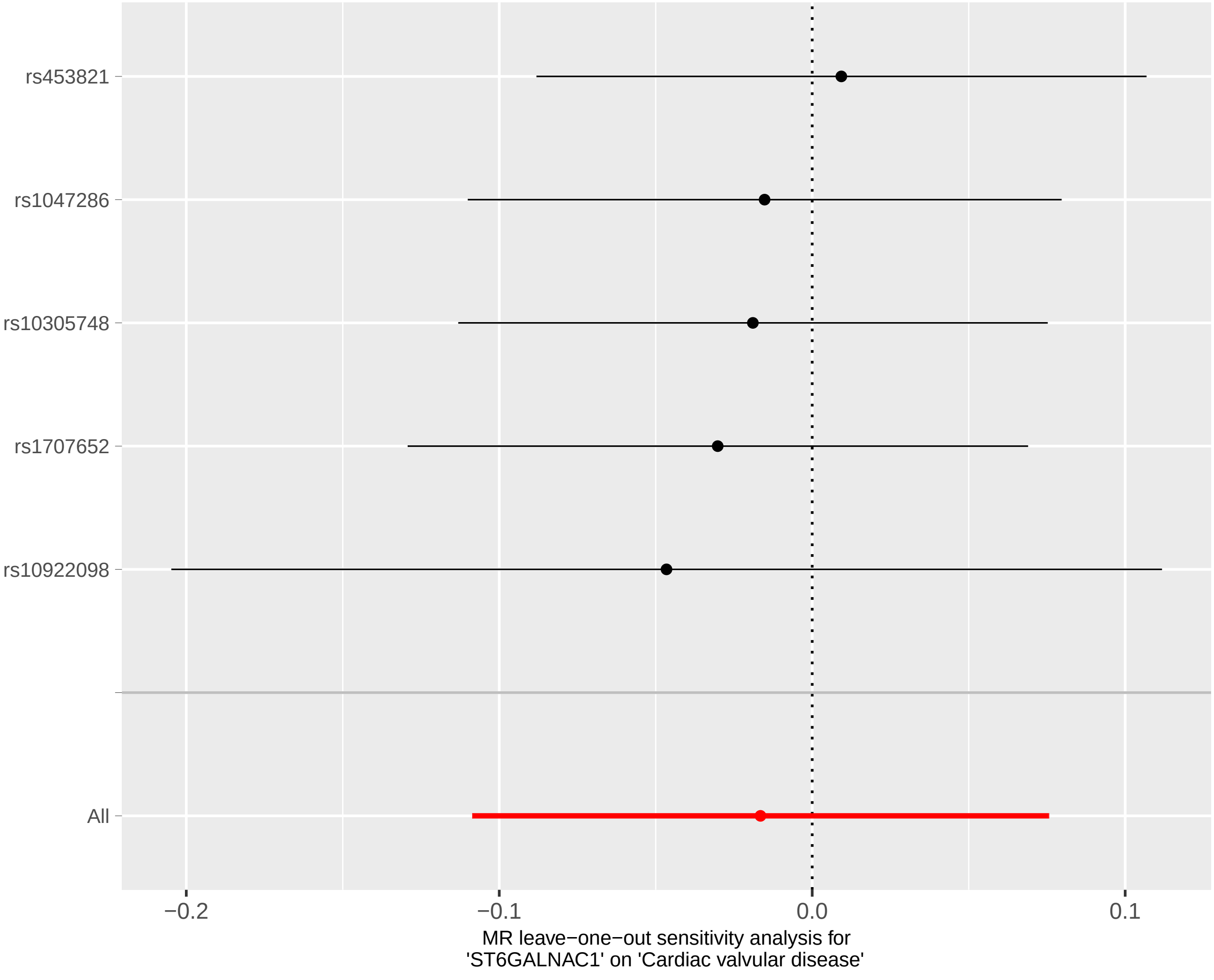
rs453821
rs1047286
rs10305748
rs1707652
rs10922098
All
−0.2
−0.1
0.0
0.1
MR leave−one−out sensitivity analysis for
'ST6GALNAC1' on 'Cardiac valvular disease'

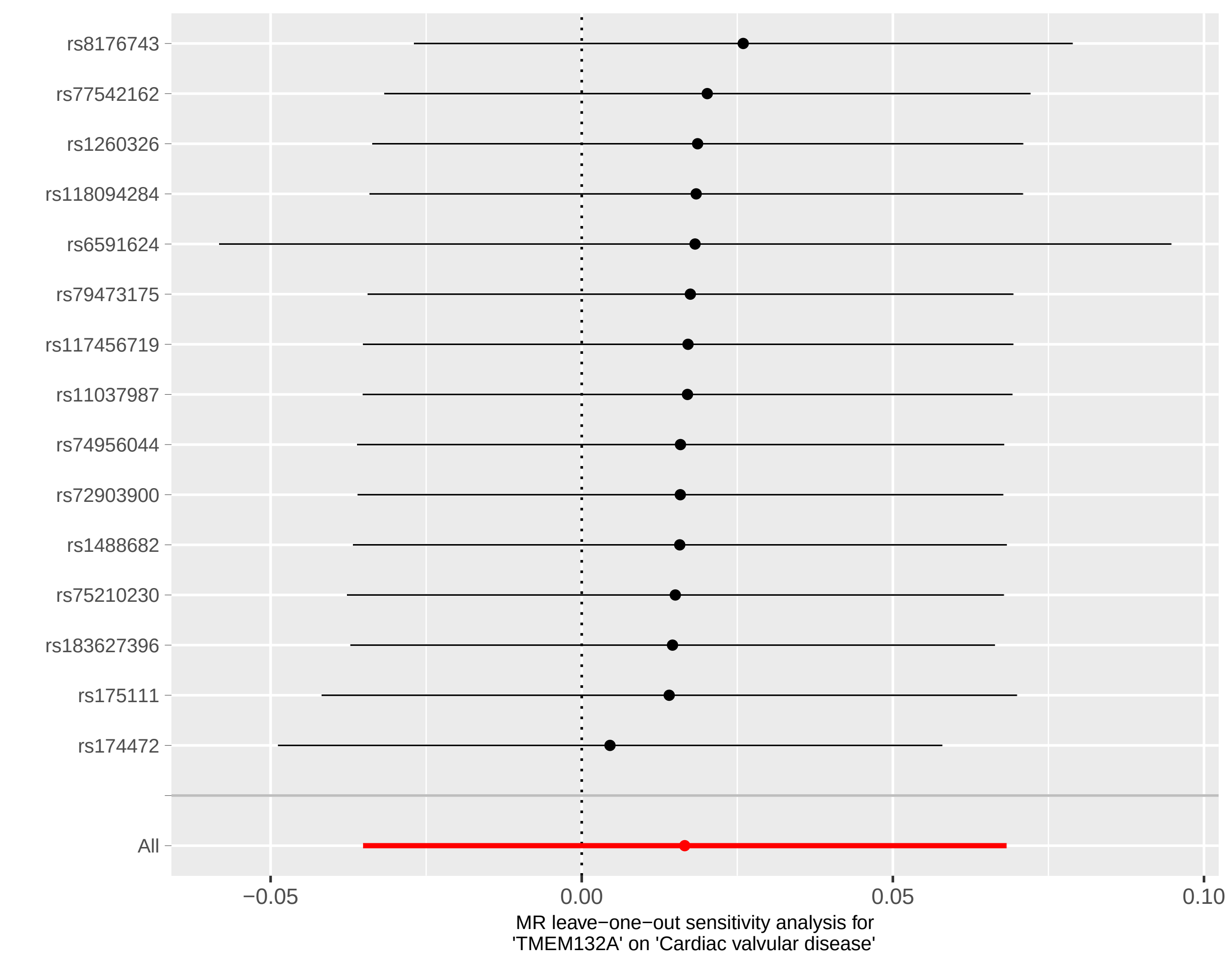
rs8176743
rs77542162
rs1260326
rs118094284
rs6591624
rs79473175
rs117456719
rs11037987
rs74956044
rs72903900
rs1488682
rs75210230
rs183627396
rs175111
rs174472
All
−0.05
0.00
0.05
0.10
MR leave−one−out sensitivity analysis for
'TMEM132A' on 'Cardiac valvular disease'

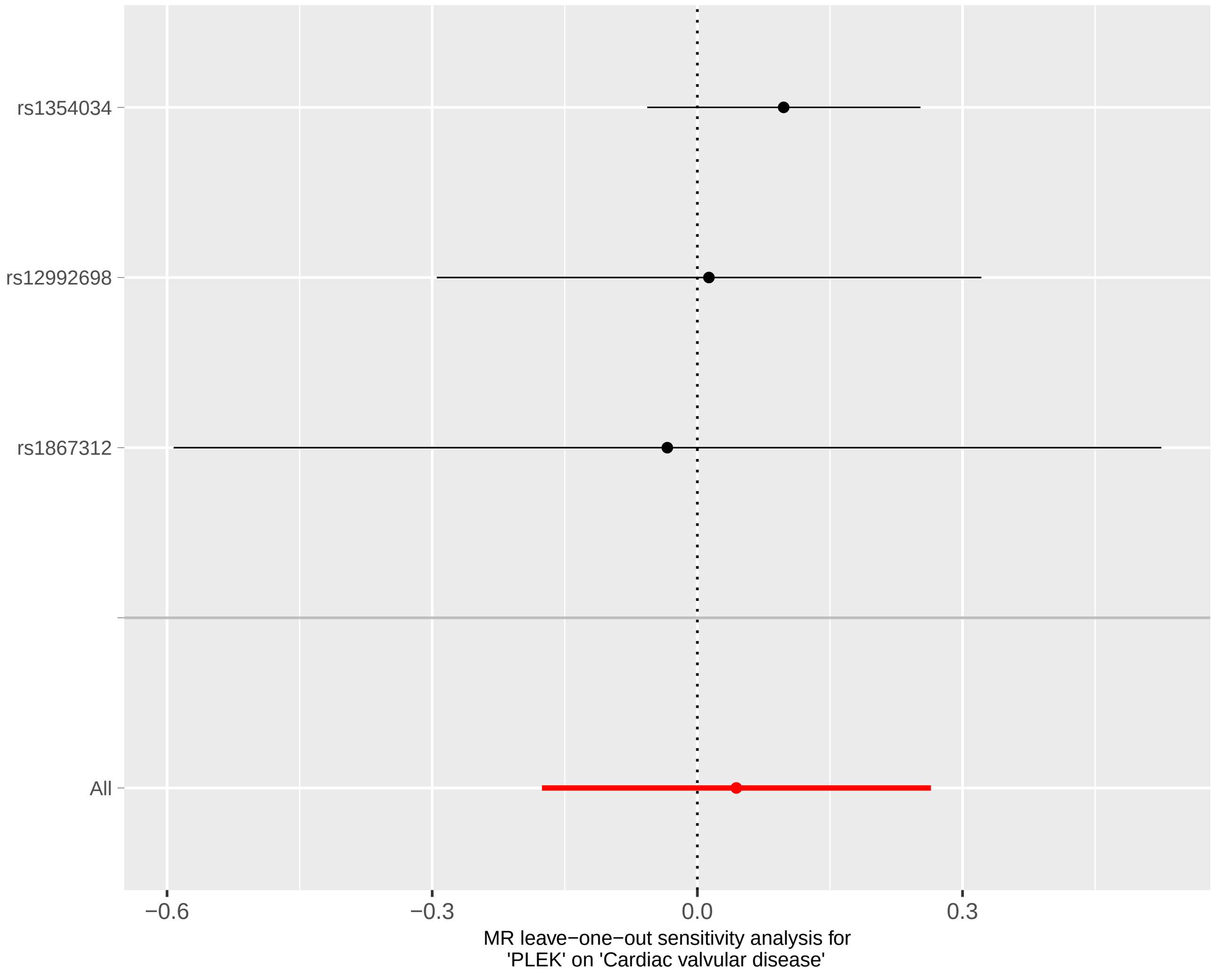
rs1354034
rs12992698
rs1867312
All
−0.6
−0.3
0.0
0.3
MR leave−one−out sensitivity analysis for
'PLEK' on 'Cardiac valvular disease'

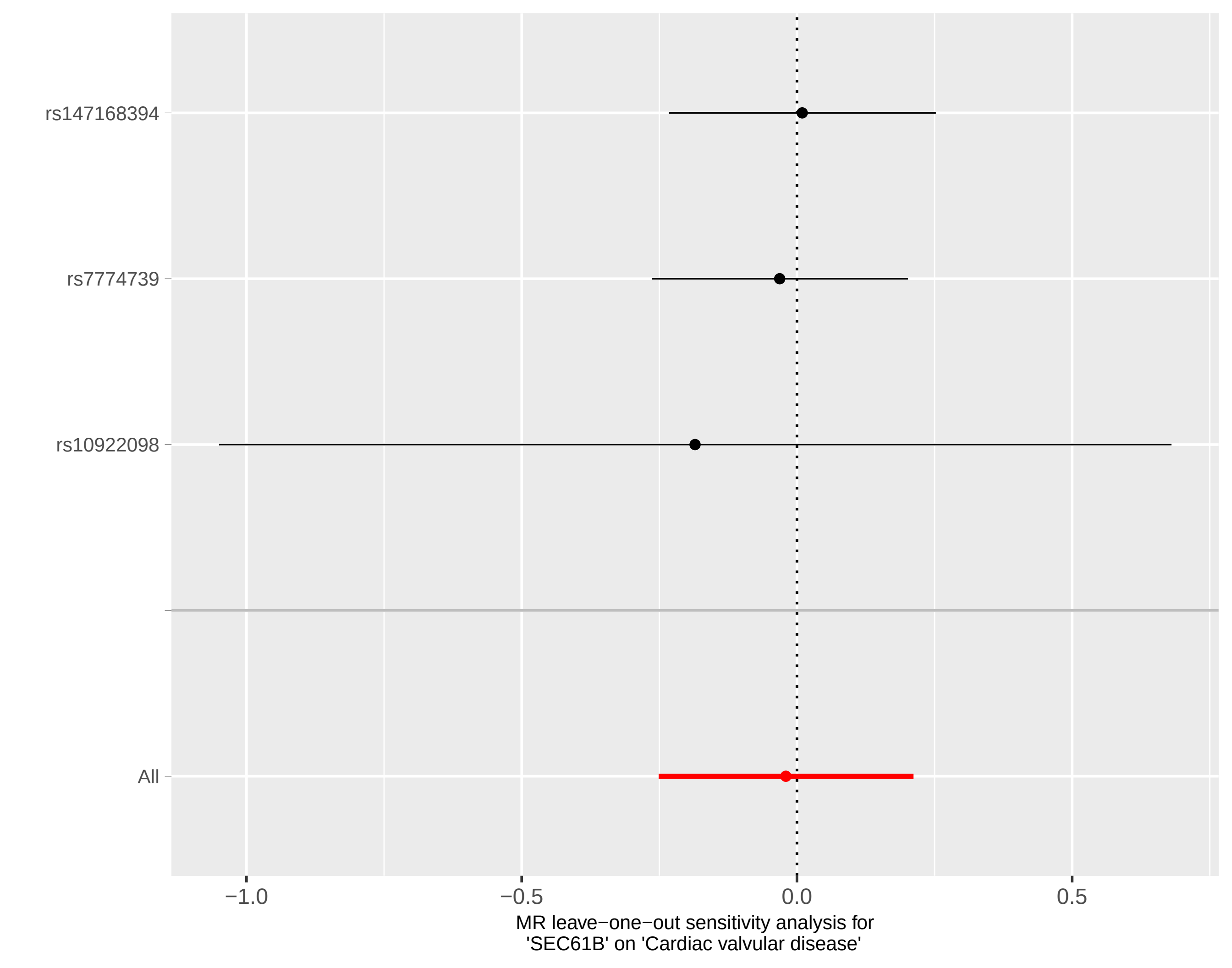
rs147168394
rs7774739
rs10922098
All
−1.0
−0.5
0.0
0.5
MR leave−one−out sensitivity analysis for
'SEC61B' on 'Cardiac valvular disease'

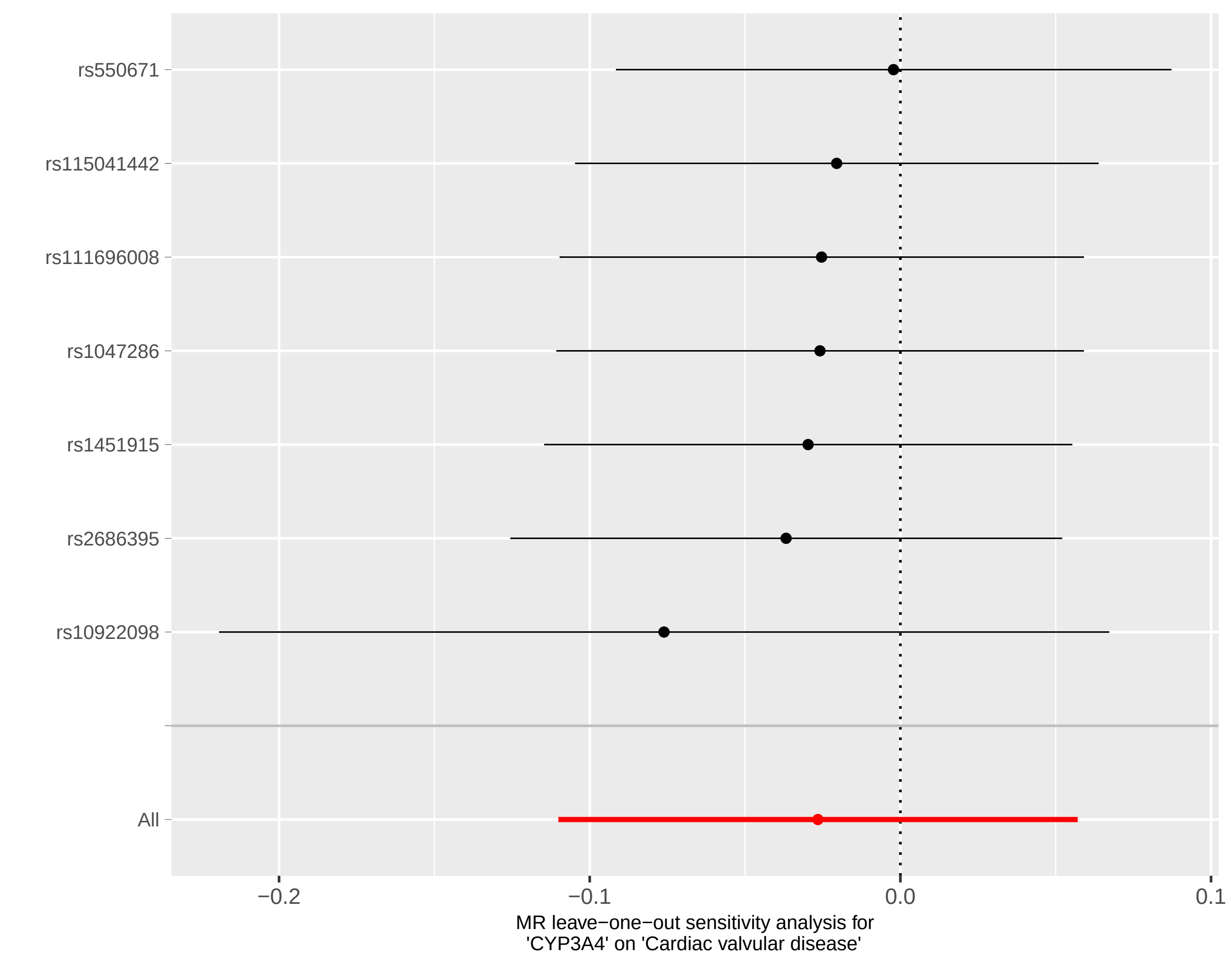

rs550671
rs115041442
rs111696008
rs1047286
rs1451915
rs2686395
rs10922098
All
−0.2
−0.1
0.0
0.1
MR leave−one−out sensitivity analysis for
'CYP3A4' on 'Cardiac valvular disease'

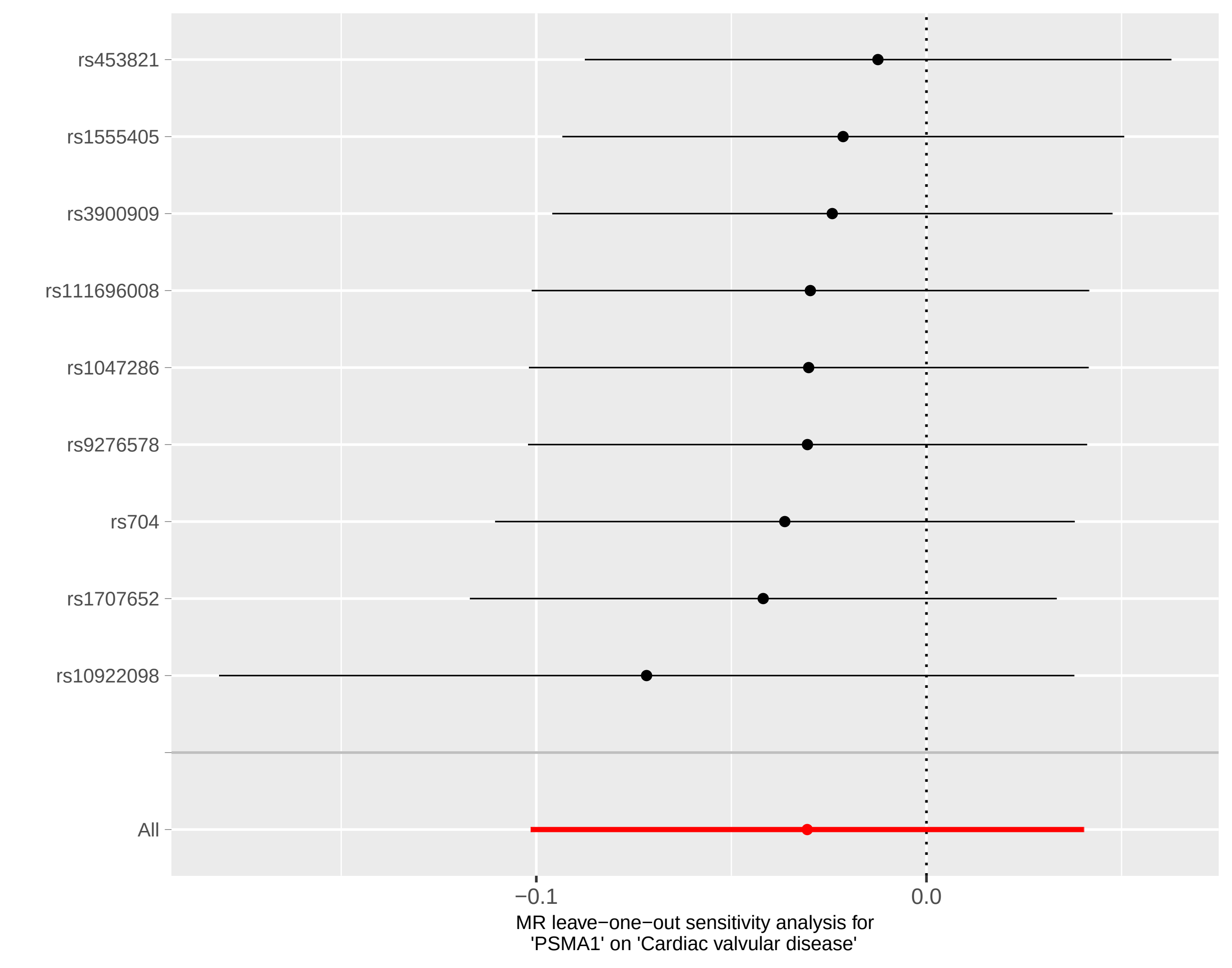
rs453821
rs1555405
rs3900909
rs111696008
rs1047286
rs9276578
rs704
rs1707652
rs10922098
All
−0.1
0.0
MR leave−one−out sensitivity analysis for
'PSMA1' on 'Cardiac valvular disease'

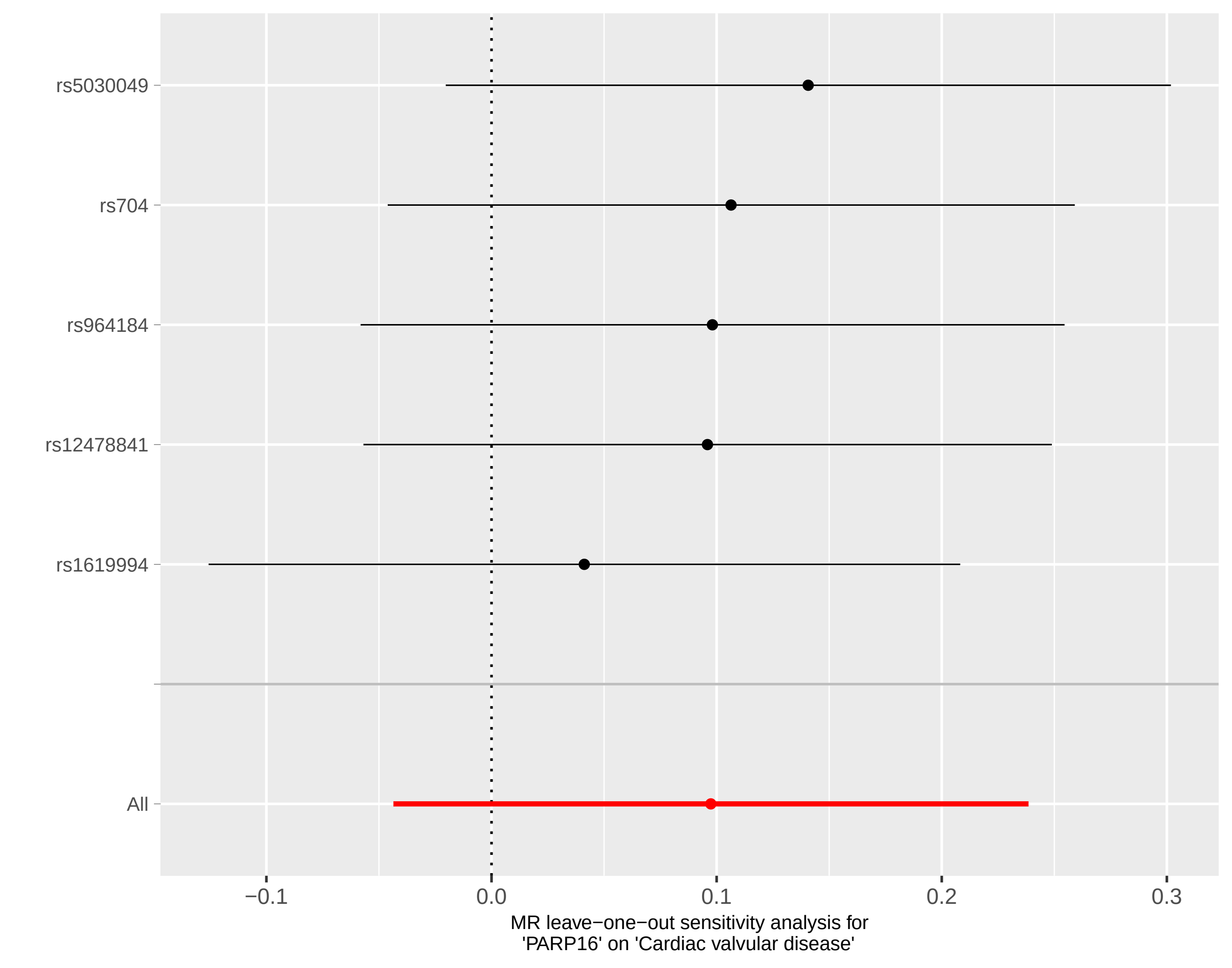

rs5030049
rs704
rs964184
rs12478841
rs1619994
All
−0.1
0.0
0.1
0.2
0.3
MR leave−one−out sensitivity analysis for
'PARP16' on 'Cardiac valvular disease'

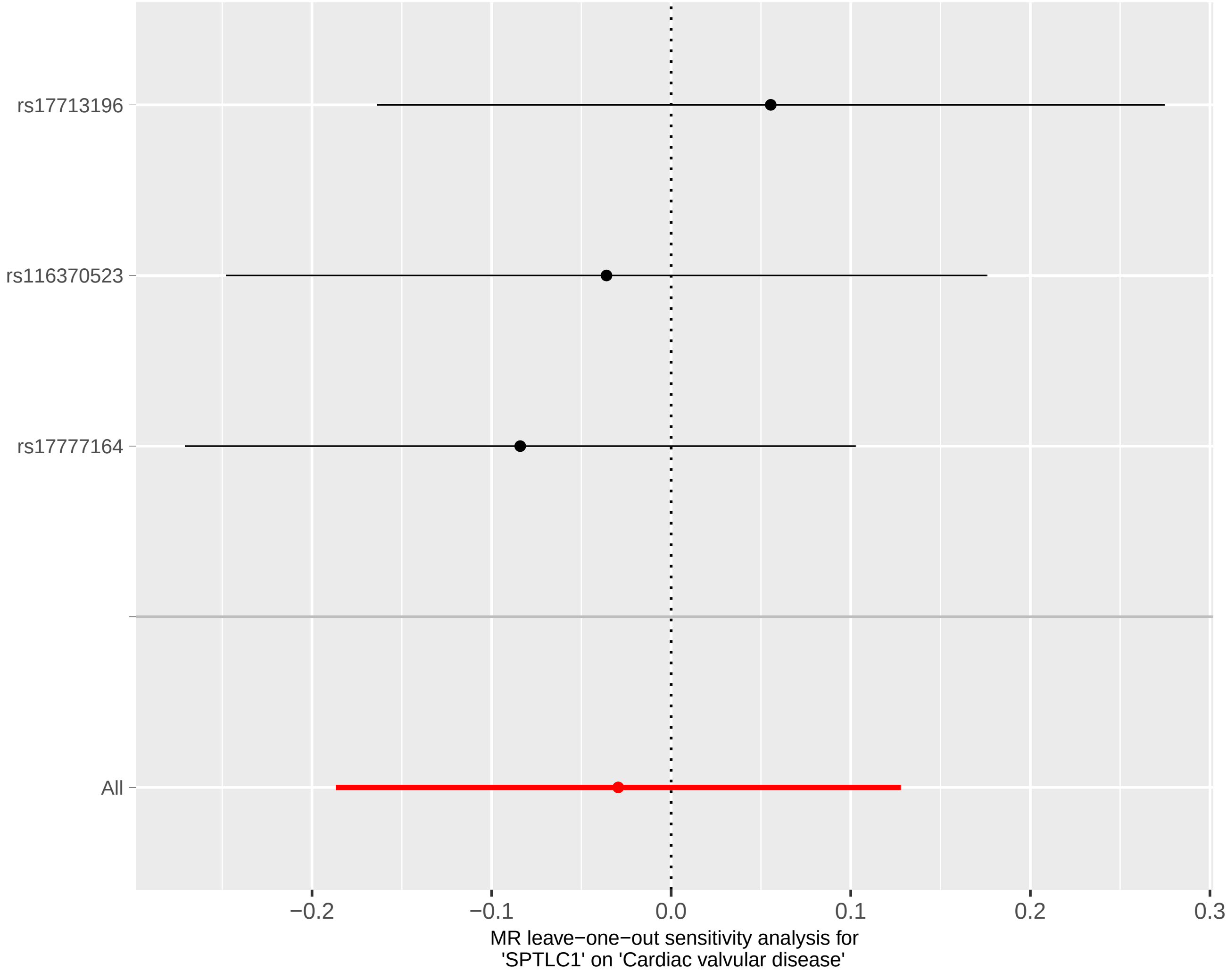
rs17713196
rs116370523
rs17777164
All
−0.2
−0.1
0.0
0.1
0.2
0.3
MR leave−one−out sensitivity analysis for
'SPTLC1' on 'Cardiac valvular disease'

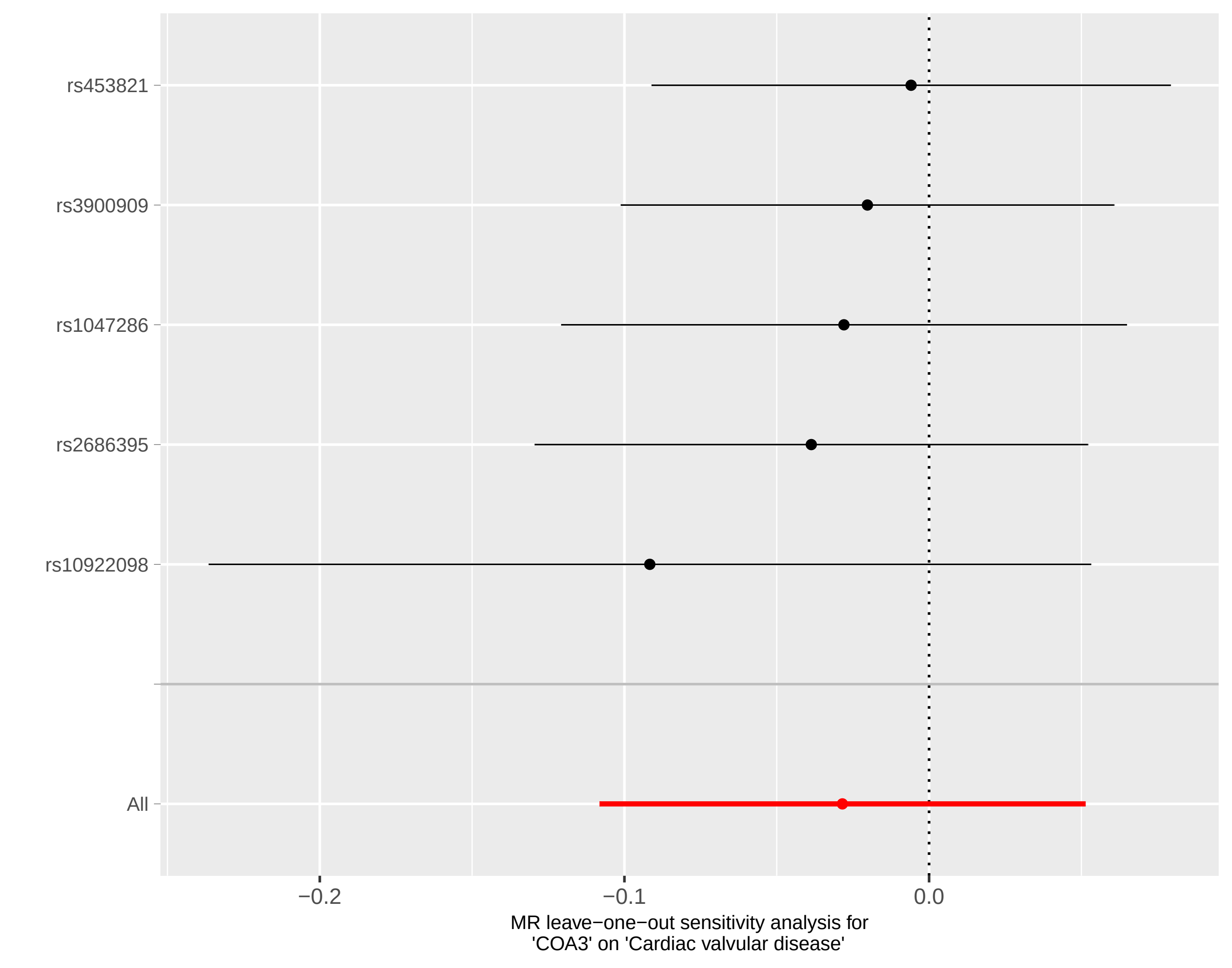
rs453821
rs3900909
rs1047286
rs2686395
rs10922098
All
−0.2
−0.1
0.0
MR leave−one−out sensitivity analysis for
'COA3' on 'Cardiac valvular disease'

Insufficient number of SNPs

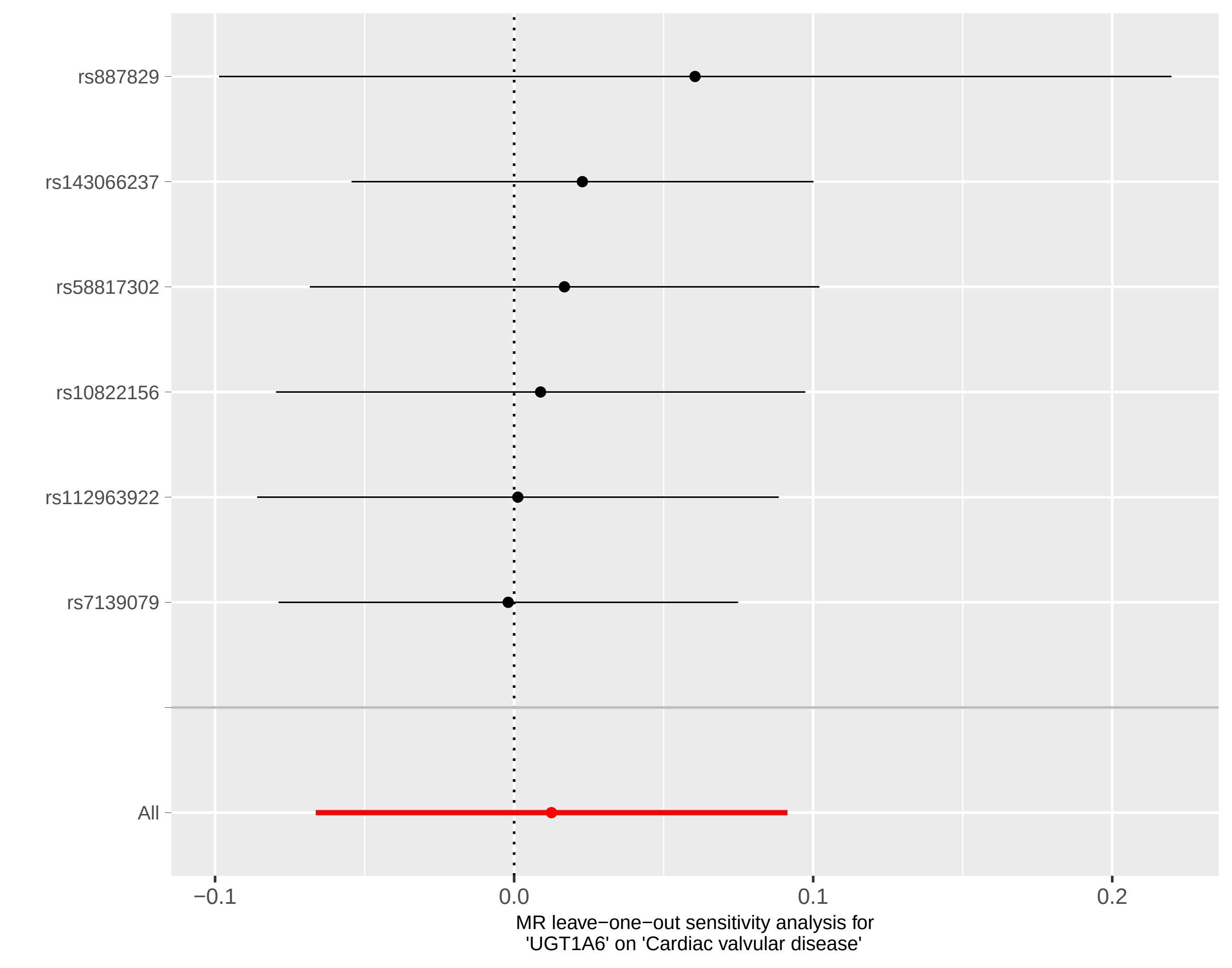
rs887829
rs143066237
rs58817302
rs10822156
rs112963922
rs7139079
All
−0.1
0.0
0.1
0.2
MR leave−one−out sensitivity analysis for
'UGT1A6' on 'Cardiac valvular disease'

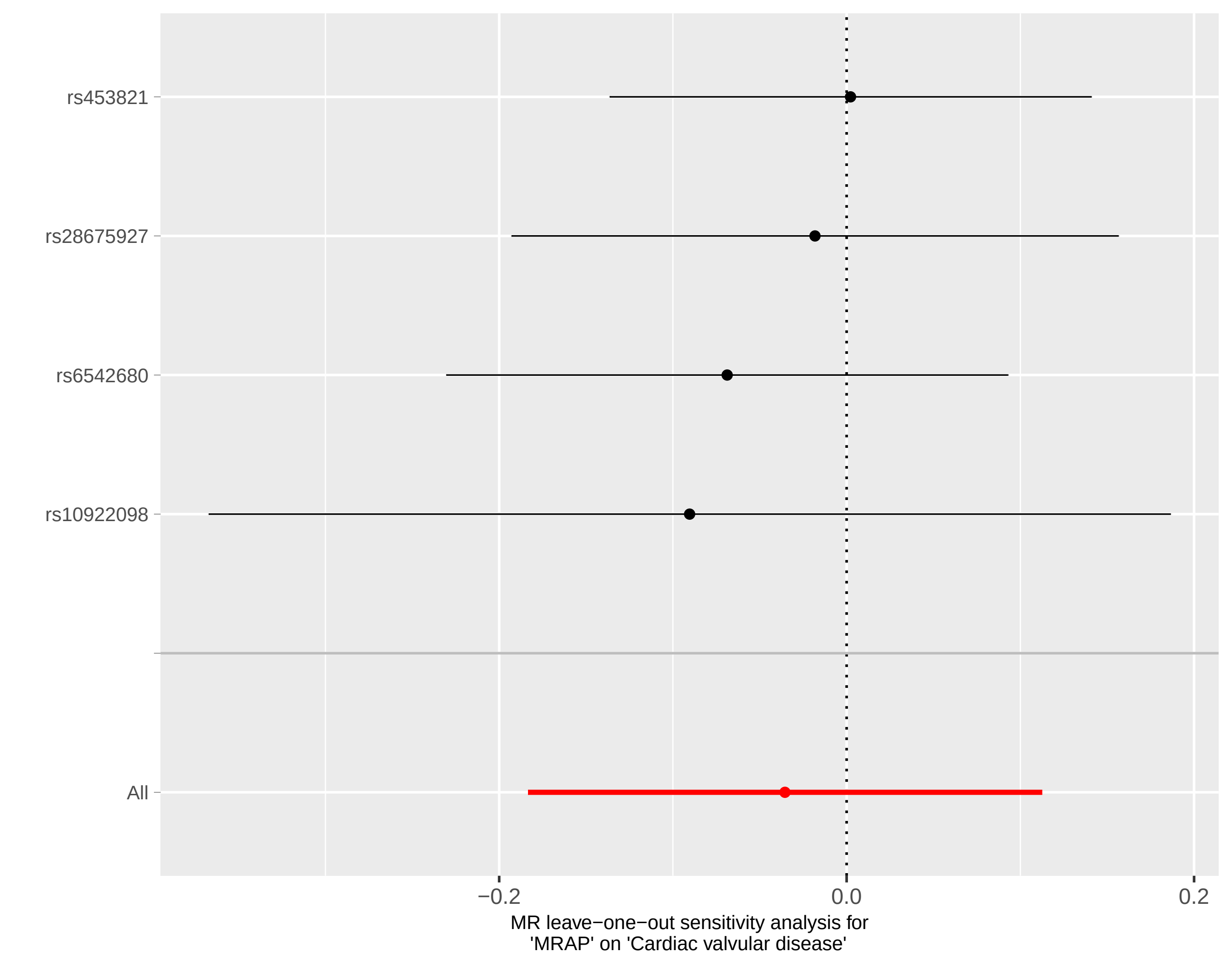

rs453821
rs28675927
rs6542680
rs10922098
All
−0.2
0.0
0.2
MR leave−one−out sensitivity analysis for
'MRAP' on 'Cardiac valvular disease'

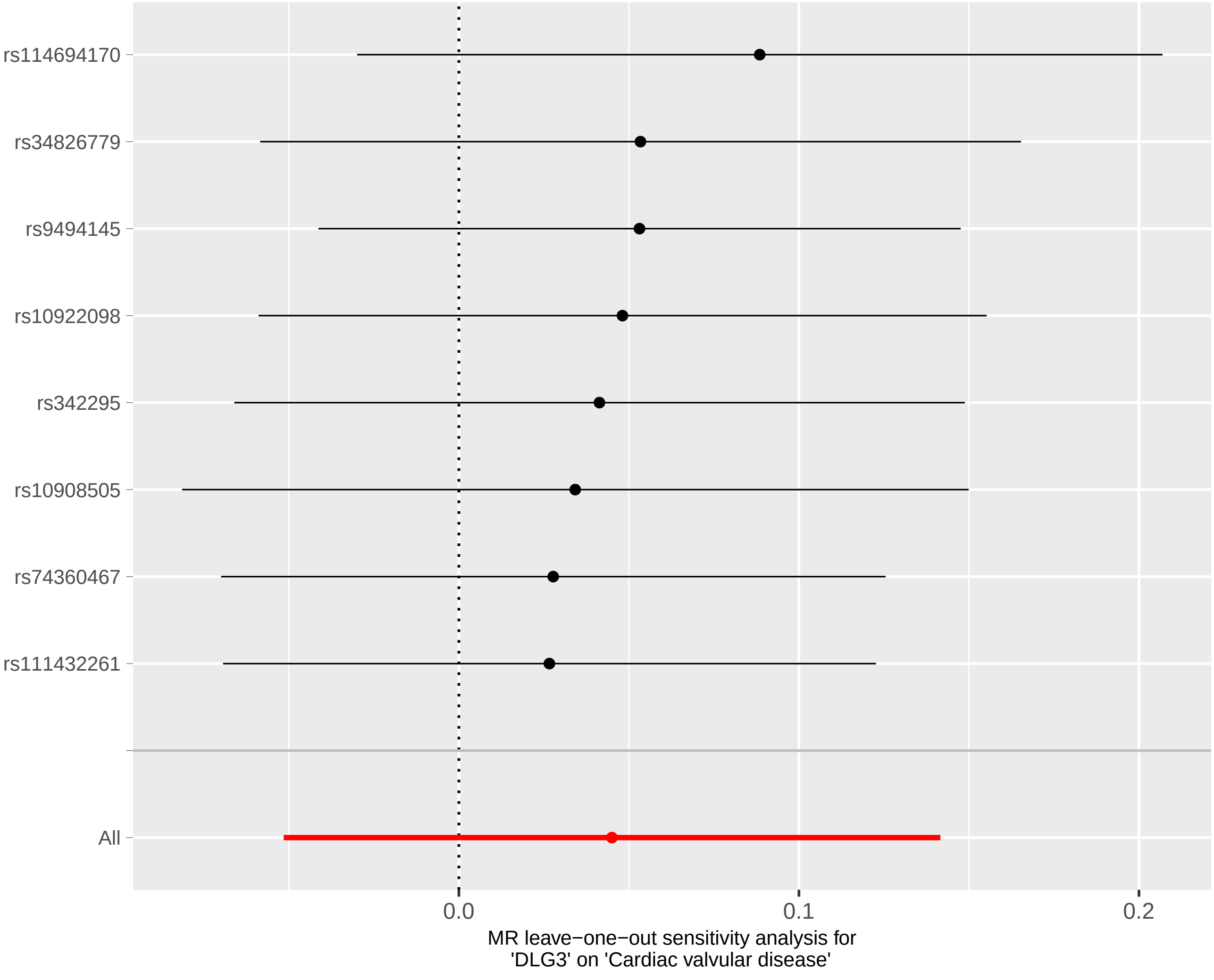
rs114694170
rs34826779
rs9494145
rs10922098
rs342295
rs10908505
rs74360467
rs111432261
All
0.0
0.1
0.2
MR leave−one−out sensitivity analysis for
'DLG3' on 'Cardiac valvular disease'

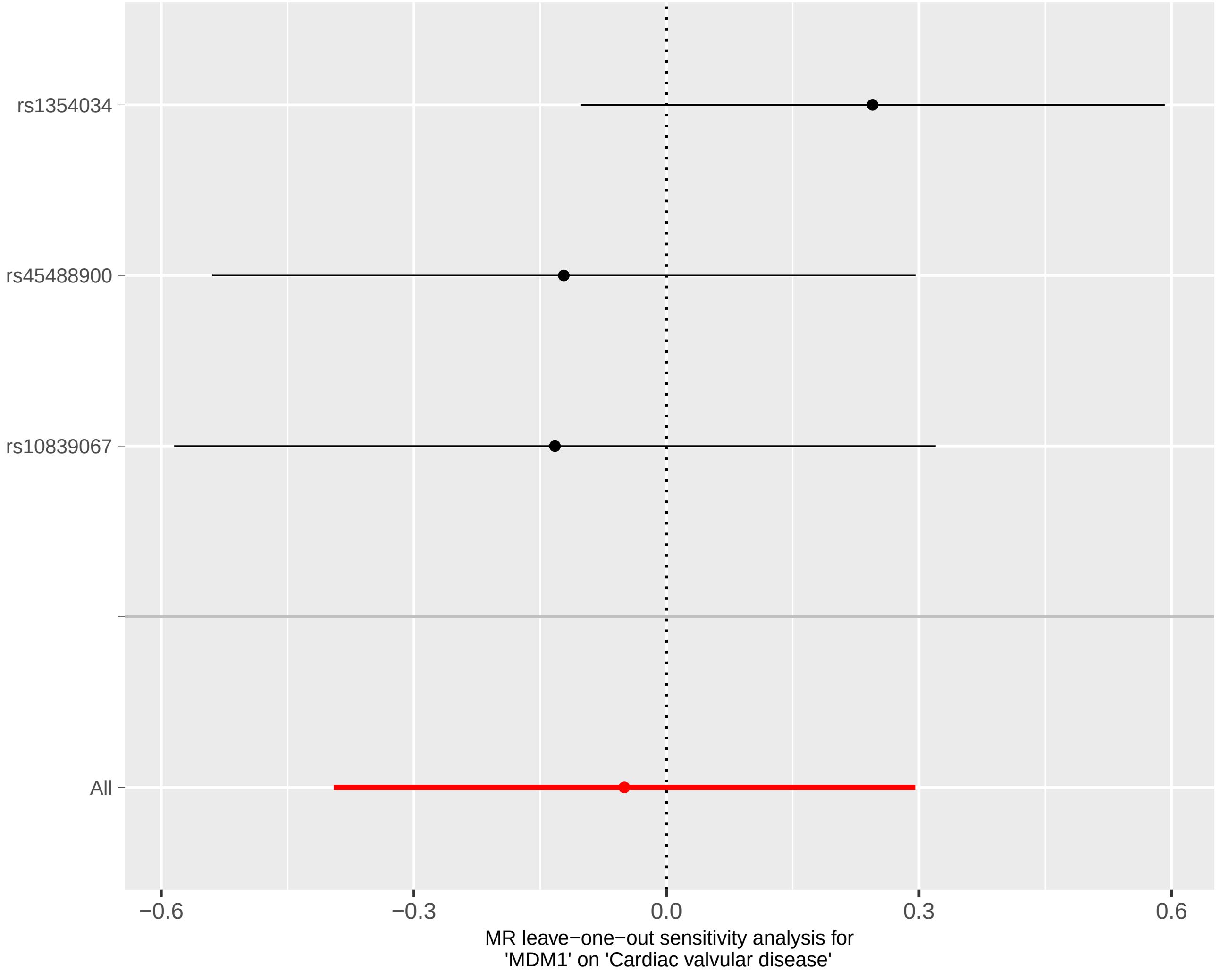
rs1354034
rs45488900
rs10839067
All
−0.6
−0.3
0.0
0.3
0.6
MR leave−one−out sensitivity analysis for
'MDM1' on 'Cardiac valvular disease'

Insufficient number of SNPs

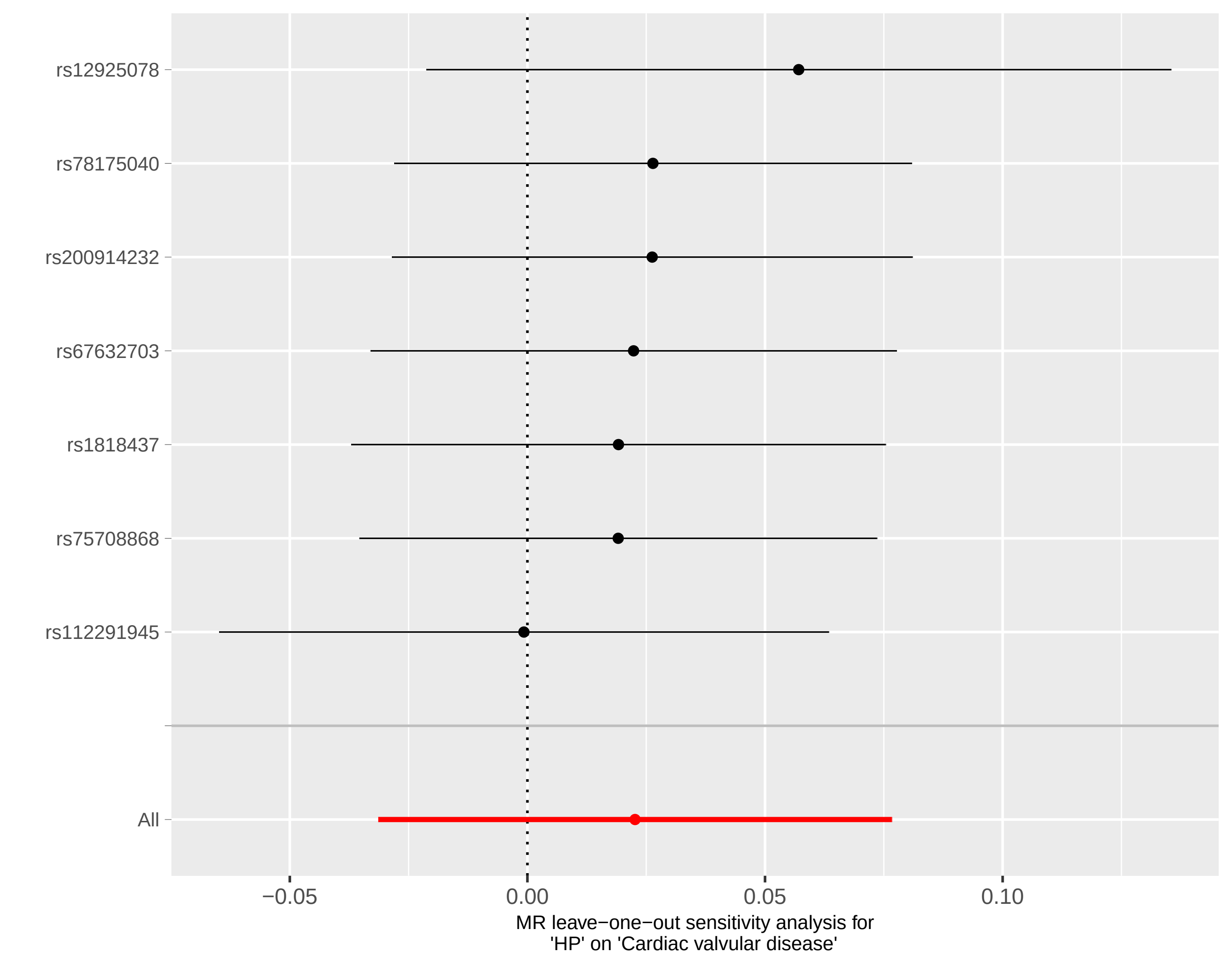
rs12925078
rs78175040
rs200914232
rs67632703
rs1818437
rs75708868
rs112291945
All
−0.05
0.00
0.05
0.10
MR leave−one−out sensitivity analysis for
'HP' on 'Cardiac valvular disease'

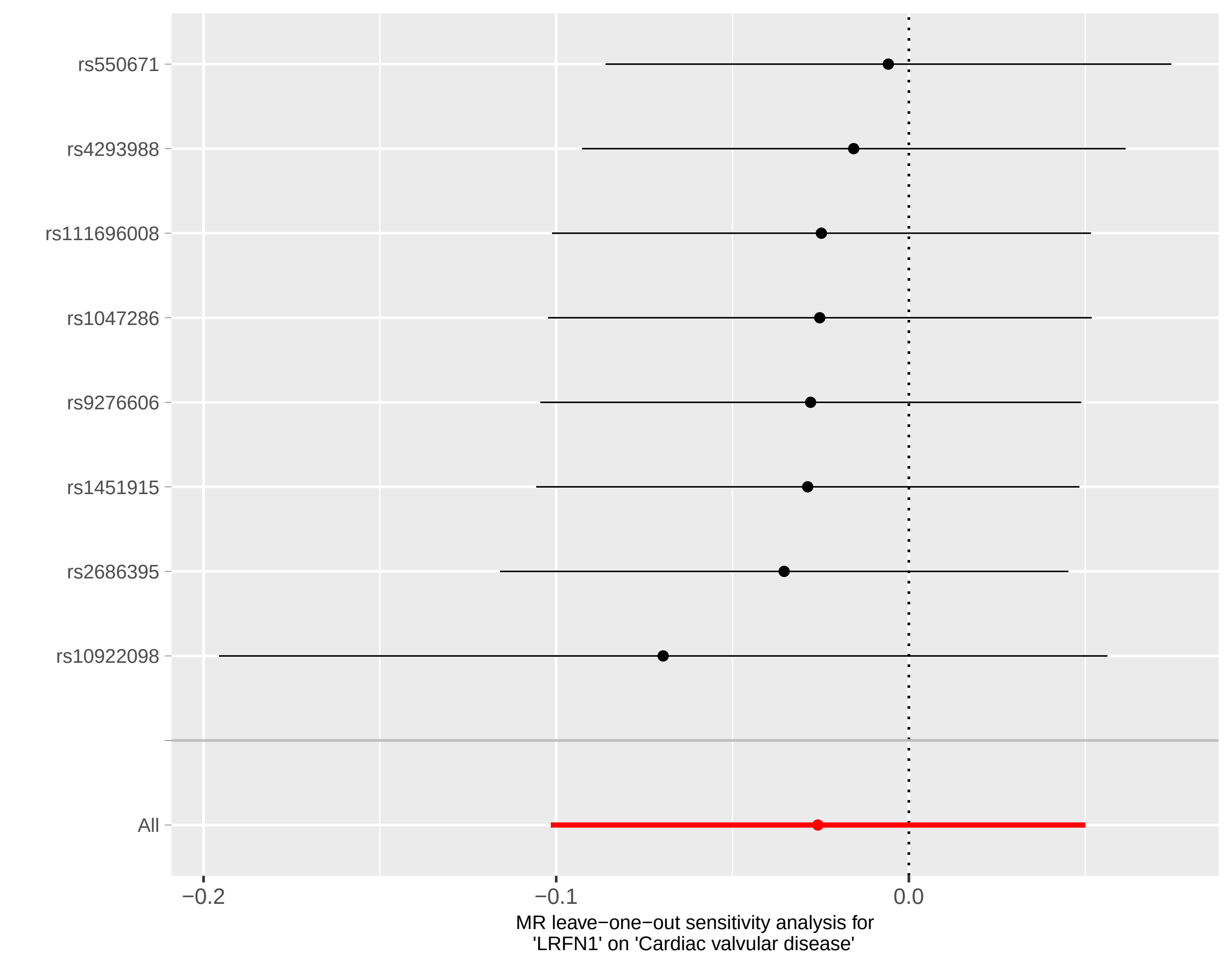
rs550671
rs4293988
rs111696008
rs1047286
rs9276606
rs1451915
rs2686395
rs10922098
All
−0.2
−0.1
0.0
MR leave−one−out sensitivity analysis for
'LRFN1' on 'Cardiac valvular disease'

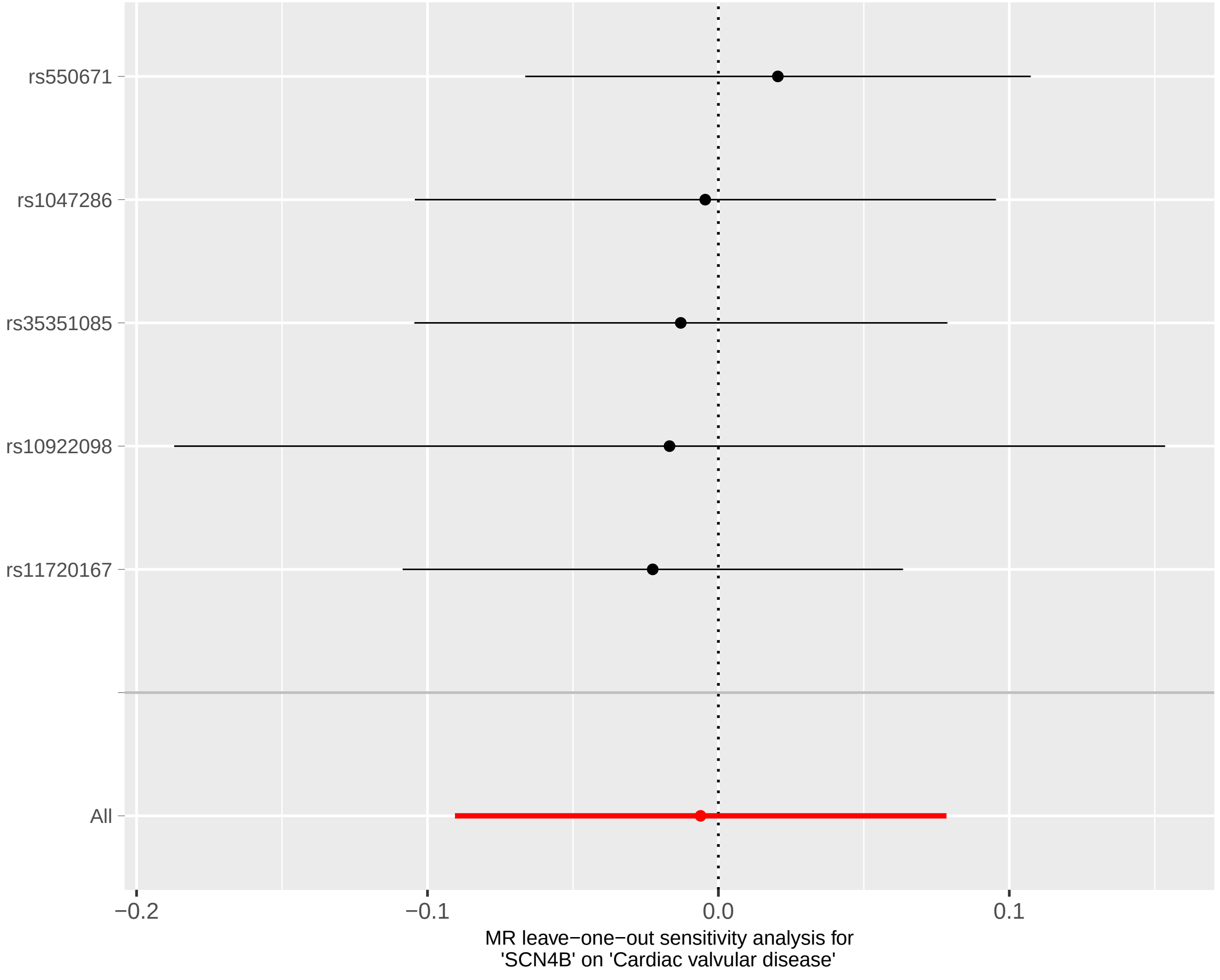
rs550671
rs1047286
rs35351085
rs10922098
rs11720167
All
−0.2
−0.1
0.0
0.1
MR leave−one−out sensitivity analysis for
'SCN4B' on 'Cardiac valvular disease'

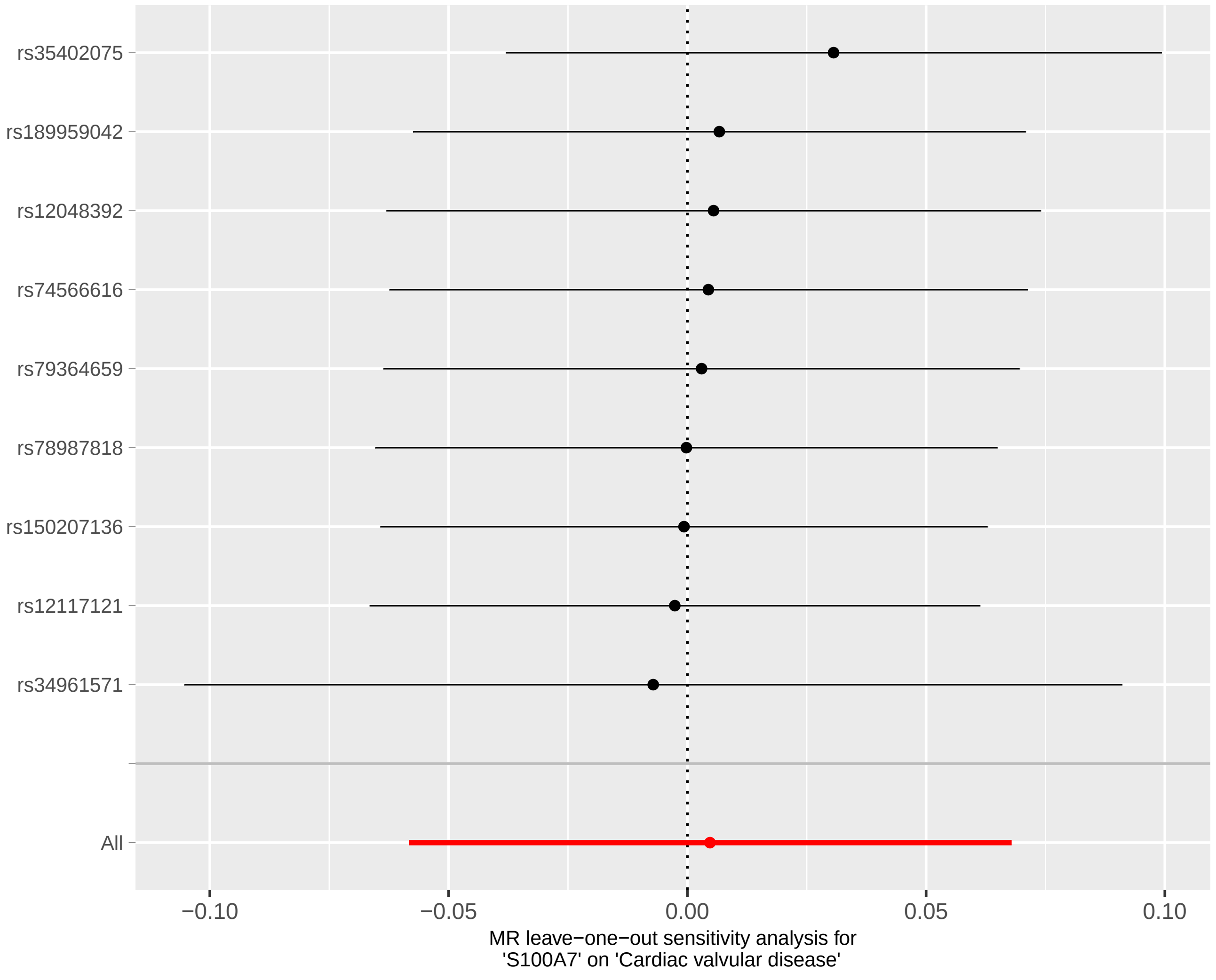

rs35402075
rs189959042
rs12048392
rs74566616
rs79364659
rs78987818
rs150207136
rs12117121
rs34961571
All
−0.10
−0.05
0.00
0.05
0.10
MR leave−one−out sensitivity analysis for
'S100A7' on 'Cardiac valvular disease'

Insufficient number of SNPs

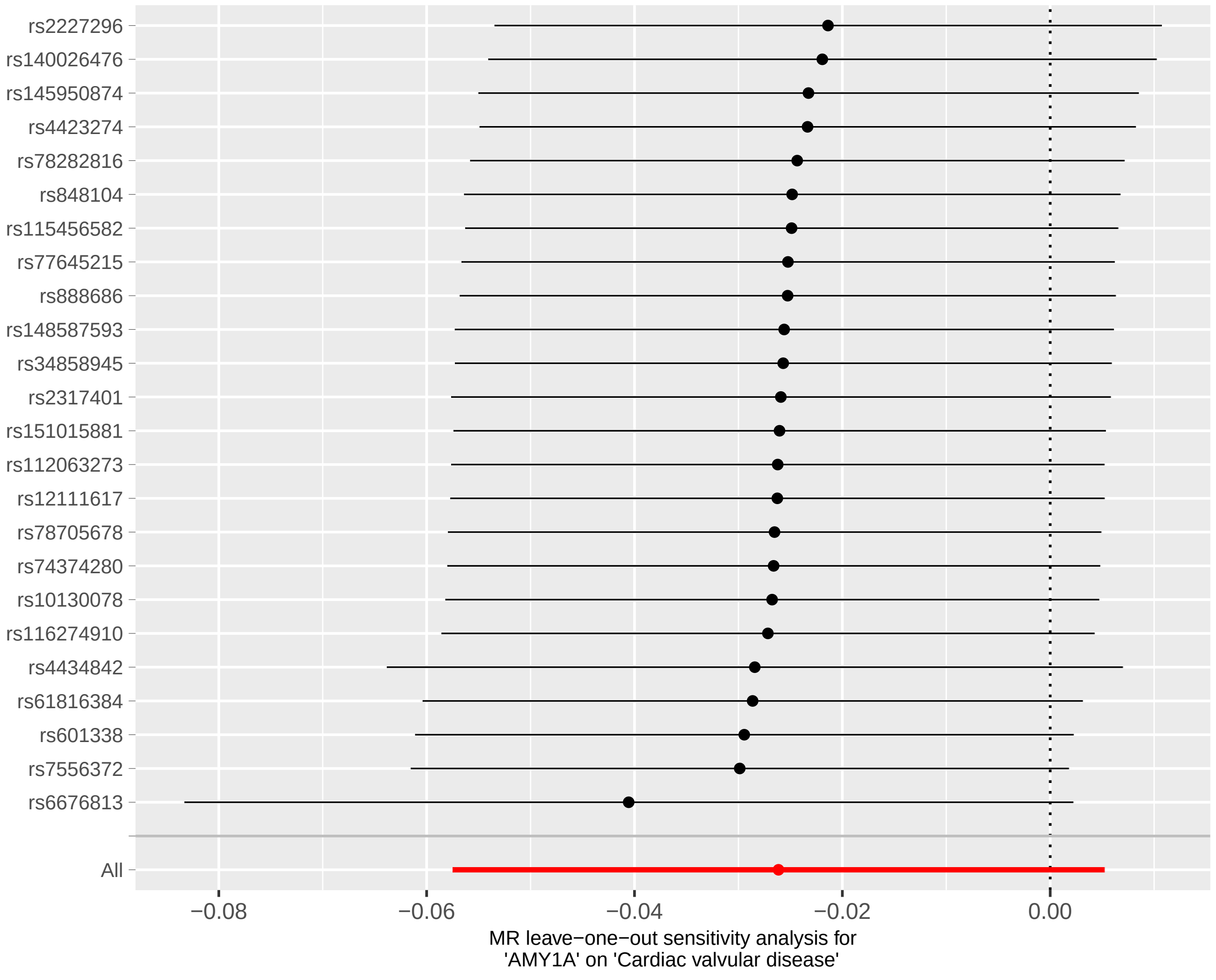
rs2227296
rs140026476
rs145950874
rs4423274
rs78282816
rs848104
rs115456582
rs77645215
rs888686
rs148587593
rs34858945
rs2317401
rs151015881
rs112063273
rs12111617
rs78705678
rs74374280
rs10130078
rs116274910
rs4434842
rs61816384
rs601338
rs7556372
rs6676813
All
−0.08
−0.06
−0.04
−0.02
0.00
MR leave−one−out sensitivity analysis for
'AMY1A' on 'Cardiac valvular disease'

Insufficient number of SNPs

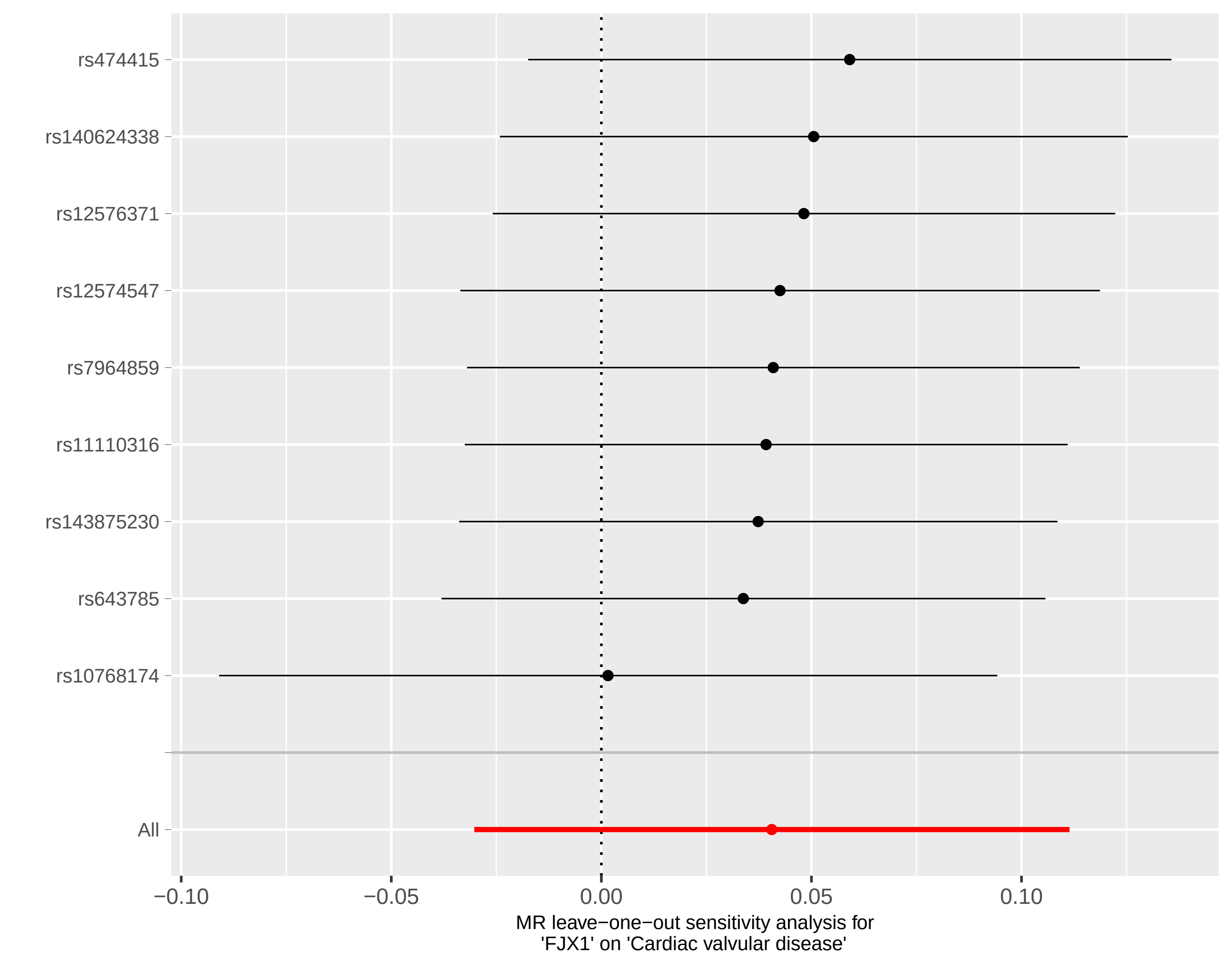

rs474415
rs140624338
rs12576371
rs12574547
rs7964859
rs11110316
rs143875230
rs643785
rs10768174
All
−0.10
−0.05
0.00
0.05
0.10
MR leave−one−out sensitivity analysis for
'FJX1' on 'Cardiac valvular disease'

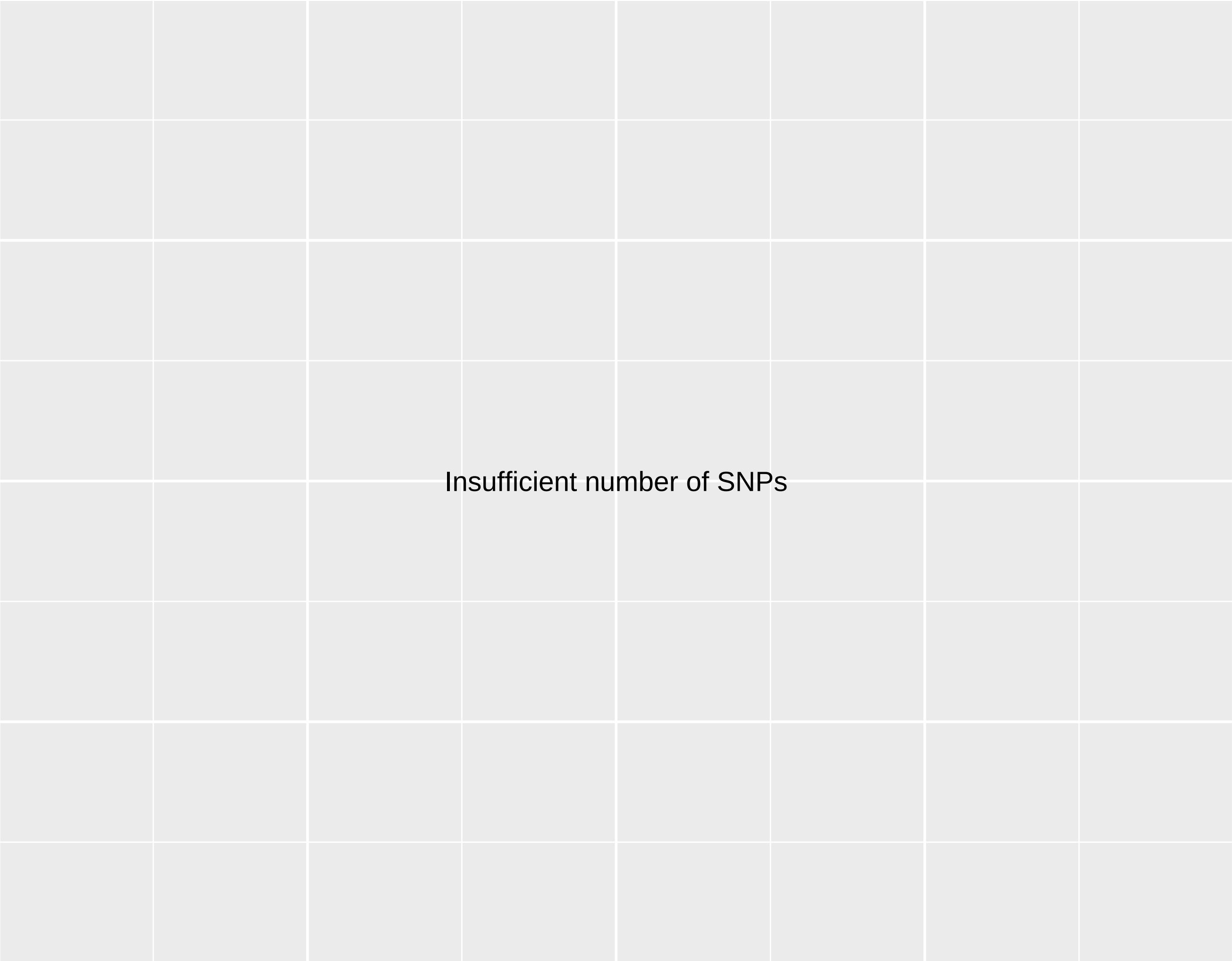
Insufficient number of SNPs

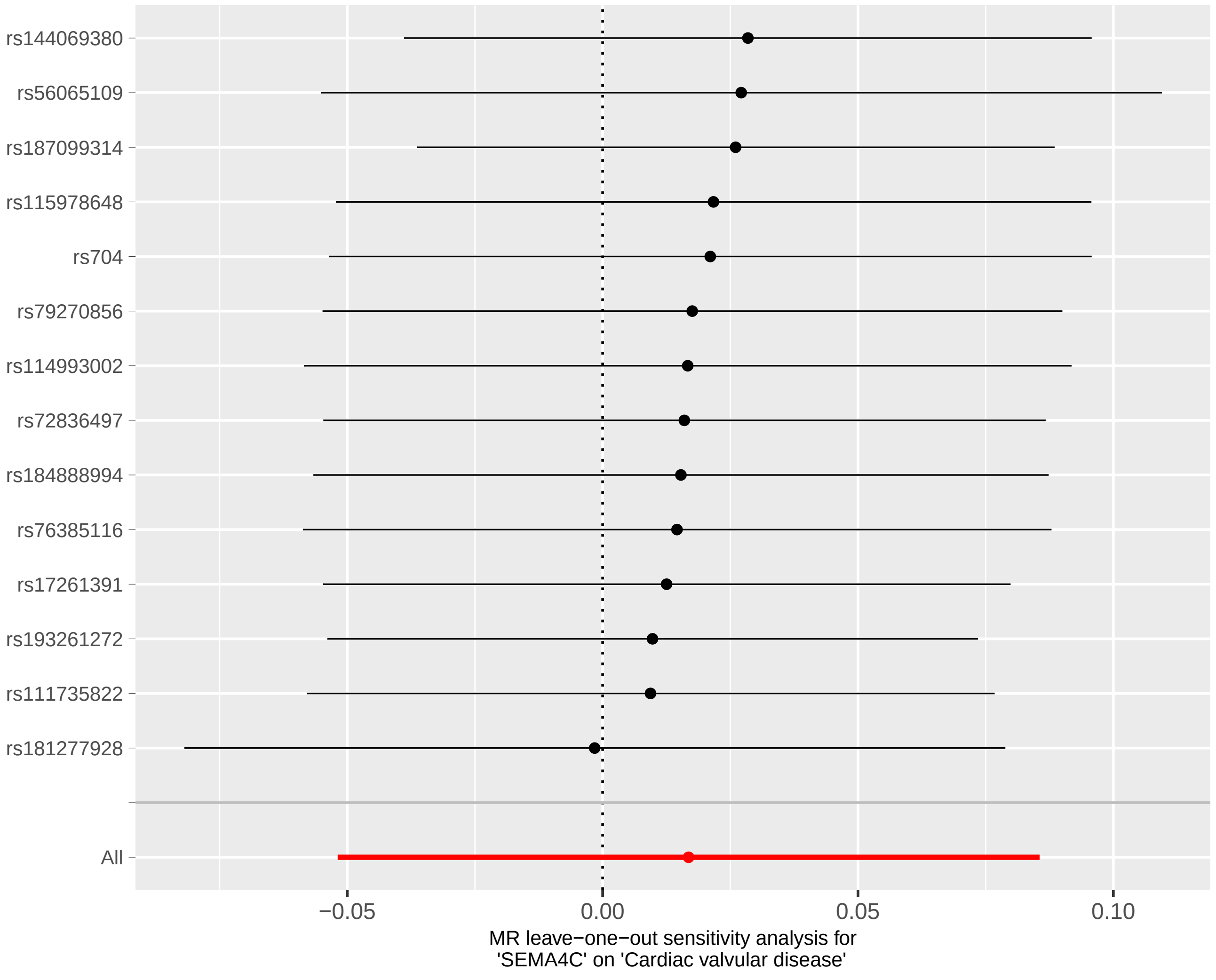
rs144069380
rs56065109
rs187099314
rs115978648
rs704
rs79270856
rs114993002
rs72836497
rs184888994
rs76385116
rs17261391
rs193261272
rs111735822
rs181277928
All
−0.05
0.00
0.05
0.10
MR leave−one−out sensitivity analysis for
'SEMA4C' on 'Cardiac valvular disease'

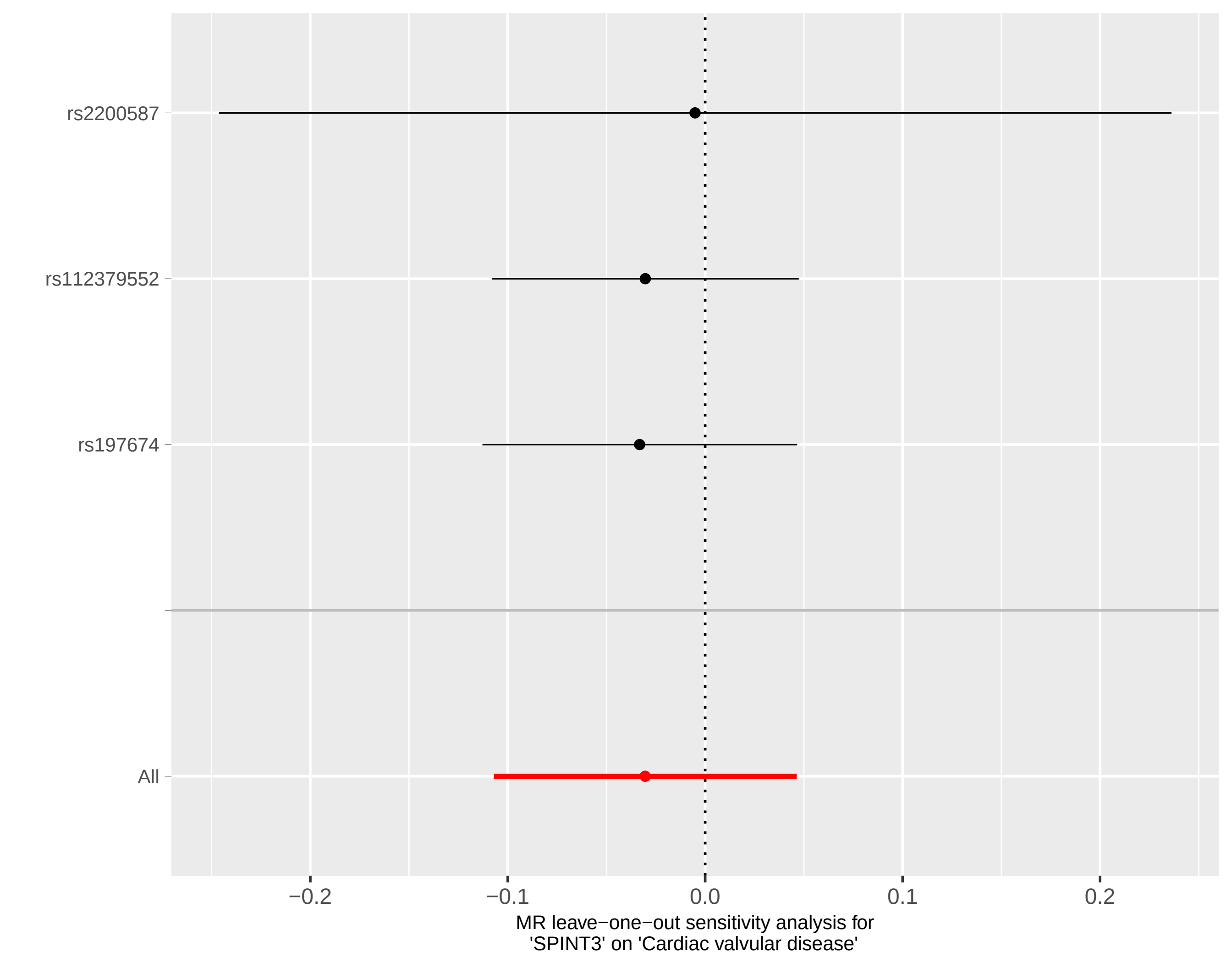
rs2200587
rs112379552
rs197674
All
−0.2
−0.1
0.0
0.1
0.2
MR leave−one−out sensitivity analysis for
'SPINT3' on 'Cardiac valvular disease'

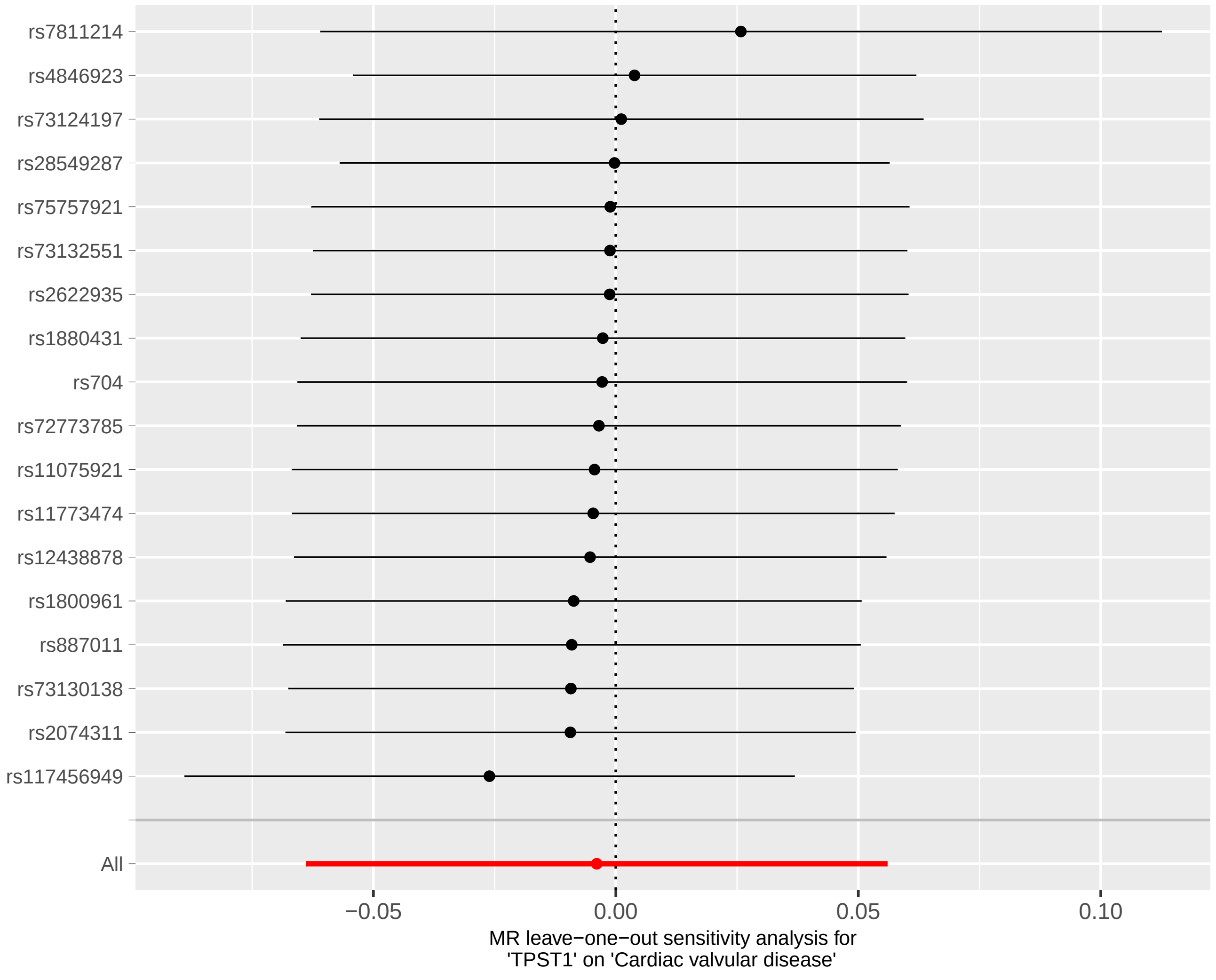
rs7811214
rs4846923
rs73124197
rs28549287
rs75757921
rs73132551
rs2622935
rs1880431
rs704
rs72773785
rs11075921
rs11773474
rs12438878
rs1800961
rs887011
rs73130138
rs2074311
rs117456949
All
−0.05
0.00
0.05
0.10
MR leave−one−out sensitivity analysis for
'TPST1' on 'Cardiac valvular disease'

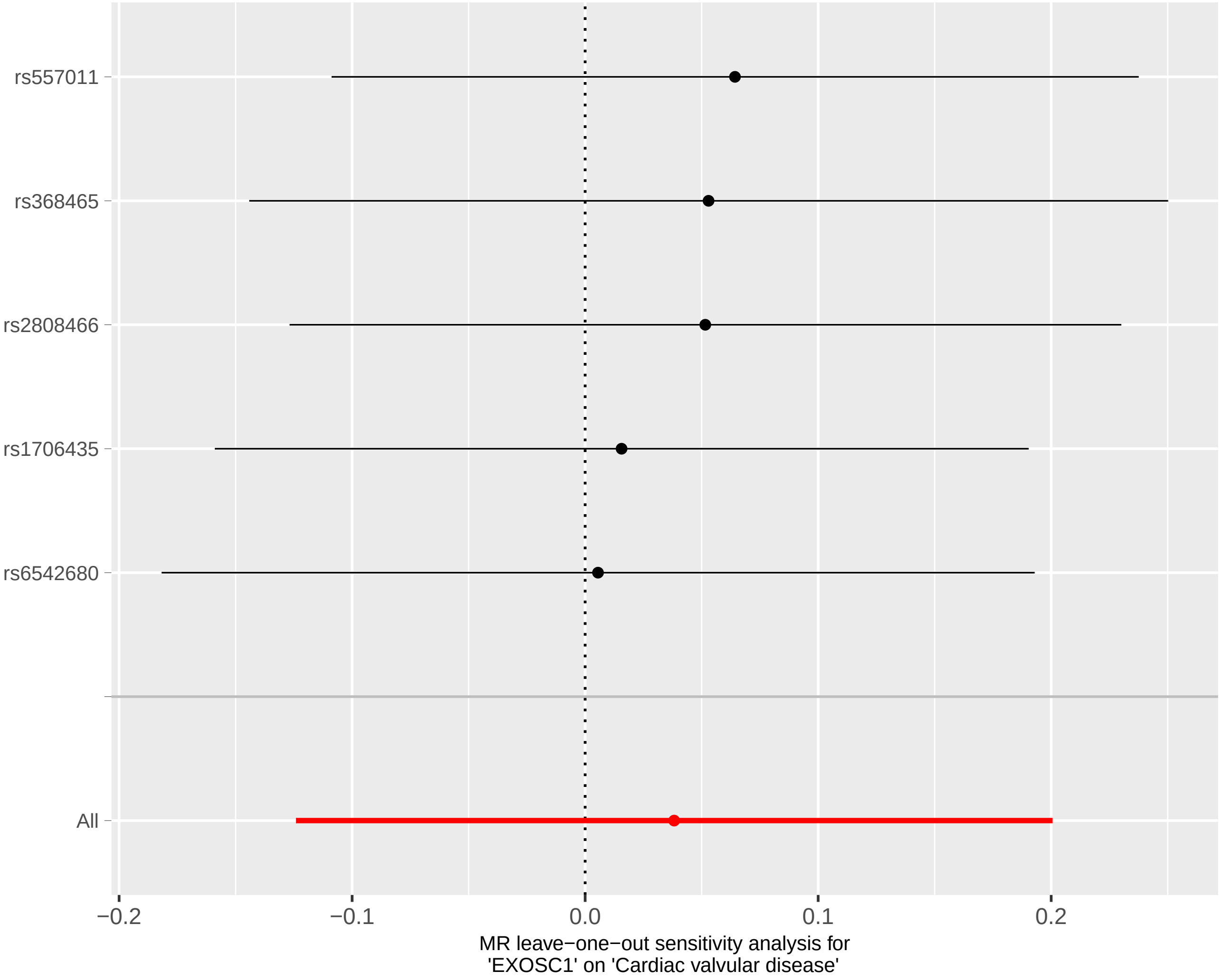
rs557011
rs368465
rs2808466
rs1706435
rs6542680
All
−0.2
−0.1
0.0
0.1
0.2
MR leave−one−out sensitivity analysis for
'EXOSC1' on 'Cardiac valvular disease'

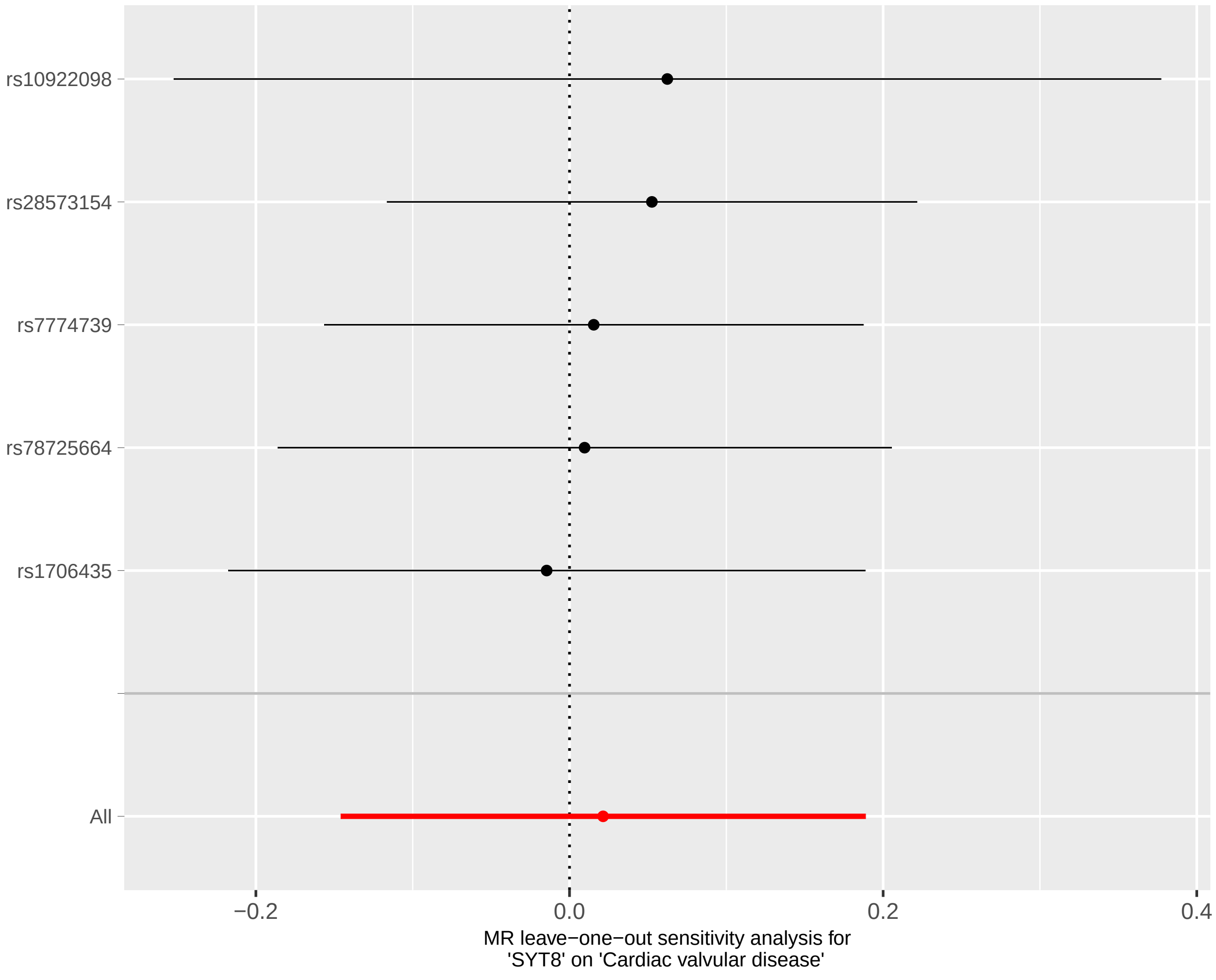
rs10922098
rs28573154
rs7774739
rs78725664
rs1706435
All
−0.2
0.0
0.2
0.4
MR leave−one−out sensitivity analysis for
'SYT8' on 'Cardiac valvular disease'

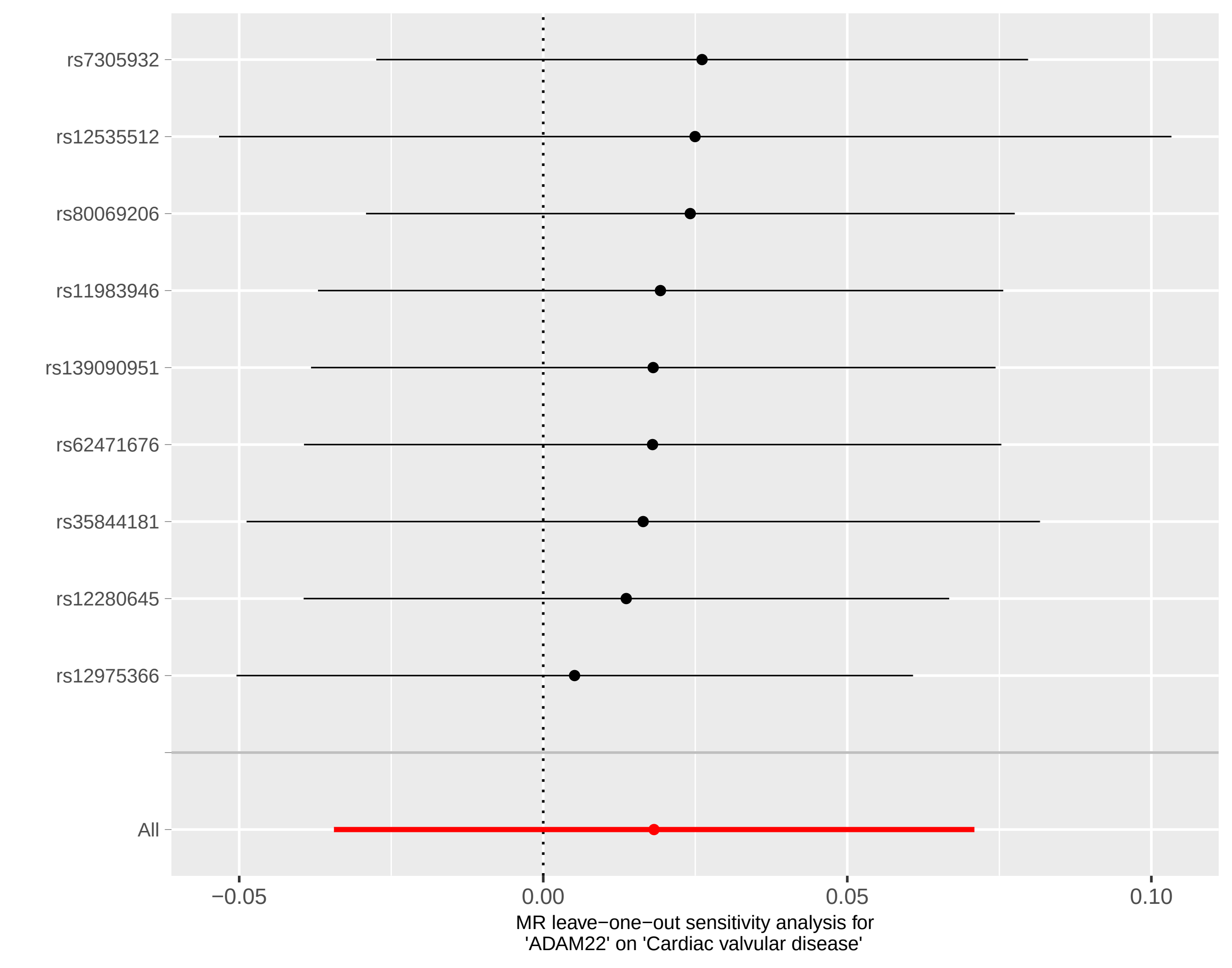
rs7305932
rs12535512
rs80069206
rs11983946
rs139090951
rs62471676
rs35844181
rs12280645
rs12975366
All
−0.05
0.00
0.05
0.10
MR leave−one−out sensitivity analysis for
'ADAM22' on 'Cardiac valvular disease'

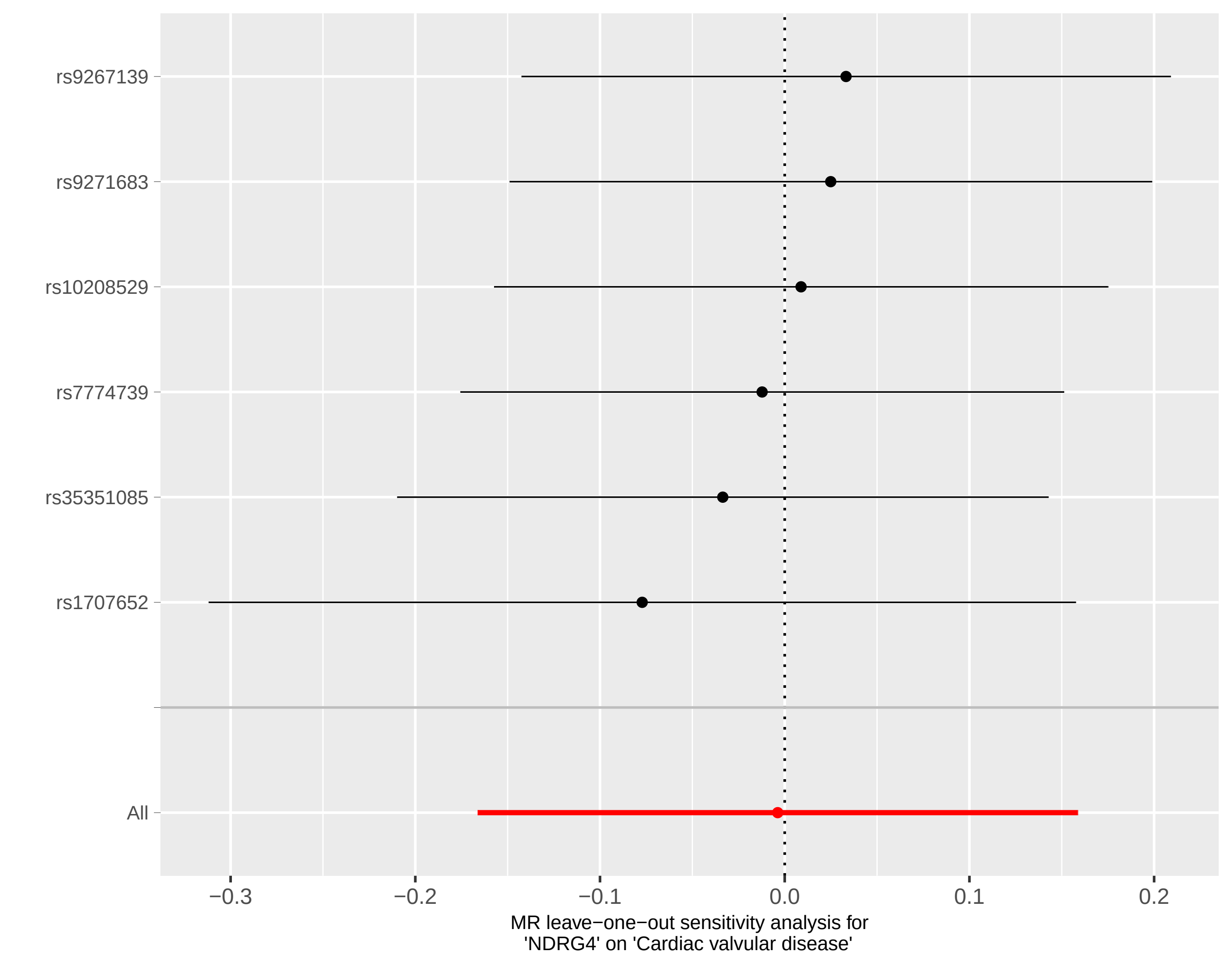
rs9267139
rs9271683
rs10208529
rs7774739
rs35351085
rs1707652
All
−0.3
−0.2
−0.1
0.0
0.1
0.2
MR leave−one−out sensitivity analysis for
'NDRG4' on 'Cardiac valvular disease'

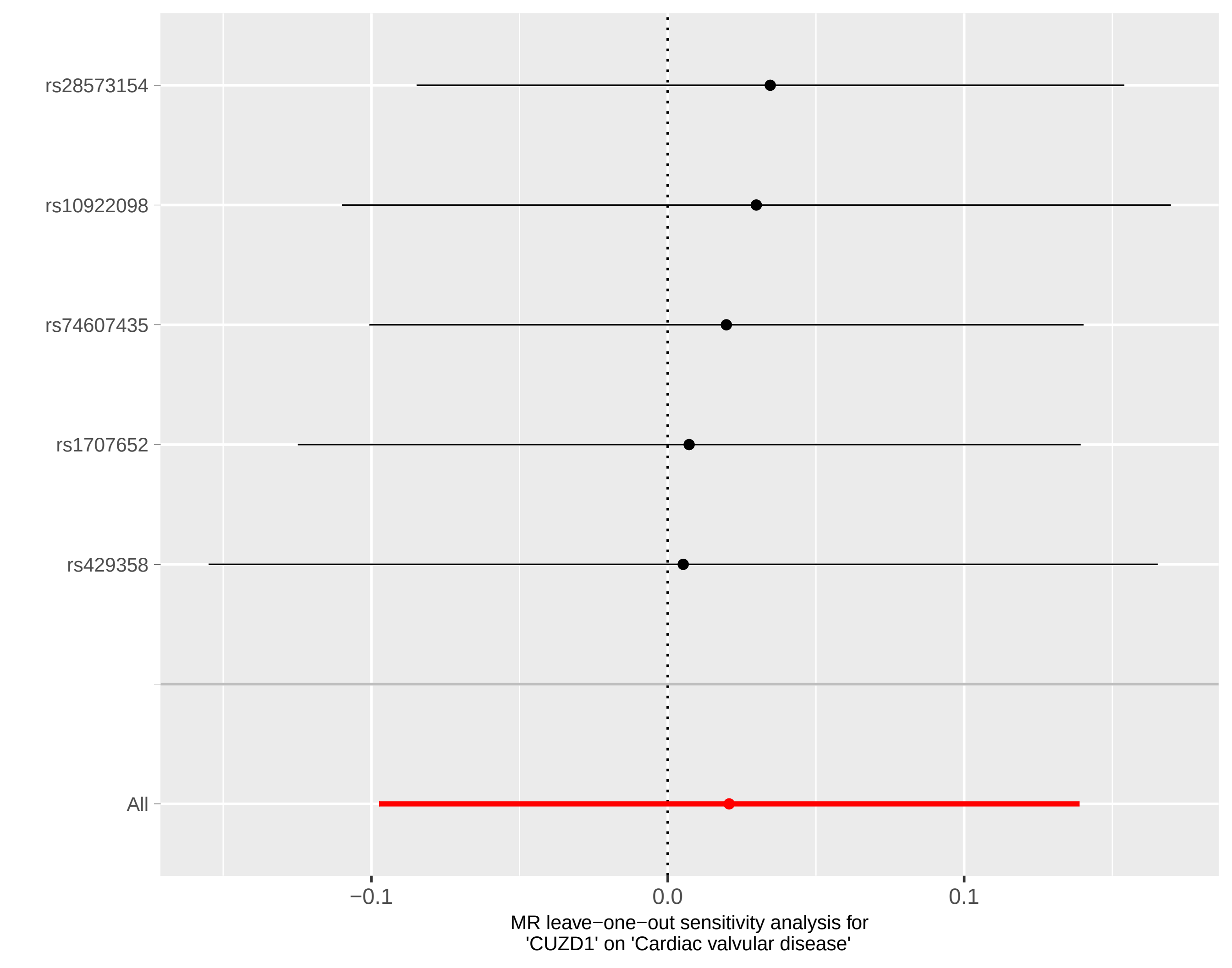
rs28573154
rs10922098
rs74607435
rs1707652
rs429358
All
−0.1
0.0
0.1
MR leave−one−out sensitivity analysis for
'CUZD1' on 'Cardiac valvular disease'

Insufficient number of SNPs

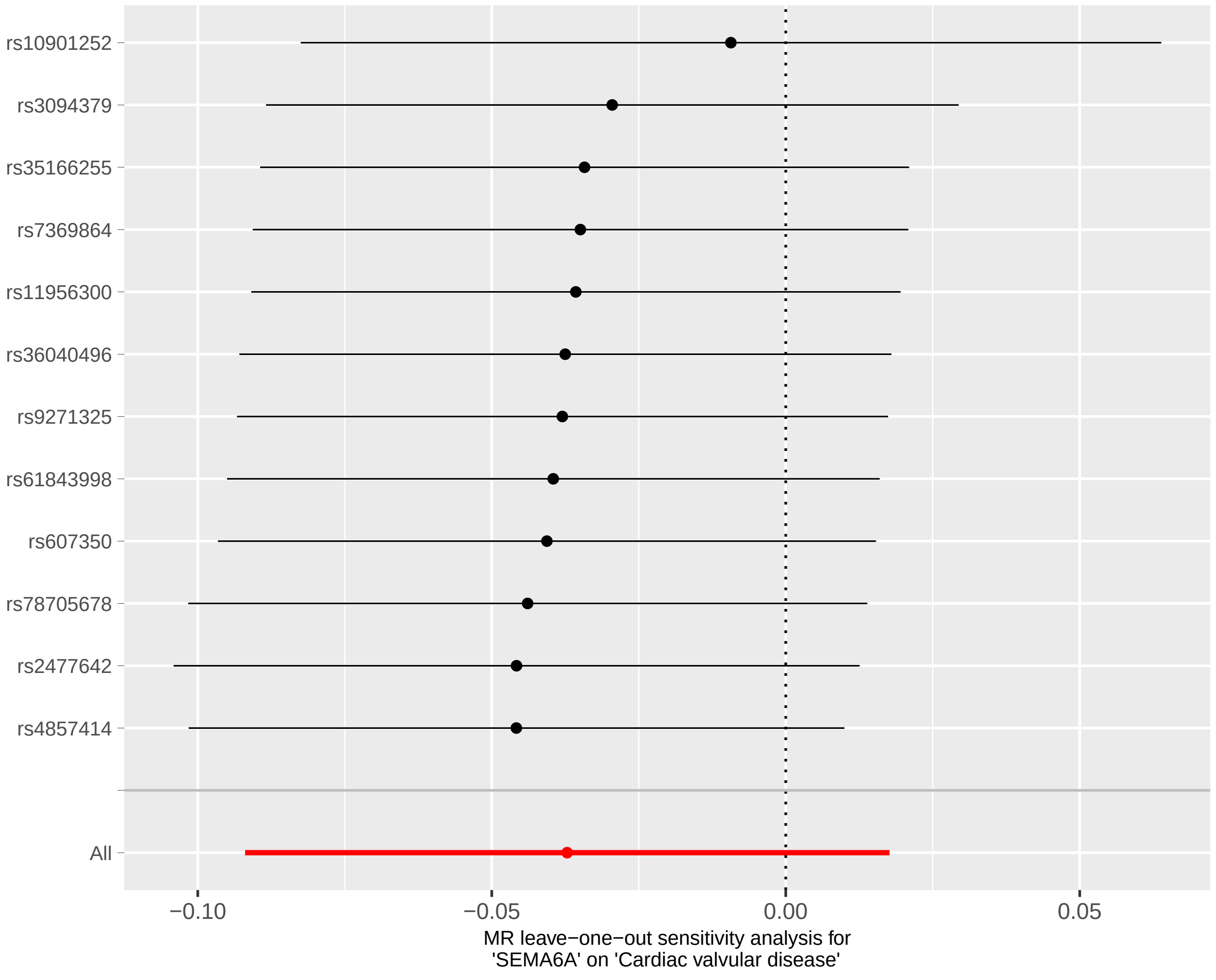

rs10901252
rs3094379
rs35166255
rs7369864
rs11956300
rs36040496
rs9271325
rs61843998
rs607350
rs78705678
rs2477642
rs4857414
All
−0.10
−0.05
0.00
0.05
MR leave−one−out sensitivity analysis for
'SEMA6A' on 'Cardiac valvular disease'

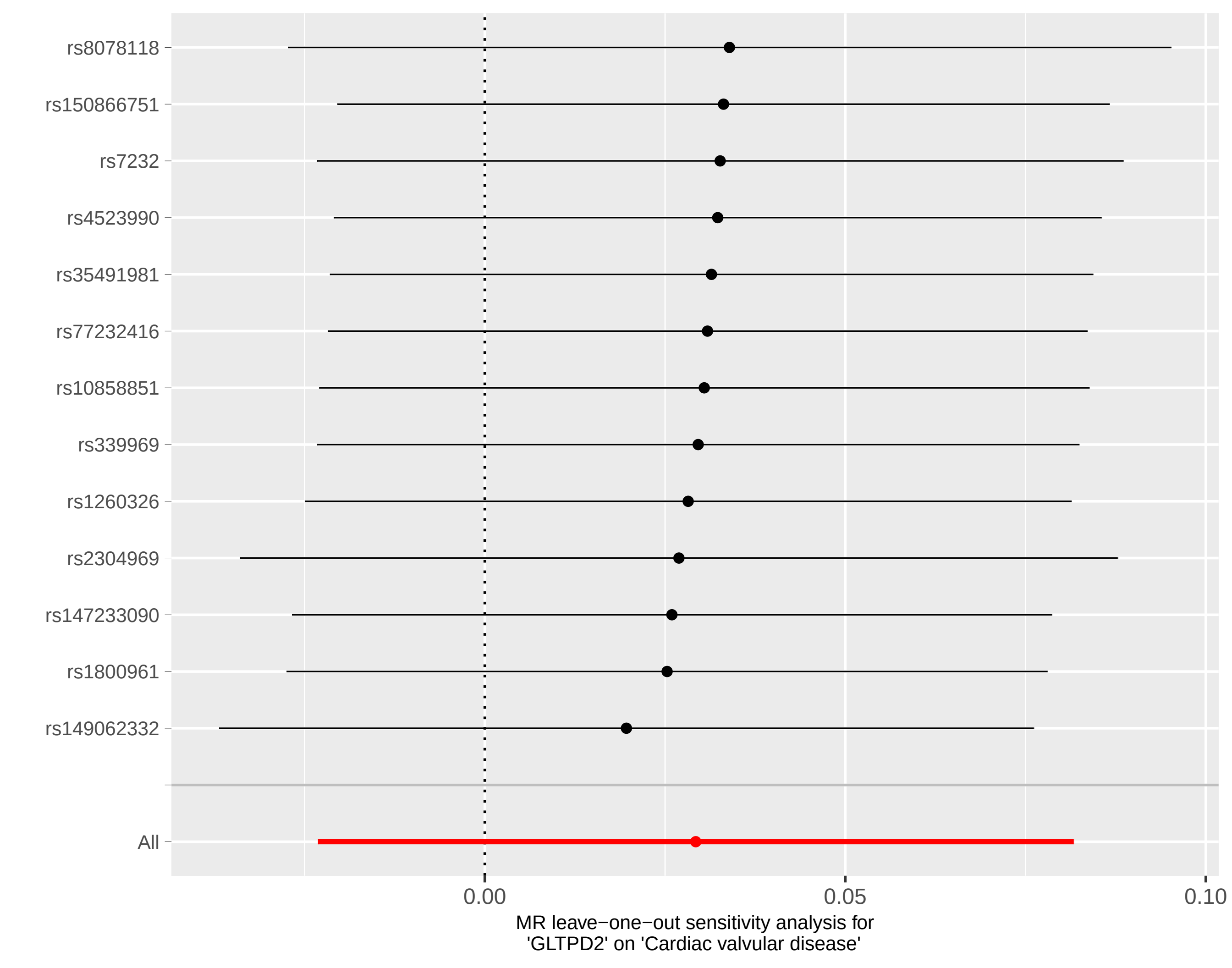
rs8078118
rs150866751
rs7232
rs4523990
rs35491981
rs77232416
rs10858851
rs339969
rs1260326
rs2304969
rs147233090
rs1800961
rs149062332
All
0.00
0.05
0.10
MR leave−one−out sensitivity analysis for
'GLTPD2' on 'Cardiac valvular disease'

Insufficient number of SNPs

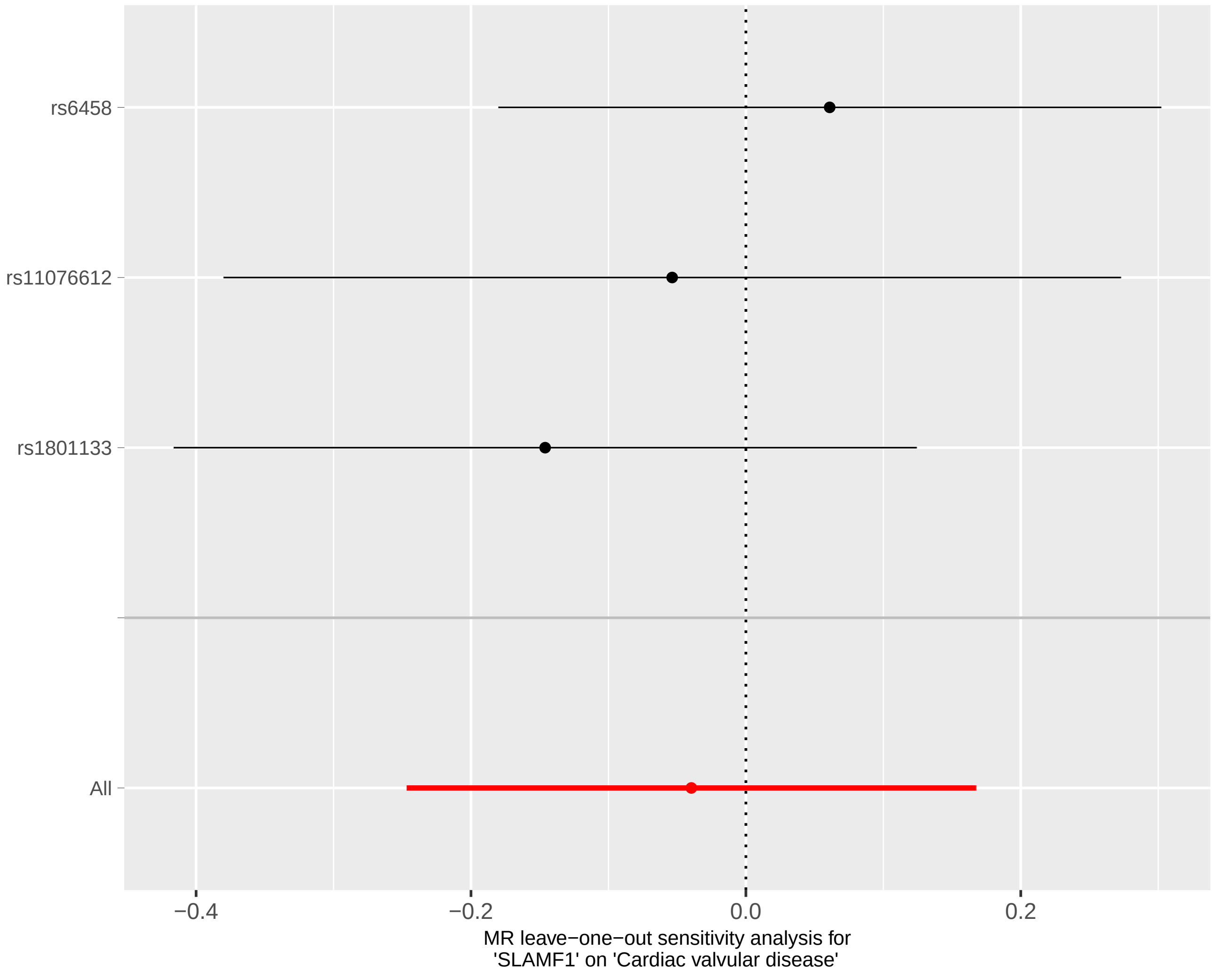
rs6458
rs11076612
rs1801133
All
−0.4
−0.2
0.0
0.2
MR leave−one−out sensitivity analysis for
'SLAMF1' on 'Cardiac valvular disease'

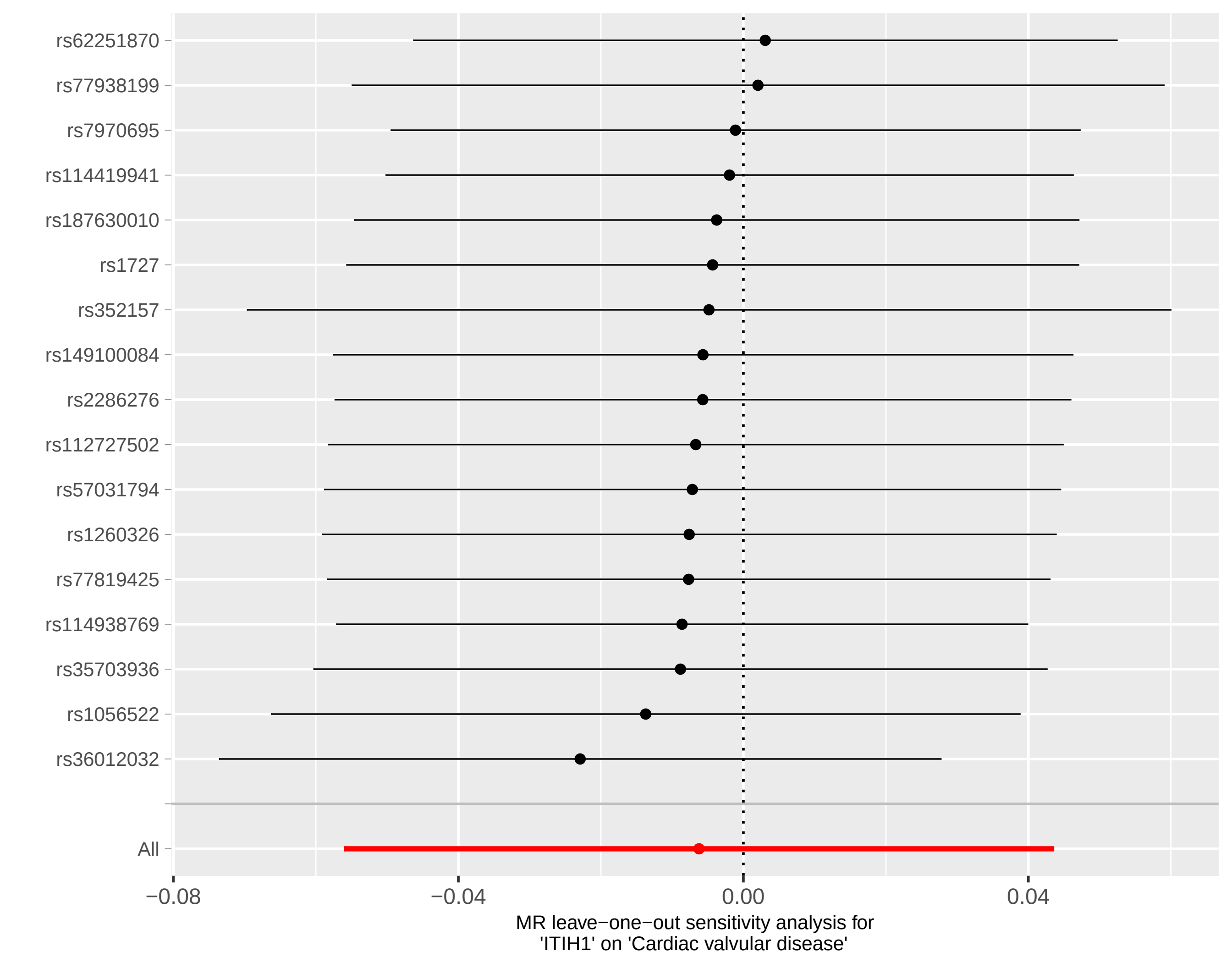

rs62251870
rs77938199
rs7970695
rs114419941
rs187630010
rs1727
rs352157
rs149100084
rs2286276
rs112727502
rs57031794
rs1260326
rs77819425
rs114938769
rs35703936
rs1056522
rs36012032
All
−0.08
−0.04
0.00
0.04
MR leave−one−out sensitivity analysis for
'ITIH1' on 'Cardiac valvular disease'

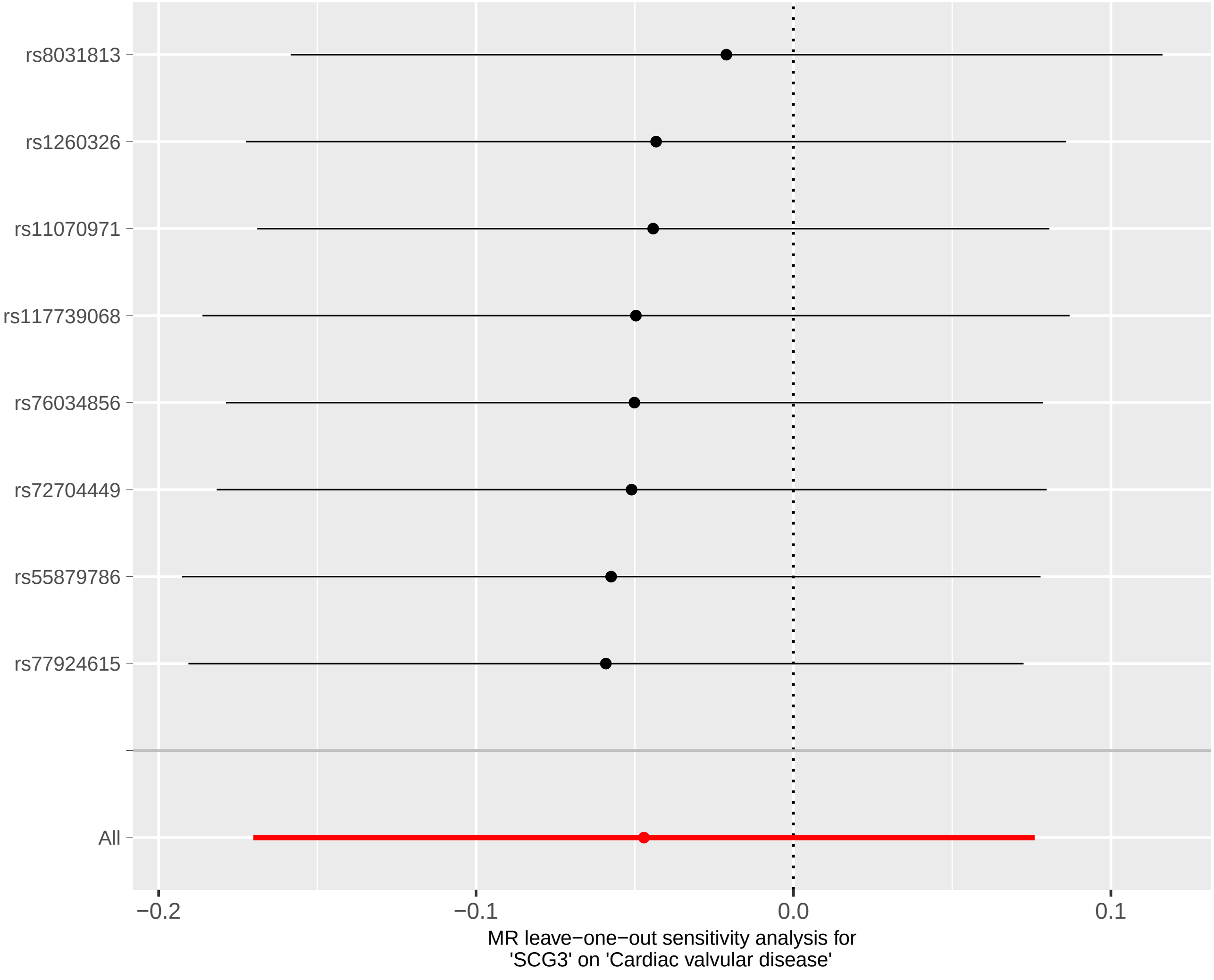
rs8031813
rs1260326
rs11070971
rs117739068
rs76034856
rs72704449
rs55879786
rs77924615
All
−0.2
−0.1
0.0
0.1
MR leave−one−out sensitivity analysis for
'SCG3' on 'Cardiac valvular disease'

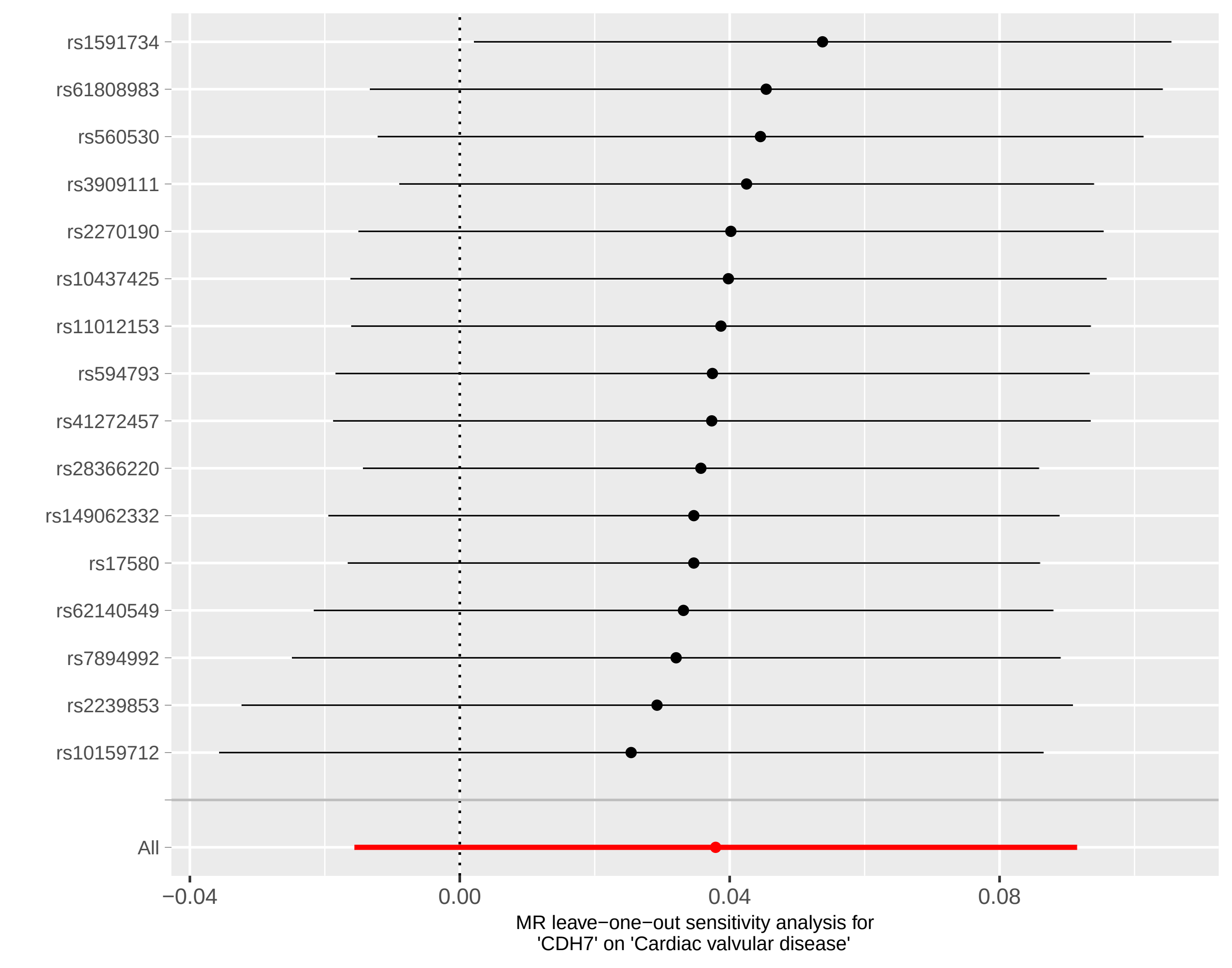
rs1591734
rs61808983
rs560530
rs3909111
rs2270190
rs10437425
rs11012153
rs594793
rs41272457
rs28366220
rs149062332
rs17580
rs62140549
rs7894992
rs2239853
rs10159712
All
−0.04
0.00
0.04
0.08
MR leave−one−out sensitivity analysis for
'CDH7' on 'Cardiac valvular disease'

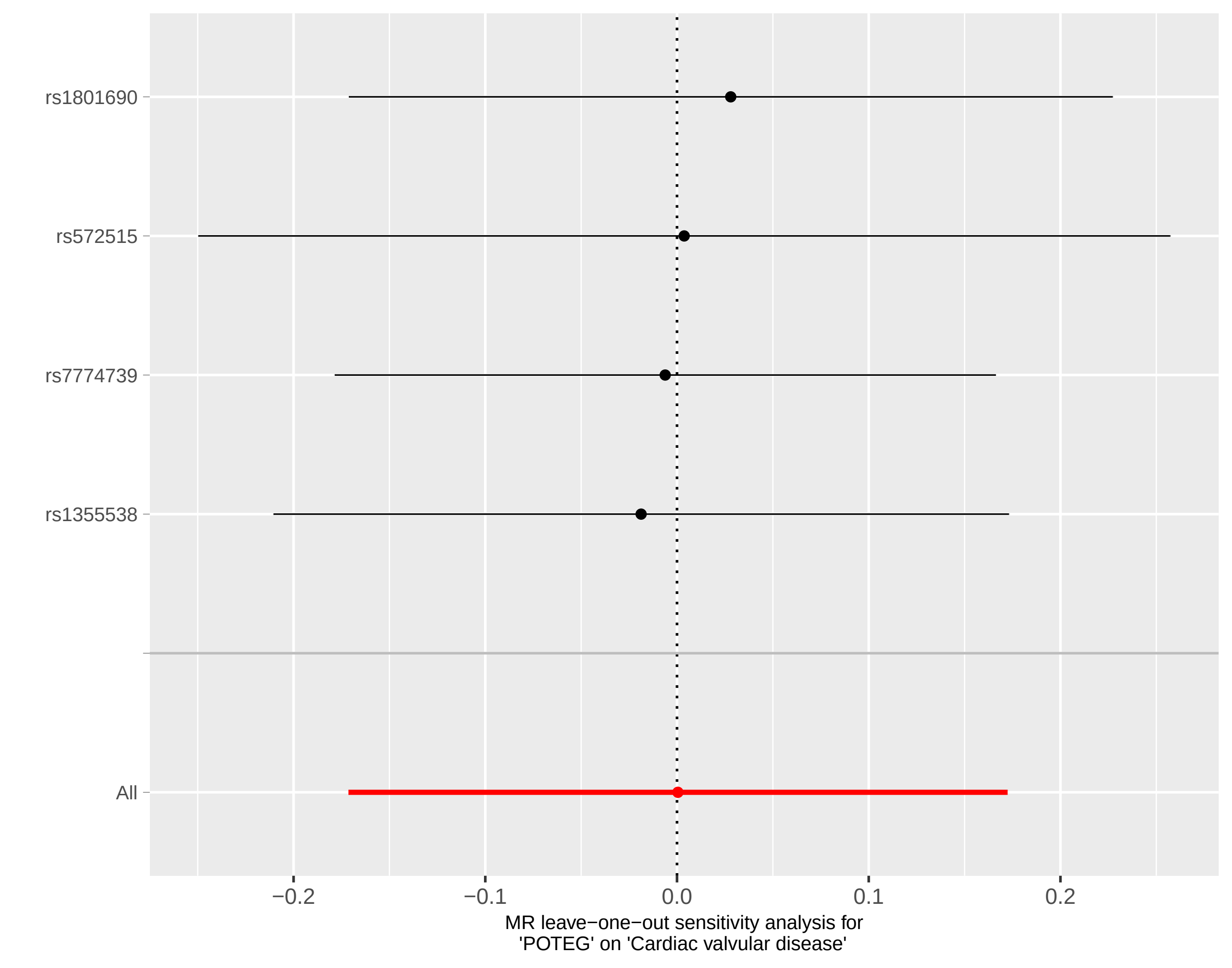
rs1801690
rs572515
rs7774739
rs1355538
All
−0.2
−0.1
0.0
0.1
0.2
MR leave−one−out sensitivity analysis for
'POTEG' on 'Cardiac valvular disease'

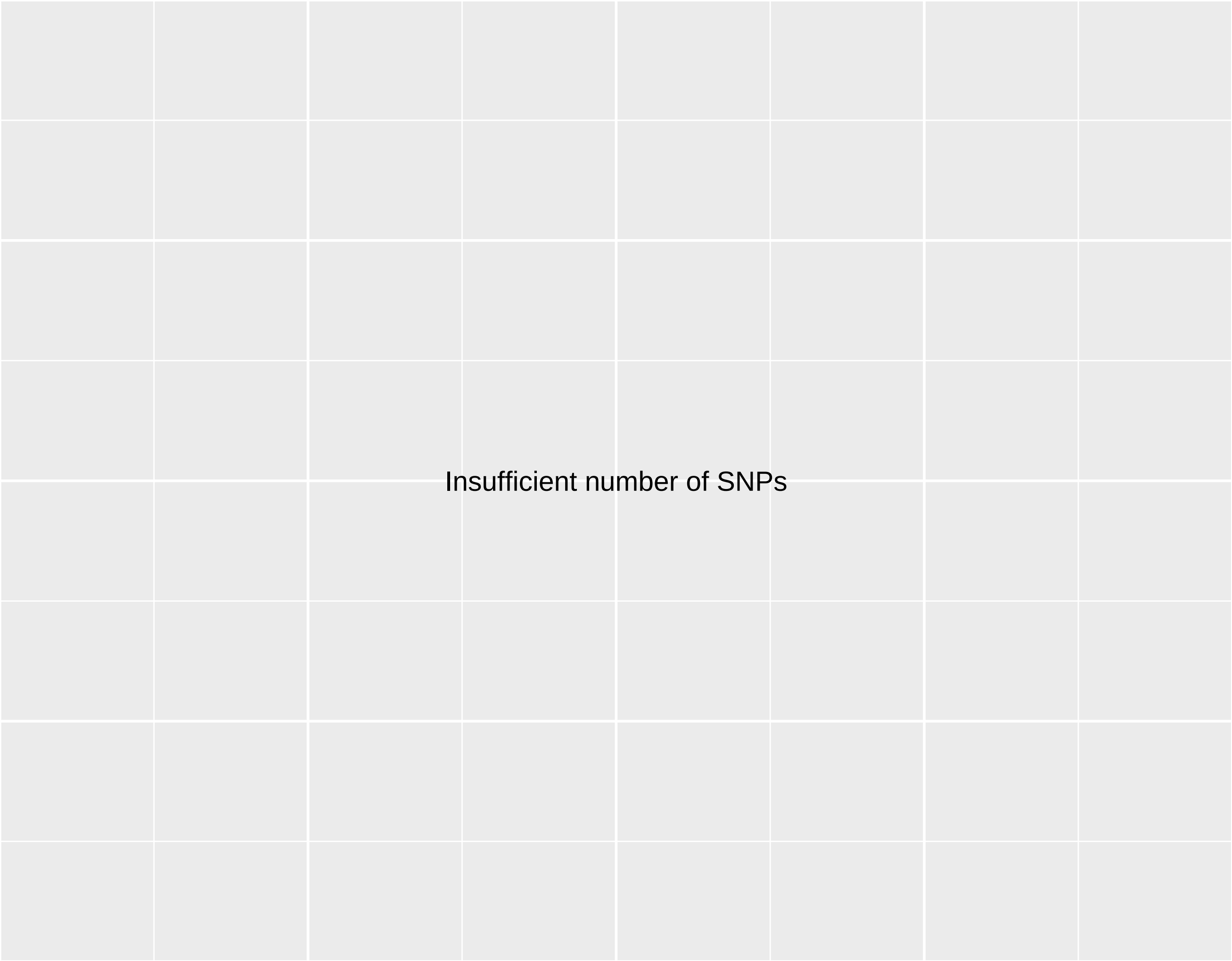
Insufficient number of SNPs

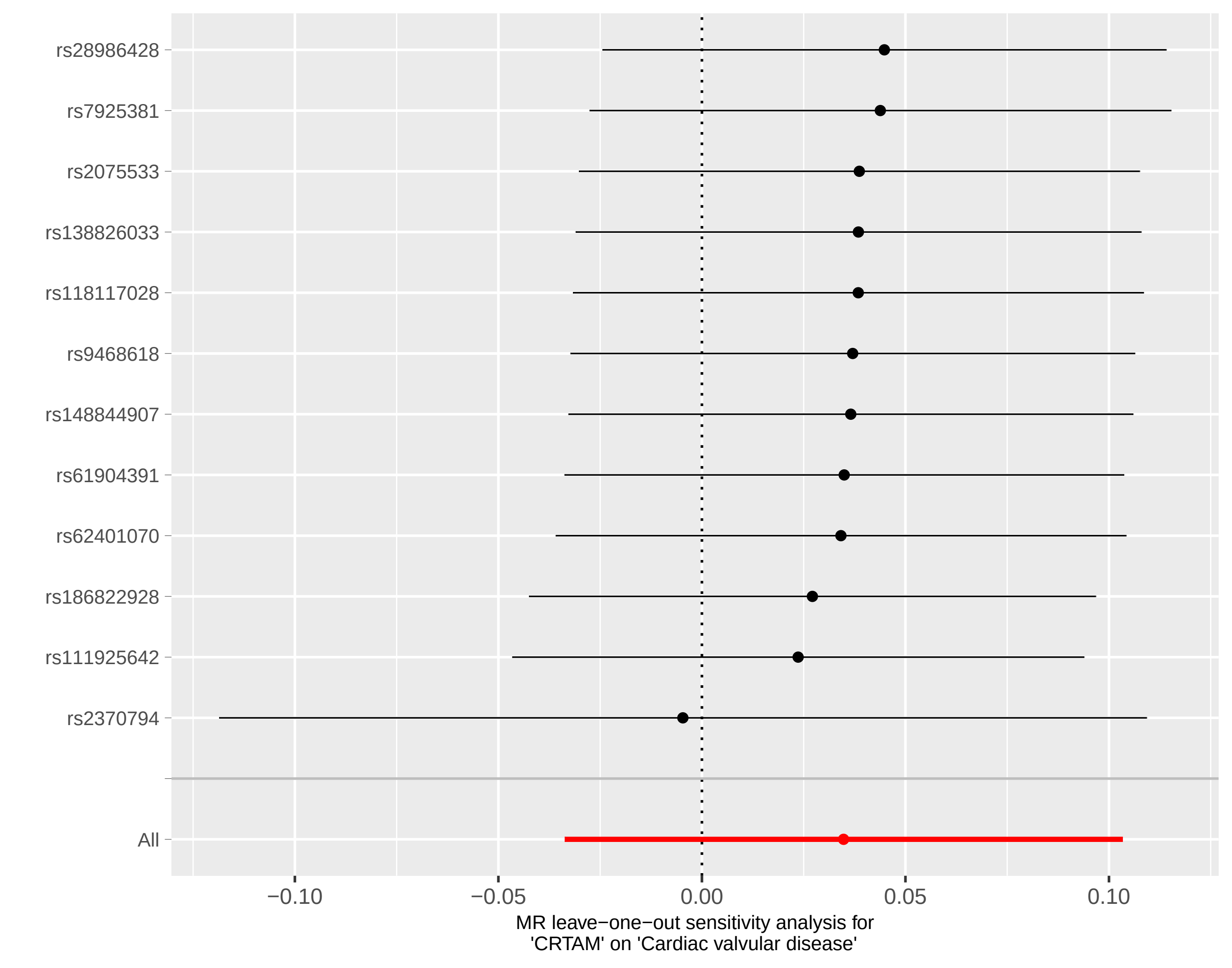
rs28986428
rs7925381
rs2075533
rs138826033
rs118117028
rs9468618
rs148844907
rs61904391
rs62401070
rs186822928
rs111925642
rs2370794
All
−0.10
−0.05
0.00
0.05
0.10
MR leave−one−out sensitivity analysis for
'CRTAM' on 'Cardiac valvular disease'

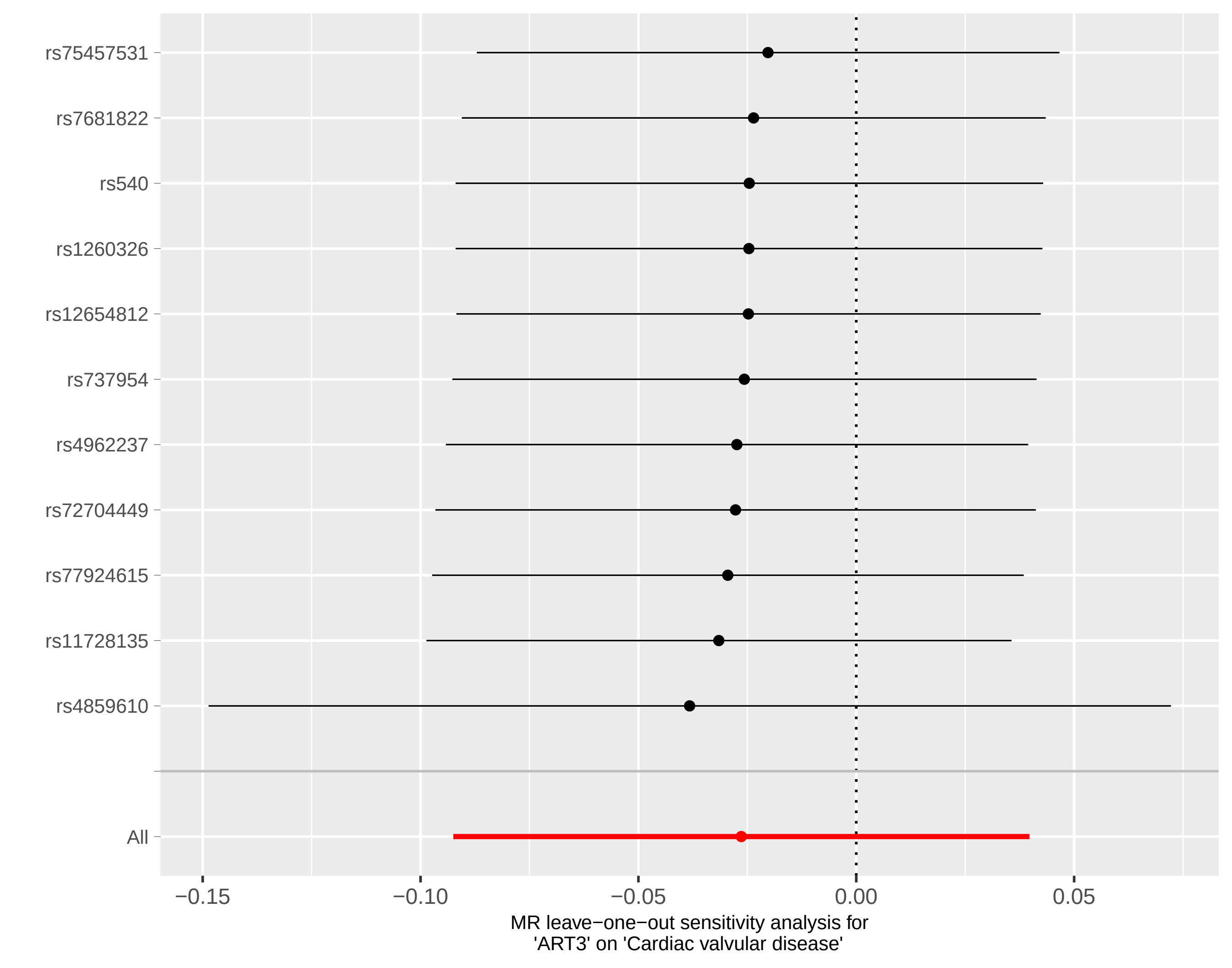
rs75457531
rs7681822
rs540
rs1260326
rs12654812
rs737954
rs4962237
rs72704449
rs77924615
rs11728135
rs4859610
All
−0.15
−0.10
−0.05
0.00
0.05
MR leave−one−out sensitivity analysis for
'ART3' on 'Cardiac valvular disease'

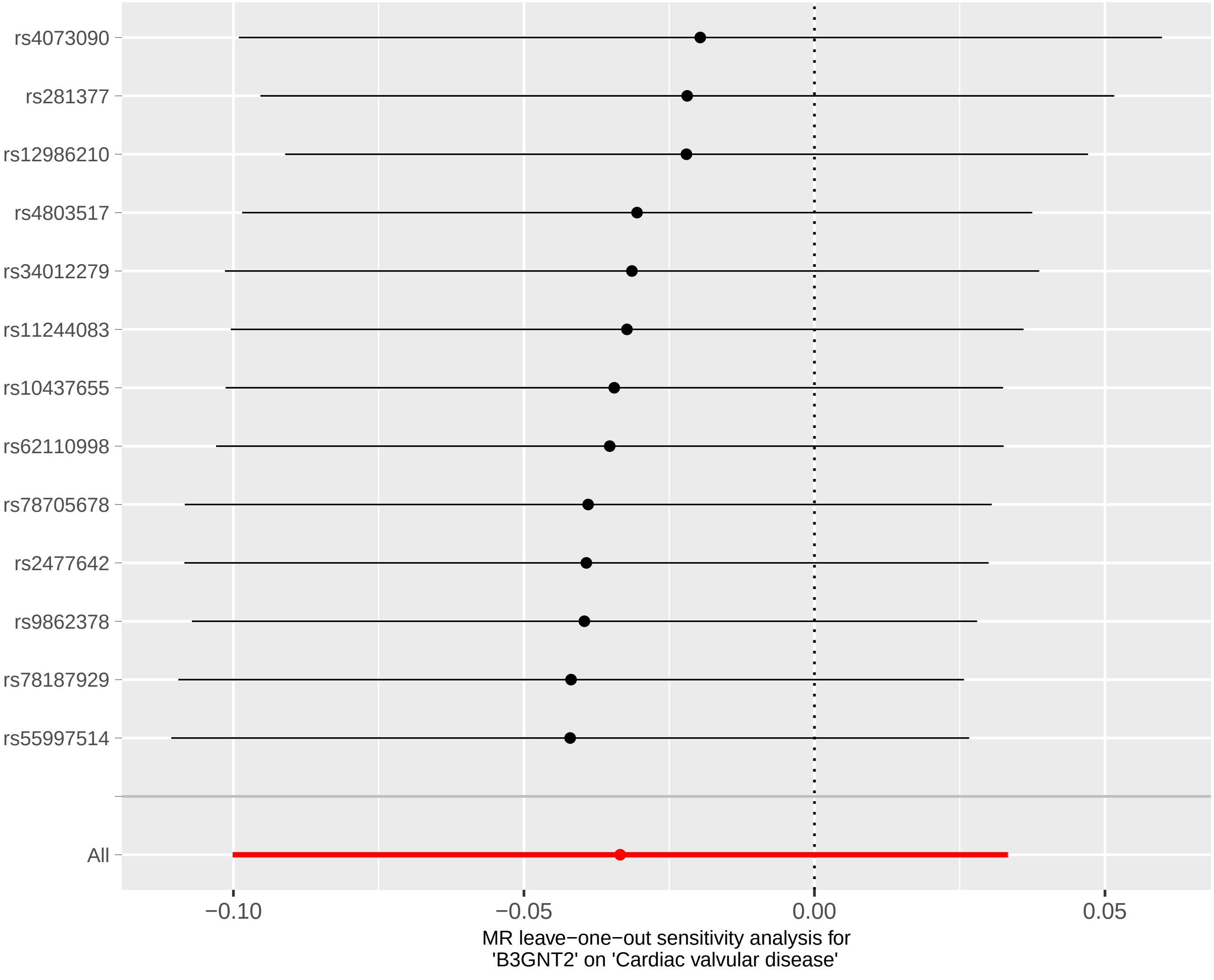

rs4073090
rs281377
rs12986210
rs4803517
rs34012279
rs11244083
rs10437655
rs62110998
rs78705678
rs2477642
rs9862378
rs78187929
rs55997514
All
−0.10
−0.05
0.00
0.05
MR leave−one−out sensitivity analysis for
'B3GNT2' on 'Cardiac valvular disease'

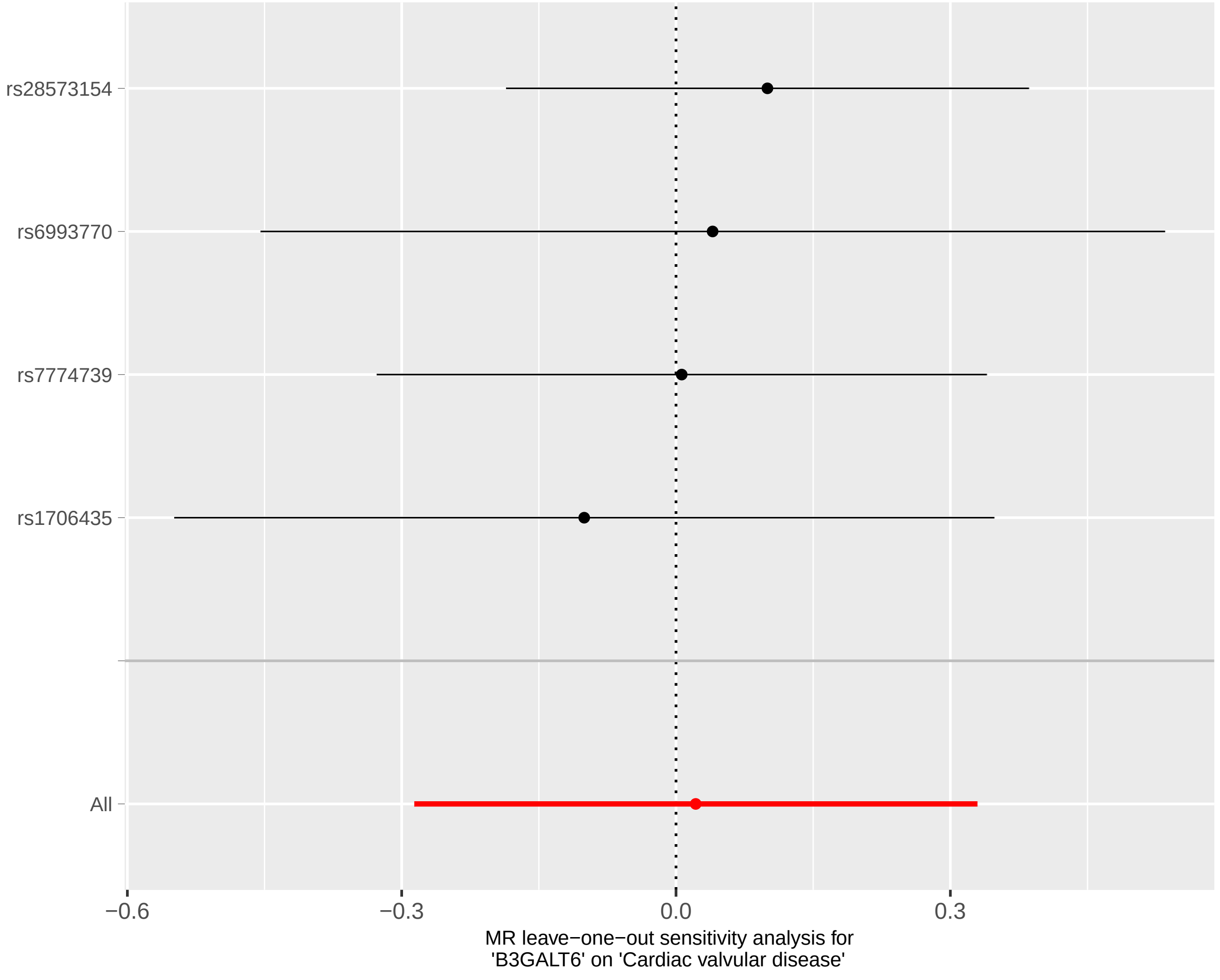

rs28573154
rs6993770
rs7774739
rs1706435
All
−0.6
−0.3
0.0
0.3
MR leave−one−out sensitivity analysis for
'B3GALT6' on 'Cardiac valvular disease'

Insufficient number of SNPs

Insufficient number of SNPs

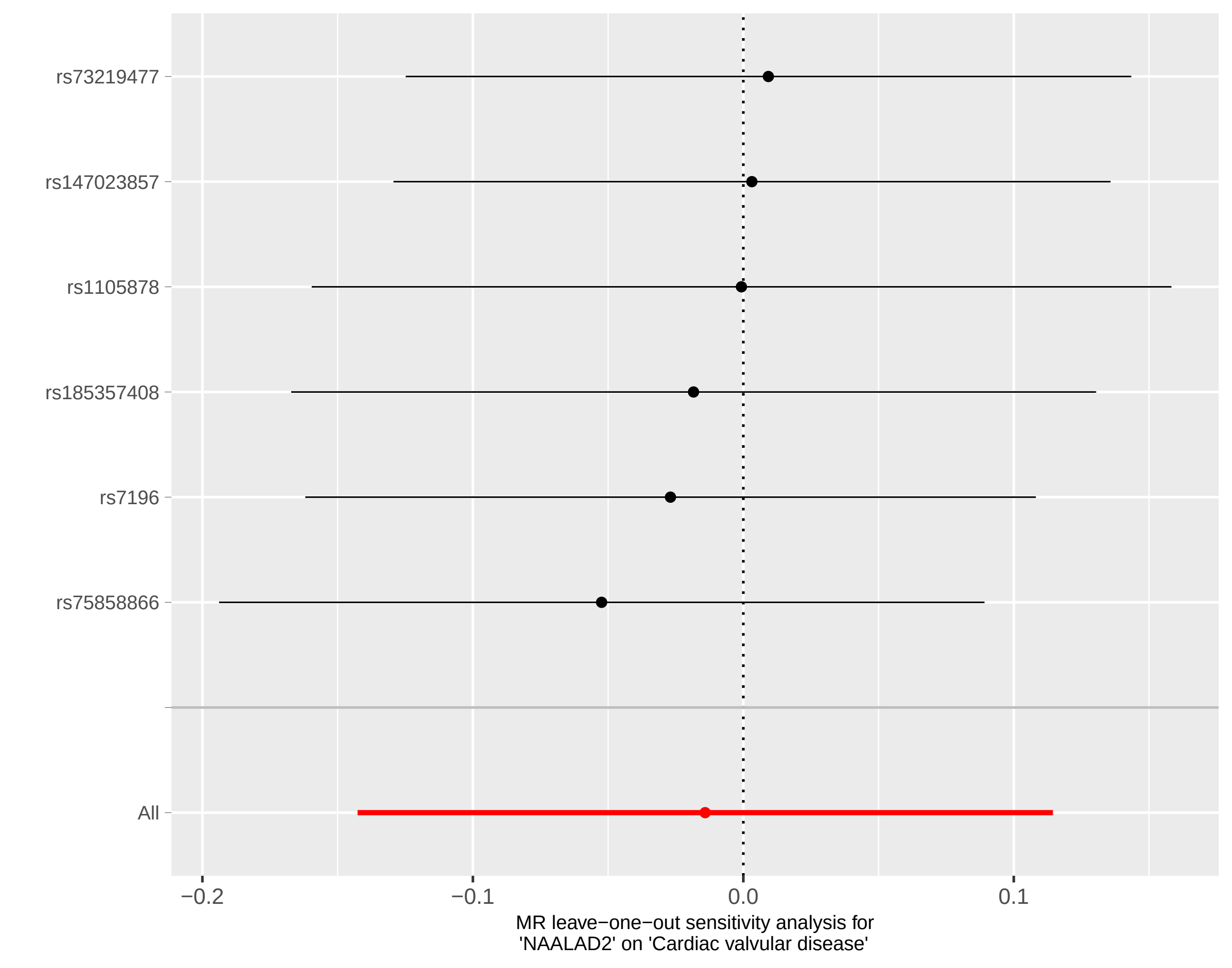

rs73219477
rs147023857
rs1105878
rs185357408
rs7196
rs75858866
All
−0.2
−0.1
0.0
0.1
MR leave−one−out sensitivity analysis for
'NAALAD2' on 'Cardiac valvular disease'

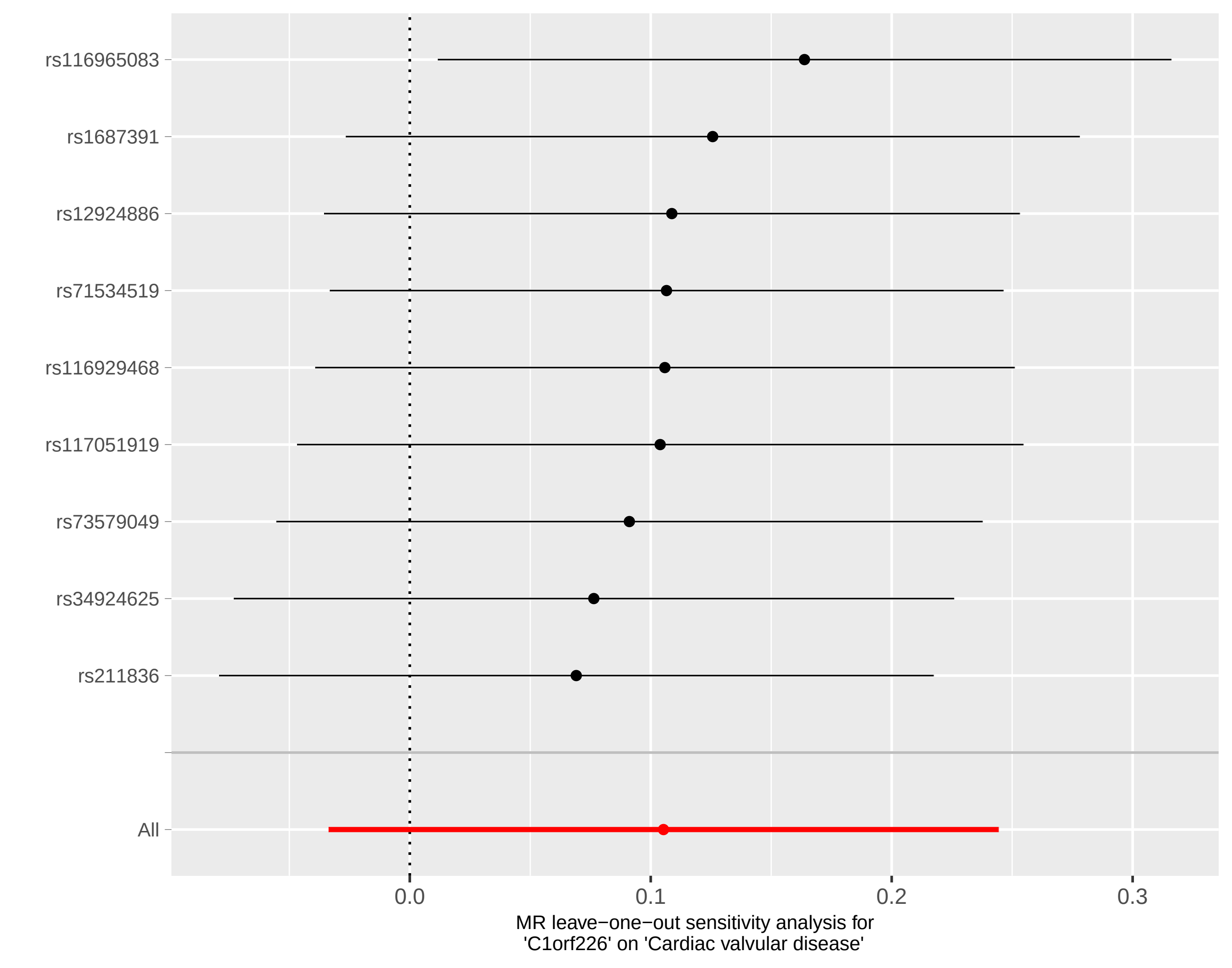
rs116965083
rs1687391
rs12924886
rs71534519
rs116929468
rs117051919
rs73579049
rs34924625
rs211836
All
0.0
0.1
0.2
0.3
MR leave−one−out sensitivity analysis for
'C1orf226' on 'Cardiac valvular disease'

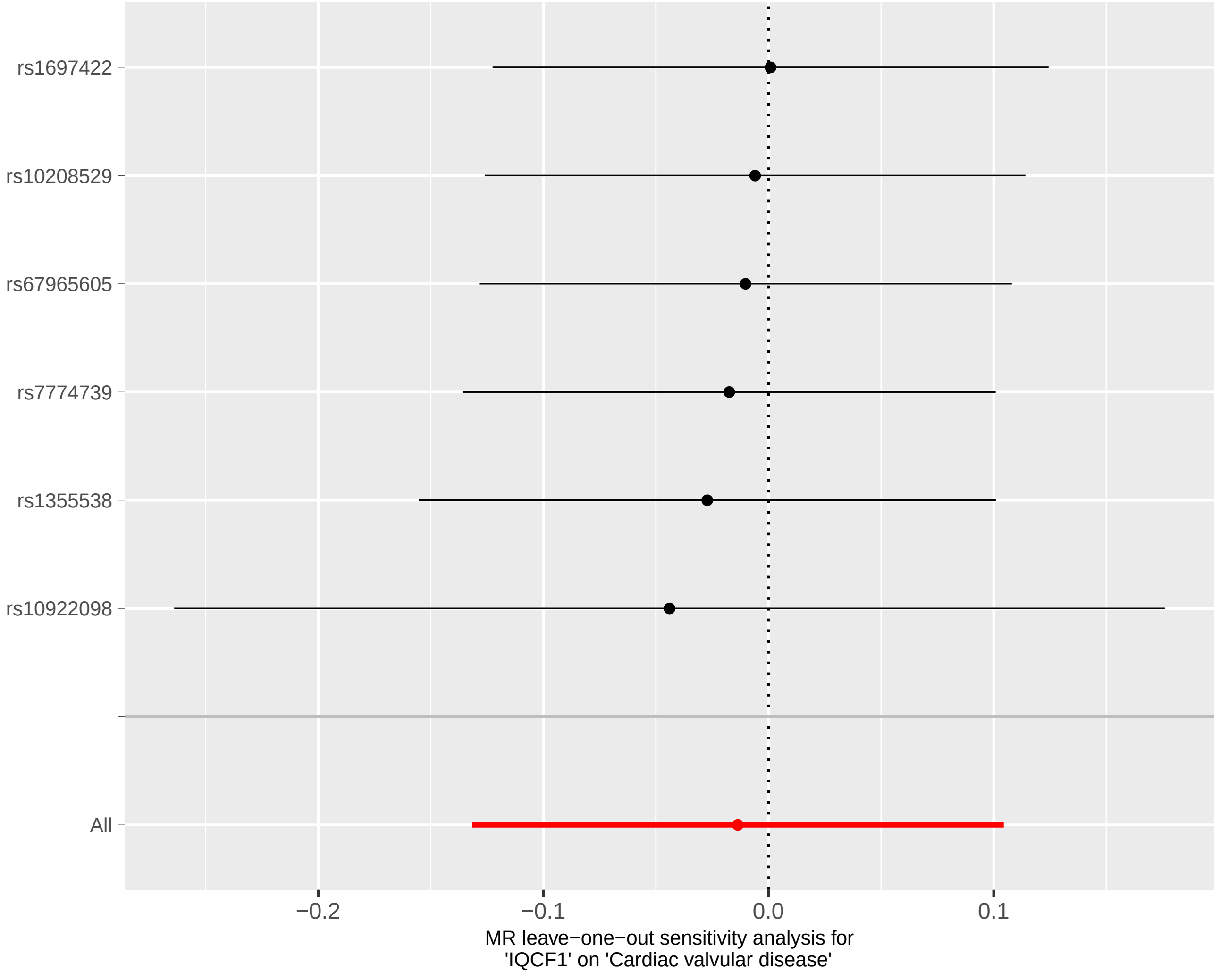

rs1697422
rs10208529
rs67965605
rs7774739
rs1355538
rs10922098
All
−0.2
−0.1
0.0
0.1
MR leave−one−out sensitivity analysis for
'IQCF1' on 'Cardiac valvular disease'

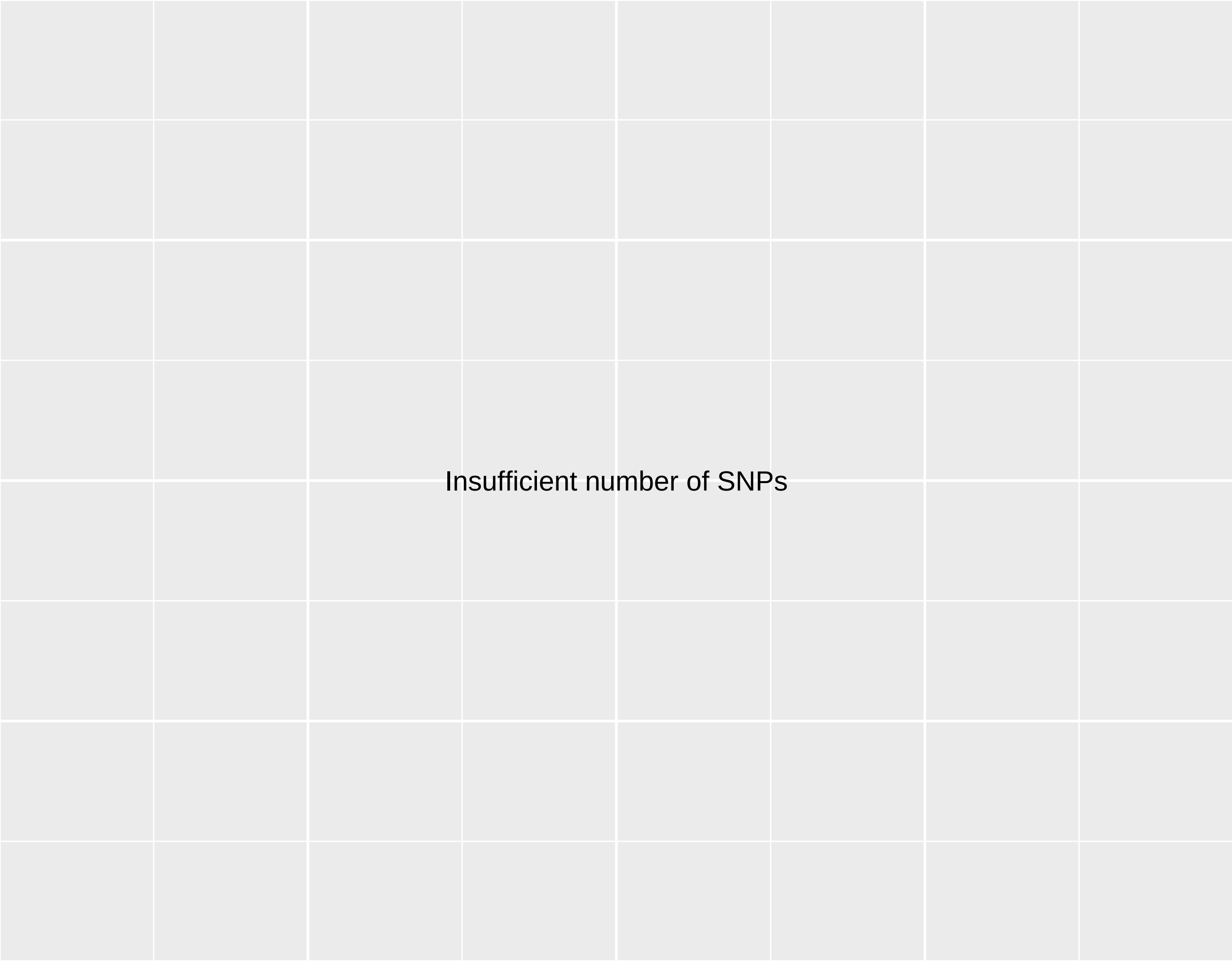
Insufficient number of SNPs

Insufficient number of SNPs

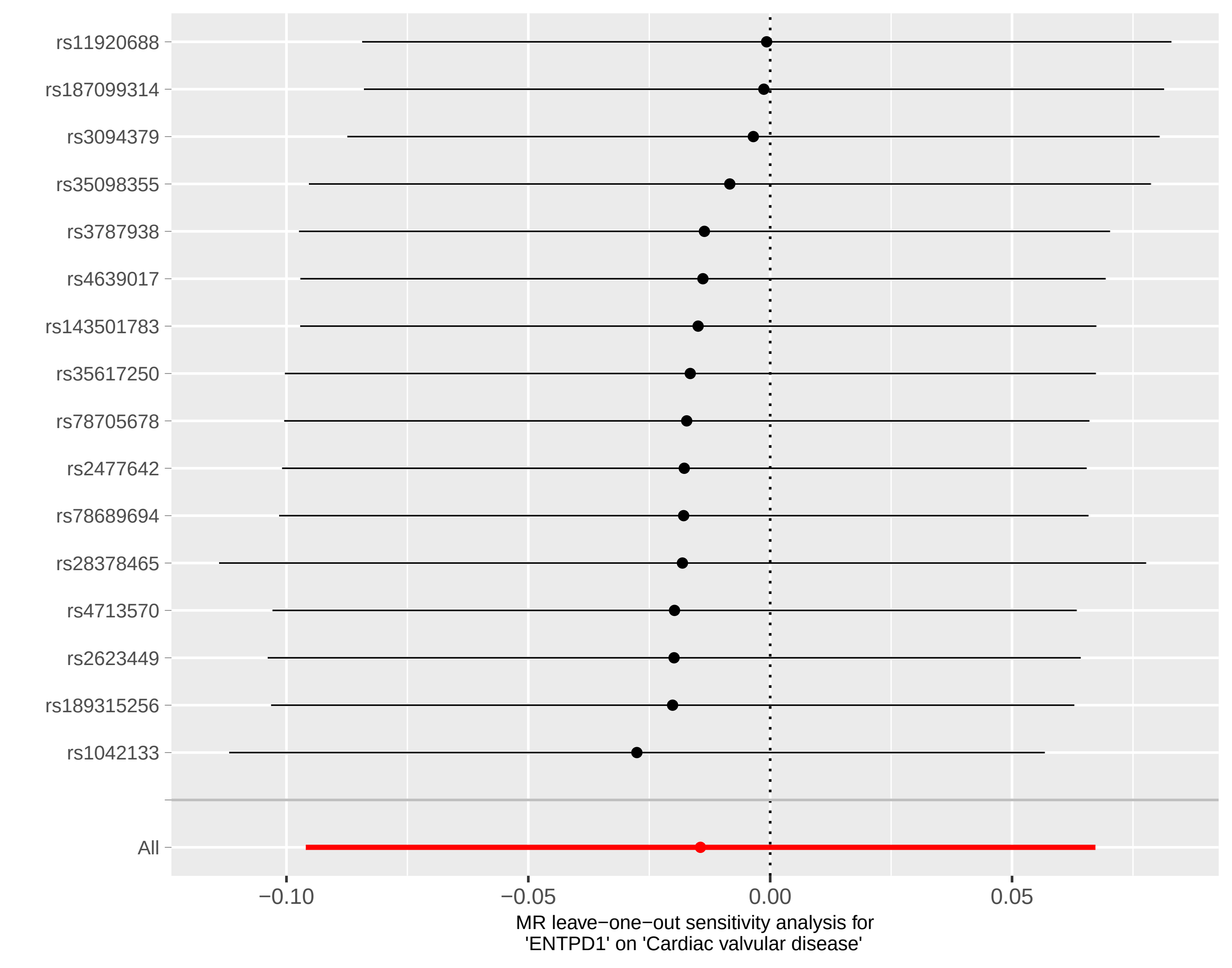
rs11920688
rs187099314
rs3094379
rs35098355
rs3787938
rs4639017
rs143501783
rs35617250
rs78705678
rs2477642
rs78689694
rs28378465
rs4713570
rs2623449
rs189315256
rs1042133
All
−0.10
−0.05
0.00
0.05
MR leave−one−out sensitivity analysis for
'ENTPD1' on 'Cardiac valvular disease'

Insufficient number of SNPs

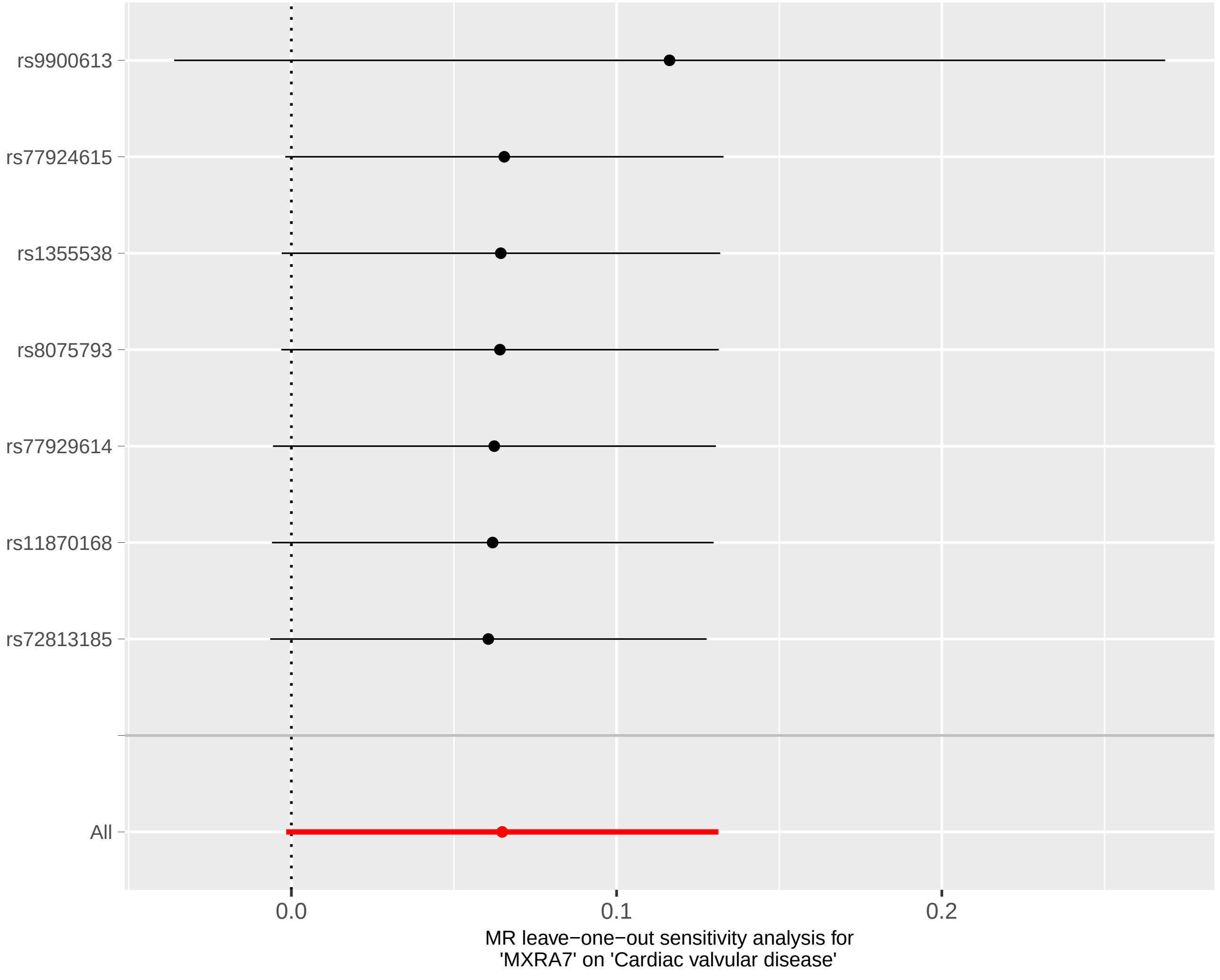
rs9900613
rs77924615
rs1355538
rs8075793
rs77929614
rs11870168
rs72813185
All
0.0
0.1
0.2
MR leave−one−out sensitivity analysis for
'MXRA7' on 'Cardiac valvular disease'

Insufficient number of SNPs

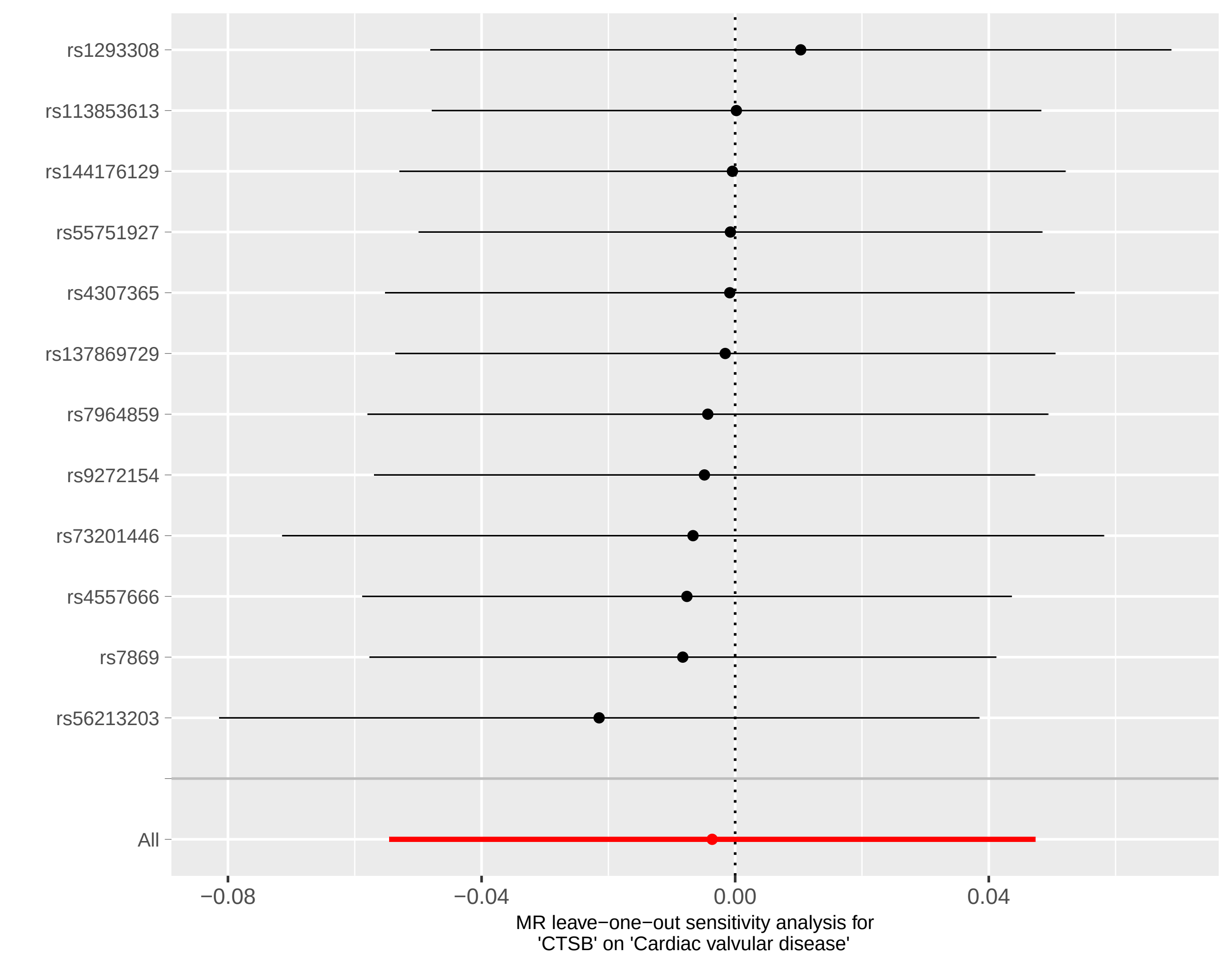
rs1293308
rs113853613
rs144176129
rs55751927
rs4307365
rs137869729
rs7964859
rs9272154
rs73201446
rs4557666
rs7869
rs56213203
All
−0.08
−0.04
0.00
0.04
MR leave−one−out sensitivity analysis for
'CTSB' on 'Cardiac valvular disease'

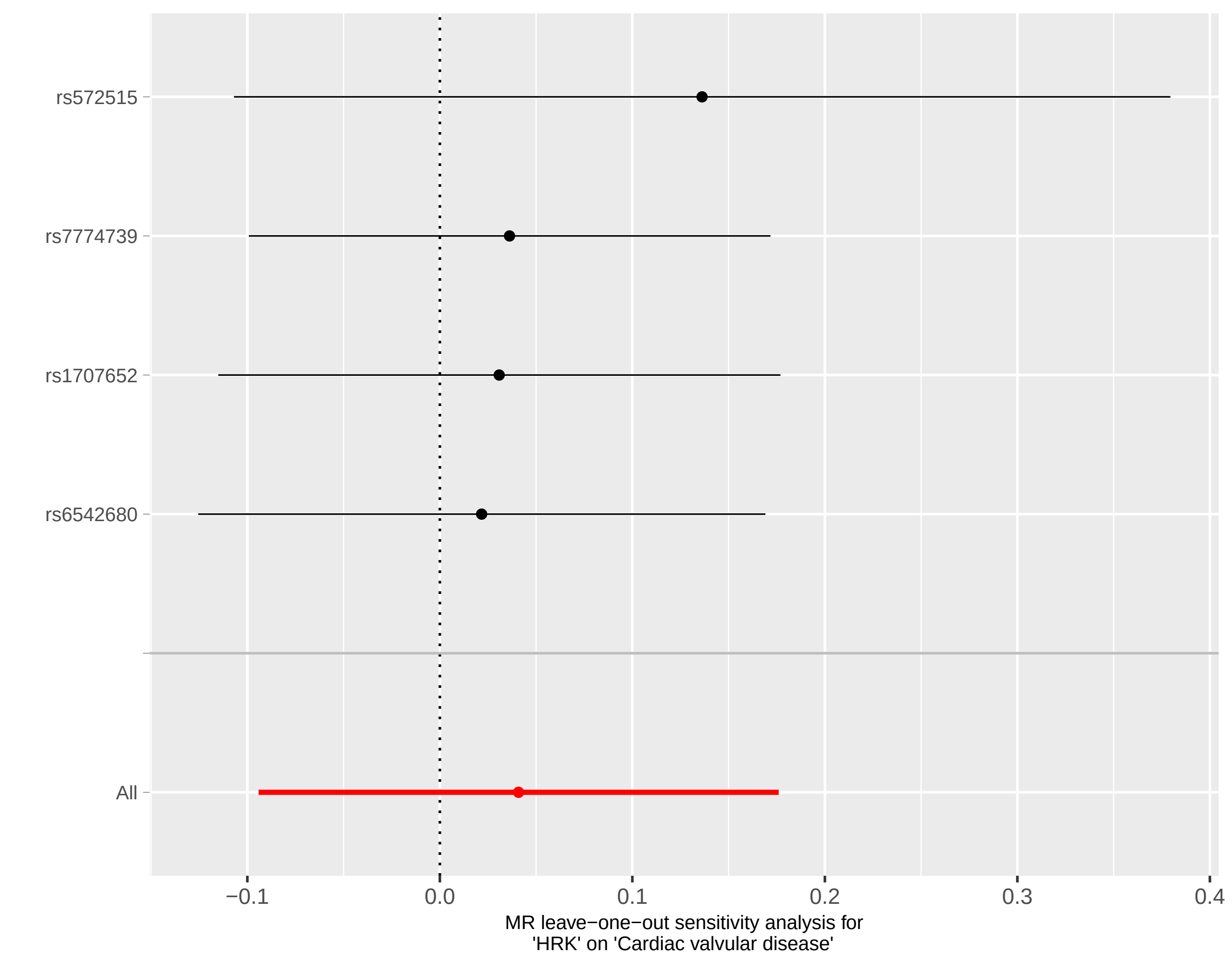
rs572515
rs7774739
rs1707652
rs6542680
All
−0.1
0.0
0.1
0.2
0.3
0.4
MR leave−one−out sensitivity analysis for
'HRK' on 'Cardiac valvular disease'

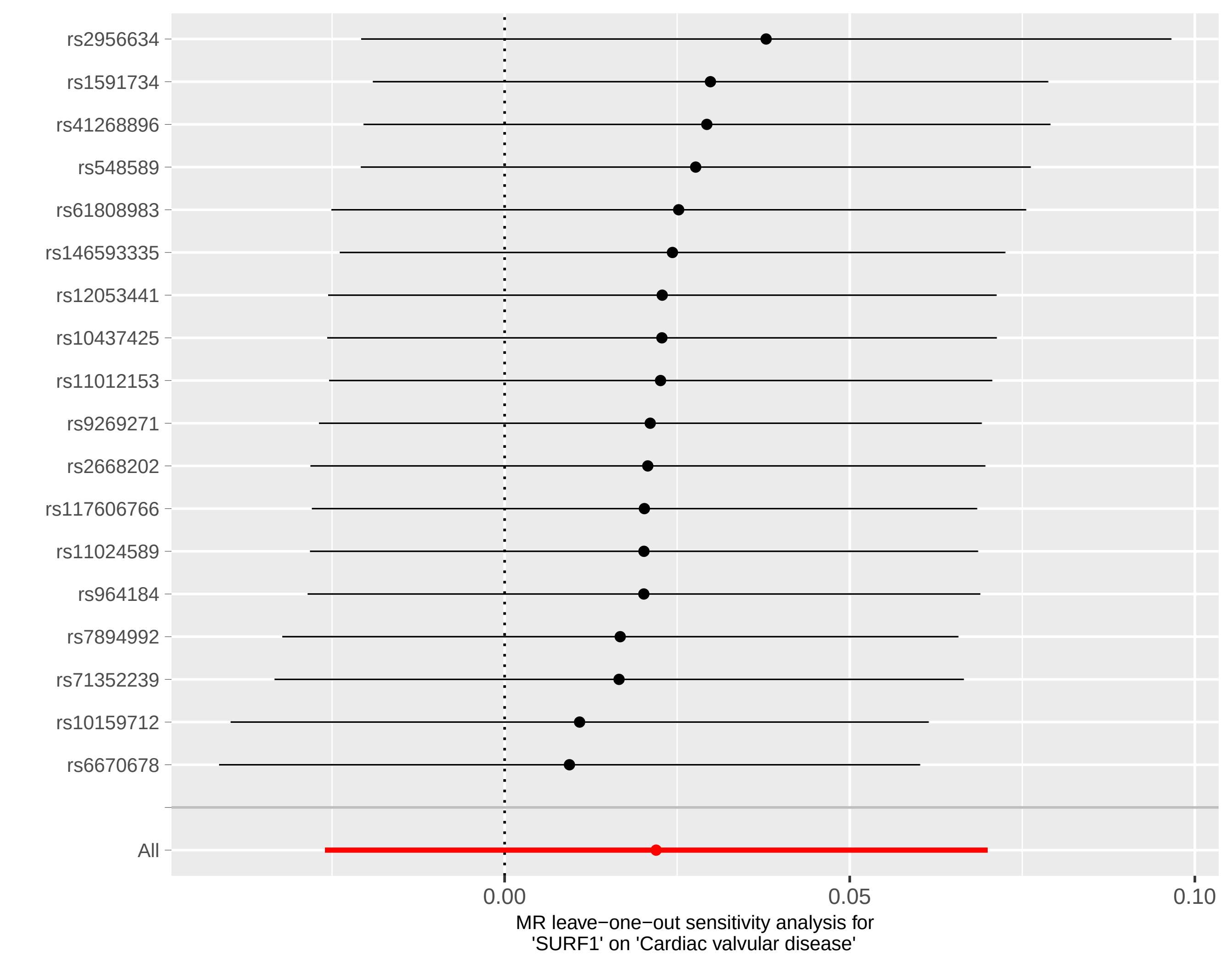
rs2956634
rs1591734
rs41268896
rs548589
rs61808983
rs146593335
rs12053441
rs10437425
rs11012153
rs9269271
rs2668202
rs117606766
rs11024589
rs964184
rs7894992
rs71352239
rs10159712
rs6670678
All
0.00
0.05
0.10
MR leave−one−out sensitivity analysis for
'SURF1' on 'Cardiac valvular disease'

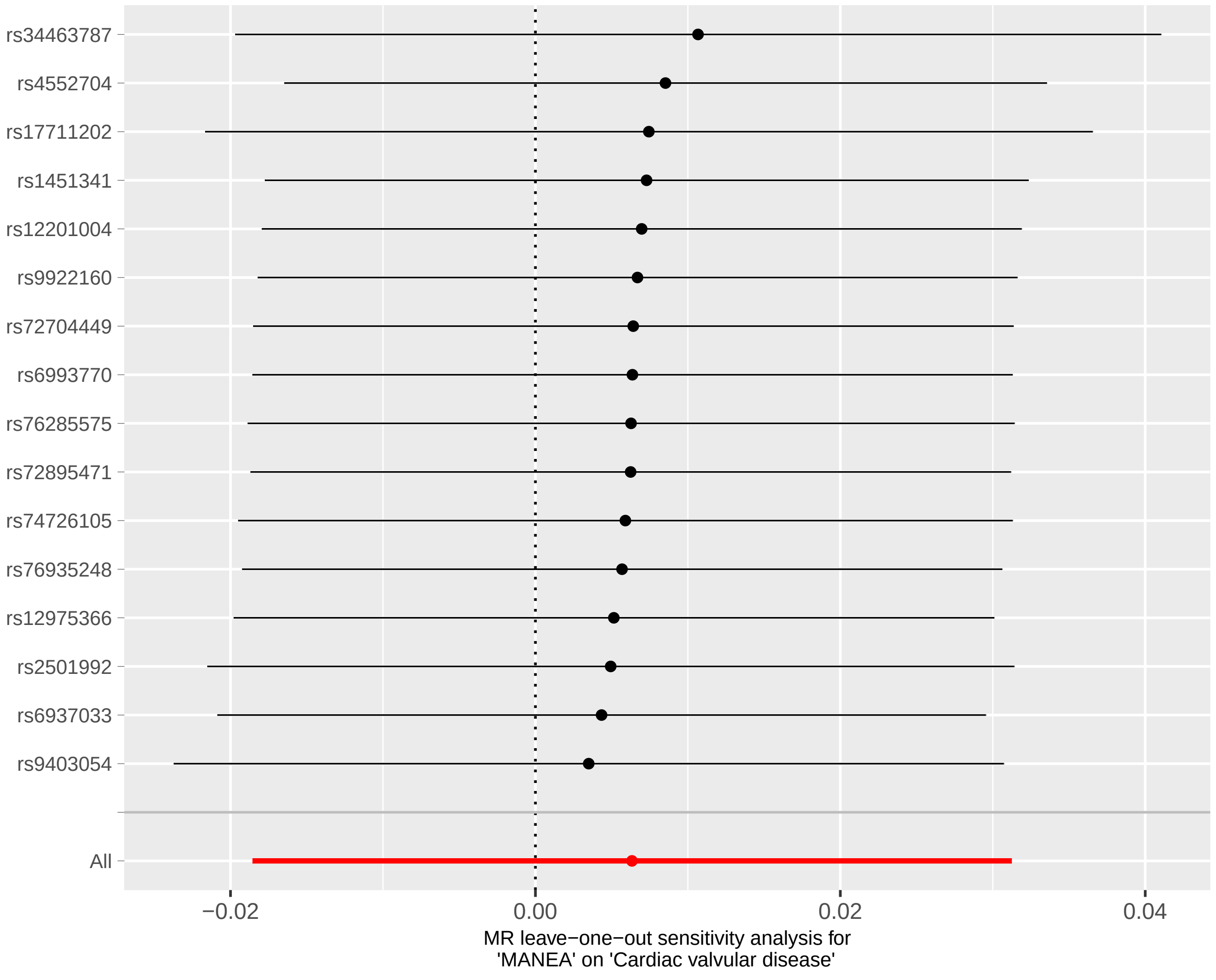

rs34463787
rs4552704
rs17711202
rs1451341
rs12201004
rs9922160
rs72704449
rs6993770
rs76285575
rs72895471
rs74726105
rs76935248
rs12975366
rs2501992
rs6937033
rs9403054
All
−0.02
0.00
0.02
0.04
MR leave−one−out sensitivity analysis for
'MANEA' on 'Cardiac valvular disease'

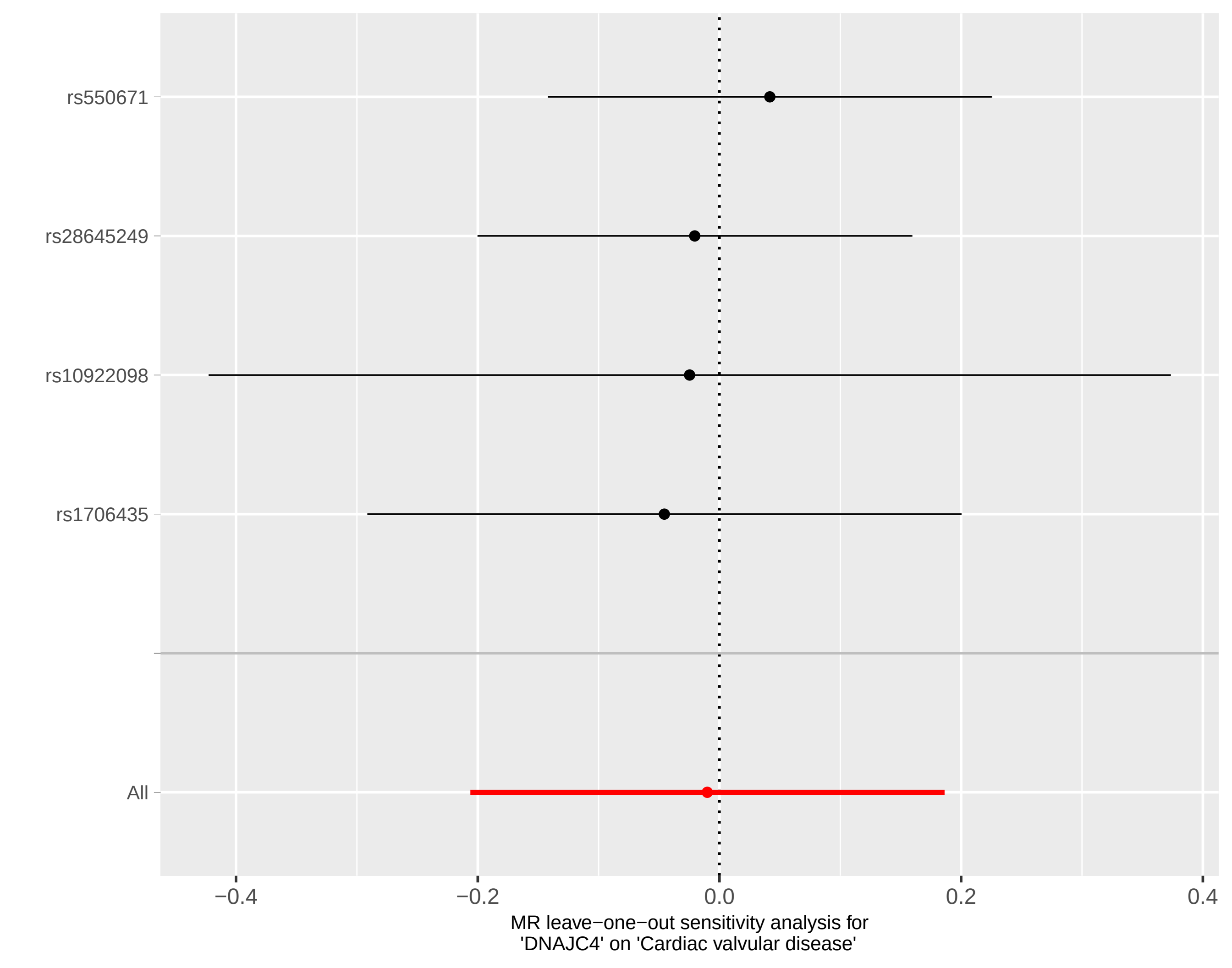

rs550671
rs28645249
rs10922098
rs1706435
All
−0.4
−0.2
0.0
0.2
0.4
MR leave−one−out sensitivity analysis for
'DNAJC4' on 'Cardiac valvular disease'

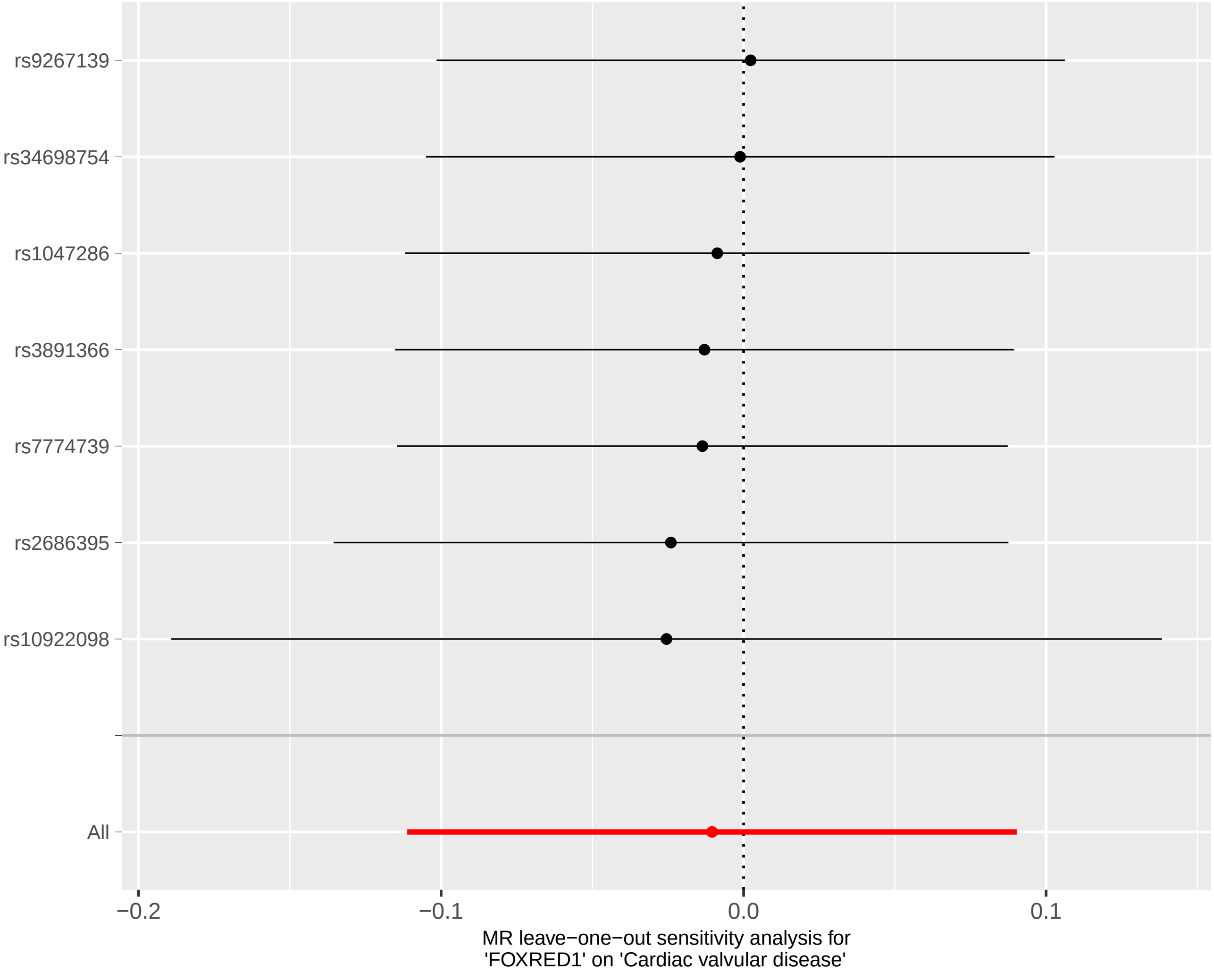
rs9267139
rs34698754
rs1047286
rs3891366
rs7774739
rs2686395
rs10922098
All
−0.2
−0.1
0.0
0.1
MR leave−one−out sensitivity analysis for
'FOXRED1' on 'Cardiac valvular disease'

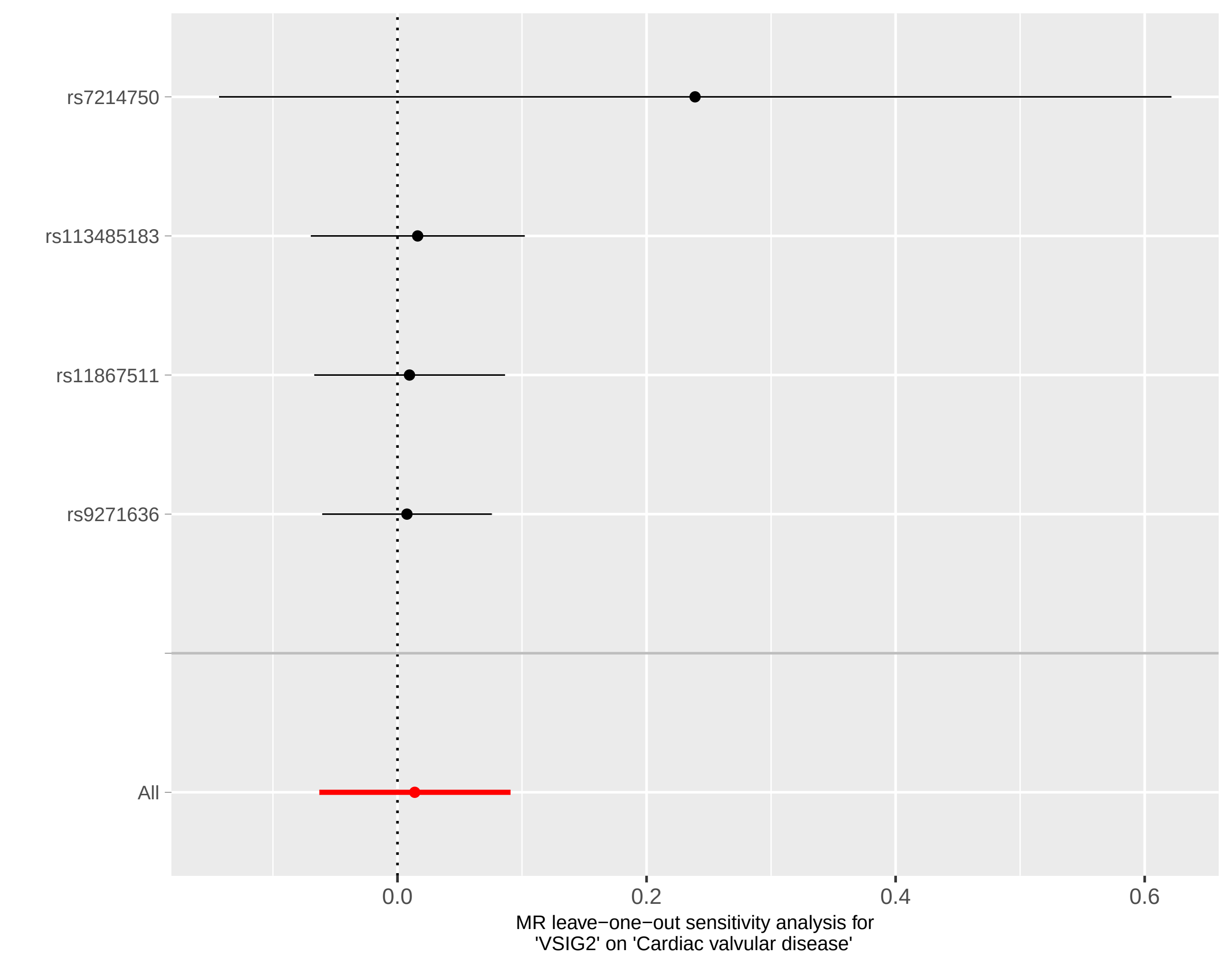

rs7214750
rs113485183
rs11867511
rs9271636
All
0.0
0.2
0.4
0.6
MR leave−one−out sensitivity analysis for
'VSIG2' on 'Cardiac valvular disease'

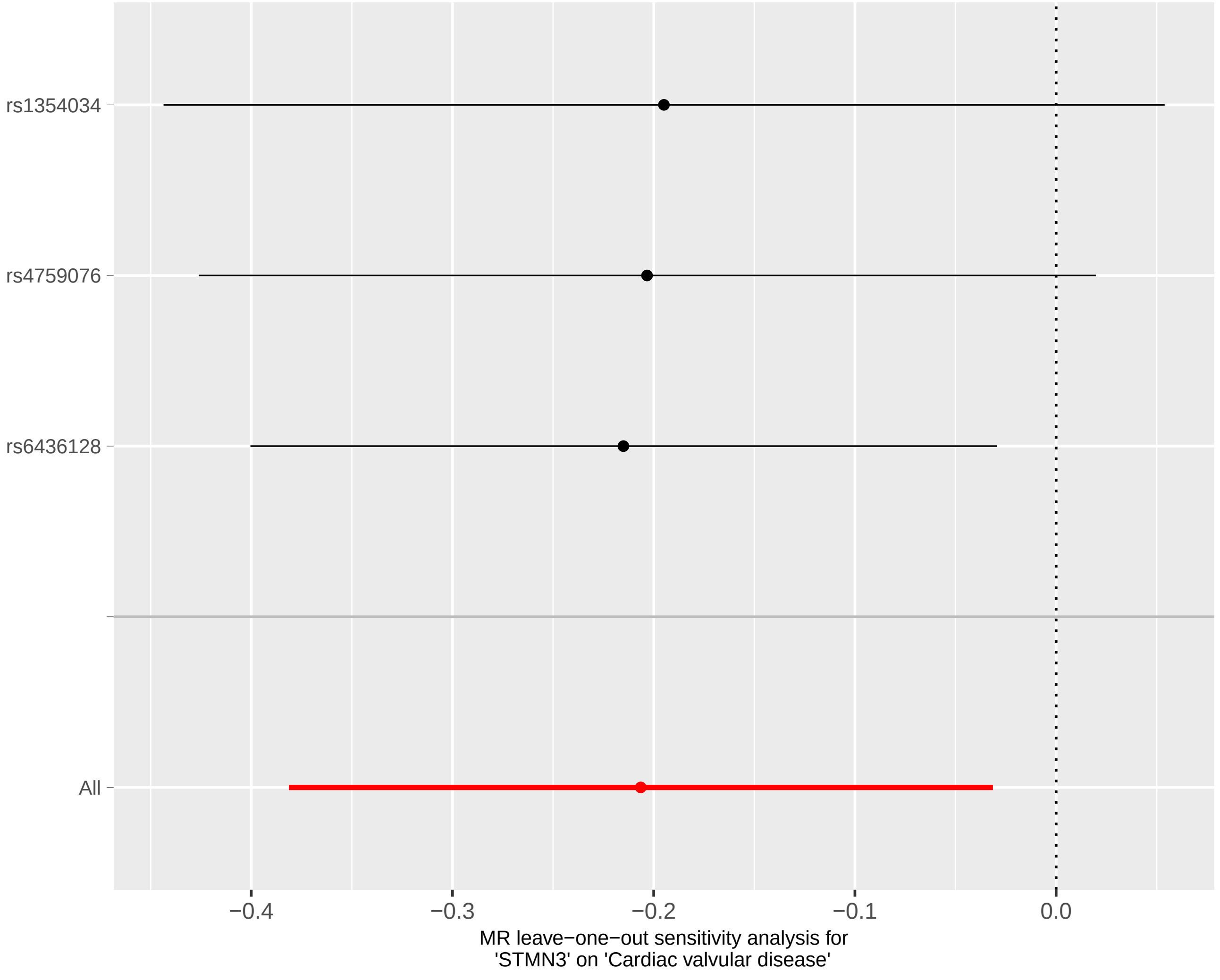
rs1354034
rs4759076
rs6436128
All
−0.4
−0.3
−0.2
−0.1
0.0
MR leave−one−out sensitivity analysis for
'STMN3' on 'Cardiac valvular disease'

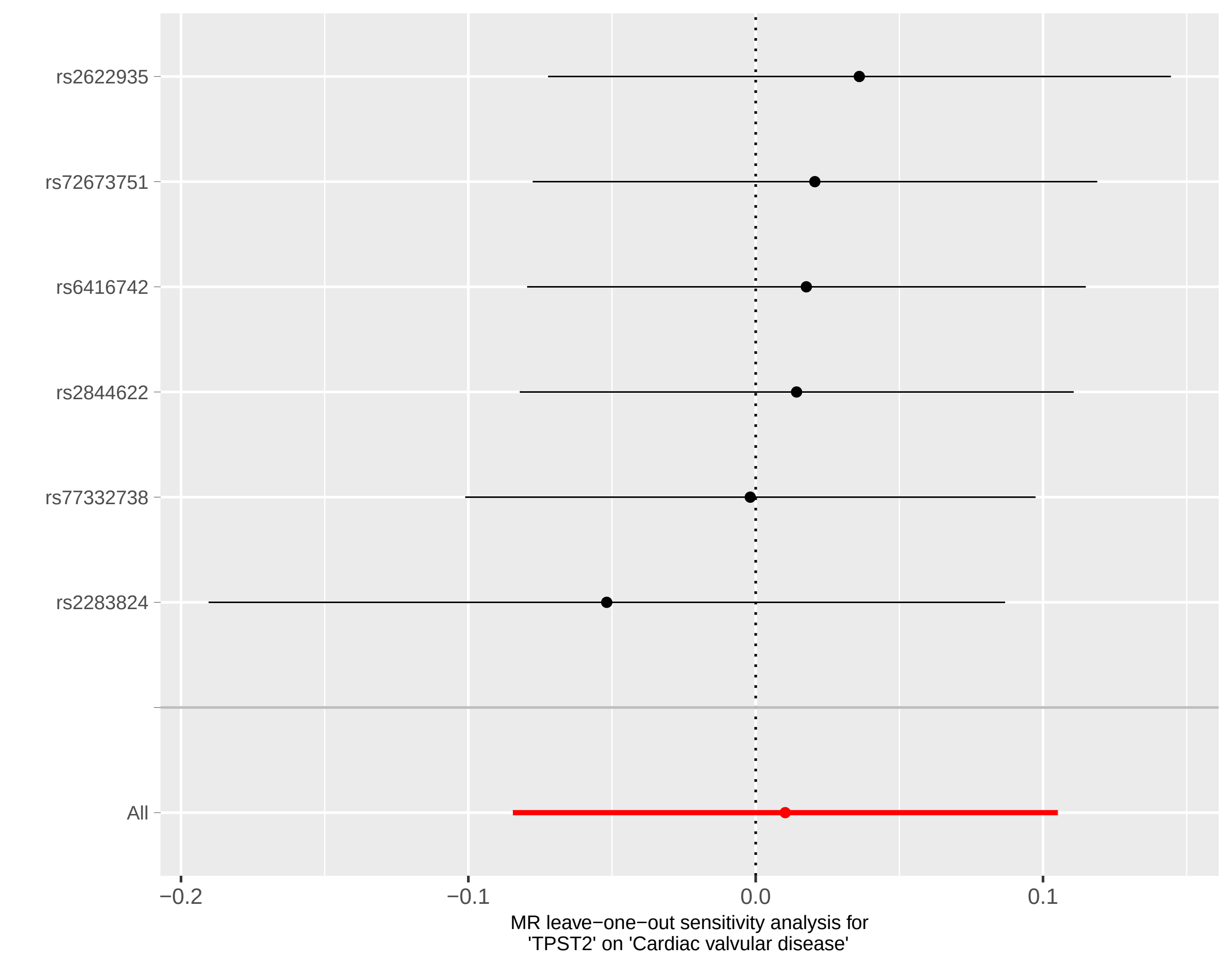
rs2622935
rs72673751
rs6416742
rs2844622
rs77332738
rs2283824
All
−0.2
−0.1
0.0
0.1
MR leave−one−out sensitivity analysis for
'TPST2' on 'Cardiac valvular disease'

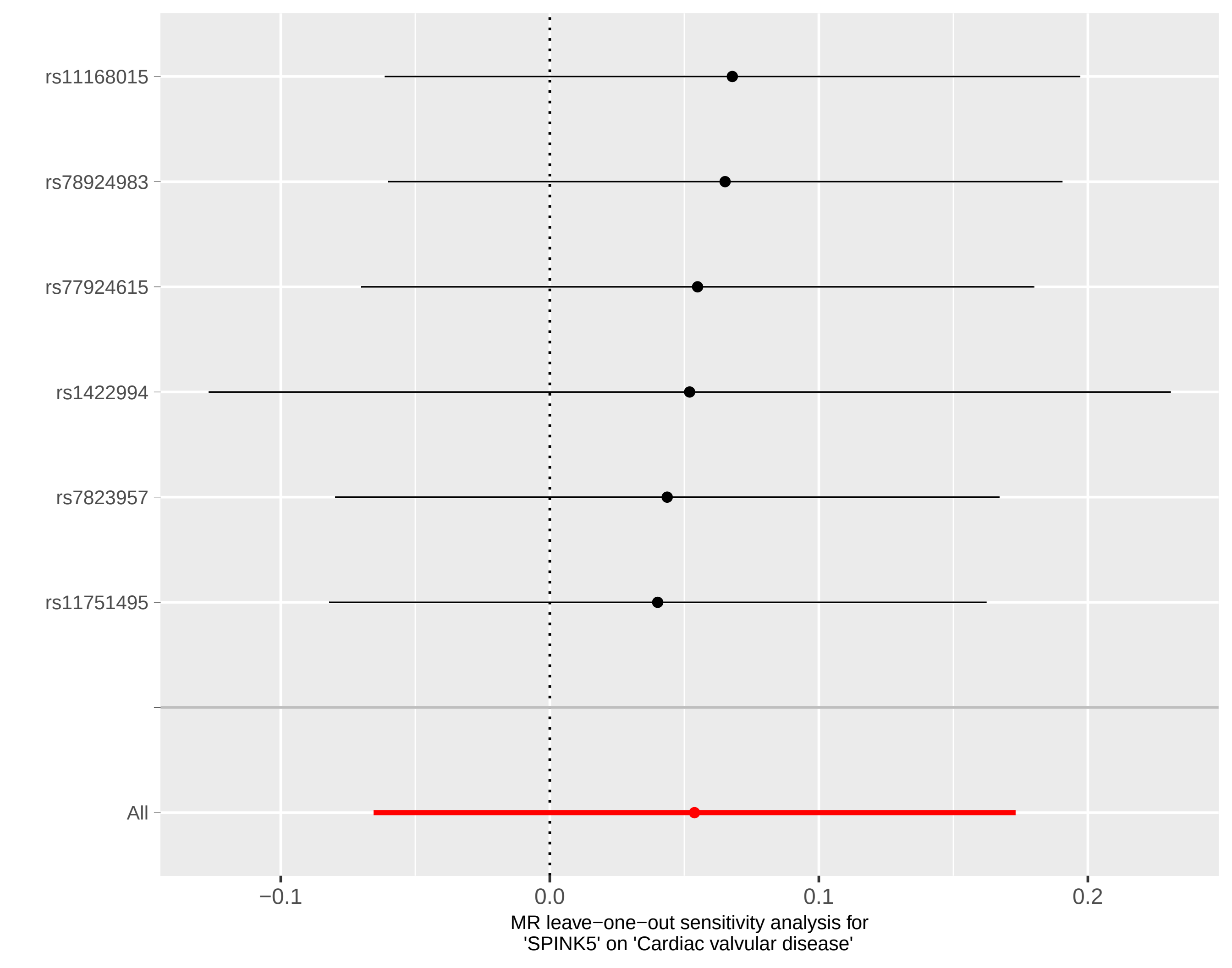
rs11168015
rs78924983
rs77924615
rs1422994
rs7823957
rs11751495
All
−0.1
0.0
0.1
0.2
MR leave−one−out sensitivity analysis for
'SPINK5' on 'Cardiac valvular disease'

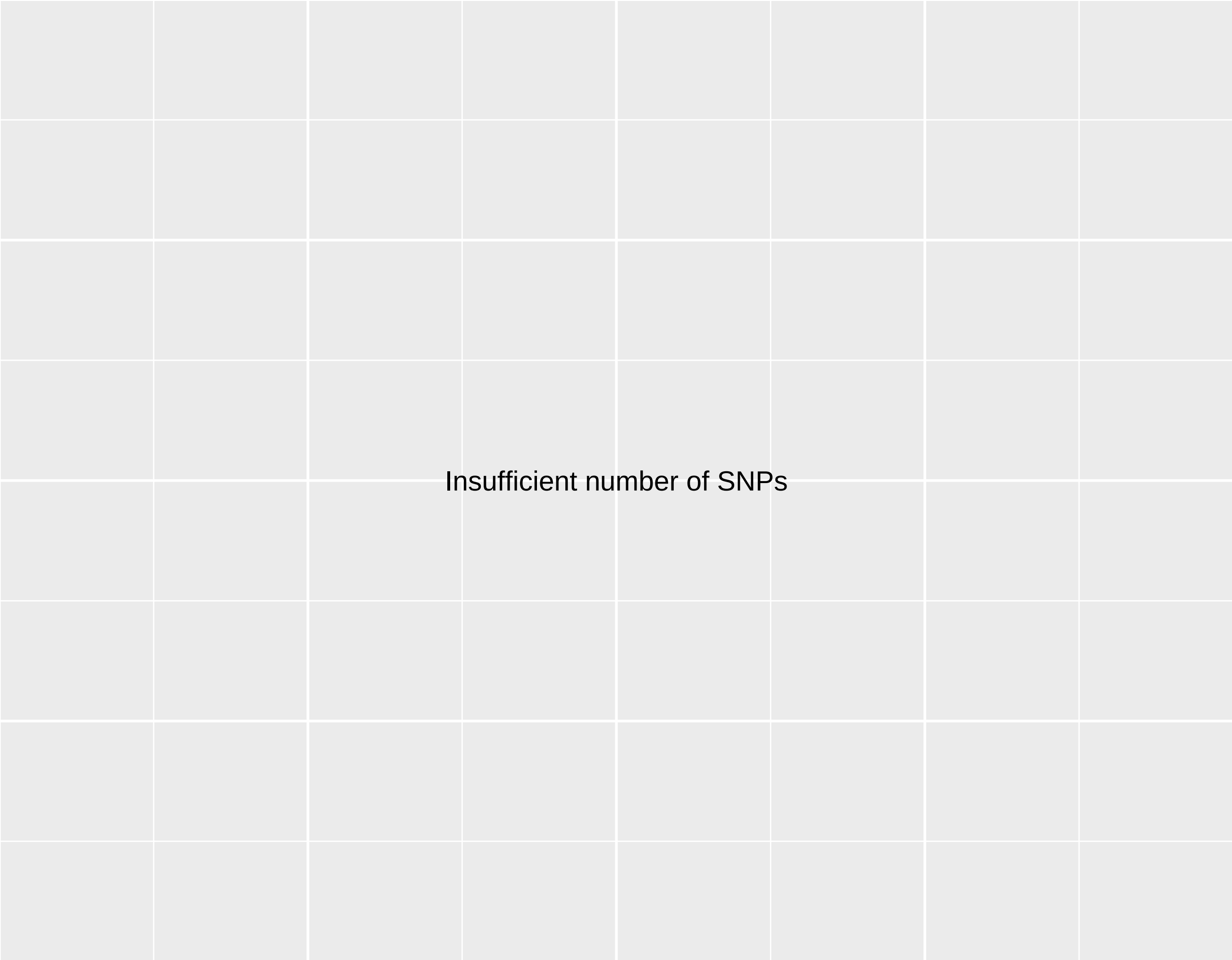
Insufficient number of SNPs

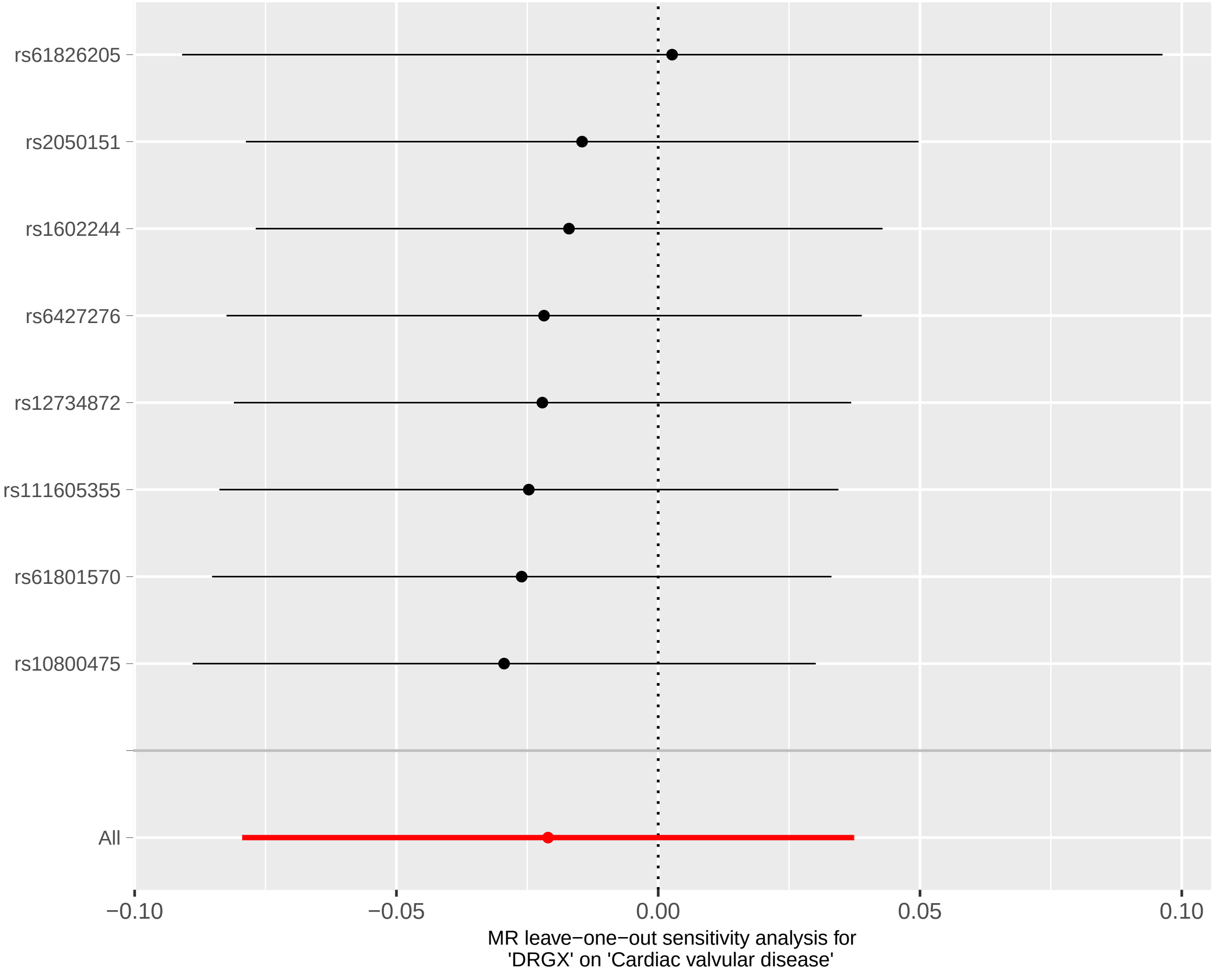

rs61826205
rs2050151
rs1602244
rs6427276
rs12734872
rs111605355
rs61801570
rs10800475
All
−0.10
−0.05
0.00
0.05
0.10
MR leave−one−out sensitivity analysis for
'DRGX' on 'Cardiac valvular disease'

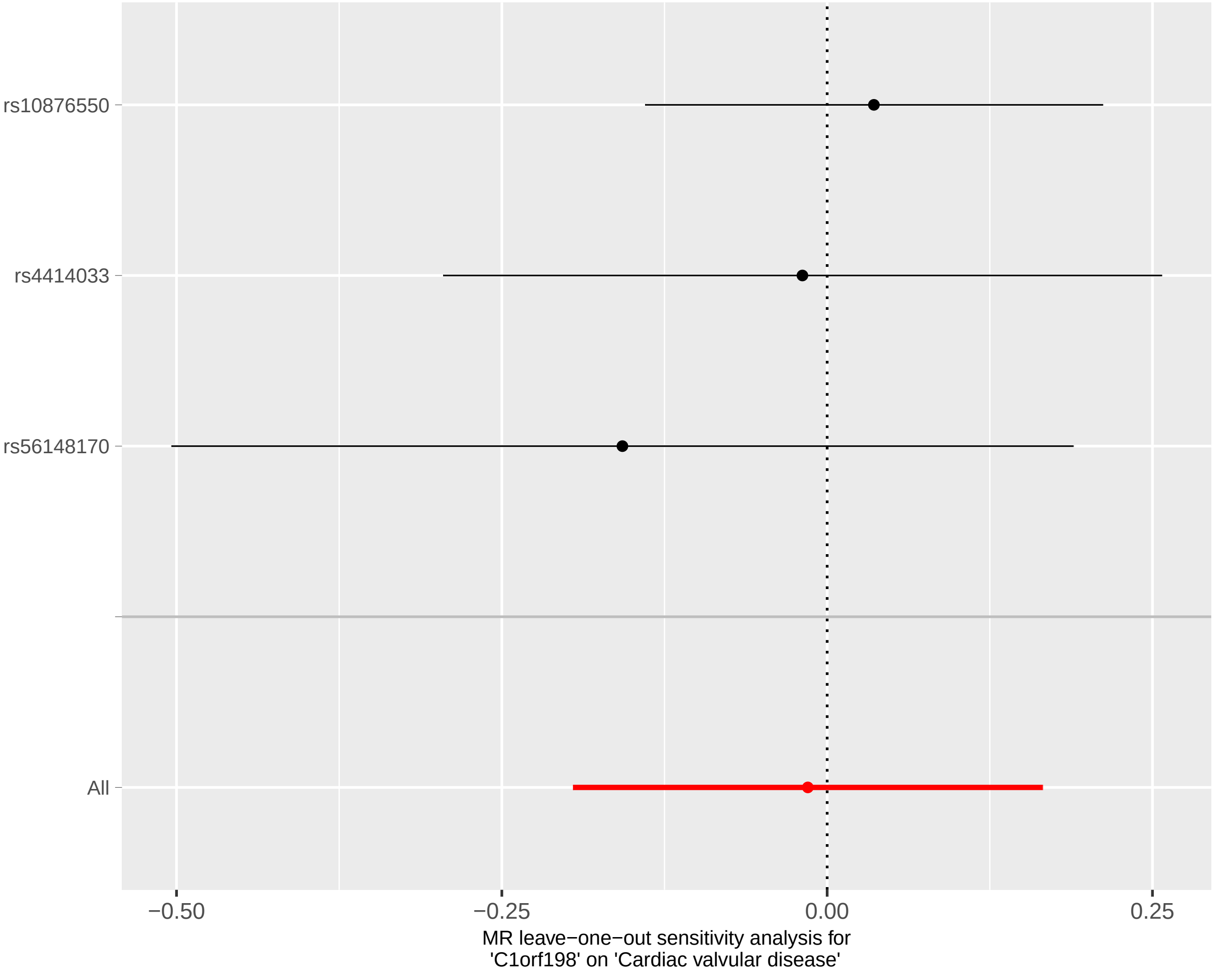
rs10876550
rs4414033
rs56148170
All
−0.50
−0.25
0.00
0.25
MR leave−one−out sensitivity analysis for
'C1orf198' on 'Cardiac valvular disease'

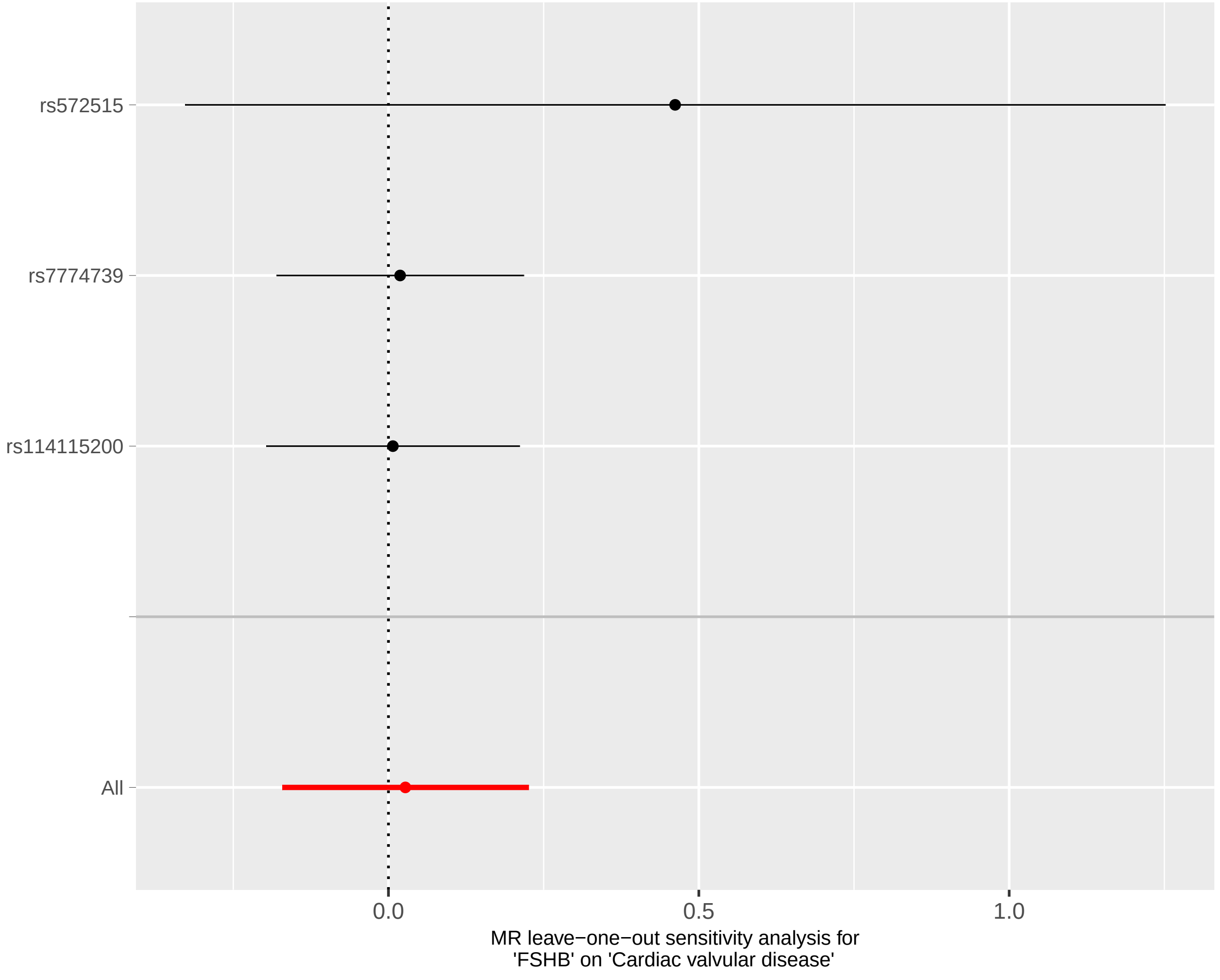
rs572515
rs7774739
rs114115200
All
0.0
0.5
1.0
MR leave−one−out sensitivity analysis for
'FSHB' on 'Cardiac valvular disease'

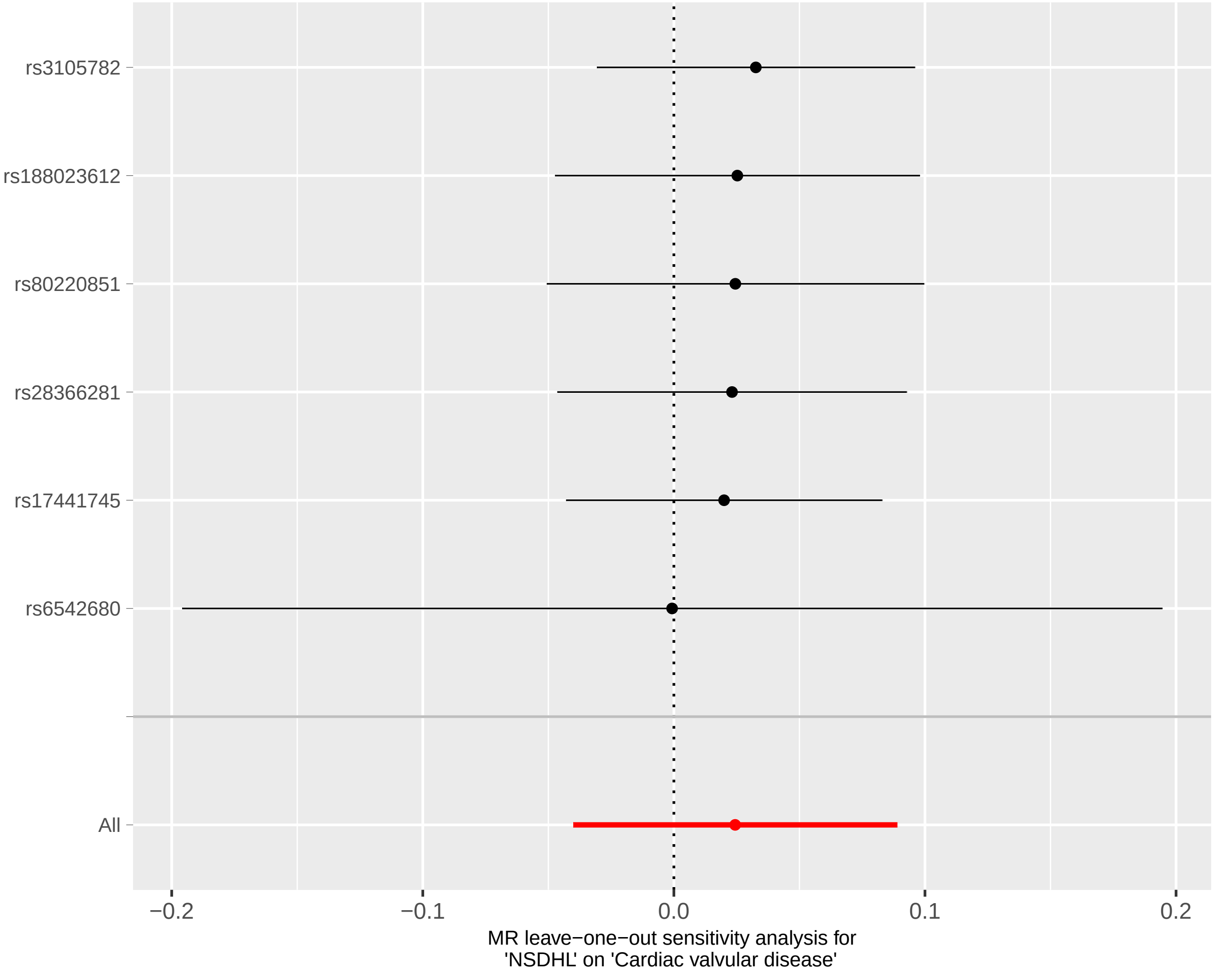

rs3105782
rs188023612
rs80220851
rs28366281
rs17441745
rs6542680
All
−0.2
−0.1
0.0
0.1
0.2
MR leave−one−out sensitivity analysis for
'NSDHL' on 'Cardiac valvular disease'

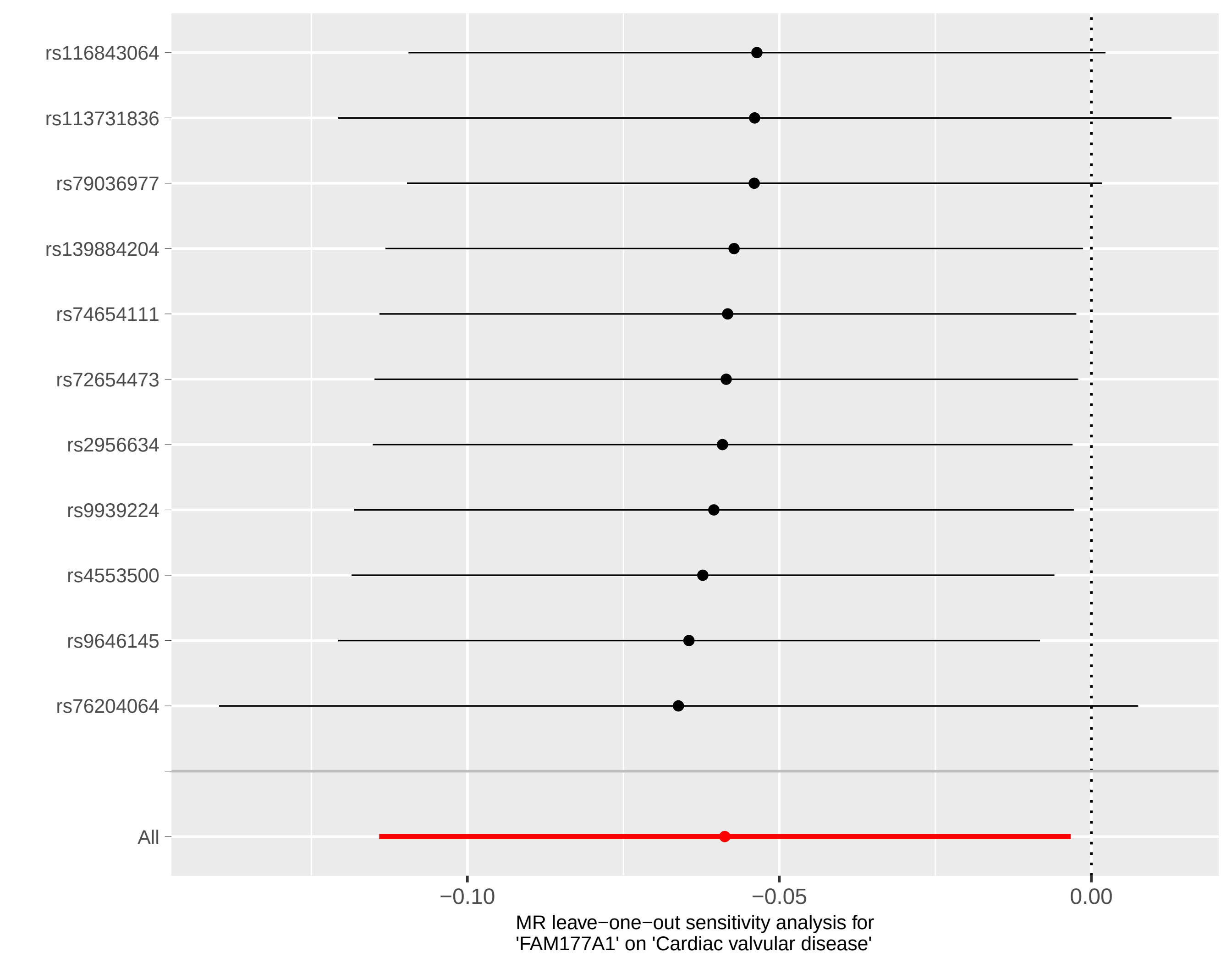

rs116843064
rs113731836
rs79036977
rs139884204
rs74654111
rs72654473
rs2956634
rs9939224
rs4553500
rs9646145
rs76204064
All
−0.10
−0.05
0.00
MR leave−one−out sensitivity analysis for
'FAM177A1' on 'Cardiac valvular disease'

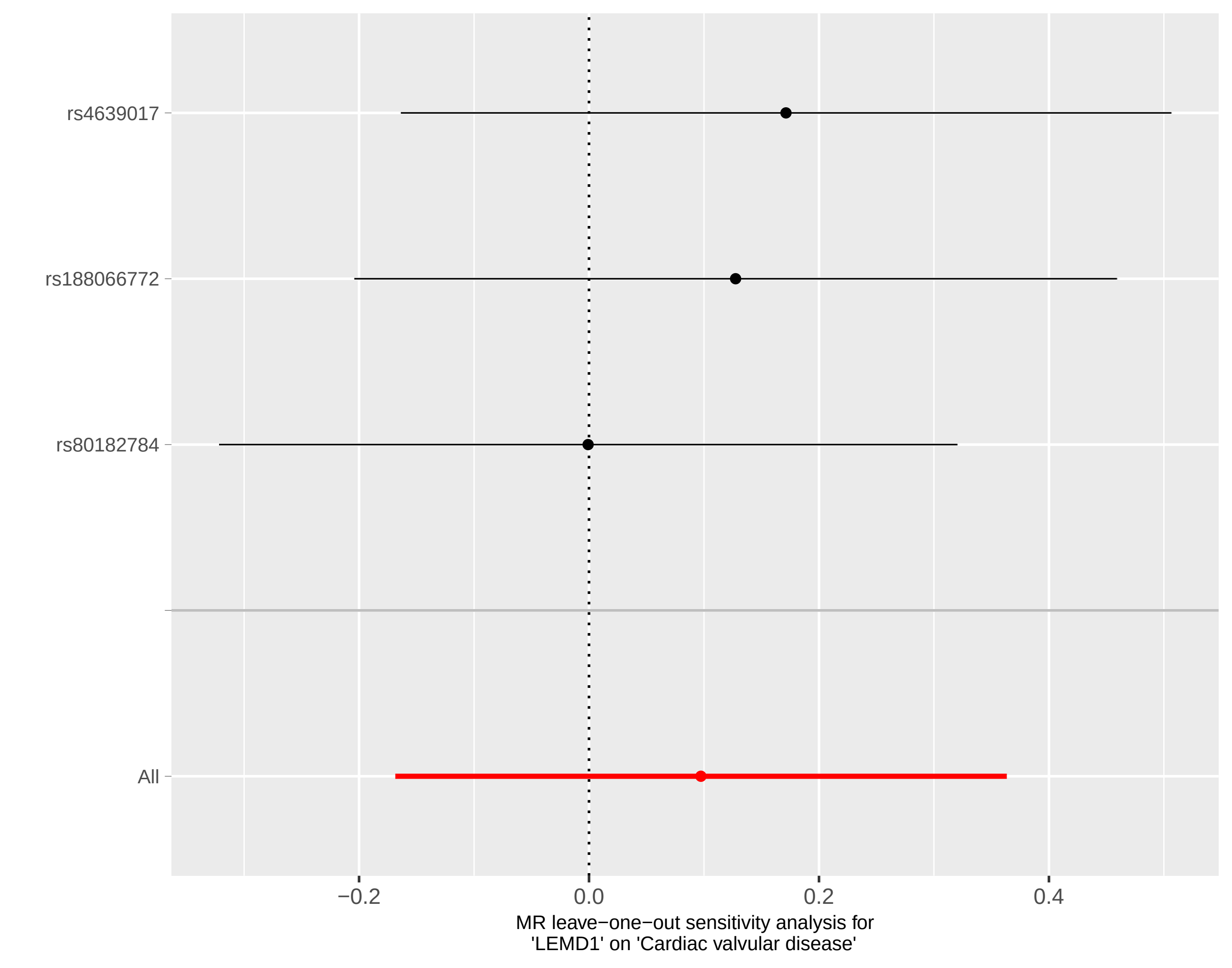
rs4639017
rs188066772
rs80182784
All
−0.2
0.0
0.2
0.4
MR leave−one−out sensitivity analysis for
'LEMD1' on 'Cardiac valvular disease'

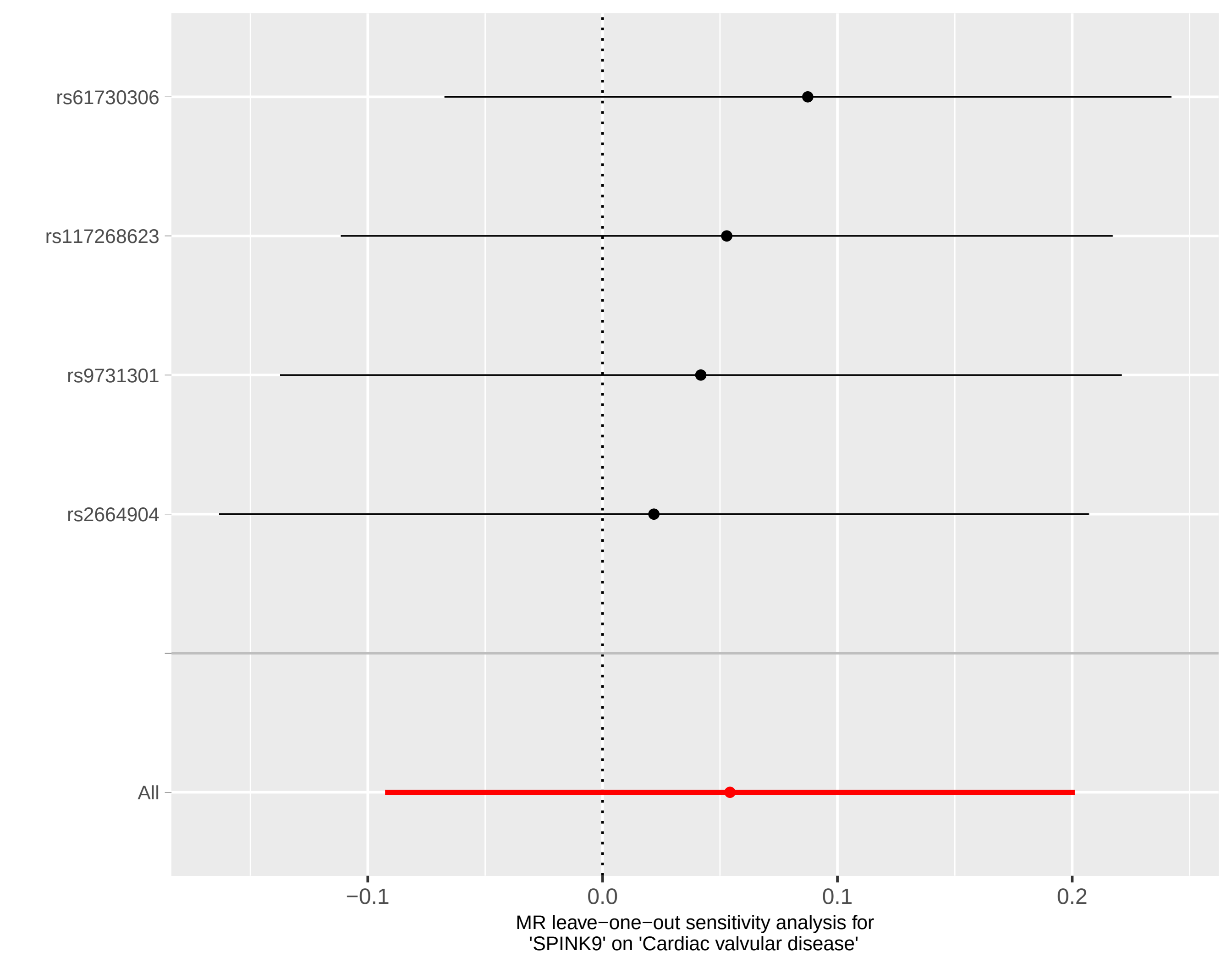

rs61730306
rs117268623
rs9731301
rs2664904
All
−0.1
0.0
0.1
0.2
MR leave−one−out sensitivity analysis for
'SPINK9' on 'Cardiac valvular disease'

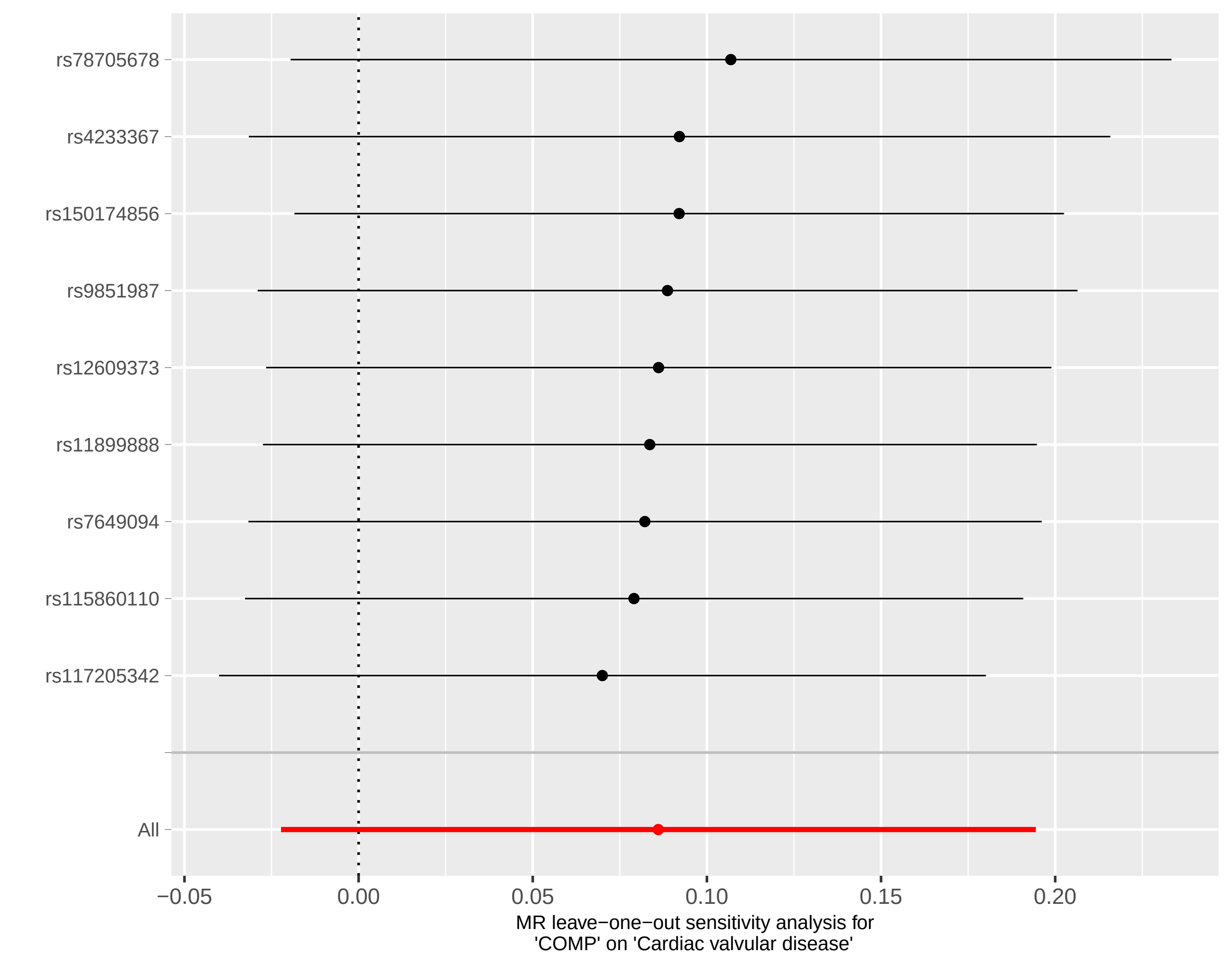

rs78705678
rs4233367
rs150174856
rs9851987
rs12609373
rs11899888
rs7649094
rs115860110
rs117205342
All
−0.05
0.00
0.05
0.10
0.15
0.20
MR leave−one−out sensitivity analysis for
'COMP' on 'Cardiac valvular disease'

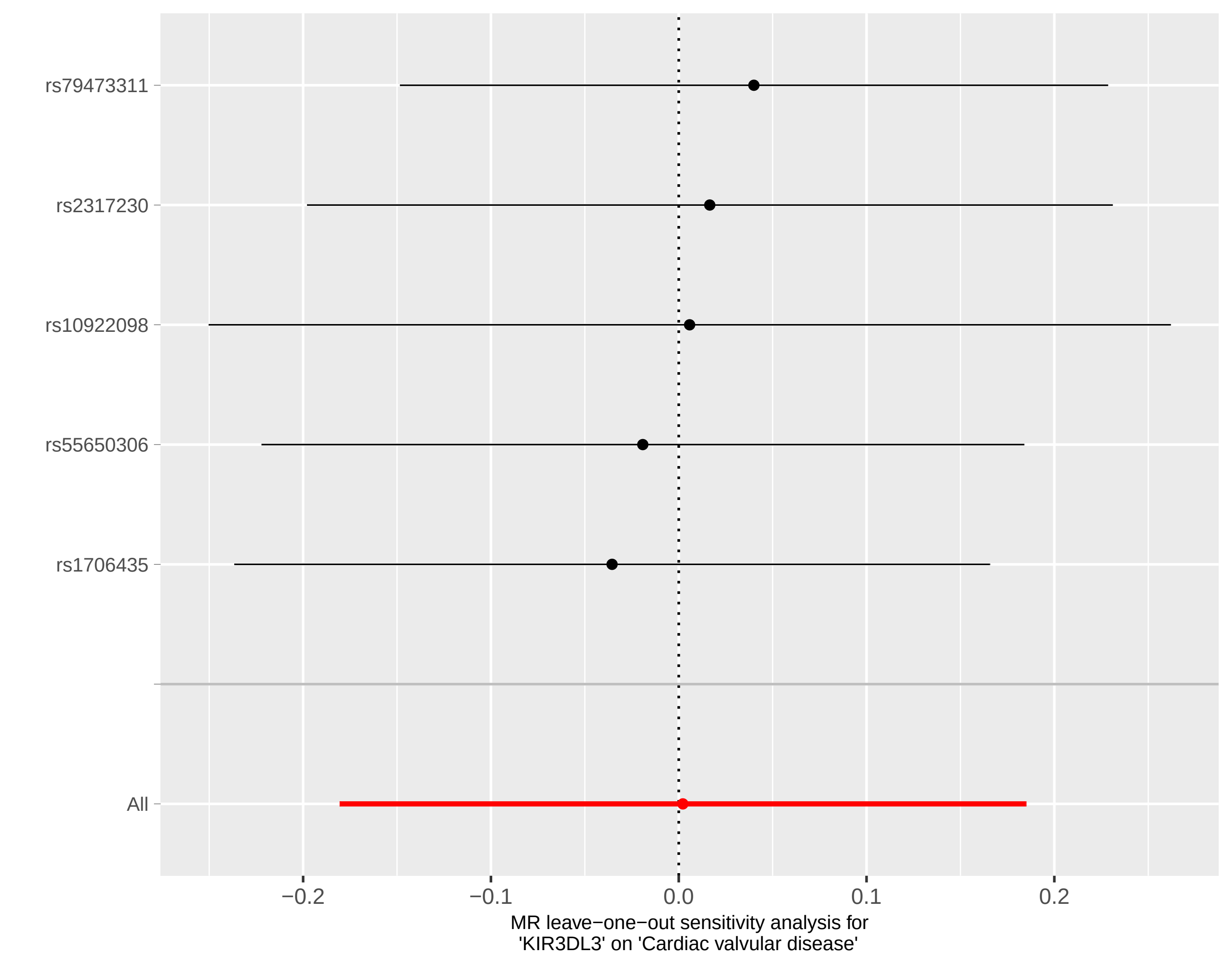

rs79473311
rs2317230
rs10922098
rs55650306
rs1706435
All
−0.2
−0.1
0.0
0.1
0.2
MR leave−one−out sensitivity analysis for
'KIR3DL3' on 'Cardiac valvular disease'

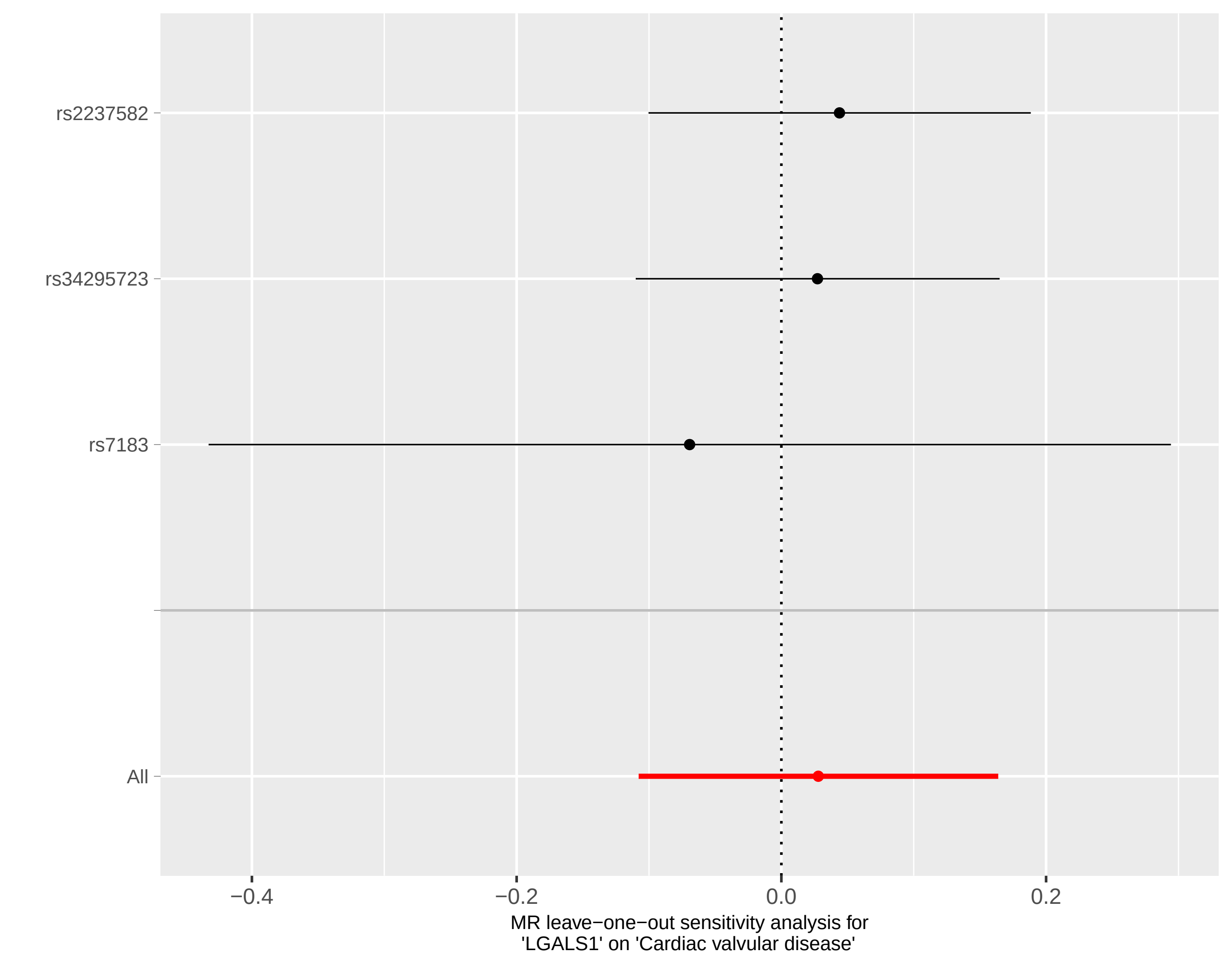

rs2237582
rs34295723
rs7183
All
−0.4
−0.2
0.0
0.2
MR leave−one−out sensitivity analysis for
'LGALS1' on 'Cardiac valvular disease'

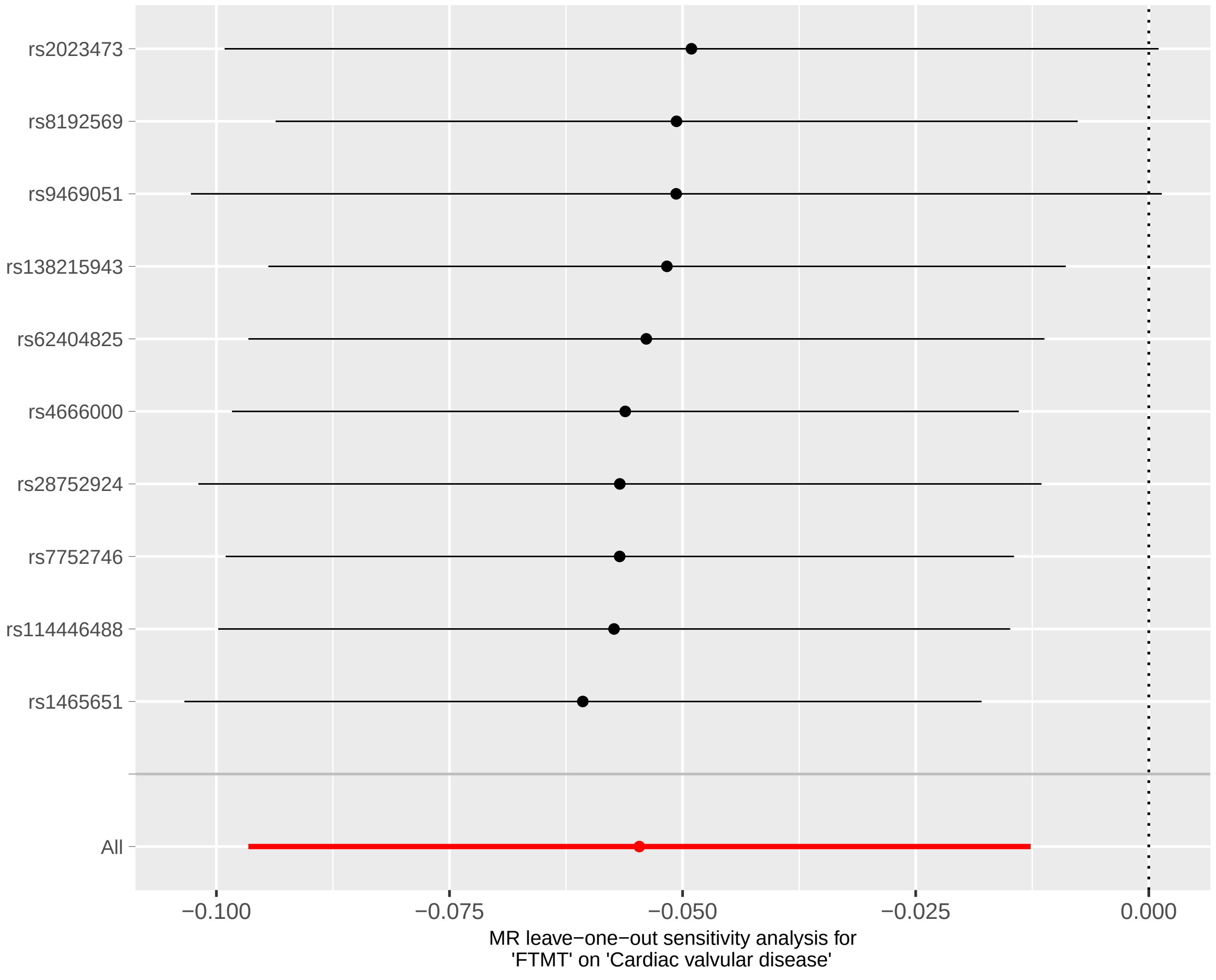
rs2023473
rs8192569
rs9469051
rs138215943
rs62404825
rs4666000
rs28752924
rs7752746
rs114446488
rs1465651
All
−0.100
−0.075
−0.050
−0.025
0.000
MR leave−one−out sensitivity analysis for
'FTMT' on 'Cardiac valvular disease'

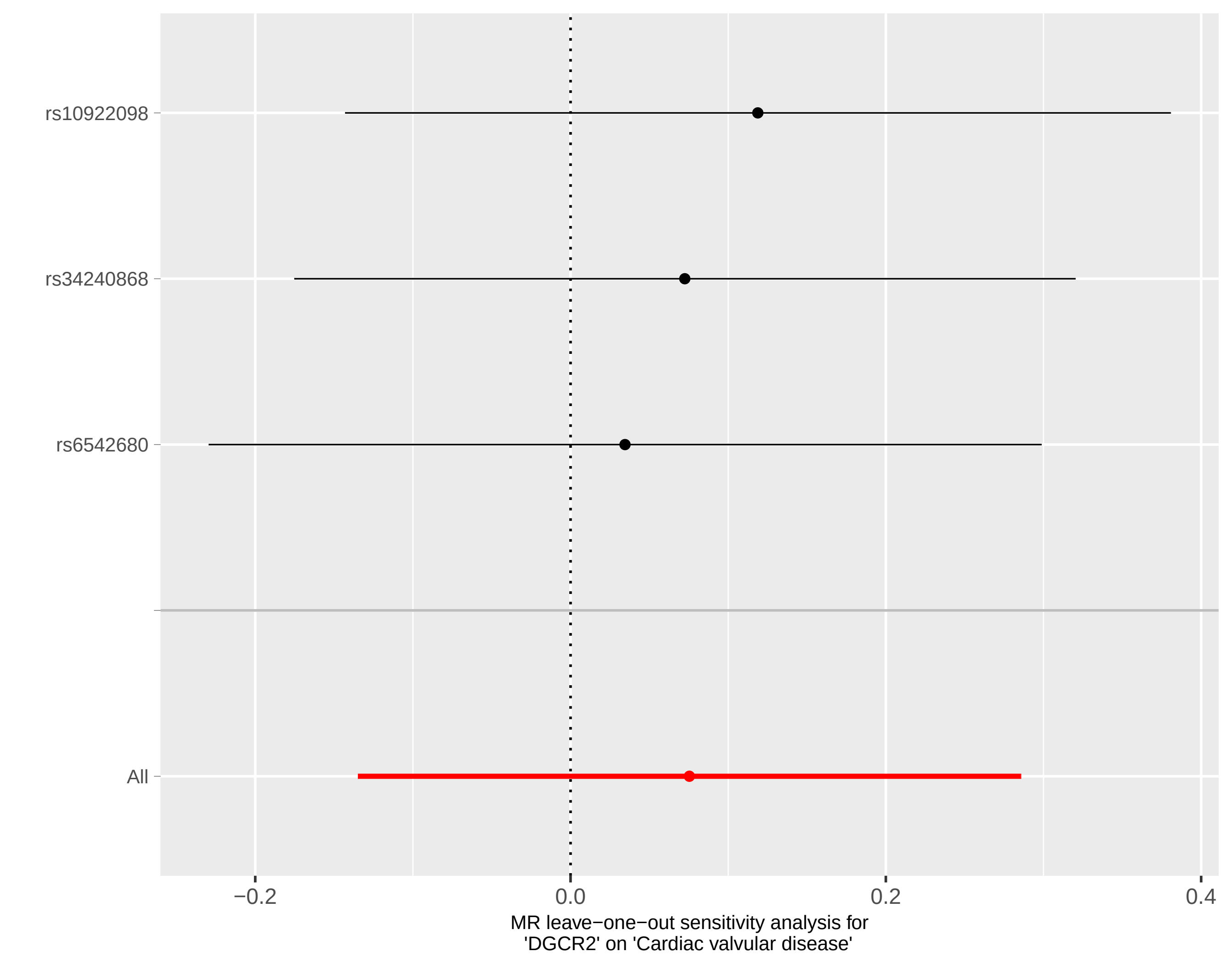

rs10922098
rs34240868
rs6542680
All
−0.2
0.0
0.2
0.4
MR leave−one−out sensitivity analysis for
'DGCR2' on 'Cardiac valvular disease'

Insufficient number of SNPs

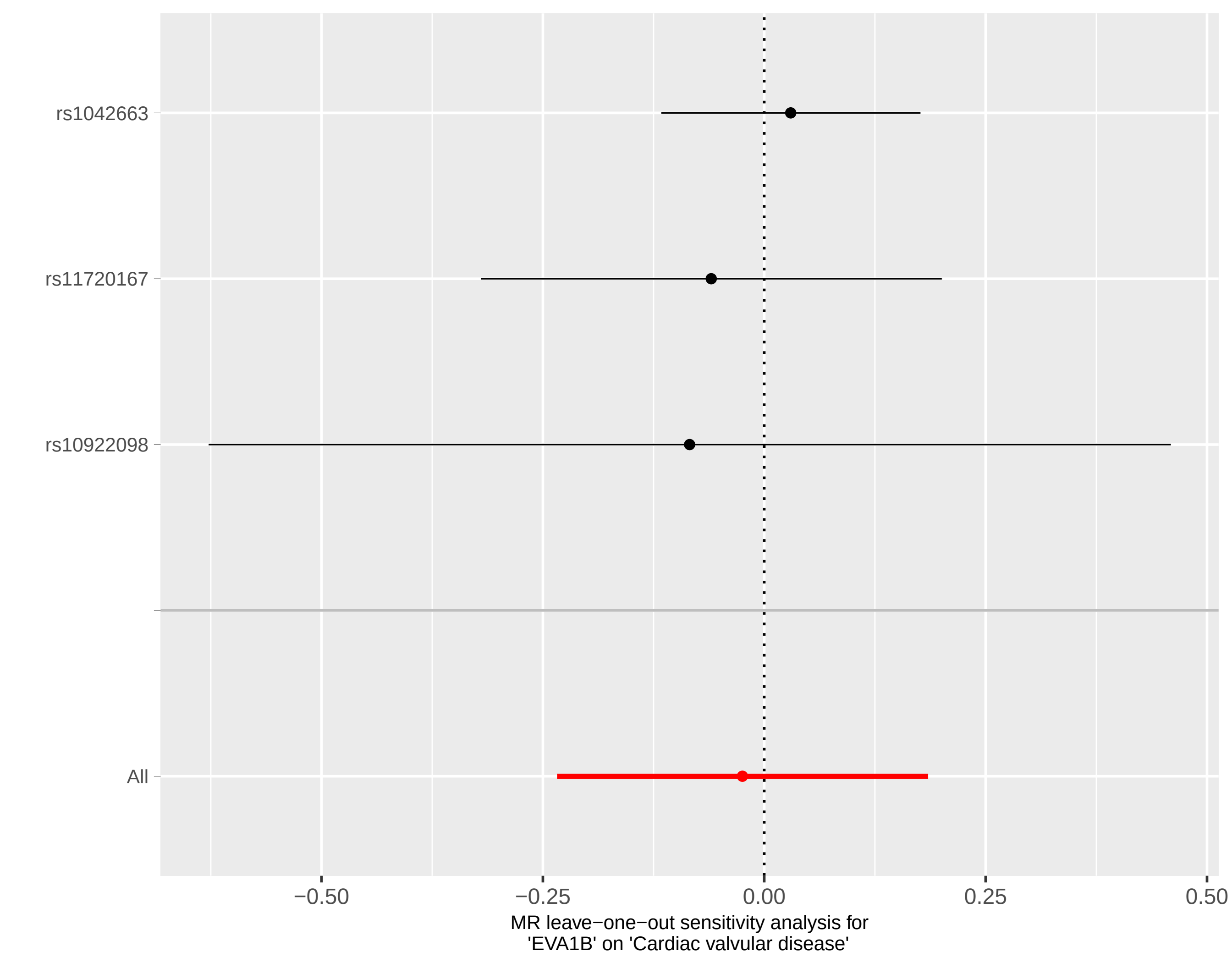
rs1042663
rs11720167
rs10922098
All
−0.50
−0.25
0.00
0.25
0.50
MR leave−one−out sensitivity analysis for
'EVA1B' on 'Cardiac valvular disease'

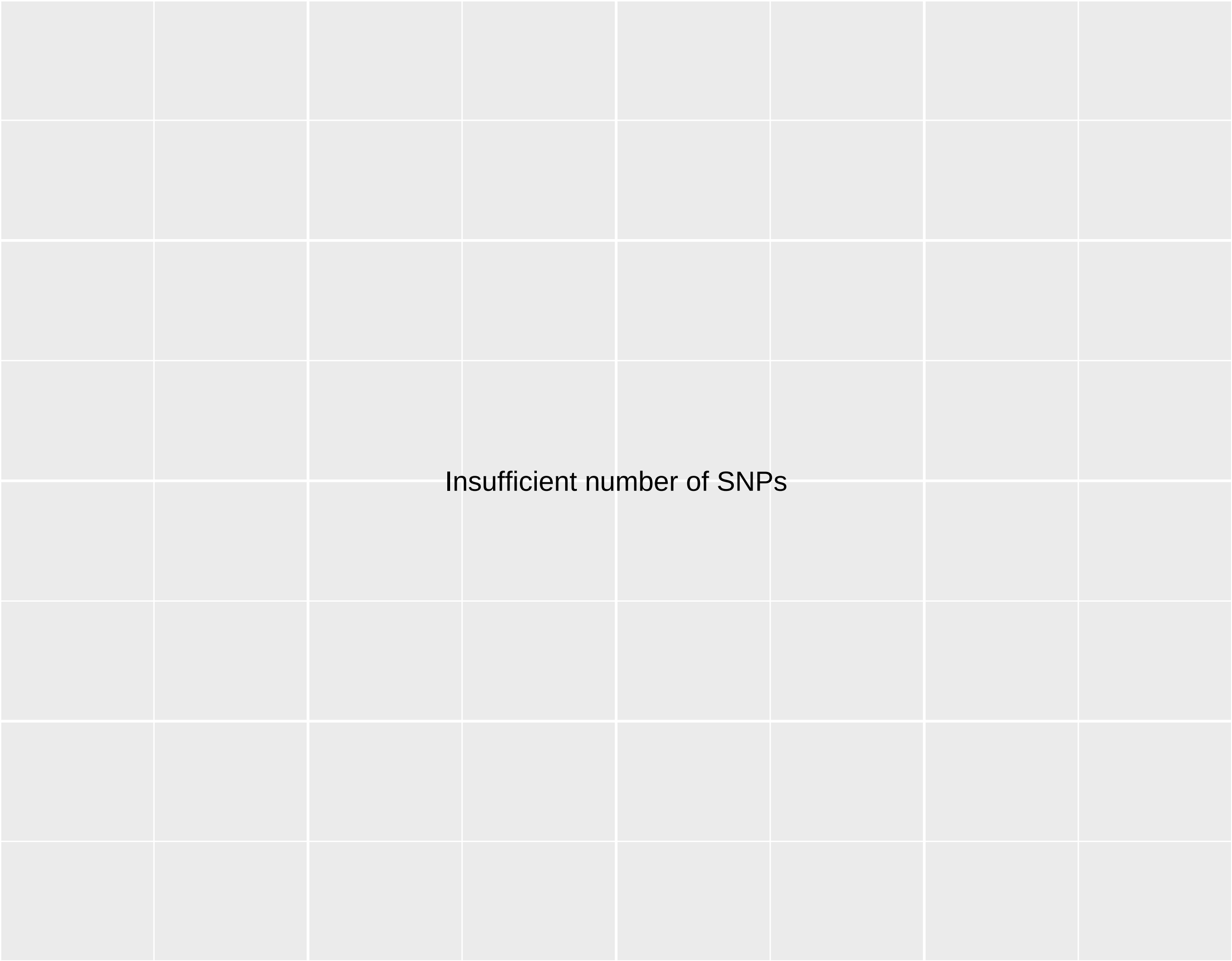
Insufficient number of SNPs

Insufficient number of SNPs

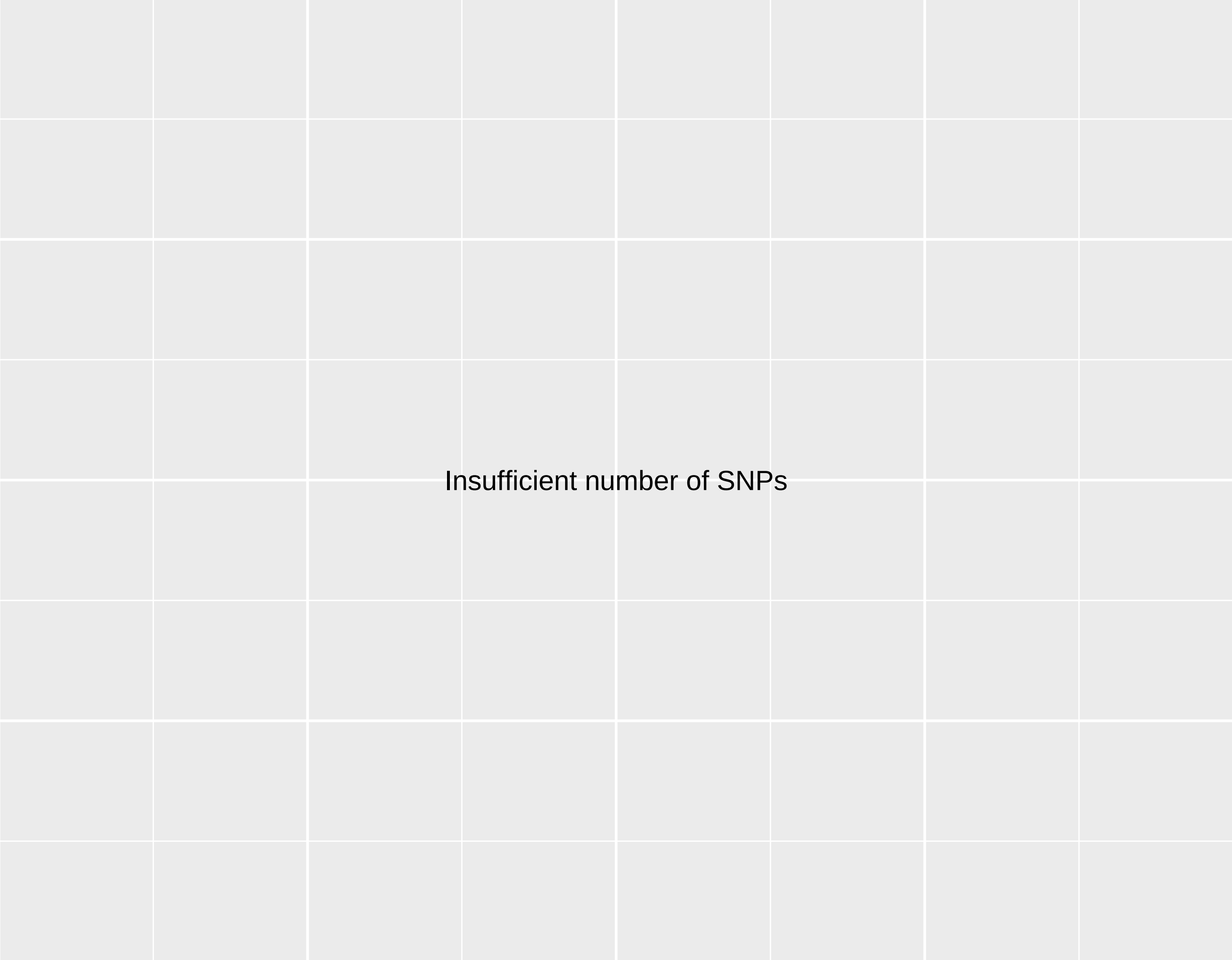
Insufficient number of SNPs

Insufficient number of SNPs

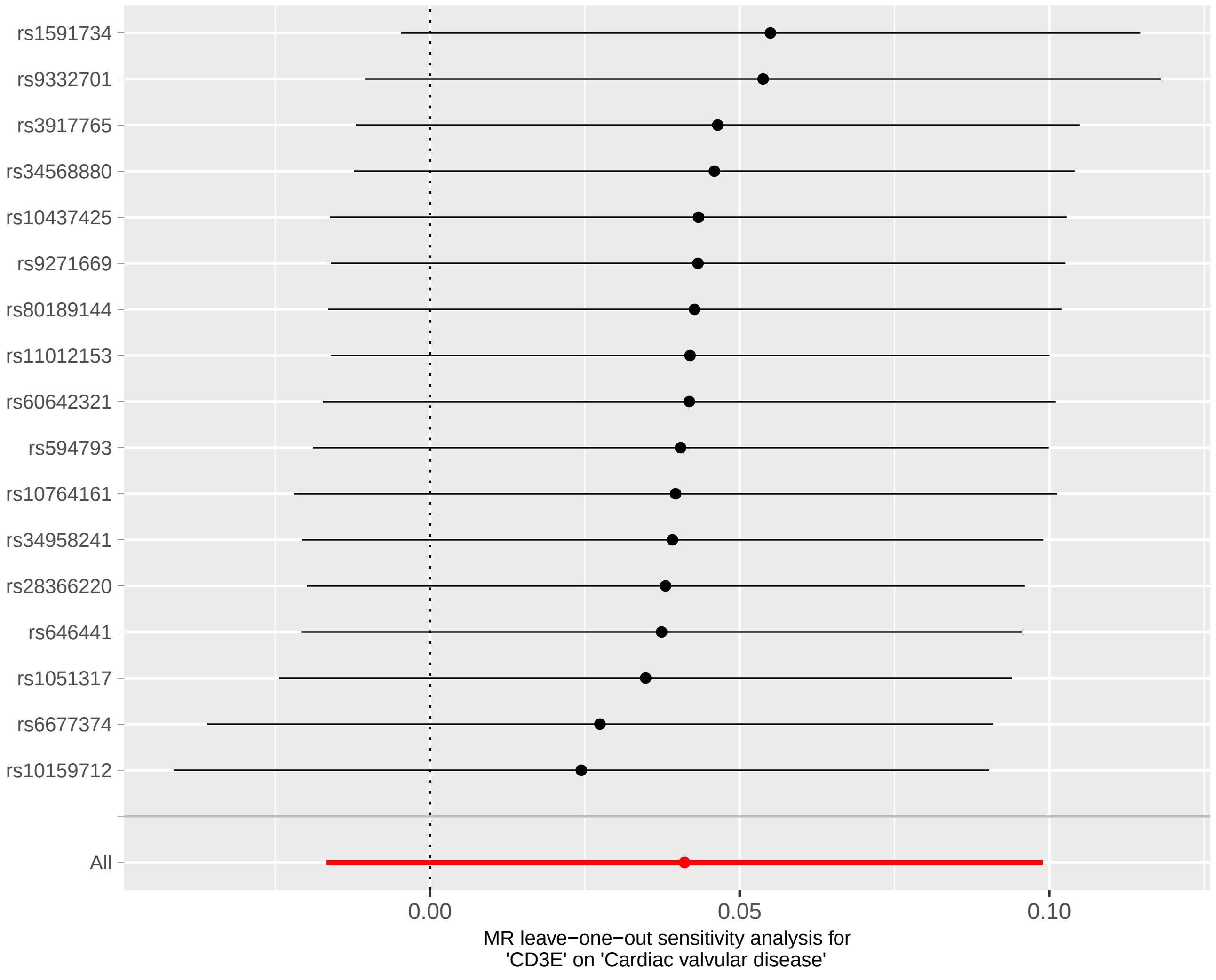

rs1591734
rs9332701
rs3917765
rs34568880
rs10437425
rs9271669
rs80189144
rs11012153
rs60642321
rs594793
rs10764161
rs34958241
rs28366220
rs646441
rs1051317
rs6677374
rs10159712
All
0.00
0.05
0.10
MR leave−one−out sensitivity analysis for
'CD3E' on 'Cardiac valvular disease'

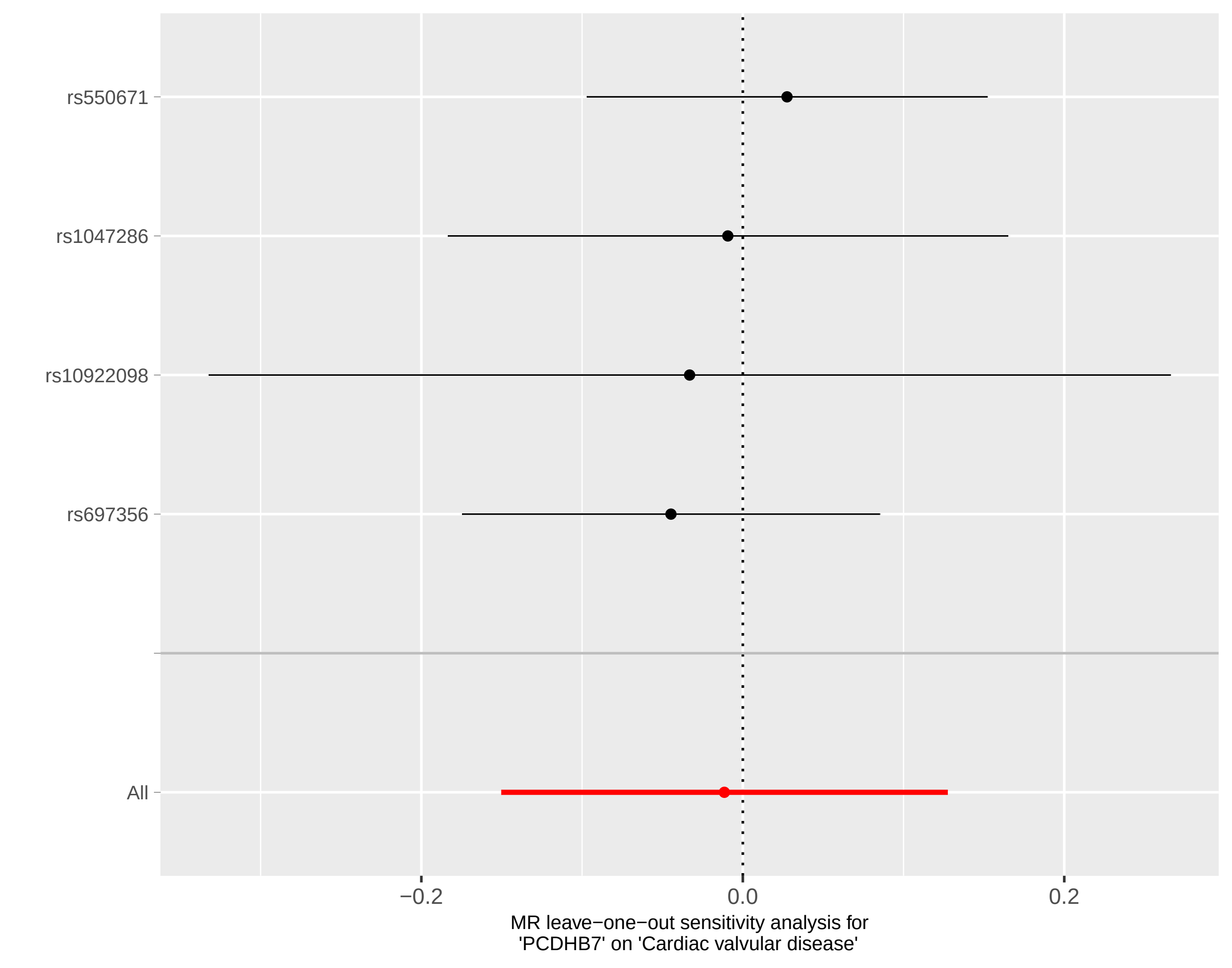
rs550671
rs1047286
rs10922098
rs697356
All
−0.2
0.0
0.2
MR leave−one−out sensitivity analysis for
'PCDHB7' on 'Cardiac valvular disease'

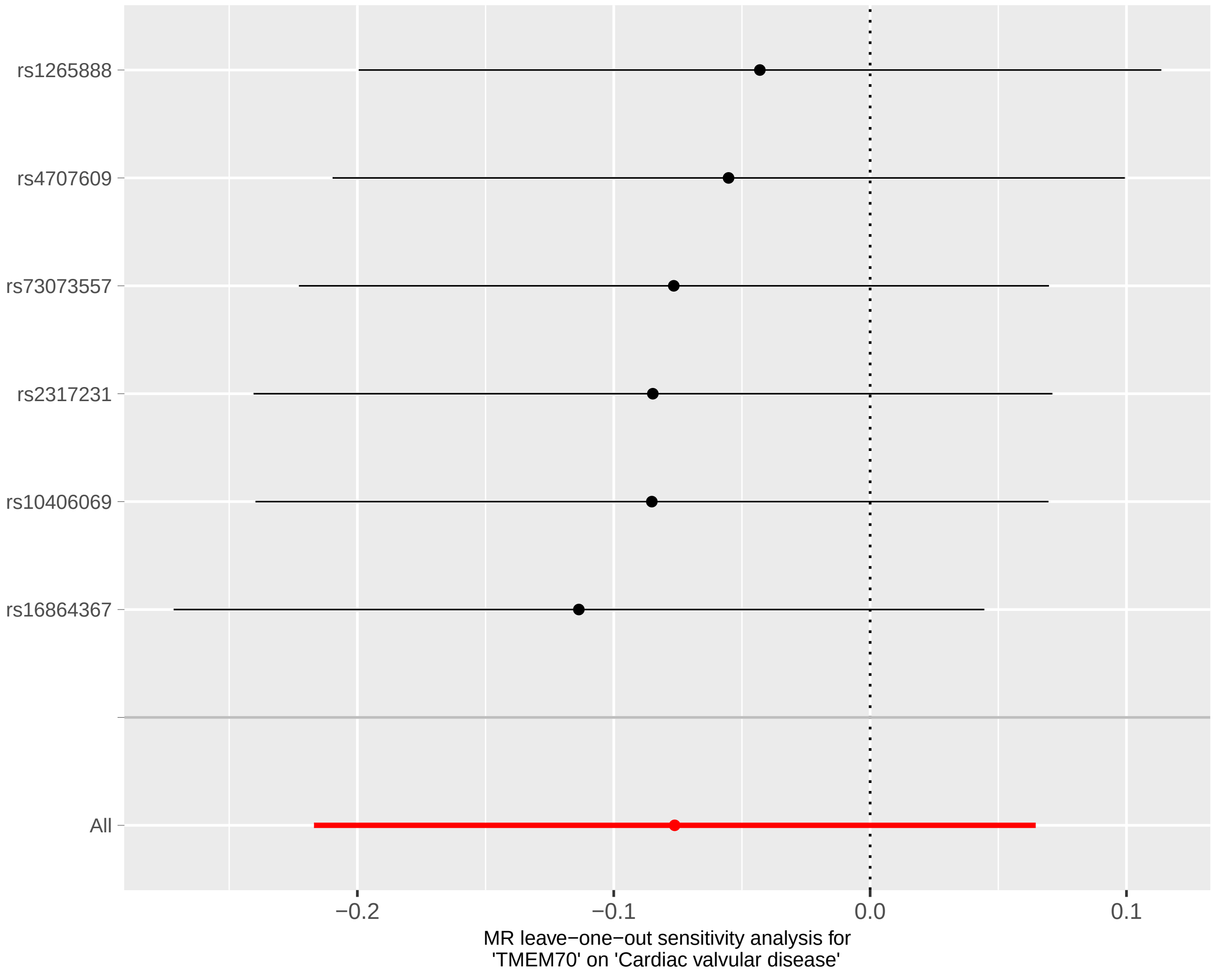
rs1265888
rs4707609
rs73073557
rs2317231
rs10406069
rs16864367
All
−0.2
−0.1
0.0
0.1
MR leave−one−out sensitivity analysis for
'TMEM70' on 'Cardiac valvular disease'

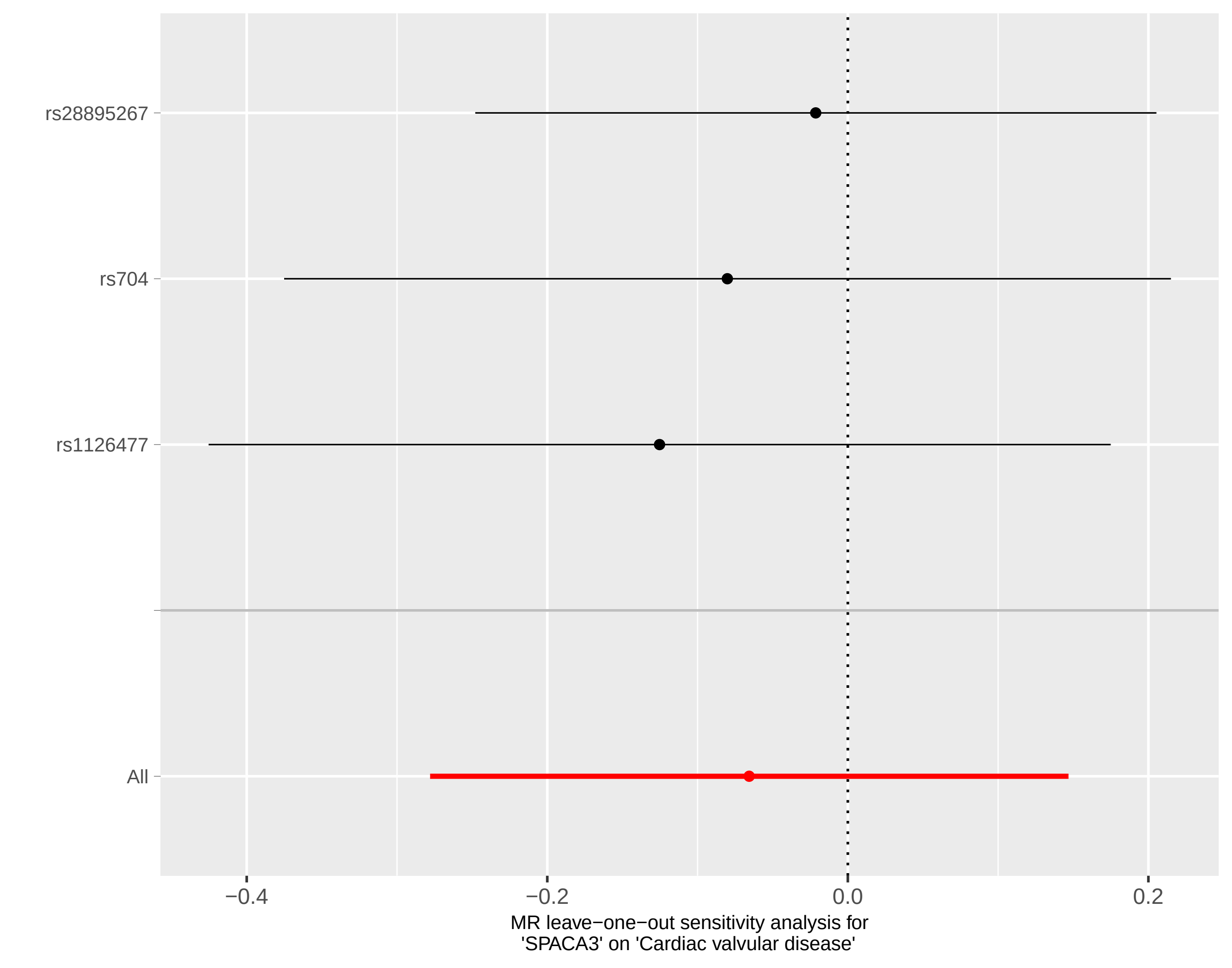

rs28895267
rs704
rs1126477
All
−0.4
−0.2
0.0
0.2
MR leave−one−out sensitivity analysis for
'SPACA3' on 'Cardiac valvular disease'

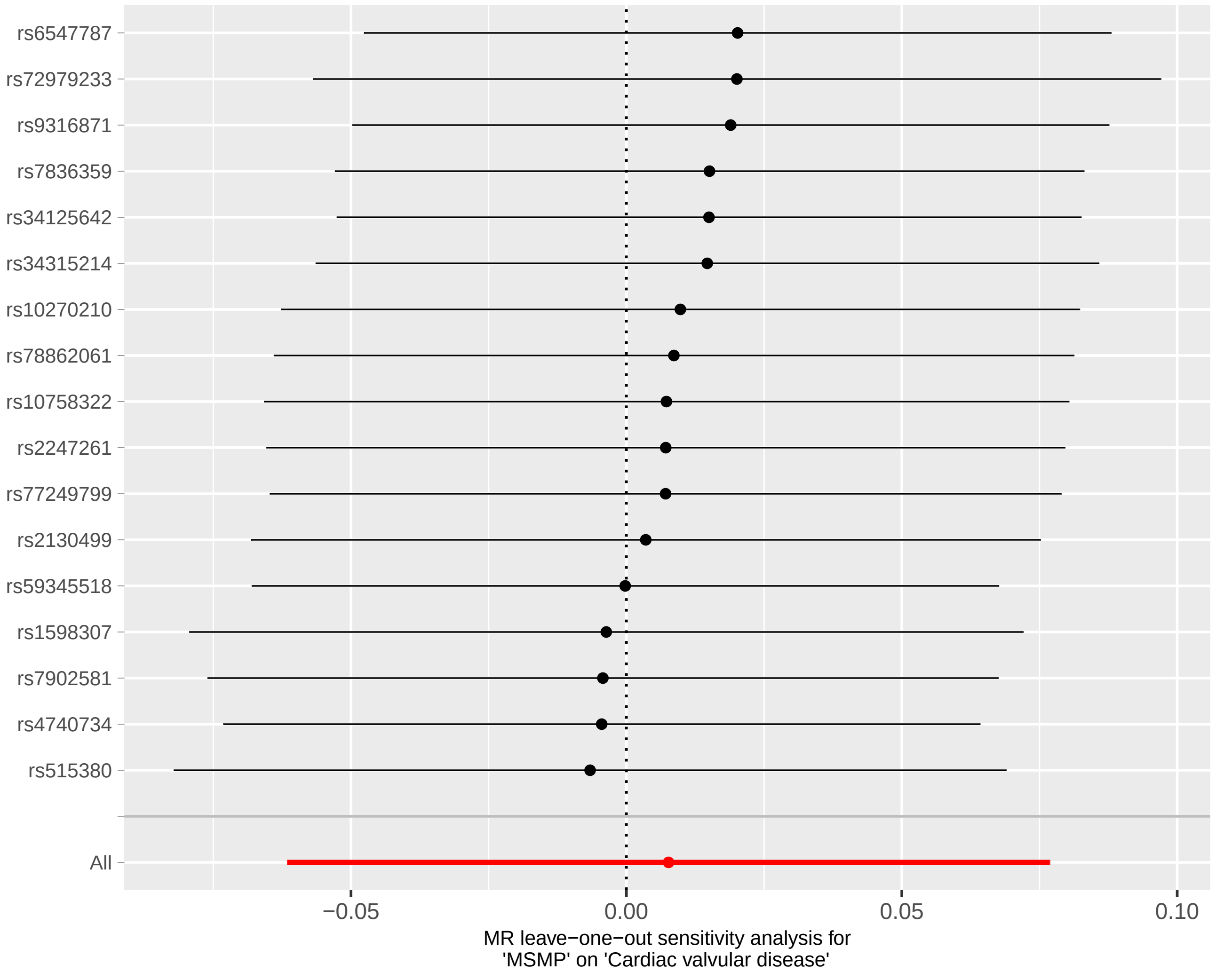
rs6547787
rs72979233
rs9316871
rs7836359
rs34125642
rs34315214
rs10270210
rs78862061
rs10758322
rs2247261
rs77249799
rs2130499
rs59345518
rs1598307
rs7902581
rs4740734
rs515380
All
−0.05
0.00
0.05
0.10
MR leave−one−out sensitivity analysis for
'MSMP' on 'Cardiac valvular disease'

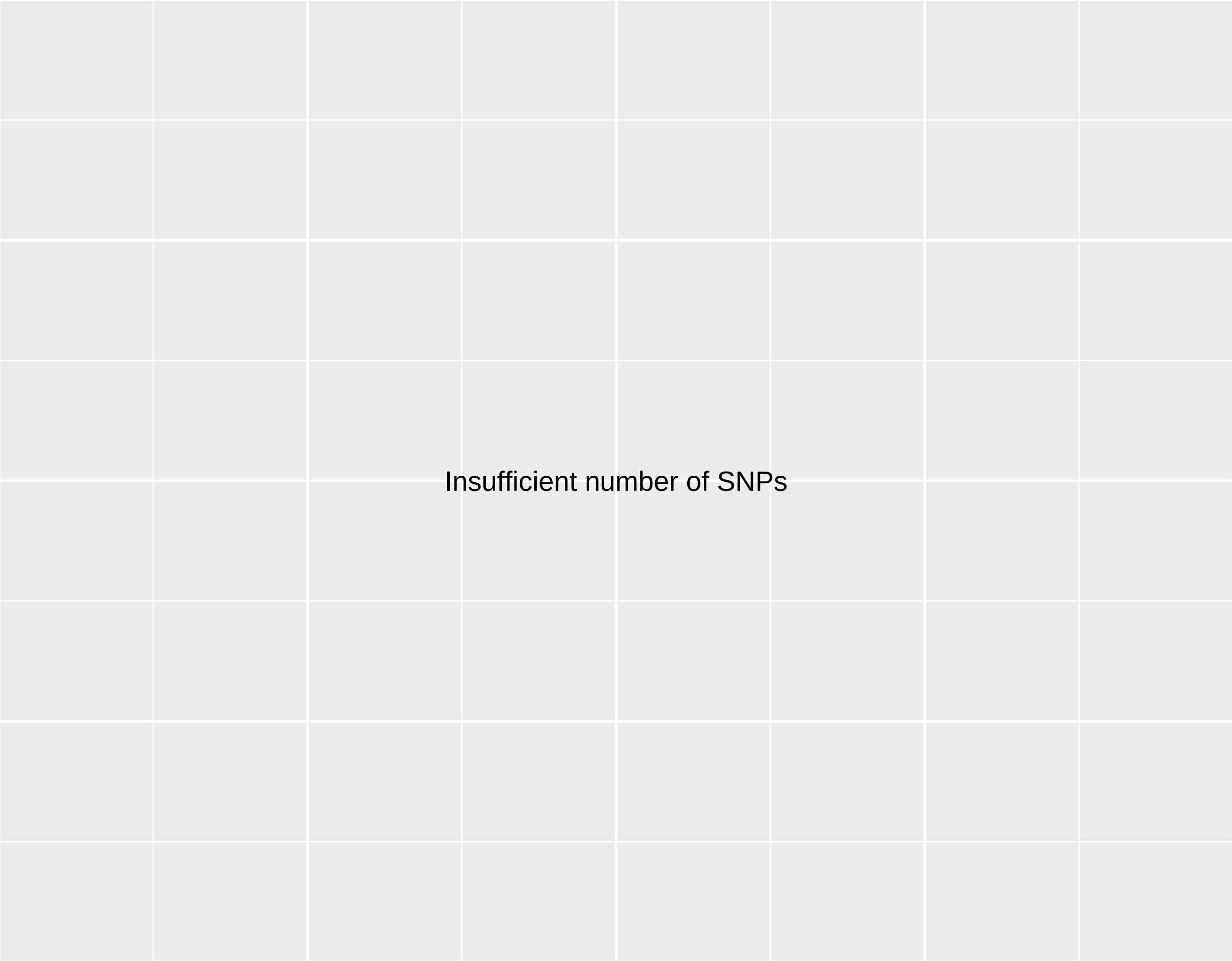
Insufficient number of SNPs

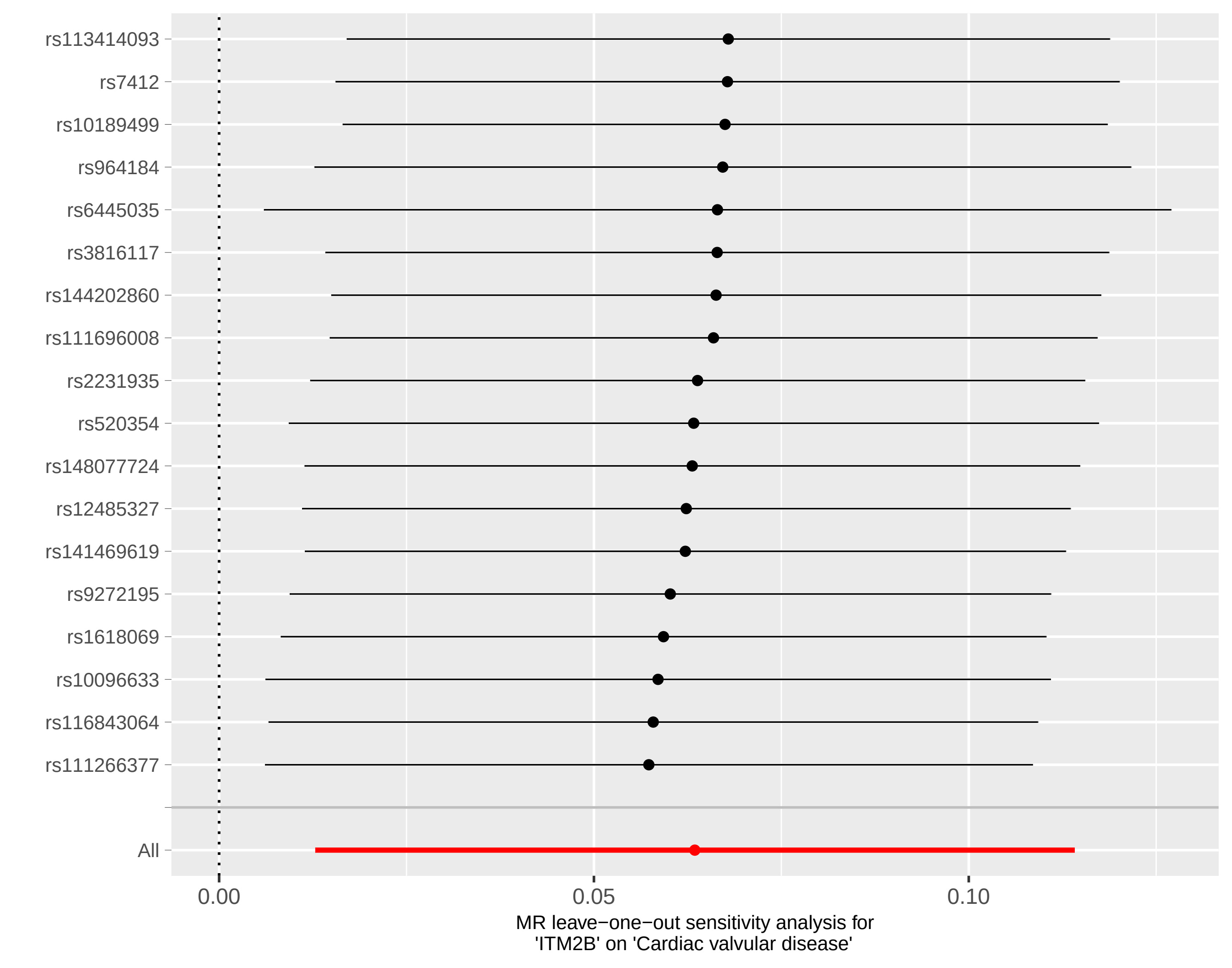
rs113414093
rs7412
rs10189499
rs964184
rs6445035
rs3816117
rs144202860
rs111696008
rs2231935
rs520354
rs148077724
rs12485327
rs141469619
rs9272195
rs1618069
rs10096633
rs116843064
rs111266377
All
0.00
0.05
0.10
MR leave−one−out sensitivity analysis for
'ITM2B' on 'Cardiac valvular disease'

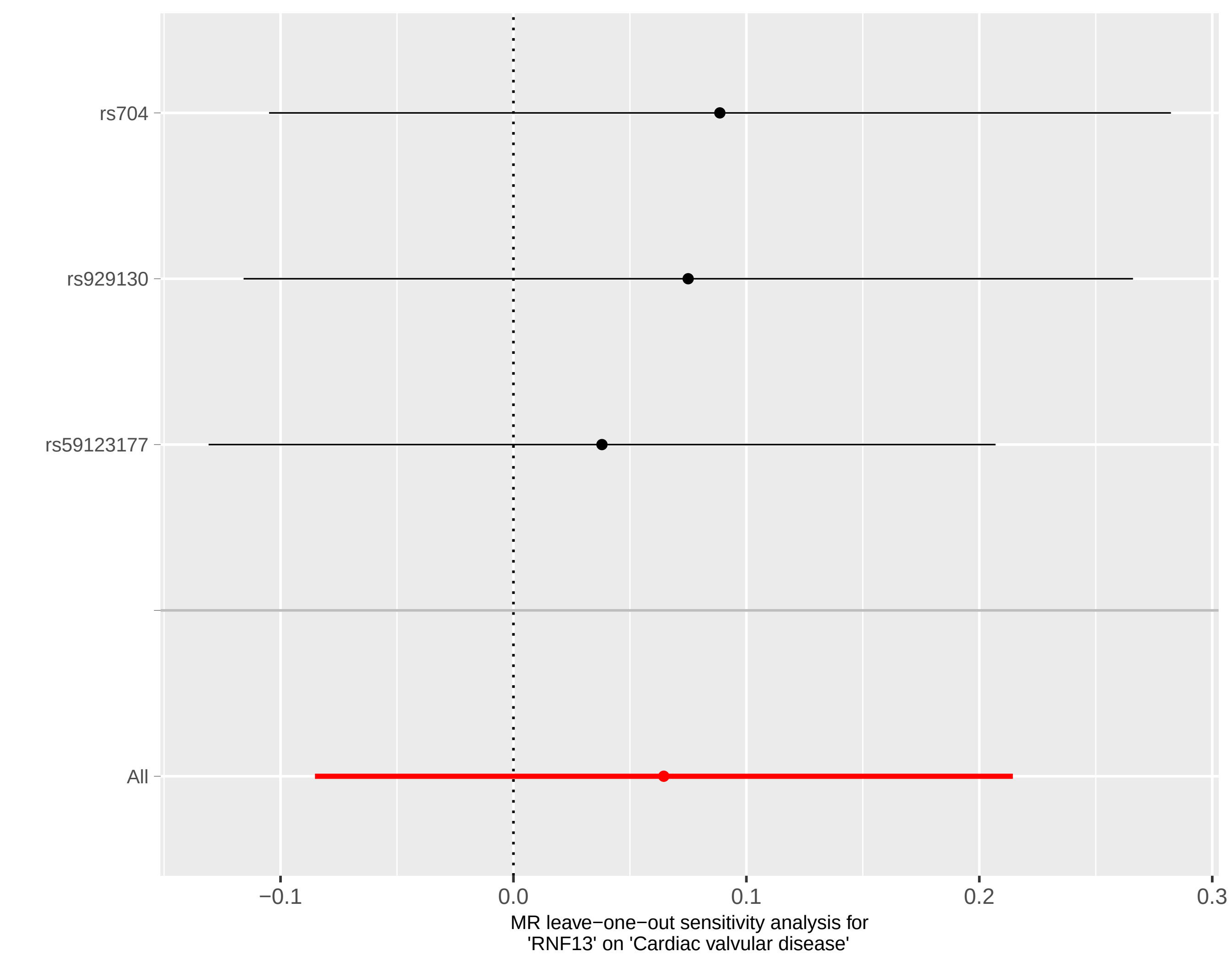

rs704
rs929130
rs59123177
All
−0.1
0.0
0.1
0.2
0.3
MR leave−one−out sensitivity analysis for
'RNF13' on 'Cardiac valvular disease'

Insufficient number of SNPs

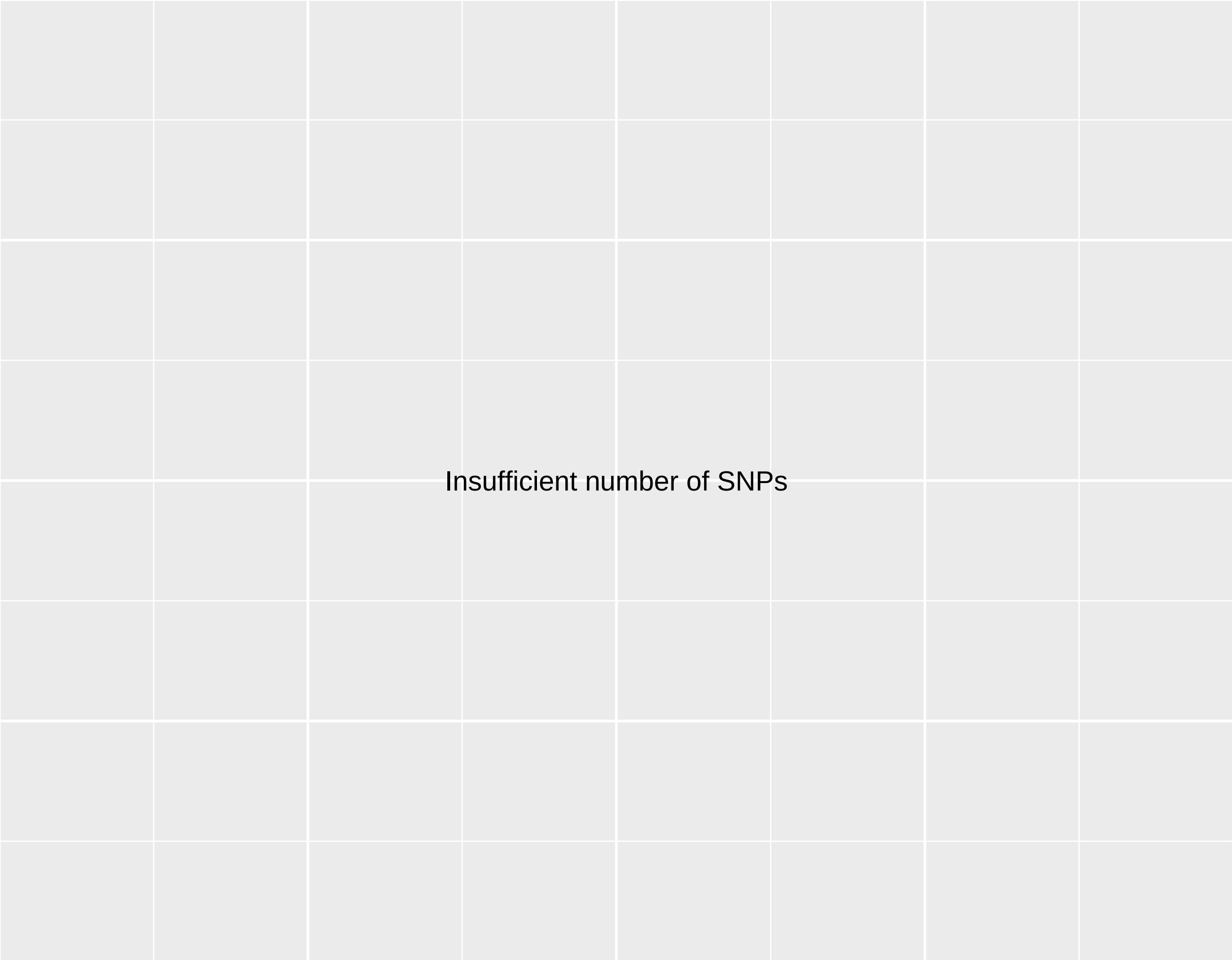
Insufficient number of SNPs

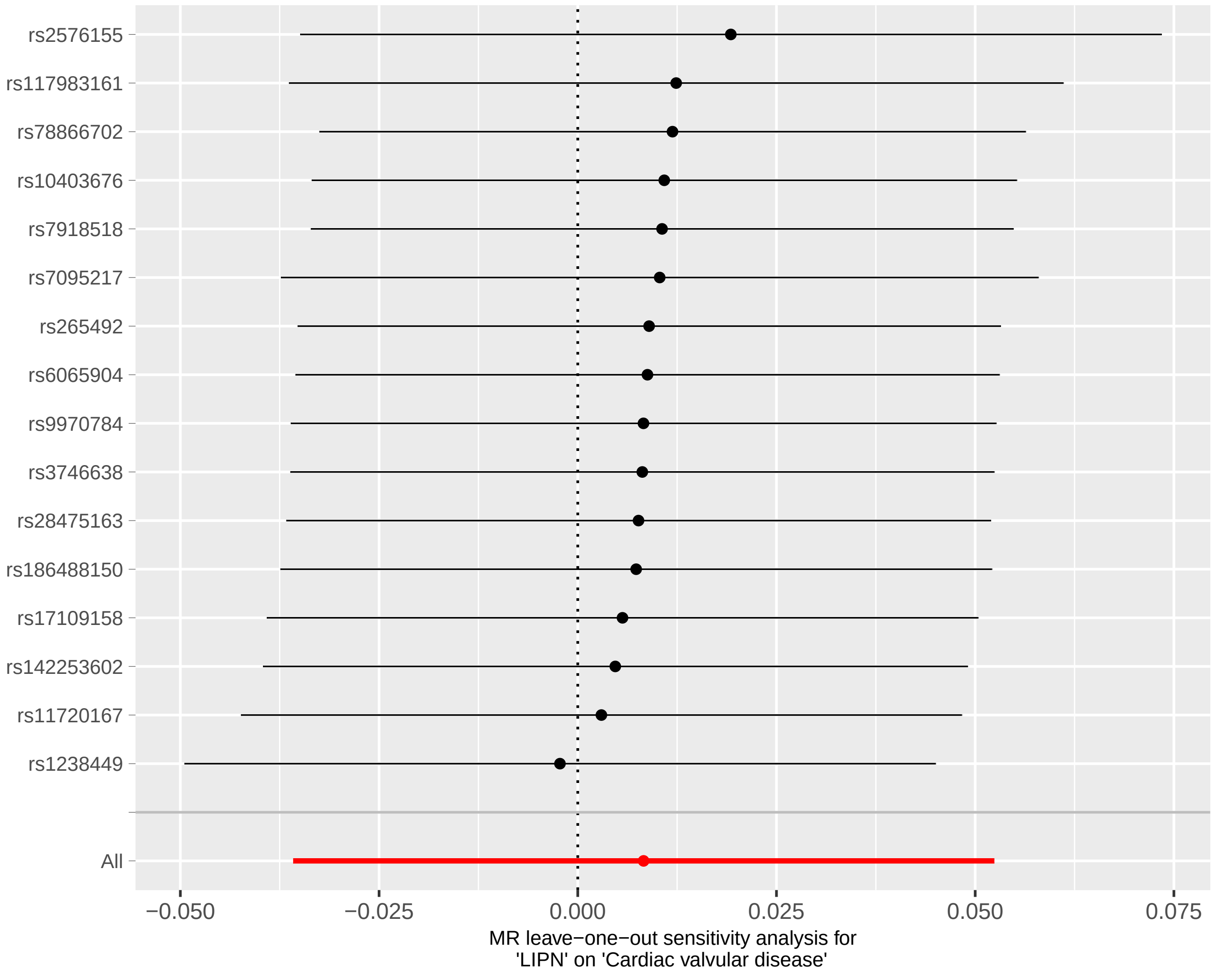

rs2576155
rs117983161
rs78866702
rs10403676
rs7918518
rs7095217
rs265492
rs6065904
rs9970784
rs3746638
rs28475163
rs186488150
rs17109158
rs142253602
rs11720167
rs1238449
All
−0.050
−0.025
0.000
0.025
0.050
0.075
MR leave−one−out sensitivity analysis for
'LIPN' on 'Cardiac valvular disease'

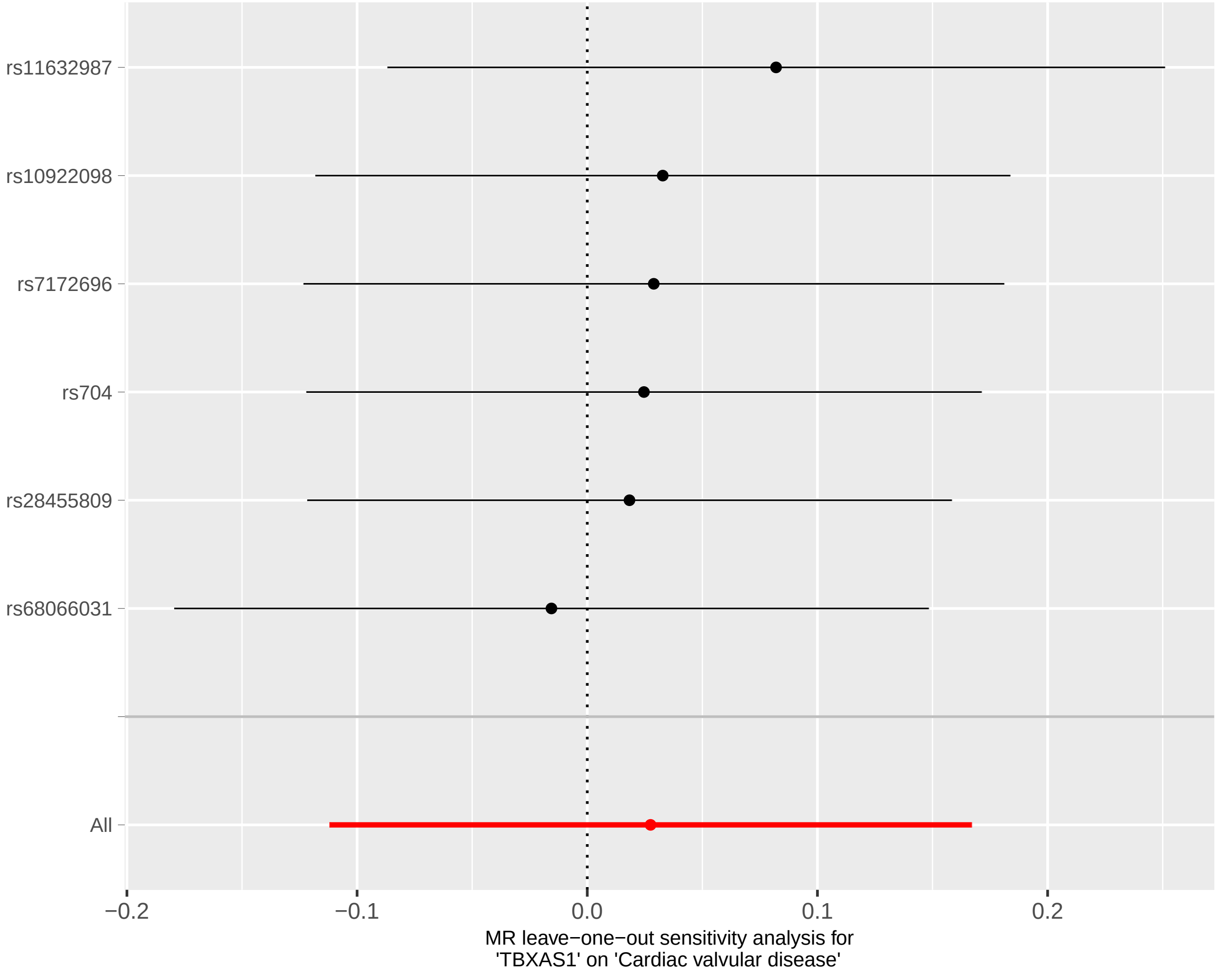
rs11632987
rs10922098
rs7172696
rs704
rs28455809
rs68066031
All
−0.2
−0.1
0.0
0.1
0.2
MR leave−one−out sensitivity analysis for
'TBXAS1' on 'Cardiac valvular disease'

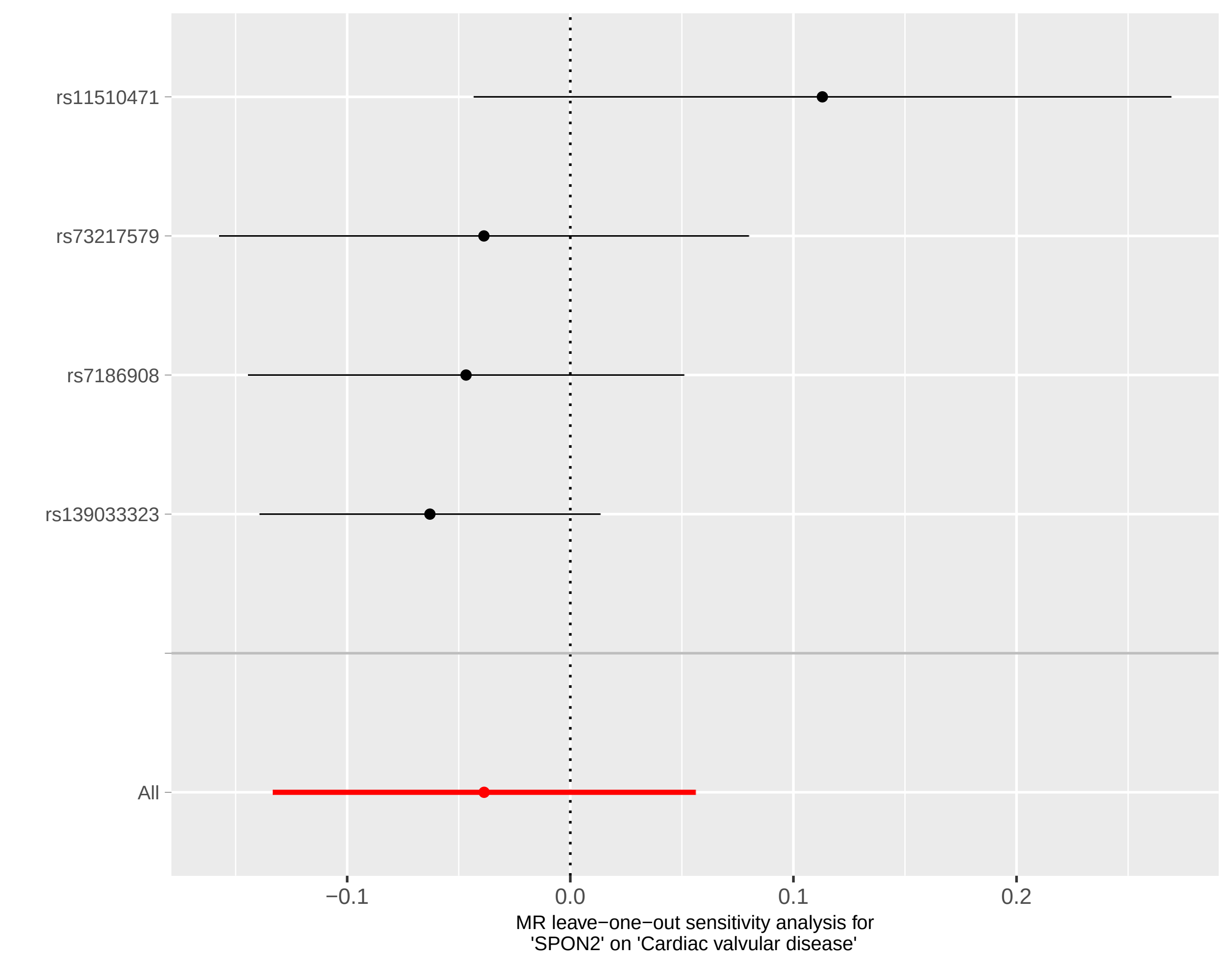
rs11510471
rs73217579
rs7186908
rs139033323
All
−0.1
0.0
0.1
0.2
MR leave−one−out sensitivity analysis for
'SPON2' on 'Cardiac valvular disease'

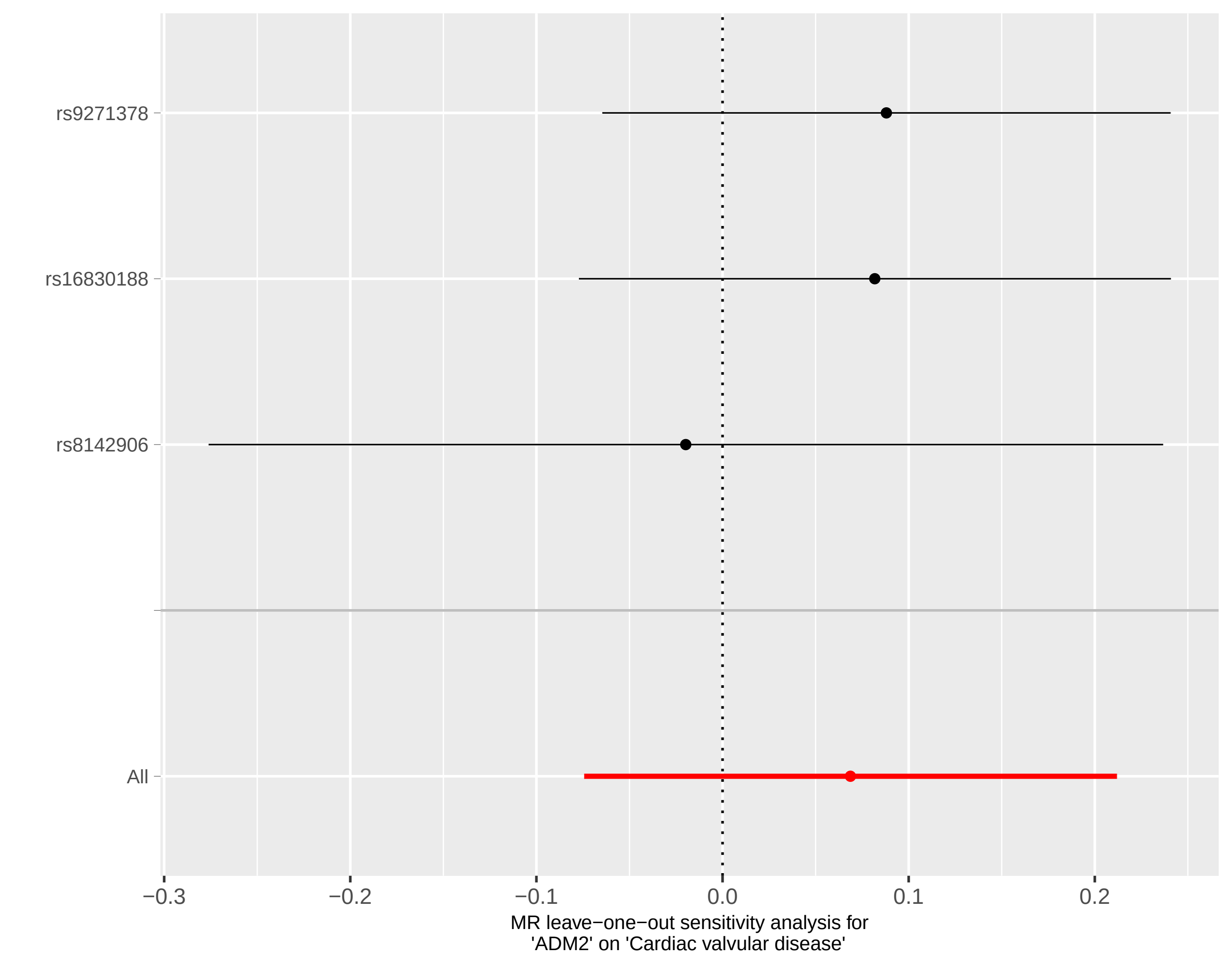
rs9271378
rs16830188
rs8142906
All
−0.3
−0.2
−0.1
0.0
0.1
0.2
MR leave−one−out sensitivity analysis for
'ADM2' on 'Cardiac valvular disease'

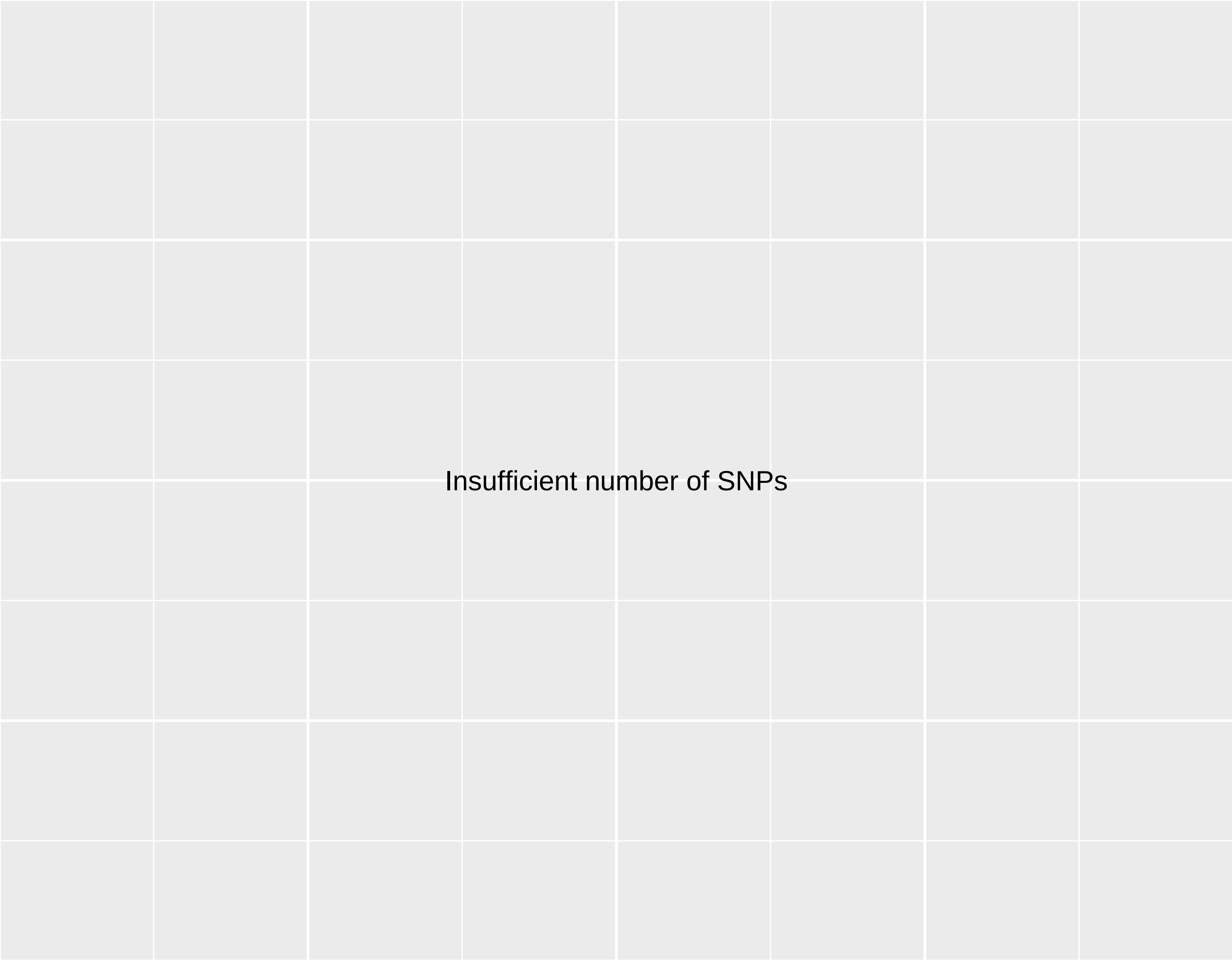
Insufficient number of SNPs

Insufficient number of SNPs

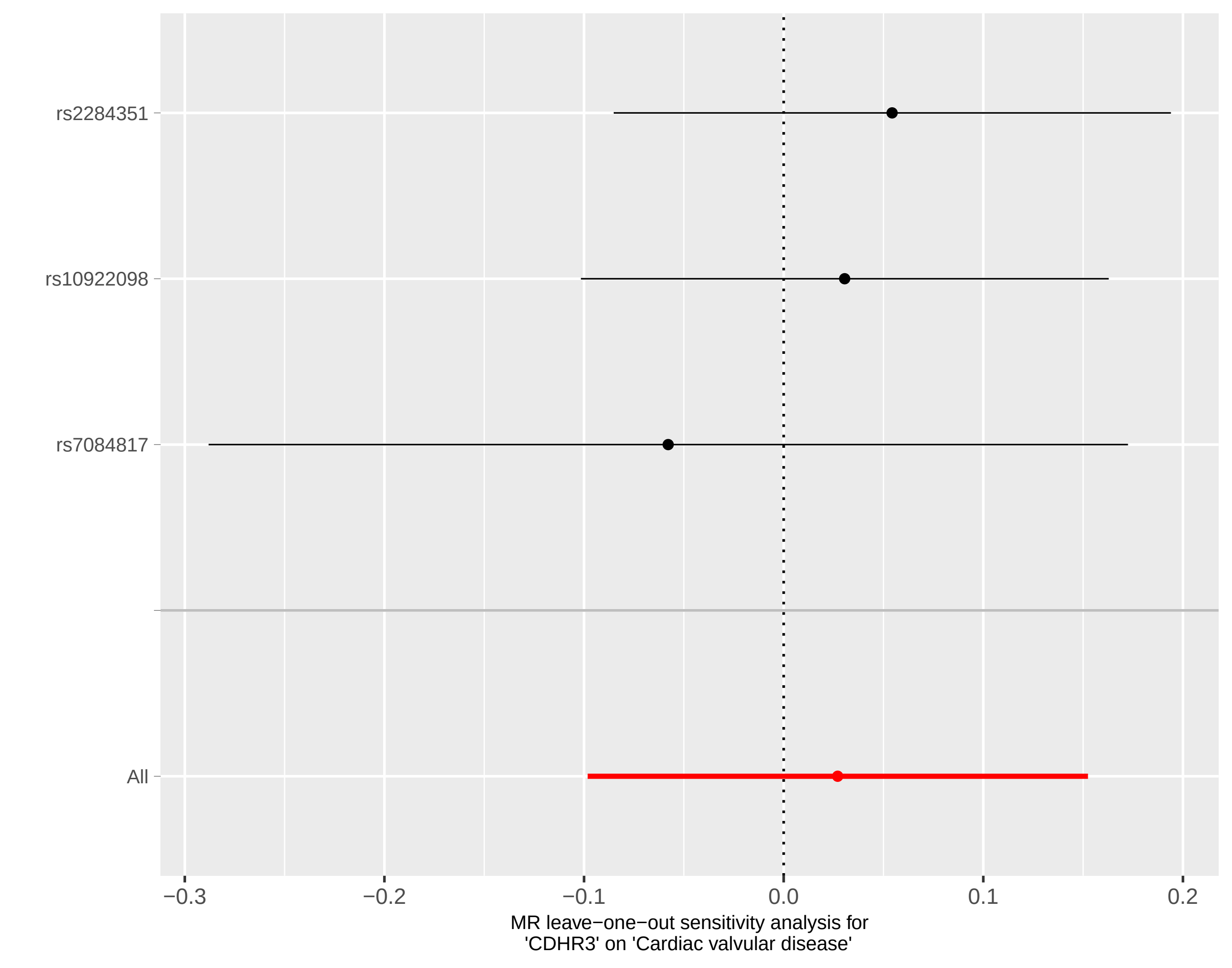

rs2284351
rs10922098
rs7084817
All
−0.3
−0.2
−0.1
0.0
0.1
0.2
MR leave−one−out sensitivity analysis for
'CDHR3' on 'Cardiac valvular disease'

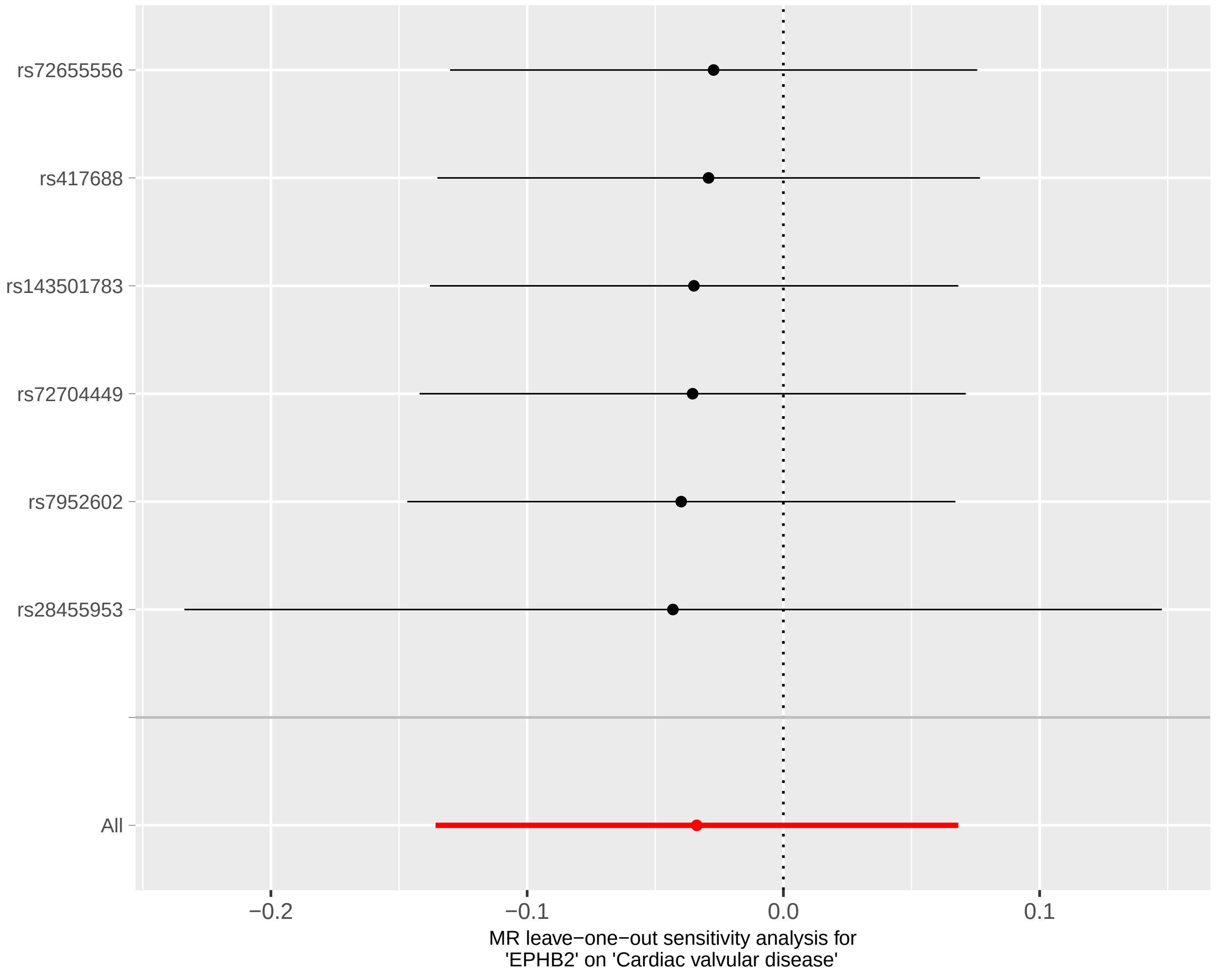
rs72655556
rs417688
rs143501783
rs72704449
rs7952602
rs28455953
All
−0.2
−0.1
0.0
0.1
MR leave−one−out sensitivity analysis for
'EPHB2' on 'Cardiac valvular disease'

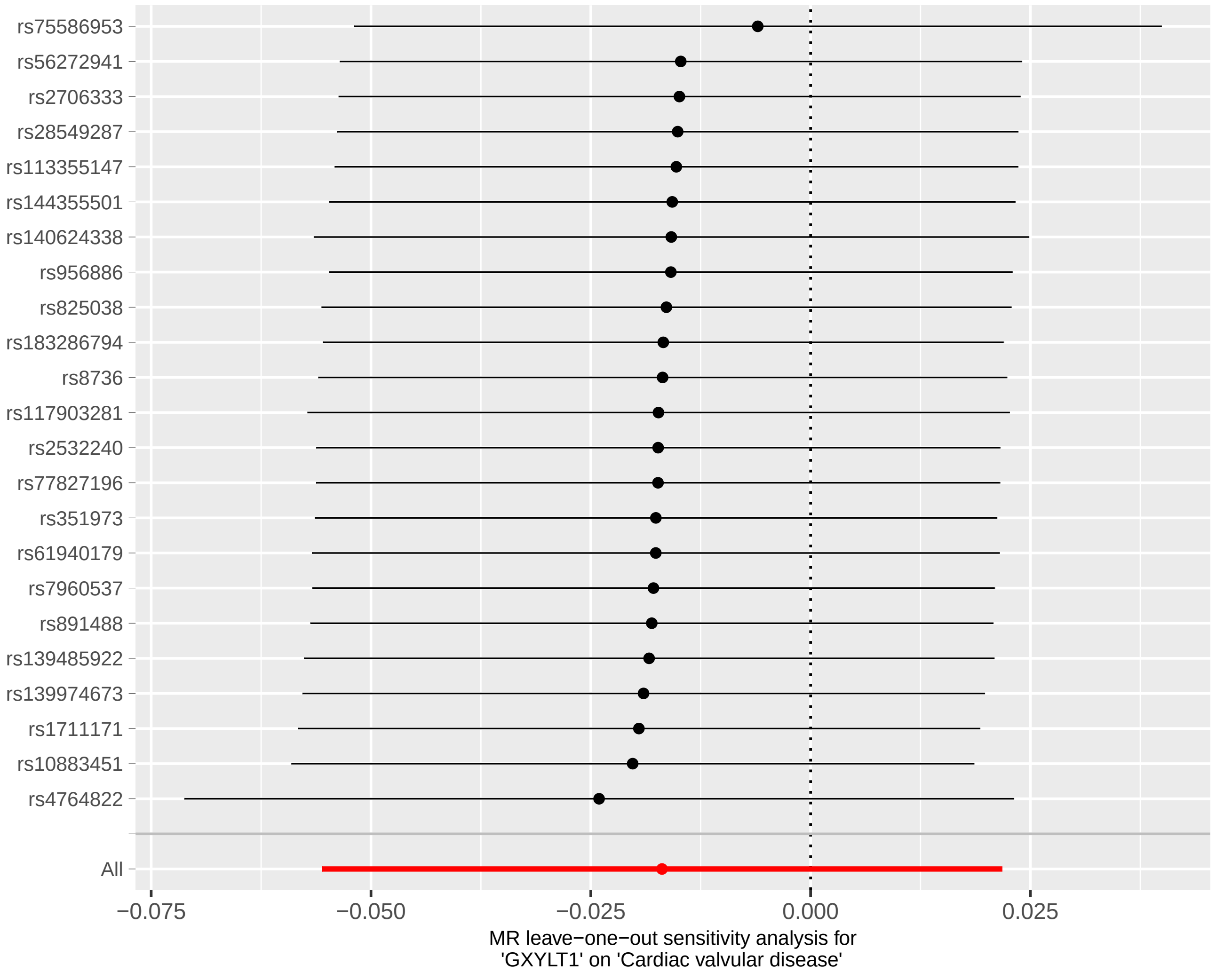
rs75586953
rs56272941
rs2706333
rs28549287
rs113355147
rs144355501
rs140624338
rs956886
rs825038
rs183286794
rs8736
rs117903281
rs2532240
rs77827196
rs351973
rs61940179
rs7960537
rs891488
rs139485922
rs139974673
rs1711171
rs10883451
rs4764822
All
−0.075
−0.050
−0.025
0.000
0.025
MR leave−one−out sensitivity analysis for
'GXYLT1' on 'Cardiac valvular disease'

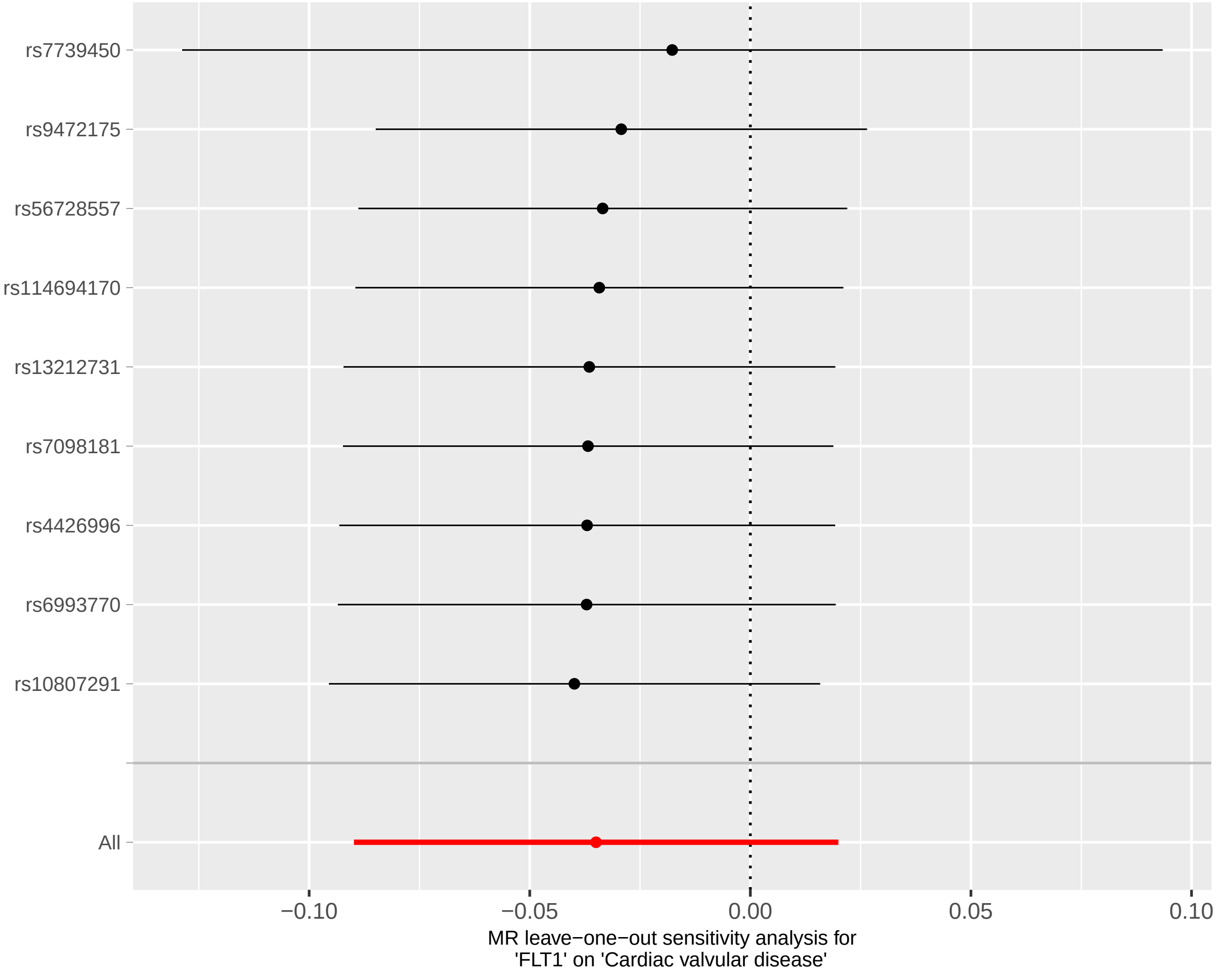

rs7739450
rs9472175
rs56728557
rs114694170
rs13212731
rs7098181
rs4426996
rs6993770
rs10807291
All
−0.10
−0.05
0.00
0.05
0.10
MR leave−one−out sensitivity analysis for
'FLT1' on 'Cardiac valvular disease'

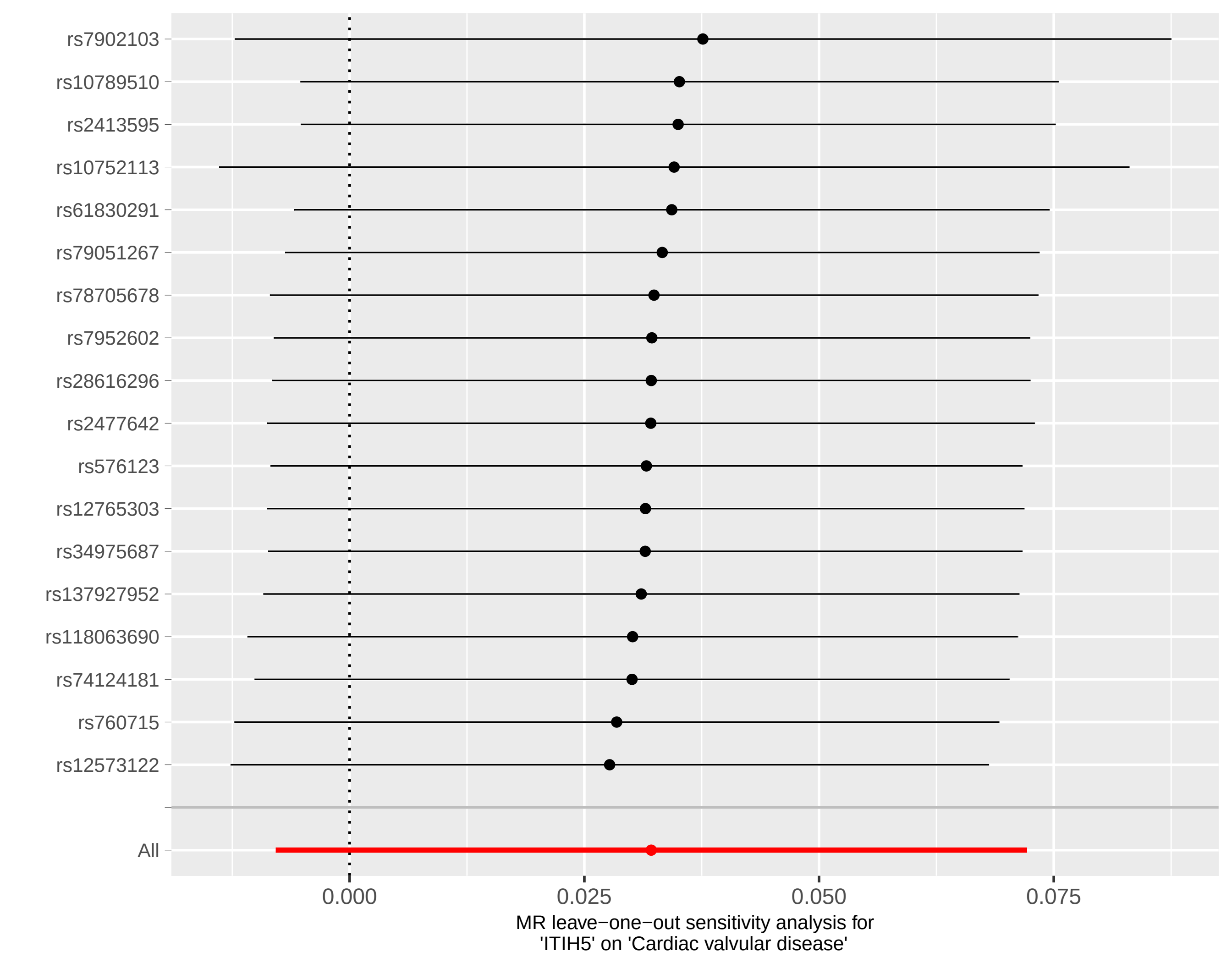
rs7902103
rs10789510
rs2413595
rs10752113
rs61830291
rs79051267
rs78705678
rs7952602
rs28616296
rs2477642
rs576123
rs12765303
rs34975687
rs137927952
rs118063690
rs74124181
rs760715
rs12573122
All
0.000
0.025
0.050
0.075
MR leave−one−out sensitivity analysis for
'ITIH5' on 'Cardiac valvular disease'

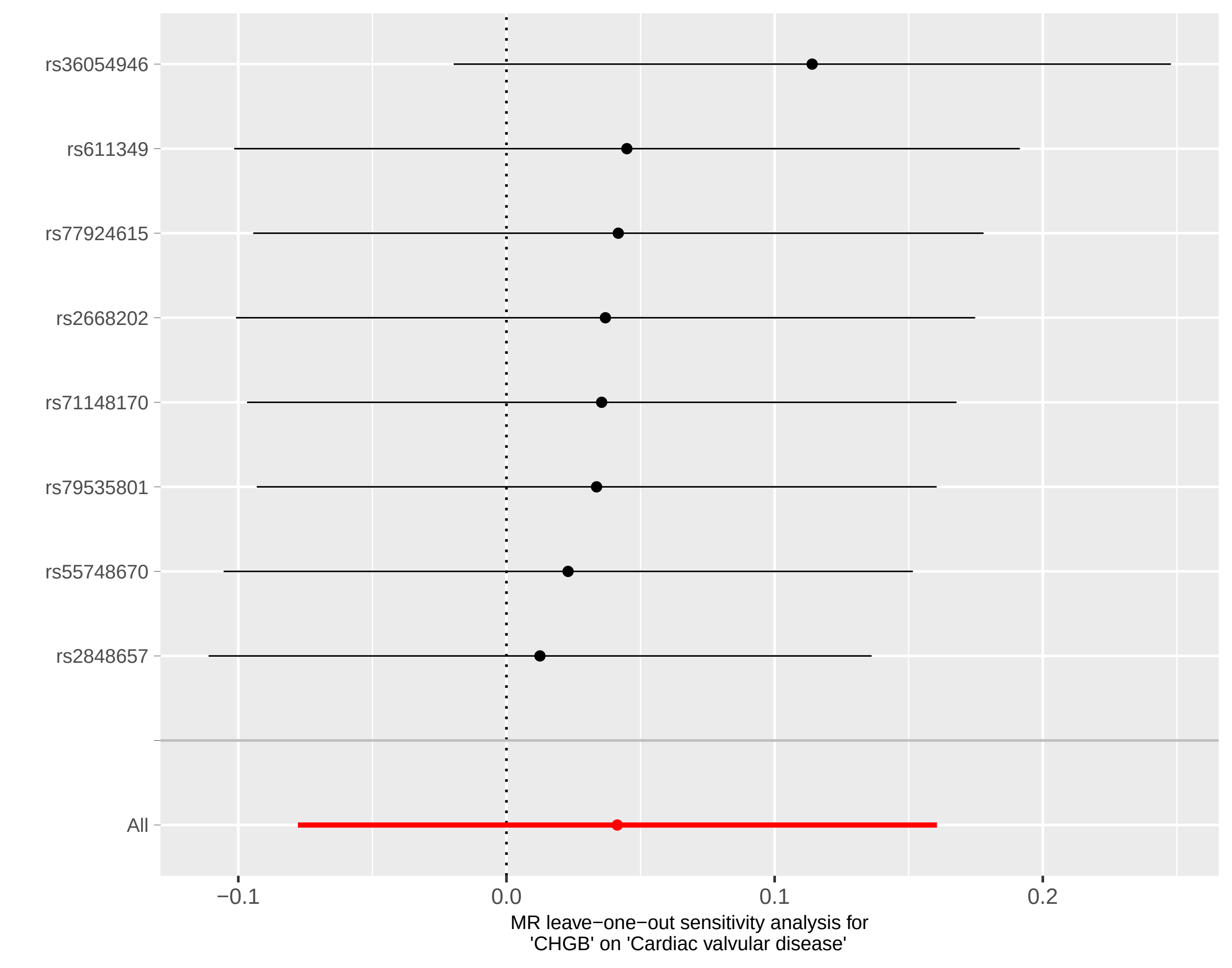
rs36054946
rs611349
rs77924615
rs2668202
rs71148170
rs79535801
rs55748670
rs2848657
All
−0.1
0.0
0.1
0.2
MR leave−one−out sensitivity analysis for
'CHGB' on 'Cardiac valvular disease'

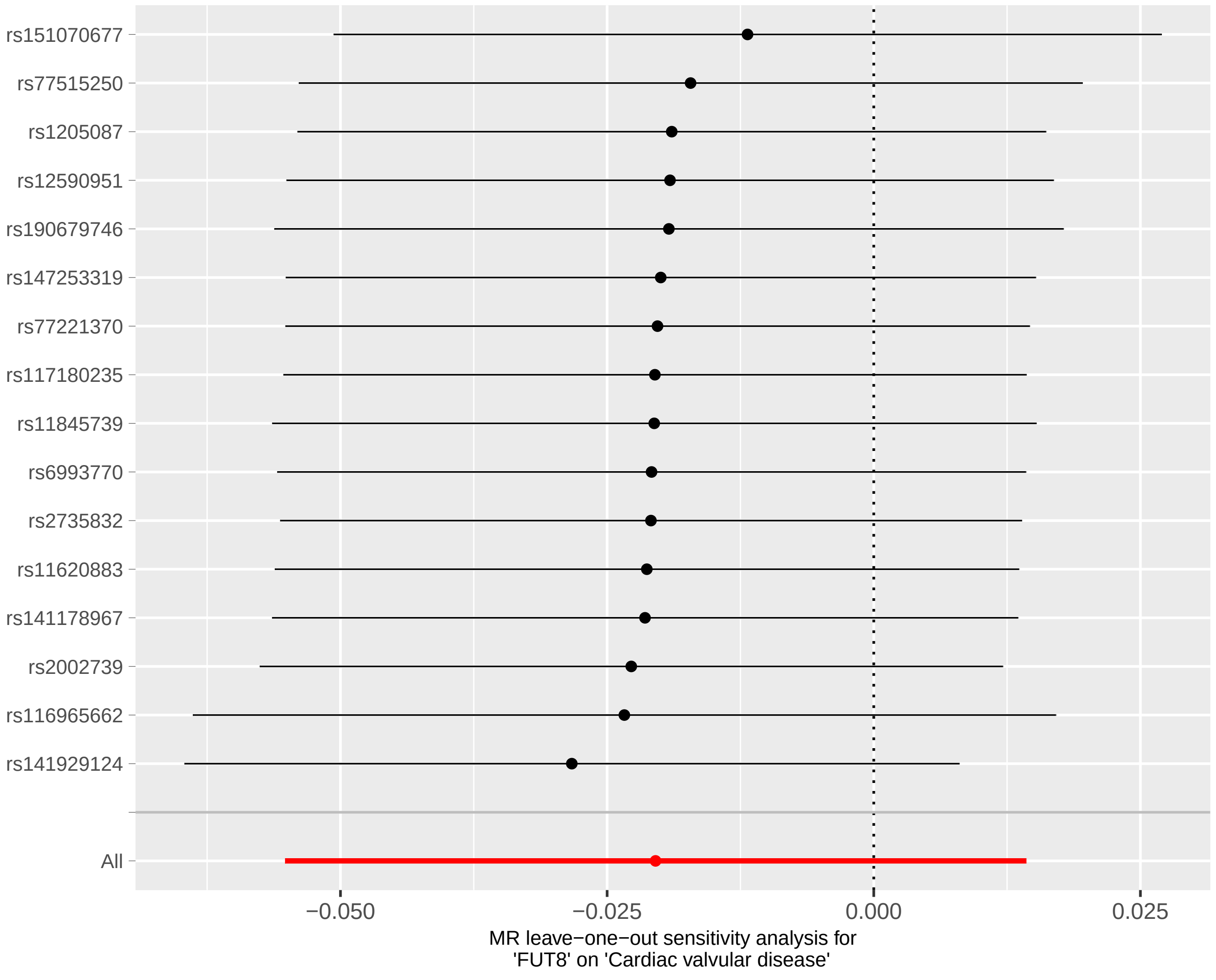
rs151070677
rs77515250
rs1205087
rs12590951
rs190679746
rs147253319
rs77221370
rs117180235
rs11845739
rs6993770
rs2735832
rs11620883
rs141178967
rs2002739
rs116965662
rs141929124
All
−0.050
−0.025
0.000
0.025
MR leave−one−out sensitivity analysis for
'FUT8' on 'Cardiac valvular disease'

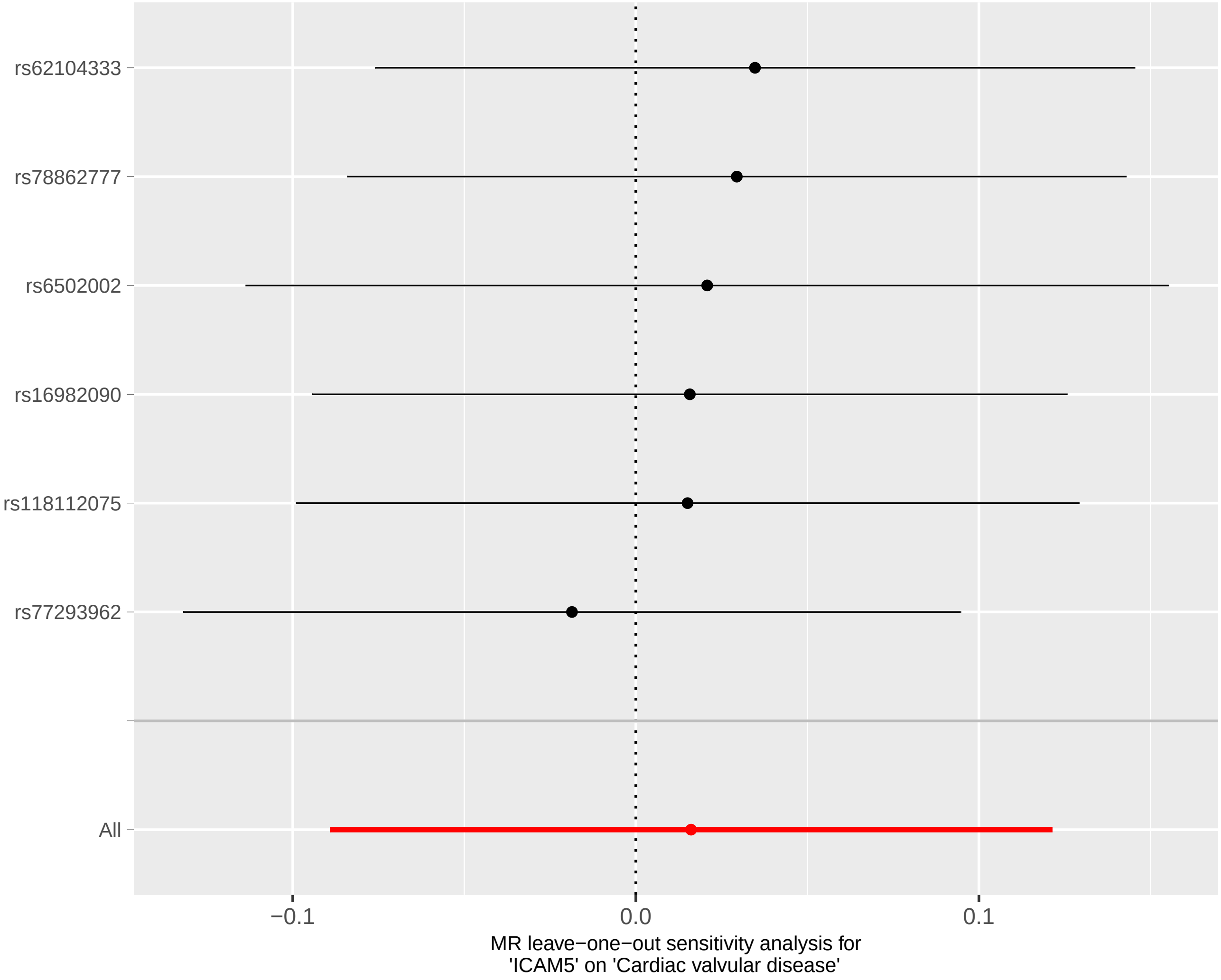

rs62104333
rs78862777
rs6502002
rs16982090
rs118112075
rs77293962
All
−0.1
0.0
0.1
MR leave−one−out sensitivity analysis for
'ICAM5' on 'Cardiac valvular disease'

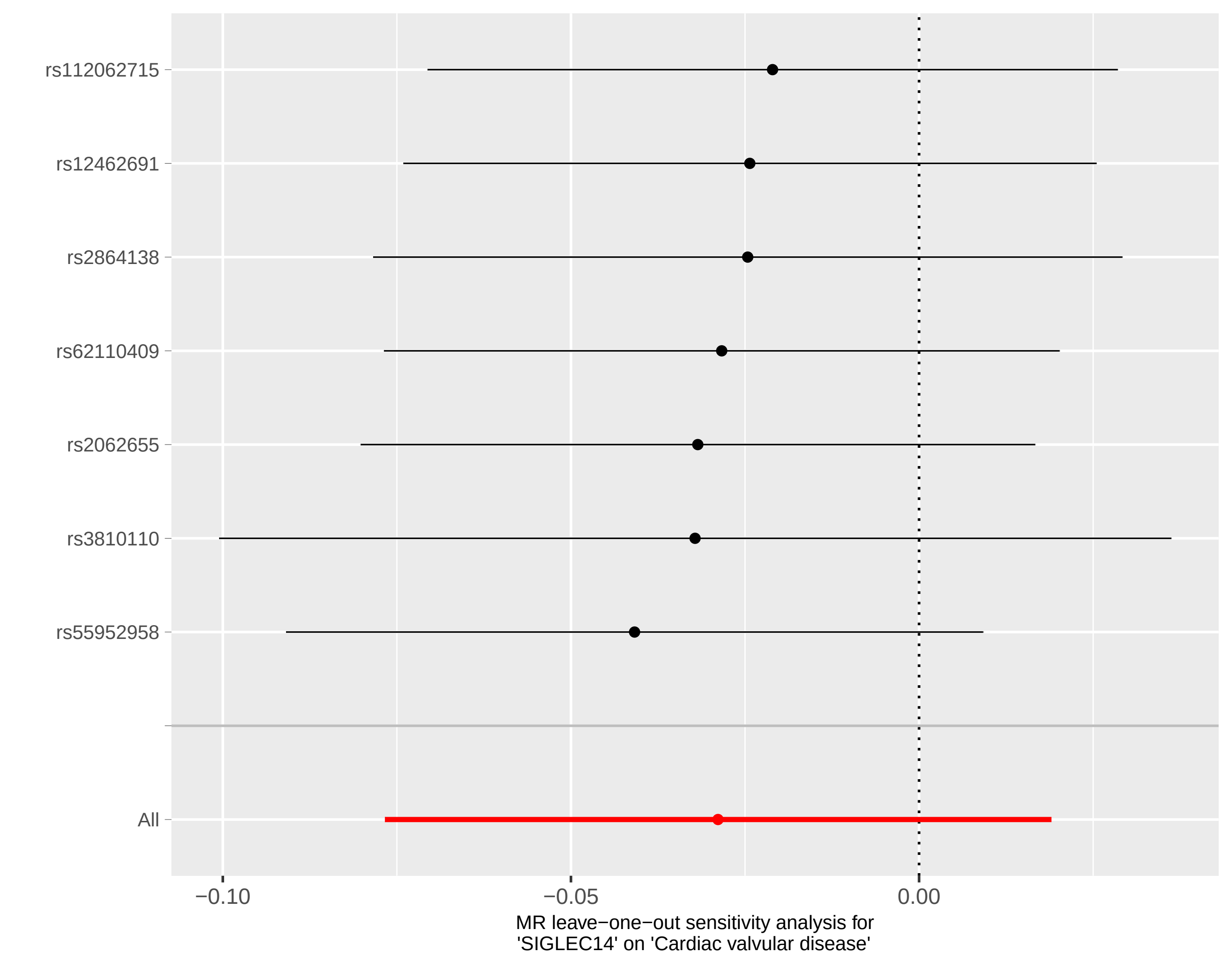

rs112062715
rs12462691
rs2864138
rs62110409
rs2062655
rs3810110
rs55952958
All
−0.10
−0.05
0.00
MR leave−one−out sensitivity analysis for
'SIGLEC14' on 'Cardiac valvular disease'

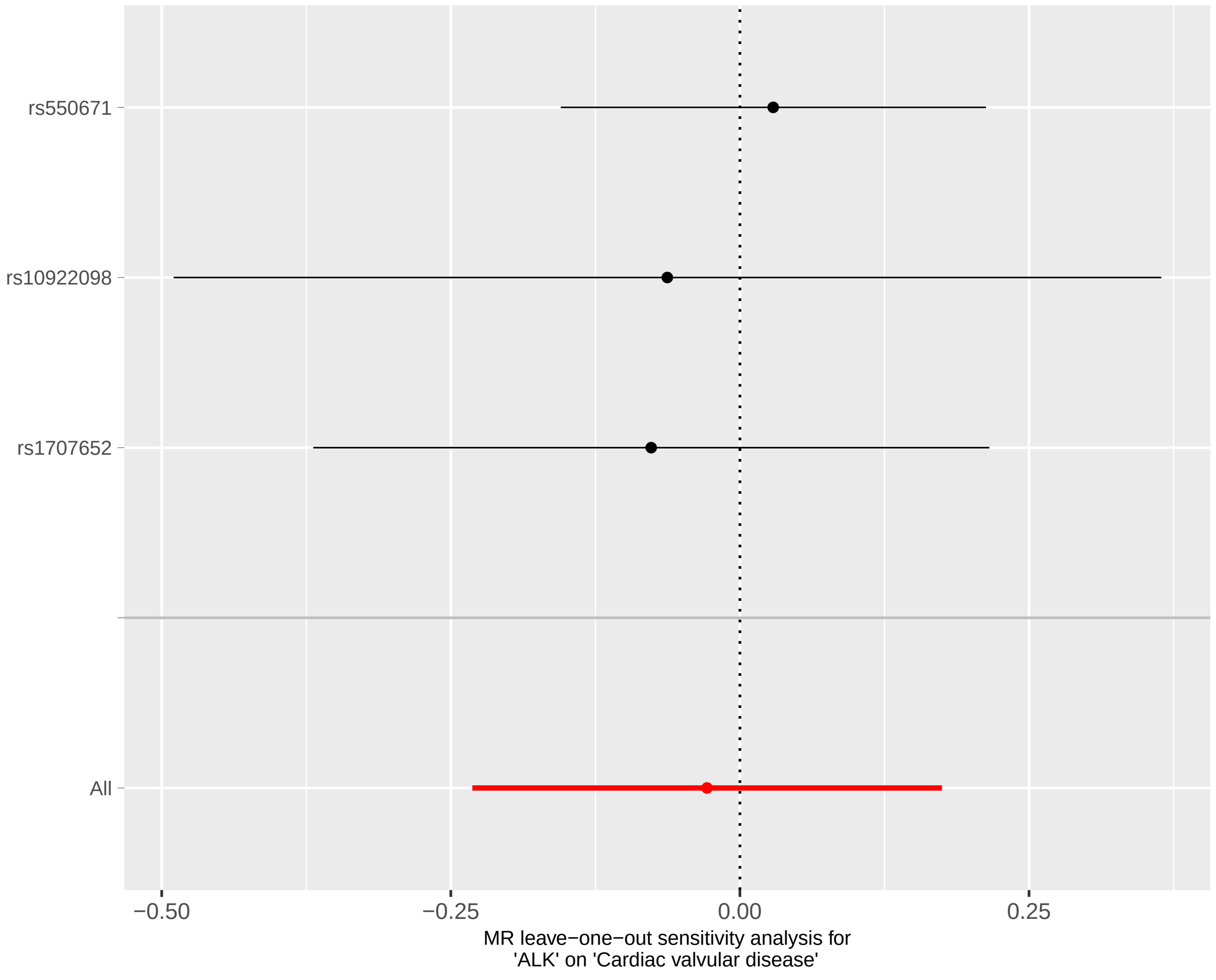
rs550671
rs10922098
rs1707652
All
−0.50
−0.25
0.00
0.25
MR leave−one−out sensitivity analysis for
'ALK' on 'Cardiac valvular disease'

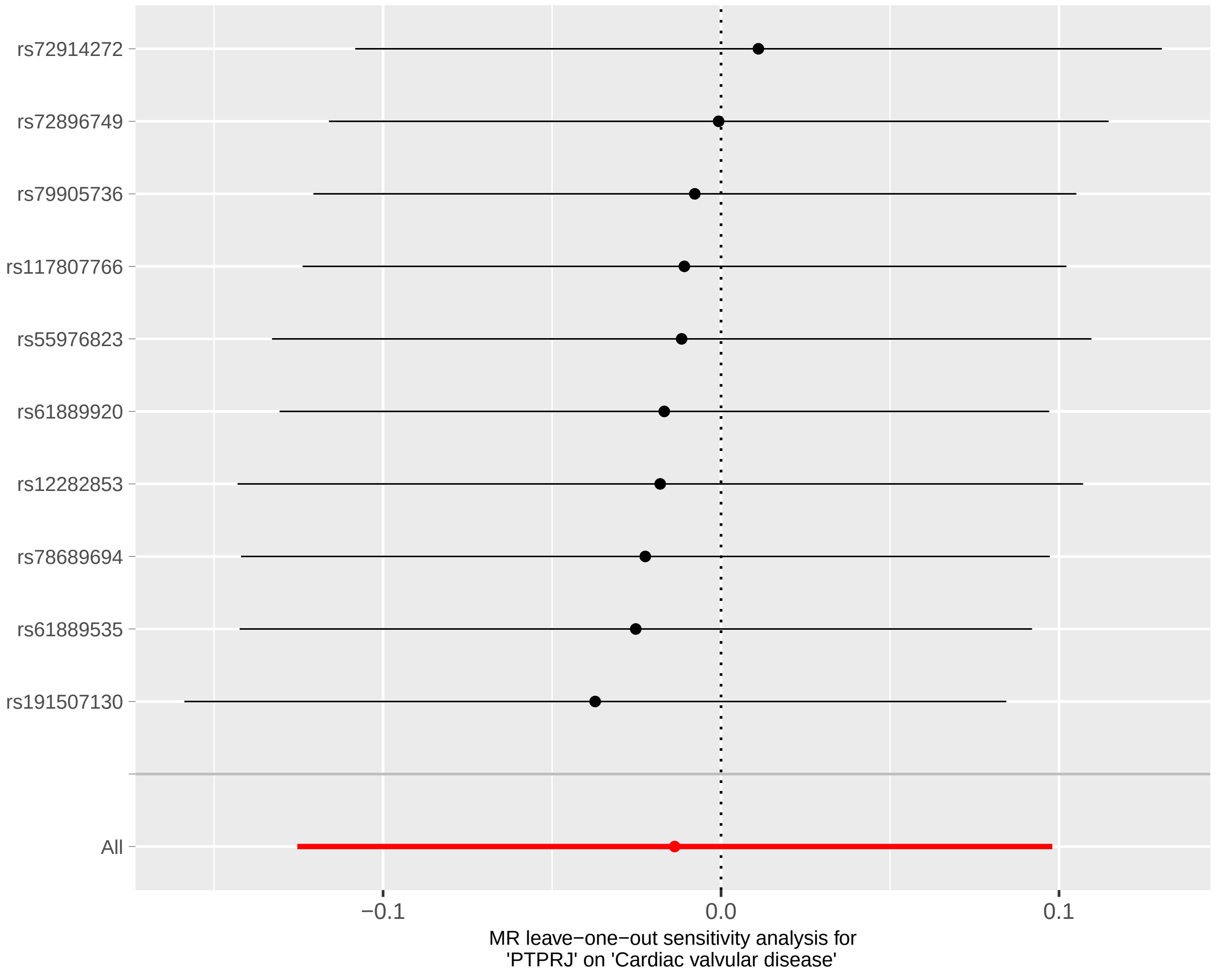
rs72914272
rs72896749
rs79905736
rs117807766
rs55976823
rs61889920
rs12282853
rs78689694
rs61889535
rs191507130
All
−0.1
0.0
0.1
MR leave−one−out sensitivity analysis for
'PTPRJ' on 'Cardiac valvular disease'

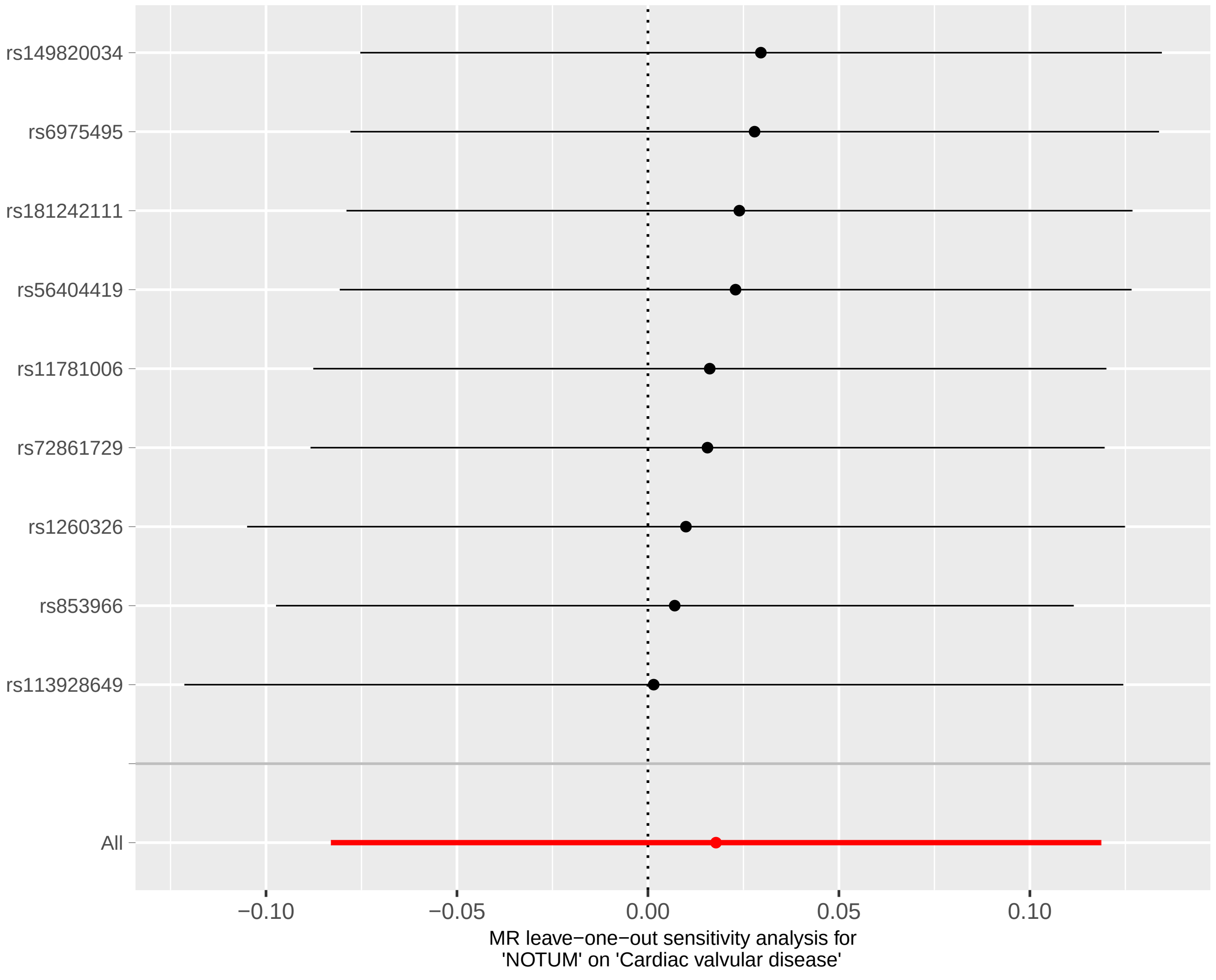

rs149820034
rs6975495
rs181242111
rs56404419
rs11781006
rs72861729
rs1260326
rs853966
rs113928649
All
−0.10
−0.05
0.00
0.05
0.10
MR leave−one−out sensitivity analysis for
'NOTUM' on 'Cardiac valvular disease'

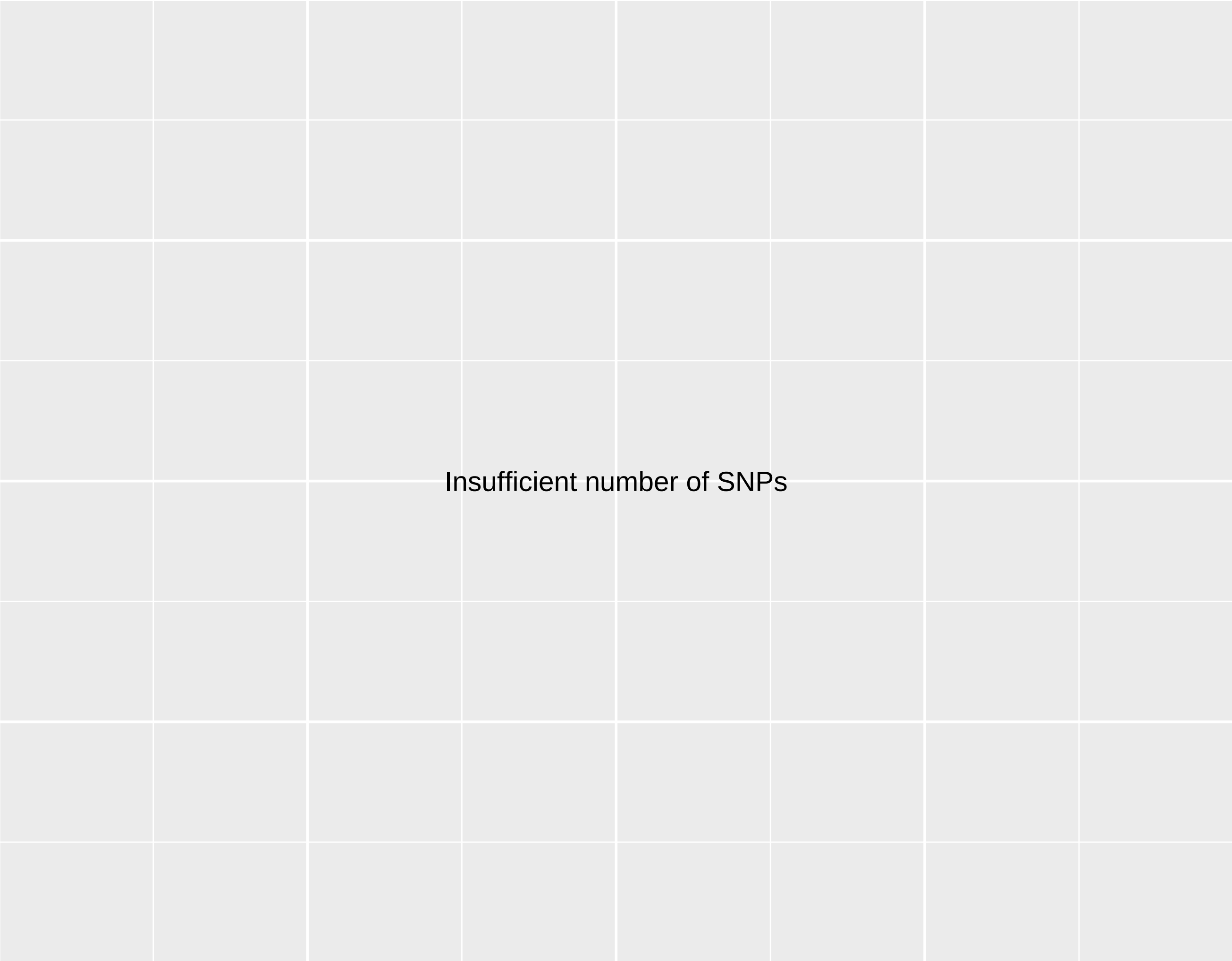
Insufficient number of SNPs

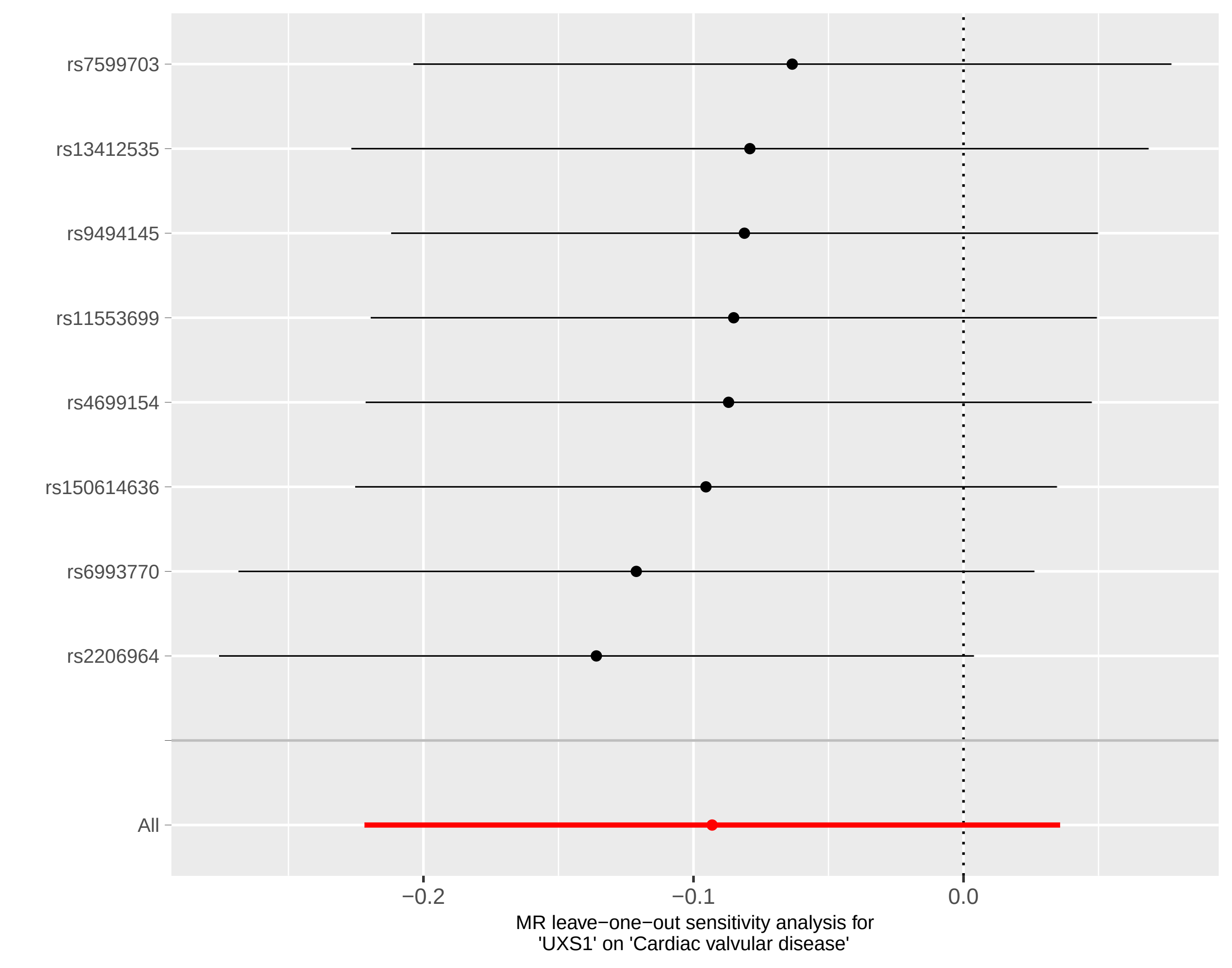
rs7599703
rs13412535
rs9494145
rs11553699
rs4699154
rs150614636
rs6993770
rs2206964
All
−0.2
−0.1
0.0
MR leave−one−out sensitivity analysis for
'UXS1' on 'Cardiac valvular disease'

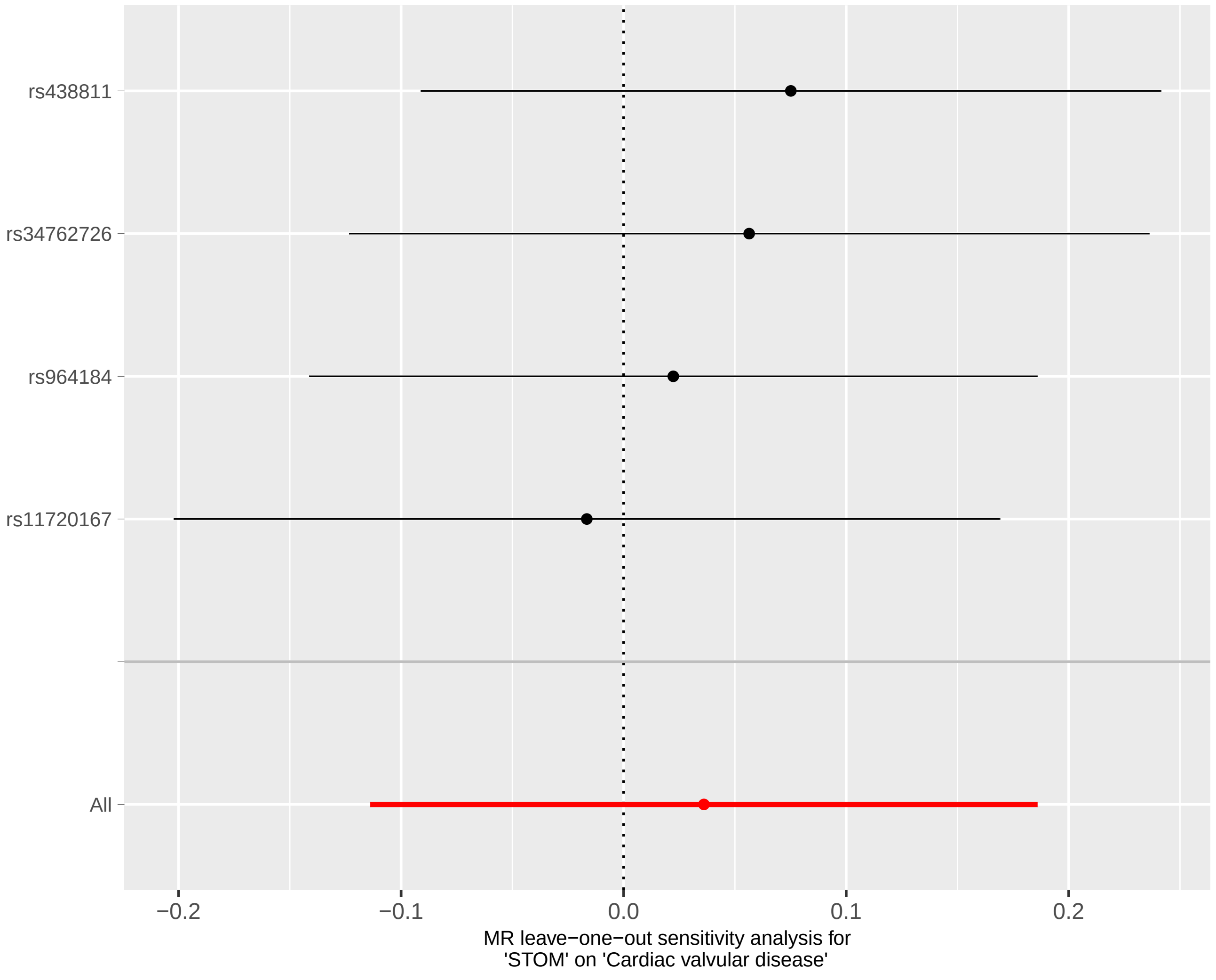
rs438811
rs34762726
rs964184
rs11720167
All
−0.2
−0.1
0.0
0.1
0.2
MR leave−one−out sensitivity analysis for
'STOM' on 'Cardiac valvular disease'

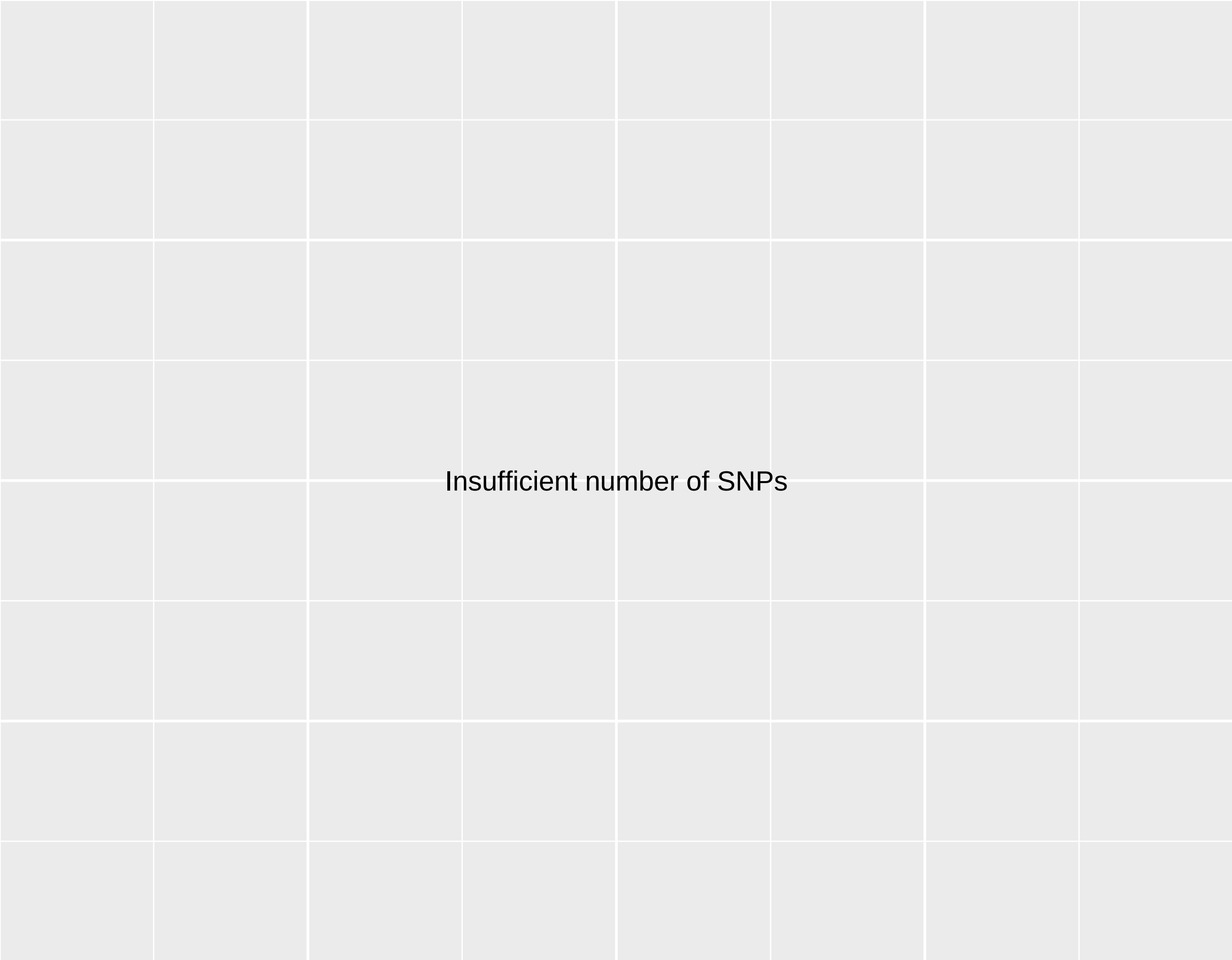
Insufficient number of SNPs

Insufficient number of SNPs

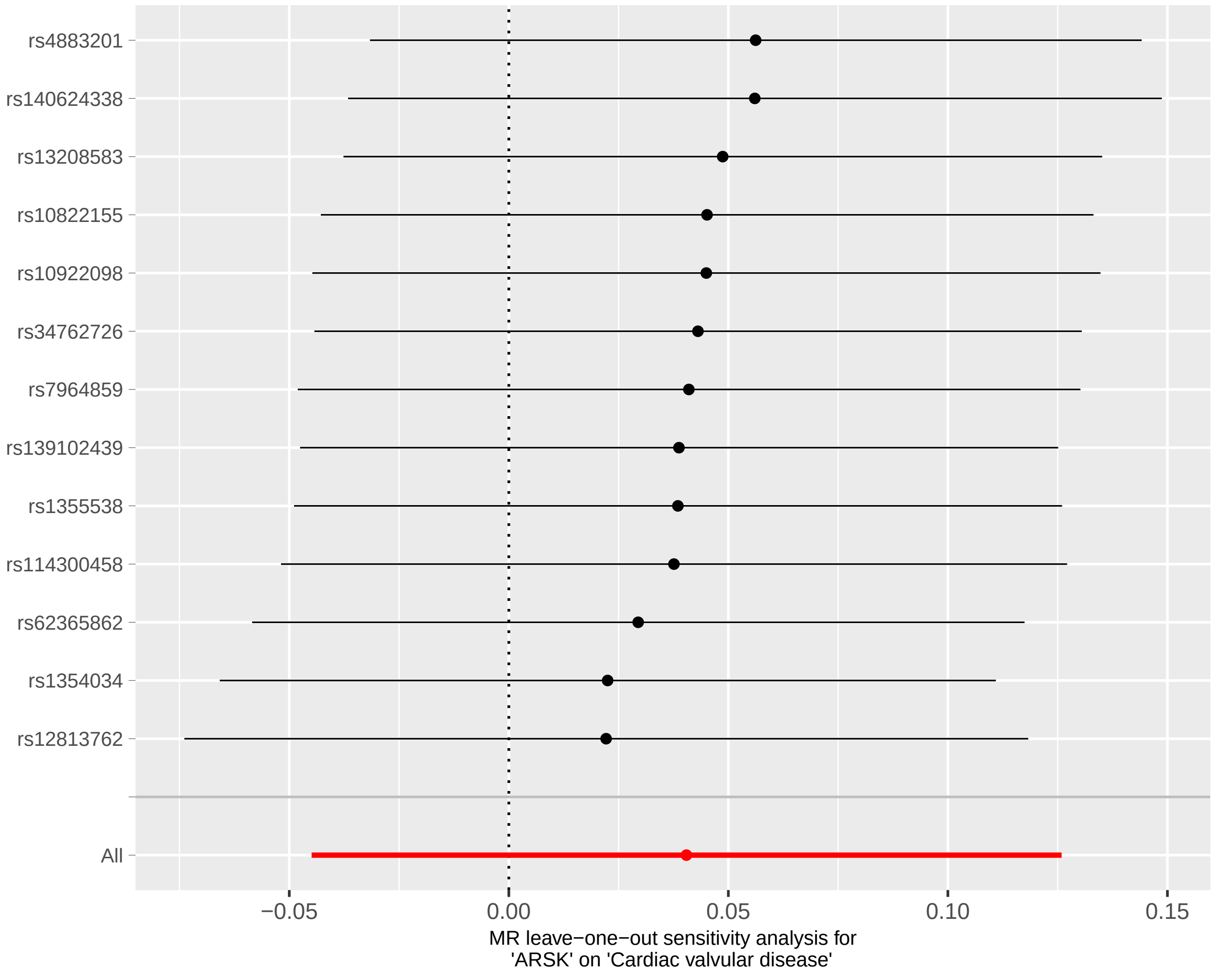

rs4883201
rs140624338
rs13208583
rs10822155
rs10922098
rs34762726
rs7964859
rs139102439
rs1355538
rs114300458
rs62365862
rs1354034
rs12813762
All
−0.05
0.00
0.05
0.10
0.15
MR leave−one−out sensitivity analysis for
'ARSK' on 'Cardiac valvular disease'

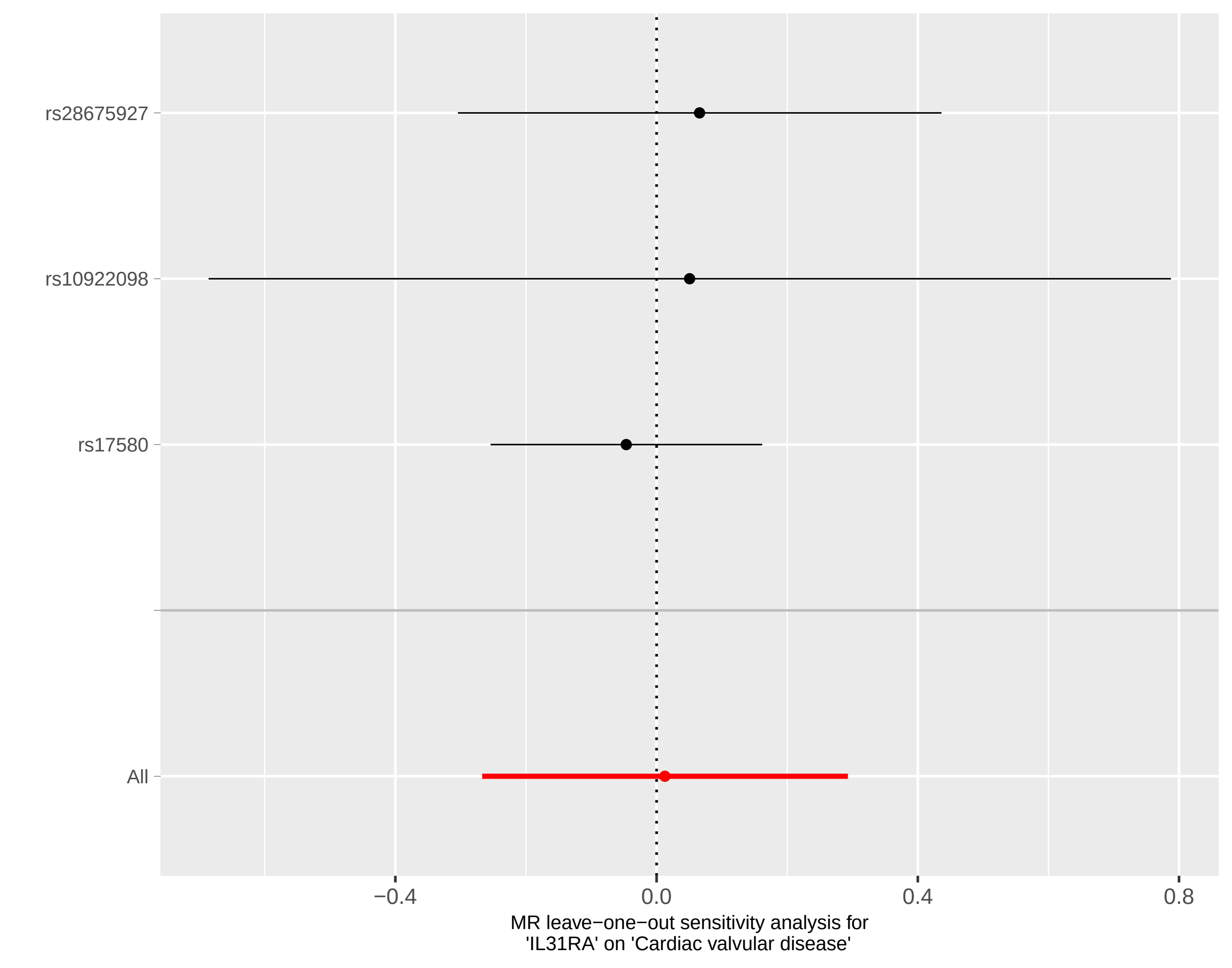
rs28675927
rs10922098
rs17580
All
−0.4
0.0
0.4
0.8
MR leave−one−out sensitivity analysis for
'IL31RA' on 'Cardiac valvular disease'

Insufficient number of SNPs

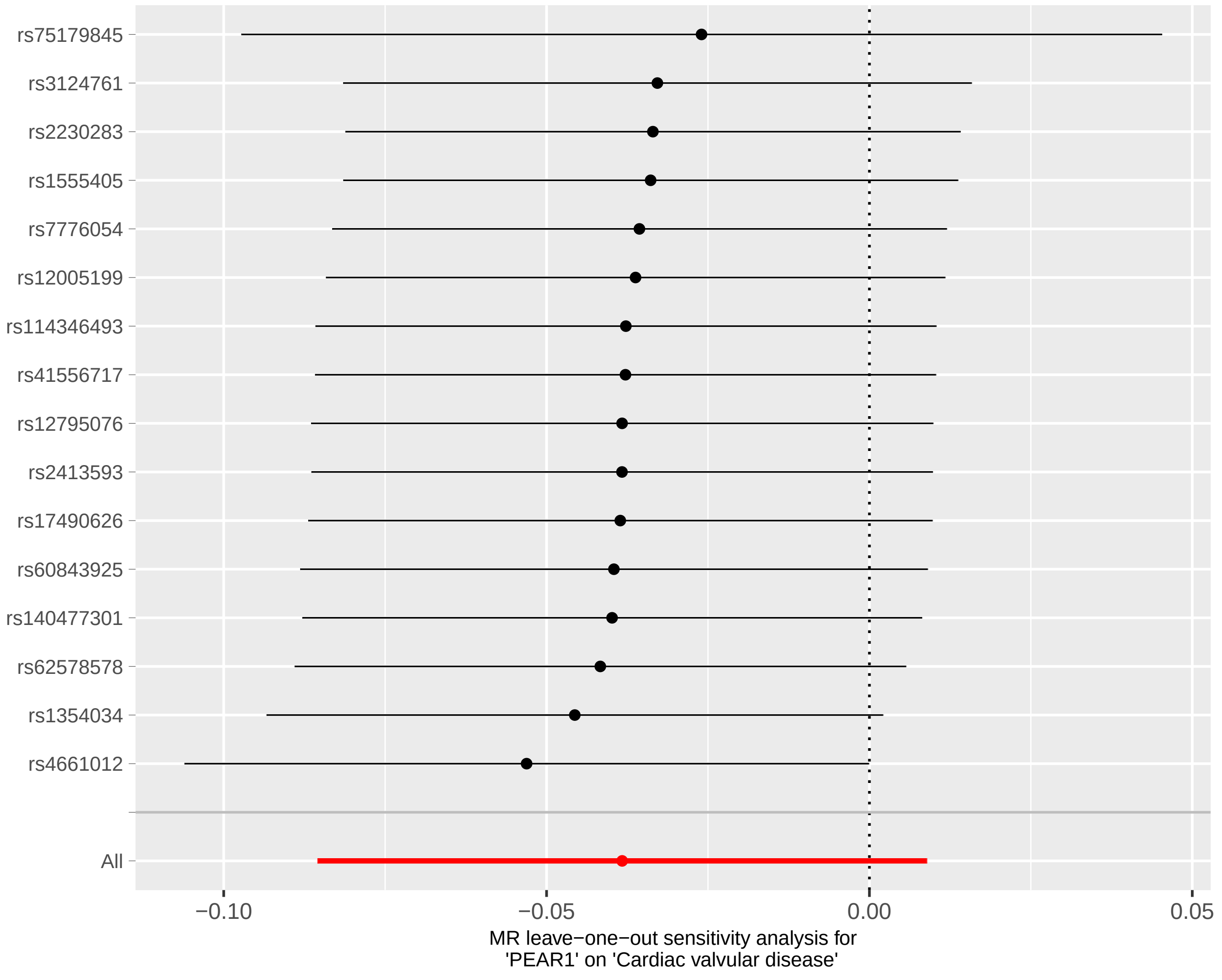
rs75179845
rs3124761
rs2230283
rs1555405
rs7776054
rs12005199
rs114346493
rs41556717
rs12795076
rs2413593
rs17490626
rs60843925
rs140477301
rs62578578
rs1354034
rs4661012
All
−0.10
−0.05
0.00
0.05
MR leave−one−out sensitivity analysis for
'PEAR1' on 'Cardiac valvular disease'

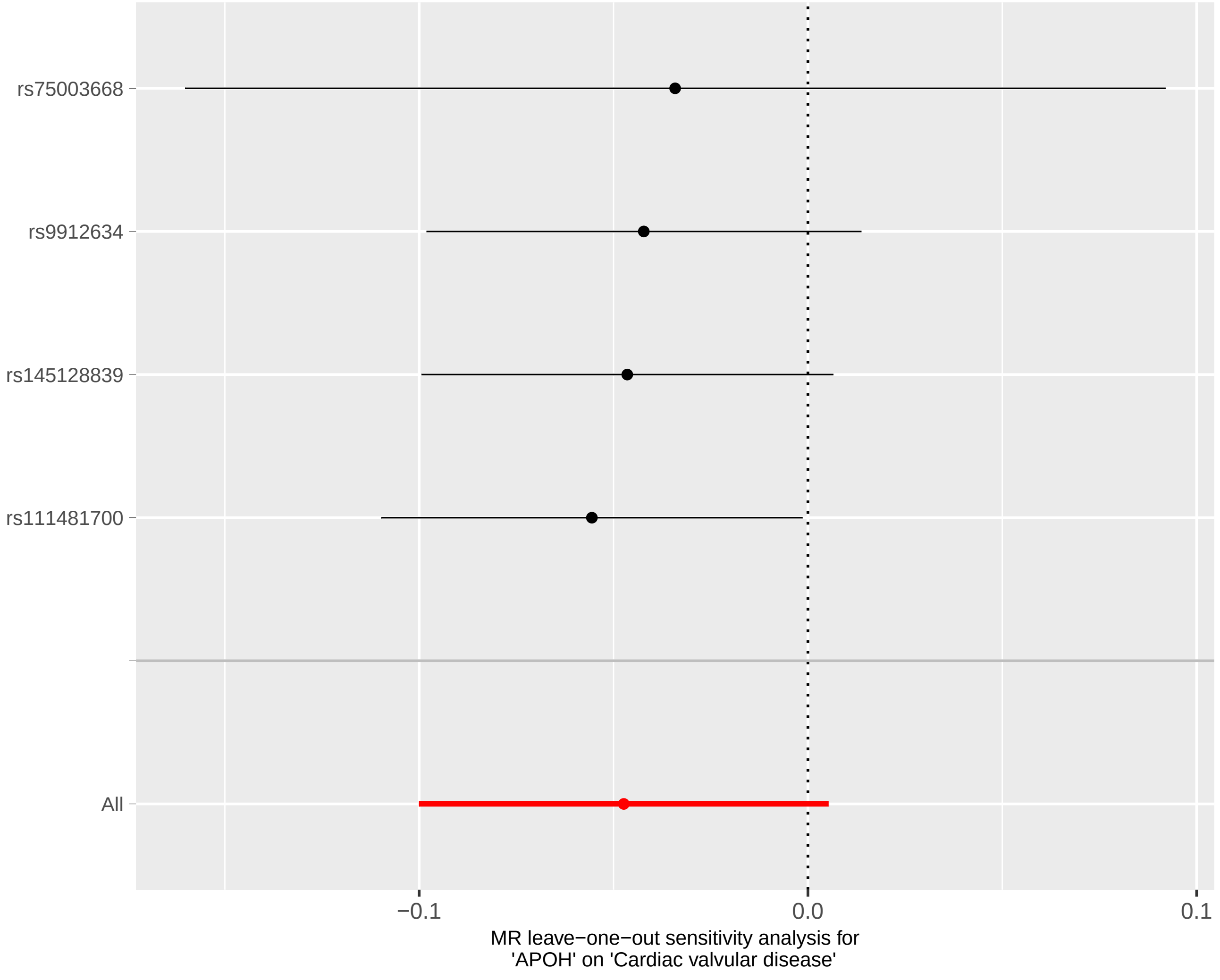

rs75003668
rs9912634
rs145128839
rs111481700
All
−0.1
0.0
0.1
MR leave−one−out sensitivity analysis for
'APOH' on 'Cardiac valvular disease'

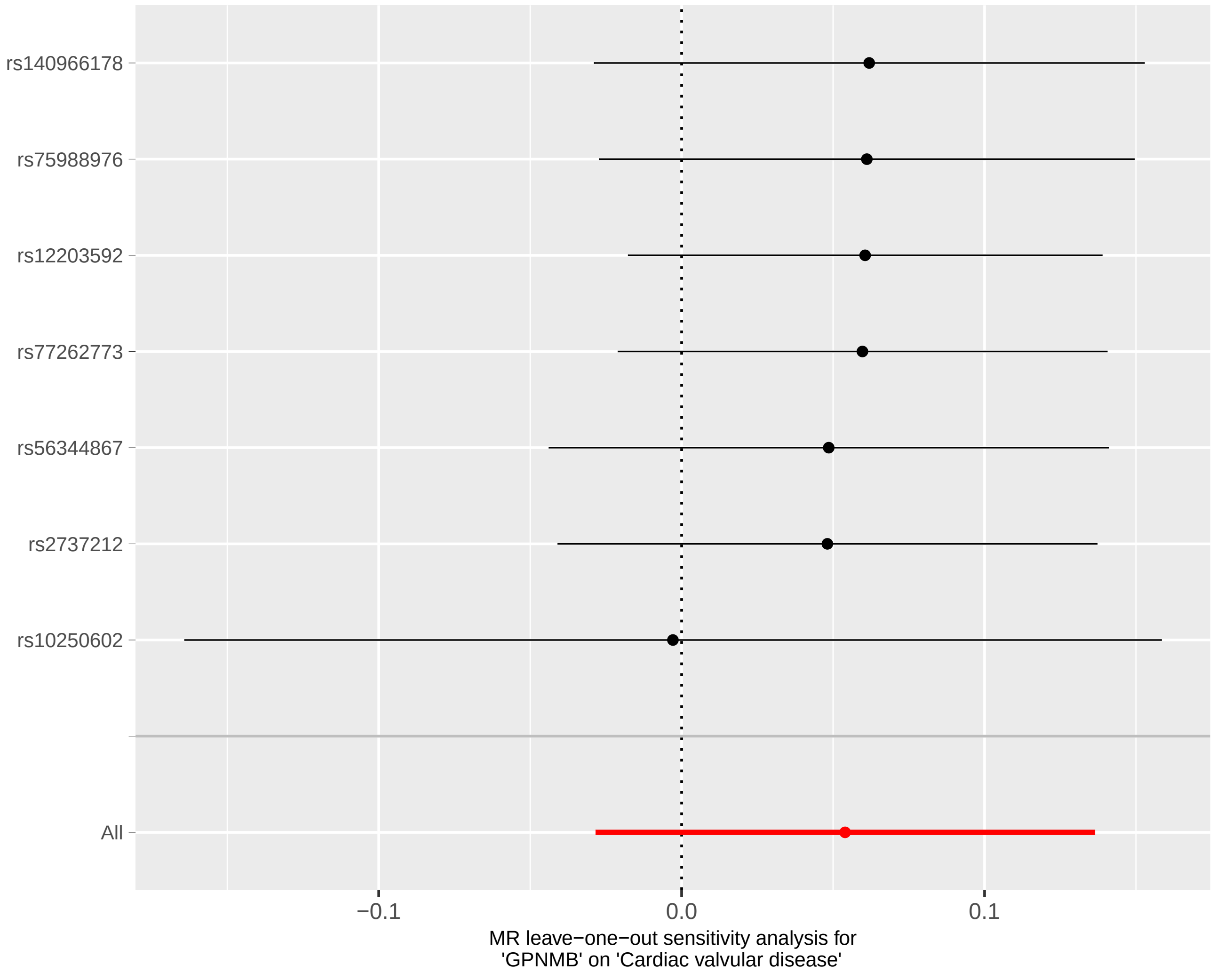

rs140966178
rs75988976
rs12203592
rs77262773
rs56344867
rs2737212
rs10250602
All
−0.1
0.0
0.1
MR leave−one−out sensitivity analysis for
'GPNMB' on 'Cardiac valvular disease'

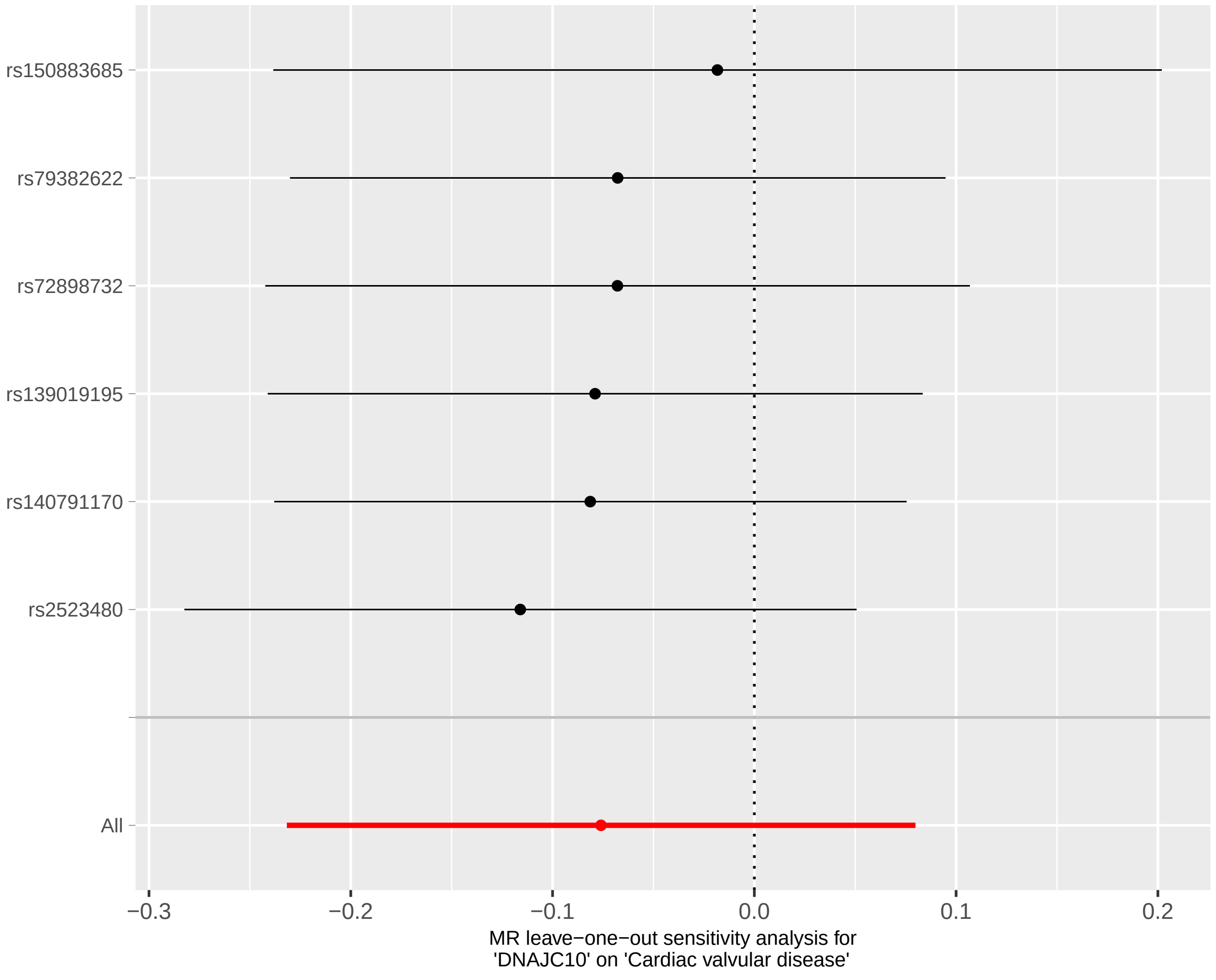
rs150883685
rs79382622
rs72898732
rs139019195
rs140791170
rs2523480
All
−0.3
−0.2
−0.1
0.0
0.1
0.2
MR leave−one−out sensitivity analysis for
'DNAJC10' on 'Cardiac valvular disease'

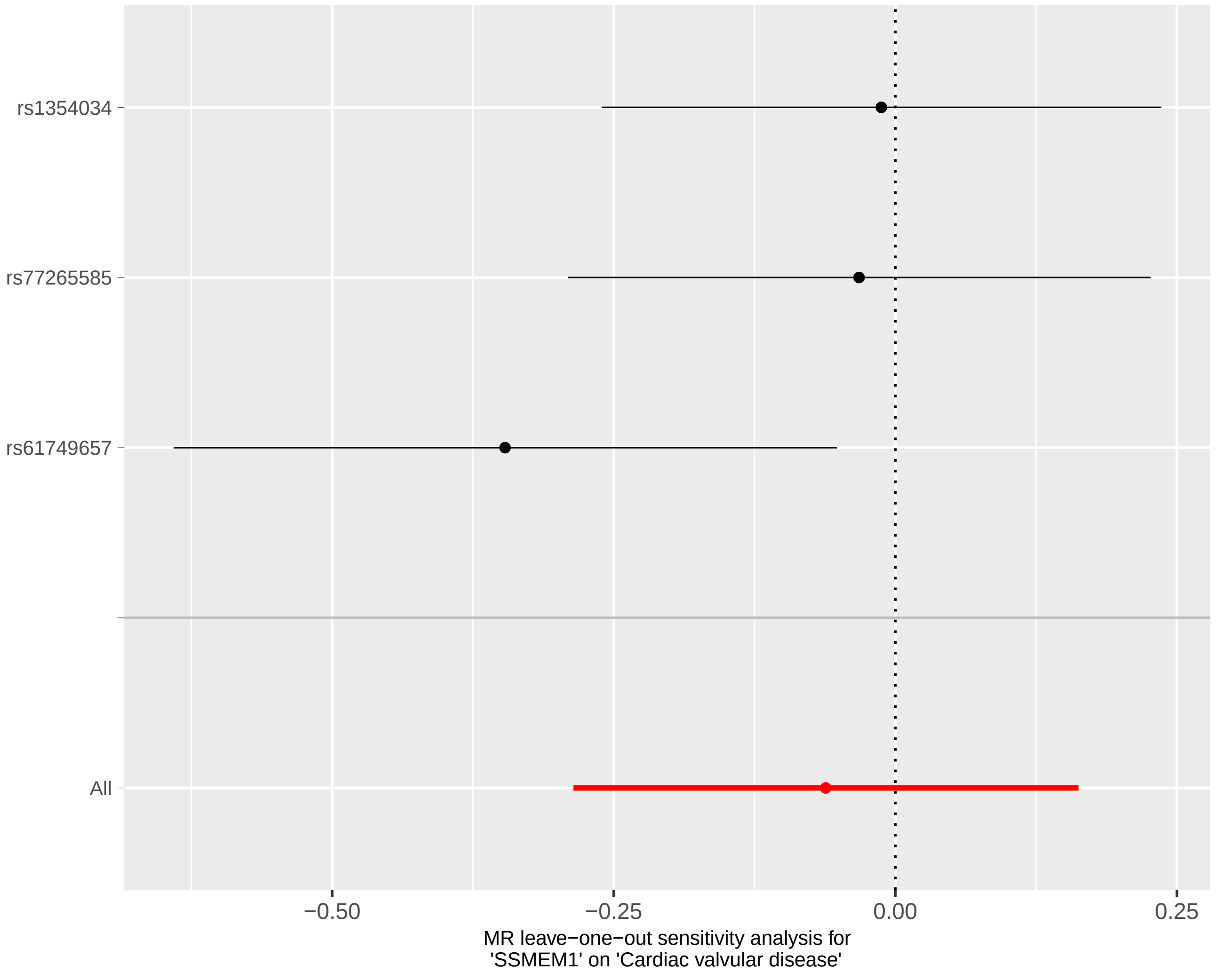
rs1354034
rs77265585
rs61749657
All
−0.50
−0.25
0.00
0.25
MR leave−one−out sensitivity analysis for
'SSMEM1' on 'Cardiac valvular disease'

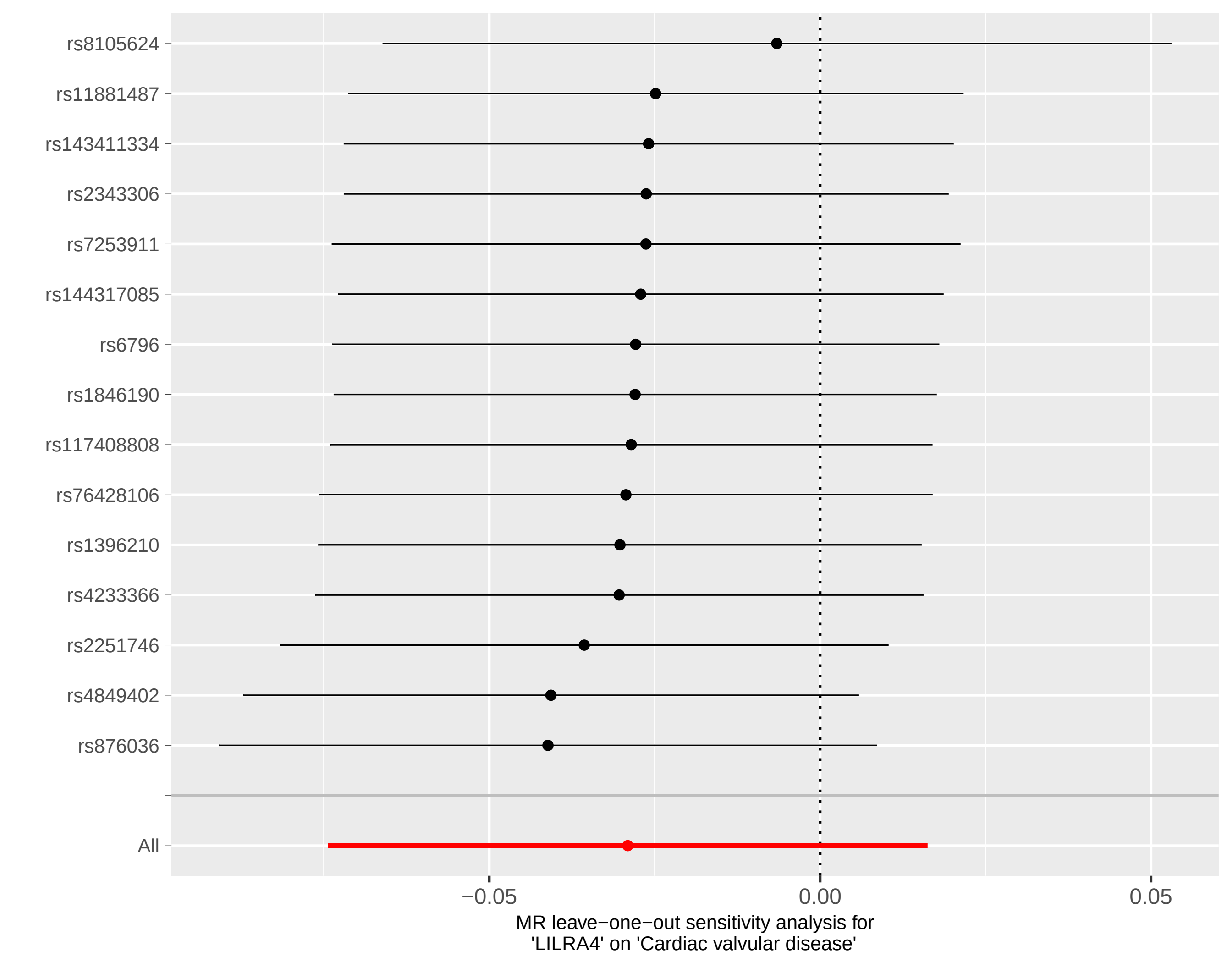
rs8105624
rs11881487
rs143411334
rs2343306
rs7253911
rs144317085
rs6796
rs1846190
rs117408808
rs76428106
rs1396210
rs4233366
rs2251746
rs4849402
rs876036
All
−0.05
0.00
0.05
MR leave−one−out sensitivity analysis for
'LILRA4' on 'Cardiac valvular disease'

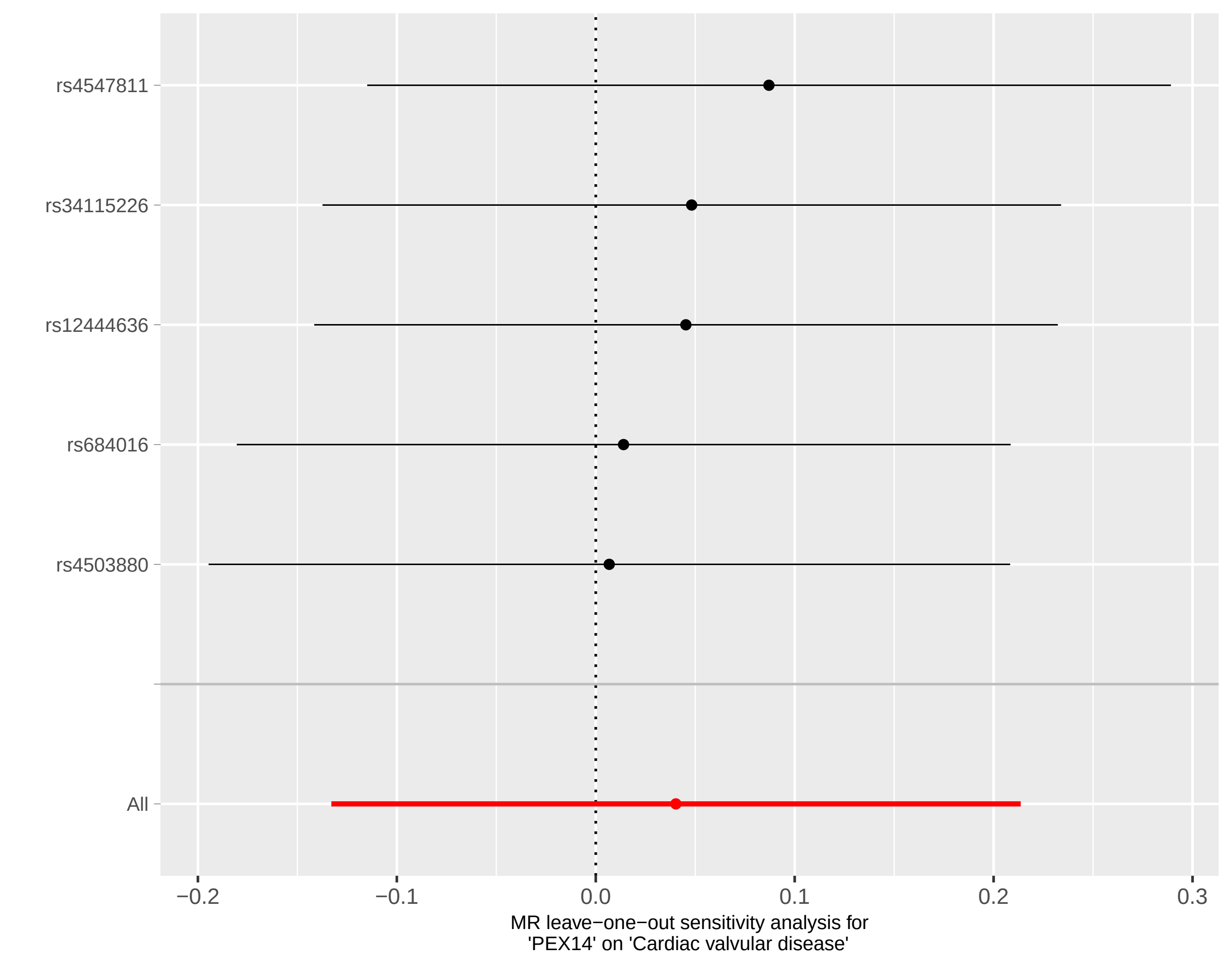
rs4547811
rs34115226
rs12444636
rs684016
rs4503880
All
−0.2
−0.1
0.0
0.1
0.2
0.3
MR leave−one−out sensitivity analysis for
'PEX14' on 'Cardiac valvular disease'

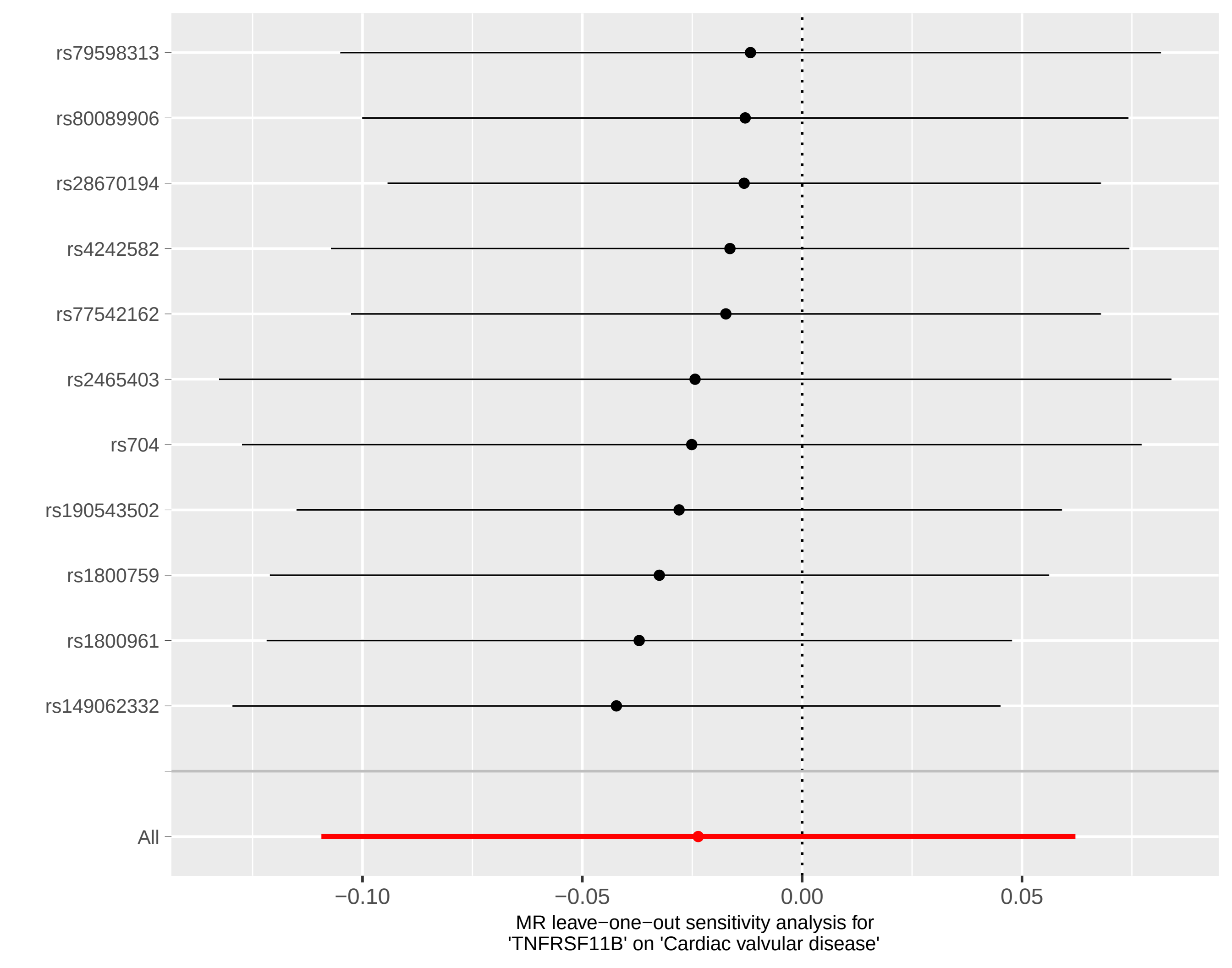
rs79598313
rs80089906
rs28670194
rs4242582
rs77542162
rs2465403
rs704
rs190543502
rs1800759
rs1800961
rs149062332
All
−0.10
−0.05
0.00
0.05
MR leave−one−out sensitivity analysis for
'TNFRSF11B' on 'Cardiac valvular disease'

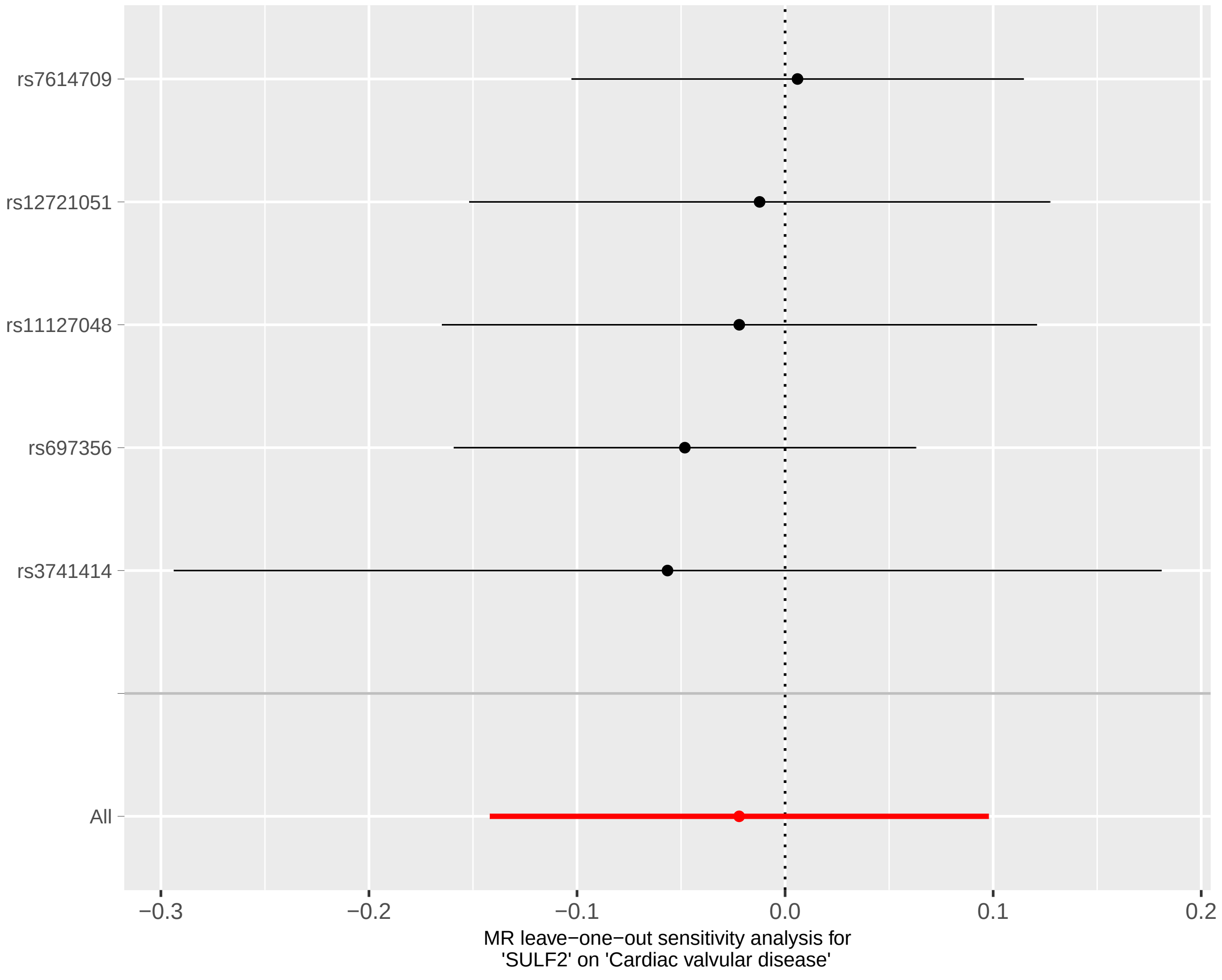

rs7614709
rs12721051
rs11127048
rs697356
rs3741414
All
−0.3
−0.2
−0.1
0.0
0.1
0.2
MR leave−one−out sensitivity analysis for
'SULF2' on 'Cardiac valvular disease'

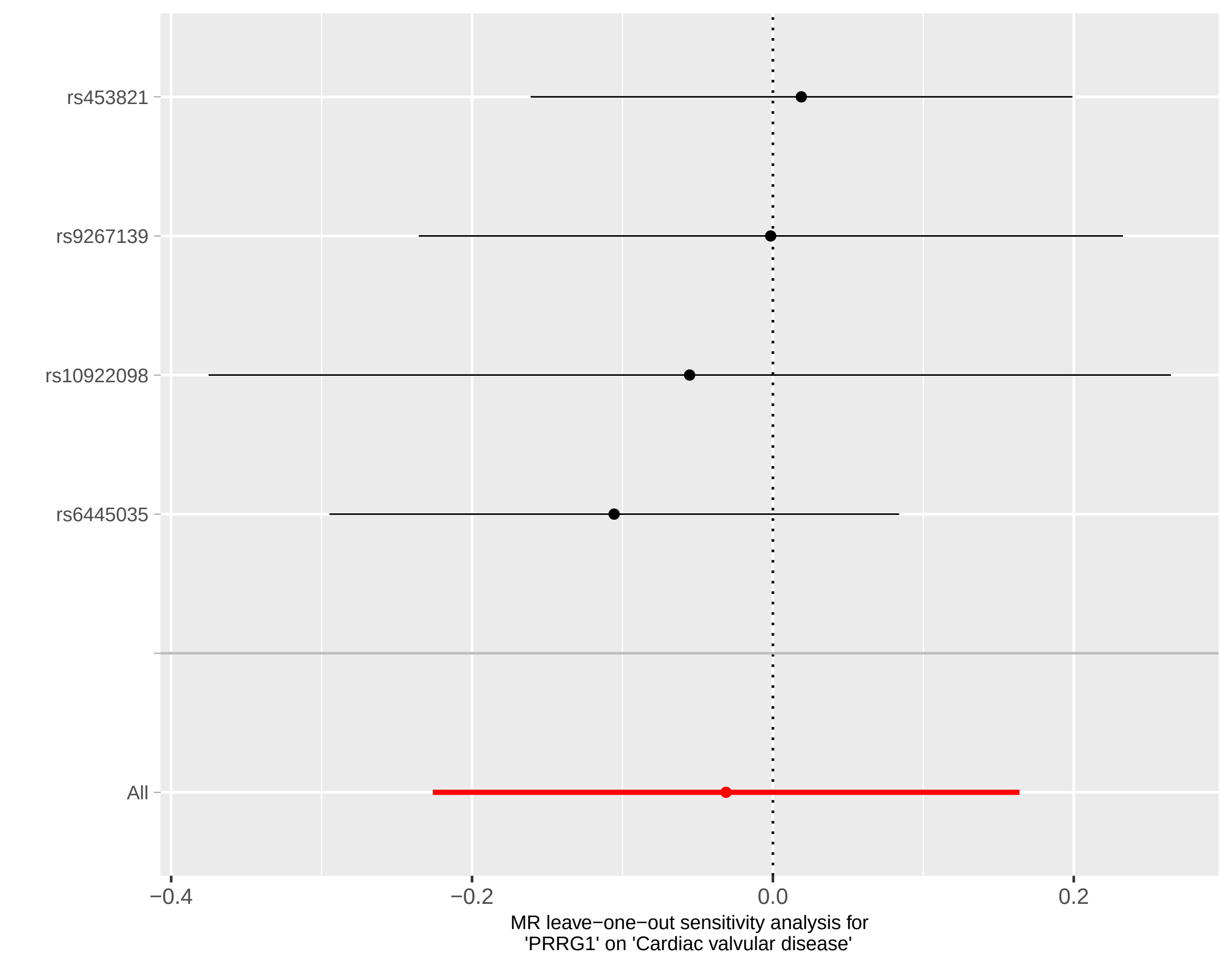
rs453821
rs9267139
rs10922098
rs6445035
All
−0.4
−0.2
0.0
0.2
MR leave−one−out sensitivity analysis for
'PRRG1' on 'Cardiac valvular disease'

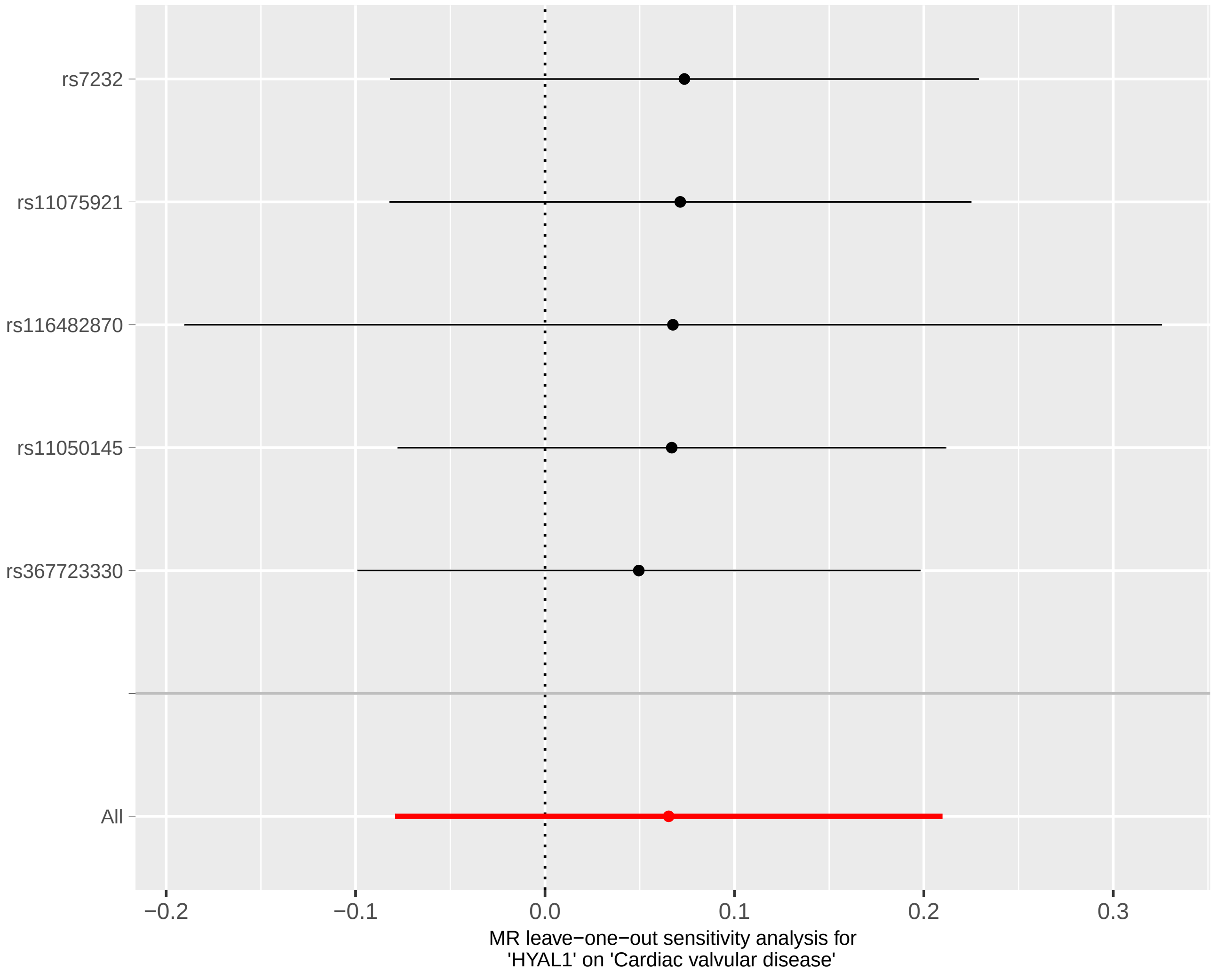

rs7232
rs11075921
rs116482870
rs11050145
rs367723330
All
−0.2
−0.1
0.0
0.1
0.2
0.3
MR leave−one−out sensitivity analysis for
'HYAL1' on 'Cardiac valvular disease'

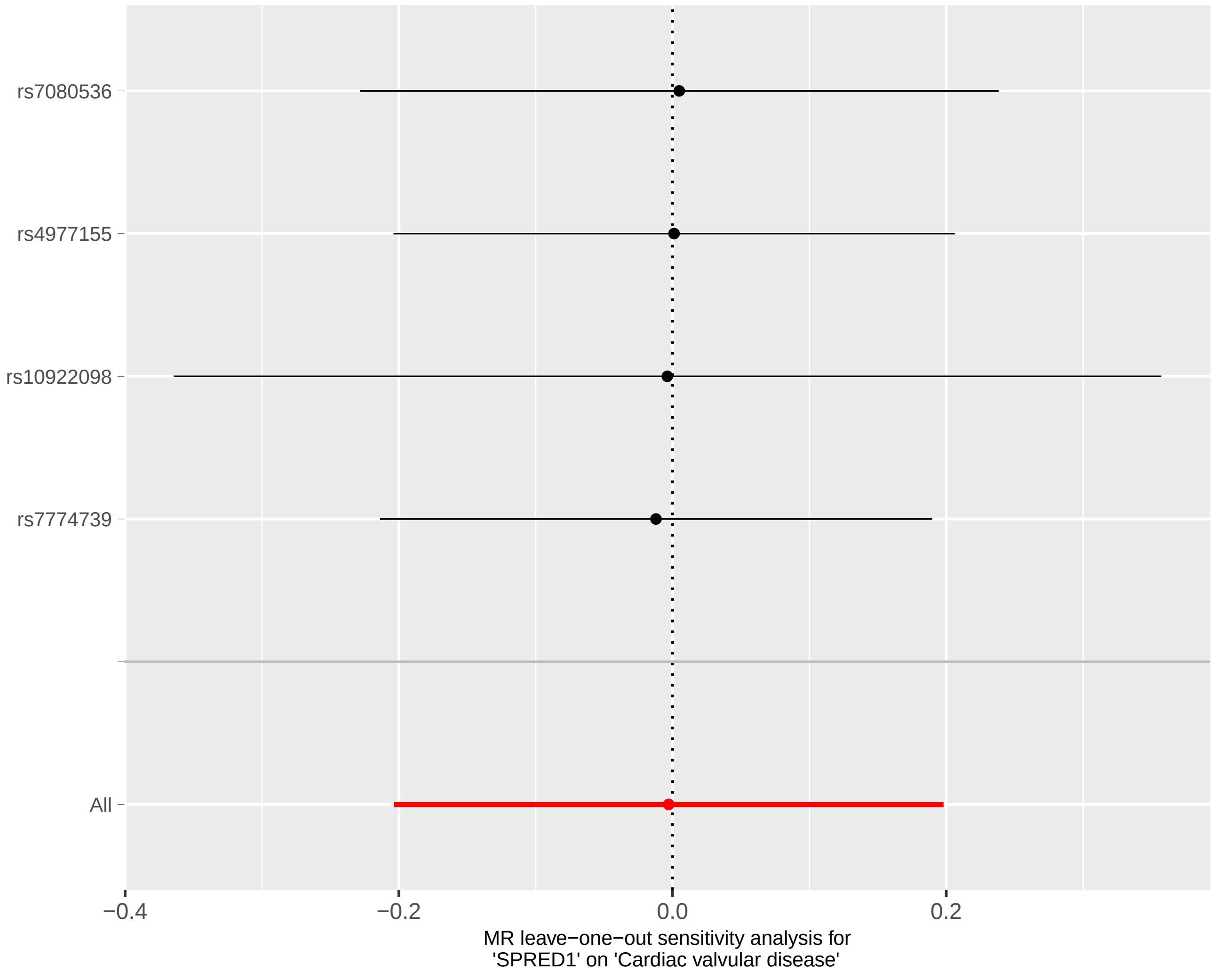

rs7080536
rs4977155
rs10922098
rs7774739
All
−0.4
−0.2
0.0
0.2
MR leave−one−out sensitivity analysis for
'SPRED1' on 'Cardiac valvular disease'

Insufficient number of SNPs

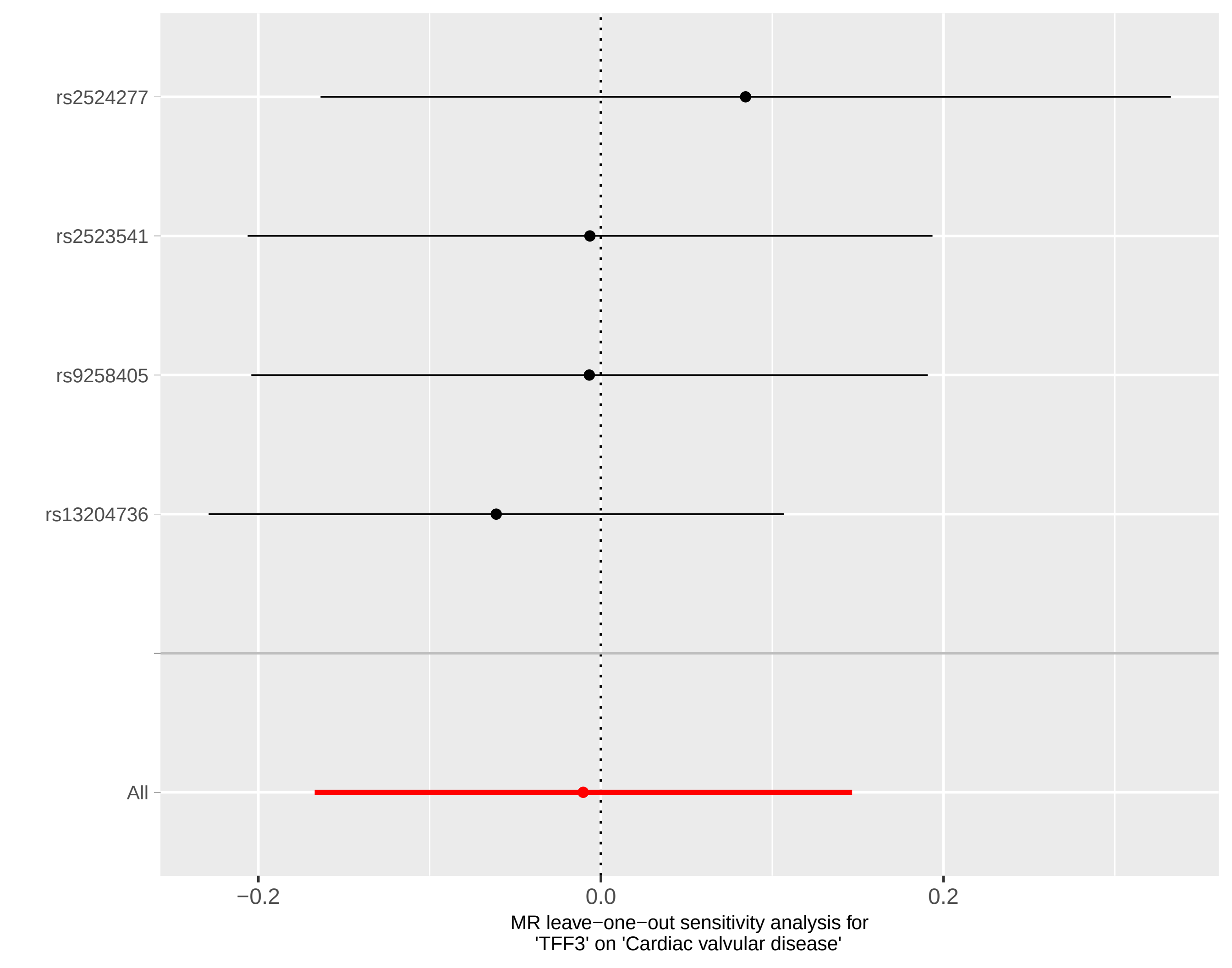
rs2524277
rs2523541
rs9258405
rs13204736
All
−0.2
0.0
0.2
MR leave−one−out sensitivity analysis for
'TFF3' on 'Cardiac valvular disease'

Insufficient number of SNPs

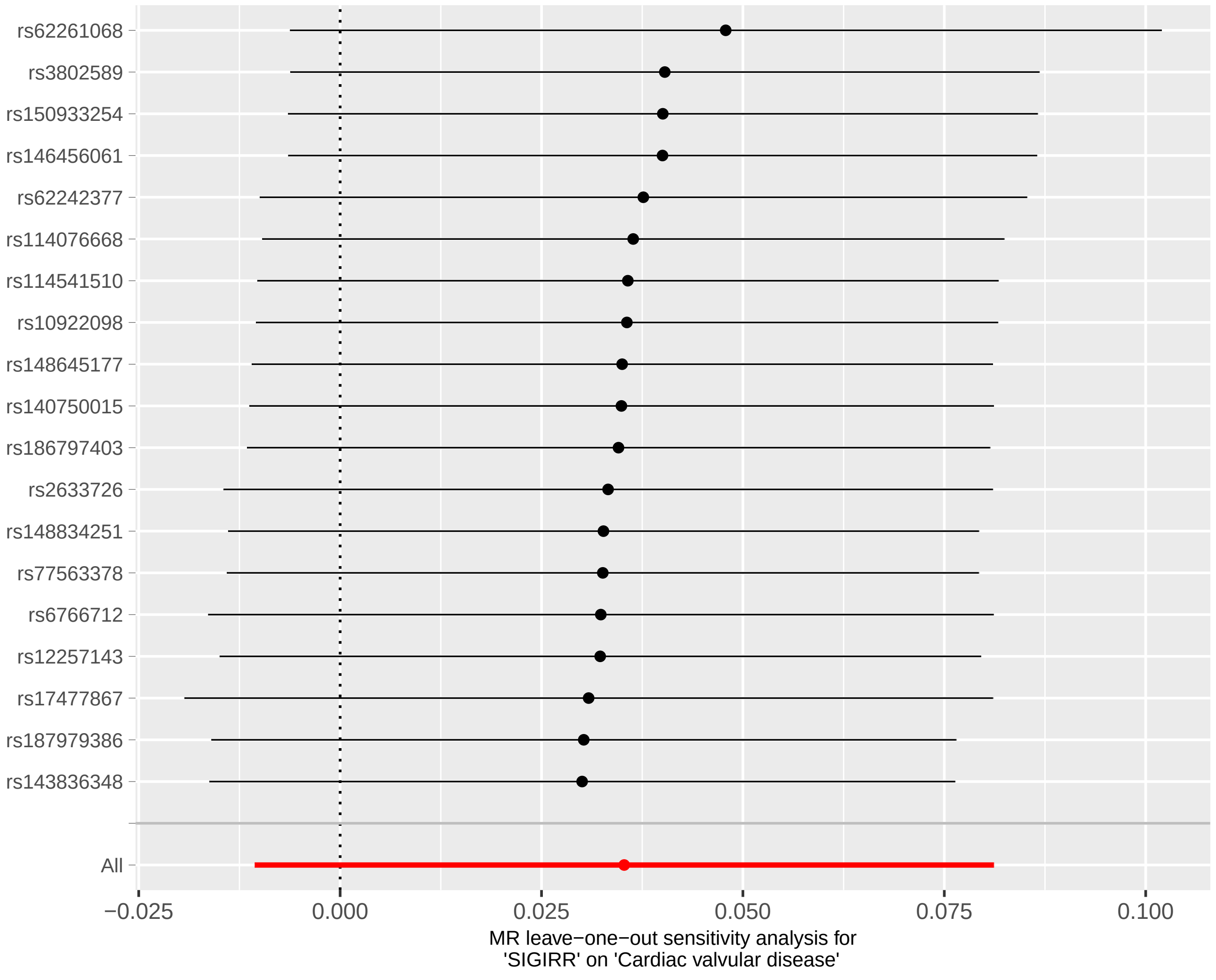

rs62261068
rs3802589
rs150933254
rs146456061
rs62242377
rs114076668
rs114541510
rs10922098
rs148645177
rs140750015
rs186797403
rs2633726
rs148834251
rs77563378
rs6766712
rs12257143
rs17477867
rs187979386
rs143836348
All
−0.025
0.000
0.025
0.050
0.075
0.100
MR leave−one−out sensitivity analysis for
'SIGIRR' on 'Cardiac valvular disease'

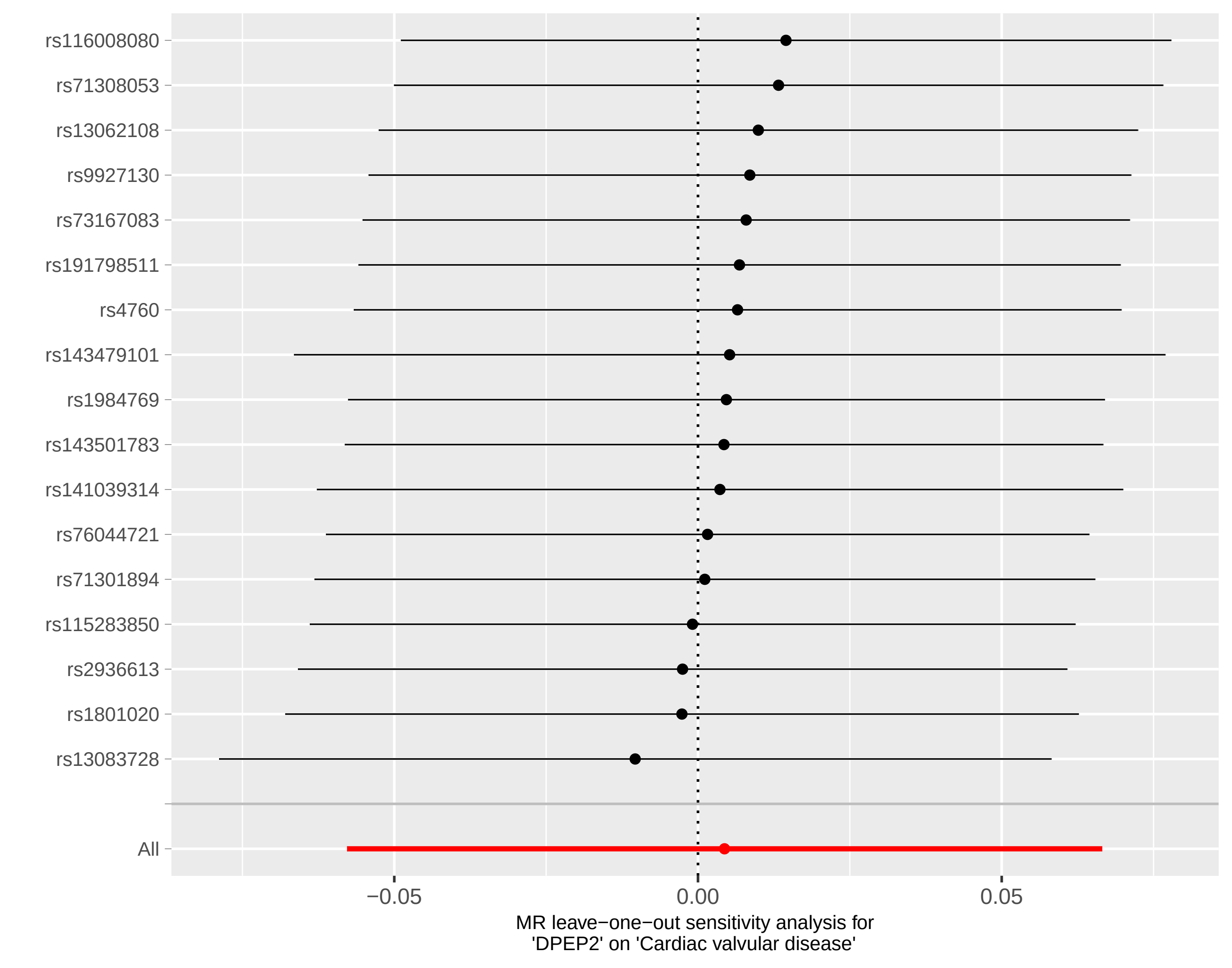

rs116008080
rs71308053
rs13062108
rs9927130
rs73167083
rs191798511
rs4760
rs143479101
rs1984769
rs143501783
rs141039314
rs76044721
rs71301894
rs115283850
rs2936613
rs1801020
rs13083728
All
−0.05
0.00
0.05
MR leave−one−out sensitivity analysis for
'DPEP2' on 'Cardiac valvular disease'

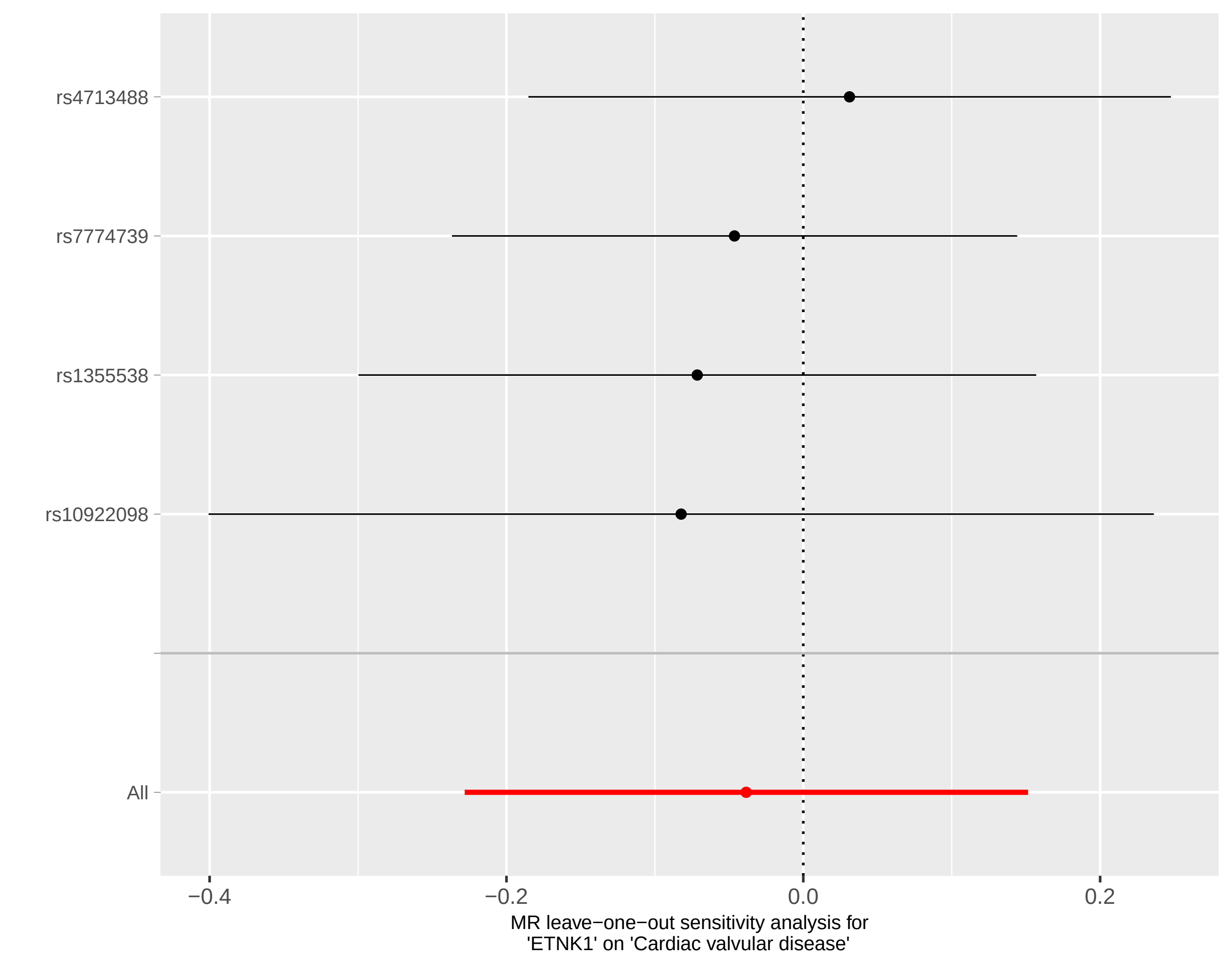

rs4713488
rs7774739
rs1355538
rs10922098
All
−0.4
−0.2
0.0
0.2
MR leave−one−out sensitivity analysis for
'ETNK1' on 'Cardiac valvular disease'

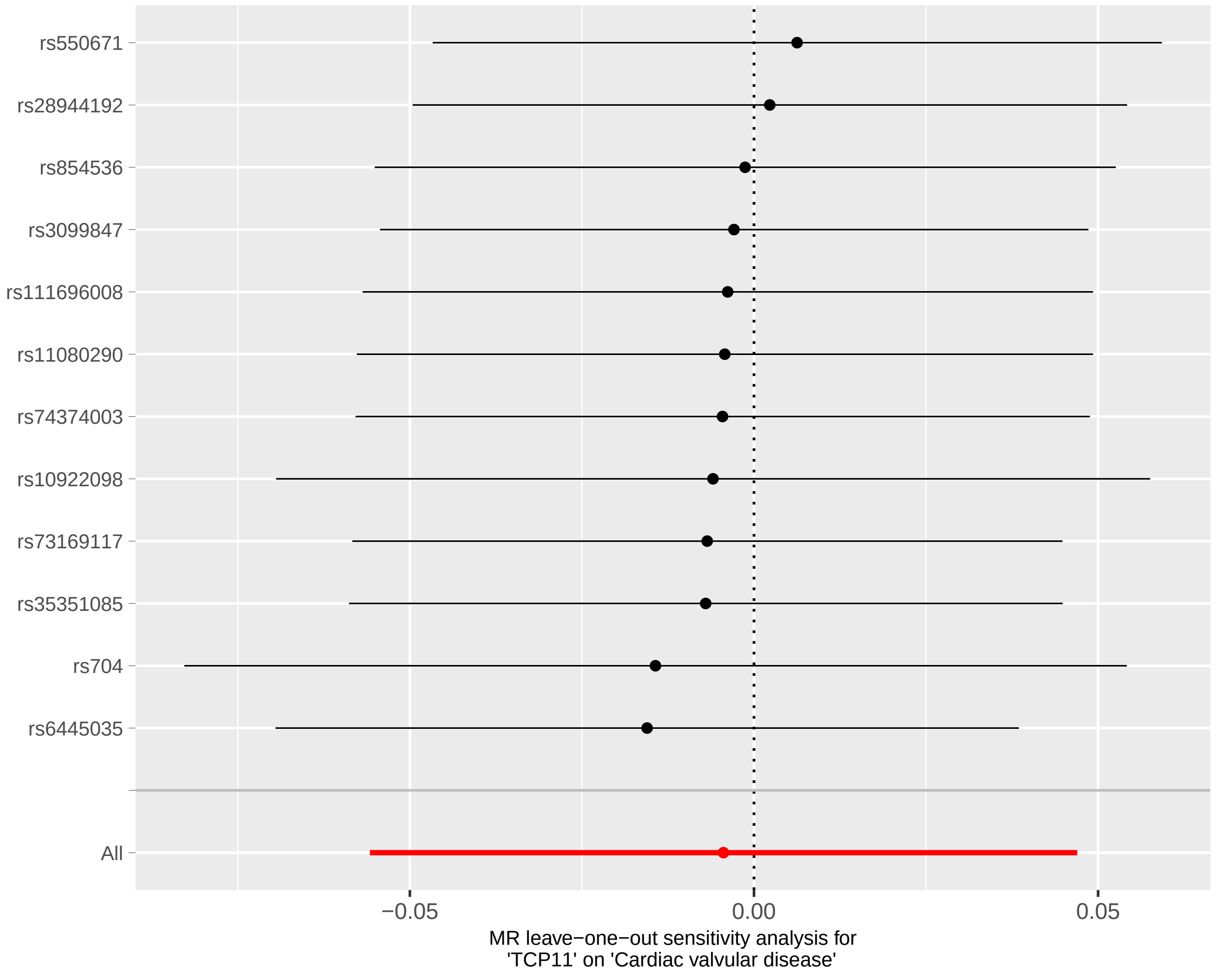
rs550671
rs28944192
rs854536
rs3099847
rs111696008
rs11080290
rs74374003
rs10922098
rs73169117
rs35351085
rs704
rs6445035
All
−0.05
0.00
0.05
MR leave−one−out sensitivity analysis for
'TCP11' on 'Cardiac valvular disease'

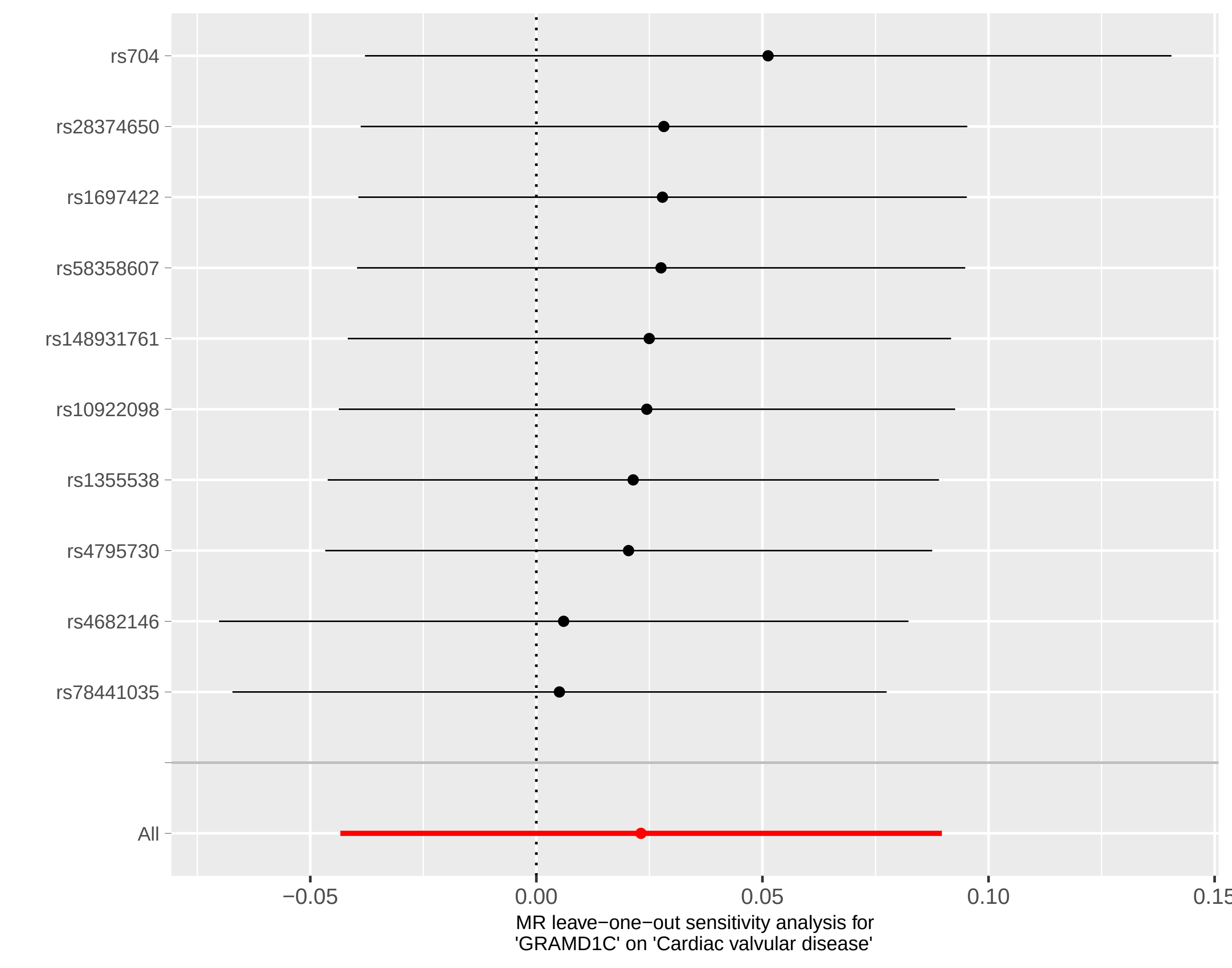
rs704
rs28374650
rs1697422
rs58358607
rs148931761
rs10922098
rs1355538
rs4795730
rs4682146
rs78441035
All
−0.05
0.00
0.05
0.10
0.15
MR leave−one−out sensitivity analysis for
'GRAMD1C' on 'Cardiac valvular disease'

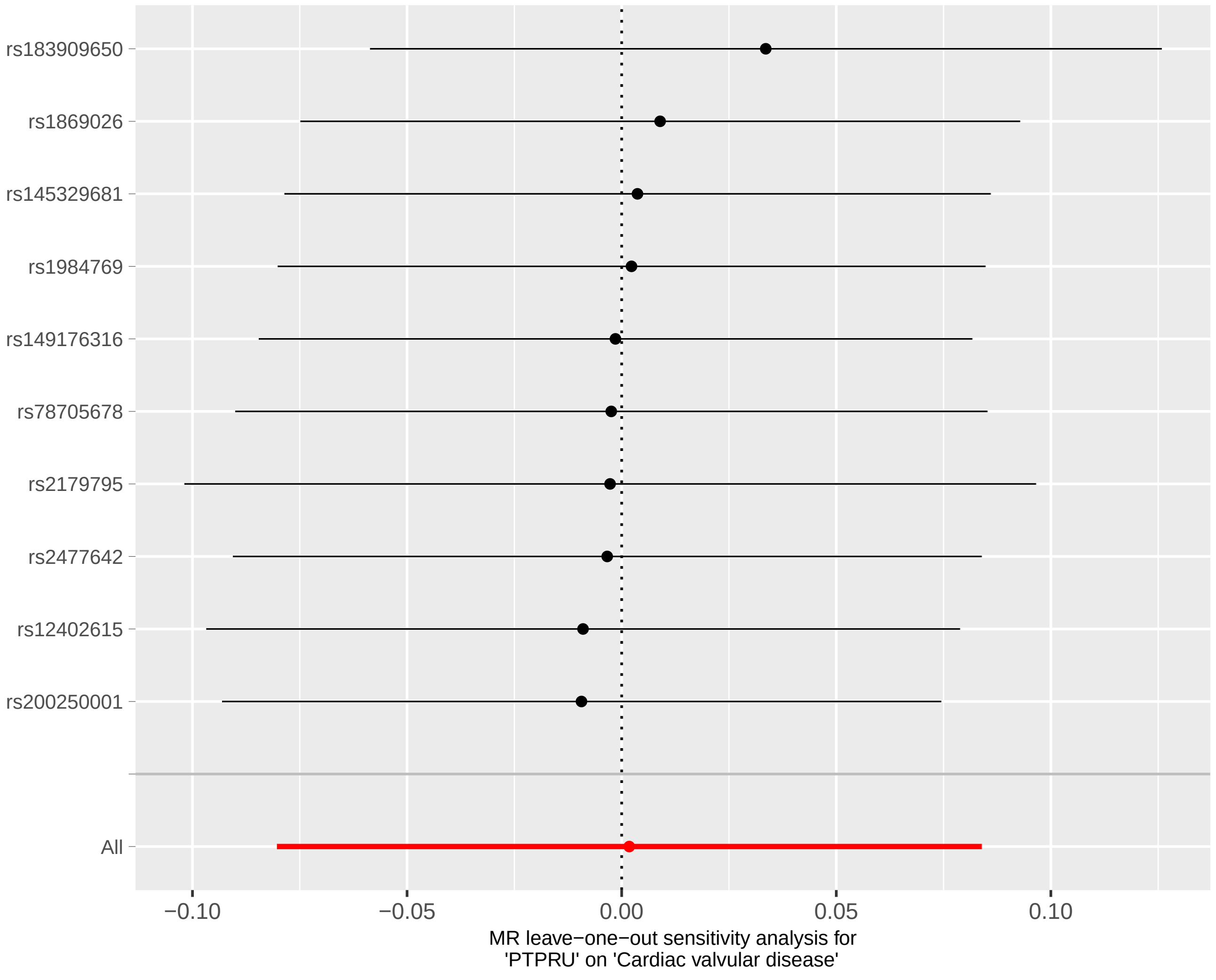

rs183909650
rs1869026
rs145329681
rs1984769
rs149176316
rs78705678
rs2179795
rs2477642
rs12402615
rs200250001
All
−0.10
−0.05
0.00
0.05
0.10
MR leave−one−out sensitivity analysis for
'PTPRU' on 'Cardiac valvular disease'

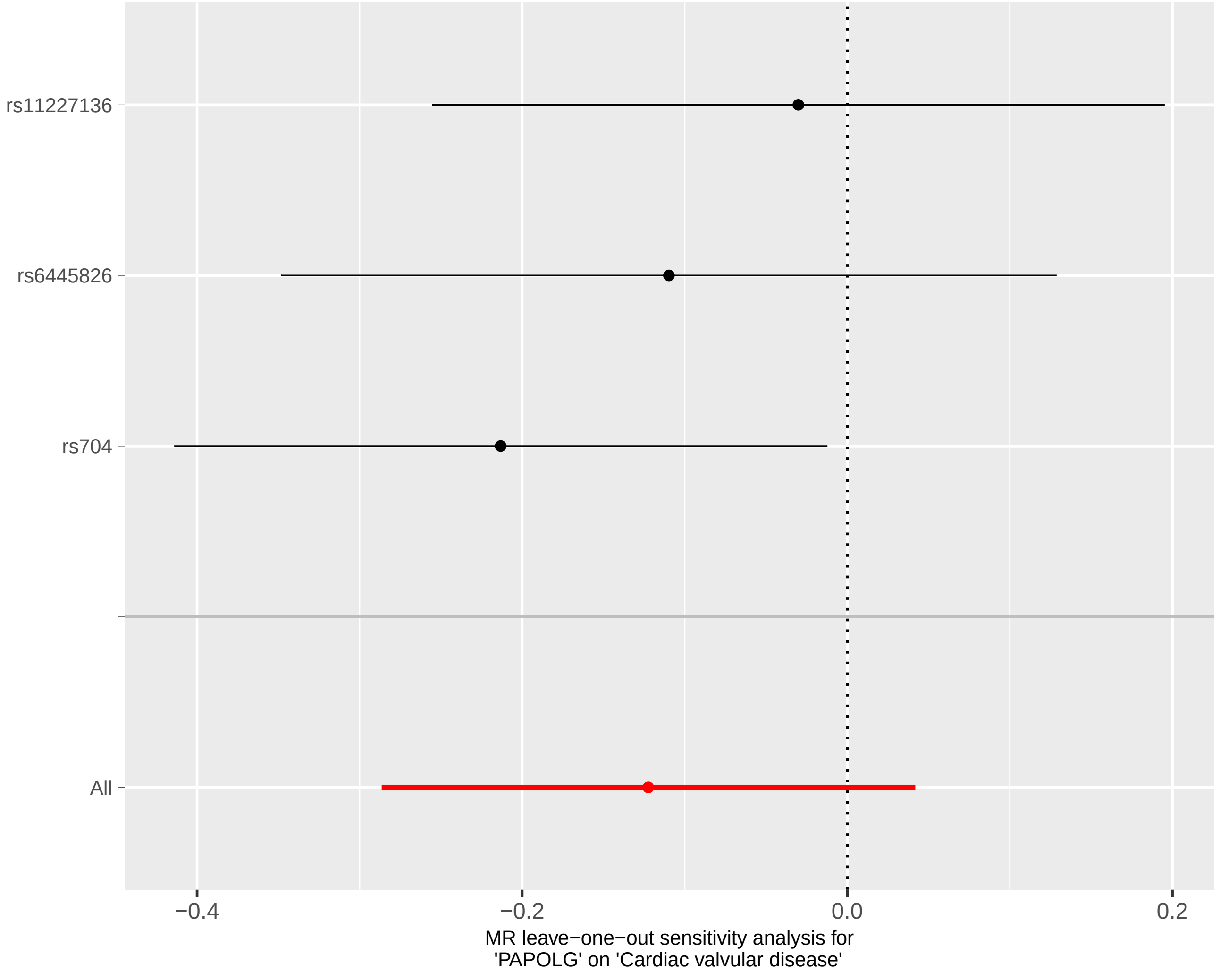
rs11227136
rs6445826
rs704
All
−0.4
−0.2
0.0
0.2
MR leave−one−out sensitivity analysis for
'PAPOLG' on 'Cardiac valvular disease'

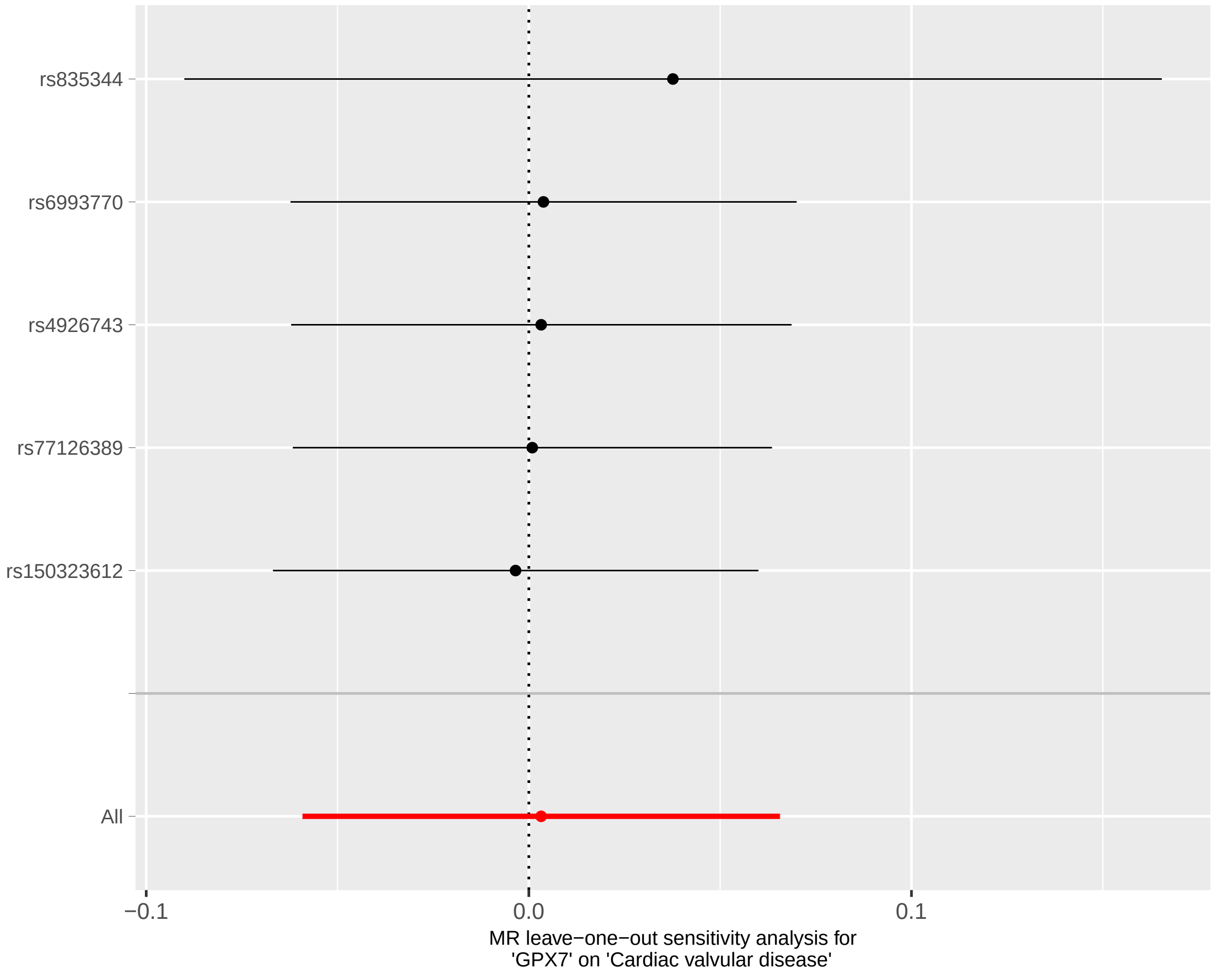
rs835344
rs6993770
rs4926743
rs77126389
rs150323612
All
−0.1
0.0
0.1
MR leave−one−out sensitivity analysis for
'GPX7' on 'Cardiac valvular disease'

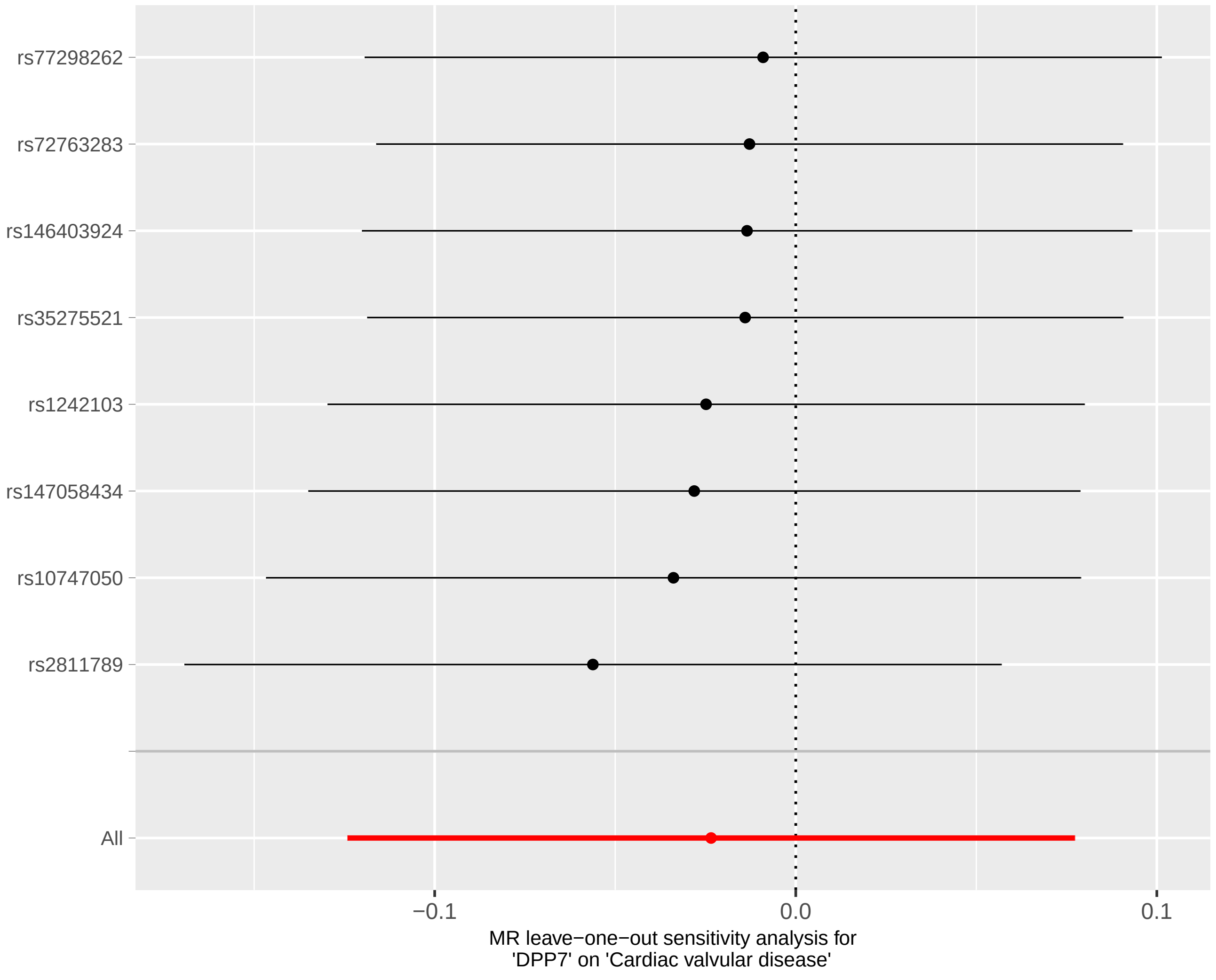
rs77298262
rs72763283
rs146403924
rs35275521
rs1242103
rs147058434
rs10747050
rs2811789
All
−0.1
0.0
0.1
MR leave−one−out sensitivity analysis for
'DPP7' on 'Cardiac valvular disease'

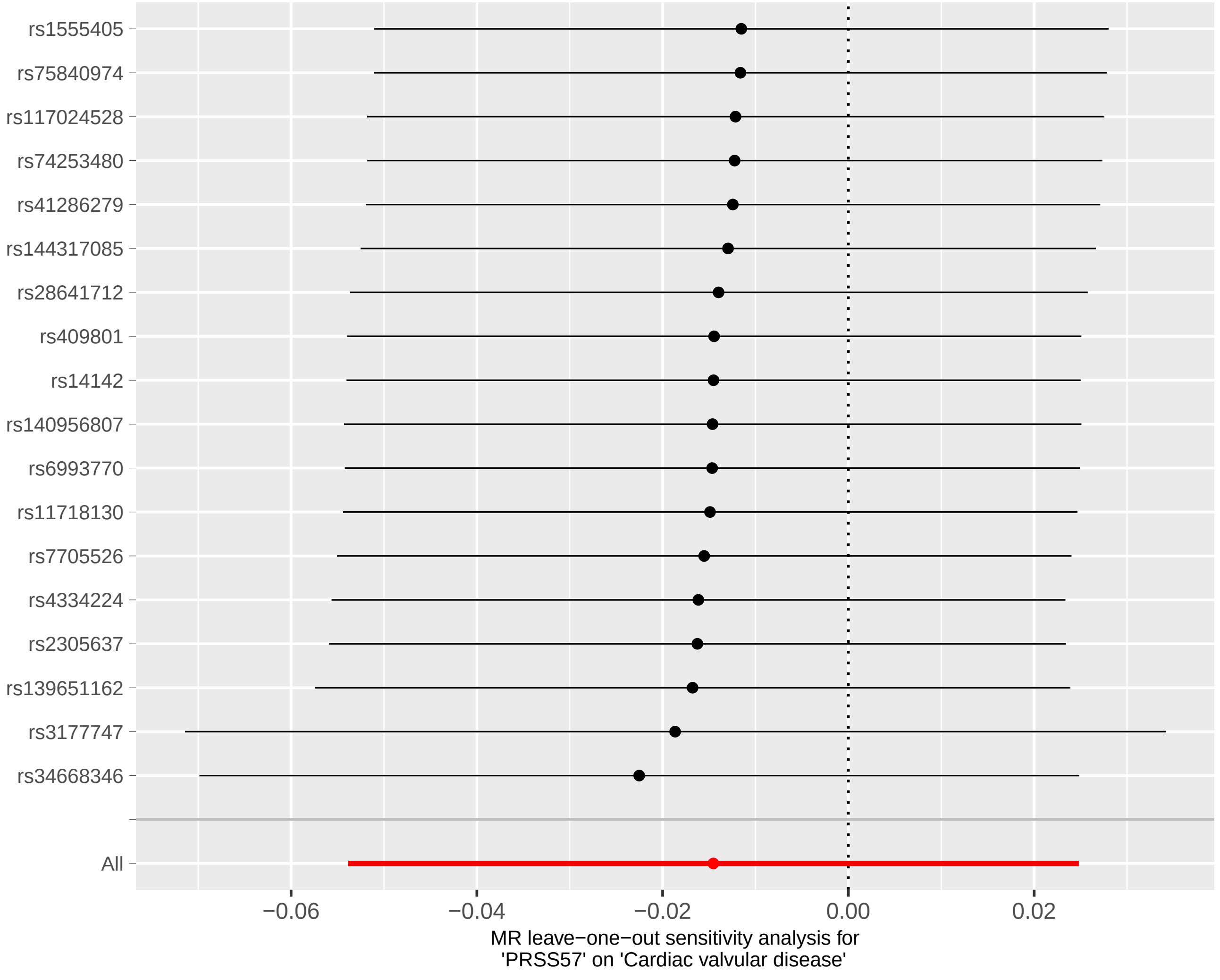
rs1555405
rs75840974
rs117024528
rs74253480
rs41286279
rs144317085
rs28641712
rs409801
rs14142
rs140956807
rs6993770
rs11718130
rs7705526
rs4334224
rs2305637
rs139651162
rs3177747
rs34668346
All
−0.06
−0.04
−0.02
0.00
0.02
MR leave−one−out sensitivity analysis for
'PRSS57' on 'Cardiac valvular disease'

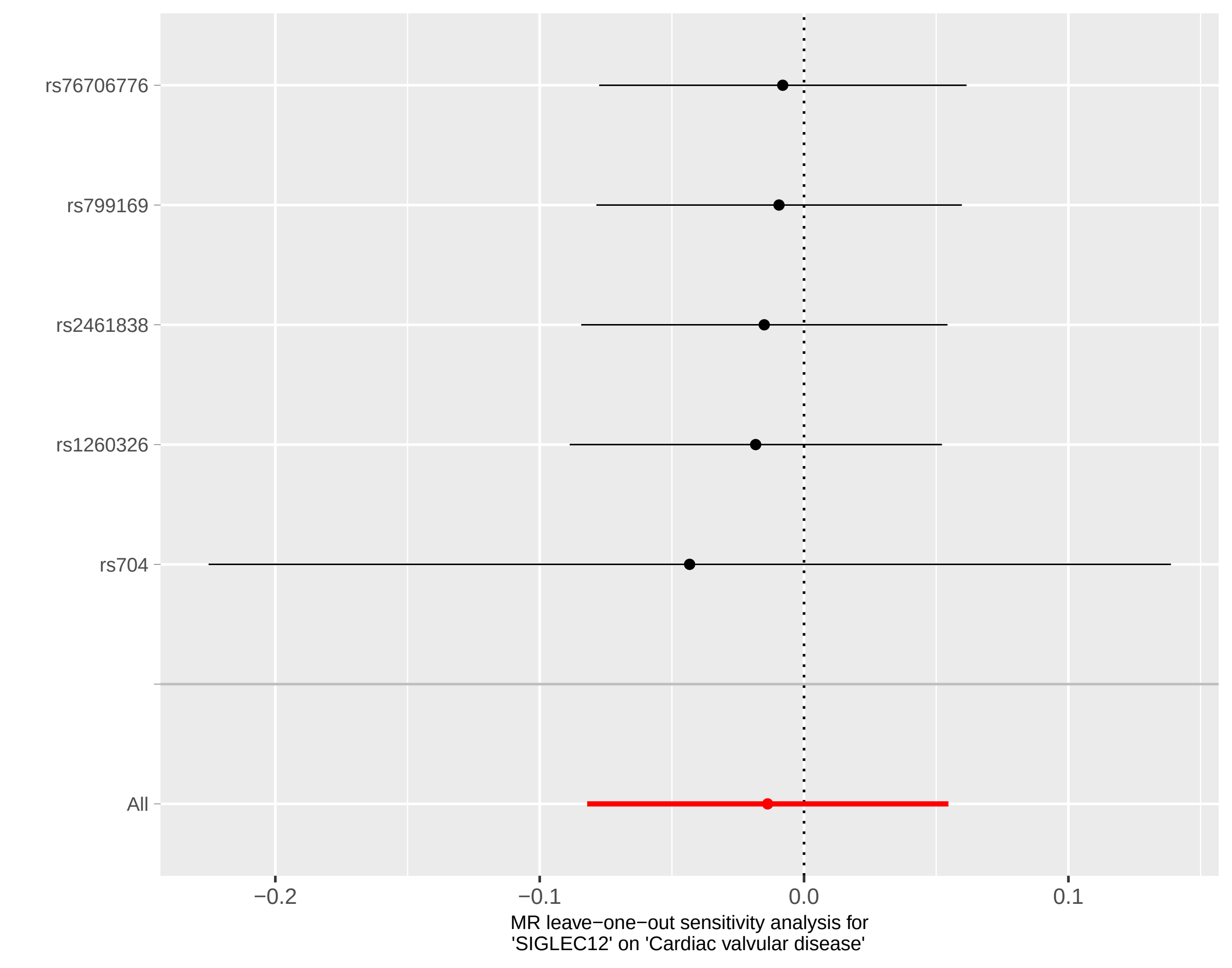
rs76706776
rs799169
rs2461838
rs1260326
rs704
All
−0.2
−0.1
0.0
0.1
MR leave−one−out sensitivity analysis for
'SIGLEC12' on 'Cardiac valvular disease'

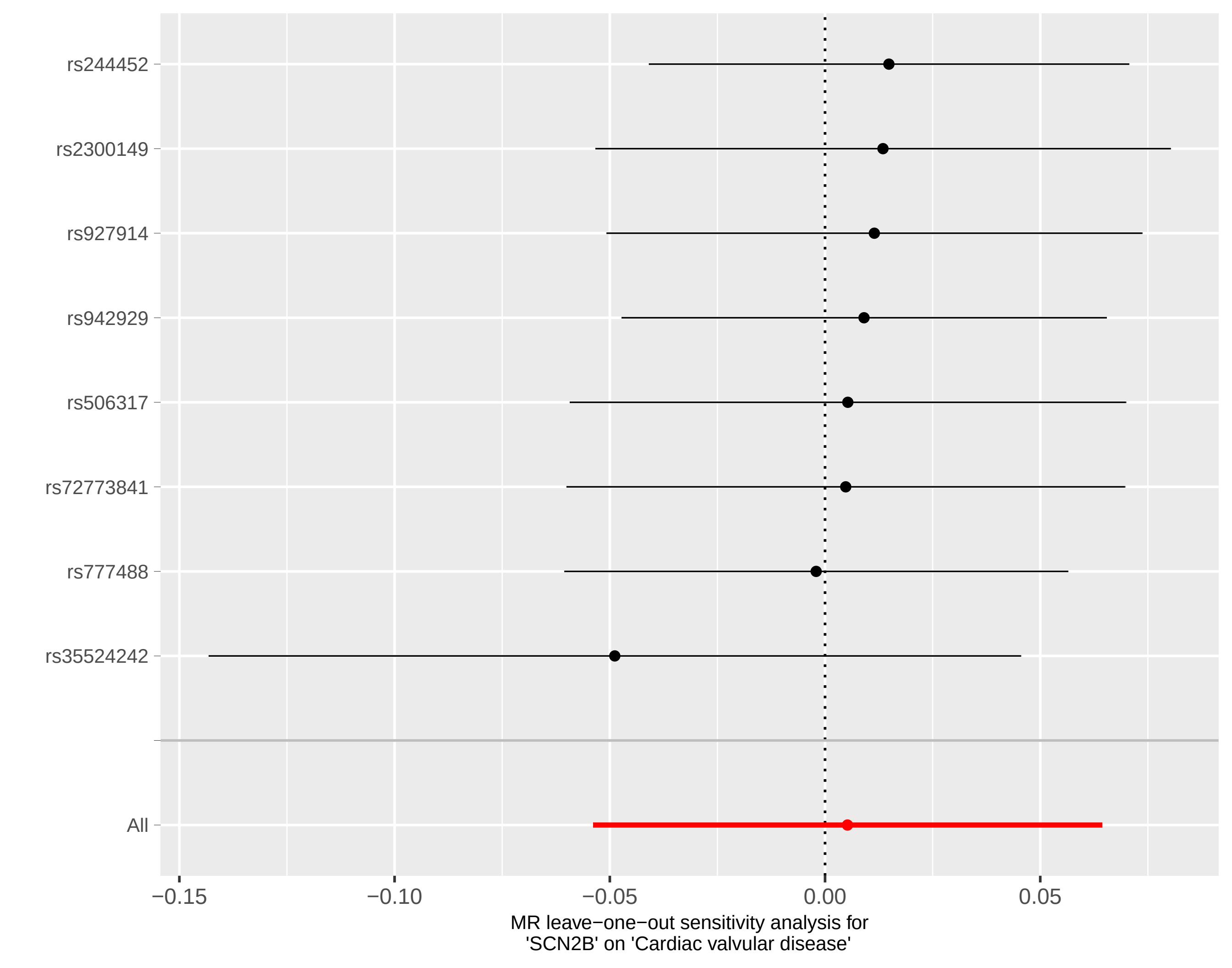
rs244452
rs2300149
rs927914
rs942929
rs506317
rs72773841
rs777488
rs35524242
All
−0.15
−0.10
−0.05
0.00
0.05
MR leave−one−out sensitivity analysis for
'SCN2B' on 'Cardiac valvular disease'

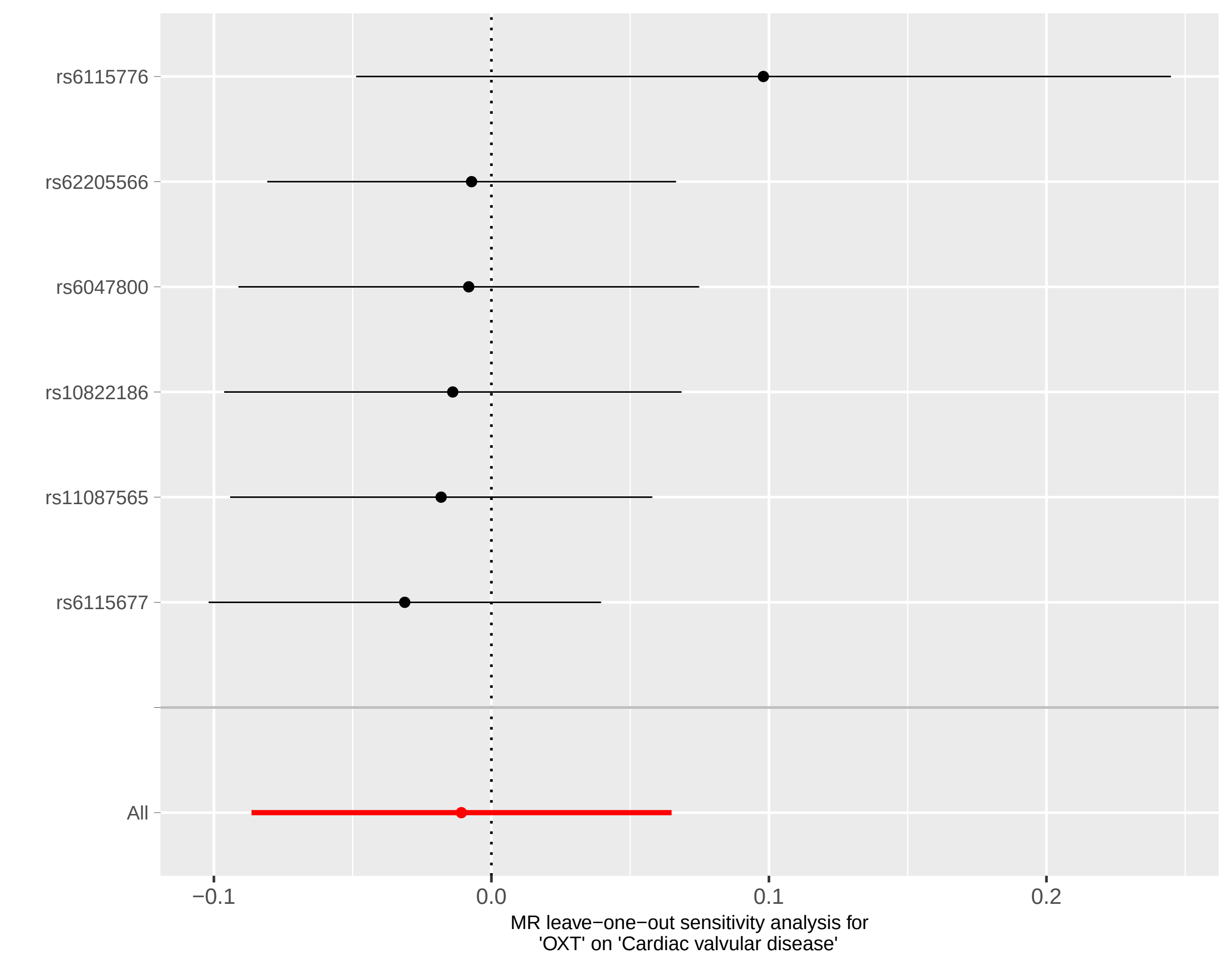
rs6115776
rs62205566
rs6047800
rs10822186
rs11087565
rs6115677
All
−0.1
0.0
0.1
0.2
MR leave−one−out sensitivity analysis for
'OXT' on 'Cardiac valvular disease'

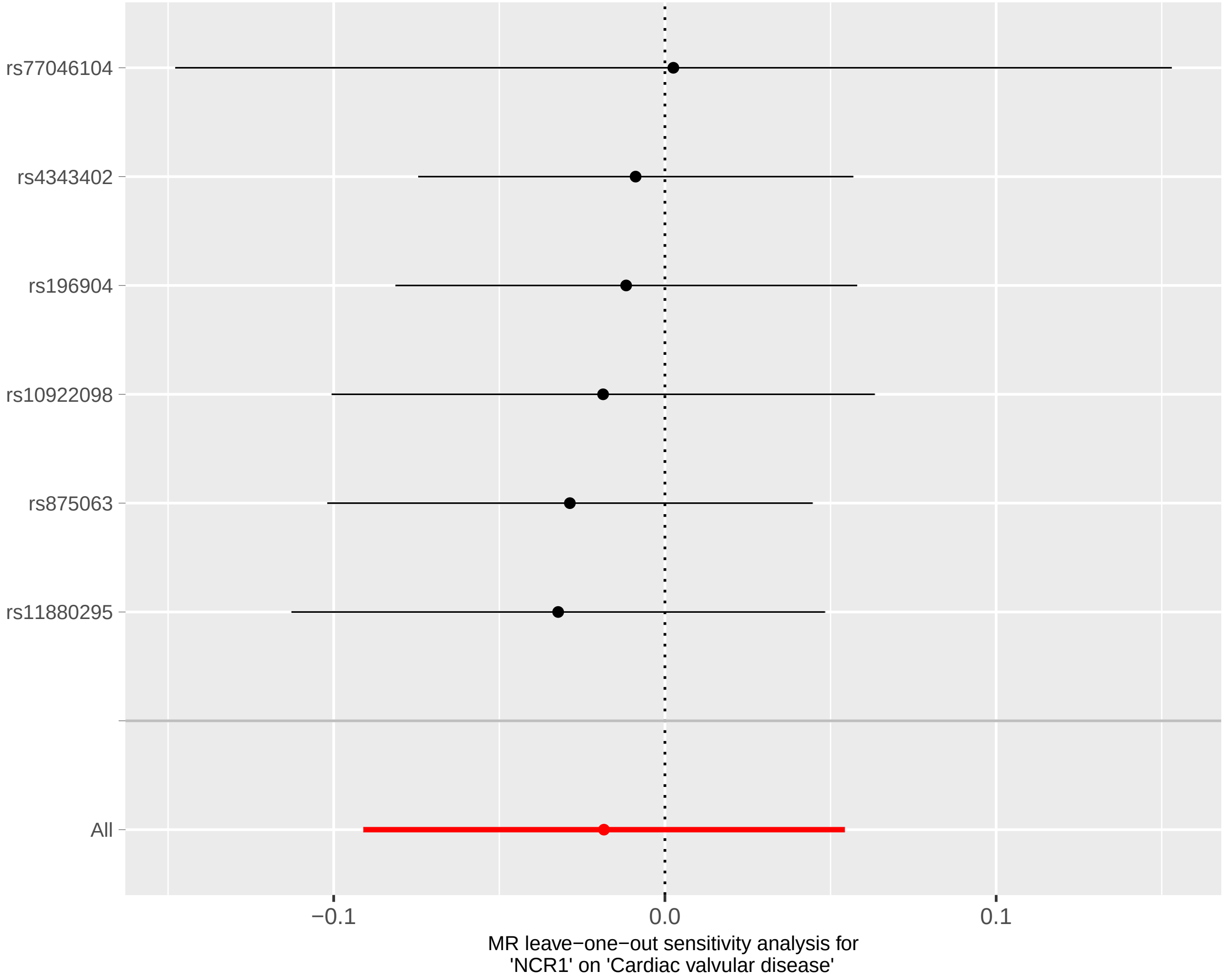
rs77046104
rs4343402
rs196904
rs10922098
rs875063
rs11880295
All
−0.1
0.0
0.1
MR leave−one−out sensitivity analysis for
'NCR1' on 'Cardiac valvular disease'

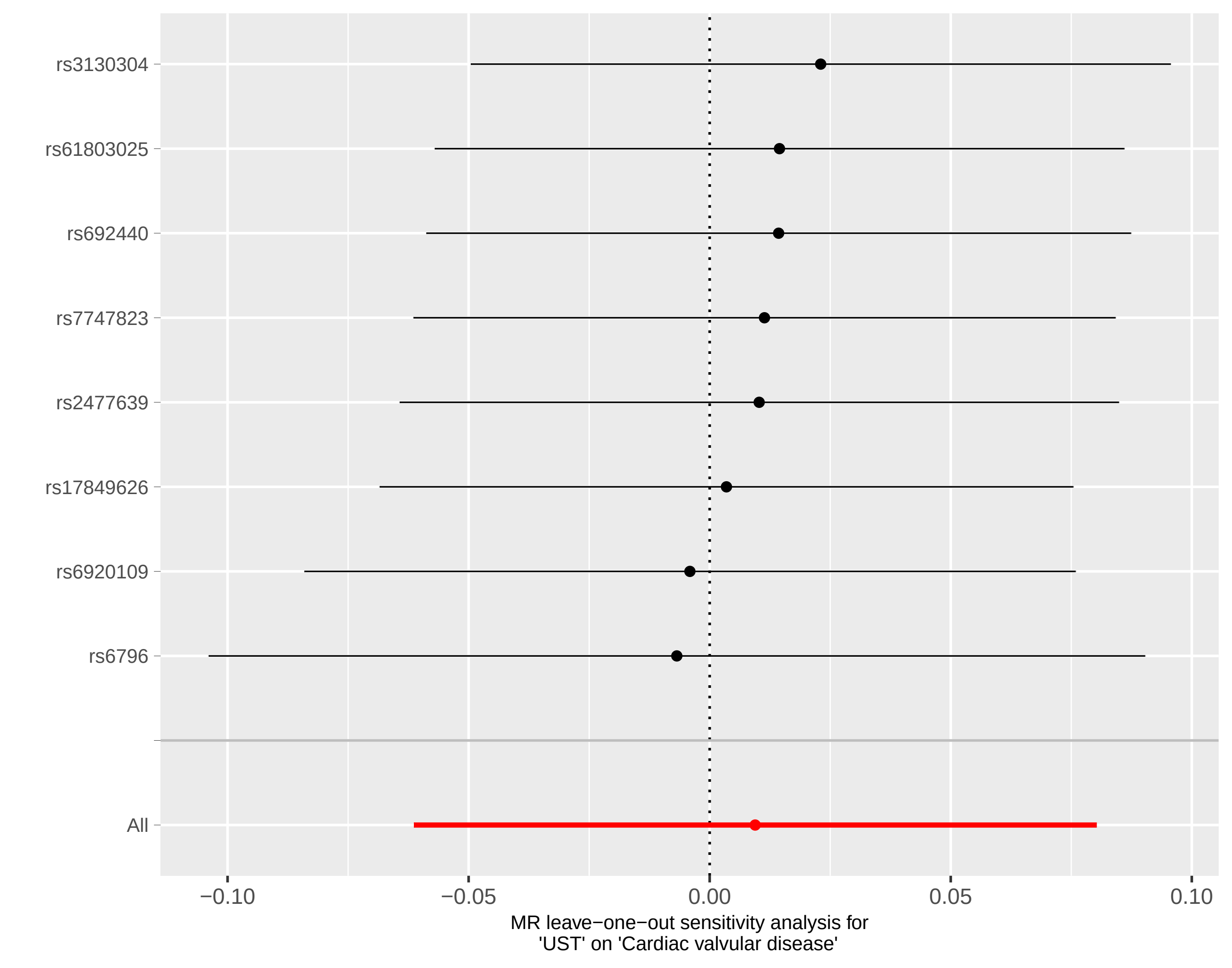

rs3130304
rs61803025
rs692440
rs7747823
rs2477639
rs17849626
rs6920109
rs6796
All
−0.10
−0.05
0.00
0.05
0.10
MR leave−one−out sensitivity analysis for
'UST' on 'Cardiac valvular disease'

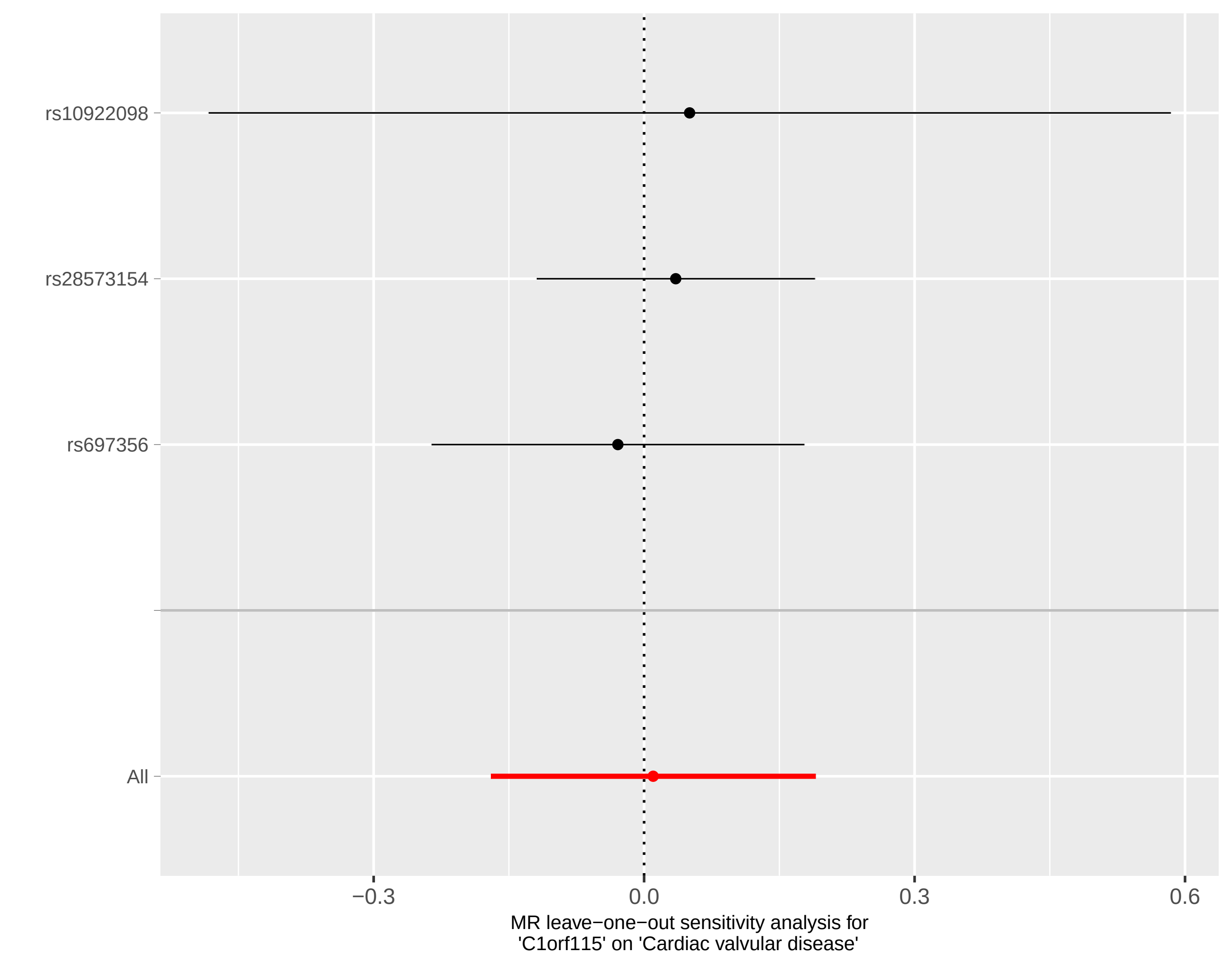
rs10922098
rs28573154
rs697356
All
−0.3
0.0
0.3
0.6
MR leave−one−out sensitivity analysis for
'C1orf115' on 'Cardiac valvular disease'

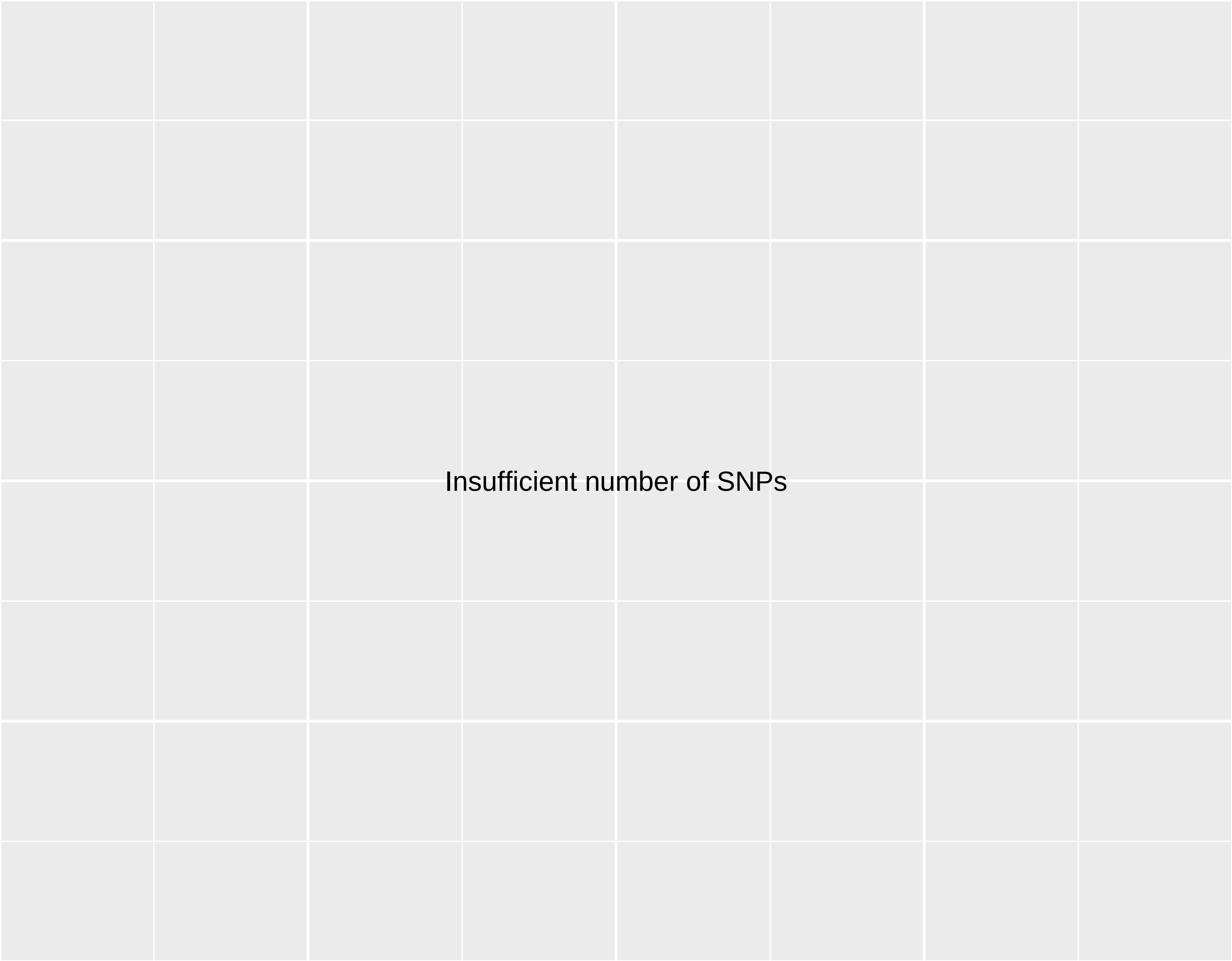
Insufficient number of SNPs

Insufficient number of SNPs

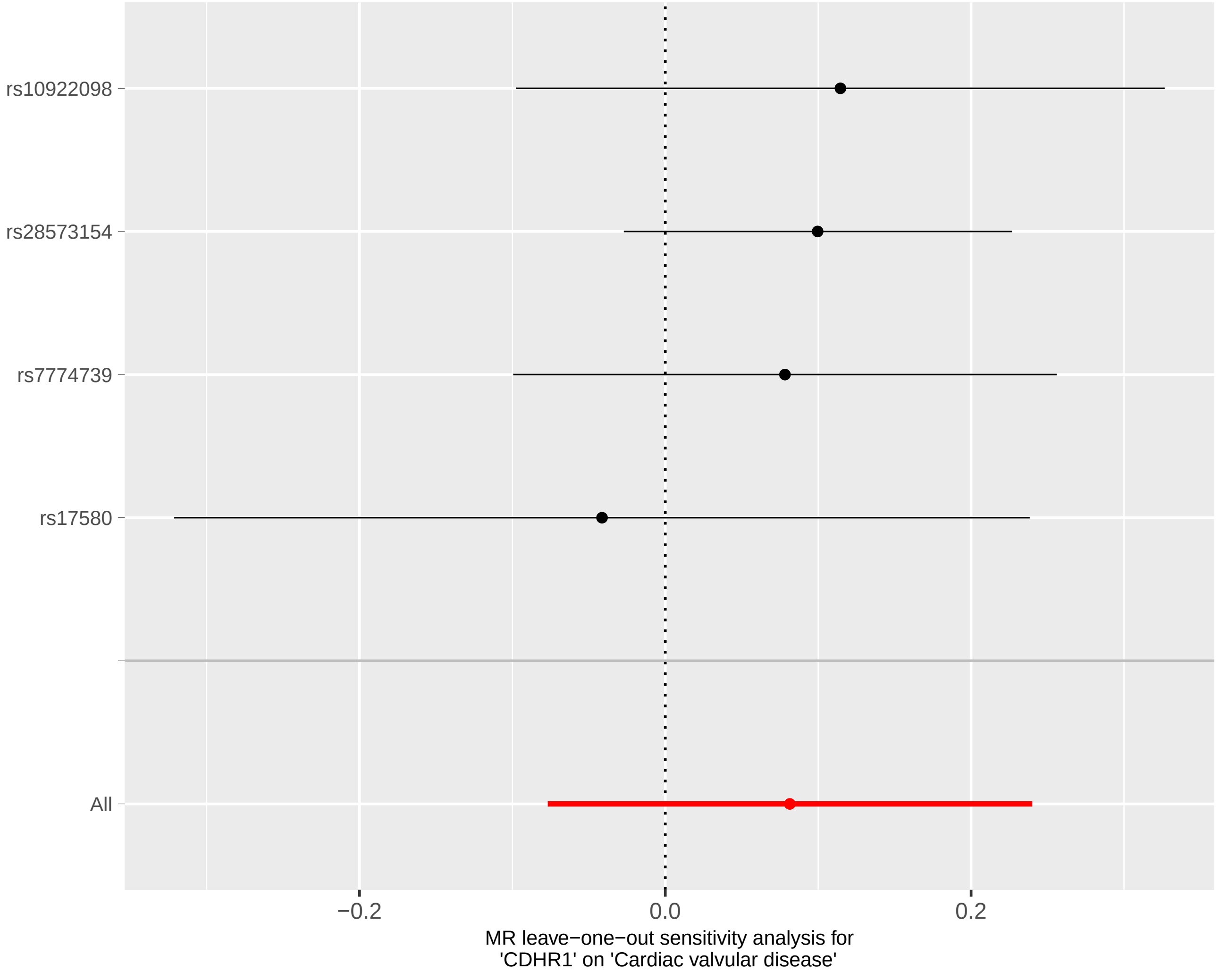
rs10922098
rs28573154
rs7774739
rs17580
All
−0.2
0.0
0.2
MR leave−one−out sensitivity analysis for
'CDHR1' on 'Cardiac valvular disease'

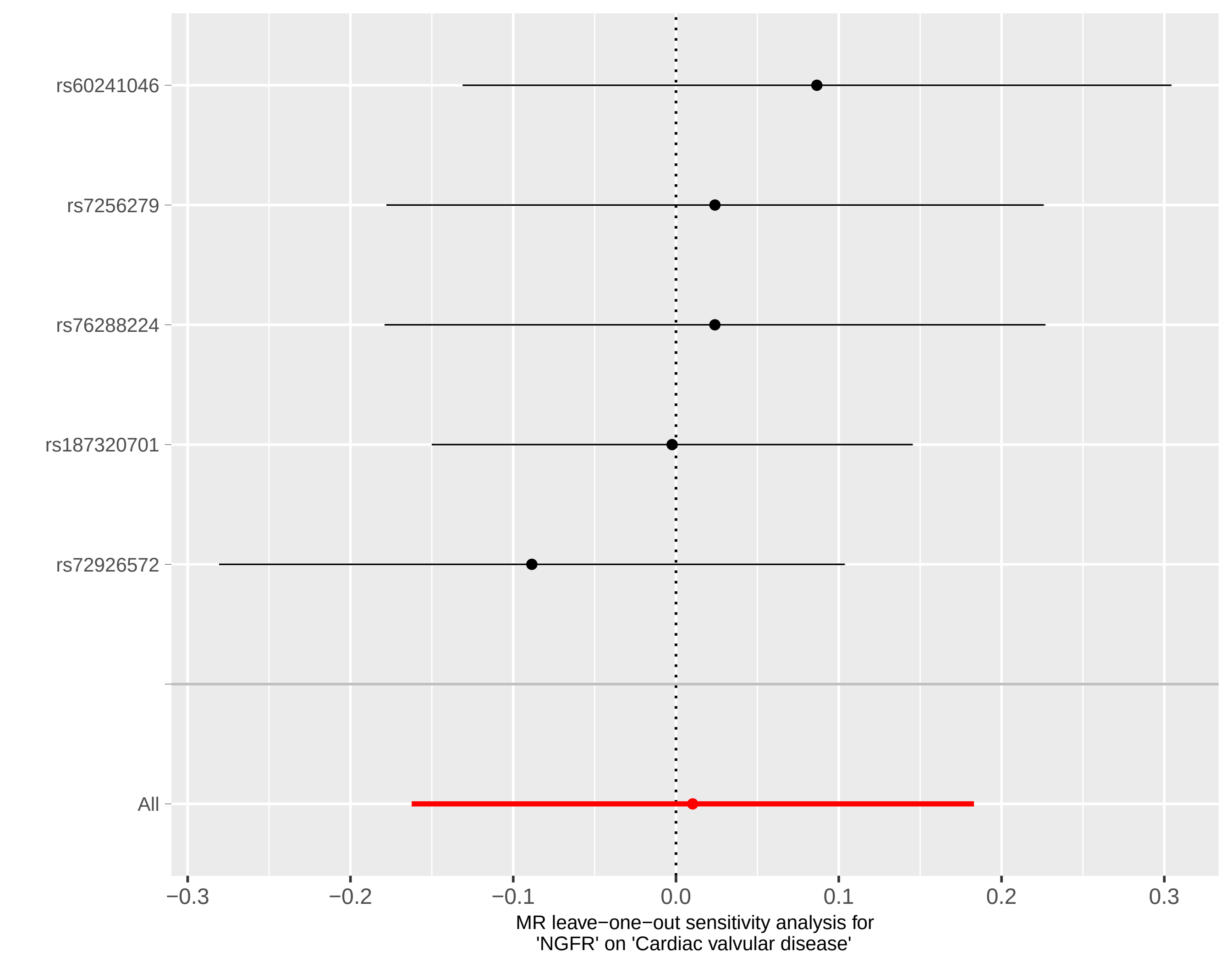
rs60241046
rs7256279
rs76288224
rs187320701
rs72926572
All
−0.3
−0.2
−0.1
0.0
0.1
0.2
0.3
MR leave−one−out sensitivity analysis for
'NGFR' on 'Cardiac valvular disease'

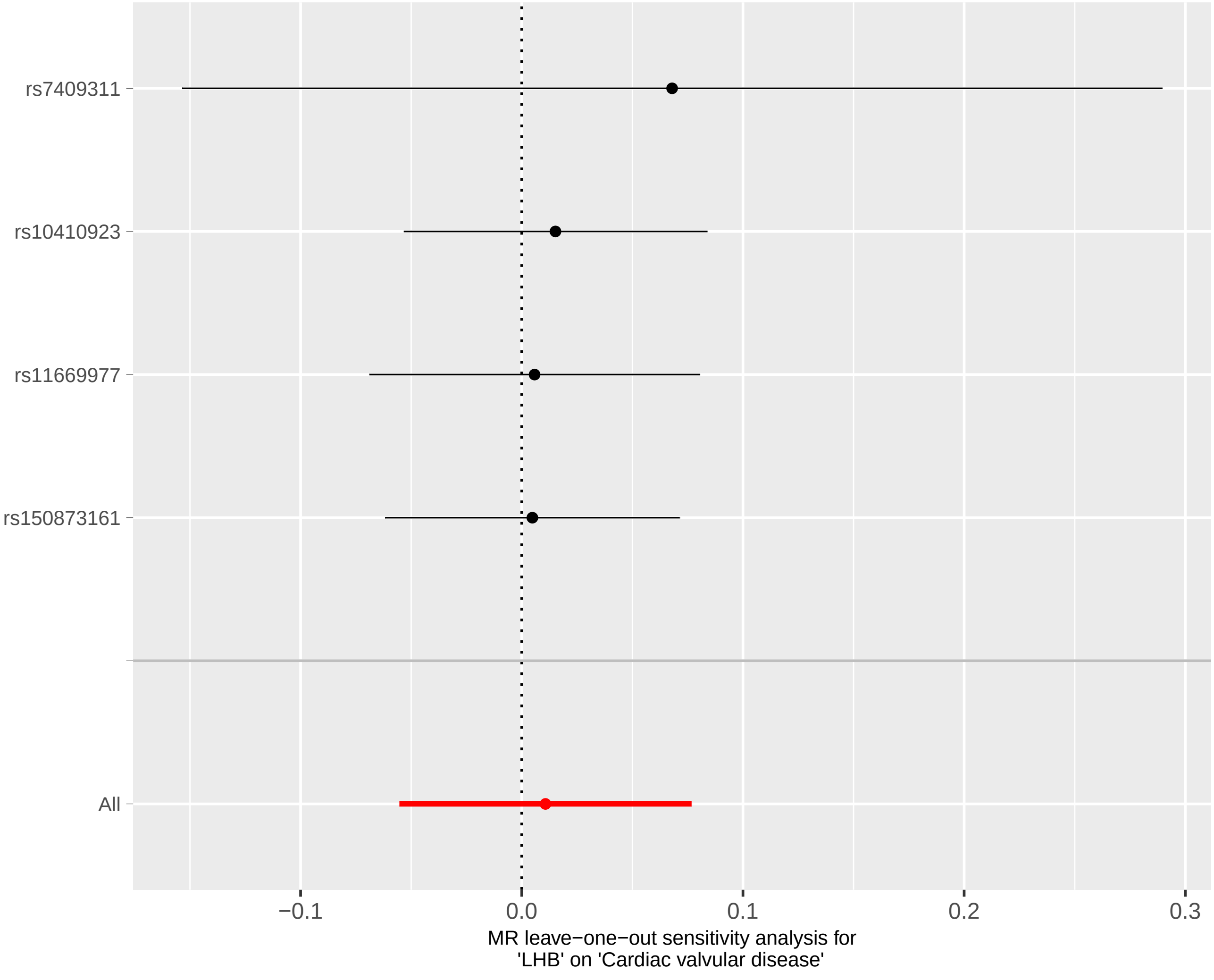

rs7409311
rs10410923
rs11669977
rs150873161
All
−0.1
0.0
0.1
0.2
0.3
MR leave−one−out sensitivity analysis for
'LHB' on 'Cardiac valvular disease'

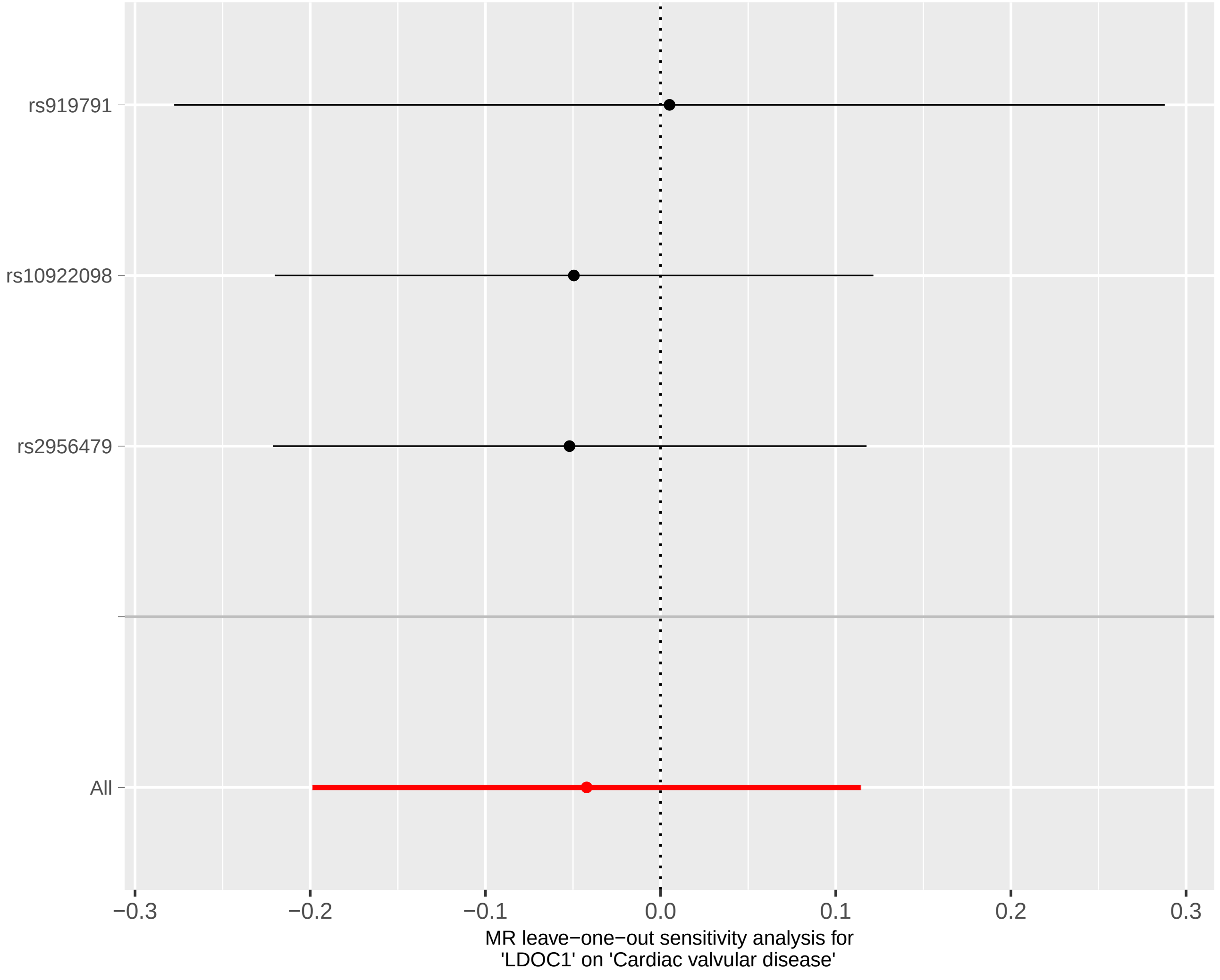
rs919791
rs10922098
rs2956479
All
−0.3
−0.2
−0.1
0.0
0.1
0.2
0.3
MR leave−one−out sensitivity analysis for
'LDOC1' on 'Cardiac valvular disease'

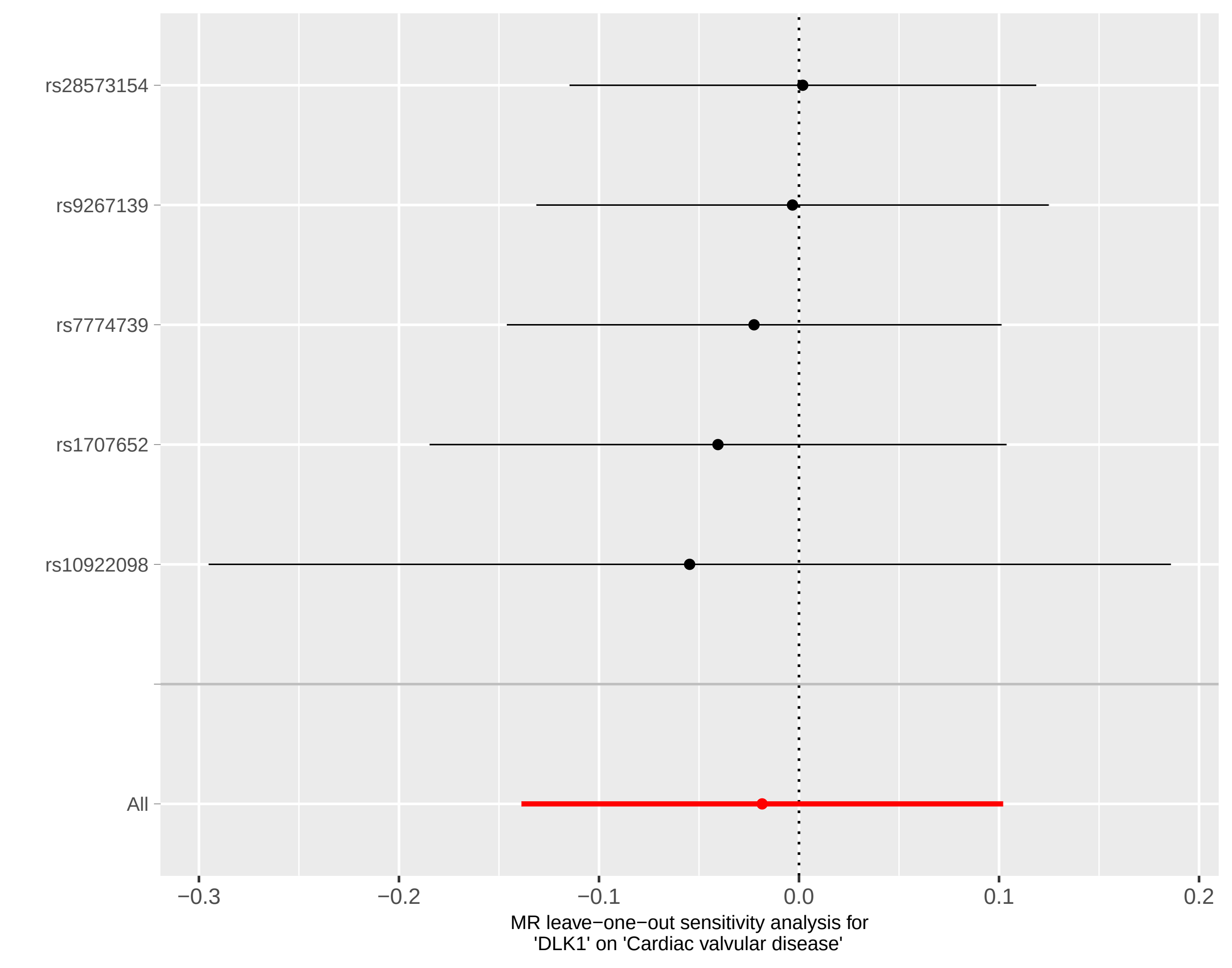
rs28573154
rs9267139
rs7774739
rs1707652
rs10922098
All
−0.3
−0.2
−0.1
0.0
0.1
0.2
MR leave−one−out sensitivity analysis for
'DLK1' on 'Cardiac valvular disease'

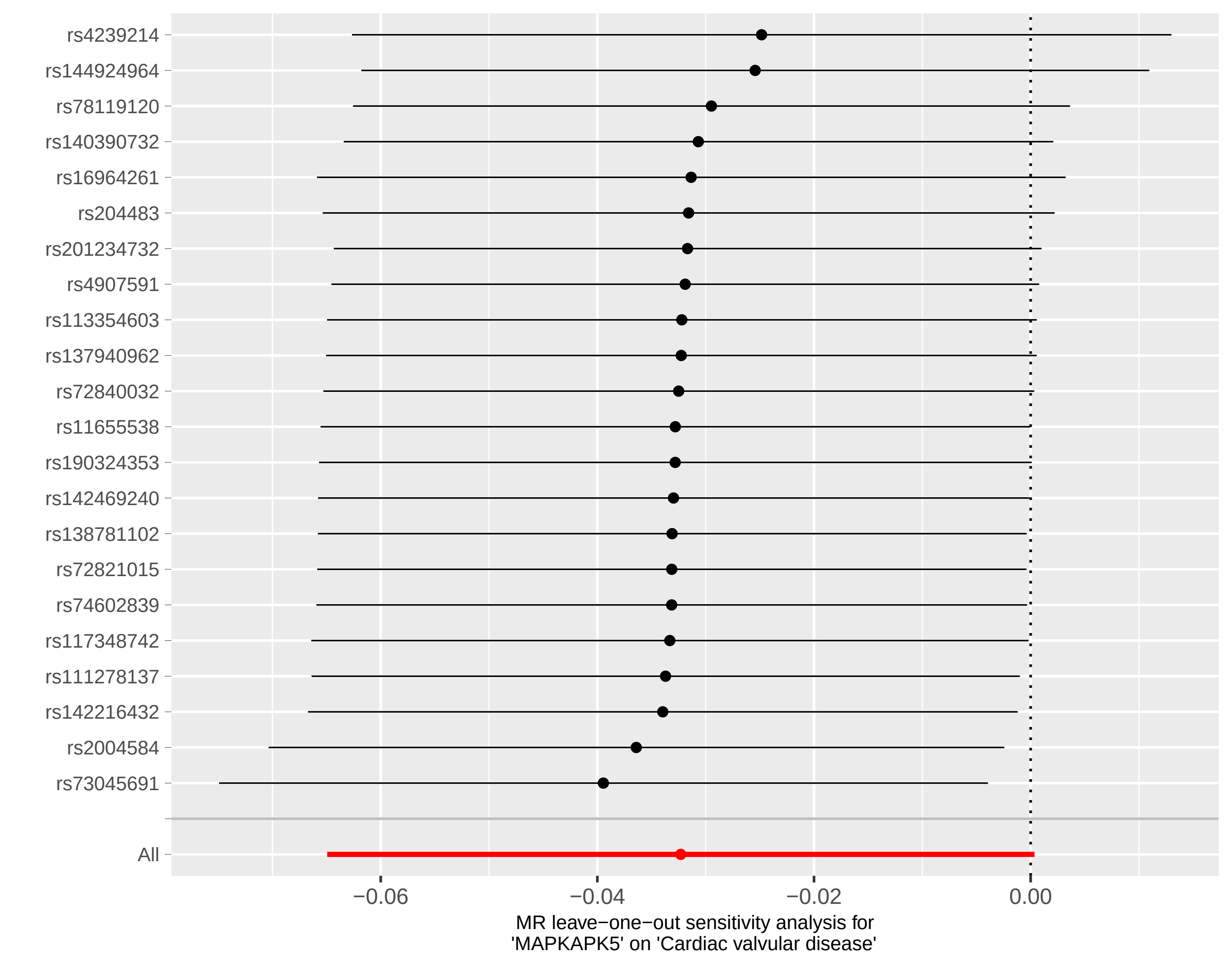
rs4239214
rs144924964
rs78119120
rs140390732
rs16964261
rs204483
rs201234732
rs4907591
rs113354603
rs137940962
rs72840032
rs11655538
rs190324353
rs142469240
rs138781102
rs72821015
rs74602839
rs117348742
rs111278137
rs142216432
rs2004584
rs73045691
All
−0.06
−0.04
−0.02
0.00
MR leave−one−out sensitivity analysis for
'MAPKAPK5' on 'Cardiac valvular disease'

Insufficient number of SNPs

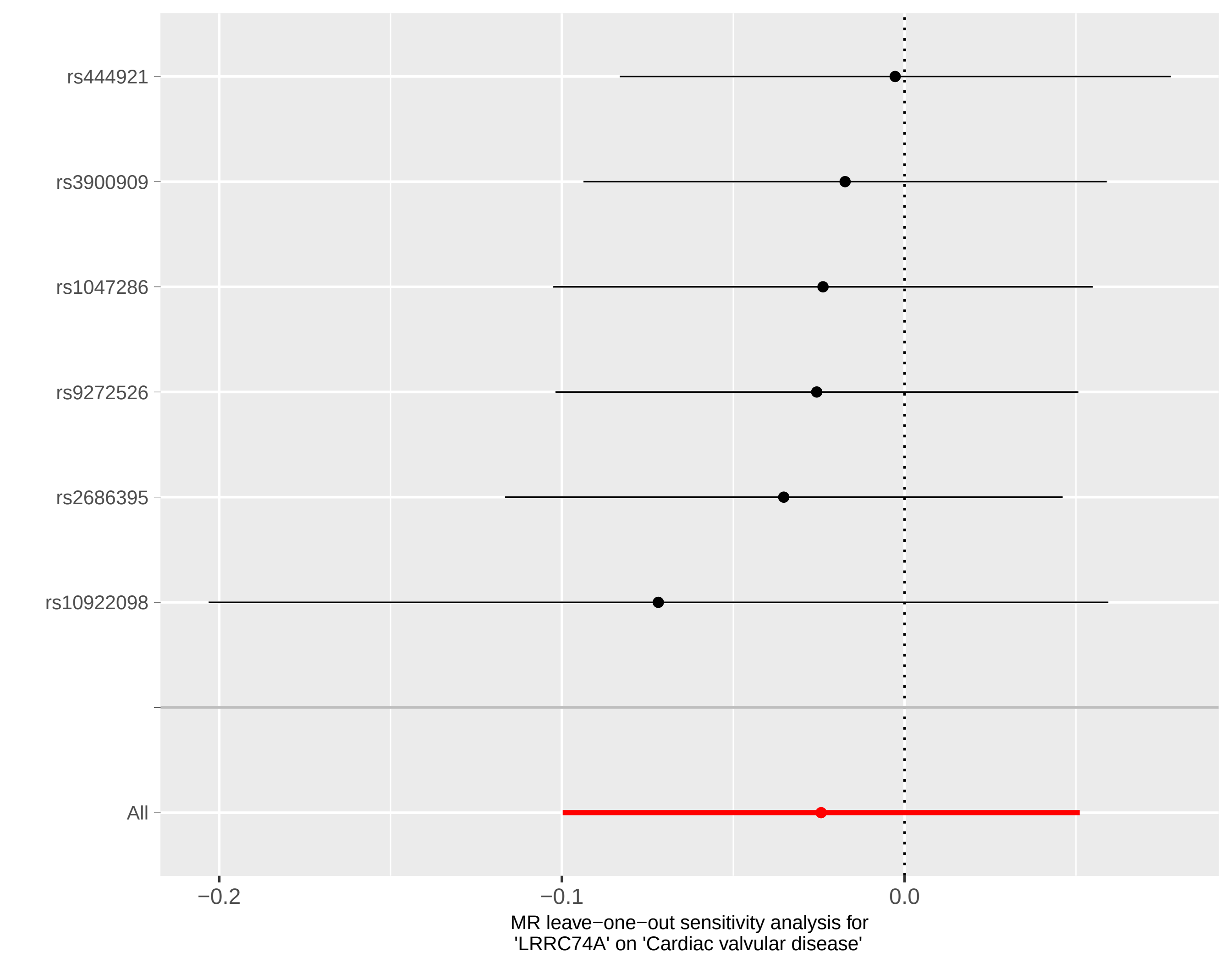
rs444921
rs3900909
rs1047286
rs9272526
rs2686395
rs10922098
All
−0.2
−0.1
0.0
MR leave−one−out sensitivity analysis for
'LRRC74A' on 'Cardiac valvular disease'

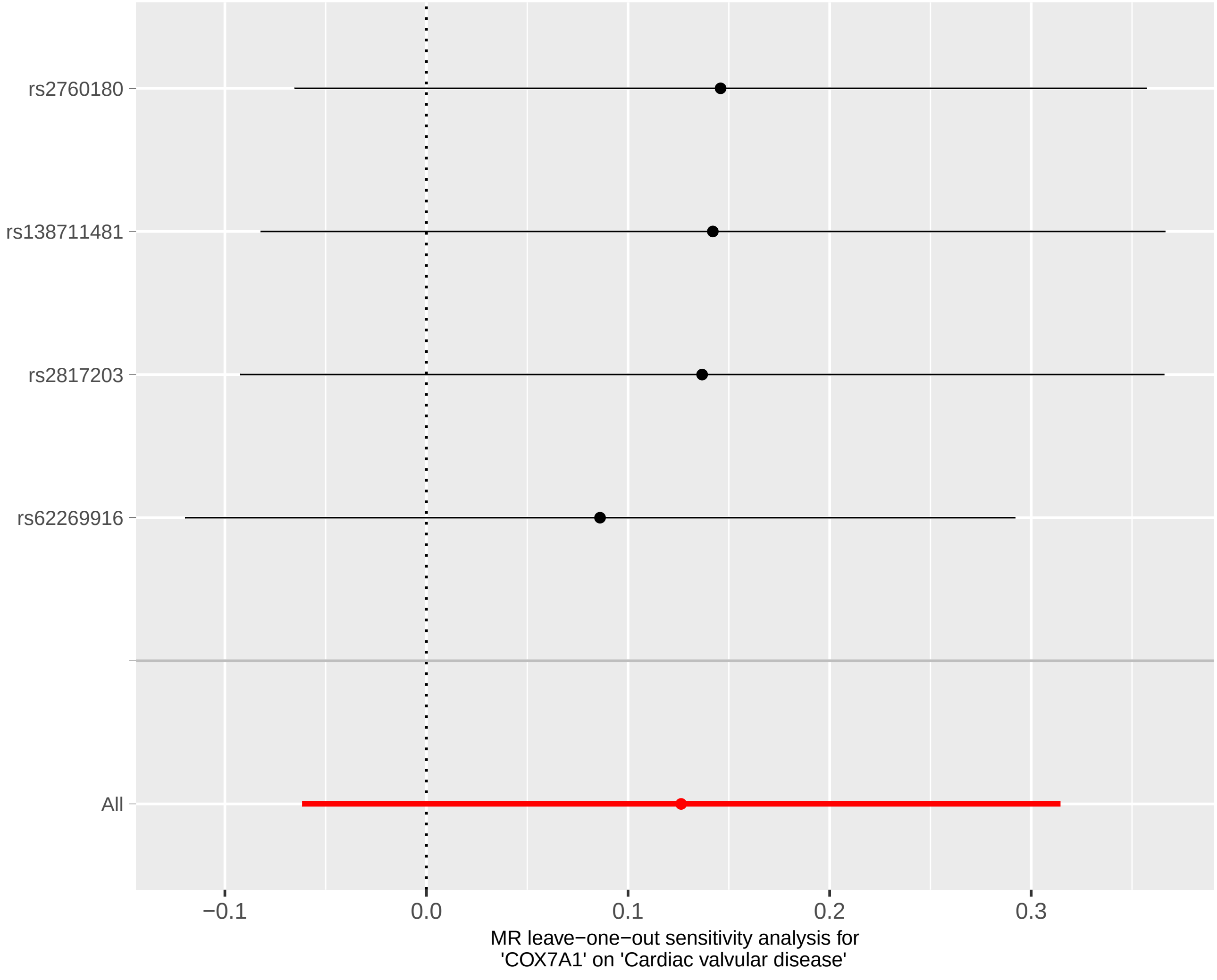
rs2760180
rs138711481
rs2817203
rs62269916
All
−0.1
0.0
0.1
0.2
0.3
MR leave−one−out sensitivity analysis for
'COX7A1' on 'Cardiac valvular disease'

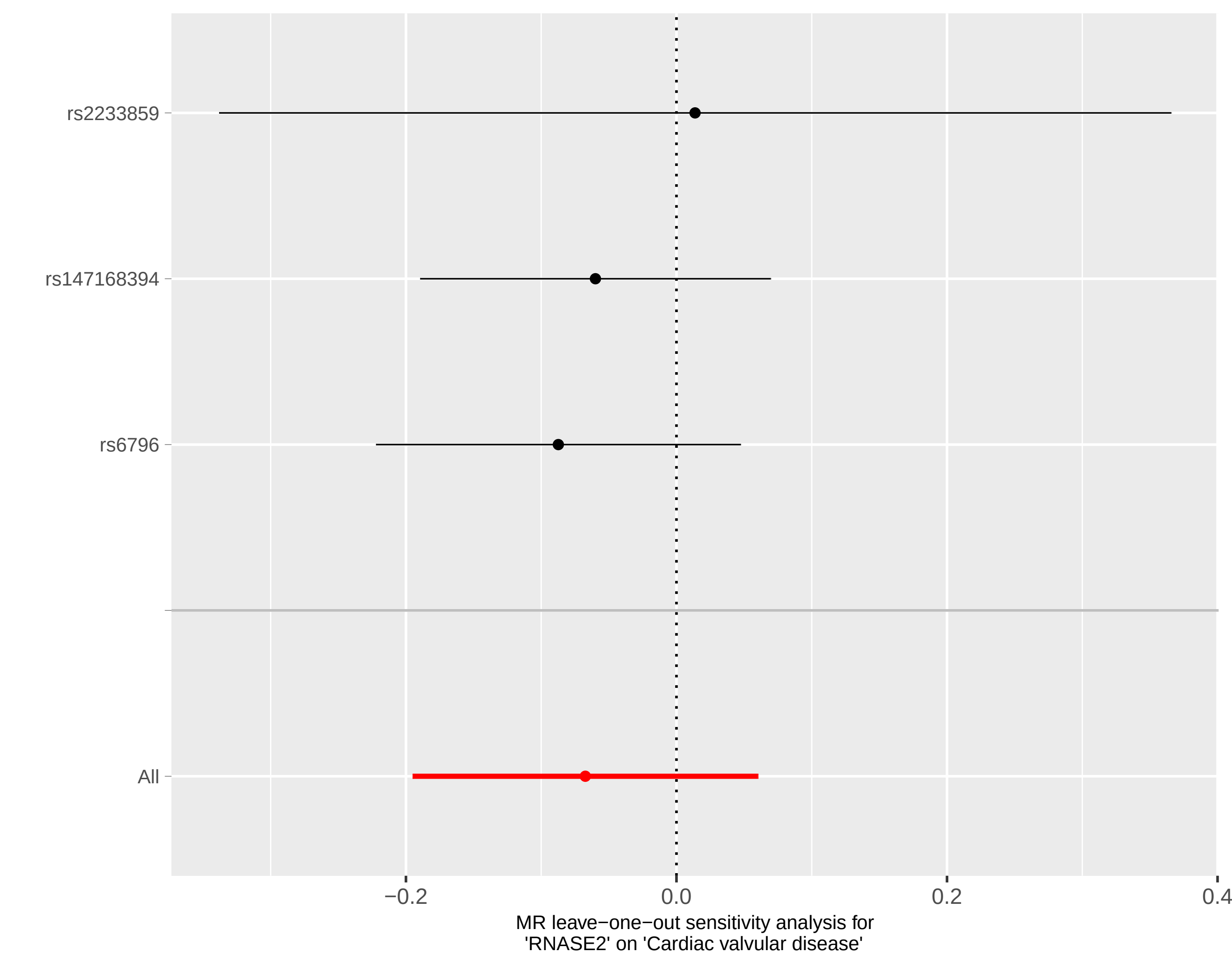
rs2233859
rs147168394
rs6796
All
−0.2
0.0
0.2
0.4
MR leave−one−out sensitivity analysis for
'RNASE2' on 'Cardiac valvular disease'

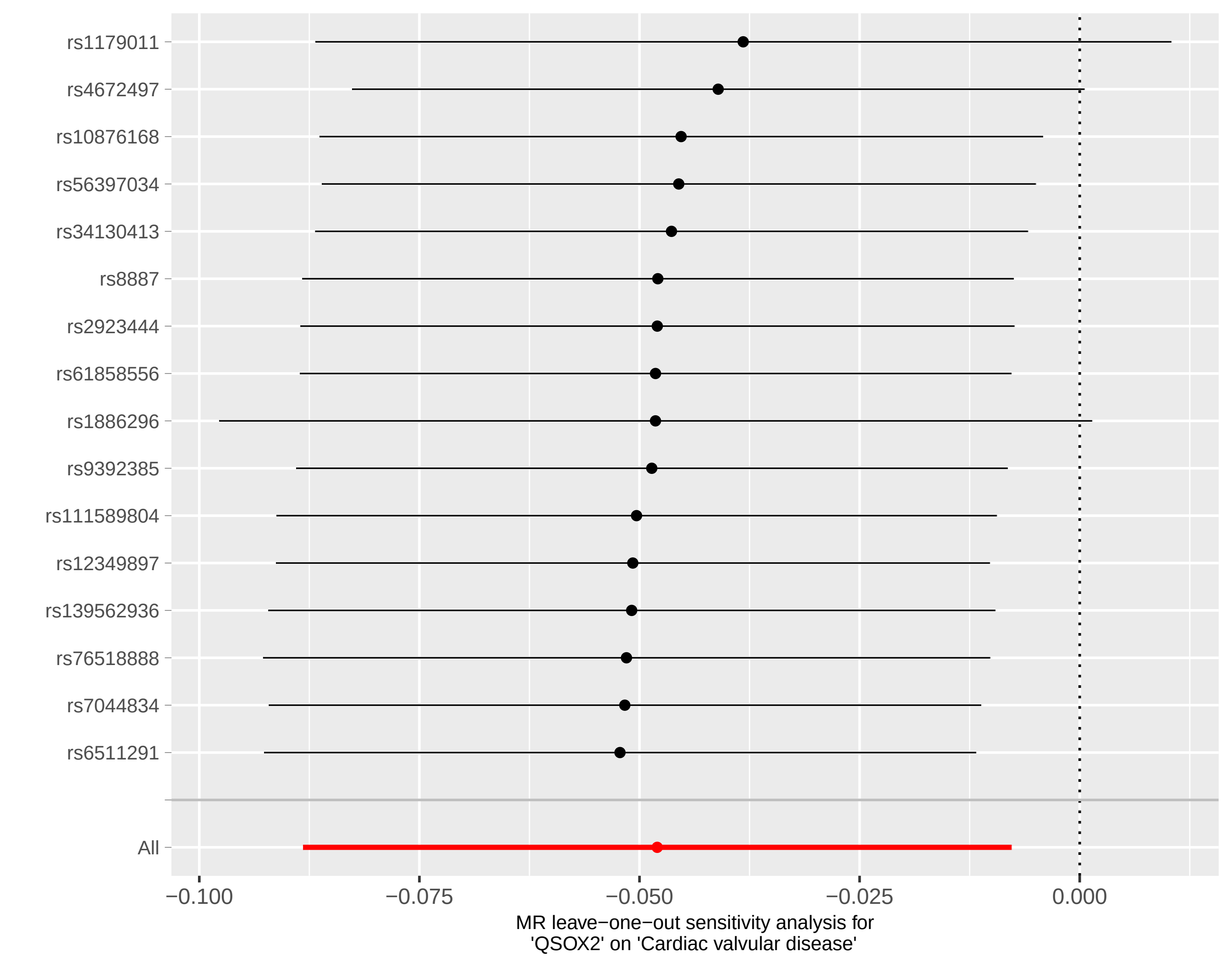
rs1179011
rs4672497
rs10876168
rs56397034
rs34130413
rs8887
rs2923444
rs61858556
rs1886296
rs9392385
rs111589804
rs12349897
rs139562936
rs76518888
rs7044834
rs6511291
All
−0.100
−0.075
−0.050
−0.025
0.000
MR leave−one−out sensitivity analysis for
'QSOX2' on 'Cardiac valvular disease'

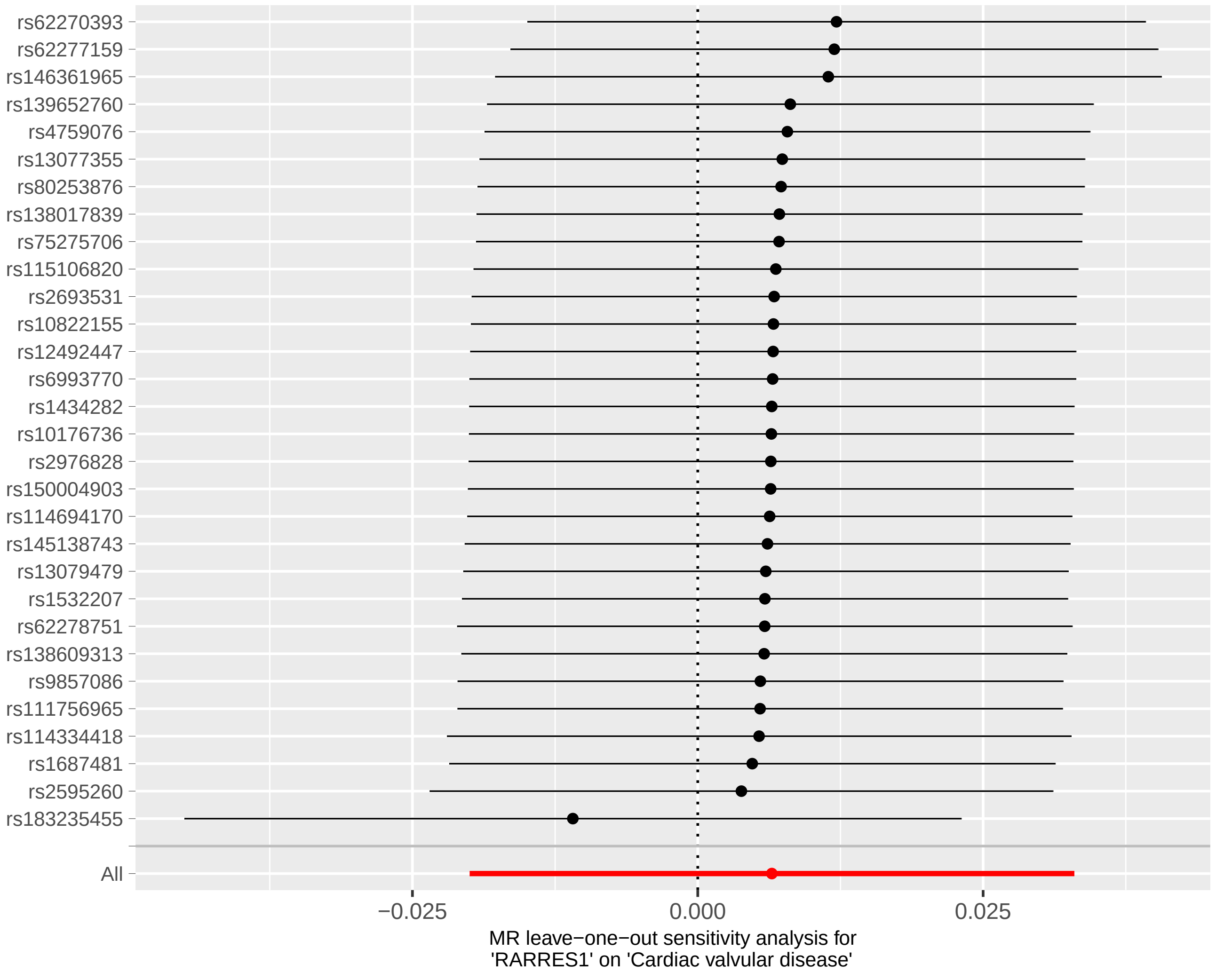
rs62270393
rs62277159
rs146361965
rs139652760
rs4759076
rs13077355
rs80253876
rs138017839
rs75275706
rs115106820
rs2693531
rs10822155
rs12492447
rs6993770
rs1434282
rs10176736
rs2976828
rs150004903
rs114694170
rs145138743
rs13079479
rs1532207
rs62278751
rs138609313
rs9857086
rs111756965
rs114334418
rs1687481
rs2595260
rs183235455
All
−0.025
0.000
0.025
MR leave−one−out sensitivity analysis for
'RARRES1' on 'Cardiac valvular disease'

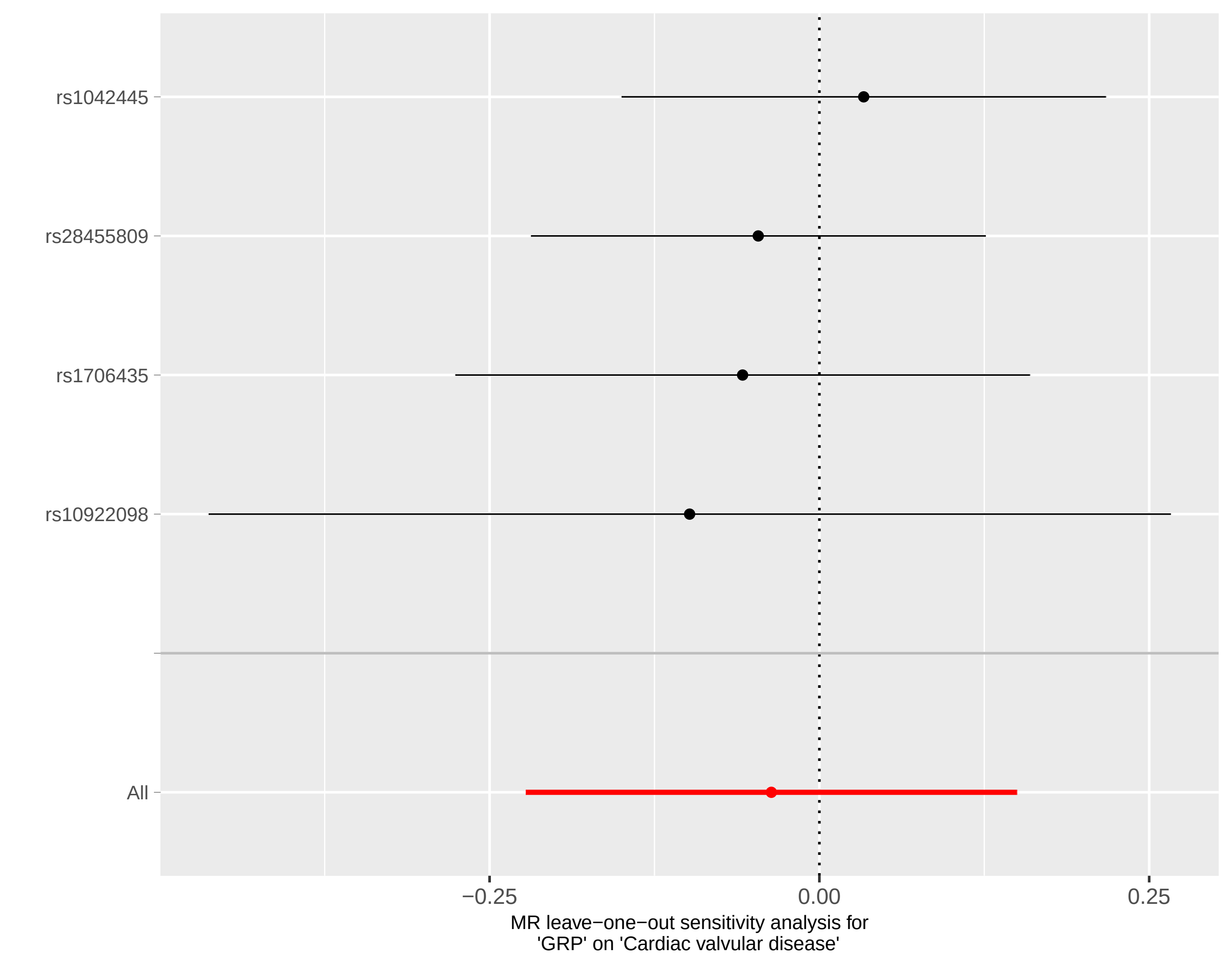
rs1042445
rs28455809
rs1706435
rs10922098
All
−0.25
0.00
0.25
MR leave−one−out sensitivity analysis for
'GRP' on 'Cardiac valvular disease'

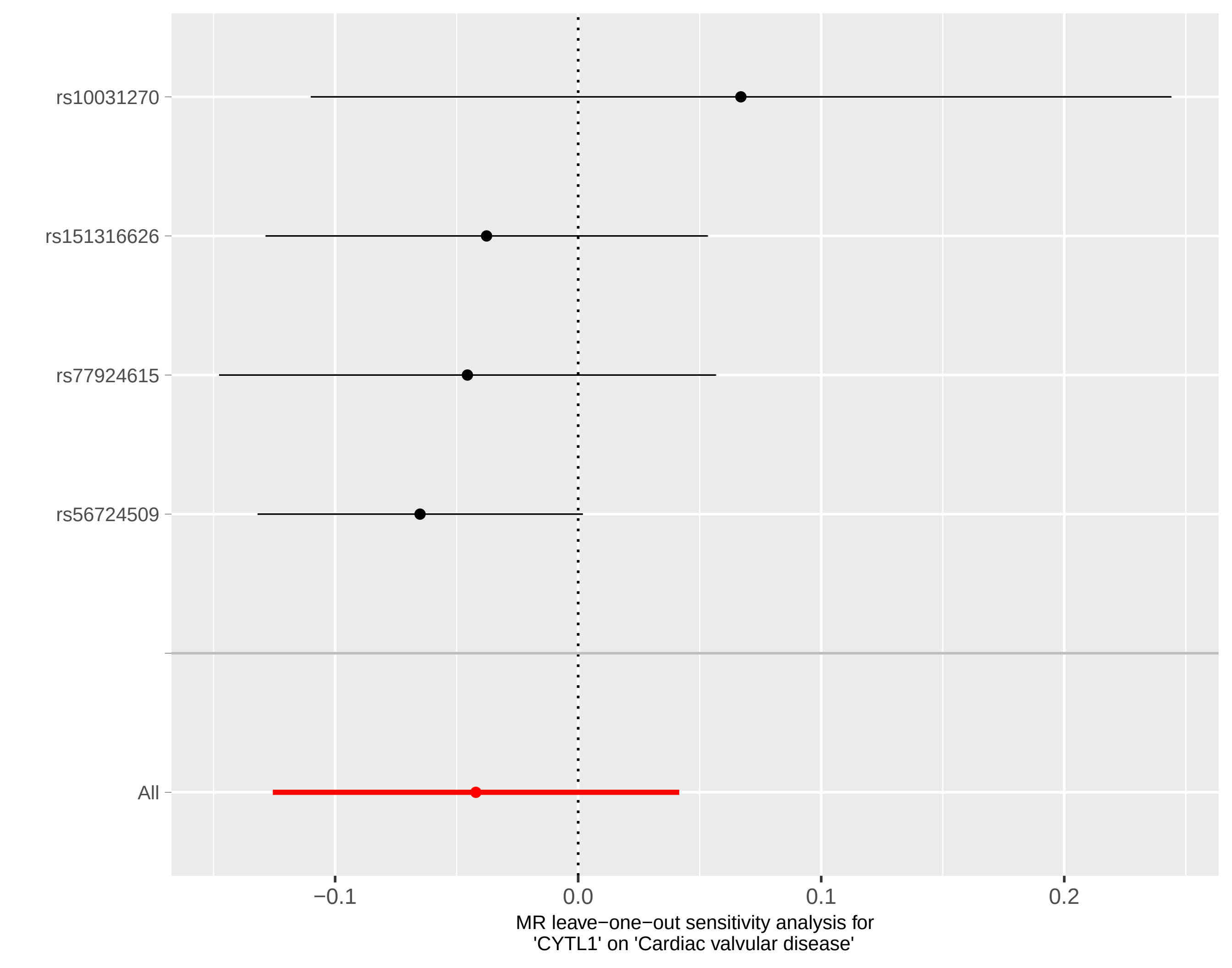

rs10031270
rs151316626
rs77924615
rs56724509
All
−0.1
0.0
0.1
0.2
MR leave−one−out sensitivity analysis for
'CYTL1' on 'Cardiac valvular disease'

Insufficient number of SNPs

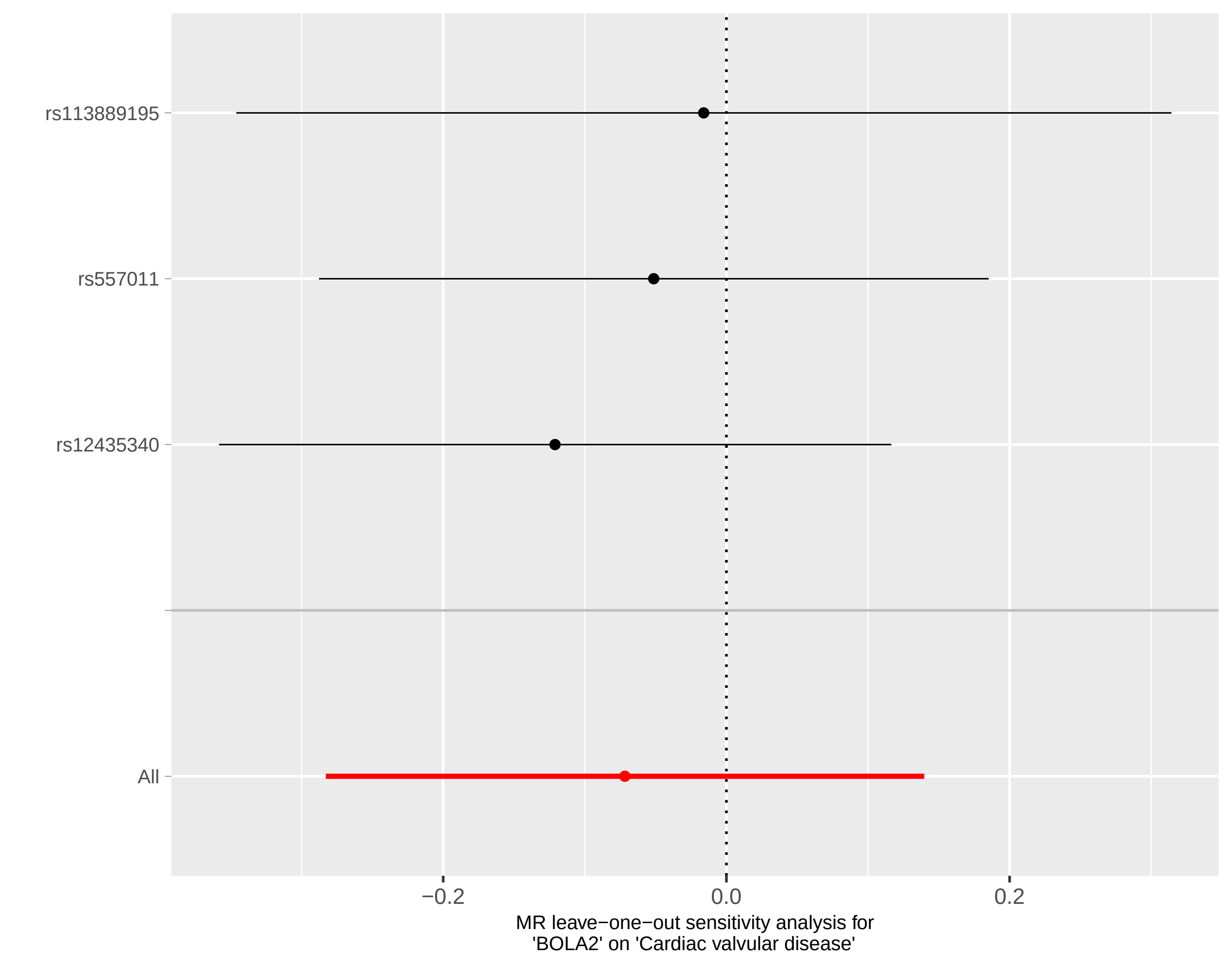

rs113889195
rs557011
rs12435340
All
−0.2
0.0
0.2
MR leave−one−out sensitivity analysis for
'BOLA2' on 'Cardiac valvular disease'

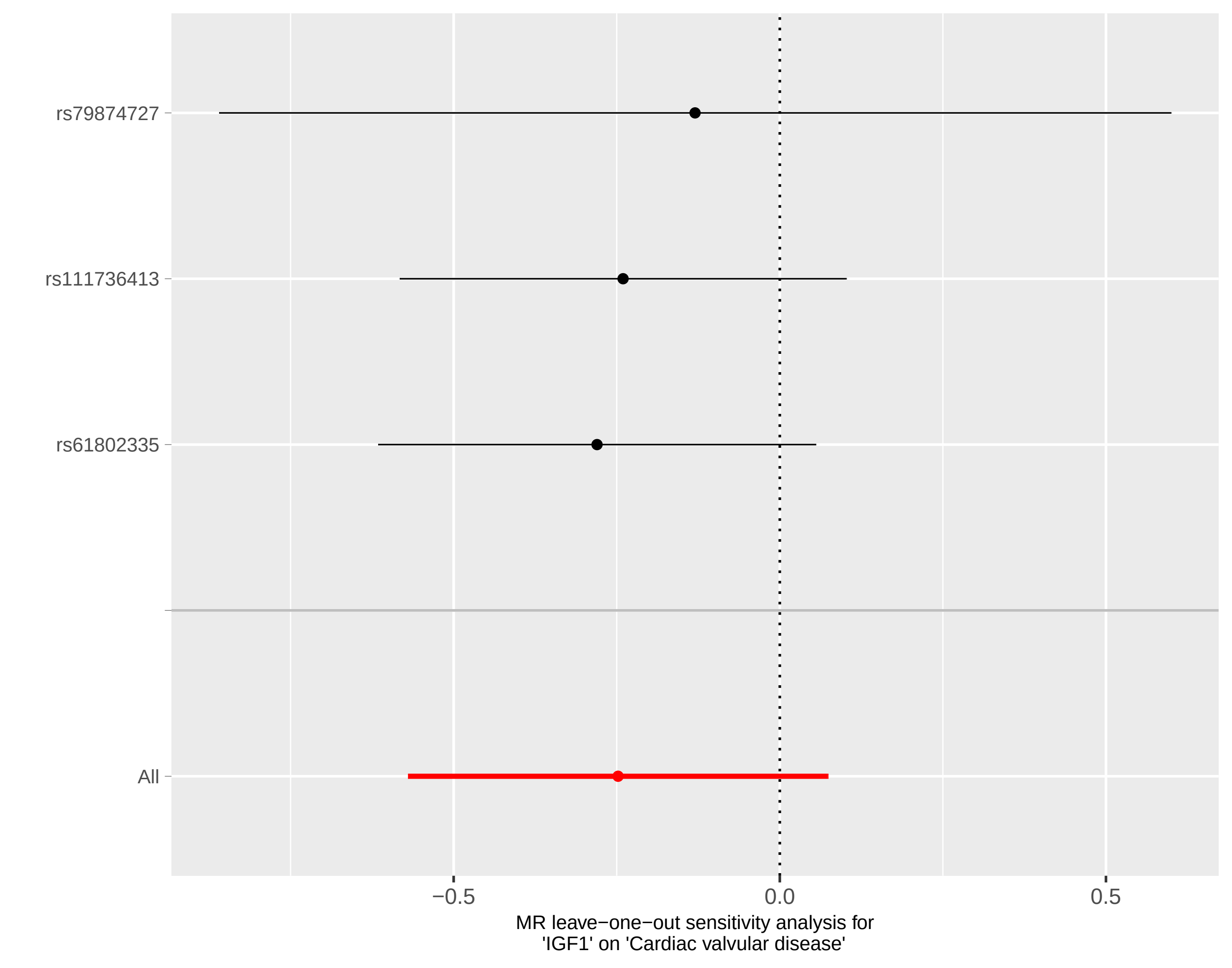
rs79874727
rs111736413
rs61802335
All
−0.5
0.0
0.5
MR leave−one−out sensitivity analysis for
'IGF1' on 'Cardiac valvular disease'

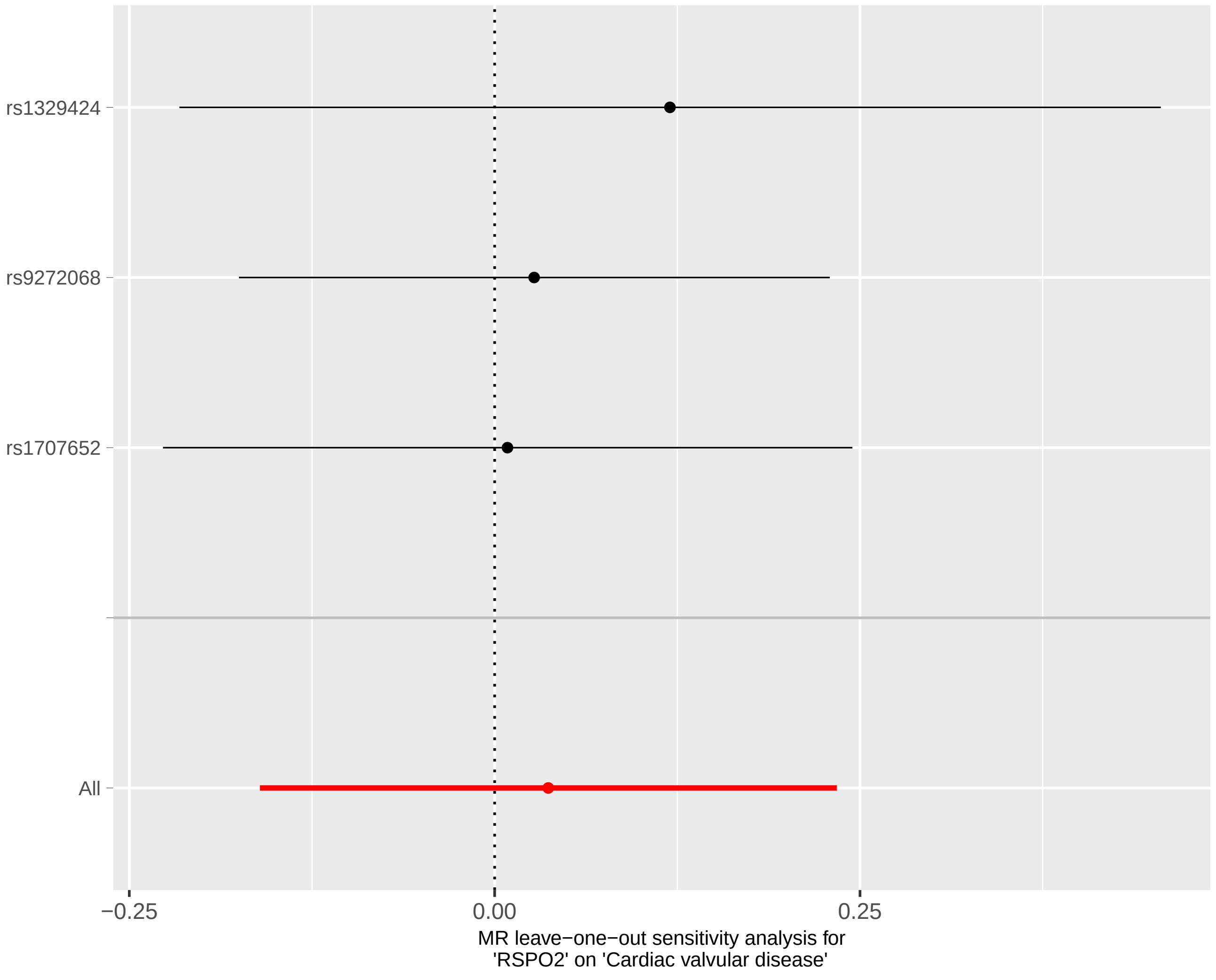
rs1329424
rs9272068
rs1707652
All
−0.25
0.00
0.25
MR leave−one−out sensitivity analysis for
'RSPO2' on 'Cardiac valvular disease'

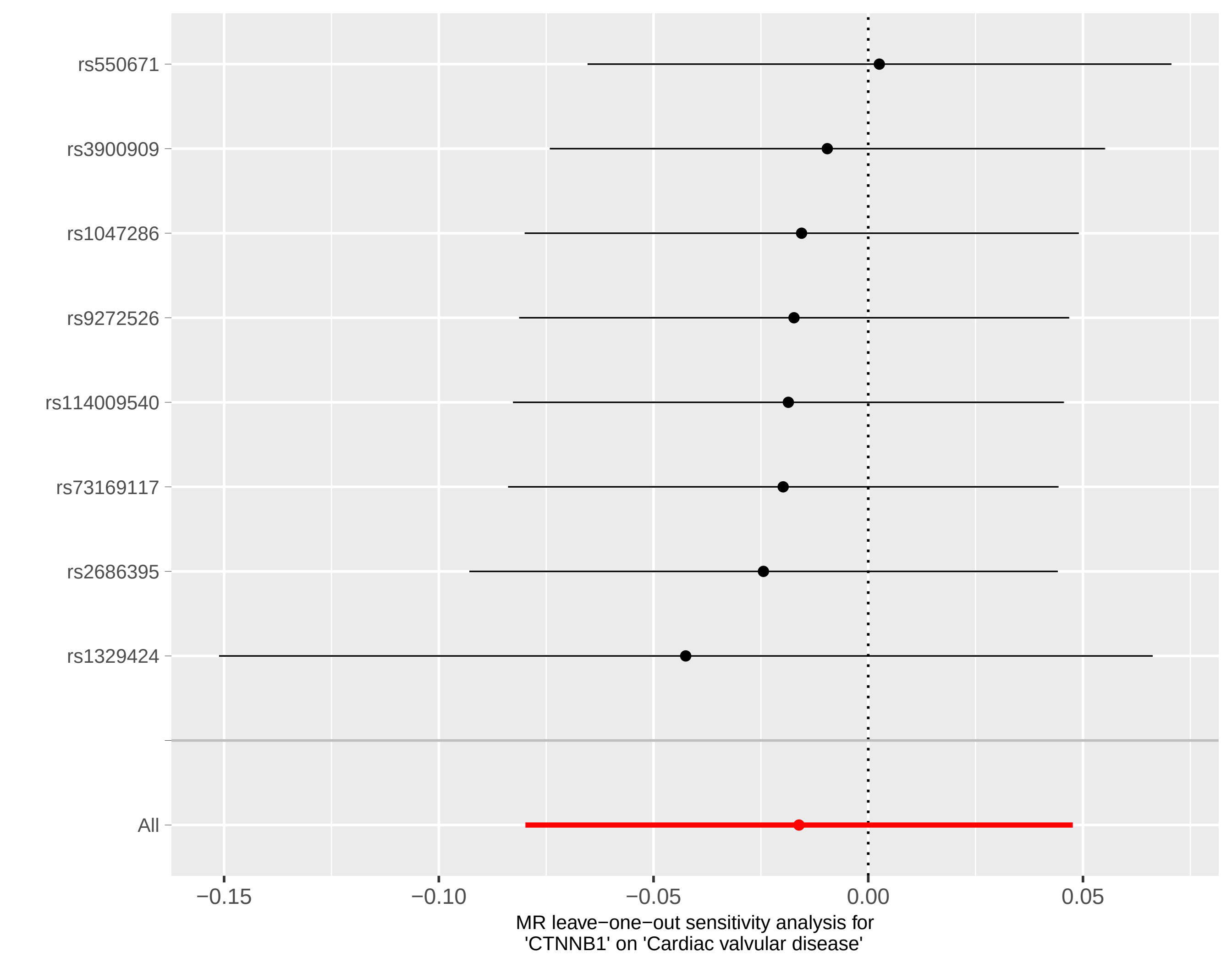

rs550671
rs3900909
rs1047286
rs9272526
rs114009540
rs73169117
rs2686395
rs1329424
All
−0.15
−0.10
−0.05
0.00
0.05
MR leave−one−out sensitivity analysis for
'CTNNB1' on 'Cardiac valvular disease'

Insufficient number of SNPs

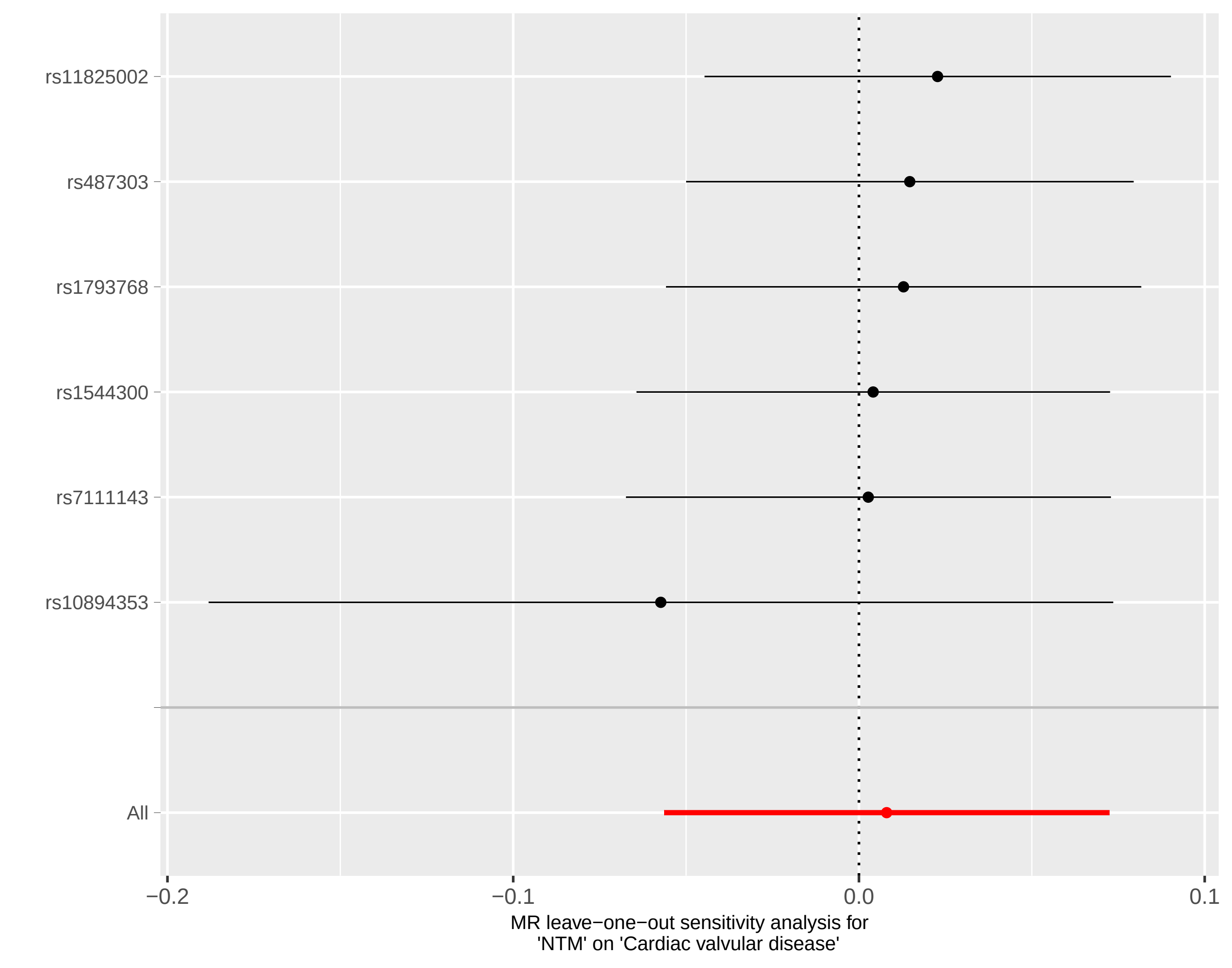
rs11825002
rs487303
rs1793768
rs1544300
rs7111143
rs10894353
All
−0.2
−0.1
0.0
0.1
MR leave−one−out sensitivity analysis for
'NTM' on 'Cardiac valvular disease'

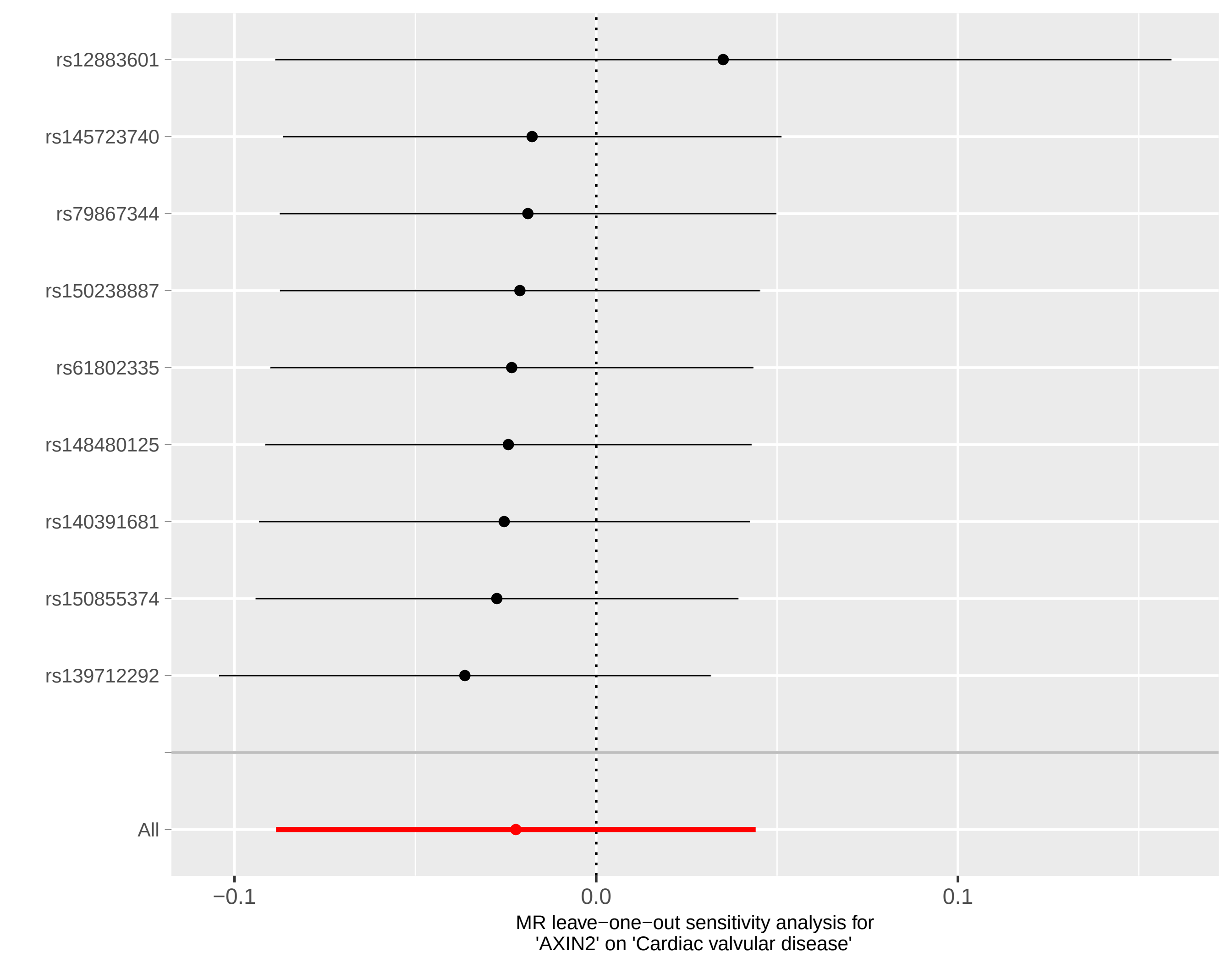
rs12883601
rs145723740
rs79867344
rs150238887
rs61802335
rs148480125
rs140391681
rs150855374
rs139712292
All
−0.1
0.0
0.1
MR leave−one−out sensitivity analysis for
'AXIN2' on 'Cardiac valvular disease'

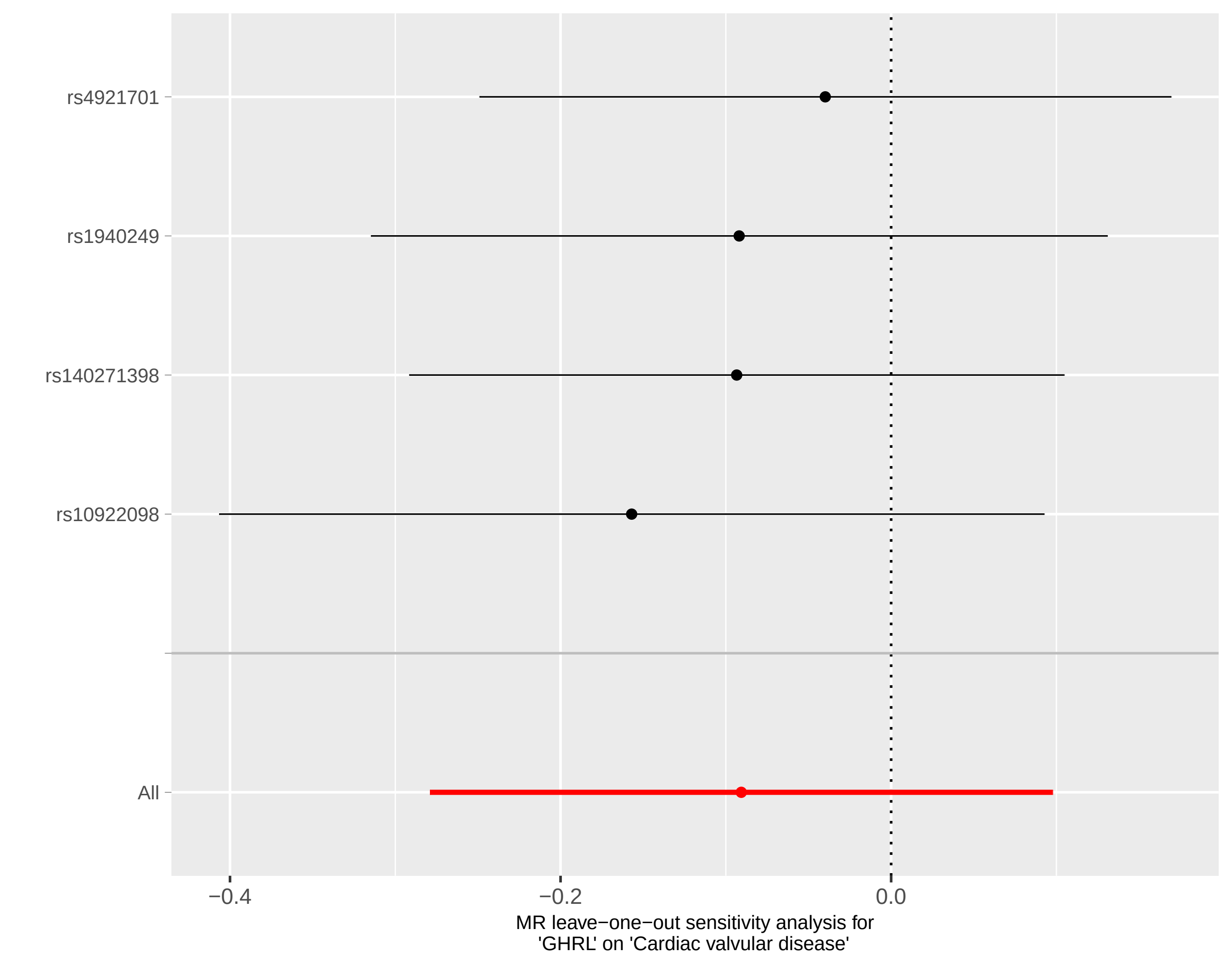
rs4921701
rs1940249
rs140271398
rs10922098
All
−0.4
−0.2
0.0
MR leave−one−out sensitivity analysis for
'GHRL' on 'Cardiac valvular disease'

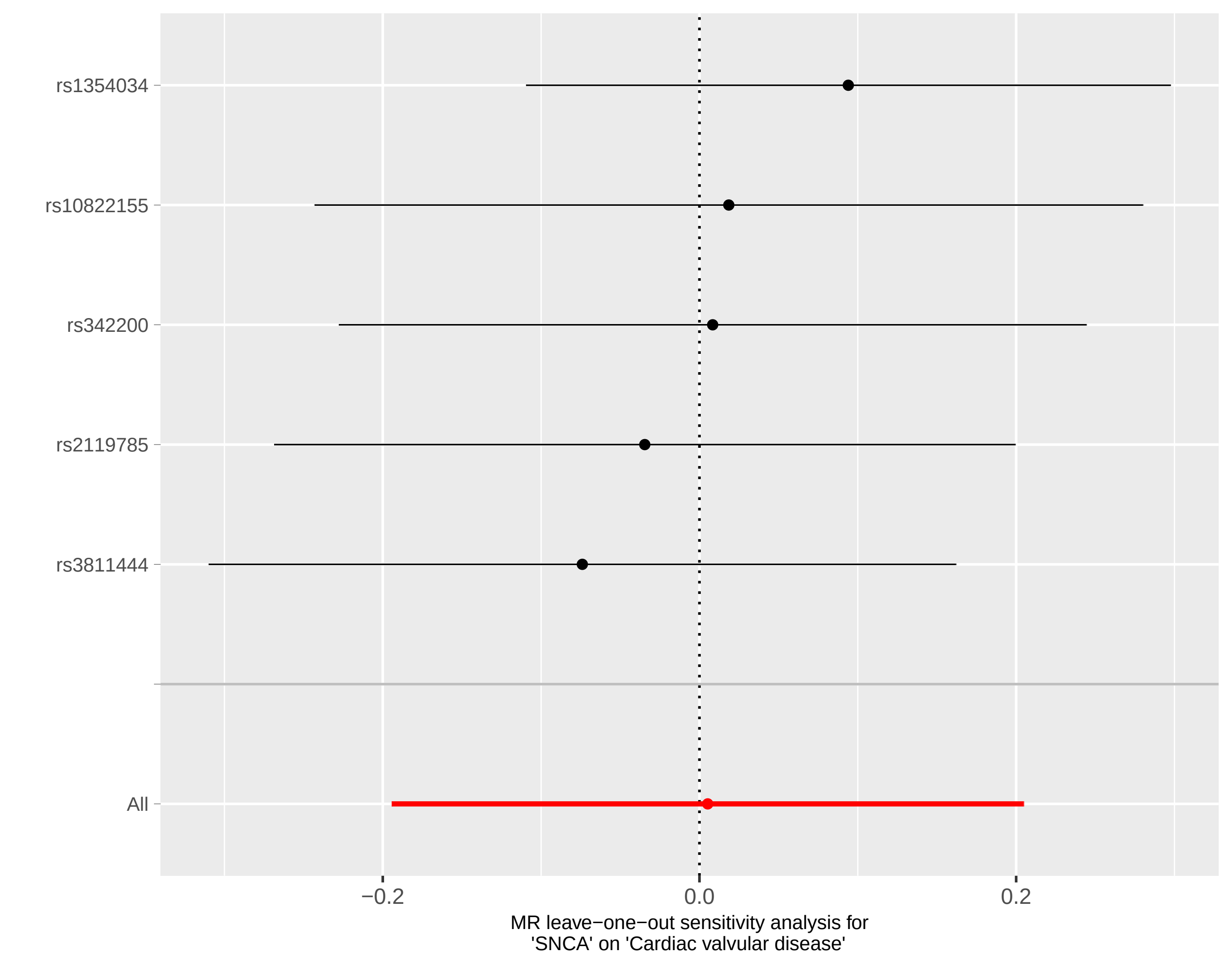
rs1354034
rs10822155
rs342200
rs2119785
rs3811444
All
−0.2
0.0
0.2
MR leave−one−out sensitivity analysis for
'SNCA' on 'Cardiac valvular disease'

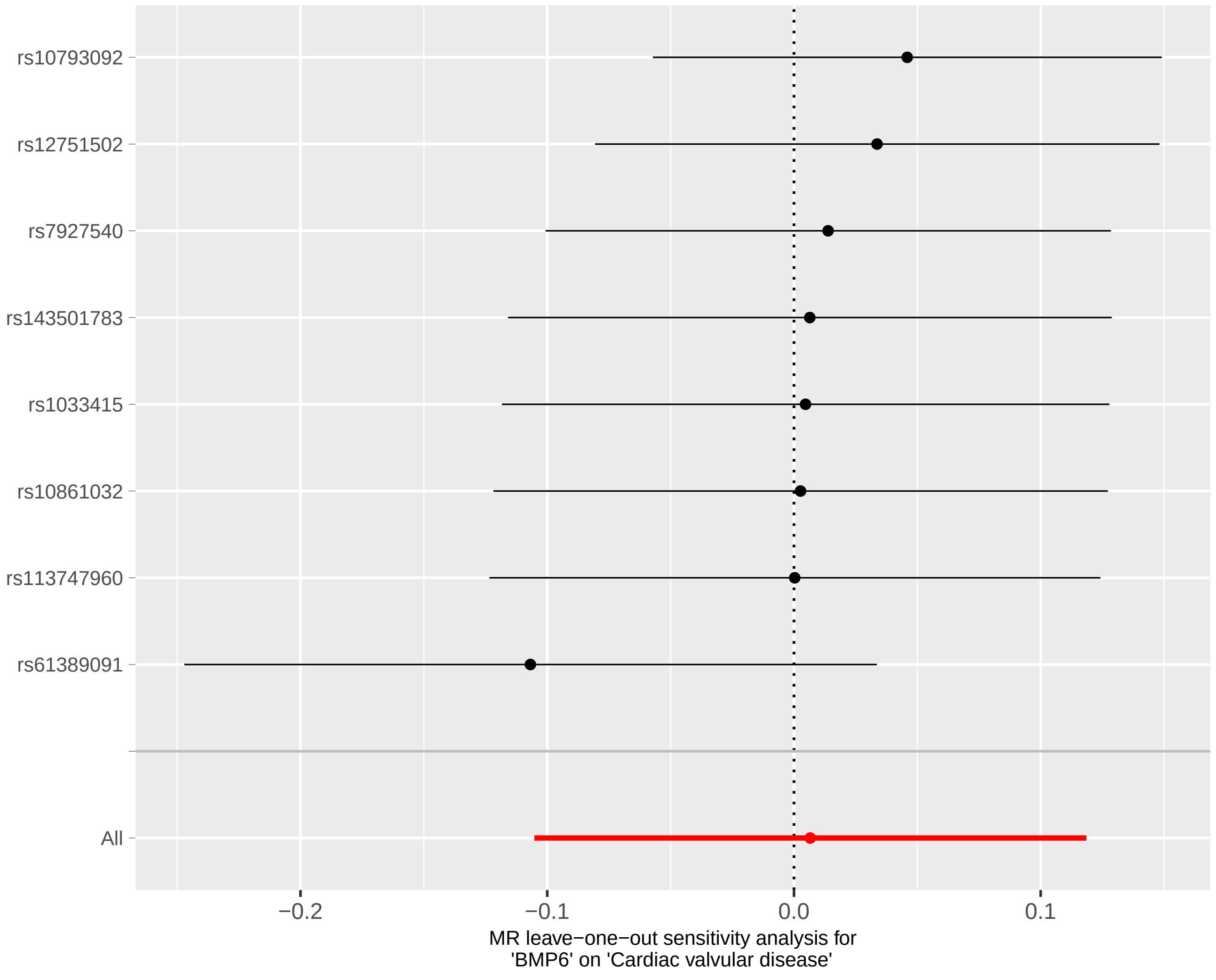
rs10793092
rs12751502
rs7927540
rs143501783
rs1033415
rs10861032
rs113747960
rs61389091
All
−0.2
−0.1
0.0
0.1
MR leave−one−out sensitivity analysis for
'BMP6' on 'Cardiac valvular disease'

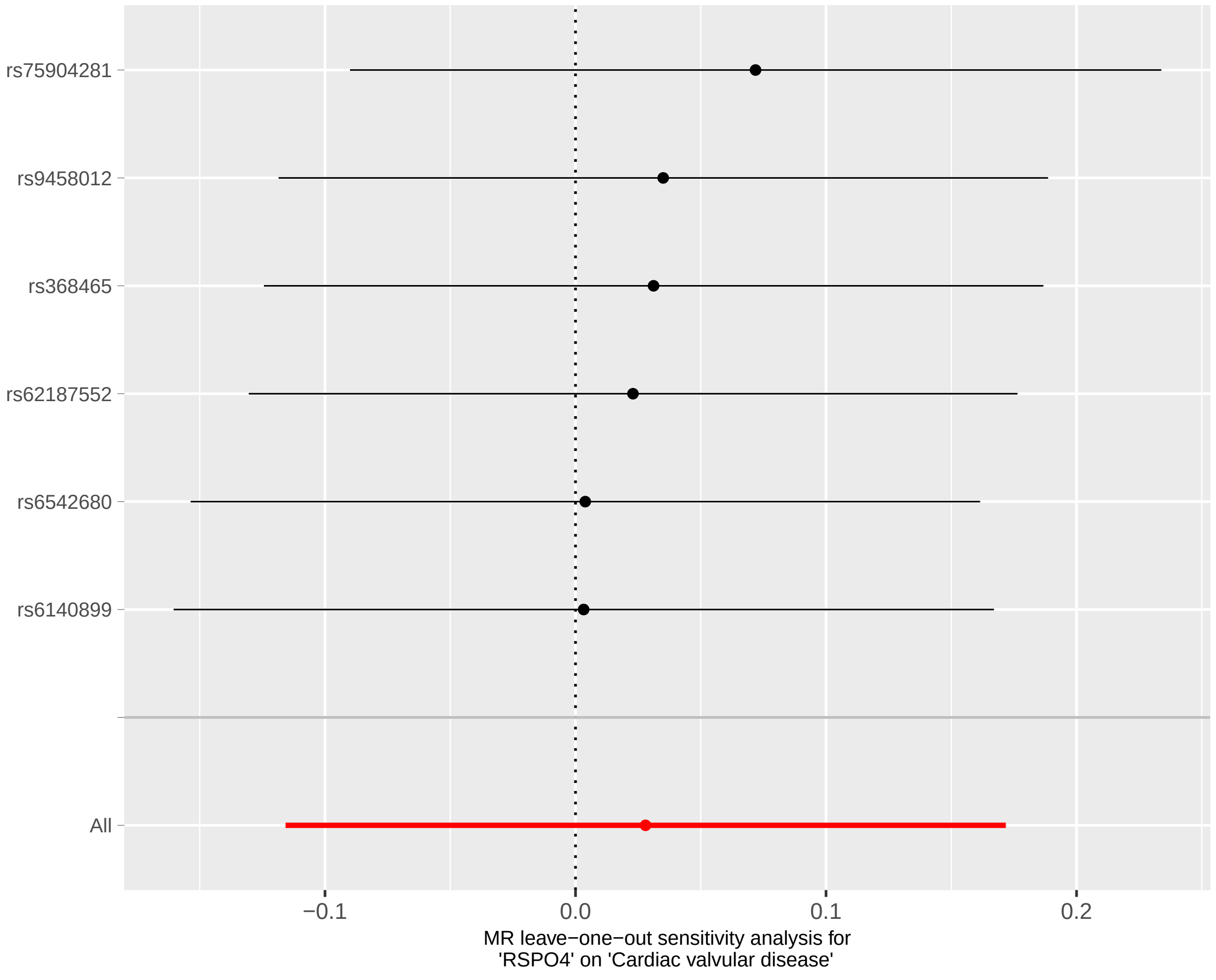
rs75904281
rs9458012
rs368465
rs62187552
rs6542680
rs6140899
All
−0.1
0.0
0.1
0.2
MR leave−one−out sensitivity analysis for
'RSPO4' on 'Cardiac valvular disease'

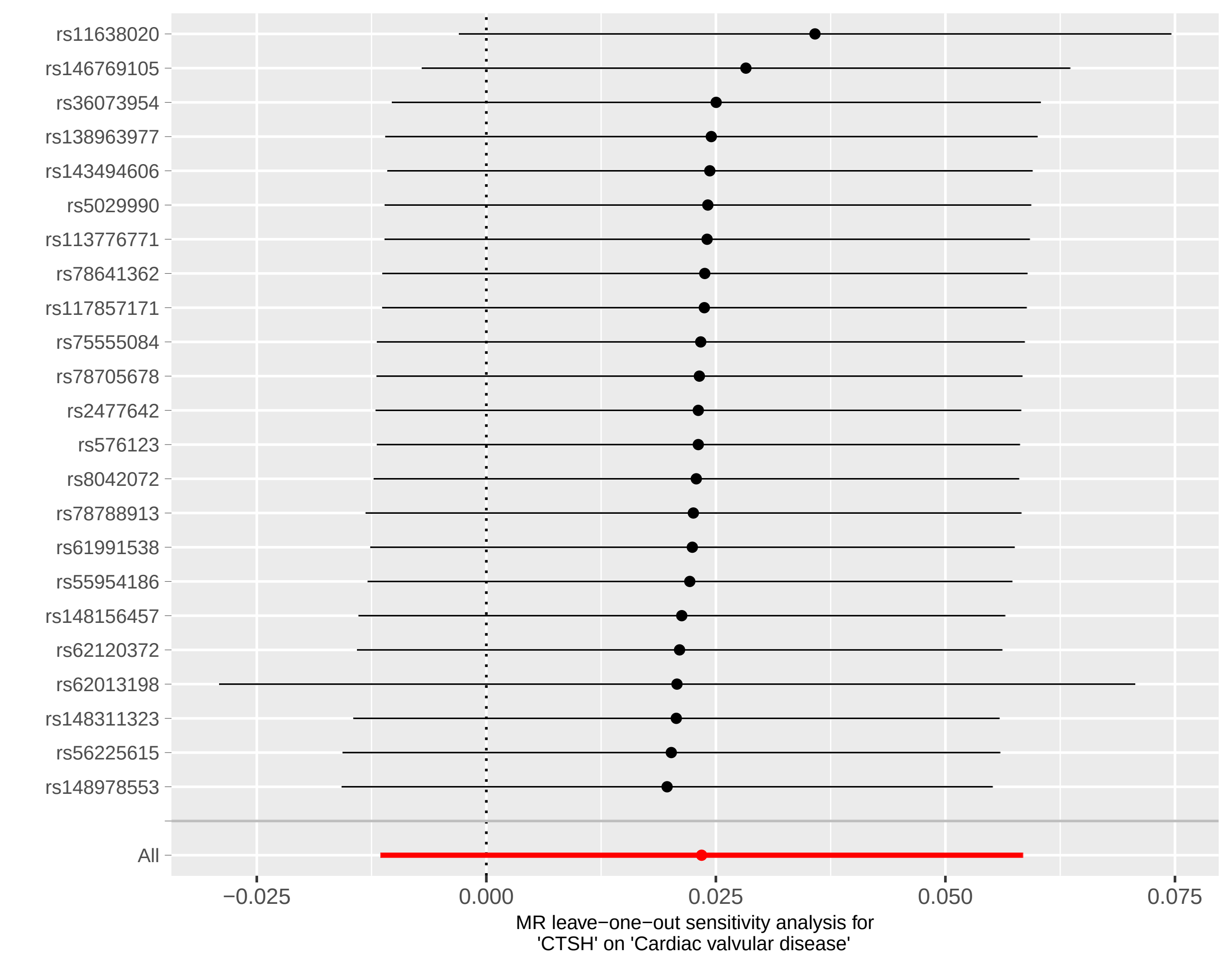
rs11638020
rs146769105
rs36073954
rs138963977
rs143494606
rs5029990
rs113776771
rs78641362
rs117857171
rs75555084
rs78705678
rs2477642
rs576123
rs8042072
rs78788913
rs61991538
rs55954186
rs148156457
rs62120372
rs62013198
rs148311323
rs56225615
rs148978553
All
−0.025
0.000
0.025
0.050
0.075
MR leave−one−out sensitivity analysis for
'CTSH' on 'Cardiac valvular disease'

Insufficient number of SNPs

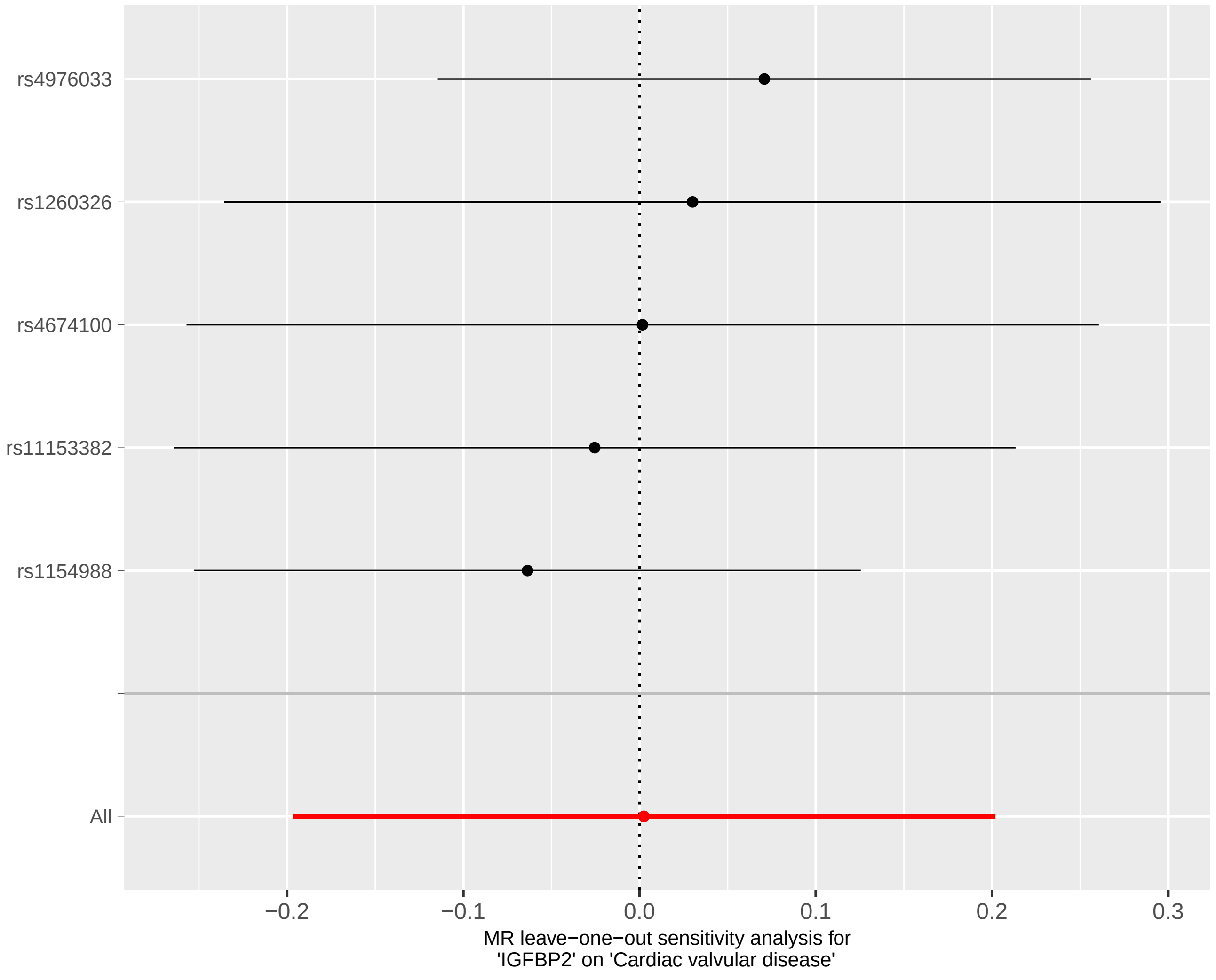
rs4976033
rs1260326
rs4674100
rs11153382
rs1154988
All
−0.2
−0.1
0.0
0.1
0.2
0.3
MR leave−one−out sensitivity analysis for
'IGFBP2' on 'Cardiac valvular disease'

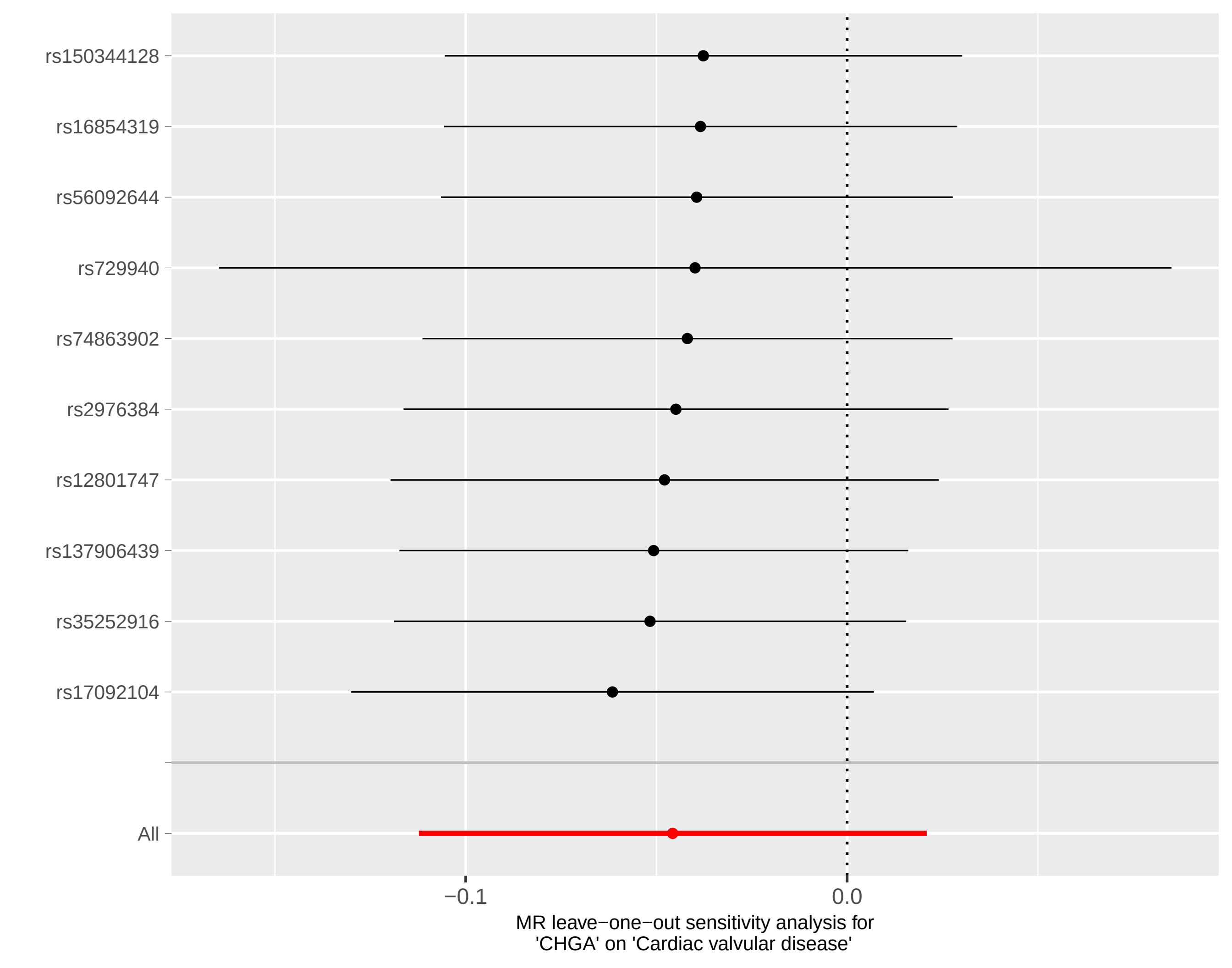
rs150344128
rs16854319
rs56092644
rs729940
rs74863902
rs2976384
rs12801747
rs137906439
rs35252916
rs17092104
All
−0.1
0.0
MR leave−one−out sensitivity analysis for
'CHGA' on 'Cardiac valvular disease'

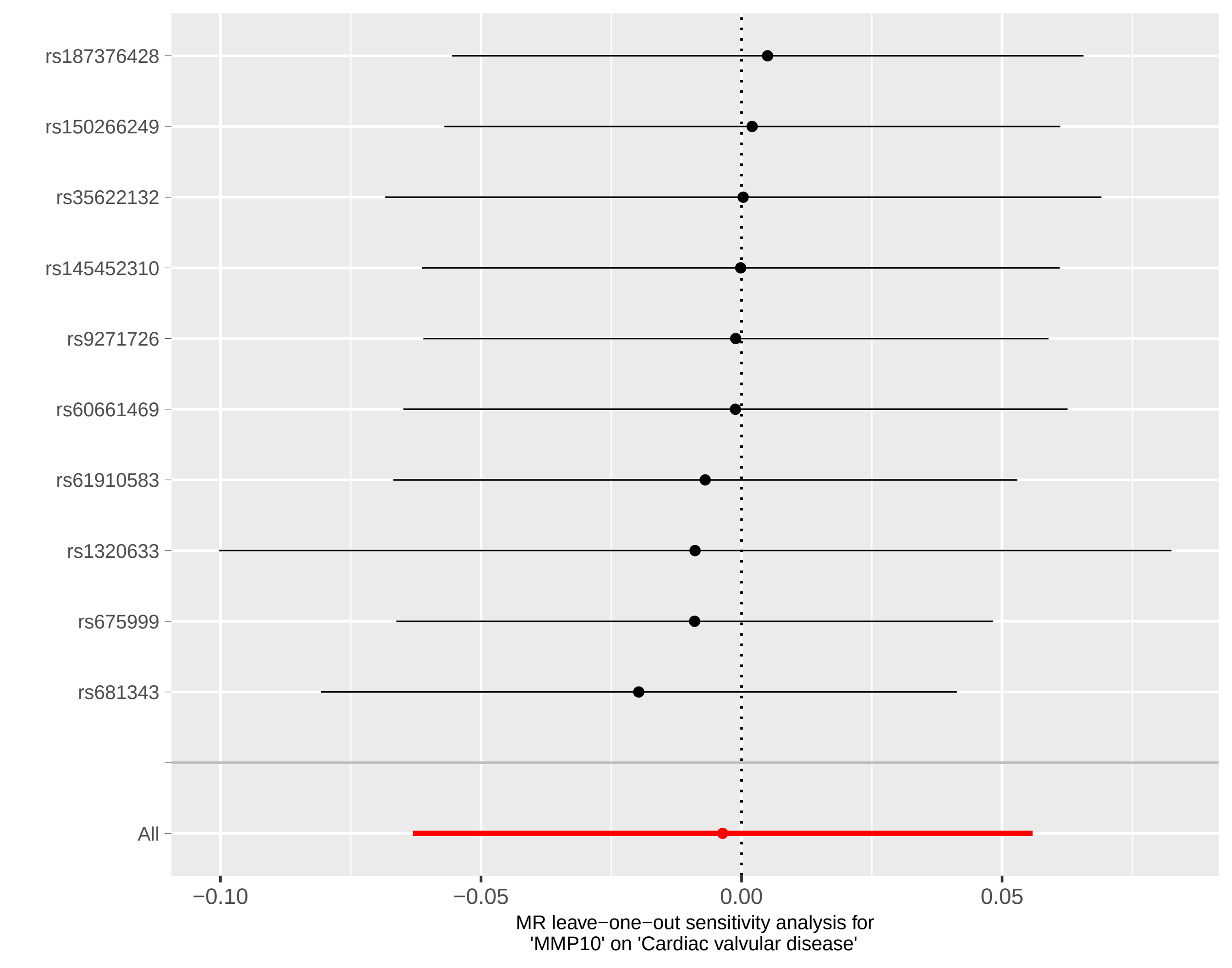

rs187376428
rs150266249
rs35622132
rs145452310
rs9271726
rs60661469
rs61910583
rs1320633
rs675999
rs681343
All
−0.10
−0.05
0.00
0.05
MR leave−one−out sensitivity analysis for
'MMP10' on 'Cardiac valvular disease'

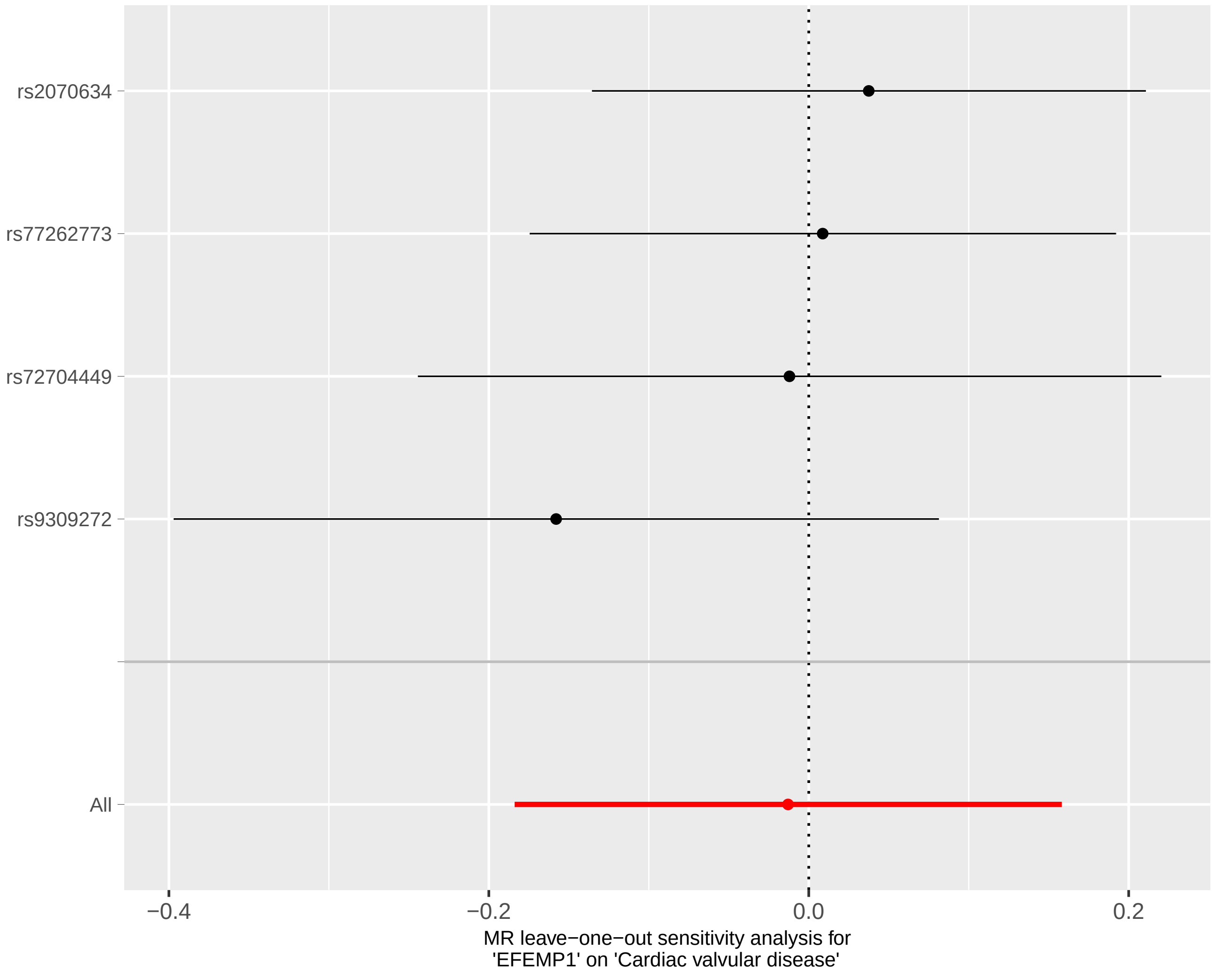
rs2070634
rs77262773
rs72704449
rs9309272
All
−0.4
−0.2
0.0
0.2
MR leave−one−out sensitivity analysis for
'EFEMP1' on 'Cardiac valvular disease'

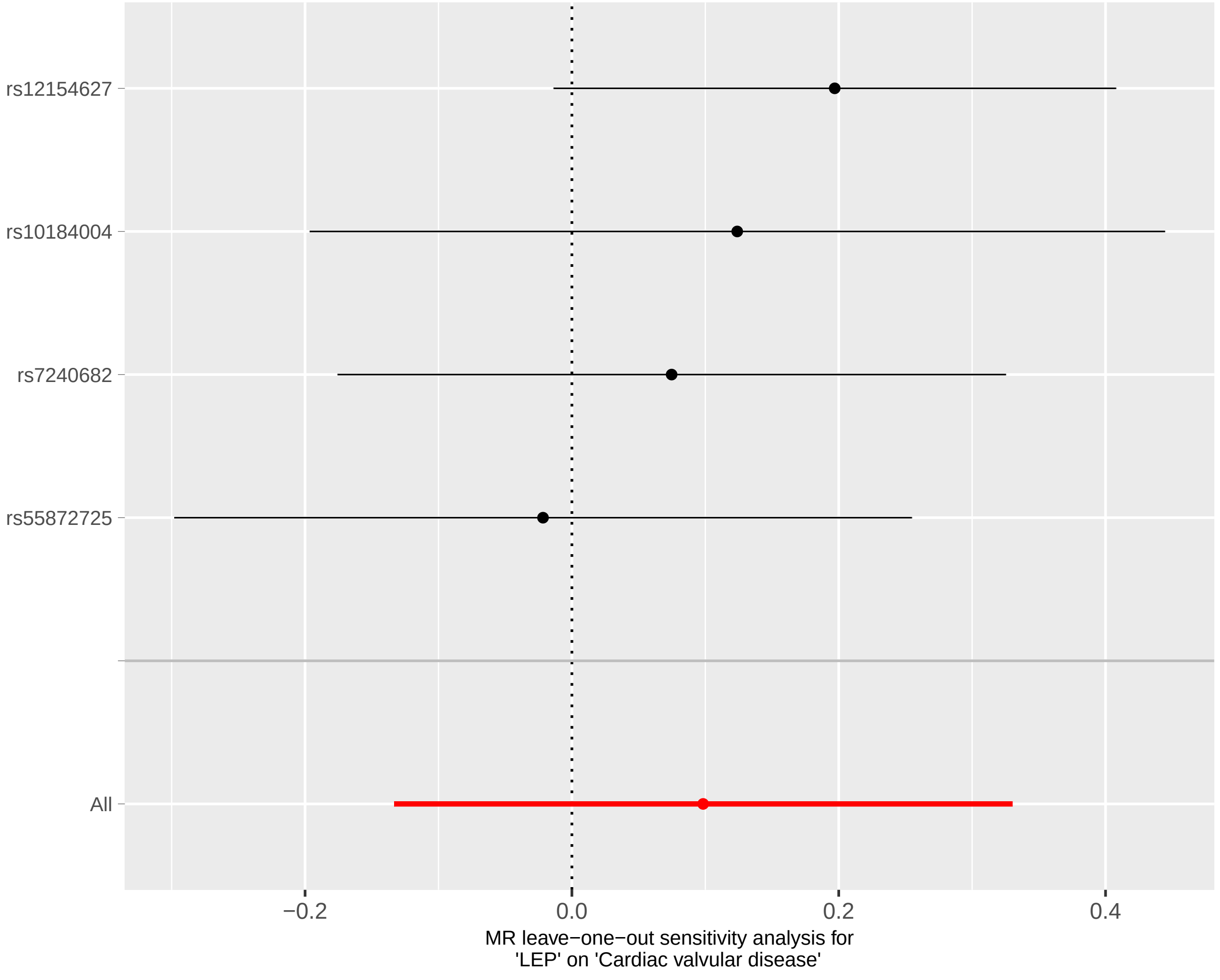

rs12154627
rs10184004
rs7240682
rs55872725
All
−0.2
0.0
0.2
0.4
MR leave−one−out sensitivity analysis for
'LEP' on 'Cardiac valvular disease'

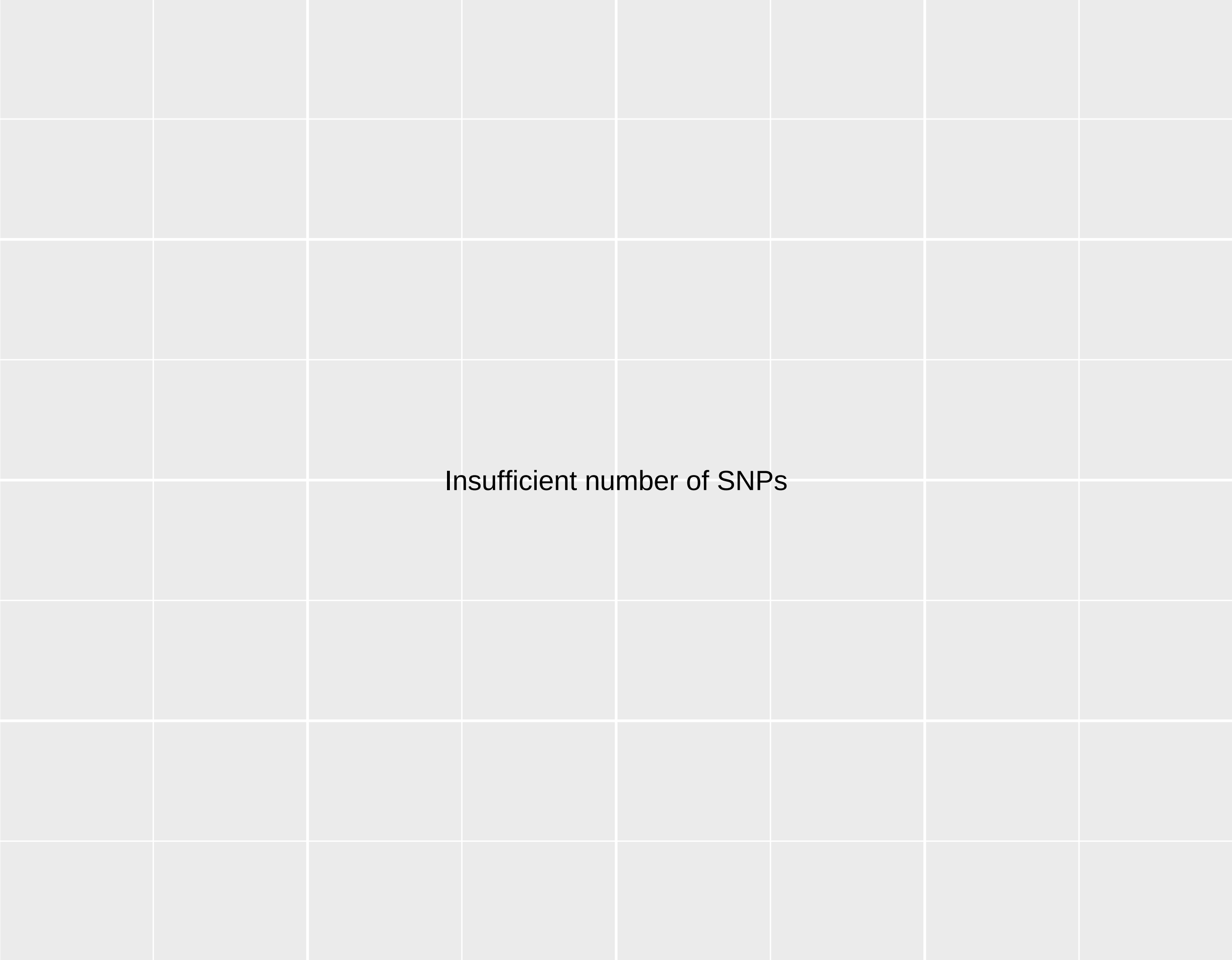
Insufficient number of SNPs

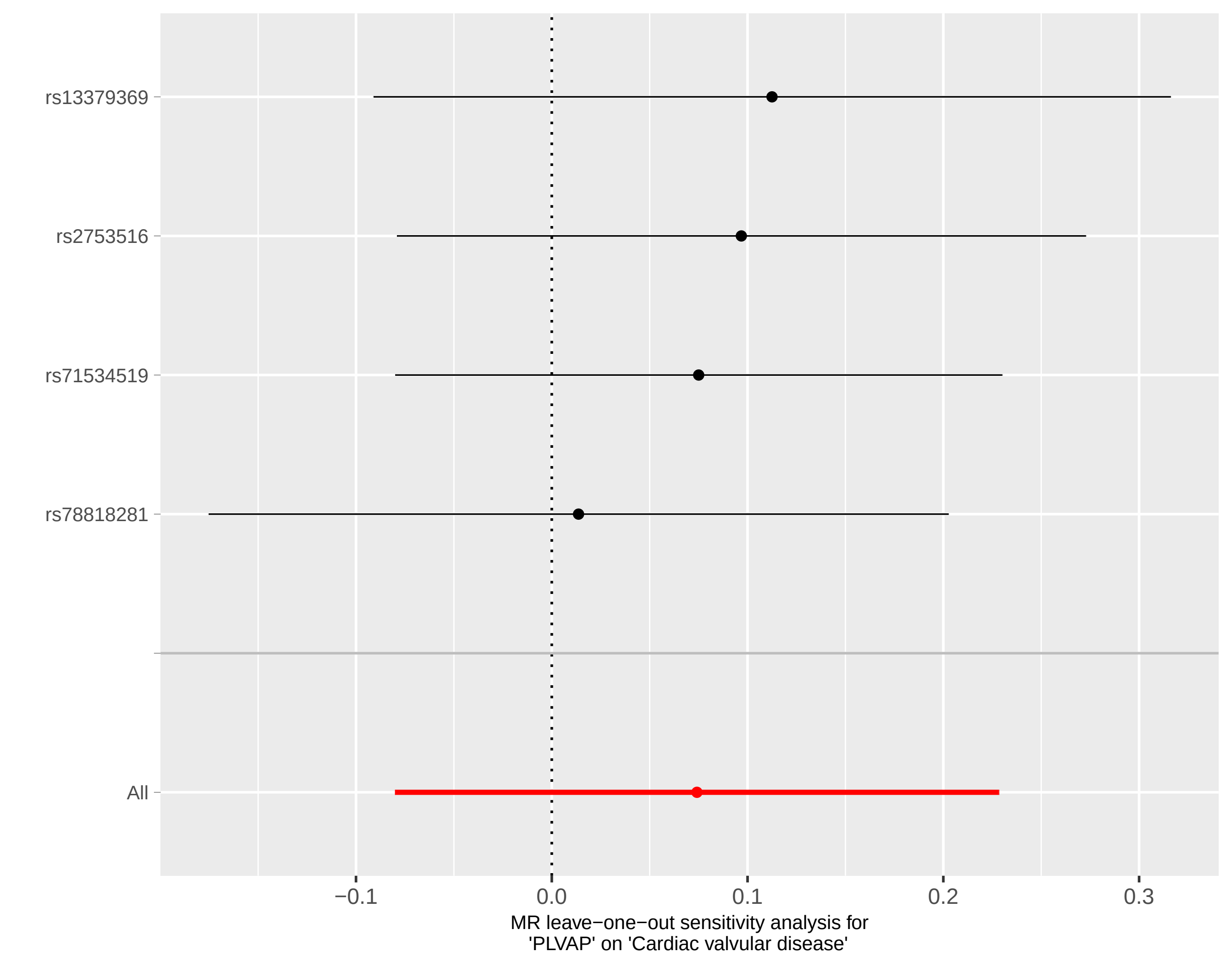
rs13379369
rs2753516
rs71534519
rs78818281
All
−0.1
0.0
0.1
0.2
0.3
MR leave−one−out sensitivity analysis for
'PLVAP' on 'Cardiac valvular disease'

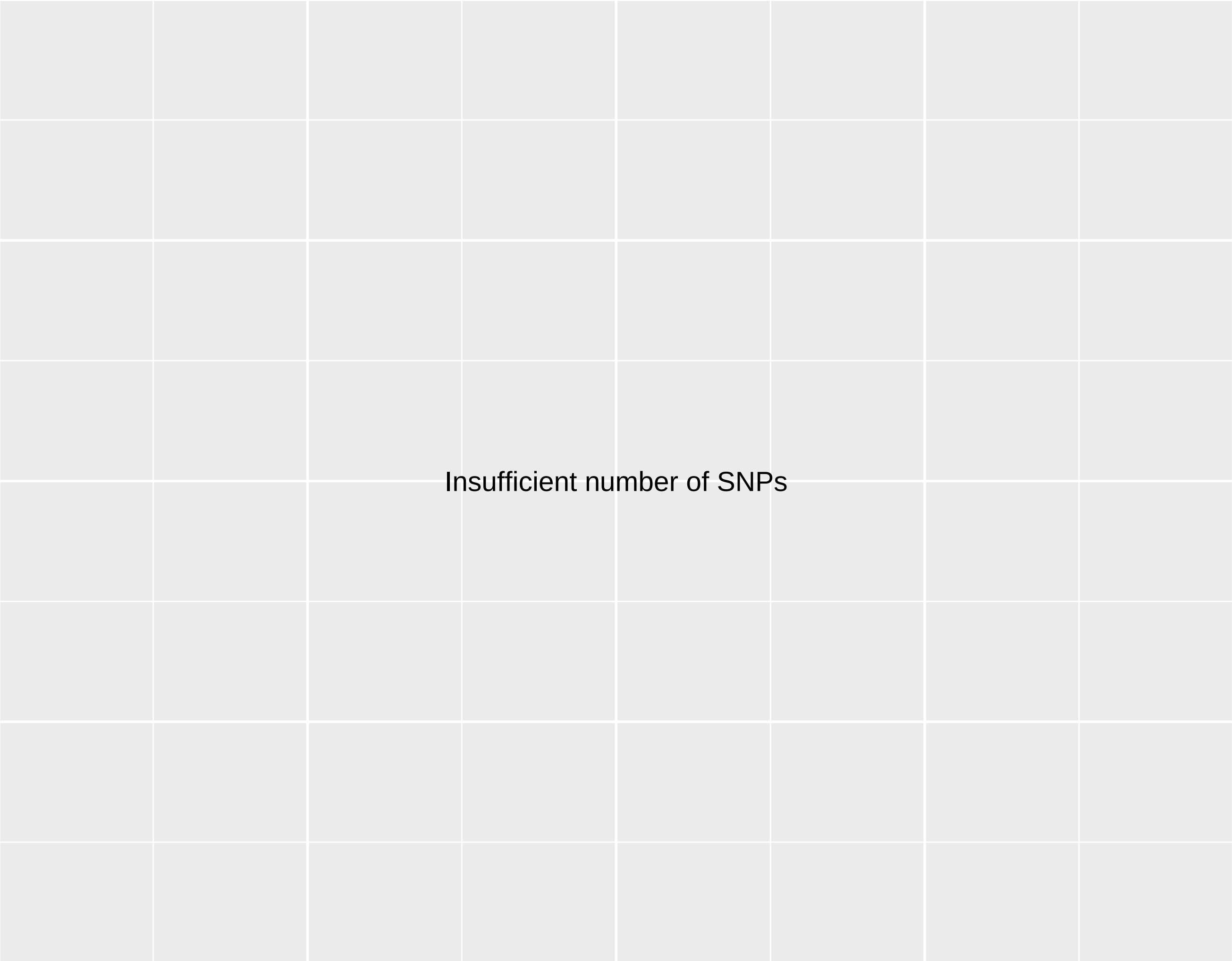
Insufficient number of SNPs

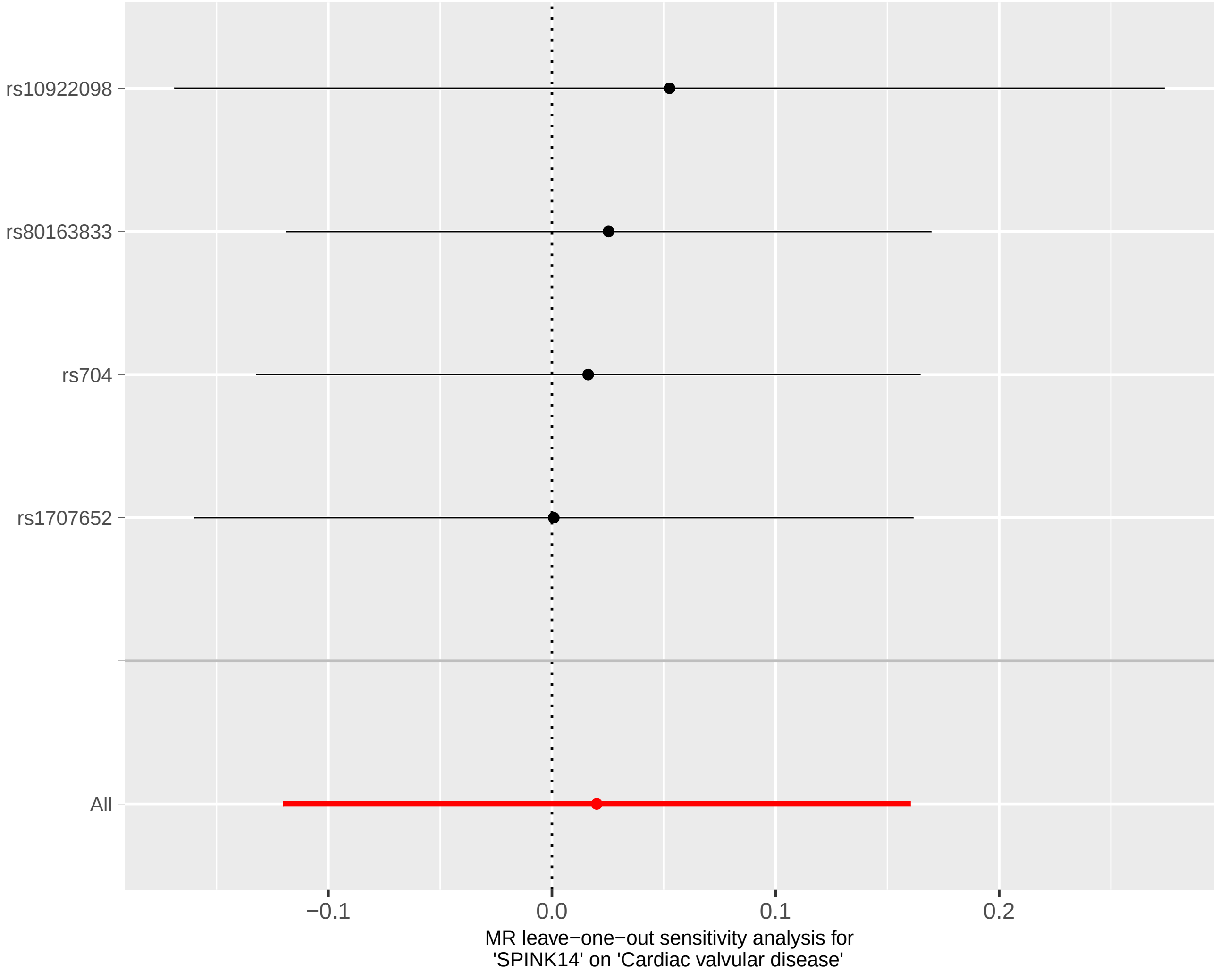
rs10922098
rs80163833
rs704
rs1707652
All
−0.1
0.0
0.1
0.2
MR leave−one−out sensitivity analysis for
'SPINK14' on 'Cardiac valvular disease'

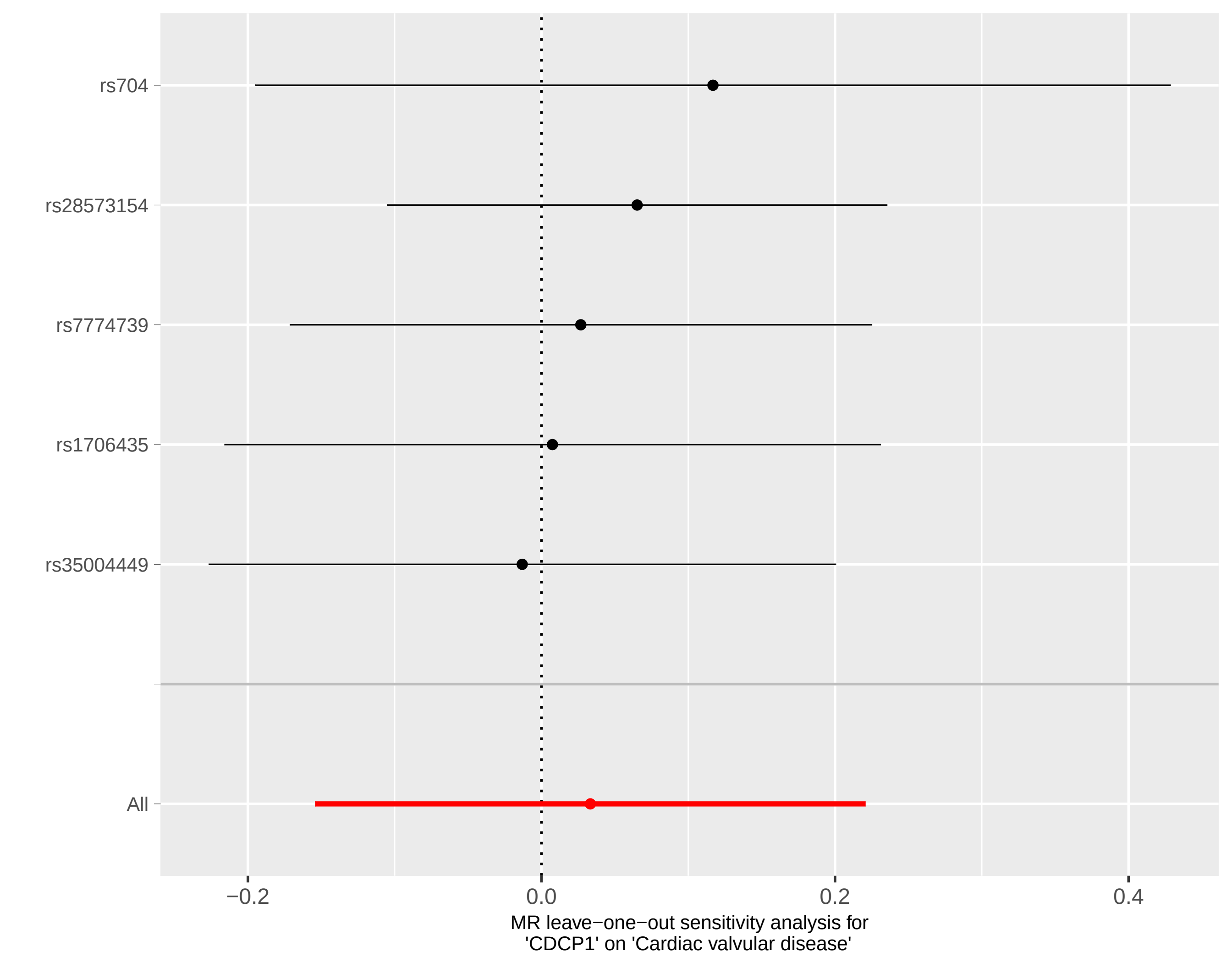

rs704
rs28573154
rs7774739
rs1706435
rs35004449
All
−0.2
0.0
0.2
0.4
MR leave−one−out sensitivity analysis for
'CDCP1' on 'Cardiac valvular disease'

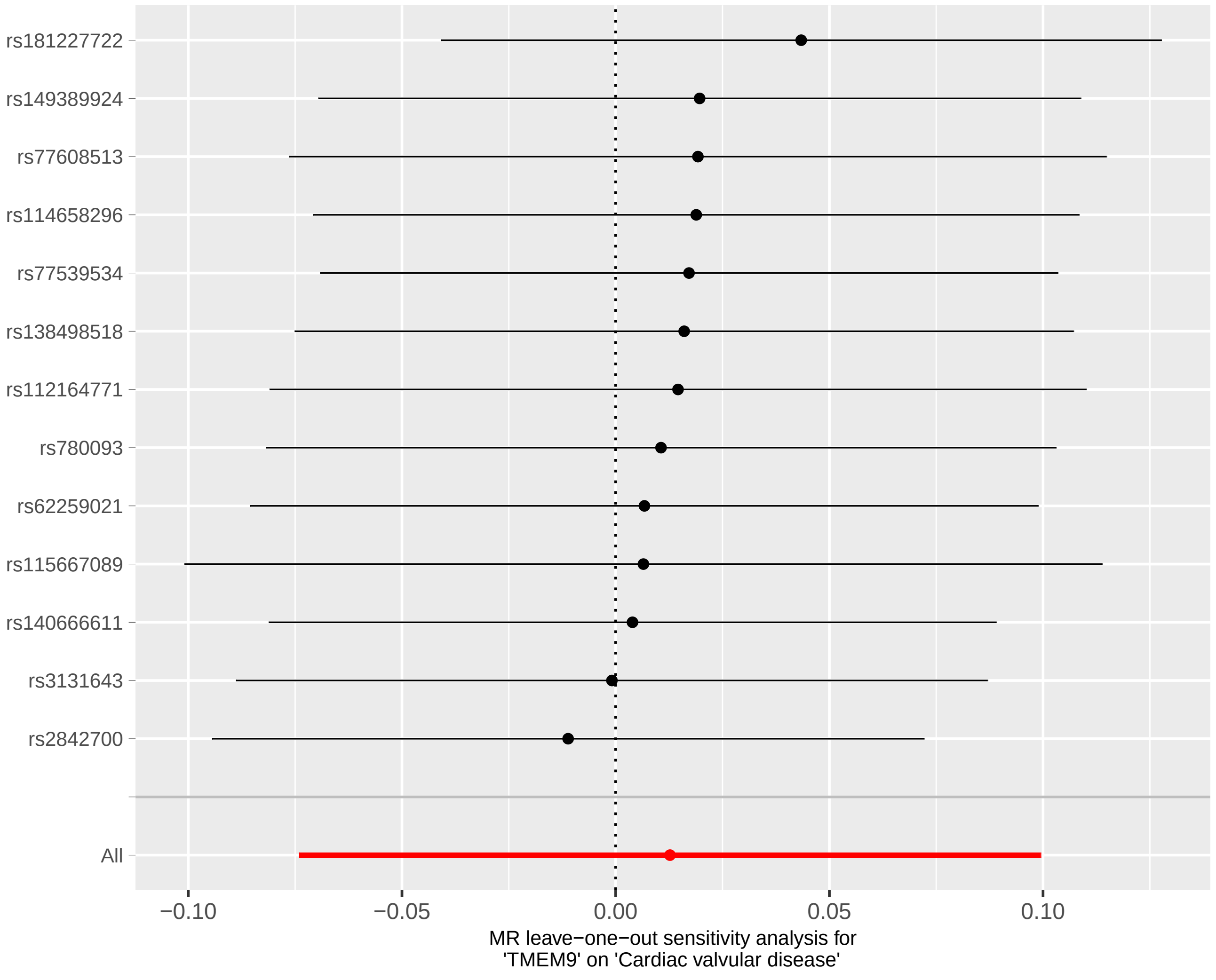
rs181227722
rs149389924
rs77608513
rs114658296
rs77539534
rs138498518
rs112164771
rs780093
rs62259021
rs115667089
rs140666611
rs3131643
rs2842700
All
−0.10
−0.05
0.00
0.05
0.10
MR leave−one−out sensitivity analysis for
'TMEM9' on 'Cardiac valvular disease'

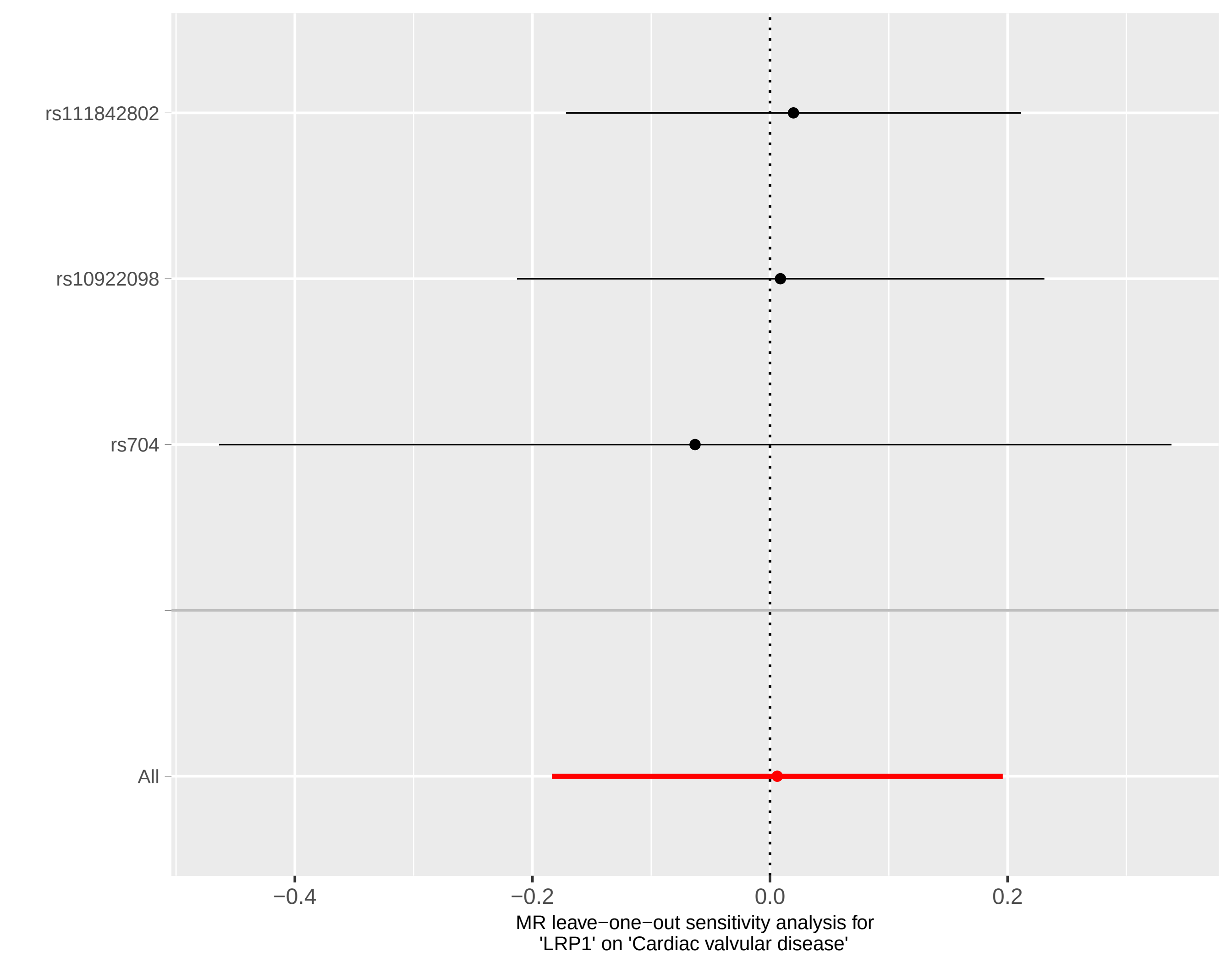
rs111842802
rs10922098
rs704
All
−0.4
−0.2
0.0
0.2
MR leave−one−out sensitivity analysis for
'LRP1' on 'Cardiac valvular disease'

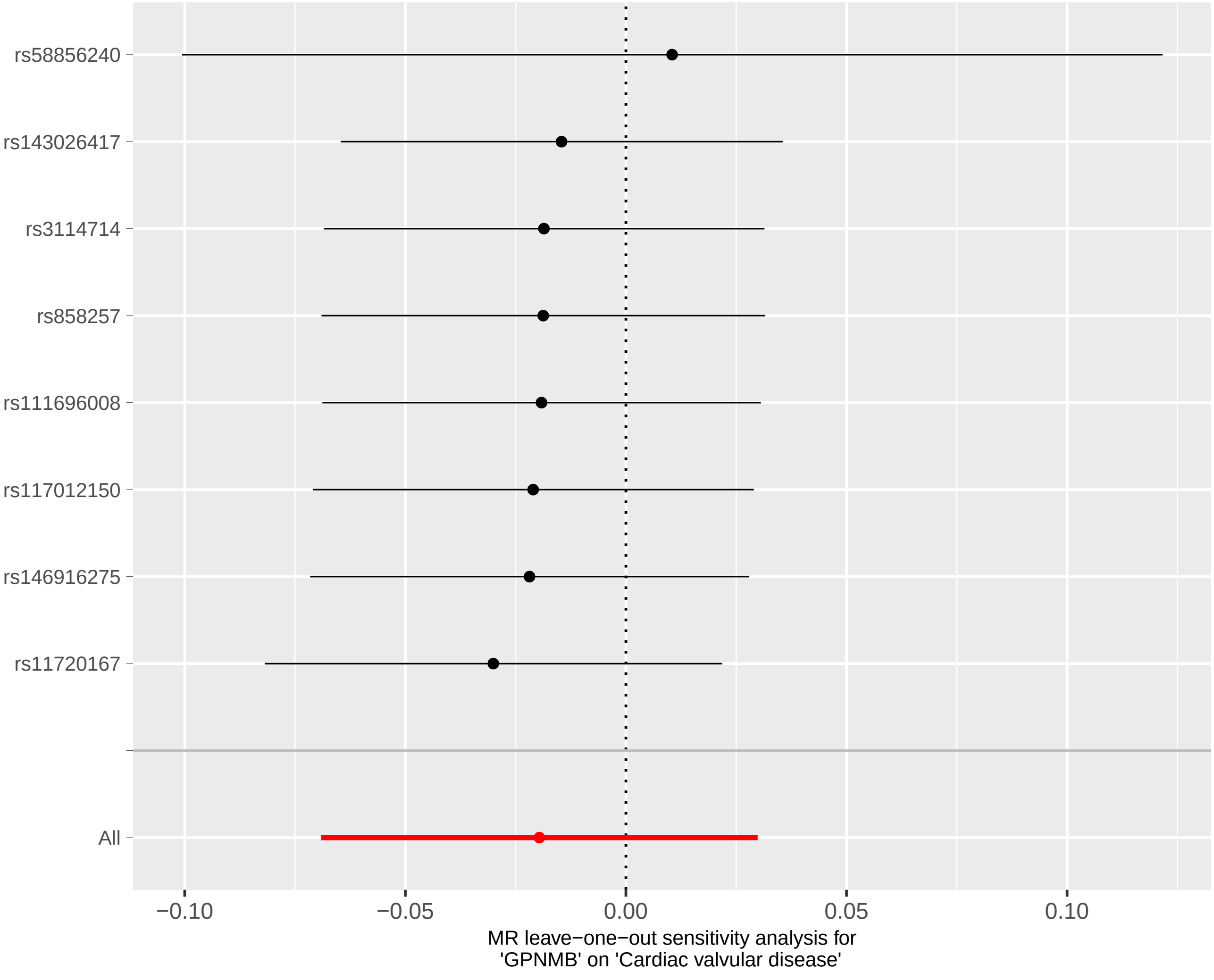
rs58856240
rs143026417
rs3114714
rs858257
rs111696008
rs117012150
rs146916275
rs11720167
All
−0.10
−0.05
0.00
0.05
0.10
MR leave−one−out sensitivity analysis for
'GPNMB' on 'Cardiac valvular disease'

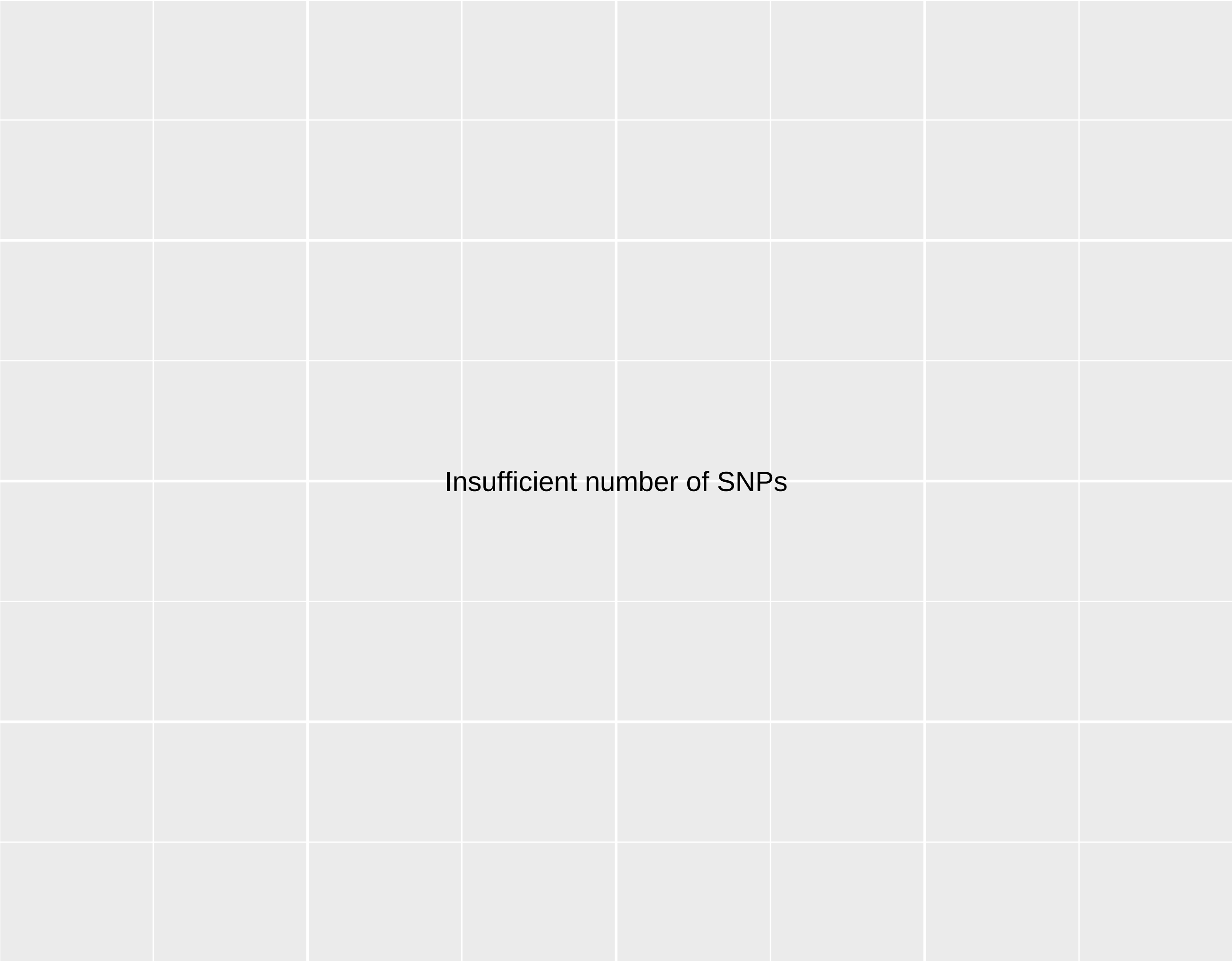
Insufficient number of SNPs

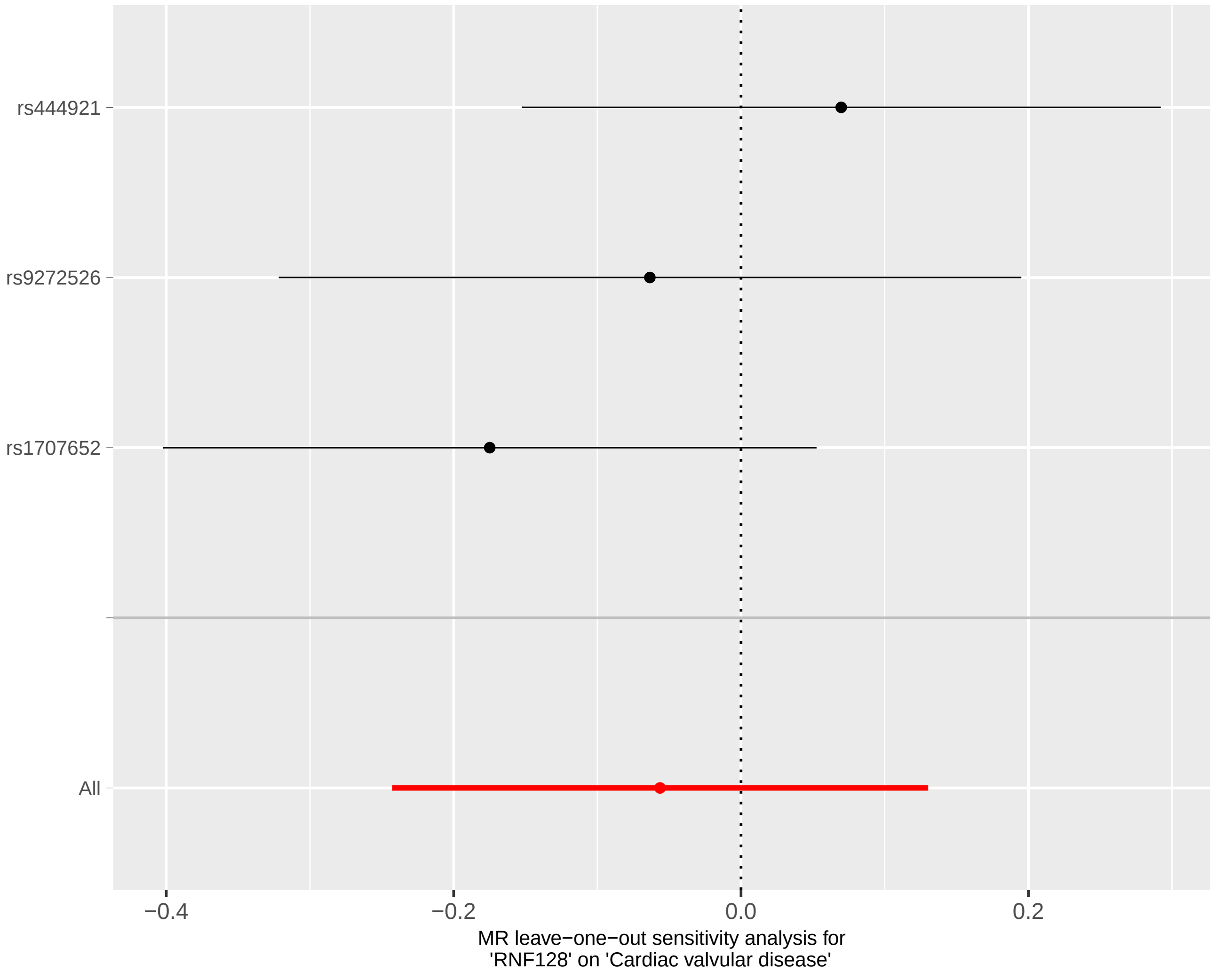
rs444921
rs9272526
rs1707652
All
−0.4
−0.2
0.0
0.2
MR leave−one−out sensitivity analysis for
'RNF128' on 'Cardiac valvular disease'

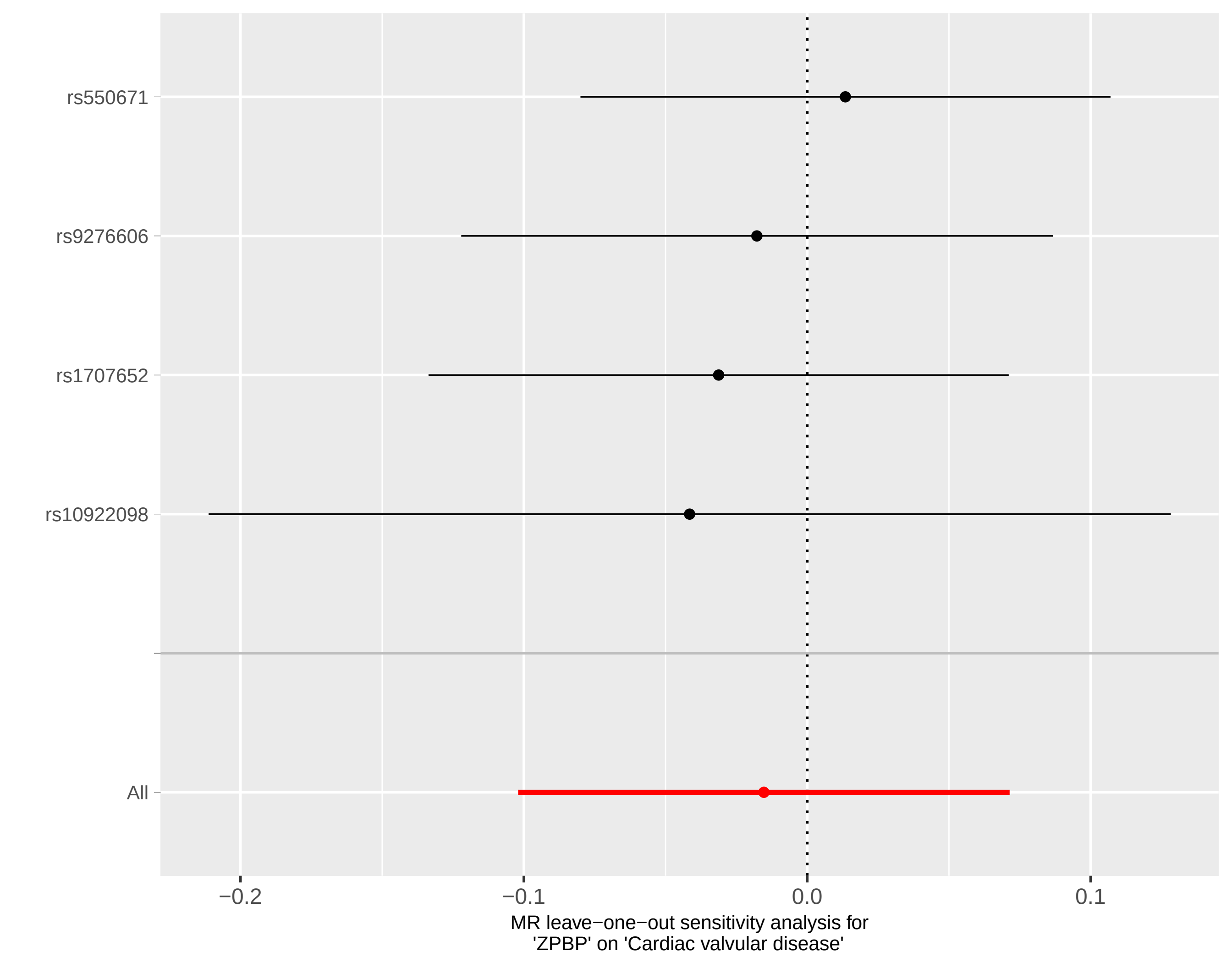
rs550671
rs9276606
rs1707652
rs10922098
All
−0.2
−0.1
0.0
0.1
MR leave−one−out sensitivity analysis for
'ZPBP' on 'Cardiac valvular disease'

Insufficient number of SNPs

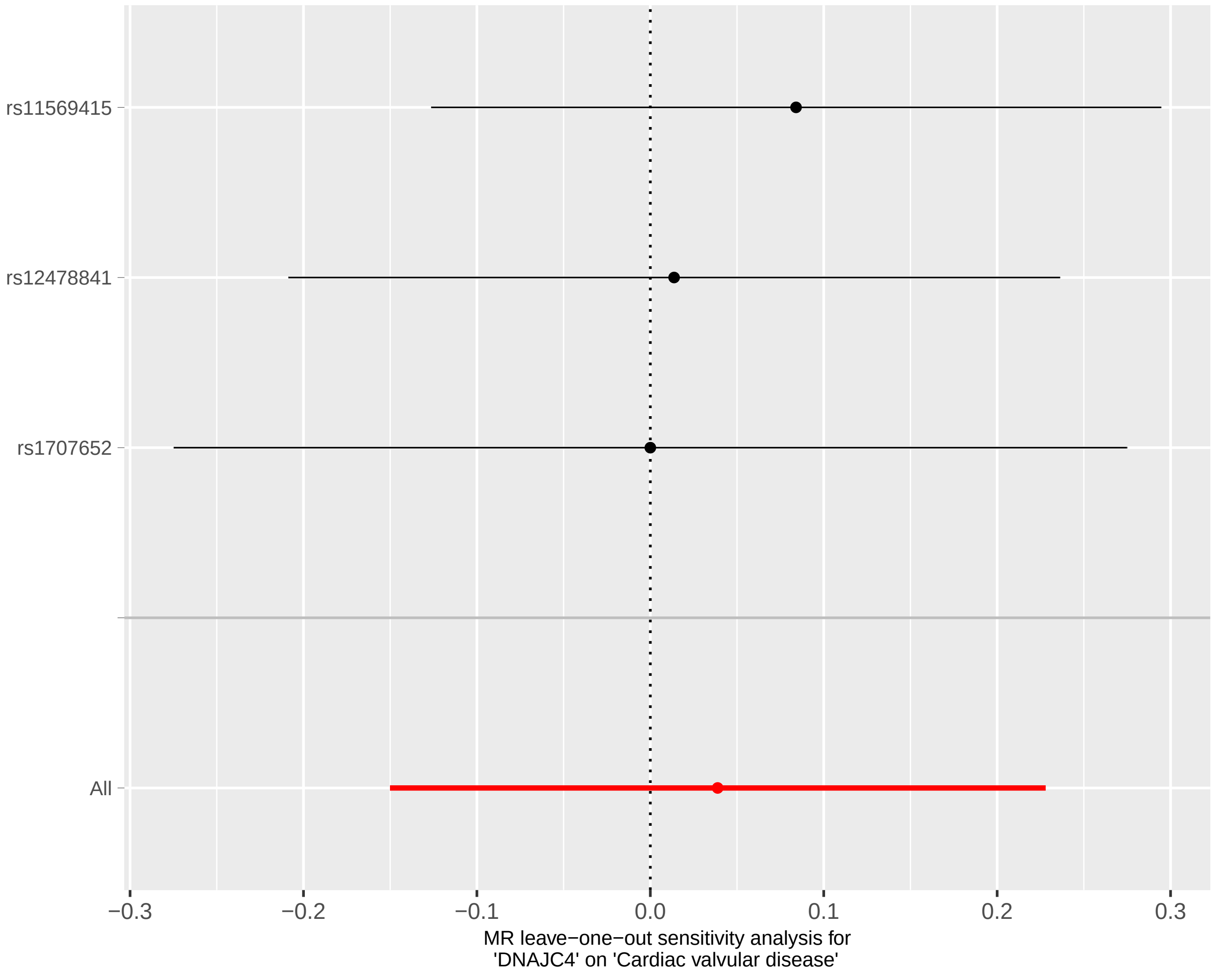

rs11569415
rs12478841
rs1707652
All
−0.3
−0.2
−0.1
0.0
0.1
0.2
0.3
MR leave−one−out sensitivity analysis for
'DNAJC4' on 'Cardiac valvular disease'

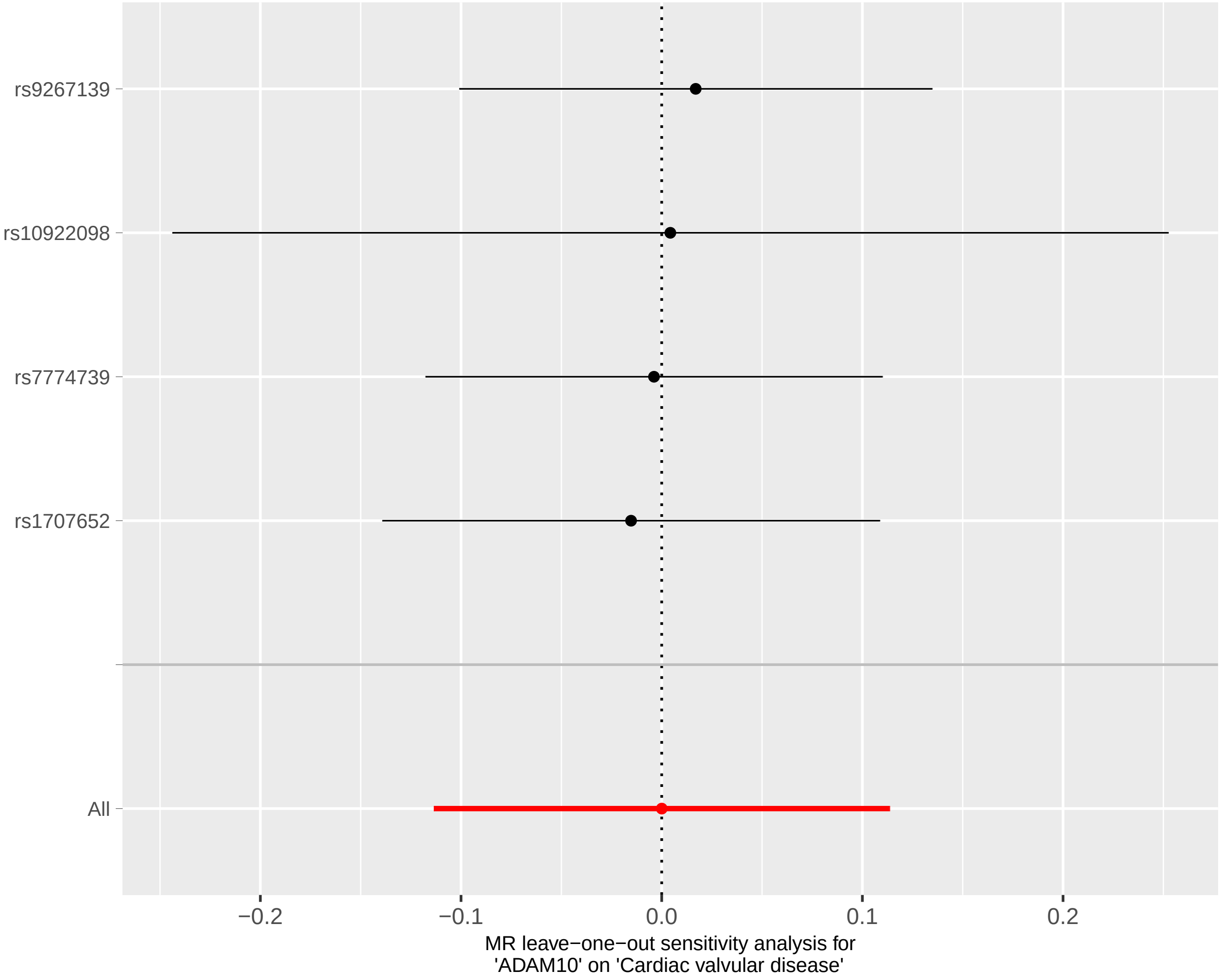

rs9267139
rs10922098
rs7774739
rs1707652
All
−0.2
−0.1
0.0
0.1
0.2
MR leave−one−out sensitivity analysis for
'ADAM10' on 'Cardiac valvular disease'

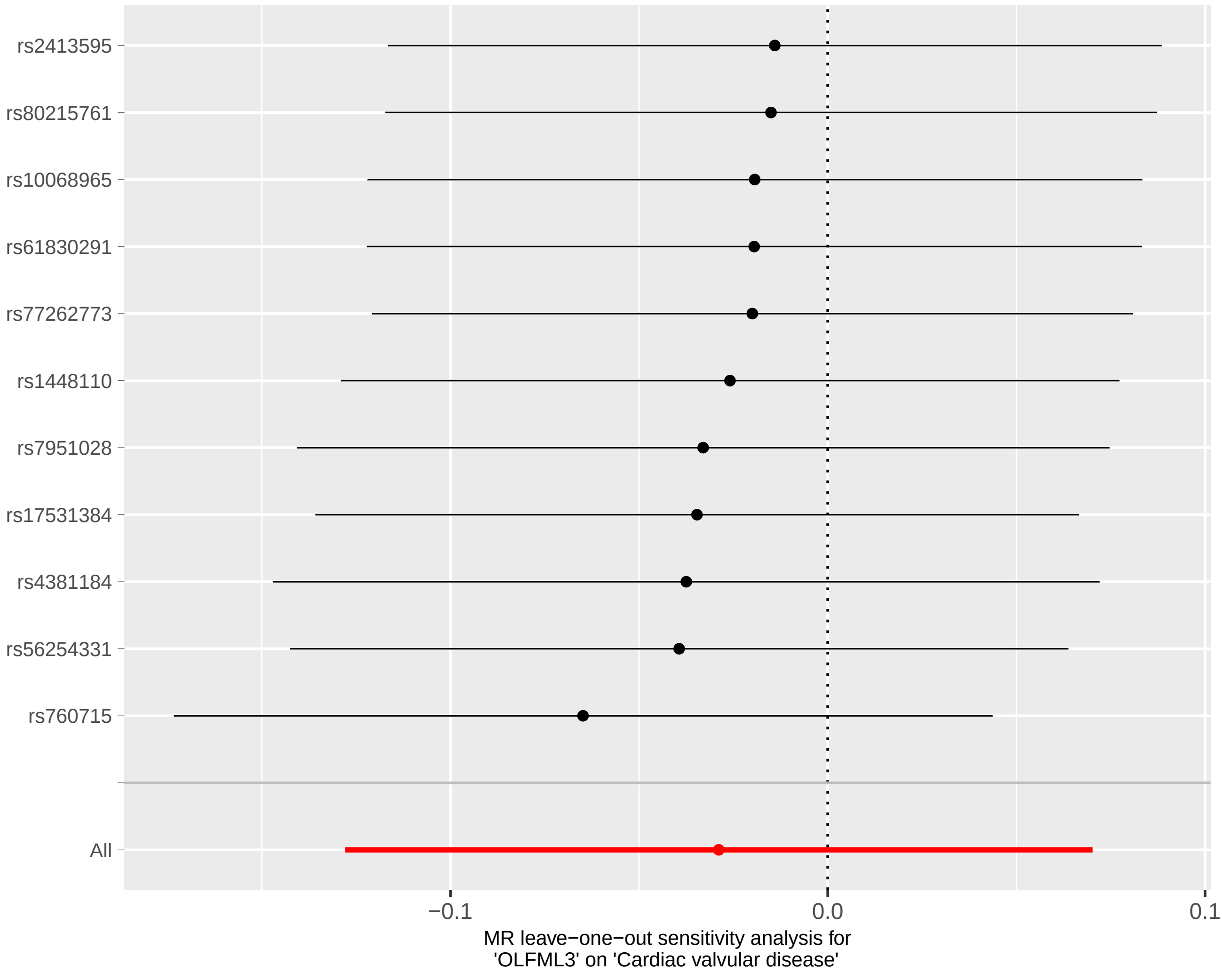
rs2413595
rs80215761
rs10068965
rs61830291
rs77262773
rs1448110
rs7951028
rs17531384
rs4381184
rs56254331
rs760715
All
−0.1
0.0
0.1
MR leave−one−out sensitivity analysis for
'OLFML3' on 'Cardiac valvular disease'

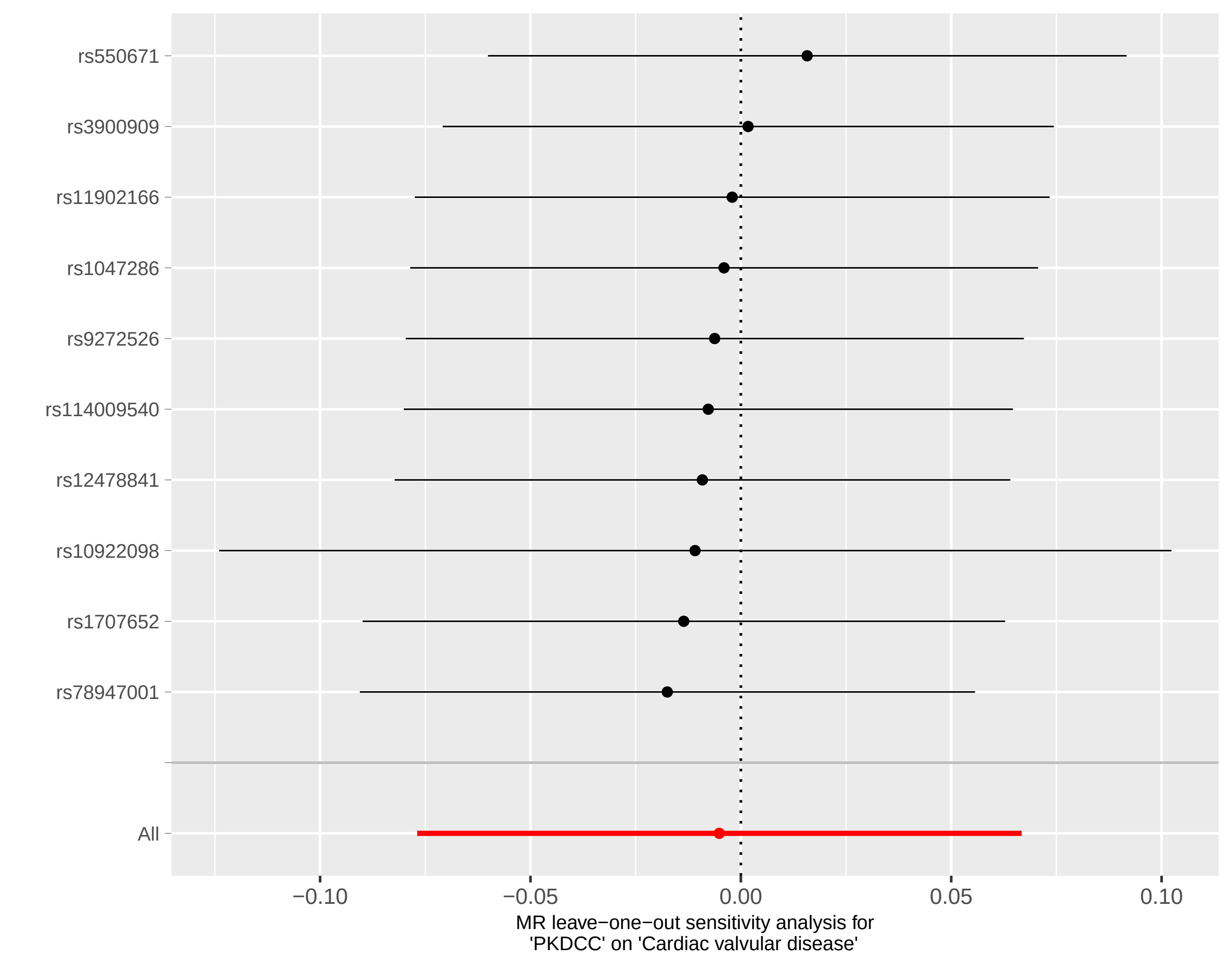
rs550671
rs3900909
rs11902166
rs1047286
rs9272526
rs114009540
rs12478841
rs10922098
rs1707652
rs78947001
All
−0.10
−0.05
0.00
0.05
0.10
MR leave−one−out sensitivity analysis for
'PKDCC' on 'Cardiac valvular disease'

Insufficient number of SNPs

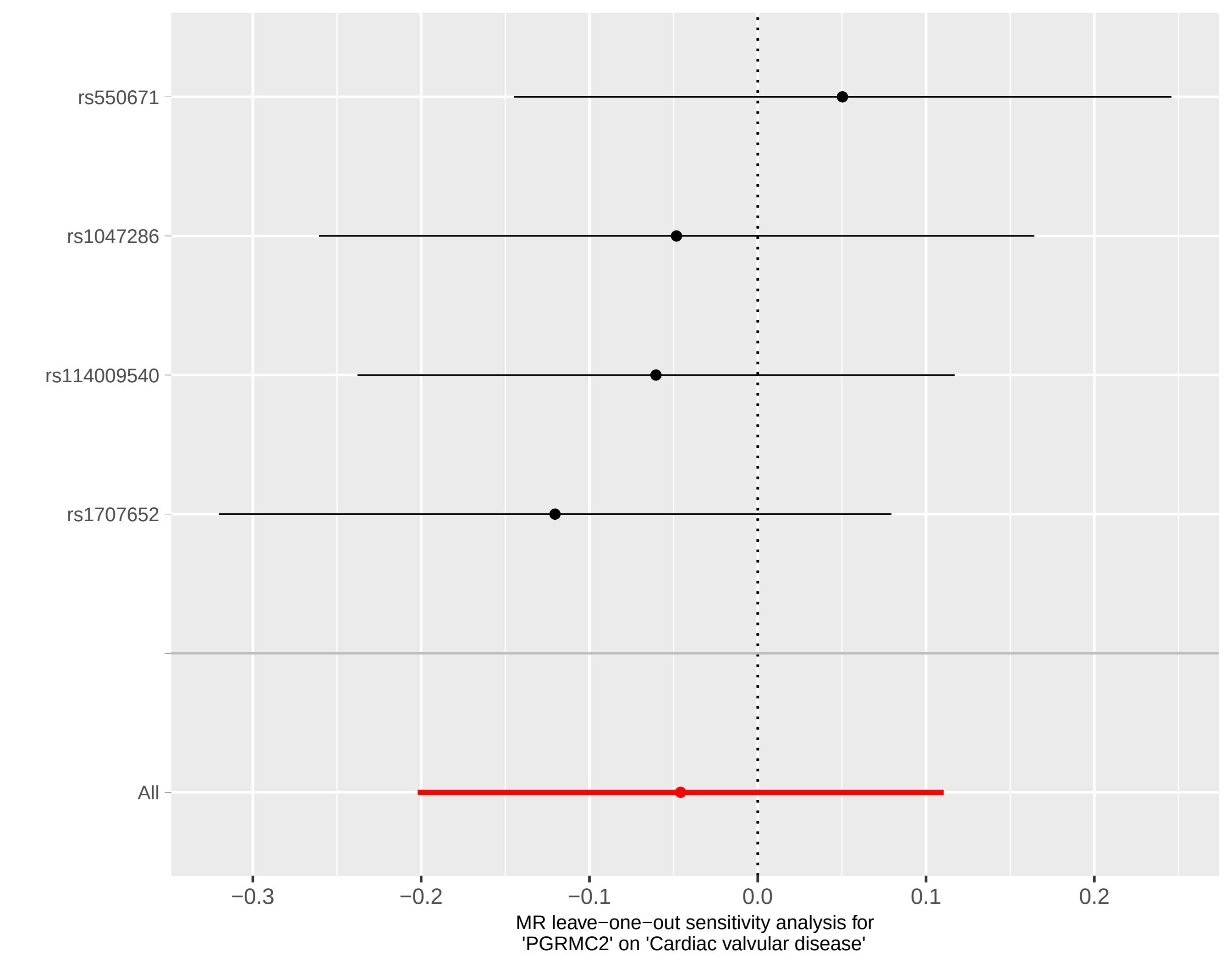

rs550671
rs1047286
rs114009540
rs1707652
All
−0.3
−0.2
−0.1
0.0
0.1
0.2
MR leave−one−out sensitivity analysis for
'PGRMC2' on 'Cardiac valvular disease'

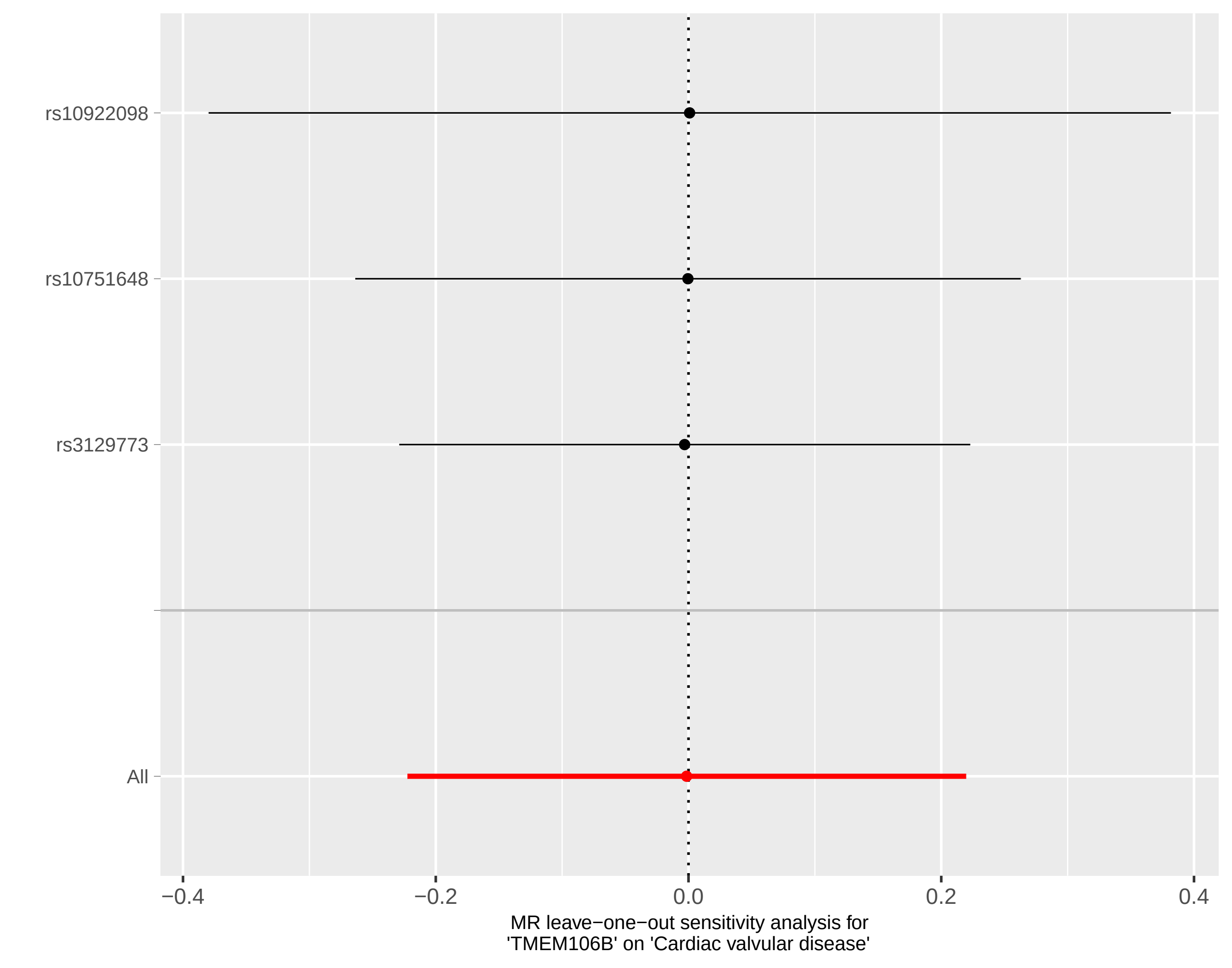
rs10922098
rs10751648
rs3129773
All
−0.4
−0.2
0.0
0.2
0.4
MR leave−one−out sensitivity analysis for
'TMEM106B' on 'Cardiac valvular disease'

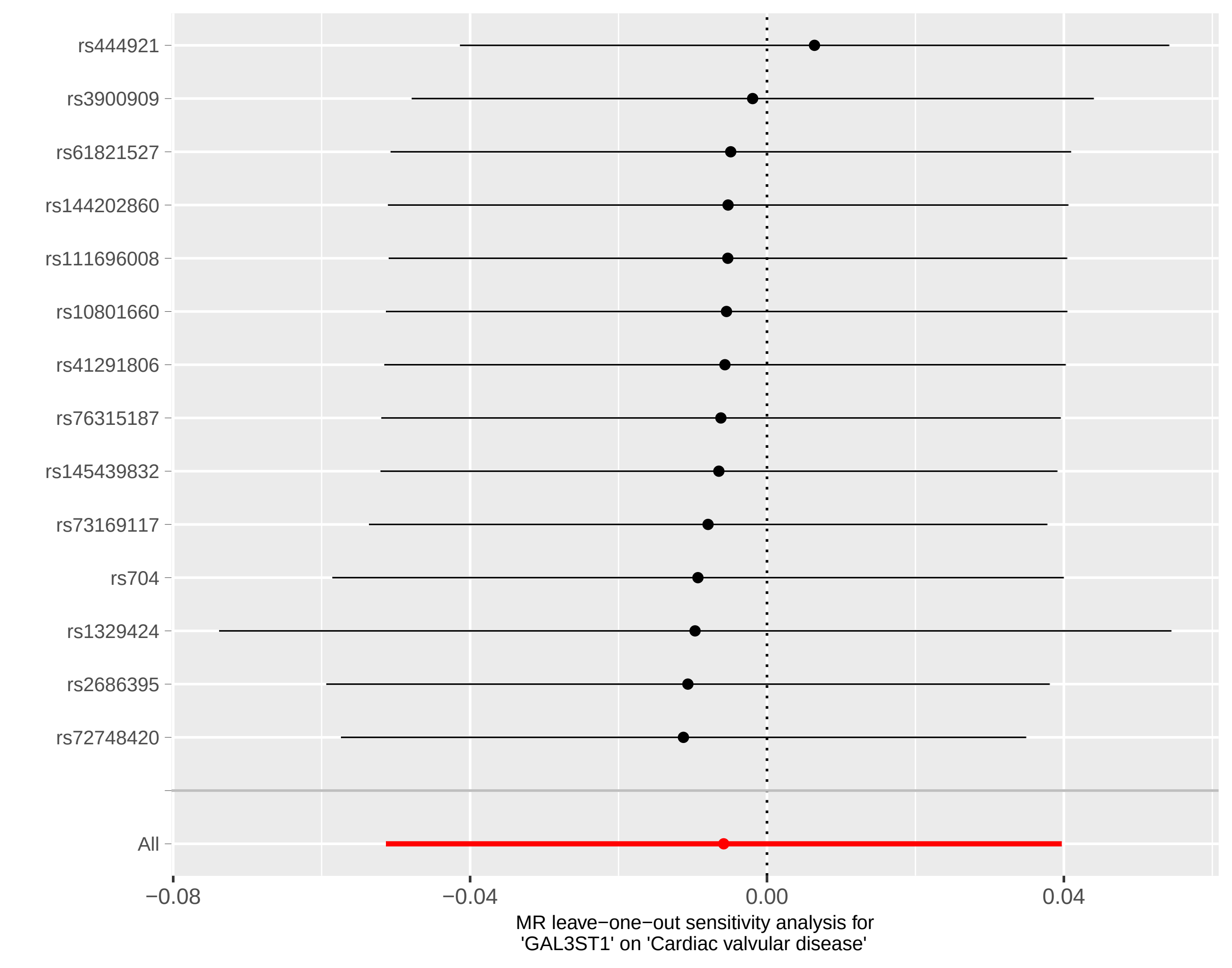
rs444921
rs3900909
rs61821527
rs144202860
rs111696008
rs10801660
rs41291806
rs76315187
rs145439832
rs73169117
rs704
rs1329424
rs2686395
rs72748420
All
−0.08
−0.04
0.00
0.04
MR leave−one−out sensitivity analysis for
'GAL3ST1' on 'Cardiac valvular disease'

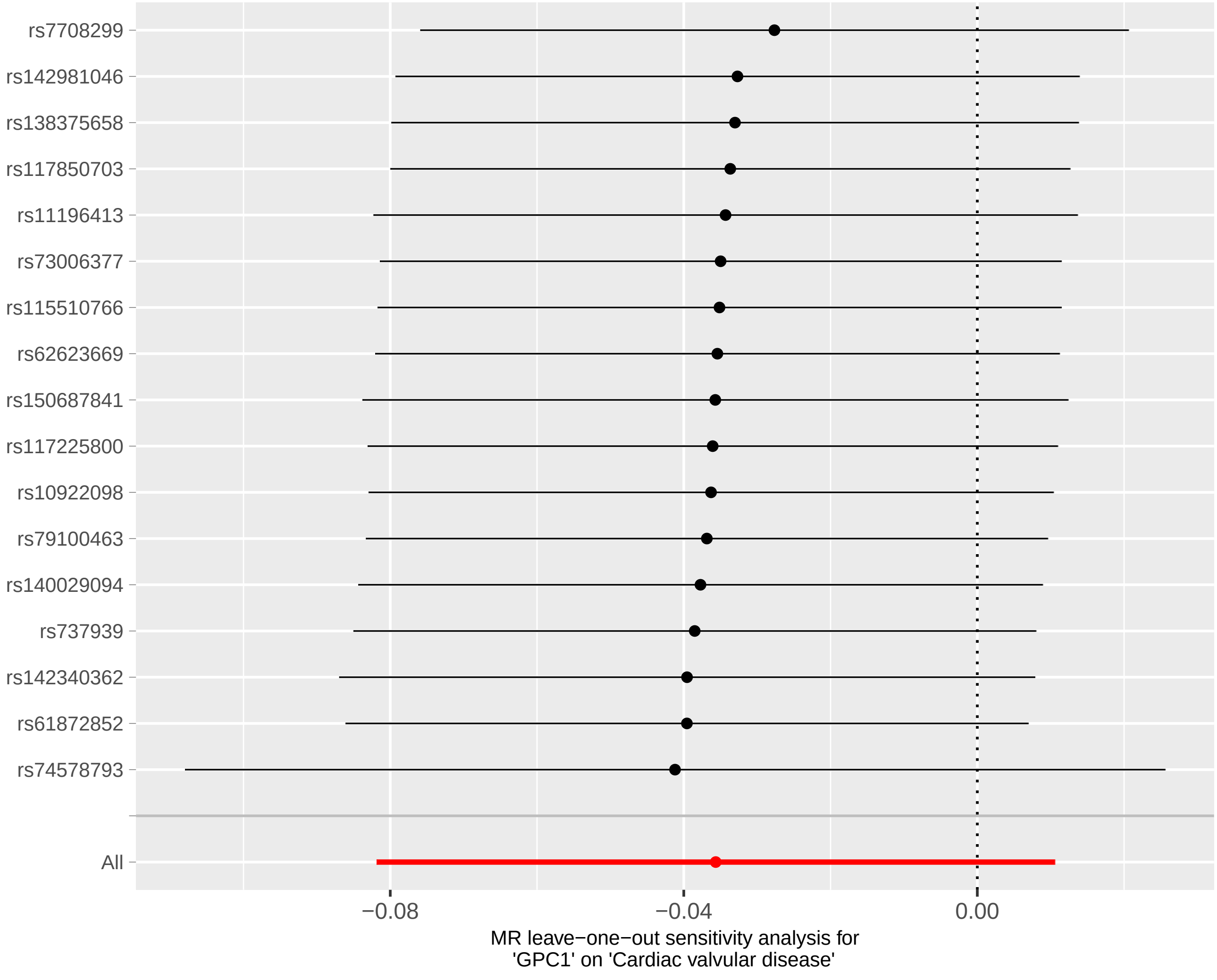
rs7708299
rs142981046
rs138375658
rs117850703
rs11196413
rs73006377
rs115510766
rs62623669
rs150687841
rs117225800
rs10922098
rs79100463
rs140029094
rs737939
rs142340362
rs61872852
rs74578793
All
−0.08
−0.04
0.00
MR leave−one−out sensitivity analysis for
'GPC1' on 'Cardiac valvular disease'

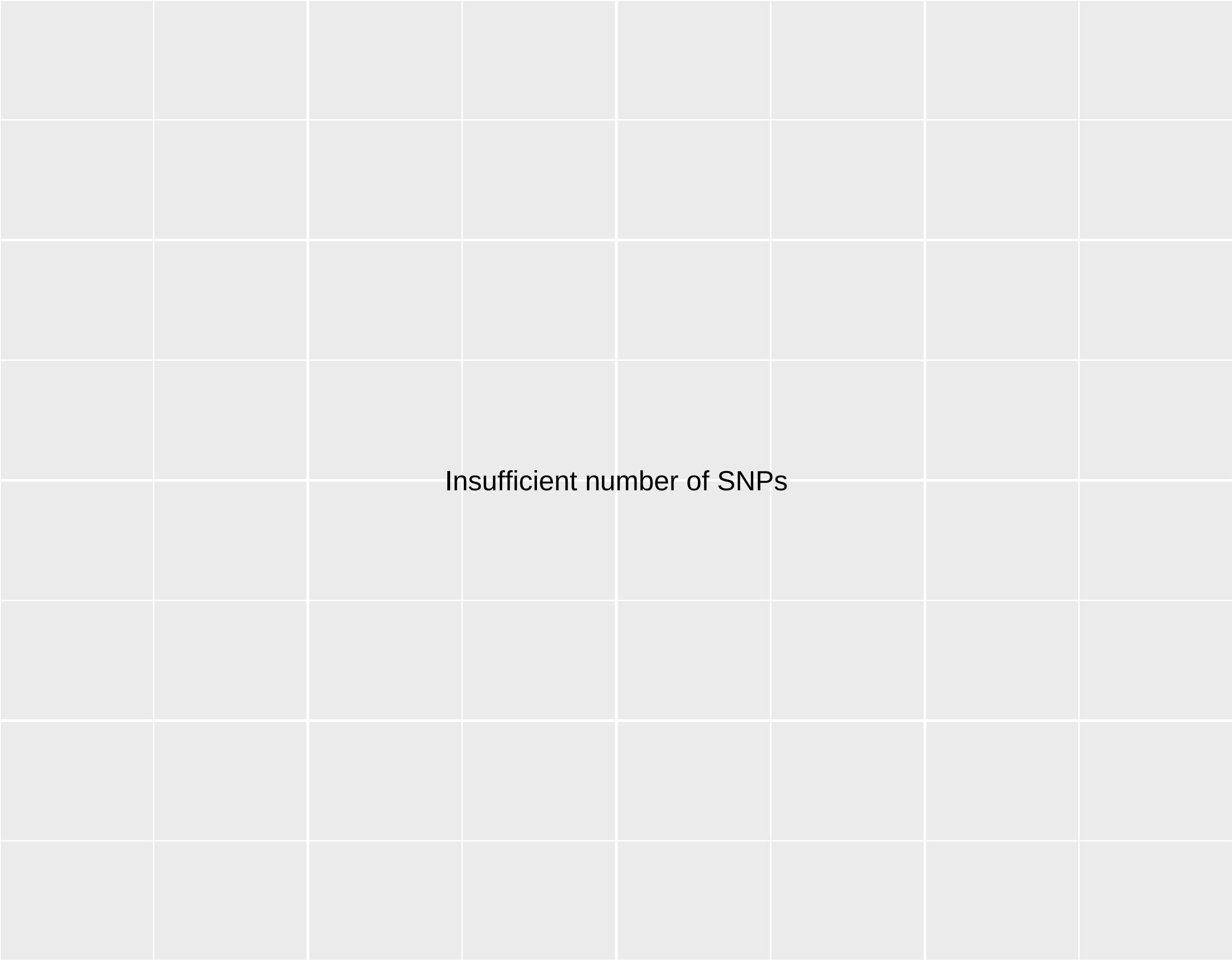
Insufficient number of SNPs

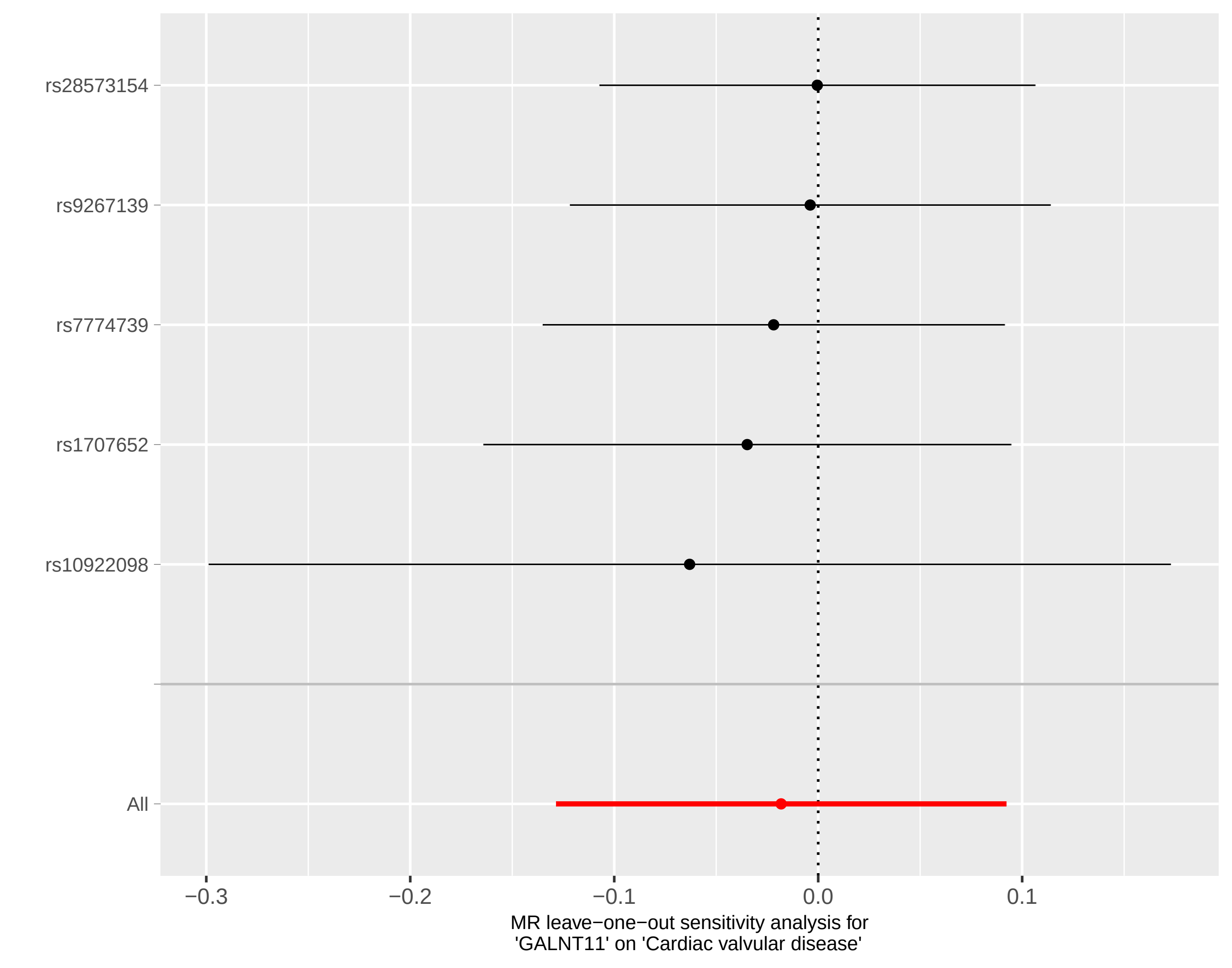
rs28573154
rs9267139
rs7774739
rs1707652
rs10922098
All
−0.3
−0.2
−0.1
0.0
0.1
MR leave−one−out sensitivity analysis for
'GALNT11' on 'Cardiac valvular disease'

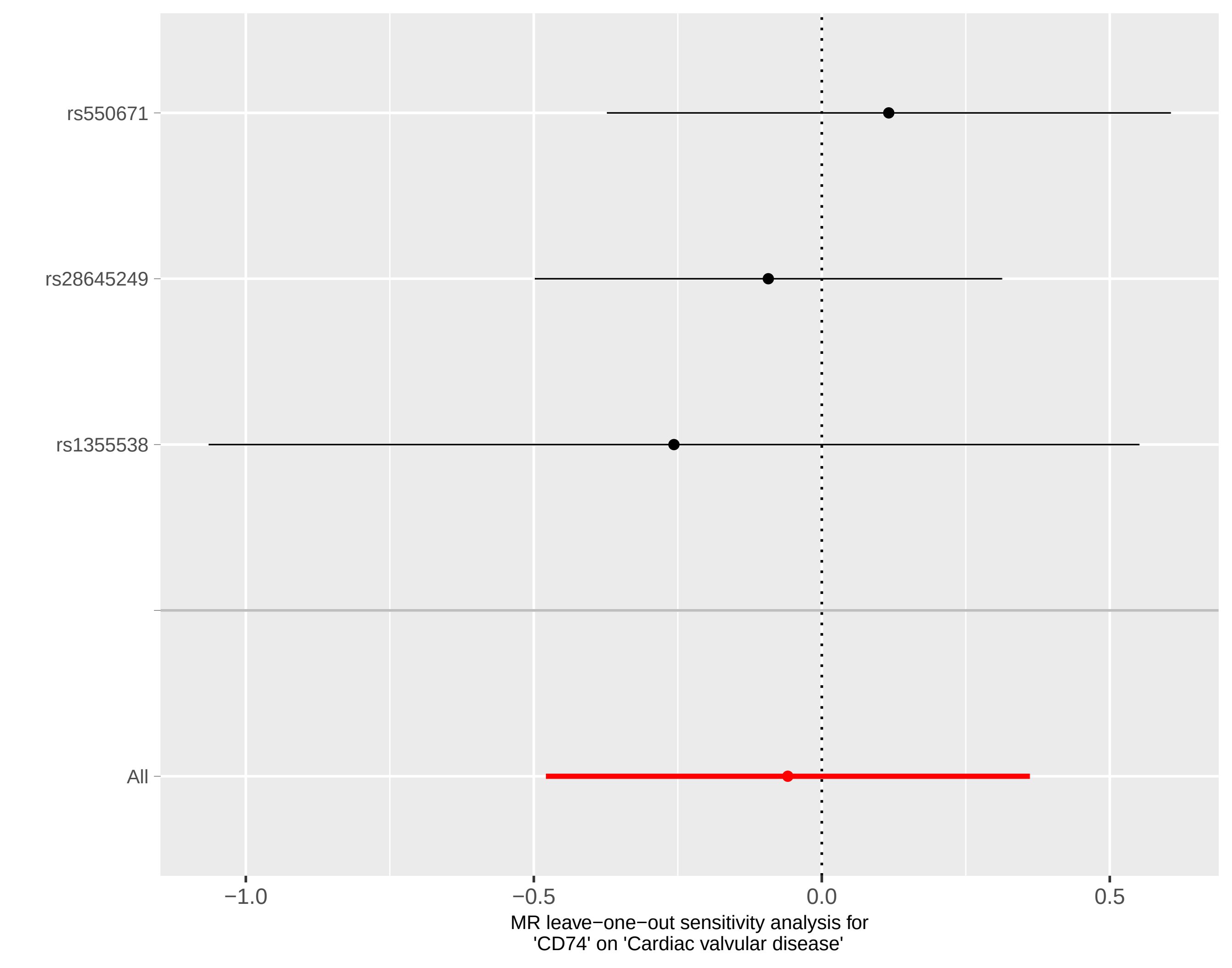

rs550671
rs28645249
rs1355538
All
−1.0
−0.5
0.0
0.5
MR leave−one−out sensitivity analysis for
'CD74' on 'Cardiac valvular disease'

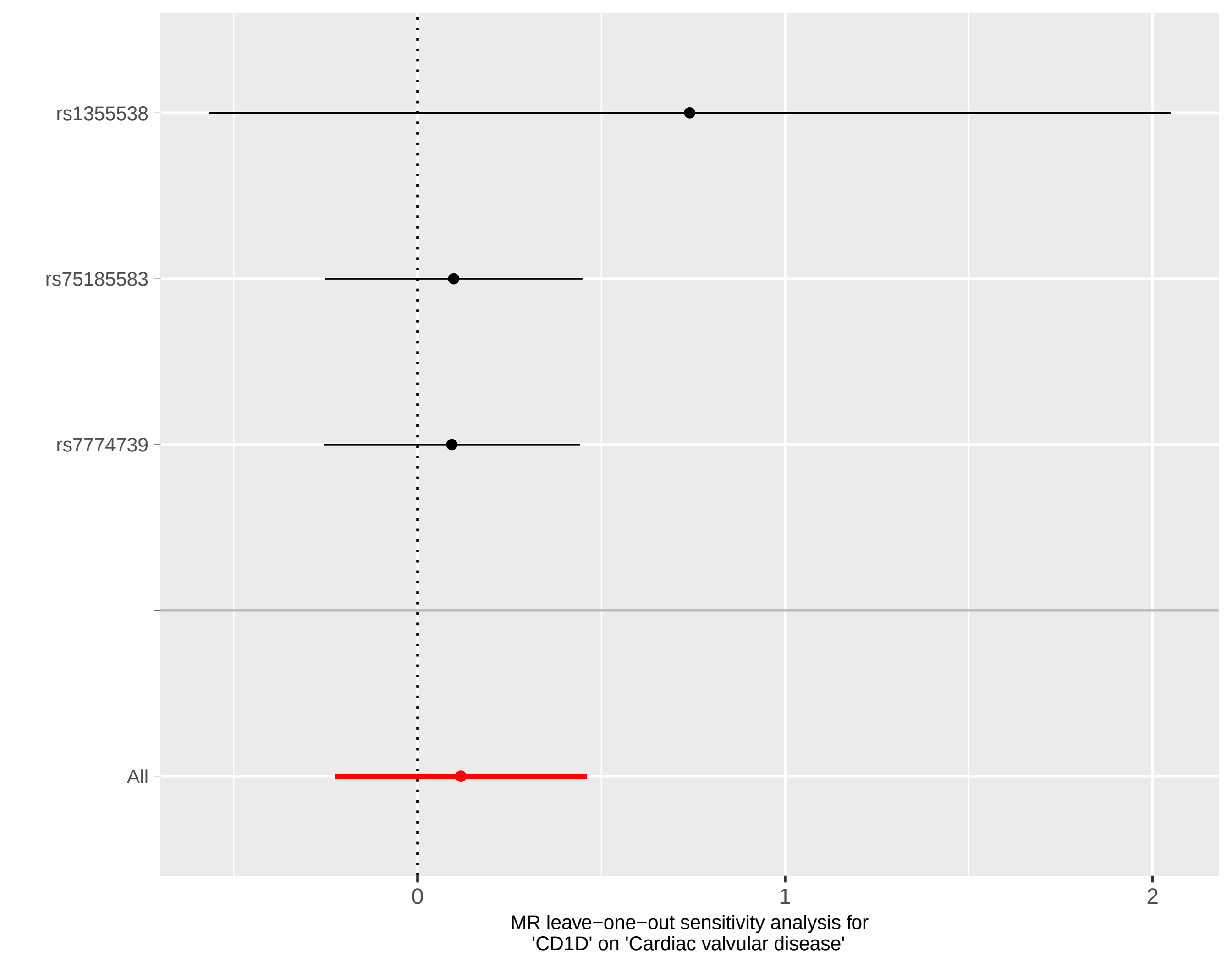
rs1355538
rs75185583
rs7774739
All
0
1
2
MR leave−one−out sensitivity analysis for
'CD1D' on 'Cardiac valvular disease'

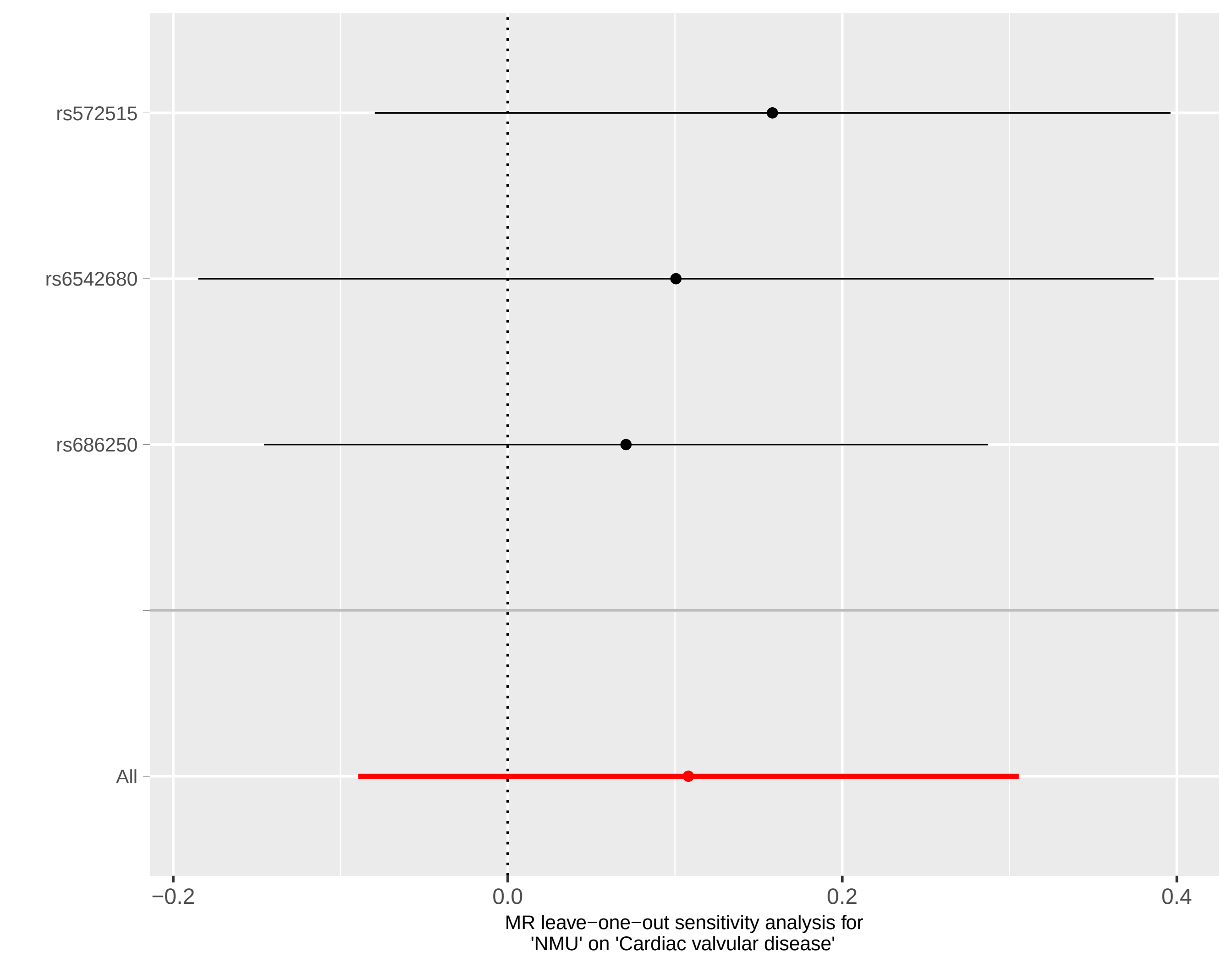
rs572515
rs6542680
rs686250
All
−0.2
0.0
0.2
0.4
MR leave−one−out sensitivity analysis for
'NMU' on 'Cardiac valvular disease'

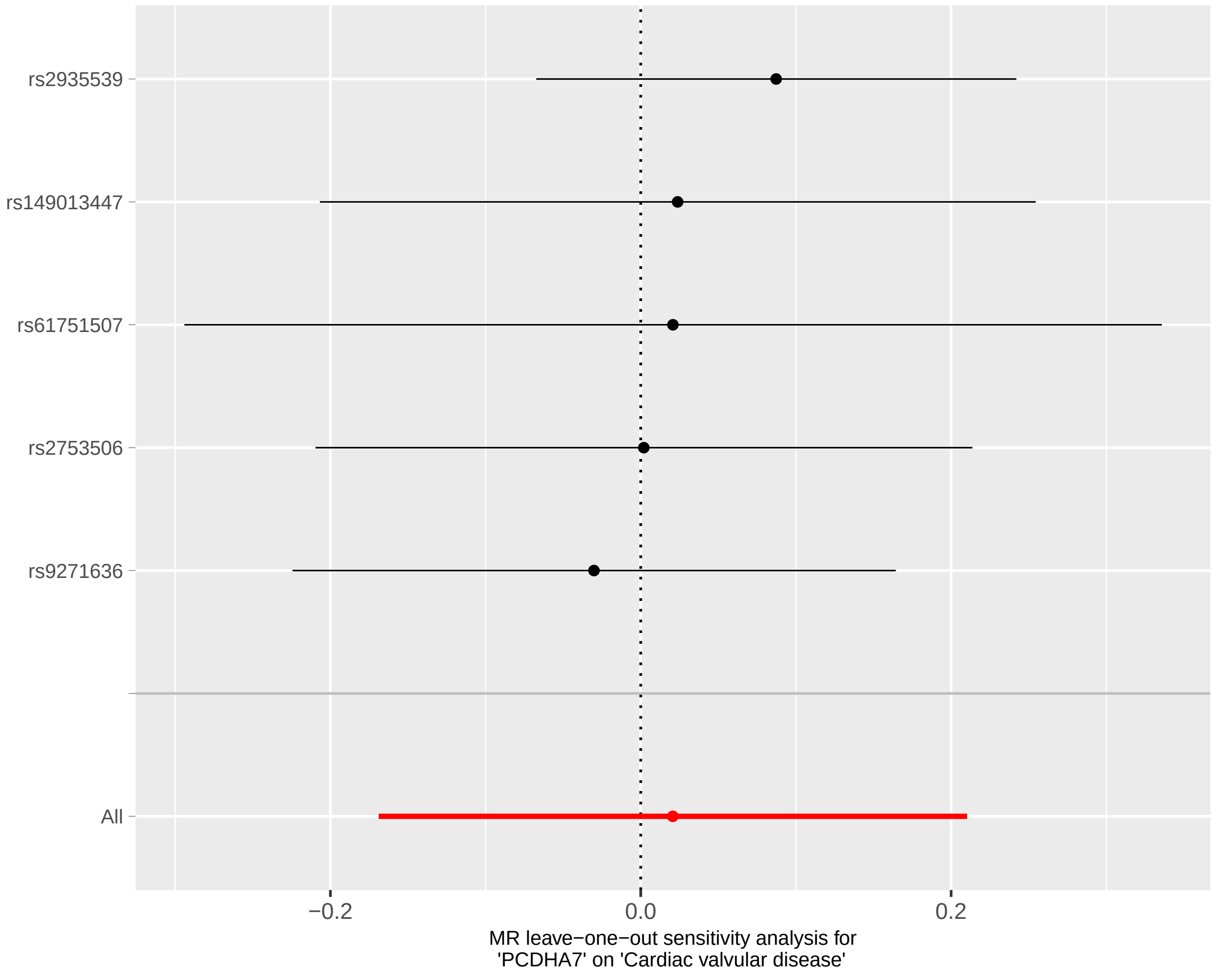
rs2935539
rs149013447
rs61751507
rs2753506
rs9271636
All
−0.2
0.0
0.2
MR leave−one−out sensitivity analysis for
'PCDHA7' on 'Cardiac valvular disease'

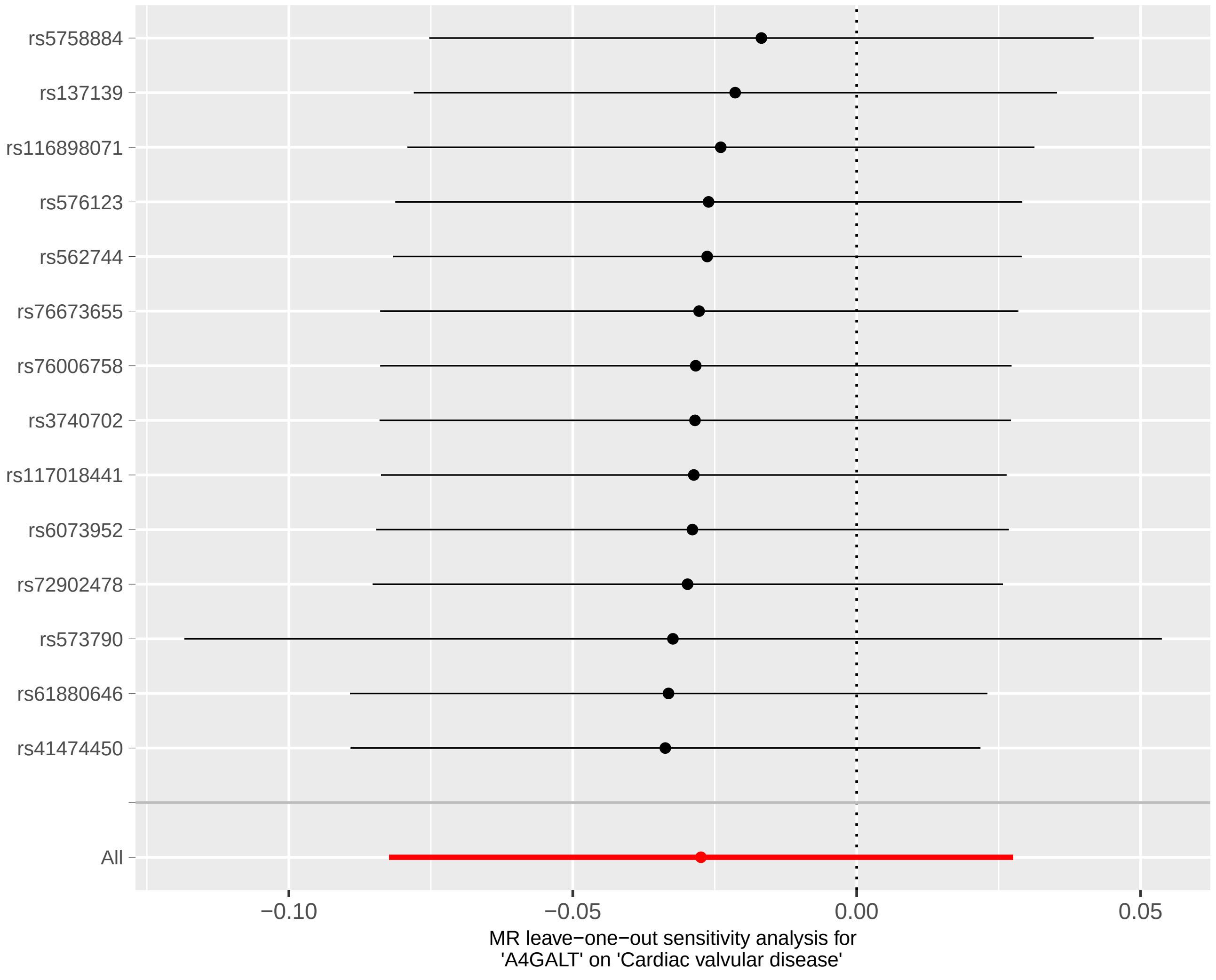
rs5758884
rs137139
rs116898071
rs576123
rs562744
rs76673655
rs76006758
rs3740702
rs117018441
rs6073952
rs72902478
rs573790
rs61880646
rs41474450
All
−0.10
−0.05
0.00
0.05
MR leave−one−out sensitivity analysis for
'A4GALT' on 'Cardiac valvular disease'

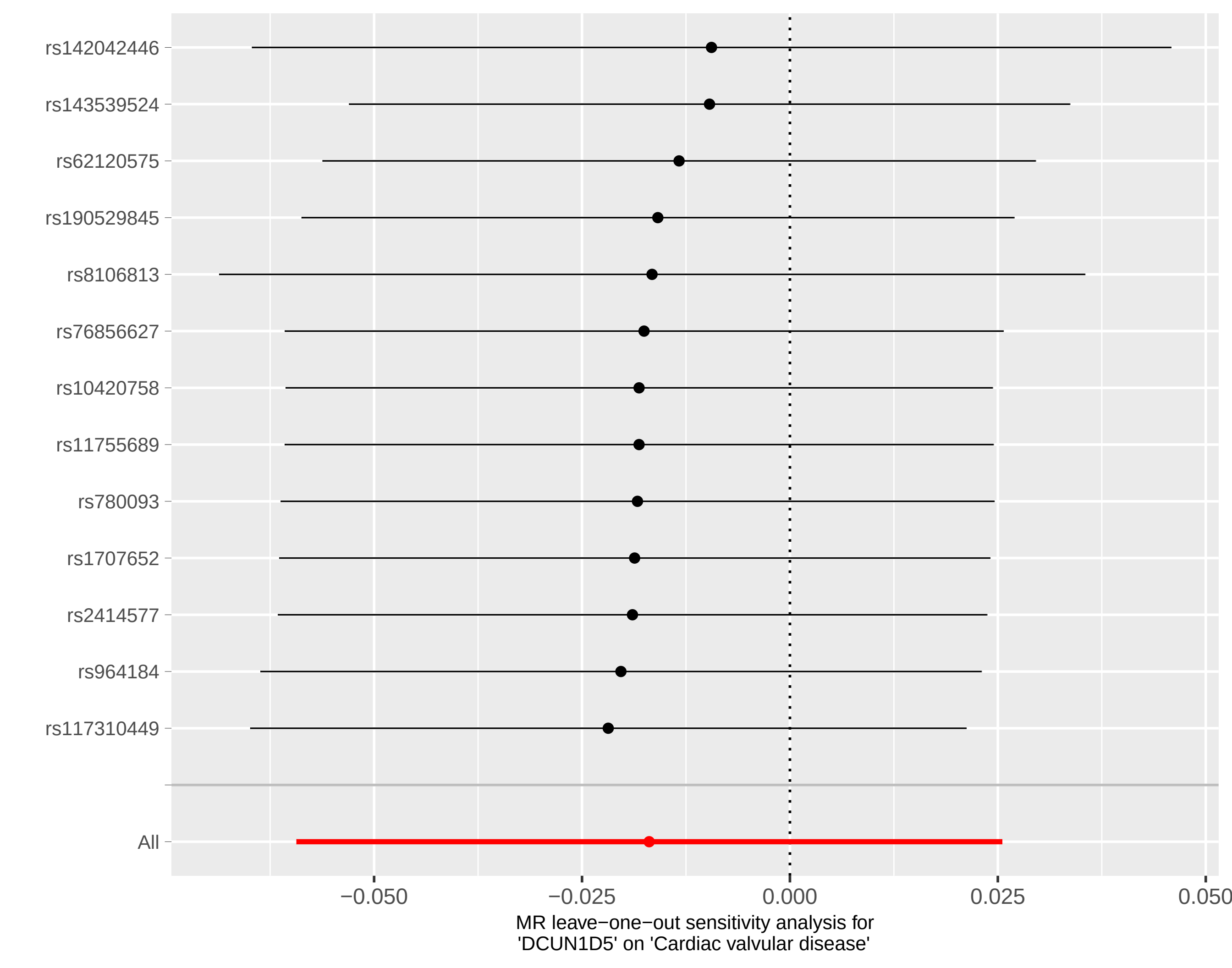
rs142042446
rs143539524
rs62120575
rs190529845
rs8106813
rs76856627
rs10420758
rs11755689
rs780093
rs1707652
rs2414577
rs964184
rs117310449
All
−0.050
−0.025
0.000
0.025
0.050
MR leave−one−out sensitivity analysis for
'DCUN1D5' on 'Cardiac valvular disease'

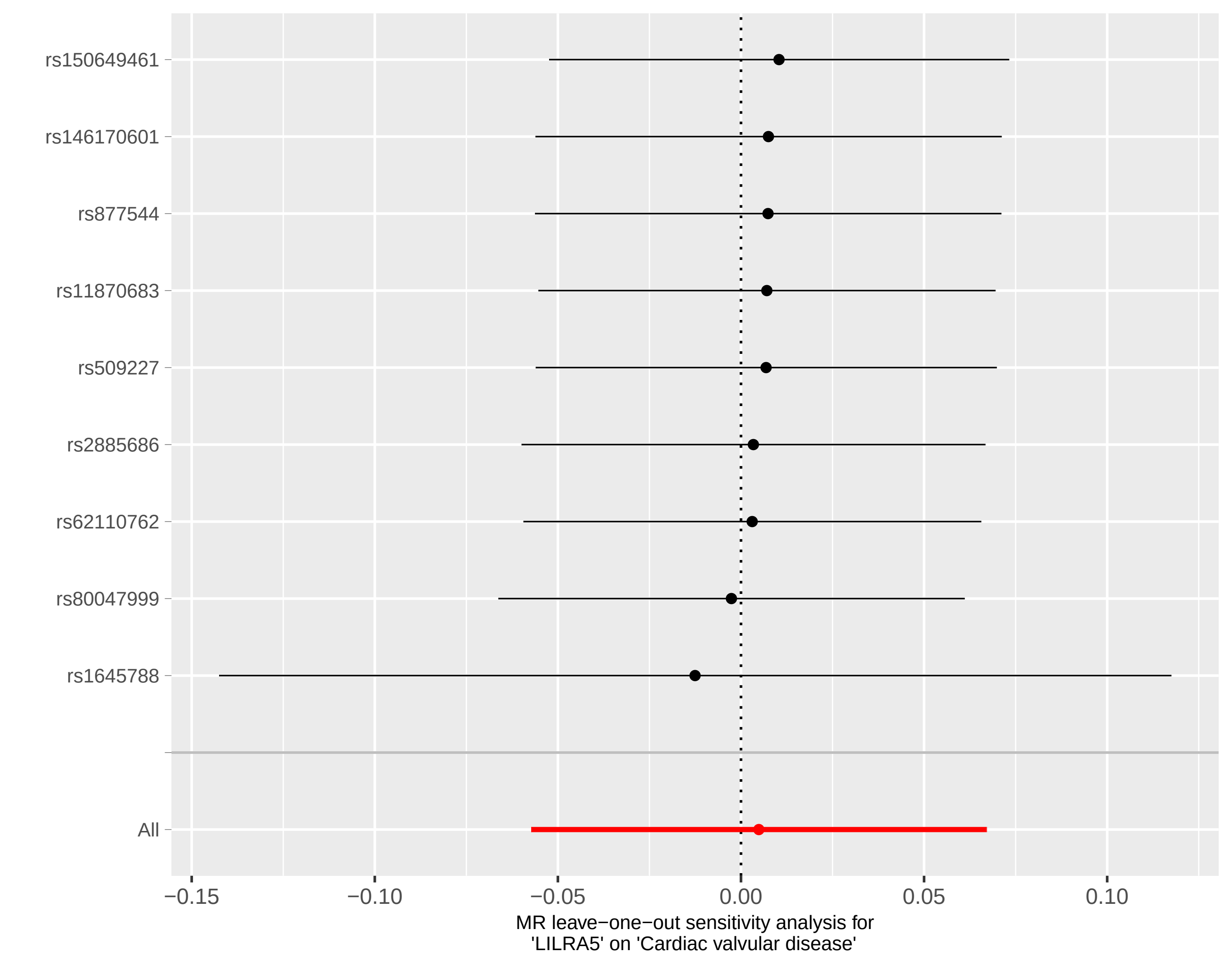
rs150649461
rs146170601
rs877544
rs11870683
rs509227
rs2885686
rs62110762
rs80047999
rs1645788
All
−0.15
−0.10
−0.05
0.00
0.05
0.10
MR leave−one−out sensitivity analysis for
'LILRA5' on 'Cardiac valvular disease'

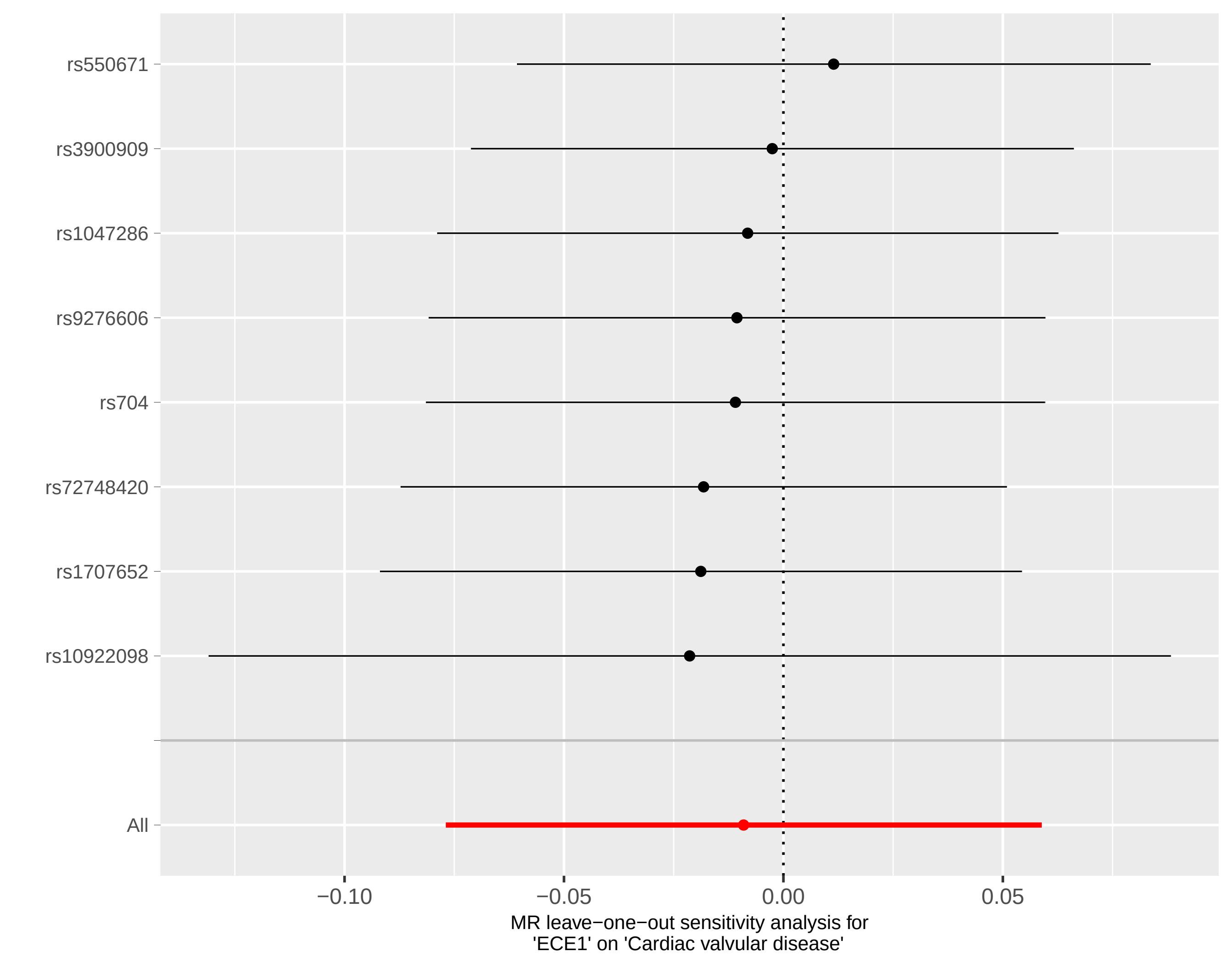
rs550671
rs3900909
rs1047286
rs9276606
rs704
rs72748420
rs1707652
rs10922098
All
−0.10
−0.05
0.00
0.05
MR leave−one−out sensitivity analysis for
'ECE1' on 'Cardiac valvular disease'

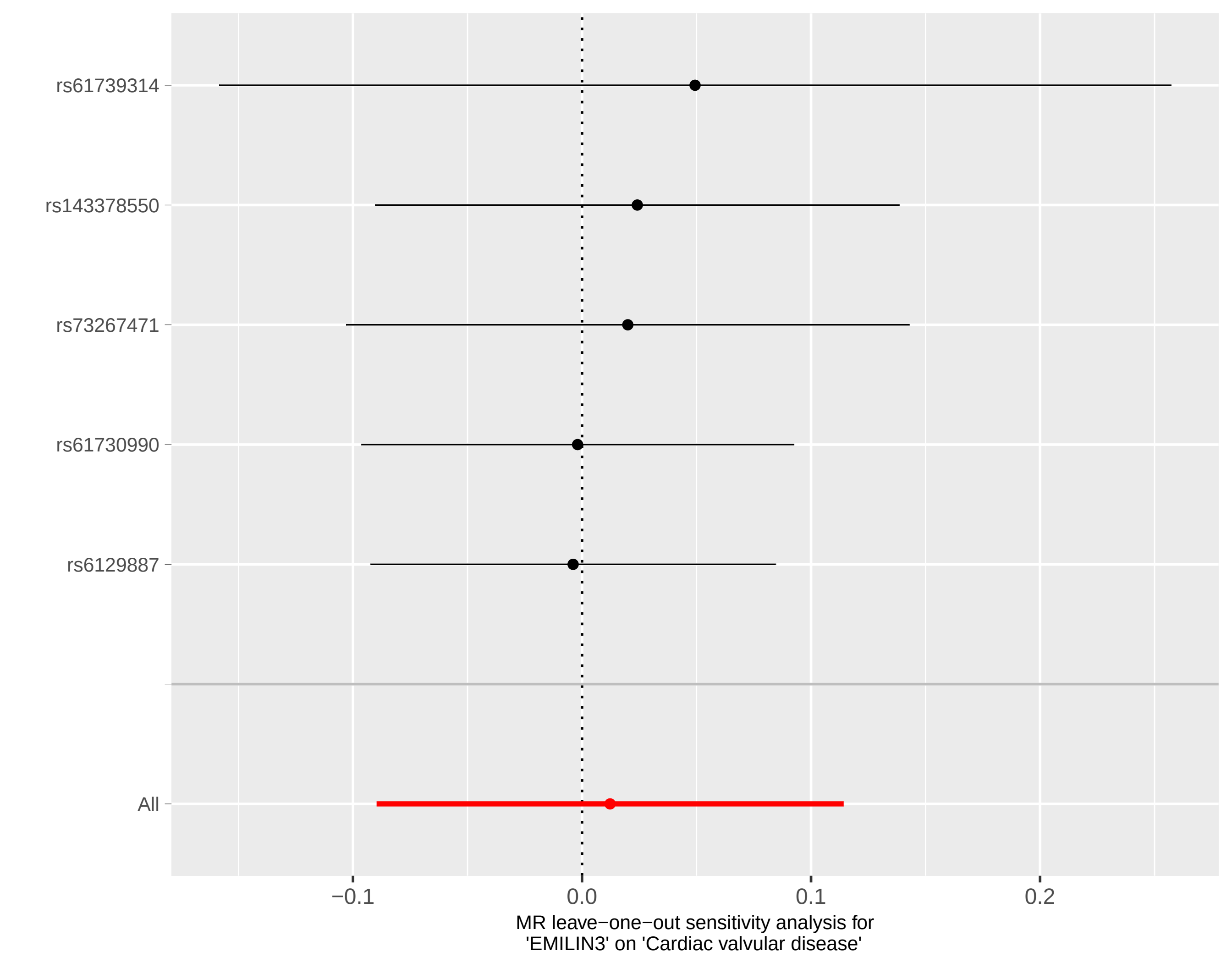
rs61739314
rs143378550
rs73267471
rs61730990
rs6129887
All
−0.1
0.0
0.1
0.2
MR leave−one−out sensitivity analysis for
'EMILIN3' on 'Cardiac valvular disease'

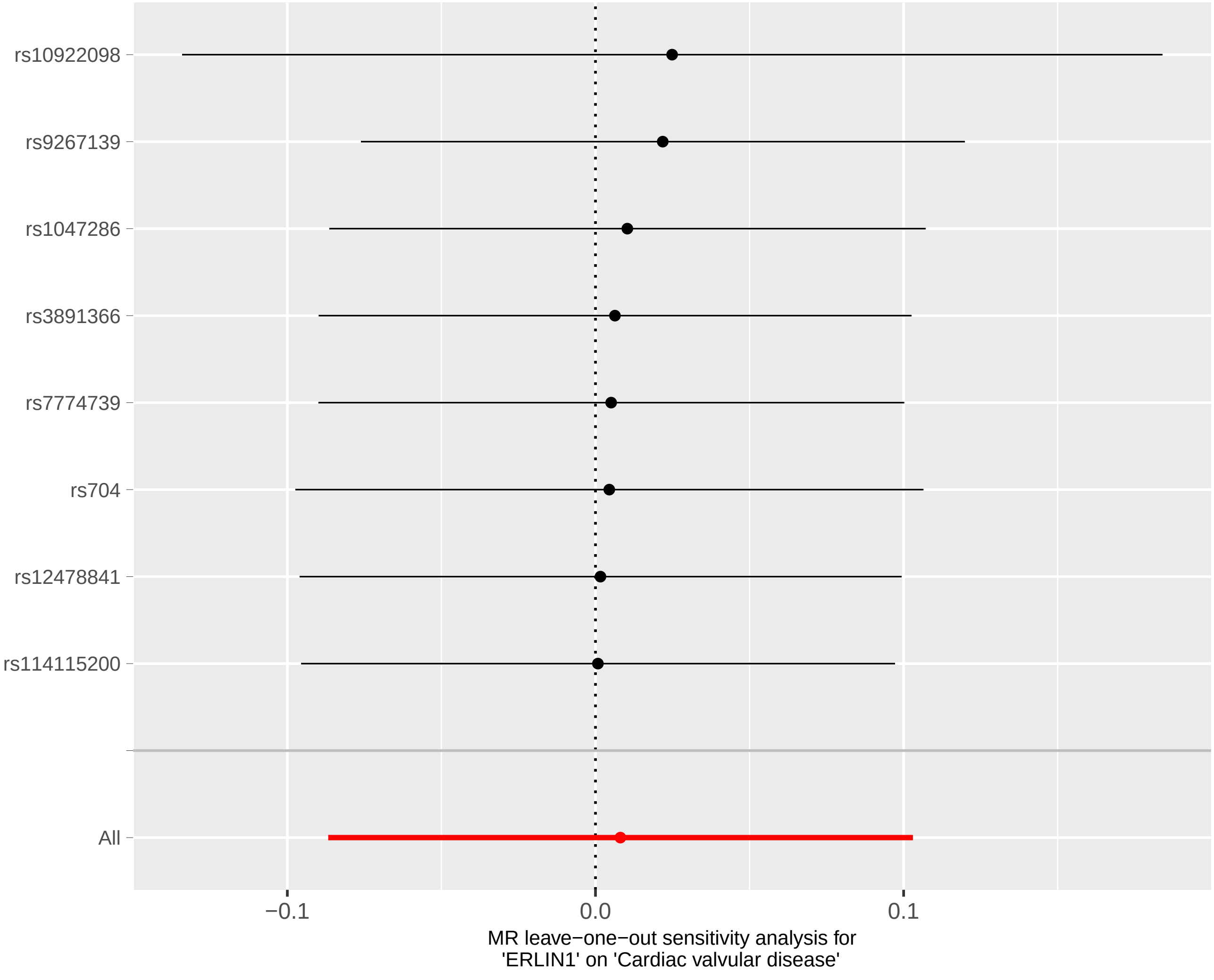
rs10922098
rs9267139
rs1047286
rs3891366
rs7774739
rs704
rs12478841
rs114115200
All
−0.1
0.0
0.1
MR leave−one−out sensitivity analysis for
'ERLIN1' on 'Cardiac valvular disease'

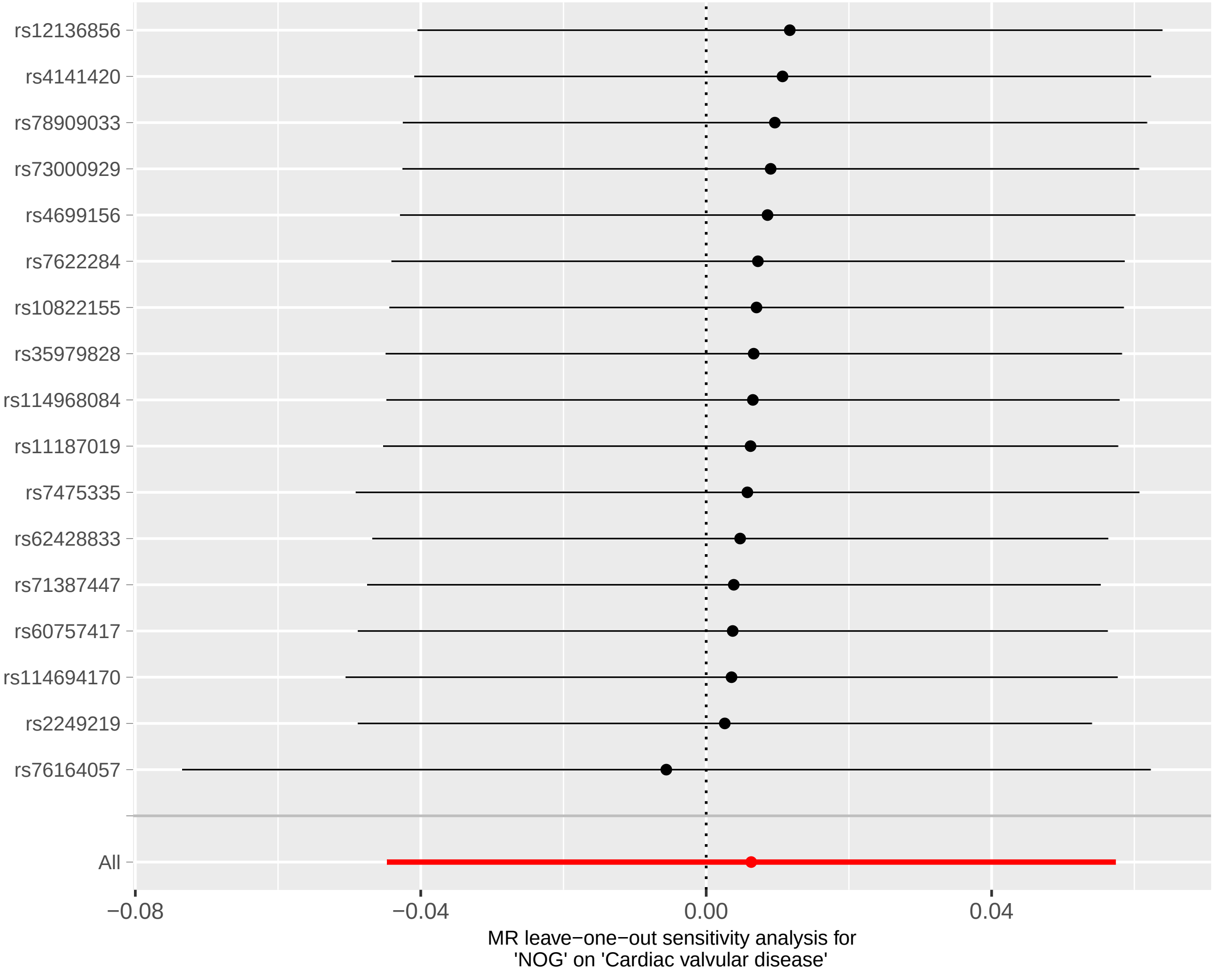
rs12136856
rs4141420
rs78909033
rs73000929
rs4699156
rs7622284
rs10822155
rs35979828
rs114968084
rs11187019
rs7475335
rs62428833
rs71387447
rs60757417
rs114694170
rs2249219
rs76164057
All
−0.08
−0.04
0.00
0.04
MR leave−one−out sensitivity analysis for
'NOG' on 'Cardiac valvular disease'

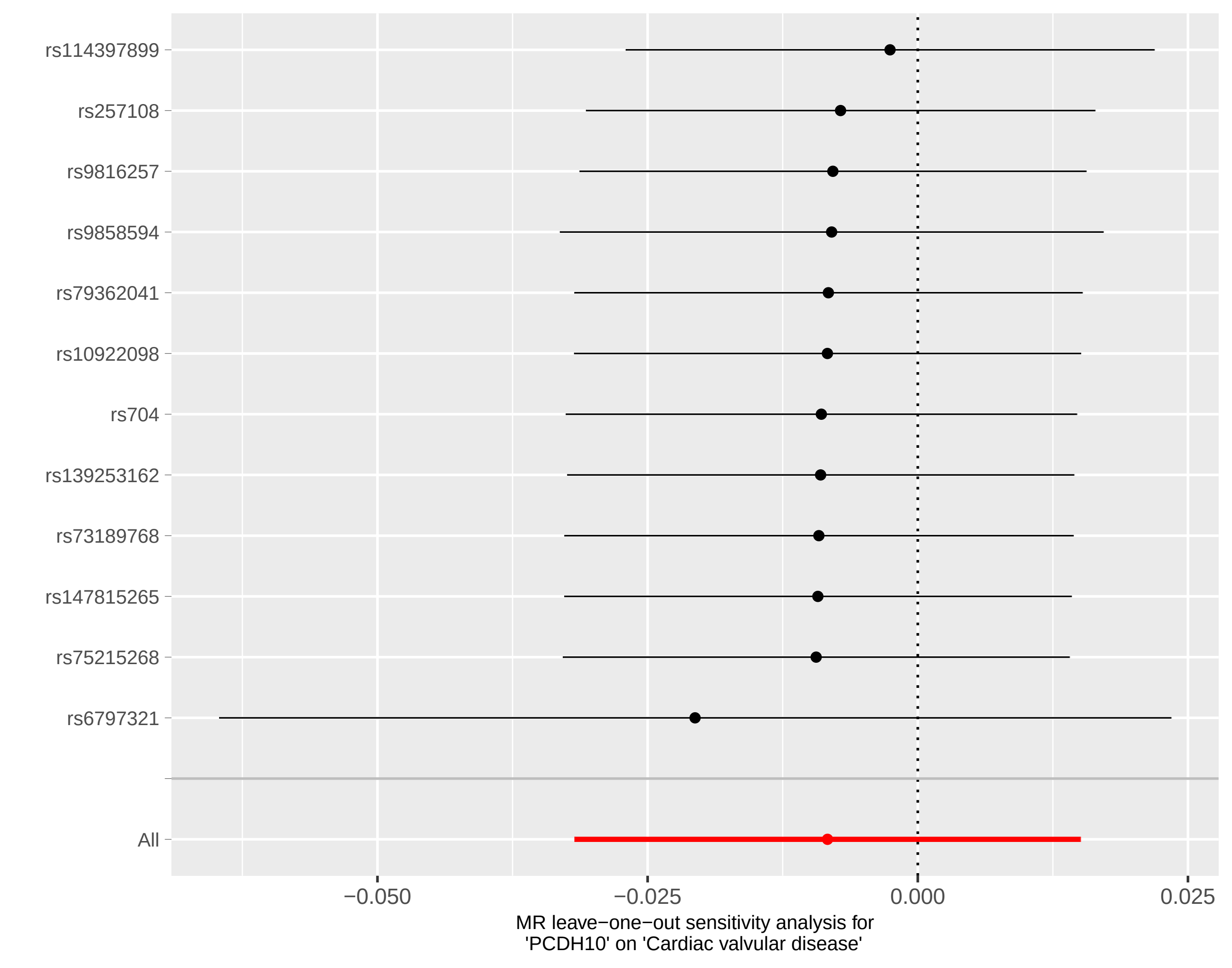
rs114397899
rs257108
rs9816257
rs9858594
rs79362041
rs10922098
rs704
rs139253162
rs73189768
rs147815265
rs75215268
rs6797321
All
−0.050
−0.025
0.000
0.025
MR leave−one−out sensitivity analysis for
'PCDH10' on 'Cardiac valvular disease'

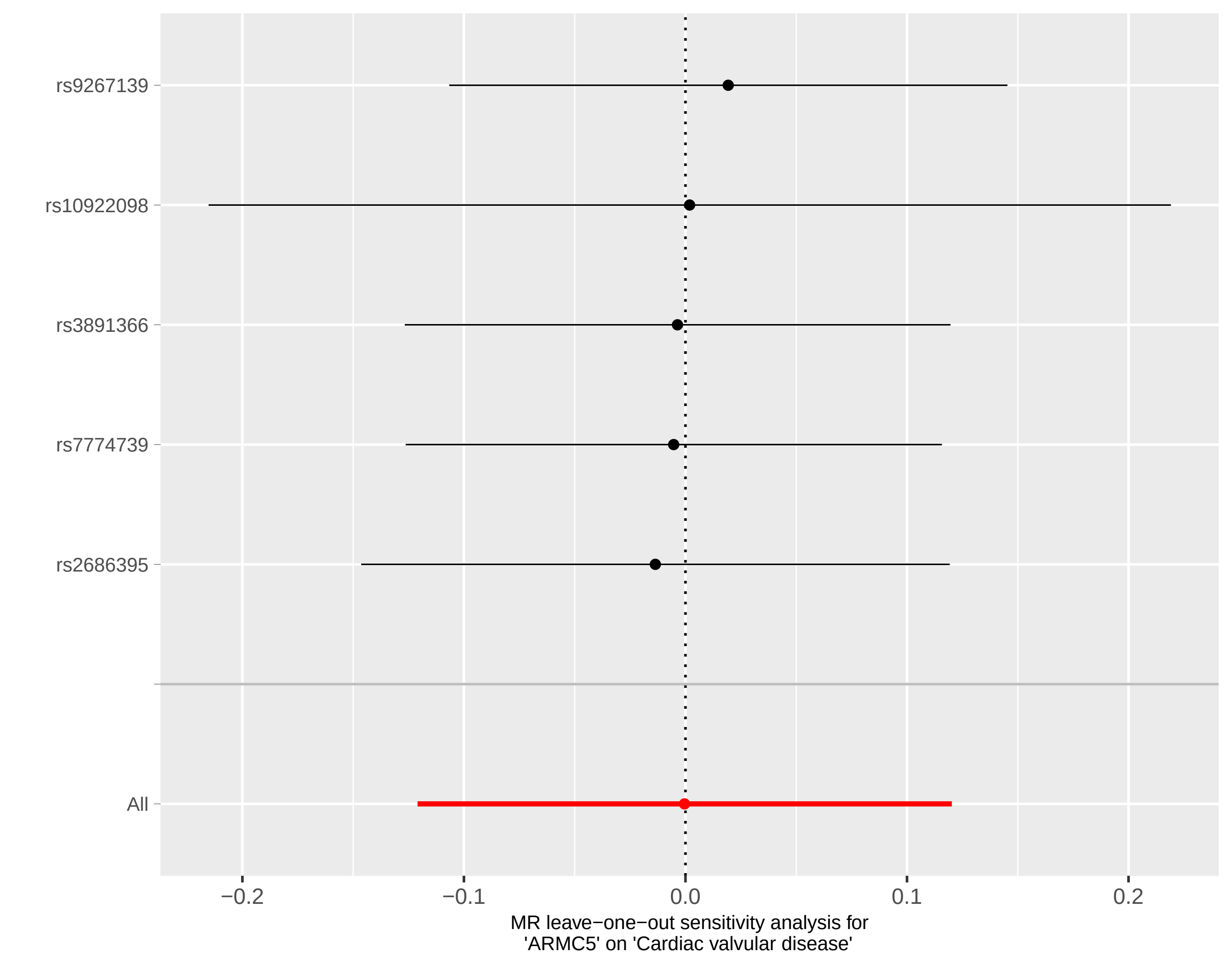

rs9267139
rs10922098
rs3891366
rs7774739
rs2686395
All
−0.2
−0.1
0.0
0.1
0.2
MR leave−one−out sensitivity analysis for
'ARMC5' on 'Cardiac valvular disease'

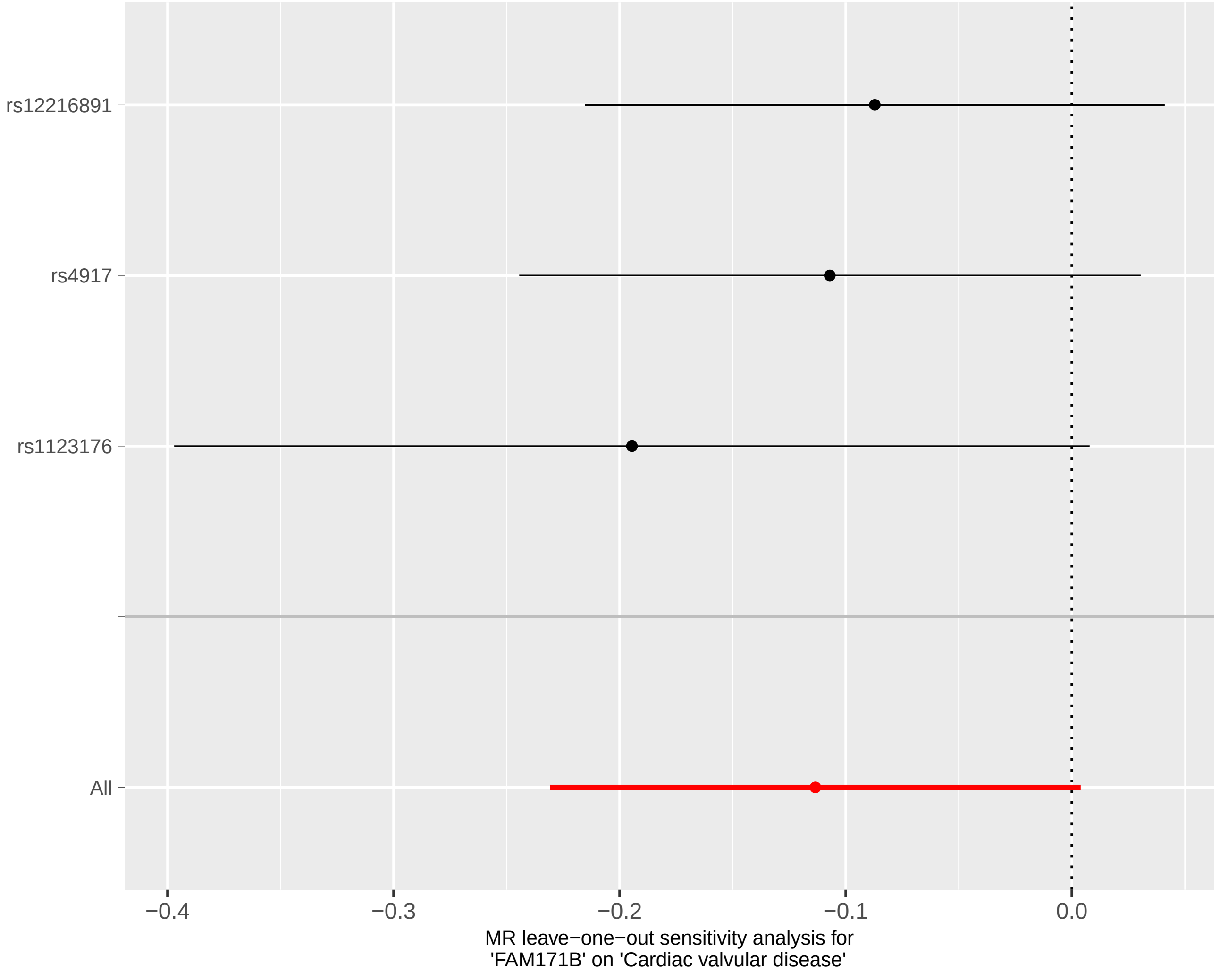
rs12216891
rs4917
rs1123176
All
−0.4
−0.3
−0.2
−0.1
0.0
MR leave−one−out sensitivity analysis for
'FAM171B' on 'Cardiac valvular disease'

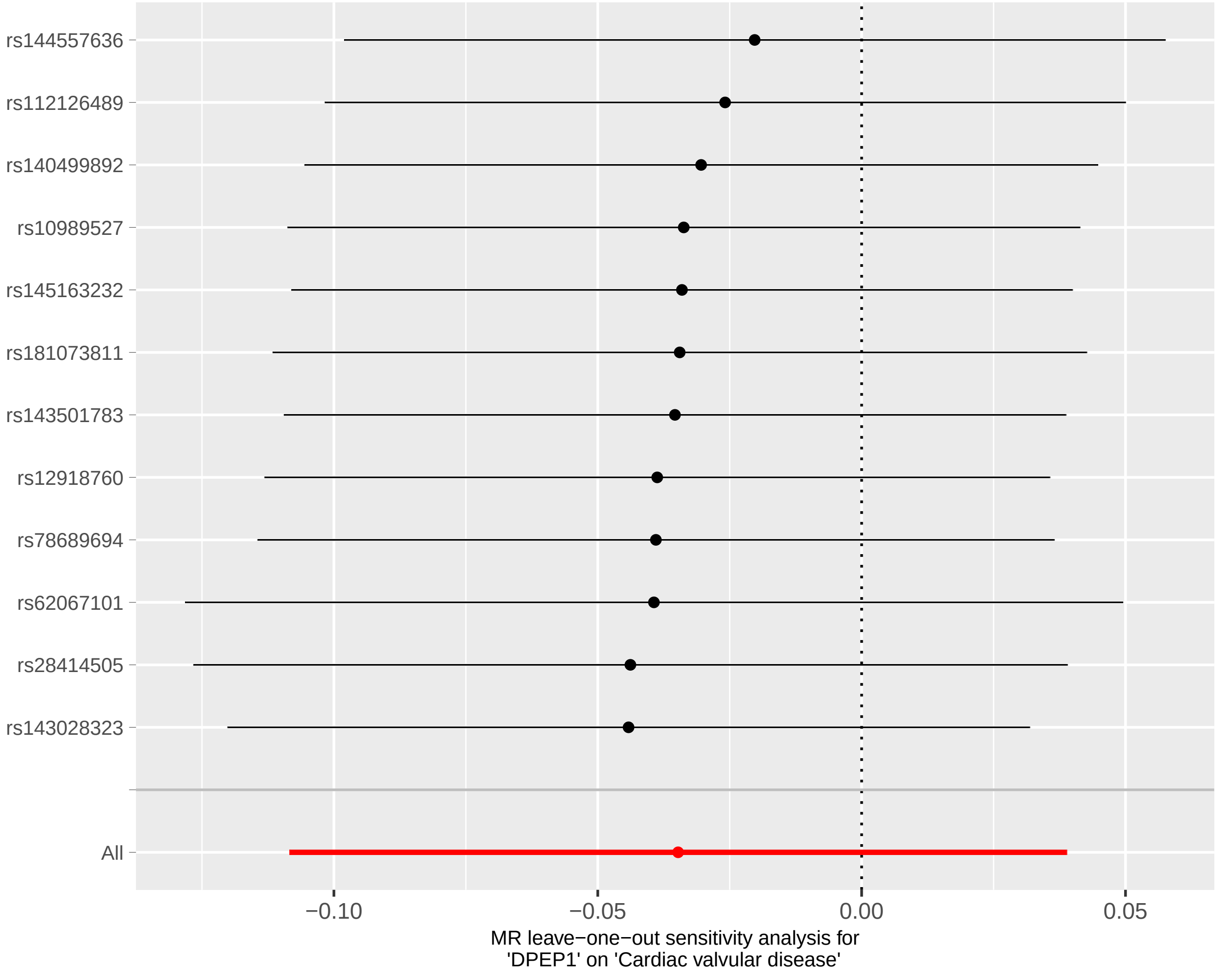
rs144557636
rs112126489
rs140499892
rs10989527
rs145163232
rs181073811
rs143501783
rs12918760
rs78689694
rs62067101
rs28414505
rs143028323
All
−0.10
−0.05
0.00
0.05
MR leave−one−out sensitivity analysis for
'DPEP1' on 'Cardiac valvular disease'

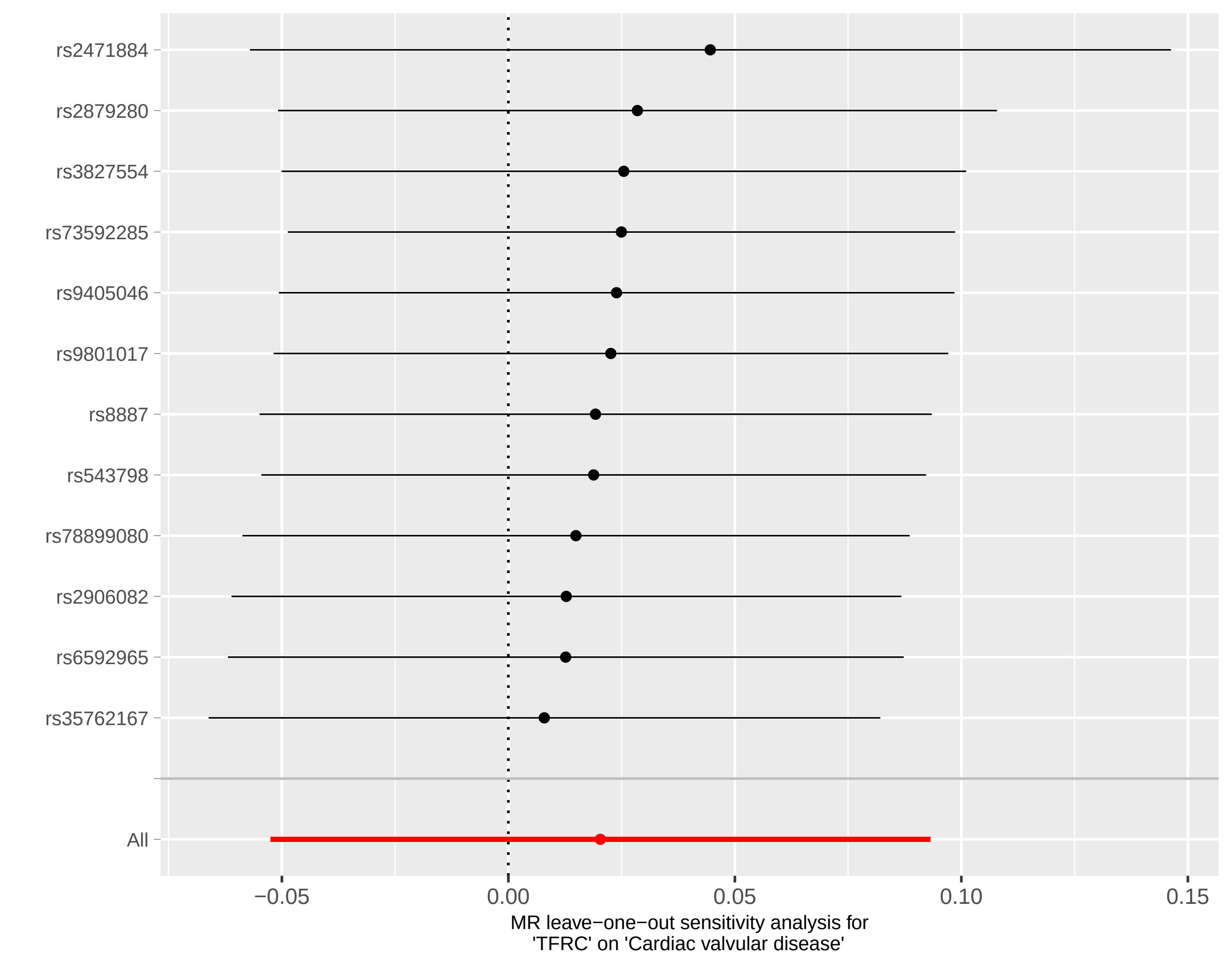
rs2471884
rs2879280
rs3827554
rs73592285
rs9405046
rs9801017
rs8887
rs543798
rs78899080
rs2906082
rs6592965
rs35762167
All
−0.05
0.00
0.05
0.10
0.15
MR leave−one−out sensitivity analysis for
'TFRC' on 'Cardiac valvular disease'

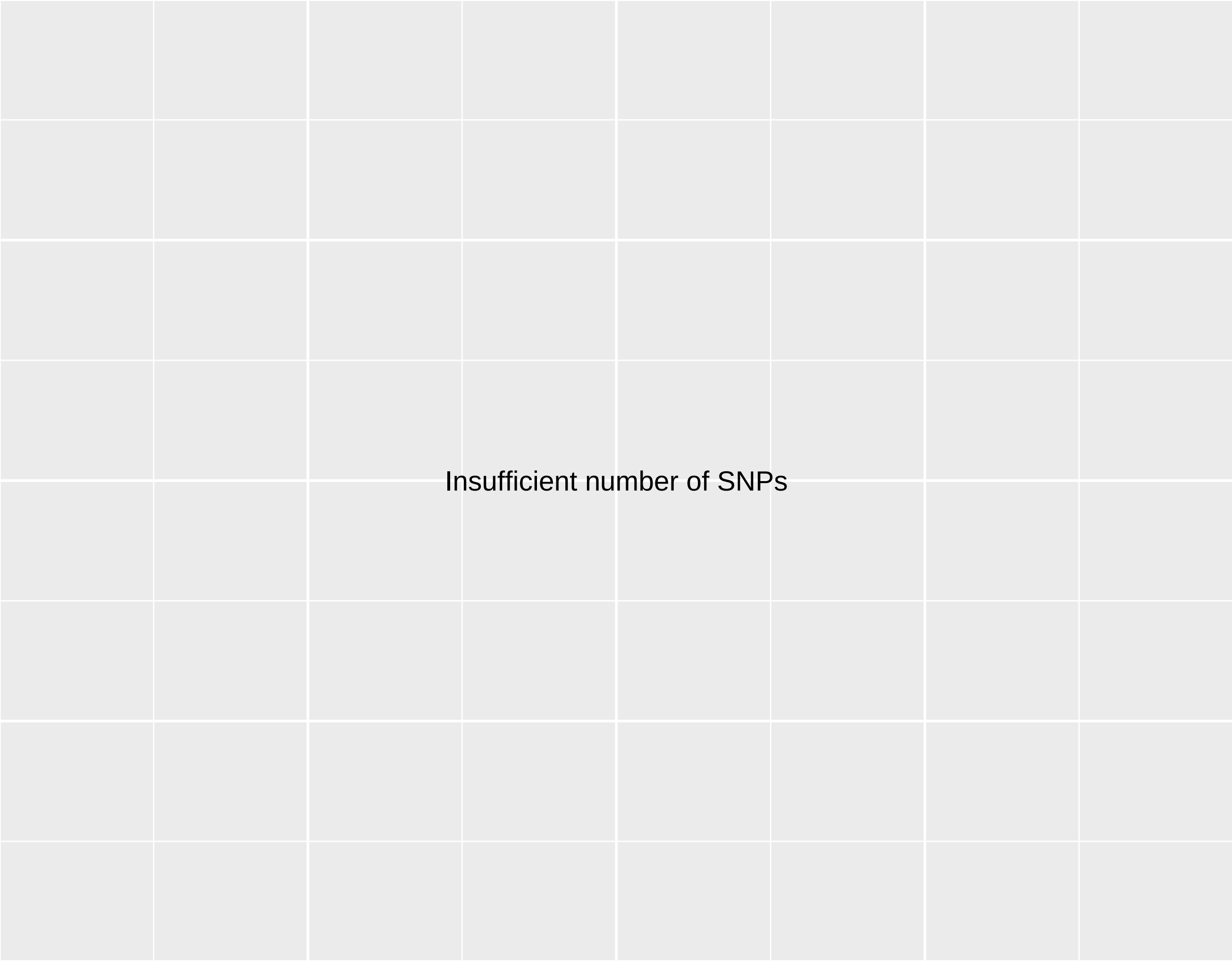
Insufficient number of SNPs

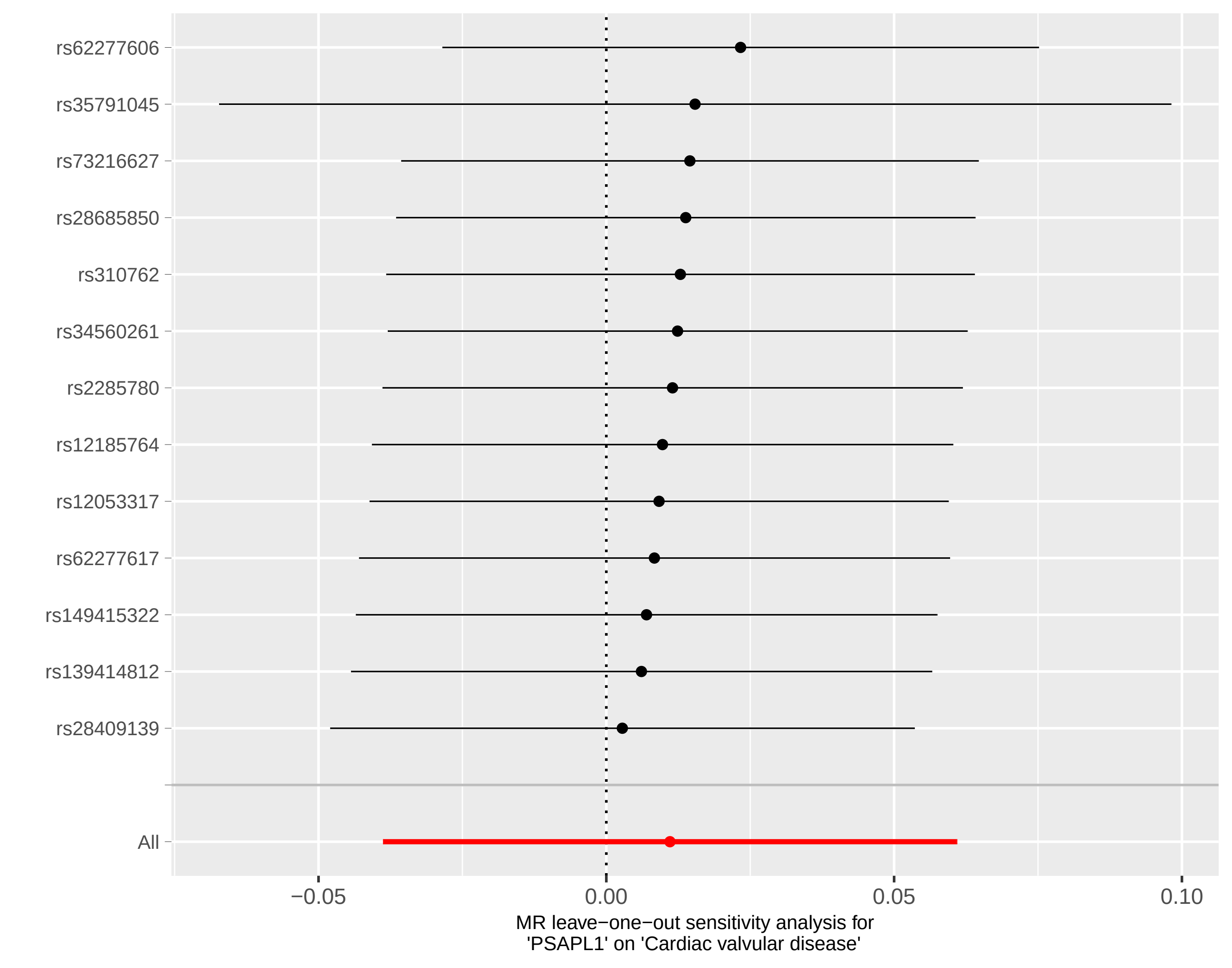

rs62277606
rs35791045
rs73216627
rs28685850
rs310762
rs34560261
rs2285780
rs12185764
rs12053317
rs62277617
rs149415322
rs139414812
rs28409139
All
−0.05
0.00
0.05
0.10
MR leave−one−out sensitivity analysis for
'PSAPL1' on 'Cardiac valvular disease'

Insufficient number of SNPs

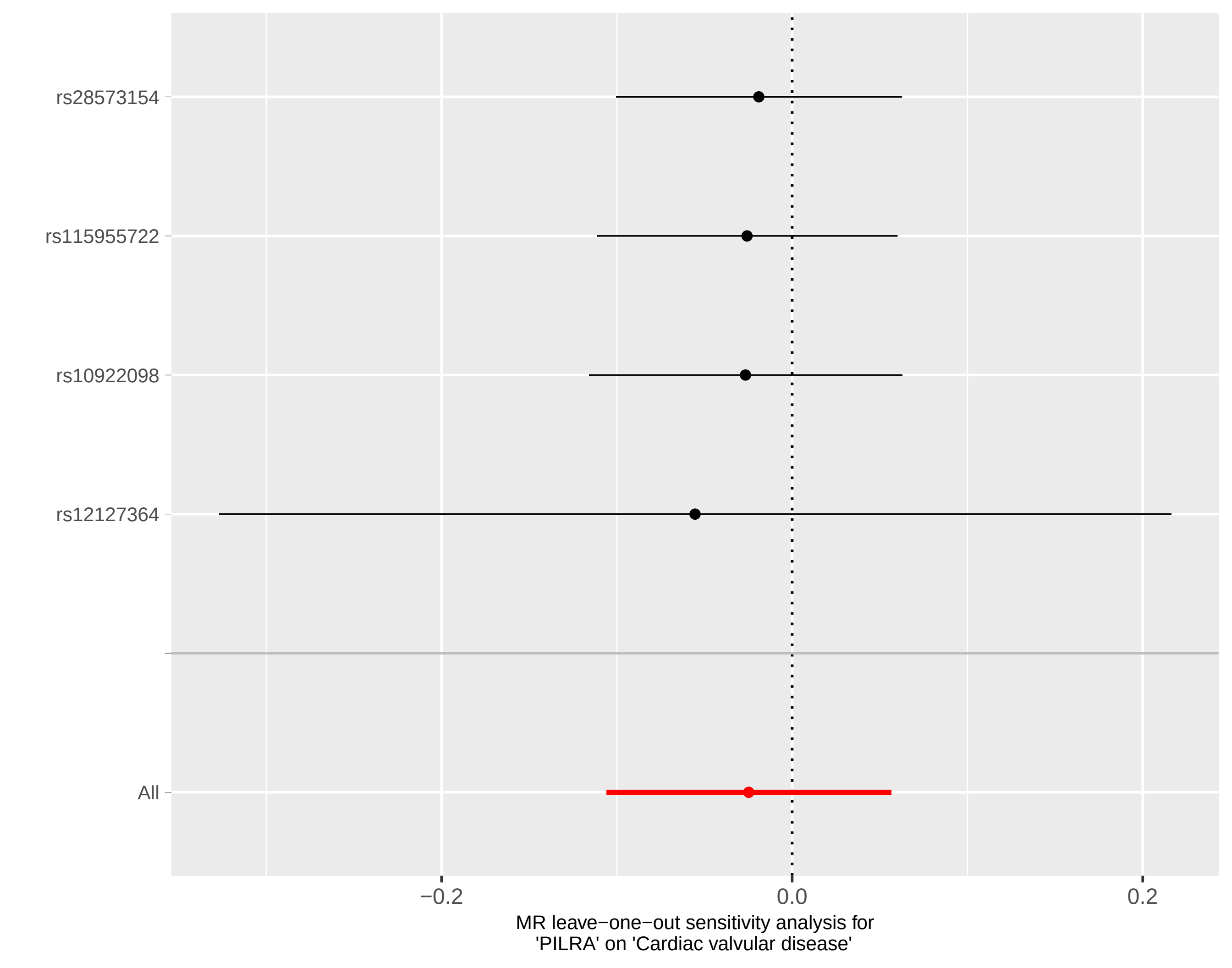
rs28573154
rs115955722
rs10922098
rs12127364
All
−0.2
0.0
0.2
MR leave−one−out sensitivity analysis for
'PILRA' on 'Cardiac valvular disease'

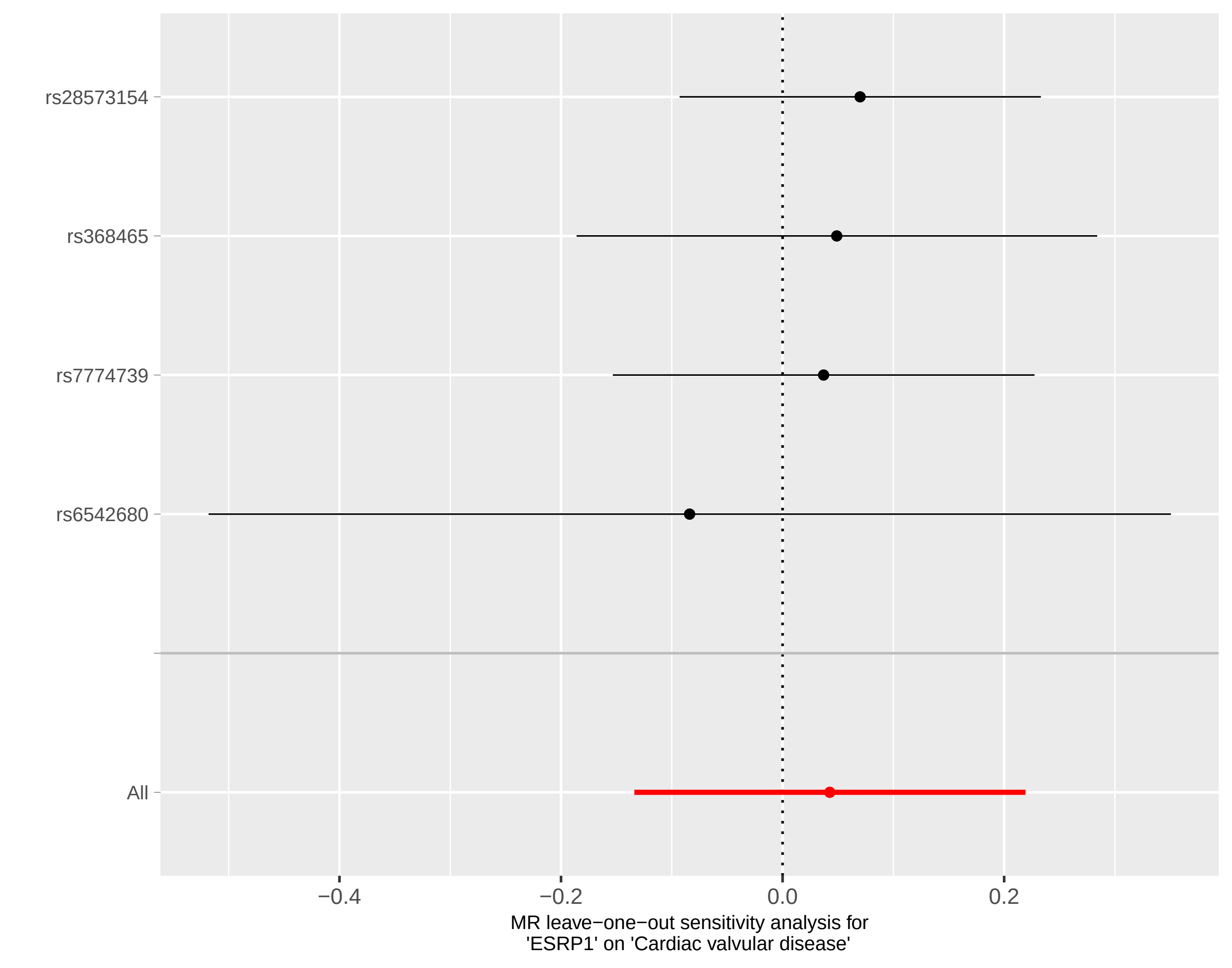

rs28573154
rs368465
rs7774739
rs6542680
All
−0.4
−0.2
0.0
0.2
MR leave−one−out sensitivity analysis for
'ESRP1' on 'Cardiac valvular disease'

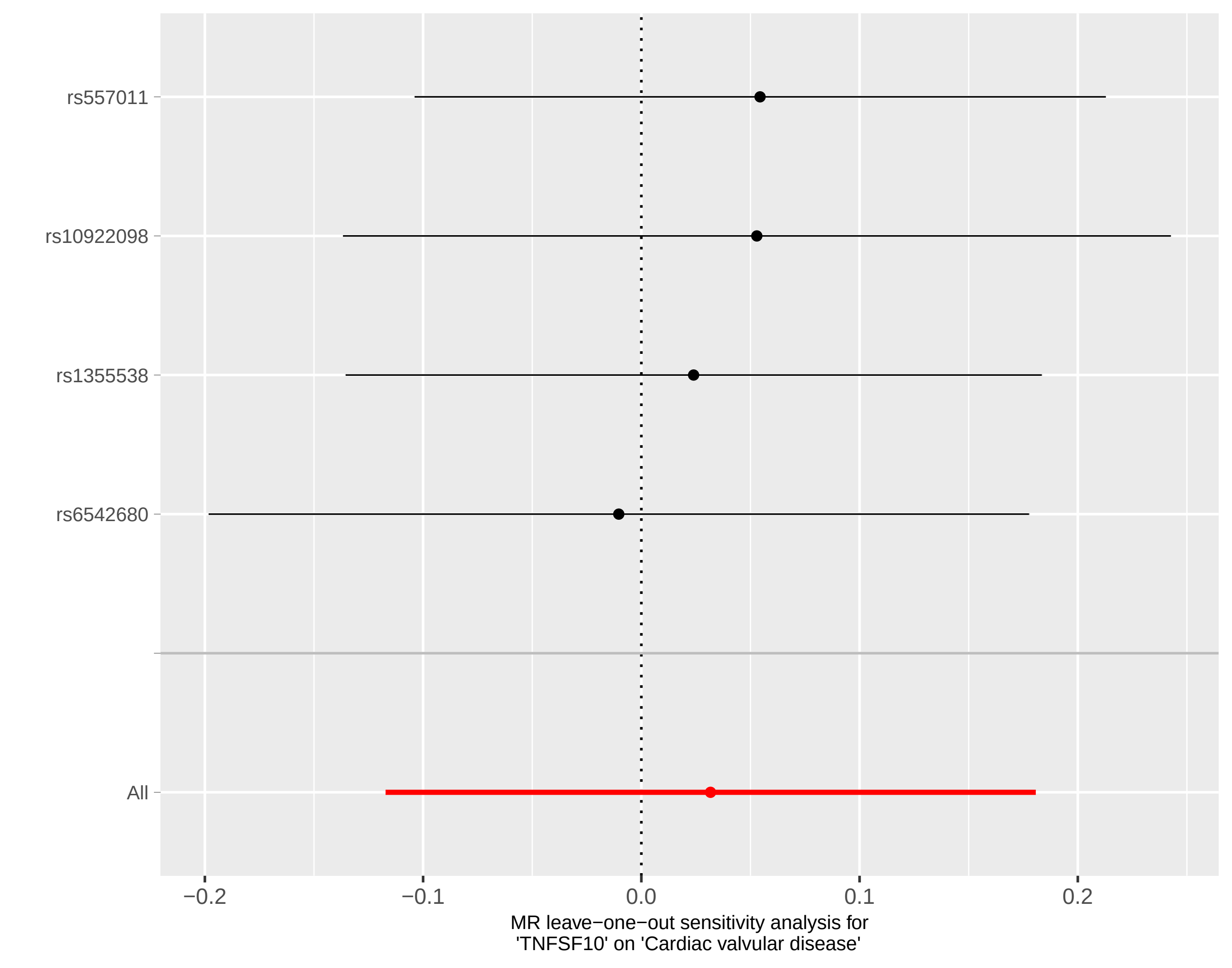
rs557011
rs10922098
rs1355538
rs6542680
All
−0.2
−0.1
0.0
0.1
0.2
MR leave−one−out sensitivity analysis for
'TNFSF10' on 'Cardiac valvular disease'

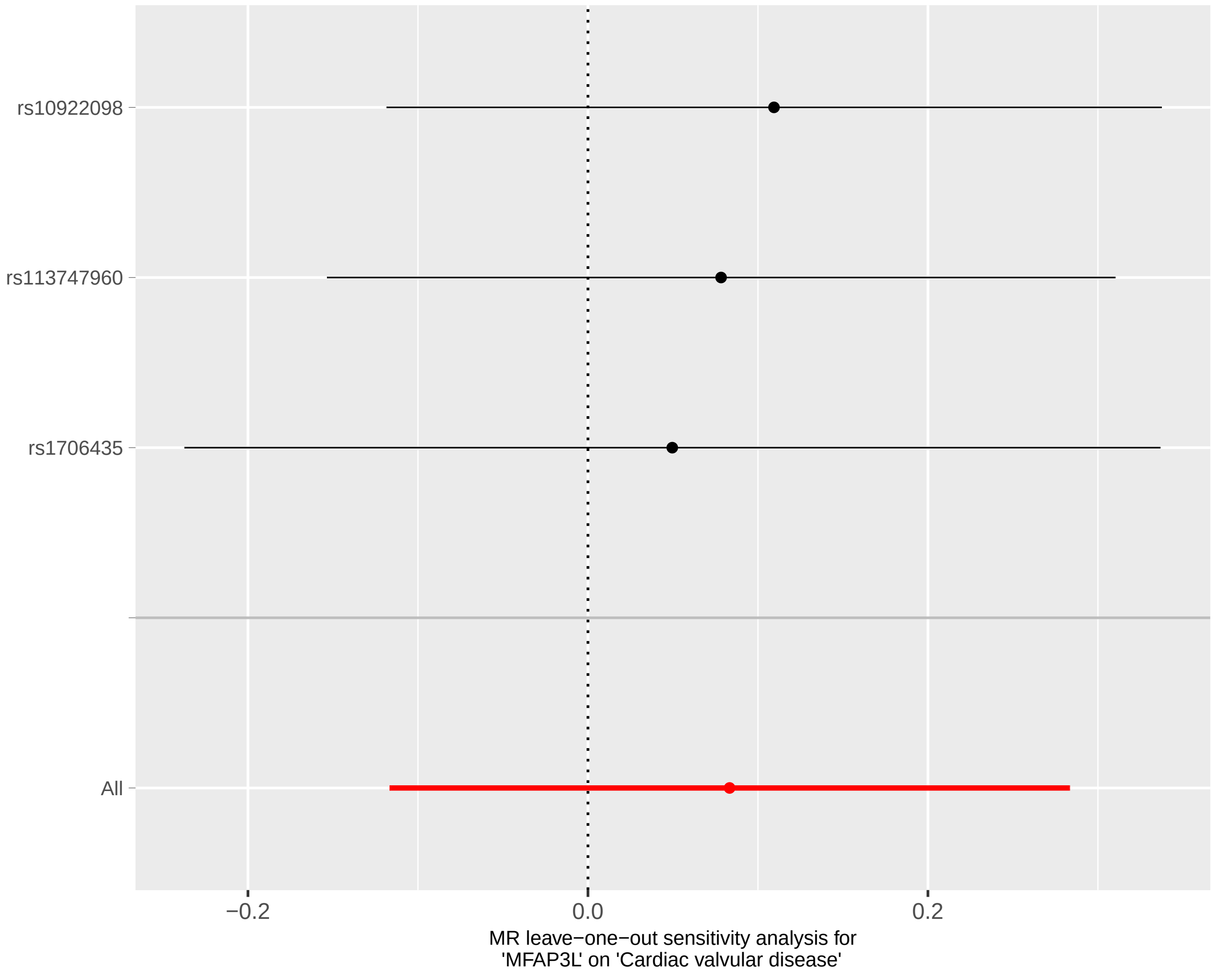
rs10922098
rs113747960
rs1706435
All
−0.2
0.0
0.2
MR leave−one−out sensitivity analysis for
'MFAP3L' on 'Cardiac valvular disease'

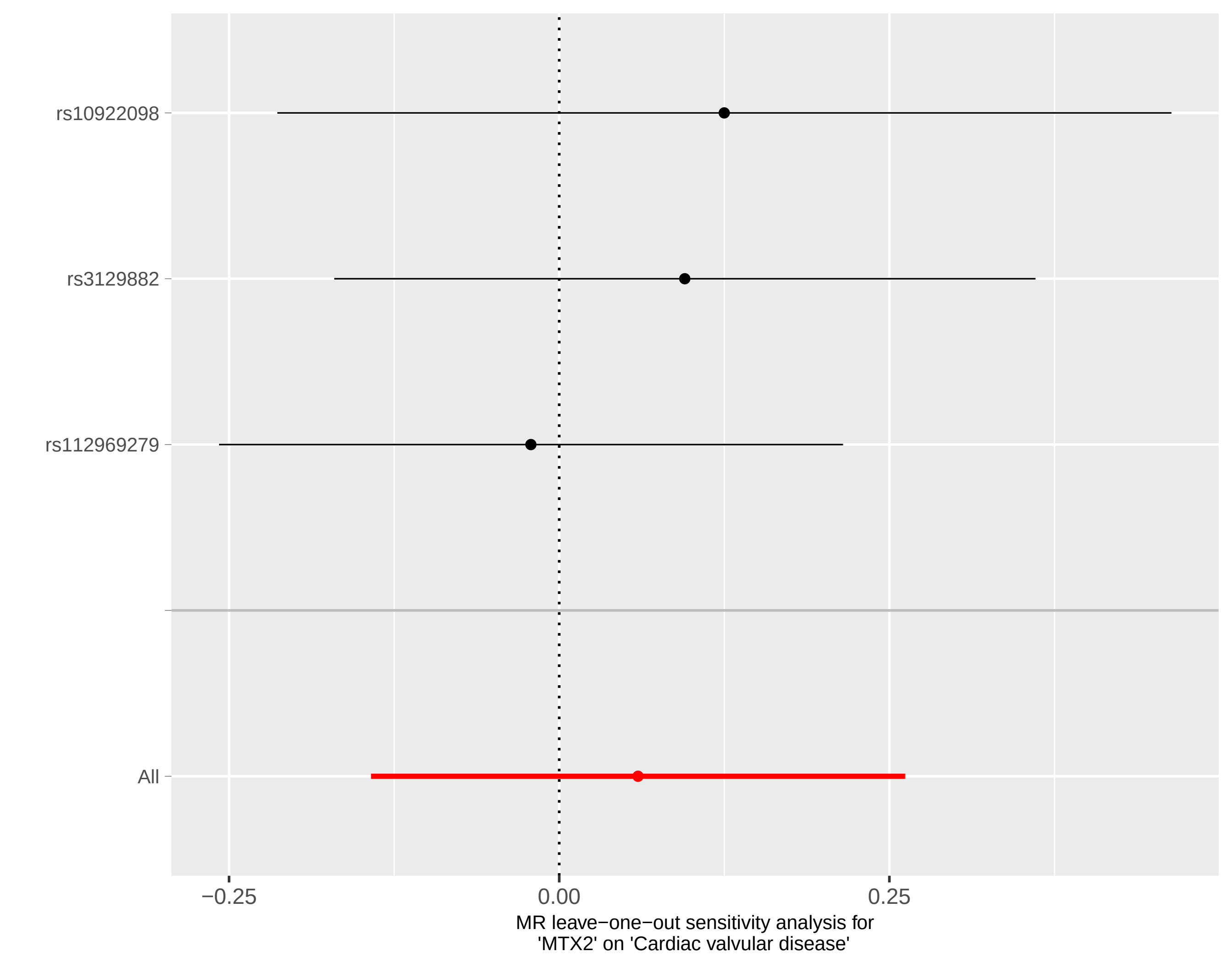
rs10922098
rs3129882
rs112969279
All
−0.25
0.00
0.25
MR leave−one−out sensitivity analysis for
'MTX2' on 'Cardiac valvular disease'

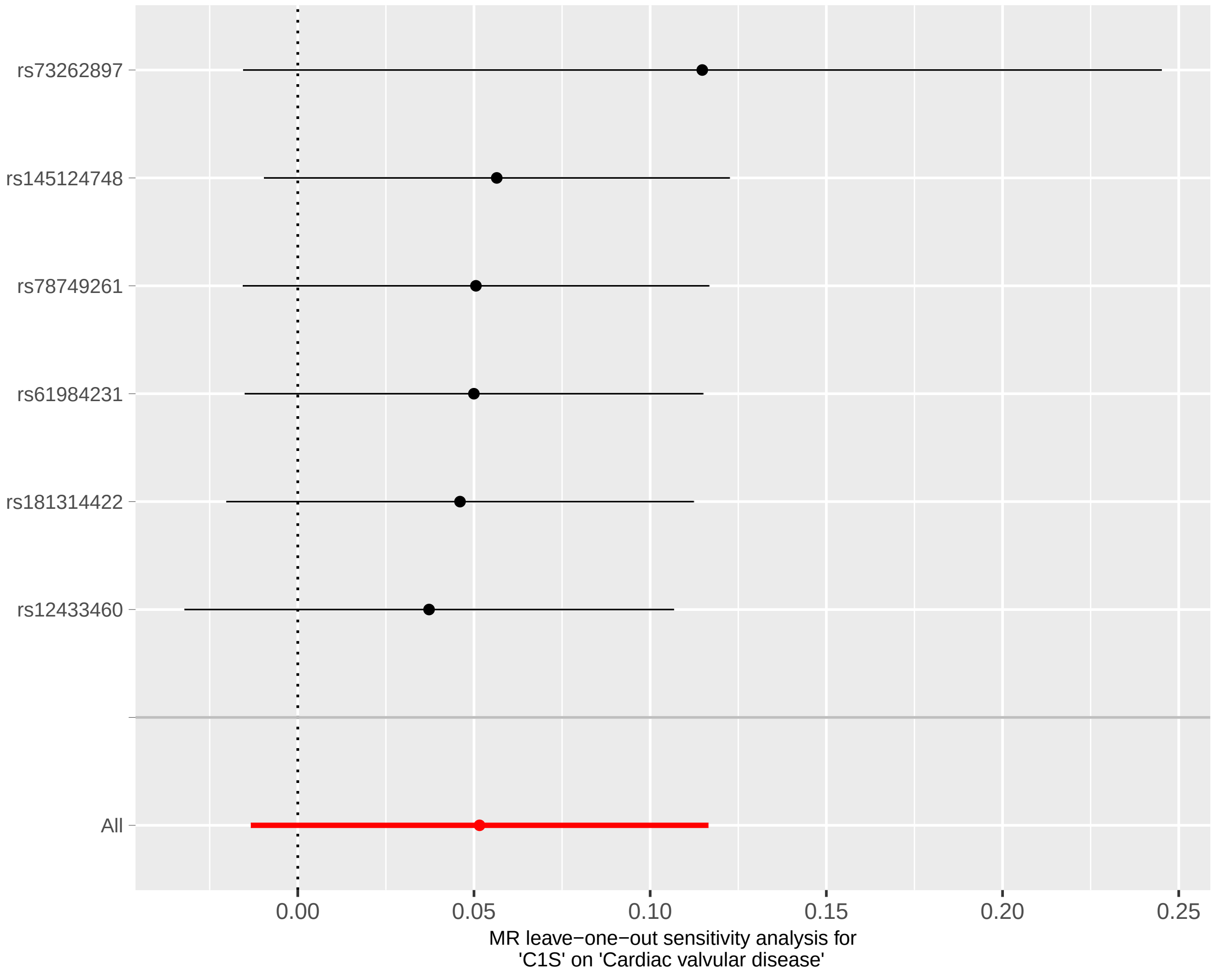
rs73262897
rs145124748
rs78749261
rs61984231
rs181314422
rs12433460
All
0.00
0.05
0.10
0.15
0.20
0.25
MR leave−one−out sensitivity analysis for
'C1S' on 'Cardiac valvular disease'

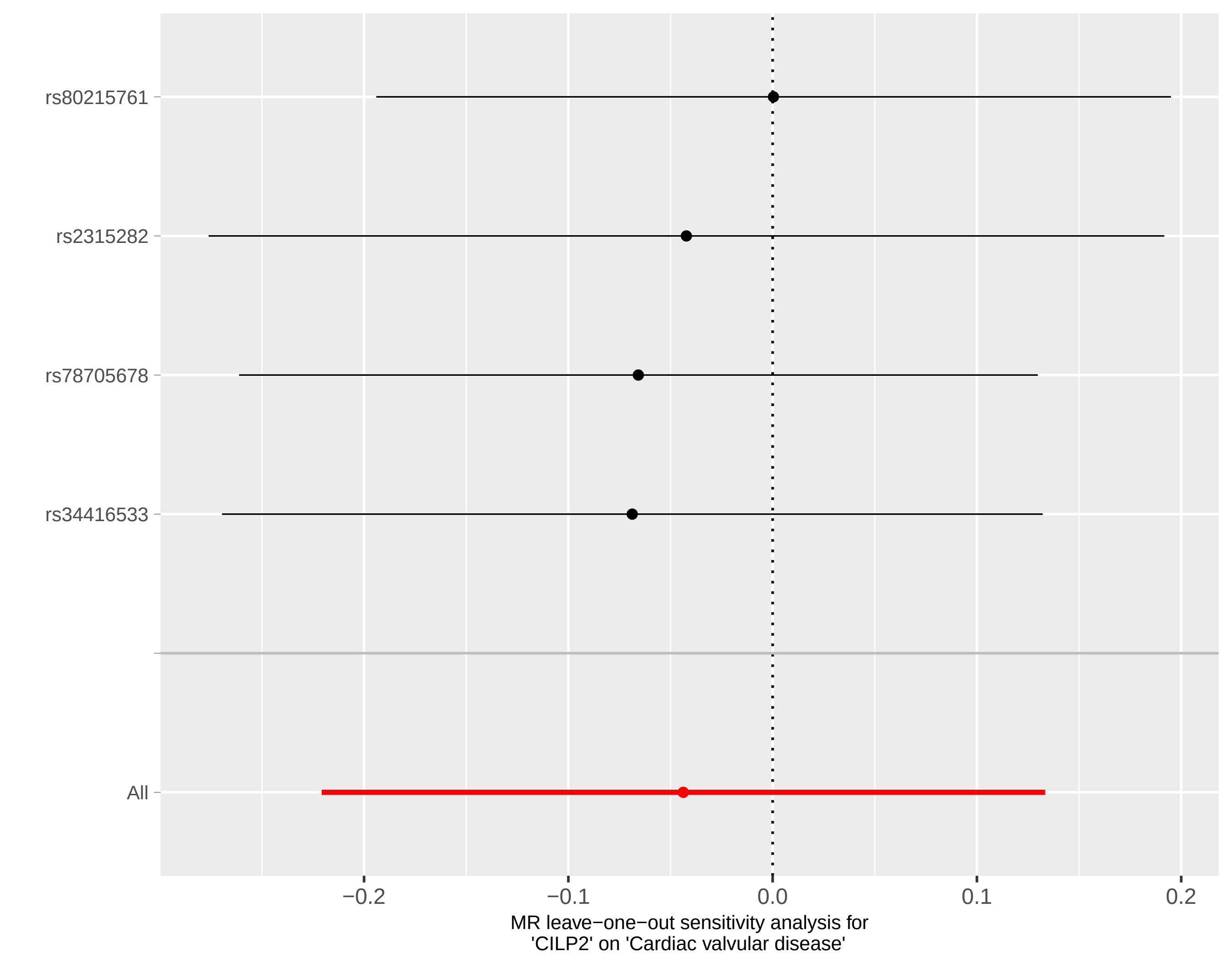
rs80215761
rs2315282
rs78705678
rs34416533
All
−0.2
−0.1
0.0
0.1
0.2
MR leave−one−out sensitivity analysis for
'CILP2' on 'Cardiac valvular disease'

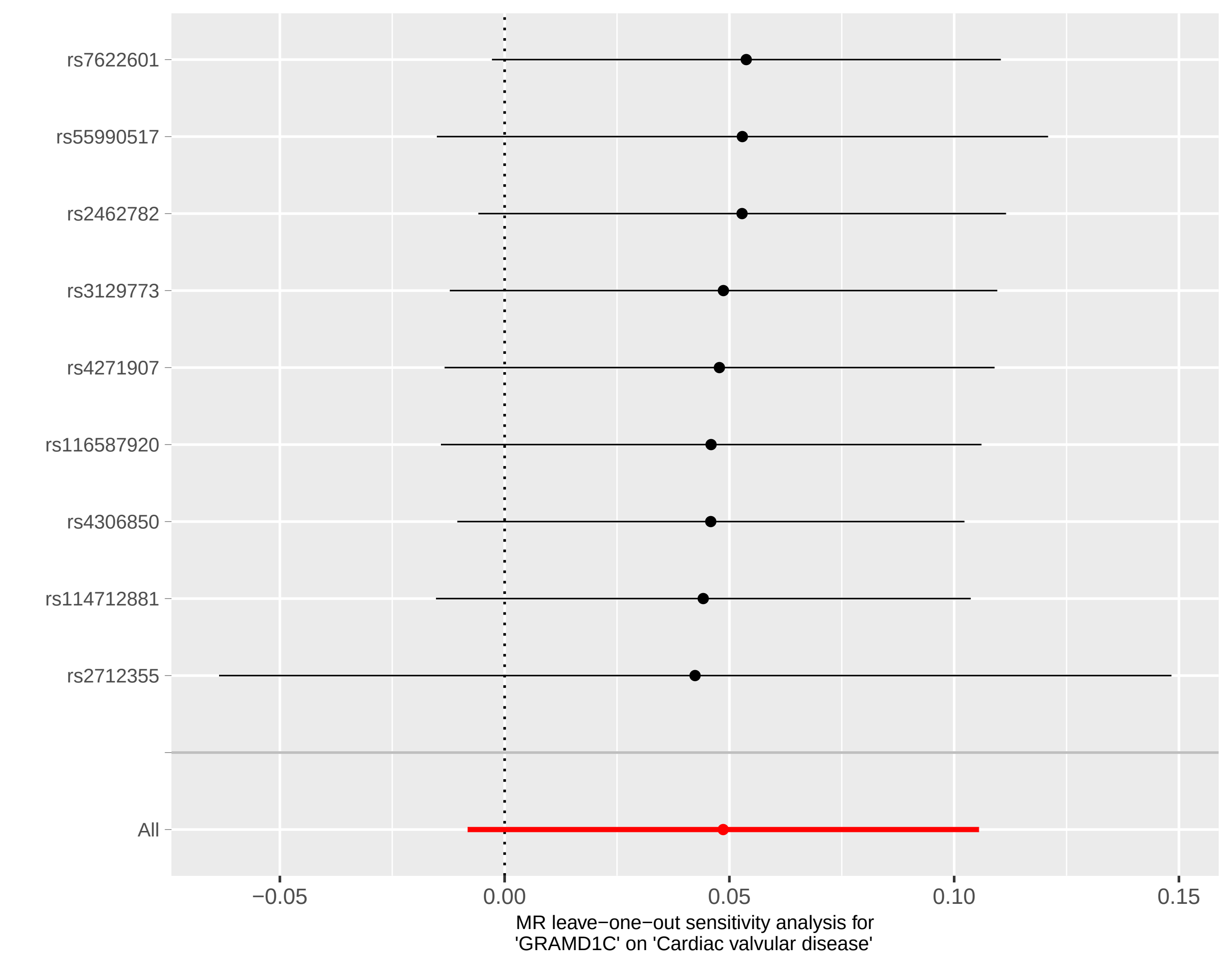
rs7622601
rs55990517
rs2462782
rs3129773
rs4271907
rs116587920
rs4306850
rs114712881
rs2712355
All
−0.05
0.00
0.05
0.10
0.15
MR leave−one−out sensitivity analysis for
'GRAMD1C' on 'Cardiac valvular disease'

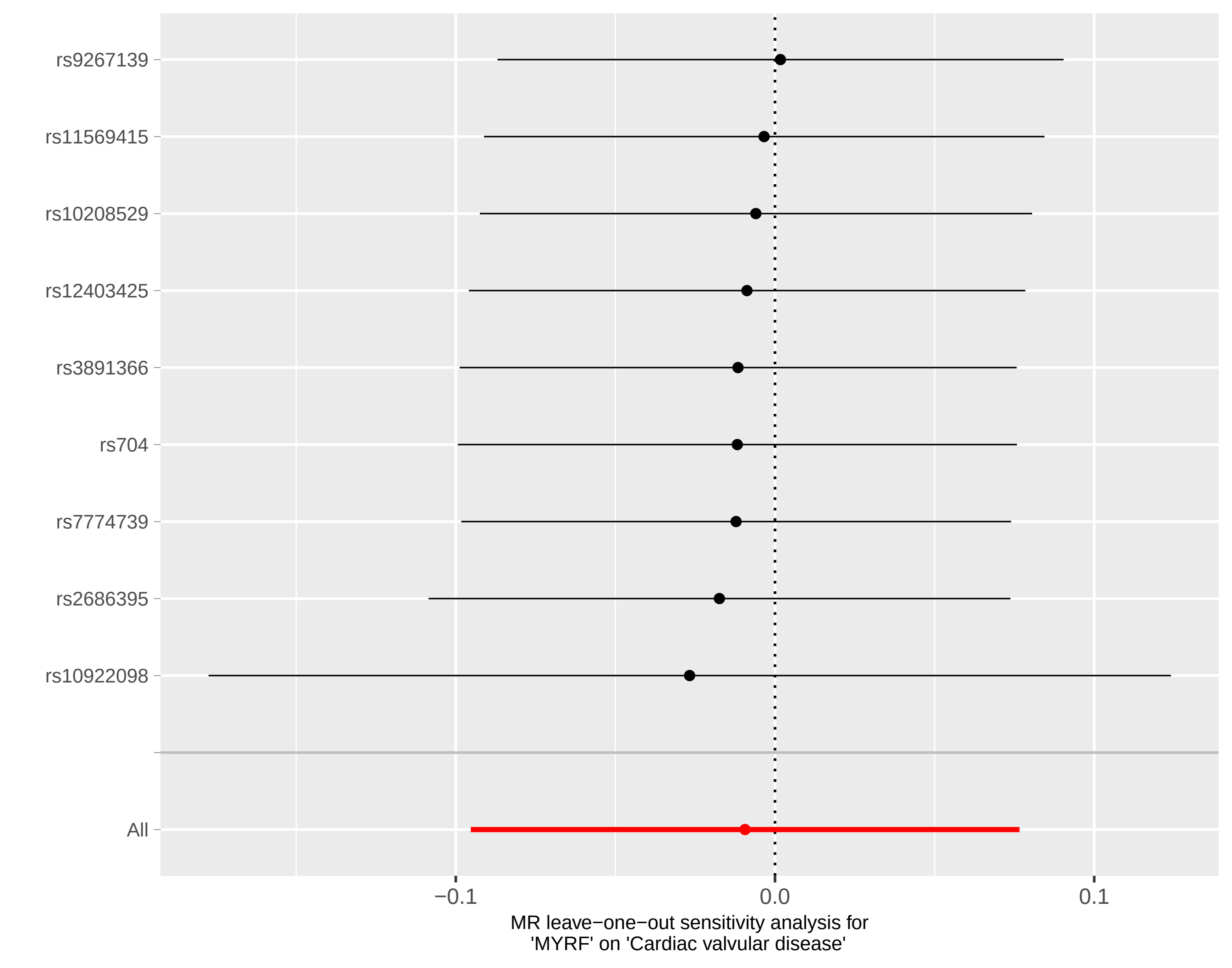

rs9267139
rs11569415
rs10208529
rs12403425
rs3891366
rs704
rs7774739
rs2686395
rs10922098
All
−0.1
0.0
0.1
MR leave−one−out sensitivity analysis for
'MYRF' on 'Cardiac valvular disease'

Insufficient number of SNPs

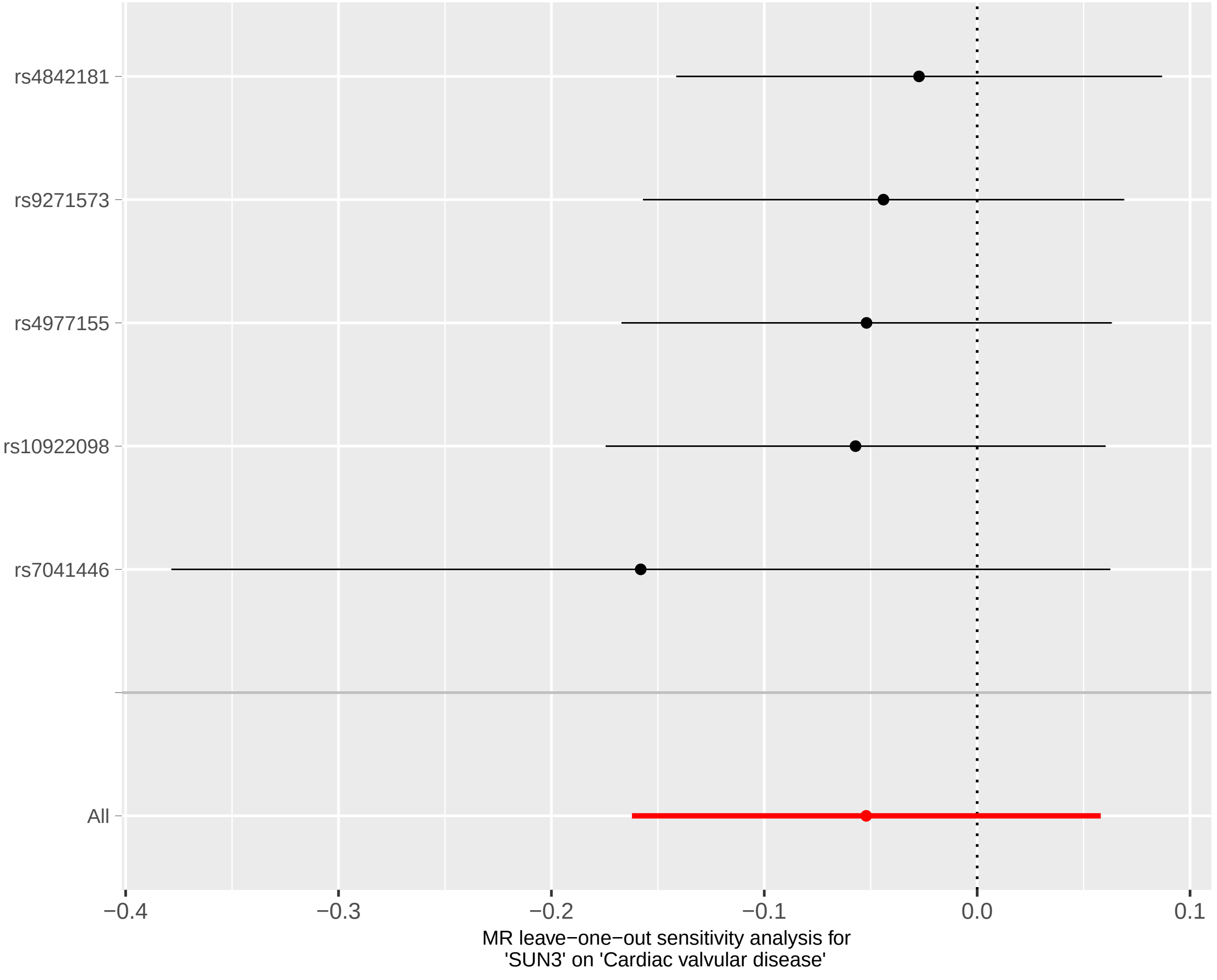
rs4842181
rs9271573
rs4977155
rs10922098
rs7041446
All
−0.4
−0.3
−0.2
−0.1
0.0
0.1
MR leave−one−out sensitivity analysis for
'SUN3' on 'Cardiac valvular disease'

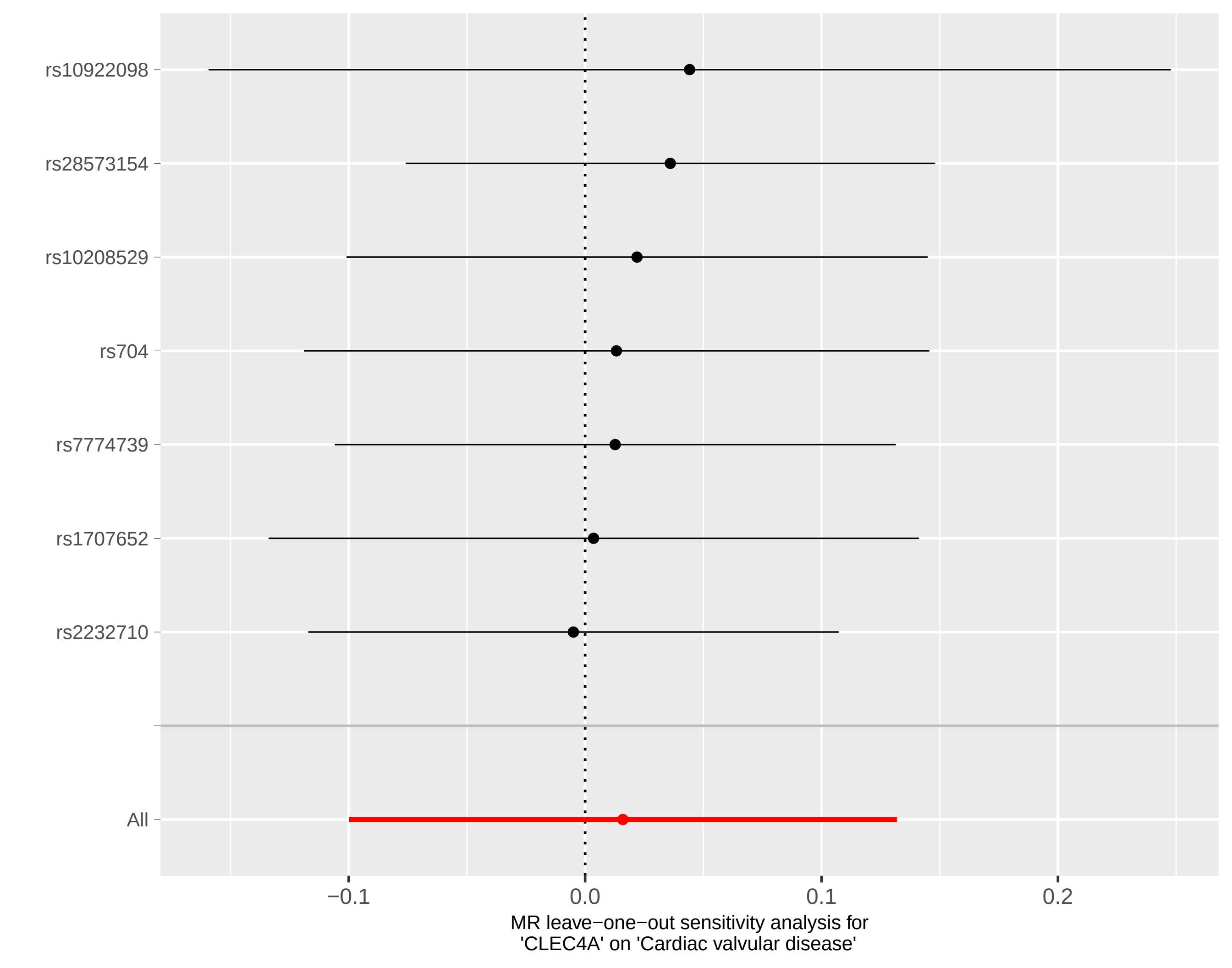
rs10922098
rs28573154
rs10208529
rs704
rs7774739
rs1707652
rs2232710
All
−0.1
0.0
0.1
0.2
MR leave−one−out sensitivity analysis for
'CLEC4A' on 'Cardiac valvular disease'

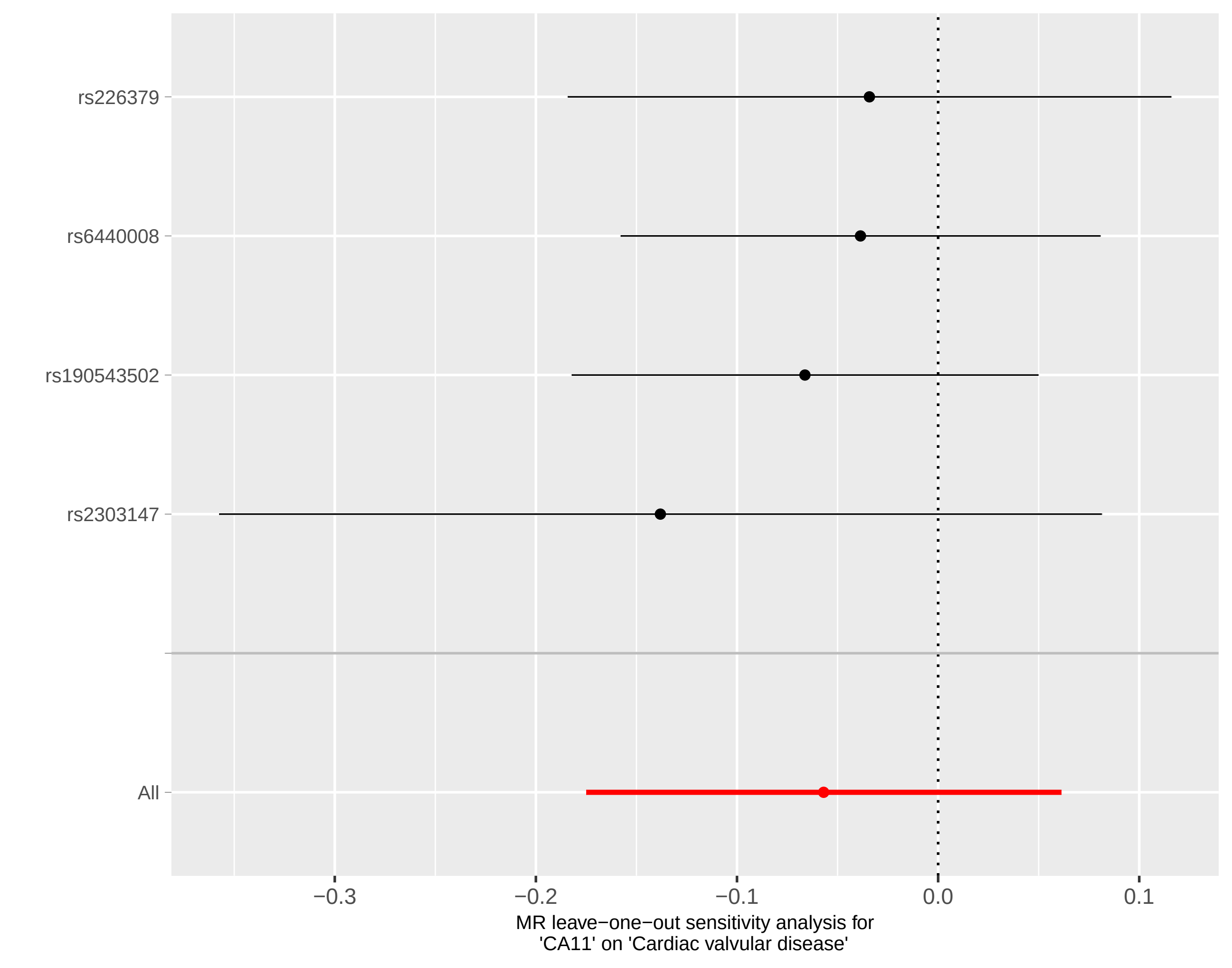

rs226379
rs6440008
rs190543502
rs2303147
All
−0.3
−0.2
−0.1
0.0
0.1
MR leave−one−out sensitivity analysis for
'CA11' on 'Cardiac valvular disease'

Insufficient number of SNPs

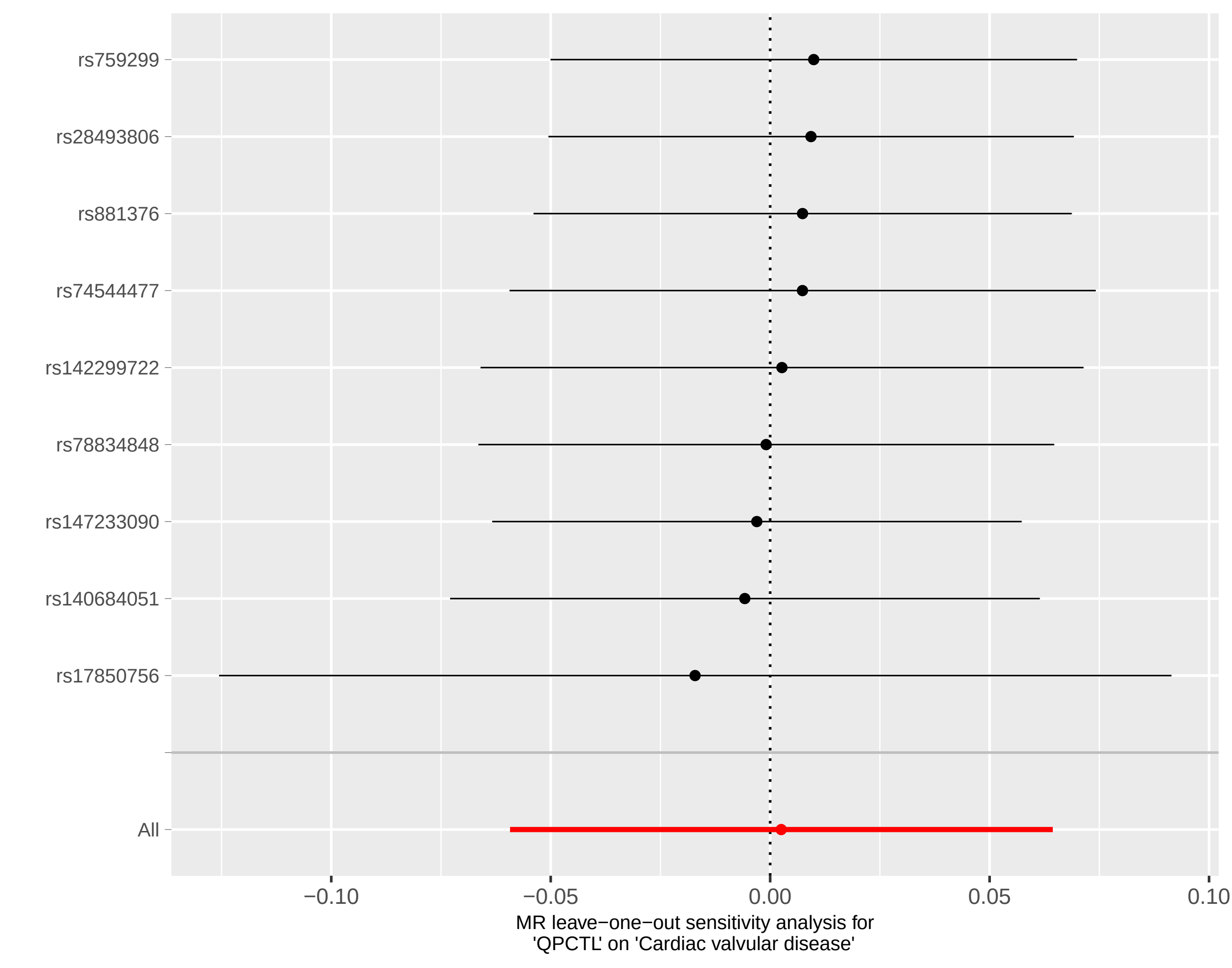

rs759299
rs28493806
rs881376
rs74544477
rs142299722
rs78834848
rs147233090
rs140684051
rs17850756
All
−0.10
−0.05
0.00
0.05
0.10
MR leave−one−out sensitivity analysis for
'QPCTL' on 'Cardiac valvular disease'

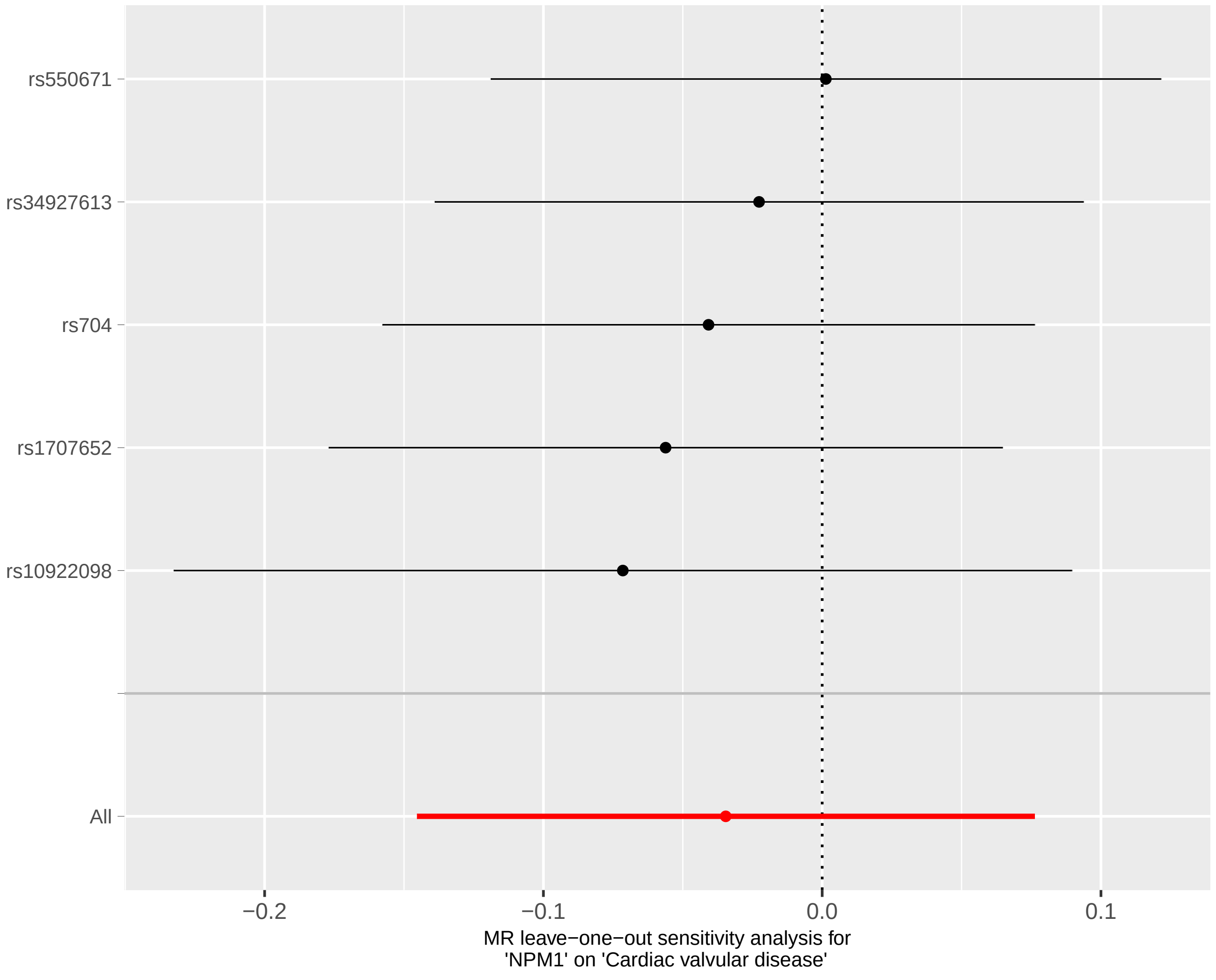

rs550671
rs34927613
rs704
rs1707652
rs10922098
All
−0.2
−0.1
0.0
0.1
MR leave−one−out sensitivity analysis for
'NPM1' on 'Cardiac valvular disease'

Insufficient number of SNPs

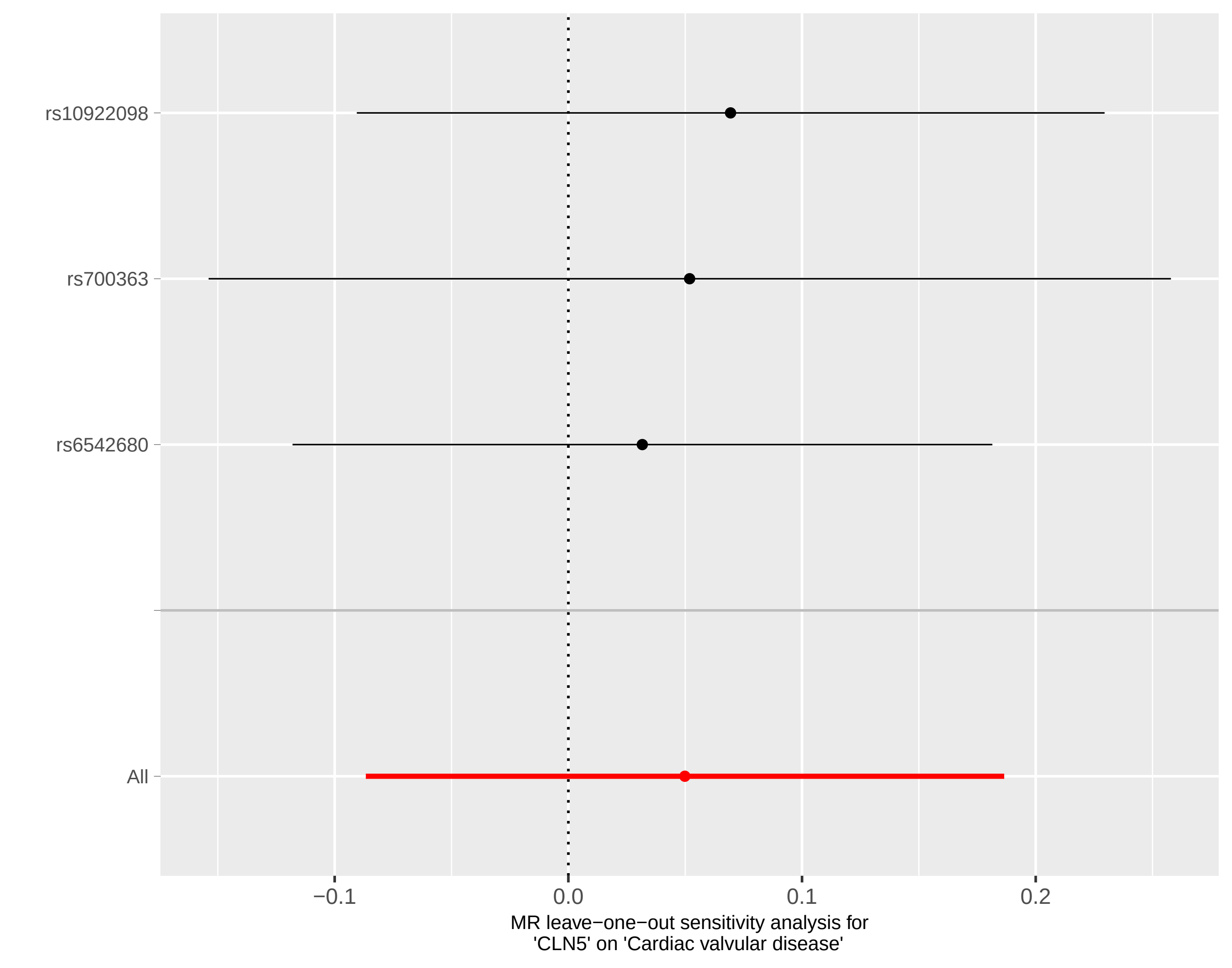

rs10922098
rs700363
rs6542680
All
−0.1
0.0
0.1
0.2
MR leave−one−out sensitivity analysis for
'CLN5' on 'Cardiac valvular disease'

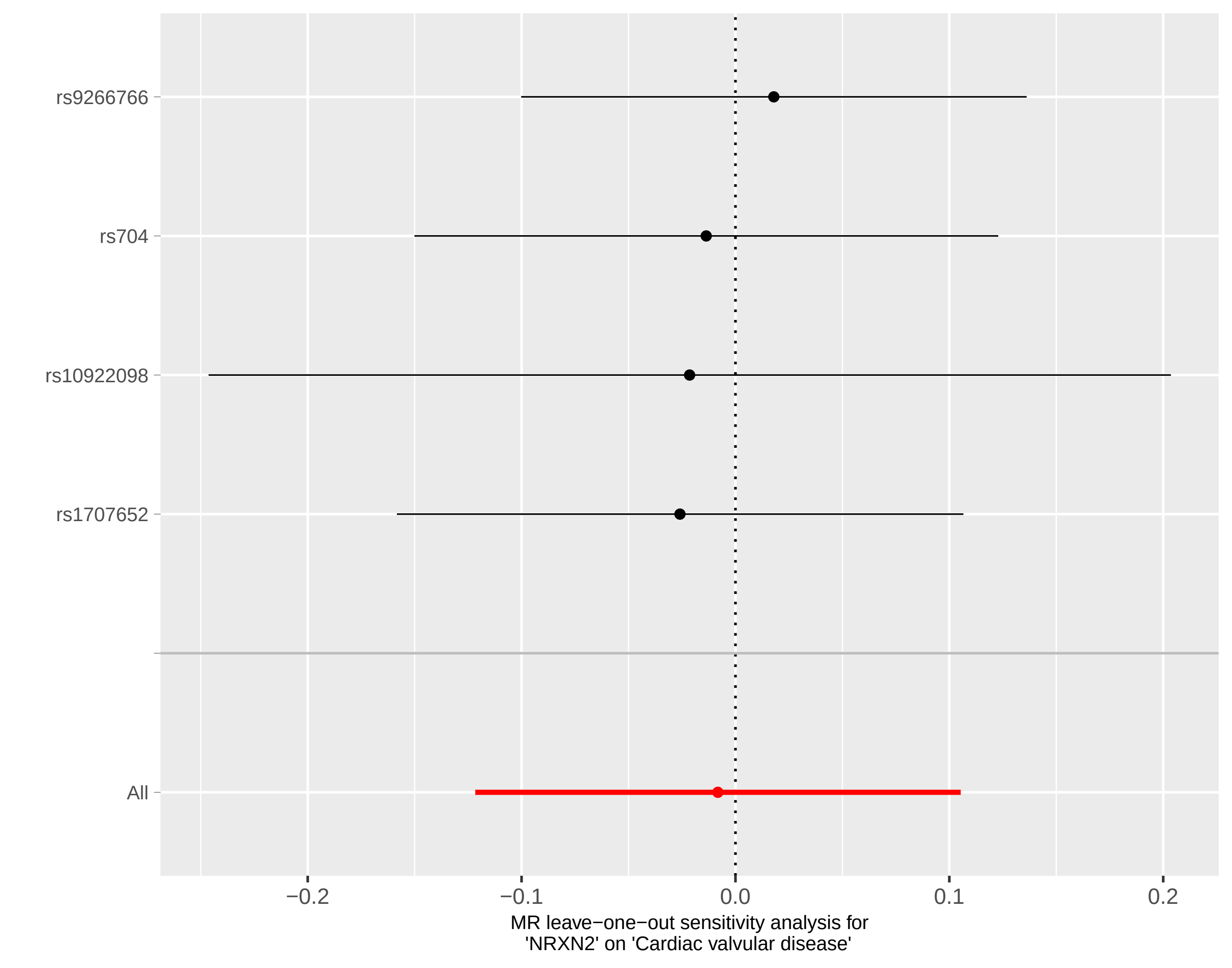

rs9266766
rs704
rs10922098
rs1707652
All
−0.2
−0.1
0.0
0.1
0.2
MR leave−one−out sensitivity analysis for
'NRXN2' on 'Cardiac valvular disease'

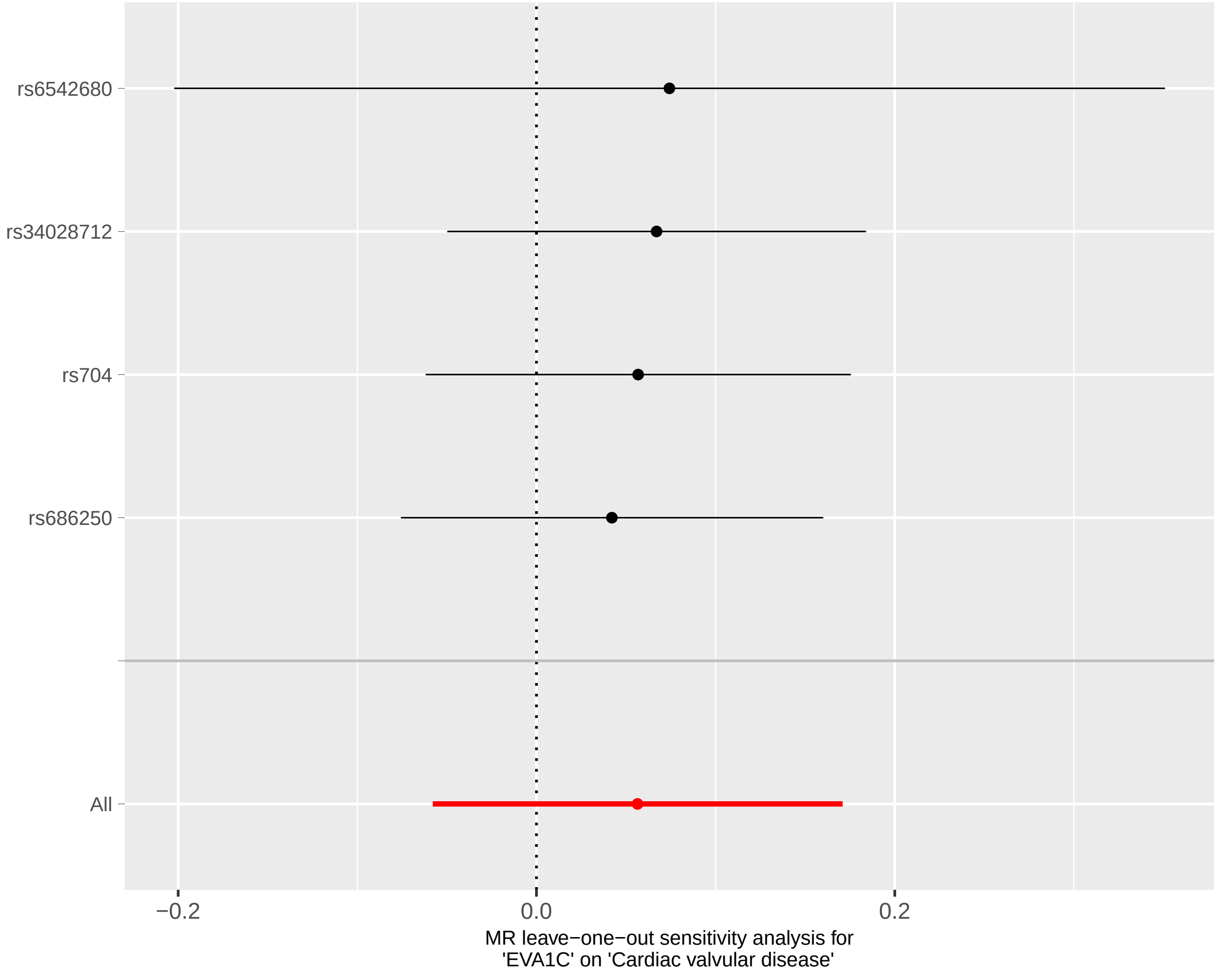
rs6542680
rs34028712
rs704
rs686250
All
−0.2
0.0
0.2
MR leave−one−out sensitivity analysis for
'EVA1C' on 'Cardiac valvular disease'

Insufficient number of SNPs

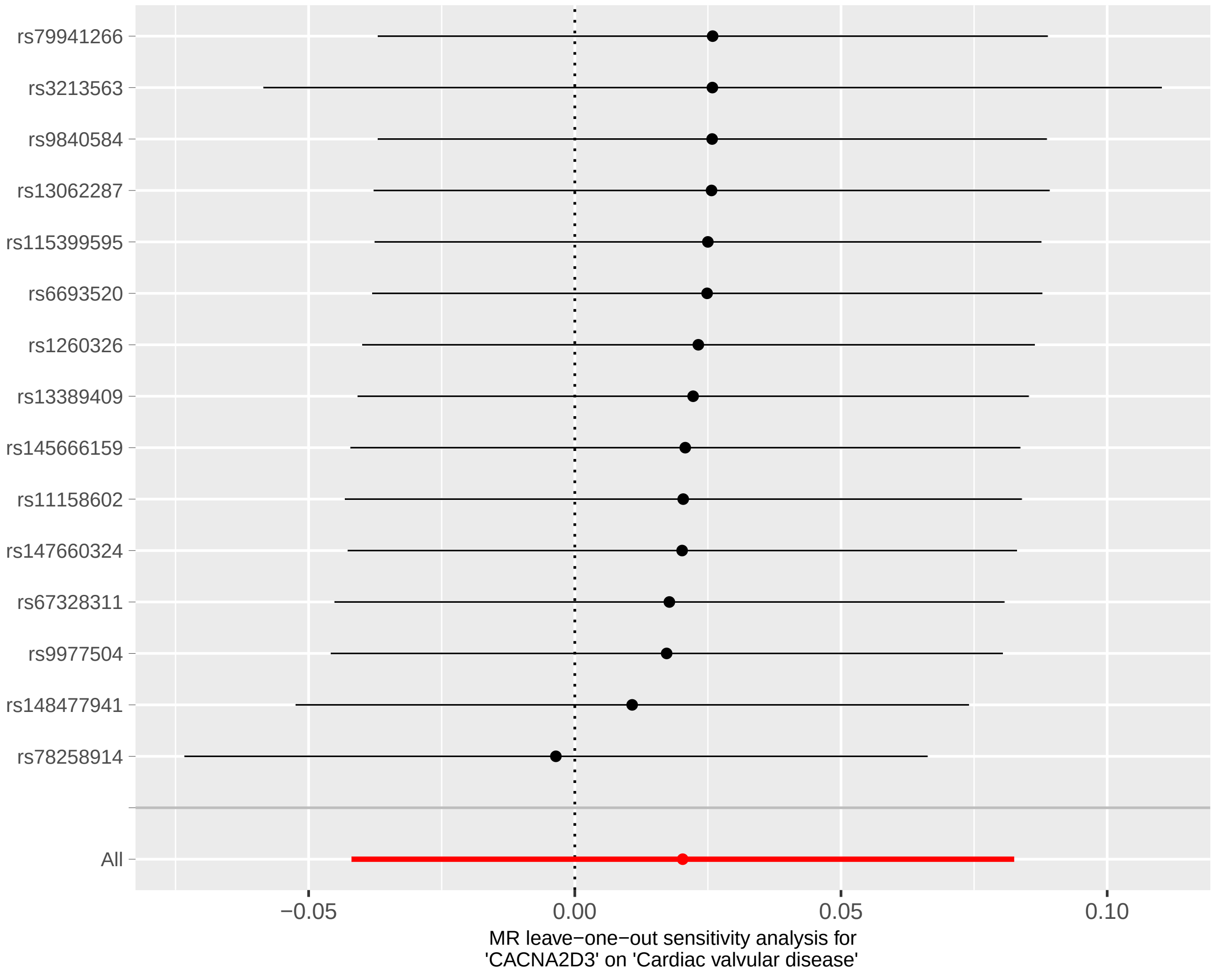
rs79941266
rs3213563
rs9840584
rs13062287
rs115399595
rs6693520
rs1260326
rs13389409
rs145666159
rs11158602
rs147660324
rs67328311
rs9977504
rs148477941
rs78258914
All
−0.05
0.00
0.05
0.10
MR leave−one−out sensitivity analysis for
'CACNA2D3' on 'Cardiac valvular disease'

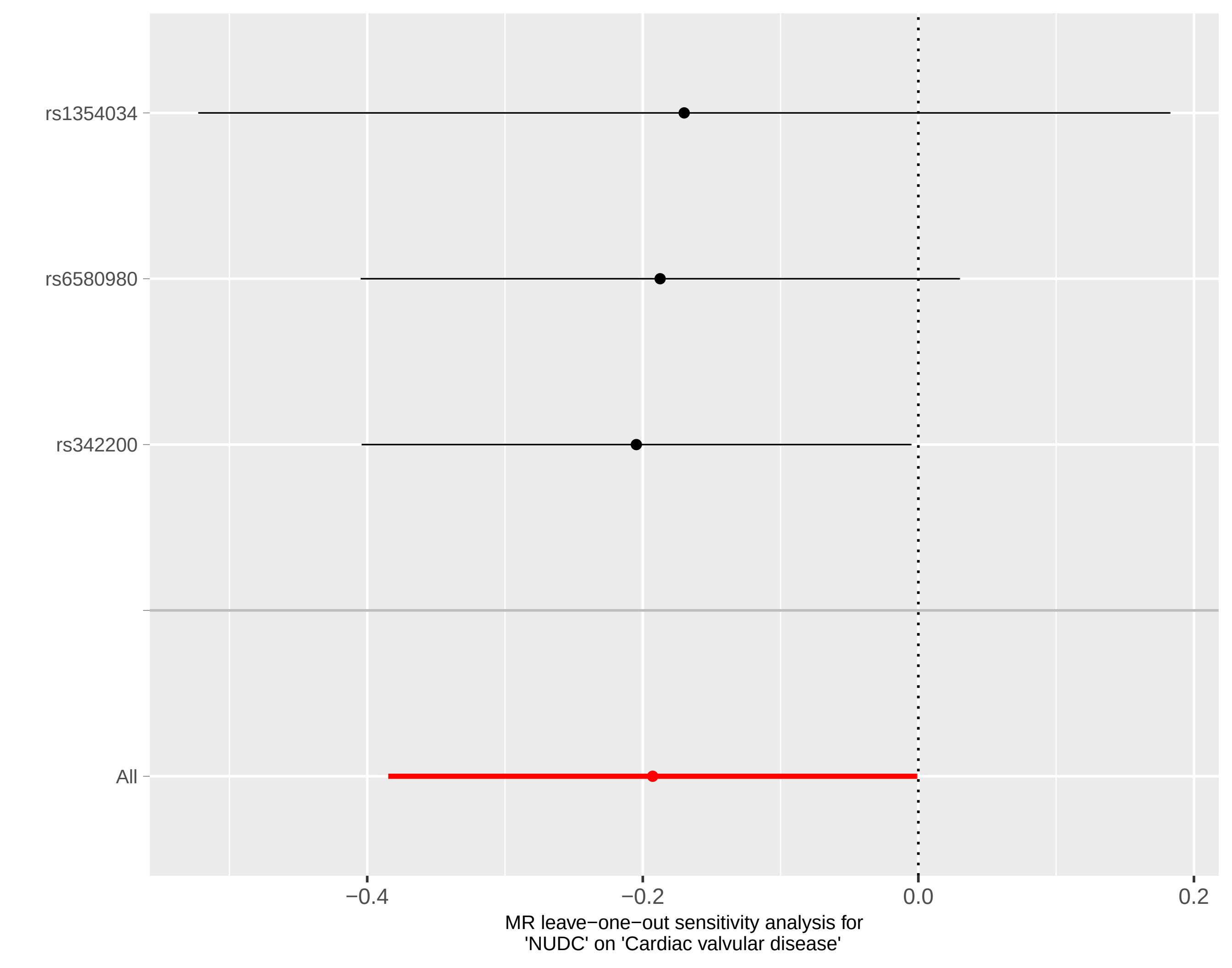

rs1354034
rs6580980
rs342200
All
−0.4
−0.2
0.0
0.2
MR leave−one−out sensitivity analysis for
'NUDC' on 'Cardiac valvular disease'

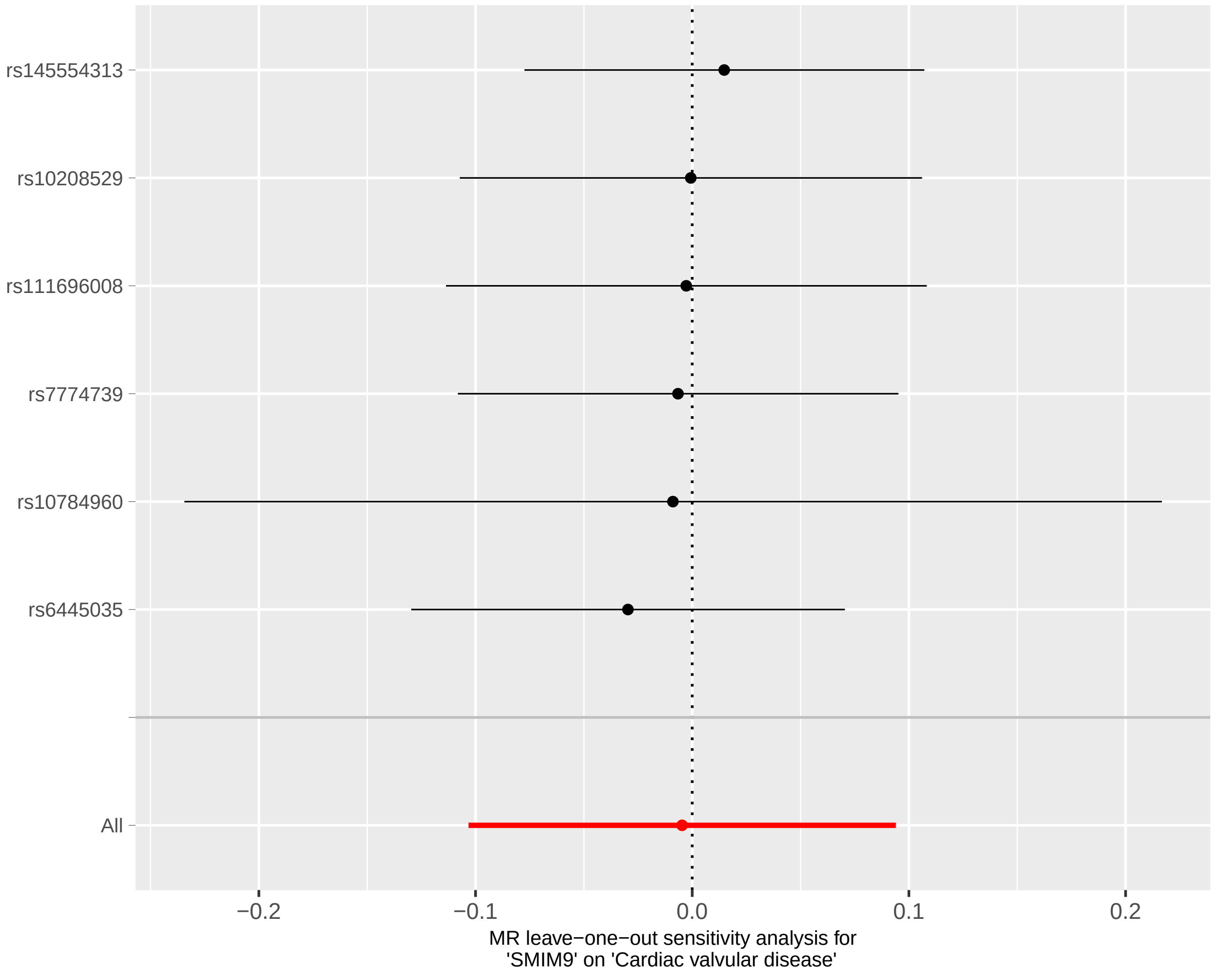

rs145554313
rs10208529
rs111696008
rs7774739
rs10784960
rs6445035
All
−0.2
−0.1
0.0
0.1
0.2
MR leave−one−out sensitivity analysis for
'SMIM9' on 'Cardiac valvular disease'

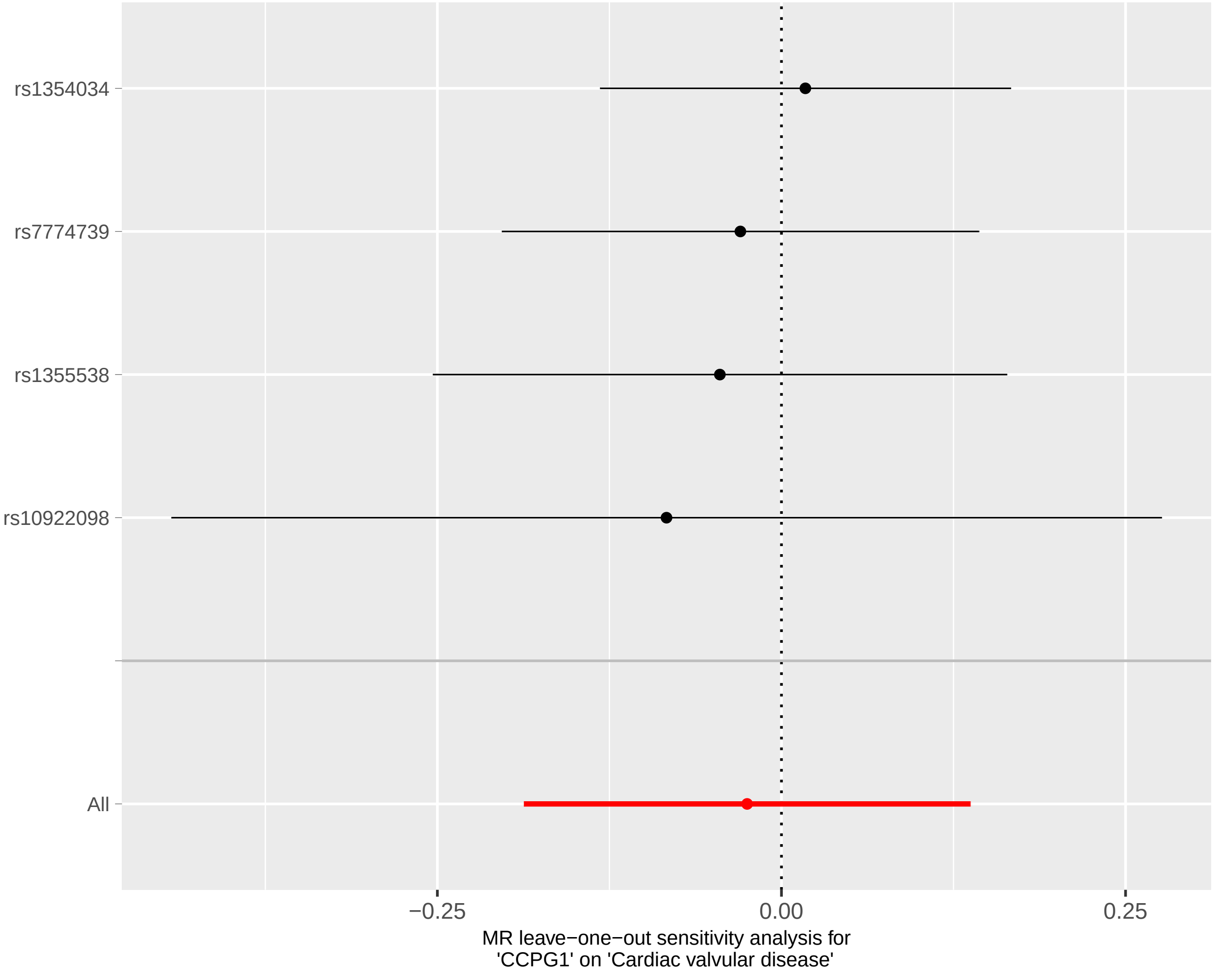

rs1354034
rs7774739
rs1355538
rs10922098
All
−0.25
0.00
0.25
MR leave−one−out sensitivity analysis for
'CCPG1' on 'Cardiac valvular disease'

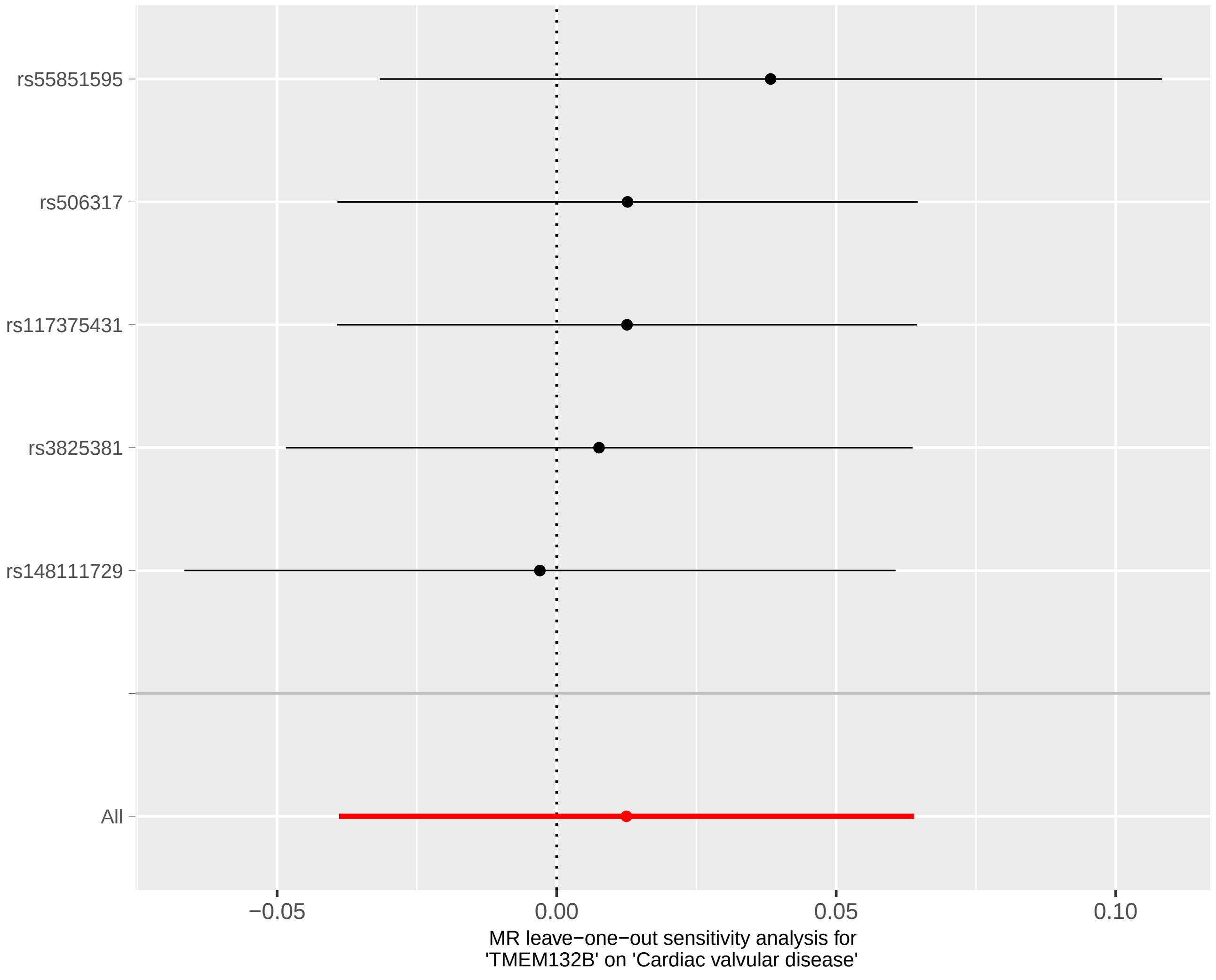
rs55851595
rs506317
rs117375431
rs3825381
rs148111729
All
−0.05
0.00
0.05
0.10
MR leave−one−out sensitivity analysis for
'TMEM132B' on 'Cardiac valvular disease'

Insufficient number of SNPs

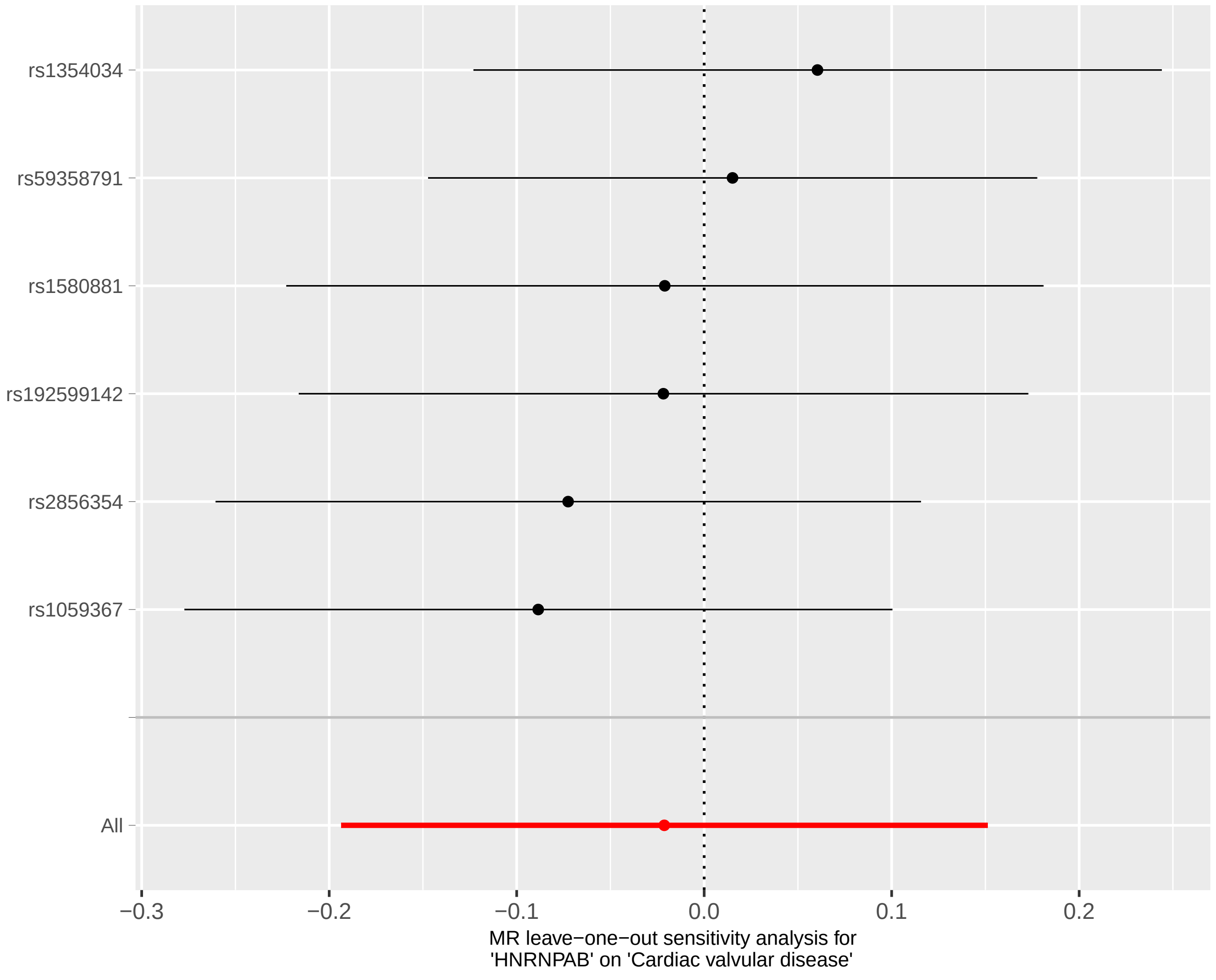
rs1354034
rs59358791
rs1580881
rs192599142
rs2856354
rs1059367
All
−0.3
−0.2
−0.1
0.0
0.1
0.2
MR leave−one−out sensitivity analysis for
'HNRNPAB' on 'Cardiac valvular disease'

Insufficient number of SNPs

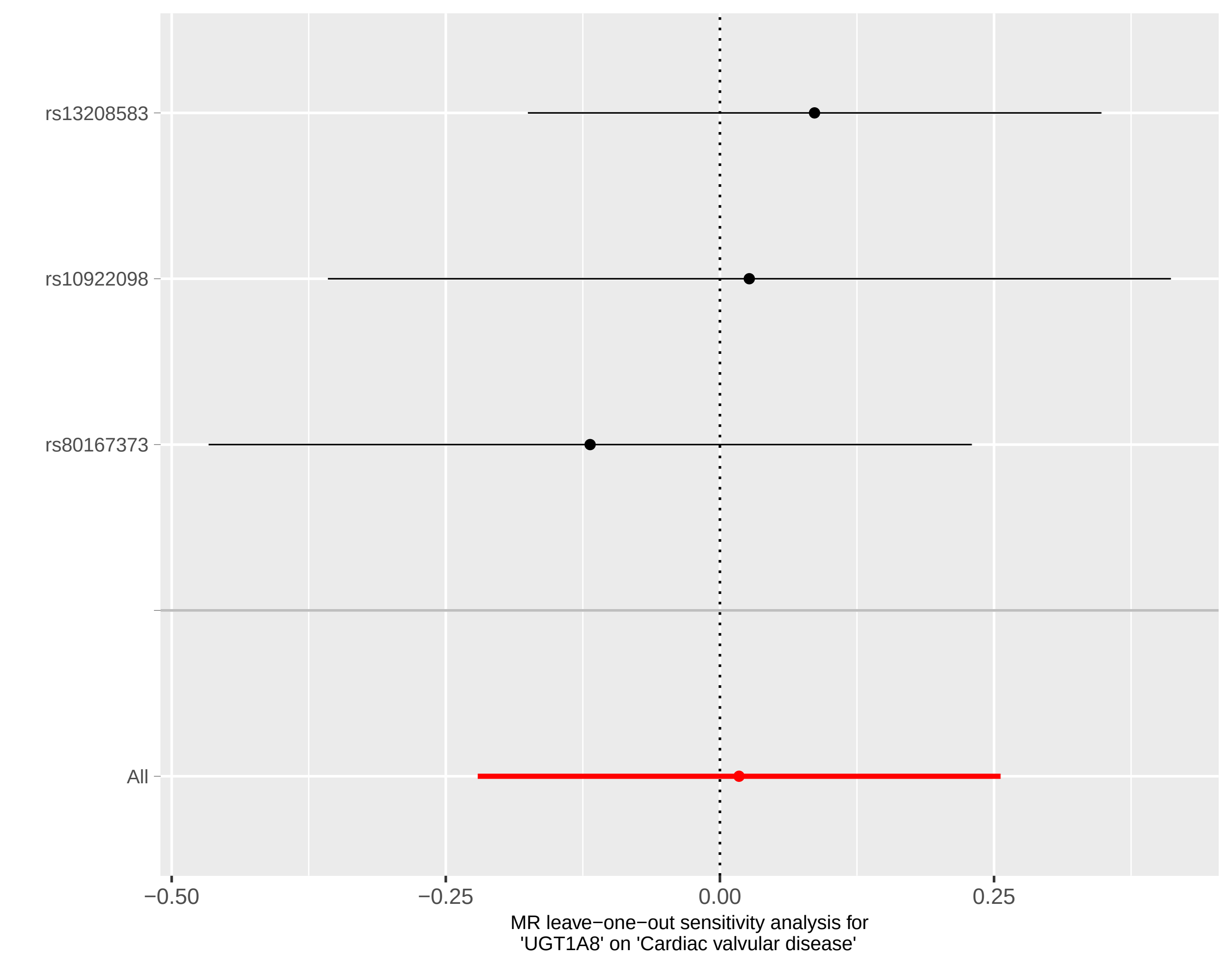
rs13208583
rs10922098
rs80167373
All
−0.50
−0.25
0.00
0.25
MR leave−one−out sensitivity analysis for
'UGT1A8' on 'Cardiac valvular disease'

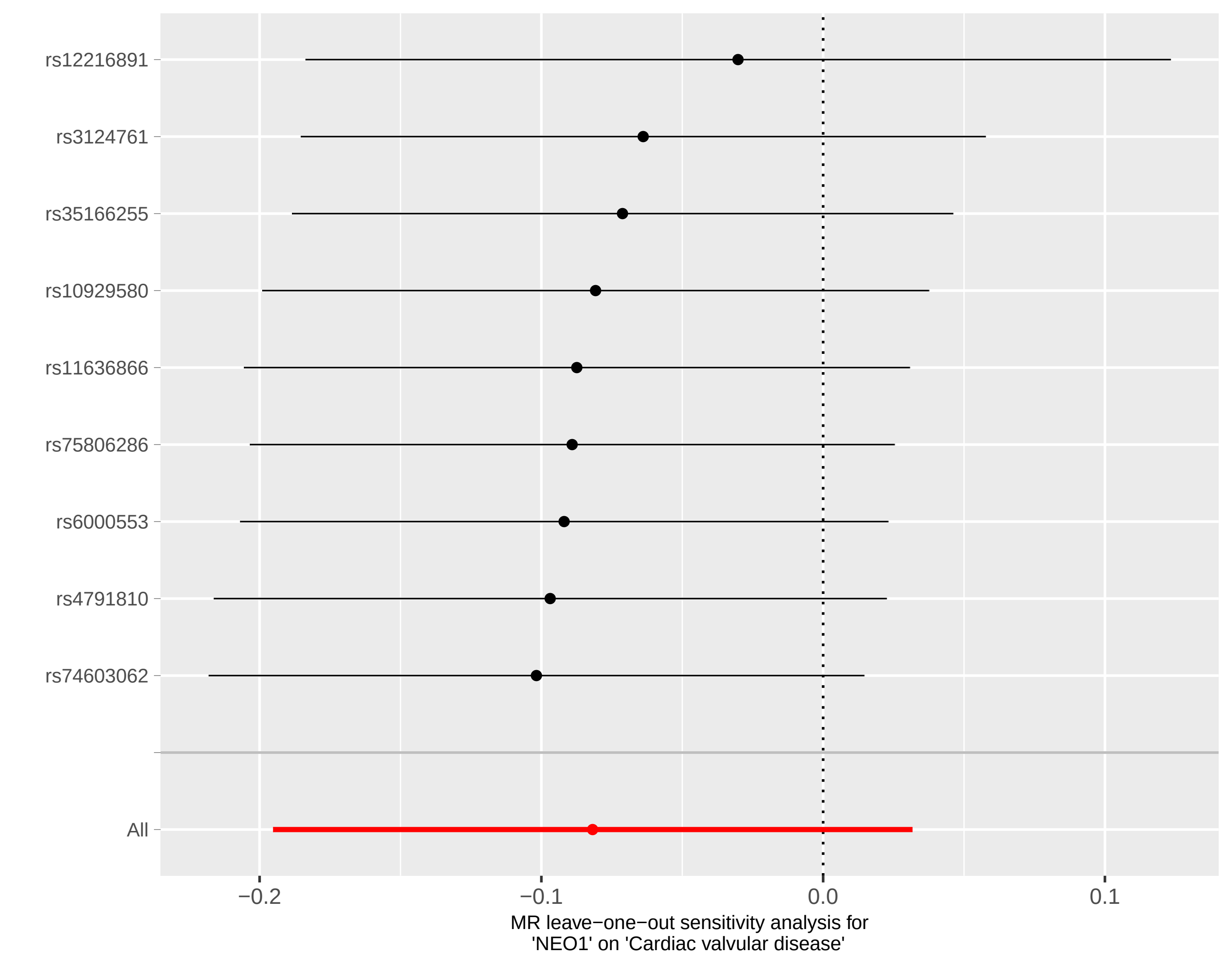
rs12216891
rs3124761
rs35166255
rs10929580
rs11636866
rs75806286
rs6000553
rs4791810
rs74603062
All
−0.2
−0.1
0.0
0.1
MR leave−one−out sensitivity analysis for
'NEO1' on 'Cardiac valvular disease'

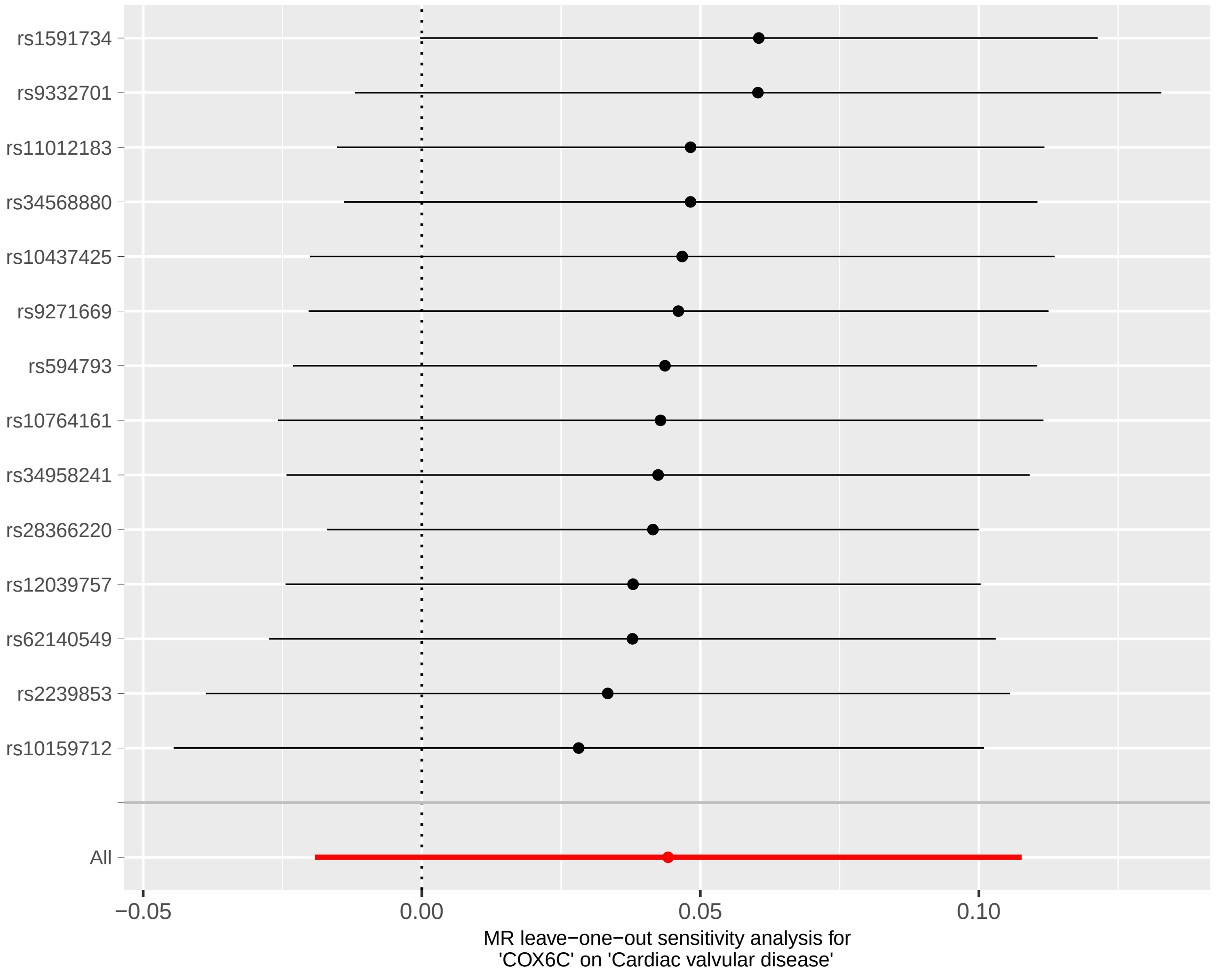

rs1591734
rs9332701
rs11012183
rs34568880
rs10437425
rs9271669
rs594793
rs10764161
rs34958241
rs28366220
rs12039757
rs62140549
rs2239853
rs10159712
All
−0.05
0.00
0.05
0.10
MR leave−one−out sensitivity analysis for
'COX6C' on 'Cardiac valvular disease'

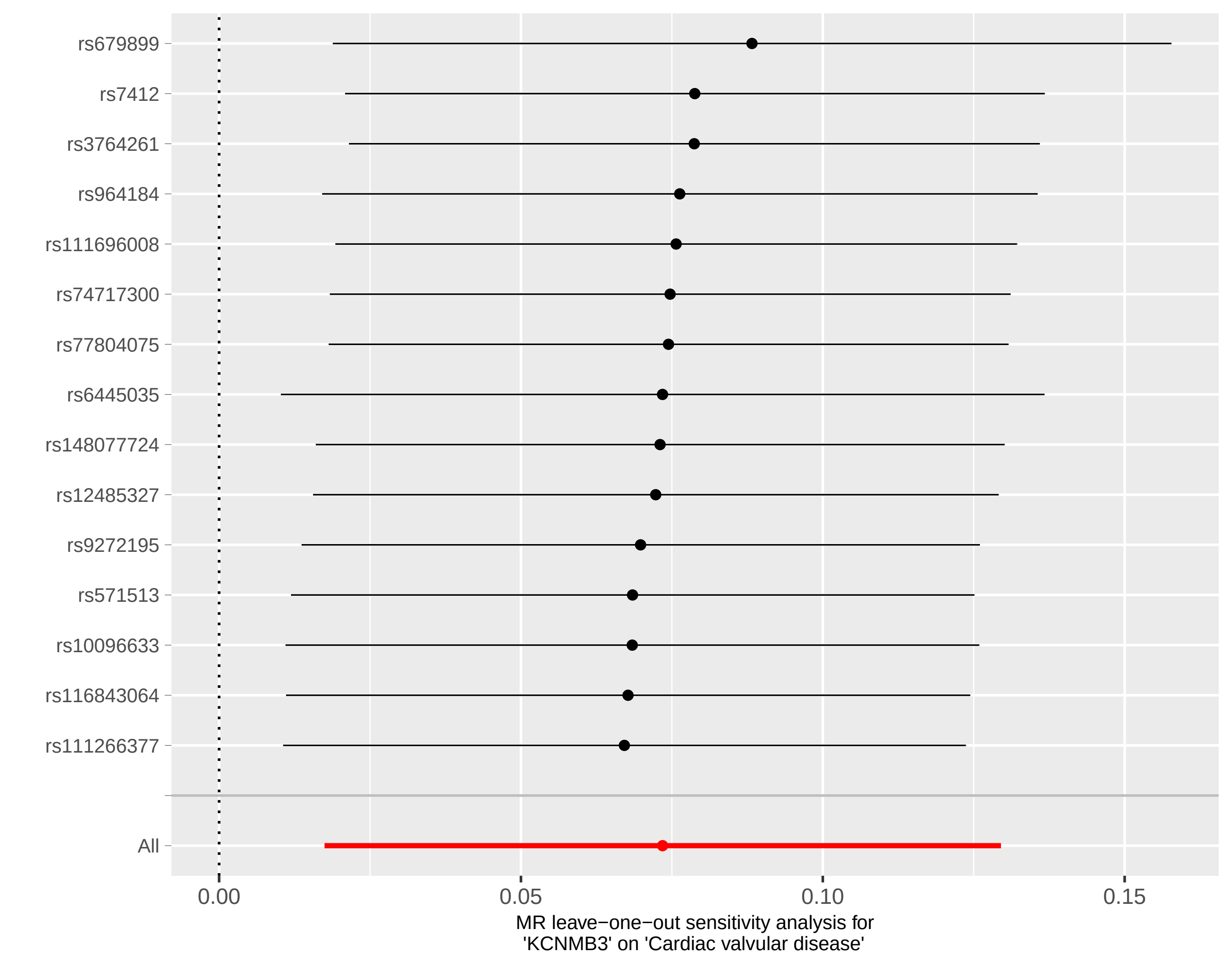

rs679899
rs7412
rs3764261
rs964184
rs111696008
rs74717300
rs77804075
rs6445035
rs148077724
rs12485327
rs9272195
rs571513
rs10096633
rs116843064
rs111266377
All
0.00
0.05
0.10
0.15
MR leave−one−out sensitivity analysis for
'KCNMB3' on 'Cardiac valvular disease'

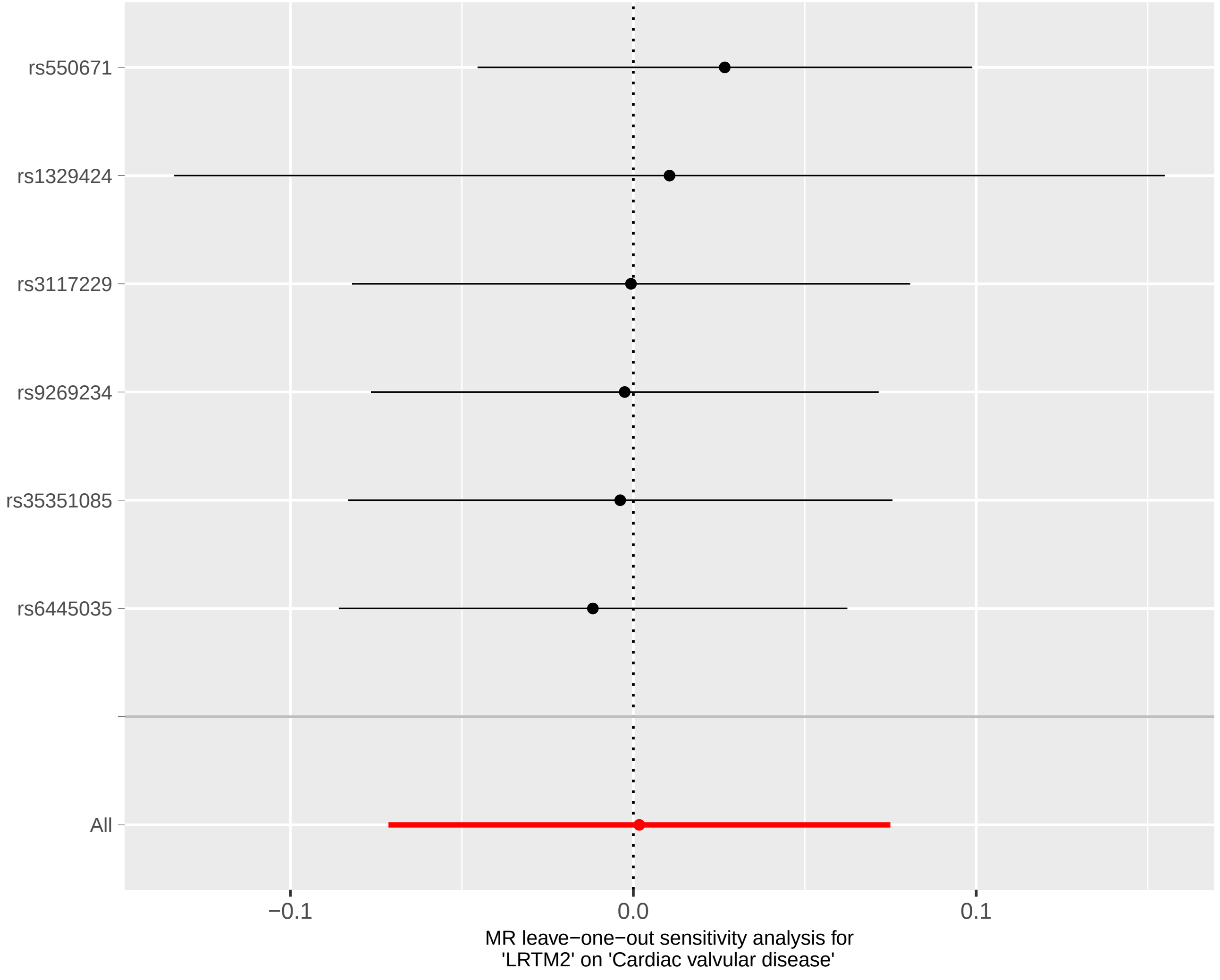
rs550671
rs1329424
rs3117229
rs9269234
rs35351085
rs6445035
All
−0.1
0.0
0.1
MR leave−one−out sensitivity analysis for
'LRTM2' on 'Cardiac valvular disease'

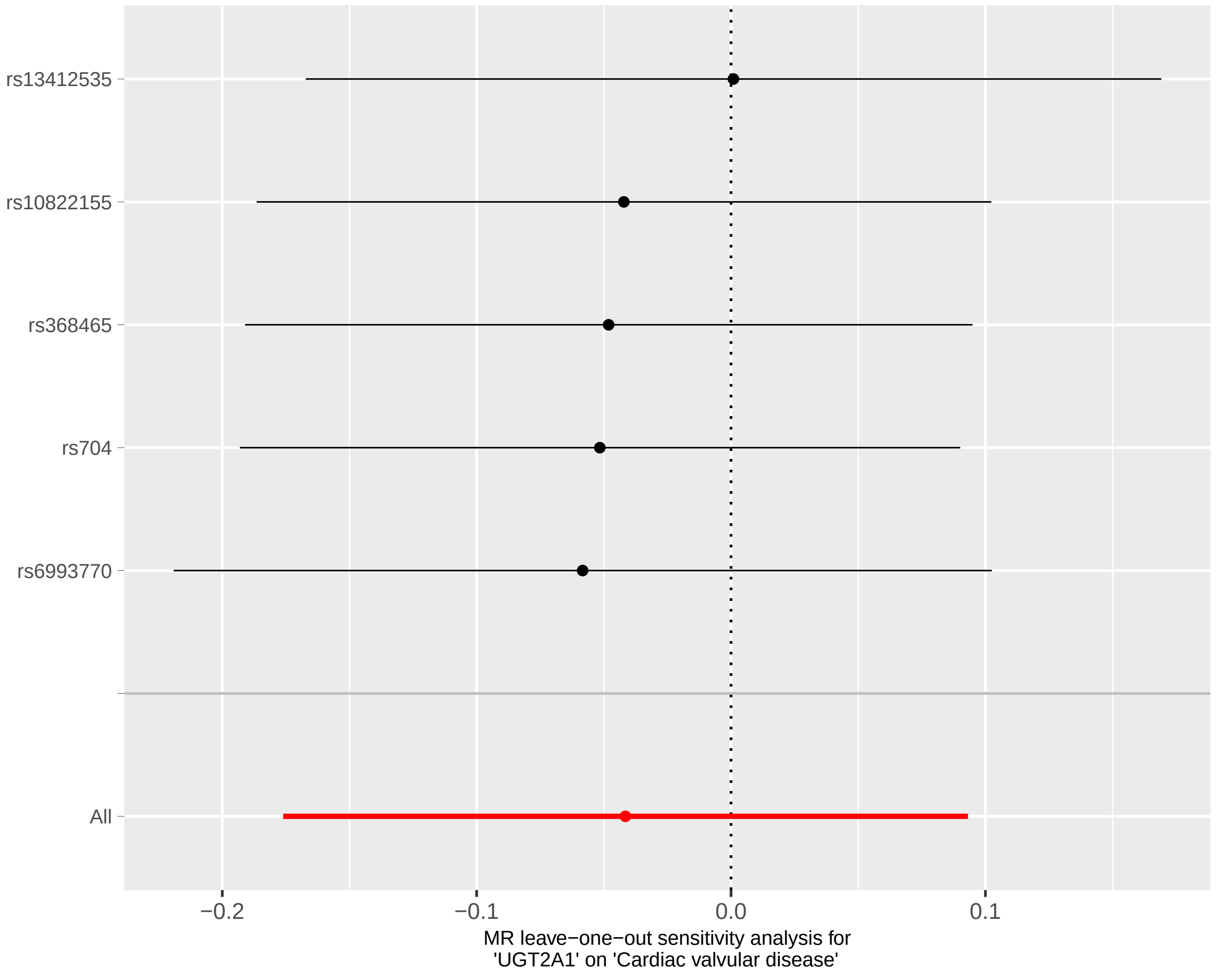
rs13412535
rs10822155
rs368465
rs704
rs6993770
All
−0.2
−0.1
0.0
0.1
MR leave−one−out sensitivity analysis for
'UGT2A1' on 'Cardiac valvular disease'

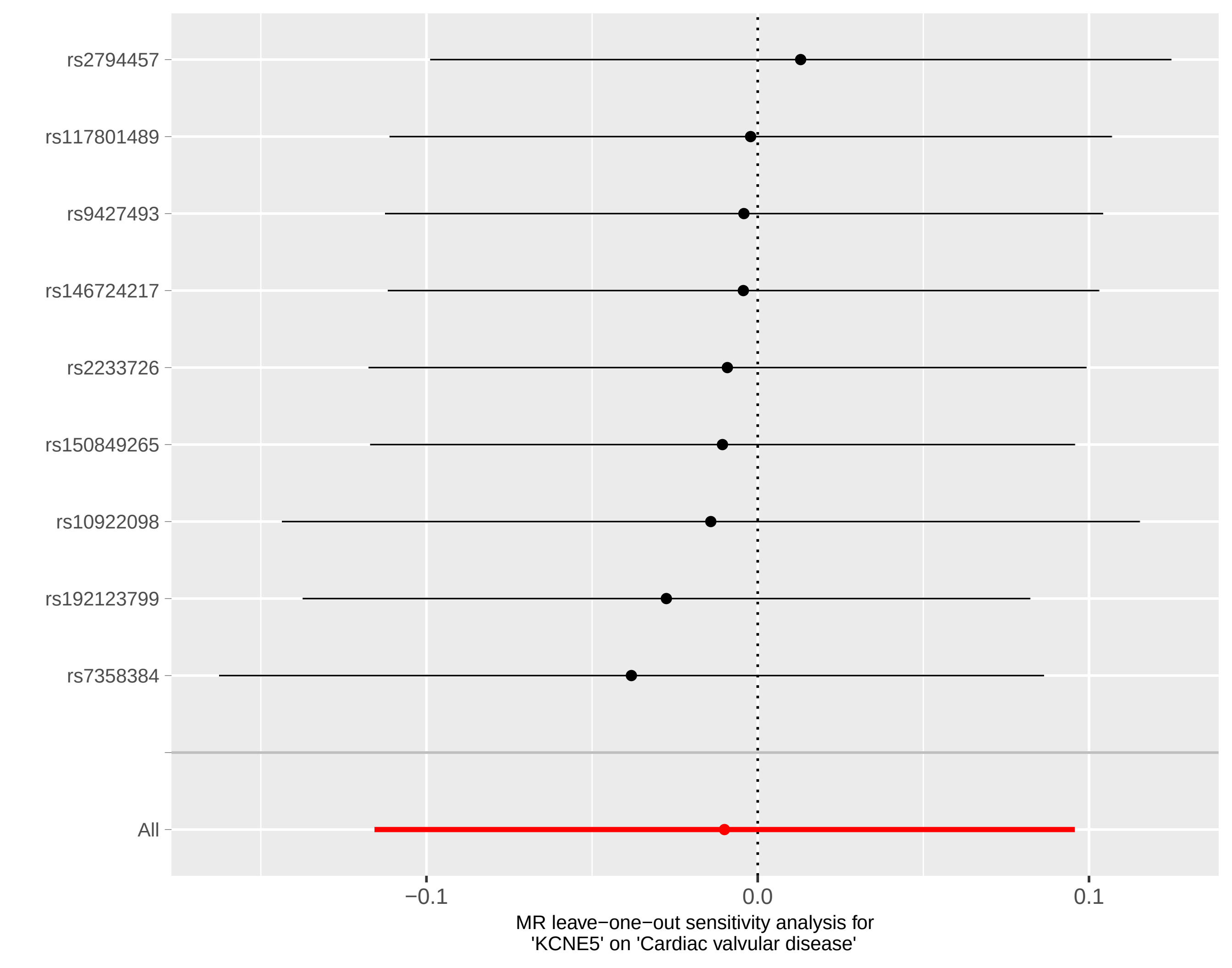

rs2794457
rs117801489
rs9427493
rs146724217
rs2233726
rs150849265
rs10922098
rs192123799
rs7358384
All
−0.1
0.0
0.1
MR leave−one−out sensitivity analysis for
'KCNE5' on 'Cardiac valvular disease'

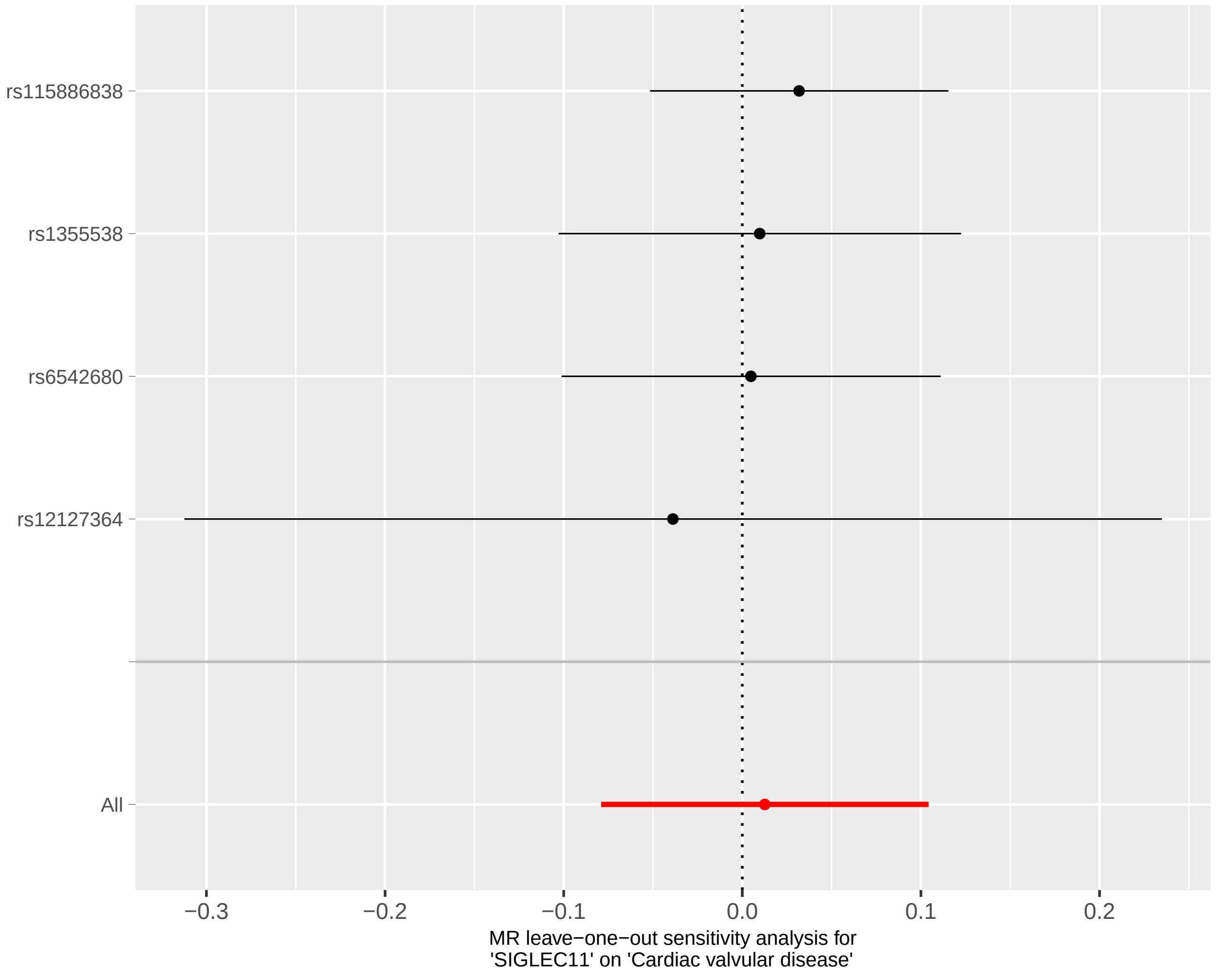

rs115886838
rs1355538
rs6542680
rs12127364
All
−0.3
−0.2
−0.1
0.0
0.1
0.2
MR leave−one−out sensitivity analysis for
'SIGLEC11' on 'Cardiac valvular disease'

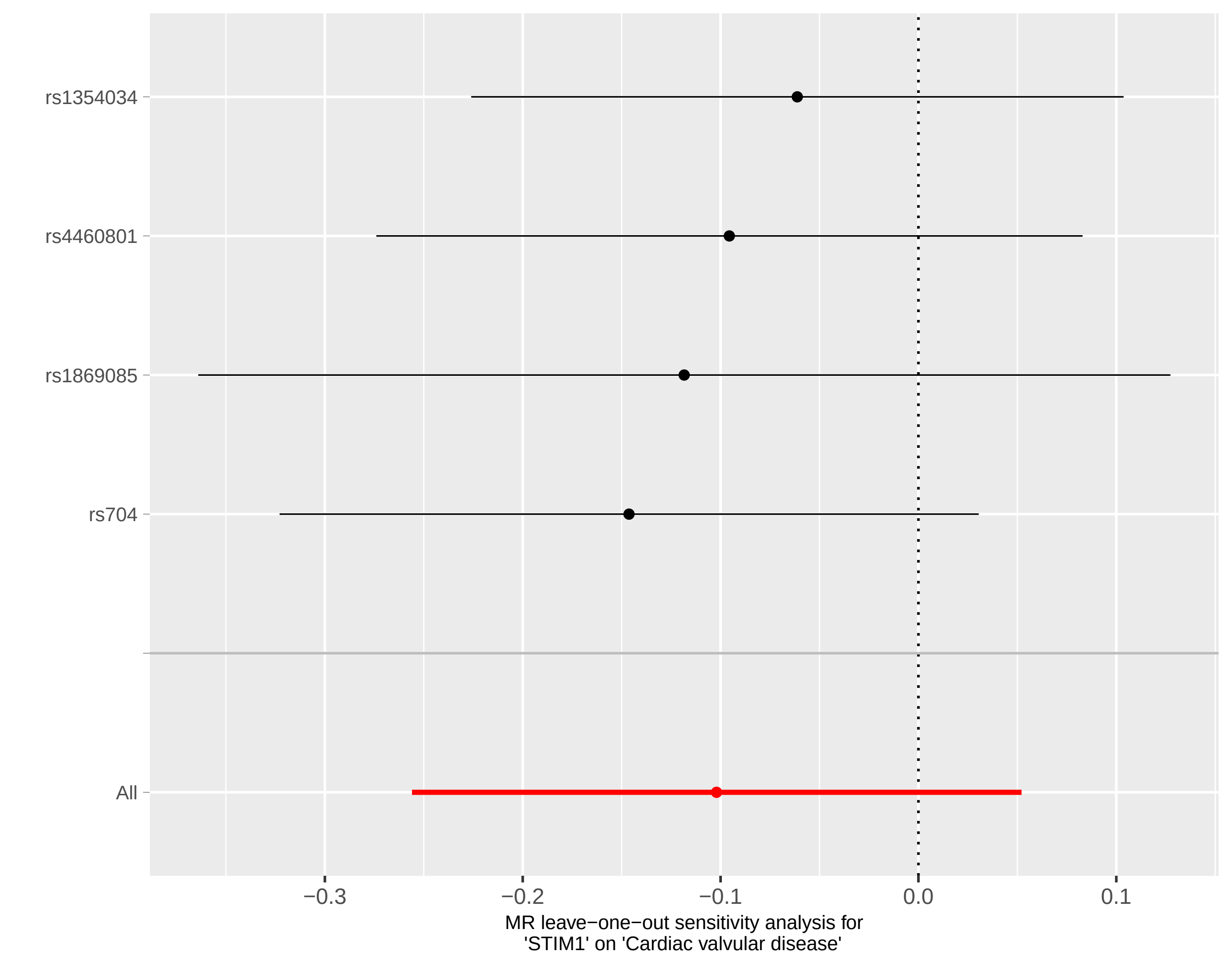

rs1354034
rs4460801
rs1869085
rs704
All
−0.3
−0.2
−0.1
0.0
0.1
MR leave−one−out sensitivity analysis for
'STIM1' on 'Cardiac valvular disease'

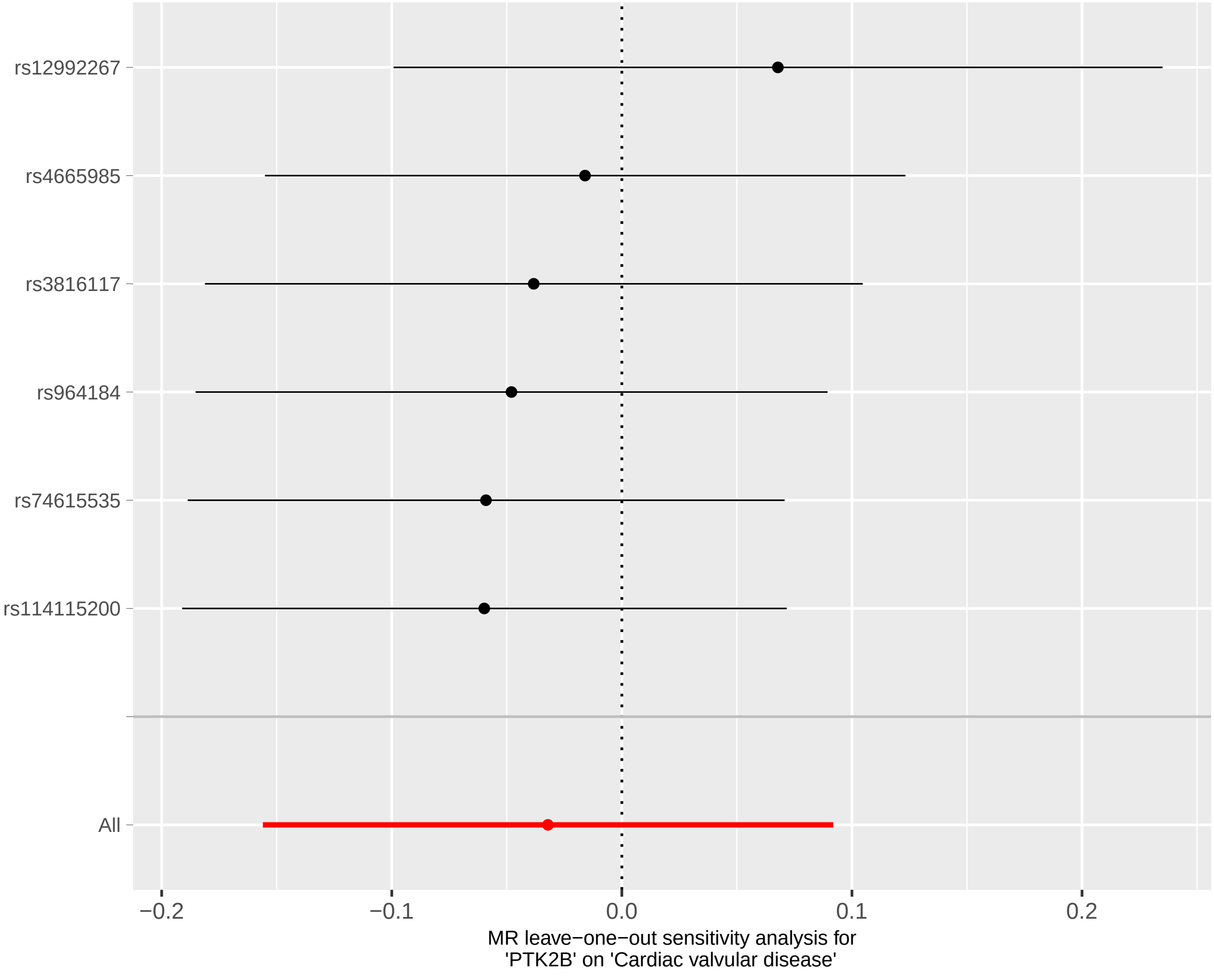
rs12992267
rs4665985
rs3816117
rs964184
rs74615535
rs114115200
All
−0.2
−0.1
0.0
0.1
0.2
MR leave−one−out sensitivity analysis for
'PTK2B' on 'Cardiac valvular disease'

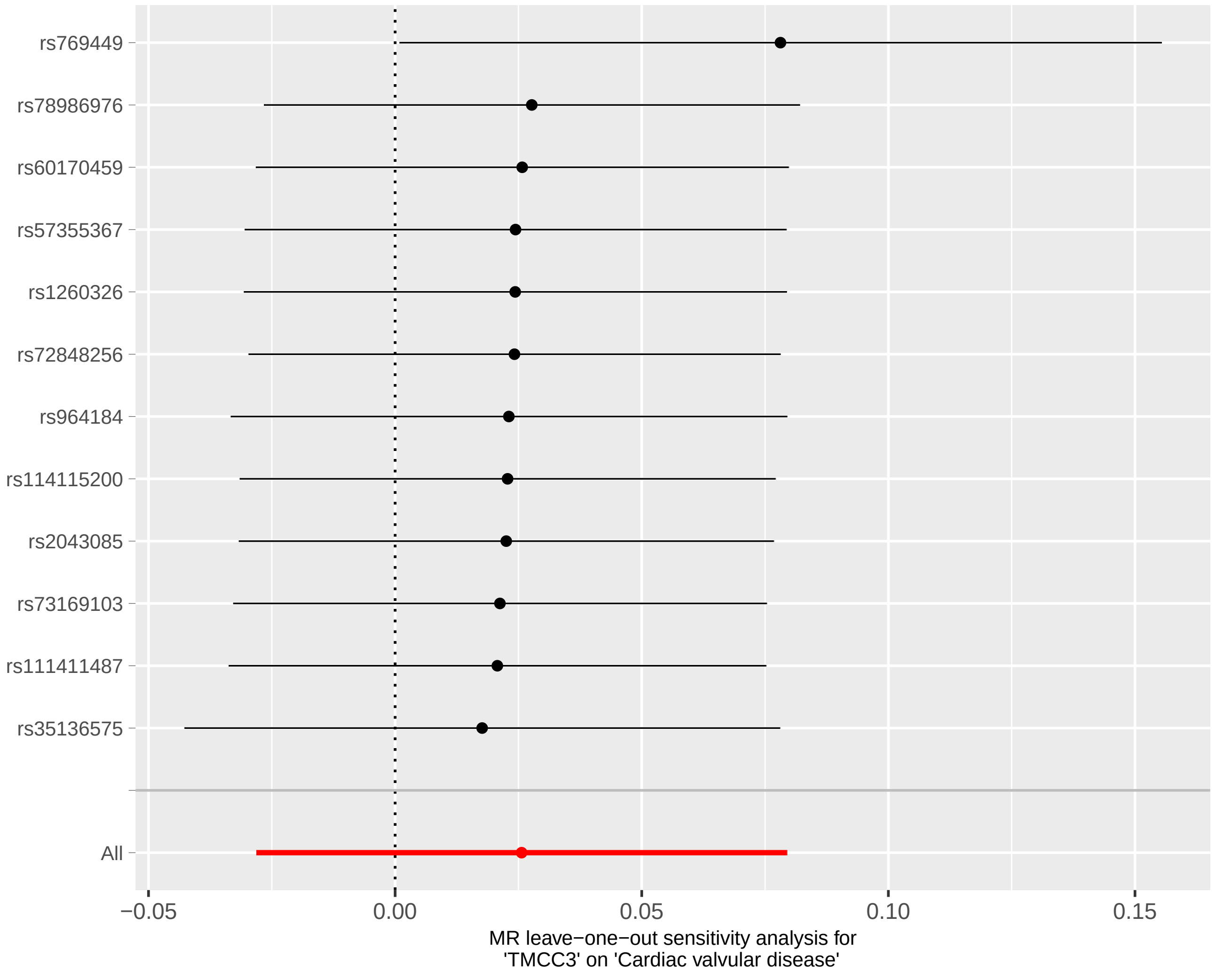
rs769449
rs78986976
rs60170459
rs57355367
rs1260326
rs72848256
rs964184
rs114115200
rs2043085
rs73169103
rs111411487
rs35136575
All
−0.05
0.00
0.05
0.10
0.15
MR leave−one−out sensitivity analysis for
'TMCC3' on 'Cardiac valvular disease'

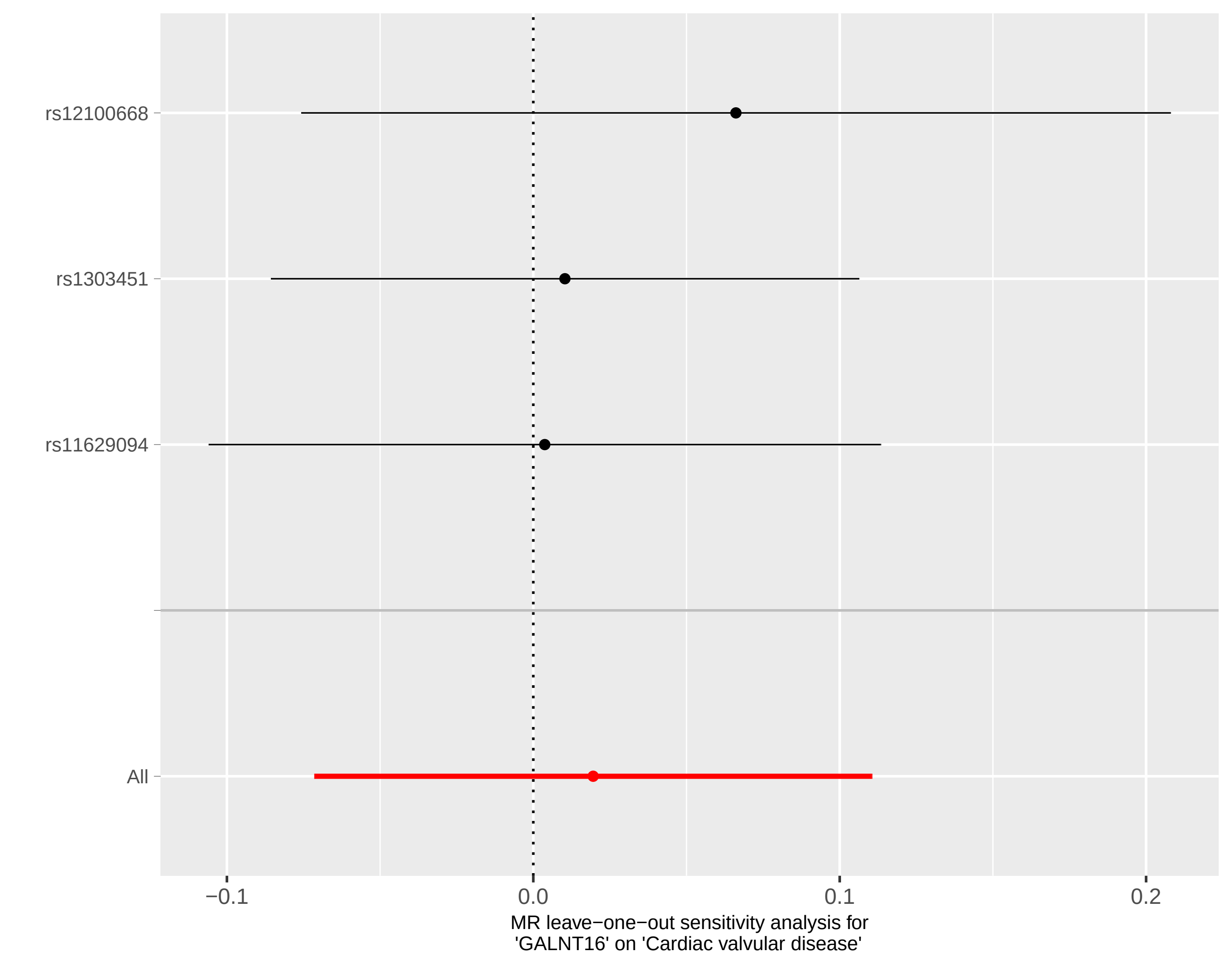
rs12100668
rs1303451
rs11629094
All
−0.1
0.0
0.1
0.2
MR leave−one−out sensitivity analysis for
'GALNT16' on 'Cardiac valvular disease'

Insufficient number of SNPs

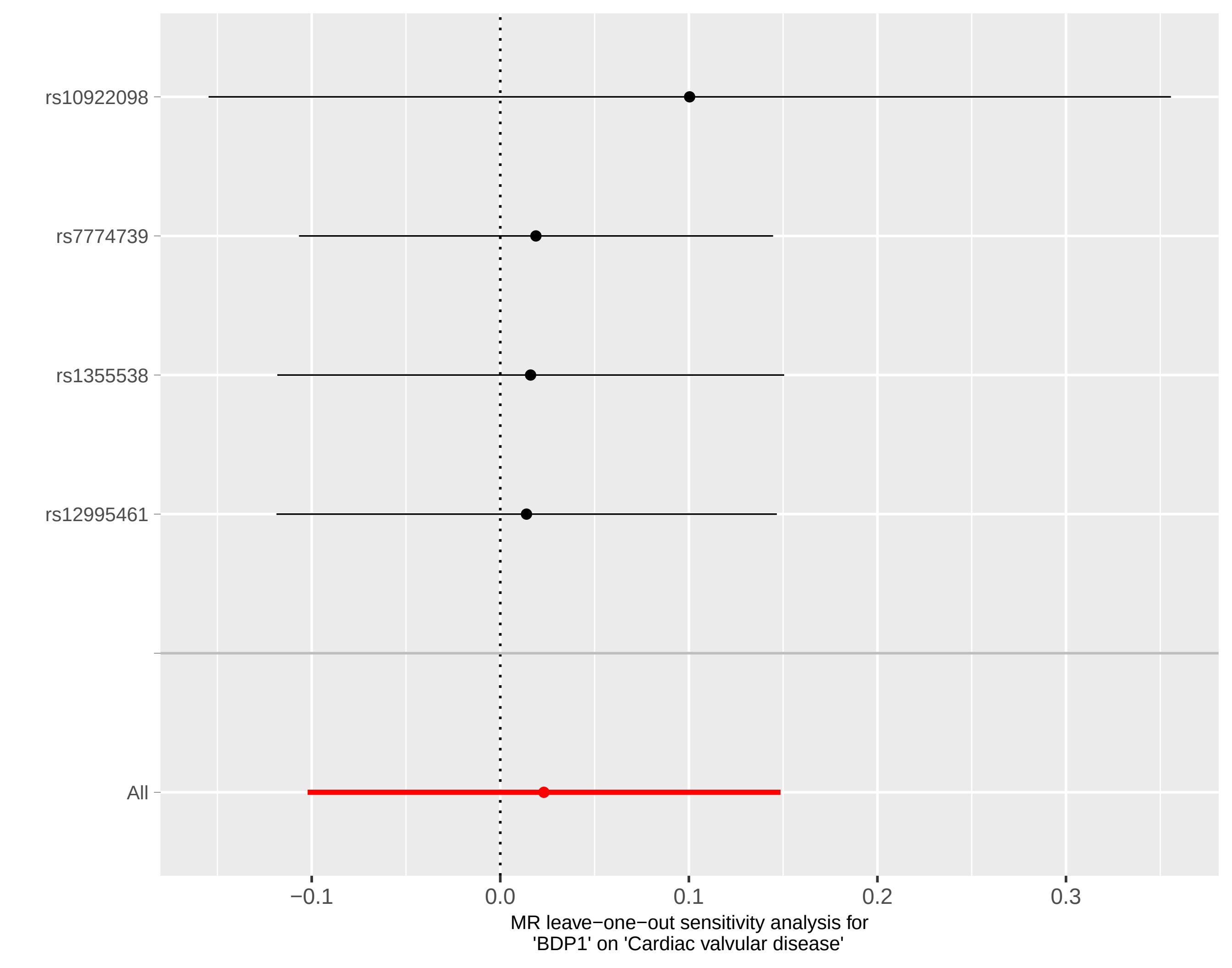

rs10922098
rs7774739
rs1355538
rs12995461
All
−0.1
0.0
0.1
0.2
0.3
MR leave−one−out sensitivity analysis for
'BDP1' on 'Cardiac valvular disease'

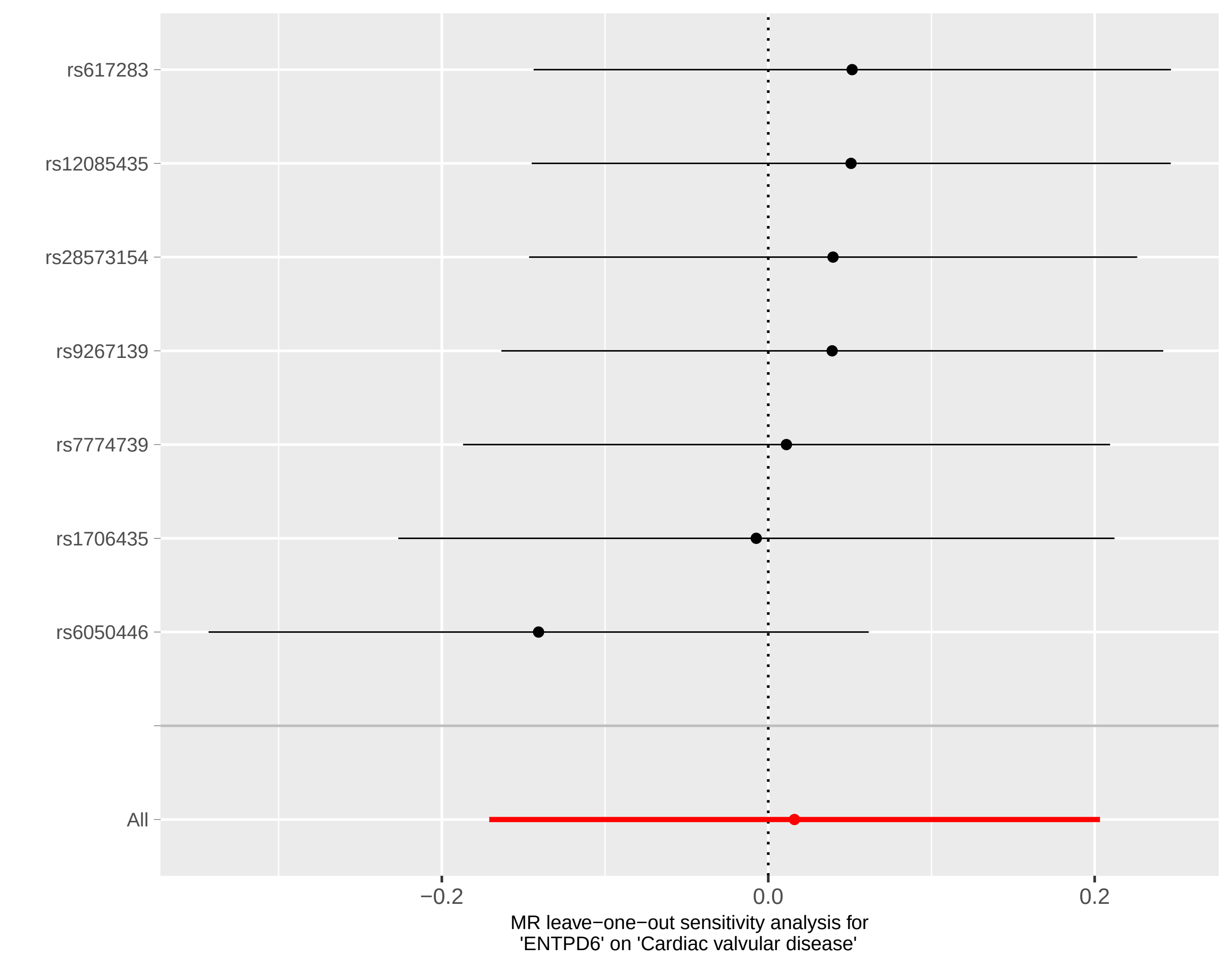
rs617283
rs12085435
rs28573154
rs9267139
rs7774739
rs1706435
rs6050446
All
−0.2
0.0
0.2
MR leave−one−out sensitivity analysis for
'ENTPD6' on 'Cardiac valvular disease'

Insufficient number of SNPs

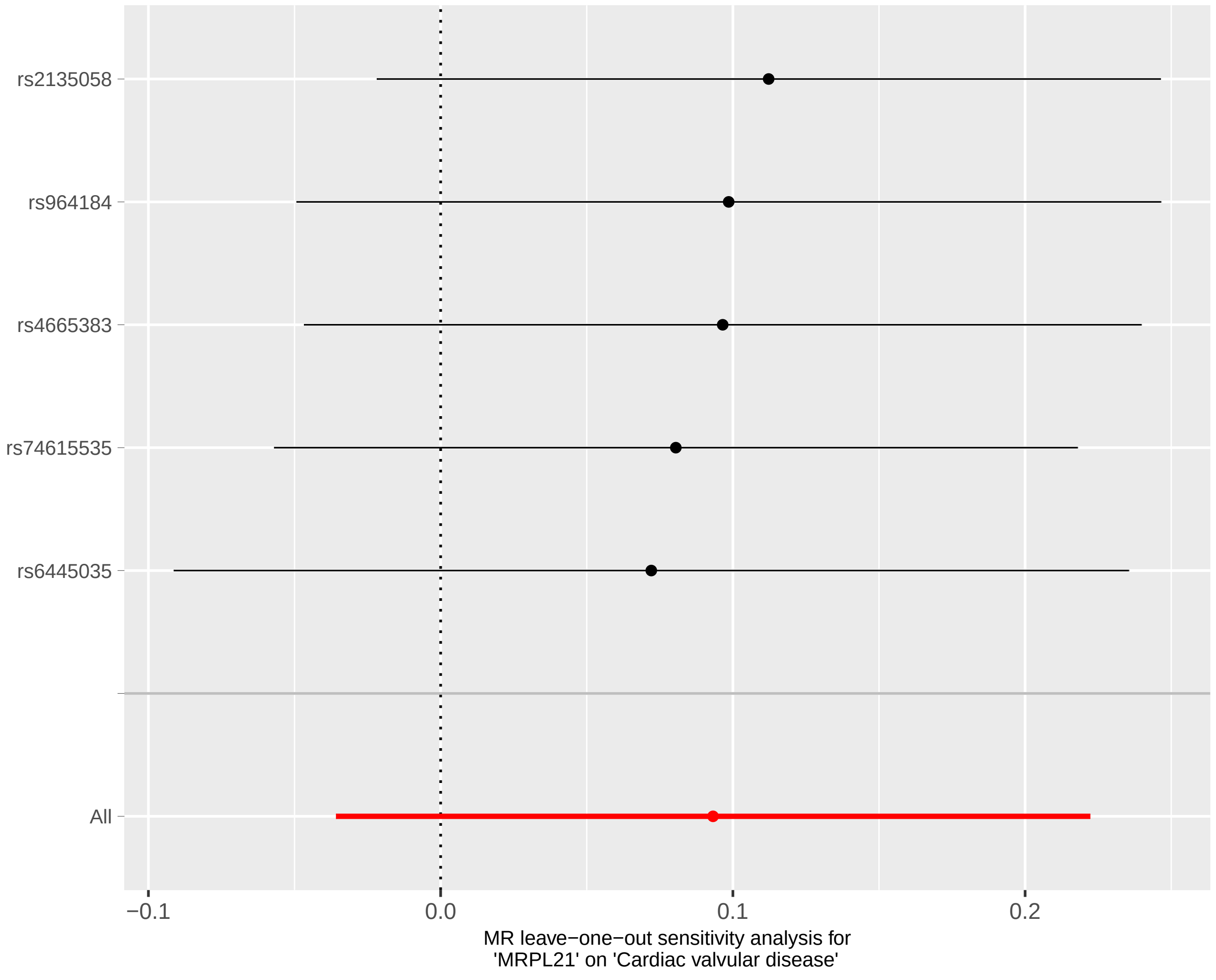

rs2135058
rs964184
rs4665383
rs74615535
rs6445035
All
−0.1
0.0
0.1
0.2
MR leave−one−out sensitivity analysis for
'MRPL21' on 'Cardiac valvular disease'

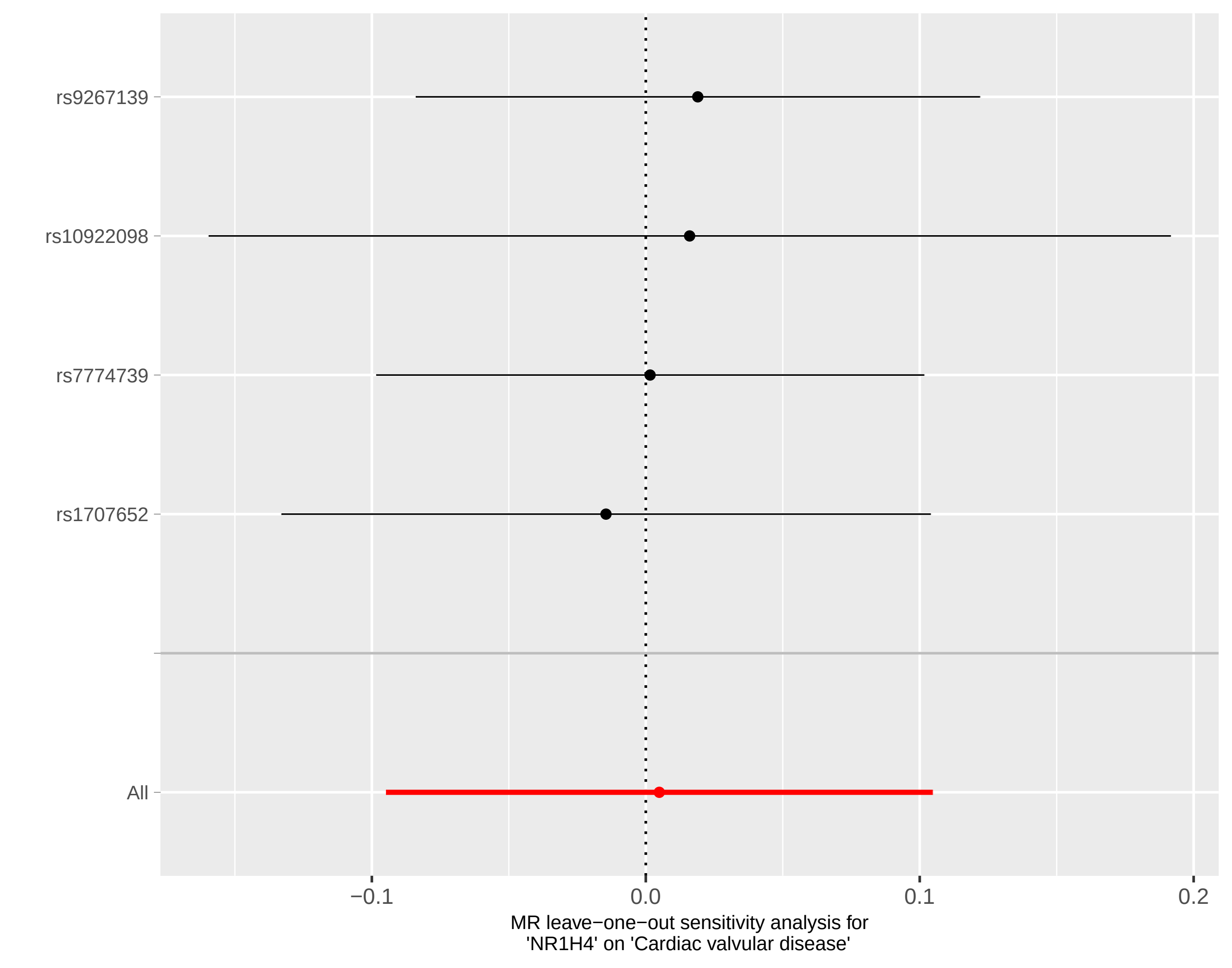
rs9267139
rs10922098
rs7774739
rs1707652
All
−0.1
0.0
0.1
0.2
MR leave−one−out sensitivity analysis for
'NR1H4' on 'Cardiac valvular disease'

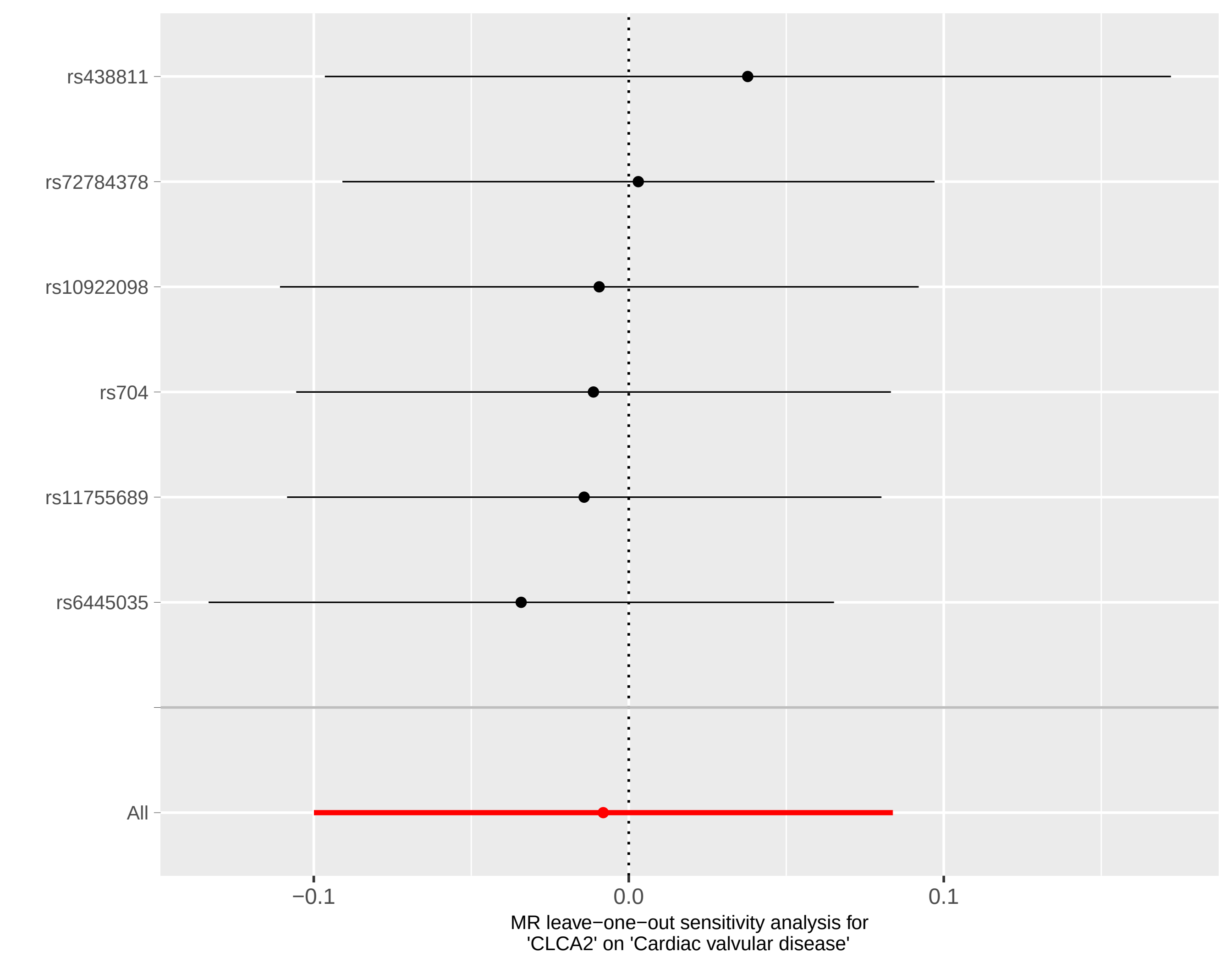
rs438811
rs72784378
rs10922098
rs704
rs11755689
rs6445035
All
−0.1
0.0
0.1
MR leave−one−out sensitivity analysis for
'CLCA2' on 'Cardiac valvular disease'

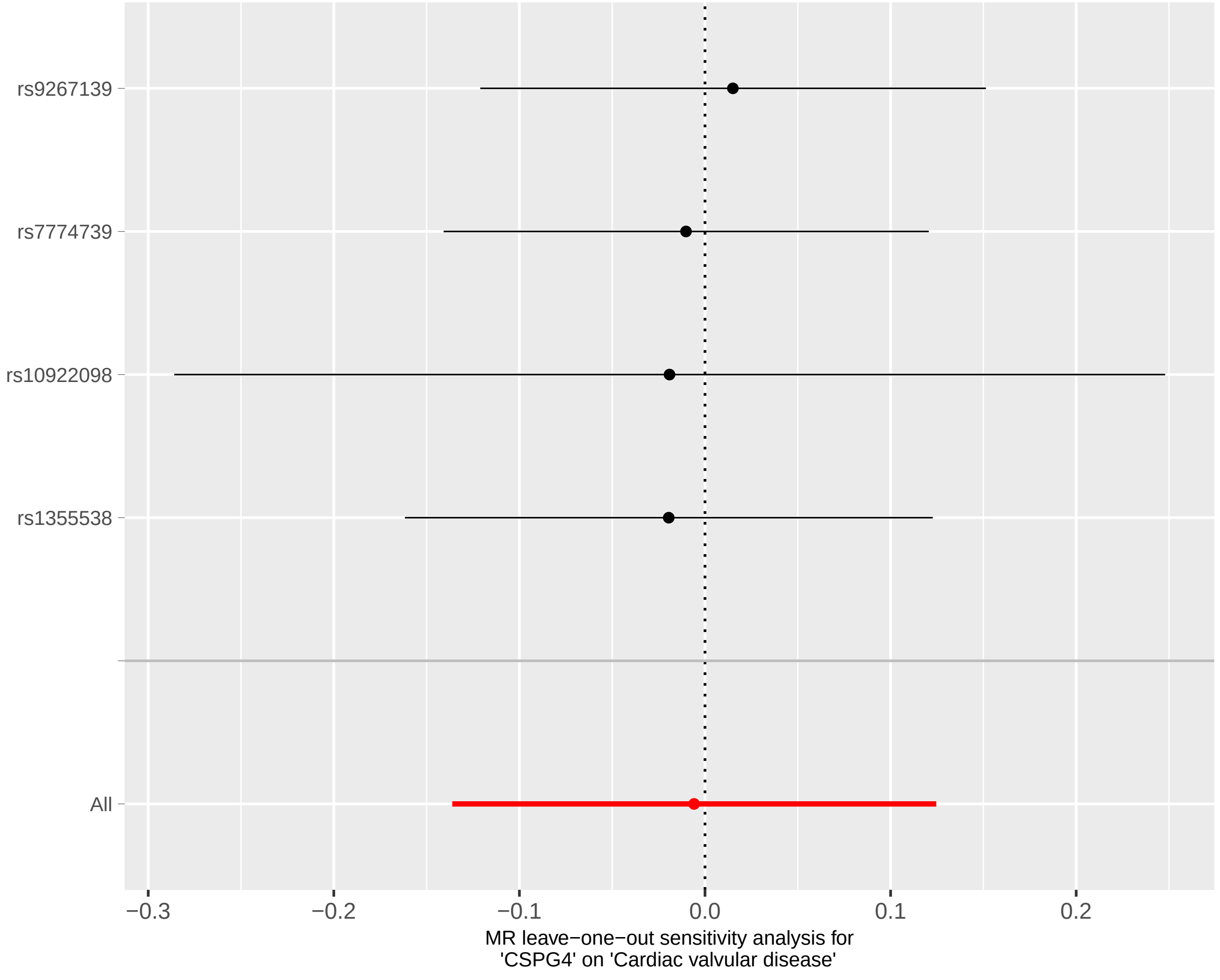

rs9267139
rs7774739
rs10922098
rs1355538
All
−0.3
−0.2
−0.1
0.0
0.1
0.2
MR leave−one−out sensitivity analysis for
'CSPG4' on 'Cardiac valvular disease'

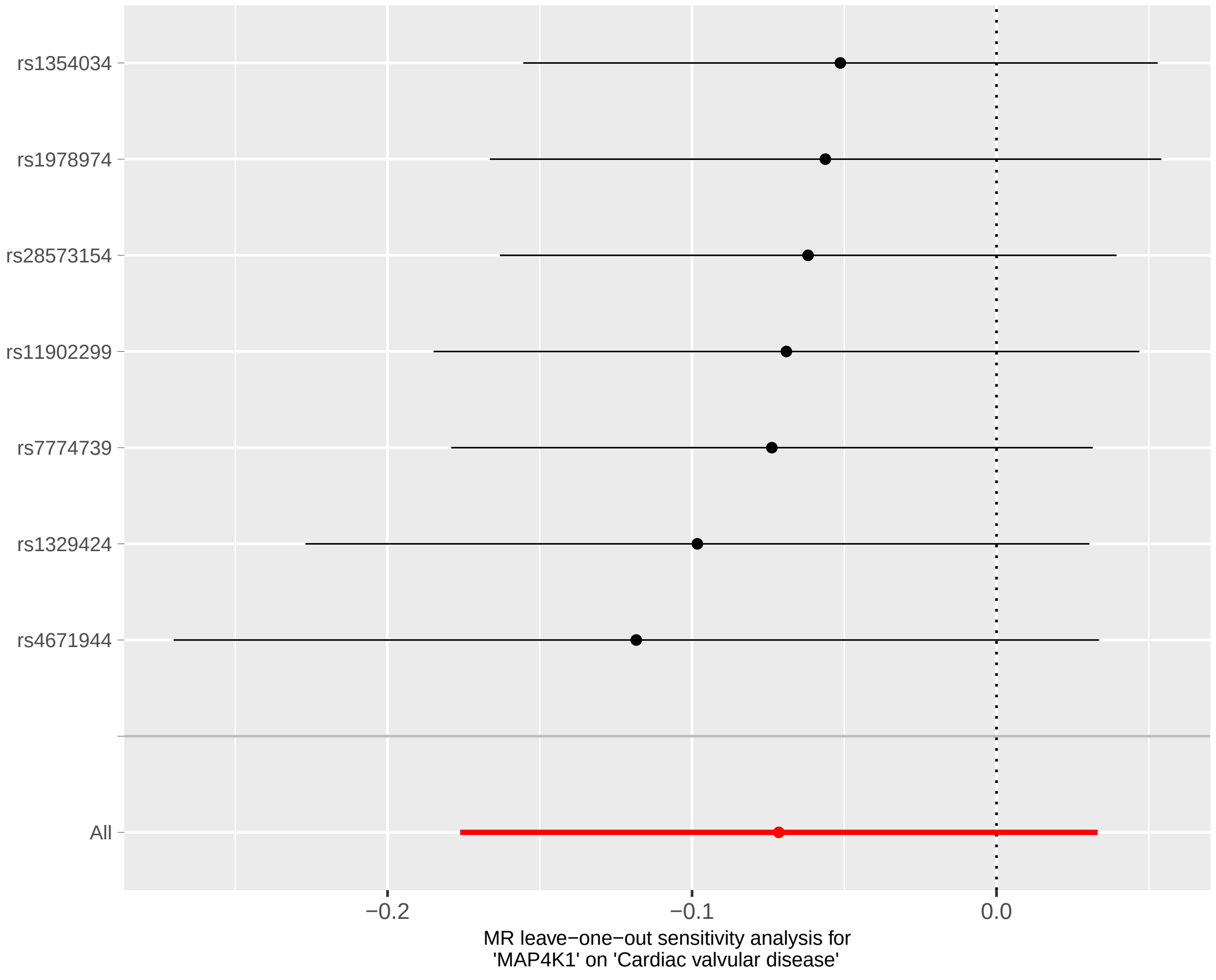
rs1354034
rs1978974
rs28573154
rs11902299
rs7774739
rs1329424
rs4671944
All
−0.2
−0.1
0.0
MR leave−one−out sensitivity analysis for
'MAP4K1' on 'Cardiac valvular disease'

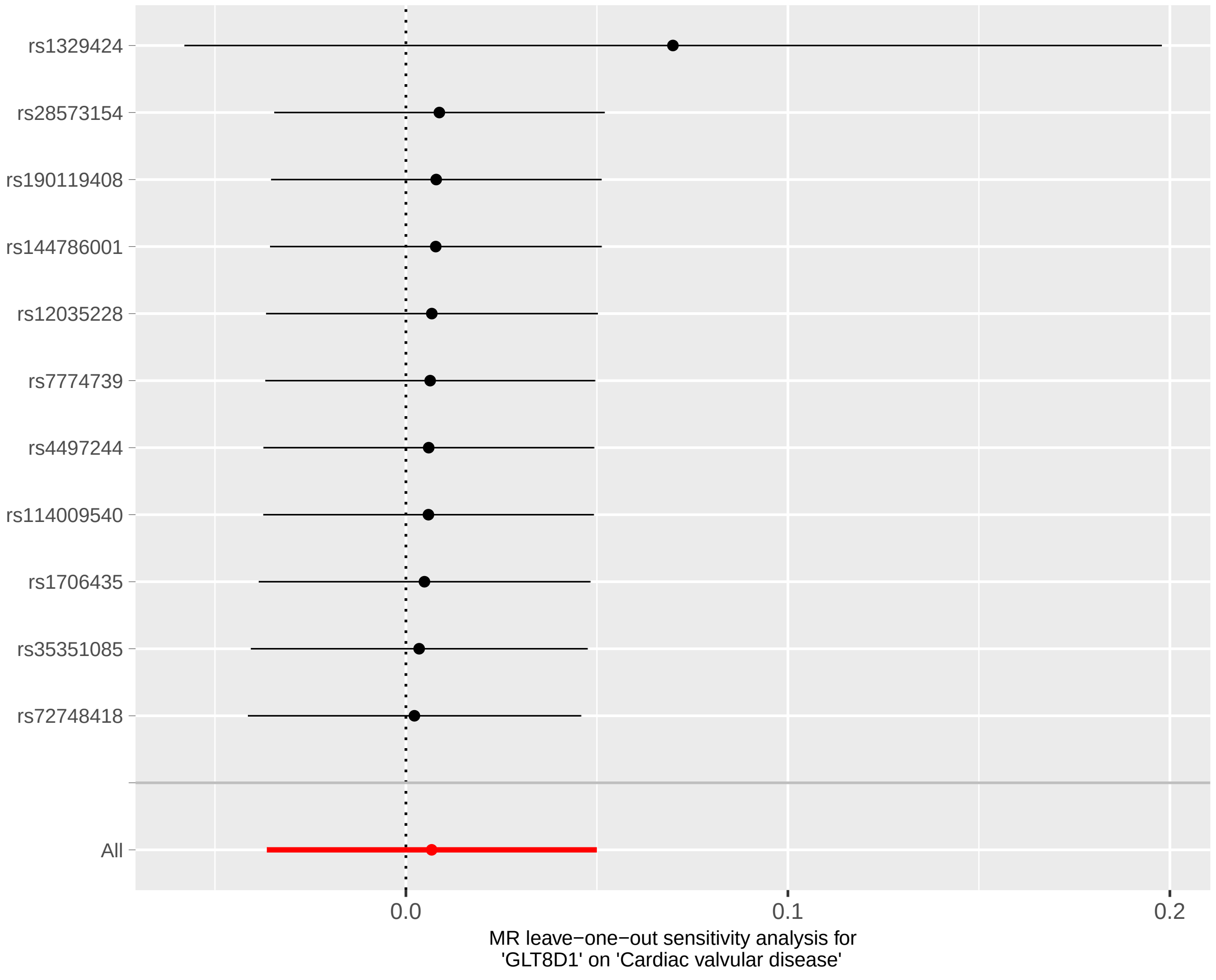
rs1329424
rs28573154
rs190119408
rs144786001
rs12035228
rs7774739
rs4497244
rs114009540
rs1706435
rs35351085
rs72748418
All
0.0
0.1
0.2
MR leave−one−out sensitivity analysis for
'GLT8D1' on 'Cardiac valvular disease'

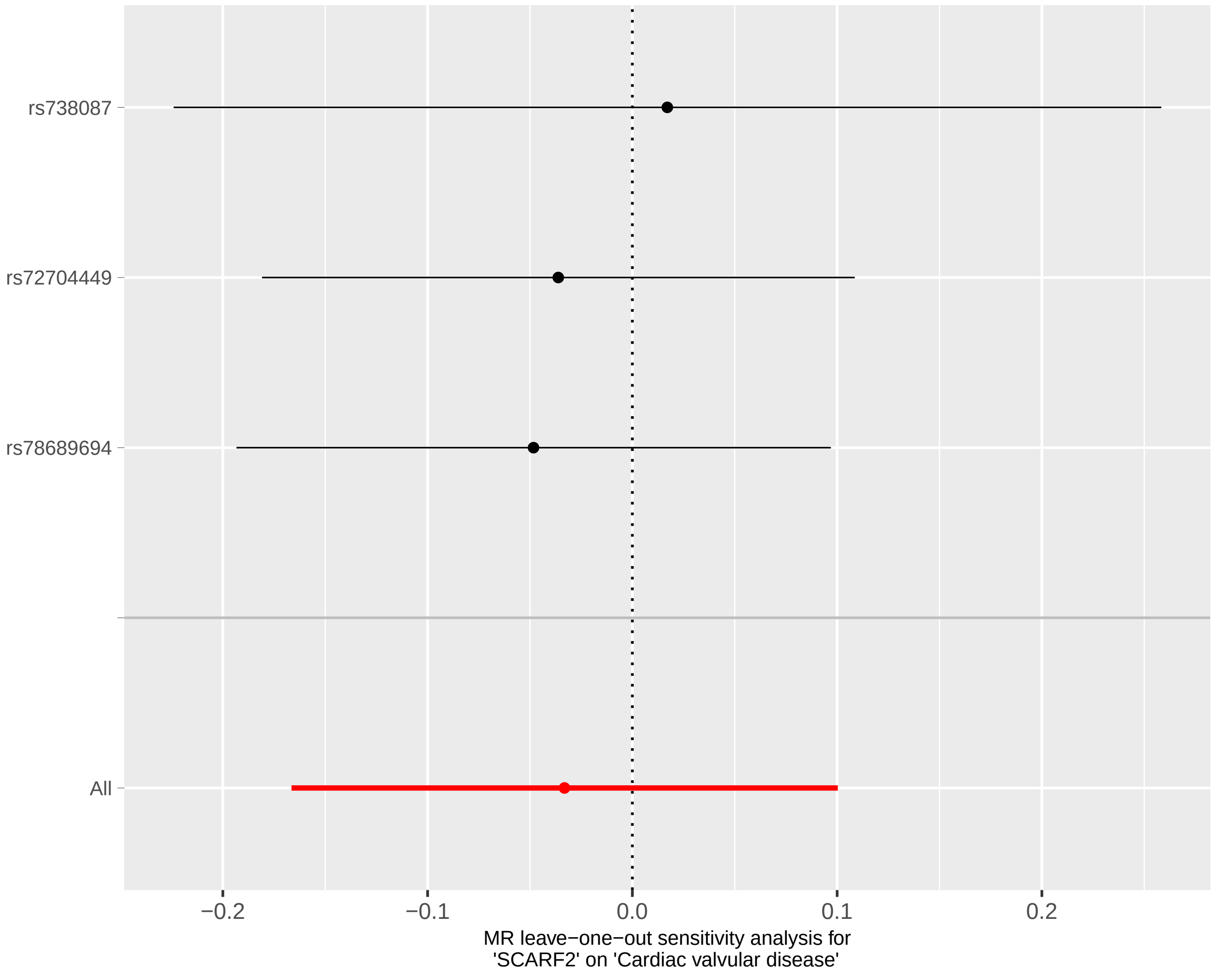
rs738087
rs72704449
rs78689694
All
−0.2
−0.1
0.0
0.1
0.2
MR leave−one−out sensitivity analysis for
'SCARF2' on 'Cardiac valvular disease'

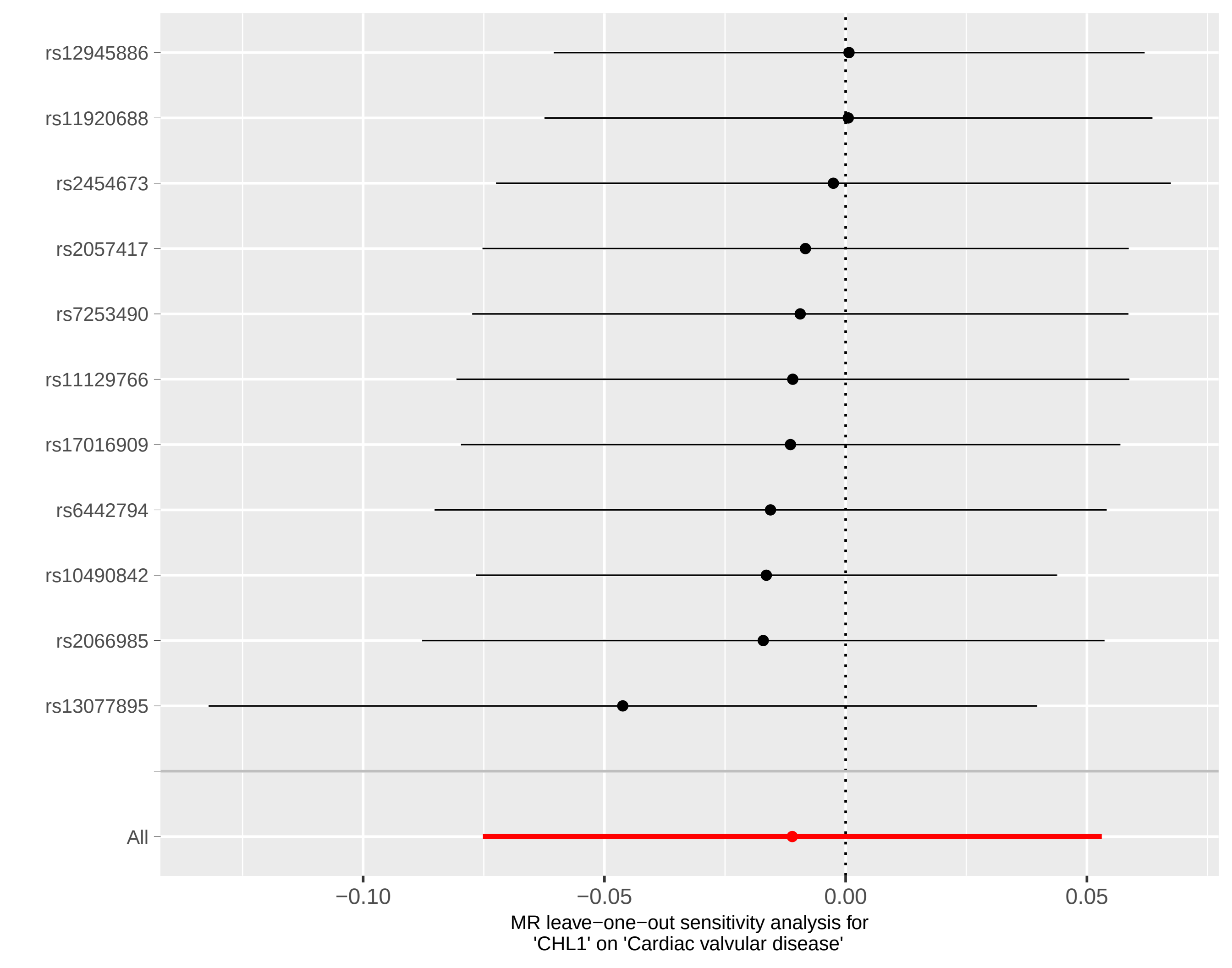
rs12945886
rs11920688
rs2454673
rs2057417
rs7253490
rs11129766
rs17016909
rs6442794
rs10490842
rs2066985
rs13077895
All
−0.10
−0.05
0.00
0.05
MR leave−one−out sensitivity analysis for
'CHL1' on 'Cardiac valvular disease'

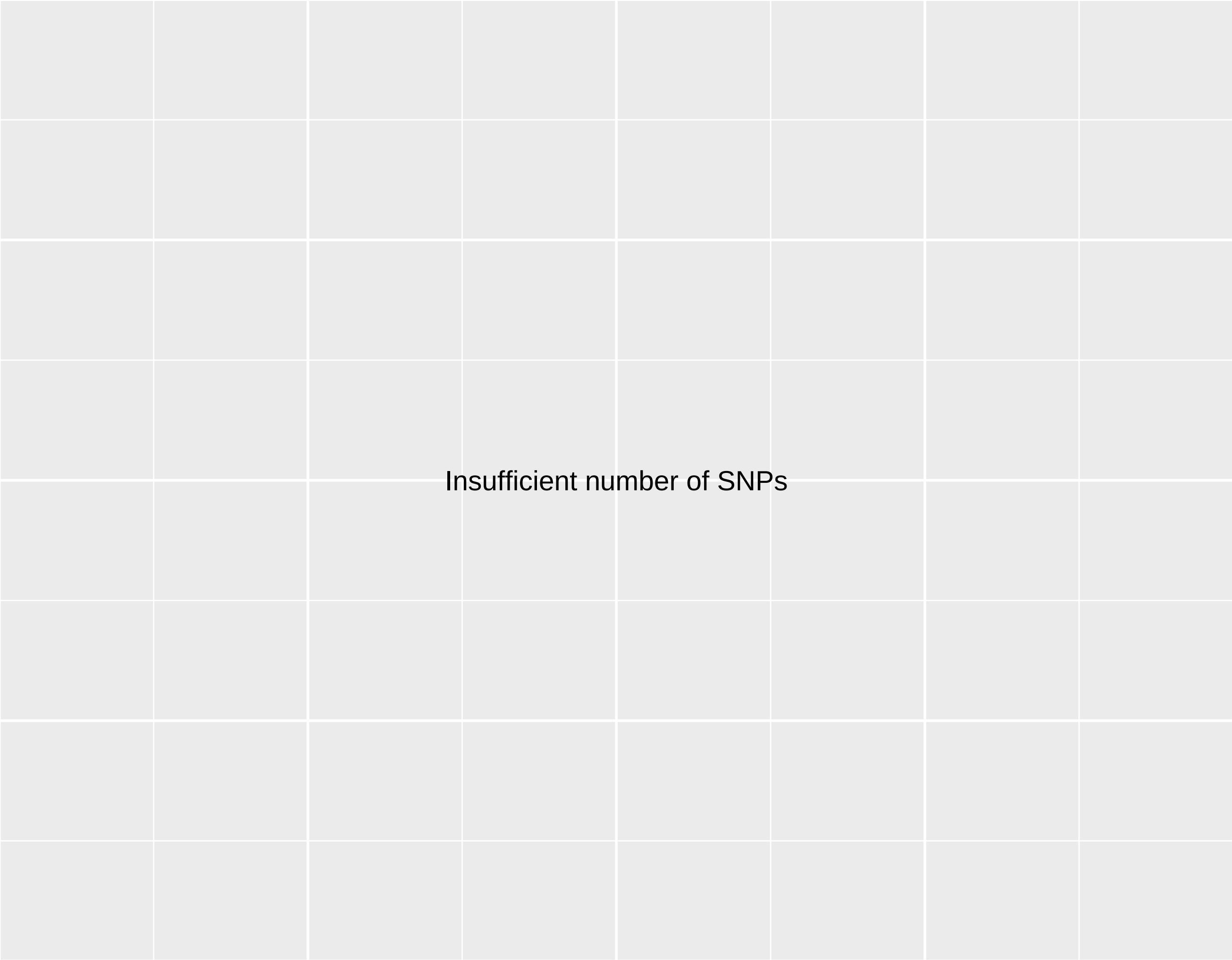
Insufficient number of SNPs

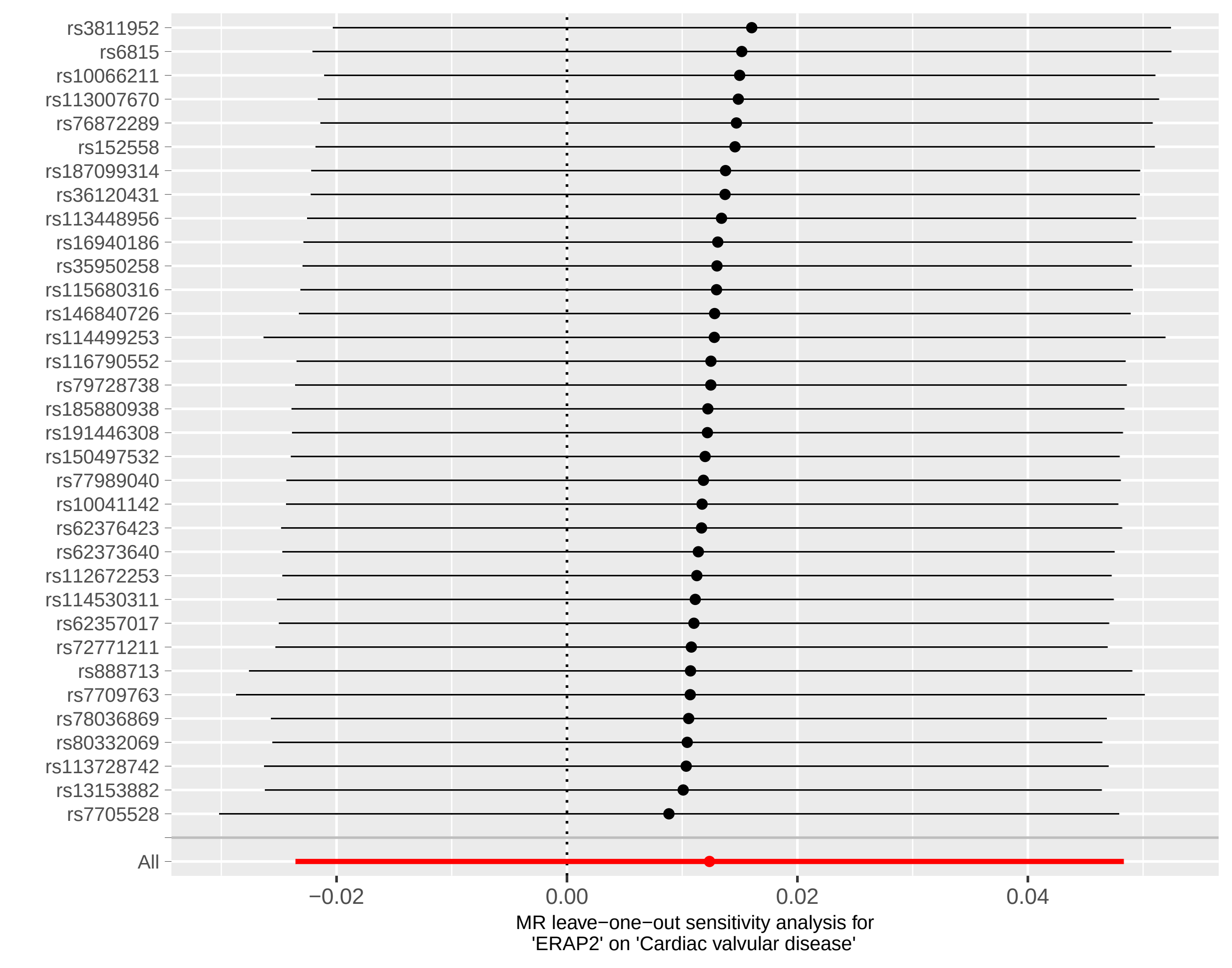
rs3811952
rs6815
rs10066211
rs113007670
rs76872289
rs152558
rs187099314
rs36120431
rs113448956
rs16940186
rs35950258
rs115680316
rs146840726
rs114499253
rs116790552
rs79728738
rs185880938
rs191446308
rs150497532
rs77989040
rs10041142
rs62376423
rs62373640
rs112672253
rs114530311
rs62357017
rs72771211
rs888713
rs7709763
rs78036869
rs80332069
rs113728742
rs13153882
rs7705528
All
−0.02
0.00
0.02
0.04
MR leave−one−out sensitivity analysis for
'ERAP2' on 'Cardiac valvular disease'

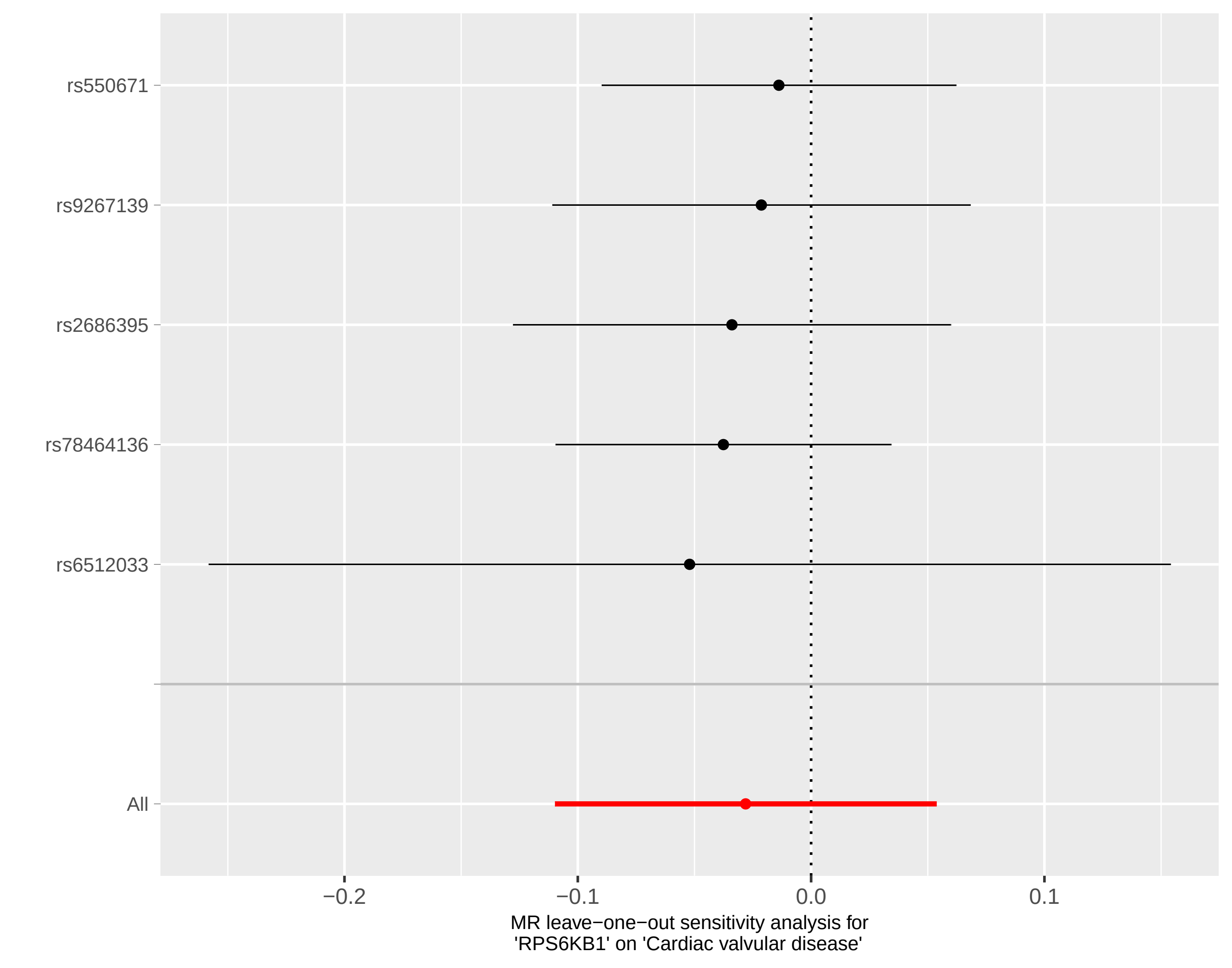
rs550671
rs9267139
rs2686395
rs78464136
rs6512033
All
−0.2
−0.1
0.0
0.1
MR leave−one−out sensitivity analysis for
'RPS6KB1' on 'Cardiac valvular disease'

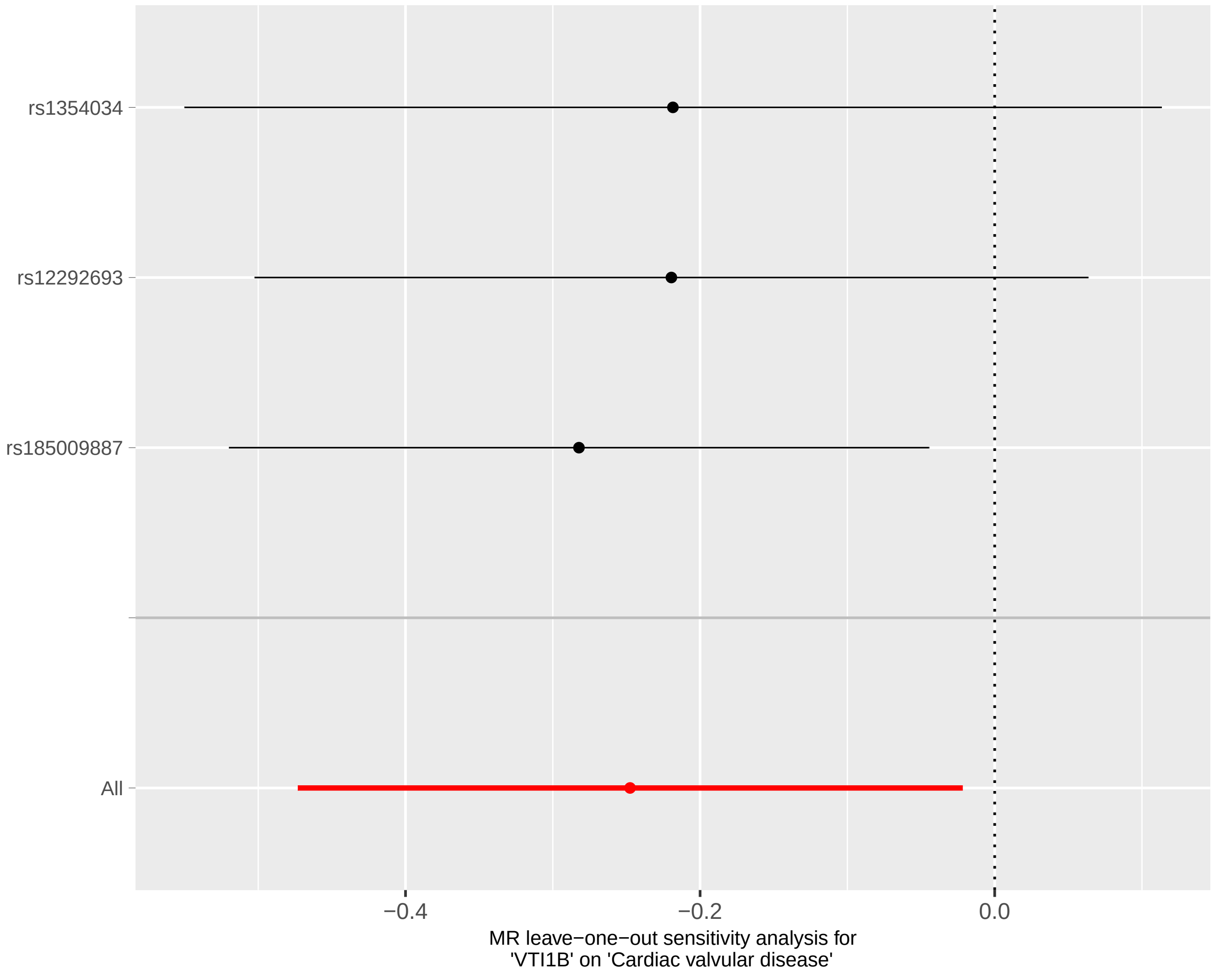
rs1354034
rs12292693
rs185009887
All
−0.4
−0.2
0.0
MR leave−one−out sensitivity analysis for
'VTI1B' on 'Cardiac valvular disease'

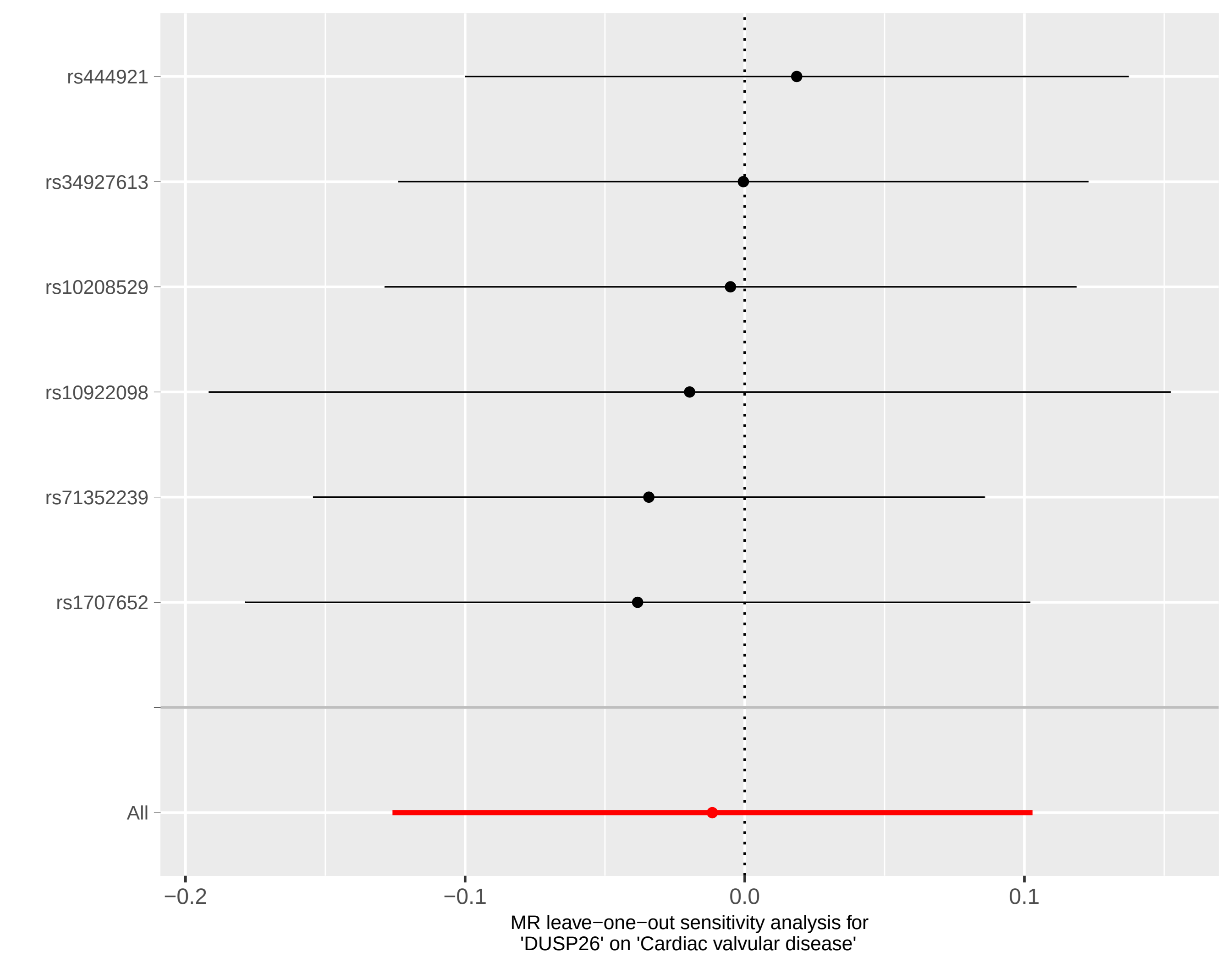
rs444921
rs34927613
rs10208529
rs10922098
rs71352239
rs1707652
All
−0.2
−0.1
0.0
0.1
MR leave−one−out sensitivity analysis for
'DUSP26' on 'Cardiac valvular disease'

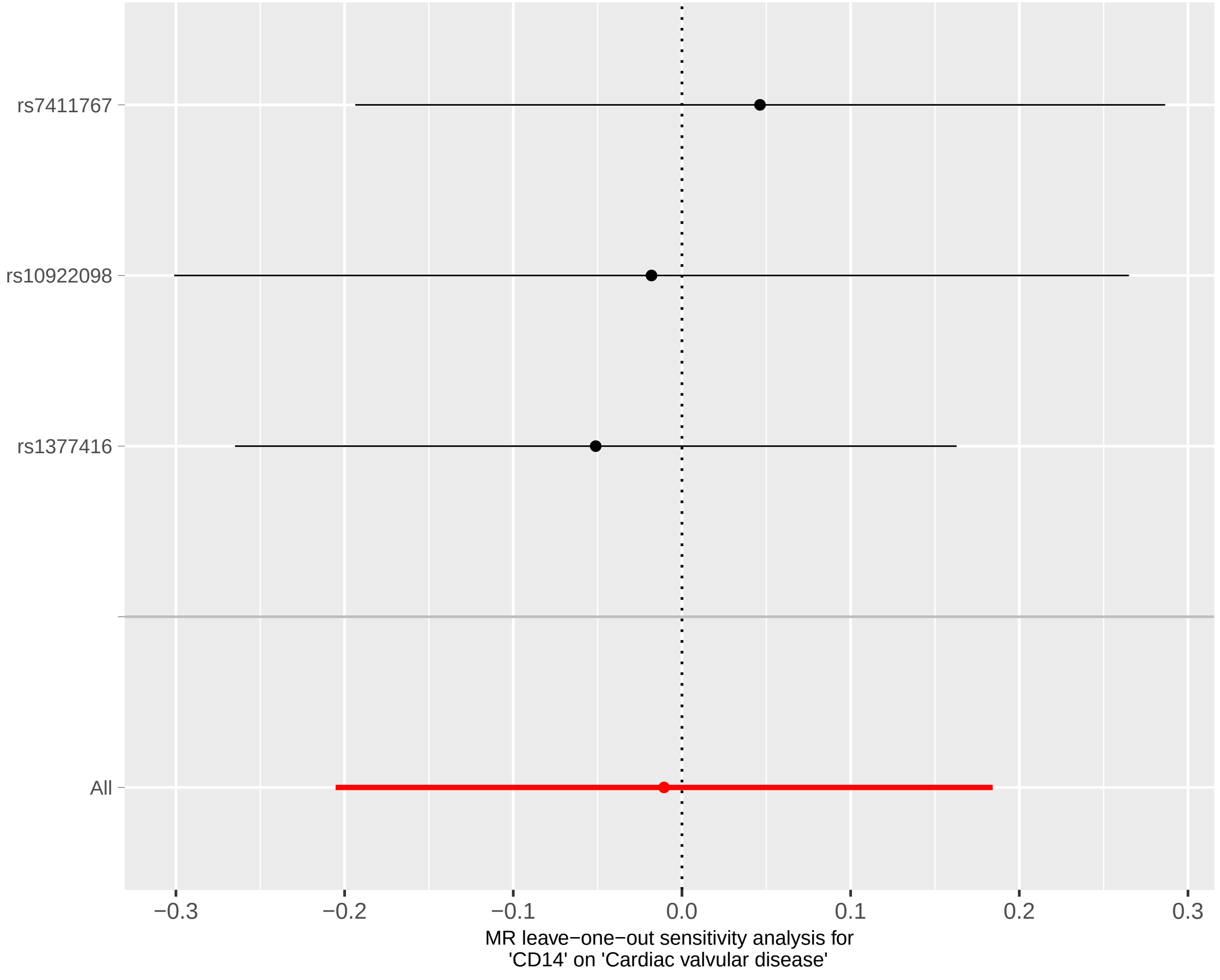
rs7411767
rs10922098
rs1377416
All
−0.3
−0.2
−0.1
0.0
0.1
0.2
0.3
MR leave−one−out sensitivity analysis for
'CD14' on 'Cardiac valvular disease'

Insufficient number of SNPs

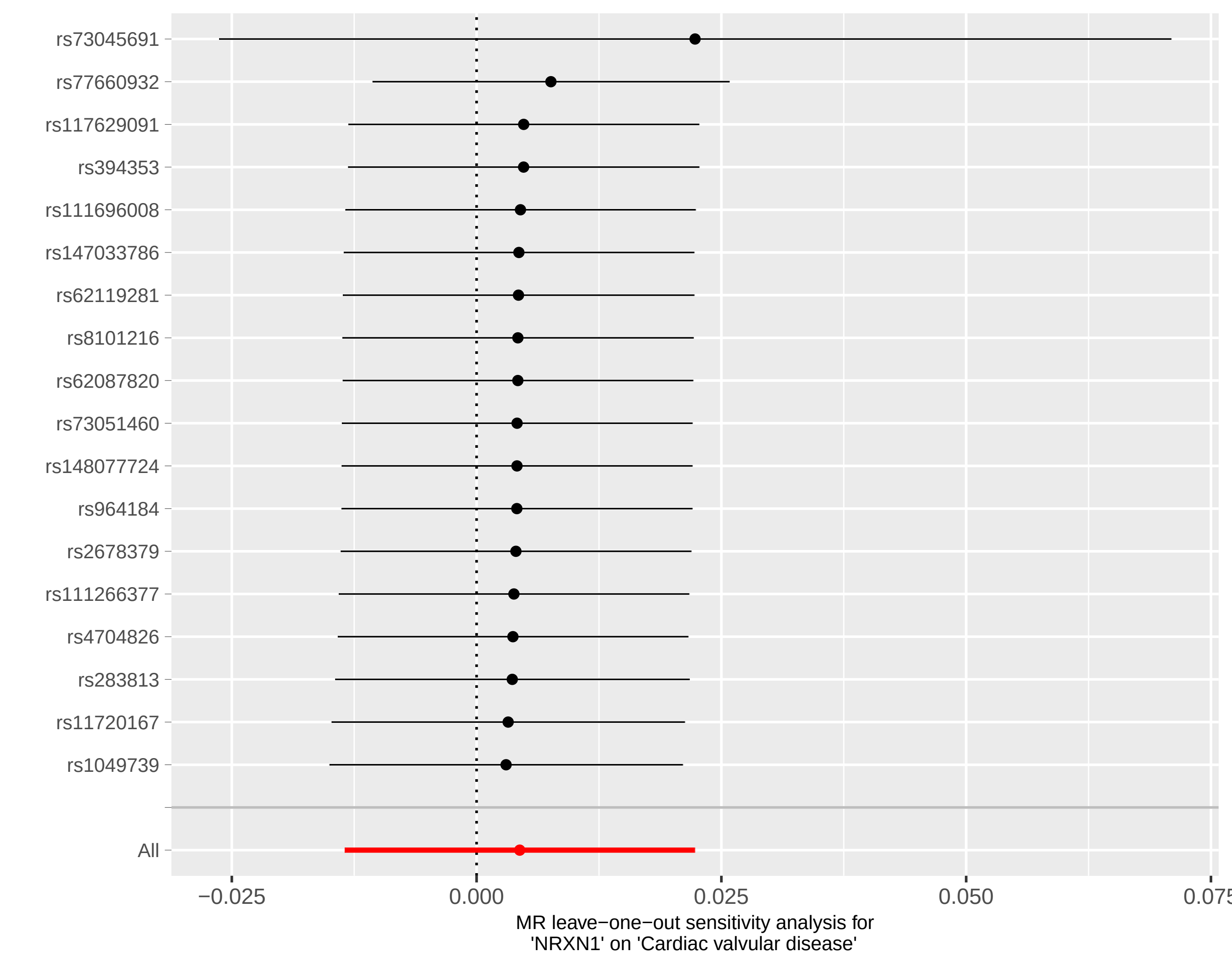

rs73045691
rs77660932
rs117629091
rs394353
rs111696008
rs147033786
rs62119281
rs8101216
rs62087820
rs73051460
rs148077724
rs964184
rs2678379
rs111266377
rs4704826
rs283813
rs11720167
rs1049739
All
−0.025
0.000
0.025
0.050
0.075
MR leave−one−out sensitivity analysis for
'NRXN1' on 'Cardiac valvular disease'

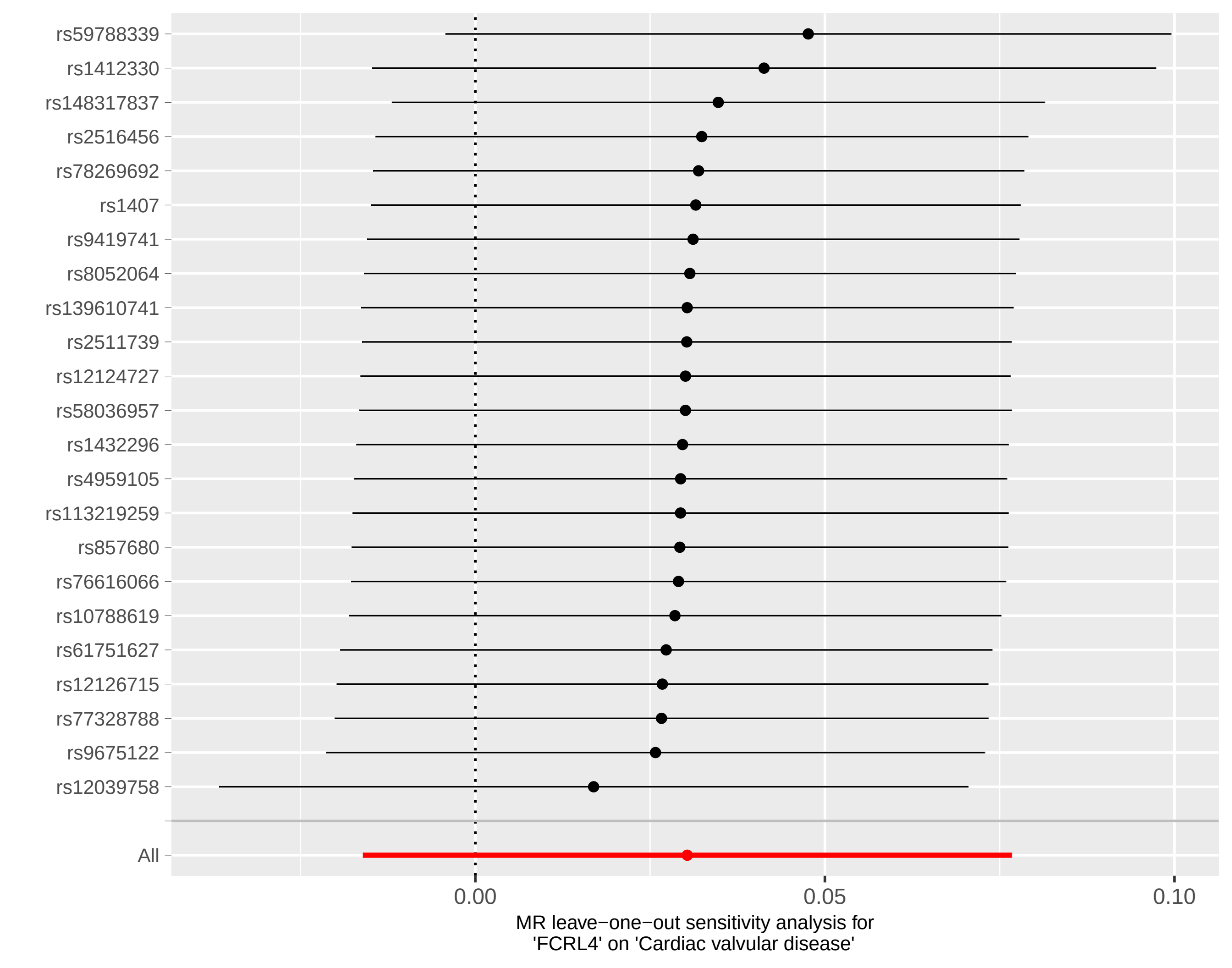
rs59788339
rs1412330
rs148317837
rs2516456
rs78269692
rs1407
rs9419741
rs8052064
rs139610741
rs2511739
rs12124727
rs58036957
rs1432296
rs4959105
rs113219259
rs857680
rs76616066
rs10788619
rs61751627
rs12126715
rs77328788
rs9675122
rs12039758
All
0.00
0.05
0.10
MR leave−one−out sensitivity analysis for
'FCRL4' on 'Cardiac valvular disease'

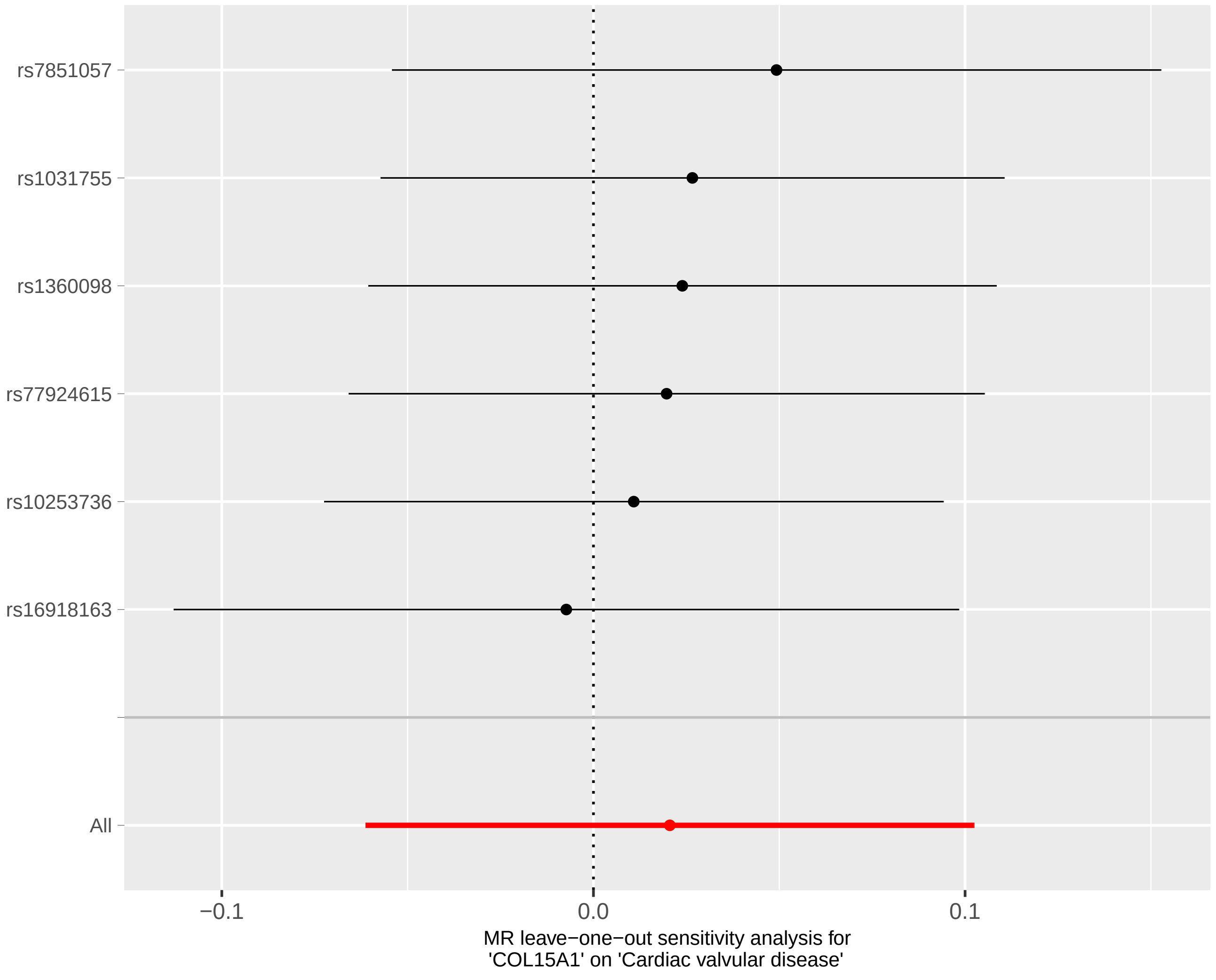
rs7851057
rs1031755
rs1360098
rs77924615
rs10253736
rs16918163
All
−0.1
0.0
0.1
MR leave−one−out sensitivity analysis for
'COL15A1' on 'Cardiac valvular disease'

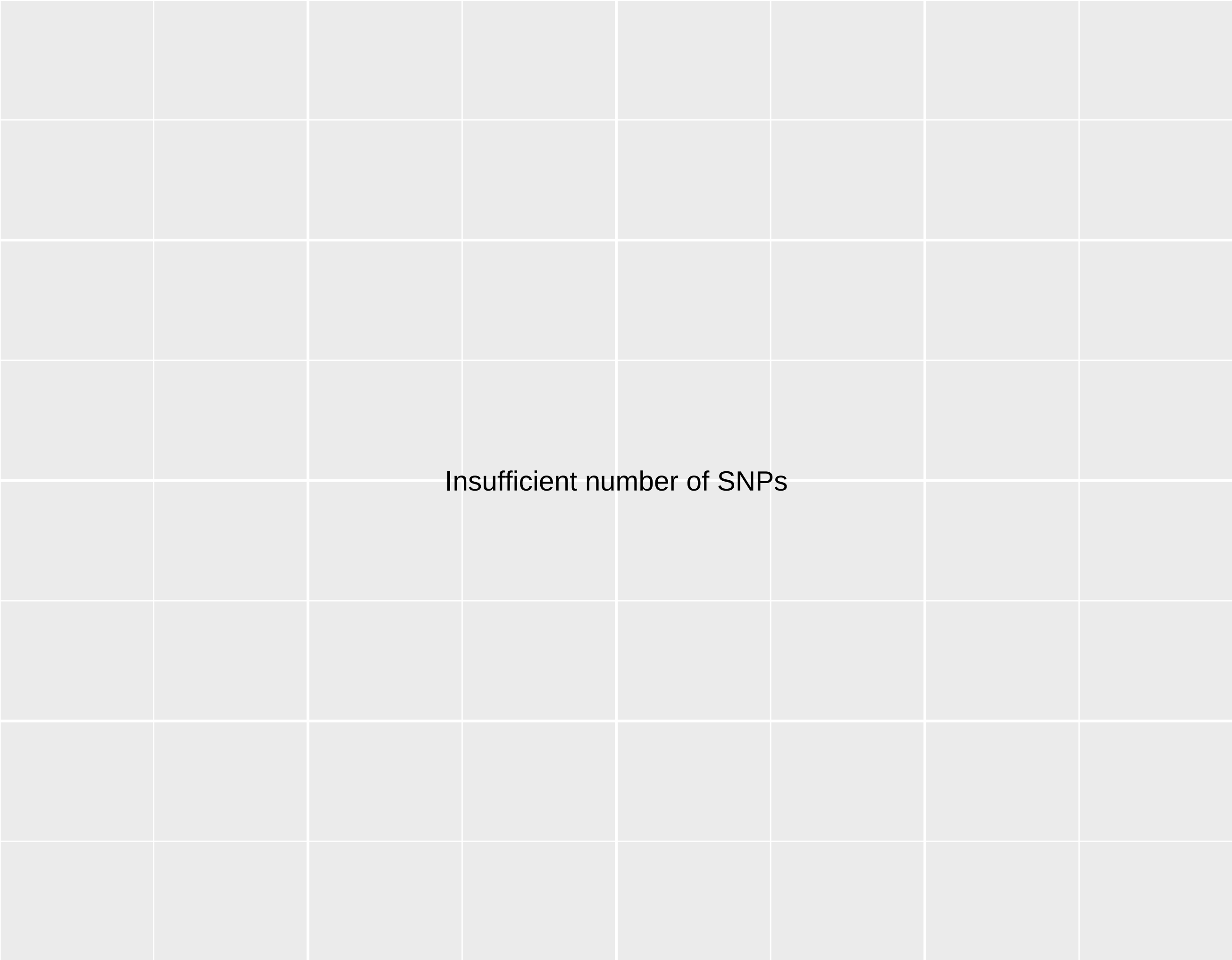
Insufficient number of SNPs

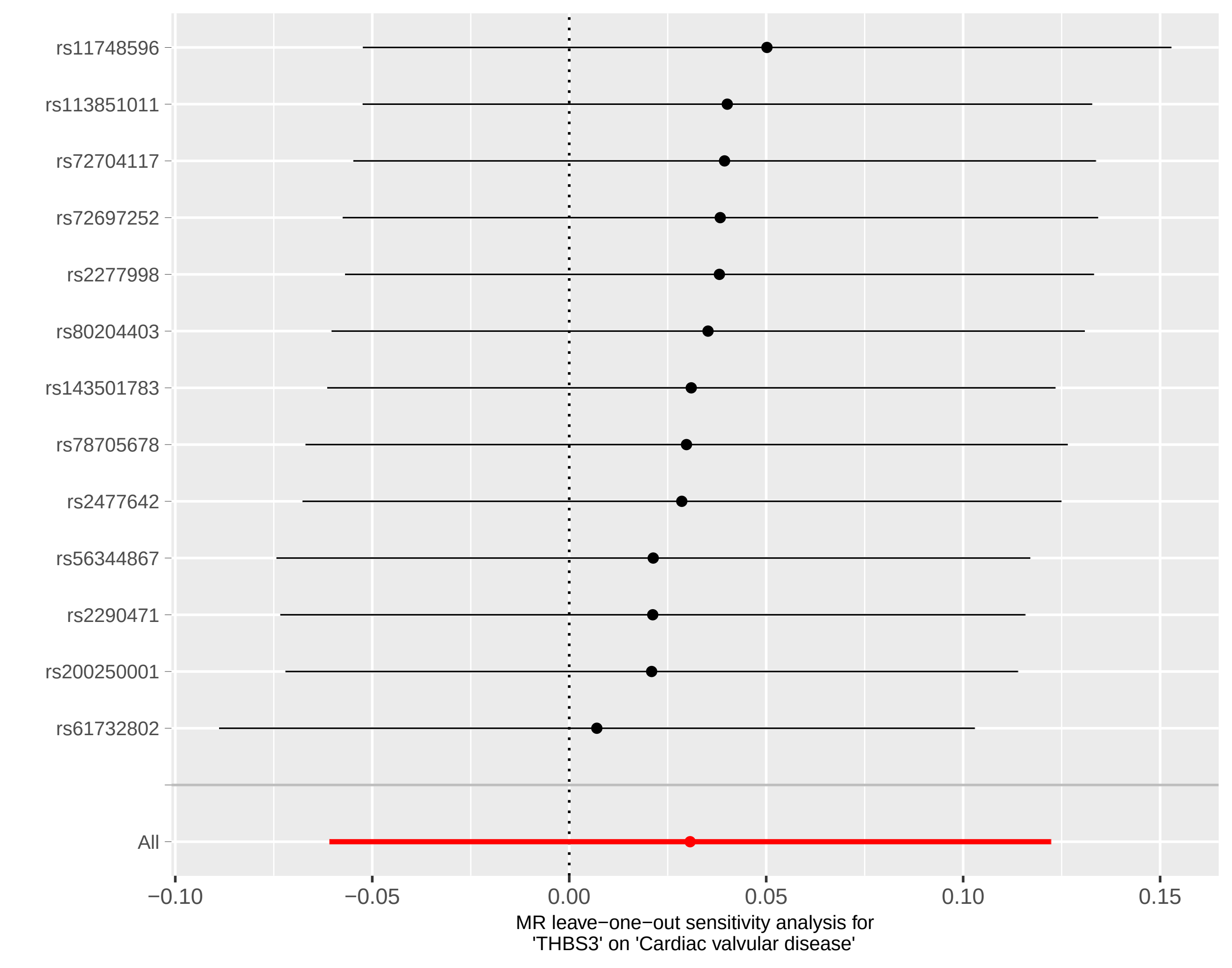
rs11748596
rs113851011
rs72704117
rs72697252
rs2277998
rs80204403
rs143501783
rs78705678
rs2477642
rs56344867
rs2290471
rs200250001
rs61732802
All
−0.10
−0.05
0.00
0.05
0.10
0.15
MR leave−one−out sensitivity analysis for
'THBS3' on 'Cardiac valvular disease'

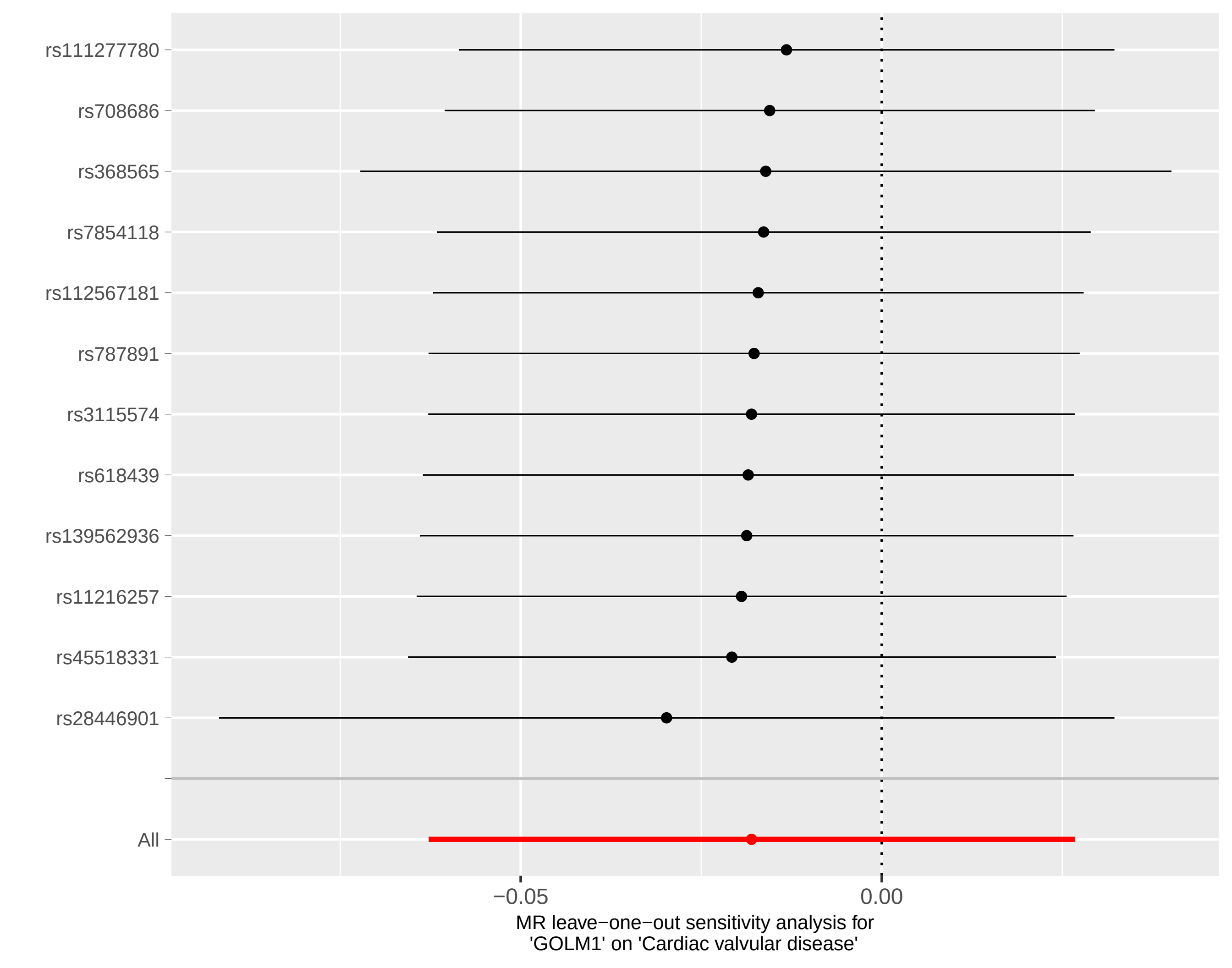

rs111277780
rs708686
rs368565
rs7854118
rs112567181
rs787891
rs3115574
rs618439
rs139562936
rs11216257
rs45518331
rs28446901
All
−0.05
0.00
MR leave−one−out sensitivity analysis for
'GOLM1' on 'Cardiac valvular disease'

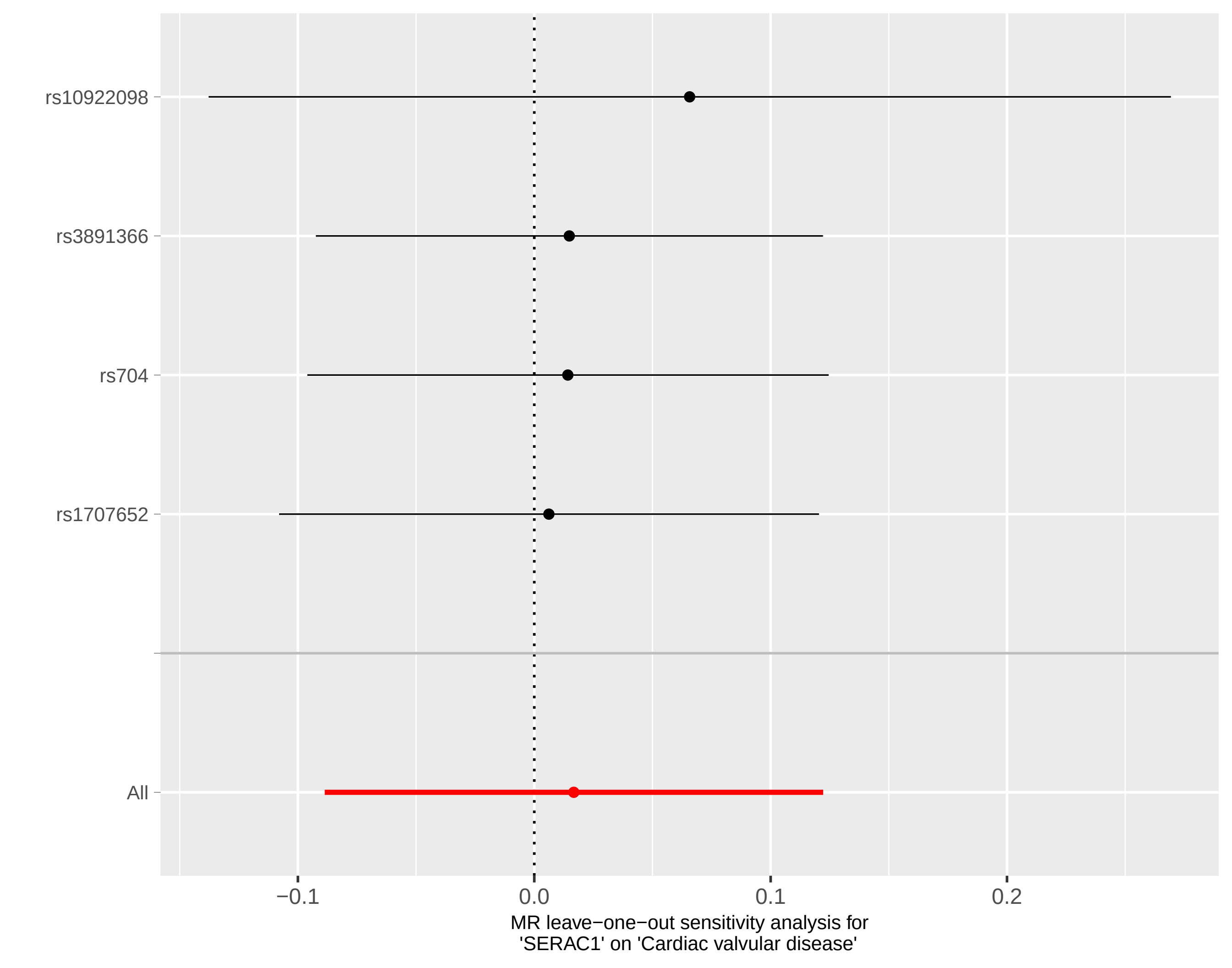
rs10922098
rs3891366
rs704
rs1707652
All
−0.1
0.0
0.1
0.2
MR leave−one−out sensitivity analysis for
'SERAC1' on 'Cardiac valvular disease'

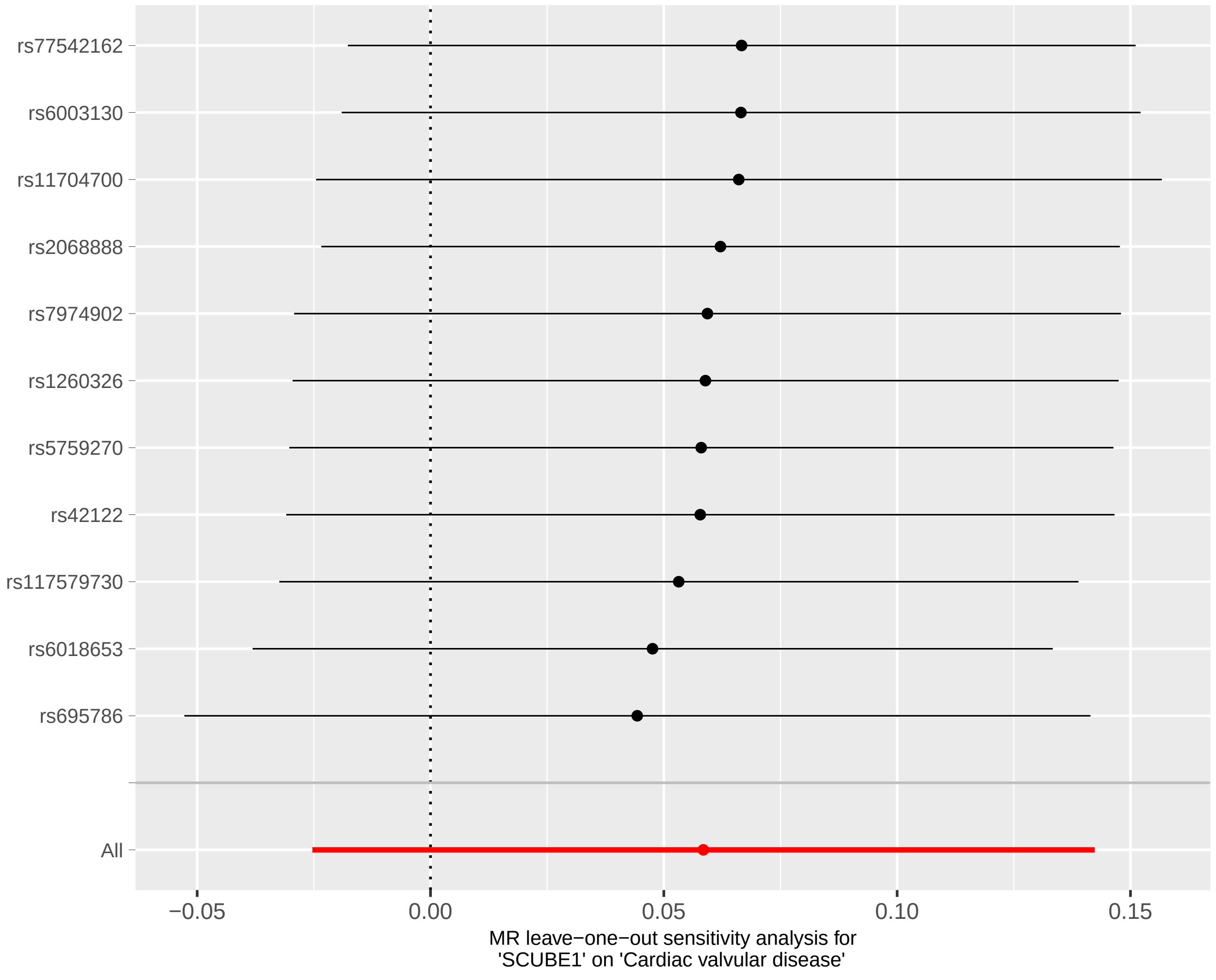
rs77542162
rs6003130
rs11704700
rs2068888
rs7974902
rs1260326
rs5759270
rs42122
rs117579730
rs6018653
rs695786
All
−0.05
0.00
0.05
0.10
0.15
MR leave−one−out sensitivity analysis for
'SCUBE1' on 'Cardiac valvular disease'

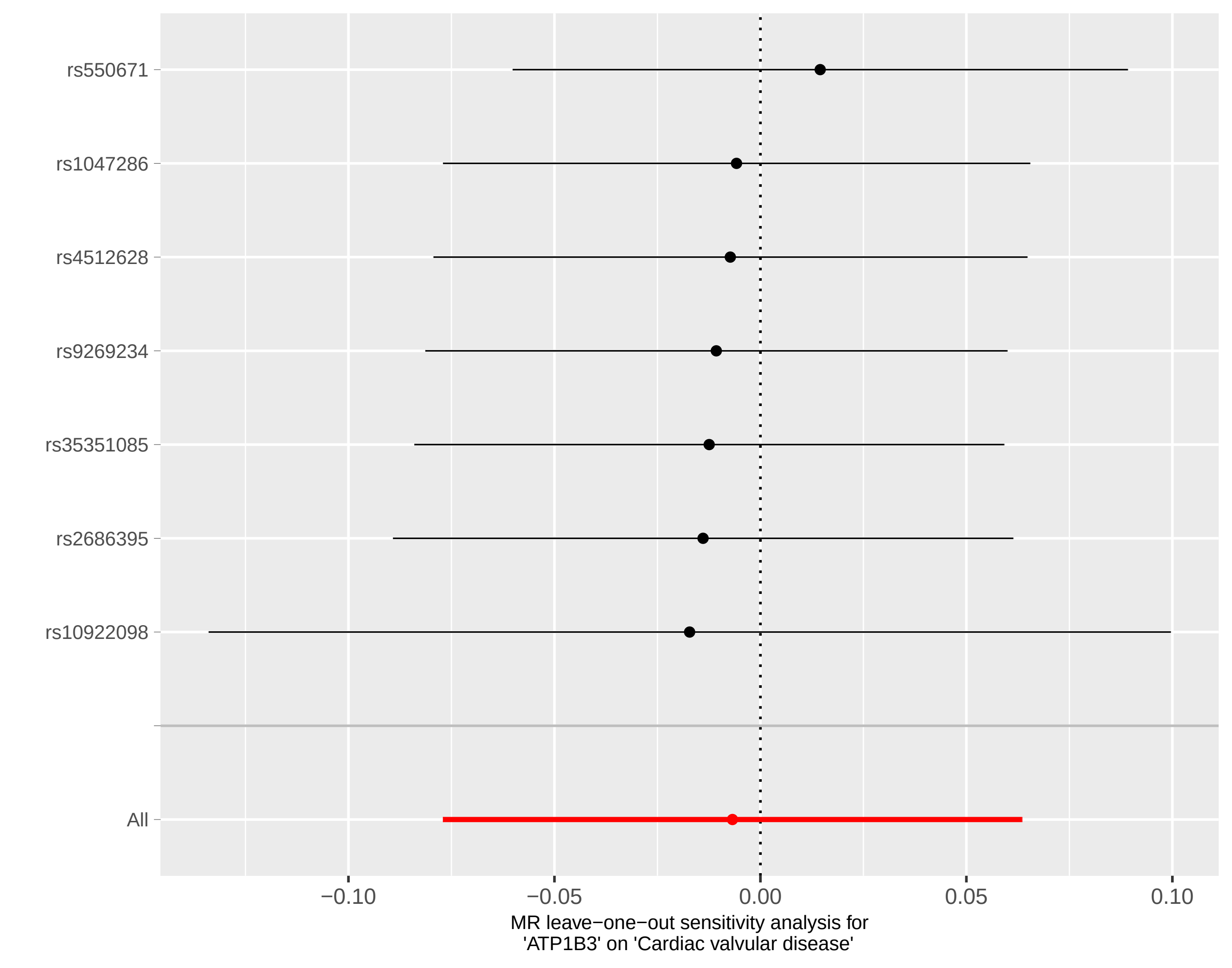
rs550671
rs1047286
rs4512628
rs9269234
rs35351085
rs2686395
rs10922098
All
−0.10
−0.05
0.00
0.05
0.10
MR leave−one−out sensitivity analysis for
'ATP1B3' on 'Cardiac valvular disease'

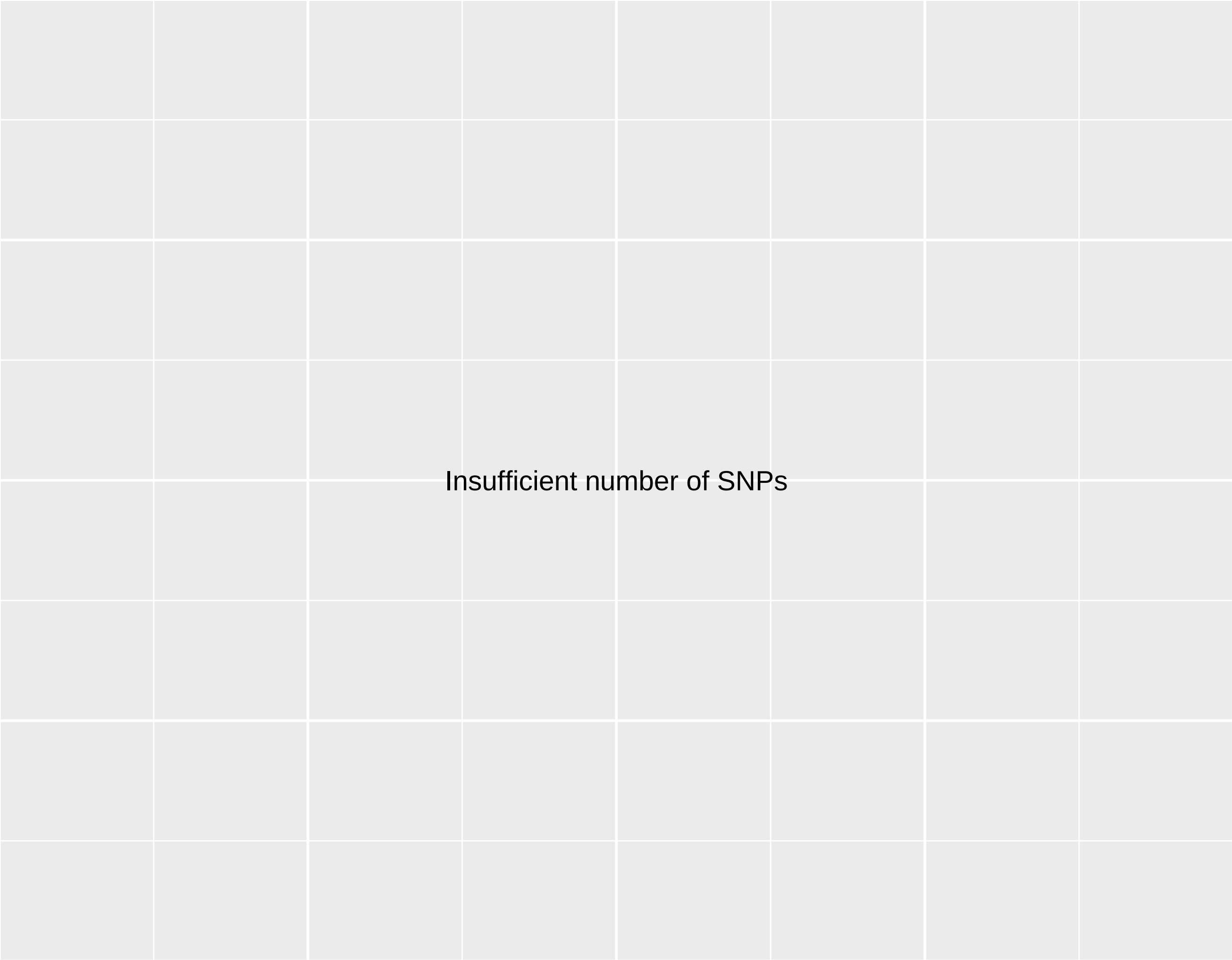
Insufficient number of SNPs

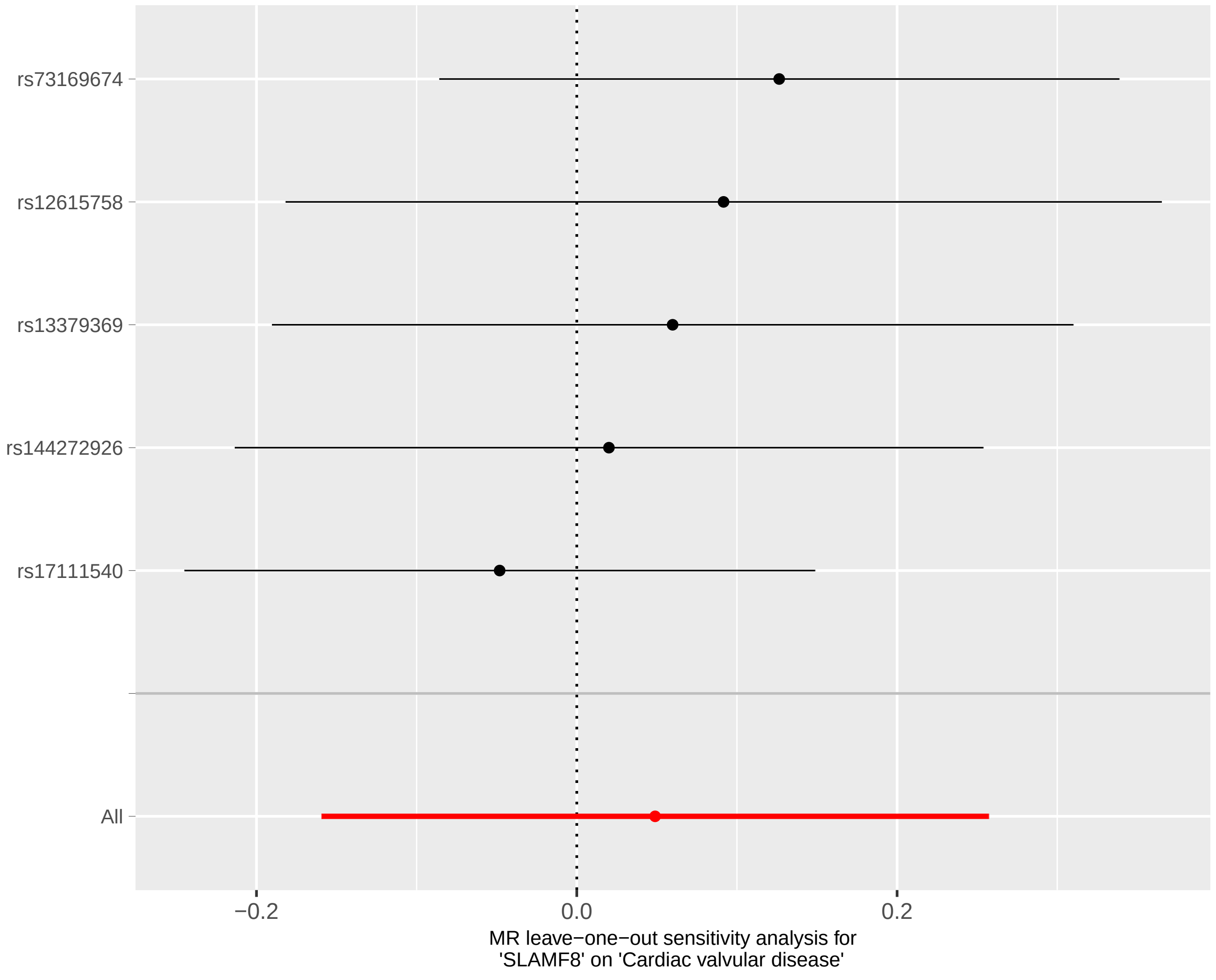
rs73169674
rs12615758
rs13379369
rs144272926
rs17111540
All
−0.2
0.0
0.2
MR leave−one−out sensitivity analysis for
'SLAMF8' on 'Cardiac valvular disease'

Insufficient number of SNPs

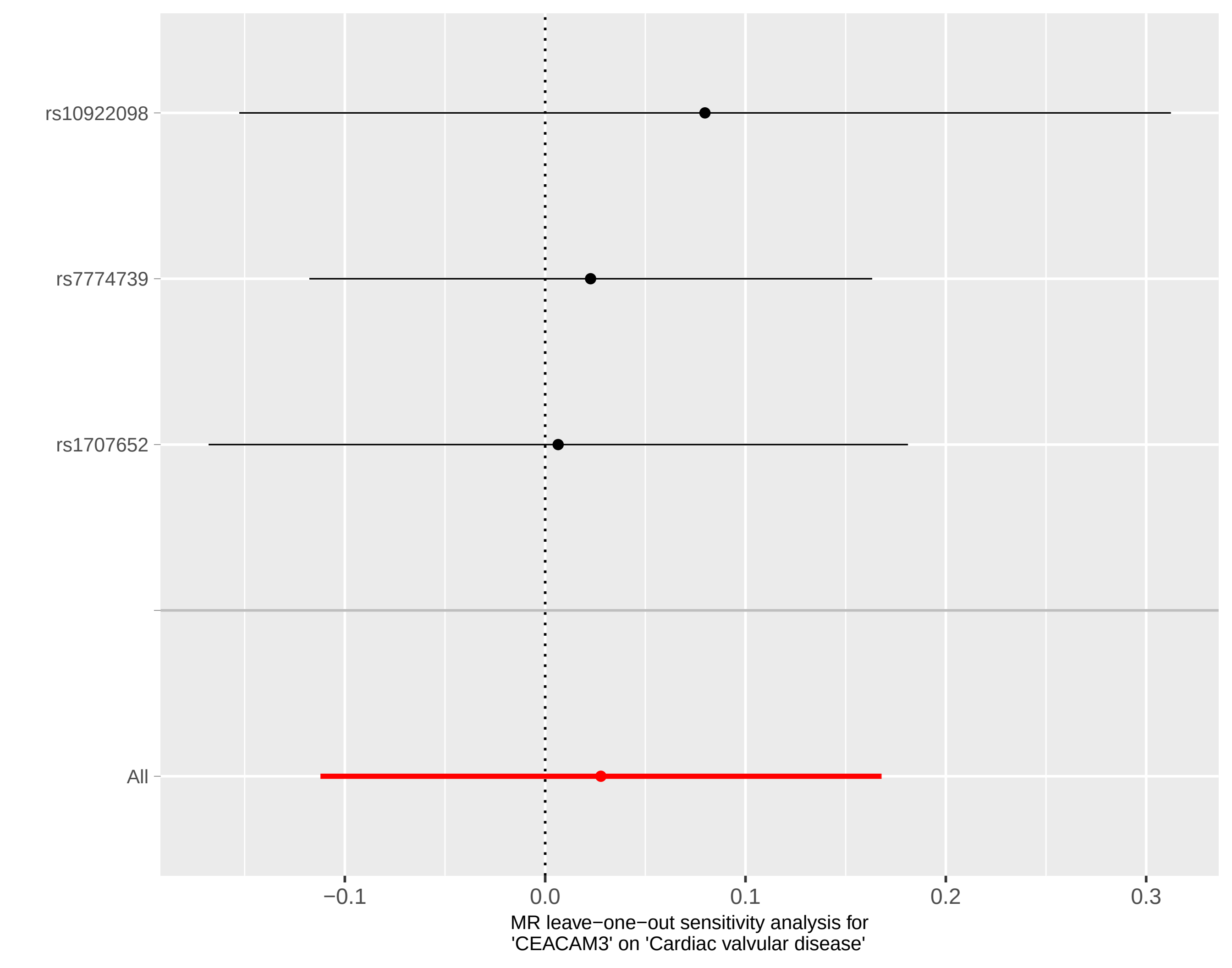
rs10922098
rs7774739
rs1707652
All
−0.1
0.0
0.1
0.2
0.3
MR leave−one−out sensitivity analysis for
'CEACAM3' on 'Cardiac valvular disease'

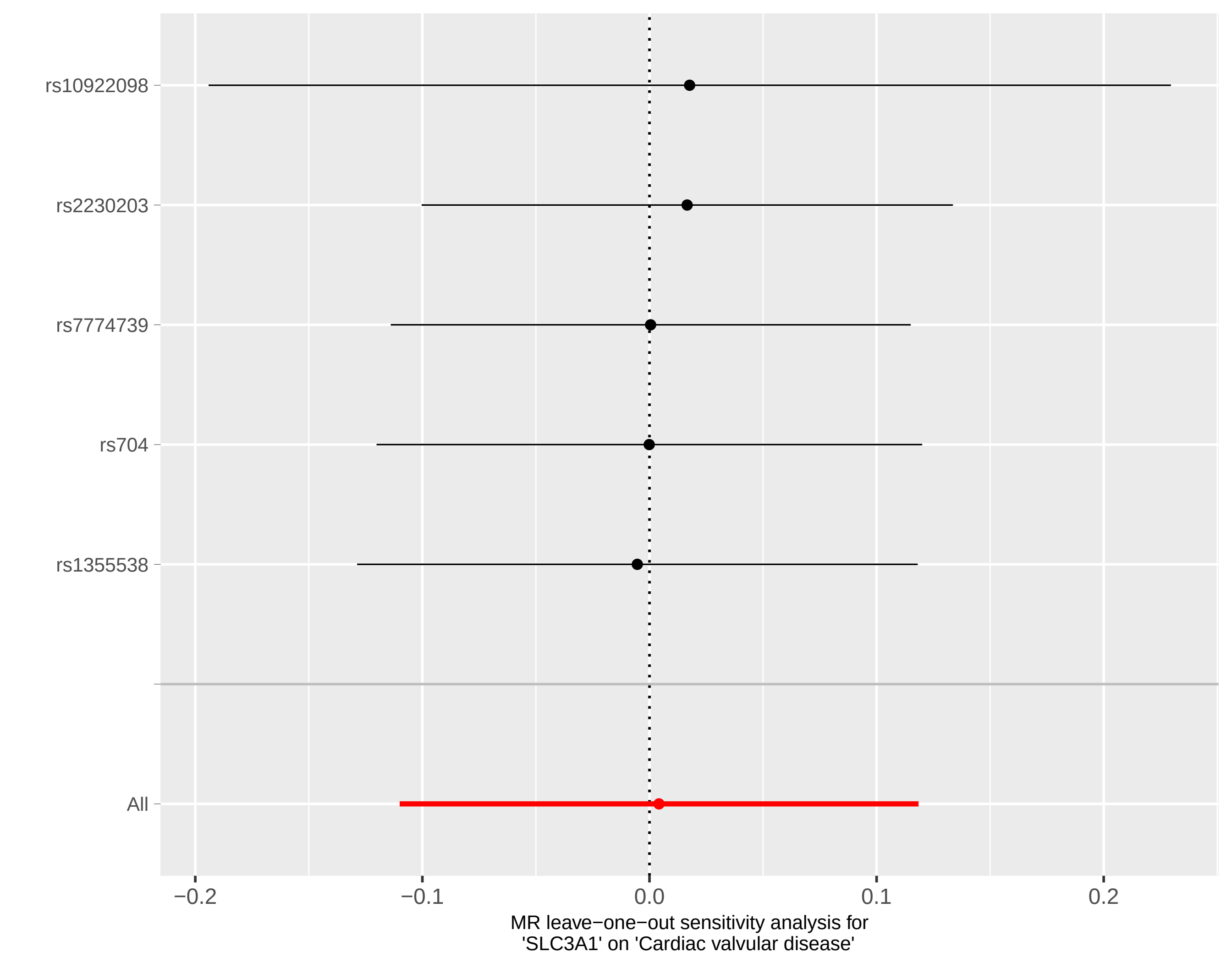
rs10922098
rs2230203
rs7774739
rs704
rs1355538
All
−0.2
−0.1
0.0
0.1
0.2
MR leave−one−out sensitivity analysis for
'SLC3A1' on 'Cardiac valvular disease'

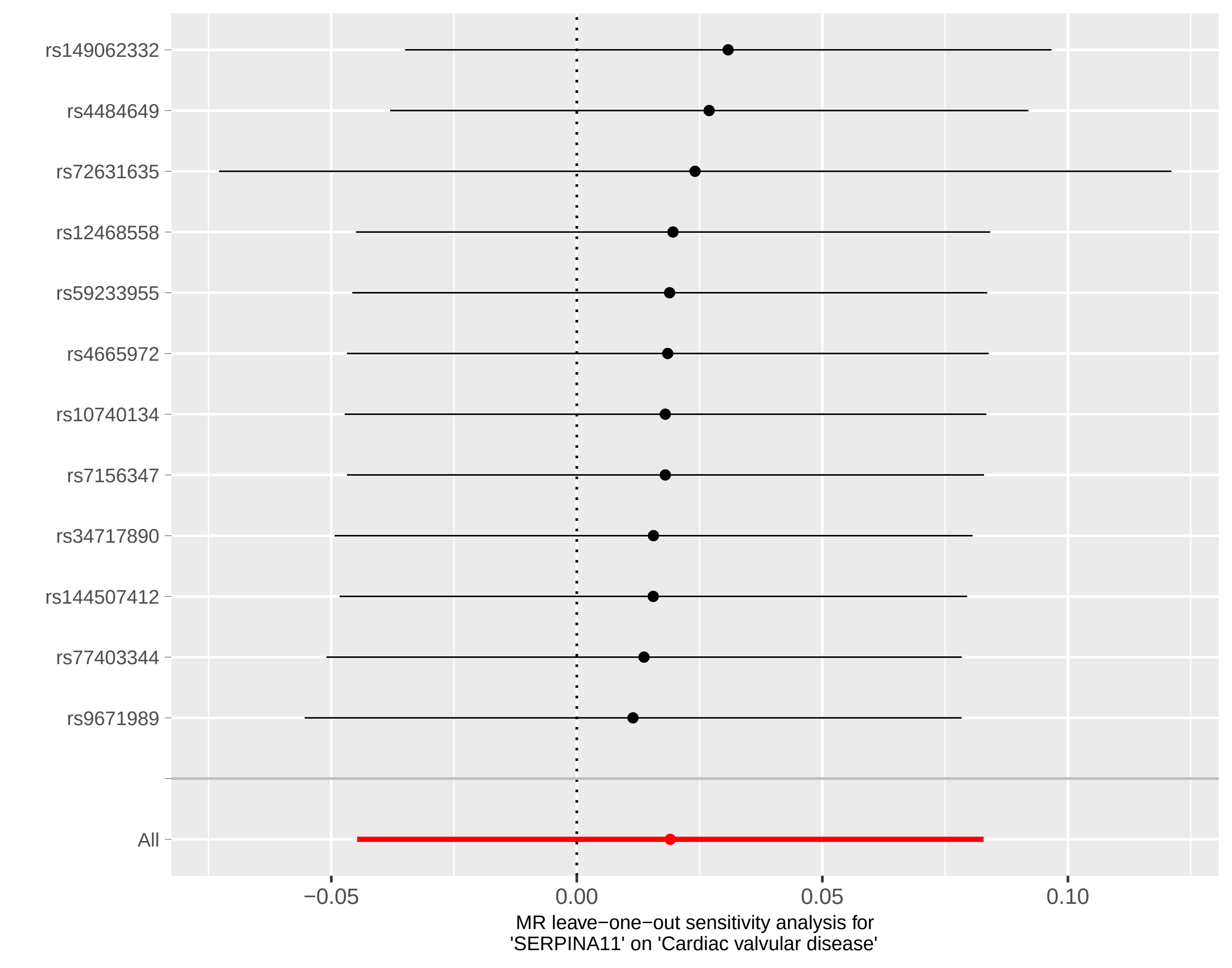

rs149062332
rs4484649
rs72631635
rs12468558
rs59233955
rs4665972
rs10740134
rs7156347
rs34717890
rs144507412
rs77403344
rs9671989
All
−0.05
0.00
0.05
0.10
MR leave−one−out sensitivity analysis for
'SERPINA11' on 'Cardiac valvular disease'

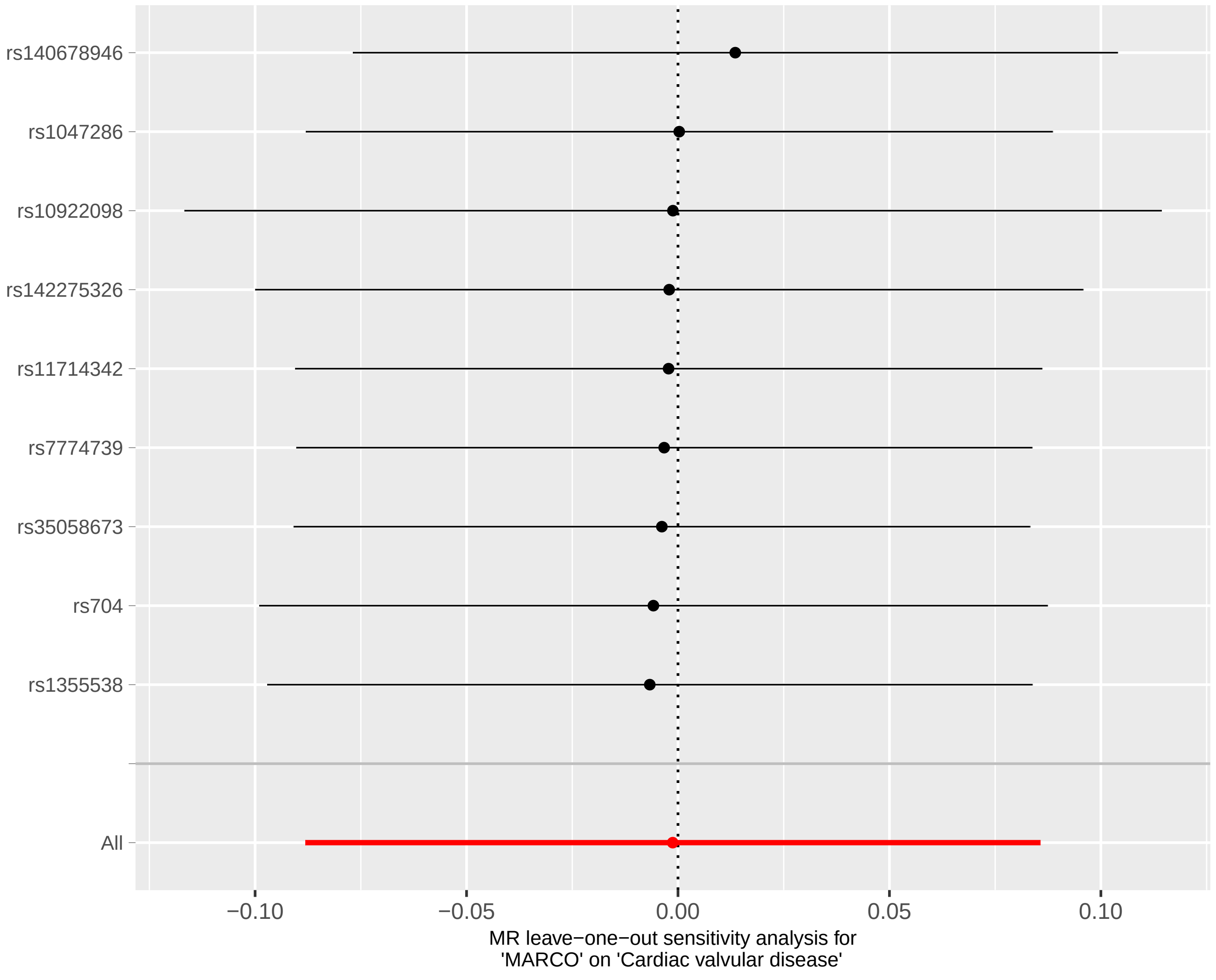

rs140678946
rs1047286
rs10922098
rs142275326
rs11714342
rs7774739
rs35058673
rs704
rs1355538
All
−0.10
−0.05
0.00
0.05
0.10
MR leave−one−out sensitivity analysis for
'MARCO' on 'Cardiac valvular disease'

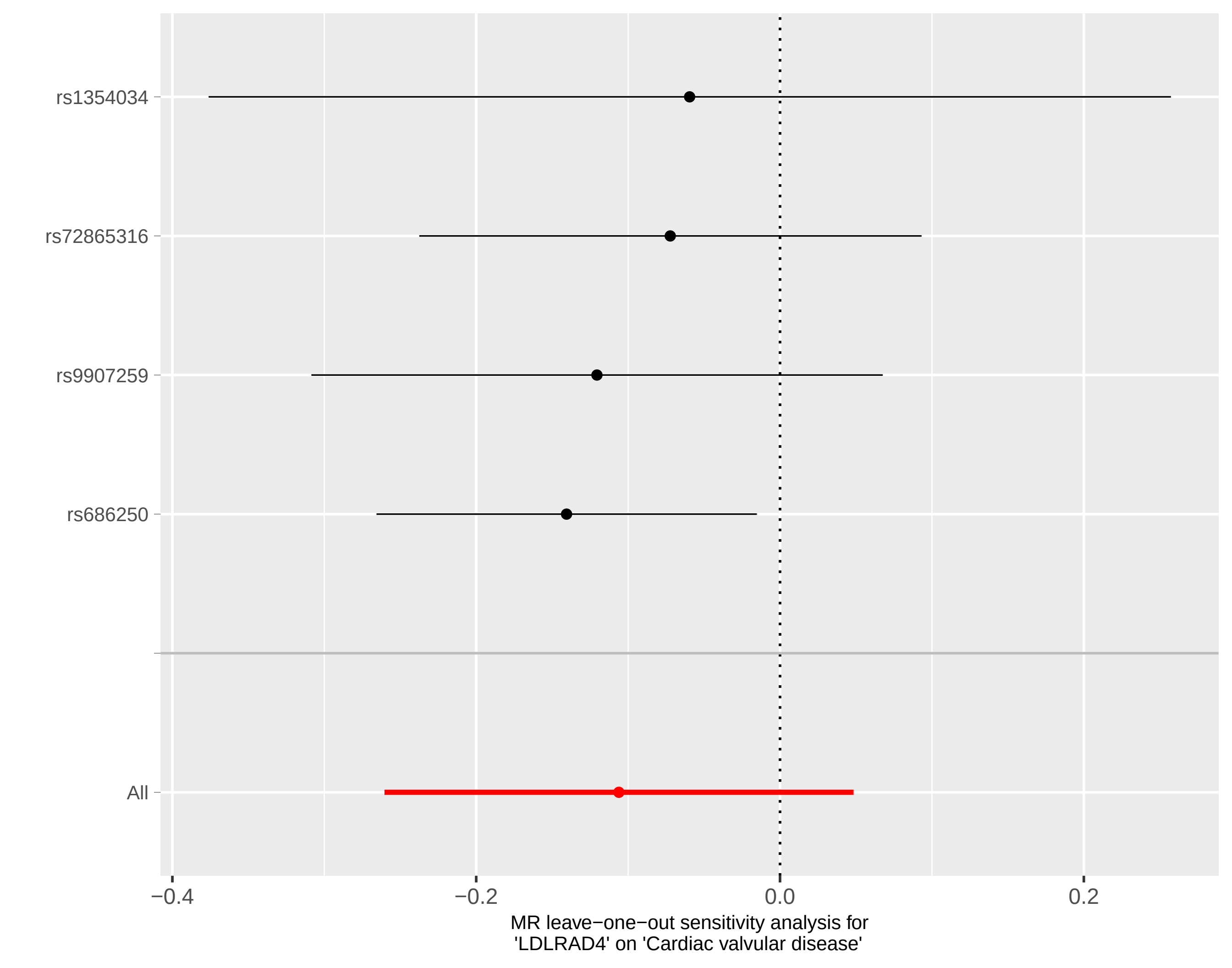
rs1354034
rs72865316
rs9907259
rs686250
All
−0.4
−0.2
0.0
0.2
MR leave−one−out sensitivity analysis for
'LDLRAD4' on 'Cardiac valvular disease'

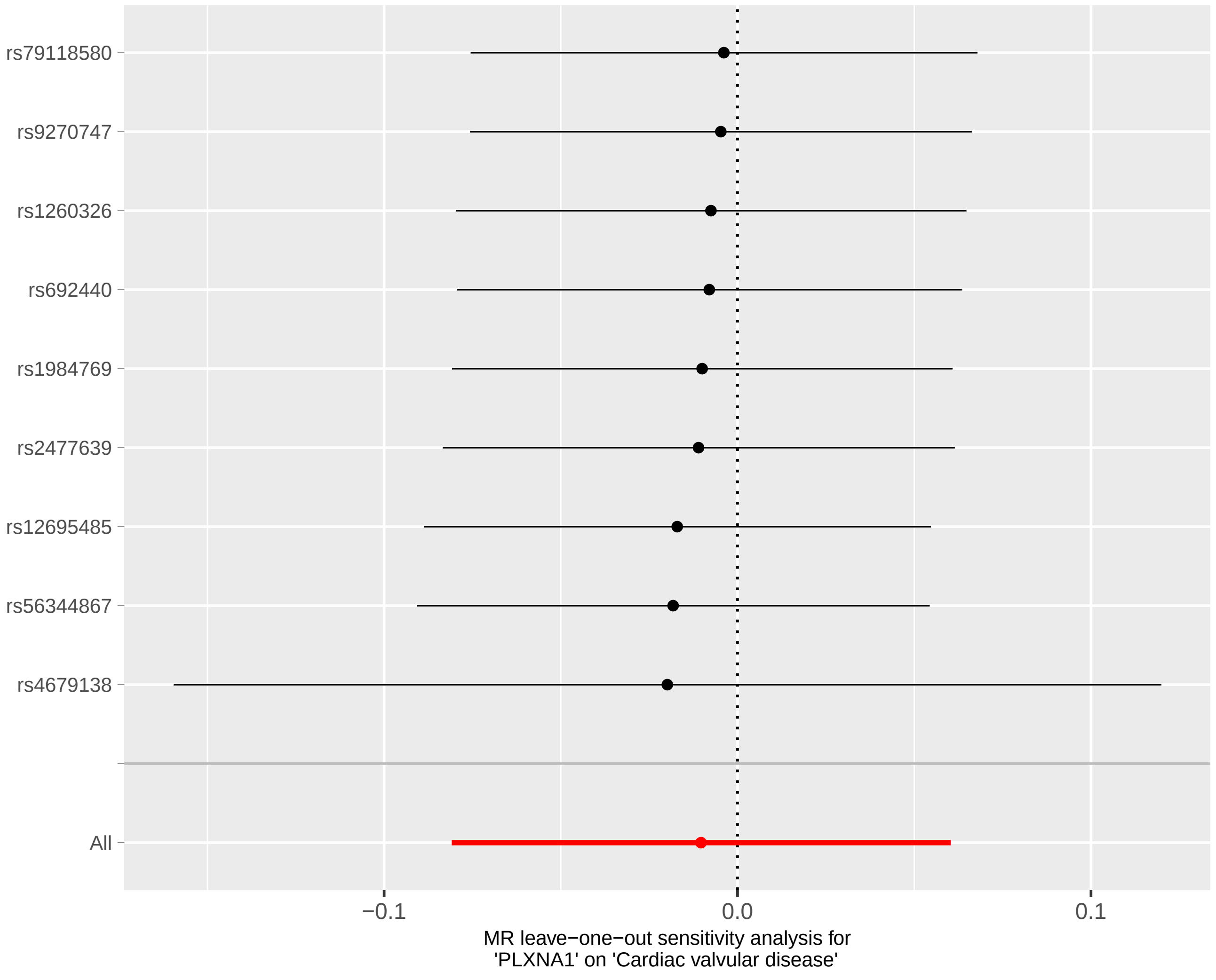
rs79118580
rs9270747
rs1260326
rs692440
rs1984769
rs2477639
rs12695485
rs56344867
rs4679138
All
−0.1
0.0
0.1
MR leave−one−out sensitivity analysis for
'PLXNA1' on 'Cardiac valvular disease'

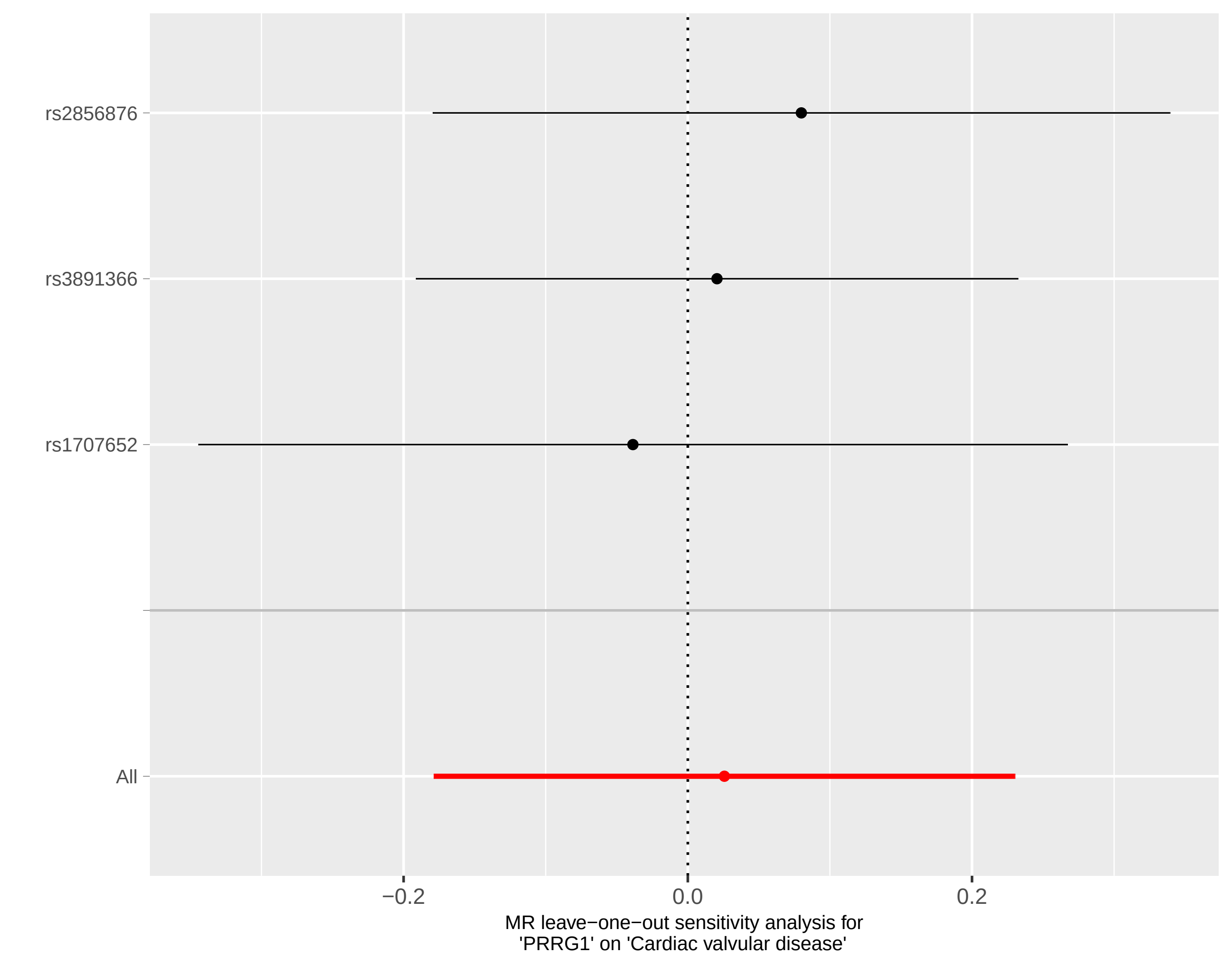
rs2856876
rs3891366
rs1707652
All
−0.2
0.0
0.2
MR leave−one−out sensitivity analysis for
'PRRG1' on 'Cardiac valvular disease'

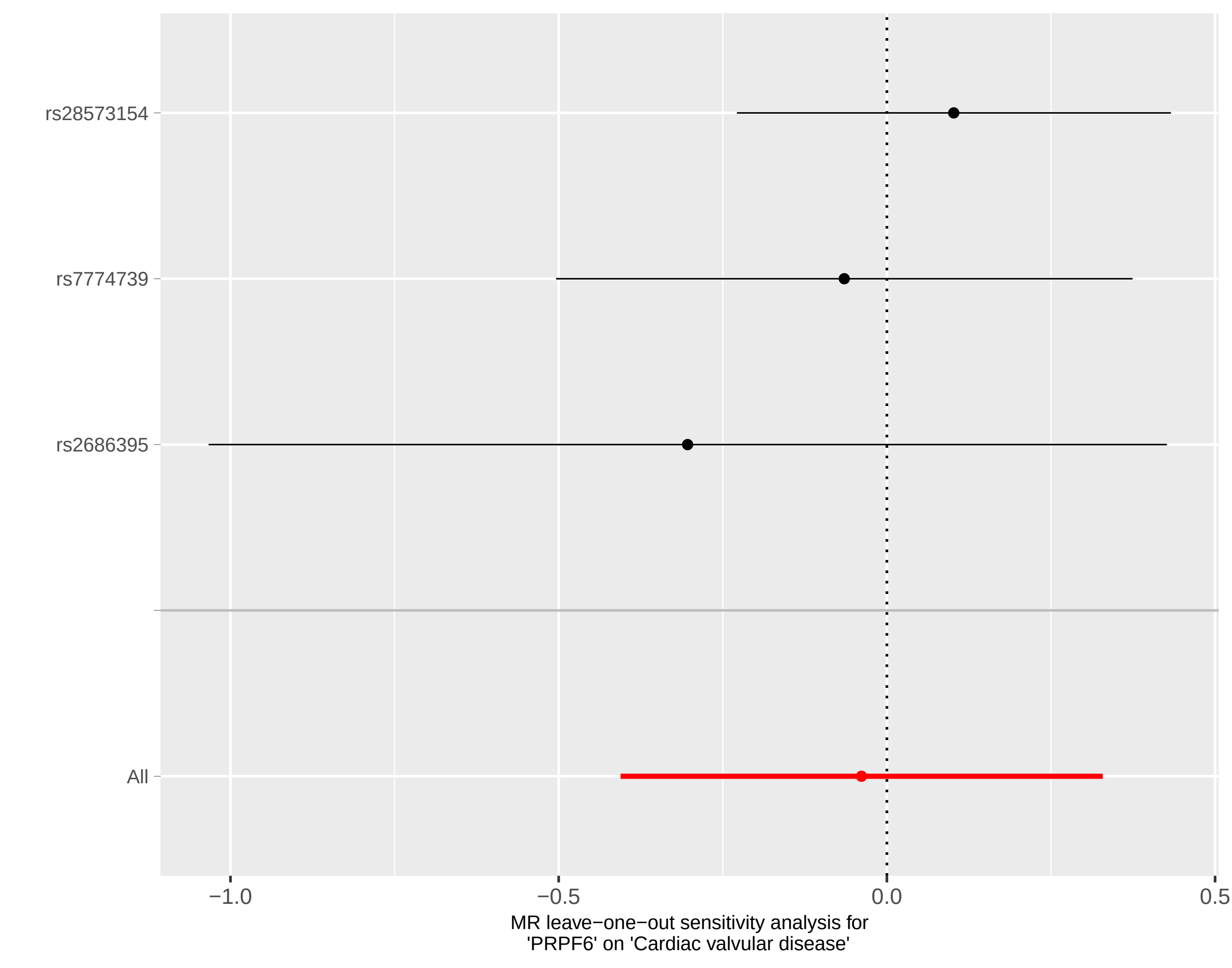
rs28573154
rs7774739
rs2686395
All
−1.0
−0.5
0.0
0.5
MR leave−one−out sensitivity analysis for
'PRPF6' on 'Cardiac valvular disease'

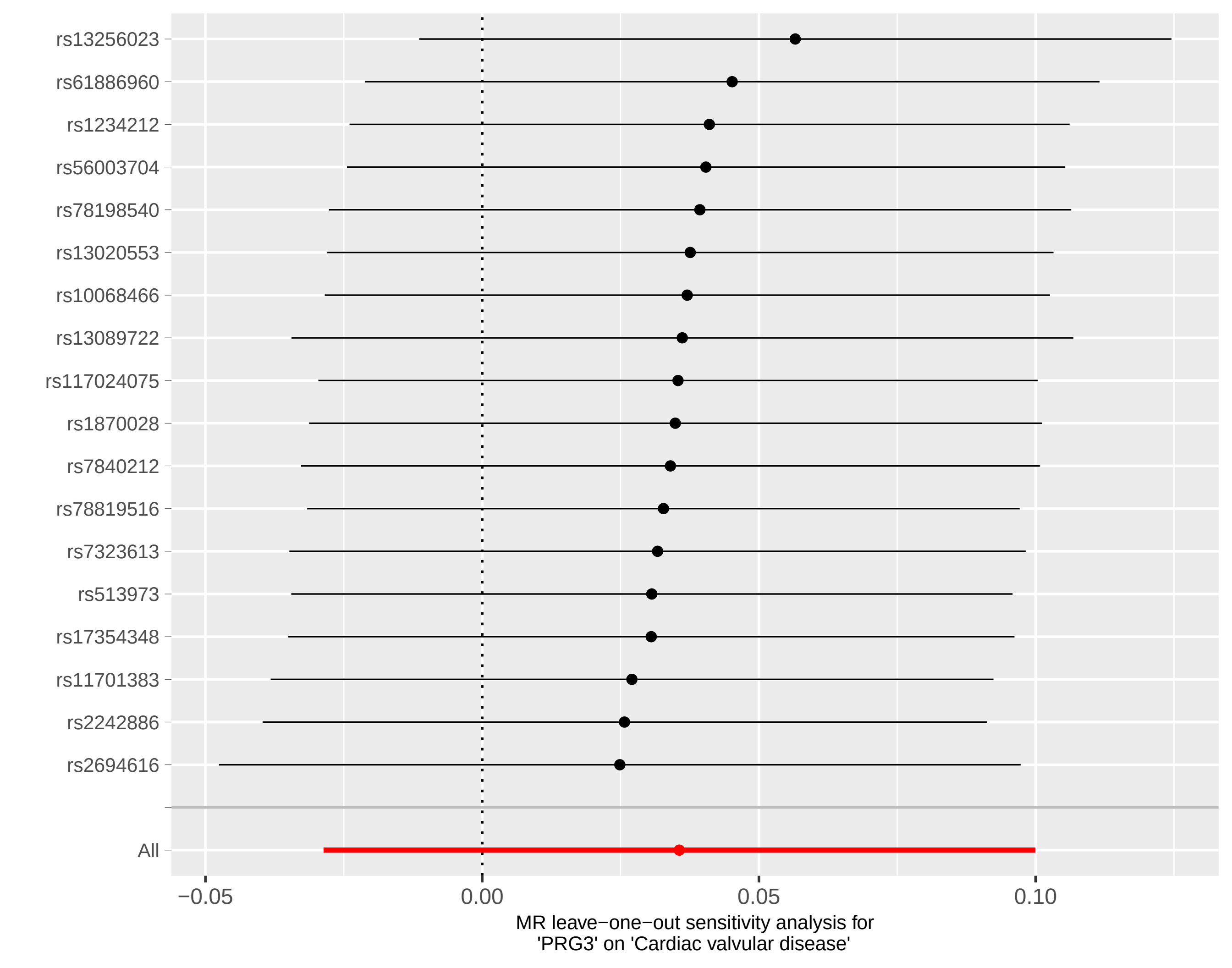

rs13256023
rs61886960
rs1234212
rs56003704
rs78198540
rs13020553
rs10068466
rs13089722
rs117024075
rs1870028
rs7840212
rs78819516
rs7323613
rs513973
rs17354348
rs11701383
rs2242886
rs2694616
All
−0.05
0.00
0.05
0.10
MR leave−one−out sensitivity analysis for
'PRG3' on 'Cardiac valvular disease'

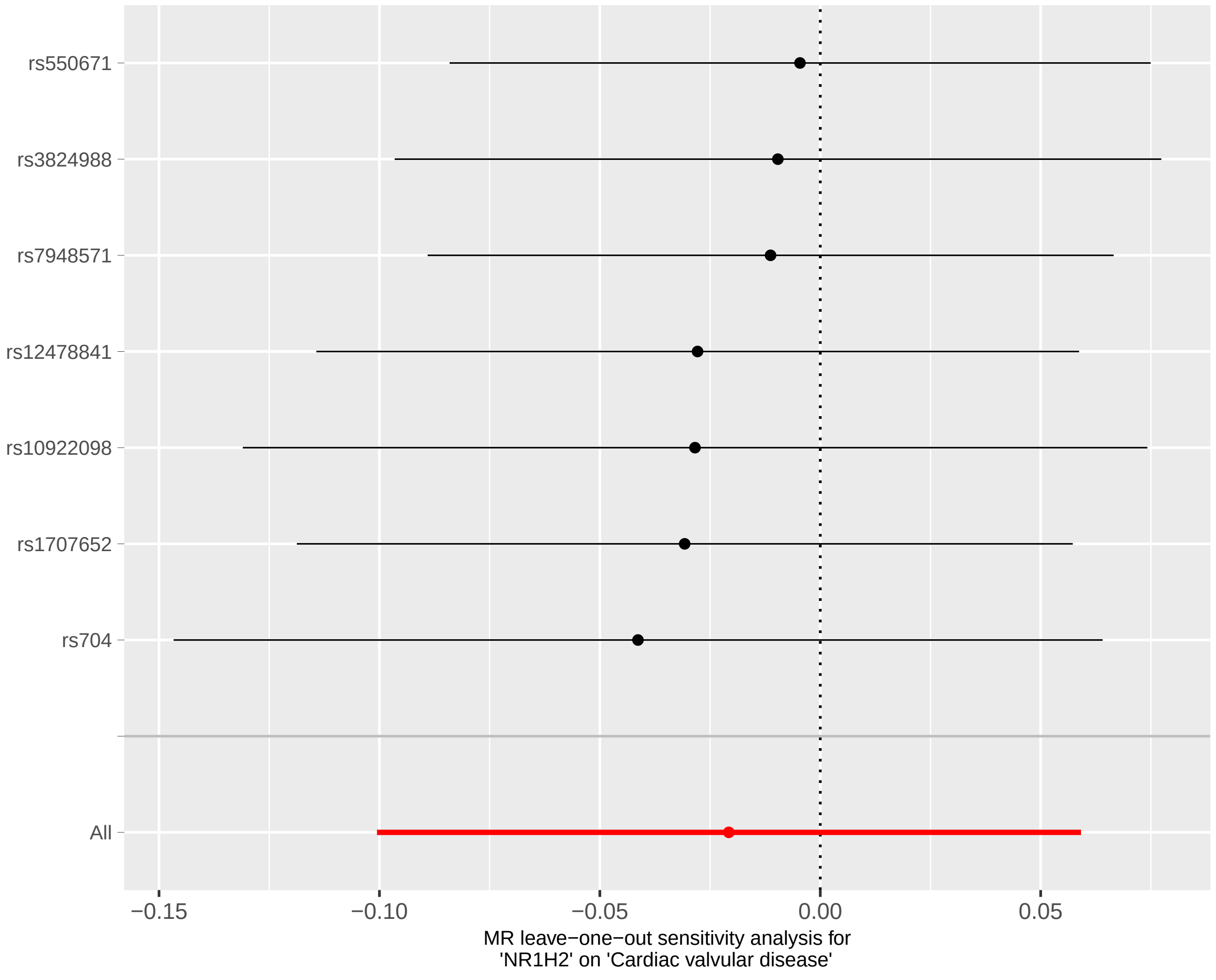
rs550671
rs3824988
rs7948571
rs12478841
rs10922098
rs1707652
rs704
All
−0.15
−0.10
−0.05
0.00
0.05
MR leave−one−out sensitivity analysis for
'NR1H2' on 'Cardiac valvular disease'

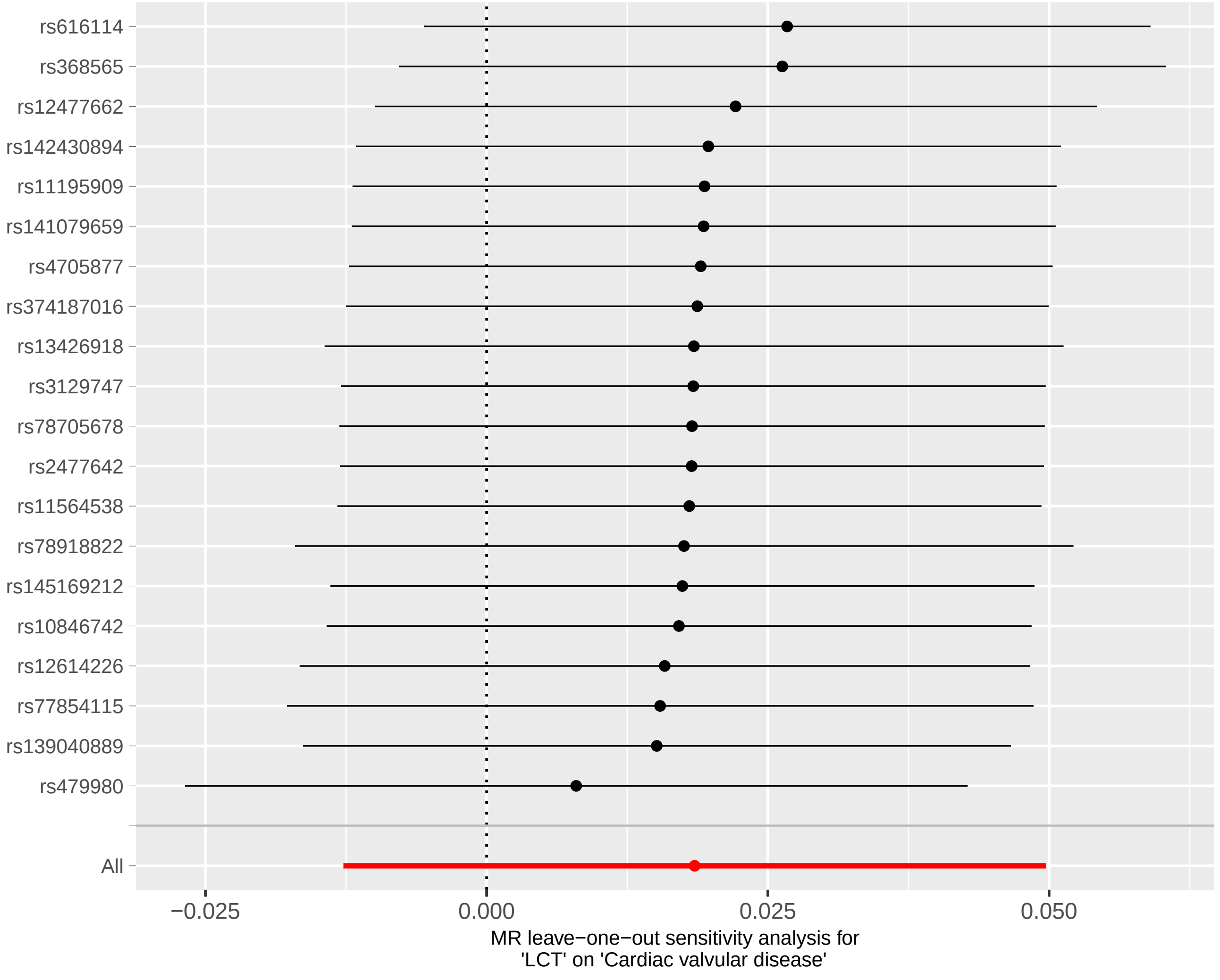

rs616114
rs368565
rs12477662
rs142430894
rs11195909
rs141079659
rs4705877
rs374187016
rs13426918
rs3129747
rs78705678
rs2477642
rs11564538
rs78918822
rs145169212
rs10846742
rs12614226
rs77854115
rs139040889
rs479980
All
−0.025
0.000
0.025
0.050
MR leave−one−out sensitivity analysis for
'LCT' on 'Cardiac valvular disease'

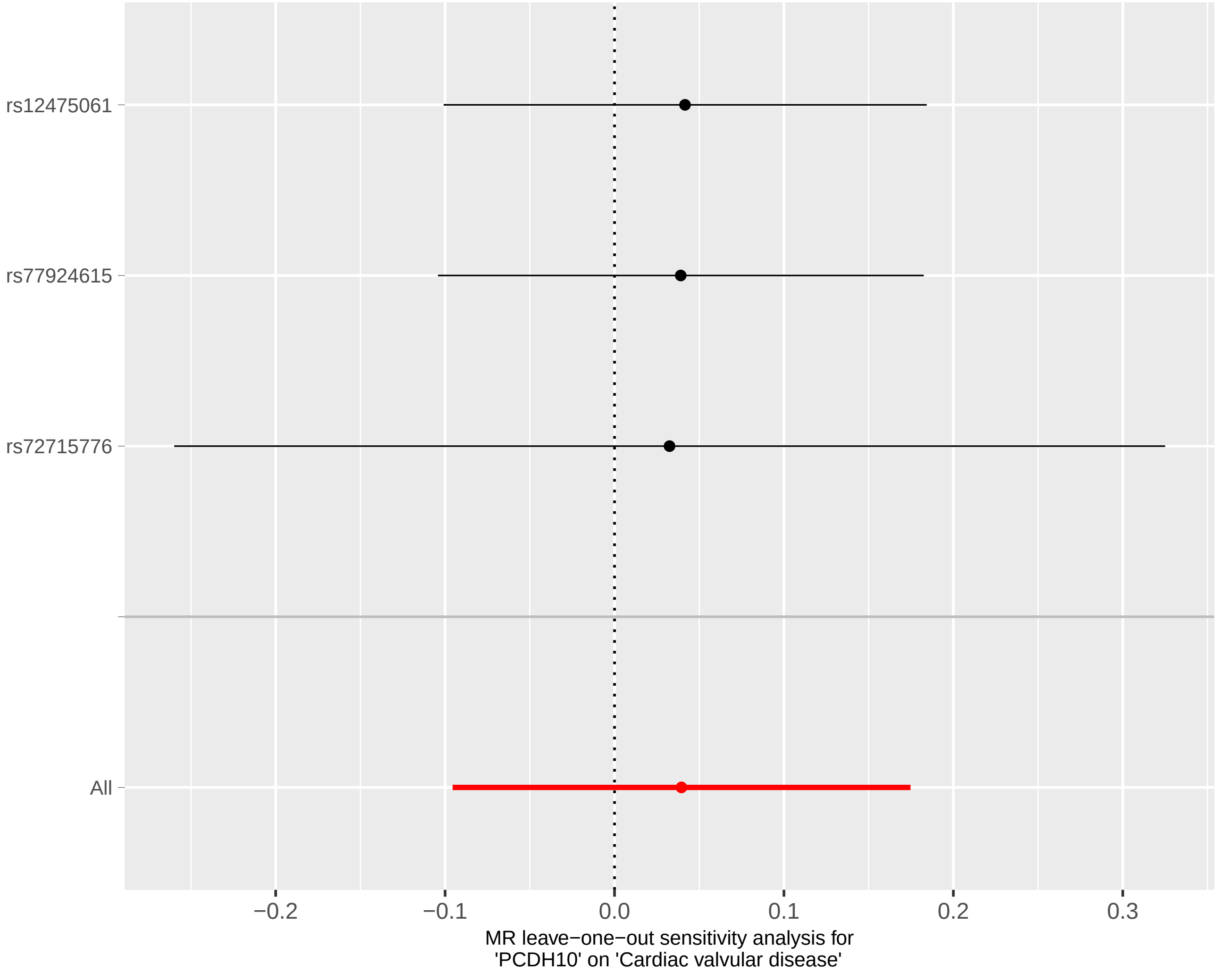
rs12475061
rs77924615
rs72715776
All
−0.2
−0.1
0.0
0.1
0.2
0.3
MR leave−one−out sensitivity analysis for
'PCDH10' on 'Cardiac valvular disease'

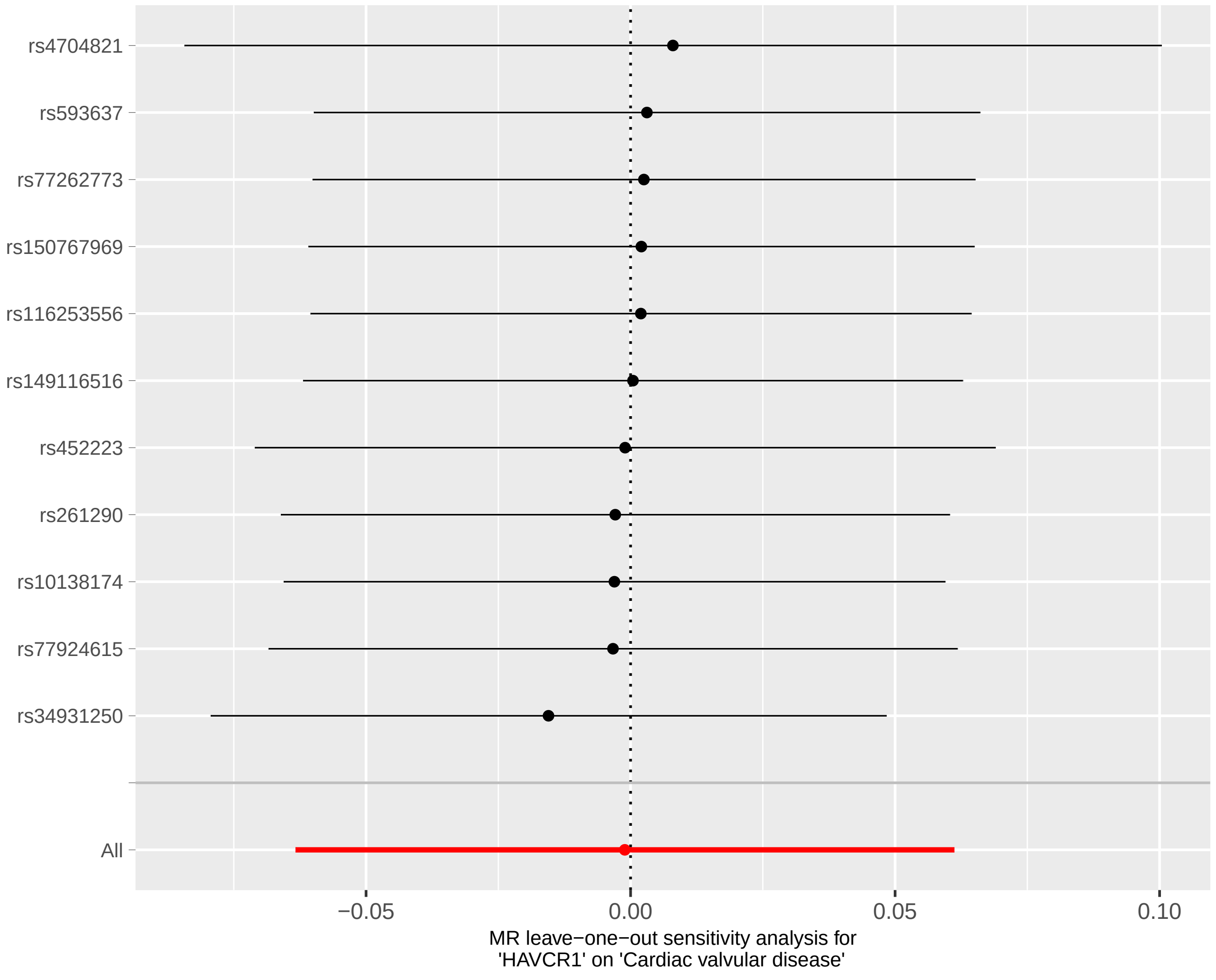
rs4704821
rs593637
rs77262773
rs150767969
rs116253556
rs149116516
rs452223
rs261290
rs10138174
rs77924615
rs34931250
All
−0.05
0.00
0.05
0.10
MR leave−one−out sensitivity analysis for
'HAVCR1' on 'Cardiac valvular disease'

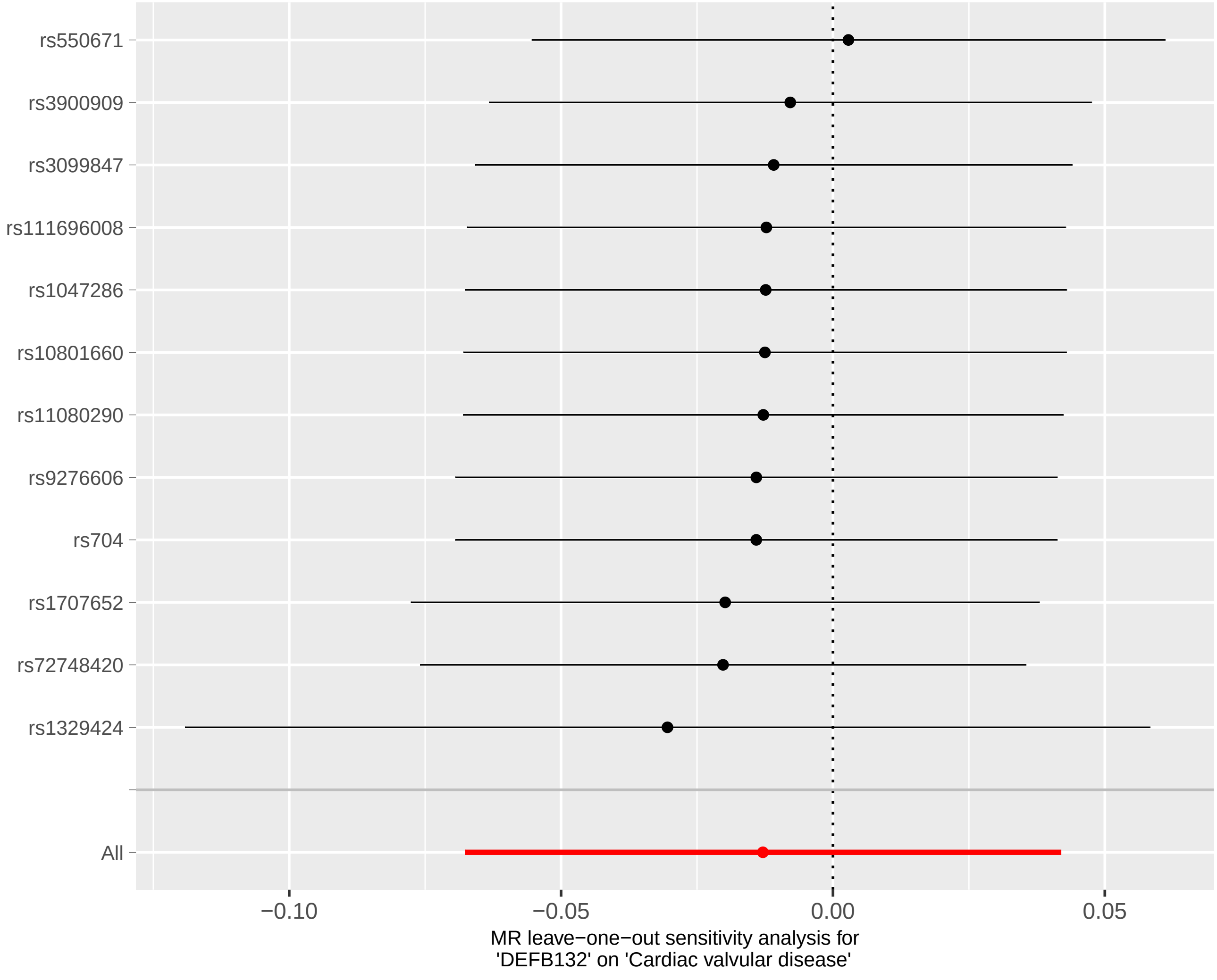
rs550671
rs3900909
rs3099847
rs111696008
rs1047286
rs10801660
rs11080290
rs9276606
rs704
rs1707652
rs72748420
rs1329424
All
−0.10
−0.05
0.00
0.05
MR leave−one−out sensitivity analysis for
'DEFB132' on 'Cardiac valvular disease'

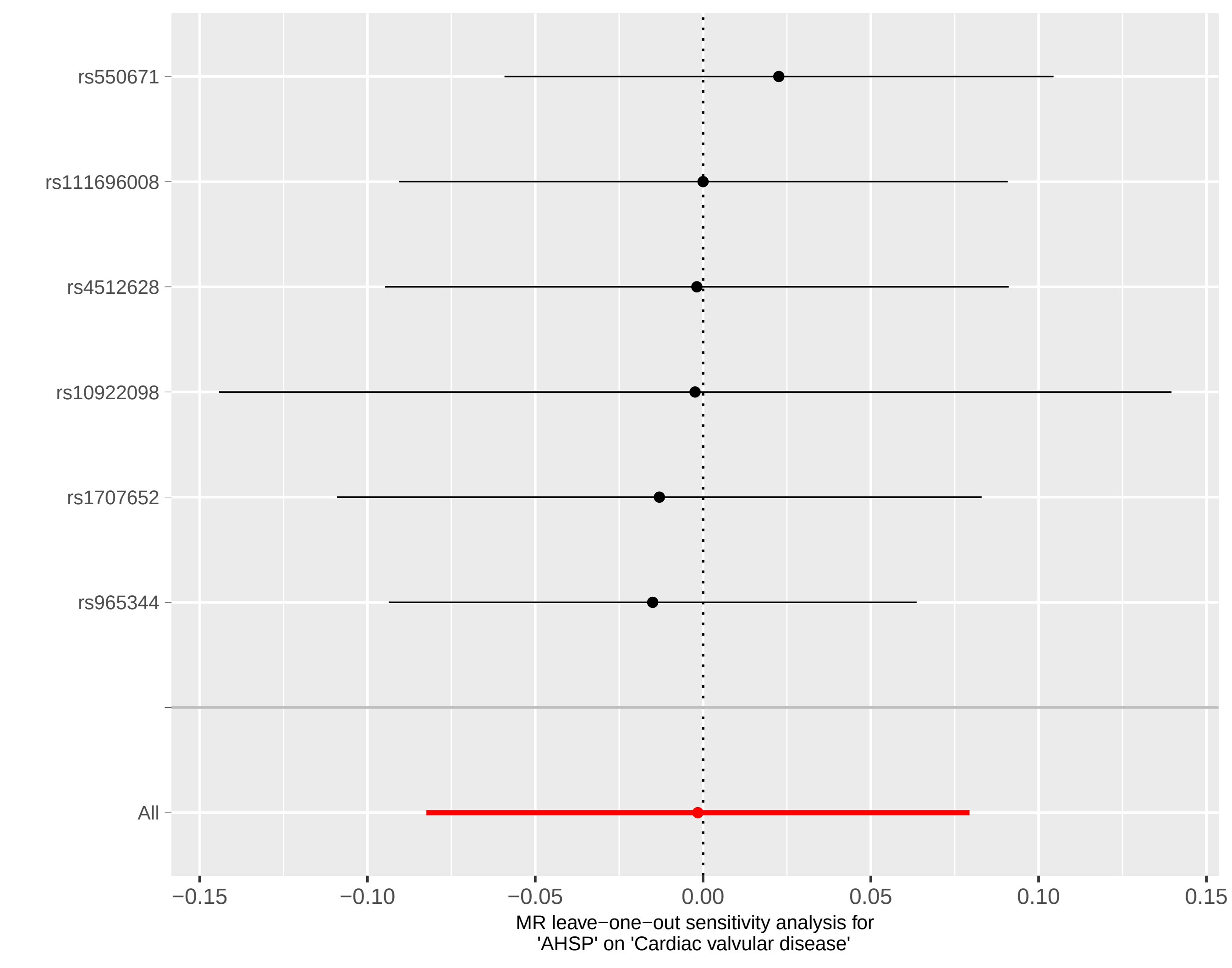

rs550671
rs111696008
rs4512628
rs10922098
rs1707652
rs965344
All
−0.15
−0.10
−0.05
0.00
0.05
0.10
0.15
MR leave−one−out sensitivity analysis for
'AHSP' on 'Cardiac valvular disease'

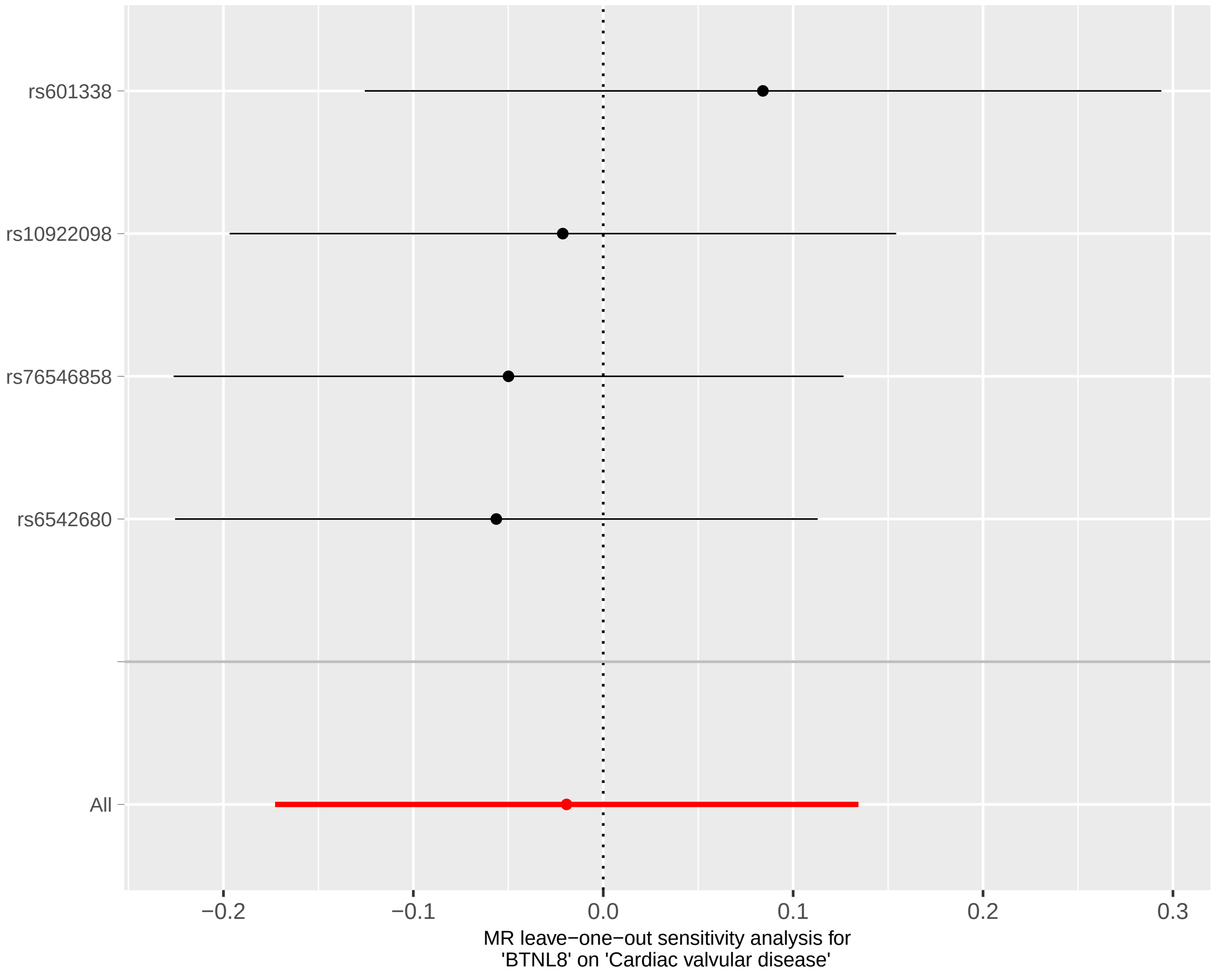
rs601338
rs10922098
rs76546858
rs6542680
All
−0.2
−0.1
0.0
0.1
0.2
0.3
MR leave−one−out sensitivity analysis for
'BTNL8' on 'Cardiac valvular disease'

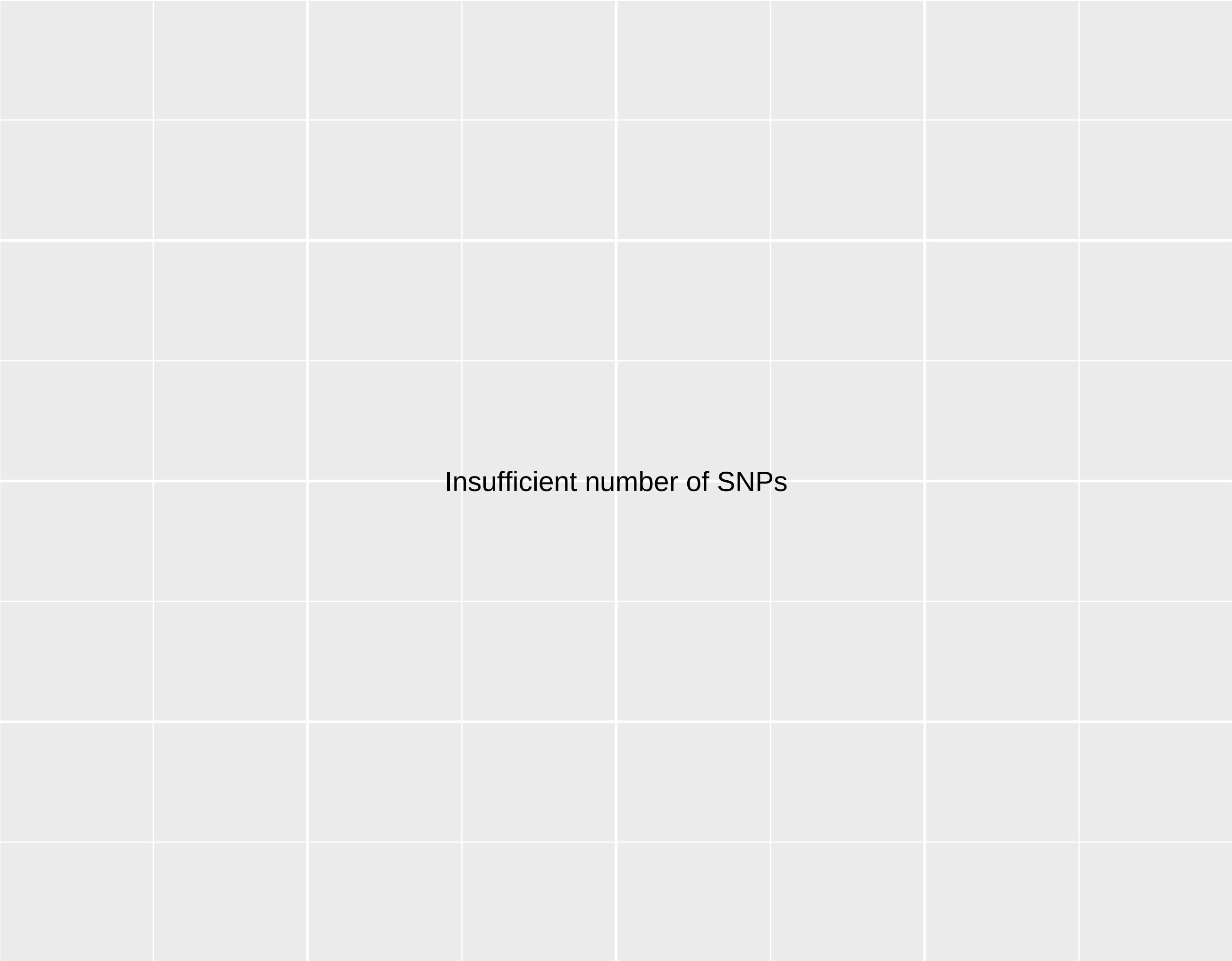
Insufficient number of SNPs

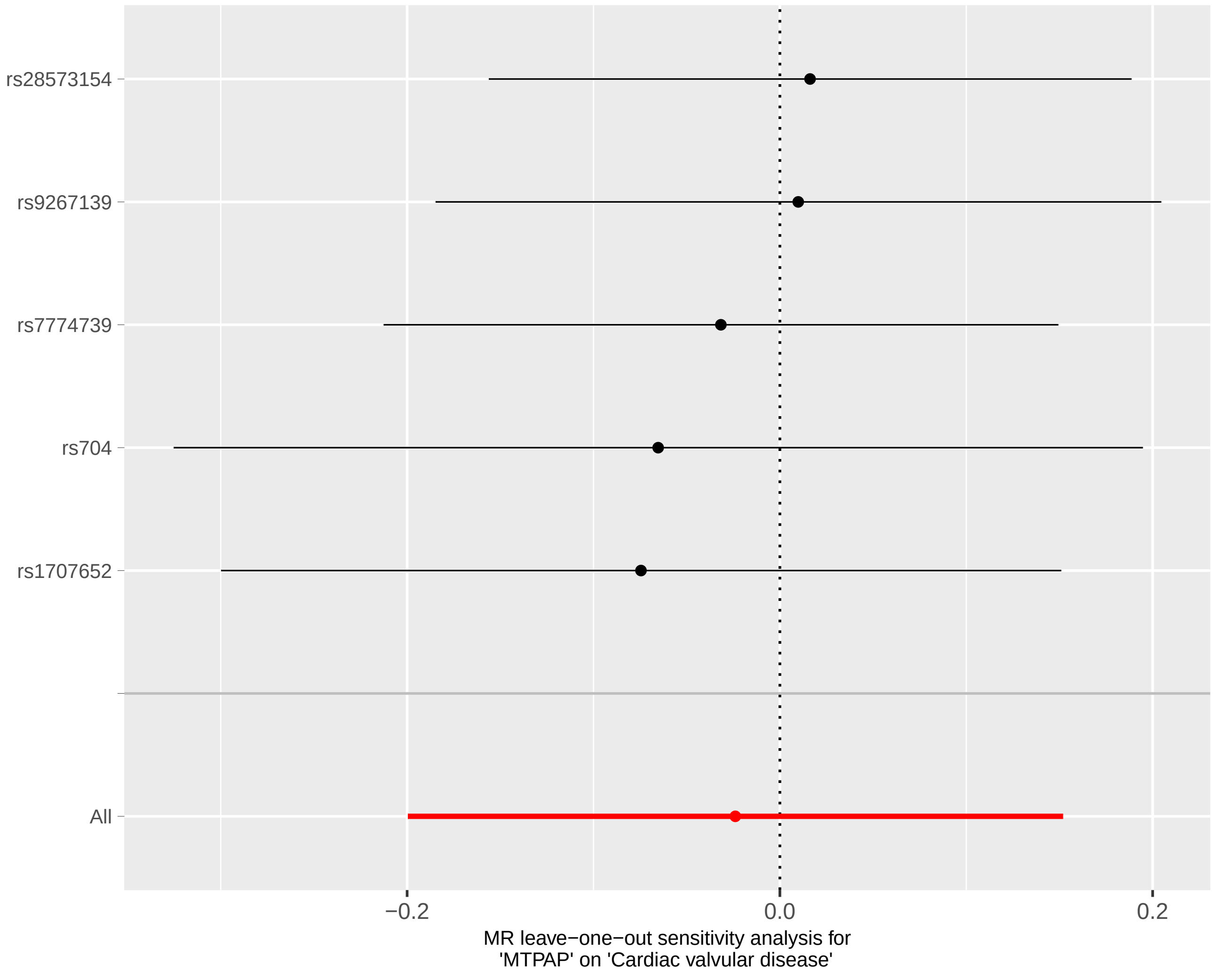
rs28573154
rs9267139
rs7774739
rs704
rs1707652
All
−0.2
0.0
0.2
MR leave−one−out sensitivity analysis for
'MTPAP' on 'Cardiac valvular disease'

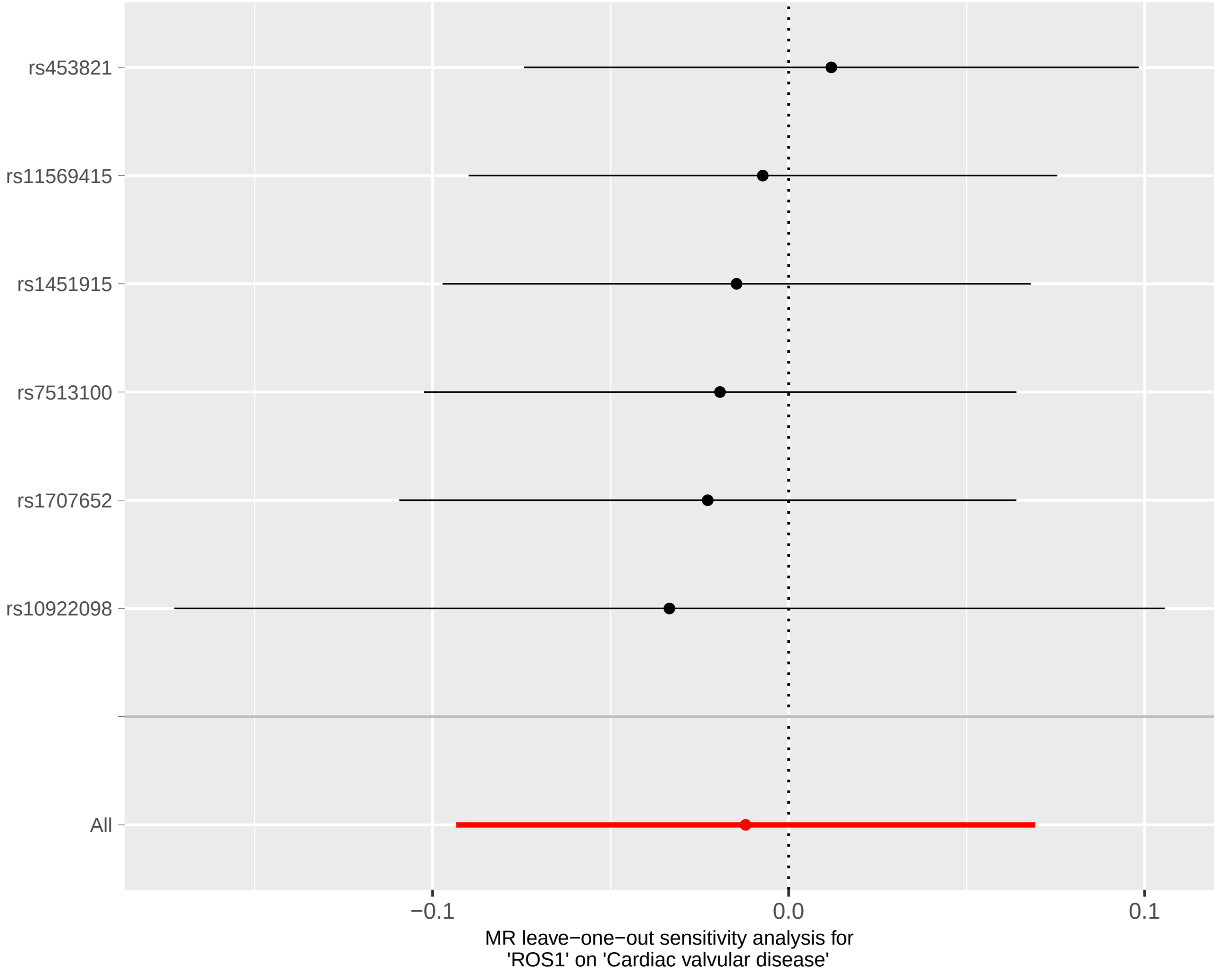
rs453821
rs11569415
rs1451915
rs7513100
rs1707652
rs10922098
All
−0.1
0.0
0.1
MR leave−one−out sensitivity analysis for
'ROS1' on 'Cardiac valvular disease'

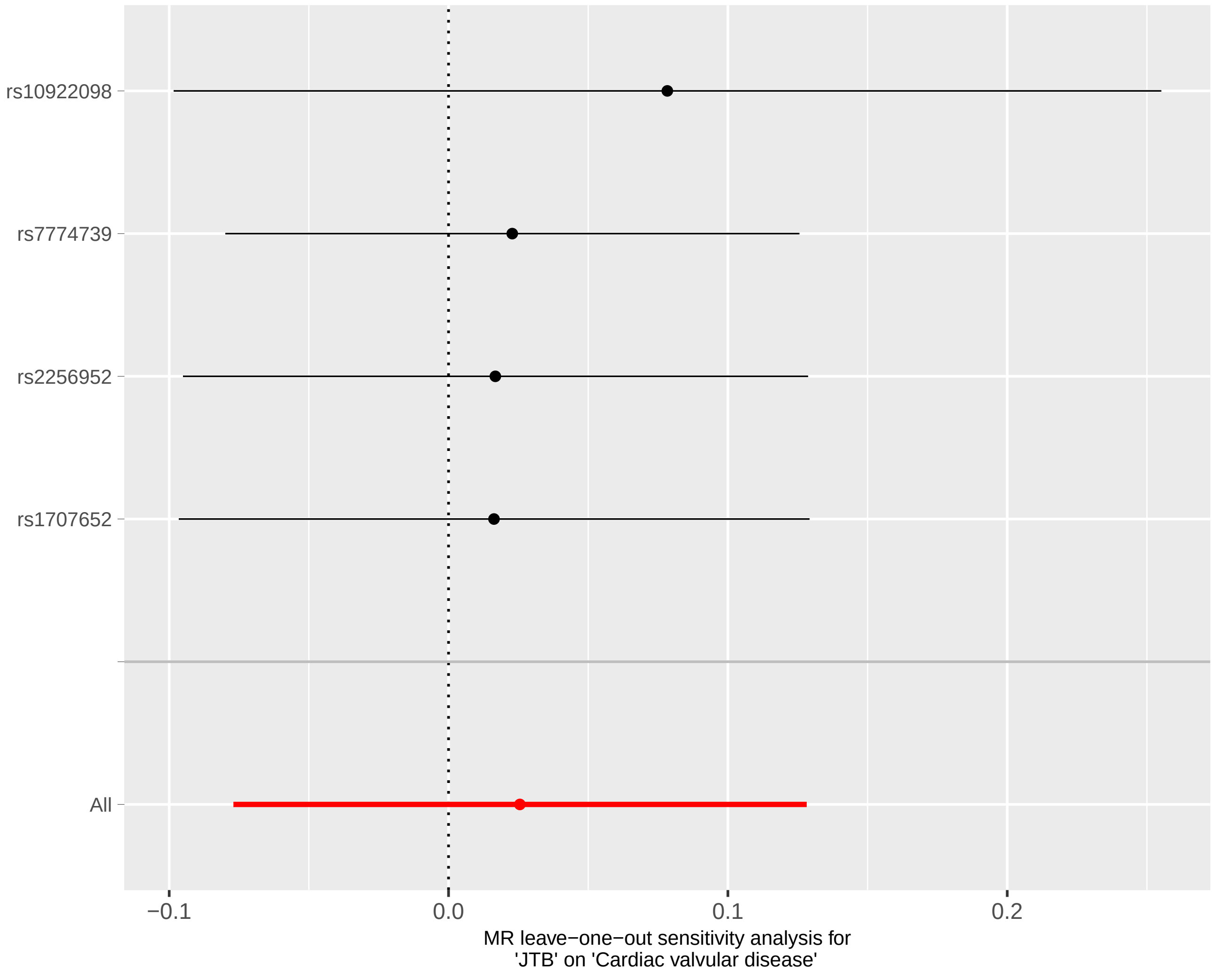
rs10922098
rs7774739
rs2256952
rs1707652
All
−0.1
0.0
0.1
0.2
MR leave−one−out sensitivity analysis for
'JTB' on 'Cardiac valvular disease'

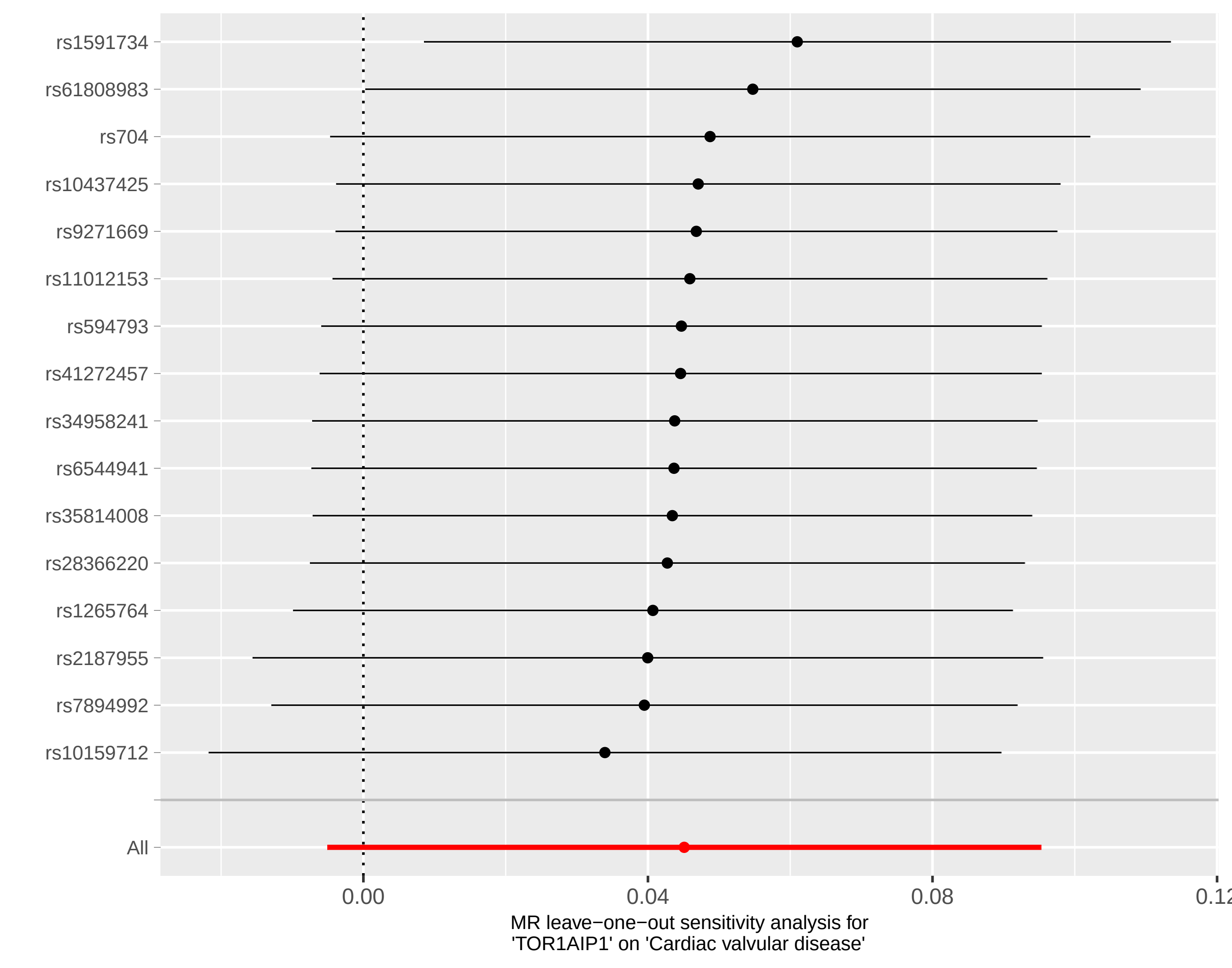
rs1591734
rs61808983
rs704
rs10437425
rs9271669
rs11012153
rs594793
rs41272457
rs34958241
rs6544941
rs35814008
rs28366220
rs1265764
rs2187955
rs7894992
rs10159712
All
0.00
0.04
0.08
MR leave−one−out sensitivity analysis for
'TOR1AIP1' on 'Cardiac valvular disease'

Insufficient number of SNPs

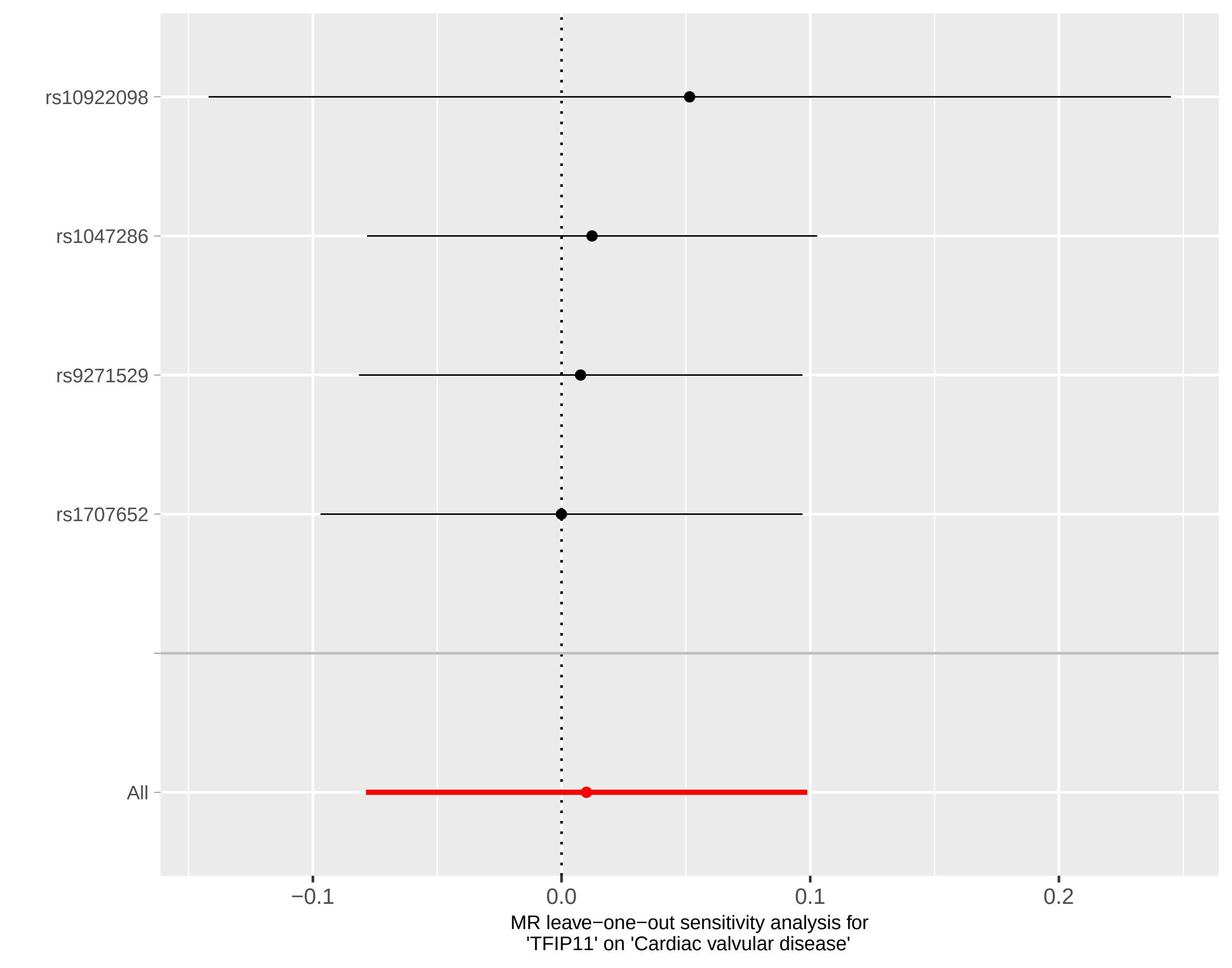

rs10922098
rs1047286
rs9271529
rs1707652
All
−0.1
0.0
0.1
0.2
MR leave−one−out sensitivity analysis for
'TFIP11' on 'Cardiac valvular disease'

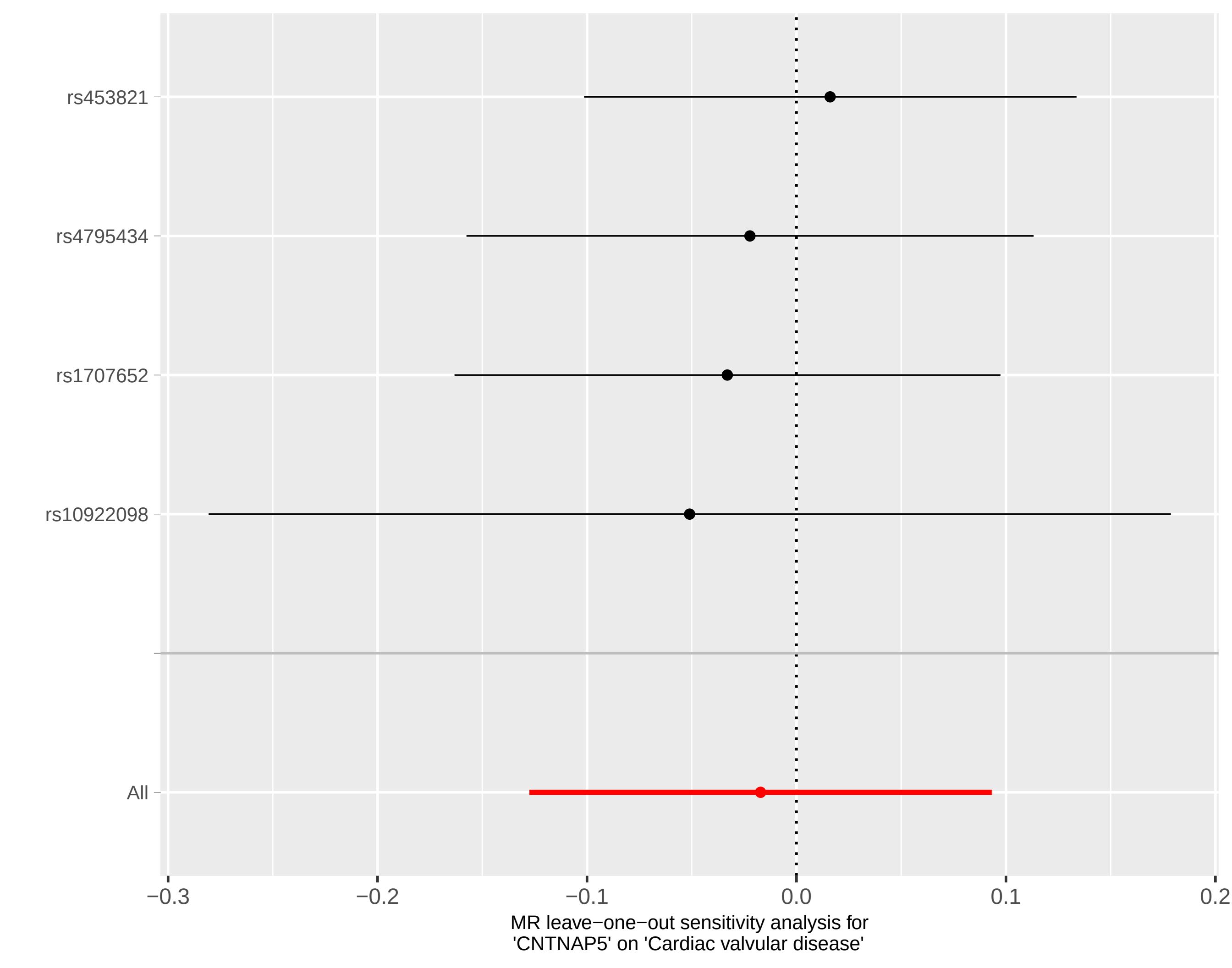
rs453821
rs4795434
rs1707652
rs10922098
All
−0.3
−0.2
−0.1
0.0
0.1
0.2
MR leave−one−out sensitivity analysis for
'CNTNAP5' on 'Cardiac valvular disease'

Insufficient number of SNPs

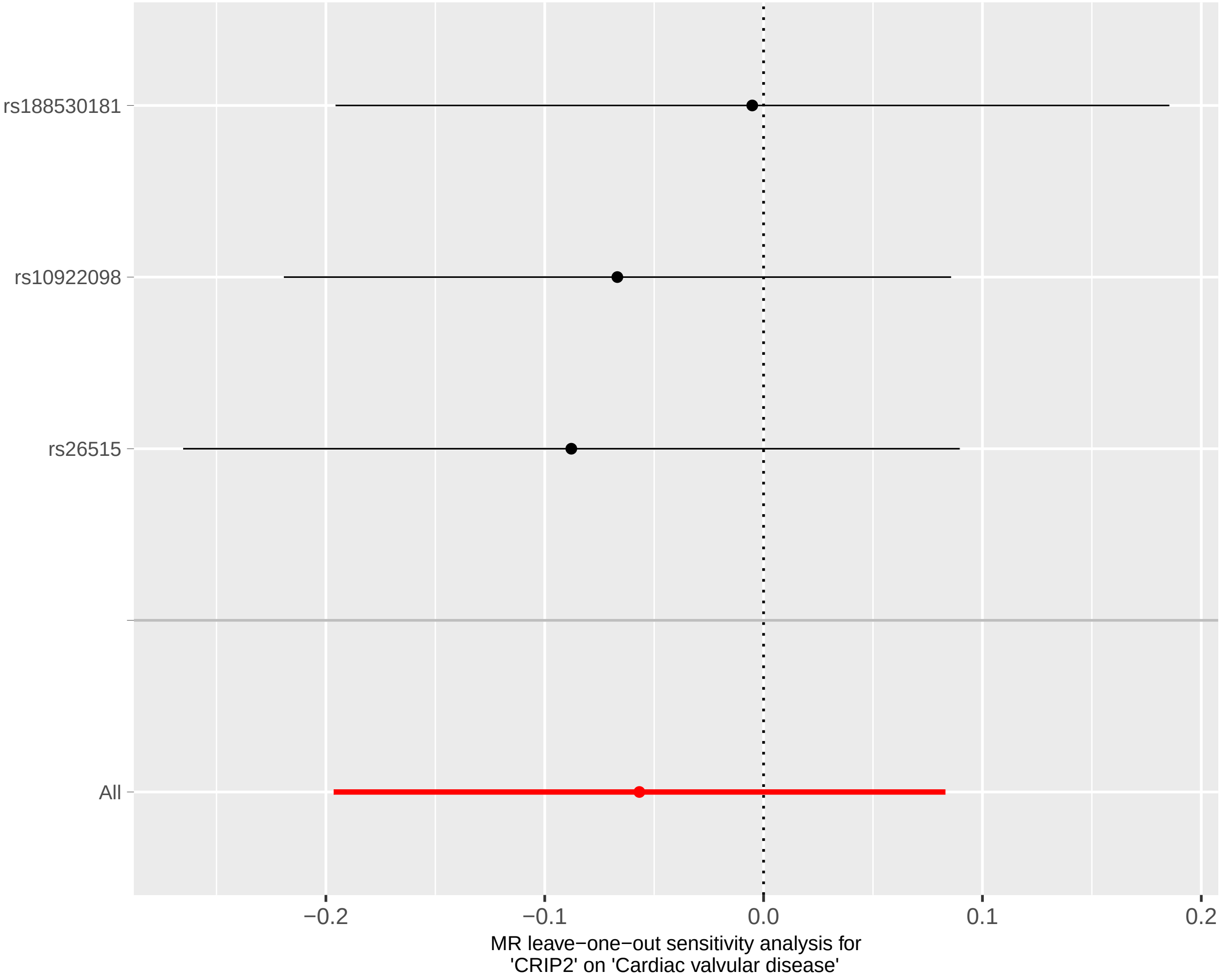

rs188530181
rs10922098
rs26515
All
−0.2
−0.1
0.0
0.1
0.2
MR leave−one−out sensitivity analysis for
'CRIP2' on 'Cardiac valvular disease'

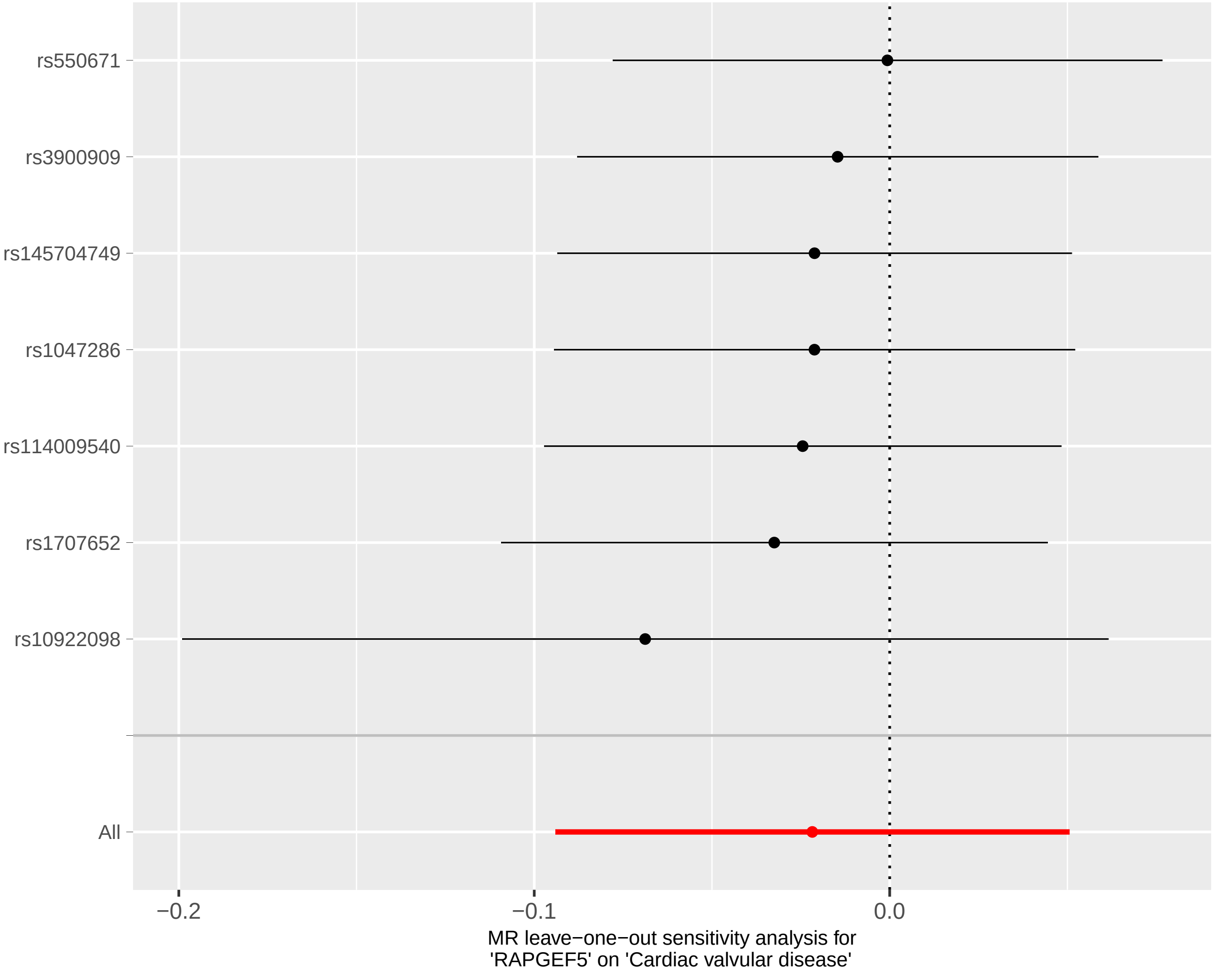
rs550671
rs3900909
rs145704749
rs1047286
rs114009540
rs1707652
rs10922098
All
−0.2
−0.1
0.0
MR leave−one−out sensitivity analysis for
'RAPGEF5' on 'Cardiac valvular disease'

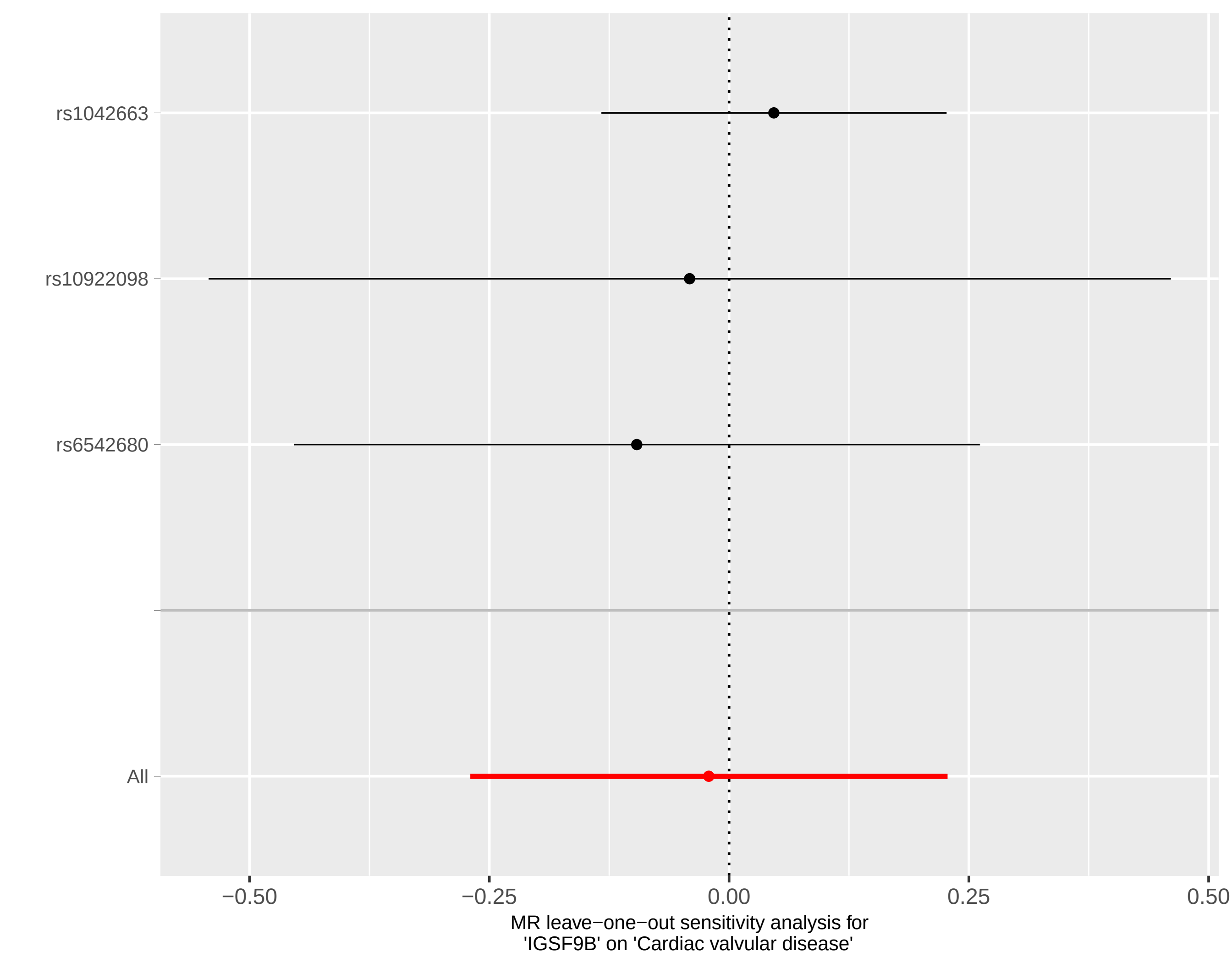

rs1042663
rs10922098
rs6542680
All
−0.50
−0.25
0.00
0.25
0.50
MR leave−one−out sensitivity analysis for
'IGSF9B' on 'Cardiac valvular disease'

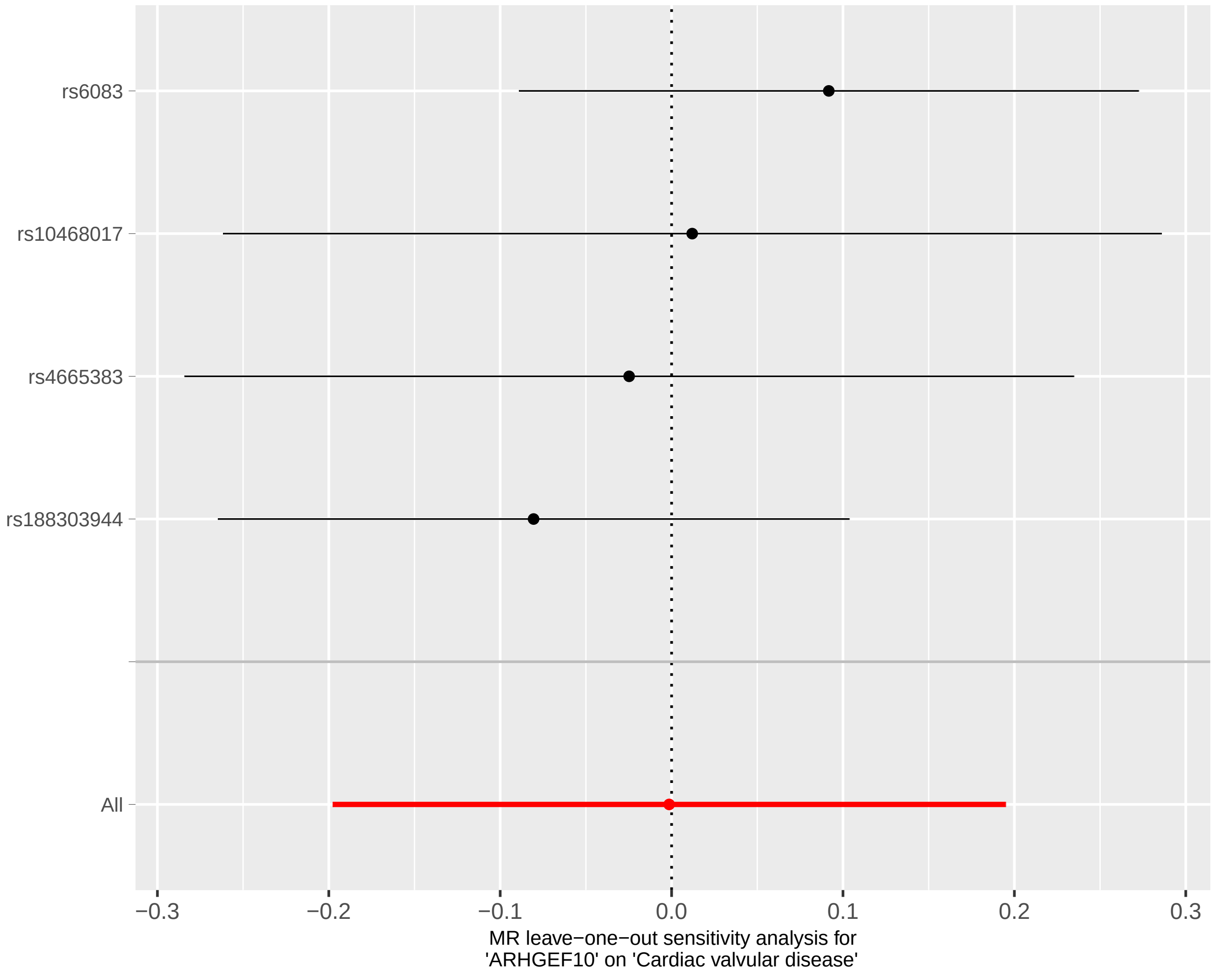

rs6083
rs10468017
rs4665383
rs188303944
All
−0.3
−0.2
−0.1
0.0
0.1
0.2
0.3
MR leave−one−out sensitivity analysis for
'ARHGEF10' on 'Cardiac valvular disease'

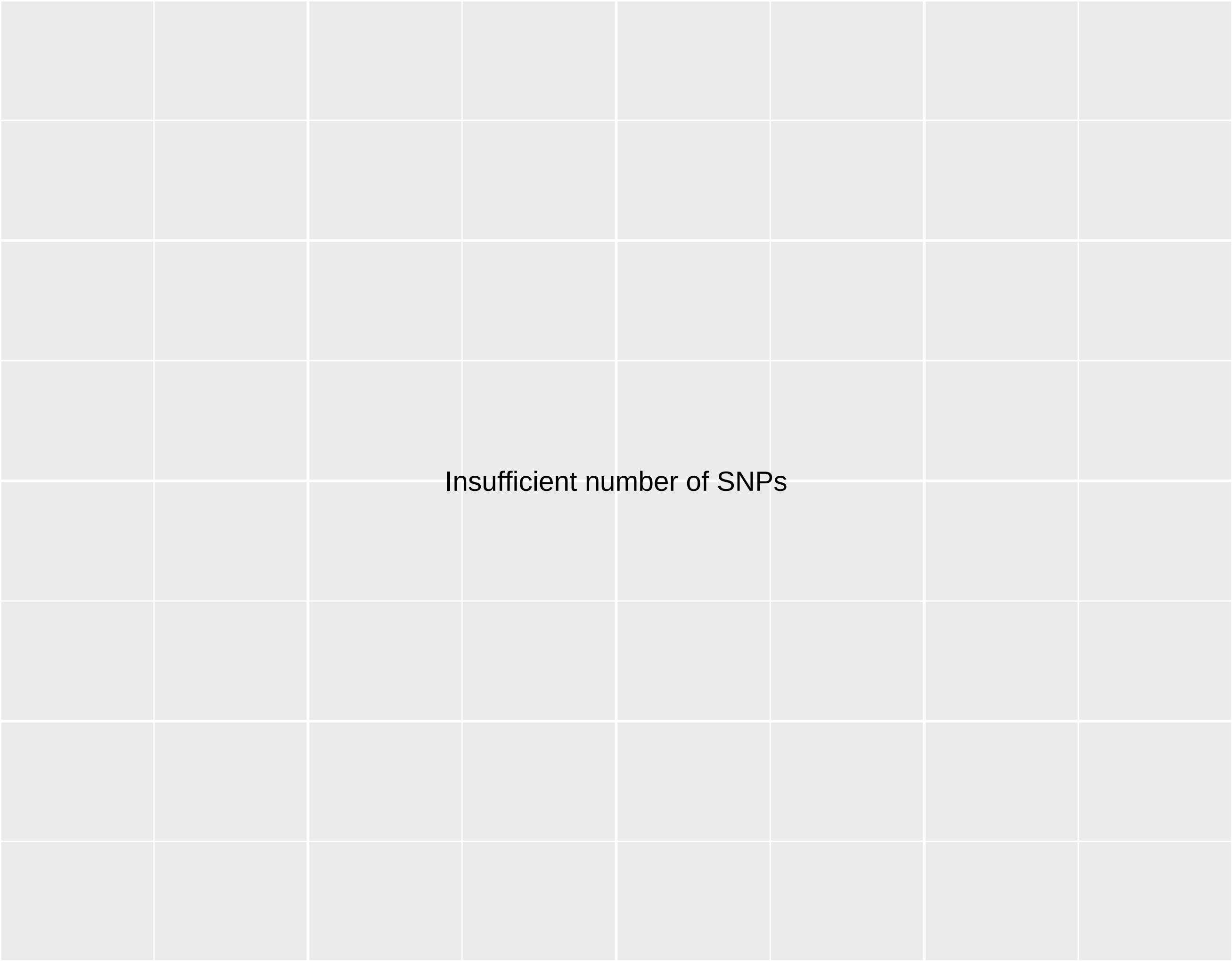
Insufficient number of SNPs

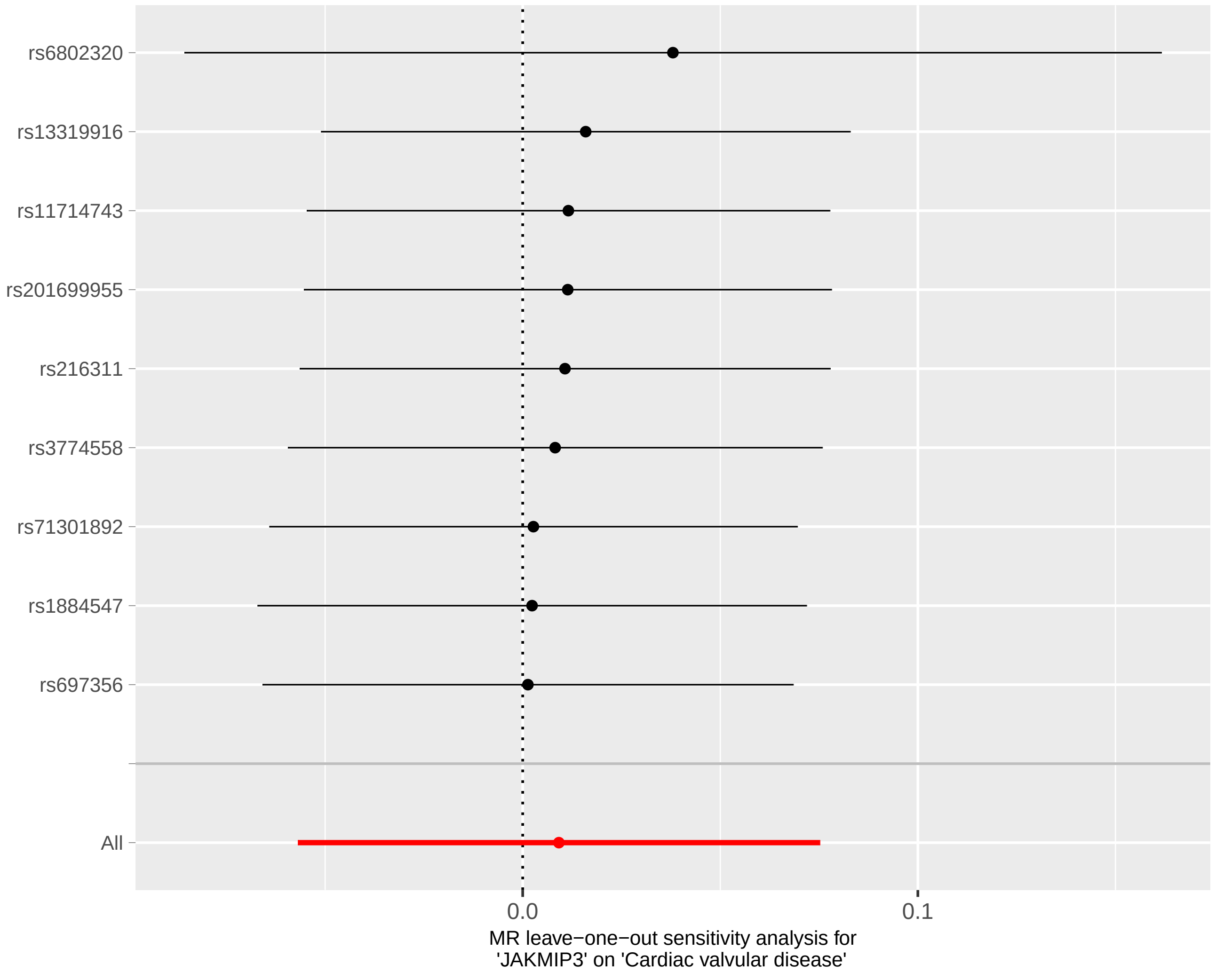

rs6802320
rs13319916
rs11714743
rs201699955
rs216311
rs3774558
rs71301892
rs1884547
rs697356
All
0.0
0.1
MR leave−one−out sensitivity analysis for
'JAKMIP3' on 'Cardiac valvular disease'

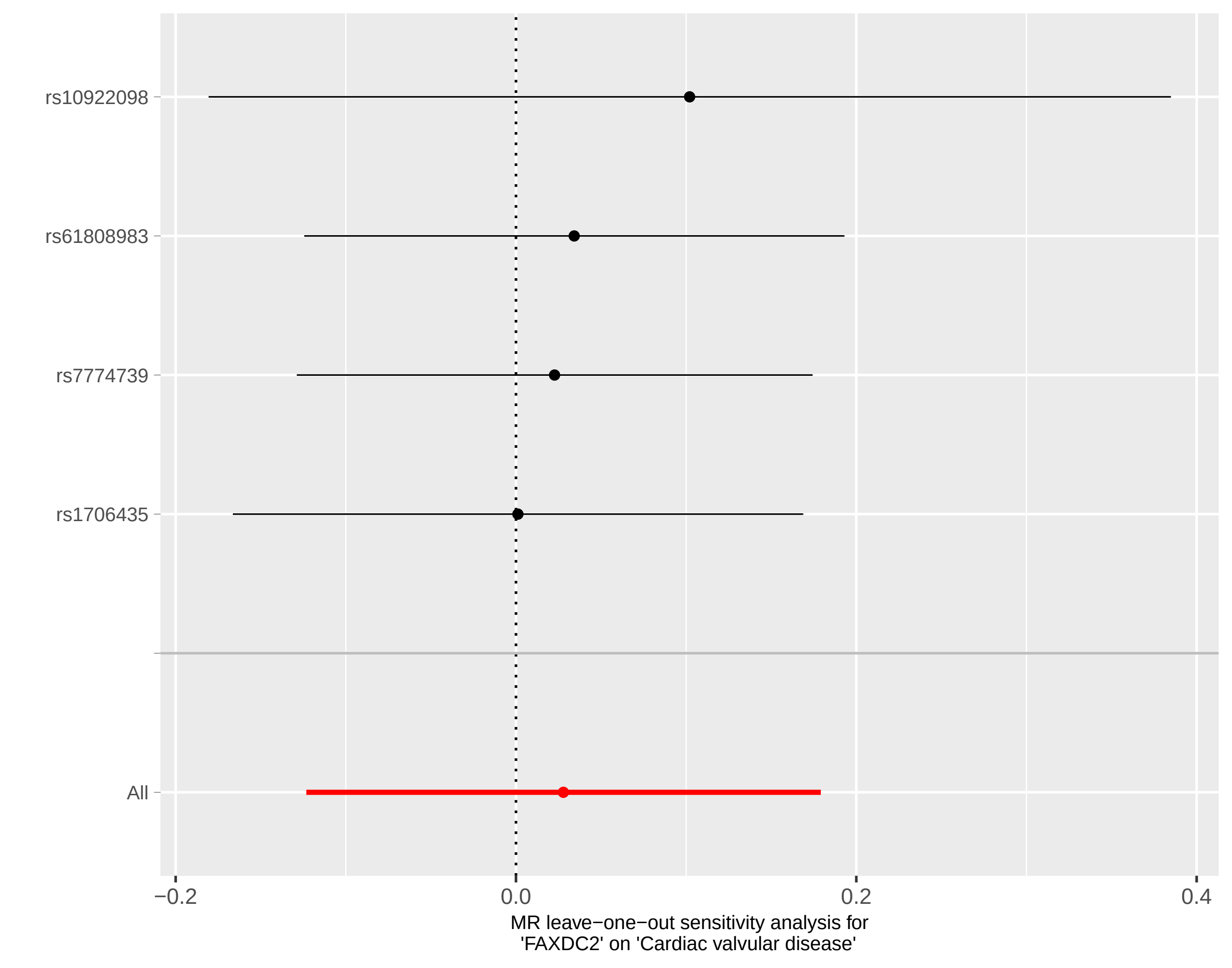

rs10922098
rs61808983
rs7774739
rs1706435
All
−0.2
0.0
0.2
0.4
MR leave−one−out sensitivity analysis for
'FAXDC2' on 'Cardiac valvular disease'

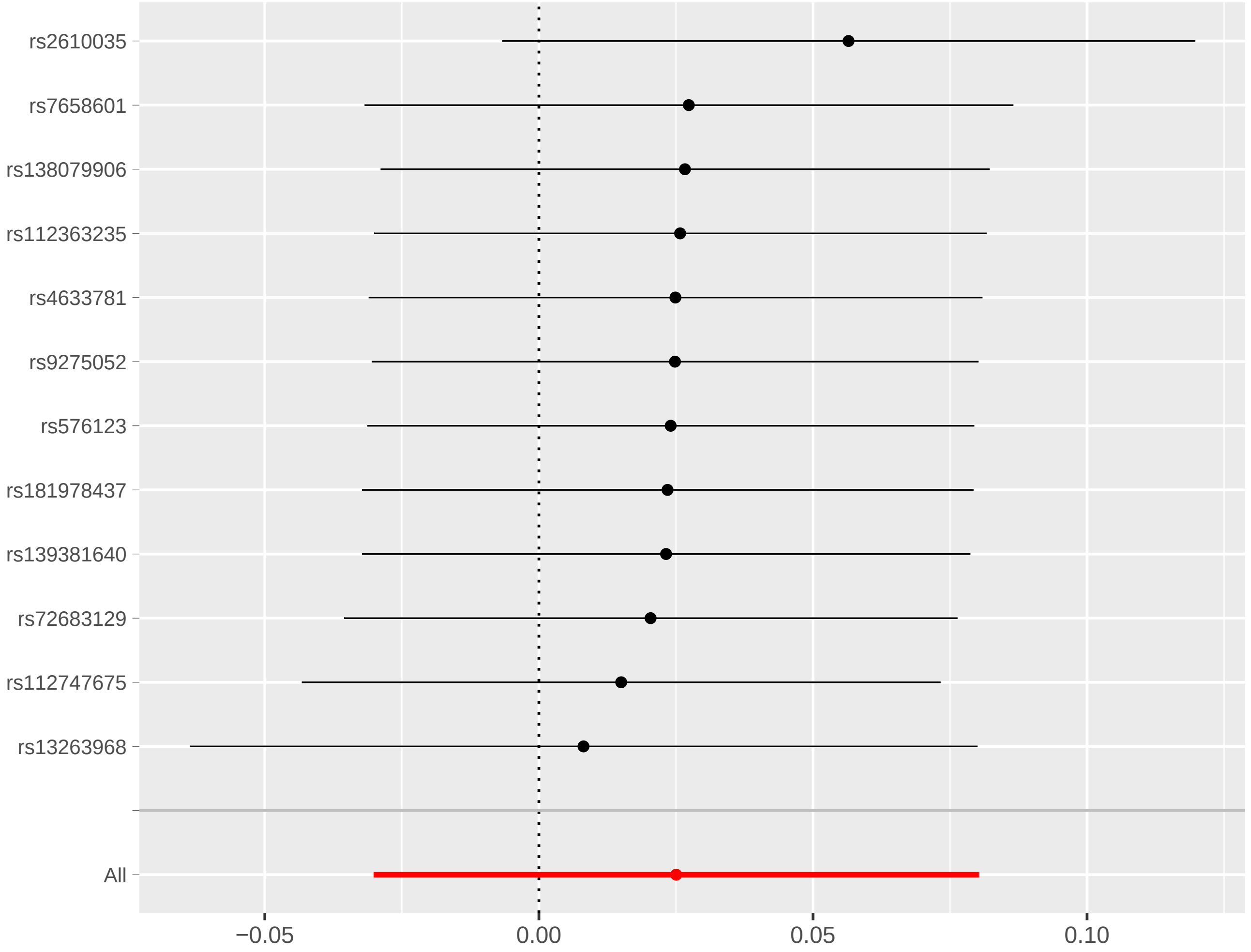

MR leave−one−out sensitivity analysis for
'PENK' on 'Cardiac valvular disease'

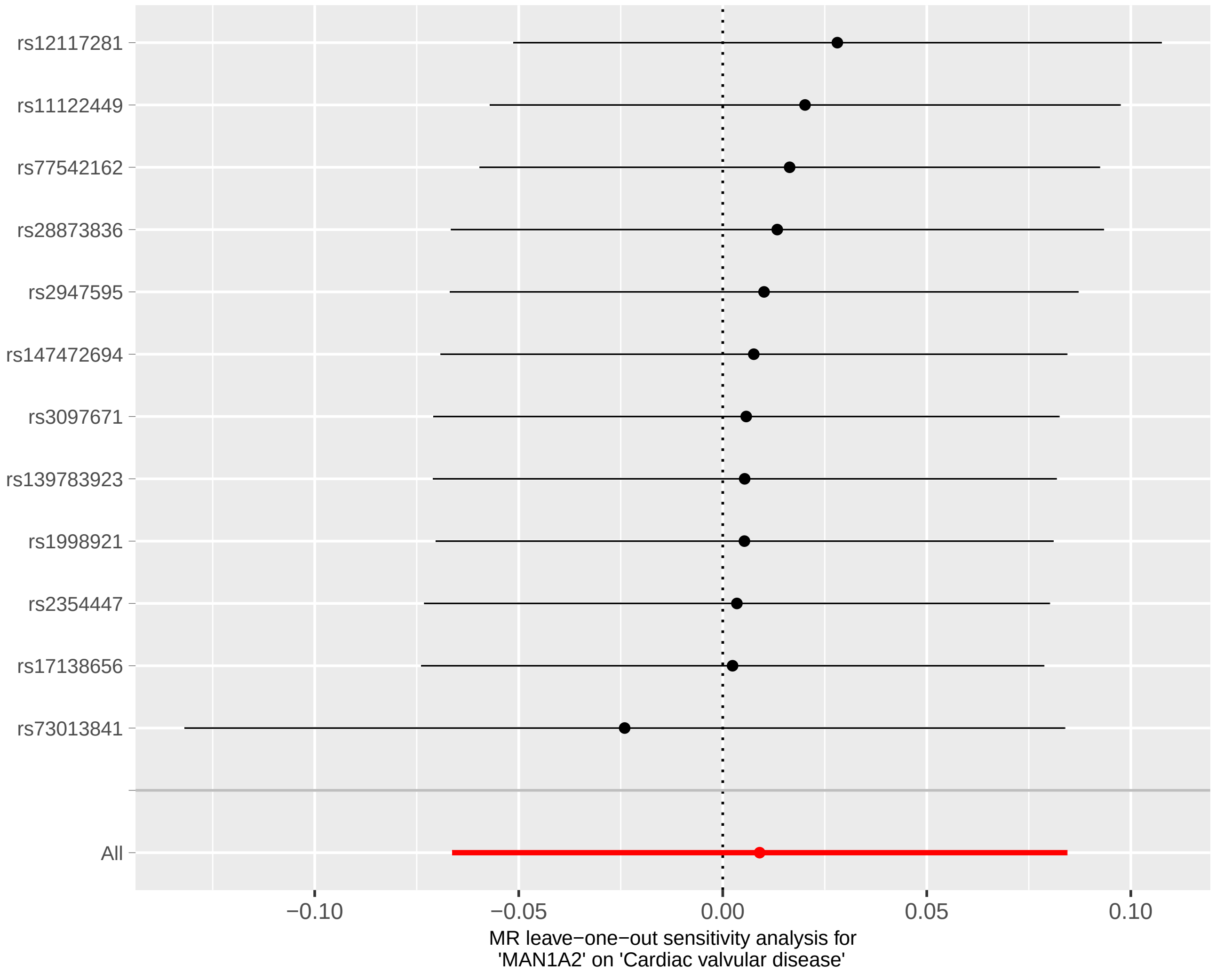
rs12117281
rs11122449
rs77542162
rs28873836
rs2947595
rs147472694
rs3097671
rs139783923
rs1998921
rs2354447
rs17138656
rs73013841
All
−0.10
−0.05
0.00
0.05
0.10
MR leave−one−out sensitivity analysis for
'MAN1A2' on 'Cardiac valvular disease'

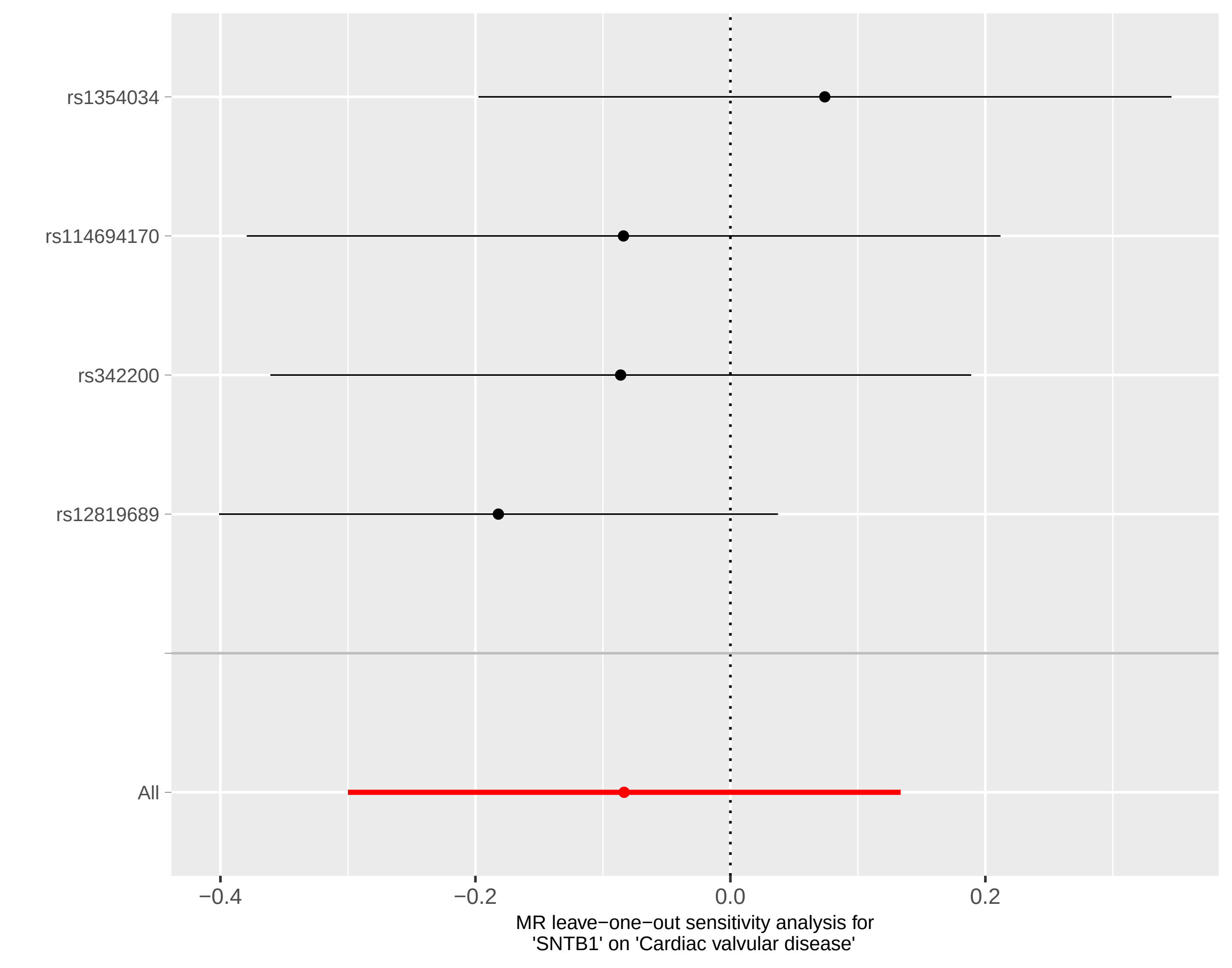

rs1354034
rs114694170
rs342200
rs12819689
All
−0.4
−0.2
0.0
0.2
MR leave−one−out sensitivity analysis for
'SNTB1' on 'Cardiac valvular disease'

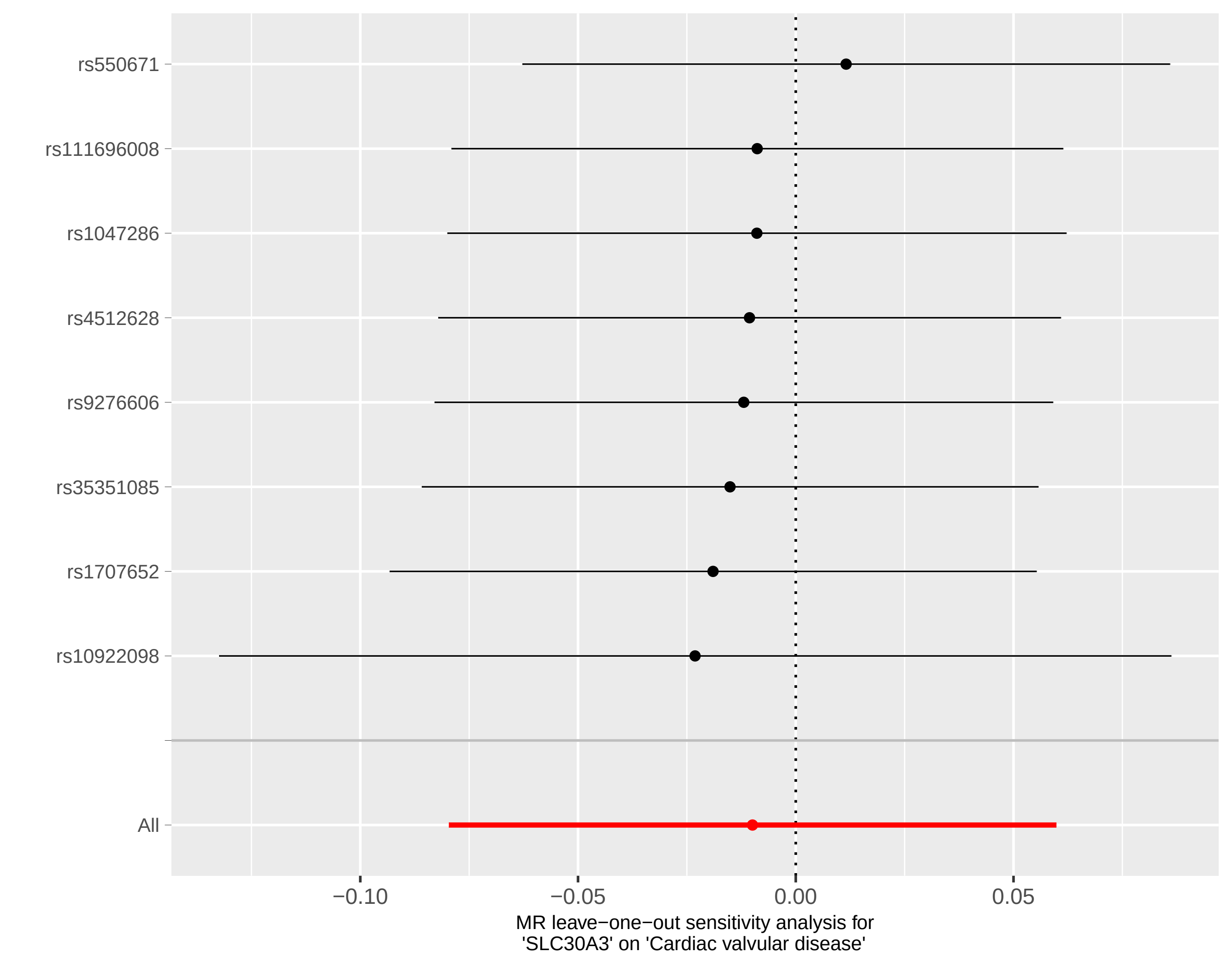
rs550671
rs111696008
rs1047286
rs4512628
rs9276606
rs35351085
rs1707652
rs10922098
All
−0.10
−0.05
0.00
0.05
MR leave−one−out sensitivity analysis for
'SLC30A3' on 'Cardiac valvular disease'

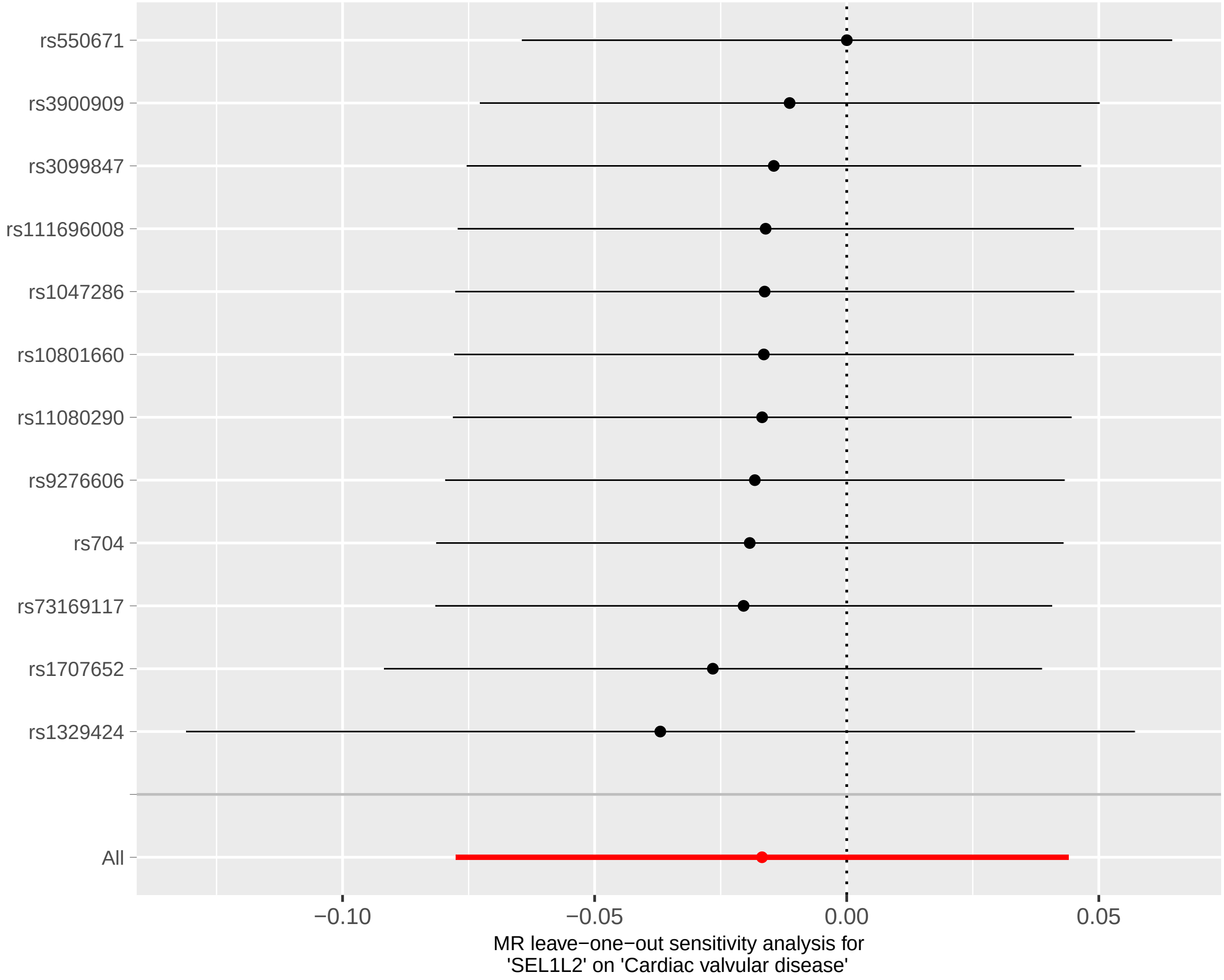

rs550671
rs3900909
rs3099847
rs111696008
rs1047286
rs10801660
rs11080290
rs9276606
rs704
rs73169117
rs1707652
rs1329424
All
−0.10
−0.05
0.00
0.05
MR leave−one−out sensitivity analysis for
'SEL1L2' on 'Cardiac valvular disease'

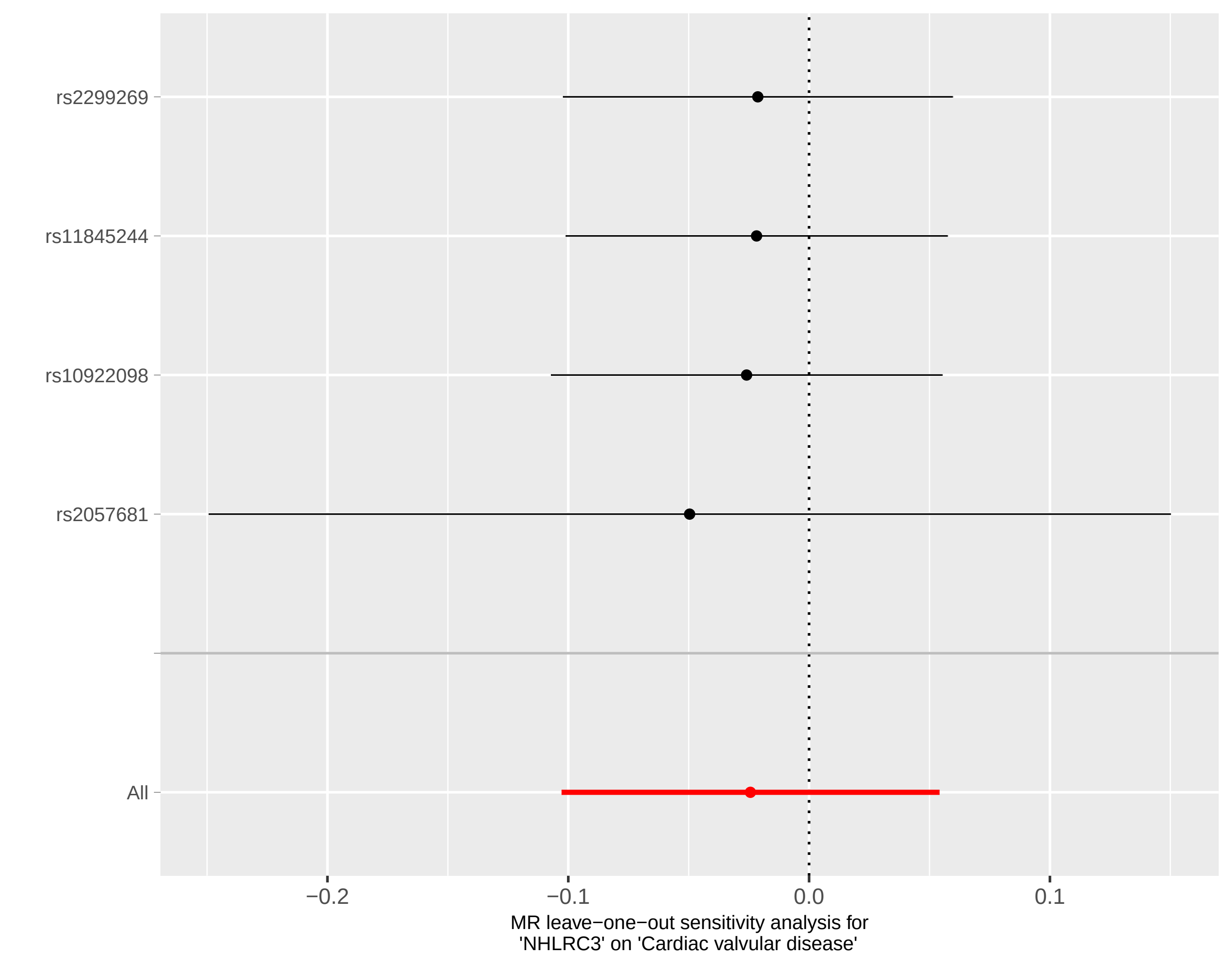

rs2299269
rs11845244
rs10922098
rs2057681
All
−0.2
−0.1
0.0
0.1
MR leave−one−out sensitivity analysis for
'NHLRC3' on 'Cardiac valvular disease'

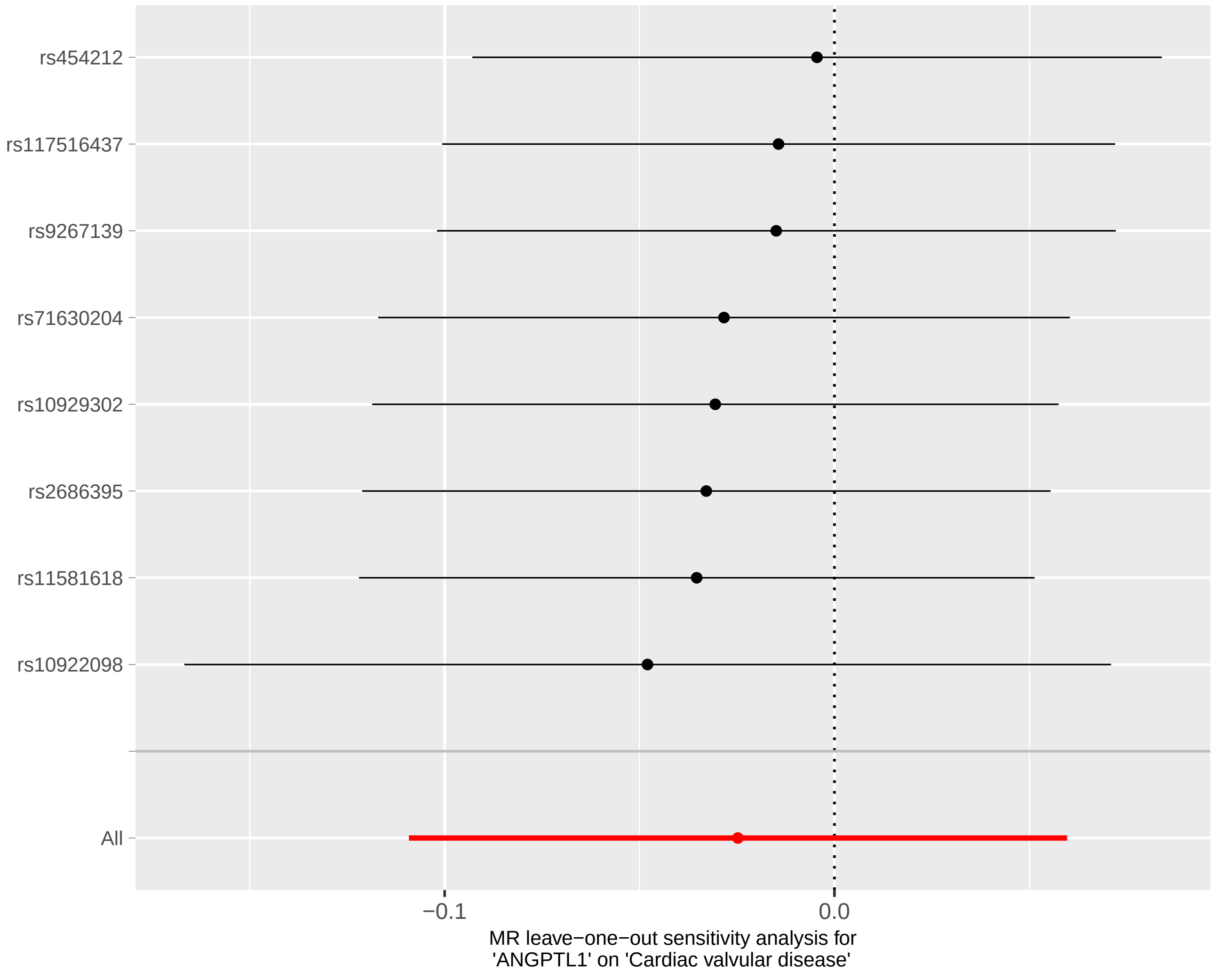
rs454212
rs117516437
rs9267139
rs71630204
rs10929302
rs2686395
rs11581618
rs10922098
All
−0.1
0.0
MR leave−one−out sensitivity analysis for
'ANGPTL1' on 'Cardiac valvular disease'

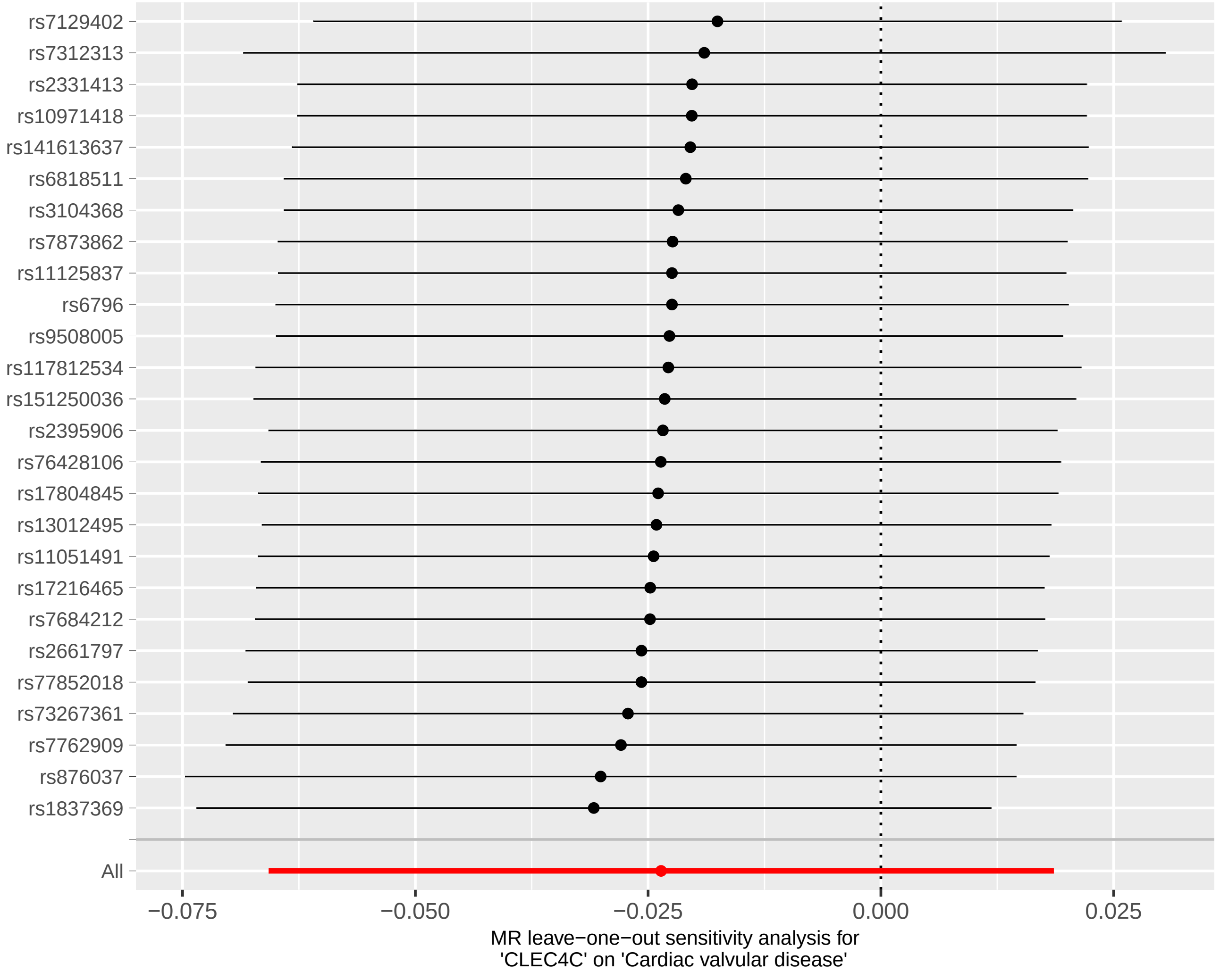

rs7129402
rs7312313
rs2331413
rs10971418
rs141613637
rs6818511
rs3104368
rs7873862
rs11125837
rs6796
rs9508005
rs117812534
rs151250036
rs2395906
rs76428106
rs17804845
rs13012495
rs11051491
rs17216465
rs7684212
rs2661797
rs77852018
rs73267361
rs7762909
rs876037
rs1837369
All
−0.075
−0.050
−0.025
0.000
0.025
MR leave−one−out sensitivity analysis for
'CLEC4C' on 'Cardiac valvular disease'

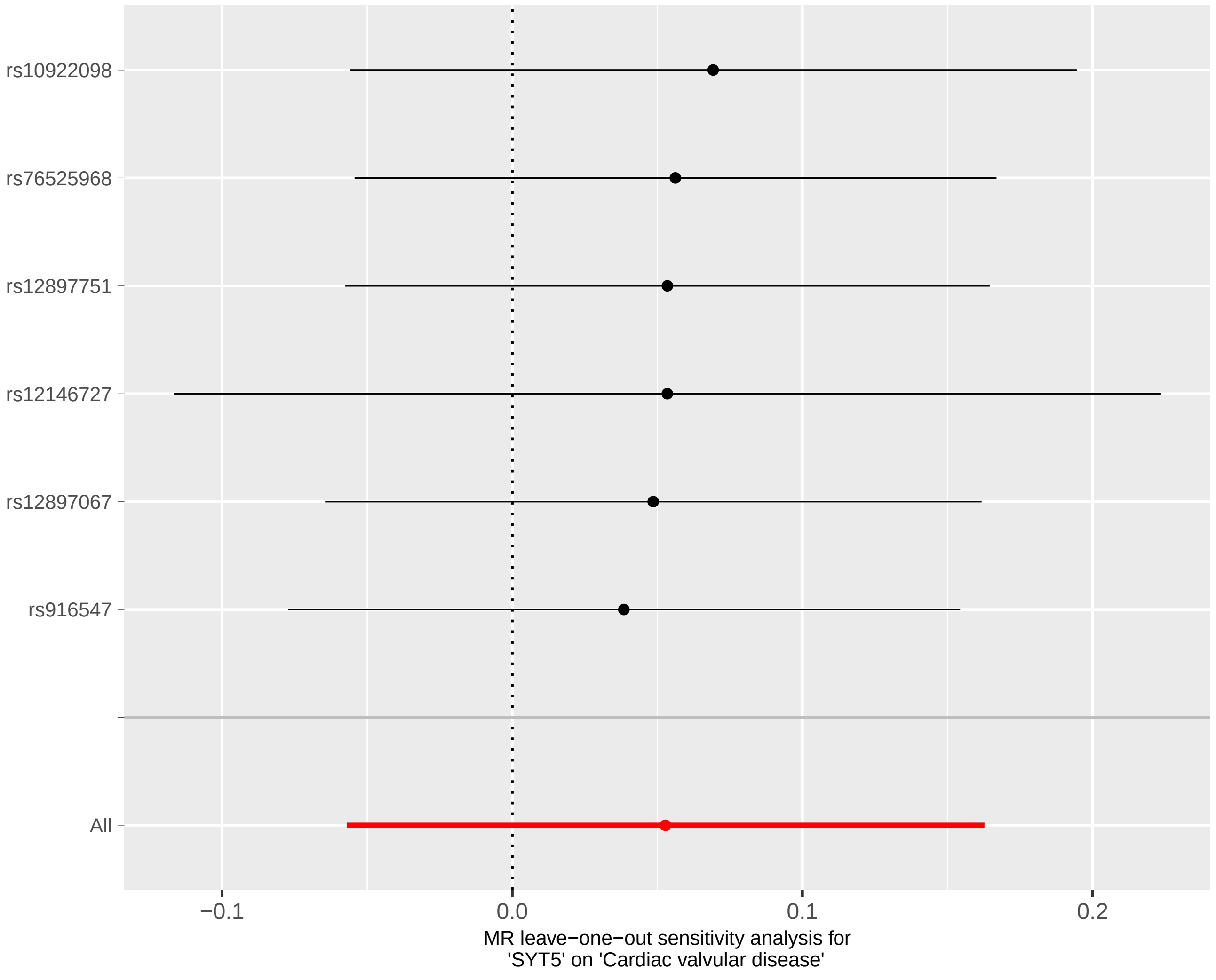
rs10922098
rs76525968
rs12897751
rs12146727
rs12897067
rs916547
All
−0.1
0.0
0.1
0.2
MR leave−one−out sensitivity analysis for
'SYT5' on 'Cardiac valvular disease'

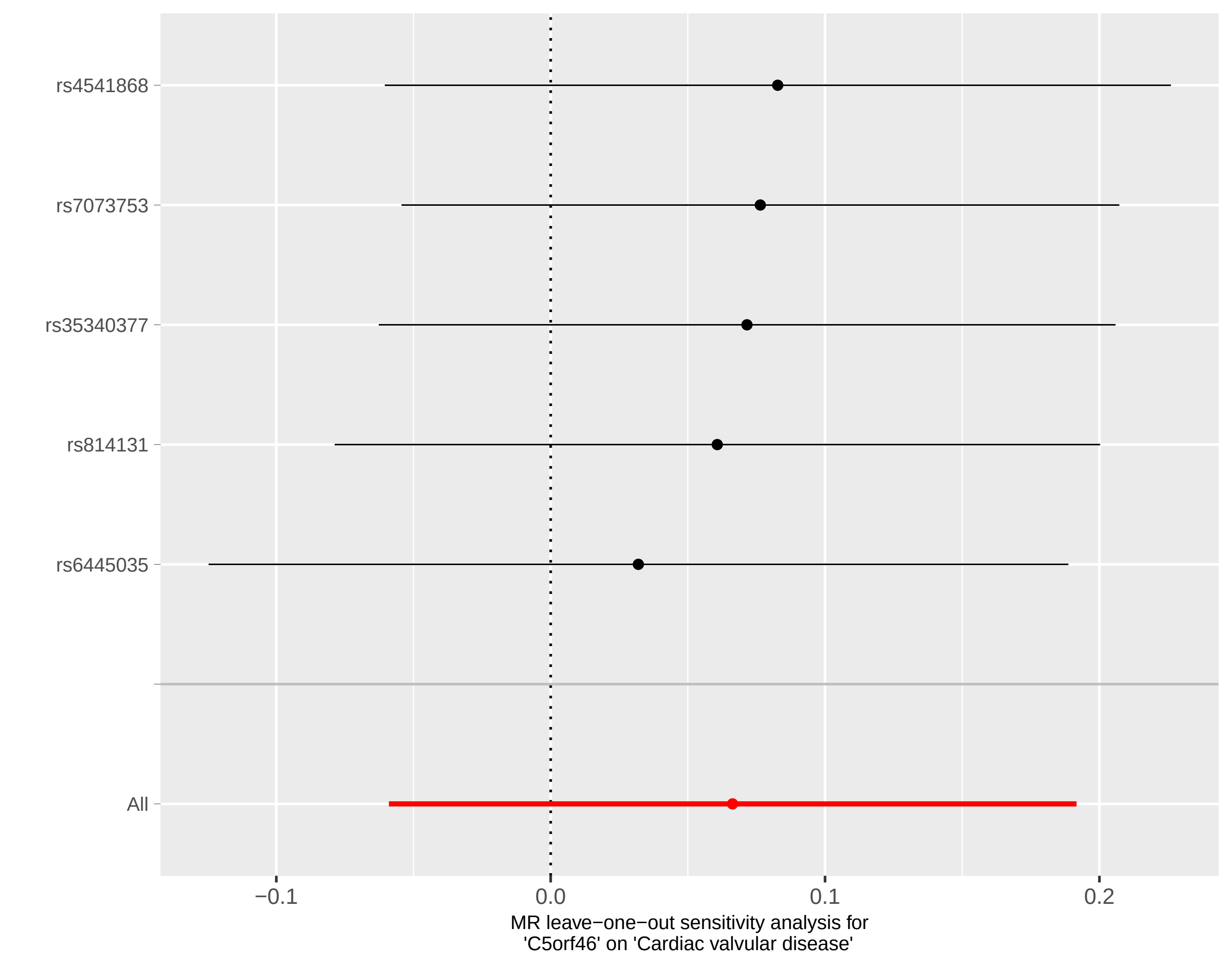
rs4541868
rs7073753
rs35340377
rs814131
rs6445035
All
−0.1
0.0
0.1
0.2
MR leave−one−out sensitivity analysis for
'C5orf46' on 'Cardiac valvular disease'

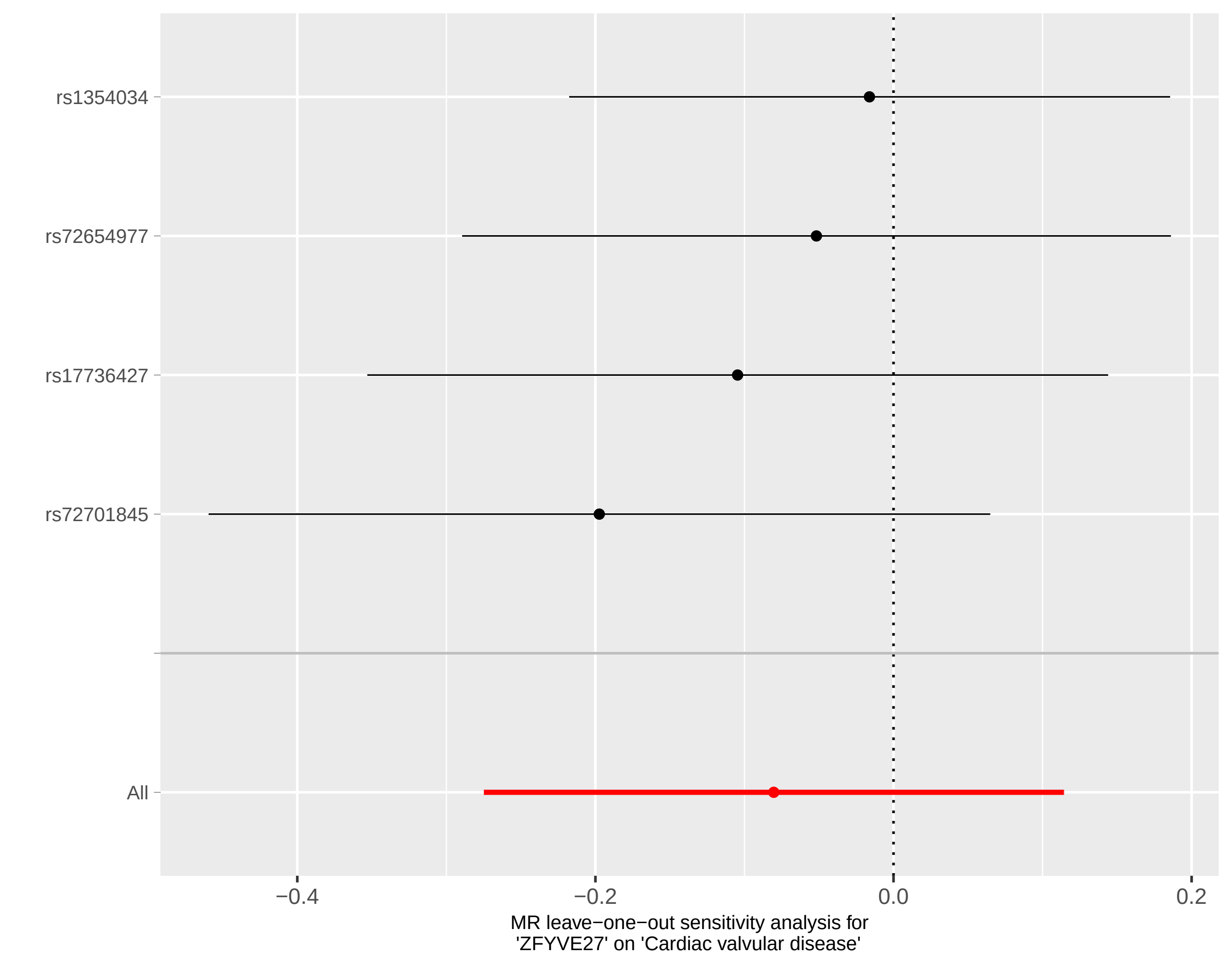
rs1354034
rs72654977
rs17736427
rs72701845
All
−0.4
−0.2
0.0
0.2
MR leave−one−out sensitivity analysis for
'ZFYVE27' on 'Cardiac valvular disease'

Insufficient number of SNPs

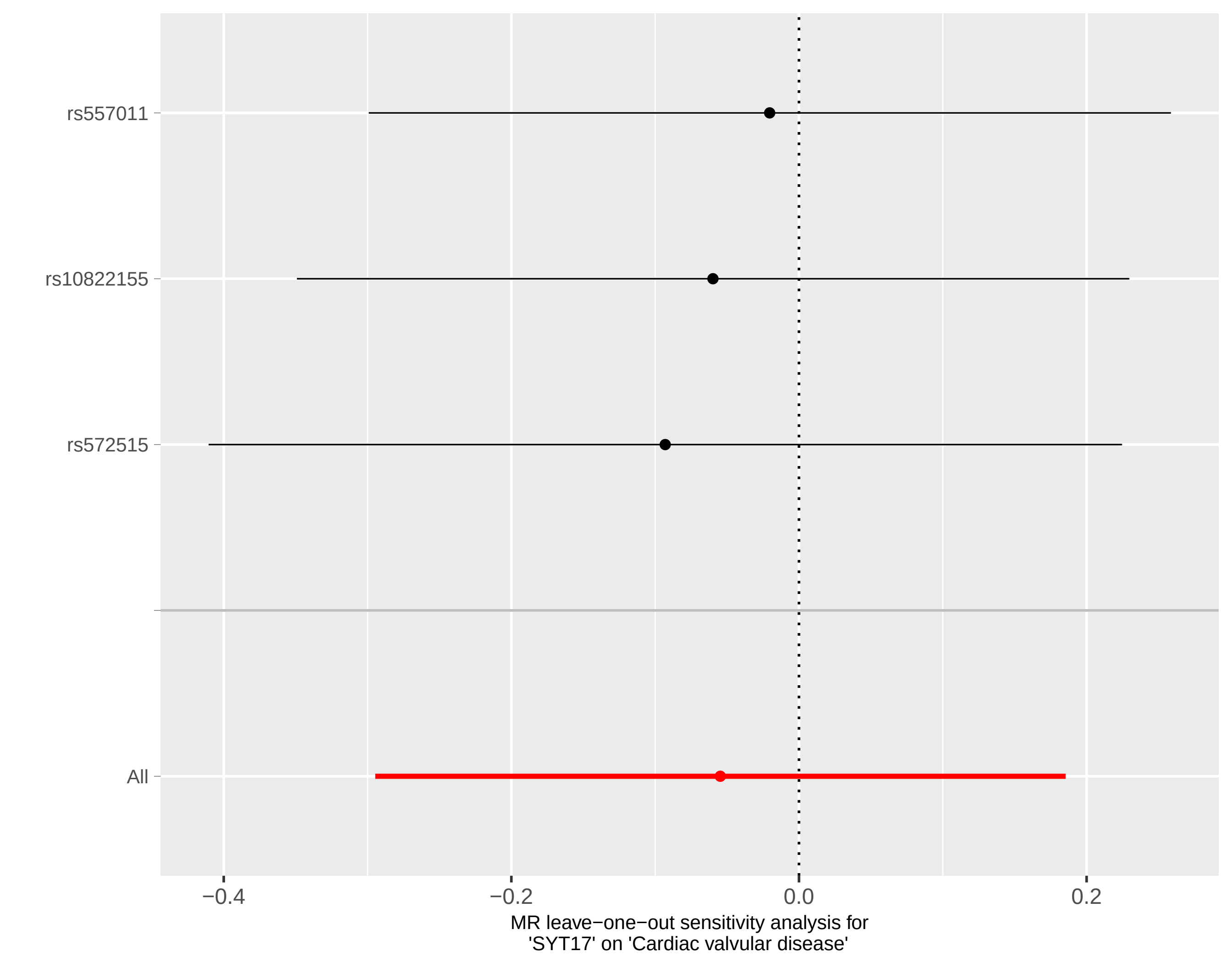
rs557011
rs10822155
rs572515
All
−0.4
−0.2
0.0
0.2
MR leave−one−out sensitivity analysis for
'SYT17' on 'Cardiac valvular disease'

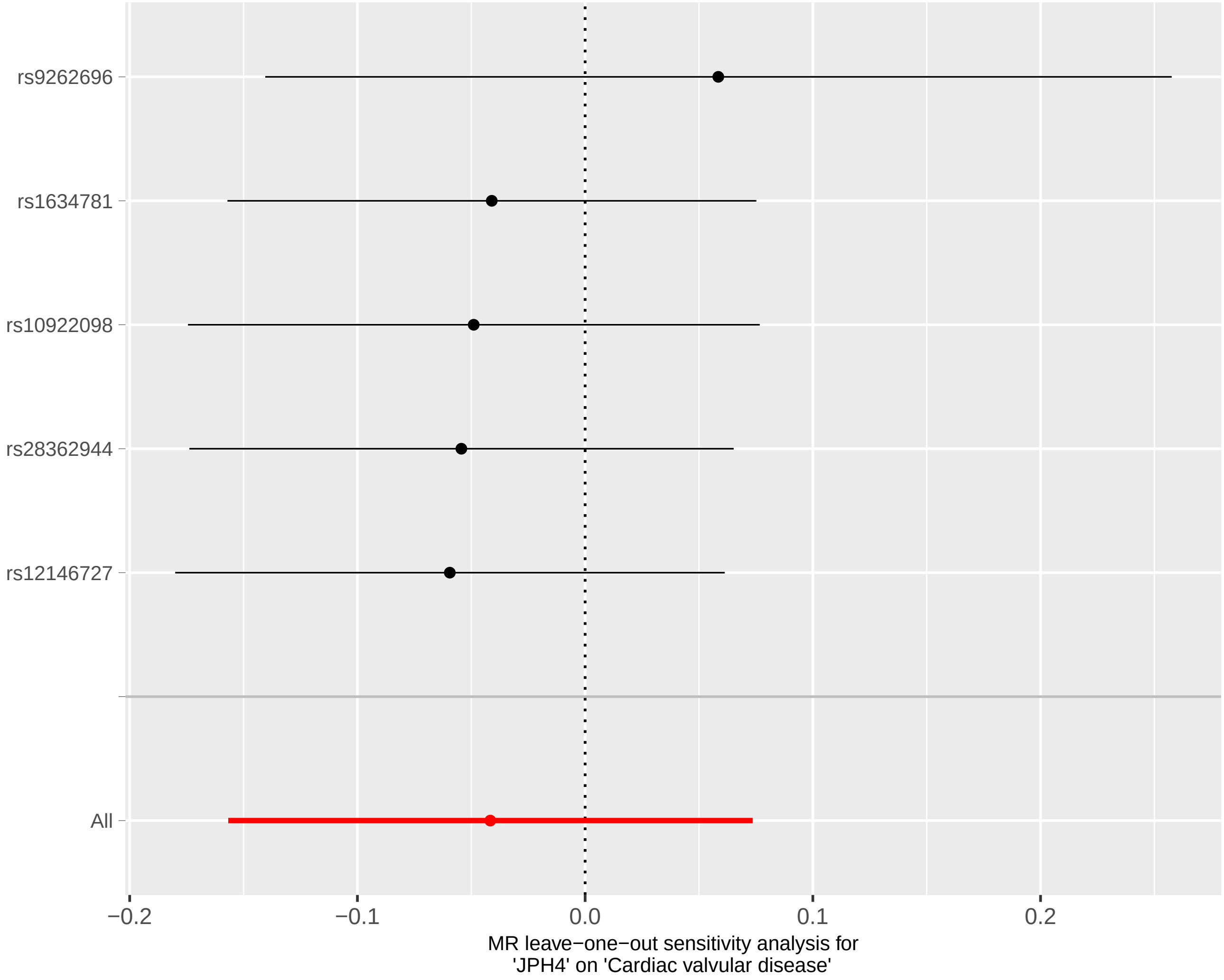
rs9262696
rs1634781
rs10922098
rs28362944
rs12146727
All
−0.2
−0.1
0.0
0.1
0.2
MR leave−one−out sensitivity analysis for
'JPH4' on 'Cardiac valvular disease'

Insufficient number of SNPs

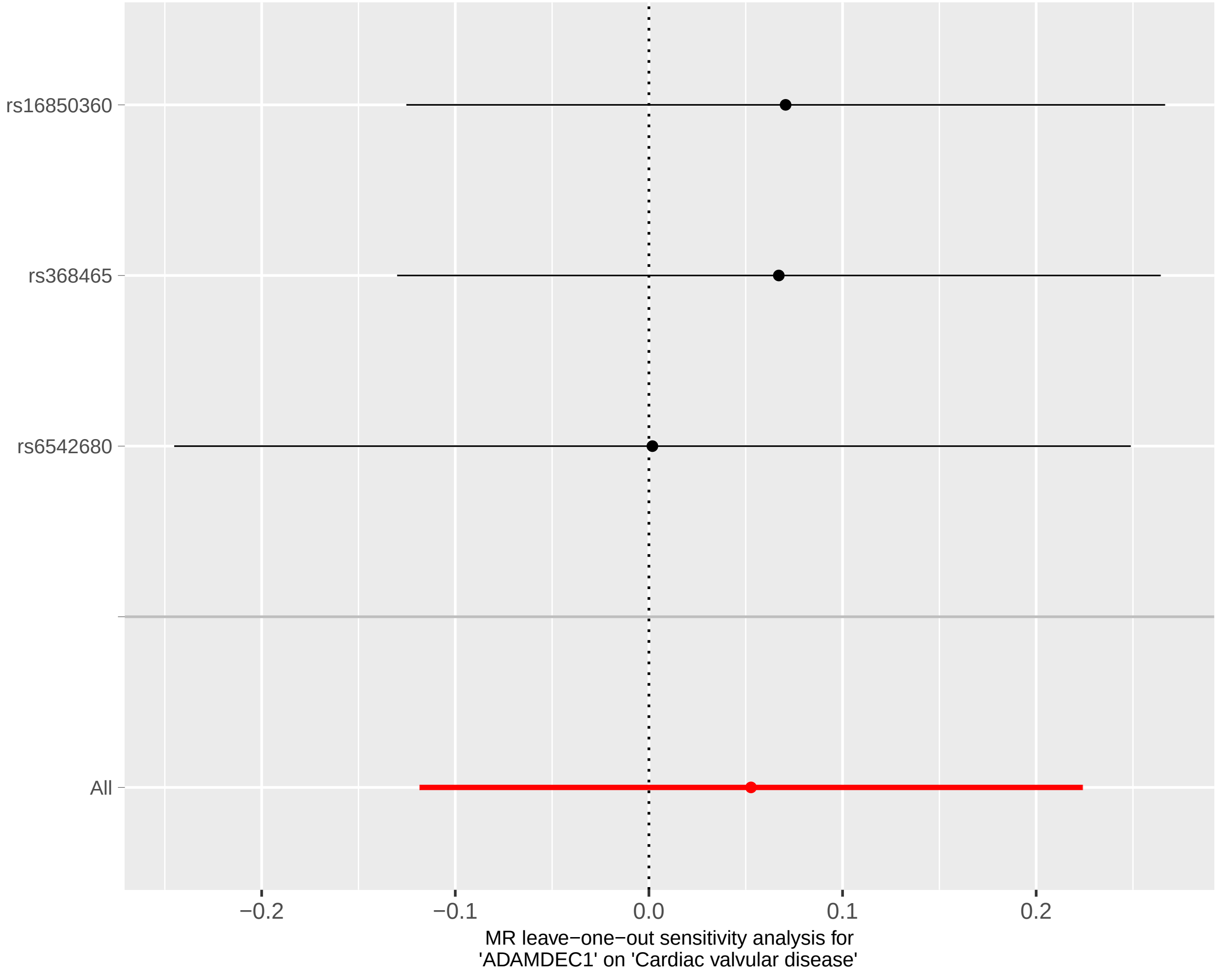
rs16850360
rs368465
rs6542680
All
−0.2
−0.1
0.0
0.1
0.2
MR leave−one−out sensitivity analysis for
'ADAMDEC1' on 'Cardiac valvular disease'

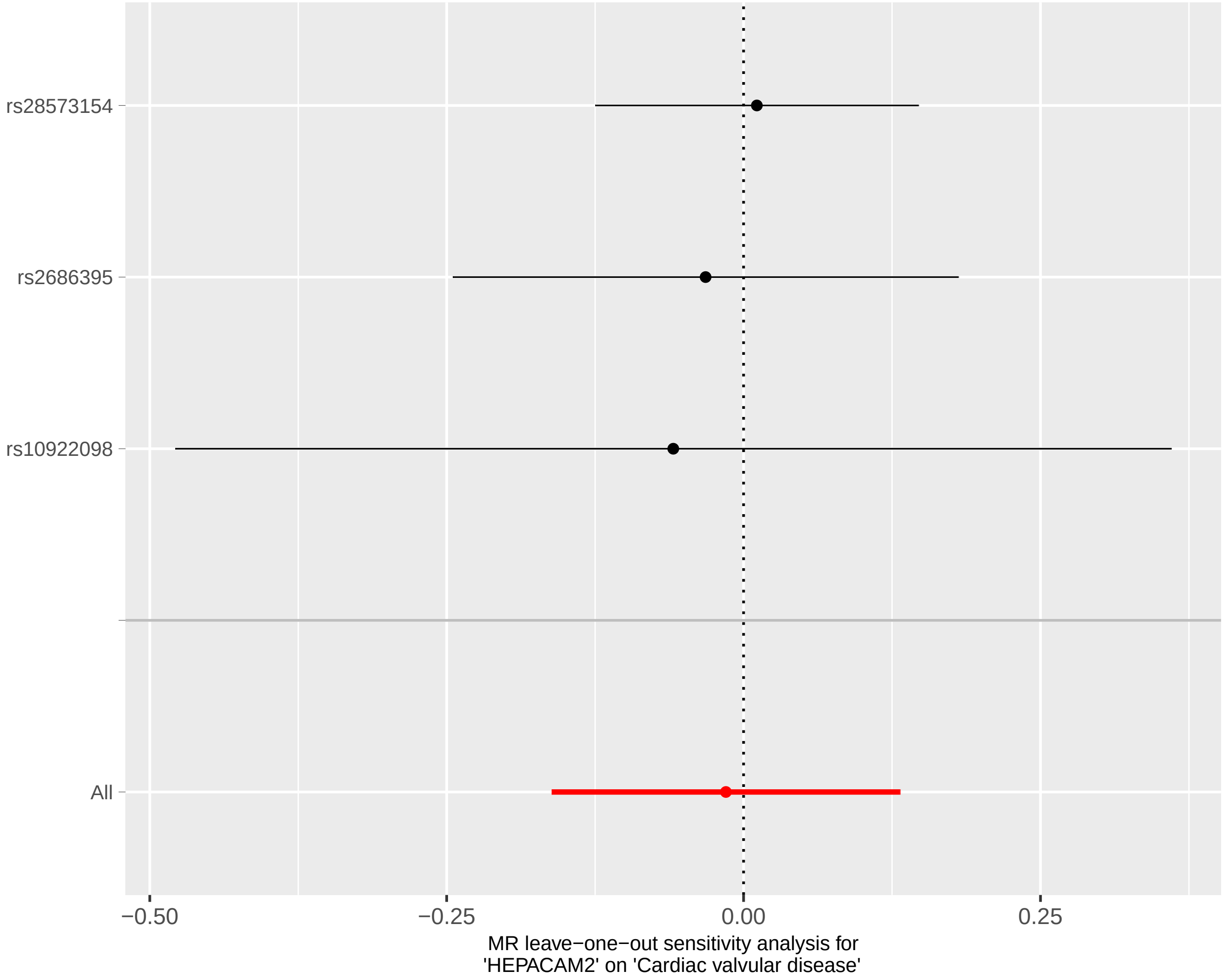

rs28573154
rs2686395
rs10922098
All
−0.50
−0.25
0.00
0.25
MR leave−one−out sensitivity analysis for
'HEPACAM2' on 'Cardiac valvular disease'

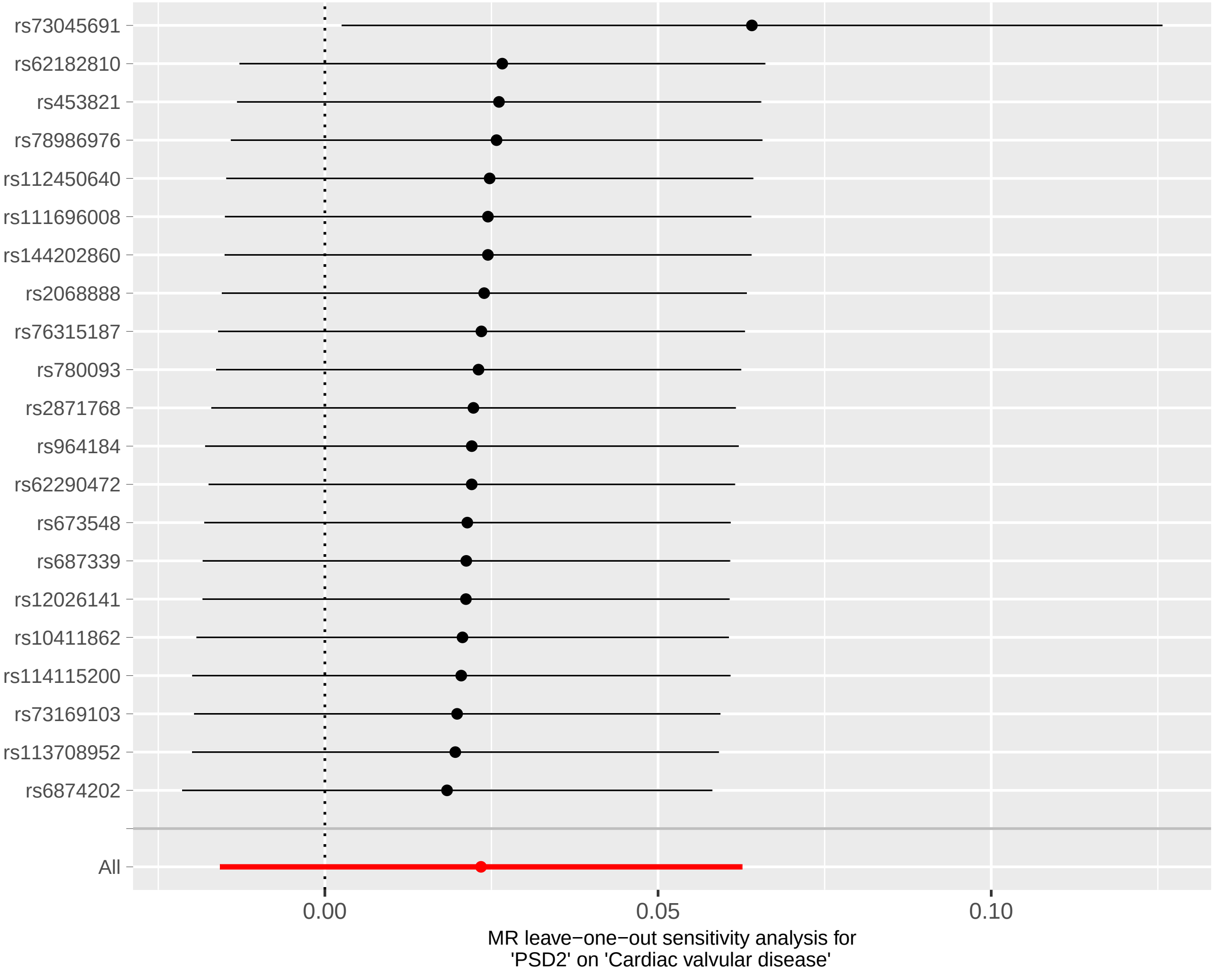
rs73045691
rs62182810
rs453821
rs78986976
rs112450640
rs111696008
rs144202860
rs2068888
rs76315187
rs780093
rs2871768
rs964184
rs62290472
rs673548
rs687339
rs12026141
rs10411862
rs114115200
rs73169103
rs113708952
rs6874202
All
0.00
0.05
0.10
MR leave−one−out sensitivity analysis for
'PSD2' on 'Cardiac valvular disease'

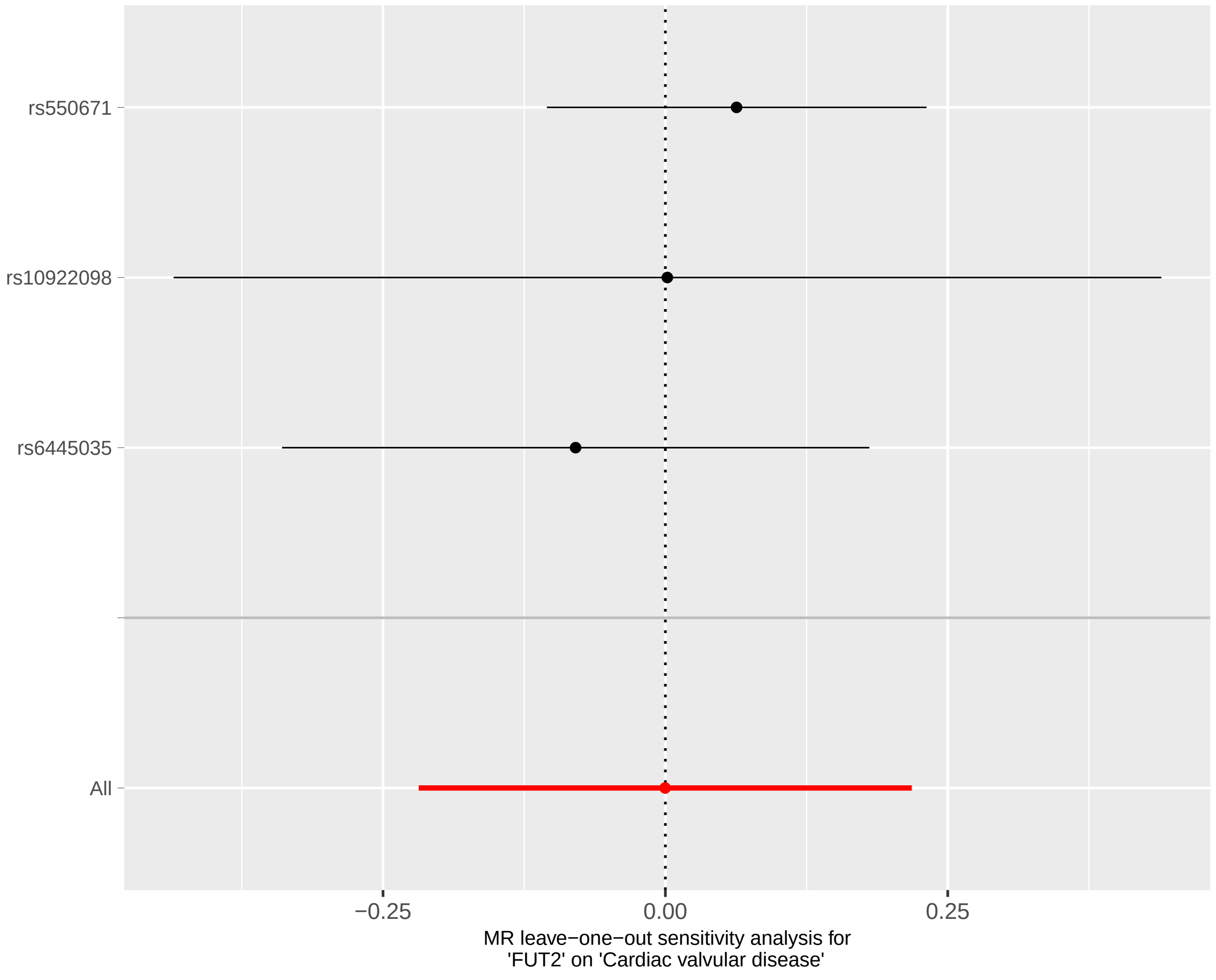

rs550671
rs10922098
rs6445035
All
−0.25
0.00
0.25
MR leave−one−out sensitivity analysis for
'FUT2' on 'Cardiac valvular disease'

Insufficient number of SNPs

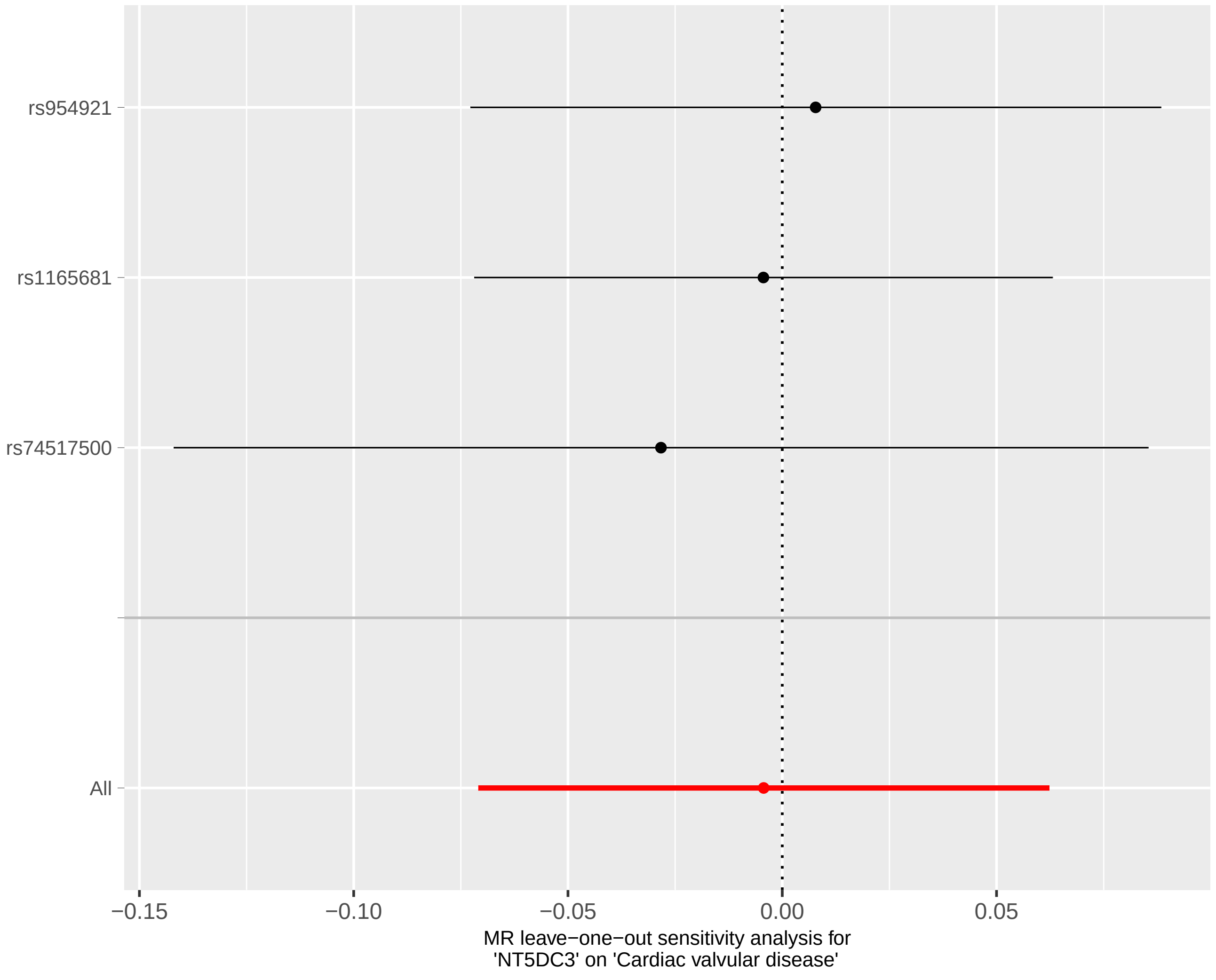
rs954921
rs1165681
rs74517500
All
−0.15
−0.10
−0.05
0.00
0.05
MR leave−one−out sensitivity analysis for
'NT5DC3' on 'Cardiac valvular disease'

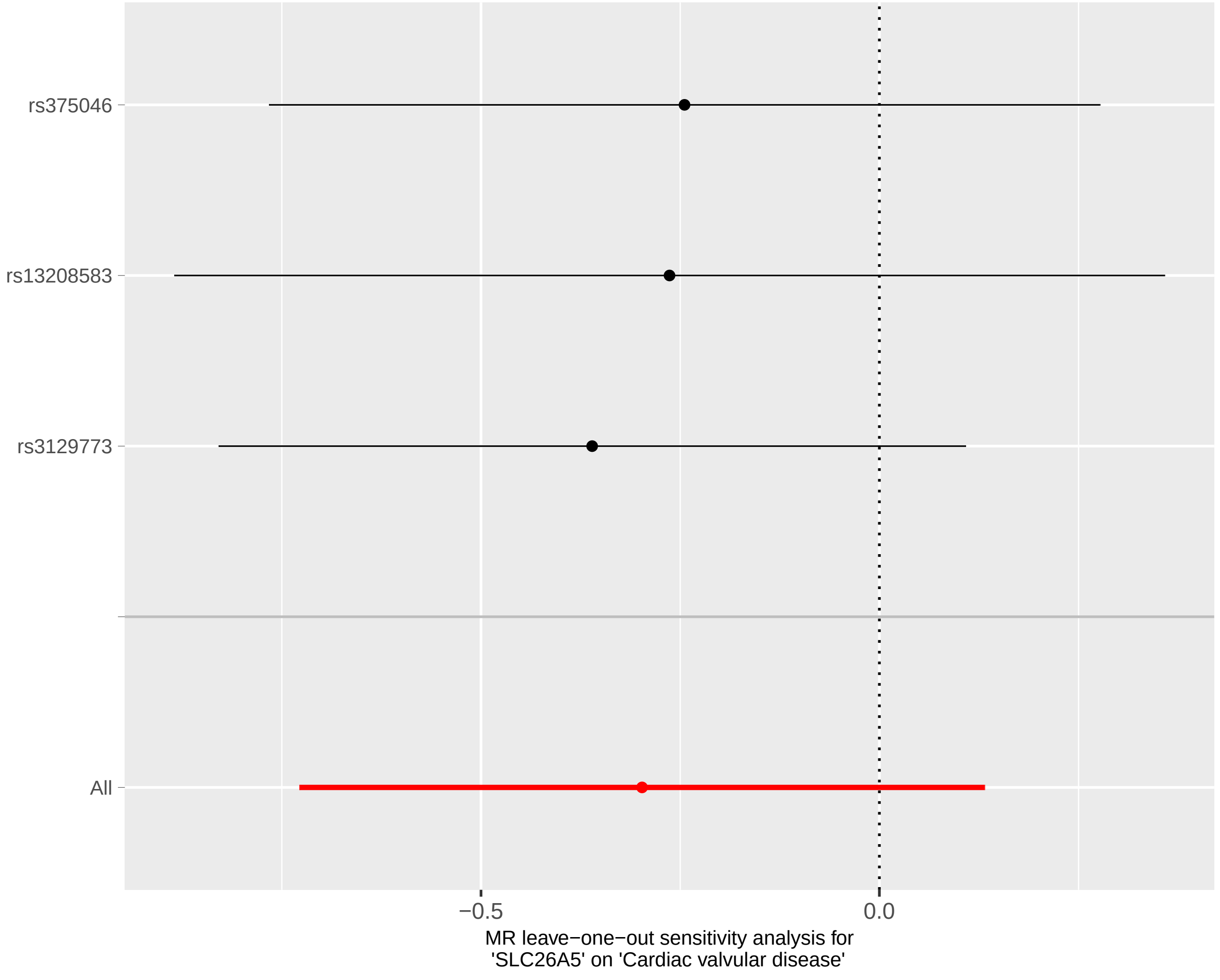
rs375046
rs13208583
rs3129773
All
−0.5
0.0
MR leave−one−out sensitivity analysis for
'SLC26A5' on 'Cardiac valvular disease'

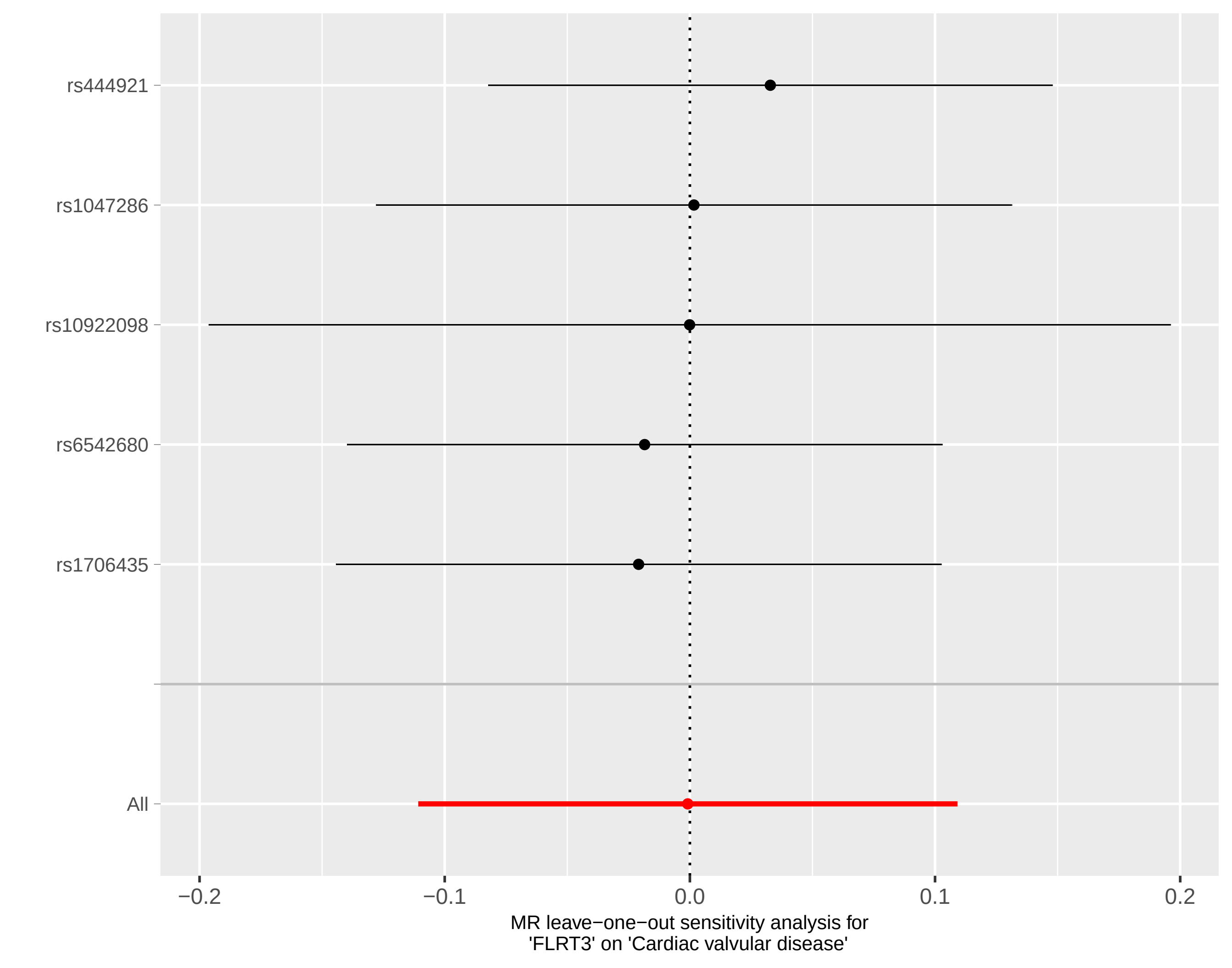

rs444921
rs1047286
rs10922098
rs6542680
rs1706435
All
−0.2
−0.1
0.0
0.1
0.2
MR leave−one−out sensitivity analysis for
'FLRT3' on 'Cardiac valvular disease'

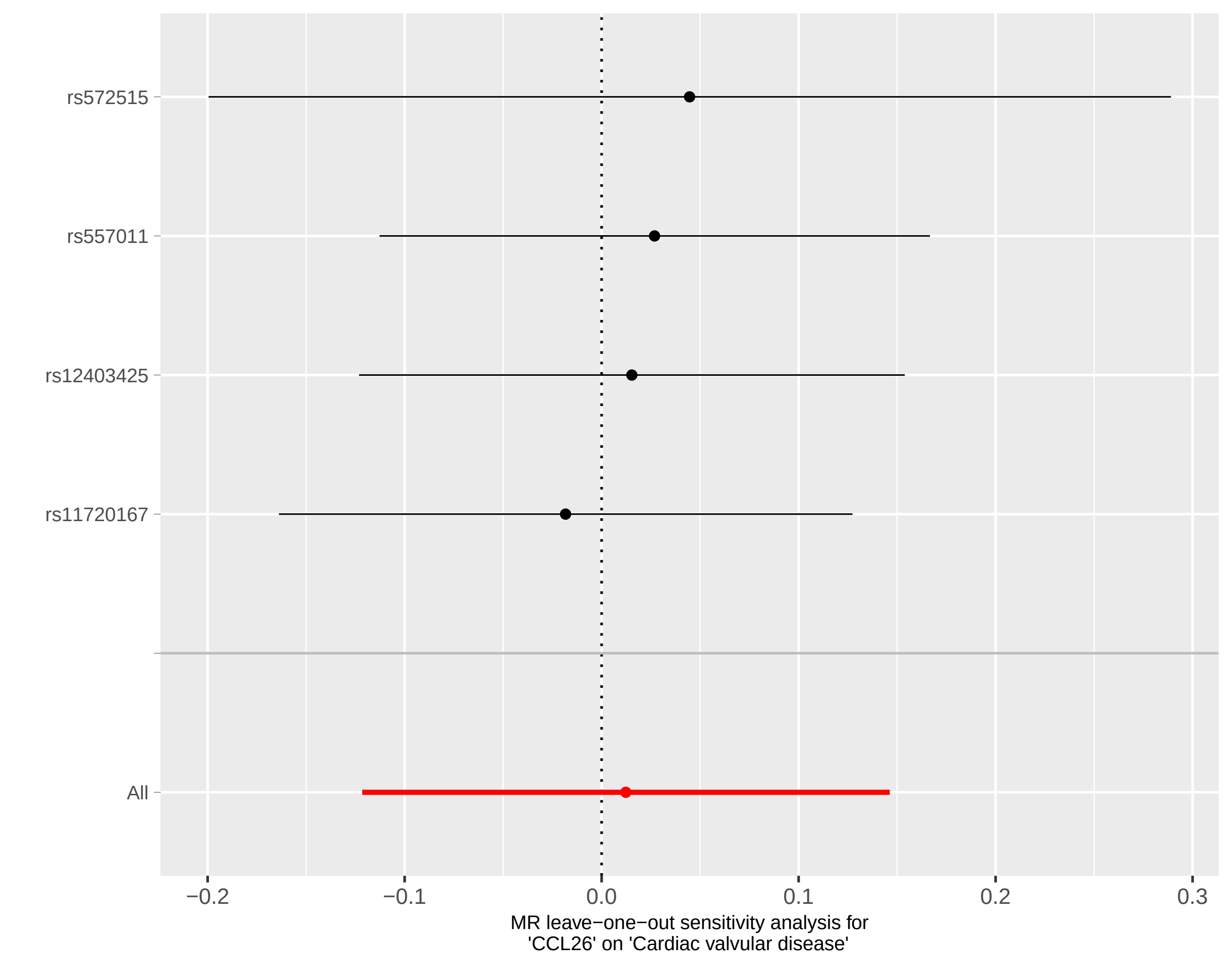
rs572515
rs557011
rs12403425
rs11720167
All
−0.2
−0.1
0.0
0.1
0.2
0.3
MR leave−one−out sensitivity analysis for
'CCL26' on 'Cardiac valvular disease'

Insufficient number of SNPs

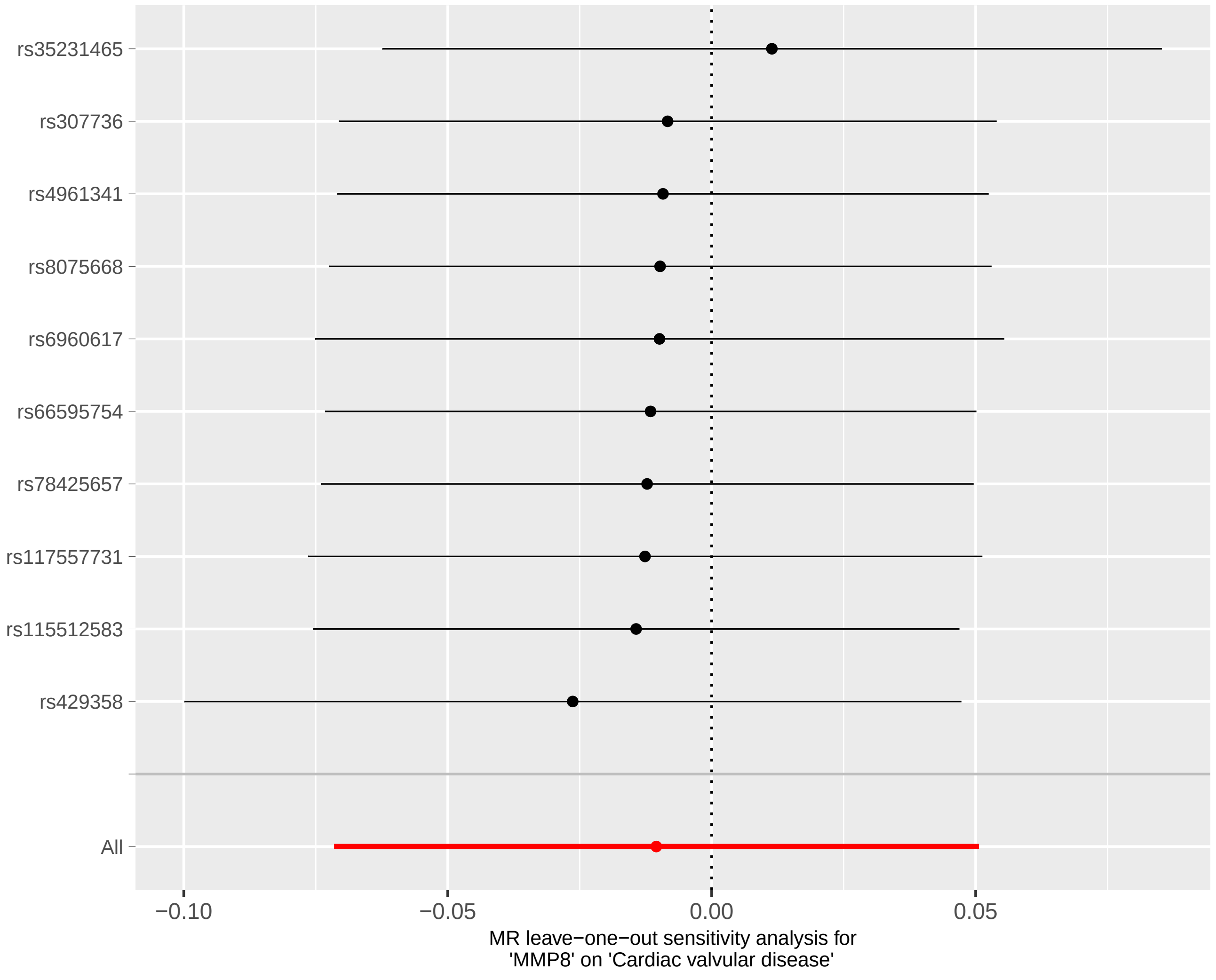
rs35231465
rs307736
rs4961341
rs8075668
rs6960617
rs66595754
rs78425657
rs117557731
rs115512583
rs429358
All
−0.10
−0.05
0.00
0.05
MR leave−one−out sensitivity analysis for
'MMP8' on 'Cardiac valvular disease'

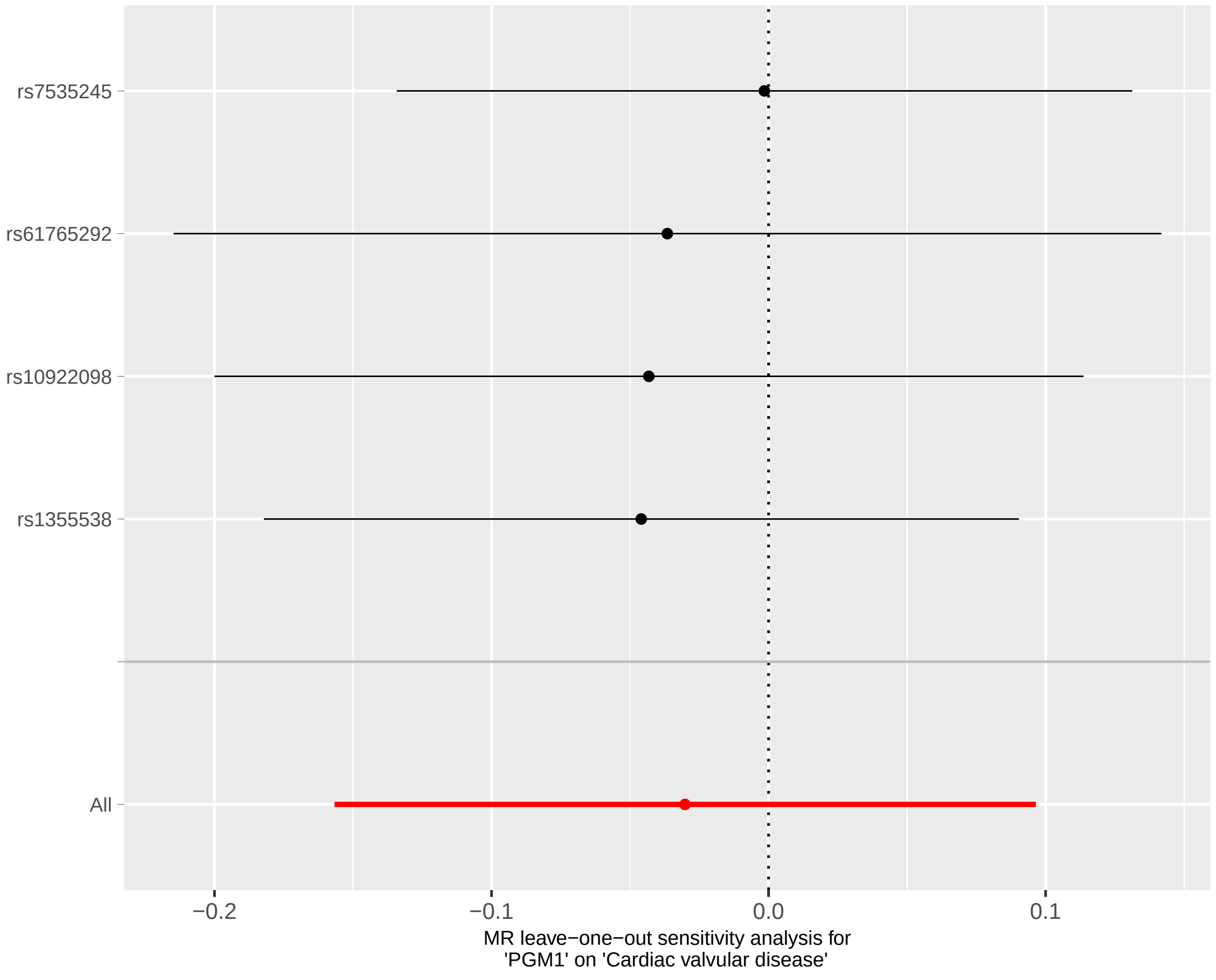

rs7535245
rs61765292
rs10922098
rs1355538
All
−0.2
−0.1
0.0
0.1
MR leave−one−out sensitivity analysis for
'PGM1' on 'Cardiac valvular disease'

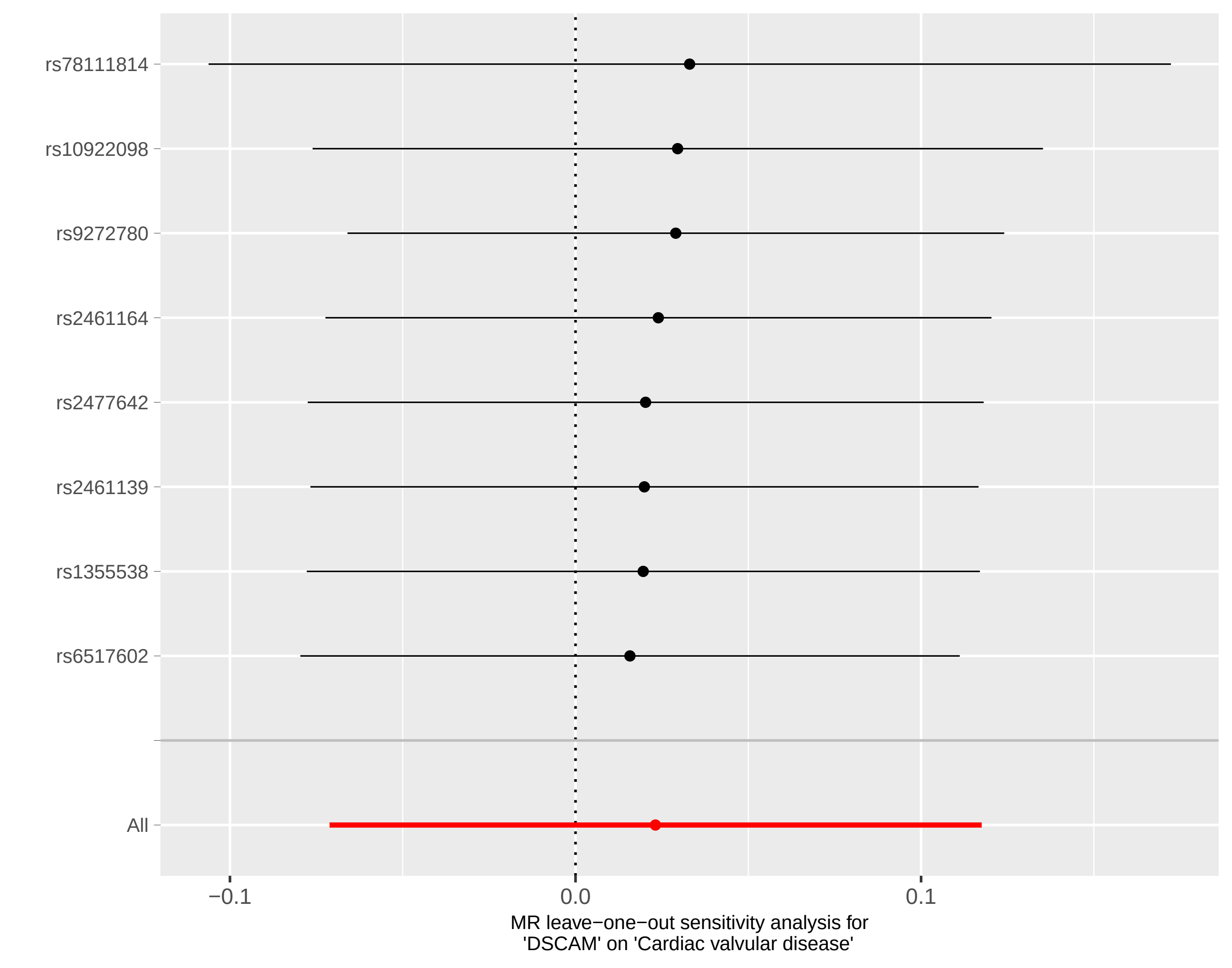
rs78111814
rs10922098
rs9272780
rs2461164
rs2477642
rs2461139
rs1355538
rs6517602
All
−0.1
0.0
0.1
MR leave−one−out sensitivity analysis for
'DSCAM' on 'Cardiac valvular disease'

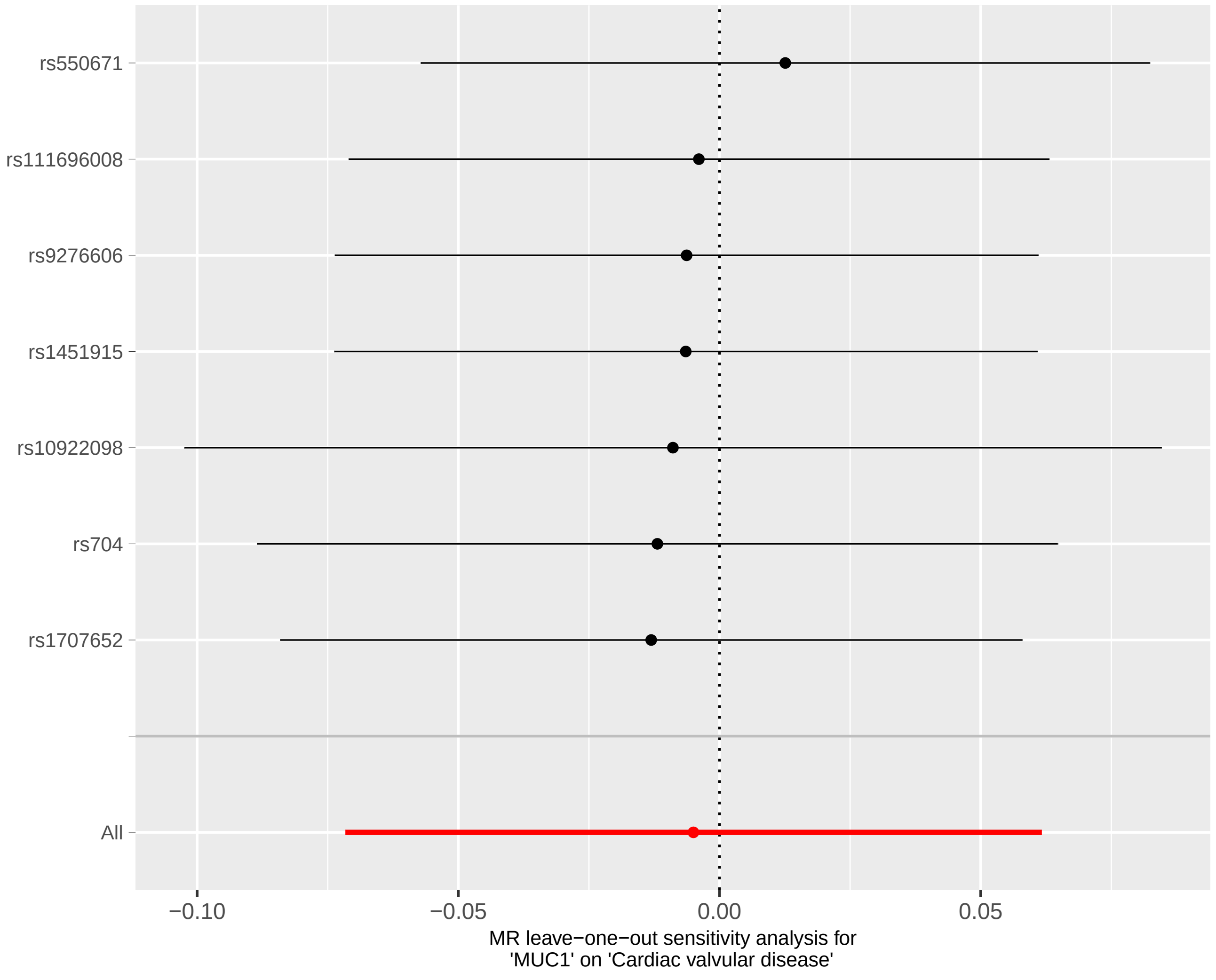
rs550671
rs111696008
rs9276606
rs1451915
rs10922098
rs704
rs1707652
All
−0.10
−0.05
0.00
0.05
MR leave−one−out sensitivity analysis for
'MUC1' on 'Cardiac valvular disease'

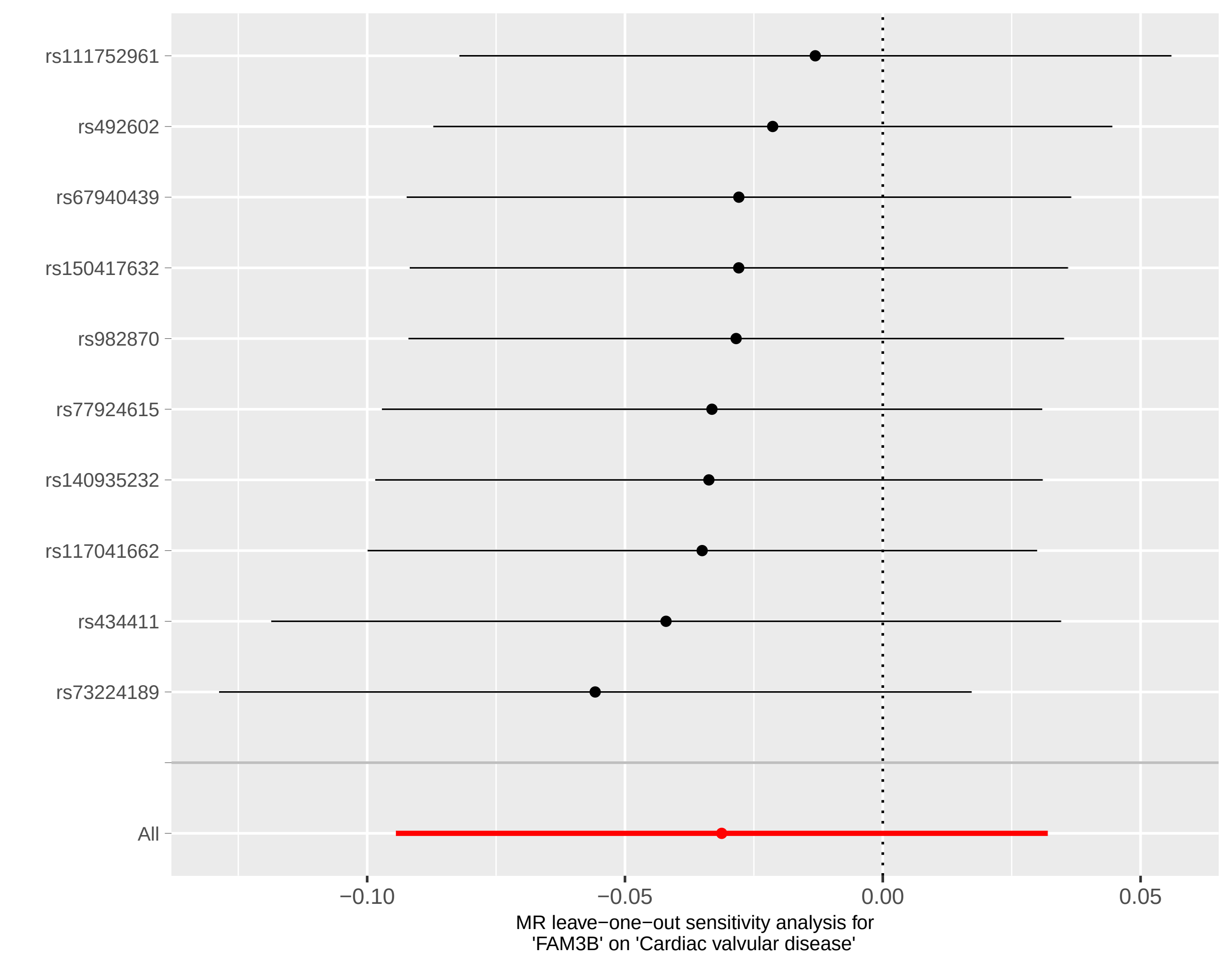
rs111752961
rs492602
rs67940439
rs150417632
rs982870
rs77924615
rs140935232
rs117041662
rs434411
rs73224189
All
−0.10
−0.05
0.00
0.05
MR leave−one−out sensitivity analysis for
'FAM3B' on 'Cardiac valvular disease'

Insufficient number of SNPs

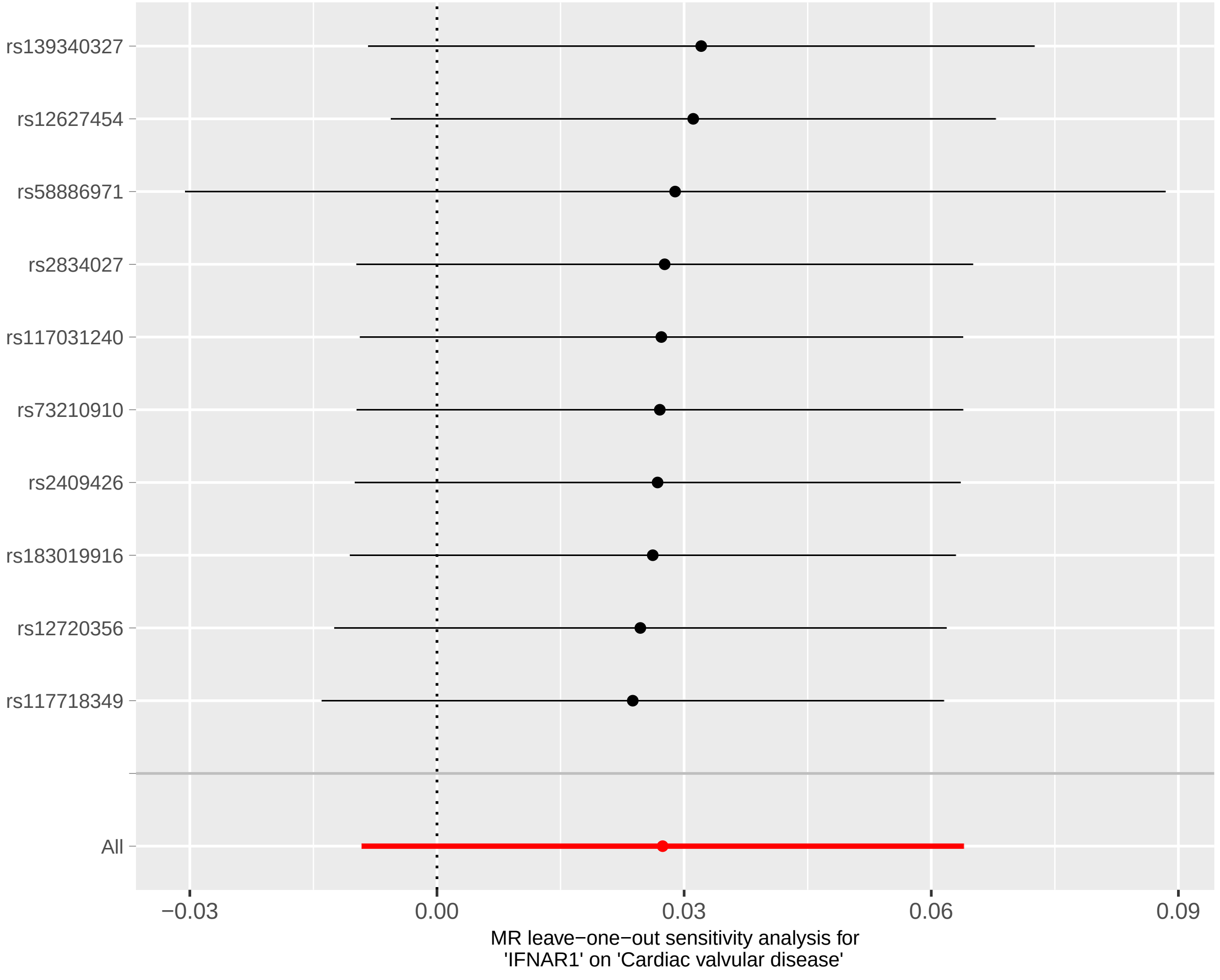

rs139340327
rs12627454
rs58886971
rs2834027
rs117031240
rs73210910
rs2409426
rs183019916
rs12720356
rs117718349
All
−0.03
0.00
0.03
0.06
0.09
MR leave−one−out sensitivity analysis for
'IFNAR1' on 'Cardiac valvular disease'

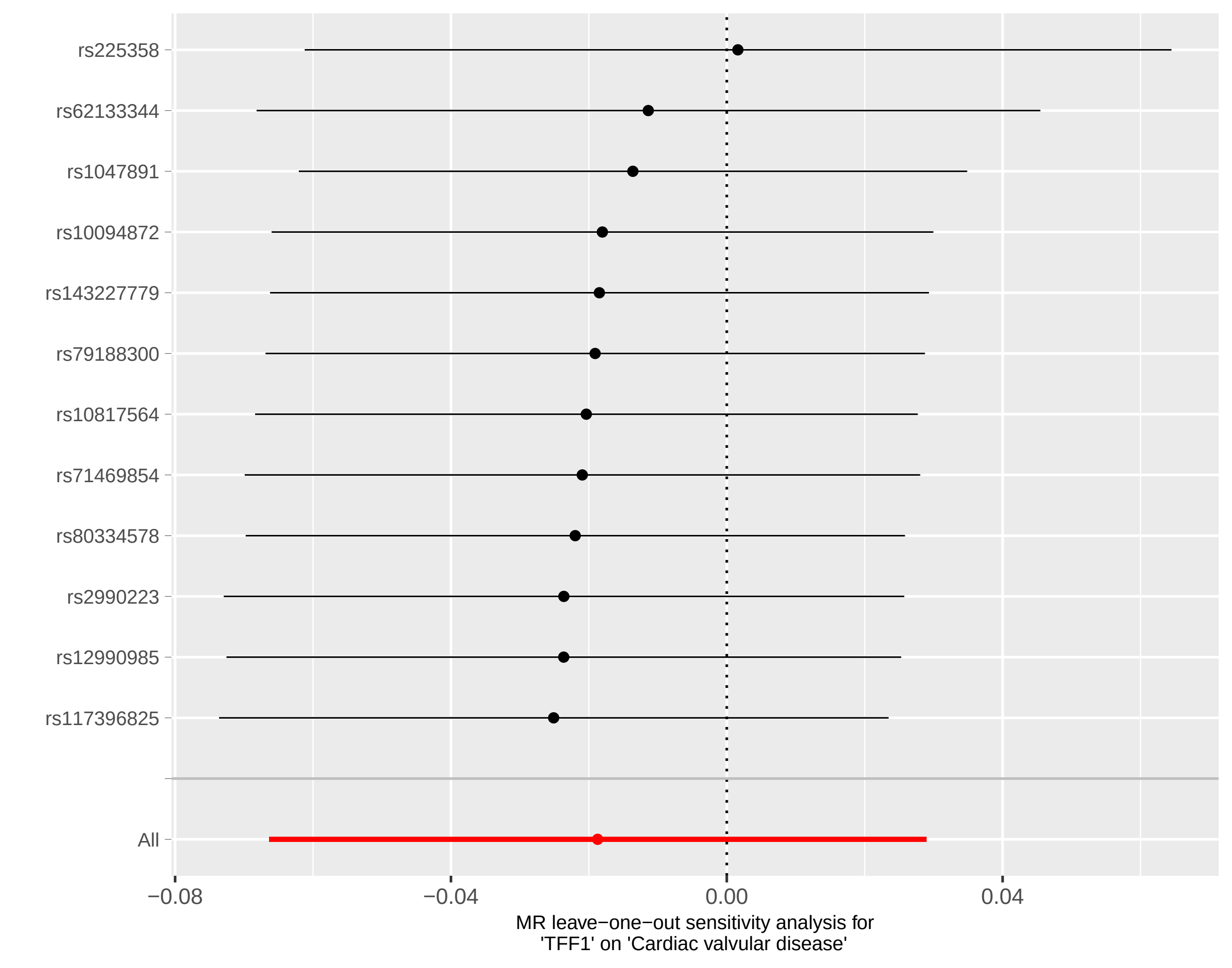
rs225358
rs62133344
rs1047891
rs10094872
rs143227779
rs79188300
rs10817564
rs71469854
rs80334578
rs2990223
rs12990985
rs117396825
All
−0.08
−0.04
0.00
0.04
MR leave−one−out sensitivity analysis for
'TFF1' on 'Cardiac valvular disease'

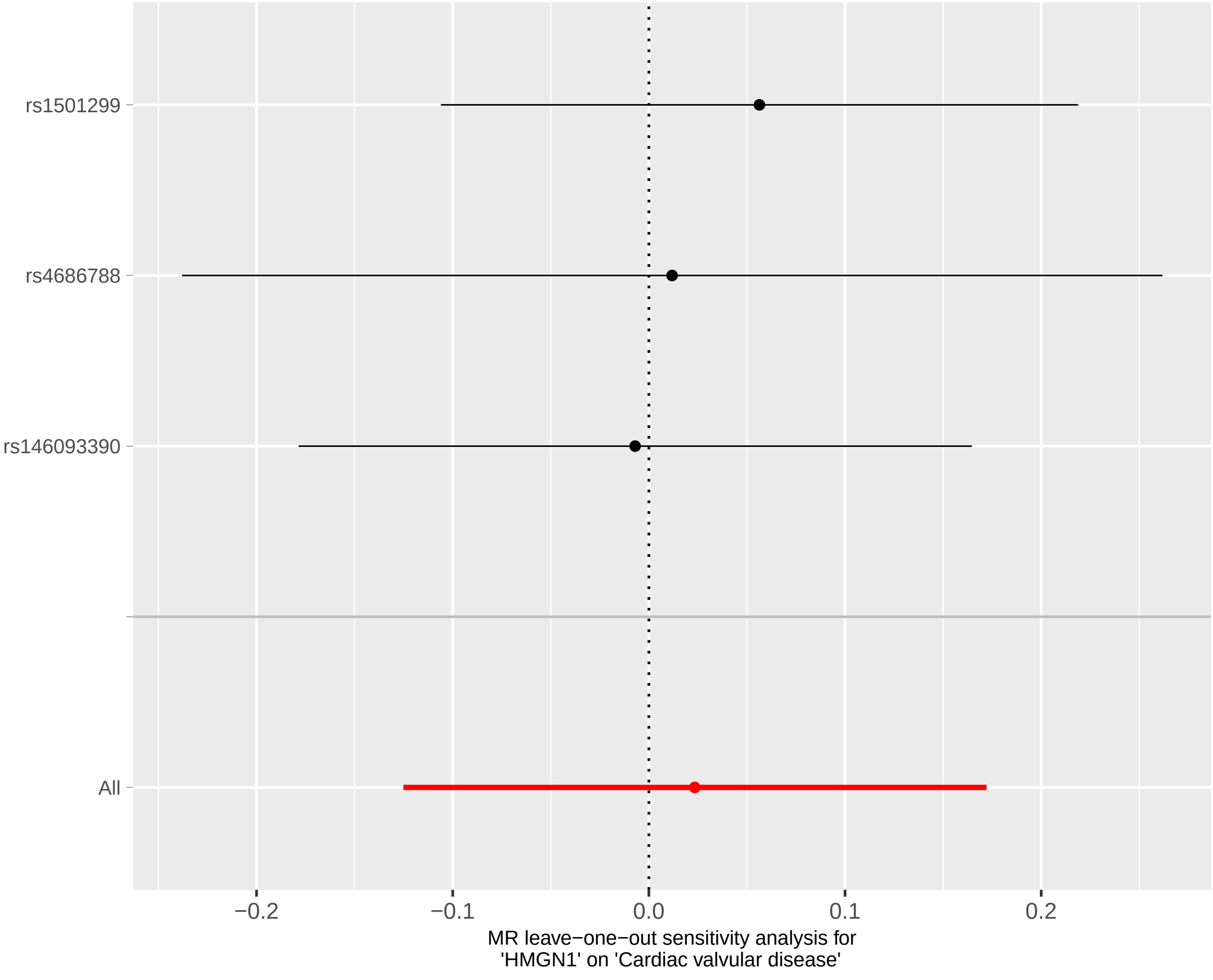
rs1501299
rs4686788
rs146093390
All
−0.2
−0.1
0.0
0.1
0.2
MR leave−one−out sensitivity analysis for
'HMGN1' on 'Cardiac valvular disease'

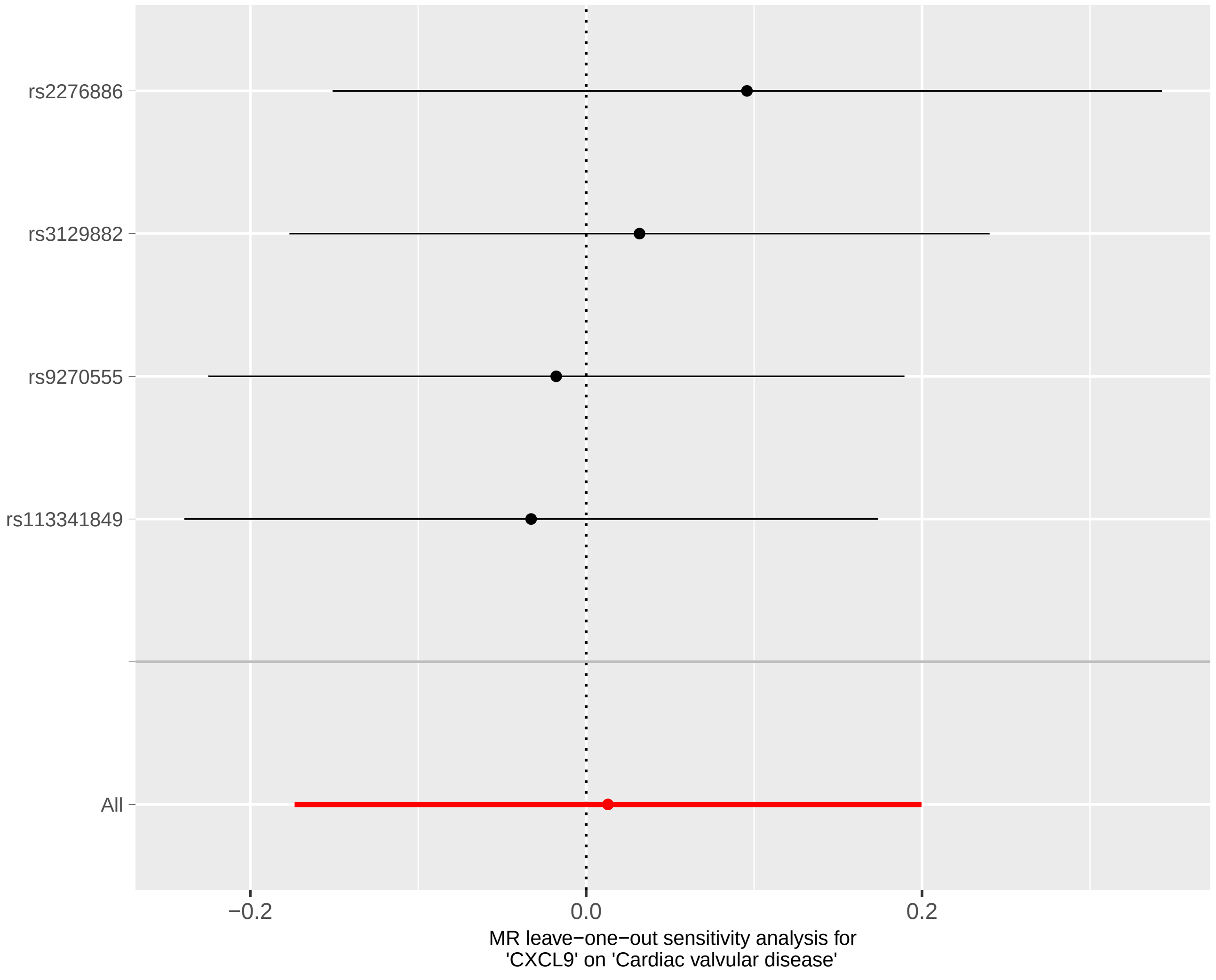
rs2276886
rs3129882
rs9270555
rs113341849
All
−0.2
0.0
0.2
MR leave−one−out sensitivity analysis for
'CXCL9' on 'Cardiac valvular disease'

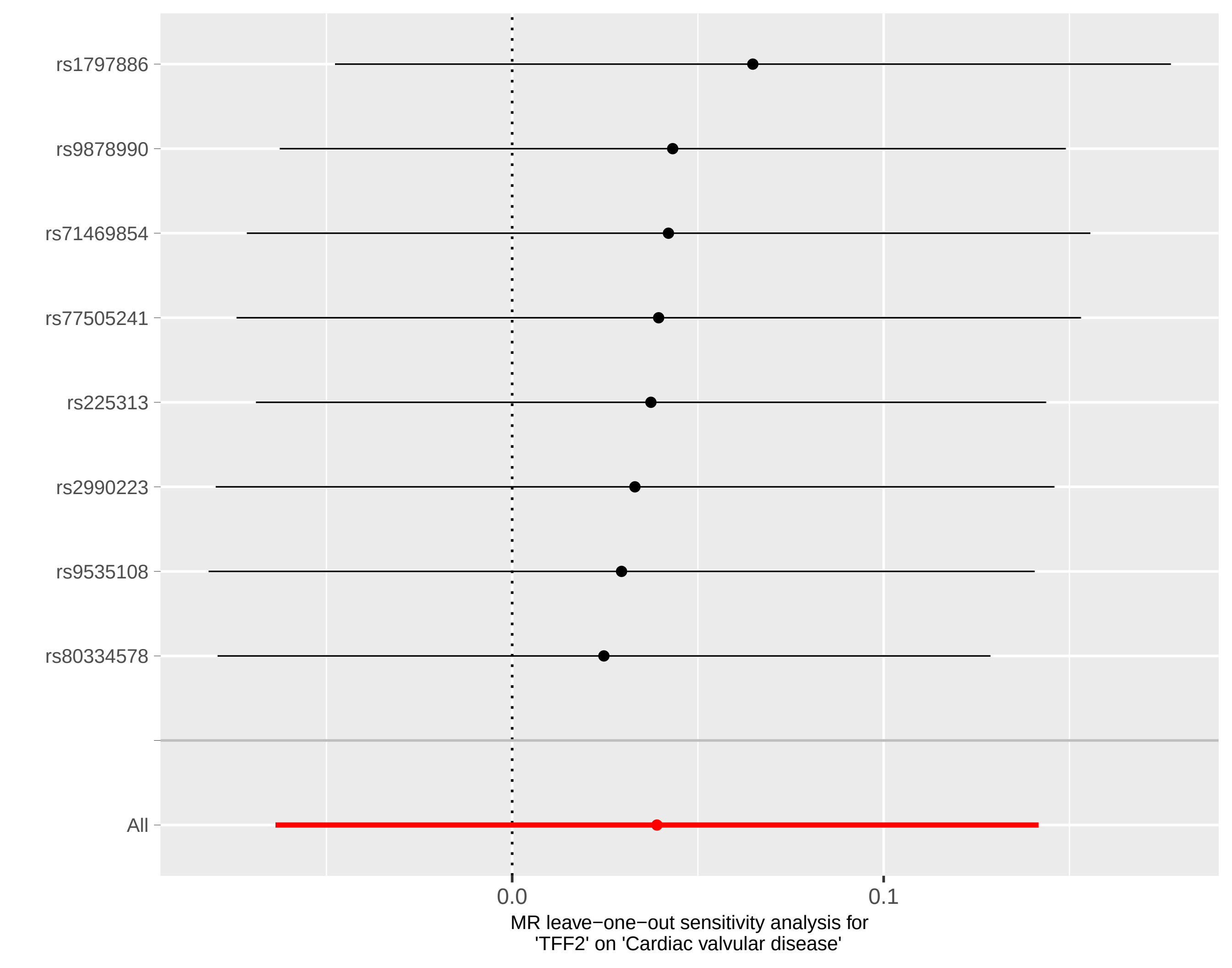
rs1797886
rs9878990
rs71469854
rs77505241
rs225313
rs2990223
rs9535108
rs80334578
All
0.0
0.1
MR leave−one−out sensitivity analysis for
'TFF2' on 'Cardiac valvular disease'

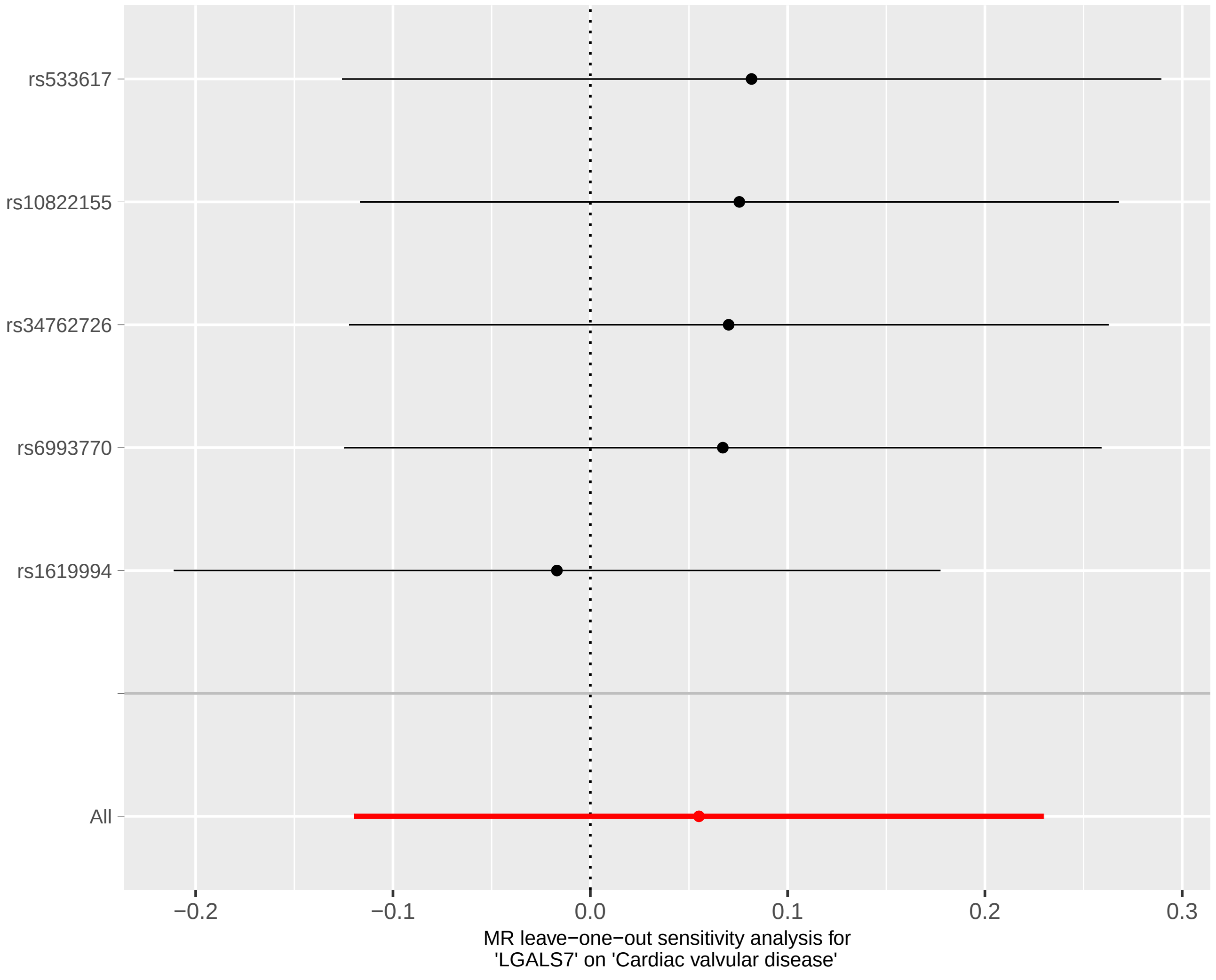

rs533617
rs10822155
rs34762726
rs6993770
rs1619994
All
−0.2
−0.1
0.0
0.1
0.2
0.3
MR leave−one−out sensitivity analysis for
'LGALS7' on 'Cardiac valvular disease'

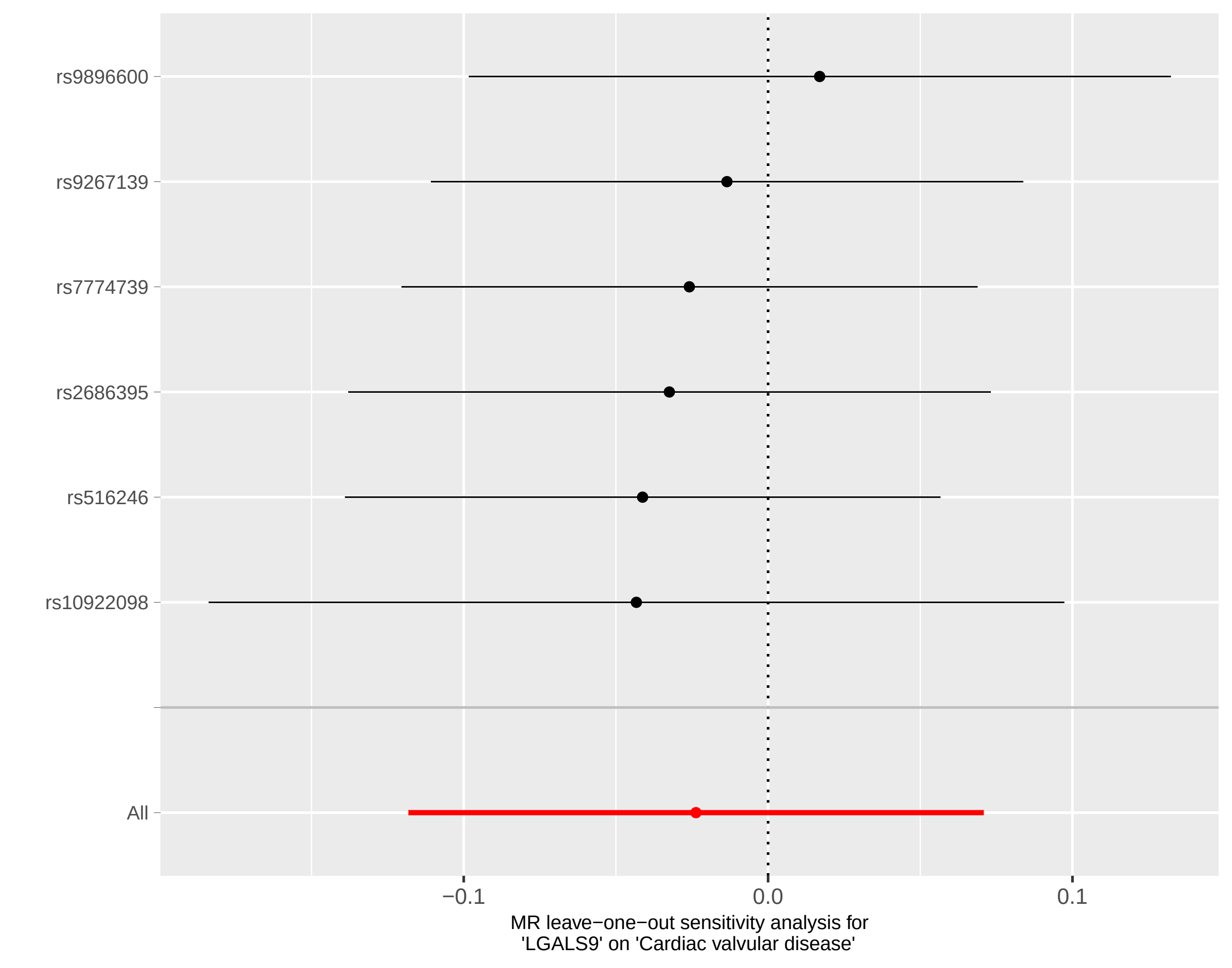
rs9896600
rs9267139
rs7774739
rs2686395
rs516246
rs10922098
All
−0.1
0.0
0.1
MR leave−one−out sensitivity analysis for
'LGALS9' on 'Cardiac valvular disease'

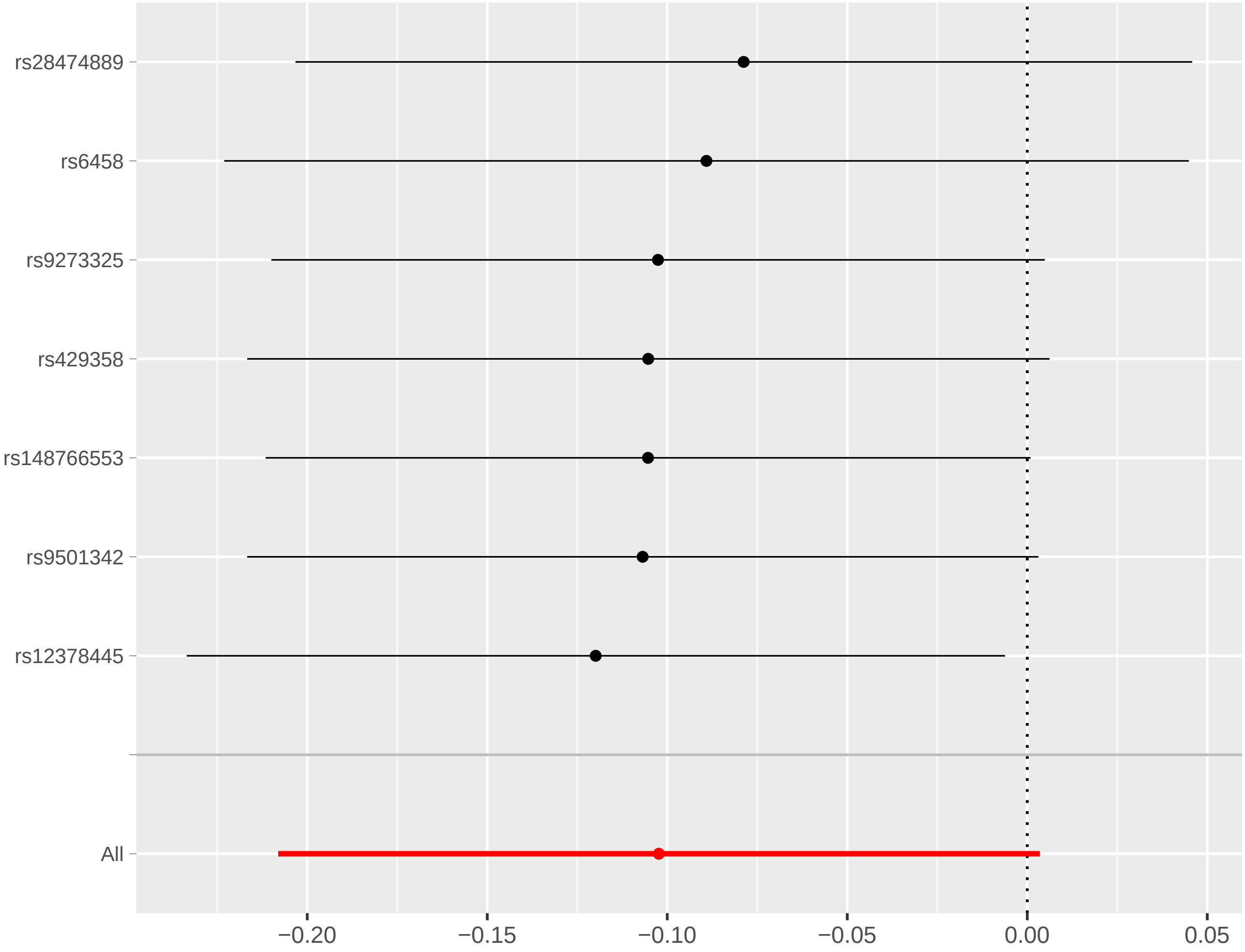

MR leave−one−out sensitivity analysis for
'UBE2G2' on 'Cardiac valvular disease'

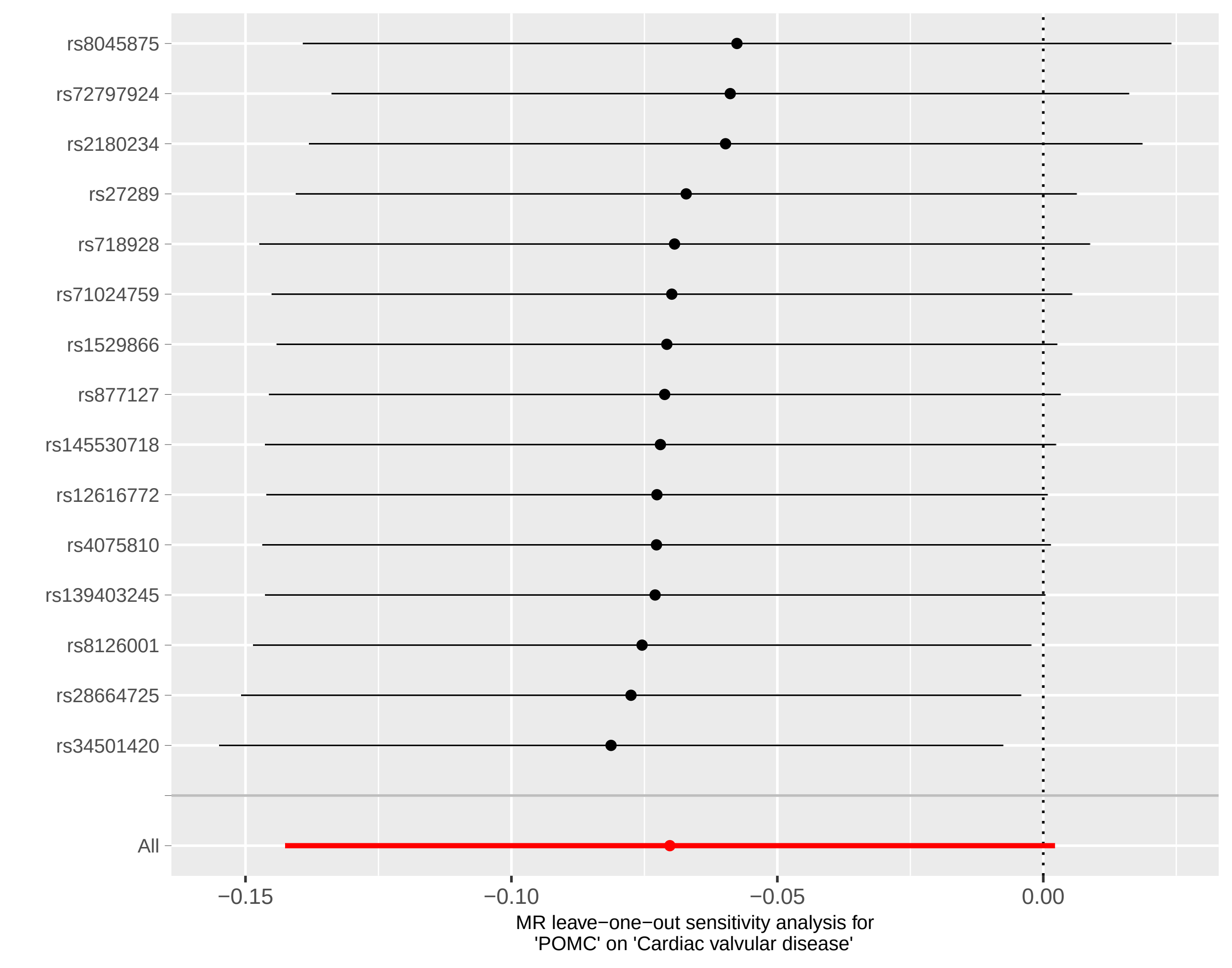
rs8045875
rs72797924
rs2180234
rs27289
rs718928
rs71024759
rs1529866
rs877127
rs145530718
rs12616772
rs4075810
rs139403245
rs8126001
rs28664725
rs34501420
All
−0.15
−0.10
−0.05
0.00
MR leave−one−out sensitivity analysis for
'POMC' on 'Cardiac valvular disease'

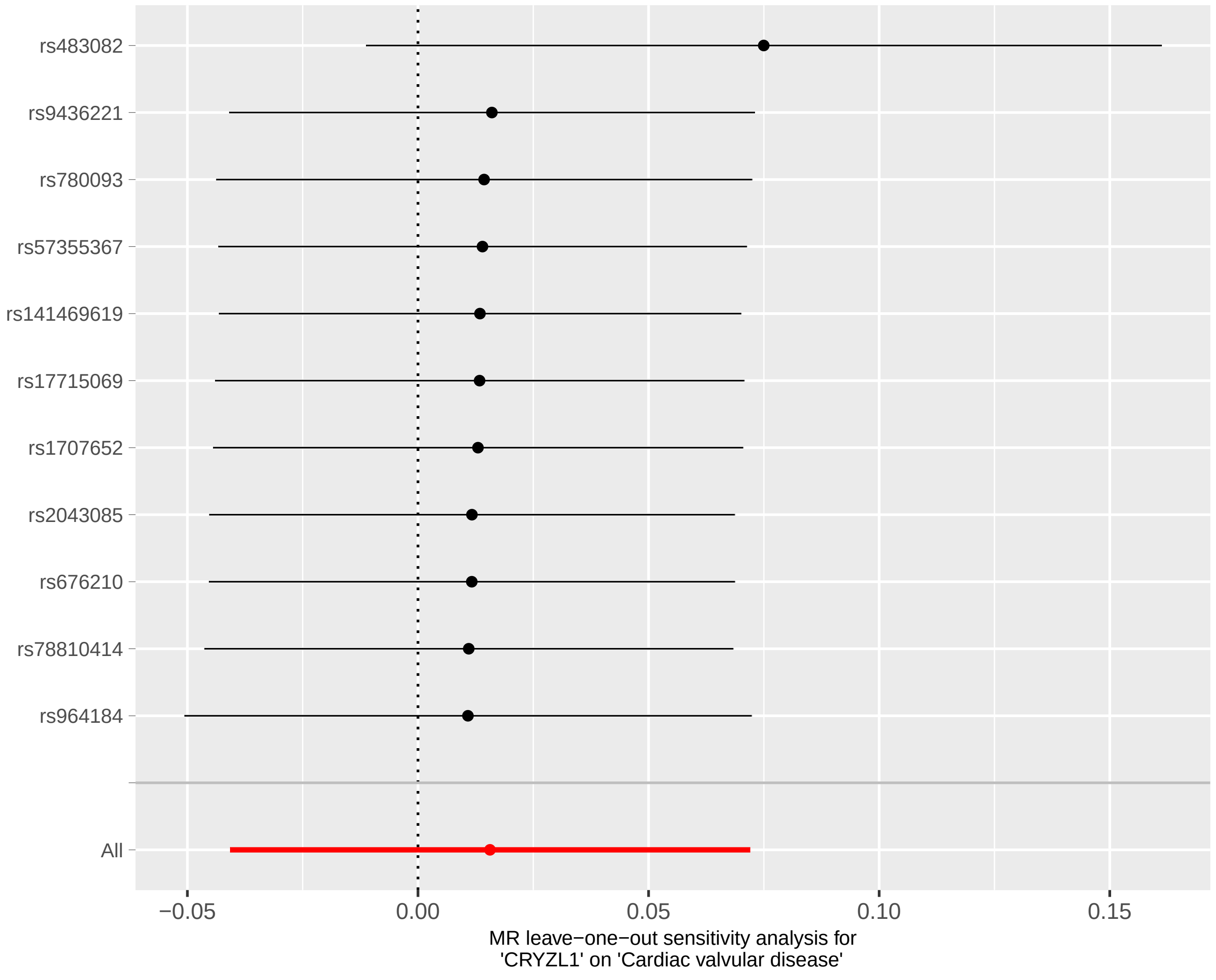
rs483082
rs9436221
rs780093
rs57355367
rs141469619
rs17715069
rs1707652
rs2043085
rs676210
rs78810414
rs964184
All
−0.05
0.00
0.05
0.10
0.15
MR leave−one−out sensitivity analysis for
'CRYZL1' on 'Cardiac valvular disease'

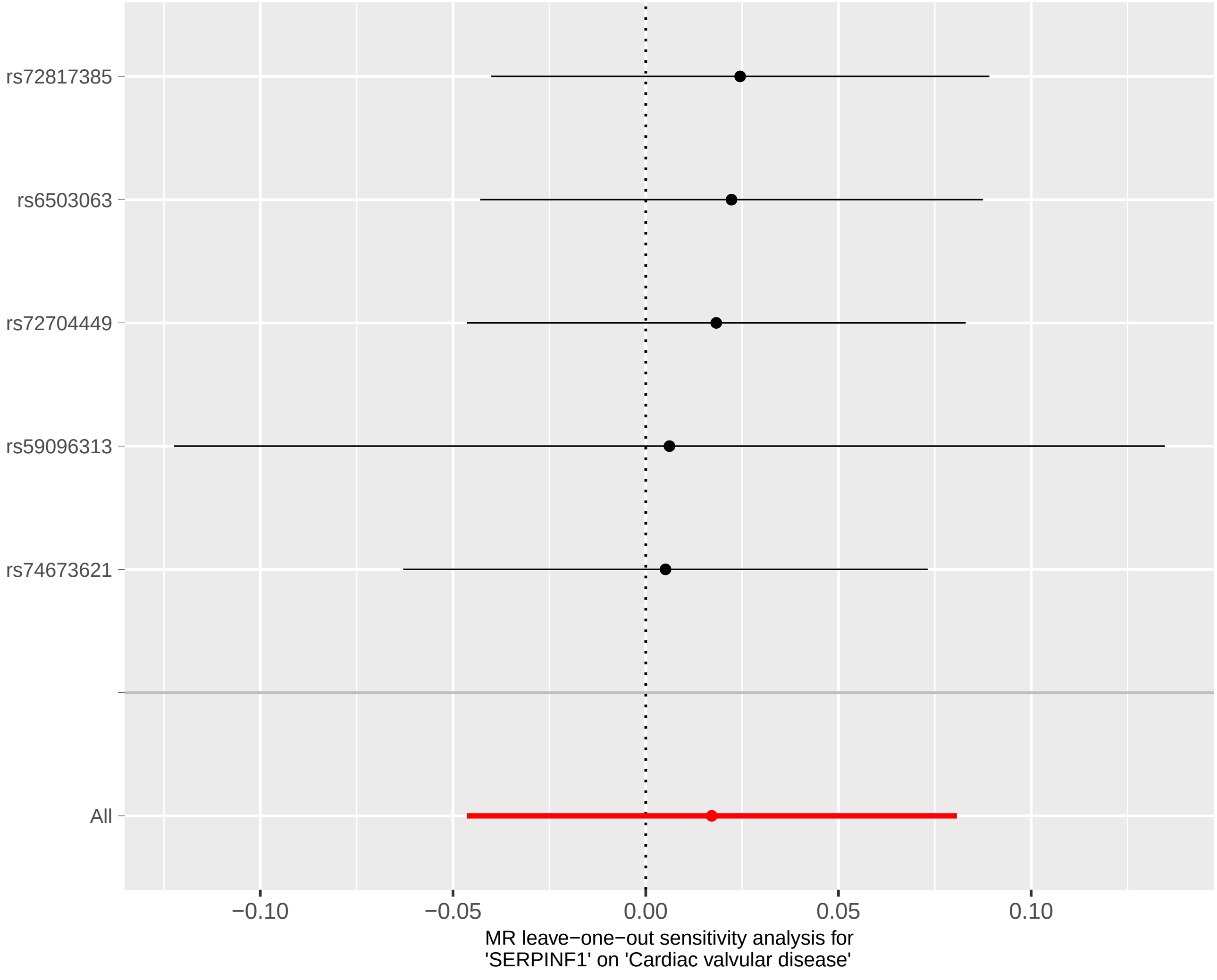
rs72817385
rs6503063
rs72704449
rs59096313
rs74673621
All
−0.10
−0.05
0.00
0.05
0.10
MR leave−one−out sensitivity analysis for
'SERPINF1' on 'Cardiac valvular disease'

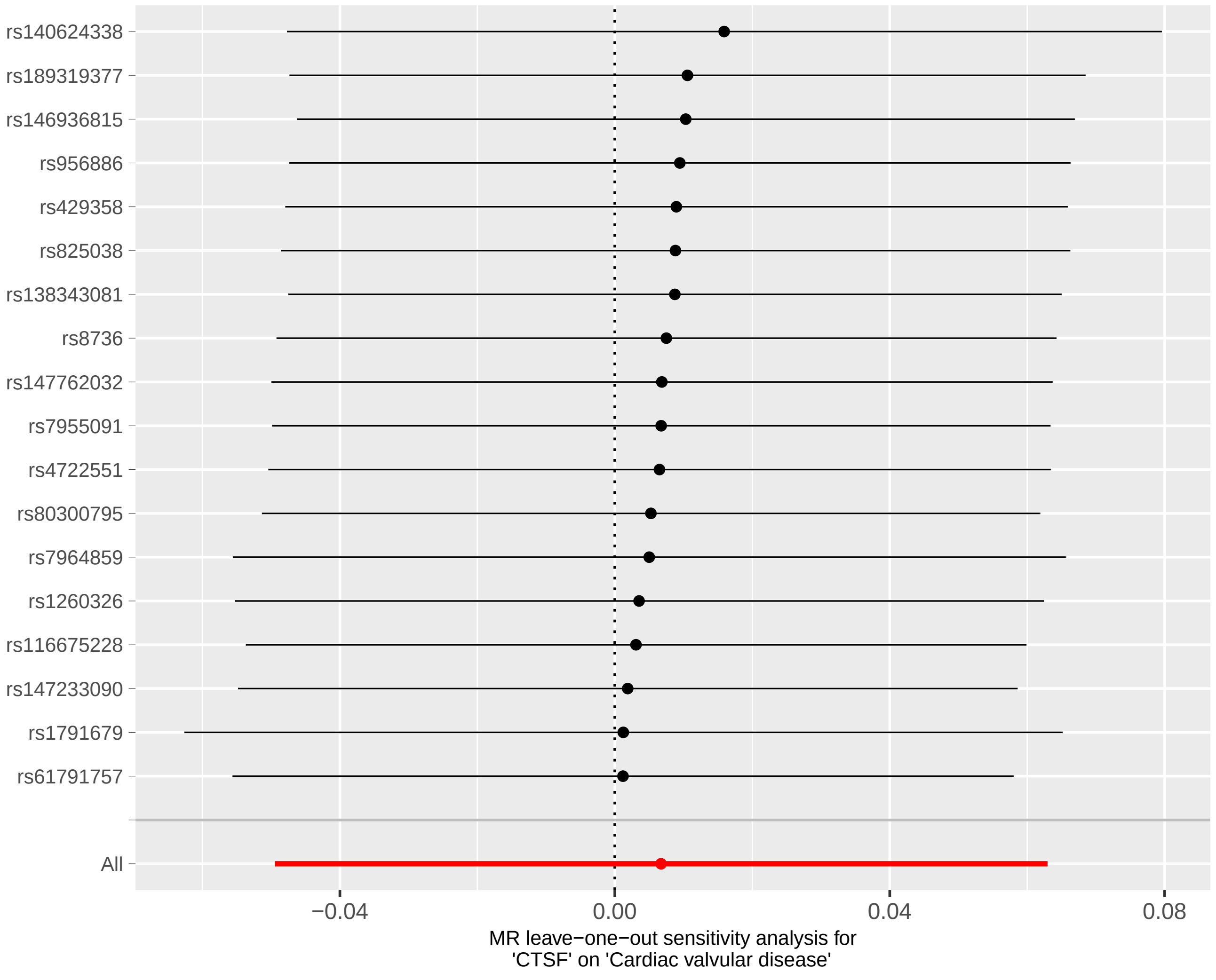
rs140624338
rs189319377
rs146936815
rs956886
rs429358
rs825038
rs138343081
rs8736
rs147762032
rs7955091
rs4722551
rs80300795
rs7964859
rs1260326
rs116675228
rs147233090
rs1791679
rs61791757
All
−0.04
0.00
0.04
0.08
MR leave−one−out sensitivity analysis for
'CTSF' on 'Cardiac valvular disease'

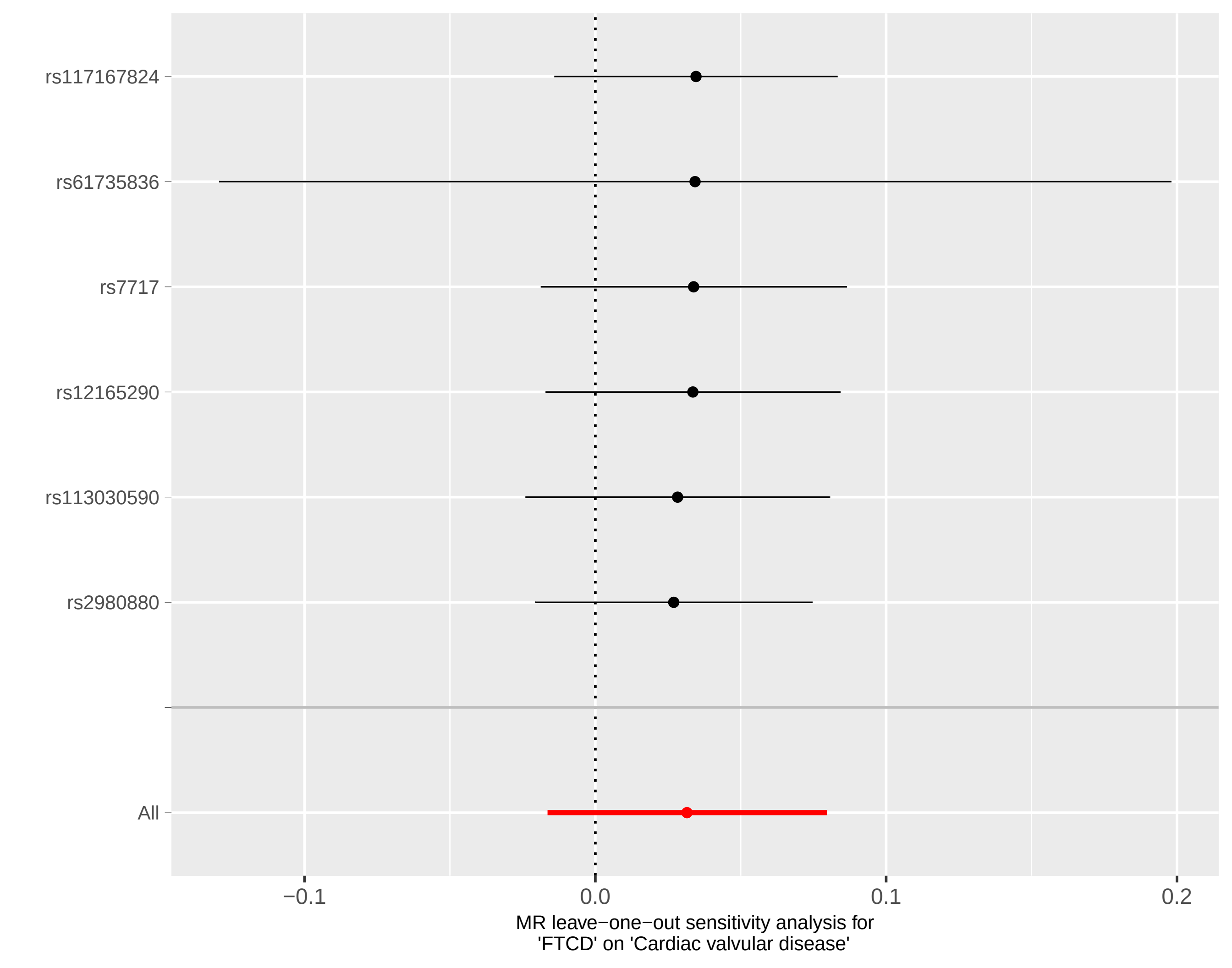
rs117167824
rs61735836
rs7717
rs12165290
rs113030590
rs2980880
All
−0.1
0.0
0.1
0.2
MR leave−one−out sensitivity analysis for
'FTCD' on 'Cardiac valvular disease'

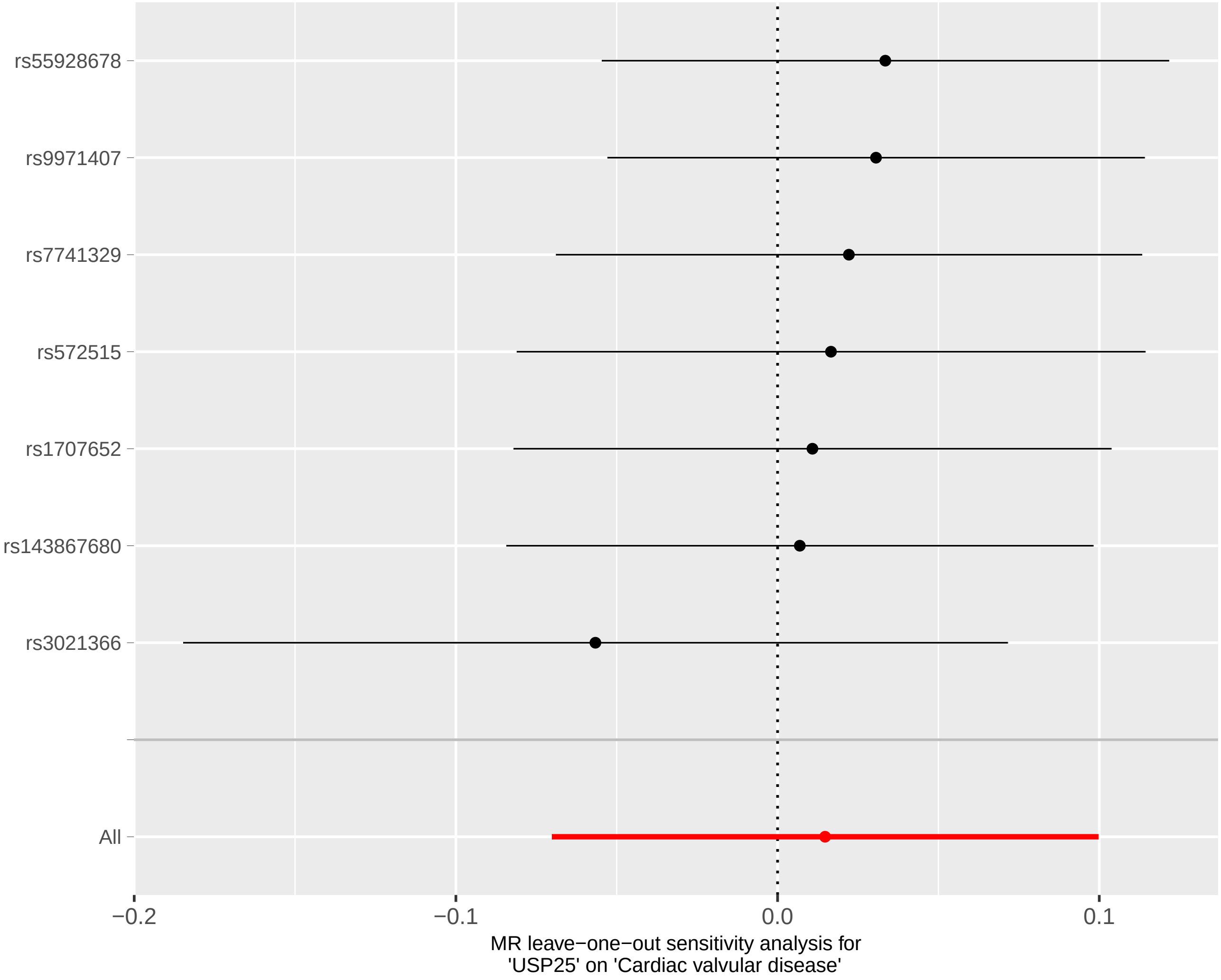

rs55928678
rs9971407
rs7741329
rs572515
rs1707652
rs143867680
rs3021366
All
−0.2
−0.1
0.0
0.1
MR leave−one−out sensitivity analysis for
'USP25' on 'Cardiac valvular disease'

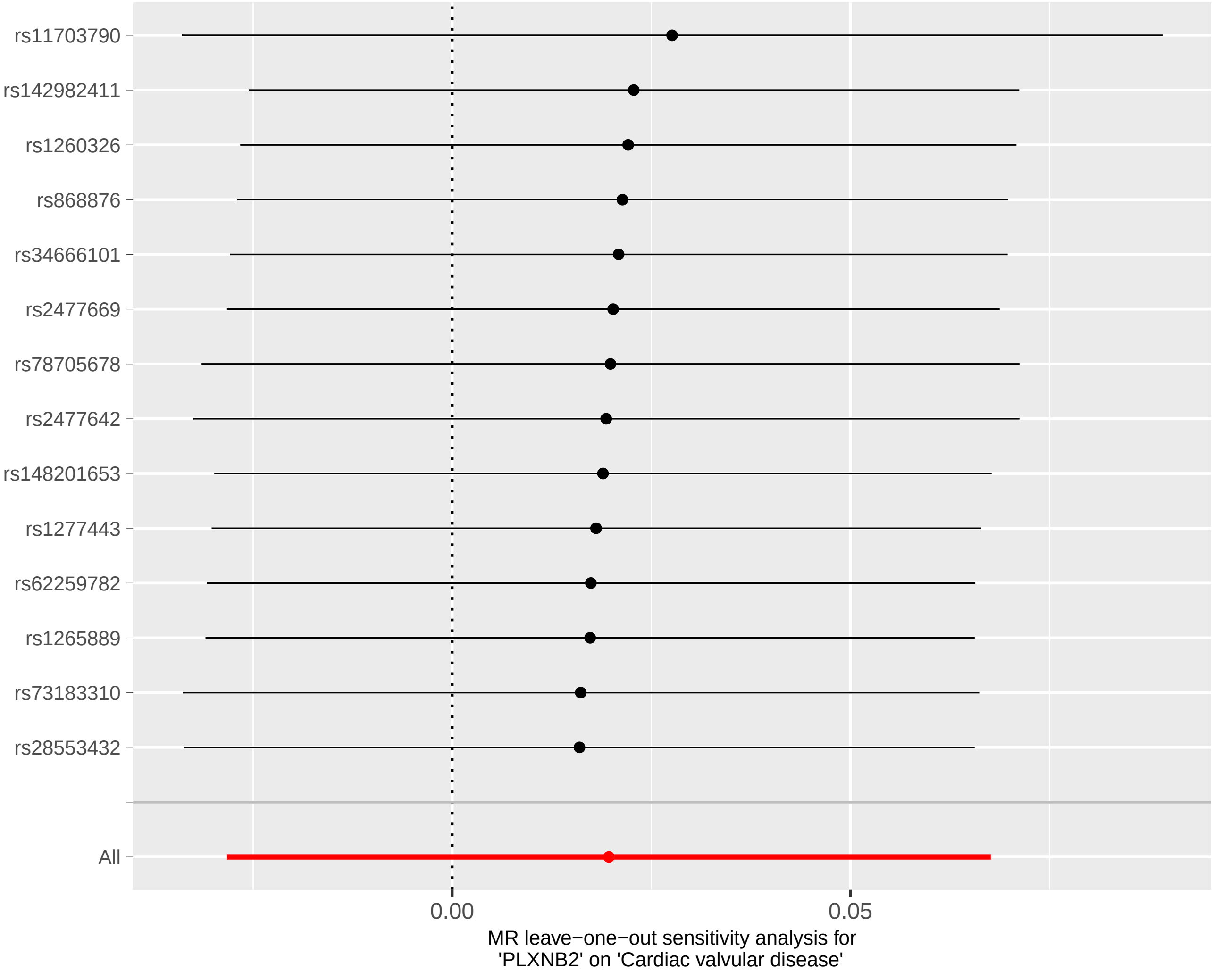
rs11703790
rs142982411
rs1260326
rs868876
rs34666101
rs2477669
rs78705678
rs2477642
rs148201653
rs1277443
rs62259782
rs1265889
rs73183310
rs28553432
All
0.00
0.05
MR leave−one−out sensitivity analysis for
'PLXNB2' on 'Cardiac valvular disease'

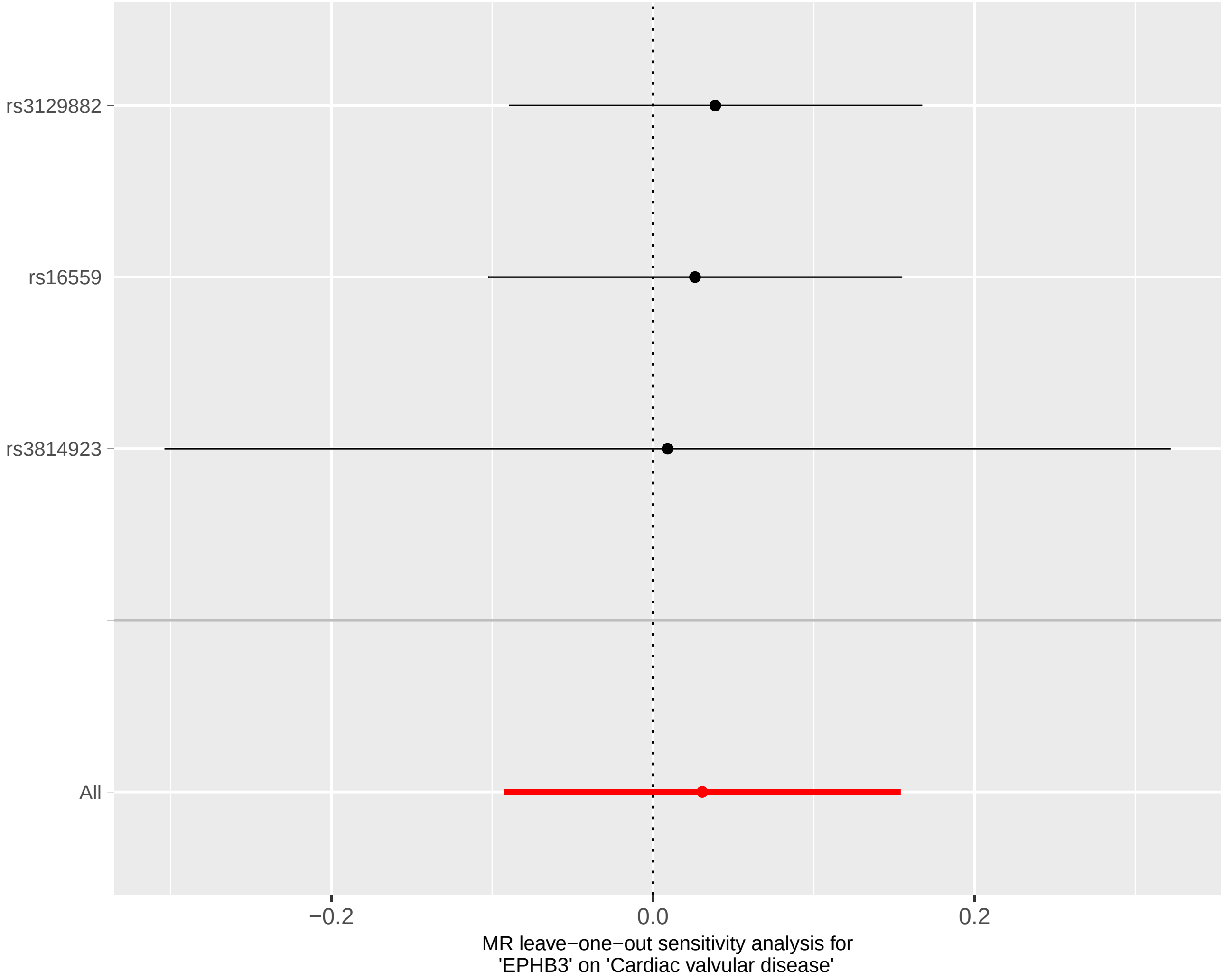

rs3129882
rs16559
rs3814923
All
−0.2
0.0
0.2
MR leave−one−out sensitivity analysis for
'EPHB3' on 'Cardiac valvular disease'

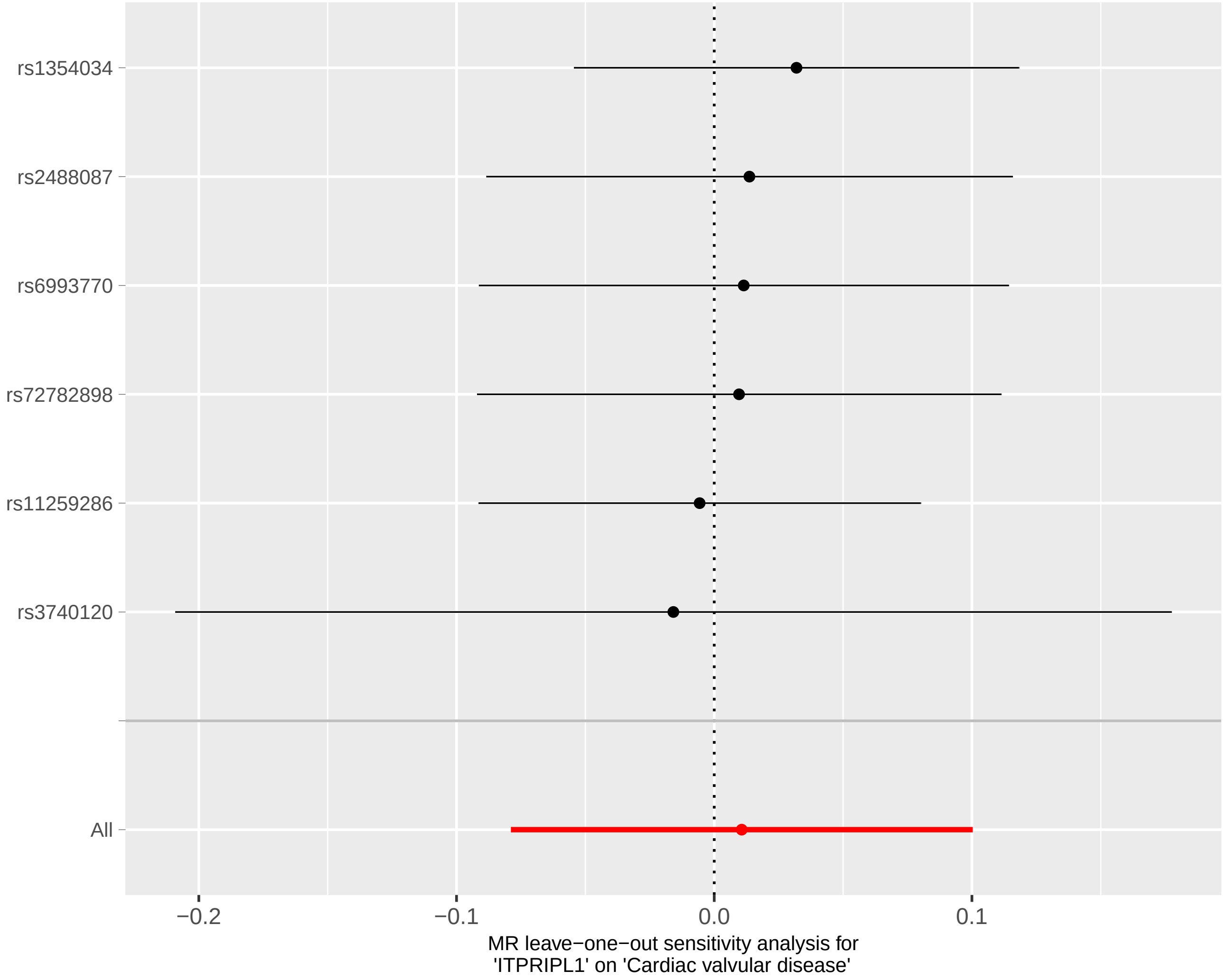
rs1354034
rs2488087
rs6993770
rs72782898
rs11259286
rs3740120
All
−0.2
−0.1
0.0
0.1
MR leave−one−out sensitivity analysis for
'ITPRIPL1' on 'Cardiac valvular disease'

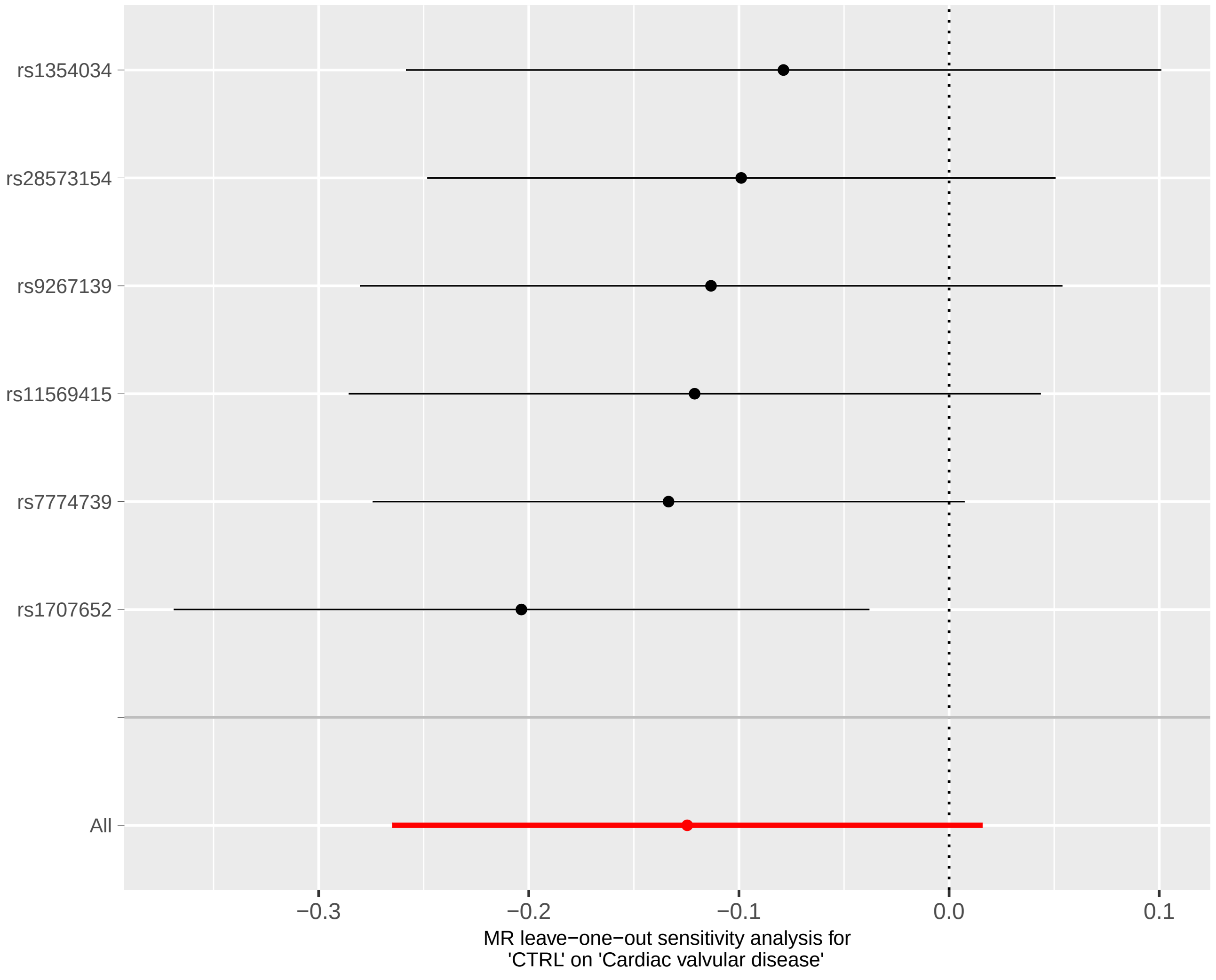
rs1354034
rs28573154
rs9267139
rs11569415
rs7774739
rs1707652
All
−0.3
−0.2
−0.1
0.0
0.1
MR leave−one−out sensitivity analysis for
'CTRL' on 'Cardiac valvular disease'

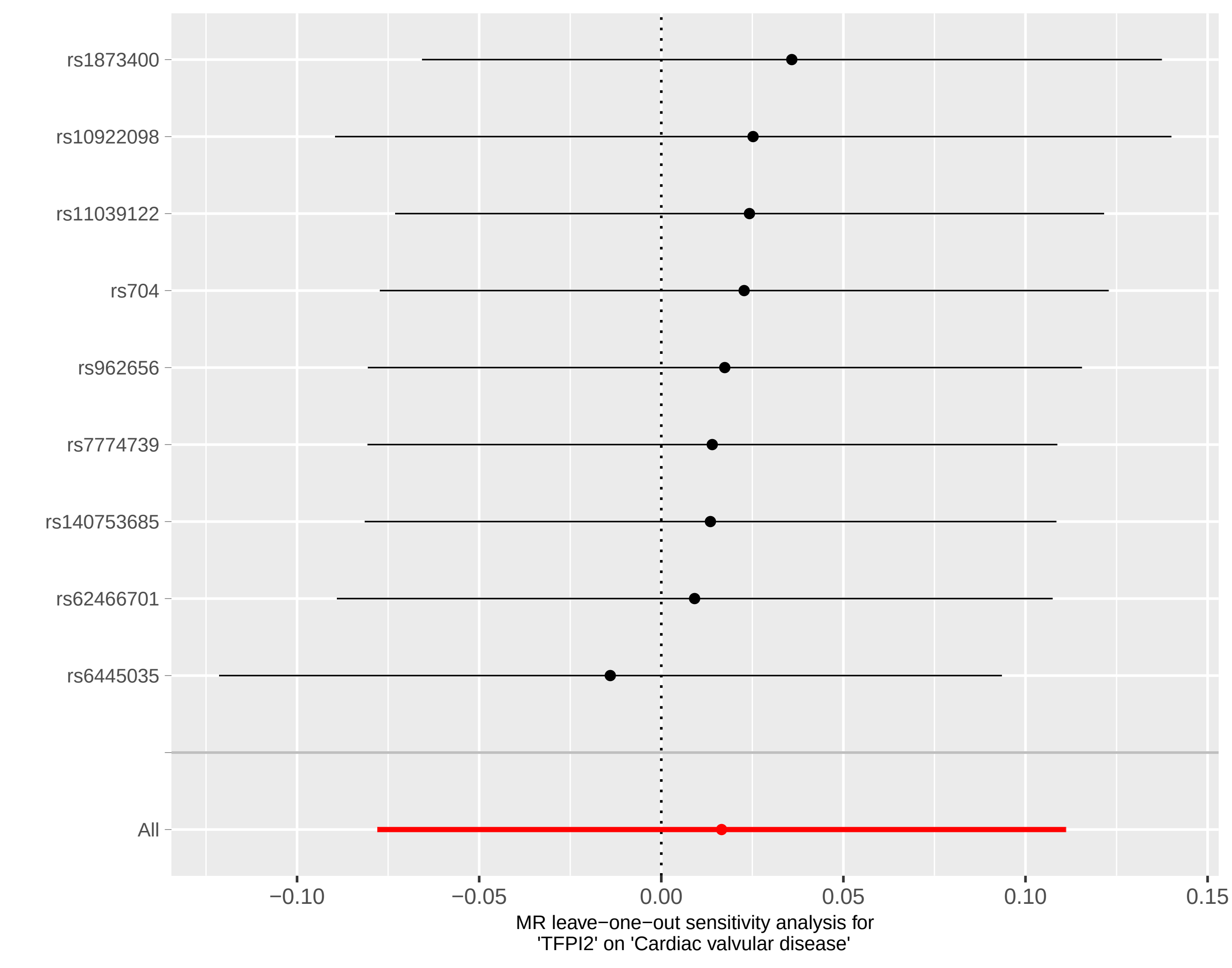

rs1873400
rs10922098
rs11039122
rs704
rs962656
rs7774739
rs140753685
rs62466701
rs6445035
All
−0.10
−0.05
0.00
0.05
0.10
0.15
MR leave−one−out sensitivity analysis for
'TFPI2' on 'Cardiac valvular disease'

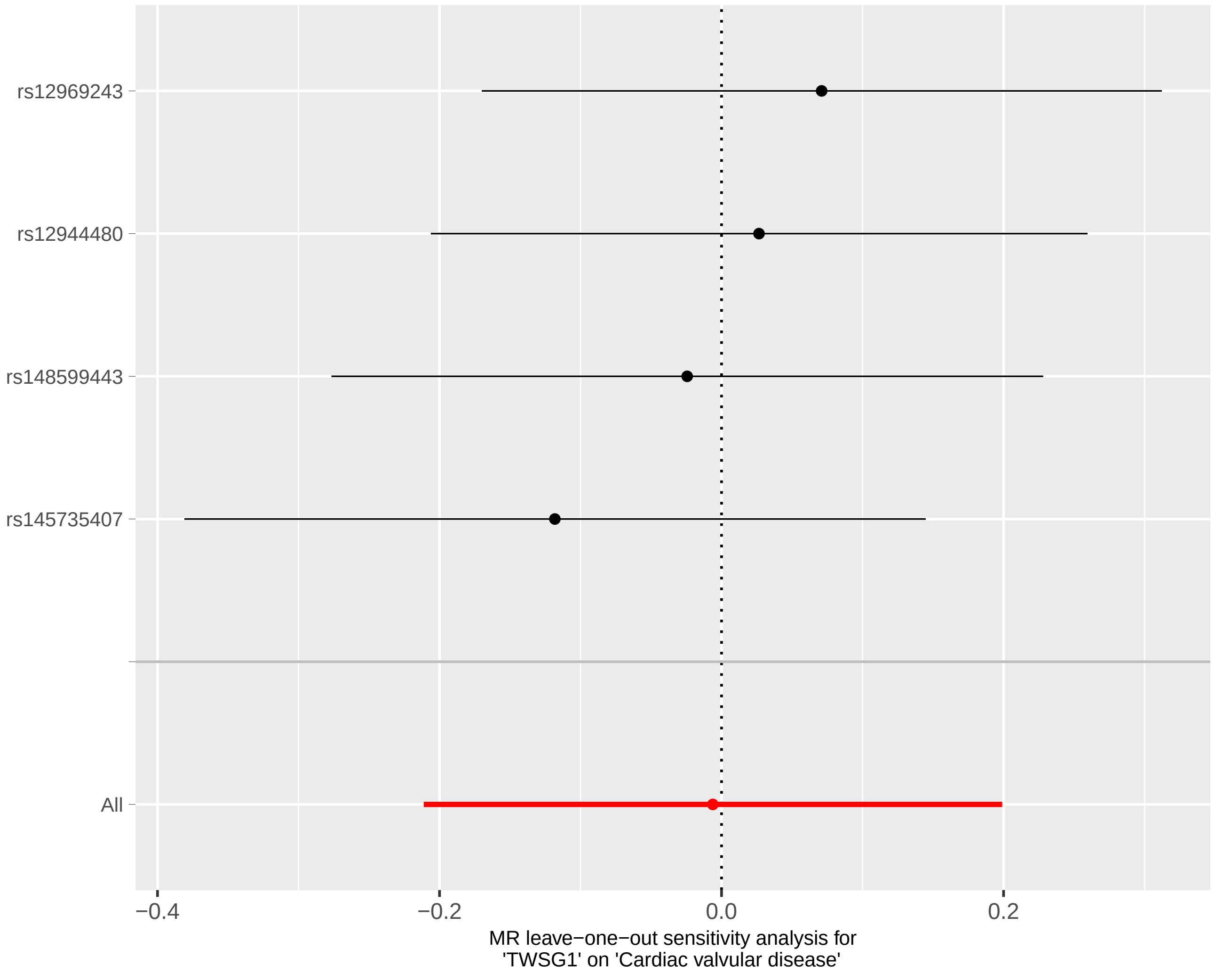
rs12969243
rs12944480
rs148599443
rs145735407
All
−0.4
−0.2
0.0
0.2
MR leave−one−out sensitivity analysis for
'TWSG1' on 'Cardiac valvular disease'

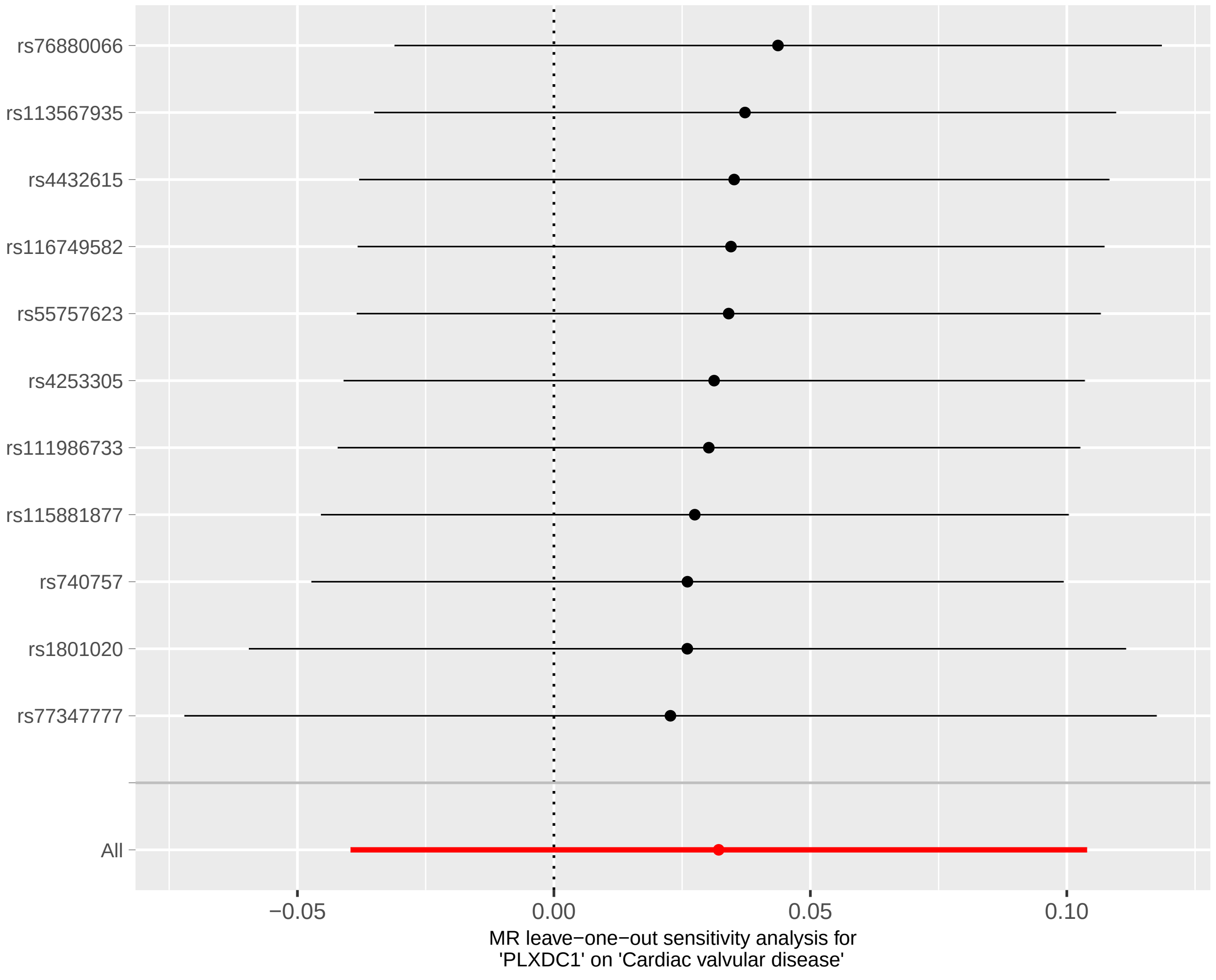
rs76880066
rs113567935
rs4432615
rs116749582
rs55757623
rs4253305
rs111986733
rs115881877
rs740757
rs1801020
rs77347777
All
−0.05
0.00
0.05
0.10
MR leave−one−out sensitivity analysis for
'PLXDC1' on 'Cardiac valvular disease'

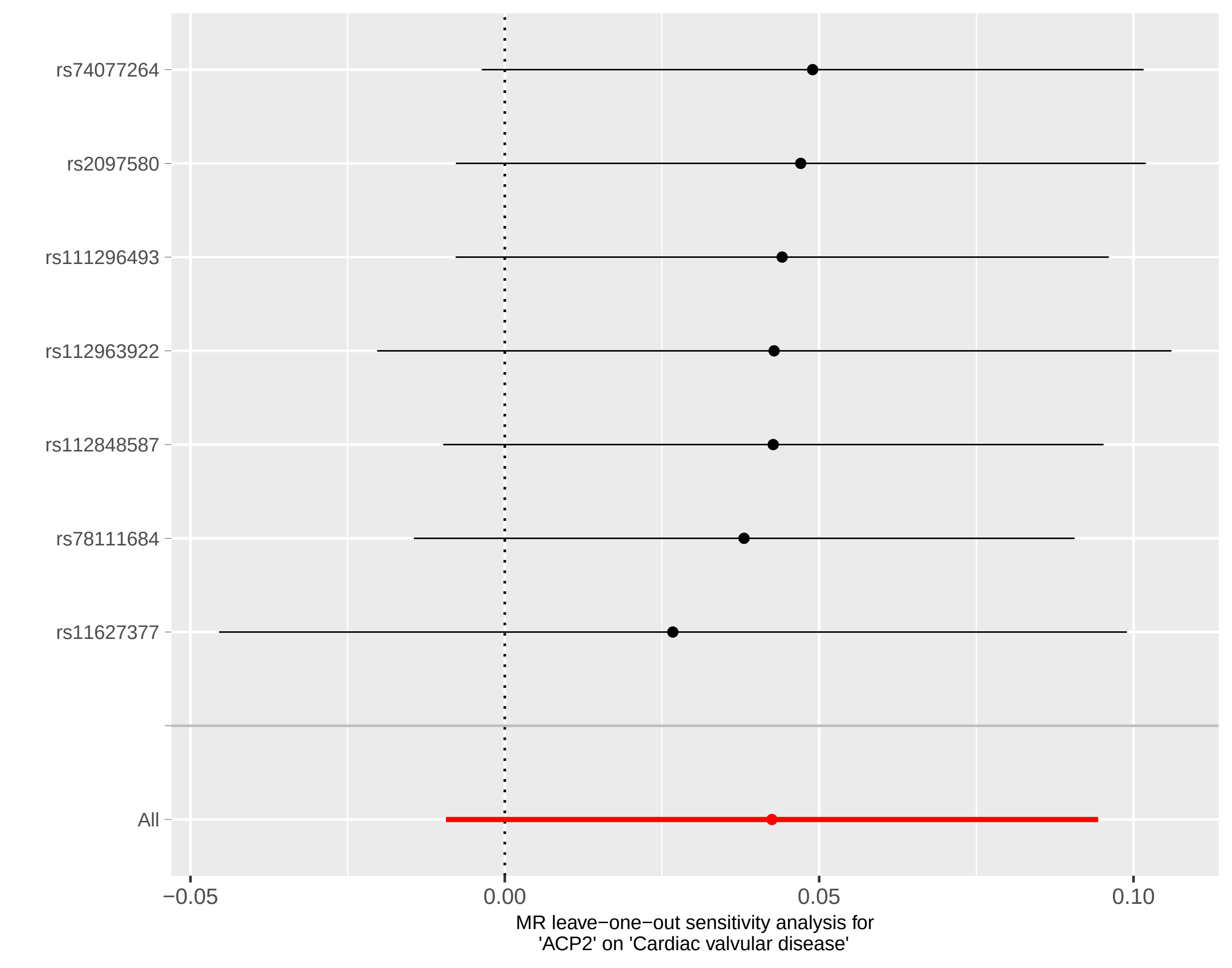
rs74077264
rs2097580
rs111296493
rs112963922
rs112848587
rs78111684
rs11627377
All
−0.05
0.00
0.05
0.10
MR leave−one−out sensitivity analysis for
'ACP2' on 'Cardiac valvular disease'

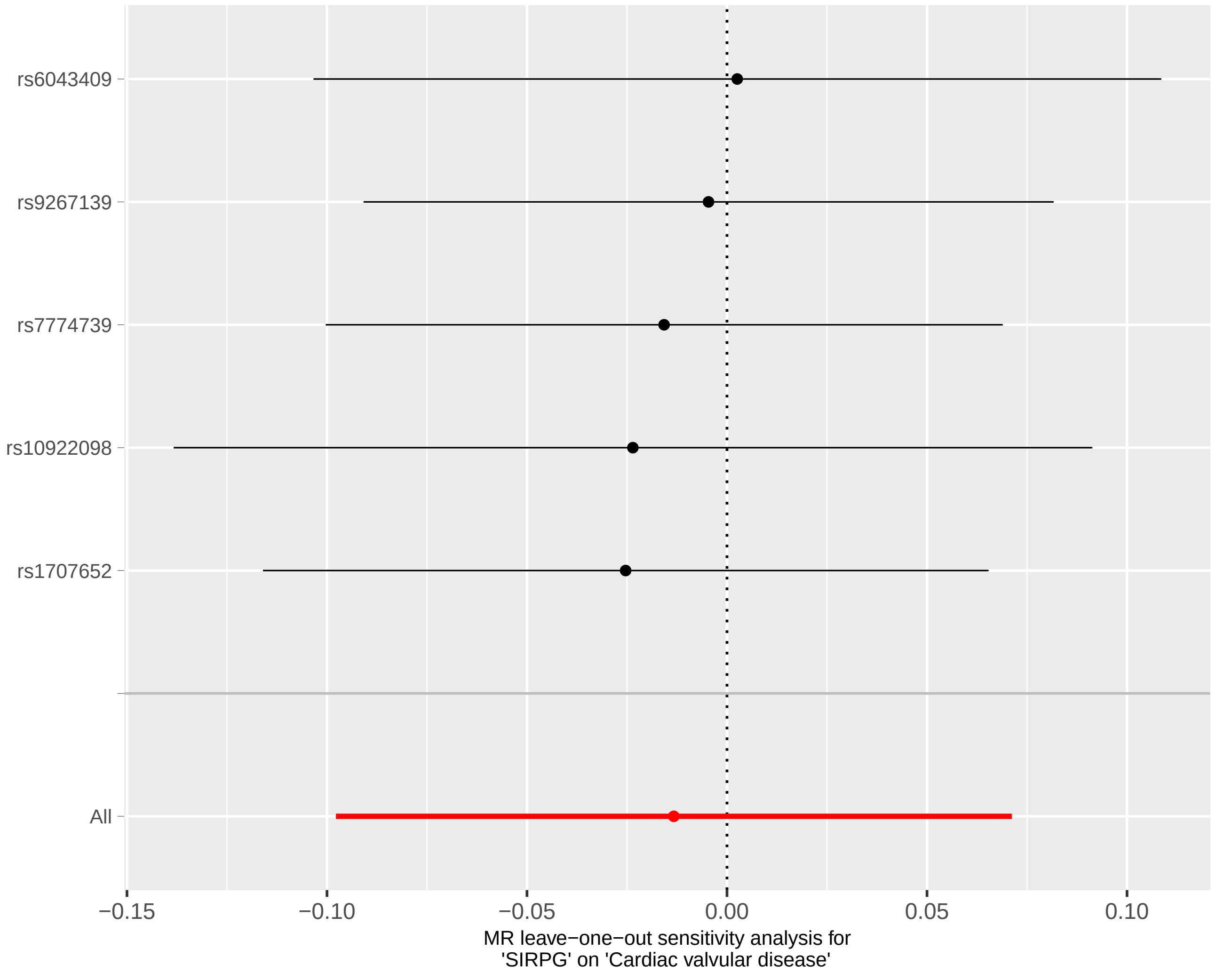
rs6043409
rs9267139
rs7774739
rs10922098
rs1707652
All
−0.15
−0.10
−0.05
0.00
0.05
0.10
MR leave−one−out sensitivity analysis for
'SIRPG' on 'Cardiac valvular disease'

Insufficient number of SNPs

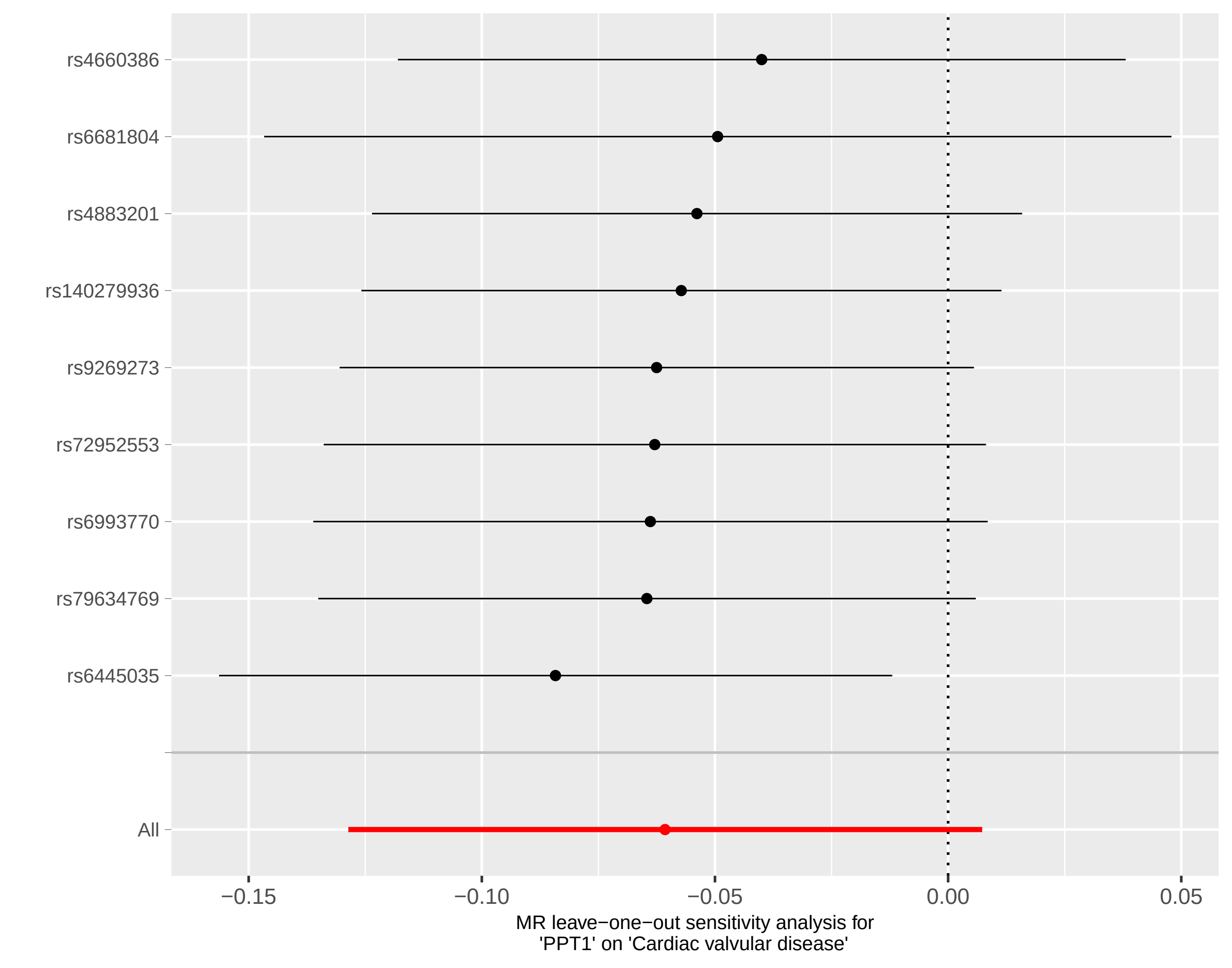
rs4660386
rs6681804
rs4883201
rs140279936
rs9269273
rs72952553
rs6993770
rs79634769
rs6445035
All
−0.15
−0.10
−0.05
0.00
0.05
MR leave−one−out sensitivity analysis for
'PPT1' on 'Cardiac valvular disease'

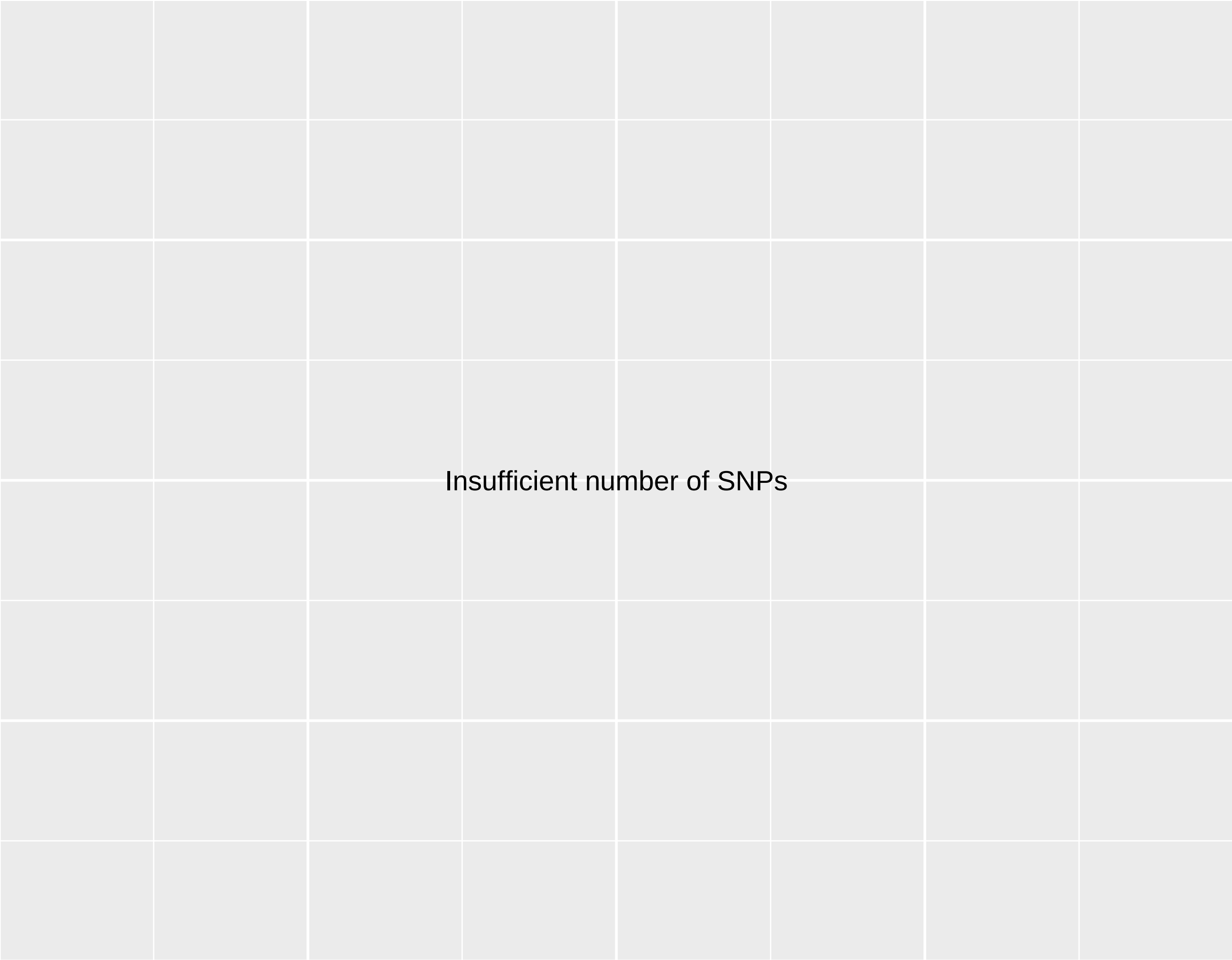
Insufficient number of SNPs

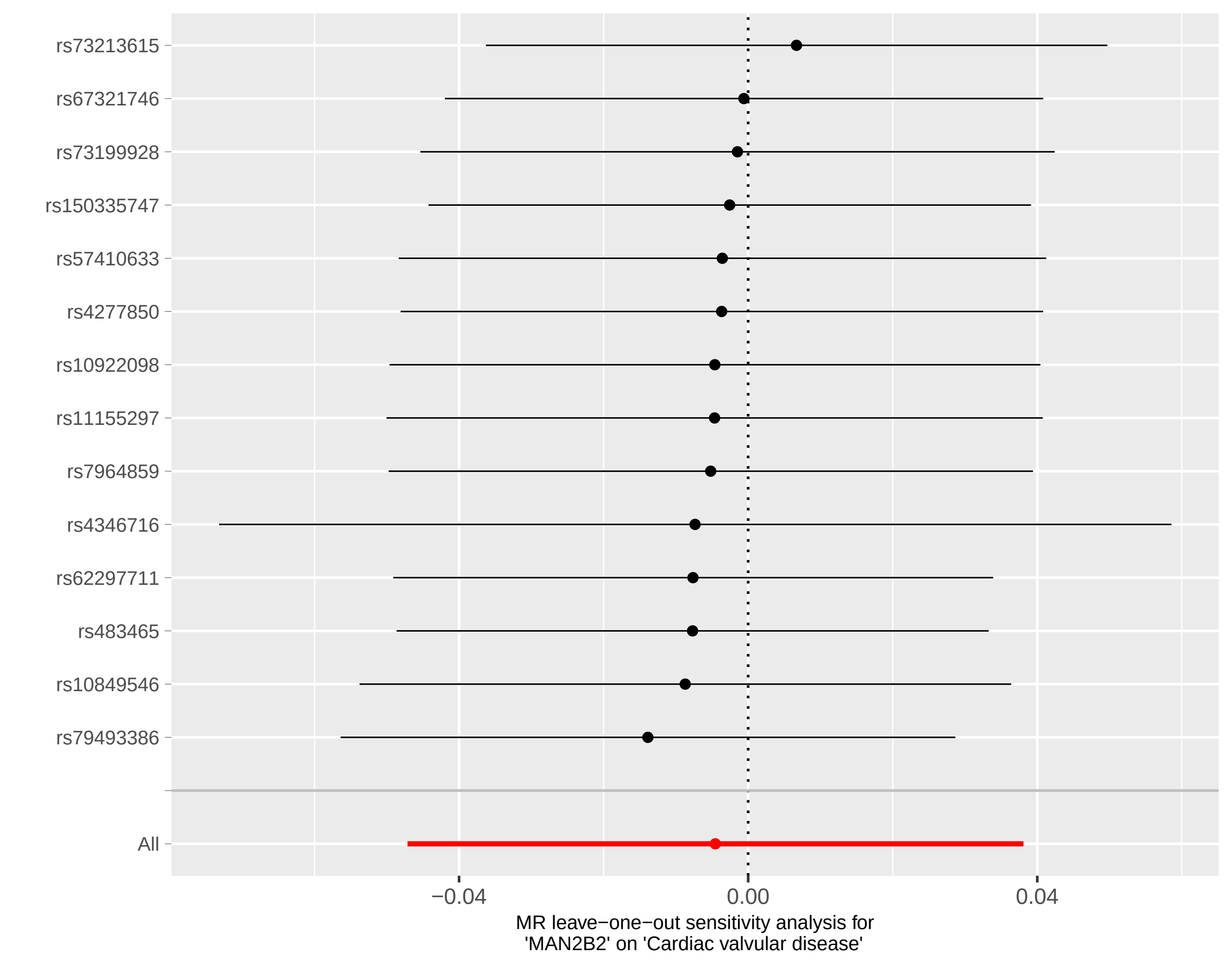
rs73213615
rs67321746
rs73199928
rs150335747
rs57410633
rs4277850
rs10922098
rs11155297
rs7964859
rs4346716
rs62297711
rs483465
rs10849546
rs79493386
All
−0.04
0.00
0.04
MR leave−one−out sensitivity analysis for
'MAN2B2' on 'Cardiac valvular disease'

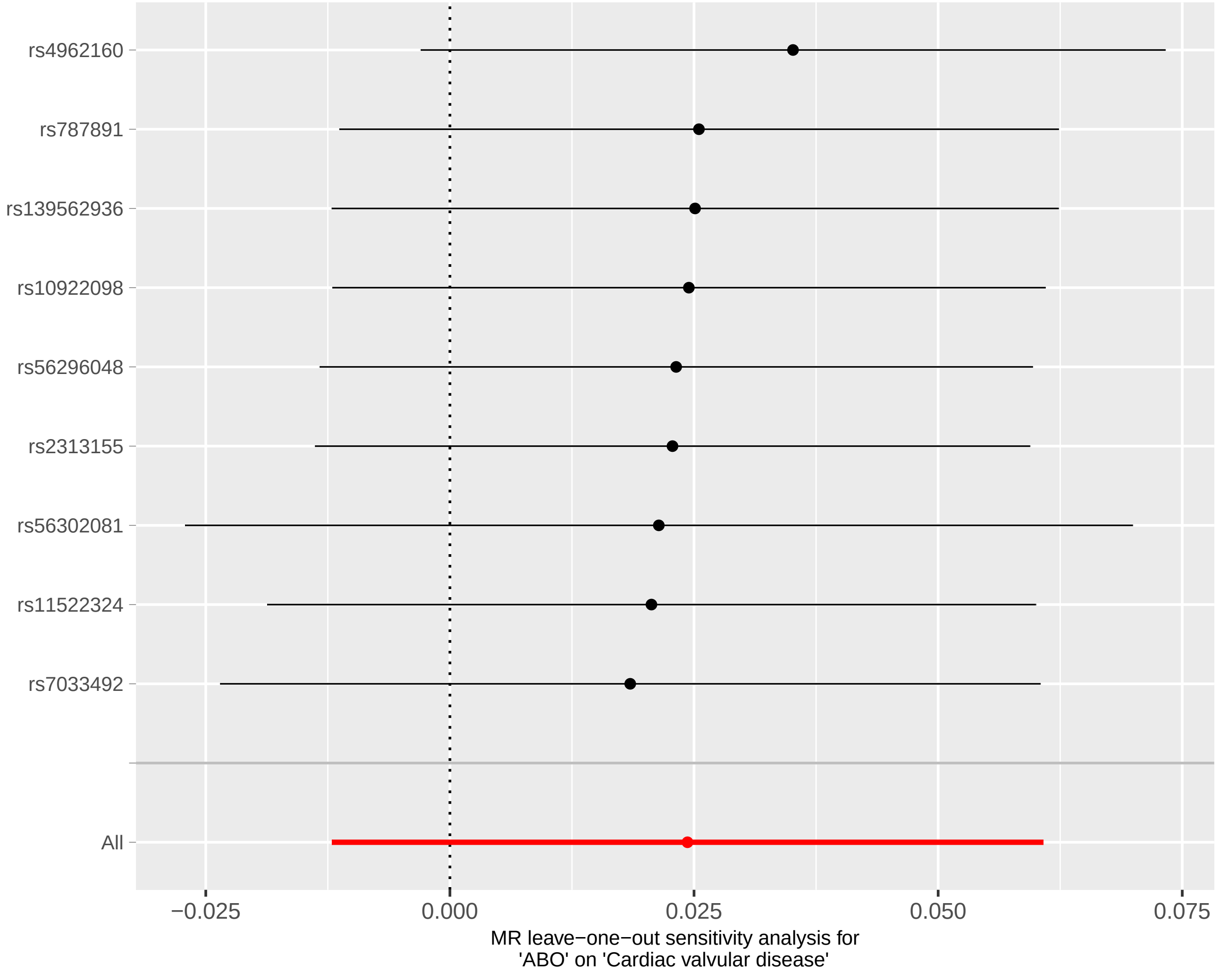

rs4962160
rs787891
rs139562936
rs10922098
rs56296048
rs2313155
rs56302081
rs11522324
rs7033492
All
−0.025
0.000
0.025
0.050
0.075
MR leave−one−out sensitivity analysis for
'ABO' on 'Cardiac valvular disease'

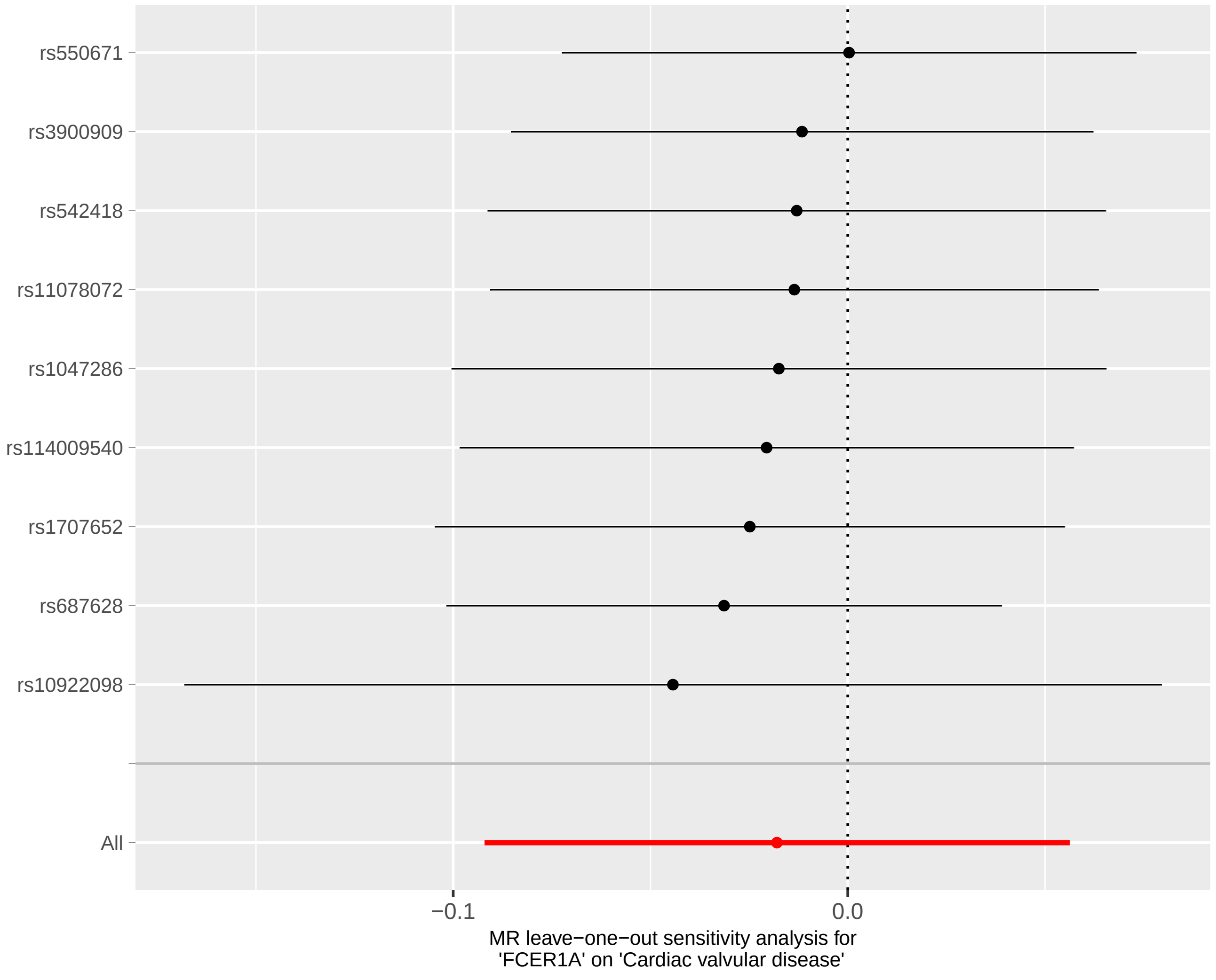

rs550671
rs3900909
rs542418
rs11078072
rs1047286
rs114009540
rs1707652
rs687628
rs10922098
All
−0.1
0.0
MR leave−one−out sensitivity analysis for
'FCER1A' on 'Cardiac valvular disease'

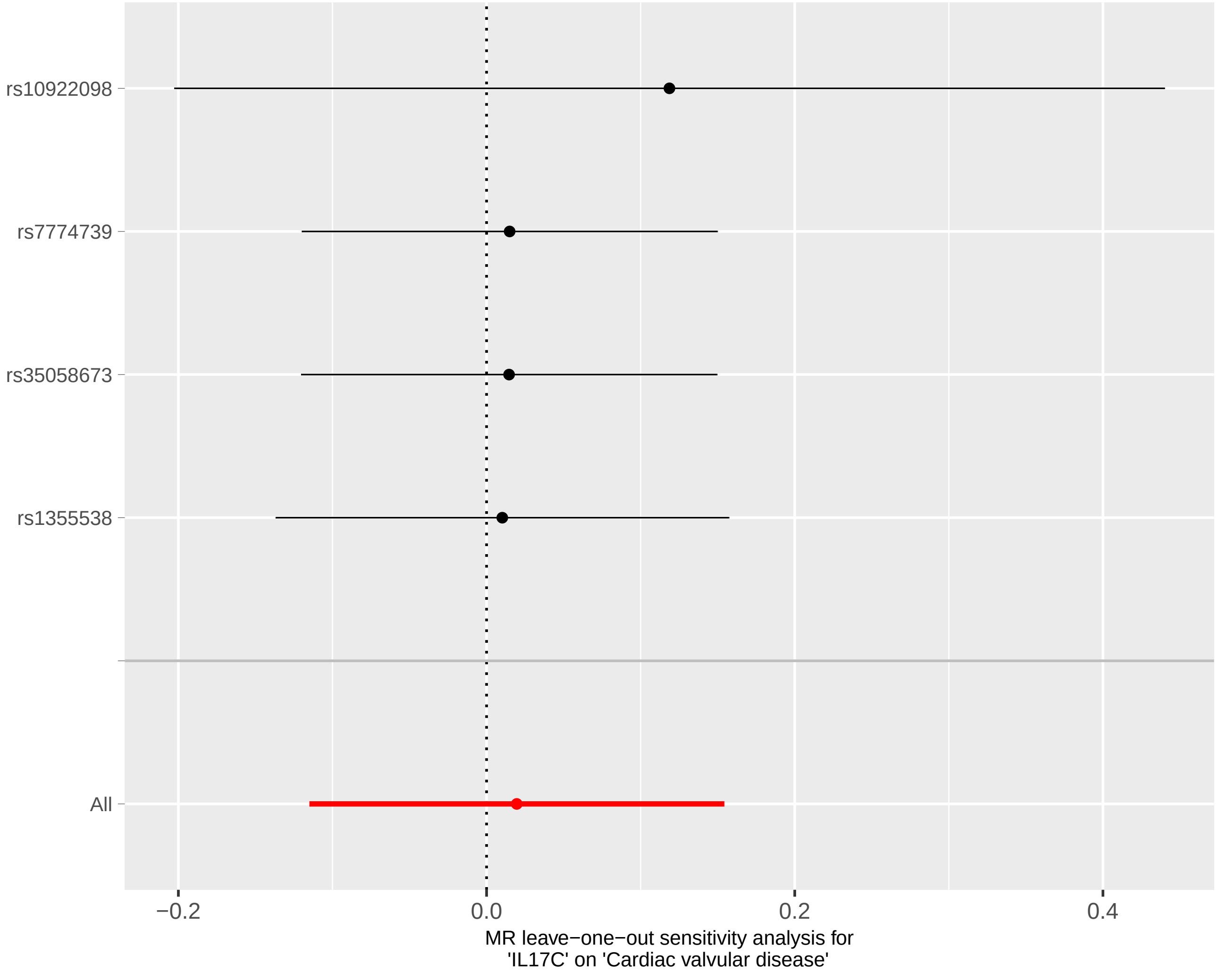
rs10922098
rs7774739
rs35058673
rs1355538
All
−0.2
0.0
0.2
0.4
MR leave−one−out sensitivity analysis for
'IL17C' on 'Cardiac valvular disease'

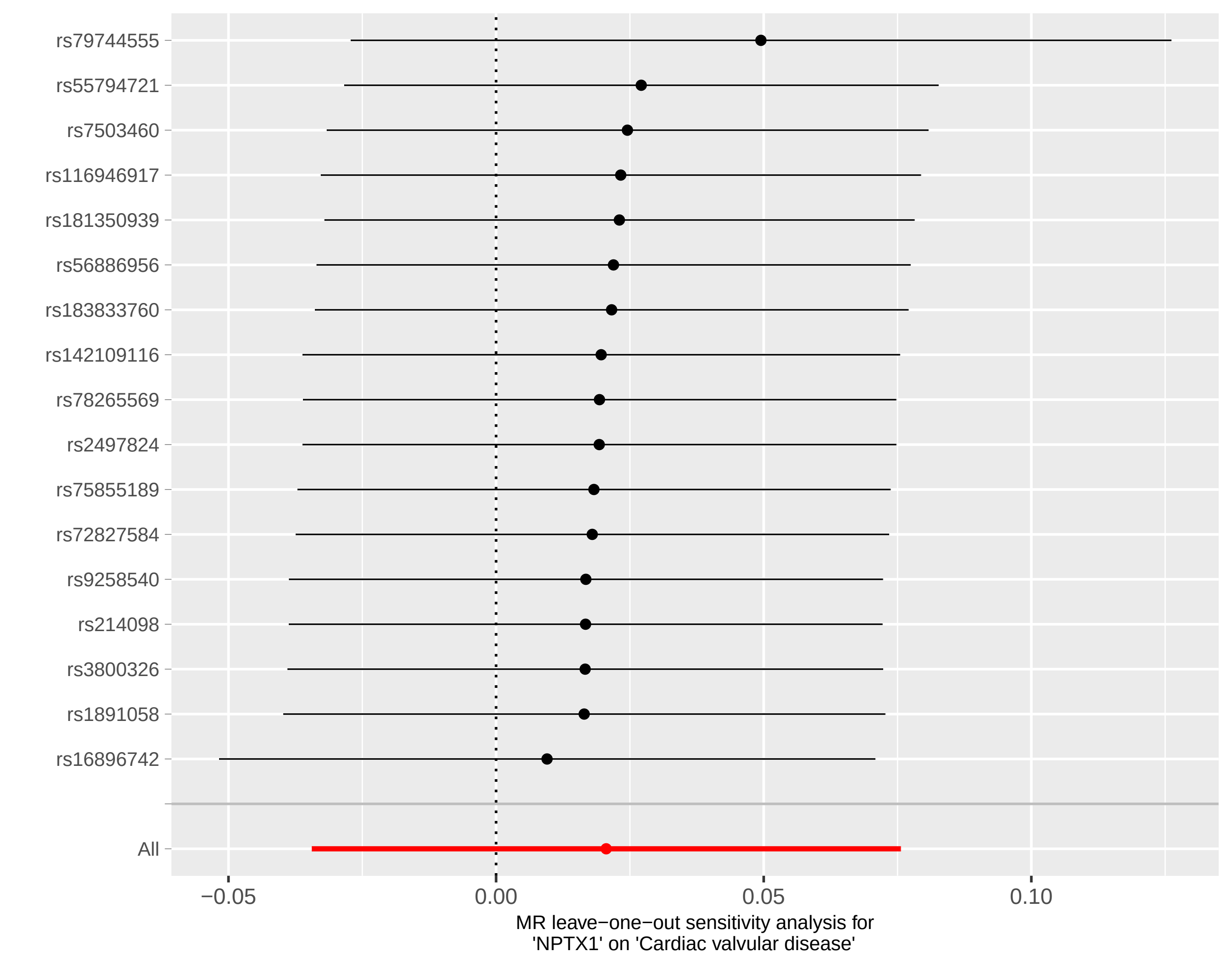
rs79744555
rs55794721
rs7503460
rs116946917
rs181350939
rs56886956
rs183833760
rs142109116
rs78265569
rs2497824
rs75855189
rs72827584
rs9258540
rs214098
rs3800326
rs1891058
rs16896742
All
−0.05
0.00
0.05
0.10
MR leave−one−out sensitivity analysis for
'NPTX1' on 'Cardiac valvular disease'

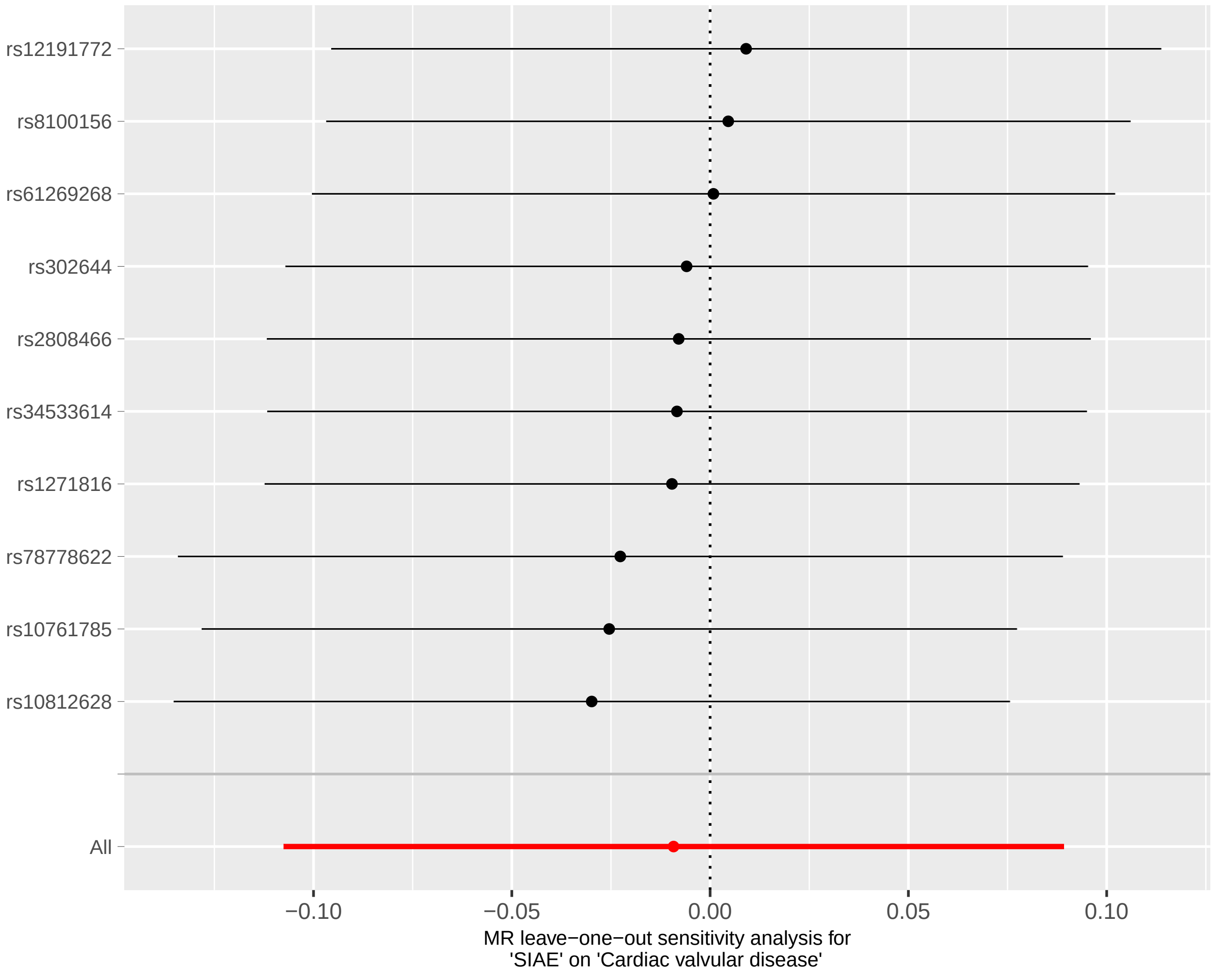
rs12191772
rs8100156
rs61269268
rs302644
rs2808466
rs34533614
rs1271816
rs78778622
rs10761785
rs10812628
All
−0.10
−0.05
0.00
0.05
0.10
MR leave−one−out sensitivity analysis for
'SIAE' on 'Cardiac valvular disease'

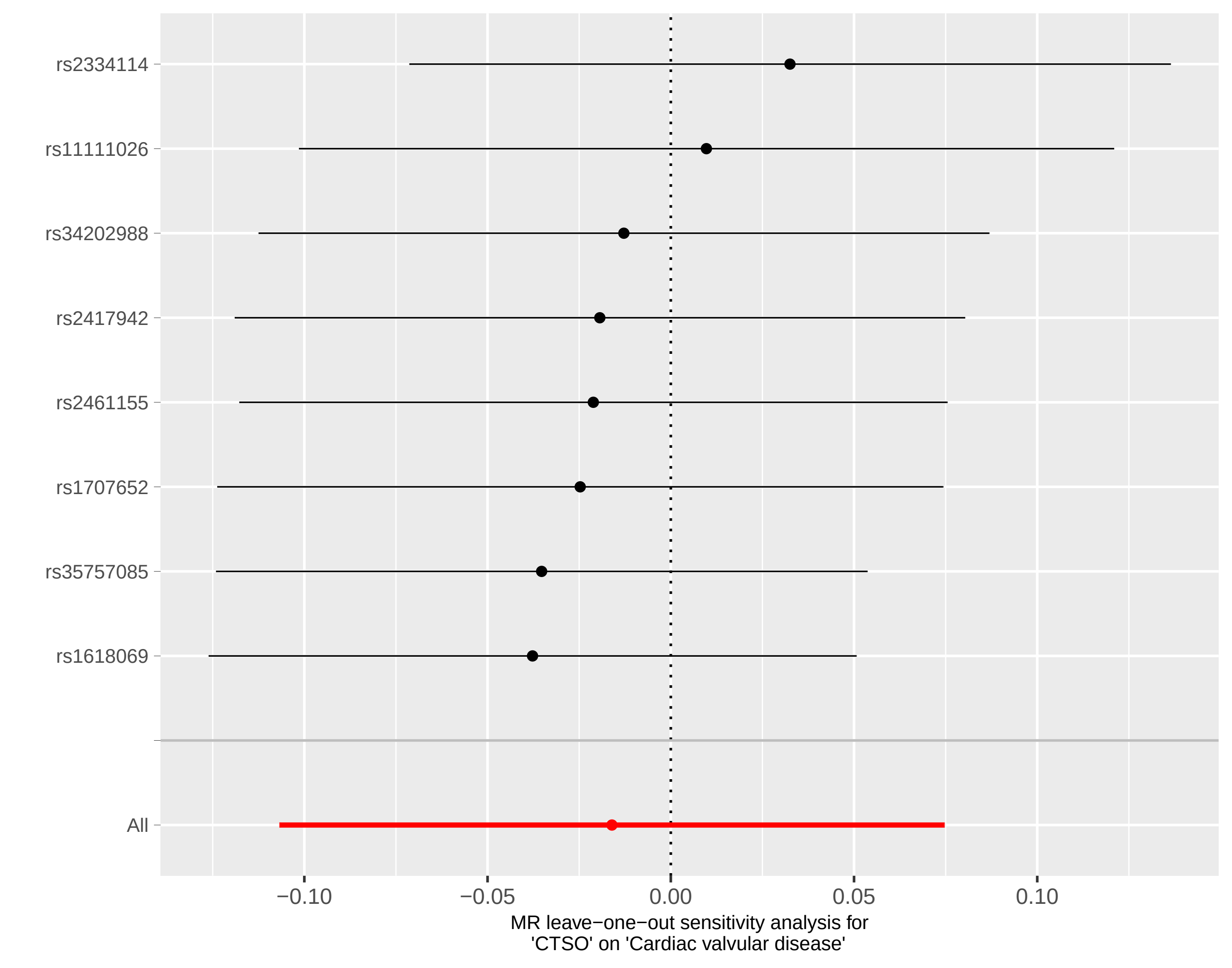

rs2334114
rs11111026
rs34202988
rs2417942
rs2461155
rs1707652
rs35757085
rs1618069
All
−0.10
−0.05
0.00
0.05
0.10
MR leave−one−out sensitivity analysis for
'CTSO' on 'Cardiac valvular disease'

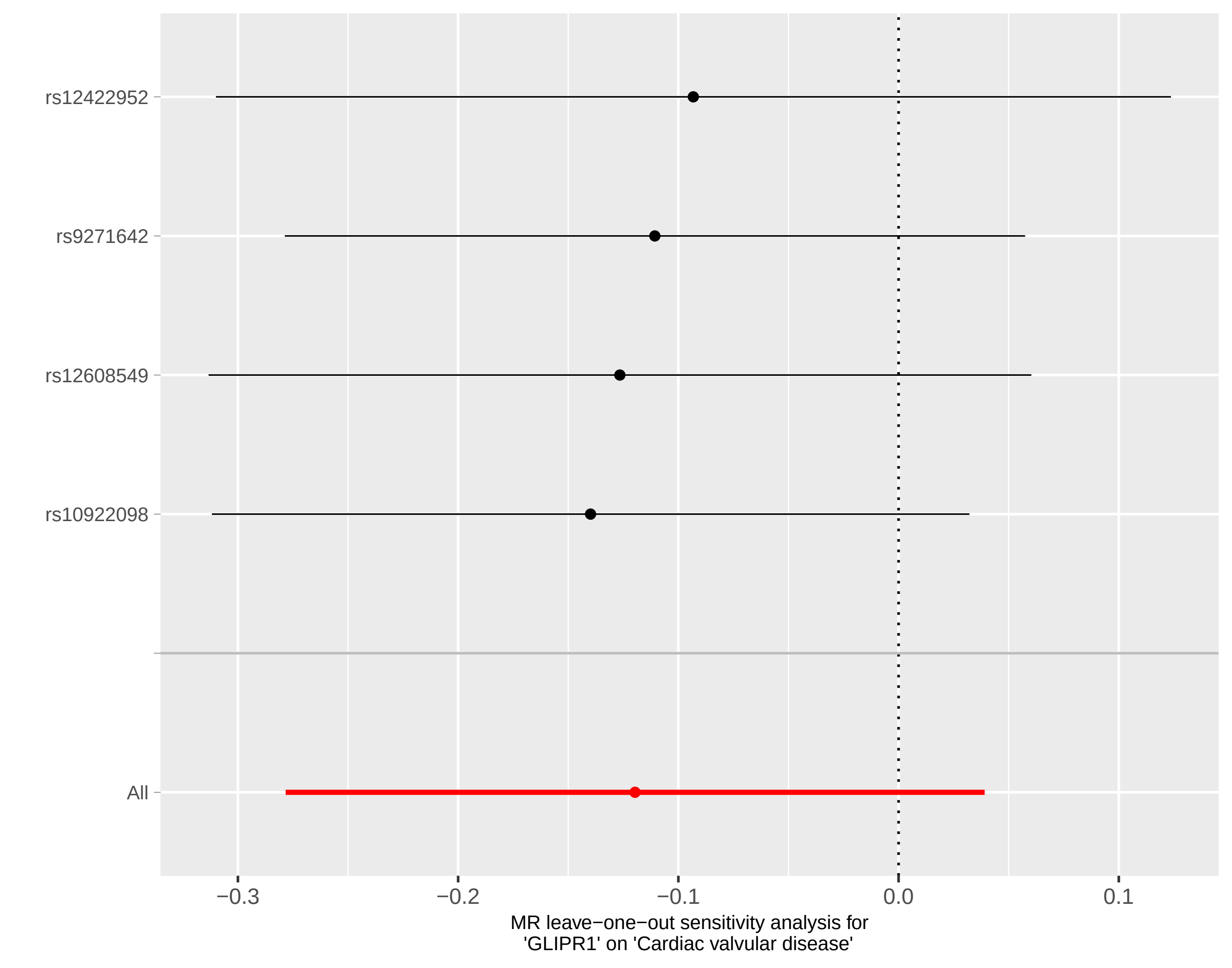
rs12422952
rs9271642
rs12608549
rs10922098
All
−0.3
−0.2
−0.1
0.0
0.1
MR leave−one−out sensitivity analysis for
'GLIPR1' on 'Cardiac valvular disease'

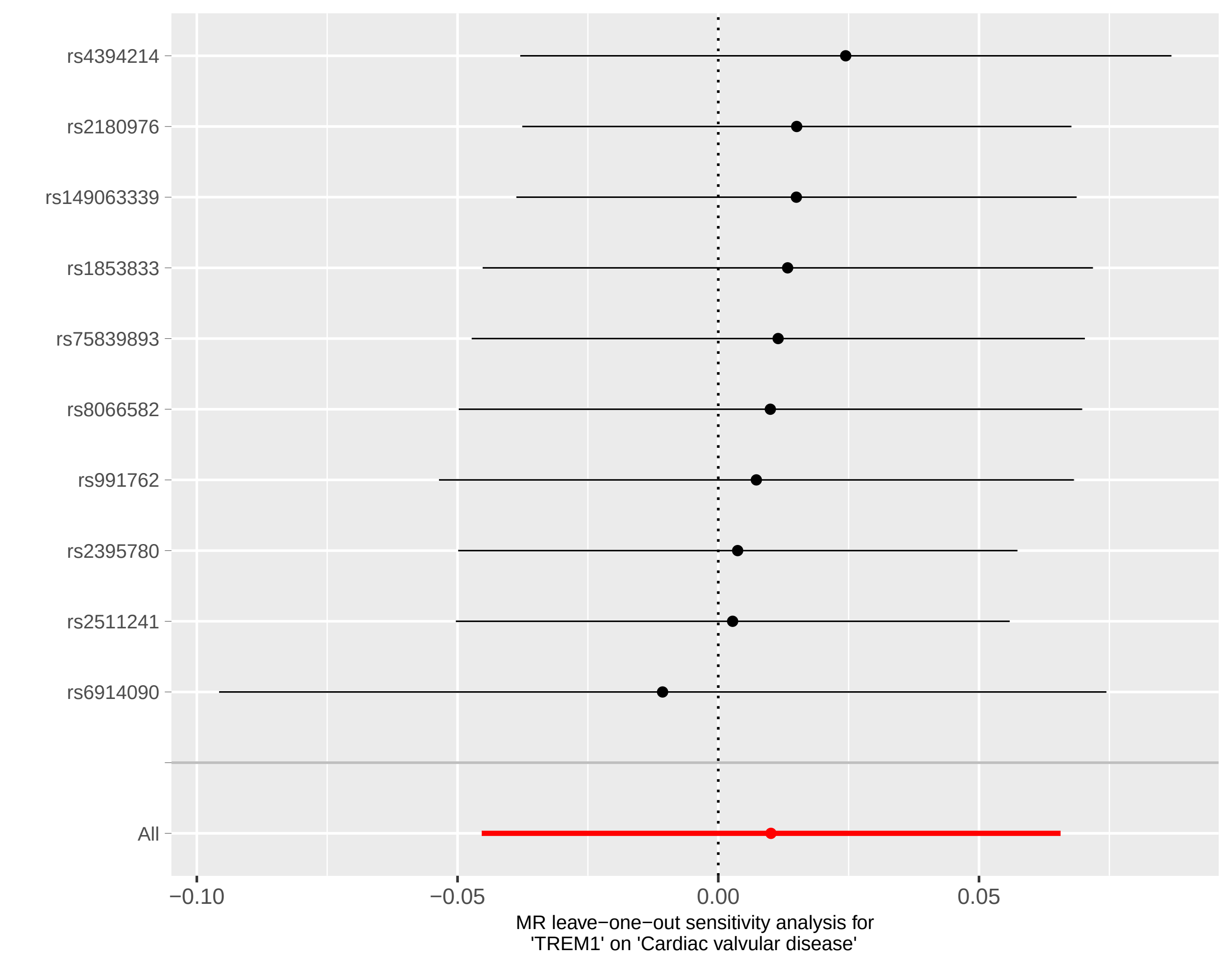
rs4394214
rs2180976
rs149063339
rs1853833
rs75839893
rs8066582
rs991762
rs2395780
rs2511241
rs6914090
All
−0.10
−0.05
0.00
0.05
MR leave−one−out sensitivity analysis for
'TREM1' on 'Cardiac valvular disease'

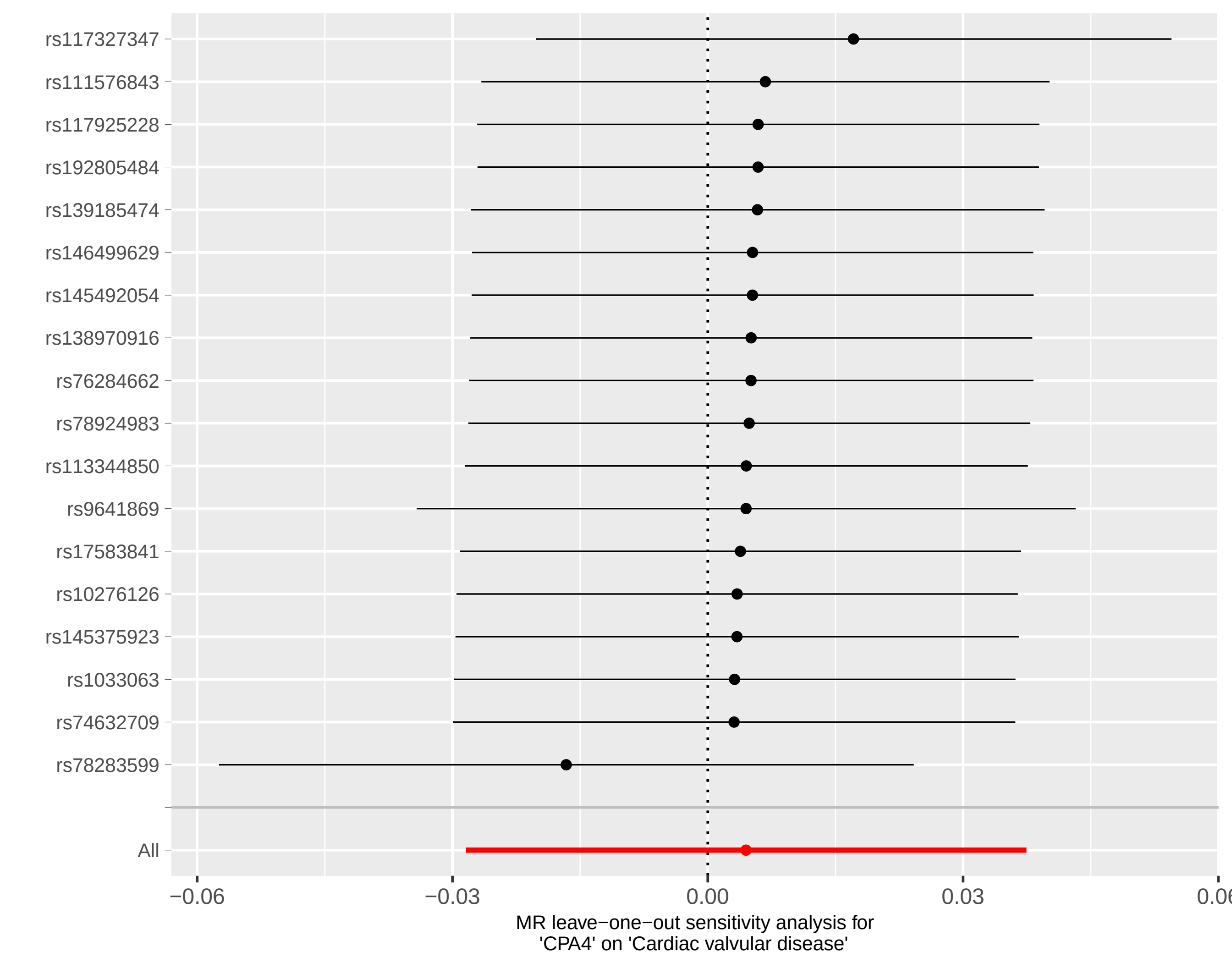
rs117327347
rs111576843
rs117925228
rs192805484
rs139185474
rs146499629
rs145492054
rs138970916
rs76284662
rs78924983
rs113344850
rs9641869
rs17583841
rs10276126
rs145375923
rs1033063
rs74632709
rs78283599
All
−0.06
−0.03
0.00
0.03
MR leave−one−out sensitivity analysis for
'CPA4' on 'Cardiac valvular disease'

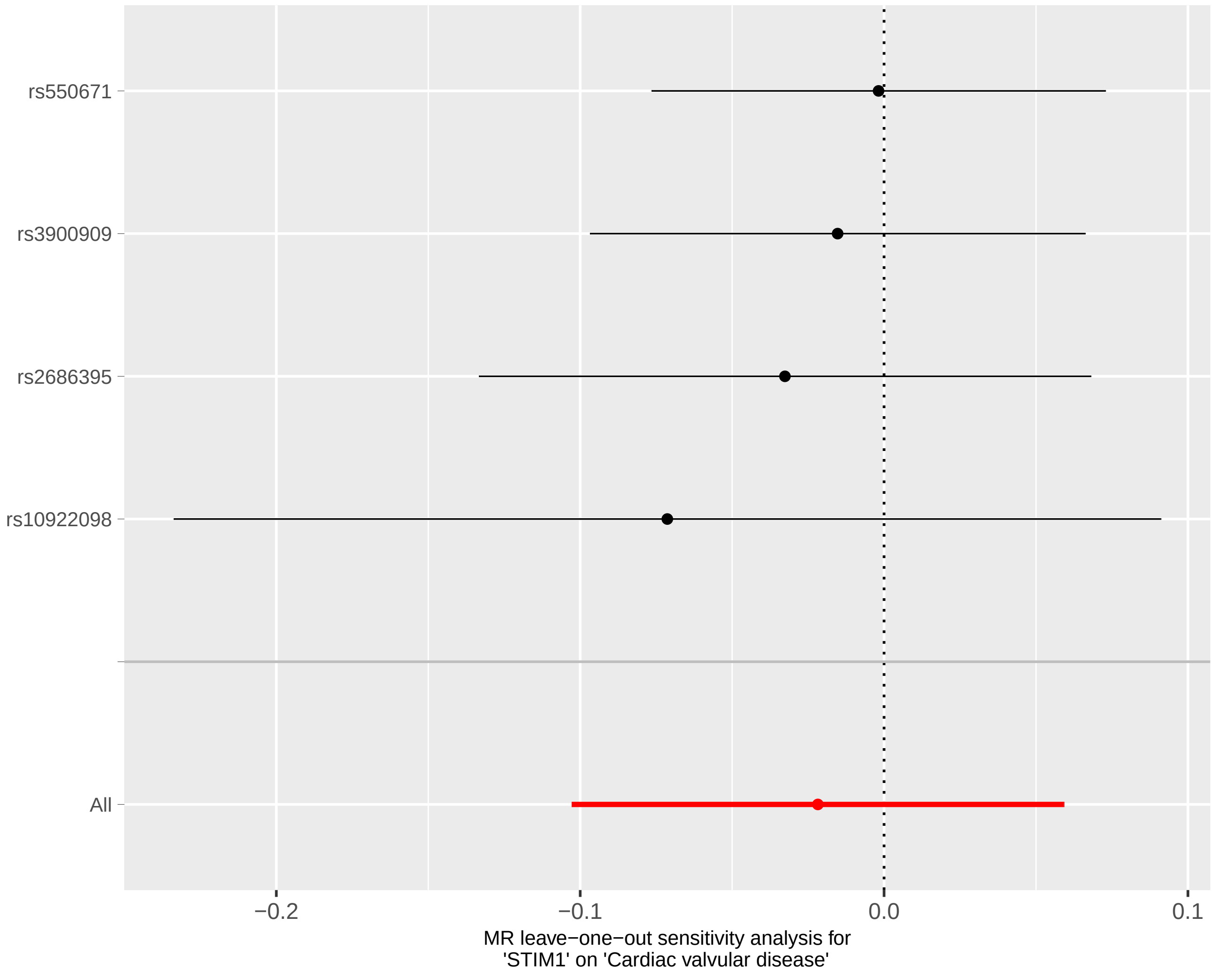
rs550671
rs3900909
rs2686395
rs10922098
All
−0.2
−0.1
0.0
0.1
MR leave−one−out sensitivity analysis for
'STIM1' on 'Cardiac valvular disease'

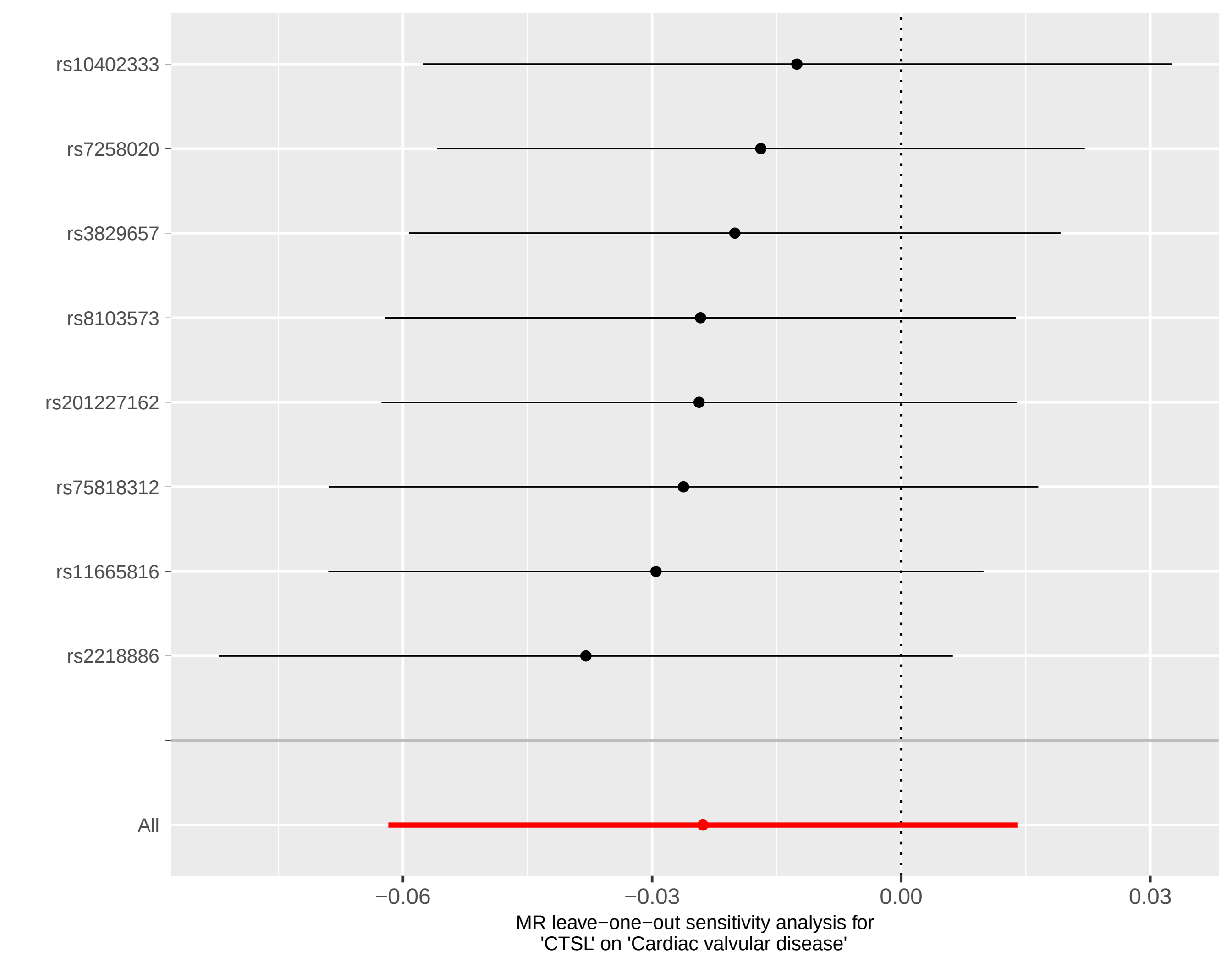
rs10402333
rs7258020
rs3829657
rs8103573
rs201227162
rs75818312
rs11665816
rs2218886
All
−0.06
−0.03
0.00
0.03
MR leave−one−out sensitivity analysis for
'CTSL' on 'Cardiac valvular disease'

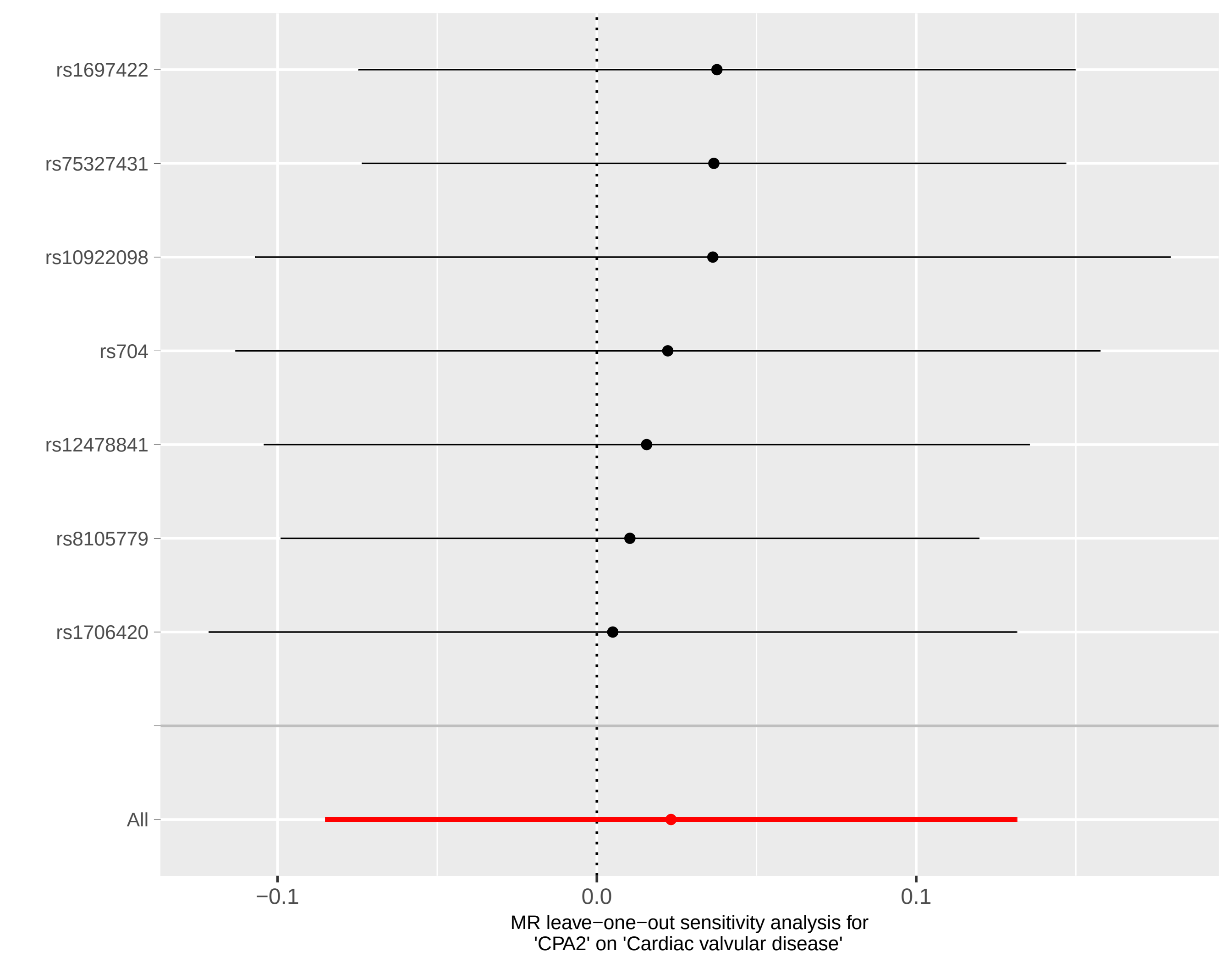
rs1697422
rs75327431
rs10922098
rs704
rs12478841
rs8105779
rs1706420
All
−0.1
0.0
0.1
MR leave−one−out sensitivity analysis for
'CPA2' on 'Cardiac valvular disease'

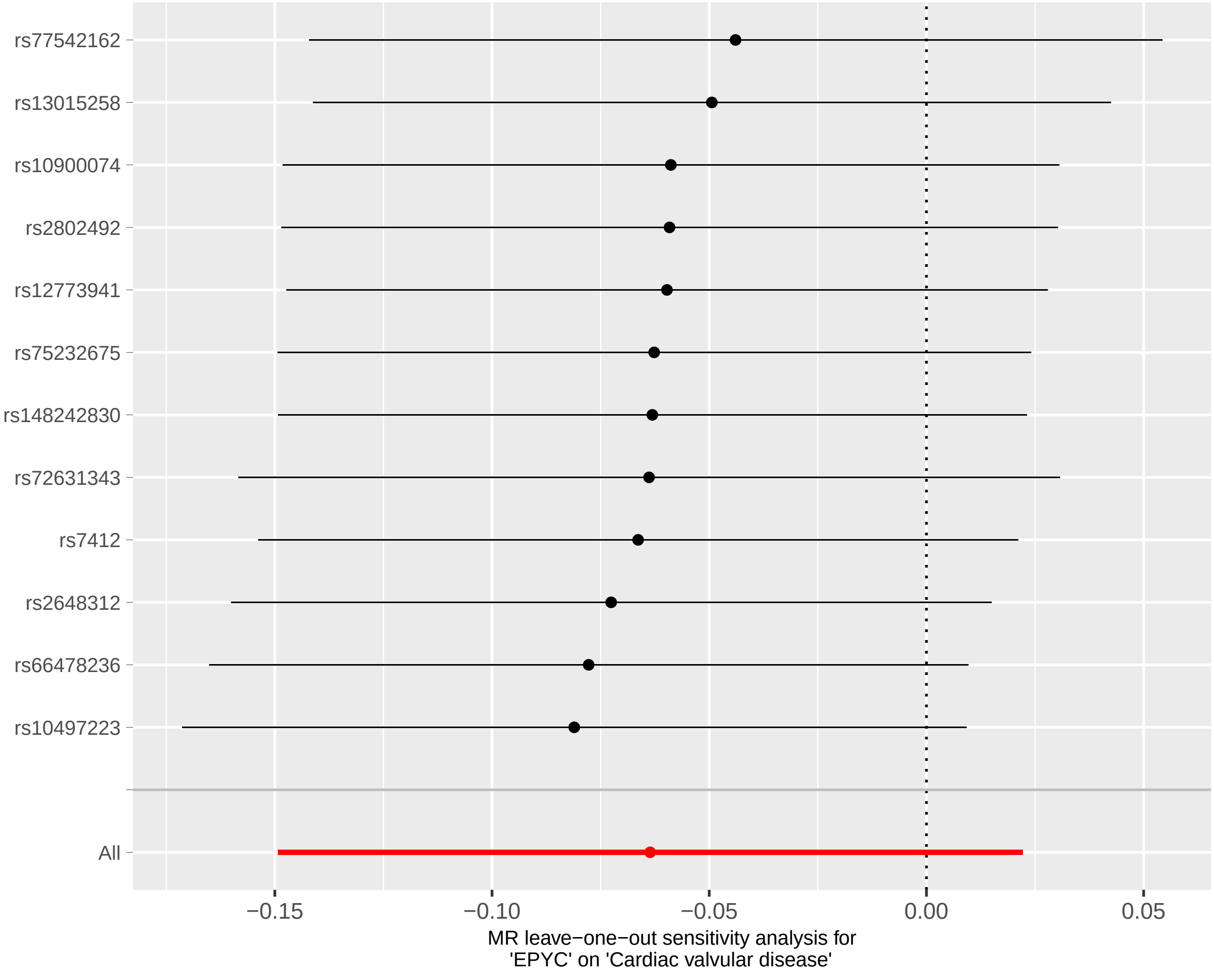
rs77542162
rs13015258
rs10900074
rs2802492
rs12773941
rs75232675
rs148242830
rs72631343
rs7412
rs2648312
rs66478236
rs10497223
All
−0.15
−0.10
−0.05
0.00
0.05
MR leave−one−out sensitivity analysis for
'EPYC' on 'Cardiac valvular disease'

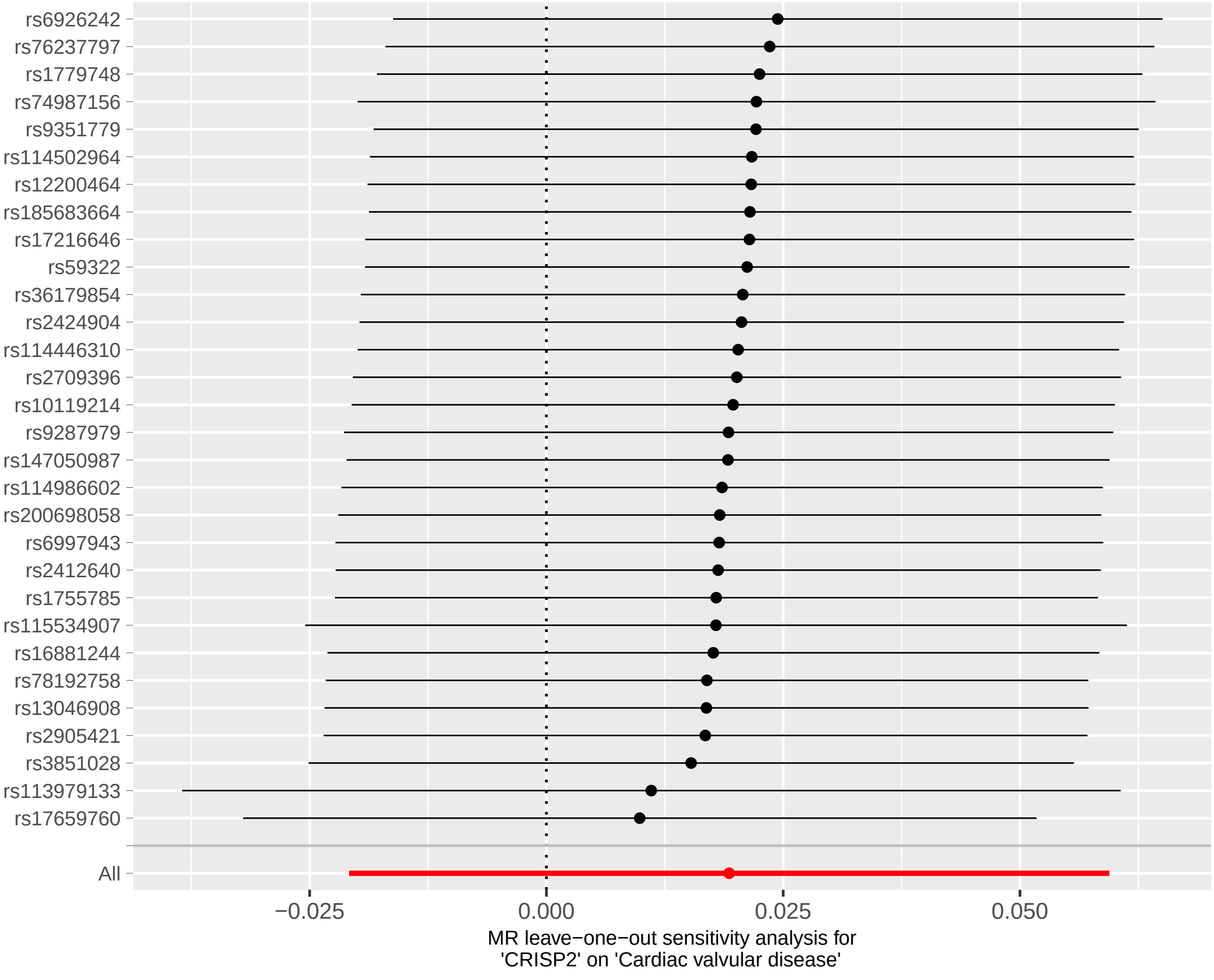
rs6926242
rs76237797
rs1779748
rs74987156
rs9351779
rs114502964
rs12200464
rs185683664
rs17216646
rs59322
rs36179854
rs2424904
rs114446310
rs2709396
rs10119214
rs9287979
rs147050987
rs114986602
rs200698058
rs6997943
rs2412640
rs1755785
rs115534907
rs16881244
rs78192758
rs13046908
rs2905421
rs3851028
rs113979133
rs17659760
All
−0.025
0.000
0.025
0.050
MR leave−one−out sensitivity analysis for
'CRISP2' on 'Cardiac valvular disease'

Insufficient number of SNPs

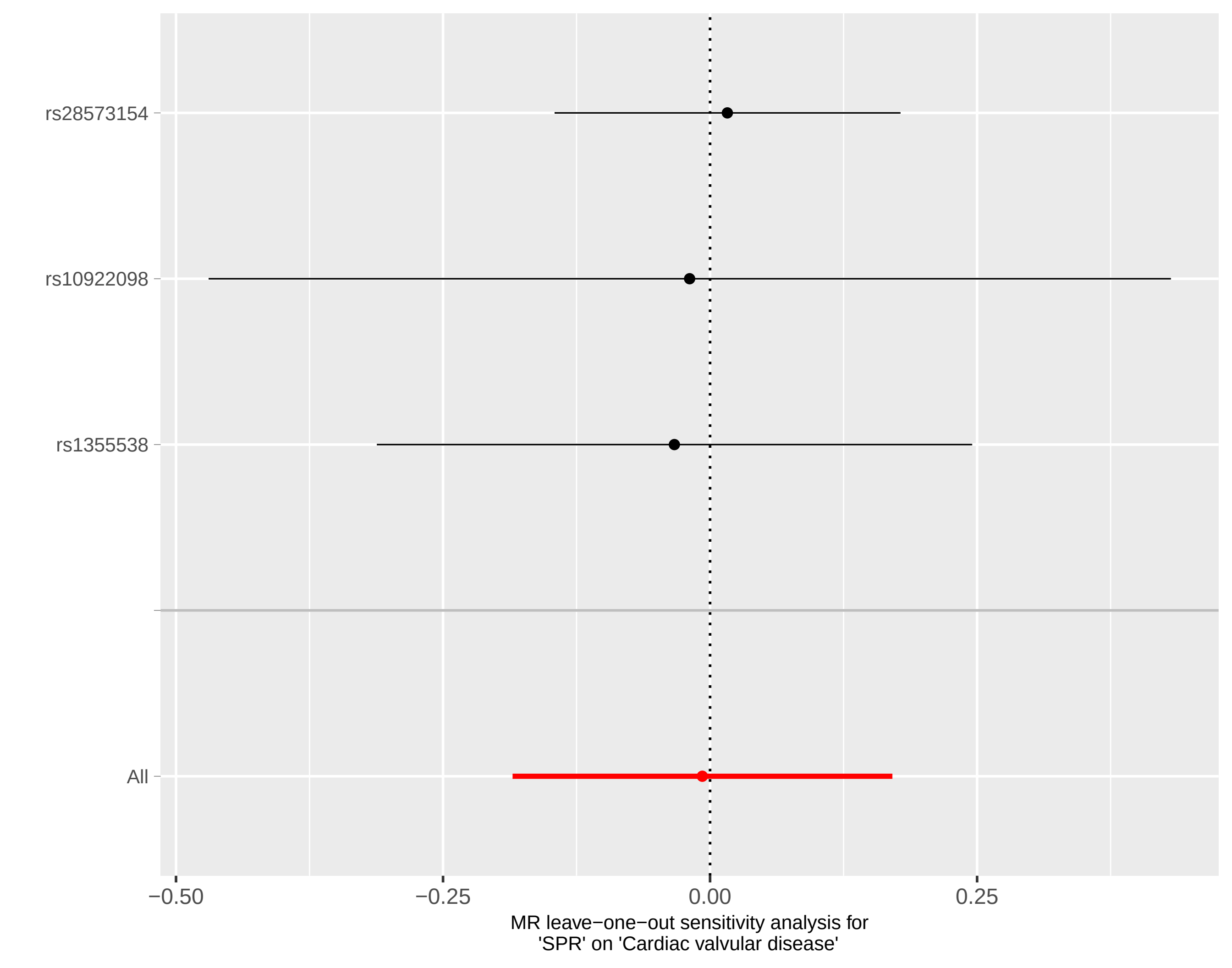

rs28573154
rs10922098
rs1355538
All
−0.50
−0.25
0.00
0.25
MR leave−one−out sensitivity analysis for
'SPR' on 'Cardiac valvular disease'

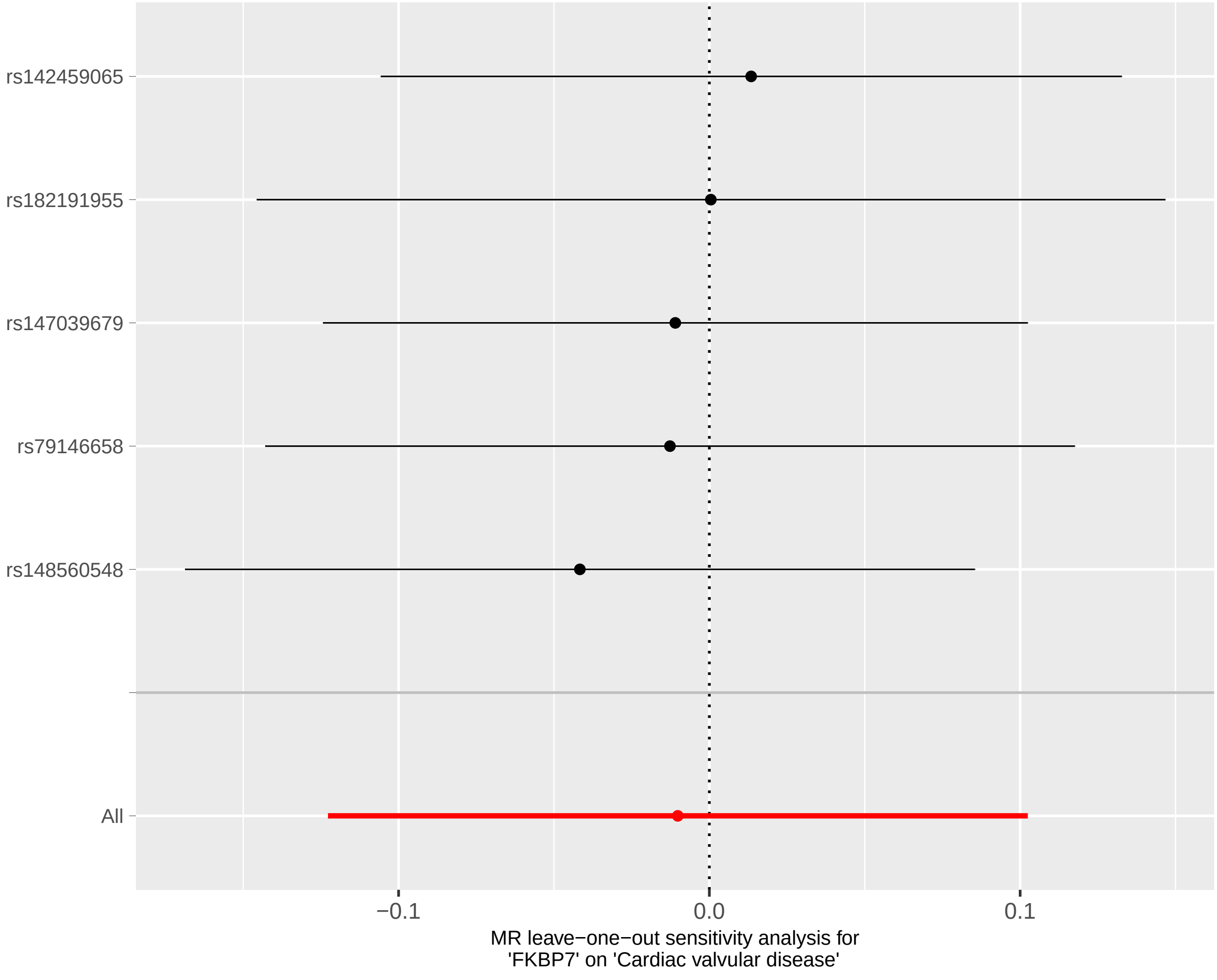
rs142459065
rs182191955
rs147039679
rs79146658
rs148560548
All
−0.1
0.0
0.1
MR leave−one−out sensitivity analysis for
'FKBP7' on 'Cardiac valvular disease'

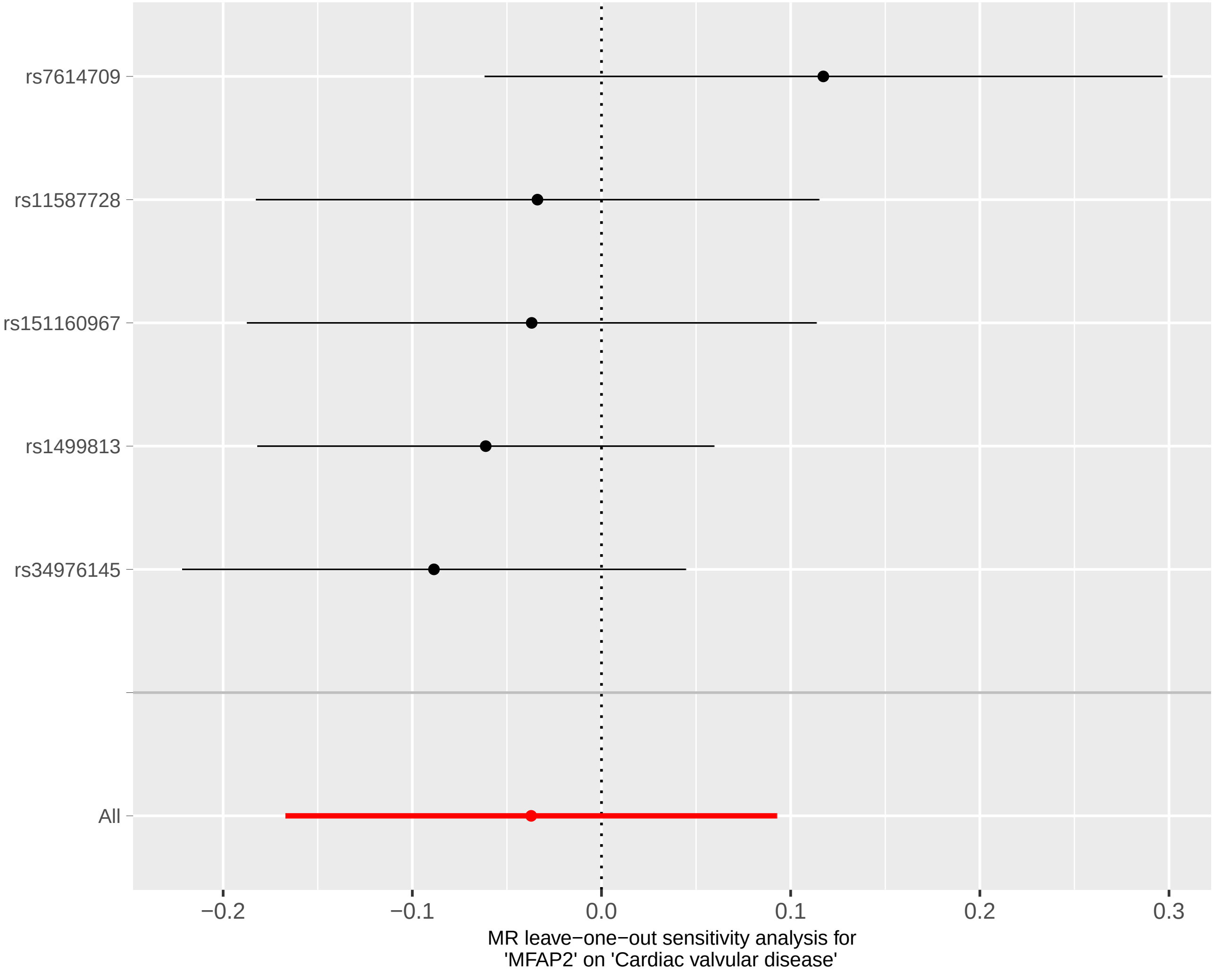

rs7614709
rs11587728
rs151160967
rs1499813
rs34976145
All
−0.2
−0.1
0.0
0.1
0.2
0.3
MR leave−one−out sensitivity analysis for
'MFAP2' on 'Cardiac valvular disease'

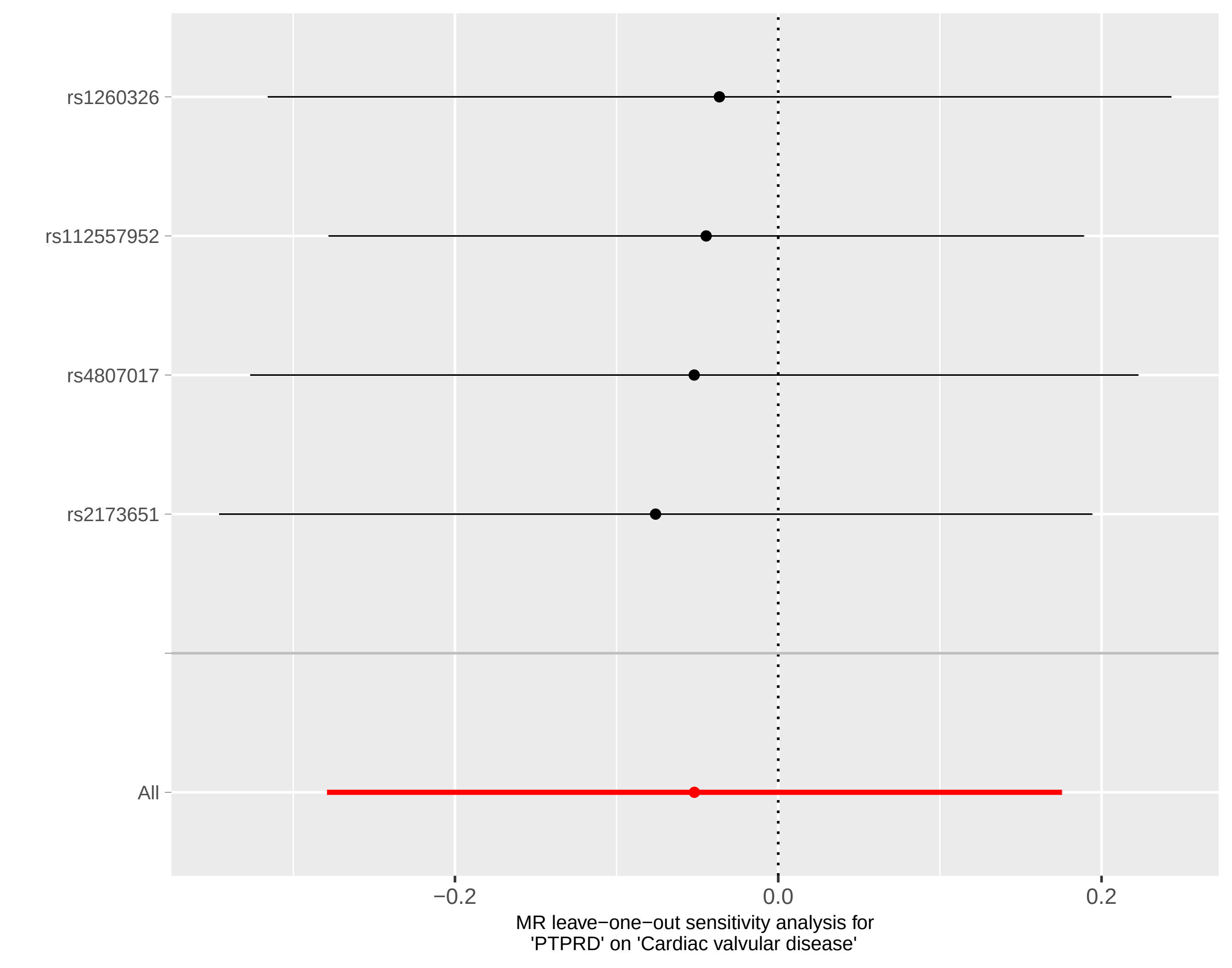
rs1260326
rs112557952
rs4807017
rs2173651
All
−0.2
0.0
0.2
MR leave−one−out sensitivity analysis for
'PTPRD' on 'Cardiac valvular disease'

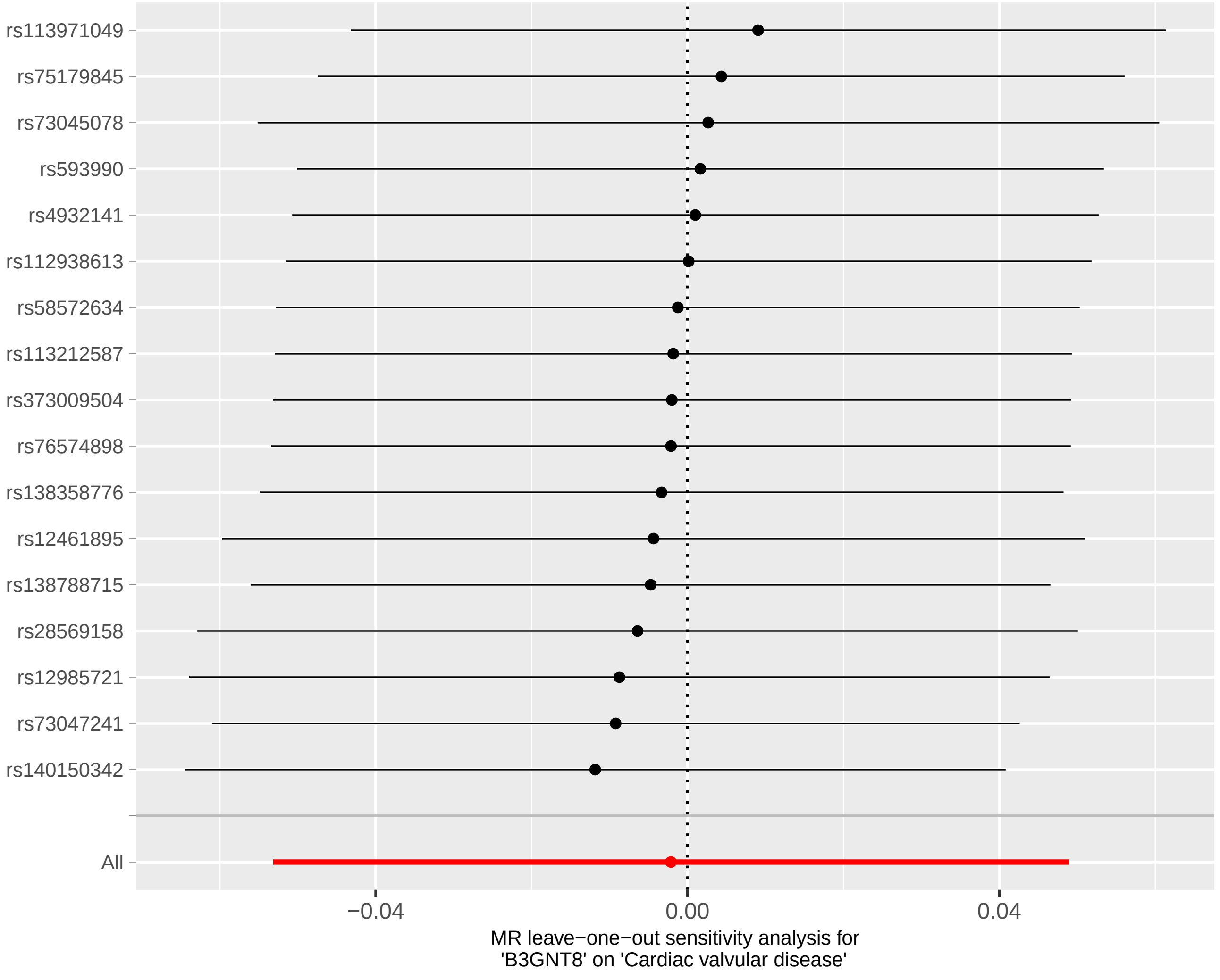

rs113971049
rs75179845
rs73045078
rs593990
rs4932141
rs112938613
rs58572634
rs113212587
rs373009504
rs76574898
rs138358776
rs12461895
rs138788715
rs28569158
rs12985721
rs73047241
rs140150342
All
−0.04
0.00
0.04
MR leave−one−out sensitivity analysis for
'B3GNT8' on 'Cardiac valvular disease'

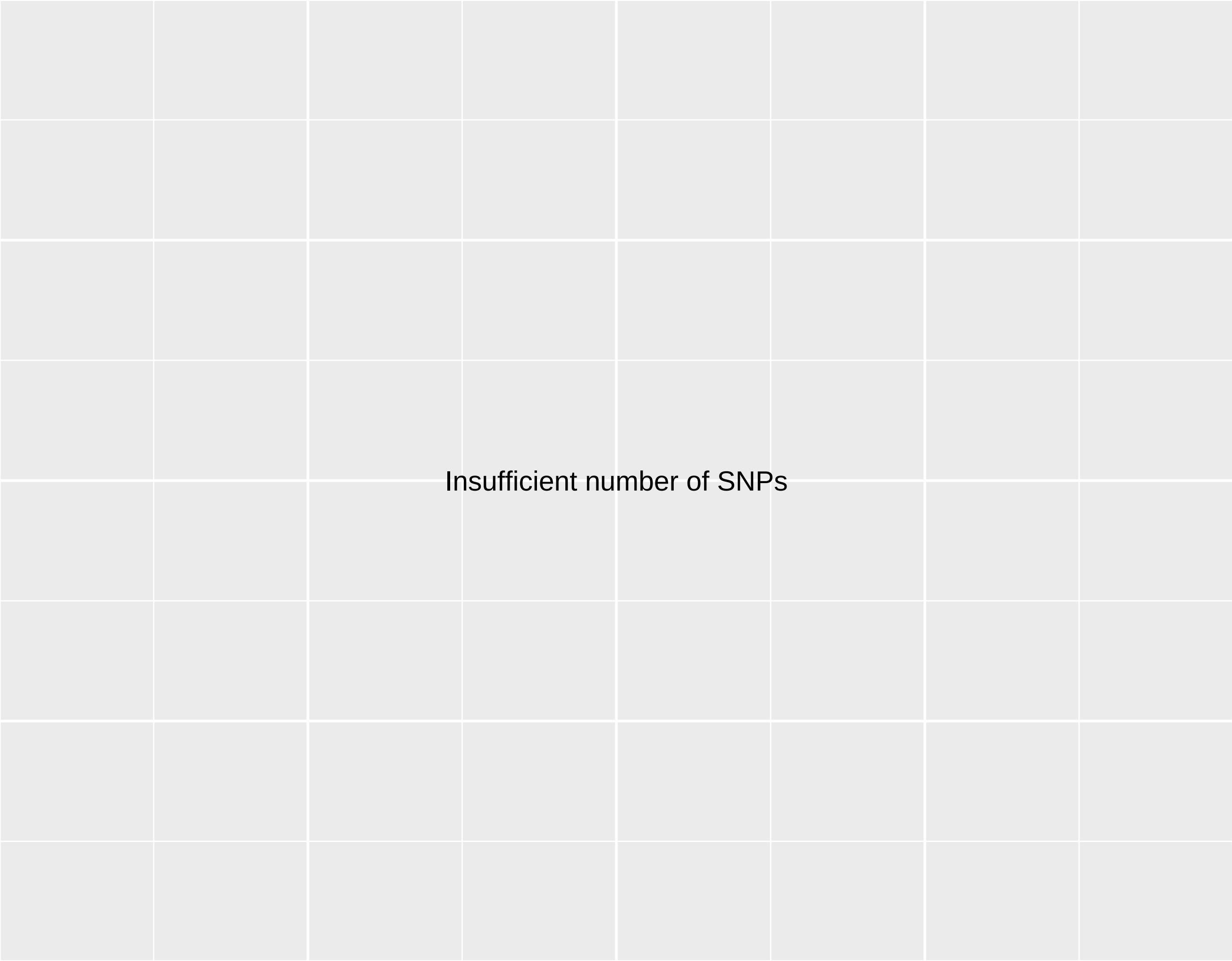
Insufficient number of SNPs

Insufficient number of SNPs

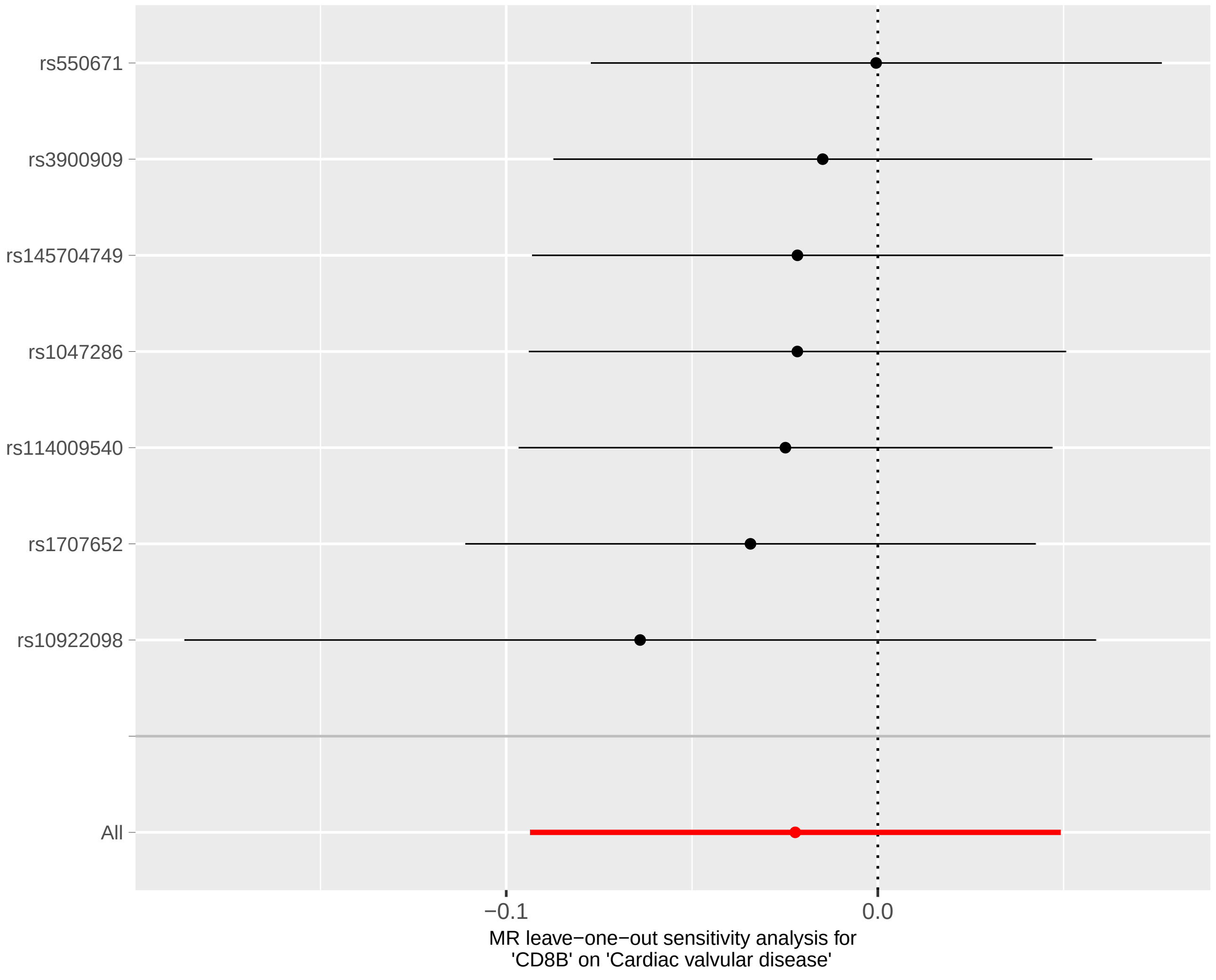
rs550671
rs3900909
rs145704749
rs1047286
rs114009540
rs1707652
rs10922098
All
−0.1
0.0
MR leave−one−out sensitivity analysis for
'CD8B' on 'Cardiac valvular disease'

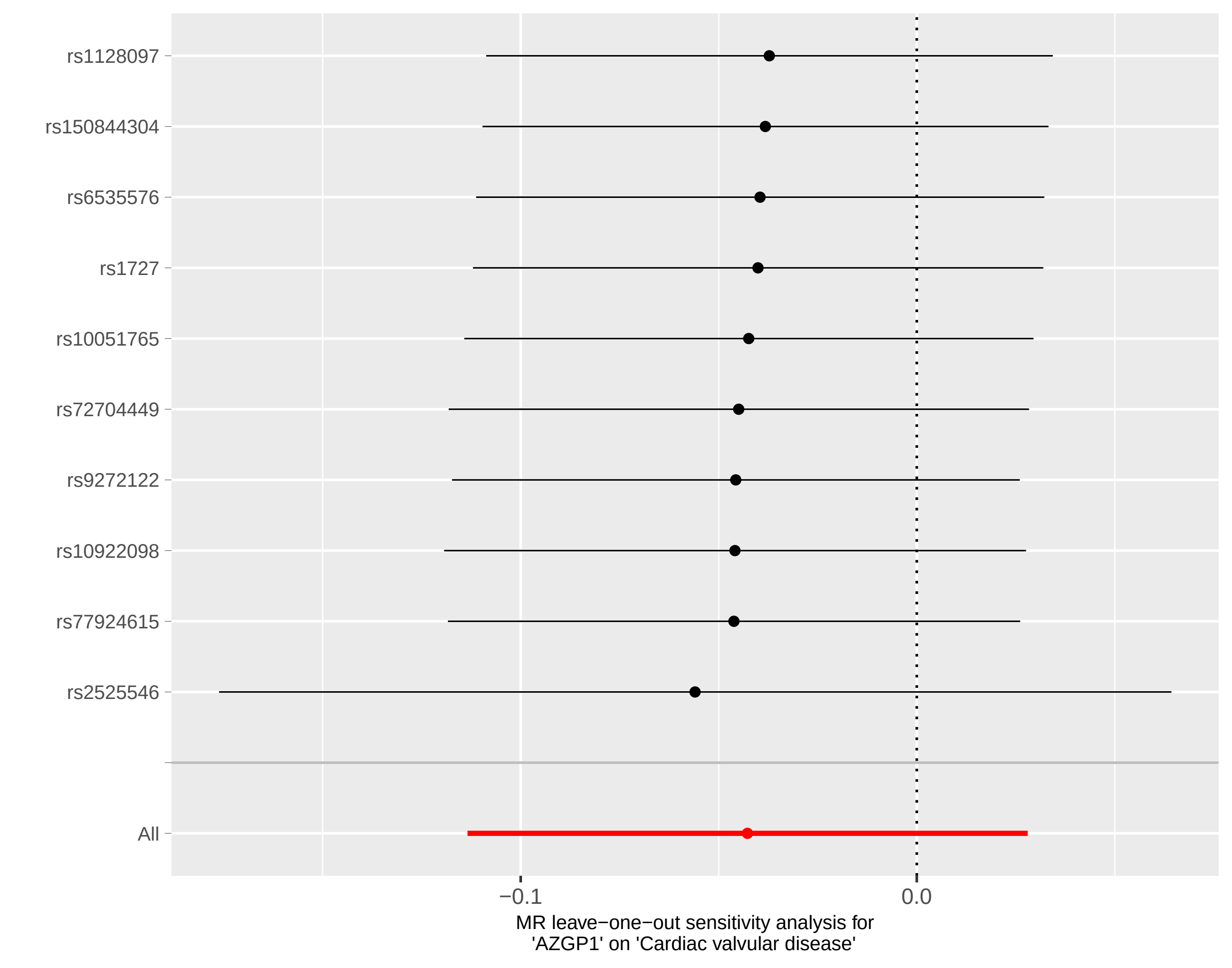
rs1128097
rs150844304
rs6535576
rs1727
rs10051765
rs72704449
rs9272122
rs10922098
rs77924615
rs2525546
All
−0.1
0.0
MR leave−one−out sensitivity analysis for
'AZGP1' on 'Cardiac valvular disease'

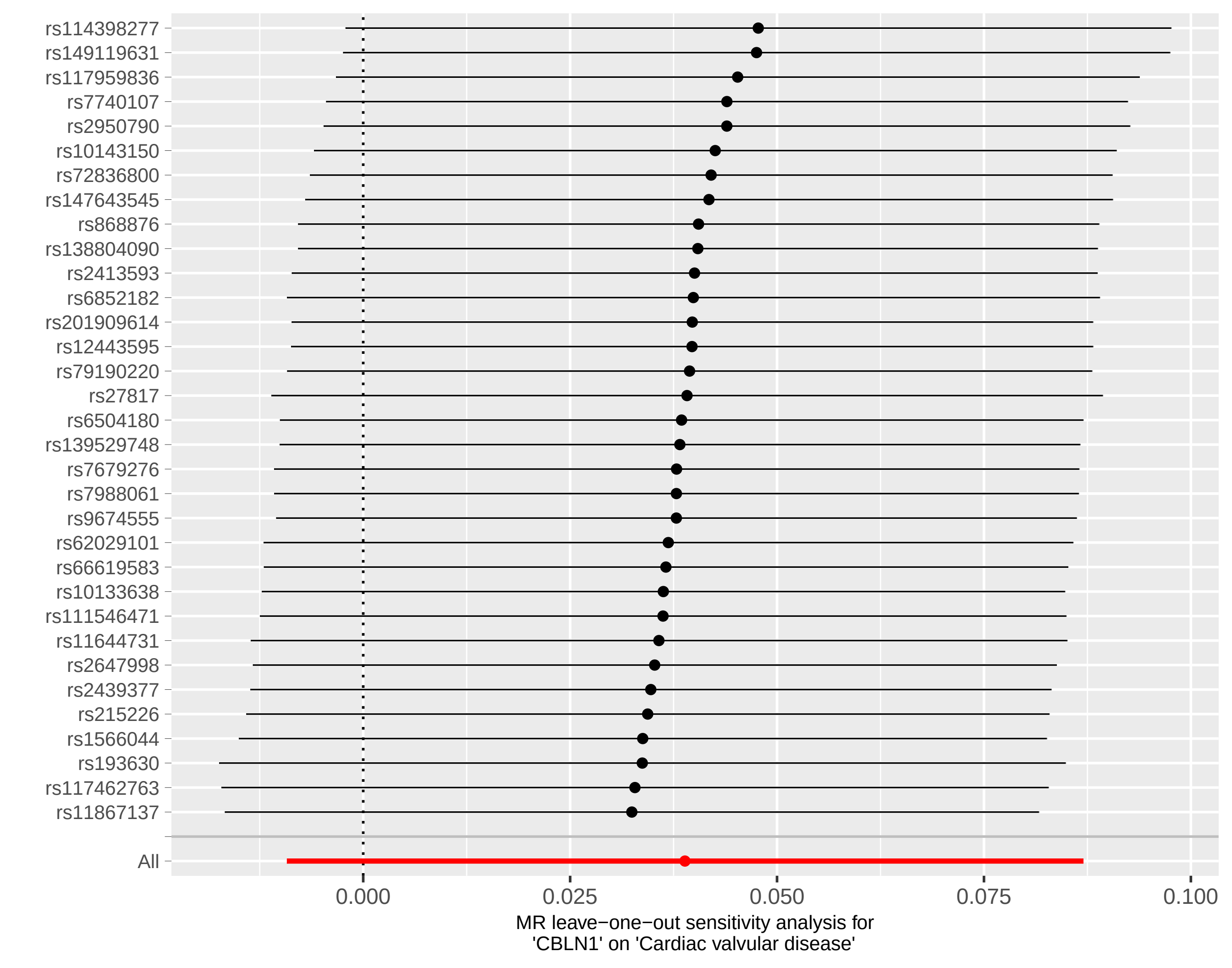
rs114398277
rs149119631
rs117959836
rs7740107
rs2950790
rs10143150
rs72836800
rs147643545
rs868876
rs138804090
rs2413593
rs6852182
rs201909614
rs12443595
rs79190220
rs27817
rs6504180
rs139529748
rs7679276
rs7988061
rs9674555
rs62029101
rs66619583
rs10133638
rs111546471
rs11644731
rs2647998
rs2439377
rs215226
rs1566044
rs193630
rs117462763
rs11867137
All
0.000
0.025
0.050
0.075
0.100
MR leave−one−out sensitivity analysis for
'CBLN1' on 'Cardiac valvular disease'

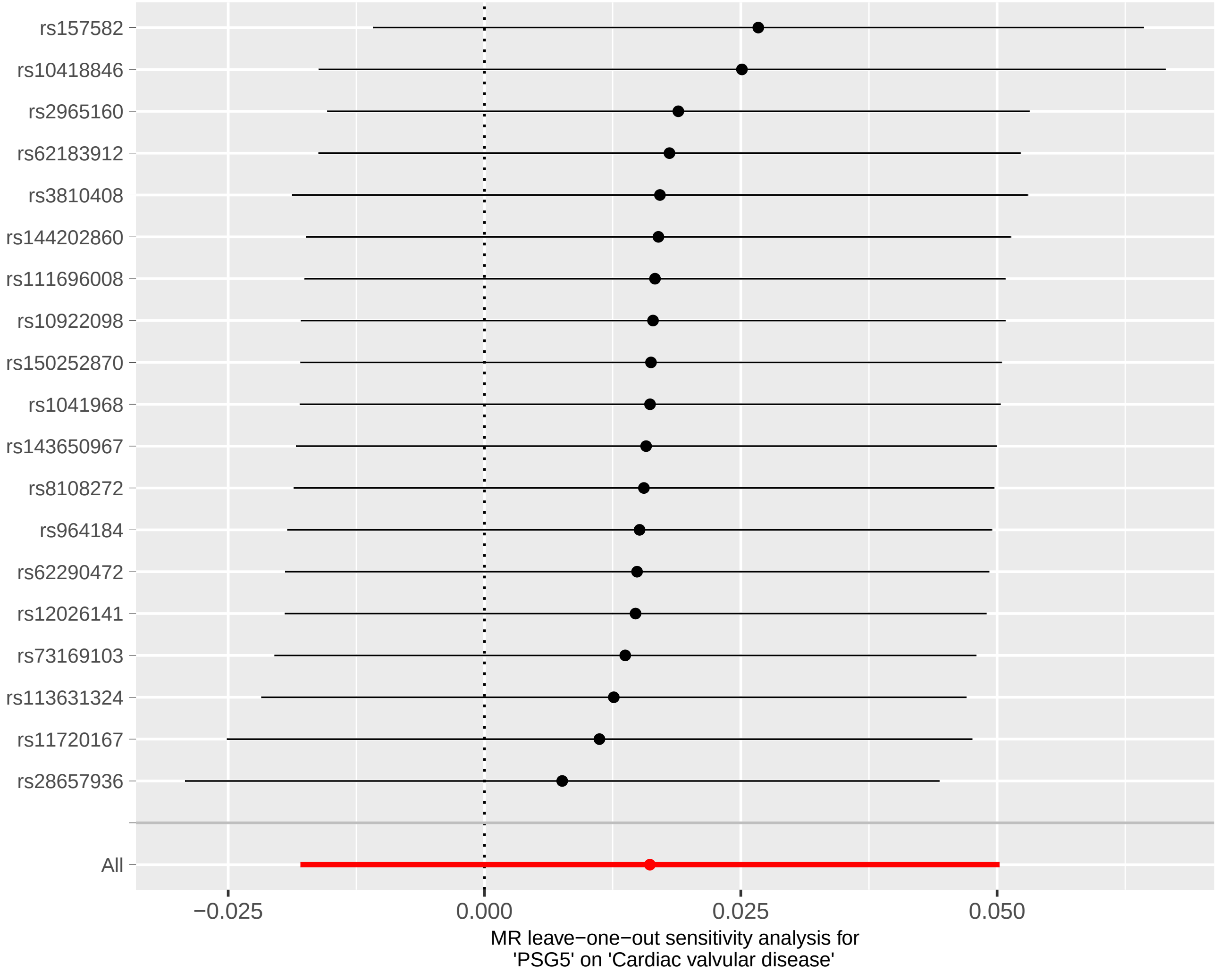
rs157582
rs10418846
rs2965160
rs62183912
rs3810408
rs144202860
rs111696008
rs10922098
rs150252870
rs1041968
rs143650967
rs8108272
rs964184
rs62290472
rs12026141
rs73169103
rs113631324
rs11720167
rs28657936
All
−0.025
0.000
0.025
0.050
MR leave−one−out sensitivity analysis for
'PSG5' on 'Cardiac valvular disease'

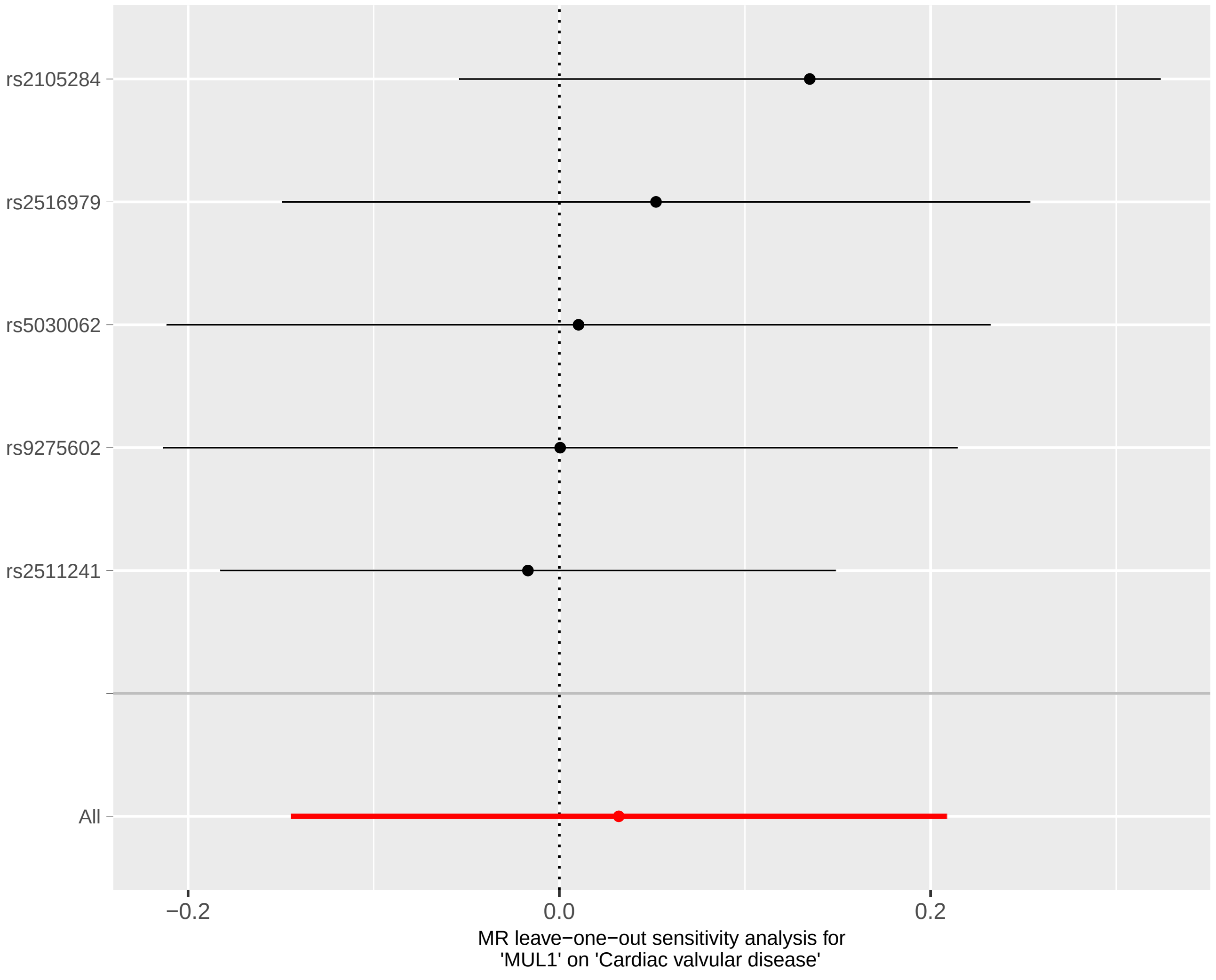

rs2105284
rs2516979
rs5030062
rs9275602
rs2511241
All
−0.2
0.0
0.2
MR leave−one−out sensitivity analysis for
'MUL1' on 'Cardiac valvular disease'

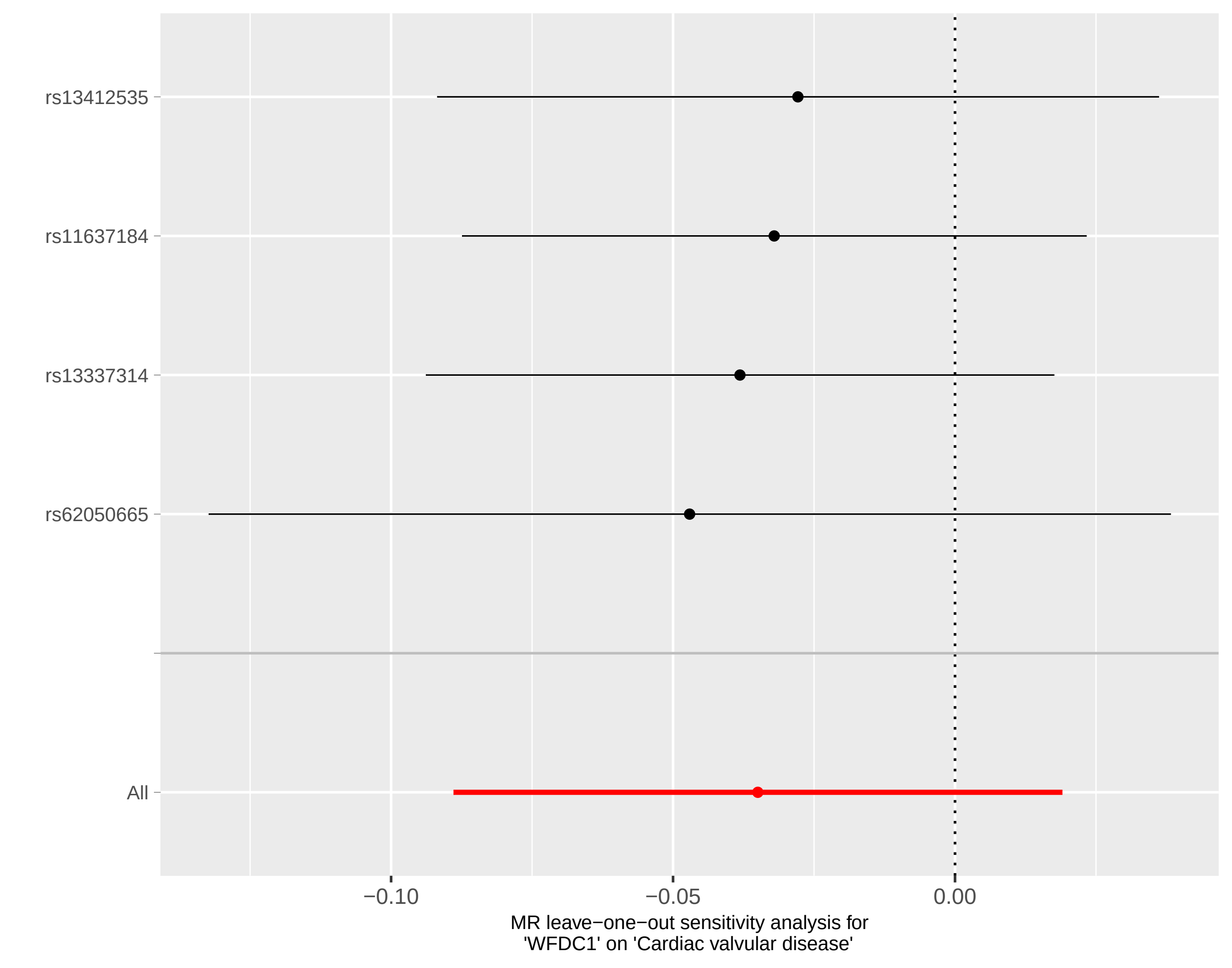
rs13412535
rs11637184
rs13337314
rs62050665
All
−0.10
−0.05
0.00
MR leave−one−out sensitivity analysis for
'WFDC1' on 'Cardiac valvular disease'

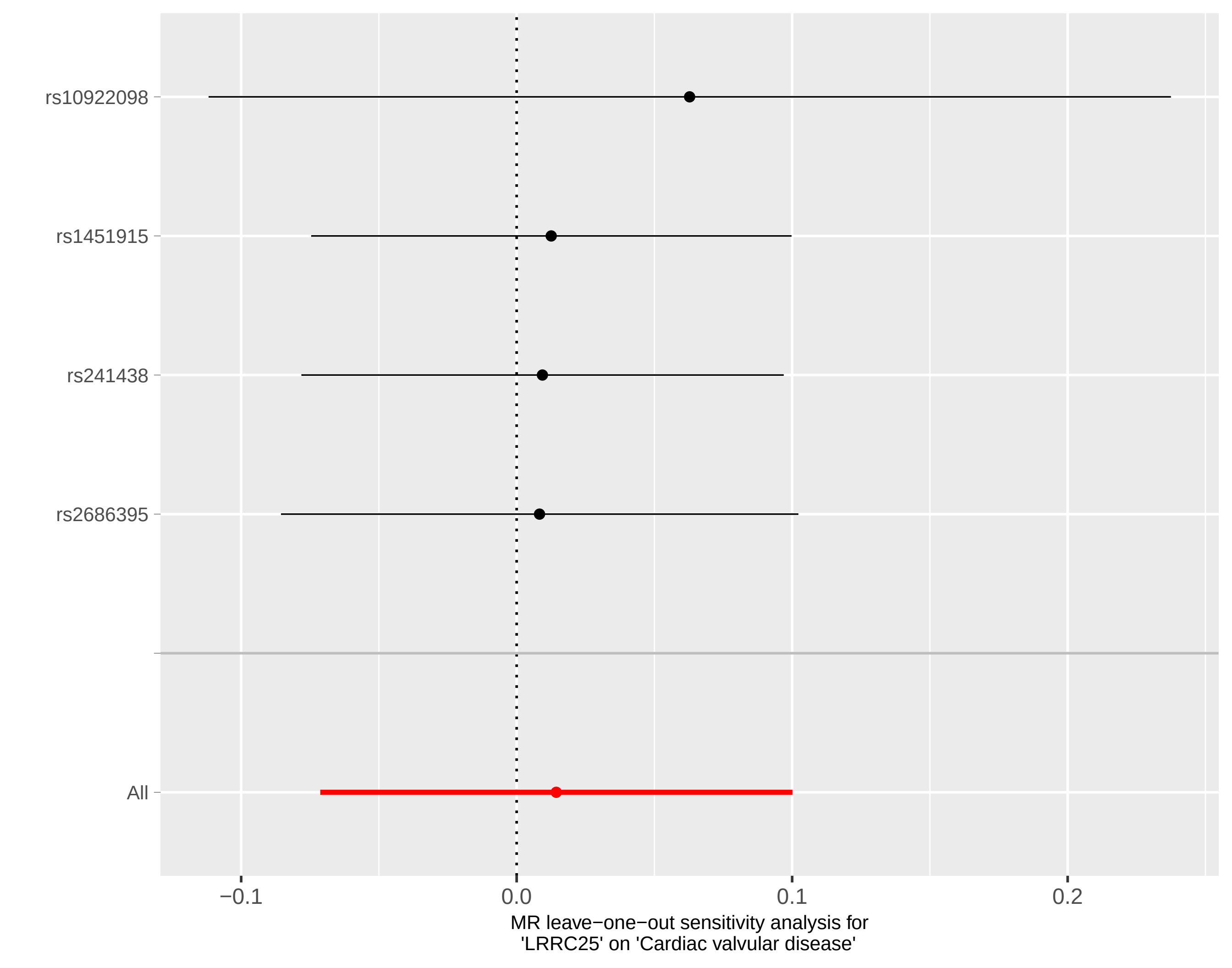

rs10922098
rs1451915
rs241438
rs2686395
All
−0.1
0.0
0.1
0.2
MR leave−one−out sensitivity analysis for
'LRRC25' on 'Cardiac valvular disease'

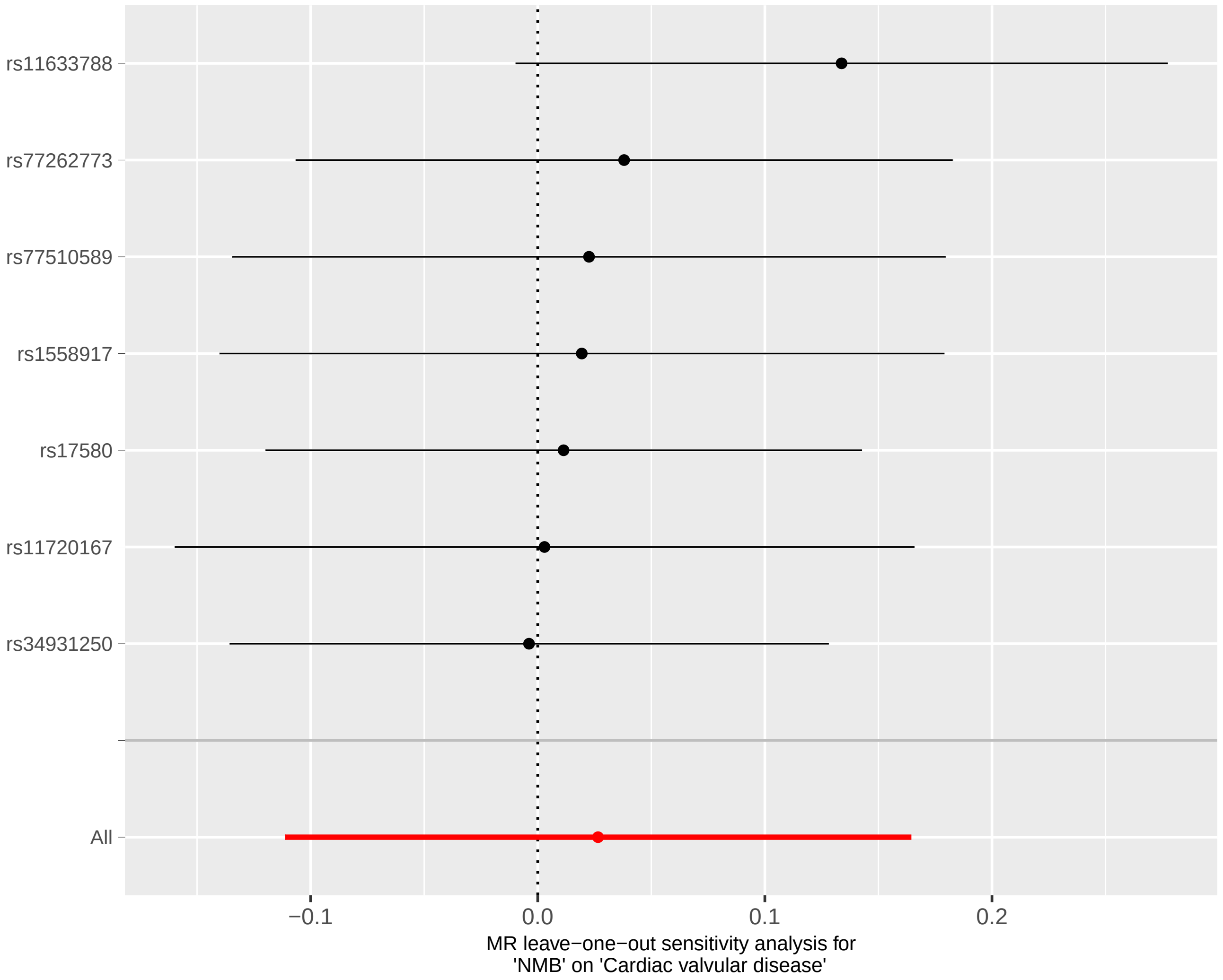
rs11633788
rs77262773
rs77510589
rs1558917
rs17580
rs11720167
rs34931250
All
−0.1
0.0
0.1
0.2
MR leave−one−out sensitivity analysis for
'NMB' on 'Cardiac valvular disease'

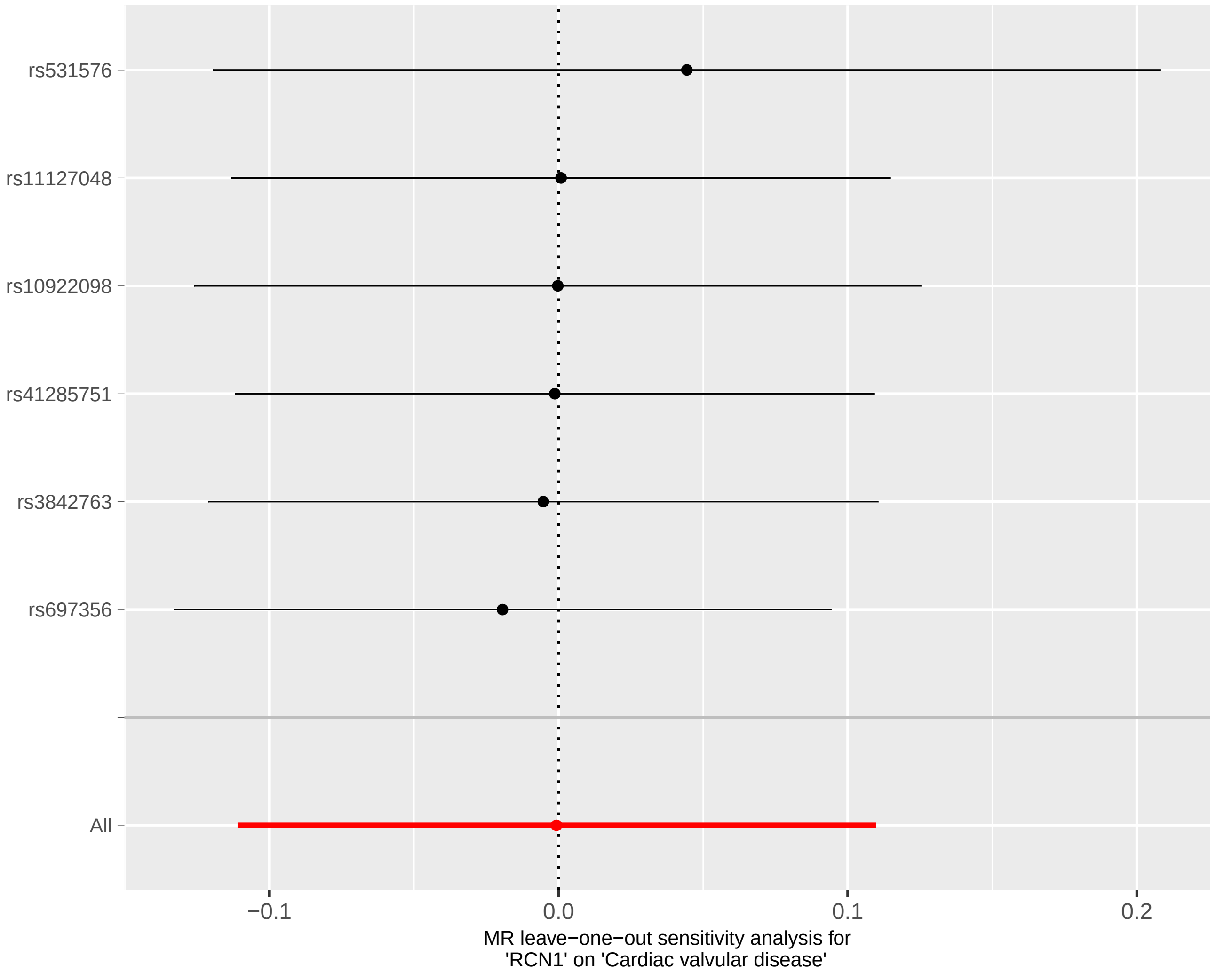

rs531576
rs11127048
rs10922098
rs41285751
rs3842763
rs697356
All
−0.1
0.0
0.1
0.2
MR leave−one−out sensitivity analysis for
'RCN1' on 'Cardiac valvular disease'

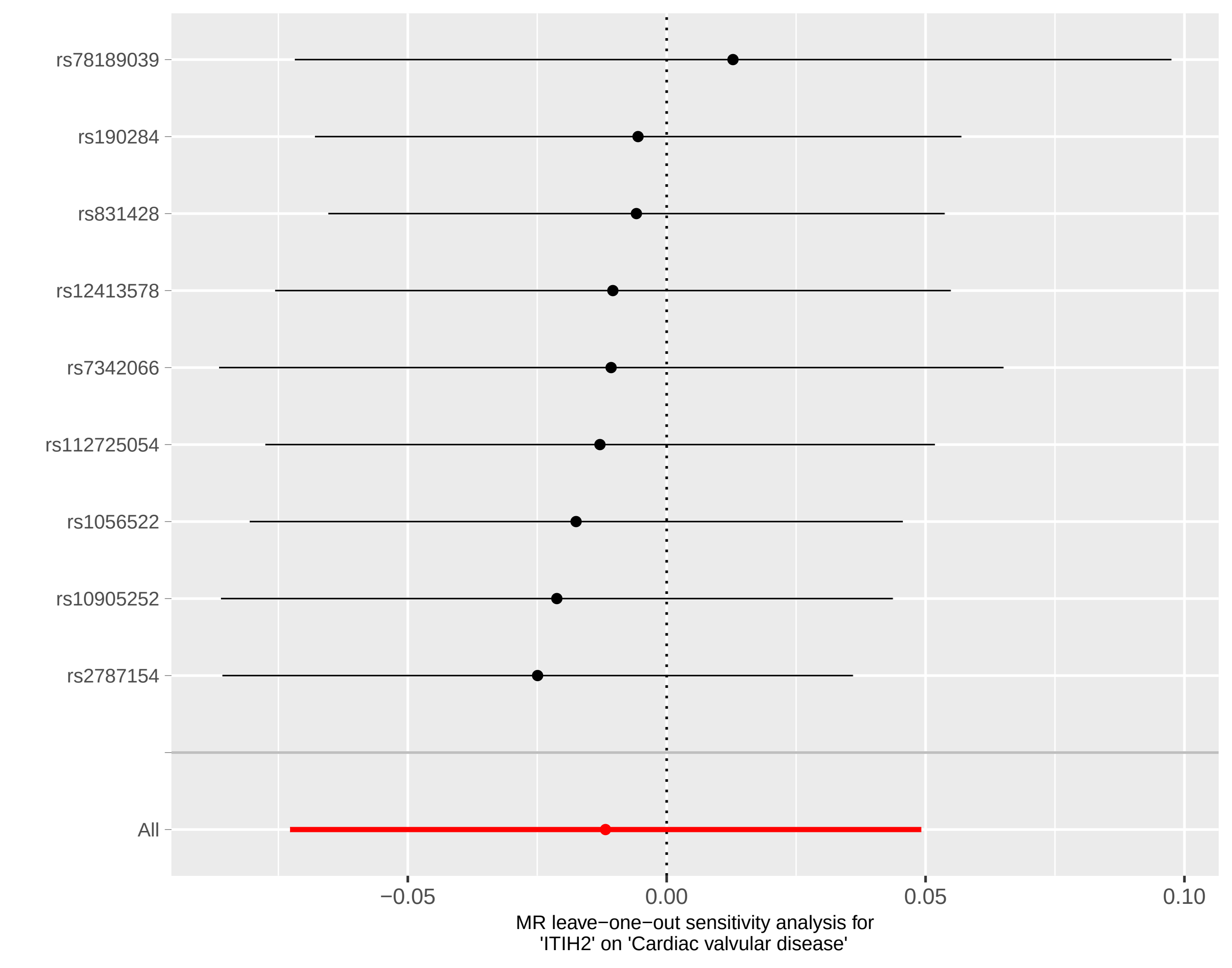
rs78189039
rs190284
rs831428
rs12413578
rs7342066
rs112725054
rs1056522
rs10905252
rs2787154
All
−0.05
0.00
0.05
0.10
MR leave−one−out sensitivity analysis for
'ITIH2' on 'Cardiac valvular disease'

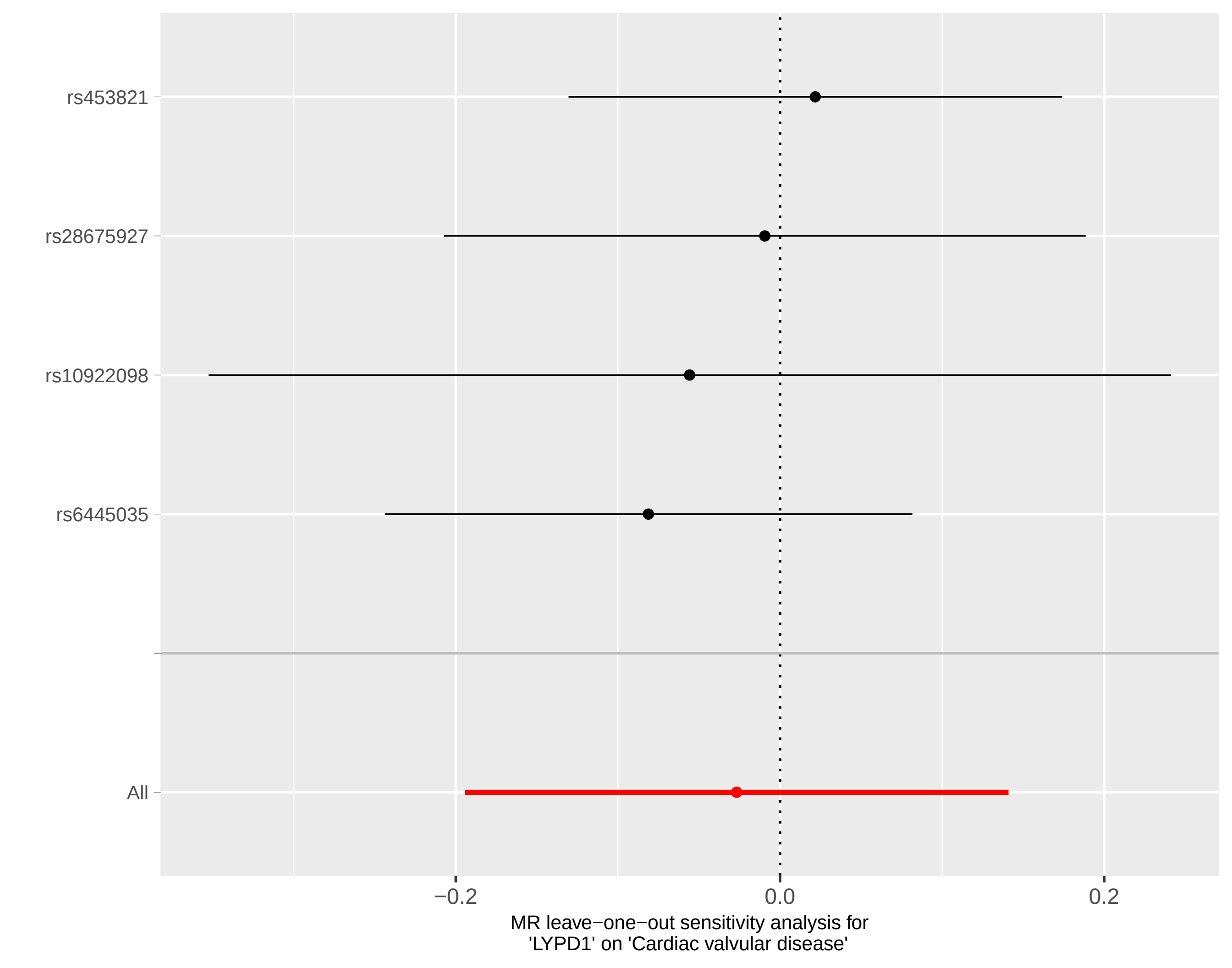

rs453821
rs28675927
rs10922098
rs6445035
All
−0.2
0.0
0.2
MR leave−one−out sensitivity analysis for
'LYPD1' on 'Cardiac valvular disease'

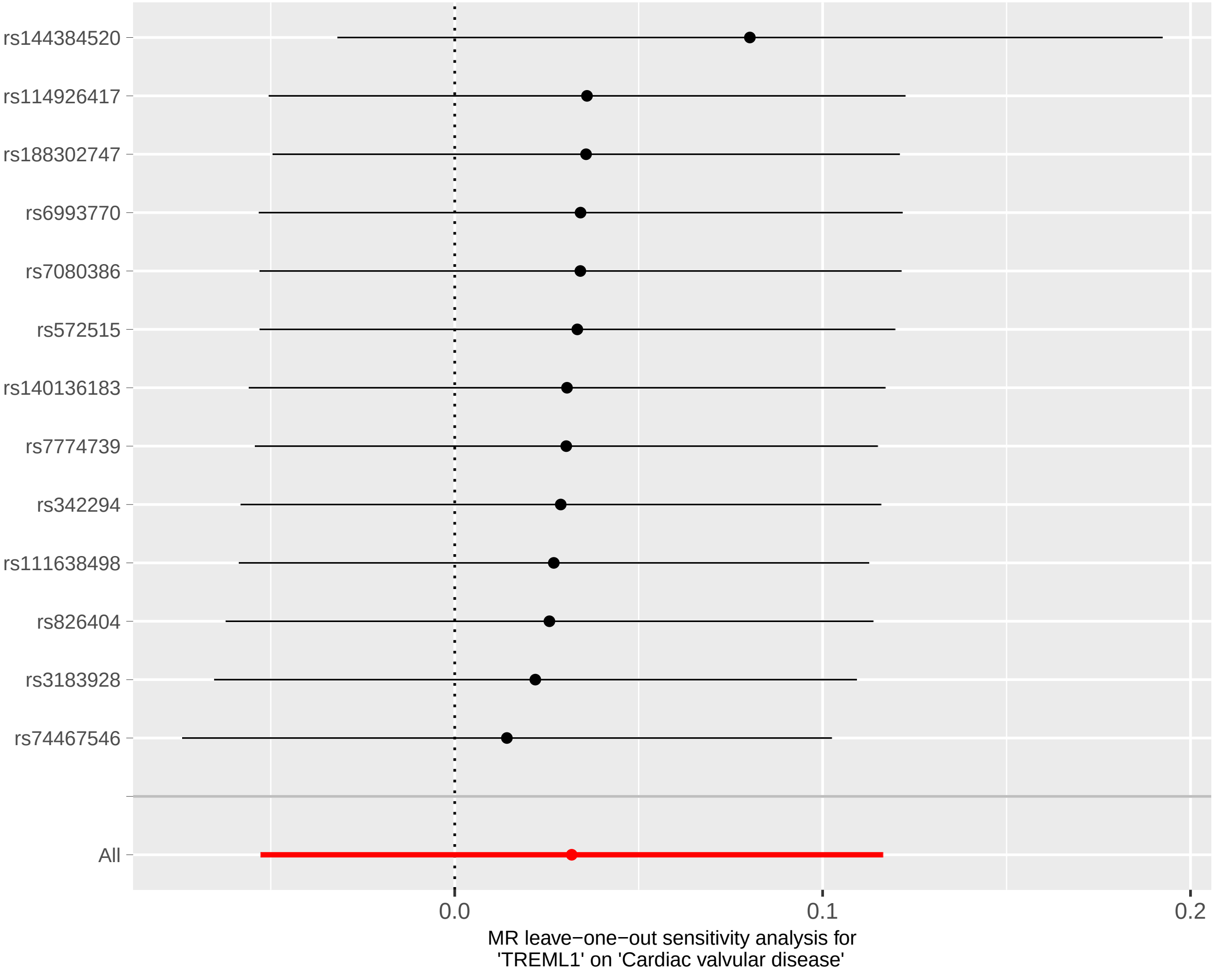
rs144384520
rs114926417
rs188302747
rs6993770
rs7080386
rs572515
rs140136183
rs7774739
rs342294
rs111638498
rs826404
rs3183928
rs74467546
All
0.0
0.1
0.2
MR leave−one−out sensitivity analysis for
'TREML1' on 'Cardiac valvular disease'

Insufficient number of SNPs

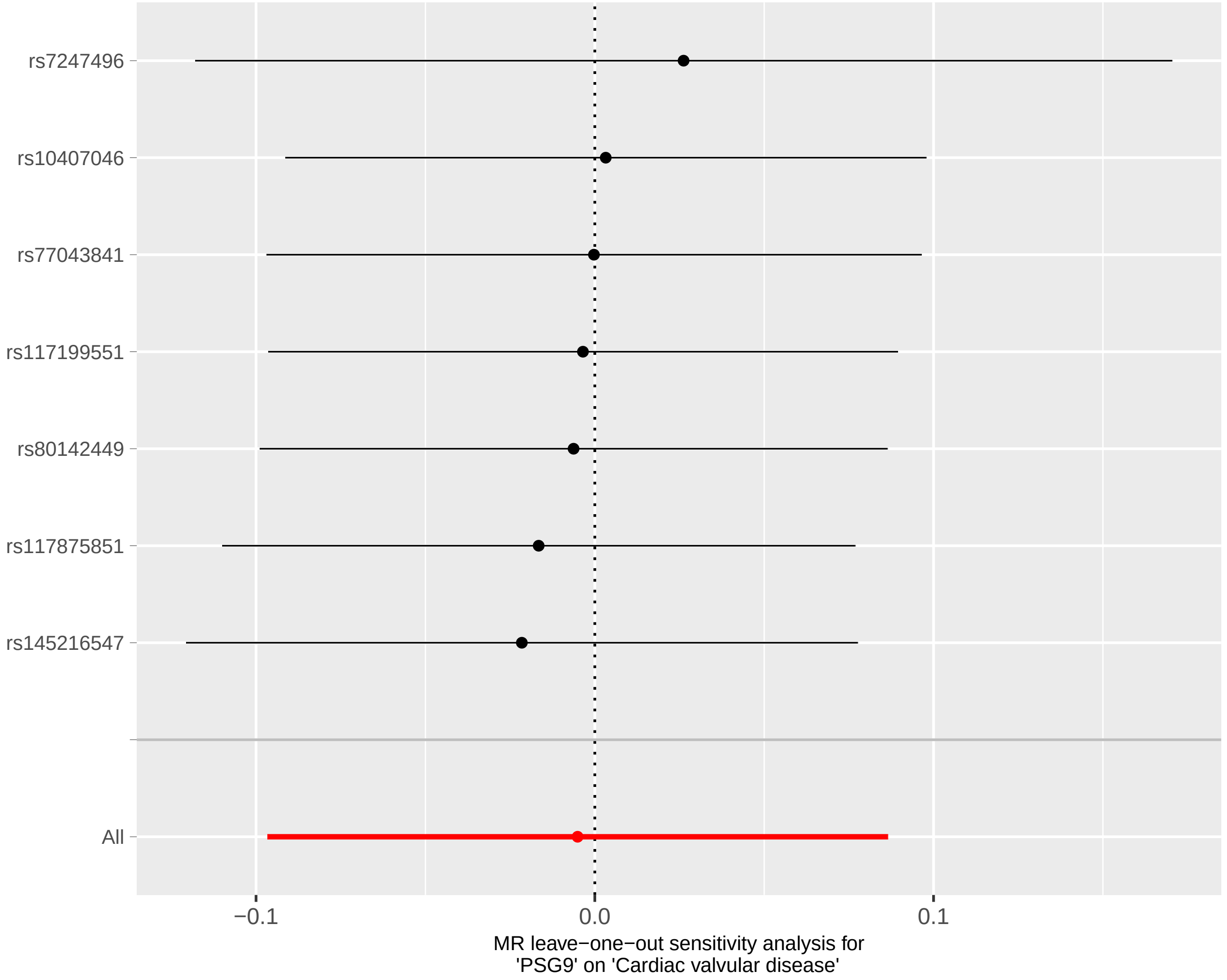
rs7247496
rs10407046
rs77043841
rs117199551
rs80142449
rs117875851
rs145216547
All
−0.1
0.0
0.1
MR leave−one−out sensitivity analysis for
'PSG9' on 'Cardiac valvular disease'

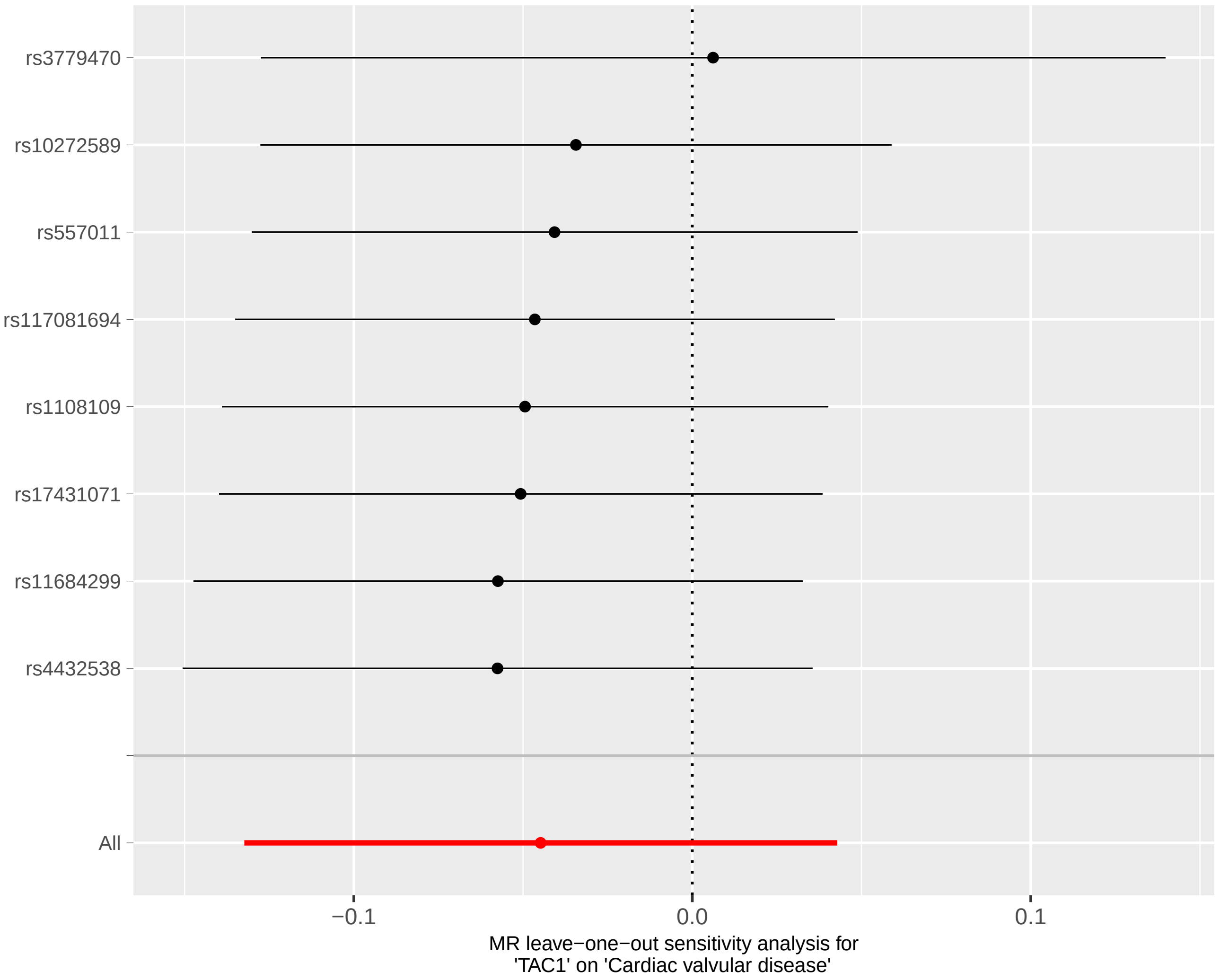
rs3779470
rs10272589
rs557011
rs117081694
rs1108109
rs17431071
rs11684299
rs4432538
All
−0.1
0.0
0.1
MR leave−one−out sensitivity analysis for
'TAC1' on 'Cardiac valvular disease'

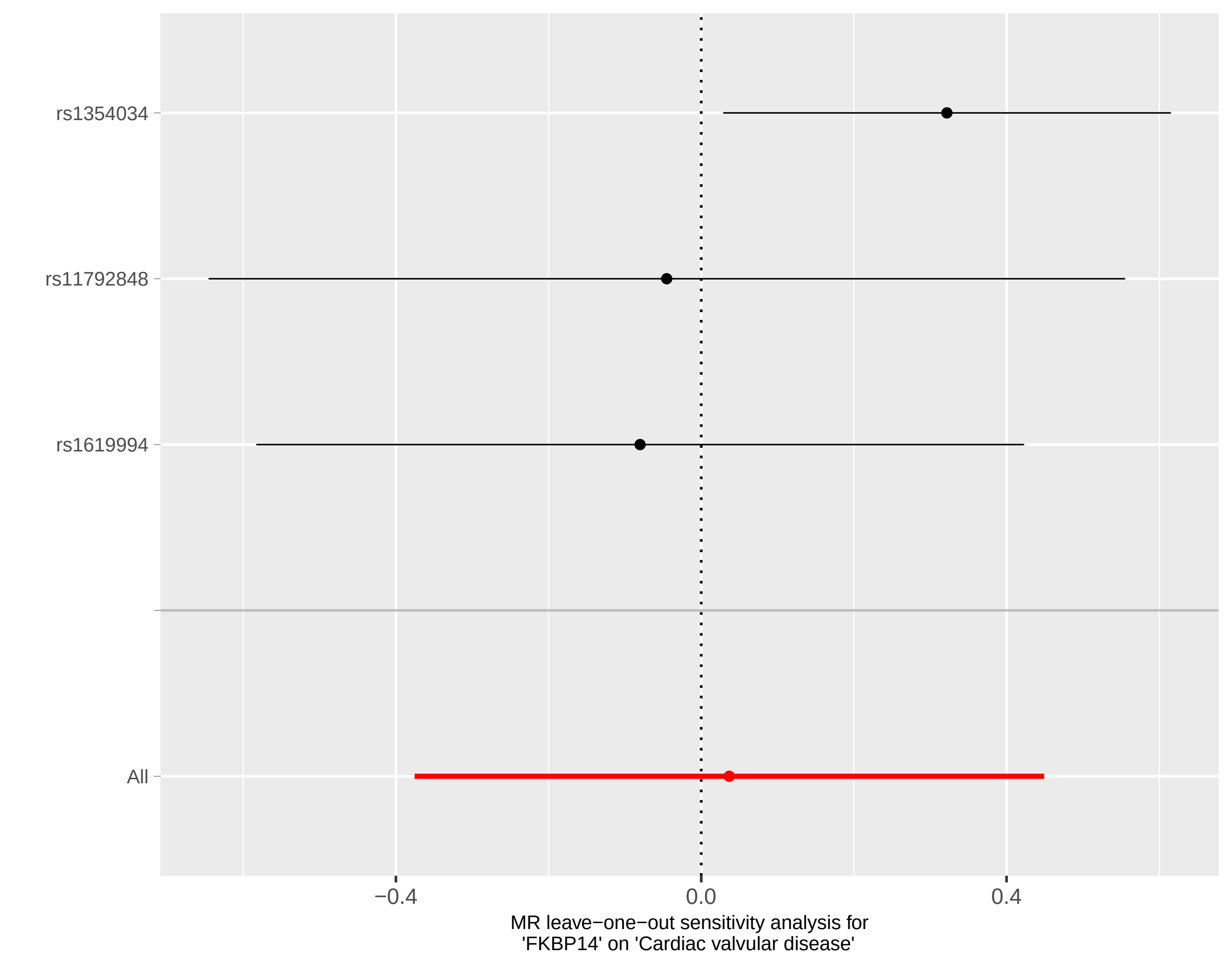

rs1354034
rs11792848
rs1619994
All
−0.4
0.0
0.4
MR leave−one−out sensitivity analysis for
'FKBP14' on 'Cardiac valvular disease'

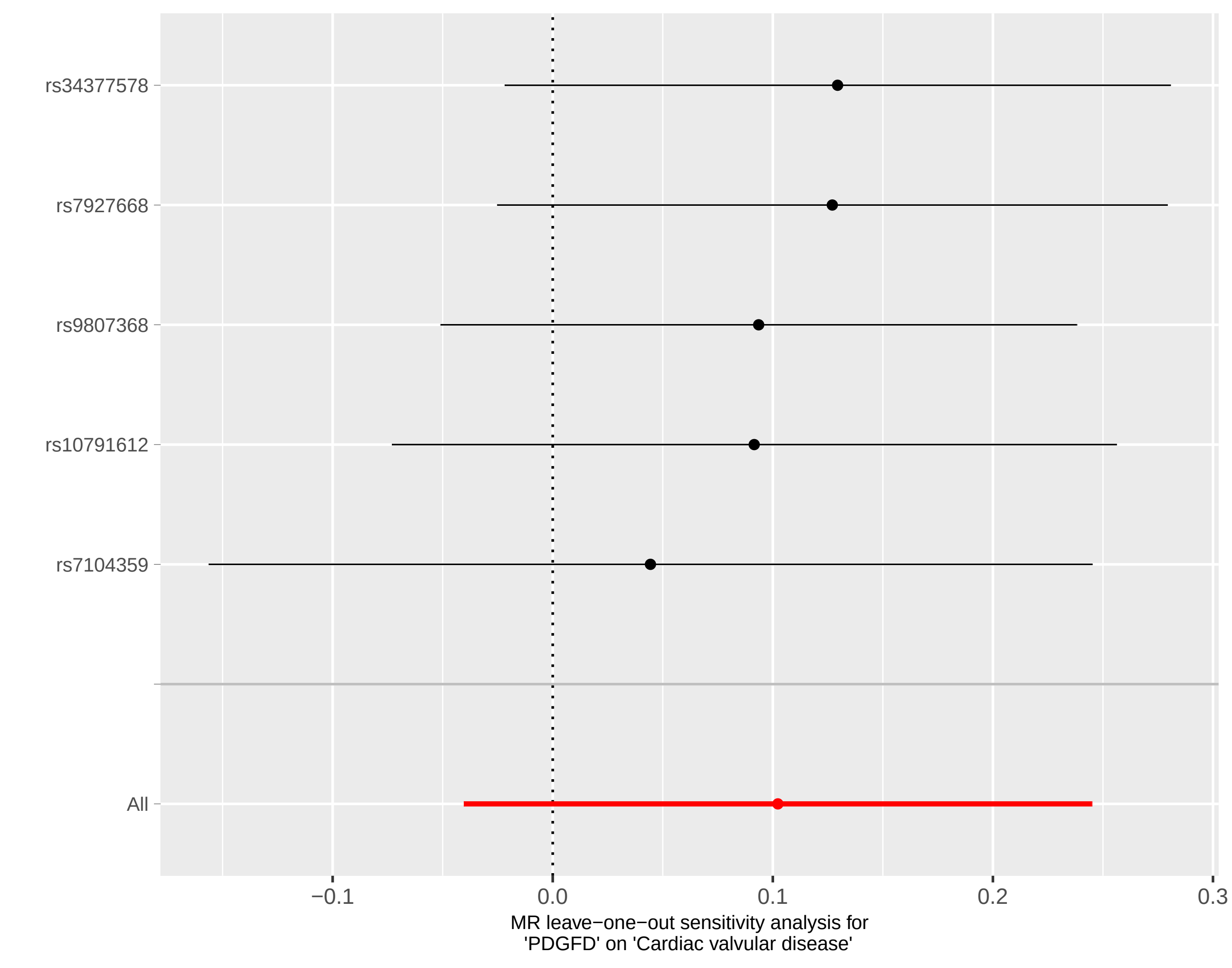

rs34377578
rs7927668
rs9807368
rs10791612
rs7104359
All
−0.1
0.0
0.1
0.2
0.3
MR leave−one−out sensitivity analysis for
'PDGFD' on 'Cardiac valvular disease'

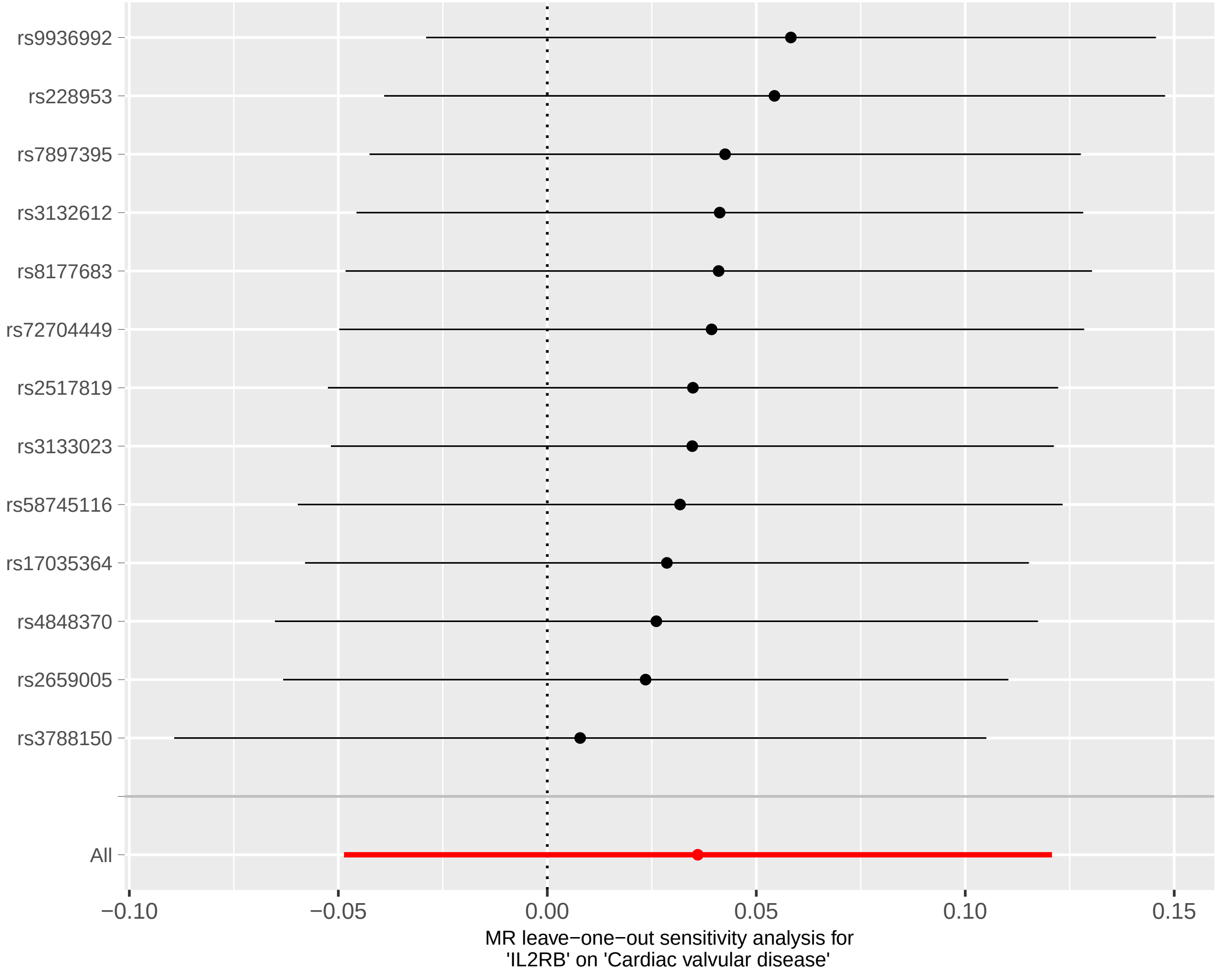
rs9936992
rs228953
rs7897395
rs3132612
rs8177683
rs72704449
rs2517819
rs3133023
rs58745116
rs17035364
rs4848370
rs2659005
rs3788150
All
−0.10
−0.05
0.00
0.05
0.10
0.15
MR leave−one−out sensitivity analysis for
'IL2RB' on 'Cardiac valvular disease'

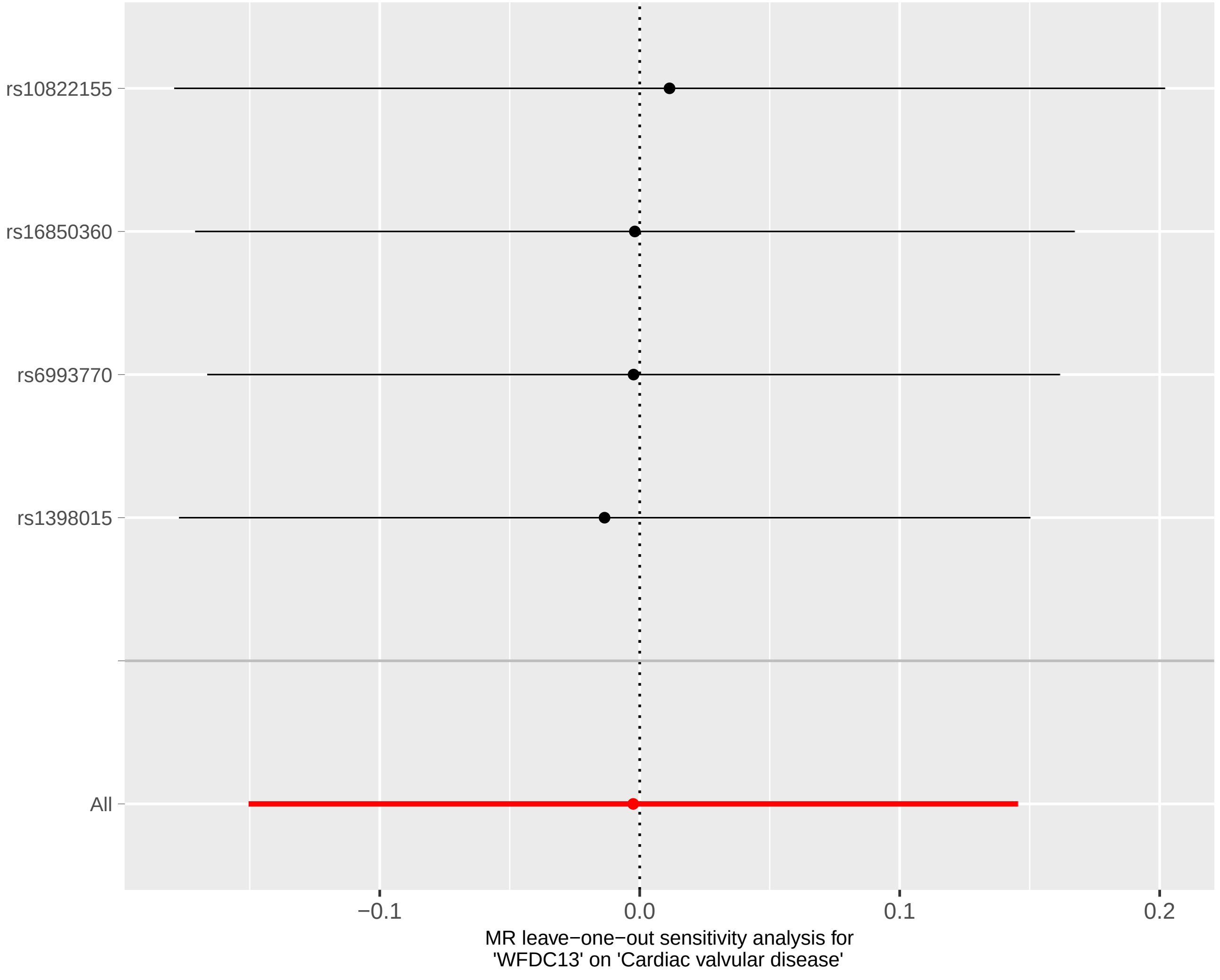
rs10822155
rs16850360
rs6993770
rs1398015
All
−0.1
0.0
0.1
0.2
MR leave−one−out sensitivity analysis for
'WFDC13' on 'Cardiac valvular disease'

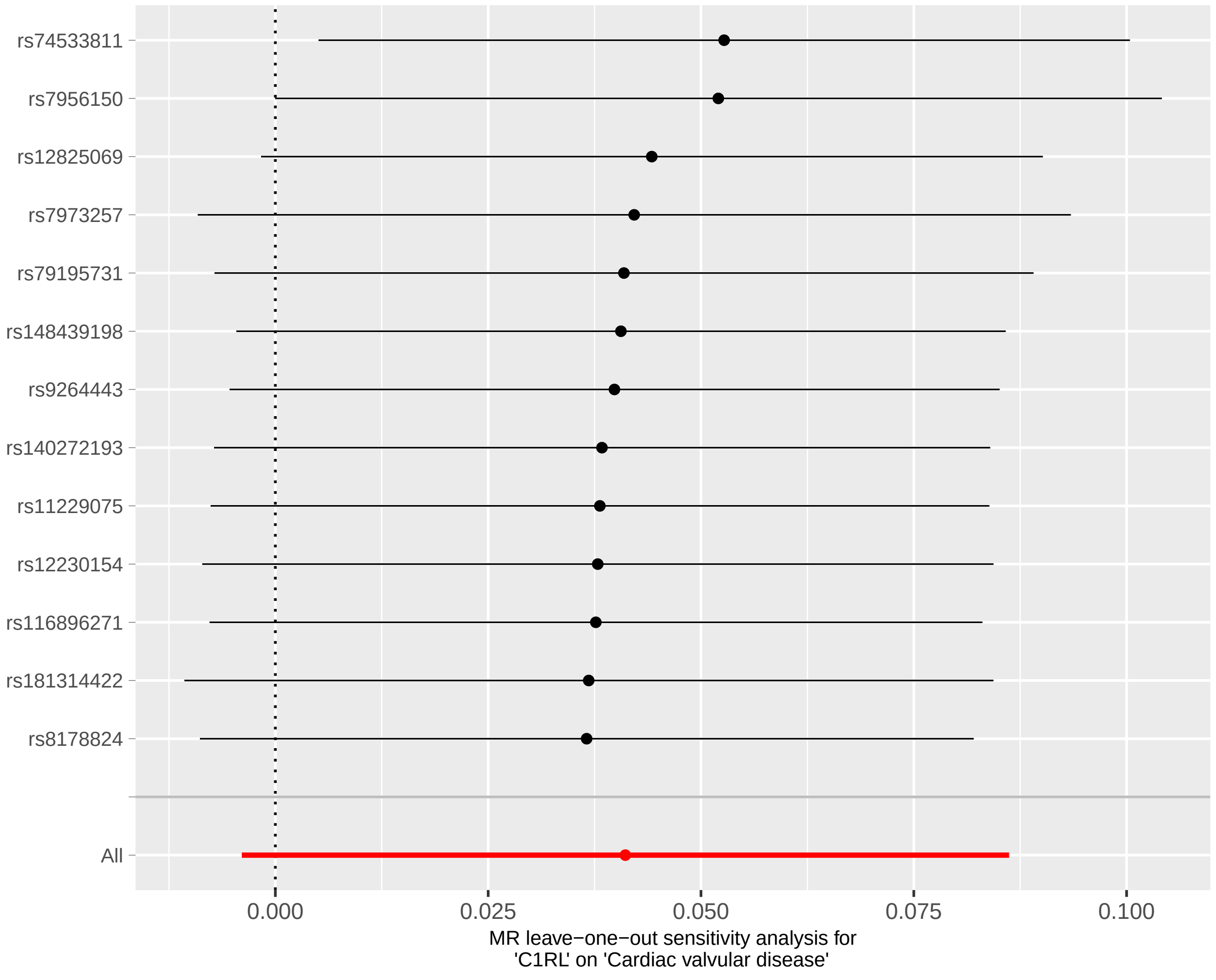
rs74533811
rs7956150
rs12825069
rs7973257
rs79195731
rs148439198
rs9264443
rs140272193
rs11229075
rs12230154
rs116896271
rs181314422
rs8178824
All
0.000
0.025
0.050
0.075
0.100
MR leave−one−out sensitivity analysis for
'C1RL' on 'Cardiac valvular disease'

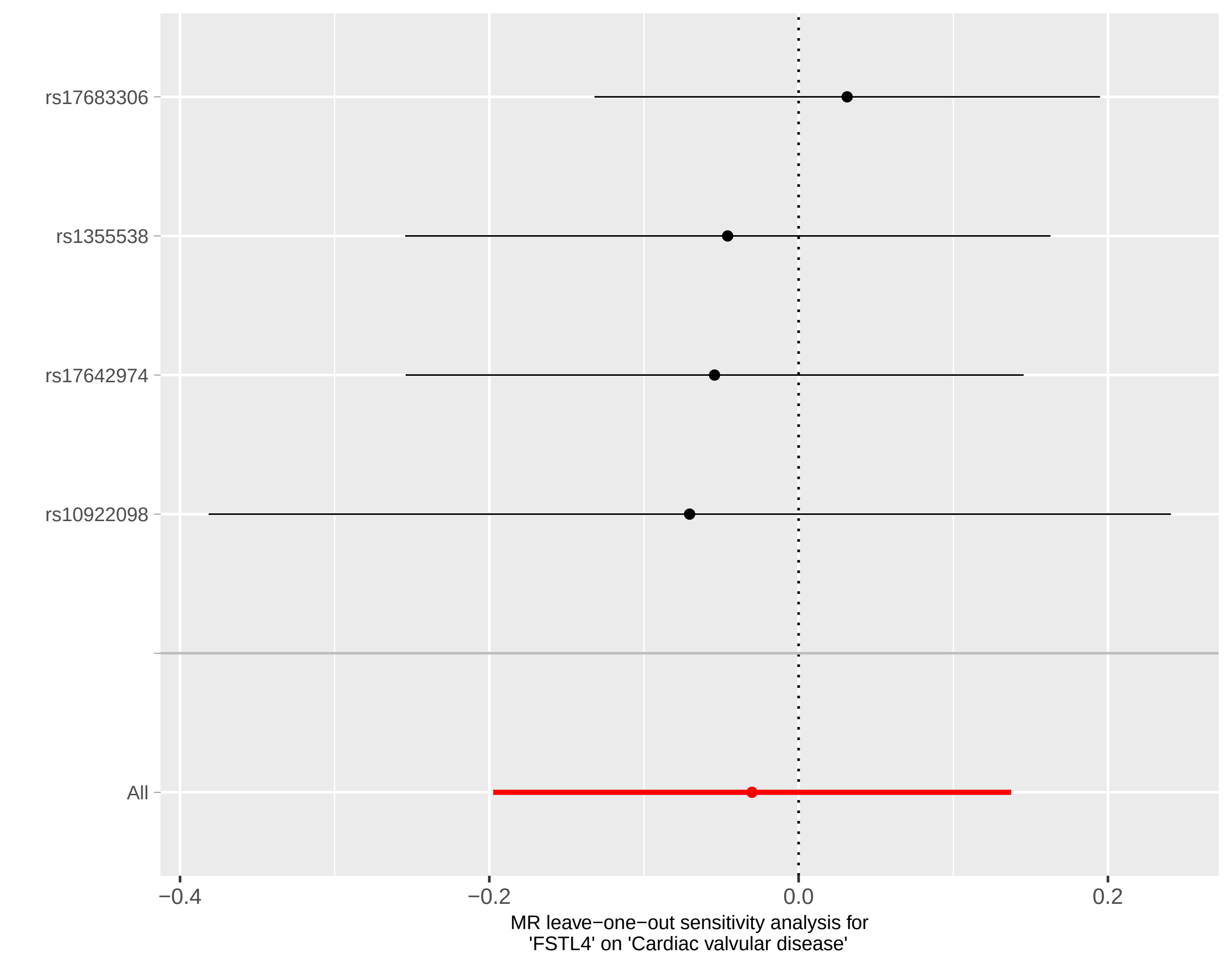
rs17683306
rs1355538
rs17642974
rs10922098
All
−0.4
−0.2
0.0
0.2
MR leave−one−out sensitivity analysis for
'FSTL4' on 'Cardiac valvular disease'

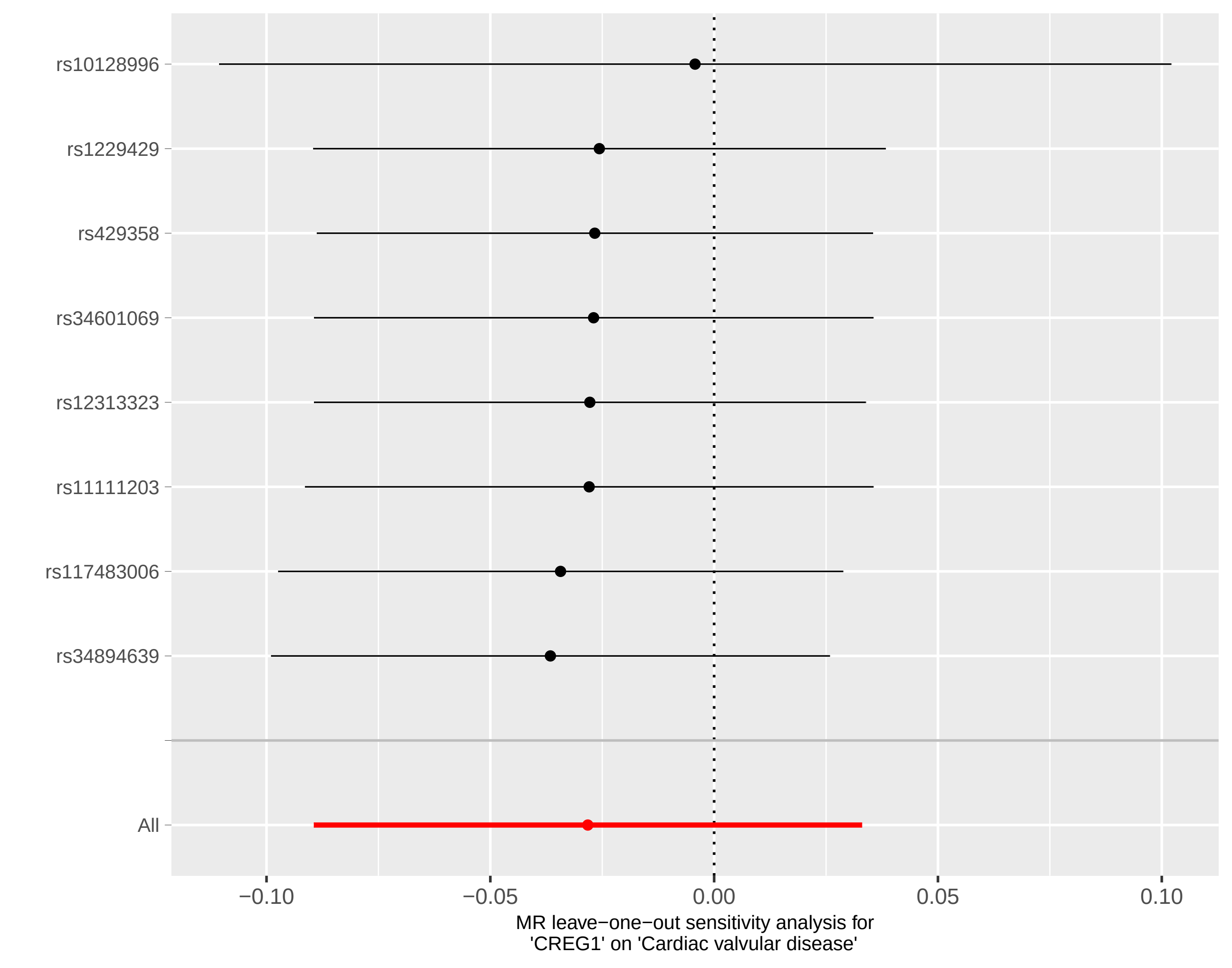

rs10128996
rs1229429
rs429358
rs34601069
rs12313323
rs11111203
rs117483006
rs34894639
All
−0.10
−0.05
0.00
0.05
0.10
MR leave−one−out sensitivity analysis for
'CREG1' on 'Cardiac valvular disease'

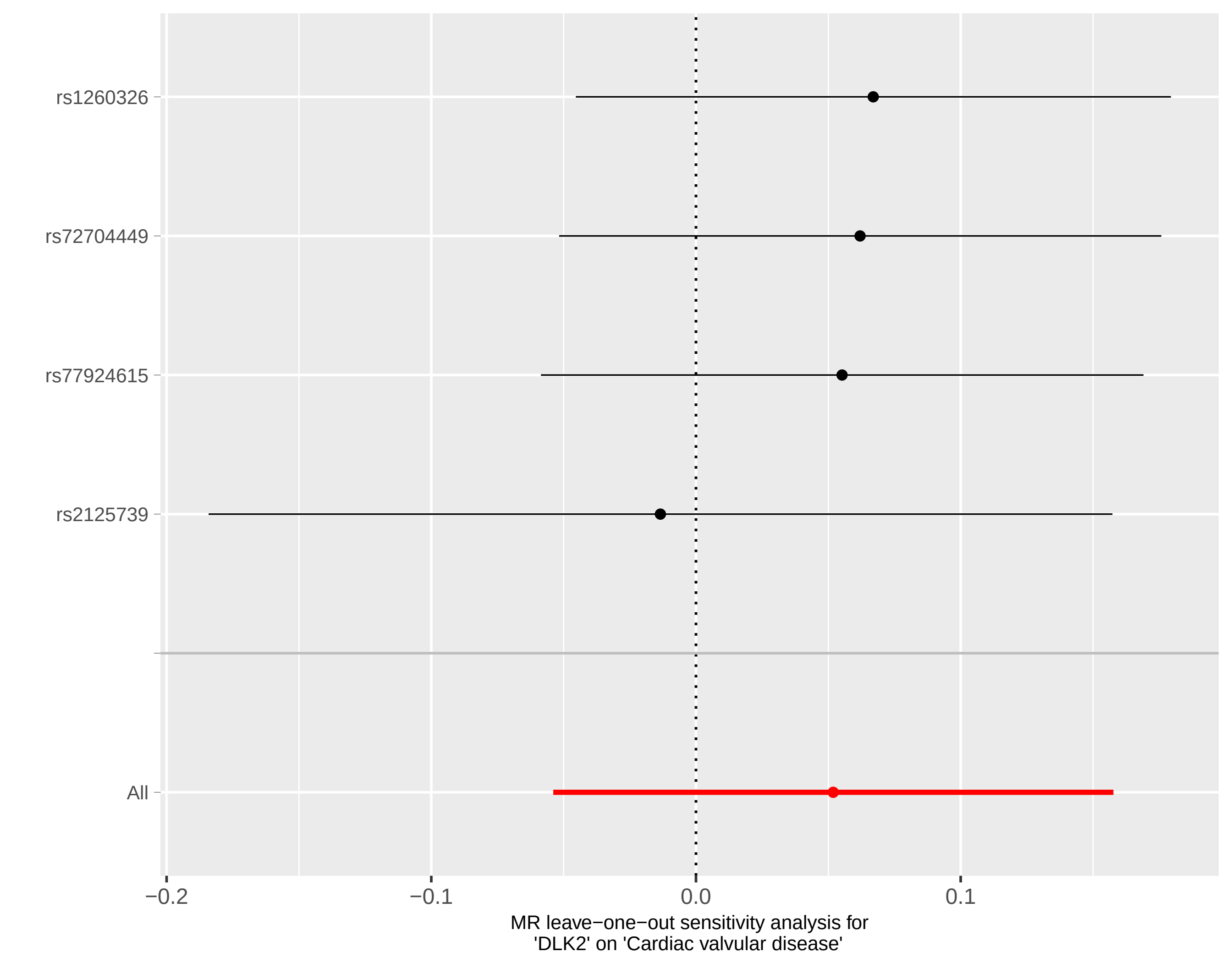
rs1260326
rs72704449
rs77924615
rs2125739
All
−0.2
−0.1
0.0
0.1
MR leave−one−out sensitivity analysis for
'DLK2' on 'Cardiac valvular disease'

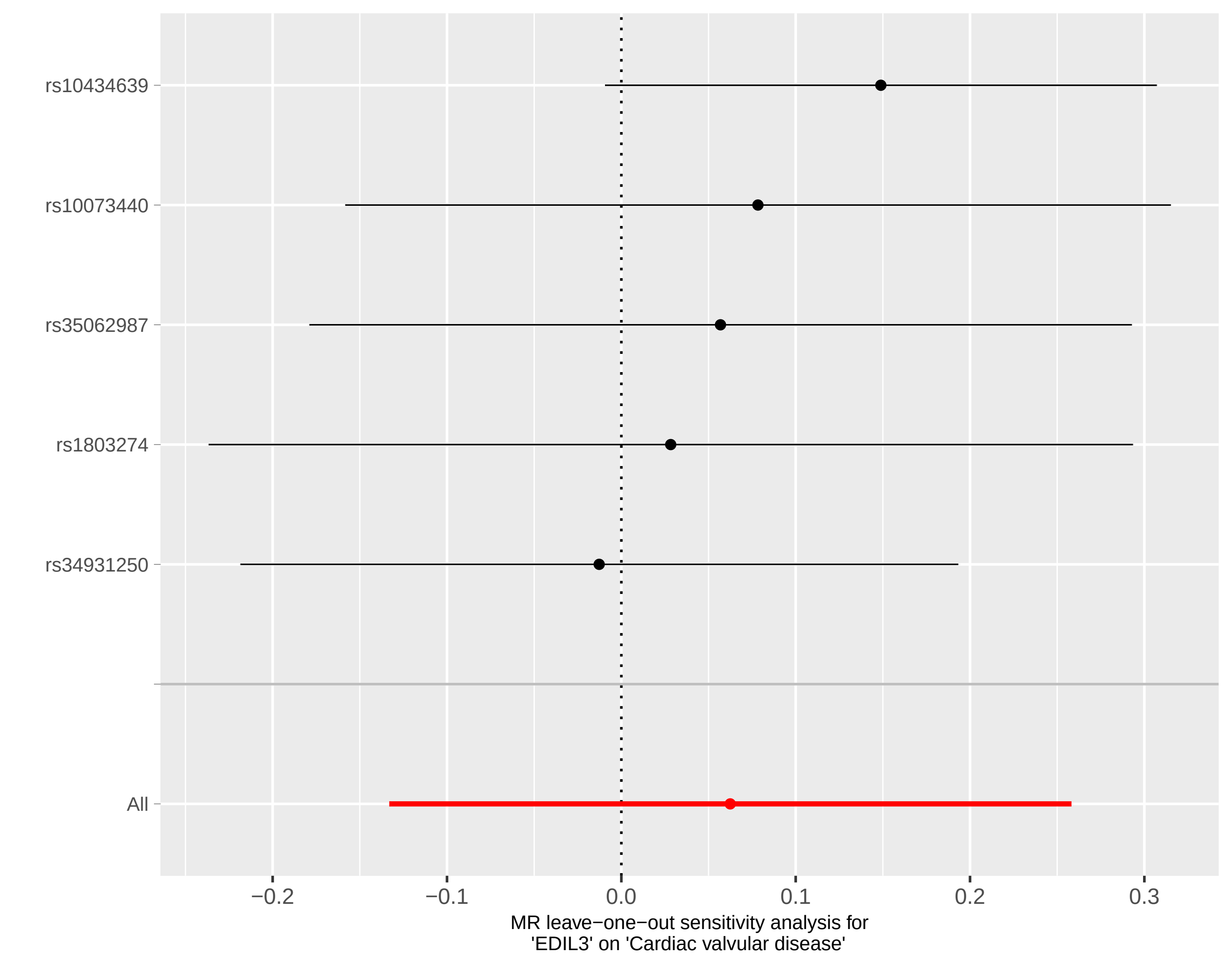

rs10434639
rs10073440
rs35062987
rs1803274
rs34931250
All
−0.2
−0.1
0.0
0.1
0.2
0.3
MR leave−one−out sensitivity analysis for
'EDIL3' on 'Cardiac valvular disease'

Insufficient number of SNPs

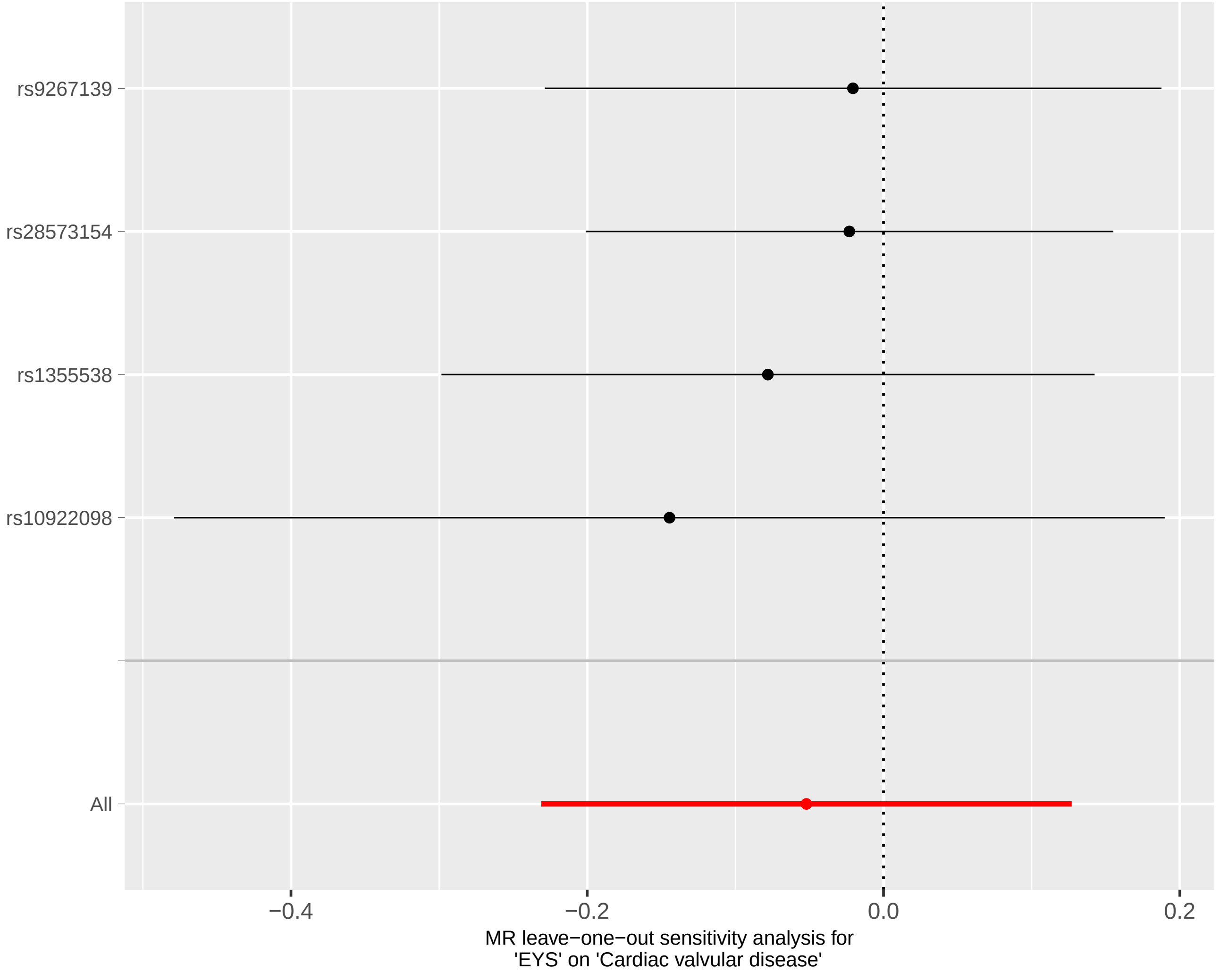
rs9267139
rs28573154
rs1355538
rs10922098
All
−0.4
−0.2
0.0
0.2
MR leave−one−out sensitivity analysis for
'EYS' on 'Cardiac valvular disease'

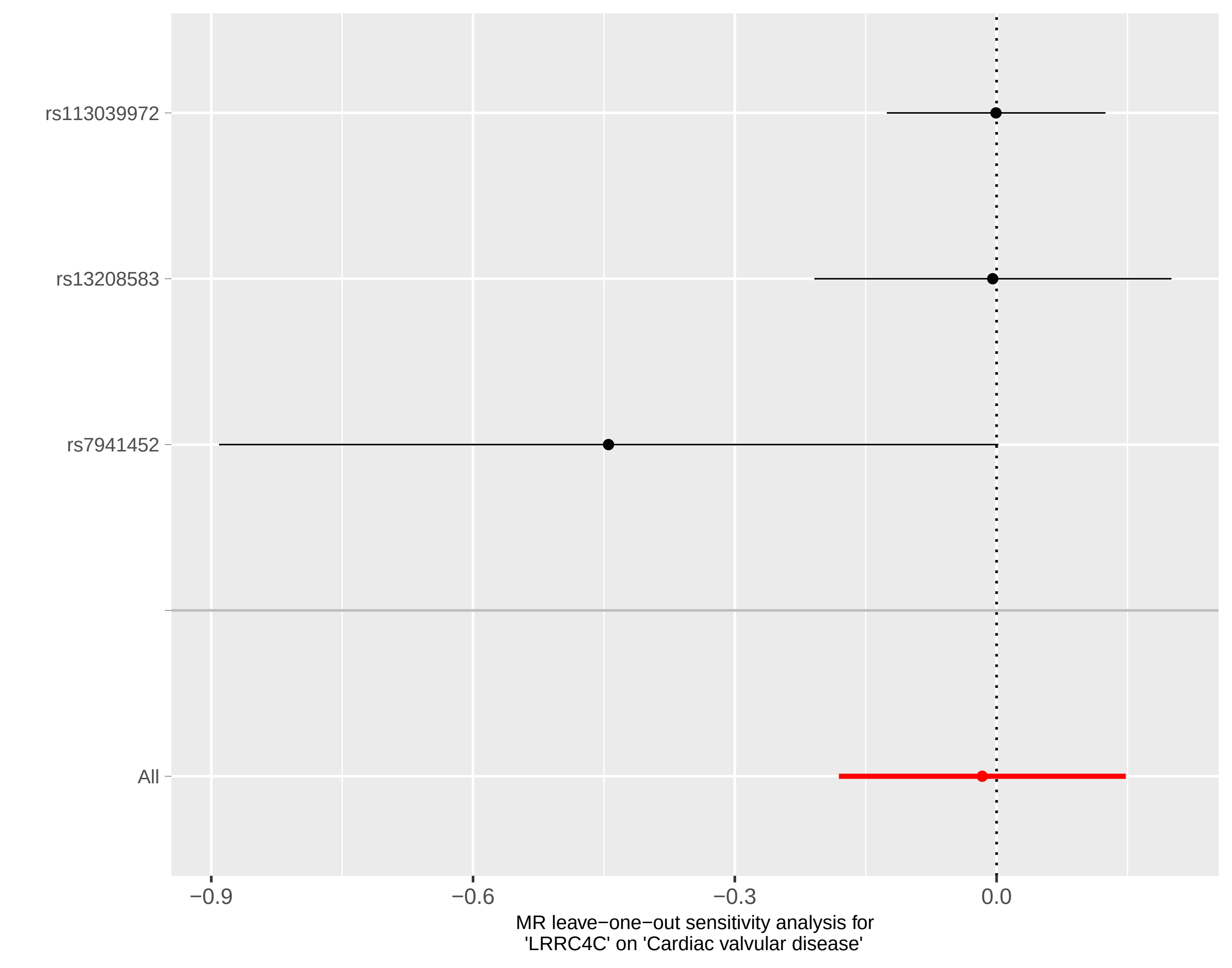

rs113039972
rs13208583
rs7941452
All
−0.9
−0.6
−0.3
0.0
MR leave−one−out sensitivity analysis for
'LRRC4C' on 'Cardiac valvular disease'

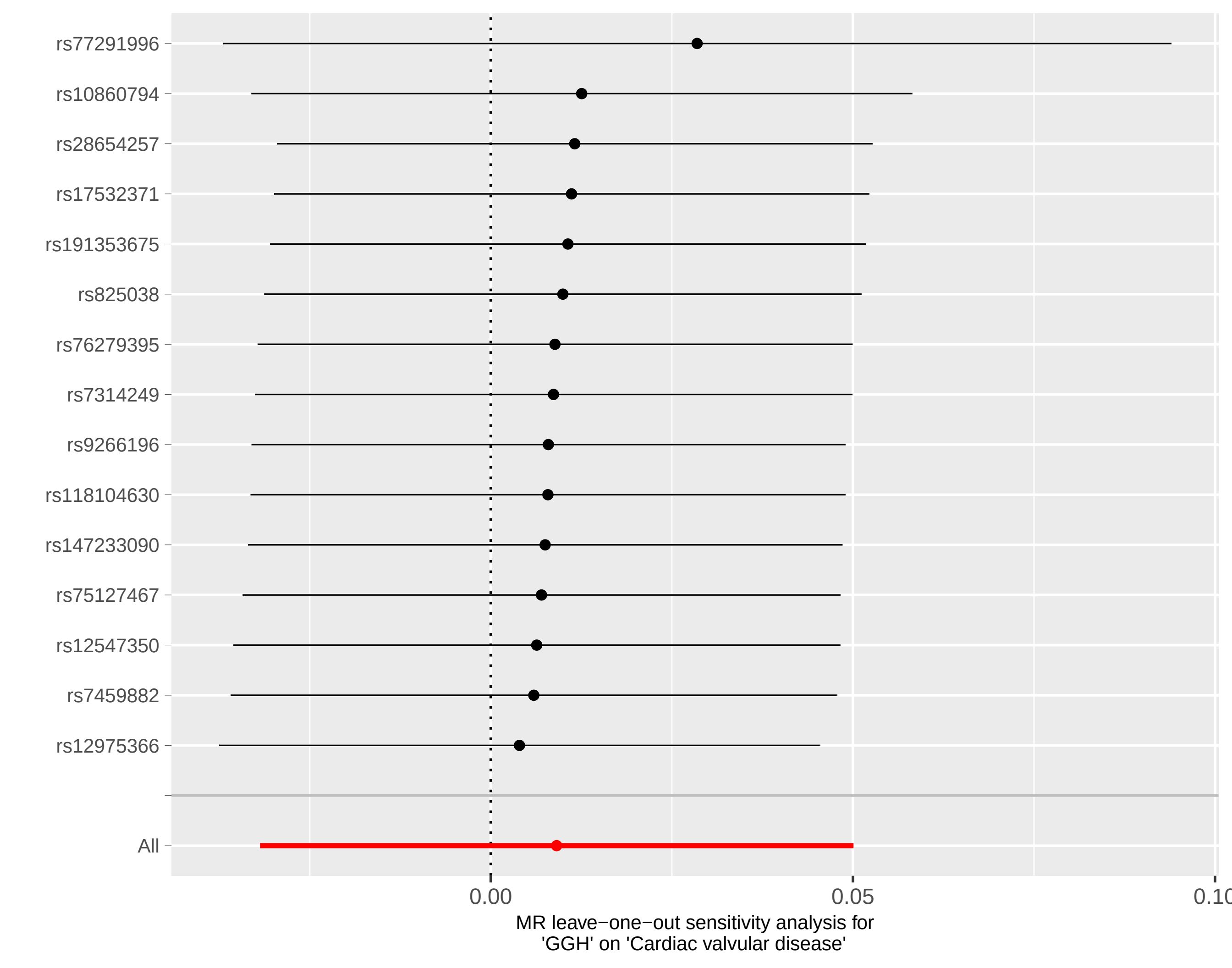
rs77291996
rs10860794
rs28654257
rs17532371
rs191353675
rs825038
rs76279395
rs7314249
rs9266196
rs118104630
rs147233090
rs75127467
rs12547350
rs7459882
rs12975366
All
0.00
0.05
0.10
MR leave−one−out sensitivity analysis for
'GGH' on 'Cardiac valvular disease'

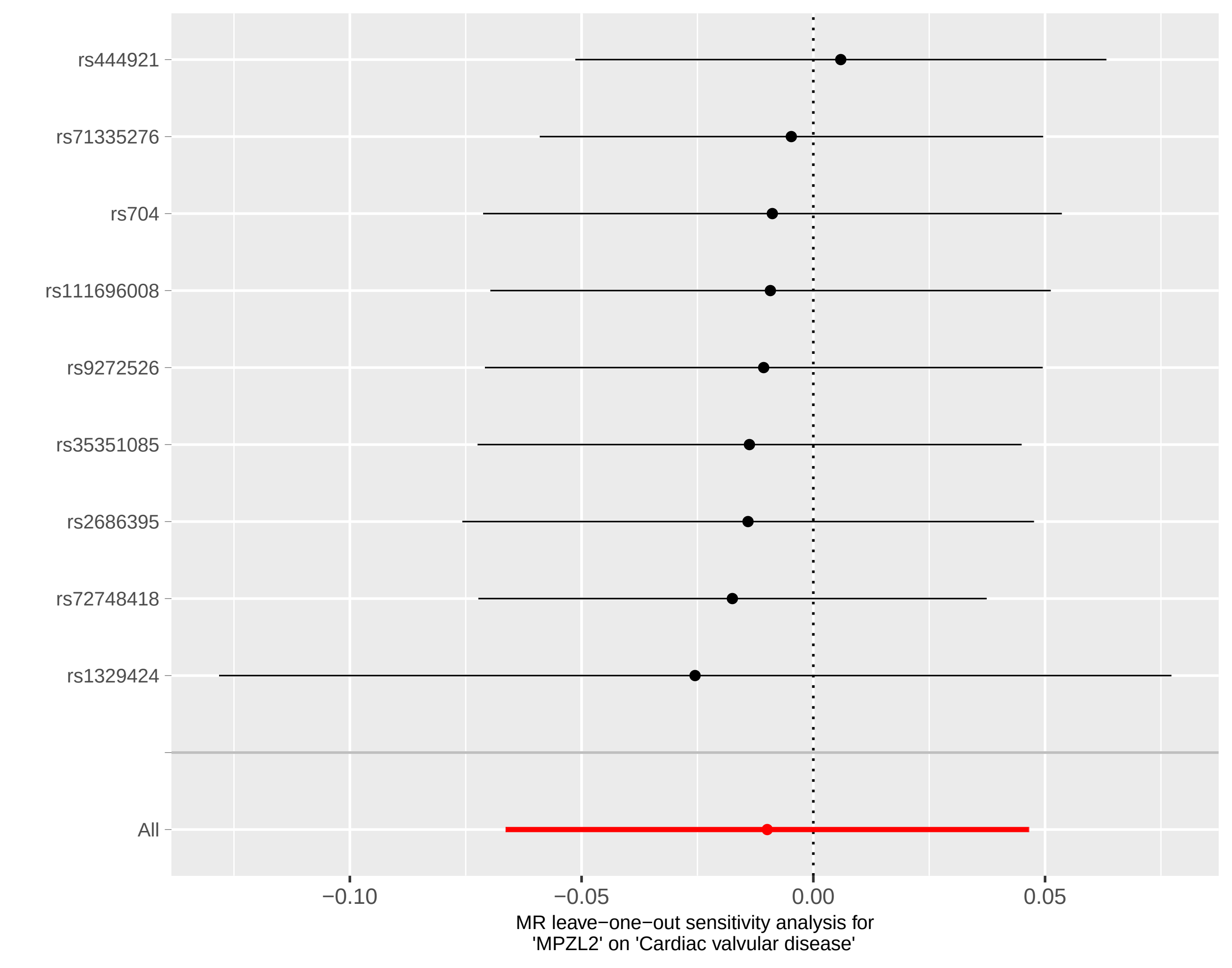
rs444921
rs71335276
rs704
rs111696008
rs9272526
rs35351085
rs2686395
rs72748418
rs1329424
All
−0.10
−0.05
0.00
0.05
MR leave−one−out sensitivity analysis for
'MPZL2' on 'Cardiac valvular disease'

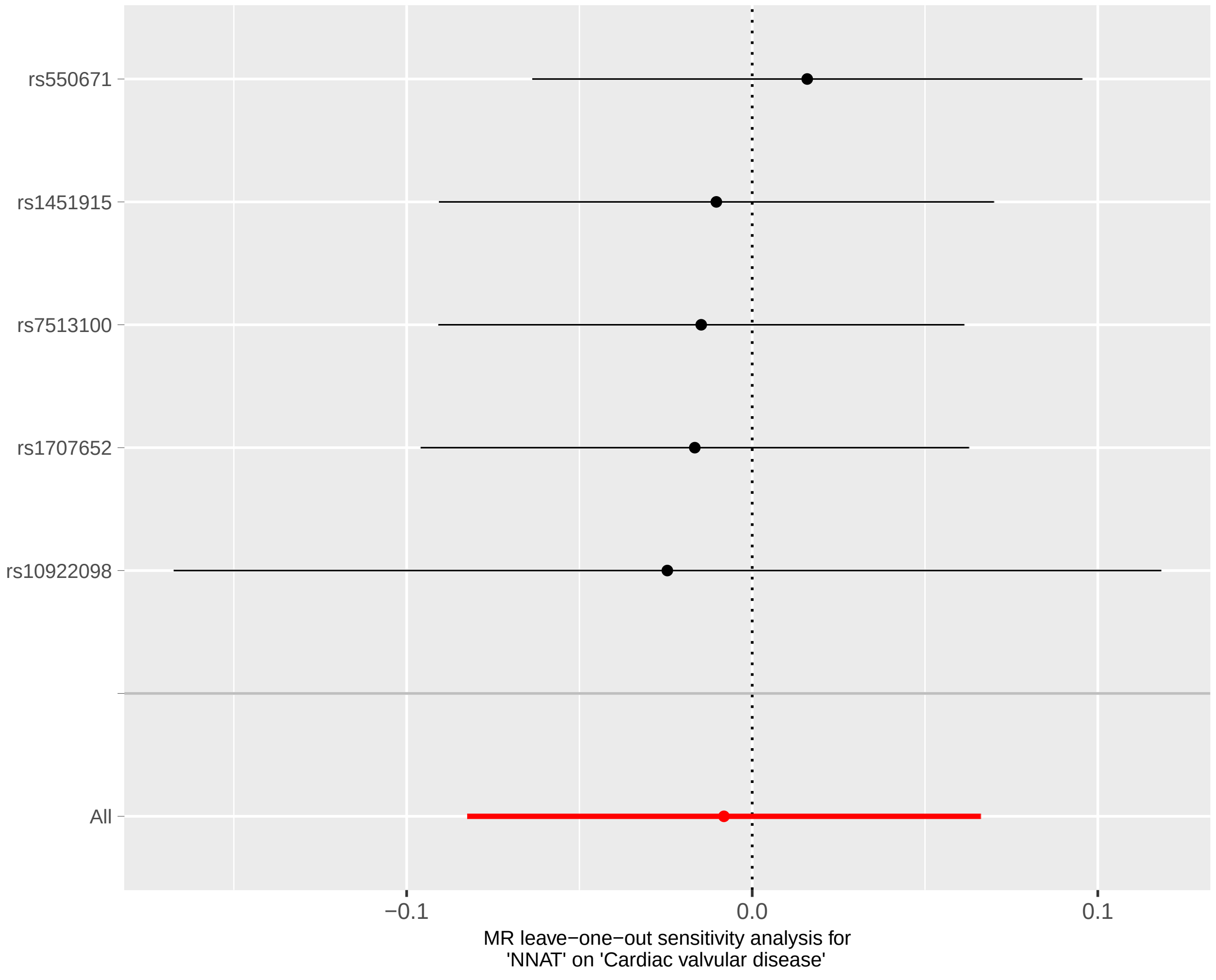
rs550671
rs1451915
rs7513100
rs1707652
rs10922098
All
−0.1
0.0
0.1
MR leave−one−out sensitivity analysis for
'NNAT' on 'Cardiac valvular disease'

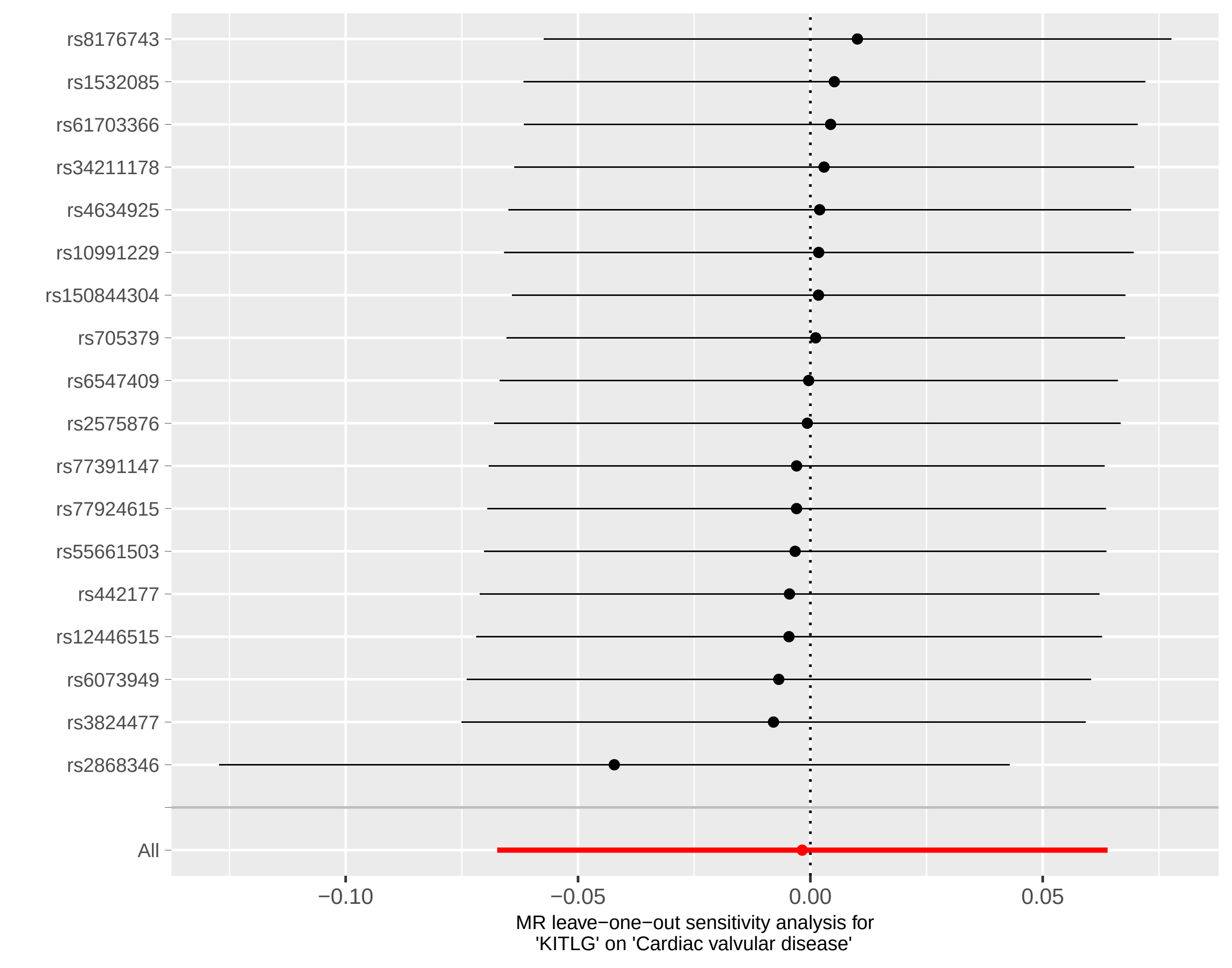
rs8176743
rs1532085
rs61703366
rs34211178
rs4634925
rs10991229
rs150844304
rs705379
rs6547409
rs2575876
rs77391147
rs77924615
rs55661503
rs442177
rs12446515
rs6073949
rs3824477
rs2868346
All
−0.10
−0.05
0.00
0.05
MR leave−one−out sensitivity analysis for
'KITLG' on 'Cardiac valvular disease'

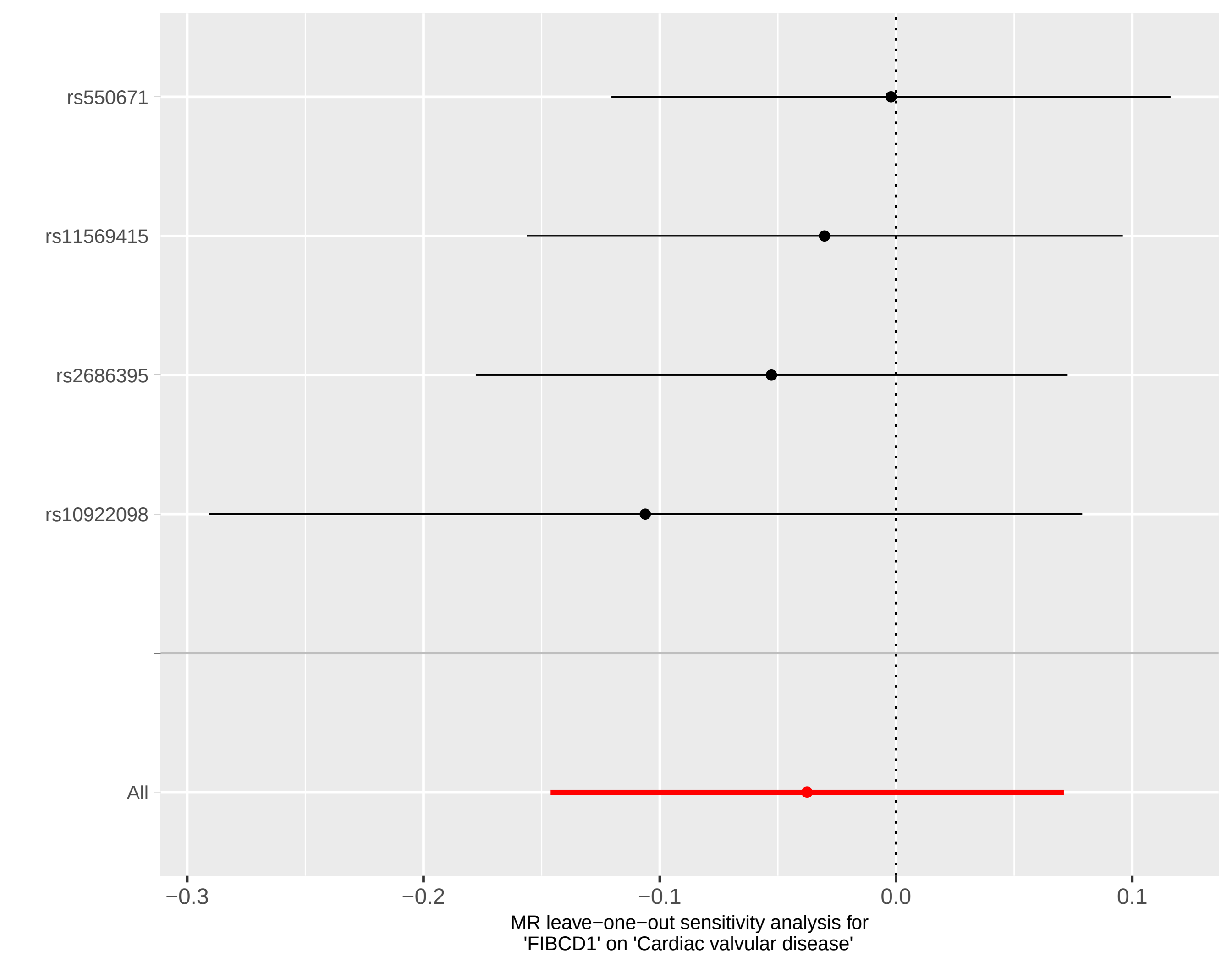
rs550671
rs11569415
rs2686395
rs10922098
All
−0.3
−0.2
−0.1
0.0
0.1
MR leave−one−out sensitivity analysis for
'FIBCD1' on 'Cardiac valvular disease'

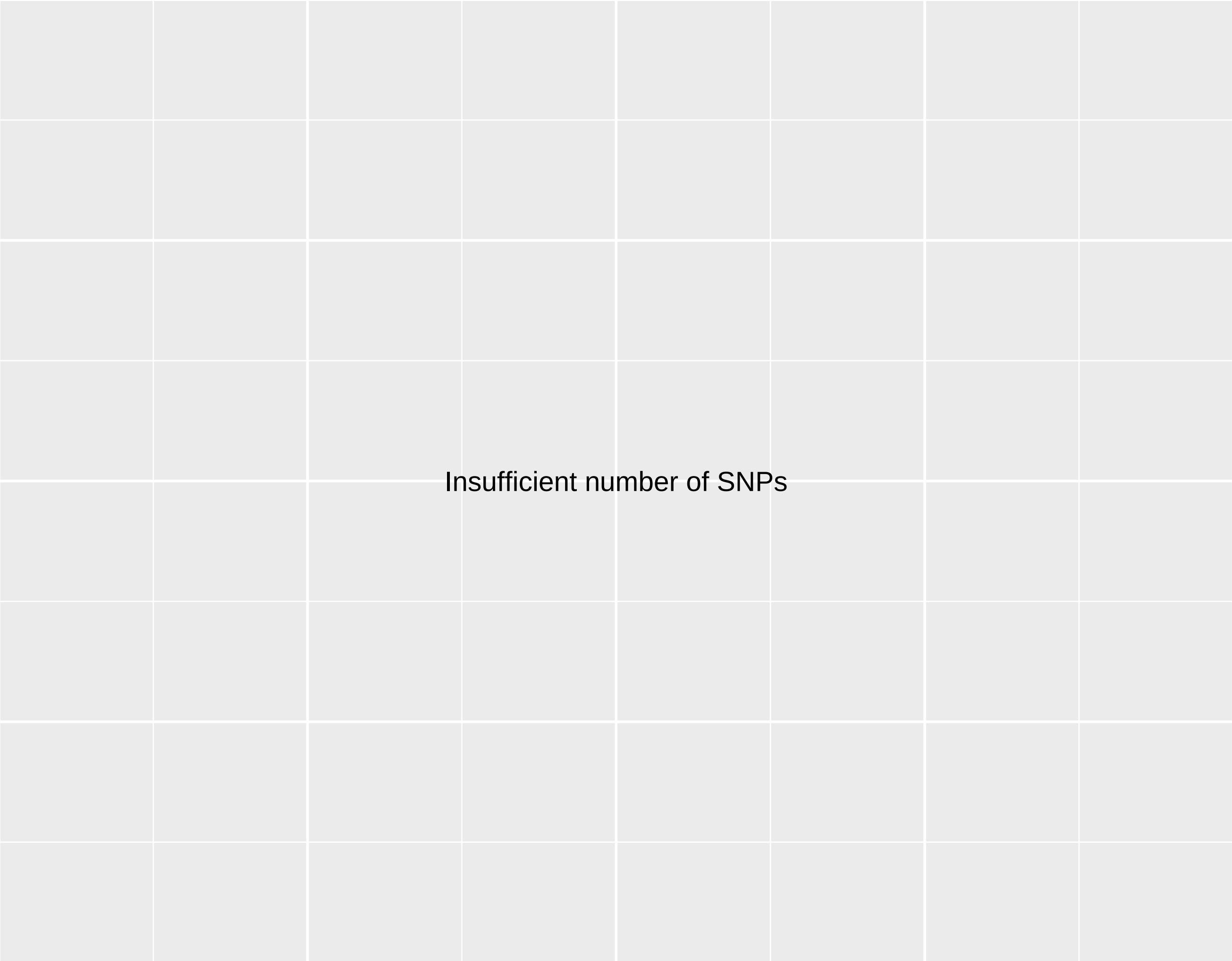
Insufficient number of SNPs

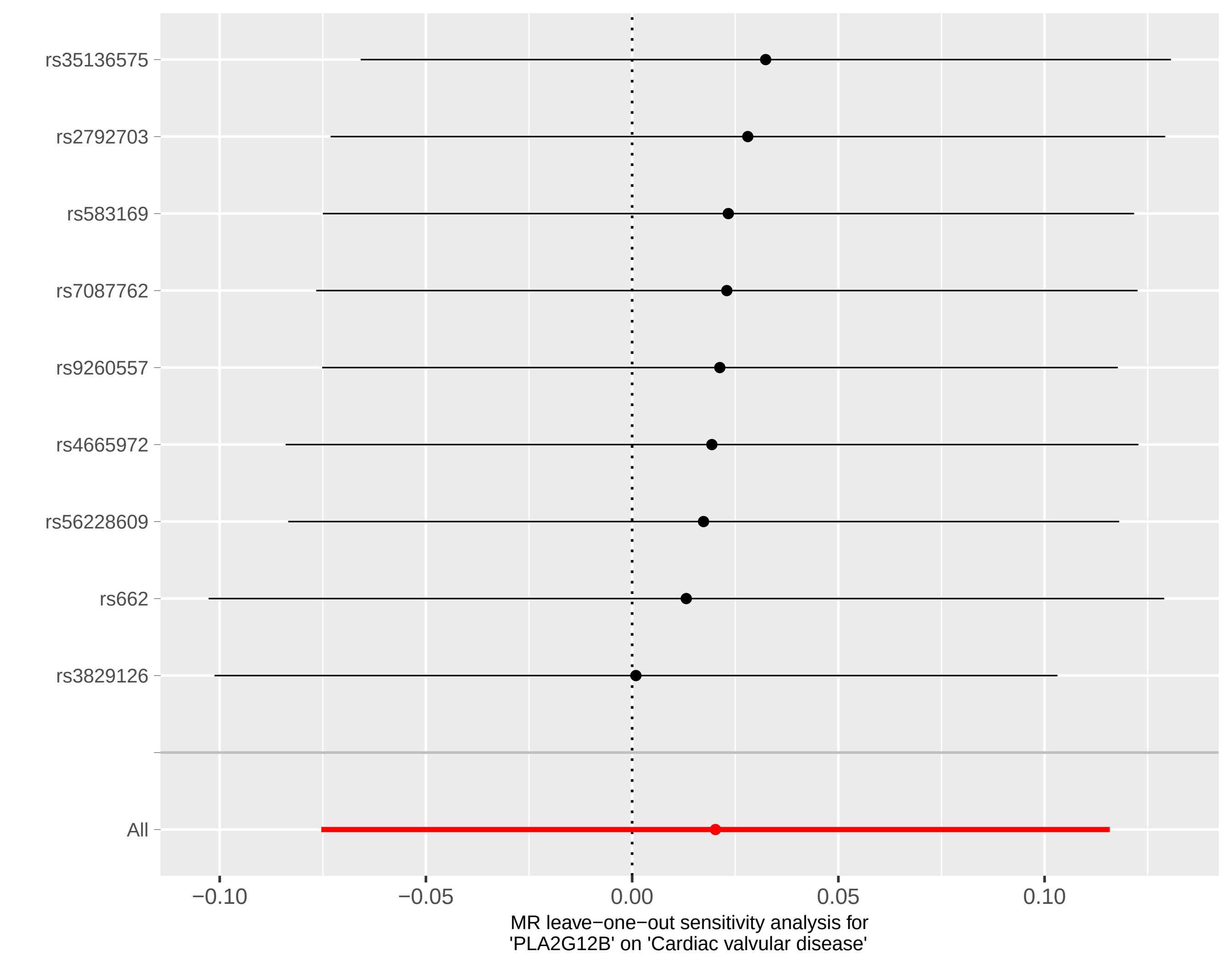

rs35136575
rs2792703
rs583169
rs7087762
rs9260557
rs4665972
rs56228609
rs662
rs3829126
All
−0.10
−0.05
0.00
0.05
0.10
MR leave−one−out sensitivity analysis for
'PLA2G12B' on 'Cardiac valvular disease'

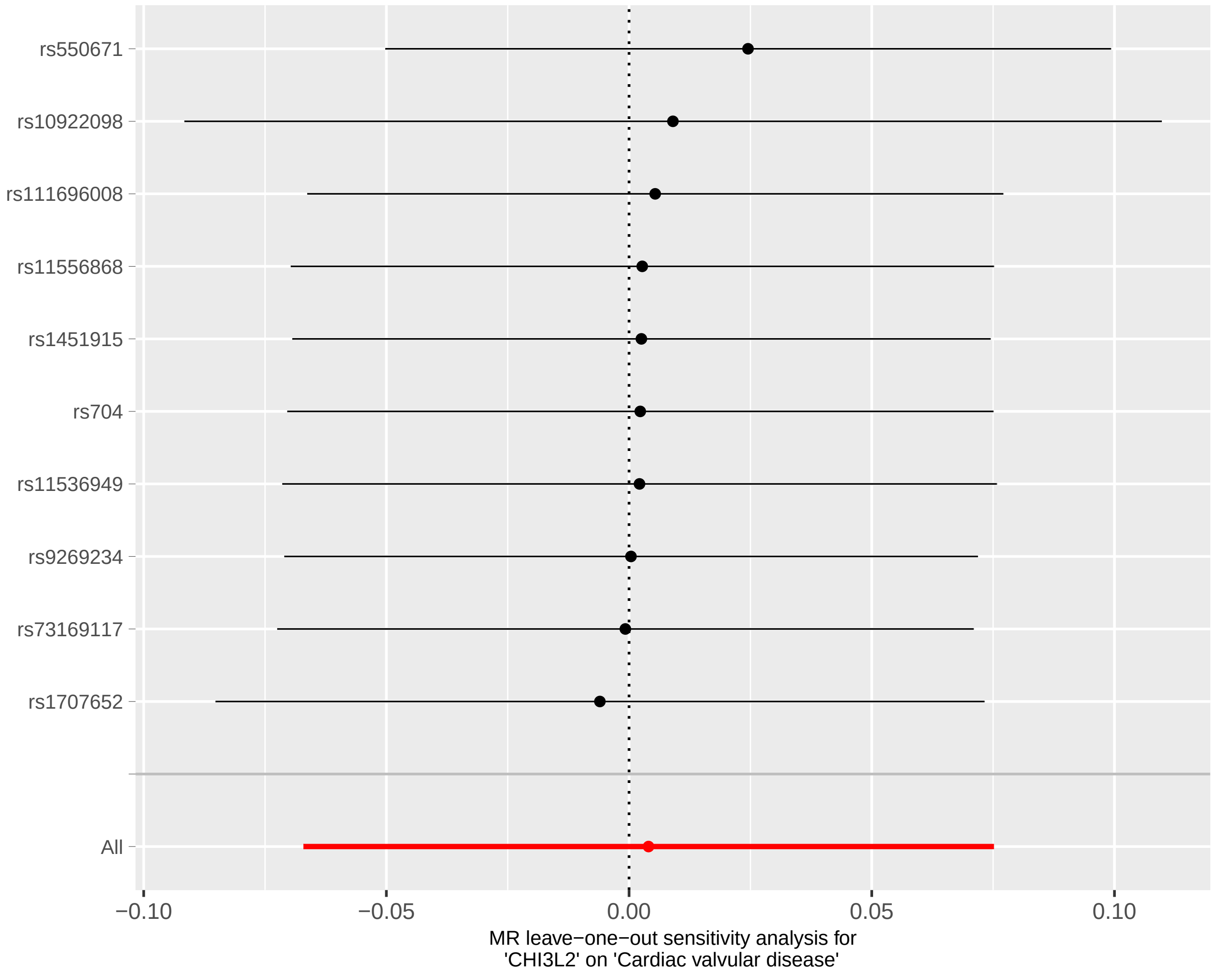
rs550671
rs10922098
rs111696008
rs11556868
rs1451915
rs704
rs11536949
rs9269234
rs73169117
rs1707652
All
−0.10
−0.05
0.00
0.05
0.10
MR leave−one−out sensitivity analysis for
'CHI3L2' on 'Cardiac valvular disease'

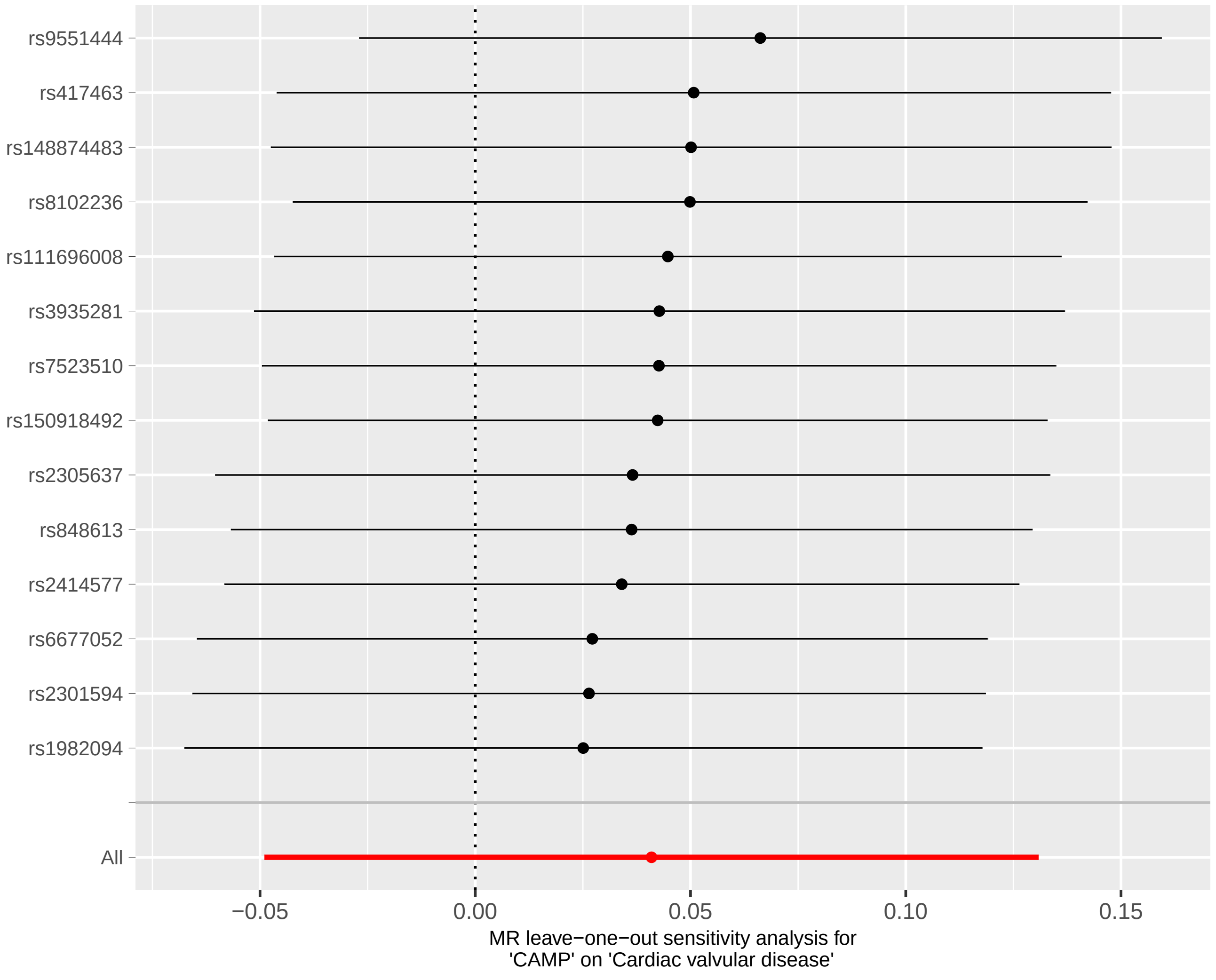

rs9551444
rs417463
rs148874483
rs8102236
rs111696008
rs3935281
rs7523510
rs150918492
rs2305637
rs848613
rs2414577
rs6677052
rs2301594
rs1982094
All
−0.05
0.00
0.05
0.10
0.15
MR leave−one−out sensitivity analysis for
'CAMP' on 'Cardiac valvular disease'

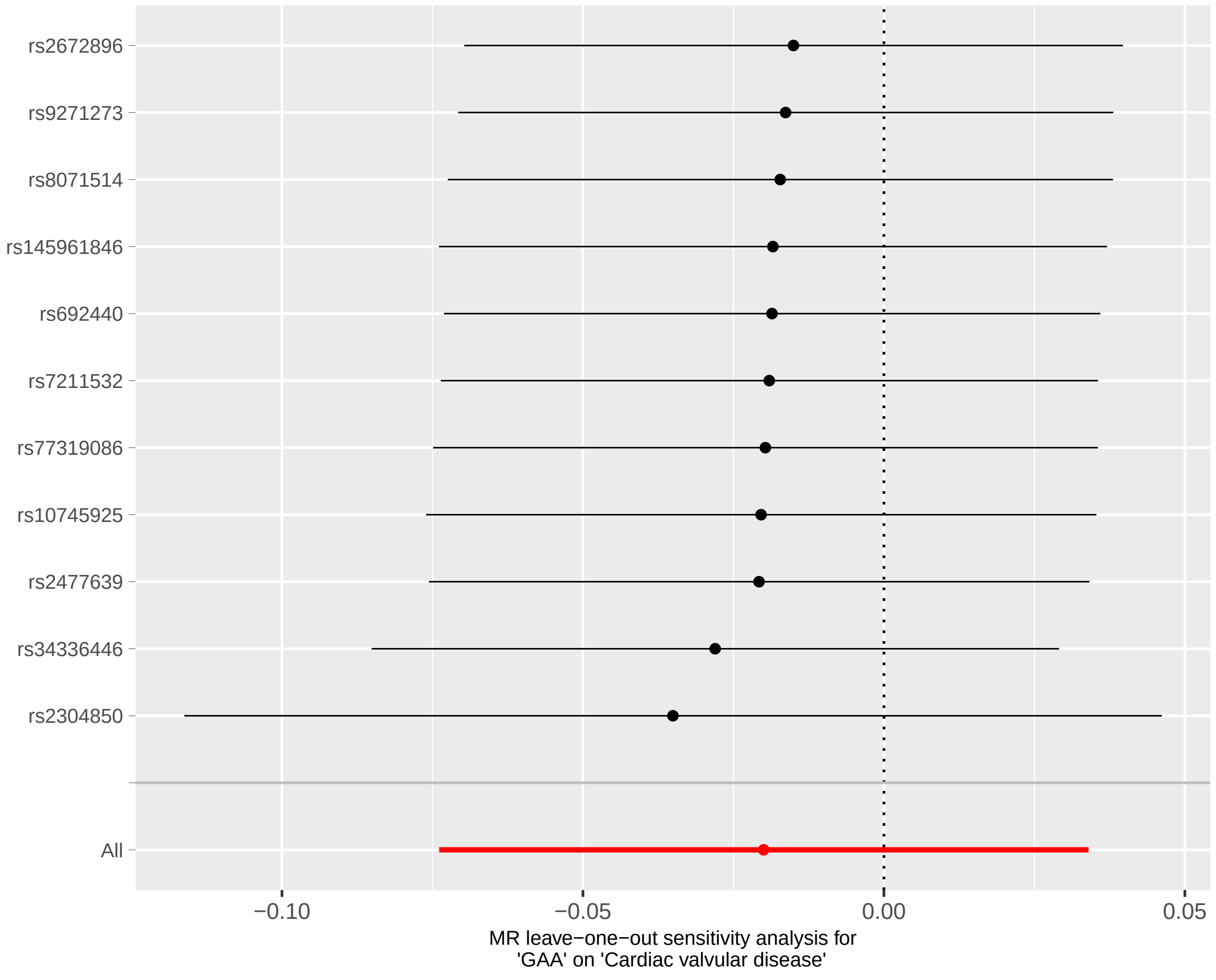
rs2672896
rs9271273
rs8071514
rs145961846
rs692440
rs7211532
rs77319086
rs10745925
rs2477639
rs34336446
rs2304850
All
−0.10
−0.05
0.00
0.05
MR leave−one−out sensitivity analysis for
'GAA' on 'Cardiac valvular disease'

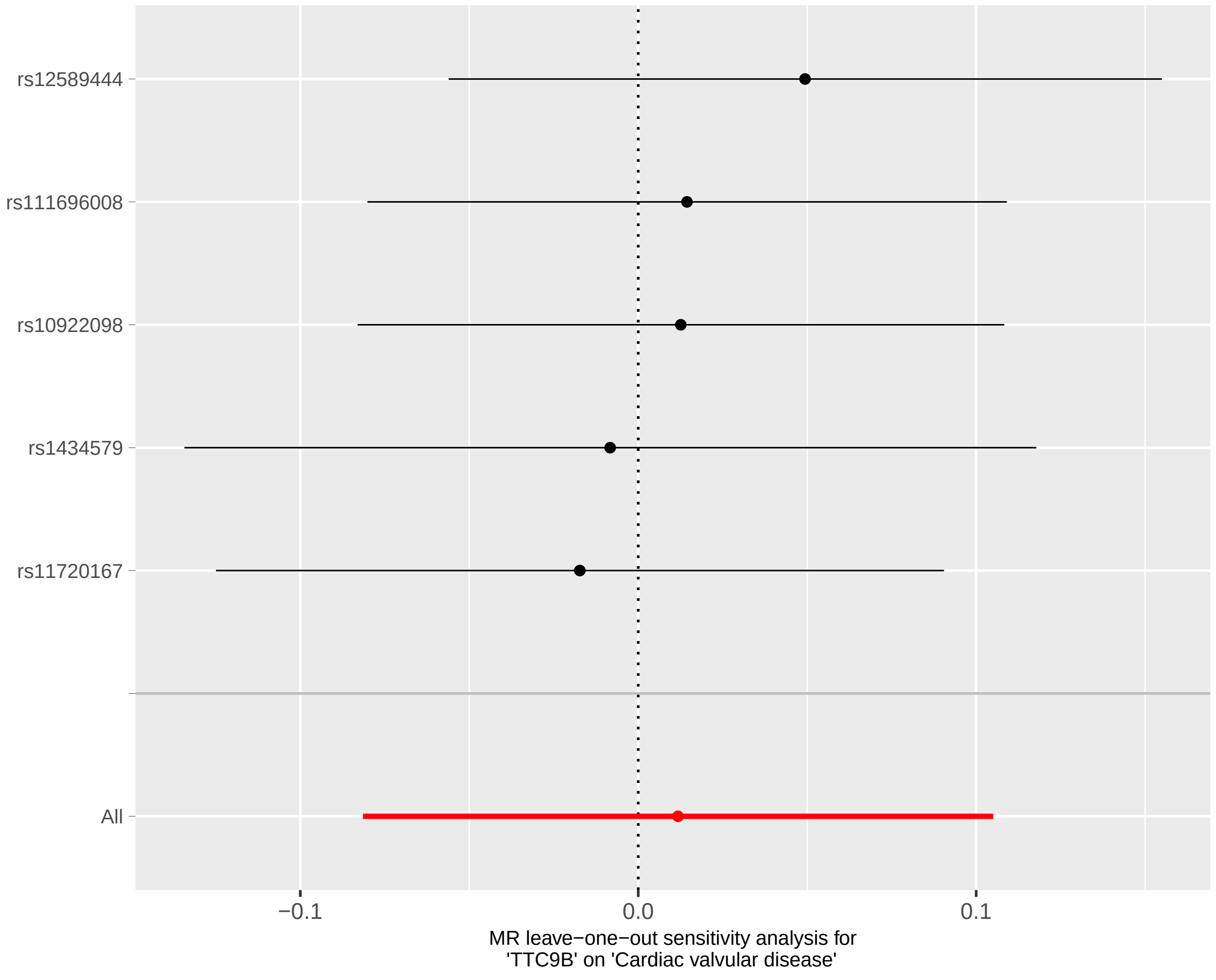

rs12589444
rs111696008
rs10922098
rs1434579
rs11720167
All
−0.1
0.0
0.1
MR leave−one−out sensitivity analysis for
'TTC9B' on 'Cardiac valvular disease'

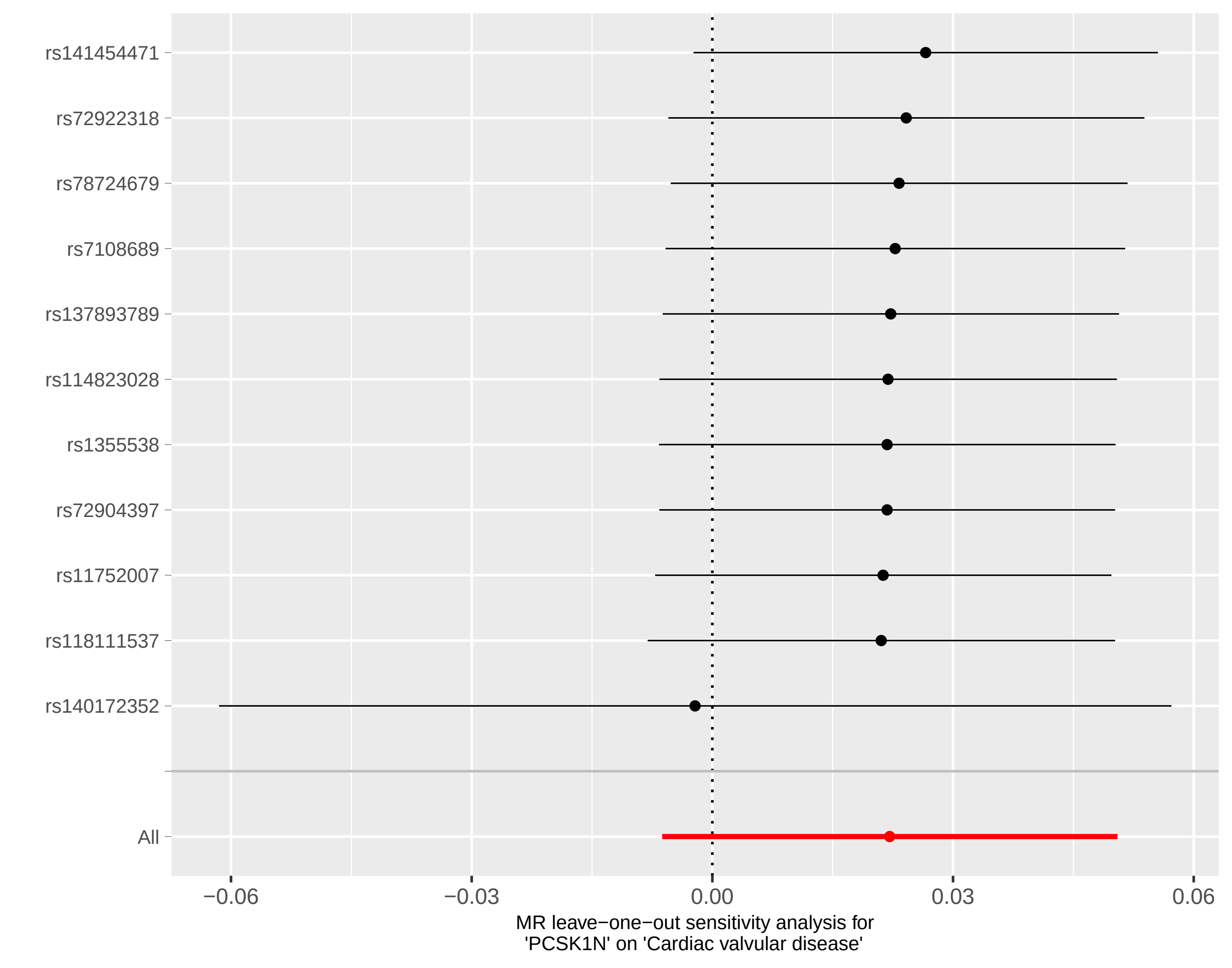
rs141454471
rs72922318
rs78724679
rs7108689
rs137893789
rs114823028
rs1355538
rs72904397
rs11752007
rs118111537
rs140172352
All
−0.06
−0.03
0.00
0.03
0.06
MR leave−one−out sensitivity analysis for
'PCSK1N' on 'Cardiac valvular disease'

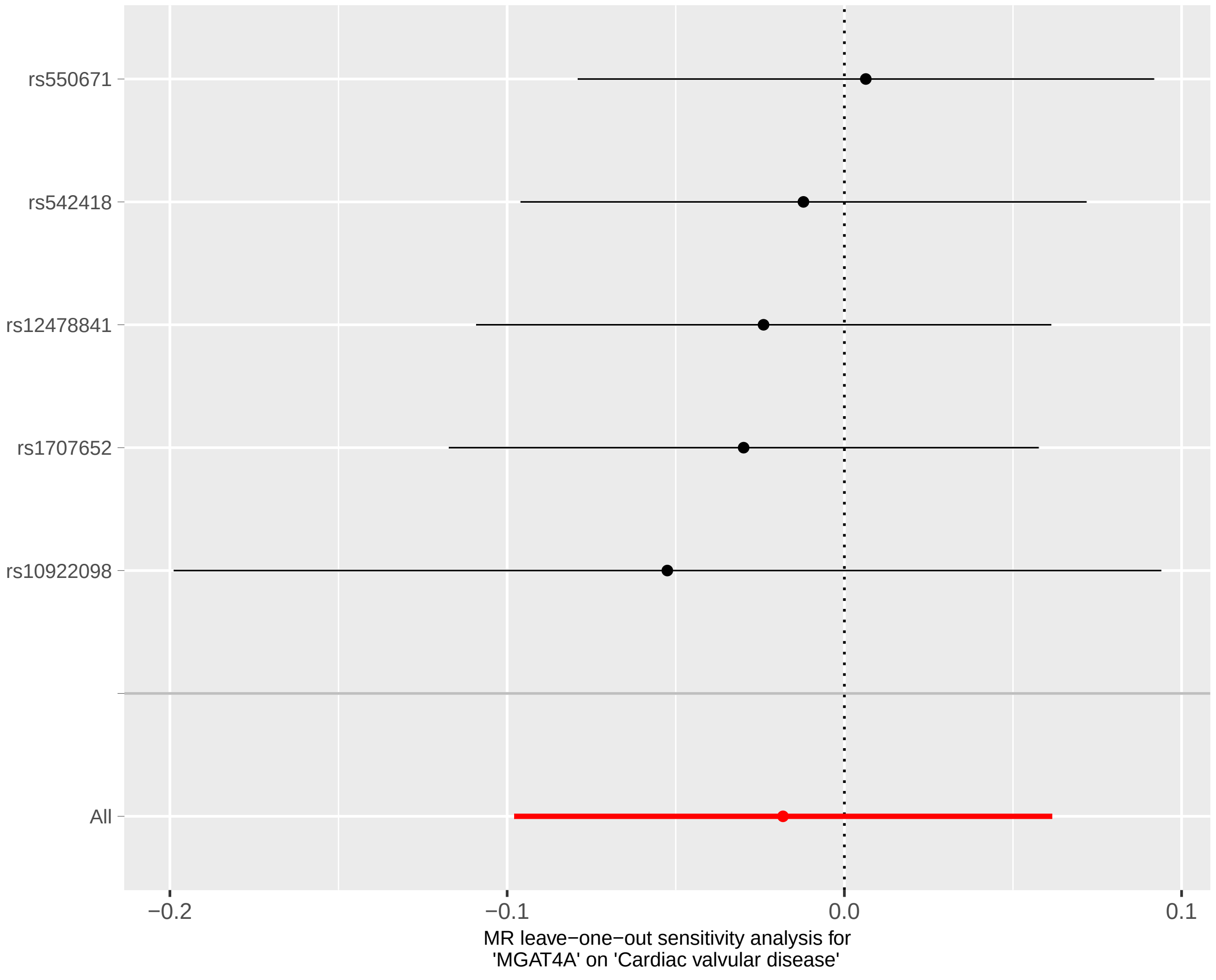
rs550671
rs542418
rs12478841
rs1707652
rs10922098
All
−0.2
−0.1
0.0
0.1
MR leave−one−out sensitivity analysis for
'MGAT4A' on 'Cardiac valvular disease'

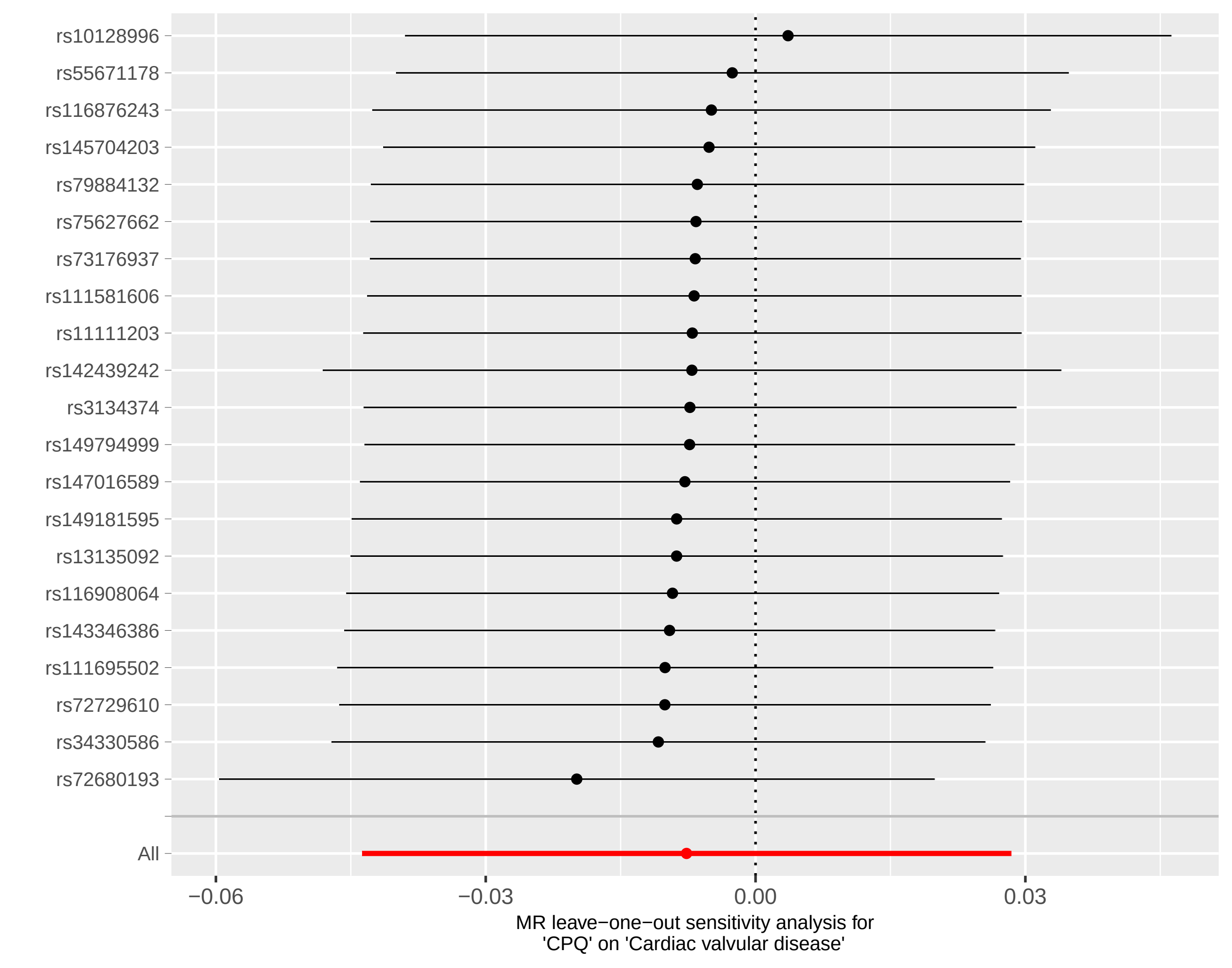

rs10128996
rs55671178
rs116876243
rs145704203
rs79884132
rs75627662
rs73176937
rs111581606
rs11111203
rs142439242
rs3134374
rs149794999
rs147016589
rs149181595
rs13135092
rs116908064
rs143346386
rs111695502
rs72729610
rs34330586
rs72680193
All
−0.06
−0.03
0.00
0.03
MR leave−one−out sensitivity analysis for
'CPQ' on 'Cardiac valvular disease'

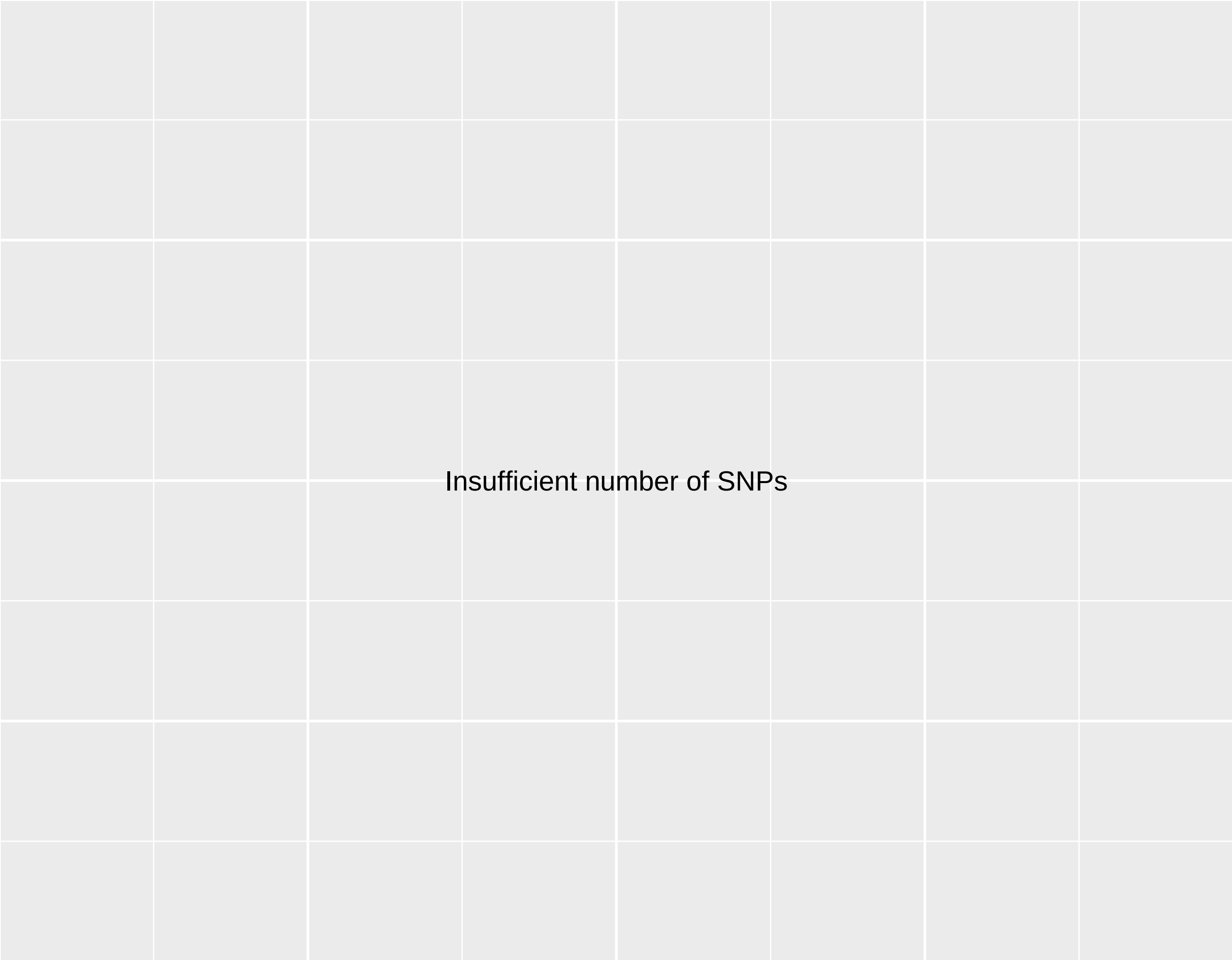
Insufficient number of SNPs

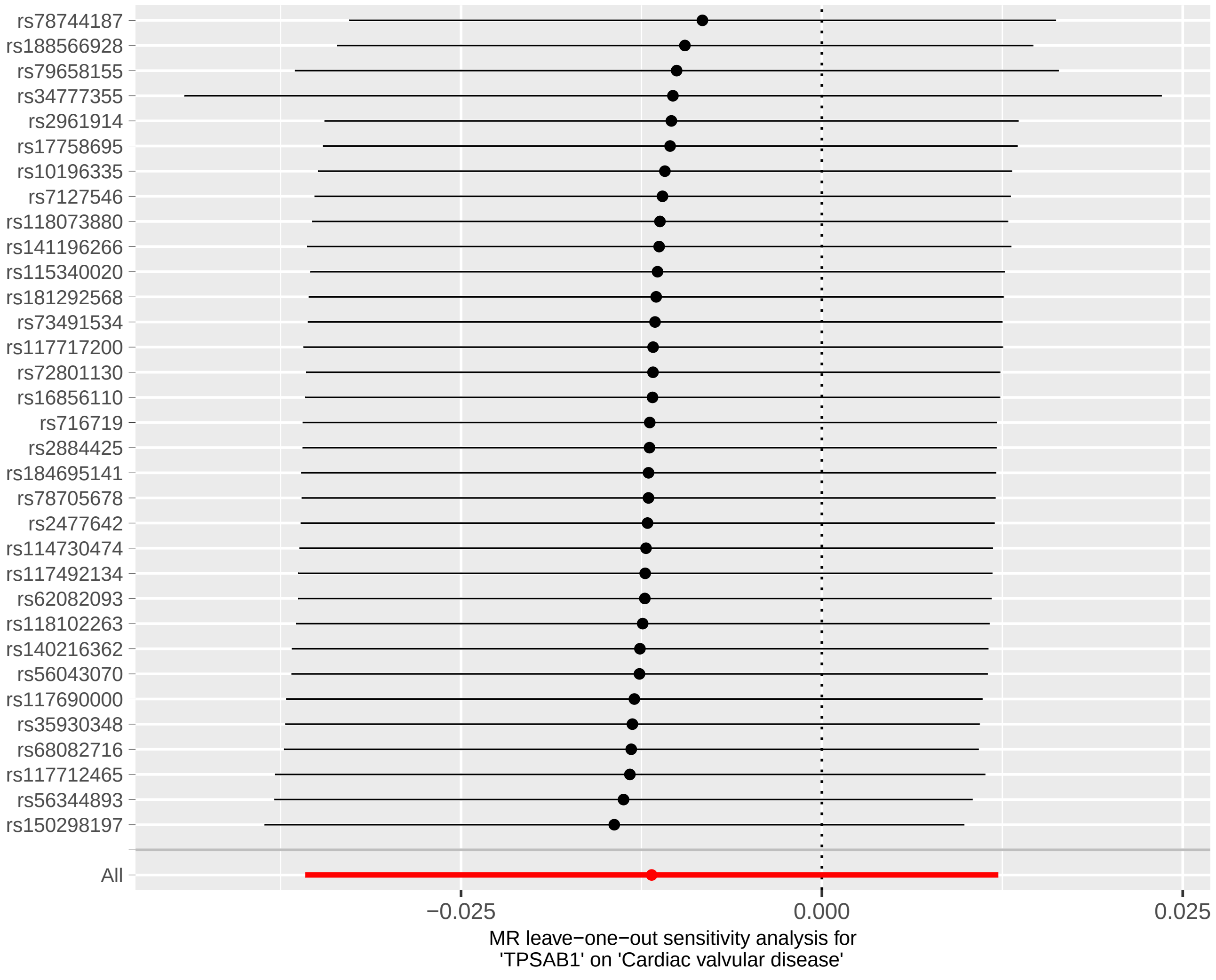
rs78744187
rs188566928
rs79658155
rs34777355
rs2961914
rs17758695
rs10196335
rs7127546
rs118073880
rs141196266
rs115340020
rs181292568
rs73491534
rs117717200
rs72801130
rs16856110
rs716719
rs2884425
rs184695141
rs78705678
rs2477642
rs114730474
rs117492134
rs62082093
rs118102263
rs140216362
rs56043070
rs117690000
rs35930348
rs68082716
rs117712465
rs56344893
rs150298197
All
−0.025
0.000
0.025
MR leave−one−out sensitivity analysis for
'TPSAB1' on 'Cardiac valvular disease'

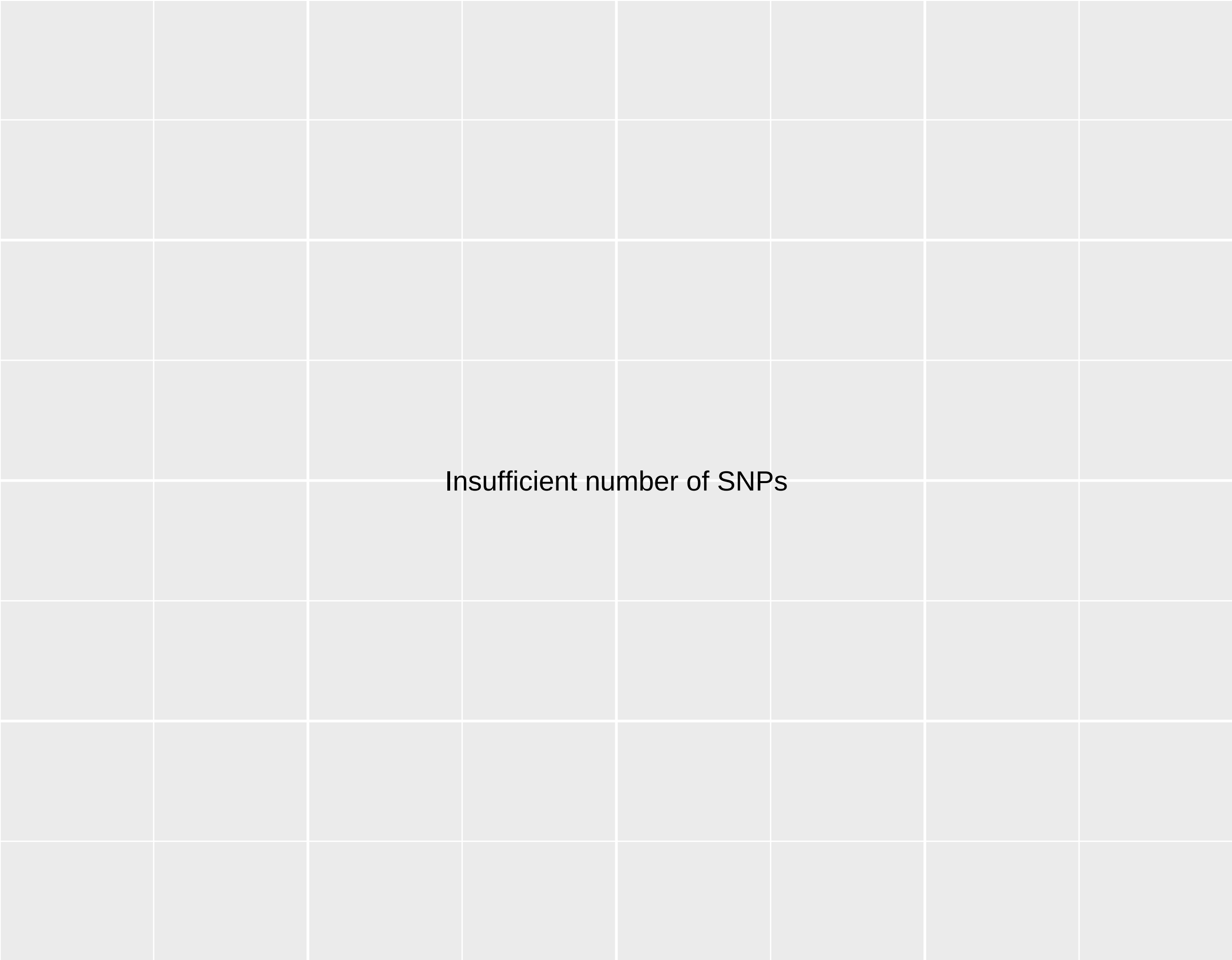
Insufficient number of SNPs

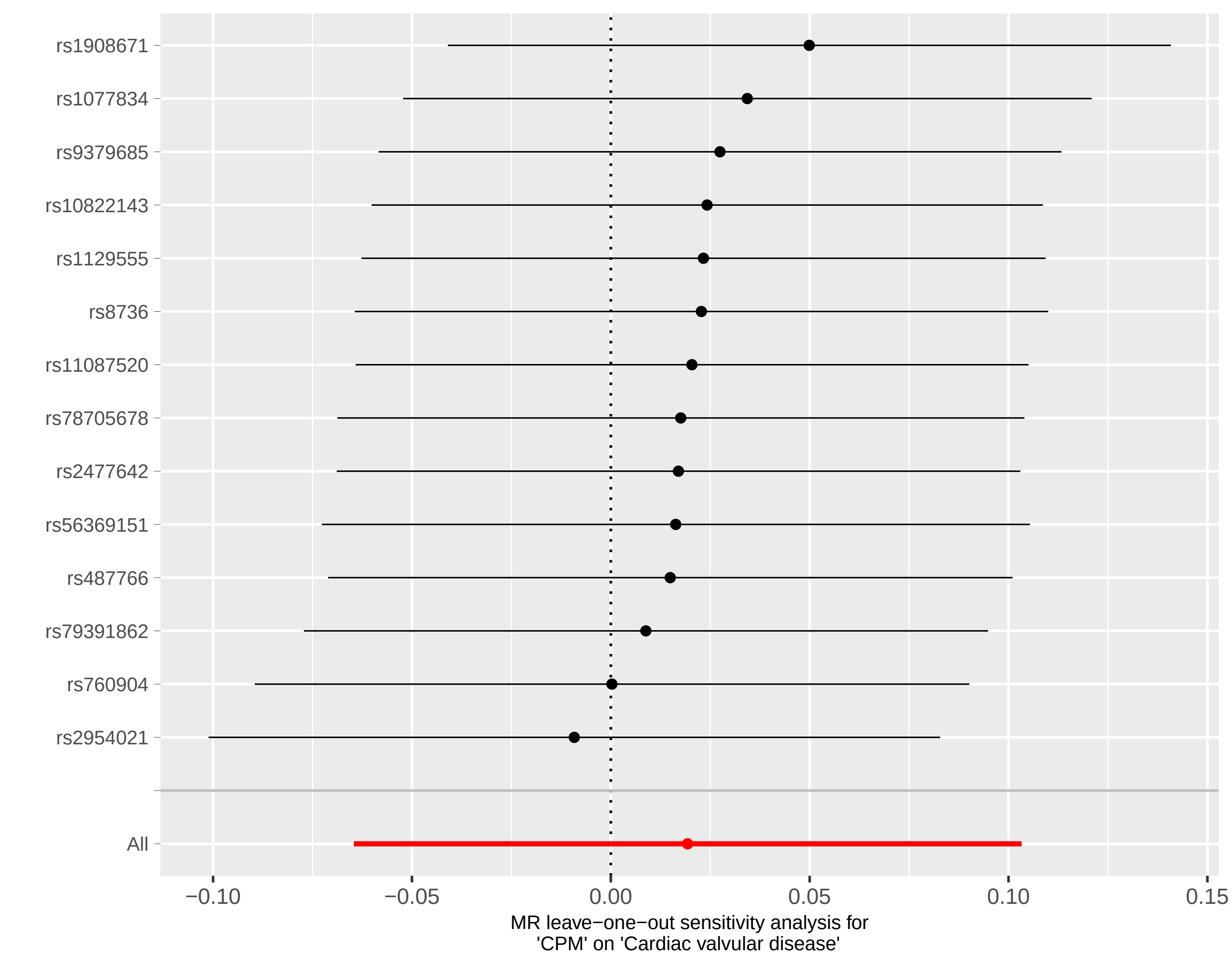
rs1908671
rs1077834
rs9379685
rs10822143
rs1129555
rs8736
rs11087520
rs78705678
rs2477642
rs56369151
rs487766
rs79391862
rs760904
rs2954021
All
−0.10
−0.05
0.00
0.05
0.10
0.15
MR leave−one−out sensitivity analysis for
'CPM' on 'Cardiac valvular disease'

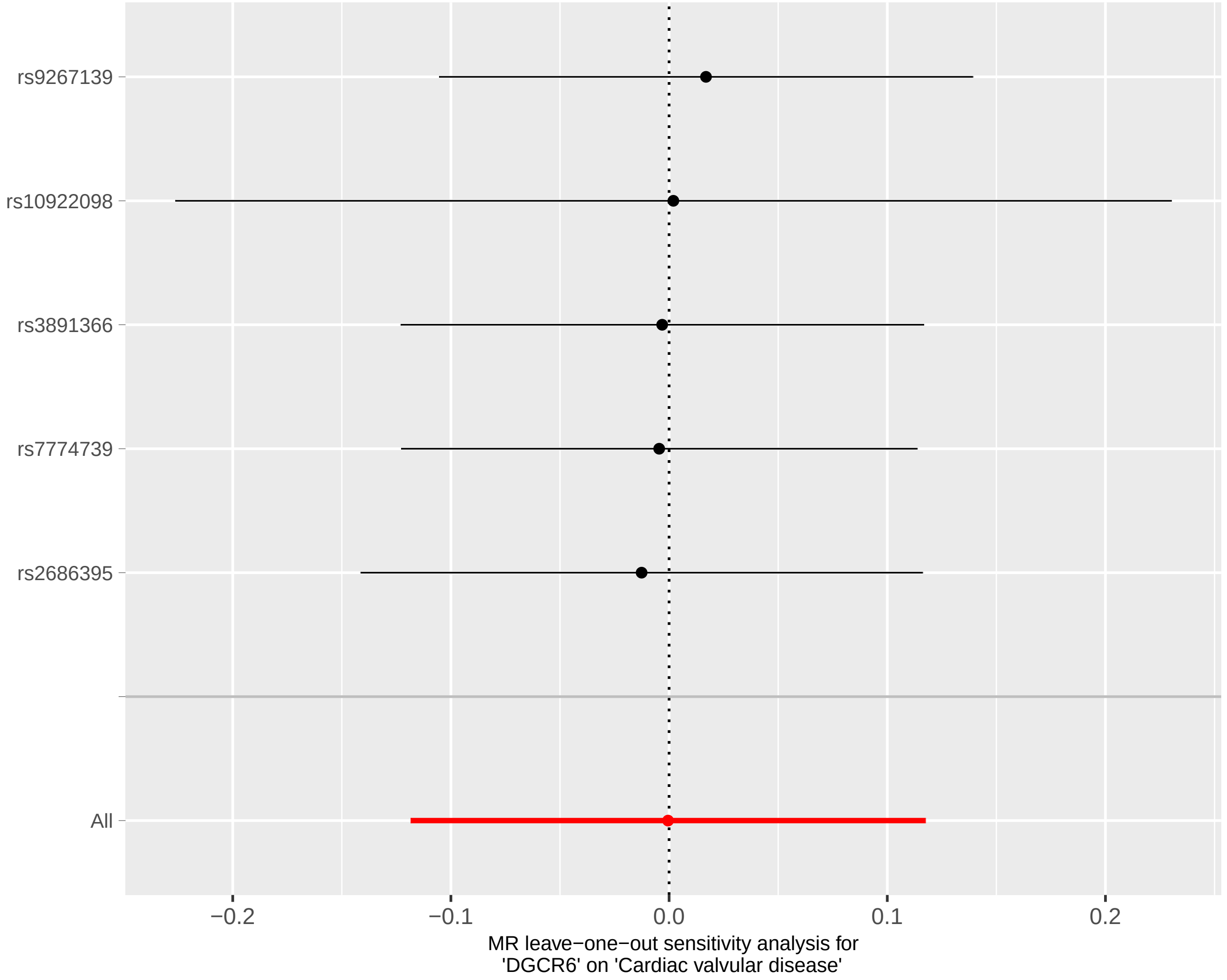

rs9267139
rs10922098
rs3891366
rs7774739
rs2686395
All
−0.2
−0.1
0.0
0.1
0.2
MR leave−one−out sensitivity analysis for
'DGCR6' on 'Cardiac valvular disease'

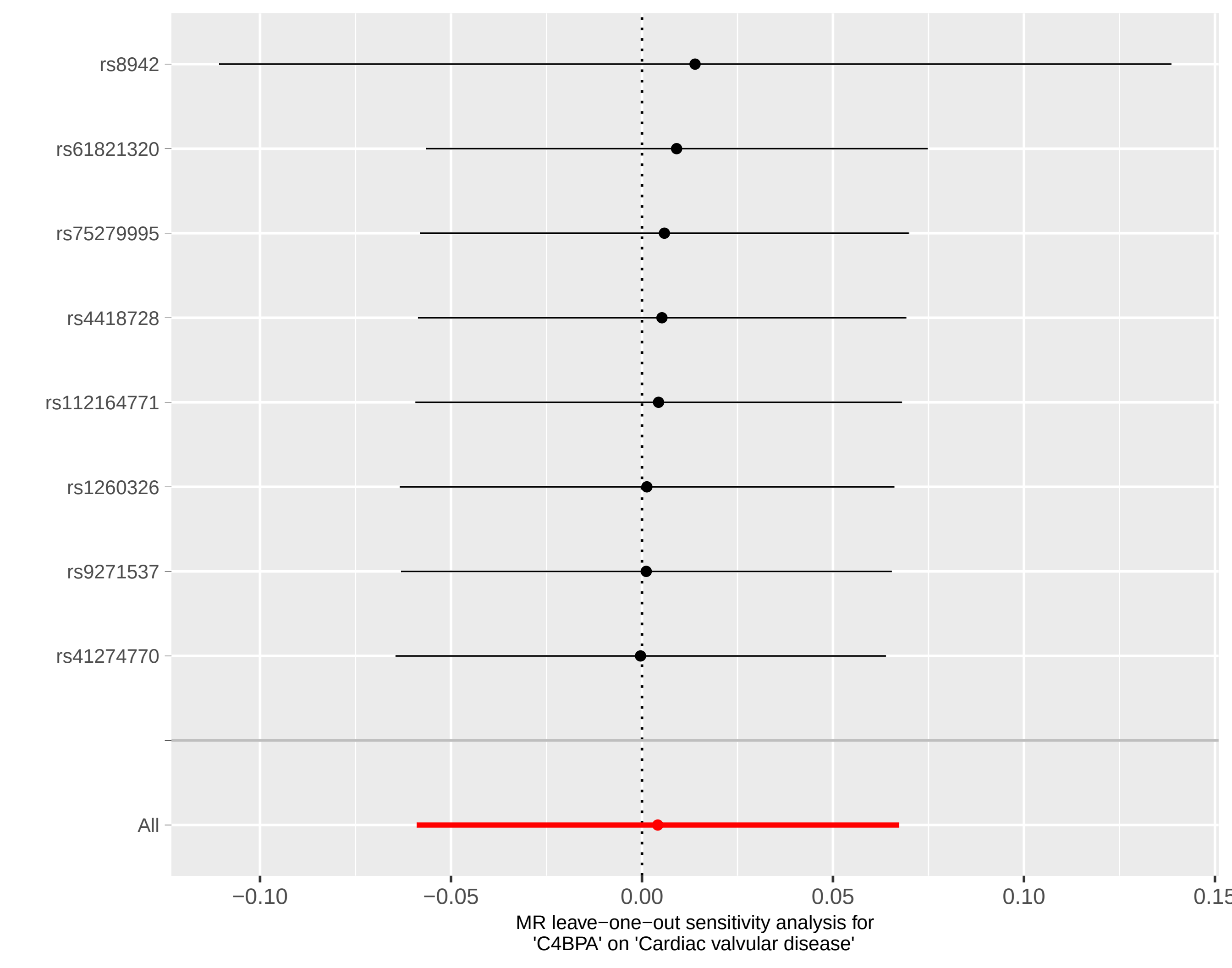

rs8942
rs61821320
rs75279995
rs4418728
rs112164771
rs1260326
rs9271537
rs41274770
All
−0.10
−0.05
0.00
0.05
0.10
0.15
MR leave−one−out sensitivity analysis for
'C4BPA' on 'Cardiac valvular disease'

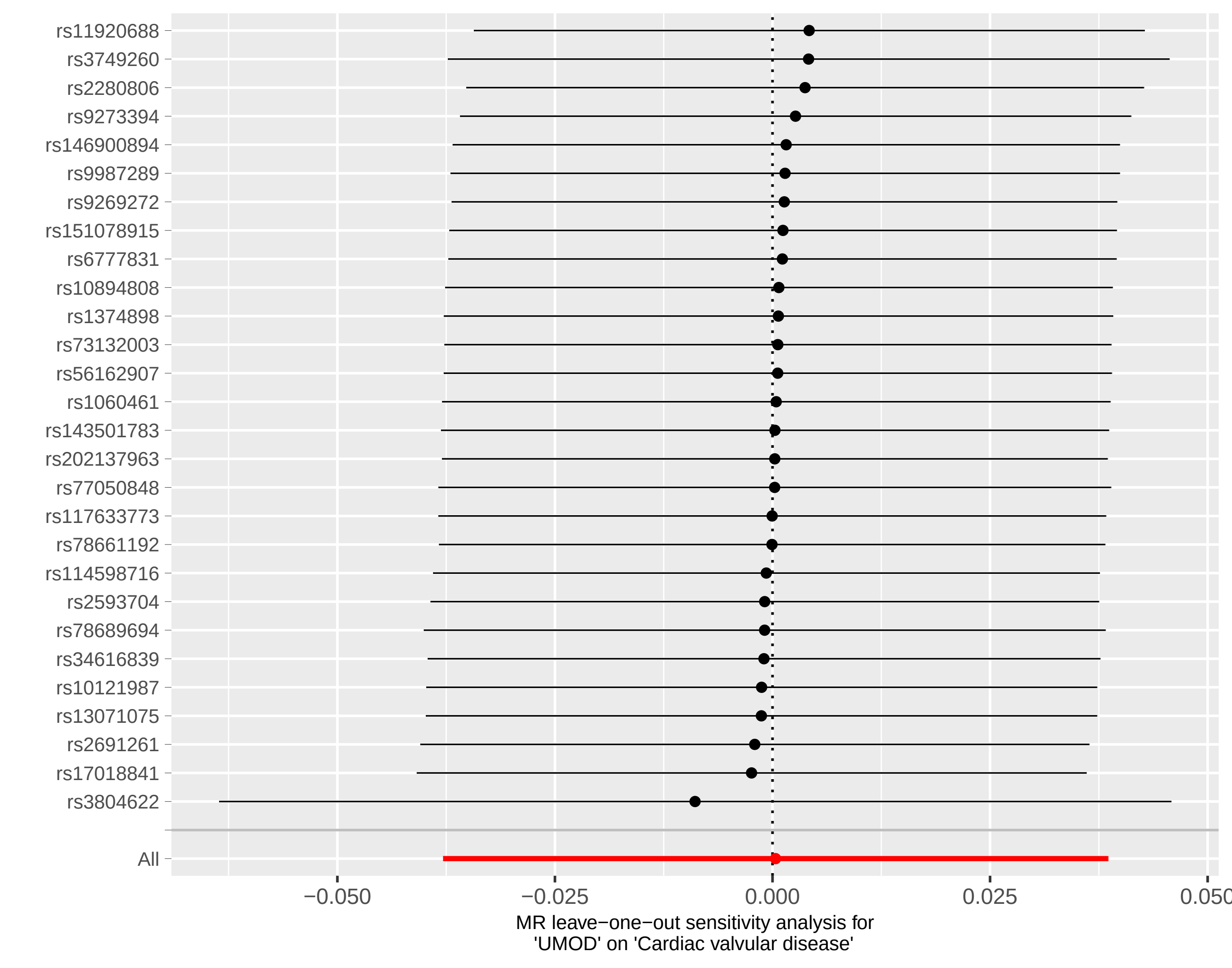
rs11920688
rs3749260
rs2280806
rs9273394
rs146900894
rs9987289
rs9269272
rs151078915
rs6777831
rs10894808
rs1374898
rs73132003
rs56162907
rs1060461
rs143501783
rs202137963
rs77050848
rs117633773
rs78661192
rs114598716
rs2593704
rs78689694
rs34616839
rs10121987
rs13071075
rs2691261
rs17018841
rs3804622
All
−0.050
−0.025
0.000
0.025
0.050
MR leave−one−out sensitivity analysis for
'UMOD' on 'Cardiac valvular disease'

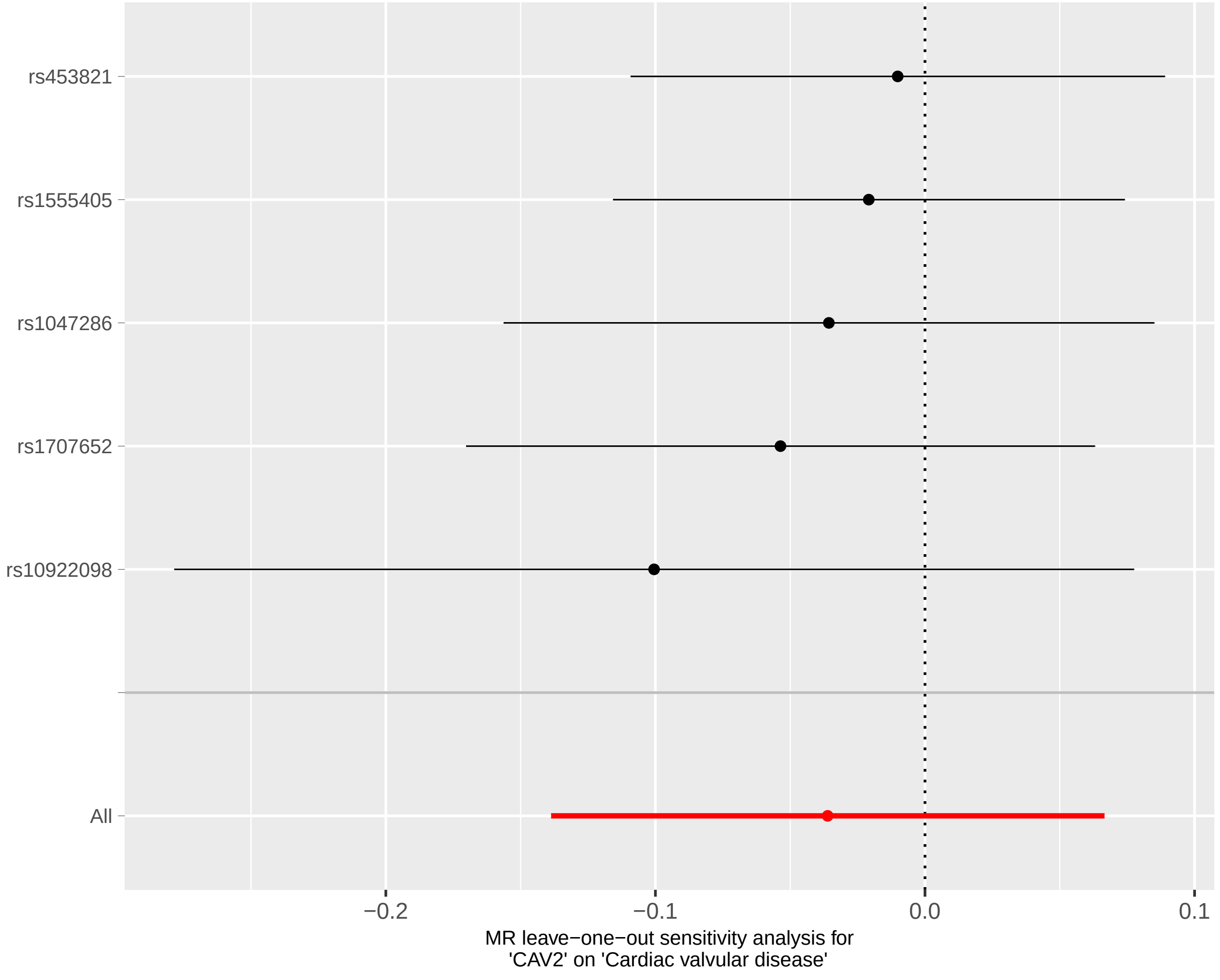

rs453821
rs1555405
rs1047286
rs1707652
rs10922098
All
−0.2
−0.1
0.0
0.1
MR leave−one−out sensitivity analysis for
'CAV2' on 'Cardiac valvular disease'

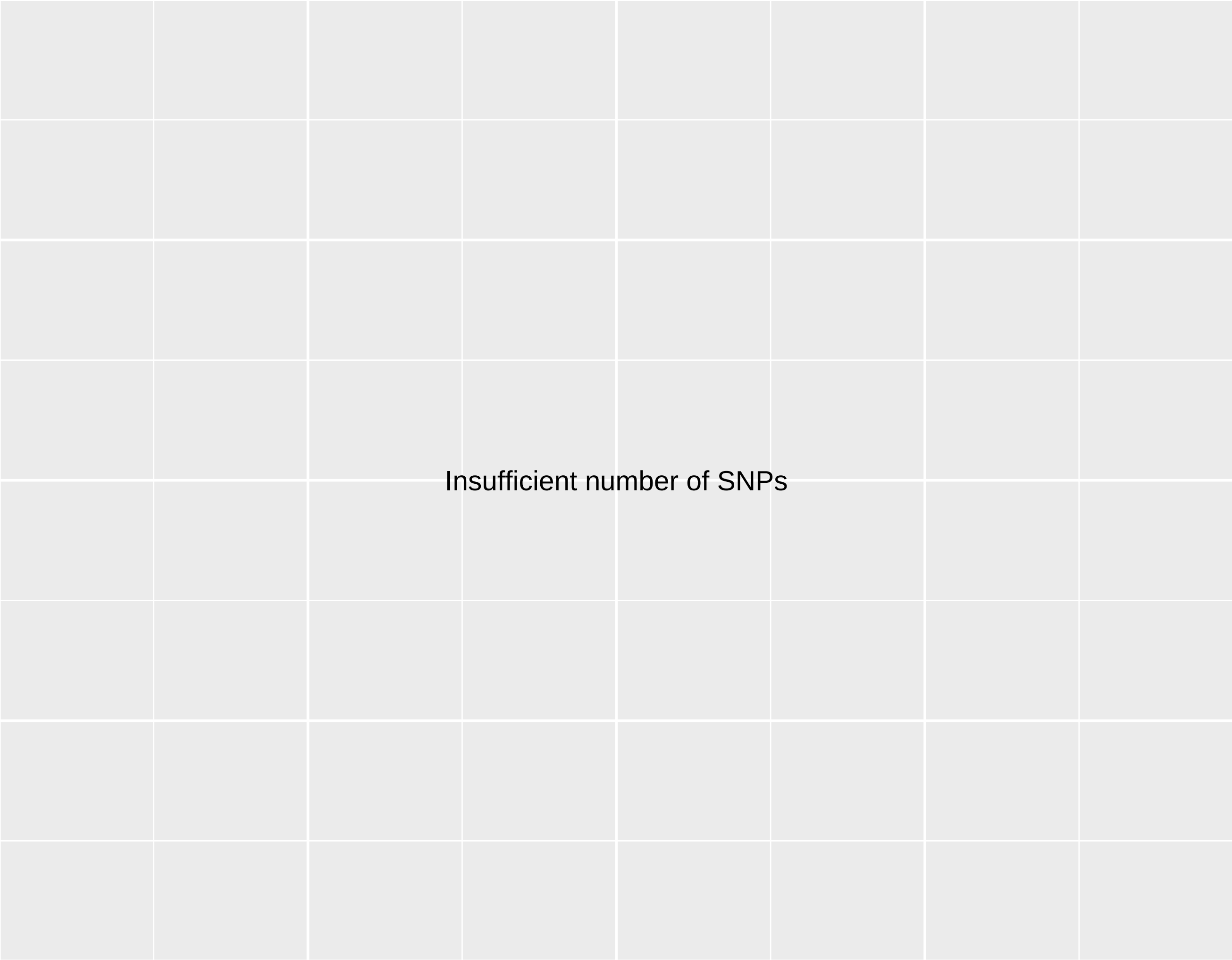
Insufficient number of SNPs

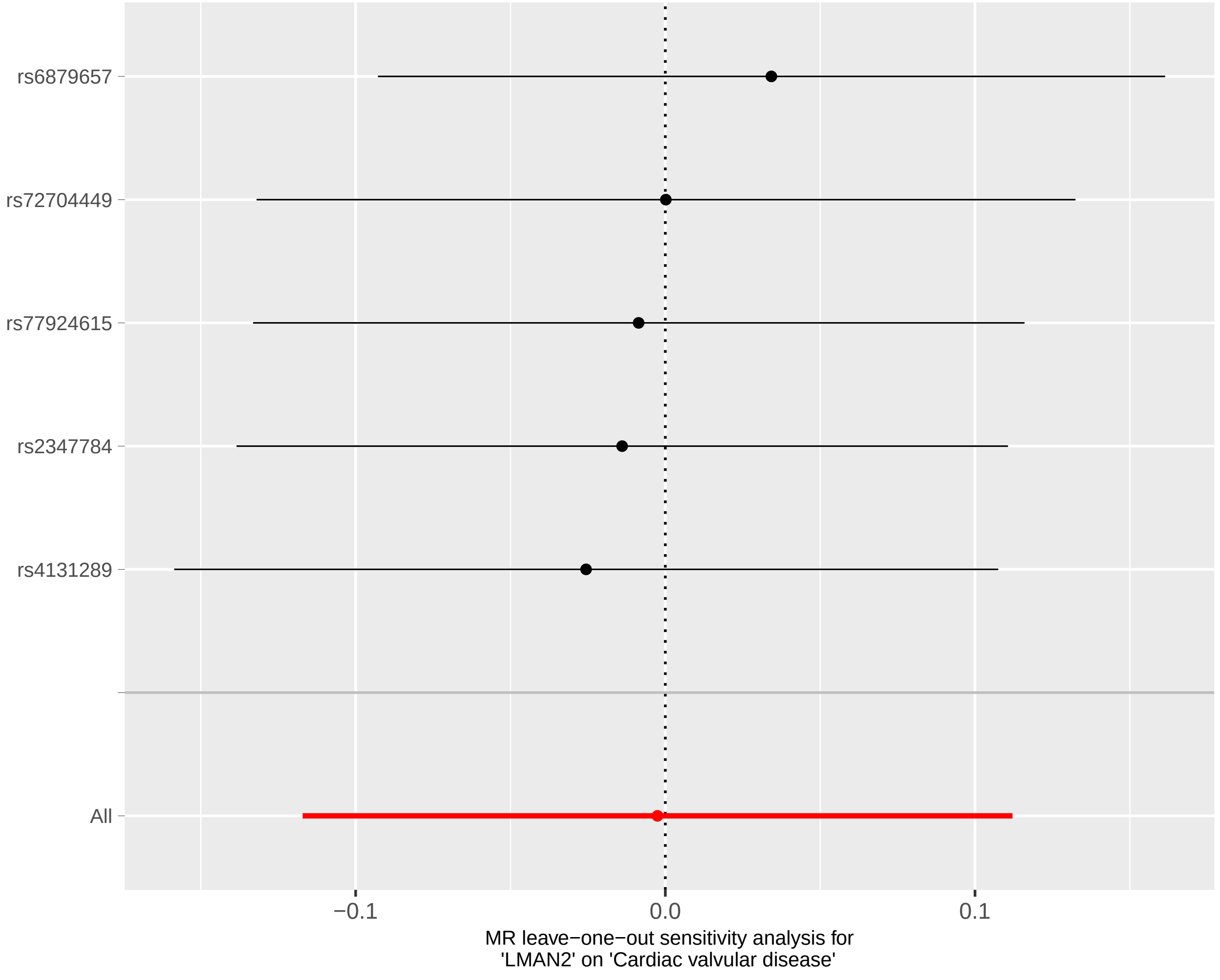
rs6879657
rs72704449
rs77924615
rs2347784
rs4131289
All
−0.1
0.0
0.1
MR leave−one−out sensitivity analysis for
'LMAN2' on 'Cardiac valvular disease'

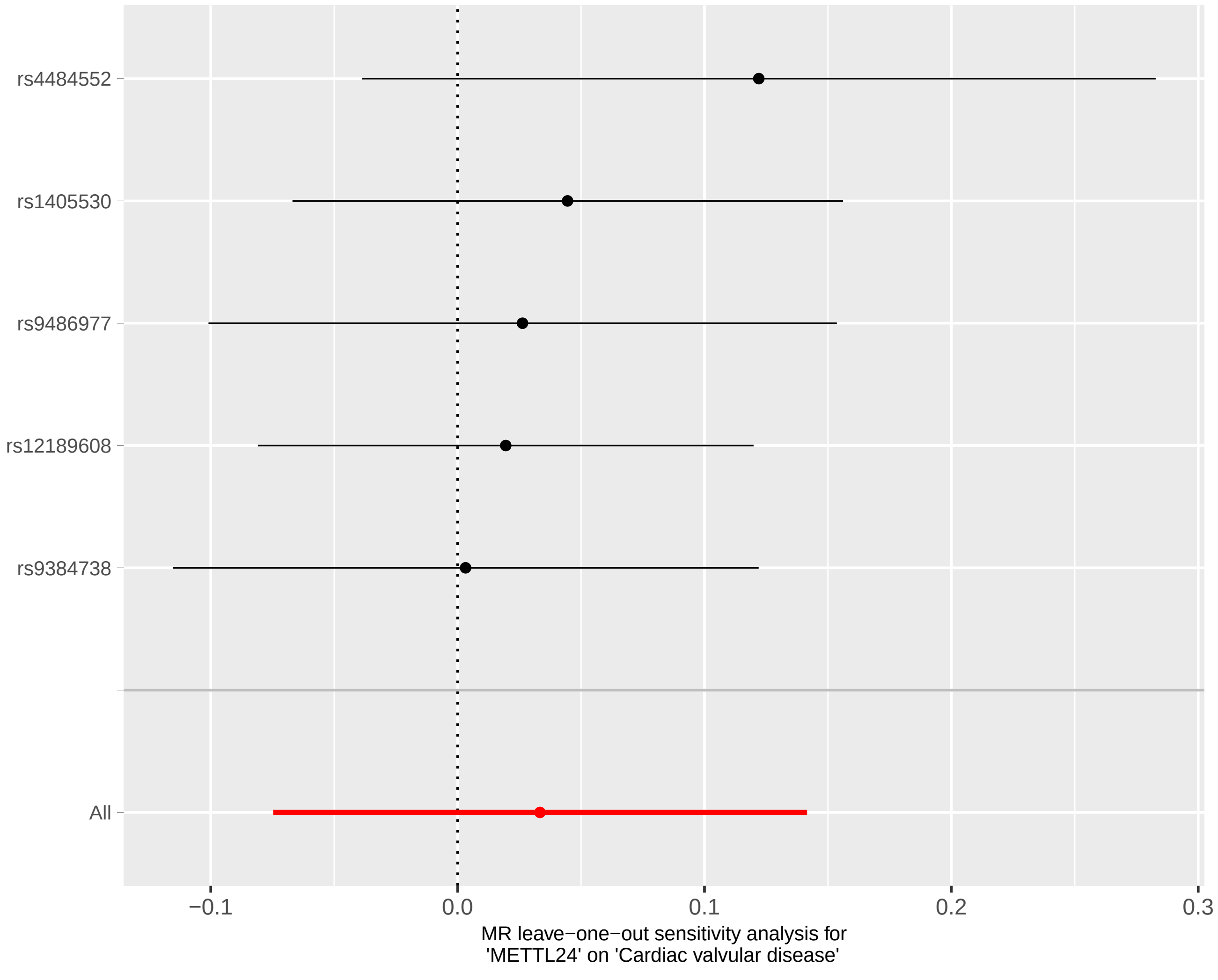

rs4484552
rs1405530
rs9486977
rs12189608
rs9384738
All
−0.1
0.0
0.1
0.2
0.3
MR leave−one−out sensitivity analysis for
'METTL24' on 'Cardiac valvular disease'

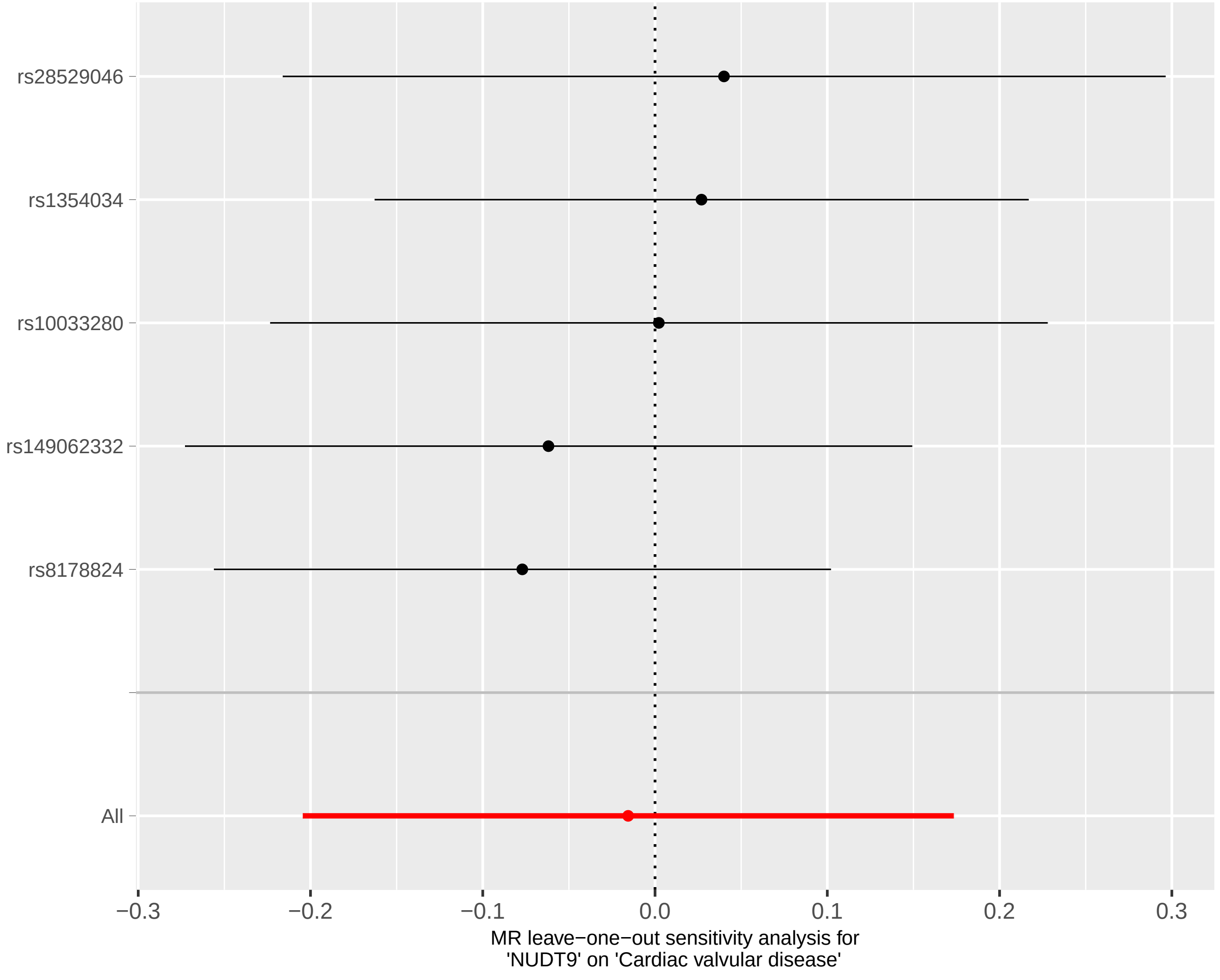

rs28529046
rs1354034
rs10033280
rs149062332
rs8178824
All
−0.3
−0.2
−0.1
0.0
0.1
0.2
0.3
MR leave−one−out sensitivity analysis for
'NUDT9' on 'Cardiac valvular disease'

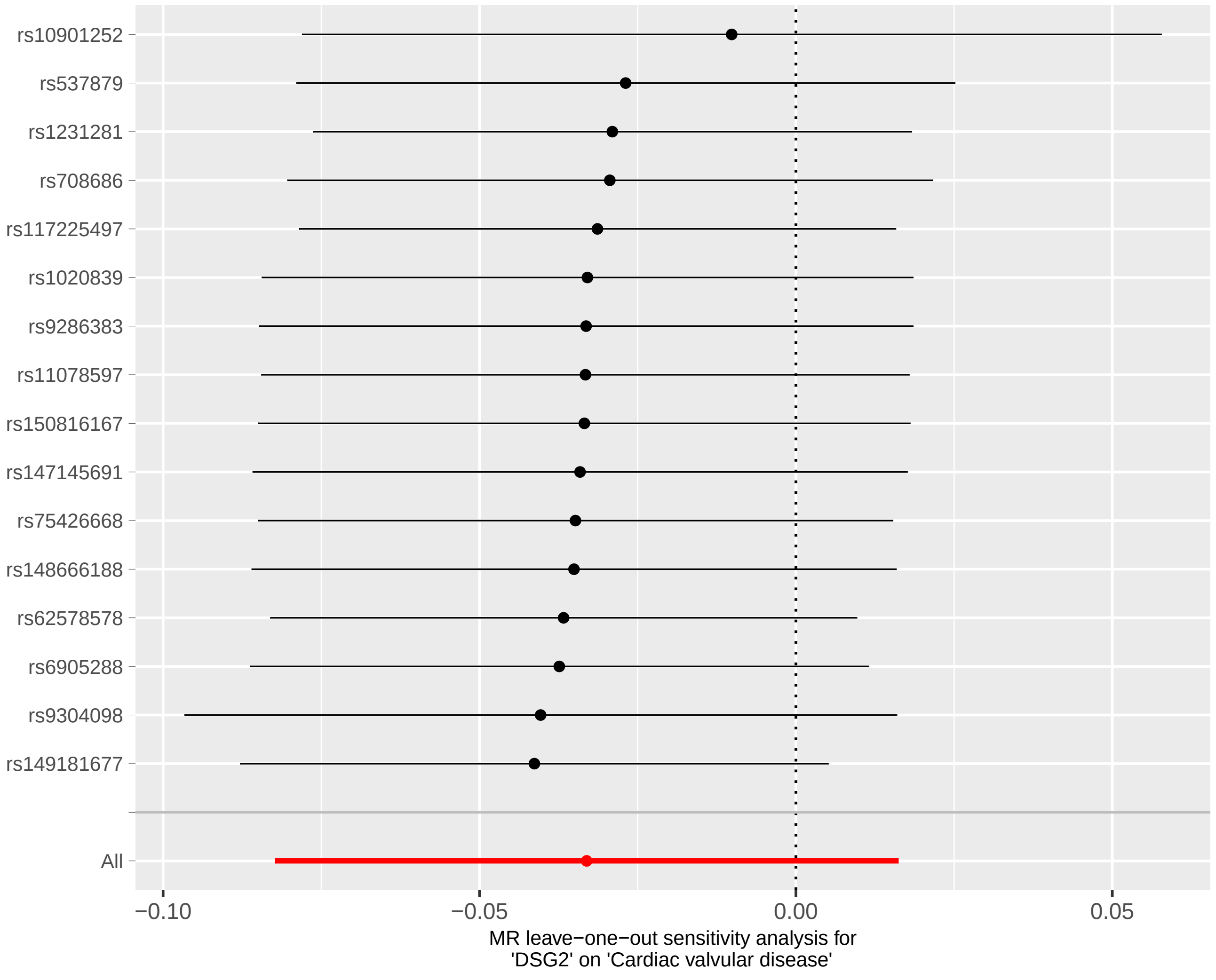
rs10901252
rs537879
rs1231281
rs708686
rs117225497
rs1020839
rs9286383
rs11078597
rs150816167
rs147145691
rs75426668
rs148666188
rs62578578
rs6905288
rs9304098
rs149181677
All
−0.10
−0.05
0.00
0.05
MR leave−one−out sensitivity analysis for
'DSG2' on 'Cardiac valvular disease'

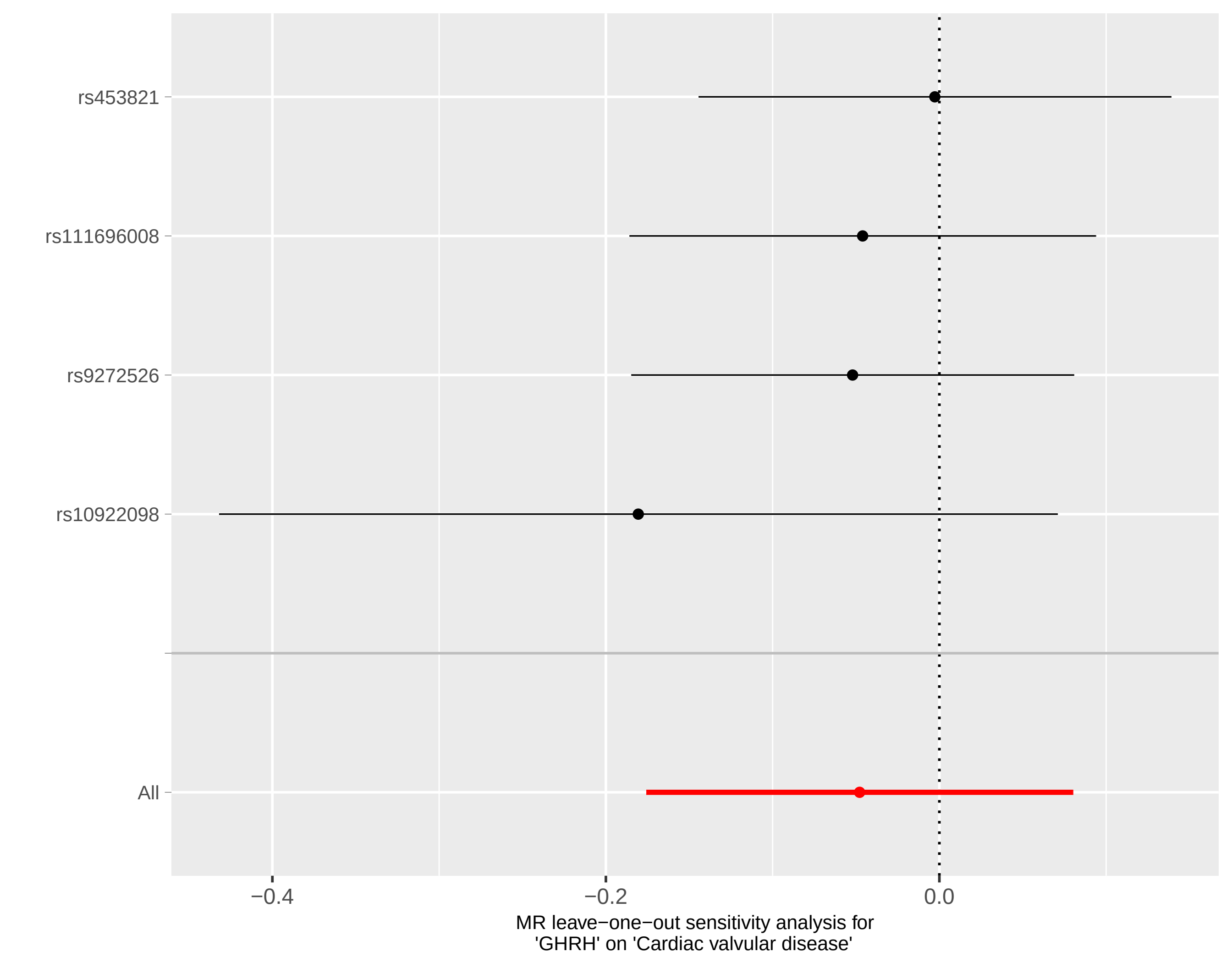
rs453821
rs111696008
rs9272526
rs10922098
All
−0.4
−0.2
0.0
MR leave−one−out sensitivity analysis for
'GHRH' on 'Cardiac valvular disease'

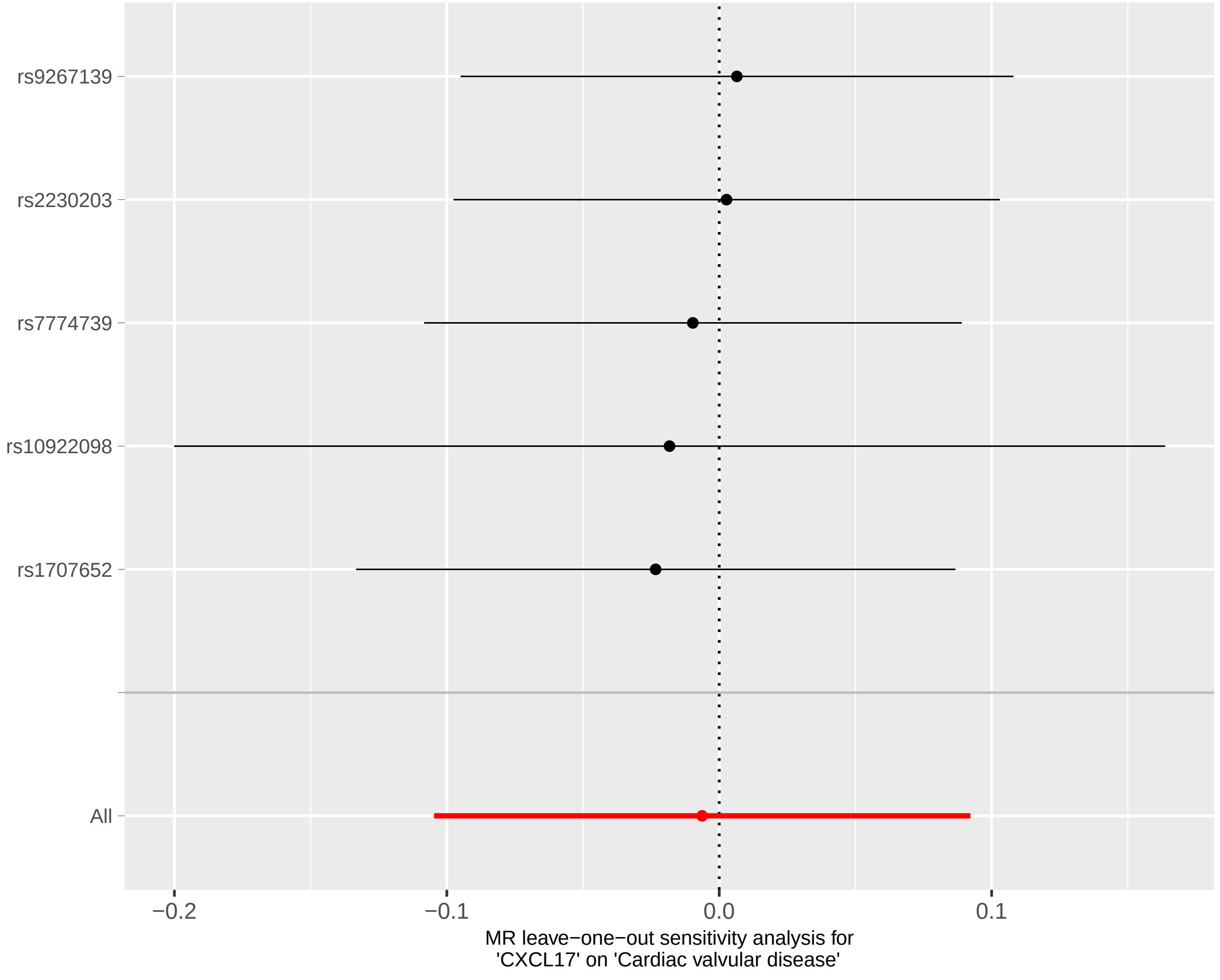
rs9267139
rs2230203
rs7774739
rs10922098
rs1707652
All
−0.2
−0.1
0.0
0.1
MR leave−one−out sensitivity analysis for
'CXCL17' on 'Cardiac valvular disease'

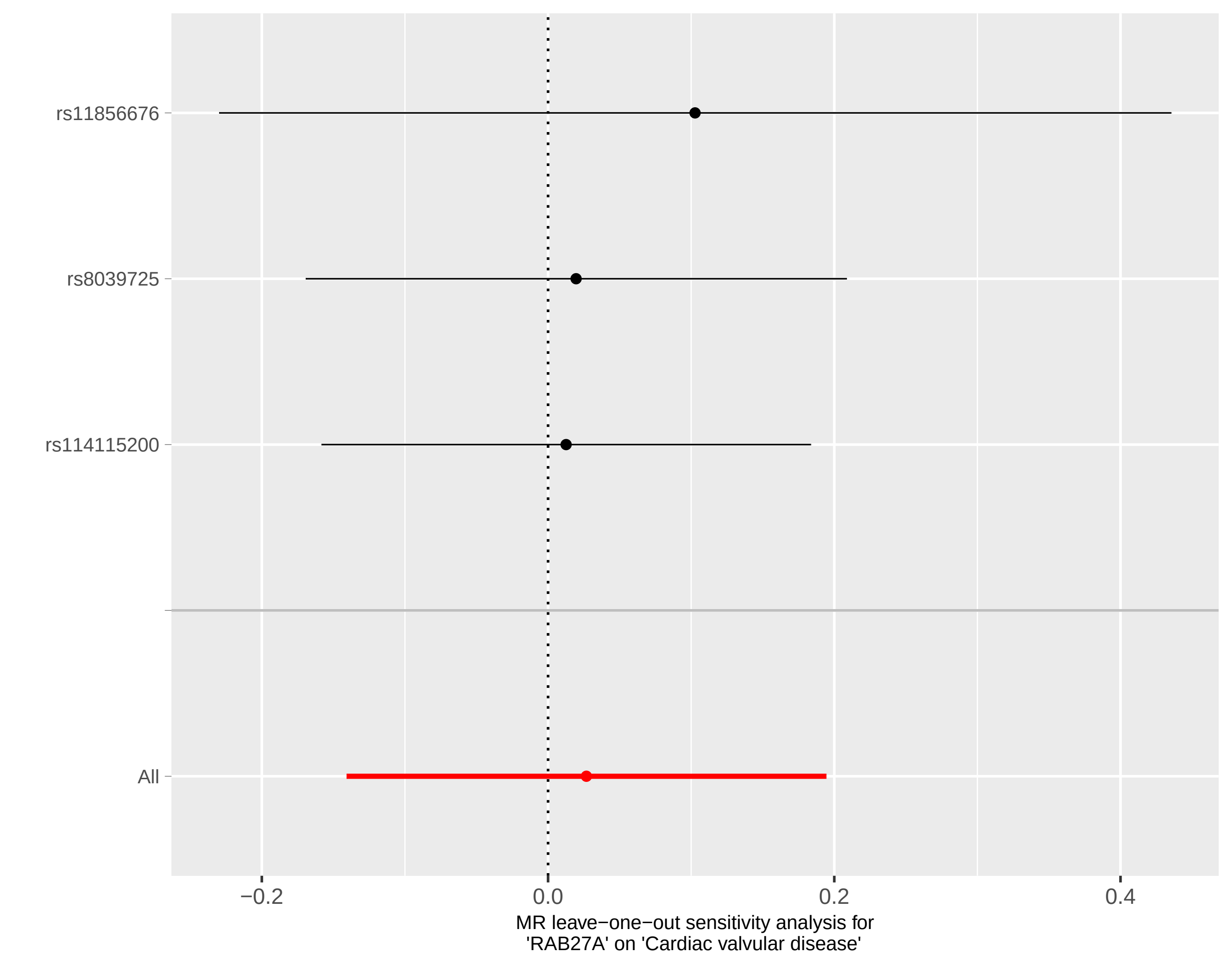
rs11856676
rs8039725
rs114115200
All
−0.2
0.0
0.2
0.4
MR leave−one−out sensitivity analysis for
'RAB27A' on 'Cardiac valvular disease'

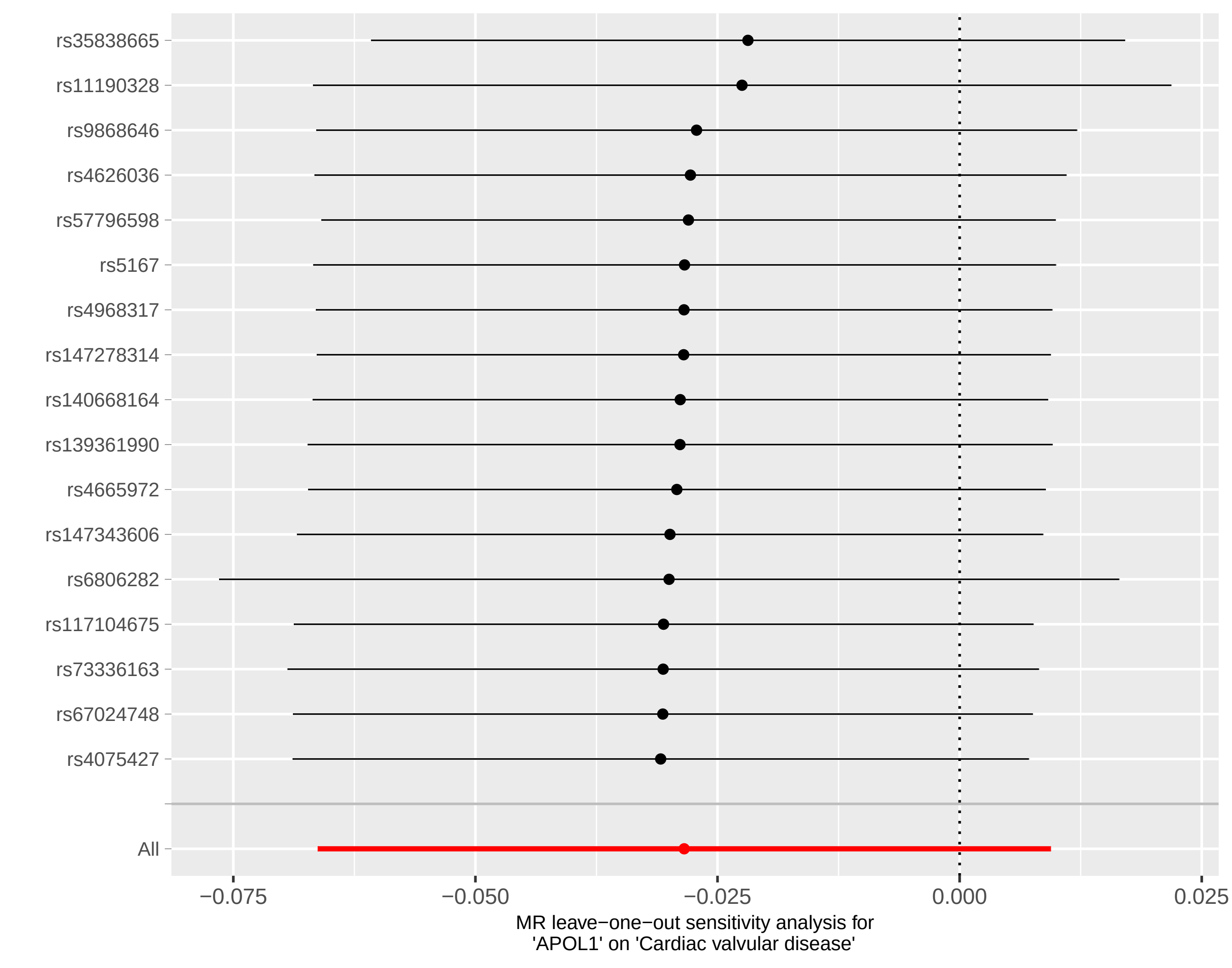

rs35838665
rs11190328
rs9868646
rs4626036
rs57796598
rs5167
rs4968317
rs147278314
rs140668164
rs139361990
rs4665972
rs147343606
rs6806282
rs117104675
rs73336163
rs67024748
rs4075427
All
−0.075
−0.050
−0.025
0.000
0.025
MR leave−one−out sensitivity analysis for
'APOL1' on 'Cardiac valvular disease'

Insufficient number of SNPs

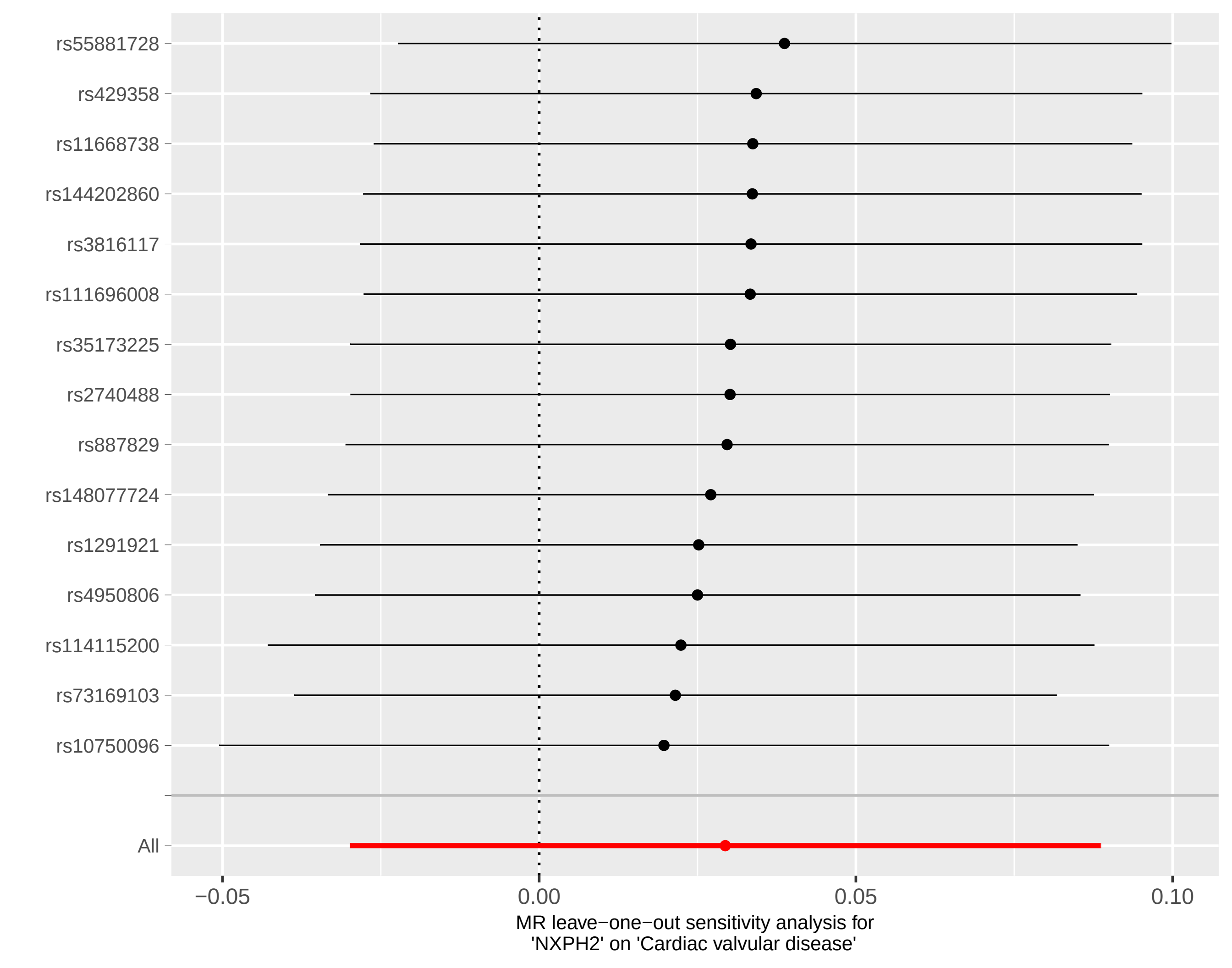
rs55881728
rs429358
rs11668738
rs144202860
rs3816117
rs111696008
rs35173225
rs2740488
rs887829
rs148077724
rs1291921
rs4950806
rs114115200
rs73169103
rs10750096
All
−0.05
0.00
0.05
0.10
MR leave−one−out sensitivity analysis for
'NXPH2' on 'Cardiac valvular disease'

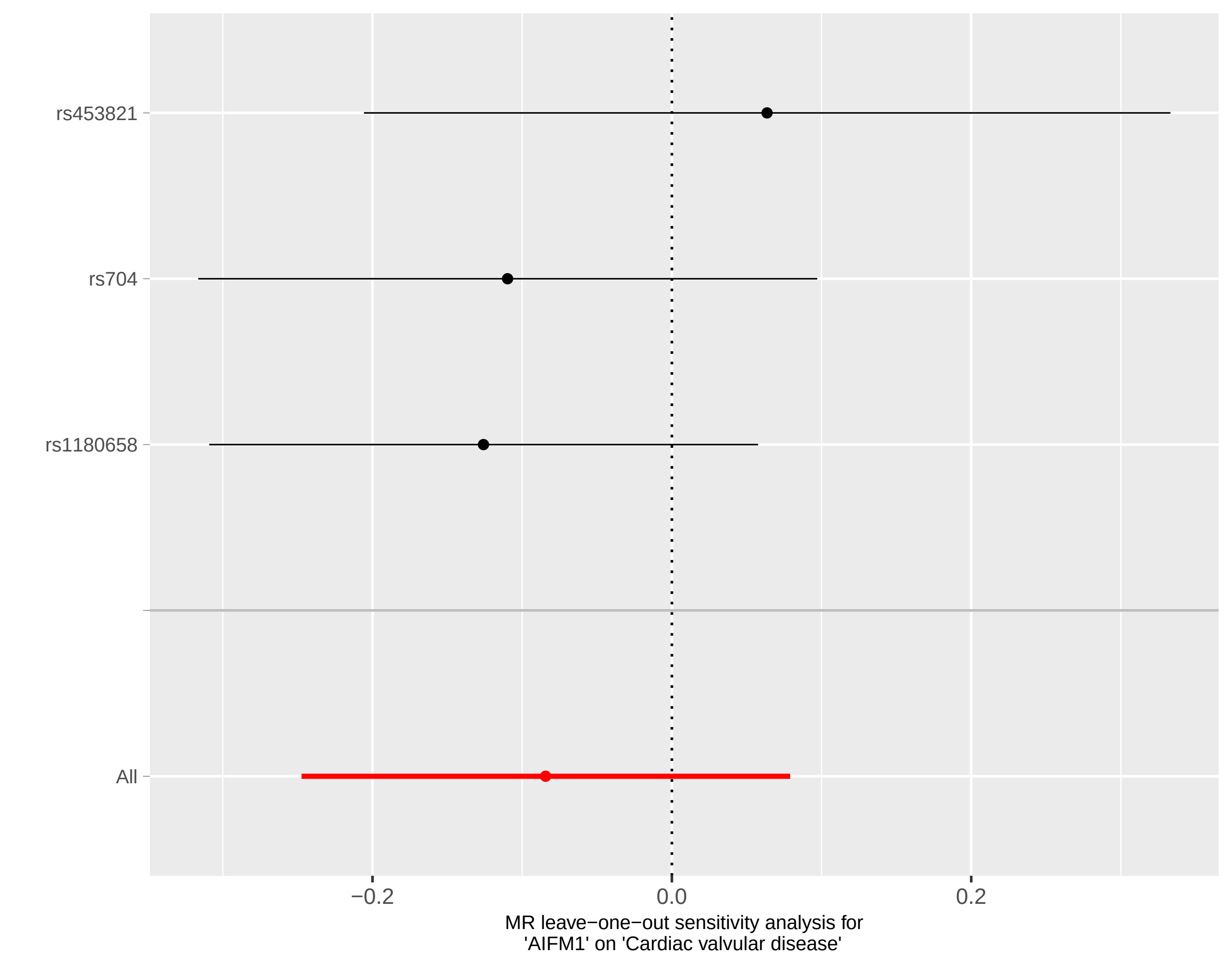
rs453821
rs704
rs1180658
All
−0.2
0.0
0.2
MR leave−one−out sensitivity analysis for
'AIFM1' on 'Cardiac valvular disease'

Insufficient number of SNPs

Insufficient number of SNPs

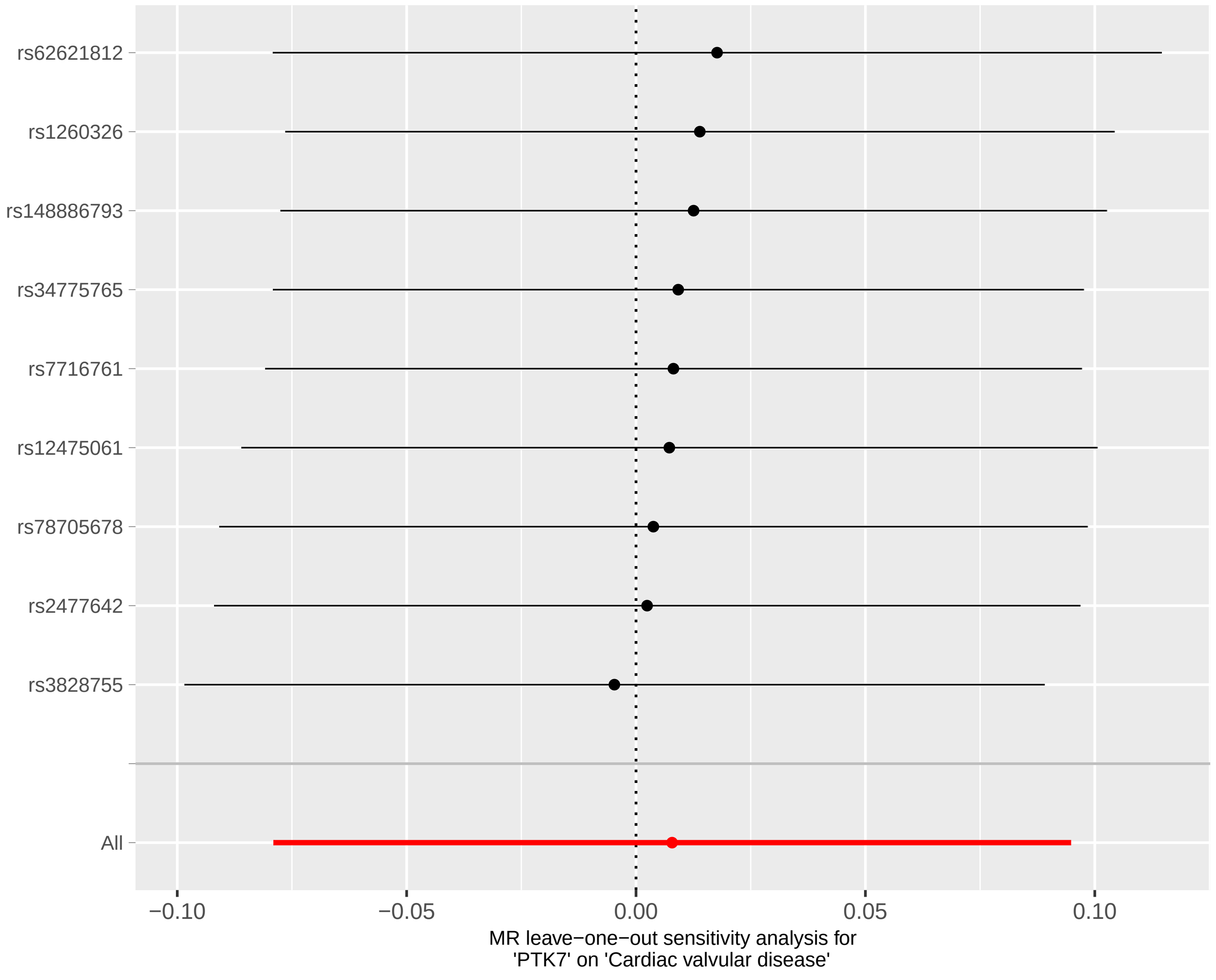
rs62621812
rs1260326
rs148886793
rs34775765
rs7716761
rs12475061
rs78705678
rs2477642
rs3828755
All
−0.10
−0.05
0.00
0.05
0.10
MR leave−one−out sensitivity analysis for
'PTK7' on 'Cardiac valvular disease'

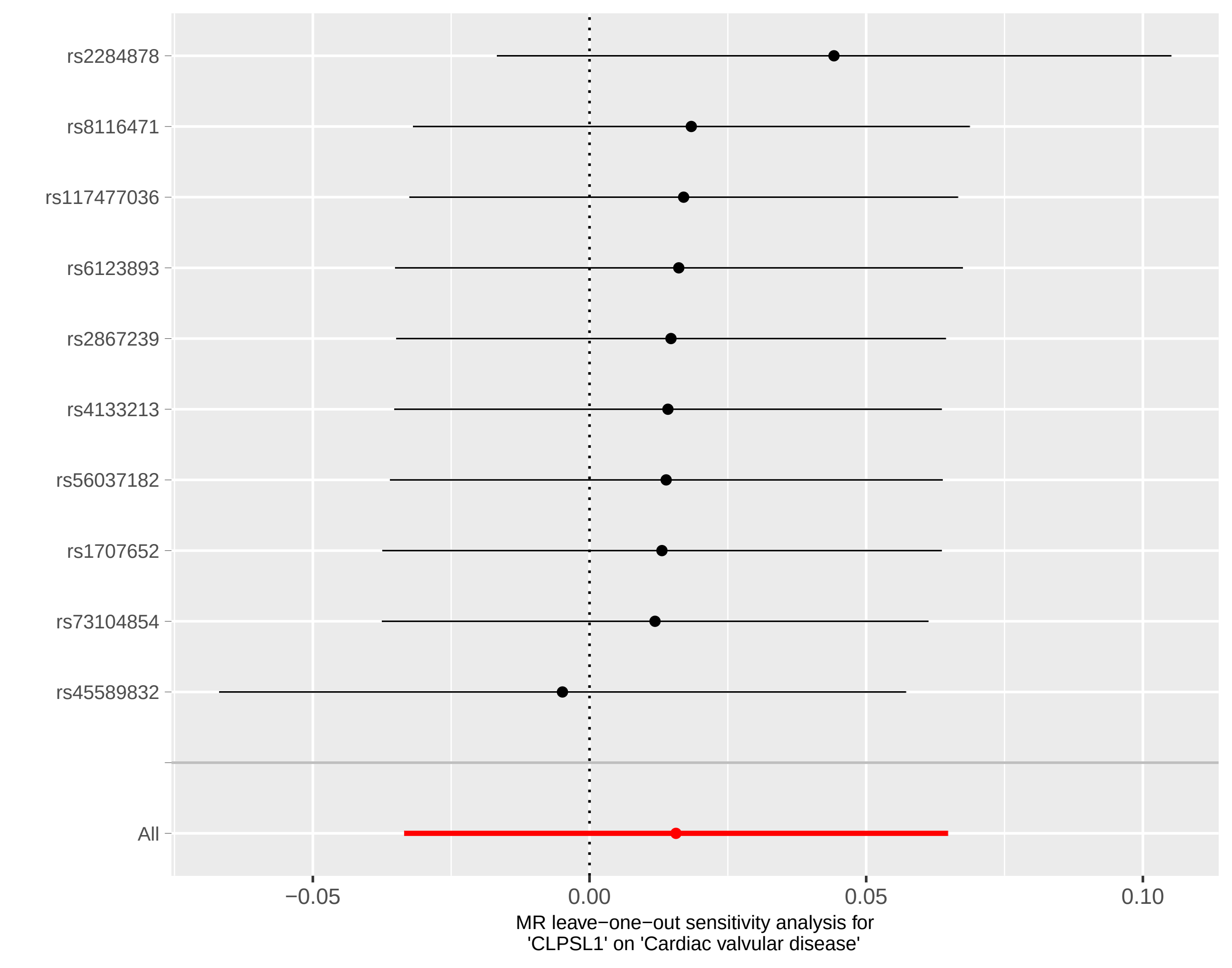
rs2284878
rs8116471
rs117477036
rs6123893
rs2867239
rs4133213
rs56037182
rs1707652
rs73104854
rs45589832
All
−0.05
0.00
0.05
0.10
MR leave−one−out sensitivity analysis for
'CLPSL1' on 'Cardiac valvular disease'

Insufficient number of SNPs

Insufficient number of SNPs

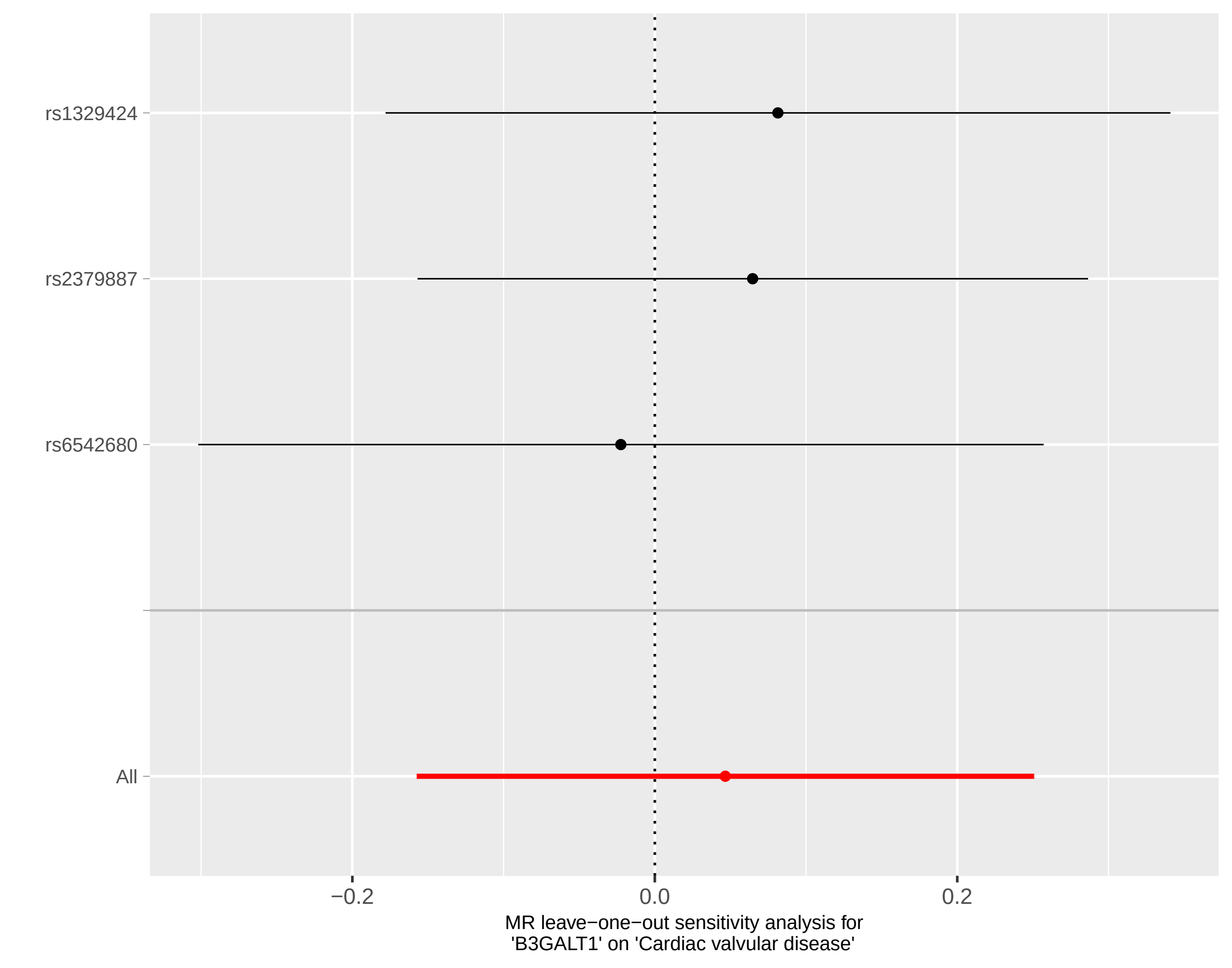
rs1329424
rs2379887
rs6542680
All
−0.2
0.0
0.2
MR leave−one−out sensitivity analysis for
'B3GALT1' on 'Cardiac valvular disease'

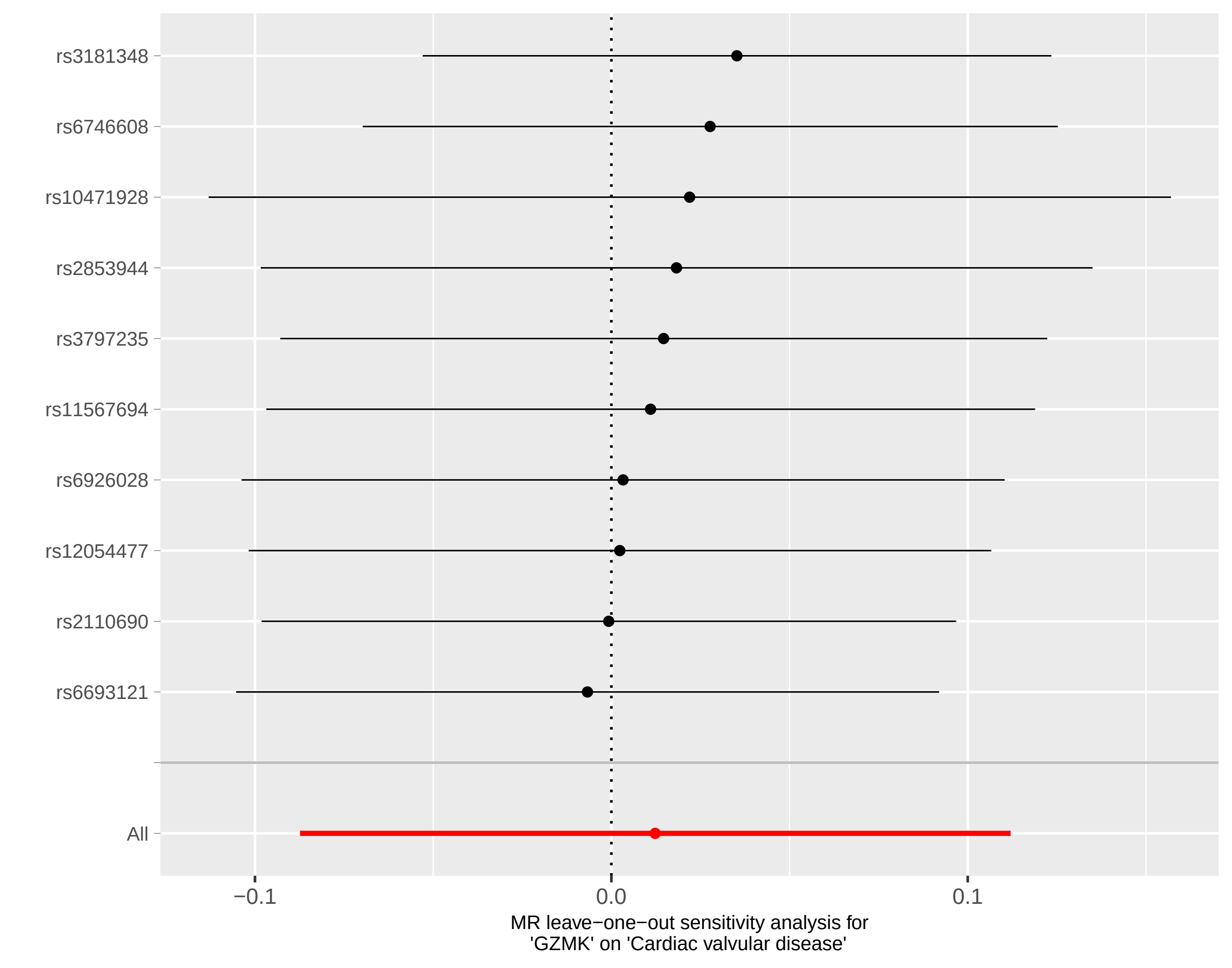
rs3181348
rs6746608
rs10471928
rs2853944
rs3797235
rs11567694
rs6926028
rs12054477
rs2110690
rs6693121
All
−0.1
0.0
0.1
MR leave−one−out sensitivity analysis for
'GZMK' on 'Cardiac valvular disease'

Insufficient number of SNPs

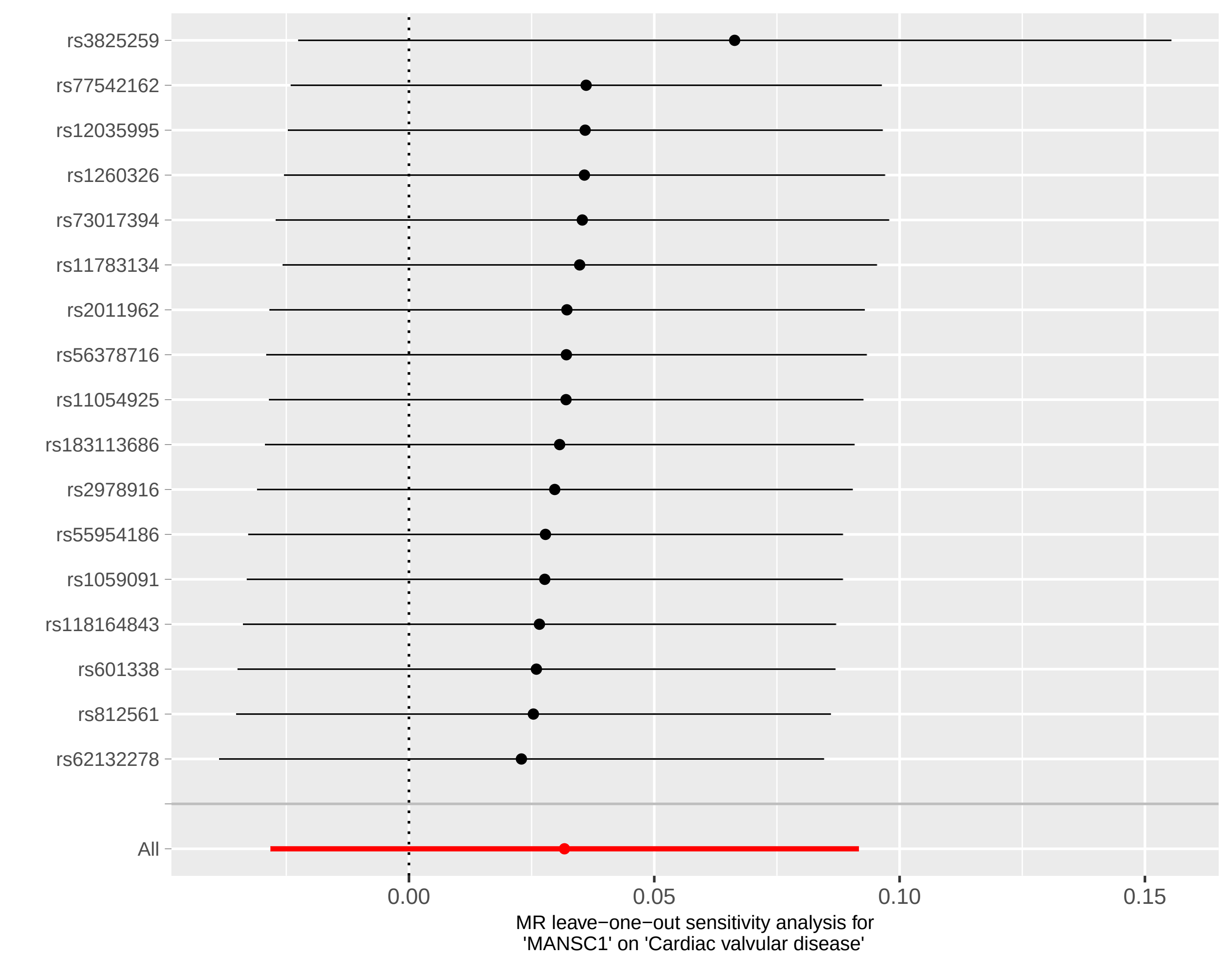
rs3825259
rs77542162
rs12035995
rs1260326
rs73017394
rs11783134
rs2011962
rs56378716
rs11054925
rs183113686
rs2978916
rs55954186
rs1059091
rs118164843
rs601338
rs812561
rs62132278
All
0.00
0.05
0.10
0.15
MR leave−one−out sensitivity analysis for
'MANSC1' on 'Cardiac valvular disease'

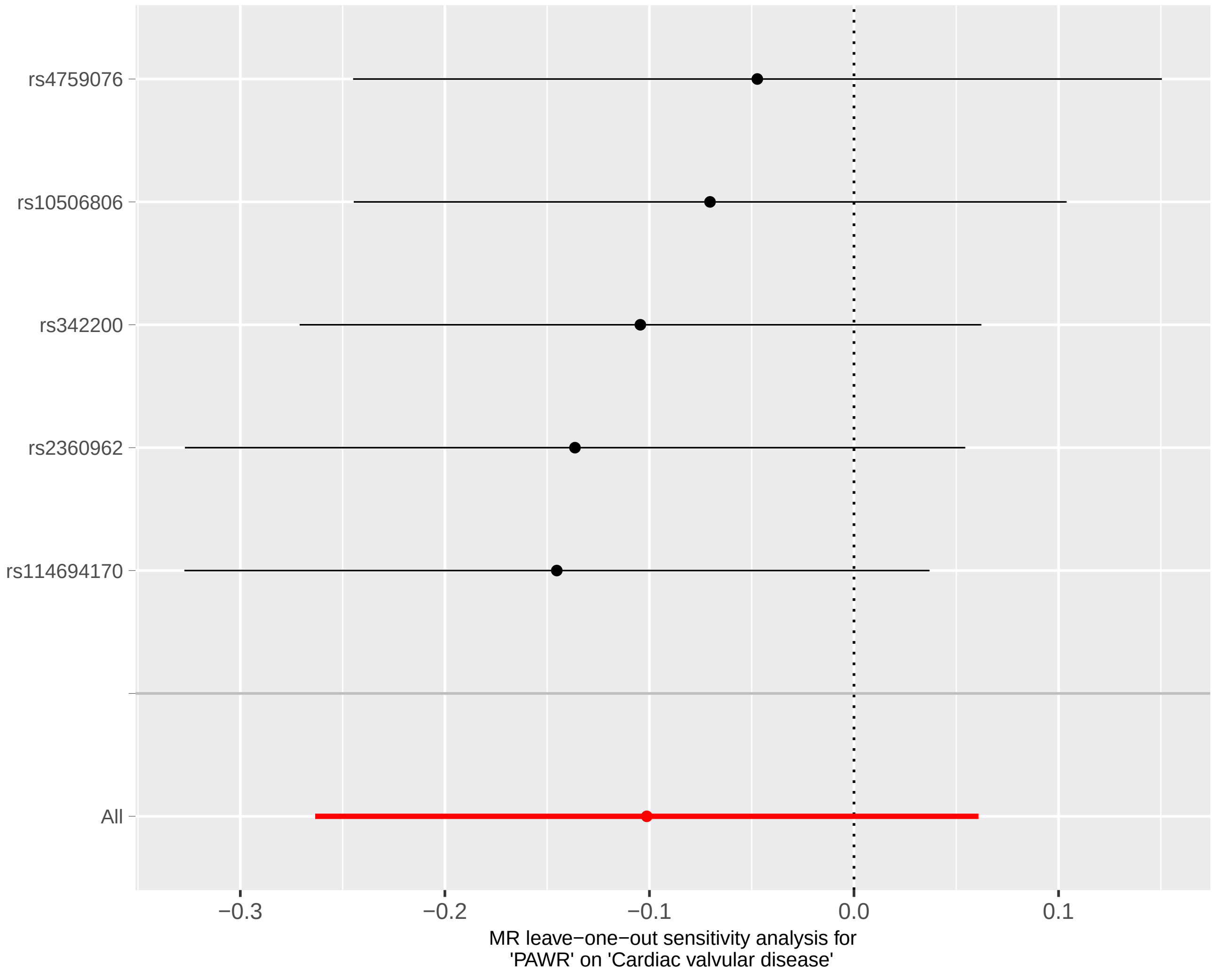
rs4759076
rs10506806
rs342200
rs2360962
rs114694170
All
−0.3
−0.2
−0.1
0.0
0.1
MR leave−one−out sensitivity analysis for
'PAWR' on 'Cardiac valvular disease'

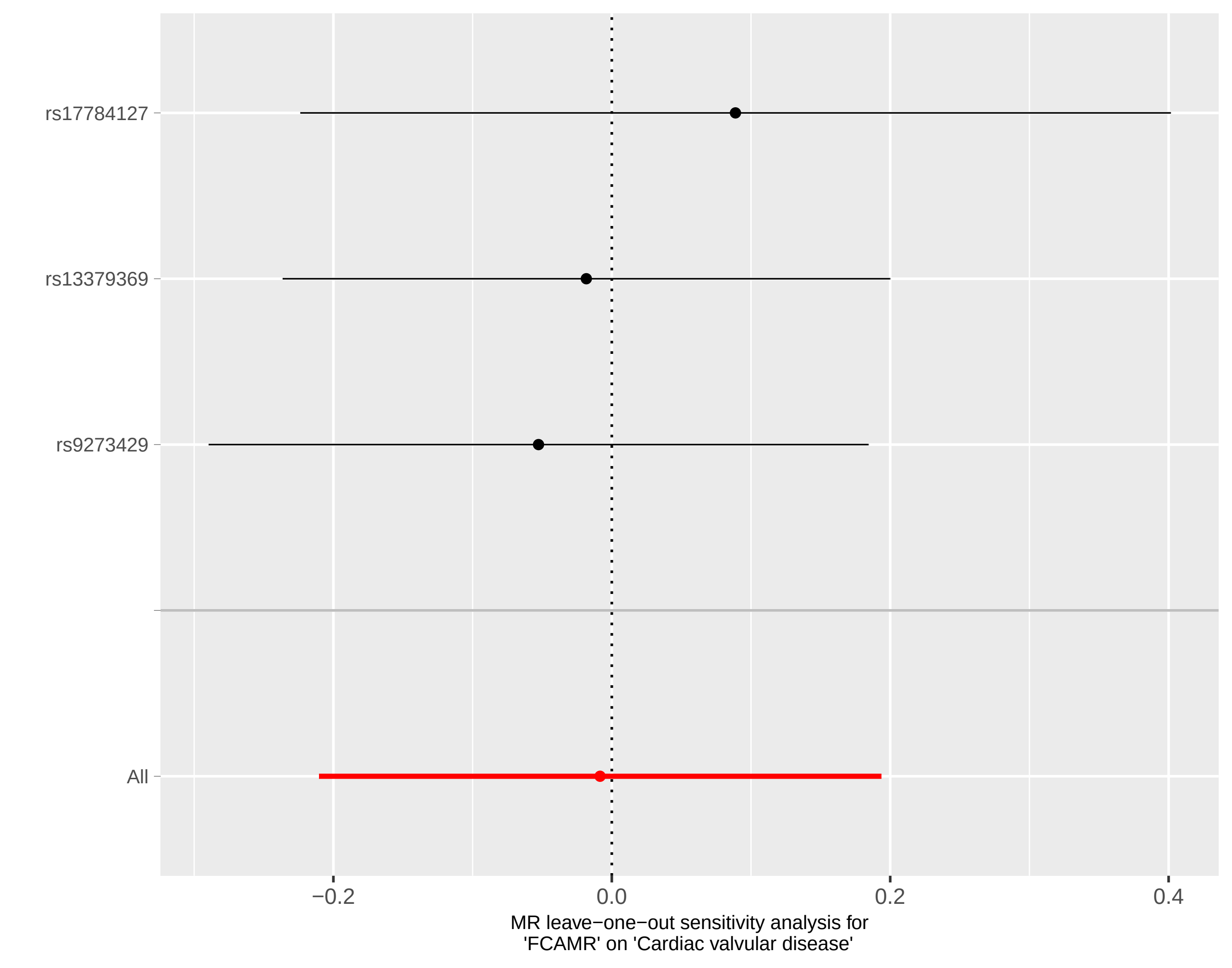

rs17784127
rs13379369
rs9273429
All
−0.2
0.0
0.2
0.4
MR leave−one−out sensitivity analysis for
'FCAMR' on 'Cardiac valvular disease'

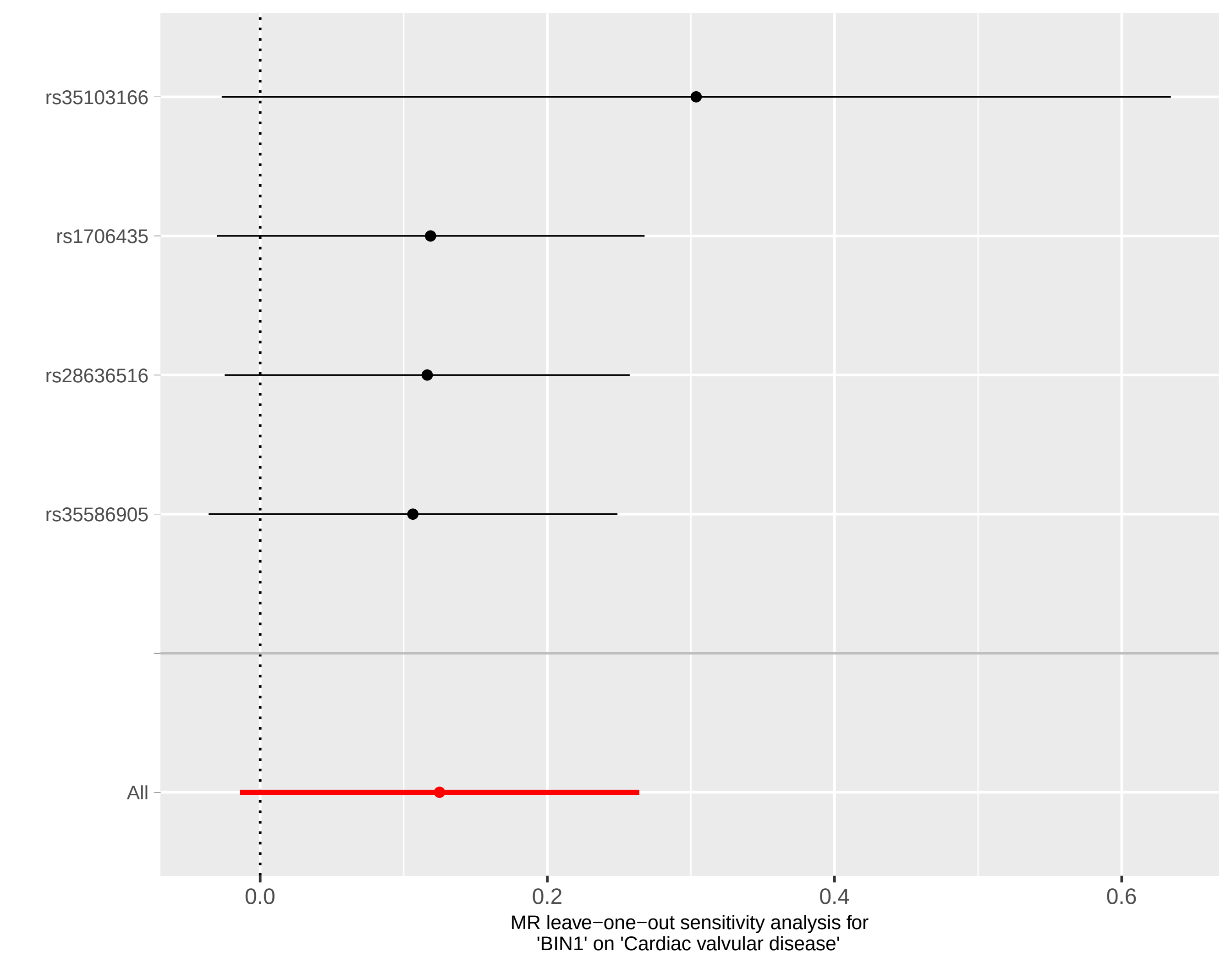
rs35103166
rs1706435
rs28636516
rs35586905
All
0.0
0.2
0.4
0.6
MR leave−one−out sensitivity analysis for
'BIN1' on 'Cardiac valvular disease'

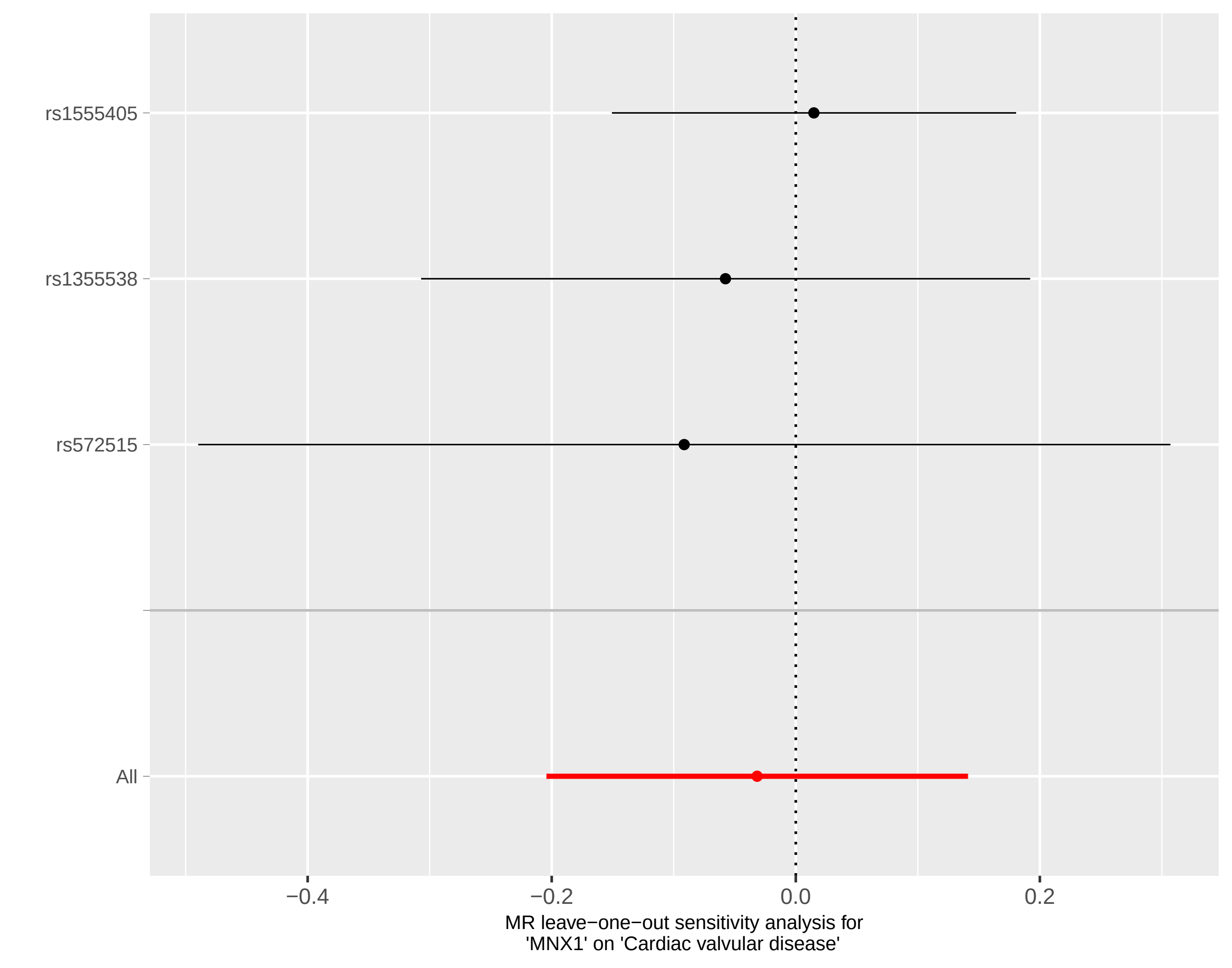

rs1555405
rs1355538
rs572515
All
−0.4
−0.2
0.0
0.2
MR leave−one−out sensitivity analysis for
'MNX1' on 'Cardiac valvular disease'

Insufficient number of SNPs

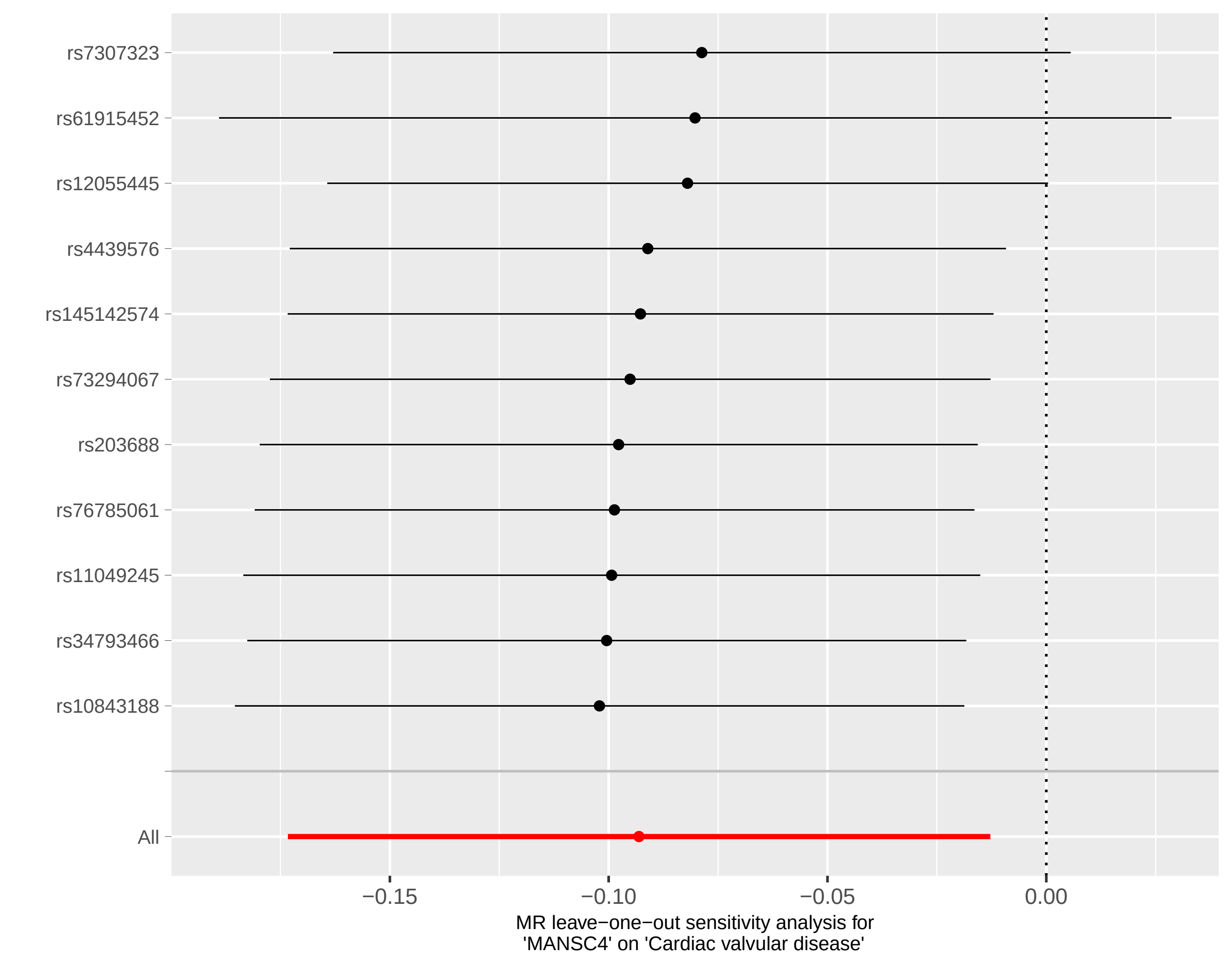
rs7307323
rs61915452
rs12055445
rs4439576
rs145142574
rs73294067
rs203688
rs76785061
rs11049245
rs34793466
rs10843188
All
−0.15
−0.10
−0.05
0.00
MR leave−one−out sensitivity analysis for
'MANSC4' on 'Cardiac valvular disease'

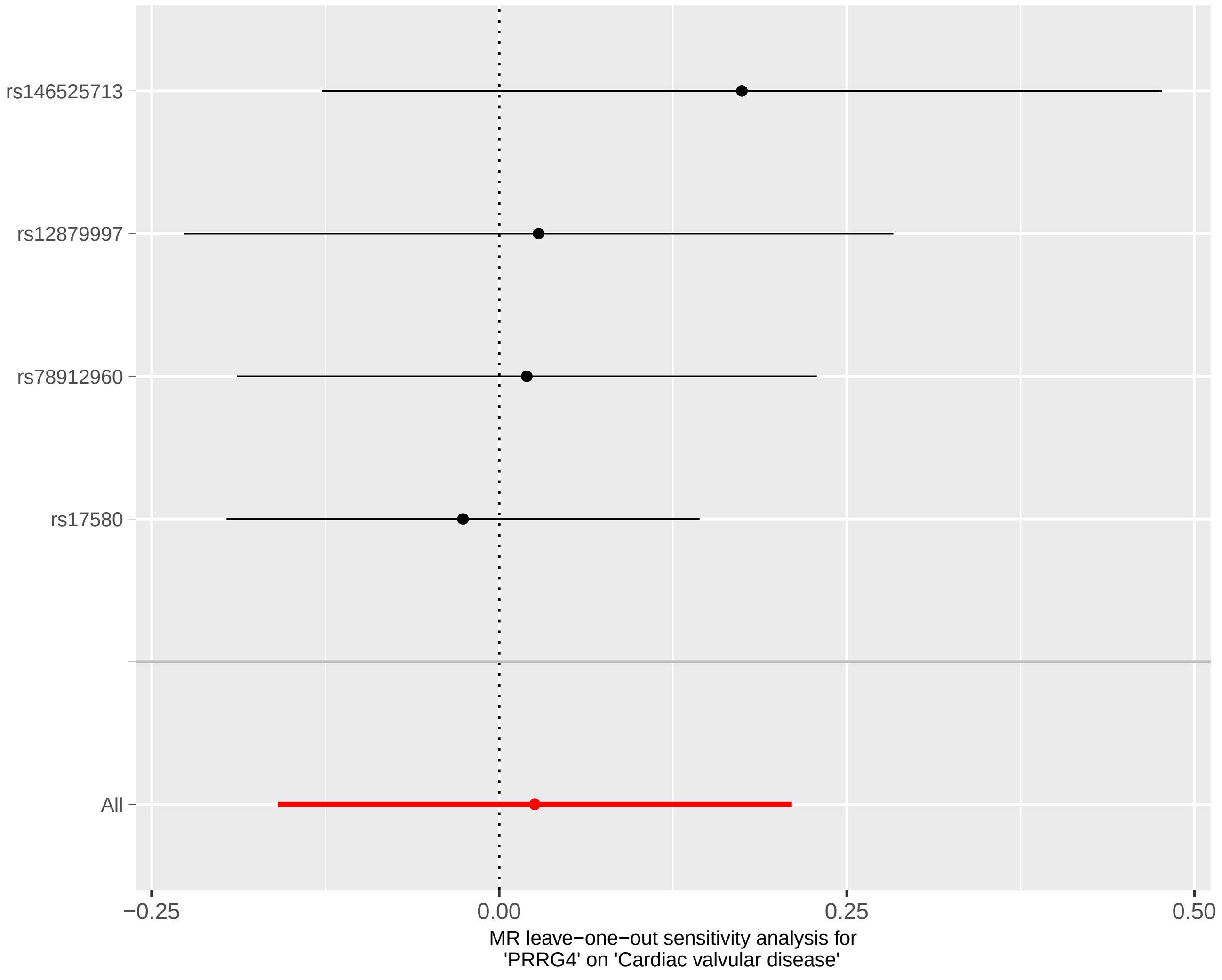
rs146525713
rs12879997
rs78912960
rs17580
All
−0.25
0.00
0.25
0.50
MR leave−one−out sensitivity analysis for
'PRRG4' on 'Cardiac valvular disease'

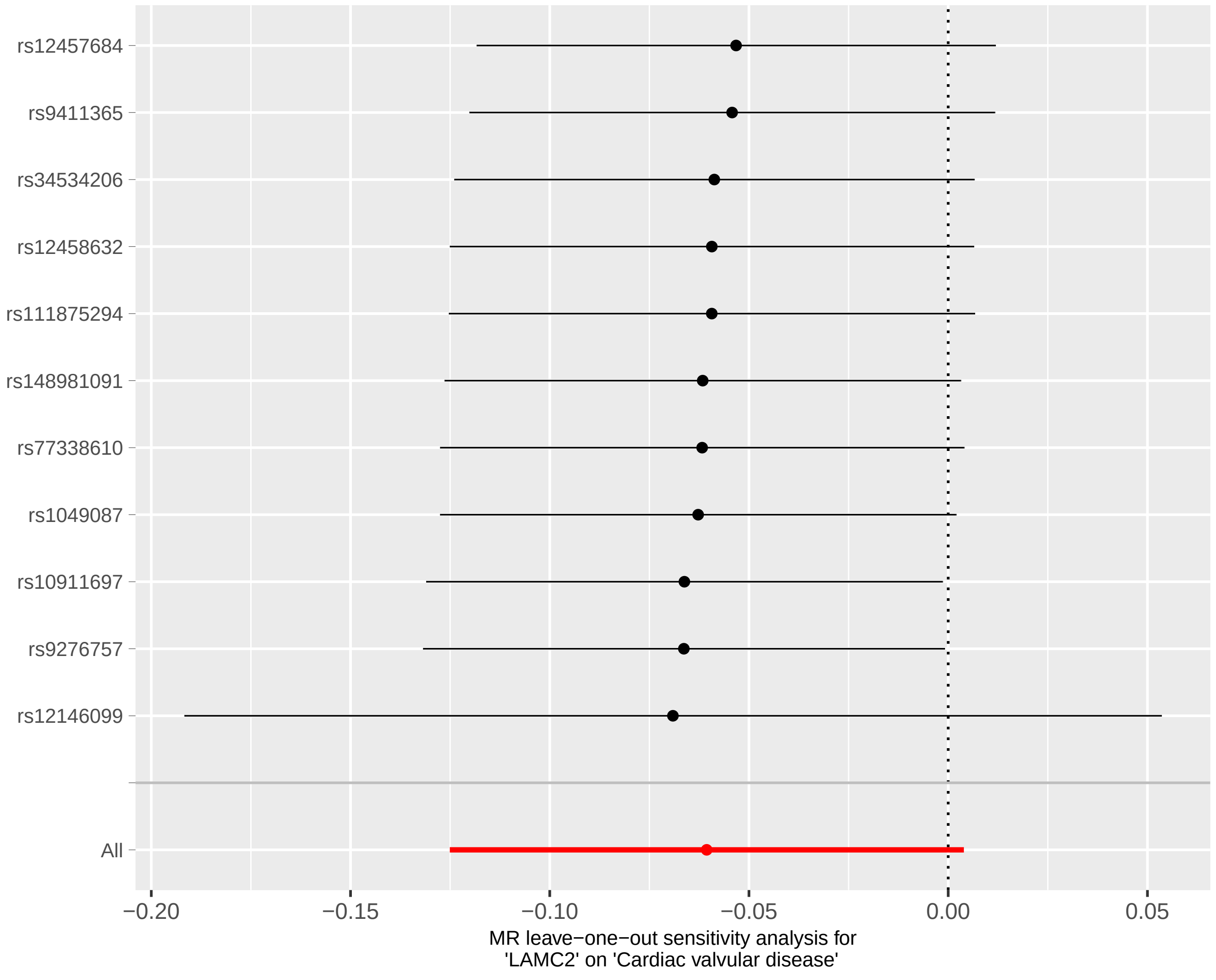
rs12457684
rs9411365
rs34534206
rs12458632
rs111875294
rs148981091
rs77338610
rs1049087
rs10911697
rs9276757
rs12146099
All
−0.20
−0.15
−0.10
−0.05
0.00
0.05
MR leave−one−out sensitivity analysis for
'LAMC2' on 'Cardiac valvular disease'

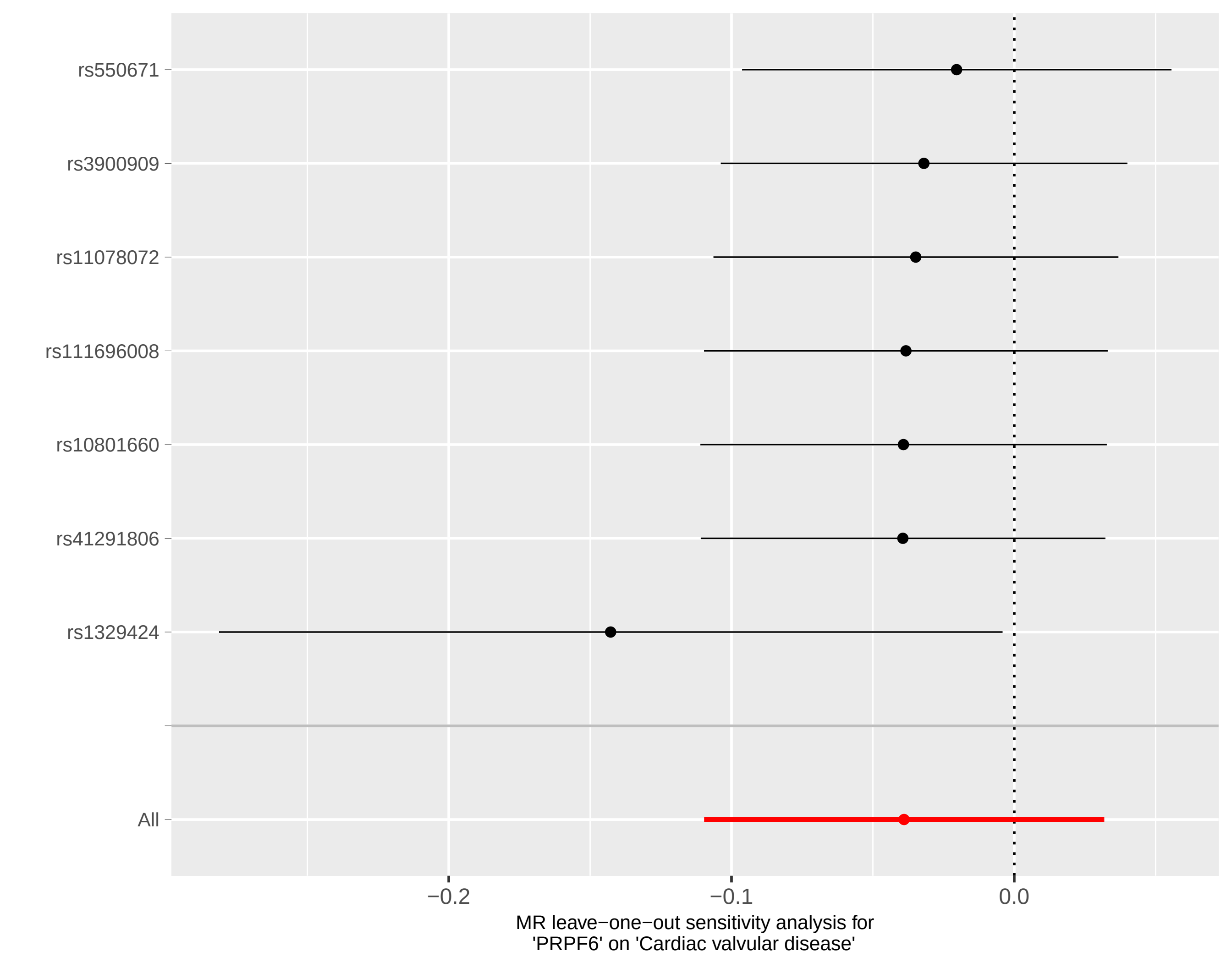

rs550671
rs3900909
rs11078072
rs111696008
rs10801660
rs41291806
rs1329424
All
−0.2
−0.1
0.0
MR leave−one−out sensitivity analysis for
'PRPF6' on 'Cardiac valvular disease'

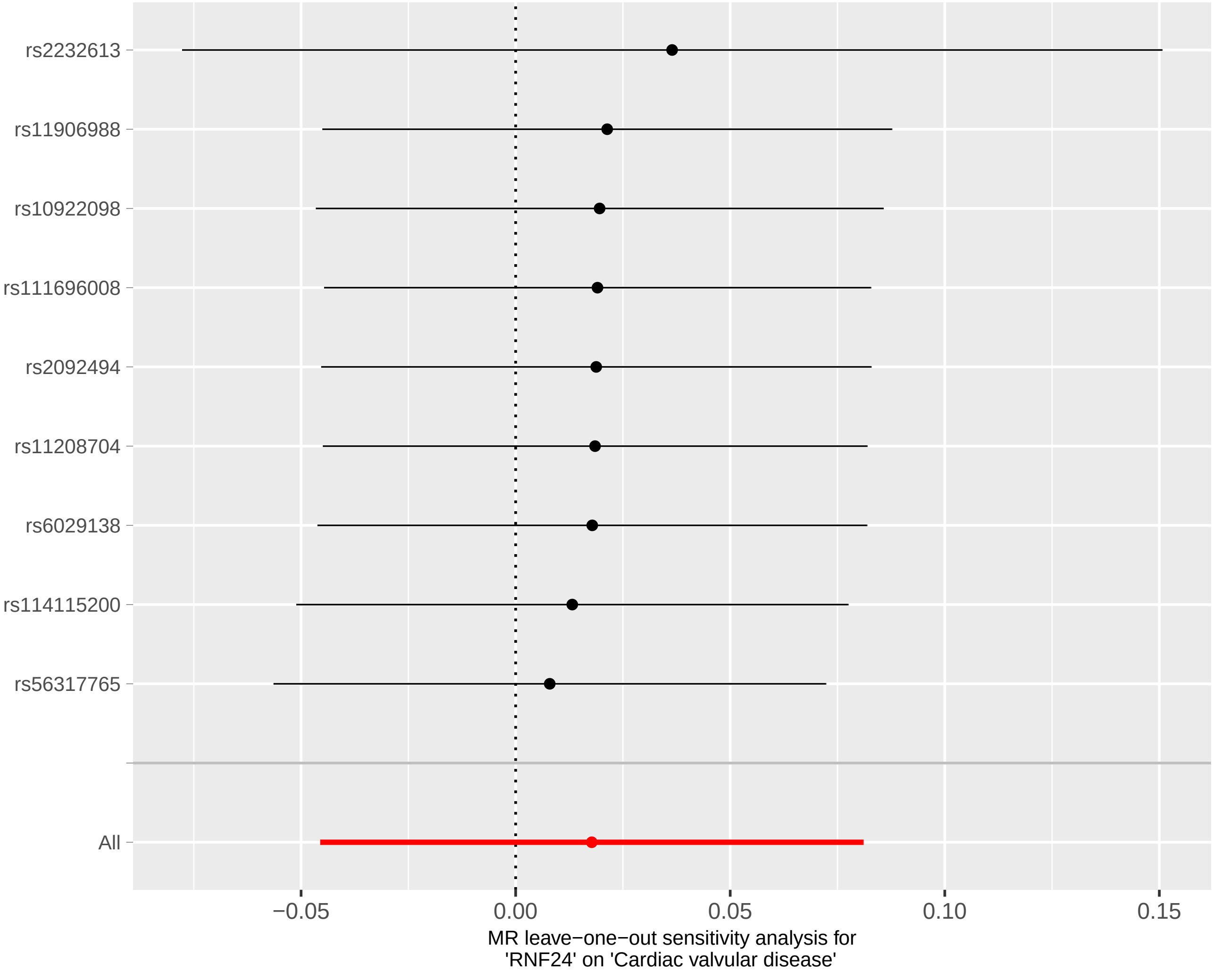
rs2232613
rs11906988
rs10922098
rs111696008
rs2092494
rs11208704
rs6029138
rs114115200
rs56317765
All
−0.05
0.00
0.05
0.10
0.15
MR leave−one−out sensitivity analysis for
'RNF24' on 'Cardiac valvular disease'

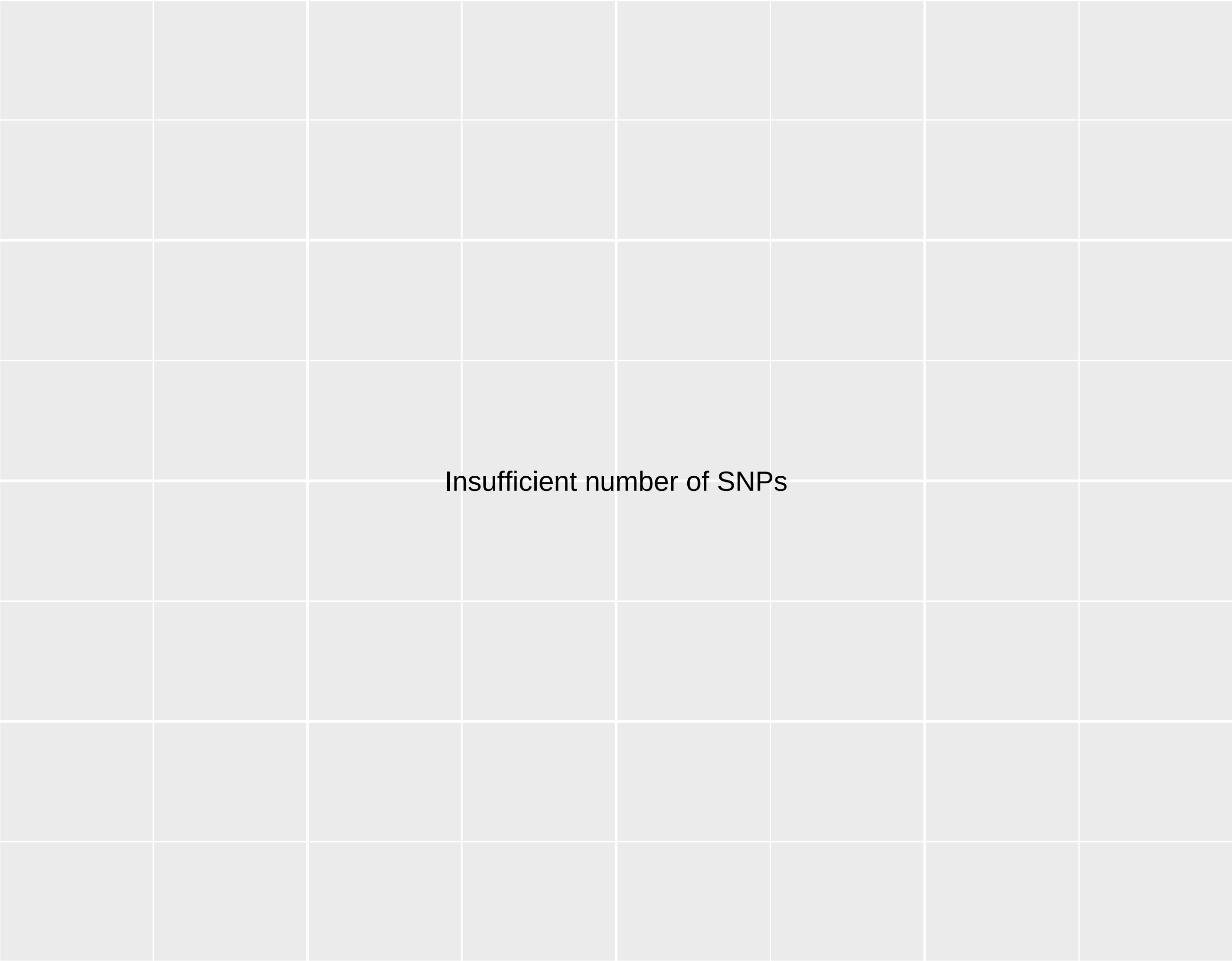
Insufficient number of SNPs

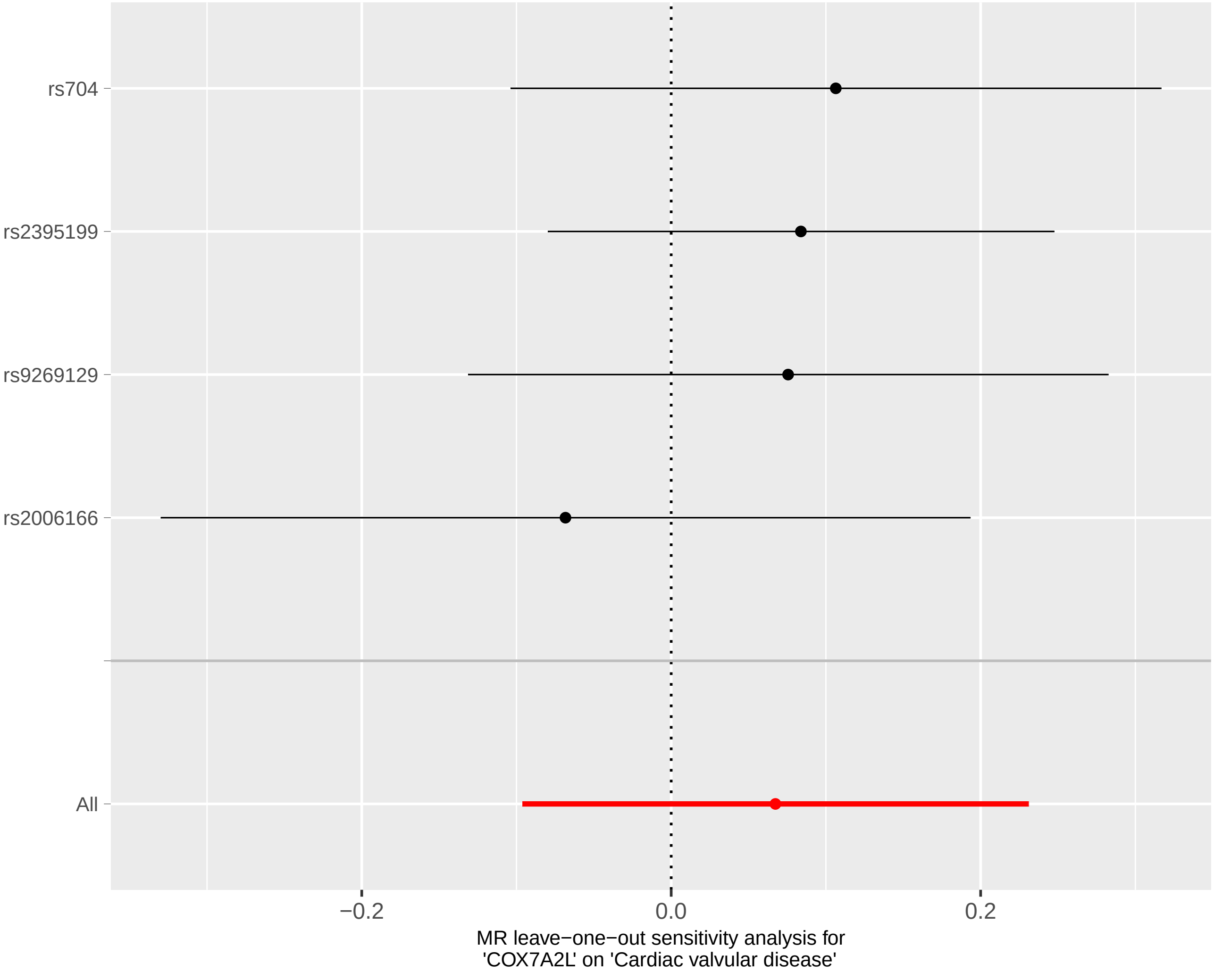

rs704
rs2395199
rs9269129
rs2006166
All
−0.2
0.0
0.2
MR leave−one−out sensitivity analysis for
'COX7A2L' on 'Cardiac valvular disease'

Insufficient number of SNPs

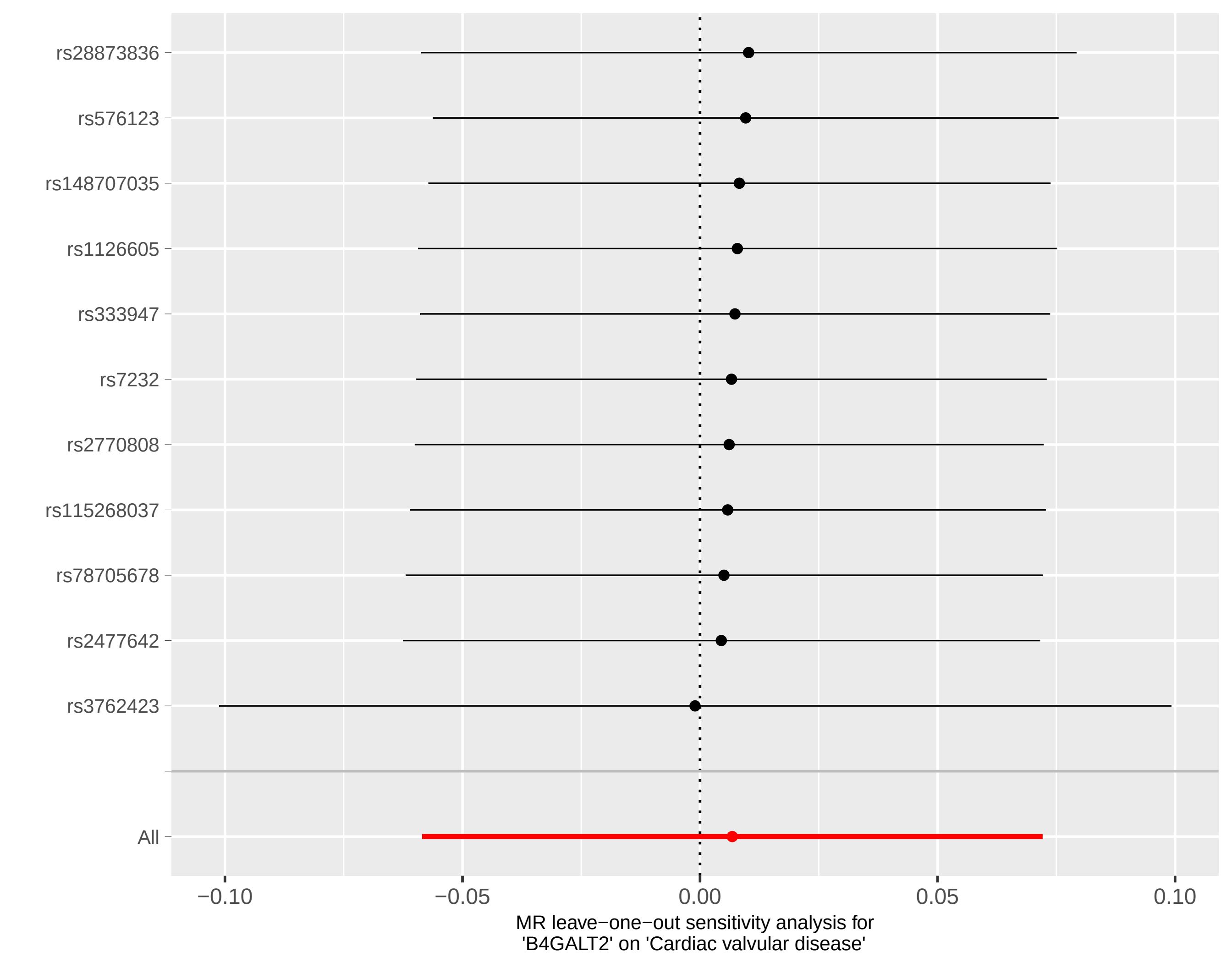
rs28873836
rs576123
rs148707035
rs1126605
rs333947
rs7232
rs2770808
rs115268037
rs78705678
rs2477642
rs3762423
All
−0.10
−0.05
0.00
0.05
0.10
MR leave−one−out sensitivity analysis for
'B4GALT2' on 'Cardiac valvular disease'

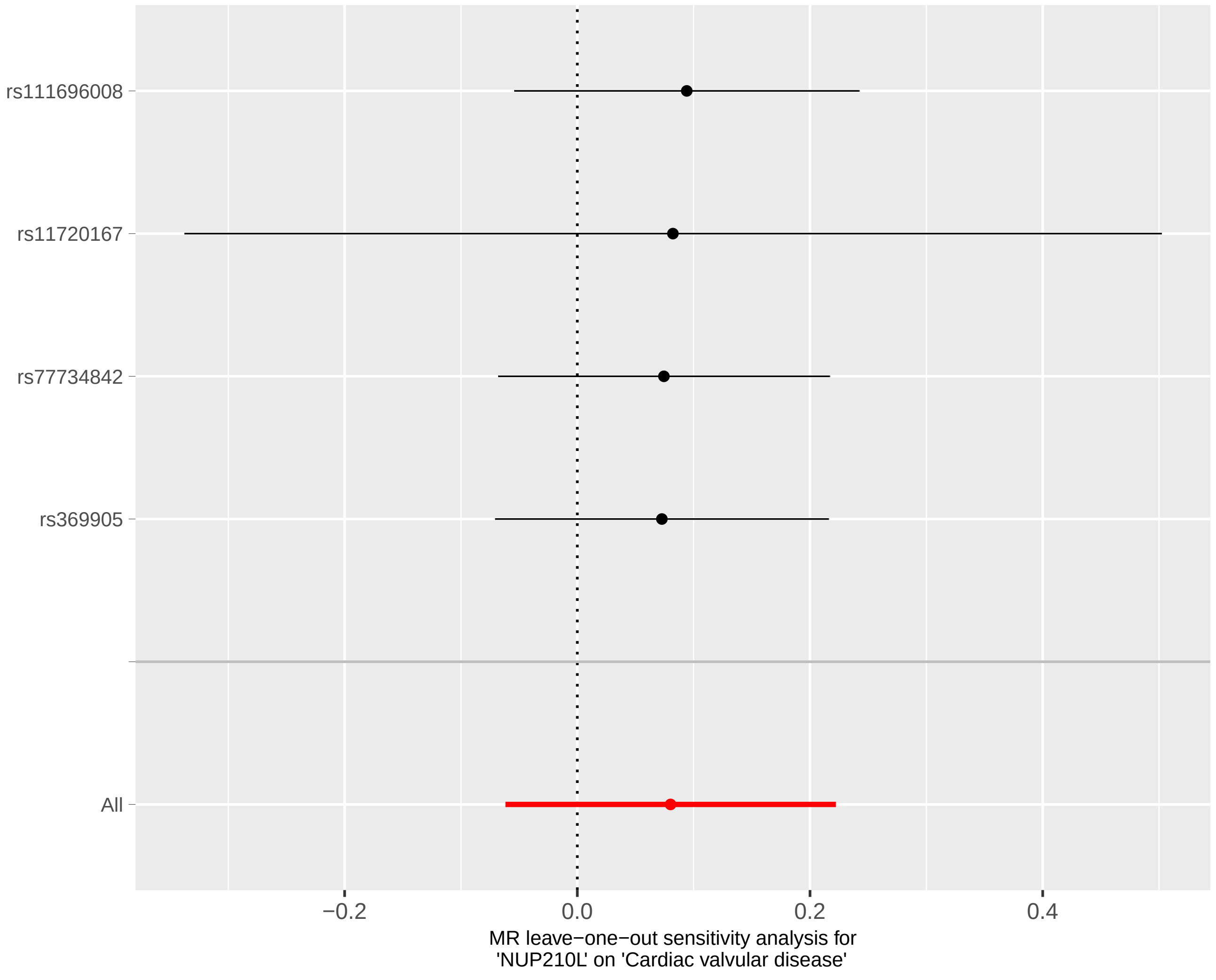
rs111696008
rs11720167
rs77734842
rs369905
All
−0.2
0.0
0.2
0.4
MR leave−one−out sensitivity analysis for
'NUP210L' on 'Cardiac valvular disease'

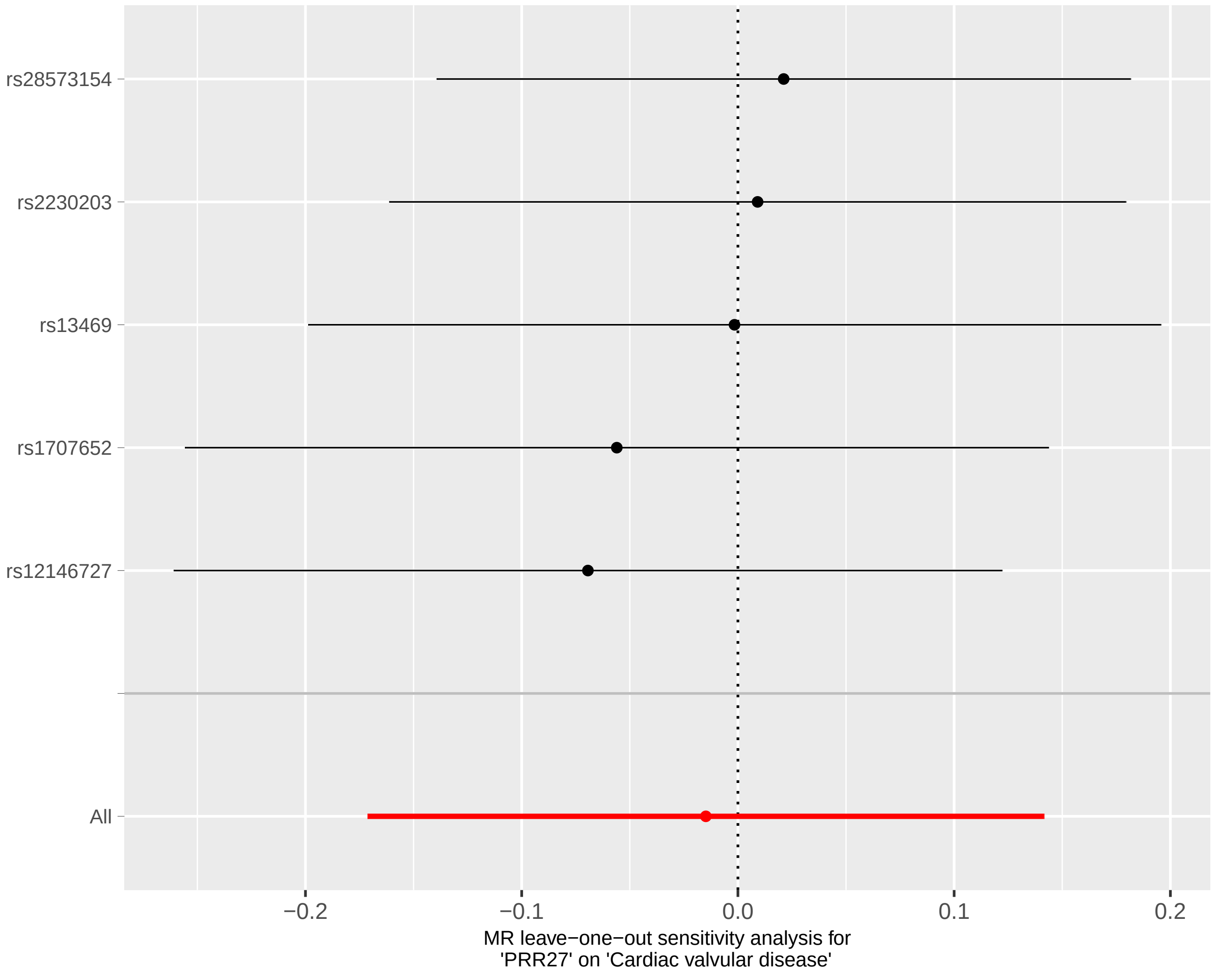

rs28573154
rs2230203
rs13469
rs1707652
rs12146727
All
−0.2
−0.1
0.0
0.1
0.2
MR leave−one−out sensitivity analysis for
'PRR27' on 'Cardiac valvular disease'

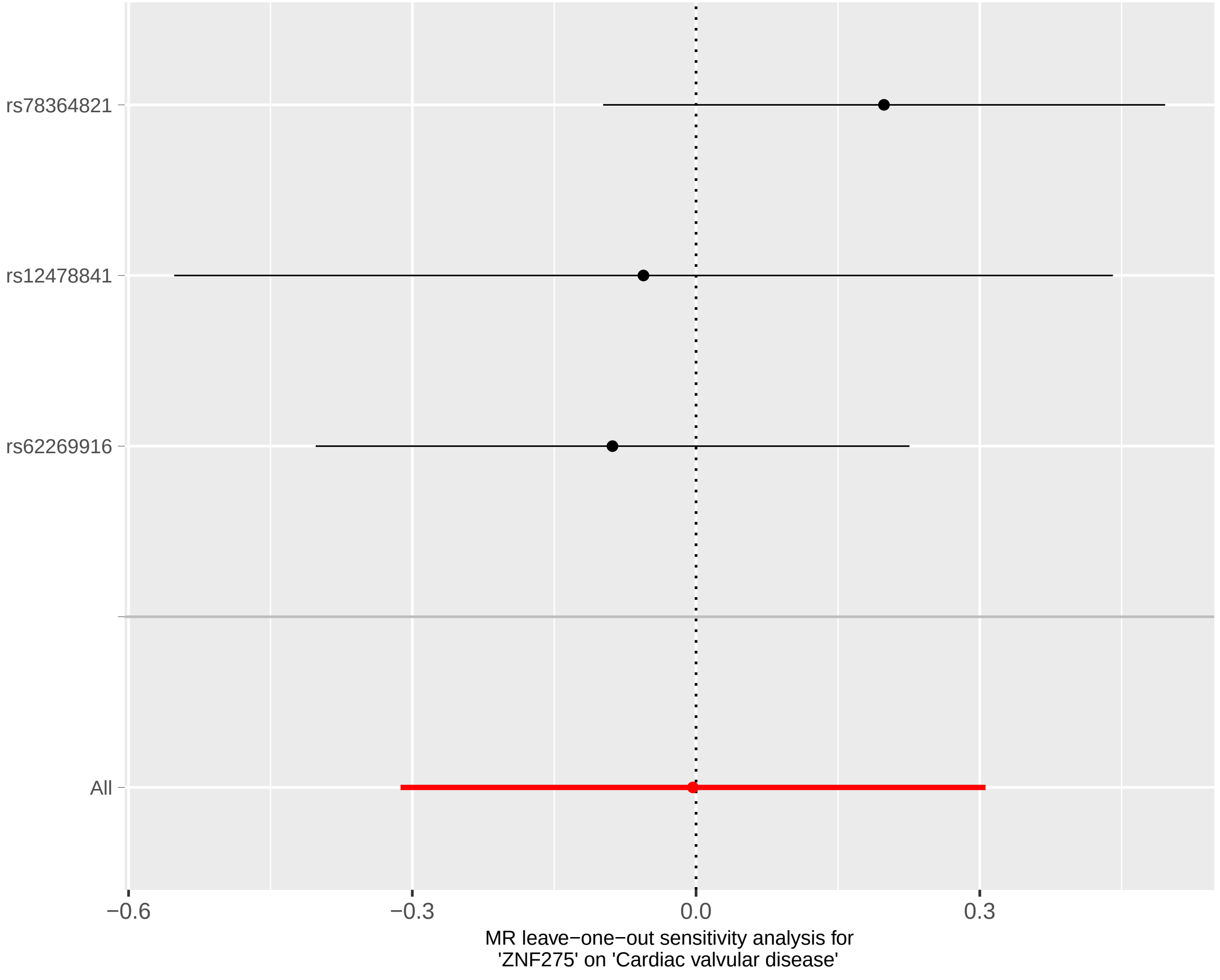
rs78364821
rs12478841
rs62269916
All
−0.6
−0.3
0.0
0.3
MR leave−one−out sensitivity analysis for
'ZNF275' on 'Cardiac valvular disease'

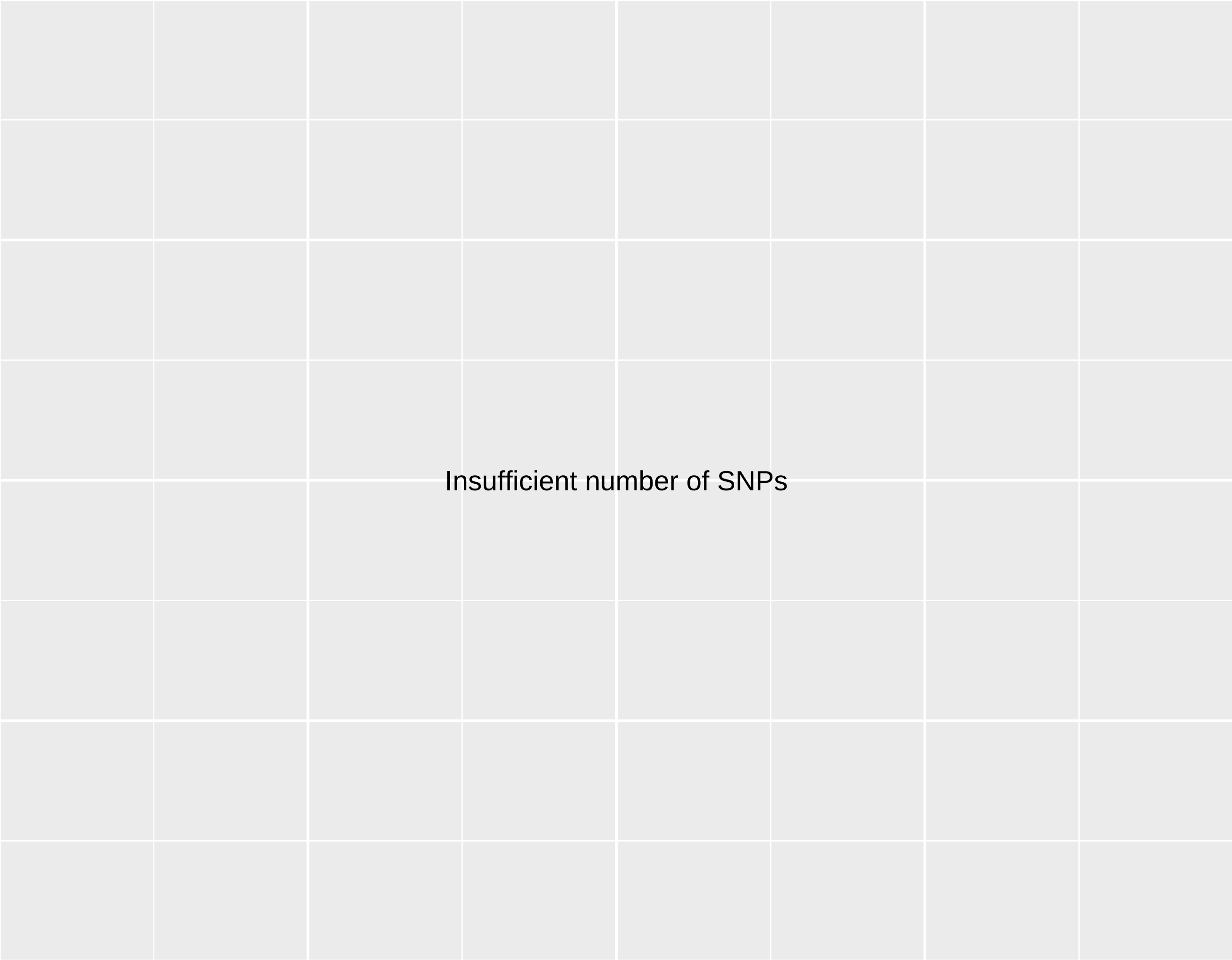
Insufficient number of SNPs

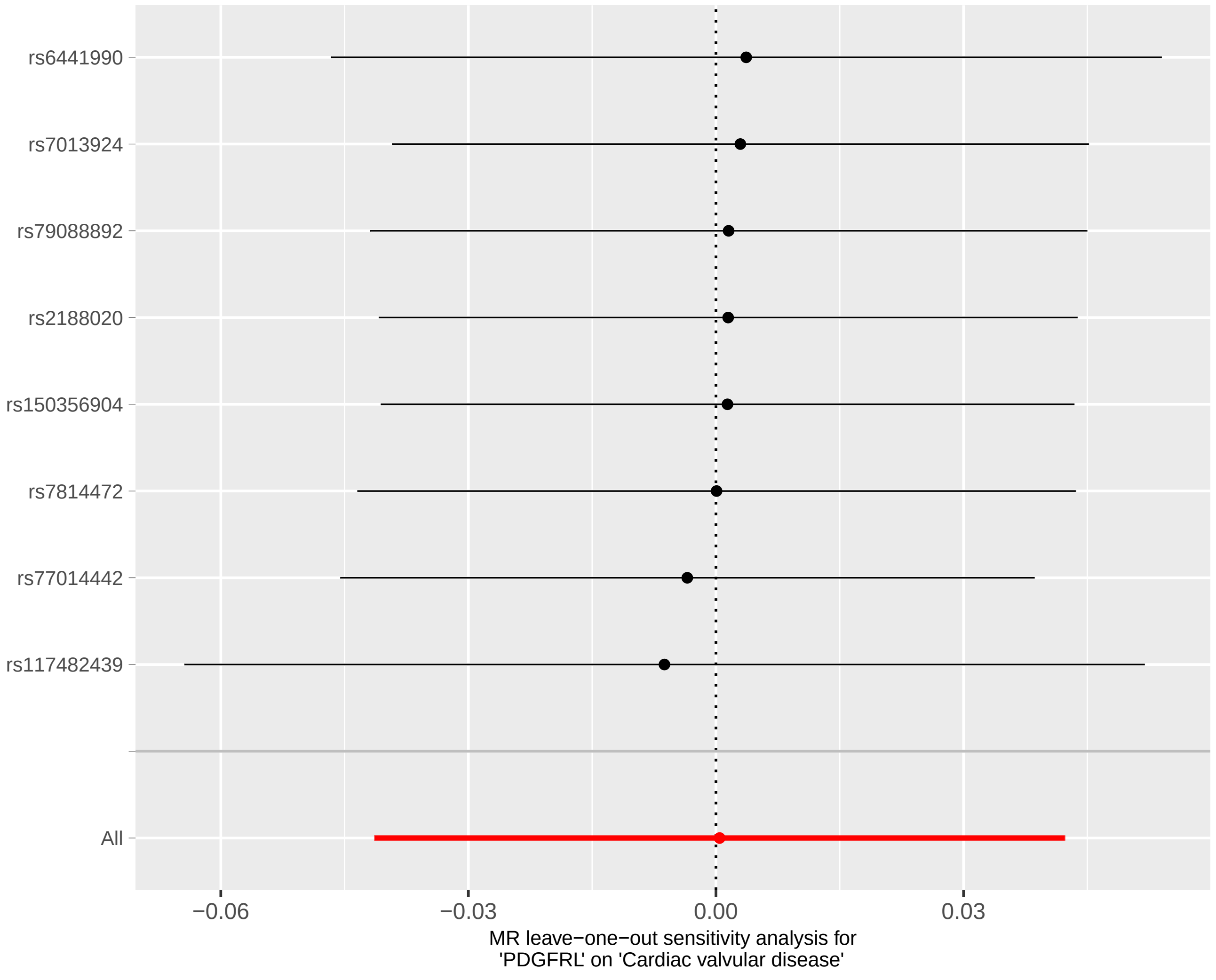

rs6441990
rs7013924
rs79088892
rs2188020
rs150356904
rs7814472
rs77014442
rs117482439
All
−0.06
−0.03
0.00
0.03
MR leave−one−out sensitivity analysis for
'PDGFRL' on 'Cardiac valvular disease'

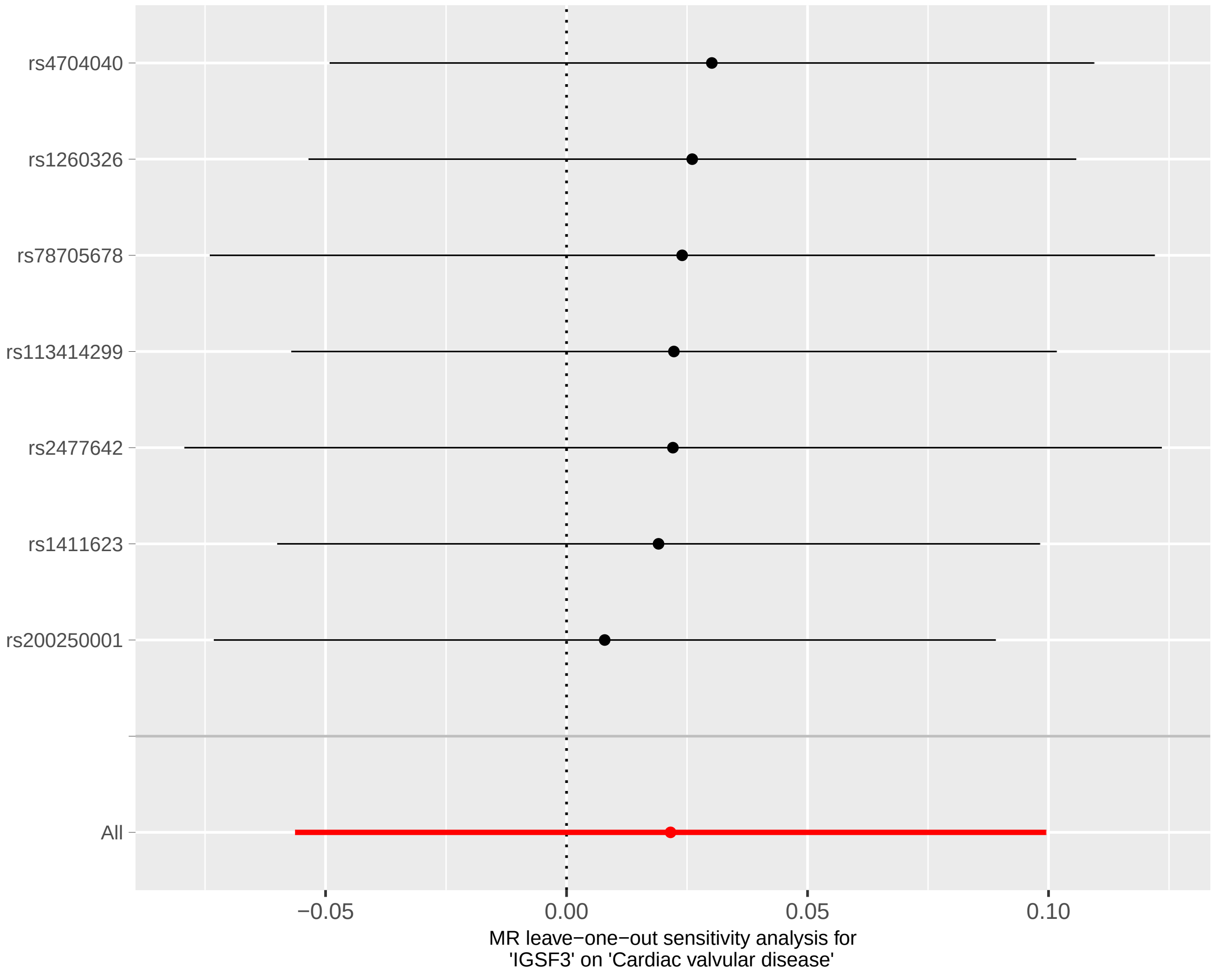
rs4704040
rs1260326
rs78705678
rs113414299
rs2477642
rs1411623
rs200250001
All
−0.05
0.00
0.05
0.10
MR leave−one−out sensitivity analysis for
'IGSF3' on 'Cardiac valvular disease'

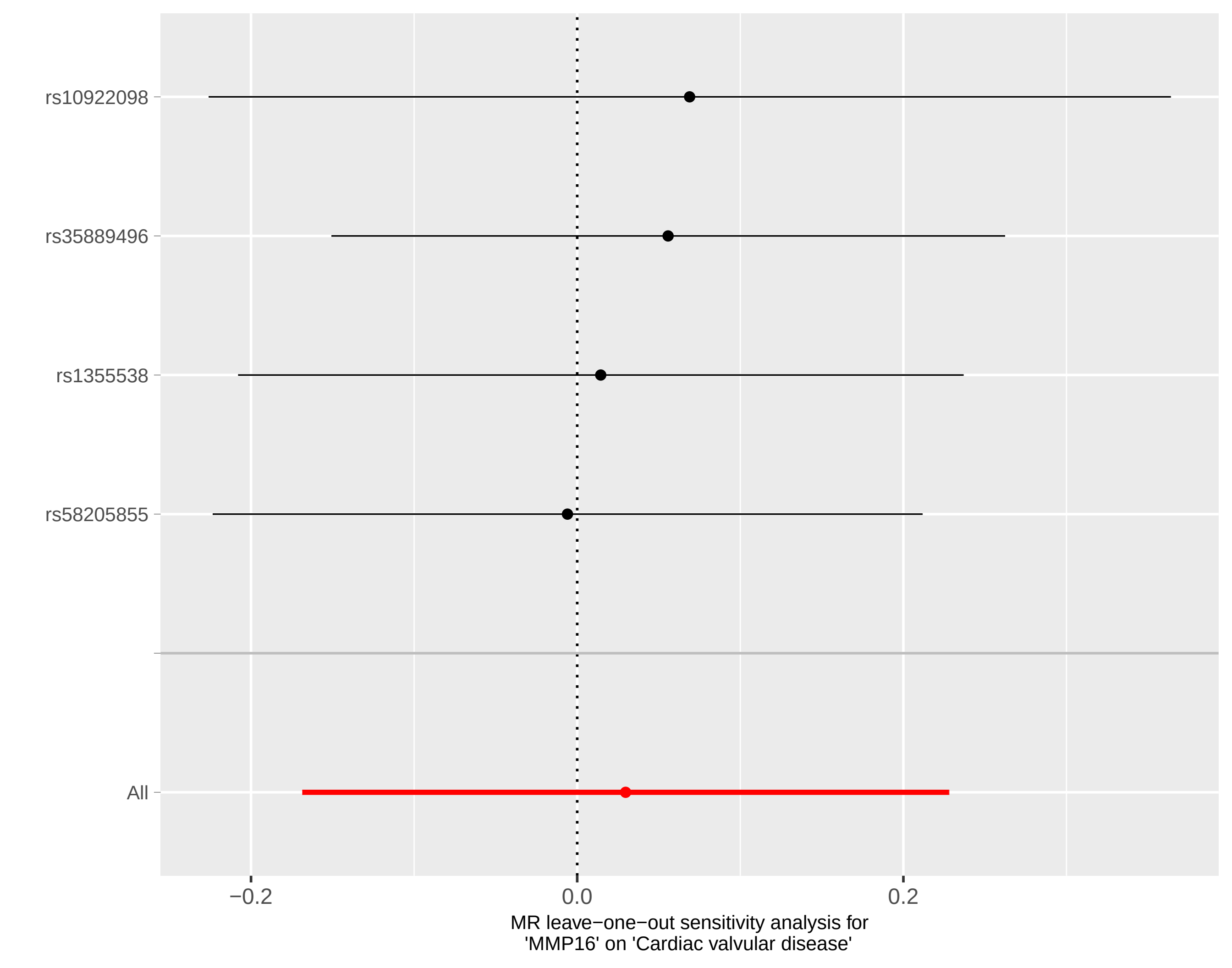
rs10922098
rs35889496
rs1355538
rs58205855
All
−0.2
0.0
0.2
MR leave−one−out sensitivity analysis for
'MMP16' on 'Cardiac valvular disease'

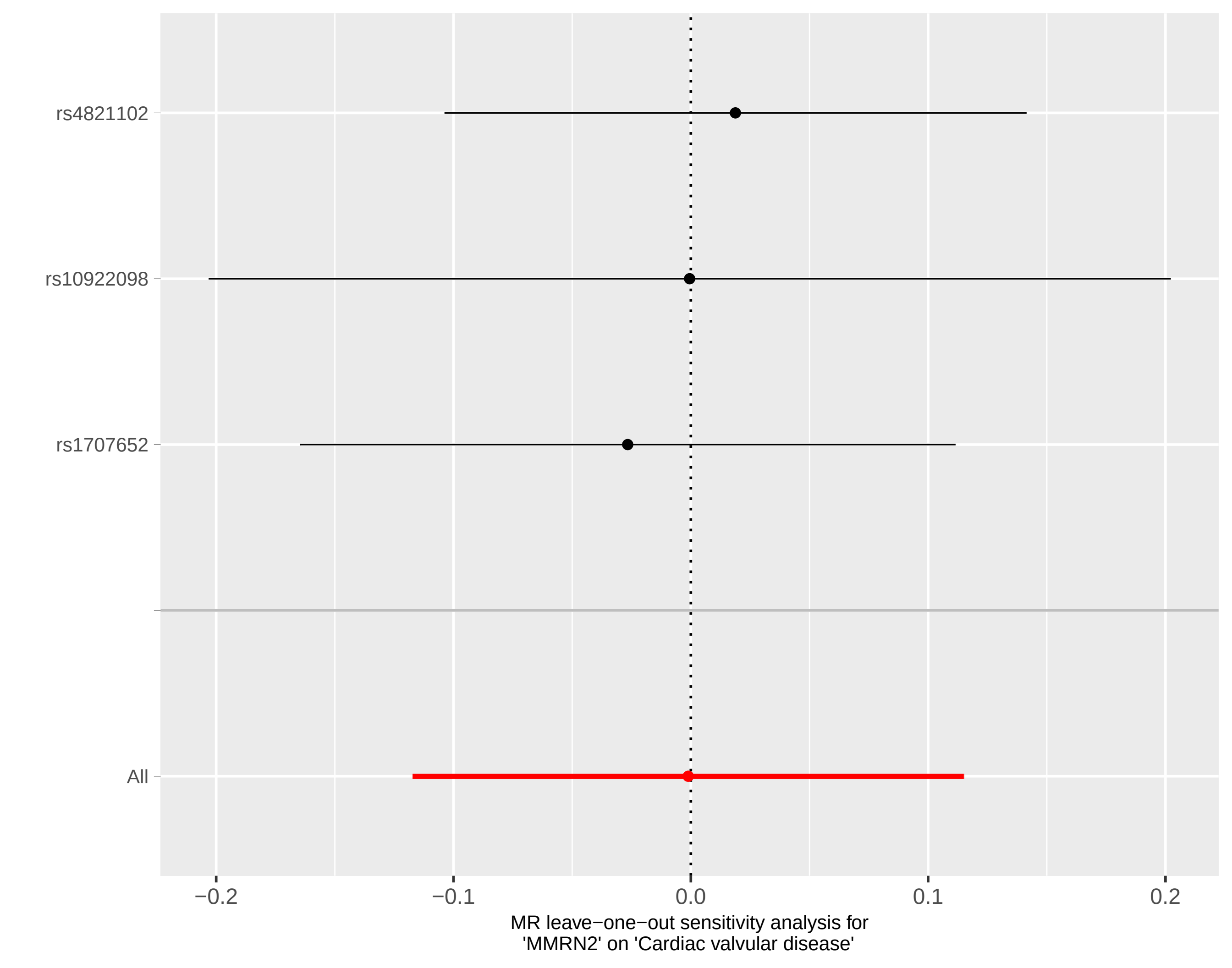
rs4821102
rs10922098
rs1707652
All
−0.2
−0.1
0.0
0.1
0.2
MR leave−one−out sensitivity analysis for
'MMRN2' on 'Cardiac valvular disease'

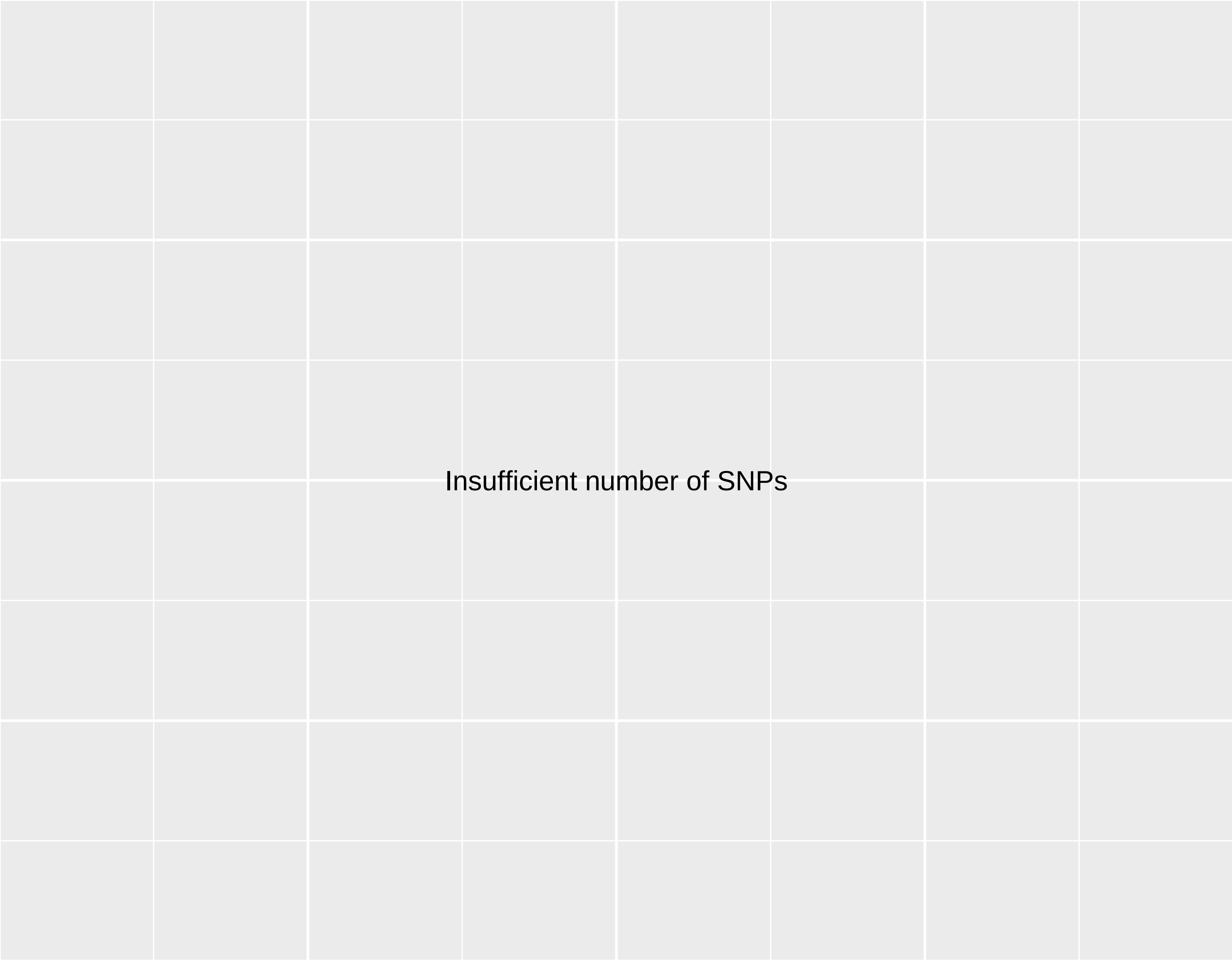
Insufficient number of SNPs

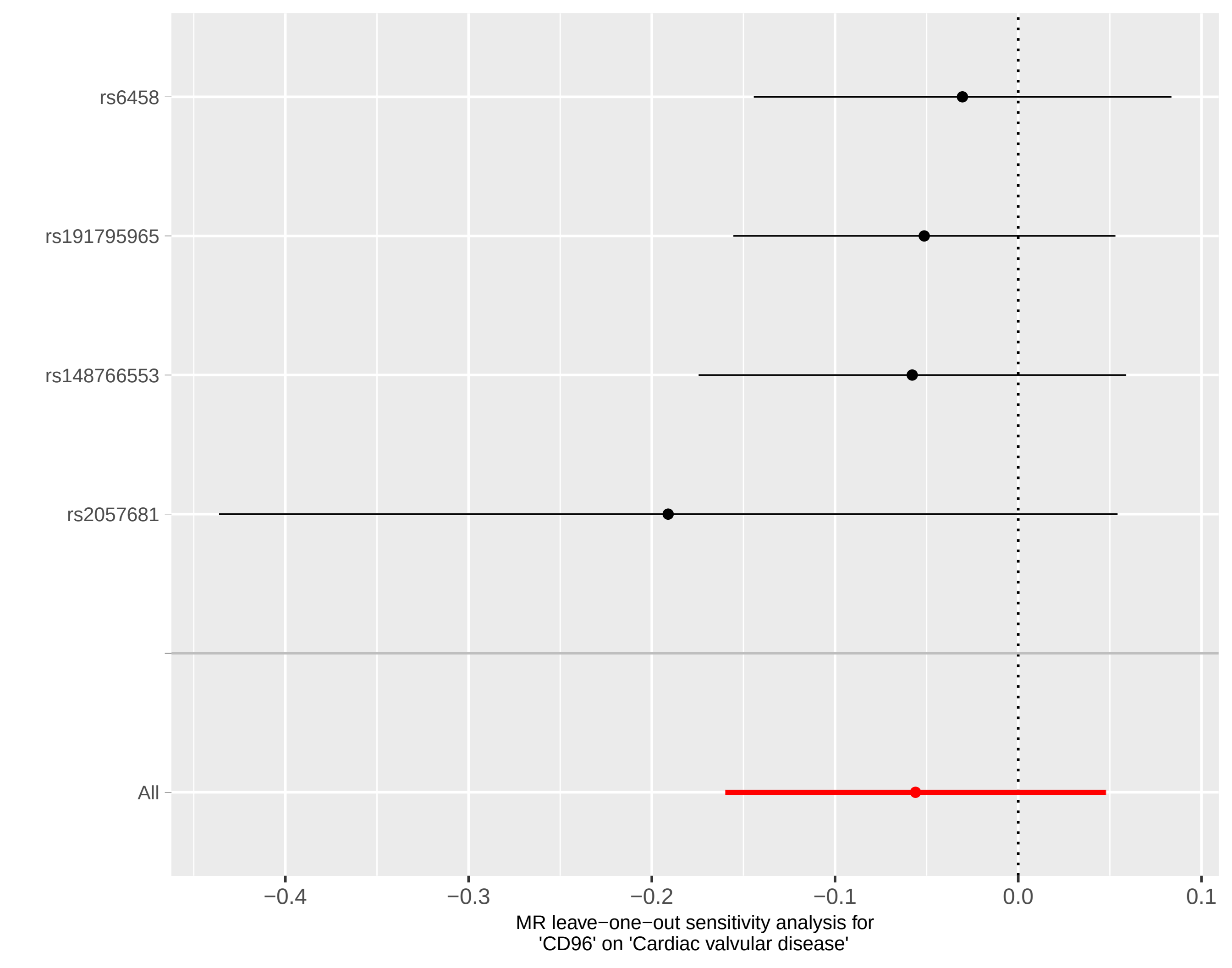

rs6458
rs191795965
rs148766553
rs2057681
All
−0.4
−0.3
−0.2
−0.1
0.0
0.1
MR leave−one−out sensitivity analysis for
'CD96' on 'Cardiac valvular disease'

Insufficient number of SNPs

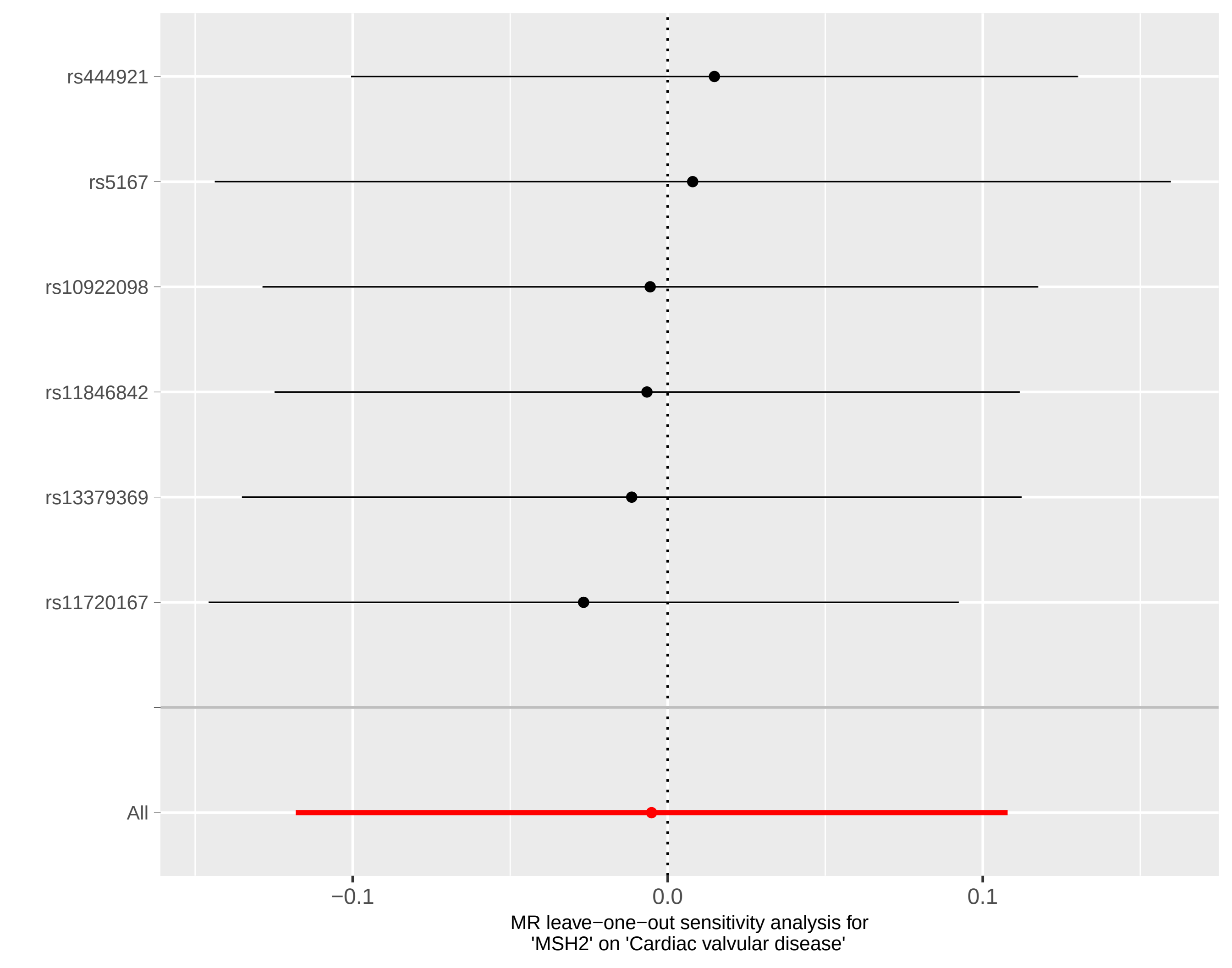
rs444921
rs5167
rs10922098
rs11846842
rs13379369
rs11720167
All
−0.1
0.0
0.1
MR leave−one−out sensitivity analysis for
'MSH2' on 'Cardiac valvular disease'

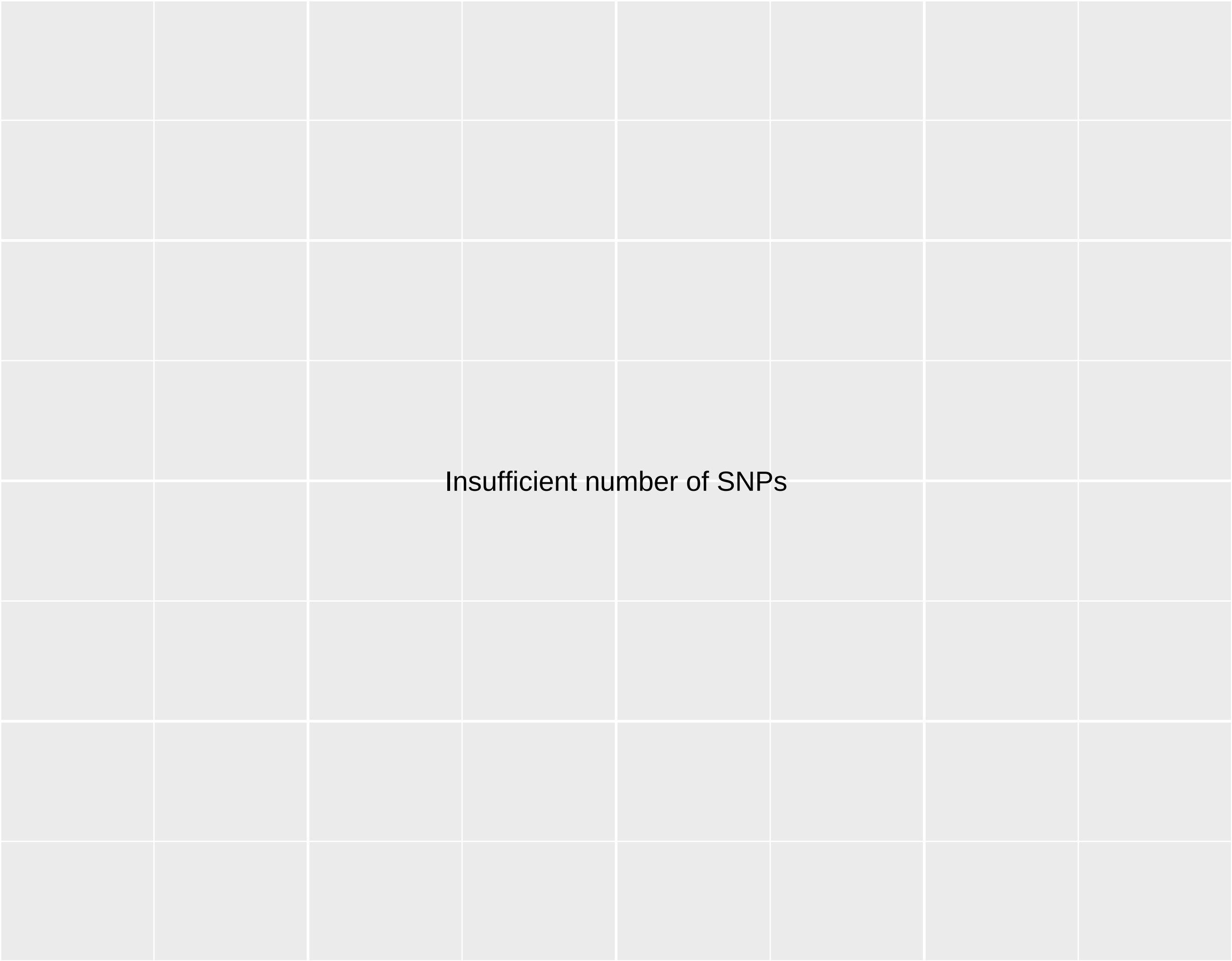
Insufficient number of SNPs

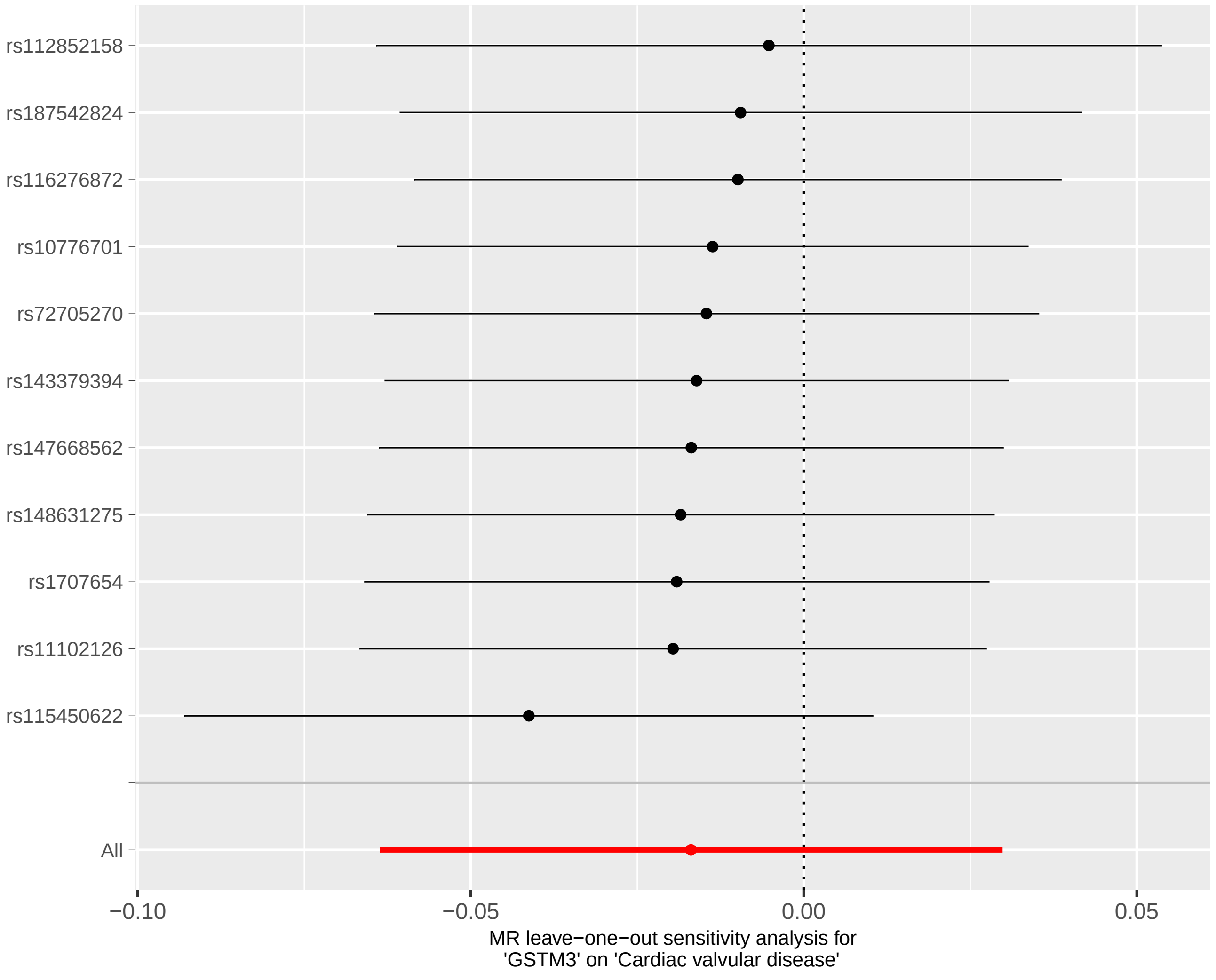

rs112852158
rs187542824
rs116276872
rs10776701
rs72705270
rs143379394
rs147668562
rs148631275
rs1707654
rs11102126
rs115450622
All
−0.10
−0.05
0.00
0.05
MR leave−one−out sensitivity analysis for
'GSTM3' on 'Cardiac valvular disease'

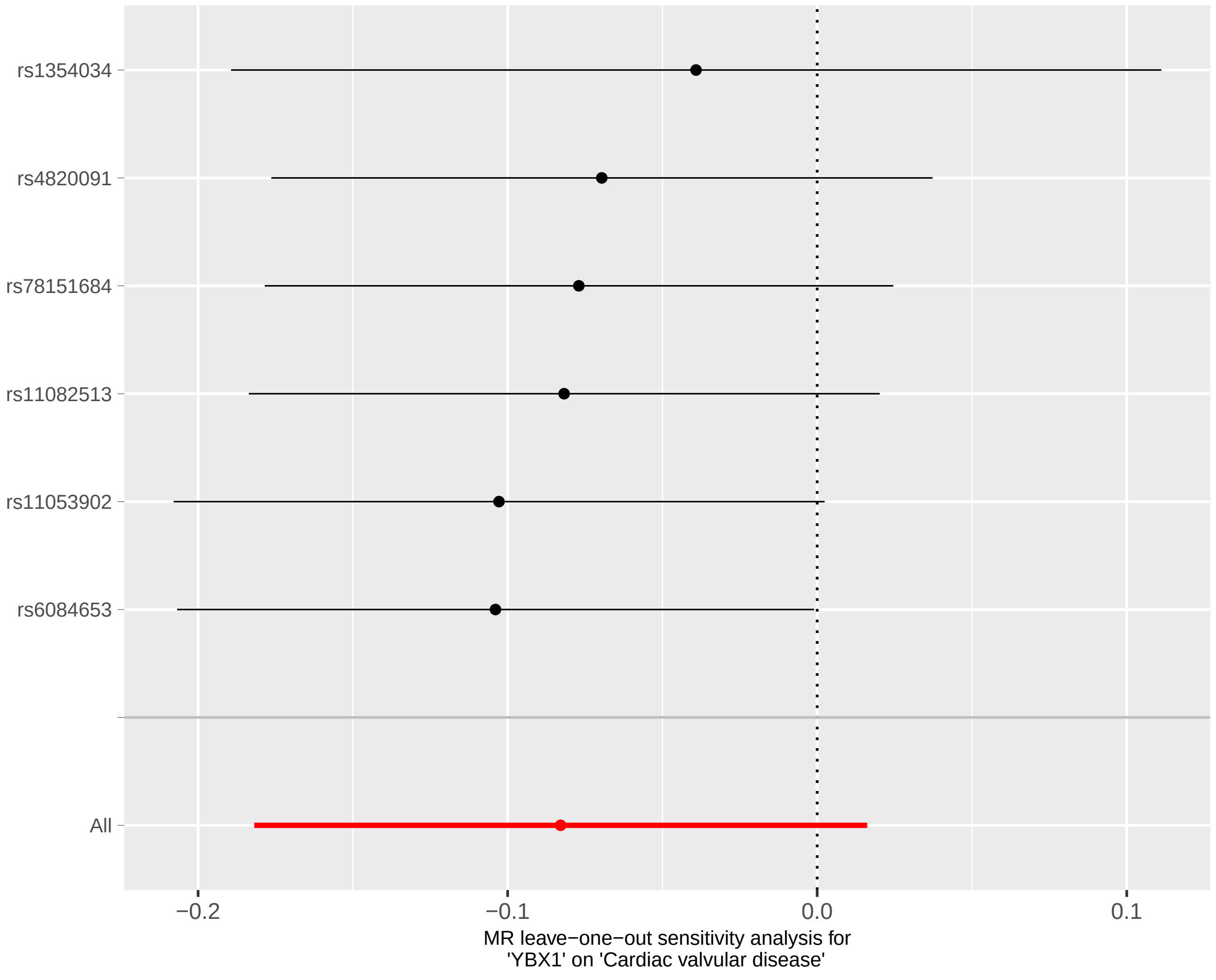
rs1354034
rs4820091
rs78151684
rs11082513
rs11053902
rs6084653
All
−0.2
−0.1
0.0
0.1
MR leave−one−out sensitivity analysis for
'YBX1' on 'Cardiac valvular disease'

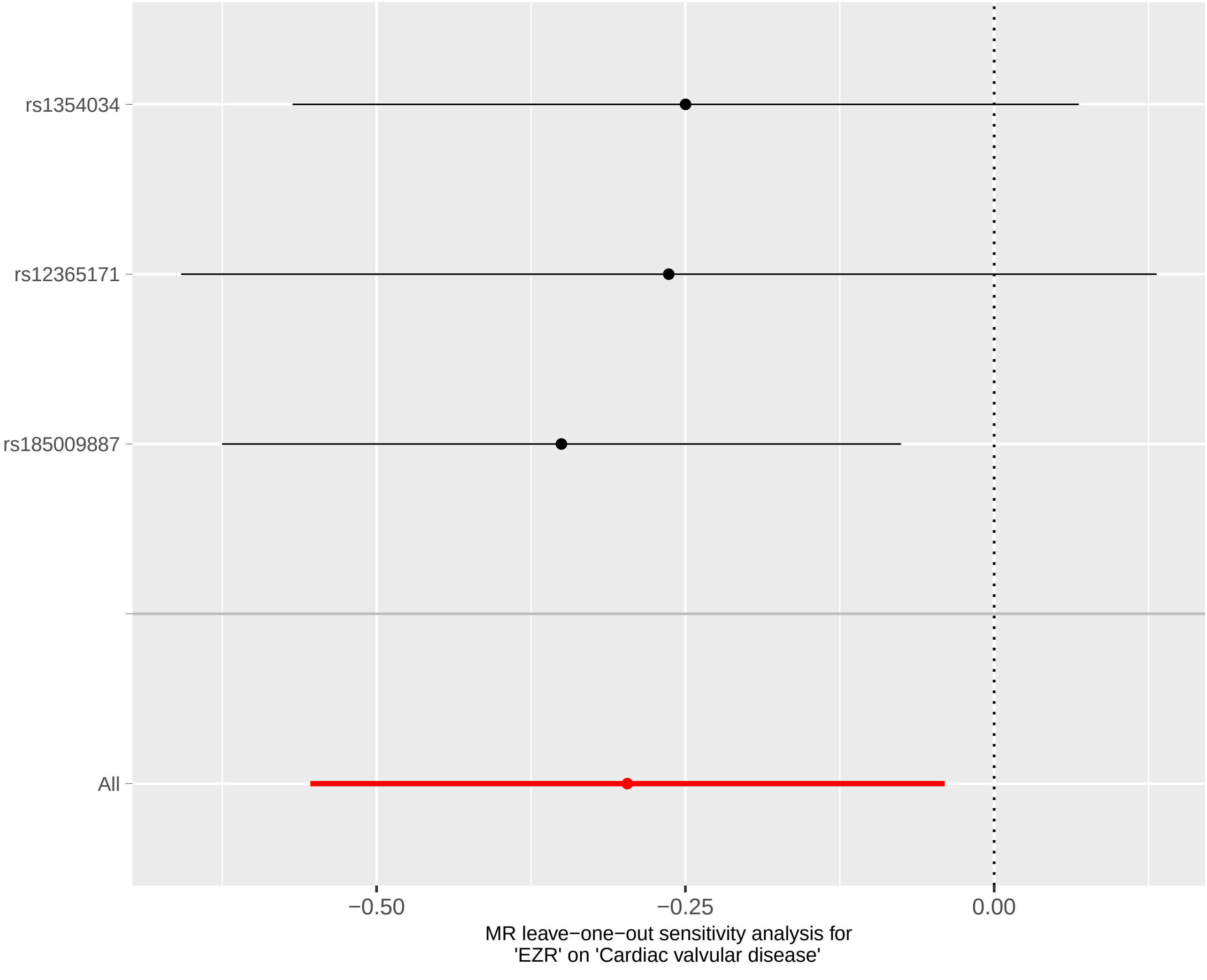
rs1354034
rs12365171
rs185009887
All
−0.50
−0.25
0.00
MR leave−one−out sensitivity analysis for
'EZR' on 'Cardiac valvular disease'

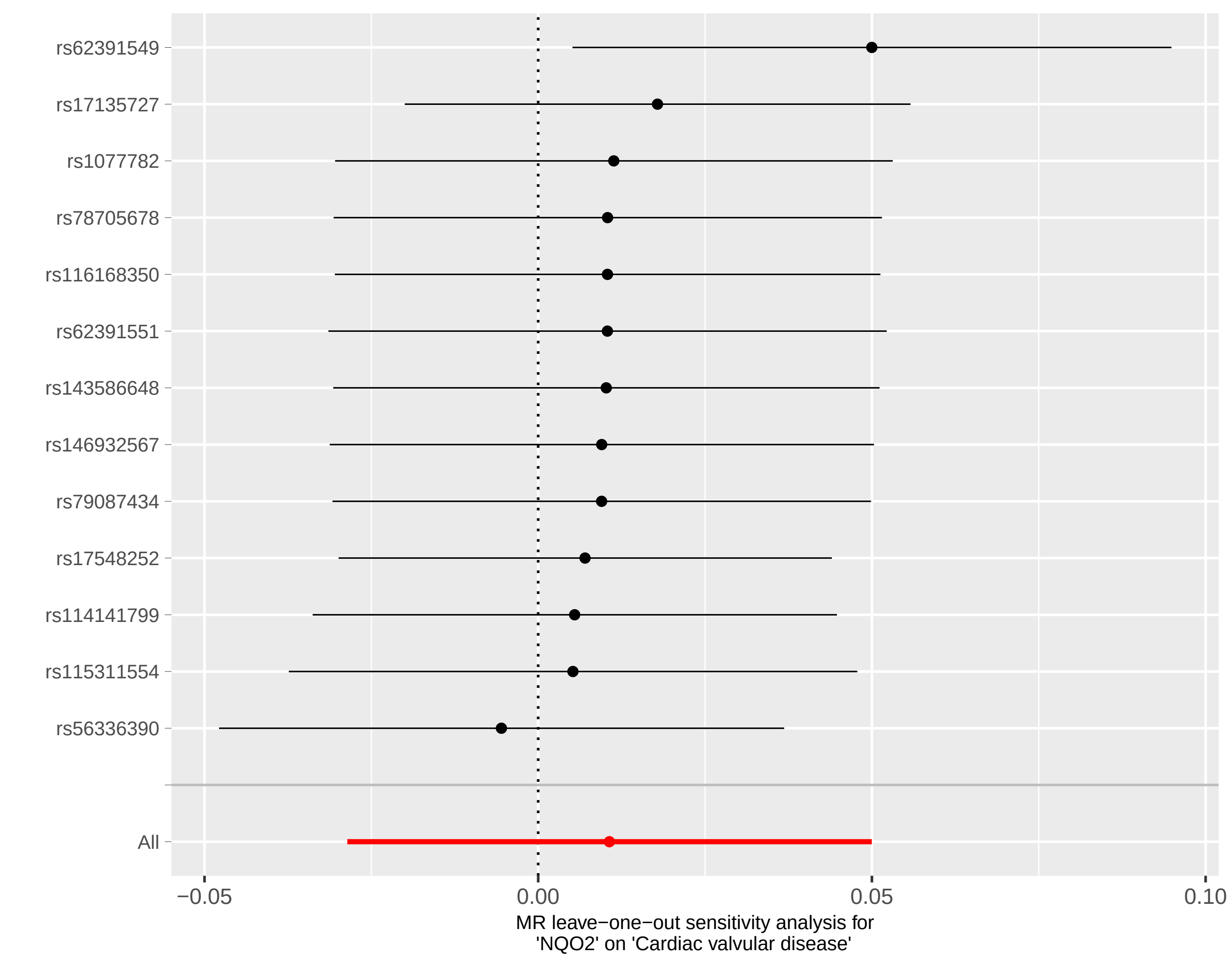
rs62391549
rs17135727
rs1077782
rs78705678
rs116168350
rs62391551
rs143586648
rs146932567
rs79087434
rs17548252
rs114141799
rs115311554
rs56336390
All
−0.05
0.00
0.05
0.10
MR leave−one−out sensitivity analysis for
'NQO2' on 'Cardiac valvular disease'

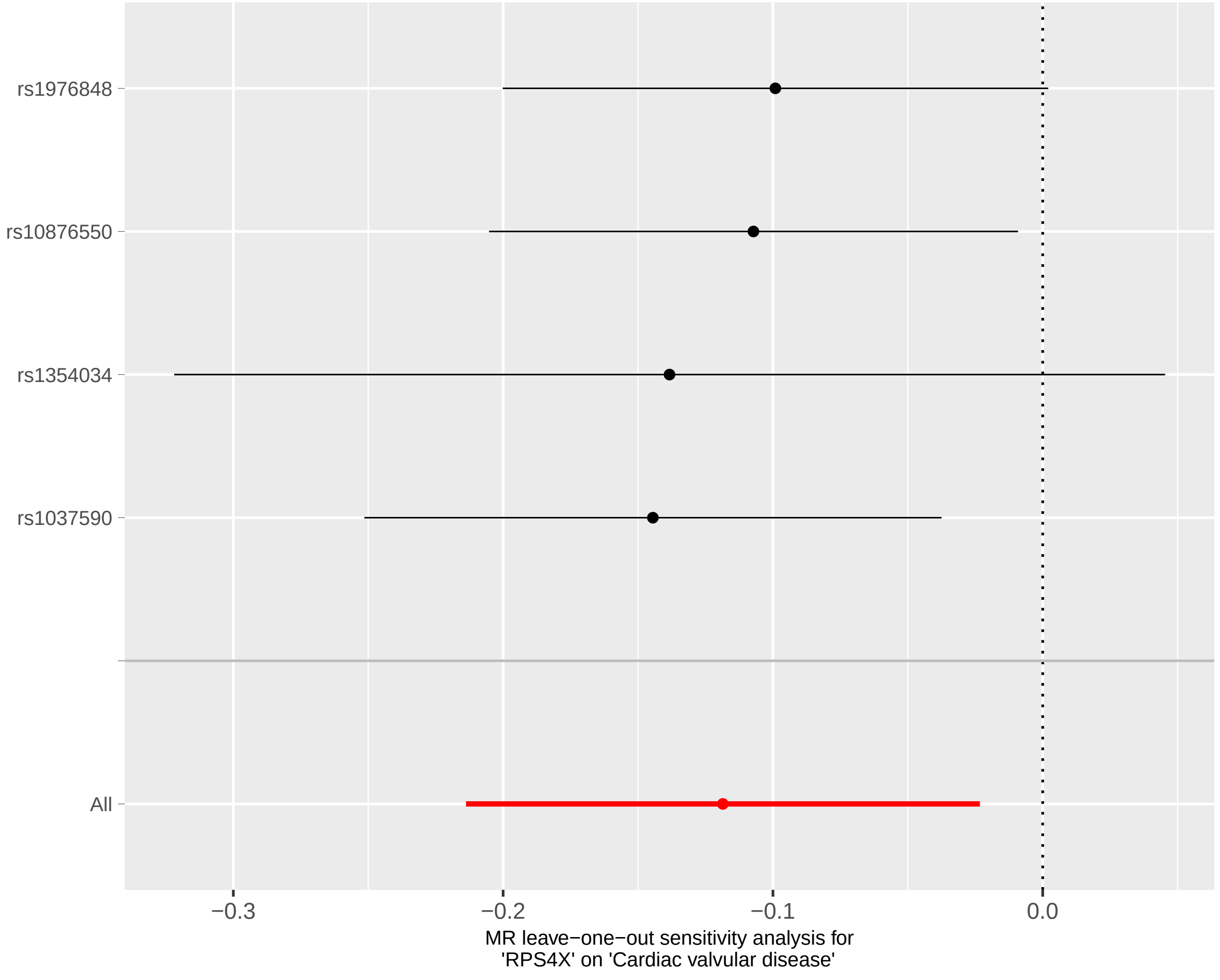
rs1976848
rs10876550
rs1354034
rs1037590
All
−0.3
−0.2
−0.1
0.0
MR leave−one−out sensitivity analysis for
'RPS4X' on 'Cardiac valvular disease'

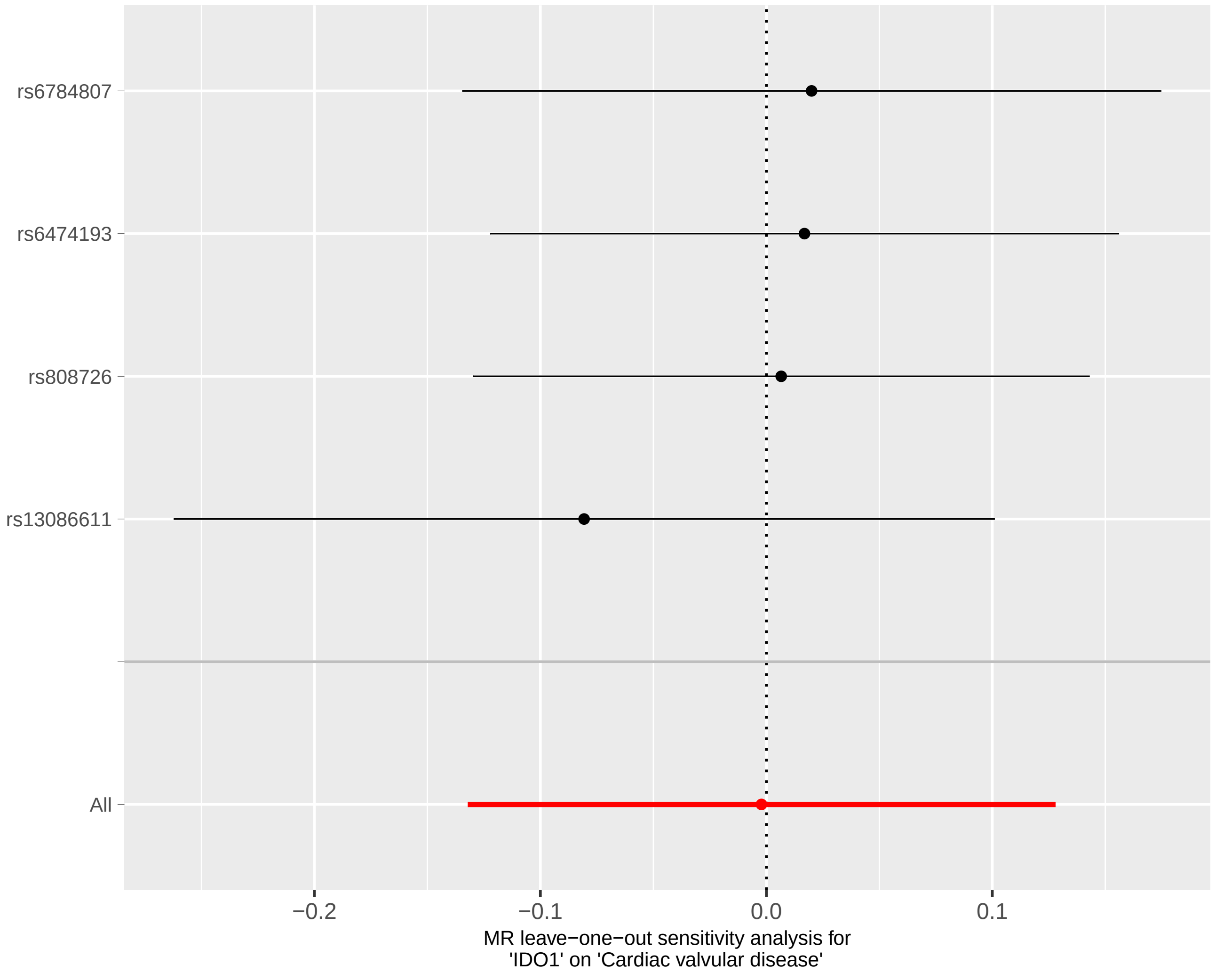
rs6784807
rs6474193
rs808726
rs13086611
All
−0.2
−0.1
0.0
0.1
MR leave−one−out sensitivity analysis for
'IDO1' on 'Cardiac valvular disease'

Insufficient number of SNPs

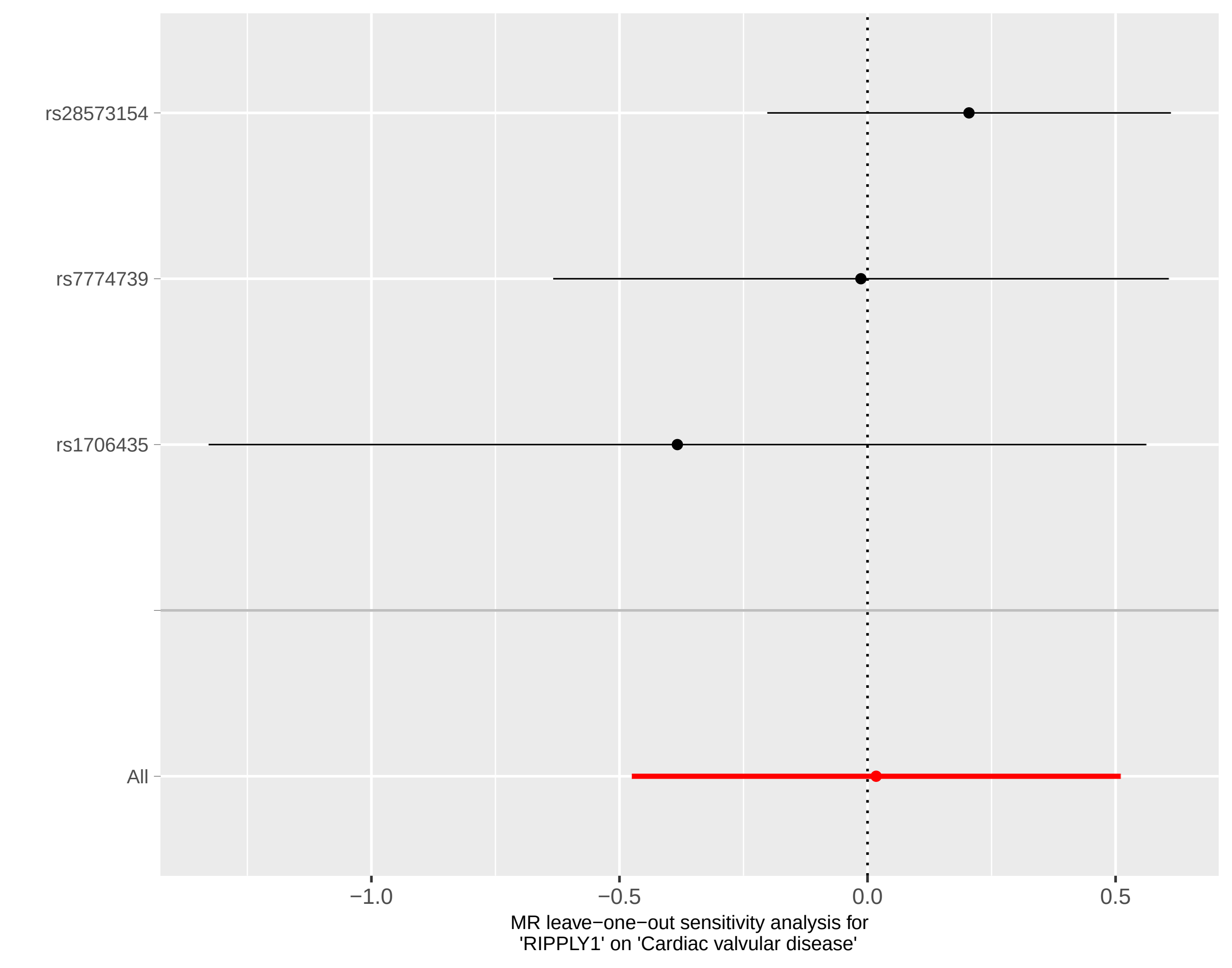

rs28573154
rs7774739
rs1706435
All
−1.0
−0.5
0.0
0.5
MR leave−one−out sensitivity analysis for
'RIPPLY1' on 'Cardiac valvular disease'

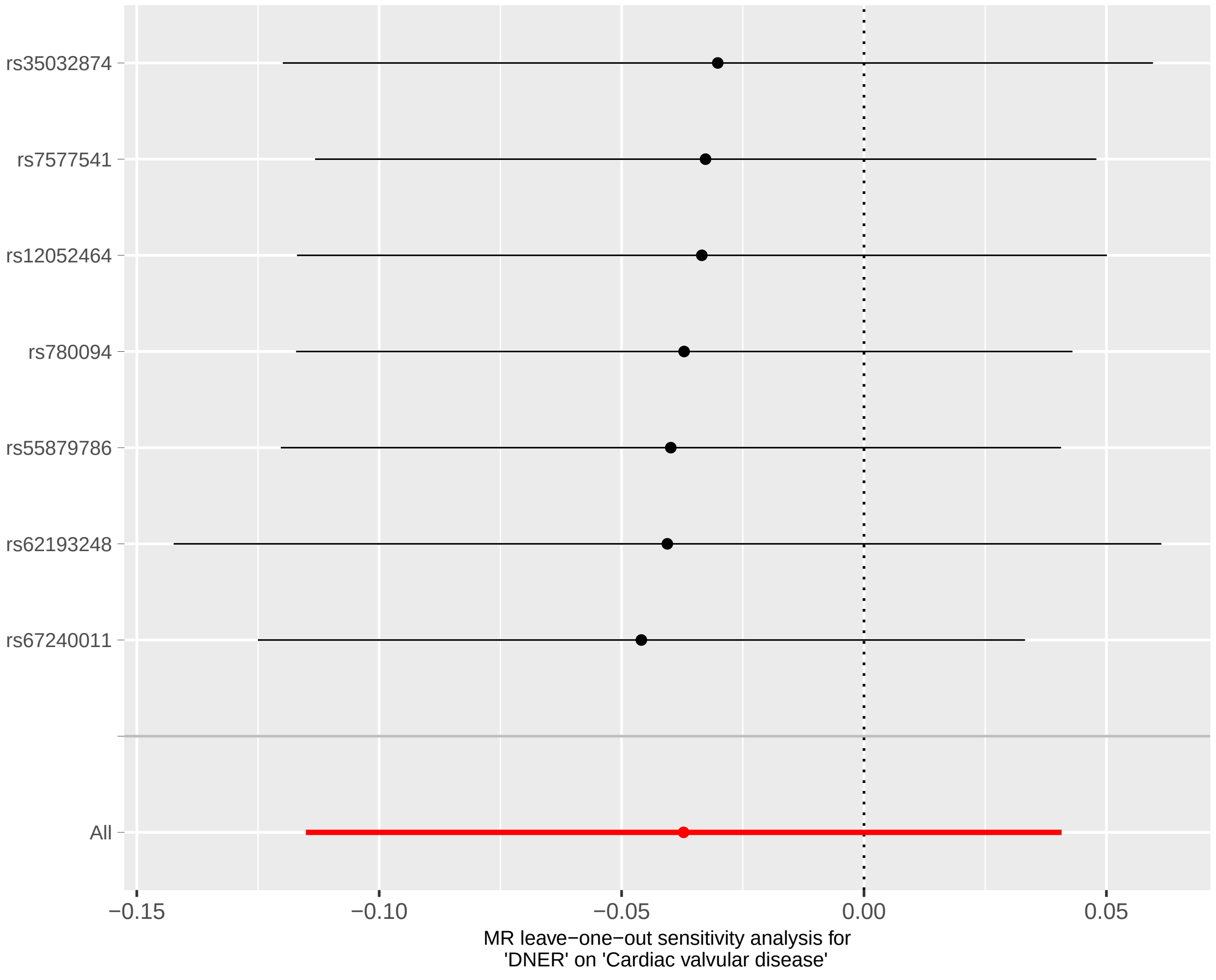
rs35032874
rs7577541
rs12052464
rs780094
rs55879786
rs62193248
rs67240011
All
−0.15
−0.10
−0.05
0.00
0.05
MR leave−one−out sensitivity analysis for
'DNER' on 'Cardiac valvular disease'

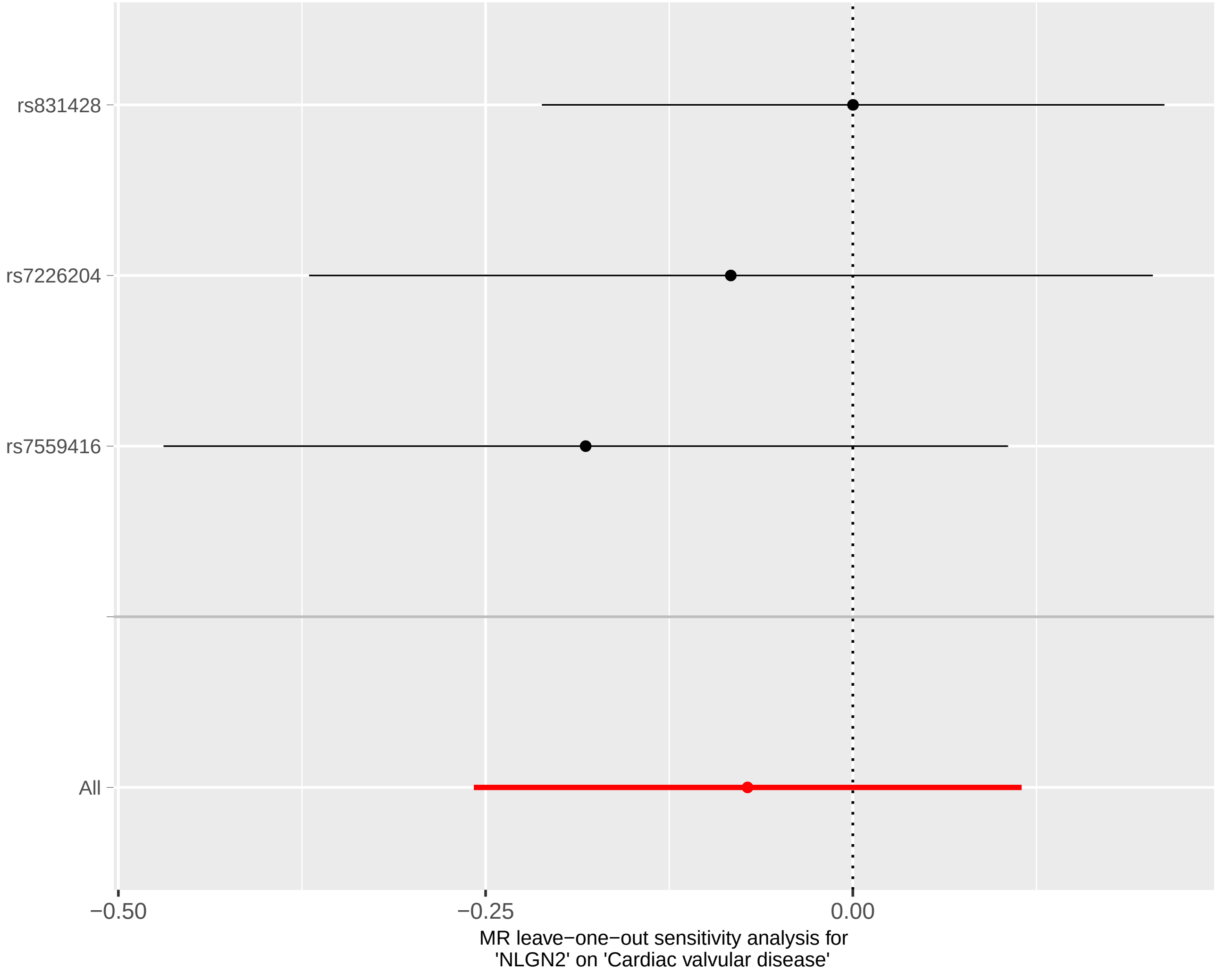
rs831428
rs7226204
rs7559416
All
−0.50
−0.25
0.00
MR leave−one−out sensitivity analysis for
'NLGN2' on 'Cardiac valvular disease'

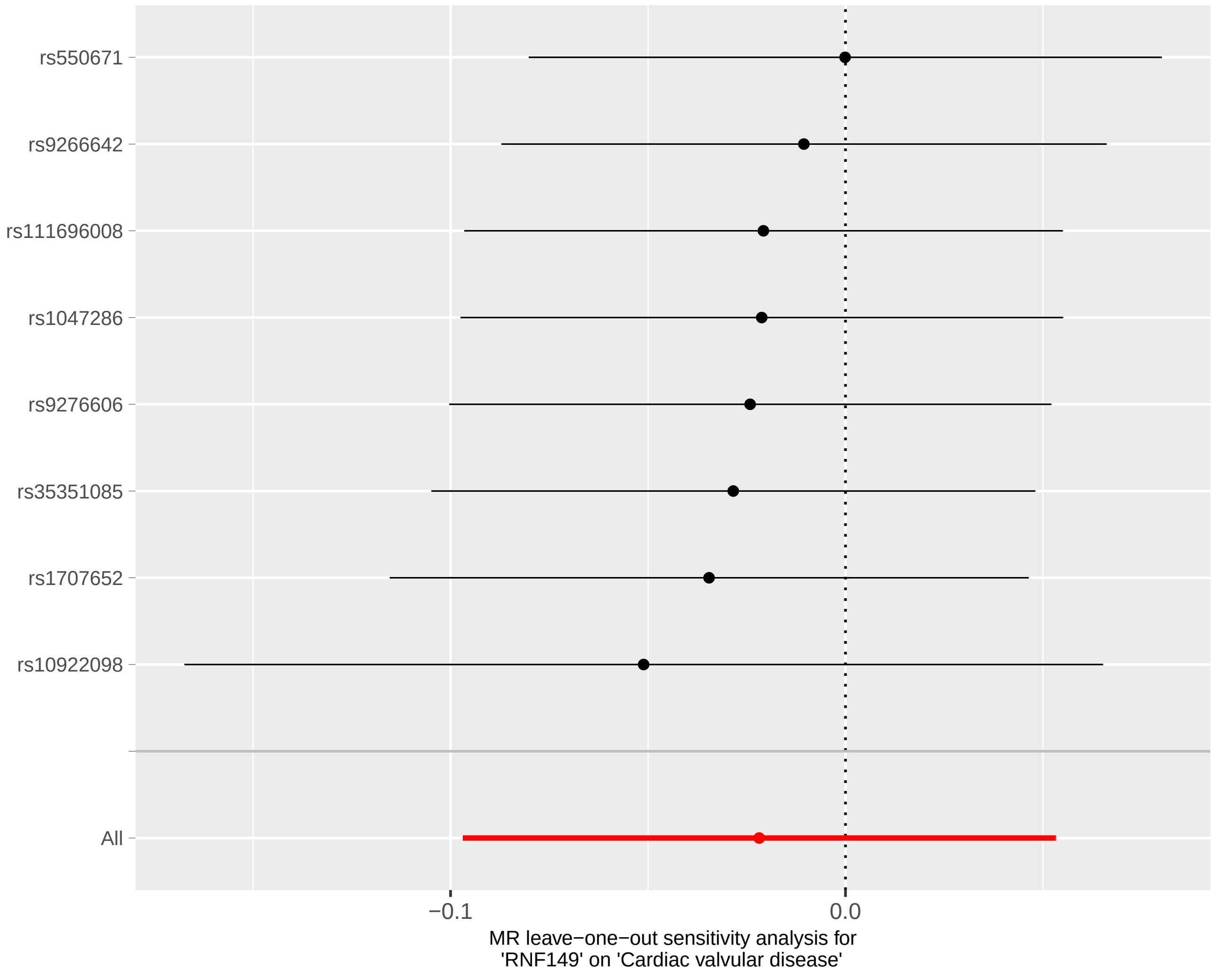
rs550671
rs9266642
rs111696008
rs1047286
rs9276606
rs35351085
rs1707652
rs10922098
All
−0.1
0.0
MR leave−one−out sensitivity analysis for
'RNF149' on 'Cardiac valvular disease'

Insufficient number of SNPs

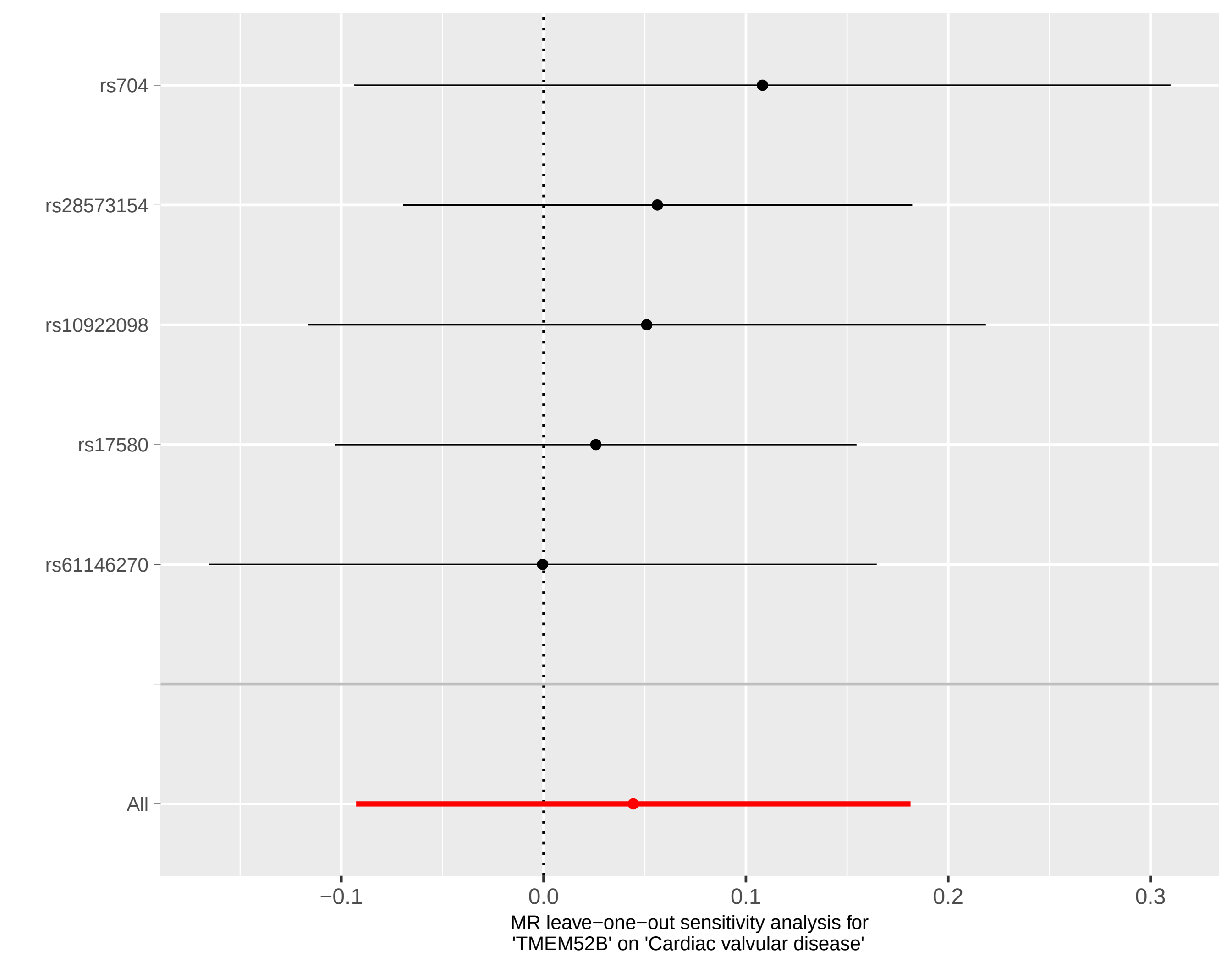
rs704
rs28573154
rs10922098
rs17580
rs61146270
All
−0.1
0.0
0.1
0.2
0.3
MR leave−one−out sensitivity analysis for
'TMEM52B' on 'Cardiac valvular disease'

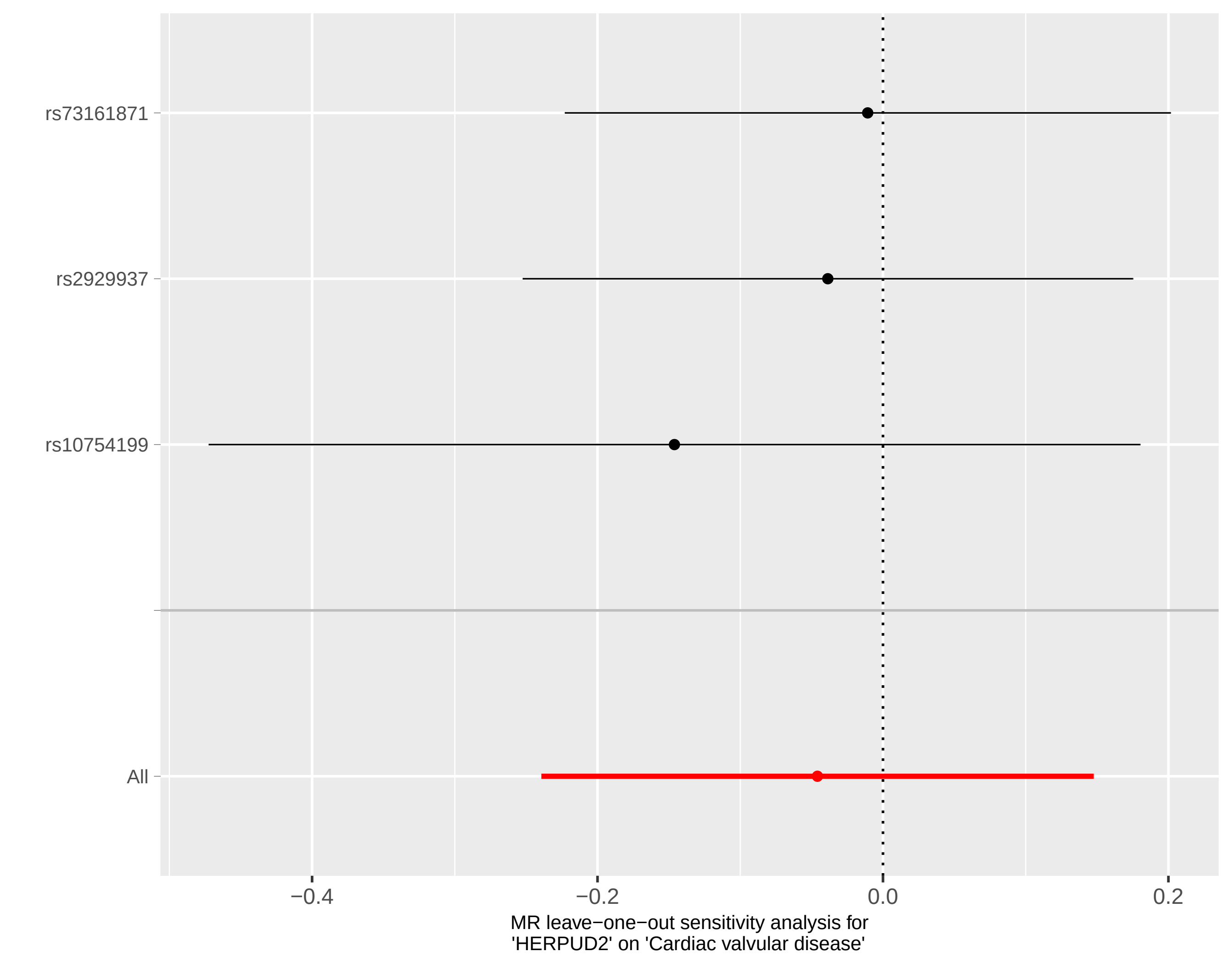
rs73161871
rs2929937
rs10754199
All
−0.4
−0.2
0.0
0.2
MR leave−one−out sensitivity analysis for
'HERPUD2' on 'Cardiac valvular disease'

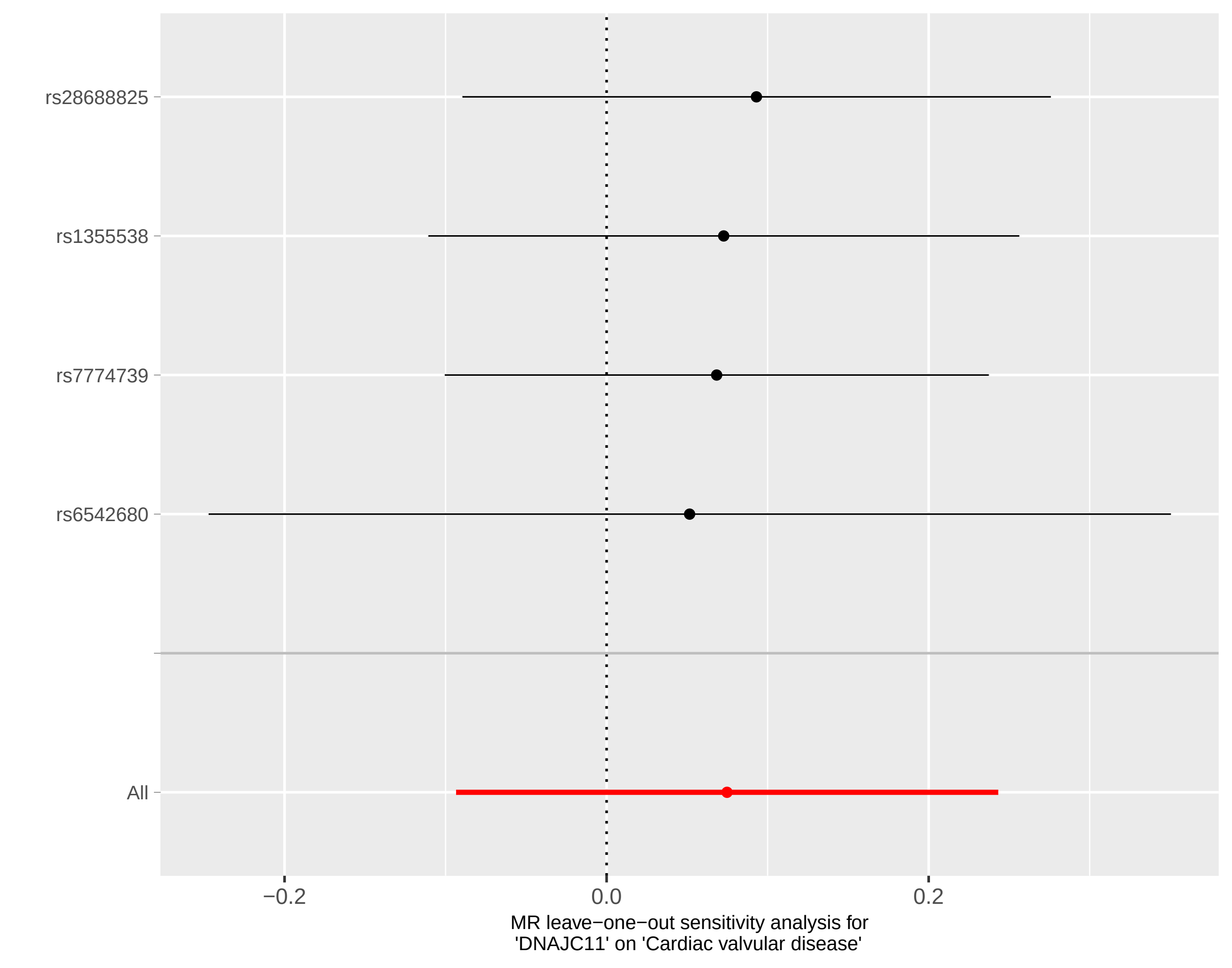
rs28688825
rs1355538
rs7774739
rs6542680
All
−0.2
0.0
0.2
MR leave−one−out sensitivity analysis for
'DNAJC11' on 'Cardiac valvular disease'

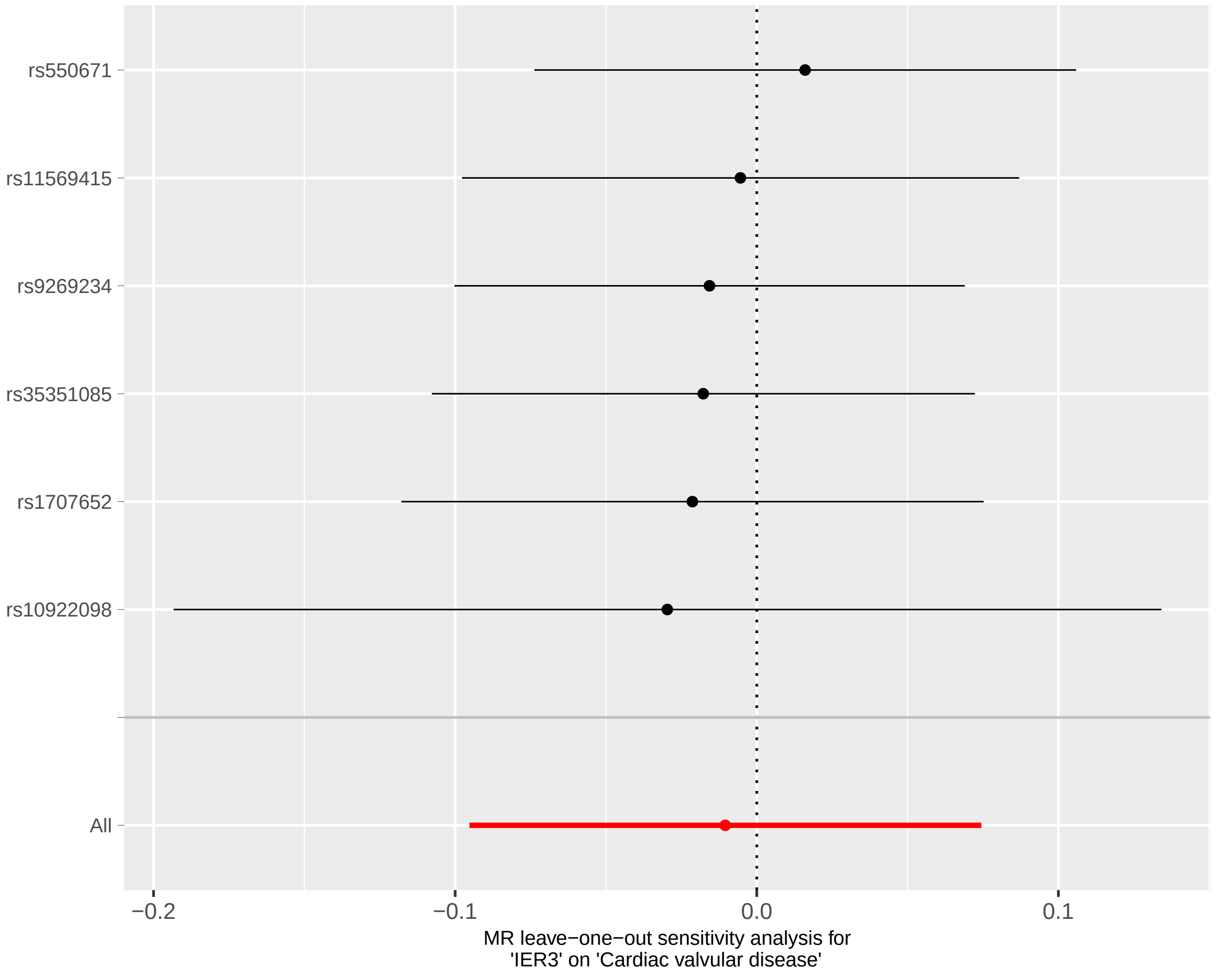

rs550671
rs11569415
rs9269234
rs35351085
rs1707652
rs10922098
All
−0.2
−0.1
0.0
0.1
MR leave−one−out sensitivity analysis for
'IER3' on 'Cardiac valvular disease'

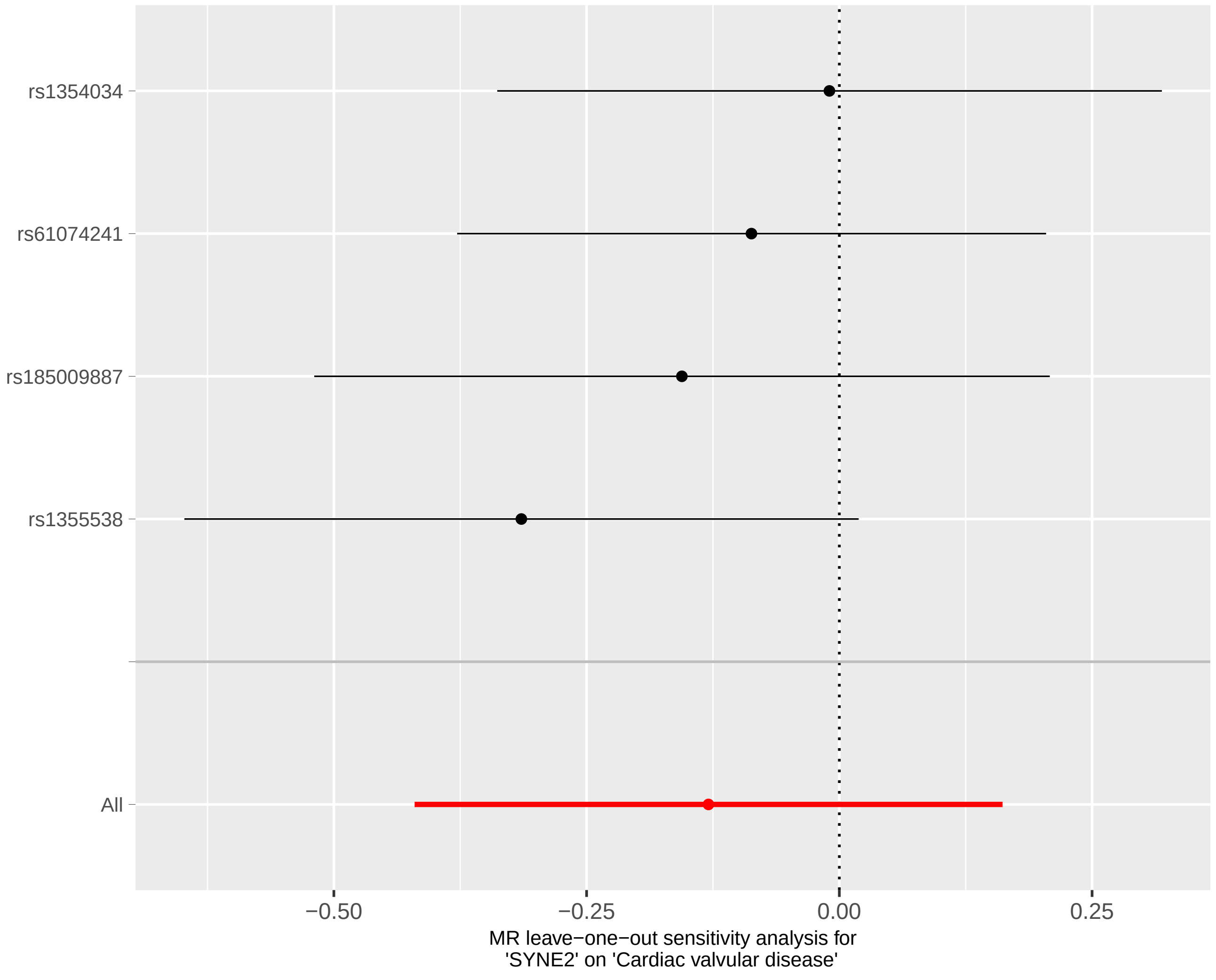
rs1354034
rs61074241
rs185009887
rs1355538
All
−0.50
−0.25
0.00
0.25
MR leave−one−out sensitivity analysis for
'SYNE2' on 'Cardiac valvular disease'

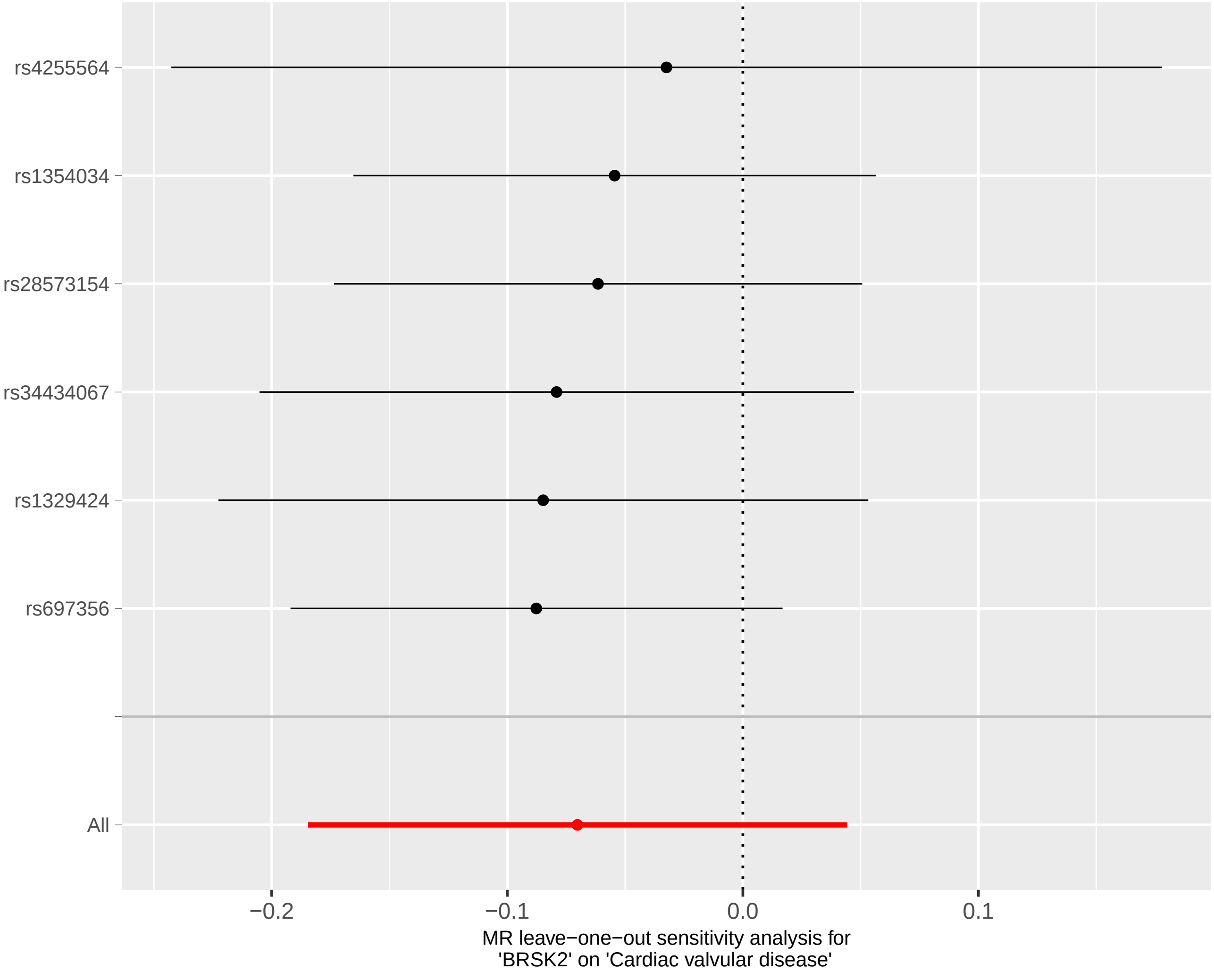
rs4255564
rs1354034
rs28573154
rs34434067
rs1329424
rs697356
All
−0.2
−0.1
0.0
0.1
MR leave−one−out sensitivity analysis for
'BRSK2' on 'Cardiac valvular disease'

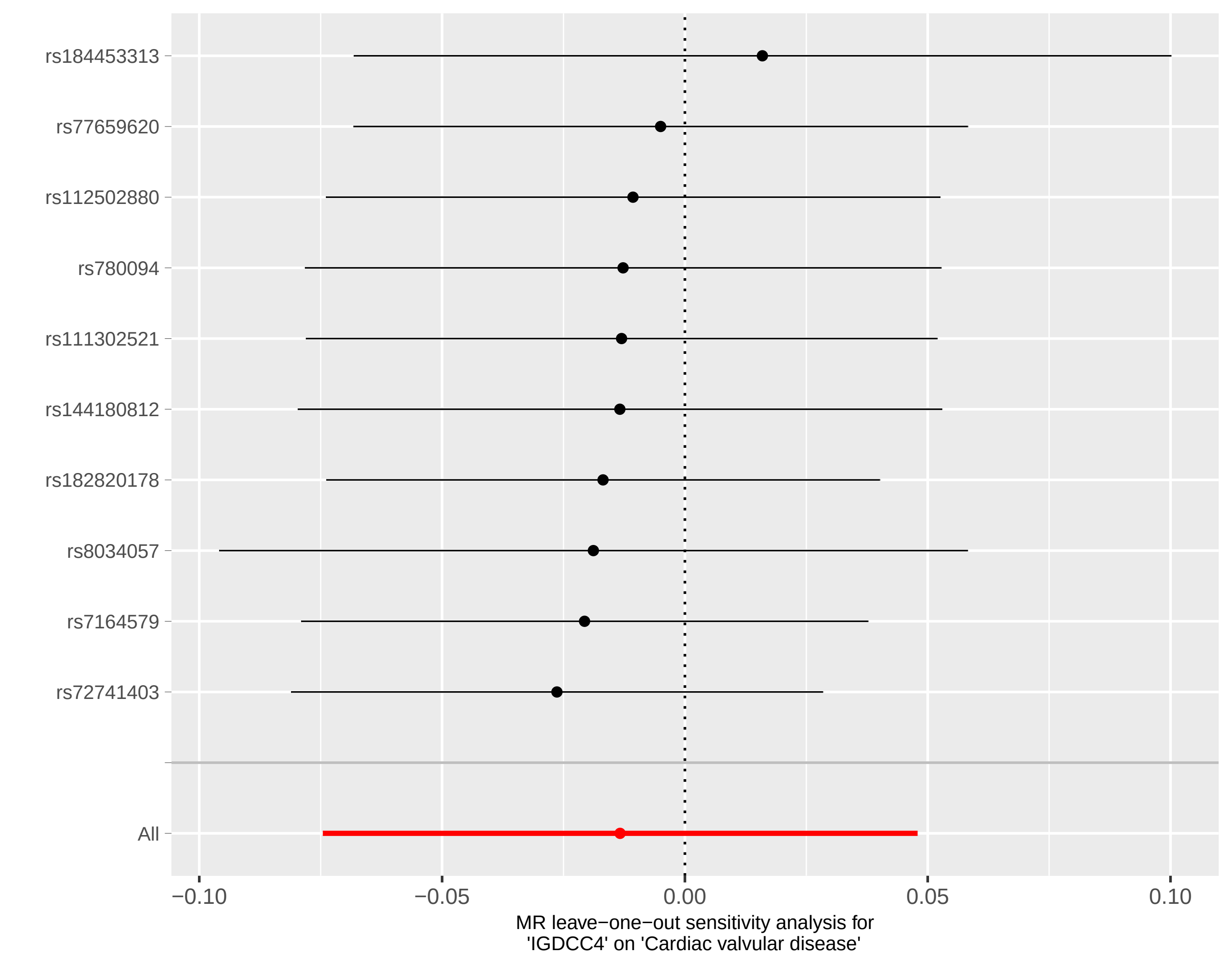
rs184453313
rs77659620
rs112502880
rs780094
rs111302521
rs144180812
rs182820178
rs8034057
rs7164579
rs72741403
All
−0.10
−0.05
0.00
0.05
0.10
MR leave−one−out sensitivity analysis for
'IGDCC4' on 'Cardiac valvular disease'

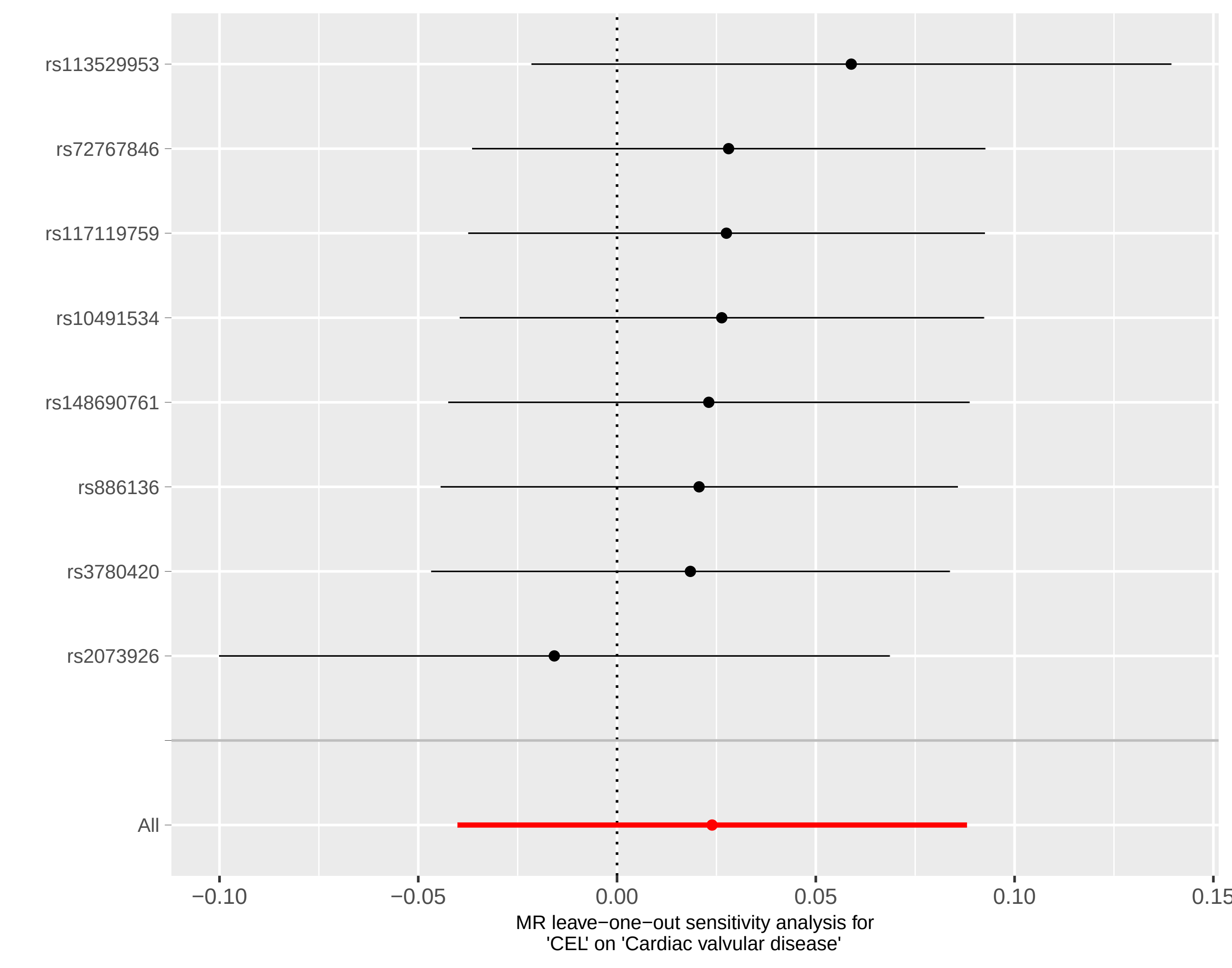
rs113529953
rs72767846
rs117119759
rs10491534
rs148690761
rs886136
rs3780420
rs2073926
All
−0.10
−0.05
0.00
0.05
0.10
MR leave−one−out sensitivity analysis for
'CEL' on 'Cardiac valvular disease'

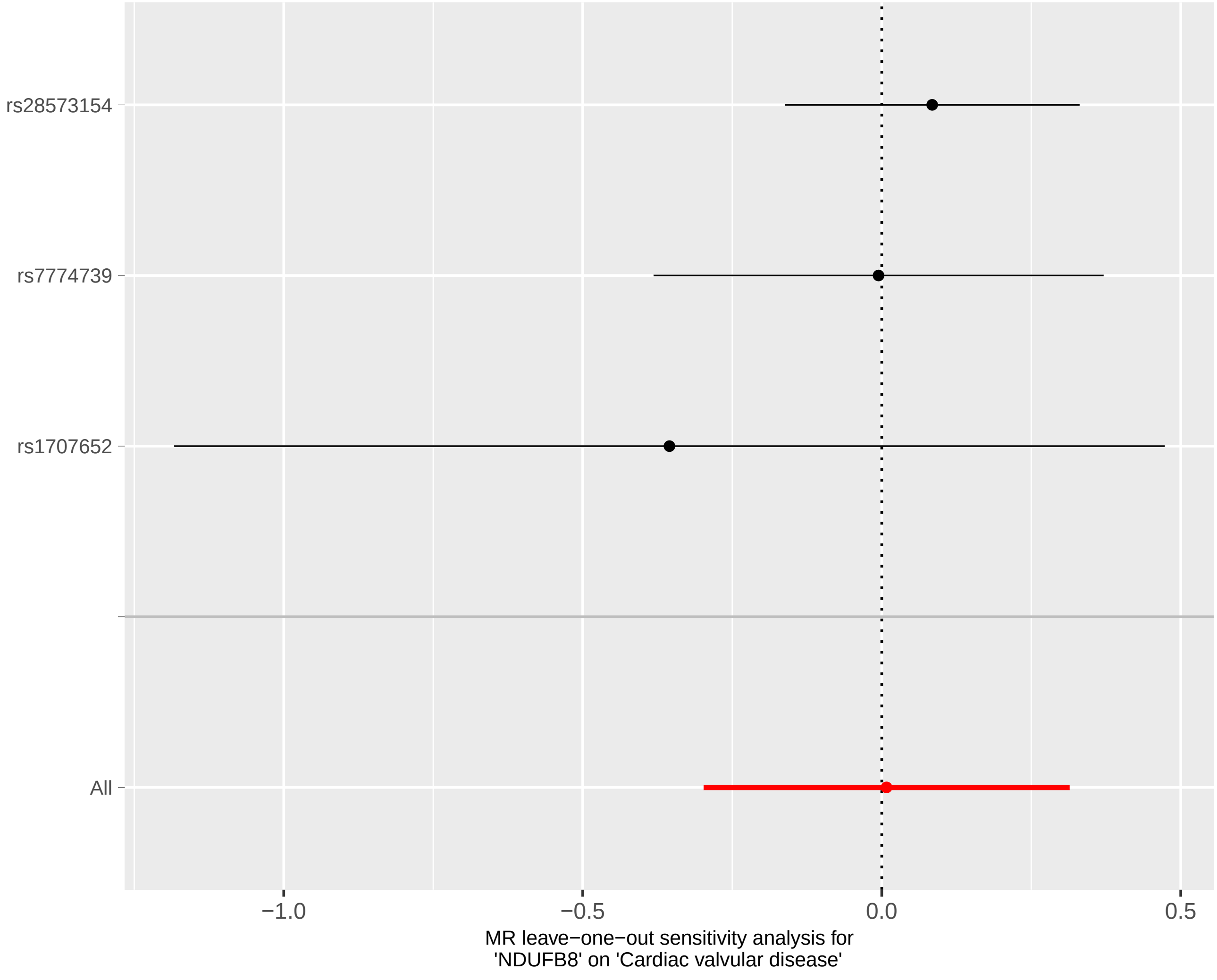

rs28573154
rs7774739
rs1707652
All
−1.0
−0.5
0.0
0.5
MR leave−one−out sensitivity analysis for
'NDUFB8' on 'Cardiac valvular disease'

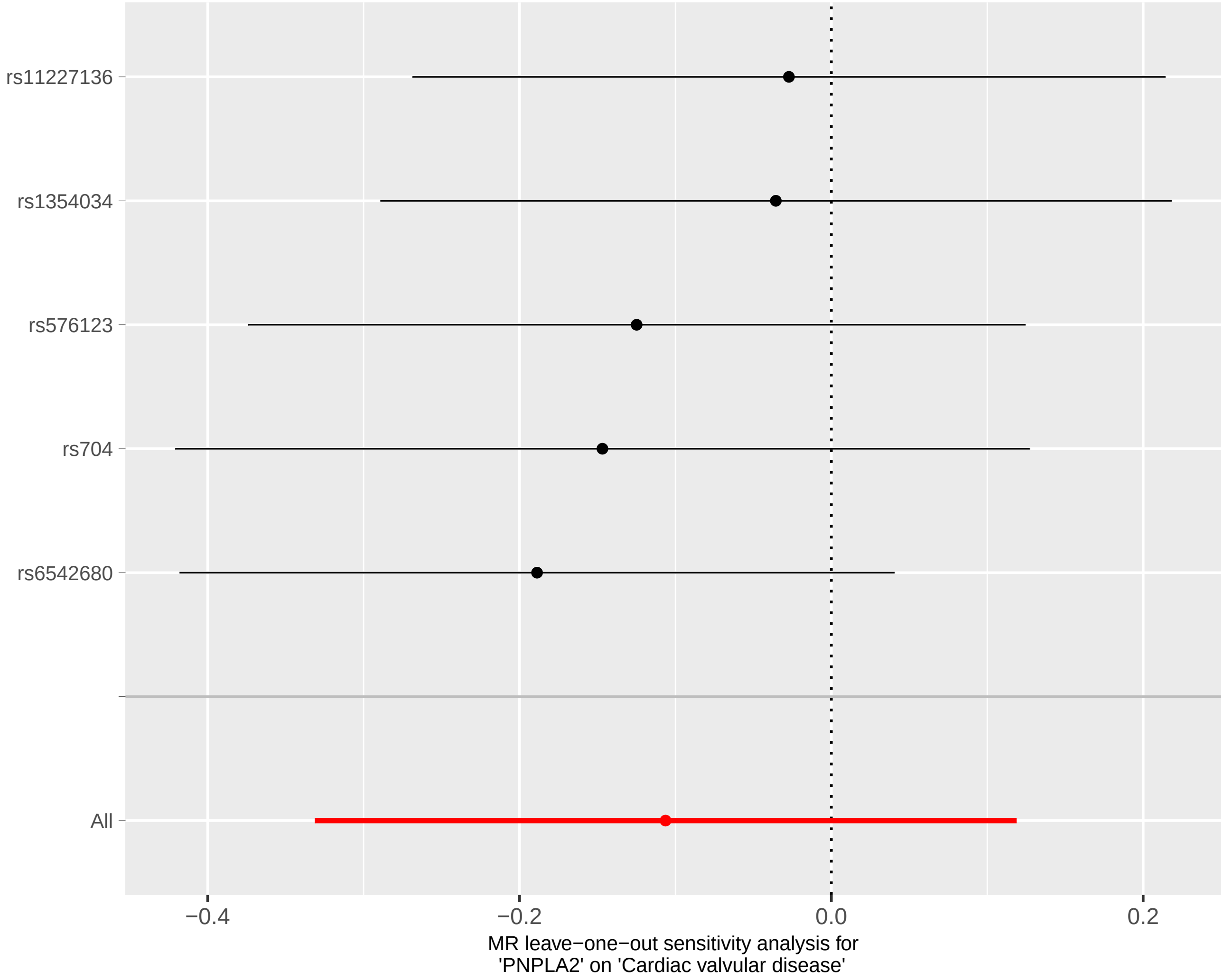
rs11227136
rs1354034
rs576123
rs704
rs6542680
All
−0.4
−0.2
0.0
0.2
MR leave−one−out sensitivity analysis for
'PNPLA2' on 'Cardiac valvular disease'

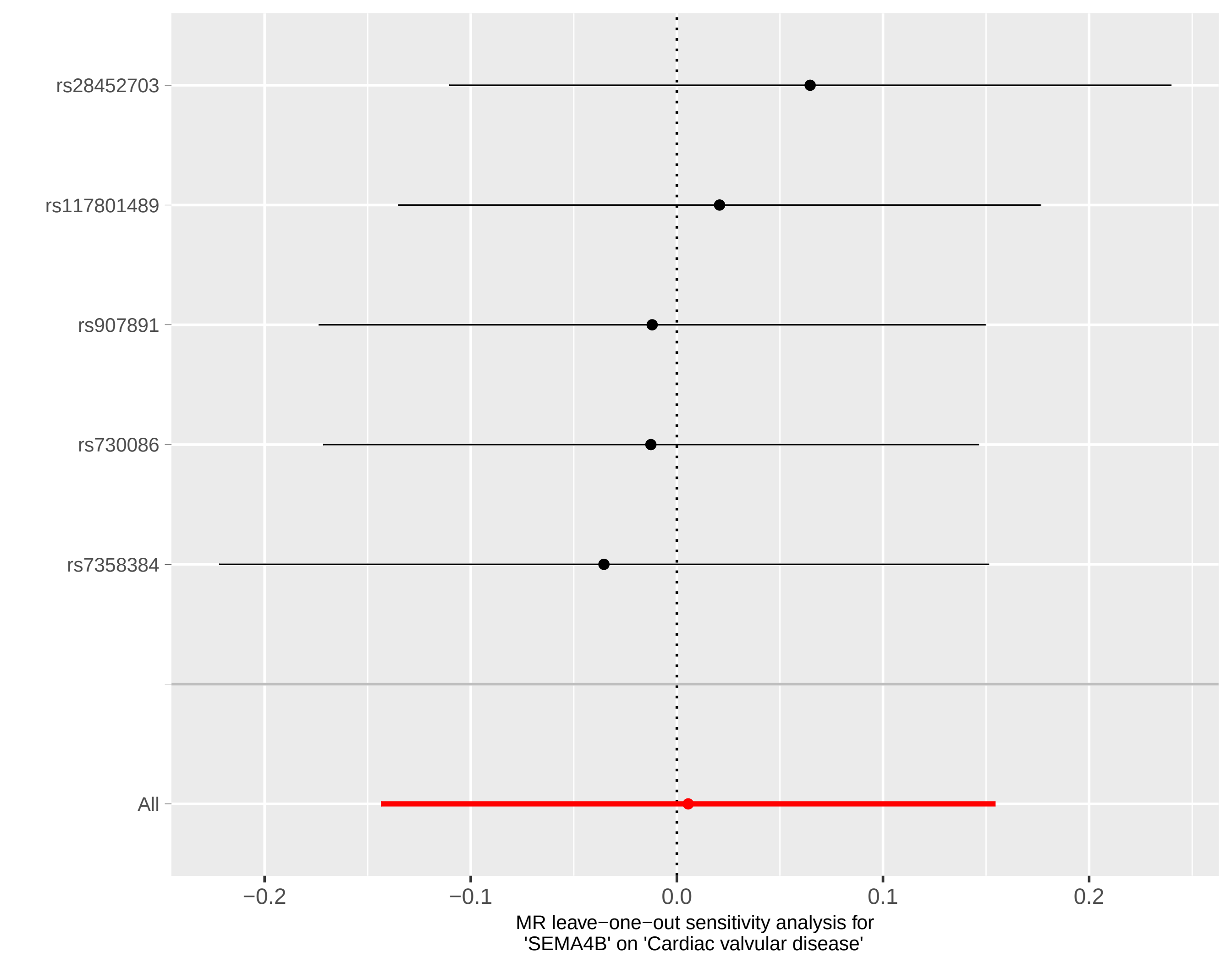
rs28452703
rs117801489
rs907891
rs730086
rs7358384
All
−0.2
−0.1
0.0
0.1
0.2
MR leave−one−out sensitivity analysis for
'SEMA4B' on 'Cardiac valvular disease'

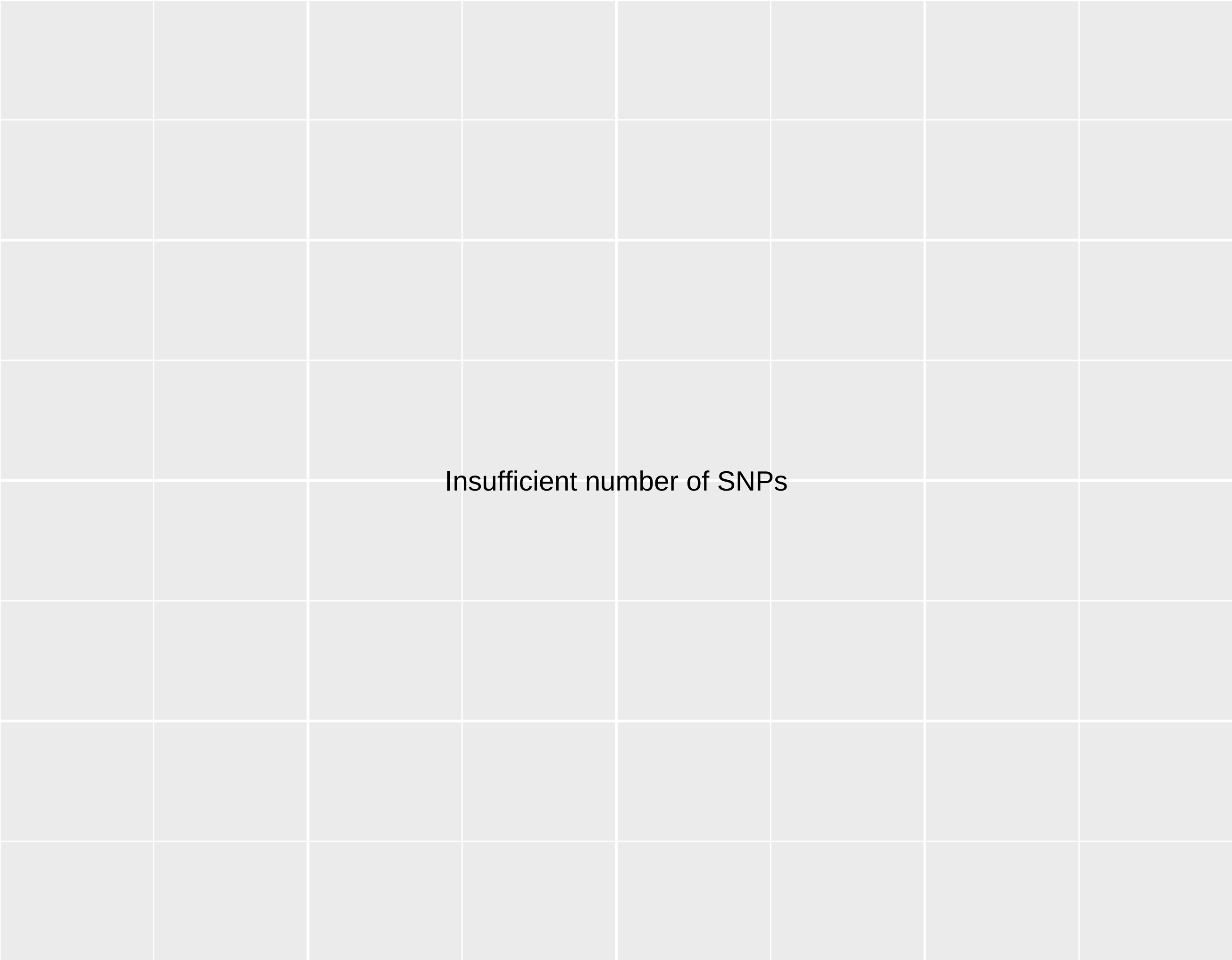
Insufficient number of SNPs

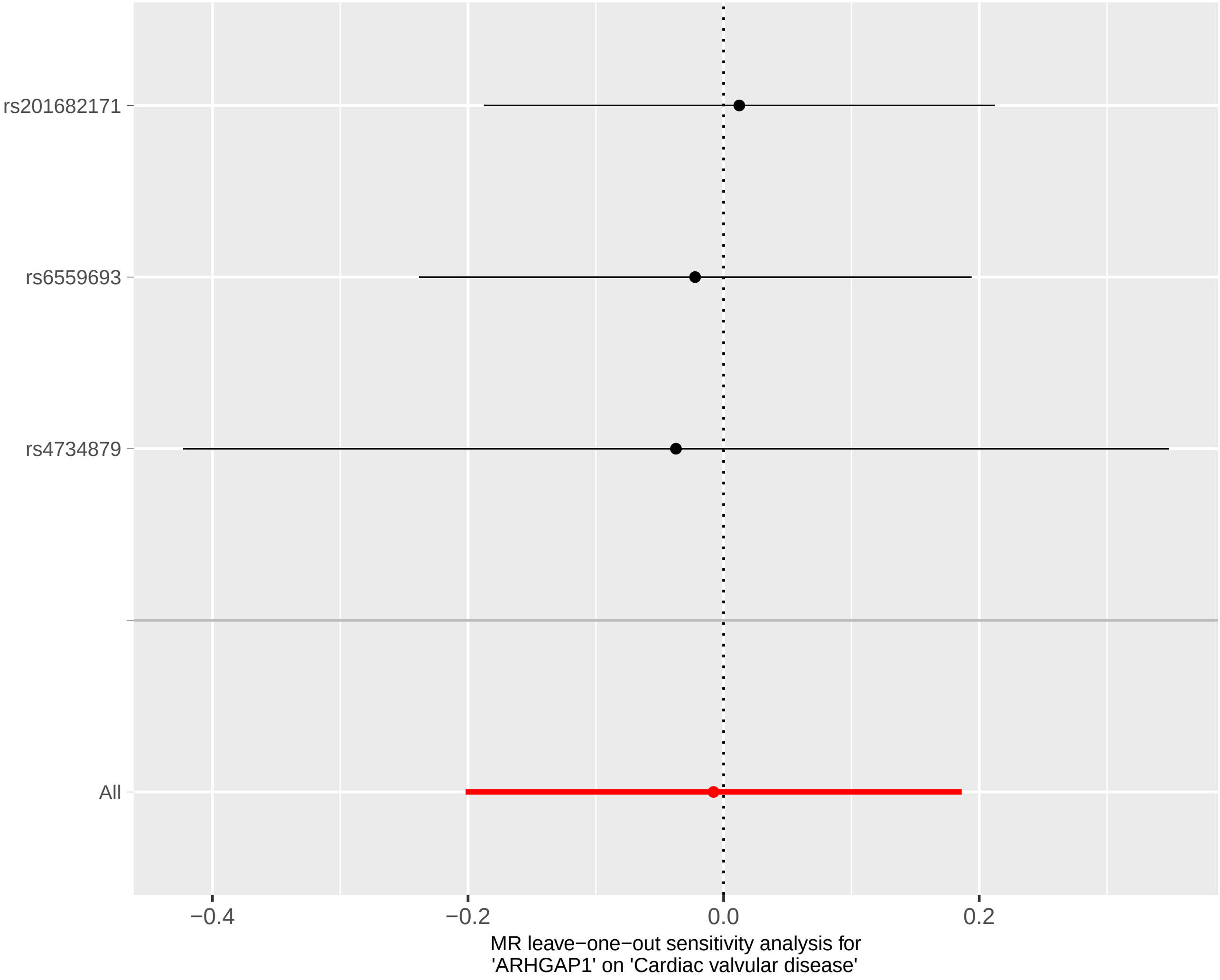
rs201682171
rs6559693
rs4734879
All
−0.4
−0.2
0.0
0.2
MR leave−one−out sensitivity analysis for
'ARHGAP1' on 'Cardiac valvular disease'

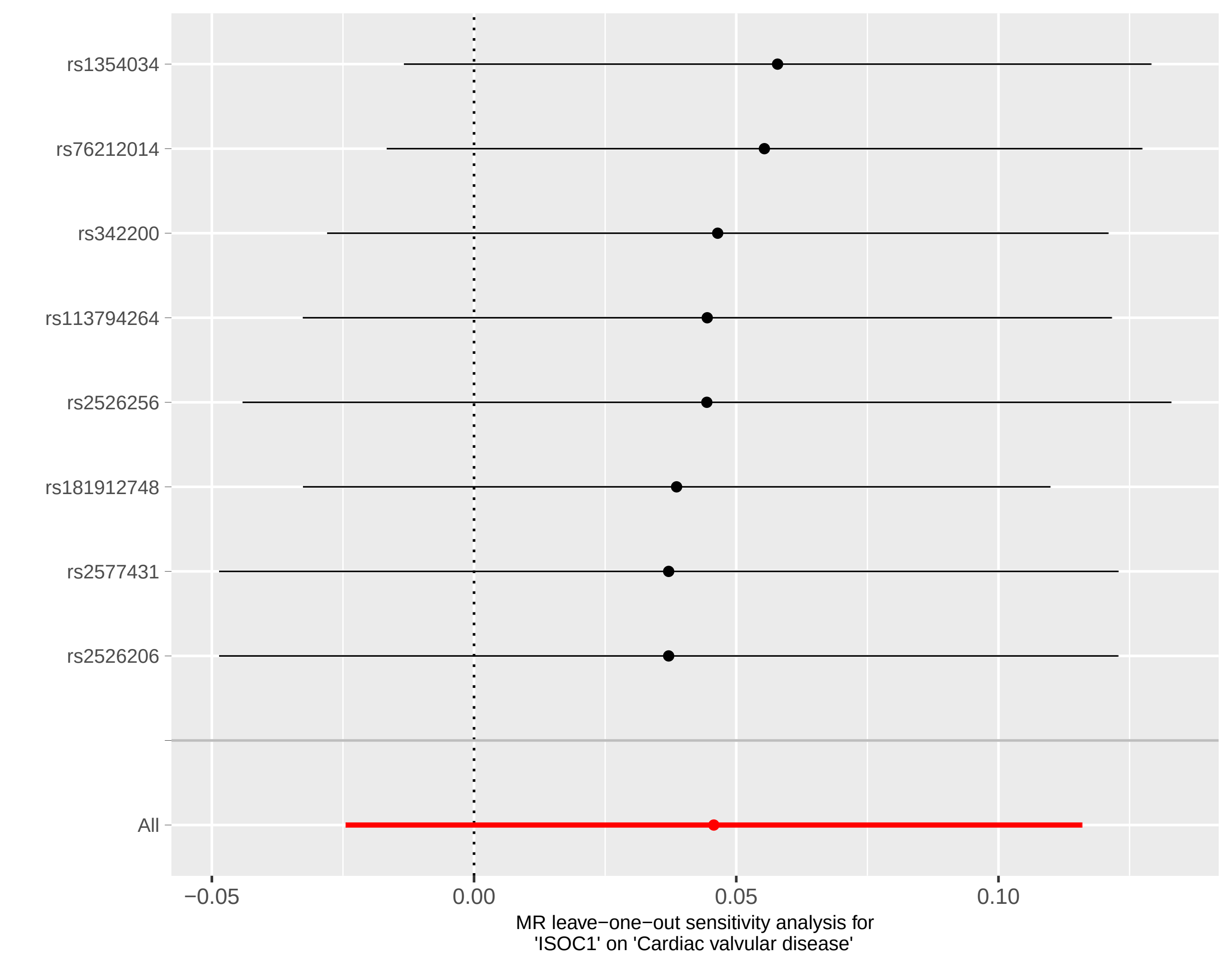

rs1354034
rs76212014
rs342200
rs113794264
rs2526256
rs181912748
rs2577431
rs2526206
All
−0.05
0.00
0.05
0.10
MR leave−one−out sensitivity analysis for
'ISOC1' on 'Cardiac valvular disease'

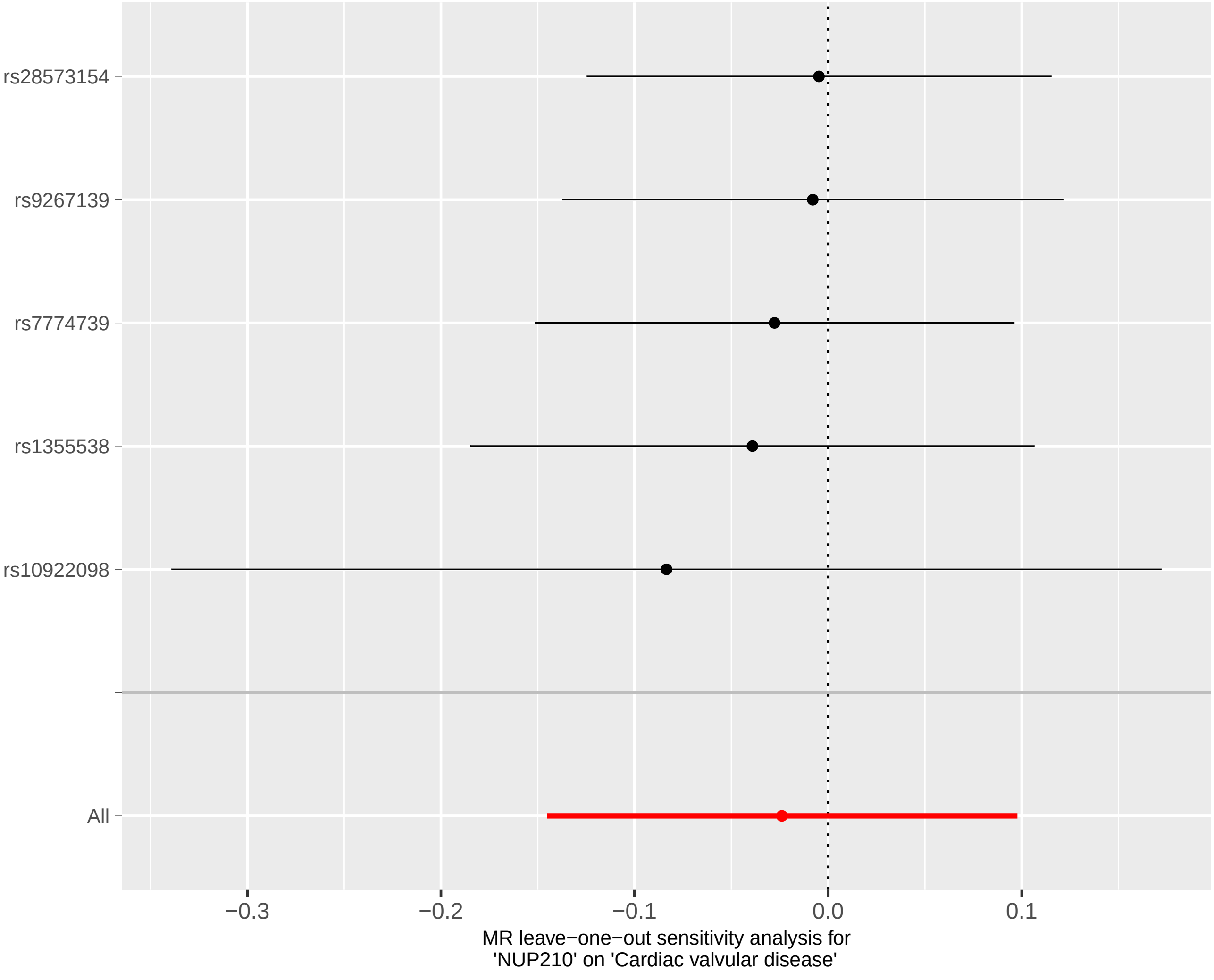
rs28573154
rs9267139
rs7774739
rs1355538
rs10922098
All
−0.3
−0.2
−0.1
0.0
0.1
MR leave−one−out sensitivity analysis for
'NUP210' on 'Cardiac valvular disease'

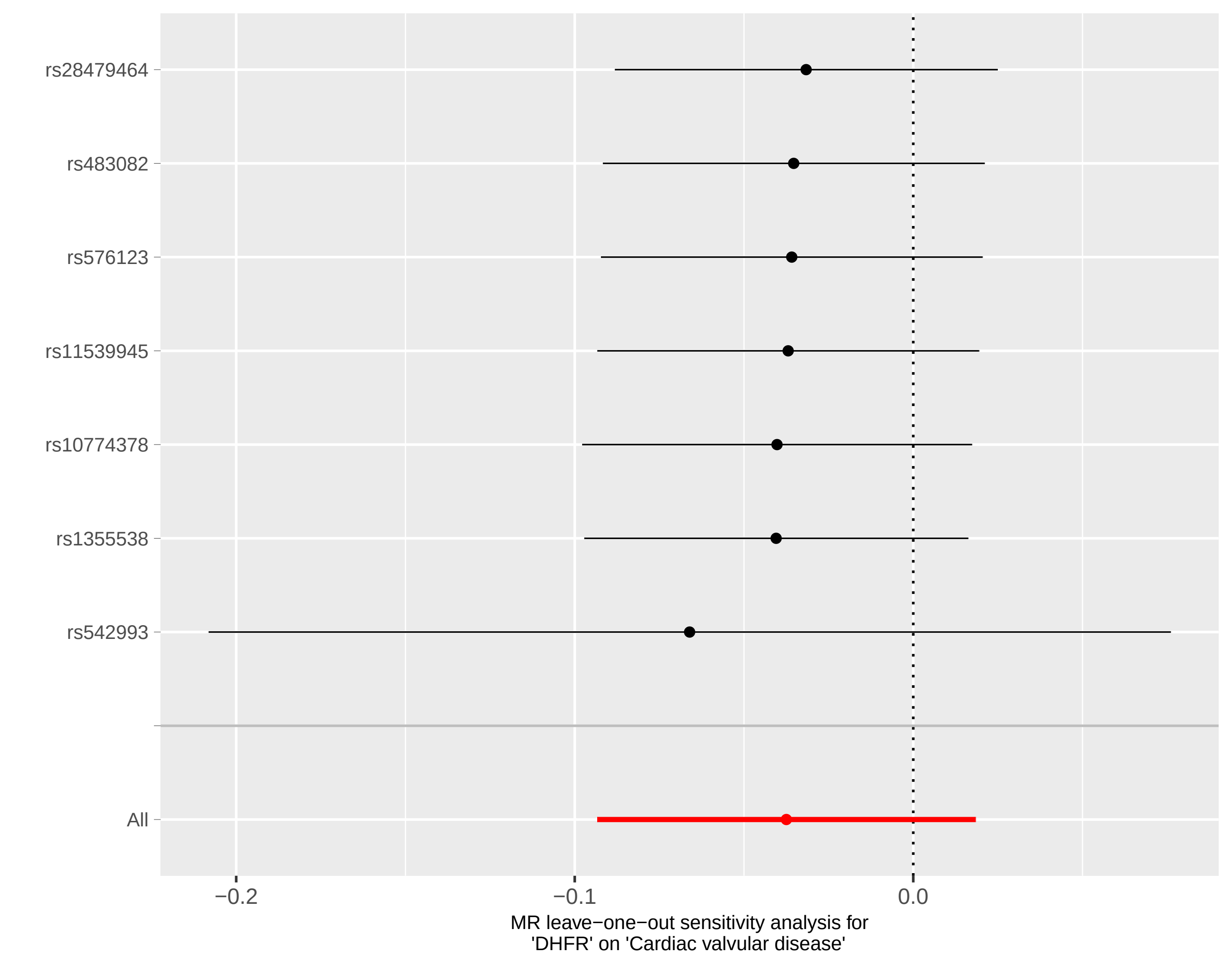

rs28479464
rs483082
rs576123
rs11539945
rs10774378
rs1355538
rs542993
All
−0.2
−0.1
0.0
MR leave−one−out sensitivity analysis for
'DHFR' on 'Cardiac valvular disease'

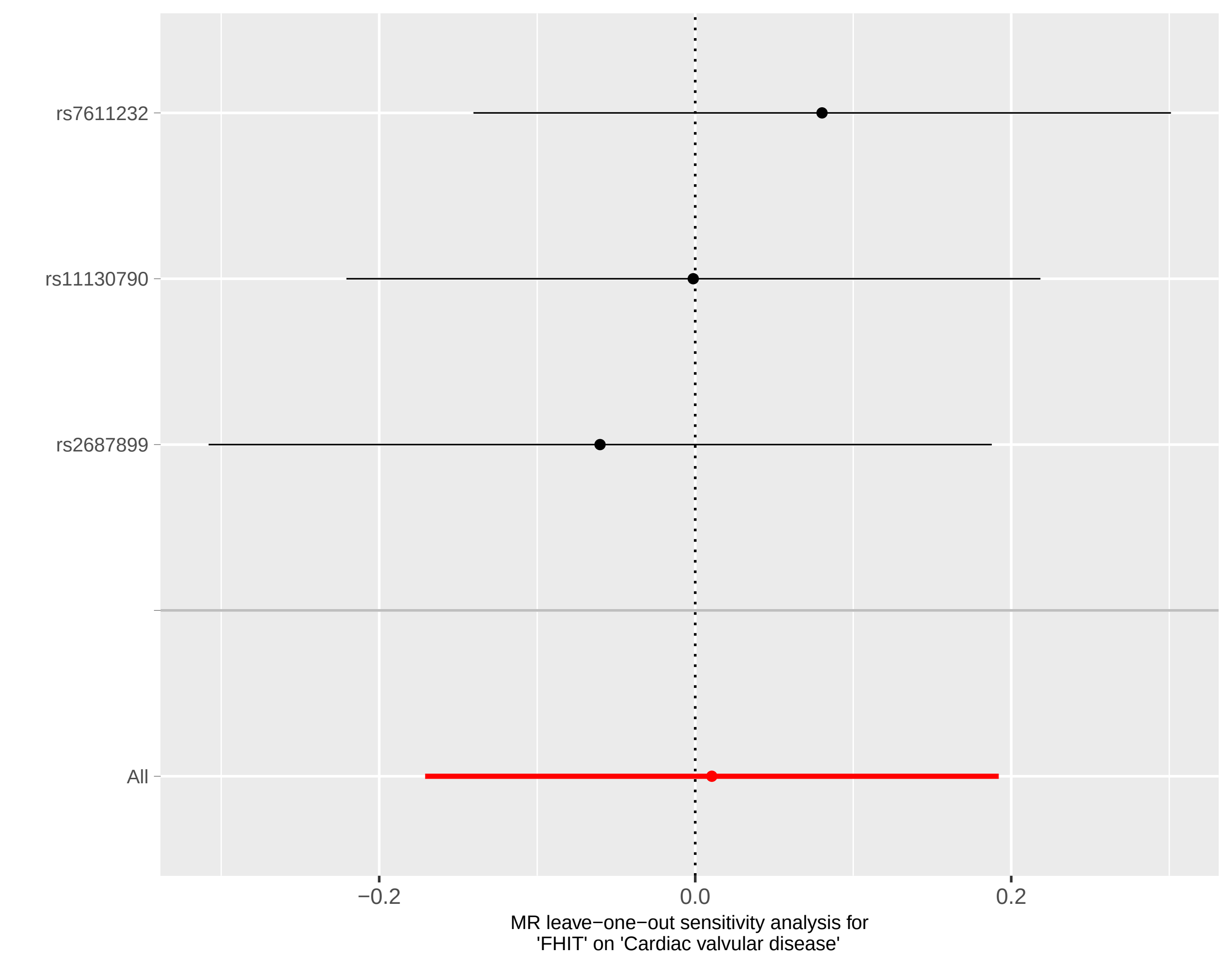
rs7611232
rs11130790
rs2687899
All
−0.2
0.0
0.2
MR leave−one−out sensitivity analysis for
'FHIT' on 'Cardiac valvular disease'

Insufficient number of SNPs

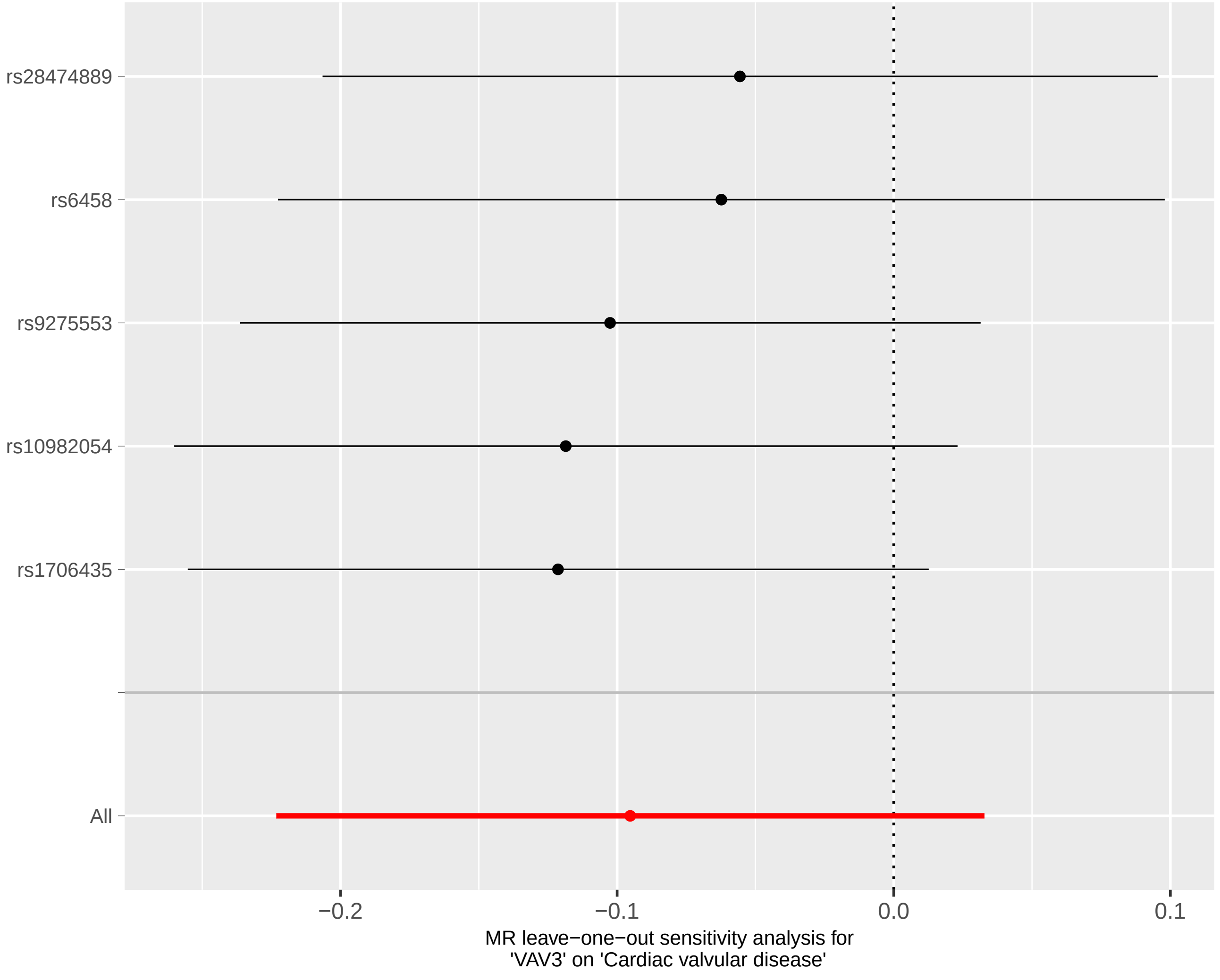

rs28474889
rs6458
rs9275553
rs10982054
rs1706435
All
−0.2
−0.1
0.0
0.1
MR leave−one−out sensitivity analysis for
'VAV3' on 'Cardiac valvular disease'

Insufficient number of SNPs

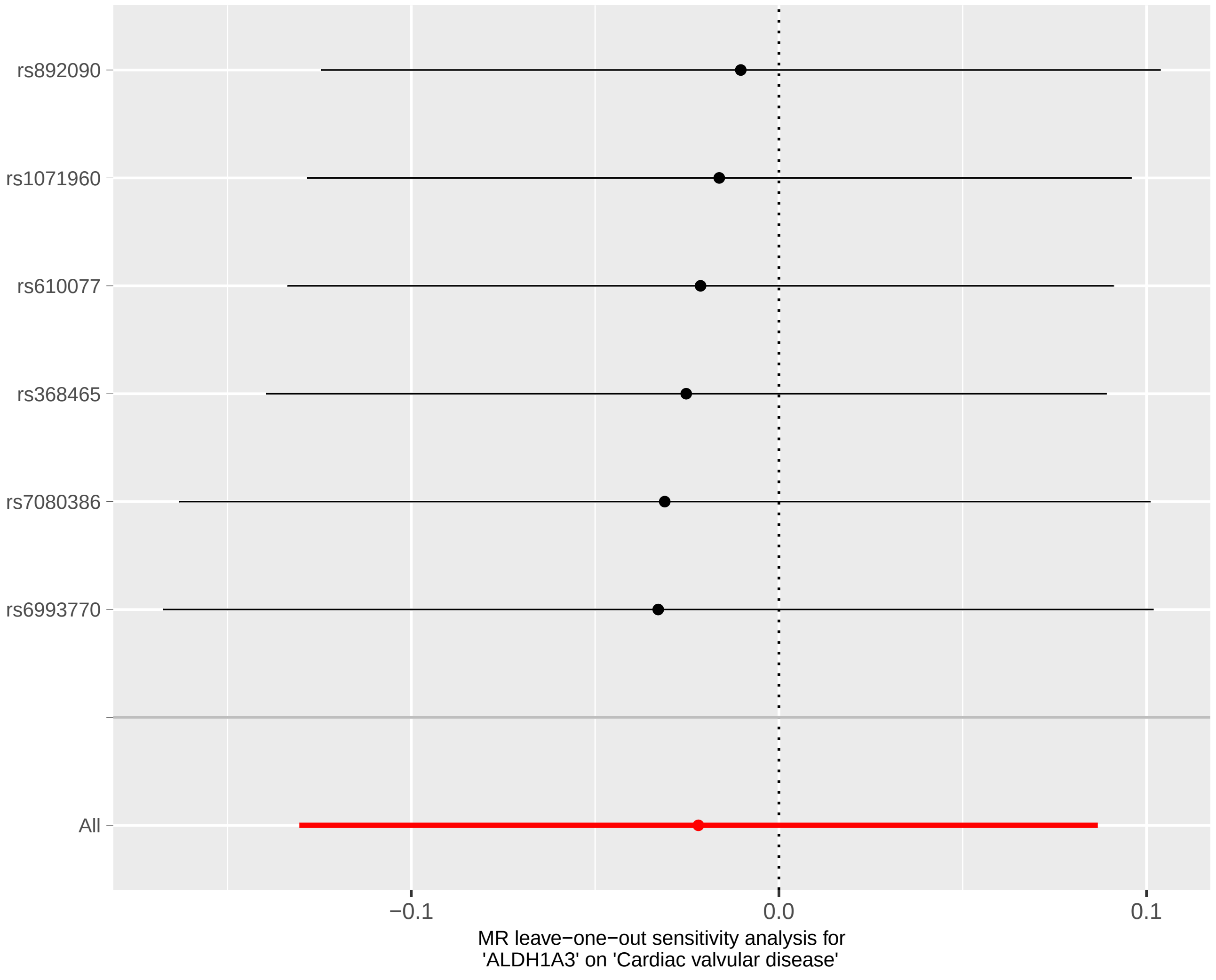
rs892090
rs1071960
rs610077
rs368465
rs7080386
rs6993770
All
−0.1
0.0
0.1
MR leave−one−out sensitivity analysis for
'ALDH1A3' on 'Cardiac valvular disease'

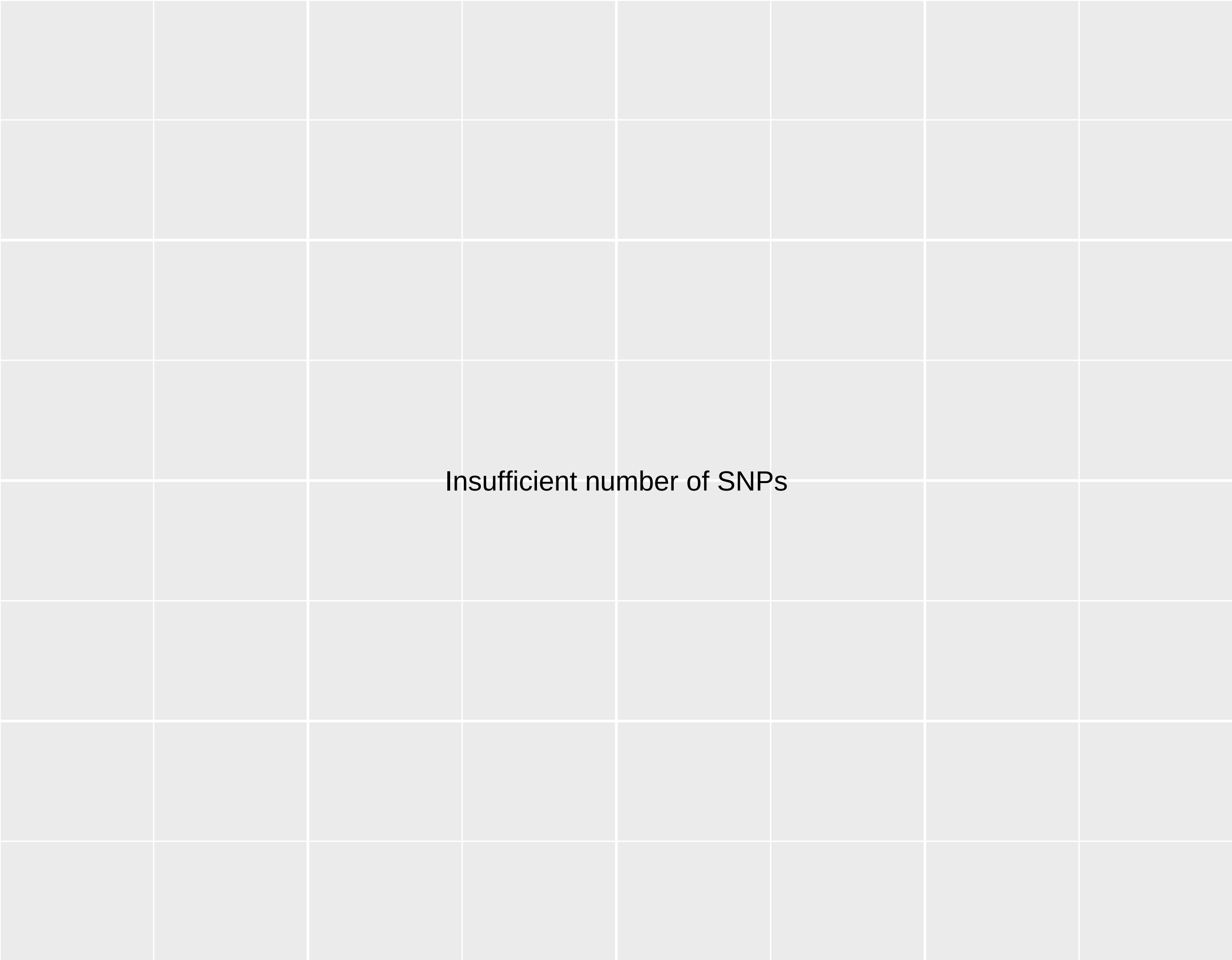
Insufficient number of SNPs

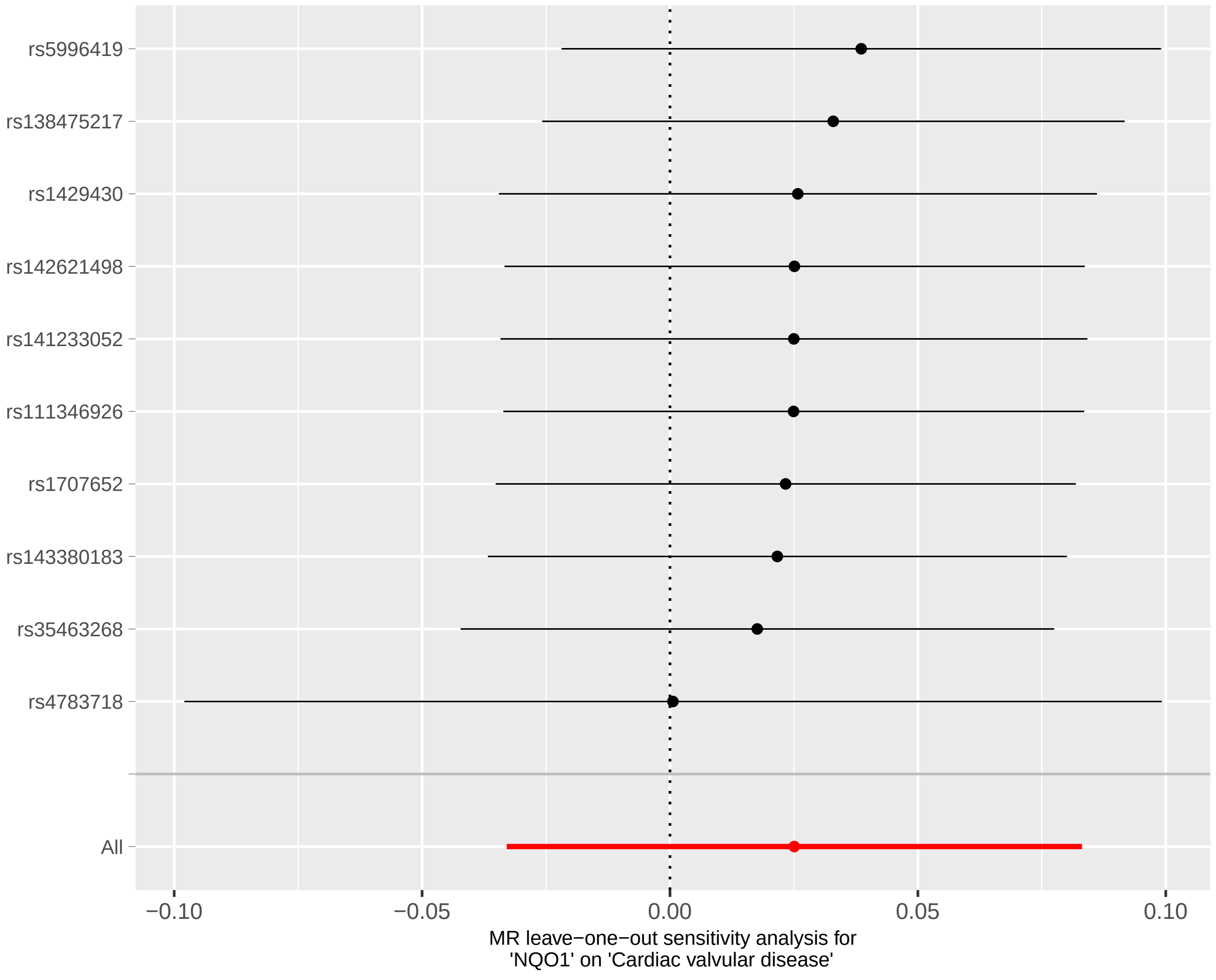
rs5996419
rs138475217
rs1429430
rs142621498
rs141233052
rs111346926
rs1707652
rs143380183
rs35463268
rs4783718
All
−0.10
−0.05
0.00
0.05
0.10
MR leave−one−out sensitivity analysis for
'NQO1' on 'Cardiac valvular disease'

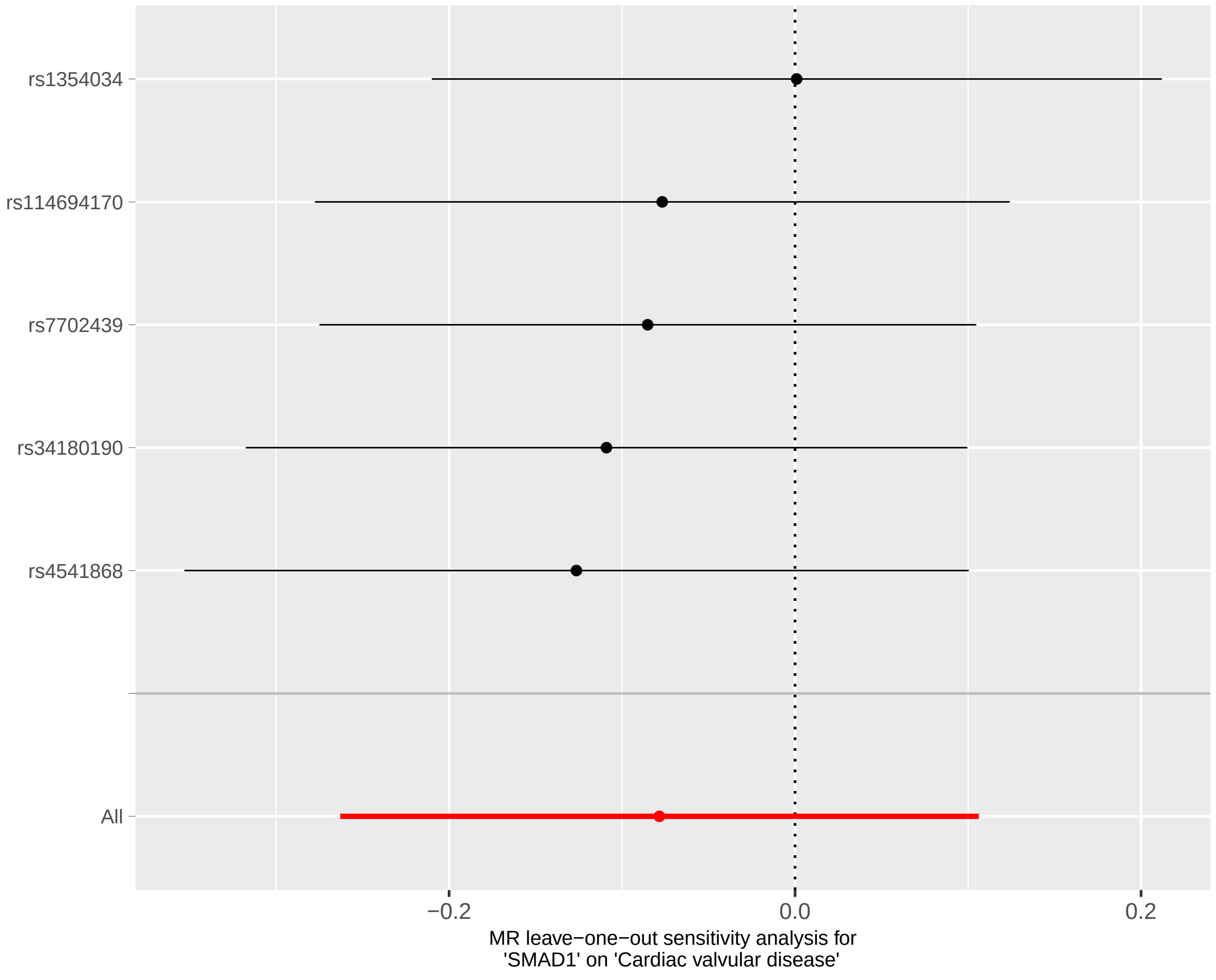

rs1354034
rs114694170
rs7702439
rs34180190
rs4541868
All
−0.2
0.0
0.2
MR leave−one−out sensitivity analysis for
'SMAD1' on 'Cardiac valvular disease'

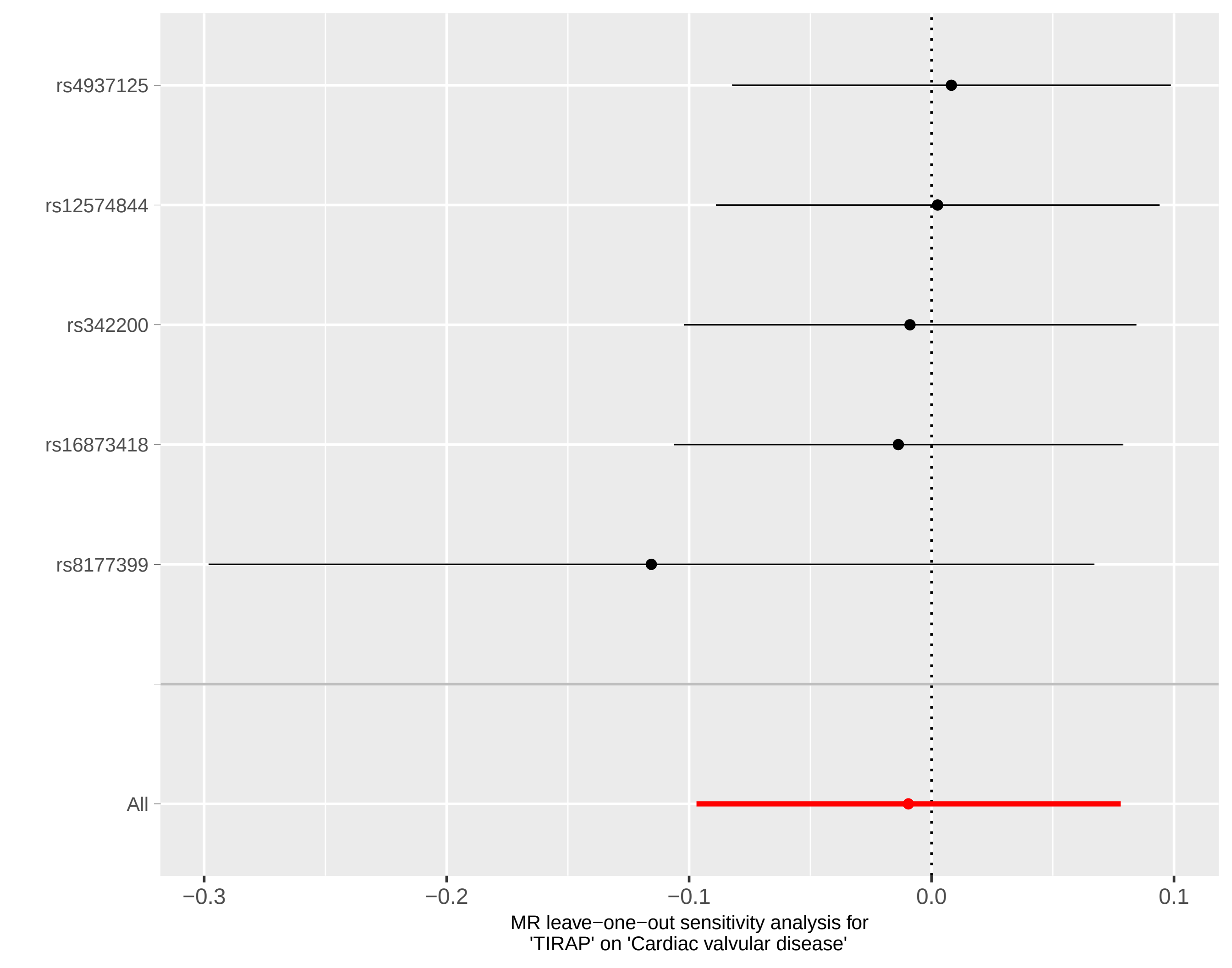

rs4937125
rs12574844
rs342200
rs16873418
rs8177399
All
−0.3
−0.2
−0.1
0.0
0.1
MR leave−one−out sensitivity analysis for
'TIRAP' on 'Cardiac valvular disease'

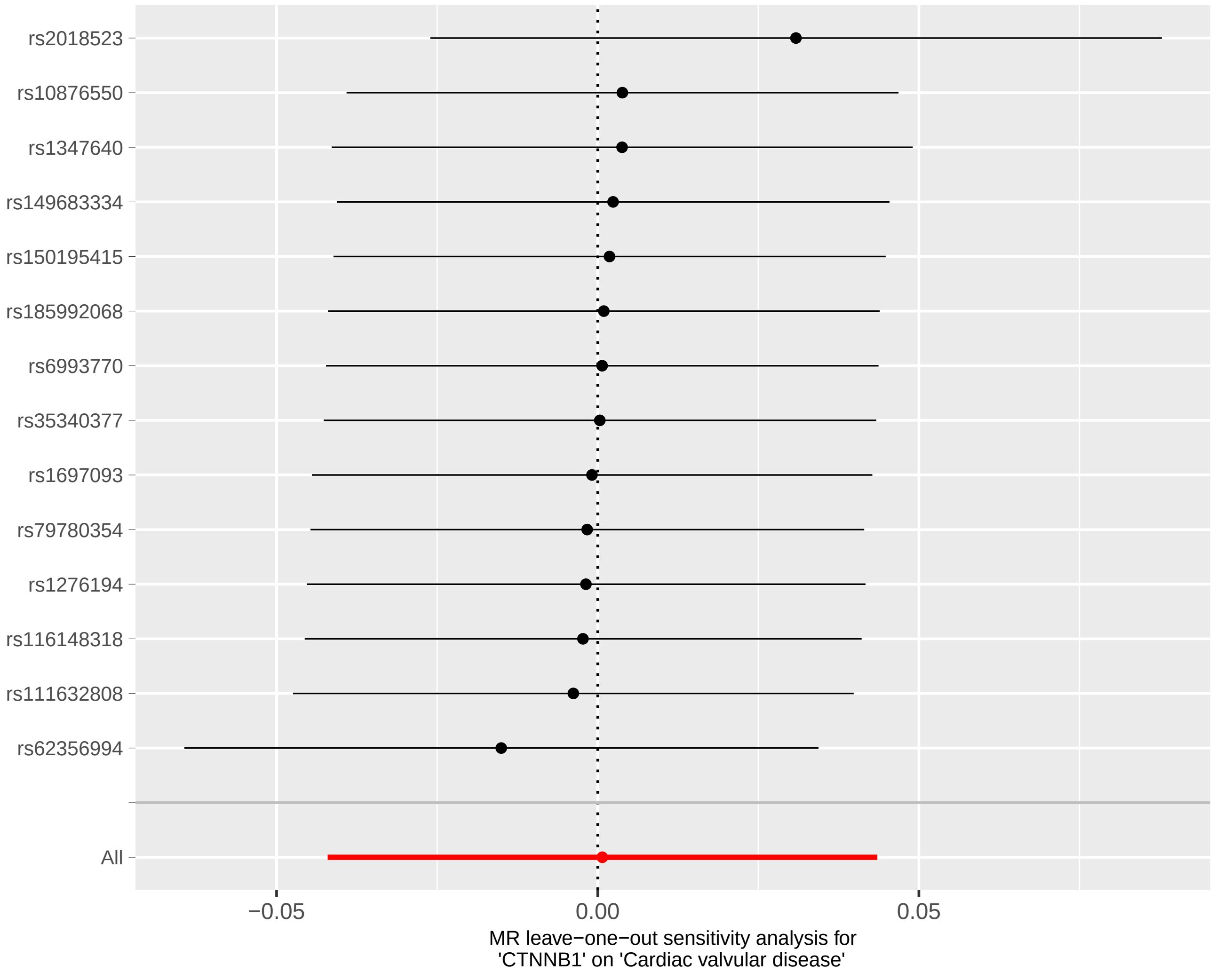
rs2018523
rs10876550
rs1347640
rs149683334
rs150195415
rs185992068
rs6993770
rs35340377
rs1697093
rs79780354
rs1276194
rs116148318
rs111632808
rs62356994
All
−0.05
0.00
0.05
MR leave−one−out sensitivity analysis for
'CTNNB1' on 'Cardiac valvular disease'

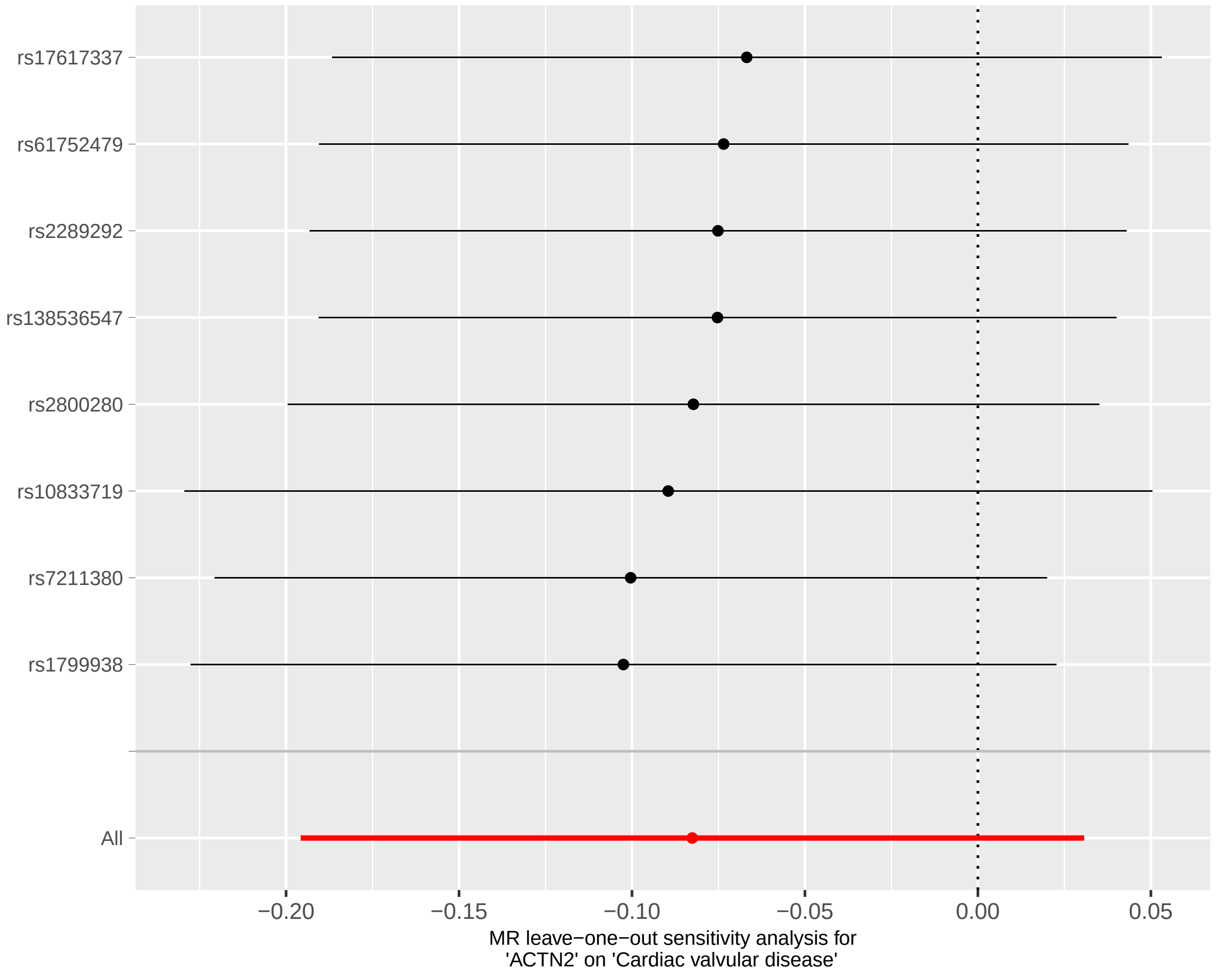
rs17617337
rs61752479
rs2289292
rs138536547
rs2800280
rs10833719
rs7211380
rs1799938
All
−0.20
−0.15
−0.10
−0.05
0.00
0.05
MR leave−one−out sensitivity analysis for
'ACTN2' on 'Cardiac valvular disease'

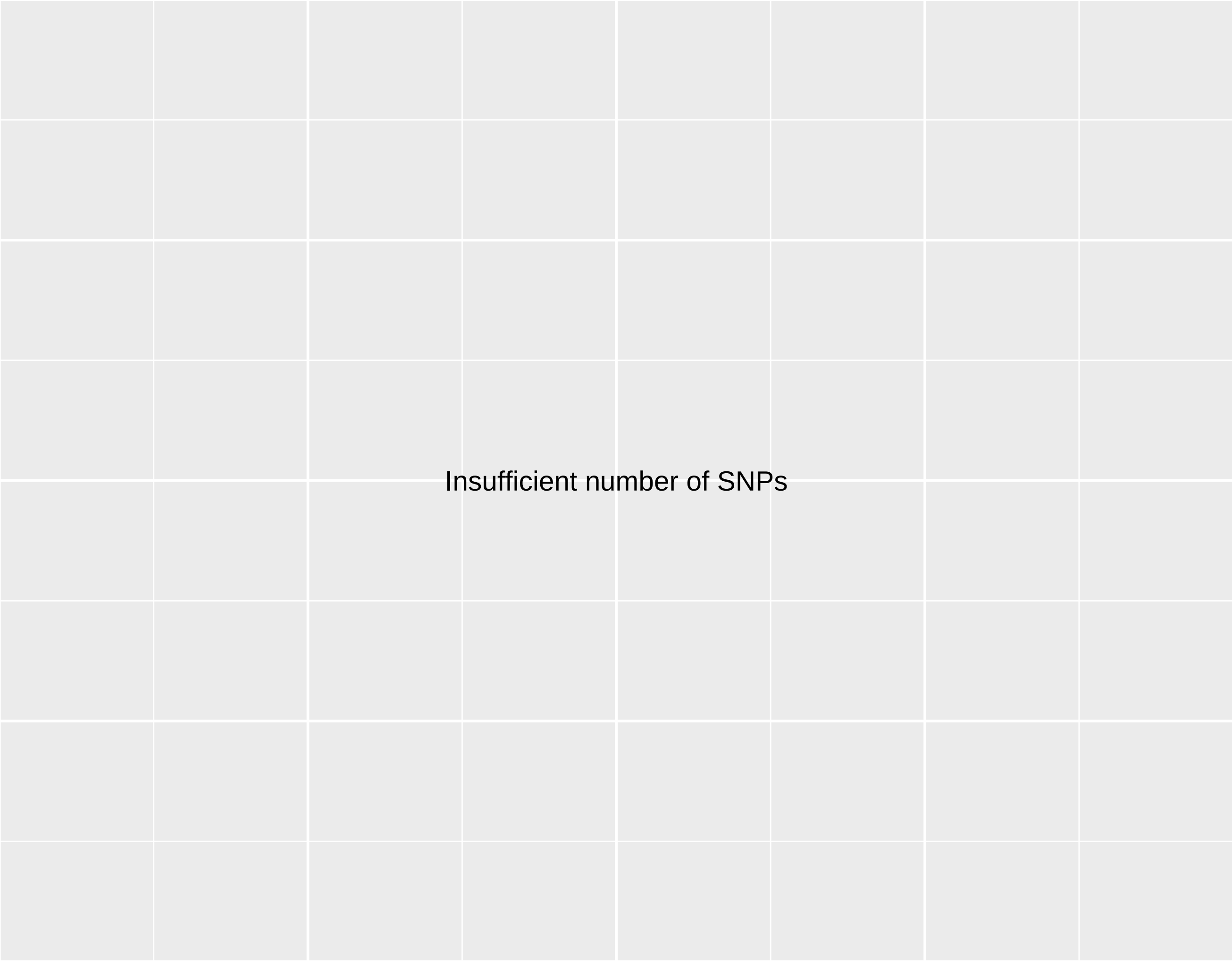
Insufficient number of SNPs

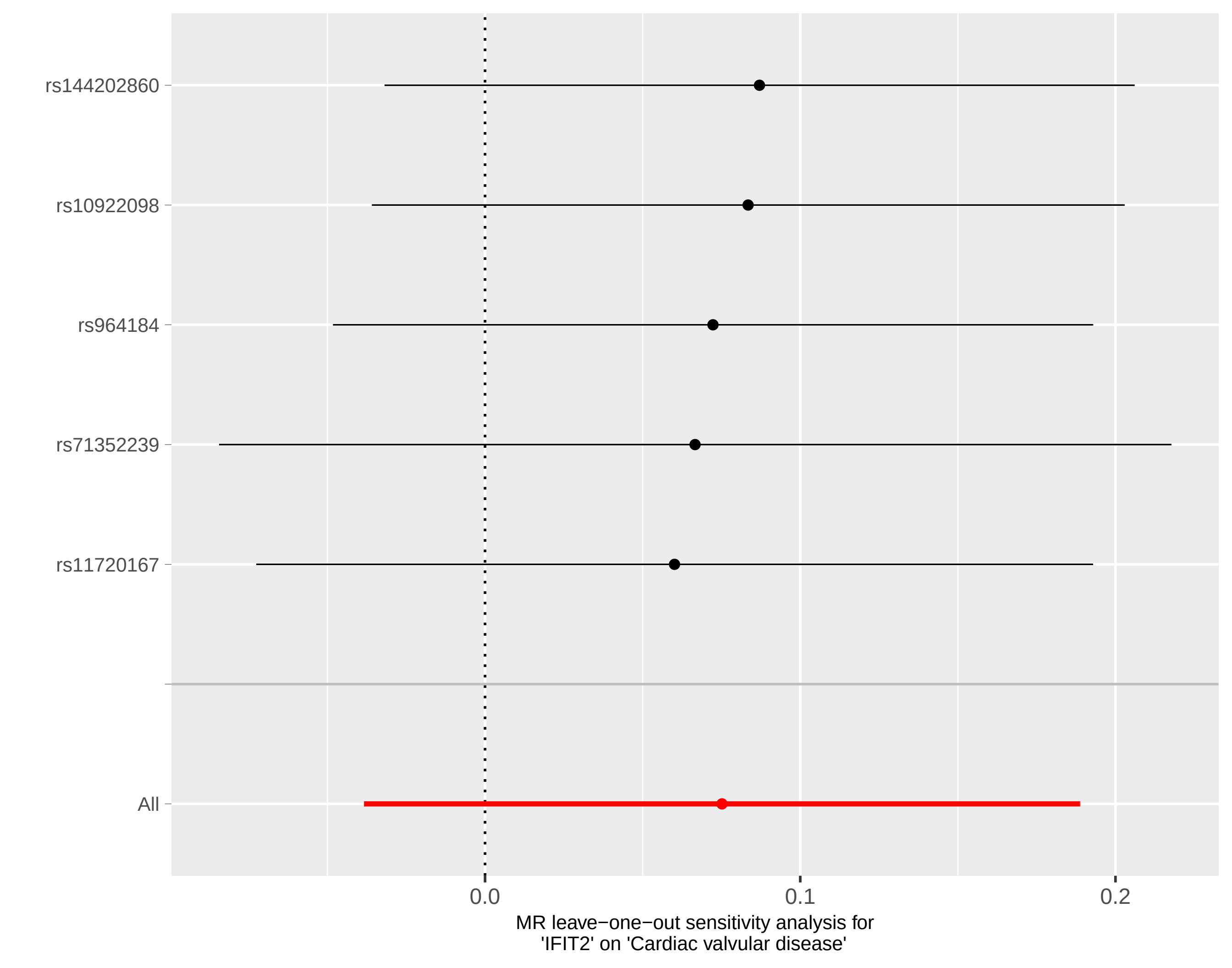

rs144202860
rs10922098
rs964184
rs71352239
rs11720167
All
0.0
0.1
0.2
MR leave−one−out sensitivity analysis for
'IFIT2' on 'Cardiac valvular disease'

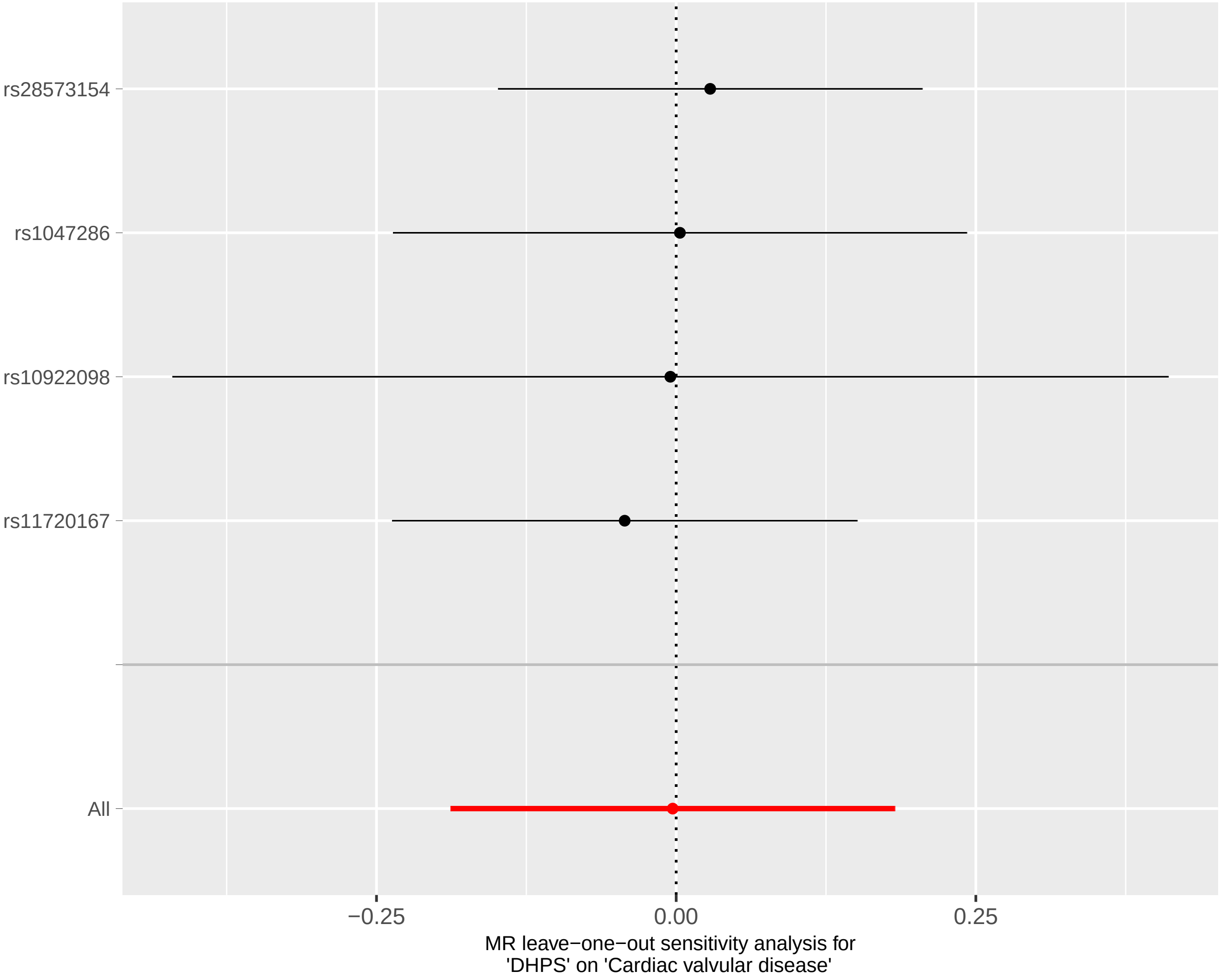
rs28573154
rs1047286
rs10922098
rs11720167
All
−0.25
0.00
0.25
MR leave−one−out sensitivity analysis for
'DHPS' on 'Cardiac valvular disease'

Insufficient number of SNPs

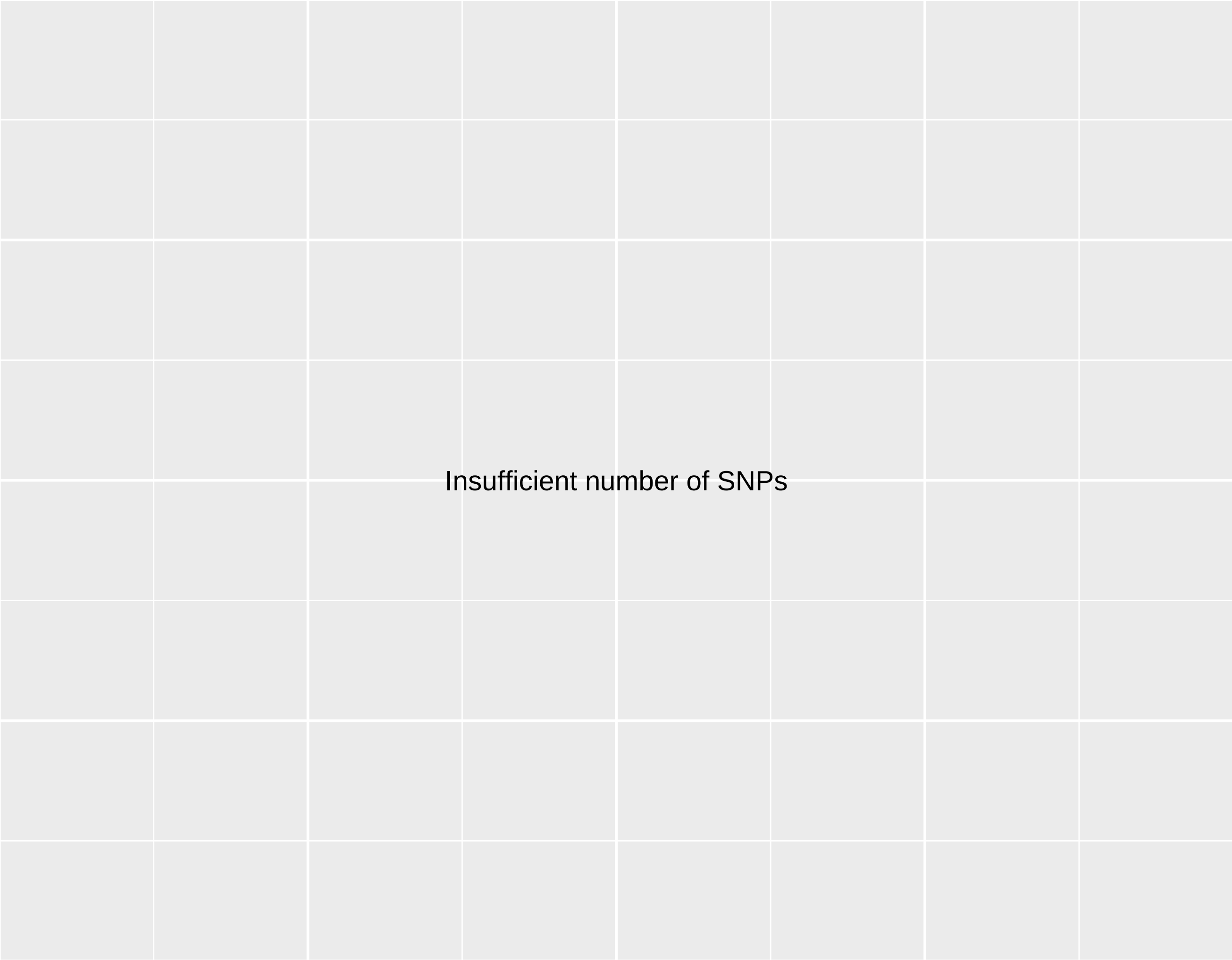
Insufficient number of SNPs

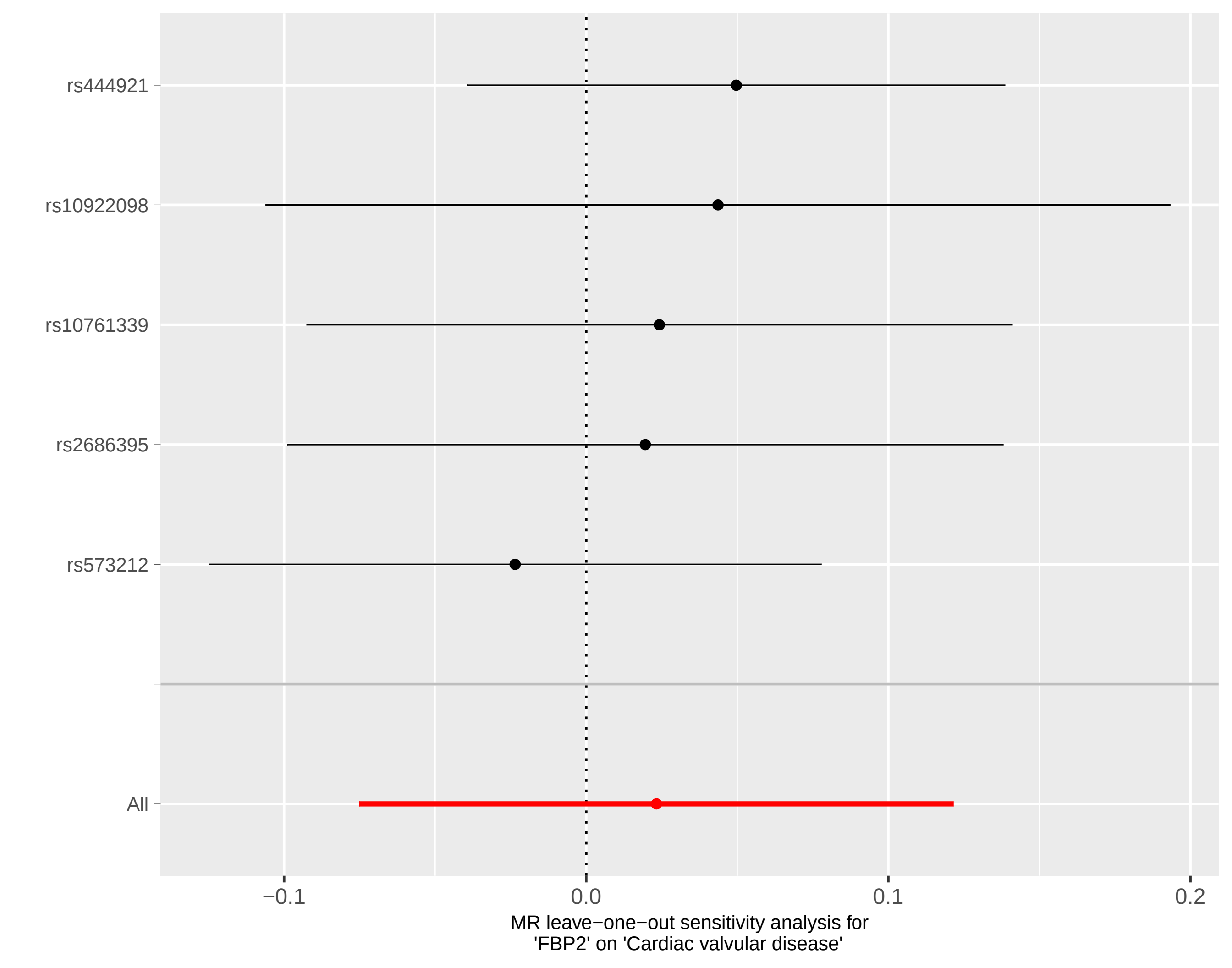
rs444921
rs10922098
rs10761339
rs2686395
rs573212
All
−0.1
0.0
0.1
0.2
MR leave−one−out sensitivity analysis for
'FBP2' on 'Cardiac valvular disease'

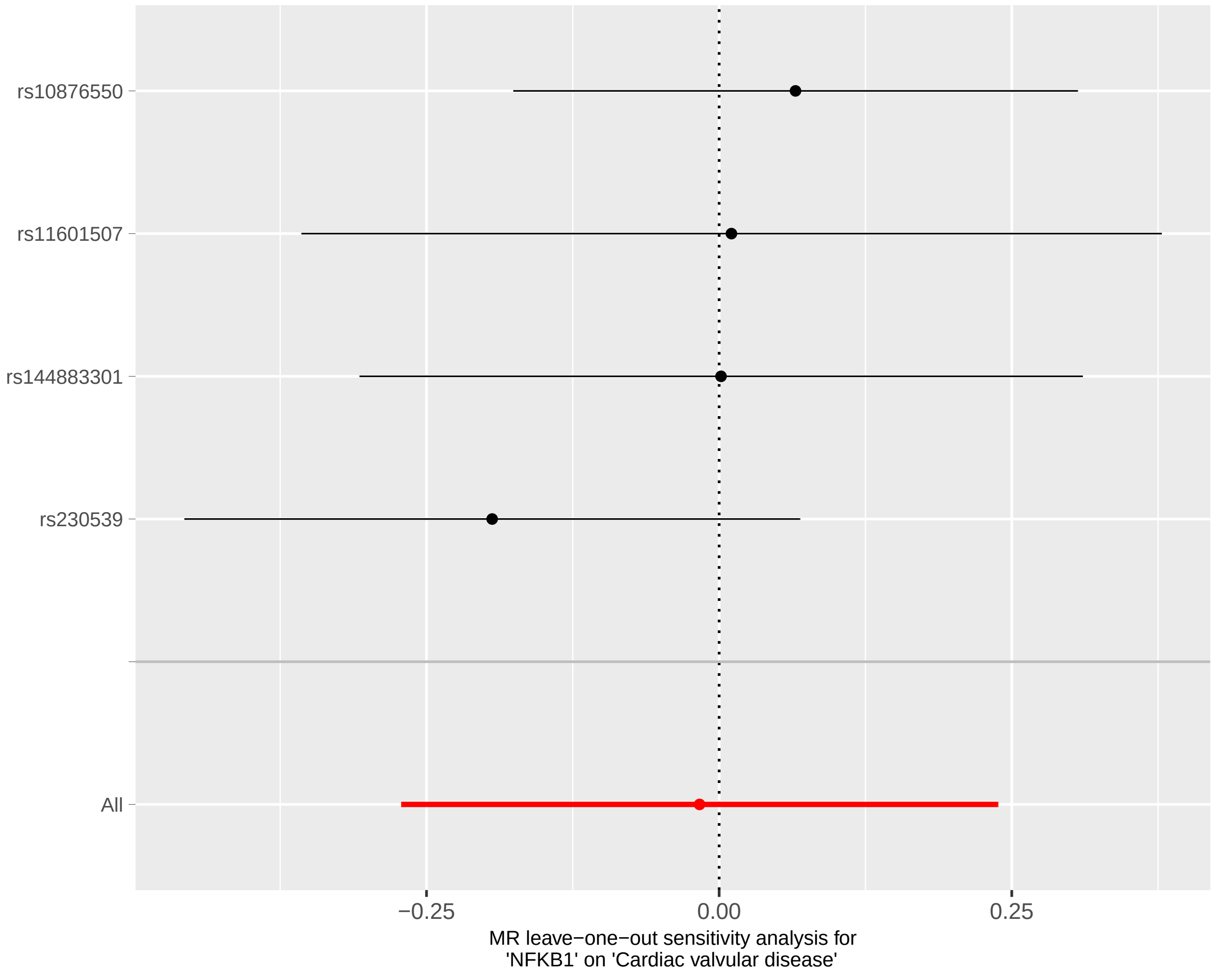
rs10876550
rs11601507
rs144883301
rs230539
All
−0.25
0.00
0.25
MR leave−one−out sensitivity analysis for
'NFKB1' on 'Cardiac valvular disease'

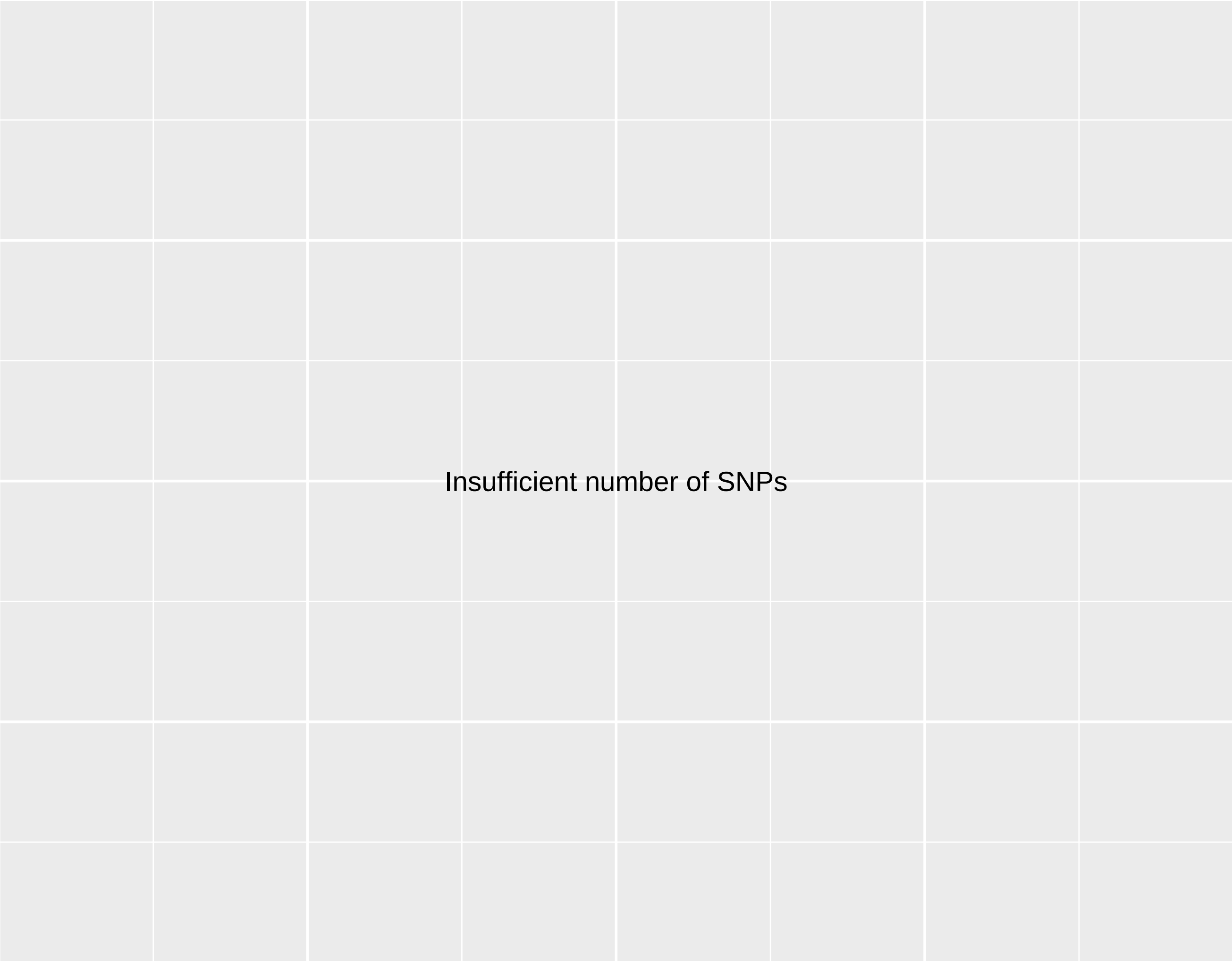
Insufficient number of SNPs

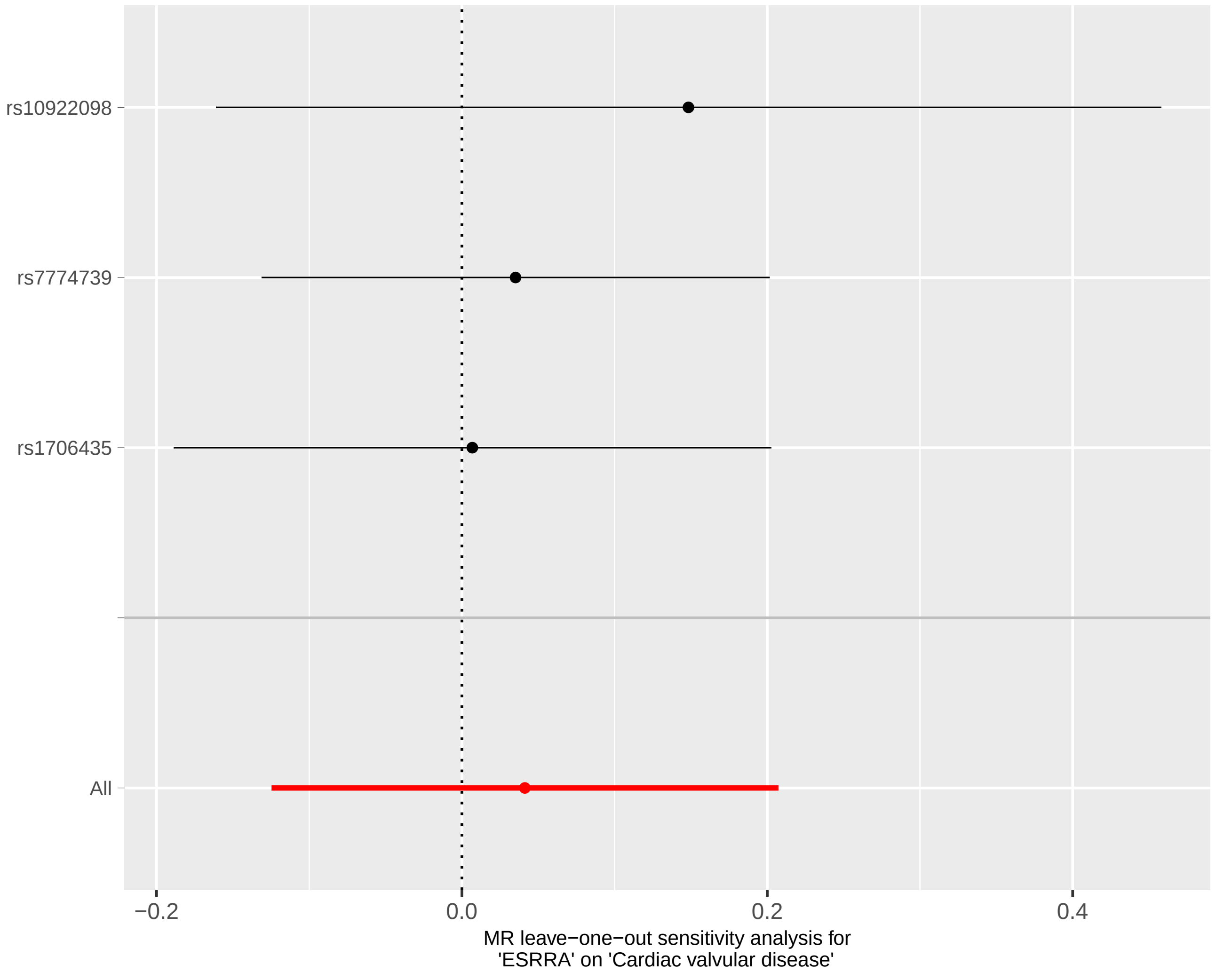
rs10922098
rs7774739
rs1706435
All
−0.2
0.0
0.2
0.4
MR leave−one−out sensitivity analysis for
'ESRRA' on 'Cardiac valvular disease'

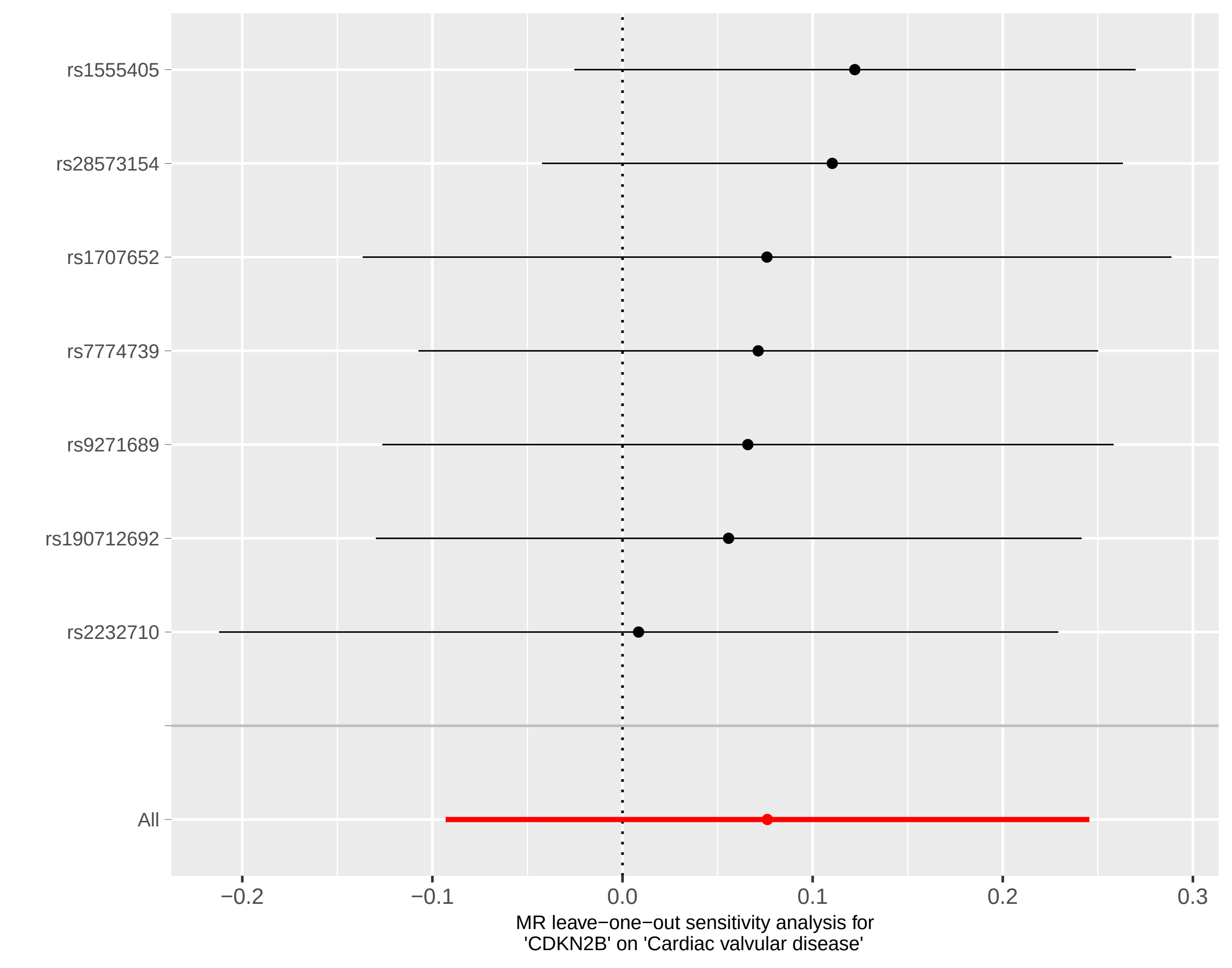
rs1555405
rs28573154
rs1707652
rs7774739
rs9271689
rs190712692
rs2232710
All
−0.2
−0.1
0.0
0.1
0.2
0.3
MR leave−one−out sensitivity analysis for
'CDKN2B' on 'Cardiac valvular disease'

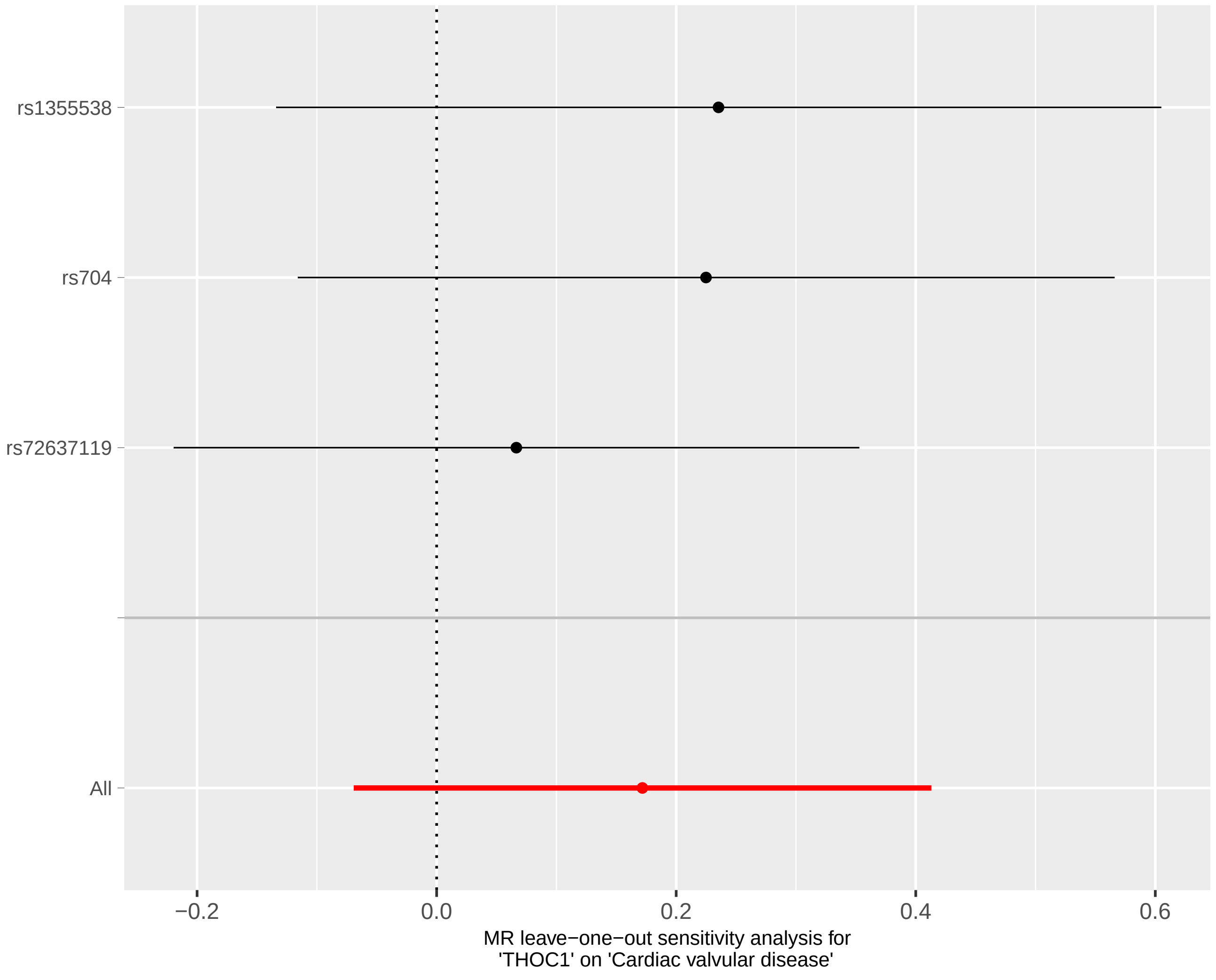

rs1355538
rs704
rs72637119
All
−0.2
0.0
0.2
0.4
0.6
MR leave−one−out sensitivity analysis for
'THOC1' on 'Cardiac valvular disease'

Insufficient number of SNPs

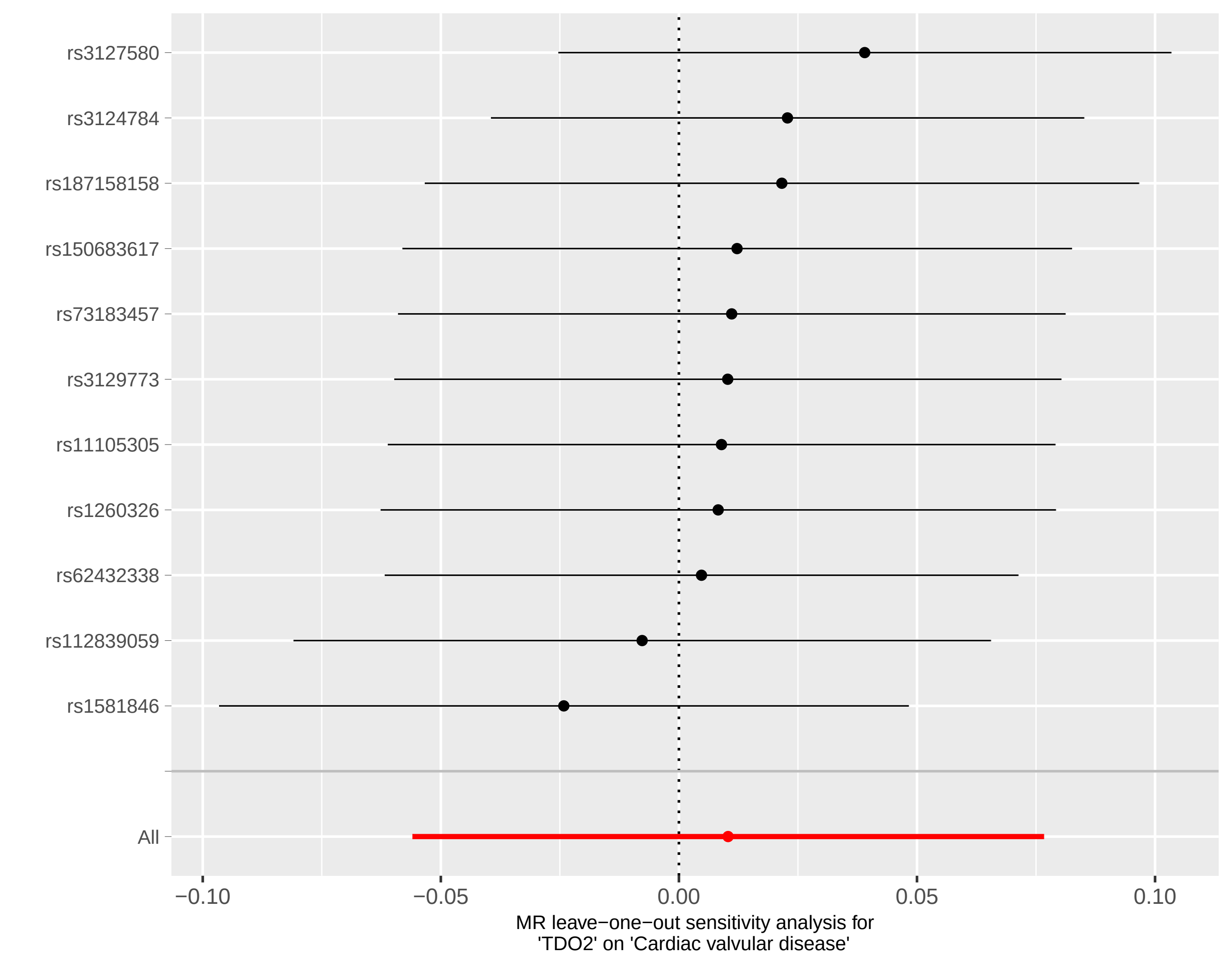
rs3127580
rs3124784
rs187158158
rs150683617
rs73183457
rs3129773
rs11105305
rs1260326
rs62432338
rs112839059
rs1581846
All
−0.10
−0.05
0.00
0.05
0.10
MR leave−one−out sensitivity analysis for
'TDO2' on 'Cardiac valvular disease'

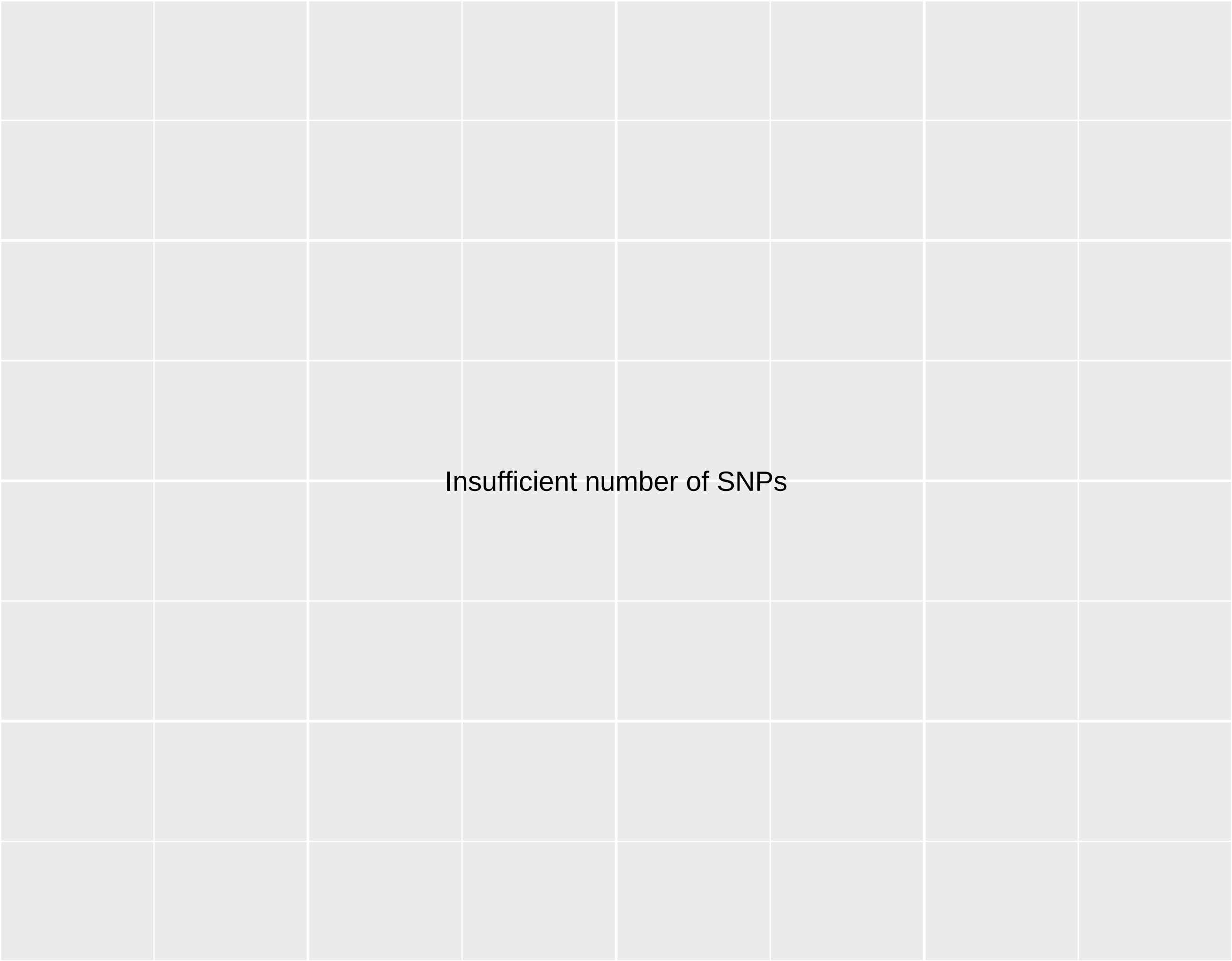
Insufficient number of SNPs

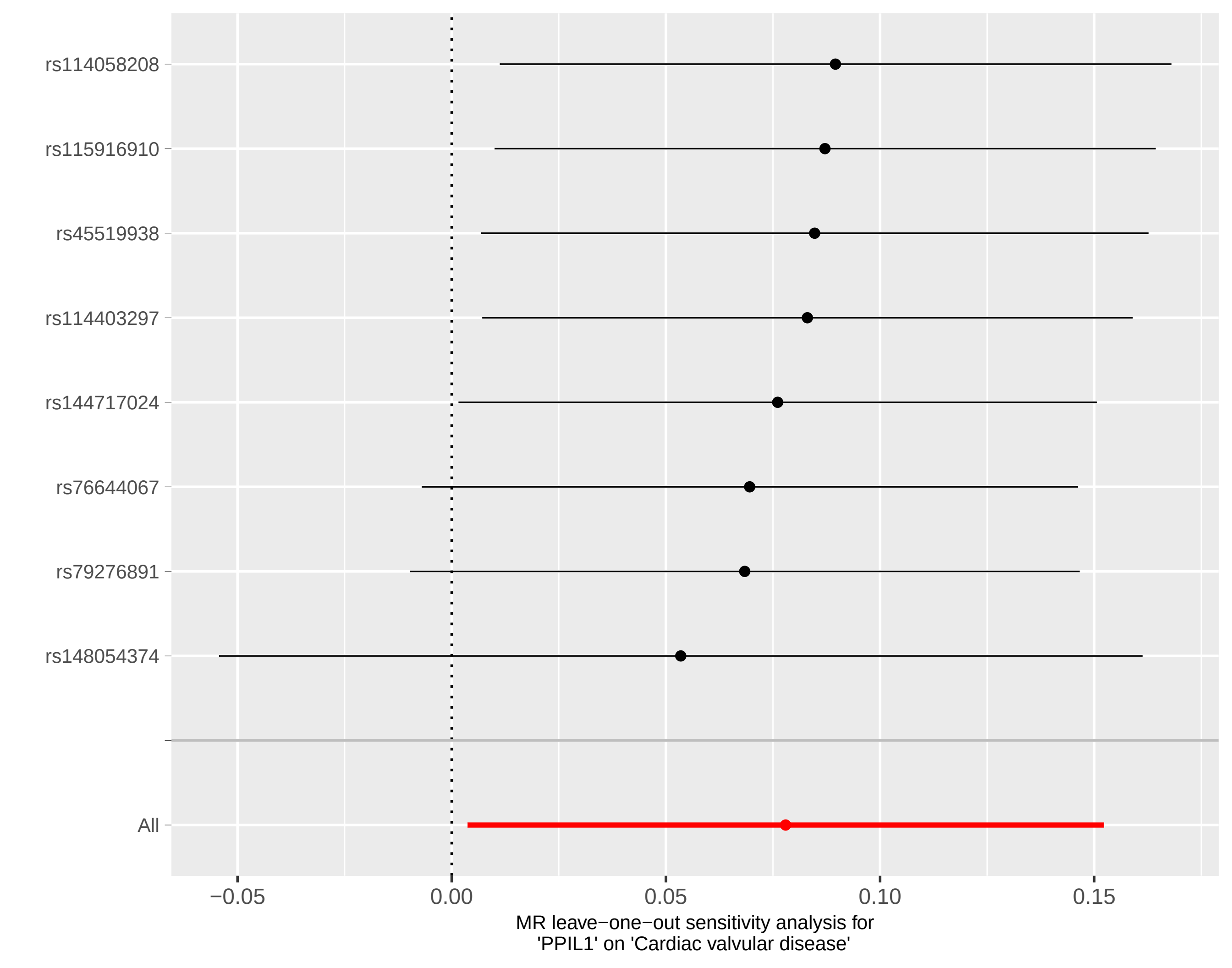
rs114058208
rs115916910
rs45519938
rs114403297
rs144717024
rs76644067
rs79276891
rs148054374
All
−0.05
0.00
0.05
0.10
0.15
MR leave−one−out sensitivity analysis for
'PPIL1' on 'Cardiac valvular disease'

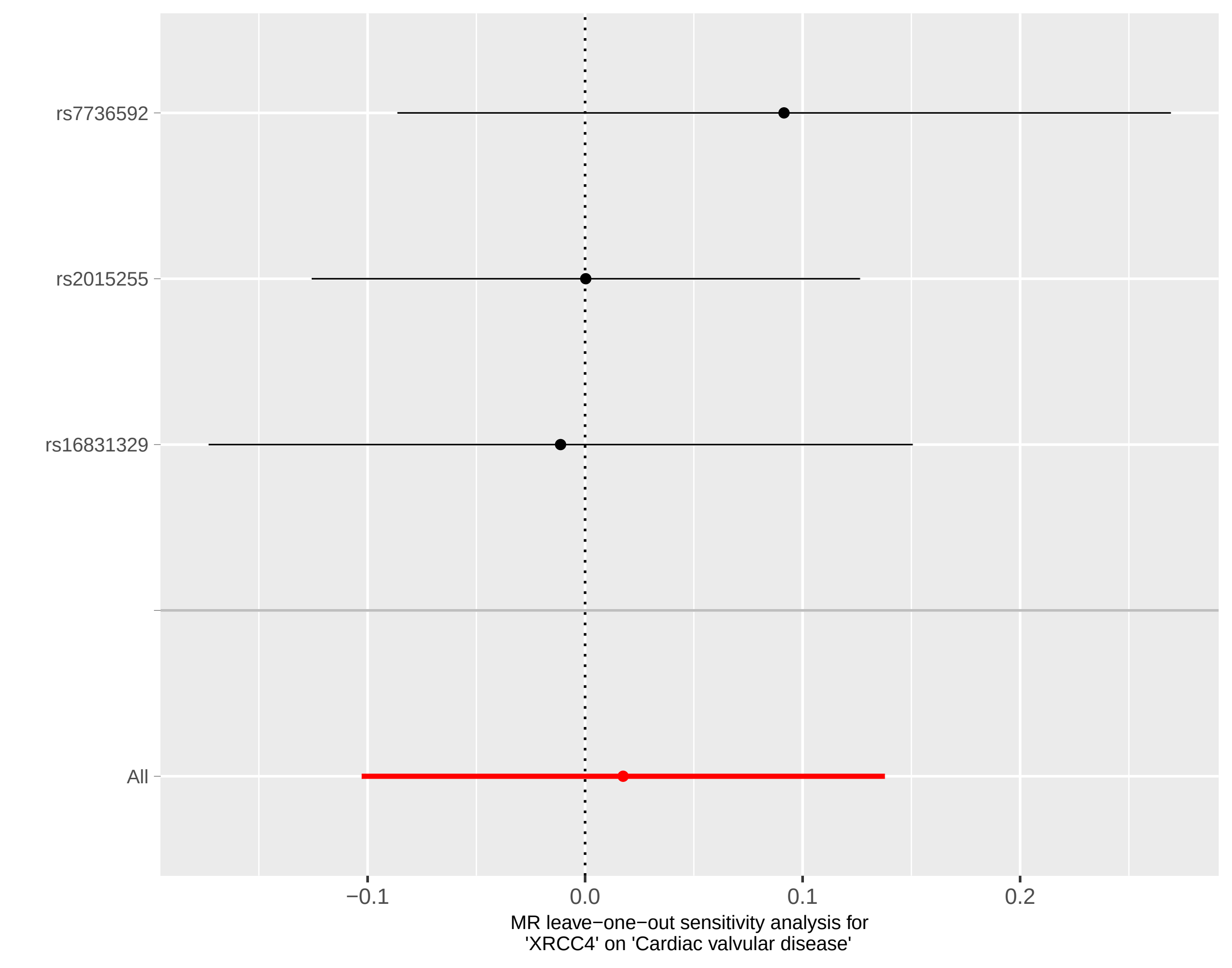
rs7736592
rs2015255
rs16831329
All
−0.1
0.0
0.1
0.2
MR leave−one−out sensitivity analysis for
'XRCC4' on 'Cardiac valvular disease'

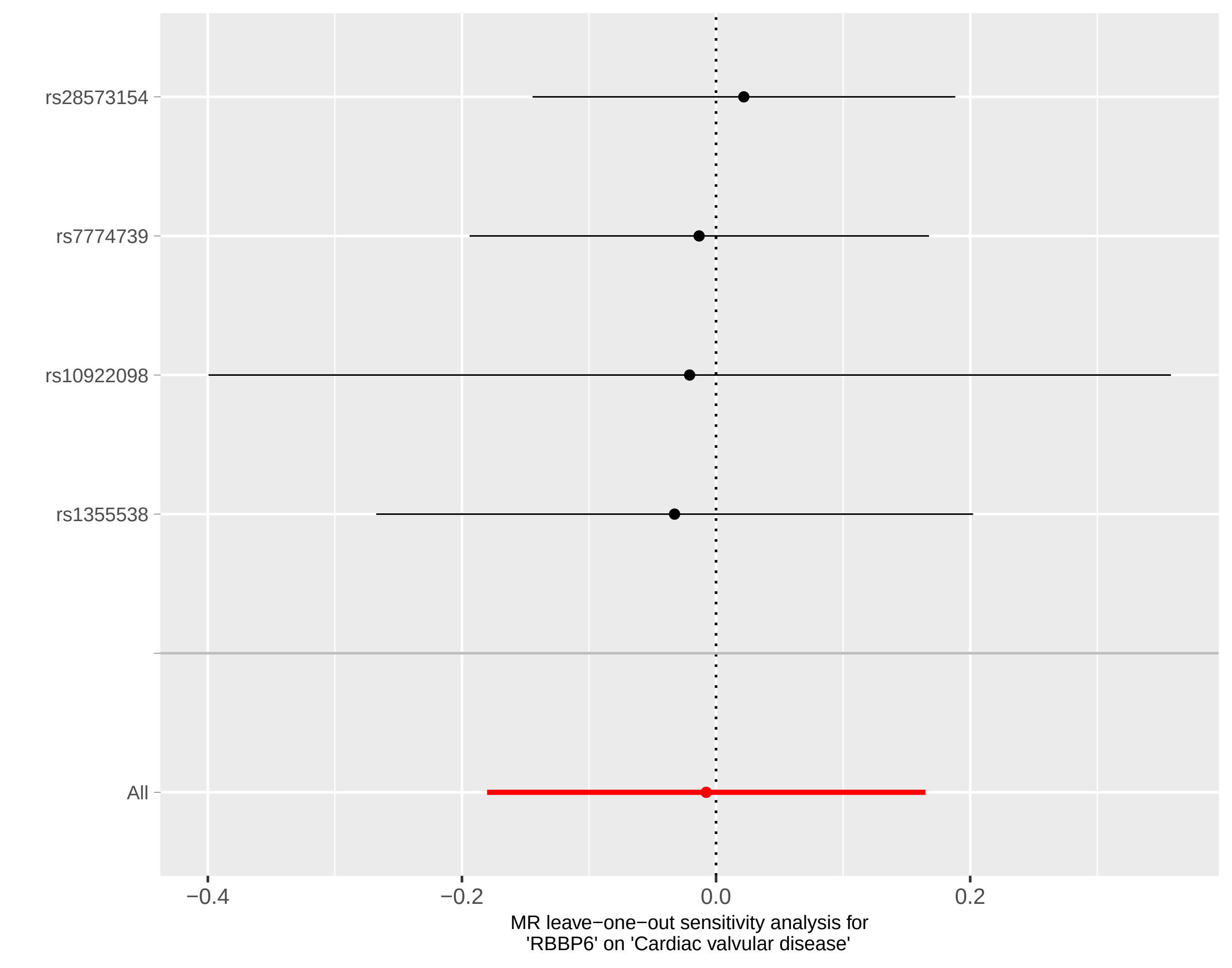
rs28573154
rs7774739
rs10922098
rs1355538
All
−0.4
−0.2
0.0
0.2
MR leave−one−out sensitivity analysis for
'RBBP6' on 'Cardiac valvular disease'

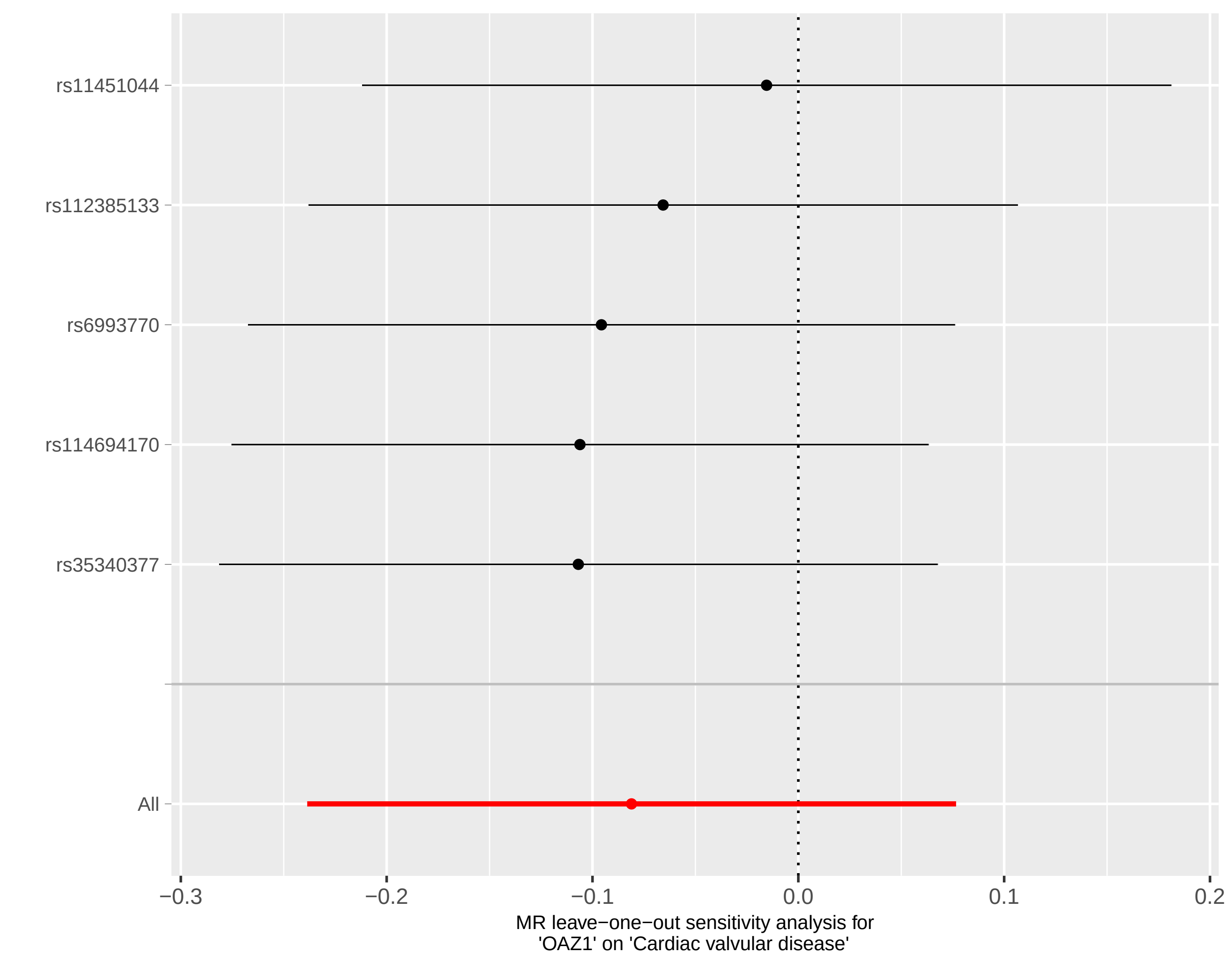
rs11451044
rs112385133
rs6993770
rs114694170
rs35340377
All
−0.3
−0.2
−0.1
0.0
0.1
0.2
MR leave−one−out sensitivity analysis for
'OAZ1' on 'Cardiac valvular disease'

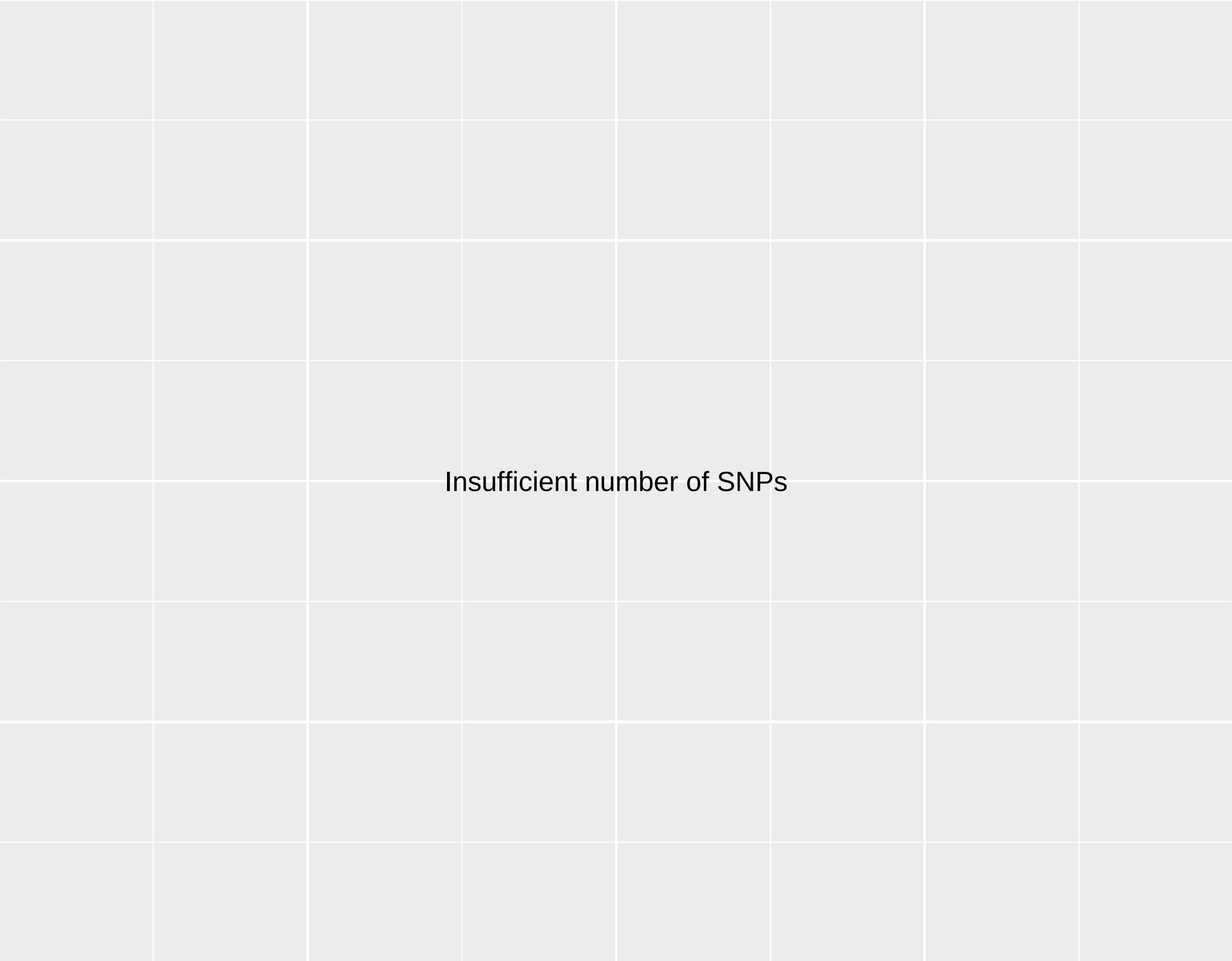
Insufficient number of SNPs

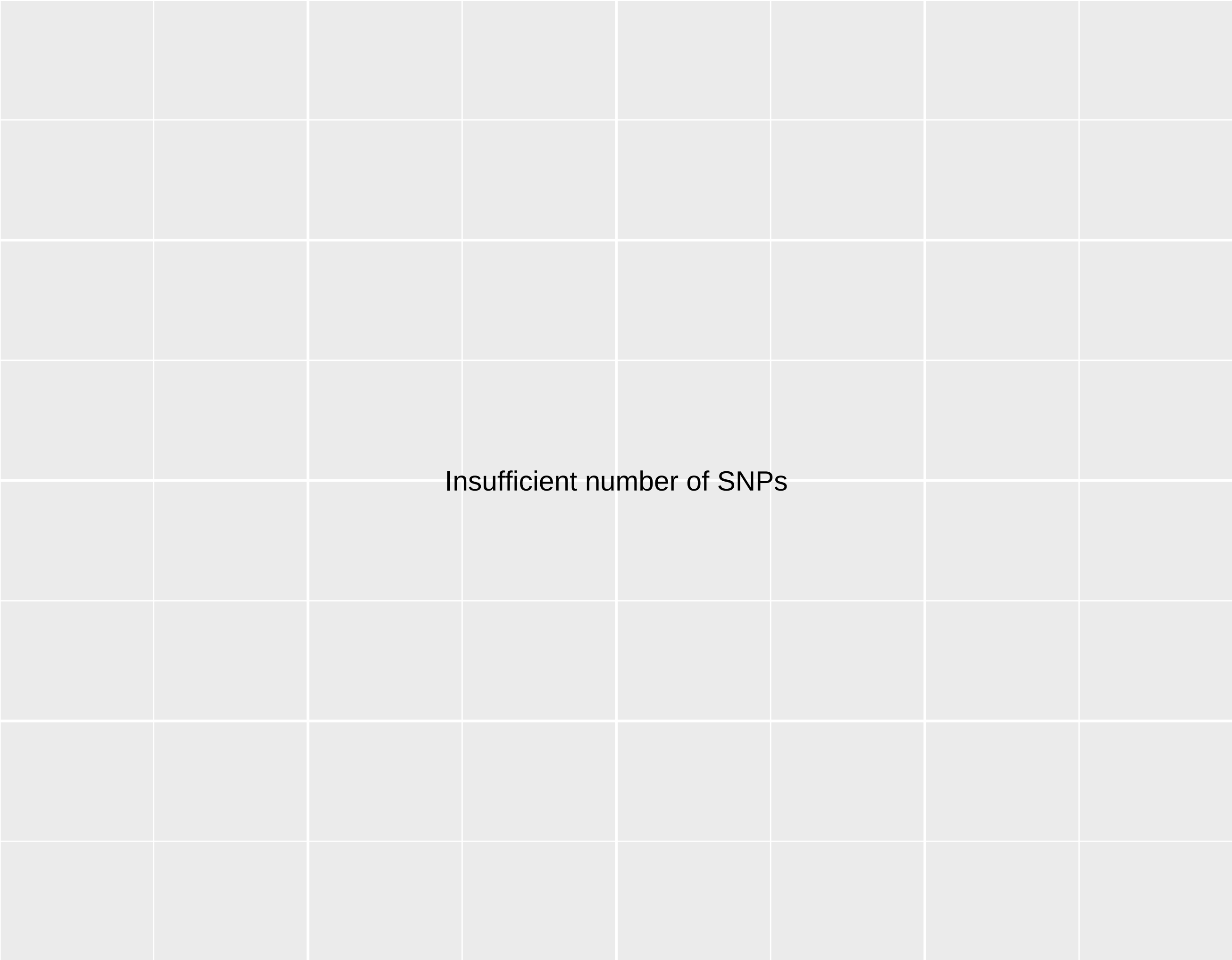
Insufficient number of SNPs

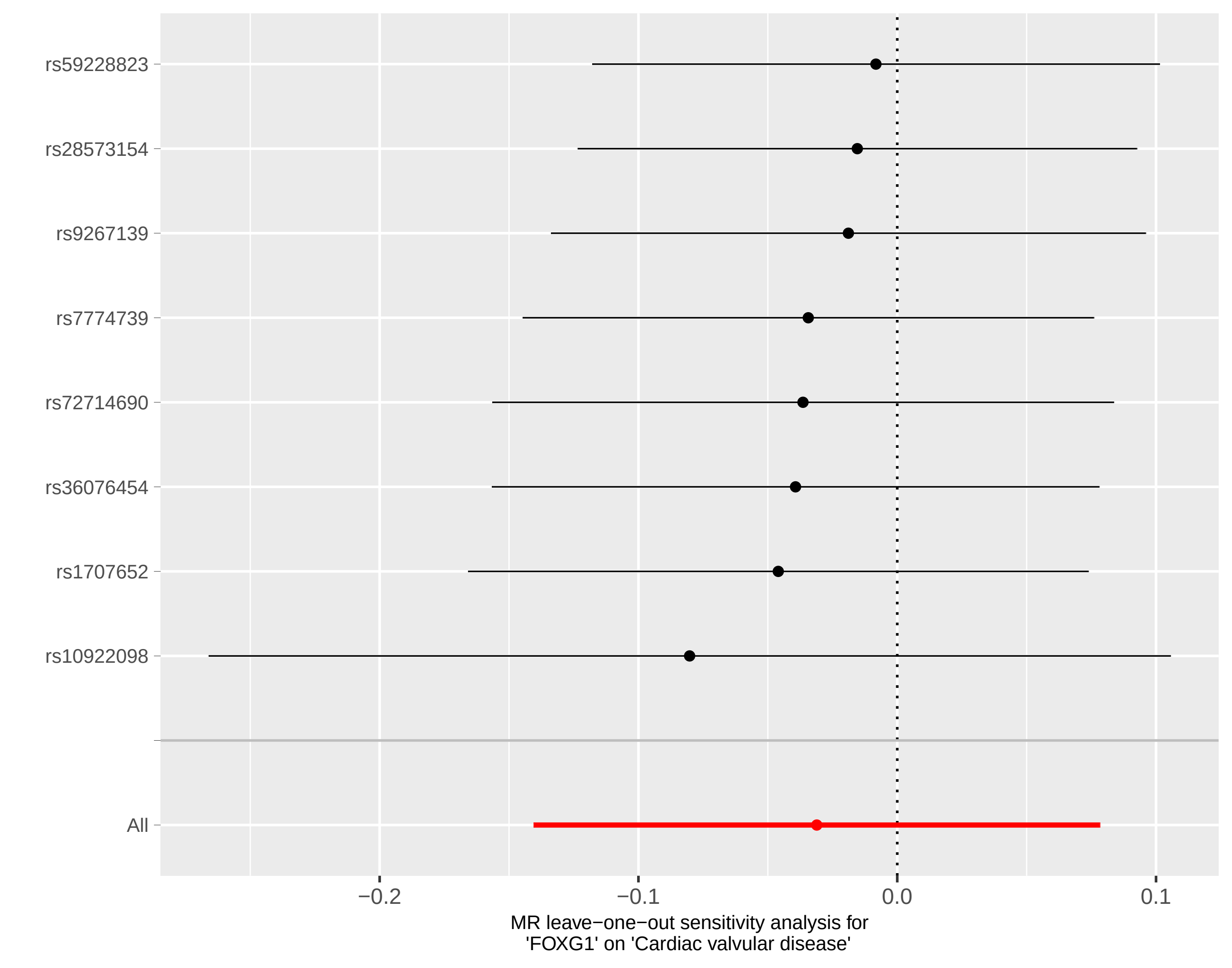
rs59228823
rs28573154
rs9267139
rs7774739
rs72714690
rs36076454
rs1707652
rs10922098
All
−0.2
−0.1
0.0
0.1
MR leave−one−out sensitivity analysis for
'FOXG1' on 'Cardiac valvular disease'

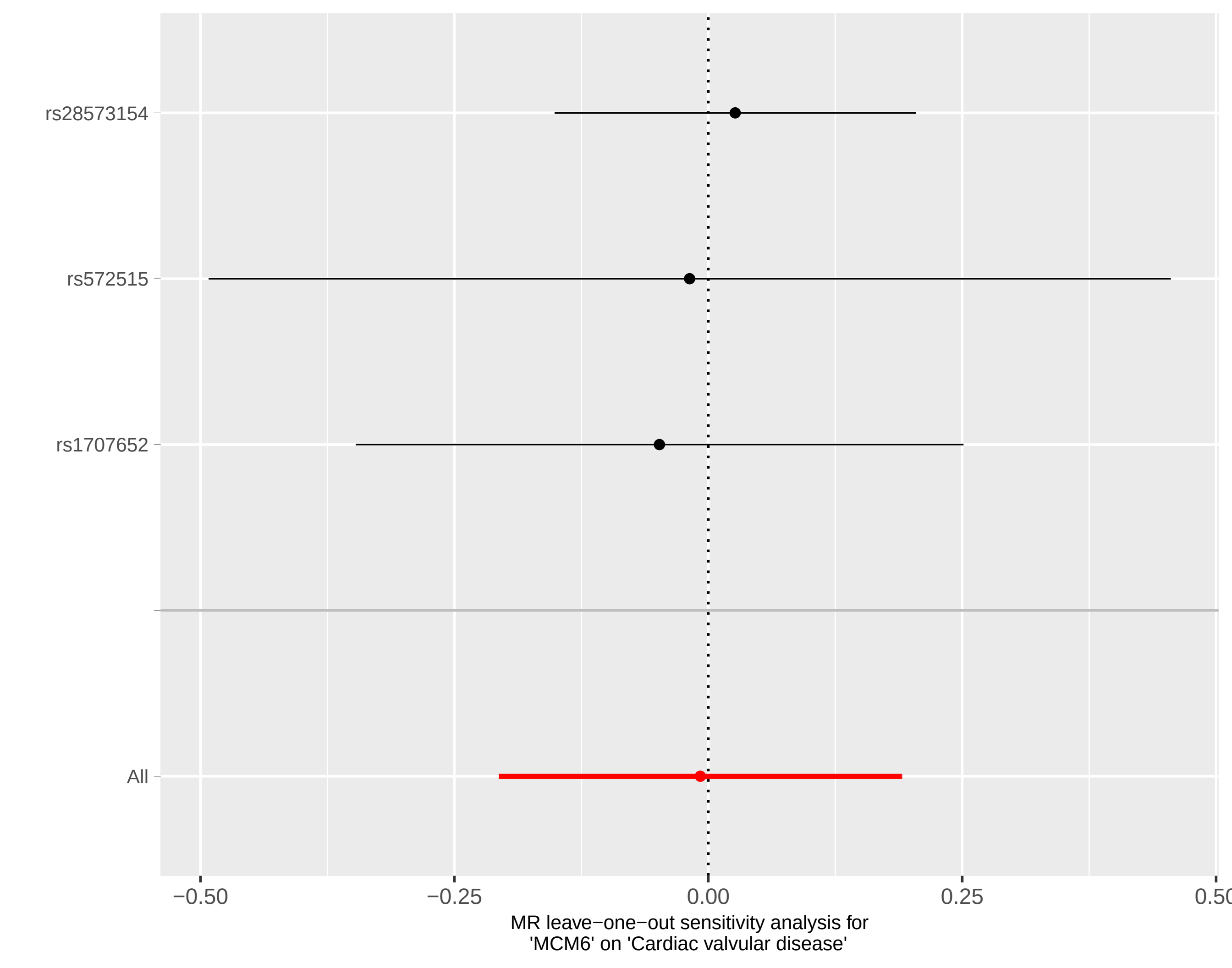
rs28573154
rs572515
rs1707652
All
−0.50
−0.25
0.00
0.25
0.50
MR leave−one−out sensitivity analysis for
'MCM6' on 'Cardiac valvular disease'

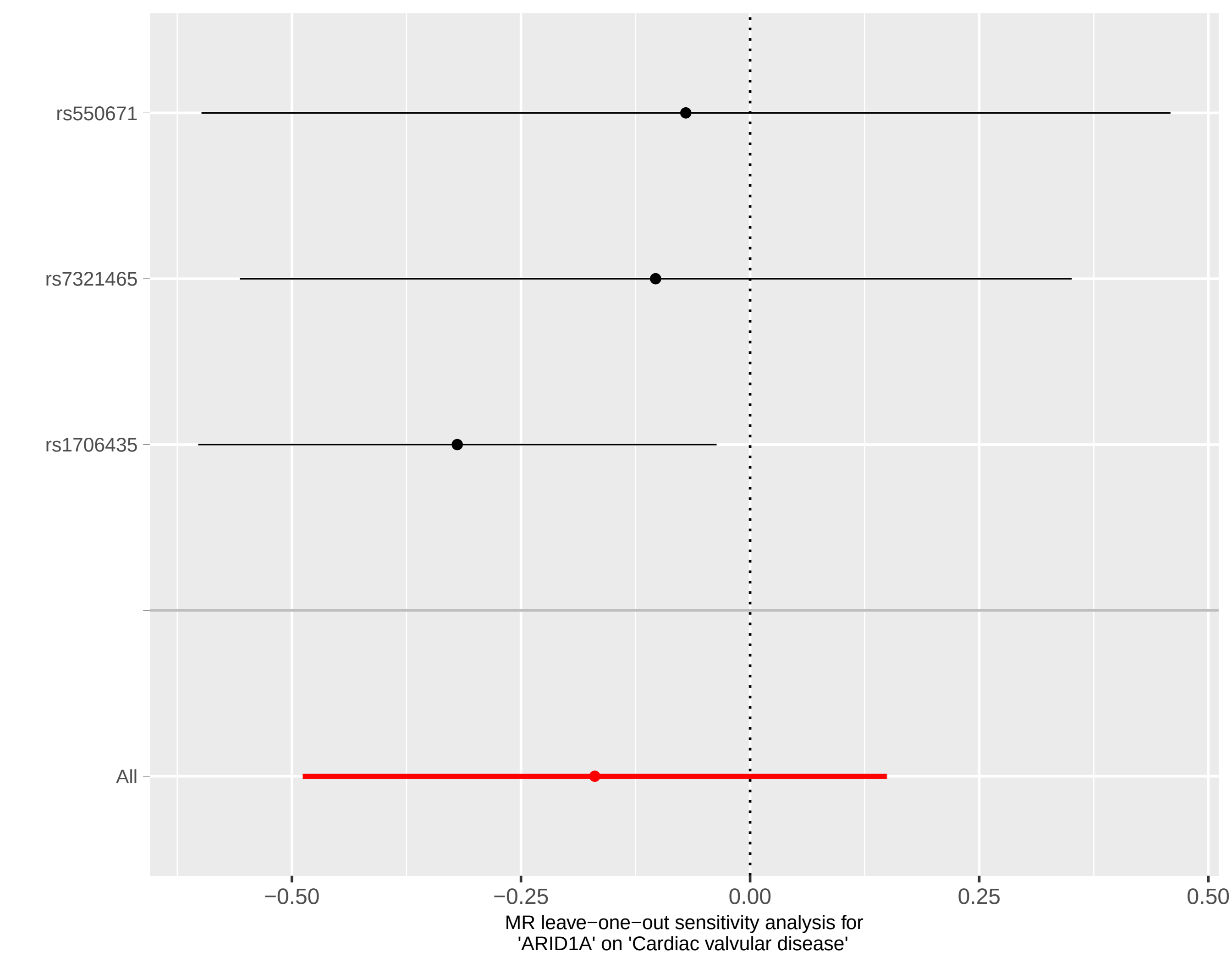
rs550671
rs7321465
rs1706435
All
−0.50
−0.25
0.00
0.25
0.50
MR leave−one−out sensitivity analysis for
'ARID1A' on 'Cardiac valvular disease'

Insufficient number of SNPs

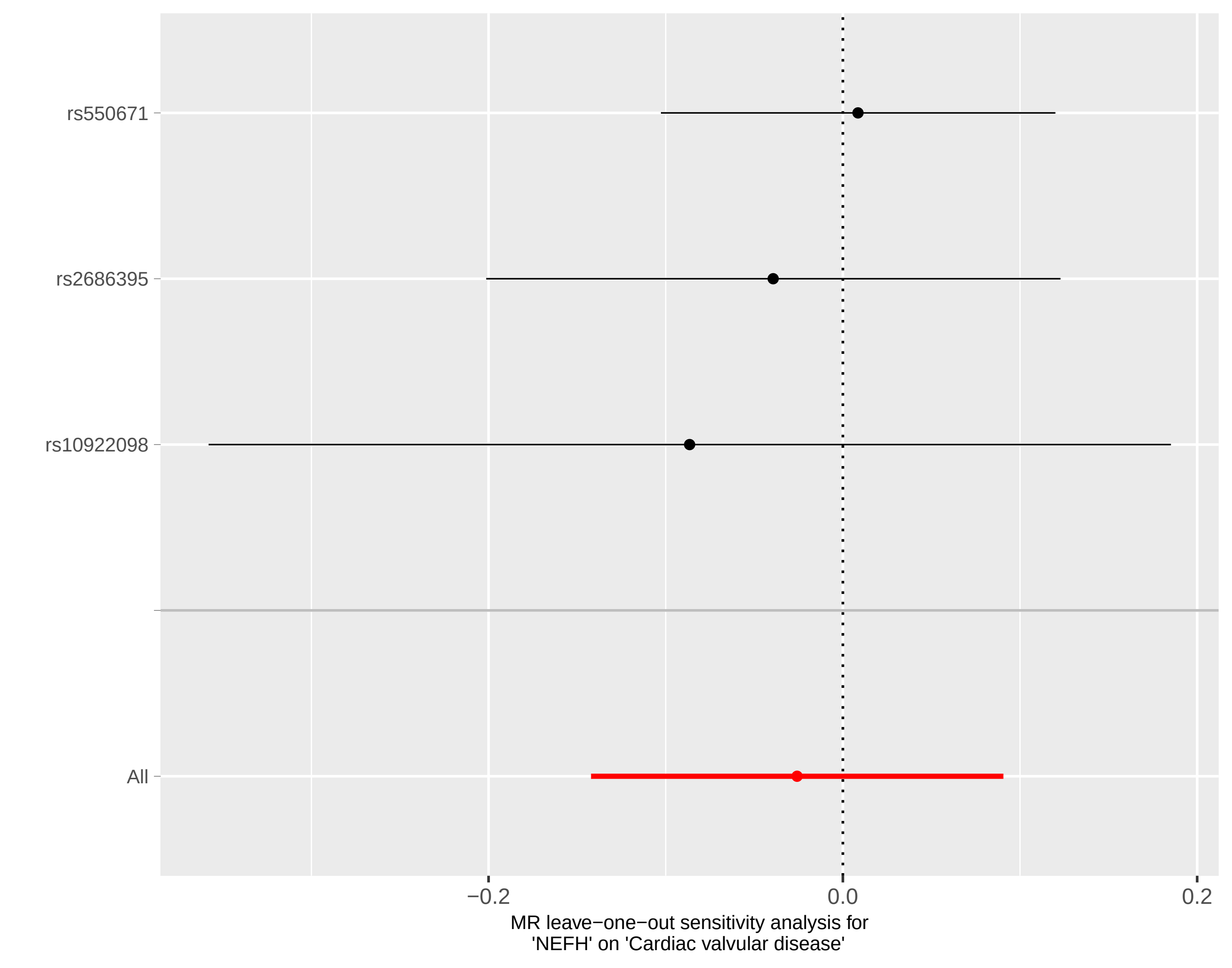
rs550671
rs2686395
rs10922098
All
−0.2
0.0
0.2
MR leave−one−out sensitivity analysis for
'NEFH' on 'Cardiac valvular disease'

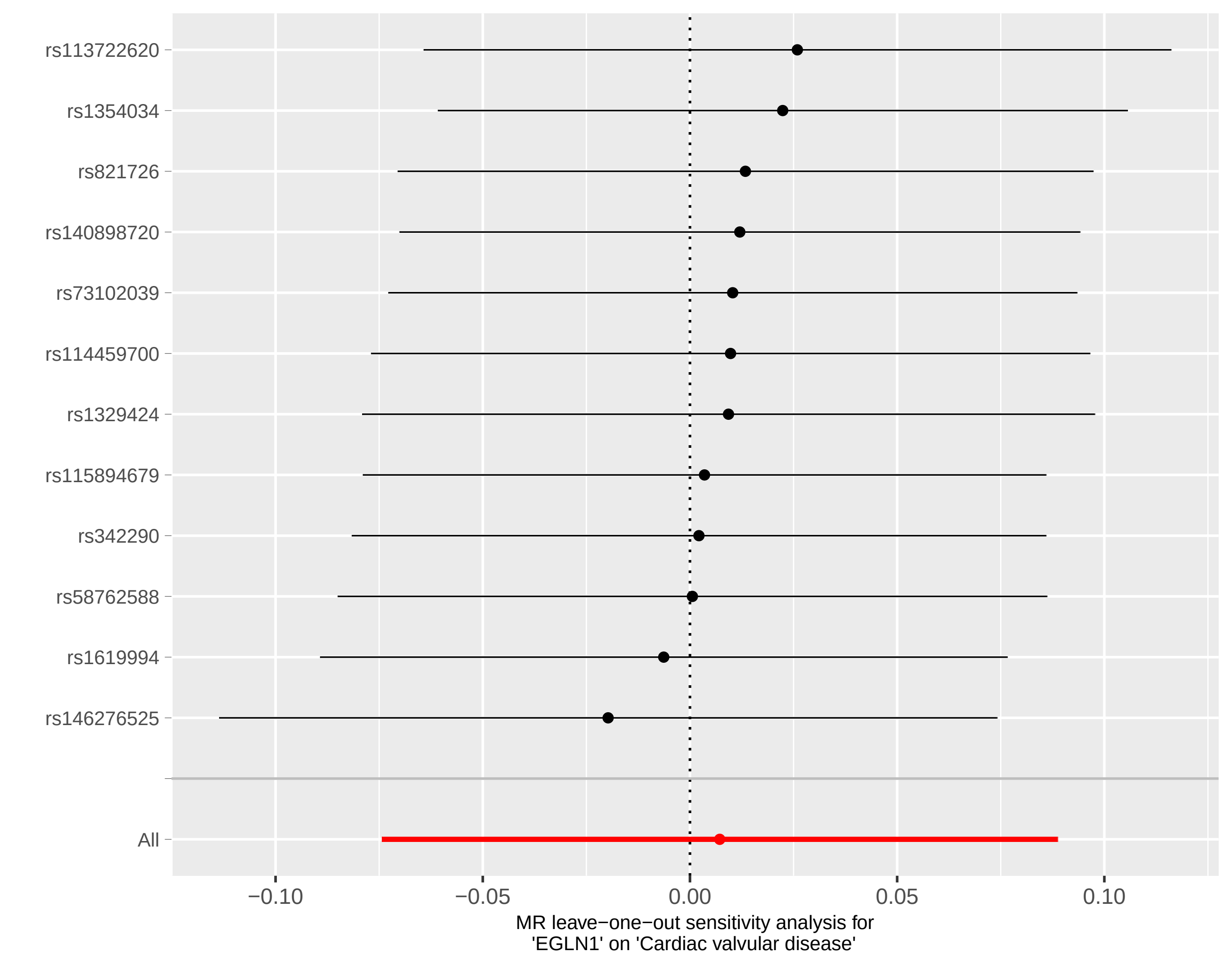

rs113722620
rs1354034
rs821726
rs140898720
rs73102039
rs114459700
rs1329424
rs115894679
rs342290
rs58762588
rs1619994
rs146276525
All
−0.10
−0.05
0.00
0.05
0.10
MR leave−one−out sensitivity analysis for
'EGLN1' on 'Cardiac valvular disease'

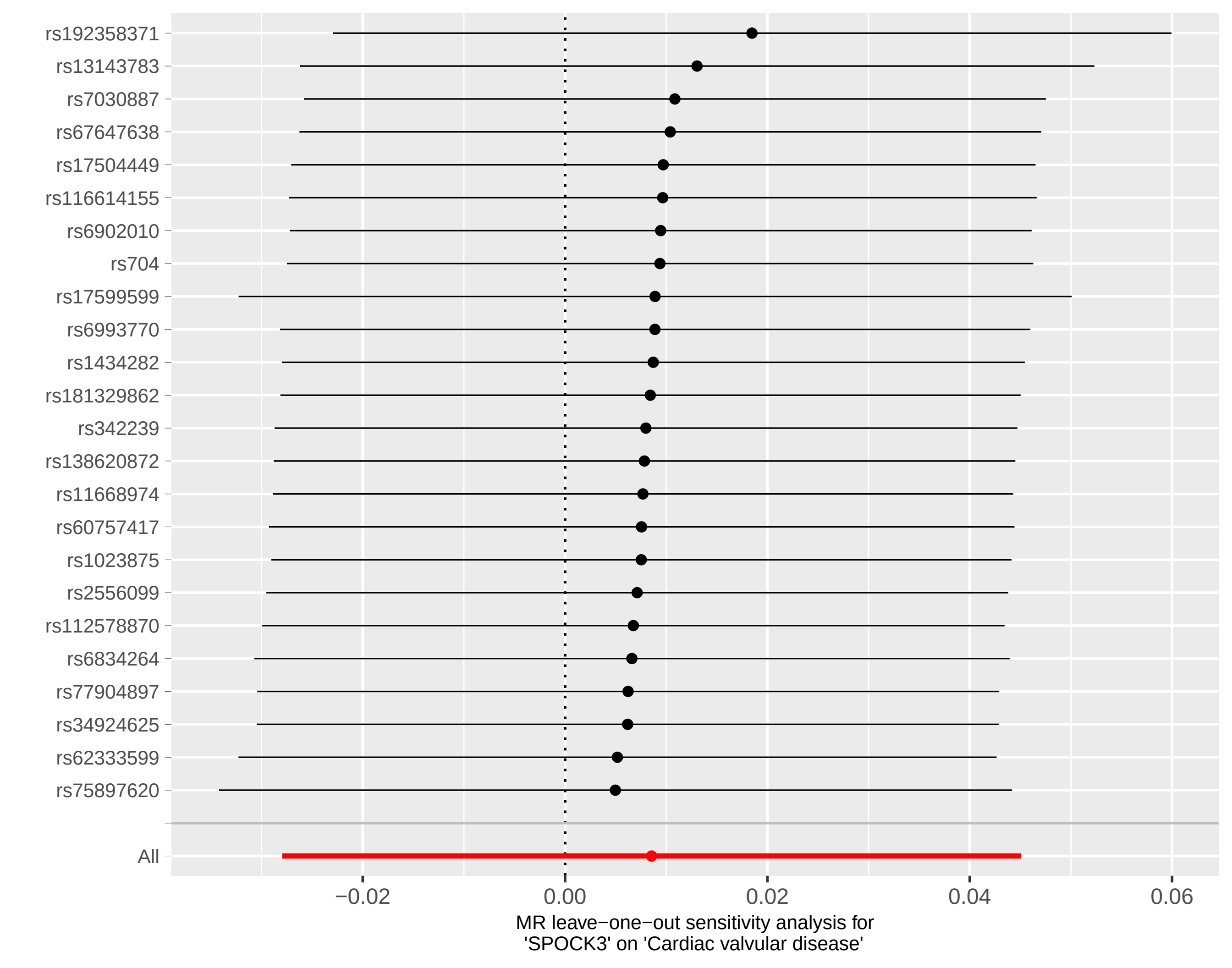
rs192358371
rs13143783
rs7030887
rs67647638
rs17504449
rs116614155
rs6902010
rs704
rs17599599
rs6993770
rs1434282
rs181329862
rs342239
rs138620872
rs11668974
rs60757417
rs1023875
rs2556099
rs112578870
rs6834264
rs77904897
rs34924625
rs62333599
rs75897620
All
−0.02
0.00
0.02
0.04
0.06
MR leave−one−out sensitivity analysis for
'SPOCK3' on 'Cardiac valvular disease'

Insufficient number of SNPs

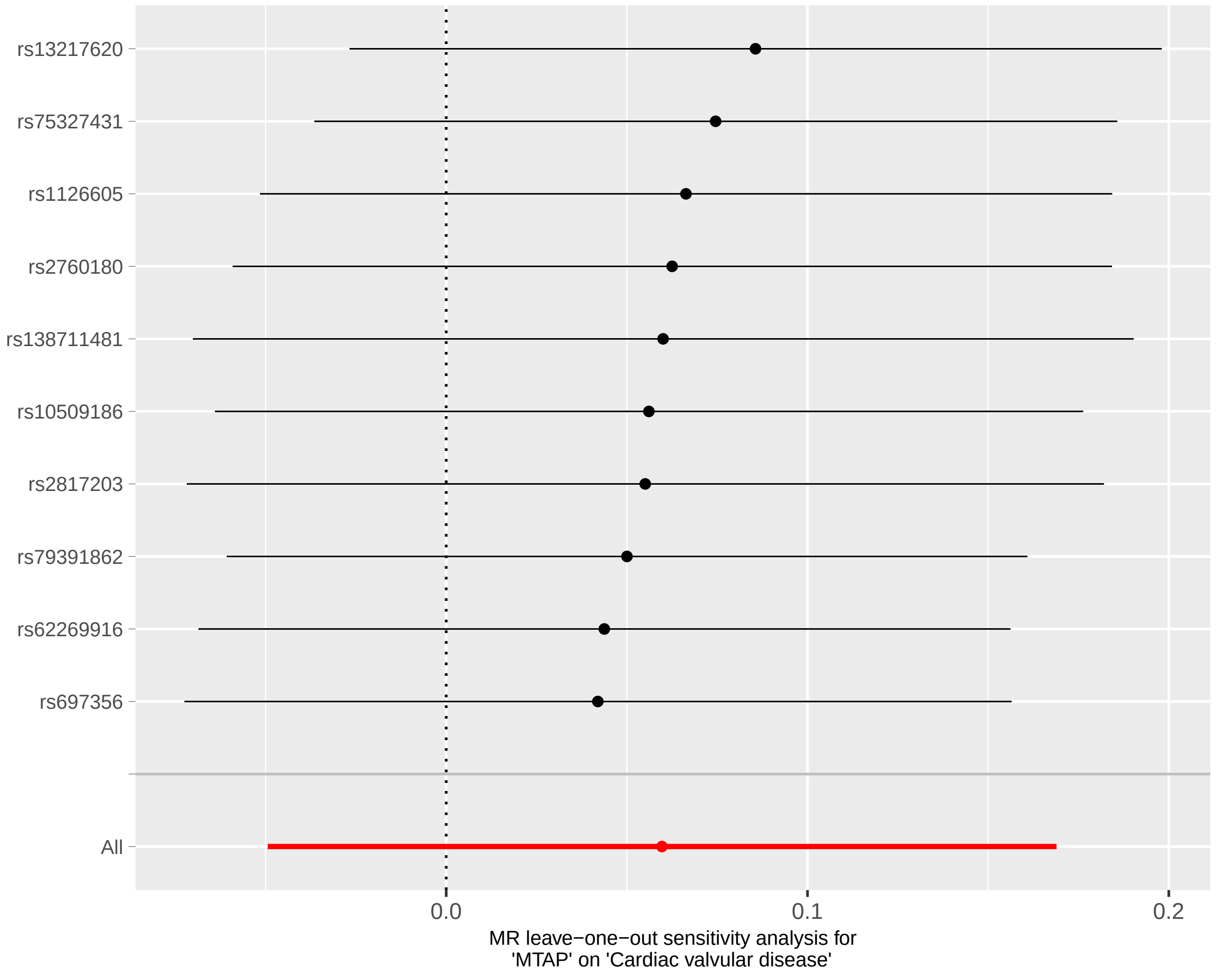

rs13217620
rs75327431
rs1126605
rs2760180
rs138711481
rs10509186
rs2817203
rs79391862
rs62269916
rs697356
All
0.0
0.1
0.2
MR leave−one−out sensitivity analysis for
'MTAP' on 'Cardiac valvular disease'

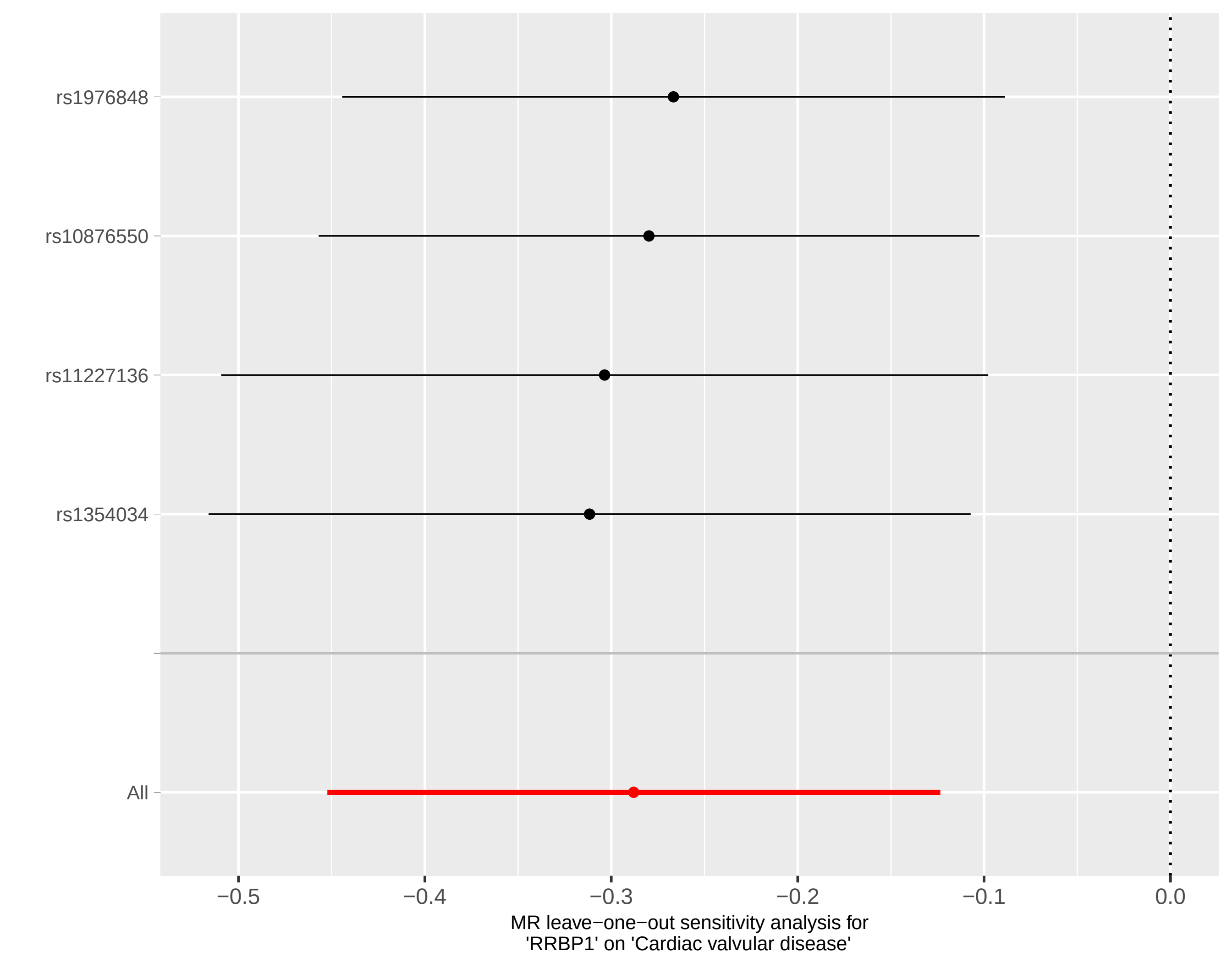
rs1976848
rs10876550
rs11227136
rs1354034
All
−0.5
−0.4
−0.3
−0.2
−0.1
0.0
MR leave−one−out sensitivity analysis for
'RRBP1' on 'Cardiac valvular disease'

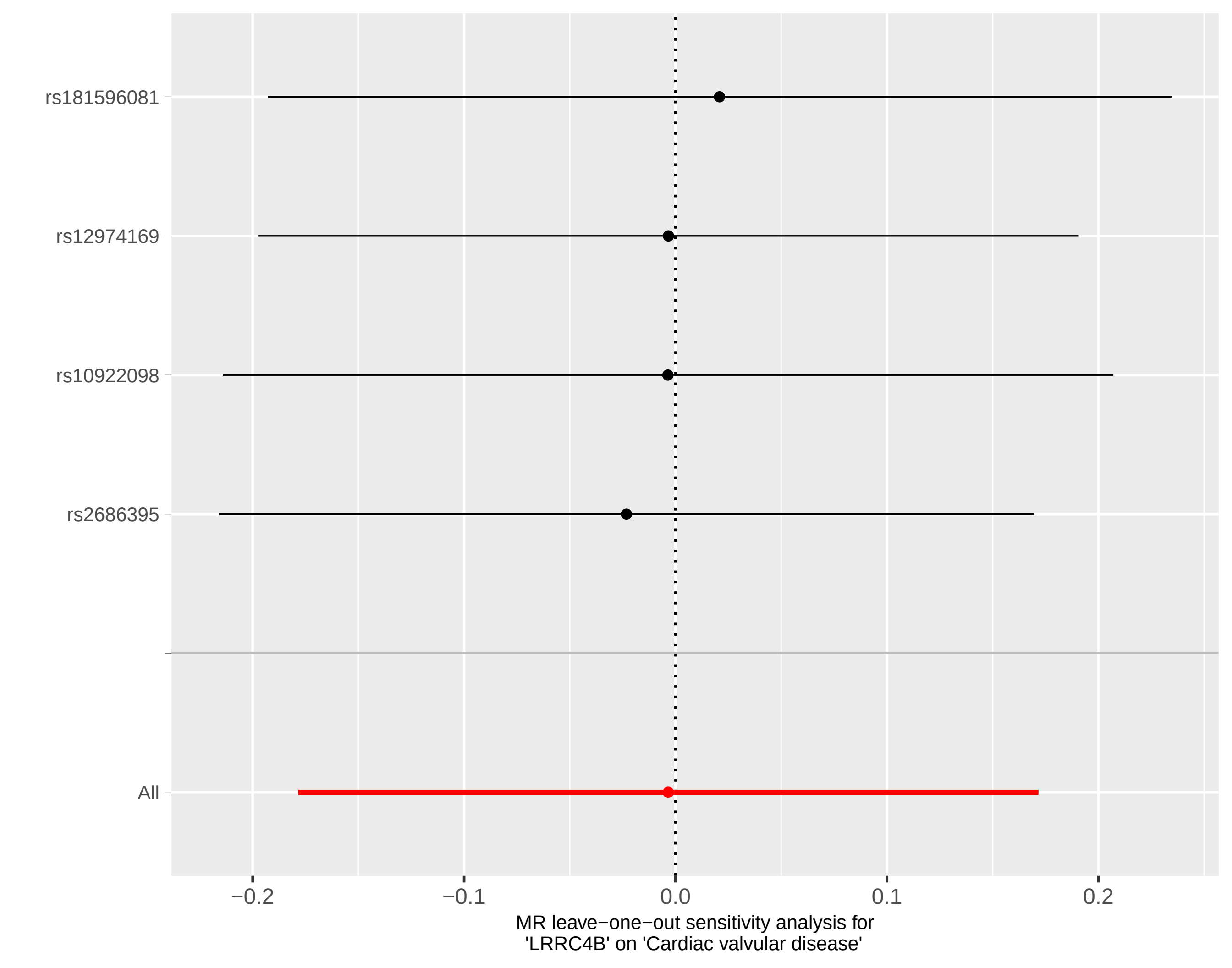
rs181596081
rs12974169
rs10922098
rs2686395
All
−0.2
−0.1
0.0
0.1
0.2
MR leave−one−out sensitivity analysis for
'LRRC4B' on 'Cardiac valvular disease'

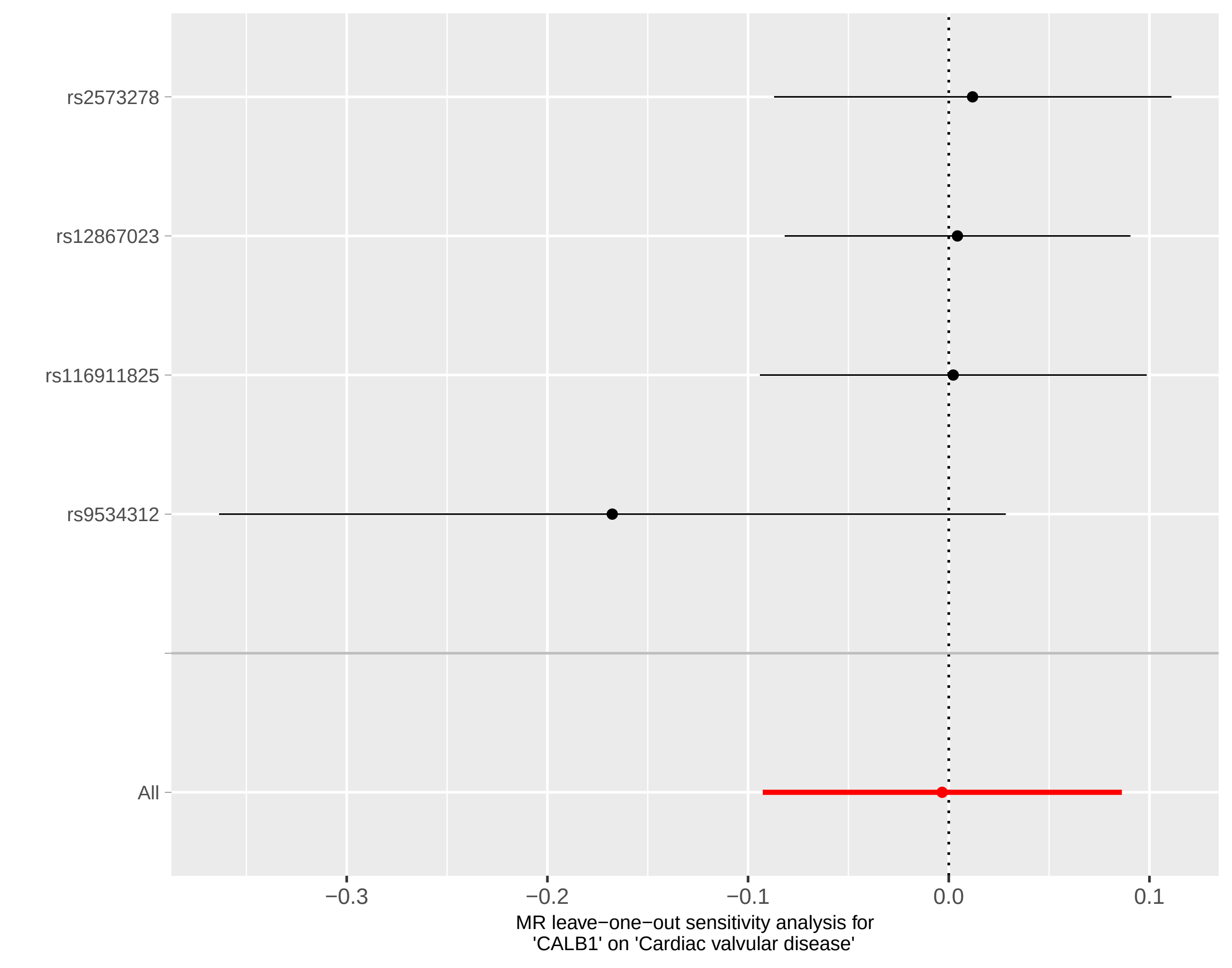
rs2573278
rs12867023
rs116911825
rs9534312
All
−0.3
−0.2
−0.1
0.0
0.1
MR leave−one−out sensitivity analysis for
'CALB1' on 'Cardiac valvular disease'

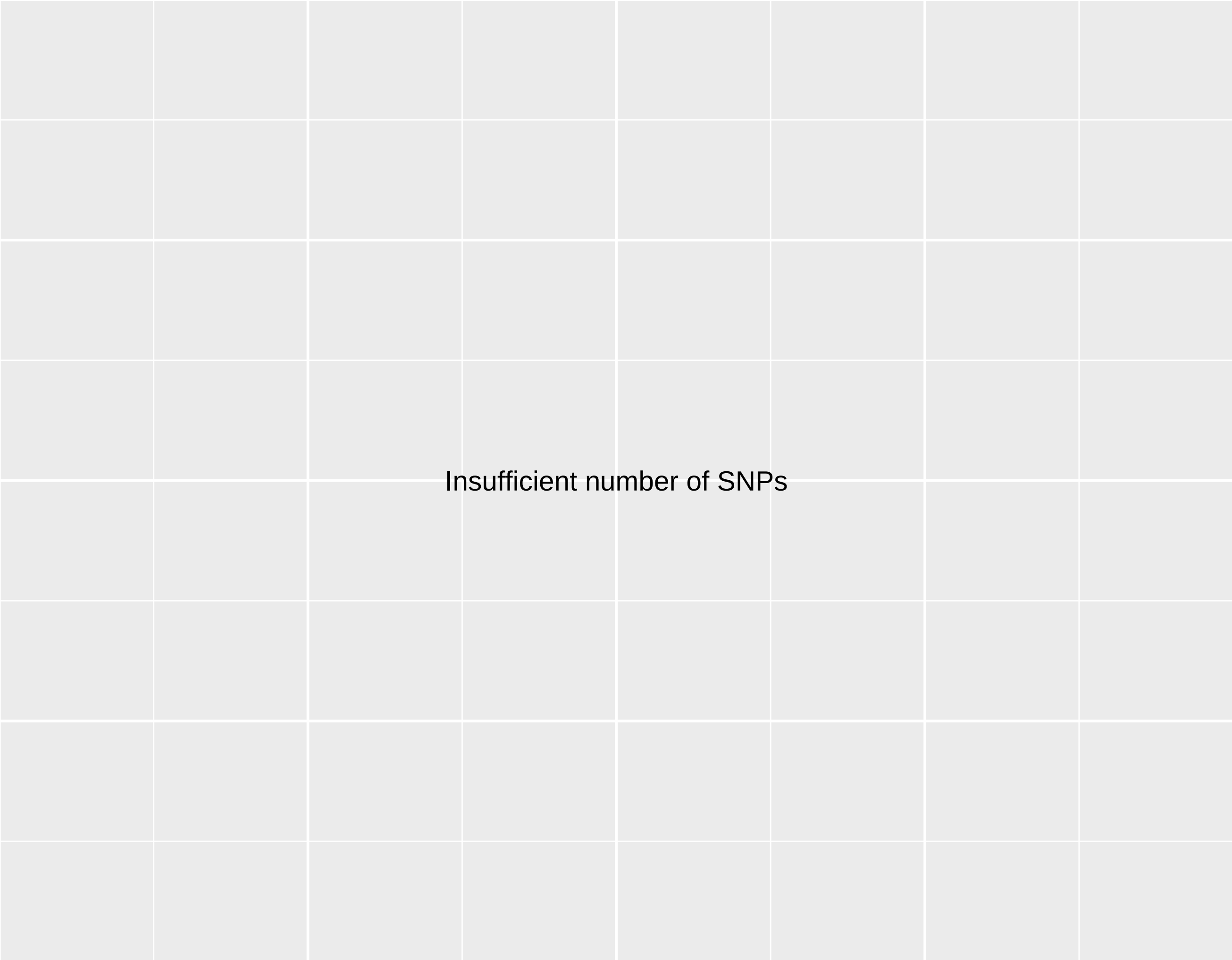
Insufficient number of SNPs

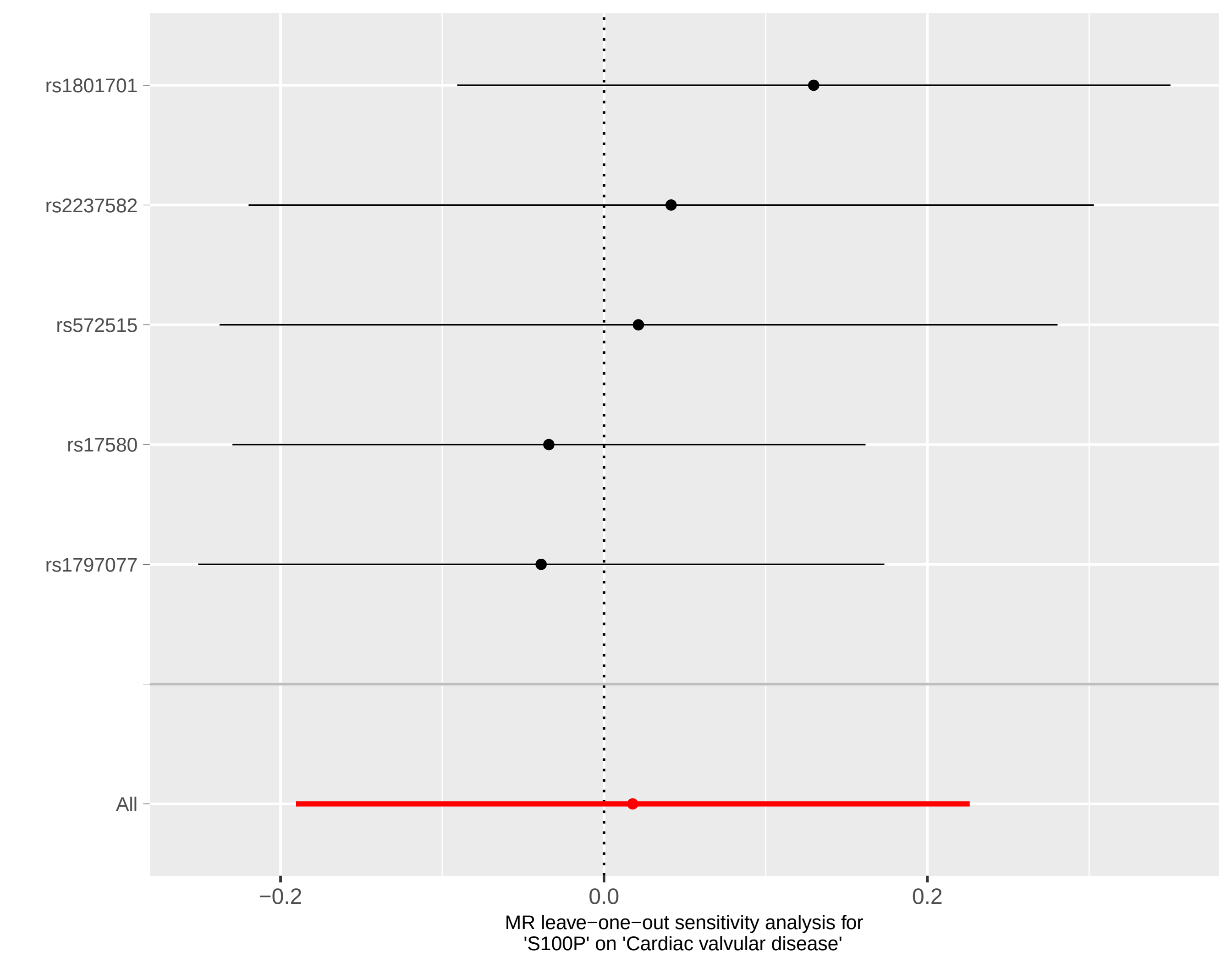
rs1801701
rs2237582
rs572515
rs17580
rs1797077
All
−0.2
0.0
0.2
MR leave−one−out sensitivity analysis for
'S100P' on 'Cardiac valvular disease'

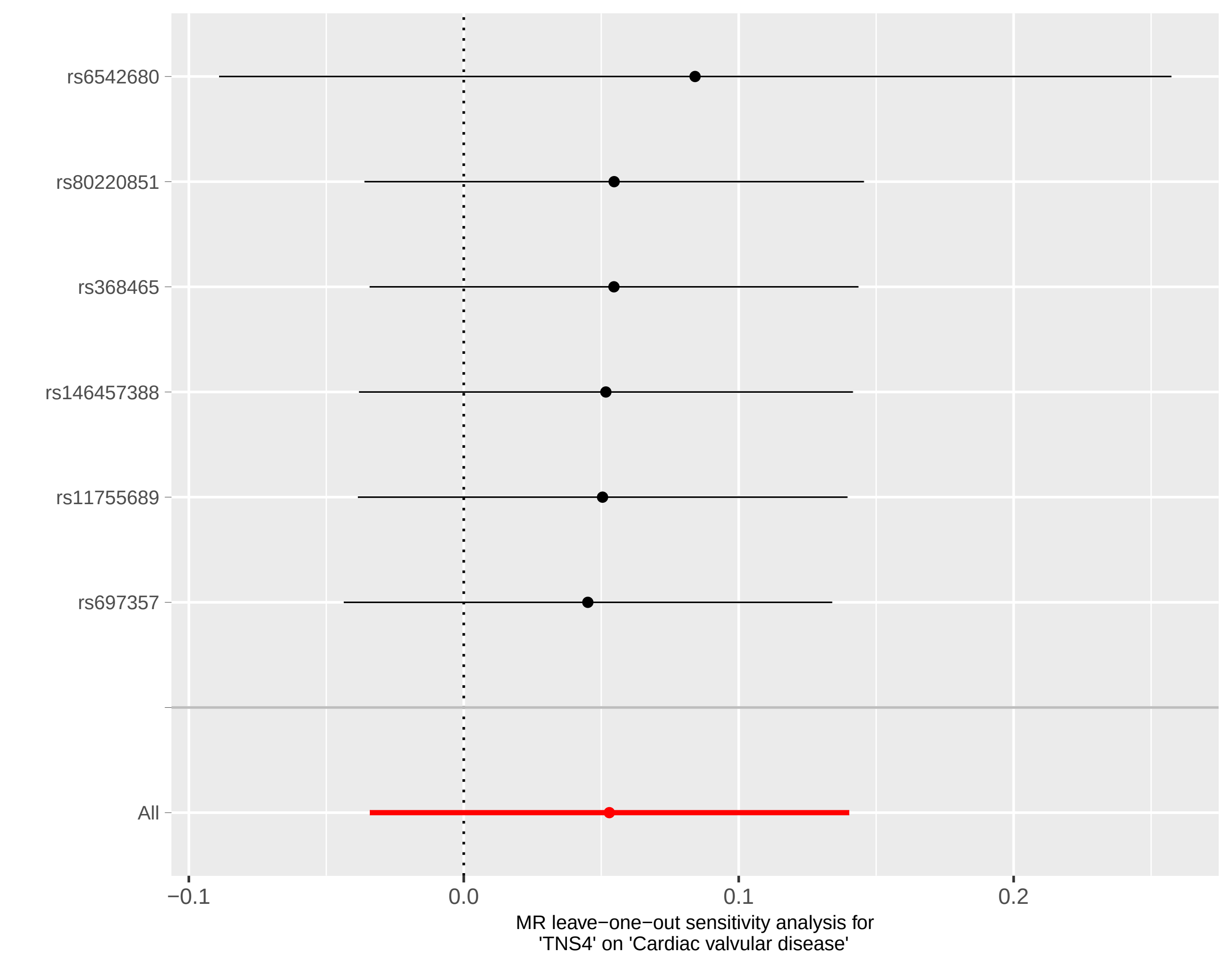
rs6542680
rs80220851
rs368465
rs146457388
rs11755689
rs697357
All
−0.1
0.0
0.1
0.2
MR leave−one−out sensitivity analysis for
'TNS4' on 'Cardiac valvular disease'

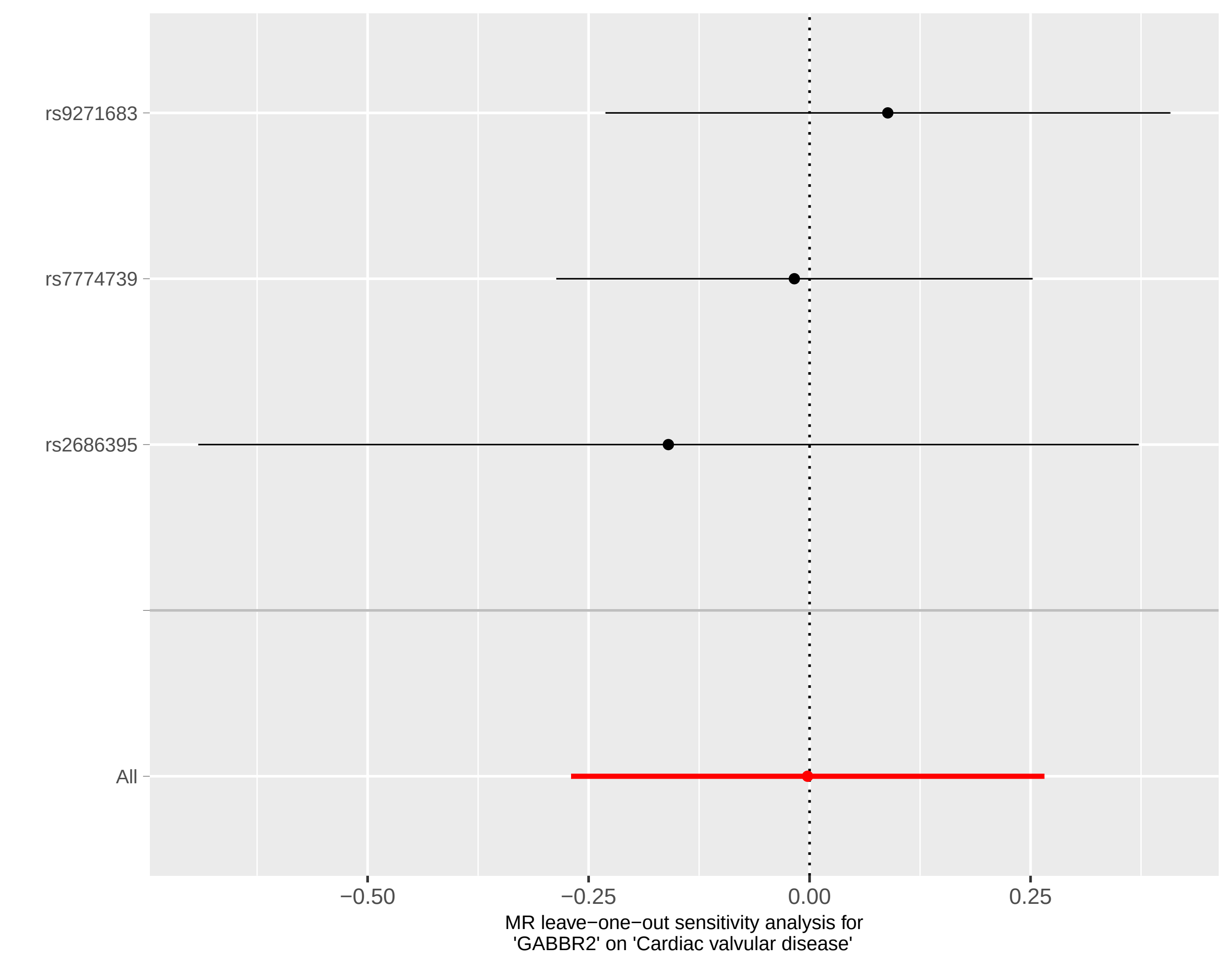
rs9271683
rs7774739
rs2686395
All
−0.50
−0.25
0.00
0.25
MR leave−one−out sensitivity analysis for
'GABBR2' on 'Cardiac valvular disease'

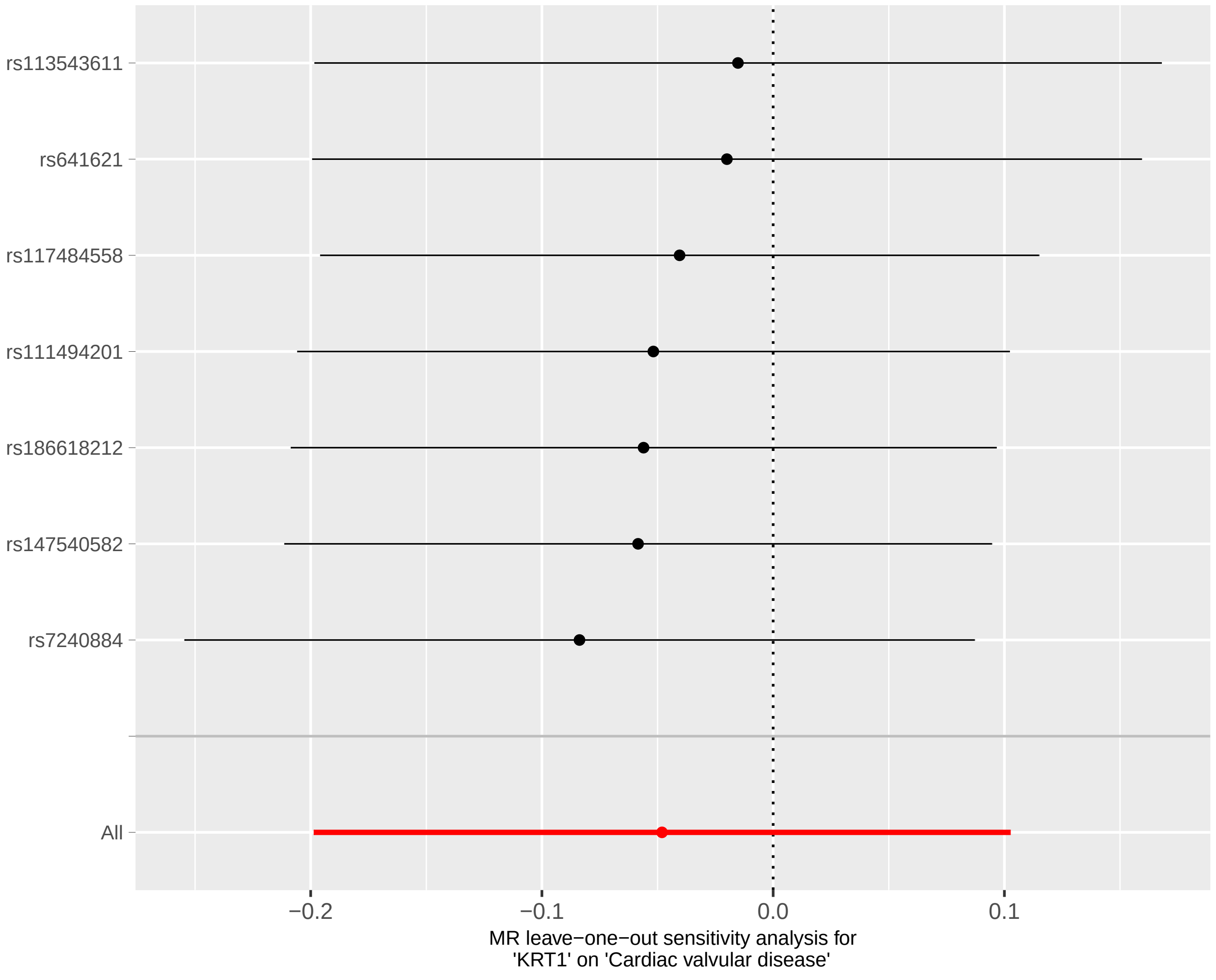

rs113543611
rs641621
rs117484558
rs111494201
rs186618212
rs147540582
rs7240884
All
−0.2
−0.1
0.0
0.1
MR leave−one−out sensitivity analysis for
'KRT1' on 'Cardiac valvular disease'

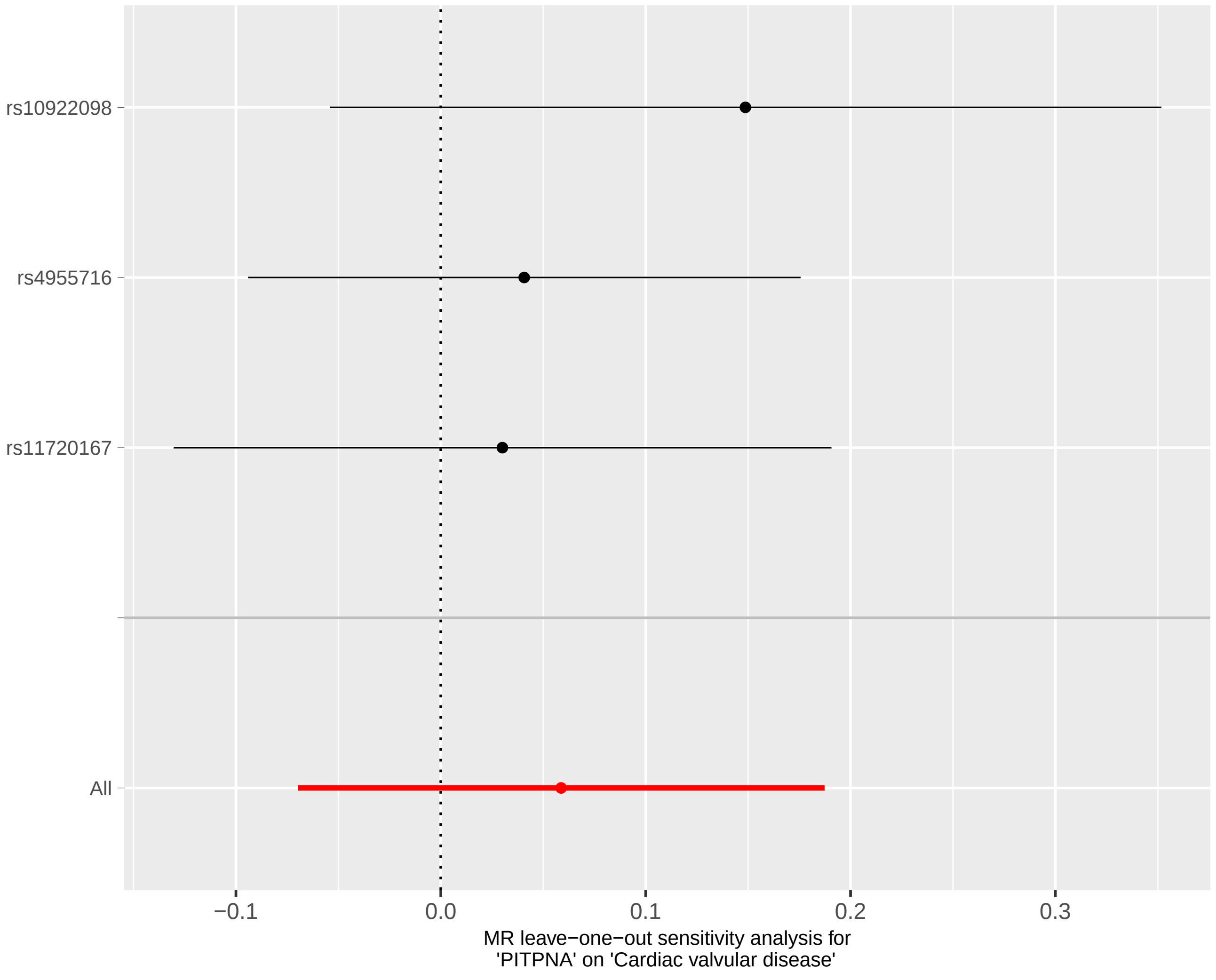
rs10922098
rs4955716
rs11720167
All
−0.1
0.0
0.1
0.2
0.3
MR leave−one−out sensitivity analysis for
'PITPNA' on 'Cardiac valvular disease'

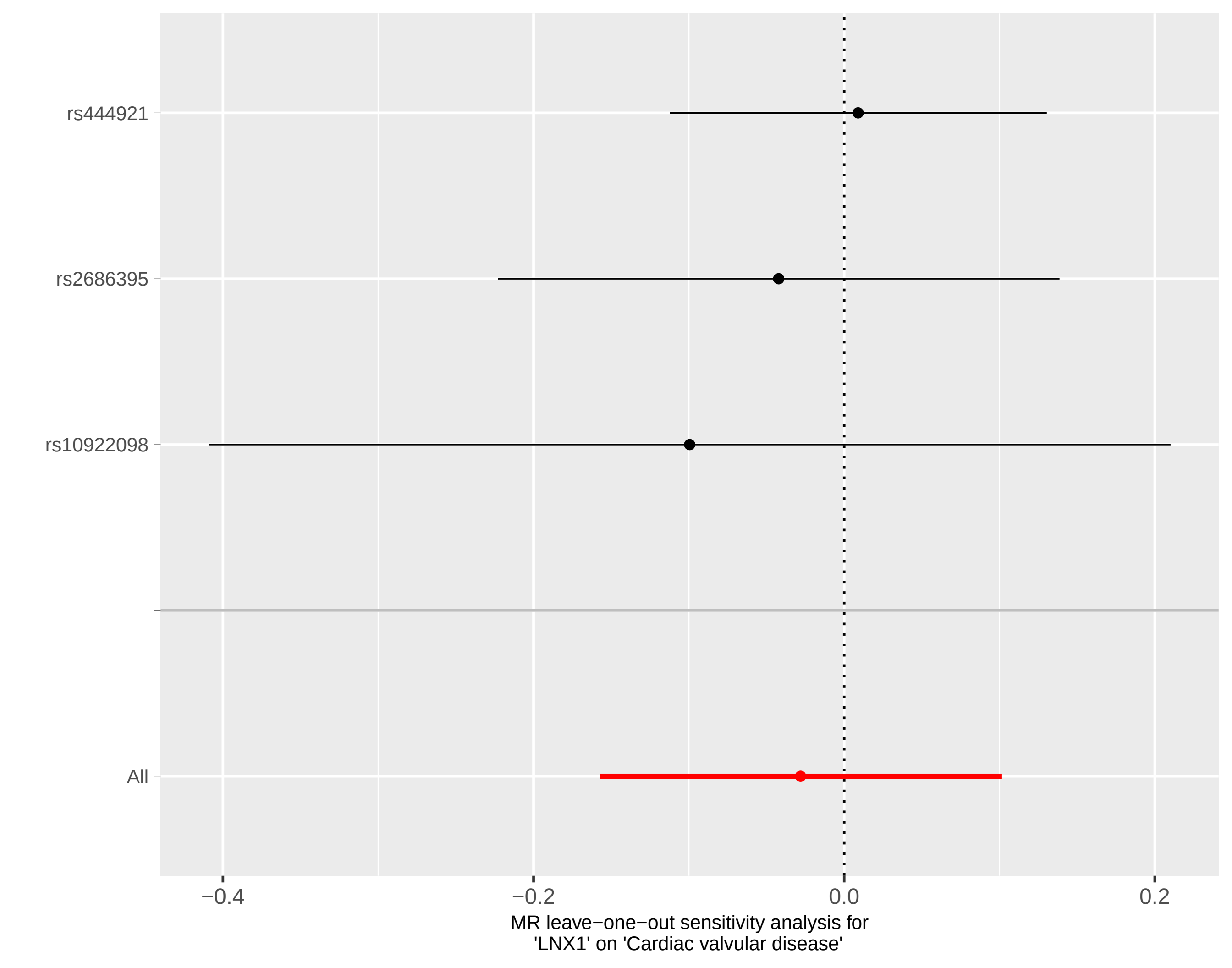
rs444921
rs2686395
rs10922098
All
−0.4
−0.2
0.0
0.2
MR leave−one−out sensitivity analysis for
'LNX1' on 'Cardiac valvular disease'

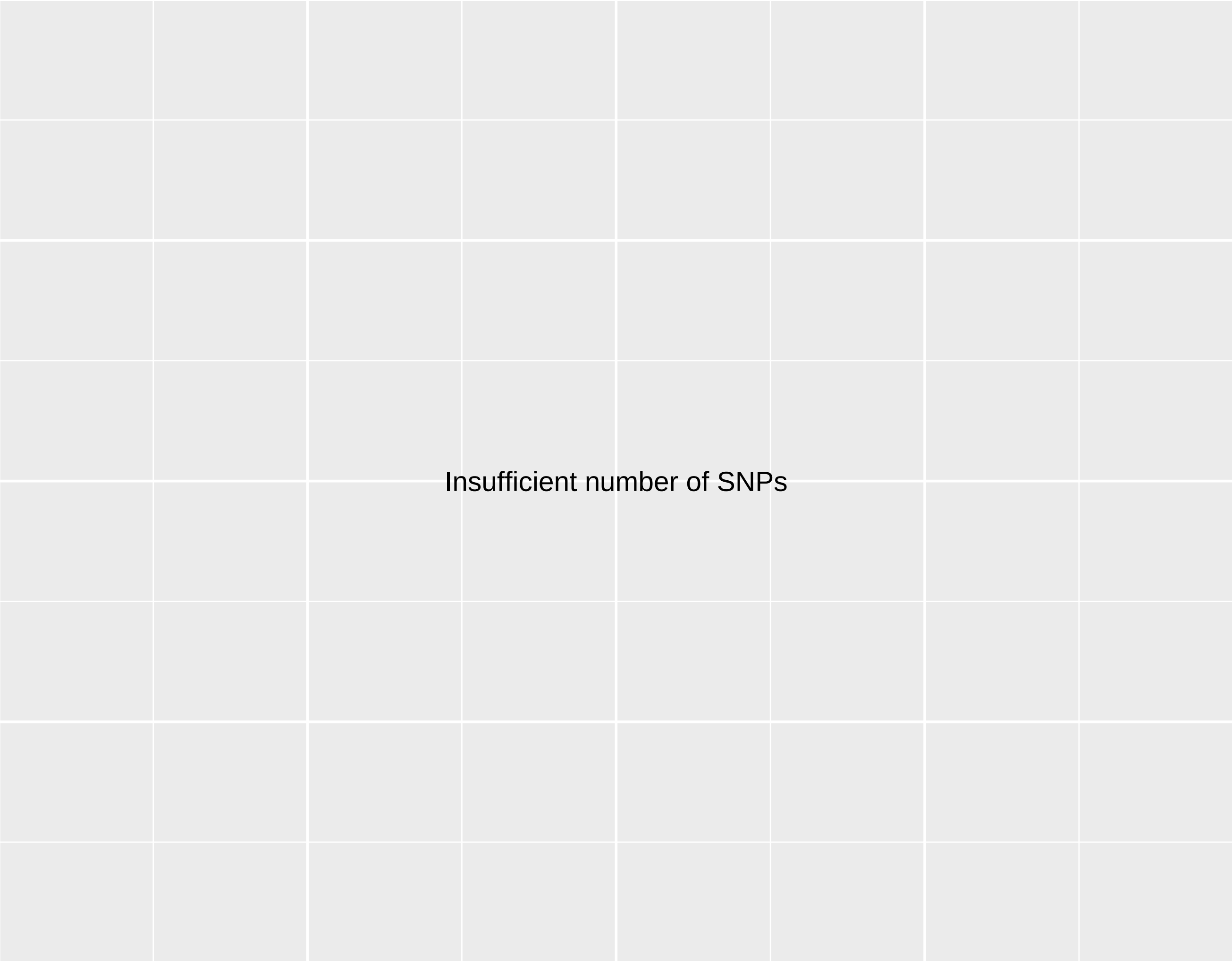
Insufficient number of SNPs

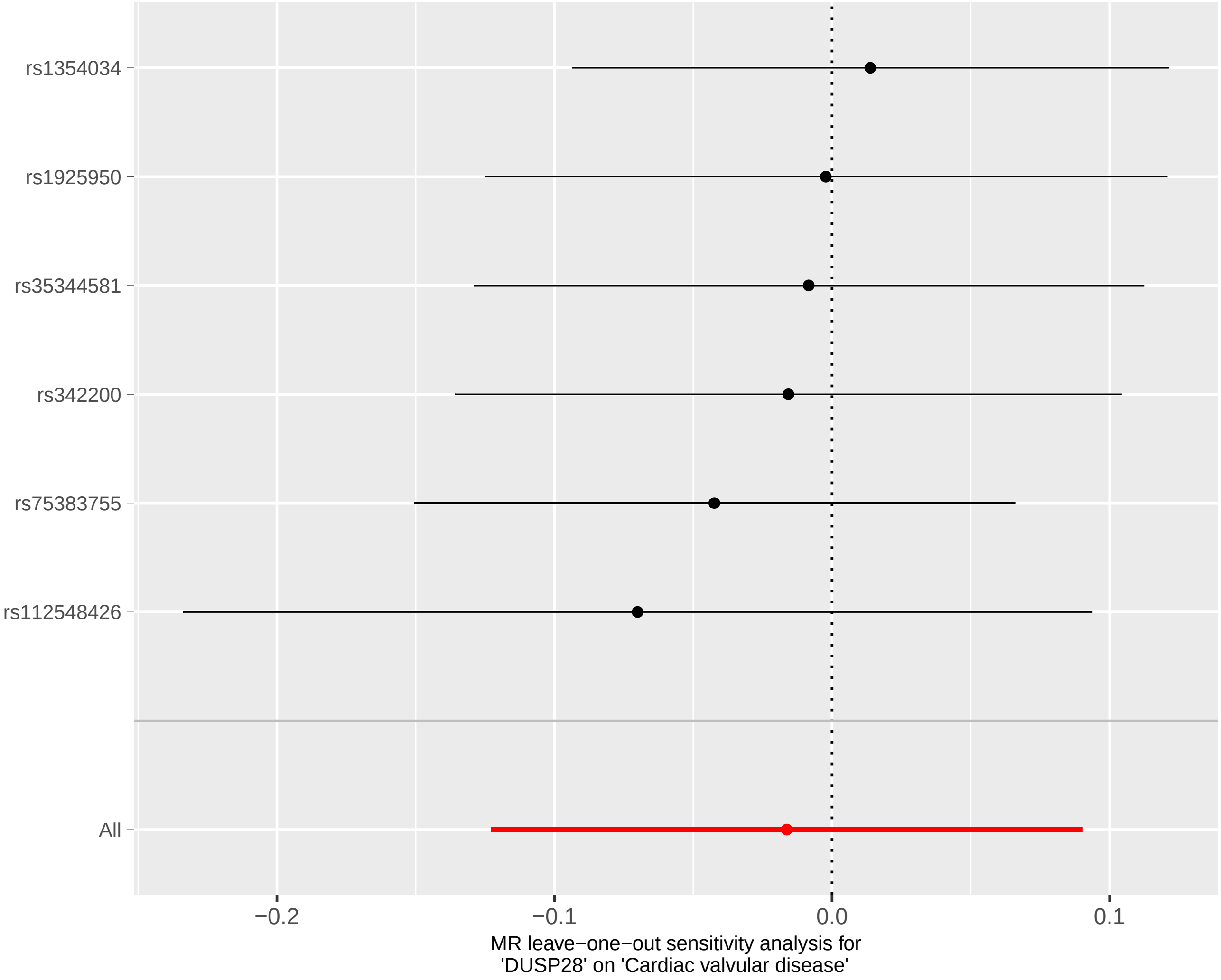

rs1354034
rs1925950
rs35344581
rs342200
rs75383755
rs112548426
All
−0.2
−0.1
0.0
0.1
MR leave−one−out sensitivity analysis for
'DUSP28' on 'Cardiac valvular disease'

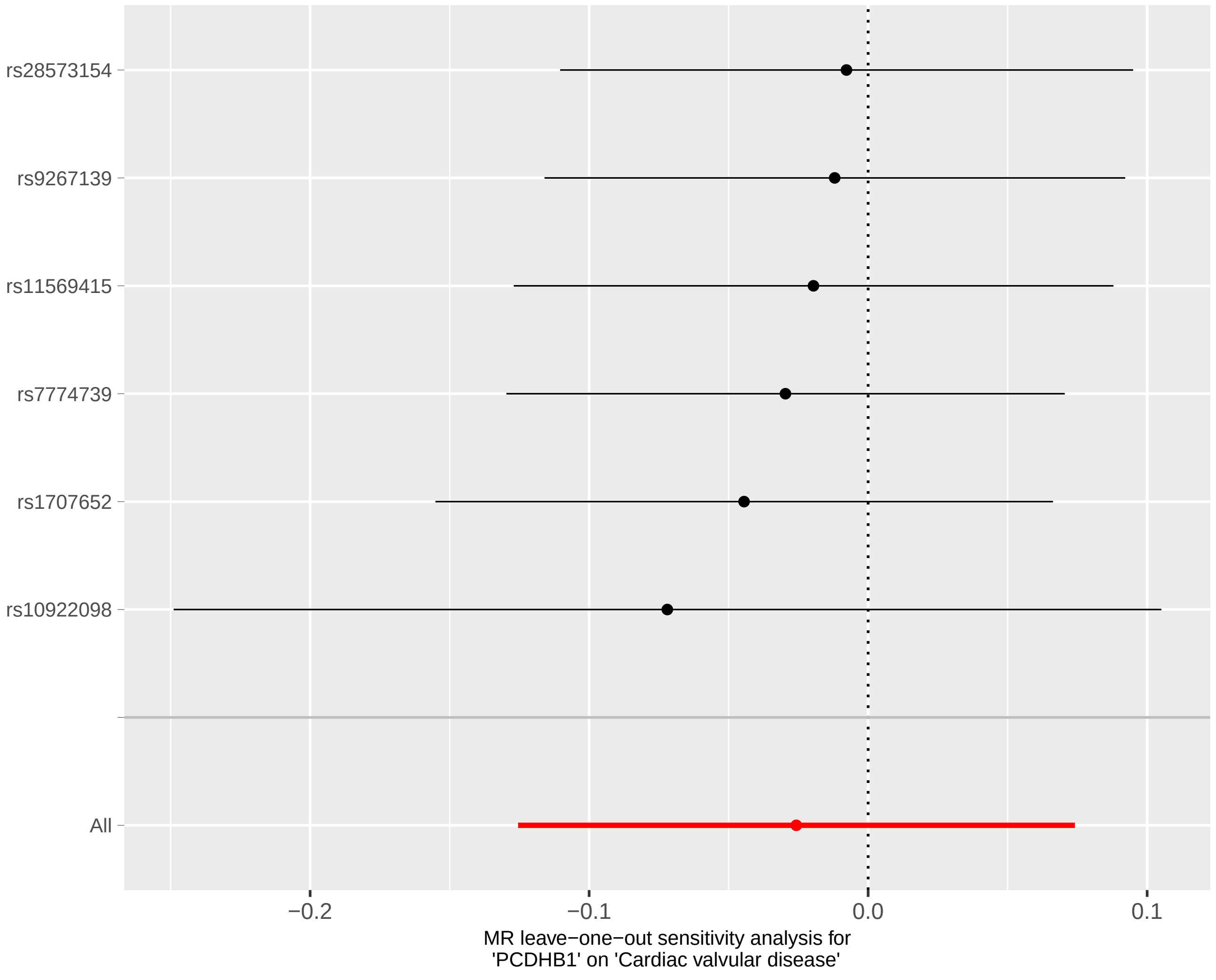
rs28573154
rs9267139
rs11569415
rs7774739
rs1707652
rs10922098
All
−0.2
−0.1
0.0
0.1
MR leave−one−out sensitivity analysis for
'PCDHB1' on 'Cardiac valvular disease'

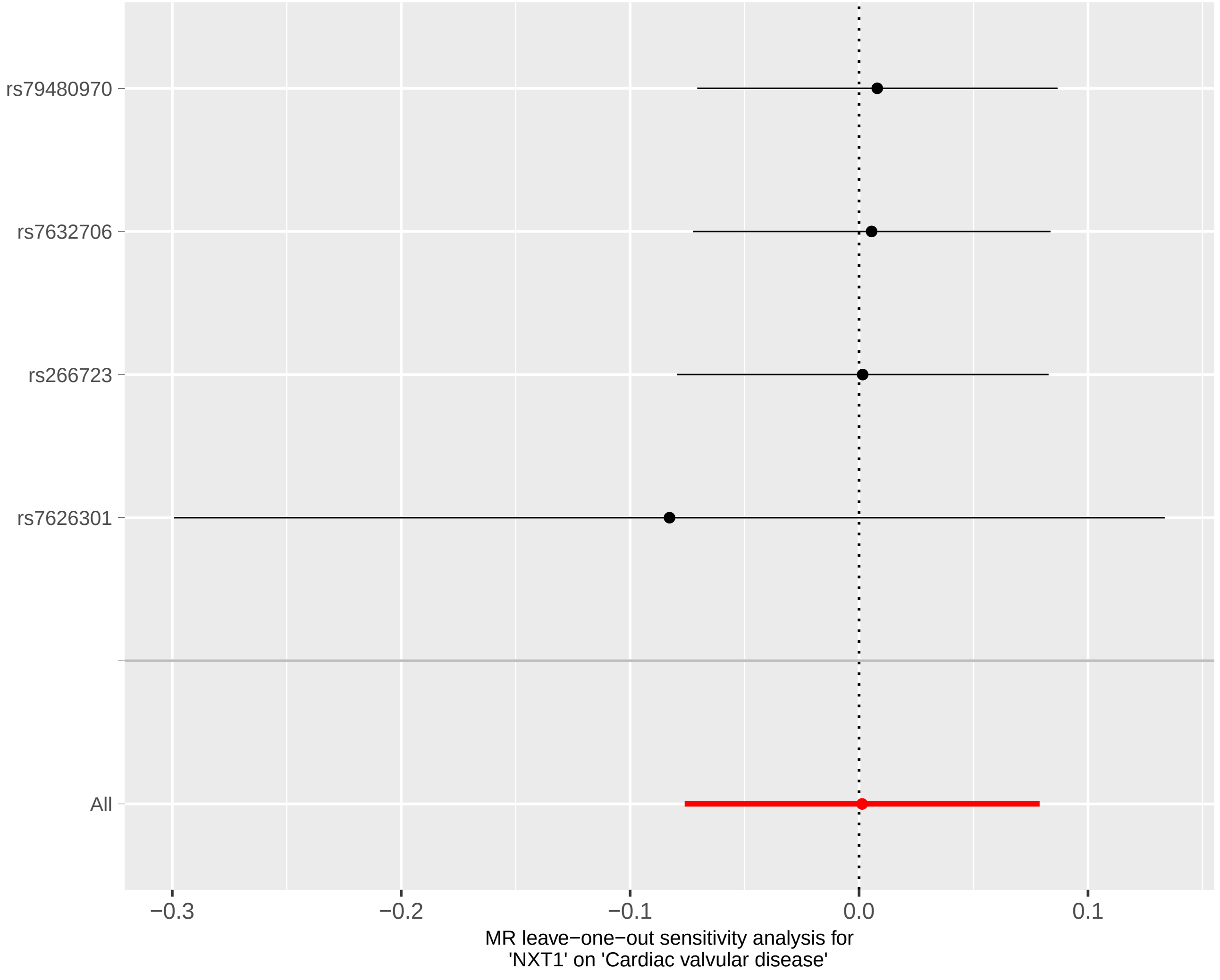

rs79480970
rs7632706
rs266723
rs7626301
All
−0.3
−0.2
−0.1
0.0
0.1
MR leave−one−out sensitivity analysis for
'NXT1' on 'Cardiac valvular disease'

Insufficient number of SNPs

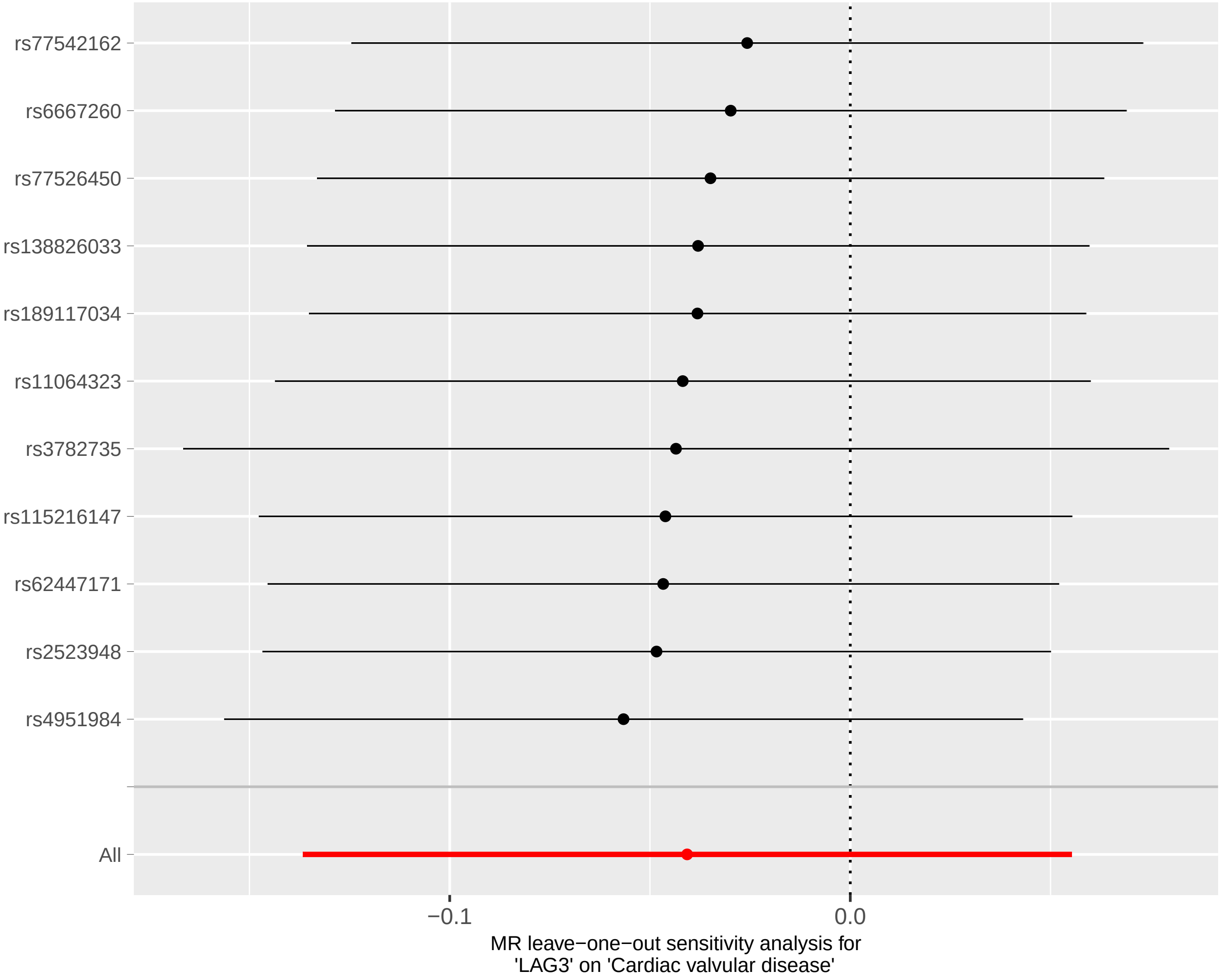
rs77542162
rs6667260
rs77526450
rs138826033
rs189117034
rs11064323
rs3782735
rs115216147
rs62447171
rs2523948
rs4951984
All
−0.1
0.0
MR leave−one−out sensitivity analysis for
'LAG3' on 'Cardiac valvular disease'

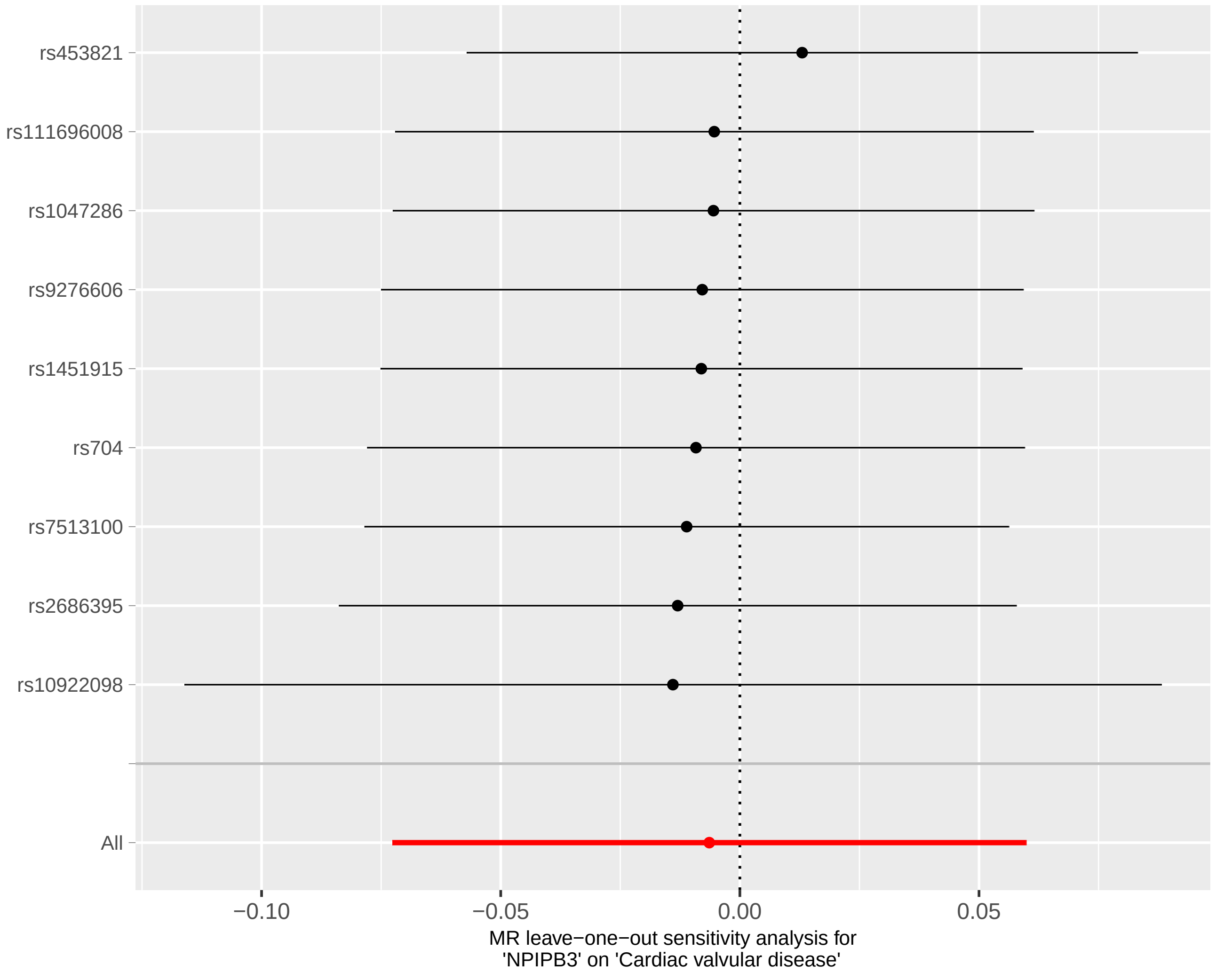

rs453821
rs111696008
rs1047286
rs9276606
rs1451915
rs704
rs7513100
rs2686395
rs10922098
All
−0.10
−0.05
0.00
0.05
MR leave−one−out sensitivity analysis for
'NPIPB3' on 'Cardiac valvular disease'

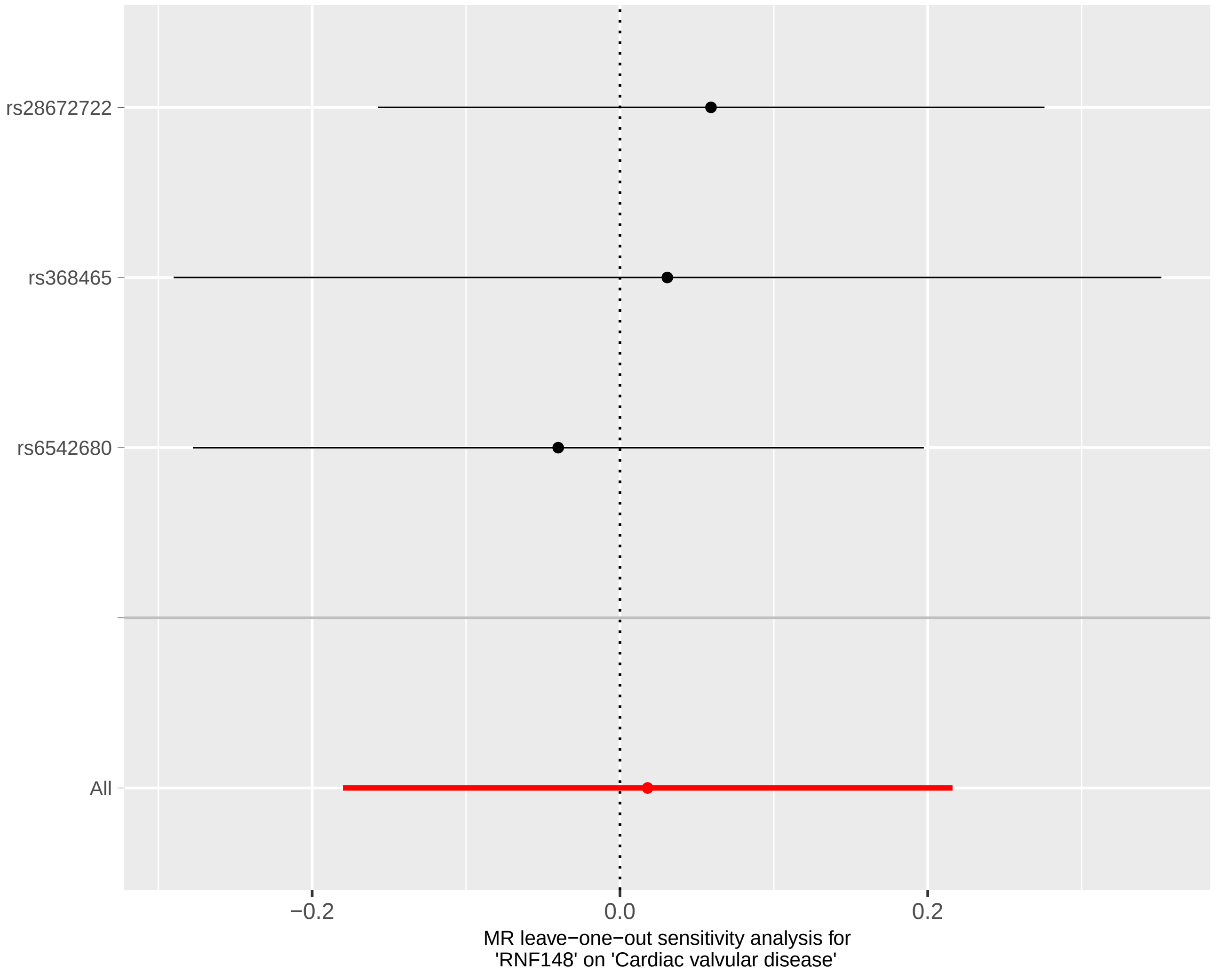
rs28672722
rs368465
rs6542680
All
−0.2
0.0
0.2
MR leave−one−out sensitivity analysis for
'RNF148' on 'Cardiac valvular disease'

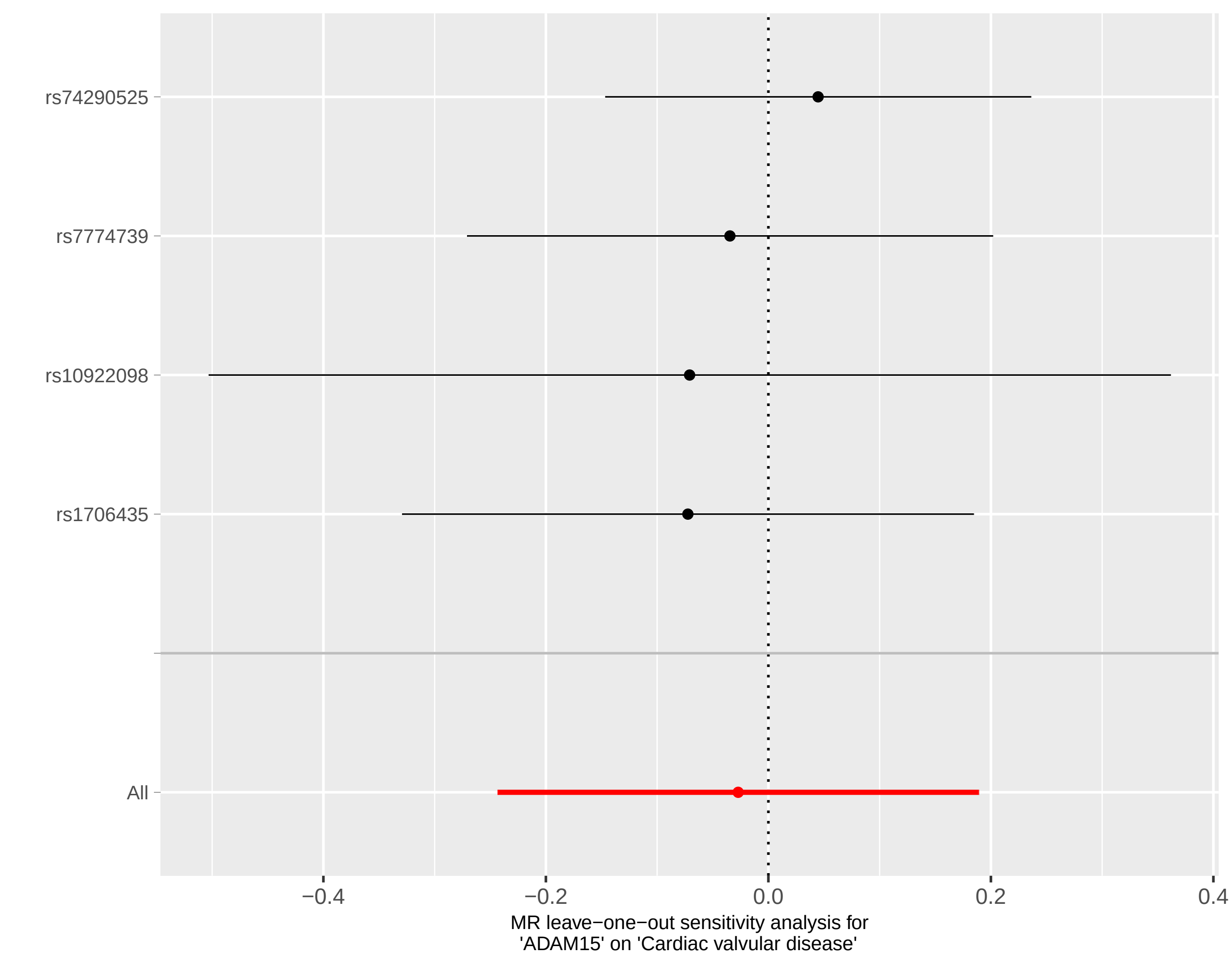
rs74290525
rs7774739
rs10922098
rs1706435
All
−0.4
−0.2
0.0
0.2
0.4
MR leave−one−out sensitivity analysis for
'ADAM15' on 'Cardiac valvular disease'

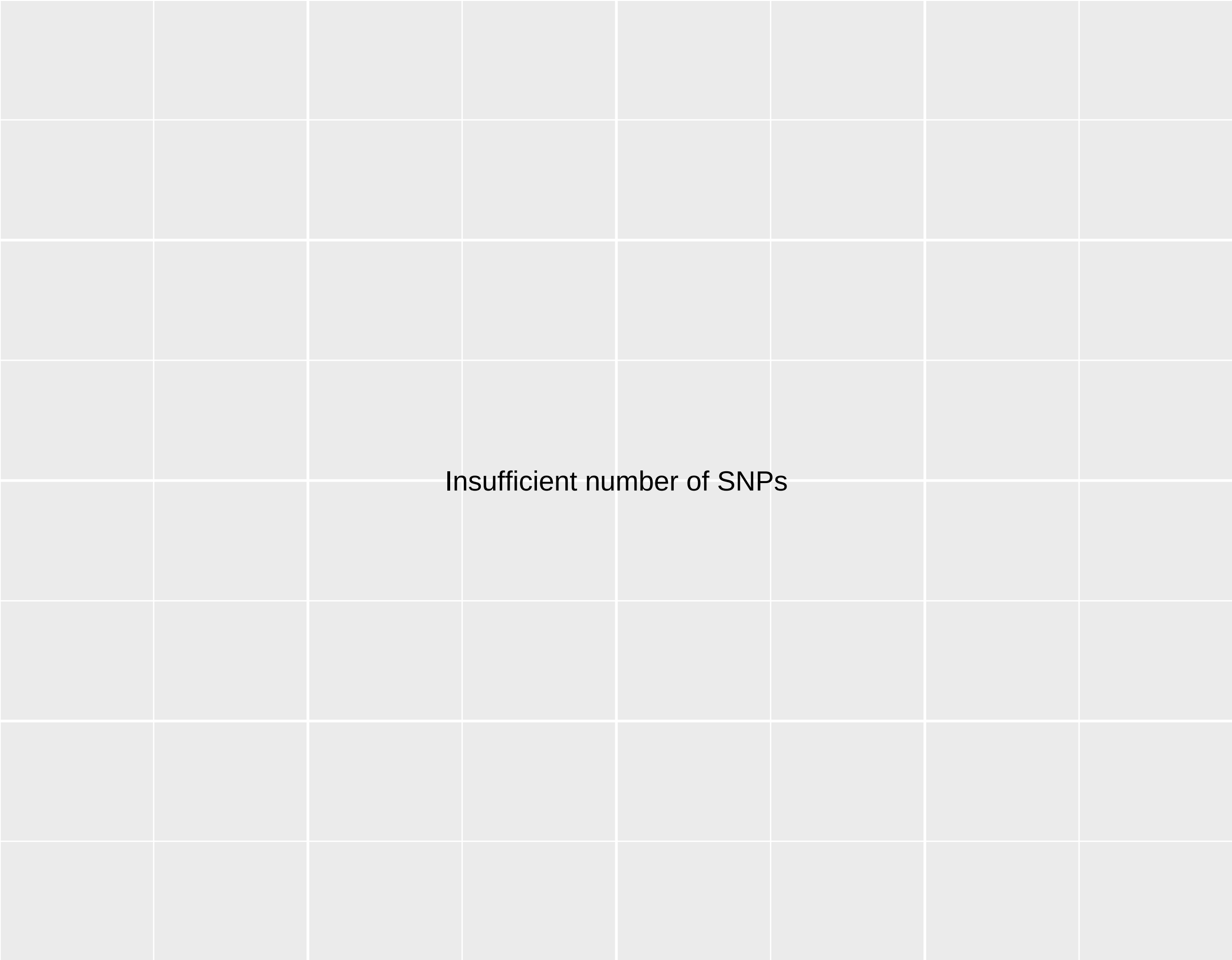
Insufficient number of SNPs

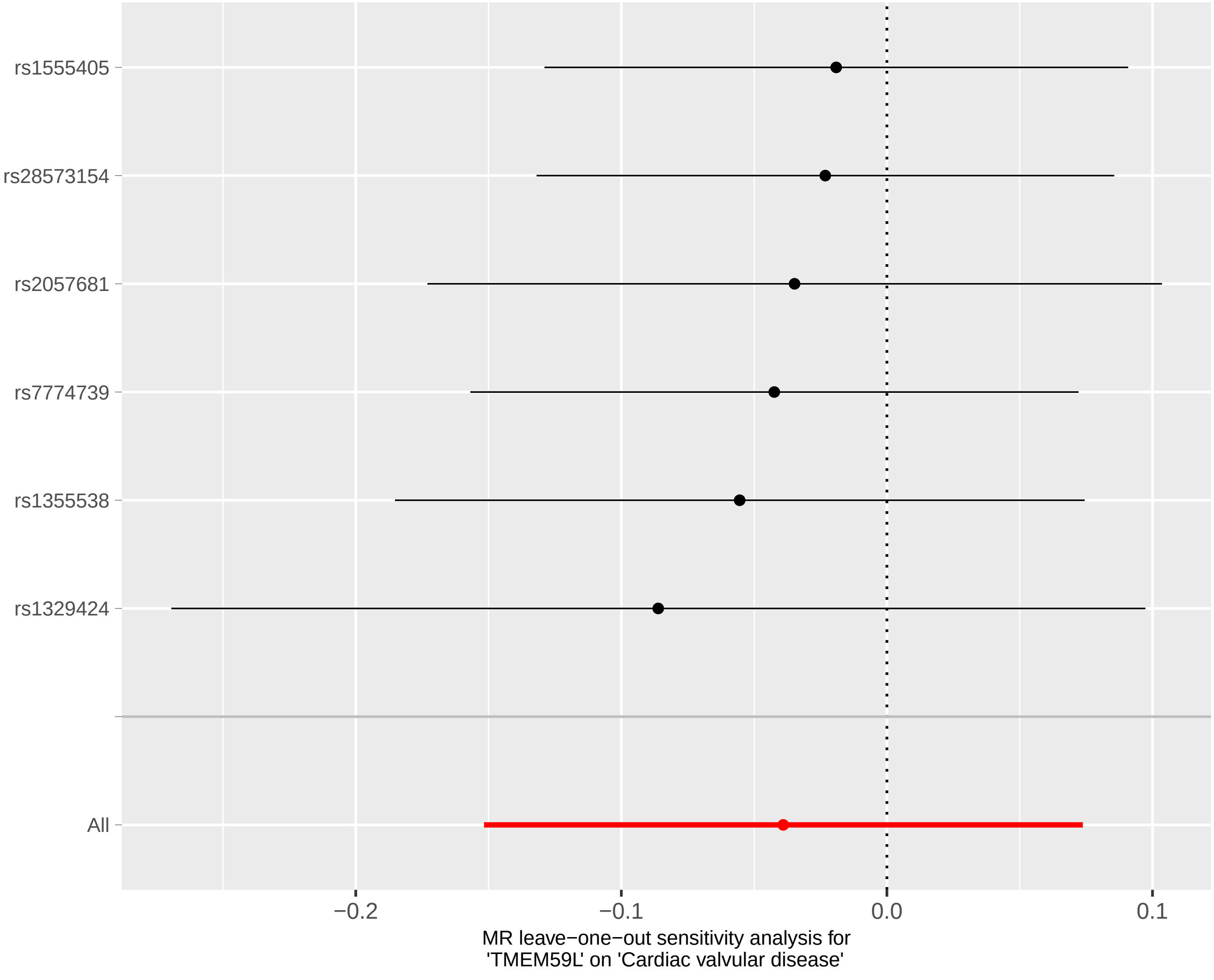
rs1555405
rs28573154
rs2057681
rs7774739
rs1355538
rs1329424
All
−0.2
−0.1
0.0
0.1
MR leave−one−out sensitivity analysis for
'TMEM59L' on 'Cardiac valvular disease'

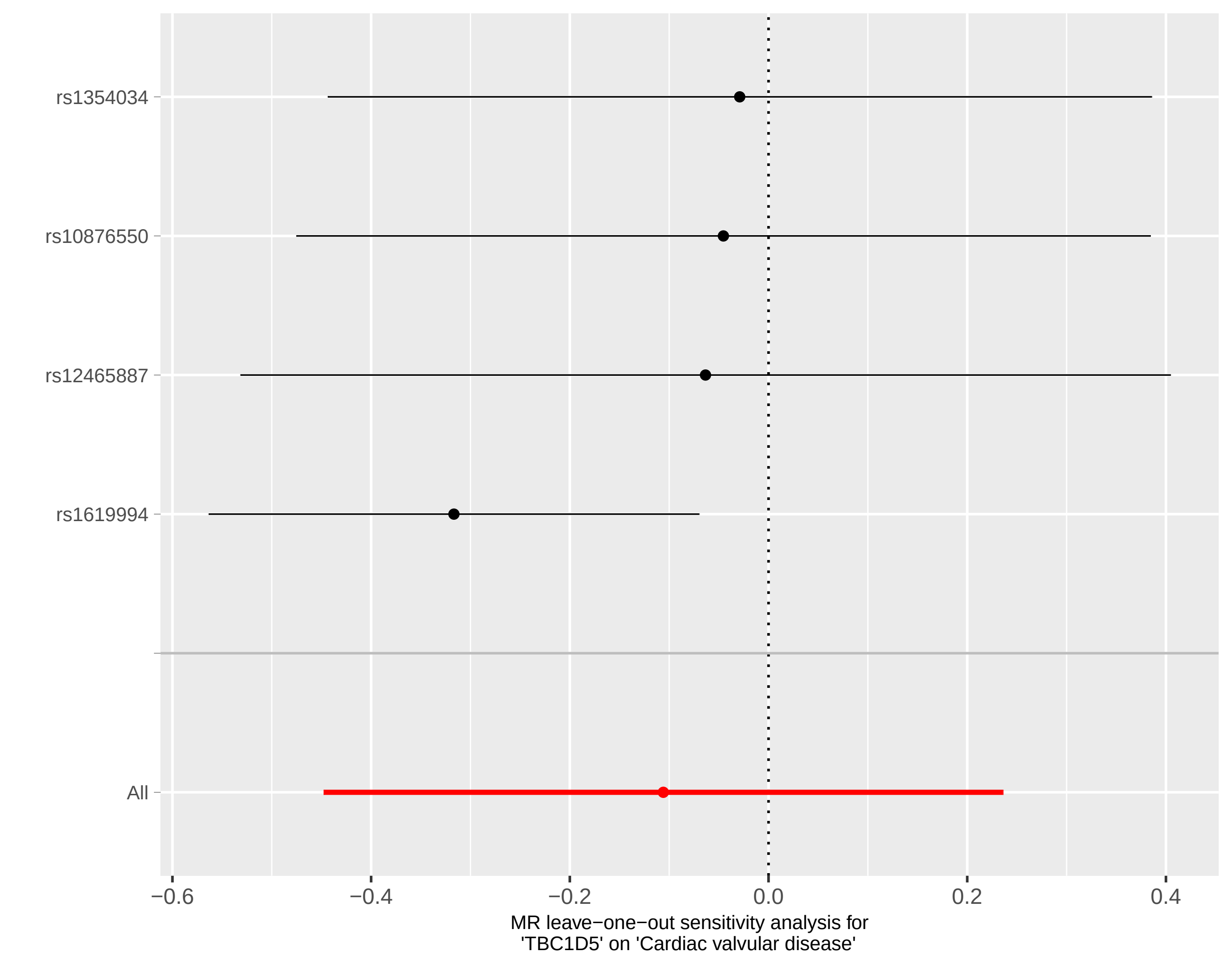

rs1354034
rs10876550
rs12465887
rs1619994
All
−0.6
−0.4
−0.2
0.0
0.2
0.4
MR leave−one−out sensitivity analysis for
'TBC1D5' on 'Cardiac valvular disease'

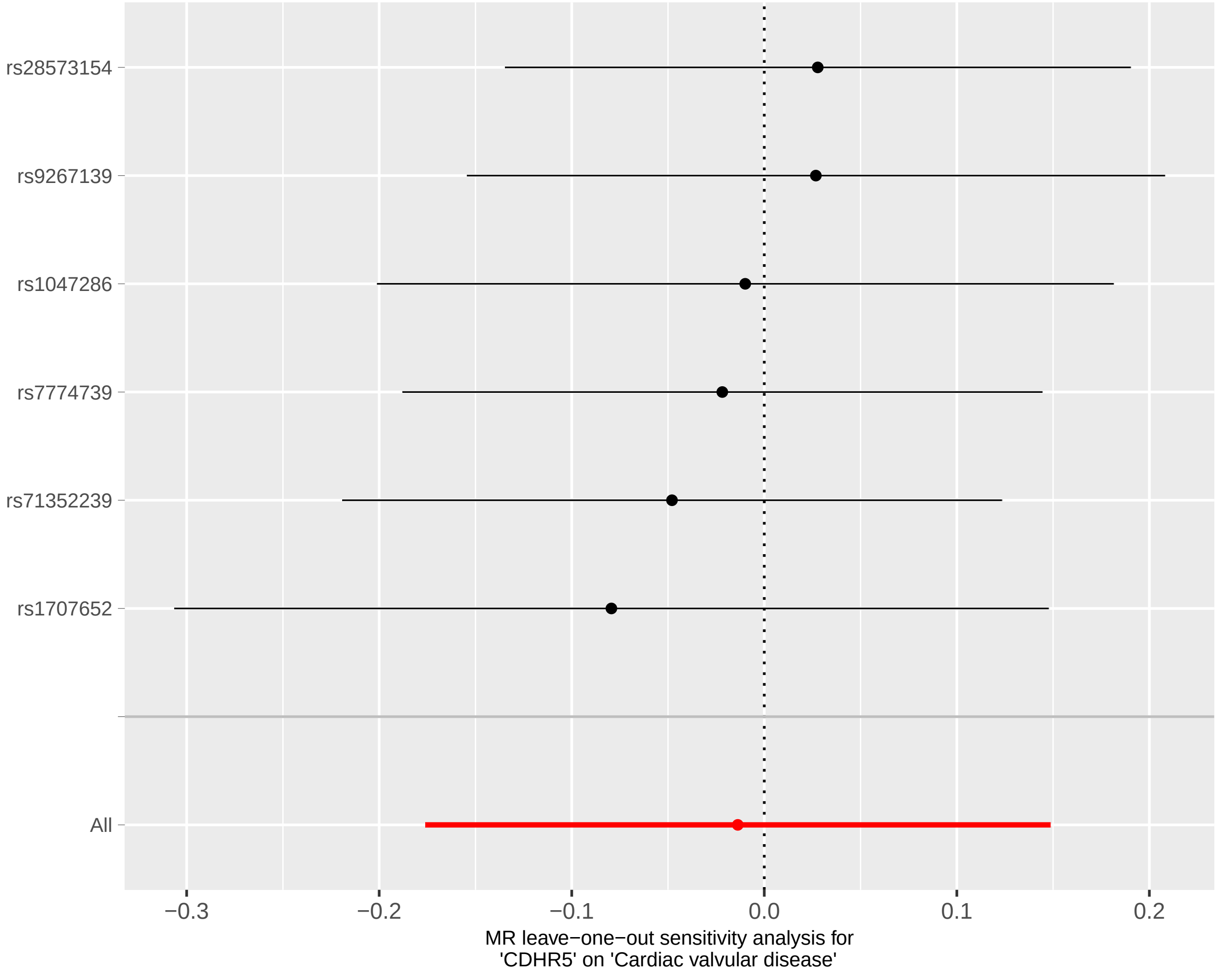
rs28573154
rs9267139
rs1047286
rs7774739
rs71352239
rs1707652
All
−0.3
−0.2
−0.1
0.0
0.1
0.2
MR leave−one−out sensitivity analysis for
'CDHR5' on 'Cardiac valvular disease'

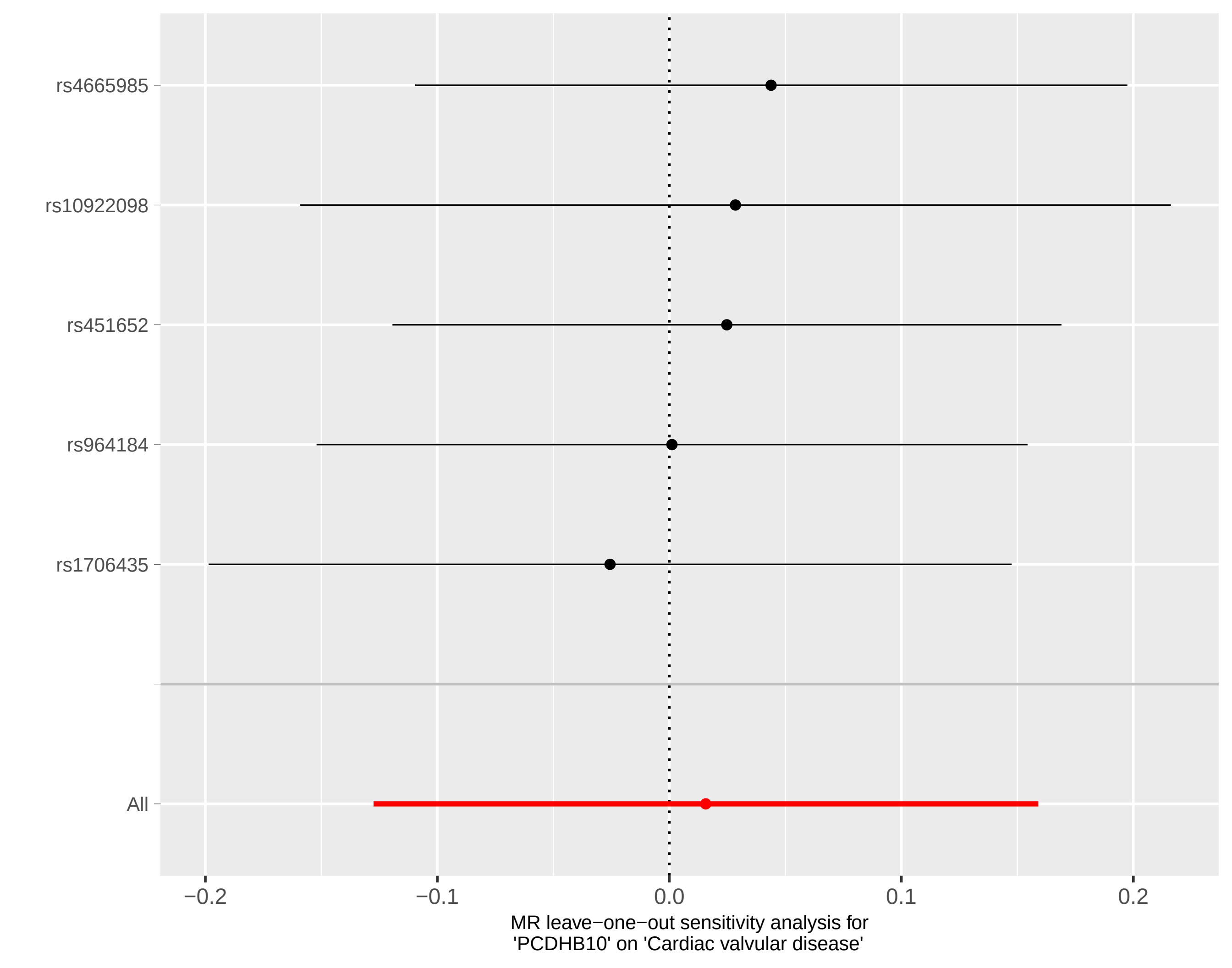

rs4665985
rs10922098
rs451652
rs964184
rs1706435
All
−0.2
−0.1
0.0
0.1
0.2
MR leave−one−out sensitivity analysis for
'PCDHB10' on 'Cardiac valvular disease'

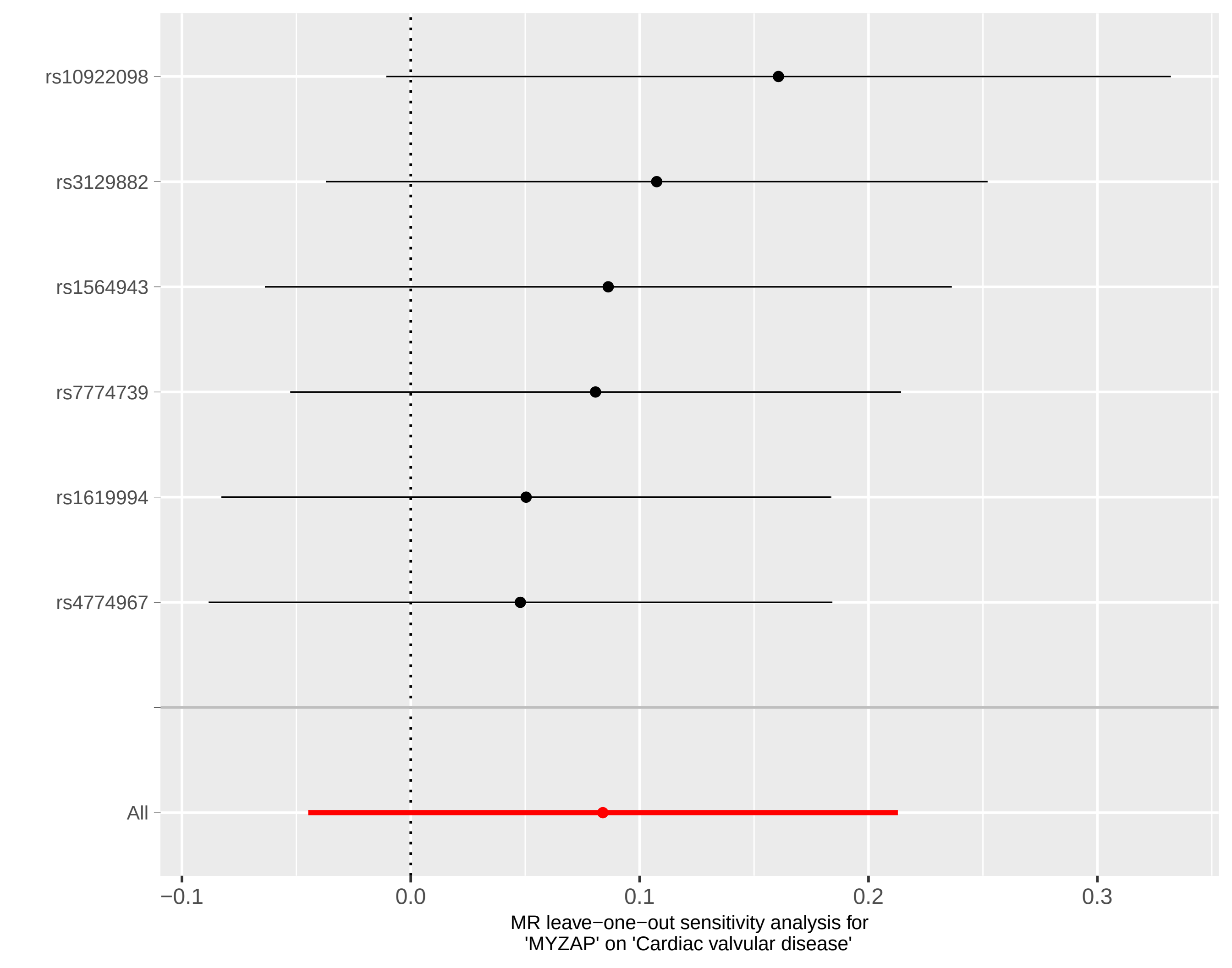
rs10922098
rs3129882
rs1564943
rs7774739
rs1619994
rs4774967
All
−0.1
0.0
0.1
0.2
0.3
MR leave−one−out sensitivity analysis for
'MYZAP' on 'Cardiac valvular disease'

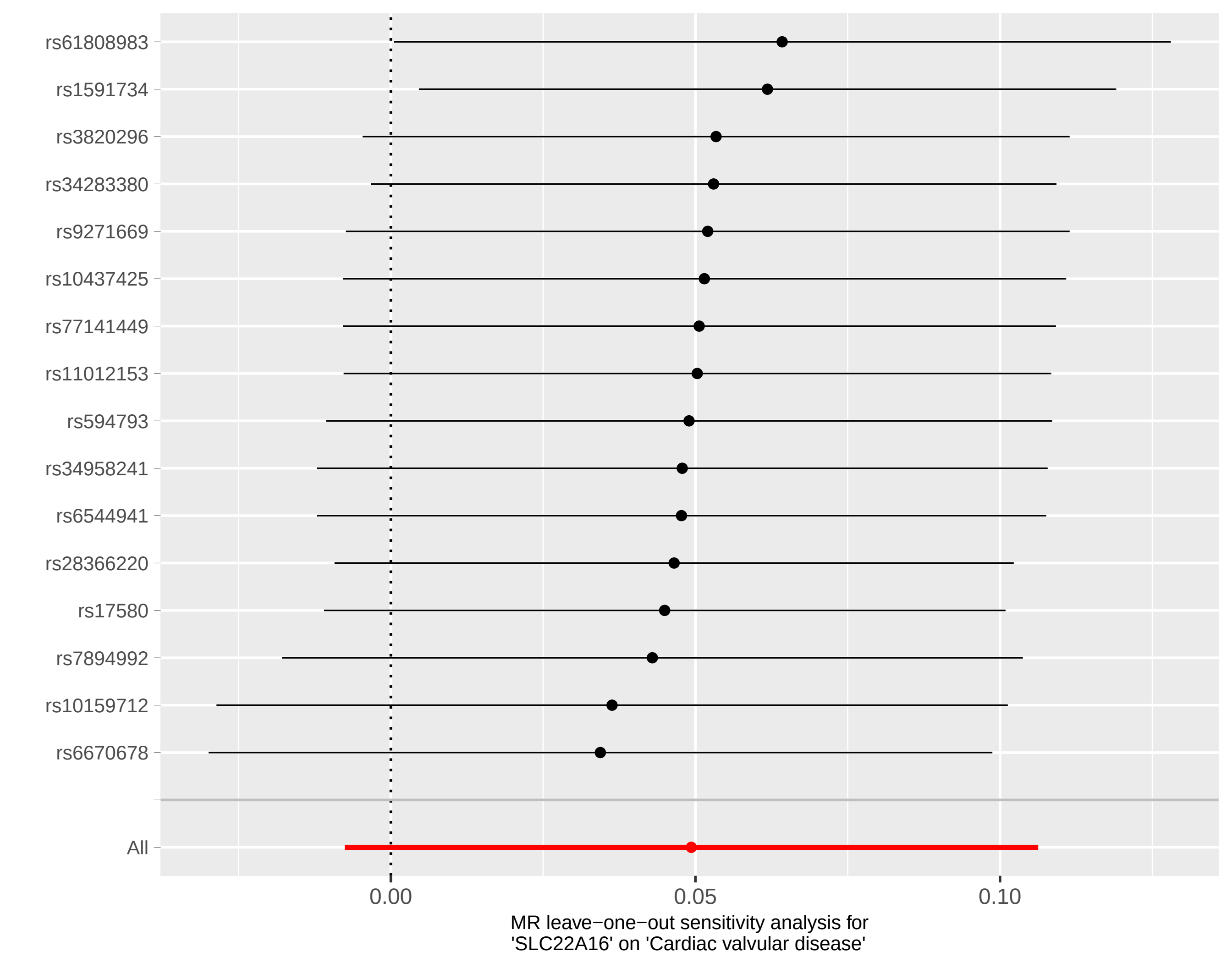
rs61808983
rs1591734
rs3820296
rs34283380
rs9271669
rs10437425
rs77141449
rs11012153
rs594793
rs34958241
rs6544941
rs28366220
rs17580
rs7894992
rs10159712
rs6670678
All
0.00
0.05
0.10
MR leave−one−out sensitivity analysis for
'SLC22A16' on 'Cardiac valvular disease'

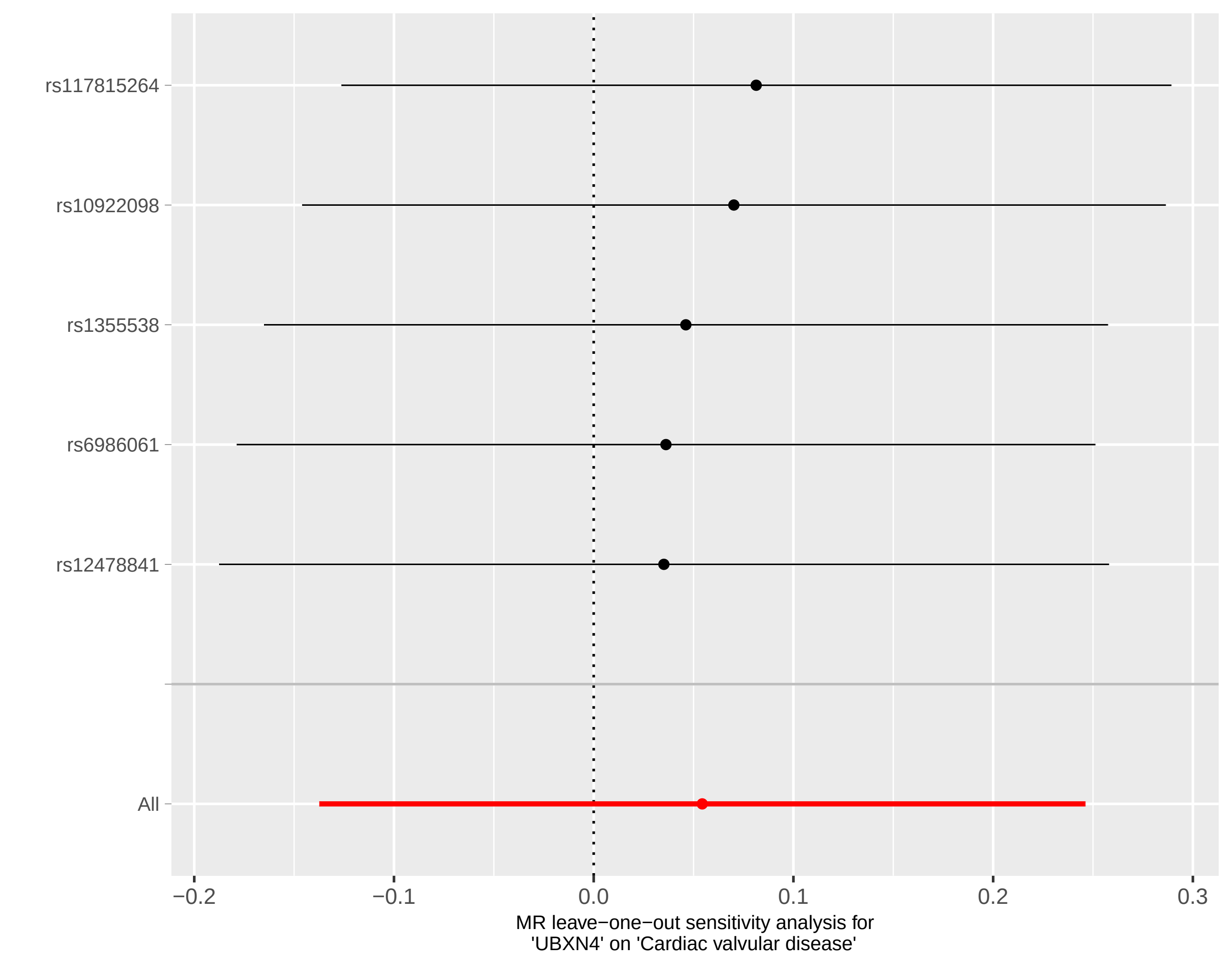

rs117815264
rs10922098
rs1355538
rs6986061
rs12478841
All
−0.2
−0.1
0.0
0.1
0.2
0.3
MR leave−one−out sensitivity analysis for
'UBXN4' on 'Cardiac valvular disease'

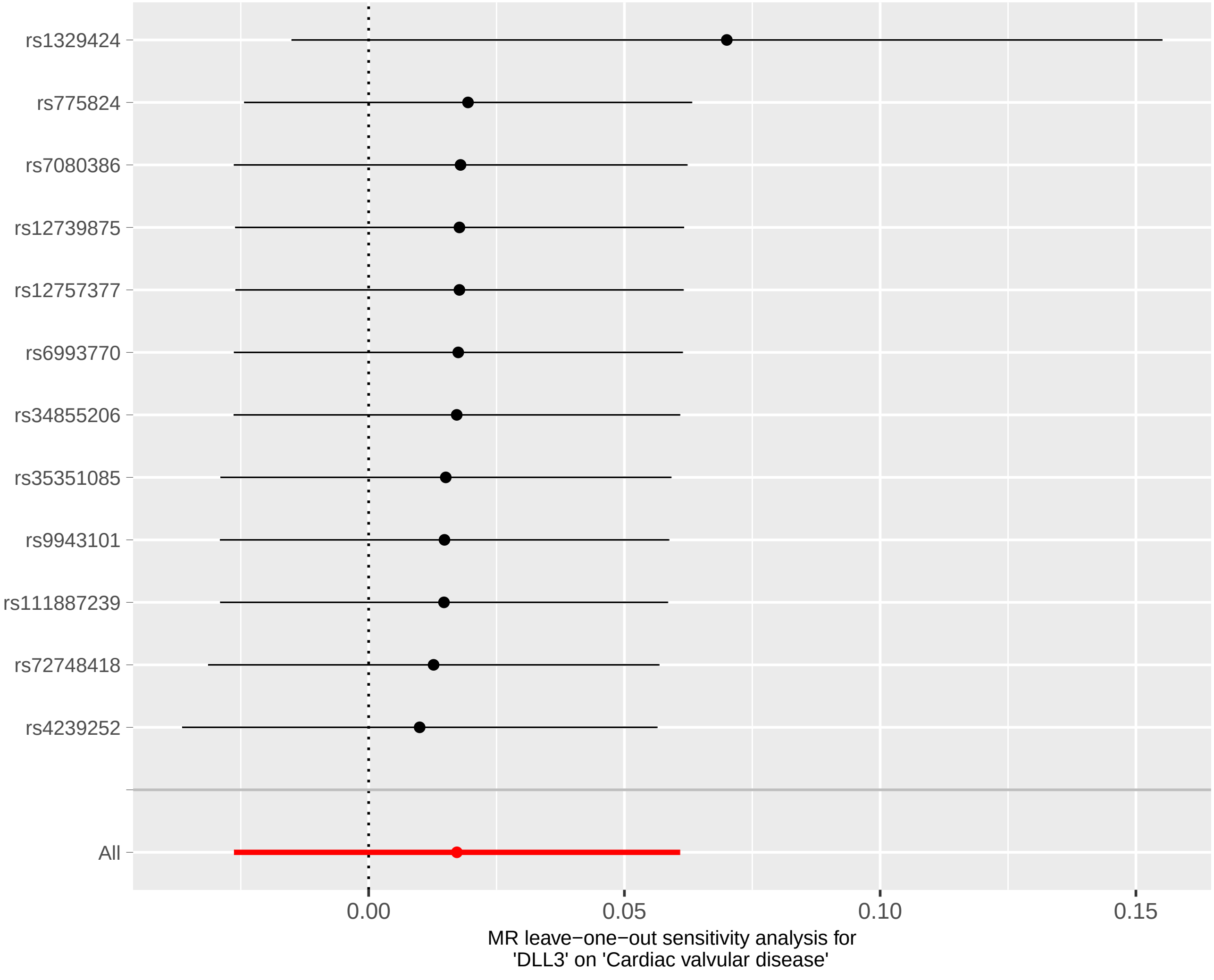
rs1329424
rs775824
rs7080386
rs12739875
rs12757377
rs6993770
rs34855206
rs35351085
rs9943101
rs111887239
rs72748418
rs4239252
All
0.00
0.05
0.10
0.15
MR leave−one−out sensitivity analysis for
'DLL3' on 'Cardiac valvular disease'

Insufficient number of SNPs

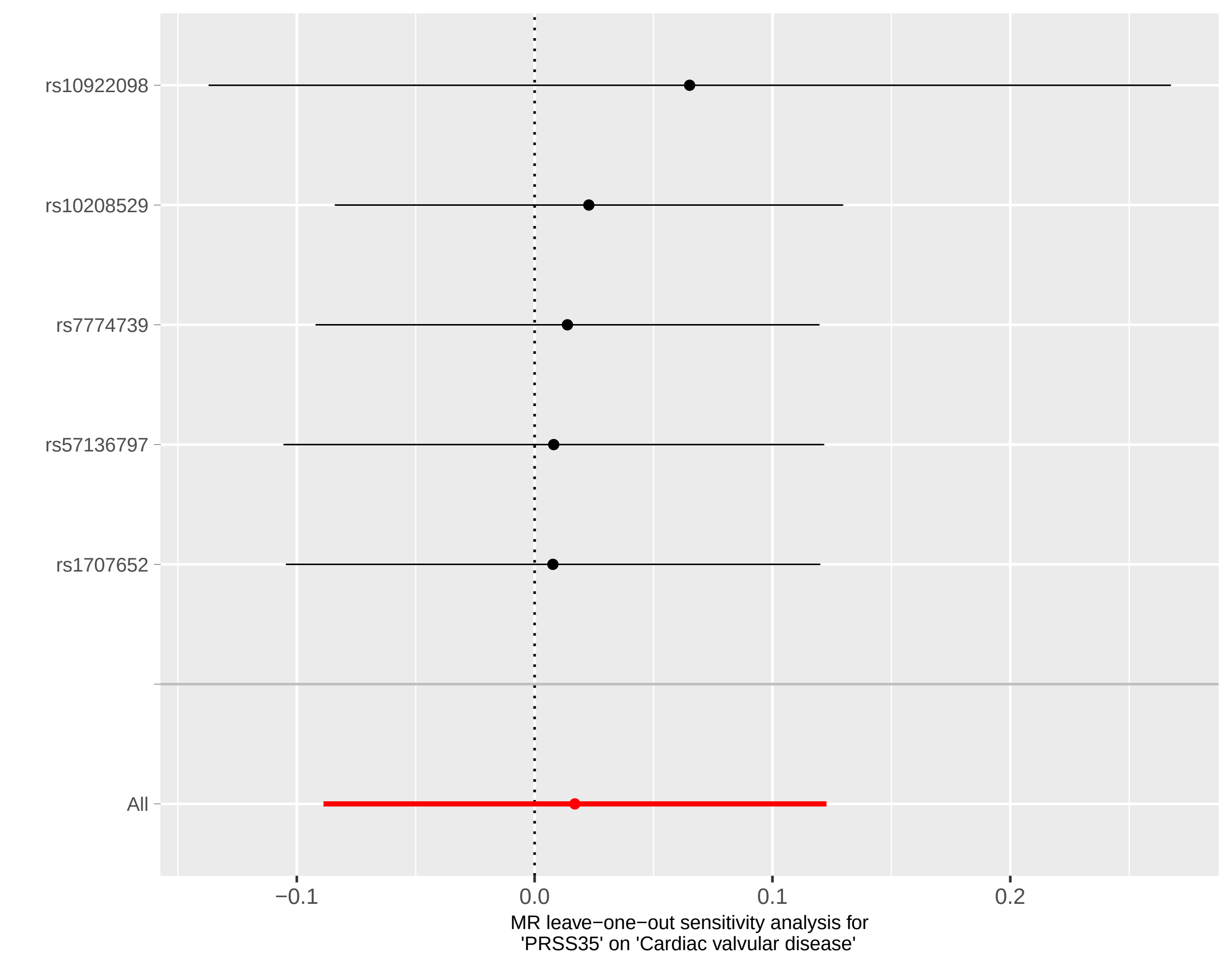
rs10922098
rs10208529
rs7774739
rs57136797
rs1707652
All
−0.1
0.0
0.1
0.2
MR leave−one−out sensitivity analysis for
'PRSS35' on 'Cardiac valvular disease'

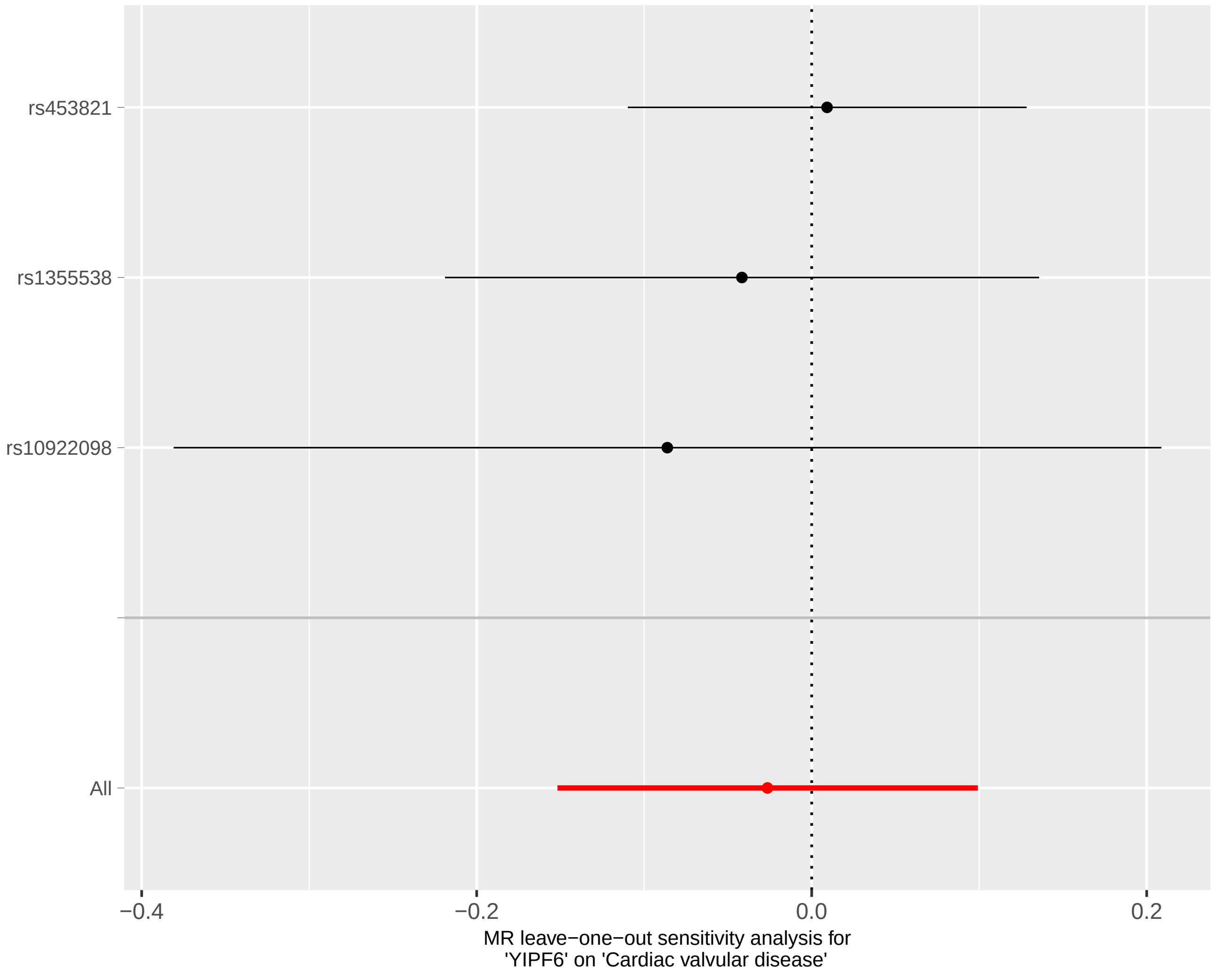
rs453821
rs1355538
rs10922098
All
−0.4
−0.2
0.0
0.2
MR leave−one−out sensitivity analysis for
'YIPF6' on 'Cardiac valvular disease'

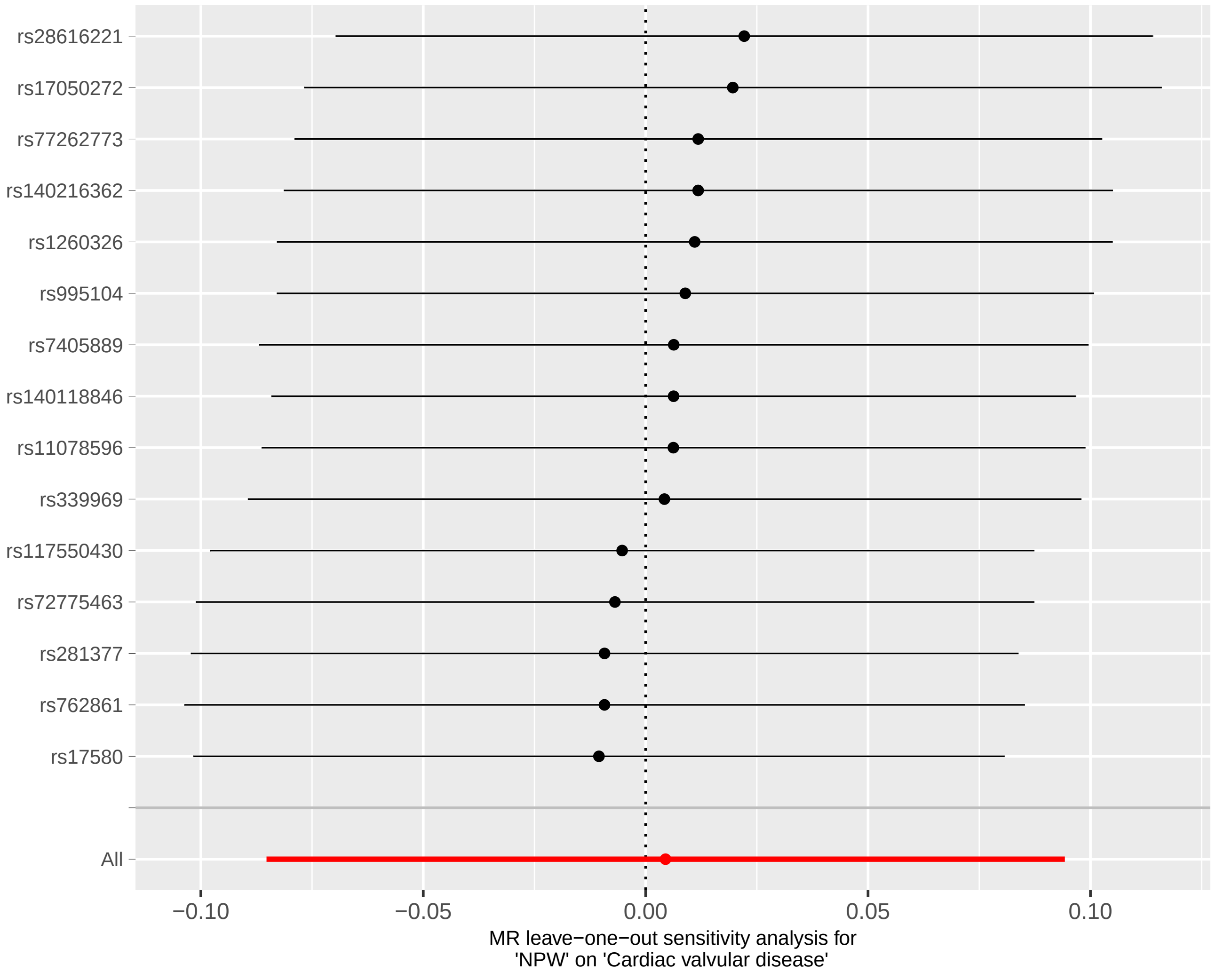
rs28616221
rs17050272
rs77262773
rs140216362
rs1260326
rs995104
rs7405889
rs140118846
rs11078596
rs339969
rs117550430
rs72775463
rs281377
rs762861
rs17580
All
−0.10
−0.05
0.00
0.05
0.10
MR leave−one−out sensitivity analysis for
'NPW' on 'Cardiac valvular disease'

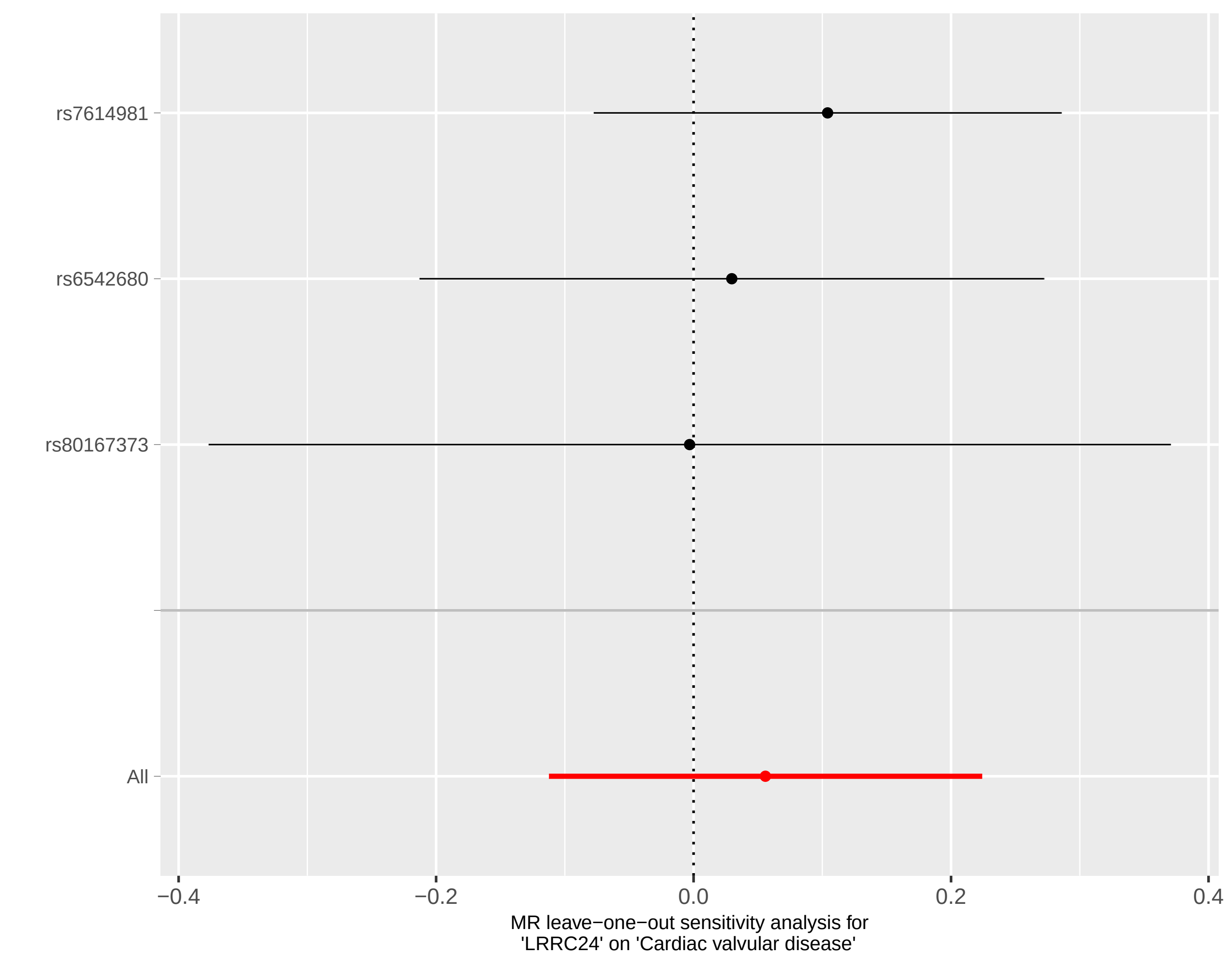
rs7614981
rs6542680
rs80167373
All
−0.4
−0.2
0.0
0.2
0.4
MR leave−one−out sensitivity analysis for
'LRRC24' on 'Cardiac valvular disease'

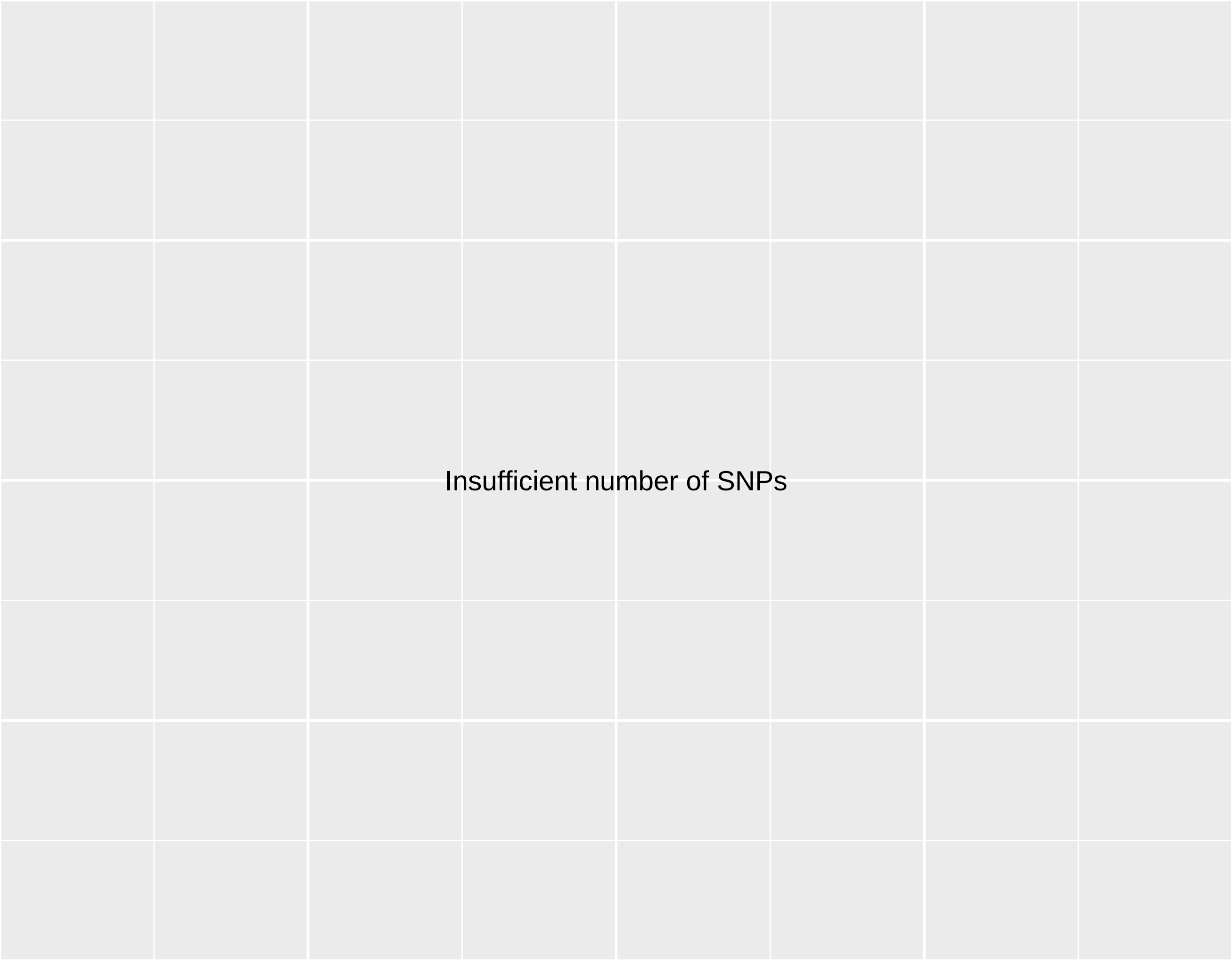
Insufficient number of SNPs

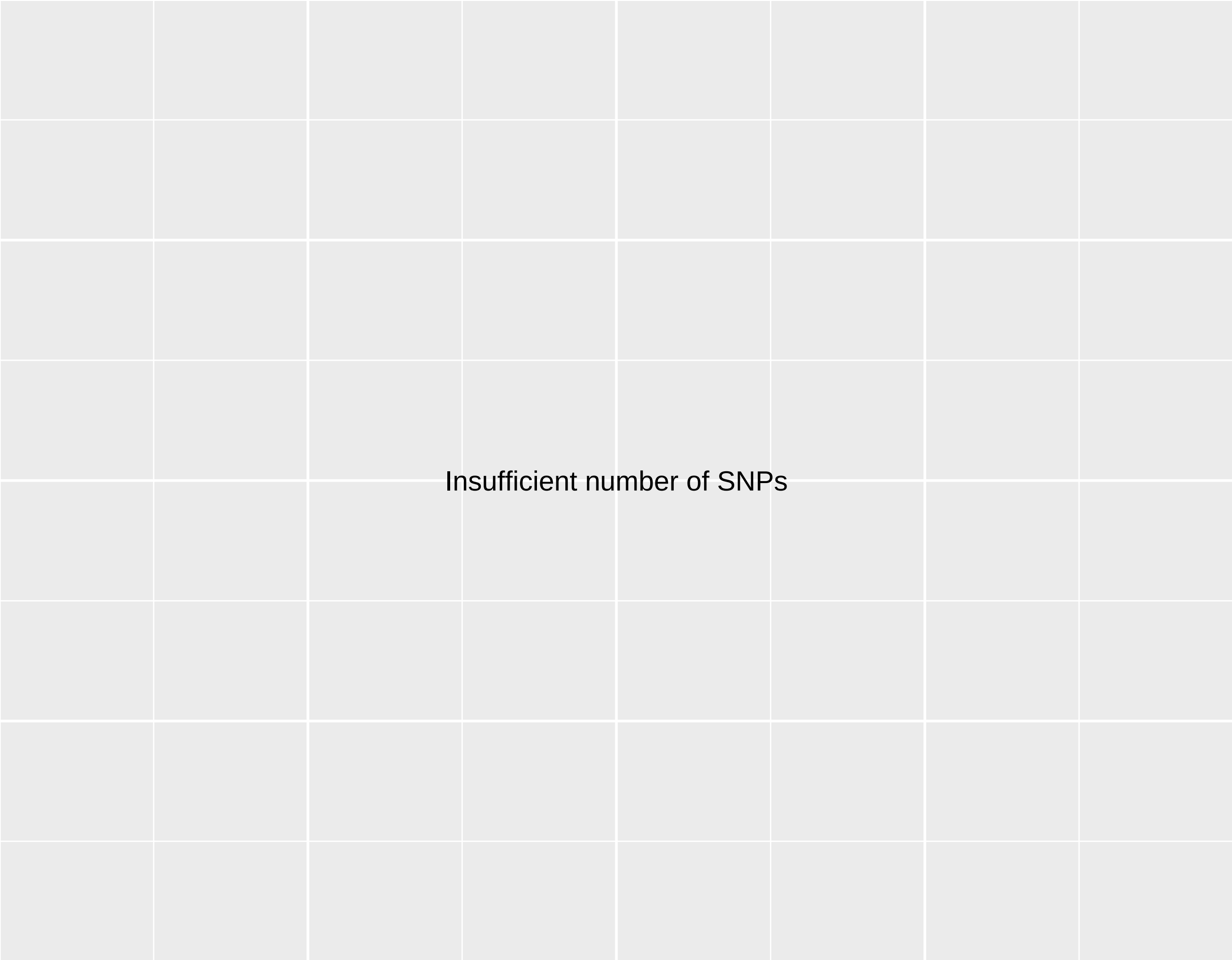
Insufficient number of SNPs

Insufficient number of SNPs

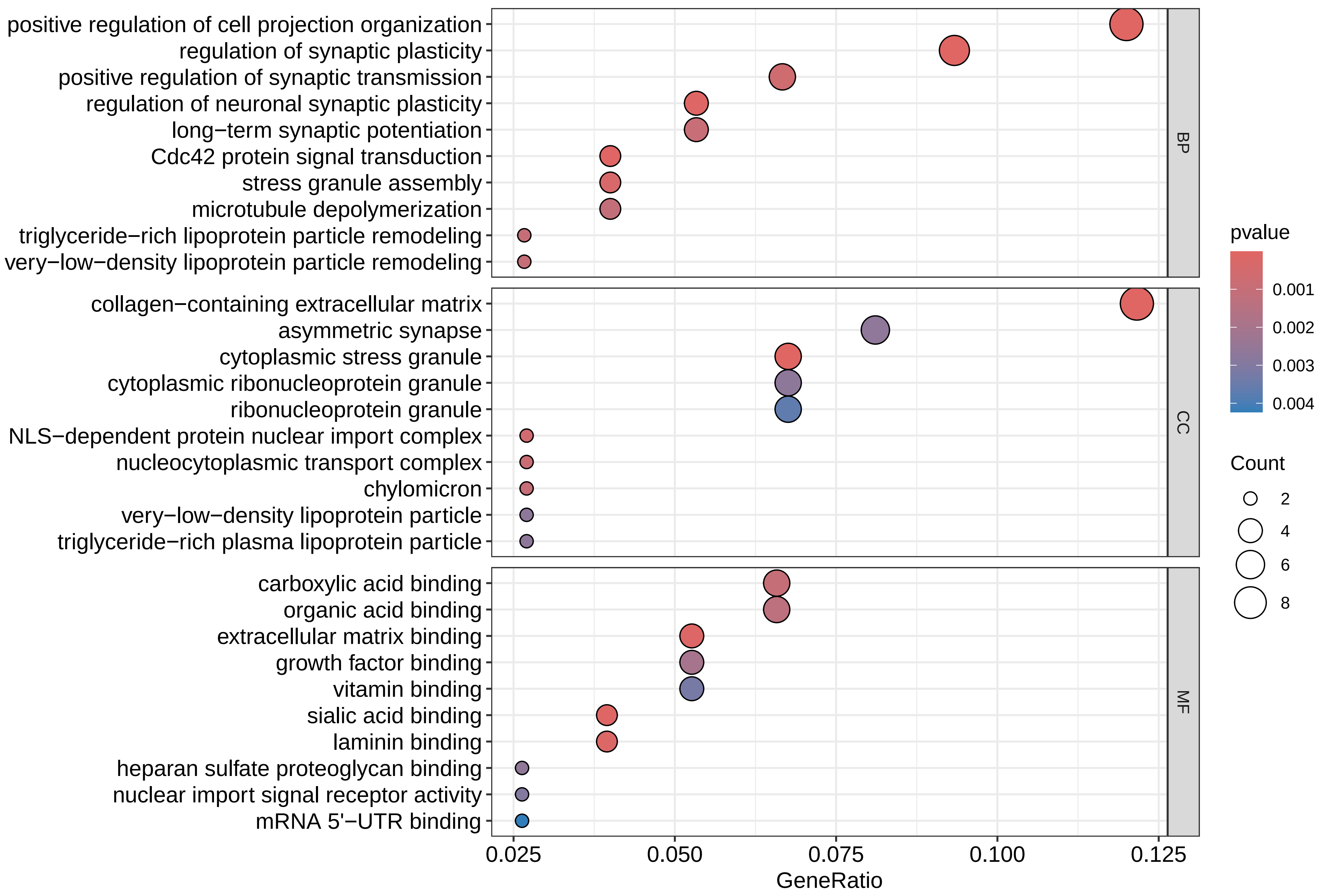
positive regulation of cell projection organization
regulation of synaptic plasticity
positive regulation of synaptic transmission
regulation of neuronal synaptic plasticity
long−term synaptic potentiation
Cdc42 protein signal transduction
stress granule assembly
microtubule depolymerization
triglyceride−rich lipoprotein particle remodeling
very−low−density lipoprotein particle remodeling
collagen−containing extracellular matrix
asymmetric synapse
cytoplasmic stress granule
cytoplasmic ribonucleoprotein granule
ribonucleoprotein granule
NLS−dependent protein nuclear import complex
nucleocytoplasmic transport complex
chylomicron
very−low−density lipoprotein particle
triglyceride−rich plasma lipoprotein particle
carboxylic acid binding
organic acid binding
extracellular matrix binding
growth factor binding
vitamin binding
sialic acid binding
laminin binding
heparan sulfate proteoglycan binding
nuclear import signal receptor activity
mRNA 5'−UTR binding
BP
CC
MF
0.025
0.050
0.075
0.100
0.125
GeneRatio
pvalue
0.001
0.002
0.003
0.004
Count
2
4
6
8

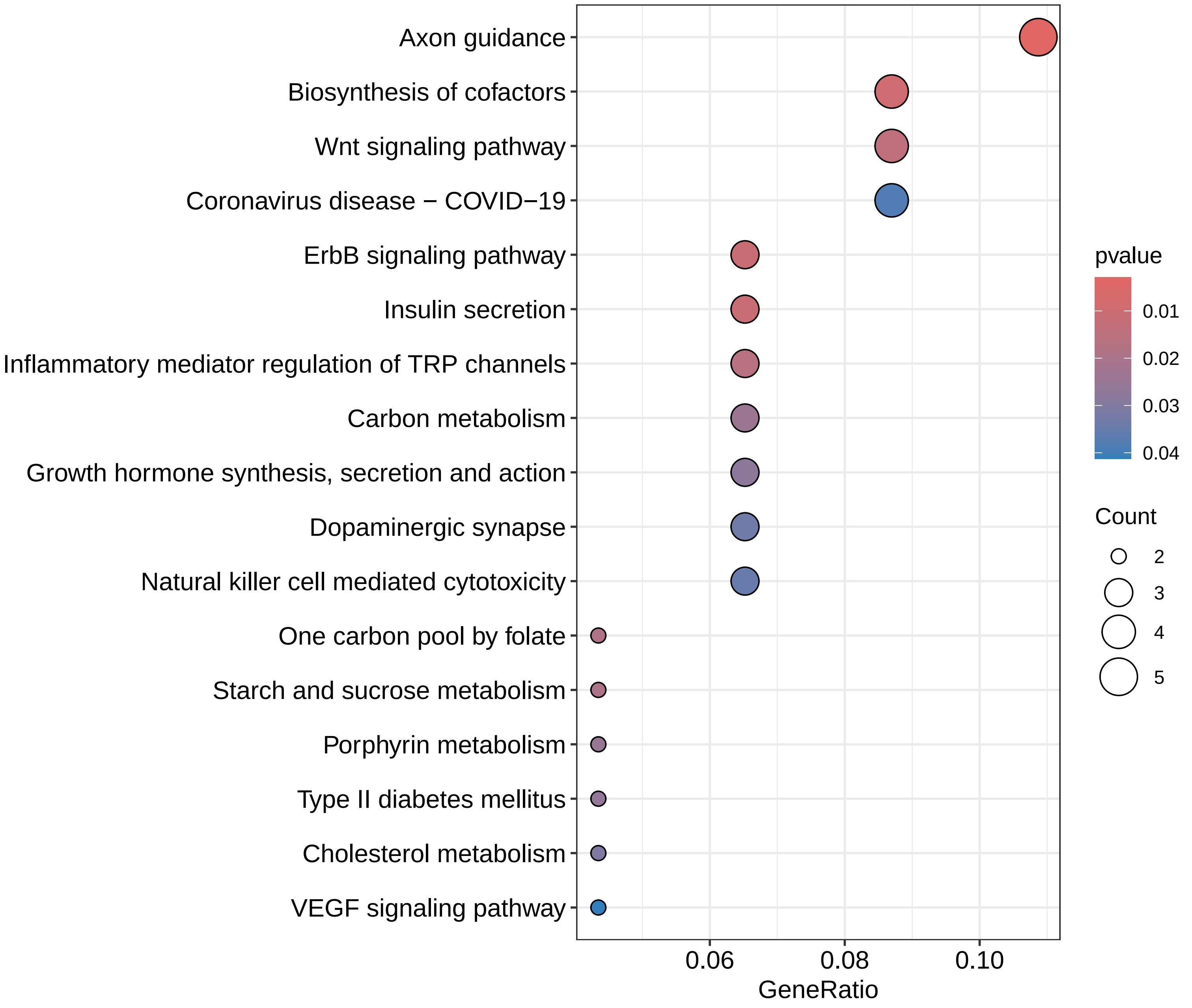

Axon guidance
Biosynthesis of cofactors
Wnt signaling pathway
Coronavirus disease − COVID−19
ErbB signaling pathway
Insulin secretion
Inflammatory mediator regulation of TRP channels
Carbon metabolism
Growth hormone synthesis, secretion and action
Dopaminergic synapse
Natural killer cell mediated cytotoxicity
One carbon pool by folate
Starch and sucrose metabolism
Porphyrin metabolism
Type II diabetes mellitus
Cholesterol metabolism
VEGF signaling pathway
0.06
0.08
0.10
GeneRatio
pvalue
0.01
0.02
0.03
0.04
Count
2
3
4
5
